# Artefact-free topography based scleral-asymmetry

**DOI:** 10.1371/journal.pone.0219789

**Published:** 2019-07-26

**Authors:** Ahmed Abass, Bernardo T. Lopes, Ashkan Eliasy, Marcella Salomao, Richard Wu, Lynn White, Steve Jones, John Clamp, Renato Ambrósio, Ahmed Elsheikh

**Affiliations:** 1 School of Engineering, University of Liverpool, Liverpool, United Kingdom; 2 Federal University of São Paulo, São Paulo, Brazil; 3 Central Taiwan University of Science and Technology, Taichung, Taiwan; 4 Pacific University, College of Optometry, Forest Grove, Oregon, United States of America; 5 UltraVision CLPL, Leighton Buzzard, United Kingdom; 6 Federal University of the State of Rio de Janeiro, RJ, Brazil; 7 National Institute for Health Research (NIHR) Biomedical Research Centre at Moorfields Eye Hospital NHS Foundation Trust and UCL Institute of Ophthalmology, London, United Kingdom; 8 School of Biological Science and Biomedical Engineering, Beihang University, Beijing, China; Aston University School of Life and Health Sciences, UNITED KINGDOM

## Abstract

**Purpose:**

To present a three-dimensional non-parametric method for detecting scleral asymmetry using corneoscleral topography data that are free of edge-effect artefacts.

**Methods:**

The study included 88 participants aged 23 to 65 years (37.7±9.7), 47 women and 41 men. The eye topography data were exported from the Eye Surface Profiler software in MATLAB binary data container format then processed by custom built MATLAB codes entirely independent from the profiler software. Scleral asymmetry was determined initially from the unprocessed topography before being determined again after removing the edge-effect noise. Topography data were levelled around the limbus, then edge-effect was eliminated using a robust statistical moving median technique. In addition to comparing raw elevation data, scleral elevation was also compared through fitting a sphere to every single scleral surface and determining the relative elevation from the best-fit sphere reference surface.

**Results:**

When considering the averaged raw topography elevation data in the scleral section of the eye at radius 8 mm, the average raw elevations of the right eyes’ sclera were -1.5±1.77, -1.87±2.12, -1.36±1.82 and -1.57±1.87 mm. In the left eyes at the same radius the average raw elevations were -1.62±1.78, -1.82±2.07, -1.28±1.76 and -1.68±1.93 mm. While, when considering the average raw elevation of the sclera after removing the edge effect, the average raw elevations of the right eyes were -3.71±0.25, -4.06±0.23, -3.95±0.19 and -3.95±0.23 mm. In the left eyes at the same radius the average raw elevations were -3.71±0.19, -3.97±0.22, -3.96±0.19 and -3.96±0.18 mm in the nasal, temporal, superior and inferior sides respectively. Maximum raw elevation asymmetry in the averaged scleral raw elevation was 1.6647±0.9015 mm in right eyes and 1.0358±0.6842 mm in left eyes, both detected at -38° to the nasal side. Best-fit sphere-based relative elevation showed that sclera is more elevated in three main meridians at angles -40°, 76°, and 170° in right eyes and -40°, 76°, and 170° in left eyes, all measured from the nasal meridian. Maximum recorded relative elevation asymmetries were 0.0844±0.0355 mm and 0.068±0.0607 mm at angular positions 76° and 63.5° for right and left eyes in turn.

**Conclusions:**

It is not possible to use corneoscleral topography data to predict the scleral shape without considering a method of removing the edge-effect from the topography data. The nasal side of the sclera is higher than the temporal side, therefore, rotationally symmetric scleral contact lenses are more likely to be translated towards the temporal side. The scleral shape is best described by levelled raw elevation rather than relative elevation.

## Introduction

The anterior ocular surface consists of two main components; the cornea and the sclera, they are different in many ways. The cornea is a refractive element that provides more than 70% of the eye’s refractive power [1, 2], while the sclera provides the mechanical strength which maintains the eye’s shape and withstands the intra-ocular pressure [3]. In addition, it guarantees that the light scattered within the eyeball does not disturb the retinal image and it also facilitates rotation of eye via muscles [4]. The bearing surfaces of scleral lenses rest, as the name suggests, on the sclera which has the advantage of being significantly less innervated than the cornea and therefore cause less discomfort than rigid corneal lenses [5]. In recent years, the scleral contact lens market has increased, leading to professional interest in fitting the anterior scleral segment more accurately. To facilitate this, and contact lens fitting in general, there is an increased demand for evaluation of the anterior scleral profile in three dimensions [6–12].

In a review, Walker considered the asymmetric sclera as a major fitting challenge associated with scleral contact lenses [13] and recommended that the even distribution of the weight of the lens around the entire circumference of the eye should be the goal in scleral contact lenses fitting.

The use of optical coherence tomography (OCT) based machines to characterise the human sclera *in-vivo* is common practice [14–17]. While OCT may provide a detailed image of the ocular structure, it has a shared disadvantage that no continuum eye surface can be measured at one time by a single measurement. Also, subjective measurement inaccuracies arise due to lack of an automated process. Moreover, as segmentation is necessary for reconstructing the eye’s components in three-dimensions in all OCT-based methods, limitations in the ability to align images accurately can pollute the measurement quality [18]. The main issue when using OCT for conjunctival mapping is the parallax generated by the necessity to have the eye turned to the right or to the left to acquire a wider scan. The process of stitching together the images causes the reference plane to be lost and consequently, the peripheral curves are mistranslated. Furthermore, as with any transmitted signal, OCT images are affected by digital noise. In the imaging area where there is a strong signal, the signal-to-noise ratio (SNR) is high and the image is truly reflecting the real world. However, in the area of weak signal, the SNR is low, and noise may predominate the image. Therefore, the resultant output cannot be considered to be an accurate representation of the ocular surfaces being imaged [19, 20]. Time-domain OCT artefact effects are not new, they have been classified since 2009 in terms of misidentification of the inner retinal layer, misidentification of the outer retinal layer, out of register artefacts, degraded image scan, cut edge artefacts and off centre artefacts [21, 22]. Recently, the development in OCT technology from time-domain to spectral-domain has allowed higher imaging resolution and more accurate segmentation [23]. Spectral-domain OCTs possess the inherent ability to autocorrelated noise and now provide complex conjugate images in their outputs. However, this can make the interpretation of the image difficult in some cases and contribute to degradation of the overall system performance [24].

The use of an eye topographer to characterise the scleral shape *in-vivo* was not possible until the past few years as most of the topographers were not able to measure the area of the eye that covers the limbus and part of the sclera [25]. The situation has changed recently, and some newly developed topographers are able to do this by capturing the exposed portion of the sclera either in a single shot measurement, as with the Eye Surface Profiler (ESP) version used in the current study, or in a series of conservative measurements, as happens when using the sMap 3D fluorescence-based structured light topographer or the Pentacam Cornea Scleral Profile (CSP) optional software. The ESP corneoscleral topographers used in the current study can cover up to a 20 mm diameter of the eye with more than 250,000 measurement points without extrapolation [26]. This development in the instrumentation capabilities encouraged researchers to start characterising the sclera using these recently developed topographers that can provide the anterior eye surface up to 5 mm beyond the limbus [27]. There are several technical limitations associated with eye topography measurements. Some of them are due to inherent system assumptions, instrument software interface, hardware features, working distance, faceplate geometry, camera resolution, edge detection limits, algorithms implemented, instrument sensitivity to focus and alignment error [28]. As a result, the evaluation of the eye measurement is varying, and the quality of the detected eye surface could be low especially around the edges of the measured surface. The artefacts around the edges are not naturally present features but appear on the measured surface as a result of the instrument limitation, the measurement protocol and the technological limits. The availability of these more advanced topographers to assess the scleral asymmetry without considering the edge-effect has motivated the authors of this paper to investigate whether the reported topography-based scleral asymmetry has been miscalculated or even imperfectly assessed as a result of ignoring the edge-effect [12].

Using topography data, Consejo *et al*. [12] reported that corneal and scleral asymmetry are highly correlated in astigmatic eyes, with the nasal area of the sclera showing less relative elevation than the temporal area, and the inferior area of the sclera was slightly less elevated than the superior area [29]. The relative elevation in their results was calculated as the difference between the scleral raw elevation data and a simple quadratic function fitted to a scleral 2 mm width ring [30]. However, a simple quadratic equation cannot be expected to be accurately fitted to the anterior surfaces of these astigmatic eyes, considering the complex mathematical characteristics involved. As a polynomial function, a quadratic function will never generate a fitted surface that looks like the anterior scleral profile when extrapolated beyond the existing ESP data points. Moreover, and as understood by the authors of this manuscript, there was no accurate localisation or levelling of the limbus which was assumed to have a diameter of 12 mm for all participants, ignoring individual differences. This was a result of the absence of any programmed limbus detection procedures which would have more accurately determined the limbal dimensions.

In addition, the limitations of topography measurements were not taken into account: (i) individual eyes do not perform identically during the fixation process [31] which is essential during the topography taking procedure, (ii) the eye is always naturally tilted during the topography scan because of fixation on close objects, such as a topographer’s target, which requires the eye to rotate to achieve focused vision [32]. In the light of these limitations, it may not be appropriate to average scleral characteristics without levelling the eye geometry around a physical landmark like the limbus.

The current study uses a novel method, free of fitted-parameters, for detecting the topography data edge-effect on corneoscleral topographers’ data in three-dimensions. It then applies this method to a set of clinical data to investigate the scleral asymmetry free of the edge-effect.

## Materials and methods

This record review study was conducted according to the tenets of the Declaration of Helsinki and was approved by the IRB (Institutional Review Board) and Human Ethics Committee of the Federal University of São Paulo (UNIFESP, SP, Brazil). All patients provided informed consent for the use of their de-identified data in scientific research. The data were anonymised at Brigthen Optix Corporation in Taiwan.

### Participants

Data were collected from patients that underwent an ophthalmological examination at the Brighten Optix Corporation (Taipei, Taiwan). The study involved 88 participants aged 23 to 65 years (37.7 mean ±9.7 STD), 47 women and 41 men examined between August 2015 and January 2016. The inclusion criterion was the absence of ocular disease other than ametropia. The exclusion criteria were a history of previous eye surgery, ocular surface disease or scarring, report of connective tissue disease and pregnant or early puerperal women. All patients had a comprehensive ophthalmic examination, including topographic measurements with the ESP (Eaglet Eye, Houten, Netherlands, b.v.). The wearing of the soft contact lens was discontinued for at least two weeks prior to the examination and rigid contact lenses were discontinued for a minimum period of four weeks.

### ESP Measurement

The ESP measurement technique involves using Moire fringes reflected from the surface of the tear film. This instrument requires instillation of a viscous solution (in this study; one drop of Lubristil, 1 mg/mL sodium hyaluronate) and fluorescein in order to achieve a measurement. The height of the table and chinrest was adjusted to optimise the head position and to ensure that the video feed from the instrument was centralised. The subject was asked to observe the fixation (red-cross) point while this was viewed by the clinician on the computer monitor. Alignment on the ESP instrument was achieved by identifying the centre point of two corneal images of lights originating from the instrument. The red-cross was then aligned with this central point and a reading initiated. Once this had been done, the subject was directed to sit back and one unpreserved lubricating drop (Lubrisitil, 1mg/mL sodium hyaluronate) was instilled into the lower fornix. This was followed by the application of fluorescein in upper and lower fornix to maximise coverage. The subjected was directed to blink a couple of times, and the level of coverage was then checked visually before proceeding further. The subject was instructed to open their eyelids as wide as possible while a measurement was being taken to ensure sufficient data were captured. The measurement of the ESP was taken three times by the machine in rapid succession within a few milliseconds then the device software allowed the user to select and save the best scans based on user experience. The data was exported from the ESP software in MATLAB (MathWorks, Natick, USA) binary data container format (*.mat). The eye surface data was processed by custom built MATLAB codes entirely independent from the built-in ESP software digital signal processing (DSP) algorithms.

### Scleral asymmetry from raw topography data

Raw elevation data for right and left eyes were analysed separately in this study and no mirror symmetry has been assumed at any stage of this investigation, as fellow eyes are not reflected images of each other during fixation process [31]. At this phase of the analysis, raw topography elevation data were considered as they were exported from the ESP without applying any DSP procedures. They were only averaged all together, hence the mean and the standard deviation of eye surface raw elevation were determined.

### Eye levelling

The limbus of each eye was calculated using the three-dimensional non-parametric method presented in a previous study [27], then each eye’s topography data was levelled to the best fit plan that passed through the detected limbus. As the ESP is able to gather the corneal surface data and a portion of the sclera, the limbus can be detected through the ESP’s raw elevation data. The limbus detection algorithm is based on the fact that the cornea and the sclera have different curvatures [33] and, regarding surface profile, the limbus is the area where the corneal curvature turns to the scleral curvature [34]. As the eye surface tangent gradient (1^st^ derivative) is changing from zero at the apex to a maximum just before the limbus before it decreases gradually at the limbus then increases again as it moves on the sclera. As the limbus is the place where the rate of change of the 1^st^ derivative is a minimum, it can be detected by locating the turning point of the raw elevation 2^nd^ derivative at each meridian. Thus, all detected limbus points on all meridians were fitted to a plane which was rotated with the surface data until it becomes horizontal. More details about this non-parametric hypothesis of limbus detection can be found in the authors’ previous study published in 2018 [27].

To achieve this levelling, the angles of the limbus plane with the horizontal and vertical axis *α*_*x*_ and *α*_*y*_ were determined by the inverse trigonometric cosine function of the dot product of the normal vector of the limbus plane (*N*_*x*_, *N*_*y*,_ *N*_*z*_) and each of the Y-axis (0,1,0) and X-axis (0,0,1) unit vectors respectively as shown in Eqs 1 and 2.

$$\alpha_{x}=\frac{-\pi }{2}+\cos^{-1}\left(\left(N_{x},N_{y},N_{z}\right)\cdot \left(0,1,0\right)\right)$$ Eq 1

$$\alpha_{y}=\frac{-\pi }{2}+\cos^{-1}\left(\left(N_{x},N_{y},N_{z}\right)\cdot \left(0,0,1\right)\right)$$ Eq 2

Then the corneal surface was rotated around the X- axes and Y-axes by the tilt angles *α*_*x*_ and *α*_*y*_, respectively in order to level each eye’s limbus plane in the XY-plane. The three-dimensional rotation was achieved by applying 3D rotation matrices [35], in which the rotation angle about the Z-axis, *α*_*z*_, was set to zero [31]. Before moving to the next processing stage, the origin position of each levelled eye’s surface was shifted to the highest point of the limbus-levelled eye surface (apex).

### Edge-effect elimination

**Fig 1:**
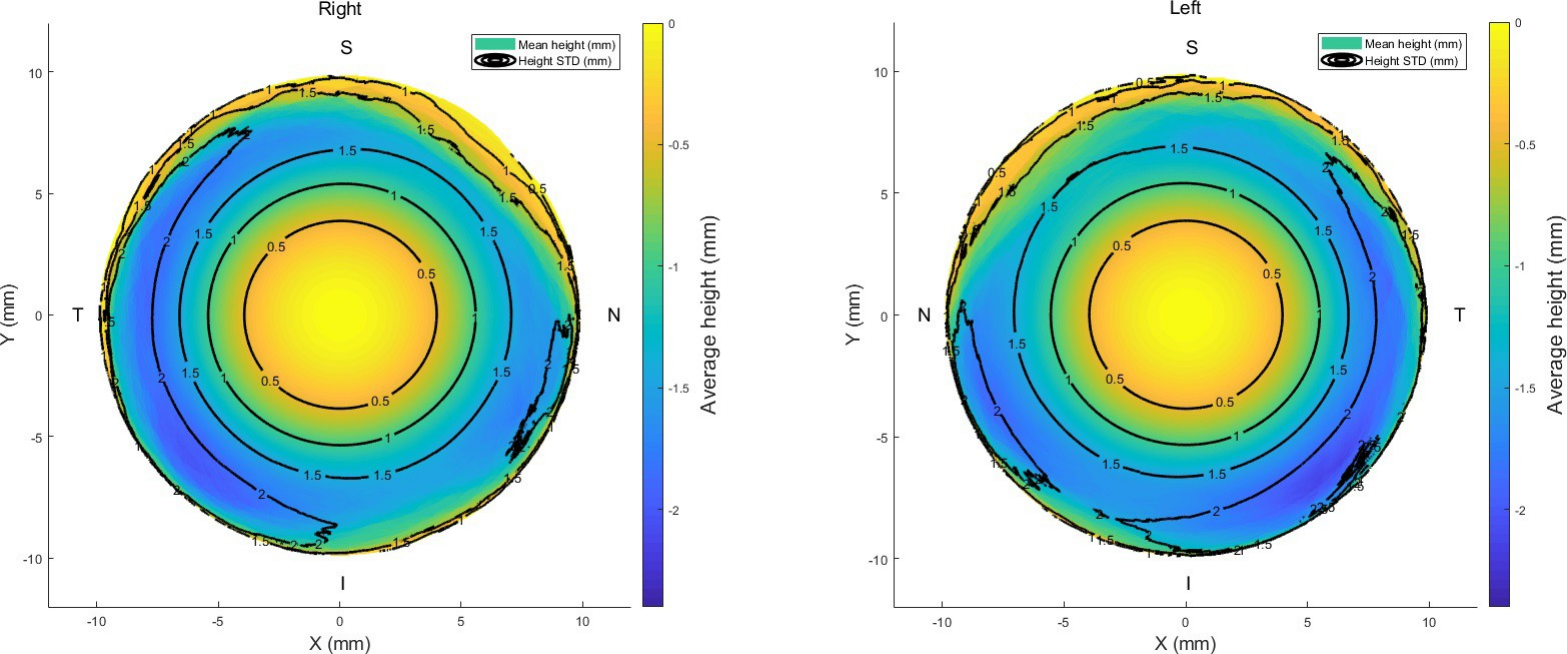
Average raw elevation maps for right and left eyes. Black contour lines represent the standard deviation of the raw elevation data.

**Fig 2:**
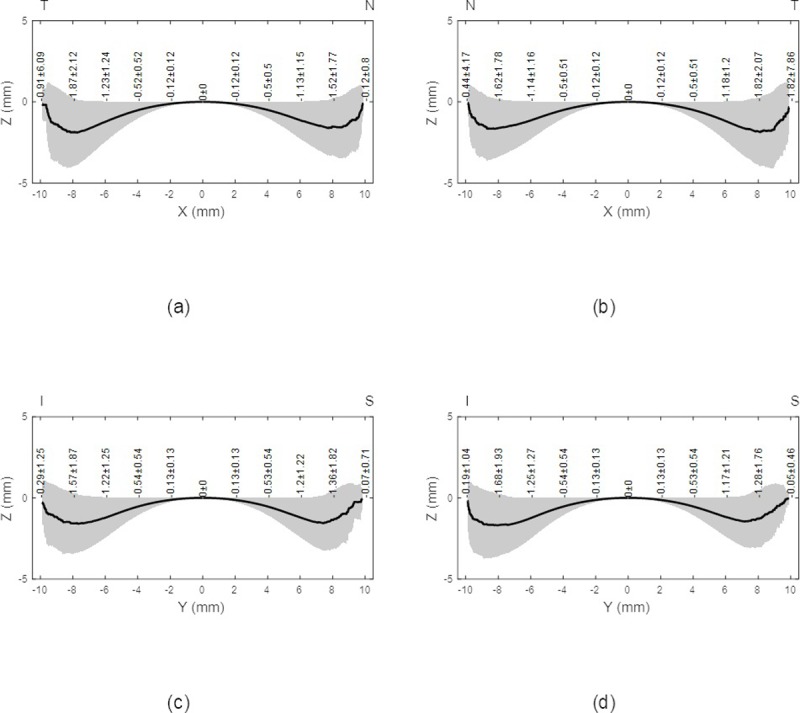
Average eyes’ raw elevation as measured by ESP with the origin at the corneal apex, (a) Right eyes temporal-nasal, (b) Left eyes nasal-temporal, (c) Right eyes inferior-superior, (d) Left eyes inferior-superior.

Considering the geometry of the human eye, it was clear that the presented records, in Figs 1 and 2, which were built from the raw topography elevation data obtained by the ESP did not match the known geometrical characteristics of the eye in their peripheral areas. However, the natural human eye anterior surface is always convex, there were changes from convex to concave surfaces at the edges of the averaged eyes (see S1, S2 and S3 Tables). Considering the pattern of the human eye as described in the literature [36–38], the shape of the anterior scleral pattern was represented by a sphere of radius 11.5 mm. Comparing this shape with the findings in Figs 1 and 2, the averaged scleral surface weakly correlated with the anterior scleral pattern with correlation coefficients 0.1841, 0.0534, 0.1502 and 0.1971 for the nasal, temporal, superior and inferior sides respectively for right eyes and 0.0526, 0.0757, 0.1448 and 0.1928 in the same order for left eyes.

**Fig 3:**
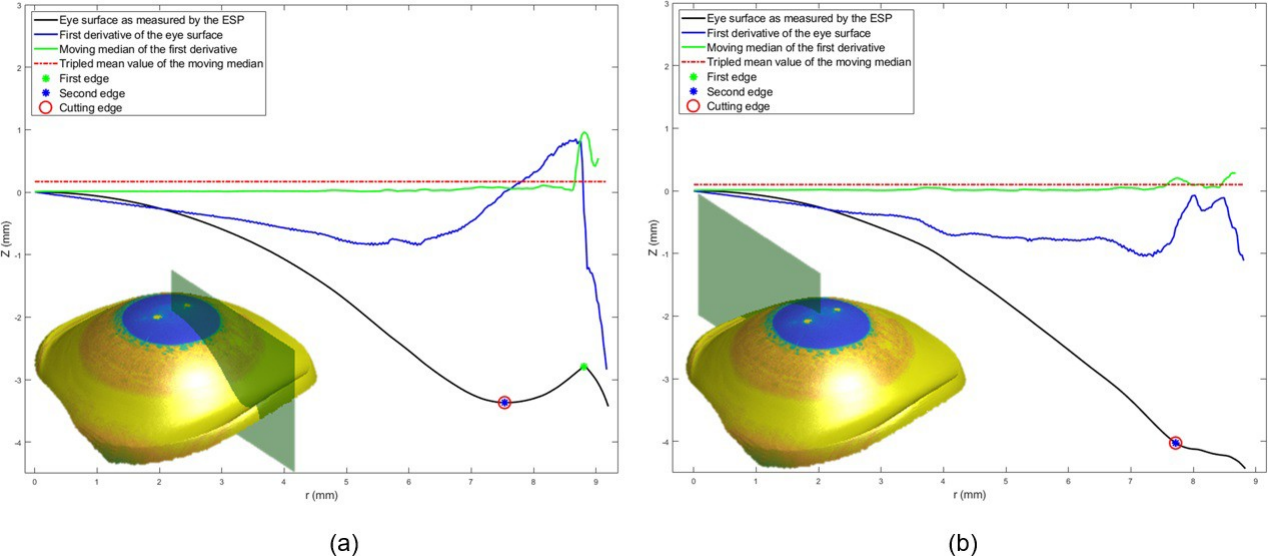
Edge effect detection example for the right eye of a 43 years old female participant, (a) Inferior meridian where two edges were detected, (b) Superior meridian where one edge was detected. The digital image of the eye as captured by the ESP was projected onto the eye surface for display purposes.

**Fig 4:**
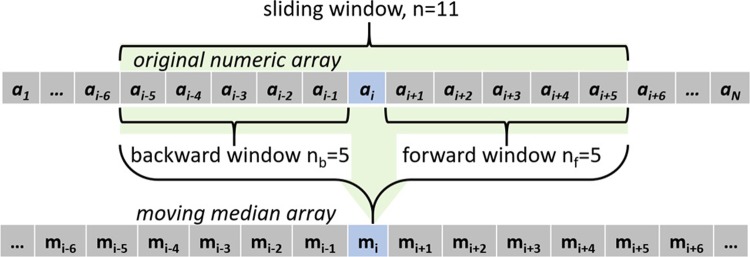
The moving median algorithm used in detecting the edge-effect.

Therefore, a method to differentiate the consistent portion of the raw elevation data and the perceived distortion caused by either the instrument hardware or software was needed. In this study, two edge detection strategies were used together to cut the edge of the eye’s surface data at the border between the authentic eye surface and the artificial boundaries. The first strategy is based on the observation of artefacts in the measured eye surface which does not follow the natural shape of the eye where the sclera comprises more than 80% of the outer tunic of the eye and is almost spherical with an average diameter of 24 mm [4, 39]. The appearance of topographical artefacts looks as if there is a sudden, unexpected and significant change in surface direction as a result of the effects of interference of tears, eyelid edges or lashes. Using the principles of robust statistics, that are not unduly affected by outliers, edge-effects can be detected by calculating the moving median of the eye raw elevation data along meridians. Firstly, the eye raw elevation data was considered meridian by meridian with one-degree polar steps, before the first derivative of the raw elevation data was calculated numerically. Then, using a window width of 0.1 mm in both radial sides (forward and backward) moving medians of the raw elevation’s first derivatives were calculated as an array corresponding to each meridian, Fig 3. As can be seen in Fig 4, the moving median array was achieved by averaging the elements in a sliding window consisting of 11 elements, however, the backward window n_b_ shrinks at the beginning of each meridian according to the available number of elements within the window width and the forward window n_f_ shrinks towards the end of each meridian. The moving meridian array elements were determined according to the following equation.

$$m_{i}=\frac{1}{n}\overset{i+n_{f}}{\underset{k=i-n_{b}}{\sum }}a_{k}$$ Eq 3

At that point, the average value of each moving medians array for each meridian was determined before its tripled value was taken as a cut-off threshold. Where the moving median value exceeds the tripled value of its mean, this indicates that the behaviour of the surface of this area is not ordinary and a first cutting edge is triggered as a result. As the first strategy is detecting the sudden unusual change in the eye surface, it may miss the right cutting edge if the measured eye surface moved from the real eye area to the edge-affected area smoothly. Therefore, the radial distance between the apex and the first cutting edge is searched to detect if there is a minimum value of the raw elevation data less than the first detected cutting edge. If there is such a minimum point, it is taken as a second cutting edge. Finally, the ultimate cutting edge was taken either as the first or the second cutting edge, whichever was closest to the apex. Fig 3 shows an edge-effect detection example where two edges were detected along the inferior meridian, however, a single edge was detected for the superior meridian.

### Scleral relative elevation map

Scleral relative elevation was determined by subtracting a spherical reference surface from the scleral raw elevation data. The reference surface (topographical static sea-level datum) was the best-fitted sphere to the scleral height data where the radius and the centre of the fitted sphere were determined by finding the values of the best fit sphere centre and radius minimising the summation of squared errors as exposed in Eq 4 for n scattered scleral height points. The best fit sphere height Z_s_ was determined as in Eq 5 before elevation surface maps for both right and left eyes groups were determined as Z_i_-Z_si_ for every point i of the n points.

$$\overset{n}{\underset{i=1}{\sum }}{\left({\left(X_{i}-X_{c}\right)}^{2}+{\left(Y_{i}-Y_{c}\right)}^{2}+{\left(Z_{i}-Z_{c}\right)}^{2}-R_{s}{}^{2}\right)}^{2}$$ Eq 4

$$Z_{si}=Z_{c}+\sqrt{R_{s}{}^{2}-{\left(X_{i}-X_{c}\right)}^{2}-{\left(Y_{i}-Y_{c}\right)}^{2}}$$ Eq 5

**Fig 5:**
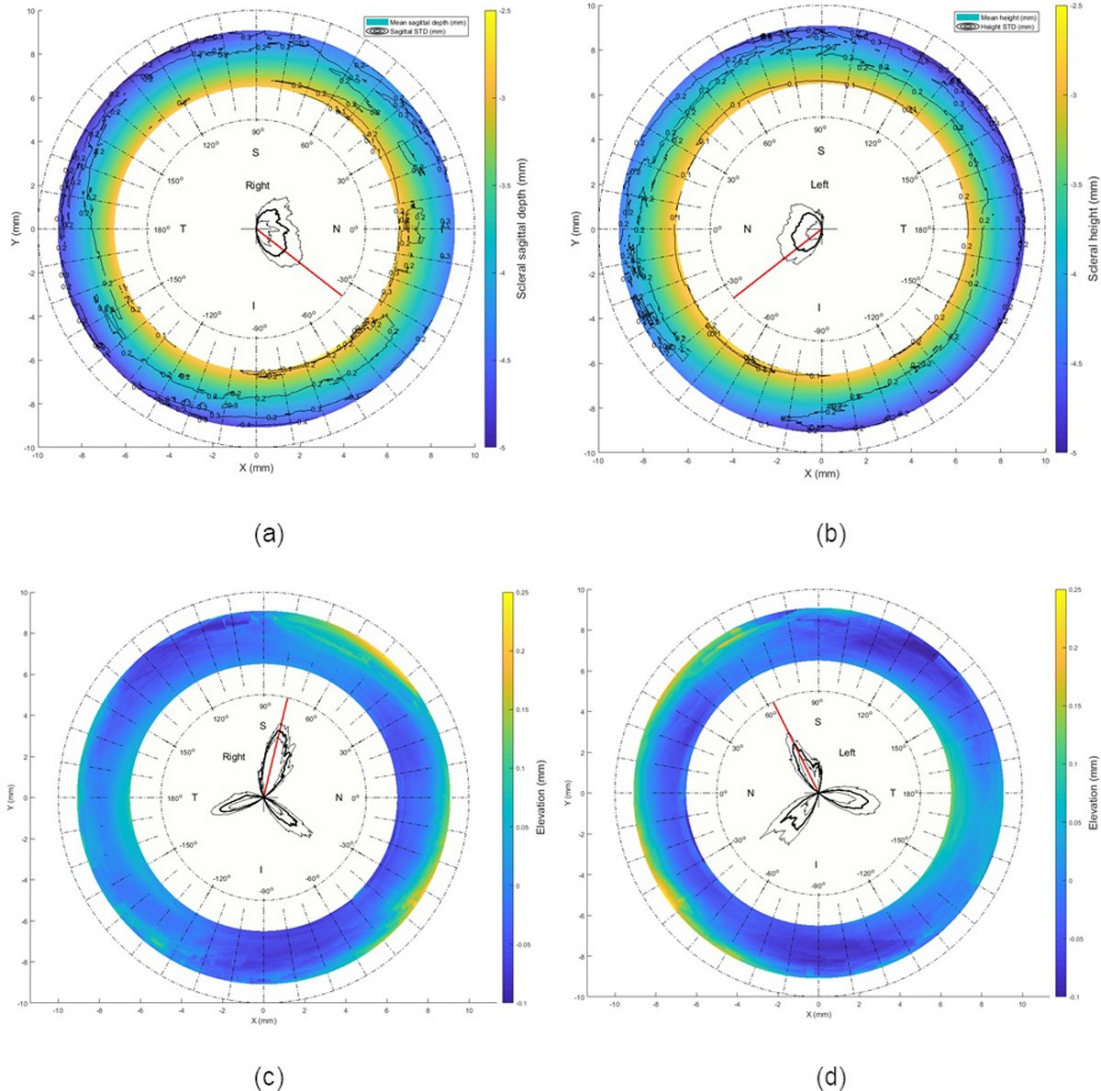
Scleral raw elevation and relative elevation as determined after levelling the eyes and eliminating the edge effect; (a) Raw elevation (right eye); (b) Raw elevation (left eye). The polar plot in the middle of subplots (a) and (a) shows the scleral raw elevation asymmetry in polar coordinates scaled 5 times their values for display purposes. The thick black line is the average asymmetry and the thin black lines are the standard deviation added and subtracted to the mean values. The red line is pointing to the angle where the asymmetry was a maximum. (c) Relative elevation (right eye); (b) Relative elevation (left eye). Elevation reference for both right and left eyes were best-fitted spheres whose radii were determined by minimising the least squares fitting error. The polar plot on the middle of subplots (c) and (d) shows the scleral relative elevation asymmetry in polar coordinates scaled 40 times their values with their standard deviation scaled up to 10 times for display purposes.

Where X_i_, Y_i_ and Z_i_ are the scleral height data, X_c_, Y_c_ and Z_c_ are the best-fitted sphere's centre three-dimensional coordinates, and R_s_ is the radius. Eventually, the difference evaluation analysis in this study was carried out at every meridian starting from the nasal side at 0°. Therefore, the difference either in scleral height or relative elevation (height minus best-fit sphere) was always calculated as the values at the meridian with an angle of [0°,1°,2°,…,179°] minus the values at the meridian with angle [-180°, -179°,178°,….,-1°]. These differences were presented as polar plots centred at the origin of the subplots of Fig 5.

### Statistical analysis

Statistical analysis was performed using MATLAB Statistics and Machine Learning Toolbox. The null hypothesis probability (p) at 95% confidence level was calculated. Two sample t-tests were used to investigate the significance between pairs of data sets to check whether the results represent independent records. The probability p is an element of the period [0, 1] where values of p higher than 0.05 indicate the validity of the null hypothesis (31). The t-test results in this study were expressed by a binary value, 1 for statistically significant and 0 for non-statistically significant.

## Results

Three-dimensional averaged raw elevation maps for right and left eyes are presented in Fig 1 with the Cartesian coordinates’ origin at the corneal apex. Considering the principal directions, the average raw elevation data in the nasal-temporal direction of both right and left eyes are presented in Fig 2A and 2B, however, the average raw elevation data in the superior-inferior directions are presented in Fig 2C and 2D, respectively. The clinical parameters that were extracted from the ESP software were only used for reporting the ESP system clinical parameters as shown in Table 1 and were not used for obtaining any results presented in this study.

**Table 1:** Average characteristics of participants’ eyes as measured by the ESP system.

Characteristic	Right eyes Mean ± STD	Left eyes Mean ± STD
Horizontal visible iris diameter HVID (mm)	11.99 ± 0.40	11.97 ± 0.41
Astigmatism (Dioptre)	-1.72 ± 0.71	-1.82 ± 0.69
Axis (°)	96.37 ± 13.95	88.79 ± 6.85
Sphere (Dioptre)	43.08 ± 1.66	43.12 ± 1.77
Sim-K astigmatism (Dioptre)	-2.68 ± 1.07	-2.95 ± 1.03
Sim-K angle (°)	93.45 ± 15.54	91.03 ± 7.00
Sim-K flat radius (mm)	8.41 ± 0.40	8.44 ± 0.40
Sim-K steep radius (mm)	7.88 ± 0.35	7.86 ± 0.37

On one hand, when considering the averaged raw topography elevation data in the scleral section of the eye, the statistics were showing relatively high standard deviations, Figs 1 and 2. For example, the average raw elevation of the right eyes at radius 8 mm was -1.5±1.77 mm on the nasal side, -1.87±2.12 mm on the temporal side, -1.36±1.82 mm and -1.57±1.87 mm on the inferior side. However, in the left eyes at the same radius average raw elevation was -1.62±1.78 mm on the nasal side and -1.82±2.07 mm on the temporal side, -1.28±1.76 mm on the superior side and -1.68±1.93 mm on the inferior side.

**Fig 6:**
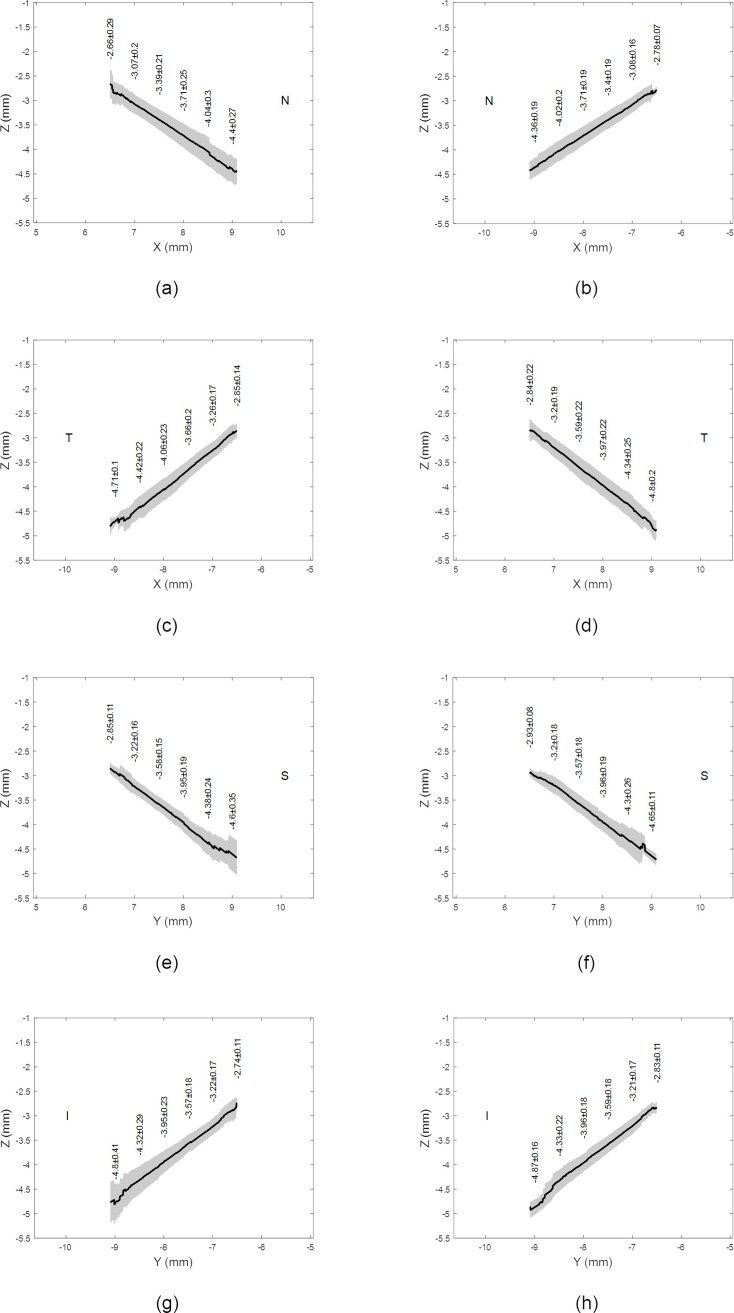
Average eyes’ raw elevation as determined after removing the edge-effect with the origin at the corneal apex, (a) Right eyes nasal side, (b) Left eyes nasal side, (c) Right eyes temporal side, (d) Left eyes temporal side, (e) Right eyes superior side, (f) Left eyes superior side, (g) Right eyes inferior side, and (h) Left eyes inferior side.

On the other hand, when considering the average raw elevation of the sclera after removing the edge effect, and at an 8 mm radius as an example, the statistics showed relatively low standard deviations compared to the unprocessed data, Figs 5 and 6. The average raw elevation of the right eyes was -3.71±0.25 mm on the nasal side, -4.06±0.23 mm on the temporal side, -3.95±0.19 mm and -3.95±0.23 mm on the inferior side. However, in the left eyes at the same radius, the average raw elevation was -3.71±0.19 mm on the nasal side and -3.97±0.22 mm on the temporal side, -3.96±0.19 mm on the superior side and -3.96±0.18 mm on the inferior side. Maximum raw elevation asymmetry in the averaged scleral raw elevation was 1.6647±0.9015 mm, detected at -38° on the nasal side in right eyes (Fig 5A) and 1.0358±0.6842 mm detected at -38° on the nasal side in left eyes (Fig 5B).

**Fig 7:**
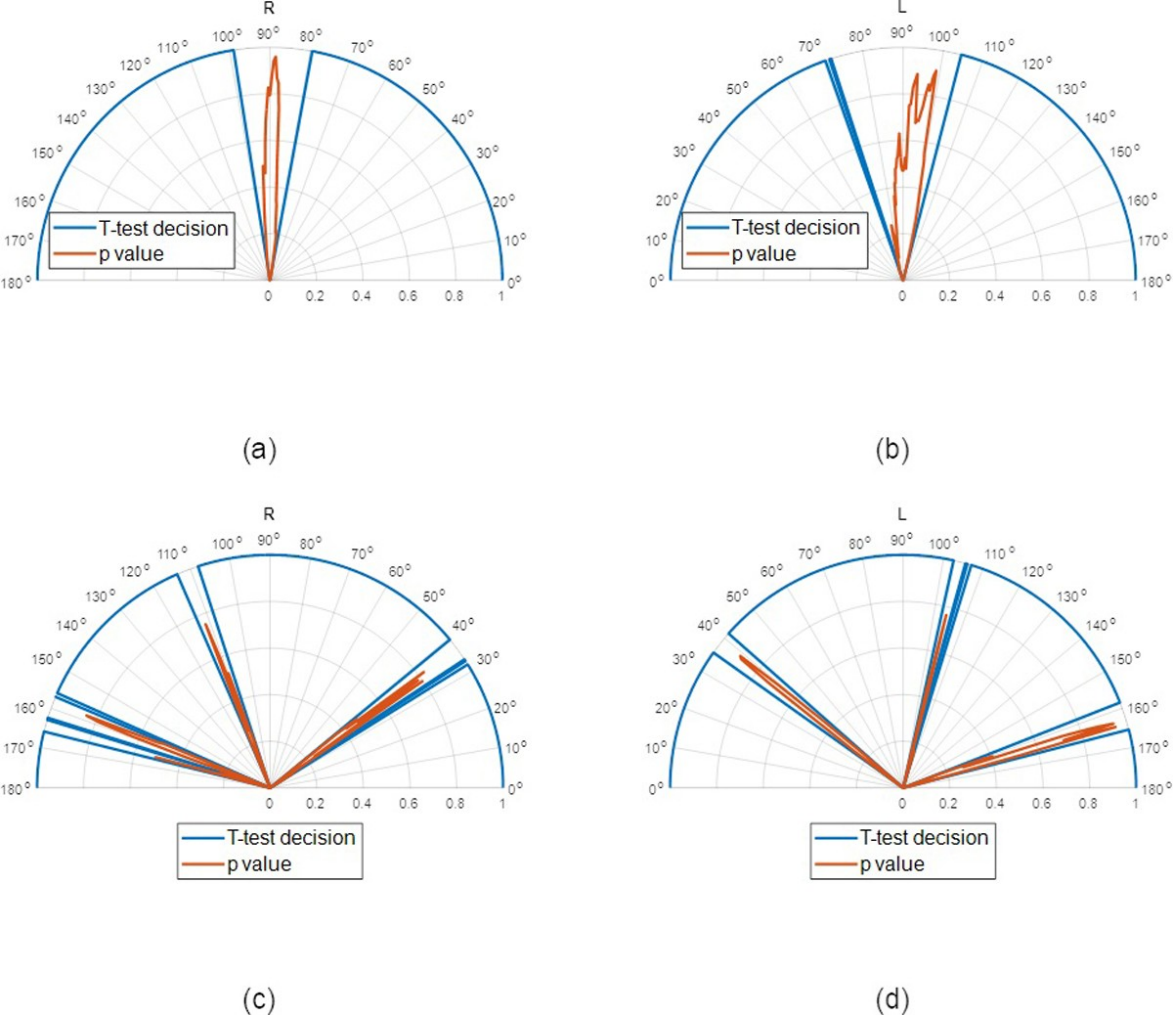
Asymmetry significance around the sclera, the value 1.0 indicates positive test decision and 0.0 indicates a negative test decision, however the significance (p-value) was presented in red (a) Raw elevation asymmetry, right eyes, (b) Raw elevation asymmetry, left eyes, (c) Relative elevation asymmetry, right eyes, (d) Relative elevation asymmetry, left eyes.

Comparing the significance in the scleral raw elevation asymmetry among right eyes on every meridian with the opposite meridian (across 180°) showed statistically significant differences in the nasal-temporal (p<0.05) direction and non-statistically significant differences in the superior-inferior direction (p = 0.794), however the insignificance in the superior-inferior angular range (79.5° to 99°) is less than the significance in the nasal-temporal direction angular range (0° to 79.5° and 99° to 180°), Fig 7A. Left eyes showed similar trends with statistically significant differences in the nasal-temporal (p<0.05) direction and non-statistically significant differences in the superior-inferior direction (p = 0.47), and yet again the insignificance in the superior-inferior angular range (70.5° to 104.5°) is less than the significance in the nasal-temporal direction angular range (0° to 70.5° and 104.5° to 180°), Fig 7B. When the asymmetry in scleral relative elevation was compared among right eyes, insignificances were observed in the angular ranges 32° to 39.5° (p_max_ = 0.8253), 108° to 113.5° (p_max_ = 0.7545) and 156° to 166° (p_max_ = 0.8464), however, there were statistically significant relative elevation differences otherwise (p<0.05), Fig 7C. When the asymmetry in scleral relative elevation was compared among left eyes, insignificances were observed in the angular ranges 35.5° to 41.5° (p_max_ = 0.9475), 102.5° to 107° (p_max_ = 0.764) and 153.5° to 165.5° (p_max_ = 0.8986), however, there were statistically significant relative elevation differences otherwise (p<0.05), Fig 7D. The best-fit sphere-based elevation showed that the sclera is mostly elevated in three main meridians at angles -40°, 76°, and 170° in right eyes and -40°, 76°, and 170° in left eyes, all measured from the nasal meridian. Maximum recorded relative elevation asymmetries were 0.0844±0.0355 mm and 0.068±0.0607 mm at angular positions 76° and 63.5° for right and left eyes, in turn. Detailed numerical representation of the relative elevation asymmetry and their significance is presented in subfigures Fig 7C and 7D.

## Discussion

**Fig 8:**
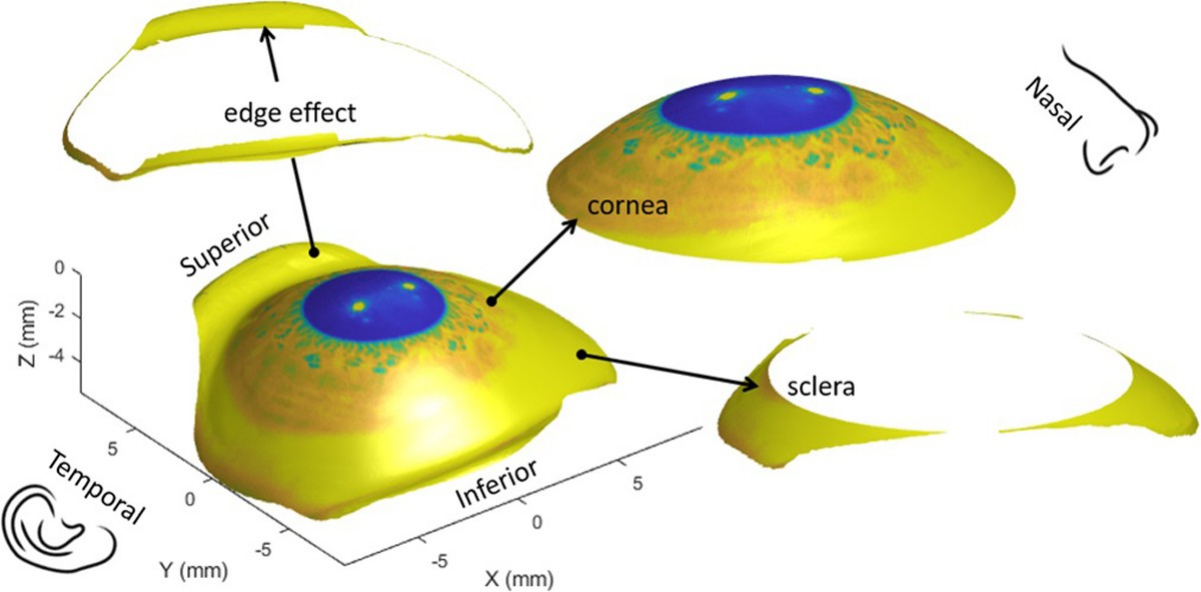
Right eye of a 33 years old male participant divided into three sections; corneal surface, scleral ring and artefact ring called the ‘edge effect’. The digital image of the eye as captured by the ESP was projected onto the eye surface for display purposes.

This study presents a three-dimensional method for detecting the topography data edge-effect on the ESP corneo-scleral topographer. It then determines the scleral asymmetry without the influence of edge effect, Fig 8. As the eye’s surface measurements by the ESP corneoscleral profilometer require the instillation of fluorescein, what is actually measured by the ESP is the viscous surface of the tears mixed with fluorescein, not the actual ocular exterior surface. This tear-fluorescein mixed surface may not only cover the corneo-scleral surface but can be transferred onto the lids. Additionally, tear pooling can occur at the lid margins, especially inferiorly, and thus can create a “false” surface causing what is called an ‘edge-effect’. In the central corneal area, the alterations produced by the tear film are known to cause surface irregularities that distort the topographic image, reduce the eye symmetry, affect power measurement and the location of the steepest point [40–42]. Therefore, it is highly likely that the combination of the eyelid edge effects and any excess tears trapped in the fornices within the measurable area is going to affect the eye surface representation as measured by an ESP corneoscleral topographer, as it is not able to measure the scleral exterior surface itself but the viscous tear-fluorescein film on the conjunctiva [43]. The recent example of Consejo’s conclusion that scleral shape undergoes changes with accommodation [44], which was rejected by Schachar [36], raises the importance of innovative methods of edge-effect detection for instruments like the non-contact ESP corneoscleral topographer. Without these methods, incorrect conclusions are likely to keep appearing in the literature that is based on using this relatively new corneoscleral topographer.

The findings of the current study confirm the belief that the natural shape of the sclera does not exhibit astigmatism patterns like the cornea, but instead is markedly more complex [13]. The results presented here suggest that the sclera is steeper not only at the temporal side as reported by Consejo [29] but also in two other meridians creating angles of nearly 120° between them (Fig 5C and 5D). When Bandlitz [11] measured the limbal scleral radii of 30 subjects, he found that median scleral radii in superior-nasal were the flattest and along temporal direction was the steepest meridian. Superior was significantly flatter than temporal radius and nasal was also significantly flatter than the temporal radius. In his study, Hall [14] also found that corneal-scleral junctions were the sharpest at the nasal but contradictory he found a progressive significant flattening at temporal, inferior and superior junctions. As the asymmetry evaluation analysis in this study was carried out at all meridians (with 1°intervals) neither at wide sectors as in [29, 30] nor cross-sectional slices as in [15, 45], it provides a detailed overview of the scleral shape up to 3 mm beyond the corneoscleral junction. Comparing the statistical figures of the scleral average raw elevation before and after removing the edge, effect showed an increase in the mean values of raw elevation and a decrease in their standard deviations as a result of removing the effect of artificial lift-off caused by the edges. The common temporal lens decentration that is often observed by practitioners [13] is likely because the nasal side of the sclera is higher than the temporal side as can be seen in Fig 5A and 5B [46]. As the sclera is known for not following the same astigmatism rules as the cornea, describing the sclera by a relative elevation map may not be as useful as it is for the cornea [13]. The main reason for this is the fact that the relative elevation map is highly dependent on the selected reference surface. Spherical reference surfaces give different elevation values from ellipsoid reference surfaces, and both are unlike quadratic function reference surfaces. Therefore, offering a levelled raw elevation map as this study does gives a direct artefact-free measurement of scleral asymmetry.

There are several methods described in the literature with different findings that are in some cases disparate and conflicting with each other [11, 12, 29]. Bandlitz reported that scleral radii measured along the (nasal superior) were significantly flatter than other directions. In addition, the nasal scleral radii along 0° (nasal side) were significant flatter than the temporal scleral radii along 180° (temporal side) [11]. Using the ESP, Consejo found that the nasal area of the sclera showed less relative elevation than the temporal area [29]. Differences between the superior and inferior areas were not statistically significant. Besides, the asymmetry of the sclera was found to increase with radial distance from the corneal apex. Tan reported that the flattest topography was in the temporal quadrant and that this value was higher in Whites than Latinos and Asians [45]. The steepest quadrant was found at the nasal side causing a larger corneoscleral angle, this angle gradually decreased among Whites, Latinos and Asians, respectively. Hall found that the mean sclera curvature was steepest in the temporal sclera contradicting the findings in this study and reported asymmetry in the horizontal sclera [9], Table 2. A recent study by Piñero compared the variations in corneoscleral between Keratoconus and healthy patients [47]. In that study, edge effect and rotation of the topographies were not considered. He concluded that the diagnostic accuracy of corneoscleral topographic data for keratoconus detection was significantly limited as his team were unable to find tangible differences between the radius of corneoscleral topography. The outcome of such a study could be influenced significantly by implementing the edge-effect elimination method proposed in this study.

**Table 2:** Scleral and corneoscleral junction shape as reported in previous studies.

Study	Measuring device	Findings
Consejo A, Rozema JJ., 2018 [12]	Eye Surface Profiler (Eaglet Eye b.v.)	Corneal and scleral asymmetry are highly correlated in astigmatic eyes, nonetheless both were independent in normal eyes; no significant decentration difference between astigmatic and normal eyes, whereas for the astigmatic eyes, the decentration differences were significant.
Consejo A, Llorens-Quintana C, Bartuzel MM, Iskander DR, Rozema JJ., 2018 [29]	Eye Surface Profiler (Eaglet Eye b.v.)	The nasal sclera was less elevated than the temporal one; no significant difference in the superior-inferior direction; scleral asymmetry was increasing with radial distance from the corneal apex; no significant difference between right and left eyes.
Bandlitz S, Baumer J, Conrad U, Wolffsohn J., 2017 [11]	OCT (Optos Inc) & Keratograph 4 (Oculus Optikgeräte GmbH)	Scleral radii along the nasal-superior direction was significantly flatter compared to other directions; nasal scleral radii were significant flatter than the temporal scleral radii; central corneal radius in flat and steep meridians were not correlated with scleral radii; no significant correlation between corneal eccentricity and scleral radii in each meridian.
Hall LA, Young G, Wolffsohn JS, Riley C., 2011 [9]	OCT (Carl Zeiss Meditec AG)	The mean corneoscleral junction angle was the sharpest (least) at the nasal side and became flatter (larger) at the inferior, temporal, and superior junctions respectively; nasal-temporal sclera was asymmetric.

These conflicting findings can be explained by the practical challenges present in the *in-vivo* evaluation of the scleral shape. The method described in this paper proved to be accurate and reliable by overcoming these conflicting factors of the scleral *in-vivo* measurements. An accurate method of evaluating the scleral asymmetries is important for scleral contact lens fitting to balance weight bearing, avoid conjunctival impingement compression and improve comfort and wearing time [13]. One of the main fitting problems with scleral contact lenses derived from trying to fit a regular spherical haptic design lens to a toric or asymmetric scleral shape [13]. Visser *et al*. reported improved comfort and wearing time of back surface toric lens when compared to the rotationally symmetric scleral lens. [48]. Inferotemporal scleral lens decentration, in accordance with the findings of scleral shape asymmetries observed in the present study, has been widely reported [46, 49–51]. Besides fitting problems, the decentration can lead to reduced optical performance, that is more evident in special lenses like custom wavefront-guided scleral lenses [52] and multifocal lenses [53]. Decentring the lens optic nasally by 1.0 to 1.5 mm or even customised based on patient-specific decentration pattern has been proposed [46]. New lens designs can also help in the centration issue. Improving the peripheral curves of posterior toric surface scleral lenses led to an increase in centration success of up to 20% [50, 51]. However, still 9% of the cases showed a decentred optical zone [50]. The new concepts introduced in this study can be used to improve the scleral lens design for better optical performance. One limitation of this study is that it considered only data from a single Asian centre, which means that its findings cannot be directly applied to different ethnic populations.

In conclusion, the edge-effect of topography data is a major confounding factor for describing the scleral topography. Analysing the levelled raw elevation data and correcting it is the most consistent way of describing the scleral asymmetries.

## Supporting information

S1 Table X and Y Cartesian coordinates of averaged raw data. (ZIP)

S2 Table Averaged raw elevation data. (ZIP)

S3 Table Standard deviation of raw elevation data. (ZIP)
