# Supplementary material for: Artefact-free topography based scleral-asymmetry
Source: PLoS One. 2019 Jul 26;14(7):e0219789. doi: 10.1371/journal.pone.0219789 (PMC6660081; doi:10.1371/journal.pone.0219789)
Supplement: S1 Table — (ZIP) [file X.pdf › X.pdf]

-12	-11.99	-11.98	-11.97	-11.96	-11.95	-11.94	-11.93	-11.92
-12	-11.99	-11.98	-11.97	-11.96	-11.95	-11.94	-11.93	-11.92
-12	-11.99	-11.98	-11.97	-11.96	-11.95	-11.94	-11.93	-11.92
-12	-11.99	-11.98	-11.97	-11.96	-11.95	-11.94	-11.93	-11.92
-12	-11.99	-11.98	-11.97	-11.96	-11.95	-11.94	-11.93	-11.92
-12	-11.99	-11.98	-11.97	-11.96	-11.95	-11.94	-11.93	-11.92
-12	-11.99	-11.98	-11.97	-11.96	-11.95	-11.94	-11.93	-11.92
-12	-11.99	-11.98	-11.97	-11.96	-11.95	-11.94	-11.93	-11.92
-12	-11.99	-11.98	-11.97	-11.96	-11.95	-11.94	-11.93	-11.92
-12	-11.99	-11.98	-11.97	-11.96	-11.95	-11.94	-11.93	-11.92
-12	-11.99	-11.98	-11.97	-11.96	-11.95	-11.94	-11.93	-11.92
-12	-11.99	-11.98	-11.97	-11.96	-11.95	-11.94	-11.93	-11.92
-12	-11.99	-11.98	-11.97	-11.96	-11.95	-11.94	-11.93	-11.92
-12	-11.99	-11.98	-11.97	-11.96	-11.95	-11.94	-11.93	-11.92
-12	-11.99	-11.98	-11.97	-11.96	-11.95	-11.94	-11.93	-11.92
-12	-11.99	-11.98	-11.97	-11.96	-11.95	-11.94	-11.93	-11.92
-12	-11.99	-11.98	-11.97	-11.96	-11.95	-11.94	-11.93	-11.92
-12	-11.99	-11.98	-11.97	-11.96	-11.95	-11.94	-11.93	-11.92
-12	-11.99	-11.98	-11.97	-11.96	-11.95	-11.94	-11.93	-11.92
-12	-11.99	-11.98	-11.97	-11.96	-11.95	-11.94	-11.93	-11.92
-12	-11.99	-11.98	-11.97	-11.96	-11.95	-11.94	-11.93	-11.92
-12	-11.99	-11.98	-11.97	-11.96	-11.95	-11.94	-11.93	-11.92
-12	-11.99	-11.98	-11.97	-11.96	-11.95	-11.94	-11.93	-11.92
-12	-11.99	-11.98	-11.97	-11.96	-11.95	-11.94	-11.93	-11.92
-12	-11.99	-11.98	-11.97	-11.96	-11.95	-11.94	-11.93	-11.92
-12	-11.99	-11.98	-11.97	-11.96	-11.95	-11.94	-11.93	-11.92
-12	-11.99	-11.98	-11.97	-11.96	-11.95	-11.94	-11.93	-11.92
-12	-11.99	-11.98	-11.97	-11.96	-11.95	-11.94	-11.93	-11.92
-12	-11.99	-11.98	-11.97	-11.96	-11.95	-11.94	-11.93	-11.92
-12	-11.99	-11.98	-11.97	-11.96	-11.95	-11.94	-11.93	-11.92
-12	-11.99	-11.98	-11.97	-11.96	-11.95	-11.94	-11.93	-11.92
-12	-11.99	-11.98	-11.97	-11.96	-11.95	-11.94	-11.93	-11.92
-12	-11.99	-11.98	-11.97	-11.96	-11.95	-11.94	-11.93	-11.92
-12	-11.99	-11.98	-11.97	-11.96	-11.95	-11.94	-11.93	-11.92
-12	-11.99	-11.98	-11.97	-11.96	-11.95	-11.94	-11.93	-11.92
-12	-11.99	-11.98	-11.97	-11.96	-11.95	-11.94	-11.93	-11.92
-12	-11.99	-11.98	-11.97	-11.96	-11.95	-11.94	-11.93	-11.92
-12	-11.99	-11.98	-11.97	-11.96	-11.95	-11.94	-11.93	-11.92
-12	-11.99	-11.98	-11.97	-11.96	-11.95	-11.94	-11.93	-11.92
-12	-11.99	-11.98	-11.97	-11.96	-11.95	-11.94	-11.93	-11.92
-12	-11.99	-11.98	-11.97	-11.96	-11.95	-11.94	-11.93	-11.92
-12	-11.99	-11.98	-11.97	-11.96	-11.95	-11.94	-11.93	-11.92
-12	-11.99	-11.98	-11.97	-11.96	-11.95	-11.94	-11.93	-11.92
-12	-11.99	-11.98	-11.97	-11.96	-11.95	-11.94	-11.93	-11.92
-12	-11.99	-11.98	-11.97	-11.96	-11.95	-11.94	-11.93	-11.92
-12	-11.99	-11.98	-11.97	-11.96	-11.95	-11.94	-11.93	-11.92
-12	-11.99	-11.98	-11.97	-11.96	-11.95	-11.94	-11.93	-11.92
-12	-11.99	-11.98	-11.97	-11.96	-11.95	-11.94	-11.93	-11.92
-12	-11.99	-11.98	-11.97	-11.96	-11.95	-11.94	-11.93	-11.92
-12	-11.99	-11.98	-11.97	-11.96	-11.95	-11.94	-11.93	-11.92

-12	-11.99	-11.98	-11.97	-11.96	-11.95	-11.94	-11.93	-11.92
-12	-11.99	-11.98	-11.97	-11.96	-11.95	-11.94	-11.93	-11.92
-12	-11.99	-11.98	-11.97	-11.96	-11.95	-11.94	-11.93	-11.92
-12	-11.99	-11.98	-11.97	-11.96	-11.95	-11.94	-11.93	-11.92
-12	-11.99	-11.98	-11.97	-11.96	-11.95	-11.94	-11.93	-11.92
-12	-11.99	-11.98	-11.97	-11.96	-11.95	-11.94	-11.93	-11.92
-12	-11.99	-11.98	-11.97	-11.96	-11.95	-11.94	-11.93	-11.92
-12	-11.99	-11.98	-11.97	-11.96	-11.95	-11.94	-11.93	-11.92
-12	-11.99	-11.98	-11.97	-11.96	-11.95	-11.94	-11.93	-11.92
-12	-11.99	-11.98	-11.97	-11.96	-11.95	-11.94	-11.93	-11.92
-12	-11.99	-11.98	-11.97	-11.96	-11.95	-11.94	-11.93	-11.92
-12	-11.99	-11.98	-11.97	-11.96	-11.95	-11.94	-11.93	-11.92
-12	-11.99	-11.98	-11.97	-11.96	-11.95	-11.94	-11.93	-11.92
-12	-11.99	-11.98	-11.97	-11.96	-11.95	-11.94	-11.93	-11.92
-12	-11.99	-11.98	-11.97	-11.96	-11.95	-11.94	-11.93	-11.92
-12	-11.99	-11.98	-11.97	-11.96	-11.95	-11.94	-11.93	-11.92
-12	-11.99	-11.98	-11.97	-11.96	-11.95	-11.94	-11.93	-11.92
-12	-11.99	-11.98	-11.97	-11.96	-11.95	-11.94	-11.93	-11.92
-12	-11.99	-11.98	-11.97	-11.96	-11.95	-11.94	-11.93	-11.92
-12	-11.99	-11.98	-11.97	-11.96	-11.95	-11.94	-11.93	-11.92
-12	-11.99	-11.98	-11.97	-11.96	-11.95	-11.94	-11.93	-11.92
-12	-11.99	-11.98	-11.97	-11.96	-11.95	-11.94	-11.93	-11.92
-12	-11.99	-11.98	-11.97	-11.96	-11.95	-11.94	-11.93	-11.92
-12	-11.99	-11.98	-11.97	-11.96	-11.95	-11.94	-11.93	-11.92
-12	-11.99	-11.98	-11.97	-11.96	-11.95	-11.94	-11.93	-11.92
-12	-11.99	-11.98	-11.97	-11.96	-11.95	-11.94	-11.93	-11.92
-12	-11.99	-11.98	-11.97	-11.96	-11.95	-11.94	-11.93	-11.92
-12	-11.99	-11.98	-11.97	-11.96	-11.95	-11.94	-11.93	-11.92
-12	-11.99	-11.98	-11.97	-11.96	-11.95	-11.94	-11.93	-11.92
-12	-11.99	-11.98	-11.97	-11.96	-11.95	-11.94	-11.93	-11.92
-12	-11.99	-11.98	-11.97	-11.96	-11.95	-11.94	-11.93	-11.92
-12	-11.99	-11.98	-11.97	-11.96	-11.95	-11.94	-11.93	-11.92
-12	-11.99	-11.98	-11.97	-11.96	-11.95	-11.94	-11.93	-11.92
-12	-11.99	-11.98	-11.97	-11.96	-11.95	-11.94	-11.93	-11.92
-12	-11.99	-11.98	-11.97	-11.96	-11.95	-11.94	-11.93	-11.92
-12	-11.99	-11.98	-11.97	-11.96	-11.95	-11.94	-11.93	-11.92
-12	-11.99	-11.98	-11.97	-11.96	-11.95	-11.94	-11.93	-11.92
-12	-11.99	-11.98	-11.97	-11.96	-11.95	-11.94	-11.93	-11.92
-12	-11.99	-11.98	-11.97	-11.96	-11.95	-11.94	-11.93	-11.92
-12	-11.99	-11.98	-11.97	-11.96	-11.95	-11.94	-11.93	-11.92
-12	-11.99	-11.98	-11.97	-11.96	-11.95	-11.94	-11.93	-11.92
-12	-11.99	-11.98	-11.97	-11.96	-11.95	-11.94	-11.93	-11.92
-12	-11.99	-11.98	-11.97	-11.96	-11.95	-11.94	-11.93	-11.92
-12	-11.99	-11.98	-11.97	-11.96	-11.95	-11.94	-11.93	-11.92
-12	-11.99	-11.98	-11.97	-11.96	-11.95	-11.94	-11.93	-11.92
-12	-11.99	-11.98	-11.97	-11.96	-11.95	-11.94	-11.93	-11.92
-12	-11.99	-11.98	-11.97	-11.96	-11.95	-11.94	-11.93	-11.92
-12	-11.99	-11.98	-11.97	-11.96	-11.95	-11.94	-11.93	-11.92
-12	-11.99	-11.98	-11.97	-11.96	-11.95	-11.94	-11.93	-11.92
-12	-11.99	-11.98	-11.97	-11.96	-11.95	-11.94	-11.93	-11.92

-12	-11.99	-11.98	-11.97	-11.96	-11.95	-11.94	-11.93	-11.92
-12	-11.99	-11.98	-11.97	-11.96	-11.95	-11.94	-11.93	-11.92
-12	-11.99	-11.98	-11.97	-11.96	-11.95	-11.94	-11.93	-11.92
-12	-11.99	-11.98	-11.97	-11.96	-11.95	-11.94	-11.93	-11.92
-12	-11.99	-11.98	-11.97	-11.96	-11.95	-11.94	-11.93	-11.92
-12	-11.99	-11.98	-11.97	-11.96	-11.95	-11.94	-11.93	-11.92
-12	-11.99	-11.98	-11.97	-11.96	-11.95	-11.94	-11.93	-11.92
-12	-11.99	-11.98	-11.97	-11.96	-11.95	-11.94	-11.93	-11.92
-12	-11.99	-11.98	-11.97	-11.96	-11.95	-11.94	-11.93	-11.92
-12	-11.99	-11.98	-11.97	-11.96	-11.95	-11.94	-11.93	-11.92
-12	-11.99	-11.98	-11.97	-11.96	-11.95	-11.94	-11.93	-11.92
-12	-11.99	-11.98	-11.97	-11.96	-11.95	-11.94	-11.93	-11.92
-12	-11.99	-11.98	-11.97	-11.96	-11.95	-11.94	-11.93	-11.92
-12	-11.99	-11.98	-11.97	-11.96	-11.95	-11.94	-11.93	-11.92
-12	-11.99	-11.98	-11.97	-11.96	-11.95	-11.94	-11.93	-11.92
-12	-11.99	-11.98	-11.97	-11.96	-11.95	-11.94	-11.93	-11.92
-12	-11.99	-11.98	-11.97	-11.96	-11.95	-11.94	-11.93	-11.92
-12	-11.99	-11.98	-11.97	-11.96	-11.95	-11.94	-11.93	-11.92
-12	-11.99	-11.98	-11.97	-11.96	-11.95	-11.94	-11.93	-11.92
-12	-11.99	-11.98	-11.97	-11.96	-11.95	-11.94	-11.93	-11.92
-12	-11.99	-11.98	-11.97	-11.96	-11.95	-11.94	-11.93	-11.92
-12	-11.99	-11.98	-11.97	-11.96	-11.95	-11.94	-11.93	-11.92
-12	-11.99	-11.98	-11.97	-11.96	-11.95	-11.94	-11.93	-11.92
-12	-11.99	-11.98	-11.97	-11.96	-11.95	-11.94	-11.93	-11.92
-12	-11.99	-11.98	-11.97	-11.96	-11.95	-11.94	-11.93	-11.92
-12	-11.99	-11.98	-11.97	-11.96	-11.95	-11.94	-11.93	-11.92
-12	-11.99	-11.98	-11.97	-11.96	-11.95	-11.94	-11.93	-11.92
-12	-11.99	-11.98	-11.97	-11.96	-11.95	-11.94	-11.93	-11.92
-12	-11.99	-11.98	-11.97	-11.96	-11.95	-11.94	-11.93	-11.92
-12	-11.99	-11.98	-11.97	-11.96	-11.95	-11.94	-11.93	-11.92
-12	-11.99	-11.98	-11.97	-11.96	-11.95	-11.94	-11.93	-11.92
-12	-11.99	-11.98	-11.97	-11.96	-11.95	-11.94	-11.93	-11.92
-12	-11.99	-11.98	-11.97	-11.96	-11.95	-11.94	-11.93	-11.92
-12	-11.99	-11.98	-11.97	-11.96	-11.95	-11.94	-11.93	-11.92
-12	-11.99	-11.98	-11.97	-11.96	-11.95	-11.94	-11.93	-11.92
-12	-11.99	-11.98	-11.97	-11.96	-11.95	-11.94	-11.93	-11.92
-12	-11.99	-11.98	-11.97	-11.96	-11.95	-11.94	-11.93	-11.92
-12	-11.99	-11.98	-11.97	-11.96	-11.95	-11.94	-11.93	-11.92
-12	-11.99	-11.98	-11.97	-11.96	-11.95	-11.94	-11.93	-11.92
-12	-11.99	-11.98	-11.97	-11.96	-11.95	-11.94	-11.93	-11.92
-12	-11.99	-11.98	-11.97	-11.96	-11.95	-11.94	-11.93	-11.92
-12	-11.99	-11.98	-11.97	-11.96	-11.95	-11.94	-11.93	-11.92
-12	-11.99	-11.98	-11.97	-11.96	-11.95	-11.94	-11.93	-11.92
-12	-11.99	-11.98	-11.97	-11.96	-11.95	-11.94	-11.93	-11.92
-12	-11.99	-11.98	-11.97	-11.96	-11.95	-11.94	-11.93	-11.92
-12	-11.99	-11.98	-11.97	-11.96	-11.95	-11.94	-11.93	-11.92
-12	-11.99	-11.98	-11.97	-11.96	-11.95	-11.94	-11.93	-11.92
-12	-11.99	-11.98	-11.97	-11.96	-11.95	-11.94	-11.93	-11.92
-12	-11.99	-11.98	-11.97	-11.96	-11.95	-11.94	-11.93	-11.92
-12	-11.99	-11.98	-11.97	-11.96	-11.95	-11.94	-11.93	-11.92

-12	-11.99	-11.98	-11.97	-11.96	-11.95	-11.94	-11.93	-11.92
-12	-11.99	-11.98	-11.97	-11.96	-11.95	-11.94	-11.93	-11.92
-12	-11.99	-11.98	-11.97	-11.96	-11.95	-11.94	-11.93	-11.92
-12	-11.99	-11.98	-11.97	-11.96	-11.95	-11.94	-11.93	-11.92
-12	-11.99	-11.98	-11.97	-11.96	-11.95	-11.94	-11.93	-11.92
-12	-11.99	-11.98	-11.97	-11.96	-11.95	-11.94	-11.93	-11.92
-12	-11.99	-11.98	-11.97	-11.96	-11.95	-11.94	-11.93	-11.92
-12	-11.99	-11.98	-11.97	-11.96	-11.95	-11.94	-11.93	-11.92
-12	-11.99	-11.98	-11.97	-11.96	-11.95	-11.94	-11.93	-11.92
-12	-11.99	-11.98	-11.97	-11.96	-11.95	-11.94	-11.93	-11.92
-12	-11.99	-11.98	-11.97	-11.96	-11.95	-11.94	-11.93	-11.92
-12	-11.99	-11.98	-11.97	-11.96	-11.95	-11.94	-11.93	-11.92
-12	-11.99	-11.98	-11.97	-11.96	-11.95	-11.94	-11.93	-11.92
-12	-11.99	-11.98	-11.97	-11.96	-11.95	-11.94	-11.93	-11.92
-12	-11.99	-11.98	-11.97	-11.96	-11.95	-11.94	-11.93	-11.92
-12	-11.99	-11.98	-11.97	-11.96	-11.95	-11.94	-11.93	-11.92
-12	-11.99	-11.98	-11.97	-11.96	-11.95	-11.94	-11.93	-11.92
-12	-11.99	-11.98	-11.97	-11.96	-11.95	-11.94	-11.93	-11.92
-12	-11.99	-11.98	-11.97	-11.96	-11.95	-11.94	-11.93	-11.92
-12	-11.99	-11.98	-11.97	-11.96	-11.95	-11.94	-11.93	-11.92
-12	-11.99	-11.98	-11.97	-11.96	-11.95	-11.94	-11.93	-11.92
-12	-11.99	-11.98	-11.97	-11.96	-11.95	-11.94	-11.93	-11.92
-12	-11.99	-11.98	-11.97	-11.96	-11.95	-11.94	-11.93	-11.92
-12	-11.99	-11.98	-11.97	-11.96	-11.95	-11.94	-11.93	-11.92
-12	-11.99	-11.98	-11.97	-11.96	-11.95	-11.94	-11.93	-11.92
-12	-11.99	-11.98	-11.97	-11.96	-11.95	-11.94	-11.93	-11.92
-12	-11.99	-11.98	-11.97	-11.96	-11.95	-11.94	-11.93	-11.92
-12	-11.99	-11.98	-11.97	-11.96	-11.95	-11.94	-11.93	-11.92
-12	-11.99	-11.98	-11.97	-11.96	-11.95	-11.94	-11.93	-11.92
-12	-11.99	-11.98	-11.97	-11.96	-11.95	-11.94	-11.93	-11.92
-12	-11.99	-11.98	-11.97	-11.96	-11.95	-11.94	-11.93	-11.92
-12	-11.99	-11.98	-11.97	-11.96	-11.95	-11.94	-11.93	-11.92
-12	-11.99	-11.98	-11.97	-11.96	-11.95	-11.94	-11.93	-11.92
-12	-11.99	-11.98	-11.97	-11.96	-11.95	-11.94	-11.93	-11.92
-12	-11.99	-11.98	-11.97	-11.96	-11.95	-11.94	-11.93	-11.92
-12	-11.99	-11.98	-11.97	-11.96	-11.95	-11.94	-11.93	-11.92
-12	-11.99	-11.98	-11.97	-11.96	-11.95	-11.94	-11.93	-11.92
-12	-11.99	-11.98	-11.97	-11.96	-11.95	-11.94	-11.93	-11.92
-12	-11.99	-11.98	-11.97	-11.96	-11.95	-11.94	-11.93	-11.92
-12	-11.99	-11.98	-11.97	-11.96	-11.95	-11.94	-11.93	-11.92
-12	-11.99	-11.98	-11.97	-11.96	-11.95	-11.94	-11.93	-11.92
-12	-11.99	-11.98	-11.97	-11.96	-11.95	-11.94	-11.93	-11.92
-12	-11.99	-11.98	-11.97	-11.96	-11.95	-11.94	-11.93	-11.92
-12	-11.99	-11.98	-11.97	-11.96	-11.95	-11.94	-11.93	-11.92
-12	-11.99	-11.98	-11.97	-11.96	-11.95	-11.94	-11.93	-11.92
-12	-11.99	-11.98	-11.97	-11.96	-11.95	-11.94	-11.93	-11.92
-12	-11.99	-11.98	-11.97	-11.96	-11.95	-11.94	-11.93	-11.92
-12	-11.99	-11.98	-11.97	-11.96	-11.95	-11.94	-11.93	-11.92
-12	-11.99	-11.98	-11.97	-11.96	-11.95	-11.94	-11.93	-11.92
-12	-11.99	-11.98	-11.97	-11.96	-11.95	-11.94	-11.93	-11.92

-12	-11.99	-11.98	-11.97	-11.96	-11.95	-11.94	-11.93	-11.92
-12	-11.99	-11.98	-11.97	-11.96	-11.95	-11.94	-11.93	-11.92
-12	-11.99	-11.98	-11.97	-11.96	-11.95	-11.94	-11.93	-11.92
-12	-11.99	-11.98	-11.97	-11.96	-11.95	-11.94	-11.93	-11.92
-12	-11.99	-11.98	-11.97	-11.96	-11.95	-11.94	-11.93	-11.92
-12	-11.99	-11.98	-11.97	-11.96	-11.95	-11.94	-11.93	-11.92
-12	-11.99	-11.98	-11.97	-11.96	-11.95	-11.94	-11.93	-11.92
-12	-11.99	-11.98	-11.97	-11.96	-11.95	-11.94	-11.93	-11.92
-12	-11.99	-11.98	-11.97	-11.96	-11.95	-11.94	-11.93	-11.92
-12	-11.99	-11.98	-11.97	-11.96	-11.95	-11.94	-11.93	-11.92
-12	-11.99	-11.98	-11.97	-11.96	-11.95	-11.94	-11.93	-11.92
-12	-11.99	-11.98	-11.97	-11.96	-11.95	-11.94	-11.93	-11.92
-12	-11.99	-11.98	-11.97	-11.96	-11.95	-11.94	-11.93	-11.92
-12	-11.99	-11.98	-11.97	-11.96	-11.95	-11.94	-11.93	-11.92
-12	-11.99	-11.98	-11.97	-11.96	-11.95	-11.94	-11.93	-11.92
-12	-11.99	-11.98	-11.97	-11.96	-11.95	-11.94	-11.93	-11.92
-12	-11.99	-11.98	-11.97	-11.96	-11.95	-11.94	-11.93	-11.92
-12	-11.99	-11.98	-11.97	-11.96	-11.95	-11.94	-11.93	-11.92
-12	-11.99	-11.98	-11.97	-11.96	-11.95	-11.94	-11.93	-11.92
-12	-11.99	-11.98	-11.97	-11.96	-11.95	-11.94	-11.93	-11.92
-12	-11.99	-11.98	-11.97	-11.96	-11.95	-11.94	-11.93	-11.92
-12	-11.99	-11.98	-11.97	-11.96	-11.95	-11.94	-11.93	-11.92
-12	-11.99	-11.98	-11.97	-11.96	-11.95	-11.94	-11.93	-11.92
-12	-11.99	-11.98	-11.97	-11.96	-11.95	-11.94	-11.93	-11.92
-12	-11.99	-11.98	-11.97	-11.96	-11.95	-11.94	-11.93	-11.92
-12	-11.99	-11.98	-11.97	-11.96	-11.95	-11.94	-11.93	-11.92
-12	-11.99	-11.98	-11.97	-11.96	-11.95	-11.94	-11.93	-11.92
-12	-11.99	-11.98	-11.97	-11.96	-11.95	-11.94	-11.93	-11.92
-12	-11.99	-11.98	-11.97	-11.96	-11.95	-11.94	-11.93	-11.92
-12	-11.99	-11.98	-11.97	-11.96	-11.95	-11.94	-11.93	-11.92
-12	-11.99	-11.98	-11.97	-11.96	-11.95	-11.94	-11.93	-11.92
-12	-11.99	-11.98	-11.97	-11.96	-11.95	-11.94	-11.93	-11.92
-12	-11.99	-11.98	-11.97	-11.96	-11.95	-11.94	-11.93	-11.92
-12	-11.99	-11.98	-11.97	-11.96	-11.95	-11.94	-11.93	-11.92
-12	-11.99	-11.98	-11.97	-11.96	-11.95	-11.94	-11.93	-11.92
-12	-11.99	-11.98	-11.97	-11.96	-11.95	-11.94	-11.93	-11.92
-12	-11.99	-11.98	-11.97	-11.96	-11.95	-11.94	-11.93	-11.92
-12	-11.99	-11.98	-11.97	-11.96	-11.95	-11.94	-11.93	-11.92
-12	-11.99	-11.98	-11.97	-11.96	-11.95	-11.94	-11.93	-11.92
-12	-11.99	-11.98	-11.97	-11.96	-11.95	-11.94	-11.93	-11.92
-12	-11.99	-11.98	-11.97	-11.96	-11.95	-11.94	-11.93	-11.92
-12	-11.99	-11.98	-11.97	-11.96	-11.95	-11.94	-11.93	-11.92
-12	-11.99	-11.98	-11.97	-11.96	-11.95	-11.94	-11.93	-11.92
-12	-11.99	-11.98	-11.97	-11.96	-11.95	-11.94	-11.93	-11.92
-12	-11.99	-11.98	-11.97	-11.96	-11.95	-11.94	-11.93	-11.92
-12	-11.99	-11.98	-11.97	-11.96	-11.95	-11.94	-11.93	-11.92
-12	-11.99	-11.98	-11.97	-11.96	-11.95	-11.94	-11.93	-11.92
-12	-11.99	-11.98	-11.97	-11.96	-11.95	-11.94	-11.93	-11.92
-12	-11.99	-11.98	-11.97	-11.96	-11.95	-11.94	-11.93	-11.92
-12	-11.99	-11.98	-11.97	-11.96	-11.95	-11.94	-11.93	-11.92

-12	-11.99	-11.98	-11.97	-11.96	-11.95	-11.94	-11.93	-11.92
-12	-11.99	-11.98	-11.97	-11.96	-11.95	-11.94	-11.93	-11.92
-12	-11.99	-11.98	-11.97	-11.96	-11.95	-11.94	-11.93	-11.92
-12	-11.99	-11.98	-11.97	-11.96	-11.95	-11.94	-11.93	-11.92
-12	-11.99	-11.98	-11.97	-11.96	-11.95	-11.94	-11.93	-11.92
-12	-11.99	-11.98	-11.97	-11.96	-11.95	-11.94	-11.93	-11.92
-12	-11.99	-11.98	-11.97	-11.96	-11.95	-11.94	-11.93	-11.92
-12	-11.99	-11.98	-11.97	-11.96	-11.95	-11.94	-11.93	-11.92
-12	-11.99	-11.98	-11.97	-11.96	-11.95	-11.94	-11.93	-11.92
-12	-11.99	-11.98	-11.97	-11.96	-11.95	-11.94	-11.93	-11.92
-12	-11.99	-11.98	-11.97	-11.96	-11.95	-11.94	-11.93	-11.92
-12	-11.99	-11.98	-11.97	-11.96	-11.95	-11.94	-11.93	-11.92
-12	-11.99	-11.98	-11.97	-11.96	-11.95	-11.94	-11.93	-11.92
-12	-11.99	-11.98	-11.97	-11.96	-11.95	-11.94	-11.93	-11.92
-12	-11.99	-11.98	-11.97	-11.96	-11.95	-11.94	-11.93	-11.92
-12	-11.99	-11.98	-11.97	-11.96	-11.95	-11.94	-11.93	-11.92
-12	-11.99	-11.98	-11.97	-11.96	-11.95	-11.94	-11.93	-11.92
-12	-11.99	-11.98	-11.97	-11.96	-11.95	-11.94	-11.93	-11.92
-12	-11.99	-11.98	-11.97	-11.96	-11.95	-11.94	-11.93	-11.92
-12	-11.99	-11.98	-11.97	-11.96	-11.95	-11.94	-11.93	-11.92
-12	-11.99	-11.98	-11.97	-11.96	-11.95	-11.94	-11.93	-11.92
-12	-11.99	-11.98	-11.97	-11.96	-11.95	-11.94	-11.93	-11.92
-12	-11.99	-11.98	-11.97	-11.96	-11.95	-11.94	-11.93	-11.92
-12	-11.99	-11.98	-11.97	-11.96	-11.95	-11.94	-11.93	-11.92
-12	-11.99	-11.98	-11.97	-11.96	-11.95	-11.94	-11.93	-11.92
-12	-11.99	-11.98	-11.97	-11.96	-11.95	-11.94	-11.93	-11.92
-12	-11.99	-11.98	-11.97	-11.96	-11.95	-11.94	-11.93	-11.92
-12	-11.99	-11.98	-11.97	-11.96	-11.95	-11.94	-11.93	-11.92
-12	-11.99	-11.98	-11.97	-11.96	-11.95	-11.94	-11.93	-11.92
-12	-11.99	-11.98	-11.97	-11.96	-11.95	-11.94	-11.93	-11.92
-12	-11.99	-11.98	-11.97	-11.96	-11.95	-11.94	-11.93	-11.92
-12	-11.99	-11.98	-11.97	-11.96	-11.95	-11.94	-11.93	-11.92
-12	-11.99	-11.98	-11.97	-11.96	-11.95	-11.94	-11.93	-11.92
-12	-11.99	-11.98	-11.97	-11.96	-11.95	-11.94	-11.93	-11.92
-12	-11.99	-11.98	-11.97	-11.96	-11.95	-11.94	-11.93	-11.92
-12	-11.99	-11.98	-11.97	-11.96	-11.95	-11.94	-11.93	-11.92
-12	-11.99	-11.98	-11.97	-11.96	-11.95	-11.94	-11.93	-11.92
-12	-11.99	-11.98	-11.97	-11.96	-11.95	-11.94	-11.93	-11.92
-12	-11.99	-11.98	-11.97	-11.96	-11.95	-11.94	-11.93	-11.92
-12	-11.99	-11.98	-11.97	-11.96	-11.95	-11.94	-11.93	-11.92
-12	-11.99	-11.98	-11.97	-11.96	-11.95	-11.94	-11.93	-11.92
-12	-11.99	-11.98	-11.97	-11.96	-11.95	-11.94	-11.93	-11.92
-12	-11.99	-11.98	-11.97	-11.96	-11.95	-11.94	-11.93	-11.92
-12	-11.99	-11.98	-11.97	-11.96	-11.95	-11.94	-11.93	-11.92
-12	-11.99	-11.98	-11.97	-11.96	-11.95	-11.94	-11.93	-11.92
-12	-11.99	-11.98	-11.97	-11.96	-11.95	-11.94	-11.93	-11.92
-12	-11.99	-11.98	-11.97	-11.96	-11.95	-11.94	-11.93	-11.92
-12	-11.99	-11.98	-11.97	-11.96	-11.95	-11.94	-11.93	-11.92
-12	-11.99	-11.98	-11.97	-11.96	-11.95	-11.94	-11.93	-11.92
-12	-11.99	-11.98	-11.97	-11.96	-11.95	-11.94	-11.93	-11.92

-12	-11.99	-11.98	-11.97	-11.96	-11.95	-11.94	-11.93	-11.92
-12	-11.99	-11.98	-11.97	-11.96	-11.95	-11.94	-11.93	-11.92
-12	-11.99	-11.98	-11.97	-11.96	-11.95	-11.94	-11.93	-11.92
-12	-11.99	-11.98	-11.97	-11.96	-11.95	-11.94	-11.93	-11.92
-12	-11.99	-11.98	-11.97	-11.96	-11.95	-11.94	-11.93	-11.92
-12	-11.99	-11.98	-11.97	-11.96	-11.95	-11.94	-11.93	-11.92
-12	-11.99	-11.98	-11.97	-11.96	-11.95	-11.94	-11.93	-11.92
-12	-11.99	-11.98	-11.97	-11.96	-11.95	-11.94	-11.93	-11.92
-12	-11.99	-11.98	-11.97	-11.96	-11.95	-11.94	-11.93	-11.92
-12	-11.99	-11.98	-11.97	-11.96	-11.95	-11.94	-11.93	-11.92
-12	-11.99	-11.98	-11.97	-11.96	-11.95	-11.94	-11.93	-11.92
-12	-11.99	-11.98	-11.97	-11.96	-11.95	-11.94	-11.93	-11.92
-12	-11.99	-11.98	-11.97	-11.96	-11.95	-11.94	-11.93	-11.92
-12	-11.99	-11.98	-11.97	-11.96	-11.95	-11.94	-11.93	-11.92
-12	-11.99	-11.98	-11.97	-11.96	-11.95	-11.94	-11.93	-11.92
-12	-11.99	-11.98	-11.97	-11.96	-11.95	-11.94	-11.93	-11.92
-12	-11.99	-11.98	-11.97	-11.96	-11.95	-11.94	-11.93	-11.92
-12	-11.99	-11.98	-11.97	-11.96	-11.95	-11.94	-11.93	-11.92
-12	-11.99	-11.98	-11.97	-11.96	-11.95	-11.94	-11.93	-11.92
-12	-11.99	-11.98	-11.97	-11.96	-11.95	-11.94	-11.93	-11.92
-12	-11.99	-11.98	-11.97	-11.96	-11.95	-11.94	-11.93	-11.92
-12	-11.99	-11.98	-11.97	-11.96	-11.95	-11.94	-11.93	-11.92
-12	-11.99	-11.98	-11.97	-11.96	-11.95	-11.94	-11.93	-11.92
-12	-11.99	-11.98	-11.97	-11.96	-11.95	-11.94	-11.93	-11.92
-12	-11.99	-11.98	-11.97	-11.96	-11.95	-11.94	-11.93	-11.92
-12	-11.99	-11.98	-11.97	-11.96	-11.95	-11.94	-11.93	-11.92
-12	-11.99	-11.98	-11.97	-11.96	-11.95	-11.94	-11.93	-11.92
-12	-11.99	-11.98	-11.97	-11.96	-11.95	-11.94	-11.93	-11.92
-12	-11.99	-11.98	-11.97	-11.96	-11.95	-11.94	-11.93	-11.92
-12	-11.99	-11.98	-11.97	-11.96	-11.95	-11.94	-11.93	-11.92
-12	-11.99	-11.98	-11.97	-11.96	-11.95	-11.94	-11.93	-11.92
-12	-11.99	-11.98	-11.97	-11.96	-11.95	-11.94	-11.93	-11.92
-12	-11.99	-11.98	-11.97	-11.96	-11.95	-11.94	-11.93	-11.92
-12	-11.99	-11.98	-11.97	-11.96	-11.95	-11.94	-11.93	-11.92
-12	-11.99	-11.98	-11.97	-11.96	-11.95	-11.94	-11.93	-11.92
-12	-11.99	-11.98	-11.97	-11.96	-11.95	-11.94	-11.93	-11.92
-12	-11.99	-11.98	-11.97	-11.96	-11.95	-11.94	-11.93	-11.92
-12	-11.99	-11.98	-11.97	-11.96	-11.95	-11.94	-11.93	-11.92
-12	-11.99	-11.98	-11.97	-11.96	-11.95	-11.94	-11.93	-11.92
-12	-11.99	-11.98	-11.97	-11.96	-11.95	-11.94	-11.93	-11.92
-12	-11.99	-11.98	-11.97	-11.96	-11.95	-11.94	-11.93	-11.92
-12	-11.99	-11.98	-11.97	-11.96	-11.95	-11.94	-11.93	-11.92
-12	-11.99	-11.98	-11.97	-11.96	-11.95	-11.94	-11.93	-11.92
-12	-11.99	-11.98	-11.97	-11.96	-11.95	-11.94	-11.93	-11.92
-12	-11.99	-11.98	-11.97	-11.96	-11.95	-11.94	-11.93	-11.92
-12	-11.99	-11.98	-11.97	-11.96	-11.95	-11.94	-11.93	-11.92
-12	-11.99	-11.98	-11.97	-11.96	-11.95	-11.94	-11.93	-11.92
-12	-11.99	-11.98	-11.97	-11.96	-11.95	-11.94	-11.93	-11.92
-12	-11.99	-11.98	-11.97	-11.96	-11.95	-11.94	-11.93	-11.92
-12	-11.99	-11.98	-11.97	-11.96	-11.95	-11.94	-11.93	-11.92

-12	-11.99	-11.98	-11.97	-11.96	-11.95	-11.94	-11.93	-11.92
-12	-11.99	-11.98	-11.97	-11.96	-11.95	-11.94	-11.93	-11.92
-12	-11.99	-11.98	-11.97	-11.96	-11.95	-11.94	-11.93	-11.92
-12	-11.99	-11.98	-11.97	-11.96	-11.95	-11.94	-11.93	-11.92
-12	-11.99	-11.98	-11.97	-11.96	-11.95	-11.94	-11.93	-11.92
-12	-11.99	-11.98	-11.97	-11.96	-11.95	-11.94	-11.93	-11.92
-12	-11.99	-11.98	-11.97	-11.96	-11.95	-11.94	-11.93	-11.92
-12	-11.99	-11.98	-11.97	-11.96	-11.95	-11.94	-11.93	-11.92
-12	-11.99	-11.98	-11.97	-11.96	-11.95	-11.94	-11.93	-11.92
-12	-11.99	-11.98	-11.97	-11.96	-11.95	-11.94	-11.93	-11.92
-12	-11.99	-11.98	-11.97	-11.96	-11.95	-11.94	-11.93	-11.92
-12	-11.99	-11.98	-11.97	-11.96	-11.95	-11.94	-11.93	-11.92
-12	-11.99	-11.98	-11.97	-11.96	-11.95	-11.94	-11.93	-11.92
-12	-11.99	-11.98	-11.97	-11.96	-11.95	-11.94	-11.93	-11.92
-12	-11.99	-11.98	-11.97	-11.96	-11.95	-11.94	-11.93	-11.92
-12	-11.99	-11.98	-11.97	-11.96	-11.95	-11.94	-11.93	-11.92
-12	-11.99	-11.98	-11.97	-11.96	-11.95	-11.94	-11.93	-11.92
-12	-11.99	-11.98	-11.97	-11.96	-11.95	-11.94	-11.93	-11.92
-12	-11.99	-11.98	-11.97	-11.96	-11.95	-11.94	-11.93	-11.92
-12	-11.99	-11.98	-11.97	-11.96	-11.95	-11.94	-11.93	-11.92
-12	-11.99	-11.98	-11.97	-11.96	-11.95	-11.94	-11.93	-11.92
-12	-11.99	-11.98	-11.97	-11.96	-11.95	-11.94	-11.93	-11.92
-12	-11.99	-11.98	-11.97	-11.96	-11.95	-11.94	-11.93	-11.92
-12	-11.99	-11.98	-11.97	-11.96	-11.95	-11.94	-11.93	-11.92
-12	-11.99	-11.98	-11.97	-11.96	-11.95	-11.94	-11.93	-11.92
-12	-11.99	-11.98	-11.97	-11.96	-11.95	-11.94	-11.93	-11.92
-12	-11.99	-11.98	-11.97	-11.96	-11.95	-11.94	-11.93	-11.92
-12	-11.99	-11.98	-11.97	-11.96	-11.95	-11.94	-11.93	-11.92
-12	-11.99	-11.98	-11.97	-11.96	-11.95	-11.94	-11.93	-11.92
-12	-11.99	-11.98	-11.97	-11.96	-11.95	-11.94	-11.93	-11.92
-12	-11.99	-11.98	-11.97	-11.96	-11.95	-11.94	-11.93	-11.92
-12	-11.99	-11.98	-11.97	-11.96	-11.95	-11.94	-11.93	-11.92
-12	-11.99	-11.98	-11.97	-11.96	-11.95	-11.94	-11.93	-11.92
-12	-11.99	-11.98	-11.97	-11.96	-11.95	-11.94	-11.93	-11.92
-12	-11.99	-11.98	-11.97	-11.96	-11.95	-11.94	-11.93	-11.92
-12	-11.99	-11.98	-11.97	-11.96	-11.95	-11.94	-11.93	-11.92
-12	-11.99	-11.98	-11.97	-11.96	-11.95	-11.94	-11.93	-11.92
-12	-11.99	-11.98	-11.97	-11.96	-11.95	-11.94	-11.93	-11.92
-12	-11.99	-11.98	-11.97	-11.96	-11.95	-11.94	-11.93	-11.92
-12	-11.99	-11.98	-11.97	-11.96	-11.95	-11.94	-11.93	-11.92
-12	-11.99	-11.98	-11.97	-11.96	-11.95	-11.94	-11.93	-11.92
-12	-11.99	-11.98	-11.97	-11.96	-11.95	-11.94	-11.93	-11.92
-12	-11.99	-11.98	-11.97	-11.96	-11.95	-11.94	-11.93	-11.92
-12	-11.99	-11.98	-11.97	-11.96	-11.95	-11.94	-11.93	-11.92
-12	-11.99	-11.98	-11.97	-11.96	-11.95	-11.94	-11.93	-11.92
-12	-11.99	-11.98	-11.97	-11.96	-11.95	-11.94	-11.93	-11.92
-12	-11.99	-11.98	-11.97	-11.96	-11.95	-11.94	-11.93	-11.92
-12	-11.99	-11.98	-11.97	-11.96	-11.95	-11.94	-11.93	-11.92
-12	-11.99	-11.98	-11.97	-11.96	-11.95	-11.94	-11.93	-11.92
-12	-11.99	-11.98	-11.97	-11.96	-11.95	-11.94	-11.93	-11.92

-12	-11.99	-11.98	-11.97	-11.96	-11.95	-11.94	-11.93	-11.92
-12	-11.99	-11.98	-11.97	-11.96	-11.95	-11.94	-11.93	-11.92
-12	-11.99	-11.98	-11.97	-11.96	-11.95	-11.94	-11.93	-11.92
-12	-11.99	-11.98	-11.97	-11.96	-11.95	-11.94	-11.93	-11.92
-12	-11.99	-11.98	-11.97	-11.96	-11.95	-11.94	-11.93	-11.92
-12	-11.99	-11.98	-11.97	-11.96	-11.95	-11.94	-11.93	-11.92
-12	-11.99	-11.98	-11.97	-11.96	-11.95	-11.94	-11.93	-11.92
-12	-11.99	-11.98	-11.97	-11.96	-11.95	-11.94	-11.93	-11.92
-12	-11.99	-11.98	-11.97	-11.96	-11.95	-11.94	-11.93	-11.92
-12	-11.99	-11.98	-11.97	-11.96	-11.95	-11.94	-11.93	-11.92
-12	-11.99	-11.98	-11.97	-11.96	-11.95	-11.94	-11.93	-11.92
-12	-11.99	-11.98	-11.97	-11.96	-11.95	-11.94	-11.93	-11.92
-12	-11.99	-11.98	-11.97	-11.96	-11.95	-11.94	-11.93	-11.92
-12	-11.99	-11.98	-11.97	-11.96	-11.95	-11.94	-11.93	-11.92
-12	-11.99	-11.98	-11.97	-11.96	-11.95	-11.94	-11.93	-11.92
-12	-11.99	-11.98	-11.97	-11.96	-11.95	-11.94	-11.93	-11.92
-12	-11.99	-11.98	-11.97	-11.96	-11.95	-11.94	-11.93	-11.92
-12	-11.99	-11.98	-11.97	-11.96	-11.95	-11.94	-11.93	-11.92
-12	-11.99	-11.98	-11.97	-11.96	-11.95	-11.94	-11.93	-11.92
-12	-11.99	-11.98	-11.97	-11.96	-11.95	-11.94	-11.93	-11.92
-12	-11.99	-11.98	-11.97	-11.96	-11.95	-11.94	-11.93	-11.92
-12	-11.99	-11.98	-11.97	-11.96	-11.95	-11.94	-11.93	-11.92
-12	-11.99	-11.98	-11.97	-11.96	-11.95	-11.94	-11.93	-11.92
-12	-11.99	-11.98	-11.97	-11.96	-11.95	-11.94	-11.93	-11.92
-12	-11.99	-11.98	-11.97	-11.96	-11.95	-11.94	-11.93	-11.92
-12	-11.99	-11.98	-11.97	-11.96	-11.95	-11.94	-11.93	-11.92
-12	-11.99	-11.98	-11.97	-11.96	-11.95	-11.94	-11.93	-11.92
-12	-11.99	-11.98	-11.97	-11.96	-11.95	-11.94	-11.93	-11.92
-12	-11.99	-11.98	-11.97	-11.96	-11.95	-11.94	-11.93	-11.92
-12	-11.99	-11.98	-11.97	-11.96	-11.95	-11.94	-11.93	-11.92
-12	-11.99	-11.98	-11.97	-11.96	-11.95	-11.94	-11.93	-11.92
-12	-11.99	-11.98	-11.97	-11.96	-11.95	-11.94	-11.93	-11.92
-12	-11.99	-11.98	-11.97	-11.96	-11.95	-11.94	-11.93	-11.92
-12	-11.99	-11.98	-11.97	-11.96	-11.95	-11.94	-11.93	-11.92
-12	-11.99	-11.98	-11.97	-11.96	-11.95	-11.94	-11.93	-11.92
-12	-11.99	-11.98	-11.97	-11.96	-11.95	-11.94	-11.93	-11.92
-12	-11.99	-11.98	-11.97	-11.96	-11.95	-11.94	-11.93	-11.92
-12	-11.99	-11.98	-11.97	-11.96	-11.95	-11.94	-11.93	-11.92
-12	-11.99	-11.98	-11.97	-11.96	-11.95	-11.94	-11.93	-11.92
-12	-11.99	-11.98	-11.97	-11.96	-11.95	-11.94	-11.93	-11.92
-12	-11.99	-11.98	-11.97	-11.96	-11.95	-11.94	-11.93	-11.92
-12	-11.99	-11.98	-11.97	-11.96	-11.95	-11.94	-11.93	-11.92
-12	-11.99	-11.98	-11.97	-11.96	-11.95	-11.94	-11.93	-11.92
-12	-11.99	-11.98	-11.97	-11.96	-11.95	-11.94	-11.93	-11.92
-12	-11.99	-11.98	-11.97	-11.96	-11.95	-11.94	-11.93	-11.92
-12	-11.99	-11.98	-11.97	-11.96	-11.95	-11.94	-11.93	-11.92
-12	-11.99	-11.98	-11.97	-11.96	-11.95	-11.94	-11.93	-11.92
-12	-11.99	-11.98	-11.97	-11.96	-11.95	-11.94	-11.93	-11.92
-12	-11.99	-11.98	-11.97	-11.96	-11.95	-11.94	-11.93	-11.92
-12	-11.99	-11.98	-11.97	-11.96	-11.95	-11.94	-11.93	-11.92

-12	-11.99	-11.98	-11.97	-11.96	-11.95	-11.94	-11.93	-11.92
-12	-11.99	-11.98	-11.97	-11.96	-11.95	-11.94	-11.93	-11.92
-12	-11.99	-11.98	-11.97	-11.96	-11.95	-11.94	-11.93	-11.92
-12	-11.99	-11.98	-11.97	-11.96	-11.95	-11.94	-11.93	-11.92
-12	-11.99	-11.98	-11.97	-11.96	-11.95	-11.94	-11.93	-11.92
-12	-11.99	-11.98	-11.97	-11.96	-11.95	-11.94	-11.93	-11.92
-12	-11.99	-11.98	-11.97	-11.96	-11.95	-11.94	-11.93	-11.92
-12	-11.99	-11.98	-11.97	-11.96	-11.95	-11.94	-11.93	-11.92
-12	-11.99	-11.98	-11.97	-11.96	-11.95	-11.94	-11.93	-11.92
-12	-11.99	-11.98	-11.97	-11.96	-11.95	-11.94	-11.93	-11.92
-12	-11.99	-11.98	-11.97	-11.96	-11.95	-11.94	-11.93	-11.92
-12	-11.99	-11.98	-11.97	-11.96	-11.95	-11.94	-11.93	-11.92
-12	-11.99	-11.98	-11.97	-11.96	-11.95	-11.94	-11.93	-11.92
-12	-11.99	-11.98	-11.97	-11.96	-11.95	-11.94	-11.93	-11.92
-12	-11.99	-11.98	-11.97	-11.96	-11.95	-11.94	-11.93	-11.92
-12	-11.99	-11.98	-11.97	-11.96	-11.95	-11.94	-11.93	-11.92
-12	-11.99	-11.98	-11.97	-11.96	-11.95	-11.94	-11.93	-11.92
-12	-11.99	-11.98	-11.97	-11.96	-11.95	-11.94	-11.93	-11.92
-12	-11.99	-11.98	-11.97	-11.96	-11.95	-11.94	-11.93	-11.92
-12	-11.99	-11.98	-11.97	-11.96	-11.95	-11.94	-11.93	-11.92
-12	-11.99	-11.98	-11.97	-11.96	-11.95	-11.94	-11.93	-11.92
-12	-11.99	-11.98	-11.97	-11.96	-11.95	-11.94	-11.93	-11.92
-12	-11.99	-11.98	-11.97	-11.96	-11.95	-11.94	-11.93	-11.92
-12	-11.99	-11.98	-11.97	-11.96	-11.95	-11.94	-11.93	-11.92
-12	-11.99	-11.98	-11.97	-11.96	-11.95	-11.94	-11.93	-11.92
-12	-11.99	-11.98	-11.97	-11.96	-11.95	-11.94	-11.93	-11.92
-12	-11.99	-11.98	-11.97	-11.96	-11.95	-11.94	-11.93	-11.92
-12	-11.99	-11.98	-11.97	-11.96	-11.95	-11.94	-11.93	-11.92
-12	-11.99	-11.98	-11.97	-11.96	-11.95	-11.94	-11.93	-11.92
-12	-11.99	-11.98	-11.97	-11.96	-11.95	-11.94	-11.93	-11.92
-12	-11.99	-11.98	-11.97	-11.96	-11.95	-11.94	-11.93	-11.92
-12	-11.99	-11.98	-11.97	-11.96	-11.95	-11.94	-11.93	-11.92
-12	-11.99	-11.98	-11.97	-11.96	-11.95	-11.94	-11.93	-11.92
-12	-11.99	-11.98	-11.97	-11.96	-11.95	-11.94	-11.93	-11.92
-12	-11.99	-11.98	-11.97	-11.96	-11.95	-11.94	-11.93	-11.92
-12	-11.99	-11.98	-11.97	-11.96	-11.95	-11.94	-11.93	-11.92
-12	-11.99	-11.98	-11.97	-11.96	-11.95	-11.94	-11.93	-11.92
-12	-11.99	-11.98	-11.97	-11.96	-11.95	-11.94	-11.93	-11.92
-12	-11.99	-11.98	-11.97	-11.96	-11.95	-11.94	-11.93	-11.92
-12	-11.99	-11.98	-11.97	-11.96	-11.95	-11.94	-11.93	-11.92
-12	-11.99	-11.98	-11.97	-11.96	-11.95	-11.94	-11.93	-11.92
-12	-11.99	-11.98	-11.97	-11.96	-11.95	-11.94	-11.93	-11.92
-12	-11.99	-11.98	-11.97	-11.96	-11.95	-11.94	-11.93	-11.92
-12	-11.99	-11.98	-11.97	-11.96	-11.95	-11.94	-11.93	-11.92
-12	-11.99	-11.98	-11.97	-11.96	-11.95	-11.94	-11.93	-11.92
-12	-11.99	-11.98	-11.97	-11.96	-11.95	-11.94	-11.93	-11.92
-12	-11.99	-11.98	-11.97	-11.96	-11.95	-11.94	-11.93	-11.92
-12	-11.99	-11.98	-11.97	-11.96	-11.95	-11.94	-11.93	-11.92
-12	-11.99	-11.98	-11.97	-11.96	-11.95	-11.94	-11.93	-11.92
-12	-11.99	-11.98	-11.97	-11.96	-11.95	-11.94	-11.93	-11.92

-12	-11.99	-11.98	-11.97	-11.96	-11.95	-11.94	-11.93	-11.92
-12	-11.99	-11.98	-11.97	-11.96	-11.95	-11.94	-11.93	-11.92
-12	-11.99	-11.98	-11.97	-11.96	-11.95	-11.94	-11.93	-11.92
-12	-11.99	-11.98	-11.97	-11.96	-11.95	-11.94	-11.93	-11.92
-12	-11.99	-11.98	-11.97	-11.96	-11.95	-11.94	-11.93	-11.92
-12	-11.99	-11.98	-11.97	-11.96	-11.95	-11.94	-11.93	-11.92
-12	-11.99	-11.98	-11.97	-11.96	-11.95	-11.94	-11.93	-11.92
-12	-11.99	-11.98	-11.97	-11.96	-11.95	-11.94	-11.93	-11.92
-12	-11.99	-11.98	-11.97	-11.96	-11.95	-11.94	-11.93	-11.92
-12	-11.99	-11.98	-11.97	-11.96	-11.95	-11.94	-11.93	-11.92
-12	-11.99	-11.98	-11.97	-11.96	-11.95	-11.94	-11.93	-11.92
-12	-11.99	-11.98	-11.97	-11.96	-11.95	-11.94	-11.93	-11.92
-12	-11.99	-11.98	-11.97	-11.96	-11.95	-11.94	-11.93	-11.92
-12	-11.99	-11.98	-11.97	-11.96	-11.95	-11.94	-11.93	-11.92
-12	-11.99	-11.98	-11.97	-11.96	-11.95	-11.94	-11.93	-11.92
-12	-11.99	-11.98	-11.97	-11.96	-11.95	-11.94	-11.93	-11.92
-12	-11.99	-11.98	-11.97	-11.96	-11.95	-11.94	-11.93	-11.92
-12	-11.99	-11.98	-11.97	-11.96	-11.95	-11.94	-11.93	-11.92
-12	-11.99	-11.98	-11.97	-11.96	-11.95	-11.94	-11.93	-11.92
-12	-11.99	-11.98	-11.97	-11.96	-11.95	-11.94	-11.93	-11.92
-12	-11.99	-11.98	-11.97	-11.96	-11.95	-11.94	-11.93	-11.92
-12	-11.99	-11.98	-11.97	-11.96	-11.95	-11.94	-11.93	-11.92
-12	-11.99	-11.98	-11.97	-11.96	-11.95	-11.94	-11.93	-11.92
-12	-11.99	-11.98	-11.97	-11.96	-11.95	-11.94	-11.93	-11.92
-12	-11.99	-11.98	-11.97	-11.96	-11.95	-11.94	-11.93	-11.92
-12	-11.99	-11.98	-11.97	-11.96	-11.95	-11.94	-11.93	-11.92
-12	-11.99	-11.98	-11.97	-11.96	-11.95	-11.94	-11.93	-11.92
-12	-11.99	-11.98	-11.97	-11.96	-11.95	-11.94	-11.93	-11.92
-12	-11.99	-11.98	-11.97	-11.96	-11.95	-11.94	-11.93	-11.92
-12	-11.99	-11.98	-11.97	-11.96	-11.95	-11.94	-11.93	-11.92
-12	-11.99	-11.98	-11.97	-11.96	-11.95	-11.94	-11.93	-11.92
-12	-11.99	-11.98	-11.97	-11.96	-11.95	-11.94	-11.93	-11.92
-12	-11.99	-11.98	-11.97	-11.96	-11.95	-11.94	-11.93	-11.92
-12	-11.99	-11.98	-11.97	-11.96	-11.95	-11.94	-11.93	-11.92
-12	-11.99	-11.98	-11.97	-11.96	-11.95	-11.94	-11.93	-11.92
-12	-11.99	-11.98	-11.97	-11.96	-11.95	-11.94	-11.93	-11.92
-12	-11.99	-11.98	-11.97	-11.96	-11.95	-11.94	-11.93	-11.92
-12	-11.99	-11.98	-11.97	-11.96	-11.95	-11.94	-11.93	-11.92
-12	-11.99	-11.98	-11.97	-11.96	-11.95	-11.94	-11.93	-11.92
-12	-11.99	-11.98	-11.97	-11.96	-11.95	-11.94	-11.93	-11.92
-12	-11.99	-11.98	-11.97	-11.96	-11.95	-11.94	-11.93	-11.92
-12	-11.99	-11.98	-11.97	-11.96	-11.95	-11.94	-11.93	-11.92
-12	-11.99	-11.98	-11.97	-11.96	-11.95	-11.94	-11.93	-11.92
-12	-11.99	-11.98	-11.97	-11.96	-11.95	-11.94	-11.93	-11.92
-12	-11.99	-11.98	-11.97	-11.96	-11.95	-11.94	-11.93	-11.92
-12	-11.99	-11.98	-11.97	-11.96	-11.95	-11.94	-11.93	-11.92
-12	-11.99	-11.98	-11.97	-11.96	-11.95	-11.94	-11.93	-11.92
-12	-11.99	-11.98	-11.97	-11.96	-11.95	-11.94	-11.93	-11.92
-12	-11.99	-11.98	-11.97	-11.96	-11.95	-11.94	-11.93	-11.92
-12	-11.99	-11.98	-11.97	-11.96	-11.95	-11.94	-11.93	-11.92

-12	-11.99	-11.98	-11.97	-11.96	-11.95	-11.94	-11.93	-11.92
-12	-11.99	-11.98	-11.97	-11.96	-11.95	-11.94	-11.93	-11.92
-12	-11.99	-11.98	-11.97	-11.96	-11.95	-11.94	-11.93	-11.92
-12	-11.99	-11.98	-11.97	-11.96	-11.95	-11.94	-11.93	-11.92
-12	-11.99	-11.98	-11.97	-11.96	-11.95	-11.94	-11.93	-11.92
-12	-11.99	-11.98	-11.97	-11.96	-11.95	-11.94	-11.93	-11.92
-12	-11.99	-11.98	-11.97	-11.96	-11.95	-11.94	-11.93	-11.92
-12	-11.99	-11.98	-11.97	-11.96	-11.95	-11.94	-11.93	-11.92
-12	-11.99	-11.98	-11.97	-11.96	-11.95	-11.94	-11.93	-11.92
-12	-11.99	-11.98	-11.97	-11.96	-11.95	-11.94	-11.93	-11.92
-12	-11.99	-11.98	-11.97	-11.96	-11.95	-11.94	-11.93	-11.92
-12	-11.99	-11.98	-11.97	-11.96	-11.95	-11.94	-11.93	-11.92
-12	-11.99	-11.98	-11.97	-11.96	-11.95	-11.94	-11.93	-11.92
-12	-11.99	-11.98	-11.97	-11.96	-11.95	-11.94	-11.93	-11.92
-12	-11.99	-11.98	-11.97	-11.96	-11.95	-11.94	-11.93	-11.92
-12	-11.99	-11.98	-11.97	-11.96	-11.95	-11.94	-11.93	-11.92
-12	-11.99	-11.98	-11.97	-11.96	-11.95	-11.94	-11.93	-11.92
-12	-11.99	-11.98	-11.97	-11.96	-11.95	-11.94	-11.93	-11.92
-12	-11.99	-11.98	-11.97	-11.96	-11.95	-11.94	-11.93	-11.92
-12	-11.99	-11.98	-11.97	-11.96	-11.95	-11.94	-11.93	-11.92
-12	-11.99	-11.98	-11.97	-11.96	-11.95	-11.94	-11.93	-11.92
-12	-11.99	-11.98	-11.97	-11.96	-11.95	-11.94	-11.93	-11.92
-12	-11.99	-11.98	-11.97	-11.96	-11.95	-11.94	-11.93	-11.92
-12	-11.99	-11.98	-11.97	-11.96	-11.95	-11.94	-11.93	-11.92
-12	-11.99	-11.98	-11.97	-11.96	-11.95	-11.94	-11.93	-11.92
-12	-11.99	-11.98	-11.97	-11.96	-11.95	-11.94	-11.93	-11.92
-12	-11.99	-11.98	-11.97	-11.96	-11.95	-11.94	-11.93	-11.92
-12	-11.99	-11.98	-11.97	-11.96	-11.95	-11.94	-11.93	-11.92
-12	-11.99	-11.98	-11.97	-11.96	-11.95	-11.94	-11.93	-11.92
-12	-11.99	-11.98	-11.97	-11.96	-11.95	-11.94	-11.93	-11.92
-12	-11.99	-11.98	-11.97	-11.96	-11.95	-11.94	-11.93	-11.92
-12	-11.99	-11.98	-11.97	-11.96	-11.95	-11.94	-11.93	-11.92
-12	-11.99	-11.98	-11.97	-11.96	-11.95	-11.94	-11.93	-11.92
-12	-11.99	-11.98	-11.97	-11.96	-11.95	-11.94	-11.93	-11.92
-12	-11.99	-11.98	-11.97	-11.96	-11.95	-11.94	-11.93	-11.92
-12	-11.99	-11.98	-11.97	-11.96	-11.95	-11.94	-11.93	-11.92
-12	-11.99	-11.98	-11.97	-11.96	-11.95	-11.94	-11.93	-11.92
-12	-11.99	-11.98	-11.97	-11.96	-11.95	-11.94	-11.93	-11.92
-12	-11.99	-11.98	-11.97	-11.96	-11.95	-11.94	-11.93	-11.92
-12	-11.99	-11.98	-11.97	-11.96	-11.95	-11.94	-11.93	-11.92
-12	-11.99	-11.98	-11.97	-11.96	-11.95	-11.94	-11.93	-11.92
-12	-11.99	-11.98	-11.97	-11.96	-11.95	-11.94	-11.93	-11.92
-12	-11.99	-11.98	-11.97	-11.96	-11.95	-11.94	-11.93	-11.92
-12	-11.99	-11.98	-11.97	-11.96	-11.95	-11.94	-11.93	-11.92
-12	-11.99	-11.98	-11.97	-11.96	-11.95	-11.94	-11.93	-11.92
-12	-11.99	-11.98	-11.97	-11.96	-11.95	-11.94	-11.93	-11.92
-12	-11.99	-11.98	-11.97	-11.96	-11.95	-11.94	-11.93	-11.92
-12	-11.99	-11.98	-11.97	-11.96	-11.95	-11.94	-11.93	-11.92
-12	-11.99	-11.98	-11.97	-11.96	-11.95	-11.94	-11.93	-11.92
-12	-11.99	-11.98	-11.97	-11.96	-11.95	-11.94	-11.93	-11.92

-12	-11.99	-11.98	-11.97	-11.96	-11.95	-11.94	-11.93	-11.92
-12	-11.99	-11.98	-11.97	-11.96	-11.95	-11.94	-11.93	-11.92
-12	-11.99	-11.98	-11.97	-11.96	-11.95	-11.94	-11.93	-11.92
-12	-11.99	-11.98	-11.97	-11.96	-11.95	-11.94	-11.93	-11.92
-12	-11.99	-11.98	-11.97	-11.96	-11.95	-11.94	-11.93	-11.92
-12	-11.99	-11.98	-11.97	-11.96	-11.95	-11.94	-11.93	-11.92
-12	-11.99	-11.98	-11.97	-11.96	-11.95	-11.94	-11.93	-11.92
-12	-11.99	-11.98	-11.97	-11.96	-11.95	-11.94	-11.93	-11.92
-12	-11.99	-11.98	-11.97	-11.96	-11.95	-11.94	-11.93	-11.92
-12	-11.99	-11.98	-11.97	-11.96	-11.95	-11.94	-11.93	-11.92
-12	-11.99	-11.98	-11.97	-11.96	-11.95	-11.94	-11.93	-11.92
-12	-11.99	-11.98	-11.97	-11.96	-11.95	-11.94	-11.93	-11.92
-12	-11.99	-11.98	-11.97	-11.96	-11.95	-11.94	-11.93	-11.92
-12	-11.99	-11.98	-11.97	-11.96	-11.95	-11.94	-11.93	-11.92
-12	-11.99	-11.98	-11.97	-11.96	-11.95	-11.94	-11.93	-11.92
-12	-11.99	-11.98	-11.97	-11.96	-11.95	-11.94	-11.93	-11.92
-12	-11.99	-11.98	-11.97	-11.96	-11.95	-11.94	-11.93	-11.92
-12	-11.99	-11.98	-11.97	-11.96	-11.95	-11.94	-11.93	-11.92
-12	-11.99	-11.98	-11.97	-11.96	-11.95	-11.94	-11.93	-11.92
-12	-11.99	-11.98	-11.97	-11.96	-11.95	-11.94	-11.93	-11.92
-12	-11.99	-11.98	-11.97	-11.96	-11.95	-11.94	-11.93	-11.92
-12	-11.99	-11.98	-11.97	-11.96	-11.95	-11.94	-11.93	-11.92
-12	-11.99	-11.98	-11.97	-11.96	-11.95	-11.94	-11.93	-11.92
-12	-11.99	-11.98	-11.97	-11.96	-11.95	-11.94	-11.93	-11.92
-12	-11.99	-11.98	-11.97	-11.96	-11.95	-11.94	-11.93	-11.92
-12	-11.99	-11.98	-11.97	-11.96	-11.95	-11.94	-11.93	-11.92
-12	-11.99	-11.98	-11.97	-11.96	-11.95	-11.94	-11.93	-11.92
-12	-11.99	-11.98	-11.97	-11.96	-11.95	-11.94	-11.93	-11.92
-12	-11.99	-11.98	-11.97	-11.96	-11.95	-11.94	-11.93	-11.92
-12	-11.99	-11.98	-11.97	-11.96	-11.95	-11.94	-11.93	-11.92
-12	-11.99	-11.98	-11.97	-11.96	-11.95	-11.94	-11.93	-11.92
-12	-11.99	-11.98	-11.97	-11.96	-11.95	-11.94	-11.93	-11.92
-12	-11.99	-11.98	-11.97	-11.96	-11.95	-11.94	-11.93	-11.92
-12	-11.99	-11.98	-11.97	-11.96	-11.95	-11.94	-11.93	-11.92
-12	-11.99	-11.98	-11.97	-11.96	-11.95	-11.94	-11.93	-11.92
-12	-11.99	-11.98	-11.97	-11.96	-11.95	-11.94	-11.93	-11.92
-12	-11.99	-11.98	-11.97	-11.96	-11.95	-11.94	-11.93	-11.92
-12	-11.99	-11.98	-11.97	-11.96	-11.95	-11.94	-11.93	-11.92
-12	-11.99	-11.98	-11.97	-11.96	-11.95	-11.94	-11.93	-11.92
-12	-11.99	-11.98	-11.97	-11.96	-11.95	-11.94	-11.93	-11.92
-12	-11.99	-11.98	-11.97	-11.96	-11.95	-11.94	-11.93	-11.92
-12	-11.99	-11.98	-11.97	-11.96	-11.95	-11.94	-11.93	-11.92
-12	-11.99	-11.98	-11.97	-11.96	-11.95	-11.94	-11.93	-11.92
-12	-11.99	-11.98	-11.97	-11.96	-11.95	-11.94	-11.93	-11.92
-12	-11.99	-11.98	-11.97	-11.96	-11.95	-11.94	-11.93	-11.92
-12	-11.99	-11.98	-11.97	-11.96	-11.95	-11.94	-11.93	-11.92
-12	-11.99	-11.98	-11.97	-11.96	-11.95	-11.94	-11.93	-11.92
-12	-11.99	-11.98	-11.97	-11.96	-11.95	-11.94	-11.93	-11.92
-12	-11.99	-11.98	-11.97	-11.96	-11.95	-11.94	-11.93	-11.92
-12	-11.99	-11.98	-11.97	-11.96	-11.95	-11.94	-11.93	-11.92

-12	-11.99	-11.98	-11.97	-11.96	-11.95	-11.94	-11.93	-11.92
-12	-11.99	-11.98	-11.97	-11.96	-11.95	-11.94	-11.93	-11.92
-12	-11.99	-11.98	-11.97	-11.96	-11.95	-11.94	-11.93	-11.92
-12	-11.99	-11.98	-11.97	-11.96	-11.95	-11.94	-11.93	-11.92
-12	-11.99	-11.98	-11.97	-11.96	-11.95	-11.94	-11.93	-11.92
-12	-11.99	-11.98	-11.97	-11.96	-11.95	-11.94	-11.93	-11.92
-12	-11.99	-11.98	-11.97	-11.96	-11.95	-11.94	-11.93	-11.92
-12	-11.99	-11.98	-11.97	-11.96	-11.95	-11.94	-11.93	-11.92
-12	-11.99	-11.98	-11.97	-11.96	-11.95	-11.94	-11.93	-11.92
-12	-11.99	-11.98	-11.97	-11.96	-11.95	-11.94	-11.93	-11.92
-12	-11.99	-11.98	-11.97	-11.96	-11.95	-11.94	-11.93	-11.92
-12	-11.99	-11.98	-11.97	-11.96	-11.95	-11.94	-11.93	-11.92
-12	-11.99	-11.98	-11.97	-11.96	-11.95	-11.94	-11.93	-11.92
-12	-11.99	-11.98	-11.97	-11.96	-11.95	-11.94	-11.93	-11.92
-12	-11.99	-11.98	-11.97	-11.96	-11.95	-11.94	-11.93	-11.92
-12	-11.99	-11.98	-11.97	-11.96	-11.95	-11.94	-11.93	-11.92
-12	-11.99	-11.98	-11.97	-11.96	-11.95	-11.94	-11.93	-11.92
-12	-11.99	-11.98	-11.97	-11.96	-11.95	-11.94	-11.93	-11.92
-12	-11.99	-11.98	-11.97	-11.96	-11.95	-11.94	-11.93	-11.92
-12	-11.99	-11.98	-11.97	-11.96	-11.95	-11.94	-11.93	-11.92
-12	-11.99	-11.98	-11.97	-11.96	-11.95	-11.94	-11.93	-11.92
-12	-11.99	-11.98	-11.97	-11.96	-11.95	-11.94	-11.93	-11.92
-12	-11.99	-11.98	-11.97	-11.96	-11.95	-11.94	-11.93	-11.92
-12	-11.99	-11.98	-11.97	-11.96	-11.95	-11.94	-11.93	-11.92
-12	-11.99	-11.98	-11.97	-11.96	-11.95	-11.94	-11.93	-11.92
-12	-11.99	-11.98	-11.97	-11.96	-11.95	-11.94	-11.93	-11.92
-12	-11.99	-11.98	-11.97	-11.96	-11.95	-11.94	-11.93	-11.92
-12	-11.99	-11.98	-11.97	-11.96	-11.95	-11.94	-11.93	-11.92
-12	-11.99	-11.98	-11.97	-11.96	-11.95	-11.94	-11.93	-11.92
-12	-11.99	-11.98	-11.97	-11.96	-11.95	-11.94	-11.93	-11.92
-12	-11.99	-11.98	-11.97	-11.96	-11.95	-11.94	-11.93	-11.92
-12	-11.99	-11.98	-11.97	-11.96	-11.95	-11.94	-11.93	-11.92
-12	-11.99	-11.98	-11.97	-11.96	-11.95	-11.94	-11.93	-11.92
-12	-11.99	-11.98	-11.97	-11.96	-11.95	-11.94	-11.93	-11.92
-12	-11.99	-11.98	-11.97	-11.96	-11.95	-11.94	-11.93	-11.92
-12	-11.99	-11.98	-11.97	-11.96	-11.95	-11.94	-11.93	-11.92
-12	-11.99	-11.98	-11.97	-11.96	-11.95	-11.94	-11.93	-11.92
-12	-11.99	-11.98	-11.97	-11.96	-11.95	-11.94	-11.93	-11.92
-12	-11.99	-11.98	-11.97	-11.96	-11.95	-11.94	-11.93	-11.92
-12	-11.99	-11.98	-11.97	-11.96	-11.95	-11.94	-11.93	-11.92
-12	-11.99	-11.98	-11.97	-11.96	-11.95	-11.94	-11.93	-11.92
-12	-11.99	-11.98	-11.97	-11.96	-11.95	-11.94	-11.93	-11.92
-12	-11.99	-11.98	-11.97	-11.96	-11.95	-11.94	-11.93	-11.92
-12	-11.99	-11.98	-11.97	-11.96	-11.95	-11.94	-11.93	-11.92
-12	-11.99	-11.98	-11.97	-11.96	-11.95	-11.94	-11.93	-11.92
-12	-11.99	-11.98	-11.97	-11.96	-11.95	-11.94	-11.93	-11.92
-12	-11.99	-11.98	-11.97	-11.96	-11.95	-11.94	-11.93	-11.92
-12	-11.99	-11.98	-11.97	-11.96	-11.95	-11.94	-11.93	-11.92
-12	-11.99	-11.98	-11.97	-11.96	-11.95	-11.94	-11.93	-11.92
-12	-11.99	-11.98	-11.97	-11.96	-11.95	-11.94	-11.93	-11.92

-12	-11.99	-11.98	-11.97	-11.96	-11.95	-11.94	-11.93	-11.92
-12	-11.99	-11.98	-11.97	-11.96	-11.95	-11.94	-11.93	-11.92
-12	-11.99	-11.98	-11.97	-11.96	-11.95	-11.94	-11.93	-11.92
-12	-11.99	-11.98	-11.97	-11.96	-11.95	-11.94	-11.93	-11.92
-12	-11.99	-11.98	-11.97	-11.96	-11.95	-11.94	-11.93	-11.92
-12	-11.99	-11.98	-11.97	-11.96	-11.95	-11.94	-11.93	-11.92
-12	-11.99	-11.98	-11.97	-11.96	-11.95	-11.94	-11.93	-11.92
-12	-11.99	-11.98	-11.97	-11.96	-11.95	-11.94	-11.93	-11.92
-12	-11.99	-11.98	-11.97	-11.96	-11.95	-11.94	-11.93	-11.92
-12	-11.99	-11.98	-11.97	-11.96	-11.95	-11.94	-11.93	-11.92
-12	-11.99	-11.98	-11.97	-11.96	-11.95	-11.94	-11.93	-11.92
-12	-11.99	-11.98	-11.97	-11.96	-11.95	-11.94	-11.93	-11.92
-12	-11.99	-11.98	-11.97	-11.96	-11.95	-11.94	-11.93	-11.92
-12	-11.99	-11.98	-11.97	-11.96	-11.95	-11.94	-11.93	-11.92
-12	-11.99	-11.98	-11.97	-11.96	-11.95	-11.94	-11.93	-11.92
-12	-11.99	-11.98	-11.97	-11.96	-11.95	-11.94	-11.93	-11.92
-12	-11.99	-11.98	-11.97	-11.96	-11.95	-11.94	-11.93	-11.92
-12	-11.99	-11.98	-11.97	-11.96	-11.95	-11.94	-11.93	-11.92
-12	-11.99	-11.98	-11.97	-11.96	-11.95	-11.94	-11.93	-11.92
-12	-11.99	-11.98	-11.97	-11.96	-11.95	-11.94	-11.93	-11.92
-12	-11.99	-11.98	-11.97	-11.96	-11.95	-11.94	-11.93	-11.92
-12	-11.99	-11.98	-11.97	-11.96	-11.95	-11.94	-11.93	-11.92
-12	-11.99	-11.98	-11.97	-11.96	-11.95	-11.94	-11.93	-11.92
-12	-11.99	-11.98	-11.97	-11.96	-11.95	-11.94	-11.93	-11.92
-12	-11.99	-11.98	-11.97	-11.96	-11.95	-11.94	-11.93	-11.92
-12	-11.99	-11.98	-11.97	-11.96	-11.95	-11.94	-11.93	-11.92
-12	-11.99	-11.98	-11.97	-11.96	-11.95	-11.94	-11.93	-11.92
-12	-11.99	-11.98	-11.97	-11.96	-11.95	-11.94	-11.93	-11.92
-12	-11.99	-11.98	-11.97	-11.96	-11.95	-11.94	-11.93	-11.92
-12	-11.99	-11.98	-11.97	-11.96	-11.95	-11.94	-11.93	-11.92
-12	-11.99	-11.98	-11.97	-11.96	-11.95	-11.94	-11.93	-11.92
-12	-11.99	-11.98	-11.97	-11.96	-11.95	-11.94	-11.93	-11.92
-12	-11.99	-11.98	-11.97	-11.96	-11.95	-11.94	-11.93	-11.92
-12	-11.99	-11.98	-11.97	-11.96	-11.95	-11.94	-11.93	-11.92
-12	-11.99	-11.98	-11.97	-11.96	-11.95	-11.94	-11.93	-11.92
-12	-11.99	-11.98	-11.97	-11.96	-11.95	-11.94	-11.93	-11.92
-12	-11.99	-11.98	-11.97	-11.96	-11.95	-11.94	-11.93	-11.92
-12	-11.99	-11.98	-11.97	-11.96	-11.95	-11.94	-11.93	-11.92
-12	-11.99	-11.98	-11.97	-11.96	-11.95	-11.94	-11.93	-11.92
-12	-11.99	-11.98	-11.97	-11.96	-11.95	-11.94	-11.93	-11.92
-12	-11.99	-11.98	-11.97	-11.96	-11.95	-11.94	-11.93	-11.92
-12	-11.99	-11.98	-11.97	-11.96	-11.95	-11.94	-11.93	-11.92
-12	-11.99	-11.98	-11.97	-11.96	-11.95	-11.94	-11.93	-11.92
-12	-11.99	-11.98	-11.97	-11.96	-11.95	-11.94	-11.93	-11.92
-12	-11.99	-11.98	-11.97	-11.96	-11.95	-11.94	-11.93	-11.92
-12	-11.99	-11.98	-11.97	-11.96	-11.95	-11.94	-11.93	-11.92
-12	-11.99	-11.98	-11.97	-11.96	-11.95	-11.94	-11.93	-11.92
-12	-11.99	-11.98	-11.97	-11.96	-11.95	-11.94	-11.93	-11.92
-12	-11.99	-11.98	-11.97	-11.96	-11.95	-11.94	-11.93	-11.92
-12	-11.99	-11.98	-11.97	-11.96	-11.95	-11.94	-11.93	-11.92

-12	-11.99	-11.98	-11.97	-11.96	-11.95	-11.94	-11.93	-11.92
-12	-11.99	-11.98	-11.97	-11.96	-11.95	-11.94	-11.93	-11.92
-12	-11.99	-11.98	-11.97	-11.96	-11.95	-11.94	-11.93	-11.92
-12	-11.99	-11.98	-11.97	-11.96	-11.95	-11.94	-11.93	-11.92
-12	-11.99	-11.98	-11.97	-11.96	-11.95	-11.94	-11.93	-11.92
-12	-11.99	-11.98	-11.97	-11.96	-11.95	-11.94	-11.93	-11.92
-12	-11.99	-11.98	-11.97	-11.96	-11.95	-11.94	-11.93	-11.92
-12	-11.99	-11.98	-11.97	-11.96	-11.95	-11.94	-11.93	-11.92
-12	-11.99	-11.98	-11.97	-11.96	-11.95	-11.94	-11.93	-11.92
-12	-11.99	-11.98	-11.97	-11.96	-11.95	-11.94	-11.93	-11.92
-12	-11.99	-11.98	-11.97	-11.96	-11.95	-11.94	-11.93	-11.92
-12	-11.99	-11.98	-11.97	-11.96	-11.95	-11.94	-11.93	-11.92
-12	-11.99	-11.98	-11.97	-11.96	-11.95	-11.94	-11.93	-11.92
-12	-11.99	-11.98	-11.97	-11.96	-11.95	-11.94	-11.93	-11.92
-12	-11.99	-11.98	-11.97	-11.96	-11.95	-11.94	-11.93	-11.92
-12	-11.99	-11.98	-11.97	-11.96	-11.95	-11.94	-11.93	-11.92
-12	-11.99	-11.98	-11.97	-11.96	-11.95	-11.94	-11.93	-11.92
-12	-11.99	-11.98	-11.97	-11.96	-11.95	-11.94	-11.93	-11.92
-12	-11.99	-11.98	-11.97	-11.96	-11.95	-11.94	-11.93	-11.92
-12	-11.99	-11.98	-11.97	-11.96	-11.95	-11.94	-11.93	-11.92
-12	-11.99	-11.98	-11.97	-11.96	-11.95	-11.94	-11.93	-11.92
-12	-11.99	-11.98	-11.97	-11.96	-11.95	-11.94	-11.93	-11.92
-12	-11.99	-11.98	-11.97	-11.96	-11.95	-11.94	-11.93	-11.92
-12	-11.99	-11.98	-11.97	-11.96	-11.95	-11.94	-11.93	-11.92
-12	-11.99	-11.98	-11.97	-11.96	-11.95	-11.94	-11.93	-11.92
-12	-11.99	-11.98	-11.97	-11.96	-11.95	-11.94	-11.93	-11.92
-12	-11.99	-11.98	-11.97	-11.96	-11.95	-11.94	-11.93	-11.92
-12	-11.99	-11.98	-11.97	-11.96	-11.95	-11.94	-11.93	-11.92
-12	-11.99	-11.98	-11.97	-11.96	-11.95	-11.94	-11.93	-11.92
-12	-11.99	-11.98	-11.97	-11.96	-11.95	-11.94	-11.93	-11.92
-12	-11.99	-11.98	-11.97	-11.96	-11.95	-11.94	-11.93	-11.92
-12	-11.99	-11.98	-11.97	-11.96	-11.95	-11.94	-11.93	-11.92
-12	-11.99	-11.98	-11.97	-11.96	-11.95	-11.94	-11.93	-11.92
-12	-11.99	-11.98	-11.97	-11.96	-11.95	-11.94	-11.93	-11.92
-12	-11.99	-11.98	-11.97	-11.96	-11.95	-11.94	-11.93	-11.92
-12	-11.99	-11.98	-11.97	-11.96	-11.95	-11.94	-11.93	-11.92
-12	-11.99	-11.98	-11.97	-11.96	-11.95	-11.94	-11.93	-11.92
-12	-11.99	-11.98	-11.97	-11.96	-11.95	-11.94	-11.93	-11.92
-12	-11.99	-11.98	-11.97	-11.96	-11.95	-11.94	-11.93	-11.92
-12	-11.99	-11.98	-11.97	-11.96	-11.95	-11.94	-11.93	-11.92
-12	-11.99	-11.98	-11.97	-11.96	-11.95	-11.94	-11.93	-11.92
-12	-11.99	-11.98	-11.97	-11.96	-11.95	-11.94	-11.93	-11.92
-12	-11.99	-11.98	-11.97	-11.96	-11.95	-11.94	-11.93	-11.92
-12	-11.99	-11.98	-11.97	-11.96	-11.95	-11.94	-11.93	-11.92
-12	-11.99	-11.98	-11.97	-11.96	-11.95	-11.94	-11.93	-11.92
-12	-11.99	-11.98	-11.97	-11.96	-11.95	-11.94	-11.93	-11.92
-12	-11.99	-11.98	-11.97	-11.96	-11.95	-11.94	-11.93	-11.92
-12	-11.99	-11.98	-11.97	-11.96	-11.95	-11.94	-11.93	-11.92
-12	-11.99	-11.98	-11.97	-11.96	-11.95	-11.94	-11.93	-11.92
-12	-11.99	-11.98	-11.97	-11.96	-11.95	-11.94	-11.93	-11.92

-12	-11.99	-11.98	-11.97	-11.96	-11.95	-11.94	-11.93	-11.92
-12	-11.99	-11.98	-11.97	-11.96	-11.95	-11.94	-11.93	-11.92
-12	-11.99	-11.98	-11.97	-11.96	-11.95	-11.94	-11.93	-11.92
-12	-11.99	-11.98	-11.97	-11.96	-11.95	-11.94	-11.93	-11.92
-12	-11.99	-11.98	-11.97	-11.96	-11.95	-11.94	-11.93	-11.92
-12	-11.99	-11.98	-11.97	-11.96	-11.95	-11.94	-11.93	-11.92
-12	-11.99	-11.98	-11.97	-11.96	-11.95	-11.94	-11.93	-11.92
-12	-11.99	-11.98	-11.97	-11.96	-11.95	-11.94	-11.93	-11.92
-12	-11.99	-11.98	-11.97	-11.96	-11.95	-11.94	-11.93	-11.92
-12	-11.99	-11.98	-11.97	-11.96	-11.95	-11.94	-11.93	-11.92
-12	-11.99	-11.98	-11.97	-11.96	-11.95	-11.94	-11.93	-11.92
-12	-11.99	-11.98	-11.97	-11.96	-11.95	-11.94	-11.93	-11.92
-12	-11.99	-11.98	-11.97	-11.96	-11.95	-11.94	-11.93	-11.92
-12	-11.99	-11.98	-11.97	-11.96	-11.95	-11.94	-11.93	-11.92
-12	-11.99	-11.98	-11.97	-11.96	-11.95	-11.94	-11.93	-11.92
-12	-11.99	-11.98	-11.97	-11.96	-11.95	-11.94	-11.93	-11.92
-12	-11.99	-11.98	-11.97	-11.96	-11.95	-11.94	-11.93	-11.92
-12	-11.99	-11.98	-11.97	-11.96	-11.95	-11.94	-11.93	-11.92
-12	-11.99	-11.98	-11.97	-11.96	-11.95	-11.94	-11.93	-11.92
-12	-11.99	-11.98	-11.97	-11.96	-11.95	-11.94	-11.93	-11.92
-12	-11.99	-11.98	-11.97	-11.96	-11.95	-11.94	-11.93	-11.92
-12	-11.99	-11.98	-11.97	-11.96	-11.95	-11.94	-11.93	-11.92
-12	-11.99	-11.98	-11.97	-11.96	-11.95	-11.94	-11.93	-11.92
-12	-11.99	-11.98	-11.97	-11.96	-11.95	-11.94	-11.93	-11.92
-12	-11.99	-11.98	-11.97	-11.96	-11.95	-11.94	-11.93	-11.92
-12	-11.99	-11.98	-11.97	-11.96	-11.95	-11.94	-11.93	-11.92
-12	-11.99	-11.98	-11.97	-11.96	-11.95	-11.94	-11.93	-11.92
-12	-11.99	-11.98	-11.97	-11.96	-11.95	-11.94	-11.93	-11.92
-12	-11.99	-11.98	-11.97	-11.96	-11.95	-11.94	-11.93	-11.92
-12	-11.99	-11.98	-11.97	-11.96	-11.95	-11.94	-11.93	-11.92
-12	-11.99	-11.98	-11.97	-11.96	-11.95	-11.94	-11.93	-11.92
-12	-11.99	-11.98	-11.97	-11.96	-11.95	-11.94	-11.93	-11.92
-12	-11.99	-11.98	-11.97	-11.96	-11.95	-11.94	-11.93	-11.92
-12	-11.99	-11.98	-11.97	-11.96	-11.95	-11.94	-11.93	-11.92
-12	-11.99	-11.98	-11.97	-11.96	-11.95	-11.94	-11.93	-11.92
-12	-11.99	-11.98	-11.97	-11.96	-11.95	-11.94	-11.93	-11.92
-12	-11.99	-11.98	-11.97	-11.96	-11.95	-11.94	-11.93	-11.92
-12	-11.99	-11.98	-11.97	-11.96	-11.95	-11.94	-11.93	-11.92
-12	-11.99	-11.98	-11.97	-11.96	-11.95	-11.94	-11.93	-11.92
-12	-11.99	-11.98	-11.97	-11.96	-11.95	-11.94	-11.93	-11.92
-12	-11.99	-11.98	-11.97	-11.96	-11.95	-11.94	-11.93	-11.92
-12	-11.99	-11.98	-11.97	-11.96	-11.95	-11.94	-11.93	-11.92
-12	-11.99	-11.98	-11.97	-11.96	-11.95	-11.94	-11.93	-11.92
-12	-11.99	-11.98	-11.97	-11.96	-11.95	-11.94	-11.93	-11.92
-12	-11.99	-11.98	-11.97	-11.96	-11.95	-11.94	-11.93	-11.92
-12	-11.99	-11.98	-11.97	-11.96	-11.95	-11.94	-11.93	-11.92
-12	-11.99	-11.98	-11.97	-11.96	-11.95	-11.94	-11.93	-11.92
-12	-11.99	-11.98	-11.97	-11.96	-11.95	-11.94	-11.93	-11.92
-12	-11.99	-11.98	-11.97	-11.96	-11.95	-11.94	-11.93	-11.92
-12	-11.99	-11.98	-11.97	-11.96	-11.95	-11.94	-11.93	-11.92

-12	-11.99	-11.98	-11.97	-11.96	-11.95	-11.94	-11.93	-11.92
-12	-11.99	-11.98	-11.97	-11.96	-11.95	-11.94	-11.93	-11.92
-12	-11.99	-11.98	-11.97	-11.96	-11.95	-11.94	-11.93	-11.92
-12	-11.99	-11.98	-11.97	-11.96	-11.95	-11.94	-11.93	-11.92
-12	-11.99	-11.98	-11.97	-11.96	-11.95	-11.94	-11.93	-11.92
-12	-11.99	-11.98	-11.97	-11.96	-11.95	-11.94	-11.93	-11.92
-12	-11.99	-11.98	-11.97	-11.96	-11.95	-11.94	-11.93	-11.92
-12	-11.99	-11.98	-11.97	-11.96	-11.95	-11.94	-11.93	-11.92
-12	-11.99	-11.98	-11.97	-11.96	-11.95	-11.94	-11.93	-11.92
-12	-11.99	-11.98	-11.97	-11.96	-11.95	-11.94	-11.93	-11.92
-12	-11.99	-11.98	-11.97	-11.96	-11.95	-11.94	-11.93	-11.92
-12	-11.99	-11.98	-11.97	-11.96	-11.95	-11.94	-11.93	-11.92
-12	-11.99	-11.98	-11.97	-11.96	-11.95	-11.94	-11.93	-11.92
-12	-11.99	-11.98	-11.97	-11.96	-11.95	-11.94	-11.93	-11.92
-12	-11.99	-11.98	-11.97	-11.96	-11.95	-11.94	-11.93	-11.92
-12	-11.99	-11.98	-11.97	-11.96	-11.95	-11.94	-11.93	-11.92
-12	-11.99	-11.98	-11.97	-11.96	-11.95	-11.94	-11.93	-11.92
-12	-11.99	-11.98	-11.97	-11.96	-11.95	-11.94	-11.93	-11.92
-12	-11.99	-11.98	-11.97	-11.96	-11.95	-11.94	-11.93	-11.92
-12	-11.99	-11.98	-11.97	-11.96	-11.95	-11.94	-11.93	-11.92
-12	-11.99	-11.98	-11.97	-11.96	-11.95	-11.94	-11.93	-11.92
-12	-11.99	-11.98	-11.97	-11.96	-11.95	-11.94	-11.93	-11.92
-12	-11.99	-11.98	-11.97	-11.96	-11.95	-11.94	-11.93	-11.92
-12	-11.99	-11.98	-11.97	-11.96	-11.95	-11.94	-11.93	-11.92
-12	-11.99	-11.98	-11.97	-11.96	-11.95	-11.94	-11.93	-11.92
-12	-11.99	-11.98	-11.97	-11.96	-11.95	-11.94	-11.93	-11.92
-12	-11.99	-11.98	-11.97	-11.96	-11.95	-11.94	-11.93	-11.92
-12	-11.99	-11.98	-11.97	-11.96	-11.95	-11.94	-11.93	-11.92
-12	-11.99	-11.98	-11.97	-11.96	-11.95	-11.94	-11.93	-11.92
-12	-11.99	-11.98	-11.97	-11.96	-11.95	-11.94	-11.93	-11.92
-12	-11.99	-11.98	-11.97	-11.96	-11.95	-11.94	-11.93	-11.92
-12	-11.99	-11.98	-11.97	-11.96	-11.95	-11.94	-11.93	-11.92
-12	-11.99	-11.98	-11.97	-11.96	-11.95	-11.94	-11.93	-11.92
-12	-11.99	-11.98	-11.97	-11.96	-11.95	-11.94	-11.93	-11.92
-12	-11.99	-11.98	-11.97	-11.96	-11.95	-11.94	-11.93	-11.92
-12	-11.99	-11.98	-11.97	-11.96	-11.95	-11.94	-11.93	-11.92
-12	-11.99	-11.98	-11.97	-11.96	-11.95	-11.94	-11.93	-11.92
-12	-11.99	-11.98	-11.97	-11.96	-11.95	-11.94	-11.93	-11.92
-12	-11.99	-11.98	-11.97	-11.96	-11.95	-11.94	-11.93	-11.92
-12	-11.99	-11.98	-11.97	-11.96	-11.95	-11.94	-11.93	-11.92
-12	-11.99	-11.98	-11.97	-11.96	-11.95	-11.94	-11.93	-11.92
-12	-11.99	-11.98	-11.97	-11.96	-11.95	-11.94	-11.93	-11.92
-12	-11.99	-11.98	-11.97	-11.96	-11.95	-11.94	-11.93	-11.92
-12	-11.99	-11.98	-11.97	-11.96	-11.95	-11.94	-11.93	-11.92
-12	-11.99	-11.98	-11.97	-11.96	-11.95	-11.94	-11.93	-11.92
-12	-11.99	-11.98	-11.97	-11.96	-11.95	-11.94	-11.93	-11.92
-12	-11.99	-11.98	-11.97	-11.96	-11.95	-11.94	-11.93	-11.92
-12	-11.99	-11.98	-11.97	-11.96	-11.95	-11.94	-11.93	-11.92
-12	-11.99	-11.98	-11.97	-11.96	-11.95	-11.94	-11.93	-11.92
-12	-11.99	-11.98	-11.97	-11.96	-11.95	-11.94	-11.93	-11.92

-12	-11.99	-11.98	-11.97	-11.96	-11.95	-11.94	-11.93	-11.92
-12	-11.99	-11.98	-11.97	-11.96	-11.95	-11.94	-11.93	-11.92
-12	-11.99	-11.98	-11.97	-11.96	-11.95	-11.94	-11.93	-11.92
-12	-11.99	-11.98	-11.97	-11.96	-11.95	-11.94	-11.93	-11.92
-12	-11.99	-11.98	-11.97	-11.96	-11.95	-11.94	-11.93	-11.92
-12	-11.99	-11.98	-11.97	-11.96	-11.95	-11.94	-11.93	-11.92
-12	-11.99	-11.98	-11.97	-11.96	-11.95	-11.94	-11.93	-11.92
-12	-11.99	-11.98	-11.97	-11.96	-11.95	-11.94	-11.93	-11.92
-12	-11.99	-11.98	-11.97	-11.96	-11.95	-11.94	-11.93	-11.92
-12	-11.99	-11.98	-11.97	-11.96	-11.95	-11.94	-11.93	-11.92
-12	-11.99	-11.98	-11.97	-11.96	-11.95	-11.94	-11.93	-11.92
-12	-11.99	-11.98	-11.97	-11.96	-11.95	-11.94	-11.93	-11.92
-12	-11.99	-11.98	-11.97	-11.96	-11.95	-11.94	-11.93	-11.92
-12	-11.99	-11.98	-11.97	-11.96	-11.95	-11.94	-11.93	-11.92
-12	-11.99	-11.98	-11.97	-11.96	-11.95	-11.94	-11.93	-11.92
-12	-11.99	-11.98	-11.97	-11.96	-11.95	-11.94	-11.93	-11.92
-12	-11.99	-11.98	-11.97	-11.96	-11.95	-11.94	-11.93	-11.92
-12	-11.99	-11.98	-11.97	-11.96	-11.95	-11.94	-11.93	-11.92
-12	-11.99	-11.98	-11.97	-11.96	-11.95	-11.94	-11.93	-11.92
-12	-11.99	-11.98	-11.97	-11.96	-11.95	-11.94	-11.93	-11.92
-12	-11.99	-11.98	-11.97	-11.96	-11.95	-11.94	-11.93	-11.92
-12	-11.99	-11.98	-11.97	-11.96	-11.95	-11.94	-11.93	-11.92
-12	-11.99	-11.98	-11.97	-11.96	-11.95	-11.94	-11.93	-11.92
-12	-11.99	-11.98	-11.97	-11.96	-11.95	-11.94	-11.93	-11.92
-12	-11.99	-11.98	-11.97	-11.96	-11.95	-11.94	-11.93	-11.92
-12	-11.99	-11.98	-11.97	-11.96	-11.95	-11.94	-11.93	-11.92
-12	-11.99	-11.98	-11.97	-11.96	-11.95	-11.94	-11.93	-11.92
-12	-11.99	-11.98	-11.97	-11.96	-11.95	-11.94	-11.93	-11.92
-12	-11.99	-11.98	-11.97	-11.96	-11.95	-11.94	-11.93	-11.92
-12	-11.99	-11.98	-11.97	-11.96	-11.95	-11.94	-11.93	-11.92
-12	-11.99	-11.98	-11.97	-11.96	-11.95	-11.94	-11.93	-11.92
-12	-11.99	-11.98	-11.97	-11.96	-11.95	-11.94	-11.93	-11.92
-12	-11.99	-11.98	-11.97	-11.96	-11.95	-11.94	-11.93	-11.92
-12	-11.99	-11.98	-11.97	-11.96	-11.95	-11.94	-11.93	-11.92
-12	-11.99	-11.98	-11.97	-11.96	-11.95	-11.94	-11.93	-11.92
-12	-11.99	-11.98	-11.97	-11.96	-11.95	-11.94	-11.93	-11.92
-12	-11.99	-11.98	-11.97	-11.96	-11.95	-11.94	-11.93	-11.92
-12	-11.99	-11.98	-11.97	-11.96	-11.95	-11.94	-11.93	-11.92
-12	-11.99	-11.98	-11.97	-11.96	-11.95	-11.94	-11.93	-11.92
-12	-11.99	-11.98	-11.97	-11.96	-11.95	-11.94	-11.93	-11.92
-12	-11.99	-11.98	-11.97	-11.96	-11.95	-11.94	-11.93	-11.92
-12	-11.99	-11.98	-11.97	-11.96	-11.95	-11.94	-11.93	-11.92
-12	-11.99	-11.98	-11.97	-11.96	-11.95	-11.94	-11.93	-11.92
-12	-11.99	-11.98	-11.97	-11.96	-11.95	-11.94	-11.93	-11.92
-12	-11.99	-11.98	-11.97	-11.96	-11.95	-11.94	-11.93	-11.92
-12	-11.99	-11.98	-11.97	-11.96	-11.95	-11.94	-11.93	-11.92
-12	-11.99	-11.98	-11.97	-11.96	-11.95	-11.94	-11.93	-11.92
-12	-11.99	-11.98	-11.97	-11.96	-11.95	-11.94	-11.93	-11.92
-12	-11.99	-11.98	-11.97	-11.96	-11.95	-11.94	-11.93	-11.92
-12	-11.99	-11.98	-11.97	-11.96	-11.95	-11.94	-11.93	-11.92

-12	-11.99	-11.98	-11.97	-11.96	-11.95	-11.94	-11.93	-11.92
-12	-11.99	-11.98	-11.97	-11.96	-11.95	-11.94	-11.93	-11.92
-12	-11.99	-11.98	-11.97	-11.96	-11.95	-11.94	-11.93	-11.92
-12	-11.99	-11.98	-11.97	-11.96	-11.95	-11.94	-11.93	-11.92
-12	-11.99	-11.98	-11.97	-11.96	-11.95	-11.94	-11.93	-11.92
-12	-11.99	-11.98	-11.97	-11.96	-11.95	-11.94	-11.93	-11.92
-12	-11.99	-11.98	-11.97	-11.96	-11.95	-11.94	-11.93	-11.92
-12	-11.99	-11.98	-11.97	-11.96	-11.95	-11.94	-11.93	-11.92
-12	-11.99	-11.98	-11.97	-11.96	-11.95	-11.94	-11.93	-11.92
-12	-11.99	-11.98	-11.97	-11.96	-11.95	-11.94	-11.93	-11.92
-12	-11.99	-11.98	-11.97	-11.96	-11.95	-11.94	-11.93	-11.92
-12	-11.99	-11.98	-11.97	-11.96	-11.95	-11.94	-11.93	-11.92
-12	-11.99	-11.98	-11.97	-11.96	-11.95	-11.94	-11.93	-11.92
-12	-11.99	-11.98	-11.97	-11.96	-11.95	-11.94	-11.93	-11.92
-12	-11.99	-11.98	-11.97	-11.96	-11.95	-11.94	-11.93	-11.92
-12	-11.99	-11.98	-11.97	-11.96	-11.95	-11.94	-11.93	-11.92
-12	-11.99	-11.98	-11.97	-11.96	-11.95	-11.94	-11.93	-11.92
-12	-11.99	-11.98	-11.97	-11.96	-11.95	-11.94	-11.93	-11.92
-12	-11.99	-11.98	-11.97	-11.96	-11.95	-11.94	-11.93	-11.92
-12	-11.99	-11.98	-11.97	-11.96	-11.95	-11.94	-11.93	-11.92
-12	-11.99	-11.98	-11.97	-11.96	-11.95	-11.94	-11.93	-11.92
-12	-11.99	-11.98	-11.97	-11.96	-11.95	-11.94	-11.93	-11.92
-12	-11.99	-11.98	-11.97	-11.96	-11.95	-11.94	-11.93	-11.92
-12	-11.99	-11.98	-11.97	-11.96	-11.95	-11.94	-11.93	-11.92
-12	-11.99	-11.98	-11.97	-11.96	-11.95	-11.94	-11.93	-11.92
-12	-11.99	-11.98	-11.97	-11.96	-11.95	-11.94	-11.93	-11.92
-12	-11.99	-11.98	-11.97	-11.96	-11.95	-11.94	-11.93	-11.92
-12	-11.99	-11.98	-11.97	-11.96	-11.95	-11.94	-11.93	-11.92
-12	-11.99	-11.98	-11.97	-11.96	-11.95	-11.94	-11.93	-11.92
-12	-11.99	-11.98	-11.97	-11.96	-11.95	-11.94	-11.93	-11.92
-12	-11.99	-11.98	-11.97	-11.96	-11.95	-11.94	-11.93	-11.92
-12	-11.99	-11.98	-11.97	-11.96	-11.95	-11.94	-11.93	-11.92
-12	-11.99	-11.98	-11.97	-11.96	-11.95	-11.94	-11.93	-11.92
-12	-11.99	-11.98	-11.97	-11.96	-11.95	-11.94	-11.93	-11.92
-12	-11.99	-11.98	-11.97	-11.96	-11.95	-11.94	-11.93	-11.92
-12	-11.99	-11.98	-11.97	-11.96	-11.95	-11.94	-11.93	-11.92
-12	-11.99	-11.98	-11.97	-11.96	-11.95	-11.94	-11.93	-11.92
-12	-11.99	-11.98	-11.97	-11.96	-11.95	-11.94	-11.93	-11.92
-12	-11.99	-11.98	-11.97	-11.96	-11.95	-11.94	-11.93	-11.92
-12	-11.99	-11.98	-11.97	-11.96	-11.95	-11.94	-11.93	-11.92
-12	-11.99	-11.98	-11.97	-11.96	-11.95	-11.94	-11.93	-11.92
-12	-11.99	-11.98	-11.97	-11.96	-11.95	-11.94	-11.93	-11.92
-12	-11.99	-11.98	-11.97	-11.96	-11.95	-11.94	-11.93	-11.92
-12	-11.99	-11.98	-11.97	-11.96	-11.95	-11.94	-11.93	-11.92
-12	-11.99	-11.98	-11.97	-11.96	-11.95	-11.94	-11.93	-11.92
-12	-11.99	-11.98	-11.97	-11.96	-11.95	-11.94	-11.93	-11.92
-12	-11.99	-11.98	-11.97	-11.96	-11.95	-11.94	-11.93	-11.92
-12	-11.99	-11.98	-11.97	-11.96	-11.95	-11.94	-11.93	-11.92
-12	-11.99	-11.98	-11.97	-11.96	-11.95	-11.94	-11.93	-11.92
-12	-11.99	-11.98	-11.97	-11.96	-11.95	-11.94	-11.93	-11.92

-12	-11.99	-11.98	-11.97	-11.96	-11.95	-11.94	-11.93	-11.92
-12	-11.99	-11.98	-11.97	-11.96	-11.95	-11.94	-11.93	-11.92
-12	-11.99	-11.98	-11.97	-11.96	-11.95	-11.94	-11.93	-11.92
-12	-11.99	-11.98	-11.97	-11.96	-11.95	-11.94	-11.93	-11.92
-12	-11.99	-11.98	-11.97	-11.96	-11.95	-11.94	-11.93	-11.92
-12	-11.99	-11.98	-11.97	-11.96	-11.95	-11.94	-11.93	-11.92
-12	-11.99	-11.98	-11.97	-11.96	-11.95	-11.94	-11.93	-11.92
-12	-11.99	-11.98	-11.97	-11.96	-11.95	-11.94	-11.93	-11.92
-12	-11.99	-11.98	-11.97	-11.96	-11.95	-11.94	-11.93	-11.92
-12	-11.99	-11.98	-11.97	-11.96	-11.95	-11.94	-11.93	-11.92
-12	-11.99	-11.98	-11.97	-11.96	-11.95	-11.94	-11.93	-11.92
-12	-11.99	-11.98	-11.97	-11.96	-11.95	-11.94	-11.93	-11.92
-12	-11.99	-11.98	-11.97	-11.96	-11.95	-11.94	-11.93	-11.92
-12	-11.99	-11.98	-11.97	-11.96	-11.95	-11.94	-11.93	-11.92
-12	-11.99	-11.98	-11.97	-11.96	-11.95	-11.94	-11.93	-11.92
-12	-11.99	-11.98	-11.97	-11.96	-11.95	-11.94	-11.93	-11.92
-12	-11.99	-11.98	-11.97	-11.96	-11.95	-11.94	-11.93	-11.92
-12	-11.99	-11.98	-11.97	-11.96	-11.95	-11.94	-11.93	-11.92
-12	-11.99	-11.98	-11.97	-11.96	-11.95	-11.94	-11.93	-11.92
-12	-11.99	-11.98	-11.97	-11.96	-11.95	-11.94	-11.93	-11.92
-12	-11.99	-11.98	-11.97	-11.96	-11.95	-11.94	-11.93	-11.92
-12	-11.99	-11.98	-11.97	-11.96	-11.95	-11.94	-11.93	-11.92
-12	-11.99	-11.98	-11.97	-11.96	-11.95	-11.94	-11.93	-11.92
-12	-11.99	-11.98	-11.97	-11.96	-11.95	-11.94	-11.93	-11.92
-12	-11.99	-11.98	-11.97	-11.96	-11.95	-11.94	-11.93	-11.92
-12	-11.99	-11.98	-11.97	-11.96	-11.95	-11.94	-11.93	-11.92
-12	-11.99	-11.98	-11.97	-11.96	-11.95	-11.94	-11.93	-11.92
-12	-11.99	-11.98	-11.97	-11.96	-11.95	-11.94	-11.93	-11.92
-12	-11.99	-11.98	-11.97	-11.96	-11.95	-11.94	-11.93	-11.92
-12	-11.99	-11.98	-11.97	-11.96	-11.95	-11.94	-11.93	-11.92
-12	-11.99	-11.98	-11.97	-11.96	-11.95	-11.94	-11.93	-11.92
-12	-11.99	-11.98	-11.97	-11.96	-11.95	-11.94	-11.93	-11.92
-12	-11.99	-11.98	-11.97	-11.96	-11.95	-11.94	-11.93	-11.92
-12	-11.99	-11.98	-11.97	-11.96	-11.95	-11.94	-11.93	-11.92
-12	-11.99	-11.98	-11.97	-11.96	-11.95	-11.94	-11.93	-11.92
-12	-11.99	-11.98	-11.97	-11.96	-11.95	-11.94	-11.93	-11.92
-12	-11.99	-11.98	-11.97	-11.96	-11.95	-11.94	-11.93	-11.92
-12	-11.99	-11.98	-11.97	-11.96	-11.95	-11.94	-11.93	-11.92
-12	-11.99	-11.98	-11.97	-11.96	-11.95	-11.94	-11.93	-11.92
-12	-11.99	-11.98	-11.97	-11.96	-11.95	-11.94	-11.93	-11.92
-12	-11.99	-11.98	-11.97	-11.96	-11.95	-11.94	-11.93	-11.92
-12	-11.99	-11.98	-11.97	-11.96	-11.95	-11.94	-11.93	-11.92
-12	-11.99	-11.98	-11.97	-11.96	-11.95	-11.94	-11.93	-11.92
-12	-11.99	-11.98	-11.97	-11.96	-11.95	-11.94	-11.93	-11.92
-12	-11.99	-11.98	-11.97	-11.96	-11.95	-11.94	-11.93	-11.92
-12	-11.99	-11.98	-11.97	-11.96	-11.95	-11.94	-11.93	-11.92
-12	-11.99	-11.98	-11.97	-11.96	-11.95	-11.94	-11.93	-11.92
-12	-11.99	-11.98	-11.97	-11.96	-11.95	-11.94	-11.93	-11.92
-12	-11.99	-11.98	-11.97	-11.96	-11.95	-11.94	-11.93	-11.92
-12	-11.99	-11.98	-11.97	-11.96	-11.95	-11.94	-11.93	-11.92

-12	-11.99	-11.98	-11.97	-11.96	-11.95	-11.94	-11.93	-11.92
-12	-11.99	-11.98	-11.97	-11.96	-11.95	-11.94	-11.93	-11.92
-12	-11.99	-11.98	-11.97	-11.96	-11.95	-11.94	-11.93	-11.92
-12	-11.99	-11.98	-11.97	-11.96	-11.95	-11.94	-11.93	-11.92
-12	-11.99	-11.98	-11.97	-11.96	-11.95	-11.94	-11.93	-11.92
-12	-11.99	-11.98	-11.97	-11.96	-11.95	-11.94	-11.93	-11.92
-12	-11.99	-11.98	-11.97	-11.96	-11.95	-11.94	-11.93	-11.92
-12	-11.99	-11.98	-11.97	-11.96	-11.95	-11.94	-11.93	-11.92
-12	-11.99	-11.98	-11.97	-11.96	-11.95	-11.94	-11.93	-11.92
-12	-11.99	-11.98	-11.97	-11.96	-11.95	-11.94	-11.93	-11.92
-12	-11.99	-11.98	-11.97	-11.96	-11.95	-11.94	-11.93	-11.92
-12	-11.99	-11.98	-11.97	-11.96	-11.95	-11.94	-11.93	-11.92
-12	-11.99	-11.98	-11.97	-11.96	-11.95	-11.94	-11.93	-11.92
-12	-11.99	-11.98	-11.97	-11.96	-11.95	-11.94	-11.93	-11.92
-12	-11.99	-11.98	-11.97	-11.96	-11.95	-11.94	-11.93	-11.92
-12	-11.99	-11.98	-11.97	-11.96	-11.95	-11.94	-11.93	-11.92
-12	-11.99	-11.98	-11.97	-11.96	-11.95	-11.94	-11.93	-11.92
-12	-11.99	-11.98	-11.97	-11.96	-11.95	-11.94	-11.93	-11.92
-12	-11.99	-11.98	-11.97	-11.96	-11.95	-11.94	-11.93	-11.92
-12	-11.99	-11.98	-11.97	-11.96	-11.95	-11.94	-11.93	-11.92
-12	-11.99	-11.98	-11.97	-11.96	-11.95	-11.94	-11.93	-11.92
-12	-11.99	-11.98	-11.97	-11.96	-11.95	-11.94	-11.93	-11.92
-12	-11.99	-11.98	-11.97	-11.96	-11.95	-11.94	-11.93	-11.92
-12	-11.99	-11.98	-11.97	-11.96	-11.95	-11.94	-11.93	-11.92
-12	-11.99	-11.98	-11.97	-11.96	-11.95	-11.94	-11.93	-11.92
-12	-11.99	-11.98	-11.97	-11.96	-11.95	-11.94	-11.93	-11.92
-12	-11.99	-11.98	-11.97	-11.96	-11.95	-11.94	-11.93	-11.92
-12	-11.99	-11.98	-11.97	-11.96	-11.95	-11.94	-11.93	-11.92
-12	-11.99	-11.98	-11.97	-11.96	-11.95	-11.94	-11.93	-11.92
-12	-11.99	-11.98	-11.97	-11.96	-11.95	-11.94	-11.93	-11.92
-12	-11.99	-11.98	-11.97	-11.96	-11.95	-11.94	-11.93	-11.92
-12	-11.99	-11.98	-11.97	-11.96	-11.95	-11.94	-11.93	-11.92
-12	-11.99	-11.98	-11.97	-11.96	-11.95	-11.94	-11.93	-11.92
-12	-11.99	-11.98	-11.97	-11.96	-11.95	-11.94	-11.93	-11.92
-12	-11.99	-11.98	-11.97	-11.96	-11.95	-11.94	-11.93	-11.92
-12	-11.99	-11.98	-11.97	-11.96	-11.95	-11.94	-11.93	-11.92
-12	-11.99	-11.98	-11.97	-11.96	-11.95	-11.94	-11.93	-11.92
-12	-11.99	-11.98	-11.97	-11.96	-11.95	-11.94	-11.93	-11.92
-12	-11.99	-11.98	-11.97	-11.96	-11.95	-11.94	-11.93	-11.92
-12	-11.99	-11.98	-11.97	-11.96	-11.95	-11.94	-11.93	-11.92
-12	-11.99	-11.98	-11.97	-11.96	-11.95	-11.94	-11.93	-11.92
-12	-11.99	-11.98	-11.97	-11.96	-11.95	-11.94	-11.93	-11.92
-12	-11.99	-11.98	-11.97	-11.96	-11.95	-11.94	-11.93	-11.92
-12	-11.99	-11.98	-11.97	-11.96	-11.95	-11.94	-11.93	-11.92
-12	-11.99	-11.98	-11.97	-11.96	-11.95	-11.94	-11.93	-11.92
-12	-11.99	-11.98	-11.97	-11.96	-11.95	-11.94	-11.93	-11.92
-12	-11.99	-11.98	-11.97	-11.96	-11.95	-11.94	-11.93	-11.92
-12	-11.99	-11.98	-11.97	-11.96	-11.95	-11.94	-11.93	-11.92
-12	-11.99	-11.98	-11.97	-11.96	-11.95	-11.94	-11.93	-11.92
-12	-11.99	-11.98	-11.97	-11.96	-11.95	-11.94	-11.93	-11.92

-12	-11.99	-11.98	-11.97	-11.96	-11.95	-11.94	-11.93	-11.92
-12	-11.99	-11.98	-11.97	-11.96	-11.95	-11.94	-11.93	-11.92
-12	-11.99	-11.98	-11.97	-11.96	-11.95	-11.94	-11.93	-11.92
-12	-11.99	-11.98	-11.97	-11.96	-11.95	-11.94	-11.93	-11.92
-12	-11.99	-11.98	-11.97	-11.96	-11.95	-11.94	-11.93	-11.92
-12	-11.99	-11.98	-11.97	-11.96	-11.95	-11.94	-11.93	-11.92
-12	-11.99	-11.98	-11.97	-11.96	-11.95	-11.94	-11.93	-11.92
-12	-11.99	-11.98	-11.97	-11.96	-11.95	-11.94	-11.93	-11.92
-12	-11.99	-11.98	-11.97	-11.96	-11.95	-11.94	-11.93	-11.92
-12	-11.99	-11.98	-11.97	-11.96	-11.95	-11.94	-11.93	-11.92
-12	-11.99	-11.98	-11.97	-11.96	-11.95	-11.94	-11.93	-11.92
-12	-11.99	-11.98	-11.97	-11.96	-11.95	-11.94	-11.93	-11.92
-12	-11.99	-11.98	-11.97	-11.96	-11.95	-11.94	-11.93	-11.92
-12	-11.99	-11.98	-11.97	-11.96	-11.95	-11.94	-11.93	-11.92
-12	-11.99	-11.98	-11.97	-11.96	-11.95	-11.94	-11.93	-11.92
-12	-11.99	-11.98	-11.97	-11.96	-11.95	-11.94	-11.93	-11.92
-12	-11.99	-11.98	-11.97	-11.96	-11.95	-11.94	-11.93	-11.92
-12	-11.99	-11.98	-11.97	-11.96	-11.95	-11.94	-11.93	-11.92
-12	-11.99	-11.98	-11.97	-11.96	-11.95	-11.94	-11.93	-11.92
-12	-11.99	-11.98	-11.97	-11.96	-11.95	-11.94	-11.93	-11.92
-12	-11.99	-11.98	-11.97	-11.96	-11.95	-11.94	-11.93	-11.92
-12	-11.99	-11.98	-11.97	-11.96	-11.95	-11.94	-11.93	-11.92
-12	-11.99	-11.98	-11.97	-11.96	-11.95	-11.94	-11.93	-11.92
-12	-11.99	-11.98	-11.97	-11.96	-11.95	-11.94	-11.93	-11.92
-12	-11.99	-11.98	-11.97	-11.96	-11.95	-11.94	-11.93	-11.92
-12	-11.99	-11.98	-11.97	-11.96	-11.95	-11.94	-11.93	-11.92
-12	-11.99	-11.98	-11.97	-11.96	-11.95	-11.94	-11.93	-11.92
-12	-11.99	-11.98	-11.97	-11.96	-11.95	-11.94	-11.93	-11.92
-12	-11.99	-11.98	-11.97	-11.96	-11.95	-11.94	-11.93	-11.92
-12	-11.99	-11.98	-11.97	-11.96	-11.95	-11.94	-11.93	-11.92
-12	-11.99	-11.98	-11.97	-11.96	-11.95	-11.94	-11.93	-11.92
-12	-11.99	-11.98	-11.97	-11.96	-11.95	-11.94	-11.93	-11.92
-12	-11.99	-11.98	-11.97	-11.96	-11.95	-11.94	-11.93	-11.92
-12	-11.99	-11.98	-11.97	-11.96	-11.95	-11.94	-11.93	-11.92
-12	-11.99	-11.98	-11.97	-11.96	-11.95	-11.94	-11.93	-11.92
-12	-11.99	-11.98	-11.97	-11.96	-11.95	-11.94	-11.93	-11.92
-12	-11.99	-11.98	-11.97	-11.96	-11.95	-11.94	-11.93	-11.92
-12	-11.99	-11.98	-11.97	-11.96	-11.95	-11.94	-11.93	-11.92
-12	-11.99	-11.98	-11.97	-11.96	-11.95	-11.94	-11.93	-11.92
-12	-11.99	-11.98	-11.97	-11.96	-11.95	-11.94	-11.93	-11.92
-12	-11.99	-11.98	-11.97	-11.96	-11.95	-11.94	-11.93	-11.92
-12	-11.99	-11.98	-11.97	-11.96	-11.95	-11.94	-11.93	-11.92
-12	-11.99	-11.98	-11.97	-11.96	-11.95	-11.94	-11.93	-11.92
-12	-11.99	-11.98	-11.97	-11.96	-11.95	-11.94	-11.93	-11.92
-12	-11.99	-11.98	-11.97	-11.96	-11.95	-11.94	-11.93	-11.92
-12	-11.99	-11.98	-11.97	-11.96	-11.95	-11.94	-11.93	-11.92
-12	-11.99	-11.98	-11.97	-11.96	-11.95	-11.94	-11.93	-11.92
-12	-11.99	-11.98	-11.97	-11.96	-11.95	-11.94	-11.93	-11.92
-12	-11.99	-11.98	-11.97	-11.96	-11.95	-11.94	-11.93	-11.92
-12	-11.99	-11.98	-11.97	-11.96	-11.95	-11.94	-11.93	-11.92

-12	-11.99	-11.98	-11.97	-11.96	-11.95	-11.94	-11.93	-11.92
-12	-11.99	-11.98	-11.97	-11.96	-11.95	-11.94	-11.93	-11.92
-12	-11.99	-11.98	-11.97	-11.96	-11.95	-11.94	-11.93	-11.92
-12	-11.99	-11.98	-11.97	-11.96	-11.95	-11.94	-11.93	-11.92
-12	-11.99	-11.98	-11.97	-11.96	-11.95	-11.94	-11.93	-11.92
-12	-11.99	-11.98	-11.97	-11.96	-11.95	-11.94	-11.93	-11.92
-12	-11.99	-11.98	-11.97	-11.96	-11.95	-11.94	-11.93	-11.92
-12	-11.99	-11.98	-11.97	-11.96	-11.95	-11.94	-11.93	-11.92
-12	-11.99	-11.98	-11.97	-11.96	-11.95	-11.94	-11.93	-11.92
-12	-11.99	-11.98	-11.97	-11.96	-11.95	-11.94	-11.93	-11.92
-12	-11.99	-11.98	-11.97	-11.96	-11.95	-11.94	-11.93	-11.92
-12	-11.99	-11.98	-11.97	-11.96	-11.95	-11.94	-11.93	-11.92
-12	-11.99	-11.98	-11.97	-11.96	-11.95	-11.94	-11.93	-11.92
-12	-11.99	-11.98	-11.97	-11.96	-11.95	-11.94	-11.93	-11.92
-12	-11.99	-11.98	-11.97	-11.96	-11.95	-11.94	-11.93	-11.92
-12	-11.99	-11.98	-11.97	-11.96	-11.95	-11.94	-11.93	-11.92
-12	-11.99	-11.98	-11.97	-11.96	-11.95	-11.94	-11.93	-11.92
-12	-11.99	-11.98	-11.97	-11.96	-11.95	-11.94	-11.93	-11.92
-12	-11.99	-11.98	-11.97	-11.96	-11.95	-11.94	-11.93	-11.92
-12	-11.99	-11.98	-11.97	-11.96	-11.95	-11.94	-11.93	-11.92
-12	-11.99	-11.98	-11.97	-11.96	-11.95	-11.94	-11.93	-11.92
-12	-11.99	-11.98	-11.97	-11.96	-11.95	-11.94	-11.93	-11.92
-12	-11.99	-11.98	-11.97	-11.96	-11.95	-11.94	-11.93	-11.92
-12	-11.99	-11.98	-11.97	-11.96	-11.95	-11.94	-11.93	-11.92
-12	-11.99	-11.98	-11.97	-11.96	-11.95	-11.94	-11.93	-11.92
-12	-11.99	-11.98	-11.97	-11.96	-11.95	-11.94	-11.93	-11.92
-12	-11.99	-11.98	-11.97	-11.96	-11.95	-11.94	-11.93	-11.92
-12	-11.99	-11.98	-11.97	-11.96	-11.95	-11.94	-11.93	-11.92
-12	-11.99	-11.98	-11.97	-11.96	-11.95	-11.94	-11.93	-11.92
-12	-11.99	-11.98	-11.97	-11.96	-11.95	-11.94	-11.93	-11.92
-12	-11.99	-11.98	-11.97	-11.96	-11.95	-11.94	-11.93	-11.92
-12	-11.99	-11.98	-11.97	-11.96	-11.95	-11.94	-11.93	-11.92
-12	-11.99	-11.98	-11.97	-11.96	-11.95	-11.94	-11.93	-11.92
-12	-11.99	-11.98	-11.97	-11.96	-11.95	-11.94	-11.93	-11.92
-12	-11.99	-11.98	-11.97	-11.96	-11.95	-11.94	-11.93	-11.92
-12	-11.99	-11.98	-11.97	-11.96	-11.95	-11.94	-11.93	-11.92
-12	-11.99	-11.98	-11.97	-11.96	-11.95	-11.94	-11.93	-11.92
-12	-11.99	-11.98	-11.97	-11.96	-11.95	-11.94	-11.93	-11.92
-12	-11.99	-11.98	-11.97	-11.96	-11.95	-11.94	-11.93	-11.92
-12	-11.99	-11.98	-11.97	-11.96	-11.95	-11.94	-11.93	-11.92
-12	-11.99	-11.98	-11.97	-11.96	-11.95	-11.94	-11.93	-11.92
-12	-11.99	-11.98	-11.97	-11.96	-11.95	-11.94	-11.93	-11.92
-12	-11.99	-11.98	-11.97	-11.96	-11.95	-11.94	-11.93	-11.92
-12	-11.99	-11.98	-11.97	-11.96	-11.95	-11.94	-11.93	-11.92
-12	-11.99	-11.98	-11.97	-11.96	-11.95	-11.94	-11.93	-11.92
-12	-11.99	-11.98	-11.97	-11.96	-11.95	-11.94	-11.93	-11.92
-12	-11.99	-11.98	-11.97	-11.96	-11.95	-11.94	-11.93	-11.92
-12	-11.99	-11.98	-11.97	-11.96	-11.95	-11.94	-11.93	-11.92
-12	-11.99	-11.98	-11.97	-11.96	-11.95	-11.94	-11.93	-11.92
-12	-11.99	-11.98	-11.97	-11.96	-11.95	-11.94	-11.93	-11.92

-12	-11.99	-11.98	-11.97	-11.96	-11.95	-11.94	-11.93	-11.92
-12	-11.99	-11.98	-11.97	-11.96	-11.95	-11.94	-11.93	-11.92
-12	-11.99	-11.98	-11.97	-11.96	-11.95	-11.94	-11.93	-11.92
-12	-11.99	-11.98	-11.97	-11.96	-11.95	-11.94	-11.93	-11.92
-12	-11.99	-11.98	-11.97	-11.96	-11.95	-11.94	-11.93	-11.92
-12	-11.99	-11.98	-11.97	-11.96	-11.95	-11.94	-11.93	-11.92
-12	-11.99	-11.98	-11.97	-11.96	-11.95	-11.94	-11.93	-11.92
-12	-11.99	-11.98	-11.97	-11.96	-11.95	-11.94	-11.93	-11.92
-12	-11.99	-11.98	-11.97	-11.96	-11.95	-11.94	-11.93	-11.92
-12	-11.99	-11.98	-11.97	-11.96	-11.95	-11.94	-11.93	-11.92
-12	-11.99	-11.98	-11.97	-11.96	-11.95	-11.94	-11.93	-11.92
-12	-11.99	-11.98	-11.97	-11.96	-11.95	-11.94	-11.93	-11.92
-12	-11.99	-11.98	-11.97	-11.96	-11.95	-11.94	-11.93	-11.92
-12	-11.99	-11.98	-11.97	-11.96	-11.95	-11.94	-11.93	-11.92
-12	-11.99	-11.98	-11.97	-11.96	-11.95	-11.94	-11.93	-11.92
-12	-11.99	-11.98	-11.97	-11.96	-11.95	-11.94	-11.93	-11.92
-12	-11.99	-11.98	-11.97	-11.96	-11.95	-11.94	-11.93	-11.92
-12	-11.99	-11.98	-11.97	-11.96	-11.95	-11.94	-11.93	-11.92
-12	-11.99	-11.98	-11.97	-11.96	-11.95	-11.94	-11.93	-11.92
-12	-11.99	-11.98	-11.97	-11.96	-11.95	-11.94	-11.93	-11.92
-12	-11.99	-11.98	-11.97	-11.96	-11.95	-11.94	-11.93	-11.92
-12	-11.99	-11.98	-11.97	-11.96	-11.95	-11.94	-11.93	-11.92
-12	-11.99	-11.98	-11.97	-11.96	-11.95	-11.94	-11.93	-11.92
-12	-11.99	-11.98	-11.97	-11.96	-11.95	-11.94	-11.93	-11.92
-12	-11.99	-11.98	-11.97	-11.96	-11.95	-11.94	-11.93	-11.92
-12	-11.99	-11.98	-11.97	-11.96	-11.95	-11.94	-11.93	-11.92
-12	-11.99	-11.98	-11.97	-11.96	-11.95	-11.94	-11.93	-11.92
-12	-11.99	-11.98	-11.97	-11.96	-11.95	-11.94	-11.93	-11.92
-12	-11.99	-11.98	-11.97	-11.96	-11.95	-11.94	-11.93	-11.92
-12	-11.99	-11.98	-11.97	-11.96	-11.95	-11.94	-11.93	-11.92
-12	-11.99	-11.98	-11.97	-11.96	-11.95	-11.94	-11.93	-11.92
-12	-11.99	-11.98	-11.97	-11.96	-11.95	-11.94	-11.93	-11.92
-12	-11.99	-11.98	-11.97	-11.96	-11.95	-11.94	-11.93	-11.92
-12	-11.99	-11.98	-11.97	-11.96	-11.95	-11.94	-11.93	-11.92
-12	-11.99	-11.98	-11.97	-11.96	-11.95	-11.94	-11.93	-11.92
-12	-11.99	-11.98	-11.97	-11.96	-11.95	-11.94	-11.93	-11.92
-12	-11.99	-11.98	-11.97	-11.96	-11.95	-11.94	-11.93	-11.92
-12	-11.99	-11.98	-11.97	-11.96	-11.95	-11.94	-11.93	-11.92
-12	-11.99	-11.98	-11.97	-11.96	-11.95	-11.94	-11.93	-11.92
-12	-11.99	-11.98	-11.97	-11.96	-11.95	-11.94	-11.93	-11.92
-12	-11.99	-11.98	-11.97	-11.96	-11.95	-11.94	-11.93	-11.92
-12	-11.99	-11.98	-11.97	-11.96	-11.95	-11.94	-11.93	-11.92
-12	-11.99	-11.98	-11.97	-11.96	-11.95	-11.94	-11.93	-11.92
-12	-11.99	-11.98	-11.97	-11.96	-11.95	-11.94	-11.93	-11.92
-12	-11.99	-11.98	-11.97	-11.96	-11.95	-11.94	-11.93	-11.92
-12	-11.99	-11.98	-11.97	-11.96	-11.95	-11.94	-11.93	-11.92
-12	-11.99	-11.98	-11.97	-11.96	-11.95	-11.94	-11.93	-11.92
-12	-11.99	-11.98	-11.97	-11.96	-11.95	-11.94	-11.93	-11.92
-12	-11.99	-11.98	-11.97	-11.96	-11.95	-11.94	-11.93	-11.92
-12	-11.99	-11.98	-11.97	-11.96	-11.95	-11.94	-11.93	-11.92

-12	-11.99	-11.98	-11.97	-11.96	-11.95	-11.94	-11.93	-11.92
-12	-11.99	-11.98	-11.97	-11.96	-11.95	-11.94	-11.93	-11.92
-12	-11.99	-11.98	-11.97	-11.96	-11.95	-11.94	-11.93	-11.92
-12	-11.99	-11.98	-11.97	-11.96	-11.95	-11.94	-11.93	-11.92
-12	-11.99	-11.98	-11.97	-11.96	-11.95	-11.94	-11.93	-11.92
-12	-11.99	-11.98	-11.97	-11.96	-11.95	-11.94	-11.93	-11.92
-12	-11.99	-11.98	-11.97	-11.96	-11.95	-11.94	-11.93	-11.92
-12	-11.99	-11.98	-11.97	-11.96	-11.95	-11.94	-11.93	-11.92
-12	-11.99	-11.98	-11.97	-11.96	-11.95	-11.94	-11.93	-11.92
-12	-11.99	-11.98	-11.97	-11.96	-11.95	-11.94	-11.93	-11.92
-12	-11.99	-11.98	-11.97	-11.96	-11.95	-11.94	-11.93	-11.92
-12	-11.99	-11.98	-11.97	-11.96	-11.95	-11.94	-11.93	-11.92
-12	-11.99	-11.98	-11.97	-11.96	-11.95	-11.94	-11.93	-11.92
-12	-11.99	-11.98	-11.97	-11.96	-11.95	-11.94	-11.93	-11.92
-12	-11.99	-11.98	-11.97	-11.96	-11.95	-11.94	-11.93	-11.92
-12	-11.99	-11.98	-11.97	-11.96	-11.95	-11.94	-11.93	-11.92
-12	-11.99	-11.98	-11.97	-11.96	-11.95	-11.94	-11.93	-11.92
-12	-11.99	-11.98	-11.97	-11.96	-11.95	-11.94	-11.93	-11.92
-12	-11.99	-11.98	-11.97	-11.96	-11.95	-11.94	-11.93	-11.92
-12	-11.99	-11.98	-11.97	-11.96	-11.95	-11.94	-11.93	-11.92
-12	-11.99	-11.98	-11.97	-11.96	-11.95	-11.94	-11.93	-11.92
-12	-11.99	-11.98	-11.97	-11.96	-11.95	-11.94	-11.93	-11.92
-12	-11.99	-11.98	-11.97	-11.96	-11.95	-11.94	-11.93	-11.92
-12	-11.99	-11.98	-11.97	-11.96	-11.95	-11.94	-11.93	-11.92
-12	-11.99	-11.98	-11.97	-11.96	-11.95	-11.94	-11.93	-11.92
-12	-11.99	-11.98	-11.97	-11.96	-11.95	-11.94	-11.93	-11.92
-12	-11.99	-11.98	-11.97	-11.96	-11.95	-11.94	-11.93	-11.92
-12	-11.99	-11.98	-11.97	-11.96	-11.95	-11.94	-11.93	-11.92
-12	-11.99	-11.98	-11.97	-11.96	-11.95	-11.94	-11.93	-11.92
-12	-11.99	-11.98	-11.97	-11.96	-11.95	-11.94	-11.93	-11.92
-12	-11.99	-11.98	-11.97	-11.96	-11.95	-11.94	-11.93	-11.92
-12	-11.99	-11.98	-11.97	-11.96	-11.95	-11.94	-11.93	-11.92
-12	-11.99	-11.98	-11.97	-11.96	-11.95	-11.94	-11.93	-11.92
-12	-11.99	-11.98	-11.97	-11.96	-11.95	-11.94	-11.93	-11.92
-12	-11.99	-11.98	-11.97	-11.96	-11.95	-11.94	-11.93	-11.92
-12	-11.99	-11.98	-11.97	-11.96	-11.95	-11.94	-11.93	-11.92
-12	-11.99	-11.98	-11.97	-11.96	-11.95	-11.94	-11.93	-11.92
-12	-11.99	-11.98	-11.97	-11.96	-11.95	-11.94	-11.93	-11.92
-12	-11.99	-11.98	-11.97	-11.96	-11.95	-11.94	-11.93	-11.92
-12	-11.99	-11.98	-11.97	-11.96	-11.95	-11.94	-11.93	-11.92
-12	-11.99	-11.98	-11.97	-11.96	-11.95	-11.94	-11.93	-11.92
-12	-11.99	-11.98	-11.97	-11.96	-11.95	-11.94	-11.93	-11.92
-12	-11.99	-11.98	-11.97	-11.96	-11.95	-11.94	-11.93	-11.92
-12	-11.99	-11.98	-11.97	-11.96	-11.95	-11.94	-11.93	-11.92
-12	-11.99	-11.98	-11.97	-11.96	-11.95	-11.94	-11.93	-11.92
-12	-11.99	-11.98	-11.97	-11.96	-11.95	-11.94	-11.93	-11.92
-12	-11.99	-11.98	-11.97	-11.96	-11.95	-11.94	-11.93	-11.92
-12	-11.99	-11.98	-11.97	-11.96	-11.95	-11.94	-11.93	-11.92
-12	-11.99	-11.98	-11.97	-11.96	-11.95	-11.94	-11.93	-11.92
-12	-11.99	-11.98	-11.97	-11.96	-11.95	-11.94	-11.93	-11.92

-12	-11.99	-11.98	-11.97	-11.96	-11.95	-11.94	-11.93	-11.92
-12	-11.99	-11.98	-11.97	-11.96	-11.95	-11.94	-11.93	-11.92
-12	-11.99	-11.98	-11.97	-11.96	-11.95	-11.94	-11.93	-11.92
-12	-11.99	-11.98	-11.97	-11.96	-11.95	-11.94	-11.93	-11.92
-12	-11.99	-11.98	-11.97	-11.96	-11.95	-11.94	-11.93	-11.92
-12	-11.99	-11.98	-11.97	-11.96	-11.95	-11.94	-11.93	-11.92
-12	-11.99	-11.98	-11.97	-11.96	-11.95	-11.94	-11.93	-11.92
-12	-11.99	-11.98	-11.97	-11.96	-11.95	-11.94	-11.93	-11.92
-12	-11.99	-11.98	-11.97	-11.96	-11.95	-11.94	-11.93	-11.92
-12	-11.99	-11.98	-11.97	-11.96	-11.95	-11.94	-11.93	-11.92
-12	-11.99	-11.98	-11.97	-11.96	-11.95	-11.94	-11.93	-11.92
-12	-11.99	-11.98	-11.97	-11.96	-11.95	-11.94	-11.93	-11.92
-12	-11.99	-11.98	-11.97	-11.96	-11.95	-11.94	-11.93	-11.92
-12	-11.99	-11.98	-11.97	-11.96	-11.95	-11.94	-11.93	-11.92
-12	-11.99	-11.98	-11.97	-11.96	-11.95	-11.94	-11.93	-11.92
-12	-11.99	-11.98	-11.97	-11.96	-11.95	-11.94	-11.93	-11.92
-12	-11.99	-11.98	-11.97	-11.96	-11.95	-11.94	-11.93	-11.92
-12	-11.99	-11.98	-11.97	-11.96	-11.95	-11.94	-11.93	-11.92
-12	-11.99	-11.98	-11.97	-11.96	-11.95	-11.94	-11.93	-11.92
-12	-11.99	-11.98	-11.97	-11.96	-11.95	-11.94	-11.93	-11.92
-12	-11.99	-11.98	-11.97	-11.96	-11.95	-11.94	-11.93	-11.92
-12	-11.99	-11.98	-11.97	-11.96	-11.95	-11.94	-11.93	-11.92
-12	-11.99	-11.98	-11.97	-11.96	-11.95	-11.94	-11.93	-11.92
-12	-11.99	-11.98	-11.97	-11.96	-11.95	-11.94	-11.93	-11.92
-12	-11.99	-11.98	-11.97	-11.96	-11.95	-11.94	-11.93	-11.92
-12	-11.99	-11.98	-11.97	-11.96	-11.95	-11.94	-11.93	-11.92
-12	-11.99	-11.98	-11.97	-11.96	-11.95	-11.94	-11.93	-11.92
-12	-11.99	-11.98	-11.97	-11.96	-11.95	-11.94	-11.93	-11.92
-12	-11.99	-11.98	-11.97	-11.96	-11.95	-11.94	-11.93	-11.92
-12	-11.99	-11.98	-11.97	-11.96	-11.95	-11.94	-11.93	-11.92
-12	-11.99	-11.98	-11.97	-11.96	-11.95	-11.94	-11.93	-11.92
-12	-11.99	-11.98	-11.97	-11.96	-11.95	-11.94	-11.93	-11.92
-12	-11.99	-11.98	-11.97	-11.96	-11.95	-11.94	-11.93	-11.92
-12	-11.99	-11.98	-11.97	-11.96	-11.95	-11.94	-11.93	-11.92
-12	-11.99	-11.98	-11.97	-11.96	-11.95	-11.94	-11.93	-11.92
-12	-11.99	-11.98	-11.97	-11.96	-11.95	-11.94	-11.93	-11.92
-12	-11.99	-11.98	-11.97	-11.96	-11.95	-11.94	-11.93	-11.92
-12	-11.99	-11.98	-11.97	-11.96	-11.95	-11.94	-11.93	-11.92
-12	-11.99	-11.98	-11.97	-11.96	-11.95	-11.94	-11.93	-11.92
-12	-11.99	-11.98	-11.97	-11.96	-11.95	-11.94	-11.93	-11.92
-12	-11.99	-11.98	-11.97	-11.96	-11.95	-11.94	-11.93	-11.92
-12	-11.99	-11.98	-11.97	-11.96	-11.95	-11.94	-11.93	-11.92
-12	-11.99	-11.98	-11.97	-11.96	-11.95	-11.94	-11.93	-11.92
-12	-11.99	-11.98	-11.97	-11.96	-11.95	-11.94	-11.93	-11.92
-12	-11.99	-11.98	-11.97	-11.96	-11.95	-11.94	-11.93	-11.92
-12	-11.99	-11.98	-11.97	-11.96	-11.95	-11.94	-11.93	-11.92
-12	-11.99	-11.98	-11.97	-11.96	-11.95	-11.94	-11.93	-11.92
-12	-11.99	-11.98	-11.97	-11.96	-11.95	-11.94	-11.93	-11.92
-12	-11.99	-11.98	-11.97	-11.96	-11.95	-11.94	-11.93	-11.92
-12	-11.99	-11.98	-11.97	-11.96	-11.95	-11.94	-11.93	-11.92

-12	-11.99	-11.98	-11.97	-11.96	-11.95	-11.94	-11.93	-11.92
-12	-11.99	-11.98	-11.97	-11.96	-11.95	-11.94	-11.93	-11.92
-12	-11.99	-11.98	-11.97	-11.96	-11.95	-11.94	-11.93	-11.92
-12	-11.99	-11.98	-11.97	-11.96	-11.95	-11.94	-11.93	-11.92
-12	-11.99	-11.98	-11.97	-11.96	-11.95	-11.94	-11.93	-11.92
-12	-11.99	-11.98	-11.97	-11.96	-11.95	-11.94	-11.93	-11.92
-12	-11.99	-11.98	-11.97	-11.96	-11.95	-11.94	-11.93	-11.92
-12	-11.99	-11.98	-11.97	-11.96	-11.95	-11.94	-11.93	-11.92
-12	-11.99	-11.98	-11.97	-11.96	-11.95	-11.94	-11.93	-11.92
-12	-11.99	-11.98	-11.97	-11.96	-11.95	-11.94	-11.93	-11.92
-12	-11.99	-11.98	-11.97	-11.96	-11.95	-11.94	-11.93	-11.92
-12	-11.99	-11.98	-11.97	-11.96	-11.95	-11.94	-11.93	-11.92
-12	-11.99	-11.98	-11.97	-11.96	-11.95	-11.94	-11.93	-11.92
-12	-11.99	-11.98	-11.97	-11.96	-11.95	-11.94	-11.93	-11.92
-12	-11.99	-11.98	-11.97	-11.96	-11.95	-11.94	-11.93	-11.92
-12	-11.99	-11.98	-11.97	-11.96	-11.95	-11.94	-11.93	-11.92
-12	-11.99	-11.98	-11.97	-11.96	-11.95	-11.94	-11.93	-11.92
-12	-11.99	-11.98	-11.97	-11.96	-11.95	-11.94	-11.93	-11.92
-12	-11.99	-11.98	-11.97	-11.96	-11.95	-11.94	-11.93	-11.92
-12	-11.99	-11.98	-11.97	-11.96	-11.95	-11.94	-11.93	-11.92
-12	-11.99	-11.98	-11.97	-11.96	-11.95	-11.94	-11.93	-11.92
-12	-11.99	-11.98	-11.97	-11.96	-11.95	-11.94	-11.93	-11.92
-12	-11.99	-11.98	-11.97	-11.96	-11.95	-11.94	-11.93	-11.92
-12	-11.99	-11.98	-11.97	-11.96	-11.95	-11.94	-11.93	-11.92
-12	-11.99	-11.98	-11.97	-11.96	-11.95	-11.94	-11.93	-11.92
-12	-11.99	-11.98	-11.97	-11.96	-11.95	-11.94	-11.93	-11.92
-12	-11.99	-11.98	-11.97	-11.96	-11.95	-11.94	-11.93	-11.92
-12	-11.99	-11.98	-11.97	-11.96	-11.95	-11.94	-11.93	-11.92
-12	-11.99	-11.98	-11.97	-11.96	-11.95	-11.94	-11.93	-11.92
-12	-11.99	-11.98	-11.97	-11.96	-11.95	-11.94	-11.93	-11.92
-12	-11.99	-11.98	-11.97	-11.96	-11.95	-11.94	-11.93	-11.92
-12	-11.99	-11.98	-11.97	-11.96	-11.95	-11.94	-11.93	-11.92
-12	-11.99	-11.98	-11.97	-11.96	-11.95	-11.94	-11.93	-11.92
-12	-11.99	-11.98	-11.97	-11.96	-11.95	-11.94	-11.93	-11.92
-12	-11.99	-11.98	-11.97	-11.96	-11.95	-11.94	-11.93	-11.92
-12	-11.99	-11.98	-11.97	-11.96	-11.95	-11.94	-11.93	-11.92
-12	-11.99	-11.98	-11.97	-11.96	-11.95	-11.94	-11.93	-11.92
-12	-11.99	-11.98	-11.97	-11.96	-11.95	-11.94	-11.93	-11.92
-12	-11.99	-11.98	-11.97	-11.96	-11.95	-11.94	-11.93	-11.92
-12	-11.99	-11.98	-11.97	-11.96	-11.95	-11.94	-11.93	-11.92
-12	-11.99	-11.98	-11.97	-11.96	-11.95	-11.94	-11.93	-11.92
-12	-11.99	-11.98	-11.97	-11.96	-11.95	-11.94	-11.93	-11.92
-12	-11.99	-11.98	-11.97	-11.96	-11.95	-11.94	-11.93	-11.92
-12	-11.99	-11.98	-11.97	-11.96	-11.95	-11.94	-11.93	-11.92
-12	-11.99	-11.98	-11.97	-11.96	-11.95	-11.94	-11.93	-11.92
-12	-11.99	-11.98	-11.97	-11.96	-11.95	-11.94	-11.93	-11.92
-12	-11.99	-11.98	-11.97	-11.96	-11.95	-11.94	-11.93	-11.92
-12	-11.99	-11.98	-11.97	-11.96	-11.95	-11.94	-11.93	-11.92
-12	-11.99	-11.98	-11.97	-11.96	-11.95	-11.94	-11.93	-11.92
-12	-11.99	-11.98	-11.97	-11.96	-11.95	-11.94	-11.93	-11.92

-12	-11.99	-11.98	-11.97	-11.96	-11.95	-11.94	-11.93	-11.92
-12	-11.99	-11.98	-11.97	-11.96	-11.95	-11.94	-11.93	-11.92
-12	-11.99	-11.98	-11.97	-11.96	-11.95	-11.94	-11.93	-11.92
-12	-11.99	-11.98	-11.97	-11.96	-11.95	-11.94	-11.93	-11.92
-12	-11.99	-11.98	-11.97	-11.96	-11.95	-11.94	-11.93	-11.92
-12	-11.99	-11.98	-11.97	-11.96	-11.95	-11.94	-11.93	-11.92
-12	-11.99	-11.98	-11.97	-11.96	-11.95	-11.94	-11.93	-11.92
-12	-11.99	-11.98	-11.97	-11.96	-11.95	-11.94	-11.93	-11.92
-12	-11.99	-11.98	-11.97	-11.96	-11.95	-11.94	-11.93	-11.92
-12	-11.99	-11.98	-11.97	-11.96	-11.95	-11.94	-11.93	-11.92
-12	-11.99	-11.98	-11.97	-11.96	-11.95	-11.94	-11.93	-11.92
-12	-11.99	-11.98	-11.97	-11.96	-11.95	-11.94	-11.93	-11.92
-12	-11.99	-11.98	-11.97	-11.96	-11.95	-11.94	-11.93	-11.92
-12	-11.99	-11.98	-11.97	-11.96	-11.95	-11.94	-11.93	-11.92
-12	-11.99	-11.98	-11.97	-11.96	-11.95	-11.94	-11.93	-11.92
-12	-11.99	-11.98	-11.97	-11.96	-11.95	-11.94	-11.93	-11.92
-12	-11.99	-11.98	-11.97	-11.96	-11.95	-11.94	-11.93	-11.92
-12	-11.99	-11.98	-11.97	-11.96	-11.95	-11.94	-11.93	-11.92
-12	-11.99	-11.98	-11.97	-11.96	-11.95	-11.94	-11.93	-11.92
-12	-11.99	-11.98	-11.97	-11.96	-11.95	-11.94	-11.93	-11.92
-12	-11.99	-11.98	-11.97	-11.96	-11.95	-11.94	-11.93	-11.92
-12	-11.99	-11.98	-11.97	-11.96	-11.95	-11.94	-11.93	-11.92
-12	-11.99	-11.98	-11.97	-11.96	-11.95	-11.94	-11.93	-11.92
-12	-11.99	-11.98	-11.97	-11.96	-11.95	-11.94	-11.93	-11.92
-12	-11.99	-11.98	-11.97	-11.96	-11.95	-11.94	-11.93	-11.92
-12	-11.99	-11.98	-11.97	-11.96	-11.95	-11.94	-11.93	-11.92
-12	-11.99	-11.98	-11.97	-11.96	-11.95	-11.94	-11.93	-11.92
-12	-11.99	-11.98	-11.97	-11.96	-11.95	-11.94	-11.93	-11.92
-12	-11.99	-11.98	-11.97	-11.96	-11.95	-11.94	-11.93	-11.92
-12	-11.99	-11.98	-11.97	-11.96	-11.95	-11.94	-11.93	-11.92
-12	-11.99	-11.98	-11.97	-11.96	-11.95	-11.94	-11.93	-11.92
-12	-11.99	-11.98	-11.97	-11.96	-11.95	-11.94	-11.93	-11.92
-12	-11.99	-11.98	-11.97	-11.96	-11.95	-11.94	-11.93	-11.92
-12	-11.99	-11.98	-11.97	-11.96	-11.95	-11.94	-11.93	-11.92
-12	-11.99	-11.98	-11.97	-11.96	-11.95	-11.94	-11.93	-11.92
-12	-11.99	-11.98	-11.97	-11.96	-11.95	-11.94	-11.93	-11.92
-12	-11.99	-11.98	-11.97	-11.96	-11.95	-11.94	-11.93	-11.92
-12	-11.99	-11.98	-11.97	-11.96	-11.95	-11.94	-11.93	-11.92
-12	-11.99	-11.98	-11.97	-11.96	-11.95	-11.94	-11.93	-11.92
-12	-11.99	-11.98	-11.97	-11.96	-11.95	-11.94	-11.93	-11.92
-12	-11.99	-11.98	-11.97	-11.96	-11.95	-11.94	-11.93	-11.92
-12	-11.99	-11.98	-11.97	-11.96	-11.95	-11.94	-11.93	-11.92
-12	-11.99	-11.98	-11.97	-11.96	-11.95	-11.94	-11.93	-11.92
-12	-11.99	-11.98	-11.97	-11.96	-11.95	-11.94	-11.93	-11.92
-12	-11.99	-11.98	-11.97	-11.96	-11.95	-11.94	-11.93	-11.92
-12	-11.99	-11.98	-11.97	-11.96	-11.95	-11.94	-11.93	-11.92
-12	-11.99	-11.98	-11.97	-11.96	-11.95	-11.94	-11.93	-11.92
-12	-11.99	-11.98	-11.97	-11.96	-11.95	-11.94	-11.93	-11.92
-12	-11.99	-11.98	-11.97	-11.96	-11.95	-11.94	-11.93	-11.92
-12	-11.99	-11.98	-11.97	-11.96	-11.95	-11.94	-11.93	-11.92

-12	-11.99	-11.98	-11.97	-11.96	-11.95	-11.94	-11.93	-11.92
-12	-11.99	-11.98	-11.97	-11.96	-11.95	-11.94	-11.93	-11.92
-12	-11.99	-11.98	-11.97	-11.96	-11.95	-11.94	-11.93	-11.92
-12	-11.99	-11.98	-11.97	-11.96	-11.95	-11.94	-11.93	-11.92
-12	-11.99	-11.98	-11.97	-11.96	-11.95	-11.94	-11.93	-11.92
-12	-11.99	-11.98	-11.97	-11.96	-11.95	-11.94	-11.93	-11.92
-12	-11.99	-11.98	-11.97	-11.96	-11.95	-11.94	-11.93	-11.92
-12	-11.99	-11.98	-11.97	-11.96	-11.95	-11.94	-11.93	-11.92
-12	-11.99	-11.98	-11.97	-11.96	-11.95	-11.94	-11.93	-11.92
-12	-11.99	-11.98	-11.97	-11.96	-11.95	-11.94	-11.93	-11.92
-12	-11.99	-11.98	-11.97	-11.96	-11.95	-11.94	-11.93	-11.92
-12	-11.99	-11.98	-11.97	-11.96	-11.95	-11.94	-11.93	-11.92
-12	-11.99	-11.98	-11.97	-11.96	-11.95	-11.94	-11.93	-11.92
-12	-11.99	-11.98	-11.97	-11.96	-11.95	-11.94	-11.93	-11.92
-12	-11.99	-11.98	-11.97	-11.96	-11.95	-11.94	-11.93	-11.92
-12	-11.99	-11.98	-11.97	-11.96	-11.95	-11.94	-11.93	-11.92
-12	-11.99	-11.98	-11.97	-11.96	-11.95	-11.94	-11.93	-11.92
-12	-11.99	-11.98	-11.97	-11.96	-11.95	-11.94	-11.93	-11.92
-12	-11.99	-11.98	-11.97	-11.96	-11.95	-11.94	-11.93	-11.92
-12	-11.99	-11.98	-11.97	-11.96	-11.95	-11.94	-11.93	-11.92
-12	-11.99	-11.98	-11.97	-11.96	-11.95	-11.94	-11.93	-11.92
-12	-11.99	-11.98	-11.97	-11.96	-11.95	-11.94	-11.93	-11.92
-12	-11.99	-11.98	-11.97	-11.96	-11.95	-11.94	-11.93	-11.92
-12	-11.99	-11.98	-11.97	-11.96	-11.95	-11.94	-11.93	-11.92
-12	-11.99	-11.98	-11.97	-11.96	-11.95	-11.94	-11.93	-11.92
-12	-11.99	-11.98	-11.97	-11.96	-11.95	-11.94	-11.93	-11.92
-12	-11.99	-11.98	-11.97	-11.96	-11.95	-11.94	-11.93	-11.92
-12	-11.99	-11.98	-11.97	-11.96	-11.95	-11.94	-11.93	-11.92
-12	-11.99	-11.98	-11.97	-11.96	-11.95	-11.94	-11.93	-11.92
-12	-11.99	-11.98	-11.97	-11.96	-11.95	-11.94	-11.93	-11.92
-12	-11.99	-11.98	-11.97	-11.96	-11.95	-11.94	-11.93	-11.92
-12	-11.99	-11.98	-11.97	-11.96	-11.95	-11.94	-11.93	-11.92
-12	-11.99	-11.98	-11.97	-11.96	-11.95	-11.94	-11.93	-11.92
-12	-11.99	-11.98	-11.97	-11.96	-11.95	-11.94	-11.93	-11.92
-12	-11.99	-11.98	-11.97	-11.96	-11.95	-11.94	-11.93	-11.92
-12	-11.99	-11.98	-11.97	-11.96	-11.95	-11.94	-11.93	-11.92
-12	-11.99	-11.98	-11.97	-11.96	-11.95	-11.94	-11.93	-11.92
-12	-11.99	-11.98	-11.97	-11.96	-11.95	-11.94	-11.93	-11.92
-12	-11.99	-11.98	-11.97	-11.96	-11.95	-11.94	-11.93	-11.92
-12	-11.99	-11.98	-11.97	-11.96	-11.95	-11.94	-11.93	-11.92
-12	-11.99	-11.98	-11.97	-11.96	-11.95	-11.94	-11.93	-11.92
-12	-11.99	-11.98	-11.97	-11.96	-11.95	-11.94	-11.93	-11.92
-12	-11.99	-11.98	-11.97	-11.96	-11.95	-11.94	-11.93	-11.92
-12	-11.99	-11.98	-11.97	-11.96	-11.95	-11.94	-11.93	-11.92
-12	-11.99	-11.98	-11.97	-11.96	-11.95	-11.94	-11.93	-11.92
-12	-11.99	-11.98	-11.97	-11.96	-11.95	-11.94	-11.93	-11.92
-12	-11.99	-11.98	-11.97	-11.96	-11.95	-11.94	-11.93	-11.92
-12	-11.99	-11.98	-11.97	-11.96	-11.95	-11.94	-11.93	-11.92
-12	-11.99	-11.98	-11.97	-11.96	-11.95	-11.94	-11.93	-11.92
-12	-11.99	-11.98	-11.97	-11.96	-11.95	-11.94	-11.93	-11.92

-12	-11.99	-11.98	-11.97	-11.96	-11.95	-11.94	-11.93	-11.92
-12	-11.99	-11.98	-11.97	-11.96	-11.95	-11.94	-11.93	-11.92
-12	-11.99	-11.98	-11.97	-11.96	-11.95	-11.94	-11.93	-11.92
-12	-11.99	-11.98	-11.97	-11.96	-11.95	-11.94	-11.93	-11.92
-12	-11.99	-11.98	-11.97	-11.96	-11.95	-11.94	-11.93	-11.92
-12	-11.99	-11.98	-11.97	-11.96	-11.95	-11.94	-11.93	-11.92
-12	-11.99	-11.98	-11.97	-11.96	-11.95	-11.94	-11.93	-11.92
-12	-11.99	-11.98	-11.97	-11.96	-11.95	-11.94	-11.93	-11.92
-12	-11.99	-11.98	-11.97	-11.96	-11.95	-11.94	-11.93	-11.92
-12	-11.99	-11.98	-11.97	-11.96	-11.95	-11.94	-11.93	-11.92
-12	-11.99	-11.98	-11.97	-11.96	-11.95	-11.94	-11.93	-11.92
-12	-11.99	-11.98	-11.97	-11.96	-11.95	-11.94	-11.93	-11.92
-12	-11.99	-11.98	-11.97	-11.96	-11.95	-11.94	-11.93	-11.92
-12	-11.99	-11.98	-11.97	-11.96	-11.95	-11.94	-11.93	-11.92
-12	-11.99	-11.98	-11.97	-11.96	-11.95	-11.94	-11.93	-11.92
-12	-11.99	-11.98	-11.97	-11.96	-11.95	-11.94	-11.93	-11.92
-12	-11.99	-11.98	-11.97	-11.96	-11.95	-11.94	-11.93	-11.92
-12	-11.99	-11.98	-11.97	-11.96	-11.95	-11.94	-11.93	-11.92
-12	-11.99	-11.98	-11.97	-11.96	-11.95	-11.94	-11.93	-11.92
-12	-11.99	-11.98	-11.97	-11.96	-11.95	-11.94	-11.93	-11.92
-12	-11.99	-11.98	-11.97	-11.96	-11.95	-11.94	-11.93	-11.92
-12	-11.99	-11.98	-11.97	-11.96	-11.95	-11.94	-11.93	-11.92
-12	-11.99	-11.98	-11.97	-11.96	-11.95	-11.94	-11.93	-11.92
-12	-11.99	-11.98	-11.97	-11.96	-11.95	-11.94	-11.93	-11.92
-12	-11.99	-11.98	-11.97	-11.96	-11.95	-11.94	-11.93	-11.92
-12	-11.99	-11.98	-11.97	-11.96	-11.95	-11.94	-11.93	-11.92
-12	-11.99	-11.98	-11.97	-11.96	-11.95	-11.94	-11.93	-11.92
-12	-11.99	-11.98	-11.97	-11.96	-11.95	-11.94	-11.93	-11.92
-12	-11.99	-11.98	-11.97	-11.96	-11.95	-11.94	-11.93	-11.92
-12	-11.99	-11.98	-11.97	-11.96	-11.95	-11.94	-11.93	-11.92
-12	-11.99	-11.98	-11.97	-11.96	-11.95	-11.94	-11.93	-11.92
-12	-11.99	-11.98	-11.97	-11.96	-11.95	-11.94	-11.93	-11.92
-12	-11.99	-11.98	-11.97	-11.96	-11.95	-11.94	-11.93	-11.92
-12	-11.99	-11.98	-11.97	-11.96	-11.95	-11.94	-11.93	-11.92
-12	-11.99	-11.98	-11.97	-11.96	-11.95	-11.94	-11.93	-11.92
-12	-11.99	-11.98	-11.97	-11.96	-11.95	-11.94	-11.93	-11.92
-12	-11.99	-11.98	-11.97	-11.96	-11.95	-11.94	-11.93	-11.92
-12	-11.99	-11.98	-11.97	-11.96	-11.95	-11.94	-11.93	-11.92
-12	-11.99	-11.98	-11.97	-11.96	-11.95	-11.94	-11.93	-11.92
-12	-11.99	-11.98	-11.97	-11.96	-11.95	-11.94	-11.93	-11.92
-12	-11.99	-11.98	-11.97	-11.96	-11.95	-11.94	-11.93	-11.92
-12	-11.99	-11.98	-11.97	-11.96	-11.95	-11.94	-11.93	-11.92
-12	-11.99	-11.98	-11.97	-11.96	-11.95	-11.94	-11.93	-11.92
-12	-11.99	-11.98	-11.97	-11.96	-11.95	-11.94	-11.93	-11.92
-12	-11.99	-11.98	-11.97	-11.96	-11.95	-11.94	-11.93	-11.92
-12	-11.99	-11.98	-11.97	-11.96	-11.95	-11.94	-11.93	-11.92
-12	-11.99	-11.98	-11.97	-11.96	-11.95	-11.94	-11.93	-11.92
-12	-11.99	-11.98	-11.97	-11.96	-11.95	-11.94	-11.93	-11.92
-12	-11.99	-11.98	-11.97	-11.96	-11.95	-11.94	-11.93	-11.92
-12	-11.99	-11.98	-11.97	-11.96	-11.95	-11.94	-11.93	-11.92

-12	-11.99	-11.98	-11.97	-11.96	-11.95	-11.94	-11.93	-11.92
-12	-11.99	-11.98	-11.97	-11.96	-11.95	-11.94	-11.93	-11.92
-12	-11.99	-11.98	-11.97	-11.96	-11.95	-11.94	-11.93	-11.92
-12	-11.99	-11.98	-11.97	-11.96	-11.95	-11.94	-11.93	-11.92
-12	-11.99	-11.98	-11.97	-11.96	-11.95	-11.94	-11.93	-11.92
-12	-11.99	-11.98	-11.97	-11.96	-11.95	-11.94	-11.93	-11.92
-12	-11.99	-11.98	-11.97	-11.96	-11.95	-11.94	-11.93	-11.92
-12	-11.99	-11.98	-11.97	-11.96	-11.95	-11.94	-11.93	-11.92
-12	-11.99	-11.98	-11.97	-11.96	-11.95	-11.94	-11.93	-11.92
-12	-11.99	-11.98	-11.97	-11.96	-11.95	-11.94	-11.93	-11.92
-12	-11.99	-11.98	-11.97	-11.96	-11.95	-11.94	-11.93	-11.92
-12	-11.99	-11.98	-11.97	-11.96	-11.95	-11.94	-11.93	-11.92
-12	-11.99	-11.98	-11.97	-11.96	-11.95	-11.94	-11.93	-11.92
-12	-11.99	-11.98	-11.97	-11.96	-11.95	-11.94	-11.93	-11.92
-12	-11.99	-11.98	-11.97	-11.96	-11.95	-11.94	-11.93	-11.92
-12	-11.99	-11.98	-11.97	-11.96	-11.95	-11.94	-11.93	-11.92
-12	-11.99	-11.98	-11.97	-11.96	-11.95	-11.94	-11.93	-11.92
-12	-11.99	-11.98	-11.97	-11.96	-11.95	-11.94	-11.93	-11.92
-12	-11.99	-11.98	-11.97	-11.96	-11.95	-11.94	-11.93	-11.92
-12	-11.99	-11.98	-11.97	-11.96	-11.95	-11.94	-11.93	-11.92
-12	-11.99	-11.98	-11.97	-11.96	-11.95	-11.94	-11.93	-11.92
-12	-11.99	-11.98	-11.97	-11.96	-11.95	-11.94	-11.93	-11.92
-12	-11.99	-11.98	-11.97	-11.96	-11.95	-11.94	-11.93	-11.92
-12	-11.99	-11.98	-11.97	-11.96	-11.95	-11.94	-11.93	-11.92
-12	-11.99	-11.98	-11.97	-11.96	-11.95	-11.94	-11.93	-11.92
-12	-11.99	-11.98	-11.97	-11.96	-11.95	-11.94	-11.93	-11.92
-12	-11.99	-11.98	-11.97	-11.96	-11.95	-11.94	-11.93	-11.92
-12	-11.99	-11.98	-11.97	-11.96	-11.95	-11.94	-11.93	-11.92
-12	-11.99	-11.98	-11.97	-11.96	-11.95	-11.94	-11.93	-11.92
-12	-11.99	-11.98	-11.97	-11.96	-11.95	-11.94	-11.93	-11.92
-12	-11.99	-11.98	-11.97	-11.96	-11.95	-11.94	-11.93	-11.92
-12	-11.99	-11.98	-11.97	-11.96	-11.95	-11.94	-11.93	-11.92
-12	-11.99	-11.98	-11.97	-11.96	-11.95	-11.94	-11.93	-11.92
-12	-11.99	-11.98	-11.97	-11.96	-11.95	-11.94	-11.93	-11.92
-12	-11.99	-11.98	-11.97	-11.96	-11.95	-11.94	-11.93	-11.92
-12	-11.99	-11.98	-11.97	-11.96	-11.95	-11.94	-11.93	-11.92
-12	-11.99	-11.98	-11.97	-11.96	-11.95	-11.94	-11.93	-11.92
-12	-11.99	-11.98	-11.97	-11.96	-11.95	-11.94	-11.93	-11.92
-12	-11.99	-11.98	-11.97	-11.96	-11.95	-11.94	-11.93	-11.92
-12	-11.99	-11.98	-11.97	-11.96	-11.95	-11.94	-11.93	-11.92
-12	-11.99	-11.98	-11.97	-11.96	-11.95	-11.94	-11.93	-11.92
-12	-11.99	-11.98	-11.97	-11.96	-11.95	-11.94	-11.93	-11.92
-12	-11.99	-11.98	-11.97	-11.96	-11.95	-11.94	-11.93	-11.92
-12	-11.99	-11.98	-11.97	-11.96	-11.95	-11.94	-11.93	-11.92
-12	-11.99	-11.98	-11.97	-11.96	-11.95	-11.94	-11.93	-11.92
-12	-11.99	-11.98	-11.97	-11.96	-11.95	-11.94	-11.93	-11.92
-12	-11.99	-11.98	-11.97	-11.96	-11.95	-11.94	-11.93	-11.92
-12	-11.99	-11.98	-11.97	-11.96	-11.95	-11.94	-11.93	-11.92
-12	-11.99	-11.98	-11.97	-11.96	-11.95	-11.94	-11.93	-11.92
-12	-11.99	-11.98	-11.97	-11.96	-11.95	-11.94	-11.93	-11.92

-12	-11.99	-11.98	-11.97	-11.96	-11.95	-11.94	-11.93	-11.92
-12	-11.99	-11.98	-11.97	-11.96	-11.95	-11.94	-11.93	-11.92
-12	-11.99	-11.98	-11.97	-11.96	-11.95	-11.94	-11.93	-11.92
-12	-11.99	-11.98	-11.97	-11.96	-11.95	-11.94	-11.93	-11.92
-12	-11.99	-11.98	-11.97	-11.96	-11.95	-11.94	-11.93	-11.92
-12	-11.99	-11.98	-11.97	-11.96	-11.95	-11.94	-11.93	-11.92
-12	-11.99	-11.98	-11.97	-11.96	-11.95	-11.94	-11.93	-11.92
-12	-11.99	-11.98	-11.97	-11.96	-11.95	-11.94	-11.93	-11.92
-12	-11.99	-11.98	-11.97	-11.96	-11.95	-11.94	-11.93	-11.92
-12	-11.99	-11.98	-11.97	-11.96	-11.95	-11.94	-11.93	-11.92
-12	-11.99	-11.98	-11.97	-11.96	-11.95	-11.94	-11.93	-11.92
-12	-11.99	-11.98	-11.97	-11.96	-11.95	-11.94	-11.93	-11.92
-12	-11.99	-11.98	-11.97	-11.96	-11.95	-11.94	-11.93	-11.92
-12	-11.99	-11.98	-11.97	-11.96	-11.95	-11.94	-11.93	-11.92
-12	-11.99	-11.98	-11.97	-11.96	-11.95	-11.94	-11.93	-11.92
-12	-11.99	-11.98	-11.97	-11.96	-11.95	-11.94	-11.93	-11.92
-12	-11.99	-11.98	-11.97	-11.96	-11.95	-11.94	-11.93	-11.92
-12	-11.99	-11.98	-11.97	-11.96	-11.95	-11.94	-11.93	-11.92
-12	-11.99	-11.98	-11.97	-11.96	-11.95	-11.94	-11.93	-11.92
-12	-11.99	-11.98	-11.97	-11.96	-11.95	-11.94	-11.93	-11.92
-12	-11.99	-11.98	-11.97	-11.96	-11.95	-11.94	-11.93	-11.92
-12	-11.99	-11.98	-11.97	-11.96	-11.95	-11.94	-11.93	-11.92
-12	-11.99	-11.98	-11.97	-11.96	-11.95	-11.94	-11.93	-11.92
-12	-11.99	-11.98	-11.97	-11.96	-11.95	-11.94	-11.93	-11.92
-12	-11.99	-11.98	-11.97	-11.96	-11.95	-11.94	-11.93	-11.92
-12	-11.99	-11.98	-11.97	-11.96	-11.95	-11.94	-11.93	-11.92
-12	-11.99	-11.98	-11.97	-11.96	-11.95	-11.94	-11.93	-11.92
-12	-11.99	-11.98	-11.97	-11.96	-11.95	-11.94	-11.93	-11.92
-12	-11.99	-11.98	-11.97	-11.96	-11.95	-11.94	-11.93	-11.92
-12	-11.99	-11.98	-11.97	-11.96	-11.95	-11.94	-11.93	-11.92
-12	-11.99	-11.98	-11.97	-11.96	-11.95	-11.94	-11.93	-11.92
-12	-11.99	-11.98	-11.97	-11.96	-11.95	-11.94	-11.93	-11.92
-12	-11.99	-11.98	-11.97	-11.96	-11.95	-11.94	-11.93	-11.92
-12	-11.99	-11.98	-11.97	-11.96	-11.95	-11.94	-11.93	-11.92
-12	-11.99	-11.98	-11.97	-11.96	-11.95	-11.94	-11.93	-11.92
-12	-11.99	-11.98	-11.97	-11.96	-11.95	-11.94	-11.93	-11.92
-12	-11.99	-11.98	-11.97	-11.96	-11.95	-11.94	-11.93	-11.92
-12	-11.99	-11.98	-11.97	-11.96	-11.95	-11.94	-11.93	-11.92
-12	-11.99	-11.98	-11.97	-11.96	-11.95	-11.94	-11.93	-11.92
-12	-11.99	-11.98	-11.97	-11.96	-11.95	-11.94	-11.93	-11.92
-12	-11.99	-11.98	-11.97	-11.96	-11.95	-11.94	-11.93	-11.92
-12	-11.99	-11.98	-11.97	-11.96	-11.95	-11.94	-11.93	-11.92
-12	-11.99	-11.98	-11.97	-11.96	-11.95	-11.94	-11.93	-11.92
-12	-11.99	-11.98	-11.97	-11.96	-11.95	-11.94	-11.93	-11.92
-12	-11.99	-11.98	-11.97	-11.96	-11.95	-11.94	-11.93	-11.92
-12	-11.99	-11.98	-11.97	-11.96	-11.95	-11.94	-11.93	-11.92
-12	-11.99	-11.98	-11.97	-11.96	-11.95	-11.94	-11.93	-11.92
-12	-11.99	-11.98	-11.97	-11.96	-11.95	-11.94	-11.93	-11.92
-12	-11.99	-11.98	-11.97	-11.96	-11.95	-11.94	-11.93	-11.92
-12	-11.99	-11.98	-11.97	-11.96	-11.95	-11.94	-11.93	-11.92

-12	-11.99	-11.98	-11.97	-11.96	-11.95	-11.94	-11.93	-11.92
-12	-11.99	-11.98	-11.97	-11.96	-11.95	-11.94	-11.93	-11.92
-12	-11.99	-11.98	-11.97	-11.96	-11.95	-11.94	-11.93	-11.92
-12	-11.99	-11.98	-11.97	-11.96	-11.95	-11.94	-11.93	-11.92
-12	-11.99	-11.98	-11.97	-11.96	-11.95	-11.94	-11.93	-11.92
-12	-11.99	-11.98	-11.97	-11.96	-11.95	-11.94	-11.93	-11.92
-12	-11.99	-11.98	-11.97	-11.96	-11.95	-11.94	-11.93	-11.92
-12	-11.99	-11.98	-11.97	-11.96	-11.95	-11.94	-11.93	-11.92
-12	-11.99	-11.98	-11.97	-11.96	-11.95	-11.94	-11.93	-11.92
-12	-11.99	-11.98	-11.97	-11.96	-11.95	-11.94	-11.93	-11.92
-12	-11.99	-11.98	-11.97	-11.96	-11.95	-11.94	-11.93	-11.92
-12	-11.99	-11.98	-11.97	-11.96	-11.95	-11.94	-11.93	-11.92
-12	-11.99	-11.98	-11.97	-11.96	-11.95	-11.94	-11.93	-11.92
-12	-11.99	-11.98	-11.97	-11.96	-11.95	-11.94	-11.93	-11.92
-12	-11.99	-11.98	-11.97	-11.96	-11.95	-11.94	-11.93	-11.92
-12	-11.99	-11.98	-11.97	-11.96	-11.95	-11.94	-11.93	-11.92
-12	-11.99	-11.98	-11.97	-11.96	-11.95	-11.94	-11.93	-11.92
-12	-11.99	-11.98	-11.97	-11.96	-11.95	-11.94	-11.93	-11.92
-12	-11.99	-11.98	-11.97	-11.96	-11.95	-11.94	-11.93	-11.92
-12	-11.99	-11.98	-11.97	-11.96	-11.95	-11.94	-11.93	-11.92
-12	-11.99	-11.98	-11.97	-11.96	-11.95	-11.94	-11.93	-11.92
-12	-11.99	-11.98	-11.97	-11.96	-11.95	-11.94	-11.93	-11.92
-12	-11.99	-11.98	-11.97	-11.96	-11.95	-11.94	-11.93	-11.92
-12	-11.99	-11.98	-11.97	-11.96	-11.95	-11.94	-11.93	-11.92
-12	-11.99	-11.98	-11.97	-11.96	-11.95	-11.94	-11.93	-11.92
-12	-11.99	-11.98	-11.97	-11.96	-11.95	-11.94	-11.93	-11.92
-12	-11.99	-11.98	-11.97	-11.96	-11.95	-11.94	-11.93	-11.92
-12	-11.99	-11.98	-11.97	-11.96	-11.95	-11.94	-11.93	-11.92
-12	-11.99	-11.98	-11.97	-11.96	-11.95	-11.94	-11.93	-11.92
-12	-11.99	-11.98	-11.97	-11.96	-11.95	-11.94	-11.93	-11.92
-12	-11.99	-11.98	-11.97	-11.96	-11.95	-11.94	-11.93	-11.92
-12	-11.99	-11.98	-11.97	-11.96	-11.95	-11.94	-11.93	-11.92
-12	-11.99	-11.98	-11.97	-11.96	-11.95	-11.94	-11.93	-11.92
-12	-11.99	-11.98	-11.97	-11.96	-11.95	-11.94	-11.93	-11.92
-12	-11.99	-11.98	-11.97	-11.96	-11.95	-11.94	-11.93	-11.92
-12	-11.99	-11.98	-11.97	-11.96	-11.95	-11.94	-11.93	-11.92
-12	-11.99	-11.98	-11.97	-11.96	-11.95	-11.94	-11.93	-11.92
-12	-11.99	-11.98	-11.97	-11.96	-11.95	-11.94	-11.93	-11.92
-12	-11.99	-11.98	-11.97	-11.96	-11.95	-11.94	-11.93	-11.92
-12	-11.99	-11.98	-11.97	-11.96	-11.95	-11.94	-11.93	-11.92
-12	-11.99	-11.98	-11.97	-11.96	-11.95	-11.94	-11.93	-11.92
-12	-11.99	-11.98	-11.97	-11.96	-11.95	-11.94	-11.93	-11.92
-12	-11.99	-11.98	-11.97	-11.96	-11.95	-11.94	-11.93	-11.92
-12	-11.99	-11.98	-11.97	-11.96	-11.95	-11.94	-11.93	-11.92
-12	-11.99	-11.98	-11.97	-11.96	-11.95	-11.94	-11.93	-11.92
-12	-11.99	-11.98	-11.97	-11.96	-11.95	-11.94	-11.93	-11.92
-12	-11.99	-11.98	-11.97	-11.96	-11.95	-11.94	-11.93	-11.92
-12	-11.99	-11.98	-11.97	-11.96	-11.95	-11.94	-11.93	-11.92
-12	-11.99	-11.98	-11.97	-11.96	-11.95	-11.94	-11.93	-11.92
-12	-11.99	-11.98	-11.97	-11.96	-11.95	-11.94	-11.93	-11.92

-12	-11.99	-11.98	-11.97	-11.96	-11.95	-11.94	-11.93	-11.92
-12	-11.99	-11.98	-11.97	-11.96	-11.95	-11.94	-11.93	-11.92
-12	-11.99	-11.98	-11.97	-11.96	-11.95	-11.94	-11.93	-11.92
-12	-11.99	-11.98	-11.97	-11.96	-11.95	-11.94	-11.93	-11.92
-12	-11.99	-11.98	-11.97	-11.96	-11.95	-11.94	-11.93	-11.92
-12	-11.99	-11.98	-11.97	-11.96	-11.95	-11.94	-11.93	-11.92
-12	-11.99	-11.98	-11.97	-11.96	-11.95	-11.94	-11.93	-11.92
-12	-11.99	-11.98	-11.97	-11.96	-11.95	-11.94	-11.93	-11.92
-12	-11.99	-11.98	-11.97	-11.96	-11.95	-11.94	-11.93	-11.92
-12	-11.99	-11.98	-11.97	-11.96	-11.95	-11.94	-11.93	-11.92
-12	-11.99	-11.98	-11.97	-11.96	-11.95	-11.94	-11.93	-11.92
-12	-11.99	-11.98	-11.97	-11.96	-11.95	-11.94	-11.93	-11.92
-12	-11.99	-11.98	-11.97	-11.96	-11.95	-11.94	-11.93	-11.92
-12	-11.99	-11.98	-11.97	-11.96	-11.95	-11.94	-11.93	-11.92
-12	-11.99	-11.98	-11.97	-11.96	-11.95	-11.94	-11.93	-11.92
-12	-11.99	-11.98	-11.97	-11.96	-11.95	-11.94	-11.93	-11.92
-12	-11.99	-11.98	-11.97	-11.96	-11.95	-11.94	-11.93	-11.92
-12	-11.99	-11.98	-11.97	-11.96	-11.95	-11.94	-11.93	-11.92
-12	-11.99	-11.98	-11.97	-11.96	-11.95	-11.94	-11.93	-11.92
-12	-11.99	-11.98	-11.97	-11.96	-11.95	-11.94	-11.93	-11.92
-12	-11.99	-11.98	-11.97	-11.96	-11.95	-11.94	-11.93	-11.92
-12	-11.99	-11.98	-11.97	-11.96	-11.95	-11.94	-11.93	-11.92
-12	-11.99	-11.98	-11.97	-11.96	-11.95	-11.94	-11.93	-11.92
-12	-11.99	-11.98	-11.97	-11.96	-11.95	-11.94	-11.93	-11.92
-12	-11.99	-11.98	-11.97	-11.96	-11.95	-11.94	-11.93	-11.92
-12	-11.99	-11.98	-11.97	-11.96	-11.95	-11.94	-11.93	-11.92
-12	-11.99	-11.98	-11.97	-11.96	-11.95	-11.94	-11.93	-11.92
-12	-11.99	-11.98	-11.97	-11.96	-11.95	-11.94	-11.93	-11.92
-12	-11.99	-11.98	-11.97	-11.96	-11.95	-11.94	-11.93	-11.92
-12	-11.99	-11.98	-11.97	-11.96	-11.95	-11.94	-11.93	-11.92
-12	-11.99	-11.98	-11.97	-11.96	-11.95	-11.94	-11.93	-11.92
-12	-11.99	-11.98	-11.97	-11.96	-11.95	-11.94	-11.93	-11.92
-12	-11.99	-11.98	-11.97	-11.96	-11.95	-11.94	-11.93	-11.92
-12	-11.99	-11.98	-11.97	-11.96	-11.95	-11.94	-11.93	-11.92
-12	-11.99	-11.98	-11.97	-11.96	-11.95	-11.94	-11.93	-11.92
-12	-11.99	-11.98	-11.97	-11.96	-11.95	-11.94	-11.93	-11.92
-12	-11.99	-11.98	-11.97	-11.96	-11.95	-11.94	-11.93	-11.92
-12	-11.99	-11.98	-11.97	-11.96	-11.95	-11.94	-11.93	-11.92
-12	-11.99	-11.98	-11.97	-11.96	-11.95	-11.94	-11.93	-11.92
-12	-11.99	-11.98	-11.97	-11.96	-11.95	-11.94	-11.93	-11.92
-12	-11.99	-11.98	-11.97	-11.96	-11.95	-11.94	-11.93	-11.92
-12	-11.99	-11.98	-11.97	-11.96	-11.95	-11.94	-11.93	-11.92
-12	-11.99	-11.98	-11.97	-11.96	-11.95	-11.94	-11.93	-11.92
-12	-11.99	-11.98	-11.97	-11.96	-11.95	-11.94	-11.93	-11.92
-12	-11.99	-11.98	-11.97	-11.96	-11.95	-11.94	-11.93	-11.92
-12	-11.99	-11.98	-11.97	-11.96	-11.95	-11.94	-11.93	-11.92
-12	-11.99	-11.98	-11.97	-11.96	-11.95	-11.94	-11.93	-11.92
-12	-11.99	-11.98	-11.97	-11.96	-11.95	-11.94	-11.93	-11.92
-12	-11.99	-11.98	-11.97	-11.96	-11.95	-11.94	-11.93	-11.92
-12	-11.99	-11.98	-11.97	-11.96	-11.95	-11.94	-11.93	-11.92

-12	-11.99	-11.98	-11.97	-11.96	-11.95	-11.94	-11.93	-11.92
-12	-11.99	-11.98	-11.97	-11.96	-11.95	-11.94	-11.93	-11.92
-12	-11.99	-11.98	-11.97	-11.96	-11.95	-11.94	-11.93	-11.92
-12	-11.99	-11.98	-11.97	-11.96	-11.95	-11.94	-11.93	-11.92
-12	-11.99	-11.98	-11.97	-11.96	-11.95	-11.94	-11.93	-11.92
-12	-11.99	-11.98	-11.97	-11.96	-11.95	-11.94	-11.93	-11.92
-12	-11.99	-11.98	-11.97	-11.96	-11.95	-11.94	-11.93	-11.92
-12	-11.99	-11.98	-11.97	-11.96	-11.95	-11.94	-11.93	-11.92
-12	-11.99	-11.98	-11.97	-11.96	-11.95	-11.94	-11.93	-11.92
-12	-11.99	-11.98	-11.97	-11.96	-11.95	-11.94	-11.93	-11.92
-12	-11.99	-11.98	-11.97	-11.96	-11.95	-11.94	-11.93	-11.92
-12	-11.99	-11.98	-11.97	-11.96	-11.95	-11.94	-11.93	-11.92
-12	-11.99	-11.98	-11.97	-11.96	-11.95	-11.94	-11.93	-11.92
-12	-11.99	-11.98	-11.97	-11.96	-11.95	-11.94	-11.93	-11.92
-12	-11.99	-11.98	-11.97	-11.96	-11.95	-11.94	-11.93	-11.92
-12	-11.99	-11.98	-11.97	-11.96	-11.95	-11.94	-11.93	-11.92
-12	-11.99	-11.98	-11.97	-11.96	-11.95	-11.94	-11.93	-11.92
-12	-11.99	-11.98	-11.97	-11.96	-11.95	-11.94	-11.93	-11.92
-12	-11.99	-11.98	-11.97	-11.96	-11.95	-11.94	-11.93	-11.92
-12	-11.99	-11.98	-11.97	-11.96	-11.95	-11.94	-11.93	-11.92
-12	-11.99	-11.98	-11.97	-11.96	-11.95	-11.94	-11.93	-11.92
-12	-11.99	-11.98	-11.97	-11.96	-11.95	-11.94	-11.93	-11.92
-12	-11.99	-11.98	-11.97	-11.96	-11.95	-11.94	-11.93	-11.92
-12	-11.99	-11.98	-11.97	-11.96	-11.95	-11.94	-11.93	-11.92
-12	-11.99	-11.98	-11.97	-11.96	-11.95	-11.94	-11.93	-11.92
-12	-11.99	-11.98	-11.97	-11.96	-11.95	-11.94	-11.93	-11.92
-12	-11.99	-11.98	-11.97	-11.96	-11.95	-11.94	-11.93	-11.92
-12	-11.99	-11.98	-11.97	-11.96	-11.95	-11.94	-11.93	-11.92
-12	-11.99	-11.98	-11.97	-11.96	-11.95	-11.94	-11.93	-11.92
-12	-11.99	-11.98	-11.97	-11.96	-11.95	-11.94	-11.93	-11.92
-12	-11.99	-11.98	-11.97	-11.96	-11.95	-11.94	-11.93	-11.92
-12	-11.99	-11.98	-11.97	-11.96	-11.95	-11.94	-11.93	-11.92
-12	-11.99	-11.98	-11.97	-11.96	-11.95	-11.94	-11.93	-11.92
-12	-11.99	-11.98	-11.97	-11.96	-11.95	-11.94	-11.93	-11.92
-12	-11.99	-11.98	-11.97	-11.96	-11.95	-11.94	-11.93	-11.92
-12	-11.99	-11.98	-11.97	-11.96	-11.95	-11.94	-11.93	-11.92
-12	-11.99	-11.98	-11.97	-11.96	-11.95	-11.94	-11.93	-11.92
-12	-11.99	-11.98	-11.97	-11.96	-11.95	-11.94	-11.93	-11.92
-12	-11.99	-11.98	-11.97	-11.96	-11.95	-11.94	-11.93	-11.92
-12	-11.99	-11.98	-11.97	-11.96	-11.95	-11.94	-11.93	-11.92
-12	-11.99	-11.98	-11.97	-11.96	-11.95	-11.94	-11.93	-11.92
-12	-11.99	-11.98	-11.97	-11.96	-11.95	-11.94	-11.93	-11.92
-12	-11.99	-11.98	-11.97	-11.96	-11.95	-11.94	-11.93	-11.92
-12	-11.99	-11.98	-11.97	-11.96	-11.95	-11.94	-11.93	-11.92
-12	-11.99	-11.98	-11.97	-11.96	-11.95	-11.94	-11.93	-11.92
-12	-11.99	-11.98	-11.97	-11.96	-11.95	-11.94	-11.93	-11.92
-12	-11.99	-11.98	-11.97	-11.96	-11.95	-11.94	-11.93	-11.92
-12	-11.99	-11.98	-11.97	-11.96	-11.95	-11.94	-11.93	-11.92
-12	-11.99	-11.98	-11.97	-11.96	-11.95	-11.94	-11.93	-11.92
-12	-11.99	-11.98	-11.97	-11.96	-11.95	-11.94	-11.93	-11.92

-12	-11.99	-11.98	-11.97	-11.96	-11.95	-11.94	-11.93	-11.92
-12	-11.99	-11.98	-11.97	-11.96	-11.95	-11.94	-11.93	-11.92
-12	-11.99	-11.98	-11.97	-11.96	-11.95	-11.94	-11.93	-11.92
-12	-11.99	-11.98	-11.97	-11.96	-11.95	-11.94	-11.93	-11.92
-12	-11.99	-11.98	-11.97	-11.96	-11.95	-11.94	-11.93	-11.92
-12	-11.99	-11.98	-11.97	-11.96	-11.95	-11.94	-11.93	-11.92
-12	-11.99	-11.98	-11.97	-11.96	-11.95	-11.94	-11.93	-11.92
-12	-11.99	-11.98	-11.97	-11.96	-11.95	-11.94	-11.93	-11.92
-12	-11.99	-11.98	-11.97	-11.96	-11.95	-11.94	-11.93	-11.92
-12	-11.99	-11.98	-11.97	-11.96	-11.95	-11.94	-11.93	-11.92
-12	-11.99	-11.98	-11.97	-11.96	-11.95	-11.94	-11.93	-11.92
-12	-11.99	-11.98	-11.97	-11.96	-11.95	-11.94	-11.93	-11.92
-12	-11.99	-11.98	-11.97	-11.96	-11.95	-11.94	-11.93	-11.92
-12	-11.99	-11.98	-11.97	-11.96	-11.95	-11.94	-11.93	-11.92
-12	-11.99	-11.98	-11.97	-11.96	-11.95	-11.94	-11.93	-11.92
-12	-11.99	-11.98	-11.97	-11.96	-11.95	-11.94	-11.93	-11.92
-12	-11.99	-11.98	-11.97	-11.96	-11.95	-11.94	-11.93	-11.92
-12	-11.99	-11.98	-11.97	-11.96	-11.95	-11.94	-11.93	-11.92
-12	-11.99	-11.98	-11.97	-11.96	-11.95	-11.94	-11.93	-11.92
-12	-11.99	-11.98	-11.97	-11.96	-11.95	-11.94	-11.93	-11.92
-12	-11.99	-11.98	-11.97	-11.96	-11.95	-11.94	-11.93	-11.92
-12	-11.99	-11.98	-11.97	-11.96	-11.95	-11.94	-11.93	-11.92
-12	-11.99	-11.98	-11.97	-11.96	-11.95	-11.94	-11.93	-11.92
-12	-11.99	-11.98	-11.97	-11.96	-11.95	-11.94	-11.93	-11.92
-12	-11.99	-11.98	-11.97	-11.96	-11.95	-11.94	-11.93	-11.92
-12	-11.99	-11.98	-11.97	-11.96	-11.95	-11.94	-11.93	-11.92
-12	-11.99	-11.98	-11.97	-11.96	-11.95	-11.94	-11.93	-11.92
-12	-11.99	-11.98	-11.97	-11.96	-11.95	-11.94	-11.93	-11.92
-12	-11.99	-11.98	-11.97	-11.96	-11.95	-11.94	-11.93	-11.92
-12	-11.99	-11.98	-11.97	-11.96	-11.95	-11.94	-11.93	-11.92
-12	-11.99	-11.98	-11.97	-11.96	-11.95	-11.94	-11.93	-11.92
-12	-11.99	-11.98	-11.97	-11.96	-11.95	-11.94	-11.93	-11.92
-12	-11.99	-11.98	-11.97	-11.96	-11.95	-11.94	-11.93	-11.92
-12	-11.99	-11.98	-11.97	-11.96	-11.95	-11.94	-11.93	-11.92
-12	-11.99	-11.98	-11.97	-11.96	-11.95	-11.94	-11.93	-11.92
-12	-11.99	-11.98	-11.97	-11.96	-11.95	-11.94	-11.93	-11.92
-12	-11.99	-11.98	-11.97	-11.96	-11.95	-11.94	-11.93	-11.92
-12	-11.99	-11.98	-11.97	-11.96	-11.95	-11.94	-11.93	-11.92
-12	-11.99	-11.98	-11.97	-11.96	-11.95	-11.94	-11.93	-11.92
-12	-11.99	-11.98	-11.97	-11.96	-11.95	-11.94	-11.93	-11.92
-12	-11.99	-11.98	-11.97	-11.96	-11.95	-11.94	-11.93	-11.92
-12	-11.99	-11.98	-11.97	-11.96	-11.95	-11.94	-11.93	-11.92
-12	-11.99	-11.98	-11.97	-11.96	-11.95	-11.94	-11.93	-11.92
-12	-11.99	-11.98	-11.97	-11.96	-11.95	-11.94	-11.93	-11.92
-12	-11.99	-11.98	-11.97	-11.96	-11.95	-11.94	-11.93	-11.92
-12	-11.99	-11.98	-11.97	-11.96	-11.95	-11.94	-11.93	-11.92
-12	-11.99	-11.98	-11.97	-11.96	-11.95	-11.94	-11.93	-11.92
-12	-11.99	-11.98	-11.97	-11.96	-11.95	-11.94	-11.93	-11.92
-12	-11.99	-11.98	-11.97	-11.96	-11.95	-11.94	-11.93	-11.92
-12	-11.99	-11.98	-11.97	-11.96	-11.95	-11.94	-11.93	-11.92

-12	-11.99	-11.98	-11.97	-11.96	-11.95	-11.94	-11.93	-11.92
-12	-11.99	-11.98	-11.97	-11.96	-11.95	-11.94	-11.93	-11.92
-12	-11.99	-11.98	-11.97	-11.96	-11.95	-11.94	-11.93	-11.92
-12	-11.99	-11.98	-11.97	-11.96	-11.95	-11.94	-11.93	-11.92
-12	-11.99	-11.98	-11.97	-11.96	-11.95	-11.94	-11.93	-11.92
-12	-11.99	-11.98	-11.97	-11.96	-11.95	-11.94	-11.93	-11.92
-12	-11.99	-11.98	-11.97	-11.96	-11.95	-11.94	-11.93	-11.92
-12	-11.99	-11.98	-11.97	-11.96	-11.95	-11.94	-11.93	-11.92
-12	-11.99	-11.98	-11.97	-11.96	-11.95	-11.94	-11.93	-11.92
-12	-11.99	-11.98	-11.97	-11.96	-11.95	-11.94	-11.93	-11.92
-12	-11.99	-11.98	-11.97	-11.96	-11.95	-11.94	-11.93	-11.92
-12	-11.99	-11.98	-11.97	-11.96	-11.95	-11.94	-11.93	-11.92
-12	-11.99	-11.98	-11.97	-11.96	-11.95	-11.94	-11.93	-11.92
-12	-11.99	-11.98	-11.97	-11.96	-11.95	-11.94	-11.93	-11.92
-12	-11.99	-11.98	-11.97	-11.96	-11.95	-11.94	-11.93	-11.92
-12	-11.99	-11.98	-11.97	-11.96	-11.95	-11.94	-11.93	-11.92
-12	-11.99	-11.98	-11.97	-11.96	-11.95	-11.94	-11.93	-11.92
-12	-11.99	-11.98	-11.97	-11.96	-11.95	-11.94	-11.93	-11.92
-12	-11.99	-11.98	-11.97	-11.96	-11.95	-11.94	-11.93	-11.92
-12	-11.99	-11.98	-11.97	-11.96	-11.95	-11.94	-11.93	-11.92
-12	-11.99	-11.98	-11.97	-11.96	-11.95	-11.94	-11.93	-11.92
-12	-11.99	-11.98	-11.97	-11.96	-11.95	-11.94	-11.93	-11.92
-12	-11.99	-11.98	-11.97	-11.96	-11.95	-11.94	-11.93	-11.92
-12	-11.99	-11.98	-11.97	-11.96	-11.95	-11.94	-11.93	-11.92
-12	-11.99	-11.98	-11.97	-11.96	-11.95	-11.94	-11.93	-11.92
-12	-11.99	-11.98	-11.97	-11.96	-11.95	-11.94	-11.93	-11.92
-12	-11.99	-11.98	-11.97	-11.96	-11.95	-11.94	-11.93	-11.92
-12	-11.99	-11.98	-11.97	-11.96	-11.95	-11.94	-11.93	-11.92
-12	-11.99	-11.98	-11.97	-11.96	-11.95	-11.94	-11.93	-11.92
-12	-11.99	-11.98	-11.97	-11.96	-11.95	-11.94	-11.93	-11.92
-12	-11.99	-11.98	-11.97	-11.96	-11.95	-11.94	-11.93	-11.92
-12	-11.99	-11.98	-11.97	-11.96	-11.95	-11.94	-11.93	-11.92
-12	-11.99	-11.98	-11.97	-11.96	-11.95	-11.94	-11.93	-11.92
-12	-11.99	-11.98	-11.97	-11.96	-11.95	-11.94	-11.93	-11.92
-12	-11.99	-11.98	-11.97	-11.96	-11.95	-11.94	-11.93	-11.92
-12	-11.99	-11.98	-11.97	-11.96	-11.95	-11.94	-11.93	-11.92
-12	-11.99	-11.98	-11.97	-11.96	-11.95	-11.94	-11.93	-11.92
-12	-11.99	-11.98	-11.97	-11.96	-11.95	-11.94	-11.93	-11.92
-12	-11.99	-11.98	-11.97	-11.96	-11.95	-11.94	-11.93	-11.92
-12	-11.99	-11.98	-11.97	-11.96	-11.95	-11.94	-11.93	-11.92
-12	-11.99	-11.98	-11.97	-11.96	-11.95	-11.94	-11.93	-11.92
-12	-11.99	-11.98	-11.97	-11.96	-11.95	-11.94	-11.93	-11.92
-12	-11.99	-11.98	-11.97	-11.96	-11.95	-11.94	-11.93	-11.92
-12	-11.99	-11.98	-11.97	-11.96	-11.95	-11.94	-11.93	-11.92
-12	-11.99	-11.98	-11.97	-11.96	-11.95	-11.94	-11.93	-11.92
-12	-11.99	-11.98	-11.97	-11.96	-11.95	-11.94	-11.93	-11.92
-12	-11.99	-11.98	-11.97	-11.96	-11.95	-11.94	-11.93	-11.92
-12	-11.99	-11.98	-11.97	-11.96	-11.95	-11.94	-11.93	-11.92
-12	-11.99	-11.98	-11.97	-11.96	-11.95	-11.94	-11.93	-11.92
-12	-11.99	-11.98	-11.97	-11.96	-11.95	-11.94	-11.93	-11.92

-12	-11.99	-11.98	-11.97	-11.96	-11.95	-11.94	-11.93	-11.92
-12	-11.99	-11.98	-11.97	-11.96	-11.95	-11.94	-11.93	-11.92
-12	-11.99	-11.98	-11.97	-11.96	-11.95	-11.94	-11.93	-11.92
-12	-11.99	-11.98	-11.97	-11.96	-11.95	-11.94	-11.93	-11.92
-12	-11.99	-11.98	-11.97	-11.96	-11.95	-11.94	-11.93	-11.92
-12	-11.99	-11.98	-11.97	-11.96	-11.95	-11.94	-11.93	-11.92
-12	-11.99	-11.98	-11.97	-11.96	-11.95	-11.94	-11.93	-11.92
-12	-11.99	-11.98	-11.97	-11.96	-11.95	-11.94	-11.93	-11.92
-12	-11.99	-11.98	-11.97	-11.96	-11.95	-11.94	-11.93	-11.92
-12	-11.99	-11.98	-11.97	-11.96	-11.95	-11.94	-11.93	-11.92
-12	-11.99	-11.98	-11.97	-11.96	-11.95	-11.94	-11.93	-11.92
-12	-11.99	-11.98	-11.97	-11.96	-11.95	-11.94	-11.93	-11.92
-12	-11.99	-11.98	-11.97	-11.96	-11.95	-11.94	-11.93	-11.92
-12	-11.99	-11.98	-11.97	-11.96	-11.95	-11.94	-11.93	-11.92
-12	-11.99	-11.98	-11.97	-11.96	-11.95	-11.94	-11.93	-11.92
-12	-11.99	-11.98	-11.97	-11.96	-11.95	-11.94	-11.93	-11.92
-12	-11.99	-11.98	-11.97	-11.96	-11.95	-11.94	-11.93	-11.92
-12	-11.99	-11.98	-11.97	-11.96	-11.95	-11.94	-11.93	-11.92
-12	-11.99	-11.98	-11.97	-11.96	-11.95	-11.94	-11.93	-11.92
-12	-11.99	-11.98	-11.97	-11.96	-11.95	-11.94	-11.93	-11.92
-12	-11.99	-11.98	-11.97	-11.96	-11.95	-11.94	-11.93	-11.92
-12	-11.99	-11.98	-11.97	-11.96	-11.95	-11.94	-11.93	-11.92
-12	-11.99	-11.98	-11.97	-11.96	-11.95	-11.94	-11.93	-11.92
-12	-11.99	-11.98	-11.97	-11.96	-11.95	-11.94	-11.93	-11.92
-12	-11.99	-11.98	-11.97	-11.96	-11.95	-11.94	-11.93	-11.92
-12	-11.99	-11.98	-11.97	-11.96	-11.95	-11.94	-11.93	-11.92
-12	-11.99	-11.98	-11.97	-11.96	-11.95	-11.94	-11.93	-11.92
-12	-11.99	-11.98	-11.97	-11.96	-11.95	-11.94	-11.93	-11.92
-12	-11.99	-11.98	-11.97	-11.96	-11.95	-11.94	-11.93	-11.92
-12	-11.99	-11.98	-11.97	-11.96	-11.95	-11.94	-11.93	-11.92
-12	-11.99	-11.98	-11.97	-11.96	-11.95	-11.94	-11.93	-11.92
-12	-11.99	-11.98	-11.97	-11.96	-11.95	-11.94	-11.93	-11.92
-12	-11.99	-11.98	-11.97	-11.96	-11.95	-11.94	-11.93	-11.92
-12	-11.99	-11.98	-11.97	-11.96	-11.95	-11.94	-11.93	-11.92
-12	-11.99	-11.98	-11.97	-11.96	-11.95	-11.94	-11.93	-11.92
-12	-11.99	-11.98	-11.97	-11.96	-11.95	-11.94	-11.93	-11.92
-12	-11.99	-11.98	-11.97	-11.96	-11.95	-11.94	-11.93	-11.92
-12	-11.99	-11.98	-11.97	-11.96	-11.95	-11.94	-11.93	-11.92
-12	-11.99	-11.98	-11.97	-11.96	-11.95	-11.94	-11.93	-11.92
-12	-11.99	-11.98	-11.97	-11.96	-11.95	-11.94	-11.93	-11.92
-12	-11.99	-11.98	-11.97	-11.96	-11.95	-11.94	-11.93	-11.92
-12	-11.99	-11.98	-11.97	-11.96	-11.95	-11.94	-11.93	-11.92
-12	-11.99	-11.98	-11.97	-11.96	-11.95	-11.94	-11.93	-11.92
-12	-11.99	-11.98	-11.97	-11.96	-11.95	-11.94	-11.93	-11.92
-12	-11.99	-11.98	-11.97	-11.96	-11.95	-11.94	-11.93	-11.92
-12	-11.99	-11.98	-11.97	-11.96	-11.95	-11.94	-11.93	-11.92
-12	-11.99	-11.98	-11.97	-11.96	-11.95	-11.94	-11.93	-11.92
-12	-11.99	-11.98	-11.97	-11.96	-11.95	-11.94	-11.93	-11.92
-12	-11.99	-11.98	-11.97	-11.96	-11.95	-11.94	-11.93	-11.92
-12	-11.99	-11.98	-11.97	-11.96	-11.95	-11.94	-11.93	-11.92

-12	-11.99	-11.98	-11.97	-11.96	-11.95	-11.94	-11.93	-11.92
-12	-11.99	-11.98	-11.97	-11.96	-11.95	-11.94	-11.93	-11.92
-12	-11.99	-11.98	-11.97	-11.96	-11.95	-11.94	-11.93	-11.92
-12	-11.99	-11.98	-11.97	-11.96	-11.95	-11.94	-11.93	-11.92
-12	-11.99	-11.98	-11.97	-11.96	-11.95	-11.94	-11.93	-11.92
-12	-11.99	-11.98	-11.97	-11.96	-11.95	-11.94	-11.93	-11.92
-12	-11.99	-11.98	-11.97	-11.96	-11.95	-11.94	-11.93	-11.92
-12	-11.99	-11.98	-11.97	-11.96	-11.95	-11.94	-11.93	-11.92
-12	-11.99	-11.98	-11.97	-11.96	-11.95	-11.94	-11.93	-11.92
-12	-11.99	-11.98	-11.97	-11.96	-11.95	-11.94	-11.93	-11.92
-12	-11.99	-11.98	-11.97	-11.96	-11.95	-11.94	-11.93	-11.92
-12	-11.99	-11.98	-11.97	-11.96	-11.95	-11.94	-11.93	-11.92
-12	-11.99	-11.98	-11.97	-11.96	-11.95	-11.94	-11.93	-11.92
-12	-11.99	-11.98	-11.97	-11.96	-11.95	-11.94	-11.93	-11.92
-12	-11.99	-11.98	-11.97	-11.96	-11.95	-11.94	-11.93	-11.92
-12	-11.99	-11.98	-11.97	-11.96	-11.95	-11.94	-11.93	-11.92
-12	-11.99	-11.98	-11.97	-11.96	-11.95	-11.94	-11.93	-11.92
-12	-11.99	-11.98	-11.97	-11.96	-11.95	-11.94	-11.93	-11.92
-12	-11.99	-11.98	-11.97	-11.96	-11.95	-11.94	-11.93	-11.92
-12	-11.99	-11.98	-11.97	-11.96	-11.95	-11.94	-11.93	-11.92
-12	-11.99	-11.98	-11.97	-11.96	-11.95	-11.94	-11.93	-11.92
-12	-11.99	-11.98	-11.97	-11.96	-11.95	-11.94	-11.93	-11.92
-12	-11.99	-11.98	-11.97	-11.96	-11.95	-11.94	-11.93	-11.92
-12	-11.99	-11.98	-11.97	-11.96	-11.95	-11.94	-11.93	-11.92
-12	-11.99	-11.98	-11.97	-11.96	-11.95	-11.94	-11.93	-11.92
-12	-11.99	-11.98	-11.97	-11.96	-11.95	-11.94	-11.93	-11.92
-12	-11.99	-11.98	-11.97	-11.96	-11.95	-11.94	-11.93	-11.92
-12	-11.99	-11.98	-11.97	-11.96	-11.95	-11.94	-11.93	-11.92
-12	-11.99	-11.98	-11.97	-11.96	-11.95	-11.94	-11.93	-11.92
-12	-11.99	-11.98	-11.97	-11.96	-11.95	-11.94	-11.93	-11.92
-12	-11.99	-11.98	-11.97	-11.96	-11.95	-11.94	-11.93	-11.92
-12	-11.99	-11.98	-11.97	-11.96	-11.95	-11.94	-11.93	-11.92
-12	-11.99	-11.98	-11.97	-11.96	-11.95	-11.94	-11.93	-11.92
-12	-11.99	-11.98	-11.97	-11.96	-11.95	-11.94	-11.93	-11.92
-12	-11.99	-11.98	-11.97	-11.96	-11.95	-11.94	-11.93	-11.92
-12	-11.99	-11.98	-11.97	-11.96	-11.95	-11.94	-11.93	-11.92
-12	-11.99	-11.98	-11.97	-11.96	-11.95	-11.94	-11.93	-11.92
-12	-11.99	-11.98	-11.97	-11.96	-11.95	-11.94	-11.93	-11.92
-12	-11.99	-11.98	-11.97	-11.96	-11.95	-11.94	-11.93	-11.92
-12	-11.99	-11.98	-11.97	-11.96	-11.95	-11.94	-11.93	-11.92
-12	-11.99	-11.98	-11.97	-11.96	-11.95	-11.94	-11.93	-11.92
-12	-11.99	-11.98	-11.97	-11.96	-11.95	-11.94	-11.93	-11.92
-12	-11.99	-11.98	-11.97	-11.96	-11.95	-11.94	-11.93	-11.92
-12	-11.99	-11.98	-11.97	-11.96	-11.95	-11.94	-11.93	-11.92
-12	-11.99	-11.98	-11.97	-11.96	-11.95	-11.94	-11.93	-11.92
-12	-11.99	-11.98	-11.97	-11.96	-11.95	-11.94	-11.93	-11.92
-12	-11.99	-11.98	-11.97	-11.96	-11.95	-11.94	-11.93	-11.92
-12	-11.99	-11.98	-11.97	-11.96	-11.95	-11.94	-11.93	-11.92
-12	-11.99	-11.98	-11.97	-11.96	-11.95	-11.94	-11.93	-11.92
-12	-11.99	-11.98	-11.97	-11.96	-11.95	-11.94	-11.93	-11.92

-12	-11.99	-11.98	-11.97	-11.96	-11.95	-11.94	-11.93	-11.92
-12	-11.99	-11.98	-11.97	-11.96	-11.95	-11.94	-11.93	-11.92
-12	-11.99	-11.98	-11.97	-11.96	-11.95	-11.94	-11.93	-11.92
-12	-11.99	-11.98	-11.97	-11.96	-11.95	-11.94	-11.93	-11.92
-12	-11.99	-11.98	-11.97	-11.96	-11.95	-11.94	-11.93	-11.92
-12	-11.99	-11.98	-11.97	-11.96	-11.95	-11.94	-11.93	-11.92
-12	-11.99	-11.98	-11.97	-11.96	-11.95	-11.94	-11.93	-11.92
-12	-11.99	-11.98	-11.97	-11.96	-11.95	-11.94	-11.93	-11.92
-12	-11.99	-11.98	-11.97	-11.96	-11.95	-11.94	-11.93	-11.92
-12	-11.99	-11.98	-11.97	-11.96	-11.95	-11.94	-11.93	-11.92
-12	-11.99	-11.98	-11.97	-11.96	-11.95	-11.94	-11.93	-11.92
-12	-11.99	-11.98	-11.97	-11.96	-11.95	-11.94	-11.93	-11.92
-12	-11.99	-11.98	-11.97	-11.96	-11.95	-11.94	-11.93	-11.92
-12	-11.99	-11.98	-11.97	-11.96	-11.95	-11.94	-11.93	-11.92
-12	-11.99	-11.98	-11.97	-11.96	-11.95	-11.94	-11.93	-11.92
-12	-11.99	-11.98	-11.97	-11.96	-11.95	-11.94	-11.93	-11.92
-12	-11.99	-11.98	-11.97	-11.96	-11.95	-11.94	-11.93	-11.92
-12	-11.99	-11.98	-11.97	-11.96	-11.95	-11.94	-11.93	-11.92
-12	-11.99	-11.98	-11.97	-11.96	-11.95	-11.94	-11.93	-11.92
-12	-11.99	-11.98	-11.97	-11.96	-11.95	-11.94	-11.93	-11.92
-12	-11.99	-11.98	-11.97	-11.96	-11.95	-11.94	-11.93	-11.92
-12	-11.99	-11.98	-11.97	-11.96	-11.95	-11.94	-11.93	-11.92
-12	-11.99	-11.98	-11.97	-11.96	-11.95	-11.94	-11.93	-11.92
-12	-11.99	-11.98	-11.97	-11.96	-11.95	-11.94	-11.93	-11.92
-12	-11.99	-11.98	-11.97	-11.96	-11.95	-11.94	-11.93	-11.92
-12	-11.99	-11.98	-11.97	-11.96	-11.95	-11.94	-11.93	-11.92
-12	-11.99	-11.98	-11.97	-11.96	-11.95	-11.94	-11.93	-11.92
-12	-11.99	-11.98	-11.97	-11.96	-11.95	-11.94	-11.93	-11.92
-12	-11.99	-11.98	-11.97	-11.96	-11.95	-11.94	-11.93	-11.92
-12	-11.99	-11.98	-11.97	-11.96	-11.95	-11.94	-11.93	-11.92
-12	-11.99	-11.98	-11.97	-11.96	-11.95	-11.94	-11.93	-11.92
-12	-11.99	-11.98	-11.97	-11.96	-11.95	-11.94	-11.93	-11.92
-12	-11.99	-11.98	-11.97	-11.96	-11.95	-11.94	-11.93	-11.92
-12	-11.99	-11.98	-11.97	-11.96	-11.95	-11.94	-11.93	-11.92
-12	-11.99	-11.98	-11.97	-11.96	-11.95	-11.94	-11.93	-11.92
-12	-11.99	-11.98	-11.97	-11.96	-11.95	-11.94	-11.93	-11.92
-12	-11.99	-11.98	-11.97	-11.96	-11.95	-11.94	-11.93	-11.92
-12	-11.99	-11.98	-11.97	-11.96	-11.95	-11.94	-11.93	-11.92
-12	-11.99	-11.98	-11.97	-11.96	-11.95	-11.94	-11.93	-11.92
-12	-11.99	-11.98	-11.97	-11.96	-11.95	-11.94	-11.93	-11.92
-12	-11.99	-11.98	-11.97	-11.96	-11.95	-11.94	-11.93	-11.92
-12	-11.99	-11.98	-11.97	-11.96	-11.95	-11.94	-11.93	-11.92
-12	-11.99	-11.98	-11.97	-11.96	-11.95	-11.94	-11.93	-11.92
-12	-11.99	-11.98	-11.97	-11.96	-11.95	-11.94	-11.93	-11.92
-12	-11.99	-11.98	-11.97	-11.96	-11.95	-11.94	-11.93	-11.92
-12	-11.99	-11.98	-11.97	-11.96	-11.95	-11.94	-11.93	-11.92
-12	-11.99	-11.98	-11.97	-11.96	-11.95	-11.94	-11.93	-11.92
-12	-11.99	-11.98	-11.97	-11.96	-11.95	-11.94	-11.93	-11.92
-12	-11.99	-11.98	-11.97	-11.96	-11.95	-11.94	-11.93	-11.92
-12	-11.99	-11.98	-11.97	-11.96	-11.95	-11.94	-11.93	-11.92

-12	-11.99	-11.98	-11.97	-11.96	-11.95	-11.94	-11.93	-11.92
-12	-11.99	-11.98	-11.97	-11.96	-11.95	-11.94	-11.93	-11.92
-12	-11.99	-11.98	-11.97	-11.96	-11.95	-11.94	-11.93	-11.92
-12	-11.99	-11.98	-11.97	-11.96	-11.95	-11.94	-11.93	-11.92
-12	-11.99	-11.98	-11.97	-11.96	-11.95	-11.94	-11.93	-11.92
-12	-11.99	-11.98	-11.97	-11.96	-11.95	-11.94	-11.93	-11.92
-12	-11.99	-11.98	-11.97	-11.96	-11.95	-11.94	-11.93	-11.92
-12	-11.99	-11.98	-11.97	-11.96	-11.95	-11.94	-11.93	-11.92
-12	-11.99	-11.98	-11.97	-11.96	-11.95	-11.94	-11.93	-11.92
-12	-11.99	-11.98	-11.97	-11.96	-11.95	-11.94	-11.93	-11.92
-12	-11.99	-11.98	-11.97	-11.96	-11.95	-11.94	-11.93	-11.92
-12	-11.99	-11.98	-11.97	-11.96	-11.95	-11.94	-11.93	-11.92
-12	-11.99	-11.98	-11.97	-11.96	-11.95	-11.94	-11.93	-11.92
-12	-11.99	-11.98	-11.97	-11.96	-11.95	-11.94	-11.93	-11.92
-12	-11.99	-11.98	-11.97	-11.96	-11.95	-11.94	-11.93	-11.92
-12	-11.99	-11.98	-11.97	-11.96	-11.95	-11.94	-11.93	-11.92
-12	-11.99	-11.98	-11.97	-11.96	-11.95	-11.94	-11.93	-11.92
-12	-11.99	-11.98	-11.97	-11.96	-11.95	-11.94	-11.93	-11.92
-12	-11.99	-11.98	-11.97	-11.96	-11.95	-11.94	-11.93	-11.92
-12	-11.99	-11.98	-11.97	-11.96	-11.95	-11.94	-11.93	-11.92
-12	-11.99	-11.98	-11.97	-11.96	-11.95	-11.94	-11.93	-11.92
-12	-11.99	-11.98	-11.97	-11.96	-11.95	-11.94	-11.93	-11.92
-12	-11.99	-11.98	-11.97	-11.96	-11.95	-11.94	-11.93	-11.92
-12	-11.99	-11.98	-11.97	-11.96	-11.95	-11.94	-11.93	-11.92
-12	-11.99	-11.98	-11.97	-11.96	-11.95	-11.94	-11.93	-11.92
-12	-11.99	-11.98	-11.97	-11.96	-11.95	-11.94	-11.93	-11.92
-12	-11.99	-11.98	-11.97	-11.96	-11.95	-11.94	-11.93	-11.92
-12	-11.99	-11.98	-11.97	-11.96	-11.95	-11.94	-11.93	-11.92
-12	-11.99	-11.98	-11.97	-11.96	-11.95	-11.94	-11.93	-11.92
-12	-11.99	-11.98	-11.97	-11.96	-11.95	-11.94	-11.93	-11.92
-12	-11.99	-11.98	-11.97	-11.96	-11.95	-11.94	-11.93	-11.92
-12	-11.99	-11.98	-11.97	-11.96	-11.95	-11.94	-11.93	-11.92
-12	-11.99	-11.98	-11.97	-11.96	-11.95	-11.94	-11.93	-11.92
-12	-11.99	-11.98	-11.97	-11.96	-11.95	-11.94	-11.93	-11.92
-12	-11.99	-11.98	-11.97	-11.96	-11.95	-11.94	-11.93	-11.92
-12	-11.99	-11.98	-11.97	-11.96	-11.95	-11.94	-11.93	-11.92
-12	-11.99	-11.98	-11.97	-11.96	-11.95	-11.94	-11.93	-11.92
-12	-11.99	-11.98	-11.97	-11.96	-11.95	-11.94	-11.93	-11.92
-12	-11.99	-11.98	-11.97	-11.96	-11.95	-11.94	-11.93	-11.92
-12	-11.99	-11.98	-11.97	-11.96	-11.95	-11.94	-11.93	-11.92
-12	-11.99	-11.98	-11.97	-11.96	-11.95	-11.94	-11.93	-11.92
-12	-11.99	-11.98	-11.97	-11.96	-11.95	-11.94	-11.93	-11.92
-12	-11.99	-11.98	-11.97	-11.96	-11.95	-11.94	-11.93	-11.92
-12	-11.99	-11.98	-11.97	-11.96	-11.95	-11.94	-11.93	-11.92
-12	-11.99	-11.98	-11.97	-11.96	-11.95	-11.94	-11.93	-11.92
-12	-11.99	-11.98	-11.97	-11.96	-11.95	-11.94	-11.93	-11.92
-12	-11.99	-11.98	-11.97	-11.96	-11.95	-11.94	-11.93	-11.92
-12	-11.99	-11.98	-11.97	-11.96	-11.95	-11.94	-11.93	-11.92
-12	-11.99	-11.98	-11.97	-11.96	-11.95	-11.94	-11.93	-11.92
-12	-11.99	-11.98	-11.97	-11.96	-11.95	-11.94	-11.93	-11.92

-12	-11.99	-11.98	-11.97	-11.96	-11.95	-11.94	-11.93	-11.92
-12	-11.99	-11.98	-11.97	-11.96	-11.95	-11.94	-11.93	-11.92
-12	-11.99	-11.98	-11.97	-11.96	-11.95	-11.94	-11.93	-11.92
-12	-11.99	-11.98	-11.97	-11.96	-11.95	-11.94	-11.93	-11.92
-12	-11.99	-11.98	-11.97	-11.96	-11.95	-11.94	-11.93	-11.92
-12	-11.99	-11.98	-11.97	-11.96	-11.95	-11.94	-11.93	-11.92
-12	-11.99	-11.98	-11.97	-11.96	-11.95	-11.94	-11.93	-11.92
-12	-11.99	-11.98	-11.97	-11.96	-11.95	-11.94	-11.93	-11.92
-12	-11.99	-11.98	-11.97	-11.96	-11.95	-11.94	-11.93	-11.92
-12	-11.99	-11.98	-11.97	-11.96	-11.95	-11.94	-11.93	-11.92
-12	-11.99	-11.98	-11.97	-11.96	-11.95	-11.94	-11.93	-11.92
-12	-11.99	-11.98	-11.97	-11.96	-11.95	-11.94	-11.93	-11.92
-12	-11.99	-11.98	-11.97	-11.96	-11.95	-11.94	-11.93	-11.92
-12	-11.99	-11.98	-11.97	-11.96	-11.95	-11.94	-11.93	-11.92
-12	-11.99	-11.98	-11.97	-11.96	-11.95	-11.94	-11.93	-11.92
-12	-11.99	-11.98	-11.97	-11.96	-11.95	-11.94	-11.93	-11.92
-12	-11.99	-11.98	-11.97	-11.96	-11.95	-11.94	-11.93	-11.92
-12	-11.99	-11.98	-11.97	-11.96	-11.95	-11.94	-11.93	-11.92
-12	-11.99	-11.98	-11.97	-11.96	-11.95	-11.94	-11.93	-11.92
-12	-11.99	-11.98	-11.97	-11.96	-11.95	-11.94	-11.93	-11.92
-12	-11.99	-11.98	-11.97	-11.96	-11.95	-11.94	-11.93	-11.92
-12	-11.99	-11.98	-11.97	-11.96	-11.95	-11.94	-11.93	-11.92
-12	-11.99	-11.98	-11.97	-11.96	-11.95	-11.94	-11.93	-11.92
-12	-11.99	-11.98	-11.97	-11.96	-11.95	-11.94	-11.93	-11.92
-12	-11.99	-11.98	-11.97	-11.96	-11.95	-11.94	-11.93	-11.92
-12	-11.99	-11.98	-11.97	-11.96	-11.95	-11.94	-11.93	-11.92
-12	-11.99	-11.98	-11.97	-11.96	-11.95	-11.94	-11.93	-11.92
-12	-11.99	-11.98	-11.97	-11.96	-11.95	-11.94	-11.93	-11.92
-12	-11.99	-11.98	-11.97	-11.96	-11.95	-11.94	-11.93	-11.92
-12	-11.99	-11.98	-11.97	-11.96	-11.95	-11.94	-11.93	-11.92
-12	-11.99	-11.98	-11.97	-11.96	-11.95	-11.94	-11.93	-11.92
-12	-11.99	-11.98	-11.97	-11.96	-11.95	-11.94	-11.93	-11.92
-12	-11.99	-11.98	-11.97	-11.96	-11.95	-11.94	-11.93	-11.92
-12	-11.99	-11.98	-11.97	-11.96	-11.95	-11.94	-11.93	-11.92
-12	-11.99	-11.98	-11.97	-11.96	-11.95	-11.94	-11.93	-11.92
-12	-11.99	-11.98	-11.97	-11.96	-11.95	-11.94	-11.93	-11.92
-12	-11.99	-11.98	-11.97	-11.96	-11.95	-11.94	-11.93	-11.92
-12	-11.99	-11.98	-11.97	-11.96	-11.95	-11.94	-11.93	-11.92
-12	-11.99	-11.98	-11.97	-11.96	-11.95	-11.94	-11.93	-11.92
-12	-11.99	-11.98	-11.97	-11.96	-11.95	-11.94	-11.93	-11.92
-12	-11.99	-11.98	-11.97	-11.96	-11.95	-11.94	-11.93	-11.92
-12	-11.99	-11.98	-11.97	-11.96	-11.95	-11.94	-11.93	-11.92
-12	-11.99	-11.98	-11.97	-11.96	-11.95	-11.94	-11.93	-11.92
-12	-11.99	-11.98	-11.97	-11.96	-11.95	-11.94	-11.93	-11.92
-12	-11.99	-11.98	-11.97	-11.96	-11.95	-11.94	-11.93	-11.92
-12	-11.99	-11.98	-11.97	-11.96	-11.95	-11.94	-11.93	-11.92
-12	-11.99	-11.98	-11.97	-11.96	-11.95	-11.94	-11.93	-11.92
-12	-11.99	-11.98	-11.97	-11.96	-11.95	-11.94	-11.93	-11.92
-12	-11.99	-11.98	-11.97	-11.96	-11.95	-11.94	-11.93	-11.92
-12	-11.99	-11.98	-11.97	-11.96	-11.95	-11.94	-11.93	-11.92

-12	-11.99	-11.98	-11.97	-11.96	-11.95	-11.94	-11.93	-11.92
-12	-11.99	-11.98	-11.97	-11.96	-11.95	-11.94	-11.93	-11.92
-12	-11.99	-11.98	-11.97	-11.96	-11.95	-11.94	-11.93	-11.92
-12	-11.99	-11.98	-11.97	-11.96	-11.95	-11.94	-11.93	-11.92
-12	-11.99	-11.98	-11.97	-11.96	-11.95	-11.94	-11.93	-11.92
-12	-11.99	-11.98	-11.97	-11.96	-11.95	-11.94	-11.93	-11.92
-12	-11.99	-11.98	-11.97	-11.96	-11.95	-11.94	-11.93	-11.92
-12	-11.99	-11.98	-11.97	-11.96	-11.95	-11.94	-11.93	-11.92
-12	-11.99	-11.98	-11.97	-11.96	-11.95	-11.94	-11.93	-11.92
-12	-11.99	-11.98	-11.97	-11.96	-11.95	-11.94	-11.93	-11.92
-12	-11.99	-11.98	-11.97	-11.96	-11.95	-11.94	-11.93	-11.92
-12	-11.99	-11.98	-11.97	-11.96	-11.95	-11.94	-11.93	-11.92
-12	-11.99	-11.98	-11.97	-11.96	-11.95	-11.94	-11.93	-11.92
-12	-11.99	-11.98	-11.97	-11.96	-11.95	-11.94	-11.93	-11.92
-12	-11.99	-11.98	-11.97	-11.96	-11.95	-11.94	-11.93	-11.92
-12	-11.99	-11.98	-11.97	-11.96	-11.95	-11.94	-11.93	-11.92
-12	-11.99	-11.98	-11.97	-11.96	-11.95	-11.94	-11.93	-11.92
-12	-11.99	-11.98	-11.97	-11.96	-11.95	-11.94	-11.93	-11.92
-12	-11.99	-11.98	-11.97	-11.96	-11.95	-11.94	-11.93	-11.92
-12	-11.99	-11.98	-11.97	-11.96	-11.95	-11.94	-11.93	-11.92
-12	-11.99	-11.98	-11.97	-11.96	-11.95	-11.94	-11.93	-11.92
-12	-11.99	-11.98	-11.97	-11.96	-11.95	-11.94	-11.93	-11.92
-12	-11.99	-11.98	-11.97	-11.96	-11.95	-11.94	-11.93	-11.92
-12	-11.99	-11.98	-11.97	-11.96	-11.95	-11.94	-11.93	-11.92
-12	-11.99	-11.98	-11.97	-11.96	-11.95	-11.94	-11.93	-11.92
-12	-11.99	-11.98	-11.97	-11.96	-11.95	-11.94	-11.93	-11.92
-12	-11.99	-11.98	-11.97	-11.96	-11.95	-11.94	-11.93	-11.92
-12	-11.99	-11.98	-11.97	-11.96	-11.95	-11.94	-11.93	-11.92
-12	-11.99	-11.98	-11.97	-11.96	-11.95	-11.94	-11.93	-11.92
-12	-11.99	-11.98	-11.97	-11.96	-11.95	-11.94	-11.93	-11.92
-12	-11.99	-11.98	-11.97	-11.96	-11.95	-11.94	-11.93	-11.92
-12	-11.99	-11.98	-11.97	-11.96	-11.95	-11.94	-11.93	-11.92
-12	-11.99	-11.98	-11.97	-11.96	-11.95	-11.94	-11.93	-11.92
-12	-11.99	-11.98	-11.97	-11.96	-11.95	-11.94	-11.93	-11.92
-12	-11.99	-11.98	-11.97	-11.96	-11.95	-11.94	-11.93	-11.92
-12	-11.99	-11.98	-11.97	-11.96	-11.95	-11.94	-11.93	-11.92
-12	-11.99	-11.98	-11.97	-11.96	-11.95	-11.94	-11.93	-11.92
-12	-11.99	-11.98	-11.97	-11.96	-11.95	-11.94	-11.93	-11.92
-12	-11.99	-11.98	-11.97	-11.96	-11.95	-11.94	-11.93	-11.92
-12	-11.99	-11.98	-11.97	-11.96	-11.95	-11.94	-11.93	-11.92
-12	-11.99	-11.98	-11.97	-11.96	-11.95	-11.94	-11.93	-11.92
-12	-11.99	-11.98	-11.97	-11.96	-11.95	-11.94	-11.93	-11.92
-12	-11.99	-11.98	-11.97	-11.96	-11.95	-11.94	-11.93	-11.92
-12	-11.99	-11.98	-11.97	-11.96	-11.95	-11.94	-11.93	-11.92
-12	-11.99	-11.98	-11.97	-11.96	-11.95	-11.94	-11.93	-11.92
-12	-11.99	-11.98	-11.97	-11.96	-11.95	-11.94	-11.93	-11.92
-12	-11.99	-11.98	-11.97	-11.96	-11.95	-11.94	-11.93	-11.92
-12	-11.99	-11.98	-11.97	-11.96	-11.95	-11.94	-11.93	-11.92
-12	-11.99	-11.98	-11.97	-11.96	-11.95	-11.94	-11.93	-11.92
-12	-11.99	-11.98	-11.97	-11.96	-11.95	-11.94	-11.93	-11.92

-12	-11.99	-11.98	-11.97	-11.96	-11.95	-11.94	-11.93	-11.92
-12	-11.99	-11.98	-11.97	-11.96	-11.95	-11.94	-11.93	-11.92
-12	-11.99	-11.98	-11.97	-11.96	-11.95	-11.94	-11.93	-11.92
-12	-11.99	-11.98	-11.97	-11.96	-11.95	-11.94	-11.93	-11.92
-12	-11.99	-11.98	-11.97	-11.96	-11.95	-11.94	-11.93	-11.92
-12	-11.99	-11.98	-11.97	-11.96	-11.95	-11.94	-11.93	-11.92
-12	-11.99	-11.98	-11.97	-11.96	-11.95	-11.94	-11.93	-11.92
-12	-11.99	-11.98	-11.97	-11.96	-11.95	-11.94	-11.93	-11.92
-12	-11.99	-11.98	-11.97	-11.96	-11.95	-11.94	-11.93	-11.92
-12	-11.99	-11.98	-11.97	-11.96	-11.95	-11.94	-11.93	-11.92
-12	-11.99	-11.98	-11.97	-11.96	-11.95	-11.94	-11.93	-11.92
-12	-11.99	-11.98	-11.97	-11.96	-11.95	-11.94	-11.93	-11.92
-12	-11.99	-11.98	-11.97	-11.96	-11.95	-11.94	-11.93	-11.92
-12	-11.99	-11.98	-11.97	-11.96	-11.95	-11.94	-11.93	-11.92
-12	-11.99	-11.98	-11.97	-11.96	-11.95	-11.94	-11.93	-11.92
-12	-11.99	-11.98	-11.97	-11.96	-11.95	-11.94	-11.93	-11.92
-12	-11.99	-11.98	-11.97	-11.96	-11.95	-11.94	-11.93	-11.92
-12	-11.99	-11.98	-11.97	-11.96	-11.95	-11.94	-11.93	-11.92
-12	-11.99	-11.98	-11.97	-11.96	-11.95	-11.94	-11.93	-11.92
-12	-11.99	-11.98	-11.97	-11.96	-11.95	-11.94	-11.93	-11.92
-12	-11.99	-11.98	-11.97	-11.96	-11.95	-11.94	-11.93	-11.92
-12	-11.99	-11.98	-11.97	-11.96	-11.95	-11.94	-11.93	-11.92
-12	-11.99	-11.98	-11.97	-11.96	-11.95	-11.94	-11.93	-11.92
-12	-11.99	-11.98	-11.97	-11.96	-11.95	-11.94	-11.93	-11.92
-12	-11.99	-11.98	-11.97	-11.96	-11.95	-11.94	-11.93	-11.92
-12	-11.99	-11.98	-11.97	-11.96	-11.95	-11.94	-11.93	-11.92
-12	-11.99	-11.98	-11.97	-11.96	-11.95	-11.94	-11.93	-11.92
-12	-11.99	-11.98	-11.97	-11.96	-11.95	-11.94	-11.93	-11.92
-12	-11.99	-11.98	-11.97	-11.96	-11.95	-11.94	-11.93	-11.92
-12	-11.99	-11.98	-11.97	-11.96	-11.95	-11.94	-11.93	-11.92
-12	-11.99	-11.98	-11.97	-11.96	-11.95	-11.94	-11.93	-11.92
-12	-11.99	-11.98	-11.97	-11.96	-11.95	-11.94	-11.93	-11.92
-12	-11.99	-11.98	-11.97	-11.96	-11.95	-11.94	-11.93	-11.92
-12	-11.99	-11.98	-11.97	-11.96	-11.95	-11.94	-11.93	-11.92
-12	-11.99	-11.98	-11.97	-11.96	-11.95	-11.94	-11.93	-11.92
-12	-11.99	-11.98	-11.97	-11.96	-11.95	-11.94	-11.93	-11.92
-12	-11.99	-11.98	-11.97	-11.96	-11.95	-11.94	-11.93	-11.92
-12	-11.99	-11.98	-11.97	-11.96	-11.95	-11.94	-11.93	-11.92
-12	-11.99	-11.98	-11.97	-11.96	-11.95	-11.94	-11.93	-11.92
-12	-11.99	-11.98	-11.97	-11.96	-11.95	-11.94	-11.93	-11.92
-12	-11.99	-11.98	-11.97	-11.96	-11.95	-11.94	-11.93	-11.92
-12	-11.99	-11.98	-11.97	-11.96	-11.95	-11.94	-11.93	-11.92
-12	-11.99	-11.98	-11.97	-11.96	-11.95	-11.94	-11.93	-11.92
-12	-11.99	-11.98	-11.97	-11.96	-11.95	-11.94	-11.93	-11.92
-12	-11.99	-11.98	-11.97	-11.96	-11.95	-11.94	-11.93	-11.92
-12	-11.99	-11.98	-11.97	-11.96	-11.95	-11.94	-11.93	-11.92
-12	-11.99	-11.98	-11.97	-11.96	-11.95	-11.94	-11.93	-11.92
-12	-11.99	-11.98	-11.97	-11.96	-11.95	-11.94	-11.93	-11.92
-12	-11.99	-11.98	-11.97	-11.96	-11.95	-11.94	-11.93	-11.92
-12	-11.99	-11.98	-11.97	-11.96	-11.95	-11.94	-11.93	-11.92

-12	-11.99	-11.98	-11.97	-11.96	-11.95	-11.94	-11.93	-11.92
-12	-11.99	-11.98	-11.97	-11.96	-11.95	-11.94	-11.93	-11.92
-12	-11.99	-11.98	-11.97	-11.96	-11.95	-11.94	-11.93	-11.92
-12	-11.99	-11.98	-11.97	-11.96	-11.95	-11.94	-11.93	-11.92
-12	-11.99	-11.98	-11.97	-11.96	-11.95	-11.94	-11.93	-11.92
-12	-11.99	-11.98	-11.97	-11.96	-11.95	-11.94	-11.93	-11.92
-12	-11.99	-11.98	-11.97	-11.96	-11.95	-11.94	-11.93	-11.92
-12	-11.99	-11.98	-11.97	-11.96	-11.95	-11.94	-11.93	-11.92
-12	-11.99	-11.98	-11.97	-11.96	-11.95	-11.94	-11.93	-11.92
-12	-11.99	-11.98	-11.97	-11.96	-11.95	-11.94	-11.93	-11.92
-12	-11.99	-11.98	-11.97	-11.96	-11.95	-11.94	-11.93	-11.92
-12	-11.99	-11.98	-11.97	-11.96	-11.95	-11.94	-11.93	-11.92
-12	-11.99	-11.98	-11.97	-11.96	-11.95	-11.94	-11.93	-11.92
-12	-11.99	-11.98	-11.97	-11.96	-11.95	-11.94	-11.93	-11.92
-12	-11.99	-11.98	-11.97	-11.96	-11.95	-11.94	-11.93	-11.92
-12	-11.99	-11.98	-11.97	-11.96	-11.95	-11.94	-11.93	-11.92
-12	-11.99	-11.98	-11.97	-11.96	-11.95	-11.94	-11.93	-11.92
-12	-11.99	-11.98	-11.97	-11.96	-11.95	-11.94	-11.93	-11.92
-12	-11.99	-11.98	-11.97	-11.96	-11.95	-11.94	-11.93	-11.92
-12	-11.99	-11.98	-11.97	-11.96	-11.95	-11.94	-11.93	-11.92
-12	-11.99	-11.98	-11.97	-11.96	-11.95	-11.94	-11.93	-11.92
-12	-11.99	-11.98	-11.97	-11.96	-11.95	-11.94	-11.93	-11.92
-12	-11.99	-11.98	-11.97	-11.96	-11.95	-11.94	-11.93	-11.92
-12	-11.99	-11.98	-11.97	-11.96	-11.95	-11.94	-11.93	-11.92
-12	-11.99	-11.98	-11.97	-11.96	-11.95	-11.94	-11.93	-11.92
-12	-11.99	-11.98	-11.97	-11.96	-11.95	-11.94	-11.93	-11.92
-12	-11.99	-11.98	-11.97	-11.96	-11.95	-11.94	-11.93	-11.92
-12	-11.99	-11.98	-11.97	-11.96	-11.95	-11.94	-11.93	-11.92
-12	-11.99	-11.98	-11.97	-11.96	-11.95	-11.94	-11.93	-11.92
-12	-11.99	-11.98	-11.97	-11.96	-11.95	-11.94	-11.93	-11.92
-12	-11.99	-11.98	-11.97	-11.96	-11.95	-11.94	-11.93	-11.92
-12	-11.99	-11.98	-11.97	-11.96	-11.95	-11.94	-11.93	-11.92
-12	-11.99	-11.98	-11.97	-11.96	-11.95	-11.94	-11.93	-11.92
-12	-11.99	-11.98	-11.97	-11.96	-11.95	-11.94	-11.93	-11.92
-12	-11.99	-11.98	-11.97	-11.96	-11.95	-11.94	-11.93	-11.92
-12	-11.99	-11.98	-11.97	-11.96	-11.95	-11.94	-11.93	-11.92
-12	-11.99	-11.98	-11.97	-11.96	-11.95	-11.94	-11.93	-11.92
-12	-11.99	-11.98	-11.97	-11.96	-11.95	-11.94	-11.93	-11.92
-12	-11.99	-11.98	-11.97	-11.96	-11.95	-11.94	-11.93	-11.92
-12	-11.99	-11.98	-11.97	-11.96	-11.95	-11.94	-11.93	-11.92
-12	-11.99	-11.98	-11.97	-11.96	-11.95	-11.94	-11.93	-11.92
-12	-11.99	-11.98	-11.97	-11.96	-11.95	-11.94	-11.93	-11.92
-12	-11.99	-11.98	-11.97	-11.96	-11.95	-11.94	-11.93	-11.92
-12	-11.99	-11.98	-11.97	-11.96	-11.95	-11.94	-11.93	-11.92
-12	-11.99	-11.98	-11.97	-11.96	-11.95	-11.94	-11.93	-11.92
-12	-11.99	-11.98	-11.97	-11.96	-11.95	-11.94	-11.93	-11.92
-12	-11.99	-11.98	-11.97	-11.96	-11.95	-11.94	-11.93	-11.92
-12	-11.99	-11.98	-11.97	-11.96	-11.95	-11.94	-11.93	-11.92
-12	-11.99	-11.98	-11.97	-11.96	-11.95	-11.94	-11.93	-11.92
-12	-11.99	-11.98	-11.97	-11.96	-11.95	-11.94	-11.93	-11.92

-12	-11.99	-11.98	-11.97	-11.96	-11.95	-11.94	-11.93	-11.92
-12	-11.99	-11.98	-11.97	-11.96	-11.95	-11.94	-11.93	-11.92
-12	-11.99	-11.98	-11.97	-11.96	-11.95	-11.94	-11.93	-11.92
-12	-11.99	-11.98	-11.97	-11.96	-11.95	-11.94	-11.93	-11.92
-12	-11.99	-11.98	-11.97	-11.96	-11.95	-11.94	-11.93	-11.92
-12	-11.99	-11.98	-11.97	-11.96	-11.95	-11.94	-11.93	-11.92
-12	-11.99	-11.98	-11.97	-11.96	-11.95	-11.94	-11.93	-11.92
-12	-11.99	-11.98	-11.97	-11.96	-11.95	-11.94	-11.93	-11.92
-12	-11.99	-11.98	-11.97	-11.96	-11.95	-11.94	-11.93	-11.92
-12	-11.99	-11.98	-11.97	-11.96	-11.95	-11.94	-11.93	-11.92
-12	-11.99	-11.98	-11.97	-11.96	-11.95	-11.94	-11.93	-11.92
-12	-11.99	-11.98	-11.97	-11.96	-11.95	-11.94	-11.93	-11.92
-12	-11.99	-11.98	-11.97	-11.96	-11.95	-11.94	-11.93	-11.92
-12	-11.99	-11.98	-11.97	-11.96	-11.95	-11.94	-11.93	-11.92
-12	-11.99	-11.98	-11.97	-11.96	-11.95	-11.94	-11.93	-11.92
-12	-11.99	-11.98	-11.97	-11.96	-11.95	-11.94	-11.93	-11.92
-12	-11.99	-11.98	-11.97	-11.96	-11.95	-11.94	-11.93	-11.92
-12	-11.99	-11.98	-11.97	-11.96	-11.95	-11.94	-11.93	-11.92
-12	-11.99	-11.98	-11.97	-11.96	-11.95	-11.94	-11.93	-11.92
-12	-11.99	-11.98	-11.97	-11.96	-11.95	-11.94	-11.93	-11.92
-12	-11.99	-11.98	-11.97	-11.96	-11.95	-11.94	-11.93	-11.92
-12	-11.99	-11.98	-11.97	-11.96	-11.95	-11.94	-11.93	-11.92
-12	-11.99	-11.98	-11.97	-11.96	-11.95	-11.94	-11.93	-11.92
-12	-11.99	-11.98	-11.97	-11.96	-11.95	-11.94	-11.93	-11.92
-12	-11.99	-11.98	-11.97	-11.96	-11.95	-11.94	-11.93	-11.92
-12	-11.99	-11.98	-11.97	-11.96	-11.95	-11.94	-11.93	-11.92
-12	-11.99	-11.98	-11.97	-11.96	-11.95	-11.94	-11.93	-11.92
-12	-11.99	-11.98	-11.97	-11.96	-11.95	-11.94	-11.93	-11.92
-12	-11.99	-11.98	-11.97	-11.96	-11.95	-11.94	-11.93	-11.92
-12	-11.99	-11.98	-11.97	-11.96	-11.95	-11.94	-11.93	-11.92
-12	-11.99	-11.98	-11.97	-11.96	-11.95	-11.94	-11.93	-11.92
-12	-11.99	-11.98	-11.97	-11.96	-11.95	-11.94	-11.93	-11.92
-12	-11.99	-11.98	-11.97	-11.96	-11.95	-11.94	-11.93	-11.92
-12	-11.99	-11.98	-11.97	-11.96	-11.95	-11.94	-11.93	-11.92
-12	-11.99	-11.98	-11.97	-11.96	-11.95	-11.94	-11.93	-11.92
-12	-11.99	-11.98	-11.97	-11.96	-11.95	-11.94	-11.93	-11.92
-12	-11.99	-11.98	-11.97	-11.96	-11.95	-11.94	-11.93	-11.92
-12	-11.99	-11.98	-11.97	-11.96	-11.95	-11.94	-11.93	-11.92
-12	-11.99	-11.98	-11.97	-11.96	-11.95	-11.94	-11.93	-11.92
-12	-11.99	-11.98	-11.97	-11.96	-11.95	-11.94	-11.93	-11.92
-12	-11.99	-11.98	-11.97	-11.96	-11.95	-11.94	-11.93	-11.92
-12	-11.99	-11.98	-11.97	-11.96	-11.95	-11.94	-11.93	-11.92
-12	-11.99	-11.98	-11.97	-11.96	-11.95	-11.94	-11.93	-11.92
-12	-11.99	-11.98	-11.97	-11.96	-11.95	-11.94	-11.93	-11.92
-12	-11.99	-11.98	-11.97	-11.96	-11.95	-11.94	-11.93	-11.92
-12	-11.99	-11.98	-11.97	-11.96	-11.95	-11.94	-11.93	-11.92
-12	-11.99	-11.98	-11.97	-11.96	-11.95	-11.94	-11.93	-11.92
-12	-11.99	-11.98	-11.97	-11.96	-11.95	-11.94	-11.93	-11.92
-12	-11.99	-11.98	-11.97	-11.96	-11.95	-11.94	-11.93	-11.92
-12	-11.99	-11.98	-11.97	-11.96	-11.95	-11.94	-11.93	-11.92

-12	-11.99	-11.98	-11.97	-11.96	-11.95	-11.94	-11.93	-11.92
-12	-11.99	-11.98	-11.97	-11.96	-11.95	-11.94	-11.93	-11.92
-12	-11.99	-11.98	-11.97	-11.96	-11.95	-11.94	-11.93	-11.92
-12	-11.99	-11.98	-11.97	-11.96	-11.95	-11.94	-11.93	-11.92
-12	-11.99	-11.98	-11.97	-11.96	-11.95	-11.94	-11.93	-11.92
-12	-11.99	-11.98	-11.97	-11.96	-11.95	-11.94	-11.93	-11.92
-12	-11.99	-11.98	-11.97	-11.96	-11.95	-11.94	-11.93	-11.92
-12	-11.99	-11.98	-11.97	-11.96	-11.95	-11.94	-11.93	-11.92
-12	-11.99	-11.98	-11.97	-11.96	-11.95	-11.94	-11.93	-11.92
-12	-11.99	-11.98	-11.97	-11.96	-11.95	-11.94	-11.93	-11.92
-12	-11.99	-11.98	-11.97	-11.96	-11.95	-11.94	-11.93	-11.92
-12	-11.99	-11.98	-11.97	-11.96	-11.95	-11.94	-11.93	-11.92
-12	-11.99	-11.98	-11.97	-11.96	-11.95	-11.94	-11.93	-11.92
-12	-11.99	-11.98	-11.97	-11.96	-11.95	-11.94	-11.93	-11.92
-12	-11.99	-11.98	-11.97	-11.96	-11.95	-11.94	-11.93	-11.92
-12	-11.99	-11.98	-11.97	-11.96	-11.95	-11.94	-11.93	-11.92
-12	-11.99	-11.98	-11.97	-11.96	-11.95	-11.94	-11.93	-11.92
-12	-11.99	-11.98	-11.97	-11.96	-11.95	-11.94	-11.93	-11.92
-12	-11.99	-11.98	-11.97	-11.96	-11.95	-11.94	-11.93	-11.92
-12	-11.99	-11.98	-11.97	-11.96	-11.95	-11.94	-11.93	-11.92
-12	-11.99	-11.98	-11.97	-11.96	-11.95	-11.94	-11.93	-11.92
-12	-11.99	-11.98	-11.97	-11.96	-11.95	-11.94	-11.93	-11.92
-12	-11.99	-11.98	-11.97	-11.96	-11.95	-11.94	-11.93	-11.92
-12	-11.99	-11.98	-11.97	-11.96	-11.95	-11.94	-11.93	-11.92
-12	-11.99	-11.98	-11.97	-11.96	-11.95	-11.94	-11.93	-11.92
-12	-11.99	-11.98	-11.97	-11.96	-11.95	-11.94	-11.93	-11.92
-12	-11.99	-11.98	-11.97	-11.96	-11.95	-11.94	-11.93	-11.92
-12	-11.99	-11.98	-11.97	-11.96	-11.95	-11.94	-11.93	-11.92
-12	-11.99	-11.98	-11.97	-11.96	-11.95	-11.94	-11.93	-11.92
-12	-11.99	-11.98	-11.97	-11.96	-11.95	-11.94	-11.93	-11.92
-12	-11.99	-11.98	-11.97	-11.96	-11.95	-11.94	-11.93	-11.92
-12	-11.99	-11.98	-11.97	-11.96	-11.95	-11.94	-11.93	-11.92
-12	-11.99	-11.98	-11.97	-11.96	-11.95	-11.94	-11.93	-11.92
-12	-11.99	-11.98	-11.97	-11.96	-11.95	-11.94	-11.93	-11.92
-12	-11.99	-11.98	-11.97	-11.96	-11.95	-11.94	-11.93	-11.92
-12	-11.99	-11.98	-11.97	-11.96	-11.95	-11.94	-11.93	-11.92
-12	-11.99	-11.98	-11.97	-11.96	-11.95	-11.94	-11.93	-11.92
-12	-11.99	-11.98	-11.97	-11.96	-11.95	-11.94	-11.93	-11.92
-12	-11.99	-11.98	-11.97	-11.96	-11.95	-11.94	-11.93	-11.92
-12	-11.99	-11.98	-11.97	-11.96	-11.95	-11.94	-11.93	-11.92
-12	-11.99	-11.98	-11.97	-11.96	-11.95	-11.94	-11.93	-11.92
-12	-11.99	-11.98	-11.97	-11.96	-11.95	-11.94	-11.93	-11.92
-12	-11.99	-11.98	-11.97	-11.96	-11.95	-11.94	-11.93	-11.92
-12	-11.99	-11.98	-11.97	-11.96	-11.95	-11.94	-11.93	-11.92
-12	-11.99	-11.98	-11.97	-11.96	-11.95	-11.94	-11.93	-11.92
-12	-11.99	-11.98	-11.97	-11.96	-11.95	-11.94	-11.93	-11.92
-12	-11.99	-11.98	-11.97	-11.96	-11.95	-11.94	-11.93	-11.92
-12	-11.99	-11.98	-11.97	-11.96	-11.95	-11.94	-11.93	-11.92
-12	-11.99	-11.98	-11.97	-11.96	-11.95	-11.94	-11.93	-11.92
-12	-11.99	-11.98	-11.97	-11.96	-11.95	-11.94	-11.93	-11.92

-12 -11.99 -11.98 -11.97 -11.96 -11.95 -11.94 -11.93 -11.92

-11.91	-11.9	-11.89	-11.88	-11.87	-11.86	-11.85	-11.84	-11.83
-11.91	-11.9	-11.89	-11.88	-11.87	-11.86	-11.85	-11.84	-11.83
-11.91	-11.9	-11.89	-11.88	-11.87	-11.86	-11.85	-11.84	-11.83
-11.91	-11.9	-11.89	-11.88	-11.87	-11.86	-11.85	-11.84	-11.83
-11.91	-11.9	-11.89	-11.88	-11.87	-11.86	-11.85	-11.84	-11.83
-11.91	-11.9	-11.89	-11.88	-11.87	-11.86	-11.85	-11.84	-11.83
-11.91	-11.9	-11.89	-11.88	-11.87	-11.86	-11.85	-11.84	-11.83
-11.91	-11.9	-11.89	-11.88	-11.87	-11.86	-11.85	-11.84	-11.83
-11.91	-11.9	-11.89	-11.88	-11.87	-11.86	-11.85	-11.84	-11.83
-11.91	-11.9	-11.89	-11.88	-11.87	-11.86	-11.85	-11.84	-11.83
-11.91	-11.9	-11.89	-11.88	-11.87	-11.86	-11.85	-11.84	-11.83
-11.91	-11.9	-11.89	-11.88	-11.87	-11.86	-11.85	-11.84	-11.83
-11.91	-11.9	-11.89	-11.88	-11.87	-11.86	-11.85	-11.84	-11.83
-11.91	-11.9	-11.89	-11.88	-11.87	-11.86	-11.85	-11.84	-11.83
-11.91	-11.9	-11.89	-11.88	-11.87	-11.86	-11.85	-11.84	-11.83
-11.91	-11.9	-11.89	-11.88	-11.87	-11.86	-11.85	-11.84	-11.83
-11.91	-11.9	-11.89	-11.88	-11.87	-11.86	-11.85	-11.84	-11.83
-11.91	-11.9	-11.89	-11.88	-11.87	-11.86	-11.85	-11.84	-11.83
-11.91	-11.9	-11.89	-11.88	-11.87	-11.86	-11.85	-11.84	-11.83
-11.91	-11.9	-11.89	-11.88	-11.87	-11.86	-11.85	-11.84	-11.83
-11.91	-11.9	-11.89	-11.88	-11.87	-11.86	-11.85	-11.84	-11.83
-11.91	-11.9	-11.89	-11.88	-11.87	-11.86	-11.85	-11.84	-11.83
-11.91	-11.9	-11.89	-11.88	-11.87	-11.86	-11.85	-11.84	-11.83
-11.91	-11.9	-11.89	-11.88	-11.87	-11.86	-11.85	-11.84	-11.83
-11.91	-11.9	-11.89	-11.88	-11.87	-11.86	-11.85	-11.84	-11.83
-11.91	-11.9	-11.89	-11.88	-11.87	-11.86	-11.85	-11.84	-11.83
-11.91	-11.9	-11.89	-11.88	-11.87	-11.86	-11.85	-11.84	-11.83
-11.91	-11.9	-11.89	-11.88	-11.87	-11.86	-11.85	-11.84	-11.83
-11.91	-11.9	-11.89	-11.88	-11.87	-11.86	-11.85	-11.84	-11.83
-11.91	-11.9	-11.89	-11.88	-11.87	-11.86	-11.85	-11.84	-11.83
-11.91	-11.9	-11.89	-11.88	-11.87	-11.86	-11.85	-11.84	-11.83
-11.91	-11.9	-11.89	-11.88	-11.87	-11.86	-11.85	-11.84	-11.83
-11.91	-11.9	-11.89	-11.88	-11.87	-11.86	-11.85	-11.84	-11.83
-11.91	-11.9	-11.89	-11.88	-11.87	-11.86	-11.85	-11.84	-11.83
-11.91	-11.9	-11.89	-11.88	-11.87	-11.86	-11.85	-11.84	-11.83
-11.91	-11.9	-11.89	-11.88	-11.87	-11.86	-11.85	-11.84	-11.83
-11.91	-11.9	-11.89	-11.88	-11.87	-11.86	-11.85	-11.84	-11.83
-11.91	-11.9	-11.89	-11.88	-11.87	-11.86	-11.85	-11.84	-11.83
-11.91	-11.9	-11.89	-11.88	-11.87	-11.86	-11.85	-11.84	-11.83
-11.91	-11.9	-11.89	-11.88	-11.87	-11.86	-11.85	-11.84	-11.83
-11.91	-11.9	-11.89	-11.88	-11.87	-11.86	-11.85	-11.84	-11.83
-11.91	-11.9	-11.89	-11.88	-11.87	-11.86	-11.85	-11.84	-11.83
-11.91	-11.9	-11.89	-11.88	-11.87	-11.86	-11.85	-11.84	-11.83
-11.91	-11.9	-11.89	-11.88	-11.87	-11.86	-11.85	-11.84	-11.83
-11.91	-11.9	-11.89	-11.88	-11.87	-11.86	-11.85	-11.84	-11.83
-11.91	-11.9	-11.89	-11.88	-11.87	-11.86	-11.85	-11.84	-11.83
-11.91	-11.9	-11.89	-11.88	-11.87	-11.86	-11.85	-11.84	-11.83
-11.91	-11.9	-11.89	-11.88	-11.87	-11.86	-11.85	-11.84	-11.83
-11.91	-11.9	-11.89	-11.88	-11.87	-11.86	-11.85	-11.84	-11.83
-11.91	-11.9	-11.89	-11.88	-11.87	-11.86	-11.85	-11.84	-11.83

-11.91	-11.9	-11.89	-11.88	-11.87	-11.86	-11.85	-11.84	-11.83
-11.91	-11.9	-11.89	-11.88	-11.87	-11.86	-11.85	-11.84	-11.83
-11.91	-11.9	-11.89	-11.88	-11.87	-11.86	-11.85	-11.84	-11.83
-11.91	-11.9	-11.89	-11.88	-11.87	-11.86	-11.85	-11.84	-11.83
-11.91	-11.9	-11.89	-11.88	-11.87	-11.86	-11.85	-11.84	-11.83
-11.91	-11.9	-11.89	-11.88	-11.87	-11.86	-11.85	-11.84	-11.83
-11.91	-11.9	-11.89	-11.88	-11.87	-11.86	-11.85	-11.84	-11.83
-11.91	-11.9	-11.89	-11.88	-11.87	-11.86	-11.85	-11.84	-11.83
-11.91	-11.9	-11.89	-11.88	-11.87	-11.86	-11.85	-11.84	-11.83
-11.91	-11.9	-11.89	-11.88	-11.87	-11.86	-11.85	-11.84	-11.83
-11.91	-11.9	-11.89	-11.88	-11.87	-11.86	-11.85	-11.84	-11.83
-11.91	-11.9	-11.89	-11.88	-11.87	-11.86	-11.85	-11.84	-11.83
-11.91	-11.9	-11.89	-11.88	-11.87	-11.86	-11.85	-11.84	-11.83
-11.91	-11.9	-11.89	-11.88	-11.87	-11.86	-11.85	-11.84	-11.83
-11.91	-11.9	-11.89	-11.88	-11.87	-11.86	-11.85	-11.84	-11.83
-11.91	-11.9	-11.89	-11.88	-11.87	-11.86	-11.85	-11.84	-11.83
-11.91	-11.9	-11.89	-11.88	-11.87	-11.86	-11.85	-11.84	-11.83
-11.91	-11.9	-11.89	-11.88	-11.87	-11.86	-11.85	-11.84	-11.83
-11.91	-11.9	-11.89	-11.88	-11.87	-11.86	-11.85	-11.84	-11.83
-11.91	-11.9	-11.89	-11.88	-11.87	-11.86	-11.85	-11.84	-11.83
-11.91	-11.9	-11.89	-11.88	-11.87	-11.86	-11.85	-11.84	-11.83
-11.91	-11.9	-11.89	-11.88	-11.87	-11.86	-11.85	-11.84	-11.83
-11.91	-11.9	-11.89	-11.88	-11.87	-11.86	-11.85	-11.84	-11.83
-11.91	-11.9	-11.89	-11.88	-11.87	-11.86	-11.85	-11.84	-11.83
-11.91	-11.9	-11.89	-11.88	-11.87	-11.86	-11.85	-11.84	-11.83
-11.91	-11.9	-11.89	-11.88	-11.87	-11.86	-11.85	-11.84	-11.83
-11.91	-11.9	-11.89	-11.88	-11.87	-11.86	-11.85	-11.84	-11.83
-11.91	-11.9	-11.89	-11.88	-11.87	-11.86	-11.85	-11.84	-11.83
-11.91	-11.9	-11.89	-11.88	-11.87	-11.86	-11.85	-11.84	-11.83
-11.91	-11.9	-11.89	-11.88	-11.87	-11.86	-11.85	-11.84	-11.83
-11.91	-11.9	-11.89	-11.88	-11.87	-11.86	-11.85	-11.84	-11.83
-11.91	-11.9	-11.89	-11.88	-11.87	-11.86	-11.85	-11.84	-11.83
-11.91	-11.9	-11.89	-11.88	-11.87	-11.86	-11.85	-11.84	-11.83
-11.91	-11.9	-11.89	-11.88	-11.87	-11.86	-11.85	-11.84	-11.83
-11.91	-11.9	-11.89	-11.88	-11.87	-11.86	-11.85	-11.84	-11.83
-11.91	-11.9	-11.89	-11.88	-11.87	-11.86	-11.85	-11.84	-11.83
-11.91	-11.9	-11.89	-11.88	-11.87	-11.86	-11.85	-11.84	-11.83
-11.91	-11.9	-11.89	-11.88	-11.87	-11.86	-11.85	-11.84	-11.83
-11.91	-11.9	-11.89	-11.88	-11.87	-11.86	-11.85	-11.84	-11.83
-11.91	-11.9	-11.89	-11.88	-11.87	-11.86	-11.85	-11.84	-11.83
-11.91	-11.9	-11.89	-11.88	-11.87	-11.86	-11.85	-11.84	-11.83
-11.91	-11.9	-11.89	-11.88	-11.87	-11.86	-11.85	-11.84	-11.83
-11.91	-11.9	-11.89	-11.88	-11.87	-11.86	-11.85	-11.84	-11.83
-11.91	-11.9	-11.89	-11.88	-11.87	-11.86	-11.85	-11.84	-11.83
-11.91	-11.9	-11.89	-11.88	-11.87	-11.86	-11.85	-11.84	-11.83
-11.91	-11.9	-11.89	-11.88	-11.87	-11.86	-11.85	-11.84	-11.83
-11.91	-11.9	-11.89	-11.88	-11.87	-11.86	-11.85	-11.84	-11.83
-11.91	-11.9	-11.89	-11.88	-11.87	-11.86	-11.85	-11.84	-11.83
-11.91	-11.9	-11.89	-11.88	-11.87	-11.86	-11.85	-11.84	-11.83
-11.91	-11.9	-11.89	-11.88	-11.87	-11.86	-11.85	-11.84	-11.83

-11.91	-11.9	-11.89	-11.88	-11.87	-11.86	-11.85	-11.84	-11.83
-11.91	-11.9	-11.89	-11.88	-11.87	-11.86	-11.85	-11.84	-11.83
-11.91	-11.9	-11.89	-11.88	-11.87	-11.86	-11.85	-11.84	-11.83
-11.91	-11.9	-11.89	-11.88	-11.87	-11.86	-11.85	-11.84	-11.83
-11.91	-11.9	-11.89	-11.88	-11.87	-11.86	-11.85	-11.84	-11.83
-11.91	-11.9	-11.89	-11.88	-11.87	-11.86	-11.85	-11.84	-11.83
-11.91	-11.9	-11.89	-11.88	-11.87	-11.86	-11.85	-11.84	-11.83
-11.91	-11.9	-11.89	-11.88	-11.87	-11.86	-11.85	-11.84	-11.83
-11.91	-11.9	-11.89	-11.88	-11.87	-11.86	-11.85	-11.84	-11.83
-11.91	-11.9	-11.89	-11.88	-11.87	-11.86	-11.85	-11.84	-11.83
-11.91	-11.9	-11.89	-11.88	-11.87	-11.86	-11.85	-11.84	-11.83
-11.91	-11.9	-11.89	-11.88	-11.87	-11.86	-11.85	-11.84	-11.83
-11.91	-11.9	-11.89	-11.88	-11.87	-11.86	-11.85	-11.84	-11.83
-11.91	-11.9	-11.89	-11.88	-11.87	-11.86	-11.85	-11.84	-11.83
-11.91	-11.9	-11.89	-11.88	-11.87	-11.86	-11.85	-11.84	-11.83
-11.91	-11.9	-11.89	-11.88	-11.87	-11.86	-11.85	-11.84	-11.83
-11.91	-11.9	-11.89	-11.88	-11.87	-11.86	-11.85	-11.84	-11.83
-11.91	-11.9	-11.89	-11.88	-11.87	-11.86	-11.85	-11.84	-11.83
-11.91	-11.9	-11.89	-11.88	-11.87	-11.86	-11.85	-11.84	-11.83
-11.91	-11.9	-11.89	-11.88	-11.87	-11.86	-11.85	-11.84	-11.83
-11.91	-11.9	-11.89	-11.88	-11.87	-11.86	-11.85	-11.84	-11.83
-11.91	-11.9	-11.89	-11.88	-11.87	-11.86	-11.85	-11.84	-11.83
-11.91	-11.9	-11.89	-11.88	-11.87	-11.86	-11.85	-11.84	-11.83
-11.91	-11.9	-11.89	-11.88	-11.87	-11.86	-11.85	-11.84	-11.83
-11.91	-11.9	-11.89	-11.88	-11.87	-11.86	-11.85	-11.84	-11.83
-11.91	-11.9	-11.89	-11.88	-11.87	-11.86	-11.85	-11.84	-11.83
-11.91	-11.9	-11.89	-11.88	-11.87	-11.86	-11.85	-11.84	-11.83
-11.91	-11.9	-11.89	-11.88	-11.87	-11.86	-11.85	-11.84	-11.83
-11.91	-11.9	-11.89	-11.88	-11.87	-11.86	-11.85	-11.84	-11.83
-11.91	-11.9	-11.89	-11.88	-11.87	-11.86	-11.85	-11.84	-11.83
-11.91	-11.9	-11.89	-11.88	-11.87	-11.86	-11.85	-11.84	-11.83
-11.91	-11.9	-11.89	-11.88	-11.87	-11.86	-11.85	-11.84	-11.83
-11.91	-11.9	-11.89	-11.88	-11.87	-11.86	-11.85	-11.84	-11.83
-11.91	-11.9	-11.89	-11.88	-11.87	-11.86	-11.85	-11.84	-11.83
-11.91	-11.9	-11.89	-11.88	-11.87	-11.86	-11.85	-11.84	-11.83
-11.91	-11.9	-11.89	-11.88	-11.87	-11.86	-11.85	-11.84	-11.83
-11.91	-11.9	-11.89	-11.88	-11.87	-11.86	-11.85	-11.84	-11.83
-11.91	-11.9	-11.89	-11.88	-11.87	-11.86	-11.85	-11.84	-11.83
-11.91	-11.9	-11.89	-11.88	-11.87	-11.86	-11.85	-11.84	-11.83
-11.91	-11.9	-11.89	-11.88	-11.87	-11.86	-11.85	-11.84	-11.83
-11.91	-11.9	-11.89	-11.88	-11.87	-11.86	-11.85	-11.84	-11.83
-11.91	-11.9	-11.89	-11.88	-11.87	-11.86	-11.85	-11.84	-11.83
-11.91	-11.9	-11.89	-11.88	-11.87	-11.86	-11.85	-11.84	-11.83
-11.91	-11.9	-11.89	-11.88	-11.87	-11.86	-11.85	-11.84	-11.83
-11.91	-11.9	-11.89	-11.88	-11.87	-11.86	-11.85	-11.84	-11.83
-11.91	-11.9	-11.89	-11.88	-11.87	-11.86	-11.85	-11.84	-11.83
-11.91	-11.9	-11.89	-11.88	-11.87	-11.86	-11.85	-11.84	-11.83
-11.91	-11.9	-11.89	-11.88	-11.87	-11.86	-11.85	-11.84	-11.83
-11.91	-11.9	-11.89	-11.88	-11.87	-11.86	-11.85	-11.84	-11.83
-11.91	-11.9	-11.89	-11.88	-11.87	-11.86	-11.85	-11.84	-11.83

-11.91	-11.9	-11.89	-11.88	-11.87	-11.86	-11.85	-11.84	-11.83
-11.91	-11.9	-11.89	-11.88	-11.87	-11.86	-11.85	-11.84	-11.83
-11.91	-11.9	-11.89	-11.88	-11.87	-11.86	-11.85	-11.84	-11.83
-11.91	-11.9	-11.89	-11.88	-11.87	-11.86	-11.85	-11.84	-11.83
-11.91	-11.9	-11.89	-11.88	-11.87	-11.86	-11.85	-11.84	-11.83
-11.91	-11.9	-11.89	-11.88	-11.87	-11.86	-11.85	-11.84	-11.83
-11.91	-11.9	-11.89	-11.88	-11.87	-11.86	-11.85	-11.84	-11.83
-11.91	-11.9	-11.89	-11.88	-11.87	-11.86	-11.85	-11.84	-11.83
-11.91	-11.9	-11.89	-11.88	-11.87	-11.86	-11.85	-11.84	-11.83
-11.91	-11.9	-11.89	-11.88	-11.87	-11.86	-11.85	-11.84	-11.83
-11.91	-11.9	-11.89	-11.88	-11.87	-11.86	-11.85	-11.84	-11.83
-11.91	-11.9	-11.89	-11.88	-11.87	-11.86	-11.85	-11.84	-11.83
-11.91	-11.9	-11.89	-11.88	-11.87	-11.86	-11.85	-11.84	-11.83
-11.91	-11.9	-11.89	-11.88	-11.87	-11.86	-11.85	-11.84	-11.83
-11.91	-11.9	-11.89	-11.88	-11.87	-11.86	-11.85	-11.84	-11.83
-11.91	-11.9	-11.89	-11.88	-11.87	-11.86	-11.85	-11.84	-11.83
-11.91	-11.9	-11.89	-11.88	-11.87	-11.86	-11.85	-11.84	-11.83
-11.91	-11.9	-11.89	-11.88	-11.87	-11.86	-11.85	-11.84	-11.83
-11.91	-11.9	-11.89	-11.88	-11.87	-11.86	-11.85	-11.84	-11.83
-11.91	-11.9	-11.89	-11.88	-11.87	-11.86	-11.85	-11.84	-11.83
-11.91	-11.9	-11.89	-11.88	-11.87	-11.86	-11.85	-11.84	-11.83
-11.91	-11.9	-11.89	-11.88	-11.87	-11.86	-11.85	-11.84	-11.83
-11.91	-11.9	-11.89	-11.88	-11.87	-11.86	-11.85	-11.84	-11.83
-11.91	-11.9	-11.89	-11.88	-11.87	-11.86	-11.85	-11.84	-11.83
-11.91	-11.9	-11.89	-11.88	-11.87	-11.86	-11.85	-11.84	-11.83
-11.91	-11.9	-11.89	-11.88	-11.87	-11.86	-11.85	-11.84	-11.83
-11.91	-11.9	-11.89	-11.88	-11.87	-11.86	-11.85	-11.84	-11.83
-11.91	-11.9	-11.89	-11.88	-11.87	-11.86	-11.85	-11.84	-11.83
-11.91	-11.9	-11.89	-11.88	-11.87	-11.86	-11.85	-11.84	-11.83
-11.91	-11.9	-11.89	-11.88	-11.87	-11.86	-11.85	-11.84	-11.83
-11.91	-11.9	-11.89	-11.88	-11.87	-11.86	-11.85	-11.84	-11.83
-11.91	-11.9	-11.89	-11.88	-11.87	-11.86	-11.85	-11.84	-11.83
-11.91	-11.9	-11.89	-11.88	-11.87	-11.86	-11.85	-11.84	-11.83
-11.91	-11.9	-11.89	-11.88	-11.87	-11.86	-11.85	-11.84	-11.83
-11.91	-11.9	-11.89	-11.88	-11.87	-11.86	-11.85	-11.84	-11.83
-11.91	-11.9	-11.89	-11.88	-11.87	-11.86	-11.85	-11.84	-11.83
-11.91	-11.9	-11.89	-11.88	-11.87	-11.86	-11.85	-11.84	-11.83
-11.91	-11.9	-11.89	-11.88	-11.87	-11.86	-11.85	-11.84	-11.83
-11.91	-11.9	-11.89	-11.88	-11.87	-11.86	-11.85	-11.84	-11.83
-11.91	-11.9	-11.89	-11.88	-11.87	-11.86	-11.85	-11.84	-11.83
-11.91	-11.9	-11.89	-11.88	-11.87	-11.86	-11.85	-11.84	-11.83
-11.91	-11.9	-11.89	-11.88	-11.87	-11.86	-11.85	-11.84	-11.83
-11.91	-11.9	-11.89	-11.88	-11.87	-11.86	-11.85	-11.84	-11.83
-11.91	-11.9	-11.89	-11.88	-11.87	-11.86	-11.85	-11.84	-11.83
-11.91	-11.9	-11.89	-11.88	-11.87	-11.86	-11.85	-11.84	-11.83
-11.91	-11.9	-11.89	-11.88	-11.87	-11.86	-11.85	-11.84	-11.83
-11.91	-11.9	-11.89	-11.88	-11.87	-11.86	-11.85	-11.84	-11.83
-11.91	-11.9	-11.89	-11.88	-11.87	-11.86	-11.85	-11.84	-11.83
-11.91	-11.9	-11.89	-11.88	-11.87	-11.86	-11.85	-11.84	-11.83
-11.91	-11.9	-11.89	-11.88	-11.87	-11.86	-11.85	-11.84	-11.83

-11.91	-11.9	-11.89	-11.88	-11.87	-11.86	-11.85	-11.84	-11.83
-11.91	-11.9	-11.89	-11.88	-11.87	-11.86	-11.85	-11.84	-11.83
-11.91	-11.9	-11.89	-11.88	-11.87	-11.86	-11.85	-11.84	-11.83
-11.91	-11.9	-11.89	-11.88	-11.87	-11.86	-11.85	-11.84	-11.83
-11.91	-11.9	-11.89	-11.88	-11.87	-11.86	-11.85	-11.84	-11.83
-11.91	-11.9	-11.89	-11.88	-11.87	-11.86	-11.85	-11.84	-11.83
-11.91	-11.9	-11.89	-11.88	-11.87	-11.86	-11.85	-11.84	-11.83
-11.91	-11.9	-11.89	-11.88	-11.87	-11.86	-11.85	-11.84	-11.83
-11.91	-11.9	-11.89	-11.88	-11.87	-11.86	-11.85	-11.84	-11.83
-11.91	-11.9	-11.89	-11.88	-11.87	-11.86	-11.85	-11.84	-11.83
-11.91	-11.9	-11.89	-11.88	-11.87	-11.86	-11.85	-11.84	-11.83
-11.91	-11.9	-11.89	-11.88	-11.87	-11.86	-11.85	-11.84	-11.83
-11.91	-11.9	-11.89	-11.88	-11.87	-11.86	-11.85	-11.84	-11.83
-11.91	-11.9	-11.89	-11.88	-11.87	-11.86	-11.85	-11.84	-11.83
-11.91	-11.9	-11.89	-11.88	-11.87	-11.86	-11.85	-11.84	-11.83
-11.91	-11.9	-11.89	-11.88	-11.87	-11.86	-11.85	-11.84	-11.83
-11.91	-11.9	-11.89	-11.88	-11.87	-11.86	-11.85	-11.84	-11.83
-11.91	-11.9	-11.89	-11.88	-11.87	-11.86	-11.85	-11.84	-11.83
-11.91	-11.9	-11.89	-11.88	-11.87	-11.86	-11.85	-11.84	-11.83
-11.91	-11.9	-11.89	-11.88	-11.87	-11.86	-11.85	-11.84	-11.83
-11.91	-11.9	-11.89	-11.88	-11.87	-11.86	-11.85	-11.84	-11.83
-11.91	-11.9	-11.89	-11.88	-11.87	-11.86	-11.85	-11.84	-11.83
-11.91	-11.9	-11.89	-11.88	-11.87	-11.86	-11.85	-11.84	-11.83
-11.91	-11.9	-11.89	-11.88	-11.87	-11.86	-11.85	-11.84	-11.83
-11.91	-11.9	-11.89	-11.88	-11.87	-11.86	-11.85	-11.84	-11.83
-11.91	-11.9	-11.89	-11.88	-11.87	-11.86	-11.85	-11.84	-11.83
-11.91	-11.9	-11.89	-11.88	-11.87	-11.86	-11.85	-11.84	-11.83
-11.91	-11.9	-11.89	-11.88	-11.87	-11.86	-11.85	-11.84	-11.83
-11.91	-11.9	-11.89	-11.88	-11.87	-11.86	-11.85	-11.84	-11.83
-11.91	-11.9	-11.89	-11.88	-11.87	-11.86	-11.85	-11.84	-11.83
-11.91	-11.9	-11.89	-11.88	-11.87	-11.86	-11.85	-11.84	-11.83
-11.91	-11.9	-11.89	-11.88	-11.87	-11.86	-11.85	-11.84	-11.83
-11.91	-11.9	-11.89	-11.88	-11.87	-11.86	-11.85	-11.84	-11.83
-11.91	-11.9	-11.89	-11.88	-11.87	-11.86	-11.85	-11.84	-11.83
-11.91	-11.9	-11.89	-11.88	-11.87	-11.86	-11.85	-11.84	-11.83
-11.91	-11.9	-11.89	-11.88	-11.87	-11.86	-11.85	-11.84	-11.83
-11.91	-11.9	-11.89	-11.88	-11.87	-11.86	-11.85	-11.84	-11.83
-11.91	-11.9	-11.89	-11.88	-11.87	-11.86	-11.85	-11.84	-11.83
-11.91	-11.9	-11.89	-11.88	-11.87	-11.86	-11.85	-11.84	-11.83
-11.91	-11.9	-11.89	-11.88	-11.87	-11.86	-11.85	-11.84	-11.83
-11.91	-11.9	-11.89	-11.88	-11.87	-11.86	-11.85	-11.84	-11.83
-11.91	-11.9	-11.89	-11.88	-11.87	-11.86	-11.85	-11.84	-11.83
-11.91	-11.9	-11.89	-11.88	-11.87	-11.86	-11.85	-11.84	-11.83
-11.91	-11.9	-11.89	-11.88	-11.87	-11.86	-11.85	-11.84	-11.83
-11.91	-11.9	-11.89	-11.88	-11.87	-11.86	-11.85	-11.84	-11.83
-11.91	-11.9	-11.89	-11.88	-11.87	-11.86	-11.85	-11.84	-11.83
-11.91	-11.9	-11.89	-11.88	-11.87	-11.86	-11.85	-11.84	-11.83
-11.91	-11.9	-11.89	-11.88	-11.87	-11.86	-11.85	-11.84	-11.83
-11.91	-11.9	-11.89	-11.88	-11.87	-11.86	-11.85	-11.84	-11.83
-11.91	-11.9	-11.89	-11.88	-11.87	-11.86	-11.85	-11.84	-11.83

-11.91	-11.9	-11.89	-11.88	-11.87	-11.86	-11.85	-11.84	-11.83
-11.91	-11.9	-11.89	-11.88	-11.87	-11.86	-11.85	-11.84	-11.83
-11.91	-11.9	-11.89	-11.88	-11.87	-11.86	-11.85	-11.84	-11.83
-11.91	-11.9	-11.89	-11.88	-11.87	-11.86	-11.85	-11.84	-11.83
-11.91	-11.9	-11.89	-11.88	-11.87	-11.86	-11.85	-11.84	-11.83
-11.91	-11.9	-11.89	-11.88	-11.87	-11.86	-11.85	-11.84	-11.83
-11.91	-11.9	-11.89	-11.88	-11.87	-11.86	-11.85	-11.84	-11.83
-11.91	-11.9	-11.89	-11.88	-11.87	-11.86	-11.85	-11.84	-11.83
-11.91	-11.9	-11.89	-11.88	-11.87	-11.86	-11.85	-11.84	-11.83
-11.91	-11.9	-11.89	-11.88	-11.87	-11.86	-11.85	-11.84	-11.83
-11.91	-11.9	-11.89	-11.88	-11.87	-11.86	-11.85	-11.84	-11.83
-11.91	-11.9	-11.89	-11.88	-11.87	-11.86	-11.85	-11.84	-11.83
-11.91	-11.9	-11.89	-11.88	-11.87	-11.86	-11.85	-11.84	-11.83
-11.91	-11.9	-11.89	-11.88	-11.87	-11.86	-11.85	-11.84	-11.83
-11.91	-11.9	-11.89	-11.88	-11.87	-11.86	-11.85	-11.84	-11.83
-11.91	-11.9	-11.89	-11.88	-11.87	-11.86	-11.85	-11.84	-11.83
-11.91	-11.9	-11.89	-11.88	-11.87	-11.86	-11.85	-11.84	-11.83
-11.91	-11.9	-11.89	-11.88	-11.87	-11.86	-11.85	-11.84	-11.83
-11.91	-11.9	-11.89	-11.88	-11.87	-11.86	-11.85	-11.84	-11.83
-11.91	-11.9	-11.89	-11.88	-11.87	-11.86	-11.85	-11.84	-11.83
-11.91	-11.9	-11.89	-11.88	-11.87	-11.86	-11.85	-11.84	-11.83
-11.91	-11.9	-11.89	-11.88	-11.87	-11.86	-11.85	-11.84	-11.83
-11.91	-11.9	-11.89	-11.88	-11.87	-11.86	-11.85	-11.84	-11.83
-11.91	-11.9	-11.89	-11.88	-11.87	-11.86	-11.85	-11.84	-11.83
-11.91	-11.9	-11.89	-11.88	-11.87	-11.86	-11.85	-11.84	-11.83
-11.91	-11.9	-11.89	-11.88	-11.87	-11.86	-11.85	-11.84	-11.83
-11.91	-11.9	-11.89	-11.88	-11.87	-11.86	-11.85	-11.84	-11.83
-11.91	-11.9	-11.89	-11.88	-11.87	-11.86	-11.85	-11.84	-11.83
-11.91	-11.9	-11.89	-11.88	-11.87	-11.86	-11.85	-11.84	-11.83
-11.91	-11.9	-11.89	-11.88	-11.87	-11.86	-11.85	-11.84	-11.83
-11.91	-11.9	-11.89	-11.88	-11.87	-11.86	-11.85	-11.84	-11.83
-11.91	-11.9	-11.89	-11.88	-11.87	-11.86	-11.85	-11.84	-11.83
-11.91	-11.9	-11.89	-11.88	-11.87	-11.86	-11.85	-11.84	-11.83
-11.91	-11.9	-11.89	-11.88	-11.87	-11.86	-11.85	-11.84	-11.83
-11.91	-11.9	-11.89	-11.88	-11.87	-11.86	-11.85	-11.84	-11.83
-11.91	-11.9	-11.89	-11.88	-11.87	-11.86	-11.85	-11.84	-11.83
-11.91	-11.9	-11.89	-11.88	-11.87	-11.86	-11.85	-11.84	-11.83
-11.91	-11.9	-11.89	-11.88	-11.87	-11.86	-11.85	-11.84	-11.83
-11.91	-11.9	-11.89	-11.88	-11.87	-11.86	-11.85	-11.84	-11.83
-11.91	-11.9	-11.89	-11.88	-11.87	-11.86	-11.85	-11.84	-11.83
-11.91	-11.9	-11.89	-11.88	-11.87	-11.86	-11.85	-11.84	-11.83
-11.91	-11.9	-11.89	-11.88	-11.87	-11.86	-11.85	-11.84	-11.83
-11.91	-11.9	-11.89	-11.88	-11.87	-11.86	-11.85	-11.84	-11.83
-11.91	-11.9	-11.89	-11.88	-11.87	-11.86	-11.85	-11.84	-11.83
-11.91	-11.9	-11.89	-11.88	-11.87	-11.86	-11.85	-11.84	-11.83
-11.91	-11.9	-11.89	-11.88	-11.87	-11.86	-11.85	-11.84	-11.83
-11.91	-11.9	-11.89	-11.88	-11.87	-11.86	-11.85	-11.84	-11.83
-11.91	-11.9	-11.89	-11.88	-11.87	-11.86	-11.85	-11.84	-11.83
-11.91	-11.9	-11.89	-11.88	-11.87	-11.86	-11.85	-11.84	-11.83
-11.91	-11.9	-11.89	-11.88	-11.87	-11.86	-11.85	-11.84	-11.83

-11.91	-11.9	-11.89	-11.88	-11.87	-11.86	-11.85	-11.84	-11.83
-11.91	-11.9	-11.89	-11.88	-11.87	-11.86	-11.85	-11.84	-11.83
-11.91	-11.9	-11.89	-11.88	-11.87	-11.86	-11.85	-11.84	-11.83
-11.91	-11.9	-11.89	-11.88	-11.87	-11.86	-11.85	-11.84	-11.83
-11.91	-11.9	-11.89	-11.88	-11.87	-11.86	-11.85	-11.84	-11.83
-11.91	-11.9	-11.89	-11.88	-11.87	-11.86	-11.85	-11.84	-11.83
-11.91	-11.9	-11.89	-11.88	-11.87	-11.86	-11.85	-11.84	-11.83
-11.91	-11.9	-11.89	-11.88	-11.87	-11.86	-11.85	-11.84	-11.83
-11.91	-11.9	-11.89	-11.88	-11.87	-11.86	-11.85	-11.84	-11.83
-11.91	-11.9	-11.89	-11.88	-11.87	-11.86	-11.85	-11.84	-11.83
-11.91	-11.9	-11.89	-11.88	-11.87	-11.86	-11.85	-11.84	-11.83
-11.91	-11.9	-11.89	-11.88	-11.87	-11.86	-11.85	-11.84	-11.83
-11.91	-11.9	-11.89	-11.88	-11.87	-11.86	-11.85	-11.84	-11.83
-11.91	-11.9	-11.89	-11.88	-11.87	-11.86	-11.85	-11.84	-11.83
-11.91	-11.9	-11.89	-11.88	-11.87	-11.86	-11.85	-11.84	-11.83
-11.91	-11.9	-11.89	-11.88	-11.87	-11.86	-11.85	-11.84	-11.83
-11.91	-11.9	-11.89	-11.88	-11.87	-11.86	-11.85	-11.84	-11.83
-11.91	-11.9	-11.89	-11.88	-11.87	-11.86	-11.85	-11.84	-11.83
-11.91	-11.9	-11.89	-11.88	-11.87	-11.86	-11.85	-11.84	-11.83
-11.91	-11.9	-11.89	-11.88	-11.87	-11.86	-11.85	-11.84	-11.83
-11.91	-11.9	-11.89	-11.88	-11.87	-11.86	-11.85	-11.84	-11.83
-11.91	-11.9	-11.89	-11.88	-11.87	-11.86	-11.85	-11.84	-11.83
-11.91	-11.9	-11.89	-11.88	-11.87	-11.86	-11.85	-11.84	-11.83
-11.91	-11.9	-11.89	-11.88	-11.87	-11.86	-11.85	-11.84	-11.83
-11.91	-11.9	-11.89	-11.88	-11.87	-11.86	-11.85	-11.84	-11.83
-11.91	-11.9	-11.89	-11.88	-11.87	-11.86	-11.85	-11.84	-11.83
-11.91	-11.9	-11.89	-11.88	-11.87	-11.86	-11.85	-11.84	-11.83
-11.91	-11.9	-11.89	-11.88	-11.87	-11.86	-11.85	-11.84	-11.83
-11.91	-11.9	-11.89	-11.88	-11.87	-11.86	-11.85	-11.84	-11.83
-11.91	-11.9	-11.89	-11.88	-11.87	-11.86	-11.85	-11.84	-11.83
-11.91	-11.9	-11.89	-11.88	-11.87	-11.86	-11.85	-11.84	-11.83
-11.91	-11.9	-11.89	-11.88	-11.87	-11.86	-11.85	-11.84	-11.83
-11.91	-11.9	-11.89	-11.88	-11.87	-11.86	-11.85	-11.84	-11.83
-11.91	-11.9	-11.89	-11.88	-11.87	-11.86	-11.85	-11.84	-11.83
-11.91	-11.9	-11.89	-11.88	-11.87	-11.86	-11.85	-11.84	-11.83
-11.91	-11.9	-11.89	-11.88	-11.87	-11.86	-11.85	-11.84	-11.83
-11.91	-11.9	-11.89	-11.88	-11.87	-11.86	-11.85	-11.84	-11.83
-11.91	-11.9	-11.89	-11.88	-11.87	-11.86	-11.85	-11.84	-11.83
-11.91	-11.9	-11.89	-11.88	-11.87	-11.86	-11.85	-11.84	-11.83
-11.91	-11.9	-11.89	-11.88	-11.87	-11.86	-11.85	-11.84	-11.83
-11.91	-11.9	-11.89	-11.88	-11.87	-11.86	-11.85	-11.84	-11.83
-11.91	-11.9	-11.89	-11.88	-11.87	-11.86	-11.85	-11.84	-11.83
-11.91	-11.9	-11.89	-11.88	-11.87	-11.86	-11.85	-11.84	-11.83
-11.91	-11.9	-11.89	-11.88	-11.87	-11.86	-11.85	-11.84	-11.83
-11.91	-11.9	-11.89	-11.88	-11.87	-11.86	-11.85	-11.84	-11.83
-11.91	-11.9	-11.89	-11.88	-11.87	-11.86	-11.85	-11.84	-11.83
-11.91	-11.9	-11.89	-11.88	-11.87	-11.86	-11.85	-11.84	-11.83
-11.91	-11.9	-11.89	-11.88	-11.87	-11.86	-11.85	-11.84	-11.83
-11.91	-11.9	-11.89	-11.88	-11.87	-11.86	-11.85	-11.84	-11.83
-11.91	-11.9	-11.89	-11.88	-11.87	-11.86	-11.85	-11.84	-11.83

-11.91	-11.9	-11.89	-11.88	-11.87	-11.86	-11.85	-11.84	-11.83
-11.91	-11.9	-11.89	-11.88	-11.87	-11.86	-11.85	-11.84	-11.83
-11.91	-11.9	-11.89	-11.88	-11.87	-11.86	-11.85	-11.84	-11.83
-11.91	-11.9	-11.89	-11.88	-11.87	-11.86	-11.85	-11.84	-11.83
-11.91	-11.9	-11.89	-11.88	-11.87	-11.86	-11.85	-11.84	-11.83
-11.91	-11.9	-11.89	-11.88	-11.87	-11.86	-11.85	-11.84	-11.83
-11.91	-11.9	-11.89	-11.88	-11.87	-11.86	-11.85	-11.84	-11.83
-11.91	-11.9	-11.89	-11.88	-11.87	-11.86	-11.85	-11.84	-11.83
-11.91	-11.9	-11.89	-11.88	-11.87	-11.86	-11.85	-11.84	-11.83
-11.91	-11.9	-11.89	-11.88	-11.87	-11.86	-11.85	-11.84	-11.83
-11.91	-11.9	-11.89	-11.88	-11.87	-11.86	-11.85	-11.84	-11.83
-11.91	-11.9	-11.89	-11.88	-11.87	-11.86	-11.85	-11.84	-11.83
-11.91	-11.9	-11.89	-11.88	-11.87	-11.86	-11.85	-11.84	-11.83
-11.91	-11.9	-11.89	-11.88	-11.87	-11.86	-11.85	-11.84	-11.83
-11.91	-11.9	-11.89	-11.88	-11.87	-11.86	-11.85	-11.84	-11.83
-11.91	-11.9	-11.89	-11.88	-11.87	-11.86	-11.85	-11.84	-11.83
-11.91	-11.9	-11.89	-11.88	-11.87	-11.86	-11.85	-11.84	-11.83
-11.91	-11.9	-11.89	-11.88	-11.87	-11.86	-11.85	-11.84	-11.83
-11.91	-11.9	-11.89	-11.88	-11.87	-11.86	-11.85	-11.84	-11.83
-11.91	-11.9	-11.89	-11.88	-11.87	-11.86	-11.85	-11.84	-11.83
-11.91	-11.9	-11.89	-11.88	-11.87	-11.86	-11.85	-11.84	-11.83
-11.91	-11.9	-11.89	-11.88	-11.87	-11.86	-11.85	-11.84	-11.83
-11.91	-11.9	-11.89	-11.88	-11.87	-11.86	-11.85	-11.84	-11.83
-11.91	-11.9	-11.89	-11.88	-11.87	-11.86	-11.85	-11.84	-11.83
-11.91	-11.9	-11.89	-11.88	-11.87	-11.86	-11.85	-11.84	-11.83
-11.91	-11.9	-11.89	-11.88	-11.87	-11.86	-11.85	-11.84	-11.83
-11.91	-11.9	-11.89	-11.88	-11.87	-11.86	-11.85	-11.84	-11.83
-11.91	-11.9	-11.89	-11.88	-11.87	-11.86	-11.85	-11.84	-11.83
-11.91	-11.9	-11.89	-11.88	-11.87	-11.86	-11.85	-11.84	-11.83
-11.91	-11.9	-11.89	-11.88	-11.87	-11.86	-11.85	-11.84	-11.83
-11.91	-11.9	-11.89	-11.88	-11.87	-11.86	-11.85	-11.84	-11.83
-11.91	-11.9	-11.89	-11.88	-11.87	-11.86	-11.85	-11.84	-11.83
-11.91	-11.9	-11.89	-11.88	-11.87	-11.86	-11.85	-11.84	-11.83
-11.91	-11.9	-11.89	-11.88	-11.87	-11.86	-11.85	-11.84	-11.83
-11.91	-11.9	-11.89	-11.88	-11.87	-11.86	-11.85	-11.84	-11.83
-11.91	-11.9	-11.89	-11.88	-11.87	-11.86	-11.85	-11.84	-11.83
-11.91	-11.9	-11.89	-11.88	-11.87	-11.86	-11.85	-11.84	-11.83
-11.91	-11.9	-11.89	-11.88	-11.87	-11.86	-11.85	-11.84	-11.83
-11.91	-11.9	-11.89	-11.88	-11.87	-11.86	-11.85	-11.84	-11.83
-11.91	-11.9	-11.89	-11.88	-11.87	-11.86	-11.85	-11.84	-11.83
-11.91	-11.9	-11.89	-11.88	-11.87	-11.86	-11.85	-11.84	-11.83
-11.91	-11.9	-11.89	-11.88	-11.87	-11.86	-11.85	-11.84	-11.83
-11.91	-11.9	-11.89	-11.88	-11.87	-11.86	-11.85	-11.84	-11.83
-11.91	-11.9	-11.89	-11.88	-11.87	-11.86	-11.85	-11.84	-11.83
-11.91	-11.9	-11.89	-11.88	-11.87	-11.86	-11.85	-11.84	-11.83
-11.91	-11.9	-11.89	-11.88	-11.87	-11.86	-11.85	-11.84	-11.83
-11.91	-11.9	-11.89	-11.88	-11.87	-11.86	-11.85	-11.84	-11.83
-11.91	-11.9	-11.89	-11.88	-11.87	-11.86	-11.85	-11.84	-11.83
-11.91	-11.9	-11.89	-11.88	-11.87	-11.86	-11.85	-11.84	-11.83
-11.91	-11.9	-11.89	-11.88	-11.87	-11.86	-11.85	-11.84	-11.83

-11.91	-11.9	-11.89	-11.88	-11.87	-11.86	-11.85	-11.84	-11.83
-11.91	-11.9	-11.89	-11.88	-11.87	-11.86	-11.85	-11.84	-11.83
-11.91	-11.9	-11.89	-11.88	-11.87	-11.86	-11.85	-11.84	-11.83
-11.91	-11.9	-11.89	-11.88	-11.87	-11.86	-11.85	-11.84	-11.83
-11.91	-11.9	-11.89	-11.88	-11.87	-11.86	-11.85	-11.84	-11.83
-11.91	-11.9	-11.89	-11.88	-11.87	-11.86	-11.85	-11.84	-11.83
-11.91	-11.9	-11.89	-11.88	-11.87	-11.86	-11.85	-11.84	-11.83
-11.91	-11.9	-11.89	-11.88	-11.87	-11.86	-11.85	-11.84	-11.83
-11.91	-11.9	-11.89	-11.88	-11.87	-11.86	-11.85	-11.84	-11.83
-11.91	-11.9	-11.89	-11.88	-11.87	-11.86	-11.85	-11.84	-11.83
-11.91	-11.9	-11.89	-11.88	-11.87	-11.86	-11.85	-11.84	-11.83
-11.91	-11.9	-11.89	-11.88	-11.87	-11.86	-11.85	-11.84	-11.83
-11.91	-11.9	-11.89	-11.88	-11.87	-11.86	-11.85	-11.84	-11.83
-11.91	-11.9	-11.89	-11.88	-11.87	-11.86	-11.85	-11.84	-11.83
-11.91	-11.9	-11.89	-11.88	-11.87	-11.86	-11.85	-11.84	-11.83
-11.91	-11.9	-11.89	-11.88	-11.87	-11.86	-11.85	-11.84	-11.83
-11.91	-11.9	-11.89	-11.88	-11.87	-11.86	-11.85	-11.84	-11.83
-11.91	-11.9	-11.89	-11.88	-11.87	-11.86	-11.85	-11.84	-11.83
-11.91	-11.9	-11.89	-11.88	-11.87	-11.86	-11.85	-11.84	-11.83
-11.91	-11.9	-11.89	-11.88	-11.87	-11.86	-11.85	-11.84	-11.83
-11.91	-11.9	-11.89	-11.88	-11.87	-11.86	-11.85	-11.84	-11.83
-11.91	-11.9	-11.89	-11.88	-11.87	-11.86	-11.85	-11.84	-11.83
-11.91	-11.9	-11.89	-11.88	-11.87	-11.86	-11.85	-11.84	-11.83
-11.91	-11.9	-11.89	-11.88	-11.87	-11.86	-11.85	-11.84	-11.83
-11.91	-11.9	-11.89	-11.88	-11.87	-11.86	-11.85	-11.84	-11.83
-11.91	-11.9	-11.89	-11.88	-11.87	-11.86	-11.85	-11.84	-11.83
-11.91	-11.9	-11.89	-11.88	-11.87	-11.86	-11.85	-11.84	-11.83
-11.91	-11.9	-11.89	-11.88	-11.87	-11.86	-11.85	-11.84	-11.83
-11.91	-11.9	-11.89	-11.88	-11.87	-11.86	-11.85	-11.84	-11.83
-11.91	-11.9	-11.89	-11.88	-11.87	-11.86	-11.85	-11.84	-11.83
-11.91	-11.9	-11.89	-11.88	-11.87	-11.86	-11.85	-11.84	-11.83
-11.91	-11.9	-11.89	-11.88	-11.87	-11.86	-11.85	-11.84	-11.83
-11.91	-11.9	-11.89	-11.88	-11.87	-11.86	-11.85	-11.84	-11.83
-11.91	-11.9	-11.89	-11.88	-11.87	-11.86	-11.85	-11.84	-11.83
-11.91	-11.9	-11.89	-11.88	-11.87	-11.86	-11.85	-11.84	-11.83
-11.91	-11.9	-11.89	-11.88	-11.87	-11.86	-11.85	-11.84	-11.83
-11.91	-11.9	-11.89	-11.88	-11.87	-11.86	-11.85	-11.84	-11.83
-11.91	-11.9	-11.89	-11.88	-11.87	-11.86	-11.85	-11.84	-11.83
-11.91	-11.9	-11.89	-11.88	-11.87	-11.86	-11.85	-11.84	-11.83
-11.91	-11.9	-11.89	-11.88	-11.87	-11.86	-11.85	-11.84	-11.83
-11.91	-11.9	-11.89	-11.88	-11.87	-11.86	-11.85	-11.84	-11.83
-11.91	-11.9	-11.89	-11.88	-11.87	-11.86	-11.85	-11.84	-11.83
-11.91	-11.9	-11.89	-11.88	-11.87	-11.86	-11.85	-11.84	-11.83
-11.91	-11.9	-11.89	-11.88	-11.87	-11.86	-11.85	-11.84	-11.83
-11.91	-11.9	-11.89	-11.88	-11.87	-11.86	-11.85	-11.84	-11.83
-11.91	-11.9	-11.89	-11.88	-11.87	-11.86	-11.85	-11.84	-11.83
-11.91	-11.9	-11.89	-11.88	-11.87	-11.86	-11.85	-11.84	-11.83
-11.91	-11.9	-11.89	-11.88	-11.87	-11.86	-11.85	-11.84	-11.83
-11.91	-11.9	-11.89	-11.88	-11.87	-11.86	-11.85	-11.84	-11.83
-11.91	-11.9	-11.89	-11.88	-11.87	-11.86	-11.85	-11.84	-11.83

-11.91	-11.9	-11.89	-11.88	-11.87	-11.86	-11.85	-11.84	-11.83
-11.91	-11.9	-11.89	-11.88	-11.87	-11.86	-11.85	-11.84	-11.83
-11.91	-11.9	-11.89	-11.88	-11.87	-11.86	-11.85	-11.84	-11.83
-11.91	-11.9	-11.89	-11.88	-11.87	-11.86	-11.85	-11.84	-11.83
-11.91	-11.9	-11.89	-11.88	-11.87	-11.86	-11.85	-11.84	-11.83
-11.91	-11.9	-11.89	-11.88	-11.87	-11.86	-11.85	-11.84	-11.83
-11.91	-11.9	-11.89	-11.88	-11.87	-11.86	-11.85	-11.84	-11.83
-11.91	-11.9	-11.89	-11.88	-11.87	-11.86	-11.85	-11.84	-11.83
-11.91	-11.9	-11.89	-11.88	-11.87	-11.86	-11.85	-11.84	-11.83
-11.91	-11.9	-11.89	-11.88	-11.87	-11.86	-11.85	-11.84	-11.83
-11.91	-11.9	-11.89	-11.88	-11.87	-11.86	-11.85	-11.84	-11.83
-11.91	-11.9	-11.89	-11.88	-11.87	-11.86	-11.85	-11.84	-11.83
-11.91	-11.9	-11.89	-11.88	-11.87	-11.86	-11.85	-11.84	-11.83
-11.91	-11.9	-11.89	-11.88	-11.87	-11.86	-11.85	-11.84	-11.83
-11.91	-11.9	-11.89	-11.88	-11.87	-11.86	-11.85	-11.84	-11.83
-11.91	-11.9	-11.89	-11.88	-11.87	-11.86	-11.85	-11.84	-11.83
-11.91	-11.9	-11.89	-11.88	-11.87	-11.86	-11.85	-11.84	-11.83
-11.91	-11.9	-11.89	-11.88	-11.87	-11.86	-11.85	-11.84	-11.83
-11.91	-11.9	-11.89	-11.88	-11.87	-11.86	-11.85	-11.84	-11.83
-11.91	-11.9	-11.89	-11.88	-11.87	-11.86	-11.85	-11.84	-11.83
-11.91	-11.9	-11.89	-11.88	-11.87	-11.86	-11.85	-11.84	-11.83
-11.91	-11.9	-11.89	-11.88	-11.87	-11.86	-11.85	-11.84	-11.83
-11.91	-11.9	-11.89	-11.88	-11.87	-11.86	-11.85	-11.84	-11.83
-11.91	-11.9	-11.89	-11.88	-11.87	-11.86	-11.85	-11.84	-11.83
-11.91	-11.9	-11.89	-11.88	-11.87	-11.86	-11.85	-11.84	-11.83
-11.91	-11.9	-11.89	-11.88	-11.87	-11.86	-11.85	-11.84	-11.83
-11.91	-11.9	-11.89	-11.88	-11.87	-11.86	-11.85	-11.84	-11.83
-11.91	-11.9	-11.89	-11.88	-11.87	-11.86	-11.85	-11.84	-11.83
-11.91	-11.9	-11.89	-11.88	-11.87	-11.86	-11.85	-11.84	-11.83
-11.91	-11.9	-11.89	-11.88	-11.87	-11.86	-11.85	-11.84	-11.83
-11.91	-11.9	-11.89	-11.88	-11.87	-11.86	-11.85	-11.84	-11.83
-11.91	-11.9	-11.89	-11.88	-11.87	-11.86	-11.85	-11.84	-11.83
-11.91	-11.9	-11.89	-11.88	-11.87	-11.86	-11.85	-11.84	-11.83
-11.91	-11.9	-11.89	-11.88	-11.87	-11.86	-11.85	-11.84	-11.83
-11.91	-11.9	-11.89	-11.88	-11.87	-11.86	-11.85	-11.84	-11.83
-11.91	-11.9	-11.89	-11.88	-11.87	-11.86	-11.85	-11.84	-11.83
-11.91	-11.9	-11.89	-11.88	-11.87	-11.86	-11.85	-11.84	-11.83
-11.91	-11.9	-11.89	-11.88	-11.87	-11.86	-11.85	-11.84	-11.83
-11.91	-11.9	-11.89	-11.88	-11.87	-11.86	-11.85	-11.84	-11.83
-11.91	-11.9	-11.89	-11.88	-11.87	-11.86	-11.85	-11.84	-11.83
-11.91	-11.9	-11.89	-11.88	-11.87	-11.86	-11.85	-11.84	-11.83
-11.91	-11.9	-11.89	-11.88	-11.87	-11.86	-11.85	-11.84	-11.83
-11.91	-11.9	-11.89	-11.88	-11.87	-11.86	-11.85	-11.84	-11.83
-11.91	-11.9	-11.89	-11.88	-11.87	-11.86	-11.85	-11.84	-11.83
-11.91	-11.9	-11.89	-11.88	-11.87	-11.86	-11.85	-11.84	-11.83
-11.91	-11.9	-11.89	-11.88	-11.87	-11.86	-11.85	-11.84	-11.83
-11.91	-11.9	-11.89	-11.88	-11.87	-11.86	-11.85	-11.84	-11.83
-11.91	-11.9	-11.89	-11.88	-11.87	-11.86	-11.85	-11.84	-11.83
-11.91	-11.9	-11.89	-11.88	-11.87	-11.86	-11.85	-11.84	-11.83
-11.91	-11.9	-11.89	-11.88	-11.87	-11.86	-11.85	-11.84	-11.83

-11.91	-11.9	-11.89	-11.88	-11.87	-11.86	-11.85	-11.84	-11.83
-11.91	-11.9	-11.89	-11.88	-11.87	-11.86	-11.85	-11.84	-11.83
-11.91	-11.9	-11.89	-11.88	-11.87	-11.86	-11.85	-11.84	-11.83
-11.91	-11.9	-11.89	-11.88	-11.87	-11.86	-11.85	-11.84	-11.83
-11.91	-11.9	-11.89	-11.88	-11.87	-11.86	-11.85	-11.84	-11.83
-11.91	-11.9	-11.89	-11.88	-11.87	-11.86	-11.85	-11.84	-11.83
-11.91	-11.9	-11.89	-11.88	-11.87	-11.86	-11.85	-11.84	-11.83
-11.91	-11.9	-11.89	-11.88	-11.87	-11.86	-11.85	-11.84	-11.83
-11.91	-11.9	-11.89	-11.88	-11.87	-11.86	-11.85	-11.84	-11.83
-11.91	-11.9	-11.89	-11.88	-11.87	-11.86	-11.85	-11.84	-11.83
-11.91	-11.9	-11.89	-11.88	-11.87	-11.86	-11.85	-11.84	-11.83
-11.91	-11.9	-11.89	-11.88	-11.87	-11.86	-11.85	-11.84	-11.83
-11.91	-11.9	-11.89	-11.88	-11.87	-11.86	-11.85	-11.84	-11.83
-11.91	-11.9	-11.89	-11.88	-11.87	-11.86	-11.85	-11.84	-11.83
-11.91	-11.9	-11.89	-11.88	-11.87	-11.86	-11.85	-11.84	-11.83
-11.91	-11.9	-11.89	-11.88	-11.87	-11.86	-11.85	-11.84	-11.83
-11.91	-11.9	-11.89	-11.88	-11.87	-11.86	-11.85	-11.84	-11.83
-11.91	-11.9	-11.89	-11.88	-11.87	-11.86	-11.85	-11.84	-11.83
-11.91	-11.9	-11.89	-11.88	-11.87	-11.86	-11.85	-11.84	-11.83
-11.91	-11.9	-11.89	-11.88	-11.87	-11.86	-11.85	-11.84	-11.83
-11.91	-11.9	-11.89	-11.88	-11.87	-11.86	-11.85	-11.84	-11.83
-11.91	-11.9	-11.89	-11.88	-11.87	-11.86	-11.85	-11.84	-11.83
-11.91	-11.9	-11.89	-11.88	-11.87	-11.86	-11.85	-11.84	-11.83
-11.91	-11.9	-11.89	-11.88	-11.87	-11.86	-11.85	-11.84	-11.83
-11.91	-11.9	-11.89	-11.88	-11.87	-11.86	-11.85	-11.84	-11.83
-11.91	-11.9	-11.89	-11.88	-11.87	-11.86	-11.85	-11.84	-11.83
-11.91	-11.9	-11.89	-11.88	-11.87	-11.86	-11.85	-11.84	-11.83
-11.91	-11.9	-11.89	-11.88	-11.87	-11.86	-11.85	-11.84	-11.83
-11.91	-11.9	-11.89	-11.88	-11.87	-11.86	-11.85	-11.84	-11.83
-11.91	-11.9	-11.89	-11.88	-11.87	-11.86	-11.85	-11.84	-11.83
-11.91	-11.9	-11.89	-11.88	-11.87	-11.86	-11.85	-11.84	-11.83
-11.91	-11.9	-11.89	-11.88	-11.87	-11.86	-11.85	-11.84	-11.83
-11.91	-11.9	-11.89	-11.88	-11.87	-11.86	-11.85	-11.84	-11.83
-11.91	-11.9	-11.89	-11.88	-11.87	-11.86	-11.85	-11.84	-11.83
-11.91	-11.9	-11.89	-11.88	-11.87	-11.86	-11.85	-11.84	-11.83
-11.91	-11.9	-11.89	-11.88	-11.87	-11.86	-11.85	-11.84	-11.83
-11.91	-11.9	-11.89	-11.88	-11.87	-11.86	-11.85	-11.84	-11.83
-11.91	-11.9	-11.89	-11.88	-11.87	-11.86	-11.85	-11.84	-11.83
-11.91	-11.9	-11.89	-11.88	-11.87	-11.86	-11.85	-11.84	-11.83
-11.91	-11.9	-11.89	-11.88	-11.87	-11.86	-11.85	-11.84	-11.83
-11.91	-11.9	-11.89	-11.88	-11.87	-11.86	-11.85	-11.84	-11.83
-11.91	-11.9	-11.89	-11.88	-11.87	-11.86	-11.85	-11.84	-11.83
-11.91	-11.9	-11.89	-11.88	-11.87	-11.86	-11.85	-11.84	-11.83
-11.91	-11.9	-11.89	-11.88	-11.87	-11.86	-11.85	-11.84	-11.83
-11.91	-11.9	-11.89	-11.88	-11.87	-11.86	-11.85	-11.84	-11.83
-11.91	-11.9	-11.89	-11.88	-11.87	-11.86	-11.85	-11.84	-11.83
-11.91	-11.9	-11.89	-11.88	-11.87	-11.86	-11.85	-11.84	-11.83
-11.91	-11.9	-11.89	-11.88	-11.87	-11.86	-11.85	-11.84	-11.83
-11.91	-11.9	-11.89	-11.88	-11.87	-11.86	-11.85	-11.84	-11.83
-11.91	-11.9	-11.89	-11.88	-11.87	-11.86	-11.85	-11.84	-11.83

-11.91	-11.9	-11.89	-11.88	-11.87	-11.86	-11.85	-11.84	-11.83
-11.91	-11.9	-11.89	-11.88	-11.87	-11.86	-11.85	-11.84	-11.83
-11.91	-11.9	-11.89	-11.88	-11.87	-11.86	-11.85	-11.84	-11.83
-11.91	-11.9	-11.89	-11.88	-11.87	-11.86	-11.85	-11.84	-11.83
-11.91	-11.9	-11.89	-11.88	-11.87	-11.86	-11.85	-11.84	-11.83
-11.91	-11.9	-11.89	-11.88	-11.87	-11.86	-11.85	-11.84	-11.83
-11.91	-11.9	-11.89	-11.88	-11.87	-11.86	-11.85	-11.84	-11.83
-11.91	-11.9	-11.89	-11.88	-11.87	-11.86	-11.85	-11.84	-11.83
-11.91	-11.9	-11.89	-11.88	-11.87	-11.86	-11.85	-11.84	-11.83
-11.91	-11.9	-11.89	-11.88	-11.87	-11.86	-11.85	-11.84	-11.83
-11.91	-11.9	-11.89	-11.88	-11.87	-11.86	-11.85	-11.84	-11.83
-11.91	-11.9	-11.89	-11.88	-11.87	-11.86	-11.85	-11.84	-11.83
-11.91	-11.9	-11.89	-11.88	-11.87	-11.86	-11.85	-11.84	-11.83
-11.91	-11.9	-11.89	-11.88	-11.87	-11.86	-11.85	-11.84	-11.83
-11.91	-11.9	-11.89	-11.88	-11.87	-11.86	-11.85	-11.84	-11.83
-11.91	-11.9	-11.89	-11.88	-11.87	-11.86	-11.85	-11.84	-11.83
-11.91	-11.9	-11.89	-11.88	-11.87	-11.86	-11.85	-11.84	-11.83
-11.91	-11.9	-11.89	-11.88	-11.87	-11.86	-11.85	-11.84	-11.83
-11.91	-11.9	-11.89	-11.88	-11.87	-11.86	-11.85	-11.84	-11.83
-11.91	-11.9	-11.89	-11.88	-11.87	-11.86	-11.85	-11.84	-11.83
-11.91	-11.9	-11.89	-11.88	-11.87	-11.86	-11.85	-11.84	-11.83
-11.91	-11.9	-11.89	-11.88	-11.87	-11.86	-11.85	-11.84	-11.83
-11.91	-11.9	-11.89	-11.88	-11.87	-11.86	-11.85	-11.84	-11.83
-11.91	-11.9	-11.89	-11.88	-11.87	-11.86	-11.85	-11.84	-11.83
-11.91	-11.9	-11.89	-11.88	-11.87	-11.86	-11.85	-11.84	-11.83
-11.91	-11.9	-11.89	-11.88	-11.87	-11.86	-11.85	-11.84	-11.83
-11.91	-11.9	-11.89	-11.88	-11.87	-11.86	-11.85	-11.84	-11.83
-11.91	-11.9	-11.89	-11.88	-11.87	-11.86	-11.85	-11.84	-11.83
-11.91	-11.9	-11.89	-11.88	-11.87	-11.86	-11.85	-11.84	-11.83
-11.91	-11.9	-11.89	-11.88	-11.87	-11.86	-11.85	-11.84	-11.83
-11.91	-11.9	-11.89	-11.88	-11.87	-11.86	-11.85	-11.84	-11.83
-11.91	-11.9	-11.89	-11.88	-11.87	-11.86	-11.85	-11.84	-11.83
-11.91	-11.9	-11.89	-11.88	-11.87	-11.86	-11.85	-11.84	-11.83
-11.91	-11.9	-11.89	-11.88	-11.87	-11.86	-11.85	-11.84	-11.83
-11.91	-11.9	-11.89	-11.88	-11.87	-11.86	-11.85	-11.84	-11.83
-11.91	-11.9	-11.89	-11.88	-11.87	-11.86	-11.85	-11.84	-11.83
-11.91	-11.9	-11.89	-11.88	-11.87	-11.86	-11.85	-11.84	-11.83
-11.91	-11.9	-11.89	-11.88	-11.87	-11.86	-11.85	-11.84	-11.83
-11.91	-11.9	-11.89	-11.88	-11.87	-11.86	-11.85	-11.84	-11.83
-11.91	-11.9	-11.89	-11.88	-11.87	-11.86	-11.85	-11.84	-11.83
-11.91	-11.9	-11.89	-11.88	-11.87	-11.86	-11.85	-11.84	-11.83
-11.91	-11.9	-11.89	-11.88	-11.87	-11.86	-11.85	-11.84	-11.83
-11.91	-11.9	-11.89	-11.88	-11.87	-11.86	-11.85	-11.84	-11.83
-11.91	-11.9	-11.89	-11.88	-11.87	-11.86	-11.85	-11.84	-11.83
-11.91	-11.9	-11.89	-11.88	-11.87	-11.86	-11.85	-11.84	-11.83
-11.91	-11.9	-11.89	-11.88	-11.87	-11.86	-11.85	-11.84	-11.83
-11.91	-11.9	-11.89	-11.88	-11.87	-11.86	-11.85	-11.84	-11.83
-11.91	-11.9	-11.89	-11.88	-11.87	-11.86	-11.85	-11.84	-11.83
-11.91	-11.9	-11.89	-11.88	-11.87	-11.86	-11.85	-11.84	-11.83
-11.91	-11.9	-11.89	-11.88	-11.87	-11.86	-11.85	-11.84	-11.83

-11.91	-11.9	-11.89	-11.88	-11.87	-11.86	-11.85	-11.84	-11.83
-11.91	-11.9	-11.89	-11.88	-11.87	-11.86	-11.85	-11.84	-11.83
-11.91	-11.9	-11.89	-11.88	-11.87	-11.86	-11.85	-11.84	-11.83
-11.91	-11.9	-11.89	-11.88	-11.87	-11.86	-11.85	-11.84	-11.83
-11.91	-11.9	-11.89	-11.88	-11.87	-11.86	-11.85	-11.84	-11.83
-11.91	-11.9	-11.89	-11.88	-11.87	-11.86	-11.85	-11.84	-11.83
-11.91	-11.9	-11.89	-11.88	-11.87	-11.86	-11.85	-11.84	-11.83
-11.91	-11.9	-11.89	-11.88	-11.87	-11.86	-11.85	-11.84	-11.83
-11.91	-11.9	-11.89	-11.88	-11.87	-11.86	-11.85	-11.84	-11.83
-11.91	-11.9	-11.89	-11.88	-11.87	-11.86	-11.85	-11.84	-11.83
-11.91	-11.9	-11.89	-11.88	-11.87	-11.86	-11.85	-11.84	-11.83
-11.91	-11.9	-11.89	-11.88	-11.87	-11.86	-11.85	-11.84	-11.83
-11.91	-11.9	-11.89	-11.88	-11.87	-11.86	-11.85	-11.84	-11.83
-11.91	-11.9	-11.89	-11.88	-11.87	-11.86	-11.85	-11.84	-11.83
-11.91	-11.9	-11.89	-11.88	-11.87	-11.86	-11.85	-11.84	-11.83
-11.91	-11.9	-11.89	-11.88	-11.87	-11.86	-11.85	-11.84	-11.83
-11.91	-11.9	-11.89	-11.88	-11.87	-11.86	-11.85	-11.84	-11.83
-11.91	-11.9	-11.89	-11.88	-11.87	-11.86	-11.85	-11.84	-11.83
-11.91	-11.9	-11.89	-11.88	-11.87	-11.86	-11.85	-11.84	-11.83
-11.91	-11.9	-11.89	-11.88	-11.87	-11.86	-11.85	-11.84	-11.83
-11.91	-11.9	-11.89	-11.88	-11.87	-11.86	-11.85	-11.84	-11.83
-11.91	-11.9	-11.89	-11.88	-11.87	-11.86	-11.85	-11.84	-11.83
-11.91	-11.9	-11.89	-11.88	-11.87	-11.86	-11.85	-11.84	-11.83
-11.91	-11.9	-11.89	-11.88	-11.87	-11.86	-11.85	-11.84	-11.83
-11.91	-11.9	-11.89	-11.88	-11.87	-11.86	-11.85	-11.84	-11.83
-11.91	-11.9	-11.89	-11.88	-11.87	-11.86	-11.85	-11.84	-11.83
-11.91	-11.9	-11.89	-11.88	-11.87	-11.86	-11.85	-11.84	-11.83
-11.91	-11.9	-11.89	-11.88	-11.87	-11.86	-11.85	-11.84	-11.83
-11.91	-11.9	-11.89	-11.88	-11.87	-11.86	-11.85	-11.84	-11.83
-11.91	-11.9	-11.89	-11.88	-11.87	-11.86	-11.85	-11.84	-11.83
-11.91	-11.9	-11.89	-11.88	-11.87	-11.86	-11.85	-11.84	-11.83
-11.91	-11.9	-11.89	-11.88	-11.87	-11.86	-11.85	-11.84	-11.83
-11.91	-11.9	-11.89	-11.88	-11.87	-11.86	-11.85	-11.84	-11.83
-11.91	-11.9	-11.89	-11.88	-11.87	-11.86	-11.85	-11.84	-11.83
-11.91	-11.9	-11.89	-11.88	-11.87	-11.86	-11.85	-11.84	-11.83
-11.91	-11.9	-11.89	-11.88	-11.87	-11.86	-11.85	-11.84	-11.83
-11.91	-11.9	-11.89	-11.88	-11.87	-11.86	-11.85	-11.84	-11.83
-11.91	-11.9	-11.89	-11.88	-11.87	-11.86	-11.85	-11.84	-11.83
-11.91	-11.9	-11.89	-11.88	-11.87	-11.86	-11.85	-11.84	-11.83
-11.91	-11.9	-11.89	-11.88	-11.87	-11.86	-11.85	-11.84	-11.83
-11.91	-11.9	-11.89	-11.88	-11.87	-11.86	-11.85	-11.84	-11.83
-11.91	-11.9	-11.89	-11.88	-11.87	-11.86	-11.85	-11.84	-11.83
-11.91	-11.9	-11.89	-11.88	-11.87	-11.86	-11.85	-11.84	-11.83
-11.91	-11.9	-11.89	-11.88	-11.87	-11.86	-11.85	-11.84	-11.83
-11.91	-11.9	-11.89	-11.88	-11.87	-11.86	-11.85	-11.84	-11.83
-11.91	-11.9	-11.89	-11.88	-11.87	-11.86	-11.85	-11.84	-11.83
-11.91	-11.9	-11.89	-11.88	-11.87	-11.86	-11.85	-11.84	-11.83
-11.91	-11.9	-11.89	-11.88	-11.87	-11.86	-11.85	-11.84	-11.83
-11.91	-11.9	-11.89	-11.88	-11.87	-11.86	-11.85	-11.84	-11.83
-11.91	-11.9	-11.89	-11.88	-11.87	-11.86	-11.85	-11.84	-11.83

-11.91	-11.9	-11.89	-11.88	-11.87	-11.86	-11.85	-11.84	-11.83
-11.91	-11.9	-11.89	-11.88	-11.87	-11.86	-11.85	-11.84	-11.83
-11.91	-11.9	-11.89	-11.88	-11.87	-11.86	-11.85	-11.84	-11.83
-11.91	-11.9	-11.89	-11.88	-11.87	-11.86	-11.85	-11.84	-11.83
-11.91	-11.9	-11.89	-11.88	-11.87	-11.86	-11.85	-11.84	-11.83
-11.91	-11.9	-11.89	-11.88	-11.87	-11.86	-11.85	-11.84	-11.83
-11.91	-11.9	-11.89	-11.88	-11.87	-11.86	-11.85	-11.84	-11.83
-11.91	-11.9	-11.89	-11.88	-11.87	-11.86	-11.85	-11.84	-11.83
-11.91	-11.9	-11.89	-11.88	-11.87	-11.86	-11.85	-11.84	-11.83
-11.91	-11.9	-11.89	-11.88	-11.87	-11.86	-11.85	-11.84	-11.83
-11.91	-11.9	-11.89	-11.88	-11.87	-11.86	-11.85	-11.84	-11.83
-11.91	-11.9	-11.89	-11.88	-11.87	-11.86	-11.85	-11.84	-11.83
-11.91	-11.9	-11.89	-11.88	-11.87	-11.86	-11.85	-11.84	-11.83
-11.91	-11.9	-11.89	-11.88	-11.87	-11.86	-11.85	-11.84	-11.83
-11.91	-11.9	-11.89	-11.88	-11.87	-11.86	-11.85	-11.84	-11.83
-11.91	-11.9	-11.89	-11.88	-11.87	-11.86	-11.85	-11.84	-11.83
-11.91	-11.9	-11.89	-11.88	-11.87	-11.86	-11.85	-11.84	-11.83
-11.91	-11.9	-11.89	-11.88	-11.87	-11.86	-11.85	-11.84	-11.83
-11.91	-11.9	-11.89	-11.88	-11.87	-11.86	-11.85	-11.84	-11.83
-11.91	-11.9	-11.89	-11.88	-11.87	-11.86	-11.85	-11.84	-11.83
-11.91	-11.9	-11.89	-11.88	-11.87	-11.86	-11.85	-11.84	-11.83
-11.91	-11.9	-11.89	-11.88	-11.87	-11.86	-11.85	-11.84	-11.83
-11.91	-11.9	-11.89	-11.88	-11.87	-11.86	-11.85	-11.84	-11.83
-11.91	-11.9	-11.89	-11.88	-11.87	-11.86	-11.85	-11.84	-11.83
-11.91	-11.9	-11.89	-11.88	-11.87	-11.86	-11.85	-11.84	-11.83
-11.91	-11.9	-11.89	-11.88	-11.87	-11.86	-11.85	-11.84	-11.83
-11.91	-11.9	-11.89	-11.88	-11.87	-11.86	-11.85	-11.84	-11.83
-11.91	-11.9	-11.89	-11.88	-11.87	-11.86	-11.85	-11.84	-11.83
-11.91	-11.9	-11.89	-11.88	-11.87	-11.86	-11.85	-11.84	-11.83
-11.91	-11.9	-11.89	-11.88	-11.87	-11.86	-11.85	-11.84	-11.83
-11.91	-11.9	-11.89	-11.88	-11.87	-11.86	-11.85	-11.84	-11.83
-11.91	-11.9	-11.89	-11.88	-11.87	-11.86	-11.85	-11.84	-11.83
-11.91	-11.9	-11.89	-11.88	-11.87	-11.86	-11.85	-11.84	-11.83
-11.91	-11.9	-11.89	-11.88	-11.87	-11.86	-11.85	-11.84	-11.83
-11.91	-11.9	-11.89	-11.88	-11.87	-11.86	-11.85	-11.84	-11.83
-11.91	-11.9	-11.89	-11.88	-11.87	-11.86	-11.85	-11.84	-11.83
-11.91	-11.9	-11.89	-11.88	-11.87	-11.86	-11.85	-11.84	-11.83
-11.91	-11.9	-11.89	-11.88	-11.87	-11.86	-11.85	-11.84	-11.83
-11.91	-11.9	-11.89	-11.88	-11.87	-11.86	-11.85	-11.84	-11.83
-11.91	-11.9	-11.89	-11.88	-11.87	-11.86	-11.85	-11.84	-11.83
-11.91	-11.9	-11.89	-11.88	-11.87	-11.86	-11.85	-11.84	-11.83
-11.91	-11.9	-11.89	-11.88	-11.87	-11.86	-11.85	-11.84	-11.83
-11.91	-11.9	-11.89	-11.88	-11.87	-11.86	-11.85	-11.84	-11.83
-11.91	-11.9	-11.89	-11.88	-11.87	-11.86	-11.85	-11.84	-11.83
-11.91	-11.9	-11.89	-11.88	-11.87	-11.86	-11.85	-11.84	-11.83
-11.91	-11.9	-11.89	-11.88	-11.87	-11.86	-11.85	-11.84	-11.83
-11.91	-11.9	-11.89	-11.88	-11.87	-11.86	-11.85	-11.84	-11.83
-11.91	-11.9	-11.89	-11.88	-11.87	-11.86	-11.85	-11.84	-11.83
-11.91	-11.9	-11.89	-11.88	-11.87	-11.86	-11.85	-11.84	-11.83
-11.91	-11.9	-11.89	-11.88	-11.87	-11.86	-11.85	-11.84	-11.83

-11.91	-11.9	-11.89	-11.88	-11.87	-11.86	-11.85	-11.84	-11.83
-11.91	-11.9	-11.89	-11.88	-11.87	-11.86	-11.85	-11.84	-11.83
-11.91	-11.9	-11.89	-11.88	-11.87	-11.86	-11.85	-11.84	-11.83
-11.91	-11.9	-11.89	-11.88	-11.87	-11.86	-11.85	-11.84	-11.83
-11.91	-11.9	-11.89	-11.88	-11.87	-11.86	-11.85	-11.84	-11.83
-11.91	-11.9	-11.89	-11.88	-11.87	-11.86	-11.85	-11.84	-11.83
-11.91	-11.9	-11.89	-11.88	-11.87	-11.86	-11.85	-11.84	-11.83
-11.91	-11.9	-11.89	-11.88	-11.87	-11.86	-11.85	-11.84	-11.83
-11.91	-11.9	-11.89	-11.88	-11.87	-11.86	-11.85	-11.84	-11.83
-11.91	-11.9	-11.89	-11.88	-11.87	-11.86	-11.85	-11.84	-11.83
-11.91	-11.9	-11.89	-11.88	-11.87	-11.86	-11.85	-11.84	-11.83
-11.91	-11.9	-11.89	-11.88	-11.87	-11.86	-11.85	-11.84	-11.83
-11.91	-11.9	-11.89	-11.88	-11.87	-11.86	-11.85	-11.84	-11.83
-11.91	-11.9	-11.89	-11.88	-11.87	-11.86	-11.85	-11.84	-11.83
-11.91	-11.9	-11.89	-11.88	-11.87	-11.86	-11.85	-11.84	-11.83
-11.91	-11.9	-11.89	-11.88	-11.87	-11.86	-11.85	-11.84	-11.83
-11.91	-11.9	-11.89	-11.88	-11.87	-11.86	-11.85	-11.84	-11.83
-11.91	-11.9	-11.89	-11.88	-11.87	-11.86	-11.85	-11.84	-11.83
-11.91	-11.9	-11.89	-11.88	-11.87	-11.86	-11.85	-11.84	-11.83
-11.91	-11.9	-11.89	-11.88	-11.87	-11.86	-11.85	-11.84	-11.83
-11.91	-11.9	-11.89	-11.88	-11.87	-11.86	-11.85	-11.84	-11.83
-11.91	-11.9	-11.89	-11.88	-11.87	-11.86	-11.85	-11.84	-11.83
-11.91	-11.9	-11.89	-11.88	-11.87	-11.86	-11.85	-11.84	-11.83
-11.91	-11.9	-11.89	-11.88	-11.87	-11.86	-11.85	-11.84	-11.83
-11.91	-11.9	-11.89	-11.88	-11.87	-11.86	-11.85	-11.84	-11.83
-11.91	-11.9	-11.89	-11.88	-11.87	-11.86	-11.85	-11.84	-11.83
-11.91	-11.9	-11.89	-11.88	-11.87	-11.86	-11.85	-11.84	-11.83
-11.91	-11.9	-11.89	-11.88	-11.87	-11.86	-11.85	-11.84	-11.83
-11.91	-11.9	-11.89	-11.88	-11.87	-11.86	-11.85	-11.84	-11.83
-11.91	-11.9	-11.89	-11.88	-11.87	-11.86	-11.85	-11.84	-11.83
-11.91	-11.9	-11.89	-11.88	-11.87	-11.86	-11.85	-11.84	-11.83
-11.91	-11.9	-11.89	-11.88	-11.87	-11.86	-11.85	-11.84	-11.83
-11.91	-11.9	-11.89	-11.88	-11.87	-11.86	-11.85	-11.84	-11.83
-11.91	-11.9	-11.89	-11.88	-11.87	-11.86	-11.85	-11.84	-11.83
-11.91	-11.9	-11.89	-11.88	-11.87	-11.86	-11.85	-11.84	-11.83
-11.91	-11.9	-11.89	-11.88	-11.87	-11.86	-11.85	-11.84	-11.83
-11.91	-11.9	-11.89	-11.88	-11.87	-11.86	-11.85	-11.84	-11.83
-11.91	-11.9	-11.89	-11.88	-11.87	-11.86	-11.85	-11.84	-11.83
-11.91	-11.9	-11.89	-11.88	-11.87	-11.86	-11.85	-11.84	-11.83
-11.91	-11.9	-11.89	-11.88	-11.87	-11.86	-11.85	-11.84	-11.83
-11.91	-11.9	-11.89	-11.88	-11.87	-11.86	-11.85	-11.84	-11.83
-11.91	-11.9	-11.89	-11.88	-11.87	-11.86	-11.85	-11.84	-11.83
-11.91	-11.9	-11.89	-11.88	-11.87	-11.86	-11.85	-11.84	-11.83
-11.91	-11.9	-11.89	-11.88	-11.87	-11.86	-11.85	-11.84	-11.83
-11.91	-11.9	-11.89	-11.88	-11.87	-11.86	-11.85	-11.84	-11.83
-11.91	-11.9	-11.89	-11.88	-11.87	-11.86	-11.85	-11.84	-11.83
-11.91	-11.9	-11.89	-11.88	-11.87	-11.86	-11.85	-11.84	-11.83
-11.91	-11.9	-11.89	-11.88	-11.87	-11.86	-11.85	-11.84	-11.83
-11.91	-11.9	-11.89	-11.88	-11.87	-11.86	-11.85	-11.84	-11.83
-11.91	-11.9	-11.89	-11.88	-11.87	-11.86	-11.85	-11.84	-11.83

-11.91	-11.9	-11.89	-11.88	-11.87	-11.86	-11.85	-11.84	-11.83
-11.91	-11.9	-11.89	-11.88	-11.87	-11.86	-11.85	-11.84	-11.83
-11.91	-11.9	-11.89	-11.88	-11.87	-11.86	-11.85	-11.84	-11.83
-11.91	-11.9	-11.89	-11.88	-11.87	-11.86	-11.85	-11.84	-11.83
-11.91	-11.9	-11.89	-11.88	-11.87	-11.86	-11.85	-11.84	-11.83
-11.91	-11.9	-11.89	-11.88	-11.87	-11.86	-11.85	-11.84	-11.83
-11.91	-11.9	-11.89	-11.88	-11.87	-11.86	-11.85	-11.84	-11.83
-11.91	-11.9	-11.89	-11.88	-11.87	-11.86	-11.85	-11.84	-11.83
-11.91	-11.9	-11.89	-11.88	-11.87	-11.86	-11.85	-11.84	-11.83
-11.91	-11.9	-11.89	-11.88	-11.87	-11.86	-11.85	-11.84	-11.83
-11.91	-11.9	-11.89	-11.88	-11.87	-11.86	-11.85	-11.84	-11.83
-11.91	-11.9	-11.89	-11.88	-11.87	-11.86	-11.85	-11.84	-11.83
-11.91	-11.9	-11.89	-11.88	-11.87	-11.86	-11.85	-11.84	-11.83
-11.91	-11.9	-11.89	-11.88	-11.87	-11.86	-11.85	-11.84	-11.83
-11.91	-11.9	-11.89	-11.88	-11.87	-11.86	-11.85	-11.84	-11.83
-11.91	-11.9	-11.89	-11.88	-11.87	-11.86	-11.85	-11.84	-11.83
-11.91	-11.9	-11.89	-11.88	-11.87	-11.86	-11.85	-11.84	-11.83
-11.91	-11.9	-11.89	-11.88	-11.87	-11.86	-11.85	-11.84	-11.83
-11.91	-11.9	-11.89	-11.88	-11.87	-11.86	-11.85	-11.84	-11.83
-11.91	-11.9	-11.89	-11.88	-11.87	-11.86	-11.85	-11.84	-11.83
-11.91	-11.9	-11.89	-11.88	-11.87	-11.86	-11.85	-11.84	-11.83
-11.91	-11.9	-11.89	-11.88	-11.87	-11.86	-11.85	-11.84	-11.83
-11.91	-11.9	-11.89	-11.88	-11.87	-11.86	-11.85	-11.84	-11.83
-11.91	-11.9	-11.89	-11.88	-11.87	-11.86	-11.85	-11.84	-11.83
-11.91	-11.9	-11.89	-11.88	-11.87	-11.86	-11.85	-11.84	-11.83
-11.91	-11.9	-11.89	-11.88	-11.87	-11.86	-11.85	-11.84	-11.83
-11.91	-11.9	-11.89	-11.88	-11.87	-11.86	-11.85	-11.84	-11.83
-11.91	-11.9	-11.89	-11.88	-11.87	-11.86	-11.85	-11.84	-11.83
-11.91	-11.9	-11.89	-11.88	-11.87	-11.86	-11.85	-11.84	-11.83
-11.91	-11.9	-11.89	-11.88	-11.87	-11.86	-11.85	-11.84	-11.83
-11.91	-11.9	-11.89	-11.88	-11.87	-11.86	-11.85	-11.84	-11.83
-11.91	-11.9	-11.89	-11.88	-11.87	-11.86	-11.85	-11.84	-11.83
-11.91	-11.9	-11.89	-11.88	-11.87	-11.86	-11.85	-11.84	-11.83
-11.91	-11.9	-11.89	-11.88	-11.87	-11.86	-11.85	-11.84	-11.83
-11.91	-11.9	-11.89	-11.88	-11.87	-11.86	-11.85	-11.84	-11.83
-11.91	-11.9	-11.89	-11.88	-11.87	-11.86	-11.85	-11.84	-11.83
-11.91	-11.9	-11.89	-11.88	-11.87	-11.86	-11.85	-11.84	-11.83
-11.91	-11.9	-11.89	-11.88	-11.87	-11.86	-11.85	-11.84	-11.83
-11.91	-11.9	-11.89	-11.88	-11.87	-11.86	-11.85	-11.84	-11.83
-11.91	-11.9	-11.89	-11.88	-11.87	-11.86	-11.85	-11.84	-11.83
-11.91	-11.9	-11.89	-11.88	-11.87	-11.86	-11.85	-11.84	-11.83
-11.91	-11.9	-11.89	-11.88	-11.87	-11.86	-11.85	-11.84	-11.83
-11.91	-11.9	-11.89	-11.88	-11.87	-11.86	-11.85	-11.84	-11.83
-11.91	-11.9	-11.89	-11.88	-11.87	-11.86	-11.85	-11.84	-11.83
-11.91	-11.9	-11.89	-11.88	-11.87	-11.86	-11.85	-11.84	-11.83
-11.91	-11.9	-11.89	-11.88	-11.87	-11.86	-11.85	-11.84	-11.83
-11.91	-11.9	-11.89	-11.88	-11.87	-11.86	-11.85	-11.84	-11.83
-11.91	-11.9	-11.89	-11.88	-11.87	-11.86	-11.85	-11.84	-11.83
-11.91	-11.9	-11.89	-11.88	-11.87	-11.86	-11.85	-11.84	-11.83
-11.91	-11.9	-11.89	-11.88	-11.87	-11.86	-11.85	-11.84	-11.83

-11.91	-11.9	-11.89	-11.88	-11.87	-11.86	-11.85	-11.84	-11.83
-11.91	-11.9	-11.89	-11.88	-11.87	-11.86	-11.85	-11.84	-11.83
-11.91	-11.9	-11.89	-11.88	-11.87	-11.86	-11.85	-11.84	-11.83
-11.91	-11.9	-11.89	-11.88	-11.87	-11.86	-11.85	-11.84	-11.83
-11.91	-11.9	-11.89	-11.88	-11.87	-11.86	-11.85	-11.84	-11.83
-11.91	-11.9	-11.89	-11.88	-11.87	-11.86	-11.85	-11.84	-11.83
-11.91	-11.9	-11.89	-11.88	-11.87	-11.86	-11.85	-11.84	-11.83
-11.91	-11.9	-11.89	-11.88	-11.87	-11.86	-11.85	-11.84	-11.83
-11.91	-11.9	-11.89	-11.88	-11.87	-11.86	-11.85	-11.84	-11.83
-11.91	-11.9	-11.89	-11.88	-11.87	-11.86	-11.85	-11.84	-11.83
-11.91	-11.9	-11.89	-11.88	-11.87	-11.86	-11.85	-11.84	-11.83
-11.91	-11.9	-11.89	-11.88	-11.87	-11.86	-11.85	-11.84	-11.83
-11.91	-11.9	-11.89	-11.88	-11.87	-11.86	-11.85	-11.84	-11.83
-11.91	-11.9	-11.89	-11.88	-11.87	-11.86	-11.85	-11.84	-11.83
-11.91	-11.9	-11.89	-11.88	-11.87	-11.86	-11.85	-11.84	-11.83
-11.91	-11.9	-11.89	-11.88	-11.87	-11.86	-11.85	-11.84	-11.83
-11.91	-11.9	-11.89	-11.88	-11.87	-11.86	-11.85	-11.84	-11.83
-11.91	-11.9	-11.89	-11.88	-11.87	-11.86	-11.85	-11.84	-11.83
-11.91	-11.9	-11.89	-11.88	-11.87	-11.86	-11.85	-11.84	-11.83
-11.91	-11.9	-11.89	-11.88	-11.87	-11.86	-11.85	-11.84	-11.83
-11.91	-11.9	-11.89	-11.88	-11.87	-11.86	-11.85	-11.84	-11.83
-11.91	-11.9	-11.89	-11.88	-11.87	-11.86	-11.85	-11.84	-11.83
-11.91	-11.9	-11.89	-11.88	-11.87	-11.86	-11.85	-11.84	-11.83
-11.91	-11.9	-11.89	-11.88	-11.87	-11.86	-11.85	-11.84	-11.83
-11.91	-11.9	-11.89	-11.88	-11.87	-11.86	-11.85	-11.84	-11.83
-11.91	-11.9	-11.89	-11.88	-11.87	-11.86	-11.85	-11.84	-11.83
-11.91	-11.9	-11.89	-11.88	-11.87	-11.86	-11.85	-11.84	-11.83
-11.91	-11.9	-11.89	-11.88	-11.87	-11.86	-11.85	-11.84	-11.83
-11.91	-11.9	-11.89	-11.88	-11.87	-11.86	-11.85	-11.84	-11.83
-11.91	-11.9	-11.89	-11.88	-11.87	-11.86	-11.85	-11.84	-11.83
-11.91	-11.9	-11.89	-11.88	-11.87	-11.86	-11.85	-11.84	-11.83
-11.91	-11.9	-11.89	-11.88	-11.87	-11.86	-11.85	-11.84	-11.83
-11.91	-11.9	-11.89	-11.88	-11.87	-11.86	-11.85	-11.84	-11.83
-11.91	-11.9	-11.89	-11.88	-11.87	-11.86	-11.85	-11.84	-11.83
-11.91	-11.9	-11.89	-11.88	-11.87	-11.86	-11.85	-11.84	-11.83
-11.91	-11.9	-11.89	-11.88	-11.87	-11.86	-11.85	-11.84	-11.83
-11.91	-11.9	-11.89	-11.88	-11.87	-11.86	-11.85	-11.84	-11.83
-11.91	-11.9	-11.89	-11.88	-11.87	-11.86	-11.85	-11.84	-11.83
-11.91	-11.9	-11.89	-11.88	-11.87	-11.86	-11.85	-11.84	-11.83
-11.91	-11.9	-11.89	-11.88	-11.87	-11.86	-11.85	-11.84	-11.83
-11.91	-11.9	-11.89	-11.88	-11.87	-11.86	-11.85	-11.84	-11.83
-11.91	-11.9	-11.89	-11.88	-11.87	-11.86	-11.85	-11.84	-11.83
-11.91	-11.9	-11.89	-11.88	-11.87	-11.86	-11.85	-11.84	-11.83
-11.91	-11.9	-11.89	-11.88	-11.87	-11.86	-11.85	-11.84	-11.83
-11.91	-11.9	-11.89	-11.88	-11.87	-11.86	-11.85	-11.84	-11.83
-11.91	-11.9	-11.89	-11.88	-11.87	-11.86	-11.85	-11.84	-11.83
-11.91	-11.9	-11.89	-11.88	-11.87	-11.86	-11.85	-11.84	-11.83
-11.91	-11.9	-11.89	-11.88	-11.87	-11.86	-11.85	-11.84	-11.83
-11.91	-11.9	-11.89	-11.88	-11.87	-11.86	-11.85	-11.84	-11.83
-11.91	-11.9	-11.89	-11.88	-11.87	-11.86	-11.85	-11.84	-11.83

-11.91	-11.9	-11.89	-11.88	-11.87	-11.86	-11.85	-11.84	-11.83
-11.91	-11.9	-11.89	-11.88	-11.87	-11.86	-11.85	-11.84	-11.83
-11.91	-11.9	-11.89	-11.88	-11.87	-11.86	-11.85	-11.84	-11.83
-11.91	-11.9	-11.89	-11.88	-11.87	-11.86	-11.85	-11.84	-11.83
-11.91	-11.9	-11.89	-11.88	-11.87	-11.86	-11.85	-11.84	-11.83
-11.91	-11.9	-11.89	-11.88	-11.87	-11.86	-11.85	-11.84	-11.83
-11.91	-11.9	-11.89	-11.88	-11.87	-11.86	-11.85	-11.84	-11.83
-11.91	-11.9	-11.89	-11.88	-11.87	-11.86	-11.85	-11.84	-11.83
-11.91	-11.9	-11.89	-11.88	-11.87	-11.86	-11.85	-11.84	-11.83
-11.91	-11.9	-11.89	-11.88	-11.87	-11.86	-11.85	-11.84	-11.83
-11.91	-11.9	-11.89	-11.88	-11.87	-11.86	-11.85	-11.84	-11.83
-11.91	-11.9	-11.89	-11.88	-11.87	-11.86	-11.85	-11.84	-11.83
-11.91	-11.9	-11.89	-11.88	-11.87	-11.86	-11.85	-11.84	-11.83
-11.91	-11.9	-11.89	-11.88	-11.87	-11.86	-11.85	-11.84	-11.83
-11.91	-11.9	-11.89	-11.88	-11.87	-11.86	-11.85	-11.84	-11.83
-11.91	-11.9	-11.89	-11.88	-11.87	-11.86	-11.85	-11.84	-11.83
-11.91	-11.9	-11.89	-11.88	-11.87	-11.86	-11.85	-11.84	-11.83
-11.91	-11.9	-11.89	-11.88	-11.87	-11.86	-11.85	-11.84	-11.83
-11.91	-11.9	-11.89	-11.88	-11.87	-11.86	-11.85	-11.84	-11.83
-11.91	-11.9	-11.89	-11.88	-11.87	-11.86	-11.85	-11.84	-11.83
-11.91	-11.9	-11.89	-11.88	-11.87	-11.86	-11.85	-11.84	-11.83
-11.91	-11.9	-11.89	-11.88	-11.87	-11.86	-11.85	-11.84	-11.83
-11.91	-11.9	-11.89	-11.88	-11.87	-11.86	-11.85	-11.84	-11.83
-11.91	-11.9	-11.89	-11.88	-11.87	-11.86	-11.85	-11.84	-11.83
-11.91	-11.9	-11.89	-11.88	-11.87	-11.86	-11.85	-11.84	-11.83
-11.91	-11.9	-11.89	-11.88	-11.87	-11.86	-11.85	-11.84	-11.83
-11.91	-11.9	-11.89	-11.88	-11.87	-11.86	-11.85	-11.84	-11.83
-11.91	-11.9	-11.89	-11.88	-11.87	-11.86	-11.85	-11.84	-11.83
-11.91	-11.9	-11.89	-11.88	-11.87	-11.86	-11.85	-11.84	-11.83
-11.91	-11.9	-11.89	-11.88	-11.87	-11.86	-11.85	-11.84	-11.83
-11.91	-11.9	-11.89	-11.88	-11.87	-11.86	-11.85	-11.84	-11.83
-11.91	-11.9	-11.89	-11.88	-11.87	-11.86	-11.85	-11.84	-11.83
-11.91	-11.9	-11.89	-11.88	-11.87	-11.86	-11.85	-11.84	-11.83
-11.91	-11.9	-11.89	-11.88	-11.87	-11.86	-11.85	-11.84	-11.83
-11.91	-11.9	-11.89	-11.88	-11.87	-11.86	-11.85	-11.84	-11.83
-11.91	-11.9	-11.89	-11.88	-11.87	-11.86	-11.85	-11.84	-11.83
-11.91	-11.9	-11.89	-11.88	-11.87	-11.86	-11.85	-11.84	-11.83
-11.91	-11.9	-11.89	-11.88	-11.87	-11.86	-11.85	-11.84	-11.83
-11.91	-11.9	-11.89	-11.88	-11.87	-11.86	-11.85	-11.84	-11.83
-11.91	-11.9	-11.89	-11.88	-11.87	-11.86	-11.85	-11.84	-11.83
-11.91	-11.9	-11.89	-11.88	-11.87	-11.86	-11.85	-11.84	-11.83
-11.91	-11.9	-11.89	-11.88	-11.87	-11.86	-11.85	-11.84	-11.83
-11.91	-11.9	-11.89	-11.88	-11.87	-11.86	-11.85	-11.84	-11.83
-11.91	-11.9	-11.89	-11.88	-11.87	-11.86	-11.85	-11.84	-11.83
-11.91	-11.9	-11.89	-11.88	-11.87	-11.86	-11.85	-11.84	-11.83
-11.91	-11.9	-11.89	-11.88	-11.87	-11.86	-11.85	-11.84	-11.83
-11.91	-11.9	-11.89	-11.88	-11.87	-11.86	-11.85	-11.84	-11.83
-11.91	-11.9	-11.89	-11.88	-11.87	-11.86	-11.85	-11.84	-11.83
-11.91	-11.9	-11.89	-11.88	-11.87	-11.86	-11.85	-11.84	-11.83
-11.91	-11.9	-11.89	-11.88	-11.87	-11.86	-11.85	-11.84	-11.83

-11.91	-11.9	-11.89	-11.88	-11.87	-11.86	-11.85	-11.84	-11.83
-11.91	-11.9	-11.89	-11.88	-11.87	-11.86	-11.85	-11.84	-11.83
-11.91	-11.9	-11.89	-11.88	-11.87	-11.86	-11.85	-11.84	-11.83
-11.91	-11.9	-11.89	-11.88	-11.87	-11.86	-11.85	-11.84	-11.83
-11.91	-11.9	-11.89	-11.88	-11.87	-11.86	-11.85	-11.84	-11.83
-11.91	-11.9	-11.89	-11.88	-11.87	-11.86	-11.85	-11.84	-11.83
-11.91	-11.9	-11.89	-11.88	-11.87	-11.86	-11.85	-11.84	-11.83
-11.91	-11.9	-11.89	-11.88	-11.87	-11.86	-11.85	-11.84	-11.83
-11.91	-11.9	-11.89	-11.88	-11.87	-11.86	-11.85	-11.84	-11.83
-11.91	-11.9	-11.89	-11.88	-11.87	-11.86	-11.85	-11.84	-11.83
-11.91	-11.9	-11.89	-11.88	-11.87	-11.86	-11.85	-11.84	-11.83
-11.91	-11.9	-11.89	-11.88	-11.87	-11.86	-11.85	-11.84	-11.83
-11.91	-11.9	-11.89	-11.88	-11.87	-11.86	-11.85	-11.84	-11.83
-11.91	-11.9	-11.89	-11.88	-11.87	-11.86	-11.85	-11.84	-11.83
-11.91	-11.9	-11.89	-11.88	-11.87	-11.86	-11.85	-11.84	-11.83
-11.91	-11.9	-11.89	-11.88	-11.87	-11.86	-11.85	-11.84	-11.83
-11.91	-11.9	-11.89	-11.88	-11.87	-11.86	-11.85	-11.84	-11.83
-11.91	-11.9	-11.89	-11.88	-11.87	-11.86	-11.85	-11.84	-11.83
-11.91	-11.9	-11.89	-11.88	-11.87	-11.86	-11.85	-11.84	-11.83
-11.91	-11.9	-11.89	-11.88	-11.87	-11.86	-11.85	-11.84	-11.83
-11.91	-11.9	-11.89	-11.88	-11.87	-11.86	-11.85	-11.84	-11.83
-11.91	-11.9	-11.89	-11.88	-11.87	-11.86	-11.85	-11.84	-11.83
-11.91	-11.9	-11.89	-11.88	-11.87	-11.86	-11.85	-11.84	-11.83
-11.91	-11.9	-11.89	-11.88	-11.87	-11.86	-11.85	-11.84	-11.83
-11.91	-11.9	-11.89	-11.88	-11.87	-11.86	-11.85	-11.84	-11.83
-11.91	-11.9	-11.89	-11.88	-11.87	-11.86	-11.85	-11.84	-11.83
-11.91	-11.9	-11.89	-11.88	-11.87	-11.86	-11.85	-11.84	-11.83
-11.91	-11.9	-11.89	-11.88	-11.87	-11.86	-11.85	-11.84	-11.83
-11.91	-11.9	-11.89	-11.88	-11.87	-11.86	-11.85	-11.84	-11.83
-11.91	-11.9	-11.89	-11.88	-11.87	-11.86	-11.85	-11.84	-11.83
-11.91	-11.9	-11.89	-11.88	-11.87	-11.86	-11.85	-11.84	-11.83
-11.91	-11.9	-11.89	-11.88	-11.87	-11.86	-11.85	-11.84	-11.83
-11.91	-11.9	-11.89	-11.88	-11.87	-11.86	-11.85	-11.84	-11.83
-11.91	-11.9	-11.89	-11.88	-11.87	-11.86	-11.85	-11.84	-11.83
-11.91	-11.9	-11.89	-11.88	-11.87	-11.86	-11.85	-11.84	-11.83
-11.91	-11.9	-11.89	-11.88	-11.87	-11.86	-11.85	-11.84	-11.83
-11.91	-11.9	-11.89	-11.88	-11.87	-11.86	-11.85	-11.84	-11.83
-11.91	-11.9	-11.89	-11.88	-11.87	-11.86	-11.85	-11.84	-11.83
-11.91	-11.9	-11.89	-11.88	-11.87	-11.86	-11.85	-11.84	-11.83
-11.91	-11.9	-11.89	-11.88	-11.87	-11.86	-11.85	-11.84	-11.83
-11.91	-11.9	-11.89	-11.88	-11.87	-11.86	-11.85	-11.84	-11.83
-11.91	-11.9	-11.89	-11.88	-11.87	-11.86	-11.85	-11.84	-11.83
-11.91	-11.9	-11.89	-11.88	-11.87	-11.86	-11.85	-11.84	-11.83
-11.91	-11.9	-11.89	-11.88	-11.87	-11.86	-11.85	-11.84	-11.83
-11.91	-11.9	-11.89	-11.88	-11.87	-11.86	-11.85	-11.84	-11.83
-11.91	-11.9	-11.89	-11.88	-11.87	-11.86	-11.85	-11.84	-11.83
-11.91	-11.9	-11.89	-11.88	-11.87	-11.86	-11.85	-11.84	-11.83
-11.91	-11.9	-11.89	-11.88	-11.87	-11.86	-11.85	-11.84	-11.83
-11.91	-11.9	-11.89	-11.88	-11.87	-11.86	-11.85	-11.84	-11.83
-11.91	-11.9	-11.89	-11.88	-11.87	-11.86	-11.85	-11.84	-11.83

-11.91	-11.9	-11.89	-11.88	-11.87	-11.86	-11.85	-11.84	-11.83
-11.91	-11.9	-11.89	-11.88	-11.87	-11.86	-11.85	-11.84	-11.83
-11.91	-11.9	-11.89	-11.88	-11.87	-11.86	-11.85	-11.84	-11.83
-11.91	-11.9	-11.89	-11.88	-11.87	-11.86	-11.85	-11.84	-11.83
-11.91	-11.9	-11.89	-11.88	-11.87	-11.86	-11.85	-11.84	-11.83
-11.91	-11.9	-11.89	-11.88	-11.87	-11.86	-11.85	-11.84	-11.83
-11.91	-11.9	-11.89	-11.88	-11.87	-11.86	-11.85	-11.84	-11.83
-11.91	-11.9	-11.89	-11.88	-11.87	-11.86	-11.85	-11.84	-11.83
-11.91	-11.9	-11.89	-11.88	-11.87	-11.86	-11.85	-11.84	-11.83
-11.91	-11.9	-11.89	-11.88	-11.87	-11.86	-11.85	-11.84	-11.83
-11.91	-11.9	-11.89	-11.88	-11.87	-11.86	-11.85	-11.84	-11.83
-11.91	-11.9	-11.89	-11.88	-11.87	-11.86	-11.85	-11.84	-11.83
-11.91	-11.9	-11.89	-11.88	-11.87	-11.86	-11.85	-11.84	-11.83
-11.91	-11.9	-11.89	-11.88	-11.87	-11.86	-11.85	-11.84	-11.83
-11.91	-11.9	-11.89	-11.88	-11.87	-11.86	-11.85	-11.84	-11.83
-11.91	-11.9	-11.89	-11.88	-11.87	-11.86	-11.85	-11.84	-11.83
-11.91	-11.9	-11.89	-11.88	-11.87	-11.86	-11.85	-11.84	-11.83
-11.91	-11.9	-11.89	-11.88	-11.87	-11.86	-11.85	-11.84	-11.83
-11.91	-11.9	-11.89	-11.88	-11.87	-11.86	-11.85	-11.84	-11.83
-11.91	-11.9	-11.89	-11.88	-11.87	-11.86	-11.85	-11.84	-11.83
-11.91	-11.9	-11.89	-11.88	-11.87	-11.86	-11.85	-11.84	-11.83
-11.91	-11.9	-11.89	-11.88	-11.87	-11.86	-11.85	-11.84	-11.83
-11.91	-11.9	-11.89	-11.88	-11.87	-11.86	-11.85	-11.84	-11.83
-11.91	-11.9	-11.89	-11.88	-11.87	-11.86	-11.85	-11.84	-11.83
-11.91	-11.9	-11.89	-11.88	-11.87	-11.86	-11.85	-11.84	-11.83
-11.91	-11.9	-11.89	-11.88	-11.87	-11.86	-11.85	-11.84	-11.83
-11.91	-11.9	-11.89	-11.88	-11.87	-11.86	-11.85	-11.84	-11.83
-11.91	-11.9	-11.89	-11.88	-11.87	-11.86	-11.85	-11.84	-11.83
-11.91	-11.9	-11.89	-11.88	-11.87	-11.86	-11.85	-11.84	-11.83
-11.91	-11.9	-11.89	-11.88	-11.87	-11.86	-11.85	-11.84	-11.83
-11.91	-11.9	-11.89	-11.88	-11.87	-11.86	-11.85	-11.84	-11.83
-11.91	-11.9	-11.89	-11.88	-11.87	-11.86	-11.85	-11.84	-11.83
-11.91	-11.9	-11.89	-11.88	-11.87	-11.86	-11.85	-11.84	-11.83
-11.91	-11.9	-11.89	-11.88	-11.87	-11.86	-11.85	-11.84	-11.83
-11.91	-11.9	-11.89	-11.88	-11.87	-11.86	-11.85	-11.84	-11.83
-11.91	-11.9	-11.89	-11.88	-11.87	-11.86	-11.85	-11.84	-11.83
-11.91	-11.9	-11.89	-11.88	-11.87	-11.86	-11.85	-11.84	-11.83
-11.91	-11.9	-11.89	-11.88	-11.87	-11.86	-11.85	-11.84	-11.83
-11.91	-11.9	-11.89	-11.88	-11.87	-11.86	-11.85	-11.84	-11.83
-11.91	-11.9	-11.89	-11.88	-11.87	-11.86	-11.85	-11.84	-11.83
-11.91	-11.9	-11.89	-11.88	-11.87	-11.86	-11.85	-11.84	-11.83
-11.91	-11.9	-11.89	-11.88	-11.87	-11.86	-11.85	-11.84	-11.83
-11.91	-11.9	-11.89	-11.88	-11.87	-11.86	-11.85	-11.84	-11.83
-11.91	-11.9	-11.89	-11.88	-11.87	-11.86	-11.85	-11.84	-11.83
-11.91	-11.9	-11.89	-11.88	-11.87	-11.86	-11.85	-11.84	-11.83
-11.91	-11.9	-11.89	-11.88	-11.87	-11.86	-11.85	-11.84	-11.83
-11.91	-11.9	-11.89	-11.88	-11.87	-11.86	-11.85	-11.84	-11.83
-11.91	-11.9	-11.89	-11.88	-11.87	-11.86	-11.85	-11.84	-11.83
-11.91	-11.9	-11.89	-11.88	-11.87	-11.86	-11.85	-11.84	-11.83
-11.91	-11.9	-11.89	-11.88	-11.87	-11.86	-11.85	-11.84	-11.83

-11.91	-11.9	-11.89	-11.88	-11.87	-11.86	-11.85	-11.84	-11.83
-11.91	-11.9	-11.89	-11.88	-11.87	-11.86	-11.85	-11.84	-11.83
-11.91	-11.9	-11.89	-11.88	-11.87	-11.86	-11.85	-11.84	-11.83
-11.91	-11.9	-11.89	-11.88	-11.87	-11.86	-11.85	-11.84	-11.83
-11.91	-11.9	-11.89	-11.88	-11.87	-11.86	-11.85	-11.84	-11.83
-11.91	-11.9	-11.89	-11.88	-11.87	-11.86	-11.85	-11.84	-11.83
-11.91	-11.9	-11.89	-11.88	-11.87	-11.86	-11.85	-11.84	-11.83
-11.91	-11.9	-11.89	-11.88	-11.87	-11.86	-11.85	-11.84	-11.83
-11.91	-11.9	-11.89	-11.88	-11.87	-11.86	-11.85	-11.84	-11.83
-11.91	-11.9	-11.89	-11.88	-11.87	-11.86	-11.85	-11.84	-11.83
-11.91	-11.9	-11.89	-11.88	-11.87	-11.86	-11.85	-11.84	-11.83
-11.91	-11.9	-11.89	-11.88	-11.87	-11.86	-11.85	-11.84	-11.83
-11.91	-11.9	-11.89	-11.88	-11.87	-11.86	-11.85	-11.84	-11.83
-11.91	-11.9	-11.89	-11.88	-11.87	-11.86	-11.85	-11.84	-11.83
-11.91	-11.9	-11.89	-11.88	-11.87	-11.86	-11.85	-11.84	-11.83
-11.91	-11.9	-11.89	-11.88	-11.87	-11.86	-11.85	-11.84	-11.83
-11.91	-11.9	-11.89	-11.88	-11.87	-11.86	-11.85	-11.84	-11.83
-11.91	-11.9	-11.89	-11.88	-11.87	-11.86	-11.85	-11.84	-11.83
-11.91	-11.9	-11.89	-11.88	-11.87	-11.86	-11.85	-11.84	-11.83
-11.91	-11.9	-11.89	-11.88	-11.87	-11.86	-11.85	-11.84	-11.83
-11.91	-11.9	-11.89	-11.88	-11.87	-11.86	-11.85	-11.84	-11.83
-11.91	-11.9	-11.89	-11.88	-11.87	-11.86	-11.85	-11.84	-11.83
-11.91	-11.9	-11.89	-11.88	-11.87	-11.86	-11.85	-11.84	-11.83
-11.91	-11.9	-11.89	-11.88	-11.87	-11.86	-11.85	-11.84	-11.83
-11.91	-11.9	-11.89	-11.88	-11.87	-11.86	-11.85	-11.84	-11.83
-11.91	-11.9	-11.89	-11.88	-11.87	-11.86	-11.85	-11.84	-11.83
-11.91	-11.9	-11.89	-11.88	-11.87	-11.86	-11.85	-11.84	-11.83
-11.91	-11.9	-11.89	-11.88	-11.87	-11.86	-11.85	-11.84	-11.83
-11.91	-11.9	-11.89	-11.88	-11.87	-11.86	-11.85	-11.84	-11.83
-11.91	-11.9	-11.89	-11.88	-11.87	-11.86	-11.85	-11.84	-11.83
-11.91	-11.9	-11.89	-11.88	-11.87	-11.86	-11.85	-11.84	-11.83
-11.91	-11.9	-11.89	-11.88	-11.87	-11.86	-11.85	-11.84	-11.83
-11.91	-11.9	-11.89	-11.88	-11.87	-11.86	-11.85	-11.84	-11.83
-11.91	-11.9	-11.89	-11.88	-11.87	-11.86	-11.85	-11.84	-11.83
-11.91	-11.9	-11.89	-11.88	-11.87	-11.86	-11.85	-11.84	-11.83
-11.91	-11.9	-11.89	-11.88	-11.87	-11.86	-11.85	-11.84	-11.83
-11.91	-11.9	-11.89	-11.88	-11.87	-11.86	-11.85	-11.84	-11.83
-11.91	-11.9	-11.89	-11.88	-11.87	-11.86	-11.85	-11.84	-11.83
-11.91	-11.9	-11.89	-11.88	-11.87	-11.86	-11.85	-11.84	-11.83
-11.91	-11.9	-11.89	-11.88	-11.87	-11.86	-11.85	-11.84	-11.83
-11.91	-11.9	-11.89	-11.88	-11.87	-11.86	-11.85	-11.84	-11.83
-11.91	-11.9	-11.89	-11.88	-11.87	-11.86	-11.85	-11.84	-11.83
-11.91	-11.9	-11.89	-11.88	-11.87	-11.86	-11.85	-11.84	-11.83
-11.91	-11.9	-11.89	-11.88	-11.87	-11.86	-11.85	-11.84	-11.83
-11.91	-11.9	-11.89	-11.88	-11.87	-11.86	-11.85	-11.84	-11.83
-11.91	-11.9	-11.89	-11.88	-11.87	-11.86	-11.85	-11.84	-11.83
-11.91	-11.9	-11.89	-11.88	-11.87	-11.86	-11.85	-11.84	-11.83
-11.91	-11.9	-11.89	-11.88	-11.87	-11.86	-11.85	-11.84	-11.83
-11.91	-11.9	-11.89	-11.88	-11.87	-11.86	-11.85	-11.84	-11.83
-11.91	-11.9	-11.89	-11.88	-11.87	-11.86	-11.85	-11.84	-11.83

-11.91	-11.9	-11.89	-11.88	-11.87	-11.86	-11.85	-11.84	-11.83
-11.91	-11.9	-11.89	-11.88	-11.87	-11.86	-11.85	-11.84	-11.83
-11.91	-11.9	-11.89	-11.88	-11.87	-11.86	-11.85	-11.84	-11.83
-11.91	-11.9	-11.89	-11.88	-11.87	-11.86	-11.85	-11.84	-11.83
-11.91	-11.9	-11.89	-11.88	-11.87	-11.86	-11.85	-11.84	-11.83
-11.91	-11.9	-11.89	-11.88	-11.87	-11.86	-11.85	-11.84	-11.83
-11.91	-11.9	-11.89	-11.88	-11.87	-11.86	-11.85	-11.84	-11.83
-11.91	-11.9	-11.89	-11.88	-11.87	-11.86	-11.85	-11.84	-11.83
-11.91	-11.9	-11.89	-11.88	-11.87	-11.86	-11.85	-11.84	-11.83
-11.91	-11.9	-11.89	-11.88	-11.87	-11.86	-11.85	-11.84	-11.83
-11.91	-11.9	-11.89	-11.88	-11.87	-11.86	-11.85	-11.84	-11.83
-11.91	-11.9	-11.89	-11.88	-11.87	-11.86	-11.85	-11.84	-11.83
-11.91	-11.9	-11.89	-11.88	-11.87	-11.86	-11.85	-11.84	-11.83
-11.91	-11.9	-11.89	-11.88	-11.87	-11.86	-11.85	-11.84	-11.83
-11.91	-11.9	-11.89	-11.88	-11.87	-11.86	-11.85	-11.84	-11.83
-11.91	-11.9	-11.89	-11.88	-11.87	-11.86	-11.85	-11.84	-11.83
-11.91	-11.9	-11.89	-11.88	-11.87	-11.86	-11.85	-11.84	-11.83
-11.91	-11.9	-11.89	-11.88	-11.87	-11.86	-11.85	-11.84	-11.83
-11.91	-11.9	-11.89	-11.88	-11.87	-11.86	-11.85	-11.84	-11.83
-11.91	-11.9	-11.89	-11.88	-11.87	-11.86	-11.85	-11.84	-11.83
-11.91	-11.9	-11.89	-11.88	-11.87	-11.86	-11.85	-11.84	-11.83
-11.91	-11.9	-11.89	-11.88	-11.87	-11.86	-11.85	-11.84	-11.83
-11.91	-11.9	-11.89	-11.88	-11.87	-11.86	-11.85	-11.84	-11.83
-11.91	-11.9	-11.89	-11.88	-11.87	-11.86	-11.85	-11.84	-11.83
-11.91	-11.9	-11.89	-11.88	-11.87	-11.86	-11.85	-11.84	-11.83
-11.91	-11.9	-11.89	-11.88	-11.87	-11.86	-11.85	-11.84	-11.83
-11.91	-11.9	-11.89	-11.88	-11.87	-11.86	-11.85	-11.84	-11.83
-11.91	-11.9	-11.89	-11.88	-11.87	-11.86	-11.85	-11.84	-11.83
-11.91	-11.9	-11.89	-11.88	-11.87	-11.86	-11.85	-11.84	-11.83
-11.91	-11.9	-11.89	-11.88	-11.87	-11.86	-11.85	-11.84	-11.83
-11.91	-11.9	-11.89	-11.88	-11.87	-11.86	-11.85	-11.84	-11.83
-11.91	-11.9	-11.89	-11.88	-11.87	-11.86	-11.85	-11.84	-11.83
-11.91	-11.9	-11.89	-11.88	-11.87	-11.86	-11.85	-11.84	-11.83
-11.91	-11.9	-11.89	-11.88	-11.87	-11.86	-11.85	-11.84	-11.83
-11.91	-11.9	-11.89	-11.88	-11.87	-11.86	-11.85	-11.84	-11.83
-11.91	-11.9	-11.89	-11.88	-11.87	-11.86	-11.85	-11.84	-11.83
-11.91	-11.9	-11.89	-11.88	-11.87	-11.86	-11.85	-11.84	-11.83
-11.91	-11.9	-11.89	-11.88	-11.87	-11.86	-11.85	-11.84	-11.83
-11.91	-11.9	-11.89	-11.88	-11.87	-11.86	-11.85	-11.84	-11.83
-11.91	-11.9	-11.89	-11.88	-11.87	-11.86	-11.85	-11.84	-11.83
-11.91	-11.9	-11.89	-11.88	-11.87	-11.86	-11.85	-11.84	-11.83
-11.91	-11.9	-11.89	-11.88	-11.87	-11.86	-11.85	-11.84	-11.83
-11.91	-11.9	-11.89	-11.88	-11.87	-11.86	-11.85	-11.84	-11.83
-11.91	-11.9	-11.89	-11.88	-11.87	-11.86	-11.85	-11.84	-11.83
-11.91	-11.9	-11.89	-11.88	-11.87	-11.86	-11.85	-11.84	-11.83
-11.91	-11.9	-11.89	-11.88	-11.87	-11.86	-11.85	-11.84	-11.83
-11.91	-11.9	-11.89	-11.88	-11.87	-11.86	-11.85	-11.84	-11.83
-11.91	-11.9	-11.89	-11.88	-11.87	-11.86	-11.85	-11.84	-11.83
-11.91	-11.9	-11.89	-11.88	-11.87	-11.86	-11.85	-11.84	-11.83
-11.91	-11.9	-11.89	-11.88	-11.87	-11.86	-11.85	-11.84	-11.83

-11.91	-11.9	-11.89	-11.88	-11.87	-11.86	-11.85	-11.84	-11.83
-11.91	-11.9	-11.89	-11.88	-11.87	-11.86	-11.85	-11.84	-11.83
-11.91	-11.9	-11.89	-11.88	-11.87	-11.86	-11.85	-11.84	-11.83
-11.91	-11.9	-11.89	-11.88	-11.87	-11.86	-11.85	-11.84	-11.83
-11.91	-11.9	-11.89	-11.88	-11.87	-11.86	-11.85	-11.84	-11.83
-11.91	-11.9	-11.89	-11.88	-11.87	-11.86	-11.85	-11.84	-11.83
-11.91	-11.9	-11.89	-11.88	-11.87	-11.86	-11.85	-11.84	-11.83
-11.91	-11.9	-11.89	-11.88	-11.87	-11.86	-11.85	-11.84	-11.83
-11.91	-11.9	-11.89	-11.88	-11.87	-11.86	-11.85	-11.84	-11.83
-11.91	-11.9	-11.89	-11.88	-11.87	-11.86	-11.85	-11.84	-11.83
-11.91	-11.9	-11.89	-11.88	-11.87	-11.86	-11.85	-11.84	-11.83
-11.91	-11.9	-11.89	-11.88	-11.87	-11.86	-11.85	-11.84	-11.83
-11.91	-11.9	-11.89	-11.88	-11.87	-11.86	-11.85	-11.84	-11.83
-11.91	-11.9	-11.89	-11.88	-11.87	-11.86	-11.85	-11.84	-11.83
-11.91	-11.9	-11.89	-11.88	-11.87	-11.86	-11.85	-11.84	-11.83
-11.91	-11.9	-11.89	-11.88	-11.87	-11.86	-11.85	-11.84	-11.83
-11.91	-11.9	-11.89	-11.88	-11.87	-11.86	-11.85	-11.84	-11.83
-11.91	-11.9	-11.89	-11.88	-11.87	-11.86	-11.85	-11.84	-11.83
-11.91	-11.9	-11.89	-11.88	-11.87	-11.86	-11.85	-11.84	-11.83
-11.91	-11.9	-11.89	-11.88	-11.87	-11.86	-11.85	-11.84	-11.83
-11.91	-11.9	-11.89	-11.88	-11.87	-11.86	-11.85	-11.84	-11.83
-11.91	-11.9	-11.89	-11.88	-11.87	-11.86	-11.85	-11.84	-11.83
-11.91	-11.9	-11.89	-11.88	-11.87	-11.86	-11.85	-11.84	-11.83
-11.91	-11.9	-11.89	-11.88	-11.87	-11.86	-11.85	-11.84	-11.83
-11.91	-11.9	-11.89	-11.88	-11.87	-11.86	-11.85	-11.84	-11.83
-11.91	-11.9	-11.89	-11.88	-11.87	-11.86	-11.85	-11.84	-11.83
-11.91	-11.9	-11.89	-11.88	-11.87	-11.86	-11.85	-11.84	-11.83
-11.91	-11.9	-11.89	-11.88	-11.87	-11.86	-11.85	-11.84	-11.83
-11.91	-11.9	-11.89	-11.88	-11.87	-11.86	-11.85	-11.84	-11.83
-11.91	-11.9	-11.89	-11.88	-11.87	-11.86	-11.85	-11.84	-11.83
-11.91	-11.9	-11.89	-11.88	-11.87	-11.86	-11.85	-11.84	-11.83
-11.91	-11.9	-11.89	-11.88	-11.87	-11.86	-11.85	-11.84	-11.83
-11.91	-11.9	-11.89	-11.88	-11.87	-11.86	-11.85	-11.84	-11.83
-11.91	-11.9	-11.89	-11.88	-11.87	-11.86	-11.85	-11.84	-11.83
-11.91	-11.9	-11.89	-11.88	-11.87	-11.86	-11.85	-11.84	-11.83
-11.91	-11.9	-11.89	-11.88	-11.87	-11.86	-11.85	-11.84	-11.83
-11.91	-11.9	-11.89	-11.88	-11.87	-11.86	-11.85	-11.84	-11.83
-11.91	-11.9	-11.89	-11.88	-11.87	-11.86	-11.85	-11.84	-11.83
-11.91	-11.9	-11.89	-11.88	-11.87	-11.86	-11.85	-11.84	-11.83
-11.91	-11.9	-11.89	-11.88	-11.87	-11.86	-11.85	-11.84	-11.83
-11.91	-11.9	-11.89	-11.88	-11.87	-11.86	-11.85	-11.84	-11.83
-11.91	-11.9	-11.89	-11.88	-11.87	-11.86	-11.85	-11.84	-11.83
-11.91	-11.9	-11.89	-11.88	-11.87	-11.86	-11.85	-11.84	-11.83
-11.91	-11.9	-11.89	-11.88	-11.87	-11.86	-11.85	-11.84	-11.83
-11.91	-11.9	-11.89	-11.88	-11.87	-11.86	-11.85	-11.84	-11.83
-11.91	-11.9	-11.89	-11.88	-11.87	-11.86	-11.85	-11.84	-11.83
-11.91	-11.9	-11.89	-11.88	-11.87	-11.86	-11.85	-11.84	-11.83
-11.91	-11.9	-11.89	-11.88	-11.87	-11.86	-11.85	-11.84	-11.83
-11.91	-11.9	-11.89	-11.88	-11.87	-11.86	-11.85	-11.84	-11.83
-11.91	-11.9	-11.89	-11.88	-11.87	-11.86	-11.85	-11.84	-11.83

-11.91	-11.9	-11.89	-11.88	-11.87	-11.86	-11.85	-11.84	-11.83
-11.91	-11.9	-11.89	-11.88	-11.87	-11.86	-11.85	-11.84	-11.83
-11.91	-11.9	-11.89	-11.88	-11.87	-11.86	-11.85	-11.84	-11.83
-11.91	-11.9	-11.89	-11.88	-11.87	-11.86	-11.85	-11.84	-11.83
-11.91	-11.9	-11.89	-11.88	-11.87	-11.86	-11.85	-11.84	-11.83
-11.91	-11.9	-11.89	-11.88	-11.87	-11.86	-11.85	-11.84	-11.83
-11.91	-11.9	-11.89	-11.88	-11.87	-11.86	-11.85	-11.84	-11.83
-11.91	-11.9	-11.89	-11.88	-11.87	-11.86	-11.85	-11.84	-11.83
-11.91	-11.9	-11.89	-11.88	-11.87	-11.86	-11.85	-11.84	-11.83
-11.91	-11.9	-11.89	-11.88	-11.87	-11.86	-11.85	-11.84	-11.83
-11.91	-11.9	-11.89	-11.88	-11.87	-11.86	-11.85	-11.84	-11.83
-11.91	-11.9	-11.89	-11.88	-11.87	-11.86	-11.85	-11.84	-11.83
-11.91	-11.9	-11.89	-11.88	-11.87	-11.86	-11.85	-11.84	-11.83
-11.91	-11.9	-11.89	-11.88	-11.87	-11.86	-11.85	-11.84	-11.83
-11.91	-11.9	-11.89	-11.88	-11.87	-11.86	-11.85	-11.84	-11.83
-11.91	-11.9	-11.89	-11.88	-11.87	-11.86	-11.85	-11.84	-11.83
-11.91	-11.9	-11.89	-11.88	-11.87	-11.86	-11.85	-11.84	-11.83
-11.91	-11.9	-11.89	-11.88	-11.87	-11.86	-11.85	-11.84	-11.83
-11.91	-11.9	-11.89	-11.88	-11.87	-11.86	-11.85	-11.84	-11.83
-11.91	-11.9	-11.89	-11.88	-11.87	-11.86	-11.85	-11.84	-11.83
-11.91	-11.9	-11.89	-11.88	-11.87	-11.86	-11.85	-11.84	-11.83
-11.91	-11.9	-11.89	-11.88	-11.87	-11.86	-11.85	-11.84	-11.83
-11.91	-11.9	-11.89	-11.88	-11.87	-11.86	-11.85	-11.84	-11.83
-11.91	-11.9	-11.89	-11.88	-11.87	-11.86	-11.85	-11.84	-11.83
-11.91	-11.9	-11.89	-11.88	-11.87	-11.86	-11.85	-11.84	-11.83
-11.91	-11.9	-11.89	-11.88	-11.87	-11.86	-11.85	-11.84	-11.83
-11.91	-11.9	-11.89	-11.88	-11.87	-11.86	-11.85	-11.84	-11.83
-11.91	-11.9	-11.89	-11.88	-11.87	-11.86	-11.85	-11.84	-11.83
-11.91	-11.9	-11.89	-11.88	-11.87	-11.86	-11.85	-11.84	-11.83
-11.91	-11.9	-11.89	-11.88	-11.87	-11.86	-11.85	-11.84	-11.83
-11.91	-11.9	-11.89	-11.88	-11.87	-11.86	-11.85	-11.84	-11.83
-11.91	-11.9	-11.89	-11.88	-11.87	-11.86	-11.85	-11.84	-11.83
-11.91	-11.9	-11.89	-11.88	-11.87	-11.86	-11.85	-11.84	-11.83
-11.91	-11.9	-11.89	-11.88	-11.87	-11.86	-11.85	-11.84	-11.83
-11.91	-11.9	-11.89	-11.88	-11.87	-11.86	-11.85	-11.84	-11.83
-11.91	-11.9	-11.89	-11.88	-11.87	-11.86	-11.85	-11.84	-11.83
-11.91	-11.9	-11.89	-11.88	-11.87	-11.86	-11.85	-11.84	-11.83
-11.91	-11.9	-11.89	-11.88	-11.87	-11.86	-11.85	-11.84	-11.83
-11.91	-11.9	-11.89	-11.88	-11.87	-11.86	-11.85	-11.84	-11.83
-11.91	-11.9	-11.89	-11.88	-11.87	-11.86	-11.85	-11.84	-11.83
-11.91	-11.9	-11.89	-11.88	-11.87	-11.86	-11.85	-11.84	-11.83
-11.91	-11.9	-11.89	-11.88	-11.87	-11.86	-11.85	-11.84	-11.83
-11.91	-11.9	-11.89	-11.88	-11.87	-11.86	-11.85	-11.84	-11.83
-11.91	-11.9	-11.89	-11.88	-11.87	-11.86	-11.85	-11.84	-11.83
-11.91	-11.9	-11.89	-11.88	-11.87	-11.86	-11.85	-11.84	-11.83
-11.91	-11.9	-11.89	-11.88	-11.87	-11.86	-11.85	-11.84	-11.83
-11.91	-11.9	-11.89	-11.88	-11.87	-11.86	-11.85	-11.84	-11.83
-11.91	-11.9	-11.89	-11.88	-11.87	-11.86	-11.85	-11.84	-11.83
-11.91	-11.9	-11.89	-11.88	-11.87	-11.86	-11.85	-11.84	-11.83
-11.91	-11.9	-11.89	-11.88	-11.87	-11.86	-11.85	-11.84	-11.83

-11.91	-11.9	-11.89	-11.88	-11.87	-11.86	-11.85	-11.84	-11.83
-11.91	-11.9	-11.89	-11.88	-11.87	-11.86	-11.85	-11.84	-11.83
-11.91	-11.9	-11.89	-11.88	-11.87	-11.86	-11.85	-11.84	-11.83
-11.91	-11.9	-11.89	-11.88	-11.87	-11.86	-11.85	-11.84	-11.83
-11.91	-11.9	-11.89	-11.88	-11.87	-11.86	-11.85	-11.84	-11.83
-11.91	-11.9	-11.89	-11.88	-11.87	-11.86	-11.85	-11.84	-11.83
-11.91	-11.9	-11.89	-11.88	-11.87	-11.86	-11.85	-11.84	-11.83
-11.91	-11.9	-11.89	-11.88	-11.87	-11.86	-11.85	-11.84	-11.83
-11.91	-11.9	-11.89	-11.88	-11.87	-11.86	-11.85	-11.84	-11.83
-11.91	-11.9	-11.89	-11.88	-11.87	-11.86	-11.85	-11.84	-11.83
-11.91	-11.9	-11.89	-11.88	-11.87	-11.86	-11.85	-11.84	-11.83
-11.91	-11.9	-11.89	-11.88	-11.87	-11.86	-11.85	-11.84	-11.83
-11.91	-11.9	-11.89	-11.88	-11.87	-11.86	-11.85	-11.84	-11.83
-11.91	-11.9	-11.89	-11.88	-11.87	-11.86	-11.85	-11.84	-11.83
-11.91	-11.9	-11.89	-11.88	-11.87	-11.86	-11.85	-11.84	-11.83
-11.91	-11.9	-11.89	-11.88	-11.87	-11.86	-11.85	-11.84	-11.83
-11.91	-11.9	-11.89	-11.88	-11.87	-11.86	-11.85	-11.84	-11.83
-11.91	-11.9	-11.89	-11.88	-11.87	-11.86	-11.85	-11.84	-11.83
-11.91	-11.9	-11.89	-11.88	-11.87	-11.86	-11.85	-11.84	-11.83
-11.91	-11.9	-11.89	-11.88	-11.87	-11.86	-11.85	-11.84	-11.83
-11.91	-11.9	-11.89	-11.88	-11.87	-11.86	-11.85	-11.84	-11.83
-11.91	-11.9	-11.89	-11.88	-11.87	-11.86	-11.85	-11.84	-11.83
-11.91	-11.9	-11.89	-11.88	-11.87	-11.86	-11.85	-11.84	-11.83
-11.91	-11.9	-11.89	-11.88	-11.87	-11.86	-11.85	-11.84	-11.83
-11.91	-11.9	-11.89	-11.88	-11.87	-11.86	-11.85	-11.84	-11.83
-11.91	-11.9	-11.89	-11.88	-11.87	-11.86	-11.85	-11.84	-11.83
-11.91	-11.9	-11.89	-11.88	-11.87	-11.86	-11.85	-11.84	-11.83
-11.91	-11.9	-11.89	-11.88	-11.87	-11.86	-11.85	-11.84	-11.83
-11.91	-11.9	-11.89	-11.88	-11.87	-11.86	-11.85	-11.84	-11.83
-11.91	-11.9	-11.89	-11.88	-11.87	-11.86	-11.85	-11.84	-11.83
-11.91	-11.9	-11.89	-11.88	-11.87	-11.86	-11.85	-11.84	-11.83
-11.91	-11.9	-11.89	-11.88	-11.87	-11.86	-11.85	-11.84	-11.83
-11.91	-11.9	-11.89	-11.88	-11.87	-11.86	-11.85	-11.84	-11.83
-11.91	-11.9	-11.89	-11.88	-11.87	-11.86	-11.85	-11.84	-11.83
-11.91	-11.9	-11.89	-11.88	-11.87	-11.86	-11.85	-11.84	-11.83
-11.91	-11.9	-11.89	-11.88	-11.87	-11.86	-11.85	-11.84	-11.83
-11.91	-11.9	-11.89	-11.88	-11.87	-11.86	-11.85	-11.84	-11.83
-11.91	-11.9	-11.89	-11.88	-11.87	-11.86	-11.85	-11.84	-11.83
-11.91	-11.9	-11.89	-11.88	-11.87	-11.86	-11.85	-11.84	-11.83
-11.91	-11.9	-11.89	-11.88	-11.87	-11.86	-11.85	-11.84	-11.83
-11.91	-11.9	-11.89	-11.88	-11.87	-11.86	-11.85	-11.84	-11.83
-11.91	-11.9	-11.89	-11.88	-11.87	-11.86	-11.85	-11.84	-11.83
-11.91	-11.9	-11.89	-11.88	-11.87	-11.86	-11.85	-11.84	-11.83
-11.91	-11.9	-11.89	-11.88	-11.87	-11.86	-11.85	-11.84	-11.83
-11.91	-11.9	-11.89	-11.88	-11.87	-11.86	-11.85	-11.84	-11.83
-11.91	-11.9	-11.89	-11.88	-11.87	-11.86	-11.85	-11.84	-11.83
-11.91	-11.9	-11.89	-11.88	-11.87	-11.86	-11.85	-11.84	-11.83
-11.91	-11.9	-11.89	-11.88	-11.87	-11.86	-11.85	-11.84	-11.83
-11.91	-11.9	-11.89	-11.88	-11.87	-11.86	-11.85	-11.84	-11.83
-11.91	-11.9	-11.89	-11.88	-11.87	-11.86	-11.85	-11.84	-11.83

-11.91	-11.9	-11.89	-11.88	-11.87	-11.86	-11.85	-11.84	-11.83
-11.91	-11.9	-11.89	-11.88	-11.87	-11.86	-11.85	-11.84	-11.83
-11.91	-11.9	-11.89	-11.88	-11.87	-11.86	-11.85	-11.84	-11.83
-11.91	-11.9	-11.89	-11.88	-11.87	-11.86	-11.85	-11.84	-11.83
-11.91	-11.9	-11.89	-11.88	-11.87	-11.86	-11.85	-11.84	-11.83
-11.91	-11.9	-11.89	-11.88	-11.87	-11.86	-11.85	-11.84	-11.83
-11.91	-11.9	-11.89	-11.88	-11.87	-11.86	-11.85	-11.84	-11.83
-11.91	-11.9	-11.89	-11.88	-11.87	-11.86	-11.85	-11.84	-11.83
-11.91	-11.9	-11.89	-11.88	-11.87	-11.86	-11.85	-11.84	-11.83
-11.91	-11.9	-11.89	-11.88	-11.87	-11.86	-11.85	-11.84	-11.83
-11.91	-11.9	-11.89	-11.88	-11.87	-11.86	-11.85	-11.84	-11.83
-11.91	-11.9	-11.89	-11.88	-11.87	-11.86	-11.85	-11.84	-11.83
-11.91	-11.9	-11.89	-11.88	-11.87	-11.86	-11.85	-11.84	-11.83
-11.91	-11.9	-11.89	-11.88	-11.87	-11.86	-11.85	-11.84	-11.83
-11.91	-11.9	-11.89	-11.88	-11.87	-11.86	-11.85	-11.84	-11.83
-11.91	-11.9	-11.89	-11.88	-11.87	-11.86	-11.85	-11.84	-11.83
-11.91	-11.9	-11.89	-11.88	-11.87	-11.86	-11.85	-11.84	-11.83
-11.91	-11.9	-11.89	-11.88	-11.87	-11.86	-11.85	-11.84	-11.83
-11.91	-11.9	-11.89	-11.88	-11.87	-11.86	-11.85	-11.84	-11.83
-11.91	-11.9	-11.89	-11.88	-11.87	-11.86	-11.85	-11.84	-11.83
-11.91	-11.9	-11.89	-11.88	-11.87	-11.86	-11.85	-11.84	-11.83
-11.91	-11.9	-11.89	-11.88	-11.87	-11.86	-11.85	-11.84	-11.83
-11.91	-11.9	-11.89	-11.88	-11.87	-11.86	-11.85	-11.84	-11.83
-11.91	-11.9	-11.89	-11.88	-11.87	-11.86	-11.85	-11.84	-11.83
-11.91	-11.9	-11.89	-11.88	-11.87	-11.86	-11.85	-11.84	-11.83
-11.91	-11.9	-11.89	-11.88	-11.87	-11.86	-11.85	-11.84	-11.83
-11.91	-11.9	-11.89	-11.88	-11.87	-11.86	-11.85	-11.84	-11.83
-11.91	-11.9	-11.89	-11.88	-11.87	-11.86	-11.85	-11.84	-11.83
-11.91	-11.9	-11.89	-11.88	-11.87	-11.86	-11.85	-11.84	-11.83
-11.91	-11.9	-11.89	-11.88	-11.87	-11.86	-11.85	-11.84	-11.83
-11.91	-11.9	-11.89	-11.88	-11.87	-11.86	-11.85	-11.84	-11.83
-11.91	-11.9	-11.89	-11.88	-11.87	-11.86	-11.85	-11.84	-11.83
-11.91	-11.9	-11.89	-11.88	-11.87	-11.86	-11.85	-11.84	-11.83
-11.91	-11.9	-11.89	-11.88	-11.87	-11.86	-11.85	-11.84	-11.83
-11.91	-11.9	-11.89	-11.88	-11.87	-11.86	-11.85	-11.84	-11.83
-11.91	-11.9	-11.89	-11.88	-11.87	-11.86	-11.85	-11.84	-11.83
-11.91	-11.9	-11.89	-11.88	-11.87	-11.86	-11.85	-11.84	-11.83
-11.91	-11.9	-11.89	-11.88	-11.87	-11.86	-11.85	-11.84	-11.83
-11.91	-11.9	-11.89	-11.88	-11.87	-11.86	-11.85	-11.84	-11.83
-11.91	-11.9	-11.89	-11.88	-11.87	-11.86	-11.85	-11.84	-11.83
-11.91	-11.9	-11.89	-11.88	-11.87	-11.86	-11.85	-11.84	-11.83
-11.91	-11.9	-11.89	-11.88	-11.87	-11.86	-11.85	-11.84	-11.83
-11.91	-11.9	-11.89	-11.88	-11.87	-11.86	-11.85	-11.84	-11.83
-11.91	-11.9	-11.89	-11.88	-11.87	-11.86	-11.85	-11.84	-11.83
-11.91	-11.9	-11.89	-11.88	-11.87	-11.86	-11.85	-11.84	-11.83
-11.91	-11.9	-11.89	-11.88	-11.87	-11.86	-11.85	-11.84	-11.83
-11.91	-11.9	-11.89	-11.88	-11.87	-11.86	-11.85	-11.84	-11.83
-11.91	-11.9	-11.89	-11.88	-11.87	-11.86	-11.85	-11.84	-11.83
-11.91	-11.9	-11.89	-11.88	-11.87	-11.86	-11.85	-11.84	-11.83
-11.91	-11.9	-11.89	-11.88	-11.87	-11.86	-11.85	-11.84	-11.83

-11.91	-11.9	-11.89	-11.88	-11.87	-11.86	-11.85	-11.84	-11.83
-11.91	-11.9	-11.89	-11.88	-11.87	-11.86	-11.85	-11.84	-11.83
-11.91	-11.9	-11.89	-11.88	-11.87	-11.86	-11.85	-11.84	-11.83
-11.91	-11.9	-11.89	-11.88	-11.87	-11.86	-11.85	-11.84	-11.83
-11.91	-11.9	-11.89	-11.88	-11.87	-11.86	-11.85	-11.84	-11.83
-11.91	-11.9	-11.89	-11.88	-11.87	-11.86	-11.85	-11.84	-11.83
-11.91	-11.9	-11.89	-11.88	-11.87	-11.86	-11.85	-11.84	-11.83
-11.91	-11.9	-11.89	-11.88	-11.87	-11.86	-11.85	-11.84	-11.83
-11.91	-11.9	-11.89	-11.88	-11.87	-11.86	-11.85	-11.84	-11.83
-11.91	-11.9	-11.89	-11.88	-11.87	-11.86	-11.85	-11.84	-11.83
-11.91	-11.9	-11.89	-11.88	-11.87	-11.86	-11.85	-11.84	-11.83
-11.91	-11.9	-11.89	-11.88	-11.87	-11.86	-11.85	-11.84	-11.83
-11.91	-11.9	-11.89	-11.88	-11.87	-11.86	-11.85	-11.84	-11.83
-11.91	-11.9	-11.89	-11.88	-11.87	-11.86	-11.85	-11.84	-11.83
-11.91	-11.9	-11.89	-11.88	-11.87	-11.86	-11.85	-11.84	-11.83
-11.91	-11.9	-11.89	-11.88	-11.87	-11.86	-11.85	-11.84	-11.83
-11.91	-11.9	-11.89	-11.88	-11.87	-11.86	-11.85	-11.84	-11.83
-11.91	-11.9	-11.89	-11.88	-11.87	-11.86	-11.85	-11.84	-11.83
-11.91	-11.9	-11.89	-11.88	-11.87	-11.86	-11.85	-11.84	-11.83
-11.91	-11.9	-11.89	-11.88	-11.87	-11.86	-11.85	-11.84	-11.83
-11.91	-11.9	-11.89	-11.88	-11.87	-11.86	-11.85	-11.84	-11.83
-11.91	-11.9	-11.89	-11.88	-11.87	-11.86	-11.85	-11.84	-11.83
-11.91	-11.9	-11.89	-11.88	-11.87	-11.86	-11.85	-11.84	-11.83
-11.91	-11.9	-11.89	-11.88	-11.87	-11.86	-11.85	-11.84	-11.83
-11.91	-11.9	-11.89	-11.88	-11.87	-11.86	-11.85	-11.84	-11.83
-11.91	-11.9	-11.89	-11.88	-11.87	-11.86	-11.85	-11.84	-11.83
-11.91	-11.9	-11.89	-11.88	-11.87	-11.86	-11.85	-11.84	-11.83
-11.91	-11.9	-11.89	-11.88	-11.87	-11.86	-11.85	-11.84	-11.83
-11.91	-11.9	-11.89	-11.88	-11.87	-11.86	-11.85	-11.84	-11.83
-11.91	-11.9	-11.89	-11.88	-11.87	-11.86	-11.85	-11.84	-11.83
-11.91	-11.9	-11.89	-11.88	-11.87	-11.86	-11.85	-11.84	-11.83
-11.91	-11.9	-11.89	-11.88	-11.87	-11.86	-11.85	-11.84	-11.83
-11.91	-11.9	-11.89	-11.88	-11.87	-11.86	-11.85	-11.84	-11.83
-11.91	-11.9	-11.89	-11.88	-11.87	-11.86	-11.85	-11.84	-11.83
-11.91	-11.9	-11.89	-11.88	-11.87	-11.86	-11.85	-11.84	-11.83
-11.91	-11.9	-11.89	-11.88	-11.87	-11.86	-11.85	-11.84	-11.83
-11.91	-11.9	-11.89	-11.88	-11.87	-11.86	-11.85	-11.84	-11.83
-11.91	-11.9	-11.89	-11.88	-11.87	-11.86	-11.85	-11.84	-11.83
-11.91	-11.9	-11.89	-11.88	-11.87	-11.86	-11.85	-11.84	-11.83
-11.91	-11.9	-11.89	-11.88	-11.87	-11.86	-11.85	-11.84	-11.83
-11.91	-11.9	-11.89	-11.88	-11.87	-11.86	-11.85	-11.84	-11.83
-11.91	-11.9	-11.89	-11.88	-11.87	-11.86	-11.85	-11.84	-11.83
-11.91	-11.9	-11.89	-11.88	-11.87	-11.86	-11.85	-11.84	-11.83
-11.91	-11.9	-11.89	-11.88	-11.87	-11.86	-11.85	-11.84	-11.83
-11.91	-11.9	-11.89	-11.88	-11.87	-11.86	-11.85	-11.84	-11.83
-11.91	-11.9	-11.89	-11.88	-11.87	-11.86	-11.85	-11.84	-11.83
-11.91	-11.9	-11.89	-11.88	-11.87	-11.86	-11.85	-11.84	-11.83
-11.91	-11.9	-11.89	-11.88	-11.87	-11.86	-11.85	-11.84	-11.83
-11.91	-11.9	-11.89	-11.88	-11.87	-11.86	-11.85	-11.84	-11.83
-11.91	-11.9	-11.89	-11.88	-11.87	-11.86	-11.85	-11.84	-11.83

-11.91	-11.9	-11.89	-11.88	-11.87	-11.86	-11.85	-11.84	-11.83
-11.91	-11.9	-11.89	-11.88	-11.87	-11.86	-11.85	-11.84	-11.83
-11.91	-11.9	-11.89	-11.88	-11.87	-11.86	-11.85	-11.84	-11.83
-11.91	-11.9	-11.89	-11.88	-11.87	-11.86	-11.85	-11.84	-11.83
-11.91	-11.9	-11.89	-11.88	-11.87	-11.86	-11.85	-11.84	-11.83
-11.91	-11.9	-11.89	-11.88	-11.87	-11.86	-11.85	-11.84	-11.83
-11.91	-11.9	-11.89	-11.88	-11.87	-11.86	-11.85	-11.84	-11.83
-11.91	-11.9	-11.89	-11.88	-11.87	-11.86	-11.85	-11.84	-11.83
-11.91	-11.9	-11.89	-11.88	-11.87	-11.86	-11.85	-11.84	-11.83
-11.91	-11.9	-11.89	-11.88	-11.87	-11.86	-11.85	-11.84	-11.83
-11.91	-11.9	-11.89	-11.88	-11.87	-11.86	-11.85	-11.84	-11.83
-11.91	-11.9	-11.89	-11.88	-11.87	-11.86	-11.85	-11.84	-11.83
-11.91	-11.9	-11.89	-11.88	-11.87	-11.86	-11.85	-11.84	-11.83
-11.91	-11.9	-11.89	-11.88	-11.87	-11.86	-11.85	-11.84	-11.83
-11.91	-11.9	-11.89	-11.88	-11.87	-11.86	-11.85	-11.84	-11.83
-11.91	-11.9	-11.89	-11.88	-11.87	-11.86	-11.85	-11.84	-11.83
-11.91	-11.9	-11.89	-11.88	-11.87	-11.86	-11.85	-11.84	-11.83
-11.91	-11.9	-11.89	-11.88	-11.87	-11.86	-11.85	-11.84	-11.83
-11.91	-11.9	-11.89	-11.88	-11.87	-11.86	-11.85	-11.84	-11.83
-11.91	-11.9	-11.89	-11.88	-11.87	-11.86	-11.85	-11.84	-11.83
-11.91	-11.9	-11.89	-11.88	-11.87	-11.86	-11.85	-11.84	-11.83
-11.91	-11.9	-11.89	-11.88	-11.87	-11.86	-11.85	-11.84	-11.83
-11.91	-11.9	-11.89	-11.88	-11.87	-11.86	-11.85	-11.84	-11.83
-11.91	-11.9	-11.89	-11.88	-11.87	-11.86	-11.85	-11.84	-11.83
-11.91	-11.9	-11.89	-11.88	-11.87	-11.86	-11.85	-11.84	-11.83
-11.91	-11.9	-11.89	-11.88	-11.87	-11.86	-11.85	-11.84	-11.83
-11.91	-11.9	-11.89	-11.88	-11.87	-11.86	-11.85	-11.84	-11.83
-11.91	-11.9	-11.89	-11.88	-11.87	-11.86	-11.85	-11.84	-11.83
-11.91	-11.9	-11.89	-11.88	-11.87	-11.86	-11.85	-11.84	-11.83
-11.91	-11.9	-11.89	-11.88	-11.87	-11.86	-11.85	-11.84	-11.83
-11.91	-11.9	-11.89	-11.88	-11.87	-11.86	-11.85	-11.84	-11.83
-11.91	-11.9	-11.89	-11.88	-11.87	-11.86	-11.85	-11.84	-11.83
-11.91	-11.9	-11.89	-11.88	-11.87	-11.86	-11.85	-11.84	-11.83
-11.91	-11.9	-11.89	-11.88	-11.87	-11.86	-11.85	-11.84	-11.83
-11.91	-11.9	-11.89	-11.88	-11.87	-11.86	-11.85	-11.84	-11.83
-11.91	-11.9	-11.89	-11.88	-11.87	-11.86	-11.85	-11.84	-11.83
-11.91	-11.9	-11.89	-11.88	-11.87	-11.86	-11.85	-11.84	-11.83
-11.91	-11.9	-11.89	-11.88	-11.87	-11.86	-11.85	-11.84	-11.83
-11.91	-11.9	-11.89	-11.88	-11.87	-11.86	-11.85	-11.84	-11.83
-11.91	-11.9	-11.89	-11.88	-11.87	-11.86	-11.85	-11.84	-11.83
-11.91	-11.9	-11.89	-11.88	-11.87	-11.86	-11.85	-11.84	-11.83
-11.91	-11.9	-11.89	-11.88	-11.87	-11.86	-11.85	-11.84	-11.83
-11.91	-11.9	-11.89	-11.88	-11.87	-11.86	-11.85	-11.84	-11.83
-11.91	-11.9	-11.89	-11.88	-11.87	-11.86	-11.85	-11.84	-11.83
-11.91	-11.9	-11.89	-11.88	-11.87	-11.86	-11.85	-11.84	-11.83
-11.91	-11.9	-11.89	-11.88	-11.87	-11.86	-11.85	-11.84	-11.83
-11.91	-11.9	-11.89	-11.88	-11.87	-11.86	-11.85	-11.84	-11.83
-11.91	-11.9	-11.89	-11.88	-11.87	-11.86	-11.85	-11.84	-11.83
-11.91	-11.9	-11.89	-11.88	-11.87	-11.86	-11.85	-11.84	-11.83
-11.91	-11.9	-11.89	-11.88	-11.87	-11.86	-11.85	-11.84	-11.83

-11.91	-11.9	-11.89	-11.88	-11.87	-11.86	-11.85	-11.84	-11.83
-11.91	-11.9	-11.89	-11.88	-11.87	-11.86	-11.85	-11.84	-11.83
-11.91	-11.9	-11.89	-11.88	-11.87	-11.86	-11.85	-11.84	-11.83
-11.91	-11.9	-11.89	-11.88	-11.87	-11.86	-11.85	-11.84	-11.83
-11.91	-11.9	-11.89	-11.88	-11.87	-11.86	-11.85	-11.84	-11.83
-11.91	-11.9	-11.89	-11.88	-11.87	-11.86	-11.85	-11.84	-11.83
-11.91	-11.9	-11.89	-11.88	-11.87	-11.86	-11.85	-11.84	-11.83
-11.91	-11.9	-11.89	-11.88	-11.87	-11.86	-11.85	-11.84	-11.83
-11.91	-11.9	-11.89	-11.88	-11.87	-11.86	-11.85	-11.84	-11.83
-11.91	-11.9	-11.89	-11.88	-11.87	-11.86	-11.85	-11.84	-11.83
-11.91	-11.9	-11.89	-11.88	-11.87	-11.86	-11.85	-11.84	-11.83
-11.91	-11.9	-11.89	-11.88	-11.87	-11.86	-11.85	-11.84	-11.83
-11.91	-11.9	-11.89	-11.88	-11.87	-11.86	-11.85	-11.84	-11.83
-11.91	-11.9	-11.89	-11.88	-11.87	-11.86	-11.85	-11.84	-11.83
-11.91	-11.9	-11.89	-11.88	-11.87	-11.86	-11.85	-11.84	-11.83
-11.91	-11.9	-11.89	-11.88	-11.87	-11.86	-11.85	-11.84	-11.83
-11.91	-11.9	-11.89	-11.88	-11.87	-11.86	-11.85	-11.84	-11.83
-11.91	-11.9	-11.89	-11.88	-11.87	-11.86	-11.85	-11.84	-11.83
-11.91	-11.9	-11.89	-11.88	-11.87	-11.86	-11.85	-11.84	-11.83
-11.91	-11.9	-11.89	-11.88	-11.87	-11.86	-11.85	-11.84	-11.83
-11.91	-11.9	-11.89	-11.88	-11.87	-11.86	-11.85	-11.84	-11.83
-11.91	-11.9	-11.89	-11.88	-11.87	-11.86	-11.85	-11.84	-11.83
-11.91	-11.9	-11.89	-11.88	-11.87	-11.86	-11.85	-11.84	-11.83
-11.91	-11.9	-11.89	-11.88	-11.87	-11.86	-11.85	-11.84	-11.83
-11.91	-11.9	-11.89	-11.88	-11.87	-11.86	-11.85	-11.84	-11.83
-11.91	-11.9	-11.89	-11.88	-11.87	-11.86	-11.85	-11.84	-11.83
-11.91	-11.9	-11.89	-11.88	-11.87	-11.86	-11.85	-11.84	-11.83
-11.91	-11.9	-11.89	-11.88	-11.87	-11.86	-11.85	-11.84	-11.83
-11.91	-11.9	-11.89	-11.88	-11.87	-11.86	-11.85	-11.84	-11.83
-11.91	-11.9	-11.89	-11.88	-11.87	-11.86	-11.85	-11.84	-11.83
-11.91	-11.9	-11.89	-11.88	-11.87	-11.86	-11.85	-11.84	-11.83
-11.91	-11.9	-11.89	-11.88	-11.87	-11.86	-11.85	-11.84	-11.83
-11.91	-11.9	-11.89	-11.88	-11.87	-11.86	-11.85	-11.84	-11.83
-11.91	-11.9	-11.89	-11.88	-11.87	-11.86	-11.85	-11.84	-11.83
-11.91	-11.9	-11.89	-11.88	-11.87	-11.86	-11.85	-11.84	-11.83
-11.91	-11.9	-11.89	-11.88	-11.87	-11.86	-11.85	-11.84	-11.83
-11.91	-11.9	-11.89	-11.88	-11.87	-11.86	-11.85	-11.84	-11.83
-11.91	-11.9	-11.89	-11.88	-11.87	-11.86	-11.85	-11.84	-11.83
-11.91	-11.9	-11.89	-11.88	-11.87	-11.86	-11.85	-11.84	-11.83
-11.91	-11.9	-11.89	-11.88	-11.87	-11.86	-11.85	-11.84	-11.83
-11.91	-11.9	-11.89	-11.88	-11.87	-11.86	-11.85	-11.84	-11.83
-11.91	-11.9	-11.89	-11.88	-11.87	-11.86	-11.85	-11.84	-11.83
-11.91	-11.9	-11.89	-11.88	-11.87	-11.86	-11.85	-11.84	-11.83
-11.91	-11.9	-11.89	-11.88	-11.87	-11.86	-11.85	-11.84	-11.83
-11.91	-11.9	-11.89	-11.88	-11.87	-11.86	-11.85	-11.84	-11.83
-11.91	-11.9	-11.89	-11.88	-11.87	-11.86	-11.85	-11.84	-11.83
-11.91	-11.9	-11.89	-11.88	-11.87	-11.86	-11.85	-11.84	-11.83
-11.91	-11.9	-11.89	-11.88	-11.87	-11.86	-11.85	-11.84	-11.83
-11.91	-11.9	-11.89	-11.88	-11.87	-11.86	-11.85	-11.84	-11.83
-11.91	-11.9	-11.89	-11.88	-11.87	-11.86	-11.85	-11.84	-11.83

-11.91	-11.9	-11.89	-11.88	-11.87	-11.86	-11.85	-11.84	-11.83
-11.91	-11.9	-11.89	-11.88	-11.87	-11.86	-11.85	-11.84	-11.83
-11.91	-11.9	-11.89	-11.88	-11.87	-11.86	-11.85	-11.84	-11.83
-11.91	-11.9	-11.89	-11.88	-11.87	-11.86	-11.85	-11.84	-11.83
-11.91	-11.9	-11.89	-11.88	-11.87	-11.86	-11.85	-11.84	-11.83
-11.91	-11.9	-11.89	-11.88	-11.87	-11.86	-11.85	-11.84	-11.83
-11.91	-11.9	-11.89	-11.88	-11.87	-11.86	-11.85	-11.84	-11.83
-11.91	-11.9	-11.89	-11.88	-11.87	-11.86	-11.85	-11.84	-11.83
-11.91	-11.9	-11.89	-11.88	-11.87	-11.86	-11.85	-11.84	-11.83
-11.91	-11.9	-11.89	-11.88	-11.87	-11.86	-11.85	-11.84	-11.83
-11.91	-11.9	-11.89	-11.88	-11.87	-11.86	-11.85	-11.84	-11.83
-11.91	-11.9	-11.89	-11.88	-11.87	-11.86	-11.85	-11.84	-11.83
-11.91	-11.9	-11.89	-11.88	-11.87	-11.86	-11.85	-11.84	-11.83
-11.91	-11.9	-11.89	-11.88	-11.87	-11.86	-11.85	-11.84	-11.83
-11.91	-11.9	-11.89	-11.88	-11.87	-11.86	-11.85	-11.84	-11.83
-11.91	-11.9	-11.89	-11.88	-11.87	-11.86	-11.85	-11.84	-11.83
-11.91	-11.9	-11.89	-11.88	-11.87	-11.86	-11.85	-11.84	-11.83
-11.91	-11.9	-11.89	-11.88	-11.87	-11.86	-11.85	-11.84	-11.83
-11.91	-11.9	-11.89	-11.88	-11.87	-11.86	-11.85	-11.84	-11.83
-11.91	-11.9	-11.89	-11.88	-11.87	-11.86	-11.85	-11.84	-11.83
-11.91	-11.9	-11.89	-11.88	-11.87	-11.86	-11.85	-11.84	-11.83
-11.91	-11.9	-11.89	-11.88	-11.87	-11.86	-11.85	-11.84	-11.83
-11.91	-11.9	-11.89	-11.88	-11.87	-11.86	-11.85	-11.84	-11.83
-11.91	-11.9	-11.89	-11.88	-11.87	-11.86	-11.85	-11.84	-11.83
-11.91	-11.9	-11.89	-11.88	-11.87	-11.86	-11.85	-11.84	-11.83
-11.91	-11.9	-11.89	-11.88	-11.87	-11.86	-11.85	-11.84	-11.83
-11.91	-11.9	-11.89	-11.88	-11.87	-11.86	-11.85	-11.84	-11.83
-11.91	-11.9	-11.89	-11.88	-11.87	-11.86	-11.85	-11.84	-11.83
-11.91	-11.9	-11.89	-11.88	-11.87	-11.86	-11.85	-11.84	-11.83
-11.91	-11.9	-11.89	-11.88	-11.87	-11.86	-11.85	-11.84	-11.83
-11.91	-11.9	-11.89	-11.88	-11.87	-11.86	-11.85	-11.84	-11.83
-11.91	-11.9	-11.89	-11.88	-11.87	-11.86	-11.85	-11.84	-11.83
-11.91	-11.9	-11.89	-11.88	-11.87	-11.86	-11.85	-11.84	-11.83
-11.91	-11.9	-11.89	-11.88	-11.87	-11.86	-11.85	-11.84	-11.83
-11.91	-11.9	-11.89	-11.88	-11.87	-11.86	-11.85	-11.84	-11.83
-11.91	-11.9	-11.89	-11.88	-11.87	-11.86	-11.85	-11.84	-11.83
-11.91	-11.9	-11.89	-11.88	-11.87	-11.86	-11.85	-11.84	-11.83
-11.91	-11.9	-11.89	-11.88	-11.87	-11.86	-11.85	-11.84	-11.83
-11.91	-11.9	-11.89	-11.88	-11.87	-11.86	-11.85	-11.84	-11.83
-11.91	-11.9	-11.89	-11.88	-11.87	-11.86	-11.85	-11.84	-11.83
-11.91	-11.9	-11.89	-11.88	-11.87	-11.86	-11.85	-11.84	-11.83
-11.91	-11.9	-11.89	-11.88	-11.87	-11.86	-11.85	-11.84	-11.83
-11.91	-11.9	-11.89	-11.88	-11.87	-11.86	-11.85	-11.84	-11.83
-11.91	-11.9	-11.89	-11.88	-11.87	-11.86	-11.85	-11.84	-11.83
-11.91	-11.9	-11.89	-11.88	-11.87	-11.86	-11.85	-11.84	-11.83
-11.91	-11.9	-11.89	-11.88	-11.87	-11.86	-11.85	-11.84	-11.83
-11.91	-11.9	-11.89	-11.88	-11.87	-11.86	-11.85	-11.84	-11.83
-11.91	-11.9	-11.89	-11.88	-11.87	-11.86	-11.85	-11.84	-11.83
-11.91	-11.9	-11.89	-11.88	-11.87	-11.86	-11.85	-11.84	-11.83
-11.91	-11.9	-11.89	-11.88	-11.87	-11.86	-11.85	-11.84	-11.83

-11.91	-11.9	-11.89	-11.88	-11.87	-11.86	-11.85	-11.84	-11.83
-11.91	-11.9	-11.89	-11.88	-11.87	-11.86	-11.85	-11.84	-11.83
-11.91	-11.9	-11.89	-11.88	-11.87	-11.86	-11.85	-11.84	-11.83
-11.91	-11.9	-11.89	-11.88	-11.87	-11.86	-11.85	-11.84	-11.83
-11.91	-11.9	-11.89	-11.88	-11.87	-11.86	-11.85	-11.84	-11.83
-11.91	-11.9	-11.89	-11.88	-11.87	-11.86	-11.85	-11.84	-11.83
-11.91	-11.9	-11.89	-11.88	-11.87	-11.86	-11.85	-11.84	-11.83
-11.91	-11.9	-11.89	-11.88	-11.87	-11.86	-11.85	-11.84	-11.83
-11.91	-11.9	-11.89	-11.88	-11.87	-11.86	-11.85	-11.84	-11.83
-11.91	-11.9	-11.89	-11.88	-11.87	-11.86	-11.85	-11.84	-11.83
-11.91	-11.9	-11.89	-11.88	-11.87	-11.86	-11.85	-11.84	-11.83
-11.91	-11.9	-11.89	-11.88	-11.87	-11.86	-11.85	-11.84	-11.83
-11.91	-11.9	-11.89	-11.88	-11.87	-11.86	-11.85	-11.84	-11.83
-11.91	-11.9	-11.89	-11.88	-11.87	-11.86	-11.85	-11.84	-11.83
-11.91	-11.9	-11.89	-11.88	-11.87	-11.86	-11.85	-11.84	-11.83
-11.91	-11.9	-11.89	-11.88	-11.87	-11.86	-11.85	-11.84	-11.83
-11.91	-11.9	-11.89	-11.88	-11.87	-11.86	-11.85	-11.84	-11.83
-11.91	-11.9	-11.89	-11.88	-11.87	-11.86	-11.85	-11.84	-11.83
-11.91	-11.9	-11.89	-11.88	-11.87	-11.86	-11.85	-11.84	-11.83
-11.91	-11.9	-11.89	-11.88	-11.87	-11.86	-11.85	-11.84	-11.83
-11.91	-11.9	-11.89	-11.88	-11.87	-11.86	-11.85	-11.84	-11.83
-11.91	-11.9	-11.89	-11.88	-11.87	-11.86	-11.85	-11.84	-11.83
-11.91	-11.9	-11.89	-11.88	-11.87	-11.86	-11.85	-11.84	-11.83
-11.91	-11.9	-11.89	-11.88	-11.87	-11.86	-11.85	-11.84	-11.83
-11.91	-11.9	-11.89	-11.88	-11.87	-11.86	-11.85	-11.84	-11.83
-11.91	-11.9	-11.89	-11.88	-11.87	-11.86	-11.85	-11.84	-11.83
-11.91	-11.9	-11.89	-11.88	-11.87	-11.86	-11.85	-11.84	-11.83
-11.91	-11.9	-11.89	-11.88	-11.87	-11.86	-11.85	-11.84	-11.83
-11.91	-11.9	-11.89	-11.88	-11.87	-11.86	-11.85	-11.84	-11.83
-11.91	-11.9	-11.89	-11.88	-11.87	-11.86	-11.85	-11.84	-11.83
-11.91	-11.9	-11.89	-11.88	-11.87	-11.86	-11.85	-11.84	-11.83
-11.91	-11.9	-11.89	-11.88	-11.87	-11.86	-11.85	-11.84	-11.83
-11.91	-11.9	-11.89	-11.88	-11.87	-11.86	-11.85	-11.84	-11.83
-11.91	-11.9	-11.89	-11.88	-11.87	-11.86	-11.85	-11.84	-11.83
-11.91	-11.9	-11.89	-11.88	-11.87	-11.86	-11.85	-11.84	-11.83
-11.91	-11.9	-11.89	-11.88	-11.87	-11.86	-11.85	-11.84	-11.83
-11.91	-11.9	-11.89	-11.88	-11.87	-11.86	-11.85	-11.84	-11.83
-11.91	-11.9	-11.89	-11.88	-11.87	-11.86	-11.85	-11.84	-11.83
-11.91	-11.9	-11.89	-11.88	-11.87	-11.86	-11.85	-11.84	-11.83
-11.91	-11.9	-11.89	-11.88	-11.87	-11.86	-11.85	-11.84	-11.83
-11.91	-11.9	-11.89	-11.88	-11.87	-11.86	-11.85	-11.84	-11.83
-11.91	-11.9	-11.89	-11.88	-11.87	-11.86	-11.85	-11.84	-11.83
-11.91	-11.9	-11.89	-11.88	-11.87	-11.86	-11.85	-11.84	-11.83
-11.91	-11.9	-11.89	-11.88	-11.87	-11.86	-11.85	-11.84	-11.83
-11.91	-11.9	-11.89	-11.88	-11.87	-11.86	-11.85	-11.84	-11.83
-11.91	-11.9	-11.89	-11.88	-11.87	-11.86	-11.85	-11.84	-11.83
-11.91	-11.9	-11.89	-11.88	-11.87	-11.86	-11.85	-11.84	-11.83
-11.91	-11.9	-11.89	-11.88	-11.87	-11.86	-11.85	-11.84	-11.83
-11.91	-11.9	-11.89	-11.88	-11.87	-11.86	-11.85	-11.84	-11.83
-11.91	-11.9	-11.89	-11.88	-11.87	-11.86	-11.85	-11.84	-11.83

-11.91	-11.9	-11.89	-11.88	-11.87	-11.86	-11.85	-11.84	-11.83
-11.91	-11.9	-11.89	-11.88	-11.87	-11.86	-11.85	-11.84	-11.83
-11.91	-11.9	-11.89	-11.88	-11.87	-11.86	-11.85	-11.84	-11.83
-11.91	-11.9	-11.89	-11.88	-11.87	-11.86	-11.85	-11.84	-11.83
-11.91	-11.9	-11.89	-11.88	-11.87	-11.86	-11.85	-11.84	-11.83
-11.91	-11.9	-11.89	-11.88	-11.87	-11.86	-11.85	-11.84	-11.83
-11.91	-11.9	-11.89	-11.88	-11.87	-11.86	-11.85	-11.84	-11.83
-11.91	-11.9	-11.89	-11.88	-11.87	-11.86	-11.85	-11.84	-11.83
-11.91	-11.9	-11.89	-11.88	-11.87	-11.86	-11.85	-11.84	-11.83
-11.91	-11.9	-11.89	-11.88	-11.87	-11.86	-11.85	-11.84	-11.83
-11.91	-11.9	-11.89	-11.88	-11.87	-11.86	-11.85	-11.84	-11.83
-11.91	-11.9	-11.89	-11.88	-11.87	-11.86	-11.85	-11.84	-11.83
-11.91	-11.9	-11.89	-11.88	-11.87	-11.86	-11.85	-11.84	-11.83
-11.91	-11.9	-11.89	-11.88	-11.87	-11.86	-11.85	-11.84	-11.83
-11.91	-11.9	-11.89	-11.88	-11.87	-11.86	-11.85	-11.84	-11.83
-11.91	-11.9	-11.89	-11.88	-11.87	-11.86	-11.85	-11.84	-11.83
-11.91	-11.9	-11.89	-11.88	-11.87	-11.86	-11.85	-11.84	-11.83
-11.91	-11.9	-11.89	-11.88	-11.87	-11.86	-11.85	-11.84	-11.83
-11.91	-11.9	-11.89	-11.88	-11.87	-11.86	-11.85	-11.84	-11.83
-11.91	-11.9	-11.89	-11.88	-11.87	-11.86	-11.85	-11.84	-11.83
-11.91	-11.9	-11.89	-11.88	-11.87	-11.86	-11.85	-11.84	-11.83
-11.91	-11.9	-11.89	-11.88	-11.87	-11.86	-11.85	-11.84	-11.83
-11.91	-11.9	-11.89	-11.88	-11.87	-11.86	-11.85	-11.84	-11.83
-11.91	-11.9	-11.89	-11.88	-11.87	-11.86	-11.85	-11.84	-11.83
-11.91	-11.9	-11.89	-11.88	-11.87	-11.86	-11.85	-11.84	-11.83
-11.91	-11.9	-11.89	-11.88	-11.87	-11.86	-11.85	-11.84	-11.83
-11.91	-11.9	-11.89	-11.88	-11.87	-11.86	-11.85	-11.84	-11.83
-11.91	-11.9	-11.89	-11.88	-11.87	-11.86	-11.85	-11.84	-11.83
-11.91	-11.9	-11.89	-11.88	-11.87	-11.86	-11.85	-11.84	-11.83
-11.91	-11.9	-11.89	-11.88	-11.87	-11.86	-11.85	-11.84	-11.83
-11.91	-11.9	-11.89	-11.88	-11.87	-11.86	-11.85	-11.84	-11.83
-11.91	-11.9	-11.89	-11.88	-11.87	-11.86	-11.85	-11.84	-11.83
-11.91	-11.9	-11.89	-11.88	-11.87	-11.86	-11.85	-11.84	-11.83
-11.91	-11.9	-11.89	-11.88	-11.87	-11.86	-11.85	-11.84	-11.83
-11.91	-11.9	-11.89	-11.88	-11.87	-11.86	-11.85	-11.84	-11.83
-11.91	-11.9	-11.89	-11.88	-11.87	-11.86	-11.85	-11.84	-11.83
-11.91	-11.9	-11.89	-11.88	-11.87	-11.86	-11.85	-11.84	-11.83
-11.91	-11.9	-11.89	-11.88	-11.87	-11.86	-11.85	-11.84	-11.83
-11.91	-11.9	-11.89	-11.88	-11.87	-11.86	-11.85	-11.84	-11.83
-11.91	-11.9	-11.89	-11.88	-11.87	-11.86	-11.85	-11.84	-11.83
-11.91	-11.9	-11.89	-11.88	-11.87	-11.86	-11.85	-11.84	-11.83
-11.91	-11.9	-11.89	-11.88	-11.87	-11.86	-11.85	-11.84	-11.83
-11.91	-11.9	-11.89	-11.88	-11.87	-11.86	-11.85	-11.84	-11.83
-11.91	-11.9	-11.89	-11.88	-11.87	-11.86	-11.85	-11.84	-11.83
-11.91	-11.9	-11.89	-11.88	-11.87	-11.86	-11.85	-11.84	-11.83
-11.91	-11.9	-11.89	-11.88	-11.87	-11.86	-11.85	-11.84	-11.83
-11.91	-11.9	-11.89	-11.88	-11.87	-11.86	-11.85	-11.84	-11.83
-11.91	-11.9	-11.89	-11.88	-11.87	-11.86	-11.85	-11.84	-11.83
-11.91	-11.9	-11.89	-11.88	-11.87	-11.86	-11.85	-11.84	-11.83
-11.91	-11.9	-11.89	-11.88	-11.87	-11.86	-11.85	-11.84	-11.83

-11.91	-11.9	-11.89	-11.88	-11.87	-11.86	-11.85	-11.84	-11.83
-11.91	-11.9	-11.89	-11.88	-11.87	-11.86	-11.85	-11.84	-11.83
-11.91	-11.9	-11.89	-11.88	-11.87	-11.86	-11.85	-11.84	-11.83
-11.91	-11.9	-11.89	-11.88	-11.87	-11.86	-11.85	-11.84	-11.83
-11.91	-11.9	-11.89	-11.88	-11.87	-11.86	-11.85	-11.84	-11.83
-11.91	-11.9	-11.89	-11.88	-11.87	-11.86	-11.85	-11.84	-11.83
-11.91	-11.9	-11.89	-11.88	-11.87	-11.86	-11.85	-11.84	-11.83
-11.91	-11.9	-11.89	-11.88	-11.87	-11.86	-11.85	-11.84	-11.83
-11.91	-11.9	-11.89	-11.88	-11.87	-11.86	-11.85	-11.84	-11.83
-11.91	-11.9	-11.89	-11.88	-11.87	-11.86	-11.85	-11.84	-11.83
-11.91	-11.9	-11.89	-11.88	-11.87	-11.86	-11.85	-11.84	-11.83
-11.91	-11.9	-11.89	-11.88	-11.87	-11.86	-11.85	-11.84	-11.83
-11.91	-11.9	-11.89	-11.88	-11.87	-11.86	-11.85	-11.84	-11.83
-11.91	-11.9	-11.89	-11.88	-11.87	-11.86	-11.85	-11.84	-11.83
-11.91	-11.9	-11.89	-11.88	-11.87	-11.86	-11.85	-11.84	-11.83
-11.91	-11.9	-11.89	-11.88	-11.87	-11.86	-11.85	-11.84	-11.83
-11.91	-11.9	-11.89	-11.88	-11.87	-11.86	-11.85	-11.84	-11.83
-11.91	-11.9	-11.89	-11.88	-11.87	-11.86	-11.85	-11.84	-11.83
-11.91	-11.9	-11.89	-11.88	-11.87	-11.86	-11.85	-11.84	-11.83
-11.91	-11.9	-11.89	-11.88	-11.87	-11.86	-11.85	-11.84	-11.83
-11.91	-11.9	-11.89	-11.88	-11.87	-11.86	-11.85	-11.84	-11.83
-11.91	-11.9	-11.89	-11.88	-11.87	-11.86	-11.85	-11.84	-11.83
-11.91	-11.9	-11.89	-11.88	-11.87	-11.86	-11.85	-11.84	-11.83
-11.91	-11.9	-11.89	-11.88	-11.87	-11.86	-11.85	-11.84	-11.83
-11.91	-11.9	-11.89	-11.88	-11.87	-11.86	-11.85	-11.84	-11.83
-11.91	-11.9	-11.89	-11.88	-11.87	-11.86	-11.85	-11.84	-11.83
-11.91	-11.9	-11.89	-11.88	-11.87	-11.86	-11.85	-11.84	-11.83
-11.91	-11.9	-11.89	-11.88	-11.87	-11.86	-11.85	-11.84	-11.83
-11.91	-11.9	-11.89	-11.88	-11.87	-11.86	-11.85	-11.84	-11.83
-11.91	-11.9	-11.89	-11.88	-11.87	-11.86	-11.85	-11.84	-11.83
-11.91	-11.9	-11.89	-11.88	-11.87	-11.86	-11.85	-11.84	-11.83
-11.91	-11.9	-11.89	-11.88	-11.87	-11.86	-11.85	-11.84	-11.83
-11.91	-11.9	-11.89	-11.88	-11.87	-11.86	-11.85	-11.84	-11.83
-11.91	-11.9	-11.89	-11.88	-11.87	-11.86	-11.85	-11.84	-11.83
-11.91	-11.9	-11.89	-11.88	-11.87	-11.86	-11.85	-11.84	-11.83
-11.91	-11.9	-11.89	-11.88	-11.87	-11.86	-11.85	-11.84	-11.83
-11.91	-11.9	-11.89	-11.88	-11.87	-11.86	-11.85	-11.84	-11.83
-11.91	-11.9	-11.89	-11.88	-11.87	-11.86	-11.85	-11.84	-11.83
-11.91	-11.9	-11.89	-11.88	-11.87	-11.86	-11.85	-11.84	-11.83
-11.91	-11.9	-11.89	-11.88	-11.87	-11.86	-11.85	-11.84	-11.83
-11.91	-11.9	-11.89	-11.88	-11.87	-11.86	-11.85	-11.84	-11.83
-11.91	-11.9	-11.89	-11.88	-11.87	-11.86	-11.85	-11.84	-11.83
-11.91	-11.9	-11.89	-11.88	-11.87	-11.86	-11.85	-11.84	-11.83
-11.91	-11.9	-11.89	-11.88	-11.87	-11.86	-11.85	-11.84	-11.83
-11.91	-11.9	-11.89	-11.88	-11.87	-11.86	-11.85	-11.84	-11.83
-11.91	-11.9	-11.89	-11.88	-11.87	-11.86	-11.85	-11.84	-11.83
-11.91	-11.9	-11.89	-11.88	-11.87	-11.86	-11.85	-11.84	-11.83
-11.91	-11.9	-11.89	-11.88	-11.87	-11.86	-11.85	-11.84	-11.83
-11.91	-11.9	-11.89	-11.88	-11.87	-11.86	-11.85	-11.84	-11.83
-11.91	-11.9	-11.89	-11.88	-11.87	-11.86	-11.85	-11.84	-11.83

-11.91	-11.9	-11.89	-11.88	-11.87	-11.86	-11.85	-11.84	-11.83
-11.91	-11.9	-11.89	-11.88	-11.87	-11.86	-11.85	-11.84	-11.83
-11.91	-11.9	-11.89	-11.88	-11.87	-11.86	-11.85	-11.84	-11.83
-11.91	-11.9	-11.89	-11.88	-11.87	-11.86	-11.85	-11.84	-11.83
-11.91	-11.9	-11.89	-11.88	-11.87	-11.86	-11.85	-11.84	-11.83
-11.91	-11.9	-11.89	-11.88	-11.87	-11.86	-11.85	-11.84	-11.83
-11.91	-11.9	-11.89	-11.88	-11.87	-11.86	-11.85	-11.84	-11.83
-11.91	-11.9	-11.89	-11.88	-11.87	-11.86	-11.85	-11.84	-11.83
-11.91	-11.9	-11.89	-11.88	-11.87	-11.86	-11.85	-11.84	-11.83
-11.91	-11.9	-11.89	-11.88	-11.87	-11.86	-11.85	-11.84	-11.83
-11.91	-11.9	-11.89	-11.88	-11.87	-11.86	-11.85	-11.84	-11.83
-11.91	-11.9	-11.89	-11.88	-11.87	-11.86	-11.85	-11.84	-11.83
-11.91	-11.9	-11.89	-11.88	-11.87	-11.86	-11.85	-11.84	-11.83
-11.91	-11.9	-11.89	-11.88	-11.87	-11.86	-11.85	-11.84	-11.83
-11.91	-11.9	-11.89	-11.88	-11.87	-11.86	-11.85	-11.84	-11.83
-11.91	-11.9	-11.89	-11.88	-11.87	-11.86	-11.85	-11.84	-11.83
-11.91	-11.9	-11.89	-11.88	-11.87	-11.86	-11.85	-11.84	-11.83
-11.91	-11.9	-11.89	-11.88	-11.87	-11.86	-11.85	-11.84	-11.83
-11.91	-11.9	-11.89	-11.88	-11.87	-11.86	-11.85	-11.84	-11.83
-11.91	-11.9	-11.89	-11.88	-11.87	-11.86	-11.85	-11.84	-11.83
-11.91	-11.9	-11.89	-11.88	-11.87	-11.86	-11.85	-11.84	-11.83
-11.91	-11.9	-11.89	-11.88	-11.87	-11.86	-11.85	-11.84	-11.83
-11.91	-11.9	-11.89	-11.88	-11.87	-11.86	-11.85	-11.84	-11.83
-11.91	-11.9	-11.89	-11.88	-11.87	-11.86	-11.85	-11.84	-11.83
-11.91	-11.9	-11.89	-11.88	-11.87	-11.86	-11.85	-11.84	-11.83
-11.91	-11.9	-11.89	-11.88	-11.87	-11.86	-11.85	-11.84	-11.83
-11.91	-11.9	-11.89	-11.88	-11.87	-11.86	-11.85	-11.84	-11.83
-11.91	-11.9	-11.89	-11.88	-11.87	-11.86	-11.85	-11.84	-11.83
-11.91	-11.9	-11.89	-11.88	-11.87	-11.86	-11.85	-11.84	-11.83
-11.91	-11.9	-11.89	-11.88	-11.87	-11.86	-11.85	-11.84	-11.83
-11.91	-11.9	-11.89	-11.88	-11.87	-11.86	-11.85	-11.84	-11.83
-11.91	-11.9	-11.89	-11.88	-11.87	-11.86	-11.85	-11.84	-11.83
-11.91	-11.9	-11.89	-11.88	-11.87	-11.86	-11.85	-11.84	-11.83
-11.91	-11.9	-11.89	-11.88	-11.87	-11.86	-11.85	-11.84	-11.83
-11.91	-11.9	-11.89	-11.88	-11.87	-11.86	-11.85	-11.84	-11.83
-11.91	-11.9	-11.89	-11.88	-11.87	-11.86	-11.85	-11.84	-11.83
-11.91	-11.9	-11.89	-11.88	-11.87	-11.86	-11.85	-11.84	-11.83
-11.91	-11.9	-11.89	-11.88	-11.87	-11.86	-11.85	-11.84	-11.83
-11.91	-11.9	-11.89	-11.88	-11.87	-11.86	-11.85	-11.84	-11.83
-11.91	-11.9	-11.89	-11.88	-11.87	-11.86	-11.85	-11.84	-11.83
-11.91	-11.9	-11.89	-11.88	-11.87	-11.86	-11.85	-11.84	-11.83
-11.91	-11.9	-11.89	-11.88	-11.87	-11.86	-11.85	-11.84	-11.83
-11.91	-11.9	-11.89	-11.88	-11.87	-11.86	-11.85	-11.84	-11.83
-11.91	-11.9	-11.89	-11.88	-11.87	-11.86	-11.85	-11.84	-11.83
-11.91	-11.9	-11.89	-11.88	-11.87	-11.86	-11.85	-11.84	-11.83
-11.91	-11.9	-11.89	-11.88	-11.87	-11.86	-11.85	-11.84	-11.83
-11.91	-11.9	-11.89	-11.88	-11.87	-11.86	-11.85	-11.84	-11.83
-11.91	-11.9	-11.89	-11.88	-11.87	-11.86	-11.85	-11.84	-11.83
-11.91	-11.9	-11.89	-11.88	-11.87	-11.86	-11.85	-11.84	-11.83
-11.91	-11.9	-11.89	-11.88	-11.87	-11.86	-11.85	-11.84	-11.83

-11.91	-11.9	-11.89	-11.88	-11.87	-11.86	-11.85	-11.84	-11.83
-11.91	-11.9	-11.89	-11.88	-11.87	-11.86	-11.85	-11.84	-11.83
-11.91	-11.9	-11.89	-11.88	-11.87	-11.86	-11.85	-11.84	-11.83
-11.91	-11.9	-11.89	-11.88	-11.87	-11.86	-11.85	-11.84	-11.83
-11.91	-11.9	-11.89	-11.88	-11.87	-11.86	-11.85	-11.84	-11.83
-11.91	-11.9	-11.89	-11.88	-11.87	-11.86	-11.85	-11.84	-11.83
-11.91	-11.9	-11.89	-11.88	-11.87	-11.86	-11.85	-11.84	-11.83
-11.91	-11.9	-11.89	-11.88	-11.87	-11.86	-11.85	-11.84	-11.83
-11.91	-11.9	-11.89	-11.88	-11.87	-11.86	-11.85	-11.84	-11.83
-11.91	-11.9	-11.89	-11.88	-11.87	-11.86	-11.85	-11.84	-11.83
-11.91	-11.9	-11.89	-11.88	-11.87	-11.86	-11.85	-11.84	-11.83
-11.91	-11.9	-11.89	-11.88	-11.87	-11.86	-11.85	-11.84	-11.83
-11.91	-11.9	-11.89	-11.88	-11.87	-11.86	-11.85	-11.84	-11.83
-11.91	-11.9	-11.89	-11.88	-11.87	-11.86	-11.85	-11.84	-11.83
-11.91	-11.9	-11.89	-11.88	-11.87	-11.86	-11.85	-11.84	-11.83
-11.91	-11.9	-11.89	-11.88	-11.87	-11.86	-11.85	-11.84	-11.83
-11.91	-11.9	-11.89	-11.88	-11.87	-11.86	-11.85	-11.84	-11.83
-11.91	-11.9	-11.89	-11.88	-11.87	-11.86	-11.85	-11.84	-11.83
-11.91	-11.9	-11.89	-11.88	-11.87	-11.86	-11.85	-11.84	-11.83
-11.91	-11.9	-11.89	-11.88	-11.87	-11.86	-11.85	-11.84	-11.83
-11.91	-11.9	-11.89	-11.88	-11.87	-11.86	-11.85	-11.84	-11.83
-11.91	-11.9	-11.89	-11.88	-11.87	-11.86	-11.85	-11.84	-11.83
-11.91	-11.9	-11.89	-11.88	-11.87	-11.86	-11.85	-11.84	-11.83
-11.91	-11.9	-11.89	-11.88	-11.87	-11.86	-11.85	-11.84	-11.83
-11.91	-11.9	-11.89	-11.88	-11.87	-11.86	-11.85	-11.84	-11.83
-11.91	-11.9	-11.89	-11.88	-11.87	-11.86	-11.85	-11.84	-11.83
-11.91	-11.9	-11.89	-11.88	-11.87	-11.86	-11.85	-11.84	-11.83
-11.91	-11.9	-11.89	-11.88	-11.87	-11.86	-11.85	-11.84	-11.83
-11.91	-11.9	-11.89	-11.88	-11.87	-11.86	-11.85	-11.84	-11.83
-11.91	-11.9	-11.89	-11.88	-11.87	-11.86	-11.85	-11.84	-11.83
-11.91	-11.9	-11.89	-11.88	-11.87	-11.86	-11.85	-11.84	-11.83
-11.91	-11.9	-11.89	-11.88	-11.87	-11.86	-11.85	-11.84	-11.83
-11.91	-11.9	-11.89	-11.88	-11.87	-11.86	-11.85	-11.84	-11.83
-11.91	-11.9	-11.89	-11.88	-11.87	-11.86	-11.85	-11.84	-11.83
-11.91	-11.9	-11.89	-11.88	-11.87	-11.86	-11.85	-11.84	-11.83
-11.91	-11.9	-11.89	-11.88	-11.87	-11.86	-11.85	-11.84	-11.83
-11.91	-11.9	-11.89	-11.88	-11.87	-11.86	-11.85	-11.84	-11.83
-11.91	-11.9	-11.89	-11.88	-11.87	-11.86	-11.85	-11.84	-11.83
-11.91	-11.9	-11.89	-11.88	-11.87	-11.86	-11.85	-11.84	-11.83
-11.91	-11.9	-11.89	-11.88	-11.87	-11.86	-11.85	-11.84	-11.83
-11.91	-11.9	-11.89	-11.88	-11.87	-11.86	-11.85	-11.84	-11.83
-11.91	-11.9	-11.89	-11.88	-11.87	-11.86	-11.85	-11.84	-11.83
-11.91	-11.9	-11.89	-11.88	-11.87	-11.86	-11.85	-11.84	-11.83
-11.91	-11.9	-11.89	-11.88	-11.87	-11.86	-11.85	-11.84	-11.83
-11.91	-11.9	-11.89	-11.88	-11.87	-11.86	-11.85	-11.84	-11.83
-11.91	-11.9	-11.89	-11.88	-11.87	-11.86	-11.85	-11.84	-11.83
-11.91	-11.9	-11.89	-11.88	-11.87	-11.86	-11.85	-11.84	-11.83
-11.91	-11.9	-11.89	-11.88	-11.87	-11.86	-11.85	-11.84	-11.83
-11.91	-11.9	-11.89	-11.88	-11.87	-11.86	-11.85	-11.84	-11.83
-11.91	-11.9	-11.89	-11.88	-11.87	-11.86	-11.85	-11.84	-11.83

-11.91	-11.9	-11.89	-11.88	-11.87	-11.86	-11.85	-11.84	-11.83
-11.91	-11.9	-11.89	-11.88	-11.87	-11.86	-11.85	-11.84	-11.83
-11.91	-11.9	-11.89	-11.88	-11.87	-11.86	-11.85	-11.84	-11.83
-11.91	-11.9	-11.89	-11.88	-11.87	-11.86	-11.85	-11.84	-11.83
-11.91	-11.9	-11.89	-11.88	-11.87	-11.86	-11.85	-11.84	-11.83
-11.91	-11.9	-11.89	-11.88	-11.87	-11.86	-11.85	-11.84	-11.83
-11.91	-11.9	-11.89	-11.88	-11.87	-11.86	-11.85	-11.84	-11.83
-11.91	-11.9	-11.89	-11.88	-11.87	-11.86	-11.85	-11.84	-11.83
-11.91	-11.9	-11.89	-11.88	-11.87	-11.86	-11.85	-11.84	-11.83
-11.91	-11.9	-11.89	-11.88	-11.87	-11.86	-11.85	-11.84	-11.83
-11.91	-11.9	-11.89	-11.88	-11.87	-11.86	-11.85	-11.84	-11.83
-11.91	-11.9	-11.89	-11.88	-11.87	-11.86	-11.85	-11.84	-11.83
-11.91	-11.9	-11.89	-11.88	-11.87	-11.86	-11.85	-11.84	-11.83
-11.91	-11.9	-11.89	-11.88	-11.87	-11.86	-11.85	-11.84	-11.83
-11.91	-11.9	-11.89	-11.88	-11.87	-11.86	-11.85	-11.84	-11.83
-11.91	-11.9	-11.89	-11.88	-11.87	-11.86	-11.85	-11.84	-11.83
-11.91	-11.9	-11.89	-11.88	-11.87	-11.86	-11.85	-11.84	-11.83
-11.91	-11.9	-11.89	-11.88	-11.87	-11.86	-11.85	-11.84	-11.83
-11.91	-11.9	-11.89	-11.88	-11.87	-11.86	-11.85	-11.84	-11.83
-11.91	-11.9	-11.89	-11.88	-11.87	-11.86	-11.85	-11.84	-11.83
-11.91	-11.9	-11.89	-11.88	-11.87	-11.86	-11.85	-11.84	-11.83
-11.91	-11.9	-11.89	-11.88	-11.87	-11.86	-11.85	-11.84	-11.83
-11.91	-11.9	-11.89	-11.88	-11.87	-11.86	-11.85	-11.84	-11.83
-11.91	-11.9	-11.89	-11.88	-11.87	-11.86	-11.85	-11.84	-11.83
-11.91	-11.9	-11.89	-11.88	-11.87	-11.86	-11.85	-11.84	-11.83
-11.91	-11.9	-11.89	-11.88	-11.87	-11.86	-11.85	-11.84	-11.83
-11.91	-11.9	-11.89	-11.88	-11.87	-11.86	-11.85	-11.84	-11.83
-11.91	-11.9	-11.89	-11.88	-11.87	-11.86	-11.85	-11.84	-11.83
-11.91	-11.9	-11.89	-11.88	-11.87	-11.86	-11.85	-11.84	-11.83
-11.91	-11.9	-11.89	-11.88	-11.87	-11.86	-11.85	-11.84	-11.83
-11.91	-11.9	-11.89	-11.88	-11.87	-11.86	-11.85	-11.84	-11.83
-11.91	-11.9	-11.89	-11.88	-11.87	-11.86	-11.85	-11.84	-11.83
-11.91	-11.9	-11.89	-11.88	-11.87	-11.86	-11.85	-11.84	-11.83
-11.91	-11.9	-11.89	-11.88	-11.87	-11.86	-11.85	-11.84	-11.83
-11.91	-11.9	-11.89	-11.88	-11.87	-11.86	-11.85	-11.84	-11.83
-11.91	-11.9	-11.89	-11.88	-11.87	-11.86	-11.85	-11.84	-11.83
-11.91	-11.9	-11.89	-11.88	-11.87	-11.86	-11.85	-11.84	-11.83
-11.91	-11.9	-11.89	-11.88	-11.87	-11.86	-11.85	-11.84	-11.83
-11.91	-11.9	-11.89	-11.88	-11.87	-11.86	-11.85	-11.84	-11.83
-11.91	-11.9	-11.89	-11.88	-11.87	-11.86	-11.85	-11.84	-11.83
-11.91	-11.9	-11.89	-11.88	-11.87	-11.86	-11.85	-11.84	-11.83
-11.91	-11.9	-11.89	-11.88	-11.87	-11.86	-11.85	-11.84	-11.83
-11.91	-11.9	-11.89	-11.88	-11.87	-11.86	-11.85	-11.84	-11.83
-11.91	-11.9	-11.89	-11.88	-11.87	-11.86	-11.85	-11.84	-11.83
-11.91	-11.9	-11.89	-11.88	-11.87	-11.86	-11.85	-11.84	-11.83
-11.91	-11.9	-11.89	-11.88	-11.87	-11.86	-11.85	-11.84	-11.83
-11.91	-11.9	-11.89	-11.88	-11.87	-11.86	-11.85	-11.84	-11.83
-11.91	-11.9	-11.89	-11.88	-11.87	-11.86	-11.85	-11.84	-11.83
-11.91	-11.9	-11.89	-11.88	-11.87	-11.86	-11.85	-11.84	-11.83
-11.91	-11.9	-11.89	-11.88	-11.87	-11.86	-11.85	-11.84	-11.83

-11.91	-11.9	-11.89	-11.88	-11.87	-11.86	-11.85	-11.84	-11.83
-11.91	-11.9	-11.89	-11.88	-11.87	-11.86	-11.85	-11.84	-11.83
-11.91	-11.9	-11.89	-11.88	-11.87	-11.86	-11.85	-11.84	-11.83
-11.91	-11.9	-11.89	-11.88	-11.87	-11.86	-11.85	-11.84	-11.83
-11.91	-11.9	-11.89	-11.88	-11.87	-11.86	-11.85	-11.84	-11.83
-11.91	-11.9	-11.89	-11.88	-11.87	-11.86	-11.85	-11.84	-11.83
-11.91	-11.9	-11.89	-11.88	-11.87	-11.86	-11.85	-11.84	-11.83
-11.91	-11.9	-11.89	-11.88	-11.87	-11.86	-11.85	-11.84	-11.83
-11.91	-11.9	-11.89	-11.88	-11.87	-11.86	-11.85	-11.84	-11.83
-11.91	-11.9	-11.89	-11.88	-11.87	-11.86	-11.85	-11.84	-11.83
-11.91	-11.9	-11.89	-11.88	-11.87	-11.86	-11.85	-11.84	-11.83
-11.91	-11.9	-11.89	-11.88	-11.87	-11.86	-11.85	-11.84	-11.83
-11.91	-11.9	-11.89	-11.88	-11.87	-11.86	-11.85	-11.84	-11.83
-11.91	-11.9	-11.89	-11.88	-11.87	-11.86	-11.85	-11.84	-11.83
-11.91	-11.9	-11.89	-11.88	-11.87	-11.86	-11.85	-11.84	-11.83
-11.91	-11.9	-11.89	-11.88	-11.87	-11.86	-11.85	-11.84	-11.83
-11.91	-11.9	-11.89	-11.88	-11.87	-11.86	-11.85	-11.84	-11.83
-11.91	-11.9	-11.89	-11.88	-11.87	-11.86	-11.85	-11.84	-11.83
-11.91	-11.9	-11.89	-11.88	-11.87	-11.86	-11.85	-11.84	-11.83
-11.91	-11.9	-11.89	-11.88	-11.87	-11.86	-11.85	-11.84	-11.83
-11.91	-11.9	-11.89	-11.88	-11.87	-11.86	-11.85	-11.84	-11.83
-11.91	-11.9	-11.89	-11.88	-11.87	-11.86	-11.85	-11.84	-11.83
-11.91	-11.9	-11.89	-11.88	-11.87	-11.86	-11.85	-11.84	-11.83
-11.91	-11.9	-11.89	-11.88	-11.87	-11.86	-11.85	-11.84	-11.83
-11.91	-11.9	-11.89	-11.88	-11.87	-11.86	-11.85	-11.84	-11.83
-11.91	-11.9	-11.89	-11.88	-11.87	-11.86	-11.85	-11.84	-11.83
-11.91	-11.9	-11.89	-11.88	-11.87	-11.86	-11.85	-11.84	-11.83
-11.91	-11.9	-11.89	-11.88	-11.87	-11.86	-11.85	-11.84	-11.83
-11.91	-11.9	-11.89	-11.88	-11.87	-11.86	-11.85	-11.84	-11.83
-11.91	-11.9	-11.89	-11.88	-11.87	-11.86	-11.85	-11.84	-11.83
-11.91	-11.9	-11.89	-11.88	-11.87	-11.86	-11.85	-11.84	-11.83
-11.91	-11.9	-11.89	-11.88	-11.87	-11.86	-11.85	-11.84	-11.83
-11.91	-11.9	-11.89	-11.88	-11.87	-11.86	-11.85	-11.84	-11.83
-11.91	-11.9	-11.89	-11.88	-11.87	-11.86	-11.85	-11.84	-11.83
-11.91	-11.9	-11.89	-11.88	-11.87	-11.86	-11.85	-11.84	-11.83
-11.91	-11.9	-11.89	-11.88	-11.87	-11.86	-11.85	-11.84	-11.83
-11.91	-11.9	-11.89	-11.88	-11.87	-11.86	-11.85	-11.84	-11.83
-11.91	-11.9	-11.89	-11.88	-11.87	-11.86	-11.85	-11.84	-11.83
-11.91	-11.9	-11.89	-11.88	-11.87	-11.86	-11.85	-11.84	-11.83
-11.91	-11.9	-11.89	-11.88	-11.87	-11.86	-11.85	-11.84	-11.83
-11.91	-11.9	-11.89	-11.88	-11.87	-11.86	-11.85	-11.84	-11.83
-11.91	-11.9	-11.89	-11.88	-11.87	-11.86	-11.85	-11.84	-11.83
-11.91	-11.9	-11.89	-11.88	-11.87	-11.86	-11.85	-11.84	-11.83
-11.91	-11.9	-11.89	-11.88	-11.87	-11.86	-11.85	-11.84	-11.83
-11.91	-11.9	-11.89	-11.88	-11.87	-11.86	-11.85	-11.84	-11.83
-11.91	-11.9	-11.89	-11.88	-11.87	-11.86	-11.85	-11.84	-11.83
-11.91	-11.9	-11.89	-11.88	-11.87	-11.86	-11.85	-11.84	-11.83
-11.91	-11.9	-11.89	-11.88	-11.87	-11.86	-11.85	-11.84	-11.83
-11.91	-11.9	-11.89	-11.88	-11.87	-11.86	-11.85	-11.84	-11.83
-11.91	-11.9	-11.89	-11.88	-11.87	-11.86	-11.85	-11.84	-11.83

-11.91	-11.9	-11.89	-11.88	-11.87	-11.86	-11.85	-11.84	-11.83
-11.91	-11.9	-11.89	-11.88	-11.87	-11.86	-11.85	-11.84	-11.83
-11.91	-11.9	-11.89	-11.88	-11.87	-11.86	-11.85	-11.84	-11.83
-11.91	-11.9	-11.89	-11.88	-11.87	-11.86	-11.85	-11.84	-11.83
-11.91	-11.9	-11.89	-11.88	-11.87	-11.86	-11.85	-11.84	-11.83
-11.91	-11.9	-11.89	-11.88	-11.87	-11.86	-11.85	-11.84	-11.83
-11.91	-11.9	-11.89	-11.88	-11.87	-11.86	-11.85	-11.84	-11.83
-11.91	-11.9	-11.89	-11.88	-11.87	-11.86	-11.85	-11.84	-11.83
-11.91	-11.9	-11.89	-11.88	-11.87	-11.86	-11.85	-11.84	-11.83
-11.91	-11.9	-11.89	-11.88	-11.87	-11.86	-11.85	-11.84	-11.83
-11.91	-11.9	-11.89	-11.88	-11.87	-11.86	-11.85	-11.84	-11.83
-11.91	-11.9	-11.89	-11.88	-11.87	-11.86	-11.85	-11.84	-11.83
-11.91	-11.9	-11.89	-11.88	-11.87	-11.86	-11.85	-11.84	-11.83
-11.91	-11.9	-11.89	-11.88	-11.87	-11.86	-11.85	-11.84	-11.83
-11.91	-11.9	-11.89	-11.88	-11.87	-11.86	-11.85	-11.84	-11.83
-11.91	-11.9	-11.89	-11.88	-11.87	-11.86	-11.85	-11.84	-11.83
-11.91	-11.9	-11.89	-11.88	-11.87	-11.86	-11.85	-11.84	-11.83
-11.91	-11.9	-11.89	-11.88	-11.87	-11.86	-11.85	-11.84	-11.83
-11.91	-11.9	-11.89	-11.88	-11.87	-11.86	-11.85	-11.84	-11.83
-11.91	-11.9	-11.89	-11.88	-11.87	-11.86	-11.85	-11.84	-11.83
-11.91	-11.9	-11.89	-11.88	-11.87	-11.86	-11.85	-11.84	-11.83
-11.91	-11.9	-11.89	-11.88	-11.87	-11.86	-11.85	-11.84	-11.83
-11.91	-11.9	-11.89	-11.88	-11.87	-11.86	-11.85	-11.84	-11.83
-11.91	-11.9	-11.89	-11.88	-11.87	-11.86	-11.85	-11.84	-11.83
-11.91	-11.9	-11.89	-11.88	-11.87	-11.86	-11.85	-11.84	-11.83
-11.91	-11.9	-11.89	-11.88	-11.87	-11.86	-11.85	-11.84	-11.83
-11.91	-11.9	-11.89	-11.88	-11.87	-11.86	-11.85	-11.84	-11.83
-11.91	-11.9	-11.89	-11.88	-11.87	-11.86	-11.85	-11.84	-11.83
-11.91	-11.9	-11.89	-11.88	-11.87	-11.86	-11.85	-11.84	-11.83
-11.91	-11.9	-11.89	-11.88	-11.87	-11.86	-11.85	-11.84	-11.83
-11.91	-11.9	-11.89	-11.88	-11.87	-11.86	-11.85	-11.84	-11.83
-11.91	-11.9	-11.89	-11.88	-11.87	-11.86	-11.85	-11.84	-11.83
-11.91	-11.9	-11.89	-11.88	-11.87	-11.86	-11.85	-11.84	-11.83
-11.91	-11.9	-11.89	-11.88	-11.87	-11.86	-11.85	-11.84	-11.83
-11.91	-11.9	-11.89	-11.88	-11.87	-11.86	-11.85	-11.84	-11.83
-11.91	-11.9	-11.89	-11.88	-11.87	-11.86	-11.85	-11.84	-11.83
-11.91	-11.9	-11.89	-11.88	-11.87	-11.86	-11.85	-11.84	-11.83
-11.91	-11.9	-11.89	-11.88	-11.87	-11.86	-11.85	-11.84	-11.83
-11.91	-11.9	-11.89	-11.88	-11.87	-11.86	-11.85	-11.84	-11.83
-11.91	-11.9	-11.89	-11.88	-11.87	-11.86	-11.85	-11.84	-11.83
-11.91	-11.9	-11.89	-11.88	-11.87	-11.86	-11.85	-11.84	-11.83
-11.91	-11.9	-11.89	-11.88	-11.87	-11.86	-11.85	-11.84	-11.83
-11.91	-11.9	-11.89	-11.88	-11.87	-11.86	-11.85	-11.84	-11.83
-11.91	-11.9	-11.89	-11.88	-11.87	-11.86	-11.85	-11.84	-11.83
-11.91	-11.9	-11.89	-11.88	-11.87	-11.86	-11.85	-11.84	-11.83
-11.91	-11.9	-11.89	-11.88	-11.87	-11.86	-11.85	-11.84	-11.83
-11.91	-11.9	-11.89	-11.88	-11.87	-11.86	-11.85	-11.84	-11.83
-11.91	-11.9	-11.89	-11.88	-11.87	-11.86	-11.85	-11.84	-11.83
-11.91	-11.9	-11.89	-11.88	-11.87	-11.86	-11.85	-11.84	-11.83
-11.91	-11.9	-11.89	-11.88	-11.87	-11.86	-11.85	-11.84	-11.83

-11.91	-11.9	-11.89	-11.88	-11.87	-11.86	-11.85	-11.84	-11.83
-11.91	-11.9	-11.89	-11.88	-11.87	-11.86	-11.85	-11.84	-11.83
-11.91	-11.9	-11.89	-11.88	-11.87	-11.86	-11.85	-11.84	-11.83
-11.91	-11.9	-11.89	-11.88	-11.87	-11.86	-11.85	-11.84	-11.83
-11.91	-11.9	-11.89	-11.88	-11.87	-11.86	-11.85	-11.84	-11.83
-11.91	-11.9	-11.89	-11.88	-11.87	-11.86	-11.85	-11.84	-11.83
-11.91	-11.9	-11.89	-11.88	-11.87	-11.86	-11.85	-11.84	-11.83
-11.91	-11.9	-11.89	-11.88	-11.87	-11.86	-11.85	-11.84	-11.83
-11.91	-11.9	-11.89	-11.88	-11.87	-11.86	-11.85	-11.84	-11.83
-11.91	-11.9	-11.89	-11.88	-11.87	-11.86	-11.85	-11.84	-11.83
-11.91	-11.9	-11.89	-11.88	-11.87	-11.86	-11.85	-11.84	-11.83
-11.91	-11.9	-11.89	-11.88	-11.87	-11.86	-11.85	-11.84	-11.83
-11.91	-11.9	-11.89	-11.88	-11.87	-11.86	-11.85	-11.84	-11.83
-11.91	-11.9	-11.89	-11.88	-11.87	-11.86	-11.85	-11.84	-11.83
-11.91	-11.9	-11.89	-11.88	-11.87	-11.86	-11.85	-11.84	-11.83
-11.91	-11.9	-11.89	-11.88	-11.87	-11.86	-11.85	-11.84	-11.83
-11.91	-11.9	-11.89	-11.88	-11.87	-11.86	-11.85	-11.84	-11.83
-11.91	-11.9	-11.89	-11.88	-11.87	-11.86	-11.85	-11.84	-11.83
-11.91	-11.9	-11.89	-11.88	-11.87	-11.86	-11.85	-11.84	-11.83
-11.91	-11.9	-11.89	-11.88	-11.87	-11.86	-11.85	-11.84	-11.83
-11.91	-11.9	-11.89	-11.88	-11.87	-11.86	-11.85	-11.84	-11.83
-11.91	-11.9	-11.89	-11.88	-11.87	-11.86	-11.85	-11.84	-11.83
-11.91	-11.9	-11.89	-11.88	-11.87	-11.86	-11.85	-11.84	-11.83
-11.91	-11.9	-11.89	-11.88	-11.87	-11.86	-11.85	-11.84	-11.83
-11.91	-11.9	-11.89	-11.88	-11.87	-11.86	-11.85	-11.84	-11.83
-11.91	-11.9	-11.89	-11.88	-11.87	-11.86	-11.85	-11.84	-11.83
-11.91	-11.9	-11.89	-11.88	-11.87	-11.86	-11.85	-11.84	-11.83
-11.91	-11.9	-11.89	-11.88	-11.87	-11.86	-11.85	-11.84	-11.83
-11.91	-11.9	-11.89	-11.88	-11.87	-11.86	-11.85	-11.84	-11.83
-11.91	-11.9	-11.89	-11.88	-11.87	-11.86	-11.85	-11.84	-11.83
-11.91	-11.9	-11.89	-11.88	-11.87	-11.86	-11.85	-11.84	-11.83
-11.91	-11.9	-11.89	-11.88	-11.87	-11.86	-11.85	-11.84	-11.83
-11.91	-11.9	-11.89	-11.88	-11.87	-11.86	-11.85	-11.84	-11.83
-11.91	-11.9	-11.89	-11.88	-11.87	-11.86	-11.85	-11.84	-11.83
-11.91	-11.9	-11.89	-11.88	-11.87	-11.86	-11.85	-11.84	-11.83
-11.91	-11.9	-11.89	-11.88	-11.87	-11.86	-11.85	-11.84	-11.83
-11.91	-11.9	-11.89	-11.88	-11.87	-11.86	-11.85	-11.84	-11.83
-11.91	-11.9	-11.89	-11.88	-11.87	-11.86	-11.85	-11.84	-11.83
-11.91	-11.9	-11.89	-11.88	-11.87	-11.86	-11.85	-11.84	-11.83
-11.91	-11.9	-11.89	-11.88	-11.87	-11.86	-11.85	-11.84	-11.83
-11.91	-11.9	-11.89	-11.88	-11.87	-11.86	-11.85	-11.84	-11.83
-11.91	-11.9	-11.89	-11.88	-11.87	-11.86	-11.85	-11.84	-11.83
-11.91	-11.9	-11.89	-11.88	-11.87	-11.86	-11.85	-11.84	-11.83
-11.91	-11.9	-11.89	-11.88	-11.87	-11.86	-11.85	-11.84	-11.83
-11.91	-11.9	-11.89	-11.88	-11.87	-11.86	-11.85	-11.84	-11.83
-11.91	-11.9	-11.89	-11.88	-11.87	-11.86	-11.85	-11.84	-11.83
-11.91	-11.9	-11.89	-11.88	-11.87	-11.86	-11.85	-11.84	-11.83
-11.91	-11.9	-11.89	-11.88	-11.87	-11.86	-11.85	-11.84	-11.83
-11.91	-11.9	-11.89	-11.88	-11.87	-11.86	-11.85	-11.84	-11.83
-11.91	-11.9	-11.89	-11.88	-11.87	-11.86	-11.85	-11.84	-11.83

-11.91	-11.9	-11.89	-11.88	-11.87	-11.86	-11.85	-11.84	-11.83
-11.91	-11.9	-11.89	-11.88	-11.87	-11.86	-11.85	-11.84	-11.83
-11.91	-11.9	-11.89	-11.88	-11.87	-11.86	-11.85	-11.84	-11.83
-11.91	-11.9	-11.89	-11.88	-11.87	-11.86	-11.85	-11.84	-11.83
-11.91	-11.9	-11.89	-11.88	-11.87	-11.86	-11.85	-11.84	-11.83
-11.91	-11.9	-11.89	-11.88	-11.87	-11.86	-11.85	-11.84	-11.83
-11.91	-11.9	-11.89	-11.88	-11.87	-11.86	-11.85	-11.84	-11.83
-11.91	-11.9	-11.89	-11.88	-11.87	-11.86	-11.85	-11.84	-11.83
-11.91	-11.9	-11.89	-11.88	-11.87	-11.86	-11.85	-11.84	-11.83
-11.91	-11.9	-11.89	-11.88	-11.87	-11.86	-11.85	-11.84	-11.83
-11.91	-11.9	-11.89	-11.88	-11.87	-11.86	-11.85	-11.84	-11.83
-11.91	-11.9	-11.89	-11.88	-11.87	-11.86	-11.85	-11.84	-11.83
-11.91	-11.9	-11.89	-11.88	-11.87	-11.86	-11.85	-11.84	-11.83
-11.91	-11.9	-11.89	-11.88	-11.87	-11.86	-11.85	-11.84	-11.83
-11.91	-11.9	-11.89	-11.88	-11.87	-11.86	-11.85	-11.84	-11.83
-11.91	-11.9	-11.89	-11.88	-11.87	-11.86	-11.85	-11.84	-11.83
-11.91	-11.9	-11.89	-11.88	-11.87	-11.86	-11.85	-11.84	-11.83
-11.91	-11.9	-11.89	-11.88	-11.87	-11.86	-11.85	-11.84	-11.83
-11.91	-11.9	-11.89	-11.88	-11.87	-11.86	-11.85	-11.84	-11.83
-11.91	-11.9	-11.89	-11.88	-11.87	-11.86	-11.85	-11.84	-11.83
-11.91	-11.9	-11.89	-11.88	-11.87	-11.86	-11.85	-11.84	-11.83
-11.91	-11.9	-11.89	-11.88	-11.87	-11.86	-11.85	-11.84	-11.83
-11.91	-11.9	-11.89	-11.88	-11.87	-11.86	-11.85	-11.84	-11.83
-11.91	-11.9	-11.89	-11.88	-11.87	-11.86	-11.85	-11.84	-11.83
-11.91	-11.9	-11.89	-11.88	-11.87	-11.86	-11.85	-11.84	-11.83
-11.91	-11.9	-11.89	-11.88	-11.87	-11.86	-11.85	-11.84	-11.83
-11.91	-11.9	-11.89	-11.88	-11.87	-11.86	-11.85	-11.84	-11.83
-11.91	-11.9	-11.89	-11.88	-11.87	-11.86	-11.85	-11.84	-11.83
-11.91	-11.9	-11.89	-11.88	-11.87	-11.86	-11.85	-11.84	-11.83
-11.91	-11.9	-11.89	-11.88	-11.87	-11.86	-11.85	-11.84	-11.83
-11.91	-11.9	-11.89	-11.88	-11.87	-11.86	-11.85	-11.84	-11.83
-11.91	-11.9	-11.89	-11.88	-11.87	-11.86	-11.85	-11.84	-11.83
-11.91	-11.9	-11.89	-11.88	-11.87	-11.86	-11.85	-11.84	-11.83
-11.91	-11.9	-11.89	-11.88	-11.87	-11.86	-11.85	-11.84	-11.83
-11.91	-11.9	-11.89	-11.88	-11.87	-11.86	-11.85	-11.84	-11.83
-11.91	-11.9	-11.89	-11.88	-11.87	-11.86	-11.85	-11.84	-11.83
-11.91	-11.9	-11.89	-11.88	-11.87	-11.86	-11.85	-11.84	-11.83
-11.91	-11.9	-11.89	-11.88	-11.87	-11.86	-11.85	-11.84	-11.83
-11.91	-11.9	-11.89	-11.88	-11.87	-11.86	-11.85	-11.84	-11.83
-11.91	-11.9	-11.89	-11.88	-11.87	-11.86	-11.85	-11.84	-11.83
-11.91	-11.9	-11.89	-11.88	-11.87	-11.86	-11.85	-11.84	-11.83
-11.91	-11.9	-11.89	-11.88	-11.87	-11.86	-11.85	-11.84	-11.83
-11.91	-11.9	-11.89	-11.88	-11.87	-11.86	-11.85	-11.84	-11.83
-11.91	-11.9	-11.89	-11.88	-11.87	-11.86	-11.85	-11.84	-11.83
-11.91	-11.9	-11.89	-11.88	-11.87	-11.86	-11.85	-11.84	-11.83
-11.91	-11.9	-11.89	-11.88	-11.87	-11.86	-11.85	-11.84	-11.83
-11.91	-11.9	-11.89	-11.88	-11.87	-11.86	-11.85	-11.84	-11.83
-11.91	-11.9	-11.89	-11.88	-11.87	-11.86	-11.85	-11.84	-11.83
-11.91	-11.9	-11.89	-11.88	-11.87	-11.86	-11.85	-11.84	-11.83
-11.91	-11.9	-11.89	-11.88	-11.87	-11.86	-11.85	-11.84	-11.83

-11.91	-11.9	-11.89	-11.88	-11.87	-11.86	-11.85	-11.84	-11.83
-11.91	-11.9	-11.89	-11.88	-11.87	-11.86	-11.85	-11.84	-11.83
-11.91	-11.9	-11.89	-11.88	-11.87	-11.86	-11.85	-11.84	-11.83
-11.91	-11.9	-11.89	-11.88	-11.87	-11.86	-11.85	-11.84	-11.83
-11.91	-11.9	-11.89	-11.88	-11.87	-11.86	-11.85	-11.84	-11.83
-11.91	-11.9	-11.89	-11.88	-11.87	-11.86	-11.85	-11.84	-11.83
-11.91	-11.9	-11.89	-11.88	-11.87	-11.86	-11.85	-11.84	-11.83
-11.91	-11.9	-11.89	-11.88	-11.87	-11.86	-11.85	-11.84	-11.83
-11.91	-11.9	-11.89	-11.88	-11.87	-11.86	-11.85	-11.84	-11.83
-11.91	-11.9	-11.89	-11.88	-11.87	-11.86	-11.85	-11.84	-11.83
-11.91	-11.9	-11.89	-11.88	-11.87	-11.86	-11.85	-11.84	-11.83
-11.91	-11.9	-11.89	-11.88	-11.87	-11.86	-11.85	-11.84	-11.83
-11.91	-11.9	-11.89	-11.88	-11.87	-11.86	-11.85	-11.84	-11.83
-11.91	-11.9	-11.89	-11.88	-11.87	-11.86	-11.85	-11.84	-11.83
-11.91	-11.9	-11.89	-11.88	-11.87	-11.86	-11.85	-11.84	-11.83
-11.91	-11.9	-11.89	-11.88	-11.87	-11.86	-11.85	-11.84	-11.83
-11.91	-11.9	-11.89	-11.88	-11.87	-11.86	-11.85	-11.84	-11.83
-11.91	-11.9	-11.89	-11.88	-11.87	-11.86	-11.85	-11.84	-11.83
-11.91	-11.9	-11.89	-11.88	-11.87	-11.86	-11.85	-11.84	-11.83
-11.91	-11.9	-11.89	-11.88	-11.87	-11.86	-11.85	-11.84	-11.83
-11.91	-11.9	-11.89	-11.88	-11.87	-11.86	-11.85	-11.84	-11.83
-11.91	-11.9	-11.89	-11.88	-11.87	-11.86	-11.85	-11.84	-11.83
-11.91	-11.9	-11.89	-11.88	-11.87	-11.86	-11.85	-11.84	-11.83
-11.91	-11.9	-11.89	-11.88	-11.87	-11.86	-11.85	-11.84	-11.83
-11.91	-11.9	-11.89	-11.88	-11.87	-11.86	-11.85	-11.84	-11.83
-11.91	-11.9	-11.89	-11.88	-11.87	-11.86	-11.85	-11.84	-11.83
-11.91	-11.9	-11.89	-11.88	-11.87	-11.86	-11.85	-11.84	-11.83
-11.91	-11.9	-11.89	-11.88	-11.87	-11.86	-11.85	-11.84	-11.83
-11.91	-11.9	-11.89	-11.88	-11.87	-11.86	-11.85	-11.84	-11.83
-11.91	-11.9	-11.89	-11.88	-11.87	-11.86	-11.85	-11.84	-11.83
-11.91	-11.9	-11.89	-11.88	-11.87	-11.86	-11.85	-11.84	-11.83
-11.91	-11.9	-11.89	-11.88	-11.87	-11.86	-11.85	-11.84	-11.83
-11.91	-11.9	-11.89	-11.88	-11.87	-11.86	-11.85	-11.84	-11.83
-11.91	-11.9	-11.89	-11.88	-11.87	-11.86	-11.85	-11.84	-11.83
-11.91	-11.9	-11.89	-11.88	-11.87	-11.86	-11.85	-11.84	-11.83
-11.91	-11.9	-11.89	-11.88	-11.87	-11.86	-11.85	-11.84	-11.83
-11.91	-11.9	-11.89	-11.88	-11.87	-11.86	-11.85	-11.84	-11.83
-11.91	-11.9	-11.89	-11.88	-11.87	-11.86	-11.85	-11.84	-11.83
-11.91	-11.9	-11.89	-11.88	-11.87	-11.86	-11.85	-11.84	-11.83
-11.91	-11.9	-11.89	-11.88	-11.87	-11.86	-11.85	-11.84	-11.83
-11.91	-11.9	-11.89	-11.88	-11.87	-11.86	-11.85	-11.84	-11.83
-11.91	-11.9	-11.89	-11.88	-11.87	-11.86	-11.85	-11.84	-11.83
-11.91	-11.9	-11.89	-11.88	-11.87	-11.86	-11.85	-11.84	-11.83
-11.91	-11.9	-11.89	-11.88	-11.87	-11.86	-11.85	-11.84	-11.83
-11.91	-11.9	-11.89	-11.88	-11.87	-11.86	-11.85	-11.84	-11.83
-11.91	-11.9	-11.89	-11.88	-11.87	-11.86	-11.85	-11.84	-11.83
-11.91	-11.9	-11.89	-11.88	-11.87	-11.86	-11.85	-11.84	-11.83
-11.91	-11.9	-11.89	-11.88	-11.87	-11.86	-11.85	-11.84	-11.83
-11.91	-11.9	-11.89	-11.88	-11.87	-11.86	-11.85	-11.84	-11.83
-11.91	-11.9	-11.89	-11.88	-11.87	-11.86	-11.85	-11.84	-11.83

-11.91	-11.9	-11.89	-11.88	-11.87	-11.86	-11.85	-11.84	-11.83
-11.91	-11.9	-11.89	-11.88	-11.87	-11.86	-11.85	-11.84	-11.83
-11.91	-11.9	-11.89	-11.88	-11.87	-11.86	-11.85	-11.84	-11.83
-11.91	-11.9	-11.89	-11.88	-11.87	-11.86	-11.85	-11.84	-11.83
-11.91	-11.9	-11.89	-11.88	-11.87	-11.86	-11.85	-11.84	-11.83
-11.91	-11.9	-11.89	-11.88	-11.87	-11.86	-11.85	-11.84	-11.83
-11.91	-11.9	-11.89	-11.88	-11.87	-11.86	-11.85	-11.84	-11.83
-11.91	-11.9	-11.89	-11.88	-11.87	-11.86	-11.85	-11.84	-11.83
-11.91	-11.9	-11.89	-11.88	-11.87	-11.86	-11.85	-11.84	-11.83
-11.91	-11.9	-11.89	-11.88	-11.87	-11.86	-11.85	-11.84	-11.83
-11.91	-11.9	-11.89	-11.88	-11.87	-11.86	-11.85	-11.84	-11.83
-11.91	-11.9	-11.89	-11.88	-11.87	-11.86	-11.85	-11.84	-11.83
-11.91	-11.9	-11.89	-11.88	-11.87	-11.86	-11.85	-11.84	-11.83
-11.91	-11.9	-11.89	-11.88	-11.87	-11.86	-11.85	-11.84	-11.83
-11.91	-11.9	-11.89	-11.88	-11.87	-11.86	-11.85	-11.84	-11.83
-11.91	-11.9	-11.89	-11.88	-11.87	-11.86	-11.85	-11.84	-11.83
-11.91	-11.9	-11.89	-11.88	-11.87	-11.86	-11.85	-11.84	-11.83
-11.91	-11.9	-11.89	-11.88	-11.87	-11.86	-11.85	-11.84	-11.83
-11.91	-11.9	-11.89	-11.88	-11.87	-11.86	-11.85	-11.84	-11.83
-11.91	-11.9	-11.89	-11.88	-11.87	-11.86	-11.85	-11.84	-11.83
-11.91	-11.9	-11.89	-11.88	-11.87	-11.86	-11.85	-11.84	-11.83
-11.91	-11.9	-11.89	-11.88	-11.87	-11.86	-11.85	-11.84	-11.83
-11.91	-11.9	-11.89	-11.88	-11.87	-11.86	-11.85	-11.84	-11.83
-11.91	-11.9	-11.89	-11.88	-11.87	-11.86	-11.85	-11.84	-11.83
-11.91	-11.9	-11.89	-11.88	-11.87	-11.86	-11.85	-11.84	-11.83
-11.91	-11.9	-11.89	-11.88	-11.87	-11.86	-11.85	-11.84	-11.83
-11.91	-11.9	-11.89	-11.88	-11.87	-11.86	-11.85	-11.84	-11.83
-11.91	-11.9	-11.89	-11.88	-11.87	-11.86	-11.85	-11.84	-11.83
-11.91	-11.9	-11.89	-11.88	-11.87	-11.86	-11.85	-11.84	-11.83
-11.91	-11.9	-11.89	-11.88	-11.87	-11.86	-11.85	-11.84	-11.83
-11.91	-11.9	-11.89	-11.88	-11.87	-11.86	-11.85	-11.84	-11.83
-11.91	-11.9	-11.89	-11.88	-11.87	-11.86	-11.85	-11.84	-11.83
-11.91	-11.9	-11.89	-11.88	-11.87	-11.86	-11.85	-11.84	-11.83
-11.91	-11.9	-11.89	-11.88	-11.87	-11.86	-11.85	-11.84	-11.83
-11.91	-11.9	-11.89	-11.88	-11.87	-11.86	-11.85	-11.84	-11.83
-11.91	-11.9	-11.89	-11.88	-11.87	-11.86	-11.85	-11.84	-11.83
-11.91	-11.9	-11.89	-11.88	-11.87	-11.86	-11.85	-11.84	-11.83
-11.91	-11.9	-11.89	-11.88	-11.87	-11.86	-11.85	-11.84	-11.83
-11.91	-11.9	-11.89	-11.88	-11.87	-11.86	-11.85	-11.84	-11.83
-11.91	-11.9	-11.89	-11.88	-11.87	-11.86	-11.85	-11.84	-11.83
-11.91	-11.9	-11.89	-11.88	-11.87	-11.86	-11.85	-11.84	-11.83
-11.91	-11.9	-11.89	-11.88	-11.87	-11.86	-11.85	-11.84	-11.83
-11.91	-11.9	-11.89	-11.88	-11.87	-11.86	-11.85	-11.84	-11.83
-11.91	-11.9	-11.89	-11.88	-11.87	-11.86	-11.85	-11.84	-11.83
-11.91	-11.9	-11.89	-11.88	-11.87	-11.86	-11.85	-11.84	-11.83
-11.91	-11.9	-11.89	-11.88	-11.87	-11.86	-11.85	-11.84	-11.83
-11.91	-11.9	-11.89	-11.88	-11.87	-11.86	-11.85	-11.84	-11.83
-11.91	-11.9	-11.89	-11.88	-11.87	-11.86	-11.85	-11.84	-11.83
-11.91	-11.9	-11.89	-11.88	-11.87	-11.86	-11.85	-11.84	-11.83
-11.91	-11.9	-11.89	-11.88	-11.87	-11.86	-11.85	-11.84	-11.83

-11.91	-11.9	-11.89	-11.88	-11.87	-11.86	-11.85	-11.84	-11.83
-11.91	-11.9	-11.89	-11.88	-11.87	-11.86	-11.85	-11.84	-11.83
-11.91	-11.9	-11.89	-11.88	-11.87	-11.86	-11.85	-11.84	-11.83
-11.91	-11.9	-11.89	-11.88	-11.87	-11.86	-11.85	-11.84	-11.83
-11.91	-11.9	-11.89	-11.88	-11.87	-11.86	-11.85	-11.84	-11.83
-11.91	-11.9	-11.89	-11.88	-11.87	-11.86	-11.85	-11.84	-11.83
-11.91	-11.9	-11.89	-11.88	-11.87	-11.86	-11.85	-11.84	-11.83
-11.91	-11.9	-11.89	-11.88	-11.87	-11.86	-11.85	-11.84	-11.83
-11.91	-11.9	-11.89	-11.88	-11.87	-11.86	-11.85	-11.84	-11.83
-11.91	-11.9	-11.89	-11.88	-11.87	-11.86	-11.85	-11.84	-11.83
-11.91	-11.9	-11.89	-11.88	-11.87	-11.86	-11.85	-11.84	-11.83
-11.91	-11.9	-11.89	-11.88	-11.87	-11.86	-11.85	-11.84	-11.83
-11.91	-11.9	-11.89	-11.88	-11.87	-11.86	-11.85	-11.84	-11.83
-11.91	-11.9	-11.89	-11.88	-11.87	-11.86	-11.85	-11.84	-11.83
-11.91	-11.9	-11.89	-11.88	-11.87	-11.86	-11.85	-11.84	-11.83
-11.91	-11.9	-11.89	-11.88	-11.87	-11.86	-11.85	-11.84	-11.83
-11.91	-11.9	-11.89	-11.88	-11.87	-11.86	-11.85	-11.84	-11.83
-11.91	-11.9	-11.89	-11.88	-11.87	-11.86	-11.85	-11.84	-11.83
-11.91	-11.9	-11.89	-11.88	-11.87	-11.86	-11.85	-11.84	-11.83
-11.91	-11.9	-11.89	-11.88	-11.87	-11.86	-11.85	-11.84	-11.83
-11.91	-11.9	-11.89	-11.88	-11.87	-11.86	-11.85	-11.84	-11.83
-11.91	-11.9	-11.89	-11.88	-11.87	-11.86	-11.85	-11.84	-11.83
-11.91	-11.9	-11.89	-11.88	-11.87	-11.86	-11.85	-11.84	-11.83
-11.91	-11.9	-11.89	-11.88	-11.87	-11.86	-11.85	-11.84	-11.83
-11.91	-11.9	-11.89	-11.88	-11.87	-11.86	-11.85	-11.84	-11.83
-11.91	-11.9	-11.89	-11.88	-11.87	-11.86	-11.85	-11.84	-11.83
-11.91	-11.9	-11.89	-11.88	-11.87	-11.86	-11.85	-11.84	-11.83
-11.91	-11.9	-11.89	-11.88	-11.87	-11.86	-11.85	-11.84	-11.83
-11.91	-11.9	-11.89	-11.88	-11.87	-11.86	-11.85	-11.84	-11.83
-11.91	-11.9	-11.89	-11.88	-11.87	-11.86	-11.85	-11.84	-11.83
-11.91	-11.9	-11.89	-11.88	-11.87	-11.86	-11.85	-11.84	-11.83
-11.91	-11.9	-11.89	-11.88	-11.87	-11.86	-11.85	-11.84	-11.83
-11.91	-11.9	-11.89	-11.88	-11.87	-11.86	-11.85	-11.84	-11.83
-11.91	-11.9	-11.89	-11.88	-11.87	-11.86	-11.85	-11.84	-11.83
-11.91	-11.9	-11.89	-11.88	-11.87	-11.86	-11.85	-11.84	-11.83
-11.91	-11.9	-11.89	-11.88	-11.87	-11.86	-11.85	-11.84	-11.83
-11.91	-11.9	-11.89	-11.88	-11.87	-11.86	-11.85	-11.84	-11.83
-11.91	-11.9	-11.89	-11.88	-11.87	-11.86	-11.85	-11.84	-11.83
-11.91	-11.9	-11.89	-11.88	-11.87	-11.86	-11.85	-11.84	-11.83
-11.91	-11.9	-11.89	-11.88	-11.87	-11.86	-11.85	-11.84	-11.83
-11.91	-11.9	-11.89	-11.88	-11.87	-11.86	-11.85	-11.84	-11.83
-11.91	-11.9	-11.89	-11.88	-11.87	-11.86	-11.85	-11.84	-11.83
-11.91	-11.9	-11.89	-11.88	-11.87	-11.86	-11.85	-11.84	-11.83
-11.91	-11.9	-11.89	-11.88	-11.87	-11.86	-11.85	-11.84	-11.83
-11.91	-11.9	-11.89	-11.88	-11.87	-11.86	-11.85	-11.84	-11.83
-11.91	-11.9	-11.89	-11.88	-11.87	-11.86	-11.85	-11.84	-11.83
-11.91	-11.9	-11.89	-11.88	-11.87	-11.86	-11.85	-11.84	-11.83
-11.91	-11.9	-11.89	-11.88	-11.87	-11.86	-11.85	-11.84	-11.83
-11.91	-11.9	-11.89	-11.88	-11.87	-11.86	-11.85	-11.84	-11.83
-11.91	-11.9	-11.89	-11.88	-11.87	-11.86	-11.85	-11.84	-11.83

-11.91	-11.9	-11.89	-11.88	-11.87	-11.86	-11.85	-11.84	-11.83
-11.91	-11.9	-11.89	-11.88	-11.87	-11.86	-11.85	-11.84	-11.83
-11.91	-11.9	-11.89	-11.88	-11.87	-11.86	-11.85	-11.84	-11.83
-11.91	-11.9	-11.89	-11.88	-11.87	-11.86	-11.85	-11.84	-11.83
-11.91	-11.9	-11.89	-11.88	-11.87	-11.86	-11.85	-11.84	-11.83
-11.91	-11.9	-11.89	-11.88	-11.87	-11.86	-11.85	-11.84	-11.83
-11.91	-11.9	-11.89	-11.88	-11.87	-11.86	-11.85	-11.84	-11.83
-11.91	-11.9	-11.89	-11.88	-11.87	-11.86	-11.85	-11.84	-11.83
-11.91	-11.9	-11.89	-11.88	-11.87	-11.86	-11.85	-11.84	-11.83
-11.91	-11.9	-11.89	-11.88	-11.87	-11.86	-11.85	-11.84	-11.83
-11.91	-11.9	-11.89	-11.88	-11.87	-11.86	-11.85	-11.84	-11.83
-11.91	-11.9	-11.89	-11.88	-11.87	-11.86	-11.85	-11.84	-11.83
-11.91	-11.9	-11.89	-11.88	-11.87	-11.86	-11.85	-11.84	-11.83
-11.91	-11.9	-11.89	-11.88	-11.87	-11.86	-11.85	-11.84	-11.83
-11.91	-11.9	-11.89	-11.88	-11.87	-11.86	-11.85	-11.84	-11.83
-11.91	-11.9	-11.89	-11.88	-11.87	-11.86	-11.85	-11.84	-11.83
-11.91	-11.9	-11.89	-11.88	-11.87	-11.86	-11.85	-11.84	-11.83
-11.91	-11.9	-11.89	-11.88	-11.87	-11.86	-11.85	-11.84	-11.83
-11.91	-11.9	-11.89	-11.88	-11.87	-11.86	-11.85	-11.84	-11.83
-11.91	-11.9	-11.89	-11.88	-11.87	-11.86	-11.85	-11.84	-11.83
-11.91	-11.9	-11.89	-11.88	-11.87	-11.86	-11.85	-11.84	-11.83
-11.91	-11.9	-11.89	-11.88	-11.87	-11.86	-11.85	-11.84	-11.83
-11.91	-11.9	-11.89	-11.88	-11.87	-11.86	-11.85	-11.84	-11.83
-11.91	-11.9	-11.89	-11.88	-11.87	-11.86	-11.85	-11.84	-11.83
-11.91	-11.9	-11.89	-11.88	-11.87	-11.86	-11.85	-11.84	-11.83
-11.91	-11.9	-11.89	-11.88	-11.87	-11.86	-11.85	-11.84	-11.83
-11.91	-11.9	-11.89	-11.88	-11.87	-11.86	-11.85	-11.84	-11.83
-11.91	-11.9	-11.89	-11.88	-11.87	-11.86	-11.85	-11.84	-11.83
-11.91	-11.9	-11.89	-11.88	-11.87	-11.86	-11.85	-11.84	-11.83
-11.91	-11.9	-11.89	-11.88	-11.87	-11.86	-11.85	-11.84	-11.83
-11.91	-11.9	-11.89	-11.88	-11.87	-11.86	-11.85	-11.84	-11.83
-11.91	-11.9	-11.89	-11.88	-11.87	-11.86	-11.85	-11.84	-11.83
-11.91	-11.9	-11.89	-11.88	-11.87	-11.86	-11.85	-11.84	-11.83
-11.91	-11.9	-11.89	-11.88	-11.87	-11.86	-11.85	-11.84	-11.83
-11.91	-11.9	-11.89	-11.88	-11.87	-11.86	-11.85	-11.84	-11.83
-11.91	-11.9	-11.89	-11.88	-11.87	-11.86	-11.85	-11.84	-11.83
-11.91	-11.9	-11.89	-11.88	-11.87	-11.86	-11.85	-11.84	-11.83
-11.91	-11.9	-11.89	-11.88	-11.87	-11.86	-11.85	-11.84	-11.83
-11.91	-11.9	-11.89	-11.88	-11.87	-11.86	-11.85	-11.84	-11.83
-11.91	-11.9	-11.89	-11.88	-11.87	-11.86	-11.85	-11.84	-11.83
-11.91	-11.9	-11.89	-11.88	-11.87	-11.86	-11.85	-11.84	-11.83
-11.91	-11.9	-11.89	-11.88	-11.87	-11.86	-11.85	-11.84	-11.83
-11.91	-11.9	-11.89	-11.88	-11.87	-11.86	-11.85	-11.84	-11.83
-11.91	-11.9	-11.89	-11.88	-11.87	-11.86	-11.85	-11.84	-11.83
-11.91	-11.9	-11.89	-11.88	-11.87	-11.86	-11.85	-11.84	-11.83
-11.91	-11.9	-11.89	-11.88	-11.87	-11.86	-11.85	-11.84	-11.83
-11.91	-11.9	-11.89	-11.88	-11.87	-11.86	-11.85	-11.84	-11.83
-11.91	-11.9	-11.89	-11.88	-11.87	-11.86	-11.85	-11.84	-11.83
-11.91	-11.9	-11.89	-11.88	-11.87	-11.86	-11.85	-11.84	-11.83
-11.91	-11.9	-11.89	-11.88	-11.87	-11.86	-11.85	-11.84	-11.83

-11.91	-11.9	-11.89	-11.88	-11.87	-11.86	-11.85	-11.84	-11.83
-11.91	-11.9	-11.89	-11.88	-11.87	-11.86	-11.85	-11.84	-11.83
-11.91	-11.9	-11.89	-11.88	-11.87	-11.86	-11.85	-11.84	-11.83
-11.91	-11.9	-11.89	-11.88	-11.87	-11.86	-11.85	-11.84	-11.83
-11.91	-11.9	-11.89	-11.88	-11.87	-11.86	-11.85	-11.84	-11.83
-11.91	-11.9	-11.89	-11.88	-11.87	-11.86	-11.85	-11.84	-11.83
-11.91	-11.9	-11.89	-11.88	-11.87	-11.86	-11.85	-11.84	-11.83
-11.91	-11.9	-11.89	-11.88	-11.87	-11.86	-11.85	-11.84	-11.83
-11.91	-11.9	-11.89	-11.88	-11.87	-11.86	-11.85	-11.84	-11.83
-11.91	-11.9	-11.89	-11.88	-11.87	-11.86	-11.85	-11.84	-11.83
-11.91	-11.9	-11.89	-11.88	-11.87	-11.86	-11.85	-11.84	-11.83
-11.91	-11.9	-11.89	-11.88	-11.87	-11.86	-11.85	-11.84	-11.83
-11.91	-11.9	-11.89	-11.88	-11.87	-11.86	-11.85	-11.84	-11.83
-11.91	-11.9	-11.89	-11.88	-11.87	-11.86	-11.85	-11.84	-11.83
-11.91	-11.9	-11.89	-11.88	-11.87	-11.86	-11.85	-11.84	-11.83
-11.91	-11.9	-11.89	-11.88	-11.87	-11.86	-11.85	-11.84	-11.83
-11.91	-11.9	-11.89	-11.88	-11.87	-11.86	-11.85	-11.84	-11.83
-11.91	-11.9	-11.89	-11.88	-11.87	-11.86	-11.85	-11.84	-11.83
-11.91	-11.9	-11.89	-11.88	-11.87	-11.86	-11.85	-11.84	-11.83
-11.91	-11.9	-11.89	-11.88	-11.87	-11.86	-11.85	-11.84	-11.83
-11.91	-11.9	-11.89	-11.88	-11.87	-11.86	-11.85	-11.84	-11.83
-11.91	-11.9	-11.89	-11.88	-11.87	-11.86	-11.85	-11.84	-11.83
-11.91	-11.9	-11.89	-11.88	-11.87	-11.86	-11.85	-11.84	-11.83
-11.91	-11.9	-11.89	-11.88	-11.87	-11.86	-11.85	-11.84	-11.83
-11.91	-11.9	-11.89	-11.88	-11.87	-11.86	-11.85	-11.84	-11.83
-11.91	-11.9	-11.89	-11.88	-11.87	-11.86	-11.85	-11.84	-11.83
-11.91	-11.9	-11.89	-11.88	-11.87	-11.86	-11.85	-11.84	-11.83
-11.91	-11.9	-11.89	-11.88	-11.87	-11.86	-11.85	-11.84	-11.83
-11.91	-11.9	-11.89	-11.88	-11.87	-11.86	-11.85	-11.84	-11.83
-11.91	-11.9	-11.89	-11.88	-11.87	-11.86	-11.85	-11.84	-11.83
-11.91	-11.9	-11.89	-11.88	-11.87	-11.86	-11.85	-11.84	-11.83
-11.91	-11.9	-11.89	-11.88	-11.87	-11.86	-11.85	-11.84	-11.83
-11.91	-11.9	-11.89	-11.88	-11.87	-11.86	-11.85	-11.84	-11.83
-11.91	-11.9	-11.89	-11.88	-11.87	-11.86	-11.85	-11.84	-11.83
-11.91	-11.9	-11.89	-11.88	-11.87	-11.86	-11.85	-11.84	-11.83
-11.91	-11.9	-11.89	-11.88	-11.87	-11.86	-11.85	-11.84	-11.83
-11.91	-11.9	-11.89	-11.88	-11.87	-11.86	-11.85	-11.84	-11.83
-11.91	-11.9	-11.89	-11.88	-11.87	-11.86	-11.85	-11.84	-11.83
-11.91	-11.9	-11.89	-11.88	-11.87	-11.86	-11.85	-11.84	-11.83
-11.91	-11.9	-11.89	-11.88	-11.87	-11.86	-11.85	-11.84	-11.83
-11.91	-11.9	-11.89	-11.88	-11.87	-11.86	-11.85	-11.84	-11.83
-11.91	-11.9	-11.89	-11.88	-11.87	-11.86	-11.85	-11.84	-11.83
-11.91	-11.9	-11.89	-11.88	-11.87	-11.86	-11.85	-11.84	-11.83
-11.91	-11.9	-11.89	-11.88	-11.87	-11.86	-11.85	-11.84	-11.83
-11.91	-11.9	-11.89	-11.88	-11.87	-11.86	-11.85	-11.84	-11.83
-11.91	-11.9	-11.89	-11.88	-11.87	-11.86	-11.85	-11.84	-11.83
-11.91	-11.9	-11.89	-11.88	-11.87	-11.86	-11.85	-11.84	-11.83
-11.91	-11.9	-11.89	-11.88	-11.87	-11.86	-11.85	-11.84	-11.83
-11.91	-11.9	-11.89	-11.88	-11.87	-11.86	-11.85	-11.84	-11.83
-11.91	-11.9	-11.89	-11.88	-11.87	-11.86	-11.85	-11.84	-11.83

-11.91	-11.9	-11.89	-11.88	-11.87	-11.86	-11.85	-11.84	-11.83
-11.91	-11.9	-11.89	-11.88	-11.87	-11.86	-11.85	-11.84	-11.83
-11.91	-11.9	-11.89	-11.88	-11.87	-11.86	-11.85	-11.84	-11.83
-11.91	-11.9	-11.89	-11.88	-11.87	-11.86	-11.85	-11.84	-11.83
-11.91	-11.9	-11.89	-11.88	-11.87	-11.86	-11.85	-11.84	-11.83
-11.91	-11.9	-11.89	-11.88	-11.87	-11.86	-11.85	-11.84	-11.83
-11.91	-11.9	-11.89	-11.88	-11.87	-11.86	-11.85	-11.84	-11.83
-11.91	-11.9	-11.89	-11.88	-11.87	-11.86	-11.85	-11.84	-11.83
-11.91	-11.9	-11.89	-11.88	-11.87	-11.86	-11.85	-11.84	-11.83
-11.91	-11.9	-11.89	-11.88	-11.87	-11.86	-11.85	-11.84	-11.83
-11.91	-11.9	-11.89	-11.88	-11.87	-11.86	-11.85	-11.84	-11.83
-11.91	-11.9	-11.89	-11.88	-11.87	-11.86	-11.85	-11.84	-11.83
-11.91	-11.9	-11.89	-11.88	-11.87	-11.86	-11.85	-11.84	-11.83
-11.91	-11.9	-11.89	-11.88	-11.87	-11.86	-11.85	-11.84	-11.83
-11.91	-11.9	-11.89	-11.88	-11.87	-11.86	-11.85	-11.84	-11.83
-11.91	-11.9	-11.89	-11.88	-11.87	-11.86	-11.85	-11.84	-11.83
-11.91	-11.9	-11.89	-11.88	-11.87	-11.86	-11.85	-11.84	-11.83
-11.91	-11.9	-11.89	-11.88	-11.87	-11.86	-11.85	-11.84	-11.83
-11.91	-11.9	-11.89	-11.88	-11.87	-11.86	-11.85	-11.84	-11.83
-11.91	-11.9	-11.89	-11.88	-11.87	-11.86	-11.85	-11.84	-11.83
-11.91	-11.9	-11.89	-11.88	-11.87	-11.86	-11.85	-11.84	-11.83
-11.91	-11.9	-11.89	-11.88	-11.87	-11.86	-11.85	-11.84	-11.83
-11.91	-11.9	-11.89	-11.88	-11.87	-11.86	-11.85	-11.84	-11.83
-11.91	-11.9	-11.89	-11.88	-11.87	-11.86	-11.85	-11.84	-11.83
-11.91	-11.9	-11.89	-11.88	-11.87	-11.86	-11.85	-11.84	-11.83
-11.91	-11.9	-11.89	-11.88	-11.87	-11.86	-11.85	-11.84	-11.83
-11.91	-11.9	-11.89	-11.88	-11.87	-11.86	-11.85	-11.84	-11.83
-11.91	-11.9	-11.89	-11.88	-11.87	-11.86	-11.85	-11.84	-11.83
-11.91	-11.9	-11.89	-11.88	-11.87	-11.86	-11.85	-11.84	-11.83
-11.91	-11.9	-11.89	-11.88	-11.87	-11.86	-11.85	-11.84	-11.83
-11.91	-11.9	-11.89	-11.88	-11.87	-11.86	-11.85	-11.84	-11.83
-11.91	-11.9	-11.89	-11.88	-11.87	-11.86	-11.85	-11.84	-11.83
-11.91	-11.9	-11.89	-11.88	-11.87	-11.86	-11.85	-11.84	-11.83
-11.91	-11.9	-11.89	-11.88	-11.87	-11.86	-11.85	-11.84	-11.83
-11.91	-11.9	-11.89	-11.88	-11.87	-11.86	-11.85	-11.84	-11.83
-11.91	-11.9	-11.89	-11.88	-11.87	-11.86	-11.85	-11.84	-11.83
-11.91	-11.9	-11.89	-11.88	-11.87	-11.86	-11.85	-11.84	-11.83
-11.91	-11.9	-11.89	-11.88	-11.87	-11.86	-11.85	-11.84	-11.83
-11.91	-11.9	-11.89	-11.88	-11.87	-11.86	-11.85	-11.84	-11.83
-11.91	-11.9	-11.89	-11.88	-11.87	-11.86	-11.85	-11.84	-11.83
-11.91	-11.9	-11.89	-11.88	-11.87	-11.86	-11.85	-11.84	-11.83
-11.91	-11.9	-11.89	-11.88	-11.87	-11.86	-11.85	-11.84	-11.83
-11.91	-11.9	-11.89	-11.88	-11.87	-11.86	-11.85	-11.84	-11.83
-11.91	-11.9	-11.89	-11.88	-11.87	-11.86	-11.85	-11.84	-11.83
-11.91	-11.9	-11.89	-11.88	-11.87	-11.86	-11.85	-11.84	-11.83
-11.91	-11.9	-11.89	-11.88	-11.87	-11.86	-11.85	-11.84	-11.83
-11.91	-11.9	-11.89	-11.88	-11.87	-11.86	-11.85	-11.84	-11.83
-11.91	-11.9	-11.89	-11.88	-11.87	-11.86	-11.85	-11.84	-11.83
-11.91	-11.9	-11.89	-11.88	-11.87	-11.86	-11.85	-11.84	-11.83
-11.91	-11.9	-11.89	-11.88	-11.87	-11.86	-11.85	-11.84	-11.83

-11.91	-11.9	-11.89	-11.88	-11.87	-11.86	-11.85	-11.84	-11.83
-11.91	-11.9	-11.89	-11.88	-11.87	-11.86	-11.85	-11.84	-11.83
-11.91	-11.9	-11.89	-11.88	-11.87	-11.86	-11.85	-11.84	-11.83
-11.91	-11.9	-11.89	-11.88	-11.87	-11.86	-11.85	-11.84	-11.83
-11.91	-11.9	-11.89	-11.88	-11.87	-11.86	-11.85	-11.84	-11.83
-11.91	-11.9	-11.89	-11.88	-11.87	-11.86	-11.85	-11.84	-11.83
-11.91	-11.9	-11.89	-11.88	-11.87	-11.86	-11.85	-11.84	-11.83
-11.91	-11.9	-11.89	-11.88	-11.87	-11.86	-11.85	-11.84	-11.83
-11.91	-11.9	-11.89	-11.88	-11.87	-11.86	-11.85	-11.84	-11.83
-11.91	-11.9	-11.89	-11.88	-11.87	-11.86	-11.85	-11.84	-11.83
-11.91	-11.9	-11.89	-11.88	-11.87	-11.86	-11.85	-11.84	-11.83
-11.91	-11.9	-11.89	-11.88	-11.87	-11.86	-11.85	-11.84	-11.83
-11.91	-11.9	-11.89	-11.88	-11.87	-11.86	-11.85	-11.84	-11.83
-11.91	-11.9	-11.89	-11.88	-11.87	-11.86	-11.85	-11.84	-11.83
-11.91	-11.9	-11.89	-11.88	-11.87	-11.86	-11.85	-11.84	-11.83
-11.91	-11.9	-11.89	-11.88	-11.87	-11.86	-11.85	-11.84	-11.83
-11.91	-11.9	-11.89	-11.88	-11.87	-11.86	-11.85	-11.84	-11.83
-11.91	-11.9	-11.89	-11.88	-11.87	-11.86	-11.85	-11.84	-11.83
-11.91	-11.9	-11.89	-11.88	-11.87	-11.86	-11.85	-11.84	-11.83
-11.91	-11.9	-11.89	-11.88	-11.87	-11.86	-11.85	-11.84	-11.83
-11.91	-11.9	-11.89	-11.88	-11.87	-11.86	-11.85	-11.84	-11.83
-11.91	-11.9	-11.89	-11.88	-11.87	-11.86	-11.85	-11.84	-11.83
-11.91	-11.9	-11.89	-11.88	-11.87	-11.86	-11.85	-11.84	-11.83
-11.91	-11.9	-11.89	-11.88	-11.87	-11.86	-11.85	-11.84	-11.83
-11.91	-11.9	-11.89	-11.88	-11.87	-11.86	-11.85	-11.84	-11.83
-11.91	-11.9	-11.89	-11.88	-11.87	-11.86	-11.85	-11.84	-11.83
-11.91	-11.9	-11.89	-11.88	-11.87	-11.86	-11.85	-11.84	-11.83
-11.91	-11.9	-11.89	-11.88	-11.87	-11.86	-11.85	-11.84	-11.83
-11.91	-11.9	-11.89	-11.88	-11.87	-11.86	-11.85	-11.84	-11.83
-11.91	-11.9	-11.89	-11.88	-11.87	-11.86	-11.85	-11.84	-11.83
-11.91	-11.9	-11.89	-11.88	-11.87	-11.86	-11.85	-11.84	-11.83
-11.91	-11.9	-11.89	-11.88	-11.87	-11.86	-11.85	-11.84	-11.83
-11.91	-11.9	-11.89	-11.88	-11.87	-11.86	-11.85	-11.84	-11.83
-11.91	-11.9	-11.89	-11.88	-11.87	-11.86	-11.85	-11.84	-11.83
-11.91	-11.9	-11.89	-11.88	-11.87	-11.86	-11.85	-11.84	-11.83
-11.91	-11.9	-11.89	-11.88	-11.87	-11.86	-11.85	-11.84	-11.83
-11.91	-11.9	-11.89	-11.88	-11.87	-11.86	-11.85	-11.84	-11.83
-11.91	-11.9	-11.89	-11.88	-11.87	-11.86	-11.85	-11.84	-11.83
-11.91	-11.9	-11.89	-11.88	-11.87	-11.86	-11.85	-11.84	-11.83
-11.91	-11.9	-11.89	-11.88	-11.87	-11.86	-11.85	-11.84	-11.83
-11.91	-11.9	-11.89	-11.88	-11.87	-11.86	-11.85	-11.84	-11.83
-11.91	-11.9	-11.89	-11.88	-11.87	-11.86	-11.85	-11.84	-11.83
-11.91	-11.9	-11.89	-11.88	-11.87	-11.86	-11.85	-11.84	-11.83
-11.91	-11.9	-11.89	-11.88	-11.87	-11.86	-11.85	-11.84	-11.83
-11.91	-11.9	-11.89	-11.88	-11.87	-11.86	-11.85	-11.84	-11.83
-11.91	-11.9	-11.89	-11.88	-11.87	-11.86	-11.85	-11.84	-11.83
-11.91	-11.9	-11.89	-11.88	-11.87	-11.86	-11.85	-11.84	-11.83
-11.91	-11.9	-11.89	-11.88	-11.87	-11.86	-11.85	-11.84	-11.83
-11.91	-11.9	-11.89	-11.88	-11.87	-11.86	-11.85	-11.84	-11.83
-11.91	-11.9	-11.89	-11.88	-11.87	-11.86	-11.85	-11.84	-11.83

-11.91	-11.9	-11.89	-11.88	-11.87	-11.86	-11.85	-11.84	-11.83
-11.91	-11.9	-11.89	-11.88	-11.87	-11.86	-11.85	-11.84	-11.83
-11.91	-11.9	-11.89	-11.88	-11.87	-11.86	-11.85	-11.84	-11.83
-11.91	-11.9	-11.89	-11.88	-11.87	-11.86	-11.85	-11.84	-11.83
-11.91	-11.9	-11.89	-11.88	-11.87	-11.86	-11.85	-11.84	-11.83
-11.91	-11.9	-11.89	-11.88	-11.87	-11.86	-11.85	-11.84	-11.83
-11.91	-11.9	-11.89	-11.88	-11.87	-11.86	-11.85	-11.84	-11.83
-11.91	-11.9	-11.89	-11.88	-11.87	-11.86	-11.85	-11.84	-11.83
-11.91	-11.9	-11.89	-11.88	-11.87	-11.86	-11.85	-11.84	-11.83
-11.91	-11.9	-11.89	-11.88	-11.87	-11.86	-11.85	-11.84	-11.83
-11.91	-11.9	-11.89	-11.88	-11.87	-11.86	-11.85	-11.84	-11.83
-11.91	-11.9	-11.89	-11.88	-11.87	-11.86	-11.85	-11.84	-11.83
-11.91	-11.9	-11.89	-11.88	-11.87	-11.86	-11.85	-11.84	-11.83
-11.91	-11.9	-11.89	-11.88	-11.87	-11.86	-11.85	-11.84	-11.83
-11.91	-11.9	-11.89	-11.88	-11.87	-11.86	-11.85	-11.84	-11.83
-11.91	-11.9	-11.89	-11.88	-11.87	-11.86	-11.85	-11.84	-11.83
-11.91	-11.9	-11.89	-11.88	-11.87	-11.86	-11.85	-11.84	-11.83
-11.91	-11.9	-11.89	-11.88	-11.87	-11.86	-11.85	-11.84	-11.83
-11.91	-11.9	-11.89	-11.88	-11.87	-11.86	-11.85	-11.84	-11.83
-11.91	-11.9	-11.89	-11.88	-11.87	-11.86	-11.85	-11.84	-11.83
-11.91	-11.9	-11.89	-11.88	-11.87	-11.86	-11.85	-11.84	-11.83
-11.91	-11.9	-11.89	-11.88	-11.87	-11.86	-11.85	-11.84	-11.83
-11.91	-11.9	-11.89	-11.88	-11.87	-11.86	-11.85	-11.84	-11.83
-11.91	-11.9	-11.89	-11.88	-11.87	-11.86	-11.85	-11.84	-11.83
-11.91	-11.9	-11.89	-11.88	-11.87	-11.86	-11.85	-11.84	-11.83
-11.91	-11.9	-11.89	-11.88	-11.87	-11.86	-11.85	-11.84	-11.83
-11.91	-11.9	-11.89	-11.88	-11.87	-11.86	-11.85	-11.84	-11.83
-11.91	-11.9	-11.89	-11.88	-11.87	-11.86	-11.85	-11.84	-11.83
-11.91	-11.9	-11.89	-11.88	-11.87	-11.86	-11.85	-11.84	-11.83
-11.91	-11.9	-11.89	-11.88	-11.87	-11.86	-11.85	-11.84	-11.83
-11.91	-11.9	-11.89	-11.88	-11.87	-11.86	-11.85	-11.84	-11.83
-11.91	-11.9	-11.89	-11.88	-11.87	-11.86	-11.85	-11.84	-11.83
-11.91	-11.9	-11.89	-11.88	-11.87	-11.86	-11.85	-11.84	-11.83
-11.91	-11.9	-11.89	-11.88	-11.87	-11.86	-11.85	-11.84	-11.83
-11.91	-11.9	-11.89	-11.88	-11.87	-11.86	-11.85	-11.84	-11.83
-11.91	-11.9	-11.89	-11.88	-11.87	-11.86	-11.85	-11.84	-11.83
-11.91	-11.9	-11.89	-11.88	-11.87	-11.86	-11.85	-11.84	-11.83
-11.91	-11.9	-11.89	-11.88	-11.87	-11.86	-11.85	-11.84	-11.83
-11.91	-11.9	-11.89	-11.88	-11.87	-11.86	-11.85	-11.84	-11.83
-11.91	-11.9	-11.89	-11.88	-11.87	-11.86	-11.85	-11.84	-11.83
-11.91	-11.9	-11.89	-11.88	-11.87	-11.86	-11.85	-11.84	-11.83
-11.91	-11.9	-11.89	-11.88	-11.87	-11.86	-11.85	-11.84	-11.83
-11.91	-11.9	-11.89	-11.88	-11.87	-11.86	-11.85	-11.84	-11.83
-11.91	-11.9	-11.89	-11.88	-11.87	-11.86	-11.85	-11.84	-11.83
-11.91	-11.9	-11.89	-11.88	-11.87	-11.86	-11.85	-11.84	-11.83
-11.91	-11.9	-11.89	-11.88	-11.87	-11.86	-11.85	-11.84	-11.83
-11.91	-11.9	-11.89	-11.88	-11.87	-11.86	-11.85	-11.84	-11.83
-11.91	-11.9	-11.89	-11.88	-11.87	-11.86	-11.85	-11.84	-11.83
-11.91	-11.9	-11.89	-11.88	-11.87	-11.86	-11.85	-11.84	-11.83
-11.91	-11.9	-11.89	-11.88	-11.87	-11.86	-11.85	-11.84	-11.83

-11.91	-11.9	-11.89	-11.88	-11.87	-11.86	-11.85	-11.84	-11.83

-11.82	-11.81	-11.8	-11.79	-11.78	-11.77	-11.76	-11.75	-11.74
-11.82	-11.81	-11.8	-11.79	-11.78	-11.77	-11.76	-11.75	-11.74
-11.82	-11.81	-11.8	-11.79	-11.78	-11.77	-11.76	-11.75	-11.74
-11.82	-11.81	-11.8	-11.79	-11.78	-11.77	-11.76	-11.75	-11.74
-11.82	-11.81	-11.8	-11.79	-11.78	-11.77	-11.76	-11.75	-11.74
-11.82	-11.81	-11.8	-11.79	-11.78	-11.77	-11.76	-11.75	-11.74
-11.82	-11.81	-11.8	-11.79	-11.78	-11.77	-11.76	-11.75	-11.74
-11.82	-11.81	-11.8	-11.79	-11.78	-11.77	-11.76	-11.75	-11.74
-11.82	-11.81	-11.8	-11.79	-11.78	-11.77	-11.76	-11.75	-11.74
-11.82	-11.81	-11.8	-11.79	-11.78	-11.77	-11.76	-11.75	-11.74
-11.82	-11.81	-11.8	-11.79	-11.78	-11.77	-11.76	-11.75	-11.74
-11.82	-11.81	-11.8	-11.79	-11.78	-11.77	-11.76	-11.75	-11.74
-11.82	-11.81	-11.8	-11.79	-11.78	-11.77	-11.76	-11.75	-11.74
-11.82	-11.81	-11.8	-11.79	-11.78	-11.77	-11.76	-11.75	-11.74
-11.82	-11.81	-11.8	-11.79	-11.78	-11.77	-11.76	-11.75	-11.74
-11.82	-11.81	-11.8	-11.79	-11.78	-11.77	-11.76	-11.75	-11.74
-11.82	-11.81	-11.8	-11.79	-11.78	-11.77	-11.76	-11.75	-11.74
-11.82	-11.81	-11.8	-11.79	-11.78	-11.77	-11.76	-11.75	-11.74
-11.82	-11.81	-11.8	-11.79	-11.78	-11.77	-11.76	-11.75	-11.74
-11.82	-11.81	-11.8	-11.79	-11.78	-11.77	-11.76	-11.75	-11.74
-11.82	-11.81	-11.8	-11.79	-11.78	-11.77	-11.76	-11.75	-11.74
-11.82	-11.81	-11.8	-11.79	-11.78	-11.77	-11.76	-11.75	-11.74
-11.82	-11.81	-11.8	-11.79	-11.78	-11.77	-11.76	-11.75	-11.74
-11.82	-11.81	-11.8	-11.79	-11.78	-11.77	-11.76	-11.75	-11.74
-11.82	-11.81	-11.8	-11.79	-11.78	-11.77	-11.76	-11.75	-11.74
-11.82	-11.81	-11.8	-11.79	-11.78	-11.77	-11.76	-11.75	-11.74
-11.82	-11.81	-11.8	-11.79	-11.78	-11.77	-11.76	-11.75	-11.74
-11.82	-11.81	-11.8	-11.79	-11.78	-11.77	-11.76	-11.75	-11.74
-11.82	-11.81	-11.8	-11.79	-11.78	-11.77	-11.76	-11.75	-11.74
-11.82	-11.81	-11.8	-11.79	-11.78	-11.77	-11.76	-11.75	-11.74
-11.82	-11.81	-11.8	-11.79	-11.78	-11.77	-11.76	-11.75	-11.74
-11.82	-11.81	-11.8	-11.79	-11.78	-11.77	-11.76	-11.75	-11.74
-11.82	-11.81	-11.8	-11.79	-11.78	-11.77	-11.76	-11.75	-11.74
-11.82	-11.81	-11.8	-11.79	-11.78	-11.77	-11.76	-11.75	-11.74
-11.82	-11.81	-11.8	-11.79	-11.78	-11.77	-11.76	-11.75	-11.74
-11.82	-11.81	-11.8	-11.79	-11.78	-11.77	-11.76	-11.75	-11.74
-11.82	-11.81	-11.8	-11.79	-11.78	-11.77	-11.76	-11.75	-11.74
-11.82	-11.81	-11.8	-11.79	-11.78	-11.77	-11.76	-11.75	-11.74
-11.82	-11.81	-11.8	-11.79	-11.78	-11.77	-11.76	-11.75	-11.74
-11.82	-11.81	-11.8	-11.79	-11.78	-11.77	-11.76	-11.75	-11.74
-11.82	-11.81	-11.8	-11.79	-11.78	-11.77	-11.76	-11.75	-11.74
-11.82	-11.81	-11.8	-11.79	-11.78	-11.77	-11.76	-11.75	-11.74
-11.82	-11.81	-11.8	-11.79	-11.78	-11.77	-11.76	-11.75	-11.74
-11.82	-11.81	-11.8	-11.79	-11.78	-11.77	-11.76	-11.75	-11.74
-11.82	-11.81	-11.8	-11.79	-11.78	-11.77	-11.76	-11.75	-11.74
-11.82	-11.81	-11.8	-11.79	-11.78	-11.77	-11.76	-11.75	-11.74
-11.82	-11.81	-11.8	-11.79	-11.78	-11.77	-11.76	-11.75	-11.74
-11.82	-11.81	-11.8	-11.79	-11.78	-11.77	-11.76	-11.75	-11.74
-11.82	-11.81	-11.8	-11.79	-11.78	-11.77	-11.76	-11.75	-11.74
-11.82	-11.81	-11.8	-11.79	-11.78	-11.77	-11.76	-11.75	-11.74

-11.82	-11.81	-11.8	-11.79	-11.78	-11.77	-11.76	-11.75	-11.74
-11.82	-11.81	-11.8	-11.79	-11.78	-11.77	-11.76	-11.75	-11.74
-11.82	-11.81	-11.8	-11.79	-11.78	-11.77	-11.76	-11.75	-11.74
-11.82	-11.81	-11.8	-11.79	-11.78	-11.77	-11.76	-11.75	-11.74
-11.82	-11.81	-11.8	-11.79	-11.78	-11.77	-11.76	-11.75	-11.74
-11.82	-11.81	-11.8	-11.79	-11.78	-11.77	-11.76	-11.75	-11.74
-11.82	-11.81	-11.8	-11.79	-11.78	-11.77	-11.76	-11.75	-11.74
-11.82	-11.81	-11.8	-11.79	-11.78	-11.77	-11.76	-11.75	-11.74
-11.82	-11.81	-11.8	-11.79	-11.78	-11.77	-11.76	-11.75	-11.74
-11.82	-11.81	-11.8	-11.79	-11.78	-11.77	-11.76	-11.75	-11.74
-11.82	-11.81	-11.8	-11.79	-11.78	-11.77	-11.76	-11.75	-11.74
-11.82	-11.81	-11.8	-11.79	-11.78	-11.77	-11.76	-11.75	-11.74
-11.82	-11.81	-11.8	-11.79	-11.78	-11.77	-11.76	-11.75	-11.74
-11.82	-11.81	-11.8	-11.79	-11.78	-11.77	-11.76	-11.75	-11.74
-11.82	-11.81	-11.8	-11.79	-11.78	-11.77	-11.76	-11.75	-11.74
-11.82	-11.81	-11.8	-11.79	-11.78	-11.77	-11.76	-11.75	-11.74
-11.82	-11.81	-11.8	-11.79	-11.78	-11.77	-11.76	-11.75	-11.74
-11.82	-11.81	-11.8	-11.79	-11.78	-11.77	-11.76	-11.75	-11.74
-11.82	-11.81	-11.8	-11.79	-11.78	-11.77	-11.76	-11.75	-11.74
-11.82	-11.81	-11.8	-11.79	-11.78	-11.77	-11.76	-11.75	-11.74
-11.82	-11.81	-11.8	-11.79	-11.78	-11.77	-11.76	-11.75	-11.74
-11.82	-11.81	-11.8	-11.79	-11.78	-11.77	-11.76	-11.75	-11.74
-11.82	-11.81	-11.8	-11.79	-11.78	-11.77	-11.76	-11.75	-11.74
-11.82	-11.81	-11.8	-11.79	-11.78	-11.77	-11.76	-11.75	-11.74
-11.82	-11.81	-11.8	-11.79	-11.78	-11.77	-11.76	-11.75	-11.74
-11.82	-11.81	-11.8	-11.79	-11.78	-11.77	-11.76	-11.75	-11.74
-11.82	-11.81	-11.8	-11.79	-11.78	-11.77	-11.76	-11.75	-11.74
-11.82	-11.81	-11.8	-11.79	-11.78	-11.77	-11.76	-11.75	-11.74
-11.82	-11.81	-11.8	-11.79	-11.78	-11.77	-11.76	-11.75	-11.74
-11.82	-11.81	-11.8	-11.79	-11.78	-11.77	-11.76	-11.75	-11.74
-11.82	-11.81	-11.8	-11.79	-11.78	-11.77	-11.76	-11.75	-11.74
-11.82	-11.81	-11.8	-11.79	-11.78	-11.77	-11.76	-11.75	-11.74
-11.82	-11.81	-11.8	-11.79	-11.78	-11.77	-11.76	-11.75	-11.74
-11.82	-11.81	-11.8	-11.79	-11.78	-11.77	-11.76	-11.75	-11.74
-11.82	-11.81	-11.8	-11.79	-11.78	-11.77	-11.76	-11.75	-11.74
-11.82	-11.81	-11.8	-11.79	-11.78	-11.77	-11.76	-11.75	-11.74
-11.82	-11.81	-11.8	-11.79	-11.78	-11.77	-11.76	-11.75	-11.74
-11.82	-11.81	-11.8	-11.79	-11.78	-11.77	-11.76	-11.75	-11.74
-11.82	-11.81	-11.8	-11.79	-11.78	-11.77	-11.76	-11.75	-11.74
-11.82	-11.81	-11.8	-11.79	-11.78	-11.77	-11.76	-11.75	-11.74
-11.82	-11.81	-11.8	-11.79	-11.78	-11.77	-11.76	-11.75	-11.74
-11.82	-11.81	-11.8	-11.79	-11.78	-11.77	-11.76	-11.75	-11.74
-11.82	-11.81	-11.8	-11.79	-11.78	-11.77	-11.76	-11.75	-11.74
-11.82	-11.81	-11.8	-11.79	-11.78	-11.77	-11.76	-11.75	-11.74
-11.82	-11.81	-11.8	-11.79	-11.78	-11.77	-11.76	-11.75	-11.74
-11.82	-11.81	-11.8	-11.79	-11.78	-11.77	-11.76	-11.75	-11.74
-11.82	-11.81	-11.8	-11.79	-11.78	-11.77	-11.76	-11.75	-11.74
-11.82	-11.81	-11.8	-11.79	-11.78	-11.77	-11.76	-11.75	-11.74
-11.82	-11.81	-11.8	-11.79	-11.78	-11.77	-11.76	-11.75	-11.74
-11.82	-11.81	-11.8	-11.79	-11.78	-11.77	-11.76	-11.75	-11.74

-11.82	-11.81	-11.8	-11.79	-11.78	-11.77	-11.76	-11.75	-11.74
-11.82	-11.81	-11.8	-11.79	-11.78	-11.77	-11.76	-11.75	-11.74
-11.82	-11.81	-11.8	-11.79	-11.78	-11.77	-11.76	-11.75	-11.74
-11.82	-11.81	-11.8	-11.79	-11.78	-11.77	-11.76	-11.75	-11.74
-11.82	-11.81	-11.8	-11.79	-11.78	-11.77	-11.76	-11.75	-11.74
-11.82	-11.81	-11.8	-11.79	-11.78	-11.77	-11.76	-11.75	-11.74
-11.82	-11.81	-11.8	-11.79	-11.78	-11.77	-11.76	-11.75	-11.74
-11.82	-11.81	-11.8	-11.79	-11.78	-11.77	-11.76	-11.75	-11.74
-11.82	-11.81	-11.8	-11.79	-11.78	-11.77	-11.76	-11.75	-11.74
-11.82	-11.81	-11.8	-11.79	-11.78	-11.77	-11.76	-11.75	-11.74
-11.82	-11.81	-11.8	-11.79	-11.78	-11.77	-11.76	-11.75	-11.74
-11.82	-11.81	-11.8	-11.79	-11.78	-11.77	-11.76	-11.75	-11.74
-11.82	-11.81	-11.8	-11.79	-11.78	-11.77	-11.76	-11.75	-11.74
-11.82	-11.81	-11.8	-11.79	-11.78	-11.77	-11.76	-11.75	-11.74
-11.82	-11.81	-11.8	-11.79	-11.78	-11.77	-11.76	-11.75	-11.74
-11.82	-11.81	-11.8	-11.79	-11.78	-11.77	-11.76	-11.75	-11.74
-11.82	-11.81	-11.8	-11.79	-11.78	-11.77	-11.76	-11.75	-11.74
-11.82	-11.81	-11.8	-11.79	-11.78	-11.77	-11.76	-11.75	-11.74
-11.82	-11.81	-11.8	-11.79	-11.78	-11.77	-11.76	-11.75	-11.74
-11.82	-11.81	-11.8	-11.79	-11.78	-11.77	-11.76	-11.75	-11.74
-11.82	-11.81	-11.8	-11.79	-11.78	-11.77	-11.76	-11.75	-11.74
-11.82	-11.81	-11.8	-11.79	-11.78	-11.77	-11.76	-11.75	-11.74
-11.82	-11.81	-11.8	-11.79	-11.78	-11.77	-11.76	-11.75	-11.74
-11.82	-11.81	-11.8	-11.79	-11.78	-11.77	-11.76	-11.75	-11.74
-11.82	-11.81	-11.8	-11.79	-11.78	-11.77	-11.76	-11.75	-11.74
-11.82	-11.81	-11.8	-11.79	-11.78	-11.77	-11.76	-11.75	-11.74
-11.82	-11.81	-11.8	-11.79	-11.78	-11.77	-11.76	-11.75	-11.74
-11.82	-11.81	-11.8	-11.79	-11.78	-11.77	-11.76	-11.75	-11.74
-11.82	-11.81	-11.8	-11.79	-11.78	-11.77	-11.76	-11.75	-11.74
-11.82	-11.81	-11.8	-11.79	-11.78	-11.77	-11.76	-11.75	-11.74
-11.82	-11.81	-11.8	-11.79	-11.78	-11.77	-11.76	-11.75	-11.74
-11.82	-11.81	-11.8	-11.79	-11.78	-11.77	-11.76	-11.75	-11.74
-11.82	-11.81	-11.8	-11.79	-11.78	-11.77	-11.76	-11.75	-11.74
-11.82	-11.81	-11.8	-11.79	-11.78	-11.77	-11.76	-11.75	-11.74
-11.82	-11.81	-11.8	-11.79	-11.78	-11.77	-11.76	-11.75	-11.74
-11.82	-11.81	-11.8	-11.79	-11.78	-11.77	-11.76	-11.75	-11.74
-11.82	-11.81	-11.8	-11.79	-11.78	-11.77	-11.76	-11.75	-11.74
-11.82	-11.81	-11.8	-11.79	-11.78	-11.77	-11.76	-11.75	-11.74
-11.82	-11.81	-11.8	-11.79	-11.78	-11.77	-11.76	-11.75	-11.74
-11.82	-11.81	-11.8	-11.79	-11.78	-11.77	-11.76	-11.75	-11.74
-11.82	-11.81	-11.8	-11.79	-11.78	-11.77	-11.76	-11.75	-11.74
-11.82	-11.81	-11.8	-11.79	-11.78	-11.77	-11.76	-11.75	-11.74
-11.82	-11.81	-11.8	-11.79	-11.78	-11.77	-11.76	-11.75	-11.74
-11.82	-11.81	-11.8	-11.79	-11.78	-11.77	-11.76	-11.75	-11.74
-11.82	-11.81	-11.8	-11.79	-11.78	-11.77	-11.76	-11.75	-11.74
-11.82	-11.81	-11.8	-11.79	-11.78	-11.77	-11.76	-11.75	-11.74
-11.82	-11.81	-11.8	-11.79	-11.78	-11.77	-11.76	-11.75	-11.74
-11.82	-11.81	-11.8	-11.79	-11.78	-11.77	-11.76	-11.75	-11.74
-11.82	-11.81	-11.8	-11.79	-11.78	-11.77	-11.76	-11.75	-11.74
-11.82	-11.81	-11.8	-11.79	-11.78	-11.77	-11.76	-11.75	-11.74

-11.82	-11.81	-11.8	-11.79	-11.78	-11.77	-11.76	-11.75	-11.74
-11.82	-11.81	-11.8	-11.79	-11.78	-11.77	-11.76	-11.75	-11.74
-11.82	-11.81	-11.8	-11.79	-11.78	-11.77	-11.76	-11.75	-11.74
-11.82	-11.81	-11.8	-11.79	-11.78	-11.77	-11.76	-11.75	-11.74
-11.82	-11.81	-11.8	-11.79	-11.78	-11.77	-11.76	-11.75	-11.74
-11.82	-11.81	-11.8	-11.79	-11.78	-11.77	-11.76	-11.75	-11.74
-11.82	-11.81	-11.8	-11.79	-11.78	-11.77	-11.76	-11.75	-11.74
-11.82	-11.81	-11.8	-11.79	-11.78	-11.77	-11.76	-11.75	-11.74
-11.82	-11.81	-11.8	-11.79	-11.78	-11.77	-11.76	-11.75	-11.74
-11.82	-11.81	-11.8	-11.79	-11.78	-11.77	-11.76	-11.75	-11.74
-11.82	-11.81	-11.8	-11.79	-11.78	-11.77	-11.76	-11.75	-11.74
-11.82	-11.81	-11.8	-11.79	-11.78	-11.77	-11.76	-11.75	-11.74
-11.82	-11.81	-11.8	-11.79	-11.78	-11.77	-11.76	-11.75	-11.74
-11.82	-11.81	-11.8	-11.79	-11.78	-11.77	-11.76	-11.75	-11.74
-11.82	-11.81	-11.8	-11.79	-11.78	-11.77	-11.76	-11.75	-11.74
-11.82	-11.81	-11.8	-11.79	-11.78	-11.77	-11.76	-11.75	-11.74
-11.82	-11.81	-11.8	-11.79	-11.78	-11.77	-11.76	-11.75	-11.74
-11.82	-11.81	-11.8	-11.79	-11.78	-11.77	-11.76	-11.75	-11.74
-11.82	-11.81	-11.8	-11.79	-11.78	-11.77	-11.76	-11.75	-11.74
-11.82	-11.81	-11.8	-11.79	-11.78	-11.77	-11.76	-11.75	-11.74
-11.82	-11.81	-11.8	-11.79	-11.78	-11.77	-11.76	-11.75	-11.74
-11.82	-11.81	-11.8	-11.79	-11.78	-11.77	-11.76	-11.75	-11.74
-11.82	-11.81	-11.8	-11.79	-11.78	-11.77	-11.76	-11.75	-11.74
-11.82	-11.81	-11.8	-11.79	-11.78	-11.77	-11.76	-11.75	-11.74
-11.82	-11.81	-11.8	-11.79	-11.78	-11.77	-11.76	-11.75	-11.74
-11.82	-11.81	-11.8	-11.79	-11.78	-11.77	-11.76	-11.75	-11.74
-11.82	-11.81	-11.8	-11.79	-11.78	-11.77	-11.76	-11.75	-11.74
-11.82	-11.81	-11.8	-11.79	-11.78	-11.77	-11.76	-11.75	-11.74
-11.82	-11.81	-11.8	-11.79	-11.78	-11.77	-11.76	-11.75	-11.74
-11.82	-11.81	-11.8	-11.79	-11.78	-11.77	-11.76	-11.75	-11.74
-11.82	-11.81	-11.8	-11.79	-11.78	-11.77	-11.76	-11.75	-11.74
-11.82	-11.81	-11.8	-11.79	-11.78	-11.77	-11.76	-11.75	-11.74
-11.82	-11.81	-11.8	-11.79	-11.78	-11.77	-11.76	-11.75	-11.74
-11.82	-11.81	-11.8	-11.79	-11.78	-11.77	-11.76	-11.75	-11.74
-11.82	-11.81	-11.8	-11.79	-11.78	-11.77	-11.76	-11.75	-11.74
-11.82	-11.81	-11.8	-11.79	-11.78	-11.77	-11.76	-11.75	-11.74
-11.82	-11.81	-11.8	-11.79	-11.78	-11.77	-11.76	-11.75	-11.74
-11.82	-11.81	-11.8	-11.79	-11.78	-11.77	-11.76	-11.75	-11.74
-11.82	-11.81	-11.8	-11.79	-11.78	-11.77	-11.76	-11.75	-11.74
-11.82	-11.81	-11.8	-11.79	-11.78	-11.77	-11.76	-11.75	-11.74
-11.82	-11.81	-11.8	-11.79	-11.78	-11.77	-11.76	-11.75	-11.74
-11.82	-11.81	-11.8	-11.79	-11.78	-11.77	-11.76	-11.75	-11.74
-11.82	-11.81	-11.8	-11.79	-11.78	-11.77	-11.76	-11.75	-11.74
-11.82	-11.81	-11.8	-11.79	-11.78	-11.77	-11.76	-11.75	-11.74
-11.82	-11.81	-11.8	-11.79	-11.78	-11.77	-11.76	-11.75	-11.74
-11.82	-11.81	-11.8	-11.79	-11.78	-11.77	-11.76	-11.75	-11.74
-11.82	-11.81	-11.8	-11.79	-11.78	-11.77	-11.76	-11.75	-11.74
-11.82	-11.81	-11.8	-11.79	-11.78	-11.77	-11.76	-11.75	-11.74
-11.82	-11.81	-11.8	-11.79	-11.78	-11.77	-11.76	-11.75	-11.74
-11.82	-11.81	-11.8	-11.79	-11.78	-11.77	-11.76	-11.75	-11.74

-11.82	-11.81	-11.8	-11.79	-11.78	-11.77	-11.76	-11.75	-11.74
-11.82	-11.81	-11.8	-11.79	-11.78	-11.77	-11.76	-11.75	-11.74
-11.82	-11.81	-11.8	-11.79	-11.78	-11.77	-11.76	-11.75	-11.74
-11.82	-11.81	-11.8	-11.79	-11.78	-11.77	-11.76	-11.75	-11.74
-11.82	-11.81	-11.8	-11.79	-11.78	-11.77	-11.76	-11.75	-11.74
-11.82	-11.81	-11.8	-11.79	-11.78	-11.77	-11.76	-11.75	-11.74
-11.82	-11.81	-11.8	-11.79	-11.78	-11.77	-11.76	-11.75	-11.74
-11.82	-11.81	-11.8	-11.79	-11.78	-11.77	-11.76	-11.75	-11.74
-11.82	-11.81	-11.8	-11.79	-11.78	-11.77	-11.76	-11.75	-11.74
-11.82	-11.81	-11.8	-11.79	-11.78	-11.77	-11.76	-11.75	-11.74
-11.82	-11.81	-11.8	-11.79	-11.78	-11.77	-11.76	-11.75	-11.74
-11.82	-11.81	-11.8	-11.79	-11.78	-11.77	-11.76	-11.75	-11.74
-11.82	-11.81	-11.8	-11.79	-11.78	-11.77	-11.76	-11.75	-11.74
-11.82	-11.81	-11.8	-11.79	-11.78	-11.77	-11.76	-11.75	-11.74
-11.82	-11.81	-11.8	-11.79	-11.78	-11.77	-11.76	-11.75	-11.74
-11.82	-11.81	-11.8	-11.79	-11.78	-11.77	-11.76	-11.75	-11.74
-11.82	-11.81	-11.8	-11.79	-11.78	-11.77	-11.76	-11.75	-11.74
-11.82	-11.81	-11.8	-11.79	-11.78	-11.77	-11.76	-11.75	-11.74
-11.82	-11.81	-11.8	-11.79	-11.78	-11.77	-11.76	-11.75	-11.74
-11.82	-11.81	-11.8	-11.79	-11.78	-11.77	-11.76	-11.75	-11.74
-11.82	-11.81	-11.8	-11.79	-11.78	-11.77	-11.76	-11.75	-11.74
-11.82	-11.81	-11.8	-11.79	-11.78	-11.77	-11.76	-11.75	-11.74
-11.82	-11.81	-11.8	-11.79	-11.78	-11.77	-11.76	-11.75	-11.74
-11.82	-11.81	-11.8	-11.79	-11.78	-11.77	-11.76	-11.75	-11.74
-11.82	-11.81	-11.8	-11.79	-11.78	-11.77	-11.76	-11.75	-11.74
-11.82	-11.81	-11.8	-11.79	-11.78	-11.77	-11.76	-11.75	-11.74
-11.82	-11.81	-11.8	-11.79	-11.78	-11.77	-11.76	-11.75	-11.74
-11.82	-11.81	-11.8	-11.79	-11.78	-11.77	-11.76	-11.75	-11.74
-11.82	-11.81	-11.8	-11.79	-11.78	-11.77	-11.76	-11.75	-11.74
-11.82	-11.81	-11.8	-11.79	-11.78	-11.77	-11.76	-11.75	-11.74
-11.82	-11.81	-11.8	-11.79	-11.78	-11.77	-11.76	-11.75	-11.74
-11.82	-11.81	-11.8	-11.79	-11.78	-11.77	-11.76	-11.75	-11.74
-11.82	-11.81	-11.8	-11.79	-11.78	-11.77	-11.76	-11.75	-11.74
-11.82	-11.81	-11.8	-11.79	-11.78	-11.77	-11.76	-11.75	-11.74
-11.82	-11.81	-11.8	-11.79	-11.78	-11.77	-11.76	-11.75	-11.74
-11.82	-11.81	-11.8	-11.79	-11.78	-11.77	-11.76	-11.75	-11.74
-11.82	-11.81	-11.8	-11.79	-11.78	-11.77	-11.76	-11.75	-11.74
-11.82	-11.81	-11.8	-11.79	-11.78	-11.77	-11.76	-11.75	-11.74
-11.82	-11.81	-11.8	-11.79	-11.78	-11.77	-11.76	-11.75	-11.74
-11.82	-11.81	-11.8	-11.79	-11.78	-11.77	-11.76	-11.75	-11.74
-11.82	-11.81	-11.8	-11.79	-11.78	-11.77	-11.76	-11.75	-11.74
-11.82	-11.81	-11.8	-11.79	-11.78	-11.77	-11.76	-11.75	-11.74
-11.82	-11.81	-11.8	-11.79	-11.78	-11.77	-11.76	-11.75	-11.74
-11.82	-11.81	-11.8	-11.79	-11.78	-11.77	-11.76	-11.75	-11.74
-11.82	-11.81	-11.8	-11.79	-11.78	-11.77	-11.76	-11.75	-11.74
-11.82	-11.81	-11.8	-11.79	-11.78	-11.77	-11.76	-11.75	-11.74
-11.82	-11.81	-11.8	-11.79	-11.78	-11.77	-11.76	-11.75	-11.74
-11.82	-11.81	-11.8	-11.79	-11.78	-11.77	-11.76	-11.75	-11.74
-11.82	-11.81	-11.8	-11.79	-11.78	-11.77	-11.76	-11.75	-11.74
-11.82	-11.81	-11.8	-11.79	-11.78	-11.77	-11.76	-11.75	-11.74

-11.82	-11.81	-11.8	-11.79	-11.78	-11.77	-11.76	-11.75	-11.74
-11.82	-11.81	-11.8	-11.79	-11.78	-11.77	-11.76	-11.75	-11.74
-11.82	-11.81	-11.8	-11.79	-11.78	-11.77	-11.76	-11.75	-11.74
-11.82	-11.81	-11.8	-11.79	-11.78	-11.77	-11.76	-11.75	-11.74
-11.82	-11.81	-11.8	-11.79	-11.78	-11.77	-11.76	-11.75	-11.74
-11.82	-11.81	-11.8	-11.79	-11.78	-11.77	-11.76	-11.75	-11.74
-11.82	-11.81	-11.8	-11.79	-11.78	-11.77	-11.76	-11.75	-11.74
-11.82	-11.81	-11.8	-11.79	-11.78	-11.77	-11.76	-11.75	-11.74
-11.82	-11.81	-11.8	-11.79	-11.78	-11.77	-11.76	-11.75	-11.74
-11.82	-11.81	-11.8	-11.79	-11.78	-11.77	-11.76	-11.75	-11.74
-11.82	-11.81	-11.8	-11.79	-11.78	-11.77	-11.76	-11.75	-11.74
-11.82	-11.81	-11.8	-11.79	-11.78	-11.77	-11.76	-11.75	-11.74
-11.82	-11.81	-11.8	-11.79	-11.78	-11.77	-11.76	-11.75	-11.74
-11.82	-11.81	-11.8	-11.79	-11.78	-11.77	-11.76	-11.75	-11.74
-11.82	-11.81	-11.8	-11.79	-11.78	-11.77	-11.76	-11.75	-11.74
-11.82	-11.81	-11.8	-11.79	-11.78	-11.77	-11.76	-11.75	-11.74
-11.82	-11.81	-11.8	-11.79	-11.78	-11.77	-11.76	-11.75	-11.74
-11.82	-11.81	-11.8	-11.79	-11.78	-11.77	-11.76	-11.75	-11.74
-11.82	-11.81	-11.8	-11.79	-11.78	-11.77	-11.76	-11.75	-11.74
-11.82	-11.81	-11.8	-11.79	-11.78	-11.77	-11.76	-11.75	-11.74
-11.82	-11.81	-11.8	-11.79	-11.78	-11.77	-11.76	-11.75	-11.74
-11.82	-11.81	-11.8	-11.79	-11.78	-11.77	-11.76	-11.75	-11.74
-11.82	-11.81	-11.8	-11.79	-11.78	-11.77	-11.76	-11.75	-11.74
-11.82	-11.81	-11.8	-11.79	-11.78	-11.77	-11.76	-11.75	-11.74
-11.82	-11.81	-11.8	-11.79	-11.78	-11.77	-11.76	-11.75	-11.74
-11.82	-11.81	-11.8	-11.79	-11.78	-11.77	-11.76	-11.75	-11.74
-11.82	-11.81	-11.8	-11.79	-11.78	-11.77	-11.76	-11.75	-11.74
-11.82	-11.81	-11.8	-11.79	-11.78	-11.77	-11.76	-11.75	-11.74
-11.82	-11.81	-11.8	-11.79	-11.78	-11.77	-11.76	-11.75	-11.74
-11.82	-11.81	-11.8	-11.79	-11.78	-11.77	-11.76	-11.75	-11.74
-11.82	-11.81	-11.8	-11.79	-11.78	-11.77	-11.76	-11.75	-11.74
-11.82	-11.81	-11.8	-11.79	-11.78	-11.77	-11.76	-11.75	-11.74
-11.82	-11.81	-11.8	-11.79	-11.78	-11.77	-11.76	-11.75	-11.74
-11.82	-11.81	-11.8	-11.79	-11.78	-11.77	-11.76	-11.75	-11.74
-11.82	-11.81	-11.8	-11.79	-11.78	-11.77	-11.76	-11.75	-11.74
-11.82	-11.81	-11.8	-11.79	-11.78	-11.77	-11.76	-11.75	-11.74
-11.82	-11.81	-11.8	-11.79	-11.78	-11.77	-11.76	-11.75	-11.74
-11.82	-11.81	-11.8	-11.79	-11.78	-11.77	-11.76	-11.75	-11.74
-11.82	-11.81	-11.8	-11.79	-11.78	-11.77	-11.76	-11.75	-11.74
-11.82	-11.81	-11.8	-11.79	-11.78	-11.77	-11.76	-11.75	-11.74
-11.82	-11.81	-11.8	-11.79	-11.78	-11.77	-11.76	-11.75	-11.74
-11.82	-11.81	-11.8	-11.79	-11.78	-11.77	-11.76	-11.75	-11.74
-11.82	-11.81	-11.8	-11.79	-11.78	-11.77	-11.76	-11.75	-11.74
-11.82	-11.81	-11.8	-11.79	-11.78	-11.77	-11.76	-11.75	-11.74
-11.82	-11.81	-11.8	-11.79	-11.78	-11.77	-11.76	-11.75	-11.74
-11.82	-11.81	-11.8	-11.79	-11.78	-11.77	-11.76	-11.75	-11.74
-11.82	-11.81	-11.8	-11.79	-11.78	-11.77	-11.76	-11.75	-11.74
-11.82	-11.81	-11.8	-11.79	-11.78	-11.77	-11.76	-11.75	-11.74
-11.82	-11.81	-11.8	-11.79	-11.78	-11.77	-11.76	-11.75	-11.74
-11.82	-11.81	-11.8	-11.79	-11.78	-11.77	-11.76	-11.75	-11.74

-11.82	-11.81	-11.8	-11.79	-11.78	-11.77	-11.76	-11.75	-11.74
-11.82	-11.81	-11.8	-11.79	-11.78	-11.77	-11.76	-11.75	-11.74
-11.82	-11.81	-11.8	-11.79	-11.78	-11.77	-11.76	-11.75	-11.74
-11.82	-11.81	-11.8	-11.79	-11.78	-11.77	-11.76	-11.75	-11.74
-11.82	-11.81	-11.8	-11.79	-11.78	-11.77	-11.76	-11.75	-11.74
-11.82	-11.81	-11.8	-11.79	-11.78	-11.77	-11.76	-11.75	-11.74
-11.82	-11.81	-11.8	-11.79	-11.78	-11.77	-11.76	-11.75	-11.74
-11.82	-11.81	-11.8	-11.79	-11.78	-11.77	-11.76	-11.75	-11.74
-11.82	-11.81	-11.8	-11.79	-11.78	-11.77	-11.76	-11.75	-11.74
-11.82	-11.81	-11.8	-11.79	-11.78	-11.77	-11.76	-11.75	-11.74
-11.82	-11.81	-11.8	-11.79	-11.78	-11.77	-11.76	-11.75	-11.74
-11.82	-11.81	-11.8	-11.79	-11.78	-11.77	-11.76	-11.75	-11.74
-11.82	-11.81	-11.8	-11.79	-11.78	-11.77	-11.76	-11.75	-11.74
-11.82	-11.81	-11.8	-11.79	-11.78	-11.77	-11.76	-11.75	-11.74
-11.82	-11.81	-11.8	-11.79	-11.78	-11.77	-11.76	-11.75	-11.74
-11.82	-11.81	-11.8	-11.79	-11.78	-11.77	-11.76	-11.75	-11.74
-11.82	-11.81	-11.8	-11.79	-11.78	-11.77	-11.76	-11.75	-11.74
-11.82	-11.81	-11.8	-11.79	-11.78	-11.77	-11.76	-11.75	-11.74
-11.82	-11.81	-11.8	-11.79	-11.78	-11.77	-11.76	-11.75	-11.74
-11.82	-11.81	-11.8	-11.79	-11.78	-11.77	-11.76	-11.75	-11.74
-11.82	-11.81	-11.8	-11.79	-11.78	-11.77	-11.76	-11.75	-11.74
-11.82	-11.81	-11.8	-11.79	-11.78	-11.77	-11.76	-11.75	-11.74
-11.82	-11.81	-11.8	-11.79	-11.78	-11.77	-11.76	-11.75	-11.74
-11.82	-11.81	-11.8	-11.79	-11.78	-11.77	-11.76	-11.75	-11.74
-11.82	-11.81	-11.8	-11.79	-11.78	-11.77	-11.76	-11.75	-11.74
-11.82	-11.81	-11.8	-11.79	-11.78	-11.77	-11.76	-11.75	-11.74
-11.82	-11.81	-11.8	-11.79	-11.78	-11.77	-11.76	-11.75	-11.74
-11.82	-11.81	-11.8	-11.79	-11.78	-11.77	-11.76	-11.75	-11.74
-11.82	-11.81	-11.8	-11.79	-11.78	-11.77	-11.76	-11.75	-11.74
-11.82	-11.81	-11.8	-11.79	-11.78	-11.77	-11.76	-11.75	-11.74
-11.82	-11.81	-11.8	-11.79	-11.78	-11.77	-11.76	-11.75	-11.74
-11.82	-11.81	-11.8	-11.79	-11.78	-11.77	-11.76	-11.75	-11.74
-11.82	-11.81	-11.8	-11.79	-11.78	-11.77	-11.76	-11.75	-11.74
-11.82	-11.81	-11.8	-11.79	-11.78	-11.77	-11.76	-11.75	-11.74
-11.82	-11.81	-11.8	-11.79	-11.78	-11.77	-11.76	-11.75	-11.74
-11.82	-11.81	-11.8	-11.79	-11.78	-11.77	-11.76	-11.75	-11.74
-11.82	-11.81	-11.8	-11.79	-11.78	-11.77	-11.76	-11.75	-11.74
-11.82	-11.81	-11.8	-11.79	-11.78	-11.77	-11.76	-11.75	-11.74
-11.82	-11.81	-11.8	-11.79	-11.78	-11.77	-11.76	-11.75	-11.74
-11.82	-11.81	-11.8	-11.79	-11.78	-11.77	-11.76	-11.75	-11.74
-11.82	-11.81	-11.8	-11.79	-11.78	-11.77	-11.76	-11.75	-11.74
-11.82	-11.81	-11.8	-11.79	-11.78	-11.77	-11.76	-11.75	-11.74
-11.82	-11.81	-11.8	-11.79	-11.78	-11.77	-11.76	-11.75	-11.74
-11.82	-11.81	-11.8	-11.79	-11.78	-11.77	-11.76	-11.75	-11.74
-11.82	-11.81	-11.8	-11.79	-11.78	-11.77	-11.76	-11.75	-11.74
-11.82	-11.81	-11.8	-11.79	-11.78	-11.77	-11.76	-11.75	-11.74
-11.82	-11.81	-11.8	-11.79	-11.78	-11.77	-11.76	-11.75	-11.74
-11.82	-11.81	-11.8	-11.79	-11.78	-11.77	-11.76	-11.75	-11.74
-11.82	-11.81	-11.8	-11.79	-11.78	-11.77	-11.76	-11.75	-11.74
-11.82	-11.81	-11.8	-11.79	-11.78	-11.77	-11.76	-11.75	-11.74

-11.82	-11.81	-11.8	-11.79	-11.78	-11.77	-11.76	-11.75	-11.74
-11.82	-11.81	-11.8	-11.79	-11.78	-11.77	-11.76	-11.75	-11.74
-11.82	-11.81	-11.8	-11.79	-11.78	-11.77	-11.76	-11.75	-11.74
-11.82	-11.81	-11.8	-11.79	-11.78	-11.77	-11.76	-11.75	-11.74
-11.82	-11.81	-11.8	-11.79	-11.78	-11.77	-11.76	-11.75	-11.74
-11.82	-11.81	-11.8	-11.79	-11.78	-11.77	-11.76	-11.75	-11.74
-11.82	-11.81	-11.8	-11.79	-11.78	-11.77	-11.76	-11.75	-11.74
-11.82	-11.81	-11.8	-11.79	-11.78	-11.77	-11.76	-11.75	-11.74
-11.82	-11.81	-11.8	-11.79	-11.78	-11.77	-11.76	-11.75	-11.74
-11.82	-11.81	-11.8	-11.79	-11.78	-11.77	-11.76	-11.75	-11.74
-11.82	-11.81	-11.8	-11.79	-11.78	-11.77	-11.76	-11.75	-11.74
-11.82	-11.81	-11.8	-11.79	-11.78	-11.77	-11.76	-11.75	-11.74
-11.82	-11.81	-11.8	-11.79	-11.78	-11.77	-11.76	-11.75	-11.74
-11.82	-11.81	-11.8	-11.79	-11.78	-11.77	-11.76	-11.75	-11.74
-11.82	-11.81	-11.8	-11.79	-11.78	-11.77	-11.76	-11.75	-11.74
-11.82	-11.81	-11.8	-11.79	-11.78	-11.77	-11.76	-11.75	-11.74
-11.82	-11.81	-11.8	-11.79	-11.78	-11.77	-11.76	-11.75	-11.74
-11.82	-11.81	-11.8	-11.79	-11.78	-11.77	-11.76	-11.75	-11.74
-11.82	-11.81	-11.8	-11.79	-11.78	-11.77	-11.76	-11.75	-11.74
-11.82	-11.81	-11.8	-11.79	-11.78	-11.77	-11.76	-11.75	-11.74
-11.82	-11.81	-11.8	-11.79	-11.78	-11.77	-11.76	-11.75	-11.74
-11.82	-11.81	-11.8	-11.79	-11.78	-11.77	-11.76	-11.75	-11.74
-11.82	-11.81	-11.8	-11.79	-11.78	-11.77	-11.76	-11.75	-11.74
-11.82	-11.81	-11.8	-11.79	-11.78	-11.77	-11.76	-11.75	-11.74
-11.82	-11.81	-11.8	-11.79	-11.78	-11.77	-11.76	-11.75	-11.74
-11.82	-11.81	-11.8	-11.79	-11.78	-11.77	-11.76	-11.75	-11.74
-11.82	-11.81	-11.8	-11.79	-11.78	-11.77	-11.76	-11.75	-11.74
-11.82	-11.81	-11.8	-11.79	-11.78	-11.77	-11.76	-11.75	-11.74
-11.82	-11.81	-11.8	-11.79	-11.78	-11.77	-11.76	-11.75	-11.74
-11.82	-11.81	-11.8	-11.79	-11.78	-11.77	-11.76	-11.75	-11.74
-11.82	-11.81	-11.8	-11.79	-11.78	-11.77	-11.76	-11.75	-11.74
-11.82	-11.81	-11.8	-11.79	-11.78	-11.77	-11.76	-11.75	-11.74
-11.82	-11.81	-11.8	-11.79	-11.78	-11.77	-11.76	-11.75	-11.74
-11.82	-11.81	-11.8	-11.79	-11.78	-11.77	-11.76	-11.75	-11.74
-11.82	-11.81	-11.8	-11.79	-11.78	-11.77	-11.76	-11.75	-11.74
-11.82	-11.81	-11.8	-11.79	-11.78	-11.77	-11.76	-11.75	-11.74
-11.82	-11.81	-11.8	-11.79	-11.78	-11.77	-11.76	-11.75	-11.74
-11.82	-11.81	-11.8	-11.79	-11.78	-11.77	-11.76	-11.75	-11.74
-11.82	-11.81	-11.8	-11.79	-11.78	-11.77	-11.76	-11.75	-11.74
-11.82	-11.81	-11.8	-11.79	-11.78	-11.77	-11.76	-11.75	-11.74
-11.82	-11.81	-11.8	-11.79	-11.78	-11.77	-11.76	-11.75	-11.74
-11.82	-11.81	-11.8	-11.79	-11.78	-11.77	-11.76	-11.75	-11.74
-11.82	-11.81	-11.8	-11.79	-11.78	-11.77	-11.76	-11.75	-11.74
-11.82	-11.81	-11.8	-11.79	-11.78	-11.77	-11.76	-11.75	-11.74
-11.82	-11.81	-11.8	-11.79	-11.78	-11.77	-11.76	-11.75	-11.74
-11.82	-11.81	-11.8	-11.79	-11.78	-11.77	-11.76	-11.75	-11.74
-11.82	-11.81	-11.8	-11.79	-11.78	-11.77	-11.76	-11.75	-11.74
-11.82	-11.81	-11.8	-11.79	-11.78	-11.77	-11.76	-11.75	-11.74
-11.82	-11.81	-11.8	-11.79	-11.78	-11.77	-11.76	-11.75	-11.74
-11.82	-11.81	-11.8	-11.79	-11.78	-11.77	-11.76	-11.75	-11.74

-11.82	-11.81	-11.8	-11.79	-11.78	-11.77	-11.76	-11.75	-11.74
-11.82	-11.81	-11.8	-11.79	-11.78	-11.77	-11.76	-11.75	-11.74
-11.82	-11.81	-11.8	-11.79	-11.78	-11.77	-11.76	-11.75	-11.74
-11.82	-11.81	-11.8	-11.79	-11.78	-11.77	-11.76	-11.75	-11.74
-11.82	-11.81	-11.8	-11.79	-11.78	-11.77	-11.76	-11.75	-11.74
-11.82	-11.81	-11.8	-11.79	-11.78	-11.77	-11.76	-11.75	-11.74
-11.82	-11.81	-11.8	-11.79	-11.78	-11.77	-11.76	-11.75	-11.74
-11.82	-11.81	-11.8	-11.79	-11.78	-11.77	-11.76	-11.75	-11.74
-11.82	-11.81	-11.8	-11.79	-11.78	-11.77	-11.76	-11.75	-11.74
-11.82	-11.81	-11.8	-11.79	-11.78	-11.77	-11.76	-11.75	-11.74
-11.82	-11.81	-11.8	-11.79	-11.78	-11.77	-11.76	-11.75	-11.74
-11.82	-11.81	-11.8	-11.79	-11.78	-11.77	-11.76	-11.75	-11.74
-11.82	-11.81	-11.8	-11.79	-11.78	-11.77	-11.76	-11.75	-11.74
-11.82	-11.81	-11.8	-11.79	-11.78	-11.77	-11.76	-11.75	-11.74
-11.82	-11.81	-11.8	-11.79	-11.78	-11.77	-11.76	-11.75	-11.74
-11.82	-11.81	-11.8	-11.79	-11.78	-11.77	-11.76	-11.75	-11.74
-11.82	-11.81	-11.8	-11.79	-11.78	-11.77	-11.76	-11.75	-11.74
-11.82	-11.81	-11.8	-11.79	-11.78	-11.77	-11.76	-11.75	-11.74
-11.82	-11.81	-11.8	-11.79	-11.78	-11.77	-11.76	-11.75	-11.74
-11.82	-11.81	-11.8	-11.79	-11.78	-11.77	-11.76	-11.75	-11.74
-11.82	-11.81	-11.8	-11.79	-11.78	-11.77	-11.76	-11.75	-11.74
-11.82	-11.81	-11.8	-11.79	-11.78	-11.77	-11.76	-11.75	-11.74
-11.82	-11.81	-11.8	-11.79	-11.78	-11.77	-11.76	-11.75	-11.74
-11.82	-11.81	-11.8	-11.79	-11.78	-11.77	-11.76	-11.75	-11.74
-11.82	-11.81	-11.8	-11.79	-11.78	-11.77	-11.76	-11.75	-11.74
-11.82	-11.81	-11.8	-11.79	-11.78	-11.77	-11.76	-11.75	-11.74
-11.82	-11.81	-11.8	-11.79	-11.78	-11.77	-11.76	-11.75	-11.74
-11.82	-11.81	-11.8	-11.79	-11.78	-11.77	-11.76	-11.75	-11.74
-11.82	-11.81	-11.8	-11.79	-11.78	-11.77	-11.76	-11.75	-11.74
-11.82	-11.81	-11.8	-11.79	-11.78	-11.77	-11.76	-11.75	-11.74
-11.82	-11.81	-11.8	-11.79	-11.78	-11.77	-11.76	-11.75	-11.74
-11.82	-11.81	-11.8	-11.79	-11.78	-11.77	-11.76	-11.75	-11.74
-11.82	-11.81	-11.8	-11.79	-11.78	-11.77	-11.76	-11.75	-11.74
-11.82	-11.81	-11.8	-11.79	-11.78	-11.77	-11.76	-11.75	-11.74
-11.82	-11.81	-11.8	-11.79	-11.78	-11.77	-11.76	-11.75	-11.74
-11.82	-11.81	-11.8	-11.79	-11.78	-11.77	-11.76	-11.75	-11.74
-11.82	-11.81	-11.8	-11.79	-11.78	-11.77	-11.76	-11.75	-11.74
-11.82	-11.81	-11.8	-11.79	-11.78	-11.77	-11.76	-11.75	-11.74
-11.82	-11.81	-11.8	-11.79	-11.78	-11.77	-11.76	-11.75	-11.74
-11.82	-11.81	-11.8	-11.79	-11.78	-11.77	-11.76	-11.75	-11.74
-11.82	-11.81	-11.8	-11.79	-11.78	-11.77	-11.76	-11.75	-11.74
-11.82	-11.81	-11.8	-11.79	-11.78	-11.77	-11.76	-11.75	-11.74
-11.82	-11.81	-11.8	-11.79	-11.78	-11.77	-11.76	-11.75	-11.74
-11.82	-11.81	-11.8	-11.79	-11.78	-11.77	-11.76	-11.75	-11.74
-11.82	-11.81	-11.8	-11.79	-11.78	-11.77	-11.76	-11.75	-11.74
-11.82	-11.81	-11.8	-11.79	-11.78	-11.77	-11.76	-11.75	-11.74
-11.82	-11.81	-11.8	-11.79	-11.78	-11.77	-11.76	-11.75	-11.74
-11.82	-11.81	-11.8	-11.79	-11.78	-11.77	-11.76	-11.75	-11.74
-11.82	-11.81	-11.8	-11.79	-11.78	-11.77	-11.76	-11.75	-11.74
-11.82	-11.81	-11.8	-11.79	-11.78	-11.77	-11.76	-11.75	-11.74

-11.82	-11.81	-11.8	-11.79	-11.78	-11.77	-11.76	-11.75	-11.74
-11.82	-11.81	-11.8	-11.79	-11.78	-11.77	-11.76	-11.75	-11.74
-11.82	-11.81	-11.8	-11.79	-11.78	-11.77	-11.76	-11.75	-11.74
-11.82	-11.81	-11.8	-11.79	-11.78	-11.77	-11.76	-11.75	-11.74
-11.82	-11.81	-11.8	-11.79	-11.78	-11.77	-11.76	-11.75	-11.74
-11.82	-11.81	-11.8	-11.79	-11.78	-11.77	-11.76	-11.75	-11.74
-11.82	-11.81	-11.8	-11.79	-11.78	-11.77	-11.76	-11.75	-11.74
-11.82	-11.81	-11.8	-11.79	-11.78	-11.77	-11.76	-11.75	-11.74
-11.82	-11.81	-11.8	-11.79	-11.78	-11.77	-11.76	-11.75	-11.74
-11.82	-11.81	-11.8	-11.79	-11.78	-11.77	-11.76	-11.75	-11.74
-11.82	-11.81	-11.8	-11.79	-11.78	-11.77	-11.76	-11.75	-11.74
-11.82	-11.81	-11.8	-11.79	-11.78	-11.77	-11.76	-11.75	-11.74
-11.82	-11.81	-11.8	-11.79	-11.78	-11.77	-11.76	-11.75	-11.74
-11.82	-11.81	-11.8	-11.79	-11.78	-11.77	-11.76	-11.75	-11.74
-11.82	-11.81	-11.8	-11.79	-11.78	-11.77	-11.76	-11.75	-11.74
-11.82	-11.81	-11.8	-11.79	-11.78	-11.77	-11.76	-11.75	-11.74
-11.82	-11.81	-11.8	-11.79	-11.78	-11.77	-11.76	-11.75	-11.74
-11.82	-11.81	-11.8	-11.79	-11.78	-11.77	-11.76	-11.75	-11.74
-11.82	-11.81	-11.8	-11.79	-11.78	-11.77	-11.76	-11.75	-11.74
-11.82	-11.81	-11.8	-11.79	-11.78	-11.77	-11.76	-11.75	-11.74
-11.82	-11.81	-11.8	-11.79	-11.78	-11.77	-11.76	-11.75	-11.74
-11.82	-11.81	-11.8	-11.79	-11.78	-11.77	-11.76	-11.75	-11.74
-11.82	-11.81	-11.8	-11.79	-11.78	-11.77	-11.76	-11.75	-11.74
-11.82	-11.81	-11.8	-11.79	-11.78	-11.77	-11.76	-11.75	-11.74
-11.82	-11.81	-11.8	-11.79	-11.78	-11.77	-11.76	-11.75	-11.74
-11.82	-11.81	-11.8	-11.79	-11.78	-11.77	-11.76	-11.75	-11.74
-11.82	-11.81	-11.8	-11.79	-11.78	-11.77	-11.76	-11.75	-11.74
-11.82	-11.81	-11.8	-11.79	-11.78	-11.77	-11.76	-11.75	-11.74
-11.82	-11.81	-11.8	-11.79	-11.78	-11.77	-11.76	-11.75	-11.74
-11.82	-11.81	-11.8	-11.79	-11.78	-11.77	-11.76	-11.75	-11.74
-11.82	-11.81	-11.8	-11.79	-11.78	-11.77	-11.76	-11.75	-11.74
-11.82	-11.81	-11.8	-11.79	-11.78	-11.77	-11.76	-11.75	-11.74
-11.82	-11.81	-11.8	-11.79	-11.78	-11.77	-11.76	-11.75	-11.74
-11.82	-11.81	-11.8	-11.79	-11.78	-11.77	-11.76	-11.75	-11.74
-11.82	-11.81	-11.8	-11.79	-11.78	-11.77	-11.76	-11.75	-11.74
-11.82	-11.81	-11.8	-11.79	-11.78	-11.77	-11.76	-11.75	-11.74
-11.82	-11.81	-11.8	-11.79	-11.78	-11.77	-11.76	-11.75	-11.74
-11.82	-11.81	-11.8	-11.79	-11.78	-11.77	-11.76	-11.75	-11.74
-11.82	-11.81	-11.8	-11.79	-11.78	-11.77	-11.76	-11.75	-11.74
-11.82	-11.81	-11.8	-11.79	-11.78	-11.77	-11.76	-11.75	-11.74
-11.82	-11.81	-11.8	-11.79	-11.78	-11.77	-11.76	-11.75	-11.74
-11.82	-11.81	-11.8	-11.79	-11.78	-11.77	-11.76	-11.75	-11.74
-11.82	-11.81	-11.8	-11.79	-11.78	-11.77	-11.76	-11.75	-11.74
-11.82	-11.81	-11.8	-11.79	-11.78	-11.77	-11.76	-11.75	-11.74
-11.82	-11.81	-11.8	-11.79	-11.78	-11.77	-11.76	-11.75	-11.74
-11.82	-11.81	-11.8	-11.79	-11.78	-11.77	-11.76	-11.75	-11.74
-11.82	-11.81	-11.8	-11.79	-11.78	-11.77	-11.76	-11.75	-11.74
-11.82	-11.81	-11.8	-11.79	-11.78	-11.77	-11.76	-11.75	-11.74
-11.82	-11.81	-11.8	-11.79	-11.78	-11.77	-11.76	-11.75	-11.74
-11.82	-11.81	-11.8	-11.79	-11.78	-11.77	-11.76	-11.75	-11.74

-11.82	-11.81	-11.8	-11.79	-11.78	-11.77	-11.76	-11.75	-11.74
-11.82	-11.81	-11.8	-11.79	-11.78	-11.77	-11.76	-11.75	-11.74
-11.82	-11.81	-11.8	-11.79	-11.78	-11.77	-11.76	-11.75	-11.74
-11.82	-11.81	-11.8	-11.79	-11.78	-11.77	-11.76	-11.75	-11.74
-11.82	-11.81	-11.8	-11.79	-11.78	-11.77	-11.76	-11.75	-11.74
-11.82	-11.81	-11.8	-11.79	-11.78	-11.77	-11.76	-11.75	-11.74
-11.82	-11.81	-11.8	-11.79	-11.78	-11.77	-11.76	-11.75	-11.74
-11.82	-11.81	-11.8	-11.79	-11.78	-11.77	-11.76	-11.75	-11.74
-11.82	-11.81	-11.8	-11.79	-11.78	-11.77	-11.76	-11.75	-11.74
-11.82	-11.81	-11.8	-11.79	-11.78	-11.77	-11.76	-11.75	-11.74
-11.82	-11.81	-11.8	-11.79	-11.78	-11.77	-11.76	-11.75	-11.74
-11.82	-11.81	-11.8	-11.79	-11.78	-11.77	-11.76	-11.75	-11.74
-11.82	-11.81	-11.8	-11.79	-11.78	-11.77	-11.76	-11.75	-11.74
-11.82	-11.81	-11.8	-11.79	-11.78	-11.77	-11.76	-11.75	-11.74
-11.82	-11.81	-11.8	-11.79	-11.78	-11.77	-11.76	-11.75	-11.74
-11.82	-11.81	-11.8	-11.79	-11.78	-11.77	-11.76	-11.75	-11.74
-11.82	-11.81	-11.8	-11.79	-11.78	-11.77	-11.76	-11.75	-11.74
-11.82	-11.81	-11.8	-11.79	-11.78	-11.77	-11.76	-11.75	-11.74
-11.82	-11.81	-11.8	-11.79	-11.78	-11.77	-11.76	-11.75	-11.74
-11.82	-11.81	-11.8	-11.79	-11.78	-11.77	-11.76	-11.75	-11.74
-11.82	-11.81	-11.8	-11.79	-11.78	-11.77	-11.76	-11.75	-11.74
-11.82	-11.81	-11.8	-11.79	-11.78	-11.77	-11.76	-11.75	-11.74
-11.82	-11.81	-11.8	-11.79	-11.78	-11.77	-11.76	-11.75	-11.74
-11.82	-11.81	-11.8	-11.79	-11.78	-11.77	-11.76	-11.75	-11.74
-11.82	-11.81	-11.8	-11.79	-11.78	-11.77	-11.76	-11.75	-11.74
-11.82	-11.81	-11.8	-11.79	-11.78	-11.77	-11.76	-11.75	-11.74
-11.82	-11.81	-11.8	-11.79	-11.78	-11.77	-11.76	-11.75	-11.74
-11.82	-11.81	-11.8	-11.79	-11.78	-11.77	-11.76	-11.75	-11.74
-11.82	-11.81	-11.8	-11.79	-11.78	-11.77	-11.76	-11.75	-11.74
-11.82	-11.81	-11.8	-11.79	-11.78	-11.77	-11.76	-11.75	-11.74
-11.82	-11.81	-11.8	-11.79	-11.78	-11.77	-11.76	-11.75	-11.74
-11.82	-11.81	-11.8	-11.79	-11.78	-11.77	-11.76	-11.75	-11.74
-11.82	-11.81	-11.8	-11.79	-11.78	-11.77	-11.76	-11.75	-11.74
-11.82	-11.81	-11.8	-11.79	-11.78	-11.77	-11.76	-11.75	-11.74
-11.82	-11.81	-11.8	-11.79	-11.78	-11.77	-11.76	-11.75	-11.74
-11.82	-11.81	-11.8	-11.79	-11.78	-11.77	-11.76	-11.75	-11.74
-11.82	-11.81	-11.8	-11.79	-11.78	-11.77	-11.76	-11.75	-11.74
-11.82	-11.81	-11.8	-11.79	-11.78	-11.77	-11.76	-11.75	-11.74
-11.82	-11.81	-11.8	-11.79	-11.78	-11.77	-11.76	-11.75	-11.74
-11.82	-11.81	-11.8	-11.79	-11.78	-11.77	-11.76	-11.75	-11.74
-11.82	-11.81	-11.8	-11.79	-11.78	-11.77	-11.76	-11.75	-11.74
-11.82	-11.81	-11.8	-11.79	-11.78	-11.77	-11.76	-11.75	-11.74
-11.82	-11.81	-11.8	-11.79	-11.78	-11.77	-11.76	-11.75	-11.74
-11.82	-11.81	-11.8	-11.79	-11.78	-11.77	-11.76	-11.75	-11.74
-11.82	-11.81	-11.8	-11.79	-11.78	-11.77	-11.76	-11.75	-11.74
-11.82	-11.81	-11.8	-11.79	-11.78	-11.77	-11.76	-11.75	-11.74
-11.82	-11.81	-11.8	-11.79	-11.78	-11.77	-11.76	-11.75	-11.74
-11.82	-11.81	-11.8	-11.79	-11.78	-11.77	-11.76	-11.75	-11.74
-11.82	-11.81	-11.8	-11.79	-11.78	-11.77	-11.76	-11.75	-11.74
-11.82	-11.81	-11.8	-11.79	-11.78	-11.77	-11.76	-11.75	-11.74

-11.82	-11.81	-11.8	-11.79	-11.78	-11.77	-11.76	-11.75	-11.74
-11.82	-11.81	-11.8	-11.79	-11.78	-11.77	-11.76	-11.75	-11.74
-11.82	-11.81	-11.8	-11.79	-11.78	-11.77	-11.76	-11.75	-11.74
-11.82	-11.81	-11.8	-11.79	-11.78	-11.77	-11.76	-11.75	-11.74
-11.82	-11.81	-11.8	-11.79	-11.78	-11.77	-11.76	-11.75	-11.74
-11.82	-11.81	-11.8	-11.79	-11.78	-11.77	-11.76	-11.75	-11.74
-11.82	-11.81	-11.8	-11.79	-11.78	-11.77	-11.76	-11.75	-11.74
-11.82	-11.81	-11.8	-11.79	-11.78	-11.77	-11.76	-11.75	-11.74
-11.82	-11.81	-11.8	-11.79	-11.78	-11.77	-11.76	-11.75	-11.74
-11.82	-11.81	-11.8	-11.79	-11.78	-11.77	-11.76	-11.75	-11.74
-11.82	-11.81	-11.8	-11.79	-11.78	-11.77	-11.76	-11.75	-11.74
-11.82	-11.81	-11.8	-11.79	-11.78	-11.77	-11.76	-11.75	-11.74
-11.82	-11.81	-11.8	-11.79	-11.78	-11.77	-11.76	-11.75	-11.74
-11.82	-11.81	-11.8	-11.79	-11.78	-11.77	-11.76	-11.75	-11.74
-11.82	-11.81	-11.8	-11.79	-11.78	-11.77	-11.76	-11.75	-11.74
-11.82	-11.81	-11.8	-11.79	-11.78	-11.77	-11.76	-11.75	-11.74
-11.82	-11.81	-11.8	-11.79	-11.78	-11.77	-11.76	-11.75	-11.74
-11.82	-11.81	-11.8	-11.79	-11.78	-11.77	-11.76	-11.75	-11.74
-11.82	-11.81	-11.8	-11.79	-11.78	-11.77	-11.76	-11.75	-11.74
-11.82	-11.81	-11.8	-11.79	-11.78	-11.77	-11.76	-11.75	-11.74
-11.82	-11.81	-11.8	-11.79	-11.78	-11.77	-11.76	-11.75	-11.74
-11.82	-11.81	-11.8	-11.79	-11.78	-11.77	-11.76	-11.75	-11.74
-11.82	-11.81	-11.8	-11.79	-11.78	-11.77	-11.76	-11.75	-11.74
-11.82	-11.81	-11.8	-11.79	-11.78	-11.77	-11.76	-11.75	-11.74
-11.82	-11.81	-11.8	-11.79	-11.78	-11.77	-11.76	-11.75	-11.74
-11.82	-11.81	-11.8	-11.79	-11.78	-11.77	-11.76	-11.75	-11.74
-11.82	-11.81	-11.8	-11.79	-11.78	-11.77	-11.76	-11.75	-11.74
-11.82	-11.81	-11.8	-11.79	-11.78	-11.77	-11.76	-11.75	-11.74
-11.82	-11.81	-11.8	-11.79	-11.78	-11.77	-11.76	-11.75	-11.74
-11.82	-11.81	-11.8	-11.79	-11.78	-11.77	-11.76	-11.75	-11.74
-11.82	-11.81	-11.8	-11.79	-11.78	-11.77	-11.76	-11.75	-11.74
-11.82	-11.81	-11.8	-11.79	-11.78	-11.77	-11.76	-11.75	-11.74
-11.82	-11.81	-11.8	-11.79	-11.78	-11.77	-11.76	-11.75	-11.74
-11.82	-11.81	-11.8	-11.79	-11.78	-11.77	-11.76	-11.75	-11.74
-11.82	-11.81	-11.8	-11.79	-11.78	-11.77	-11.76	-11.75	-11.74
-11.82	-11.81	-11.8	-11.79	-11.78	-11.77	-11.76	-11.75	-11.74
-11.82	-11.81	-11.8	-11.79	-11.78	-11.77	-11.76	-11.75	-11.74
-11.82	-11.81	-11.8	-11.79	-11.78	-11.77	-11.76	-11.75	-11.74
-11.82	-11.81	-11.8	-11.79	-11.78	-11.77	-11.76	-11.75	-11.74
-11.82	-11.81	-11.8	-11.79	-11.78	-11.77	-11.76	-11.75	-11.74
-11.82	-11.81	-11.8	-11.79	-11.78	-11.77	-11.76	-11.75	-11.74
-11.82	-11.81	-11.8	-11.79	-11.78	-11.77	-11.76	-11.75	-11.74
-11.82	-11.81	-11.8	-11.79	-11.78	-11.77	-11.76	-11.75	-11.74
-11.82	-11.81	-11.8	-11.79	-11.78	-11.77	-11.76	-11.75	-11.74
-11.82	-11.81	-11.8	-11.79	-11.78	-11.77	-11.76	-11.75	-11.74
-11.82	-11.81	-11.8	-11.79	-11.78	-11.77	-11.76	-11.75	-11.74
-11.82	-11.81	-11.8	-11.79	-11.78	-11.77	-11.76	-11.75	-11.74
-11.82	-11.81	-11.8	-11.79	-11.78	-11.77	-11.76	-11.75	-11.74
-11.82	-11.81	-11.8	-11.79	-11.78	-11.77	-11.76	-11.75	-11.74
-11.82	-11.81	-11.8	-11.79	-11.78	-11.77	-11.76	-11.75	-11.74

-11.82	-11.81	-11.8	-11.79	-11.78	-11.77	-11.76	-11.75	-11.74
-11.82	-11.81	-11.8	-11.79	-11.78	-11.77	-11.76	-11.75	-11.74
-11.82	-11.81	-11.8	-11.79	-11.78	-11.77	-11.76	-11.75	-11.74
-11.82	-11.81	-11.8	-11.79	-11.78	-11.77	-11.76	-11.75	-11.74
-11.82	-11.81	-11.8	-11.79	-11.78	-11.77	-11.76	-11.75	-11.74
-11.82	-11.81	-11.8	-11.79	-11.78	-11.77	-11.76	-11.75	-11.74
-11.82	-11.81	-11.8	-11.79	-11.78	-11.77	-11.76	-11.75	-11.74
-11.82	-11.81	-11.8	-11.79	-11.78	-11.77	-11.76	-11.75	-11.74
-11.82	-11.81	-11.8	-11.79	-11.78	-11.77	-11.76	-11.75	-11.74
-11.82	-11.81	-11.8	-11.79	-11.78	-11.77	-11.76	-11.75	-11.74
-11.82	-11.81	-11.8	-11.79	-11.78	-11.77	-11.76	-11.75	-11.74
-11.82	-11.81	-11.8	-11.79	-11.78	-11.77	-11.76	-11.75	-11.74
-11.82	-11.81	-11.8	-11.79	-11.78	-11.77	-11.76	-11.75	-11.74
-11.82	-11.81	-11.8	-11.79	-11.78	-11.77	-11.76	-11.75	-11.74
-11.82	-11.81	-11.8	-11.79	-11.78	-11.77	-11.76	-11.75	-11.74
-11.82	-11.81	-11.8	-11.79	-11.78	-11.77	-11.76	-11.75	-11.74
-11.82	-11.81	-11.8	-11.79	-11.78	-11.77	-11.76	-11.75	-11.74
-11.82	-11.81	-11.8	-11.79	-11.78	-11.77	-11.76	-11.75	-11.74
-11.82	-11.81	-11.8	-11.79	-11.78	-11.77	-11.76	-11.75	-11.74
-11.82	-11.81	-11.8	-11.79	-11.78	-11.77	-11.76	-11.75	-11.74
-11.82	-11.81	-11.8	-11.79	-11.78	-11.77	-11.76	-11.75	-11.74
-11.82	-11.81	-11.8	-11.79	-11.78	-11.77	-11.76	-11.75	-11.74
-11.82	-11.81	-11.8	-11.79	-11.78	-11.77	-11.76	-11.75	-11.74
-11.82	-11.81	-11.8	-11.79	-11.78	-11.77	-11.76	-11.75	-11.74
-11.82	-11.81	-11.8	-11.79	-11.78	-11.77	-11.76	-11.75	-11.74
-11.82	-11.81	-11.8	-11.79	-11.78	-11.77	-11.76	-11.75	-11.74
-11.82	-11.81	-11.8	-11.79	-11.78	-11.77	-11.76	-11.75	-11.74
-11.82	-11.81	-11.8	-11.79	-11.78	-11.77	-11.76	-11.75	-11.74
-11.82	-11.81	-11.8	-11.79	-11.78	-11.77	-11.76	-11.75	-11.74
-11.82	-11.81	-11.8	-11.79	-11.78	-11.77	-11.76	-11.75	-11.74
-11.82	-11.81	-11.8	-11.79	-11.78	-11.77	-11.76	-11.75	-11.74
-11.82	-11.81	-11.8	-11.79	-11.78	-11.77	-11.76	-11.75	-11.74
-11.82	-11.81	-11.8	-11.79	-11.78	-11.77	-11.76	-11.75	-11.74
-11.82	-11.81	-11.8	-11.79	-11.78	-11.77	-11.76	-11.75	-11.74
-11.82	-11.81	-11.8	-11.79	-11.78	-11.77	-11.76	-11.75	-11.74
-11.82	-11.81	-11.8	-11.79	-11.78	-11.77	-11.76	-11.75	-11.74
-11.82	-11.81	-11.8	-11.79	-11.78	-11.77	-11.76	-11.75	-11.74
-11.82	-11.81	-11.8	-11.79	-11.78	-11.77	-11.76	-11.75	-11.74
-11.82	-11.81	-11.8	-11.79	-11.78	-11.77	-11.76	-11.75	-11.74
-11.82	-11.81	-11.8	-11.79	-11.78	-11.77	-11.76	-11.75	-11.74
-11.82	-11.81	-11.8	-11.79	-11.78	-11.77	-11.76	-11.75	-11.74
-11.82	-11.81	-11.8	-11.79	-11.78	-11.77	-11.76	-11.75	-11.74
-11.82	-11.81	-11.8	-11.79	-11.78	-11.77	-11.76	-11.75	-11.74
-11.82	-11.81	-11.8	-11.79	-11.78	-11.77	-11.76	-11.75	-11.74
-11.82	-11.81	-11.8	-11.79	-11.78	-11.77	-11.76	-11.75	-11.74
-11.82	-11.81	-11.8	-11.79	-11.78	-11.77	-11.76	-11.75	-11.74
-11.82	-11.81	-11.8	-11.79	-11.78	-11.77	-11.76	-11.75	-11.74
-11.82	-11.81	-11.8	-11.79	-11.78	-11.77	-11.76	-11.75	-11.74
-11.82	-11.81	-11.8	-11.79	-11.78	-11.77	-11.76	-11.75	-11.74
-11.82	-11.81	-11.8	-11.79	-11.78	-11.77	-11.76	-11.75	-11.74

-11.82	-11.81	-11.8	-11.79	-11.78	-11.77	-11.76	-11.75	-11.74
-11.82	-11.81	-11.8	-11.79	-11.78	-11.77	-11.76	-11.75	-11.74
-11.82	-11.81	-11.8	-11.79	-11.78	-11.77	-11.76	-11.75	-11.74
-11.82	-11.81	-11.8	-11.79	-11.78	-11.77	-11.76	-11.75	-11.74
-11.82	-11.81	-11.8	-11.79	-11.78	-11.77	-11.76	-11.75	-11.74
-11.82	-11.81	-11.8	-11.79	-11.78	-11.77	-11.76	-11.75	-11.74
-11.82	-11.81	-11.8	-11.79	-11.78	-11.77	-11.76	-11.75	-11.74
-11.82	-11.81	-11.8	-11.79	-11.78	-11.77	-11.76	-11.75	-11.74
-11.82	-11.81	-11.8	-11.79	-11.78	-11.77	-11.76	-11.75	-11.74
-11.82	-11.81	-11.8	-11.79	-11.78	-11.77	-11.76	-11.75	-11.74
-11.82	-11.81	-11.8	-11.79	-11.78	-11.77	-11.76	-11.75	-11.74
-11.82	-11.81	-11.8	-11.79	-11.78	-11.77	-11.76	-11.75	-11.74
-11.82	-11.81	-11.8	-11.79	-11.78	-11.77	-11.76	-11.75	-11.74
-11.82	-11.81	-11.8	-11.79	-11.78	-11.77	-11.76	-11.75	-11.74
-11.82	-11.81	-11.8	-11.79	-11.78	-11.77	-11.76	-11.75	-11.74
-11.82	-11.81	-11.8	-11.79	-11.78	-11.77	-11.76	-11.75	-11.74
-11.82	-11.81	-11.8	-11.79	-11.78	-11.77	-11.76	-11.75	-11.74
-11.82	-11.81	-11.8	-11.79	-11.78	-11.77	-11.76	-11.75	-11.74
-11.82	-11.81	-11.8	-11.79	-11.78	-11.77	-11.76	-11.75	-11.74
-11.82	-11.81	-11.8	-11.79	-11.78	-11.77	-11.76	-11.75	-11.74
-11.82	-11.81	-11.8	-11.79	-11.78	-11.77	-11.76	-11.75	-11.74
-11.82	-11.81	-11.8	-11.79	-11.78	-11.77	-11.76	-11.75	-11.74
-11.82	-11.81	-11.8	-11.79	-11.78	-11.77	-11.76	-11.75	-11.74
-11.82	-11.81	-11.8	-11.79	-11.78	-11.77	-11.76	-11.75	-11.74
-11.82	-11.81	-11.8	-11.79	-11.78	-11.77	-11.76	-11.75	-11.74
-11.82	-11.81	-11.8	-11.79	-11.78	-11.77	-11.76	-11.75	-11.74
-11.82	-11.81	-11.8	-11.79	-11.78	-11.77	-11.76	-11.75	-11.74
-11.82	-11.81	-11.8	-11.79	-11.78	-11.77	-11.76	-11.75	-11.74
-11.82	-11.81	-11.8	-11.79	-11.78	-11.77	-11.76	-11.75	-11.74
-11.82	-11.81	-11.8	-11.79	-11.78	-11.77	-11.76	-11.75	-11.74
-11.82	-11.81	-11.8	-11.79	-11.78	-11.77	-11.76	-11.75	-11.74
-11.82	-11.81	-11.8	-11.79	-11.78	-11.77	-11.76	-11.75	-11.74
-11.82	-11.81	-11.8	-11.79	-11.78	-11.77	-11.76	-11.75	-11.74
-11.82	-11.81	-11.8	-11.79	-11.78	-11.77	-11.76	-11.75	-11.74
-11.82	-11.81	-11.8	-11.79	-11.78	-11.77	-11.76	-11.75	-11.74
-11.82	-11.81	-11.8	-11.79	-11.78	-11.77	-11.76	-11.75	-11.74
-11.82	-11.81	-11.8	-11.79	-11.78	-11.77	-11.76	-11.75	-11.74
-11.82	-11.81	-11.8	-11.79	-11.78	-11.77	-11.76	-11.75	-11.74
-11.82	-11.81	-11.8	-11.79	-11.78	-11.77	-11.76	-11.75	-11.74
-11.82	-11.81	-11.8	-11.79	-11.78	-11.77	-11.76	-11.75	-11.74
-11.82	-11.81	-11.8	-11.79	-11.78	-11.77	-11.76	-11.75	-11.74
-11.82	-11.81	-11.8	-11.79	-11.78	-11.77	-11.76	-11.75	-11.74
-11.82	-11.81	-11.8	-11.79	-11.78	-11.77	-11.76	-11.75	-11.74
-11.82	-11.81	-11.8	-11.79	-11.78	-11.77	-11.76	-11.75	-11.74
-11.82	-11.81	-11.8	-11.79	-11.78	-11.77	-11.76	-11.75	-11.74
-11.82	-11.81	-11.8	-11.79	-11.78	-11.77	-11.76	-11.75	-11.74
-11.82	-11.81	-11.8	-11.79	-11.78	-11.77	-11.76	-11.75	-11.74
-11.82	-11.81	-11.8	-11.79	-11.78	-11.77	-11.76	-11.75	-11.74
-11.82	-11.81	-11.8	-11.79	-11.78	-11.77	-11.76	-11.75	-11.74
-11.82	-11.81	-11.8	-11.79	-11.78	-11.77	-11.76	-11.75	-11.74

-11.82	-11.81	-11.8	-11.79	-11.78	-11.77	-11.76	-11.75	-11.74
-11.82	-11.81	-11.8	-11.79	-11.78	-11.77	-11.76	-11.75	-11.74
-11.82	-11.81	-11.8	-11.79	-11.78	-11.77	-11.76	-11.75	-11.74
-11.82	-11.81	-11.8	-11.79	-11.78	-11.77	-11.76	-11.75	-11.74
-11.82	-11.81	-11.8	-11.79	-11.78	-11.77	-11.76	-11.75	-11.74
-11.82	-11.81	-11.8	-11.79	-11.78	-11.77	-11.76	-11.75	-11.74
-11.82	-11.81	-11.8	-11.79	-11.78	-11.77	-11.76	-11.75	-11.74
-11.82	-11.81	-11.8	-11.79	-11.78	-11.77	-11.76	-11.75	-11.74
-11.82	-11.81	-11.8	-11.79	-11.78	-11.77	-11.76	-11.75	-11.74
-11.82	-11.81	-11.8	-11.79	-11.78	-11.77	-11.76	-11.75	-11.74
-11.82	-11.81	-11.8	-11.79	-11.78	-11.77	-11.76	-11.75	-11.74
-11.82	-11.81	-11.8	-11.79	-11.78	-11.77	-11.76	-11.75	-11.74
-11.82	-11.81	-11.8	-11.79	-11.78	-11.77	-11.76	-11.75	-11.74
-11.82	-11.81	-11.8	-11.79	-11.78	-11.77	-11.76	-11.75	-11.74
-11.82	-11.81	-11.8	-11.79	-11.78	-11.77	-11.76	-11.75	-11.74
-11.82	-11.81	-11.8	-11.79	-11.78	-11.77	-11.76	-11.75	-11.74
-11.82	-11.81	-11.8	-11.79	-11.78	-11.77	-11.76	-11.75	-11.74
-11.82	-11.81	-11.8	-11.79	-11.78	-11.77	-11.76	-11.75	-11.74
-11.82	-11.81	-11.8	-11.79	-11.78	-11.77	-11.76	-11.75	-11.74
-11.82	-11.81	-11.8	-11.79	-11.78	-11.77	-11.76	-11.75	-11.74
-11.82	-11.81	-11.8	-11.79	-11.78	-11.77	-11.76	-11.75	-11.74
-11.82	-11.81	-11.8	-11.79	-11.78	-11.77	-11.76	-11.75	-11.74
-11.82	-11.81	-11.8	-11.79	-11.78	-11.77	-11.76	-11.75	-11.74
-11.82	-11.81	-11.8	-11.79	-11.78	-11.77	-11.76	-11.75	-11.74
-11.82	-11.81	-11.8	-11.79	-11.78	-11.77	-11.76	-11.75	-11.74
-11.82	-11.81	-11.8	-11.79	-11.78	-11.77	-11.76	-11.75	-11.74
-11.82	-11.81	-11.8	-11.79	-11.78	-11.77	-11.76	-11.75	-11.74
-11.82	-11.81	-11.8	-11.79	-11.78	-11.77	-11.76	-11.75	-11.74
-11.82	-11.81	-11.8	-11.79	-11.78	-11.77	-11.76	-11.75	-11.74
-11.82	-11.81	-11.8	-11.79	-11.78	-11.77	-11.76	-11.75	-11.74
-11.82	-11.81	-11.8	-11.79	-11.78	-11.77	-11.76	-11.75	-11.74
-11.82	-11.81	-11.8	-11.79	-11.78	-11.77	-11.76	-11.75	-11.74
-11.82	-11.81	-11.8	-11.79	-11.78	-11.77	-11.76	-11.75	-11.74
-11.82	-11.81	-11.8	-11.79	-11.78	-11.77	-11.76	-11.75	-11.74
-11.82	-11.81	-11.8	-11.79	-11.78	-11.77	-11.76	-11.75	-11.74
-11.82	-11.81	-11.8	-11.79	-11.78	-11.77	-11.76	-11.75	-11.74
-11.82	-11.81	-11.8	-11.79	-11.78	-11.77	-11.76	-11.75	-11.74
-11.82	-11.81	-11.8	-11.79	-11.78	-11.77	-11.76	-11.75	-11.74
-11.82	-11.81	-11.8	-11.79	-11.78	-11.77	-11.76	-11.75	-11.74
-11.82	-11.81	-11.8	-11.79	-11.78	-11.77	-11.76	-11.75	-11.74
-11.82	-11.81	-11.8	-11.79	-11.78	-11.77	-11.76	-11.75	-11.74
-11.82	-11.81	-11.8	-11.79	-11.78	-11.77	-11.76	-11.75	-11.74
-11.82	-11.81	-11.8	-11.79	-11.78	-11.77	-11.76	-11.75	-11.74
-11.82	-11.81	-11.8	-11.79	-11.78	-11.77	-11.76	-11.75	-11.74
-11.82	-11.81	-11.8	-11.79	-11.78	-11.77	-11.76	-11.75	-11.74
-11.82	-11.81	-11.8	-11.79	-11.78	-11.77	-11.76	-11.75	-11.74
-11.82	-11.81	-11.8	-11.79	-11.78	-11.77	-11.76	-11.75	-11.74
-11.82	-11.81	-11.8	-11.79	-11.78	-11.77	-11.76	-11.75	-11.74
-11.82	-11.81	-11.8	-11.79	-11.78	-11.77	-11.76	-11.75	-11.74
-11.82	-11.81	-11.8	-11.79	-11.78	-11.77	-11.76	-11.75	-11.74

-11.82	-11.81	-11.8	-11.79	-11.78	-11.77	-11.76	-11.75	-11.74
-11.82	-11.81	-11.8	-11.79	-11.78	-11.77	-11.76	-11.75	-11.74
-11.82	-11.81	-11.8	-11.79	-11.78	-11.77	-11.76	-11.75	-11.74
-11.82	-11.81	-11.8	-11.79	-11.78	-11.77	-11.76	-11.75	-11.74
-11.82	-11.81	-11.8	-11.79	-11.78	-11.77	-11.76	-11.75	-11.74
-11.82	-11.81	-11.8	-11.79	-11.78	-11.77	-11.76	-11.75	-11.74
-11.82	-11.81	-11.8	-11.79	-11.78	-11.77	-11.76	-11.75	-11.74
-11.82	-11.81	-11.8	-11.79	-11.78	-11.77	-11.76	-11.75	-11.74
-11.82	-11.81	-11.8	-11.79	-11.78	-11.77	-11.76	-11.75	-11.74
-11.82	-11.81	-11.8	-11.79	-11.78	-11.77	-11.76	-11.75	-11.74
-11.82	-11.81	-11.8	-11.79	-11.78	-11.77	-11.76	-11.75	-11.74
-11.82	-11.81	-11.8	-11.79	-11.78	-11.77	-11.76	-11.75	-11.74
-11.82	-11.81	-11.8	-11.79	-11.78	-11.77	-11.76	-11.75	-11.74
-11.82	-11.81	-11.8	-11.79	-11.78	-11.77	-11.76	-11.75	-11.74
-11.82	-11.81	-11.8	-11.79	-11.78	-11.77	-11.76	-11.75	-11.74
-11.82	-11.81	-11.8	-11.79	-11.78	-11.77	-11.76	-11.75	-11.74
-11.82	-11.81	-11.8	-11.79	-11.78	-11.77	-11.76	-11.75	-11.74
-11.82	-11.81	-11.8	-11.79	-11.78	-11.77	-11.76	-11.75	-11.74
-11.82	-11.81	-11.8	-11.79	-11.78	-11.77	-11.76	-11.75	-11.74
-11.82	-11.81	-11.8	-11.79	-11.78	-11.77	-11.76	-11.75	-11.74
-11.82	-11.81	-11.8	-11.79	-11.78	-11.77	-11.76	-11.75	-11.74
-11.82	-11.81	-11.8	-11.79	-11.78	-11.77	-11.76	-11.75	-11.74
-11.82	-11.81	-11.8	-11.79	-11.78	-11.77	-11.76	-11.75	-11.74
-11.82	-11.81	-11.8	-11.79	-11.78	-11.77	-11.76	-11.75	-11.74
-11.82	-11.81	-11.8	-11.79	-11.78	-11.77	-11.76	-11.75	-11.74
-11.82	-11.81	-11.8	-11.79	-11.78	-11.77	-11.76	-11.75	-11.74
-11.82	-11.81	-11.8	-11.79	-11.78	-11.77	-11.76	-11.75	-11.74
-11.82	-11.81	-11.8	-11.79	-11.78	-11.77	-11.76	-11.75	-11.74
-11.82	-11.81	-11.8	-11.79	-11.78	-11.77	-11.76	-11.75	-11.74
-11.82	-11.81	-11.8	-11.79	-11.78	-11.77	-11.76	-11.75	-11.74
-11.82	-11.81	-11.8	-11.79	-11.78	-11.77	-11.76	-11.75	-11.74
-11.82	-11.81	-11.8	-11.79	-11.78	-11.77	-11.76	-11.75	-11.74
-11.82	-11.81	-11.8	-11.79	-11.78	-11.77	-11.76	-11.75	-11.74
-11.82	-11.81	-11.8	-11.79	-11.78	-11.77	-11.76	-11.75	-11.74
-11.82	-11.81	-11.8	-11.79	-11.78	-11.77	-11.76	-11.75	-11.74
-11.82	-11.81	-11.8	-11.79	-11.78	-11.77	-11.76	-11.75	-11.74
-11.82	-11.81	-11.8	-11.79	-11.78	-11.77	-11.76	-11.75	-11.74
-11.82	-11.81	-11.8	-11.79	-11.78	-11.77	-11.76	-11.75	-11.74
-11.82	-11.81	-11.8	-11.79	-11.78	-11.77	-11.76	-11.75	-11.74
-11.82	-11.81	-11.8	-11.79	-11.78	-11.77	-11.76	-11.75	-11.74
-11.82	-11.81	-11.8	-11.79	-11.78	-11.77	-11.76	-11.75	-11.74
-11.82	-11.81	-11.8	-11.79	-11.78	-11.77	-11.76	-11.75	-11.74
-11.82	-11.81	-11.8	-11.79	-11.78	-11.77	-11.76	-11.75	-11.74
-11.82	-11.81	-11.8	-11.79	-11.78	-11.77	-11.76	-11.75	-11.74
-11.82	-11.81	-11.8	-11.79	-11.78	-11.77	-11.76	-11.75	-11.74
-11.82	-11.81	-11.8	-11.79	-11.78	-11.77	-11.76	-11.75	-11.74
-11.82	-11.81	-11.8	-11.79	-11.78	-11.77	-11.76	-11.75	-11.74
-11.82	-11.81	-11.8	-11.79	-11.78	-11.77	-11.76	-11.75	-11.74
-11.82	-11.81	-11.8	-11.79	-11.78	-11.77	-11.76	-11.75	-11.74
-11.82	-11.81	-11.8	-11.79	-11.78	-11.77	-11.76	-11.75	-11.74

-11.82	-11.81	-11.8	-11.79	-11.78	-11.77	-11.76	-11.75	-11.74
-11.82	-11.81	-11.8	-11.79	-11.78	-11.77	-11.76	-11.75	-11.74
-11.82	-11.81	-11.8	-11.79	-11.78	-11.77	-11.76	-11.75	-11.74
-11.82	-11.81	-11.8	-11.79	-11.78	-11.77	-11.76	-11.75	-11.74
-11.82	-11.81	-11.8	-11.79	-11.78	-11.77	-11.76	-11.75	-11.74
-11.82	-11.81	-11.8	-11.79	-11.78	-11.77	-11.76	-11.75	-11.74
-11.82	-11.81	-11.8	-11.79	-11.78	-11.77	-11.76	-11.75	-11.74
-11.82	-11.81	-11.8	-11.79	-11.78	-11.77	-11.76	-11.75	-11.74
-11.82	-11.81	-11.8	-11.79	-11.78	-11.77	-11.76	-11.75	-11.74
-11.82	-11.81	-11.8	-11.79	-11.78	-11.77	-11.76	-11.75	-11.74
-11.82	-11.81	-11.8	-11.79	-11.78	-11.77	-11.76	-11.75	-11.74
-11.82	-11.81	-11.8	-11.79	-11.78	-11.77	-11.76	-11.75	-11.74
-11.82	-11.81	-11.8	-11.79	-11.78	-11.77	-11.76	-11.75	-11.74
-11.82	-11.81	-11.8	-11.79	-11.78	-11.77	-11.76	-11.75	-11.74
-11.82	-11.81	-11.8	-11.79	-11.78	-11.77	-11.76	-11.75	-11.74
-11.82	-11.81	-11.8	-11.79	-11.78	-11.77	-11.76	-11.75	-11.74
-11.82	-11.81	-11.8	-11.79	-11.78	-11.77	-11.76	-11.75	-11.74
-11.82	-11.81	-11.8	-11.79	-11.78	-11.77	-11.76	-11.75	-11.74
-11.82	-11.81	-11.8	-11.79	-11.78	-11.77	-11.76	-11.75	-11.74
-11.82	-11.81	-11.8	-11.79	-11.78	-11.77	-11.76	-11.75	-11.74
-11.82	-11.81	-11.8	-11.79	-11.78	-11.77	-11.76	-11.75	-11.74
-11.82	-11.81	-11.8	-11.79	-11.78	-11.77	-11.76	-11.75	-11.74
-11.82	-11.81	-11.8	-11.79	-11.78	-11.77	-11.76	-11.75	-11.74
-11.82	-11.81	-11.8	-11.79	-11.78	-11.77	-11.76	-11.75	-11.74
-11.82	-11.81	-11.8	-11.79	-11.78	-11.77	-11.76	-11.75	-11.74
-11.82	-11.81	-11.8	-11.79	-11.78	-11.77	-11.76	-11.75	-11.74
-11.82	-11.81	-11.8	-11.79	-11.78	-11.77	-11.76	-11.75	-11.74
-11.82	-11.81	-11.8	-11.79	-11.78	-11.77	-11.76	-11.75	-11.74
-11.82	-11.81	-11.8	-11.79	-11.78	-11.77	-11.76	-11.75	-11.74
-11.82	-11.81	-11.8	-11.79	-11.78	-11.77	-11.76	-11.75	-11.74
-11.82	-11.81	-11.8	-11.79	-11.78	-11.77	-11.76	-11.75	-11.74
-11.82	-11.81	-11.8	-11.79	-11.78	-11.77	-11.76	-11.75	-11.74
-11.82	-11.81	-11.8	-11.79	-11.78	-11.77	-11.76	-11.75	-11.74
-11.82	-11.81	-11.8	-11.79	-11.78	-11.77	-11.76	-11.75	-11.74
-11.82	-11.81	-11.8	-11.79	-11.78	-11.77	-11.76	-11.75	-11.74
-11.82	-11.81	-11.8	-11.79	-11.78	-11.77	-11.76	-11.75	-11.74
-11.82	-11.81	-11.8	-11.79	-11.78	-11.77	-11.76	-11.75	-11.74
-11.82	-11.81	-11.8	-11.79	-11.78	-11.77	-11.76	-11.75	-11.74
-11.82	-11.81	-11.8	-11.79	-11.78	-11.77	-11.76	-11.75	-11.74
-11.82	-11.81	-11.8	-11.79	-11.78	-11.77	-11.76	-11.75	-11.74
-11.82	-11.81	-11.8	-11.79	-11.78	-11.77	-11.76	-11.75	-11.74
-11.82	-11.81	-11.8	-11.79	-11.78	-11.77	-11.76	-11.75	-11.74
-11.82	-11.81	-11.8	-11.79	-11.78	-11.77	-11.76	-11.75	-11.74
-11.82	-11.81	-11.8	-11.79	-11.78	-11.77	-11.76	-11.75	-11.74
-11.82	-11.81	-11.8	-11.79	-11.78	-11.77	-11.76	-11.75	-11.74
-11.82	-11.81	-11.8	-11.79	-11.78	-11.77	-11.76	-11.75	-11.74
-11.82	-11.81	-11.8	-11.79	-11.78	-11.77	-11.76	-11.75	-11.74
-11.82	-11.81	-11.8	-11.79	-11.78	-11.77	-11.76	-11.75	-11.74
-11.82	-11.81	-11.8	-11.79	-11.78	-11.77	-11.76	-11.75	-11.74
-11.82	-11.81	-11.8	-11.79	-11.78	-11.77	-11.76	-11.75	-11.74

-11.82	-11.81	-11.8	-11.79	-11.78	-11.77	-11.76	-11.75	-11.74
-11.82	-11.81	-11.8	-11.79	-11.78	-11.77	-11.76	-11.75	-11.74
-11.82	-11.81	-11.8	-11.79	-11.78	-11.77	-11.76	-11.75	-11.74
-11.82	-11.81	-11.8	-11.79	-11.78	-11.77	-11.76	-11.75	-11.74
-11.82	-11.81	-11.8	-11.79	-11.78	-11.77	-11.76	-11.75	-11.74
-11.82	-11.81	-11.8	-11.79	-11.78	-11.77	-11.76	-11.75	-11.74
-11.82	-11.81	-11.8	-11.79	-11.78	-11.77	-11.76	-11.75	-11.74
-11.82	-11.81	-11.8	-11.79	-11.78	-11.77	-11.76	-11.75	-11.74
-11.82	-11.81	-11.8	-11.79	-11.78	-11.77	-11.76	-11.75	-11.74
-11.82	-11.81	-11.8	-11.79	-11.78	-11.77	-11.76	-11.75	-11.74
-11.82	-11.81	-11.8	-11.79	-11.78	-11.77	-11.76	-11.75	-11.74
-11.82	-11.81	-11.8	-11.79	-11.78	-11.77	-11.76	-11.75	-11.74
-11.82	-11.81	-11.8	-11.79	-11.78	-11.77	-11.76	-11.75	-11.74
-11.82	-11.81	-11.8	-11.79	-11.78	-11.77	-11.76	-11.75	-11.74
-11.82	-11.81	-11.8	-11.79	-11.78	-11.77	-11.76	-11.75	-11.74
-11.82	-11.81	-11.8	-11.79	-11.78	-11.77	-11.76	-11.75	-11.74
-11.82	-11.81	-11.8	-11.79	-11.78	-11.77	-11.76	-11.75	-11.74
-11.82	-11.81	-11.8	-11.79	-11.78	-11.77	-11.76	-11.75	-11.74
-11.82	-11.81	-11.8	-11.79	-11.78	-11.77	-11.76	-11.75	-11.74
-11.82	-11.81	-11.8	-11.79	-11.78	-11.77	-11.76	-11.75	-11.74
-11.82	-11.81	-11.8	-11.79	-11.78	-11.77	-11.76	-11.75	-11.74
-11.82	-11.81	-11.8	-11.79	-11.78	-11.77	-11.76	-11.75	-11.74
-11.82	-11.81	-11.8	-11.79	-11.78	-11.77	-11.76	-11.75	-11.74
-11.82	-11.81	-11.8	-11.79	-11.78	-11.77	-11.76	-11.75	-11.74
-11.82	-11.81	-11.8	-11.79	-11.78	-11.77	-11.76	-11.75	-11.74
-11.82	-11.81	-11.8	-11.79	-11.78	-11.77	-11.76	-11.75	-11.74
-11.82	-11.81	-11.8	-11.79	-11.78	-11.77	-11.76	-11.75	-11.74
-11.82	-11.81	-11.8	-11.79	-11.78	-11.77	-11.76	-11.75	-11.74
-11.82	-11.81	-11.8	-11.79	-11.78	-11.77	-11.76	-11.75	-11.74
-11.82	-11.81	-11.8	-11.79	-11.78	-11.77	-11.76	-11.75	-11.74
-11.82	-11.81	-11.8	-11.79	-11.78	-11.77	-11.76	-11.75	-11.74
-11.82	-11.81	-11.8	-11.79	-11.78	-11.77	-11.76	-11.75	-11.74
-11.82	-11.81	-11.8	-11.79	-11.78	-11.77	-11.76	-11.75	-11.74
-11.82	-11.81	-11.8	-11.79	-11.78	-11.77	-11.76	-11.75	-11.74
-11.82	-11.81	-11.8	-11.79	-11.78	-11.77	-11.76	-11.75	-11.74
-11.82	-11.81	-11.8	-11.79	-11.78	-11.77	-11.76	-11.75	-11.74
-11.82	-11.81	-11.8	-11.79	-11.78	-11.77	-11.76	-11.75	-11.74
-11.82	-11.81	-11.8	-11.79	-11.78	-11.77	-11.76	-11.75	-11.74
-11.82	-11.81	-11.8	-11.79	-11.78	-11.77	-11.76	-11.75	-11.74
-11.82	-11.81	-11.8	-11.79	-11.78	-11.77	-11.76	-11.75	-11.74
-11.82	-11.81	-11.8	-11.79	-11.78	-11.77	-11.76	-11.75	-11.74
-11.82	-11.81	-11.8	-11.79	-11.78	-11.77	-11.76	-11.75	-11.74
-11.82	-11.81	-11.8	-11.79	-11.78	-11.77	-11.76	-11.75	-11.74
-11.82	-11.81	-11.8	-11.79	-11.78	-11.77	-11.76	-11.75	-11.74
-11.82	-11.81	-11.8	-11.79	-11.78	-11.77	-11.76	-11.75	-11.74
-11.82	-11.81	-11.8	-11.79	-11.78	-11.77	-11.76	-11.75	-11.74
-11.82	-11.81	-11.8	-11.79	-11.78	-11.77	-11.76	-11.75	-11.74
-11.82	-11.81	-11.8	-11.79	-11.78	-11.77	-11.76	-11.75	-11.74
-11.82	-11.81	-11.8	-11.79	-11.78	-11.77	-11.76	-11.75	-11.74
-11.82	-11.81	-11.8	-11.79	-11.78	-11.77	-11.76	-11.75	-11.74

-11.82	-11.81	-11.8	-11.79	-11.78	-11.77	-11.76	-11.75	-11.74
-11.82	-11.81	-11.8	-11.79	-11.78	-11.77	-11.76	-11.75	-11.74
-11.82	-11.81	-11.8	-11.79	-11.78	-11.77	-11.76	-11.75	-11.74
-11.82	-11.81	-11.8	-11.79	-11.78	-11.77	-11.76	-11.75	-11.74
-11.82	-11.81	-11.8	-11.79	-11.78	-11.77	-11.76	-11.75	-11.74
-11.82	-11.81	-11.8	-11.79	-11.78	-11.77	-11.76	-11.75	-11.74
-11.82	-11.81	-11.8	-11.79	-11.78	-11.77	-11.76	-11.75	-11.74
-11.82	-11.81	-11.8	-11.79	-11.78	-11.77	-11.76	-11.75	-11.74
-11.82	-11.81	-11.8	-11.79	-11.78	-11.77	-11.76	-11.75	-11.74
-11.82	-11.81	-11.8	-11.79	-11.78	-11.77	-11.76	-11.75	-11.74
-11.82	-11.81	-11.8	-11.79	-11.78	-11.77	-11.76	-11.75	-11.74
-11.82	-11.81	-11.8	-11.79	-11.78	-11.77	-11.76	-11.75	-11.74
-11.82	-11.81	-11.8	-11.79	-11.78	-11.77	-11.76	-11.75	-11.74
-11.82	-11.81	-11.8	-11.79	-11.78	-11.77	-11.76	-11.75	-11.74
-11.82	-11.81	-11.8	-11.79	-11.78	-11.77	-11.76	-11.75	-11.74
-11.82	-11.81	-11.8	-11.79	-11.78	-11.77	-11.76	-11.75	-11.74
-11.82	-11.81	-11.8	-11.79	-11.78	-11.77	-11.76	-11.75	-11.74
-11.82	-11.81	-11.8	-11.79	-11.78	-11.77	-11.76	-11.75	-11.74
-11.82	-11.81	-11.8	-11.79	-11.78	-11.77	-11.76	-11.75	-11.74
-11.82	-11.81	-11.8	-11.79	-11.78	-11.77	-11.76	-11.75	-11.74
-11.82	-11.81	-11.8	-11.79	-11.78	-11.77	-11.76	-11.75	-11.74
-11.82	-11.81	-11.8	-11.79	-11.78	-11.77	-11.76	-11.75	-11.74
-11.82	-11.81	-11.8	-11.79	-11.78	-11.77	-11.76	-11.75	-11.74
-11.82	-11.81	-11.8	-11.79	-11.78	-11.77	-11.76	-11.75	-11.74
-11.82	-11.81	-11.8	-11.79	-11.78	-11.77	-11.76	-11.75	-11.74
-11.82	-11.81	-11.8	-11.79	-11.78	-11.77	-11.76	-11.75	-11.74
-11.82	-11.81	-11.8	-11.79	-11.78	-11.77	-11.76	-11.75	-11.74
-11.82	-11.81	-11.8	-11.79	-11.78	-11.77	-11.76	-11.75	-11.74
-11.82	-11.81	-11.8	-11.79	-11.78	-11.77	-11.76	-11.75	-11.74
-11.82	-11.81	-11.8	-11.79	-11.78	-11.77	-11.76	-11.75	-11.74
-11.82	-11.81	-11.8	-11.79	-11.78	-11.77	-11.76	-11.75	-11.74
-11.82	-11.81	-11.8	-11.79	-11.78	-11.77	-11.76	-11.75	-11.74
-11.82	-11.81	-11.8	-11.79	-11.78	-11.77	-11.76	-11.75	-11.74
-11.82	-11.81	-11.8	-11.79	-11.78	-11.77	-11.76	-11.75	-11.74
-11.82	-11.81	-11.8	-11.79	-11.78	-11.77	-11.76	-11.75	-11.74
-11.82	-11.81	-11.8	-11.79	-11.78	-11.77	-11.76	-11.75	-11.74
-11.82	-11.81	-11.8	-11.79	-11.78	-11.77	-11.76	-11.75	-11.74
-11.82	-11.81	-11.8	-11.79	-11.78	-11.77	-11.76	-11.75	-11.74
-11.82	-11.81	-11.8	-11.79	-11.78	-11.77	-11.76	-11.75	-11.74
-11.82	-11.81	-11.8	-11.79	-11.78	-11.77	-11.76	-11.75	-11.74
-11.82	-11.81	-11.8	-11.79	-11.78	-11.77	-11.76	-11.75	-11.74
-11.82	-11.81	-11.8	-11.79	-11.78	-11.77	-11.76	-11.75	-11.74
-11.82	-11.81	-11.8	-11.79	-11.78	-11.77	-11.76	-11.75	-11.74
-11.82	-11.81	-11.8	-11.79	-11.78	-11.77	-11.76	-11.75	-11.74
-11.82	-11.81	-11.8	-11.79	-11.78	-11.77	-11.76	-11.75	-11.74
-11.82	-11.81	-11.8	-11.79	-11.78	-11.77	-11.76	-11.75	-11.74
-11.82	-11.81	-11.8	-11.79	-11.78	-11.77	-11.76	-11.75	-11.74
-11.82	-11.81	-11.8	-11.79	-11.78	-11.77	-11.76	-11.75	-11.74
-11.82	-11.81	-11.8	-11.79	-11.78	-11.77	-11.76	-11.75	-11.74
-11.82	-11.81	-11.8	-11.79	-11.78	-11.77	-11.76	-11.75	-11.74

-11.82	-11.81	-11.8	-11.79	-11.78	-11.77	-11.76	-11.75	-11.74
-11.82	-11.81	-11.8	-11.79	-11.78	-11.77	-11.76	-11.75	-11.74
-11.82	-11.81	-11.8	-11.79	-11.78	-11.77	-11.76	-11.75	-11.74
-11.82	-11.81	-11.8	-11.79	-11.78	-11.77	-11.76	-11.75	-11.74
-11.82	-11.81	-11.8	-11.79	-11.78	-11.77	-11.76	-11.75	-11.74
-11.82	-11.81	-11.8	-11.79	-11.78	-11.77	-11.76	-11.75	-11.74
-11.82	-11.81	-11.8	-11.79	-11.78	-11.77	-11.76	-11.75	-11.74
-11.82	-11.81	-11.8	-11.79	-11.78	-11.77	-11.76	-11.75	-11.74
-11.82	-11.81	-11.8	-11.79	-11.78	-11.77	-11.76	-11.75	-11.74
-11.82	-11.81	-11.8	-11.79	-11.78	-11.77	-11.76	-11.75	-11.74
-11.82	-11.81	-11.8	-11.79	-11.78	-11.77	-11.76	-11.75	-11.74
-11.82	-11.81	-11.8	-11.79	-11.78	-11.77	-11.76	-11.75	-11.74
-11.82	-11.81	-11.8	-11.79	-11.78	-11.77	-11.76	-11.75	-11.74
-11.82	-11.81	-11.8	-11.79	-11.78	-11.77	-11.76	-11.75	-11.74
-11.82	-11.81	-11.8	-11.79	-11.78	-11.77	-11.76	-11.75	-11.74
-11.82	-11.81	-11.8	-11.79	-11.78	-11.77	-11.76	-11.75	-11.74
-11.82	-11.81	-11.8	-11.79	-11.78	-11.77	-11.76	-11.75	-11.74
-11.82	-11.81	-11.8	-11.79	-11.78	-11.77	-11.76	-11.75	-11.74
-11.82	-11.81	-11.8	-11.79	-11.78	-11.77	-11.76	-11.75	-11.74
-11.82	-11.81	-11.8	-11.79	-11.78	-11.77	-11.76	-11.75	-11.74
-11.82	-11.81	-11.8	-11.79	-11.78	-11.77	-11.76	-11.75	-11.74
-11.82	-11.81	-11.8	-11.79	-11.78	-11.77	-11.76	-11.75	-11.74
-11.82	-11.81	-11.8	-11.79	-11.78	-11.77	-11.76	-11.75	-11.74
-11.82	-11.81	-11.8	-11.79	-11.78	-11.77	-11.76	-11.75	-11.74
-11.82	-11.81	-11.8	-11.79	-11.78	-11.77	-11.76	-11.75	-11.74
-11.82	-11.81	-11.8	-11.79	-11.78	-11.77	-11.76	-11.75	-11.74
-11.82	-11.81	-11.8	-11.79	-11.78	-11.77	-11.76	-11.75	-11.74
-11.82	-11.81	-11.8	-11.79	-11.78	-11.77	-11.76	-11.75	-11.74
-11.82	-11.81	-11.8	-11.79	-11.78	-11.77	-11.76	-11.75	-11.74
-11.82	-11.81	-11.8	-11.79	-11.78	-11.77	-11.76	-11.75	-11.74
-11.82	-11.81	-11.8	-11.79	-11.78	-11.77	-11.76	-11.75	-11.74
-11.82	-11.81	-11.8	-11.79	-11.78	-11.77	-11.76	-11.75	-11.74
-11.82	-11.81	-11.8	-11.79	-11.78	-11.77	-11.76	-11.75	-11.74
-11.82	-11.81	-11.8	-11.79	-11.78	-11.77	-11.76	-11.75	-11.74
-11.82	-11.81	-11.8	-11.79	-11.78	-11.77	-11.76	-11.75	-11.74
-11.82	-11.81	-11.8	-11.79	-11.78	-11.77	-11.76	-11.75	-11.74
-11.82	-11.81	-11.8	-11.79	-11.78	-11.77	-11.76	-11.75	-11.74
-11.82	-11.81	-11.8	-11.79	-11.78	-11.77	-11.76	-11.75	-11.74
-11.82	-11.81	-11.8	-11.79	-11.78	-11.77	-11.76	-11.75	-11.74
-11.82	-11.81	-11.8	-11.79	-11.78	-11.77	-11.76	-11.75	-11.74
-11.82	-11.81	-11.8	-11.79	-11.78	-11.77	-11.76	-11.75	-11.74
-11.82	-11.81	-11.8	-11.79	-11.78	-11.77	-11.76	-11.75	-11.74
-11.82	-11.81	-11.8	-11.79	-11.78	-11.77	-11.76	-11.75	-11.74
-11.82	-11.81	-11.8	-11.79	-11.78	-11.77	-11.76	-11.75	-11.74
-11.82	-11.81	-11.8	-11.79	-11.78	-11.77	-11.76	-11.75	-11.74
-11.82	-11.81	-11.8	-11.79	-11.78	-11.77	-11.76	-11.75	-11.74
-11.82	-11.81	-11.8	-11.79	-11.78	-11.77	-11.76	-11.75	-11.74
-11.82	-11.81	-11.8	-11.79	-11.78	-11.77	-11.76	-11.75	-11.74
-11.82	-11.81	-11.8	-11.79	-11.78	-11.77	-11.76	-11.75	-11.74
-11.82	-11.81	-11.8	-11.79	-11.78	-11.77	-11.76	-11.75	-11.74

-11.82	-11.81	-11.8	-11.79	-11.78	-11.77	-11.76	-11.75	-11.74
-11.82	-11.81	-11.8	-11.79	-11.78	-11.77	-11.76	-11.75	-11.74
-11.82	-11.81	-11.8	-11.79	-11.78	-11.77	-11.76	-11.75	-11.74
-11.82	-11.81	-11.8	-11.79	-11.78	-11.77	-11.76	-11.75	-11.74
-11.82	-11.81	-11.8	-11.79	-11.78	-11.77	-11.76	-11.75	-11.74
-11.82	-11.81	-11.8	-11.79	-11.78	-11.77	-11.76	-11.75	-11.74
-11.82	-11.81	-11.8	-11.79	-11.78	-11.77	-11.76	-11.75	-11.74
-11.82	-11.81	-11.8	-11.79	-11.78	-11.77	-11.76	-11.75	-11.74
-11.82	-11.81	-11.8	-11.79	-11.78	-11.77	-11.76	-11.75	-11.74
-11.82	-11.81	-11.8	-11.79	-11.78	-11.77	-11.76	-11.75	-11.74
-11.82	-11.81	-11.8	-11.79	-11.78	-11.77	-11.76	-11.75	-11.74
-11.82	-11.81	-11.8	-11.79	-11.78	-11.77	-11.76	-11.75	-11.74
-11.82	-11.81	-11.8	-11.79	-11.78	-11.77	-11.76	-11.75	-11.74
-11.82	-11.81	-11.8	-11.79	-11.78	-11.77	-11.76	-11.75	-11.74
-11.82	-11.81	-11.8	-11.79	-11.78	-11.77	-11.76	-11.75	-11.74
-11.82	-11.81	-11.8	-11.79	-11.78	-11.77	-11.76	-11.75	-11.74
-11.82	-11.81	-11.8	-11.79	-11.78	-11.77	-11.76	-11.75	-11.74
-11.82	-11.81	-11.8	-11.79	-11.78	-11.77	-11.76	-11.75	-11.74
-11.82	-11.81	-11.8	-11.79	-11.78	-11.77	-11.76	-11.75	-11.74
-11.82	-11.81	-11.8	-11.79	-11.78	-11.77	-11.76	-11.75	-11.74
-11.82	-11.81	-11.8	-11.79	-11.78	-11.77	-11.76	-11.75	-11.74
-11.82	-11.81	-11.8	-11.79	-11.78	-11.77	-11.76	-11.75	-11.74
-11.82	-11.81	-11.8	-11.79	-11.78	-11.77	-11.76	-11.75	-11.74
-11.82	-11.81	-11.8	-11.79	-11.78	-11.77	-11.76	-11.75	-11.74
-11.82	-11.81	-11.8	-11.79	-11.78	-11.77	-11.76	-11.75	-11.74
-11.82	-11.81	-11.8	-11.79	-11.78	-11.77	-11.76	-11.75	-11.74
-11.82	-11.81	-11.8	-11.79	-11.78	-11.77	-11.76	-11.75	-11.74
-11.82	-11.81	-11.8	-11.79	-11.78	-11.77	-11.76	-11.75	-11.74
-11.82	-11.81	-11.8	-11.79	-11.78	-11.77	-11.76	-11.75	-11.74
-11.82	-11.81	-11.8	-11.79	-11.78	-11.77	-11.76	-11.75	-11.74
-11.82	-11.81	-11.8	-11.79	-11.78	-11.77	-11.76	-11.75	-11.74
-11.82	-11.81	-11.8	-11.79	-11.78	-11.77	-11.76	-11.75	-11.74
-11.82	-11.81	-11.8	-11.79	-11.78	-11.77	-11.76	-11.75	-11.74
-11.82	-11.81	-11.8	-11.79	-11.78	-11.77	-11.76	-11.75	-11.74
-11.82	-11.81	-11.8	-11.79	-11.78	-11.77	-11.76	-11.75	-11.74
-11.82	-11.81	-11.8	-11.79	-11.78	-11.77	-11.76	-11.75	-11.74
-11.82	-11.81	-11.8	-11.79	-11.78	-11.77	-11.76	-11.75	-11.74
-11.82	-11.81	-11.8	-11.79	-11.78	-11.77	-11.76	-11.75	-11.74
-11.82	-11.81	-11.8	-11.79	-11.78	-11.77	-11.76	-11.75	-11.74
-11.82	-11.81	-11.8	-11.79	-11.78	-11.77	-11.76	-11.75	-11.74
-11.82	-11.81	-11.8	-11.79	-11.78	-11.77	-11.76	-11.75	-11.74
-11.82	-11.81	-11.8	-11.79	-11.78	-11.77	-11.76	-11.75	-11.74
-11.82	-11.81	-11.8	-11.79	-11.78	-11.77	-11.76	-11.75	-11.74
-11.82	-11.81	-11.8	-11.79	-11.78	-11.77	-11.76	-11.75	-11.74
-11.82	-11.81	-11.8	-11.79	-11.78	-11.77	-11.76	-11.75	-11.74
-11.82	-11.81	-11.8	-11.79	-11.78	-11.77	-11.76	-11.75	-11.74
-11.82	-11.81	-11.8	-11.79	-11.78	-11.77	-11.76	-11.75	-11.74
-11.82	-11.81	-11.8	-11.79	-11.78	-11.77	-11.76	-11.75	-11.74
-11.82	-11.81	-11.8	-11.79	-11.78	-11.77	-11.76	-11.75	-11.74
-11.82	-11.81	-11.8	-11.79	-11.78	-11.77	-11.76	-11.75	-11.74

-11.82	-11.81	-11.8	-11.79	-11.78	-11.77	-11.76	-11.75	-11.74
-11.82	-11.81	-11.8	-11.79	-11.78	-11.77	-11.76	-11.75	-11.74
-11.82	-11.81	-11.8	-11.79	-11.78	-11.77	-11.76	-11.75	-11.74
-11.82	-11.81	-11.8	-11.79	-11.78	-11.77	-11.76	-11.75	-11.74
-11.82	-11.81	-11.8	-11.79	-11.78	-11.77	-11.76	-11.75	-11.74
-11.82	-11.81	-11.8	-11.79	-11.78	-11.77	-11.76	-11.75	-11.74
-11.82	-11.81	-11.8	-11.79	-11.78	-11.77	-11.76	-11.75	-11.74
-11.82	-11.81	-11.8	-11.79	-11.78	-11.77	-11.76	-11.75	-11.74
-11.82	-11.81	-11.8	-11.79	-11.78	-11.77	-11.76	-11.75	-11.74
-11.82	-11.81	-11.8	-11.79	-11.78	-11.77	-11.76	-11.75	-11.74
-11.82	-11.81	-11.8	-11.79	-11.78	-11.77	-11.76	-11.75	-11.74
-11.82	-11.81	-11.8	-11.79	-11.78	-11.77	-11.76	-11.75	-11.74
-11.82	-11.81	-11.8	-11.79	-11.78	-11.77	-11.76	-11.75	-11.74
-11.82	-11.81	-11.8	-11.79	-11.78	-11.77	-11.76	-11.75	-11.74
-11.82	-11.81	-11.8	-11.79	-11.78	-11.77	-11.76	-11.75	-11.74
-11.82	-11.81	-11.8	-11.79	-11.78	-11.77	-11.76	-11.75	-11.74
-11.82	-11.81	-11.8	-11.79	-11.78	-11.77	-11.76	-11.75	-11.74
-11.82	-11.81	-11.8	-11.79	-11.78	-11.77	-11.76	-11.75	-11.74
-11.82	-11.81	-11.8	-11.79	-11.78	-11.77	-11.76	-11.75	-11.74
-11.82	-11.81	-11.8	-11.79	-11.78	-11.77	-11.76	-11.75	-11.74
-11.82	-11.81	-11.8	-11.79	-11.78	-11.77	-11.76	-11.75	-11.74
-11.82	-11.81	-11.8	-11.79	-11.78	-11.77	-11.76	-11.75	-11.74
-11.82	-11.81	-11.8	-11.79	-11.78	-11.77	-11.76	-11.75	-11.74
-11.82	-11.81	-11.8	-11.79	-11.78	-11.77	-11.76	-11.75	-11.74
-11.82	-11.81	-11.8	-11.79	-11.78	-11.77	-11.76	-11.75	-11.74
-11.82	-11.81	-11.8	-11.79	-11.78	-11.77	-11.76	-11.75	-11.74
-11.82	-11.81	-11.8	-11.79	-11.78	-11.77	-11.76	-11.75	-11.74
-11.82	-11.81	-11.8	-11.79	-11.78	-11.77	-11.76	-11.75	-11.74
-11.82	-11.81	-11.8	-11.79	-11.78	-11.77	-11.76	-11.75	-11.74
-11.82	-11.81	-11.8	-11.79	-11.78	-11.77	-11.76	-11.75	-11.74
-11.82	-11.81	-11.8	-11.79	-11.78	-11.77	-11.76	-11.75	-11.74
-11.82	-11.81	-11.8	-11.79	-11.78	-11.77	-11.76	-11.75	-11.74
-11.82	-11.81	-11.8	-11.79	-11.78	-11.77	-11.76	-11.75	-11.74
-11.82	-11.81	-11.8	-11.79	-11.78	-11.77	-11.76	-11.75	-11.74
-11.82	-11.81	-11.8	-11.79	-11.78	-11.77	-11.76	-11.75	-11.74
-11.82	-11.81	-11.8	-11.79	-11.78	-11.77	-11.76	-11.75	-11.74
-11.82	-11.81	-11.8	-11.79	-11.78	-11.77	-11.76	-11.75	-11.74
-11.82	-11.81	-11.8	-11.79	-11.78	-11.77	-11.76	-11.75	-11.74
-11.82	-11.81	-11.8	-11.79	-11.78	-11.77	-11.76	-11.75	-11.74
-11.82	-11.81	-11.8	-11.79	-11.78	-11.77	-11.76	-11.75	-11.74
-11.82	-11.81	-11.8	-11.79	-11.78	-11.77	-11.76	-11.75	-11.74
-11.82	-11.81	-11.8	-11.79	-11.78	-11.77	-11.76	-11.75	-11.74
-11.82	-11.81	-11.8	-11.79	-11.78	-11.77	-11.76	-11.75	-11.74
-11.82	-11.81	-11.8	-11.79	-11.78	-11.77	-11.76	-11.75	-11.74
-11.82	-11.81	-11.8	-11.79	-11.78	-11.77	-11.76	-11.75	-11.74
-11.82	-11.81	-11.8	-11.79	-11.78	-11.77	-11.76	-11.75	-11.74
-11.82	-11.81	-11.8	-11.79	-11.78	-11.77	-11.76	-11.75	-11.74
-11.82	-11.81	-11.8	-11.79	-11.78	-11.77	-11.76	-11.75	-11.74
-11.82	-11.81	-11.8	-11.79	-11.78	-11.77	-11.76	-11.75	-11.74
-11.82	-11.81	-11.8	-11.79	-11.78	-11.77	-11.76	-11.75	-11.74

-11.82	-11.81	-11.8	-11.79	-11.78	-11.77	-11.76	-11.75	-11.74
-11.82	-11.81	-11.8	-11.79	-11.78	-11.77	-11.76	-11.75	-11.74
-11.82	-11.81	-11.8	-11.79	-11.78	-11.77	-11.76	-11.75	-11.74
-11.82	-11.81	-11.8	-11.79	-11.78	-11.77	-11.76	-11.75	-11.74
-11.82	-11.81	-11.8	-11.79	-11.78	-11.77	-11.76	-11.75	-11.74
-11.82	-11.81	-11.8	-11.79	-11.78	-11.77	-11.76	-11.75	-11.74
-11.82	-11.81	-11.8	-11.79	-11.78	-11.77	-11.76	-11.75	-11.74
-11.82	-11.81	-11.8	-11.79	-11.78	-11.77	-11.76	-11.75	-11.74
-11.82	-11.81	-11.8	-11.79	-11.78	-11.77	-11.76	-11.75	-11.74
-11.82	-11.81	-11.8	-11.79	-11.78	-11.77	-11.76	-11.75	-11.74
-11.82	-11.81	-11.8	-11.79	-11.78	-11.77	-11.76	-11.75	-11.74
-11.82	-11.81	-11.8	-11.79	-11.78	-11.77	-11.76	-11.75	-11.74
-11.82	-11.81	-11.8	-11.79	-11.78	-11.77	-11.76	-11.75	-11.74
-11.82	-11.81	-11.8	-11.79	-11.78	-11.77	-11.76	-11.75	-11.74
-11.82	-11.81	-11.8	-11.79	-11.78	-11.77	-11.76	-11.75	-11.74
-11.82	-11.81	-11.8	-11.79	-11.78	-11.77	-11.76	-11.75	-11.74
-11.82	-11.81	-11.8	-11.79	-11.78	-11.77	-11.76	-11.75	-11.74
-11.82	-11.81	-11.8	-11.79	-11.78	-11.77	-11.76	-11.75	-11.74
-11.82	-11.81	-11.8	-11.79	-11.78	-11.77	-11.76	-11.75	-11.74
-11.82	-11.81	-11.8	-11.79	-11.78	-11.77	-11.76	-11.75	-11.74
-11.82	-11.81	-11.8	-11.79	-11.78	-11.77	-11.76	-11.75	-11.74
-11.82	-11.81	-11.8	-11.79	-11.78	-11.77	-11.76	-11.75	-11.74
-11.82	-11.81	-11.8	-11.79	-11.78	-11.77	-11.76	-11.75	-11.74
-11.82	-11.81	-11.8	-11.79	-11.78	-11.77	-11.76	-11.75	-11.74
-11.82	-11.81	-11.8	-11.79	-11.78	-11.77	-11.76	-11.75	-11.74
-11.82	-11.81	-11.8	-11.79	-11.78	-11.77	-11.76	-11.75	-11.74
-11.82	-11.81	-11.8	-11.79	-11.78	-11.77	-11.76	-11.75	-11.74
-11.82	-11.81	-11.8	-11.79	-11.78	-11.77	-11.76	-11.75	-11.74
-11.82	-11.81	-11.8	-11.79	-11.78	-11.77	-11.76	-11.75	-11.74
-11.82	-11.81	-11.8	-11.79	-11.78	-11.77	-11.76	-11.75	-11.74
-11.82	-11.81	-11.8	-11.79	-11.78	-11.77	-11.76	-11.75	-11.74
-11.82	-11.81	-11.8	-11.79	-11.78	-11.77	-11.76	-11.75	-11.74
-11.82	-11.81	-11.8	-11.79	-11.78	-11.77	-11.76	-11.75	-11.74
-11.82	-11.81	-11.8	-11.79	-11.78	-11.77	-11.76	-11.75	-11.74
-11.82	-11.81	-11.8	-11.79	-11.78	-11.77	-11.76	-11.75	-11.74
-11.82	-11.81	-11.8	-11.79	-11.78	-11.77	-11.76	-11.75	-11.74
-11.82	-11.81	-11.8	-11.79	-11.78	-11.77	-11.76	-11.75	-11.74
-11.82	-11.81	-11.8	-11.79	-11.78	-11.77	-11.76	-11.75	-11.74
-11.82	-11.81	-11.8	-11.79	-11.78	-11.77	-11.76	-11.75	-11.74
-11.82	-11.81	-11.8	-11.79	-11.78	-11.77	-11.76	-11.75	-11.74
-11.82	-11.81	-11.8	-11.79	-11.78	-11.77	-11.76	-11.75	-11.74
-11.82	-11.81	-11.8	-11.79	-11.78	-11.77	-11.76	-11.75	-11.74
-11.82	-11.81	-11.8	-11.79	-11.78	-11.77	-11.76	-11.75	-11.74
-11.82	-11.81	-11.8	-11.79	-11.78	-11.77	-11.76	-11.75	-11.74
-11.82	-11.81	-11.8	-11.79	-11.78	-11.77	-11.76	-11.75	-11.74
-11.82	-11.81	-11.8	-11.79	-11.78	-11.77	-11.76	-11.75	-11.74
-11.82	-11.81	-11.8	-11.79	-11.78	-11.77	-11.76	-11.75	-11.74
-11.82	-11.81	-11.8	-11.79	-11.78	-11.77	-11.76	-11.75	-11.74
-11.82	-11.81	-11.8	-11.79	-11.78	-11.77	-11.76	-11.75	-11.74
-11.82	-11.81	-11.8	-11.79	-11.78	-11.77	-11.76	-11.75	-11.74

-11.82	-11.81	-11.8	-11.79	-11.78	-11.77	-11.76	-11.75	-11.74
-11.82	-11.81	-11.8	-11.79	-11.78	-11.77	-11.76	-11.75	-11.74
-11.82	-11.81	-11.8	-11.79	-11.78	-11.77	-11.76	-11.75	-11.74
-11.82	-11.81	-11.8	-11.79	-11.78	-11.77	-11.76	-11.75	-11.74
-11.82	-11.81	-11.8	-11.79	-11.78	-11.77	-11.76	-11.75	-11.74
-11.82	-11.81	-11.8	-11.79	-11.78	-11.77	-11.76	-11.75	-11.74
-11.82	-11.81	-11.8	-11.79	-11.78	-11.77	-11.76	-11.75	-11.74
-11.82	-11.81	-11.8	-11.79	-11.78	-11.77	-11.76	-11.75	-11.74
-11.82	-11.81	-11.8	-11.79	-11.78	-11.77	-11.76	-11.75	-11.74
-11.82	-11.81	-11.8	-11.79	-11.78	-11.77	-11.76	-11.75	-11.74
-11.82	-11.81	-11.8	-11.79	-11.78	-11.77	-11.76	-11.75	-11.74
-11.82	-11.81	-11.8	-11.79	-11.78	-11.77	-11.76	-11.75	-11.74
-11.82	-11.81	-11.8	-11.79	-11.78	-11.77	-11.76	-11.75	-11.74
-11.82	-11.81	-11.8	-11.79	-11.78	-11.77	-11.76	-11.75	-11.74
-11.82	-11.81	-11.8	-11.79	-11.78	-11.77	-11.76	-11.75	-11.74
-11.82	-11.81	-11.8	-11.79	-11.78	-11.77	-11.76	-11.75	-11.74
-11.82	-11.81	-11.8	-11.79	-11.78	-11.77	-11.76	-11.75	-11.74
-11.82	-11.81	-11.8	-11.79	-11.78	-11.77	-11.76	-11.75	-11.74
-11.82	-11.81	-11.8	-11.79	-11.78	-11.77	-11.76	-11.75	-11.74
-11.82	-11.81	-11.8	-11.79	-11.78	-11.77	-11.76	-11.75	-11.74
-11.82	-11.81	-11.8	-11.79	-11.78	-11.77	-11.76	-11.75	-11.74
-11.82	-11.81	-11.8	-11.79	-11.78	-11.77	-11.76	-11.75	-11.74
-11.82	-11.81	-11.8	-11.79	-11.78	-11.77	-11.76	-11.75	-11.74
-11.82	-11.81	-11.8	-11.79	-11.78	-11.77	-11.76	-11.75	-11.74
-11.82	-11.81	-11.8	-11.79	-11.78	-11.77	-11.76	-11.75	-11.74
-11.82	-11.81	-11.8	-11.79	-11.78	-11.77	-11.76	-11.75	-11.74
-11.82	-11.81	-11.8	-11.79	-11.78	-11.77	-11.76	-11.75	-11.74
-11.82	-11.81	-11.8	-11.79	-11.78	-11.77	-11.76	-11.75	-11.74
-11.82	-11.81	-11.8	-11.79	-11.78	-11.77	-11.76	-11.75	-11.74
-11.82	-11.81	-11.8	-11.79	-11.78	-11.77	-11.76	-11.75	-11.74
-11.82	-11.81	-11.8	-11.79	-11.78	-11.77	-11.76	-11.75	-11.74
-11.82	-11.81	-11.8	-11.79	-11.78	-11.77	-11.76	-11.75	-11.74
-11.82	-11.81	-11.8	-11.79	-11.78	-11.77	-11.76	-11.75	-11.74
-11.82	-11.81	-11.8	-11.79	-11.78	-11.77	-11.76	-11.75	-11.74
-11.82	-11.81	-11.8	-11.79	-11.78	-11.77	-11.76	-11.75	-11.74
-11.82	-11.81	-11.8	-11.79	-11.78	-11.77	-11.76	-11.75	-11.74
-11.82	-11.81	-11.8	-11.79	-11.78	-11.77	-11.76	-11.75	-11.74
-11.82	-11.81	-11.8	-11.79	-11.78	-11.77	-11.76	-11.75	-11.74
-11.82	-11.81	-11.8	-11.79	-11.78	-11.77	-11.76	-11.75	-11.74
-11.82	-11.81	-11.8	-11.79	-11.78	-11.77	-11.76	-11.75	-11.74
-11.82	-11.81	-11.8	-11.79	-11.78	-11.77	-11.76	-11.75	-11.74
-11.82	-11.81	-11.8	-11.79	-11.78	-11.77	-11.76	-11.75	-11.74
-11.82	-11.81	-11.8	-11.79	-11.78	-11.77	-11.76	-11.75	-11.74
-11.82	-11.81	-11.8	-11.79	-11.78	-11.77	-11.76	-11.75	-11.74
-11.82	-11.81	-11.8	-11.79	-11.78	-11.77	-11.76	-11.75	-11.74
-11.82	-11.81	-11.8	-11.79	-11.78	-11.77	-11.76	-11.75	-11.74
-11.82	-11.81	-11.8	-11.79	-11.78	-11.77	-11.76	-11.75	-11.74
-11.82	-11.81	-11.8	-11.79	-11.78	-11.77	-11.76	-11.75	-11.74
-11.82	-11.81	-11.8	-11.79	-11.78	-11.77	-11.76	-11.75	-11.74
-11.82	-11.81	-11.8	-11.79	-11.78	-11.77	-11.76	-11.75	-11.74

-11.82	-11.81	-11.8	-11.79	-11.78	-11.77	-11.76	-11.75	-11.74
-11.82	-11.81	-11.8	-11.79	-11.78	-11.77	-11.76	-11.75	-11.74
-11.82	-11.81	-11.8	-11.79	-11.78	-11.77	-11.76	-11.75	-11.74
-11.82	-11.81	-11.8	-11.79	-11.78	-11.77	-11.76	-11.75	-11.74
-11.82	-11.81	-11.8	-11.79	-11.78	-11.77	-11.76	-11.75	-11.74
-11.82	-11.81	-11.8	-11.79	-11.78	-11.77	-11.76	-11.75	-11.74
-11.82	-11.81	-11.8	-11.79	-11.78	-11.77	-11.76	-11.75	-11.74
-11.82	-11.81	-11.8	-11.79	-11.78	-11.77	-11.76	-11.75	-11.74
-11.82	-11.81	-11.8	-11.79	-11.78	-11.77	-11.76	-11.75	-11.74
-11.82	-11.81	-11.8	-11.79	-11.78	-11.77	-11.76	-11.75	-11.74
-11.82	-11.81	-11.8	-11.79	-11.78	-11.77	-11.76	-11.75	-11.74
-11.82	-11.81	-11.8	-11.79	-11.78	-11.77	-11.76	-11.75	-11.74
-11.82	-11.81	-11.8	-11.79	-11.78	-11.77	-11.76	-11.75	-11.74
-11.82	-11.81	-11.8	-11.79	-11.78	-11.77	-11.76	-11.75	-11.74
-11.82	-11.81	-11.8	-11.79	-11.78	-11.77	-11.76	-11.75	-11.74
-11.82	-11.81	-11.8	-11.79	-11.78	-11.77	-11.76	-11.75	-11.74
-11.82	-11.81	-11.8	-11.79	-11.78	-11.77	-11.76	-11.75	-11.74
-11.82	-11.81	-11.8	-11.79	-11.78	-11.77	-11.76	-11.75	-11.74
-11.82	-11.81	-11.8	-11.79	-11.78	-11.77	-11.76	-11.75	-11.74
-11.82	-11.81	-11.8	-11.79	-11.78	-11.77	-11.76	-11.75	-11.74
-11.82	-11.81	-11.8	-11.79	-11.78	-11.77	-11.76	-11.75	-11.74
-11.82	-11.81	-11.8	-11.79	-11.78	-11.77	-11.76	-11.75	-11.74
-11.82	-11.81	-11.8	-11.79	-11.78	-11.77	-11.76	-11.75	-11.74
-11.82	-11.81	-11.8	-11.79	-11.78	-11.77	-11.76	-11.75	-11.74
-11.82	-11.81	-11.8	-11.79	-11.78	-11.77	-11.76	-11.75	-11.74
-11.82	-11.81	-11.8	-11.79	-11.78	-11.77	-11.76	-11.75	-11.74
-11.82	-11.81	-11.8	-11.79	-11.78	-11.77	-11.76	-11.75	-11.74
-11.82	-11.81	-11.8	-11.79	-11.78	-11.77	-11.76	-11.75	-11.74
-11.82	-11.81	-11.8	-11.79	-11.78	-11.77	-11.76	-11.75	-11.74
-11.82	-11.81	-11.8	-11.79	-11.78	-11.77	-11.76	-11.75	-11.74
-11.82	-11.81	-11.8	-11.79	-11.78	-11.77	-11.76	-11.75	-11.74
-11.82	-11.81	-11.8	-11.79	-11.78	-11.77	-11.76	-11.75	-11.74
-11.82	-11.81	-11.8	-11.79	-11.78	-11.77	-11.76	-11.75	-11.74
-11.82	-11.81	-11.8	-11.79	-11.78	-11.77	-11.76	-11.75	-11.74
-11.82	-11.81	-11.8	-11.79	-11.78	-11.77	-11.76	-11.75	-11.74
-11.82	-11.81	-11.8	-11.79	-11.78	-11.77	-11.76	-11.75	-11.74
-11.82	-11.81	-11.8	-11.79	-11.78	-11.77	-11.76	-11.75	-11.74
-11.82	-11.81	-11.8	-11.79	-11.78	-11.77	-11.76	-11.75	-11.74
-11.82	-11.81	-11.8	-11.79	-11.78	-11.77	-11.76	-11.75	-11.74
-11.82	-11.81	-11.8	-11.79	-11.78	-11.77	-11.76	-11.75	-11.74
-11.82	-11.81	-11.8	-11.79	-11.78	-11.77	-11.76	-11.75	-11.74
-11.82	-11.81	-11.8	-11.79	-11.78	-11.77	-11.76	-11.75	-11.74
-11.82	-11.81	-11.8	-11.79	-11.78	-11.77	-11.76	-11.75	-11.74
-11.82	-11.81	-11.8	-11.79	-11.78	-11.77	-11.76	-11.75	-11.74
-11.82	-11.81	-11.8	-11.79	-11.78	-11.77	-11.76	-11.75	-11.74
-11.82	-11.81	-11.8	-11.79	-11.78	-11.77	-11.76	-11.75	-11.74
-11.82	-11.81	-11.8	-11.79	-11.78	-11.77	-11.76	-11.75	-11.74
-11.82	-11.81	-11.8	-11.79	-11.78	-11.77	-11.76	-11.75	-11.74
-11.82	-11.81	-11.8	-11.79	-11.78	-11.77	-11.76	-11.75	-11.74
-11.82	-11.81	-11.8	-11.79	-11.78	-11.77	-11.76	-11.75	-11.74

-11.82	-11.81	-11.8	-11.79	-11.78	-11.77	-11.76	-11.75	-11.74
-11.82	-11.81	-11.8	-11.79	-11.78	-11.77	-11.76	-11.75	-11.74
-11.82	-11.81	-11.8	-11.79	-11.78	-11.77	-11.76	-11.75	-11.74
-11.82	-11.81	-11.8	-11.79	-11.78	-11.77	-11.76	-11.75	-11.74
-11.82	-11.81	-11.8	-11.79	-11.78	-11.77	-11.76	-11.75	-11.74
-11.82	-11.81	-11.8	-11.79	-11.78	-11.77	-11.76	-11.75	-11.74
-11.82	-11.81	-11.8	-11.79	-11.78	-11.77	-11.76	-11.75	-11.74
-11.82	-11.81	-11.8	-11.79	-11.78	-11.77	-11.76	-11.75	-11.74
-11.82	-11.81	-11.8	-11.79	-11.78	-11.77	-11.76	-11.75	-11.74
-11.82	-11.81	-11.8	-11.79	-11.78	-11.77	-11.76	-11.75	-11.74
-11.82	-11.81	-11.8	-11.79	-11.78	-11.77	-11.76	-11.75	-11.74
-11.82	-11.81	-11.8	-11.79	-11.78	-11.77	-11.76	-11.75	-11.74
-11.82	-11.81	-11.8	-11.79	-11.78	-11.77	-11.76	-11.75	-11.74
-11.82	-11.81	-11.8	-11.79	-11.78	-11.77	-11.76	-11.75	-11.74
-11.82	-11.81	-11.8	-11.79	-11.78	-11.77	-11.76	-11.75	-11.74
-11.82	-11.81	-11.8	-11.79	-11.78	-11.77	-11.76	-11.75	-11.74
-11.82	-11.81	-11.8	-11.79	-11.78	-11.77	-11.76	-11.75	-11.74
-11.82	-11.81	-11.8	-11.79	-11.78	-11.77	-11.76	-11.75	-11.74
-11.82	-11.81	-11.8	-11.79	-11.78	-11.77	-11.76	-11.75	-11.74
-11.82	-11.81	-11.8	-11.79	-11.78	-11.77	-11.76	-11.75	-11.74
-11.82	-11.81	-11.8	-11.79	-11.78	-11.77	-11.76	-11.75	-11.74
-11.82	-11.81	-11.8	-11.79	-11.78	-11.77	-11.76	-11.75	-11.74
-11.82	-11.81	-11.8	-11.79	-11.78	-11.77	-11.76	-11.75	-11.74
-11.82	-11.81	-11.8	-11.79	-11.78	-11.77	-11.76	-11.75	-11.74
-11.82	-11.81	-11.8	-11.79	-11.78	-11.77	-11.76	-11.75	-11.74
-11.82	-11.81	-11.8	-11.79	-11.78	-11.77	-11.76	-11.75	-11.74
-11.82	-11.81	-11.8	-11.79	-11.78	-11.77	-11.76	-11.75	-11.74
-11.82	-11.81	-11.8	-11.79	-11.78	-11.77	-11.76	-11.75	-11.74
-11.82	-11.81	-11.8	-11.79	-11.78	-11.77	-11.76	-11.75	-11.74
-11.82	-11.81	-11.8	-11.79	-11.78	-11.77	-11.76	-11.75	-11.74
-11.82	-11.81	-11.8	-11.79	-11.78	-11.77	-11.76	-11.75	-11.74
-11.82	-11.81	-11.8	-11.79	-11.78	-11.77	-11.76	-11.75	-11.74
-11.82	-11.81	-11.8	-11.79	-11.78	-11.77	-11.76	-11.75	-11.74
-11.82	-11.81	-11.8	-11.79	-11.78	-11.77	-11.76	-11.75	-11.74
-11.82	-11.81	-11.8	-11.79	-11.78	-11.77	-11.76	-11.75	-11.74
-11.82	-11.81	-11.8	-11.79	-11.78	-11.77	-11.76	-11.75	-11.74
-11.82	-11.81	-11.8	-11.79	-11.78	-11.77	-11.76	-11.75	-11.74
-11.82	-11.81	-11.8	-11.79	-11.78	-11.77	-11.76	-11.75	-11.74
-11.82	-11.81	-11.8	-11.79	-11.78	-11.77	-11.76	-11.75	-11.74
-11.82	-11.81	-11.8	-11.79	-11.78	-11.77	-11.76	-11.75	-11.74
-11.82	-11.81	-11.8	-11.79	-11.78	-11.77	-11.76	-11.75	-11.74
-11.82	-11.81	-11.8	-11.79	-11.78	-11.77	-11.76	-11.75	-11.74
-11.82	-11.81	-11.8	-11.79	-11.78	-11.77	-11.76	-11.75	-11.74
-11.82	-11.81	-11.8	-11.79	-11.78	-11.77	-11.76	-11.75	-11.74
-11.82	-11.81	-11.8	-11.79	-11.78	-11.77	-11.76	-11.75	-11.74
-11.82	-11.81	-11.8	-11.79	-11.78	-11.77	-11.76	-11.75	-11.74
-11.82	-11.81	-11.8	-11.79	-11.78	-11.77	-11.76	-11.75	-11.74
-11.82	-11.81	-11.8	-11.79	-11.78	-11.77	-11.76	-11.75	-11.74
-11.82	-11.81	-11.8	-11.79	-11.78	-11.77	-11.76	-11.75	-11.74
-11.82	-11.81	-11.8	-11.79	-11.78	-11.77	-11.76	-11.75	-11.74

-11.82	-11.81	-11.8	-11.79	-11.78	-11.77	-11.76	-11.75	-11.74
-11.82	-11.81	-11.8	-11.79	-11.78	-11.77	-11.76	-11.75	-11.74
-11.82	-11.81	-11.8	-11.79	-11.78	-11.77	-11.76	-11.75	-11.74
-11.82	-11.81	-11.8	-11.79	-11.78	-11.77	-11.76	-11.75	-11.74
-11.82	-11.81	-11.8	-11.79	-11.78	-11.77	-11.76	-11.75	-11.74
-11.82	-11.81	-11.8	-11.79	-11.78	-11.77	-11.76	-11.75	-11.74
-11.82	-11.81	-11.8	-11.79	-11.78	-11.77	-11.76	-11.75	-11.74
-11.82	-11.81	-11.8	-11.79	-11.78	-11.77	-11.76	-11.75	-11.74
-11.82	-11.81	-11.8	-11.79	-11.78	-11.77	-11.76	-11.75	-11.74
-11.82	-11.81	-11.8	-11.79	-11.78	-11.77	-11.76	-11.75	-11.74
-11.82	-11.81	-11.8	-11.79	-11.78	-11.77	-11.76	-11.75	-11.74
-11.82	-11.81	-11.8	-11.79	-11.78	-11.77	-11.76	-11.75	-11.74
-11.82	-11.81	-11.8	-11.79	-11.78	-11.77	-11.76	-11.75	-11.74
-11.82	-11.81	-11.8	-11.79	-11.78	-11.77	-11.76	-11.75	-11.74
-11.82	-11.81	-11.8	-11.79	-11.78	-11.77	-11.76	-11.75	-11.74
-11.82	-11.81	-11.8	-11.79	-11.78	-11.77	-11.76	-11.75	-11.74
-11.82	-11.81	-11.8	-11.79	-11.78	-11.77	-11.76	-11.75	-11.74
-11.82	-11.81	-11.8	-11.79	-11.78	-11.77	-11.76	-11.75	-11.74
-11.82	-11.81	-11.8	-11.79	-11.78	-11.77	-11.76	-11.75	-11.74
-11.82	-11.81	-11.8	-11.79	-11.78	-11.77	-11.76	-11.75	-11.74
-11.82	-11.81	-11.8	-11.79	-11.78	-11.77	-11.76	-11.75	-11.74
-11.82	-11.81	-11.8	-11.79	-11.78	-11.77	-11.76	-11.75	-11.74
-11.82	-11.81	-11.8	-11.79	-11.78	-11.77	-11.76	-11.75	-11.74
-11.82	-11.81	-11.8	-11.79	-11.78	-11.77	-11.76	-11.75	-11.74
-11.82	-11.81	-11.8	-11.79	-11.78	-11.77	-11.76	-11.75	-11.74
-11.82	-11.81	-11.8	-11.79	-11.78	-11.77	-11.76	-11.75	-11.74
-11.82	-11.81	-11.8	-11.79	-11.78	-11.77	-11.76	-11.75	-11.74
-11.82	-11.81	-11.8	-11.79	-11.78	-11.77	-11.76	-11.75	-11.74
-11.82	-11.81	-11.8	-11.79	-11.78	-11.77	-11.76	-11.75	-11.74
-11.82	-11.81	-11.8	-11.79	-11.78	-11.77	-11.76	-11.75	-11.74
-11.82	-11.81	-11.8	-11.79	-11.78	-11.77	-11.76	-11.75	-11.74
-11.82	-11.81	-11.8	-11.79	-11.78	-11.77	-11.76	-11.75	-11.74
-11.82	-11.81	-11.8	-11.79	-11.78	-11.77	-11.76	-11.75	-11.74
-11.82	-11.81	-11.8	-11.79	-11.78	-11.77	-11.76	-11.75	-11.74
-11.82	-11.81	-11.8	-11.79	-11.78	-11.77	-11.76	-11.75	-11.74
-11.82	-11.81	-11.8	-11.79	-11.78	-11.77	-11.76	-11.75	-11.74
-11.82	-11.81	-11.8	-11.79	-11.78	-11.77	-11.76	-11.75	-11.74
-11.82	-11.81	-11.8	-11.79	-11.78	-11.77	-11.76	-11.75	-11.74
-11.82	-11.81	-11.8	-11.79	-11.78	-11.77	-11.76	-11.75	-11.74
-11.82	-11.81	-11.8	-11.79	-11.78	-11.77	-11.76	-11.75	-11.74
-11.82	-11.81	-11.8	-11.79	-11.78	-11.77	-11.76	-11.75	-11.74
-11.82	-11.81	-11.8	-11.79	-11.78	-11.77	-11.76	-11.75	-11.74
-11.82	-11.81	-11.8	-11.79	-11.78	-11.77	-11.76	-11.75	-11.74
-11.82	-11.81	-11.8	-11.79	-11.78	-11.77	-11.76	-11.75	-11.74
-11.82	-11.81	-11.8	-11.79	-11.78	-11.77	-11.76	-11.75	-11.74
-11.82	-11.81	-11.8	-11.79	-11.78	-11.77	-11.76	-11.75	-11.74
-11.82	-11.81	-11.8	-11.79	-11.78	-11.77	-11.76	-11.75	-11.74
-11.82	-11.81	-11.8	-11.79	-11.78	-11.77	-11.76	-11.75	-11.74
-11.82	-11.81	-11.8	-11.79	-11.78	-11.77	-11.76	-11.75	-11.74
-11.82	-11.81	-11.8	-11.79	-11.78	-11.77	-11.76	-11.75	-11.74

-11.82	-11.81	-11.8	-11.79	-11.78	-11.77	-11.76	-11.75	-11.74
-11.82	-11.81	-11.8	-11.79	-11.78	-11.77	-11.76	-11.75	-11.74
-11.82	-11.81	-11.8	-11.79	-11.78	-11.77	-11.76	-11.75	-11.74
-11.82	-11.81	-11.8	-11.79	-11.78	-11.77	-11.76	-11.75	-11.74
-11.82	-11.81	-11.8	-11.79	-11.78	-11.77	-11.76	-11.75	-11.74
-11.82	-11.81	-11.8	-11.79	-11.78	-11.77	-11.76	-11.75	-11.74
-11.82	-11.81	-11.8	-11.79	-11.78	-11.77	-11.76	-11.75	-11.74
-11.82	-11.81	-11.8	-11.79	-11.78	-11.77	-11.76	-11.75	-11.74
-11.82	-11.81	-11.8	-11.79	-11.78	-11.77	-11.76	-11.75	-11.74
-11.82	-11.81	-11.8	-11.79	-11.78	-11.77	-11.76	-11.75	-11.74
-11.82	-11.81	-11.8	-11.79	-11.78	-11.77	-11.76	-11.75	-11.74
-11.82	-11.81	-11.8	-11.79	-11.78	-11.77	-11.76	-11.75	-11.74
-11.82	-11.81	-11.8	-11.79	-11.78	-11.77	-11.76	-11.75	-11.74
-11.82	-11.81	-11.8	-11.79	-11.78	-11.77	-11.76	-11.75	-11.74
-11.82	-11.81	-11.8	-11.79	-11.78	-11.77	-11.76	-11.75	-11.74
-11.82	-11.81	-11.8	-11.79	-11.78	-11.77	-11.76	-11.75	-11.74
-11.82	-11.81	-11.8	-11.79	-11.78	-11.77	-11.76	-11.75	-11.74
-11.82	-11.81	-11.8	-11.79	-11.78	-11.77	-11.76	-11.75	-11.74
-11.82	-11.81	-11.8	-11.79	-11.78	-11.77	-11.76	-11.75	-11.74
-11.82	-11.81	-11.8	-11.79	-11.78	-11.77	-11.76	-11.75	-11.74
-11.82	-11.81	-11.8	-11.79	-11.78	-11.77	-11.76	-11.75	-11.74
-11.82	-11.81	-11.8	-11.79	-11.78	-11.77	-11.76	-11.75	-11.74
-11.82	-11.81	-11.8	-11.79	-11.78	-11.77	-11.76	-11.75	-11.74
-11.82	-11.81	-11.8	-11.79	-11.78	-11.77	-11.76	-11.75	-11.74
-11.82	-11.81	-11.8	-11.79	-11.78	-11.77	-11.76	-11.75	-11.74
-11.82	-11.81	-11.8	-11.79	-11.78	-11.77	-11.76	-11.75	-11.74
-11.82	-11.81	-11.8	-11.79	-11.78	-11.77	-11.76	-11.75	-11.74
-11.82	-11.81	-11.8	-11.79	-11.78	-11.77	-11.76	-11.75	-11.74
-11.82	-11.81	-11.8	-11.79	-11.78	-11.77	-11.76	-11.75	-11.74
-11.82	-11.81	-11.8	-11.79	-11.78	-11.77	-11.76	-11.75	-11.74
-11.82	-11.81	-11.8	-11.79	-11.78	-11.77	-11.76	-11.75	-11.74
-11.82	-11.81	-11.8	-11.79	-11.78	-11.77	-11.76	-11.75	-11.74
-11.82	-11.81	-11.8	-11.79	-11.78	-11.77	-11.76	-11.75	-11.74
-11.82	-11.81	-11.8	-11.79	-11.78	-11.77	-11.76	-11.75	-11.74
-11.82	-11.81	-11.8	-11.79	-11.78	-11.77	-11.76	-11.75	-11.74
-11.82	-11.81	-11.8	-11.79	-11.78	-11.77	-11.76	-11.75	-11.74
-11.82	-11.81	-11.8	-11.79	-11.78	-11.77	-11.76	-11.75	-11.74
-11.82	-11.81	-11.8	-11.79	-11.78	-11.77	-11.76	-11.75	-11.74
-11.82	-11.81	-11.8	-11.79	-11.78	-11.77	-11.76	-11.75	-11.74
-11.82	-11.81	-11.8	-11.79	-11.78	-11.77	-11.76	-11.75	-11.74
-11.82	-11.81	-11.8	-11.79	-11.78	-11.77	-11.76	-11.75	-11.74
-11.82	-11.81	-11.8	-11.79	-11.78	-11.77	-11.76	-11.75	-11.74
-11.82	-11.81	-11.8	-11.79	-11.78	-11.77	-11.76	-11.75	-11.74
-11.82	-11.81	-11.8	-11.79	-11.78	-11.77	-11.76	-11.75	-11.74
-11.82	-11.81	-11.8	-11.79	-11.78	-11.77	-11.76	-11.75	-11.74
-11.82	-11.81	-11.8	-11.79	-11.78	-11.77	-11.76	-11.75	-11.74
-11.82	-11.81	-11.8	-11.79	-11.78	-11.77	-11.76	-11.75	-11.74
-11.82	-11.81	-11.8	-11.79	-11.78	-11.77	-11.76	-11.75	-11.74
-11.82	-11.81	-11.8	-11.79	-11.78	-11.77	-11.76	-11.75	-11.74
-11.82	-11.81	-11.8	-11.79	-11.78	-11.77	-11.76	-11.75	-11.74

-11.82	-11.81	-11.8	-11.79	-11.78	-11.77	-11.76	-11.75	-11.74
-11.82	-11.81	-11.8	-11.79	-11.78	-11.77	-11.76	-11.75	-11.74
-11.82	-11.81	-11.8	-11.79	-11.78	-11.77	-11.76	-11.75	-11.74
-11.82	-11.81	-11.8	-11.79	-11.78	-11.77	-11.76	-11.75	-11.74
-11.82	-11.81	-11.8	-11.79	-11.78	-11.77	-11.76	-11.75	-11.74
-11.82	-11.81	-11.8	-11.79	-11.78	-11.77	-11.76	-11.75	-11.74
-11.82	-11.81	-11.8	-11.79	-11.78	-11.77	-11.76	-11.75	-11.74
-11.82	-11.81	-11.8	-11.79	-11.78	-11.77	-11.76	-11.75	-11.74
-11.82	-11.81	-11.8	-11.79	-11.78	-11.77	-11.76	-11.75	-11.74
-11.82	-11.81	-11.8	-11.79	-11.78	-11.77	-11.76	-11.75	-11.74
-11.82	-11.81	-11.8	-11.79	-11.78	-11.77	-11.76	-11.75	-11.74
-11.82	-11.81	-11.8	-11.79	-11.78	-11.77	-11.76	-11.75	-11.74
-11.82	-11.81	-11.8	-11.79	-11.78	-11.77	-11.76	-11.75	-11.74
-11.82	-11.81	-11.8	-11.79	-11.78	-11.77	-11.76	-11.75	-11.74
-11.82	-11.81	-11.8	-11.79	-11.78	-11.77	-11.76	-11.75	-11.74
-11.82	-11.81	-11.8	-11.79	-11.78	-11.77	-11.76	-11.75	-11.74
-11.82	-11.81	-11.8	-11.79	-11.78	-11.77	-11.76	-11.75	-11.74
-11.82	-11.81	-11.8	-11.79	-11.78	-11.77	-11.76	-11.75	-11.74
-11.82	-11.81	-11.8	-11.79	-11.78	-11.77	-11.76	-11.75	-11.74
-11.82	-11.81	-11.8	-11.79	-11.78	-11.77	-11.76	-11.75	-11.74
-11.82	-11.81	-11.8	-11.79	-11.78	-11.77	-11.76	-11.75	-11.74
-11.82	-11.81	-11.8	-11.79	-11.78	-11.77	-11.76	-11.75	-11.74
-11.82	-11.81	-11.8	-11.79	-11.78	-11.77	-11.76	-11.75	-11.74
-11.82	-11.81	-11.8	-11.79	-11.78	-11.77	-11.76	-11.75	-11.74
-11.82	-11.81	-11.8	-11.79	-11.78	-11.77	-11.76	-11.75	-11.74
-11.82	-11.81	-11.8	-11.79	-11.78	-11.77	-11.76	-11.75	-11.74
-11.82	-11.81	-11.8	-11.79	-11.78	-11.77	-11.76	-11.75	-11.74
-11.82	-11.81	-11.8	-11.79	-11.78	-11.77	-11.76	-11.75	-11.74
-11.82	-11.81	-11.8	-11.79	-11.78	-11.77	-11.76	-11.75	-11.74
-11.82	-11.81	-11.8	-11.79	-11.78	-11.77	-11.76	-11.75	-11.74
-11.82	-11.81	-11.8	-11.79	-11.78	-11.77	-11.76	-11.75	-11.74
-11.82	-11.81	-11.8	-11.79	-11.78	-11.77	-11.76	-11.75	-11.74
-11.82	-11.81	-11.8	-11.79	-11.78	-11.77	-11.76	-11.75	-11.74
-11.82	-11.81	-11.8	-11.79	-11.78	-11.77	-11.76	-11.75	-11.74
-11.82	-11.81	-11.8	-11.79	-11.78	-11.77	-11.76	-11.75	-11.74
-11.82	-11.81	-11.8	-11.79	-11.78	-11.77	-11.76	-11.75	-11.74
-11.82	-11.81	-11.8	-11.79	-11.78	-11.77	-11.76	-11.75	-11.74
-11.82	-11.81	-11.8	-11.79	-11.78	-11.77	-11.76	-11.75	-11.74
-11.82	-11.81	-11.8	-11.79	-11.78	-11.77	-11.76	-11.75	-11.74
-11.82	-11.81	-11.8	-11.79	-11.78	-11.77	-11.76	-11.75	-11.74
-11.82	-11.81	-11.8	-11.79	-11.78	-11.77	-11.76	-11.75	-11.74
-11.82	-11.81	-11.8	-11.79	-11.78	-11.77	-11.76	-11.75	-11.74
-11.82	-11.81	-11.8	-11.79	-11.78	-11.77	-11.76	-11.75	-11.74
-11.82	-11.81	-11.8	-11.79	-11.78	-11.77	-11.76	-11.75	-11.74
-11.82	-11.81	-11.8	-11.79	-11.78	-11.77	-11.76	-11.75	-11.74
-11.82	-11.81	-11.8	-11.79	-11.78	-11.77	-11.76	-11.75	-11.74
-11.82	-11.81	-11.8	-11.79	-11.78	-11.77	-11.76	-11.75	-11.74
-11.82	-11.81	-11.8	-11.79	-11.78	-11.77	-11.76	-11.75	-11.74
-11.82	-11.81	-11.8	-11.79	-11.78	-11.77	-11.76	-11.75	-11.74
-11.82	-11.81	-11.8	-11.79	-11.78	-11.77	-11.76	-11.75	-11.74

-11.82	-11.81	-11.8	-11.79	-11.78	-11.77	-11.76	-11.75	-11.74
-11.82	-11.81	-11.8	-11.79	-11.78	-11.77	-11.76	-11.75	-11.74
-11.82	-11.81	-11.8	-11.79	-11.78	-11.77	-11.76	-11.75	-11.74
-11.82	-11.81	-11.8	-11.79	-11.78	-11.77	-11.76	-11.75	-11.74
-11.82	-11.81	-11.8	-11.79	-11.78	-11.77	-11.76	-11.75	-11.74
-11.82	-11.81	-11.8	-11.79	-11.78	-11.77	-11.76	-11.75	-11.74
-11.82	-11.81	-11.8	-11.79	-11.78	-11.77	-11.76	-11.75	-11.74
-11.82	-11.81	-11.8	-11.79	-11.78	-11.77	-11.76	-11.75	-11.74
-11.82	-11.81	-11.8	-11.79	-11.78	-11.77	-11.76	-11.75	-11.74
-11.82	-11.81	-11.8	-11.79	-11.78	-11.77	-11.76	-11.75	-11.74
-11.82	-11.81	-11.8	-11.79	-11.78	-11.77	-11.76	-11.75	-11.74
-11.82	-11.81	-11.8	-11.79	-11.78	-11.77	-11.76	-11.75	-11.74
-11.82	-11.81	-11.8	-11.79	-11.78	-11.77	-11.76	-11.75	-11.74
-11.82	-11.81	-11.8	-11.79	-11.78	-11.77	-11.76	-11.75	-11.74
-11.82	-11.81	-11.8	-11.79	-11.78	-11.77	-11.76	-11.75	-11.74
-11.82	-11.81	-11.8	-11.79	-11.78	-11.77	-11.76	-11.75	-11.74
-11.82	-11.81	-11.8	-11.79	-11.78	-11.77	-11.76	-11.75	-11.74
-11.82	-11.81	-11.8	-11.79	-11.78	-11.77	-11.76	-11.75	-11.74
-11.82	-11.81	-11.8	-11.79	-11.78	-11.77	-11.76	-11.75	-11.74
-11.82	-11.81	-11.8	-11.79	-11.78	-11.77	-11.76	-11.75	-11.74
-11.82	-11.81	-11.8	-11.79	-11.78	-11.77	-11.76	-11.75	-11.74
-11.82	-11.81	-11.8	-11.79	-11.78	-11.77	-11.76	-11.75	-11.74
-11.82	-11.81	-11.8	-11.79	-11.78	-11.77	-11.76	-11.75	-11.74
-11.82	-11.81	-11.8	-11.79	-11.78	-11.77	-11.76	-11.75	-11.74
-11.82	-11.81	-11.8	-11.79	-11.78	-11.77	-11.76	-11.75	-11.74
-11.82	-11.81	-11.8	-11.79	-11.78	-11.77	-11.76	-11.75	-11.74
-11.82	-11.81	-11.8	-11.79	-11.78	-11.77	-11.76	-11.75	-11.74
-11.82	-11.81	-11.8	-11.79	-11.78	-11.77	-11.76	-11.75	-11.74
-11.82	-11.81	-11.8	-11.79	-11.78	-11.77	-11.76	-11.75	-11.74
-11.82	-11.81	-11.8	-11.79	-11.78	-11.77	-11.76	-11.75	-11.74
-11.82	-11.81	-11.8	-11.79	-11.78	-11.77	-11.76	-11.75	-11.74
-11.82	-11.81	-11.8	-11.79	-11.78	-11.77	-11.76	-11.75	-11.74
-11.82	-11.81	-11.8	-11.79	-11.78	-11.77	-11.76	-11.75	-11.74
-11.82	-11.81	-11.8	-11.79	-11.78	-11.77	-11.76	-11.75	-11.74
-11.82	-11.81	-11.8	-11.79	-11.78	-11.77	-11.76	-11.75	-11.74
-11.82	-11.81	-11.8	-11.79	-11.78	-11.77	-11.76	-11.75	-11.74
-11.82	-11.81	-11.8	-11.79	-11.78	-11.77	-11.76	-11.75	-11.74
-11.82	-11.81	-11.8	-11.79	-11.78	-11.77	-11.76	-11.75	-11.74
-11.82	-11.81	-11.8	-11.79	-11.78	-11.77	-11.76	-11.75	-11.74
-11.82	-11.81	-11.8	-11.79	-11.78	-11.77	-11.76	-11.75	-11.74
-11.82	-11.81	-11.8	-11.79	-11.78	-11.77	-11.76	-11.75	-11.74
-11.82	-11.81	-11.8	-11.79	-11.78	-11.77	-11.76	-11.75	-11.74
-11.82	-11.81	-11.8	-11.79	-11.78	-11.77	-11.76	-11.75	-11.74
-11.82	-11.81	-11.8	-11.79	-11.78	-11.77	-11.76	-11.75	-11.74
-11.82	-11.81	-11.8	-11.79	-11.78	-11.77	-11.76	-11.75	-11.74
-11.82	-11.81	-11.8	-11.79	-11.78	-11.77	-11.76	-11.75	-11.74
-11.82	-11.81	-11.8	-11.79	-11.78	-11.77	-11.76	-11.75	-11.74
-11.82	-11.81	-11.8	-11.79	-11.78	-11.77	-11.76	-11.75	-11.74
-11.82	-11.81	-11.8	-11.79	-11.78	-11.77	-11.76	-11.75	-11.74
-11.82	-11.81	-11.8	-11.79	-11.78	-11.77	-11.76	-11.75	-11.74

-11.82	-11.81	-11.8	-11.79	-11.78	-11.77	-11.76	-11.75	-11.74
-11.82	-11.81	-11.8	-11.79	-11.78	-11.77	-11.76	-11.75	-11.74
-11.82	-11.81	-11.8	-11.79	-11.78	-11.77	-11.76	-11.75	-11.74
-11.82	-11.81	-11.8	-11.79	-11.78	-11.77	-11.76	-11.75	-11.74
-11.82	-11.81	-11.8	-11.79	-11.78	-11.77	-11.76	-11.75	-11.74
-11.82	-11.81	-11.8	-11.79	-11.78	-11.77	-11.76	-11.75	-11.74
-11.82	-11.81	-11.8	-11.79	-11.78	-11.77	-11.76	-11.75	-11.74
-11.82	-11.81	-11.8	-11.79	-11.78	-11.77	-11.76	-11.75	-11.74
-11.82	-11.81	-11.8	-11.79	-11.78	-11.77	-11.76	-11.75	-11.74
-11.82	-11.81	-11.8	-11.79	-11.78	-11.77	-11.76	-11.75	-11.74
-11.82	-11.81	-11.8	-11.79	-11.78	-11.77	-11.76	-11.75	-11.74
-11.82	-11.81	-11.8	-11.79	-11.78	-11.77	-11.76	-11.75	-11.74
-11.82	-11.81	-11.8	-11.79	-11.78	-11.77	-11.76	-11.75	-11.74
-11.82	-11.81	-11.8	-11.79	-11.78	-11.77	-11.76	-11.75	-11.74
-11.82	-11.81	-11.8	-11.79	-11.78	-11.77	-11.76	-11.75	-11.74
-11.82	-11.81	-11.8	-11.79	-11.78	-11.77	-11.76	-11.75	-11.74
-11.82	-11.81	-11.8	-11.79	-11.78	-11.77	-11.76	-11.75	-11.74
-11.82	-11.81	-11.8	-11.79	-11.78	-11.77	-11.76	-11.75	-11.74
-11.82	-11.81	-11.8	-11.79	-11.78	-11.77	-11.76	-11.75	-11.74
-11.82	-11.81	-11.8	-11.79	-11.78	-11.77	-11.76	-11.75	-11.74
-11.82	-11.81	-11.8	-11.79	-11.78	-11.77	-11.76	-11.75	-11.74
-11.82	-11.81	-11.8	-11.79	-11.78	-11.77	-11.76	-11.75	-11.74
-11.82	-11.81	-11.8	-11.79	-11.78	-11.77	-11.76	-11.75	-11.74
-11.82	-11.81	-11.8	-11.79	-11.78	-11.77	-11.76	-11.75	-11.74
-11.82	-11.81	-11.8	-11.79	-11.78	-11.77	-11.76	-11.75	-11.74
-11.82	-11.81	-11.8	-11.79	-11.78	-11.77	-11.76	-11.75	-11.74
-11.82	-11.81	-11.8	-11.79	-11.78	-11.77	-11.76	-11.75	-11.74
-11.82	-11.81	-11.8	-11.79	-11.78	-11.77	-11.76	-11.75	-11.74
-11.82	-11.81	-11.8	-11.79	-11.78	-11.77	-11.76	-11.75	-11.74
-11.82	-11.81	-11.8	-11.79	-11.78	-11.77	-11.76	-11.75	-11.74
-11.82	-11.81	-11.8	-11.79	-11.78	-11.77	-11.76	-11.75	-11.74
-11.82	-11.81	-11.8	-11.79	-11.78	-11.77	-11.76	-11.75	-11.74
-11.82	-11.81	-11.8	-11.79	-11.78	-11.77	-11.76	-11.75	-11.74
-11.82	-11.81	-11.8	-11.79	-11.78	-11.77	-11.76	-11.75	-11.74
-11.82	-11.81	-11.8	-11.79	-11.78	-11.77	-11.76	-11.75	-11.74
-11.82	-11.81	-11.8	-11.79	-11.78	-11.77	-11.76	-11.75	-11.74
-11.82	-11.81	-11.8	-11.79	-11.78	-11.77	-11.76	-11.75	-11.74
-11.82	-11.81	-11.8	-11.79	-11.78	-11.77	-11.76	-11.75	-11.74
-11.82	-11.81	-11.8	-11.79	-11.78	-11.77	-11.76	-11.75	-11.74
-11.82	-11.81	-11.8	-11.79	-11.78	-11.77	-11.76	-11.75	-11.74
-11.82	-11.81	-11.8	-11.79	-11.78	-11.77	-11.76	-11.75	-11.74
-11.82	-11.81	-11.8	-11.79	-11.78	-11.77	-11.76	-11.75	-11.74
-11.82	-11.81	-11.8	-11.79	-11.78	-11.77	-11.76	-11.75	-11.74
-11.82	-11.81	-11.8	-11.79	-11.78	-11.77	-11.76	-11.75	-11.74
-11.82	-11.81	-11.8	-11.79	-11.78	-11.77	-11.76	-11.75	-11.74
-11.82	-11.81	-11.8	-11.79	-11.78	-11.77	-11.76	-11.75	-11.74
-11.82	-11.81	-11.8	-11.79	-11.78	-11.77	-11.76	-11.75	-11.74
-11.82	-11.81	-11.8	-11.79	-11.78	-11.77	-11.76	-11.75	-11.74
-11.82	-11.81	-11.8	-11.79	-11.78	-11.77	-11.76	-11.75	-11.74
-11.82	-11.81	-11.8	-11.79	-11.78	-11.77	-11.76	-11.75	-11.74

-11.82	-11.81	-11.8	-11.79	-11.78	-11.77	-11.76	-11.75	-11.74
-11.82	-11.81	-11.8	-11.79	-11.78	-11.77	-11.76	-11.75	-11.74
-11.82	-11.81	-11.8	-11.79	-11.78	-11.77	-11.76	-11.75	-11.74
-11.82	-11.81	-11.8	-11.79	-11.78	-11.77	-11.76	-11.75	-11.74
-11.82	-11.81	-11.8	-11.79	-11.78	-11.77	-11.76	-11.75	-11.74
-11.82	-11.81	-11.8	-11.79	-11.78	-11.77	-11.76	-11.75	-11.74
-11.82	-11.81	-11.8	-11.79	-11.78	-11.77	-11.76	-11.75	-11.74
-11.82	-11.81	-11.8	-11.79	-11.78	-11.77	-11.76	-11.75	-11.74
-11.82	-11.81	-11.8	-11.79	-11.78	-11.77	-11.76	-11.75	-11.74
-11.82	-11.81	-11.8	-11.79	-11.78	-11.77	-11.76	-11.75	-11.74
-11.82	-11.81	-11.8	-11.79	-11.78	-11.77	-11.76	-11.75	-11.74
-11.82	-11.81	-11.8	-11.79	-11.78	-11.77	-11.76	-11.75	-11.74
-11.82	-11.81	-11.8	-11.79	-11.78	-11.77	-11.76	-11.75	-11.74
-11.82	-11.81	-11.8	-11.79	-11.78	-11.77	-11.76	-11.75	-11.74
-11.82	-11.81	-11.8	-11.79	-11.78	-11.77	-11.76	-11.75	-11.74
-11.82	-11.81	-11.8	-11.79	-11.78	-11.77	-11.76	-11.75	-11.74
-11.82	-11.81	-11.8	-11.79	-11.78	-11.77	-11.76	-11.75	-11.74
-11.82	-11.81	-11.8	-11.79	-11.78	-11.77	-11.76	-11.75	-11.74
-11.82	-11.81	-11.8	-11.79	-11.78	-11.77	-11.76	-11.75	-11.74
-11.82	-11.81	-11.8	-11.79	-11.78	-11.77	-11.76	-11.75	-11.74
-11.82	-11.81	-11.8	-11.79	-11.78	-11.77	-11.76	-11.75	-11.74
-11.82	-11.81	-11.8	-11.79	-11.78	-11.77	-11.76	-11.75	-11.74
-11.82	-11.81	-11.8	-11.79	-11.78	-11.77	-11.76	-11.75	-11.74
-11.82	-11.81	-11.8	-11.79	-11.78	-11.77	-11.76	-11.75	-11.74
-11.82	-11.81	-11.8	-11.79	-11.78	-11.77	-11.76	-11.75	-11.74
-11.82	-11.81	-11.8	-11.79	-11.78	-11.77	-11.76	-11.75	-11.74
-11.82	-11.81	-11.8	-11.79	-11.78	-11.77	-11.76	-11.75	-11.74
-11.82	-11.81	-11.8	-11.79	-11.78	-11.77	-11.76	-11.75	-11.74
-11.82	-11.81	-11.8	-11.79	-11.78	-11.77	-11.76	-11.75	-11.74
-11.82	-11.81	-11.8	-11.79	-11.78	-11.77	-11.76	-11.75	-11.74
-11.82	-11.81	-11.8	-11.79	-11.78	-11.77	-11.76	-11.75	-11.74
-11.82	-11.81	-11.8	-11.79	-11.78	-11.77	-11.76	-11.75	-11.74
-11.82	-11.81	-11.8	-11.79	-11.78	-11.77	-11.76	-11.75	-11.74
-11.82	-11.81	-11.8	-11.79	-11.78	-11.77	-11.76	-11.75	-11.74
-11.82	-11.81	-11.8	-11.79	-11.78	-11.77	-11.76	-11.75	-11.74
-11.82	-11.81	-11.8	-11.79	-11.78	-11.77	-11.76	-11.75	-11.74
-11.82	-11.81	-11.8	-11.79	-11.78	-11.77	-11.76	-11.75	-11.74
-11.82	-11.81	-11.8	-11.79	-11.78	-11.77	-11.76	-11.75	-11.74
-11.82	-11.81	-11.8	-11.79	-11.78	-11.77	-11.76	-11.75	-11.74
-11.82	-11.81	-11.8	-11.79	-11.78	-11.77	-11.76	-11.75	-11.74
-11.82	-11.81	-11.8	-11.79	-11.78	-11.77	-11.76	-11.75	-11.74
-11.82	-11.81	-11.8	-11.79	-11.78	-11.77	-11.76	-11.75	-11.74
-11.82	-11.81	-11.8	-11.79	-11.78	-11.77	-11.76	-11.75	-11.74
-11.82	-11.81	-11.8	-11.79	-11.78	-11.77	-11.76	-11.75	-11.74
-11.82	-11.81	-11.8	-11.79	-11.78	-11.77	-11.76	-11.75	-11.74
-11.82	-11.81	-11.8	-11.79	-11.78	-11.77	-11.76	-11.75	-11.74
-11.82	-11.81	-11.8	-11.79	-11.78	-11.77	-11.76	-11.75	-11.74
-11.82	-11.81	-11.8	-11.79	-11.78	-11.77	-11.76	-11.75	-11.74
-11.82	-11.81	-11.8	-11.79	-11.78	-11.77	-11.76	-11.75	-11.74
-11.82	-11.81	-11.8	-11.79	-11.78	-11.77	-11.76	-11.75	-11.74

-11.82	-11.81	-11.8	-11.79	-11.78	-11.77	-11.76	-11.75	-11.74
-11.82	-11.81	-11.8	-11.79	-11.78	-11.77	-11.76	-11.75	-11.74
-11.82	-11.81	-11.8	-11.79	-11.78	-11.77	-11.76	-11.75	-11.74
-11.82	-11.81	-11.8	-11.79	-11.78	-11.77	-11.76	-11.75	-11.74
-11.82	-11.81	-11.8	-11.79	-11.78	-11.77	-11.76	-11.75	-11.74
-11.82	-11.81	-11.8	-11.79	-11.78	-11.77	-11.76	-11.75	-11.74
-11.82	-11.81	-11.8	-11.79	-11.78	-11.77	-11.76	-11.75	-11.74
-11.82	-11.81	-11.8	-11.79	-11.78	-11.77	-11.76	-11.75	-11.74
-11.82	-11.81	-11.8	-11.79	-11.78	-11.77	-11.76	-11.75	-11.74
-11.82	-11.81	-11.8	-11.79	-11.78	-11.77	-11.76	-11.75	-11.74
-11.82	-11.81	-11.8	-11.79	-11.78	-11.77	-11.76	-11.75	-11.74
-11.82	-11.81	-11.8	-11.79	-11.78	-11.77	-11.76	-11.75	-11.74
-11.82	-11.81	-11.8	-11.79	-11.78	-11.77	-11.76	-11.75	-11.74
-11.82	-11.81	-11.8	-11.79	-11.78	-11.77	-11.76	-11.75	-11.74
-11.82	-11.81	-11.8	-11.79	-11.78	-11.77	-11.76	-11.75	-11.74
-11.82	-11.81	-11.8	-11.79	-11.78	-11.77	-11.76	-11.75	-11.74
-11.82	-11.81	-11.8	-11.79	-11.78	-11.77	-11.76	-11.75	-11.74
-11.82	-11.81	-11.8	-11.79	-11.78	-11.77	-11.76	-11.75	-11.74
-11.82	-11.81	-11.8	-11.79	-11.78	-11.77	-11.76	-11.75	-11.74
-11.82	-11.81	-11.8	-11.79	-11.78	-11.77	-11.76	-11.75	-11.74
-11.82	-11.81	-11.8	-11.79	-11.78	-11.77	-11.76	-11.75	-11.74
-11.82	-11.81	-11.8	-11.79	-11.78	-11.77	-11.76	-11.75	-11.74
-11.82	-11.81	-11.8	-11.79	-11.78	-11.77	-11.76	-11.75	-11.74
-11.82	-11.81	-11.8	-11.79	-11.78	-11.77	-11.76	-11.75	-11.74
-11.82	-11.81	-11.8	-11.79	-11.78	-11.77	-11.76	-11.75	-11.74
-11.82	-11.81	-11.8	-11.79	-11.78	-11.77	-11.76	-11.75	-11.74
-11.82	-11.81	-11.8	-11.79	-11.78	-11.77	-11.76	-11.75	-11.74
-11.82	-11.81	-11.8	-11.79	-11.78	-11.77	-11.76	-11.75	-11.74
-11.82	-11.81	-11.8	-11.79	-11.78	-11.77	-11.76	-11.75	-11.74
-11.82	-11.81	-11.8	-11.79	-11.78	-11.77	-11.76	-11.75	-11.74
-11.82	-11.81	-11.8	-11.79	-11.78	-11.77	-11.76	-11.75	-11.74
-11.82	-11.81	-11.8	-11.79	-11.78	-11.77	-11.76	-11.75	-11.74
-11.82	-11.81	-11.8	-11.79	-11.78	-11.77	-11.76	-11.75	-11.74
-11.82	-11.81	-11.8	-11.79	-11.78	-11.77	-11.76	-11.75	-11.74
-11.82	-11.81	-11.8	-11.79	-11.78	-11.77	-11.76	-11.75	-11.74
-11.82	-11.81	-11.8	-11.79	-11.78	-11.77	-11.76	-11.75	-11.74
-11.82	-11.81	-11.8	-11.79	-11.78	-11.77	-11.76	-11.75	-11.74
-11.82	-11.81	-11.8	-11.79	-11.78	-11.77	-11.76	-11.75	-11.74
-11.82	-11.81	-11.8	-11.79	-11.78	-11.77	-11.76	-11.75	-11.74
-11.82	-11.81	-11.8	-11.79	-11.78	-11.77	-11.76	-11.75	-11.74
-11.82	-11.81	-11.8	-11.79	-11.78	-11.77	-11.76	-11.75	-11.74
-11.82	-11.81	-11.8	-11.79	-11.78	-11.77	-11.76	-11.75	-11.74
-11.82	-11.81	-11.8	-11.79	-11.78	-11.77	-11.76	-11.75	-11.74
-11.82	-11.81	-11.8	-11.79	-11.78	-11.77	-11.76	-11.75	-11.74
-11.82	-11.81	-11.8	-11.79	-11.78	-11.77	-11.76	-11.75	-11.74
-11.82	-11.81	-11.8	-11.79	-11.78	-11.77	-11.76	-11.75	-11.74
-11.82	-11.81	-11.8	-11.79	-11.78	-11.77	-11.76	-11.75	-11.74
-11.82	-11.81	-11.8	-11.79	-11.78	-11.77	-11.76	-11.75	-11.74
-11.82	-11.81	-11.8	-11.79	-11.78	-11.77	-11.76	-11.75	-11.74
-11.82	-11.81	-11.8	-11.79	-11.78	-11.77	-11.76	-11.75	-11.74

-11.82	-11.81	-11.8	-11.79	-11.78	-11.77	-11.76	-11.75	-11.74
-11.82	-11.81	-11.8	-11.79	-11.78	-11.77	-11.76	-11.75	-11.74
-11.82	-11.81	-11.8	-11.79	-11.78	-11.77	-11.76	-11.75	-11.74
-11.82	-11.81	-11.8	-11.79	-11.78	-11.77	-11.76	-11.75	-11.74
-11.82	-11.81	-11.8	-11.79	-11.78	-11.77	-11.76	-11.75	-11.74
-11.82	-11.81	-11.8	-11.79	-11.78	-11.77	-11.76	-11.75	-11.74
-11.82	-11.81	-11.8	-11.79	-11.78	-11.77	-11.76	-11.75	-11.74
-11.82	-11.81	-11.8	-11.79	-11.78	-11.77	-11.76	-11.75	-11.74
-11.82	-11.81	-11.8	-11.79	-11.78	-11.77	-11.76	-11.75	-11.74
-11.82	-11.81	-11.8	-11.79	-11.78	-11.77	-11.76	-11.75	-11.74
-11.82	-11.81	-11.8	-11.79	-11.78	-11.77	-11.76	-11.75	-11.74
-11.82	-11.81	-11.8	-11.79	-11.78	-11.77	-11.76	-11.75	-11.74
-11.82	-11.81	-11.8	-11.79	-11.78	-11.77	-11.76	-11.75	-11.74
-11.82	-11.81	-11.8	-11.79	-11.78	-11.77	-11.76	-11.75	-11.74
-11.82	-11.81	-11.8	-11.79	-11.78	-11.77	-11.76	-11.75	-11.74
-11.82	-11.81	-11.8	-11.79	-11.78	-11.77	-11.76	-11.75	-11.74
-11.82	-11.81	-11.8	-11.79	-11.78	-11.77	-11.76	-11.75	-11.74
-11.82	-11.81	-11.8	-11.79	-11.78	-11.77	-11.76	-11.75	-11.74
-11.82	-11.81	-11.8	-11.79	-11.78	-11.77	-11.76	-11.75	-11.74
-11.82	-11.81	-11.8	-11.79	-11.78	-11.77	-11.76	-11.75	-11.74
-11.82	-11.81	-11.8	-11.79	-11.78	-11.77	-11.76	-11.75	-11.74
-11.82	-11.81	-11.8	-11.79	-11.78	-11.77	-11.76	-11.75	-11.74
-11.82	-11.81	-11.8	-11.79	-11.78	-11.77	-11.76	-11.75	-11.74
-11.82	-11.81	-11.8	-11.79	-11.78	-11.77	-11.76	-11.75	-11.74
-11.82	-11.81	-11.8	-11.79	-11.78	-11.77	-11.76	-11.75	-11.74
-11.82	-11.81	-11.8	-11.79	-11.78	-11.77	-11.76	-11.75	-11.74
-11.82	-11.81	-11.8	-11.79	-11.78	-11.77	-11.76	-11.75	-11.74
-11.82	-11.81	-11.8	-11.79	-11.78	-11.77	-11.76	-11.75	-11.74
-11.82	-11.81	-11.8	-11.79	-11.78	-11.77	-11.76	-11.75	-11.74
-11.82	-11.81	-11.8	-11.79	-11.78	-11.77	-11.76	-11.75	-11.74
-11.82	-11.81	-11.8	-11.79	-11.78	-11.77	-11.76	-11.75	-11.74
-11.82	-11.81	-11.8	-11.79	-11.78	-11.77	-11.76	-11.75	-11.74
-11.82	-11.81	-11.8	-11.79	-11.78	-11.77	-11.76	-11.75	-11.74
-11.82	-11.81	-11.8	-11.79	-11.78	-11.77	-11.76	-11.75	-11.74
-11.82	-11.81	-11.8	-11.79	-11.78	-11.77	-11.76	-11.75	-11.74
-11.82	-11.81	-11.8	-11.79	-11.78	-11.77	-11.76	-11.75	-11.74
-11.82	-11.81	-11.8	-11.79	-11.78	-11.77	-11.76	-11.75	-11.74
-11.82	-11.81	-11.8	-11.79	-11.78	-11.77	-11.76	-11.75	-11.74
-11.82	-11.81	-11.8	-11.79	-11.78	-11.77	-11.76	-11.75	-11.74
-11.82	-11.81	-11.8	-11.79	-11.78	-11.77	-11.76	-11.75	-11.74
-11.82	-11.81	-11.8	-11.79	-11.78	-11.77	-11.76	-11.75	-11.74
-11.82	-11.81	-11.8	-11.79	-11.78	-11.77	-11.76	-11.75	-11.74
-11.82	-11.81	-11.8	-11.79	-11.78	-11.77	-11.76	-11.75	-11.74
-11.82	-11.81	-11.8	-11.79	-11.78	-11.77	-11.76	-11.75	-11.74
-11.82	-11.81	-11.8	-11.79	-11.78	-11.77	-11.76	-11.75	-11.74
-11.82	-11.81	-11.8	-11.79	-11.78	-11.77	-11.76	-11.75	-11.74
-11.82	-11.81	-11.8	-11.79	-11.78	-11.77	-11.76	-11.75	-11.74
-11.82	-11.81	-11.8	-11.79	-11.78	-11.77	-11.76	-11.75	-11.74
-11.82	-11.81	-11.8	-11.79	-11.78	-11.77	-11.76	-11.75	-11.74
-11.82	-11.81	-11.8	-11.79	-11.78	-11.77	-11.76	-11.75	-11.74

-11.82	-11.81	-11.8	-11.79	-11.78	-11.77	-11.76	-11.75	-11.74
-11.82	-11.81	-11.8	-11.79	-11.78	-11.77	-11.76	-11.75	-11.74
-11.82	-11.81	-11.8	-11.79	-11.78	-11.77	-11.76	-11.75	-11.74
-11.82	-11.81	-11.8	-11.79	-11.78	-11.77	-11.76	-11.75	-11.74
-11.82	-11.81	-11.8	-11.79	-11.78	-11.77	-11.76	-11.75	-11.74
-11.82	-11.81	-11.8	-11.79	-11.78	-11.77	-11.76	-11.75	-11.74
-11.82	-11.81	-11.8	-11.79	-11.78	-11.77	-11.76	-11.75	-11.74
-11.82	-11.81	-11.8	-11.79	-11.78	-11.77	-11.76	-11.75	-11.74
-11.82	-11.81	-11.8	-11.79	-11.78	-11.77	-11.76	-11.75	-11.74
-11.82	-11.81	-11.8	-11.79	-11.78	-11.77	-11.76	-11.75	-11.74
-11.82	-11.81	-11.8	-11.79	-11.78	-11.77	-11.76	-11.75	-11.74
-11.82	-11.81	-11.8	-11.79	-11.78	-11.77	-11.76	-11.75	-11.74
-11.82	-11.81	-11.8	-11.79	-11.78	-11.77	-11.76	-11.75	-11.74
-11.82	-11.81	-11.8	-11.79	-11.78	-11.77	-11.76	-11.75	-11.74
-11.82	-11.81	-11.8	-11.79	-11.78	-11.77	-11.76	-11.75	-11.74
-11.82	-11.81	-11.8	-11.79	-11.78	-11.77	-11.76	-11.75	-11.74
-11.82	-11.81	-11.8	-11.79	-11.78	-11.77	-11.76	-11.75	-11.74
-11.82	-11.81	-11.8	-11.79	-11.78	-11.77	-11.76	-11.75	-11.74
-11.82	-11.81	-11.8	-11.79	-11.78	-11.77	-11.76	-11.75	-11.74
-11.82	-11.81	-11.8	-11.79	-11.78	-11.77	-11.76	-11.75	-11.74
-11.82	-11.81	-11.8	-11.79	-11.78	-11.77	-11.76	-11.75	-11.74
-11.82	-11.81	-11.8	-11.79	-11.78	-11.77	-11.76	-11.75	-11.74
-11.82	-11.81	-11.8	-11.79	-11.78	-11.77	-11.76	-11.75	-11.74
-11.82	-11.81	-11.8	-11.79	-11.78	-11.77	-11.76	-11.75	-11.74
-11.82	-11.81	-11.8	-11.79	-11.78	-11.77	-11.76	-11.75	-11.74
-11.82	-11.81	-11.8	-11.79	-11.78	-11.77	-11.76	-11.75	-11.74
-11.82	-11.81	-11.8	-11.79	-11.78	-11.77	-11.76	-11.75	-11.74
-11.82	-11.81	-11.8	-11.79	-11.78	-11.77	-11.76	-11.75	-11.74
-11.82	-11.81	-11.8	-11.79	-11.78	-11.77	-11.76	-11.75	-11.74
-11.82	-11.81	-11.8	-11.79	-11.78	-11.77	-11.76	-11.75	-11.74
-11.82	-11.81	-11.8	-11.79	-11.78	-11.77	-11.76	-11.75	-11.74
-11.82	-11.81	-11.8	-11.79	-11.78	-11.77	-11.76	-11.75	-11.74
-11.82	-11.81	-11.8	-11.79	-11.78	-11.77	-11.76	-11.75	-11.74
-11.82	-11.81	-11.8	-11.79	-11.78	-11.77	-11.76	-11.75	-11.74
-11.82	-11.81	-11.8	-11.79	-11.78	-11.77	-11.76	-11.75	-11.74
-11.82	-11.81	-11.8	-11.79	-11.78	-11.77	-11.76	-11.75	-11.74
-11.82	-11.81	-11.8	-11.79	-11.78	-11.77	-11.76	-11.75	-11.74
-11.82	-11.81	-11.8	-11.79	-11.78	-11.77	-11.76	-11.75	-11.74
-11.82	-11.81	-11.8	-11.79	-11.78	-11.77	-11.76	-11.75	-11.74
-11.82	-11.81	-11.8	-11.79	-11.78	-11.77	-11.76	-11.75	-11.74
-11.82	-11.81	-11.8	-11.79	-11.78	-11.77	-11.76	-11.75	-11.74
-11.82	-11.81	-11.8	-11.79	-11.78	-11.77	-11.76	-11.75	-11.74
-11.82	-11.81	-11.8	-11.79	-11.78	-11.77	-11.76	-11.75	-11.74
-11.82	-11.81	-11.8	-11.79	-11.78	-11.77	-11.76	-11.75	-11.74
-11.82	-11.81	-11.8	-11.79	-11.78	-11.77	-11.76	-11.75	-11.74
-11.82	-11.81	-11.8	-11.79	-11.78	-11.77	-11.76	-11.75	-11.74
-11.82	-11.81	-11.8	-11.79	-11.78	-11.77	-11.76	-11.75	-11.74
-11.82	-11.81	-11.8	-11.79	-11.78	-11.77	-11.76	-11.75	-11.74
-11.82	-11.81	-11.8	-11.79	-11.78	-11.77	-11.76	-11.75	-11.74
-11.82	-11.81	-11.8	-11.79	-11.78	-11.77	-11.76	-11.75	-11.74

-11.82	-11.81	-11.8	-11.79	-11.78	-11.77	-11.76	-11.75	-11.74
-11.82	-11.81	-11.8	-11.79	-11.78	-11.77	-11.76	-11.75	-11.74
-11.82	-11.81	-11.8	-11.79	-11.78	-11.77	-11.76	-11.75	-11.74
-11.82	-11.81	-11.8	-11.79	-11.78	-11.77	-11.76	-11.75	-11.74
-11.82	-11.81	-11.8	-11.79	-11.78	-11.77	-11.76	-11.75	-11.74
-11.82	-11.81	-11.8	-11.79	-11.78	-11.77	-11.76	-11.75	-11.74
-11.82	-11.81	-11.8	-11.79	-11.78	-11.77	-11.76	-11.75	-11.74
-11.82	-11.81	-11.8	-11.79	-11.78	-11.77	-11.76	-11.75	-11.74
-11.82	-11.81	-11.8	-11.79	-11.78	-11.77	-11.76	-11.75	-11.74
-11.82	-11.81	-11.8	-11.79	-11.78	-11.77	-11.76	-11.75	-11.74
-11.82	-11.81	-11.8	-11.79	-11.78	-11.77	-11.76	-11.75	-11.74
-11.82	-11.81	-11.8	-11.79	-11.78	-11.77	-11.76	-11.75	-11.74
-11.82	-11.81	-11.8	-11.79	-11.78	-11.77	-11.76	-11.75	-11.74
-11.82	-11.81	-11.8	-11.79	-11.78	-11.77	-11.76	-11.75	-11.74
-11.82	-11.81	-11.8	-11.79	-11.78	-11.77	-11.76	-11.75	-11.74
-11.82	-11.81	-11.8	-11.79	-11.78	-11.77	-11.76	-11.75	-11.74
-11.82	-11.81	-11.8	-11.79	-11.78	-11.77	-11.76	-11.75	-11.74
-11.82	-11.81	-11.8	-11.79	-11.78	-11.77	-11.76	-11.75	-11.74
-11.82	-11.81	-11.8	-11.79	-11.78	-11.77	-11.76	-11.75	-11.74
-11.82	-11.81	-11.8	-11.79	-11.78	-11.77	-11.76	-11.75	-11.74
-11.82	-11.81	-11.8	-11.79	-11.78	-11.77	-11.76	-11.75	-11.74
-11.82	-11.81	-11.8	-11.79	-11.78	-11.77	-11.76	-11.75	-11.74
-11.82	-11.81	-11.8	-11.79	-11.78	-11.77	-11.76	-11.75	-11.74
-11.82	-11.81	-11.8	-11.79	-11.78	-11.77	-11.76	-11.75	-11.74
-11.82	-11.81	-11.8	-11.79	-11.78	-11.77	-11.76	-11.75	-11.74
-11.82	-11.81	-11.8	-11.79	-11.78	-11.77	-11.76	-11.75	-11.74
-11.82	-11.81	-11.8	-11.79	-11.78	-11.77	-11.76	-11.75	-11.74
-11.82	-11.81	-11.8	-11.79	-11.78	-11.77	-11.76	-11.75	-11.74
-11.82	-11.81	-11.8	-11.79	-11.78	-11.77	-11.76	-11.75	-11.74
-11.82	-11.81	-11.8	-11.79	-11.78	-11.77	-11.76	-11.75	-11.74
-11.82	-11.81	-11.8	-11.79	-11.78	-11.77	-11.76	-11.75	-11.74
-11.82	-11.81	-11.8	-11.79	-11.78	-11.77	-11.76	-11.75	-11.74
-11.82	-11.81	-11.8	-11.79	-11.78	-11.77	-11.76	-11.75	-11.74
-11.82	-11.81	-11.8	-11.79	-11.78	-11.77	-11.76	-11.75	-11.74
-11.82	-11.81	-11.8	-11.79	-11.78	-11.77	-11.76	-11.75	-11.74
-11.82	-11.81	-11.8	-11.79	-11.78	-11.77	-11.76	-11.75	-11.74
-11.82	-11.81	-11.8	-11.79	-11.78	-11.77	-11.76	-11.75	-11.74
-11.82	-11.81	-11.8	-11.79	-11.78	-11.77	-11.76	-11.75	-11.74
-11.82	-11.81	-11.8	-11.79	-11.78	-11.77	-11.76	-11.75	-11.74
-11.82	-11.81	-11.8	-11.79	-11.78	-11.77	-11.76	-11.75	-11.74
-11.82	-11.81	-11.8	-11.79	-11.78	-11.77	-11.76	-11.75	-11.74
-11.82	-11.81	-11.8	-11.79	-11.78	-11.77	-11.76	-11.75	-11.74
-11.82	-11.81	-11.8	-11.79	-11.78	-11.77	-11.76	-11.75	-11.74
-11.82	-11.81	-11.8	-11.79	-11.78	-11.77	-11.76	-11.75	-11.74
-11.82	-11.81	-11.8	-11.79	-11.78	-11.77	-11.76	-11.75	-11.74
-11.82	-11.81	-11.8	-11.79	-11.78	-11.77	-11.76	-11.75	-11.74
-11.82	-11.81	-11.8	-11.79	-11.78	-11.77	-11.76	-11.75	-11.74
-11.82	-11.81	-11.8	-11.79	-11.78	-11.77	-11.76	-11.75	-11.74
-11.82	-11.81	-11.8	-11.79	-11.78	-11.77	-11.76	-11.75	-11.74
-11.82	-11.81	-11.8	-11.79	-11.78	-11.77	-11.76	-11.75	-11.74

-11.82	-11.81	-11.8	-11.79	-11.78	-11.77	-11.76	-11.75	-11.74
-11.82	-11.81	-11.8	-11.79	-11.78	-11.77	-11.76	-11.75	-11.74
-11.82	-11.81	-11.8	-11.79	-11.78	-11.77	-11.76	-11.75	-11.74
-11.82	-11.81	-11.8	-11.79	-11.78	-11.77	-11.76	-11.75	-11.74
-11.82	-11.81	-11.8	-11.79	-11.78	-11.77	-11.76	-11.75	-11.74
-11.82	-11.81	-11.8	-11.79	-11.78	-11.77	-11.76	-11.75	-11.74
-11.82	-11.81	-11.8	-11.79	-11.78	-11.77	-11.76	-11.75	-11.74
-11.82	-11.81	-11.8	-11.79	-11.78	-11.77	-11.76	-11.75	-11.74
-11.82	-11.81	-11.8	-11.79	-11.78	-11.77	-11.76	-11.75	-11.74
-11.82	-11.81	-11.8	-11.79	-11.78	-11.77	-11.76	-11.75	-11.74
-11.82	-11.81	-11.8	-11.79	-11.78	-11.77	-11.76	-11.75	-11.74
-11.82	-11.81	-11.8	-11.79	-11.78	-11.77	-11.76	-11.75	-11.74
-11.82	-11.81	-11.8	-11.79	-11.78	-11.77	-11.76	-11.75	-11.74
-11.82	-11.81	-11.8	-11.79	-11.78	-11.77	-11.76	-11.75	-11.74
-11.82	-11.81	-11.8	-11.79	-11.78	-11.77	-11.76	-11.75	-11.74
-11.82	-11.81	-11.8	-11.79	-11.78	-11.77	-11.76	-11.75	-11.74
-11.82	-11.81	-11.8	-11.79	-11.78	-11.77	-11.76	-11.75	-11.74
-11.82	-11.81	-11.8	-11.79	-11.78	-11.77	-11.76	-11.75	-11.74
-11.82	-11.81	-11.8	-11.79	-11.78	-11.77	-11.76	-11.75	-11.74
-11.82	-11.81	-11.8	-11.79	-11.78	-11.77	-11.76	-11.75	-11.74
-11.82	-11.81	-11.8	-11.79	-11.78	-11.77	-11.76	-11.75	-11.74
-11.82	-11.81	-11.8	-11.79	-11.78	-11.77	-11.76	-11.75	-11.74
-11.82	-11.81	-11.8	-11.79	-11.78	-11.77	-11.76	-11.75	-11.74
-11.82	-11.81	-11.8	-11.79	-11.78	-11.77	-11.76	-11.75	-11.74
-11.82	-11.81	-11.8	-11.79	-11.78	-11.77	-11.76	-11.75	-11.74
-11.82	-11.81	-11.8	-11.79	-11.78	-11.77	-11.76	-11.75	-11.74
-11.82	-11.81	-11.8	-11.79	-11.78	-11.77	-11.76	-11.75	-11.74
-11.82	-11.81	-11.8	-11.79	-11.78	-11.77	-11.76	-11.75	-11.74
-11.82	-11.81	-11.8	-11.79	-11.78	-11.77	-11.76	-11.75	-11.74
-11.82	-11.81	-11.8	-11.79	-11.78	-11.77	-11.76	-11.75	-11.74
-11.82	-11.81	-11.8	-11.79	-11.78	-11.77	-11.76	-11.75	-11.74
-11.82	-11.81	-11.8	-11.79	-11.78	-11.77	-11.76	-11.75	-11.74
-11.82	-11.81	-11.8	-11.79	-11.78	-11.77	-11.76	-11.75	-11.74
-11.82	-11.81	-11.8	-11.79	-11.78	-11.77	-11.76	-11.75	-11.74
-11.82	-11.81	-11.8	-11.79	-11.78	-11.77	-11.76	-11.75	-11.74
-11.82	-11.81	-11.8	-11.79	-11.78	-11.77	-11.76	-11.75	-11.74
-11.82	-11.81	-11.8	-11.79	-11.78	-11.77	-11.76	-11.75	-11.74
-11.82	-11.81	-11.8	-11.79	-11.78	-11.77	-11.76	-11.75	-11.74
-11.82	-11.81	-11.8	-11.79	-11.78	-11.77	-11.76	-11.75	-11.74
-11.82	-11.81	-11.8	-11.79	-11.78	-11.77	-11.76	-11.75	-11.74
-11.82	-11.81	-11.8	-11.79	-11.78	-11.77	-11.76	-11.75	-11.74
-11.82	-11.81	-11.8	-11.79	-11.78	-11.77	-11.76	-11.75	-11.74
-11.82	-11.81	-11.8	-11.79	-11.78	-11.77	-11.76	-11.75	-11.74
-11.82	-11.81	-11.8	-11.79	-11.78	-11.77	-11.76	-11.75	-11.74
-11.82	-11.81	-11.8	-11.79	-11.78	-11.77	-11.76	-11.75	-11.74
-11.82	-11.81	-11.8	-11.79	-11.78	-11.77	-11.76	-11.75	-11.74
-11.82	-11.81	-11.8	-11.79	-11.78	-11.77	-11.76	-11.75	-11.74
-11.82	-11.81	-11.8	-11.79	-11.78	-11.77	-11.76	-11.75	-11.74
-11.82	-11.81	-11.8	-11.79	-11.78	-11.77	-11.76	-11.75	-11.74
-11.82	-11.81	-11.8	-11.79	-11.78	-11.77	-11.76	-11.75	-11.74

-11.82	-11.81	-11.8	-11.79	-11.78	-11.77	-11.76	-11.75	-11.74
-11.82	-11.81	-11.8	-11.79	-11.78	-11.77	-11.76	-11.75	-11.74
-11.82	-11.81	-11.8	-11.79	-11.78	-11.77	-11.76	-11.75	-11.74
-11.82	-11.81	-11.8	-11.79	-11.78	-11.77	-11.76	-11.75	-11.74
-11.82	-11.81	-11.8	-11.79	-11.78	-11.77	-11.76	-11.75	-11.74
-11.82	-11.81	-11.8	-11.79	-11.78	-11.77	-11.76	-11.75	-11.74
-11.82	-11.81	-11.8	-11.79	-11.78	-11.77	-11.76	-11.75	-11.74
-11.82	-11.81	-11.8	-11.79	-11.78	-11.77	-11.76	-11.75	-11.74
-11.82	-11.81	-11.8	-11.79	-11.78	-11.77	-11.76	-11.75	-11.74
-11.82	-11.81	-11.8	-11.79	-11.78	-11.77	-11.76	-11.75	-11.74
-11.82	-11.81	-11.8	-11.79	-11.78	-11.77	-11.76	-11.75	-11.74
-11.82	-11.81	-11.8	-11.79	-11.78	-11.77	-11.76	-11.75	-11.74
-11.82	-11.81	-11.8	-11.79	-11.78	-11.77	-11.76	-11.75	-11.74
-11.82	-11.81	-11.8	-11.79	-11.78	-11.77	-11.76	-11.75	-11.74
-11.82	-11.81	-11.8	-11.79	-11.78	-11.77	-11.76	-11.75	-11.74
-11.82	-11.81	-11.8	-11.79	-11.78	-11.77	-11.76	-11.75	-11.74
-11.82	-11.81	-11.8	-11.79	-11.78	-11.77	-11.76	-11.75	-11.74
-11.82	-11.81	-11.8	-11.79	-11.78	-11.77	-11.76	-11.75	-11.74
-11.82	-11.81	-11.8	-11.79	-11.78	-11.77	-11.76	-11.75	-11.74
-11.82	-11.81	-11.8	-11.79	-11.78	-11.77	-11.76	-11.75	-11.74
-11.82	-11.81	-11.8	-11.79	-11.78	-11.77	-11.76	-11.75	-11.74
-11.82	-11.81	-11.8	-11.79	-11.78	-11.77	-11.76	-11.75	-11.74
-11.82	-11.81	-11.8	-11.79	-11.78	-11.77	-11.76	-11.75	-11.74
-11.82	-11.81	-11.8	-11.79	-11.78	-11.77	-11.76	-11.75	-11.74
-11.82	-11.81	-11.8	-11.79	-11.78	-11.77	-11.76	-11.75	-11.74
-11.82	-11.81	-11.8	-11.79	-11.78	-11.77	-11.76	-11.75	-11.74
-11.82	-11.81	-11.8	-11.79	-11.78	-11.77	-11.76	-11.75	-11.74
-11.82	-11.81	-11.8	-11.79	-11.78	-11.77	-11.76	-11.75	-11.74
-11.82	-11.81	-11.8	-11.79	-11.78	-11.77	-11.76	-11.75	-11.74
-11.82	-11.81	-11.8	-11.79	-11.78	-11.77	-11.76	-11.75	-11.74
-11.82	-11.81	-11.8	-11.79	-11.78	-11.77	-11.76	-11.75	-11.74
-11.82	-11.81	-11.8	-11.79	-11.78	-11.77	-11.76	-11.75	-11.74
-11.82	-11.81	-11.8	-11.79	-11.78	-11.77	-11.76	-11.75	-11.74
-11.82	-11.81	-11.8	-11.79	-11.78	-11.77	-11.76	-11.75	-11.74
-11.82	-11.81	-11.8	-11.79	-11.78	-11.77	-11.76	-11.75	-11.74
-11.82	-11.81	-11.8	-11.79	-11.78	-11.77	-11.76	-11.75	-11.74
-11.82	-11.81	-11.8	-11.79	-11.78	-11.77	-11.76	-11.75	-11.74
-11.82	-11.81	-11.8	-11.79	-11.78	-11.77	-11.76	-11.75	-11.74
-11.82	-11.81	-11.8	-11.79	-11.78	-11.77	-11.76	-11.75	-11.74
-11.82	-11.81	-11.8	-11.79	-11.78	-11.77	-11.76	-11.75	-11.74
-11.82	-11.81	-11.8	-11.79	-11.78	-11.77	-11.76	-11.75	-11.74
-11.82	-11.81	-11.8	-11.79	-11.78	-11.77	-11.76	-11.75	-11.74
-11.82	-11.81	-11.8	-11.79	-11.78	-11.77	-11.76	-11.75	-11.74
-11.82	-11.81	-11.8	-11.79	-11.78	-11.77	-11.76	-11.75	-11.74
-11.82	-11.81	-11.8	-11.79	-11.78	-11.77	-11.76	-11.75	-11.74
-11.82	-11.81	-11.8	-11.79	-11.78	-11.77	-11.76	-11.75	-11.74
-11.82	-11.81	-11.8	-11.79	-11.78	-11.77	-11.76	-11.75	-11.74
-11.82	-11.81	-11.8	-11.79	-11.78	-11.77	-11.76	-11.75	-11.74
-11.82	-11.81	-11.8	-11.79	-11.78	-11.77	-11.76	-11.75	-11.74
-11.82	-11.81	-11.8	-11.79	-11.78	-11.77	-11.76	-11.75	-11.74

-11.82	-11.81	-11.8	-11.79	-11.78	-11.77	-11.76	-11.75	-11.74
-11.82	-11.81	-11.8	-11.79	-11.78	-11.77	-11.76	-11.75	-11.74
-11.82	-11.81	-11.8	-11.79	-11.78	-11.77	-11.76	-11.75	-11.74
-11.82	-11.81	-11.8	-11.79	-11.78	-11.77	-11.76	-11.75	-11.74
-11.82	-11.81	-11.8	-11.79	-11.78	-11.77	-11.76	-11.75	-11.74
-11.82	-11.81	-11.8	-11.79	-11.78	-11.77	-11.76	-11.75	-11.74
-11.82	-11.81	-11.8	-11.79	-11.78	-11.77	-11.76	-11.75	-11.74
-11.82	-11.81	-11.8	-11.79	-11.78	-11.77	-11.76	-11.75	-11.74
-11.82	-11.81	-11.8	-11.79	-11.78	-11.77	-11.76	-11.75	-11.74
-11.82	-11.81	-11.8	-11.79	-11.78	-11.77	-11.76	-11.75	-11.74
-11.82	-11.81	-11.8	-11.79	-11.78	-11.77	-11.76	-11.75	-11.74
-11.82	-11.81	-11.8	-11.79	-11.78	-11.77	-11.76	-11.75	-11.74
-11.82	-11.81	-11.8	-11.79	-11.78	-11.77	-11.76	-11.75	-11.74
-11.82	-11.81	-11.8	-11.79	-11.78	-11.77	-11.76	-11.75	-11.74
-11.82	-11.81	-11.8	-11.79	-11.78	-11.77	-11.76	-11.75	-11.74
-11.82	-11.81	-11.8	-11.79	-11.78	-11.77	-11.76	-11.75	-11.74
-11.82	-11.81	-11.8	-11.79	-11.78	-11.77	-11.76	-11.75	-11.74
-11.82	-11.81	-11.8	-11.79	-11.78	-11.77	-11.76	-11.75	-11.74
-11.82	-11.81	-11.8	-11.79	-11.78	-11.77	-11.76	-11.75	-11.74
-11.82	-11.81	-11.8	-11.79	-11.78	-11.77	-11.76	-11.75	-11.74
-11.82	-11.81	-11.8	-11.79	-11.78	-11.77	-11.76	-11.75	-11.74
-11.82	-11.81	-11.8	-11.79	-11.78	-11.77	-11.76	-11.75	-11.74
-11.82	-11.81	-11.8	-11.79	-11.78	-11.77	-11.76	-11.75	-11.74
-11.82	-11.81	-11.8	-11.79	-11.78	-11.77	-11.76	-11.75	-11.74
-11.82	-11.81	-11.8	-11.79	-11.78	-11.77	-11.76	-11.75	-11.74
-11.82	-11.81	-11.8	-11.79	-11.78	-11.77	-11.76	-11.75	-11.74
-11.82	-11.81	-11.8	-11.79	-11.78	-11.77	-11.76	-11.75	-11.74
-11.82	-11.81	-11.8	-11.79	-11.78	-11.77	-11.76	-11.75	-11.74
-11.82	-11.81	-11.8	-11.79	-11.78	-11.77	-11.76	-11.75	-11.74
-11.82	-11.81	-11.8	-11.79	-11.78	-11.77	-11.76	-11.75	-11.74
-11.82	-11.81	-11.8	-11.79	-11.78	-11.77	-11.76	-11.75	-11.74
-11.82	-11.81	-11.8	-11.79	-11.78	-11.77	-11.76	-11.75	-11.74
-11.82	-11.81	-11.8	-11.79	-11.78	-11.77	-11.76	-11.75	-11.74
-11.82	-11.81	-11.8	-11.79	-11.78	-11.77	-11.76	-11.75	-11.74
-11.82	-11.81	-11.8	-11.79	-11.78	-11.77	-11.76	-11.75	-11.74
-11.82	-11.81	-11.8	-11.79	-11.78	-11.77	-11.76	-11.75	-11.74
-11.82	-11.81	-11.8	-11.79	-11.78	-11.77	-11.76	-11.75	-11.74
-11.82	-11.81	-11.8	-11.79	-11.78	-11.77	-11.76	-11.75	-11.74
-11.82	-11.81	-11.8	-11.79	-11.78	-11.77	-11.76	-11.75	-11.74
-11.82	-11.81	-11.8	-11.79	-11.78	-11.77	-11.76	-11.75	-11.74
-11.82	-11.81	-11.8	-11.79	-11.78	-11.77	-11.76	-11.75	-11.74
-11.82	-11.81	-11.8	-11.79	-11.78	-11.77	-11.76	-11.75	-11.74
-11.82	-11.81	-11.8	-11.79	-11.78	-11.77	-11.76	-11.75	-11.74
-11.82	-11.81	-11.8	-11.79	-11.78	-11.77	-11.76	-11.75	-11.74
-11.82	-11.81	-11.8	-11.79	-11.78	-11.77	-11.76	-11.75	-11.74
-11.82	-11.81	-11.8	-11.79	-11.78	-11.77	-11.76	-11.75	-11.74
-11.82	-11.81	-11.8	-11.79	-11.78	-11.77	-11.76	-11.75	-11.74
-11.82	-11.81	-11.8	-11.79	-11.78	-11.77	-11.76	-11.75	-11.74
-11.82	-11.81	-11.8	-11.79	-11.78	-11.77	-11.76	-11.75	-11.74
-11.82	-11.81	-11.8	-11.79	-11.78	-11.77	-11.76	-11.75	-11.74

-11.82	-11.81	-11.8	-11.79	-11.78	-11.77	-11.76	-11.75	-11.74
-11.82	-11.81	-11.8	-11.79	-11.78	-11.77	-11.76	-11.75	-11.74
-11.82	-11.81	-11.8	-11.79	-11.78	-11.77	-11.76	-11.75	-11.74
-11.82	-11.81	-11.8	-11.79	-11.78	-11.77	-11.76	-11.75	-11.74
-11.82	-11.81	-11.8	-11.79	-11.78	-11.77	-11.76	-11.75	-11.74
-11.82	-11.81	-11.8	-11.79	-11.78	-11.77	-11.76	-11.75	-11.74
-11.82	-11.81	-11.8	-11.79	-11.78	-11.77	-11.76	-11.75	-11.74
-11.82	-11.81	-11.8	-11.79	-11.78	-11.77	-11.76	-11.75	-11.74
-11.82	-11.81	-11.8	-11.79	-11.78	-11.77	-11.76	-11.75	-11.74
-11.82	-11.81	-11.8	-11.79	-11.78	-11.77	-11.76	-11.75	-11.74
-11.82	-11.81	-11.8	-11.79	-11.78	-11.77	-11.76	-11.75	-11.74
-11.82	-11.81	-11.8	-11.79	-11.78	-11.77	-11.76	-11.75	-11.74
-11.82	-11.81	-11.8	-11.79	-11.78	-11.77	-11.76	-11.75	-11.74
-11.82	-11.81	-11.8	-11.79	-11.78	-11.77	-11.76	-11.75	-11.74
-11.82	-11.81	-11.8	-11.79	-11.78	-11.77	-11.76	-11.75	-11.74
-11.82	-11.81	-11.8	-11.79	-11.78	-11.77	-11.76	-11.75	-11.74
-11.82	-11.81	-11.8	-11.79	-11.78	-11.77	-11.76	-11.75	-11.74
-11.82	-11.81	-11.8	-11.79	-11.78	-11.77	-11.76	-11.75	-11.74
-11.82	-11.81	-11.8	-11.79	-11.78	-11.77	-11.76	-11.75	-11.74
-11.82	-11.81	-11.8	-11.79	-11.78	-11.77	-11.76	-11.75	-11.74
-11.82	-11.81	-11.8	-11.79	-11.78	-11.77	-11.76	-11.75	-11.74
-11.82	-11.81	-11.8	-11.79	-11.78	-11.77	-11.76	-11.75	-11.74
-11.82	-11.81	-11.8	-11.79	-11.78	-11.77	-11.76	-11.75	-11.74
-11.82	-11.81	-11.8	-11.79	-11.78	-11.77	-11.76	-11.75	-11.74
-11.82	-11.81	-11.8	-11.79	-11.78	-11.77	-11.76	-11.75	-11.74
-11.82	-11.81	-11.8	-11.79	-11.78	-11.77	-11.76	-11.75	-11.74
-11.82	-11.81	-11.8	-11.79	-11.78	-11.77	-11.76	-11.75	-11.74
-11.82	-11.81	-11.8	-11.79	-11.78	-11.77	-11.76	-11.75	-11.74
-11.82	-11.81	-11.8	-11.79	-11.78	-11.77	-11.76	-11.75	-11.74
-11.82	-11.81	-11.8	-11.79	-11.78	-11.77	-11.76	-11.75	-11.74
-11.82	-11.81	-11.8	-11.79	-11.78	-11.77	-11.76	-11.75	-11.74
-11.82	-11.81	-11.8	-11.79	-11.78	-11.77	-11.76	-11.75	-11.74
-11.82	-11.81	-11.8	-11.79	-11.78	-11.77	-11.76	-11.75	-11.74
-11.82	-11.81	-11.8	-11.79	-11.78	-11.77	-11.76	-11.75	-11.74
-11.82	-11.81	-11.8	-11.79	-11.78	-11.77	-11.76	-11.75	-11.74
-11.82	-11.81	-11.8	-11.79	-11.78	-11.77	-11.76	-11.75	-11.74
-11.82	-11.81	-11.8	-11.79	-11.78	-11.77	-11.76	-11.75	-11.74
-11.82	-11.81	-11.8	-11.79	-11.78	-11.77	-11.76	-11.75	-11.74
-11.82	-11.81	-11.8	-11.79	-11.78	-11.77	-11.76	-11.75	-11.74
-11.82	-11.81	-11.8	-11.79	-11.78	-11.77	-11.76	-11.75	-11.74
-11.82	-11.81	-11.8	-11.79	-11.78	-11.77	-11.76	-11.75	-11.74
-11.82	-11.81	-11.8	-11.79	-11.78	-11.77	-11.76	-11.75	-11.74
-11.82	-11.81	-11.8	-11.79	-11.78	-11.77	-11.76	-11.75	-11.74
-11.82	-11.81	-11.8	-11.79	-11.78	-11.77	-11.76	-11.75	-11.74
-11.82	-11.81	-11.8	-11.79	-11.78	-11.77	-11.76	-11.75	-11.74
-11.82	-11.81	-11.8	-11.79	-11.78	-11.77	-11.76	-11.75	-11.74
-11.82	-11.81	-11.8	-11.79	-11.78	-11.77	-11.76	-11.75	-11.74
-11.82	-11.81	-11.8	-11.79	-11.78	-11.77	-11.76	-11.75	-11.74
-11.82	-11.81	-11.8	-11.79	-11.78	-11.77	-11.76	-11.75	-11.74
-11.82	-11.81	-11.8	-11.79	-11.78	-11.77	-11.76	-11.75	-11.74

-11.82	-11.81	-11.8	-11.79	-11.78	-11.77	-11.76	-11.75	-11.74
-11.82	-11.81	-11.8	-11.79	-11.78	-11.77	-11.76	-11.75	-11.74
-11.82	-11.81	-11.8	-11.79	-11.78	-11.77	-11.76	-11.75	-11.74
-11.82	-11.81	-11.8	-11.79	-11.78	-11.77	-11.76	-11.75	-11.74
-11.82	-11.81	-11.8	-11.79	-11.78	-11.77	-11.76	-11.75	-11.74
-11.82	-11.81	-11.8	-11.79	-11.78	-11.77	-11.76	-11.75	-11.74
-11.82	-11.81	-11.8	-11.79	-11.78	-11.77	-11.76	-11.75	-11.74
-11.82	-11.81	-11.8	-11.79	-11.78	-11.77	-11.76	-11.75	-11.74
-11.82	-11.81	-11.8	-11.79	-11.78	-11.77	-11.76	-11.75	-11.74
-11.82	-11.81	-11.8	-11.79	-11.78	-11.77	-11.76	-11.75	-11.74
-11.82	-11.81	-11.8	-11.79	-11.78	-11.77	-11.76	-11.75	-11.74
-11.82	-11.81	-11.8	-11.79	-11.78	-11.77	-11.76	-11.75	-11.74
-11.82	-11.81	-11.8	-11.79	-11.78	-11.77	-11.76	-11.75	-11.74
-11.82	-11.81	-11.8	-11.79	-11.78	-11.77	-11.76	-11.75	-11.74
-11.82	-11.81	-11.8	-11.79	-11.78	-11.77	-11.76	-11.75	-11.74
-11.82	-11.81	-11.8	-11.79	-11.78	-11.77	-11.76	-11.75	-11.74
-11.82	-11.81	-11.8	-11.79	-11.78	-11.77	-11.76	-11.75	-11.74
-11.82	-11.81	-11.8	-11.79	-11.78	-11.77	-11.76	-11.75	-11.74
-11.82	-11.81	-11.8	-11.79	-11.78	-11.77	-11.76	-11.75	-11.74
-11.82	-11.81	-11.8	-11.79	-11.78	-11.77	-11.76	-11.75	-11.74
-11.82	-11.81	-11.8	-11.79	-11.78	-11.77	-11.76	-11.75	-11.74
-11.82	-11.81	-11.8	-11.79	-11.78	-11.77	-11.76	-11.75	-11.74
-11.82	-11.81	-11.8	-11.79	-11.78	-11.77	-11.76	-11.75	-11.74
-11.82	-11.81	-11.8	-11.79	-11.78	-11.77	-11.76	-11.75	-11.74
-11.82	-11.81	-11.8	-11.79	-11.78	-11.77	-11.76	-11.75	-11.74
-11.82	-11.81	-11.8	-11.79	-11.78	-11.77	-11.76	-11.75	-11.74
-11.82	-11.81	-11.8	-11.79	-11.78	-11.77	-11.76	-11.75	-11.74
-11.82	-11.81	-11.8	-11.79	-11.78	-11.77	-11.76	-11.75	-11.74
-11.82	-11.81	-11.8	-11.79	-11.78	-11.77	-11.76	-11.75	-11.74
-11.82	-11.81	-11.8	-11.79	-11.78	-11.77	-11.76	-11.75	-11.74
-11.82	-11.81	-11.8	-11.79	-11.78	-11.77	-11.76	-11.75	-11.74
-11.82	-11.81	-11.8	-11.79	-11.78	-11.77	-11.76	-11.75	-11.74
-11.82	-11.81	-11.8	-11.79	-11.78	-11.77	-11.76	-11.75	-11.74
-11.82	-11.81	-11.8	-11.79	-11.78	-11.77	-11.76	-11.75	-11.74
-11.82	-11.81	-11.8	-11.79	-11.78	-11.77	-11.76	-11.75	-11.74
-11.82	-11.81	-11.8	-11.79	-11.78	-11.77	-11.76	-11.75	-11.74
-11.82	-11.81	-11.8	-11.79	-11.78	-11.77	-11.76	-11.75	-11.74
-11.82	-11.81	-11.8	-11.79	-11.78	-11.77	-11.76	-11.75	-11.74
-11.82	-11.81	-11.8	-11.79	-11.78	-11.77	-11.76	-11.75	-11.74
-11.82	-11.81	-11.8	-11.79	-11.78	-11.77	-11.76	-11.75	-11.74
-11.82	-11.81	-11.8	-11.79	-11.78	-11.77	-11.76	-11.75	-11.74
-11.82	-11.81	-11.8	-11.79	-11.78	-11.77	-11.76	-11.75	-11.74
-11.82	-11.81	-11.8	-11.79	-11.78	-11.77	-11.76	-11.75	-11.74
-11.82	-11.81	-11.8	-11.79	-11.78	-11.77	-11.76	-11.75	-11.74
-11.82	-11.81	-11.8	-11.79	-11.78	-11.77	-11.76	-11.75	-11.74
-11.82	-11.81	-11.8	-11.79	-11.78	-11.77	-11.76	-11.75	-11.74
-11.82	-11.81	-11.8	-11.79	-11.78	-11.77	-11.76	-11.75	-11.74
-11.82	-11.81	-11.8	-11.79	-11.78	-11.77	-11.76	-11.75	-11.74
-11.82	-11.81	-11.8	-11.79	-11.78	-11.77	-11.76	-11.75	-11.74
-11.82	-11.81	-11.8	-11.79	-11.78	-11.77	-11.76	-11.75	-11.74

-11.82	-11.81	-11.8	-11.79	-11.78	-11.77	-11.76	-11.75	-11.74
-11.82	-11.81	-11.8	-11.79	-11.78	-11.77	-11.76	-11.75	-11.74
-11.82	-11.81	-11.8	-11.79	-11.78	-11.77	-11.76	-11.75	-11.74
-11.82	-11.81	-11.8	-11.79	-11.78	-11.77	-11.76	-11.75	-11.74
-11.82	-11.81	-11.8	-11.79	-11.78	-11.77	-11.76	-11.75	-11.74
-11.82	-11.81	-11.8	-11.79	-11.78	-11.77	-11.76	-11.75	-11.74
-11.82	-11.81	-11.8	-11.79	-11.78	-11.77	-11.76	-11.75	-11.74
-11.82	-11.81	-11.8	-11.79	-11.78	-11.77	-11.76	-11.75	-11.74
-11.82	-11.81	-11.8	-11.79	-11.78	-11.77	-11.76	-11.75	-11.74
-11.82	-11.81	-11.8	-11.79	-11.78	-11.77	-11.76	-11.75	-11.74
-11.82	-11.81	-11.8	-11.79	-11.78	-11.77	-11.76	-11.75	-11.74
-11.82	-11.81	-11.8	-11.79	-11.78	-11.77	-11.76	-11.75	-11.74
-11.82	-11.81	-11.8	-11.79	-11.78	-11.77	-11.76	-11.75	-11.74
-11.82	-11.81	-11.8	-11.79	-11.78	-11.77	-11.76	-11.75	-11.74
-11.82	-11.81	-11.8	-11.79	-11.78	-11.77	-11.76	-11.75	-11.74
-11.82	-11.81	-11.8	-11.79	-11.78	-11.77	-11.76	-11.75	-11.74
-11.82	-11.81	-11.8	-11.79	-11.78	-11.77	-11.76	-11.75	-11.74
-11.82	-11.81	-11.8	-11.79	-11.78	-11.77	-11.76	-11.75	-11.74
-11.82	-11.81	-11.8	-11.79	-11.78	-11.77	-11.76	-11.75	-11.74
-11.82	-11.81	-11.8	-11.79	-11.78	-11.77	-11.76	-11.75	-11.74
-11.82	-11.81	-11.8	-11.79	-11.78	-11.77	-11.76	-11.75	-11.74
-11.82	-11.81	-11.8	-11.79	-11.78	-11.77	-11.76	-11.75	-11.74
-11.82	-11.81	-11.8	-11.79	-11.78	-11.77	-11.76	-11.75	-11.74
-11.82	-11.81	-11.8	-11.79	-11.78	-11.77	-11.76	-11.75	-11.74
-11.82	-11.81	-11.8	-11.79	-11.78	-11.77	-11.76	-11.75	-11.74
-11.82	-11.81	-11.8	-11.79	-11.78	-11.77	-11.76	-11.75	-11.74
-11.82	-11.81	-11.8	-11.79	-11.78	-11.77	-11.76	-11.75	-11.74
-11.82	-11.81	-11.8	-11.79	-11.78	-11.77	-11.76	-11.75	-11.74
-11.82	-11.81	-11.8	-11.79	-11.78	-11.77	-11.76	-11.75	-11.74
-11.82	-11.81	-11.8	-11.79	-11.78	-11.77	-11.76	-11.75	-11.74
-11.82	-11.81	-11.8	-11.79	-11.78	-11.77	-11.76	-11.75	-11.74
-11.82	-11.81	-11.8	-11.79	-11.78	-11.77	-11.76	-11.75	-11.74
-11.82	-11.81	-11.8	-11.79	-11.78	-11.77	-11.76	-11.75	-11.74
-11.82	-11.81	-11.8	-11.79	-11.78	-11.77	-11.76	-11.75	-11.74
-11.82	-11.81	-11.8	-11.79	-11.78	-11.77	-11.76	-11.75	-11.74
-11.82	-11.81	-11.8	-11.79	-11.78	-11.77	-11.76	-11.75	-11.74
-11.82	-11.81	-11.8	-11.79	-11.78	-11.77	-11.76	-11.75	-11.74
-11.82	-11.81	-11.8	-11.79	-11.78	-11.77	-11.76	-11.75	-11.74
-11.82	-11.81	-11.8	-11.79	-11.78	-11.77	-11.76	-11.75	-11.74
-11.82	-11.81	-11.8	-11.79	-11.78	-11.77	-11.76	-11.75	-11.74
-11.82	-11.81	-11.8	-11.79	-11.78	-11.77	-11.76	-11.75	-11.74
-11.82	-11.81	-11.8	-11.79	-11.78	-11.77	-11.76	-11.75	-11.74
-11.82	-11.81	-11.8	-11.79	-11.78	-11.77	-11.76	-11.75	-11.74
-11.82	-11.81	-11.8	-11.79	-11.78	-11.77	-11.76	-11.75	-11.74
-11.82	-11.81	-11.8	-11.79	-11.78	-11.77	-11.76	-11.75	-11.74
-11.82	-11.81	-11.8	-11.79	-11.78	-11.77	-11.76	-11.75	-11.74
-11.82	-11.81	-11.8	-11.79	-11.78	-11.77	-11.76	-11.75	-11.74
-11.82	-11.81	-11.8	-11.79	-11.78	-11.77	-11.76	-11.75	-11.74
-11.82	-11.81	-11.8	-11.79	-11.78	-11.77	-11.76	-11.75	-11.74
-11.82	-11.81	-11.8	-11.79	-11.78	-11.77	-11.76	-11.75	-11.74

-11.82	-11.81	-11.8	-11.79	-11.78	-11.77	-11.76	-11.75	-11.74
-11.82	-11.81	-11.8	-11.79	-11.78	-11.77	-11.76	-11.75	-11.74
-11.82	-11.81	-11.8	-11.79	-11.78	-11.77	-11.76	-11.75	-11.74
-11.82	-11.81	-11.8	-11.79	-11.78	-11.77	-11.76	-11.75	-11.74
-11.82	-11.81	-11.8	-11.79	-11.78	-11.77	-11.76	-11.75	-11.74
-11.82	-11.81	-11.8	-11.79	-11.78	-11.77	-11.76	-11.75	-11.74
-11.82	-11.81	-11.8	-11.79	-11.78	-11.77	-11.76	-11.75	-11.74
-11.82	-11.81	-11.8	-11.79	-11.78	-11.77	-11.76	-11.75	-11.74
-11.82	-11.81	-11.8	-11.79	-11.78	-11.77	-11.76	-11.75	-11.74
-11.82	-11.81	-11.8	-11.79	-11.78	-11.77	-11.76	-11.75	-11.74
-11.82	-11.81	-11.8	-11.79	-11.78	-11.77	-11.76	-11.75	-11.74
-11.82	-11.81	-11.8	-11.79	-11.78	-11.77	-11.76	-11.75	-11.74
-11.82	-11.81	-11.8	-11.79	-11.78	-11.77	-11.76	-11.75	-11.74
-11.82	-11.81	-11.8	-11.79	-11.78	-11.77	-11.76	-11.75	-11.74
-11.82	-11.81	-11.8	-11.79	-11.78	-11.77	-11.76	-11.75	-11.74
-11.82	-11.81	-11.8	-11.79	-11.78	-11.77	-11.76	-11.75	-11.74
-11.82	-11.81	-11.8	-11.79	-11.78	-11.77	-11.76	-11.75	-11.74
-11.82	-11.81	-11.8	-11.79	-11.78	-11.77	-11.76	-11.75	-11.74
-11.82	-11.81	-11.8	-11.79	-11.78	-11.77	-11.76	-11.75	-11.74
-11.82	-11.81	-11.8	-11.79	-11.78	-11.77	-11.76	-11.75	-11.74
-11.82	-11.81	-11.8	-11.79	-11.78	-11.77	-11.76	-11.75	-11.74
-11.82	-11.81	-11.8	-11.79	-11.78	-11.77	-11.76	-11.75	-11.74
-11.82	-11.81	-11.8	-11.79	-11.78	-11.77	-11.76	-11.75	-11.74
-11.82	-11.81	-11.8	-11.79	-11.78	-11.77	-11.76	-11.75	-11.74
-11.82	-11.81	-11.8	-11.79	-11.78	-11.77	-11.76	-11.75	-11.74
-11.82	-11.81	-11.8	-11.79	-11.78	-11.77	-11.76	-11.75	-11.74
-11.82	-11.81	-11.8	-11.79	-11.78	-11.77	-11.76	-11.75	-11.74
-11.82	-11.81	-11.8	-11.79	-11.78	-11.77	-11.76	-11.75	-11.74
-11.82	-11.81	-11.8	-11.79	-11.78	-11.77	-11.76	-11.75	-11.74
-11.82	-11.81	-11.8	-11.79	-11.78	-11.77	-11.76	-11.75	-11.74
-11.82	-11.81	-11.8	-11.79	-11.78	-11.77	-11.76	-11.75	-11.74
-11.82	-11.81	-11.8	-11.79	-11.78	-11.77	-11.76	-11.75	-11.74
-11.82	-11.81	-11.8	-11.79	-11.78	-11.77	-11.76	-11.75	-11.74
-11.82	-11.81	-11.8	-11.79	-11.78	-11.77	-11.76	-11.75	-11.74
-11.82	-11.81	-11.8	-11.79	-11.78	-11.77	-11.76	-11.75	-11.74
-11.82	-11.81	-11.8	-11.79	-11.78	-11.77	-11.76	-11.75	-11.74
-11.82	-11.81	-11.8	-11.79	-11.78	-11.77	-11.76	-11.75	-11.74
-11.82	-11.81	-11.8	-11.79	-11.78	-11.77	-11.76	-11.75	-11.74
-11.82	-11.81	-11.8	-11.79	-11.78	-11.77	-11.76	-11.75	-11.74
-11.82	-11.81	-11.8	-11.79	-11.78	-11.77	-11.76	-11.75	-11.74
-11.82	-11.81	-11.8	-11.79	-11.78	-11.77	-11.76	-11.75	-11.74
-11.82	-11.81	-11.8	-11.79	-11.78	-11.77	-11.76	-11.75	-11.74
-11.82	-11.81	-11.8	-11.79	-11.78	-11.77	-11.76	-11.75	-11.74
-11.82	-11.81	-11.8	-11.79	-11.78	-11.77	-11.76	-11.75	-11.74
-11.82	-11.81	-11.8	-11.79	-11.78	-11.77	-11.76	-11.75	-11.74
-11.82	-11.81	-11.8	-11.79	-11.78	-11.77	-11.76	-11.75	-11.74
-11.82	-11.81	-11.8	-11.79	-11.78	-11.77	-11.76	-11.75	-11.74
-11.82	-11.81	-11.8	-11.79	-11.78	-11.77	-11.76	-11.75	-11.74
-11.82	-11.81	-11.8	-11.79	-11.78	-11.77	-11.76	-11.75	-11.74
-11.82	-11.81	-11.8	-11.79	-11.78	-11.77	-11.76	-11.75	-11.74

-11.82	-11.81	-11.8	-11.79	-11.78	-11.77	-11.76	-11.75	-11.74
-11.82	-11.81	-11.8	-11.79	-11.78	-11.77	-11.76	-11.75	-11.74
-11.82	-11.81	-11.8	-11.79	-11.78	-11.77	-11.76	-11.75	-11.74
-11.82	-11.81	-11.8	-11.79	-11.78	-11.77	-11.76	-11.75	-11.74
-11.82	-11.81	-11.8	-11.79	-11.78	-11.77	-11.76	-11.75	-11.74
-11.82	-11.81	-11.8	-11.79	-11.78	-11.77	-11.76	-11.75	-11.74
-11.82	-11.81	-11.8	-11.79	-11.78	-11.77	-11.76	-11.75	-11.74
-11.82	-11.81	-11.8	-11.79	-11.78	-11.77	-11.76	-11.75	-11.74
-11.82	-11.81	-11.8	-11.79	-11.78	-11.77	-11.76	-11.75	-11.74
-11.82	-11.81	-11.8	-11.79	-11.78	-11.77	-11.76	-11.75	-11.74
-11.82	-11.81	-11.8	-11.79	-11.78	-11.77	-11.76	-11.75	-11.74
-11.82	-11.81	-11.8	-11.79	-11.78	-11.77	-11.76	-11.75	-11.74
-11.82	-11.81	-11.8	-11.79	-11.78	-11.77	-11.76	-11.75	-11.74
-11.82	-11.81	-11.8	-11.79	-11.78	-11.77	-11.76	-11.75	-11.74
-11.82	-11.81	-11.8	-11.79	-11.78	-11.77	-11.76	-11.75	-11.74
-11.82	-11.81	-11.8	-11.79	-11.78	-11.77	-11.76	-11.75	-11.74
-11.82	-11.81	-11.8	-11.79	-11.78	-11.77	-11.76	-11.75	-11.74
-11.82	-11.81	-11.8	-11.79	-11.78	-11.77	-11.76	-11.75	-11.74
-11.82	-11.81	-11.8	-11.79	-11.78	-11.77	-11.76	-11.75	-11.74
-11.82	-11.81	-11.8	-11.79	-11.78	-11.77	-11.76	-11.75	-11.74
-11.82	-11.81	-11.8	-11.79	-11.78	-11.77	-11.76	-11.75	-11.74
-11.82	-11.81	-11.8	-11.79	-11.78	-11.77	-11.76	-11.75	-11.74
-11.82	-11.81	-11.8	-11.79	-11.78	-11.77	-11.76	-11.75	-11.74
-11.82	-11.81	-11.8	-11.79	-11.78	-11.77	-11.76	-11.75	-11.74
-11.82	-11.81	-11.8	-11.79	-11.78	-11.77	-11.76	-11.75	-11.74
-11.82	-11.81	-11.8	-11.79	-11.78	-11.77	-11.76	-11.75	-11.74
-11.82	-11.81	-11.8	-11.79	-11.78	-11.77	-11.76	-11.75	-11.74
-11.82	-11.81	-11.8	-11.79	-11.78	-11.77	-11.76	-11.75	-11.74
-11.82	-11.81	-11.8	-11.79	-11.78	-11.77	-11.76	-11.75	-11.74
-11.82	-11.81	-11.8	-11.79	-11.78	-11.77	-11.76	-11.75	-11.74
-11.82	-11.81	-11.8	-11.79	-11.78	-11.77	-11.76	-11.75	-11.74
-11.82	-11.81	-11.8	-11.79	-11.78	-11.77	-11.76	-11.75	-11.74
-11.82	-11.81	-11.8	-11.79	-11.78	-11.77	-11.76	-11.75	-11.74
-11.82	-11.81	-11.8	-11.79	-11.78	-11.77	-11.76	-11.75	-11.74
-11.82	-11.81	-11.8	-11.79	-11.78	-11.77	-11.76	-11.75	-11.74
-11.82	-11.81	-11.8	-11.79	-11.78	-11.77	-11.76	-11.75	-11.74
-11.82	-11.81	-11.8	-11.79	-11.78	-11.77	-11.76	-11.75	-11.74
-11.82	-11.81	-11.8	-11.79	-11.78	-11.77	-11.76	-11.75	-11.74
-11.82	-11.81	-11.8	-11.79	-11.78	-11.77	-11.76	-11.75	-11.74
-11.82	-11.81	-11.8	-11.79	-11.78	-11.77	-11.76	-11.75	-11.74
-11.82	-11.81	-11.8	-11.79	-11.78	-11.77	-11.76	-11.75	-11.74
-11.82	-11.81	-11.8	-11.79	-11.78	-11.77	-11.76	-11.75	-11.74
-11.82	-11.81	-11.8	-11.79	-11.78	-11.77	-11.76	-11.75	-11.74
-11.82	-11.81	-11.8	-11.79	-11.78	-11.77	-11.76	-11.75	-11.74
-11.82	-11.81	-11.8	-11.79	-11.78	-11.77	-11.76	-11.75	-11.74
-11.82	-11.81	-11.8	-11.79	-11.78	-11.77	-11.76	-11.75	-11.74
-11.82	-11.81	-11.8	-11.79	-11.78	-11.77	-11.76	-11.75	-11.74
-11.82	-11.81	-11.8	-11.79	-11.78	-11.77	-11.76	-11.75	-11.74
-11.82	-11.81	-11.8	-11.79	-11.78	-11.77	-11.76	-11.75	-11.74
-11.82	-11.81	-11.8	-11.79	-11.78	-11.77	-11.76	-11.75	-11.74

-11.82	-11.81	-11.8	-11.79	-11.78	-11.77	-11.76	-11.75	-11.74
-11.82	-11.81	-11.8	-11.79	-11.78	-11.77	-11.76	-11.75	-11.74
-11.82	-11.81	-11.8	-11.79	-11.78	-11.77	-11.76	-11.75	-11.74
-11.82	-11.81	-11.8	-11.79	-11.78	-11.77	-11.76	-11.75	-11.74
-11.82	-11.81	-11.8	-11.79	-11.78	-11.77	-11.76	-11.75	-11.74
-11.82	-11.81	-11.8	-11.79	-11.78	-11.77	-11.76	-11.75	-11.74
-11.82	-11.81	-11.8	-11.79	-11.78	-11.77	-11.76	-11.75	-11.74
-11.82	-11.81	-11.8	-11.79	-11.78	-11.77	-11.76	-11.75	-11.74
-11.82	-11.81	-11.8	-11.79	-11.78	-11.77	-11.76	-11.75	-11.74
-11.82	-11.81	-11.8	-11.79	-11.78	-11.77	-11.76	-11.75	-11.74
-11.82	-11.81	-11.8	-11.79	-11.78	-11.77	-11.76	-11.75	-11.74
-11.82	-11.81	-11.8	-11.79	-11.78	-11.77	-11.76	-11.75	-11.74
-11.82	-11.81	-11.8	-11.79	-11.78	-11.77	-11.76	-11.75	-11.74
-11.82	-11.81	-11.8	-11.79	-11.78	-11.77	-11.76	-11.75	-11.74
-11.82	-11.81	-11.8	-11.79	-11.78	-11.77	-11.76	-11.75	-11.74
-11.82	-11.81	-11.8	-11.79	-11.78	-11.77	-11.76	-11.75	-11.74
-11.82	-11.81	-11.8	-11.79	-11.78	-11.77	-11.76	-11.75	-11.74
-11.82	-11.81	-11.8	-11.79	-11.78	-11.77	-11.76	-11.75	-11.74
-11.82	-11.81	-11.8	-11.79	-11.78	-11.77	-11.76	-11.75	-11.74
-11.82	-11.81	-11.8	-11.79	-11.78	-11.77	-11.76	-11.75	-11.74
-11.82	-11.81	-11.8	-11.79	-11.78	-11.77	-11.76	-11.75	-11.74
-11.82	-11.81	-11.8	-11.79	-11.78	-11.77	-11.76	-11.75	-11.74
-11.82	-11.81	-11.8	-11.79	-11.78	-11.77	-11.76	-11.75	-11.74
-11.82	-11.81	-11.8	-11.79	-11.78	-11.77	-11.76	-11.75	-11.74
-11.82	-11.81	-11.8	-11.79	-11.78	-11.77	-11.76	-11.75	-11.74
-11.82	-11.81	-11.8	-11.79	-11.78	-11.77	-11.76	-11.75	-11.74
-11.82	-11.81	-11.8	-11.79	-11.78	-11.77	-11.76	-11.75	-11.74
-11.82	-11.81	-11.8	-11.79	-11.78	-11.77	-11.76	-11.75	-11.74
-11.82	-11.81	-11.8	-11.79	-11.78	-11.77	-11.76	-11.75	-11.74
-11.82	-11.81	-11.8	-11.79	-11.78	-11.77	-11.76	-11.75	-11.74
-11.82	-11.81	-11.8	-11.79	-11.78	-11.77	-11.76	-11.75	-11.74
-11.82	-11.81	-11.8	-11.79	-11.78	-11.77	-11.76	-11.75	-11.74
-11.82	-11.81	-11.8	-11.79	-11.78	-11.77	-11.76	-11.75	-11.74
-11.82	-11.81	-11.8	-11.79	-11.78	-11.77	-11.76	-11.75	-11.74
-11.82	-11.81	-11.8	-11.79	-11.78	-11.77	-11.76	-11.75	-11.74
-11.82	-11.81	-11.8	-11.79	-11.78	-11.77	-11.76	-11.75	-11.74
-11.82	-11.81	-11.8	-11.79	-11.78	-11.77	-11.76	-11.75	-11.74
-11.82	-11.81	-11.8	-11.79	-11.78	-11.77	-11.76	-11.75	-11.74
-11.82	-11.81	-11.8	-11.79	-11.78	-11.77	-11.76	-11.75	-11.74
-11.82	-11.81	-11.8	-11.79	-11.78	-11.77	-11.76	-11.75	-11.74
-11.82	-11.81	-11.8	-11.79	-11.78	-11.77	-11.76	-11.75	-11.74
-11.82	-11.81	-11.8	-11.79	-11.78	-11.77	-11.76	-11.75	-11.74
-11.82	-11.81	-11.8	-11.79	-11.78	-11.77	-11.76	-11.75	-11.74
-11.82	-11.81	-11.8	-11.79	-11.78	-11.77	-11.76	-11.75	-11.74
-11.82	-11.81	-11.8	-11.79	-11.78	-11.77	-11.76	-11.75	-11.74
-11.82	-11.81	-11.8	-11.79	-11.78	-11.77	-11.76	-11.75	-11.74
-11.82	-11.81	-11.8	-11.79	-11.78	-11.77	-11.76	-11.75	-11.74
-11.82	-11.81	-11.8	-11.79	-11.78	-11.77	-11.76	-11.75	-11.74
-11.82	-11.81	-11.8	-11.79	-11.78	-11.77	-11.76	-11.75	-11.74
-11.82	-11.81	-11.8	-11.79	-11.78	-11.77	-11.76	-11.75	-11.74

-11.82	-11.81	-11.8	-11.79	-11.78	-11.77	-11.76	-11.75	-11.74
-11.82	-11.81	-11.8	-11.79	-11.78	-11.77	-11.76	-11.75	-11.74
-11.82	-11.81	-11.8	-11.79	-11.78	-11.77	-11.76	-11.75	-11.74
-11.82	-11.81	-11.8	-11.79	-11.78	-11.77	-11.76	-11.75	-11.74
-11.82	-11.81	-11.8	-11.79	-11.78	-11.77	-11.76	-11.75	-11.74
-11.82	-11.81	-11.8	-11.79	-11.78	-11.77	-11.76	-11.75	-11.74
-11.82	-11.81	-11.8	-11.79	-11.78	-11.77	-11.76	-11.75	-11.74
-11.82	-11.81	-11.8	-11.79	-11.78	-11.77	-11.76	-11.75	-11.74
-11.82	-11.81	-11.8	-11.79	-11.78	-11.77	-11.76	-11.75	-11.74
-11.82	-11.81	-11.8	-11.79	-11.78	-11.77	-11.76	-11.75	-11.74
-11.82	-11.81	-11.8	-11.79	-11.78	-11.77	-11.76	-11.75	-11.74
-11.82	-11.81	-11.8	-11.79	-11.78	-11.77	-11.76	-11.75	-11.74
-11.82	-11.81	-11.8	-11.79	-11.78	-11.77	-11.76	-11.75	-11.74
-11.82	-11.81	-11.8	-11.79	-11.78	-11.77	-11.76	-11.75	-11.74
-11.82	-11.81	-11.8	-11.79	-11.78	-11.77	-11.76	-11.75	-11.74
-11.82	-11.81	-11.8	-11.79	-11.78	-11.77	-11.76	-11.75	-11.74
-11.82	-11.81	-11.8	-11.79	-11.78	-11.77	-11.76	-11.75	-11.74
-11.82	-11.81	-11.8	-11.79	-11.78	-11.77	-11.76	-11.75	-11.74
-11.82	-11.81	-11.8	-11.79	-11.78	-11.77	-11.76	-11.75	-11.74
-11.82	-11.81	-11.8	-11.79	-11.78	-11.77	-11.76	-11.75	-11.74
-11.82	-11.81	-11.8	-11.79	-11.78	-11.77	-11.76	-11.75	-11.74
-11.82	-11.81	-11.8	-11.79	-11.78	-11.77	-11.76	-11.75	-11.74
-11.82	-11.81	-11.8	-11.79	-11.78	-11.77	-11.76	-11.75	-11.74
-11.82	-11.81	-11.8	-11.79	-11.78	-11.77	-11.76	-11.75	-11.74
-11.82	-11.81	-11.8	-11.79	-11.78	-11.77	-11.76	-11.75	-11.74
-11.82	-11.81	-11.8	-11.79	-11.78	-11.77	-11.76	-11.75	-11.74
-11.82	-11.81	-11.8	-11.79	-11.78	-11.77	-11.76	-11.75	-11.74
-11.82	-11.81	-11.8	-11.79	-11.78	-11.77	-11.76	-11.75	-11.74
-11.82	-11.81	-11.8	-11.79	-11.78	-11.77	-11.76	-11.75	-11.74
-11.82	-11.81	-11.8	-11.79	-11.78	-11.77	-11.76	-11.75	-11.74
-11.82	-11.81	-11.8	-11.79	-11.78	-11.77	-11.76	-11.75	-11.74
-11.82	-11.81	-11.8	-11.79	-11.78	-11.77	-11.76	-11.75	-11.74
-11.82	-11.81	-11.8	-11.79	-11.78	-11.77	-11.76	-11.75	-11.74
-11.82	-11.81	-11.8	-11.79	-11.78	-11.77	-11.76	-11.75	-11.74
-11.82	-11.81	-11.8	-11.79	-11.78	-11.77	-11.76	-11.75	-11.74
-11.82	-11.81	-11.8	-11.79	-11.78	-11.77	-11.76	-11.75	-11.74
-11.82	-11.81	-11.8	-11.79	-11.78	-11.77	-11.76	-11.75	-11.74
-11.82	-11.81	-11.8	-11.79	-11.78	-11.77	-11.76	-11.75	-11.74
-11.82	-11.81	-11.8	-11.79	-11.78	-11.77	-11.76	-11.75	-11.74
-11.82	-11.81	-11.8	-11.79	-11.78	-11.77	-11.76	-11.75	-11.74
-11.82	-11.81	-11.8	-11.79	-11.78	-11.77	-11.76	-11.75	-11.74
-11.82	-11.81	-11.8	-11.79	-11.78	-11.77	-11.76	-11.75	-11.74
-11.82	-11.81	-11.8	-11.79	-11.78	-11.77	-11.76	-11.75	-11.74
-11.82	-11.81	-11.8	-11.79	-11.78	-11.77	-11.76	-11.75	-11.74
-11.82	-11.81	-11.8	-11.79	-11.78	-11.77	-11.76	-11.75	-11.74
-11.82	-11.81	-11.8	-11.79	-11.78	-11.77	-11.76	-11.75	-11.74
-11.82	-11.81	-11.8	-11.79	-11.78	-11.77	-11.76	-11.75	-11.74
-11.82	-11.81	-11.8	-11.79	-11.78	-11.77	-11.76	-11.75	-11.74
-11.82	-11.81	-11.8	-11.79	-11.78	-11.77	-11.76	-11.75	-11.74
-11.82	-11.81	-11.8	-11.79	-11.78	-11.77	-11.76	-11.75	-11.74

-11.82	-11.81	-11.8	-11.79	-11.78	-11.77	-11.76	-11.75	-11.74
-11.82	-11.81	-11.8	-11.79	-11.78	-11.77	-11.76	-11.75	-11.74
-11.82	-11.81	-11.8	-11.79	-11.78	-11.77	-11.76	-11.75	-11.74
-11.82	-11.81	-11.8	-11.79	-11.78	-11.77	-11.76	-11.75	-11.74
-11.82	-11.81	-11.8	-11.79	-11.78	-11.77	-11.76	-11.75	-11.74
-11.82	-11.81	-11.8	-11.79	-11.78	-11.77	-11.76	-11.75	-11.74
-11.82	-11.81	-11.8	-11.79	-11.78	-11.77	-11.76	-11.75	-11.74
-11.82	-11.81	-11.8	-11.79	-11.78	-11.77	-11.76	-11.75	-11.74
-11.82	-11.81	-11.8	-11.79	-11.78	-11.77	-11.76	-11.75	-11.74
-11.82	-11.81	-11.8	-11.79	-11.78	-11.77	-11.76	-11.75	-11.74
-11.82	-11.81	-11.8	-11.79	-11.78	-11.77	-11.76	-11.75	-11.74
-11.82	-11.81	-11.8	-11.79	-11.78	-11.77	-11.76	-11.75	-11.74
-11.82	-11.81	-11.8	-11.79	-11.78	-11.77	-11.76	-11.75	-11.74
-11.82	-11.81	-11.8	-11.79	-11.78	-11.77	-11.76	-11.75	-11.74
-11.82	-11.81	-11.8	-11.79	-11.78	-11.77	-11.76	-11.75	-11.74
-11.82	-11.81	-11.8	-11.79	-11.78	-11.77	-11.76	-11.75	-11.74
-11.82	-11.81	-11.8	-11.79	-11.78	-11.77	-11.76	-11.75	-11.74
-11.82	-11.81	-11.8	-11.79	-11.78	-11.77	-11.76	-11.75	-11.74
-11.82	-11.81	-11.8	-11.79	-11.78	-11.77	-11.76	-11.75	-11.74
-11.82	-11.81	-11.8	-11.79	-11.78	-11.77	-11.76	-11.75	-11.74
-11.82	-11.81	-11.8	-11.79	-11.78	-11.77	-11.76	-11.75	-11.74
-11.82	-11.81	-11.8	-11.79	-11.78	-11.77	-11.76	-11.75	-11.74
-11.82	-11.81	-11.8	-11.79	-11.78	-11.77	-11.76	-11.75	-11.74
-11.82	-11.81	-11.8	-11.79	-11.78	-11.77	-11.76	-11.75	-11.74
-11.82	-11.81	-11.8	-11.79	-11.78	-11.77	-11.76	-11.75	-11.74
-11.82	-11.81	-11.8	-11.79	-11.78	-11.77	-11.76	-11.75	-11.74
-11.82	-11.81	-11.8	-11.79	-11.78	-11.77	-11.76	-11.75	-11.74
-11.82	-11.81	-11.8	-11.79	-11.78	-11.77	-11.76	-11.75	-11.74
-11.82	-11.81	-11.8	-11.79	-11.78	-11.77	-11.76	-11.75	-11.74
-11.82	-11.81	-11.8	-11.79	-11.78	-11.77	-11.76	-11.75	-11.74
-11.82	-11.81	-11.8	-11.79	-11.78	-11.77	-11.76	-11.75	-11.74
-11.82	-11.81	-11.8	-11.79	-11.78	-11.77	-11.76	-11.75	-11.74
-11.82	-11.81	-11.8	-11.79	-11.78	-11.77	-11.76	-11.75	-11.74
-11.82	-11.81	-11.8	-11.79	-11.78	-11.77	-11.76	-11.75	-11.74
-11.82	-11.81	-11.8	-11.79	-11.78	-11.77	-11.76	-11.75	-11.74
-11.82	-11.81	-11.8	-11.79	-11.78	-11.77	-11.76	-11.75	-11.74
-11.82	-11.81	-11.8	-11.79	-11.78	-11.77	-11.76	-11.75	-11.74
-11.82	-11.81	-11.8	-11.79	-11.78	-11.77	-11.76	-11.75	-11.74
-11.82	-11.81	-11.8	-11.79	-11.78	-11.77	-11.76	-11.75	-11.74
-11.82	-11.81	-11.8	-11.79	-11.78	-11.77	-11.76	-11.75	-11.74
-11.82	-11.81	-11.8	-11.79	-11.78	-11.77	-11.76	-11.75	-11.74
-11.82	-11.81	-11.8	-11.79	-11.78	-11.77	-11.76	-11.75	-11.74
-11.82	-11.81	-11.8	-11.79	-11.78	-11.77	-11.76	-11.75	-11.74
-11.82	-11.81	-11.8	-11.79	-11.78	-11.77	-11.76	-11.75	-11.74
-11.82	-11.81	-11.8	-11.79	-11.78	-11.77	-11.76	-11.75	-11.74
-11.82	-11.81	-11.8	-11.79	-11.78	-11.77	-11.76	-11.75	-11.74
-11.82	-11.81	-11.8	-11.79	-11.78	-11.77	-11.76	-11.75	-11.74
-11.82	-11.81	-11.8	-11.79	-11.78	-11.77	-11.76	-11.75	-11.74
-11.82	-11.81	-11.8	-11.79	-11.78	-11.77	-11.76	-11.75	-11.74
-11.82	-11.81	-11.8	-11.79	-11.78	-11.77	-11.76	-11.75	-11.74

-11.82	-11.81	-11.8	-11.79	-11.78	-11.77	-11.76	-11.75	-11.74
-11.82	-11.81	-11.8	-11.79	-11.78	-11.77	-11.76	-11.75	-11.74
-11.82	-11.81	-11.8	-11.79	-11.78	-11.77	-11.76	-11.75	-11.74
-11.82	-11.81	-11.8	-11.79	-11.78	-11.77	-11.76	-11.75	-11.74
-11.82	-11.81	-11.8	-11.79	-11.78	-11.77	-11.76	-11.75	-11.74
-11.82	-11.81	-11.8	-11.79	-11.78	-11.77	-11.76	-11.75	-11.74
-11.82	-11.81	-11.8	-11.79	-11.78	-11.77	-11.76	-11.75	-11.74
-11.82	-11.81	-11.8	-11.79	-11.78	-11.77	-11.76	-11.75	-11.74
-11.82	-11.81	-11.8	-11.79	-11.78	-11.77	-11.76	-11.75	-11.74
-11.82	-11.81	-11.8	-11.79	-11.78	-11.77	-11.76	-11.75	-11.74
-11.82	-11.81	-11.8	-11.79	-11.78	-11.77	-11.76	-11.75	-11.74
-11.82	-11.81	-11.8	-11.79	-11.78	-11.77	-11.76	-11.75	-11.74
-11.82	-11.81	-11.8	-11.79	-11.78	-11.77	-11.76	-11.75	-11.74
-11.82	-11.81	-11.8	-11.79	-11.78	-11.77	-11.76	-11.75	-11.74
-11.82	-11.81	-11.8	-11.79	-11.78	-11.77	-11.76	-11.75	-11.74
-11.82	-11.81	-11.8	-11.79	-11.78	-11.77	-11.76	-11.75	-11.74
-11.82	-11.81	-11.8	-11.79	-11.78	-11.77	-11.76	-11.75	-11.74
-11.82	-11.81	-11.8	-11.79	-11.78	-11.77	-11.76	-11.75	-11.74
-11.82	-11.81	-11.8	-11.79	-11.78	-11.77	-11.76	-11.75	-11.74
-11.82	-11.81	-11.8	-11.79	-11.78	-11.77	-11.76	-11.75	-11.74
-11.82	-11.81	-11.8	-11.79	-11.78	-11.77	-11.76	-11.75	-11.74
-11.82	-11.81	-11.8	-11.79	-11.78	-11.77	-11.76	-11.75	-11.74
-11.82	-11.81	-11.8	-11.79	-11.78	-11.77	-11.76	-11.75	-11.74
-11.82	-11.81	-11.8	-11.79	-11.78	-11.77	-11.76	-11.75	-11.74
-11.82	-11.81	-11.8	-11.79	-11.78	-11.77	-11.76	-11.75	-11.74
-11.82	-11.81	-11.8	-11.79	-11.78	-11.77	-11.76	-11.75	-11.74
-11.82	-11.81	-11.8	-11.79	-11.78	-11.77	-11.76	-11.75	-11.74
-11.82	-11.81	-11.8	-11.79	-11.78	-11.77	-11.76	-11.75	-11.74
-11.82	-11.81	-11.8	-11.79	-11.78	-11.77	-11.76	-11.75	-11.74
-11.82	-11.81	-11.8	-11.79	-11.78	-11.77	-11.76	-11.75	-11.74
-11.82	-11.81	-11.8	-11.79	-11.78	-11.77	-11.76	-11.75	-11.74
-11.82	-11.81	-11.8	-11.79	-11.78	-11.77	-11.76	-11.75	-11.74
-11.82	-11.81	-11.8	-11.79	-11.78	-11.77	-11.76	-11.75	-11.74
-11.82	-11.81	-11.8	-11.79	-11.78	-11.77	-11.76	-11.75	-11.74
-11.82	-11.81	-11.8	-11.79	-11.78	-11.77	-11.76	-11.75	-11.74
-11.82	-11.81	-11.8	-11.79	-11.78	-11.77	-11.76	-11.75	-11.74
-11.82	-11.81	-11.8	-11.79	-11.78	-11.77	-11.76	-11.75	-11.74
-11.82	-11.81	-11.8	-11.79	-11.78	-11.77	-11.76	-11.75	-11.74
-11.82	-11.81	-11.8	-11.79	-11.78	-11.77	-11.76	-11.75	-11.74
-11.82	-11.81	-11.8	-11.79	-11.78	-11.77	-11.76	-11.75	-11.74
-11.82	-11.81	-11.8	-11.79	-11.78	-11.77	-11.76	-11.75	-11.74
-11.82	-11.81	-11.8	-11.79	-11.78	-11.77	-11.76	-11.75	-11.74
-11.82	-11.81	-11.8	-11.79	-11.78	-11.77	-11.76	-11.75	-11.74
-11.82	-11.81	-11.8	-11.79	-11.78	-11.77	-11.76	-11.75	-11.74
-11.82	-11.81	-11.8	-11.79	-11.78	-11.77	-11.76	-11.75	-11.74
-11.82	-11.81	-11.8	-11.79	-11.78	-11.77	-11.76	-11.75	-11.74
-11.82	-11.81	-11.8	-11.79	-11.78	-11.77	-11.76	-11.75	-11.74
-11.82	-11.81	-11.8	-11.79	-11.78	-11.77	-11.76	-11.75	-11.74
-11.82	-11.81	-11.8	-11.79	-11.78	-11.77	-11.76	-11.75	-11.74
-11.82	-11.81	-11.8	-11.79	-11.78	-11.77	-11.76	-11.75	-11.74

-11.82	-11.81	-11.8	-11.79	-11.78	-11.77	-11.76	-11.75	-11.74

-11.73	-11.72	-11.71	-11.7	-11.69	-11.68	-11.67	-11.66	-11.65
-11.73	-11.72	-11.71	-11.7	-11.69	-11.68	-11.67	-11.66	-11.65
-11.73	-11.72	-11.71	-11.7	-11.69	-11.68	-11.67	-11.66	-11.65
-11.73	-11.72	-11.71	-11.7	-11.69	-11.68	-11.67	-11.66	-11.65
-11.73	-11.72	-11.71	-11.7	-11.69	-11.68	-11.67	-11.66	-11.65
-11.73	-11.72	-11.71	-11.7	-11.69	-11.68	-11.67	-11.66	-11.65
-11.73	-11.72	-11.71	-11.7	-11.69	-11.68	-11.67	-11.66	-11.65
-11.73	-11.72	-11.71	-11.7	-11.69	-11.68	-11.67	-11.66	-11.65
-11.73	-11.72	-11.71	-11.7	-11.69	-11.68	-11.67	-11.66	-11.65
-11.73	-11.72	-11.71	-11.7	-11.69	-11.68	-11.67	-11.66	-11.65
-11.73	-11.72	-11.71	-11.7	-11.69	-11.68	-11.67	-11.66	-11.65
-11.73	-11.72	-11.71	-11.7	-11.69	-11.68	-11.67	-11.66	-11.65
-11.73	-11.72	-11.71	-11.7	-11.69	-11.68	-11.67	-11.66	-11.65
-11.73	-11.72	-11.71	-11.7	-11.69	-11.68	-11.67	-11.66	-11.65
-11.73	-11.72	-11.71	-11.7	-11.69	-11.68	-11.67	-11.66	-11.65
-11.73	-11.72	-11.71	-11.7	-11.69	-11.68	-11.67	-11.66	-11.65
-11.73	-11.72	-11.71	-11.7	-11.69	-11.68	-11.67	-11.66	-11.65
-11.73	-11.72	-11.71	-11.7	-11.69	-11.68	-11.67	-11.66	-11.65
-11.73	-11.72	-11.71	-11.7	-11.69	-11.68	-11.67	-11.66	-11.65
-11.73	-11.72	-11.71	-11.7	-11.69	-11.68	-11.67	-11.66	-11.65
-11.73	-11.72	-11.71	-11.7	-11.69	-11.68	-11.67	-11.66	-11.65
-11.73	-11.72	-11.71	-11.7	-11.69	-11.68	-11.67	-11.66	-11.65
-11.73	-11.72	-11.71	-11.7	-11.69	-11.68	-11.67	-11.66	-11.65
-11.73	-11.72	-11.71	-11.7	-11.69	-11.68	-11.67	-11.66	-11.65
-11.73	-11.72	-11.71	-11.7	-11.69	-11.68	-11.67	-11.66	-11.65
-11.73	-11.72	-11.71	-11.7	-11.69	-11.68	-11.67	-11.66	-11.65
-11.73	-11.72	-11.71	-11.7	-11.69	-11.68	-11.67	-11.66	-11.65
-11.73	-11.72	-11.71	-11.7	-11.69	-11.68	-11.67	-11.66	-11.65
-11.73	-11.72	-11.71	-11.7	-11.69	-11.68	-11.67	-11.66	-11.65
-11.73	-11.72	-11.71	-11.7	-11.69	-11.68	-11.67	-11.66	-11.65
-11.73	-11.72	-11.71	-11.7	-11.69	-11.68	-11.67	-11.66	-11.65
-11.73	-11.72	-11.71	-11.7	-11.69	-11.68	-11.67	-11.66	-11.65
-11.73	-11.72	-11.71	-11.7	-11.69	-11.68	-11.67	-11.66	-11.65
-11.73	-11.72	-11.71	-11.7	-11.69	-11.68	-11.67	-11.66	-11.65
-11.73	-11.72	-11.71	-11.7	-11.69	-11.68	-11.67	-11.66	-11.65
-11.73	-11.72	-11.71	-11.7	-11.69	-11.68	-11.67	-11.66	-11.65
-11.73	-11.72	-11.71	-11.7	-11.69	-11.68	-11.67	-11.66	-11.65
-11.73	-11.72	-11.71	-11.7	-11.69	-11.68	-11.67	-11.66	-11.65
-11.73	-11.72	-11.71	-11.7	-11.69	-11.68	-11.67	-11.66	-11.65
-11.73	-11.72	-11.71	-11.7	-11.69	-11.68	-11.67	-11.66	-11.65
-11.73	-11.72	-11.71	-11.7	-11.69	-11.68	-11.67	-11.66	-11.65
-11.73	-11.72	-11.71	-11.7	-11.69	-11.68	-11.67	-11.66	-11.65
-11.73	-11.72	-11.71	-11.7	-11.69	-11.68	-11.67	-11.66	-11.65
-11.73	-11.72	-11.71	-11.7	-11.69	-11.68	-11.67	-11.66	-11.65
-11.73	-11.72	-11.71	-11.7	-11.69	-11.68	-11.67	-11.66	-11.65
-11.73	-11.72	-11.71	-11.7	-11.69	-11.68	-11.67	-11.66	-11.65
-11.73	-11.72	-11.71	-11.7	-11.69	-11.68	-11.67	-11.66	-11.65
-11.73	-11.72	-11.71	-11.7	-11.69	-11.68	-11.67	-11.66	-11.65
-11.73	-11.72	-11.71	-11.7	-11.69	-11.68	-11.67	-11.66	-11.65
-11.73	-11.72	-11.71	-11.7	-11.69	-11.68	-11.67	-11.66	-11.65

-11.73	-11.72	-11.71	-11.7	-11.69	-11.68	-11.67	-11.66	-11.65
-11.73	-11.72	-11.71	-11.7	-11.69	-11.68	-11.67	-11.66	-11.65
-11.73	-11.72	-11.71	-11.7	-11.69	-11.68	-11.67	-11.66	-11.65
-11.73	-11.72	-11.71	-11.7	-11.69	-11.68	-11.67	-11.66	-11.65
-11.73	-11.72	-11.71	-11.7	-11.69	-11.68	-11.67	-11.66	-11.65
-11.73	-11.72	-11.71	-11.7	-11.69	-11.68	-11.67	-11.66	-11.65
-11.73	-11.72	-11.71	-11.7	-11.69	-11.68	-11.67	-11.66	-11.65
-11.73	-11.72	-11.71	-11.7	-11.69	-11.68	-11.67	-11.66	-11.65
-11.73	-11.72	-11.71	-11.7	-11.69	-11.68	-11.67	-11.66	-11.65
-11.73	-11.72	-11.71	-11.7	-11.69	-11.68	-11.67	-11.66	-11.65
-11.73	-11.72	-11.71	-11.7	-11.69	-11.68	-11.67	-11.66	-11.65
-11.73	-11.72	-11.71	-11.7	-11.69	-11.68	-11.67	-11.66	-11.65
-11.73	-11.72	-11.71	-11.7	-11.69	-11.68	-11.67	-11.66	-11.65
-11.73	-11.72	-11.71	-11.7	-11.69	-11.68	-11.67	-11.66	-11.65
-11.73	-11.72	-11.71	-11.7	-11.69	-11.68	-11.67	-11.66	-11.65
-11.73	-11.72	-11.71	-11.7	-11.69	-11.68	-11.67	-11.66	-11.65
-11.73	-11.72	-11.71	-11.7	-11.69	-11.68	-11.67	-11.66	-11.65
-11.73	-11.72	-11.71	-11.7	-11.69	-11.68	-11.67	-11.66	-11.65
-11.73	-11.72	-11.71	-11.7	-11.69	-11.68	-11.67	-11.66	-11.65
-11.73	-11.72	-11.71	-11.7	-11.69	-11.68	-11.67	-11.66	-11.65
-11.73	-11.72	-11.71	-11.7	-11.69	-11.68	-11.67	-11.66	-11.65
-11.73	-11.72	-11.71	-11.7	-11.69	-11.68	-11.67	-11.66	-11.65
-11.73	-11.72	-11.71	-11.7	-11.69	-11.68	-11.67	-11.66	-11.65
-11.73	-11.72	-11.71	-11.7	-11.69	-11.68	-11.67	-11.66	-11.65
-11.73	-11.72	-11.71	-11.7	-11.69	-11.68	-11.67	-11.66	-11.65
-11.73	-11.72	-11.71	-11.7	-11.69	-11.68	-11.67	-11.66	-11.65
-11.73	-11.72	-11.71	-11.7	-11.69	-11.68	-11.67	-11.66	-11.65
-11.73	-11.72	-11.71	-11.7	-11.69	-11.68	-11.67	-11.66	-11.65
-11.73	-11.72	-11.71	-11.7	-11.69	-11.68	-11.67	-11.66	-11.65
-11.73	-11.72	-11.71	-11.7	-11.69	-11.68	-11.67	-11.66	-11.65
-11.73	-11.72	-11.71	-11.7	-11.69	-11.68	-11.67	-11.66	-11.65
-11.73	-11.72	-11.71	-11.7	-11.69	-11.68	-11.67	-11.66	-11.65
-11.73	-11.72	-11.71	-11.7	-11.69	-11.68	-11.67	-11.66	-11.65
-11.73	-11.72	-11.71	-11.7	-11.69	-11.68	-11.67	-11.66	-11.65
-11.73	-11.72	-11.71	-11.7	-11.69	-11.68	-11.67	-11.66	-11.65
-11.73	-11.72	-11.71	-11.7	-11.69	-11.68	-11.67	-11.66	-11.65
-11.73	-11.72	-11.71	-11.7	-11.69	-11.68	-11.67	-11.66	-11.65
-11.73	-11.72	-11.71	-11.7	-11.69	-11.68	-11.67	-11.66	-11.65
-11.73	-11.72	-11.71	-11.7	-11.69	-11.68	-11.67	-11.66	-11.65
-11.73	-11.72	-11.71	-11.7	-11.69	-11.68	-11.67	-11.66	-11.65
-11.73	-11.72	-11.71	-11.7	-11.69	-11.68	-11.67	-11.66	-11.65
-11.73	-11.72	-11.71	-11.7	-11.69	-11.68	-11.67	-11.66	-11.65
-11.73	-11.72	-11.71	-11.7	-11.69	-11.68	-11.67	-11.66	-11.65
-11.73	-11.72	-11.71	-11.7	-11.69	-11.68	-11.67	-11.66	-11.65
-11.73	-11.72	-11.71	-11.7	-11.69	-11.68	-11.67	-11.66	-11.65
-11.73	-11.72	-11.71	-11.7	-11.69	-11.68	-11.67	-11.66	-11.65
-11.73	-11.72	-11.71	-11.7	-11.69	-11.68	-11.67	-11.66	-11.65
-11.73	-11.72	-11.71	-11.7	-11.69	-11.68	-11.67	-11.66	-11.65
-11.73	-11.72	-11.71	-11.7	-11.69	-11.68	-11.67	-11.66	-11.65
-11.73	-11.72	-11.71	-11.7	-11.69	-11.68	-11.67	-11.66	-11.65

-11.73	-11.72	-11.71	-11.7	-11.69	-11.68	-11.67	-11.66	-11.65
-11.73	-11.72	-11.71	-11.7	-11.69	-11.68	-11.67	-11.66	-11.65
-11.73	-11.72	-11.71	-11.7	-11.69	-11.68	-11.67	-11.66	-11.65
-11.73	-11.72	-11.71	-11.7	-11.69	-11.68	-11.67	-11.66	-11.65
-11.73	-11.72	-11.71	-11.7	-11.69	-11.68	-11.67	-11.66	-11.65
-11.73	-11.72	-11.71	-11.7	-11.69	-11.68	-11.67	-11.66	-11.65
-11.73	-11.72	-11.71	-11.7	-11.69	-11.68	-11.67	-11.66	-11.65
-11.73	-11.72	-11.71	-11.7	-11.69	-11.68	-11.67	-11.66	-11.65
-11.73	-11.72	-11.71	-11.7	-11.69	-11.68	-11.67	-11.66	-11.65
-11.73	-11.72	-11.71	-11.7	-11.69	-11.68	-11.67	-11.66	-11.65
-11.73	-11.72	-11.71	-11.7	-11.69	-11.68	-11.67	-11.66	-11.65
-11.73	-11.72	-11.71	-11.7	-11.69	-11.68	-11.67	-11.66	-11.65
-11.73	-11.72	-11.71	-11.7	-11.69	-11.68	-11.67	-11.66	-11.65
-11.73	-11.72	-11.71	-11.7	-11.69	-11.68	-11.67	-11.66	-11.65
-11.73	-11.72	-11.71	-11.7	-11.69	-11.68	-11.67	-11.66	-11.65
-11.73	-11.72	-11.71	-11.7	-11.69	-11.68	-11.67	-11.66	-11.65
-11.73	-11.72	-11.71	-11.7	-11.69	-11.68	-11.67	-11.66	-11.65
-11.73	-11.72	-11.71	-11.7	-11.69	-11.68	-11.67	-11.66	-11.65
-11.73	-11.72	-11.71	-11.7	-11.69	-11.68	-11.67	-11.66	-11.65
-11.73	-11.72	-11.71	-11.7	-11.69	-11.68	-11.67	-11.66	-11.65
-11.73	-11.72	-11.71	-11.7	-11.69	-11.68	-11.67	-11.66	-11.65
-11.73	-11.72	-11.71	-11.7	-11.69	-11.68	-11.67	-11.66	-11.65
-11.73	-11.72	-11.71	-11.7	-11.69	-11.68	-11.67	-11.66	-11.65
-11.73	-11.72	-11.71	-11.7	-11.69	-11.68	-11.67	-11.66	-11.65
-11.73	-11.72	-11.71	-11.7	-11.69	-11.68	-11.67	-11.66	-11.65
-11.73	-11.72	-11.71	-11.7	-11.69	-11.68	-11.67	-11.66	-11.65
-11.73	-11.72	-11.71	-11.7	-11.69	-11.68	-11.67	-11.66	-11.65
-11.73	-11.72	-11.71	-11.7	-11.69	-11.68	-11.67	-11.66	-11.65
-11.73	-11.72	-11.71	-11.7	-11.69	-11.68	-11.67	-11.66	-11.65
-11.73	-11.72	-11.71	-11.7	-11.69	-11.68	-11.67	-11.66	-11.65
-11.73	-11.72	-11.71	-11.7	-11.69	-11.68	-11.67	-11.66	-11.65
-11.73	-11.72	-11.71	-11.7	-11.69	-11.68	-11.67	-11.66	-11.65
-11.73	-11.72	-11.71	-11.7	-11.69	-11.68	-11.67	-11.66	-11.65
-11.73	-11.72	-11.71	-11.7	-11.69	-11.68	-11.67	-11.66	-11.65
-11.73	-11.72	-11.71	-11.7	-11.69	-11.68	-11.67	-11.66	-11.65
-11.73	-11.72	-11.71	-11.7	-11.69	-11.68	-11.67	-11.66	-11.65
-11.73	-11.72	-11.71	-11.7	-11.69	-11.68	-11.67	-11.66	-11.65
-11.73	-11.72	-11.71	-11.7	-11.69	-11.68	-11.67	-11.66	-11.65
-11.73	-11.72	-11.71	-11.7	-11.69	-11.68	-11.67	-11.66	-11.65
-11.73	-11.72	-11.71	-11.7	-11.69	-11.68	-11.67	-11.66	-11.65
-11.73	-11.72	-11.71	-11.7	-11.69	-11.68	-11.67	-11.66	-11.65
-11.73	-11.72	-11.71	-11.7	-11.69	-11.68	-11.67	-11.66	-11.65
-11.73	-11.72	-11.71	-11.7	-11.69	-11.68	-11.67	-11.66	-11.65
-11.73	-11.72	-11.71	-11.7	-11.69	-11.68	-11.67	-11.66	-11.65
-11.73	-11.72	-11.71	-11.7	-11.69	-11.68	-11.67	-11.66	-11.65
-11.73	-11.72	-11.71	-11.7	-11.69	-11.68	-11.67	-11.66	-11.65
-11.73	-11.72	-11.71	-11.7	-11.69	-11.68	-11.67	-11.66	-11.65
-11.73	-11.72	-11.71	-11.7	-11.69	-11.68	-11.67	-11.66	-11.65
-11.73	-11.72	-11.71	-11.7	-11.69	-11.68	-11.67	-11.66	-11.65
-11.73	-11.72	-11.71	-11.7	-11.69	-11.68	-11.67	-11.66	-11.65

-11.73	-11.72	-11.71	-11.7	-11.69	-11.68	-11.67	-11.66	-11.65
-11.73	-11.72	-11.71	-11.7	-11.69	-11.68	-11.67	-11.66	-11.65
-11.73	-11.72	-11.71	-11.7	-11.69	-11.68	-11.67	-11.66	-11.65
-11.73	-11.72	-11.71	-11.7	-11.69	-11.68	-11.67	-11.66	-11.65
-11.73	-11.72	-11.71	-11.7	-11.69	-11.68	-11.67	-11.66	-11.65
-11.73	-11.72	-11.71	-11.7	-11.69	-11.68	-11.67	-11.66	-11.65
-11.73	-11.72	-11.71	-11.7	-11.69	-11.68	-11.67	-11.66	-11.65
-11.73	-11.72	-11.71	-11.7	-11.69	-11.68	-11.67	-11.66	-11.65
-11.73	-11.72	-11.71	-11.7	-11.69	-11.68	-11.67	-11.66	-11.65
-11.73	-11.72	-11.71	-11.7	-11.69	-11.68	-11.67	-11.66	-11.65
-11.73	-11.72	-11.71	-11.7	-11.69	-11.68	-11.67	-11.66	-11.65
-11.73	-11.72	-11.71	-11.7	-11.69	-11.68	-11.67	-11.66	-11.65
-11.73	-11.72	-11.71	-11.7	-11.69	-11.68	-11.67	-11.66	-11.65
-11.73	-11.72	-11.71	-11.7	-11.69	-11.68	-11.67	-11.66	-11.65
-11.73	-11.72	-11.71	-11.7	-11.69	-11.68	-11.67	-11.66	-11.65
-11.73	-11.72	-11.71	-11.7	-11.69	-11.68	-11.67	-11.66	-11.65
-11.73	-11.72	-11.71	-11.7	-11.69	-11.68	-11.67	-11.66	-11.65
-11.73	-11.72	-11.71	-11.7	-11.69	-11.68	-11.67	-11.66	-11.65
-11.73	-11.72	-11.71	-11.7	-11.69	-11.68	-11.67	-11.66	-11.65
-11.73	-11.72	-11.71	-11.7	-11.69	-11.68	-11.67	-11.66	-11.65
-11.73	-11.72	-11.71	-11.7	-11.69	-11.68	-11.67	-11.66	-11.65
-11.73	-11.72	-11.71	-11.7	-11.69	-11.68	-11.67	-11.66	-11.65
-11.73	-11.72	-11.71	-11.7	-11.69	-11.68	-11.67	-11.66	-11.65
-11.73	-11.72	-11.71	-11.7	-11.69	-11.68	-11.67	-11.66	-11.65
-11.73	-11.72	-11.71	-11.7	-11.69	-11.68	-11.67	-11.66	-11.65
-11.73	-11.72	-11.71	-11.7	-11.69	-11.68	-11.67	-11.66	-11.65
-11.73	-11.72	-11.71	-11.7	-11.69	-11.68	-11.67	-11.66	-11.65
-11.73	-11.72	-11.71	-11.7	-11.69	-11.68	-11.67	-11.66	-11.65
-11.73	-11.72	-11.71	-11.7	-11.69	-11.68	-11.67	-11.66	-11.65
-11.73	-11.72	-11.71	-11.7	-11.69	-11.68	-11.67	-11.66	-11.65
-11.73	-11.72	-11.71	-11.7	-11.69	-11.68	-11.67	-11.66	-11.65
-11.73	-11.72	-11.71	-11.7	-11.69	-11.68	-11.67	-11.66	-11.65
-11.73	-11.72	-11.71	-11.7	-11.69	-11.68	-11.67	-11.66	-11.65
-11.73	-11.72	-11.71	-11.7	-11.69	-11.68	-11.67	-11.66	-11.65
-11.73	-11.72	-11.71	-11.7	-11.69	-11.68	-11.67	-11.66	-11.65
-11.73	-11.72	-11.71	-11.7	-11.69	-11.68	-11.67	-11.66	-11.65
-11.73	-11.72	-11.71	-11.7	-11.69	-11.68	-11.67	-11.66	-11.65
-11.73	-11.72	-11.71	-11.7	-11.69	-11.68	-11.67	-11.66	-11.65
-11.73	-11.72	-11.71	-11.7	-11.69	-11.68	-11.67	-11.66	-11.65
-11.73	-11.72	-11.71	-11.7	-11.69	-11.68	-11.67	-11.66	-11.65
-11.73	-11.72	-11.71	-11.7	-11.69	-11.68	-11.67	-11.66	-11.65
-11.73	-11.72	-11.71	-11.7	-11.69	-11.68	-11.67	-11.66	-11.65
-11.73	-11.72	-11.71	-11.7	-11.69	-11.68	-11.67	-11.66	-11.65
-11.73	-11.72	-11.71	-11.7	-11.69	-11.68	-11.67	-11.66	-11.65
-11.73	-11.72	-11.71	-11.7	-11.69	-11.68	-11.67	-11.66	-11.65
-11.73	-11.72	-11.71	-11.7	-11.69	-11.68	-11.67	-11.66	-11.65
-11.73	-11.72	-11.71	-11.7	-11.69	-11.68	-11.67	-11.66	-11.65
-11.73	-11.72	-11.71	-11.7	-11.69	-11.68	-11.67	-11.66	-11.65
-11.73	-11.72	-11.71	-11.7	-11.69	-11.68	-11.67	-11.66	-11.65
-11.73	-11.72	-11.71	-11.7	-11.69	-11.68	-11.67	-11.66	-11.65

-11.73	-11.72	-11.71	-11.7	-11.69	-11.68	-11.67	-11.66	-11.65
-11.73	-11.72	-11.71	-11.7	-11.69	-11.68	-11.67	-11.66	-11.65
-11.73	-11.72	-11.71	-11.7	-11.69	-11.68	-11.67	-11.66	-11.65
-11.73	-11.72	-11.71	-11.7	-11.69	-11.68	-11.67	-11.66	-11.65
-11.73	-11.72	-11.71	-11.7	-11.69	-11.68	-11.67	-11.66	-11.65
-11.73	-11.72	-11.71	-11.7	-11.69	-11.68	-11.67	-11.66	-11.65
-11.73	-11.72	-11.71	-11.7	-11.69	-11.68	-11.67	-11.66	-11.65
-11.73	-11.72	-11.71	-11.7	-11.69	-11.68	-11.67	-11.66	-11.65
-11.73	-11.72	-11.71	-11.7	-11.69	-11.68	-11.67	-11.66	-11.65
-11.73	-11.72	-11.71	-11.7	-11.69	-11.68	-11.67	-11.66	-11.65
-11.73	-11.72	-11.71	-11.7	-11.69	-11.68	-11.67	-11.66	-11.65
-11.73	-11.72	-11.71	-11.7	-11.69	-11.68	-11.67	-11.66	-11.65
-11.73	-11.72	-11.71	-11.7	-11.69	-11.68	-11.67	-11.66	-11.65
-11.73	-11.72	-11.71	-11.7	-11.69	-11.68	-11.67	-11.66	-11.65
-11.73	-11.72	-11.71	-11.7	-11.69	-11.68	-11.67	-11.66	-11.65
-11.73	-11.72	-11.71	-11.7	-11.69	-11.68	-11.67	-11.66	-11.65
-11.73	-11.72	-11.71	-11.7	-11.69	-11.68	-11.67	-11.66	-11.65
-11.73	-11.72	-11.71	-11.7	-11.69	-11.68	-11.67	-11.66	-11.65
-11.73	-11.72	-11.71	-11.7	-11.69	-11.68	-11.67	-11.66	-11.65
-11.73	-11.72	-11.71	-11.7	-11.69	-11.68	-11.67	-11.66	-11.65
-11.73	-11.72	-11.71	-11.7	-11.69	-11.68	-11.67	-11.66	-11.65
-11.73	-11.72	-11.71	-11.7	-11.69	-11.68	-11.67	-11.66	-11.65
-11.73	-11.72	-11.71	-11.7	-11.69	-11.68	-11.67	-11.66	-11.65
-11.73	-11.72	-11.71	-11.7	-11.69	-11.68	-11.67	-11.66	-11.65
-11.73	-11.72	-11.71	-11.7	-11.69	-11.68	-11.67	-11.66	-11.65
-11.73	-11.72	-11.71	-11.7	-11.69	-11.68	-11.67	-11.66	-11.65
-11.73	-11.72	-11.71	-11.7	-11.69	-11.68	-11.67	-11.66	-11.65
-11.73	-11.72	-11.71	-11.7	-11.69	-11.68	-11.67	-11.66	-11.65
-11.73	-11.72	-11.71	-11.7	-11.69	-11.68	-11.67	-11.66	-11.65
-11.73	-11.72	-11.71	-11.7	-11.69	-11.68	-11.67	-11.66	-11.65
-11.73	-11.72	-11.71	-11.7	-11.69	-11.68	-11.67	-11.66	-11.65
-11.73	-11.72	-11.71	-11.7	-11.69	-11.68	-11.67	-11.66	-11.65
-11.73	-11.72	-11.71	-11.7	-11.69	-11.68	-11.67	-11.66	-11.65
-11.73	-11.72	-11.71	-11.7	-11.69	-11.68	-11.67	-11.66	-11.65
-11.73	-11.72	-11.71	-11.7	-11.69	-11.68	-11.67	-11.66	-11.65
-11.73	-11.72	-11.71	-11.7	-11.69	-11.68	-11.67	-11.66	-11.65
-11.73	-11.72	-11.71	-11.7	-11.69	-11.68	-11.67	-11.66	-11.65
-11.73	-11.72	-11.71	-11.7	-11.69	-11.68	-11.67	-11.66	-11.65
-11.73	-11.72	-11.71	-11.7	-11.69	-11.68	-11.67	-11.66	-11.65
-11.73	-11.72	-11.71	-11.7	-11.69	-11.68	-11.67	-11.66	-11.65
-11.73	-11.72	-11.71	-11.7	-11.69	-11.68	-11.67	-11.66	-11.65
-11.73	-11.72	-11.71	-11.7	-11.69	-11.68	-11.67	-11.66	-11.65
-11.73	-11.72	-11.71	-11.7	-11.69	-11.68	-11.67	-11.66	-11.65
-11.73	-11.72	-11.71	-11.7	-11.69	-11.68	-11.67	-11.66	-11.65
-11.73	-11.72	-11.71	-11.7	-11.69	-11.68	-11.67	-11.66	-11.65
-11.73	-11.72	-11.71	-11.7	-11.69	-11.68	-11.67	-11.66	-11.65
-11.73	-11.72	-11.71	-11.7	-11.69	-11.68	-11.67	-11.66	-11.65
-11.73	-11.72	-11.71	-11.7	-11.69	-11.68	-11.67	-11.66	-11.65
-11.73	-11.72	-11.71	-11.7	-11.69	-11.68	-11.67	-11.66	-11.65
-11.73	-11.72	-11.71	-11.7	-11.69	-11.68	-11.67	-11.66	-11.65

-11.73	-11.72	-11.71	-11.7	-11.69	-11.68	-11.67	-11.66	-11.65
-11.73	-11.72	-11.71	-11.7	-11.69	-11.68	-11.67	-11.66	-11.65
-11.73	-11.72	-11.71	-11.7	-11.69	-11.68	-11.67	-11.66	-11.65
-11.73	-11.72	-11.71	-11.7	-11.69	-11.68	-11.67	-11.66	-11.65
-11.73	-11.72	-11.71	-11.7	-11.69	-11.68	-11.67	-11.66	-11.65
-11.73	-11.72	-11.71	-11.7	-11.69	-11.68	-11.67	-11.66	-11.65
-11.73	-11.72	-11.71	-11.7	-11.69	-11.68	-11.67	-11.66	-11.65
-11.73	-11.72	-11.71	-11.7	-11.69	-11.68	-11.67	-11.66	-11.65
-11.73	-11.72	-11.71	-11.7	-11.69	-11.68	-11.67	-11.66	-11.65
-11.73	-11.72	-11.71	-11.7	-11.69	-11.68	-11.67	-11.66	-11.65
-11.73	-11.72	-11.71	-11.7	-11.69	-11.68	-11.67	-11.66	-11.65
-11.73	-11.72	-11.71	-11.7	-11.69	-11.68	-11.67	-11.66	-11.65
-11.73	-11.72	-11.71	-11.7	-11.69	-11.68	-11.67	-11.66	-11.65
-11.73	-11.72	-11.71	-11.7	-11.69	-11.68	-11.67	-11.66	-11.65
-11.73	-11.72	-11.71	-11.7	-11.69	-11.68	-11.67	-11.66	-11.65
-11.73	-11.72	-11.71	-11.7	-11.69	-11.68	-11.67	-11.66	-11.65
-11.73	-11.72	-11.71	-11.7	-11.69	-11.68	-11.67	-11.66	-11.65
-11.73	-11.72	-11.71	-11.7	-11.69	-11.68	-11.67	-11.66	-11.65
-11.73	-11.72	-11.71	-11.7	-11.69	-11.68	-11.67	-11.66	-11.65
-11.73	-11.72	-11.71	-11.7	-11.69	-11.68	-11.67	-11.66	-11.65
-11.73	-11.72	-11.71	-11.7	-11.69	-11.68	-11.67	-11.66	-11.65
-11.73	-11.72	-11.71	-11.7	-11.69	-11.68	-11.67	-11.66	-11.65
-11.73	-11.72	-11.71	-11.7	-11.69	-11.68	-11.67	-11.66	-11.65
-11.73	-11.72	-11.71	-11.7	-11.69	-11.68	-11.67	-11.66	-11.65
-11.73	-11.72	-11.71	-11.7	-11.69	-11.68	-11.67	-11.66	-11.65
-11.73	-11.72	-11.71	-11.7	-11.69	-11.68	-11.67	-11.66	-11.65
-11.73	-11.72	-11.71	-11.7	-11.69	-11.68	-11.67	-11.66	-11.65
-11.73	-11.72	-11.71	-11.7	-11.69	-11.68	-11.67	-11.66	-11.65
-11.73	-11.72	-11.71	-11.7	-11.69	-11.68	-11.67	-11.66	-11.65
-11.73	-11.72	-11.71	-11.7	-11.69	-11.68	-11.67	-11.66	-11.65
-11.73	-11.72	-11.71	-11.7	-11.69	-11.68	-11.67	-11.66	-11.65
-11.73	-11.72	-11.71	-11.7	-11.69	-11.68	-11.67	-11.66	-11.65
-11.73	-11.72	-11.71	-11.7	-11.69	-11.68	-11.67	-11.66	-11.65
-11.73	-11.72	-11.71	-11.7	-11.69	-11.68	-11.67	-11.66	-11.65
-11.73	-11.72	-11.71	-11.7	-11.69	-11.68	-11.67	-11.66	-11.65
-11.73	-11.72	-11.71	-11.7	-11.69	-11.68	-11.67	-11.66	-11.65
-11.73	-11.72	-11.71	-11.7	-11.69	-11.68	-11.67	-11.66	-11.65
-11.73	-11.72	-11.71	-11.7	-11.69	-11.68	-11.67	-11.66	-11.65
-11.73	-11.72	-11.71	-11.7	-11.69	-11.68	-11.67	-11.66	-11.65
-11.73	-11.72	-11.71	-11.7	-11.69	-11.68	-11.67	-11.66	-11.65
-11.73	-11.72	-11.71	-11.7	-11.69	-11.68	-11.67	-11.66	-11.65
-11.73	-11.72	-11.71	-11.7	-11.69	-11.68	-11.67	-11.66	-11.65
-11.73	-11.72	-11.71	-11.7	-11.69	-11.68	-11.67	-11.66	-11.65
-11.73	-11.72	-11.71	-11.7	-11.69	-11.68	-11.67	-11.66	-11.65
-11.73	-11.72	-11.71	-11.7	-11.69	-11.68	-11.67	-11.66	-11.65
-11.73	-11.72	-11.71	-11.7	-11.69	-11.68	-11.67	-11.66	-11.65
-11.73	-11.72	-11.71	-11.7	-11.69	-11.68	-11.67	-11.66	-11.65
-11.73	-11.72	-11.71	-11.7	-11.69	-11.68	-11.67	-11.66	-11.65
-11.73	-11.72	-11.71	-11.7	-11.69	-11.68	-11.67	-11.66	-11.65
-11.73	-11.72	-11.71	-11.7	-11.69	-11.68	-11.67	-11.66	-11.65

-11.73	-11.72	-11.71	-11.7	-11.69	-11.68	-11.67	-11.66	-11.65
-11.73	-11.72	-11.71	-11.7	-11.69	-11.68	-11.67	-11.66	-11.65
-11.73	-11.72	-11.71	-11.7	-11.69	-11.68	-11.67	-11.66	-11.65
-11.73	-11.72	-11.71	-11.7	-11.69	-11.68	-11.67	-11.66	-11.65
-11.73	-11.72	-11.71	-11.7	-11.69	-11.68	-11.67	-11.66	-11.65
-11.73	-11.72	-11.71	-11.7	-11.69	-11.68	-11.67	-11.66	-11.65
-11.73	-11.72	-11.71	-11.7	-11.69	-11.68	-11.67	-11.66	-11.65
-11.73	-11.72	-11.71	-11.7	-11.69	-11.68	-11.67	-11.66	-11.65
-11.73	-11.72	-11.71	-11.7	-11.69	-11.68	-11.67	-11.66	-11.65
-11.73	-11.72	-11.71	-11.7	-11.69	-11.68	-11.67	-11.66	-11.65
-11.73	-11.72	-11.71	-11.7	-11.69	-11.68	-11.67	-11.66	-11.65
-11.73	-11.72	-11.71	-11.7	-11.69	-11.68	-11.67	-11.66	-11.65
-11.73	-11.72	-11.71	-11.7	-11.69	-11.68	-11.67	-11.66	-11.65
-11.73	-11.72	-11.71	-11.7	-11.69	-11.68	-11.67	-11.66	-11.65
-11.73	-11.72	-11.71	-11.7	-11.69	-11.68	-11.67	-11.66	-11.65
-11.73	-11.72	-11.71	-11.7	-11.69	-11.68	-11.67	-11.66	-11.65
-11.73	-11.72	-11.71	-11.7	-11.69	-11.68	-11.67	-11.66	-11.65
-11.73	-11.72	-11.71	-11.7	-11.69	-11.68	-11.67	-11.66	-11.65
-11.73	-11.72	-11.71	-11.7	-11.69	-11.68	-11.67	-11.66	-11.65
-11.73	-11.72	-11.71	-11.7	-11.69	-11.68	-11.67	-11.66	-11.65
-11.73	-11.72	-11.71	-11.7	-11.69	-11.68	-11.67	-11.66	-11.65
-11.73	-11.72	-11.71	-11.7	-11.69	-11.68	-11.67	-11.66	-11.65
-11.73	-11.72	-11.71	-11.7	-11.69	-11.68	-11.67	-11.66	-11.65
-11.73	-11.72	-11.71	-11.7	-11.69	-11.68	-11.67	-11.66	-11.65
-11.73	-11.72	-11.71	-11.7	-11.69	-11.68	-11.67	-11.66	-11.65
-11.73	-11.72	-11.71	-11.7	-11.69	-11.68	-11.67	-11.66	-11.65
-11.73	-11.72	-11.71	-11.7	-11.69	-11.68	-11.67	-11.66	-11.65
-11.73	-11.72	-11.71	-11.7	-11.69	-11.68	-11.67	-11.66	-11.65
-11.73	-11.72	-11.71	-11.7	-11.69	-11.68	-11.67	-11.66	-11.65
-11.73	-11.72	-11.71	-11.7	-11.69	-11.68	-11.67	-11.66	-11.65
-11.73	-11.72	-11.71	-11.7	-11.69	-11.68	-11.67	-11.66	-11.65
-11.73	-11.72	-11.71	-11.7	-11.69	-11.68	-11.67	-11.66	-11.65
-11.73	-11.72	-11.71	-11.7	-11.69	-11.68	-11.67	-11.66	-11.65
-11.73	-11.72	-11.71	-11.7	-11.69	-11.68	-11.67	-11.66	-11.65
-11.73	-11.72	-11.71	-11.7	-11.69	-11.68	-11.67	-11.66	-11.65
-11.73	-11.72	-11.71	-11.7	-11.69	-11.68	-11.67	-11.66	-11.65
-11.73	-11.72	-11.71	-11.7	-11.69	-11.68	-11.67	-11.66	-11.65
-11.73	-11.72	-11.71	-11.7	-11.69	-11.68	-11.67	-11.66	-11.65
-11.73	-11.72	-11.71	-11.7	-11.69	-11.68	-11.67	-11.66	-11.65
-11.73	-11.72	-11.71	-11.7	-11.69	-11.68	-11.67	-11.66	-11.65
-11.73	-11.72	-11.71	-11.7	-11.69	-11.68	-11.67	-11.66	-11.65
-11.73	-11.72	-11.71	-11.7	-11.69	-11.68	-11.67	-11.66	-11.65
-11.73	-11.72	-11.71	-11.7	-11.69	-11.68	-11.67	-11.66	-11.65
-11.73	-11.72	-11.71	-11.7	-11.69	-11.68	-11.67	-11.66	-11.65
-11.73	-11.72	-11.71	-11.7	-11.69	-11.68	-11.67	-11.66	-11.65
-11.73	-11.72	-11.71	-11.7	-11.69	-11.68	-11.67	-11.66	-11.65
-11.73	-11.72	-11.71	-11.7	-11.69	-11.68	-11.67	-11.66	-11.65
-11.73	-11.72	-11.71	-11.7	-11.69	-11.68	-11.67	-11.66	-11.65
-11.73	-11.72	-11.71	-11.7	-11.69	-11.68	-11.67	-11.66	-11.65
-11.73	-11.72	-11.71	-11.7	-11.69	-11.68	-11.67	-11.66	-11.65

-11.73	-11.72	-11.71	-11.7	-11.69	-11.68	-11.67	-11.66	-11.65
-11.73	-11.72	-11.71	-11.7	-11.69	-11.68	-11.67	-11.66	-11.65
-11.73	-11.72	-11.71	-11.7	-11.69	-11.68	-11.67	-11.66	-11.65
-11.73	-11.72	-11.71	-11.7	-11.69	-11.68	-11.67	-11.66	-11.65
-11.73	-11.72	-11.71	-11.7	-11.69	-11.68	-11.67	-11.66	-11.65
-11.73	-11.72	-11.71	-11.7	-11.69	-11.68	-11.67	-11.66	-11.65
-11.73	-11.72	-11.71	-11.7	-11.69	-11.68	-11.67	-11.66	-11.65
-11.73	-11.72	-11.71	-11.7	-11.69	-11.68	-11.67	-11.66	-11.65
-11.73	-11.72	-11.71	-11.7	-11.69	-11.68	-11.67	-11.66	-11.65
-11.73	-11.72	-11.71	-11.7	-11.69	-11.68	-11.67	-11.66	-11.65
-11.73	-11.72	-11.71	-11.7	-11.69	-11.68	-11.67	-11.66	-11.65
-11.73	-11.72	-11.71	-11.7	-11.69	-11.68	-11.67	-11.66	-11.65
-11.73	-11.72	-11.71	-11.7	-11.69	-11.68	-11.67	-11.66	-11.65
-11.73	-11.72	-11.71	-11.7	-11.69	-11.68	-11.67	-11.66	-11.65
-11.73	-11.72	-11.71	-11.7	-11.69	-11.68	-11.67	-11.66	-11.65
-11.73	-11.72	-11.71	-11.7	-11.69	-11.68	-11.67	-11.66	-11.65
-11.73	-11.72	-11.71	-11.7	-11.69	-11.68	-11.67	-11.66	-11.65
-11.73	-11.72	-11.71	-11.7	-11.69	-11.68	-11.67	-11.66	-11.65
-11.73	-11.72	-11.71	-11.7	-11.69	-11.68	-11.67	-11.66	-11.65
-11.73	-11.72	-11.71	-11.7	-11.69	-11.68	-11.67	-11.66	-11.65
-11.73	-11.72	-11.71	-11.7	-11.69	-11.68	-11.67	-11.66	-11.65
-11.73	-11.72	-11.71	-11.7	-11.69	-11.68	-11.67	-11.66	-11.65
-11.73	-11.72	-11.71	-11.7	-11.69	-11.68	-11.67	-11.66	-11.65
-11.73	-11.72	-11.71	-11.7	-11.69	-11.68	-11.67	-11.66	-11.65
-11.73	-11.72	-11.71	-11.7	-11.69	-11.68	-11.67	-11.66	-11.65
-11.73	-11.72	-11.71	-11.7	-11.69	-11.68	-11.67	-11.66	-11.65
-11.73	-11.72	-11.71	-11.7	-11.69	-11.68	-11.67	-11.66	-11.65
-11.73	-11.72	-11.71	-11.7	-11.69	-11.68	-11.67	-11.66	-11.65
-11.73	-11.72	-11.71	-11.7	-11.69	-11.68	-11.67	-11.66	-11.65
-11.73	-11.72	-11.71	-11.7	-11.69	-11.68	-11.67	-11.66	-11.65
-11.73	-11.72	-11.71	-11.7	-11.69	-11.68	-11.67	-11.66	-11.65
-11.73	-11.72	-11.71	-11.7	-11.69	-11.68	-11.67	-11.66	-11.65
-11.73	-11.72	-11.71	-11.7	-11.69	-11.68	-11.67	-11.66	-11.65
-11.73	-11.72	-11.71	-11.7	-11.69	-11.68	-11.67	-11.66	-11.65
-11.73	-11.72	-11.71	-11.7	-11.69	-11.68	-11.67	-11.66	-11.65
-11.73	-11.72	-11.71	-11.7	-11.69	-11.68	-11.67	-11.66	-11.65
-11.73	-11.72	-11.71	-11.7	-11.69	-11.68	-11.67	-11.66	-11.65
-11.73	-11.72	-11.71	-11.7	-11.69	-11.68	-11.67	-11.66	-11.65
-11.73	-11.72	-11.71	-11.7	-11.69	-11.68	-11.67	-11.66	-11.65
-11.73	-11.72	-11.71	-11.7	-11.69	-11.68	-11.67	-11.66	-11.65
-11.73	-11.72	-11.71	-11.7	-11.69	-11.68	-11.67	-11.66	-11.65
-11.73	-11.72	-11.71	-11.7	-11.69	-11.68	-11.67	-11.66	-11.65
-11.73	-11.72	-11.71	-11.7	-11.69	-11.68	-11.67	-11.66	-11.65
-11.73	-11.72	-11.71	-11.7	-11.69	-11.68	-11.67	-11.66	-11.65
-11.73	-11.72	-11.71	-11.7	-11.69	-11.68	-11.67	-11.66	-11.65
-11.73	-11.72	-11.71	-11.7	-11.69	-11.68	-11.67	-11.66	-11.65
-11.73	-11.72	-11.71	-11.7	-11.69	-11.68	-11.67	-11.66	-11.65
-11.73	-11.72	-11.71	-11.7	-11.69	-11.68	-11.67	-11.66	-11.65
-11.73	-11.72	-11.71	-11.7	-11.69	-11.68	-11.67	-11.66	-11.65
-11.73	-11.72	-11.71	-11.7	-11.69	-11.68	-11.67	-11.66	-11.65

-11.73	-11.72	-11.71	-11.7	-11.69	-11.68	-11.67	-11.66	-11.65
-11.73	-11.72	-11.71	-11.7	-11.69	-11.68	-11.67	-11.66	-11.65
-11.73	-11.72	-11.71	-11.7	-11.69	-11.68	-11.67	-11.66	-11.65
-11.73	-11.72	-11.71	-11.7	-11.69	-11.68	-11.67	-11.66	-11.65
-11.73	-11.72	-11.71	-11.7	-11.69	-11.68	-11.67	-11.66	-11.65
-11.73	-11.72	-11.71	-11.7	-11.69	-11.68	-11.67	-11.66	-11.65
-11.73	-11.72	-11.71	-11.7	-11.69	-11.68	-11.67	-11.66	-11.65
-11.73	-11.72	-11.71	-11.7	-11.69	-11.68	-11.67	-11.66	-11.65
-11.73	-11.72	-11.71	-11.7	-11.69	-11.68	-11.67	-11.66	-11.65
-11.73	-11.72	-11.71	-11.7	-11.69	-11.68	-11.67	-11.66	-11.65
-11.73	-11.72	-11.71	-11.7	-11.69	-11.68	-11.67	-11.66	-11.65
-11.73	-11.72	-11.71	-11.7	-11.69	-11.68	-11.67	-11.66	-11.65
-11.73	-11.72	-11.71	-11.7	-11.69	-11.68	-11.67	-11.66	-11.65
-11.73	-11.72	-11.71	-11.7	-11.69	-11.68	-11.67	-11.66	-11.65
-11.73	-11.72	-11.71	-11.7	-11.69	-11.68	-11.67	-11.66	-11.65
-11.73	-11.72	-11.71	-11.7	-11.69	-11.68	-11.67	-11.66	-11.65
-11.73	-11.72	-11.71	-11.7	-11.69	-11.68	-11.67	-11.66	-11.65
-11.73	-11.72	-11.71	-11.7	-11.69	-11.68	-11.67	-11.66	-11.65
-11.73	-11.72	-11.71	-11.7	-11.69	-11.68	-11.67	-11.66	-11.65
-11.73	-11.72	-11.71	-11.7	-11.69	-11.68	-11.67	-11.66	-11.65
-11.73	-11.72	-11.71	-11.7	-11.69	-11.68	-11.67	-11.66	-11.65
-11.73	-11.72	-11.71	-11.7	-11.69	-11.68	-11.67	-11.66	-11.65
-11.73	-11.72	-11.71	-11.7	-11.69	-11.68	-11.67	-11.66	-11.65
-11.73	-11.72	-11.71	-11.7	-11.69	-11.68	-11.67	-11.66	-11.65
-11.73	-11.72	-11.71	-11.7	-11.69	-11.68	-11.67	-11.66	-11.65
-11.73	-11.72	-11.71	-11.7	-11.69	-11.68	-11.67	-11.66	-11.65
-11.73	-11.72	-11.71	-11.7	-11.69	-11.68	-11.67	-11.66	-11.65
-11.73	-11.72	-11.71	-11.7	-11.69	-11.68	-11.67	-11.66	-11.65
-11.73	-11.72	-11.71	-11.7	-11.69	-11.68	-11.67	-11.66	-11.65
-11.73	-11.72	-11.71	-11.7	-11.69	-11.68	-11.67	-11.66	-11.65
-11.73	-11.72	-11.71	-11.7	-11.69	-11.68	-11.67	-11.66	-11.65
-11.73	-11.72	-11.71	-11.7	-11.69	-11.68	-11.67	-11.66	-11.65
-11.73	-11.72	-11.71	-11.7	-11.69	-11.68	-11.67	-11.66	-11.65
-11.73	-11.72	-11.71	-11.7	-11.69	-11.68	-11.67	-11.66	-11.65
-11.73	-11.72	-11.71	-11.7	-11.69	-11.68	-11.67	-11.66	-11.65
-11.73	-11.72	-11.71	-11.7	-11.69	-11.68	-11.67	-11.66	-11.65
-11.73	-11.72	-11.71	-11.7	-11.69	-11.68	-11.67	-11.66	-11.65
-11.73	-11.72	-11.71	-11.7	-11.69	-11.68	-11.67	-11.66	-11.65
-11.73	-11.72	-11.71	-11.7	-11.69	-11.68	-11.67	-11.66	-11.65
-11.73	-11.72	-11.71	-11.7	-11.69	-11.68	-11.67	-11.66	-11.65
-11.73	-11.72	-11.71	-11.7	-11.69	-11.68	-11.67	-11.66	-11.65
-11.73	-11.72	-11.71	-11.7	-11.69	-11.68	-11.67	-11.66	-11.65
-11.73	-11.72	-11.71	-11.7	-11.69	-11.68	-11.67	-11.66	-11.65
-11.73	-11.72	-11.71	-11.7	-11.69	-11.68	-11.67	-11.66	-11.65
-11.73	-11.72	-11.71	-11.7	-11.69	-11.68	-11.67	-11.66	-11.65
-11.73	-11.72	-11.71	-11.7	-11.69	-11.68	-11.67	-11.66	-11.65
-11.73	-11.72	-11.71	-11.7	-11.69	-11.68	-11.67	-11.66	-11.65
-11.73	-11.72	-11.71	-11.7	-11.69	-11.68	-11.67	-11.66	-11.65
-11.73	-11.72	-11.71	-11.7	-11.69	-11.68	-11.67	-11.66	-11.65
-11.73	-11.72	-11.71	-11.7	-11.69	-11.68	-11.67	-11.66	-11.65

-11.73	-11.72	-11.71	-11.7	-11.69	-11.68	-11.67	-11.66	-11.65
-11.73	-11.72	-11.71	-11.7	-11.69	-11.68	-11.67	-11.66	-11.65
-11.73	-11.72	-11.71	-11.7	-11.69	-11.68	-11.67	-11.66	-11.65
-11.73	-11.72	-11.71	-11.7	-11.69	-11.68	-11.67	-11.66	-11.65
-11.73	-11.72	-11.71	-11.7	-11.69	-11.68	-11.67	-11.66	-11.65
-11.73	-11.72	-11.71	-11.7	-11.69	-11.68	-11.67	-11.66	-11.65
-11.73	-11.72	-11.71	-11.7	-11.69	-11.68	-11.67	-11.66	-11.65
-11.73	-11.72	-11.71	-11.7	-11.69	-11.68	-11.67	-11.66	-11.65
-11.73	-11.72	-11.71	-11.7	-11.69	-11.68	-11.67	-11.66	-11.65
-11.73	-11.72	-11.71	-11.7	-11.69	-11.68	-11.67	-11.66	-11.65
-11.73	-11.72	-11.71	-11.7	-11.69	-11.68	-11.67	-11.66	-11.65
-11.73	-11.72	-11.71	-11.7	-11.69	-11.68	-11.67	-11.66	-11.65
-11.73	-11.72	-11.71	-11.7	-11.69	-11.68	-11.67	-11.66	-11.65
-11.73	-11.72	-11.71	-11.7	-11.69	-11.68	-11.67	-11.66	-11.65
-11.73	-11.72	-11.71	-11.7	-11.69	-11.68	-11.67	-11.66	-11.65
-11.73	-11.72	-11.71	-11.7	-11.69	-11.68	-11.67	-11.66	-11.65
-11.73	-11.72	-11.71	-11.7	-11.69	-11.68	-11.67	-11.66	-11.65
-11.73	-11.72	-11.71	-11.7	-11.69	-11.68	-11.67	-11.66	-11.65
-11.73	-11.72	-11.71	-11.7	-11.69	-11.68	-11.67	-11.66	-11.65
-11.73	-11.72	-11.71	-11.7	-11.69	-11.68	-11.67	-11.66	-11.65
-11.73	-11.72	-11.71	-11.7	-11.69	-11.68	-11.67	-11.66	-11.65
-11.73	-11.72	-11.71	-11.7	-11.69	-11.68	-11.67	-11.66	-11.65
-11.73	-11.72	-11.71	-11.7	-11.69	-11.68	-11.67	-11.66	-11.65
-11.73	-11.72	-11.71	-11.7	-11.69	-11.68	-11.67	-11.66	-11.65
-11.73	-11.72	-11.71	-11.7	-11.69	-11.68	-11.67	-11.66	-11.65
-11.73	-11.72	-11.71	-11.7	-11.69	-11.68	-11.67	-11.66	-11.65
-11.73	-11.72	-11.71	-11.7	-11.69	-11.68	-11.67	-11.66	-11.65
-11.73	-11.72	-11.71	-11.7	-11.69	-11.68	-11.67	-11.66	-11.65
-11.73	-11.72	-11.71	-11.7	-11.69	-11.68	-11.67	-11.66	-11.65
-11.73	-11.72	-11.71	-11.7	-11.69	-11.68	-11.67	-11.66	-11.65
-11.73	-11.72	-11.71	-11.7	-11.69	-11.68	-11.67	-11.66	-11.65
-11.73	-11.72	-11.71	-11.7	-11.69	-11.68	-11.67	-11.66	-11.65
-11.73	-11.72	-11.71	-11.7	-11.69	-11.68	-11.67	-11.66	-11.65
-11.73	-11.72	-11.71	-11.7	-11.69	-11.68	-11.67	-11.66	-11.65
-11.73	-11.72	-11.71	-11.7	-11.69	-11.68	-11.67	-11.66	-11.65
-11.73	-11.72	-11.71	-11.7	-11.69	-11.68	-11.67	-11.66	-11.65
-11.73	-11.72	-11.71	-11.7	-11.69	-11.68	-11.67	-11.66	-11.65
-11.73	-11.72	-11.71	-11.7	-11.69	-11.68	-11.67	-11.66	-11.65
-11.73	-11.72	-11.71	-11.7	-11.69	-11.68	-11.67	-11.66	-11.65
-11.73	-11.72	-11.71	-11.7	-11.69	-11.68	-11.67	-11.66	-11.65
-11.73	-11.72	-11.71	-11.7	-11.69	-11.68	-11.67	-11.66	-11.65
-11.73	-11.72	-11.71	-11.7	-11.69	-11.68	-11.67	-11.66	-11.65
-11.73	-11.72	-11.71	-11.7	-11.69	-11.68	-11.67	-11.66	-11.65
-11.73	-11.72	-11.71	-11.7	-11.69	-11.68	-11.67	-11.66	-11.65
-11.73	-11.72	-11.71	-11.7	-11.69	-11.68	-11.67	-11.66	-11.65
-11.73	-11.72	-11.71	-11.7	-11.69	-11.68	-11.67	-11.66	-11.65
-11.73	-11.72	-11.71	-11.7	-11.69	-11.68	-11.67	-11.66	-11.65
-11.73	-11.72	-11.71	-11.7	-11.69	-11.68	-11.67	-11.66	-11.65
-11.73	-11.72	-11.71	-11.7	-11.69	-11.68	-11.67	-11.66	-11.65
-11.73	-11.72	-11.71	-11.7	-11.69	-11.68	-11.67	-11.66	-11.65

-11.73	-11.72	-11.71	-11.7	-11.69	-11.68	-11.67	-11.66	-11.65
-11.73	-11.72	-11.71	-11.7	-11.69	-11.68	-11.67	-11.66	-11.65
-11.73	-11.72	-11.71	-11.7	-11.69	-11.68	-11.67	-11.66	-11.65
-11.73	-11.72	-11.71	-11.7	-11.69	-11.68	-11.67	-11.66	-11.65
-11.73	-11.72	-11.71	-11.7	-11.69	-11.68	-11.67	-11.66	-11.65
-11.73	-11.72	-11.71	-11.7	-11.69	-11.68	-11.67	-11.66	-11.65
-11.73	-11.72	-11.71	-11.7	-11.69	-11.68	-11.67	-11.66	-11.65
-11.73	-11.72	-11.71	-11.7	-11.69	-11.68	-11.67	-11.66	-11.65
-11.73	-11.72	-11.71	-11.7	-11.69	-11.68	-11.67	-11.66	-11.65
-11.73	-11.72	-11.71	-11.7	-11.69	-11.68	-11.67	-11.66	-11.65
-11.73	-11.72	-11.71	-11.7	-11.69	-11.68	-11.67	-11.66	-11.65
-11.73	-11.72	-11.71	-11.7	-11.69	-11.68	-11.67	-11.66	-11.65
-11.73	-11.72	-11.71	-11.7	-11.69	-11.68	-11.67	-11.66	-11.65
-11.73	-11.72	-11.71	-11.7	-11.69	-11.68	-11.67	-11.66	-11.65
-11.73	-11.72	-11.71	-11.7	-11.69	-11.68	-11.67	-11.66	-11.65
-11.73	-11.72	-11.71	-11.7	-11.69	-11.68	-11.67	-11.66	-11.65
-11.73	-11.72	-11.71	-11.7	-11.69	-11.68	-11.67	-11.66	-11.65
-11.73	-11.72	-11.71	-11.7	-11.69	-11.68	-11.67	-11.66	-11.65
-11.73	-11.72	-11.71	-11.7	-11.69	-11.68	-11.67	-11.66	-11.65
-11.73	-11.72	-11.71	-11.7	-11.69	-11.68	-11.67	-11.66	-11.65
-11.73	-11.72	-11.71	-11.7	-11.69	-11.68	-11.67	-11.66	-11.65
-11.73	-11.72	-11.71	-11.7	-11.69	-11.68	-11.67	-11.66	-11.65
-11.73	-11.72	-11.71	-11.7	-11.69	-11.68	-11.67	-11.66	-11.65
-11.73	-11.72	-11.71	-11.7	-11.69	-11.68	-11.67	-11.66	-11.65
-11.73	-11.72	-11.71	-11.7	-11.69	-11.68	-11.67	-11.66	-11.65
-11.73	-11.72	-11.71	-11.7	-11.69	-11.68	-11.67	-11.66	-11.65
-11.73	-11.72	-11.71	-11.7	-11.69	-11.68	-11.67	-11.66	-11.65
-11.73	-11.72	-11.71	-11.7	-11.69	-11.68	-11.67	-11.66	-11.65
-11.73	-11.72	-11.71	-11.7	-11.69	-11.68	-11.67	-11.66	-11.65
-11.73	-11.72	-11.71	-11.7	-11.69	-11.68	-11.67	-11.66	-11.65
-11.73	-11.72	-11.71	-11.7	-11.69	-11.68	-11.67	-11.66	-11.65
-11.73	-11.72	-11.71	-11.7	-11.69	-11.68	-11.67	-11.66	-11.65
-11.73	-11.72	-11.71	-11.7	-11.69	-11.68	-11.67	-11.66	-11.65
-11.73	-11.72	-11.71	-11.7	-11.69	-11.68	-11.67	-11.66	-11.65
-11.73	-11.72	-11.71	-11.7	-11.69	-11.68	-11.67	-11.66	-11.65
-11.73	-11.72	-11.71	-11.7	-11.69	-11.68	-11.67	-11.66	-11.65
-11.73	-11.72	-11.71	-11.7	-11.69	-11.68	-11.67	-11.66	-11.65
-11.73	-11.72	-11.71	-11.7	-11.69	-11.68	-11.67	-11.66	-11.65
-11.73	-11.72	-11.71	-11.7	-11.69	-11.68	-11.67	-11.66	-11.65
-11.73	-11.72	-11.71	-11.7	-11.69	-11.68	-11.67	-11.66	-11.65
-11.73	-11.72	-11.71	-11.7	-11.69	-11.68	-11.67	-11.66	-11.65
-11.73	-11.72	-11.71	-11.7	-11.69	-11.68	-11.67	-11.66	-11.65
-11.73	-11.72	-11.71	-11.7	-11.69	-11.68	-11.67	-11.66	-11.65
-11.73	-11.72	-11.71	-11.7	-11.69	-11.68	-11.67	-11.66	-11.65
-11.73	-11.72	-11.71	-11.7	-11.69	-11.68	-11.67	-11.66	-11.65
-11.73	-11.72	-11.71	-11.7	-11.69	-11.68	-11.67	-11.66	-11.65
-11.73	-11.72	-11.71	-11.7	-11.69	-11.68	-11.67	-11.66	-11.65
-11.73	-11.72	-11.71	-11.7	-11.69	-11.68	-11.67	-11.66	-11.65
-11.73	-11.72	-11.71	-11.7	-11.69	-11.68	-11.67	-11.66	-11.65
-11.73	-11.72	-11.71	-11.7	-11.69	-11.68	-11.67	-11.66	-11.65

-11.73	-11.72	-11.71	-11.7	-11.69	-11.68	-11.67	-11.66	-11.65
-11.73	-11.72	-11.71	-11.7	-11.69	-11.68	-11.67	-11.66	-11.65
-11.73	-11.72	-11.71	-11.7	-11.69	-11.68	-11.67	-11.66	-11.65
-11.73	-11.72	-11.71	-11.7	-11.69	-11.68	-11.67	-11.66	-11.65
-11.73	-11.72	-11.71	-11.7	-11.69	-11.68	-11.67	-11.66	-11.65
-11.73	-11.72	-11.71	-11.7	-11.69	-11.68	-11.67	-11.66	-11.65
-11.73	-11.72	-11.71	-11.7	-11.69	-11.68	-11.67	-11.66	-11.65
-11.73	-11.72	-11.71	-11.7	-11.69	-11.68	-11.67	-11.66	-11.65
-11.73	-11.72	-11.71	-11.7	-11.69	-11.68	-11.67	-11.66	-11.65
-11.73	-11.72	-11.71	-11.7	-11.69	-11.68	-11.67	-11.66	-11.65
-11.73	-11.72	-11.71	-11.7	-11.69	-11.68	-11.67	-11.66	-11.65
-11.73	-11.72	-11.71	-11.7	-11.69	-11.68	-11.67	-11.66	-11.65
-11.73	-11.72	-11.71	-11.7	-11.69	-11.68	-11.67	-11.66	-11.65
-11.73	-11.72	-11.71	-11.7	-11.69	-11.68	-11.67	-11.66	-11.65
-11.73	-11.72	-11.71	-11.7	-11.69	-11.68	-11.67	-11.66	-11.65
-11.73	-11.72	-11.71	-11.7	-11.69	-11.68	-11.67	-11.66	-11.65
-11.73	-11.72	-11.71	-11.7	-11.69	-11.68	-11.67	-11.66	-11.65
-11.73	-11.72	-11.71	-11.7	-11.69	-11.68	-11.67	-11.66	-11.65
-11.73	-11.72	-11.71	-11.7	-11.69	-11.68	-11.67	-11.66	-11.65
-11.73	-11.72	-11.71	-11.7	-11.69	-11.68	-11.67	-11.66	-11.65
-11.73	-11.72	-11.71	-11.7	-11.69	-11.68	-11.67	-11.66	-11.65
-11.73	-11.72	-11.71	-11.7	-11.69	-11.68	-11.67	-11.66	-11.65
-11.73	-11.72	-11.71	-11.7	-11.69	-11.68	-11.67	-11.66	-11.65
-11.73	-11.72	-11.71	-11.7	-11.69	-11.68	-11.67	-11.66	-11.65
-11.73	-11.72	-11.71	-11.7	-11.69	-11.68	-11.67	-11.66	-11.65
-11.73	-11.72	-11.71	-11.7	-11.69	-11.68	-11.67	-11.66	-11.65
-11.73	-11.72	-11.71	-11.7	-11.69	-11.68	-11.67	-11.66	-11.65
-11.73	-11.72	-11.71	-11.7	-11.69	-11.68	-11.67	-11.66	-11.65
-11.73	-11.72	-11.71	-11.7	-11.69	-11.68	-11.67	-11.66	-11.65
-11.73	-11.72	-11.71	-11.7	-11.69	-11.68	-11.67	-11.66	-11.65
-11.73	-11.72	-11.71	-11.7	-11.69	-11.68	-11.67	-11.66	-11.65
-11.73	-11.72	-11.71	-11.7	-11.69	-11.68	-11.67	-11.66	-11.65
-11.73	-11.72	-11.71	-11.7	-11.69	-11.68	-11.67	-11.66	-11.65
-11.73	-11.72	-11.71	-11.7	-11.69	-11.68	-11.67	-11.66	-11.65
-11.73	-11.72	-11.71	-11.7	-11.69	-11.68	-11.67	-11.66	-11.65
-11.73	-11.72	-11.71	-11.7	-11.69	-11.68	-11.67	-11.66	-11.65
-11.73	-11.72	-11.71	-11.7	-11.69	-11.68	-11.67	-11.66	-11.65
-11.73	-11.72	-11.71	-11.7	-11.69	-11.68	-11.67	-11.66	-11.65
-11.73	-11.72	-11.71	-11.7	-11.69	-11.68	-11.67	-11.66	-11.65
-11.73	-11.72	-11.71	-11.7	-11.69	-11.68	-11.67	-11.66	-11.65
-11.73	-11.72	-11.71	-11.7	-11.69	-11.68	-11.67	-11.66	-11.65
-11.73	-11.72	-11.71	-11.7	-11.69	-11.68	-11.67	-11.66	-11.65
-11.73	-11.72	-11.71	-11.7	-11.69	-11.68	-11.67	-11.66	-11.65
-11.73	-11.72	-11.71	-11.7	-11.69	-11.68	-11.67	-11.66	-11.65
-11.73	-11.72	-11.71	-11.7	-11.69	-11.68	-11.67	-11.66	-11.65
-11.73	-11.72	-11.71	-11.7	-11.69	-11.68	-11.67	-11.66	-11.65
-11.73	-11.72	-11.71	-11.7	-11.69	-11.68	-11.67	-11.66	-11.65
-11.73	-11.72	-11.71	-11.7	-11.69	-11.68	-11.67	-11.66	-11.65
-11.73	-11.72	-11.71	-11.7	-11.69	-11.68	-11.67	-11.66	-11.65
-11.73	-11.72	-11.71	-11.7	-11.69	-11.68	-11.67	-11.66	-11.65

-11.73	-11.72	-11.71	-11.7	-11.69	-11.68	-11.67	-11.66	-11.65
-11.73	-11.72	-11.71	-11.7	-11.69	-11.68	-11.67	-11.66	-11.65
-11.73	-11.72	-11.71	-11.7	-11.69	-11.68	-11.67	-11.66	-11.65
-11.73	-11.72	-11.71	-11.7	-11.69	-11.68	-11.67	-11.66	-11.65
-11.73	-11.72	-11.71	-11.7	-11.69	-11.68	-11.67	-11.66	-11.65
-11.73	-11.72	-11.71	-11.7	-11.69	-11.68	-11.67	-11.66	-11.65
-11.73	-11.72	-11.71	-11.7	-11.69	-11.68	-11.67	-11.66	-11.65
-11.73	-11.72	-11.71	-11.7	-11.69	-11.68	-11.67	-11.66	-11.65
-11.73	-11.72	-11.71	-11.7	-11.69	-11.68	-11.67	-11.66	-11.65
-11.73	-11.72	-11.71	-11.7	-11.69	-11.68	-11.67	-11.66	-11.65
-11.73	-11.72	-11.71	-11.7	-11.69	-11.68	-11.67	-11.66	-11.65
-11.73	-11.72	-11.71	-11.7	-11.69	-11.68	-11.67	-11.66	-11.65
-11.73	-11.72	-11.71	-11.7	-11.69	-11.68	-11.67	-11.66	-11.65
-11.73	-11.72	-11.71	-11.7	-11.69	-11.68	-11.67	-11.66	-11.65
-11.73	-11.72	-11.71	-11.7	-11.69	-11.68	-11.67	-11.66	-11.65
-11.73	-11.72	-11.71	-11.7	-11.69	-11.68	-11.67	-11.66	-11.65
-11.73	-11.72	-11.71	-11.7	-11.69	-11.68	-11.67	-11.66	-11.65
-11.73	-11.72	-11.71	-11.7	-11.69	-11.68	-11.67	-11.66	-11.65
-11.73	-11.72	-11.71	-11.7	-11.69	-11.68	-11.67	-11.66	-11.65
-11.73	-11.72	-11.71	-11.7	-11.69	-11.68	-11.67	-11.66	-11.65
-11.73	-11.72	-11.71	-11.7	-11.69	-11.68	-11.67	-11.66	-11.65
-11.73	-11.72	-11.71	-11.7	-11.69	-11.68	-11.67	-11.66	-11.65
-11.73	-11.72	-11.71	-11.7	-11.69	-11.68	-11.67	-11.66	-11.65
-11.73	-11.72	-11.71	-11.7	-11.69	-11.68	-11.67	-11.66	-11.65
-11.73	-11.72	-11.71	-11.7	-11.69	-11.68	-11.67	-11.66	-11.65
-11.73	-11.72	-11.71	-11.7	-11.69	-11.68	-11.67	-11.66	-11.65
-11.73	-11.72	-11.71	-11.7	-11.69	-11.68	-11.67	-11.66	-11.65
-11.73	-11.72	-11.71	-11.7	-11.69	-11.68	-11.67	-11.66	-11.65
-11.73	-11.72	-11.71	-11.7	-11.69	-11.68	-11.67	-11.66	-11.65
-11.73	-11.72	-11.71	-11.7	-11.69	-11.68	-11.67	-11.66	-11.65
-11.73	-11.72	-11.71	-11.7	-11.69	-11.68	-11.67	-11.66	-11.65
-11.73	-11.72	-11.71	-11.7	-11.69	-11.68	-11.67	-11.66	-11.65
-11.73	-11.72	-11.71	-11.7	-11.69	-11.68	-11.67	-11.66	-11.65
-11.73	-11.72	-11.71	-11.7	-11.69	-11.68	-11.67	-11.66	-11.65
-11.73	-11.72	-11.71	-11.7	-11.69	-11.68	-11.67	-11.66	-11.65
-11.73	-11.72	-11.71	-11.7	-11.69	-11.68	-11.67	-11.66	-11.65
-11.73	-11.72	-11.71	-11.7	-11.69	-11.68	-11.67	-11.66	-11.65
-11.73	-11.72	-11.71	-11.7	-11.69	-11.68	-11.67	-11.66	-11.65
-11.73	-11.72	-11.71	-11.7	-11.69	-11.68	-11.67	-11.66	-11.65
-11.73	-11.72	-11.71	-11.7	-11.69	-11.68	-11.67	-11.66	-11.65
-11.73	-11.72	-11.71	-11.7	-11.69	-11.68	-11.67	-11.66	-11.65
-11.73	-11.72	-11.71	-11.7	-11.69	-11.68	-11.67	-11.66	-11.65
-11.73	-11.72	-11.71	-11.7	-11.69	-11.68	-11.67	-11.66	-11.65
-11.73	-11.72	-11.71	-11.7	-11.69	-11.68	-11.67	-11.66	-11.65
-11.73	-11.72	-11.71	-11.7	-11.69	-11.68	-11.67	-11.66	-11.65
-11.73	-11.72	-11.71	-11.7	-11.69	-11.68	-11.67	-11.66	-11.65
-11.73	-11.72	-11.71	-11.7	-11.69	-11.68	-11.67	-11.66	-11.65
-11.73	-11.72	-11.71	-11.7	-11.69	-11.68	-11.67	-11.66	-11.65
-11.73	-11.72	-11.71	-11.7	-11.69	-11.68	-11.67	-11.66	-11.65
-11.73	-11.72	-11.71	-11.7	-11.69	-11.68	-11.67	-11.66	-11.65

-11.73	-11.72	-11.71	-11.7	-11.69	-11.68	-11.67	-11.66	-11.65
-11.73	-11.72	-11.71	-11.7	-11.69	-11.68	-11.67	-11.66	-11.65
-11.73	-11.72	-11.71	-11.7	-11.69	-11.68	-11.67	-11.66	-11.65
-11.73	-11.72	-11.71	-11.7	-11.69	-11.68	-11.67	-11.66	-11.65
-11.73	-11.72	-11.71	-11.7	-11.69	-11.68	-11.67	-11.66	-11.65
-11.73	-11.72	-11.71	-11.7	-11.69	-11.68	-11.67	-11.66	-11.65
-11.73	-11.72	-11.71	-11.7	-11.69	-11.68	-11.67	-11.66	-11.65
-11.73	-11.72	-11.71	-11.7	-11.69	-11.68	-11.67	-11.66	-11.65
-11.73	-11.72	-11.71	-11.7	-11.69	-11.68	-11.67	-11.66	-11.65
-11.73	-11.72	-11.71	-11.7	-11.69	-11.68	-11.67	-11.66	-11.65
-11.73	-11.72	-11.71	-11.7	-11.69	-11.68	-11.67	-11.66	-11.65
-11.73	-11.72	-11.71	-11.7	-11.69	-11.68	-11.67	-11.66	-11.65
-11.73	-11.72	-11.71	-11.7	-11.69	-11.68	-11.67	-11.66	-11.65
-11.73	-11.72	-11.71	-11.7	-11.69	-11.68	-11.67	-11.66	-11.65
-11.73	-11.72	-11.71	-11.7	-11.69	-11.68	-11.67	-11.66	-11.65
-11.73	-11.72	-11.71	-11.7	-11.69	-11.68	-11.67	-11.66	-11.65
-11.73	-11.72	-11.71	-11.7	-11.69	-11.68	-11.67	-11.66	-11.65
-11.73	-11.72	-11.71	-11.7	-11.69	-11.68	-11.67	-11.66	-11.65
-11.73	-11.72	-11.71	-11.7	-11.69	-11.68	-11.67	-11.66	-11.65
-11.73	-11.72	-11.71	-11.7	-11.69	-11.68	-11.67	-11.66	-11.65
-11.73	-11.72	-11.71	-11.7	-11.69	-11.68	-11.67	-11.66	-11.65
-11.73	-11.72	-11.71	-11.7	-11.69	-11.68	-11.67	-11.66	-11.65
-11.73	-11.72	-11.71	-11.7	-11.69	-11.68	-11.67	-11.66	-11.65
-11.73	-11.72	-11.71	-11.7	-11.69	-11.68	-11.67	-11.66	-11.65
-11.73	-11.72	-11.71	-11.7	-11.69	-11.68	-11.67	-11.66	-11.65
-11.73	-11.72	-11.71	-11.7	-11.69	-11.68	-11.67	-11.66	-11.65
-11.73	-11.72	-11.71	-11.7	-11.69	-11.68	-11.67	-11.66	-11.65
-11.73	-11.72	-11.71	-11.7	-11.69	-11.68	-11.67	-11.66	-11.65
-11.73	-11.72	-11.71	-11.7	-11.69	-11.68	-11.67	-11.66	-11.65
-11.73	-11.72	-11.71	-11.7	-11.69	-11.68	-11.67	-11.66	-11.65
-11.73	-11.72	-11.71	-11.7	-11.69	-11.68	-11.67	-11.66	-11.65
-11.73	-11.72	-11.71	-11.7	-11.69	-11.68	-11.67	-11.66	-11.65
-11.73	-11.72	-11.71	-11.7	-11.69	-11.68	-11.67	-11.66	-11.65
-11.73	-11.72	-11.71	-11.7	-11.69	-11.68	-11.67	-11.66	-11.65
-11.73	-11.72	-11.71	-11.7	-11.69	-11.68	-11.67	-11.66	-11.65
-11.73	-11.72	-11.71	-11.7	-11.69	-11.68	-11.67	-11.66	-11.65
-11.73	-11.72	-11.71	-11.7	-11.69	-11.68	-11.67	-11.66	-11.65
-11.73	-11.72	-11.71	-11.7	-11.69	-11.68	-11.67	-11.66	-11.65
-11.73	-11.72	-11.71	-11.7	-11.69	-11.68	-11.67	-11.66	-11.65
-11.73	-11.72	-11.71	-11.7	-11.69	-11.68	-11.67	-11.66	-11.65
-11.73	-11.72	-11.71	-11.7	-11.69	-11.68	-11.67	-11.66	-11.65
-11.73	-11.72	-11.71	-11.7	-11.69	-11.68	-11.67	-11.66	-11.65
-11.73	-11.72	-11.71	-11.7	-11.69	-11.68	-11.67	-11.66	-11.65
-11.73	-11.72	-11.71	-11.7	-11.69	-11.68	-11.67	-11.66	-11.65
-11.73	-11.72	-11.71	-11.7	-11.69	-11.68	-11.67	-11.66	-11.65
-11.73	-11.72	-11.71	-11.7	-11.69	-11.68	-11.67	-11.66	-11.65
-11.73	-11.72	-11.71	-11.7	-11.69	-11.68	-11.67	-11.66	-11.65
-11.73	-11.72	-11.71	-11.7	-11.69	-11.68	-11.67	-11.66	-11.65
-11.73	-11.72	-11.71	-11.7	-11.69	-11.68	-11.67	-11.66	-11.65
-11.73	-11.72	-11.71	-11.7	-11.69	-11.68	-11.67	-11.66	-11.65

-11.73	-11.72	-11.71	-11.7	-11.69	-11.68	-11.67	-11.66	-11.65
-11.73	-11.72	-11.71	-11.7	-11.69	-11.68	-11.67	-11.66	-11.65
-11.73	-11.72	-11.71	-11.7	-11.69	-11.68	-11.67	-11.66	-11.65
-11.73	-11.72	-11.71	-11.7	-11.69	-11.68	-11.67	-11.66	-11.65
-11.73	-11.72	-11.71	-11.7	-11.69	-11.68	-11.67	-11.66	-11.65
-11.73	-11.72	-11.71	-11.7	-11.69	-11.68	-11.67	-11.66	-11.65
-11.73	-11.72	-11.71	-11.7	-11.69	-11.68	-11.67	-11.66	-11.65
-11.73	-11.72	-11.71	-11.7	-11.69	-11.68	-11.67	-11.66	-11.65
-11.73	-11.72	-11.71	-11.7	-11.69	-11.68	-11.67	-11.66	-11.65
-11.73	-11.72	-11.71	-11.7	-11.69	-11.68	-11.67	-11.66	-11.65
-11.73	-11.72	-11.71	-11.7	-11.69	-11.68	-11.67	-11.66	-11.65
-11.73	-11.72	-11.71	-11.7	-11.69	-11.68	-11.67	-11.66	-11.65
-11.73	-11.72	-11.71	-11.7	-11.69	-11.68	-11.67	-11.66	-11.65
-11.73	-11.72	-11.71	-11.7	-11.69	-11.68	-11.67	-11.66	-11.65
-11.73	-11.72	-11.71	-11.7	-11.69	-11.68	-11.67	-11.66	-11.65
-11.73	-11.72	-11.71	-11.7	-11.69	-11.68	-11.67	-11.66	-11.65
-11.73	-11.72	-11.71	-11.7	-11.69	-11.68	-11.67	-11.66	-11.65
-11.73	-11.72	-11.71	-11.7	-11.69	-11.68	-11.67	-11.66	-11.65
-11.73	-11.72	-11.71	-11.7	-11.69	-11.68	-11.67	-11.66	-11.65
-11.73	-11.72	-11.71	-11.7	-11.69	-11.68	-11.67	-11.66	-11.65
-11.73	-11.72	-11.71	-11.7	-11.69	-11.68	-11.67	-11.66	-11.65
-11.73	-11.72	-11.71	-11.7	-11.69	-11.68	-11.67	-11.66	-11.65
-11.73	-11.72	-11.71	-11.7	-11.69	-11.68	-11.67	-11.66	-11.65
-11.73	-11.72	-11.71	-11.7	-11.69	-11.68	-11.67	-11.66	-11.65
-11.73	-11.72	-11.71	-11.7	-11.69	-11.68	-11.67	-11.66	-11.65
-11.73	-11.72	-11.71	-11.7	-11.69	-11.68	-11.67	-11.66	-11.65
-11.73	-11.72	-11.71	-11.7	-11.69	-11.68	-11.67	-11.66	-11.65
-11.73	-11.72	-11.71	-11.7	-11.69	-11.68	-11.67	-11.66	-11.65
-11.73	-11.72	-11.71	-11.7	-11.69	-11.68	-11.67	-11.66	-11.65
-11.73	-11.72	-11.71	-11.7	-11.69	-11.68	-11.67	-11.66	-11.65
-11.73	-11.72	-11.71	-11.7	-11.69	-11.68	-11.67	-11.66	-11.65
-11.73	-11.72	-11.71	-11.7	-11.69	-11.68	-11.67	-11.66	-11.65
-11.73	-11.72	-11.71	-11.7	-11.69	-11.68	-11.67	-11.66	-11.65
-11.73	-11.72	-11.71	-11.7	-11.69	-11.68	-11.67	-11.66	-11.65
-11.73	-11.72	-11.71	-11.7	-11.69	-11.68	-11.67	-11.66	-11.65
-11.73	-11.72	-11.71	-11.7	-11.69	-11.68	-11.67	-11.66	-11.65
-11.73	-11.72	-11.71	-11.7	-11.69	-11.68	-11.67	-11.66	-11.65
-11.73	-11.72	-11.71	-11.7	-11.69	-11.68	-11.67	-11.66	-11.65
-11.73	-11.72	-11.71	-11.7	-11.69	-11.68	-11.67	-11.66	-11.65
-11.73	-11.72	-11.71	-11.7	-11.69	-11.68	-11.67	-11.66	-11.65
-11.73	-11.72	-11.71	-11.7	-11.69	-11.68	-11.67	-11.66	-11.65
-11.73	-11.72	-11.71	-11.7	-11.69	-11.68	-11.67	-11.66	-11.65
-11.73	-11.72	-11.71	-11.7	-11.69	-11.68	-11.67	-11.66	-11.65
-11.73	-11.72	-11.71	-11.7	-11.69	-11.68	-11.67	-11.66	-11.65
-11.73	-11.72	-11.71	-11.7	-11.69	-11.68	-11.67	-11.66	-11.65
-11.73	-11.72	-11.71	-11.7	-11.69	-11.68	-11.67	-11.66	-11.65
-11.73	-11.72	-11.71	-11.7	-11.69	-11.68	-11.67	-11.66	-11.65
-11.73	-11.72	-11.71	-11.7	-11.69	-11.68	-11.67	-11.66	-11.65
-11.73	-11.72	-11.71	-11.7	-11.69	-11.68	-11.67	-11.66	-11.65
-11.73	-11.72	-11.71	-11.7	-11.69	-11.68	-11.67	-11.66	-11.65

-11.73	-11.72	-11.71	-11.7	-11.69	-11.68	-11.67	-11.66	-11.65
-11.73	-11.72	-11.71	-11.7	-11.69	-11.68	-11.67	-11.66	-11.65
-11.73	-11.72	-11.71	-11.7	-11.69	-11.68	-11.67	-11.66	-11.65
-11.73	-11.72	-11.71	-11.7	-11.69	-11.68	-11.67	-11.66	-11.65
-11.73	-11.72	-11.71	-11.7	-11.69	-11.68	-11.67	-11.66	-11.65
-11.73	-11.72	-11.71	-11.7	-11.69	-11.68	-11.67	-11.66	-11.65
-11.73	-11.72	-11.71	-11.7	-11.69	-11.68	-11.67	-11.66	-11.65
-11.73	-11.72	-11.71	-11.7	-11.69	-11.68	-11.67	-11.66	-11.65
-11.73	-11.72	-11.71	-11.7	-11.69	-11.68	-11.67	-11.66	-11.65
-11.73	-11.72	-11.71	-11.7	-11.69	-11.68	-11.67	-11.66	-11.65
-11.73	-11.72	-11.71	-11.7	-11.69	-11.68	-11.67	-11.66	-11.65
-11.73	-11.72	-11.71	-11.7	-11.69	-11.68	-11.67	-11.66	-11.65
-11.73	-11.72	-11.71	-11.7	-11.69	-11.68	-11.67	-11.66	-11.65
-11.73	-11.72	-11.71	-11.7	-11.69	-11.68	-11.67	-11.66	-11.65
-11.73	-11.72	-11.71	-11.7	-11.69	-11.68	-11.67	-11.66	-11.65
-11.73	-11.72	-11.71	-11.7	-11.69	-11.68	-11.67	-11.66	-11.65
-11.73	-11.72	-11.71	-11.7	-11.69	-11.68	-11.67	-11.66	-11.65
-11.73	-11.72	-11.71	-11.7	-11.69	-11.68	-11.67	-11.66	-11.65
-11.73	-11.72	-11.71	-11.7	-11.69	-11.68	-11.67	-11.66	-11.65
-11.73	-11.72	-11.71	-11.7	-11.69	-11.68	-11.67	-11.66	-11.65
-11.73	-11.72	-11.71	-11.7	-11.69	-11.68	-11.67	-11.66	-11.65
-11.73	-11.72	-11.71	-11.7	-11.69	-11.68	-11.67	-11.66	-11.65
-11.73	-11.72	-11.71	-11.7	-11.69	-11.68	-11.67	-11.66	-11.65
-11.73	-11.72	-11.71	-11.7	-11.69	-11.68	-11.67	-11.66	-11.65
-11.73	-11.72	-11.71	-11.7	-11.69	-11.68	-11.67	-11.66	-11.65
-11.73	-11.72	-11.71	-11.7	-11.69	-11.68	-11.67	-11.66	-11.65
-11.73	-11.72	-11.71	-11.7	-11.69	-11.68	-11.67	-11.66	-11.65
-11.73	-11.72	-11.71	-11.7	-11.69	-11.68	-11.67	-11.66	-11.65
-11.73	-11.72	-11.71	-11.7	-11.69	-11.68	-11.67	-11.66	-11.65
-11.73	-11.72	-11.71	-11.7	-11.69	-11.68	-11.67	-11.66	-11.65
-11.73	-11.72	-11.71	-11.7	-11.69	-11.68	-11.67	-11.66	-11.65
-11.73	-11.72	-11.71	-11.7	-11.69	-11.68	-11.67	-11.66	-11.65
-11.73	-11.72	-11.71	-11.7	-11.69	-11.68	-11.67	-11.66	-11.65
-11.73	-11.72	-11.71	-11.7	-11.69	-11.68	-11.67	-11.66	-11.65
-11.73	-11.72	-11.71	-11.7	-11.69	-11.68	-11.67	-11.66	-11.65
-11.73	-11.72	-11.71	-11.7	-11.69	-11.68	-11.67	-11.66	-11.65
-11.73	-11.72	-11.71	-11.7	-11.69	-11.68	-11.67	-11.66	-11.65
-11.73	-11.72	-11.71	-11.7	-11.69	-11.68	-11.67	-11.66	-11.65
-11.73	-11.72	-11.71	-11.7	-11.69	-11.68	-11.67	-11.66	-11.65
-11.73	-11.72	-11.71	-11.7	-11.69	-11.68	-11.67	-11.66	-11.65
-11.73	-11.72	-11.71	-11.7	-11.69	-11.68	-11.67	-11.66	-11.65
-11.73	-11.72	-11.71	-11.7	-11.69	-11.68	-11.67	-11.66	-11.65
-11.73	-11.72	-11.71	-11.7	-11.69	-11.68	-11.67	-11.66	-11.65
-11.73	-11.72	-11.71	-11.7	-11.69	-11.68	-11.67	-11.66	-11.65
-11.73	-11.72	-11.71	-11.7	-11.69	-11.68	-11.67	-11.66	-11.65
-11.73	-11.72	-11.71	-11.7	-11.69	-11.68	-11.67	-11.66	-11.65
-11.73	-11.72	-11.71	-11.7	-11.69	-11.68	-11.67	-11.66	-11.65
-11.73	-11.72	-11.71	-11.7	-11.69	-11.68	-11.67	-11.66	-11.65
-11.73	-11.72	-11.71	-11.7	-11.69	-11.68	-11.67	-11.66	-11.65
-11.73	-11.72	-11.71	-11.7	-11.69	-11.68	-11.67	-11.66	-11.65

-11.73	-11.72	-11.71	-11.7	-11.69	-11.68	-11.67	-11.66	-11.65
-11.73	-11.72	-11.71	-11.7	-11.69	-11.68	-11.67	-11.66	-11.65
-11.73	-11.72	-11.71	-11.7	-11.69	-11.68	-11.67	-11.66	-11.65
-11.73	-11.72	-11.71	-11.7	-11.69	-11.68	-11.67	-11.66	-11.65
-11.73	-11.72	-11.71	-11.7	-11.69	-11.68	-11.67	-11.66	-11.65
-11.73	-11.72	-11.71	-11.7	-11.69	-11.68	-11.67	-11.66	-11.65
-11.73	-11.72	-11.71	-11.7	-11.69	-11.68	-11.67	-11.66	-11.65
-11.73	-11.72	-11.71	-11.7	-11.69	-11.68	-11.67	-11.66	-11.65
-11.73	-11.72	-11.71	-11.7	-11.69	-11.68	-11.67	-11.66	-11.65
-11.73	-11.72	-11.71	-11.7	-11.69	-11.68	-11.67	-11.66	-11.65
-11.73	-11.72	-11.71	-11.7	-11.69	-11.68	-11.67	-11.66	-11.65
-11.73	-11.72	-11.71	-11.7	-11.69	-11.68	-11.67	-11.66	-11.65
-11.73	-11.72	-11.71	-11.7	-11.69	-11.68	-11.67	-11.66	-11.65
-11.73	-11.72	-11.71	-11.7	-11.69	-11.68	-11.67	-11.66	-11.65
-11.73	-11.72	-11.71	-11.7	-11.69	-11.68	-11.67	-11.66	-11.65
-11.73	-11.72	-11.71	-11.7	-11.69	-11.68	-11.67	-11.66	-11.65
-11.73	-11.72	-11.71	-11.7	-11.69	-11.68	-11.67	-11.66	-11.65
-11.73	-11.72	-11.71	-11.7	-11.69	-11.68	-11.67	-11.66	-11.65
-11.73	-11.72	-11.71	-11.7	-11.69	-11.68	-11.67	-11.66	-11.65
-11.73	-11.72	-11.71	-11.7	-11.69	-11.68	-11.67	-11.66	-11.65
-11.73	-11.72	-11.71	-11.7	-11.69	-11.68	-11.67	-11.66	-11.65
-11.73	-11.72	-11.71	-11.7	-11.69	-11.68	-11.67	-11.66	-11.65
-11.73	-11.72	-11.71	-11.7	-11.69	-11.68	-11.67	-11.66	-11.65
-11.73	-11.72	-11.71	-11.7	-11.69	-11.68	-11.67	-11.66	-11.65
-11.73	-11.72	-11.71	-11.7	-11.69	-11.68	-11.67	-11.66	-11.65
-11.73	-11.72	-11.71	-11.7	-11.69	-11.68	-11.67	-11.66	-11.65
-11.73	-11.72	-11.71	-11.7	-11.69	-11.68	-11.67	-11.66	-11.65
-11.73	-11.72	-11.71	-11.7	-11.69	-11.68	-11.67	-11.66	-11.65
-11.73	-11.72	-11.71	-11.7	-11.69	-11.68	-11.67	-11.66	-11.65
-11.73	-11.72	-11.71	-11.7	-11.69	-11.68	-11.67	-11.66	-11.65
-11.73	-11.72	-11.71	-11.7	-11.69	-11.68	-11.67	-11.66	-11.65
-11.73	-11.72	-11.71	-11.7	-11.69	-11.68	-11.67	-11.66	-11.65
-11.73	-11.72	-11.71	-11.7	-11.69	-11.68	-11.67	-11.66	-11.65
-11.73	-11.72	-11.71	-11.7	-11.69	-11.68	-11.67	-11.66	-11.65
-11.73	-11.72	-11.71	-11.7	-11.69	-11.68	-11.67	-11.66	-11.65
-11.73	-11.72	-11.71	-11.7	-11.69	-11.68	-11.67	-11.66	-11.65
-11.73	-11.72	-11.71	-11.7	-11.69	-11.68	-11.67	-11.66	-11.65
-11.73	-11.72	-11.71	-11.7	-11.69	-11.68	-11.67	-11.66	-11.65
-11.73	-11.72	-11.71	-11.7	-11.69	-11.68	-11.67	-11.66	-11.65
-11.73	-11.72	-11.71	-11.7	-11.69	-11.68	-11.67	-11.66	-11.65
-11.73	-11.72	-11.71	-11.7	-11.69	-11.68	-11.67	-11.66	-11.65
-11.73	-11.72	-11.71	-11.7	-11.69	-11.68	-11.67	-11.66	-11.65
-11.73	-11.72	-11.71	-11.7	-11.69	-11.68	-11.67	-11.66	-11.65
-11.73	-11.72	-11.71	-11.7	-11.69	-11.68	-11.67	-11.66	-11.65
-11.73	-11.72	-11.71	-11.7	-11.69	-11.68	-11.67	-11.66	-11.65
-11.73	-11.72	-11.71	-11.7	-11.69	-11.68	-11.67	-11.66	-11.65
-11.73	-11.72	-11.71	-11.7	-11.69	-11.68	-11.67	-11.66	-11.65
-11.73	-11.72	-11.71	-11.7	-11.69	-11.68	-11.67	-11.66	-11.65
-11.73	-11.72	-11.71	-11.7	-11.69	-11.68	-11.67	-11.66	-11.65
-11.73	-11.72	-11.71	-11.7	-11.69	-11.68	-11.67	-11.66	-11.65

-11.73	-11.72	-11.71	-11.7	-11.69	-11.68	-11.67	-11.66	-11.65
-11.73	-11.72	-11.71	-11.7	-11.69	-11.68	-11.67	-11.66	-11.65
-11.73	-11.72	-11.71	-11.7	-11.69	-11.68	-11.67	-11.66	-11.65
-11.73	-11.72	-11.71	-11.7	-11.69	-11.68	-11.67	-11.66	-11.65
-11.73	-11.72	-11.71	-11.7	-11.69	-11.68	-11.67	-11.66	-11.65
-11.73	-11.72	-11.71	-11.7	-11.69	-11.68	-11.67	-11.66	-11.65
-11.73	-11.72	-11.71	-11.7	-11.69	-11.68	-11.67	-11.66	-11.65
-11.73	-11.72	-11.71	-11.7	-11.69	-11.68	-11.67	-11.66	-11.65
-11.73	-11.72	-11.71	-11.7	-11.69	-11.68	-11.67	-11.66	-11.65
-11.73	-11.72	-11.71	-11.7	-11.69	-11.68	-11.67	-11.66	-11.65
-11.73	-11.72	-11.71	-11.7	-11.69	-11.68	-11.67	-11.66	-11.65
-11.73	-11.72	-11.71	-11.7	-11.69	-11.68	-11.67	-11.66	-11.65
-11.73	-11.72	-11.71	-11.7	-11.69	-11.68	-11.67	-11.66	-11.65
-11.73	-11.72	-11.71	-11.7	-11.69	-11.68	-11.67	-11.66	-11.65
-11.73	-11.72	-11.71	-11.7	-11.69	-11.68	-11.67	-11.66	-11.65
-11.73	-11.72	-11.71	-11.7	-11.69	-11.68	-11.67	-11.66	-11.65
-11.73	-11.72	-11.71	-11.7	-11.69	-11.68	-11.67	-11.66	-11.65
-11.73	-11.72	-11.71	-11.7	-11.69	-11.68	-11.67	-11.66	-11.65
-11.73	-11.72	-11.71	-11.7	-11.69	-11.68	-11.67	-11.66	-11.65
-11.73	-11.72	-11.71	-11.7	-11.69	-11.68	-11.67	-11.66	-11.65
-11.73	-11.72	-11.71	-11.7	-11.69	-11.68	-11.67	-11.66	-11.65
-11.73	-11.72	-11.71	-11.7	-11.69	-11.68	-11.67	-11.66	-11.65
-11.73	-11.72	-11.71	-11.7	-11.69	-11.68	-11.67	-11.66	-11.65
-11.73	-11.72	-11.71	-11.7	-11.69	-11.68	-11.67	-11.66	-11.65
-11.73	-11.72	-11.71	-11.7	-11.69	-11.68	-11.67	-11.66	-11.65
-11.73	-11.72	-11.71	-11.7	-11.69	-11.68	-11.67	-11.66	-11.65
-11.73	-11.72	-11.71	-11.7	-11.69	-11.68	-11.67	-11.66	-11.65
-11.73	-11.72	-11.71	-11.7	-11.69	-11.68	-11.67	-11.66	-11.65
-11.73	-11.72	-11.71	-11.7	-11.69	-11.68	-11.67	-11.66	-11.65
-11.73	-11.72	-11.71	-11.7	-11.69	-11.68	-11.67	-11.66	-11.65
-11.73	-11.72	-11.71	-11.7	-11.69	-11.68	-11.67	-11.66	-11.65
-11.73	-11.72	-11.71	-11.7	-11.69	-11.68	-11.67	-11.66	-11.65
-11.73	-11.72	-11.71	-11.7	-11.69	-11.68	-11.67	-11.66	-11.65
-11.73	-11.72	-11.71	-11.7	-11.69	-11.68	-11.67	-11.66	-11.65
-11.73	-11.72	-11.71	-11.7	-11.69	-11.68	-11.67	-11.66	-11.65
-11.73	-11.72	-11.71	-11.7	-11.69	-11.68	-11.67	-11.66	-11.65
-11.73	-11.72	-11.71	-11.7	-11.69	-11.68	-11.67	-11.66	-11.65
-11.73	-11.72	-11.71	-11.7	-11.69	-11.68	-11.67	-11.66	-11.65
-11.73	-11.72	-11.71	-11.7	-11.69	-11.68	-11.67	-11.66	-11.65
-11.73	-11.72	-11.71	-11.7	-11.69	-11.68	-11.67	-11.66	-11.65
-11.73	-11.72	-11.71	-11.7	-11.69	-11.68	-11.67	-11.66	-11.65
-11.73	-11.72	-11.71	-11.7	-11.69	-11.68	-11.67	-11.66	-11.65
-11.73	-11.72	-11.71	-11.7	-11.69	-11.68	-11.67	-11.66	-11.65
-11.73	-11.72	-11.71	-11.7	-11.69	-11.68	-11.67	-11.66	-11.65
-11.73	-11.72	-11.71	-11.7	-11.69	-11.68	-11.67	-11.66	-11.65
-11.73	-11.72	-11.71	-11.7	-11.69	-11.68	-11.67	-11.66	-11.65
-11.73	-11.72	-11.71	-11.7	-11.69	-11.68	-11.67	-11.66	-11.65
-11.73	-11.72	-11.71	-11.7	-11.69	-11.68	-11.67	-11.66	-11.65
-11.73	-11.72	-11.71	-11.7	-11.69	-11.68	-11.67	-11.66	-11.65
-11.73	-11.72	-11.71	-11.7	-11.69	-11.68	-11.67	-11.66	-11.65

-11.73	-11.72	-11.71	-11.7	-11.69	-11.68	-11.67	-11.66	-11.65
-11.73	-11.72	-11.71	-11.7	-11.69	-11.68	-11.67	-11.66	-11.65
-11.73	-11.72	-11.71	-11.7	-11.69	-11.68	-11.67	-11.66	-11.65
-11.73	-11.72	-11.71	-11.7	-11.69	-11.68	-11.67	-11.66	-11.65
-11.73	-11.72	-11.71	-11.7	-11.69	-11.68	-11.67	-11.66	-11.65
-11.73	-11.72	-11.71	-11.7	-11.69	-11.68	-11.67	-11.66	-11.65
-11.73	-11.72	-11.71	-11.7	-11.69	-11.68	-11.67	-11.66	-11.65
-11.73	-11.72	-11.71	-11.7	-11.69	-11.68	-11.67	-11.66	-11.65
-11.73	-11.72	-11.71	-11.7	-11.69	-11.68	-11.67	-11.66	-11.65
-11.73	-11.72	-11.71	-11.7	-11.69	-11.68	-11.67	-11.66	-11.65
-11.73	-11.72	-11.71	-11.7	-11.69	-11.68	-11.67	-11.66	-11.65
-11.73	-11.72	-11.71	-11.7	-11.69	-11.68	-11.67	-11.66	-11.65
-11.73	-11.72	-11.71	-11.7	-11.69	-11.68	-11.67	-11.66	-11.65
-11.73	-11.72	-11.71	-11.7	-11.69	-11.68	-11.67	-11.66	-11.65
-11.73	-11.72	-11.71	-11.7	-11.69	-11.68	-11.67	-11.66	-11.65
-11.73	-11.72	-11.71	-11.7	-11.69	-11.68	-11.67	-11.66	-11.65
-11.73	-11.72	-11.71	-11.7	-11.69	-11.68	-11.67	-11.66	-11.65
-11.73	-11.72	-11.71	-11.7	-11.69	-11.68	-11.67	-11.66	-11.65
-11.73	-11.72	-11.71	-11.7	-11.69	-11.68	-11.67	-11.66	-11.65
-11.73	-11.72	-11.71	-11.7	-11.69	-11.68	-11.67	-11.66	-11.65
-11.73	-11.72	-11.71	-11.7	-11.69	-11.68	-11.67	-11.66	-11.65
-11.73	-11.72	-11.71	-11.7	-11.69	-11.68	-11.67	-11.66	-11.65
-11.73	-11.72	-11.71	-11.7	-11.69	-11.68	-11.67	-11.66	-11.65
-11.73	-11.72	-11.71	-11.7	-11.69	-11.68	-11.67	-11.66	-11.65
-11.73	-11.72	-11.71	-11.7	-11.69	-11.68	-11.67	-11.66	-11.65
-11.73	-11.72	-11.71	-11.7	-11.69	-11.68	-11.67	-11.66	-11.65
-11.73	-11.72	-11.71	-11.7	-11.69	-11.68	-11.67	-11.66	-11.65
-11.73	-11.72	-11.71	-11.7	-11.69	-11.68	-11.67	-11.66	-11.65
-11.73	-11.72	-11.71	-11.7	-11.69	-11.68	-11.67	-11.66	-11.65
-11.73	-11.72	-11.71	-11.7	-11.69	-11.68	-11.67	-11.66	-11.65
-11.73	-11.72	-11.71	-11.7	-11.69	-11.68	-11.67	-11.66	-11.65
-11.73	-11.72	-11.71	-11.7	-11.69	-11.68	-11.67	-11.66	-11.65
-11.73	-11.72	-11.71	-11.7	-11.69	-11.68	-11.67	-11.66	-11.65
-11.73	-11.72	-11.71	-11.7	-11.69	-11.68	-11.67	-11.66	-11.65
-11.73	-11.72	-11.71	-11.7	-11.69	-11.68	-11.67	-11.66	-11.65
-11.73	-11.72	-11.71	-11.7	-11.69	-11.68	-11.67	-11.66	-11.65
-11.73	-11.72	-11.71	-11.7	-11.69	-11.68	-11.67	-11.66	-11.65
-11.73	-11.72	-11.71	-11.7	-11.69	-11.68	-11.67	-11.66	-11.65
-11.73	-11.72	-11.71	-11.7	-11.69	-11.68	-11.67	-11.66	-11.65
-11.73	-11.72	-11.71	-11.7	-11.69	-11.68	-11.67	-11.66	-11.65
-11.73	-11.72	-11.71	-11.7	-11.69	-11.68	-11.67	-11.66	-11.65
-11.73	-11.72	-11.71	-11.7	-11.69	-11.68	-11.67	-11.66	-11.65
-11.73	-11.72	-11.71	-11.7	-11.69	-11.68	-11.67	-11.66	-11.65
-11.73	-11.72	-11.71	-11.7	-11.69	-11.68	-11.67	-11.66	-11.65
-11.73	-11.72	-11.71	-11.7	-11.69	-11.68	-11.67	-11.66	-11.65
-11.73	-11.72	-11.71	-11.7	-11.69	-11.68	-11.67	-11.66	-11.65
-11.73	-11.72	-11.71	-11.7	-11.69	-11.68	-11.67	-11.66	-11.65
-11.73	-11.72	-11.71	-11.7	-11.69	-11.68	-11.67	-11.66	-11.65
-11.73	-11.72	-11.71	-11.7	-11.69	-11.68	-11.67	-11.66	-11.65
-11.73	-11.72	-11.71	-11.7	-11.69	-11.68	-11.67	-11.66	-11.65

-11.73	-11.72	-11.71	-11.7	-11.69	-11.68	-11.67	-11.66	-11.65
-11.73	-11.72	-11.71	-11.7	-11.69	-11.68	-11.67	-11.66	-11.65
-11.73	-11.72	-11.71	-11.7	-11.69	-11.68	-11.67	-11.66	-11.65
-11.73	-11.72	-11.71	-11.7	-11.69	-11.68	-11.67	-11.66	-11.65
-11.73	-11.72	-11.71	-11.7	-11.69	-11.68	-11.67	-11.66	-11.65
-11.73	-11.72	-11.71	-11.7	-11.69	-11.68	-11.67	-11.66	-11.65
-11.73	-11.72	-11.71	-11.7	-11.69	-11.68	-11.67	-11.66	-11.65
-11.73	-11.72	-11.71	-11.7	-11.69	-11.68	-11.67	-11.66	-11.65
-11.73	-11.72	-11.71	-11.7	-11.69	-11.68	-11.67	-11.66	-11.65
-11.73	-11.72	-11.71	-11.7	-11.69	-11.68	-11.67	-11.66	-11.65
-11.73	-11.72	-11.71	-11.7	-11.69	-11.68	-11.67	-11.66	-11.65
-11.73	-11.72	-11.71	-11.7	-11.69	-11.68	-11.67	-11.66	-11.65
-11.73	-11.72	-11.71	-11.7	-11.69	-11.68	-11.67	-11.66	-11.65
-11.73	-11.72	-11.71	-11.7	-11.69	-11.68	-11.67	-11.66	-11.65
-11.73	-11.72	-11.71	-11.7	-11.69	-11.68	-11.67	-11.66	-11.65
-11.73	-11.72	-11.71	-11.7	-11.69	-11.68	-11.67	-11.66	-11.65
-11.73	-11.72	-11.71	-11.7	-11.69	-11.68	-11.67	-11.66	-11.65
-11.73	-11.72	-11.71	-11.7	-11.69	-11.68	-11.67	-11.66	-11.65
-11.73	-11.72	-11.71	-11.7	-11.69	-11.68	-11.67	-11.66	-11.65
-11.73	-11.72	-11.71	-11.7	-11.69	-11.68	-11.67	-11.66	-11.65
-11.73	-11.72	-11.71	-11.7	-11.69	-11.68	-11.67	-11.66	-11.65
-11.73	-11.72	-11.71	-11.7	-11.69	-11.68	-11.67	-11.66	-11.65
-11.73	-11.72	-11.71	-11.7	-11.69	-11.68	-11.67	-11.66	-11.65
-11.73	-11.72	-11.71	-11.7	-11.69	-11.68	-11.67	-11.66	-11.65
-11.73	-11.72	-11.71	-11.7	-11.69	-11.68	-11.67	-11.66	-11.65
-11.73	-11.72	-11.71	-11.7	-11.69	-11.68	-11.67	-11.66	-11.65
-11.73	-11.72	-11.71	-11.7	-11.69	-11.68	-11.67	-11.66	-11.65
-11.73	-11.72	-11.71	-11.7	-11.69	-11.68	-11.67	-11.66	-11.65
-11.73	-11.72	-11.71	-11.7	-11.69	-11.68	-11.67	-11.66	-11.65
-11.73	-11.72	-11.71	-11.7	-11.69	-11.68	-11.67	-11.66	-11.65
-11.73	-11.72	-11.71	-11.7	-11.69	-11.68	-11.67	-11.66	-11.65
-11.73	-11.72	-11.71	-11.7	-11.69	-11.68	-11.67	-11.66	-11.65
-11.73	-11.72	-11.71	-11.7	-11.69	-11.68	-11.67	-11.66	-11.65
-11.73	-11.72	-11.71	-11.7	-11.69	-11.68	-11.67	-11.66	-11.65
-11.73	-11.72	-11.71	-11.7	-11.69	-11.68	-11.67	-11.66	-11.65
-11.73	-11.72	-11.71	-11.7	-11.69	-11.68	-11.67	-11.66	-11.65
-11.73	-11.72	-11.71	-11.7	-11.69	-11.68	-11.67	-11.66	-11.65
-11.73	-11.72	-11.71	-11.7	-11.69	-11.68	-11.67	-11.66	-11.65
-11.73	-11.72	-11.71	-11.7	-11.69	-11.68	-11.67	-11.66	-11.65
-11.73	-11.72	-11.71	-11.7	-11.69	-11.68	-11.67	-11.66	-11.65
-11.73	-11.72	-11.71	-11.7	-11.69	-11.68	-11.67	-11.66	-11.65
-11.73	-11.72	-11.71	-11.7	-11.69	-11.68	-11.67	-11.66	-11.65
-11.73	-11.72	-11.71	-11.7	-11.69	-11.68	-11.67	-11.66	-11.65
-11.73	-11.72	-11.71	-11.7	-11.69	-11.68	-11.67	-11.66	-11.65
-11.73	-11.72	-11.71	-11.7	-11.69	-11.68	-11.67	-11.66	-11.65
-11.73	-11.72	-11.71	-11.7	-11.69	-11.68	-11.67	-11.66	-11.65
-11.73	-11.72	-11.71	-11.7	-11.69	-11.68	-11.67	-11.66	-11.65
-11.73	-11.72	-11.71	-11.7	-11.69	-11.68	-11.67	-11.66	-11.65
-11.73	-11.72	-11.71	-11.7	-11.69	-11.68	-11.67	-11.66	-11.65
-11.73	-11.72	-11.71	-11.7	-11.69	-11.68	-11.67	-11.66	-11.65

-11.73	-11.72	-11.71	-11.7	-11.69	-11.68	-11.67	-11.66	-11.65
-11.73	-11.72	-11.71	-11.7	-11.69	-11.68	-11.67	-11.66	-11.65
-11.73	-11.72	-11.71	-11.7	-11.69	-11.68	-11.67	-11.66	-11.65
-11.73	-11.72	-11.71	-11.7	-11.69	-11.68	-11.67	-11.66	-11.65
-11.73	-11.72	-11.71	-11.7	-11.69	-11.68	-11.67	-11.66	-11.65
-11.73	-11.72	-11.71	-11.7	-11.69	-11.68	-11.67	-11.66	-11.65
-11.73	-11.72	-11.71	-11.7	-11.69	-11.68	-11.67	-11.66	-11.65
-11.73	-11.72	-11.71	-11.7	-11.69	-11.68	-11.67	-11.66	-11.65
-11.73	-11.72	-11.71	-11.7	-11.69	-11.68	-11.67	-11.66	-11.65
-11.73	-11.72	-11.71	-11.7	-11.69	-11.68	-11.67	-11.66	-11.65
-11.73	-11.72	-11.71	-11.7	-11.69	-11.68	-11.67	-11.66	-11.65
-11.73	-11.72	-11.71	-11.7	-11.69	-11.68	-11.67	-11.66	-11.65
-11.73	-11.72	-11.71	-11.7	-11.69	-11.68	-11.67	-11.66	-11.65
-11.73	-11.72	-11.71	-11.7	-11.69	-11.68	-11.67	-11.66	-11.65
-11.73	-11.72	-11.71	-11.7	-11.69	-11.68	-11.67	-11.66	-11.65
-11.73	-11.72	-11.71	-11.7	-11.69	-11.68	-11.67	-11.66	-11.65
-11.73	-11.72	-11.71	-11.7	-11.69	-11.68	-11.67	-11.66	-11.65
-11.73	-11.72	-11.71	-11.7	-11.69	-11.68	-11.67	-11.66	-11.65
-11.73	-11.72	-11.71	-11.7	-11.69	-11.68	-11.67	-11.66	-11.65
-11.73	-11.72	-11.71	-11.7	-11.69	-11.68	-11.67	-11.66	-11.65
-11.73	-11.72	-11.71	-11.7	-11.69	-11.68	-11.67	-11.66	-11.65
-11.73	-11.72	-11.71	-11.7	-11.69	-11.68	-11.67	-11.66	-11.65
-11.73	-11.72	-11.71	-11.7	-11.69	-11.68	-11.67	-11.66	-11.65
-11.73	-11.72	-11.71	-11.7	-11.69	-11.68	-11.67	-11.66	-11.65
-11.73	-11.72	-11.71	-11.7	-11.69	-11.68	-11.67	-11.66	-11.65
-11.73	-11.72	-11.71	-11.7	-11.69	-11.68	-11.67	-11.66	-11.65
-11.73	-11.72	-11.71	-11.7	-11.69	-11.68	-11.67	-11.66	-11.65
-11.73	-11.72	-11.71	-11.7	-11.69	-11.68	-11.67	-11.66	-11.65
-11.73	-11.72	-11.71	-11.7	-11.69	-11.68	-11.67	-11.66	-11.65
-11.73	-11.72	-11.71	-11.7	-11.69	-11.68	-11.67	-11.66	-11.65
-11.73	-11.72	-11.71	-11.7	-11.69	-11.68	-11.67	-11.66	-11.65
-11.73	-11.72	-11.71	-11.7	-11.69	-11.68	-11.67	-11.66	-11.65
-11.73	-11.72	-11.71	-11.7	-11.69	-11.68	-11.67	-11.66	-11.65
-11.73	-11.72	-11.71	-11.7	-11.69	-11.68	-11.67	-11.66	-11.65
-11.73	-11.72	-11.71	-11.7	-11.69	-11.68	-11.67	-11.66	-11.65
-11.73	-11.72	-11.71	-11.7	-11.69	-11.68	-11.67	-11.66	-11.65
-11.73	-11.72	-11.71	-11.7	-11.69	-11.68	-11.67	-11.66	-11.65
-11.73	-11.72	-11.71	-11.7	-11.69	-11.68	-11.67	-11.66	-11.65
-11.73	-11.72	-11.71	-11.7	-11.69	-11.68	-11.67	-11.66	-11.65
-11.73	-11.72	-11.71	-11.7	-11.69	-11.68	-11.67	-11.66	-11.65
-11.73	-11.72	-11.71	-11.7	-11.69	-11.68	-11.67	-11.66	-11.65
-11.73	-11.72	-11.71	-11.7	-11.69	-11.68	-11.67	-11.66	-11.65
-11.73	-11.72	-11.71	-11.7	-11.69	-11.68	-11.67	-11.66	-11.65
-11.73	-11.72	-11.71	-11.7	-11.69	-11.68	-11.67	-11.66	-11.65
-11.73	-11.72	-11.71	-11.7	-11.69	-11.68	-11.67	-11.66	-11.65
-11.73	-11.72	-11.71	-11.7	-11.69	-11.68	-11.67	-11.66	-11.65
-11.73	-11.72	-11.71	-11.7	-11.69	-11.68	-11.67	-11.66	-11.65
-11.73	-11.72	-11.71	-11.7	-11.69	-11.68	-11.67	-11.66	-11.65
-11.73	-11.72	-11.71	-11.7	-11.69	-11.68	-11.67	-11.66	-11.65
-11.73	-11.72	-11.71	-11.7	-11.69	-11.68	-11.67	-11.66	-11.65

-11.73	-11.72	-11.71	-11.7	-11.69	-11.68	-11.67	-11.66	-11.65
-11.73	-11.72	-11.71	-11.7	-11.69	-11.68	-11.67	-11.66	-11.65
-11.73	-11.72	-11.71	-11.7	-11.69	-11.68	-11.67	-11.66	-11.65
-11.73	-11.72	-11.71	-11.7	-11.69	-11.68	-11.67	-11.66	-11.65
-11.73	-11.72	-11.71	-11.7	-11.69	-11.68	-11.67	-11.66	-11.65
-11.73	-11.72	-11.71	-11.7	-11.69	-11.68	-11.67	-11.66	-11.65
-11.73	-11.72	-11.71	-11.7	-11.69	-11.68	-11.67	-11.66	-11.65
-11.73	-11.72	-11.71	-11.7	-11.69	-11.68	-11.67	-11.66	-11.65
-11.73	-11.72	-11.71	-11.7	-11.69	-11.68	-11.67	-11.66	-11.65
-11.73	-11.72	-11.71	-11.7	-11.69	-11.68	-11.67	-11.66	-11.65
-11.73	-11.72	-11.71	-11.7	-11.69	-11.68	-11.67	-11.66	-11.65
-11.73	-11.72	-11.71	-11.7	-11.69	-11.68	-11.67	-11.66	-11.65
-11.73	-11.72	-11.71	-11.7	-11.69	-11.68	-11.67	-11.66	-11.65
-11.73	-11.72	-11.71	-11.7	-11.69	-11.68	-11.67	-11.66	-11.65
-11.73	-11.72	-11.71	-11.7	-11.69	-11.68	-11.67	-11.66	-11.65
-11.73	-11.72	-11.71	-11.7	-11.69	-11.68	-11.67	-11.66	-11.65
-11.73	-11.72	-11.71	-11.7	-11.69	-11.68	-11.67	-11.66	-11.65
-11.73	-11.72	-11.71	-11.7	-11.69	-11.68	-11.67	-11.66	-11.65
-11.73	-11.72	-11.71	-11.7	-11.69	-11.68	-11.67	-11.66	-11.65
-11.73	-11.72	-11.71	-11.7	-11.69	-11.68	-11.67	-11.66	-11.65
-11.73	-11.72	-11.71	-11.7	-11.69	-11.68	-11.67	-11.66	-11.65
-11.73	-11.72	-11.71	-11.7	-11.69	-11.68	-11.67	-11.66	-11.65
-11.73	-11.72	-11.71	-11.7	-11.69	-11.68	-11.67	-11.66	-11.65
-11.73	-11.72	-11.71	-11.7	-11.69	-11.68	-11.67	-11.66	-11.65
-11.73	-11.72	-11.71	-11.7	-11.69	-11.68	-11.67	-11.66	-11.65
-11.73	-11.72	-11.71	-11.7	-11.69	-11.68	-11.67	-11.66	-11.65
-11.73	-11.72	-11.71	-11.7	-11.69	-11.68	-11.67	-11.66	-11.65
-11.73	-11.72	-11.71	-11.7	-11.69	-11.68	-11.67	-11.66	-11.65
-11.73	-11.72	-11.71	-11.7	-11.69	-11.68	-11.67	-11.66	-11.65
-11.73	-11.72	-11.71	-11.7	-11.69	-11.68	-11.67	-11.66	-11.65
-11.73	-11.72	-11.71	-11.7	-11.69	-11.68	-11.67	-11.66	-11.65
-11.73	-11.72	-11.71	-11.7	-11.69	-11.68	-11.67	-11.66	-11.65
-11.73	-11.72	-11.71	-11.7	-11.69	-11.68	-11.67	-11.66	-11.65
-11.73	-11.72	-11.71	-11.7	-11.69	-11.68	-11.67	-11.66	-11.65
-11.73	-11.72	-11.71	-11.7	-11.69	-11.68	-11.67	-11.66	-11.65
-11.73	-11.72	-11.71	-11.7	-11.69	-11.68	-11.67	-11.66	-11.65
-11.73	-11.72	-11.71	-11.7	-11.69	-11.68	-11.67	-11.66	-11.65
-11.73	-11.72	-11.71	-11.7	-11.69	-11.68	-11.67	-11.66	-11.65
-11.73	-11.72	-11.71	-11.7	-11.69	-11.68	-11.67	-11.66	-11.65
-11.73	-11.72	-11.71	-11.7	-11.69	-11.68	-11.67	-11.66	-11.65
-11.73	-11.72	-11.71	-11.7	-11.69	-11.68	-11.67	-11.66	-11.65
-11.73	-11.72	-11.71	-11.7	-11.69	-11.68	-11.67	-11.66	-11.65
-11.73	-11.72	-11.71	-11.7	-11.69	-11.68	-11.67	-11.66	-11.65
-11.73	-11.72	-11.71	-11.7	-11.69	-11.68	-11.67	-11.66	-11.65
-11.73	-11.72	-11.71	-11.7	-11.69	-11.68	-11.67	-11.66	-11.65
-11.73	-11.72	-11.71	-11.7	-11.69	-11.68	-11.67	-11.66	-11.65
-11.73	-11.72	-11.71	-11.7	-11.69	-11.68	-11.67	-11.66	-11.65
-11.73	-11.72	-11.71	-11.7	-11.69	-11.68	-11.67	-11.66	-11.65
-11.73	-11.72	-11.71	-11.7	-11.69	-11.68	-11.67	-11.66	-11.65
-11.73	-11.72	-11.71	-11.7	-11.69	-11.68	-11.67	-11.66	-11.65

-11.73	-11.72	-11.71	-11.7	-11.69	-11.68	-11.67	-11.66	-11.65
-11.73	-11.72	-11.71	-11.7	-11.69	-11.68	-11.67	-11.66	-11.65
-11.73	-11.72	-11.71	-11.7	-11.69	-11.68	-11.67	-11.66	-11.65
-11.73	-11.72	-11.71	-11.7	-11.69	-11.68	-11.67	-11.66	-11.65
-11.73	-11.72	-11.71	-11.7	-11.69	-11.68	-11.67	-11.66	-11.65
-11.73	-11.72	-11.71	-11.7	-11.69	-11.68	-11.67	-11.66	-11.65
-11.73	-11.72	-11.71	-11.7	-11.69	-11.68	-11.67	-11.66	-11.65
-11.73	-11.72	-11.71	-11.7	-11.69	-11.68	-11.67	-11.66	-11.65
-11.73	-11.72	-11.71	-11.7	-11.69	-11.68	-11.67	-11.66	-11.65
-11.73	-11.72	-11.71	-11.7	-11.69	-11.68	-11.67	-11.66	-11.65
-11.73	-11.72	-11.71	-11.7	-11.69	-11.68	-11.67	-11.66	-11.65
-11.73	-11.72	-11.71	-11.7	-11.69	-11.68	-11.67	-11.66	-11.65
-11.73	-11.72	-11.71	-11.7	-11.69	-11.68	-11.67	-11.66	-11.65
-11.73	-11.72	-11.71	-11.7	-11.69	-11.68	-11.67	-11.66	-11.65
-11.73	-11.72	-11.71	-11.7	-11.69	-11.68	-11.67	-11.66	-11.65
-11.73	-11.72	-11.71	-11.7	-11.69	-11.68	-11.67	-11.66	-11.65
-11.73	-11.72	-11.71	-11.7	-11.69	-11.68	-11.67	-11.66	-11.65
-11.73	-11.72	-11.71	-11.7	-11.69	-11.68	-11.67	-11.66	-11.65
-11.73	-11.72	-11.71	-11.7	-11.69	-11.68	-11.67	-11.66	-11.65
-11.73	-11.72	-11.71	-11.7	-11.69	-11.68	-11.67	-11.66	-11.65
-11.73	-11.72	-11.71	-11.7	-11.69	-11.68	-11.67	-11.66	-11.65
-11.73	-11.72	-11.71	-11.7	-11.69	-11.68	-11.67	-11.66	-11.65
-11.73	-11.72	-11.71	-11.7	-11.69	-11.68	-11.67	-11.66	-11.65
-11.73	-11.72	-11.71	-11.7	-11.69	-11.68	-11.67	-11.66	-11.65
-11.73	-11.72	-11.71	-11.7	-11.69	-11.68	-11.67	-11.66	-11.65
-11.73	-11.72	-11.71	-11.7	-11.69	-11.68	-11.67	-11.66	-11.65
-11.73	-11.72	-11.71	-11.7	-11.69	-11.68	-11.67	-11.66	-11.65
-11.73	-11.72	-11.71	-11.7	-11.69	-11.68	-11.67	-11.66	-11.65
-11.73	-11.72	-11.71	-11.7	-11.69	-11.68	-11.67	-11.66	-11.65
-11.73	-11.72	-11.71	-11.7	-11.69	-11.68	-11.67	-11.66	-11.65
-11.73	-11.72	-11.71	-11.7	-11.69	-11.68	-11.67	-11.66	-11.65
-11.73	-11.72	-11.71	-11.7	-11.69	-11.68	-11.67	-11.66	-11.65
-11.73	-11.72	-11.71	-11.7	-11.69	-11.68	-11.67	-11.66	-11.65
-11.73	-11.72	-11.71	-11.7	-11.69	-11.68	-11.67	-11.66	-11.65
-11.73	-11.72	-11.71	-11.7	-11.69	-11.68	-11.67	-11.66	-11.65
-11.73	-11.72	-11.71	-11.7	-11.69	-11.68	-11.67	-11.66	-11.65
-11.73	-11.72	-11.71	-11.7	-11.69	-11.68	-11.67	-11.66	-11.65
-11.73	-11.72	-11.71	-11.7	-11.69	-11.68	-11.67	-11.66	-11.65
-11.73	-11.72	-11.71	-11.7	-11.69	-11.68	-11.67	-11.66	-11.65
-11.73	-11.72	-11.71	-11.7	-11.69	-11.68	-11.67	-11.66	-11.65
-11.73	-11.72	-11.71	-11.7	-11.69	-11.68	-11.67	-11.66	-11.65
-11.73	-11.72	-11.71	-11.7	-11.69	-11.68	-11.67	-11.66	-11.65
-11.73	-11.72	-11.71	-11.7	-11.69	-11.68	-11.67	-11.66	-11.65
-11.73	-11.72	-11.71	-11.7	-11.69	-11.68	-11.67	-11.66	-11.65
-11.73	-11.72	-11.71	-11.7	-11.69	-11.68	-11.67	-11.66	-11.65
-11.73	-11.72	-11.71	-11.7	-11.69	-11.68	-11.67	-11.66	-11.65
-11.73	-11.72	-11.71	-11.7	-11.69	-11.68	-11.67	-11.66	-11.65
-11.73	-11.72	-11.71	-11.7	-11.69	-11.68	-11.67	-11.66	-11.65
-11.73	-11.72	-11.71	-11.7	-11.69	-11.68	-11.67	-11.66	-11.65
-11.73	-11.72	-11.71	-11.7	-11.69	-11.68	-11.67	-11.66	-11.65

-11.73	-11.72	-11.71	-11.7	-11.69	-11.68	-11.67	-11.66	-11.65
-11.73	-11.72	-11.71	-11.7	-11.69	-11.68	-11.67	-11.66	-11.65
-11.73	-11.72	-11.71	-11.7	-11.69	-11.68	-11.67	-11.66	-11.65
-11.73	-11.72	-11.71	-11.7	-11.69	-11.68	-11.67	-11.66	-11.65
-11.73	-11.72	-11.71	-11.7	-11.69	-11.68	-11.67	-11.66	-11.65
-11.73	-11.72	-11.71	-11.7	-11.69	-11.68	-11.67	-11.66	-11.65
-11.73	-11.72	-11.71	-11.7	-11.69	-11.68	-11.67	-11.66	-11.65
-11.73	-11.72	-11.71	-11.7	-11.69	-11.68	-11.67	-11.66	-11.65
-11.73	-11.72	-11.71	-11.7	-11.69	-11.68	-11.67	-11.66	-11.65
-11.73	-11.72	-11.71	-11.7	-11.69	-11.68	-11.67	-11.66	-11.65
-11.73	-11.72	-11.71	-11.7	-11.69	-11.68	-11.67	-11.66	-11.65
-11.73	-11.72	-11.71	-11.7	-11.69	-11.68	-11.67	-11.66	-11.65
-11.73	-11.72	-11.71	-11.7	-11.69	-11.68	-11.67	-11.66	-11.65
-11.73	-11.72	-11.71	-11.7	-11.69	-11.68	-11.67	-11.66	-11.65
-11.73	-11.72	-11.71	-11.7	-11.69	-11.68	-11.67	-11.66	-11.65
-11.73	-11.72	-11.71	-11.7	-11.69	-11.68	-11.67	-11.66	-11.65
-11.73	-11.72	-11.71	-11.7	-11.69	-11.68	-11.67	-11.66	-11.65
-11.73	-11.72	-11.71	-11.7	-11.69	-11.68	-11.67	-11.66	-11.65
-11.73	-11.72	-11.71	-11.7	-11.69	-11.68	-11.67	-11.66	-11.65
-11.73	-11.72	-11.71	-11.7	-11.69	-11.68	-11.67	-11.66	-11.65
-11.73	-11.72	-11.71	-11.7	-11.69	-11.68	-11.67	-11.66	-11.65
-11.73	-11.72	-11.71	-11.7	-11.69	-11.68	-11.67	-11.66	-11.65
-11.73	-11.72	-11.71	-11.7	-11.69	-11.68	-11.67	-11.66	-11.65
-11.73	-11.72	-11.71	-11.7	-11.69	-11.68	-11.67	-11.66	-11.65
-11.73	-11.72	-11.71	-11.7	-11.69	-11.68	-11.67	-11.66	-11.65
-11.73	-11.72	-11.71	-11.7	-11.69	-11.68	-11.67	-11.66	-11.65
-11.73	-11.72	-11.71	-11.7	-11.69	-11.68	-11.67	-11.66	-11.65
-11.73	-11.72	-11.71	-11.7	-11.69	-11.68	-11.67	-11.66	-11.65
-11.73	-11.72	-11.71	-11.7	-11.69	-11.68	-11.67	-11.66	-11.65
-11.73	-11.72	-11.71	-11.7	-11.69	-11.68	-11.67	-11.66	-11.65
-11.73	-11.72	-11.71	-11.7	-11.69	-11.68	-11.67	-11.66	-11.65
-11.73	-11.72	-11.71	-11.7	-11.69	-11.68	-11.67	-11.66	-11.65
-11.73	-11.72	-11.71	-11.7	-11.69	-11.68	-11.67	-11.66	-11.65
-11.73	-11.72	-11.71	-11.7	-11.69	-11.68	-11.67	-11.66	-11.65
-11.73	-11.72	-11.71	-11.7	-11.69	-11.68	-11.67	-11.66	-11.65
-11.73	-11.72	-11.71	-11.7	-11.69	-11.68	-11.67	-11.66	-11.65
-11.73	-11.72	-11.71	-11.7	-11.69	-11.68	-11.67	-11.66	-11.65
-11.73	-11.72	-11.71	-11.7	-11.69	-11.68	-11.67	-11.66	-11.65
-11.73	-11.72	-11.71	-11.7	-11.69	-11.68	-11.67	-11.66	-11.65
-11.73	-11.72	-11.71	-11.7	-11.69	-11.68	-11.67	-11.66	-11.65
-11.73	-11.72	-11.71	-11.7	-11.69	-11.68	-11.67	-11.66	-11.65
-11.73	-11.72	-11.71	-11.7	-11.69	-11.68	-11.67	-11.66	-11.65
-11.73	-11.72	-11.71	-11.7	-11.69	-11.68	-11.67	-11.66	-11.65
-11.73	-11.72	-11.71	-11.7	-11.69	-11.68	-11.67	-11.66	-11.65
-11.73	-11.72	-11.71	-11.7	-11.69	-11.68	-11.67	-11.66	-11.65
-11.73	-11.72	-11.71	-11.7	-11.69	-11.68	-11.67	-11.66	-11.65
-11.73	-11.72	-11.71	-11.7	-11.69	-11.68	-11.67	-11.66	-11.65
-11.73	-11.72	-11.71	-11.7	-11.69	-11.68	-11.67	-11.66	-11.65
-11.73	-11.72	-11.71	-11.7	-11.69	-11.68	-11.67	-11.66	-11.65
-11.73	-11.72	-11.71	-11.7	-11.69	-11.68	-11.67	-11.66	-11.65

-11.73	-11.72	-11.71	-11.7	-11.69	-11.68	-11.67	-11.66	-11.65
-11.73	-11.72	-11.71	-11.7	-11.69	-11.68	-11.67	-11.66	-11.65
-11.73	-11.72	-11.71	-11.7	-11.69	-11.68	-11.67	-11.66	-11.65
-11.73	-11.72	-11.71	-11.7	-11.69	-11.68	-11.67	-11.66	-11.65
-11.73	-11.72	-11.71	-11.7	-11.69	-11.68	-11.67	-11.66	-11.65
-11.73	-11.72	-11.71	-11.7	-11.69	-11.68	-11.67	-11.66	-11.65
-11.73	-11.72	-11.71	-11.7	-11.69	-11.68	-11.67	-11.66	-11.65
-11.73	-11.72	-11.71	-11.7	-11.69	-11.68	-11.67	-11.66	-11.65
-11.73	-11.72	-11.71	-11.7	-11.69	-11.68	-11.67	-11.66	-11.65
-11.73	-11.72	-11.71	-11.7	-11.69	-11.68	-11.67	-11.66	-11.65
-11.73	-11.72	-11.71	-11.7	-11.69	-11.68	-11.67	-11.66	-11.65
-11.73	-11.72	-11.71	-11.7	-11.69	-11.68	-11.67	-11.66	-11.65
-11.73	-11.72	-11.71	-11.7	-11.69	-11.68	-11.67	-11.66	-11.65
-11.73	-11.72	-11.71	-11.7	-11.69	-11.68	-11.67	-11.66	-11.65
-11.73	-11.72	-11.71	-11.7	-11.69	-11.68	-11.67	-11.66	-11.65
-11.73	-11.72	-11.71	-11.7	-11.69	-11.68	-11.67	-11.66	-11.65
-11.73	-11.72	-11.71	-11.7	-11.69	-11.68	-11.67	-11.66	-11.65
-11.73	-11.72	-11.71	-11.7	-11.69	-11.68	-11.67	-11.66	-11.65
-11.73	-11.72	-11.71	-11.7	-11.69	-11.68	-11.67	-11.66	-11.65
-11.73	-11.72	-11.71	-11.7	-11.69	-11.68	-11.67	-11.66	-11.65
-11.73	-11.72	-11.71	-11.7	-11.69	-11.68	-11.67	-11.66	-11.65
-11.73	-11.72	-11.71	-11.7	-11.69	-11.68	-11.67	-11.66	-11.65
-11.73	-11.72	-11.71	-11.7	-11.69	-11.68	-11.67	-11.66	-11.65
-11.73	-11.72	-11.71	-11.7	-11.69	-11.68	-11.67	-11.66	-11.65
-11.73	-11.72	-11.71	-11.7	-11.69	-11.68	-11.67	-11.66	-11.65
-11.73	-11.72	-11.71	-11.7	-11.69	-11.68	-11.67	-11.66	-11.65
-11.73	-11.72	-11.71	-11.7	-11.69	-11.68	-11.67	-11.66	-11.65
-11.73	-11.72	-11.71	-11.7	-11.69	-11.68	-11.67	-11.66	-11.65
-11.73	-11.72	-11.71	-11.7	-11.69	-11.68	-11.67	-11.66	-11.65
-11.73	-11.72	-11.71	-11.7	-11.69	-11.68	-11.67	-11.66	-11.65
-11.73	-11.72	-11.71	-11.7	-11.69	-11.68	-11.67	-11.66	-11.65
-11.73	-11.72	-11.71	-11.7	-11.69	-11.68	-11.67	-11.66	-11.65
-11.73	-11.72	-11.71	-11.7	-11.69	-11.68	-11.67	-11.66	-11.65
-11.73	-11.72	-11.71	-11.7	-11.69	-11.68	-11.67	-11.66	-11.65
-11.73	-11.72	-11.71	-11.7	-11.69	-11.68	-11.67	-11.66	-11.65
-11.73	-11.72	-11.71	-11.7	-11.69	-11.68	-11.67	-11.66	-11.65
-11.73	-11.72	-11.71	-11.7	-11.69	-11.68	-11.67	-11.66	-11.65
-11.73	-11.72	-11.71	-11.7	-11.69	-11.68	-11.67	-11.66	-11.65
-11.73	-11.72	-11.71	-11.7	-11.69	-11.68	-11.67	-11.66	-11.65
-11.73	-11.72	-11.71	-11.7	-11.69	-11.68	-11.67	-11.66	-11.65
-11.73	-11.72	-11.71	-11.7	-11.69	-11.68	-11.67	-11.66	-11.65
-11.73	-11.72	-11.71	-11.7	-11.69	-11.68	-11.67	-11.66	-11.65
-11.73	-11.72	-11.71	-11.7	-11.69	-11.68	-11.67	-11.66	-11.65
-11.73	-11.72	-11.71	-11.7	-11.69	-11.68	-11.67	-11.66	-11.65
-11.73	-11.72	-11.71	-11.7	-11.69	-11.68	-11.67	-11.66	-11.65
-11.73	-11.72	-11.71	-11.7	-11.69	-11.68	-11.67	-11.66	-11.65
-11.73	-11.72	-11.71	-11.7	-11.69	-11.68	-11.67	-11.66	-11.65
-11.73	-11.72	-11.71	-11.7	-11.69	-11.68	-11.67	-11.66	-11.65
-11.73	-11.72	-11.71	-11.7	-11.69	-11.68	-11.67	-11.66	-11.65
-11.73	-11.72	-11.71	-11.7	-11.69	-11.68	-11.67	-11.66	-11.65

-11.73	-11.72	-11.71	-11.7	-11.69	-11.68	-11.67	-11.66	-11.65
-11.73	-11.72	-11.71	-11.7	-11.69	-11.68	-11.67	-11.66	-11.65
-11.73	-11.72	-11.71	-11.7	-11.69	-11.68	-11.67	-11.66	-11.65
-11.73	-11.72	-11.71	-11.7	-11.69	-11.68	-11.67	-11.66	-11.65
-11.73	-11.72	-11.71	-11.7	-11.69	-11.68	-11.67	-11.66	-11.65
-11.73	-11.72	-11.71	-11.7	-11.69	-11.68	-11.67	-11.66	-11.65
-11.73	-11.72	-11.71	-11.7	-11.69	-11.68	-11.67	-11.66	-11.65
-11.73	-11.72	-11.71	-11.7	-11.69	-11.68	-11.67	-11.66	-11.65
-11.73	-11.72	-11.71	-11.7	-11.69	-11.68	-11.67	-11.66	-11.65
-11.73	-11.72	-11.71	-11.7	-11.69	-11.68	-11.67	-11.66	-11.65
-11.73	-11.72	-11.71	-11.7	-11.69	-11.68	-11.67	-11.66	-11.65
-11.73	-11.72	-11.71	-11.7	-11.69	-11.68	-11.67	-11.66	-11.65
-11.73	-11.72	-11.71	-11.7	-11.69	-11.68	-11.67	-11.66	-11.65
-11.73	-11.72	-11.71	-11.7	-11.69	-11.68	-11.67	-11.66	-11.65
-11.73	-11.72	-11.71	-11.7	-11.69	-11.68	-11.67	-11.66	-11.65
-11.73	-11.72	-11.71	-11.7	-11.69	-11.68	-11.67	-11.66	-11.65
-11.73	-11.72	-11.71	-11.7	-11.69	-11.68	-11.67	-11.66	-11.65
-11.73	-11.72	-11.71	-11.7	-11.69	-11.68	-11.67	-11.66	-11.65
-11.73	-11.72	-11.71	-11.7	-11.69	-11.68	-11.67	-11.66	-11.65
-11.73	-11.72	-11.71	-11.7	-11.69	-11.68	-11.67	-11.66	-11.65
-11.73	-11.72	-11.71	-11.7	-11.69	-11.68	-11.67	-11.66	-11.65
-11.73	-11.72	-11.71	-11.7	-11.69	-11.68	-11.67	-11.66	-11.65
-11.73	-11.72	-11.71	-11.7	-11.69	-11.68	-11.67	-11.66	-11.65
-11.73	-11.72	-11.71	-11.7	-11.69	-11.68	-11.67	-11.66	-11.65
-11.73	-11.72	-11.71	-11.7	-11.69	-11.68	-11.67	-11.66	-11.65
-11.73	-11.72	-11.71	-11.7	-11.69	-11.68	-11.67	-11.66	-11.65
-11.73	-11.72	-11.71	-11.7	-11.69	-11.68	-11.67	-11.66	-11.65
-11.73	-11.72	-11.71	-11.7	-11.69	-11.68	-11.67	-11.66	-11.65
-11.73	-11.72	-11.71	-11.7	-11.69	-11.68	-11.67	-11.66	-11.65
-11.73	-11.72	-11.71	-11.7	-11.69	-11.68	-11.67	-11.66	-11.65
-11.73	-11.72	-11.71	-11.7	-11.69	-11.68	-11.67	-11.66	-11.65
-11.73	-11.72	-11.71	-11.7	-11.69	-11.68	-11.67	-11.66	-11.65
-11.73	-11.72	-11.71	-11.7	-11.69	-11.68	-11.67	-11.66	-11.65
-11.73	-11.72	-11.71	-11.7	-11.69	-11.68	-11.67	-11.66	-11.65
-11.73	-11.72	-11.71	-11.7	-11.69	-11.68	-11.67	-11.66	-11.65
-11.73	-11.72	-11.71	-11.7	-11.69	-11.68	-11.67	-11.66	-11.65
-11.73	-11.72	-11.71	-11.7	-11.69	-11.68	-11.67	-11.66	-11.65
-11.73	-11.72	-11.71	-11.7	-11.69	-11.68	-11.67	-11.66	-11.65
-11.73	-11.72	-11.71	-11.7	-11.69	-11.68	-11.67	-11.66	-11.65
-11.73	-11.72	-11.71	-11.7	-11.69	-11.68	-11.67	-11.66	-11.65
-11.73	-11.72	-11.71	-11.7	-11.69	-11.68	-11.67	-11.66	-11.65
-11.73	-11.72	-11.71	-11.7	-11.69	-11.68	-11.67	-11.66	-11.65
-11.73	-11.72	-11.71	-11.7	-11.69	-11.68	-11.67	-11.66	-11.65
-11.73	-11.72	-11.71	-11.7	-11.69	-11.68	-11.67	-11.66	-11.65
-11.73	-11.72	-11.71	-11.7	-11.69	-11.68	-11.67	-11.66	-11.65
-11.73	-11.72	-11.71	-11.7	-11.69	-11.68	-11.67	-11.66	-11.65
-11.73	-11.72	-11.71	-11.7	-11.69	-11.68	-11.67	-11.66	-11.65
-11.73	-11.72	-11.71	-11.7	-11.69	-11.68	-11.67	-11.66	-11.65
-11.73	-11.72	-11.71	-11.7	-11.69	-11.68	-11.67	-11.66	-11.65
-11.73	-11.72	-11.71	-11.7	-11.69	-11.68	-11.67	-11.66	-11.65

-11.73	-11.72	-11.71	-11.7	-11.69	-11.68	-11.67	-11.66	-11.65
-11.73	-11.72	-11.71	-11.7	-11.69	-11.68	-11.67	-11.66	-11.65
-11.73	-11.72	-11.71	-11.7	-11.69	-11.68	-11.67	-11.66	-11.65
-11.73	-11.72	-11.71	-11.7	-11.69	-11.68	-11.67	-11.66	-11.65
-11.73	-11.72	-11.71	-11.7	-11.69	-11.68	-11.67	-11.66	-11.65
-11.73	-11.72	-11.71	-11.7	-11.69	-11.68	-11.67	-11.66	-11.65
-11.73	-11.72	-11.71	-11.7	-11.69	-11.68	-11.67	-11.66	-11.65
-11.73	-11.72	-11.71	-11.7	-11.69	-11.68	-11.67	-11.66	-11.65
-11.73	-11.72	-11.71	-11.7	-11.69	-11.68	-11.67	-11.66	-11.65
-11.73	-11.72	-11.71	-11.7	-11.69	-11.68	-11.67	-11.66	-11.65
-11.73	-11.72	-11.71	-11.7	-11.69	-11.68	-11.67	-11.66	-11.65
-11.73	-11.72	-11.71	-11.7	-11.69	-11.68	-11.67	-11.66	-11.65
-11.73	-11.72	-11.71	-11.7	-11.69	-11.68	-11.67	-11.66	-11.65
-11.73	-11.72	-11.71	-11.7	-11.69	-11.68	-11.67	-11.66	-11.65
-11.73	-11.72	-11.71	-11.7	-11.69	-11.68	-11.67	-11.66	-11.65
-11.73	-11.72	-11.71	-11.7	-11.69	-11.68	-11.67	-11.66	-11.65
-11.73	-11.72	-11.71	-11.7	-11.69	-11.68	-11.67	-11.66	-11.65
-11.73	-11.72	-11.71	-11.7	-11.69	-11.68	-11.67	-11.66	-11.65
-11.73	-11.72	-11.71	-11.7	-11.69	-11.68	-11.67	-11.66	-11.65
-11.73	-11.72	-11.71	-11.7	-11.69	-11.68	-11.67	-11.66	-11.65
-11.73	-11.72	-11.71	-11.7	-11.69	-11.68	-11.67	-11.66	-11.65
-11.73	-11.72	-11.71	-11.7	-11.69	-11.68	-11.67	-11.66	-11.65
-11.73	-11.72	-11.71	-11.7	-11.69	-11.68	-11.67	-11.66	-11.65
-11.73	-11.72	-11.71	-11.7	-11.69	-11.68	-11.67	-11.66	-11.65
-11.73	-11.72	-11.71	-11.7	-11.69	-11.68	-11.67	-11.66	-11.65
-11.73	-11.72	-11.71	-11.7	-11.69	-11.68	-11.67	-11.66	-11.65
-11.73	-11.72	-11.71	-11.7	-11.69	-11.68	-11.67	-11.66	-11.65
-11.73	-11.72	-11.71	-11.7	-11.69	-11.68	-11.67	-11.66	-11.65
-11.73	-11.72	-11.71	-11.7	-11.69	-11.68	-11.67	-11.66	-11.65
-11.73	-11.72	-11.71	-11.7	-11.69	-11.68	-11.67	-11.66	-11.65
-11.73	-11.72	-11.71	-11.7	-11.69	-11.68	-11.67	-11.66	-11.65
-11.73	-11.72	-11.71	-11.7	-11.69	-11.68	-11.67	-11.66	-11.65
-11.73	-11.72	-11.71	-11.7	-11.69	-11.68	-11.67	-11.66	-11.65
-11.73	-11.72	-11.71	-11.7	-11.69	-11.68	-11.67	-11.66	-11.65
-11.73	-11.72	-11.71	-11.7	-11.69	-11.68	-11.67	-11.66	-11.65
-11.73	-11.72	-11.71	-11.7	-11.69	-11.68	-11.67	-11.66	-11.65
-11.73	-11.72	-11.71	-11.7	-11.69	-11.68	-11.67	-11.66	-11.65
-11.73	-11.72	-11.71	-11.7	-11.69	-11.68	-11.67	-11.66	-11.65
-11.73	-11.72	-11.71	-11.7	-11.69	-11.68	-11.67	-11.66	-11.65
-11.73	-11.72	-11.71	-11.7	-11.69	-11.68	-11.67	-11.66	-11.65
-11.73	-11.72	-11.71	-11.7	-11.69	-11.68	-11.67	-11.66	-11.65
-11.73	-11.72	-11.71	-11.7	-11.69	-11.68	-11.67	-11.66	-11.65
-11.73	-11.72	-11.71	-11.7	-11.69	-11.68	-11.67	-11.66	-11.65
-11.73	-11.72	-11.71	-11.7	-11.69	-11.68	-11.67	-11.66	-11.65
-11.73	-11.72	-11.71	-11.7	-11.69	-11.68	-11.67	-11.66	-11.65
-11.73	-11.72	-11.71	-11.7	-11.69	-11.68	-11.67	-11.66	-11.65
-11.73	-11.72	-11.71	-11.7	-11.69	-11.68	-11.67	-11.66	-11.65
-11.73	-11.72	-11.71	-11.7	-11.69	-11.68	-11.67	-11.66	-11.65
-11.73	-11.72	-11.71	-11.7	-11.69	-11.68	-11.67	-11.66	-11.65
-11.73	-11.72	-11.71	-11.7	-11.69	-11.68	-11.67	-11.66	-11.65

-11.73	-11.72	-11.71	-11.7	-11.69	-11.68	-11.67	-11.66	-11.65
-11.73	-11.72	-11.71	-11.7	-11.69	-11.68	-11.67	-11.66	-11.65
-11.73	-11.72	-11.71	-11.7	-11.69	-11.68	-11.67	-11.66	-11.65
-11.73	-11.72	-11.71	-11.7	-11.69	-11.68	-11.67	-11.66	-11.65
-11.73	-11.72	-11.71	-11.7	-11.69	-11.68	-11.67	-11.66	-11.65
-11.73	-11.72	-11.71	-11.7	-11.69	-11.68	-11.67	-11.66	-11.65
-11.73	-11.72	-11.71	-11.7	-11.69	-11.68	-11.67	-11.66	-11.65
-11.73	-11.72	-11.71	-11.7	-11.69	-11.68	-11.67	-11.66	-11.65
-11.73	-11.72	-11.71	-11.7	-11.69	-11.68	-11.67	-11.66	-11.65
-11.73	-11.72	-11.71	-11.7	-11.69	-11.68	-11.67	-11.66	-11.65
-11.73	-11.72	-11.71	-11.7	-11.69	-11.68	-11.67	-11.66	-11.65
-11.73	-11.72	-11.71	-11.7	-11.69	-11.68	-11.67	-11.66	-11.65
-11.73	-11.72	-11.71	-11.7	-11.69	-11.68	-11.67	-11.66	-11.65
-11.73	-11.72	-11.71	-11.7	-11.69	-11.68	-11.67	-11.66	-11.65
-11.73	-11.72	-11.71	-11.7	-11.69	-11.68	-11.67	-11.66	-11.65
-11.73	-11.72	-11.71	-11.7	-11.69	-11.68	-11.67	-11.66	-11.65
-11.73	-11.72	-11.71	-11.7	-11.69	-11.68	-11.67	-11.66	-11.65
-11.73	-11.72	-11.71	-11.7	-11.69	-11.68	-11.67	-11.66	-11.65
-11.73	-11.72	-11.71	-11.7	-11.69	-11.68	-11.67	-11.66	-11.65
-11.73	-11.72	-11.71	-11.7	-11.69	-11.68	-11.67	-11.66	-11.65
-11.73	-11.72	-11.71	-11.7	-11.69	-11.68	-11.67	-11.66	-11.65
-11.73	-11.72	-11.71	-11.7	-11.69	-11.68	-11.67	-11.66	-11.65
-11.73	-11.72	-11.71	-11.7	-11.69	-11.68	-11.67	-11.66	-11.65
-11.73	-11.72	-11.71	-11.7	-11.69	-11.68	-11.67	-11.66	-11.65
-11.73	-11.72	-11.71	-11.7	-11.69	-11.68	-11.67	-11.66	-11.65
-11.73	-11.72	-11.71	-11.7	-11.69	-11.68	-11.67	-11.66	-11.65
-11.73	-11.72	-11.71	-11.7	-11.69	-11.68	-11.67	-11.66	-11.65
-11.73	-11.72	-11.71	-11.7	-11.69	-11.68	-11.67	-11.66	-11.65
-11.73	-11.72	-11.71	-11.7	-11.69	-11.68	-11.67	-11.66	-11.65
-11.73	-11.72	-11.71	-11.7	-11.69	-11.68	-11.67	-11.66	-11.65
-11.73	-11.72	-11.71	-11.7	-11.69	-11.68	-11.67	-11.66	-11.65
-11.73	-11.72	-11.71	-11.7	-11.69	-11.68	-11.67	-11.66	-11.65
-11.73	-11.72	-11.71	-11.7	-11.69	-11.68	-11.67	-11.66	-11.65
-11.73	-11.72	-11.71	-11.7	-11.69	-11.68	-11.67	-11.66	-11.65
-11.73	-11.72	-11.71	-11.7	-11.69	-11.68	-11.67	-11.66	-11.65
-11.73	-11.72	-11.71	-11.7	-11.69	-11.68	-11.67	-11.66	-11.65
-11.73	-11.72	-11.71	-11.7	-11.69	-11.68	-11.67	-11.66	-11.65
-11.73	-11.72	-11.71	-11.7	-11.69	-11.68	-11.67	-11.66	-11.65
-11.73	-11.72	-11.71	-11.7	-11.69	-11.68	-11.67	-11.66	-11.65
-11.73	-11.72	-11.71	-11.7	-11.69	-11.68	-11.67	-11.66	-11.65
-11.73	-11.72	-11.71	-11.7	-11.69	-11.68	-11.67	-11.66	-11.65
-11.73	-11.72	-11.71	-11.7	-11.69	-11.68	-11.67	-11.66	-11.65
-11.73	-11.72	-11.71	-11.7	-11.69	-11.68	-11.67	-11.66	-11.65
-11.73	-11.72	-11.71	-11.7	-11.69	-11.68	-11.67	-11.66	-11.65
-11.73	-11.72	-11.71	-11.7	-11.69	-11.68	-11.67	-11.66	-11.65
-11.73	-11.72	-11.71	-11.7	-11.69	-11.68	-11.67	-11.66	-11.65
-11.73	-11.72	-11.71	-11.7	-11.69	-11.68	-11.67	-11.66	-11.65
-11.73	-11.72	-11.71	-11.7	-11.69	-11.68	-11.67	-11.66	-11.65
-11.73	-11.72	-11.71	-11.7	-11.69	-11.68	-11.67	-11.66	-11.65
-11.73	-11.72	-11.71	-11.7	-11.69	-11.68	-11.67	-11.66	-11.65

-11.73	-11.72	-11.71	-11.7	-11.69	-11.68	-11.67	-11.66	-11.65
-11.73	-11.72	-11.71	-11.7	-11.69	-11.68	-11.67	-11.66	-11.65
-11.73	-11.72	-11.71	-11.7	-11.69	-11.68	-11.67	-11.66	-11.65
-11.73	-11.72	-11.71	-11.7	-11.69	-11.68	-11.67	-11.66	-11.65
-11.73	-11.72	-11.71	-11.7	-11.69	-11.68	-11.67	-11.66	-11.65
-11.73	-11.72	-11.71	-11.7	-11.69	-11.68	-11.67	-11.66	-11.65
-11.73	-11.72	-11.71	-11.7	-11.69	-11.68	-11.67	-11.66	-11.65
-11.73	-11.72	-11.71	-11.7	-11.69	-11.68	-11.67	-11.66	-11.65
-11.73	-11.72	-11.71	-11.7	-11.69	-11.68	-11.67	-11.66	-11.65
-11.73	-11.72	-11.71	-11.7	-11.69	-11.68	-11.67	-11.66	-11.65
-11.73	-11.72	-11.71	-11.7	-11.69	-11.68	-11.67	-11.66	-11.65
-11.73	-11.72	-11.71	-11.7	-11.69	-11.68	-11.67	-11.66	-11.65
-11.73	-11.72	-11.71	-11.7	-11.69	-11.68	-11.67	-11.66	-11.65
-11.73	-11.72	-11.71	-11.7	-11.69	-11.68	-11.67	-11.66	-11.65
-11.73	-11.72	-11.71	-11.7	-11.69	-11.68	-11.67	-11.66	-11.65
-11.73	-11.72	-11.71	-11.7	-11.69	-11.68	-11.67	-11.66	-11.65
-11.73	-11.72	-11.71	-11.7	-11.69	-11.68	-11.67	-11.66	-11.65
-11.73	-11.72	-11.71	-11.7	-11.69	-11.68	-11.67	-11.66	-11.65
-11.73	-11.72	-11.71	-11.7	-11.69	-11.68	-11.67	-11.66	-11.65
-11.73	-11.72	-11.71	-11.7	-11.69	-11.68	-11.67	-11.66	-11.65
-11.73	-11.72	-11.71	-11.7	-11.69	-11.68	-11.67	-11.66	-11.65
-11.73	-11.72	-11.71	-11.7	-11.69	-11.68	-11.67	-11.66	-11.65
-11.73	-11.72	-11.71	-11.7	-11.69	-11.68	-11.67	-11.66	-11.65
-11.73	-11.72	-11.71	-11.7	-11.69	-11.68	-11.67	-11.66	-11.65
-11.73	-11.72	-11.71	-11.7	-11.69	-11.68	-11.67	-11.66	-11.65
-11.73	-11.72	-11.71	-11.7	-11.69	-11.68	-11.67	-11.66	-11.65
-11.73	-11.72	-11.71	-11.7	-11.69	-11.68	-11.67	-11.66	-11.65
-11.73	-11.72	-11.71	-11.7	-11.69	-11.68	-11.67	-11.66	-11.65
-11.73	-11.72	-11.71	-11.7	-11.69	-11.68	-11.67	-11.66	-11.65
-11.73	-11.72	-11.71	-11.7	-11.69	-11.68	-11.67	-11.66	-11.65
-11.73	-11.72	-11.71	-11.7	-11.69	-11.68	-11.67	-11.66	-11.65
-11.73	-11.72	-11.71	-11.7	-11.69	-11.68	-11.67	-11.66	-11.65
-11.73	-11.72	-11.71	-11.7	-11.69	-11.68	-11.67	-11.66	-11.65
-11.73	-11.72	-11.71	-11.7	-11.69	-11.68	-11.67	-11.66	-11.65
-11.73	-11.72	-11.71	-11.7	-11.69	-11.68	-11.67	-11.66	-11.65
-11.73	-11.72	-11.71	-11.7	-11.69	-11.68	-11.67	-11.66	-11.65
-11.73	-11.72	-11.71	-11.7	-11.69	-11.68	-11.67	-11.66	-11.65
-11.73	-11.72	-11.71	-11.7	-11.69	-11.68	-11.67	-11.66	-11.65
-11.73	-11.72	-11.71	-11.7	-11.69	-11.68	-11.67	-11.66	-11.65
-11.73	-11.72	-11.71	-11.7	-11.69	-11.68	-11.67	-11.66	-11.65
-11.73	-11.72	-11.71	-11.7	-11.69	-11.68	-11.67	-11.66	-11.65
-11.73	-11.72	-11.71	-11.7	-11.69	-11.68	-11.67	-11.66	-11.65
-11.73	-11.72	-11.71	-11.7	-11.69	-11.68	-11.67	-11.66	-11.65
-11.73	-11.72	-11.71	-11.7	-11.69	-11.68	-11.67	-11.66	-11.65
-11.73	-11.72	-11.71	-11.7	-11.69	-11.68	-11.67	-11.66	-11.65
-11.73	-11.72	-11.71	-11.7	-11.69	-11.68	-11.67	-11.66	-11.65
-11.73	-11.72	-11.71	-11.7	-11.69	-11.68	-11.67	-11.66	-11.65
-11.73	-11.72	-11.71	-11.7	-11.69	-11.68	-11.67	-11.66	-11.65
-11.73	-11.72	-11.71	-11.7	-11.69	-11.68	-11.67	-11.66	-11.65
-11.73	-11.72	-11.71	-11.7	-11.69	-11.68	-11.67	-11.66	-11.65

-11.73	-11.72	-11.71	-11.7	-11.69	-11.68	-11.67	-11.66	-11.65
-11.73	-11.72	-11.71	-11.7	-11.69	-11.68	-11.67	-11.66	-11.65
-11.73	-11.72	-11.71	-11.7	-11.69	-11.68	-11.67	-11.66	-11.65
-11.73	-11.72	-11.71	-11.7	-11.69	-11.68	-11.67	-11.66	-11.65
-11.73	-11.72	-11.71	-11.7	-11.69	-11.68	-11.67	-11.66	-11.65
-11.73	-11.72	-11.71	-11.7	-11.69	-11.68	-11.67	-11.66	-11.65
-11.73	-11.72	-11.71	-11.7	-11.69	-11.68	-11.67	-11.66	-11.65
-11.73	-11.72	-11.71	-11.7	-11.69	-11.68	-11.67	-11.66	-11.65
-11.73	-11.72	-11.71	-11.7	-11.69	-11.68	-11.67	-11.66	-11.65
-11.73	-11.72	-11.71	-11.7	-11.69	-11.68	-11.67	-11.66	-11.65
-11.73	-11.72	-11.71	-11.7	-11.69	-11.68	-11.67	-11.66	-11.65
-11.73	-11.72	-11.71	-11.7	-11.69	-11.68	-11.67	-11.66	-11.65
-11.73	-11.72	-11.71	-11.7	-11.69	-11.68	-11.67	-11.66	-11.65
-11.73	-11.72	-11.71	-11.7	-11.69	-11.68	-11.67	-11.66	-11.65
-11.73	-11.72	-11.71	-11.7	-11.69	-11.68	-11.67	-11.66	-11.65
-11.73	-11.72	-11.71	-11.7	-11.69	-11.68	-11.67	-11.66	-11.65
-11.73	-11.72	-11.71	-11.7	-11.69	-11.68	-11.67	-11.66	-11.65
-11.73	-11.72	-11.71	-11.7	-11.69	-11.68	-11.67	-11.66	-11.65
-11.73	-11.72	-11.71	-11.7	-11.69	-11.68	-11.67	-11.66	-11.65
-11.73	-11.72	-11.71	-11.7	-11.69	-11.68	-11.67	-11.66	-11.65
-11.73	-11.72	-11.71	-11.7	-11.69	-11.68	-11.67	-11.66	-11.65
-11.73	-11.72	-11.71	-11.7	-11.69	-11.68	-11.67	-11.66	-11.65
-11.73	-11.72	-11.71	-11.7	-11.69	-11.68	-11.67	-11.66	-11.65
-11.73	-11.72	-11.71	-11.7	-11.69	-11.68	-11.67	-11.66	-11.65
-11.73	-11.72	-11.71	-11.7	-11.69	-11.68	-11.67	-11.66	-11.65
-11.73	-11.72	-11.71	-11.7	-11.69	-11.68	-11.67	-11.66	-11.65
-11.73	-11.72	-11.71	-11.7	-11.69	-11.68	-11.67	-11.66	-11.65
-11.73	-11.72	-11.71	-11.7	-11.69	-11.68	-11.67	-11.66	-11.65
-11.73	-11.72	-11.71	-11.7	-11.69	-11.68	-11.67	-11.66	-11.65
-11.73	-11.72	-11.71	-11.7	-11.69	-11.68	-11.67	-11.66	-11.65
-11.73	-11.72	-11.71	-11.7	-11.69	-11.68	-11.67	-11.66	-11.65
-11.73	-11.72	-11.71	-11.7	-11.69	-11.68	-11.67	-11.66	-11.65
-11.73	-11.72	-11.71	-11.7	-11.69	-11.68	-11.67	-11.66	-11.65
-11.73	-11.72	-11.71	-11.7	-11.69	-11.68	-11.67	-11.66	-11.65
-11.73	-11.72	-11.71	-11.7	-11.69	-11.68	-11.67	-11.66	-11.65
-11.73	-11.72	-11.71	-11.7	-11.69	-11.68	-11.67	-11.66	-11.65
-11.73	-11.72	-11.71	-11.7	-11.69	-11.68	-11.67	-11.66	-11.65
-11.73	-11.72	-11.71	-11.7	-11.69	-11.68	-11.67	-11.66	-11.65
-11.73	-11.72	-11.71	-11.7	-11.69	-11.68	-11.67	-11.66	-11.65
-11.73	-11.72	-11.71	-11.7	-11.69	-11.68	-11.67	-11.66	-11.65
-11.73	-11.72	-11.71	-11.7	-11.69	-11.68	-11.67	-11.66	-11.65
-11.73	-11.72	-11.71	-11.7	-11.69	-11.68	-11.67	-11.66	-11.65
-11.73	-11.72	-11.71	-11.7	-11.69	-11.68	-11.67	-11.66	-11.65
-11.73	-11.72	-11.71	-11.7	-11.69	-11.68	-11.67	-11.66	-11.65
-11.73	-11.72	-11.71	-11.7	-11.69	-11.68	-11.67	-11.66	-11.65
-11.73	-11.72	-11.71	-11.7	-11.69	-11.68	-11.67	-11.66	-11.65
-11.73	-11.72	-11.71	-11.7	-11.69	-11.68	-11.67	-11.66	-11.65
-11.73	-11.72	-11.71	-11.7	-11.69	-11.68	-11.67	-11.66	-11.65
-11.73	-11.72	-11.71	-11.7	-11.69	-11.68	-11.67	-11.66	-11.65
-11.73	-11.72	-11.71	-11.7	-11.69	-11.68	-11.67	-11.66	-11.65

-11.73	-11.72	-11.71	-11.7	-11.69	-11.68	-11.67	-11.66	-11.65
-11.73	-11.72	-11.71	-11.7	-11.69	-11.68	-11.67	-11.66	-11.65
-11.73	-11.72	-11.71	-11.7	-11.69	-11.68	-11.67	-11.66	-11.65
-11.73	-11.72	-11.71	-11.7	-11.69	-11.68	-11.67	-11.66	-11.65
-11.73	-11.72	-11.71	-11.7	-11.69	-11.68	-11.67	-11.66	-11.65
-11.73	-11.72	-11.71	-11.7	-11.69	-11.68	-11.67	-11.66	-11.65
-11.73	-11.72	-11.71	-11.7	-11.69	-11.68	-11.67	-11.66	-11.65
-11.73	-11.72	-11.71	-11.7	-11.69	-11.68	-11.67	-11.66	-11.65
-11.73	-11.72	-11.71	-11.7	-11.69	-11.68	-11.67	-11.66	-11.65
-11.73	-11.72	-11.71	-11.7	-11.69	-11.68	-11.67	-11.66	-11.65
-11.73	-11.72	-11.71	-11.7	-11.69	-11.68	-11.67	-11.66	-11.65
-11.73	-11.72	-11.71	-11.7	-11.69	-11.68	-11.67	-11.66	-11.65
-11.73	-11.72	-11.71	-11.7	-11.69	-11.68	-11.67	-11.66	-11.65
-11.73	-11.72	-11.71	-11.7	-11.69	-11.68	-11.67	-11.66	-11.65
-11.73	-11.72	-11.71	-11.7	-11.69	-11.68	-11.67	-11.66	-11.65
-11.73	-11.72	-11.71	-11.7	-11.69	-11.68	-11.67	-11.66	-11.65
-11.73	-11.72	-11.71	-11.7	-11.69	-11.68	-11.67	-11.66	-11.65
-11.73	-11.72	-11.71	-11.7	-11.69	-11.68	-11.67	-11.66	-11.65
-11.73	-11.72	-11.71	-11.7	-11.69	-11.68	-11.67	-11.66	-11.65
-11.73	-11.72	-11.71	-11.7	-11.69	-11.68	-11.67	-11.66	-11.65
-11.73	-11.72	-11.71	-11.7	-11.69	-11.68	-11.67	-11.66	-11.65
-11.73	-11.72	-11.71	-11.7	-11.69	-11.68	-11.67	-11.66	-11.65
-11.73	-11.72	-11.71	-11.7	-11.69	-11.68	-11.67	-11.66	-11.65
-11.73	-11.72	-11.71	-11.7	-11.69	-11.68	-11.67	-11.66	-11.65
-11.73	-11.72	-11.71	-11.7	-11.69	-11.68	-11.67	-11.66	-11.65
-11.73	-11.72	-11.71	-11.7	-11.69	-11.68	-11.67	-11.66	-11.65
-11.73	-11.72	-11.71	-11.7	-11.69	-11.68	-11.67	-11.66	-11.65
-11.73	-11.72	-11.71	-11.7	-11.69	-11.68	-11.67	-11.66	-11.65
-11.73	-11.72	-11.71	-11.7	-11.69	-11.68	-11.67	-11.66	-11.65
-11.73	-11.72	-11.71	-11.7	-11.69	-11.68	-11.67	-11.66	-11.65
-11.73	-11.72	-11.71	-11.7	-11.69	-11.68	-11.67	-11.66	-11.65
-11.73	-11.72	-11.71	-11.7	-11.69	-11.68	-11.67	-11.66	-11.65
-11.73	-11.72	-11.71	-11.7	-11.69	-11.68	-11.67	-11.66	-11.65
-11.73	-11.72	-11.71	-11.7	-11.69	-11.68	-11.67	-11.66	-11.65
-11.73	-11.72	-11.71	-11.7	-11.69	-11.68	-11.67	-11.66	-11.65
-11.73	-11.72	-11.71	-11.7	-11.69	-11.68	-11.67	-11.66	-11.65
-11.73	-11.72	-11.71	-11.7	-11.69	-11.68	-11.67	-11.66	-11.65
-11.73	-11.72	-11.71	-11.7	-11.69	-11.68	-11.67	-11.66	-11.65
-11.73	-11.72	-11.71	-11.7	-11.69	-11.68	-11.67	-11.66	-11.65
-11.73	-11.72	-11.71	-11.7	-11.69	-11.68	-11.67	-11.66	-11.65
-11.73	-11.72	-11.71	-11.7	-11.69	-11.68	-11.67	-11.66	-11.65
-11.73	-11.72	-11.71	-11.7	-11.69	-11.68	-11.67	-11.66	-11.65
-11.73	-11.72	-11.71	-11.7	-11.69	-11.68	-11.67	-11.66	-11.65
-11.73	-11.72	-11.71	-11.7	-11.69	-11.68	-11.67	-11.66	-11.65
-11.73	-11.72	-11.71	-11.7	-11.69	-11.68	-11.67	-11.66	-11.65
-11.73	-11.72	-11.71	-11.7	-11.69	-11.68	-11.67	-11.66	-11.65
-11.73	-11.72	-11.71	-11.7	-11.69	-11.68	-11.67	-11.66	-11.65
-11.73	-11.72	-11.71	-11.7	-11.69	-11.68	-11.67	-11.66	-11.65
-11.73	-11.72	-11.71	-11.7	-11.69	-11.68	-11.67	-11.66	-11.65
-11.73	-11.72	-11.71	-11.7	-11.69	-11.68	-11.67	-11.66	-11.65

-11.73	-11.72	-11.71	-11.7	-11.69	-11.68	-11.67	-11.66	-11.65
-11.73	-11.72	-11.71	-11.7	-11.69	-11.68	-11.67	-11.66	-11.65
-11.73	-11.72	-11.71	-11.7	-11.69	-11.68	-11.67	-11.66	-11.65
-11.73	-11.72	-11.71	-11.7	-11.69	-11.68	-11.67	-11.66	-11.65
-11.73	-11.72	-11.71	-11.7	-11.69	-11.68	-11.67	-11.66	-11.65
-11.73	-11.72	-11.71	-11.7	-11.69	-11.68	-11.67	-11.66	-11.65
-11.73	-11.72	-11.71	-11.7	-11.69	-11.68	-11.67	-11.66	-11.65
-11.73	-11.72	-11.71	-11.7	-11.69	-11.68	-11.67	-11.66	-11.65
-11.73	-11.72	-11.71	-11.7	-11.69	-11.68	-11.67	-11.66	-11.65
-11.73	-11.72	-11.71	-11.7	-11.69	-11.68	-11.67	-11.66	-11.65
-11.73	-11.72	-11.71	-11.7	-11.69	-11.68	-11.67	-11.66	-11.65
-11.73	-11.72	-11.71	-11.7	-11.69	-11.68	-11.67	-11.66	-11.65
-11.73	-11.72	-11.71	-11.7	-11.69	-11.68	-11.67	-11.66	-11.65
-11.73	-11.72	-11.71	-11.7	-11.69	-11.68	-11.67	-11.66	-11.65
-11.73	-11.72	-11.71	-11.7	-11.69	-11.68	-11.67	-11.66	-11.65
-11.73	-11.72	-11.71	-11.7	-11.69	-11.68	-11.67	-11.66	-11.65
-11.73	-11.72	-11.71	-11.7	-11.69	-11.68	-11.67	-11.66	-11.65
-11.73	-11.72	-11.71	-11.7	-11.69	-11.68	-11.67	-11.66	-11.65
-11.73	-11.72	-11.71	-11.7	-11.69	-11.68	-11.67	-11.66	-11.65
-11.73	-11.72	-11.71	-11.7	-11.69	-11.68	-11.67	-11.66	-11.65
-11.73	-11.72	-11.71	-11.7	-11.69	-11.68	-11.67	-11.66	-11.65
-11.73	-11.72	-11.71	-11.7	-11.69	-11.68	-11.67	-11.66	-11.65
-11.73	-11.72	-11.71	-11.7	-11.69	-11.68	-11.67	-11.66	-11.65
-11.73	-11.72	-11.71	-11.7	-11.69	-11.68	-11.67	-11.66	-11.65
-11.73	-11.72	-11.71	-11.7	-11.69	-11.68	-11.67	-11.66	-11.65
-11.73	-11.72	-11.71	-11.7	-11.69	-11.68	-11.67	-11.66	-11.65
-11.73	-11.72	-11.71	-11.7	-11.69	-11.68	-11.67	-11.66	-11.65
-11.73	-11.72	-11.71	-11.7	-11.69	-11.68	-11.67	-11.66	-11.65
-11.73	-11.72	-11.71	-11.7	-11.69	-11.68	-11.67	-11.66	-11.65
-11.73	-11.72	-11.71	-11.7	-11.69	-11.68	-11.67	-11.66	-11.65
-11.73	-11.72	-11.71	-11.7	-11.69	-11.68	-11.67	-11.66	-11.65
-11.73	-11.72	-11.71	-11.7	-11.69	-11.68	-11.67	-11.66	-11.65
-11.73	-11.72	-11.71	-11.7	-11.69	-11.68	-11.67	-11.66	-11.65
-11.73	-11.72	-11.71	-11.7	-11.69	-11.68	-11.67	-11.66	-11.65
-11.73	-11.72	-11.71	-11.7	-11.69	-11.68	-11.67	-11.66	-11.65
-11.73	-11.72	-11.71	-11.7	-11.69	-11.68	-11.67	-11.66	-11.65
-11.73	-11.72	-11.71	-11.7	-11.69	-11.68	-11.67	-11.66	-11.65
-11.73	-11.72	-11.71	-11.7	-11.69	-11.68	-11.67	-11.66	-11.65
-11.73	-11.72	-11.71	-11.7	-11.69	-11.68	-11.67	-11.66	-11.65
-11.73	-11.72	-11.71	-11.7	-11.69	-11.68	-11.67	-11.66	-11.65
-11.73	-11.72	-11.71	-11.7	-11.69	-11.68	-11.67	-11.66	-11.65
-11.73	-11.72	-11.71	-11.7	-11.69	-11.68	-11.67	-11.66	-11.65
-11.73	-11.72	-11.71	-11.7	-11.69	-11.68	-11.67	-11.66	-11.65
-11.73	-11.72	-11.71	-11.7	-11.69	-11.68	-11.67	-11.66	-11.65
-11.73	-11.72	-11.71	-11.7	-11.69	-11.68	-11.67	-11.66	-11.65
-11.73	-11.72	-11.71	-11.7	-11.69	-11.68	-11.67	-11.66	-11.65
-11.73	-11.72	-11.71	-11.7	-11.69	-11.68	-11.67	-11.66	-11.65
-11.73	-11.72	-11.71	-11.7	-11.69	-11.68	-11.67	-11.66	-11.65
-11.73	-11.72	-11.71	-11.7	-11.69	-11.68	-11.67	-11.66	-11.65
-11.73	-11.72	-11.71	-11.7	-11.69	-11.68	-11.67	-11.66	-11.65

-11.73	-11.72	-11.71	-11.7	-11.69	-11.68	-11.67	-11.66	-11.65
-11.73	-11.72	-11.71	-11.7	-11.69	-11.68	-11.67	-11.66	-11.65
-11.73	-11.72	-11.71	-11.7	-11.69	-11.68	-11.67	-11.66	-11.65
-11.73	-11.72	-11.71	-11.7	-11.69	-11.68	-11.67	-11.66	-11.65
-11.73	-11.72	-11.71	-11.7	-11.69	-11.68	-11.67	-11.66	-11.65
-11.73	-11.72	-11.71	-11.7	-11.69	-11.68	-11.67	-11.66	-11.65
-11.73	-11.72	-11.71	-11.7	-11.69	-11.68	-11.67	-11.66	-11.65
-11.73	-11.72	-11.71	-11.7	-11.69	-11.68	-11.67	-11.66	-11.65
-11.73	-11.72	-11.71	-11.7	-11.69	-11.68	-11.67	-11.66	-11.65
-11.73	-11.72	-11.71	-11.7	-11.69	-11.68	-11.67	-11.66	-11.65
-11.73	-11.72	-11.71	-11.7	-11.69	-11.68	-11.67	-11.66	-11.65
-11.73	-11.72	-11.71	-11.7	-11.69	-11.68	-11.67	-11.66	-11.65
-11.73	-11.72	-11.71	-11.7	-11.69	-11.68	-11.67	-11.66	-11.65
-11.73	-11.72	-11.71	-11.7	-11.69	-11.68	-11.67	-11.66	-11.65
-11.73	-11.72	-11.71	-11.7	-11.69	-11.68	-11.67	-11.66	-11.65
-11.73	-11.72	-11.71	-11.7	-11.69	-11.68	-11.67	-11.66	-11.65
-11.73	-11.72	-11.71	-11.7	-11.69	-11.68	-11.67	-11.66	-11.65
-11.73	-11.72	-11.71	-11.7	-11.69	-11.68	-11.67	-11.66	-11.65
-11.73	-11.72	-11.71	-11.7	-11.69	-11.68	-11.67	-11.66	-11.65
-11.73	-11.72	-11.71	-11.7	-11.69	-11.68	-11.67	-11.66	-11.65
-11.73	-11.72	-11.71	-11.7	-11.69	-11.68	-11.67	-11.66	-11.65
-11.73	-11.72	-11.71	-11.7	-11.69	-11.68	-11.67	-11.66	-11.65
-11.73	-11.72	-11.71	-11.7	-11.69	-11.68	-11.67	-11.66	-11.65
-11.73	-11.72	-11.71	-11.7	-11.69	-11.68	-11.67	-11.66	-11.65
-11.73	-11.72	-11.71	-11.7	-11.69	-11.68	-11.67	-11.66	-11.65
-11.73	-11.72	-11.71	-11.7	-11.69	-11.68	-11.67	-11.66	-11.65
-11.73	-11.72	-11.71	-11.7	-11.69	-11.68	-11.67	-11.66	-11.65
-11.73	-11.72	-11.71	-11.7	-11.69	-11.68	-11.67	-11.66	-11.65
-11.73	-11.72	-11.71	-11.7	-11.69	-11.68	-11.67	-11.66	-11.65
-11.73	-11.72	-11.71	-11.7	-11.69	-11.68	-11.67	-11.66	-11.65
-11.73	-11.72	-11.71	-11.7	-11.69	-11.68	-11.67	-11.66	-11.65
-11.73	-11.72	-11.71	-11.7	-11.69	-11.68	-11.67	-11.66	-11.65
-11.73	-11.72	-11.71	-11.7	-11.69	-11.68	-11.67	-11.66	-11.65
-11.73	-11.72	-11.71	-11.7	-11.69	-11.68	-11.67	-11.66	-11.65
-11.73	-11.72	-11.71	-11.7	-11.69	-11.68	-11.67	-11.66	-11.65
-11.73	-11.72	-11.71	-11.7	-11.69	-11.68	-11.67	-11.66	-11.65
-11.73	-11.72	-11.71	-11.7	-11.69	-11.68	-11.67	-11.66	-11.65
-11.73	-11.72	-11.71	-11.7	-11.69	-11.68	-11.67	-11.66	-11.65
-11.73	-11.72	-11.71	-11.7	-11.69	-11.68	-11.67	-11.66	-11.65
-11.73	-11.72	-11.71	-11.7	-11.69	-11.68	-11.67	-11.66	-11.65
-11.73	-11.72	-11.71	-11.7	-11.69	-11.68	-11.67	-11.66	-11.65
-11.73	-11.72	-11.71	-11.7	-11.69	-11.68	-11.67	-11.66	-11.65
-11.73	-11.72	-11.71	-11.7	-11.69	-11.68	-11.67	-11.66	-11.65
-11.73	-11.72	-11.71	-11.7	-11.69	-11.68	-11.67	-11.66	-11.65
-11.73	-11.72	-11.71	-11.7	-11.69	-11.68	-11.67	-11.66	-11.65
-11.73	-11.72	-11.71	-11.7	-11.69	-11.68	-11.67	-11.66	-11.65
-11.73	-11.72	-11.71	-11.7	-11.69	-11.68	-11.67	-11.66	-11.65
-11.73	-11.72	-11.71	-11.7	-11.69	-11.68	-11.67	-11.66	-11.65
-11.73	-11.72	-11.71	-11.7	-11.69	-11.68	-11.67	-11.66	-11.65
-11.73	-11.72	-11.71	-11.7	-11.69	-11.68	-11.67	-11.66	-11.65

-11.73	-11.72	-11.71	-11.7	-11.69	-11.68	-11.67	-11.66	-11.65
-11.73	-11.72	-11.71	-11.7	-11.69	-11.68	-11.67	-11.66	-11.65
-11.73	-11.72	-11.71	-11.7	-11.69	-11.68	-11.67	-11.66	-11.65
-11.73	-11.72	-11.71	-11.7	-11.69	-11.68	-11.67	-11.66	-11.65
-11.73	-11.72	-11.71	-11.7	-11.69	-11.68	-11.67	-11.66	-11.65
-11.73	-11.72	-11.71	-11.7	-11.69	-11.68	-11.67	-11.66	-11.65
-11.73	-11.72	-11.71	-11.7	-11.69	-11.68	-11.67	-11.66	-11.65
-11.73	-11.72	-11.71	-11.7	-11.69	-11.68	-11.67	-11.66	-11.65
-11.73	-11.72	-11.71	-11.7	-11.69	-11.68	-11.67	-11.66	-11.65
-11.73	-11.72	-11.71	-11.7	-11.69	-11.68	-11.67	-11.66	-11.65
-11.73	-11.72	-11.71	-11.7	-11.69	-11.68	-11.67	-11.66	-11.65
-11.73	-11.72	-11.71	-11.7	-11.69	-11.68	-11.67	-11.66	-11.65
-11.73	-11.72	-11.71	-11.7	-11.69	-11.68	-11.67	-11.66	-11.65
-11.73	-11.72	-11.71	-11.7	-11.69	-11.68	-11.67	-11.66	-11.65
-11.73	-11.72	-11.71	-11.7	-11.69	-11.68	-11.67	-11.66	-11.65
-11.73	-11.72	-11.71	-11.7	-11.69	-11.68	-11.67	-11.66	-11.65
-11.73	-11.72	-11.71	-11.7	-11.69	-11.68	-11.67	-11.66	-11.65
-11.73	-11.72	-11.71	-11.7	-11.69	-11.68	-11.67	-11.66	-11.65
-11.73	-11.72	-11.71	-11.7	-11.69	-11.68	-11.67	-11.66	-11.65
-11.73	-11.72	-11.71	-11.7	-11.69	-11.68	-11.67	-11.66	-11.65
-11.73	-11.72	-11.71	-11.7	-11.69	-11.68	-11.67	-11.66	-11.65
-11.73	-11.72	-11.71	-11.7	-11.69	-11.68	-11.67	-11.66	-11.65
-11.73	-11.72	-11.71	-11.7	-11.69	-11.68	-11.67	-11.66	-11.65
-11.73	-11.72	-11.71	-11.7	-11.69	-11.68	-11.67	-11.66	-11.65
-11.73	-11.72	-11.71	-11.7	-11.69	-11.68	-11.67	-11.66	-11.65
-11.73	-11.72	-11.71	-11.7	-11.69	-11.68	-11.67	-11.66	-11.65
-11.73	-11.72	-11.71	-11.7	-11.69	-11.68	-11.67	-11.66	-11.65
-11.73	-11.72	-11.71	-11.7	-11.69	-11.68	-11.67	-11.66	-11.65
-11.73	-11.72	-11.71	-11.7	-11.69	-11.68	-11.67	-11.66	-11.65
-11.73	-11.72	-11.71	-11.7	-11.69	-11.68	-11.67	-11.66	-11.65
-11.73	-11.72	-11.71	-11.7	-11.69	-11.68	-11.67	-11.66	-11.65
-11.73	-11.72	-11.71	-11.7	-11.69	-11.68	-11.67	-11.66	-11.65
-11.73	-11.72	-11.71	-11.7	-11.69	-11.68	-11.67	-11.66	-11.65
-11.73	-11.72	-11.71	-11.7	-11.69	-11.68	-11.67	-11.66	-11.65
-11.73	-11.72	-11.71	-11.7	-11.69	-11.68	-11.67	-11.66	-11.65
-11.73	-11.72	-11.71	-11.7	-11.69	-11.68	-11.67	-11.66	-11.65
-11.73	-11.72	-11.71	-11.7	-11.69	-11.68	-11.67	-11.66	-11.65
-11.73	-11.72	-11.71	-11.7	-11.69	-11.68	-11.67	-11.66	-11.65
-11.73	-11.72	-11.71	-11.7	-11.69	-11.68	-11.67	-11.66	-11.65
-11.73	-11.72	-11.71	-11.7	-11.69	-11.68	-11.67	-11.66	-11.65
-11.73	-11.72	-11.71	-11.7	-11.69	-11.68	-11.67	-11.66	-11.65
-11.73	-11.72	-11.71	-11.7	-11.69	-11.68	-11.67	-11.66	-11.65
-11.73	-11.72	-11.71	-11.7	-11.69	-11.68	-11.67	-11.66	-11.65
-11.73	-11.72	-11.71	-11.7	-11.69	-11.68	-11.67	-11.66	-11.65
-11.73	-11.72	-11.71	-11.7	-11.69	-11.68	-11.67	-11.66	-11.65
-11.73	-11.72	-11.71	-11.7	-11.69	-11.68	-11.67	-11.66	-11.65
-11.73	-11.72	-11.71	-11.7	-11.69	-11.68	-11.67	-11.66	-11.65
-11.73	-11.72	-11.71	-11.7	-11.69	-11.68	-11.67	-11.66	-11.65
-11.73	-11.72	-11.71	-11.7	-11.69	-11.68	-11.67	-11.66	-11.65
-11.73	-11.72	-11.71	-11.7	-11.69	-11.68	-11.67	-11.66	-11.65

-11.73	-11.72	-11.71	-11.7	-11.69	-11.68	-11.67	-11.66	-11.65
-11.73	-11.72	-11.71	-11.7	-11.69	-11.68	-11.67	-11.66	-11.65
-11.73	-11.72	-11.71	-11.7	-11.69	-11.68	-11.67	-11.66	-11.65
-11.73	-11.72	-11.71	-11.7	-11.69	-11.68	-11.67	-11.66	-11.65
-11.73	-11.72	-11.71	-11.7	-11.69	-11.68	-11.67	-11.66	-11.65
-11.73	-11.72	-11.71	-11.7	-11.69	-11.68	-11.67	-11.66	-11.65
-11.73	-11.72	-11.71	-11.7	-11.69	-11.68	-11.67	-11.66	-11.65
-11.73	-11.72	-11.71	-11.7	-11.69	-11.68	-11.67	-11.66	-11.65
-11.73	-11.72	-11.71	-11.7	-11.69	-11.68	-11.67	-11.66	-11.65
-11.73	-11.72	-11.71	-11.7	-11.69	-11.68	-11.67	-11.66	-11.65
-11.73	-11.72	-11.71	-11.7	-11.69	-11.68	-11.67	-11.66	-11.65
-11.73	-11.72	-11.71	-11.7	-11.69	-11.68	-11.67	-11.66	-11.65
-11.73	-11.72	-11.71	-11.7	-11.69	-11.68	-11.67	-11.66	-11.65
-11.73	-11.72	-11.71	-11.7	-11.69	-11.68	-11.67	-11.66	-11.65
-11.73	-11.72	-11.71	-11.7	-11.69	-11.68	-11.67	-11.66	-11.65
-11.73	-11.72	-11.71	-11.7	-11.69	-11.68	-11.67	-11.66	-11.65
-11.73	-11.72	-11.71	-11.7	-11.69	-11.68	-11.67	-11.66	-11.65
-11.73	-11.72	-11.71	-11.7	-11.69	-11.68	-11.67	-11.66	-11.65
-11.73	-11.72	-11.71	-11.7	-11.69	-11.68	-11.67	-11.66	-11.65
-11.73	-11.72	-11.71	-11.7	-11.69	-11.68	-11.67	-11.66	-11.65
-11.73	-11.72	-11.71	-11.7	-11.69	-11.68	-11.67	-11.66	-11.65
-11.73	-11.72	-11.71	-11.7	-11.69	-11.68	-11.67	-11.66	-11.65
-11.73	-11.72	-11.71	-11.7	-11.69	-11.68	-11.67	-11.66	-11.65
-11.73	-11.72	-11.71	-11.7	-11.69	-11.68	-11.67	-11.66	-11.65
-11.73	-11.72	-11.71	-11.7	-11.69	-11.68	-11.67	-11.66	-11.65
-11.73	-11.72	-11.71	-11.7	-11.69	-11.68	-11.67	-11.66	-11.65
-11.73	-11.72	-11.71	-11.7	-11.69	-11.68	-11.67	-11.66	-11.65
-11.73	-11.72	-11.71	-11.7	-11.69	-11.68	-11.67	-11.66	-11.65
-11.73	-11.72	-11.71	-11.7	-11.69	-11.68	-11.67	-11.66	-11.65
-11.73	-11.72	-11.71	-11.7	-11.69	-11.68	-11.67	-11.66	-11.65
-11.73	-11.72	-11.71	-11.7	-11.69	-11.68	-11.67	-11.66	-11.65
-11.73	-11.72	-11.71	-11.7	-11.69	-11.68	-11.67	-11.66	-11.65
-11.73	-11.72	-11.71	-11.7	-11.69	-11.68	-11.67	-11.66	-11.65
-11.73	-11.72	-11.71	-11.7	-11.69	-11.68	-11.67	-11.66	-11.65
-11.73	-11.72	-11.71	-11.7	-11.69	-11.68	-11.67	-11.66	-11.65
-11.73	-11.72	-11.71	-11.7	-11.69	-11.68	-11.67	-11.66	-11.65
-11.73	-11.72	-11.71	-11.7	-11.69	-11.68	-11.67	-11.66	-11.65
-11.73	-11.72	-11.71	-11.7	-11.69	-11.68	-11.67	-11.66	-11.65
-11.73	-11.72	-11.71	-11.7	-11.69	-11.68	-11.67	-11.66	-11.65
-11.73	-11.72	-11.71	-11.7	-11.69	-11.68	-11.67	-11.66	-11.65
-11.73	-11.72	-11.71	-11.7	-11.69	-11.68	-11.67	-11.66	-11.65
-11.73	-11.72	-11.71	-11.7	-11.69	-11.68	-11.67	-11.66	-11.65
-11.73	-11.72	-11.71	-11.7	-11.69	-11.68	-11.67	-11.66	-11.65
-11.73	-11.72	-11.71	-11.7	-11.69	-11.68	-11.67	-11.66	-11.65
-11.73	-11.72	-11.71	-11.7	-11.69	-11.68	-11.67	-11.66	-11.65
-11.73	-11.72	-11.71	-11.7	-11.69	-11.68	-11.67	-11.66	-11.65
-11.73	-11.72	-11.71	-11.7	-11.69	-11.68	-11.67	-11.66	-11.65
-11.73	-11.72	-11.71	-11.7	-11.69	-11.68	-11.67	-11.66	-11.65
-11.73	-11.72	-11.71	-11.7	-11.69	-11.68	-11.67	-11.66	-11.65
-11.73	-11.72	-11.71	-11.7	-11.69	-11.68	-11.67	-11.66	-11.65

-11.73	-11.72	-11.71	-11.7	-11.69	-11.68	-11.67	-11.66	-11.65
-11.73	-11.72	-11.71	-11.7	-11.69	-11.68	-11.67	-11.66	-11.65
-11.73	-11.72	-11.71	-11.7	-11.69	-11.68	-11.67	-11.66	-11.65
-11.73	-11.72	-11.71	-11.7	-11.69	-11.68	-11.67	-11.66	-11.65
-11.73	-11.72	-11.71	-11.7	-11.69	-11.68	-11.67	-11.66	-11.65
-11.73	-11.72	-11.71	-11.7	-11.69	-11.68	-11.67	-11.66	-11.65
-11.73	-11.72	-11.71	-11.7	-11.69	-11.68	-11.67	-11.66	-11.65
-11.73	-11.72	-11.71	-11.7	-11.69	-11.68	-11.67	-11.66	-11.65
-11.73	-11.72	-11.71	-11.7	-11.69	-11.68	-11.67	-11.66	-11.65
-11.73	-11.72	-11.71	-11.7	-11.69	-11.68	-11.67	-11.66	-11.65
-11.73	-11.72	-11.71	-11.7	-11.69	-11.68	-11.67	-11.66	-11.65
-11.73	-11.72	-11.71	-11.7	-11.69	-11.68	-11.67	-11.66	-11.65
-11.73	-11.72	-11.71	-11.7	-11.69	-11.68	-11.67	-11.66	-11.65
-11.73	-11.72	-11.71	-11.7	-11.69	-11.68	-11.67	-11.66	-11.65
-11.73	-11.72	-11.71	-11.7	-11.69	-11.68	-11.67	-11.66	-11.65
-11.73	-11.72	-11.71	-11.7	-11.69	-11.68	-11.67	-11.66	-11.65
-11.73	-11.72	-11.71	-11.7	-11.69	-11.68	-11.67	-11.66	-11.65
-11.73	-11.72	-11.71	-11.7	-11.69	-11.68	-11.67	-11.66	-11.65
-11.73	-11.72	-11.71	-11.7	-11.69	-11.68	-11.67	-11.66	-11.65
-11.73	-11.72	-11.71	-11.7	-11.69	-11.68	-11.67	-11.66	-11.65
-11.73	-11.72	-11.71	-11.7	-11.69	-11.68	-11.67	-11.66	-11.65
-11.73	-11.72	-11.71	-11.7	-11.69	-11.68	-11.67	-11.66	-11.65
-11.73	-11.72	-11.71	-11.7	-11.69	-11.68	-11.67	-11.66	-11.65
-11.73	-11.72	-11.71	-11.7	-11.69	-11.68	-11.67	-11.66	-11.65
-11.73	-11.72	-11.71	-11.7	-11.69	-11.68	-11.67	-11.66	-11.65
-11.73	-11.72	-11.71	-11.7	-11.69	-11.68	-11.67	-11.66	-11.65
-11.73	-11.72	-11.71	-11.7	-11.69	-11.68	-11.67	-11.66	-11.65
-11.73	-11.72	-11.71	-11.7	-11.69	-11.68	-11.67	-11.66	-11.65
-11.73	-11.72	-11.71	-11.7	-11.69	-11.68	-11.67	-11.66	-11.65
-11.73	-11.72	-11.71	-11.7	-11.69	-11.68	-11.67	-11.66	-11.65
-11.73	-11.72	-11.71	-11.7	-11.69	-11.68	-11.67	-11.66	-11.65
-11.73	-11.72	-11.71	-11.7	-11.69	-11.68	-11.67	-11.66	-11.65
-11.73	-11.72	-11.71	-11.7	-11.69	-11.68	-11.67	-11.66	-11.65
-11.73	-11.72	-11.71	-11.7	-11.69	-11.68	-11.67	-11.66	-11.65
-11.73	-11.72	-11.71	-11.7	-11.69	-11.68	-11.67	-11.66	-11.65
-11.73	-11.72	-11.71	-11.7	-11.69	-11.68	-11.67	-11.66	-11.65
-11.73	-11.72	-11.71	-11.7	-11.69	-11.68	-11.67	-11.66	-11.65
-11.73	-11.72	-11.71	-11.7	-11.69	-11.68	-11.67	-11.66	-11.65
-11.73	-11.72	-11.71	-11.7	-11.69	-11.68	-11.67	-11.66	-11.65
-11.73	-11.72	-11.71	-11.7	-11.69	-11.68	-11.67	-11.66	-11.65
-11.73	-11.72	-11.71	-11.7	-11.69	-11.68	-11.67	-11.66	-11.65
-11.73	-11.72	-11.71	-11.7	-11.69	-11.68	-11.67	-11.66	-11.65
-11.73	-11.72	-11.71	-11.7	-11.69	-11.68	-11.67	-11.66	-11.65
-11.73	-11.72	-11.71	-11.7	-11.69	-11.68	-11.67	-11.66	-11.65
-11.73	-11.72	-11.71	-11.7	-11.69	-11.68	-11.67	-11.66	-11.65
-11.73	-11.72	-11.71	-11.7	-11.69	-11.68	-11.67	-11.66	-11.65
-11.73	-11.72	-11.71	-11.7	-11.69	-11.68	-11.67	-11.66	-11.65
-11.73	-11.72	-11.71	-11.7	-11.69	-11.68	-11.67	-11.66	-11.65
-11.73	-11.72	-11.71	-11.7	-11.69	-11.68	-11.67	-11.66	-11.65
-11.73	-11.72	-11.71	-11.7	-11.69	-11.68	-11.67	-11.66	-11.65

-11.73	-11.72	-11.71	-11.7	-11.69	-11.68	-11.67	-11.66	-11.65
-11.73	-11.72	-11.71	-11.7	-11.69	-11.68	-11.67	-11.66	-11.65
-11.73	-11.72	-11.71	-11.7	-11.69	-11.68	-11.67	-11.66	-11.65
-11.73	-11.72	-11.71	-11.7	-11.69	-11.68	-11.67	-11.66	-11.65
-11.73	-11.72	-11.71	-11.7	-11.69	-11.68	-11.67	-11.66	-11.65
-11.73	-11.72	-11.71	-11.7	-11.69	-11.68	-11.67	-11.66	-11.65
-11.73	-11.72	-11.71	-11.7	-11.69	-11.68	-11.67	-11.66	-11.65
-11.73	-11.72	-11.71	-11.7	-11.69	-11.68	-11.67	-11.66	-11.65
-11.73	-11.72	-11.71	-11.7	-11.69	-11.68	-11.67	-11.66	-11.65
-11.73	-11.72	-11.71	-11.7	-11.69	-11.68	-11.67	-11.66	-11.65
-11.73	-11.72	-11.71	-11.7	-11.69	-11.68	-11.67	-11.66	-11.65
-11.73	-11.72	-11.71	-11.7	-11.69	-11.68	-11.67	-11.66	-11.65
-11.73	-11.72	-11.71	-11.7	-11.69	-11.68	-11.67	-11.66	-11.65
-11.73	-11.72	-11.71	-11.7	-11.69	-11.68	-11.67	-11.66	-11.65
-11.73	-11.72	-11.71	-11.7	-11.69	-11.68	-11.67	-11.66	-11.65
-11.73	-11.72	-11.71	-11.7	-11.69	-11.68	-11.67	-11.66	-11.65
-11.73	-11.72	-11.71	-11.7	-11.69	-11.68	-11.67	-11.66	-11.65
-11.73	-11.72	-11.71	-11.7	-11.69	-11.68	-11.67	-11.66	-11.65
-11.73	-11.72	-11.71	-11.7	-11.69	-11.68	-11.67	-11.66	-11.65
-11.73	-11.72	-11.71	-11.7	-11.69	-11.68	-11.67	-11.66	-11.65
-11.73	-11.72	-11.71	-11.7	-11.69	-11.68	-11.67	-11.66	-11.65
-11.73	-11.72	-11.71	-11.7	-11.69	-11.68	-11.67	-11.66	-11.65
-11.73	-11.72	-11.71	-11.7	-11.69	-11.68	-11.67	-11.66	-11.65
-11.73	-11.72	-11.71	-11.7	-11.69	-11.68	-11.67	-11.66	-11.65
-11.73	-11.72	-11.71	-11.7	-11.69	-11.68	-11.67	-11.66	-11.65
-11.73	-11.72	-11.71	-11.7	-11.69	-11.68	-11.67	-11.66	-11.65
-11.73	-11.72	-11.71	-11.7	-11.69	-11.68	-11.67	-11.66	-11.65
-11.73	-11.72	-11.71	-11.7	-11.69	-11.68	-11.67	-11.66	-11.65
-11.73	-11.72	-11.71	-11.7	-11.69	-11.68	-11.67	-11.66	-11.65
-11.73	-11.72	-11.71	-11.7	-11.69	-11.68	-11.67	-11.66	-11.65
-11.73	-11.72	-11.71	-11.7	-11.69	-11.68	-11.67	-11.66	-11.65
-11.73	-11.72	-11.71	-11.7	-11.69	-11.68	-11.67	-11.66	-11.65
-11.73	-11.72	-11.71	-11.7	-11.69	-11.68	-11.67	-11.66	-11.65
-11.73	-11.72	-11.71	-11.7	-11.69	-11.68	-11.67	-11.66	-11.65
-11.73	-11.72	-11.71	-11.7	-11.69	-11.68	-11.67	-11.66	-11.65
-11.73	-11.72	-11.71	-11.7	-11.69	-11.68	-11.67	-11.66	-11.65
-11.73	-11.72	-11.71	-11.7	-11.69	-11.68	-11.67	-11.66	-11.65
-11.73	-11.72	-11.71	-11.7	-11.69	-11.68	-11.67	-11.66	-11.65
-11.73	-11.72	-11.71	-11.7	-11.69	-11.68	-11.67	-11.66	-11.65
-11.73	-11.72	-11.71	-11.7	-11.69	-11.68	-11.67	-11.66	-11.65
-11.73	-11.72	-11.71	-11.7	-11.69	-11.68	-11.67	-11.66	-11.65
-11.73	-11.72	-11.71	-11.7	-11.69	-11.68	-11.67	-11.66	-11.65
-11.73	-11.72	-11.71	-11.7	-11.69	-11.68	-11.67	-11.66	-11.65
-11.73	-11.72	-11.71	-11.7	-11.69	-11.68	-11.67	-11.66	-11.65
-11.73	-11.72	-11.71	-11.7	-11.69	-11.68	-11.67	-11.66	-11.65
-11.73	-11.72	-11.71	-11.7	-11.69	-11.68	-11.67	-11.66	-11.65
-11.73	-11.72	-11.71	-11.7	-11.69	-11.68	-11.67	-11.66	-11.65
-11.73	-11.72	-11.71	-11.7	-11.69	-11.68	-11.67	-11.66	-11.65
-11.73	-11.72	-11.71	-11.7	-11.69	-11.68	-11.67	-11.66	-11.65
-11.73	-11.72	-11.71	-11.7	-11.69	-11.68	-11.67	-11.66	-11.65

-11.73	-11.72	-11.71	-11.7	-11.69	-11.68	-11.67	-11.66	-11.65
-11.73	-11.72	-11.71	-11.7	-11.69	-11.68	-11.67	-11.66	-11.65
-11.73	-11.72	-11.71	-11.7	-11.69	-11.68	-11.67	-11.66	-11.65
-11.73	-11.72	-11.71	-11.7	-11.69	-11.68	-11.67	-11.66	-11.65
-11.73	-11.72	-11.71	-11.7	-11.69	-11.68	-11.67	-11.66	-11.65
-11.73	-11.72	-11.71	-11.7	-11.69	-11.68	-11.67	-11.66	-11.65
-11.73	-11.72	-11.71	-11.7	-11.69	-11.68	-11.67	-11.66	-11.65
-11.73	-11.72	-11.71	-11.7	-11.69	-11.68	-11.67	-11.66	-11.65
-11.73	-11.72	-11.71	-11.7	-11.69	-11.68	-11.67	-11.66	-11.65
-11.73	-11.72	-11.71	-11.7	-11.69	-11.68	-11.67	-11.66	-11.65
-11.73	-11.72	-11.71	-11.7	-11.69	-11.68	-11.67	-11.66	-11.65
-11.73	-11.72	-11.71	-11.7	-11.69	-11.68	-11.67	-11.66	-11.65
-11.73	-11.72	-11.71	-11.7	-11.69	-11.68	-11.67	-11.66	-11.65
-11.73	-11.72	-11.71	-11.7	-11.69	-11.68	-11.67	-11.66	-11.65
-11.73	-11.72	-11.71	-11.7	-11.69	-11.68	-11.67	-11.66	-11.65
-11.73	-11.72	-11.71	-11.7	-11.69	-11.68	-11.67	-11.66	-11.65
-11.73	-11.72	-11.71	-11.7	-11.69	-11.68	-11.67	-11.66	-11.65
-11.73	-11.72	-11.71	-11.7	-11.69	-11.68	-11.67	-11.66	-11.65
-11.73	-11.72	-11.71	-11.7	-11.69	-11.68	-11.67	-11.66	-11.65
-11.73	-11.72	-11.71	-11.7	-11.69	-11.68	-11.67	-11.66	-11.65
-11.73	-11.72	-11.71	-11.7	-11.69	-11.68	-11.67	-11.66	-11.65
-11.73	-11.72	-11.71	-11.7	-11.69	-11.68	-11.67	-11.66	-11.65
-11.73	-11.72	-11.71	-11.7	-11.69	-11.68	-11.67	-11.66	-11.65
-11.73	-11.72	-11.71	-11.7	-11.69	-11.68	-11.67	-11.66	-11.65
-11.73	-11.72	-11.71	-11.7	-11.69	-11.68	-11.67	-11.66	-11.65
-11.73	-11.72	-11.71	-11.7	-11.69	-11.68	-11.67	-11.66	-11.65
-11.73	-11.72	-11.71	-11.7	-11.69	-11.68	-11.67	-11.66	-11.65
-11.73	-11.72	-11.71	-11.7	-11.69	-11.68	-11.67	-11.66	-11.65
-11.73	-11.72	-11.71	-11.7	-11.69	-11.68	-11.67	-11.66	-11.65
-11.73	-11.72	-11.71	-11.7	-11.69	-11.68	-11.67	-11.66	-11.65
-11.73	-11.72	-11.71	-11.7	-11.69	-11.68	-11.67	-11.66	-11.65
-11.73	-11.72	-11.71	-11.7	-11.69	-11.68	-11.67	-11.66	-11.65
-11.73	-11.72	-11.71	-11.7	-11.69	-11.68	-11.67	-11.66	-11.65
-11.73	-11.72	-11.71	-11.7	-11.69	-11.68	-11.67	-11.66	-11.65
-11.73	-11.72	-11.71	-11.7	-11.69	-11.68	-11.67	-11.66	-11.65
-11.73	-11.72	-11.71	-11.7	-11.69	-11.68	-11.67	-11.66	-11.65
-11.73	-11.72	-11.71	-11.7	-11.69	-11.68	-11.67	-11.66	-11.65
-11.73	-11.72	-11.71	-11.7	-11.69	-11.68	-11.67	-11.66	-11.65
-11.73	-11.72	-11.71	-11.7	-11.69	-11.68	-11.67	-11.66	-11.65
-11.73	-11.72	-11.71	-11.7	-11.69	-11.68	-11.67	-11.66	-11.65
-11.73	-11.72	-11.71	-11.7	-11.69	-11.68	-11.67	-11.66	-11.65
-11.73	-11.72	-11.71	-11.7	-11.69	-11.68	-11.67	-11.66	-11.65
-11.73	-11.72	-11.71	-11.7	-11.69	-11.68	-11.67	-11.66	-11.65
-11.73	-11.72	-11.71	-11.7	-11.69	-11.68	-11.67	-11.66	-11.65
-11.73	-11.72	-11.71	-11.7	-11.69	-11.68	-11.67	-11.66	-11.65
-11.73	-11.72	-11.71	-11.7	-11.69	-11.68	-11.67	-11.66	-11.65
-11.73	-11.72	-11.71	-11.7	-11.69	-11.68	-11.67	-11.66	-11.65
-11.73	-11.72	-11.71	-11.7	-11.69	-11.68	-11.67	-11.66	-11.65
-11.73	-11.72	-11.71	-11.7	-11.69	-11.68	-11.67	-11.66	-11.65
-11.73	-11.72	-11.71	-11.7	-11.69	-11.68	-11.67	-11.66	-11.65

-11.73	-11.72	-11.71	-11.7	-11.69	-11.68	-11.67	-11.66	-11.65
-11.73	-11.72	-11.71	-11.7	-11.69	-11.68	-11.67	-11.66	-11.65
-11.73	-11.72	-11.71	-11.7	-11.69	-11.68	-11.67	-11.66	-11.65
-11.73	-11.72	-11.71	-11.7	-11.69	-11.68	-11.67	-11.66	-11.65
-11.73	-11.72	-11.71	-11.7	-11.69	-11.68	-11.67	-11.66	-11.65
-11.73	-11.72	-11.71	-11.7	-11.69	-11.68	-11.67	-11.66	-11.65
-11.73	-11.72	-11.71	-11.7	-11.69	-11.68	-11.67	-11.66	-11.65
-11.73	-11.72	-11.71	-11.7	-11.69	-11.68	-11.67	-11.66	-11.65
-11.73	-11.72	-11.71	-11.7	-11.69	-11.68	-11.67	-11.66	-11.65
-11.73	-11.72	-11.71	-11.7	-11.69	-11.68	-11.67	-11.66	-11.65
-11.73	-11.72	-11.71	-11.7	-11.69	-11.68	-11.67	-11.66	-11.65
-11.73	-11.72	-11.71	-11.7	-11.69	-11.68	-11.67	-11.66	-11.65
-11.73	-11.72	-11.71	-11.7	-11.69	-11.68	-11.67	-11.66	-11.65
-11.73	-11.72	-11.71	-11.7	-11.69	-11.68	-11.67	-11.66	-11.65
-11.73	-11.72	-11.71	-11.7	-11.69	-11.68	-11.67	-11.66	-11.65
-11.73	-11.72	-11.71	-11.7	-11.69	-11.68	-11.67	-11.66	-11.65
-11.73	-11.72	-11.71	-11.7	-11.69	-11.68	-11.67	-11.66	-11.65
-11.73	-11.72	-11.71	-11.7	-11.69	-11.68	-11.67	-11.66	-11.65
-11.73	-11.72	-11.71	-11.7	-11.69	-11.68	-11.67	-11.66	-11.65
-11.73	-11.72	-11.71	-11.7	-11.69	-11.68	-11.67	-11.66	-11.65
-11.73	-11.72	-11.71	-11.7	-11.69	-11.68	-11.67	-11.66	-11.65
-11.73	-11.72	-11.71	-11.7	-11.69	-11.68	-11.67	-11.66	-11.65
-11.73	-11.72	-11.71	-11.7	-11.69	-11.68	-11.67	-11.66	-11.65
-11.73	-11.72	-11.71	-11.7	-11.69	-11.68	-11.67	-11.66	-11.65
-11.73	-11.72	-11.71	-11.7	-11.69	-11.68	-11.67	-11.66	-11.65
-11.73	-11.72	-11.71	-11.7	-11.69	-11.68	-11.67	-11.66	-11.65
-11.73	-11.72	-11.71	-11.7	-11.69	-11.68	-11.67	-11.66	-11.65
-11.73	-11.72	-11.71	-11.7	-11.69	-11.68	-11.67	-11.66	-11.65
-11.73	-11.72	-11.71	-11.7	-11.69	-11.68	-11.67	-11.66	-11.65
-11.73	-11.72	-11.71	-11.7	-11.69	-11.68	-11.67	-11.66	-11.65
-11.73	-11.72	-11.71	-11.7	-11.69	-11.68	-11.67	-11.66	-11.65
-11.73	-11.72	-11.71	-11.7	-11.69	-11.68	-11.67	-11.66	-11.65
-11.73	-11.72	-11.71	-11.7	-11.69	-11.68	-11.67	-11.66	-11.65
-11.73	-11.72	-11.71	-11.7	-11.69	-11.68	-11.67	-11.66	-11.65
-11.73	-11.72	-11.71	-11.7	-11.69	-11.68	-11.67	-11.66	-11.65
-11.73	-11.72	-11.71	-11.7	-11.69	-11.68	-11.67	-11.66	-11.65
-11.73	-11.72	-11.71	-11.7	-11.69	-11.68	-11.67	-11.66	-11.65
-11.73	-11.72	-11.71	-11.7	-11.69	-11.68	-11.67	-11.66	-11.65
-11.73	-11.72	-11.71	-11.7	-11.69	-11.68	-11.67	-11.66	-11.65
-11.73	-11.72	-11.71	-11.7	-11.69	-11.68	-11.67	-11.66	-11.65
-11.73	-11.72	-11.71	-11.7	-11.69	-11.68	-11.67	-11.66	-11.65
-11.73	-11.72	-11.71	-11.7	-11.69	-11.68	-11.67	-11.66	-11.65
-11.73	-11.72	-11.71	-11.7	-11.69	-11.68	-11.67	-11.66	-11.65
-11.73	-11.72	-11.71	-11.7	-11.69	-11.68	-11.67	-11.66	-11.65
-11.73	-11.72	-11.71	-11.7	-11.69	-11.68	-11.67	-11.66	-11.65
-11.73	-11.72	-11.71	-11.7	-11.69	-11.68	-11.67	-11.66	-11.65
-11.73	-11.72	-11.71	-11.7	-11.69	-11.68	-11.67	-11.66	-11.65
-11.73	-11.72	-11.71	-11.7	-11.69	-11.68	-11.67	-11.66	-11.65
-11.73	-11.72	-11.71	-11.7	-11.69	-11.68	-11.67	-11.66	-11.65
-11.73	-11.72	-11.71	-11.7	-11.69	-11.68	-11.67	-11.66	-11.65

-11.73	-11.72	-11.71	-11.7	-11.69	-11.68	-11.67	-11.66	-11.65
-11.73	-11.72	-11.71	-11.7	-11.69	-11.68	-11.67	-11.66	-11.65
-11.73	-11.72	-11.71	-11.7	-11.69	-11.68	-11.67	-11.66	-11.65
-11.73	-11.72	-11.71	-11.7	-11.69	-11.68	-11.67	-11.66	-11.65
-11.73	-11.72	-11.71	-11.7	-11.69	-11.68	-11.67	-11.66	-11.65
-11.73	-11.72	-11.71	-11.7	-11.69	-11.68	-11.67	-11.66	-11.65
-11.73	-11.72	-11.71	-11.7	-11.69	-11.68	-11.67	-11.66	-11.65
-11.73	-11.72	-11.71	-11.7	-11.69	-11.68	-11.67	-11.66	-11.65
-11.73	-11.72	-11.71	-11.7	-11.69	-11.68	-11.67	-11.66	-11.65
-11.73	-11.72	-11.71	-11.7	-11.69	-11.68	-11.67	-11.66	-11.65
-11.73	-11.72	-11.71	-11.7	-11.69	-11.68	-11.67	-11.66	-11.65
-11.73	-11.72	-11.71	-11.7	-11.69	-11.68	-11.67	-11.66	-11.65
-11.73	-11.72	-11.71	-11.7	-11.69	-11.68	-11.67	-11.66	-11.65
-11.73	-11.72	-11.71	-11.7	-11.69	-11.68	-11.67	-11.66	-11.65
-11.73	-11.72	-11.71	-11.7	-11.69	-11.68	-11.67	-11.66	-11.65
-11.73	-11.72	-11.71	-11.7	-11.69	-11.68	-11.67	-11.66	-11.65
-11.73	-11.72	-11.71	-11.7	-11.69	-11.68	-11.67	-11.66	-11.65
-11.73	-11.72	-11.71	-11.7	-11.69	-11.68	-11.67	-11.66	-11.65
-11.73	-11.72	-11.71	-11.7	-11.69	-11.68	-11.67	-11.66	-11.65
-11.73	-11.72	-11.71	-11.7	-11.69	-11.68	-11.67	-11.66	-11.65
-11.73	-11.72	-11.71	-11.7	-11.69	-11.68	-11.67	-11.66	-11.65
-11.73	-11.72	-11.71	-11.7	-11.69	-11.68	-11.67	-11.66	-11.65
-11.73	-11.72	-11.71	-11.7	-11.69	-11.68	-11.67	-11.66	-11.65
-11.73	-11.72	-11.71	-11.7	-11.69	-11.68	-11.67	-11.66	-11.65
-11.73	-11.72	-11.71	-11.7	-11.69	-11.68	-11.67	-11.66	-11.65
-11.73	-11.72	-11.71	-11.7	-11.69	-11.68	-11.67	-11.66	-11.65
-11.73	-11.72	-11.71	-11.7	-11.69	-11.68	-11.67	-11.66	-11.65
-11.73	-11.72	-11.71	-11.7	-11.69	-11.68	-11.67	-11.66	-11.65
-11.73	-11.72	-11.71	-11.7	-11.69	-11.68	-11.67	-11.66	-11.65
-11.73	-11.72	-11.71	-11.7	-11.69	-11.68	-11.67	-11.66	-11.65
-11.73	-11.72	-11.71	-11.7	-11.69	-11.68	-11.67	-11.66	-11.65
-11.73	-11.72	-11.71	-11.7	-11.69	-11.68	-11.67	-11.66	-11.65
-11.73	-11.72	-11.71	-11.7	-11.69	-11.68	-11.67	-11.66	-11.65
-11.73	-11.72	-11.71	-11.7	-11.69	-11.68	-11.67	-11.66	-11.65
-11.73	-11.72	-11.71	-11.7	-11.69	-11.68	-11.67	-11.66	-11.65
-11.73	-11.72	-11.71	-11.7	-11.69	-11.68	-11.67	-11.66	-11.65
-11.73	-11.72	-11.71	-11.7	-11.69	-11.68	-11.67	-11.66	-11.65
-11.73	-11.72	-11.71	-11.7	-11.69	-11.68	-11.67	-11.66	-11.65
-11.73	-11.72	-11.71	-11.7	-11.69	-11.68	-11.67	-11.66	-11.65
-11.73	-11.72	-11.71	-11.7	-11.69	-11.68	-11.67	-11.66	-11.65
-11.73	-11.72	-11.71	-11.7	-11.69	-11.68	-11.67	-11.66	-11.65
-11.73	-11.72	-11.71	-11.7	-11.69	-11.68	-11.67	-11.66	-11.65
-11.73	-11.72	-11.71	-11.7	-11.69	-11.68	-11.67	-11.66	-11.65
-11.73	-11.72	-11.71	-11.7	-11.69	-11.68	-11.67	-11.66	-11.65
-11.73	-11.72	-11.71	-11.7	-11.69	-11.68	-11.67	-11.66	-11.65
-11.73	-11.72	-11.71	-11.7	-11.69	-11.68	-11.67	-11.66	-11.65
-11.73	-11.72	-11.71	-11.7	-11.69	-11.68	-11.67	-11.66	-11.65
-11.73	-11.72	-11.71	-11.7	-11.69	-11.68	-11.67	-11.66	-11.65
-11.73	-11.72	-11.71	-11.7	-11.69	-11.68	-11.67	-11.66	-11.65
-11.73	-11.72	-11.71	-11.7	-11.69	-11.68	-11.67	-11.66	-11.65

-11.73	-11.72	-11.71	-11.7	-11.69	-11.68	-11.67	-11.66	-11.65
-11.73	-11.72	-11.71	-11.7	-11.69	-11.68	-11.67	-11.66	-11.65
-11.73	-11.72	-11.71	-11.7	-11.69	-11.68	-11.67	-11.66	-11.65
-11.73	-11.72	-11.71	-11.7	-11.69	-11.68	-11.67	-11.66	-11.65
-11.73	-11.72	-11.71	-11.7	-11.69	-11.68	-11.67	-11.66	-11.65
-11.73	-11.72	-11.71	-11.7	-11.69	-11.68	-11.67	-11.66	-11.65
-11.73	-11.72	-11.71	-11.7	-11.69	-11.68	-11.67	-11.66	-11.65
-11.73	-11.72	-11.71	-11.7	-11.69	-11.68	-11.67	-11.66	-11.65
-11.73	-11.72	-11.71	-11.7	-11.69	-11.68	-11.67	-11.66	-11.65
-11.73	-11.72	-11.71	-11.7	-11.69	-11.68	-11.67	-11.66	-11.65
-11.73	-11.72	-11.71	-11.7	-11.69	-11.68	-11.67	-11.66	-11.65
-11.73	-11.72	-11.71	-11.7	-11.69	-11.68	-11.67	-11.66	-11.65
-11.73	-11.72	-11.71	-11.7	-11.69	-11.68	-11.67	-11.66	-11.65
-11.73	-11.72	-11.71	-11.7	-11.69	-11.68	-11.67	-11.66	-11.65
-11.73	-11.72	-11.71	-11.7	-11.69	-11.68	-11.67	-11.66	-11.65
-11.73	-11.72	-11.71	-11.7	-11.69	-11.68	-11.67	-11.66	-11.65
-11.73	-11.72	-11.71	-11.7	-11.69	-11.68	-11.67	-11.66	-11.65
-11.73	-11.72	-11.71	-11.7	-11.69	-11.68	-11.67	-11.66	-11.65
-11.73	-11.72	-11.71	-11.7	-11.69	-11.68	-11.67	-11.66	-11.65
-11.73	-11.72	-11.71	-11.7	-11.69	-11.68	-11.67	-11.66	-11.65
-11.73	-11.72	-11.71	-11.7	-11.69	-11.68	-11.67	-11.66	-11.65
-11.73	-11.72	-11.71	-11.7	-11.69	-11.68	-11.67	-11.66	-11.65
-11.73	-11.72	-11.71	-11.7	-11.69	-11.68	-11.67	-11.66	-11.65
-11.73	-11.72	-11.71	-11.7	-11.69	-11.68	-11.67	-11.66	-11.65
-11.73	-11.72	-11.71	-11.7	-11.69	-11.68	-11.67	-11.66	-11.65
-11.73	-11.72	-11.71	-11.7	-11.69	-11.68	-11.67	-11.66	-11.65
-11.73	-11.72	-11.71	-11.7	-11.69	-11.68	-11.67	-11.66	-11.65
-11.73	-11.72	-11.71	-11.7	-11.69	-11.68	-11.67	-11.66	-11.65
-11.73	-11.72	-11.71	-11.7	-11.69	-11.68	-11.67	-11.66	-11.65
-11.73	-11.72	-11.71	-11.7	-11.69	-11.68	-11.67	-11.66	-11.65
-11.73	-11.72	-11.71	-11.7	-11.69	-11.68	-11.67	-11.66	-11.65
-11.73	-11.72	-11.71	-11.7	-11.69	-11.68	-11.67	-11.66	-11.65
-11.73	-11.72	-11.71	-11.7	-11.69	-11.68	-11.67	-11.66	-11.65
-11.73	-11.72	-11.71	-11.7	-11.69	-11.68	-11.67	-11.66	-11.65
-11.73	-11.72	-11.71	-11.7	-11.69	-11.68	-11.67	-11.66	-11.65
-11.73	-11.72	-11.71	-11.7	-11.69	-11.68	-11.67	-11.66	-11.65
-11.73	-11.72	-11.71	-11.7	-11.69	-11.68	-11.67	-11.66	-11.65
-11.73	-11.72	-11.71	-11.7	-11.69	-11.68	-11.67	-11.66	-11.65
-11.73	-11.72	-11.71	-11.7	-11.69	-11.68	-11.67	-11.66	-11.65
-11.73	-11.72	-11.71	-11.7	-11.69	-11.68	-11.67	-11.66	-11.65
-11.73	-11.72	-11.71	-11.7	-11.69	-11.68	-11.67	-11.66	-11.65
-11.73	-11.72	-11.71	-11.7	-11.69	-11.68	-11.67	-11.66	-11.65
-11.73	-11.72	-11.71	-11.7	-11.69	-11.68	-11.67	-11.66	-11.65
-11.73	-11.72	-11.71	-11.7	-11.69	-11.68	-11.67	-11.66	-11.65
-11.73	-11.72	-11.71	-11.7	-11.69	-11.68	-11.67	-11.66	-11.65
-11.73	-11.72	-11.71	-11.7	-11.69	-11.68	-11.67	-11.66	-11.65
-11.73	-11.72	-11.71	-11.7	-11.69	-11.68	-11.67	-11.66	-11.65
-11.73	-11.72	-11.71	-11.7	-11.69	-11.68	-11.67	-11.66	-11.65
-11.73	-11.72	-11.71	-11.7	-11.69	-11.68	-11.67	-11.66	-11.65
-11.73	-11.72	-11.71	-11.7	-11.69	-11.68	-11.67	-11.66	-11.65

-11.73	-11.72	-11.71	-11.7	-11.69	-11.68	-11.67	-11.66	-11.65
-11.73	-11.72	-11.71	-11.7	-11.69	-11.68	-11.67	-11.66	-11.65
-11.73	-11.72	-11.71	-11.7	-11.69	-11.68	-11.67	-11.66	-11.65
-11.73	-11.72	-11.71	-11.7	-11.69	-11.68	-11.67	-11.66	-11.65
-11.73	-11.72	-11.71	-11.7	-11.69	-11.68	-11.67	-11.66	-11.65
-11.73	-11.72	-11.71	-11.7	-11.69	-11.68	-11.67	-11.66	-11.65
-11.73	-11.72	-11.71	-11.7	-11.69	-11.68	-11.67	-11.66	-11.65
-11.73	-11.72	-11.71	-11.7	-11.69	-11.68	-11.67	-11.66	-11.65
-11.73	-11.72	-11.71	-11.7	-11.69	-11.68	-11.67	-11.66	-11.65
-11.73	-11.72	-11.71	-11.7	-11.69	-11.68	-11.67	-11.66	-11.65
-11.73	-11.72	-11.71	-11.7	-11.69	-11.68	-11.67	-11.66	-11.65
-11.73	-11.72	-11.71	-11.7	-11.69	-11.68	-11.67	-11.66	-11.65
-11.73	-11.72	-11.71	-11.7	-11.69	-11.68	-11.67	-11.66	-11.65
-11.73	-11.72	-11.71	-11.7	-11.69	-11.68	-11.67	-11.66	-11.65
-11.73	-11.72	-11.71	-11.7	-11.69	-11.68	-11.67	-11.66	-11.65
-11.73	-11.72	-11.71	-11.7	-11.69	-11.68	-11.67	-11.66	-11.65
-11.73	-11.72	-11.71	-11.7	-11.69	-11.68	-11.67	-11.66	-11.65
-11.73	-11.72	-11.71	-11.7	-11.69	-11.68	-11.67	-11.66	-11.65
-11.73	-11.72	-11.71	-11.7	-11.69	-11.68	-11.67	-11.66	-11.65
-11.73	-11.72	-11.71	-11.7	-11.69	-11.68	-11.67	-11.66	-11.65
-11.73	-11.72	-11.71	-11.7	-11.69	-11.68	-11.67	-11.66	-11.65
-11.73	-11.72	-11.71	-11.7	-11.69	-11.68	-11.67	-11.66	-11.65
-11.73	-11.72	-11.71	-11.7	-11.69	-11.68	-11.67	-11.66	-11.65
-11.73	-11.72	-11.71	-11.7	-11.69	-11.68	-11.67	-11.66	-11.65
-11.73	-11.72	-11.71	-11.7	-11.69	-11.68	-11.67	-11.66	-11.65
-11.73	-11.72	-11.71	-11.7	-11.69	-11.68	-11.67	-11.66	-11.65
-11.73	-11.72	-11.71	-11.7	-11.69	-11.68	-11.67	-11.66	-11.65
-11.73	-11.72	-11.71	-11.7	-11.69	-11.68	-11.67	-11.66	-11.65
-11.73	-11.72	-11.71	-11.7	-11.69	-11.68	-11.67	-11.66	-11.65
-11.73	-11.72	-11.71	-11.7	-11.69	-11.68	-11.67	-11.66	-11.65
-11.73	-11.72	-11.71	-11.7	-11.69	-11.68	-11.67	-11.66	-11.65
-11.73	-11.72	-11.71	-11.7	-11.69	-11.68	-11.67	-11.66	-11.65
-11.73	-11.72	-11.71	-11.7	-11.69	-11.68	-11.67	-11.66	-11.65
-11.73	-11.72	-11.71	-11.7	-11.69	-11.68	-11.67	-11.66	-11.65
-11.73	-11.72	-11.71	-11.7	-11.69	-11.68	-11.67	-11.66	-11.65
-11.73	-11.72	-11.71	-11.7	-11.69	-11.68	-11.67	-11.66	-11.65
-11.73	-11.72	-11.71	-11.7	-11.69	-11.68	-11.67	-11.66	-11.65
-11.73	-11.72	-11.71	-11.7	-11.69	-11.68	-11.67	-11.66	-11.65
-11.73	-11.72	-11.71	-11.7	-11.69	-11.68	-11.67	-11.66	-11.65
-11.73	-11.72	-11.71	-11.7	-11.69	-11.68	-11.67	-11.66	-11.65
-11.73	-11.72	-11.71	-11.7	-11.69	-11.68	-11.67	-11.66	-11.65
-11.73	-11.72	-11.71	-11.7	-11.69	-11.68	-11.67	-11.66	-11.65
-11.73	-11.72	-11.71	-11.7	-11.69	-11.68	-11.67	-11.66	-11.65
-11.73	-11.72	-11.71	-11.7	-11.69	-11.68	-11.67	-11.66	-11.65
-11.73	-11.72	-11.71	-11.7	-11.69	-11.68	-11.67	-11.66	-11.65
-11.73	-11.72	-11.71	-11.7	-11.69	-11.68	-11.67	-11.66	-11.65
-11.73	-11.72	-11.71	-11.7	-11.69	-11.68	-11.67	-11.66	-11.65
-11.73	-11.72	-11.71	-11.7	-11.69	-11.68	-11.67	-11.66	-11.65
-11.73	-11.72	-11.71	-11.7	-11.69	-11.68	-11.67	-11.66	-11.65
-11.73	-11.72	-11.71	-11.7	-11.69	-11.68	-11.67	-11.66	-11.65

-11.73	-11.72	-11.71	-11.7	-11.69	-11.68	-11.67	-11.66	-11.65
-11.73	-11.72	-11.71	-11.7	-11.69	-11.68	-11.67	-11.66	-11.65
-11.73	-11.72	-11.71	-11.7	-11.69	-11.68	-11.67	-11.66	-11.65
-11.73	-11.72	-11.71	-11.7	-11.69	-11.68	-11.67	-11.66	-11.65
-11.73	-11.72	-11.71	-11.7	-11.69	-11.68	-11.67	-11.66	-11.65
-11.73	-11.72	-11.71	-11.7	-11.69	-11.68	-11.67	-11.66	-11.65
-11.73	-11.72	-11.71	-11.7	-11.69	-11.68	-11.67	-11.66	-11.65
-11.73	-11.72	-11.71	-11.7	-11.69	-11.68	-11.67	-11.66	-11.65
-11.73	-11.72	-11.71	-11.7	-11.69	-11.68	-11.67	-11.66	-11.65
-11.73	-11.72	-11.71	-11.7	-11.69	-11.68	-11.67	-11.66	-11.65
-11.73	-11.72	-11.71	-11.7	-11.69	-11.68	-11.67	-11.66	-11.65
-11.73	-11.72	-11.71	-11.7	-11.69	-11.68	-11.67	-11.66	-11.65
-11.73	-11.72	-11.71	-11.7	-11.69	-11.68	-11.67	-11.66	-11.65
-11.73	-11.72	-11.71	-11.7	-11.69	-11.68	-11.67	-11.66	-11.65
-11.73	-11.72	-11.71	-11.7	-11.69	-11.68	-11.67	-11.66	-11.65
-11.73	-11.72	-11.71	-11.7	-11.69	-11.68	-11.67	-11.66	-11.65
-11.73	-11.72	-11.71	-11.7	-11.69	-11.68	-11.67	-11.66	-11.65
-11.73	-11.72	-11.71	-11.7	-11.69	-11.68	-11.67	-11.66	-11.65
-11.73	-11.72	-11.71	-11.7	-11.69	-11.68	-11.67	-11.66	-11.65
-11.73	-11.72	-11.71	-11.7	-11.69	-11.68	-11.67	-11.66	-11.65
-11.73	-11.72	-11.71	-11.7	-11.69	-11.68	-11.67	-11.66	-11.65
-11.73	-11.72	-11.71	-11.7	-11.69	-11.68	-11.67	-11.66	-11.65
-11.73	-11.72	-11.71	-11.7	-11.69	-11.68	-11.67	-11.66	-11.65
-11.73	-11.72	-11.71	-11.7	-11.69	-11.68	-11.67	-11.66	-11.65
-11.73	-11.72	-11.71	-11.7	-11.69	-11.68	-11.67	-11.66	-11.65
-11.73	-11.72	-11.71	-11.7	-11.69	-11.68	-11.67	-11.66	-11.65
-11.73	-11.72	-11.71	-11.7	-11.69	-11.68	-11.67	-11.66	-11.65
-11.73	-11.72	-11.71	-11.7	-11.69	-11.68	-11.67	-11.66	-11.65
-11.73	-11.72	-11.71	-11.7	-11.69	-11.68	-11.67	-11.66	-11.65
-11.73	-11.72	-11.71	-11.7	-11.69	-11.68	-11.67	-11.66	-11.65
-11.73	-11.72	-11.71	-11.7	-11.69	-11.68	-11.67	-11.66	-11.65
-11.73	-11.72	-11.71	-11.7	-11.69	-11.68	-11.67	-11.66	-11.65
-11.73	-11.72	-11.71	-11.7	-11.69	-11.68	-11.67	-11.66	-11.65
-11.73	-11.72	-11.71	-11.7	-11.69	-11.68	-11.67	-11.66	-11.65
-11.73	-11.72	-11.71	-11.7	-11.69	-11.68	-11.67	-11.66	-11.65
-11.73	-11.72	-11.71	-11.7	-11.69	-11.68	-11.67	-11.66	-11.65
-11.73	-11.72	-11.71	-11.7	-11.69	-11.68	-11.67	-11.66	-11.65
-11.73	-11.72	-11.71	-11.7	-11.69	-11.68	-11.67	-11.66	-11.65
-11.73	-11.72	-11.71	-11.7	-11.69	-11.68	-11.67	-11.66	-11.65
-11.73	-11.72	-11.71	-11.7	-11.69	-11.68	-11.67	-11.66	-11.65
-11.73	-11.72	-11.71	-11.7	-11.69	-11.68	-11.67	-11.66	-11.65
-11.73	-11.72	-11.71	-11.7	-11.69	-11.68	-11.67	-11.66	-11.65
-11.73	-11.72	-11.71	-11.7	-11.69	-11.68	-11.67	-11.66	-11.65
-11.73	-11.72	-11.71	-11.7	-11.69	-11.68	-11.67	-11.66	-11.65
-11.73	-11.72	-11.71	-11.7	-11.69	-11.68	-11.67	-11.66	-11.65
-11.73	-11.72	-11.71	-11.7	-11.69	-11.68	-11.67	-11.66	-11.65
-11.73	-11.72	-11.71	-11.7	-11.69	-11.68	-11.67	-11.66	-11.65
-11.73	-11.72	-11.71	-11.7	-11.69	-11.68	-11.67	-11.66	-11.65
-11.73	-11.72	-11.71	-11.7	-11.69	-11.68	-11.67	-11.66	-11.65
-11.73	-11.72	-11.71	-11.7	-11.69	-11.68	-11.67	-11.66	-11.65

-11.73	-11.72	-11.71	-11.7	-11.69	-11.68	-11.67	-11.66	-11.65
-11.73	-11.72	-11.71	-11.7	-11.69	-11.68	-11.67	-11.66	-11.65
-11.73	-11.72	-11.71	-11.7	-11.69	-11.68	-11.67	-11.66	-11.65
-11.73	-11.72	-11.71	-11.7	-11.69	-11.68	-11.67	-11.66	-11.65
-11.73	-11.72	-11.71	-11.7	-11.69	-11.68	-11.67	-11.66	-11.65
-11.73	-11.72	-11.71	-11.7	-11.69	-11.68	-11.67	-11.66	-11.65
-11.73	-11.72	-11.71	-11.7	-11.69	-11.68	-11.67	-11.66	-11.65
-11.73	-11.72	-11.71	-11.7	-11.69	-11.68	-11.67	-11.66	-11.65
-11.73	-11.72	-11.71	-11.7	-11.69	-11.68	-11.67	-11.66	-11.65
-11.73	-11.72	-11.71	-11.7	-11.69	-11.68	-11.67	-11.66	-11.65
-11.73	-11.72	-11.71	-11.7	-11.69	-11.68	-11.67	-11.66	-11.65
-11.73	-11.72	-11.71	-11.7	-11.69	-11.68	-11.67	-11.66	-11.65
-11.73	-11.72	-11.71	-11.7	-11.69	-11.68	-11.67	-11.66	-11.65
-11.73	-11.72	-11.71	-11.7	-11.69	-11.68	-11.67	-11.66	-11.65
-11.73	-11.72	-11.71	-11.7	-11.69	-11.68	-11.67	-11.66	-11.65
-11.73	-11.72	-11.71	-11.7	-11.69	-11.68	-11.67	-11.66	-11.65
-11.73	-11.72	-11.71	-11.7	-11.69	-11.68	-11.67	-11.66	-11.65
-11.73	-11.72	-11.71	-11.7	-11.69	-11.68	-11.67	-11.66	-11.65
-11.73	-11.72	-11.71	-11.7	-11.69	-11.68	-11.67	-11.66	-11.65
-11.73	-11.72	-11.71	-11.7	-11.69	-11.68	-11.67	-11.66	-11.65
-11.73	-11.72	-11.71	-11.7	-11.69	-11.68	-11.67	-11.66	-11.65
-11.73	-11.72	-11.71	-11.7	-11.69	-11.68	-11.67	-11.66	-11.65
-11.73	-11.72	-11.71	-11.7	-11.69	-11.68	-11.67	-11.66	-11.65
-11.73	-11.72	-11.71	-11.7	-11.69	-11.68	-11.67	-11.66	-11.65
-11.73	-11.72	-11.71	-11.7	-11.69	-11.68	-11.67	-11.66	-11.65
-11.73	-11.72	-11.71	-11.7	-11.69	-11.68	-11.67	-11.66	-11.65
-11.73	-11.72	-11.71	-11.7	-11.69	-11.68	-11.67	-11.66	-11.65
-11.73	-11.72	-11.71	-11.7	-11.69	-11.68	-11.67	-11.66	-11.65
-11.73	-11.72	-11.71	-11.7	-11.69	-11.68	-11.67	-11.66	-11.65
-11.73	-11.72	-11.71	-11.7	-11.69	-11.68	-11.67	-11.66	-11.65
-11.73	-11.72	-11.71	-11.7	-11.69	-11.68	-11.67	-11.66	-11.65
-11.73	-11.72	-11.71	-11.7	-11.69	-11.68	-11.67	-11.66	-11.65
-11.73	-11.72	-11.71	-11.7	-11.69	-11.68	-11.67	-11.66	-11.65
-11.73	-11.72	-11.71	-11.7	-11.69	-11.68	-11.67	-11.66	-11.65
-11.73	-11.72	-11.71	-11.7	-11.69	-11.68	-11.67	-11.66	-11.65
-11.73	-11.72	-11.71	-11.7	-11.69	-11.68	-11.67	-11.66	-11.65
-11.73	-11.72	-11.71	-11.7	-11.69	-11.68	-11.67	-11.66	-11.65
-11.73	-11.72	-11.71	-11.7	-11.69	-11.68	-11.67	-11.66	-11.65
-11.73	-11.72	-11.71	-11.7	-11.69	-11.68	-11.67	-11.66	-11.65
-11.73	-11.72	-11.71	-11.7	-11.69	-11.68	-11.67	-11.66	-11.65
-11.73	-11.72	-11.71	-11.7	-11.69	-11.68	-11.67	-11.66	-11.65
-11.73	-11.72	-11.71	-11.7	-11.69	-11.68	-11.67	-11.66	-11.65
-11.73	-11.72	-11.71	-11.7	-11.69	-11.68	-11.67	-11.66	-11.65
-11.73	-11.72	-11.71	-11.7	-11.69	-11.68	-11.67	-11.66	-11.65
-11.73	-11.72	-11.71	-11.7	-11.69	-11.68	-11.67	-11.66	-11.65
-11.73	-11.72	-11.71	-11.7	-11.69	-11.68	-11.67	-11.66	-11.65
-11.73	-11.72	-11.71	-11.7	-11.69	-11.68	-11.67	-11.66	-11.65
-11.73	-11.72	-11.71	-11.7	-11.69	-11.68	-11.67	-11.66	-11.65
-11.73	-11.72	-11.71	-11.7	-11.69	-11.68	-11.67	-11.66	-11.65
-11.73	-11.72	-11.71	-11.7	-11.69	-11.68	-11.67	-11.66	-11.65

-11.73	-11.72	-11.71	-11.7	-11.69	-11.68	-11.67	-11.66	-11.65
-11.73	-11.72	-11.71	-11.7	-11.69	-11.68	-11.67	-11.66	-11.65
-11.73	-11.72	-11.71	-11.7	-11.69	-11.68	-11.67	-11.66	-11.65
-11.73	-11.72	-11.71	-11.7	-11.69	-11.68	-11.67	-11.66	-11.65
-11.73	-11.72	-11.71	-11.7	-11.69	-11.68	-11.67	-11.66	-11.65
-11.73	-11.72	-11.71	-11.7	-11.69	-11.68	-11.67	-11.66	-11.65
-11.73	-11.72	-11.71	-11.7	-11.69	-11.68	-11.67	-11.66	-11.65
-11.73	-11.72	-11.71	-11.7	-11.69	-11.68	-11.67	-11.66	-11.65
-11.73	-11.72	-11.71	-11.7	-11.69	-11.68	-11.67	-11.66	-11.65
-11.73	-11.72	-11.71	-11.7	-11.69	-11.68	-11.67	-11.66	-11.65
-11.73	-11.72	-11.71	-11.7	-11.69	-11.68	-11.67	-11.66	-11.65
-11.73	-11.72	-11.71	-11.7	-11.69	-11.68	-11.67	-11.66	-11.65
-11.73	-11.72	-11.71	-11.7	-11.69	-11.68	-11.67	-11.66	-11.65
-11.73	-11.72	-11.71	-11.7	-11.69	-11.68	-11.67	-11.66	-11.65
-11.73	-11.72	-11.71	-11.7	-11.69	-11.68	-11.67	-11.66	-11.65
-11.73	-11.72	-11.71	-11.7	-11.69	-11.68	-11.67	-11.66	-11.65
-11.73	-11.72	-11.71	-11.7	-11.69	-11.68	-11.67	-11.66	-11.65
-11.73	-11.72	-11.71	-11.7	-11.69	-11.68	-11.67	-11.66	-11.65
-11.73	-11.72	-11.71	-11.7	-11.69	-11.68	-11.67	-11.66	-11.65
-11.73	-11.72	-11.71	-11.7	-11.69	-11.68	-11.67	-11.66	-11.65
-11.73	-11.72	-11.71	-11.7	-11.69	-11.68	-11.67	-11.66	-11.65
-11.73	-11.72	-11.71	-11.7	-11.69	-11.68	-11.67	-11.66	-11.65
-11.73	-11.72	-11.71	-11.7	-11.69	-11.68	-11.67	-11.66	-11.65
-11.73	-11.72	-11.71	-11.7	-11.69	-11.68	-11.67	-11.66	-11.65
-11.73	-11.72	-11.71	-11.7	-11.69	-11.68	-11.67	-11.66	-11.65
-11.73	-11.72	-11.71	-11.7	-11.69	-11.68	-11.67	-11.66	-11.65
-11.73	-11.72	-11.71	-11.7	-11.69	-11.68	-11.67	-11.66	-11.65
-11.73	-11.72	-11.71	-11.7	-11.69	-11.68	-11.67	-11.66	-11.65
-11.73	-11.72	-11.71	-11.7	-11.69	-11.68	-11.67	-11.66	-11.65
-11.73	-11.72	-11.71	-11.7	-11.69	-11.68	-11.67	-11.66	-11.65
-11.73	-11.72	-11.71	-11.7	-11.69	-11.68	-11.67	-11.66	-11.65
-11.73	-11.72	-11.71	-11.7	-11.69	-11.68	-11.67	-11.66	-11.65
-11.73	-11.72	-11.71	-11.7	-11.69	-11.68	-11.67	-11.66	-11.65
-11.73	-11.72	-11.71	-11.7	-11.69	-11.68	-11.67	-11.66	-11.65
-11.73	-11.72	-11.71	-11.7	-11.69	-11.68	-11.67	-11.66	-11.65
-11.73	-11.72	-11.71	-11.7	-11.69	-11.68	-11.67	-11.66	-11.65
-11.73	-11.72	-11.71	-11.7	-11.69	-11.68	-11.67	-11.66	-11.65
-11.73	-11.72	-11.71	-11.7	-11.69	-11.68	-11.67	-11.66	-11.65
-11.73	-11.72	-11.71	-11.7	-11.69	-11.68	-11.67	-11.66	-11.65
-11.73	-11.72	-11.71	-11.7	-11.69	-11.68	-11.67	-11.66	-11.65
-11.73	-11.72	-11.71	-11.7	-11.69	-11.68	-11.67	-11.66	-11.65
-11.73	-11.72	-11.71	-11.7	-11.69	-11.68	-11.67	-11.66	-11.65
-11.73	-11.72	-11.71	-11.7	-11.69	-11.68	-11.67	-11.66	-11.65
-11.73	-11.72	-11.71	-11.7	-11.69	-11.68	-11.67	-11.66	-11.65
-11.73	-11.72	-11.71	-11.7	-11.69	-11.68	-11.67	-11.66	-11.65
-11.73	-11.72	-11.71	-11.7	-11.69	-11.68	-11.67	-11.66	-11.65
-11.73	-11.72	-11.71	-11.7	-11.69	-11.68	-11.67	-11.66	-11.65
-11.73	-11.72	-11.71	-11.7	-11.69	-11.68	-11.67	-11.66	-11.65
-11.73	-11.72	-11.71	-11.7	-11.69	-11.68	-11.67	-11.66	-11.65
-11.73	-11.72	-11.71	-11.7	-11.69	-11.68	-11.67	-11.66	-11.65

-11.73	-11.72	-11.71	-11.7	-11.69	-11.68	-11.67	-11.66	-11.65
-11.73	-11.72	-11.71	-11.7	-11.69	-11.68	-11.67	-11.66	-11.65
-11.73	-11.72	-11.71	-11.7	-11.69	-11.68	-11.67	-11.66	-11.65
-11.73	-11.72	-11.71	-11.7	-11.69	-11.68	-11.67	-11.66	-11.65
-11.73	-11.72	-11.71	-11.7	-11.69	-11.68	-11.67	-11.66	-11.65
-11.73	-11.72	-11.71	-11.7	-11.69	-11.68	-11.67	-11.66	-11.65
-11.73	-11.72	-11.71	-11.7	-11.69	-11.68	-11.67	-11.66	-11.65
-11.73	-11.72	-11.71	-11.7	-11.69	-11.68	-11.67	-11.66	-11.65
-11.73	-11.72	-11.71	-11.7	-11.69	-11.68	-11.67	-11.66	-11.65
-11.73	-11.72	-11.71	-11.7	-11.69	-11.68	-11.67	-11.66	-11.65
-11.73	-11.72	-11.71	-11.7	-11.69	-11.68	-11.67	-11.66	-11.65
-11.73	-11.72	-11.71	-11.7	-11.69	-11.68	-11.67	-11.66	-11.65
-11.73	-11.72	-11.71	-11.7	-11.69	-11.68	-11.67	-11.66	-11.65
-11.73	-11.72	-11.71	-11.7	-11.69	-11.68	-11.67	-11.66	-11.65
-11.73	-11.72	-11.71	-11.7	-11.69	-11.68	-11.67	-11.66	-11.65
-11.73	-11.72	-11.71	-11.7	-11.69	-11.68	-11.67	-11.66	-11.65
-11.73	-11.72	-11.71	-11.7	-11.69	-11.68	-11.67	-11.66	-11.65
-11.73	-11.72	-11.71	-11.7	-11.69	-11.68	-11.67	-11.66	-11.65
-11.73	-11.72	-11.71	-11.7	-11.69	-11.68	-11.67	-11.66	-11.65
-11.73	-11.72	-11.71	-11.7	-11.69	-11.68	-11.67	-11.66	-11.65
-11.73	-11.72	-11.71	-11.7	-11.69	-11.68	-11.67	-11.66	-11.65
-11.73	-11.72	-11.71	-11.7	-11.69	-11.68	-11.67	-11.66	-11.65
-11.73	-11.72	-11.71	-11.7	-11.69	-11.68	-11.67	-11.66	-11.65
-11.73	-11.72	-11.71	-11.7	-11.69	-11.68	-11.67	-11.66	-11.65
-11.73	-11.72	-11.71	-11.7	-11.69	-11.68	-11.67	-11.66	-11.65
-11.73	-11.72	-11.71	-11.7	-11.69	-11.68	-11.67	-11.66	-11.65
-11.73	-11.72	-11.71	-11.7	-11.69	-11.68	-11.67	-11.66	-11.65
-11.73	-11.72	-11.71	-11.7	-11.69	-11.68	-11.67	-11.66	-11.65
-11.73	-11.72	-11.71	-11.7	-11.69	-11.68	-11.67	-11.66	-11.65
-11.73	-11.72	-11.71	-11.7	-11.69	-11.68	-11.67	-11.66	-11.65
-11.73	-11.72	-11.71	-11.7	-11.69	-11.68	-11.67	-11.66	-11.65
-11.73	-11.72	-11.71	-11.7	-11.69	-11.68	-11.67	-11.66	-11.65
-11.73	-11.72	-11.71	-11.7	-11.69	-11.68	-11.67	-11.66	-11.65
-11.73	-11.72	-11.71	-11.7	-11.69	-11.68	-11.67	-11.66	-11.65
-11.73	-11.72	-11.71	-11.7	-11.69	-11.68	-11.67	-11.66	-11.65
-11.73	-11.72	-11.71	-11.7	-11.69	-11.68	-11.67	-11.66	-11.65
-11.73	-11.72	-11.71	-11.7	-11.69	-11.68	-11.67	-11.66	-11.65
-11.73	-11.72	-11.71	-11.7	-11.69	-11.68	-11.67	-11.66	-11.65
-11.73	-11.72	-11.71	-11.7	-11.69	-11.68	-11.67	-11.66	-11.65
-11.73	-11.72	-11.71	-11.7	-11.69	-11.68	-11.67	-11.66	-11.65
-11.73	-11.72	-11.71	-11.7	-11.69	-11.68	-11.67	-11.66	-11.65
-11.73	-11.72	-11.71	-11.7	-11.69	-11.68	-11.67	-11.66	-11.65
-11.73	-11.72	-11.71	-11.7	-11.69	-11.68	-11.67	-11.66	-11.65
-11.73	-11.72	-11.71	-11.7	-11.69	-11.68	-11.67	-11.66	-11.65
-11.73	-11.72	-11.71	-11.7	-11.69	-11.68	-11.67	-11.66	-11.65
-11.73	-11.72	-11.71	-11.7	-11.69	-11.68	-11.67	-11.66	-11.65
-11.73	-11.72	-11.71	-11.7	-11.69	-11.68	-11.67	-11.66	-11.65
-11.73	-11.72	-11.71	-11.7	-11.69	-11.68	-11.67	-11.66	-11.65
-11.73	-11.72	-11.71	-11.7	-11.69	-11.68	-11.67	-11.66	-11.65
-11.73	-11.72	-11.71	-11.7	-11.69	-11.68	-11.67	-11.66	-11.65

-11.73	-11.72	-11.71	-11.7	-11.69	-11.68	-11.67	-11.66	-11.65
-11.73	-11.72	-11.71	-11.7	-11.69	-11.68	-11.67	-11.66	-11.65
-11.73	-11.72	-11.71	-11.7	-11.69	-11.68	-11.67	-11.66	-11.65
-11.73	-11.72	-11.71	-11.7	-11.69	-11.68	-11.67	-11.66	-11.65
-11.73	-11.72	-11.71	-11.7	-11.69	-11.68	-11.67	-11.66	-11.65
-11.73	-11.72	-11.71	-11.7	-11.69	-11.68	-11.67	-11.66	-11.65
-11.73	-11.72	-11.71	-11.7	-11.69	-11.68	-11.67	-11.66	-11.65
-11.73	-11.72	-11.71	-11.7	-11.69	-11.68	-11.67	-11.66	-11.65
-11.73	-11.72	-11.71	-11.7	-11.69	-11.68	-11.67	-11.66	-11.65
-11.73	-11.72	-11.71	-11.7	-11.69	-11.68	-11.67	-11.66	-11.65
-11.73	-11.72	-11.71	-11.7	-11.69	-11.68	-11.67	-11.66	-11.65
-11.73	-11.72	-11.71	-11.7	-11.69	-11.68	-11.67	-11.66	-11.65
-11.73	-11.72	-11.71	-11.7	-11.69	-11.68	-11.67	-11.66	-11.65
-11.73	-11.72	-11.71	-11.7	-11.69	-11.68	-11.67	-11.66	-11.65
-11.73	-11.72	-11.71	-11.7	-11.69	-11.68	-11.67	-11.66	-11.65
-11.73	-11.72	-11.71	-11.7	-11.69	-11.68	-11.67	-11.66	-11.65
-11.73	-11.72	-11.71	-11.7	-11.69	-11.68	-11.67	-11.66	-11.65
-11.73	-11.72	-11.71	-11.7	-11.69	-11.68	-11.67	-11.66	-11.65
-11.73	-11.72	-11.71	-11.7	-11.69	-11.68	-11.67	-11.66	-11.65
-11.73	-11.72	-11.71	-11.7	-11.69	-11.68	-11.67	-11.66	-11.65
-11.73	-11.72	-11.71	-11.7	-11.69	-11.68	-11.67	-11.66	-11.65
-11.73	-11.72	-11.71	-11.7	-11.69	-11.68	-11.67	-11.66	-11.65
-11.73	-11.72	-11.71	-11.7	-11.69	-11.68	-11.67	-11.66	-11.65
-11.73	-11.72	-11.71	-11.7	-11.69	-11.68	-11.67	-11.66	-11.65
-11.73	-11.72	-11.71	-11.7	-11.69	-11.68	-11.67	-11.66	-11.65
-11.73	-11.72	-11.71	-11.7	-11.69	-11.68	-11.67	-11.66	-11.65
-11.73	-11.72	-11.71	-11.7	-11.69	-11.68	-11.67	-11.66	-11.65
-11.73	-11.72	-11.71	-11.7	-11.69	-11.68	-11.67	-11.66	-11.65
-11.73	-11.72	-11.71	-11.7	-11.69	-11.68	-11.67	-11.66	-11.65
-11.73	-11.72	-11.71	-11.7	-11.69	-11.68	-11.67	-11.66	-11.65
-11.73	-11.72	-11.71	-11.7	-11.69	-11.68	-11.67	-11.66	-11.65
-11.73	-11.72	-11.71	-11.7	-11.69	-11.68	-11.67	-11.66	-11.65
-11.73	-11.72	-11.71	-11.7	-11.69	-11.68	-11.67	-11.66	-11.65
-11.73	-11.72	-11.71	-11.7	-11.69	-11.68	-11.67	-11.66	-11.65
-11.73	-11.72	-11.71	-11.7	-11.69	-11.68	-11.67	-11.66	-11.65
-11.73	-11.72	-11.71	-11.7	-11.69	-11.68	-11.67	-11.66	-11.65
-11.73	-11.72	-11.71	-11.7	-11.69	-11.68	-11.67	-11.66	-11.65
-11.73	-11.72	-11.71	-11.7	-11.69	-11.68	-11.67	-11.66	-11.65
-11.73	-11.72	-11.71	-11.7	-11.69	-11.68	-11.67	-11.66	-11.65
-11.73	-11.72	-11.71	-11.7	-11.69	-11.68	-11.67	-11.66	-11.65
-11.73	-11.72	-11.71	-11.7	-11.69	-11.68	-11.67	-11.66	-11.65
-11.73	-11.72	-11.71	-11.7	-11.69	-11.68	-11.67	-11.66	-11.65
-11.73	-11.72	-11.71	-11.7	-11.69	-11.68	-11.67	-11.66	-11.65
-11.73	-11.72	-11.71	-11.7	-11.69	-11.68	-11.67	-11.66	-11.65
-11.73	-11.72	-11.71	-11.7	-11.69	-11.68	-11.67	-11.66	-11.65
-11.73	-11.72	-11.71	-11.7	-11.69	-11.68	-11.67	-11.66	-11.65
-11.73	-11.72	-11.71	-11.7	-11.69	-11.68	-11.67	-11.66	-11.65
-11.73	-11.72	-11.71	-11.7	-11.69	-11.68	-11.67	-11.66	-11.65
-11.73	-11.72	-11.71	-11.7	-11.69	-11.68	-11.67	-11.66	-11.65
-11.73	-11.72	-11.71	-11.7	-11.69	-11.68	-11.67	-11.66	-11.65

-11.73	-11.72	-11.71	-11.7	-11.69	-11.68	-11.67	-11.66	-11.65
-11.73	-11.72	-11.71	-11.7	-11.69	-11.68	-11.67	-11.66	-11.65
-11.73	-11.72	-11.71	-11.7	-11.69	-11.68	-11.67	-11.66	-11.65
-11.73	-11.72	-11.71	-11.7	-11.69	-11.68	-11.67	-11.66	-11.65
-11.73	-11.72	-11.71	-11.7	-11.69	-11.68	-11.67	-11.66	-11.65
-11.73	-11.72	-11.71	-11.7	-11.69	-11.68	-11.67	-11.66	-11.65
-11.73	-11.72	-11.71	-11.7	-11.69	-11.68	-11.67	-11.66	-11.65
-11.73	-11.72	-11.71	-11.7	-11.69	-11.68	-11.67	-11.66	-11.65
-11.73	-11.72	-11.71	-11.7	-11.69	-11.68	-11.67	-11.66	-11.65
-11.73	-11.72	-11.71	-11.7	-11.69	-11.68	-11.67	-11.66	-11.65
-11.73	-11.72	-11.71	-11.7	-11.69	-11.68	-11.67	-11.66	-11.65
-11.73	-11.72	-11.71	-11.7	-11.69	-11.68	-11.67	-11.66	-11.65
-11.73	-11.72	-11.71	-11.7	-11.69	-11.68	-11.67	-11.66	-11.65
-11.73	-11.72	-11.71	-11.7	-11.69	-11.68	-11.67	-11.66	-11.65
-11.73	-11.72	-11.71	-11.7	-11.69	-11.68	-11.67	-11.66	-11.65
-11.73	-11.72	-11.71	-11.7	-11.69	-11.68	-11.67	-11.66	-11.65
-11.73	-11.72	-11.71	-11.7	-11.69	-11.68	-11.67	-11.66	-11.65
-11.73	-11.72	-11.71	-11.7	-11.69	-11.68	-11.67	-11.66	-11.65
-11.73	-11.72	-11.71	-11.7	-11.69	-11.68	-11.67	-11.66	-11.65
-11.73	-11.72	-11.71	-11.7	-11.69	-11.68	-11.67	-11.66	-11.65
-11.73	-11.72	-11.71	-11.7	-11.69	-11.68	-11.67	-11.66	-11.65
-11.73	-11.72	-11.71	-11.7	-11.69	-11.68	-11.67	-11.66	-11.65
-11.73	-11.72	-11.71	-11.7	-11.69	-11.68	-11.67	-11.66	-11.65
-11.73	-11.72	-11.71	-11.7	-11.69	-11.68	-11.67	-11.66	-11.65
-11.73	-11.72	-11.71	-11.7	-11.69	-11.68	-11.67	-11.66	-11.65
-11.73	-11.72	-11.71	-11.7	-11.69	-11.68	-11.67	-11.66	-11.65
-11.73	-11.72	-11.71	-11.7	-11.69	-11.68	-11.67	-11.66	-11.65
-11.73	-11.72	-11.71	-11.7	-11.69	-11.68	-11.67	-11.66	-11.65
-11.73	-11.72	-11.71	-11.7	-11.69	-11.68	-11.67	-11.66	-11.65
-11.73	-11.72	-11.71	-11.7	-11.69	-11.68	-11.67	-11.66	-11.65
-11.73	-11.72	-11.71	-11.7	-11.69	-11.68	-11.67	-11.66	-11.65
-11.73	-11.72	-11.71	-11.7	-11.69	-11.68	-11.67	-11.66	-11.65
-11.73	-11.72	-11.71	-11.7	-11.69	-11.68	-11.67	-11.66	-11.65
-11.73	-11.72	-11.71	-11.7	-11.69	-11.68	-11.67	-11.66	-11.65
-11.73	-11.72	-11.71	-11.7	-11.69	-11.68	-11.67	-11.66	-11.65
-11.73	-11.72	-11.71	-11.7	-11.69	-11.68	-11.67	-11.66	-11.65
-11.73	-11.72	-11.71	-11.7	-11.69	-11.68	-11.67	-11.66	-11.65
-11.73	-11.72	-11.71	-11.7	-11.69	-11.68	-11.67	-11.66	-11.65
-11.73	-11.72	-11.71	-11.7	-11.69	-11.68	-11.67	-11.66	-11.65
-11.73	-11.72	-11.71	-11.7	-11.69	-11.68	-11.67	-11.66	-11.65
-11.73	-11.72	-11.71	-11.7	-11.69	-11.68	-11.67	-11.66	-11.65
-11.73	-11.72	-11.71	-11.7	-11.69	-11.68	-11.67	-11.66	-11.65
-11.73	-11.72	-11.71	-11.7	-11.69	-11.68	-11.67	-11.66	-11.65
-11.73	-11.72	-11.71	-11.7	-11.69	-11.68	-11.67	-11.66	-11.65
-11.73	-11.72	-11.71	-11.7	-11.69	-11.68	-11.67	-11.66	-11.65
-11.73	-11.72	-11.71	-11.7	-11.69	-11.68	-11.67	-11.66	-11.65
-11.73	-11.72	-11.71	-11.7	-11.69	-11.68	-11.67	-11.66	-11.65
-11.73	-11.72	-11.71	-11.7	-11.69	-11.68	-11.67	-11.66	-11.65
-11.73	-11.72	-11.71	-11.7	-11.69	-11.68	-11.67	-11.66	-11.65
-11.73	-11.72	-11.71	-11.7	-11.69	-11.68	-11.67	-11.66	-11.65

-11.73	-11.72	-11.71	-11.7	-11.69	-11.68	-11.67	-11.66	-11.65

-11.64	-11.63	-11.62	-11.61	-11.6	-11.59	-11.58	-11.57	-11.56
-11.64	-11.63	-11.62	-11.61	-11.6	-11.59	-11.58	-11.57	-11.56
-11.64	-11.63	-11.62	-11.61	-11.6	-11.59	-11.58	-11.57	-11.56
-11.64	-11.63	-11.62	-11.61	-11.6	-11.59	-11.58	-11.57	-11.56
-11.64	-11.63	-11.62	-11.61	-11.6	-11.59	-11.58	-11.57	-11.56
-11.64	-11.63	-11.62	-11.61	-11.6	-11.59	-11.58	-11.57	-11.56
-11.64	-11.63	-11.62	-11.61	-11.6	-11.59	-11.58	-11.57	-11.56
-11.64	-11.63	-11.62	-11.61	-11.6	-11.59	-11.58	-11.57	-11.56
-11.64	-11.63	-11.62	-11.61	-11.6	-11.59	-11.58	-11.57	-11.56
-11.64	-11.63	-11.62	-11.61	-11.6	-11.59	-11.58	-11.57	-11.56
-11.64	-11.63	-11.62	-11.61	-11.6	-11.59	-11.58	-11.57	-11.56
-11.64	-11.63	-11.62	-11.61	-11.6	-11.59	-11.58	-11.57	-11.56
-11.64	-11.63	-11.62	-11.61	-11.6	-11.59	-11.58	-11.57	-11.56
-11.64	-11.63	-11.62	-11.61	-11.6	-11.59	-11.58	-11.57	-11.56
-11.64	-11.63	-11.62	-11.61	-11.6	-11.59	-11.58	-11.57	-11.56
-11.64	-11.63	-11.62	-11.61	-11.6	-11.59	-11.58	-11.57	-11.56
-11.64	-11.63	-11.62	-11.61	-11.6	-11.59	-11.58	-11.57	-11.56
-11.64	-11.63	-11.62	-11.61	-11.6	-11.59	-11.58	-11.57	-11.56
-11.64	-11.63	-11.62	-11.61	-11.6	-11.59	-11.58	-11.57	-11.56
-11.64	-11.63	-11.62	-11.61	-11.6	-11.59	-11.58	-11.57	-11.56
-11.64	-11.63	-11.62	-11.61	-11.6	-11.59	-11.58	-11.57	-11.56
-11.64	-11.63	-11.62	-11.61	-11.6	-11.59	-11.58	-11.57	-11.56
-11.64	-11.63	-11.62	-11.61	-11.6	-11.59	-11.58	-11.57	-11.56
-11.64	-11.63	-11.62	-11.61	-11.6	-11.59	-11.58	-11.57	-11.56
-11.64	-11.63	-11.62	-11.61	-11.6	-11.59	-11.58	-11.57	-11.56
-11.64	-11.63	-11.62	-11.61	-11.6	-11.59	-11.58	-11.57	-11.56
-11.64	-11.63	-11.62	-11.61	-11.6	-11.59	-11.58	-11.57	-11.56
-11.64	-11.63	-11.62	-11.61	-11.6	-11.59	-11.58	-11.57	-11.56
-11.64	-11.63	-11.62	-11.61	-11.6	-11.59	-11.58	-11.57	-11.56
-11.64	-11.63	-11.62	-11.61	-11.6	-11.59	-11.58	-11.57	-11.56
-11.64	-11.63	-11.62	-11.61	-11.6	-11.59	-11.58	-11.57	-11.56
-11.64	-11.63	-11.62	-11.61	-11.6	-11.59	-11.58	-11.57	-11.56
-11.64	-11.63	-11.62	-11.61	-11.6	-11.59	-11.58	-11.57	-11.56
-11.64	-11.63	-11.62	-11.61	-11.6	-11.59	-11.58	-11.57	-11.56
-11.64	-11.63	-11.62	-11.61	-11.6	-11.59	-11.58	-11.57	-11.56
-11.64	-11.63	-11.62	-11.61	-11.6	-11.59	-11.58	-11.57	-11.56
-11.64	-11.63	-11.62	-11.61	-11.6	-11.59	-11.58	-11.57	-11.56
-11.64	-11.63	-11.62	-11.61	-11.6	-11.59	-11.58	-11.57	-11.56
-11.64	-11.63	-11.62	-11.61	-11.6	-11.59	-11.58	-11.57	-11.56
-11.64	-11.63	-11.62	-11.61	-11.6	-11.59	-11.58	-11.57	-11.56
-11.64	-11.63	-11.62	-11.61	-11.6	-11.59	-11.58	-11.57	-11.56
-11.64	-11.63	-11.62	-11.61	-11.6	-11.59	-11.58	-11.57	-11.56
-11.64	-11.63	-11.62	-11.61	-11.6	-11.59	-11.58	-11.57	-11.56
-11.64	-11.63	-11.62	-11.61	-11.6	-11.59	-11.58	-11.57	-11.56
-11.64	-11.63	-11.62	-11.61	-11.6	-11.59	-11.58	-11.57	-11.56
-11.64	-11.63	-11.62	-11.61	-11.6	-11.59	-11.58	-11.57	-11.56
-11.64	-11.63	-11.62	-11.61	-11.6	-11.59	-11.58	-11.57	-11.56
-11.64	-11.63	-11.62	-11.61	-11.6	-11.59	-11.58	-11.57	-11.56
-11.64	-11.63	-11.62	-11.61	-11.6	-11.59	-11.58	-11.57	-11.56
-11.64	-11.63	-11.62	-11.61	-11.6	-11.59	-11.58	-11.57	-11.56

-11.64	-11.63	-11.62	-11.61	-11.6	-11.59	-11.58	-11.57	-11.56
-11.64	-11.63	-11.62	-11.61	-11.6	-11.59	-11.58	-11.57	-11.56
-11.64	-11.63	-11.62	-11.61	-11.6	-11.59	-11.58	-11.57	-11.56
-11.64	-11.63	-11.62	-11.61	-11.6	-11.59	-11.58	-11.57	-11.56
-11.64	-11.63	-11.62	-11.61	-11.6	-11.59	-11.58	-11.57	-11.56
-11.64	-11.63	-11.62	-11.61	-11.6	-11.59	-11.58	-11.57	-11.56
-11.64	-11.63	-11.62	-11.61	-11.6	-11.59	-11.58	-11.57	-11.56
-11.64	-11.63	-11.62	-11.61	-11.6	-11.59	-11.58	-11.57	-11.56
-11.64	-11.63	-11.62	-11.61	-11.6	-11.59	-11.58	-11.57	-11.56
-11.64	-11.63	-11.62	-11.61	-11.6	-11.59	-11.58	-11.57	-11.56
-11.64	-11.63	-11.62	-11.61	-11.6	-11.59	-11.58	-11.57	-11.56
-11.64	-11.63	-11.62	-11.61	-11.6	-11.59	-11.58	-11.57	-11.56
-11.64	-11.63	-11.62	-11.61	-11.6	-11.59	-11.58	-11.57	-11.56
-11.64	-11.63	-11.62	-11.61	-11.6	-11.59	-11.58	-11.57	-11.56
-11.64	-11.63	-11.62	-11.61	-11.6	-11.59	-11.58	-11.57	-11.56
-11.64	-11.63	-11.62	-11.61	-11.6	-11.59	-11.58	-11.57	-11.56
-11.64	-11.63	-11.62	-11.61	-11.6	-11.59	-11.58	-11.57	-11.56
-11.64	-11.63	-11.62	-11.61	-11.6	-11.59	-11.58	-11.57	-11.56
-11.64	-11.63	-11.62	-11.61	-11.6	-11.59	-11.58	-11.57	-11.56
-11.64	-11.63	-11.62	-11.61	-11.6	-11.59	-11.58	-11.57	-11.56
-11.64	-11.63	-11.62	-11.61	-11.6	-11.59	-11.58	-11.57	-11.56
-11.64	-11.63	-11.62	-11.61	-11.6	-11.59	-11.58	-11.57	-11.56
-11.64	-11.63	-11.62	-11.61	-11.6	-11.59	-11.58	-11.57	-11.56
-11.64	-11.63	-11.62	-11.61	-11.6	-11.59	-11.58	-11.57	-11.56
-11.64	-11.63	-11.62	-11.61	-11.6	-11.59	-11.58	-11.57	-11.56
-11.64	-11.63	-11.62	-11.61	-11.6	-11.59	-11.58	-11.57	-11.56
-11.64	-11.63	-11.62	-11.61	-11.6	-11.59	-11.58	-11.57	-11.56
-11.64	-11.63	-11.62	-11.61	-11.6	-11.59	-11.58	-11.57	-11.56
-11.64	-11.63	-11.62	-11.61	-11.6	-11.59	-11.58	-11.57	-11.56
-11.64	-11.63	-11.62	-11.61	-11.6	-11.59	-11.58	-11.57	-11.56
-11.64	-11.63	-11.62	-11.61	-11.6	-11.59	-11.58	-11.57	-11.56
-11.64	-11.63	-11.62	-11.61	-11.6	-11.59	-11.58	-11.57	-11.56
-11.64	-11.63	-11.62	-11.61	-11.6	-11.59	-11.58	-11.57	-11.56
-11.64	-11.63	-11.62	-11.61	-11.6	-11.59	-11.58	-11.57	-11.56
-11.64	-11.63	-11.62	-11.61	-11.6	-11.59	-11.58	-11.57	-11.56
-11.64	-11.63	-11.62	-11.61	-11.6	-11.59	-11.58	-11.57	-11.56
-11.64	-11.63	-11.62	-11.61	-11.6	-11.59	-11.58	-11.57	-11.56
-11.64	-11.63	-11.62	-11.61	-11.6	-11.59	-11.58	-11.57	-11.56
-11.64	-11.63	-11.62	-11.61	-11.6	-11.59	-11.58	-11.57	-11.56
-11.64	-11.63	-11.62	-11.61	-11.6	-11.59	-11.58	-11.57	-11.56
-11.64	-11.63	-11.62	-11.61	-11.6	-11.59	-11.58	-11.57	-11.56
-11.64	-11.63	-11.62	-11.61	-11.6	-11.59	-11.58	-11.57	-11.56
-11.64	-11.63	-11.62	-11.61	-11.6	-11.59	-11.58	-11.57	-11.56
-11.64	-11.63	-11.62	-11.61	-11.6	-11.59	-11.58	-11.57	-11.56
-11.64	-11.63	-11.62	-11.61	-11.6	-11.59	-11.58	-11.57	-11.56
-11.64	-11.63	-11.62	-11.61	-11.6	-11.59	-11.58	-11.57	-11.56
-11.64	-11.63	-11.62	-11.61	-11.6	-11.59	-11.58	-11.57	-11.56
-11.64	-11.63	-11.62	-11.61	-11.6	-11.59	-11.58	-11.57	-11.56
-11.64	-11.63	-11.62	-11.61	-11.6	-11.59	-11.58	-11.57	-11.56
-11.64	-11.63	-11.62	-11.61	-11.6	-11.59	-11.58	-11.57	-11.56

-11.64	-11.63	-11.62	-11.61	-11.6	-11.59	-11.58	-11.57	-11.56
-11.64	-11.63	-11.62	-11.61	-11.6	-11.59	-11.58	-11.57	-11.56
-11.64	-11.63	-11.62	-11.61	-11.6	-11.59	-11.58	-11.57	-11.56
-11.64	-11.63	-11.62	-11.61	-11.6	-11.59	-11.58	-11.57	-11.56
-11.64	-11.63	-11.62	-11.61	-11.6	-11.59	-11.58	-11.57	-11.56
-11.64	-11.63	-11.62	-11.61	-11.6	-11.59	-11.58	-11.57	-11.56
-11.64	-11.63	-11.62	-11.61	-11.6	-11.59	-11.58	-11.57	-11.56
-11.64	-11.63	-11.62	-11.61	-11.6	-11.59	-11.58	-11.57	-11.56
-11.64	-11.63	-11.62	-11.61	-11.6	-11.59	-11.58	-11.57	-11.56
-11.64	-11.63	-11.62	-11.61	-11.6	-11.59	-11.58	-11.57	-11.56
-11.64	-11.63	-11.62	-11.61	-11.6	-11.59	-11.58	-11.57	-11.56
-11.64	-11.63	-11.62	-11.61	-11.6	-11.59	-11.58	-11.57	-11.56
-11.64	-11.63	-11.62	-11.61	-11.6	-11.59	-11.58	-11.57	-11.56
-11.64	-11.63	-11.62	-11.61	-11.6	-11.59	-11.58	-11.57	-11.56
-11.64	-11.63	-11.62	-11.61	-11.6	-11.59	-11.58	-11.57	-11.56
-11.64	-11.63	-11.62	-11.61	-11.6	-11.59	-11.58	-11.57	-11.56
-11.64	-11.63	-11.62	-11.61	-11.6	-11.59	-11.58	-11.57	-11.56
-11.64	-11.63	-11.62	-11.61	-11.6	-11.59	-11.58	-11.57	-11.56
-11.64	-11.63	-11.62	-11.61	-11.6	-11.59	-11.58	-11.57	-11.56
-11.64	-11.63	-11.62	-11.61	-11.6	-11.59	-11.58	-11.57	-11.56
-11.64	-11.63	-11.62	-11.61	-11.6	-11.59	-11.58	-11.57	-11.56
-11.64	-11.63	-11.62	-11.61	-11.6	-11.59	-11.58	-11.57	-11.56
-11.64	-11.63	-11.62	-11.61	-11.6	-11.59	-11.58	-11.57	-11.56
-11.64	-11.63	-11.62	-11.61	-11.6	-11.59	-11.58	-11.57	-11.56
-11.64	-11.63	-11.62	-11.61	-11.6	-11.59	-11.58	-11.57	-11.56
-11.64	-11.63	-11.62	-11.61	-11.6	-11.59	-11.58	-11.57	-11.56
-11.64	-11.63	-11.62	-11.61	-11.6	-11.59	-11.58	-11.57	-11.56
-11.64	-11.63	-11.62	-11.61	-11.6	-11.59	-11.58	-11.57	-11.56
-11.64	-11.63	-11.62	-11.61	-11.6	-11.59	-11.58	-11.57	-11.56
-11.64	-11.63	-11.62	-11.61	-11.6	-11.59	-11.58	-11.57	-11.56
-11.64	-11.63	-11.62	-11.61	-11.6	-11.59	-11.58	-11.57	-11.56
-11.64	-11.63	-11.62	-11.61	-11.6	-11.59	-11.58	-11.57	-11.56
-11.64	-11.63	-11.62	-11.61	-11.6	-11.59	-11.58	-11.57	-11.56
-11.64	-11.63	-11.62	-11.61	-11.6	-11.59	-11.58	-11.57	-11.56
-11.64	-11.63	-11.62	-11.61	-11.6	-11.59	-11.58	-11.57	-11.56
-11.64	-11.63	-11.62	-11.61	-11.6	-11.59	-11.58	-11.57	-11.56
-11.64	-11.63	-11.62	-11.61	-11.6	-11.59	-11.58	-11.57	-11.56
-11.64	-11.63	-11.62	-11.61	-11.6	-11.59	-11.58	-11.57	-11.56
-11.64	-11.63	-11.62	-11.61	-11.6	-11.59	-11.58	-11.57	-11.56
-11.64	-11.63	-11.62	-11.61	-11.6	-11.59	-11.58	-11.57	-11.56
-11.64	-11.63	-11.62	-11.61	-11.6	-11.59	-11.58	-11.57	-11.56
-11.64	-11.63	-11.62	-11.61	-11.6	-11.59	-11.58	-11.57	-11.56
-11.64	-11.63	-11.62	-11.61	-11.6	-11.59	-11.58	-11.57	-11.56
-11.64	-11.63	-11.62	-11.61	-11.6	-11.59	-11.58	-11.57	-11.56
-11.64	-11.63	-11.62	-11.61	-11.6	-11.59	-11.58	-11.57	-11.56
-11.64	-11.63	-11.62	-11.61	-11.6	-11.59	-11.58	-11.57	-11.56
-11.64	-11.63	-11.62	-11.61	-11.6	-11.59	-11.58	-11.57	-11.56
-11.64	-11.63	-11.62	-11.61	-11.6	-11.59	-11.58	-11.57	-11.56
-11.64	-11.63	-11.62	-11.61	-11.6	-11.59	-11.58	-11.57	-11.56
-11.64	-11.63	-11.62	-11.61	-11.6	-11.59	-11.58	-11.57	-11.56

-11.64	-11.63	-11.62	-11.61	-11.6	-11.59	-11.58	-11.57	-11.56
-11.64	-11.63	-11.62	-11.61	-11.6	-11.59	-11.58	-11.57	-11.56
-11.64	-11.63	-11.62	-11.61	-11.6	-11.59	-11.58	-11.57	-11.56
-11.64	-11.63	-11.62	-11.61	-11.6	-11.59	-11.58	-11.57	-11.56
-11.64	-11.63	-11.62	-11.61	-11.6	-11.59	-11.58	-11.57	-11.56
-11.64	-11.63	-11.62	-11.61	-11.6	-11.59	-11.58	-11.57	-11.56
-11.64	-11.63	-11.62	-11.61	-11.6	-11.59	-11.58	-11.57	-11.56
-11.64	-11.63	-11.62	-11.61	-11.6	-11.59	-11.58	-11.57	-11.56
-11.64	-11.63	-11.62	-11.61	-11.6	-11.59	-11.58	-11.57	-11.56
-11.64	-11.63	-11.62	-11.61	-11.6	-11.59	-11.58	-11.57	-11.56
-11.64	-11.63	-11.62	-11.61	-11.6	-11.59	-11.58	-11.57	-11.56
-11.64	-11.63	-11.62	-11.61	-11.6	-11.59	-11.58	-11.57	-11.56
-11.64	-11.63	-11.62	-11.61	-11.6	-11.59	-11.58	-11.57	-11.56
-11.64	-11.63	-11.62	-11.61	-11.6	-11.59	-11.58	-11.57	-11.56
-11.64	-11.63	-11.62	-11.61	-11.6	-11.59	-11.58	-11.57	-11.56
-11.64	-11.63	-11.62	-11.61	-11.6	-11.59	-11.58	-11.57	-11.56
-11.64	-11.63	-11.62	-11.61	-11.6	-11.59	-11.58	-11.57	-11.56
-11.64	-11.63	-11.62	-11.61	-11.6	-11.59	-11.58	-11.57	-11.56
-11.64	-11.63	-11.62	-11.61	-11.6	-11.59	-11.58	-11.57	-11.56
-11.64	-11.63	-11.62	-11.61	-11.6	-11.59	-11.58	-11.57	-11.56
-11.64	-11.63	-11.62	-11.61	-11.6	-11.59	-11.58	-11.57	-11.56
-11.64	-11.63	-11.62	-11.61	-11.6	-11.59	-11.58	-11.57	-11.56
-11.64	-11.63	-11.62	-11.61	-11.6	-11.59	-11.58	-11.57	-11.56
-11.64	-11.63	-11.62	-11.61	-11.6	-11.59	-11.58	-11.57	-11.56
-11.64	-11.63	-11.62	-11.61	-11.6	-11.59	-11.58	-11.57	-11.56
-11.64	-11.63	-11.62	-11.61	-11.6	-11.59	-11.58	-11.57	-11.56
-11.64	-11.63	-11.62	-11.61	-11.6	-11.59	-11.58	-11.57	-11.56
-11.64	-11.63	-11.62	-11.61	-11.6	-11.59	-11.58	-11.57	-11.56
-11.64	-11.63	-11.62	-11.61	-11.6	-11.59	-11.58	-11.57	-11.56
-11.64	-11.63	-11.62	-11.61	-11.6	-11.59	-11.58	-11.57	-11.56
-11.64	-11.63	-11.62	-11.61	-11.6	-11.59	-11.58	-11.57	-11.56
-11.64	-11.63	-11.62	-11.61	-11.6	-11.59	-11.58	-11.57	-11.56
-11.64	-11.63	-11.62	-11.61	-11.6	-11.59	-11.58	-11.57	-11.56
-11.64	-11.63	-11.62	-11.61	-11.6	-11.59	-11.58	-11.57	-11.56
-11.64	-11.63	-11.62	-11.61	-11.6	-11.59	-11.58	-11.57	-11.56
-11.64	-11.63	-11.62	-11.61	-11.6	-11.59	-11.58	-11.57	-11.56
-11.64	-11.63	-11.62	-11.61	-11.6	-11.59	-11.58	-11.57	-11.56
-11.64	-11.63	-11.62	-11.61	-11.6	-11.59	-11.58	-11.57	-11.56
-11.64	-11.63	-11.62	-11.61	-11.6	-11.59	-11.58	-11.57	-11.56
-11.64	-11.63	-11.62	-11.61	-11.6	-11.59	-11.58	-11.57	-11.56
-11.64	-11.63	-11.62	-11.61	-11.6	-11.59	-11.58	-11.57	-11.56
-11.64	-11.63	-11.62	-11.61	-11.6	-11.59	-11.58	-11.57	-11.56
-11.64	-11.63	-11.62	-11.61	-11.6	-11.59	-11.58	-11.57	-11.56
-11.64	-11.63	-11.62	-11.61	-11.6	-11.59	-11.58	-11.57	-11.56
-11.64	-11.63	-11.62	-11.61	-11.6	-11.59	-11.58	-11.57	-11.56
-11.64	-11.63	-11.62	-11.61	-11.6	-11.59	-11.58	-11.57	-11.56
-11.64	-11.63	-11.62	-11.61	-11.6	-11.59	-11.58	-11.57	-11.56
-11.64	-11.63	-11.62	-11.61	-11.6	-11.59	-11.58	-11.57	-11.56
-11.64	-11.63	-11.62	-11.61	-11.6	-11.59	-11.58	-11.57	-11.56
-11.64	-11.63	-11.62	-11.61	-11.6	-11.59	-11.58	-11.57	-11.56

-11.64	-11.63	-11.62	-11.61	-11.6	-11.59	-11.58	-11.57	-11.56
-11.64	-11.63	-11.62	-11.61	-11.6	-11.59	-11.58	-11.57	-11.56
-11.64	-11.63	-11.62	-11.61	-11.6	-11.59	-11.58	-11.57	-11.56
-11.64	-11.63	-11.62	-11.61	-11.6	-11.59	-11.58	-11.57	-11.56
-11.64	-11.63	-11.62	-11.61	-11.6	-11.59	-11.58	-11.57	-11.56
-11.64	-11.63	-11.62	-11.61	-11.6	-11.59	-11.58	-11.57	-11.56
-11.64	-11.63	-11.62	-11.61	-11.6	-11.59	-11.58	-11.57	-11.56
-11.64	-11.63	-11.62	-11.61	-11.6	-11.59	-11.58	-11.57	-11.56
-11.64	-11.63	-11.62	-11.61	-11.6	-11.59	-11.58	-11.57	-11.56
-11.64	-11.63	-11.62	-11.61	-11.6	-11.59	-11.58	-11.57	-11.56
-11.64	-11.63	-11.62	-11.61	-11.6	-11.59	-11.58	-11.57	-11.56
-11.64	-11.63	-11.62	-11.61	-11.6	-11.59	-11.58	-11.57	-11.56
-11.64	-11.63	-11.62	-11.61	-11.6	-11.59	-11.58	-11.57	-11.56
-11.64	-11.63	-11.62	-11.61	-11.6	-11.59	-11.58	-11.57	-11.56
-11.64	-11.63	-11.62	-11.61	-11.6	-11.59	-11.58	-11.57	-11.56
-11.64	-11.63	-11.62	-11.61	-11.6	-11.59	-11.58	-11.57	-11.56
-11.64	-11.63	-11.62	-11.61	-11.6	-11.59	-11.58	-11.57	-11.56
-11.64	-11.63	-11.62	-11.61	-11.6	-11.59	-11.58	-11.57	-11.56
-11.64	-11.63	-11.62	-11.61	-11.6	-11.59	-11.58	-11.57	-11.56
-11.64	-11.63	-11.62	-11.61	-11.6	-11.59	-11.58	-11.57	-11.56
-11.64	-11.63	-11.62	-11.61	-11.6	-11.59	-11.58	-11.57	-11.56
-11.64	-11.63	-11.62	-11.61	-11.6	-11.59	-11.58	-11.57	-11.56
-11.64	-11.63	-11.62	-11.61	-11.6	-11.59	-11.58	-11.57	-11.56
-11.64	-11.63	-11.62	-11.61	-11.6	-11.59	-11.58	-11.57	-11.56
-11.64	-11.63	-11.62	-11.61	-11.6	-11.59	-11.58	-11.57	-11.56
-11.64	-11.63	-11.62	-11.61	-11.6	-11.59	-11.58	-11.57	-11.56
-11.64	-11.63	-11.62	-11.61	-11.6	-11.59	-11.58	-11.57	-11.56
-11.64	-11.63	-11.62	-11.61	-11.6	-11.59	-11.58	-11.57	-11.56
-11.64	-11.63	-11.62	-11.61	-11.6	-11.59	-11.58	-11.57	-11.56
-11.64	-11.63	-11.62	-11.61	-11.6	-11.59	-11.58	-11.57	-11.56
-11.64	-11.63	-11.62	-11.61	-11.6	-11.59	-11.58	-11.57	-11.56
-11.64	-11.63	-11.62	-11.61	-11.6	-11.59	-11.58	-11.57	-11.56
-11.64	-11.63	-11.62	-11.61	-11.6	-11.59	-11.58	-11.57	-11.56
-11.64	-11.63	-11.62	-11.61	-11.6	-11.59	-11.58	-11.57	-11.56
-11.64	-11.63	-11.62	-11.61	-11.6	-11.59	-11.58	-11.57	-11.56
-11.64	-11.63	-11.62	-11.61	-11.6	-11.59	-11.58	-11.57	-11.56
-11.64	-11.63	-11.62	-11.61	-11.6	-11.59	-11.58	-11.57	-11.56
-11.64	-11.63	-11.62	-11.61	-11.6	-11.59	-11.58	-11.57	-11.56
-11.64	-11.63	-11.62	-11.61	-11.6	-11.59	-11.58	-11.57	-11.56
-11.64	-11.63	-11.62	-11.61	-11.6	-11.59	-11.58	-11.57	-11.56
-11.64	-11.63	-11.62	-11.61	-11.6	-11.59	-11.58	-11.57	-11.56
-11.64	-11.63	-11.62	-11.61	-11.6	-11.59	-11.58	-11.57	-11.56
-11.64	-11.63	-11.62	-11.61	-11.6	-11.59	-11.58	-11.57	-11.56
-11.64	-11.63	-11.62	-11.61	-11.6	-11.59	-11.58	-11.57	-11.56
-11.64	-11.63	-11.62	-11.61	-11.6	-11.59	-11.58	-11.57	-11.56
-11.64	-11.63	-11.62	-11.61	-11.6	-11.59	-11.58	-11.57	-11.56
-11.64	-11.63	-11.62	-11.61	-11.6	-11.59	-11.58	-11.57	-11.56
-11.64	-11.63	-11.62	-11.61	-11.6	-11.59	-11.58	-11.57	-11.56
-11.64	-11.63	-11.62	-11.61	-11.6	-11.59	-11.58	-11.57	-11.56
-11.64	-11.63	-11.62	-11.61	-11.6	-11.59	-11.58	-11.57	-11.56

-11.64	-11.63	-11.62	-11.61	-11.6	-11.59	-11.58	-11.57	-11.56
-11.64	-11.63	-11.62	-11.61	-11.6	-11.59	-11.58	-11.57	-11.56
-11.64	-11.63	-11.62	-11.61	-11.6	-11.59	-11.58	-11.57	-11.56
-11.64	-11.63	-11.62	-11.61	-11.6	-11.59	-11.58	-11.57	-11.56
-11.64	-11.63	-11.62	-11.61	-11.6	-11.59	-11.58	-11.57	-11.56
-11.64	-11.63	-11.62	-11.61	-11.6	-11.59	-11.58	-11.57	-11.56
-11.64	-11.63	-11.62	-11.61	-11.6	-11.59	-11.58	-11.57	-11.56
-11.64	-11.63	-11.62	-11.61	-11.6	-11.59	-11.58	-11.57	-11.56
-11.64	-11.63	-11.62	-11.61	-11.6	-11.59	-11.58	-11.57	-11.56
-11.64	-11.63	-11.62	-11.61	-11.6	-11.59	-11.58	-11.57	-11.56
-11.64	-11.63	-11.62	-11.61	-11.6	-11.59	-11.58	-11.57	-11.56
-11.64	-11.63	-11.62	-11.61	-11.6	-11.59	-11.58	-11.57	-11.56
-11.64	-11.63	-11.62	-11.61	-11.6	-11.59	-11.58	-11.57	-11.56
-11.64	-11.63	-11.62	-11.61	-11.6	-11.59	-11.58	-11.57	-11.56
-11.64	-11.63	-11.62	-11.61	-11.6	-11.59	-11.58	-11.57	-11.56
-11.64	-11.63	-11.62	-11.61	-11.6	-11.59	-11.58	-11.57	-11.56
-11.64	-11.63	-11.62	-11.61	-11.6	-11.59	-11.58	-11.57	-11.56
-11.64	-11.63	-11.62	-11.61	-11.6	-11.59	-11.58	-11.57	-11.56
-11.64	-11.63	-11.62	-11.61	-11.6	-11.59	-11.58	-11.57	-11.56
-11.64	-11.63	-11.62	-11.61	-11.6	-11.59	-11.58	-11.57	-11.56
-11.64	-11.63	-11.62	-11.61	-11.6	-11.59	-11.58	-11.57	-11.56
-11.64	-11.63	-11.62	-11.61	-11.6	-11.59	-11.58	-11.57	-11.56
-11.64	-11.63	-11.62	-11.61	-11.6	-11.59	-11.58	-11.57	-11.56
-11.64	-11.63	-11.62	-11.61	-11.6	-11.59	-11.58	-11.57	-11.56
-11.64	-11.63	-11.62	-11.61	-11.6	-11.59	-11.58	-11.57	-11.56
-11.64	-11.63	-11.62	-11.61	-11.6	-11.59	-11.58	-11.57	-11.56
-11.64	-11.63	-11.62	-11.61	-11.6	-11.59	-11.58	-11.57	-11.56
-11.64	-11.63	-11.62	-11.61	-11.6	-11.59	-11.58	-11.57	-11.56
-11.64	-11.63	-11.62	-11.61	-11.6	-11.59	-11.58	-11.57	-11.56
-11.64	-11.63	-11.62	-11.61	-11.6	-11.59	-11.58	-11.57	-11.56
-11.64	-11.63	-11.62	-11.61	-11.6	-11.59	-11.58	-11.57	-11.56
-11.64	-11.63	-11.62	-11.61	-11.6	-11.59	-11.58	-11.57	-11.56
-11.64	-11.63	-11.62	-11.61	-11.6	-11.59	-11.58	-11.57	-11.56
-11.64	-11.63	-11.62	-11.61	-11.6	-11.59	-11.58	-11.57	-11.56
-11.64	-11.63	-11.62	-11.61	-11.6	-11.59	-11.58	-11.57	-11.56
-11.64	-11.63	-11.62	-11.61	-11.6	-11.59	-11.58	-11.57	-11.56
-11.64	-11.63	-11.62	-11.61	-11.6	-11.59	-11.58	-11.57	-11.56
-11.64	-11.63	-11.62	-11.61	-11.6	-11.59	-11.58	-11.57	-11.56
-11.64	-11.63	-11.62	-11.61	-11.6	-11.59	-11.58	-11.57	-11.56
-11.64	-11.63	-11.62	-11.61	-11.6	-11.59	-11.58	-11.57	-11.56
-11.64	-11.63	-11.62	-11.61	-11.6	-11.59	-11.58	-11.57	-11.56
-11.64	-11.63	-11.62	-11.61	-11.6	-11.59	-11.58	-11.57	-11.56
-11.64	-11.63	-11.62	-11.61	-11.6	-11.59	-11.58	-11.57	-11.56
-11.64	-11.63	-11.62	-11.61	-11.6	-11.59	-11.58	-11.57	-11.56
-11.64	-11.63	-11.62	-11.61	-11.6	-11.59	-11.58	-11.57	-11.56
-11.64	-11.63	-11.62	-11.61	-11.6	-11.59	-11.58	-11.57	-11.56
-11.64	-11.63	-11.62	-11.61	-11.6	-11.59	-11.58	-11.57	-11.56
-11.64	-11.63	-11.62	-11.61	-11.6	-11.59	-11.58	-11.57	-11.56
-11.64	-11.63	-11.62	-11.61	-11.6	-11.59	-11.58	-11.57	-11.56
-11.64	-11.63	-11.62	-11.61	-11.6	-11.59	-11.58	-11.57	-11.56

-11.64	-11.63	-11.62	-11.61	-11.6	-11.59	-11.58	-11.57	-11.56
-11.64	-11.63	-11.62	-11.61	-11.6	-11.59	-11.58	-11.57	-11.56
-11.64	-11.63	-11.62	-11.61	-11.6	-11.59	-11.58	-11.57	-11.56
-11.64	-11.63	-11.62	-11.61	-11.6	-11.59	-11.58	-11.57	-11.56
-11.64	-11.63	-11.62	-11.61	-11.6	-11.59	-11.58	-11.57	-11.56
-11.64	-11.63	-11.62	-11.61	-11.6	-11.59	-11.58	-11.57	-11.56
-11.64	-11.63	-11.62	-11.61	-11.6	-11.59	-11.58	-11.57	-11.56
-11.64	-11.63	-11.62	-11.61	-11.6	-11.59	-11.58	-11.57	-11.56
-11.64	-11.63	-11.62	-11.61	-11.6	-11.59	-11.58	-11.57	-11.56
-11.64	-11.63	-11.62	-11.61	-11.6	-11.59	-11.58	-11.57	-11.56
-11.64	-11.63	-11.62	-11.61	-11.6	-11.59	-11.58	-11.57	-11.56
-11.64	-11.63	-11.62	-11.61	-11.6	-11.59	-11.58	-11.57	-11.56
-11.64	-11.63	-11.62	-11.61	-11.6	-11.59	-11.58	-11.57	-11.56
-11.64	-11.63	-11.62	-11.61	-11.6	-11.59	-11.58	-11.57	-11.56
-11.64	-11.63	-11.62	-11.61	-11.6	-11.59	-11.58	-11.57	-11.56
-11.64	-11.63	-11.62	-11.61	-11.6	-11.59	-11.58	-11.57	-11.56
-11.64	-11.63	-11.62	-11.61	-11.6	-11.59	-11.58	-11.57	-11.56
-11.64	-11.63	-11.62	-11.61	-11.6	-11.59	-11.58	-11.57	-11.56
-11.64	-11.63	-11.62	-11.61	-11.6	-11.59	-11.58	-11.57	-11.56
-11.64	-11.63	-11.62	-11.61	-11.6	-11.59	-11.58	-11.57	-11.56
-11.64	-11.63	-11.62	-11.61	-11.6	-11.59	-11.58	-11.57	-11.56
-11.64	-11.63	-11.62	-11.61	-11.6	-11.59	-11.58	-11.57	-11.56
-11.64	-11.63	-11.62	-11.61	-11.6	-11.59	-11.58	-11.57	-11.56
-11.64	-11.63	-11.62	-11.61	-11.6	-11.59	-11.58	-11.57	-11.56
-11.64	-11.63	-11.62	-11.61	-11.6	-11.59	-11.58	-11.57	-11.56
-11.64	-11.63	-11.62	-11.61	-11.6	-11.59	-11.58	-11.57	-11.56
-11.64	-11.63	-11.62	-11.61	-11.6	-11.59	-11.58	-11.57	-11.56
-11.64	-11.63	-11.62	-11.61	-11.6	-11.59	-11.58	-11.57	-11.56
-11.64	-11.63	-11.62	-11.61	-11.6	-11.59	-11.58	-11.57	-11.56
-11.64	-11.63	-11.62	-11.61	-11.6	-11.59	-11.58	-11.57	-11.56
-11.64	-11.63	-11.62	-11.61	-11.6	-11.59	-11.58	-11.57	-11.56
-11.64	-11.63	-11.62	-11.61	-11.6	-11.59	-11.58	-11.57	-11.56
-11.64	-11.63	-11.62	-11.61	-11.6	-11.59	-11.58	-11.57	-11.56
-11.64	-11.63	-11.62	-11.61	-11.6	-11.59	-11.58	-11.57	-11.56
-11.64	-11.63	-11.62	-11.61	-11.6	-11.59	-11.58	-11.57	-11.56
-11.64	-11.63	-11.62	-11.61	-11.6	-11.59	-11.58	-11.57	-11.56
-11.64	-11.63	-11.62	-11.61	-11.6	-11.59	-11.58	-11.57	-11.56
-11.64	-11.63	-11.62	-11.61	-11.6	-11.59	-11.58	-11.57	-11.56
-11.64	-11.63	-11.62	-11.61	-11.6	-11.59	-11.58	-11.57	-11.56
-11.64	-11.63	-11.62	-11.61	-11.6	-11.59	-11.58	-11.57	-11.56
-11.64	-11.63	-11.62	-11.61	-11.6	-11.59	-11.58	-11.57	-11.56
-11.64	-11.63	-11.62	-11.61	-11.6	-11.59	-11.58	-11.57	-11.56
-11.64	-11.63	-11.62	-11.61	-11.6	-11.59	-11.58	-11.57	-11.56
-11.64	-11.63	-11.62	-11.61	-11.6	-11.59	-11.58	-11.57	-11.56
-11.64	-11.63	-11.62	-11.61	-11.6	-11.59	-11.58	-11.57	-11.56
-11.64	-11.63	-11.62	-11.61	-11.6	-11.59	-11.58	-11.57	-11.56
-11.64	-11.63	-11.62	-11.61	-11.6	-11.59	-11.58	-11.57	-11.56
-11.64	-11.63	-11.62	-11.61	-11.6	-11.59	-11.58	-11.57	-11.56
-11.64	-11.63	-11.62	-11.61	-11.6	-11.59	-11.58	-11.57	-11.56
-11.64	-11.63	-11.62	-11.61	-11.6	-11.59	-11.58	-11.57	-11.56

-11.64	-11.63	-11.62	-11.61	-11.6	-11.59	-11.58	-11.57	-11.56
-11.64	-11.63	-11.62	-11.61	-11.6	-11.59	-11.58	-11.57	-11.56
-11.64	-11.63	-11.62	-11.61	-11.6	-11.59	-11.58	-11.57	-11.56
-11.64	-11.63	-11.62	-11.61	-11.6	-11.59	-11.58	-11.57	-11.56
-11.64	-11.63	-11.62	-11.61	-11.6	-11.59	-11.58	-11.57	-11.56
-11.64	-11.63	-11.62	-11.61	-11.6	-11.59	-11.58	-11.57	-11.56
-11.64	-11.63	-11.62	-11.61	-11.6	-11.59	-11.58	-11.57	-11.56
-11.64	-11.63	-11.62	-11.61	-11.6	-11.59	-11.58	-11.57	-11.56
-11.64	-11.63	-11.62	-11.61	-11.6	-11.59	-11.58	-11.57	-11.56
-11.64	-11.63	-11.62	-11.61	-11.6	-11.59	-11.58	-11.57	-11.56
-11.64	-11.63	-11.62	-11.61	-11.6	-11.59	-11.58	-11.57	-11.56
-11.64	-11.63	-11.62	-11.61	-11.6	-11.59	-11.58	-11.57	-11.56
-11.64	-11.63	-11.62	-11.61	-11.6	-11.59	-11.58	-11.57	-11.56
-11.64	-11.63	-11.62	-11.61	-11.6	-11.59	-11.58	-11.57	-11.56
-11.64	-11.63	-11.62	-11.61	-11.6	-11.59	-11.58	-11.57	-11.56
-11.64	-11.63	-11.62	-11.61	-11.6	-11.59	-11.58	-11.57	-11.56
-11.64	-11.63	-11.62	-11.61	-11.6	-11.59	-11.58	-11.57	-11.56
-11.64	-11.63	-11.62	-11.61	-11.6	-11.59	-11.58	-11.57	-11.56
-11.64	-11.63	-11.62	-11.61	-11.6	-11.59	-11.58	-11.57	-11.56
-11.64	-11.63	-11.62	-11.61	-11.6	-11.59	-11.58	-11.57	-11.56
-11.64	-11.63	-11.62	-11.61	-11.6	-11.59	-11.58	-11.57	-11.56
-11.64	-11.63	-11.62	-11.61	-11.6	-11.59	-11.58	-11.57	-11.56
-11.64	-11.63	-11.62	-11.61	-11.6	-11.59	-11.58	-11.57	-11.56
-11.64	-11.63	-11.62	-11.61	-11.6	-11.59	-11.58	-11.57	-11.56
-11.64	-11.63	-11.62	-11.61	-11.6	-11.59	-11.58	-11.57	-11.56
-11.64	-11.63	-11.62	-11.61	-11.6	-11.59	-11.58	-11.57	-11.56
-11.64	-11.63	-11.62	-11.61	-11.6	-11.59	-11.58	-11.57	-11.56
-11.64	-11.63	-11.62	-11.61	-11.6	-11.59	-11.58	-11.57	-11.56
-11.64	-11.63	-11.62	-11.61	-11.6	-11.59	-11.58	-11.57	-11.56
-11.64	-11.63	-11.62	-11.61	-11.6	-11.59	-11.58	-11.57	-11.56
-11.64	-11.63	-11.62	-11.61	-11.6	-11.59	-11.58	-11.57	-11.56
-11.64	-11.63	-11.62	-11.61	-11.6	-11.59	-11.58	-11.57	-11.56
-11.64	-11.63	-11.62	-11.61	-11.6	-11.59	-11.58	-11.57	-11.56
-11.64	-11.63	-11.62	-11.61	-11.6	-11.59	-11.58	-11.57	-11.56
-11.64	-11.63	-11.62	-11.61	-11.6	-11.59	-11.58	-11.57	-11.56
-11.64	-11.63	-11.62	-11.61	-11.6	-11.59	-11.58	-11.57	-11.56
-11.64	-11.63	-11.62	-11.61	-11.6	-11.59	-11.58	-11.57	-11.56
-11.64	-11.63	-11.62	-11.61	-11.6	-11.59	-11.58	-11.57	-11.56
-11.64	-11.63	-11.62	-11.61	-11.6	-11.59	-11.58	-11.57	-11.56
-11.64	-11.63	-11.62	-11.61	-11.6	-11.59	-11.58	-11.57	-11.56
-11.64	-11.63	-11.62	-11.61	-11.6	-11.59	-11.58	-11.57	-11.56
-11.64	-11.63	-11.62	-11.61	-11.6	-11.59	-11.58	-11.57	-11.56
-11.64	-11.63	-11.62	-11.61	-11.6	-11.59	-11.58	-11.57	-11.56
-11.64	-11.63	-11.62	-11.61	-11.6	-11.59	-11.58	-11.57	-11.56
-11.64	-11.63	-11.62	-11.61	-11.6	-11.59	-11.58	-11.57	-11.56
-11.64	-11.63	-11.62	-11.61	-11.6	-11.59	-11.58	-11.57	-11.56
-11.64	-11.63	-11.62	-11.61	-11.6	-11.59	-11.58	-11.57	-11.56
-11.64	-11.63	-11.62	-11.61	-11.6	-11.59	-11.58	-11.57	-11.56
-11.64	-11.63	-11.62	-11.61	-11.6	-11.59	-11.58	-11.57	-11.56
-11.64	-11.63	-11.62	-11.61	-11.6	-11.59	-11.58	-11.57	-11.56

-11.64	-11.63	-11.62	-11.61	-11.6	-11.59	-11.58	-11.57	-11.56
-11.64	-11.63	-11.62	-11.61	-11.6	-11.59	-11.58	-11.57	-11.56
-11.64	-11.63	-11.62	-11.61	-11.6	-11.59	-11.58	-11.57	-11.56
-11.64	-11.63	-11.62	-11.61	-11.6	-11.59	-11.58	-11.57	-11.56
-11.64	-11.63	-11.62	-11.61	-11.6	-11.59	-11.58	-11.57	-11.56
-11.64	-11.63	-11.62	-11.61	-11.6	-11.59	-11.58	-11.57	-11.56
-11.64	-11.63	-11.62	-11.61	-11.6	-11.59	-11.58	-11.57	-11.56
-11.64	-11.63	-11.62	-11.61	-11.6	-11.59	-11.58	-11.57	-11.56
-11.64	-11.63	-11.62	-11.61	-11.6	-11.59	-11.58	-11.57	-11.56
-11.64	-11.63	-11.62	-11.61	-11.6	-11.59	-11.58	-11.57	-11.56
-11.64	-11.63	-11.62	-11.61	-11.6	-11.59	-11.58	-11.57	-11.56
-11.64	-11.63	-11.62	-11.61	-11.6	-11.59	-11.58	-11.57	-11.56
-11.64	-11.63	-11.62	-11.61	-11.6	-11.59	-11.58	-11.57	-11.56
-11.64	-11.63	-11.62	-11.61	-11.6	-11.59	-11.58	-11.57	-11.56
-11.64	-11.63	-11.62	-11.61	-11.6	-11.59	-11.58	-11.57	-11.56
-11.64	-11.63	-11.62	-11.61	-11.6	-11.59	-11.58	-11.57	-11.56
-11.64	-11.63	-11.62	-11.61	-11.6	-11.59	-11.58	-11.57	-11.56
-11.64	-11.63	-11.62	-11.61	-11.6	-11.59	-11.58	-11.57	-11.56
-11.64	-11.63	-11.62	-11.61	-11.6	-11.59	-11.58	-11.57	-11.56
-11.64	-11.63	-11.62	-11.61	-11.6	-11.59	-11.58	-11.57	-11.56
-11.64	-11.63	-11.62	-11.61	-11.6	-11.59	-11.58	-11.57	-11.56
-11.64	-11.63	-11.62	-11.61	-11.6	-11.59	-11.58	-11.57	-11.56
-11.64	-11.63	-11.62	-11.61	-11.6	-11.59	-11.58	-11.57	-11.56
-11.64	-11.63	-11.62	-11.61	-11.6	-11.59	-11.58	-11.57	-11.56
-11.64	-11.63	-11.62	-11.61	-11.6	-11.59	-11.58	-11.57	-11.56
-11.64	-11.63	-11.62	-11.61	-11.6	-11.59	-11.58	-11.57	-11.56
-11.64	-11.63	-11.62	-11.61	-11.6	-11.59	-11.58	-11.57	-11.56
-11.64	-11.63	-11.62	-11.61	-11.6	-11.59	-11.58	-11.57	-11.56
-11.64	-11.63	-11.62	-11.61	-11.6	-11.59	-11.58	-11.57	-11.56
-11.64	-11.63	-11.62	-11.61	-11.6	-11.59	-11.58	-11.57	-11.56
-11.64	-11.63	-11.62	-11.61	-11.6	-11.59	-11.58	-11.57	-11.56
-11.64	-11.63	-11.62	-11.61	-11.6	-11.59	-11.58	-11.57	-11.56
-11.64	-11.63	-11.62	-11.61	-11.6	-11.59	-11.58	-11.57	-11.56
-11.64	-11.63	-11.62	-11.61	-11.6	-11.59	-11.58	-11.57	-11.56
-11.64	-11.63	-11.62	-11.61	-11.6	-11.59	-11.58	-11.57	-11.56
-11.64	-11.63	-11.62	-11.61	-11.6	-11.59	-11.58	-11.57	-11.56
-11.64	-11.63	-11.62	-11.61	-11.6	-11.59	-11.58	-11.57	-11.56
-11.64	-11.63	-11.62	-11.61	-11.6	-11.59	-11.58	-11.57	-11.56
-11.64	-11.63	-11.62	-11.61	-11.6	-11.59	-11.58	-11.57	-11.56
-11.64	-11.63	-11.62	-11.61	-11.6	-11.59	-11.58	-11.57	-11.56
-11.64	-11.63	-11.62	-11.61	-11.6	-11.59	-11.58	-11.57	-11.56
-11.64	-11.63	-11.62	-11.61	-11.6	-11.59	-11.58	-11.57	-11.56
-11.64	-11.63	-11.62	-11.61	-11.6	-11.59	-11.58	-11.57	-11.56
-11.64	-11.63	-11.62	-11.61	-11.6	-11.59	-11.58	-11.57	-11.56
-11.64	-11.63	-11.62	-11.61	-11.6	-11.59	-11.58	-11.57	-11.56
-11.64	-11.63	-11.62	-11.61	-11.6	-11.59	-11.58	-11.57	-11.56
-11.64	-11.63	-11.62	-11.61	-11.6	-11.59	-11.58	-11.57	-11.56
-11.64	-11.63	-11.62	-11.61	-11.6	-11.59	-11.58	-11.57	-11.56
-11.64	-11.63	-11.62	-11.61	-11.6	-11.59	-11.58	-11.57	-11.56
-11.64	-11.63	-11.62	-11.61	-11.6	-11.59	-11.58	-11.57	-11.56

-11.64	-11.63	-11.62	-11.61	-11.6	-11.59	-11.58	-11.57	-11.56
-11.64	-11.63	-11.62	-11.61	-11.6	-11.59	-11.58	-11.57	-11.56
-11.64	-11.63	-11.62	-11.61	-11.6	-11.59	-11.58	-11.57	-11.56
-11.64	-11.63	-11.62	-11.61	-11.6	-11.59	-11.58	-11.57	-11.56
-11.64	-11.63	-11.62	-11.61	-11.6	-11.59	-11.58	-11.57	-11.56
-11.64	-11.63	-11.62	-11.61	-11.6	-11.59	-11.58	-11.57	-11.56
-11.64	-11.63	-11.62	-11.61	-11.6	-11.59	-11.58	-11.57	-11.56
-11.64	-11.63	-11.62	-11.61	-11.6	-11.59	-11.58	-11.57	-11.56
-11.64	-11.63	-11.62	-11.61	-11.6	-11.59	-11.58	-11.57	-11.56
-11.64	-11.63	-11.62	-11.61	-11.6	-11.59	-11.58	-11.57	-11.56
-11.64	-11.63	-11.62	-11.61	-11.6	-11.59	-11.58	-11.57	-11.56
-11.64	-11.63	-11.62	-11.61	-11.6	-11.59	-11.58	-11.57	-11.56
-11.64	-11.63	-11.62	-11.61	-11.6	-11.59	-11.58	-11.57	-11.56
-11.64	-11.63	-11.62	-11.61	-11.6	-11.59	-11.58	-11.57	-11.56
-11.64	-11.63	-11.62	-11.61	-11.6	-11.59	-11.58	-11.57	-11.56
-11.64	-11.63	-11.62	-11.61	-11.6	-11.59	-11.58	-11.57	-11.56
-11.64	-11.63	-11.62	-11.61	-11.6	-11.59	-11.58	-11.57	-11.56
-11.64	-11.63	-11.62	-11.61	-11.6	-11.59	-11.58	-11.57	-11.56
-11.64	-11.63	-11.62	-11.61	-11.6	-11.59	-11.58	-11.57	-11.56
-11.64	-11.63	-11.62	-11.61	-11.6	-11.59	-11.58	-11.57	-11.56
-11.64	-11.63	-11.62	-11.61	-11.6	-11.59	-11.58	-11.57	-11.56
-11.64	-11.63	-11.62	-11.61	-11.6	-11.59	-11.58	-11.57	-11.56
-11.64	-11.63	-11.62	-11.61	-11.6	-11.59	-11.58	-11.57	-11.56
-11.64	-11.63	-11.62	-11.61	-11.6	-11.59	-11.58	-11.57	-11.56
-11.64	-11.63	-11.62	-11.61	-11.6	-11.59	-11.58	-11.57	-11.56
-11.64	-11.63	-11.62	-11.61	-11.6	-11.59	-11.58	-11.57	-11.56
-11.64	-11.63	-11.62	-11.61	-11.6	-11.59	-11.58	-11.57	-11.56
-11.64	-11.63	-11.62	-11.61	-11.6	-11.59	-11.58	-11.57	-11.56
-11.64	-11.63	-11.62	-11.61	-11.6	-11.59	-11.58	-11.57	-11.56
-11.64	-11.63	-11.62	-11.61	-11.6	-11.59	-11.58	-11.57	-11.56
-11.64	-11.63	-11.62	-11.61	-11.6	-11.59	-11.58	-11.57	-11.56
-11.64	-11.63	-11.62	-11.61	-11.6	-11.59	-11.58	-11.57	-11.56
-11.64	-11.63	-11.62	-11.61	-11.6	-11.59	-11.58	-11.57	-11.56
-11.64	-11.63	-11.62	-11.61	-11.6	-11.59	-11.58	-11.57	-11.56
-11.64	-11.63	-11.62	-11.61	-11.6	-11.59	-11.58	-11.57	-11.56
-11.64	-11.63	-11.62	-11.61	-11.6	-11.59	-11.58	-11.57	-11.56
-11.64	-11.63	-11.62	-11.61	-11.6	-11.59	-11.58	-11.57	-11.56
-11.64	-11.63	-11.62	-11.61	-11.6	-11.59	-11.58	-11.57	-11.56
-11.64	-11.63	-11.62	-11.61	-11.6	-11.59	-11.58	-11.57	-11.56
-11.64	-11.63	-11.62	-11.61	-11.6	-11.59	-11.58	-11.57	-11.56
-11.64	-11.63	-11.62	-11.61	-11.6	-11.59	-11.58	-11.57	-11.56
-11.64	-11.63	-11.62	-11.61	-11.6	-11.59	-11.58	-11.57	-11.56
-11.64	-11.63	-11.62	-11.61	-11.6	-11.59	-11.58	-11.57	-11.56
-11.64	-11.63	-11.62	-11.61	-11.6	-11.59	-11.58	-11.57	-11.56
-11.64	-11.63	-11.62	-11.61	-11.6	-11.59	-11.58	-11.57	-11.56
-11.64	-11.63	-11.62	-11.61	-11.6	-11.59	-11.58	-11.57	-11.56
-11.64	-11.63	-11.62	-11.61	-11.6	-11.59	-11.58	-11.57	-11.56
-11.64	-11.63	-11.62	-11.61	-11.6	-11.59	-11.58	-11.57	-11.56
-11.64	-11.63	-11.62	-11.61	-11.6	-11.59	-11.58	-11.57	-11.56
-11.64	-11.63	-11.62	-11.61	-11.6	-11.59	-11.58	-11.57	-11.56

-11.64	-11.63	-11.62	-11.61	-11.6	-11.59	-11.58	-11.57	-11.56
-11.64	-11.63	-11.62	-11.61	-11.6	-11.59	-11.58	-11.57	-11.56
-11.64	-11.63	-11.62	-11.61	-11.6	-11.59	-11.58	-11.57	-11.56
-11.64	-11.63	-11.62	-11.61	-11.6	-11.59	-11.58	-11.57	-11.56
-11.64	-11.63	-11.62	-11.61	-11.6	-11.59	-11.58	-11.57	-11.56
-11.64	-11.63	-11.62	-11.61	-11.6	-11.59	-11.58	-11.57	-11.56
-11.64	-11.63	-11.62	-11.61	-11.6	-11.59	-11.58	-11.57	-11.56
-11.64	-11.63	-11.62	-11.61	-11.6	-11.59	-11.58	-11.57	-11.56
-11.64	-11.63	-11.62	-11.61	-11.6	-11.59	-11.58	-11.57	-11.56
-11.64	-11.63	-11.62	-11.61	-11.6	-11.59	-11.58	-11.57	-11.56
-11.64	-11.63	-11.62	-11.61	-11.6	-11.59	-11.58	-11.57	-11.56
-11.64	-11.63	-11.62	-11.61	-11.6	-11.59	-11.58	-11.57	-11.56
-11.64	-11.63	-11.62	-11.61	-11.6	-11.59	-11.58	-11.57	-11.56
-11.64	-11.63	-11.62	-11.61	-11.6	-11.59	-11.58	-11.57	-11.56
-11.64	-11.63	-11.62	-11.61	-11.6	-11.59	-11.58	-11.57	-11.56
-11.64	-11.63	-11.62	-11.61	-11.6	-11.59	-11.58	-11.57	-11.56
-11.64	-11.63	-11.62	-11.61	-11.6	-11.59	-11.58	-11.57	-11.56
-11.64	-11.63	-11.62	-11.61	-11.6	-11.59	-11.58	-11.57	-11.56
-11.64	-11.63	-11.62	-11.61	-11.6	-11.59	-11.58	-11.57	-11.56
-11.64	-11.63	-11.62	-11.61	-11.6	-11.59	-11.58	-11.57	-11.56
-11.64	-11.63	-11.62	-11.61	-11.6	-11.59	-11.58	-11.57	-11.56
-11.64	-11.63	-11.62	-11.61	-11.6	-11.59	-11.58	-11.57	-11.56
-11.64	-11.63	-11.62	-11.61	-11.6	-11.59	-11.58	-11.57	-11.56
-11.64	-11.63	-11.62	-11.61	-11.6	-11.59	-11.58	-11.57	-11.56
-11.64	-11.63	-11.62	-11.61	-11.6	-11.59	-11.58	-11.57	-11.56
-11.64	-11.63	-11.62	-11.61	-11.6	-11.59	-11.58	-11.57	-11.56
-11.64	-11.63	-11.62	-11.61	-11.6	-11.59	-11.58	-11.57	-11.56
-11.64	-11.63	-11.62	-11.61	-11.6	-11.59	-11.58	-11.57	-11.56
-11.64	-11.63	-11.62	-11.61	-11.6	-11.59	-11.58	-11.57	-11.56
-11.64	-11.63	-11.62	-11.61	-11.6	-11.59	-11.58	-11.57	-11.56
-11.64	-11.63	-11.62	-11.61	-11.6	-11.59	-11.58	-11.57	-11.56
-11.64	-11.63	-11.62	-11.61	-11.6	-11.59	-11.58	-11.57	-11.56
-11.64	-11.63	-11.62	-11.61	-11.6	-11.59	-11.58	-11.57	-11.56
-11.64	-11.63	-11.62	-11.61	-11.6	-11.59	-11.58	-11.57	-11.56
-11.64	-11.63	-11.62	-11.61	-11.6	-11.59	-11.58	-11.57	-11.56
-11.64	-11.63	-11.62	-11.61	-11.6	-11.59	-11.58	-11.57	-11.56
-11.64	-11.63	-11.62	-11.61	-11.6	-11.59	-11.58	-11.57	-11.56
-11.64	-11.63	-11.62	-11.61	-11.6	-11.59	-11.58	-11.57	-11.56
-11.64	-11.63	-11.62	-11.61	-11.6	-11.59	-11.58	-11.57	-11.56
-11.64	-11.63	-11.62	-11.61	-11.6	-11.59	-11.58	-11.57	-11.56
-11.64	-11.63	-11.62	-11.61	-11.6	-11.59	-11.58	-11.57	-11.56
-11.64	-11.63	-11.62	-11.61	-11.6	-11.59	-11.58	-11.57	-11.56
-11.64	-11.63	-11.62	-11.61	-11.6	-11.59	-11.58	-11.57	-11.56
-11.64	-11.63	-11.62	-11.61	-11.6	-11.59	-11.58	-11.57	-11.56
-11.64	-11.63	-11.62	-11.61	-11.6	-11.59	-11.58	-11.57	-11.56
-11.64	-11.63	-11.62	-11.61	-11.6	-11.59	-11.58	-11.57	-11.56
-11.64	-11.63	-11.62	-11.61	-11.6	-11.59	-11.58	-11.57	-11.56
-11.64	-11.63	-11.62	-11.61	-11.6	-11.59	-11.58	-11.57	-11.56
-11.64	-11.63	-11.62	-11.61	-11.6	-11.59	-11.58	-11.57	-11.56
-11.64	-11.63	-11.62	-11.61	-11.6	-11.59	-11.58	-11.57	-11.56

-11.64	-11.63	-11.62	-11.61	-11.6	-11.59	-11.58	-11.57	-11.56
-11.64	-11.63	-11.62	-11.61	-11.6	-11.59	-11.58	-11.57	-11.56
-11.64	-11.63	-11.62	-11.61	-11.6	-11.59	-11.58	-11.57	-11.56
-11.64	-11.63	-11.62	-11.61	-11.6	-11.59	-11.58	-11.57	-11.56
-11.64	-11.63	-11.62	-11.61	-11.6	-11.59	-11.58	-11.57	-11.56
-11.64	-11.63	-11.62	-11.61	-11.6	-11.59	-11.58	-11.57	-11.56
-11.64	-11.63	-11.62	-11.61	-11.6	-11.59	-11.58	-11.57	-11.56
-11.64	-11.63	-11.62	-11.61	-11.6	-11.59	-11.58	-11.57	-11.56
-11.64	-11.63	-11.62	-11.61	-11.6	-11.59	-11.58	-11.57	-11.56
-11.64	-11.63	-11.62	-11.61	-11.6	-11.59	-11.58	-11.57	-11.56
-11.64	-11.63	-11.62	-11.61	-11.6	-11.59	-11.58	-11.57	-11.56
-11.64	-11.63	-11.62	-11.61	-11.6	-11.59	-11.58	-11.57	-11.56
-11.64	-11.63	-11.62	-11.61	-11.6	-11.59	-11.58	-11.57	-11.56
-11.64	-11.63	-11.62	-11.61	-11.6	-11.59	-11.58	-11.57	-11.56
-11.64	-11.63	-11.62	-11.61	-11.6	-11.59	-11.58	-11.57	-11.56
-11.64	-11.63	-11.62	-11.61	-11.6	-11.59	-11.58	-11.57	-11.56
-11.64	-11.63	-11.62	-11.61	-11.6	-11.59	-11.58	-11.57	-11.56
-11.64	-11.63	-11.62	-11.61	-11.6	-11.59	-11.58	-11.57	-11.56
-11.64	-11.63	-11.62	-11.61	-11.6	-11.59	-11.58	-11.57	-11.56
-11.64	-11.63	-11.62	-11.61	-11.6	-11.59	-11.58	-11.57	-11.56
-11.64	-11.63	-11.62	-11.61	-11.6	-11.59	-11.58	-11.57	-11.56
-11.64	-11.63	-11.62	-11.61	-11.6	-11.59	-11.58	-11.57	-11.56
-11.64	-11.63	-11.62	-11.61	-11.6	-11.59	-11.58	-11.57	-11.56
-11.64	-11.63	-11.62	-11.61	-11.6	-11.59	-11.58	-11.57	-11.56
-11.64	-11.63	-11.62	-11.61	-11.6	-11.59	-11.58	-11.57	-11.56
-11.64	-11.63	-11.62	-11.61	-11.6	-11.59	-11.58	-11.57	-11.56
-11.64	-11.63	-11.62	-11.61	-11.6	-11.59	-11.58	-11.57	-11.56
-11.64	-11.63	-11.62	-11.61	-11.6	-11.59	-11.58	-11.57	-11.56
-11.64	-11.63	-11.62	-11.61	-11.6	-11.59	-11.58	-11.57	-11.56
-11.64	-11.63	-11.62	-11.61	-11.6	-11.59	-11.58	-11.57	-11.56
-11.64	-11.63	-11.62	-11.61	-11.6	-11.59	-11.58	-11.57	-11.56
-11.64	-11.63	-11.62	-11.61	-11.6	-11.59	-11.58	-11.57	-11.56
-11.64	-11.63	-11.62	-11.61	-11.6	-11.59	-11.58	-11.57	-11.56
-11.64	-11.63	-11.62	-11.61	-11.6	-11.59	-11.58	-11.57	-11.56
-11.64	-11.63	-11.62	-11.61	-11.6	-11.59	-11.58	-11.57	-11.56
-11.64	-11.63	-11.62	-11.61	-11.6	-11.59	-11.58	-11.57	-11.56
-11.64	-11.63	-11.62	-11.61	-11.6	-11.59	-11.58	-11.57	-11.56
-11.64	-11.63	-11.62	-11.61	-11.6	-11.59	-11.58	-11.57	-11.56
-11.64	-11.63	-11.62	-11.61	-11.6	-11.59	-11.58	-11.57	-11.56
-11.64	-11.63	-11.62	-11.61	-11.6	-11.59	-11.58	-11.57	-11.56
-11.64	-11.63	-11.62	-11.61	-11.6	-11.59	-11.58	-11.57	-11.56
-11.64	-11.63	-11.62	-11.61	-11.6	-11.59	-11.58	-11.57	-11.56
-11.64	-11.63	-11.62	-11.61	-11.6	-11.59	-11.58	-11.57	-11.56
-11.64	-11.63	-11.62	-11.61	-11.6	-11.59	-11.58	-11.57	-11.56
-11.64	-11.63	-11.62	-11.61	-11.6	-11.59	-11.58	-11.57	-11.56
-11.64	-11.63	-11.62	-11.61	-11.6	-11.59	-11.58	-11.57	-11.56
-11.64	-11.63	-11.62	-11.61	-11.6	-11.59	-11.58	-11.57	-11.56
-11.64	-11.63	-11.62	-11.61	-11.6	-11.59	-11.58	-11.57	-11.56
-11.64	-11.63	-11.62	-11.61	-11.6	-11.59	-11.58	-11.57	-11.56
-11.64	-11.63	-11.62	-11.61	-11.6	-11.59	-11.58	-11.57	-11.56

-11.64	-11.63	-11.62	-11.61	-11.6	-11.59	-11.58	-11.57	-11.56
-11.64	-11.63	-11.62	-11.61	-11.6	-11.59	-11.58	-11.57	-11.56
-11.64	-11.63	-11.62	-11.61	-11.6	-11.59	-11.58	-11.57	-11.56
-11.64	-11.63	-11.62	-11.61	-11.6	-11.59	-11.58	-11.57	-11.56
-11.64	-11.63	-11.62	-11.61	-11.6	-11.59	-11.58	-11.57	-11.56
-11.64	-11.63	-11.62	-11.61	-11.6	-11.59	-11.58	-11.57	-11.56
-11.64	-11.63	-11.62	-11.61	-11.6	-11.59	-11.58	-11.57	-11.56
-11.64	-11.63	-11.62	-11.61	-11.6	-11.59	-11.58	-11.57	-11.56
-11.64	-11.63	-11.62	-11.61	-11.6	-11.59	-11.58	-11.57	-11.56
-11.64	-11.63	-11.62	-11.61	-11.6	-11.59	-11.58	-11.57	-11.56
-11.64	-11.63	-11.62	-11.61	-11.6	-11.59	-11.58	-11.57	-11.56
-11.64	-11.63	-11.62	-11.61	-11.6	-11.59	-11.58	-11.57	-11.56
-11.64	-11.63	-11.62	-11.61	-11.6	-11.59	-11.58	-11.57	-11.56
-11.64	-11.63	-11.62	-11.61	-11.6	-11.59	-11.58	-11.57	-11.56
-11.64	-11.63	-11.62	-11.61	-11.6	-11.59	-11.58	-11.57	-11.56
-11.64	-11.63	-11.62	-11.61	-11.6	-11.59	-11.58	-11.57	-11.56
-11.64	-11.63	-11.62	-11.61	-11.6	-11.59	-11.58	-11.57	-11.56
-11.64	-11.63	-11.62	-11.61	-11.6	-11.59	-11.58	-11.57	-11.56
-11.64	-11.63	-11.62	-11.61	-11.6	-11.59	-11.58	-11.57	-11.56
-11.64	-11.63	-11.62	-11.61	-11.6	-11.59	-11.58	-11.57	-11.56
-11.64	-11.63	-11.62	-11.61	-11.6	-11.59	-11.58	-11.57	-11.56
-11.64	-11.63	-11.62	-11.61	-11.6	-11.59	-11.58	-11.57	-11.56
-11.64	-11.63	-11.62	-11.61	-11.6	-11.59	-11.58	-11.57	-11.56
-11.64	-11.63	-11.62	-11.61	-11.6	-11.59	-11.58	-11.57	-11.56
-11.64	-11.63	-11.62	-11.61	-11.6	-11.59	-11.58	-11.57	-11.56
-11.64	-11.63	-11.62	-11.61	-11.6	-11.59	-11.58	-11.57	-11.56
-11.64	-11.63	-11.62	-11.61	-11.6	-11.59	-11.58	-11.57	-11.56
-11.64	-11.63	-11.62	-11.61	-11.6	-11.59	-11.58	-11.57	-11.56
-11.64	-11.63	-11.62	-11.61	-11.6	-11.59	-11.58	-11.57	-11.56
-11.64	-11.63	-11.62	-11.61	-11.6	-11.59	-11.58	-11.57	-11.56
-11.64	-11.63	-11.62	-11.61	-11.6	-11.59	-11.58	-11.57	-11.56
-11.64	-11.63	-11.62	-11.61	-11.6	-11.59	-11.58	-11.57	-11.56
-11.64	-11.63	-11.62	-11.61	-11.6	-11.59	-11.58	-11.57	-11.56
-11.64	-11.63	-11.62	-11.61	-11.6	-11.59	-11.58	-11.57	-11.56
-11.64	-11.63	-11.62	-11.61	-11.6	-11.59	-11.58	-11.57	-11.56
-11.64	-11.63	-11.62	-11.61	-11.6	-11.59	-11.58	-11.57	-11.56
-11.64	-11.63	-11.62	-11.61	-11.6	-11.59	-11.58	-11.57	-11.56
-11.64	-11.63	-11.62	-11.61	-11.6	-11.59	-11.58	-11.57	-11.56
-11.64	-11.63	-11.62	-11.61	-11.6	-11.59	-11.58	-11.57	-11.56
-11.64	-11.63	-11.62	-11.61	-11.6	-11.59	-11.58	-11.57	-11.56
-11.64	-11.63	-11.62	-11.61	-11.6	-11.59	-11.58	-11.57	-11.56
-11.64	-11.63	-11.62	-11.61	-11.6	-11.59	-11.58	-11.57	-11.56
-11.64	-11.63	-11.62	-11.61	-11.6	-11.59	-11.58	-11.57	-11.56
-11.64	-11.63	-11.62	-11.61	-11.6	-11.59	-11.58	-11.57	-11.56
-11.64	-11.63	-11.62	-11.61	-11.6	-11.59	-11.58	-11.57	-11.56
-11.64	-11.63	-11.62	-11.61	-11.6	-11.59	-11.58	-11.57	-11.56
-11.64	-11.63	-11.62	-11.61	-11.6	-11.59	-11.58	-11.57	-11.56
-11.64	-11.63	-11.62	-11.61	-11.6	-11.59	-11.58	-11.57	-11.56
-11.64	-11.63	-11.62	-11.61	-11.6	-11.59	-11.58	-11.57	-11.56
-11.64	-11.63	-11.62	-11.61	-11.6	-11.59	-11.58	-11.57	-11.56

-11.64	-11.63	-11.62	-11.61	-11.6	-11.59	-11.58	-11.57	-11.56
-11.64	-11.63	-11.62	-11.61	-11.6	-11.59	-11.58	-11.57	-11.56
-11.64	-11.63	-11.62	-11.61	-11.6	-11.59	-11.58	-11.57	-11.56
-11.64	-11.63	-11.62	-11.61	-11.6	-11.59	-11.58	-11.57	-11.56
-11.64	-11.63	-11.62	-11.61	-11.6	-11.59	-11.58	-11.57	-11.56
-11.64	-11.63	-11.62	-11.61	-11.6	-11.59	-11.58	-11.57	-11.56
-11.64	-11.63	-11.62	-11.61	-11.6	-11.59	-11.58	-11.57	-11.56
-11.64	-11.63	-11.62	-11.61	-11.6	-11.59	-11.58	-11.57	-11.56
-11.64	-11.63	-11.62	-11.61	-11.6	-11.59	-11.58	-11.57	-11.56
-11.64	-11.63	-11.62	-11.61	-11.6	-11.59	-11.58	-11.57	-11.56
-11.64	-11.63	-11.62	-11.61	-11.6	-11.59	-11.58	-11.57	-11.56
-11.64	-11.63	-11.62	-11.61	-11.6	-11.59	-11.58	-11.57	-11.56
-11.64	-11.63	-11.62	-11.61	-11.6	-11.59	-11.58	-11.57	-11.56
-11.64	-11.63	-11.62	-11.61	-11.6	-11.59	-11.58	-11.57	-11.56
-11.64	-11.63	-11.62	-11.61	-11.6	-11.59	-11.58	-11.57	-11.56
-11.64	-11.63	-11.62	-11.61	-11.6	-11.59	-11.58	-11.57	-11.56
-11.64	-11.63	-11.62	-11.61	-11.6	-11.59	-11.58	-11.57	-11.56
-11.64	-11.63	-11.62	-11.61	-11.6	-11.59	-11.58	-11.57	-11.56
-11.64	-11.63	-11.62	-11.61	-11.6	-11.59	-11.58	-11.57	-11.56
-11.64	-11.63	-11.62	-11.61	-11.6	-11.59	-11.58	-11.57	-11.56
-11.64	-11.63	-11.62	-11.61	-11.6	-11.59	-11.58	-11.57	-11.56
-11.64	-11.63	-11.62	-11.61	-11.6	-11.59	-11.58	-11.57	-11.56
-11.64	-11.63	-11.62	-11.61	-11.6	-11.59	-11.58	-11.57	-11.56
-11.64	-11.63	-11.62	-11.61	-11.6	-11.59	-11.58	-11.57	-11.56
-11.64	-11.63	-11.62	-11.61	-11.6	-11.59	-11.58	-11.57	-11.56
-11.64	-11.63	-11.62	-11.61	-11.6	-11.59	-11.58	-11.57	-11.56
-11.64	-11.63	-11.62	-11.61	-11.6	-11.59	-11.58	-11.57	-11.56
-11.64	-11.63	-11.62	-11.61	-11.6	-11.59	-11.58	-11.57	-11.56
-11.64	-11.63	-11.62	-11.61	-11.6	-11.59	-11.58	-11.57	-11.56
-11.64	-11.63	-11.62	-11.61	-11.6	-11.59	-11.58	-11.57	-11.56
-11.64	-11.63	-11.62	-11.61	-11.6	-11.59	-11.58	-11.57	-11.56
-11.64	-11.63	-11.62	-11.61	-11.6	-11.59	-11.58	-11.57	-11.56
-11.64	-11.63	-11.62	-11.61	-11.6	-11.59	-11.58	-11.57	-11.56
-11.64	-11.63	-11.62	-11.61	-11.6	-11.59	-11.58	-11.57	-11.56
-11.64	-11.63	-11.62	-11.61	-11.6	-11.59	-11.58	-11.57	-11.56
-11.64	-11.63	-11.62	-11.61	-11.6	-11.59	-11.58	-11.57	-11.56
-11.64	-11.63	-11.62	-11.61	-11.6	-11.59	-11.58	-11.57	-11.56
-11.64	-11.63	-11.62	-11.61	-11.6	-11.59	-11.58	-11.57	-11.56
-11.64	-11.63	-11.62	-11.61	-11.6	-11.59	-11.58	-11.57	-11.56
-11.64	-11.63	-11.62	-11.61	-11.6	-11.59	-11.58	-11.57	-11.56
-11.64	-11.63	-11.62	-11.61	-11.6	-11.59	-11.58	-11.57	-11.56
-11.64	-11.63	-11.62	-11.61	-11.6	-11.59	-11.58	-11.57	-11.56
-11.64	-11.63	-11.62	-11.61	-11.6	-11.59	-11.58	-11.57	-11.56
-11.64	-11.63	-11.62	-11.61	-11.6	-11.59	-11.58	-11.57	-11.56
-11.64	-11.63	-11.62	-11.61	-11.6	-11.59	-11.58	-11.57	-11.56
-11.64	-11.63	-11.62	-11.61	-11.6	-11.59	-11.58	-11.57	-11.56
-11.64	-11.63	-11.62	-11.61	-11.6	-11.59	-11.58	-11.57	-11.56
-11.64	-11.63	-11.62	-11.61	-11.6	-11.59	-11.58	-11.57	-11.56
-11.64	-11.63	-11.62	-11.61	-11.6	-11.59	-11.58	-11.57	-11.56
-11.64	-11.63	-11.62	-11.61	-11.6	-11.59	-11.58	-11.57	-11.56

-11.64	-11.63	-11.62	-11.61	-11.6	-11.59	-11.58	-11.57	-11.56
-11.64	-11.63	-11.62	-11.61	-11.6	-11.59	-11.58	-11.57	-11.56
-11.64	-11.63	-11.62	-11.61	-11.6	-11.59	-11.58	-11.57	-11.56
-11.64	-11.63	-11.62	-11.61	-11.6	-11.59	-11.58	-11.57	-11.56
-11.64	-11.63	-11.62	-11.61	-11.6	-11.59	-11.58	-11.57	-11.56
-11.64	-11.63	-11.62	-11.61	-11.6	-11.59	-11.58	-11.57	-11.56
-11.64	-11.63	-11.62	-11.61	-11.6	-11.59	-11.58	-11.57	-11.56
-11.64	-11.63	-11.62	-11.61	-11.6	-11.59	-11.58	-11.57	-11.56
-11.64	-11.63	-11.62	-11.61	-11.6	-11.59	-11.58	-11.57	-11.56
-11.64	-11.63	-11.62	-11.61	-11.6	-11.59	-11.58	-11.57	-11.56
-11.64	-11.63	-11.62	-11.61	-11.6	-11.59	-11.58	-11.57	-11.56
-11.64	-11.63	-11.62	-11.61	-11.6	-11.59	-11.58	-11.57	-11.56
-11.64	-11.63	-11.62	-11.61	-11.6	-11.59	-11.58	-11.57	-11.56
-11.64	-11.63	-11.62	-11.61	-11.6	-11.59	-11.58	-11.57	-11.56
-11.64	-11.63	-11.62	-11.61	-11.6	-11.59	-11.58	-11.57	-11.56
-11.64	-11.63	-11.62	-11.61	-11.6	-11.59	-11.58	-11.57	-11.56
-11.64	-11.63	-11.62	-11.61	-11.6	-11.59	-11.58	-11.57	-11.56
-11.64	-11.63	-11.62	-11.61	-11.6	-11.59	-11.58	-11.57	-11.56
-11.64	-11.63	-11.62	-11.61	-11.6	-11.59	-11.58	-11.57	-11.56
-11.64	-11.63	-11.62	-11.61	-11.6	-11.59	-11.58	-11.57	-11.56
-11.64	-11.63	-11.62	-11.61	-11.6	-11.59	-11.58	-11.57	-11.56
-11.64	-11.63	-11.62	-11.61	-11.6	-11.59	-11.58	-11.57	-11.56
-11.64	-11.63	-11.62	-11.61	-11.6	-11.59	-11.58	-11.57	-11.56
-11.64	-11.63	-11.62	-11.61	-11.6	-11.59	-11.58	-11.57	-11.56
-11.64	-11.63	-11.62	-11.61	-11.6	-11.59	-11.58	-11.57	-11.56
-11.64	-11.63	-11.62	-11.61	-11.6	-11.59	-11.58	-11.57	-11.56
-11.64	-11.63	-11.62	-11.61	-11.6	-11.59	-11.58	-11.57	-11.56
-11.64	-11.63	-11.62	-11.61	-11.6	-11.59	-11.58	-11.57	-11.56
-11.64	-11.63	-11.62	-11.61	-11.6	-11.59	-11.58	-11.57	-11.56
-11.64	-11.63	-11.62	-11.61	-11.6	-11.59	-11.58	-11.57	-11.56
-11.64	-11.63	-11.62	-11.61	-11.6	-11.59	-11.58	-11.57	-11.56
-11.64	-11.63	-11.62	-11.61	-11.6	-11.59	-11.58	-11.57	-11.56
-11.64	-11.63	-11.62	-11.61	-11.6	-11.59	-11.58	-11.57	-11.56
-11.64	-11.63	-11.62	-11.61	-11.6	-11.59	-11.58	-11.57	-11.56
-11.64	-11.63	-11.62	-11.61	-11.6	-11.59	-11.58	-11.57	-11.56
-11.64	-11.63	-11.62	-11.61	-11.6	-11.59	-11.58	-11.57	-11.56
-11.64	-11.63	-11.62	-11.61	-11.6	-11.59	-11.58	-11.57	-11.56
-11.64	-11.63	-11.62	-11.61	-11.6	-11.59	-11.58	-11.57	-11.56
-11.64	-11.63	-11.62	-11.61	-11.6	-11.59	-11.58	-11.57	-11.56
-11.64	-11.63	-11.62	-11.61	-11.6	-11.59	-11.58	-11.57	-11.56
-11.64	-11.63	-11.62	-11.61	-11.6	-11.59	-11.58	-11.57	-11.56
-11.64	-11.63	-11.62	-11.61	-11.6	-11.59	-11.58	-11.57	-11.56
-11.64	-11.63	-11.62	-11.61	-11.6	-11.59	-11.58	-11.57	-11.56
-11.64	-11.63	-11.62	-11.61	-11.6	-11.59	-11.58	-11.57	-11.56
-11.64	-11.63	-11.62	-11.61	-11.6	-11.59	-11.58	-11.57	-11.56
-11.64	-11.63	-11.62	-11.61	-11.6	-11.59	-11.58	-11.57	-11.56
-11.64	-11.63	-11.62	-11.61	-11.6	-11.59	-11.58	-11.57	-11.56
-11.64	-11.63	-11.62	-11.61	-11.6	-11.59	-11.58	-11.57	-11.56
-11.64	-11.63	-11.62	-11.61	-11.6	-11.59	-11.58	-11.57	-11.56
-11.64	-11.63	-11.62	-11.61	-11.6	-11.59	-11.58	-11.57	-11.56

-11.64	-11.63	-11.62	-11.61	-11.6	-11.59	-11.58	-11.57	-11.56
-11.64	-11.63	-11.62	-11.61	-11.6	-11.59	-11.58	-11.57	-11.56
-11.64	-11.63	-11.62	-11.61	-11.6	-11.59	-11.58	-11.57	-11.56
-11.64	-11.63	-11.62	-11.61	-11.6	-11.59	-11.58	-11.57	-11.56
-11.64	-11.63	-11.62	-11.61	-11.6	-11.59	-11.58	-11.57	-11.56
-11.64	-11.63	-11.62	-11.61	-11.6	-11.59	-11.58	-11.57	-11.56
-11.64	-11.63	-11.62	-11.61	-11.6	-11.59	-11.58	-11.57	-11.56
-11.64	-11.63	-11.62	-11.61	-11.6	-11.59	-11.58	-11.57	-11.56
-11.64	-11.63	-11.62	-11.61	-11.6	-11.59	-11.58	-11.57	-11.56
-11.64	-11.63	-11.62	-11.61	-11.6	-11.59	-11.58	-11.57	-11.56
-11.64	-11.63	-11.62	-11.61	-11.6	-11.59	-11.58	-11.57	-11.56
-11.64	-11.63	-11.62	-11.61	-11.6	-11.59	-11.58	-11.57	-11.56
-11.64	-11.63	-11.62	-11.61	-11.6	-11.59	-11.58	-11.57	-11.56
-11.64	-11.63	-11.62	-11.61	-11.6	-11.59	-11.58	-11.57	-11.56
-11.64	-11.63	-11.62	-11.61	-11.6	-11.59	-11.58	-11.57	-11.56
-11.64	-11.63	-11.62	-11.61	-11.6	-11.59	-11.58	-11.57	-11.56
-11.64	-11.63	-11.62	-11.61	-11.6	-11.59	-11.58	-11.57	-11.56
-11.64	-11.63	-11.62	-11.61	-11.6	-11.59	-11.58	-11.57	-11.56
-11.64	-11.63	-11.62	-11.61	-11.6	-11.59	-11.58	-11.57	-11.56
-11.64	-11.63	-11.62	-11.61	-11.6	-11.59	-11.58	-11.57	-11.56
-11.64	-11.63	-11.62	-11.61	-11.6	-11.59	-11.58	-11.57	-11.56
-11.64	-11.63	-11.62	-11.61	-11.6	-11.59	-11.58	-11.57	-11.56
-11.64	-11.63	-11.62	-11.61	-11.6	-11.59	-11.58	-11.57	-11.56
-11.64	-11.63	-11.62	-11.61	-11.6	-11.59	-11.58	-11.57	-11.56
-11.64	-11.63	-11.62	-11.61	-11.6	-11.59	-11.58	-11.57	-11.56
-11.64	-11.63	-11.62	-11.61	-11.6	-11.59	-11.58	-11.57	-11.56
-11.64	-11.63	-11.62	-11.61	-11.6	-11.59	-11.58	-11.57	-11.56
-11.64	-11.63	-11.62	-11.61	-11.6	-11.59	-11.58	-11.57	-11.56
-11.64	-11.63	-11.62	-11.61	-11.6	-11.59	-11.58	-11.57	-11.56
-11.64	-11.63	-11.62	-11.61	-11.6	-11.59	-11.58	-11.57	-11.56
-11.64	-11.63	-11.62	-11.61	-11.6	-11.59	-11.58	-11.57	-11.56
-11.64	-11.63	-11.62	-11.61	-11.6	-11.59	-11.58	-11.57	-11.56
-11.64	-11.63	-11.62	-11.61	-11.6	-11.59	-11.58	-11.57	-11.56
-11.64	-11.63	-11.62	-11.61	-11.6	-11.59	-11.58	-11.57	-11.56
-11.64	-11.63	-11.62	-11.61	-11.6	-11.59	-11.58	-11.57	-11.56
-11.64	-11.63	-11.62	-11.61	-11.6	-11.59	-11.58	-11.57	-11.56
-11.64	-11.63	-11.62	-11.61	-11.6	-11.59	-11.58	-11.57	-11.56
-11.64	-11.63	-11.62	-11.61	-11.6	-11.59	-11.58	-11.57	-11.56
-11.64	-11.63	-11.62	-11.61	-11.6	-11.59	-11.58	-11.57	-11.56
-11.64	-11.63	-11.62	-11.61	-11.6	-11.59	-11.58	-11.57	-11.56
-11.64	-11.63	-11.62	-11.61	-11.6	-11.59	-11.58	-11.57	-11.56
-11.64	-11.63	-11.62	-11.61	-11.6	-11.59	-11.58	-11.57	-11.56
-11.64	-11.63	-11.62	-11.61	-11.6	-11.59	-11.58	-11.57	-11.56
-11.64	-11.63	-11.62	-11.61	-11.6	-11.59	-11.58	-11.57	-11.56
-11.64	-11.63	-11.62	-11.61	-11.6	-11.59	-11.58	-11.57	-11.56
-11.64	-11.63	-11.62	-11.61	-11.6	-11.59	-11.58	-11.57	-11.56
-11.64	-11.63	-11.62	-11.61	-11.6	-11.59	-11.58	-11.57	-11.56
-11.64	-11.63	-11.62	-11.61	-11.6	-11.59	-11.58	-11.57	-11.56
-11.64	-11.63	-11.62	-11.61	-11.6	-11.59	-11.58	-11.57	-11.56
-11.64	-11.63	-11.62	-11.61	-11.6	-11.59	-11.58	-11.57	-11.56

-11.64	-11.63	-11.62	-11.61	-11.6	-11.59	-11.58	-11.57	-11.56
-11.64	-11.63	-11.62	-11.61	-11.6	-11.59	-11.58	-11.57	-11.56
-11.64	-11.63	-11.62	-11.61	-11.6	-11.59	-11.58	-11.57	-11.56
-11.64	-11.63	-11.62	-11.61	-11.6	-11.59	-11.58	-11.57	-11.56
-11.64	-11.63	-11.62	-11.61	-11.6	-11.59	-11.58	-11.57	-11.56
-11.64	-11.63	-11.62	-11.61	-11.6	-11.59	-11.58	-11.57	-11.56
-11.64	-11.63	-11.62	-11.61	-11.6	-11.59	-11.58	-11.57	-11.56
-11.64	-11.63	-11.62	-11.61	-11.6	-11.59	-11.58	-11.57	-11.56
-11.64	-11.63	-11.62	-11.61	-11.6	-11.59	-11.58	-11.57	-11.56
-11.64	-11.63	-11.62	-11.61	-11.6	-11.59	-11.58	-11.57	-11.56
-11.64	-11.63	-11.62	-11.61	-11.6	-11.59	-11.58	-11.57	-11.56
-11.64	-11.63	-11.62	-11.61	-11.6	-11.59	-11.58	-11.57	-11.56
-11.64	-11.63	-11.62	-11.61	-11.6	-11.59	-11.58	-11.57	-11.56
-11.64	-11.63	-11.62	-11.61	-11.6	-11.59	-11.58	-11.57	-11.56
-11.64	-11.63	-11.62	-11.61	-11.6	-11.59	-11.58	-11.57	-11.56
-11.64	-11.63	-11.62	-11.61	-11.6	-11.59	-11.58	-11.57	-11.56
-11.64	-11.63	-11.62	-11.61	-11.6	-11.59	-11.58	-11.57	-11.56
-11.64	-11.63	-11.62	-11.61	-11.6	-11.59	-11.58	-11.57	-11.56
-11.64	-11.63	-11.62	-11.61	-11.6	-11.59	-11.58	-11.57	-11.56
-11.64	-11.63	-11.62	-11.61	-11.6	-11.59	-11.58	-11.57	-11.56
-11.64	-11.63	-11.62	-11.61	-11.6	-11.59	-11.58	-11.57	-11.56
-11.64	-11.63	-11.62	-11.61	-11.6	-11.59	-11.58	-11.57	-11.56
-11.64	-11.63	-11.62	-11.61	-11.6	-11.59	-11.58	-11.57	-11.56
-11.64	-11.63	-11.62	-11.61	-11.6	-11.59	-11.58	-11.57	-11.56
-11.64	-11.63	-11.62	-11.61	-11.6	-11.59	-11.58	-11.57	-11.56
-11.64	-11.63	-11.62	-11.61	-11.6	-11.59	-11.58	-11.57	-11.56
-11.64	-11.63	-11.62	-11.61	-11.6	-11.59	-11.58	-11.57	-11.56
-11.64	-11.63	-11.62	-11.61	-11.6	-11.59	-11.58	-11.57	-11.56
-11.64	-11.63	-11.62	-11.61	-11.6	-11.59	-11.58	-11.57	-11.56
-11.64	-11.63	-11.62	-11.61	-11.6	-11.59	-11.58	-11.57	-11.56
-11.64	-11.63	-11.62	-11.61	-11.6	-11.59	-11.58	-11.57	-11.56
-11.64	-11.63	-11.62	-11.61	-11.6	-11.59	-11.58	-11.57	-11.56
-11.64	-11.63	-11.62	-11.61	-11.6	-11.59	-11.58	-11.57	-11.56
-11.64	-11.63	-11.62	-11.61	-11.6	-11.59	-11.58	-11.57	-11.56
-11.64	-11.63	-11.62	-11.61	-11.6	-11.59	-11.58	-11.57	-11.56
-11.64	-11.63	-11.62	-11.61	-11.6	-11.59	-11.58	-11.57	-11.56
-11.64	-11.63	-11.62	-11.61	-11.6	-11.59	-11.58	-11.57	-11.56
-11.64	-11.63	-11.62	-11.61	-11.6	-11.59	-11.58	-11.57	-11.56
-11.64	-11.63	-11.62	-11.61	-11.6	-11.59	-11.58	-11.57	-11.56
-11.64	-11.63	-11.62	-11.61	-11.6	-11.59	-11.58	-11.57	-11.56
-11.64	-11.63	-11.62	-11.61	-11.6	-11.59	-11.58	-11.57	-11.56
-11.64	-11.63	-11.62	-11.61	-11.6	-11.59	-11.58	-11.57	-11.56
-11.64	-11.63	-11.62	-11.61	-11.6	-11.59	-11.58	-11.57	-11.56
-11.64	-11.63	-11.62	-11.61	-11.6	-11.59	-11.58	-11.57	-11.56
-11.64	-11.63	-11.62	-11.61	-11.6	-11.59	-11.58	-11.57	-11.56
-11.64	-11.63	-11.62	-11.61	-11.6	-11.59	-11.58	-11.57	-11.56
-11.64	-11.63	-11.62	-11.61	-11.6	-11.59	-11.58	-11.57	-11.56
-11.64	-11.63	-11.62	-11.61	-11.6	-11.59	-11.58	-11.57	-11.56
-11.64	-11.63	-11.62	-11.61	-11.6	-11.59	-11.58	-11.57	-11.56
-11.64	-11.63	-11.62	-11.61	-11.6	-11.59	-11.58	-11.57	-11.56

-11.64	-11.63	-11.62	-11.61	-11.6	-11.59	-11.58	-11.57	-11.56
-11.64	-11.63	-11.62	-11.61	-11.6	-11.59	-11.58	-11.57	-11.56
-11.64	-11.63	-11.62	-11.61	-11.6	-11.59	-11.58	-11.57	-11.56
-11.64	-11.63	-11.62	-11.61	-11.6	-11.59	-11.58	-11.57	-11.56
-11.64	-11.63	-11.62	-11.61	-11.6	-11.59	-11.58	-11.57	-11.56
-11.64	-11.63	-11.62	-11.61	-11.6	-11.59	-11.58	-11.57	-11.56
-11.64	-11.63	-11.62	-11.61	-11.6	-11.59	-11.58	-11.57	-11.56
-11.64	-11.63	-11.62	-11.61	-11.6	-11.59	-11.58	-11.57	-11.56
-11.64	-11.63	-11.62	-11.61	-11.6	-11.59	-11.58	-11.57	-11.56
-11.64	-11.63	-11.62	-11.61	-11.6	-11.59	-11.58	-11.57	-11.56
-11.64	-11.63	-11.62	-11.61	-11.6	-11.59	-11.58	-11.57	-11.56
-11.64	-11.63	-11.62	-11.61	-11.6	-11.59	-11.58	-11.57	-11.56
-11.64	-11.63	-11.62	-11.61	-11.6	-11.59	-11.58	-11.57	-11.56
-11.64	-11.63	-11.62	-11.61	-11.6	-11.59	-11.58	-11.57	-11.56
-11.64	-11.63	-11.62	-11.61	-11.6	-11.59	-11.58	-11.57	-11.56
-11.64	-11.63	-11.62	-11.61	-11.6	-11.59	-11.58	-11.57	-11.56
-11.64	-11.63	-11.62	-11.61	-11.6	-11.59	-11.58	-11.57	-11.56
-11.64	-11.63	-11.62	-11.61	-11.6	-11.59	-11.58	-11.57	-11.56
-11.64	-11.63	-11.62	-11.61	-11.6	-11.59	-11.58	-11.57	-11.56
-11.64	-11.63	-11.62	-11.61	-11.6	-11.59	-11.58	-11.57	-11.56
-11.64	-11.63	-11.62	-11.61	-11.6	-11.59	-11.58	-11.57	-11.56
-11.64	-11.63	-11.62	-11.61	-11.6	-11.59	-11.58	-11.57	-11.56
-11.64	-11.63	-11.62	-11.61	-11.6	-11.59	-11.58	-11.57	-11.56
-11.64	-11.63	-11.62	-11.61	-11.6	-11.59	-11.58	-11.57	-11.56
-11.64	-11.63	-11.62	-11.61	-11.6	-11.59	-11.58	-11.57	-11.56
-11.64	-11.63	-11.62	-11.61	-11.6	-11.59	-11.58	-11.57	-11.56
-11.64	-11.63	-11.62	-11.61	-11.6	-11.59	-11.58	-11.57	-11.56
-11.64	-11.63	-11.62	-11.61	-11.6	-11.59	-11.58	-11.57	-11.56
-11.64	-11.63	-11.62	-11.61	-11.6	-11.59	-11.58	-11.57	-11.56
-11.64	-11.63	-11.62	-11.61	-11.6	-11.59	-11.58	-11.57	-11.56
-11.64	-11.63	-11.62	-11.61	-11.6	-11.59	-11.58	-11.57	-11.56
-11.64	-11.63	-11.62	-11.61	-11.6	-11.59	-11.58	-11.57	-11.56
-11.64	-11.63	-11.62	-11.61	-11.6	-11.59	-11.58	-11.57	-11.56
-11.64	-11.63	-11.62	-11.61	-11.6	-11.59	-11.58	-11.57	-11.56
-11.64	-11.63	-11.62	-11.61	-11.6	-11.59	-11.58	-11.57	-11.56
-11.64	-11.63	-11.62	-11.61	-11.6	-11.59	-11.58	-11.57	-11.56
-11.64	-11.63	-11.62	-11.61	-11.6	-11.59	-11.58	-11.57	-11.56
-11.64	-11.63	-11.62	-11.61	-11.6	-11.59	-11.58	-11.57	-11.56
-11.64	-11.63	-11.62	-11.61	-11.6	-11.59	-11.58	-11.57	-11.56
-11.64	-11.63	-11.62	-11.61	-11.6	-11.59	-11.58	-11.57	-11.56
-11.64	-11.63	-11.62	-11.61	-11.6	-11.59	-11.58	-11.57	-11.56
-11.64	-11.63	-11.62	-11.61	-11.6	-11.59	-11.58	-11.57	-11.56
-11.64	-11.63	-11.62	-11.61	-11.6	-11.59	-11.58	-11.57	-11.56
-11.64	-11.63	-11.62	-11.61	-11.6	-11.59	-11.58	-11.57	-11.56
-11.64	-11.63	-11.62	-11.61	-11.6	-11.59	-11.58	-11.57	-11.56
-11.64	-11.63	-11.62	-11.61	-11.6	-11.59	-11.58	-11.57	-11.56
-11.64	-11.63	-11.62	-11.61	-11.6	-11.59	-11.58	-11.57	-11.56
-11.64	-11.63	-11.62	-11.61	-11.6	-11.59	-11.58	-11.57	-11.56
-11.64	-11.63	-11.62	-11.61	-11.6	-11.59	-11.58	-11.57	-11.56
-11.64	-11.63	-11.62	-11.61	-11.6	-11.59	-11.58	-11.57	-11.56

-11.64	-11.63	-11.62	-11.61	-11.6	-11.59	-11.58	-11.57	-11.56
-11.64	-11.63	-11.62	-11.61	-11.6	-11.59	-11.58	-11.57	-11.56
-11.64	-11.63	-11.62	-11.61	-11.6	-11.59	-11.58	-11.57	-11.56
-11.64	-11.63	-11.62	-11.61	-11.6	-11.59	-11.58	-11.57	-11.56
-11.64	-11.63	-11.62	-11.61	-11.6	-11.59	-11.58	-11.57	-11.56
-11.64	-11.63	-11.62	-11.61	-11.6	-11.59	-11.58	-11.57	-11.56
-11.64	-11.63	-11.62	-11.61	-11.6	-11.59	-11.58	-11.57	-11.56
-11.64	-11.63	-11.62	-11.61	-11.6	-11.59	-11.58	-11.57	-11.56
-11.64	-11.63	-11.62	-11.61	-11.6	-11.59	-11.58	-11.57	-11.56
-11.64	-11.63	-11.62	-11.61	-11.6	-11.59	-11.58	-11.57	-11.56
-11.64	-11.63	-11.62	-11.61	-11.6	-11.59	-11.58	-11.57	-11.56
-11.64	-11.63	-11.62	-11.61	-11.6	-11.59	-11.58	-11.57	-11.56
-11.64	-11.63	-11.62	-11.61	-11.6	-11.59	-11.58	-11.57	-11.56
-11.64	-11.63	-11.62	-11.61	-11.6	-11.59	-11.58	-11.57	-11.56
-11.64	-11.63	-11.62	-11.61	-11.6	-11.59	-11.58	-11.57	-11.56
-11.64	-11.63	-11.62	-11.61	-11.6	-11.59	-11.58	-11.57	-11.56
-11.64	-11.63	-11.62	-11.61	-11.6	-11.59	-11.58	-11.57	-11.56
-11.64	-11.63	-11.62	-11.61	-11.6	-11.59	-11.58	-11.57	-11.56
-11.64	-11.63	-11.62	-11.61	-11.6	-11.59	-11.58	-11.57	-11.56
-11.64	-11.63	-11.62	-11.61	-11.6	-11.59	-11.58	-11.57	-11.56
-11.64	-11.63	-11.62	-11.61	-11.6	-11.59	-11.58	-11.57	-11.56
-11.64	-11.63	-11.62	-11.61	-11.6	-11.59	-11.58	-11.57	-11.56
-11.64	-11.63	-11.62	-11.61	-11.6	-11.59	-11.58	-11.57	-11.56
-11.64	-11.63	-11.62	-11.61	-11.6	-11.59	-11.58	-11.57	-11.56
-11.64	-11.63	-11.62	-11.61	-11.6	-11.59	-11.58	-11.57	-11.56
-11.64	-11.63	-11.62	-11.61	-11.6	-11.59	-11.58	-11.57	-11.56
-11.64	-11.63	-11.62	-11.61	-11.6	-11.59	-11.58	-11.57	-11.56
-11.64	-11.63	-11.62	-11.61	-11.6	-11.59	-11.58	-11.57	-11.56
-11.64	-11.63	-11.62	-11.61	-11.6	-11.59	-11.58	-11.57	-11.56
-11.64	-11.63	-11.62	-11.61	-11.6	-11.59	-11.58	-11.57	-11.56
-11.64	-11.63	-11.62	-11.61	-11.6	-11.59	-11.58	-11.57	-11.56
-11.64	-11.63	-11.62	-11.61	-11.6	-11.59	-11.58	-11.57	-11.56
-11.64	-11.63	-11.62	-11.61	-11.6	-11.59	-11.58	-11.57	-11.56
-11.64	-11.63	-11.62	-11.61	-11.6	-11.59	-11.58	-11.57	-11.56
-11.64	-11.63	-11.62	-11.61	-11.6	-11.59	-11.58	-11.57	-11.56
-11.64	-11.63	-11.62	-11.61	-11.6	-11.59	-11.58	-11.57	-11.56
-11.64	-11.63	-11.62	-11.61	-11.6	-11.59	-11.58	-11.57	-11.56
-11.64	-11.63	-11.62	-11.61	-11.6	-11.59	-11.58	-11.57	-11.56
-11.64	-11.63	-11.62	-11.61	-11.6	-11.59	-11.58	-11.57	-11.56
-11.64	-11.63	-11.62	-11.61	-11.6	-11.59	-11.58	-11.57	-11.56
-11.64	-11.63	-11.62	-11.61	-11.6	-11.59	-11.58	-11.57	-11.56
-11.64	-11.63	-11.62	-11.61	-11.6	-11.59	-11.58	-11.57	-11.56
-11.64	-11.63	-11.62	-11.61	-11.6	-11.59	-11.58	-11.57	-11.56
-11.64	-11.63	-11.62	-11.61	-11.6	-11.59	-11.58	-11.57	-11.56
-11.64	-11.63	-11.62	-11.61	-11.6	-11.59	-11.58	-11.57	-11.56
-11.64	-11.63	-11.62	-11.61	-11.6	-11.59	-11.58	-11.57	-11.56
-11.64	-11.63	-11.62	-11.61	-11.6	-11.59	-11.58	-11.57	-11.56
-11.64	-11.63	-11.62	-11.61	-11.6	-11.59	-11.58	-11.57	-11.56
-11.64	-11.63	-11.62	-11.61	-11.6	-11.59	-11.58	-11.57	-11.56
-11.64	-11.63	-11.62	-11.61	-11.6	-11.59	-11.58	-11.57	-11.56

-11.64	-11.63	-11.62	-11.61	-11.6	-11.59	-11.58	-11.57	-11.56
-11.64	-11.63	-11.62	-11.61	-11.6	-11.59	-11.58	-11.57	-11.56
-11.64	-11.63	-11.62	-11.61	-11.6	-11.59	-11.58	-11.57	-11.56
-11.64	-11.63	-11.62	-11.61	-11.6	-11.59	-11.58	-11.57	-11.56
-11.64	-11.63	-11.62	-11.61	-11.6	-11.59	-11.58	-11.57	-11.56
-11.64	-11.63	-11.62	-11.61	-11.6	-11.59	-11.58	-11.57	-11.56
-11.64	-11.63	-11.62	-11.61	-11.6	-11.59	-11.58	-11.57	-11.56
-11.64	-11.63	-11.62	-11.61	-11.6	-11.59	-11.58	-11.57	-11.56
-11.64	-11.63	-11.62	-11.61	-11.6	-11.59	-11.58	-11.57	-11.56
-11.64	-11.63	-11.62	-11.61	-11.6	-11.59	-11.58	-11.57	-11.56
-11.64	-11.63	-11.62	-11.61	-11.6	-11.59	-11.58	-11.57	-11.56
-11.64	-11.63	-11.62	-11.61	-11.6	-11.59	-11.58	-11.57	-11.56
-11.64	-11.63	-11.62	-11.61	-11.6	-11.59	-11.58	-11.57	-11.56
-11.64	-11.63	-11.62	-11.61	-11.6	-11.59	-11.58	-11.57	-11.56
-11.64	-11.63	-11.62	-11.61	-11.6	-11.59	-11.58	-11.57	-11.56
-11.64	-11.63	-11.62	-11.61	-11.6	-11.59	-11.58	-11.57	-11.56
-11.64	-11.63	-11.62	-11.61	-11.6	-11.59	-11.58	-11.57	-11.56
-11.64	-11.63	-11.62	-11.61	-11.6	-11.59	-11.58	-11.57	-11.56
-11.64	-11.63	-11.62	-11.61	-11.6	-11.59	-11.58	-11.57	-11.56
-11.64	-11.63	-11.62	-11.61	-11.6	-11.59	-11.58	-11.57	-11.56
-11.64	-11.63	-11.62	-11.61	-11.6	-11.59	-11.58	-11.57	-11.56
-11.64	-11.63	-11.62	-11.61	-11.6	-11.59	-11.58	-11.57	-11.56
-11.64	-11.63	-11.62	-11.61	-11.6	-11.59	-11.58	-11.57	-11.56
-11.64	-11.63	-11.62	-11.61	-11.6	-11.59	-11.58	-11.57	-11.56
-11.64	-11.63	-11.62	-11.61	-11.6	-11.59	-11.58	-11.57	-11.56
-11.64	-11.63	-11.62	-11.61	-11.6	-11.59	-11.58	-11.57	-11.56
-11.64	-11.63	-11.62	-11.61	-11.6	-11.59	-11.58	-11.57	-11.56
-11.64	-11.63	-11.62	-11.61	-11.6	-11.59	-11.58	-11.57	-11.56
-11.64	-11.63	-11.62	-11.61	-11.6	-11.59	-11.58	-11.57	-11.56
-11.64	-11.63	-11.62	-11.61	-11.6	-11.59	-11.58	-11.57	-11.56
-11.64	-11.63	-11.62	-11.61	-11.6	-11.59	-11.58	-11.57	-11.56
-11.64	-11.63	-11.62	-11.61	-11.6	-11.59	-11.58	-11.57	-11.56
-11.64	-11.63	-11.62	-11.61	-11.6	-11.59	-11.58	-11.57	-11.56
-11.64	-11.63	-11.62	-11.61	-11.6	-11.59	-11.58	-11.57	-11.56
-11.64	-11.63	-11.62	-11.61	-11.6	-11.59	-11.58	-11.57	-11.56
-11.64	-11.63	-11.62	-11.61	-11.6	-11.59	-11.58	-11.57	-11.56
-11.64	-11.63	-11.62	-11.61	-11.6	-11.59	-11.58	-11.57	-11.56
-11.64	-11.63	-11.62	-11.61	-11.6	-11.59	-11.58	-11.57	-11.56
-11.64	-11.63	-11.62	-11.61	-11.6	-11.59	-11.58	-11.57	-11.56
-11.64	-11.63	-11.62	-11.61	-11.6	-11.59	-11.58	-11.57	-11.56
-11.64	-11.63	-11.62	-11.61	-11.6	-11.59	-11.58	-11.57	-11.56
-11.64	-11.63	-11.62	-11.61	-11.6	-11.59	-11.58	-11.57	-11.56
-11.64	-11.63	-11.62	-11.61	-11.6	-11.59	-11.58	-11.57	-11.56
-11.64	-11.63	-11.62	-11.61	-11.6	-11.59	-11.58	-11.57	-11.56
-11.64	-11.63	-11.62	-11.61	-11.6	-11.59	-11.58	-11.57	-11.56
-11.64	-11.63	-11.62	-11.61	-11.6	-11.59	-11.58	-11.57	-11.56
-11.64	-11.63	-11.62	-11.61	-11.6	-11.59	-11.58	-11.57	-11.56
-11.64	-11.63	-11.62	-11.61	-11.6	-11.59	-11.58	-11.57	-11.56
-11.64	-11.63	-11.62	-11.61	-11.6	-11.59	-11.58	-11.57	-11.56
-11.64	-11.63	-11.62	-11.61	-11.6	-11.59	-11.58	-11.57	-11.56

-11.64	-11.63	-11.62	-11.61	-11.6	-11.59	-11.58	-11.57	-11.56
-11.64	-11.63	-11.62	-11.61	-11.6	-11.59	-11.58	-11.57	-11.56
-11.64	-11.63	-11.62	-11.61	-11.6	-11.59	-11.58	-11.57	-11.56
-11.64	-11.63	-11.62	-11.61	-11.6	-11.59	-11.58	-11.57	-11.56
-11.64	-11.63	-11.62	-11.61	-11.6	-11.59	-11.58	-11.57	-11.56
-11.64	-11.63	-11.62	-11.61	-11.6	-11.59	-11.58	-11.57	-11.56
-11.64	-11.63	-11.62	-11.61	-11.6	-11.59	-11.58	-11.57	-11.56
-11.64	-11.63	-11.62	-11.61	-11.6	-11.59	-11.58	-11.57	-11.56
-11.64	-11.63	-11.62	-11.61	-11.6	-11.59	-11.58	-11.57	-11.56
-11.64	-11.63	-11.62	-11.61	-11.6	-11.59	-11.58	-11.57	-11.56
-11.64	-11.63	-11.62	-11.61	-11.6	-11.59	-11.58	-11.57	-11.56
-11.64	-11.63	-11.62	-11.61	-11.6	-11.59	-11.58	-11.57	-11.56
-11.64	-11.63	-11.62	-11.61	-11.6	-11.59	-11.58	-11.57	-11.56
-11.64	-11.63	-11.62	-11.61	-11.6	-11.59	-11.58	-11.57	-11.56
-11.64	-11.63	-11.62	-11.61	-11.6	-11.59	-11.58	-11.57	-11.56
-11.64	-11.63	-11.62	-11.61	-11.6	-11.59	-11.58	-11.57	-11.56
-11.64	-11.63	-11.62	-11.61	-11.6	-11.59	-11.58	-11.57	-11.56
-11.64	-11.63	-11.62	-11.61	-11.6	-11.59	-11.58	-11.57	-11.56
-11.64	-11.63	-11.62	-11.61	-11.6	-11.59	-11.58	-11.57	-11.56
-11.64	-11.63	-11.62	-11.61	-11.6	-11.59	-11.58	-11.57	-11.56
-11.64	-11.63	-11.62	-11.61	-11.6	-11.59	-11.58	-11.57	-11.56
-11.64	-11.63	-11.62	-11.61	-11.6	-11.59	-11.58	-11.57	-11.56
-11.64	-11.63	-11.62	-11.61	-11.6	-11.59	-11.58	-11.57	-11.56
-11.64	-11.63	-11.62	-11.61	-11.6	-11.59	-11.58	-11.57	-11.56
-11.64	-11.63	-11.62	-11.61	-11.6	-11.59	-11.58	-11.57	-11.56
-11.64	-11.63	-11.62	-11.61	-11.6	-11.59	-11.58	-11.57	-11.56
-11.64	-11.63	-11.62	-11.61	-11.6	-11.59	-11.58	-11.57	-11.56
-11.64	-11.63	-11.62	-11.61	-11.6	-11.59	-11.58	-11.57	-11.56
-11.64	-11.63	-11.62	-11.61	-11.6	-11.59	-11.58	-11.57	-11.56
-11.64	-11.63	-11.62	-11.61	-11.6	-11.59	-11.58	-11.57	-11.56
-11.64	-11.63	-11.62	-11.61	-11.6	-11.59	-11.58	-11.57	-11.56
-11.64	-11.63	-11.62	-11.61	-11.6	-11.59	-11.58	-11.57	-11.56
-11.64	-11.63	-11.62	-11.61	-11.6	-11.59	-11.58	-11.57	-11.56
-11.64	-11.63	-11.62	-11.61	-11.6	-11.59	-11.58	-11.57	-11.56
-11.64	-11.63	-11.62	-11.61	-11.6	-11.59	-11.58	-11.57	-11.56
-11.64	-11.63	-11.62	-11.61	-11.6	-11.59	-11.58	-11.57	-11.56
-11.64	-11.63	-11.62	-11.61	-11.6	-11.59	-11.58	-11.57	-11.56
-11.64	-11.63	-11.62	-11.61	-11.6	-11.59	-11.58	-11.57	-11.56
-11.64	-11.63	-11.62	-11.61	-11.6	-11.59	-11.58	-11.57	-11.56
-11.64	-11.63	-11.62	-11.61	-11.6	-11.59	-11.58	-11.57	-11.56
-11.64	-11.63	-11.62	-11.61	-11.6	-11.59	-11.58	-11.57	-11.56
-11.64	-11.63	-11.62	-11.61	-11.6	-11.59	-11.58	-11.57	-11.56
-11.64	-11.63	-11.62	-11.61	-11.6	-11.59	-11.58	-11.57	-11.56
-11.64	-11.63	-11.62	-11.61	-11.6	-11.59	-11.58	-11.57	-11.56
-11.64	-11.63	-11.62	-11.61	-11.6	-11.59	-11.58	-11.57	-11.56
-11.64	-11.63	-11.62	-11.61	-11.6	-11.59	-11.58	-11.57	-11.56
-11.64	-11.63	-11.62	-11.61	-11.6	-11.59	-11.58	-11.57	-11.56
-11.64	-11.63	-11.62	-11.61	-11.6	-11.59	-11.58	-11.57	-11.56
-11.64	-11.63	-11.62	-11.61	-11.6	-11.59	-11.58	-11.57	-11.56
-11.64	-11.63	-11.62	-11.61	-11.6	-11.59	-11.58	-11.57	-11.56

-11.64	-11.63	-11.62	-11.61	-11.6	-11.59	-11.58	-11.57	-11.56
-11.64	-11.63	-11.62	-11.61	-11.6	-11.59	-11.58	-11.57	-11.56
-11.64	-11.63	-11.62	-11.61	-11.6	-11.59	-11.58	-11.57	-11.56
-11.64	-11.63	-11.62	-11.61	-11.6	-11.59	-11.58	-11.57	-11.56
-11.64	-11.63	-11.62	-11.61	-11.6	-11.59	-11.58	-11.57	-11.56
-11.64	-11.63	-11.62	-11.61	-11.6	-11.59	-11.58	-11.57	-11.56
-11.64	-11.63	-11.62	-11.61	-11.6	-11.59	-11.58	-11.57	-11.56
-11.64	-11.63	-11.62	-11.61	-11.6	-11.59	-11.58	-11.57	-11.56
-11.64	-11.63	-11.62	-11.61	-11.6	-11.59	-11.58	-11.57	-11.56
-11.64	-11.63	-11.62	-11.61	-11.6	-11.59	-11.58	-11.57	-11.56
-11.64	-11.63	-11.62	-11.61	-11.6	-11.59	-11.58	-11.57	-11.56
-11.64	-11.63	-11.62	-11.61	-11.6	-11.59	-11.58	-11.57	-11.56
-11.64	-11.63	-11.62	-11.61	-11.6	-11.59	-11.58	-11.57	-11.56
-11.64	-11.63	-11.62	-11.61	-11.6	-11.59	-11.58	-11.57	-11.56
-11.64	-11.63	-11.62	-11.61	-11.6	-11.59	-11.58	-11.57	-11.56
-11.64	-11.63	-11.62	-11.61	-11.6	-11.59	-11.58	-11.57	-11.56
-11.64	-11.63	-11.62	-11.61	-11.6	-11.59	-11.58	-11.57	-11.56
-11.64	-11.63	-11.62	-11.61	-11.6	-11.59	-11.58	-11.57	-11.56
-11.64	-11.63	-11.62	-11.61	-11.6	-11.59	-11.58	-11.57	-11.56
-11.64	-11.63	-11.62	-11.61	-11.6	-11.59	-11.58	-11.57	-11.56
-11.64	-11.63	-11.62	-11.61	-11.6	-11.59	-11.58	-11.57	-11.56
-11.64	-11.63	-11.62	-11.61	-11.6	-11.59	-11.58	-11.57	-11.56
-11.64	-11.63	-11.62	-11.61	-11.6	-11.59	-11.58	-11.57	-11.56
-11.64	-11.63	-11.62	-11.61	-11.6	-11.59	-11.58	-11.57	-11.56
-11.64	-11.63	-11.62	-11.61	-11.6	-11.59	-11.58	-11.57	-11.56
-11.64	-11.63	-11.62	-11.61	-11.6	-11.59	-11.58	-11.57	-11.56
-11.64	-11.63	-11.62	-11.61	-11.6	-11.59	-11.58	-11.57	-11.56
-11.64	-11.63	-11.62	-11.61	-11.6	-11.59	-11.58	-11.57	-11.56
-11.64	-11.63	-11.62	-11.61	-11.6	-11.59	-11.58	-11.57	-11.56
-11.64	-11.63	-11.62	-11.61	-11.6	-11.59	-11.58	-11.57	-11.56
-11.64	-11.63	-11.62	-11.61	-11.6	-11.59	-11.58	-11.57	-11.56
-11.64	-11.63	-11.62	-11.61	-11.6	-11.59	-11.58	-11.57	-11.56
-11.64	-11.63	-11.62	-11.61	-11.6	-11.59	-11.58	-11.57	-11.56
-11.64	-11.63	-11.62	-11.61	-11.6	-11.59	-11.58	-11.57	-11.56
-11.64	-11.63	-11.62	-11.61	-11.6	-11.59	-11.58	-11.57	-11.56
-11.64	-11.63	-11.62	-11.61	-11.6	-11.59	-11.58	-11.57	-11.56
-11.64	-11.63	-11.62	-11.61	-11.6	-11.59	-11.58	-11.57	-11.56
-11.64	-11.63	-11.62	-11.61	-11.6	-11.59	-11.58	-11.57	-11.56
-11.64	-11.63	-11.62	-11.61	-11.6	-11.59	-11.58	-11.57	-11.56
-11.64	-11.63	-11.62	-11.61	-11.6	-11.59	-11.58	-11.57	-11.56
-11.64	-11.63	-11.62	-11.61	-11.6	-11.59	-11.58	-11.57	-11.56
-11.64	-11.63	-11.62	-11.61	-11.6	-11.59	-11.58	-11.57	-11.56
-11.64	-11.63	-11.62	-11.61	-11.6	-11.59	-11.58	-11.57	-11.56
-11.64	-11.63	-11.62	-11.61	-11.6	-11.59	-11.58	-11.57	-11.56
-11.64	-11.63	-11.62	-11.61	-11.6	-11.59	-11.58	-11.57	-11.56
-11.64	-11.63	-11.62	-11.61	-11.6	-11.59	-11.58	-11.57	-11.56
-11.64	-11.63	-11.62	-11.61	-11.6	-11.59	-11.58	-11.57	-11.56
-11.64	-11.63	-11.62	-11.61	-11.6	-11.59	-11.58	-11.57	-11.56
-11.64	-11.63	-11.62	-11.61	-11.6	-11.59	-11.58	-11.57	-11.56
-11.64	-11.63	-11.62	-11.61	-11.6	-11.59	-11.58	-11.57	-11.56

-11.64	-11.63	-11.62	-11.61	-11.6	-11.59	-11.58	-11.57	-11.56
-11.64	-11.63	-11.62	-11.61	-11.6	-11.59	-11.58	-11.57	-11.56
-11.64	-11.63	-11.62	-11.61	-11.6	-11.59	-11.58	-11.57	-11.56
-11.64	-11.63	-11.62	-11.61	-11.6	-11.59	-11.58	-11.57	-11.56
-11.64	-11.63	-11.62	-11.61	-11.6	-11.59	-11.58	-11.57	-11.56
-11.64	-11.63	-11.62	-11.61	-11.6	-11.59	-11.58	-11.57	-11.56
-11.64	-11.63	-11.62	-11.61	-11.6	-11.59	-11.58	-11.57	-11.56
-11.64	-11.63	-11.62	-11.61	-11.6	-11.59	-11.58	-11.57	-11.56
-11.64	-11.63	-11.62	-11.61	-11.6	-11.59	-11.58	-11.57	-11.56
-11.64	-11.63	-11.62	-11.61	-11.6	-11.59	-11.58	-11.57	-11.56
-11.64	-11.63	-11.62	-11.61	-11.6	-11.59	-11.58	-11.57	-11.56
-11.64	-11.63	-11.62	-11.61	-11.6	-11.59	-11.58	-11.57	-11.56
-11.64	-11.63	-11.62	-11.61	-11.6	-11.59	-11.58	-11.57	-11.56
-11.64	-11.63	-11.62	-11.61	-11.6	-11.59	-11.58	-11.57	-11.56
-11.64	-11.63	-11.62	-11.61	-11.6	-11.59	-11.58	-11.57	-11.56
-11.64	-11.63	-11.62	-11.61	-11.6	-11.59	-11.58	-11.57	-11.56
-11.64	-11.63	-11.62	-11.61	-11.6	-11.59	-11.58	-11.57	-11.56
-11.64	-11.63	-11.62	-11.61	-11.6	-11.59	-11.58	-11.57	-11.56
-11.64	-11.63	-11.62	-11.61	-11.6	-11.59	-11.58	-11.57	-11.56
-11.64	-11.63	-11.62	-11.61	-11.6	-11.59	-11.58	-11.57	-11.56
-11.64	-11.63	-11.62	-11.61	-11.6	-11.59	-11.58	-11.57	-11.56
-11.64	-11.63	-11.62	-11.61	-11.6	-11.59	-11.58	-11.57	-11.56
-11.64	-11.63	-11.62	-11.61	-11.6	-11.59	-11.58	-11.57	-11.56
-11.64	-11.63	-11.62	-11.61	-11.6	-11.59	-11.58	-11.57	-11.56
-11.64	-11.63	-11.62	-11.61	-11.6	-11.59	-11.58	-11.57	-11.56
-11.64	-11.63	-11.62	-11.61	-11.6	-11.59	-11.58	-11.57	-11.56
-11.64	-11.63	-11.62	-11.61	-11.6	-11.59	-11.58	-11.57	-11.56
-11.64	-11.63	-11.62	-11.61	-11.6	-11.59	-11.58	-11.57	-11.56
-11.64	-11.63	-11.62	-11.61	-11.6	-11.59	-11.58	-11.57	-11.56
-11.64	-11.63	-11.62	-11.61	-11.6	-11.59	-11.58	-11.57	-11.56
-11.64	-11.63	-11.62	-11.61	-11.6	-11.59	-11.58	-11.57	-11.56
-11.64	-11.63	-11.62	-11.61	-11.6	-11.59	-11.58	-11.57	-11.56
-11.64	-11.63	-11.62	-11.61	-11.6	-11.59	-11.58	-11.57	-11.56
-11.64	-11.63	-11.62	-11.61	-11.6	-11.59	-11.58	-11.57	-11.56
-11.64	-11.63	-11.62	-11.61	-11.6	-11.59	-11.58	-11.57	-11.56
-11.64	-11.63	-11.62	-11.61	-11.6	-11.59	-11.58	-11.57	-11.56
-11.64	-11.63	-11.62	-11.61	-11.6	-11.59	-11.58	-11.57	-11.56
-11.64	-11.63	-11.62	-11.61	-11.6	-11.59	-11.58	-11.57	-11.56
-11.64	-11.63	-11.62	-11.61	-11.6	-11.59	-11.58	-11.57	-11.56
-11.64	-11.63	-11.62	-11.61	-11.6	-11.59	-11.58	-11.57	-11.56
-11.64	-11.63	-11.62	-11.61	-11.6	-11.59	-11.58	-11.57	-11.56
-11.64	-11.63	-11.62	-11.61	-11.6	-11.59	-11.58	-11.57	-11.56
-11.64	-11.63	-11.62	-11.61	-11.6	-11.59	-11.58	-11.57	-11.56
-11.64	-11.63	-11.62	-11.61	-11.6	-11.59	-11.58	-11.57	-11.56
-11.64	-11.63	-11.62	-11.61	-11.6	-11.59	-11.58	-11.57	-11.56
-11.64	-11.63	-11.62	-11.61	-11.6	-11.59	-11.58	-11.57	-11.56
-11.64	-11.63	-11.62	-11.61	-11.6	-11.59	-11.58	-11.57	-11.56
-11.64	-11.63	-11.62	-11.61	-11.6	-11.59	-11.58	-11.57	-11.56
-11.64	-11.63	-11.62	-11.61	-11.6	-11.59	-11.58	-11.57	-11.56
-11.64	-11.63	-11.62	-11.61	-11.6	-11.59	-11.58	-11.57	-11.56

-11.64	-11.63	-11.62	-11.61	-11.6	-11.59	-11.58	-11.57	-11.56
-11.64	-11.63	-11.62	-11.61	-11.6	-11.59	-11.58	-11.57	-11.56
-11.64	-11.63	-11.62	-11.61	-11.6	-11.59	-11.58	-11.57	-11.56
-11.64	-11.63	-11.62	-11.61	-11.6	-11.59	-11.58	-11.57	-11.56
-11.64	-11.63	-11.62	-11.61	-11.6	-11.59	-11.58	-11.57	-11.56
-11.64	-11.63	-11.62	-11.61	-11.6	-11.59	-11.58	-11.57	-11.56
-11.64	-11.63	-11.62	-11.61	-11.6	-11.59	-11.58	-11.57	-11.56
-11.64	-11.63	-11.62	-11.61	-11.6	-11.59	-11.58	-11.57	-11.56
-11.64	-11.63	-11.62	-11.61	-11.6	-11.59	-11.58	-11.57	-11.56
-11.64	-11.63	-11.62	-11.61	-11.6	-11.59	-11.58	-11.57	-11.56
-11.64	-11.63	-11.62	-11.61	-11.6	-11.59	-11.58	-11.57	-11.56
-11.64	-11.63	-11.62	-11.61	-11.6	-11.59	-11.58	-11.57	-11.56
-11.64	-11.63	-11.62	-11.61	-11.6	-11.59	-11.58	-11.57	-11.56
-11.64	-11.63	-11.62	-11.61	-11.6	-11.59	-11.58	-11.57	-11.56
-11.64	-11.63	-11.62	-11.61	-11.6	-11.59	-11.58	-11.57	-11.56
-11.64	-11.63	-11.62	-11.61	-11.6	-11.59	-11.58	-11.57	-11.56
-11.64	-11.63	-11.62	-11.61	-11.6	-11.59	-11.58	-11.57	-11.56
-11.64	-11.63	-11.62	-11.61	-11.6	-11.59	-11.58	-11.57	-11.56
-11.64	-11.63	-11.62	-11.61	-11.6	-11.59	-11.58	-11.57	-11.56
-11.64	-11.63	-11.62	-11.61	-11.6	-11.59	-11.58	-11.57	-11.56
-11.64	-11.63	-11.62	-11.61	-11.6	-11.59	-11.58	-11.57	-11.56
-11.64	-11.63	-11.62	-11.61	-11.6	-11.59	-11.58	-11.57	-11.56
-11.64	-11.63	-11.62	-11.61	-11.6	-11.59	-11.58	-11.57	-11.56
-11.64	-11.63	-11.62	-11.61	-11.6	-11.59	-11.58	-11.57	-11.56
-11.64	-11.63	-11.62	-11.61	-11.6	-11.59	-11.58	-11.57	-11.56
-11.64	-11.63	-11.62	-11.61	-11.6	-11.59	-11.58	-11.57	-11.56
-11.64	-11.63	-11.62	-11.61	-11.6	-11.59	-11.58	-11.57	-11.56
-11.64	-11.63	-11.62	-11.61	-11.6	-11.59	-11.58	-11.57	-11.56
-11.64	-11.63	-11.62	-11.61	-11.6	-11.59	-11.58	-11.57	-11.56
-11.64	-11.63	-11.62	-11.61	-11.6	-11.59	-11.58	-11.57	-11.56
-11.64	-11.63	-11.62	-11.61	-11.6	-11.59	-11.58	-11.57	-11.56
-11.64	-11.63	-11.62	-11.61	-11.6	-11.59	-11.58	-11.57	-11.56
-11.64	-11.63	-11.62	-11.61	-11.6	-11.59	-11.58	-11.57	-11.56
-11.64	-11.63	-11.62	-11.61	-11.6	-11.59	-11.58	-11.57	-11.56
-11.64	-11.63	-11.62	-11.61	-11.6	-11.59	-11.58	-11.57	-11.56
-11.64	-11.63	-11.62	-11.61	-11.6	-11.59	-11.58	-11.57	-11.56
-11.64	-11.63	-11.62	-11.61	-11.6	-11.59	-11.58	-11.57	-11.56
-11.64	-11.63	-11.62	-11.61	-11.6	-11.59	-11.58	-11.57	-11.56
-11.64	-11.63	-11.62	-11.61	-11.6	-11.59	-11.58	-11.57	-11.56
-11.64	-11.63	-11.62	-11.61	-11.6	-11.59	-11.58	-11.57	-11.56
-11.64	-11.63	-11.62	-11.61	-11.6	-11.59	-11.58	-11.57	-11.56
-11.64	-11.63	-11.62	-11.61	-11.6	-11.59	-11.58	-11.57	-11.56
-11.64	-11.63	-11.62	-11.61	-11.6	-11.59	-11.58	-11.57	-11.56
-11.64	-11.63	-11.62	-11.61	-11.6	-11.59	-11.58	-11.57	-11.56
-11.64	-11.63	-11.62	-11.61	-11.6	-11.59	-11.58	-11.57	-11.56
-11.64	-11.63	-11.62	-11.61	-11.6	-11.59	-11.58	-11.57	-11.56
-11.64	-11.63	-11.62	-11.61	-11.6	-11.59	-11.58	-11.57	-11.56
-11.64	-11.63	-11.62	-11.61	-11.6	-11.59	-11.58	-11.57	-11.56
-11.64	-11.63	-11.62	-11.61	-11.6	-11.59	-11.58	-11.57	-11.56
-11.64	-11.63	-11.62	-11.61	-11.6	-11.59	-11.58	-11.57	-11.56

-11.64	-11.63	-11.62	-11.61	-11.6	-11.59	-11.58	-11.57	-11.56
-11.64	-11.63	-11.62	-11.61	-11.6	-11.59	-11.58	-11.57	-11.56
-11.64	-11.63	-11.62	-11.61	-11.6	-11.59	-11.58	-11.57	-11.56
-11.64	-11.63	-11.62	-11.61	-11.6	-11.59	-11.58	-11.57	-11.56
-11.64	-11.63	-11.62	-11.61	-11.6	-11.59	-11.58	-11.57	-11.56
-11.64	-11.63	-11.62	-11.61	-11.6	-11.59	-11.58	-11.57	-11.56
-11.64	-11.63	-11.62	-11.61	-11.6	-11.59	-11.58	-11.57	-11.56
-11.64	-11.63	-11.62	-11.61	-11.6	-11.59	-11.58	-11.57	-11.56
-11.64	-11.63	-11.62	-11.61	-11.6	-11.59	-11.58	-11.57	-11.56
-11.64	-11.63	-11.62	-11.61	-11.6	-11.59	-11.58	-11.57	-11.56
-11.64	-11.63	-11.62	-11.61	-11.6	-11.59	-11.58	-11.57	-11.56
-11.64	-11.63	-11.62	-11.61	-11.6	-11.59	-11.58	-11.57	-11.56
-11.64	-11.63	-11.62	-11.61	-11.6	-11.59	-11.58	-11.57	-11.56
-11.64	-11.63	-11.62	-11.61	-11.6	-11.59	-11.58	-11.57	-11.56
-11.64	-11.63	-11.62	-11.61	-11.6	-11.59	-11.58	-11.57	-11.56
-11.64	-11.63	-11.62	-11.61	-11.6	-11.59	-11.58	-11.57	-11.56
-11.64	-11.63	-11.62	-11.61	-11.6	-11.59	-11.58	-11.57	-11.56
-11.64	-11.63	-11.62	-11.61	-11.6	-11.59	-11.58	-11.57	-11.56
-11.64	-11.63	-11.62	-11.61	-11.6	-11.59	-11.58	-11.57	-11.56
-11.64	-11.63	-11.62	-11.61	-11.6	-11.59	-11.58	-11.57	-11.56
-11.64	-11.63	-11.62	-11.61	-11.6	-11.59	-11.58	-11.57	-11.56
-11.64	-11.63	-11.62	-11.61	-11.6	-11.59	-11.58	-11.57	-11.56
-11.64	-11.63	-11.62	-11.61	-11.6	-11.59	-11.58	-11.57	-11.56
-11.64	-11.63	-11.62	-11.61	-11.6	-11.59	-11.58	-11.57	-11.56
-11.64	-11.63	-11.62	-11.61	-11.6	-11.59	-11.58	-11.57	-11.56
-11.64	-11.63	-11.62	-11.61	-11.6	-11.59	-11.58	-11.57	-11.56
-11.64	-11.63	-11.62	-11.61	-11.6	-11.59	-11.58	-11.57	-11.56
-11.64	-11.63	-11.62	-11.61	-11.6	-11.59	-11.58	-11.57	-11.56
-11.64	-11.63	-11.62	-11.61	-11.6	-11.59	-11.58	-11.57	-11.56
-11.64	-11.63	-11.62	-11.61	-11.6	-11.59	-11.58	-11.57	-11.56
-11.64	-11.63	-11.62	-11.61	-11.6	-11.59	-11.58	-11.57	-11.56
-11.64	-11.63	-11.62	-11.61	-11.6	-11.59	-11.58	-11.57	-11.56
-11.64	-11.63	-11.62	-11.61	-11.6	-11.59	-11.58	-11.57	-11.56
-11.64	-11.63	-11.62	-11.61	-11.6	-11.59	-11.58	-11.57	-11.56
-11.64	-11.63	-11.62	-11.61	-11.6	-11.59	-11.58	-11.57	-11.56
-11.64	-11.63	-11.62	-11.61	-11.6	-11.59	-11.58	-11.57	-11.56
-11.64	-11.63	-11.62	-11.61	-11.6	-11.59	-11.58	-11.57	-11.56
-11.64	-11.63	-11.62	-11.61	-11.6	-11.59	-11.58	-11.57	-11.56
-11.64	-11.63	-11.62	-11.61	-11.6	-11.59	-11.58	-11.57	-11.56
-11.64	-11.63	-11.62	-11.61	-11.6	-11.59	-11.58	-11.57	-11.56
-11.64	-11.63	-11.62	-11.61	-11.6	-11.59	-11.58	-11.57	-11.56
-11.64	-11.63	-11.62	-11.61	-11.6	-11.59	-11.58	-11.57	-11.56
-11.64	-11.63	-11.62	-11.61	-11.6	-11.59	-11.58	-11.57	-11.56
-11.64	-11.63	-11.62	-11.61	-11.6	-11.59	-11.58	-11.57	-11.56
-11.64	-11.63	-11.62	-11.61	-11.6	-11.59	-11.58	-11.57	-11.56
-11.64	-11.63	-11.62	-11.61	-11.6	-11.59	-11.58	-11.57	-11.56
-11.64	-11.63	-11.62	-11.61	-11.6	-11.59	-11.58	-11.57	-11.56
-11.64	-11.63	-11.62	-11.61	-11.6	-11.59	-11.58	-11.57	-11.56
-11.64	-11.63	-11.62	-11.61	-11.6	-11.59	-11.58	-11.57	-11.56
-11.64	-11.63	-11.62	-11.61	-11.6	-11.59	-11.58	-11.57	-11.56

-11.64	-11.63	-11.62	-11.61	-11.6	-11.59	-11.58	-11.57	-11.56
-11.64	-11.63	-11.62	-11.61	-11.6	-11.59	-11.58	-11.57	-11.56
-11.64	-11.63	-11.62	-11.61	-11.6	-11.59	-11.58	-11.57	-11.56
-11.64	-11.63	-11.62	-11.61	-11.6	-11.59	-11.58	-11.57	-11.56
-11.64	-11.63	-11.62	-11.61	-11.6	-11.59	-11.58	-11.57	-11.56
-11.64	-11.63	-11.62	-11.61	-11.6	-11.59	-11.58	-11.57	-11.56
-11.64	-11.63	-11.62	-11.61	-11.6	-11.59	-11.58	-11.57	-11.56
-11.64	-11.63	-11.62	-11.61	-11.6	-11.59	-11.58	-11.57	-11.56
-11.64	-11.63	-11.62	-11.61	-11.6	-11.59	-11.58	-11.57	-11.56
-11.64	-11.63	-11.62	-11.61	-11.6	-11.59	-11.58	-11.57	-11.56
-11.64	-11.63	-11.62	-11.61	-11.6	-11.59	-11.58	-11.57	-11.56
-11.64	-11.63	-11.62	-11.61	-11.6	-11.59	-11.58	-11.57	-11.56
-11.64	-11.63	-11.62	-11.61	-11.6	-11.59	-11.58	-11.57	-11.56
-11.64	-11.63	-11.62	-11.61	-11.6	-11.59	-11.58	-11.57	-11.56
-11.64	-11.63	-11.62	-11.61	-11.6	-11.59	-11.58	-11.57	-11.56
-11.64	-11.63	-11.62	-11.61	-11.6	-11.59	-11.58	-11.57	-11.56
-11.64	-11.63	-11.62	-11.61	-11.6	-11.59	-11.58	-11.57	-11.56
-11.64	-11.63	-11.62	-11.61	-11.6	-11.59	-11.58	-11.57	-11.56
-11.64	-11.63	-11.62	-11.61	-11.6	-11.59	-11.58	-11.57	-11.56
-11.64	-11.63	-11.62	-11.61	-11.6	-11.59	-11.58	-11.57	-11.56
-11.64	-11.63	-11.62	-11.61	-11.6	-11.59	-11.58	-11.57	-11.56
-11.64	-11.63	-11.62	-11.61	-11.6	-11.59	-11.58	-11.57	-11.56
-11.64	-11.63	-11.62	-11.61	-11.6	-11.59	-11.58	-11.57	-11.56
-11.64	-11.63	-11.62	-11.61	-11.6	-11.59	-11.58	-11.57	-11.56
-11.64	-11.63	-11.62	-11.61	-11.6	-11.59	-11.58	-11.57	-11.56
-11.64	-11.63	-11.62	-11.61	-11.6	-11.59	-11.58	-11.57	-11.56
-11.64	-11.63	-11.62	-11.61	-11.6	-11.59	-11.58	-11.57	-11.56
-11.64	-11.63	-11.62	-11.61	-11.6	-11.59	-11.58	-11.57	-11.56
-11.64	-11.63	-11.62	-11.61	-11.6	-11.59	-11.58	-11.57	-11.56
-11.64	-11.63	-11.62	-11.61	-11.6	-11.59	-11.58	-11.57	-11.56
-11.64	-11.63	-11.62	-11.61	-11.6	-11.59	-11.58	-11.57	-11.56
-11.64	-11.63	-11.62	-11.61	-11.6	-11.59	-11.58	-11.57	-11.56
-11.64	-11.63	-11.62	-11.61	-11.6	-11.59	-11.58	-11.57	-11.56
-11.64	-11.63	-11.62	-11.61	-11.6	-11.59	-11.58	-11.57	-11.56
-11.64	-11.63	-11.62	-11.61	-11.6	-11.59	-11.58	-11.57	-11.56
-11.64	-11.63	-11.62	-11.61	-11.6	-11.59	-11.58	-11.57	-11.56
-11.64	-11.63	-11.62	-11.61	-11.6	-11.59	-11.58	-11.57	-11.56
-11.64	-11.63	-11.62	-11.61	-11.6	-11.59	-11.58	-11.57	-11.56
-11.64	-11.63	-11.62	-11.61	-11.6	-11.59	-11.58	-11.57	-11.56
-11.64	-11.63	-11.62	-11.61	-11.6	-11.59	-11.58	-11.57	-11.56
-11.64	-11.63	-11.62	-11.61	-11.6	-11.59	-11.58	-11.57	-11.56
-11.64	-11.63	-11.62	-11.61	-11.6	-11.59	-11.58	-11.57	-11.56
-11.64	-11.63	-11.62	-11.61	-11.6	-11.59	-11.58	-11.57	-11.56
-11.64	-11.63	-11.62	-11.61	-11.6	-11.59	-11.58	-11.57	-11.56
-11.64	-11.63	-11.62	-11.61	-11.6	-11.59	-11.58	-11.57	-11.56
-11.64	-11.63	-11.62	-11.61	-11.6	-11.59	-11.58	-11.57	-11.56
-11.64	-11.63	-11.62	-11.61	-11.6	-11.59	-11.58	-11.57	-11.56
-11.64	-11.63	-11.62	-11.61	-11.6	-11.59	-11.58	-11.57	-11.56
-11.64	-11.63	-11.62	-11.61	-11.6	-11.59	-11.58	-11.57	-11.56
-11.64	-11.63	-11.62	-11.61	-11.6	-11.59	-11.58	-11.57	-11.56

-11.64	-11.63	-11.62	-11.61	-11.6	-11.59	-11.58	-11.57	-11.56
-11.64	-11.63	-11.62	-11.61	-11.6	-11.59	-11.58	-11.57	-11.56
-11.64	-11.63	-11.62	-11.61	-11.6	-11.59	-11.58	-11.57	-11.56
-11.64	-11.63	-11.62	-11.61	-11.6	-11.59	-11.58	-11.57	-11.56
-11.64	-11.63	-11.62	-11.61	-11.6	-11.59	-11.58	-11.57	-11.56
-11.64	-11.63	-11.62	-11.61	-11.6	-11.59	-11.58	-11.57	-11.56
-11.64	-11.63	-11.62	-11.61	-11.6	-11.59	-11.58	-11.57	-11.56
-11.64	-11.63	-11.62	-11.61	-11.6	-11.59	-11.58	-11.57	-11.56
-11.64	-11.63	-11.62	-11.61	-11.6	-11.59	-11.58	-11.57	-11.56
-11.64	-11.63	-11.62	-11.61	-11.6	-11.59	-11.58	-11.57	-11.56
-11.64	-11.63	-11.62	-11.61	-11.6	-11.59	-11.58	-11.57	-11.56
-11.64	-11.63	-11.62	-11.61	-11.6	-11.59	-11.58	-11.57	-11.56
-11.64	-11.63	-11.62	-11.61	-11.6	-11.59	-11.58	-11.57	-11.56
-11.64	-11.63	-11.62	-11.61	-11.6	-11.59	-11.58	-11.57	-11.56
-11.64	-11.63	-11.62	-11.61	-11.6	-11.59	-11.58	-11.57	-11.56
-11.64	-11.63	-11.62	-11.61	-11.6	-11.59	-11.58	-11.57	-11.56
-11.64	-11.63	-11.62	-11.61	-11.6	-11.59	-11.58	-11.57	-11.56
-11.64	-11.63	-11.62	-11.61	-11.6	-11.59	-11.58	-11.57	-11.56
-11.64	-11.63	-11.62	-11.61	-11.6	-11.59	-11.58	-11.57	-11.56
-11.64	-11.63	-11.62	-11.61	-11.6	-11.59	-11.58	-11.57	-11.56
-11.64	-11.63	-11.62	-11.61	-11.6	-11.59	-11.58	-11.57	-11.56
-11.64	-11.63	-11.62	-11.61	-11.6	-11.59	-11.58	-11.57	-11.56
-11.64	-11.63	-11.62	-11.61	-11.6	-11.59	-11.58	-11.57	-11.56
-11.64	-11.63	-11.62	-11.61	-11.6	-11.59	-11.58	-11.57	-11.56
-11.64	-11.63	-11.62	-11.61	-11.6	-11.59	-11.58	-11.57	-11.56
-11.64	-11.63	-11.62	-11.61	-11.6	-11.59	-11.58	-11.57	-11.56
-11.64	-11.63	-11.62	-11.61	-11.6	-11.59	-11.58	-11.57	-11.56
-11.64	-11.63	-11.62	-11.61	-11.6	-11.59	-11.58	-11.57	-11.56
-11.64	-11.63	-11.62	-11.61	-11.6	-11.59	-11.58	-11.57	-11.56
-11.64	-11.63	-11.62	-11.61	-11.6	-11.59	-11.58	-11.57	-11.56
-11.64	-11.63	-11.62	-11.61	-11.6	-11.59	-11.58	-11.57	-11.56
-11.64	-11.63	-11.62	-11.61	-11.6	-11.59	-11.58	-11.57	-11.56
-11.64	-11.63	-11.62	-11.61	-11.6	-11.59	-11.58	-11.57	-11.56
-11.64	-11.63	-11.62	-11.61	-11.6	-11.59	-11.58	-11.57	-11.56
-11.64	-11.63	-11.62	-11.61	-11.6	-11.59	-11.58	-11.57	-11.56
-11.64	-11.63	-11.62	-11.61	-11.6	-11.59	-11.58	-11.57	-11.56
-11.64	-11.63	-11.62	-11.61	-11.6	-11.59	-11.58	-11.57	-11.56
-11.64	-11.63	-11.62	-11.61	-11.6	-11.59	-11.58	-11.57	-11.56
-11.64	-11.63	-11.62	-11.61	-11.6	-11.59	-11.58	-11.57	-11.56
-11.64	-11.63	-11.62	-11.61	-11.6	-11.59	-11.58	-11.57	-11.56
-11.64	-11.63	-11.62	-11.61	-11.6	-11.59	-11.58	-11.57	-11.56
-11.64	-11.63	-11.62	-11.61	-11.6	-11.59	-11.58	-11.57	-11.56
-11.64	-11.63	-11.62	-11.61	-11.6	-11.59	-11.58	-11.57	-11.56
-11.64	-11.63	-11.62	-11.61	-11.6	-11.59	-11.58	-11.57	-11.56
-11.64	-11.63	-11.62	-11.61	-11.6	-11.59	-11.58	-11.57	-11.56
-11.64	-11.63	-11.62	-11.61	-11.6	-11.59	-11.58	-11.57	-11.56
-11.64	-11.63	-11.62	-11.61	-11.6	-11.59	-11.58	-11.57	-11.56
-11.64	-11.63	-11.62	-11.61	-11.6	-11.59	-11.58	-11.57	-11.56
-11.64	-11.63	-11.62	-11.61	-11.6	-11.59	-11.58	-11.57	-11.56
-11.64	-11.63	-11.62	-11.61	-11.6	-11.59	-11.58	-11.57	-11.56

-11.64	-11.63	-11.62	-11.61	-11.6	-11.59	-11.58	-11.57	-11.56
-11.64	-11.63	-11.62	-11.61	-11.6	-11.59	-11.58	-11.57	-11.56
-11.64	-11.63	-11.62	-11.61	-11.6	-11.59	-11.58	-11.57	-11.56
-11.64	-11.63	-11.62	-11.61	-11.6	-11.59	-11.58	-11.57	-11.56
-11.64	-11.63	-11.62	-11.61	-11.6	-11.59	-11.58	-11.57	-11.56
-11.64	-11.63	-11.62	-11.61	-11.6	-11.59	-11.58	-11.57	-11.56
-11.64	-11.63	-11.62	-11.61	-11.6	-11.59	-11.58	-11.57	-11.56
-11.64	-11.63	-11.62	-11.61	-11.6	-11.59	-11.58	-11.57	-11.56
-11.64	-11.63	-11.62	-11.61	-11.6	-11.59	-11.58	-11.57	-11.56
-11.64	-11.63	-11.62	-11.61	-11.6	-11.59	-11.58	-11.57	-11.56
-11.64	-11.63	-11.62	-11.61	-11.6	-11.59	-11.58	-11.57	-11.56
-11.64	-11.63	-11.62	-11.61	-11.6	-11.59	-11.58	-11.57	-11.56
-11.64	-11.63	-11.62	-11.61	-11.6	-11.59	-11.58	-11.57	-11.56
-11.64	-11.63	-11.62	-11.61	-11.6	-11.59	-11.58	-11.57	-11.56
-11.64	-11.63	-11.62	-11.61	-11.6	-11.59	-11.58	-11.57	-11.56
-11.64	-11.63	-11.62	-11.61	-11.6	-11.59	-11.58	-11.57	-11.56
-11.64	-11.63	-11.62	-11.61	-11.6	-11.59	-11.58	-11.57	-11.56
-11.64	-11.63	-11.62	-11.61	-11.6	-11.59	-11.58	-11.57	-11.56
-11.64	-11.63	-11.62	-11.61	-11.6	-11.59	-11.58	-11.57	-11.56
-11.64	-11.63	-11.62	-11.61	-11.6	-11.59	-11.58	-11.57	-11.56
-11.64	-11.63	-11.62	-11.61	-11.6	-11.59	-11.58	-11.57	-11.56
-11.64	-11.63	-11.62	-11.61	-11.6	-11.59	-11.58	-11.57	-11.56
-11.64	-11.63	-11.62	-11.61	-11.6	-11.59	-11.58	-11.57	-11.56
-11.64	-11.63	-11.62	-11.61	-11.6	-11.59	-11.58	-11.57	-11.56
-11.64	-11.63	-11.62	-11.61	-11.6	-11.59	-11.58	-11.57	-11.56
-11.64	-11.63	-11.62	-11.61	-11.6	-11.59	-11.58	-11.57	-11.56
-11.64	-11.63	-11.62	-11.61	-11.6	-11.59	-11.58	-11.57	-11.56
-11.64	-11.63	-11.62	-11.61	-11.6	-11.59	-11.58	-11.57	-11.56
-11.64	-11.63	-11.62	-11.61	-11.6	-11.59	-11.58	-11.57	-11.56
-11.64	-11.63	-11.62	-11.61	-11.6	-11.59	-11.58	-11.57	-11.56
-11.64	-11.63	-11.62	-11.61	-11.6	-11.59	-11.58	-11.57	-11.56
-11.64	-11.63	-11.62	-11.61	-11.6	-11.59	-11.58	-11.57	-11.56
-11.64	-11.63	-11.62	-11.61	-11.6	-11.59	-11.58	-11.57	-11.56
-11.64	-11.63	-11.62	-11.61	-11.6	-11.59	-11.58	-11.57	-11.56
-11.64	-11.63	-11.62	-11.61	-11.6	-11.59	-11.58	-11.57	-11.56
-11.64	-11.63	-11.62	-11.61	-11.6	-11.59	-11.58	-11.57	-11.56
-11.64	-11.63	-11.62	-11.61	-11.6	-11.59	-11.58	-11.57	-11.56
-11.64	-11.63	-11.62	-11.61	-11.6	-11.59	-11.58	-11.57	-11.56
-11.64	-11.63	-11.62	-11.61	-11.6	-11.59	-11.58	-11.57	-11.56
-11.64	-11.63	-11.62	-11.61	-11.6	-11.59	-11.58	-11.57	-11.56
-11.64	-11.63	-11.62	-11.61	-11.6	-11.59	-11.58	-11.57	-11.56
-11.64	-11.63	-11.62	-11.61	-11.6	-11.59	-11.58	-11.57	-11.56
-11.64	-11.63	-11.62	-11.61	-11.6	-11.59	-11.58	-11.57	-11.56
-11.64	-11.63	-11.62	-11.61	-11.6	-11.59	-11.58	-11.57	-11.56
-11.64	-11.63	-11.62	-11.61	-11.6	-11.59	-11.58	-11.57	-11.56
-11.64	-11.63	-11.62	-11.61	-11.6	-11.59	-11.58	-11.57	-11.56
-11.64	-11.63	-11.62	-11.61	-11.6	-11.59	-11.58	-11.57	-11.56
-11.64	-11.63	-11.62	-11.61	-11.6	-11.59	-11.58	-11.57	-11.56
-11.64	-11.63	-11.62	-11.61	-11.6	-11.59	-11.58	-11.57	-11.56
-11.64	-11.63	-11.62	-11.61	-11.6	-11.59	-11.58	-11.57	-11.56

-11.64	-11.63	-11.62	-11.61	-11.6	-11.59	-11.58	-11.57	-11.56
-11.64	-11.63	-11.62	-11.61	-11.6	-11.59	-11.58	-11.57	-11.56
-11.64	-11.63	-11.62	-11.61	-11.6	-11.59	-11.58	-11.57	-11.56
-11.64	-11.63	-11.62	-11.61	-11.6	-11.59	-11.58	-11.57	-11.56
-11.64	-11.63	-11.62	-11.61	-11.6	-11.59	-11.58	-11.57	-11.56
-11.64	-11.63	-11.62	-11.61	-11.6	-11.59	-11.58	-11.57	-11.56
-11.64	-11.63	-11.62	-11.61	-11.6	-11.59	-11.58	-11.57	-11.56
-11.64	-11.63	-11.62	-11.61	-11.6	-11.59	-11.58	-11.57	-11.56
-11.64	-11.63	-11.62	-11.61	-11.6	-11.59	-11.58	-11.57	-11.56
-11.64	-11.63	-11.62	-11.61	-11.6	-11.59	-11.58	-11.57	-11.56
-11.64	-11.63	-11.62	-11.61	-11.6	-11.59	-11.58	-11.57	-11.56
-11.64	-11.63	-11.62	-11.61	-11.6	-11.59	-11.58	-11.57	-11.56
-11.64	-11.63	-11.62	-11.61	-11.6	-11.59	-11.58	-11.57	-11.56
-11.64	-11.63	-11.62	-11.61	-11.6	-11.59	-11.58	-11.57	-11.56
-11.64	-11.63	-11.62	-11.61	-11.6	-11.59	-11.58	-11.57	-11.56
-11.64	-11.63	-11.62	-11.61	-11.6	-11.59	-11.58	-11.57	-11.56
-11.64	-11.63	-11.62	-11.61	-11.6	-11.59	-11.58	-11.57	-11.56
-11.64	-11.63	-11.62	-11.61	-11.6	-11.59	-11.58	-11.57	-11.56
-11.64	-11.63	-11.62	-11.61	-11.6	-11.59	-11.58	-11.57	-11.56
-11.64	-11.63	-11.62	-11.61	-11.6	-11.59	-11.58	-11.57	-11.56
-11.64	-11.63	-11.62	-11.61	-11.6	-11.59	-11.58	-11.57	-11.56
-11.64	-11.63	-11.62	-11.61	-11.6	-11.59	-11.58	-11.57	-11.56
-11.64	-11.63	-11.62	-11.61	-11.6	-11.59	-11.58	-11.57	-11.56
-11.64	-11.63	-11.62	-11.61	-11.6	-11.59	-11.58	-11.57	-11.56
-11.64	-11.63	-11.62	-11.61	-11.6	-11.59	-11.58	-11.57	-11.56
-11.64	-11.63	-11.62	-11.61	-11.6	-11.59	-11.58	-11.57	-11.56
-11.64	-11.63	-11.62	-11.61	-11.6	-11.59	-11.58	-11.57	-11.56
-11.64	-11.63	-11.62	-11.61	-11.6	-11.59	-11.58	-11.57	-11.56
-11.64	-11.63	-11.62	-11.61	-11.6	-11.59	-11.58	-11.57	-11.56
-11.64	-11.63	-11.62	-11.61	-11.6	-11.59	-11.58	-11.57	-11.56
-11.64	-11.63	-11.62	-11.61	-11.6	-11.59	-11.58	-11.57	-11.56
-11.64	-11.63	-11.62	-11.61	-11.6	-11.59	-11.58	-11.57	-11.56
-11.64	-11.63	-11.62	-11.61	-11.6	-11.59	-11.58	-11.57	-11.56
-11.64	-11.63	-11.62	-11.61	-11.6	-11.59	-11.58	-11.57	-11.56
-11.64	-11.63	-11.62	-11.61	-11.6	-11.59	-11.58	-11.57	-11.56
-11.64	-11.63	-11.62	-11.61	-11.6	-11.59	-11.58	-11.57	-11.56
-11.64	-11.63	-11.62	-11.61	-11.6	-11.59	-11.58	-11.57	-11.56
-11.64	-11.63	-11.62	-11.61	-11.6	-11.59	-11.58	-11.57	-11.56
-11.64	-11.63	-11.62	-11.61	-11.6	-11.59	-11.58	-11.57	-11.56
-11.64	-11.63	-11.62	-11.61	-11.6	-11.59	-11.58	-11.57	-11.56
-11.64	-11.63	-11.62	-11.61	-11.6	-11.59	-11.58	-11.57	-11.56
-11.64	-11.63	-11.62	-11.61	-11.6	-11.59	-11.58	-11.57	-11.56
-11.64	-11.63	-11.62	-11.61	-11.6	-11.59	-11.58	-11.57	-11.56
-11.64	-11.63	-11.62	-11.61	-11.6	-11.59	-11.58	-11.57	-11.56
-11.64	-11.63	-11.62	-11.61	-11.6	-11.59	-11.58	-11.57	-11.56
-11.64	-11.63	-11.62	-11.61	-11.6	-11.59	-11.58	-11.57	-11.56
-11.64	-11.63	-11.62	-11.61	-11.6	-11.59	-11.58	-11.57	-11.56
-11.64	-11.63	-11.62	-11.61	-11.6	-11.59	-11.58	-11.57	-11.56
-11.64	-11.63	-11.62	-11.61	-11.6	-11.59	-11.58	-11.57	-11.56
-11.64	-11.63	-11.62	-11.61	-11.6	-11.59	-11.58	-11.57	-11.56

-11.64	-11.63	-11.62	-11.61	-11.6	-11.59	-11.58	-11.57	-11.56
-11.64	-11.63	-11.62	-11.61	-11.6	-11.59	-11.58	-11.57	-11.56
-11.64	-11.63	-11.62	-11.61	-11.6	-11.59	-11.58	-11.57	-11.56
-11.64	-11.63	-11.62	-11.61	-11.6	-11.59	-11.58	-11.57	-11.56
-11.64	-11.63	-11.62	-11.61	-11.6	-11.59	-11.58	-11.57	-11.56
-11.64	-11.63	-11.62	-11.61	-11.6	-11.59	-11.58	-11.57	-11.56
-11.64	-11.63	-11.62	-11.61	-11.6	-11.59	-11.58	-11.57	-11.56
-11.64	-11.63	-11.62	-11.61	-11.6	-11.59	-11.58	-11.57	-11.56
-11.64	-11.63	-11.62	-11.61	-11.6	-11.59	-11.58	-11.57	-11.56
-11.64	-11.63	-11.62	-11.61	-11.6	-11.59	-11.58	-11.57	-11.56
-11.64	-11.63	-11.62	-11.61	-11.6	-11.59	-11.58	-11.57	-11.56
-11.64	-11.63	-11.62	-11.61	-11.6	-11.59	-11.58	-11.57	-11.56
-11.64	-11.63	-11.62	-11.61	-11.6	-11.59	-11.58	-11.57	-11.56
-11.64	-11.63	-11.62	-11.61	-11.6	-11.59	-11.58	-11.57	-11.56
-11.64	-11.63	-11.62	-11.61	-11.6	-11.59	-11.58	-11.57	-11.56
-11.64	-11.63	-11.62	-11.61	-11.6	-11.59	-11.58	-11.57	-11.56
-11.64	-11.63	-11.62	-11.61	-11.6	-11.59	-11.58	-11.57	-11.56
-11.64	-11.63	-11.62	-11.61	-11.6	-11.59	-11.58	-11.57	-11.56
-11.64	-11.63	-11.62	-11.61	-11.6	-11.59	-11.58	-11.57	-11.56
-11.64	-11.63	-11.62	-11.61	-11.6	-11.59	-11.58	-11.57	-11.56
-11.64	-11.63	-11.62	-11.61	-11.6	-11.59	-11.58	-11.57	-11.56
-11.64	-11.63	-11.62	-11.61	-11.6	-11.59	-11.58	-11.57	-11.56
-11.64	-11.63	-11.62	-11.61	-11.6	-11.59	-11.58	-11.57	-11.56
-11.64	-11.63	-11.62	-11.61	-11.6	-11.59	-11.58	-11.57	-11.56
-11.64	-11.63	-11.62	-11.61	-11.6	-11.59	-11.58	-11.57	-11.56
-11.64	-11.63	-11.62	-11.61	-11.6	-11.59	-11.58	-11.57	-11.56
-11.64	-11.63	-11.62	-11.61	-11.6	-11.59	-11.58	-11.57	-11.56
-11.64	-11.63	-11.62	-11.61	-11.6	-11.59	-11.58	-11.57	-11.56
-11.64	-11.63	-11.62	-11.61	-11.6	-11.59	-11.58	-11.57	-11.56
-11.64	-11.63	-11.62	-11.61	-11.6	-11.59	-11.58	-11.57	-11.56
-11.64	-11.63	-11.62	-11.61	-11.6	-11.59	-11.58	-11.57	-11.56
-11.64	-11.63	-11.62	-11.61	-11.6	-11.59	-11.58	-11.57	-11.56
-11.64	-11.63	-11.62	-11.61	-11.6	-11.59	-11.58	-11.57	-11.56
-11.64	-11.63	-11.62	-11.61	-11.6	-11.59	-11.58	-11.57	-11.56
-11.64	-11.63	-11.62	-11.61	-11.6	-11.59	-11.58	-11.57	-11.56
-11.64	-11.63	-11.62	-11.61	-11.6	-11.59	-11.58	-11.57	-11.56
-11.64	-11.63	-11.62	-11.61	-11.6	-11.59	-11.58	-11.57	-11.56
-11.64	-11.63	-11.62	-11.61	-11.6	-11.59	-11.58	-11.57	-11.56
-11.64	-11.63	-11.62	-11.61	-11.6	-11.59	-11.58	-11.57	-11.56
-11.64	-11.63	-11.62	-11.61	-11.6	-11.59	-11.58	-11.57	-11.56
-11.64	-11.63	-11.62	-11.61	-11.6	-11.59	-11.58	-11.57	-11.56
-11.64	-11.63	-11.62	-11.61	-11.6	-11.59	-11.58	-11.57	-11.56
-11.64	-11.63	-11.62	-11.61	-11.6	-11.59	-11.58	-11.57	-11.56
-11.64	-11.63	-11.62	-11.61	-11.6	-11.59	-11.58	-11.57	-11.56
-11.64	-11.63	-11.62	-11.61	-11.6	-11.59	-11.58	-11.57	-11.56
-11.64	-11.63	-11.62	-11.61	-11.6	-11.59	-11.58	-11.57	-11.56
-11.64	-11.63	-11.62	-11.61	-11.6	-11.59	-11.58	-11.57	-11.56
-11.64	-11.63	-11.62	-11.61	-11.6	-11.59	-11.58	-11.57	-11.56
-11.64	-11.63	-11.62	-11.61	-11.6	-11.59	-11.58	-11.57	-11.56
-11.64	-11.63	-11.62	-11.61	-11.6	-11.59	-11.58	-11.57	-11.56

-11.64	-11.63	-11.62	-11.61	-11.6	-11.59	-11.58	-11.57	-11.56
-11.64	-11.63	-11.62	-11.61	-11.6	-11.59	-11.58	-11.57	-11.56
-11.64	-11.63	-11.62	-11.61	-11.6	-11.59	-11.58	-11.57	-11.56
-11.64	-11.63	-11.62	-11.61	-11.6	-11.59	-11.58	-11.57	-11.56
-11.64	-11.63	-11.62	-11.61	-11.6	-11.59	-11.58	-11.57	-11.56
-11.64	-11.63	-11.62	-11.61	-11.6	-11.59	-11.58	-11.57	-11.56
-11.64	-11.63	-11.62	-11.61	-11.6	-11.59	-11.58	-11.57	-11.56
-11.64	-11.63	-11.62	-11.61	-11.6	-11.59	-11.58	-11.57	-11.56
-11.64	-11.63	-11.62	-11.61	-11.6	-11.59	-11.58	-11.57	-11.56
-11.64	-11.63	-11.62	-11.61	-11.6	-11.59	-11.58	-11.57	-11.56
-11.64	-11.63	-11.62	-11.61	-11.6	-11.59	-11.58	-11.57	-11.56
-11.64	-11.63	-11.62	-11.61	-11.6	-11.59	-11.58	-11.57	-11.56
-11.64	-11.63	-11.62	-11.61	-11.6	-11.59	-11.58	-11.57	-11.56
-11.64	-11.63	-11.62	-11.61	-11.6	-11.59	-11.58	-11.57	-11.56
-11.64	-11.63	-11.62	-11.61	-11.6	-11.59	-11.58	-11.57	-11.56
-11.64	-11.63	-11.62	-11.61	-11.6	-11.59	-11.58	-11.57	-11.56
-11.64	-11.63	-11.62	-11.61	-11.6	-11.59	-11.58	-11.57	-11.56
-11.64	-11.63	-11.62	-11.61	-11.6	-11.59	-11.58	-11.57	-11.56
-11.64	-11.63	-11.62	-11.61	-11.6	-11.59	-11.58	-11.57	-11.56
-11.64	-11.63	-11.62	-11.61	-11.6	-11.59	-11.58	-11.57	-11.56
-11.64	-11.63	-11.62	-11.61	-11.6	-11.59	-11.58	-11.57	-11.56
-11.64	-11.63	-11.62	-11.61	-11.6	-11.59	-11.58	-11.57	-11.56
-11.64	-11.63	-11.62	-11.61	-11.6	-11.59	-11.58	-11.57	-11.56
-11.64	-11.63	-11.62	-11.61	-11.6	-11.59	-11.58	-11.57	-11.56
-11.64	-11.63	-11.62	-11.61	-11.6	-11.59	-11.58	-11.57	-11.56
-11.64	-11.63	-11.62	-11.61	-11.6	-11.59	-11.58	-11.57	-11.56
-11.64	-11.63	-11.62	-11.61	-11.6	-11.59	-11.58	-11.57	-11.56
-11.64	-11.63	-11.62	-11.61	-11.6	-11.59	-11.58	-11.57	-11.56
-11.64	-11.63	-11.62	-11.61	-11.6	-11.59	-11.58	-11.57	-11.56
-11.64	-11.63	-11.62	-11.61	-11.6	-11.59	-11.58	-11.57	-11.56
-11.64	-11.63	-11.62	-11.61	-11.6	-11.59	-11.58	-11.57	-11.56
-11.64	-11.63	-11.62	-11.61	-11.6	-11.59	-11.58	-11.57	-11.56
-11.64	-11.63	-11.62	-11.61	-11.6	-11.59	-11.58	-11.57	-11.56
-11.64	-11.63	-11.62	-11.61	-11.6	-11.59	-11.58	-11.57	-11.56
-11.64	-11.63	-11.62	-11.61	-11.6	-11.59	-11.58	-11.57	-11.56
-11.64	-11.63	-11.62	-11.61	-11.6	-11.59	-11.58	-11.57	-11.56
-11.64	-11.63	-11.62	-11.61	-11.6	-11.59	-11.58	-11.57	-11.56
-11.64	-11.63	-11.62	-11.61	-11.6	-11.59	-11.58	-11.57	-11.56
-11.64	-11.63	-11.62	-11.61	-11.6	-11.59	-11.58	-11.57	-11.56
-11.64	-11.63	-11.62	-11.61	-11.6	-11.59	-11.58	-11.57	-11.56
-11.64	-11.63	-11.62	-11.61	-11.6	-11.59	-11.58	-11.57	-11.56
-11.64	-11.63	-11.62	-11.61	-11.6	-11.59	-11.58	-11.57	-11.56
-11.64	-11.63	-11.62	-11.61	-11.6	-11.59	-11.58	-11.57	-11.56
-11.64	-11.63	-11.62	-11.61	-11.6	-11.59	-11.58	-11.57	-11.56
-11.64	-11.63	-11.62	-11.61	-11.6	-11.59	-11.58	-11.57	-11.56
-11.64	-11.63	-11.62	-11.61	-11.6	-11.59	-11.58	-11.57	-11.56
-11.64	-11.63	-11.62	-11.61	-11.6	-11.59	-11.58	-11.57	-11.56
-11.64	-11.63	-11.62	-11.61	-11.6	-11.59	-11.58	-11.57	-11.56
-11.64	-11.63	-11.62	-11.61	-11.6	-11.59	-11.58	-11.57	-11.56
-11.64	-11.63	-11.62	-11.61	-11.6	-11.59	-11.58	-11.57	-11.56

-11.64	-11.63	-11.62	-11.61	-11.6	-11.59	-11.58	-11.57	-11.56
-11.64	-11.63	-11.62	-11.61	-11.6	-11.59	-11.58	-11.57	-11.56
-11.64	-11.63	-11.62	-11.61	-11.6	-11.59	-11.58	-11.57	-11.56
-11.64	-11.63	-11.62	-11.61	-11.6	-11.59	-11.58	-11.57	-11.56
-11.64	-11.63	-11.62	-11.61	-11.6	-11.59	-11.58	-11.57	-11.56
-11.64	-11.63	-11.62	-11.61	-11.6	-11.59	-11.58	-11.57	-11.56
-11.64	-11.63	-11.62	-11.61	-11.6	-11.59	-11.58	-11.57	-11.56
-11.64	-11.63	-11.62	-11.61	-11.6	-11.59	-11.58	-11.57	-11.56
-11.64	-11.63	-11.62	-11.61	-11.6	-11.59	-11.58	-11.57	-11.56
-11.64	-11.63	-11.62	-11.61	-11.6	-11.59	-11.58	-11.57	-11.56
-11.64	-11.63	-11.62	-11.61	-11.6	-11.59	-11.58	-11.57	-11.56
-11.64	-11.63	-11.62	-11.61	-11.6	-11.59	-11.58	-11.57	-11.56
-11.64	-11.63	-11.62	-11.61	-11.6	-11.59	-11.58	-11.57	-11.56
-11.64	-11.63	-11.62	-11.61	-11.6	-11.59	-11.58	-11.57	-11.56
-11.64	-11.63	-11.62	-11.61	-11.6	-11.59	-11.58	-11.57	-11.56
-11.64	-11.63	-11.62	-11.61	-11.6	-11.59	-11.58	-11.57	-11.56
-11.64	-11.63	-11.62	-11.61	-11.6	-11.59	-11.58	-11.57	-11.56
-11.64	-11.63	-11.62	-11.61	-11.6	-11.59	-11.58	-11.57	-11.56
-11.64	-11.63	-11.62	-11.61	-11.6	-11.59	-11.58	-11.57	-11.56
-11.64	-11.63	-11.62	-11.61	-11.6	-11.59	-11.58	-11.57	-11.56
-11.64	-11.63	-11.62	-11.61	-11.6	-11.59	-11.58	-11.57	-11.56
-11.64	-11.63	-11.62	-11.61	-11.6	-11.59	-11.58	-11.57	-11.56
-11.64	-11.63	-11.62	-11.61	-11.6	-11.59	-11.58	-11.57	-11.56
-11.64	-11.63	-11.62	-11.61	-11.6	-11.59	-11.58	-11.57	-11.56
-11.64	-11.63	-11.62	-11.61	-11.6	-11.59	-11.58	-11.57	-11.56
-11.64	-11.63	-11.62	-11.61	-11.6	-11.59	-11.58	-11.57	-11.56
-11.64	-11.63	-11.62	-11.61	-11.6	-11.59	-11.58	-11.57	-11.56
-11.64	-11.63	-11.62	-11.61	-11.6	-11.59	-11.58	-11.57	-11.56
-11.64	-11.63	-11.62	-11.61	-11.6	-11.59	-11.58	-11.57	-11.56
-11.64	-11.63	-11.62	-11.61	-11.6	-11.59	-11.58	-11.57	-11.56
-11.64	-11.63	-11.62	-11.61	-11.6	-11.59	-11.58	-11.57	-11.56
-11.64	-11.63	-11.62	-11.61	-11.6	-11.59	-11.58	-11.57	-11.56
-11.64	-11.63	-11.62	-11.61	-11.6	-11.59	-11.58	-11.57	-11.56
-11.64	-11.63	-11.62	-11.61	-11.6	-11.59	-11.58	-11.57	-11.56
-11.64	-11.63	-11.62	-11.61	-11.6	-11.59	-11.58	-11.57	-11.56
-11.64	-11.63	-11.62	-11.61	-11.6	-11.59	-11.58	-11.57	-11.56
-11.64	-11.63	-11.62	-11.61	-11.6	-11.59	-11.58	-11.57	-11.56
-11.64	-11.63	-11.62	-11.61	-11.6	-11.59	-11.58	-11.57	-11.56
-11.64	-11.63	-11.62	-11.61	-11.6	-11.59	-11.58	-11.57	-11.56
-11.64	-11.63	-11.62	-11.61	-11.6	-11.59	-11.58	-11.57	-11.56
-11.64	-11.63	-11.62	-11.61	-11.6	-11.59	-11.58	-11.57	-11.56
-11.64	-11.63	-11.62	-11.61	-11.6	-11.59	-11.58	-11.57	-11.56
-11.64	-11.63	-11.62	-11.61	-11.6	-11.59	-11.58	-11.57	-11.56
-11.64	-11.63	-11.62	-11.61	-11.6	-11.59	-11.58	-11.57	-11.56
-11.64	-11.63	-11.62	-11.61	-11.6	-11.59	-11.58	-11.57	-11.56
-11.64	-11.63	-11.62	-11.61	-11.6	-11.59	-11.58	-11.57	-11.56
-11.64	-11.63	-11.62	-11.61	-11.6	-11.59	-11.58	-11.57	-11.56
-11.64	-11.63	-11.62	-11.61	-11.6	-11.59	-11.58	-11.57	-11.56
-11.64	-11.63	-11.62	-11.61	-11.6	-11.59	-11.58	-11.57	-11.56
-11.64	-11.63	-11.62	-11.61	-11.6	-11.59	-11.58	-11.57	-11.56

-11.64	-11.63	-11.62	-11.61	-11.6	-11.59	-11.58	-11.57	-11.56
-11.64	-11.63	-11.62	-11.61	-11.6	-11.59	-11.58	-11.57	-11.56
-11.64	-11.63	-11.62	-11.61	-11.6	-11.59	-11.58	-11.57	-11.56
-11.64	-11.63	-11.62	-11.61	-11.6	-11.59	-11.58	-11.57	-11.56
-11.64	-11.63	-11.62	-11.61	-11.6	-11.59	-11.58	-11.57	-11.56
-11.64	-11.63	-11.62	-11.61	-11.6	-11.59	-11.58	-11.57	-11.56
-11.64	-11.63	-11.62	-11.61	-11.6	-11.59	-11.58	-11.57	-11.56
-11.64	-11.63	-11.62	-11.61	-11.6	-11.59	-11.58	-11.57	-11.56
-11.64	-11.63	-11.62	-11.61	-11.6	-11.59	-11.58	-11.57	-11.56
-11.64	-11.63	-11.62	-11.61	-11.6	-11.59	-11.58	-11.57	-11.56
-11.64	-11.63	-11.62	-11.61	-11.6	-11.59	-11.58	-11.57	-11.56
-11.64	-11.63	-11.62	-11.61	-11.6	-11.59	-11.58	-11.57	-11.56
-11.64	-11.63	-11.62	-11.61	-11.6	-11.59	-11.58	-11.57	-11.56
-11.64	-11.63	-11.62	-11.61	-11.6	-11.59	-11.58	-11.57	-11.56
-11.64	-11.63	-11.62	-11.61	-11.6	-11.59	-11.58	-11.57	-11.56
-11.64	-11.63	-11.62	-11.61	-11.6	-11.59	-11.58	-11.57	-11.56
-11.64	-11.63	-11.62	-11.61	-11.6	-11.59	-11.58	-11.57	-11.56
-11.64	-11.63	-11.62	-11.61	-11.6	-11.59	-11.58	-11.57	-11.56
-11.64	-11.63	-11.62	-11.61	-11.6	-11.59	-11.58	-11.57	-11.56
-11.64	-11.63	-11.62	-11.61	-11.6	-11.59	-11.58	-11.57	-11.56
-11.64	-11.63	-11.62	-11.61	-11.6	-11.59	-11.58	-11.57	-11.56
-11.64	-11.63	-11.62	-11.61	-11.6	-11.59	-11.58	-11.57	-11.56
-11.64	-11.63	-11.62	-11.61	-11.6	-11.59	-11.58	-11.57	-11.56
-11.64	-11.63	-11.62	-11.61	-11.6	-11.59	-11.58	-11.57	-11.56
-11.64	-11.63	-11.62	-11.61	-11.6	-11.59	-11.58	-11.57	-11.56
-11.64	-11.63	-11.62	-11.61	-11.6	-11.59	-11.58	-11.57	-11.56
-11.64	-11.63	-11.62	-11.61	-11.6	-11.59	-11.58	-11.57	-11.56
-11.64	-11.63	-11.62	-11.61	-11.6	-11.59	-11.58	-11.57	-11.56
-11.64	-11.63	-11.62	-11.61	-11.6	-11.59	-11.58	-11.57	-11.56
-11.64	-11.63	-11.62	-11.61	-11.6	-11.59	-11.58	-11.57	-11.56
-11.64	-11.63	-11.62	-11.61	-11.6	-11.59	-11.58	-11.57	-11.56
-11.64	-11.63	-11.62	-11.61	-11.6	-11.59	-11.58	-11.57	-11.56
-11.64	-11.63	-11.62	-11.61	-11.6	-11.59	-11.58	-11.57	-11.56
-11.64	-11.63	-11.62	-11.61	-11.6	-11.59	-11.58	-11.57	-11.56
-11.64	-11.63	-11.62	-11.61	-11.6	-11.59	-11.58	-11.57	-11.56
-11.64	-11.63	-11.62	-11.61	-11.6	-11.59	-11.58	-11.57	-11.56
-11.64	-11.63	-11.62	-11.61	-11.6	-11.59	-11.58	-11.57	-11.56
-11.64	-11.63	-11.62	-11.61	-11.6	-11.59	-11.58	-11.57	-11.56
-11.64	-11.63	-11.62	-11.61	-11.6	-11.59	-11.58	-11.57	-11.56
-11.64	-11.63	-11.62	-11.61	-11.6	-11.59	-11.58	-11.57	-11.56
-11.64	-11.63	-11.62	-11.61	-11.6	-11.59	-11.58	-11.57	-11.56
-11.64	-11.63	-11.62	-11.61	-11.6	-11.59	-11.58	-11.57	-11.56
-11.64	-11.63	-11.62	-11.61	-11.6	-11.59	-11.58	-11.57	-11.56
-11.64	-11.63	-11.62	-11.61	-11.6	-11.59	-11.58	-11.57	-11.56
-11.64	-11.63	-11.62	-11.61	-11.6	-11.59	-11.58	-11.57	-11.56
-11.64	-11.63	-11.62	-11.61	-11.6	-11.59	-11.58	-11.57	-11.56
-11.64	-11.63	-11.62	-11.61	-11.6	-11.59	-11.58	-11.57	-11.56
-11.64	-11.63	-11.62	-11.61	-11.6	-11.59	-11.58	-11.57	-11.56
-11.64	-11.63	-11.62	-11.61	-11.6	-11.59	-11.58	-11.57	-11.56
-11.64	-11.63	-11.62	-11.61	-11.6	-11.59	-11.58	-11.57	-11.56

-11.64	-11.63	-11.62	-11.61	-11.6	-11.59	-11.58	-11.57	-11.56
-11.64	-11.63	-11.62	-11.61	-11.6	-11.59	-11.58	-11.57	-11.56
-11.64	-11.63	-11.62	-11.61	-11.6	-11.59	-11.58	-11.57	-11.56
-11.64	-11.63	-11.62	-11.61	-11.6	-11.59	-11.58	-11.57	-11.56
-11.64	-11.63	-11.62	-11.61	-11.6	-11.59	-11.58	-11.57	-11.56
-11.64	-11.63	-11.62	-11.61	-11.6	-11.59	-11.58	-11.57	-11.56
-11.64	-11.63	-11.62	-11.61	-11.6	-11.59	-11.58	-11.57	-11.56
-11.64	-11.63	-11.62	-11.61	-11.6	-11.59	-11.58	-11.57	-11.56
-11.64	-11.63	-11.62	-11.61	-11.6	-11.59	-11.58	-11.57	-11.56
-11.64	-11.63	-11.62	-11.61	-11.6	-11.59	-11.58	-11.57	-11.56
-11.64	-11.63	-11.62	-11.61	-11.6	-11.59	-11.58	-11.57	-11.56
-11.64	-11.63	-11.62	-11.61	-11.6	-11.59	-11.58	-11.57	-11.56
-11.64	-11.63	-11.62	-11.61	-11.6	-11.59	-11.58	-11.57	-11.56
-11.64	-11.63	-11.62	-11.61	-11.6	-11.59	-11.58	-11.57	-11.56
-11.64	-11.63	-11.62	-11.61	-11.6	-11.59	-11.58	-11.57	-11.56
-11.64	-11.63	-11.62	-11.61	-11.6	-11.59	-11.58	-11.57	-11.56
-11.64	-11.63	-11.62	-11.61	-11.6	-11.59	-11.58	-11.57	-11.56
-11.64	-11.63	-11.62	-11.61	-11.6	-11.59	-11.58	-11.57	-11.56
-11.64	-11.63	-11.62	-11.61	-11.6	-11.59	-11.58	-11.57	-11.56
-11.64	-11.63	-11.62	-11.61	-11.6	-11.59	-11.58	-11.57	-11.56
-11.64	-11.63	-11.62	-11.61	-11.6	-11.59	-11.58	-11.57	-11.56
-11.64	-11.63	-11.62	-11.61	-11.6	-11.59	-11.58	-11.57	-11.56
-11.64	-11.63	-11.62	-11.61	-11.6	-11.59	-11.58	-11.57	-11.56
-11.64	-11.63	-11.62	-11.61	-11.6	-11.59	-11.58	-11.57	-11.56
-11.64	-11.63	-11.62	-11.61	-11.6	-11.59	-11.58	-11.57	-11.56
-11.64	-11.63	-11.62	-11.61	-11.6	-11.59	-11.58	-11.57	-11.56
-11.64	-11.63	-11.62	-11.61	-11.6	-11.59	-11.58	-11.57	-11.56
-11.64	-11.63	-11.62	-11.61	-11.6	-11.59	-11.58	-11.57	-11.56
-11.64	-11.63	-11.62	-11.61	-11.6	-11.59	-11.58	-11.57	-11.56
-11.64	-11.63	-11.62	-11.61	-11.6	-11.59	-11.58	-11.57	-11.56
-11.64	-11.63	-11.62	-11.61	-11.6	-11.59	-11.58	-11.57	-11.56
-11.64	-11.63	-11.62	-11.61	-11.6	-11.59	-11.58	-11.57	-11.56
-11.64	-11.63	-11.62	-11.61	-11.6	-11.59	-11.58	-11.57	-11.56
-11.64	-11.63	-11.62	-11.61	-11.6	-11.59	-11.58	-11.57	-11.56
-11.64	-11.63	-11.62	-11.61	-11.6	-11.59	-11.58	-11.57	-11.56
-11.64	-11.63	-11.62	-11.61	-11.6	-11.59	-11.58	-11.57	-11.56
-11.64	-11.63	-11.62	-11.61	-11.6	-11.59	-11.58	-11.57	-11.56
-11.64	-11.63	-11.62	-11.61	-11.6	-11.59	-11.58	-11.57	-11.56
-11.64	-11.63	-11.62	-11.61	-11.6	-11.59	-11.58	-11.57	-11.56
-11.64	-11.63	-11.62	-11.61	-11.6	-11.59	-11.58	-11.57	-11.56
-11.64	-11.63	-11.62	-11.61	-11.6	-11.59	-11.58	-11.57	-11.56
-11.64	-11.63	-11.62	-11.61	-11.6	-11.59	-11.58	-11.57	-11.56
-11.64	-11.63	-11.62	-11.61	-11.6	-11.59	-11.58	-11.57	-11.56
-11.64	-11.63	-11.62	-11.61	-11.6	-11.59	-11.58	-11.57	-11.56
-11.64	-11.63	-11.62	-11.61	-11.6	-11.59	-11.58	-11.57	-11.56
-11.64	-11.63	-11.62	-11.61	-11.6	-11.59	-11.58	-11.57	-11.56
-11.64	-11.63	-11.62	-11.61	-11.6	-11.59	-11.58	-11.57	-11.56
-11.64	-11.63	-11.62	-11.61	-11.6	-11.59	-11.58	-11.57	-11.56
-11.64	-11.63	-11.62	-11.61	-11.6	-11.59	-11.58	-11.57	-11.56
-11.64	-11.63	-11.62	-11.61	-11.6	-11.59	-11.58	-11.57	-11.56

-11.64	-11.63	-11.62	-11.61	-11.6	-11.59	-11.58	-11.57	-11.56
-11.64	-11.63	-11.62	-11.61	-11.6	-11.59	-11.58	-11.57	-11.56
-11.64	-11.63	-11.62	-11.61	-11.6	-11.59	-11.58	-11.57	-11.56
-11.64	-11.63	-11.62	-11.61	-11.6	-11.59	-11.58	-11.57	-11.56
-11.64	-11.63	-11.62	-11.61	-11.6	-11.59	-11.58	-11.57	-11.56
-11.64	-11.63	-11.62	-11.61	-11.6	-11.59	-11.58	-11.57	-11.56
-11.64	-11.63	-11.62	-11.61	-11.6	-11.59	-11.58	-11.57	-11.56
-11.64	-11.63	-11.62	-11.61	-11.6	-11.59	-11.58	-11.57	-11.56
-11.64	-11.63	-11.62	-11.61	-11.6	-11.59	-11.58	-11.57	-11.56
-11.64	-11.63	-11.62	-11.61	-11.6	-11.59	-11.58	-11.57	-11.56
-11.64	-11.63	-11.62	-11.61	-11.6	-11.59	-11.58	-11.57	-11.56
-11.64	-11.63	-11.62	-11.61	-11.6	-11.59	-11.58	-11.57	-11.56
-11.64	-11.63	-11.62	-11.61	-11.6	-11.59	-11.58	-11.57	-11.56
-11.64	-11.63	-11.62	-11.61	-11.6	-11.59	-11.58	-11.57	-11.56
-11.64	-11.63	-11.62	-11.61	-11.6	-11.59	-11.58	-11.57	-11.56
-11.64	-11.63	-11.62	-11.61	-11.6	-11.59	-11.58	-11.57	-11.56
-11.64	-11.63	-11.62	-11.61	-11.6	-11.59	-11.58	-11.57	-11.56
-11.64	-11.63	-11.62	-11.61	-11.6	-11.59	-11.58	-11.57	-11.56
-11.64	-11.63	-11.62	-11.61	-11.6	-11.59	-11.58	-11.57	-11.56
-11.64	-11.63	-11.62	-11.61	-11.6	-11.59	-11.58	-11.57	-11.56
-11.64	-11.63	-11.62	-11.61	-11.6	-11.59	-11.58	-11.57	-11.56
-11.64	-11.63	-11.62	-11.61	-11.6	-11.59	-11.58	-11.57	-11.56
-11.64	-11.63	-11.62	-11.61	-11.6	-11.59	-11.58	-11.57	-11.56
-11.64	-11.63	-11.62	-11.61	-11.6	-11.59	-11.58	-11.57	-11.56
-11.64	-11.63	-11.62	-11.61	-11.6	-11.59	-11.58	-11.57	-11.56
-11.64	-11.63	-11.62	-11.61	-11.6	-11.59	-11.58	-11.57	-11.56
-11.64	-11.63	-11.62	-11.61	-11.6	-11.59	-11.58	-11.57	-11.56
-11.64	-11.63	-11.62	-11.61	-11.6	-11.59	-11.58	-11.57	-11.56
-11.64	-11.63	-11.62	-11.61	-11.6	-11.59	-11.58	-11.57	-11.56
-11.64	-11.63	-11.62	-11.61	-11.6	-11.59	-11.58	-11.57	-11.56
-11.64	-11.63	-11.62	-11.61	-11.6	-11.59	-11.58	-11.57	-11.56
-11.64	-11.63	-11.62	-11.61	-11.6	-11.59	-11.58	-11.57	-11.56
-11.64	-11.63	-11.62	-11.61	-11.6	-11.59	-11.58	-11.57	-11.56
-11.64	-11.63	-11.62	-11.61	-11.6	-11.59	-11.58	-11.57	-11.56
-11.64	-11.63	-11.62	-11.61	-11.6	-11.59	-11.58	-11.57	-11.56
-11.64	-11.63	-11.62	-11.61	-11.6	-11.59	-11.58	-11.57	-11.56
-11.64	-11.63	-11.62	-11.61	-11.6	-11.59	-11.58	-11.57	-11.56
-11.64	-11.63	-11.62	-11.61	-11.6	-11.59	-11.58	-11.57	-11.56
-11.64	-11.63	-11.62	-11.61	-11.6	-11.59	-11.58	-11.57	-11.56
-11.64	-11.63	-11.62	-11.61	-11.6	-11.59	-11.58	-11.57	-11.56
-11.64	-11.63	-11.62	-11.61	-11.6	-11.59	-11.58	-11.57	-11.56
-11.64	-11.63	-11.62	-11.61	-11.6	-11.59	-11.58	-11.57	-11.56
-11.64	-11.63	-11.62	-11.61	-11.6	-11.59	-11.58	-11.57	-11.56
-11.64	-11.63	-11.62	-11.61	-11.6	-11.59	-11.58	-11.57	-11.56
-11.64	-11.63	-11.62	-11.61	-11.6	-11.59	-11.58	-11.57	-11.56
-11.64	-11.63	-11.62	-11.61	-11.6	-11.59	-11.58	-11.57	-11.56
-11.64	-11.63	-11.62	-11.61	-11.6	-11.59	-11.58	-11.57	-11.56
-11.64	-11.63	-11.62	-11.61	-11.6	-11.59	-11.58	-11.57	-11.56
-11.64	-11.63	-11.62	-11.61	-11.6	-11.59	-11.58	-11.57	-11.56
-11.64	-11.63	-11.62	-11.61	-11.6	-11.59	-11.58	-11.57	-11.56

-11.64	-11.63	-11.62	-11.61	-11.6	-11.59	-11.58	-11.57	-11.56
-11.64	-11.63	-11.62	-11.61	-11.6	-11.59	-11.58	-11.57	-11.56
-11.64	-11.63	-11.62	-11.61	-11.6	-11.59	-11.58	-11.57	-11.56
-11.64	-11.63	-11.62	-11.61	-11.6	-11.59	-11.58	-11.57	-11.56
-11.64	-11.63	-11.62	-11.61	-11.6	-11.59	-11.58	-11.57	-11.56
-11.64	-11.63	-11.62	-11.61	-11.6	-11.59	-11.58	-11.57	-11.56
-11.64	-11.63	-11.62	-11.61	-11.6	-11.59	-11.58	-11.57	-11.56
-11.64	-11.63	-11.62	-11.61	-11.6	-11.59	-11.58	-11.57	-11.56
-11.64	-11.63	-11.62	-11.61	-11.6	-11.59	-11.58	-11.57	-11.56
-11.64	-11.63	-11.62	-11.61	-11.6	-11.59	-11.58	-11.57	-11.56
-11.64	-11.63	-11.62	-11.61	-11.6	-11.59	-11.58	-11.57	-11.56
-11.64	-11.63	-11.62	-11.61	-11.6	-11.59	-11.58	-11.57	-11.56
-11.64	-11.63	-11.62	-11.61	-11.6	-11.59	-11.58	-11.57	-11.56
-11.64	-11.63	-11.62	-11.61	-11.6	-11.59	-11.58	-11.57	-11.56
-11.64	-11.63	-11.62	-11.61	-11.6	-11.59	-11.58	-11.57	-11.56
-11.64	-11.63	-11.62	-11.61	-11.6	-11.59	-11.58	-11.57	-11.56
-11.64	-11.63	-11.62	-11.61	-11.6	-11.59	-11.58	-11.57	-11.56
-11.64	-11.63	-11.62	-11.61	-11.6	-11.59	-11.58	-11.57	-11.56
-11.64	-11.63	-11.62	-11.61	-11.6	-11.59	-11.58	-11.57	-11.56
-11.64	-11.63	-11.62	-11.61	-11.6	-11.59	-11.58	-11.57	-11.56
-11.64	-11.63	-11.62	-11.61	-11.6	-11.59	-11.58	-11.57	-11.56
-11.64	-11.63	-11.62	-11.61	-11.6	-11.59	-11.58	-11.57	-11.56
-11.64	-11.63	-11.62	-11.61	-11.6	-11.59	-11.58	-11.57	-11.56
-11.64	-11.63	-11.62	-11.61	-11.6	-11.59	-11.58	-11.57	-11.56
-11.64	-11.63	-11.62	-11.61	-11.6	-11.59	-11.58	-11.57	-11.56
-11.64	-11.63	-11.62	-11.61	-11.6	-11.59	-11.58	-11.57	-11.56
-11.64	-11.63	-11.62	-11.61	-11.6	-11.59	-11.58	-11.57	-11.56
-11.64	-11.63	-11.62	-11.61	-11.6	-11.59	-11.58	-11.57	-11.56
-11.64	-11.63	-11.62	-11.61	-11.6	-11.59	-11.58	-11.57	-11.56
-11.64	-11.63	-11.62	-11.61	-11.6	-11.59	-11.58	-11.57	-11.56
-11.64	-11.63	-11.62	-11.61	-11.6	-11.59	-11.58	-11.57	-11.56
-11.64	-11.63	-11.62	-11.61	-11.6	-11.59	-11.58	-11.57	-11.56
-11.64	-11.63	-11.62	-11.61	-11.6	-11.59	-11.58	-11.57	-11.56
-11.64	-11.63	-11.62	-11.61	-11.6	-11.59	-11.58	-11.57	-11.56
-11.64	-11.63	-11.62	-11.61	-11.6	-11.59	-11.58	-11.57	-11.56
-11.64	-11.63	-11.62	-11.61	-11.6	-11.59	-11.58	-11.57	-11.56
-11.64	-11.63	-11.62	-11.61	-11.6	-11.59	-11.58	-11.57	-11.56
-11.64	-11.63	-11.62	-11.61	-11.6	-11.59	-11.58	-11.57	-11.56
-11.64	-11.63	-11.62	-11.61	-11.6	-11.59	-11.58	-11.57	-11.56
-11.64	-11.63	-11.62	-11.61	-11.6	-11.59	-11.58	-11.57	-11.56
-11.64	-11.63	-11.62	-11.61	-11.6	-11.59	-11.58	-11.57	-11.56
-11.64	-11.63	-11.62	-11.61	-11.6	-11.59	-11.58	-11.57	-11.56
-11.64	-11.63	-11.62	-11.61	-11.6	-11.59	-11.58	-11.57	-11.56
-11.64	-11.63	-11.62	-11.61	-11.6	-11.59	-11.58	-11.57	-11.56
-11.64	-11.63	-11.62	-11.61	-11.6	-11.59	-11.58	-11.57	-11.56
-11.64	-11.63	-11.62	-11.61	-11.6	-11.59	-11.58	-11.57	-11.56
-11.64	-11.63	-11.62	-11.61	-11.6	-11.59	-11.58	-11.57	-11.56
-11.64	-11.63	-11.62	-11.61	-11.6	-11.59	-11.58	-11.57	-11.56
-11.64	-11.63	-11.62	-11.61	-11.6	-11.59	-11.58	-11.57	-11.56
-11.64	-11.63	-11.62	-11.61	-11.6	-11.59	-11.58	-11.57	-11.56

-11.64	-11.63	-11.62	-11.61	-11.6	-11.59	-11.58	-11.57	-11.56
-11.64	-11.63	-11.62	-11.61	-11.6	-11.59	-11.58	-11.57	-11.56
-11.64	-11.63	-11.62	-11.61	-11.6	-11.59	-11.58	-11.57	-11.56
-11.64	-11.63	-11.62	-11.61	-11.6	-11.59	-11.58	-11.57	-11.56
-11.64	-11.63	-11.62	-11.61	-11.6	-11.59	-11.58	-11.57	-11.56
-11.64	-11.63	-11.62	-11.61	-11.6	-11.59	-11.58	-11.57	-11.56
-11.64	-11.63	-11.62	-11.61	-11.6	-11.59	-11.58	-11.57	-11.56
-11.64	-11.63	-11.62	-11.61	-11.6	-11.59	-11.58	-11.57	-11.56
-11.64	-11.63	-11.62	-11.61	-11.6	-11.59	-11.58	-11.57	-11.56
-11.64	-11.63	-11.62	-11.61	-11.6	-11.59	-11.58	-11.57	-11.56
-11.64	-11.63	-11.62	-11.61	-11.6	-11.59	-11.58	-11.57	-11.56
-11.64	-11.63	-11.62	-11.61	-11.6	-11.59	-11.58	-11.57	-11.56
-11.64	-11.63	-11.62	-11.61	-11.6	-11.59	-11.58	-11.57	-11.56
-11.64	-11.63	-11.62	-11.61	-11.6	-11.59	-11.58	-11.57	-11.56
-11.64	-11.63	-11.62	-11.61	-11.6	-11.59	-11.58	-11.57	-11.56
-11.64	-11.63	-11.62	-11.61	-11.6	-11.59	-11.58	-11.57	-11.56
-11.64	-11.63	-11.62	-11.61	-11.6	-11.59	-11.58	-11.57	-11.56
-11.64	-11.63	-11.62	-11.61	-11.6	-11.59	-11.58	-11.57	-11.56
-11.64	-11.63	-11.62	-11.61	-11.6	-11.59	-11.58	-11.57	-11.56
-11.64	-11.63	-11.62	-11.61	-11.6	-11.59	-11.58	-11.57	-11.56
-11.64	-11.63	-11.62	-11.61	-11.6	-11.59	-11.58	-11.57	-11.56
-11.64	-11.63	-11.62	-11.61	-11.6	-11.59	-11.58	-11.57	-11.56
-11.64	-11.63	-11.62	-11.61	-11.6	-11.59	-11.58	-11.57	-11.56
-11.64	-11.63	-11.62	-11.61	-11.6	-11.59	-11.58	-11.57	-11.56
-11.64	-11.63	-11.62	-11.61	-11.6	-11.59	-11.58	-11.57	-11.56
-11.64	-11.63	-11.62	-11.61	-11.6	-11.59	-11.58	-11.57	-11.56
-11.64	-11.63	-11.62	-11.61	-11.6	-11.59	-11.58	-11.57	-11.56
-11.64	-11.63	-11.62	-11.61	-11.6	-11.59	-11.58	-11.57	-11.56
-11.64	-11.63	-11.62	-11.61	-11.6	-11.59	-11.58	-11.57	-11.56
-11.64	-11.63	-11.62	-11.61	-11.6	-11.59	-11.58	-11.57	-11.56
-11.64	-11.63	-11.62	-11.61	-11.6	-11.59	-11.58	-11.57	-11.56
-11.64	-11.63	-11.62	-11.61	-11.6	-11.59	-11.58	-11.57	-11.56
-11.64	-11.63	-11.62	-11.61	-11.6	-11.59	-11.58	-11.57	-11.56
-11.64	-11.63	-11.62	-11.61	-11.6	-11.59	-11.58	-11.57	-11.56
-11.64	-11.63	-11.62	-11.61	-11.6	-11.59	-11.58	-11.57	-11.56
-11.64	-11.63	-11.62	-11.61	-11.6	-11.59	-11.58	-11.57	-11.56
-11.64	-11.63	-11.62	-11.61	-11.6	-11.59	-11.58	-11.57	-11.56
-11.64	-11.63	-11.62	-11.61	-11.6	-11.59	-11.58	-11.57	-11.56
-11.64	-11.63	-11.62	-11.61	-11.6	-11.59	-11.58	-11.57	-11.56
-11.64	-11.63	-11.62	-11.61	-11.6	-11.59	-11.58	-11.57	-11.56
-11.64	-11.63	-11.62	-11.61	-11.6	-11.59	-11.58	-11.57	-11.56
-11.64	-11.63	-11.62	-11.61	-11.6	-11.59	-11.58	-11.57	-11.56
-11.64	-11.63	-11.62	-11.61	-11.6	-11.59	-11.58	-11.57	-11.56
-11.64	-11.63	-11.62	-11.61	-11.6	-11.59	-11.58	-11.57	-11.56
-11.64	-11.63	-11.62	-11.61	-11.6	-11.59	-11.58	-11.57	-11.56
-11.64	-11.63	-11.62	-11.61	-11.6	-11.59	-11.58	-11.57	-11.56
-11.64	-11.63	-11.62	-11.61	-11.6	-11.59	-11.58	-11.57	-11.56
-11.64	-11.63	-11.62	-11.61	-11.6	-11.59	-11.58	-11.57	-11.56
-11.64	-11.63	-11.62	-11.61	-11.6	-11.59	-11.58	-11.57	-11.56
-11.64	-11.63	-11.62	-11.61	-11.6	-11.59	-11.58	-11.57	-11.56

-11.64	-11.63	-11.62	-11.61	-11.6	-11.59	-11.58	-11.57	-11.56
-11.64	-11.63	-11.62	-11.61	-11.6	-11.59	-11.58	-11.57	-11.56
-11.64	-11.63	-11.62	-11.61	-11.6	-11.59	-11.58	-11.57	-11.56
-11.64	-11.63	-11.62	-11.61	-11.6	-11.59	-11.58	-11.57	-11.56
-11.64	-11.63	-11.62	-11.61	-11.6	-11.59	-11.58	-11.57	-11.56
-11.64	-11.63	-11.62	-11.61	-11.6	-11.59	-11.58	-11.57	-11.56
-11.64	-11.63	-11.62	-11.61	-11.6	-11.59	-11.58	-11.57	-11.56
-11.64	-11.63	-11.62	-11.61	-11.6	-11.59	-11.58	-11.57	-11.56
-11.64	-11.63	-11.62	-11.61	-11.6	-11.59	-11.58	-11.57	-11.56
-11.64	-11.63	-11.62	-11.61	-11.6	-11.59	-11.58	-11.57	-11.56
-11.64	-11.63	-11.62	-11.61	-11.6	-11.59	-11.58	-11.57	-11.56
-11.64	-11.63	-11.62	-11.61	-11.6	-11.59	-11.58	-11.57	-11.56
-11.64	-11.63	-11.62	-11.61	-11.6	-11.59	-11.58	-11.57	-11.56
-11.64	-11.63	-11.62	-11.61	-11.6	-11.59	-11.58	-11.57	-11.56
-11.64	-11.63	-11.62	-11.61	-11.6	-11.59	-11.58	-11.57	-11.56
-11.64	-11.63	-11.62	-11.61	-11.6	-11.59	-11.58	-11.57	-11.56
-11.64	-11.63	-11.62	-11.61	-11.6	-11.59	-11.58	-11.57	-11.56
-11.64	-11.63	-11.62	-11.61	-11.6	-11.59	-11.58	-11.57	-11.56
-11.64	-11.63	-11.62	-11.61	-11.6	-11.59	-11.58	-11.57	-11.56
-11.64	-11.63	-11.62	-11.61	-11.6	-11.59	-11.58	-11.57	-11.56
-11.64	-11.63	-11.62	-11.61	-11.6	-11.59	-11.58	-11.57	-11.56
-11.64	-11.63	-11.62	-11.61	-11.6	-11.59	-11.58	-11.57	-11.56
-11.64	-11.63	-11.62	-11.61	-11.6	-11.59	-11.58	-11.57	-11.56
-11.64	-11.63	-11.62	-11.61	-11.6	-11.59	-11.58	-11.57	-11.56
-11.64	-11.63	-11.62	-11.61	-11.6	-11.59	-11.58	-11.57	-11.56
-11.64	-11.63	-11.62	-11.61	-11.6	-11.59	-11.58	-11.57	-11.56
-11.64	-11.63	-11.62	-11.61	-11.6	-11.59	-11.58	-11.57	-11.56
-11.64	-11.63	-11.62	-11.61	-11.6	-11.59	-11.58	-11.57	-11.56
-11.64	-11.63	-11.62	-11.61	-11.6	-11.59	-11.58	-11.57	-11.56
-11.64	-11.63	-11.62	-11.61	-11.6	-11.59	-11.58	-11.57	-11.56
-11.64	-11.63	-11.62	-11.61	-11.6	-11.59	-11.58	-11.57	-11.56
-11.64	-11.63	-11.62	-11.61	-11.6	-11.59	-11.58	-11.57	-11.56
-11.64	-11.63	-11.62	-11.61	-11.6	-11.59	-11.58	-11.57	-11.56
-11.64	-11.63	-11.62	-11.61	-11.6	-11.59	-11.58	-11.57	-11.56
-11.64	-11.63	-11.62	-11.61	-11.6	-11.59	-11.58	-11.57	-11.56
-11.64	-11.63	-11.62	-11.61	-11.6	-11.59	-11.58	-11.57	-11.56
-11.64	-11.63	-11.62	-11.61	-11.6	-11.59	-11.58	-11.57	-11.56
-11.64	-11.63	-11.62	-11.61	-11.6	-11.59	-11.58	-11.57	-11.56
-11.64	-11.63	-11.62	-11.61	-11.6	-11.59	-11.58	-11.57	-11.56
-11.64	-11.63	-11.62	-11.61	-11.6	-11.59	-11.58	-11.57	-11.56
-11.64	-11.63	-11.62	-11.61	-11.6	-11.59	-11.58	-11.57	-11.56
-11.64	-11.63	-11.62	-11.61	-11.6	-11.59	-11.58	-11.57	-11.56
-11.64	-11.63	-11.62	-11.61	-11.6	-11.59	-11.58	-11.57	-11.56
-11.64	-11.63	-11.62	-11.61	-11.6	-11.59	-11.58	-11.57	-11.56
-11.64	-11.63	-11.62	-11.61	-11.6	-11.59	-11.58	-11.57	-11.56
-11.64	-11.63	-11.62	-11.61	-11.6	-11.59	-11.58	-11.57	-11.56
-11.64	-11.63	-11.62	-11.61	-11.6	-11.59	-11.58	-11.57	-11.56
-11.64	-11.63	-11.62	-11.61	-11.6	-11.59	-11.58	-11.57	-11.56
-11.64	-11.63	-11.62	-11.61	-11.6	-11.59	-11.58	-11.57	-11.56
-11.64	-11.63	-11.62	-11.61	-11.6	-11.59	-11.58	-11.57	-11.56

-11.64	-11.63	-11.62	-11.61	-11.6	-11.59	-11.58	-11.57	-11.56
-11.64	-11.63	-11.62	-11.61	-11.6	-11.59	-11.58	-11.57	-11.56
-11.64	-11.63	-11.62	-11.61	-11.6	-11.59	-11.58	-11.57	-11.56
-11.64	-11.63	-11.62	-11.61	-11.6	-11.59	-11.58	-11.57	-11.56
-11.64	-11.63	-11.62	-11.61	-11.6	-11.59	-11.58	-11.57	-11.56
-11.64	-11.63	-11.62	-11.61	-11.6	-11.59	-11.58	-11.57	-11.56
-11.64	-11.63	-11.62	-11.61	-11.6	-11.59	-11.58	-11.57	-11.56
-11.64	-11.63	-11.62	-11.61	-11.6	-11.59	-11.58	-11.57	-11.56
-11.64	-11.63	-11.62	-11.61	-11.6	-11.59	-11.58	-11.57	-11.56
-11.64	-11.63	-11.62	-11.61	-11.6	-11.59	-11.58	-11.57	-11.56
-11.64	-11.63	-11.62	-11.61	-11.6	-11.59	-11.58	-11.57	-11.56
-11.64	-11.63	-11.62	-11.61	-11.6	-11.59	-11.58	-11.57	-11.56
-11.64	-11.63	-11.62	-11.61	-11.6	-11.59	-11.58	-11.57	-11.56
-11.64	-11.63	-11.62	-11.61	-11.6	-11.59	-11.58	-11.57	-11.56
-11.64	-11.63	-11.62	-11.61	-11.6	-11.59	-11.58	-11.57	-11.56
-11.64	-11.63	-11.62	-11.61	-11.6	-11.59	-11.58	-11.57	-11.56
-11.64	-11.63	-11.62	-11.61	-11.6	-11.59	-11.58	-11.57	-11.56
-11.64	-11.63	-11.62	-11.61	-11.6	-11.59	-11.58	-11.57	-11.56
-11.64	-11.63	-11.62	-11.61	-11.6	-11.59	-11.58	-11.57	-11.56
-11.64	-11.63	-11.62	-11.61	-11.6	-11.59	-11.58	-11.57	-11.56
-11.64	-11.63	-11.62	-11.61	-11.6	-11.59	-11.58	-11.57	-11.56
-11.64	-11.63	-11.62	-11.61	-11.6	-11.59	-11.58	-11.57	-11.56
-11.64	-11.63	-11.62	-11.61	-11.6	-11.59	-11.58	-11.57	-11.56
-11.64	-11.63	-11.62	-11.61	-11.6	-11.59	-11.58	-11.57	-11.56
-11.64	-11.63	-11.62	-11.61	-11.6	-11.59	-11.58	-11.57	-11.56
-11.64	-11.63	-11.62	-11.61	-11.6	-11.59	-11.58	-11.57	-11.56
-11.64	-11.63	-11.62	-11.61	-11.6	-11.59	-11.58	-11.57	-11.56
-11.64	-11.63	-11.62	-11.61	-11.6	-11.59	-11.58	-11.57	-11.56
-11.64	-11.63	-11.62	-11.61	-11.6	-11.59	-11.58	-11.57	-11.56
-11.64	-11.63	-11.62	-11.61	-11.6	-11.59	-11.58	-11.57	-11.56
-11.64	-11.63	-11.62	-11.61	-11.6	-11.59	-11.58	-11.57	-11.56
-11.64	-11.63	-11.62	-11.61	-11.6	-11.59	-11.58	-11.57	-11.56
-11.64	-11.63	-11.62	-11.61	-11.6	-11.59	-11.58	-11.57	-11.56
-11.64	-11.63	-11.62	-11.61	-11.6	-11.59	-11.58	-11.57	-11.56
-11.64	-11.63	-11.62	-11.61	-11.6	-11.59	-11.58	-11.57	-11.56
-11.64	-11.63	-11.62	-11.61	-11.6	-11.59	-11.58	-11.57	-11.56
-11.64	-11.63	-11.62	-11.61	-11.6	-11.59	-11.58	-11.57	-11.56
-11.64	-11.63	-11.62	-11.61	-11.6	-11.59	-11.58	-11.57	-11.56
-11.64	-11.63	-11.62	-11.61	-11.6	-11.59	-11.58	-11.57	-11.56
-11.64	-11.63	-11.62	-11.61	-11.6	-11.59	-11.58	-11.57	-11.56
-11.64	-11.63	-11.62	-11.61	-11.6	-11.59	-11.58	-11.57	-11.56
-11.64	-11.63	-11.62	-11.61	-11.6	-11.59	-11.58	-11.57	-11.56
-11.64	-11.63	-11.62	-11.61	-11.6	-11.59	-11.58	-11.57	-11.56
-11.64	-11.63	-11.62	-11.61	-11.6	-11.59	-11.58	-11.57	-11.56
-11.64	-11.63	-11.62	-11.61	-11.6	-11.59	-11.58	-11.57	-11.56
-11.64	-11.63	-11.62	-11.61	-11.6	-11.59	-11.58	-11.57	-11.56
-11.64	-11.63	-11.62	-11.61	-11.6	-11.59	-11.58	-11.57	-11.56
-11.64	-11.63	-11.62	-11.61	-11.6	-11.59	-11.58	-11.57	-11.56
-11.64	-11.63	-11.62	-11.61	-11.6	-11.59	-11.58	-11.57	-11.56
-11.64	-11.63	-11.62	-11.61	-11.6	-11.59	-11.58	-11.57	-11.56

-11.64	-11.63	-11.62	-11.61	-11.6	-11.59	-11.58	-11.57	-11.56
-11.64	-11.63	-11.62	-11.61	-11.6	-11.59	-11.58	-11.57	-11.56
-11.64	-11.63	-11.62	-11.61	-11.6	-11.59	-11.58	-11.57	-11.56
-11.64	-11.63	-11.62	-11.61	-11.6	-11.59	-11.58	-11.57	-11.56
-11.64	-11.63	-11.62	-11.61	-11.6	-11.59	-11.58	-11.57	-11.56
-11.64	-11.63	-11.62	-11.61	-11.6	-11.59	-11.58	-11.57	-11.56
-11.64	-11.63	-11.62	-11.61	-11.6	-11.59	-11.58	-11.57	-11.56
-11.64	-11.63	-11.62	-11.61	-11.6	-11.59	-11.58	-11.57	-11.56
-11.64	-11.63	-11.62	-11.61	-11.6	-11.59	-11.58	-11.57	-11.56
-11.64	-11.63	-11.62	-11.61	-11.6	-11.59	-11.58	-11.57	-11.56
-11.64	-11.63	-11.62	-11.61	-11.6	-11.59	-11.58	-11.57	-11.56
-11.64	-11.63	-11.62	-11.61	-11.6	-11.59	-11.58	-11.57	-11.56
-11.64	-11.63	-11.62	-11.61	-11.6	-11.59	-11.58	-11.57	-11.56
-11.64	-11.63	-11.62	-11.61	-11.6	-11.59	-11.58	-11.57	-11.56
-11.64	-11.63	-11.62	-11.61	-11.6	-11.59	-11.58	-11.57	-11.56
-11.64	-11.63	-11.62	-11.61	-11.6	-11.59	-11.58	-11.57	-11.56
-11.64	-11.63	-11.62	-11.61	-11.6	-11.59	-11.58	-11.57	-11.56
-11.64	-11.63	-11.62	-11.61	-11.6	-11.59	-11.58	-11.57	-11.56
-11.64	-11.63	-11.62	-11.61	-11.6	-11.59	-11.58	-11.57	-11.56
-11.64	-11.63	-11.62	-11.61	-11.6	-11.59	-11.58	-11.57	-11.56
-11.64	-11.63	-11.62	-11.61	-11.6	-11.59	-11.58	-11.57	-11.56
-11.64	-11.63	-11.62	-11.61	-11.6	-11.59	-11.58	-11.57	-11.56
-11.64	-11.63	-11.62	-11.61	-11.6	-11.59	-11.58	-11.57	-11.56
-11.64	-11.63	-11.62	-11.61	-11.6	-11.59	-11.58	-11.57	-11.56
-11.64	-11.63	-11.62	-11.61	-11.6	-11.59	-11.58	-11.57	-11.56
-11.64	-11.63	-11.62	-11.61	-11.6	-11.59	-11.58	-11.57	-11.56
-11.64	-11.63	-11.62	-11.61	-11.6	-11.59	-11.58	-11.57	-11.56
-11.64	-11.63	-11.62	-11.61	-11.6	-11.59	-11.58	-11.57	-11.56
-11.64	-11.63	-11.62	-11.61	-11.6	-11.59	-11.58	-11.57	-11.56
-11.64	-11.63	-11.62	-11.61	-11.6	-11.59	-11.58	-11.57	-11.56
-11.64	-11.63	-11.62	-11.61	-11.6	-11.59	-11.58	-11.57	-11.56
-11.64	-11.63	-11.62	-11.61	-11.6	-11.59	-11.58	-11.57	-11.56
-11.64	-11.63	-11.62	-11.61	-11.6	-11.59	-11.58	-11.57	-11.56
-11.64	-11.63	-11.62	-11.61	-11.6	-11.59	-11.58	-11.57	-11.56
-11.64	-11.63	-11.62	-11.61	-11.6	-11.59	-11.58	-11.57	-11.56
-11.64	-11.63	-11.62	-11.61	-11.6	-11.59	-11.58	-11.57	-11.56
-11.64	-11.63	-11.62	-11.61	-11.6	-11.59	-11.58	-11.57	-11.56
-11.64	-11.63	-11.62	-11.61	-11.6	-11.59	-11.58	-11.57	-11.56
-11.64	-11.63	-11.62	-11.61	-11.6	-11.59	-11.58	-11.57	-11.56
-11.64	-11.63	-11.62	-11.61	-11.6	-11.59	-11.58	-11.57	-11.56
-11.64	-11.63	-11.62	-11.61	-11.6	-11.59	-11.58	-11.57	-11.56
-11.64	-11.63	-11.62	-11.61	-11.6	-11.59	-11.58	-11.57	-11.56
-11.64	-11.63	-11.62	-11.61	-11.6	-11.59	-11.58	-11.57	-11.56
-11.64	-11.63	-11.62	-11.61	-11.6	-11.59	-11.58	-11.57	-11.56
-11.64	-11.63	-11.62	-11.61	-11.6	-11.59	-11.58	-11.57	-11.56
-11.64	-11.63	-11.62	-11.61	-11.6	-11.59	-11.58	-11.57	-11.56
-11.64	-11.63	-11.62	-11.61	-11.6	-11.59	-11.58	-11.57	-11.56
-11.64	-11.63	-11.62	-11.61	-11.6	-11.59	-11.58	-11.57	-11.56
-11.64	-11.63	-11.62	-11.61	-11.6	-11.59	-11.58	-11.57	-11.56
-11.64	-11.63	-11.62	-11.61	-11.6	-11.59	-11.58	-11.57	-11.56

-11.64	-11.63	-11.62	-11.61	-11.6	-11.59	-11.58	-11.57	-11.56
-11.64	-11.63	-11.62	-11.61	-11.6	-11.59	-11.58	-11.57	-11.56
-11.64	-11.63	-11.62	-11.61	-11.6	-11.59	-11.58	-11.57	-11.56
-11.64	-11.63	-11.62	-11.61	-11.6	-11.59	-11.58	-11.57	-11.56
-11.64	-11.63	-11.62	-11.61	-11.6	-11.59	-11.58	-11.57	-11.56
-11.64	-11.63	-11.62	-11.61	-11.6	-11.59	-11.58	-11.57	-11.56
-11.64	-11.63	-11.62	-11.61	-11.6	-11.59	-11.58	-11.57	-11.56
-11.64	-11.63	-11.62	-11.61	-11.6	-11.59	-11.58	-11.57	-11.56
-11.64	-11.63	-11.62	-11.61	-11.6	-11.59	-11.58	-11.57	-11.56
-11.64	-11.63	-11.62	-11.61	-11.6	-11.59	-11.58	-11.57	-11.56
-11.64	-11.63	-11.62	-11.61	-11.6	-11.59	-11.58	-11.57	-11.56
-11.64	-11.63	-11.62	-11.61	-11.6	-11.59	-11.58	-11.57	-11.56
-11.64	-11.63	-11.62	-11.61	-11.6	-11.59	-11.58	-11.57	-11.56
-11.64	-11.63	-11.62	-11.61	-11.6	-11.59	-11.58	-11.57	-11.56
-11.64	-11.63	-11.62	-11.61	-11.6	-11.59	-11.58	-11.57	-11.56
-11.64	-11.63	-11.62	-11.61	-11.6	-11.59	-11.58	-11.57	-11.56
-11.64	-11.63	-11.62	-11.61	-11.6	-11.59	-11.58	-11.57	-11.56
-11.64	-11.63	-11.62	-11.61	-11.6	-11.59	-11.58	-11.57	-11.56
-11.64	-11.63	-11.62	-11.61	-11.6	-11.59	-11.58	-11.57	-11.56
-11.64	-11.63	-11.62	-11.61	-11.6	-11.59	-11.58	-11.57	-11.56
-11.64	-11.63	-11.62	-11.61	-11.6	-11.59	-11.58	-11.57	-11.56
-11.64	-11.63	-11.62	-11.61	-11.6	-11.59	-11.58	-11.57	-11.56
-11.64	-11.63	-11.62	-11.61	-11.6	-11.59	-11.58	-11.57	-11.56
-11.64	-11.63	-11.62	-11.61	-11.6	-11.59	-11.58	-11.57	-11.56
-11.64	-11.63	-11.62	-11.61	-11.6	-11.59	-11.58	-11.57	-11.56
-11.64	-11.63	-11.62	-11.61	-11.6	-11.59	-11.58	-11.57	-11.56
-11.64	-11.63	-11.62	-11.61	-11.6	-11.59	-11.58	-11.57	-11.56
-11.64	-11.63	-11.62	-11.61	-11.6	-11.59	-11.58	-11.57	-11.56
-11.64	-11.63	-11.62	-11.61	-11.6	-11.59	-11.58	-11.57	-11.56
-11.64	-11.63	-11.62	-11.61	-11.6	-11.59	-11.58	-11.57	-11.56
-11.64	-11.63	-11.62	-11.61	-11.6	-11.59	-11.58	-11.57	-11.56
-11.64	-11.63	-11.62	-11.61	-11.6	-11.59	-11.58	-11.57	-11.56
-11.64	-11.63	-11.62	-11.61	-11.6	-11.59	-11.58	-11.57	-11.56
-11.64	-11.63	-11.62	-11.61	-11.6	-11.59	-11.58	-11.57	-11.56
-11.64	-11.63	-11.62	-11.61	-11.6	-11.59	-11.58	-11.57	-11.56
-11.64	-11.63	-11.62	-11.61	-11.6	-11.59	-11.58	-11.57	-11.56
-11.64	-11.63	-11.62	-11.61	-11.6	-11.59	-11.58	-11.57	-11.56
-11.64	-11.63	-11.62	-11.61	-11.6	-11.59	-11.58	-11.57	-11.56
-11.64	-11.63	-11.62	-11.61	-11.6	-11.59	-11.58	-11.57	-11.56
-11.64	-11.63	-11.62	-11.61	-11.6	-11.59	-11.58	-11.57	-11.56
-11.64	-11.63	-11.62	-11.61	-11.6	-11.59	-11.58	-11.57	-11.56
-11.64	-11.63	-11.62	-11.61	-11.6	-11.59	-11.58	-11.57	-11.56
-11.64	-11.63	-11.62	-11.61	-11.6	-11.59	-11.58	-11.57	-11.56
-11.64	-11.63	-11.62	-11.61	-11.6	-11.59	-11.58	-11.57	-11.56
-11.64	-11.63	-11.62	-11.61	-11.6	-11.59	-11.58	-11.57	-11.56
-11.64	-11.63	-11.62	-11.61	-11.6	-11.59	-11.58	-11.57	-11.56
-11.64	-11.63	-11.62	-11.61	-11.6	-11.59	-11.58	-11.57	-11.56
-11.64	-11.63	-11.62	-11.61	-11.6	-11.59	-11.58	-11.57	-11.56
-11.64	-11.63	-11.62	-11.61	-11.6	-11.59	-11.58	-11.57	-11.56
-11.64	-11.63	-11.62	-11.61	-11.6	-11.59	-11.58	-11.57	-11.56

-11.64	-11.63	-11.62	-11.61	-11.6	-11.59	-11.58	-11.57	-11.56
-11.64	-11.63	-11.62	-11.61	-11.6	-11.59	-11.58	-11.57	-11.56
-11.64	-11.63	-11.62	-11.61	-11.6	-11.59	-11.58	-11.57	-11.56
-11.64	-11.63	-11.62	-11.61	-11.6	-11.59	-11.58	-11.57	-11.56
-11.64	-11.63	-11.62	-11.61	-11.6	-11.59	-11.58	-11.57	-11.56
-11.64	-11.63	-11.62	-11.61	-11.6	-11.59	-11.58	-11.57	-11.56
-11.64	-11.63	-11.62	-11.61	-11.6	-11.59	-11.58	-11.57	-11.56
-11.64	-11.63	-11.62	-11.61	-11.6	-11.59	-11.58	-11.57	-11.56
-11.64	-11.63	-11.62	-11.61	-11.6	-11.59	-11.58	-11.57	-11.56
-11.64	-11.63	-11.62	-11.61	-11.6	-11.59	-11.58	-11.57	-11.56
-11.64	-11.63	-11.62	-11.61	-11.6	-11.59	-11.58	-11.57	-11.56
-11.64	-11.63	-11.62	-11.61	-11.6	-11.59	-11.58	-11.57	-11.56
-11.64	-11.63	-11.62	-11.61	-11.6	-11.59	-11.58	-11.57	-11.56
-11.64	-11.63	-11.62	-11.61	-11.6	-11.59	-11.58	-11.57	-11.56
-11.64	-11.63	-11.62	-11.61	-11.6	-11.59	-11.58	-11.57	-11.56
-11.64	-11.63	-11.62	-11.61	-11.6	-11.59	-11.58	-11.57	-11.56
-11.64	-11.63	-11.62	-11.61	-11.6	-11.59	-11.58	-11.57	-11.56
-11.64	-11.63	-11.62	-11.61	-11.6	-11.59	-11.58	-11.57	-11.56
-11.64	-11.63	-11.62	-11.61	-11.6	-11.59	-11.58	-11.57	-11.56
-11.64	-11.63	-11.62	-11.61	-11.6	-11.59	-11.58	-11.57	-11.56
-11.64	-11.63	-11.62	-11.61	-11.6	-11.59	-11.58	-11.57	-11.56
-11.64	-11.63	-11.62	-11.61	-11.6	-11.59	-11.58	-11.57	-11.56
-11.64	-11.63	-11.62	-11.61	-11.6	-11.59	-11.58	-11.57	-11.56
-11.64	-11.63	-11.62	-11.61	-11.6	-11.59	-11.58	-11.57	-11.56
-11.64	-11.63	-11.62	-11.61	-11.6	-11.59	-11.58	-11.57	-11.56
-11.64	-11.63	-11.62	-11.61	-11.6	-11.59	-11.58	-11.57	-11.56
-11.64	-11.63	-11.62	-11.61	-11.6	-11.59	-11.58	-11.57	-11.56
-11.64	-11.63	-11.62	-11.61	-11.6	-11.59	-11.58	-11.57	-11.56
-11.64	-11.63	-11.62	-11.61	-11.6	-11.59	-11.58	-11.57	-11.56
-11.64	-11.63	-11.62	-11.61	-11.6	-11.59	-11.58	-11.57	-11.56
-11.64	-11.63	-11.62	-11.61	-11.6	-11.59	-11.58	-11.57	-11.56
-11.64	-11.63	-11.62	-11.61	-11.6	-11.59	-11.58	-11.57	-11.56
-11.64	-11.63	-11.62	-11.61	-11.6	-11.59	-11.58	-11.57	-11.56
-11.64	-11.63	-11.62	-11.61	-11.6	-11.59	-11.58	-11.57	-11.56
-11.64	-11.63	-11.62	-11.61	-11.6	-11.59	-11.58	-11.57	-11.56
-11.64	-11.63	-11.62	-11.61	-11.6	-11.59	-11.58	-11.57	-11.56
-11.64	-11.63	-11.62	-11.61	-11.6	-11.59	-11.58	-11.57	-11.56
-11.64	-11.63	-11.62	-11.61	-11.6	-11.59	-11.58	-11.57	-11.56
-11.64	-11.63	-11.62	-11.61	-11.6	-11.59	-11.58	-11.57	-11.56
-11.64	-11.63	-11.62	-11.61	-11.6	-11.59	-11.58	-11.57	-11.56
-11.64	-11.63	-11.62	-11.61	-11.6	-11.59	-11.58	-11.57	-11.56
-11.64	-11.63	-11.62	-11.61	-11.6	-11.59	-11.58	-11.57	-11.56
-11.64	-11.63	-11.62	-11.61	-11.6	-11.59	-11.58	-11.57	-11.56
-11.64	-11.63	-11.62	-11.61	-11.6	-11.59	-11.58	-11.57	-11.56
-11.64	-11.63	-11.62	-11.61	-11.6	-11.59	-11.58	-11.57	-11.56
-11.64	-11.63	-11.62	-11.61	-11.6	-11.59	-11.58	-11.57	-11.56
-11.64	-11.63	-11.62	-11.61	-11.6	-11.59	-11.58	-11.57	-11.56
-11.64	-11.63	-11.62	-11.61	-11.6	-11.59	-11.58	-11.57	-11.56
-11.64	-11.63	-11.62	-11.61	-11.6	-11.59	-11.58	-11.57	-11.56
-11.64	-11.63	-11.62	-11.61	-11.6	-11.59	-11.58	-11.57	-11.56

-11.64	-11.63	-11.62	-11.61	-11.6	-11.59	-11.58	-11.57	-11.56
-11.64	-11.63	-11.62	-11.61	-11.6	-11.59	-11.58	-11.57	-11.56
-11.64	-11.63	-11.62	-11.61	-11.6	-11.59	-11.58	-11.57	-11.56
-11.64	-11.63	-11.62	-11.61	-11.6	-11.59	-11.58	-11.57	-11.56
-11.64	-11.63	-11.62	-11.61	-11.6	-11.59	-11.58	-11.57	-11.56
-11.64	-11.63	-11.62	-11.61	-11.6	-11.59	-11.58	-11.57	-11.56
-11.64	-11.63	-11.62	-11.61	-11.6	-11.59	-11.58	-11.57	-11.56
-11.64	-11.63	-11.62	-11.61	-11.6	-11.59	-11.58	-11.57	-11.56
-11.64	-11.63	-11.62	-11.61	-11.6	-11.59	-11.58	-11.57	-11.56
-11.64	-11.63	-11.62	-11.61	-11.6	-11.59	-11.58	-11.57	-11.56
-11.64	-11.63	-11.62	-11.61	-11.6	-11.59	-11.58	-11.57	-11.56
-11.64	-11.63	-11.62	-11.61	-11.6	-11.59	-11.58	-11.57	-11.56
-11.64	-11.63	-11.62	-11.61	-11.6	-11.59	-11.58	-11.57	-11.56
-11.64	-11.63	-11.62	-11.61	-11.6	-11.59	-11.58	-11.57	-11.56
-11.64	-11.63	-11.62	-11.61	-11.6	-11.59	-11.58	-11.57	-11.56
-11.64	-11.63	-11.62	-11.61	-11.6	-11.59	-11.58	-11.57	-11.56
-11.64	-11.63	-11.62	-11.61	-11.6	-11.59	-11.58	-11.57	-11.56
-11.64	-11.63	-11.62	-11.61	-11.6	-11.59	-11.58	-11.57	-11.56
-11.64	-11.63	-11.62	-11.61	-11.6	-11.59	-11.58	-11.57	-11.56
-11.64	-11.63	-11.62	-11.61	-11.6	-11.59	-11.58	-11.57	-11.56
-11.64	-11.63	-11.62	-11.61	-11.6	-11.59	-11.58	-11.57	-11.56
-11.64	-11.63	-11.62	-11.61	-11.6	-11.59	-11.58	-11.57	-11.56
-11.64	-11.63	-11.62	-11.61	-11.6	-11.59	-11.58	-11.57	-11.56
-11.64	-11.63	-11.62	-11.61	-11.6	-11.59	-11.58	-11.57	-11.56
-11.64	-11.63	-11.62	-11.61	-11.6	-11.59	-11.58	-11.57	-11.56
-11.64	-11.63	-11.62	-11.61	-11.6	-11.59	-11.58	-11.57	-11.56
-11.64	-11.63	-11.62	-11.61	-11.6	-11.59	-11.58	-11.57	-11.56
-11.64	-11.63	-11.62	-11.61	-11.6	-11.59	-11.58	-11.57	-11.56
-11.64	-11.63	-11.62	-11.61	-11.6	-11.59	-11.58	-11.57	-11.56
-11.64	-11.63	-11.62	-11.61	-11.6	-11.59	-11.58	-11.57	-11.56
-11.64	-11.63	-11.62	-11.61	-11.6	-11.59	-11.58	-11.57	-11.56
-11.64	-11.63	-11.62	-11.61	-11.6	-11.59	-11.58	-11.57	-11.56
-11.64	-11.63	-11.62	-11.61	-11.6	-11.59	-11.58	-11.57	-11.56
-11.64	-11.63	-11.62	-11.61	-11.6	-11.59	-11.58	-11.57	-11.56
-11.64	-11.63	-11.62	-11.61	-11.6	-11.59	-11.58	-11.57	-11.56
-11.64	-11.63	-11.62	-11.61	-11.6	-11.59	-11.58	-11.57	-11.56
-11.64	-11.63	-11.62	-11.61	-11.6	-11.59	-11.58	-11.57	-11.56
-11.64	-11.63	-11.62	-11.61	-11.6	-11.59	-11.58	-11.57	-11.56
-11.64	-11.63	-11.62	-11.61	-11.6	-11.59	-11.58	-11.57	-11.56
-11.64	-11.63	-11.62	-11.61	-11.6	-11.59	-11.58	-11.57	-11.56
-11.64	-11.63	-11.62	-11.61	-11.6	-11.59	-11.58	-11.57	-11.56
-11.64	-11.63	-11.62	-11.61	-11.6	-11.59	-11.58	-11.57	-11.56
-11.64	-11.63	-11.62	-11.61	-11.6	-11.59	-11.58	-11.57	-11.56
-11.64	-11.63	-11.62	-11.61	-11.6	-11.59	-11.58	-11.57	-11.56
-11.64	-11.63	-11.62	-11.61	-11.6	-11.59	-11.58	-11.57	-11.56
-11.64	-11.63	-11.62	-11.61	-11.6	-11.59	-11.58	-11.57	-11.56
-11.64	-11.63	-11.62	-11.61	-11.6	-11.59	-11.58	-11.57	-11.56
-11.64	-11.63	-11.62	-11.61	-11.6	-11.59	-11.58	-11.57	-11.56
-11.64	-11.63	-11.62	-11.61	-11.6	-11.59	-11.58	-11.57	-11.56
-11.64	-11.63	-11.62	-11.61	-11.6	-11.59	-11.58	-11.57	-11.56

-11.64	-11.63	-11.62	-11.61	-11.6	-11.59	-11.58	-11.57	-11.56
-11.64	-11.63	-11.62	-11.61	-11.6	-11.59	-11.58	-11.57	-11.56
-11.64	-11.63	-11.62	-11.61	-11.6	-11.59	-11.58	-11.57	-11.56
-11.64	-11.63	-11.62	-11.61	-11.6	-11.59	-11.58	-11.57	-11.56
-11.64	-11.63	-11.62	-11.61	-11.6	-11.59	-11.58	-11.57	-11.56
-11.64	-11.63	-11.62	-11.61	-11.6	-11.59	-11.58	-11.57	-11.56
-11.64	-11.63	-11.62	-11.61	-11.6	-11.59	-11.58	-11.57	-11.56
-11.64	-11.63	-11.62	-11.61	-11.6	-11.59	-11.58	-11.57	-11.56
-11.64	-11.63	-11.62	-11.61	-11.6	-11.59	-11.58	-11.57	-11.56
-11.64	-11.63	-11.62	-11.61	-11.6	-11.59	-11.58	-11.57	-11.56
-11.64	-11.63	-11.62	-11.61	-11.6	-11.59	-11.58	-11.57	-11.56
-11.64	-11.63	-11.62	-11.61	-11.6	-11.59	-11.58	-11.57	-11.56
-11.64	-11.63	-11.62	-11.61	-11.6	-11.59	-11.58	-11.57	-11.56
-11.64	-11.63	-11.62	-11.61	-11.6	-11.59	-11.58	-11.57	-11.56
-11.64	-11.63	-11.62	-11.61	-11.6	-11.59	-11.58	-11.57	-11.56
-11.64	-11.63	-11.62	-11.61	-11.6	-11.59	-11.58	-11.57	-11.56
-11.64	-11.63	-11.62	-11.61	-11.6	-11.59	-11.58	-11.57	-11.56
-11.64	-11.63	-11.62	-11.61	-11.6	-11.59	-11.58	-11.57	-11.56
-11.64	-11.63	-11.62	-11.61	-11.6	-11.59	-11.58	-11.57	-11.56
-11.64	-11.63	-11.62	-11.61	-11.6	-11.59	-11.58	-11.57	-11.56
-11.64	-11.63	-11.62	-11.61	-11.6	-11.59	-11.58	-11.57	-11.56
-11.64	-11.63	-11.62	-11.61	-11.6	-11.59	-11.58	-11.57	-11.56
-11.64	-11.63	-11.62	-11.61	-11.6	-11.59	-11.58	-11.57	-11.56
-11.64	-11.63	-11.62	-11.61	-11.6	-11.59	-11.58	-11.57	-11.56
-11.64	-11.63	-11.62	-11.61	-11.6	-11.59	-11.58	-11.57	-11.56
-11.64	-11.63	-11.62	-11.61	-11.6	-11.59	-11.58	-11.57	-11.56
-11.64	-11.63	-11.62	-11.61	-11.6	-11.59	-11.58	-11.57	-11.56
-11.64	-11.63	-11.62	-11.61	-11.6	-11.59	-11.58	-11.57	-11.56
-11.64	-11.63	-11.62	-11.61	-11.6	-11.59	-11.58	-11.57	-11.56
-11.64	-11.63	-11.62	-11.61	-11.6	-11.59	-11.58	-11.57	-11.56
-11.64	-11.63	-11.62	-11.61	-11.6	-11.59	-11.58	-11.57	-11.56
-11.64	-11.63	-11.62	-11.61	-11.6	-11.59	-11.58	-11.57	-11.56
-11.64	-11.63	-11.62	-11.61	-11.6	-11.59	-11.58	-11.57	-11.56
-11.64	-11.63	-11.62	-11.61	-11.6	-11.59	-11.58	-11.57	-11.56
-11.64	-11.63	-11.62	-11.61	-11.6	-11.59	-11.58	-11.57	-11.56
-11.64	-11.63	-11.62	-11.61	-11.6	-11.59	-11.58	-11.57	-11.56
-11.64	-11.63	-11.62	-11.61	-11.6	-11.59	-11.58	-11.57	-11.56
-11.64	-11.63	-11.62	-11.61	-11.6	-11.59	-11.58	-11.57	-11.56
-11.64	-11.63	-11.62	-11.61	-11.6	-11.59	-11.58	-11.57	-11.56
-11.64	-11.63	-11.62	-11.61	-11.6	-11.59	-11.58	-11.57	-11.56
-11.64	-11.63	-11.62	-11.61	-11.6	-11.59	-11.58	-11.57	-11.56
-11.64	-11.63	-11.62	-11.61	-11.6	-11.59	-11.58	-11.57	-11.56
-11.64	-11.63	-11.62	-11.61	-11.6	-11.59	-11.58	-11.57	-11.56
-11.64	-11.63	-11.62	-11.61	-11.6	-11.59	-11.58	-11.57	-11.56
-11.64	-11.63	-11.62	-11.61	-11.6	-11.59	-11.58	-11.57	-11.56
-11.64	-11.63	-11.62	-11.61	-11.6	-11.59	-11.58	-11.57	-11.56
-11.64	-11.63	-11.62	-11.61	-11.6	-11.59	-11.58	-11.57	-11.56
-11.64	-11.63	-11.62	-11.61	-11.6	-11.59	-11.58	-11.57	-11.56
-11.64	-11.63	-11.62	-11.61	-11.6	-11.59	-11.58	-11.57	-11.56
-11.64	-11.63	-11.62	-11.61	-11.6	-11.59	-11.58	-11.57	-11.56

-11.64	-11.63	-11.62	-11.61	-11.6	-11.59	-11.58	-11.57	-11.56
-11.64	-11.63	-11.62	-11.61	-11.6	-11.59	-11.58	-11.57	-11.56
-11.64	-11.63	-11.62	-11.61	-11.6	-11.59	-11.58	-11.57	-11.56
-11.64	-11.63	-11.62	-11.61	-11.6	-11.59	-11.58	-11.57	-11.56
-11.64	-11.63	-11.62	-11.61	-11.6	-11.59	-11.58	-11.57	-11.56
-11.64	-11.63	-11.62	-11.61	-11.6	-11.59	-11.58	-11.57	-11.56
-11.64	-11.63	-11.62	-11.61	-11.6	-11.59	-11.58	-11.57	-11.56
-11.64	-11.63	-11.62	-11.61	-11.6	-11.59	-11.58	-11.57	-11.56
-11.64	-11.63	-11.62	-11.61	-11.6	-11.59	-11.58	-11.57	-11.56
-11.64	-11.63	-11.62	-11.61	-11.6	-11.59	-11.58	-11.57	-11.56
-11.64	-11.63	-11.62	-11.61	-11.6	-11.59	-11.58	-11.57	-11.56
-11.64	-11.63	-11.62	-11.61	-11.6	-11.59	-11.58	-11.57	-11.56
-11.64	-11.63	-11.62	-11.61	-11.6	-11.59	-11.58	-11.57	-11.56
-11.64	-11.63	-11.62	-11.61	-11.6	-11.59	-11.58	-11.57	-11.56
-11.64	-11.63	-11.62	-11.61	-11.6	-11.59	-11.58	-11.57	-11.56
-11.64	-11.63	-11.62	-11.61	-11.6	-11.59	-11.58	-11.57	-11.56
-11.64	-11.63	-11.62	-11.61	-11.6	-11.59	-11.58	-11.57	-11.56
-11.64	-11.63	-11.62	-11.61	-11.6	-11.59	-11.58	-11.57	-11.56
-11.64	-11.63	-11.62	-11.61	-11.6	-11.59	-11.58	-11.57	-11.56
-11.64	-11.63	-11.62	-11.61	-11.6	-11.59	-11.58	-11.57	-11.56
-11.64	-11.63	-11.62	-11.61	-11.6	-11.59	-11.58	-11.57	-11.56
-11.64	-11.63	-11.62	-11.61	-11.6	-11.59	-11.58	-11.57	-11.56
-11.64	-11.63	-11.62	-11.61	-11.6	-11.59	-11.58	-11.57	-11.56
-11.64	-11.63	-11.62	-11.61	-11.6	-11.59	-11.58	-11.57	-11.56
-11.64	-11.63	-11.62	-11.61	-11.6	-11.59	-11.58	-11.57	-11.56
-11.64	-11.63	-11.62	-11.61	-11.6	-11.59	-11.58	-11.57	-11.56
-11.64	-11.63	-11.62	-11.61	-11.6	-11.59	-11.58	-11.57	-11.56
-11.64	-11.63	-11.62	-11.61	-11.6	-11.59	-11.58	-11.57	-11.56
-11.64	-11.63	-11.62	-11.61	-11.6	-11.59	-11.58	-11.57	-11.56
-11.64	-11.63	-11.62	-11.61	-11.6	-11.59	-11.58	-11.57	-11.56
-11.64	-11.63	-11.62	-11.61	-11.6	-11.59	-11.58	-11.57	-11.56
-11.64	-11.63	-11.62	-11.61	-11.6	-11.59	-11.58	-11.57	-11.56
-11.64	-11.63	-11.62	-11.61	-11.6	-11.59	-11.58	-11.57	-11.56
-11.64	-11.63	-11.62	-11.61	-11.6	-11.59	-11.58	-11.57	-11.56
-11.64	-11.63	-11.62	-11.61	-11.6	-11.59	-11.58	-11.57	-11.56
-11.64	-11.63	-11.62	-11.61	-11.6	-11.59	-11.58	-11.57	-11.56
-11.64	-11.63	-11.62	-11.61	-11.6	-11.59	-11.58	-11.57	-11.56
-11.64	-11.63	-11.62	-11.61	-11.6	-11.59	-11.58	-11.57	-11.56
-11.64	-11.63	-11.62	-11.61	-11.6	-11.59	-11.58	-11.57	-11.56
-11.64	-11.63	-11.62	-11.61	-11.6	-11.59	-11.58	-11.57	-11.56
-11.64	-11.63	-11.62	-11.61	-11.6	-11.59	-11.58	-11.57	-11.56
-11.64	-11.63	-11.62	-11.61	-11.6	-11.59	-11.58	-11.57	-11.56
-11.64	-11.63	-11.62	-11.61	-11.6	-11.59	-11.58	-11.57	-11.56
-11.64	-11.63	-11.62	-11.61	-11.6	-11.59	-11.58	-11.57	-11.56
-11.64	-11.63	-11.62	-11.61	-11.6	-11.59	-11.58	-11.57	-11.56
-11.64	-11.63	-11.62	-11.61	-11.6	-11.59	-11.58	-11.57	-11.56
-11.64	-11.63	-11.62	-11.61	-11.6	-11.59	-11.58	-11.57	-11.56
-11.64	-11.63	-11.62	-11.61	-11.6	-11.59	-11.58	-11.57	-11.56
-11.64	-11.63	-11.62	-11.61	-11.6	-11.59	-11.58	-11.57	-11.56
-11.64	-11.63	-11.62	-11.61	-11.6	-11.59	-11.58	-11.57	-11.56

-11.64	-11.63	-11.62	-11.61	-11.6	-11.59	-11.58	-11.57	-11.56
-11.64	-11.63	-11.62	-11.61	-11.6	-11.59	-11.58	-11.57	-11.56
-11.64	-11.63	-11.62	-11.61	-11.6	-11.59	-11.58	-11.57	-11.56
-11.64	-11.63	-11.62	-11.61	-11.6	-11.59	-11.58	-11.57	-11.56
-11.64	-11.63	-11.62	-11.61	-11.6	-11.59	-11.58	-11.57	-11.56
-11.64	-11.63	-11.62	-11.61	-11.6	-11.59	-11.58	-11.57	-11.56
-11.64	-11.63	-11.62	-11.61	-11.6	-11.59	-11.58	-11.57	-11.56
-11.64	-11.63	-11.62	-11.61	-11.6	-11.59	-11.58	-11.57	-11.56
-11.64	-11.63	-11.62	-11.61	-11.6	-11.59	-11.58	-11.57	-11.56
-11.64	-11.63	-11.62	-11.61	-11.6	-11.59	-11.58	-11.57	-11.56
-11.64	-11.63	-11.62	-11.61	-11.6	-11.59	-11.58	-11.57	-11.56
-11.64	-11.63	-11.62	-11.61	-11.6	-11.59	-11.58	-11.57	-11.56
-11.64	-11.63	-11.62	-11.61	-11.6	-11.59	-11.58	-11.57	-11.56
-11.64	-11.63	-11.62	-11.61	-11.6	-11.59	-11.58	-11.57	-11.56
-11.64	-11.63	-11.62	-11.61	-11.6	-11.59	-11.58	-11.57	-11.56
-11.64	-11.63	-11.62	-11.61	-11.6	-11.59	-11.58	-11.57	-11.56
-11.64	-11.63	-11.62	-11.61	-11.6	-11.59	-11.58	-11.57	-11.56
-11.64	-11.63	-11.62	-11.61	-11.6	-11.59	-11.58	-11.57	-11.56
-11.64	-11.63	-11.62	-11.61	-11.6	-11.59	-11.58	-11.57	-11.56
-11.64	-11.63	-11.62	-11.61	-11.6	-11.59	-11.58	-11.57	-11.56
-11.64	-11.63	-11.62	-11.61	-11.6	-11.59	-11.58	-11.57	-11.56
-11.64	-11.63	-11.62	-11.61	-11.6	-11.59	-11.58	-11.57	-11.56
-11.64	-11.63	-11.62	-11.61	-11.6	-11.59	-11.58	-11.57	-11.56
-11.64	-11.63	-11.62	-11.61	-11.6	-11.59	-11.58	-11.57	-11.56
-11.64	-11.63	-11.62	-11.61	-11.6	-11.59	-11.58	-11.57	-11.56
-11.64	-11.63	-11.62	-11.61	-11.6	-11.59	-11.58	-11.57	-11.56
-11.64	-11.63	-11.62	-11.61	-11.6	-11.59	-11.58	-11.57	-11.56
-11.64	-11.63	-11.62	-11.61	-11.6	-11.59	-11.58	-11.57	-11.56
-11.64	-11.63	-11.62	-11.61	-11.6	-11.59	-11.58	-11.57	-11.56
-11.64	-11.63	-11.62	-11.61	-11.6	-11.59	-11.58	-11.57	-11.56
-11.64	-11.63	-11.62	-11.61	-11.6	-11.59	-11.58	-11.57	-11.56
-11.64	-11.63	-11.62	-11.61	-11.6	-11.59	-11.58	-11.57	-11.56
-11.64	-11.63	-11.62	-11.61	-11.6	-11.59	-11.58	-11.57	-11.56
-11.64	-11.63	-11.62	-11.61	-11.6	-11.59	-11.58	-11.57	-11.56
-11.64	-11.63	-11.62	-11.61	-11.6	-11.59	-11.58	-11.57	-11.56
-11.64	-11.63	-11.62	-11.61	-11.6	-11.59	-11.58	-11.57	-11.56
-11.64	-11.63	-11.62	-11.61	-11.6	-11.59	-11.58	-11.57	-11.56
-11.64	-11.63	-11.62	-11.61	-11.6	-11.59	-11.58	-11.57	-11.56
-11.64	-11.63	-11.62	-11.61	-11.6	-11.59	-11.58	-11.57	-11.56
-11.64	-11.63	-11.62	-11.61	-11.6	-11.59	-11.58	-11.57	-11.56
-11.64	-11.63	-11.62	-11.61	-11.6	-11.59	-11.58	-11.57	-11.56
-11.64	-11.63	-11.62	-11.61	-11.6	-11.59	-11.58	-11.57	-11.56
-11.64	-11.63	-11.62	-11.61	-11.6	-11.59	-11.58	-11.57	-11.56
-11.64	-11.63	-11.62	-11.61	-11.6	-11.59	-11.58	-11.57	-11.56
-11.64	-11.63	-11.62	-11.61	-11.6	-11.59	-11.58	-11.57	-11.56
-11.64	-11.63	-11.62	-11.61	-11.6	-11.59	-11.58	-11.57	-11.56
-11.64	-11.63	-11.62	-11.61	-11.6	-11.59	-11.58	-11.57	-11.56
-11.64	-11.63	-11.62	-11.61	-11.6	-11.59	-11.58	-11.57	-11.56
-11.64	-11.63	-11.62	-11.61	-11.6	-11.59	-11.58	-11.57	-11.56
-11.64	-11.63	-11.62	-11.61	-11.6	-11.59	-11.58	-11.57	-11.56

-11.64	-11.63	-11.62	-11.61	-11.6	-11.59	-11.58	-11.57	-11.56
-11.64	-11.63	-11.62	-11.61	-11.6	-11.59	-11.58	-11.57	-11.56
-11.64	-11.63	-11.62	-11.61	-11.6	-11.59	-11.58	-11.57	-11.56
-11.64	-11.63	-11.62	-11.61	-11.6	-11.59	-11.58	-11.57	-11.56
-11.64	-11.63	-11.62	-11.61	-11.6	-11.59	-11.58	-11.57	-11.56
-11.64	-11.63	-11.62	-11.61	-11.6	-11.59	-11.58	-11.57	-11.56
-11.64	-11.63	-11.62	-11.61	-11.6	-11.59	-11.58	-11.57	-11.56
-11.64	-11.63	-11.62	-11.61	-11.6	-11.59	-11.58	-11.57	-11.56
-11.64	-11.63	-11.62	-11.61	-11.6	-11.59	-11.58	-11.57	-11.56
-11.64	-11.63	-11.62	-11.61	-11.6	-11.59	-11.58	-11.57	-11.56
-11.64	-11.63	-11.62	-11.61	-11.6	-11.59	-11.58	-11.57	-11.56
-11.64	-11.63	-11.62	-11.61	-11.6	-11.59	-11.58	-11.57	-11.56
-11.64	-11.63	-11.62	-11.61	-11.6	-11.59	-11.58	-11.57	-11.56
-11.64	-11.63	-11.62	-11.61	-11.6	-11.59	-11.58	-11.57	-11.56
-11.64	-11.63	-11.62	-11.61	-11.6	-11.59	-11.58	-11.57	-11.56
-11.64	-11.63	-11.62	-11.61	-11.6	-11.59	-11.58	-11.57	-11.56
-11.64	-11.63	-11.62	-11.61	-11.6	-11.59	-11.58	-11.57	-11.56
-11.64	-11.63	-11.62	-11.61	-11.6	-11.59	-11.58	-11.57	-11.56
-11.64	-11.63	-11.62	-11.61	-11.6	-11.59	-11.58	-11.57	-11.56
-11.64	-11.63	-11.62	-11.61	-11.6	-11.59	-11.58	-11.57	-11.56
-11.64	-11.63	-11.62	-11.61	-11.6	-11.59	-11.58	-11.57	-11.56
-11.64	-11.63	-11.62	-11.61	-11.6	-11.59	-11.58	-11.57	-11.56
-11.64	-11.63	-11.62	-11.61	-11.6	-11.59	-11.58	-11.57	-11.56
-11.64	-11.63	-11.62	-11.61	-11.6	-11.59	-11.58	-11.57	-11.56
-11.64	-11.63	-11.62	-11.61	-11.6	-11.59	-11.58	-11.57	-11.56
-11.64	-11.63	-11.62	-11.61	-11.6	-11.59	-11.58	-11.57	-11.56
-11.64	-11.63	-11.62	-11.61	-11.6	-11.59	-11.58	-11.57	-11.56
-11.64	-11.63	-11.62	-11.61	-11.6	-11.59	-11.58	-11.57	-11.56
-11.64	-11.63	-11.62	-11.61	-11.6	-11.59	-11.58	-11.57	-11.56
-11.64	-11.63	-11.62	-11.61	-11.6	-11.59	-11.58	-11.57	-11.56
-11.64	-11.63	-11.62	-11.61	-11.6	-11.59	-11.58	-11.57	-11.56
-11.64	-11.63	-11.62	-11.61	-11.6	-11.59	-11.58	-11.57	-11.56
-11.64	-11.63	-11.62	-11.61	-11.6	-11.59	-11.58	-11.57	-11.56
-11.64	-11.63	-11.62	-11.61	-11.6	-11.59	-11.58	-11.57	-11.56
-11.64	-11.63	-11.62	-11.61	-11.6	-11.59	-11.58	-11.57	-11.56
-11.64	-11.63	-11.62	-11.61	-11.6	-11.59	-11.58	-11.57	-11.56
-11.64	-11.63	-11.62	-11.61	-11.6	-11.59	-11.58	-11.57	-11.56
-11.64	-11.63	-11.62	-11.61	-11.6	-11.59	-11.58	-11.57	-11.56
-11.64	-11.63	-11.62	-11.61	-11.6	-11.59	-11.58	-11.57	-11.56
-11.64	-11.63	-11.62	-11.61	-11.6	-11.59	-11.58	-11.57	-11.56
-11.64	-11.63	-11.62	-11.61	-11.6	-11.59	-11.58	-11.57	-11.56
-11.64	-11.63	-11.62	-11.61	-11.6	-11.59	-11.58	-11.57	-11.56
-11.64	-11.63	-11.62	-11.61	-11.6	-11.59	-11.58	-11.57	-11.56
-11.64	-11.63	-11.62	-11.61	-11.6	-11.59	-11.58	-11.57	-11.56
-11.64	-11.63	-11.62	-11.61	-11.6	-11.59	-11.58	-11.57	-11.56
-11.64	-11.63	-11.62	-11.61	-11.6	-11.59	-11.58	-11.57	-11.56
-11.64	-11.63	-11.62	-11.61	-11.6	-11.59	-11.58	-11.57	-11.56
-11.64	-11.63	-11.62	-11.61	-11.6	-11.59	-11.58	-11.57	-11.56
-11.64	-11.63	-11.62	-11.61	-11.6	-11.59	-11.58	-11.57	-11.56
-11.64	-11.63	-11.62	-11.61	-11.6	-11.59	-11.58	-11.57	-11.56

-11.64	-11.63	-11.62	-11.61	-11.6	-11.59	-11.58	-11.57	-11.56
-11.64	-11.63	-11.62	-11.61	-11.6	-11.59	-11.58	-11.57	-11.56
-11.64	-11.63	-11.62	-11.61	-11.6	-11.59	-11.58	-11.57	-11.56
-11.64	-11.63	-11.62	-11.61	-11.6	-11.59	-11.58	-11.57	-11.56
-11.64	-11.63	-11.62	-11.61	-11.6	-11.59	-11.58	-11.57	-11.56
-11.64	-11.63	-11.62	-11.61	-11.6	-11.59	-11.58	-11.57	-11.56
-11.64	-11.63	-11.62	-11.61	-11.6	-11.59	-11.58	-11.57	-11.56
-11.64	-11.63	-11.62	-11.61	-11.6	-11.59	-11.58	-11.57	-11.56
-11.64	-11.63	-11.62	-11.61	-11.6	-11.59	-11.58	-11.57	-11.56
-11.64	-11.63	-11.62	-11.61	-11.6	-11.59	-11.58	-11.57	-11.56
-11.64	-11.63	-11.62	-11.61	-11.6	-11.59	-11.58	-11.57	-11.56
-11.64	-11.63	-11.62	-11.61	-11.6	-11.59	-11.58	-11.57	-11.56
-11.64	-11.63	-11.62	-11.61	-11.6	-11.59	-11.58	-11.57	-11.56
-11.64	-11.63	-11.62	-11.61	-11.6	-11.59	-11.58	-11.57	-11.56
-11.64	-11.63	-11.62	-11.61	-11.6	-11.59	-11.58	-11.57	-11.56
-11.64	-11.63	-11.62	-11.61	-11.6	-11.59	-11.58	-11.57	-11.56
-11.64	-11.63	-11.62	-11.61	-11.6	-11.59	-11.58	-11.57	-11.56
-11.64	-11.63	-11.62	-11.61	-11.6	-11.59	-11.58	-11.57	-11.56
-11.64	-11.63	-11.62	-11.61	-11.6	-11.59	-11.58	-11.57	-11.56
-11.64	-11.63	-11.62	-11.61	-11.6	-11.59	-11.58	-11.57	-11.56
-11.64	-11.63	-11.62	-11.61	-11.6	-11.59	-11.58	-11.57	-11.56
-11.64	-11.63	-11.62	-11.61	-11.6	-11.59	-11.58	-11.57	-11.56
-11.64	-11.63	-11.62	-11.61	-11.6	-11.59	-11.58	-11.57	-11.56
-11.64	-11.63	-11.62	-11.61	-11.6	-11.59	-11.58	-11.57	-11.56
-11.64	-11.63	-11.62	-11.61	-11.6	-11.59	-11.58	-11.57	-11.56
-11.64	-11.63	-11.62	-11.61	-11.6	-11.59	-11.58	-11.57	-11.56
-11.64	-11.63	-11.62	-11.61	-11.6	-11.59	-11.58	-11.57	-11.56
-11.64	-11.63	-11.62	-11.61	-11.6	-11.59	-11.58	-11.57	-11.56
-11.64	-11.63	-11.62	-11.61	-11.6	-11.59	-11.58	-11.57	-11.56
-11.64	-11.63	-11.62	-11.61	-11.6	-11.59	-11.58	-11.57	-11.56
-11.64	-11.63	-11.62	-11.61	-11.6	-11.59	-11.58	-11.57	-11.56
-11.64	-11.63	-11.62	-11.61	-11.6	-11.59	-11.58	-11.57	-11.56
-11.64	-11.63	-11.62	-11.61	-11.6	-11.59	-11.58	-11.57	-11.56
-11.64	-11.63	-11.62	-11.61	-11.6	-11.59	-11.58	-11.57	-11.56
-11.64	-11.63	-11.62	-11.61	-11.6	-11.59	-11.58	-11.57	-11.56
-11.64	-11.63	-11.62	-11.61	-11.6	-11.59	-11.58	-11.57	-11.56
-11.64	-11.63	-11.62	-11.61	-11.6	-11.59	-11.58	-11.57	-11.56
-11.64	-11.63	-11.62	-11.61	-11.6	-11.59	-11.58	-11.57	-11.56
-11.64	-11.63	-11.62	-11.61	-11.6	-11.59	-11.58	-11.57	-11.56
-11.64	-11.63	-11.62	-11.61	-11.6	-11.59	-11.58	-11.57	-11.56
-11.64	-11.63	-11.62	-11.61	-11.6	-11.59	-11.58	-11.57	-11.56
-11.64	-11.63	-11.62	-11.61	-11.6	-11.59	-11.58	-11.57	-11.56
-11.64	-11.63	-11.62	-11.61	-11.6	-11.59	-11.58	-11.57	-11.56
-11.64	-11.63	-11.62	-11.61	-11.6	-11.59	-11.58	-11.57	-11.56
-11.64	-11.63	-11.62	-11.61	-11.6	-11.59	-11.58	-11.57	-11.56
-11.64	-11.63	-11.62	-11.61	-11.6	-11.59	-11.58	-11.57	-11.56
-11.64	-11.63	-11.62	-11.61	-11.6	-11.59	-11.58	-11.57	-11.56
-11.64	-11.63	-11.62	-11.61	-11.6	-11.59	-11.58	-11.57	-11.56
-11.64	-11.63	-11.62	-11.61	-11.6	-11.59	-11.58	-11.57	-11.56
-11.64	-11.63	-11.62	-11.61	-11.6	-11.59	-11.58	-11.57	-11.56

-11.64	-11.63	-11.62	-11.61	-11.6	-11.59	-11.58	-11.57	-11.56

-11.55	-11.54	-11.53	-11.52	-11.51	-11.5	-11.49	-11.48	-11.47
-11.55	-11.54	-11.53	-11.52	-11.51	-11.5	-11.49	-11.48	-11.47
-11.55	-11.54	-11.53	-11.52	-11.51	-11.5	-11.49	-11.48	-11.47
-11.55	-11.54	-11.53	-11.52	-11.51	-11.5	-11.49	-11.48	-11.47
-11.55	-11.54	-11.53	-11.52	-11.51	-11.5	-11.49	-11.48	-11.47
-11.55	-11.54	-11.53	-11.52	-11.51	-11.5	-11.49	-11.48	-11.47
-11.55	-11.54	-11.53	-11.52	-11.51	-11.5	-11.49	-11.48	-11.47
-11.55	-11.54	-11.53	-11.52	-11.51	-11.5	-11.49	-11.48	-11.47
-11.55	-11.54	-11.53	-11.52	-11.51	-11.5	-11.49	-11.48	-11.47
-11.55	-11.54	-11.53	-11.52	-11.51	-11.5	-11.49	-11.48	-11.47
-11.55	-11.54	-11.53	-11.52	-11.51	-11.5	-11.49	-11.48	-11.47
-11.55	-11.54	-11.53	-11.52	-11.51	-11.5	-11.49	-11.48	-11.47
-11.55	-11.54	-11.53	-11.52	-11.51	-11.5	-11.49	-11.48	-11.47
-11.55	-11.54	-11.53	-11.52	-11.51	-11.5	-11.49	-11.48	-11.47
-11.55	-11.54	-11.53	-11.52	-11.51	-11.5	-11.49	-11.48	-11.47
-11.55	-11.54	-11.53	-11.52	-11.51	-11.5	-11.49	-11.48	-11.47
-11.55	-11.54	-11.53	-11.52	-11.51	-11.5	-11.49	-11.48	-11.47
-11.55	-11.54	-11.53	-11.52	-11.51	-11.5	-11.49	-11.48	-11.47
-11.55	-11.54	-11.53	-11.52	-11.51	-11.5	-11.49	-11.48	-11.47
-11.55	-11.54	-11.53	-11.52	-11.51	-11.5	-11.49	-11.48	-11.47
-11.55	-11.54	-11.53	-11.52	-11.51	-11.5	-11.49	-11.48	-11.47
-11.55	-11.54	-11.53	-11.52	-11.51	-11.5	-11.49	-11.48	-11.47
-11.55	-11.54	-11.53	-11.52	-11.51	-11.5	-11.49	-11.48	-11.47
-11.55	-11.54	-11.53	-11.52	-11.51	-11.5	-11.49	-11.48	-11.47
-11.55	-11.54	-11.53	-11.52	-11.51	-11.5	-11.49	-11.48	-11.47
-11.55	-11.54	-11.53	-11.52	-11.51	-11.5	-11.49	-11.48	-11.47
-11.55	-11.54	-11.53	-11.52	-11.51	-11.5	-11.49	-11.48	-11.47
-11.55	-11.54	-11.53	-11.52	-11.51	-11.5	-11.49	-11.48	-11.47
-11.55	-11.54	-11.53	-11.52	-11.51	-11.5	-11.49	-11.48	-11.47
-11.55	-11.54	-11.53	-11.52	-11.51	-11.5	-11.49	-11.48	-11.47
-11.55	-11.54	-11.53	-11.52	-11.51	-11.5	-11.49	-11.48	-11.47
-11.55	-11.54	-11.53	-11.52	-11.51	-11.5	-11.49	-11.48	-11.47
-11.55	-11.54	-11.53	-11.52	-11.51	-11.5	-11.49	-11.48	-11.47
-11.55	-11.54	-11.53	-11.52	-11.51	-11.5	-11.49	-11.48	-11.47
-11.55	-11.54	-11.53	-11.52	-11.51	-11.5	-11.49	-11.48	-11.47
-11.55	-11.54	-11.53	-11.52	-11.51	-11.5	-11.49	-11.48	-11.47
-11.55	-11.54	-11.53	-11.52	-11.51	-11.5	-11.49	-11.48	-11.47
-11.55	-11.54	-11.53	-11.52	-11.51	-11.5	-11.49	-11.48	-11.47
-11.55	-11.54	-11.53	-11.52	-11.51	-11.5	-11.49	-11.48	-11.47
-11.55	-11.54	-11.53	-11.52	-11.51	-11.5	-11.49	-11.48	-11.47
-11.55	-11.54	-11.53	-11.52	-11.51	-11.5	-11.49	-11.48	-11.47
-11.55	-11.54	-11.53	-11.52	-11.51	-11.5	-11.49	-11.48	-11.47
-11.55	-11.54	-11.53	-11.52	-11.51	-11.5	-11.49	-11.48	-11.47
-11.55	-11.54	-11.53	-11.52	-11.51	-11.5	-11.49	-11.48	-11.47
-11.55	-11.54	-11.53	-11.52	-11.51	-11.5	-11.49	-11.48	-11.47
-11.55	-11.54	-11.53	-11.52	-11.51	-11.5	-11.49	-11.48	-11.47
-11.55	-11.54	-11.53	-11.52	-11.51	-11.5	-11.49	-11.48	-11.47
-11.55	-11.54	-11.53	-11.52	-11.51	-11.5	-11.49	-11.48	-11.47
-11.55	-11.54	-11.53	-11.52	-11.51	-11.5	-11.49	-11.48	-11.47
-11.55	-11.54	-11.53	-11.52	-11.51	-11.5	-11.49	-11.48	-11.47

-11.55	-11.54	-11.53	-11.52	-11.51	-11.5	-11.49	-11.48	-11.47
-11.55	-11.54	-11.53	-11.52	-11.51	-11.5	-11.49	-11.48	-11.47
-11.55	-11.54	-11.53	-11.52	-11.51	-11.5	-11.49	-11.48	-11.47
-11.55	-11.54	-11.53	-11.52	-11.51	-11.5	-11.49	-11.48	-11.47
-11.55	-11.54	-11.53	-11.52	-11.51	-11.5	-11.49	-11.48	-11.47
-11.55	-11.54	-11.53	-11.52	-11.51	-11.5	-11.49	-11.48	-11.47
-11.55	-11.54	-11.53	-11.52	-11.51	-11.5	-11.49	-11.48	-11.47
-11.55	-11.54	-11.53	-11.52	-11.51	-11.5	-11.49	-11.48	-11.47
-11.55	-11.54	-11.53	-11.52	-11.51	-11.5	-11.49	-11.48	-11.47
-11.55	-11.54	-11.53	-11.52	-11.51	-11.5	-11.49	-11.48	-11.47
-11.55	-11.54	-11.53	-11.52	-11.51	-11.5	-11.49	-11.48	-11.47
-11.55	-11.54	-11.53	-11.52	-11.51	-11.5	-11.49	-11.48	-11.47
-11.55	-11.54	-11.53	-11.52	-11.51	-11.5	-11.49	-11.48	-11.47
-11.55	-11.54	-11.53	-11.52	-11.51	-11.5	-11.49	-11.48	-11.47
-11.55	-11.54	-11.53	-11.52	-11.51	-11.5	-11.49	-11.48	-11.47
-11.55	-11.54	-11.53	-11.52	-11.51	-11.5	-11.49	-11.48	-11.47
-11.55	-11.54	-11.53	-11.52	-11.51	-11.5	-11.49	-11.48	-11.47
-11.55	-11.54	-11.53	-11.52	-11.51	-11.5	-11.49	-11.48	-11.47
-11.55	-11.54	-11.53	-11.52	-11.51	-11.5	-11.49	-11.48	-11.47
-11.55	-11.54	-11.53	-11.52	-11.51	-11.5	-11.49	-11.48	-11.47
-11.55	-11.54	-11.53	-11.52	-11.51	-11.5	-11.49	-11.48	-11.47
-11.55	-11.54	-11.53	-11.52	-11.51	-11.5	-11.49	-11.48	-11.47
-11.55	-11.54	-11.53	-11.52	-11.51	-11.5	-11.49	-11.48	-11.47
-11.55	-11.54	-11.53	-11.52	-11.51	-11.5	-11.49	-11.48	-11.47
-11.55	-11.54	-11.53	-11.52	-11.51	-11.5	-11.49	-11.48	-11.47
-11.55	-11.54	-11.53	-11.52	-11.51	-11.5	-11.49	-11.48	-11.47
-11.55	-11.54	-11.53	-11.52	-11.51	-11.5	-11.49	-11.48	-11.47
-11.55	-11.54	-11.53	-11.52	-11.51	-11.5	-11.49	-11.48	-11.47
-11.55	-11.54	-11.53	-11.52	-11.51	-11.5	-11.49	-11.48	-11.47
-11.55	-11.54	-11.53	-11.52	-11.51	-11.5	-11.49	-11.48	-11.47
-11.55	-11.54	-11.53	-11.52	-11.51	-11.5	-11.49	-11.48	-11.47
-11.55	-11.54	-11.53	-11.52	-11.51	-11.5	-11.49	-11.48	-11.47
-11.55	-11.54	-11.53	-11.52	-11.51	-11.5	-11.49	-11.48	-11.47
-11.55	-11.54	-11.53	-11.52	-11.51	-11.5	-11.49	-11.48	-11.47
-11.55	-11.54	-11.53	-11.52	-11.51	-11.5	-11.49	-11.48	-11.47
-11.55	-11.54	-11.53	-11.52	-11.51	-11.5	-11.49	-11.48	-11.47
-11.55	-11.54	-11.53	-11.52	-11.51	-11.5	-11.49	-11.48	-11.47
-11.55	-11.54	-11.53	-11.52	-11.51	-11.5	-11.49	-11.48	-11.47
-11.55	-11.54	-11.53	-11.52	-11.51	-11.5	-11.49	-11.48	-11.47
-11.55	-11.54	-11.53	-11.52	-11.51	-11.5	-11.49	-11.48	-11.47
-11.55	-11.54	-11.53	-11.52	-11.51	-11.5	-11.49	-11.48	-11.47
-11.55	-11.54	-11.53	-11.52	-11.51	-11.5	-11.49	-11.48	-11.47
-11.55	-11.54	-11.53	-11.52	-11.51	-11.5	-11.49	-11.48	-11.47
-11.55	-11.54	-11.53	-11.52	-11.51	-11.5	-11.49	-11.48	-11.47
-11.55	-11.54	-11.53	-11.52	-11.51	-11.5	-11.49	-11.48	-11.47
-11.55	-11.54	-11.53	-11.52	-11.51	-11.5	-11.49	-11.48	-11.47
-11.55	-11.54	-11.53	-11.52	-11.51	-11.5	-11.49	-11.48	-11.47
-11.55	-11.54	-11.53	-11.52	-11.51	-11.5	-11.49	-11.48	-11.47
-11.55	-11.54	-11.53	-11.52	-11.51	-11.5	-11.49	-11.48	-11.47
-11.55	-11.54	-11.53	-11.52	-11.51	-11.5	-11.49	-11.48	-11.47

-11.55	-11.54	-11.53	-11.52	-11.51	-11.5	-11.49	-11.48	-11.47
-11.55	-11.54	-11.53	-11.52	-11.51	-11.5	-11.49	-11.48	-11.47
-11.55	-11.54	-11.53	-11.52	-11.51	-11.5	-11.49	-11.48	-11.47
-11.55	-11.54	-11.53	-11.52	-11.51	-11.5	-11.49	-11.48	-11.47
-11.55	-11.54	-11.53	-11.52	-11.51	-11.5	-11.49	-11.48	-11.47
-11.55	-11.54	-11.53	-11.52	-11.51	-11.5	-11.49	-11.48	-11.47
-11.55	-11.54	-11.53	-11.52	-11.51	-11.5	-11.49	-11.48	-11.47
-11.55	-11.54	-11.53	-11.52	-11.51	-11.5	-11.49	-11.48	-11.47
-11.55	-11.54	-11.53	-11.52	-11.51	-11.5	-11.49	-11.48	-11.47
-11.55	-11.54	-11.53	-11.52	-11.51	-11.5	-11.49	-11.48	-11.47
-11.55	-11.54	-11.53	-11.52	-11.51	-11.5	-11.49	-11.48	-11.47
-11.55	-11.54	-11.53	-11.52	-11.51	-11.5	-11.49	-11.48	-11.47
-11.55	-11.54	-11.53	-11.52	-11.51	-11.5	-11.49	-11.48	-11.47
-11.55	-11.54	-11.53	-11.52	-11.51	-11.5	-11.49	-11.48	-11.47
-11.55	-11.54	-11.53	-11.52	-11.51	-11.5	-11.49	-11.48	-11.47
-11.55	-11.54	-11.53	-11.52	-11.51	-11.5	-11.49	-11.48	-11.47
-11.55	-11.54	-11.53	-11.52	-11.51	-11.5	-11.49	-11.48	-11.47
-11.55	-11.54	-11.53	-11.52	-11.51	-11.5	-11.49	-11.48	-11.47
-11.55	-11.54	-11.53	-11.52	-11.51	-11.5	-11.49	-11.48	-11.47
-11.55	-11.54	-11.53	-11.52	-11.51	-11.5	-11.49	-11.48	-11.47
-11.55	-11.54	-11.53	-11.52	-11.51	-11.5	-11.49	-11.48	-11.47
-11.55	-11.54	-11.53	-11.52	-11.51	-11.5	-11.49	-11.48	-11.47
-11.55	-11.54	-11.53	-11.52	-11.51	-11.5	-11.49	-11.48	-11.47
-11.55	-11.54	-11.53	-11.52	-11.51	-11.5	-11.49	-11.48	-11.47
-11.55	-11.54	-11.53	-11.52	-11.51	-11.5	-11.49	-11.48	-11.47
-11.55	-11.54	-11.53	-11.52	-11.51	-11.5	-11.49	-11.48	-11.47
-11.55	-11.54	-11.53	-11.52	-11.51	-11.5	-11.49	-11.48	-11.47
-11.55	-11.54	-11.53	-11.52	-11.51	-11.5	-11.49	-11.48	-11.47
-11.55	-11.54	-11.53	-11.52	-11.51	-11.5	-11.49	-11.48	-11.47
-11.55	-11.54	-11.53	-11.52	-11.51	-11.5	-11.49	-11.48	-11.47
-11.55	-11.54	-11.53	-11.52	-11.51	-11.5	-11.49	-11.48	-11.47
-11.55	-11.54	-11.53	-11.52	-11.51	-11.5	-11.49	-11.48	-11.47
-11.55	-11.54	-11.53	-11.52	-11.51	-11.5	-11.49	-11.48	-11.47
-11.55	-11.54	-11.53	-11.52	-11.51	-11.5	-11.49	-11.48	-11.47
-11.55	-11.54	-11.53	-11.52	-11.51	-11.5	-11.49	-11.48	-11.47
-11.55	-11.54	-11.53	-11.52	-11.51	-11.5	-11.49	-11.48	-11.47
-11.55	-11.54	-11.53	-11.52	-11.51	-11.5	-11.49	-11.48	-11.47
-11.55	-11.54	-11.53	-11.52	-11.51	-11.5	-11.49	-11.48	-11.47
-11.55	-11.54	-11.53	-11.52	-11.51	-11.5	-11.49	-11.48	-11.47
-11.55	-11.54	-11.53	-11.52	-11.51	-11.5	-11.49	-11.48	-11.47
-11.55	-11.54	-11.53	-11.52	-11.51	-11.5	-11.49	-11.48	-11.47
-11.55	-11.54	-11.53	-11.52	-11.51	-11.5	-11.49	-11.48	-11.47
-11.55	-11.54	-11.53	-11.52	-11.51	-11.5	-11.49	-11.48	-11.47
-11.55	-11.54	-11.53	-11.52	-11.51	-11.5	-11.49	-11.48	-11.47
-11.55	-11.54	-11.53	-11.52	-11.51	-11.5	-11.49	-11.48	-11.47
-11.55	-11.54	-11.53	-11.52	-11.51	-11.5	-11.49	-11.48	-11.47
-11.55	-11.54	-11.53	-11.52	-11.51	-11.5	-11.49	-11.48	-11.47
-11.55	-11.54	-11.53	-11.52	-11.51	-11.5	-11.49	-11.48	-11.47
-11.55	-11.54	-11.53	-11.52	-11.51	-11.5	-11.49	-11.48	-11.47
-11.55	-11.54	-11.53	-11.52	-11.51	-11.5	-11.49	-11.48	-11.47

-11.55	-11.54	-11.53	-11.52	-11.51	-11.5	-11.49	-11.48	-11.47
-11.55	-11.54	-11.53	-11.52	-11.51	-11.5	-11.49	-11.48	-11.47
-11.55	-11.54	-11.53	-11.52	-11.51	-11.5	-11.49	-11.48	-11.47
-11.55	-11.54	-11.53	-11.52	-11.51	-11.5	-11.49	-11.48	-11.47
-11.55	-11.54	-11.53	-11.52	-11.51	-11.5	-11.49	-11.48	-11.47
-11.55	-11.54	-11.53	-11.52	-11.51	-11.5	-11.49	-11.48	-11.47
-11.55	-11.54	-11.53	-11.52	-11.51	-11.5	-11.49	-11.48	-11.47
-11.55	-11.54	-11.53	-11.52	-11.51	-11.5	-11.49	-11.48	-11.47
-11.55	-11.54	-11.53	-11.52	-11.51	-11.5	-11.49	-11.48	-11.47
-11.55	-11.54	-11.53	-11.52	-11.51	-11.5	-11.49	-11.48	-11.47
-11.55	-11.54	-11.53	-11.52	-11.51	-11.5	-11.49	-11.48	-11.47
-11.55	-11.54	-11.53	-11.52	-11.51	-11.5	-11.49	-11.48	-11.47
-11.55	-11.54	-11.53	-11.52	-11.51	-11.5	-11.49	-11.48	-11.47
-11.55	-11.54	-11.53	-11.52	-11.51	-11.5	-11.49	-11.48	-11.47
-11.55	-11.54	-11.53	-11.52	-11.51	-11.5	-11.49	-11.48	-11.47
-11.55	-11.54	-11.53	-11.52	-11.51	-11.5	-11.49	-11.48	-11.47
-11.55	-11.54	-11.53	-11.52	-11.51	-11.5	-11.49	-11.48	-11.47
-11.55	-11.54	-11.53	-11.52	-11.51	-11.5	-11.49	-11.48	-11.47
-11.55	-11.54	-11.53	-11.52	-11.51	-11.5	-11.49	-11.48	-11.47
-11.55	-11.54	-11.53	-11.52	-11.51	-11.5	-11.49	-11.48	-11.47
-11.55	-11.54	-11.53	-11.52	-11.51	-11.5	-11.49	-11.48	-11.47
-11.55	-11.54	-11.53	-11.52	-11.51	-11.5	-11.49	-11.48	-11.47
-11.55	-11.54	-11.53	-11.52	-11.51	-11.5	-11.49	-11.48	-11.47
-11.55	-11.54	-11.53	-11.52	-11.51	-11.5	-11.49	-11.48	-11.47
-11.55	-11.54	-11.53	-11.52	-11.51	-11.5	-11.49	-11.48	-11.47
-11.55	-11.54	-11.53	-11.52	-11.51	-11.5	-11.49	-11.48	-11.47
-11.55	-11.54	-11.53	-11.52	-11.51	-11.5	-11.49	-11.48	-11.47
-11.55	-11.54	-11.53	-11.52	-11.51	-11.5	-11.49	-11.48	-11.47
-11.55	-11.54	-11.53	-11.52	-11.51	-11.5	-11.49	-11.48	-11.47
-11.55	-11.54	-11.53	-11.52	-11.51	-11.5	-11.49	-11.48	-11.47
-11.55	-11.54	-11.53	-11.52	-11.51	-11.5	-11.49	-11.48	-11.47
-11.55	-11.54	-11.53	-11.52	-11.51	-11.5	-11.49	-11.48	-11.47
-11.55	-11.54	-11.53	-11.52	-11.51	-11.5	-11.49	-11.48	-11.47
-11.55	-11.54	-11.53	-11.52	-11.51	-11.5	-11.49	-11.48	-11.47
-11.55	-11.54	-11.53	-11.52	-11.51	-11.5	-11.49	-11.48	-11.47
-11.55	-11.54	-11.53	-11.52	-11.51	-11.5	-11.49	-11.48	-11.47
-11.55	-11.54	-11.53	-11.52	-11.51	-11.5	-11.49	-11.48	-11.47
-11.55	-11.54	-11.53	-11.52	-11.51	-11.5	-11.49	-11.48	-11.47
-11.55	-11.54	-11.53	-11.52	-11.51	-11.5	-11.49	-11.48	-11.47
-11.55	-11.54	-11.53	-11.52	-11.51	-11.5	-11.49	-11.48	-11.47
-11.55	-11.54	-11.53	-11.52	-11.51	-11.5	-11.49	-11.48	-11.47
-11.55	-11.54	-11.53	-11.52	-11.51	-11.5	-11.49	-11.48	-11.47
-11.55	-11.54	-11.53	-11.52	-11.51	-11.5	-11.49	-11.48	-11.47
-11.55	-11.54	-11.53	-11.52	-11.51	-11.5	-11.49	-11.48	-11.47
-11.55	-11.54	-11.53	-11.52	-11.51	-11.5	-11.49	-11.48	-11.47
-11.55	-11.54	-11.53	-11.52	-11.51	-11.5	-11.49	-11.48	-11.47
-11.55	-11.54	-11.53	-11.52	-11.51	-11.5	-11.49	-11.48	-11.47
-11.55	-11.54	-11.53	-11.52	-11.51	-11.5	-11.49	-11.48	-11.47
-11.55	-11.54	-11.53	-11.52	-11.51	-11.5	-11.49	-11.48	-11.47
-11.55	-11.54	-11.53	-11.52	-11.51	-11.5	-11.49	-11.48	-11.47

-11.55	-11.54	-11.53	-11.52	-11.51	-11.5	-11.49	-11.48	-11.47
-11.55	-11.54	-11.53	-11.52	-11.51	-11.5	-11.49	-11.48	-11.47
-11.55	-11.54	-11.53	-11.52	-11.51	-11.5	-11.49	-11.48	-11.47
-11.55	-11.54	-11.53	-11.52	-11.51	-11.5	-11.49	-11.48	-11.47
-11.55	-11.54	-11.53	-11.52	-11.51	-11.5	-11.49	-11.48	-11.47
-11.55	-11.54	-11.53	-11.52	-11.51	-11.5	-11.49	-11.48	-11.47
-11.55	-11.54	-11.53	-11.52	-11.51	-11.5	-11.49	-11.48	-11.47
-11.55	-11.54	-11.53	-11.52	-11.51	-11.5	-11.49	-11.48	-11.47
-11.55	-11.54	-11.53	-11.52	-11.51	-11.5	-11.49	-11.48	-11.47
-11.55	-11.54	-11.53	-11.52	-11.51	-11.5	-11.49	-11.48	-11.47
-11.55	-11.54	-11.53	-11.52	-11.51	-11.5	-11.49	-11.48	-11.47
-11.55	-11.54	-11.53	-11.52	-11.51	-11.5	-11.49	-11.48	-11.47
-11.55	-11.54	-11.53	-11.52	-11.51	-11.5	-11.49	-11.48	-11.47
-11.55	-11.54	-11.53	-11.52	-11.51	-11.5	-11.49	-11.48	-11.47
-11.55	-11.54	-11.53	-11.52	-11.51	-11.5	-11.49	-11.48	-11.47
-11.55	-11.54	-11.53	-11.52	-11.51	-11.5	-11.49	-11.48	-11.47
-11.55	-11.54	-11.53	-11.52	-11.51	-11.5	-11.49	-11.48	-11.47
-11.55	-11.54	-11.53	-11.52	-11.51	-11.5	-11.49	-11.48	-11.47
-11.55	-11.54	-11.53	-11.52	-11.51	-11.5	-11.49	-11.48	-11.47
-11.55	-11.54	-11.53	-11.52	-11.51	-11.5	-11.49	-11.48	-11.47
-11.55	-11.54	-11.53	-11.52	-11.51	-11.5	-11.49	-11.48	-11.47
-11.55	-11.54	-11.53	-11.52	-11.51	-11.5	-11.49	-11.48	-11.47
-11.55	-11.54	-11.53	-11.52	-11.51	-11.5	-11.49	-11.48	-11.47
-11.55	-11.54	-11.53	-11.52	-11.51	-11.5	-11.49	-11.48	-11.47
-11.55	-11.54	-11.53	-11.52	-11.51	-11.5	-11.49	-11.48	-11.47
-11.55	-11.54	-11.53	-11.52	-11.51	-11.5	-11.49	-11.48	-11.47
-11.55	-11.54	-11.53	-11.52	-11.51	-11.5	-11.49	-11.48	-11.47
-11.55	-11.54	-11.53	-11.52	-11.51	-11.5	-11.49	-11.48	-11.47
-11.55	-11.54	-11.53	-11.52	-11.51	-11.5	-11.49	-11.48	-11.47
-11.55	-11.54	-11.53	-11.52	-11.51	-11.5	-11.49	-11.48	-11.47
-11.55	-11.54	-11.53	-11.52	-11.51	-11.5	-11.49	-11.48	-11.47
-11.55	-11.54	-11.53	-11.52	-11.51	-11.5	-11.49	-11.48	-11.47
-11.55	-11.54	-11.53	-11.52	-11.51	-11.5	-11.49	-11.48	-11.47
-11.55	-11.54	-11.53	-11.52	-11.51	-11.5	-11.49	-11.48	-11.47
-11.55	-11.54	-11.53	-11.52	-11.51	-11.5	-11.49	-11.48	-11.47
-11.55	-11.54	-11.53	-11.52	-11.51	-11.5	-11.49	-11.48	-11.47
-11.55	-11.54	-11.53	-11.52	-11.51	-11.5	-11.49	-11.48	-11.47
-11.55	-11.54	-11.53	-11.52	-11.51	-11.5	-11.49	-11.48	-11.47
-11.55	-11.54	-11.53	-11.52	-11.51	-11.5	-11.49	-11.48	-11.47
-11.55	-11.54	-11.53	-11.52	-11.51	-11.5	-11.49	-11.48	-11.47
-11.55	-11.54	-11.53	-11.52	-11.51	-11.5	-11.49	-11.48	-11.47
-11.55	-11.54	-11.53	-11.52	-11.51	-11.5	-11.49	-11.48	-11.47
-11.55	-11.54	-11.53	-11.52	-11.51	-11.5	-11.49	-11.48	-11.47
-11.55	-11.54	-11.53	-11.52	-11.51	-11.5	-11.49	-11.48	-11.47
-11.55	-11.54	-11.53	-11.52	-11.51	-11.5	-11.49	-11.48	-11.47
-11.55	-11.54	-11.53	-11.52	-11.51	-11.5	-11.49	-11.48	-11.47
-11.55	-11.54	-11.53	-11.52	-11.51	-11.5	-11.49	-11.48	-11.47
-11.55	-11.54	-11.53	-11.52	-11.51	-11.5	-11.49	-11.48	-11.47
-11.55	-11.54	-11.53	-11.52	-11.51	-11.5	-11.49	-11.48	-11.47
-11.55	-11.54	-11.53	-11.52	-11.51	-11.5	-11.49	-11.48	-11.47

-11.55	-11.54	-11.53	-11.52	-11.51	-11.5	-11.49	-11.48	-11.47
-11.55	-11.54	-11.53	-11.52	-11.51	-11.5	-11.49	-11.48	-11.47
-11.55	-11.54	-11.53	-11.52	-11.51	-11.5	-11.49	-11.48	-11.47
-11.55	-11.54	-11.53	-11.52	-11.51	-11.5	-11.49	-11.48	-11.47
-11.55	-11.54	-11.53	-11.52	-11.51	-11.5	-11.49	-11.48	-11.47
-11.55	-11.54	-11.53	-11.52	-11.51	-11.5	-11.49	-11.48	-11.47
-11.55	-11.54	-11.53	-11.52	-11.51	-11.5	-11.49	-11.48	-11.47
-11.55	-11.54	-11.53	-11.52	-11.51	-11.5	-11.49	-11.48	-11.47
-11.55	-11.54	-11.53	-11.52	-11.51	-11.5	-11.49	-11.48	-11.47
-11.55	-11.54	-11.53	-11.52	-11.51	-11.5	-11.49	-11.48	-11.47
-11.55	-11.54	-11.53	-11.52	-11.51	-11.5	-11.49	-11.48	-11.47
-11.55	-11.54	-11.53	-11.52	-11.51	-11.5	-11.49	-11.48	-11.47
-11.55	-11.54	-11.53	-11.52	-11.51	-11.5	-11.49	-11.48	-11.47
-11.55	-11.54	-11.53	-11.52	-11.51	-11.5	-11.49	-11.48	-11.47
-11.55	-11.54	-11.53	-11.52	-11.51	-11.5	-11.49	-11.48	-11.47
-11.55	-11.54	-11.53	-11.52	-11.51	-11.5	-11.49	-11.48	-11.47
-11.55	-11.54	-11.53	-11.52	-11.51	-11.5	-11.49	-11.48	-11.47
-11.55	-11.54	-11.53	-11.52	-11.51	-11.5	-11.49	-11.48	-11.47
-11.55	-11.54	-11.53	-11.52	-11.51	-11.5	-11.49	-11.48	-11.47
-11.55	-11.54	-11.53	-11.52	-11.51	-11.5	-11.49	-11.48	-11.47
-11.55	-11.54	-11.53	-11.52	-11.51	-11.5	-11.49	-11.48	-11.47
-11.55	-11.54	-11.53	-11.52	-11.51	-11.5	-11.49	-11.48	-11.47
-11.55	-11.54	-11.53	-11.52	-11.51	-11.5	-11.49	-11.48	-11.47
-11.55	-11.54	-11.53	-11.52	-11.51	-11.5	-11.49	-11.48	-11.47
-11.55	-11.54	-11.53	-11.52	-11.51	-11.5	-11.49	-11.48	-11.47
-11.55	-11.54	-11.53	-11.52	-11.51	-11.5	-11.49	-11.48	-11.47
-11.55	-11.54	-11.53	-11.52	-11.51	-11.5	-11.49	-11.48	-11.47
-11.55	-11.54	-11.53	-11.52	-11.51	-11.5	-11.49	-11.48	-11.47
-11.55	-11.54	-11.53	-11.52	-11.51	-11.5	-11.49	-11.48	-11.47
-11.55	-11.54	-11.53	-11.52	-11.51	-11.5	-11.49	-11.48	-11.47
-11.55	-11.54	-11.53	-11.52	-11.51	-11.5	-11.49	-11.48	-11.47
-11.55	-11.54	-11.53	-11.52	-11.51	-11.5	-11.49	-11.48	-11.47
-11.55	-11.54	-11.53	-11.52	-11.51	-11.5	-11.49	-11.48	-11.47
-11.55	-11.54	-11.53	-11.52	-11.51	-11.5	-11.49	-11.48	-11.47
-11.55	-11.54	-11.53	-11.52	-11.51	-11.5	-11.49	-11.48	-11.47
-11.55	-11.54	-11.53	-11.52	-11.51	-11.5	-11.49	-11.48	-11.47
-11.55	-11.54	-11.53	-11.52	-11.51	-11.5	-11.49	-11.48	-11.47
-11.55	-11.54	-11.53	-11.52	-11.51	-11.5	-11.49	-11.48	-11.47
-11.55	-11.54	-11.53	-11.52	-11.51	-11.5	-11.49	-11.48	-11.47
-11.55	-11.54	-11.53	-11.52	-11.51	-11.5	-11.49	-11.48	-11.47
-11.55	-11.54	-11.53	-11.52	-11.51	-11.5	-11.49	-11.48	-11.47
-11.55	-11.54	-11.53	-11.52	-11.51	-11.5	-11.49	-11.48	-11.47
-11.55	-11.54	-11.53	-11.52	-11.51	-11.5	-11.49	-11.48	-11.47
-11.55	-11.54	-11.53	-11.52	-11.51	-11.5	-11.49	-11.48	-11.47
-11.55	-11.54	-11.53	-11.52	-11.51	-11.5	-11.49	-11.48	-11.47
-11.55	-11.54	-11.53	-11.52	-11.51	-11.5	-11.49	-11.48	-11.47
-11.55	-11.54	-11.53	-11.52	-11.51	-11.5	-11.49	-11.48	-11.47
-11.55	-11.54	-11.53	-11.52	-11.51	-11.5	-11.49	-11.48	-11.47
-11.55	-11.54	-11.53	-11.52	-11.51	-11.5	-11.49	-11.48	-11.47
-11.55	-11.54	-11.53	-11.52	-11.51	-11.5	-11.49	-11.48	-11.47

-11.55	-11.54	-11.53	-11.52	-11.51	-11.5	-11.49	-11.48	-11.47
-11.55	-11.54	-11.53	-11.52	-11.51	-11.5	-11.49	-11.48	-11.47
-11.55	-11.54	-11.53	-11.52	-11.51	-11.5	-11.49	-11.48	-11.47
-11.55	-11.54	-11.53	-11.52	-11.51	-11.5	-11.49	-11.48	-11.47
-11.55	-11.54	-11.53	-11.52	-11.51	-11.5	-11.49	-11.48	-11.47
-11.55	-11.54	-11.53	-11.52	-11.51	-11.5	-11.49	-11.48	-11.47
-11.55	-11.54	-11.53	-11.52	-11.51	-11.5	-11.49	-11.48	-11.47
-11.55	-11.54	-11.53	-11.52	-11.51	-11.5	-11.49	-11.48	-11.47
-11.55	-11.54	-11.53	-11.52	-11.51	-11.5	-11.49	-11.48	-11.47
-11.55	-11.54	-11.53	-11.52	-11.51	-11.5	-11.49	-11.48	-11.47
-11.55	-11.54	-11.53	-11.52	-11.51	-11.5	-11.49	-11.48	-11.47
-11.55	-11.54	-11.53	-11.52	-11.51	-11.5	-11.49	-11.48	-11.47
-11.55	-11.54	-11.53	-11.52	-11.51	-11.5	-11.49	-11.48	-11.47
-11.55	-11.54	-11.53	-11.52	-11.51	-11.5	-11.49	-11.48	-11.47
-11.55	-11.54	-11.53	-11.52	-11.51	-11.5	-11.49	-11.48	-11.47
-11.55	-11.54	-11.53	-11.52	-11.51	-11.5	-11.49	-11.48	-11.47
-11.55	-11.54	-11.53	-11.52	-11.51	-11.5	-11.49	-11.48	-11.47
-11.55	-11.54	-11.53	-11.52	-11.51	-11.5	-11.49	-11.48	-11.47
-11.55	-11.54	-11.53	-11.52	-11.51	-11.5	-11.49	-11.48	-11.47
-11.55	-11.54	-11.53	-11.52	-11.51	-11.5	-11.49	-11.48	-11.47
-11.55	-11.54	-11.53	-11.52	-11.51	-11.5	-11.49	-11.48	-11.47
-11.55	-11.54	-11.53	-11.52	-11.51	-11.5	-11.49	-11.48	-11.47
-11.55	-11.54	-11.53	-11.52	-11.51	-11.5	-11.49	-11.48	-11.47
-11.55	-11.54	-11.53	-11.52	-11.51	-11.5	-11.49	-11.48	-11.47
-11.55	-11.54	-11.53	-11.52	-11.51	-11.5	-11.49	-11.48	-11.47
-11.55	-11.54	-11.53	-11.52	-11.51	-11.5	-11.49	-11.48	-11.47
-11.55	-11.54	-11.53	-11.52	-11.51	-11.5	-11.49	-11.48	-11.47
-11.55	-11.54	-11.53	-11.52	-11.51	-11.5	-11.49	-11.48	-11.47
-11.55	-11.54	-11.53	-11.52	-11.51	-11.5	-11.49	-11.48	-11.47
-11.55	-11.54	-11.53	-11.52	-11.51	-11.5	-11.49	-11.48	-11.47
-11.55	-11.54	-11.53	-11.52	-11.51	-11.5	-11.49	-11.48	-11.47
-11.55	-11.54	-11.53	-11.52	-11.51	-11.5	-11.49	-11.48	-11.47
-11.55	-11.54	-11.53	-11.52	-11.51	-11.5	-11.49	-11.48	-11.47
-11.55	-11.54	-11.53	-11.52	-11.51	-11.5	-11.49	-11.48	-11.47
-11.55	-11.54	-11.53	-11.52	-11.51	-11.5	-11.49	-11.48	-11.47
-11.55	-11.54	-11.53	-11.52	-11.51	-11.5	-11.49	-11.48	-11.47
-11.55	-11.54	-11.53	-11.52	-11.51	-11.5	-11.49	-11.48	-11.47
-11.55	-11.54	-11.53	-11.52	-11.51	-11.5	-11.49	-11.48	-11.47
-11.55	-11.54	-11.53	-11.52	-11.51	-11.5	-11.49	-11.48	-11.47
-11.55	-11.54	-11.53	-11.52	-11.51	-11.5	-11.49	-11.48	-11.47
-11.55	-11.54	-11.53	-11.52	-11.51	-11.5	-11.49	-11.48	-11.47
-11.55	-11.54	-11.53	-11.52	-11.51	-11.5	-11.49	-11.48	-11.47
-11.55	-11.54	-11.53	-11.52	-11.51	-11.5	-11.49	-11.48	-11.47
-11.55	-11.54	-11.53	-11.52	-11.51	-11.5	-11.49	-11.48	-11.47
-11.55	-11.54	-11.53	-11.52	-11.51	-11.5	-11.49	-11.48	-11.47
-11.55	-11.54	-11.53	-11.52	-11.51	-11.5	-11.49	-11.48	-11.47
-11.55	-11.54	-11.53	-11.52	-11.51	-11.5	-11.49	-11.48	-11.47
-11.55	-11.54	-11.53	-11.52	-11.51	-11.5	-11.49	-11.48	-11.47
-11.55	-11.54	-11.53	-11.52	-11.51	-11.5	-11.49	-11.48	-11.47
-11.55	-11.54	-11.53	-11.52	-11.51	-11.5	-11.49	-11.48	-11.47

-11.55	-11.54	-11.53	-11.52	-11.51	-11.5	-11.49	-11.48	-11.47
-11.55	-11.54	-11.53	-11.52	-11.51	-11.5	-11.49	-11.48	-11.47
-11.55	-11.54	-11.53	-11.52	-11.51	-11.5	-11.49	-11.48	-11.47
-11.55	-11.54	-11.53	-11.52	-11.51	-11.5	-11.49	-11.48	-11.47
-11.55	-11.54	-11.53	-11.52	-11.51	-11.5	-11.49	-11.48	-11.47
-11.55	-11.54	-11.53	-11.52	-11.51	-11.5	-11.49	-11.48	-11.47
-11.55	-11.54	-11.53	-11.52	-11.51	-11.5	-11.49	-11.48	-11.47
-11.55	-11.54	-11.53	-11.52	-11.51	-11.5	-11.49	-11.48	-11.47
-11.55	-11.54	-11.53	-11.52	-11.51	-11.5	-11.49	-11.48	-11.47
-11.55	-11.54	-11.53	-11.52	-11.51	-11.5	-11.49	-11.48	-11.47
-11.55	-11.54	-11.53	-11.52	-11.51	-11.5	-11.49	-11.48	-11.47
-11.55	-11.54	-11.53	-11.52	-11.51	-11.5	-11.49	-11.48	-11.47
-11.55	-11.54	-11.53	-11.52	-11.51	-11.5	-11.49	-11.48	-11.47
-11.55	-11.54	-11.53	-11.52	-11.51	-11.5	-11.49	-11.48	-11.47
-11.55	-11.54	-11.53	-11.52	-11.51	-11.5	-11.49	-11.48	-11.47
-11.55	-11.54	-11.53	-11.52	-11.51	-11.5	-11.49	-11.48	-11.47
-11.55	-11.54	-11.53	-11.52	-11.51	-11.5	-11.49	-11.48	-11.47
-11.55	-11.54	-11.53	-11.52	-11.51	-11.5	-11.49	-11.48	-11.47
-11.55	-11.54	-11.53	-11.52	-11.51	-11.5	-11.49	-11.48	-11.47
-11.55	-11.54	-11.53	-11.52	-11.51	-11.5	-11.49	-11.48	-11.47
-11.55	-11.54	-11.53	-11.52	-11.51	-11.5	-11.49	-11.48	-11.47
-11.55	-11.54	-11.53	-11.52	-11.51	-11.5	-11.49	-11.48	-11.47
-11.55	-11.54	-11.53	-11.52	-11.51	-11.5	-11.49	-11.48	-11.47
-11.55	-11.54	-11.53	-11.52	-11.51	-11.5	-11.49	-11.48	-11.47
-11.55	-11.54	-11.53	-11.52	-11.51	-11.5	-11.49	-11.48	-11.47
-11.55	-11.54	-11.53	-11.52	-11.51	-11.5	-11.49	-11.48	-11.47
-11.55	-11.54	-11.53	-11.52	-11.51	-11.5	-11.49	-11.48	-11.47
-11.55	-11.54	-11.53	-11.52	-11.51	-11.5	-11.49	-11.48	-11.47
-11.55	-11.54	-11.53	-11.52	-11.51	-11.5	-11.49	-11.48	-11.47
-11.55	-11.54	-11.53	-11.52	-11.51	-11.5	-11.49	-11.48	-11.47
-11.55	-11.54	-11.53	-11.52	-11.51	-11.5	-11.49	-11.48	-11.47
-11.55	-11.54	-11.53	-11.52	-11.51	-11.5	-11.49	-11.48	-11.47
-11.55	-11.54	-11.53	-11.52	-11.51	-11.5	-11.49	-11.48	-11.47
-11.55	-11.54	-11.53	-11.52	-11.51	-11.5	-11.49	-11.48	-11.47
-11.55	-11.54	-11.53	-11.52	-11.51	-11.5	-11.49	-11.48	-11.47
-11.55	-11.54	-11.53	-11.52	-11.51	-11.5	-11.49	-11.48	-11.47
-11.55	-11.54	-11.53	-11.52	-11.51	-11.5	-11.49	-11.48	-11.47
-11.55	-11.54	-11.53	-11.52	-11.51	-11.5	-11.49	-11.48	-11.47
-11.55	-11.54	-11.53	-11.52	-11.51	-11.5	-11.49	-11.48	-11.47
-11.55	-11.54	-11.53	-11.52	-11.51	-11.5	-11.49	-11.48	-11.47
-11.55	-11.54	-11.53	-11.52	-11.51	-11.5	-11.49	-11.48	-11.47
-11.55	-11.54	-11.53	-11.52	-11.51	-11.5	-11.49	-11.48	-11.47
-11.55	-11.54	-11.53	-11.52	-11.51	-11.5	-11.49	-11.48	-11.47
-11.55	-11.54	-11.53	-11.52	-11.51	-11.5	-11.49	-11.48	-11.47
-11.55	-11.54	-11.53	-11.52	-11.51	-11.5	-11.49	-11.48	-11.47
-11.55	-11.54	-11.53	-11.52	-11.51	-11.5	-11.49	-11.48	-11.47
-11.55	-11.54	-11.53	-11.52	-11.51	-11.5	-11.49	-11.48	-11.47
-11.55	-11.54	-11.53	-11.52	-11.51	-11.5	-11.49	-11.48	-11.47
-11.55	-11.54	-11.53	-11.52	-11.51	-11.5	-11.49	-11.48	-11.47
-11.55	-11.54	-11.53	-11.52	-11.51	-11.5	-11.49	-11.48	-11.47

-11.55	-11.54	-11.53	-11.52	-11.51	-11.5	-11.49	-11.48	-11.47
-11.55	-11.54	-11.53	-11.52	-11.51	-11.5	-11.49	-11.48	-11.47
-11.55	-11.54	-11.53	-11.52	-11.51	-11.5	-11.49	-11.48	-11.47
-11.55	-11.54	-11.53	-11.52	-11.51	-11.5	-11.49	-11.48	-11.47
-11.55	-11.54	-11.53	-11.52	-11.51	-11.5	-11.49	-11.48	-11.47
-11.55	-11.54	-11.53	-11.52	-11.51	-11.5	-11.49	-11.48	-11.47
-11.55	-11.54	-11.53	-11.52	-11.51	-11.5	-11.49	-11.48	-11.47
-11.55	-11.54	-11.53	-11.52	-11.51	-11.5	-11.49	-11.48	-11.47
-11.55	-11.54	-11.53	-11.52	-11.51	-11.5	-11.49	-11.48	-11.47
-11.55	-11.54	-11.53	-11.52	-11.51	-11.5	-11.49	-11.48	-11.47
-11.55	-11.54	-11.53	-11.52	-11.51	-11.5	-11.49	-11.48	-11.47
-11.55	-11.54	-11.53	-11.52	-11.51	-11.5	-11.49	-11.48	-11.47
-11.55	-11.54	-11.53	-11.52	-11.51	-11.5	-11.49	-11.48	-11.47
-11.55	-11.54	-11.53	-11.52	-11.51	-11.5	-11.49	-11.48	-11.47
-11.55	-11.54	-11.53	-11.52	-11.51	-11.5	-11.49	-11.48	-11.47
-11.55	-11.54	-11.53	-11.52	-11.51	-11.5	-11.49	-11.48	-11.47
-11.55	-11.54	-11.53	-11.52	-11.51	-11.5	-11.49	-11.48	-11.47
-11.55	-11.54	-11.53	-11.52	-11.51	-11.5	-11.49	-11.48	-11.47
-11.55	-11.54	-11.53	-11.52	-11.51	-11.5	-11.49	-11.48	-11.47
-11.55	-11.54	-11.53	-11.52	-11.51	-11.5	-11.49	-11.48	-11.47
-11.55	-11.54	-11.53	-11.52	-11.51	-11.5	-11.49	-11.48	-11.47
-11.55	-11.54	-11.53	-11.52	-11.51	-11.5	-11.49	-11.48	-11.47
-11.55	-11.54	-11.53	-11.52	-11.51	-11.5	-11.49	-11.48	-11.47
-11.55	-11.54	-11.53	-11.52	-11.51	-11.5	-11.49	-11.48	-11.47
-11.55	-11.54	-11.53	-11.52	-11.51	-11.5	-11.49	-11.48	-11.47
-11.55	-11.54	-11.53	-11.52	-11.51	-11.5	-11.49	-11.48	-11.47
-11.55	-11.54	-11.53	-11.52	-11.51	-11.5	-11.49	-11.48	-11.47
-11.55	-11.54	-11.53	-11.52	-11.51	-11.5	-11.49	-11.48	-11.47
-11.55	-11.54	-11.53	-11.52	-11.51	-11.5	-11.49	-11.48	-11.47
-11.55	-11.54	-11.53	-11.52	-11.51	-11.5	-11.49	-11.48	-11.47
-11.55	-11.54	-11.53	-11.52	-11.51	-11.5	-11.49	-11.48	-11.47
-11.55	-11.54	-11.53	-11.52	-11.51	-11.5	-11.49	-11.48	-11.47
-11.55	-11.54	-11.53	-11.52	-11.51	-11.5	-11.49	-11.48	-11.47
-11.55	-11.54	-11.53	-11.52	-11.51	-11.5	-11.49	-11.48	-11.47
-11.55	-11.54	-11.53	-11.52	-11.51	-11.5	-11.49	-11.48	-11.47
-11.55	-11.54	-11.53	-11.52	-11.51	-11.5	-11.49	-11.48	-11.47
-11.55	-11.54	-11.53	-11.52	-11.51	-11.5	-11.49	-11.48	-11.47
-11.55	-11.54	-11.53	-11.52	-11.51	-11.5	-11.49	-11.48	-11.47
-11.55	-11.54	-11.53	-11.52	-11.51	-11.5	-11.49	-11.48	-11.47
-11.55	-11.54	-11.53	-11.52	-11.51	-11.5	-11.49	-11.48	-11.47
-11.55	-11.54	-11.53	-11.52	-11.51	-11.5	-11.49	-11.48	-11.47
-11.55	-11.54	-11.53	-11.52	-11.51	-11.5	-11.49	-11.48	-11.47
-11.55	-11.54	-11.53	-11.52	-11.51	-11.5	-11.49	-11.48	-11.47
-11.55	-11.54	-11.53	-11.52	-11.51	-11.5	-11.49	-11.48	-11.47
-11.55	-11.54	-11.53	-11.52	-11.51	-11.5	-11.49	-11.48	-11.47
-11.55	-11.54	-11.53	-11.52	-11.51	-11.5	-11.49	-11.48	-11.47
-11.55	-11.54	-11.53	-11.52	-11.51	-11.5	-11.49	-11.48	-11.47
-11.55	-11.54	-11.53	-11.52	-11.51	-11.5	-11.49	-11.48	-11.47
-11.55	-11.54	-11.53	-11.52	-11.51	-11.5	-11.49	-11.48	-11.47
-11.55	-11.54	-11.53	-11.52	-11.51	-11.5	-11.49	-11.48	-11.47

-11.55	-11.54	-11.53	-11.52	-11.51	-11.5	-11.49	-11.48	-11.47
-11.55	-11.54	-11.53	-11.52	-11.51	-11.5	-11.49	-11.48	-11.47
-11.55	-11.54	-11.53	-11.52	-11.51	-11.5	-11.49	-11.48	-11.47
-11.55	-11.54	-11.53	-11.52	-11.51	-11.5	-11.49	-11.48	-11.47
-11.55	-11.54	-11.53	-11.52	-11.51	-11.5	-11.49	-11.48	-11.47
-11.55	-11.54	-11.53	-11.52	-11.51	-11.5	-11.49	-11.48	-11.47
-11.55	-11.54	-11.53	-11.52	-11.51	-11.5	-11.49	-11.48	-11.47
-11.55	-11.54	-11.53	-11.52	-11.51	-11.5	-11.49	-11.48	-11.47
-11.55	-11.54	-11.53	-11.52	-11.51	-11.5	-11.49	-11.48	-11.47
-11.55	-11.54	-11.53	-11.52	-11.51	-11.5	-11.49	-11.48	-11.47
-11.55	-11.54	-11.53	-11.52	-11.51	-11.5	-11.49	-11.48	-11.47
-11.55	-11.54	-11.53	-11.52	-11.51	-11.5	-11.49	-11.48	-11.47
-11.55	-11.54	-11.53	-11.52	-11.51	-11.5	-11.49	-11.48	-11.47
-11.55	-11.54	-11.53	-11.52	-11.51	-11.5	-11.49	-11.48	-11.47
-11.55	-11.54	-11.53	-11.52	-11.51	-11.5	-11.49	-11.48	-11.47
-11.55	-11.54	-11.53	-11.52	-11.51	-11.5	-11.49	-11.48	-11.47
-11.55	-11.54	-11.53	-11.52	-11.51	-11.5	-11.49	-11.48	-11.47
-11.55	-11.54	-11.53	-11.52	-11.51	-11.5	-11.49	-11.48	-11.47
-11.55	-11.54	-11.53	-11.52	-11.51	-11.5	-11.49	-11.48	-11.47
-11.55	-11.54	-11.53	-11.52	-11.51	-11.5	-11.49	-11.48	-11.47
-11.55	-11.54	-11.53	-11.52	-11.51	-11.5	-11.49	-11.48	-11.47
-11.55	-11.54	-11.53	-11.52	-11.51	-11.5	-11.49	-11.48	-11.47
-11.55	-11.54	-11.53	-11.52	-11.51	-11.5	-11.49	-11.48	-11.47
-11.55	-11.54	-11.53	-11.52	-11.51	-11.5	-11.49	-11.48	-11.47
-11.55	-11.54	-11.53	-11.52	-11.51	-11.5	-11.49	-11.48	-11.47
-11.55	-11.54	-11.53	-11.52	-11.51	-11.5	-11.49	-11.48	-11.47
-11.55	-11.54	-11.53	-11.52	-11.51	-11.5	-11.49	-11.48	-11.47
-11.55	-11.54	-11.53	-11.52	-11.51	-11.5	-11.49	-11.48	-11.47
-11.55	-11.54	-11.53	-11.52	-11.51	-11.5	-11.49	-11.48	-11.47
-11.55	-11.54	-11.53	-11.52	-11.51	-11.5	-11.49	-11.48	-11.47
-11.55	-11.54	-11.53	-11.52	-11.51	-11.5	-11.49	-11.48	-11.47
-11.55	-11.54	-11.53	-11.52	-11.51	-11.5	-11.49	-11.48	-11.47
-11.55	-11.54	-11.53	-11.52	-11.51	-11.5	-11.49	-11.48	-11.47
-11.55	-11.54	-11.53	-11.52	-11.51	-11.5	-11.49	-11.48	-11.47
-11.55	-11.54	-11.53	-11.52	-11.51	-11.5	-11.49	-11.48	-11.47
-11.55	-11.54	-11.53	-11.52	-11.51	-11.5	-11.49	-11.48	-11.47
-11.55	-11.54	-11.53	-11.52	-11.51	-11.5	-11.49	-11.48	-11.47
-11.55	-11.54	-11.53	-11.52	-11.51	-11.5	-11.49	-11.48	-11.47
-11.55	-11.54	-11.53	-11.52	-11.51	-11.5	-11.49	-11.48	-11.47
-11.55	-11.54	-11.53	-11.52	-11.51	-11.5	-11.49	-11.48	-11.47
-11.55	-11.54	-11.53	-11.52	-11.51	-11.5	-11.49	-11.48	-11.47
-11.55	-11.54	-11.53	-11.52	-11.51	-11.5	-11.49	-11.48	-11.47
-11.55	-11.54	-11.53	-11.52	-11.51	-11.5	-11.49	-11.48	-11.47
-11.55	-11.54	-11.53	-11.52	-11.51	-11.5	-11.49	-11.48	-11.47
-11.55	-11.54	-11.53	-11.52	-11.51	-11.5	-11.49	-11.48	-11.47
-11.55	-11.54	-11.53	-11.52	-11.51	-11.5	-11.49	-11.48	-11.47
-11.55	-11.54	-11.53	-11.52	-11.51	-11.5	-11.49	-11.48	-11.47
-11.55	-11.54	-11.53	-11.52	-11.51	-11.5	-11.49	-11.48	-11.47
-11.55	-11.54	-11.53	-11.52	-11.51	-11.5	-11.49	-11.48	-11.47
-11.55	-11.54	-11.53	-11.52	-11.51	-11.5	-11.49	-11.48	-11.47

-11.55	-11.54	-11.53	-11.52	-11.51	-11.5	-11.49	-11.48	-11.47
-11.55	-11.54	-11.53	-11.52	-11.51	-11.5	-11.49	-11.48	-11.47
-11.55	-11.54	-11.53	-11.52	-11.51	-11.5	-11.49	-11.48	-11.47
-11.55	-11.54	-11.53	-11.52	-11.51	-11.5	-11.49	-11.48	-11.47
-11.55	-11.54	-11.53	-11.52	-11.51	-11.5	-11.49	-11.48	-11.47
-11.55	-11.54	-11.53	-11.52	-11.51	-11.5	-11.49	-11.48	-11.47
-11.55	-11.54	-11.53	-11.52	-11.51	-11.5	-11.49	-11.48	-11.47
-11.55	-11.54	-11.53	-11.52	-11.51	-11.5	-11.49	-11.48	-11.47
-11.55	-11.54	-11.53	-11.52	-11.51	-11.5	-11.49	-11.48	-11.47
-11.55	-11.54	-11.53	-11.52	-11.51	-11.5	-11.49	-11.48	-11.47
-11.55	-11.54	-11.53	-11.52	-11.51	-11.5	-11.49	-11.48	-11.47
-11.55	-11.54	-11.53	-11.52	-11.51	-11.5	-11.49	-11.48	-11.47
-11.55	-11.54	-11.53	-11.52	-11.51	-11.5	-11.49	-11.48	-11.47
-11.55	-11.54	-11.53	-11.52	-11.51	-11.5	-11.49	-11.48	-11.47
-11.55	-11.54	-11.53	-11.52	-11.51	-11.5	-11.49	-11.48	-11.47
-11.55	-11.54	-11.53	-11.52	-11.51	-11.5	-11.49	-11.48	-11.47
-11.55	-11.54	-11.53	-11.52	-11.51	-11.5	-11.49	-11.48	-11.47
-11.55	-11.54	-11.53	-11.52	-11.51	-11.5	-11.49	-11.48	-11.47
-11.55	-11.54	-11.53	-11.52	-11.51	-11.5	-11.49	-11.48	-11.47
-11.55	-11.54	-11.53	-11.52	-11.51	-11.5	-11.49	-11.48	-11.47
-11.55	-11.54	-11.53	-11.52	-11.51	-11.5	-11.49	-11.48	-11.47
-11.55	-11.54	-11.53	-11.52	-11.51	-11.5	-11.49	-11.48	-11.47
-11.55	-11.54	-11.53	-11.52	-11.51	-11.5	-11.49	-11.48	-11.47
-11.55	-11.54	-11.53	-11.52	-11.51	-11.5	-11.49	-11.48	-11.47
-11.55	-11.54	-11.53	-11.52	-11.51	-11.5	-11.49	-11.48	-11.47
-11.55	-11.54	-11.53	-11.52	-11.51	-11.5	-11.49	-11.48	-11.47
-11.55	-11.54	-11.53	-11.52	-11.51	-11.5	-11.49	-11.48	-11.47
-11.55	-11.54	-11.53	-11.52	-11.51	-11.5	-11.49	-11.48	-11.47
-11.55	-11.54	-11.53	-11.52	-11.51	-11.5	-11.49	-11.48	-11.47
-11.55	-11.54	-11.53	-11.52	-11.51	-11.5	-11.49	-11.48	-11.47
-11.55	-11.54	-11.53	-11.52	-11.51	-11.5	-11.49	-11.48	-11.47
-11.55	-11.54	-11.53	-11.52	-11.51	-11.5	-11.49	-11.48	-11.47
-11.55	-11.54	-11.53	-11.52	-11.51	-11.5	-11.49	-11.48	-11.47
-11.55	-11.54	-11.53	-11.52	-11.51	-11.5	-11.49	-11.48	-11.47
-11.55	-11.54	-11.53	-11.52	-11.51	-11.5	-11.49	-11.48	-11.47
-11.55	-11.54	-11.53	-11.52	-11.51	-11.5	-11.49	-11.48	-11.47
-11.55	-11.54	-11.53	-11.52	-11.51	-11.5	-11.49	-11.48	-11.47
-11.55	-11.54	-11.53	-11.52	-11.51	-11.5	-11.49	-11.48	-11.47
-11.55	-11.54	-11.53	-11.52	-11.51	-11.5	-11.49	-11.48	-11.47
-11.55	-11.54	-11.53	-11.52	-11.51	-11.5	-11.49	-11.48	-11.47
-11.55	-11.54	-11.53	-11.52	-11.51	-11.5	-11.49	-11.48	-11.47
-11.55	-11.54	-11.53	-11.52	-11.51	-11.5	-11.49	-11.48	-11.47
-11.55	-11.54	-11.53	-11.52	-11.51	-11.5	-11.49	-11.48	-11.47
-11.55	-11.54	-11.53	-11.52	-11.51	-11.5	-11.49	-11.48	-11.47
-11.55	-11.54	-11.53	-11.52	-11.51	-11.5	-11.49	-11.48	-11.47
-11.55	-11.54	-11.53	-11.52	-11.51	-11.5	-11.49	-11.48	-11.47
-11.55	-11.54	-11.53	-11.52	-11.51	-11.5	-11.49	-11.48	-11.47
-11.55	-11.54	-11.53	-11.52	-11.51	-11.5	-11.49	-11.48	-11.47
-11.55	-11.54	-11.53	-11.52	-11.51	-11.5	-11.49	-11.48	-11.47
-11.55	-11.54	-11.53	-11.52	-11.51	-11.5	-11.49	-11.48	-11.47

-11.55	-11.54	-11.53	-11.52	-11.51	-11.5	-11.49	-11.48	-11.47
-11.55	-11.54	-11.53	-11.52	-11.51	-11.5	-11.49	-11.48	-11.47
-11.55	-11.54	-11.53	-11.52	-11.51	-11.5	-11.49	-11.48	-11.47
-11.55	-11.54	-11.53	-11.52	-11.51	-11.5	-11.49	-11.48	-11.47
-11.55	-11.54	-11.53	-11.52	-11.51	-11.5	-11.49	-11.48	-11.47
-11.55	-11.54	-11.53	-11.52	-11.51	-11.5	-11.49	-11.48	-11.47
-11.55	-11.54	-11.53	-11.52	-11.51	-11.5	-11.49	-11.48	-11.47
-11.55	-11.54	-11.53	-11.52	-11.51	-11.5	-11.49	-11.48	-11.47
-11.55	-11.54	-11.53	-11.52	-11.51	-11.5	-11.49	-11.48	-11.47
-11.55	-11.54	-11.53	-11.52	-11.51	-11.5	-11.49	-11.48	-11.47
-11.55	-11.54	-11.53	-11.52	-11.51	-11.5	-11.49	-11.48	-11.47
-11.55	-11.54	-11.53	-11.52	-11.51	-11.5	-11.49	-11.48	-11.47
-11.55	-11.54	-11.53	-11.52	-11.51	-11.5	-11.49	-11.48	-11.47
-11.55	-11.54	-11.53	-11.52	-11.51	-11.5	-11.49	-11.48	-11.47
-11.55	-11.54	-11.53	-11.52	-11.51	-11.5	-11.49	-11.48	-11.47
-11.55	-11.54	-11.53	-11.52	-11.51	-11.5	-11.49	-11.48	-11.47
-11.55	-11.54	-11.53	-11.52	-11.51	-11.5	-11.49	-11.48	-11.47
-11.55	-11.54	-11.53	-11.52	-11.51	-11.5	-11.49	-11.48	-11.47
-11.55	-11.54	-11.53	-11.52	-11.51	-11.5	-11.49	-11.48	-11.47
-11.55	-11.54	-11.53	-11.52	-11.51	-11.5	-11.49	-11.48	-11.47
-11.55	-11.54	-11.53	-11.52	-11.51	-11.5	-11.49	-11.48	-11.47
-11.55	-11.54	-11.53	-11.52	-11.51	-11.5	-11.49	-11.48	-11.47
-11.55	-11.54	-11.53	-11.52	-11.51	-11.5	-11.49	-11.48	-11.47
-11.55	-11.54	-11.53	-11.52	-11.51	-11.5	-11.49	-11.48	-11.47
-11.55	-11.54	-11.53	-11.52	-11.51	-11.5	-11.49	-11.48	-11.47
-11.55	-11.54	-11.53	-11.52	-11.51	-11.5	-11.49	-11.48	-11.47
-11.55	-11.54	-11.53	-11.52	-11.51	-11.5	-11.49	-11.48	-11.47
-11.55	-11.54	-11.53	-11.52	-11.51	-11.5	-11.49	-11.48	-11.47
-11.55	-11.54	-11.53	-11.52	-11.51	-11.5	-11.49	-11.48	-11.47
-11.55	-11.54	-11.53	-11.52	-11.51	-11.5	-11.49	-11.48	-11.47
-11.55	-11.54	-11.53	-11.52	-11.51	-11.5	-11.49	-11.48	-11.47
-11.55	-11.54	-11.53	-11.52	-11.51	-11.5	-11.49	-11.48	-11.47
-11.55	-11.54	-11.53	-11.52	-11.51	-11.5	-11.49	-11.48	-11.47
-11.55	-11.54	-11.53	-11.52	-11.51	-11.5	-11.49	-11.48	-11.47
-11.55	-11.54	-11.53	-11.52	-11.51	-11.5	-11.49	-11.48	-11.47
-11.55	-11.54	-11.53	-11.52	-11.51	-11.5	-11.49	-11.48	-11.47
-11.55	-11.54	-11.53	-11.52	-11.51	-11.5	-11.49	-11.48	-11.47
-11.55	-11.54	-11.53	-11.52	-11.51	-11.5	-11.49	-11.48	-11.47
-11.55	-11.54	-11.53	-11.52	-11.51	-11.5	-11.49	-11.48	-11.47
-11.55	-11.54	-11.53	-11.52	-11.51	-11.5	-11.49	-11.48	-11.47
-11.55	-11.54	-11.53	-11.52	-11.51	-11.5	-11.49	-11.48	-11.47
-11.55	-11.54	-11.53	-11.52	-11.51	-11.5	-11.49	-11.48	-11.47
-11.55	-11.54	-11.53	-11.52	-11.51	-11.5	-11.49	-11.48	-11.47
-11.55	-11.54	-11.53	-11.52	-11.51	-11.5	-11.49	-11.48	-11.47
-11.55	-11.54	-11.53	-11.52	-11.51	-11.5	-11.49	-11.48	-11.47
-11.55	-11.54	-11.53	-11.52	-11.51	-11.5	-11.49	-11.48	-11.47
-11.55	-11.54	-11.53	-11.52	-11.51	-11.5	-11.49	-11.48	-11.47
-11.55	-11.54	-11.53	-11.52	-11.51	-11.5	-11.49	-11.48	-11.47
-11.55	-11.54	-11.53	-11.52	-11.51	-11.5	-11.49	-11.48	-11.47
-11.55	-11.54	-11.53	-11.52	-11.51	-11.5	-11.49	-11.48	-11.47

-11.55	-11.54	-11.53	-11.52	-11.51	-11.5	-11.49	-11.48	-11.47
-11.55	-11.54	-11.53	-11.52	-11.51	-11.5	-11.49	-11.48	-11.47
-11.55	-11.54	-11.53	-11.52	-11.51	-11.5	-11.49	-11.48	-11.47
-11.55	-11.54	-11.53	-11.52	-11.51	-11.5	-11.49	-11.48	-11.47
-11.55	-11.54	-11.53	-11.52	-11.51	-11.5	-11.49	-11.48	-11.47
-11.55	-11.54	-11.53	-11.52	-11.51	-11.5	-11.49	-11.48	-11.47
-11.55	-11.54	-11.53	-11.52	-11.51	-11.5	-11.49	-11.48	-11.47
-11.55	-11.54	-11.53	-11.52	-11.51	-11.5	-11.49	-11.48	-11.47
-11.55	-11.54	-11.53	-11.52	-11.51	-11.5	-11.49	-11.48	-11.47
-11.55	-11.54	-11.53	-11.52	-11.51	-11.5	-11.49	-11.48	-11.47
-11.55	-11.54	-11.53	-11.52	-11.51	-11.5	-11.49	-11.48	-11.47
-11.55	-11.54	-11.53	-11.52	-11.51	-11.5	-11.49	-11.48	-11.47
-11.55	-11.54	-11.53	-11.52	-11.51	-11.5	-11.49	-11.48	-11.47
-11.55	-11.54	-11.53	-11.52	-11.51	-11.5	-11.49	-11.48	-11.47
-11.55	-11.54	-11.53	-11.52	-11.51	-11.5	-11.49	-11.48	-11.47
-11.55	-11.54	-11.53	-11.52	-11.51	-11.5	-11.49	-11.48	-11.47
-11.55	-11.54	-11.53	-11.52	-11.51	-11.5	-11.49	-11.48	-11.47
-11.55	-11.54	-11.53	-11.52	-11.51	-11.5	-11.49	-11.48	-11.47
-11.55	-11.54	-11.53	-11.52	-11.51	-11.5	-11.49	-11.48	-11.47
-11.55	-11.54	-11.53	-11.52	-11.51	-11.5	-11.49	-11.48	-11.47
-11.55	-11.54	-11.53	-11.52	-11.51	-11.5	-11.49	-11.48	-11.47
-11.55	-11.54	-11.53	-11.52	-11.51	-11.5	-11.49	-11.48	-11.47
-11.55	-11.54	-11.53	-11.52	-11.51	-11.5	-11.49	-11.48	-11.47
-11.55	-11.54	-11.53	-11.52	-11.51	-11.5	-11.49	-11.48	-11.47
-11.55	-11.54	-11.53	-11.52	-11.51	-11.5	-11.49	-11.48	-11.47
-11.55	-11.54	-11.53	-11.52	-11.51	-11.5	-11.49	-11.48	-11.47
-11.55	-11.54	-11.53	-11.52	-11.51	-11.5	-11.49	-11.48	-11.47
-11.55	-11.54	-11.53	-11.52	-11.51	-11.5	-11.49	-11.48	-11.47
-11.55	-11.54	-11.53	-11.52	-11.51	-11.5	-11.49	-11.48	-11.47
-11.55	-11.54	-11.53	-11.52	-11.51	-11.5	-11.49	-11.48	-11.47
-11.55	-11.54	-11.53	-11.52	-11.51	-11.5	-11.49	-11.48	-11.47
-11.55	-11.54	-11.53	-11.52	-11.51	-11.5	-11.49	-11.48	-11.47
-11.55	-11.54	-11.53	-11.52	-11.51	-11.5	-11.49	-11.48	-11.47
-11.55	-11.54	-11.53	-11.52	-11.51	-11.5	-11.49	-11.48	-11.47
-11.55	-11.54	-11.53	-11.52	-11.51	-11.5	-11.49	-11.48	-11.47
-11.55	-11.54	-11.53	-11.52	-11.51	-11.5	-11.49	-11.48	-11.47
-11.55	-11.54	-11.53	-11.52	-11.51	-11.5	-11.49	-11.48	-11.47
-11.55	-11.54	-11.53	-11.52	-11.51	-11.5	-11.49	-11.48	-11.47
-11.55	-11.54	-11.53	-11.52	-11.51	-11.5	-11.49	-11.48	-11.47
-11.55	-11.54	-11.53	-11.52	-11.51	-11.5	-11.49	-11.48	-11.47
-11.55	-11.54	-11.53	-11.52	-11.51	-11.5	-11.49	-11.48	-11.47
-11.55	-11.54	-11.53	-11.52	-11.51	-11.5	-11.49	-11.48	-11.47
-11.55	-11.54	-11.53	-11.52	-11.51	-11.5	-11.49	-11.48	-11.47
-11.55	-11.54	-11.53	-11.52	-11.51	-11.5	-11.49	-11.48	-11.47
-11.55	-11.54	-11.53	-11.52	-11.51	-11.5	-11.49	-11.48	-11.47
-11.55	-11.54	-11.53	-11.52	-11.51	-11.5	-11.49	-11.48	-11.47
-11.55	-11.54	-11.53	-11.52	-11.51	-11.5	-11.49	-11.48	-11.47
-11.55	-11.54	-11.53	-11.52	-11.51	-11.5	-11.49	-11.48	-11.47
-11.55	-11.54	-11.53	-11.52	-11.51	-11.5	-11.49	-11.48	-11.47
-11.55	-11.54	-11.53	-11.52	-11.51	-11.5	-11.49	-11.48	-11.47

-11.55	-11.54	-11.53	-11.52	-11.51	-11.5	-11.49	-11.48	-11.47
-11.55	-11.54	-11.53	-11.52	-11.51	-11.5	-11.49	-11.48	-11.47
-11.55	-11.54	-11.53	-11.52	-11.51	-11.5	-11.49	-11.48	-11.47
-11.55	-11.54	-11.53	-11.52	-11.51	-11.5	-11.49	-11.48	-11.47
-11.55	-11.54	-11.53	-11.52	-11.51	-11.5	-11.49	-11.48	-11.47
-11.55	-11.54	-11.53	-11.52	-11.51	-11.5	-11.49	-11.48	-11.47
-11.55	-11.54	-11.53	-11.52	-11.51	-11.5	-11.49	-11.48	-11.47
-11.55	-11.54	-11.53	-11.52	-11.51	-11.5	-11.49	-11.48	-11.47
-11.55	-11.54	-11.53	-11.52	-11.51	-11.5	-11.49	-11.48	-11.47
-11.55	-11.54	-11.53	-11.52	-11.51	-11.5	-11.49	-11.48	-11.47
-11.55	-11.54	-11.53	-11.52	-11.51	-11.5	-11.49	-11.48	-11.47
-11.55	-11.54	-11.53	-11.52	-11.51	-11.5	-11.49	-11.48	-11.47
-11.55	-11.54	-11.53	-11.52	-11.51	-11.5	-11.49	-11.48	-11.47
-11.55	-11.54	-11.53	-11.52	-11.51	-11.5	-11.49	-11.48	-11.47
-11.55	-11.54	-11.53	-11.52	-11.51	-11.5	-11.49	-11.48	-11.47
-11.55	-11.54	-11.53	-11.52	-11.51	-11.5	-11.49	-11.48	-11.47
-11.55	-11.54	-11.53	-11.52	-11.51	-11.5	-11.49	-11.48	-11.47
-11.55	-11.54	-11.53	-11.52	-11.51	-11.5	-11.49	-11.48	-11.47
-11.55	-11.54	-11.53	-11.52	-11.51	-11.5	-11.49	-11.48	-11.47
-11.55	-11.54	-11.53	-11.52	-11.51	-11.5	-11.49	-11.48	-11.47
-11.55	-11.54	-11.53	-11.52	-11.51	-11.5	-11.49	-11.48	-11.47
-11.55	-11.54	-11.53	-11.52	-11.51	-11.5	-11.49	-11.48	-11.47
-11.55	-11.54	-11.53	-11.52	-11.51	-11.5	-11.49	-11.48	-11.47
-11.55	-11.54	-11.53	-11.52	-11.51	-11.5	-11.49	-11.48	-11.47
-11.55	-11.54	-11.53	-11.52	-11.51	-11.5	-11.49	-11.48	-11.47
-11.55	-11.54	-11.53	-11.52	-11.51	-11.5	-11.49	-11.48	-11.47
-11.55	-11.54	-11.53	-11.52	-11.51	-11.5	-11.49	-11.48	-11.47
-11.55	-11.54	-11.53	-11.52	-11.51	-11.5	-11.49	-11.48	-11.47
-11.55	-11.54	-11.53	-11.52	-11.51	-11.5	-11.49	-11.48	-11.47
-11.55	-11.54	-11.53	-11.52	-11.51	-11.5	-11.49	-11.48	-11.47
-11.55	-11.54	-11.53	-11.52	-11.51	-11.5	-11.49	-11.48	-11.47
-11.55	-11.54	-11.53	-11.52	-11.51	-11.5	-11.49	-11.48	-11.47
-11.55	-11.54	-11.53	-11.52	-11.51	-11.5	-11.49	-11.48	-11.47
-11.55	-11.54	-11.53	-11.52	-11.51	-11.5	-11.49	-11.48	-11.47
-11.55	-11.54	-11.53	-11.52	-11.51	-11.5	-11.49	-11.48	-11.47
-11.55	-11.54	-11.53	-11.52	-11.51	-11.5	-11.49	-11.48	-11.47
-11.55	-11.54	-11.53	-11.52	-11.51	-11.5	-11.49	-11.48	-11.47
-11.55	-11.54	-11.53	-11.52	-11.51	-11.5	-11.49	-11.48	-11.47
-11.55	-11.54	-11.53	-11.52	-11.51	-11.5	-11.49	-11.48	-11.47
-11.55	-11.54	-11.53	-11.52	-11.51	-11.5	-11.49	-11.48	-11.47
-11.55	-11.54	-11.53	-11.52	-11.51	-11.5	-11.49	-11.48	-11.47
-11.55	-11.54	-11.53	-11.52	-11.51	-11.5	-11.49	-11.48	-11.47
-11.55	-11.54	-11.53	-11.52	-11.51	-11.5	-11.49	-11.48	-11.47
-11.55	-11.54	-11.53	-11.52	-11.51	-11.5	-11.49	-11.48	-11.47
-11.55	-11.54	-11.53	-11.52	-11.51	-11.5	-11.49	-11.48	-11.47
-11.55	-11.54	-11.53	-11.52	-11.51	-11.5	-11.49	-11.48	-11.47
-11.55	-11.54	-11.53	-11.52	-11.51	-11.5	-11.49	-11.48	-11.47
-11.55	-11.54	-11.53	-11.52	-11.51	-11.5	-11.49	-11.48	-11.47
-11.55	-11.54	-11.53	-11.52	-11.51	-11.5	-11.49	-11.48	-11.47
-11.55	-11.54	-11.53	-11.52	-11.51	-11.5	-11.49	-11.48	-11.47

-11.55	-11.54	-11.53	-11.52	-11.51	-11.5	-11.49	-11.48	-11.47
-11.55	-11.54	-11.53	-11.52	-11.51	-11.5	-11.49	-11.48	-11.47
-11.55	-11.54	-11.53	-11.52	-11.51	-11.5	-11.49	-11.48	-11.47
-11.55	-11.54	-11.53	-11.52	-11.51	-11.5	-11.49	-11.48	-11.47
-11.55	-11.54	-11.53	-11.52	-11.51	-11.5	-11.49	-11.48	-11.47
-11.55	-11.54	-11.53	-11.52	-11.51	-11.5	-11.49	-11.48	-11.47
-11.55	-11.54	-11.53	-11.52	-11.51	-11.5	-11.49	-11.48	-11.47
-11.55	-11.54	-11.53	-11.52	-11.51	-11.5	-11.49	-11.48	-11.47
-11.55	-11.54	-11.53	-11.52	-11.51	-11.5	-11.49	-11.48	-11.47
-11.55	-11.54	-11.53	-11.52	-11.51	-11.5	-11.49	-11.48	-11.47
-11.55	-11.54	-11.53	-11.52	-11.51	-11.5	-11.49	-11.48	-11.47
-11.55	-11.54	-11.53	-11.52	-11.51	-11.5	-11.49	-11.48	-11.47
-11.55	-11.54	-11.53	-11.52	-11.51	-11.5	-11.49	-11.48	-11.47
-11.55	-11.54	-11.53	-11.52	-11.51	-11.5	-11.49	-11.48	-11.47
-11.55	-11.54	-11.53	-11.52	-11.51	-11.5	-11.49	-11.48	-11.47
-11.55	-11.54	-11.53	-11.52	-11.51	-11.5	-11.49	-11.48	-11.47
-11.55	-11.54	-11.53	-11.52	-11.51	-11.5	-11.49	-11.48	-11.47
-11.55	-11.54	-11.53	-11.52	-11.51	-11.5	-11.49	-11.48	-11.47
-11.55	-11.54	-11.53	-11.52	-11.51	-11.5	-11.49	-11.48	-11.47
-11.55	-11.54	-11.53	-11.52	-11.51	-11.5	-11.49	-11.48	-11.47
-11.55	-11.54	-11.53	-11.52	-11.51	-11.5	-11.49	-11.48	-11.47
-11.55	-11.54	-11.53	-11.52	-11.51	-11.5	-11.49	-11.48	-11.47
-11.55	-11.54	-11.53	-11.52	-11.51	-11.5	-11.49	-11.48	-11.47
-11.55	-11.54	-11.53	-11.52	-11.51	-11.5	-11.49	-11.48	-11.47
-11.55	-11.54	-11.53	-11.52	-11.51	-11.5	-11.49	-11.48	-11.47
-11.55	-11.54	-11.53	-11.52	-11.51	-11.5	-11.49	-11.48	-11.47
-11.55	-11.54	-11.53	-11.52	-11.51	-11.5	-11.49	-11.48	-11.47
-11.55	-11.54	-11.53	-11.52	-11.51	-11.5	-11.49	-11.48	-11.47
-11.55	-11.54	-11.53	-11.52	-11.51	-11.5	-11.49	-11.48	-11.47
-11.55	-11.54	-11.53	-11.52	-11.51	-11.5	-11.49	-11.48	-11.47
-11.55	-11.54	-11.53	-11.52	-11.51	-11.5	-11.49	-11.48	-11.47
-11.55	-11.54	-11.53	-11.52	-11.51	-11.5	-11.49	-11.48	-11.47
-11.55	-11.54	-11.53	-11.52	-11.51	-11.5	-11.49	-11.48	-11.47
-11.55	-11.54	-11.53	-11.52	-11.51	-11.5	-11.49	-11.48	-11.47
-11.55	-11.54	-11.53	-11.52	-11.51	-11.5	-11.49	-11.48	-11.47
-11.55	-11.54	-11.53	-11.52	-11.51	-11.5	-11.49	-11.48	-11.47
-11.55	-11.54	-11.53	-11.52	-11.51	-11.5	-11.49	-11.48	-11.47
-11.55	-11.54	-11.53	-11.52	-11.51	-11.5	-11.49	-11.48	-11.47
-11.55	-11.54	-11.53	-11.52	-11.51	-11.5	-11.49	-11.48	-11.47
-11.55	-11.54	-11.53	-11.52	-11.51	-11.5	-11.49	-11.48	-11.47
-11.55	-11.54	-11.53	-11.52	-11.51	-11.5	-11.49	-11.48	-11.47
-11.55	-11.54	-11.53	-11.52	-11.51	-11.5	-11.49	-11.48	-11.47
-11.55	-11.54	-11.53	-11.52	-11.51	-11.5	-11.49	-11.48	-11.47
-11.55	-11.54	-11.53	-11.52	-11.51	-11.5	-11.49	-11.48	-11.47
-11.55	-11.54	-11.53	-11.52	-11.51	-11.5	-11.49	-11.48	-11.47
-11.55	-11.54	-11.53	-11.52	-11.51	-11.5	-11.49	-11.48	-11.47
-11.55	-11.54	-11.53	-11.52	-11.51	-11.5	-11.49	-11.48	-11.47
-11.55	-11.54	-11.53	-11.52	-11.51	-11.5	-11.49	-11.48	-11.47
-11.55	-11.54	-11.53	-11.52	-11.51	-11.5	-11.49	-11.48	-11.47
-11.55	-11.54	-11.53	-11.52	-11.51	-11.5	-11.49	-11.48	-11.47

-11.55	-11.54	-11.53	-11.52	-11.51	-11.5	-11.49	-11.48	-11.47
-11.55	-11.54	-11.53	-11.52	-11.51	-11.5	-11.49	-11.48	-11.47
-11.55	-11.54	-11.53	-11.52	-11.51	-11.5	-11.49	-11.48	-11.47
-11.55	-11.54	-11.53	-11.52	-11.51	-11.5	-11.49	-11.48	-11.47
-11.55	-11.54	-11.53	-11.52	-11.51	-11.5	-11.49	-11.48	-11.47
-11.55	-11.54	-11.53	-11.52	-11.51	-11.5	-11.49	-11.48	-11.47
-11.55	-11.54	-11.53	-11.52	-11.51	-11.5	-11.49	-11.48	-11.47
-11.55	-11.54	-11.53	-11.52	-11.51	-11.5	-11.49	-11.48	-11.47
-11.55	-11.54	-11.53	-11.52	-11.51	-11.5	-11.49	-11.48	-11.47
-11.55	-11.54	-11.53	-11.52	-11.51	-11.5	-11.49	-11.48	-11.47
-11.55	-11.54	-11.53	-11.52	-11.51	-11.5	-11.49	-11.48	-11.47
-11.55	-11.54	-11.53	-11.52	-11.51	-11.5	-11.49	-11.48	-11.47
-11.55	-11.54	-11.53	-11.52	-11.51	-11.5	-11.49	-11.48	-11.47
-11.55	-11.54	-11.53	-11.52	-11.51	-11.5	-11.49	-11.48	-11.47
-11.55	-11.54	-11.53	-11.52	-11.51	-11.5	-11.49	-11.48	-11.47
-11.55	-11.54	-11.53	-11.52	-11.51	-11.5	-11.49	-11.48	-11.47
-11.55	-11.54	-11.53	-11.52	-11.51	-11.5	-11.49	-11.48	-11.47
-11.55	-11.54	-11.53	-11.52	-11.51	-11.5	-11.49	-11.48	-11.47
-11.55	-11.54	-11.53	-11.52	-11.51	-11.5	-11.49	-11.48	-11.47
-11.55	-11.54	-11.53	-11.52	-11.51	-11.5	-11.49	-11.48	-11.47
-11.55	-11.54	-11.53	-11.52	-11.51	-11.5	-11.49	-11.48	-11.47
-11.55	-11.54	-11.53	-11.52	-11.51	-11.5	-11.49	-11.48	-11.47
-11.55	-11.54	-11.53	-11.52	-11.51	-11.5	-11.49	-11.48	-11.47
-11.55	-11.54	-11.53	-11.52	-11.51	-11.5	-11.49	-11.48	-11.47
-11.55	-11.54	-11.53	-11.52	-11.51	-11.5	-11.49	-11.48	-11.47
-11.55	-11.54	-11.53	-11.52	-11.51	-11.5	-11.49	-11.48	-11.47
-11.55	-11.54	-11.53	-11.52	-11.51	-11.5	-11.49	-11.48	-11.47
-11.55	-11.54	-11.53	-11.52	-11.51	-11.5	-11.49	-11.48	-11.47
-11.55	-11.54	-11.53	-11.52	-11.51	-11.5	-11.49	-11.48	-11.47
-11.55	-11.54	-11.53	-11.52	-11.51	-11.5	-11.49	-11.48	-11.47
-11.55	-11.54	-11.53	-11.52	-11.51	-11.5	-11.49	-11.48	-11.47
-11.55	-11.54	-11.53	-11.52	-11.51	-11.5	-11.49	-11.48	-11.47
-11.55	-11.54	-11.53	-11.52	-11.51	-11.5	-11.49	-11.48	-11.47
-11.55	-11.54	-11.53	-11.52	-11.51	-11.5	-11.49	-11.48	-11.47
-11.55	-11.54	-11.53	-11.52	-11.51	-11.5	-11.49	-11.48	-11.47
-11.55	-11.54	-11.53	-11.52	-11.51	-11.5	-11.49	-11.48	-11.47
-11.55	-11.54	-11.53	-11.52	-11.51	-11.5	-11.49	-11.48	-11.47
-11.55	-11.54	-11.53	-11.52	-11.51	-11.5	-11.49	-11.48	-11.47
-11.55	-11.54	-11.53	-11.52	-11.51	-11.5	-11.49	-11.48	-11.47
-11.55	-11.54	-11.53	-11.52	-11.51	-11.5	-11.49	-11.48	-11.47
-11.55	-11.54	-11.53	-11.52	-11.51	-11.5	-11.49	-11.48	-11.47
-11.55	-11.54	-11.53	-11.52	-11.51	-11.5	-11.49	-11.48	-11.47
-11.55	-11.54	-11.53	-11.52	-11.51	-11.5	-11.49	-11.48	-11.47
-11.55	-11.54	-11.53	-11.52	-11.51	-11.5	-11.49	-11.48	-11.47
-11.55	-11.54	-11.53	-11.52	-11.51	-11.5	-11.49	-11.48	-11.47
-11.55	-11.54	-11.53	-11.52	-11.51	-11.5	-11.49	-11.48	-11.47
-11.55	-11.54	-11.53	-11.52	-11.51	-11.5	-11.49	-11.48	-11.47
-11.55	-11.54	-11.53	-11.52	-11.51	-11.5	-11.49	-11.48	-11.47
-11.55	-11.54	-11.53	-11.52	-11.51	-11.5	-11.49	-11.48	-11.47
-11.55	-11.54	-11.53	-11.52	-11.51	-11.5	-11.49	-11.48	-11.47

-11.55	-11.54	-11.53	-11.52	-11.51	-11.5	-11.49	-11.48	-11.47
-11.55	-11.54	-11.53	-11.52	-11.51	-11.5	-11.49	-11.48	-11.47
-11.55	-11.54	-11.53	-11.52	-11.51	-11.5	-11.49	-11.48	-11.47
-11.55	-11.54	-11.53	-11.52	-11.51	-11.5	-11.49	-11.48	-11.47
-11.55	-11.54	-11.53	-11.52	-11.51	-11.5	-11.49	-11.48	-11.47
-11.55	-11.54	-11.53	-11.52	-11.51	-11.5	-11.49	-11.48	-11.47
-11.55	-11.54	-11.53	-11.52	-11.51	-11.5	-11.49	-11.48	-11.47
-11.55	-11.54	-11.53	-11.52	-11.51	-11.5	-11.49	-11.48	-11.47
-11.55	-11.54	-11.53	-11.52	-11.51	-11.5	-11.49	-11.48	-11.47
-11.55	-11.54	-11.53	-11.52	-11.51	-11.5	-11.49	-11.48	-11.47
-11.55	-11.54	-11.53	-11.52	-11.51	-11.5	-11.49	-11.48	-11.47
-11.55	-11.54	-11.53	-11.52	-11.51	-11.5	-11.49	-11.48	-11.47
-11.55	-11.54	-11.53	-11.52	-11.51	-11.5	-11.49	-11.48	-11.47
-11.55	-11.54	-11.53	-11.52	-11.51	-11.5	-11.49	-11.48	-11.47
-11.55	-11.54	-11.53	-11.52	-11.51	-11.5	-11.49	-11.48	-11.47
-11.55	-11.54	-11.53	-11.52	-11.51	-11.5	-11.49	-11.48	-11.47
-11.55	-11.54	-11.53	-11.52	-11.51	-11.5	-11.49	-11.48	-11.47
-11.55	-11.54	-11.53	-11.52	-11.51	-11.5	-11.49	-11.48	-11.47
-11.55	-11.54	-11.53	-11.52	-11.51	-11.5	-11.49	-11.48	-11.47
-11.55	-11.54	-11.53	-11.52	-11.51	-11.5	-11.49	-11.48	-11.47
-11.55	-11.54	-11.53	-11.52	-11.51	-11.5	-11.49	-11.48	-11.47
-11.55	-11.54	-11.53	-11.52	-11.51	-11.5	-11.49	-11.48	-11.47
-11.55	-11.54	-11.53	-11.52	-11.51	-11.5	-11.49	-11.48	-11.47
-11.55	-11.54	-11.53	-11.52	-11.51	-11.5	-11.49	-11.48	-11.47
-11.55	-11.54	-11.53	-11.52	-11.51	-11.5	-11.49	-11.48	-11.47
-11.55	-11.54	-11.53	-11.52	-11.51	-11.5	-11.49	-11.48	-11.47
-11.55	-11.54	-11.53	-11.52	-11.51	-11.5	-11.49	-11.48	-11.47
-11.55	-11.54	-11.53	-11.52	-11.51	-11.5	-11.49	-11.48	-11.47
-11.55	-11.54	-11.53	-11.52	-11.51	-11.5	-11.49	-11.48	-11.47
-11.55	-11.54	-11.53	-11.52	-11.51	-11.5	-11.49	-11.48	-11.47
-11.55	-11.54	-11.53	-11.52	-11.51	-11.5	-11.49	-11.48	-11.47
-11.55	-11.54	-11.53	-11.52	-11.51	-11.5	-11.49	-11.48	-11.47
-11.55	-11.54	-11.53	-11.52	-11.51	-11.5	-11.49	-11.48	-11.47
-11.55	-11.54	-11.53	-11.52	-11.51	-11.5	-11.49	-11.48	-11.47
-11.55	-11.54	-11.53	-11.52	-11.51	-11.5	-11.49	-11.48	-11.47
-11.55	-11.54	-11.53	-11.52	-11.51	-11.5	-11.49	-11.48	-11.47
-11.55	-11.54	-11.53	-11.52	-11.51	-11.5	-11.49	-11.48	-11.47
-11.55	-11.54	-11.53	-11.52	-11.51	-11.5	-11.49	-11.48	-11.47
-11.55	-11.54	-11.53	-11.52	-11.51	-11.5	-11.49	-11.48	-11.47
-11.55	-11.54	-11.53	-11.52	-11.51	-11.5	-11.49	-11.48	-11.47
-11.55	-11.54	-11.53	-11.52	-11.51	-11.5	-11.49	-11.48	-11.47
-11.55	-11.54	-11.53	-11.52	-11.51	-11.5	-11.49	-11.48	-11.47
-11.55	-11.54	-11.53	-11.52	-11.51	-11.5	-11.49	-11.48	-11.47
-11.55	-11.54	-11.53	-11.52	-11.51	-11.5	-11.49	-11.48	-11.47
-11.55	-11.54	-11.53	-11.52	-11.51	-11.5	-11.49	-11.48	-11.47
-11.55	-11.54	-11.53	-11.52	-11.51	-11.5	-11.49	-11.48	-11.47
-11.55	-11.54	-11.53	-11.52	-11.51	-11.5	-11.49	-11.48	-11.47
-11.55	-11.54	-11.53	-11.52	-11.51	-11.5	-11.49	-11.48	-11.47
-11.55	-11.54	-11.53	-11.52	-11.51	-11.5	-11.49	-11.48	-11.47
-11.55	-11.54	-11.53	-11.52	-11.51	-11.5	-11.49	-11.48	-11.47

-11.55	-11.54	-11.53	-11.52	-11.51	-11.5	-11.49	-11.48	-11.47
-11.55	-11.54	-11.53	-11.52	-11.51	-11.5	-11.49	-11.48	-11.47
-11.55	-11.54	-11.53	-11.52	-11.51	-11.5	-11.49	-11.48	-11.47
-11.55	-11.54	-11.53	-11.52	-11.51	-11.5	-11.49	-11.48	-11.47
-11.55	-11.54	-11.53	-11.52	-11.51	-11.5	-11.49	-11.48	-11.47
-11.55	-11.54	-11.53	-11.52	-11.51	-11.5	-11.49	-11.48	-11.47
-11.55	-11.54	-11.53	-11.52	-11.51	-11.5	-11.49	-11.48	-11.47
-11.55	-11.54	-11.53	-11.52	-11.51	-11.5	-11.49	-11.48	-11.47
-11.55	-11.54	-11.53	-11.52	-11.51	-11.5	-11.49	-11.48	-11.47
-11.55	-11.54	-11.53	-11.52	-11.51	-11.5	-11.49	-11.48	-11.47
-11.55	-11.54	-11.53	-11.52	-11.51	-11.5	-11.49	-11.48	-11.47
-11.55	-11.54	-11.53	-11.52	-11.51	-11.5	-11.49	-11.48	-11.47
-11.55	-11.54	-11.53	-11.52	-11.51	-11.5	-11.49	-11.48	-11.47
-11.55	-11.54	-11.53	-11.52	-11.51	-11.5	-11.49	-11.48	-11.47
-11.55	-11.54	-11.53	-11.52	-11.51	-11.5	-11.49	-11.48	-11.47
-11.55	-11.54	-11.53	-11.52	-11.51	-11.5	-11.49	-11.48	-11.47
-11.55	-11.54	-11.53	-11.52	-11.51	-11.5	-11.49	-11.48	-11.47
-11.55	-11.54	-11.53	-11.52	-11.51	-11.5	-11.49	-11.48	-11.47
-11.55	-11.54	-11.53	-11.52	-11.51	-11.5	-11.49	-11.48	-11.47
-11.55	-11.54	-11.53	-11.52	-11.51	-11.5	-11.49	-11.48	-11.47
-11.55	-11.54	-11.53	-11.52	-11.51	-11.5	-11.49	-11.48	-11.47
-11.55	-11.54	-11.53	-11.52	-11.51	-11.5	-11.49	-11.48	-11.47
-11.55	-11.54	-11.53	-11.52	-11.51	-11.5	-11.49	-11.48	-11.47
-11.55	-11.54	-11.53	-11.52	-11.51	-11.5	-11.49	-11.48	-11.47
-11.55	-11.54	-11.53	-11.52	-11.51	-11.5	-11.49	-11.48	-11.47
-11.55	-11.54	-11.53	-11.52	-11.51	-11.5	-11.49	-11.48	-11.47
-11.55	-11.54	-11.53	-11.52	-11.51	-11.5	-11.49	-11.48	-11.47
-11.55	-11.54	-11.53	-11.52	-11.51	-11.5	-11.49	-11.48	-11.47
-11.55	-11.54	-11.53	-11.52	-11.51	-11.5	-11.49	-11.48	-11.47
-11.55	-11.54	-11.53	-11.52	-11.51	-11.5	-11.49	-11.48	-11.47
-11.55	-11.54	-11.53	-11.52	-11.51	-11.5	-11.49	-11.48	-11.47
-11.55	-11.54	-11.53	-11.52	-11.51	-11.5	-11.49	-11.48	-11.47
-11.55	-11.54	-11.53	-11.52	-11.51	-11.5	-11.49	-11.48	-11.47
-11.55	-11.54	-11.53	-11.52	-11.51	-11.5	-11.49	-11.48	-11.47
-11.55	-11.54	-11.53	-11.52	-11.51	-11.5	-11.49	-11.48	-11.47
-11.55	-11.54	-11.53	-11.52	-11.51	-11.5	-11.49	-11.48	-11.47
-11.55	-11.54	-11.53	-11.52	-11.51	-11.5	-11.49	-11.48	-11.47
-11.55	-11.54	-11.53	-11.52	-11.51	-11.5	-11.49	-11.48	-11.47
-11.55	-11.54	-11.53	-11.52	-11.51	-11.5	-11.49	-11.48	-11.47
-11.55	-11.54	-11.53	-11.52	-11.51	-11.5	-11.49	-11.48	-11.47
-11.55	-11.54	-11.53	-11.52	-11.51	-11.5	-11.49	-11.48	-11.47
-11.55	-11.54	-11.53	-11.52	-11.51	-11.5	-11.49	-11.48	-11.47
-11.55	-11.54	-11.53	-11.52	-11.51	-11.5	-11.49	-11.48	-11.47
-11.55	-11.54	-11.53	-11.52	-11.51	-11.5	-11.49	-11.48	-11.47
-11.55	-11.54	-11.53	-11.52	-11.51	-11.5	-11.49	-11.48	-11.47
-11.55	-11.54	-11.53	-11.52	-11.51	-11.5	-11.49	-11.48	-11.47
-11.55	-11.54	-11.53	-11.52	-11.51	-11.5	-11.49	-11.48	-11.47
-11.55	-11.54	-11.53	-11.52	-11.51	-11.5	-11.49	-11.48	-11.47
-11.55	-11.54	-11.53	-11.52	-11.51	-11.5	-11.49	-11.48	-11.47
-11.55	-11.54	-11.53	-11.52	-11.51	-11.5	-11.49	-11.48	-11.47

-11.55	-11.54	-11.53	-11.52	-11.51	-11.5	-11.49	-11.48	-11.47
-11.55	-11.54	-11.53	-11.52	-11.51	-11.5	-11.49	-11.48	-11.47
-11.55	-11.54	-11.53	-11.52	-11.51	-11.5	-11.49	-11.48	-11.47
-11.55	-11.54	-11.53	-11.52	-11.51	-11.5	-11.49	-11.48	-11.47
-11.55	-11.54	-11.53	-11.52	-11.51	-11.5	-11.49	-11.48	-11.47
-11.55	-11.54	-11.53	-11.52	-11.51	-11.5	-11.49	-11.48	-11.47
-11.55	-11.54	-11.53	-11.52	-11.51	-11.5	-11.49	-11.48	-11.47
-11.55	-11.54	-11.53	-11.52	-11.51	-11.5	-11.49	-11.48	-11.47
-11.55	-11.54	-11.53	-11.52	-11.51	-11.5	-11.49	-11.48	-11.47
-11.55	-11.54	-11.53	-11.52	-11.51	-11.5	-11.49	-11.48	-11.47
-11.55	-11.54	-11.53	-11.52	-11.51	-11.5	-11.49	-11.48	-11.47
-11.55	-11.54	-11.53	-11.52	-11.51	-11.5	-11.49	-11.48	-11.47
-11.55	-11.54	-11.53	-11.52	-11.51	-11.5	-11.49	-11.48	-11.47
-11.55	-11.54	-11.53	-11.52	-11.51	-11.5	-11.49	-11.48	-11.47
-11.55	-11.54	-11.53	-11.52	-11.51	-11.5	-11.49	-11.48	-11.47
-11.55	-11.54	-11.53	-11.52	-11.51	-11.5	-11.49	-11.48	-11.47
-11.55	-11.54	-11.53	-11.52	-11.51	-11.5	-11.49	-11.48	-11.47
-11.55	-11.54	-11.53	-11.52	-11.51	-11.5	-11.49	-11.48	-11.47
-11.55	-11.54	-11.53	-11.52	-11.51	-11.5	-11.49	-11.48	-11.47
-11.55	-11.54	-11.53	-11.52	-11.51	-11.5	-11.49	-11.48	-11.47
-11.55	-11.54	-11.53	-11.52	-11.51	-11.5	-11.49	-11.48	-11.47
-11.55	-11.54	-11.53	-11.52	-11.51	-11.5	-11.49	-11.48	-11.47
-11.55	-11.54	-11.53	-11.52	-11.51	-11.5	-11.49	-11.48	-11.47
-11.55	-11.54	-11.53	-11.52	-11.51	-11.5	-11.49	-11.48	-11.47
-11.55	-11.54	-11.53	-11.52	-11.51	-11.5	-11.49	-11.48	-11.47
-11.55	-11.54	-11.53	-11.52	-11.51	-11.5	-11.49	-11.48	-11.47
-11.55	-11.54	-11.53	-11.52	-11.51	-11.5	-11.49	-11.48	-11.47
-11.55	-11.54	-11.53	-11.52	-11.51	-11.5	-11.49	-11.48	-11.47
-11.55	-11.54	-11.53	-11.52	-11.51	-11.5	-11.49	-11.48	-11.47
-11.55	-11.54	-11.53	-11.52	-11.51	-11.5	-11.49	-11.48	-11.47
-11.55	-11.54	-11.53	-11.52	-11.51	-11.5	-11.49	-11.48	-11.47
-11.55	-11.54	-11.53	-11.52	-11.51	-11.5	-11.49	-11.48	-11.47
-11.55	-11.54	-11.53	-11.52	-11.51	-11.5	-11.49	-11.48	-11.47
-11.55	-11.54	-11.53	-11.52	-11.51	-11.5	-11.49	-11.48	-11.47
-11.55	-11.54	-11.53	-11.52	-11.51	-11.5	-11.49	-11.48	-11.47
-11.55	-11.54	-11.53	-11.52	-11.51	-11.5	-11.49	-11.48	-11.47
-11.55	-11.54	-11.53	-11.52	-11.51	-11.5	-11.49	-11.48	-11.47
-11.55	-11.54	-11.53	-11.52	-11.51	-11.5	-11.49	-11.48	-11.47
-11.55	-11.54	-11.53	-11.52	-11.51	-11.5	-11.49	-11.48	-11.47
-11.55	-11.54	-11.53	-11.52	-11.51	-11.5	-11.49	-11.48	-11.47
-11.55	-11.54	-11.53	-11.52	-11.51	-11.5	-11.49	-11.48	-11.47
-11.55	-11.54	-11.53	-11.52	-11.51	-11.5	-11.49	-11.48	-11.47
-11.55	-11.54	-11.53	-11.52	-11.51	-11.5	-11.49	-11.48	-11.47
-11.55	-11.54	-11.53	-11.52	-11.51	-11.5	-11.49	-11.48	-11.47
-11.55	-11.54	-11.53	-11.52	-11.51	-11.5	-11.49	-11.48	-11.47
-11.55	-11.54	-11.53	-11.52	-11.51	-11.5	-11.49	-11.48	-11.47
-11.55	-11.54	-11.53	-11.52	-11.51	-11.5	-11.49	-11.48	-11.47
-11.55	-11.54	-11.53	-11.52	-11.51	-11.5	-11.49	-11.48	-11.47
-11.55	-11.54	-11.53	-11.52	-11.51	-11.5	-11.49	-11.48	-11.47
-11.55	-11.54	-11.53	-11.52	-11.51	-11.5	-11.49	-11.48	-11.47

-11.55	-11.54	-11.53	-11.52	-11.51	-11.5	-11.49	-11.48	-11.47
-11.55	-11.54	-11.53	-11.52	-11.51	-11.5	-11.49	-11.48	-11.47
-11.55	-11.54	-11.53	-11.52	-11.51	-11.5	-11.49	-11.48	-11.47
-11.55	-11.54	-11.53	-11.52	-11.51	-11.5	-11.49	-11.48	-11.47
-11.55	-11.54	-11.53	-11.52	-11.51	-11.5	-11.49	-11.48	-11.47
-11.55	-11.54	-11.53	-11.52	-11.51	-11.5	-11.49	-11.48	-11.47
-11.55	-11.54	-11.53	-11.52	-11.51	-11.5	-11.49	-11.48	-11.47
-11.55	-11.54	-11.53	-11.52	-11.51	-11.5	-11.49	-11.48	-11.47
-11.55	-11.54	-11.53	-11.52	-11.51	-11.5	-11.49	-11.48	-11.47
-11.55	-11.54	-11.53	-11.52	-11.51	-11.5	-11.49	-11.48	-11.47
-11.55	-11.54	-11.53	-11.52	-11.51	-11.5	-11.49	-11.48	-11.47
-11.55	-11.54	-11.53	-11.52	-11.51	-11.5	-11.49	-11.48	-11.47
-11.55	-11.54	-11.53	-11.52	-11.51	-11.5	-11.49	-11.48	-11.47
-11.55	-11.54	-11.53	-11.52	-11.51	-11.5	-11.49	-11.48	-11.47
-11.55	-11.54	-11.53	-11.52	-11.51	-11.5	-11.49	-11.48	-11.47
-11.55	-11.54	-11.53	-11.52	-11.51	-11.5	-11.49	-11.48	-11.47
-11.55	-11.54	-11.53	-11.52	-11.51	-11.5	-11.49	-11.48	-11.47
-11.55	-11.54	-11.53	-11.52	-11.51	-11.5	-11.49	-11.48	-11.47
-11.55	-11.54	-11.53	-11.52	-11.51	-11.5	-11.49	-11.48	-11.47
-11.55	-11.54	-11.53	-11.52	-11.51	-11.5	-11.49	-11.48	-11.47
-11.55	-11.54	-11.53	-11.52	-11.51	-11.5	-11.49	-11.48	-11.47
-11.55	-11.54	-11.53	-11.52	-11.51	-11.5	-11.49	-11.48	-11.47
-11.55	-11.54	-11.53	-11.52	-11.51	-11.5	-11.49	-11.48	-11.47
-11.55	-11.54	-11.53	-11.52	-11.51	-11.5	-11.49	-11.48	-11.47
-11.55	-11.54	-11.53	-11.52	-11.51	-11.5	-11.49	-11.48	-11.47
-11.55	-11.54	-11.53	-11.52	-11.51	-11.5	-11.49	-11.48	-11.47
-11.55	-11.54	-11.53	-11.52	-11.51	-11.5	-11.49	-11.48	-11.47
-11.55	-11.54	-11.53	-11.52	-11.51	-11.5	-11.49	-11.48	-11.47
-11.55	-11.54	-11.53	-11.52	-11.51	-11.5	-11.49	-11.48	-11.47
-11.55	-11.54	-11.53	-11.52	-11.51	-11.5	-11.49	-11.48	-11.47
-11.55	-11.54	-11.53	-11.52	-11.51	-11.5	-11.49	-11.48	-11.47
-11.55	-11.54	-11.53	-11.52	-11.51	-11.5	-11.49	-11.48	-11.47
-11.55	-11.54	-11.53	-11.52	-11.51	-11.5	-11.49	-11.48	-11.47
-11.55	-11.54	-11.53	-11.52	-11.51	-11.5	-11.49	-11.48	-11.47
-11.55	-11.54	-11.53	-11.52	-11.51	-11.5	-11.49	-11.48	-11.47
-11.55	-11.54	-11.53	-11.52	-11.51	-11.5	-11.49	-11.48	-11.47
-11.55	-11.54	-11.53	-11.52	-11.51	-11.5	-11.49	-11.48	-11.47
-11.55	-11.54	-11.53	-11.52	-11.51	-11.5	-11.49	-11.48	-11.47
-11.55	-11.54	-11.53	-11.52	-11.51	-11.5	-11.49	-11.48	-11.47
-11.55	-11.54	-11.53	-11.52	-11.51	-11.5	-11.49	-11.48	-11.47
-11.55	-11.54	-11.53	-11.52	-11.51	-11.5	-11.49	-11.48	-11.47
-11.55	-11.54	-11.53	-11.52	-11.51	-11.5	-11.49	-11.48	-11.47
-11.55	-11.54	-11.53	-11.52	-11.51	-11.5	-11.49	-11.48	-11.47
-11.55	-11.54	-11.53	-11.52	-11.51	-11.5	-11.49	-11.48	-11.47
-11.55	-11.54	-11.53	-11.52	-11.51	-11.5	-11.49	-11.48	-11.47
-11.55	-11.54	-11.53	-11.52	-11.51	-11.5	-11.49	-11.48	-11.47
-11.55	-11.54	-11.53	-11.52	-11.51	-11.5	-11.49	-11.48	-11.47
-11.55	-11.54	-11.53	-11.52	-11.51	-11.5	-11.49	-11.48	-11.47
-11.55	-11.54	-11.53	-11.52	-11.51	-11.5	-11.49	-11.48	-11.47
-11.55	-11.54	-11.53	-11.52	-11.51	-11.5	-11.49	-11.48	-11.47

-11.55	-11.54	-11.53	-11.52	-11.51	-11.5	-11.49	-11.48	-11.47
-11.55	-11.54	-11.53	-11.52	-11.51	-11.5	-11.49	-11.48	-11.47
-11.55	-11.54	-11.53	-11.52	-11.51	-11.5	-11.49	-11.48	-11.47
-11.55	-11.54	-11.53	-11.52	-11.51	-11.5	-11.49	-11.48	-11.47
-11.55	-11.54	-11.53	-11.52	-11.51	-11.5	-11.49	-11.48	-11.47
-11.55	-11.54	-11.53	-11.52	-11.51	-11.5	-11.49	-11.48	-11.47
-11.55	-11.54	-11.53	-11.52	-11.51	-11.5	-11.49	-11.48	-11.47
-11.55	-11.54	-11.53	-11.52	-11.51	-11.5	-11.49	-11.48	-11.47
-11.55	-11.54	-11.53	-11.52	-11.51	-11.5	-11.49	-11.48	-11.47
-11.55	-11.54	-11.53	-11.52	-11.51	-11.5	-11.49	-11.48	-11.47
-11.55	-11.54	-11.53	-11.52	-11.51	-11.5	-11.49	-11.48	-11.47
-11.55	-11.54	-11.53	-11.52	-11.51	-11.5	-11.49	-11.48	-11.47
-11.55	-11.54	-11.53	-11.52	-11.51	-11.5	-11.49	-11.48	-11.47
-11.55	-11.54	-11.53	-11.52	-11.51	-11.5	-11.49	-11.48	-11.47
-11.55	-11.54	-11.53	-11.52	-11.51	-11.5	-11.49	-11.48	-11.47
-11.55	-11.54	-11.53	-11.52	-11.51	-11.5	-11.49	-11.48	-11.47
-11.55	-11.54	-11.53	-11.52	-11.51	-11.5	-11.49	-11.48	-11.47
-11.55	-11.54	-11.53	-11.52	-11.51	-11.5	-11.49	-11.48	-11.47
-11.55	-11.54	-11.53	-11.52	-11.51	-11.5	-11.49	-11.48	-11.47
-11.55	-11.54	-11.53	-11.52	-11.51	-11.5	-11.49	-11.48	-11.47
-11.55	-11.54	-11.53	-11.52	-11.51	-11.5	-11.49	-11.48	-11.47
-11.55	-11.54	-11.53	-11.52	-11.51	-11.5	-11.49	-11.48	-11.47
-11.55	-11.54	-11.53	-11.52	-11.51	-11.5	-11.49	-11.48	-11.47
-11.55	-11.54	-11.53	-11.52	-11.51	-11.5	-11.49	-11.48	-11.47
-11.55	-11.54	-11.53	-11.52	-11.51	-11.5	-11.49	-11.48	-11.47
-11.55	-11.54	-11.53	-11.52	-11.51	-11.5	-11.49	-11.48	-11.47
-11.55	-11.54	-11.53	-11.52	-11.51	-11.5	-11.49	-11.48	-11.47
-11.55	-11.54	-11.53	-11.52	-11.51	-11.5	-11.49	-11.48	-11.47
-11.55	-11.54	-11.53	-11.52	-11.51	-11.5	-11.49	-11.48	-11.47
-11.55	-11.54	-11.53	-11.52	-11.51	-11.5	-11.49	-11.48	-11.47
-11.55	-11.54	-11.53	-11.52	-11.51	-11.5	-11.49	-11.48	-11.47
-11.55	-11.54	-11.53	-11.52	-11.51	-11.5	-11.49	-11.48	-11.47
-11.55	-11.54	-11.53	-11.52	-11.51	-11.5	-11.49	-11.48	-11.47
-11.55	-11.54	-11.53	-11.52	-11.51	-11.5	-11.49	-11.48	-11.47
-11.55	-11.54	-11.53	-11.52	-11.51	-11.5	-11.49	-11.48	-11.47
-11.55	-11.54	-11.53	-11.52	-11.51	-11.5	-11.49	-11.48	-11.47
-11.55	-11.54	-11.53	-11.52	-11.51	-11.5	-11.49	-11.48	-11.47
-11.55	-11.54	-11.53	-11.52	-11.51	-11.5	-11.49	-11.48	-11.47
-11.55	-11.54	-11.53	-11.52	-11.51	-11.5	-11.49	-11.48	-11.47
-11.55	-11.54	-11.53	-11.52	-11.51	-11.5	-11.49	-11.48	-11.47
-11.55	-11.54	-11.53	-11.52	-11.51	-11.5	-11.49	-11.48	-11.47
-11.55	-11.54	-11.53	-11.52	-11.51	-11.5	-11.49	-11.48	-11.47
-11.55	-11.54	-11.53	-11.52	-11.51	-11.5	-11.49	-11.48	-11.47
-11.55	-11.54	-11.53	-11.52	-11.51	-11.5	-11.49	-11.48	-11.47
-11.55	-11.54	-11.53	-11.52	-11.51	-11.5	-11.49	-11.48	-11.47
-11.55	-11.54	-11.53	-11.52	-11.51	-11.5	-11.49	-11.48	-11.47
-11.55	-11.54	-11.53	-11.52	-11.51	-11.5	-11.49	-11.48	-11.47
-11.55	-11.54	-11.53	-11.52	-11.51	-11.5	-11.49	-11.48	-11.47
-11.55	-11.54	-11.53	-11.52	-11.51	-11.5	-11.49	-11.48	-11.47
-11.55	-11.54	-11.53	-11.52	-11.51	-11.5	-11.49	-11.48	-11.47

-11.55	-11.54	-11.53	-11.52	-11.51	-11.5	-11.49	-11.48	-11.47
-11.55	-11.54	-11.53	-11.52	-11.51	-11.5	-11.49	-11.48	-11.47
-11.55	-11.54	-11.53	-11.52	-11.51	-11.5	-11.49	-11.48	-11.47
-11.55	-11.54	-11.53	-11.52	-11.51	-11.5	-11.49	-11.48	-11.47
-11.55	-11.54	-11.53	-11.52	-11.51	-11.5	-11.49	-11.48	-11.47
-11.55	-11.54	-11.53	-11.52	-11.51	-11.5	-11.49	-11.48	-11.47
-11.55	-11.54	-11.53	-11.52	-11.51	-11.5	-11.49	-11.48	-11.47
-11.55	-11.54	-11.53	-11.52	-11.51	-11.5	-11.49	-11.48	-11.47
-11.55	-11.54	-11.53	-11.52	-11.51	-11.5	-11.49	-11.48	-11.47
-11.55	-11.54	-11.53	-11.52	-11.51	-11.5	-11.49	-11.48	-11.47
-11.55	-11.54	-11.53	-11.52	-11.51	-11.5	-11.49	-11.48	-11.47
-11.55	-11.54	-11.53	-11.52	-11.51	-11.5	-11.49	-11.48	-11.47
-11.55	-11.54	-11.53	-11.52	-11.51	-11.5	-11.49	-11.48	-11.47
-11.55	-11.54	-11.53	-11.52	-11.51	-11.5	-11.49	-11.48	-11.47
-11.55	-11.54	-11.53	-11.52	-11.51	-11.5	-11.49	-11.48	-11.47
-11.55	-11.54	-11.53	-11.52	-11.51	-11.5	-11.49	-11.48	-11.47
-11.55	-11.54	-11.53	-11.52	-11.51	-11.5	-11.49	-11.48	-11.47
-11.55	-11.54	-11.53	-11.52	-11.51	-11.5	-11.49	-11.48	-11.47
-11.55	-11.54	-11.53	-11.52	-11.51	-11.5	-11.49	-11.48	-11.47
-11.55	-11.54	-11.53	-11.52	-11.51	-11.5	-11.49	-11.48	-11.47
-11.55	-11.54	-11.53	-11.52	-11.51	-11.5	-11.49	-11.48	-11.47
-11.55	-11.54	-11.53	-11.52	-11.51	-11.5	-11.49	-11.48	-11.47
-11.55	-11.54	-11.53	-11.52	-11.51	-11.5	-11.49	-11.48	-11.47
-11.55	-11.54	-11.53	-11.52	-11.51	-11.5	-11.49	-11.48	-11.47
-11.55	-11.54	-11.53	-11.52	-11.51	-11.5	-11.49	-11.48	-11.47
-11.55	-11.54	-11.53	-11.52	-11.51	-11.5	-11.49	-11.48	-11.47
-11.55	-11.54	-11.53	-11.52	-11.51	-11.5	-11.49	-11.48	-11.47
-11.55	-11.54	-11.53	-11.52	-11.51	-11.5	-11.49	-11.48	-11.47
-11.55	-11.54	-11.53	-11.52	-11.51	-11.5	-11.49	-11.48	-11.47
-11.55	-11.54	-11.53	-11.52	-11.51	-11.5	-11.49	-11.48	-11.47
-11.55	-11.54	-11.53	-11.52	-11.51	-11.5	-11.49	-11.48	-11.47
-11.55	-11.54	-11.53	-11.52	-11.51	-11.5	-11.49	-11.48	-11.47
-11.55	-11.54	-11.53	-11.52	-11.51	-11.5	-11.49	-11.48	-11.47
-11.55	-11.54	-11.53	-11.52	-11.51	-11.5	-11.49	-11.48	-11.47
-11.55	-11.54	-11.53	-11.52	-11.51	-11.5	-11.49	-11.48	-11.47
-11.55	-11.54	-11.53	-11.52	-11.51	-11.5	-11.49	-11.48	-11.47
-11.55	-11.54	-11.53	-11.52	-11.51	-11.5	-11.49	-11.48	-11.47
-11.55	-11.54	-11.53	-11.52	-11.51	-11.5	-11.49	-11.48	-11.47
-11.55	-11.54	-11.53	-11.52	-11.51	-11.5	-11.49	-11.48	-11.47
-11.55	-11.54	-11.53	-11.52	-11.51	-11.5	-11.49	-11.48	-11.47
-11.55	-11.54	-11.53	-11.52	-11.51	-11.5	-11.49	-11.48	-11.47
-11.55	-11.54	-11.53	-11.52	-11.51	-11.5	-11.49	-11.48	-11.47
-11.55	-11.54	-11.53	-11.52	-11.51	-11.5	-11.49	-11.48	-11.47
-11.55	-11.54	-11.53	-11.52	-11.51	-11.5	-11.49	-11.48	-11.47
-11.55	-11.54	-11.53	-11.52	-11.51	-11.5	-11.49	-11.48	-11.47
-11.55	-11.54	-11.53	-11.52	-11.51	-11.5	-11.49	-11.48	-11.47
-11.55	-11.54	-11.53	-11.52	-11.51	-11.5	-11.49	-11.48	-11.47
-11.55	-11.54	-11.53	-11.52	-11.51	-11.5	-11.49	-11.48	-11.47
-11.55	-11.54	-11.53	-11.52	-11.51	-11.5	-11.49	-11.48	-11.47
-11.55	-11.54	-11.53	-11.52	-11.51	-11.5	-11.49	-11.48	-11.47

-11.55	-11.54	-11.53	-11.52	-11.51	-11.5	-11.49	-11.48	-11.47
-11.55	-11.54	-11.53	-11.52	-11.51	-11.5	-11.49	-11.48	-11.47
-11.55	-11.54	-11.53	-11.52	-11.51	-11.5	-11.49	-11.48	-11.47
-11.55	-11.54	-11.53	-11.52	-11.51	-11.5	-11.49	-11.48	-11.47
-11.55	-11.54	-11.53	-11.52	-11.51	-11.5	-11.49	-11.48	-11.47
-11.55	-11.54	-11.53	-11.52	-11.51	-11.5	-11.49	-11.48	-11.47
-11.55	-11.54	-11.53	-11.52	-11.51	-11.5	-11.49	-11.48	-11.47
-11.55	-11.54	-11.53	-11.52	-11.51	-11.5	-11.49	-11.48	-11.47
-11.55	-11.54	-11.53	-11.52	-11.51	-11.5	-11.49	-11.48	-11.47
-11.55	-11.54	-11.53	-11.52	-11.51	-11.5	-11.49	-11.48	-11.47
-11.55	-11.54	-11.53	-11.52	-11.51	-11.5	-11.49	-11.48	-11.47
-11.55	-11.54	-11.53	-11.52	-11.51	-11.5	-11.49	-11.48	-11.47
-11.55	-11.54	-11.53	-11.52	-11.51	-11.5	-11.49	-11.48	-11.47
-11.55	-11.54	-11.53	-11.52	-11.51	-11.5	-11.49	-11.48	-11.47
-11.55	-11.54	-11.53	-11.52	-11.51	-11.5	-11.49	-11.48	-11.47
-11.55	-11.54	-11.53	-11.52	-11.51	-11.5	-11.49	-11.48	-11.47
-11.55	-11.54	-11.53	-11.52	-11.51	-11.5	-11.49	-11.48	-11.47
-11.55	-11.54	-11.53	-11.52	-11.51	-11.5	-11.49	-11.48	-11.47
-11.55	-11.54	-11.53	-11.52	-11.51	-11.5	-11.49	-11.48	-11.47
-11.55	-11.54	-11.53	-11.52	-11.51	-11.5	-11.49	-11.48	-11.47
-11.55	-11.54	-11.53	-11.52	-11.51	-11.5	-11.49	-11.48	-11.47
-11.55	-11.54	-11.53	-11.52	-11.51	-11.5	-11.49	-11.48	-11.47
-11.55	-11.54	-11.53	-11.52	-11.51	-11.5	-11.49	-11.48	-11.47
-11.55	-11.54	-11.53	-11.52	-11.51	-11.5	-11.49	-11.48	-11.47
-11.55	-11.54	-11.53	-11.52	-11.51	-11.5	-11.49	-11.48	-11.47
-11.55	-11.54	-11.53	-11.52	-11.51	-11.5	-11.49	-11.48	-11.47
-11.55	-11.54	-11.53	-11.52	-11.51	-11.5	-11.49	-11.48	-11.47
-11.55	-11.54	-11.53	-11.52	-11.51	-11.5	-11.49	-11.48	-11.47
-11.55	-11.54	-11.53	-11.52	-11.51	-11.5	-11.49	-11.48	-11.47
-11.55	-11.54	-11.53	-11.52	-11.51	-11.5	-11.49	-11.48	-11.47
-11.55	-11.54	-11.53	-11.52	-11.51	-11.5	-11.49	-11.48	-11.47
-11.55	-11.54	-11.53	-11.52	-11.51	-11.5	-11.49	-11.48	-11.47
-11.55	-11.54	-11.53	-11.52	-11.51	-11.5	-11.49	-11.48	-11.47
-11.55	-11.54	-11.53	-11.52	-11.51	-11.5	-11.49	-11.48	-11.47
-11.55	-11.54	-11.53	-11.52	-11.51	-11.5	-11.49	-11.48	-11.47
-11.55	-11.54	-11.53	-11.52	-11.51	-11.5	-11.49	-11.48	-11.47
-11.55	-11.54	-11.53	-11.52	-11.51	-11.5	-11.49	-11.48	-11.47
-11.55	-11.54	-11.53	-11.52	-11.51	-11.5	-11.49	-11.48	-11.47
-11.55	-11.54	-11.53	-11.52	-11.51	-11.5	-11.49	-11.48	-11.47
-11.55	-11.54	-11.53	-11.52	-11.51	-11.5	-11.49	-11.48	-11.47
-11.55	-11.54	-11.53	-11.52	-11.51	-11.5	-11.49	-11.48	-11.47
-11.55	-11.54	-11.53	-11.52	-11.51	-11.5	-11.49	-11.48	-11.47
-11.55	-11.54	-11.53	-11.52	-11.51	-11.5	-11.49	-11.48	-11.47
-11.55	-11.54	-11.53	-11.52	-11.51	-11.5	-11.49	-11.48	-11.47
-11.55	-11.54	-11.53	-11.52	-11.51	-11.5	-11.49	-11.48	-11.47
-11.55	-11.54	-11.53	-11.52	-11.51	-11.5	-11.49	-11.48	-11.47
-11.55	-11.54	-11.53	-11.52	-11.51	-11.5	-11.49	-11.48	-11.47
-11.55	-11.54	-11.53	-11.52	-11.51	-11.5	-11.49	-11.48	-11.47
-11.55	-11.54	-11.53	-11.52	-11.51	-11.5	-11.49	-11.48	-11.47
-11.55	-11.54	-11.53	-11.52	-11.51	-11.5	-11.49	-11.48	-11.47

-11.55	-11.54	-11.53	-11.52	-11.51	-11.5	-11.49	-11.48	-11.47
-11.55	-11.54	-11.53	-11.52	-11.51	-11.5	-11.49	-11.48	-11.47
-11.55	-11.54	-11.53	-11.52	-11.51	-11.5	-11.49	-11.48	-11.47
-11.55	-11.54	-11.53	-11.52	-11.51	-11.5	-11.49	-11.48	-11.47
-11.55	-11.54	-11.53	-11.52	-11.51	-11.5	-11.49	-11.48	-11.47
-11.55	-11.54	-11.53	-11.52	-11.51	-11.5	-11.49	-11.48	-11.47
-11.55	-11.54	-11.53	-11.52	-11.51	-11.5	-11.49	-11.48	-11.47
-11.55	-11.54	-11.53	-11.52	-11.51	-11.5	-11.49	-11.48	-11.47
-11.55	-11.54	-11.53	-11.52	-11.51	-11.5	-11.49	-11.48	-11.47
-11.55	-11.54	-11.53	-11.52	-11.51	-11.5	-11.49	-11.48	-11.47
-11.55	-11.54	-11.53	-11.52	-11.51	-11.5	-11.49	-11.48	-11.47
-11.55	-11.54	-11.53	-11.52	-11.51	-11.5	-11.49	-11.48	-11.47
-11.55	-11.54	-11.53	-11.52	-11.51	-11.5	-11.49	-11.48	-11.47
-11.55	-11.54	-11.53	-11.52	-11.51	-11.5	-11.49	-11.48	-11.47
-11.55	-11.54	-11.53	-11.52	-11.51	-11.5	-11.49	-11.48	-11.47
-11.55	-11.54	-11.53	-11.52	-11.51	-11.5	-11.49	-11.48	-11.47
-11.55	-11.54	-11.53	-11.52	-11.51	-11.5	-11.49	-11.48	-11.47
-11.55	-11.54	-11.53	-11.52	-11.51	-11.5	-11.49	-11.48	-11.47
-11.55	-11.54	-11.53	-11.52	-11.51	-11.5	-11.49	-11.48	-11.47
-11.55	-11.54	-11.53	-11.52	-11.51	-11.5	-11.49	-11.48	-11.47
-11.55	-11.54	-11.53	-11.52	-11.51	-11.5	-11.49	-11.48	-11.47
-11.55	-11.54	-11.53	-11.52	-11.51	-11.5	-11.49	-11.48	-11.47
-11.55	-11.54	-11.53	-11.52	-11.51	-11.5	-11.49	-11.48	-11.47
-11.55	-11.54	-11.53	-11.52	-11.51	-11.5	-11.49	-11.48	-11.47
-11.55	-11.54	-11.53	-11.52	-11.51	-11.5	-11.49	-11.48	-11.47
-11.55	-11.54	-11.53	-11.52	-11.51	-11.5	-11.49	-11.48	-11.47
-11.55	-11.54	-11.53	-11.52	-11.51	-11.5	-11.49	-11.48	-11.47
-11.55	-11.54	-11.53	-11.52	-11.51	-11.5	-11.49	-11.48	-11.47
-11.55	-11.54	-11.53	-11.52	-11.51	-11.5	-11.49	-11.48	-11.47
-11.55	-11.54	-11.53	-11.52	-11.51	-11.5	-11.49	-11.48	-11.47
-11.55	-11.54	-11.53	-11.52	-11.51	-11.5	-11.49	-11.48	-11.47
-11.55	-11.54	-11.53	-11.52	-11.51	-11.5	-11.49	-11.48	-11.47
-11.55	-11.54	-11.53	-11.52	-11.51	-11.5	-11.49	-11.48	-11.47
-11.55	-11.54	-11.53	-11.52	-11.51	-11.5	-11.49	-11.48	-11.47
-11.55	-11.54	-11.53	-11.52	-11.51	-11.5	-11.49	-11.48	-11.47
-11.55	-11.54	-11.53	-11.52	-11.51	-11.5	-11.49	-11.48	-11.47
-11.55	-11.54	-11.53	-11.52	-11.51	-11.5	-11.49	-11.48	-11.47
-11.55	-11.54	-11.53	-11.52	-11.51	-11.5	-11.49	-11.48	-11.47
-11.55	-11.54	-11.53	-11.52	-11.51	-11.5	-11.49	-11.48	-11.47
-11.55	-11.54	-11.53	-11.52	-11.51	-11.5	-11.49	-11.48	-11.47
-11.55	-11.54	-11.53	-11.52	-11.51	-11.5	-11.49	-11.48	-11.47
-11.55	-11.54	-11.53	-11.52	-11.51	-11.5	-11.49	-11.48	-11.47
-11.55	-11.54	-11.53	-11.52	-11.51	-11.5	-11.49	-11.48	-11.47
-11.55	-11.54	-11.53	-11.52	-11.51	-11.5	-11.49	-11.48	-11.47
-11.55	-11.54	-11.53	-11.52	-11.51	-11.5	-11.49	-11.48	-11.47
-11.55	-11.54	-11.53	-11.52	-11.51	-11.5	-11.49	-11.48	-11.47
-11.55	-11.54	-11.53	-11.52	-11.51	-11.5	-11.49	-11.48	-11.47
-11.55	-11.54	-11.53	-11.52	-11.51	-11.5	-11.49	-11.48	-11.47
-11.55	-11.54	-11.53	-11.52	-11.51	-11.5	-11.49	-11.48	-11.47
-11.55	-11.54	-11.53	-11.52	-11.51	-11.5	-11.49	-11.48	-11.47

-11.55	-11.54	-11.53	-11.52	-11.51	-11.5	-11.49	-11.48	-11.47
-11.55	-11.54	-11.53	-11.52	-11.51	-11.5	-11.49	-11.48	-11.47
-11.55	-11.54	-11.53	-11.52	-11.51	-11.5	-11.49	-11.48	-11.47
-11.55	-11.54	-11.53	-11.52	-11.51	-11.5	-11.49	-11.48	-11.47
-11.55	-11.54	-11.53	-11.52	-11.51	-11.5	-11.49	-11.48	-11.47
-11.55	-11.54	-11.53	-11.52	-11.51	-11.5	-11.49	-11.48	-11.47
-11.55	-11.54	-11.53	-11.52	-11.51	-11.5	-11.49	-11.48	-11.47
-11.55	-11.54	-11.53	-11.52	-11.51	-11.5	-11.49	-11.48	-11.47
-11.55	-11.54	-11.53	-11.52	-11.51	-11.5	-11.49	-11.48	-11.47
-11.55	-11.54	-11.53	-11.52	-11.51	-11.5	-11.49	-11.48	-11.47
-11.55	-11.54	-11.53	-11.52	-11.51	-11.5	-11.49	-11.48	-11.47
-11.55	-11.54	-11.53	-11.52	-11.51	-11.5	-11.49	-11.48	-11.47
-11.55	-11.54	-11.53	-11.52	-11.51	-11.5	-11.49	-11.48	-11.47
-11.55	-11.54	-11.53	-11.52	-11.51	-11.5	-11.49	-11.48	-11.47
-11.55	-11.54	-11.53	-11.52	-11.51	-11.5	-11.49	-11.48	-11.47
-11.55	-11.54	-11.53	-11.52	-11.51	-11.5	-11.49	-11.48	-11.47
-11.55	-11.54	-11.53	-11.52	-11.51	-11.5	-11.49	-11.48	-11.47
-11.55	-11.54	-11.53	-11.52	-11.51	-11.5	-11.49	-11.48	-11.47
-11.55	-11.54	-11.53	-11.52	-11.51	-11.5	-11.49	-11.48	-11.47
-11.55	-11.54	-11.53	-11.52	-11.51	-11.5	-11.49	-11.48	-11.47
-11.55	-11.54	-11.53	-11.52	-11.51	-11.5	-11.49	-11.48	-11.47
-11.55	-11.54	-11.53	-11.52	-11.51	-11.5	-11.49	-11.48	-11.47
-11.55	-11.54	-11.53	-11.52	-11.51	-11.5	-11.49	-11.48	-11.47
-11.55	-11.54	-11.53	-11.52	-11.51	-11.5	-11.49	-11.48	-11.47
-11.55	-11.54	-11.53	-11.52	-11.51	-11.5	-11.49	-11.48	-11.47
-11.55	-11.54	-11.53	-11.52	-11.51	-11.5	-11.49	-11.48	-11.47
-11.55	-11.54	-11.53	-11.52	-11.51	-11.5	-11.49	-11.48	-11.47
-11.55	-11.54	-11.53	-11.52	-11.51	-11.5	-11.49	-11.48	-11.47
-11.55	-11.54	-11.53	-11.52	-11.51	-11.5	-11.49	-11.48	-11.47
-11.55	-11.54	-11.53	-11.52	-11.51	-11.5	-11.49	-11.48	-11.47
-11.55	-11.54	-11.53	-11.52	-11.51	-11.5	-11.49	-11.48	-11.47
-11.55	-11.54	-11.53	-11.52	-11.51	-11.5	-11.49	-11.48	-11.47
-11.55	-11.54	-11.53	-11.52	-11.51	-11.5	-11.49	-11.48	-11.47
-11.55	-11.54	-11.53	-11.52	-11.51	-11.5	-11.49	-11.48	-11.47
-11.55	-11.54	-11.53	-11.52	-11.51	-11.5	-11.49	-11.48	-11.47
-11.55	-11.54	-11.53	-11.52	-11.51	-11.5	-11.49	-11.48	-11.47
-11.55	-11.54	-11.53	-11.52	-11.51	-11.5	-11.49	-11.48	-11.47
-11.55	-11.54	-11.53	-11.52	-11.51	-11.5	-11.49	-11.48	-11.47
-11.55	-11.54	-11.53	-11.52	-11.51	-11.5	-11.49	-11.48	-11.47
-11.55	-11.54	-11.53	-11.52	-11.51	-11.5	-11.49	-11.48	-11.47
-11.55	-11.54	-11.53	-11.52	-11.51	-11.5	-11.49	-11.48	-11.47
-11.55	-11.54	-11.53	-11.52	-11.51	-11.5	-11.49	-11.48	-11.47
-11.55	-11.54	-11.53	-11.52	-11.51	-11.5	-11.49	-11.48	-11.47
-11.55	-11.54	-11.53	-11.52	-11.51	-11.5	-11.49	-11.48	-11.47
-11.55	-11.54	-11.53	-11.52	-11.51	-11.5	-11.49	-11.48	-11.47
-11.55	-11.54	-11.53	-11.52	-11.51	-11.5	-11.49	-11.48	-11.47
-11.55	-11.54	-11.53	-11.52	-11.51	-11.5	-11.49	-11.48	-11.47
-11.55	-11.54	-11.53	-11.52	-11.51	-11.5	-11.49	-11.48	-11.47
-11.55	-11.54	-11.53	-11.52	-11.51	-11.5	-11.49	-11.48	-11.47
-11.55	-11.54	-11.53	-11.52	-11.51	-11.5	-11.49	-11.48	-11.47

-11.55	-11.54	-11.53	-11.52	-11.51	-11.5	-11.49	-11.48	-11.47
-11.55	-11.54	-11.53	-11.52	-11.51	-11.5	-11.49	-11.48	-11.47
-11.55	-11.54	-11.53	-11.52	-11.51	-11.5	-11.49	-11.48	-11.47
-11.55	-11.54	-11.53	-11.52	-11.51	-11.5	-11.49	-11.48	-11.47
-11.55	-11.54	-11.53	-11.52	-11.51	-11.5	-11.49	-11.48	-11.47
-11.55	-11.54	-11.53	-11.52	-11.51	-11.5	-11.49	-11.48	-11.47
-11.55	-11.54	-11.53	-11.52	-11.51	-11.5	-11.49	-11.48	-11.47
-11.55	-11.54	-11.53	-11.52	-11.51	-11.5	-11.49	-11.48	-11.47
-11.55	-11.54	-11.53	-11.52	-11.51	-11.5	-11.49	-11.48	-11.47
-11.55	-11.54	-11.53	-11.52	-11.51	-11.5	-11.49	-11.48	-11.47
-11.55	-11.54	-11.53	-11.52	-11.51	-11.5	-11.49	-11.48	-11.47
-11.55	-11.54	-11.53	-11.52	-11.51	-11.5	-11.49	-11.48	-11.47
-11.55	-11.54	-11.53	-11.52	-11.51	-11.5	-11.49	-11.48	-11.47
-11.55	-11.54	-11.53	-11.52	-11.51	-11.5	-11.49	-11.48	-11.47
-11.55	-11.54	-11.53	-11.52	-11.51	-11.5	-11.49	-11.48	-11.47
-11.55	-11.54	-11.53	-11.52	-11.51	-11.5	-11.49	-11.48	-11.47
-11.55	-11.54	-11.53	-11.52	-11.51	-11.5	-11.49	-11.48	-11.47
-11.55	-11.54	-11.53	-11.52	-11.51	-11.5	-11.49	-11.48	-11.47
-11.55	-11.54	-11.53	-11.52	-11.51	-11.5	-11.49	-11.48	-11.47
-11.55	-11.54	-11.53	-11.52	-11.51	-11.5	-11.49	-11.48	-11.47
-11.55	-11.54	-11.53	-11.52	-11.51	-11.5	-11.49	-11.48	-11.47
-11.55	-11.54	-11.53	-11.52	-11.51	-11.5	-11.49	-11.48	-11.47
-11.55	-11.54	-11.53	-11.52	-11.51	-11.5	-11.49	-11.48	-11.47
-11.55	-11.54	-11.53	-11.52	-11.51	-11.5	-11.49	-11.48	-11.47
-11.55	-11.54	-11.53	-11.52	-11.51	-11.5	-11.49	-11.48	-11.47
-11.55	-11.54	-11.53	-11.52	-11.51	-11.5	-11.49	-11.48	-11.47
-11.55	-11.54	-11.53	-11.52	-11.51	-11.5	-11.49	-11.48	-11.47
-11.55	-11.54	-11.53	-11.52	-11.51	-11.5	-11.49	-11.48	-11.47
-11.55	-11.54	-11.53	-11.52	-11.51	-11.5	-11.49	-11.48	-11.47
-11.55	-11.54	-11.53	-11.52	-11.51	-11.5	-11.49	-11.48	-11.47
-11.55	-11.54	-11.53	-11.52	-11.51	-11.5	-11.49	-11.48	-11.47
-11.55	-11.54	-11.53	-11.52	-11.51	-11.5	-11.49	-11.48	-11.47
-11.55	-11.54	-11.53	-11.52	-11.51	-11.5	-11.49	-11.48	-11.47
-11.55	-11.54	-11.53	-11.52	-11.51	-11.5	-11.49	-11.48	-11.47
-11.55	-11.54	-11.53	-11.52	-11.51	-11.5	-11.49	-11.48	-11.47
-11.55	-11.54	-11.53	-11.52	-11.51	-11.5	-11.49	-11.48	-11.47
-11.55	-11.54	-11.53	-11.52	-11.51	-11.5	-11.49	-11.48	-11.47
-11.55	-11.54	-11.53	-11.52	-11.51	-11.5	-11.49	-11.48	-11.47
-11.55	-11.54	-11.53	-11.52	-11.51	-11.5	-11.49	-11.48	-11.47
-11.55	-11.54	-11.53	-11.52	-11.51	-11.5	-11.49	-11.48	-11.47
-11.55	-11.54	-11.53	-11.52	-11.51	-11.5	-11.49	-11.48	-11.47
-11.55	-11.54	-11.53	-11.52	-11.51	-11.5	-11.49	-11.48	-11.47
-11.55	-11.54	-11.53	-11.52	-11.51	-11.5	-11.49	-11.48	-11.47
-11.55	-11.54	-11.53	-11.52	-11.51	-11.5	-11.49	-11.48	-11.47
-11.55	-11.54	-11.53	-11.52	-11.51	-11.5	-11.49	-11.48	-11.47
-11.55	-11.54	-11.53	-11.52	-11.51	-11.5	-11.49	-11.48	-11.47
-11.55	-11.54	-11.53	-11.52	-11.51	-11.5	-11.49	-11.48	-11.47
-11.55	-11.54	-11.53	-11.52	-11.51	-11.5	-11.49	-11.48	-11.47
-11.55	-11.54	-11.53	-11.52	-11.51	-11.5	-11.49	-11.48	-11.47
-11.55	-11.54	-11.53	-11.52	-11.51	-11.5	-11.49	-11.48	-11.47

-11.55	-11.54	-11.53	-11.52	-11.51	-11.5	-11.49	-11.48	-11.47
-11.55	-11.54	-11.53	-11.52	-11.51	-11.5	-11.49	-11.48	-11.47
-11.55	-11.54	-11.53	-11.52	-11.51	-11.5	-11.49	-11.48	-11.47
-11.55	-11.54	-11.53	-11.52	-11.51	-11.5	-11.49	-11.48	-11.47
-11.55	-11.54	-11.53	-11.52	-11.51	-11.5	-11.49	-11.48	-11.47
-11.55	-11.54	-11.53	-11.52	-11.51	-11.5	-11.49	-11.48	-11.47
-11.55	-11.54	-11.53	-11.52	-11.51	-11.5	-11.49	-11.48	-11.47
-11.55	-11.54	-11.53	-11.52	-11.51	-11.5	-11.49	-11.48	-11.47
-11.55	-11.54	-11.53	-11.52	-11.51	-11.5	-11.49	-11.48	-11.47
-11.55	-11.54	-11.53	-11.52	-11.51	-11.5	-11.49	-11.48	-11.47
-11.55	-11.54	-11.53	-11.52	-11.51	-11.5	-11.49	-11.48	-11.47
-11.55	-11.54	-11.53	-11.52	-11.51	-11.5	-11.49	-11.48	-11.47
-11.55	-11.54	-11.53	-11.52	-11.51	-11.5	-11.49	-11.48	-11.47
-11.55	-11.54	-11.53	-11.52	-11.51	-11.5	-11.49	-11.48	-11.47
-11.55	-11.54	-11.53	-11.52	-11.51	-11.5	-11.49	-11.48	-11.47
-11.55	-11.54	-11.53	-11.52	-11.51	-11.5	-11.49	-11.48	-11.47
-11.55	-11.54	-11.53	-11.52	-11.51	-11.5	-11.49	-11.48	-11.47
-11.55	-11.54	-11.53	-11.52	-11.51	-11.5	-11.49	-11.48	-11.47
-11.55	-11.54	-11.53	-11.52	-11.51	-11.5	-11.49	-11.48	-11.47
-11.55	-11.54	-11.53	-11.52	-11.51	-11.5	-11.49	-11.48	-11.47
-11.55	-11.54	-11.53	-11.52	-11.51	-11.5	-11.49	-11.48	-11.47
-11.55	-11.54	-11.53	-11.52	-11.51	-11.5	-11.49	-11.48	-11.47
-11.55	-11.54	-11.53	-11.52	-11.51	-11.5	-11.49	-11.48	-11.47
-11.55	-11.54	-11.53	-11.52	-11.51	-11.5	-11.49	-11.48	-11.47
-11.55	-11.54	-11.53	-11.52	-11.51	-11.5	-11.49	-11.48	-11.47
-11.55	-11.54	-11.53	-11.52	-11.51	-11.5	-11.49	-11.48	-11.47
-11.55	-11.54	-11.53	-11.52	-11.51	-11.5	-11.49	-11.48	-11.47
-11.55	-11.54	-11.53	-11.52	-11.51	-11.5	-11.49	-11.48	-11.47
-11.55	-11.54	-11.53	-11.52	-11.51	-11.5	-11.49	-11.48	-11.47
-11.55	-11.54	-11.53	-11.52	-11.51	-11.5	-11.49	-11.48	-11.47
-11.55	-11.54	-11.53	-11.52	-11.51	-11.5	-11.49	-11.48	-11.47
-11.55	-11.54	-11.53	-11.52	-11.51	-11.5	-11.49	-11.48	-11.47
-11.55	-11.54	-11.53	-11.52	-11.51	-11.5	-11.49	-11.48	-11.47
-11.55	-11.54	-11.53	-11.52	-11.51	-11.5	-11.49	-11.48	-11.47
-11.55	-11.54	-11.53	-11.52	-11.51	-11.5	-11.49	-11.48	-11.47
-11.55	-11.54	-11.53	-11.52	-11.51	-11.5	-11.49	-11.48	-11.47
-11.55	-11.54	-11.53	-11.52	-11.51	-11.5	-11.49	-11.48	-11.47
-11.55	-11.54	-11.53	-11.52	-11.51	-11.5	-11.49	-11.48	-11.47
-11.55	-11.54	-11.53	-11.52	-11.51	-11.5	-11.49	-11.48	-11.47
-11.55	-11.54	-11.53	-11.52	-11.51	-11.5	-11.49	-11.48	-11.47
-11.55	-11.54	-11.53	-11.52	-11.51	-11.5	-11.49	-11.48	-11.47
-11.55	-11.54	-11.53	-11.52	-11.51	-11.5	-11.49	-11.48	-11.47
-11.55	-11.54	-11.53	-11.52	-11.51	-11.5	-11.49	-11.48	-11.47
-11.55	-11.54	-11.53	-11.52	-11.51	-11.5	-11.49	-11.48	-11.47
-11.55	-11.54	-11.53	-11.52	-11.51	-11.5	-11.49	-11.48	-11.47
-11.55	-11.54	-11.53	-11.52	-11.51	-11.5	-11.49	-11.48	-11.47
-11.55	-11.54	-11.53	-11.52	-11.51	-11.5	-11.49	-11.48	-11.47
-11.55	-11.54	-11.53	-11.52	-11.51	-11.5	-11.49	-11.48	-11.47
-11.55	-11.54	-11.53	-11.52	-11.51	-11.5	-11.49	-11.48	-11.47
-11.55	-11.54	-11.53	-11.52	-11.51	-11.5	-11.49	-11.48	-11.47

-11.55	-11.54	-11.53	-11.52	-11.51	-11.5	-11.49	-11.48	-11.47
-11.55	-11.54	-11.53	-11.52	-11.51	-11.5	-11.49	-11.48	-11.47
-11.55	-11.54	-11.53	-11.52	-11.51	-11.5	-11.49	-11.48	-11.47
-11.55	-11.54	-11.53	-11.52	-11.51	-11.5	-11.49	-11.48	-11.47
-11.55	-11.54	-11.53	-11.52	-11.51	-11.5	-11.49	-11.48	-11.47
-11.55	-11.54	-11.53	-11.52	-11.51	-11.5	-11.49	-11.48	-11.47
-11.55	-11.54	-11.53	-11.52	-11.51	-11.5	-11.49	-11.48	-11.47
-11.55	-11.54	-11.53	-11.52	-11.51	-11.5	-11.49	-11.48	-11.47
-11.55	-11.54	-11.53	-11.52	-11.51	-11.5	-11.49	-11.48	-11.47
-11.55	-11.54	-11.53	-11.52	-11.51	-11.5	-11.49	-11.48	-11.47
-11.55	-11.54	-11.53	-11.52	-11.51	-11.5	-11.49	-11.48	-11.47
-11.55	-11.54	-11.53	-11.52	-11.51	-11.5	-11.49	-11.48	-11.47
-11.55	-11.54	-11.53	-11.52	-11.51	-11.5	-11.49	-11.48	-11.47
-11.55	-11.54	-11.53	-11.52	-11.51	-11.5	-11.49	-11.48	-11.47
-11.55	-11.54	-11.53	-11.52	-11.51	-11.5	-11.49	-11.48	-11.47
-11.55	-11.54	-11.53	-11.52	-11.51	-11.5	-11.49	-11.48	-11.47
-11.55	-11.54	-11.53	-11.52	-11.51	-11.5	-11.49	-11.48	-11.47
-11.55	-11.54	-11.53	-11.52	-11.51	-11.5	-11.49	-11.48	-11.47
-11.55	-11.54	-11.53	-11.52	-11.51	-11.5	-11.49	-11.48	-11.47
-11.55	-11.54	-11.53	-11.52	-11.51	-11.5	-11.49	-11.48	-11.47
-11.55	-11.54	-11.53	-11.52	-11.51	-11.5	-11.49	-11.48	-11.47
-11.55	-11.54	-11.53	-11.52	-11.51	-11.5	-11.49	-11.48	-11.47
-11.55	-11.54	-11.53	-11.52	-11.51	-11.5	-11.49	-11.48	-11.47
-11.55	-11.54	-11.53	-11.52	-11.51	-11.5	-11.49	-11.48	-11.47
-11.55	-11.54	-11.53	-11.52	-11.51	-11.5	-11.49	-11.48	-11.47
-11.55	-11.54	-11.53	-11.52	-11.51	-11.5	-11.49	-11.48	-11.47
-11.55	-11.54	-11.53	-11.52	-11.51	-11.5	-11.49	-11.48	-11.47
-11.55	-11.54	-11.53	-11.52	-11.51	-11.5	-11.49	-11.48	-11.47
-11.55	-11.54	-11.53	-11.52	-11.51	-11.5	-11.49	-11.48	-11.47
-11.55	-11.54	-11.53	-11.52	-11.51	-11.5	-11.49	-11.48	-11.47
-11.55	-11.54	-11.53	-11.52	-11.51	-11.5	-11.49	-11.48	-11.47
-11.55	-11.54	-11.53	-11.52	-11.51	-11.5	-11.49	-11.48	-11.47
-11.55	-11.54	-11.53	-11.52	-11.51	-11.5	-11.49	-11.48	-11.47
-11.55	-11.54	-11.53	-11.52	-11.51	-11.5	-11.49	-11.48	-11.47
-11.55	-11.54	-11.53	-11.52	-11.51	-11.5	-11.49	-11.48	-11.47
-11.55	-11.54	-11.53	-11.52	-11.51	-11.5	-11.49	-11.48	-11.47
-11.55	-11.54	-11.53	-11.52	-11.51	-11.5	-11.49	-11.48	-11.47
-11.55	-11.54	-11.53	-11.52	-11.51	-11.5	-11.49	-11.48	-11.47
-11.55	-11.54	-11.53	-11.52	-11.51	-11.5	-11.49	-11.48	-11.47
-11.55	-11.54	-11.53	-11.52	-11.51	-11.5	-11.49	-11.48	-11.47
-11.55	-11.54	-11.53	-11.52	-11.51	-11.5	-11.49	-11.48	-11.47
-11.55	-11.54	-11.53	-11.52	-11.51	-11.5	-11.49	-11.48	-11.47
-11.55	-11.54	-11.53	-11.52	-11.51	-11.5	-11.49	-11.48	-11.47
-11.55	-11.54	-11.53	-11.52	-11.51	-11.5	-11.49	-11.48	-11.47
-11.55	-11.54	-11.53	-11.52	-11.51	-11.5	-11.49	-11.48	-11.47
-11.55	-11.54	-11.53	-11.52	-11.51	-11.5	-11.49	-11.48	-11.47
-11.55	-11.54	-11.53	-11.52	-11.51	-11.5	-11.49	-11.48	-11.47
-11.55	-11.54	-11.53	-11.52	-11.51	-11.5	-11.49	-11.48	-11.47
-11.55	-11.54	-11.53	-11.52	-11.51	-11.5	-11.49	-11.48	-11.47
-11.55	-11.54	-11.53	-11.52	-11.51	-11.5	-11.49	-11.48	-11.47

-11.55	-11.54	-11.53	-11.52	-11.51	-11.5	-11.49	-11.48	-11.47
-11.55	-11.54	-11.53	-11.52	-11.51	-11.5	-11.49	-11.48	-11.47
-11.55	-11.54	-11.53	-11.52	-11.51	-11.5	-11.49	-11.48	-11.47
-11.55	-11.54	-11.53	-11.52	-11.51	-11.5	-11.49	-11.48	-11.47
-11.55	-11.54	-11.53	-11.52	-11.51	-11.5	-11.49	-11.48	-11.47
-11.55	-11.54	-11.53	-11.52	-11.51	-11.5	-11.49	-11.48	-11.47
-11.55	-11.54	-11.53	-11.52	-11.51	-11.5	-11.49	-11.48	-11.47
-11.55	-11.54	-11.53	-11.52	-11.51	-11.5	-11.49	-11.48	-11.47
-11.55	-11.54	-11.53	-11.52	-11.51	-11.5	-11.49	-11.48	-11.47
-11.55	-11.54	-11.53	-11.52	-11.51	-11.5	-11.49	-11.48	-11.47
-11.55	-11.54	-11.53	-11.52	-11.51	-11.5	-11.49	-11.48	-11.47
-11.55	-11.54	-11.53	-11.52	-11.51	-11.5	-11.49	-11.48	-11.47
-11.55	-11.54	-11.53	-11.52	-11.51	-11.5	-11.49	-11.48	-11.47
-11.55	-11.54	-11.53	-11.52	-11.51	-11.5	-11.49	-11.48	-11.47
-11.55	-11.54	-11.53	-11.52	-11.51	-11.5	-11.49	-11.48	-11.47
-11.55	-11.54	-11.53	-11.52	-11.51	-11.5	-11.49	-11.48	-11.47
-11.55	-11.54	-11.53	-11.52	-11.51	-11.5	-11.49	-11.48	-11.47
-11.55	-11.54	-11.53	-11.52	-11.51	-11.5	-11.49	-11.48	-11.47
-11.55	-11.54	-11.53	-11.52	-11.51	-11.5	-11.49	-11.48	-11.47
-11.55	-11.54	-11.53	-11.52	-11.51	-11.5	-11.49	-11.48	-11.47
-11.55	-11.54	-11.53	-11.52	-11.51	-11.5	-11.49	-11.48	-11.47
-11.55	-11.54	-11.53	-11.52	-11.51	-11.5	-11.49	-11.48	-11.47
-11.55	-11.54	-11.53	-11.52	-11.51	-11.5	-11.49	-11.48	-11.47
-11.55	-11.54	-11.53	-11.52	-11.51	-11.5	-11.49	-11.48	-11.47
-11.55	-11.54	-11.53	-11.52	-11.51	-11.5	-11.49	-11.48	-11.47
-11.55	-11.54	-11.53	-11.52	-11.51	-11.5	-11.49	-11.48	-11.47
-11.55	-11.54	-11.53	-11.52	-11.51	-11.5	-11.49	-11.48	-11.47
-11.55	-11.54	-11.53	-11.52	-11.51	-11.5	-11.49	-11.48	-11.47
-11.55	-11.54	-11.53	-11.52	-11.51	-11.5	-11.49	-11.48	-11.47
-11.55	-11.54	-11.53	-11.52	-11.51	-11.5	-11.49	-11.48	-11.47
-11.55	-11.54	-11.53	-11.52	-11.51	-11.5	-11.49	-11.48	-11.47
-11.55	-11.54	-11.53	-11.52	-11.51	-11.5	-11.49	-11.48	-11.47
-11.55	-11.54	-11.53	-11.52	-11.51	-11.5	-11.49	-11.48	-11.47
-11.55	-11.54	-11.53	-11.52	-11.51	-11.5	-11.49	-11.48	-11.47
-11.55	-11.54	-11.53	-11.52	-11.51	-11.5	-11.49	-11.48	-11.47
-11.55	-11.54	-11.53	-11.52	-11.51	-11.5	-11.49	-11.48	-11.47
-11.55	-11.54	-11.53	-11.52	-11.51	-11.5	-11.49	-11.48	-11.47
-11.55	-11.54	-11.53	-11.52	-11.51	-11.5	-11.49	-11.48	-11.47
-11.55	-11.54	-11.53	-11.52	-11.51	-11.5	-11.49	-11.48	-11.47
-11.55	-11.54	-11.53	-11.52	-11.51	-11.5	-11.49	-11.48	-11.47
-11.55	-11.54	-11.53	-11.52	-11.51	-11.5	-11.49	-11.48	-11.47
-11.55	-11.54	-11.53	-11.52	-11.51	-11.5	-11.49	-11.48	-11.47
-11.55	-11.54	-11.53	-11.52	-11.51	-11.5	-11.49	-11.48	-11.47
-11.55	-11.54	-11.53	-11.52	-11.51	-11.5	-11.49	-11.48	-11.47
-11.55	-11.54	-11.53	-11.52	-11.51	-11.5	-11.49	-11.48	-11.47
-11.55	-11.54	-11.53	-11.52	-11.51	-11.5	-11.49	-11.48	-11.47
-11.55	-11.54	-11.53	-11.52	-11.51	-11.5	-11.49	-11.48	-11.47
-11.55	-11.54	-11.53	-11.52	-11.51	-11.5	-11.49	-11.48	-11.47
-11.55	-11.54	-11.53	-11.52	-11.51	-11.5	-11.49	-11.48	-11.47
-11.55	-11.54	-11.53	-11.52	-11.51	-11.5	-11.49	-11.48	-11.47

-11.55	-11.54	-11.53	-11.52	-11.51	-11.5	-11.49	-11.48	-11.47
-11.55	-11.54	-11.53	-11.52	-11.51	-11.5	-11.49	-11.48	-11.47
-11.55	-11.54	-11.53	-11.52	-11.51	-11.5	-11.49	-11.48	-11.47
-11.55	-11.54	-11.53	-11.52	-11.51	-11.5	-11.49	-11.48	-11.47
-11.55	-11.54	-11.53	-11.52	-11.51	-11.5	-11.49	-11.48	-11.47
-11.55	-11.54	-11.53	-11.52	-11.51	-11.5	-11.49	-11.48	-11.47
-11.55	-11.54	-11.53	-11.52	-11.51	-11.5	-11.49	-11.48	-11.47
-11.55	-11.54	-11.53	-11.52	-11.51	-11.5	-11.49	-11.48	-11.47
-11.55	-11.54	-11.53	-11.52	-11.51	-11.5	-11.49	-11.48	-11.47
-11.55	-11.54	-11.53	-11.52	-11.51	-11.5	-11.49	-11.48	-11.47
-11.55	-11.54	-11.53	-11.52	-11.51	-11.5	-11.49	-11.48	-11.47
-11.55	-11.54	-11.53	-11.52	-11.51	-11.5	-11.49	-11.48	-11.47
-11.55	-11.54	-11.53	-11.52	-11.51	-11.5	-11.49	-11.48	-11.47
-11.55	-11.54	-11.53	-11.52	-11.51	-11.5	-11.49	-11.48	-11.47
-11.55	-11.54	-11.53	-11.52	-11.51	-11.5	-11.49	-11.48	-11.47
-11.55	-11.54	-11.53	-11.52	-11.51	-11.5	-11.49	-11.48	-11.47
-11.55	-11.54	-11.53	-11.52	-11.51	-11.5	-11.49	-11.48	-11.47
-11.55	-11.54	-11.53	-11.52	-11.51	-11.5	-11.49	-11.48	-11.47
-11.55	-11.54	-11.53	-11.52	-11.51	-11.5	-11.49	-11.48	-11.47
-11.55	-11.54	-11.53	-11.52	-11.51	-11.5	-11.49	-11.48	-11.47
-11.55	-11.54	-11.53	-11.52	-11.51	-11.5	-11.49	-11.48	-11.47
-11.55	-11.54	-11.53	-11.52	-11.51	-11.5	-11.49	-11.48	-11.47
-11.55	-11.54	-11.53	-11.52	-11.51	-11.5	-11.49	-11.48	-11.47
-11.55	-11.54	-11.53	-11.52	-11.51	-11.5	-11.49	-11.48	-11.47
-11.55	-11.54	-11.53	-11.52	-11.51	-11.5	-11.49	-11.48	-11.47
-11.55	-11.54	-11.53	-11.52	-11.51	-11.5	-11.49	-11.48	-11.47
-11.55	-11.54	-11.53	-11.52	-11.51	-11.5	-11.49	-11.48	-11.47
-11.55	-11.54	-11.53	-11.52	-11.51	-11.5	-11.49	-11.48	-11.47
-11.55	-11.54	-11.53	-11.52	-11.51	-11.5	-11.49	-11.48	-11.47
-11.55	-11.54	-11.53	-11.52	-11.51	-11.5	-11.49	-11.48	-11.47
-11.55	-11.54	-11.53	-11.52	-11.51	-11.5	-11.49	-11.48	-11.47
-11.55	-11.54	-11.53	-11.52	-11.51	-11.5	-11.49	-11.48	-11.47
-11.55	-11.54	-11.53	-11.52	-11.51	-11.5	-11.49	-11.48	-11.47
-11.55	-11.54	-11.53	-11.52	-11.51	-11.5	-11.49	-11.48	-11.47
-11.55	-11.54	-11.53	-11.52	-11.51	-11.5	-11.49	-11.48	-11.47
-11.55	-11.54	-11.53	-11.52	-11.51	-11.5	-11.49	-11.48	-11.47
-11.55	-11.54	-11.53	-11.52	-11.51	-11.5	-11.49	-11.48	-11.47
-11.55	-11.54	-11.53	-11.52	-11.51	-11.5	-11.49	-11.48	-11.47
-11.55	-11.54	-11.53	-11.52	-11.51	-11.5	-11.49	-11.48	-11.47
-11.55	-11.54	-11.53	-11.52	-11.51	-11.5	-11.49	-11.48	-11.47
-11.55	-11.54	-11.53	-11.52	-11.51	-11.5	-11.49	-11.48	-11.47
-11.55	-11.54	-11.53	-11.52	-11.51	-11.5	-11.49	-11.48	-11.47
-11.55	-11.54	-11.53	-11.52	-11.51	-11.5	-11.49	-11.48	-11.47
-11.55	-11.54	-11.53	-11.52	-11.51	-11.5	-11.49	-11.48	-11.47
-11.55	-11.54	-11.53	-11.52	-11.51	-11.5	-11.49	-11.48	-11.47
-11.55	-11.54	-11.53	-11.52	-11.51	-11.5	-11.49	-11.48	-11.47
-11.55	-11.54	-11.53	-11.52	-11.51	-11.5	-11.49	-11.48	-11.47
-11.55	-11.54	-11.53	-11.52	-11.51	-11.5	-11.49	-11.48	-11.47
-11.55	-11.54	-11.53	-11.52	-11.51	-11.5	-11.49	-11.48	-11.47
-11.55	-11.54	-11.53	-11.52	-11.51	-11.5	-11.49	-11.48	-11.47

-11.55	-11.54	-11.53	-11.52	-11.51	-11.5	-11.49	-11.48	-11.47
-11.55	-11.54	-11.53	-11.52	-11.51	-11.5	-11.49	-11.48	-11.47
-11.55	-11.54	-11.53	-11.52	-11.51	-11.5	-11.49	-11.48	-11.47
-11.55	-11.54	-11.53	-11.52	-11.51	-11.5	-11.49	-11.48	-11.47
-11.55	-11.54	-11.53	-11.52	-11.51	-11.5	-11.49	-11.48	-11.47
-11.55	-11.54	-11.53	-11.52	-11.51	-11.5	-11.49	-11.48	-11.47
-11.55	-11.54	-11.53	-11.52	-11.51	-11.5	-11.49	-11.48	-11.47
-11.55	-11.54	-11.53	-11.52	-11.51	-11.5	-11.49	-11.48	-11.47
-11.55	-11.54	-11.53	-11.52	-11.51	-11.5	-11.49	-11.48	-11.47
-11.55	-11.54	-11.53	-11.52	-11.51	-11.5	-11.49	-11.48	-11.47
-11.55	-11.54	-11.53	-11.52	-11.51	-11.5	-11.49	-11.48	-11.47
-11.55	-11.54	-11.53	-11.52	-11.51	-11.5	-11.49	-11.48	-11.47
-11.55	-11.54	-11.53	-11.52	-11.51	-11.5	-11.49	-11.48	-11.47
-11.55	-11.54	-11.53	-11.52	-11.51	-11.5	-11.49	-11.48	-11.47
-11.55	-11.54	-11.53	-11.52	-11.51	-11.5	-11.49	-11.48	-11.47
-11.55	-11.54	-11.53	-11.52	-11.51	-11.5	-11.49	-11.48	-11.47
-11.55	-11.54	-11.53	-11.52	-11.51	-11.5	-11.49	-11.48	-11.47
-11.55	-11.54	-11.53	-11.52	-11.51	-11.5	-11.49	-11.48	-11.47
-11.55	-11.54	-11.53	-11.52	-11.51	-11.5	-11.49	-11.48	-11.47
-11.55	-11.54	-11.53	-11.52	-11.51	-11.5	-11.49	-11.48	-11.47
-11.55	-11.54	-11.53	-11.52	-11.51	-11.5	-11.49	-11.48	-11.47
-11.55	-11.54	-11.53	-11.52	-11.51	-11.5	-11.49	-11.48	-11.47
-11.55	-11.54	-11.53	-11.52	-11.51	-11.5	-11.49	-11.48	-11.47
-11.55	-11.54	-11.53	-11.52	-11.51	-11.5	-11.49	-11.48	-11.47
-11.55	-11.54	-11.53	-11.52	-11.51	-11.5	-11.49	-11.48	-11.47
-11.55	-11.54	-11.53	-11.52	-11.51	-11.5	-11.49	-11.48	-11.47
-11.55	-11.54	-11.53	-11.52	-11.51	-11.5	-11.49	-11.48	-11.47
-11.55	-11.54	-11.53	-11.52	-11.51	-11.5	-11.49	-11.48	-11.47
-11.55	-11.54	-11.53	-11.52	-11.51	-11.5	-11.49	-11.48	-11.47
-11.55	-11.54	-11.53	-11.52	-11.51	-11.5	-11.49	-11.48	-11.47
-11.55	-11.54	-11.53	-11.52	-11.51	-11.5	-11.49	-11.48	-11.47
-11.55	-11.54	-11.53	-11.52	-11.51	-11.5	-11.49	-11.48	-11.47
-11.55	-11.54	-11.53	-11.52	-11.51	-11.5	-11.49	-11.48	-11.47
-11.55	-11.54	-11.53	-11.52	-11.51	-11.5	-11.49	-11.48	-11.47
-11.55	-11.54	-11.53	-11.52	-11.51	-11.5	-11.49	-11.48	-11.47
-11.55	-11.54	-11.53	-11.52	-11.51	-11.5	-11.49	-11.48	-11.47
-11.55	-11.54	-11.53	-11.52	-11.51	-11.5	-11.49	-11.48	-11.47
-11.55	-11.54	-11.53	-11.52	-11.51	-11.5	-11.49	-11.48	-11.47
-11.55	-11.54	-11.53	-11.52	-11.51	-11.5	-11.49	-11.48	-11.47
-11.55	-11.54	-11.53	-11.52	-11.51	-11.5	-11.49	-11.48	-11.47
-11.55	-11.54	-11.53	-11.52	-11.51	-11.5	-11.49	-11.48	-11.47
-11.55	-11.54	-11.53	-11.52	-11.51	-11.5	-11.49	-11.48	-11.47
-11.55	-11.54	-11.53	-11.52	-11.51	-11.5	-11.49	-11.48	-11.47
-11.55	-11.54	-11.53	-11.52	-11.51	-11.5	-11.49	-11.48	-11.47
-11.55	-11.54	-11.53	-11.52	-11.51	-11.5	-11.49	-11.48	-11.47
-11.55	-11.54	-11.53	-11.52	-11.51	-11.5	-11.49	-11.48	-11.47
-11.55	-11.54	-11.53	-11.52	-11.51	-11.5	-11.49	-11.48	-11.47
-11.55	-11.54	-11.53	-11.52	-11.51	-11.5	-11.49	-11.48	-11.47
-11.55	-11.54	-11.53	-11.52	-11.51	-11.5	-11.49	-11.48	-11.47
-11.55	-11.54	-11.53	-11.52	-11.51	-11.5	-11.49	-11.48	-11.47

-11.55	-11.54	-11.53	-11.52	-11.51	-11.5	-11.49	-11.48	-11.47
-11.55	-11.54	-11.53	-11.52	-11.51	-11.5	-11.49	-11.48	-11.47
-11.55	-11.54	-11.53	-11.52	-11.51	-11.5	-11.49	-11.48	-11.47
-11.55	-11.54	-11.53	-11.52	-11.51	-11.5	-11.49	-11.48	-11.47
-11.55	-11.54	-11.53	-11.52	-11.51	-11.5	-11.49	-11.48	-11.47
-11.55	-11.54	-11.53	-11.52	-11.51	-11.5	-11.49	-11.48	-11.47
-11.55	-11.54	-11.53	-11.52	-11.51	-11.5	-11.49	-11.48	-11.47
-11.55	-11.54	-11.53	-11.52	-11.51	-11.5	-11.49	-11.48	-11.47
-11.55	-11.54	-11.53	-11.52	-11.51	-11.5	-11.49	-11.48	-11.47
-11.55	-11.54	-11.53	-11.52	-11.51	-11.5	-11.49	-11.48	-11.47
-11.55	-11.54	-11.53	-11.52	-11.51	-11.5	-11.49	-11.48	-11.47
-11.55	-11.54	-11.53	-11.52	-11.51	-11.5	-11.49	-11.48	-11.47
-11.55	-11.54	-11.53	-11.52	-11.51	-11.5	-11.49	-11.48	-11.47
-11.55	-11.54	-11.53	-11.52	-11.51	-11.5	-11.49	-11.48	-11.47
-11.55	-11.54	-11.53	-11.52	-11.51	-11.5	-11.49	-11.48	-11.47
-11.55	-11.54	-11.53	-11.52	-11.51	-11.5	-11.49	-11.48	-11.47
-11.55	-11.54	-11.53	-11.52	-11.51	-11.5	-11.49	-11.48	-11.47
-11.55	-11.54	-11.53	-11.52	-11.51	-11.5	-11.49	-11.48	-11.47
-11.55	-11.54	-11.53	-11.52	-11.51	-11.5	-11.49	-11.48	-11.47
-11.55	-11.54	-11.53	-11.52	-11.51	-11.5	-11.49	-11.48	-11.47
-11.55	-11.54	-11.53	-11.52	-11.51	-11.5	-11.49	-11.48	-11.47
-11.55	-11.54	-11.53	-11.52	-11.51	-11.5	-11.49	-11.48	-11.47
-11.55	-11.54	-11.53	-11.52	-11.51	-11.5	-11.49	-11.48	-11.47
-11.55	-11.54	-11.53	-11.52	-11.51	-11.5	-11.49	-11.48	-11.47
-11.55	-11.54	-11.53	-11.52	-11.51	-11.5	-11.49	-11.48	-11.47
-11.55	-11.54	-11.53	-11.52	-11.51	-11.5	-11.49	-11.48	-11.47
-11.55	-11.54	-11.53	-11.52	-11.51	-11.5	-11.49	-11.48	-11.47
-11.55	-11.54	-11.53	-11.52	-11.51	-11.5	-11.49	-11.48	-11.47
-11.55	-11.54	-11.53	-11.52	-11.51	-11.5	-11.49	-11.48	-11.47
-11.55	-11.54	-11.53	-11.52	-11.51	-11.5	-11.49	-11.48	-11.47
-11.55	-11.54	-11.53	-11.52	-11.51	-11.5	-11.49	-11.48	-11.47
-11.55	-11.54	-11.53	-11.52	-11.51	-11.5	-11.49	-11.48	-11.47
-11.55	-11.54	-11.53	-11.52	-11.51	-11.5	-11.49	-11.48	-11.47
-11.55	-11.54	-11.53	-11.52	-11.51	-11.5	-11.49	-11.48	-11.47
-11.55	-11.54	-11.53	-11.52	-11.51	-11.5	-11.49	-11.48	-11.47
-11.55	-11.54	-11.53	-11.52	-11.51	-11.5	-11.49	-11.48	-11.47
-11.55	-11.54	-11.53	-11.52	-11.51	-11.5	-11.49	-11.48	-11.47
-11.55	-11.54	-11.53	-11.52	-11.51	-11.5	-11.49	-11.48	-11.47
-11.55	-11.54	-11.53	-11.52	-11.51	-11.5	-11.49	-11.48	-11.47
-11.55	-11.54	-11.53	-11.52	-11.51	-11.5	-11.49	-11.48	-11.47
-11.55	-11.54	-11.53	-11.52	-11.51	-11.5	-11.49	-11.48	-11.47
-11.55	-11.54	-11.53	-11.52	-11.51	-11.5	-11.49	-11.48	-11.47
-11.55	-11.54	-11.53	-11.52	-11.51	-11.5	-11.49	-11.48	-11.47
-11.55	-11.54	-11.53	-11.52	-11.51	-11.5	-11.49	-11.48	-11.47
-11.55	-11.54	-11.53	-11.52	-11.51	-11.5	-11.49	-11.48	-11.47
-11.55	-11.54	-11.53	-11.52	-11.51	-11.5	-11.49	-11.48	-11.47
-11.55	-11.54	-11.53	-11.52	-11.51	-11.5	-11.49	-11.48	-11.47
-11.55	-11.54	-11.53	-11.52	-11.51	-11.5	-11.49	-11.48	-11.47
-11.55	-11.54	-11.53	-11.52	-11.51	-11.5	-11.49	-11.48	-11.47
-11.55	-11.54	-11.53	-11.52	-11.51	-11.5	-11.49	-11.48	-11.47

-11.55	-11.54	-11.53	-11.52	-11.51	-11.5	-11.49	-11.48	-11.47
-11.55	-11.54	-11.53	-11.52	-11.51	-11.5	-11.49	-11.48	-11.47
-11.55	-11.54	-11.53	-11.52	-11.51	-11.5	-11.49	-11.48	-11.47
-11.55	-11.54	-11.53	-11.52	-11.51	-11.5	-11.49	-11.48	-11.47
-11.55	-11.54	-11.53	-11.52	-11.51	-11.5	-11.49	-11.48	-11.47
-11.55	-11.54	-11.53	-11.52	-11.51	-11.5	-11.49	-11.48	-11.47
-11.55	-11.54	-11.53	-11.52	-11.51	-11.5	-11.49	-11.48	-11.47
-11.55	-11.54	-11.53	-11.52	-11.51	-11.5	-11.49	-11.48	-11.47
-11.55	-11.54	-11.53	-11.52	-11.51	-11.5	-11.49	-11.48	-11.47
-11.55	-11.54	-11.53	-11.52	-11.51	-11.5	-11.49	-11.48	-11.47
-11.55	-11.54	-11.53	-11.52	-11.51	-11.5	-11.49	-11.48	-11.47
-11.55	-11.54	-11.53	-11.52	-11.51	-11.5	-11.49	-11.48	-11.47
-11.55	-11.54	-11.53	-11.52	-11.51	-11.5	-11.49	-11.48	-11.47
-11.55	-11.54	-11.53	-11.52	-11.51	-11.5	-11.49	-11.48	-11.47
-11.55	-11.54	-11.53	-11.52	-11.51	-11.5	-11.49	-11.48	-11.47
-11.55	-11.54	-11.53	-11.52	-11.51	-11.5	-11.49	-11.48	-11.47
-11.55	-11.54	-11.53	-11.52	-11.51	-11.5	-11.49	-11.48	-11.47
-11.55	-11.54	-11.53	-11.52	-11.51	-11.5	-11.49	-11.48	-11.47
-11.55	-11.54	-11.53	-11.52	-11.51	-11.5	-11.49	-11.48	-11.47
-11.55	-11.54	-11.53	-11.52	-11.51	-11.5	-11.49	-11.48	-11.47
-11.55	-11.54	-11.53	-11.52	-11.51	-11.5	-11.49	-11.48	-11.47
-11.55	-11.54	-11.53	-11.52	-11.51	-11.5	-11.49	-11.48	-11.47
-11.55	-11.54	-11.53	-11.52	-11.51	-11.5	-11.49	-11.48	-11.47
-11.55	-11.54	-11.53	-11.52	-11.51	-11.5	-11.49	-11.48	-11.47
-11.55	-11.54	-11.53	-11.52	-11.51	-11.5	-11.49	-11.48	-11.47
-11.55	-11.54	-11.53	-11.52	-11.51	-11.5	-11.49	-11.48	-11.47
-11.55	-11.54	-11.53	-11.52	-11.51	-11.5	-11.49	-11.48	-11.47
-11.55	-11.54	-11.53	-11.52	-11.51	-11.5	-11.49	-11.48	-11.47
-11.55	-11.54	-11.53	-11.52	-11.51	-11.5	-11.49	-11.48	-11.47
-11.55	-11.54	-11.53	-11.52	-11.51	-11.5	-11.49	-11.48	-11.47
-11.55	-11.54	-11.53	-11.52	-11.51	-11.5	-11.49	-11.48	-11.47
-11.55	-11.54	-11.53	-11.52	-11.51	-11.5	-11.49	-11.48	-11.47
-11.55	-11.54	-11.53	-11.52	-11.51	-11.5	-11.49	-11.48	-11.47
-11.55	-11.54	-11.53	-11.52	-11.51	-11.5	-11.49	-11.48	-11.47
-11.55	-11.54	-11.53	-11.52	-11.51	-11.5	-11.49	-11.48	-11.47
-11.55	-11.54	-11.53	-11.52	-11.51	-11.5	-11.49	-11.48	-11.47
-11.55	-11.54	-11.53	-11.52	-11.51	-11.5	-11.49	-11.48	-11.47
-11.55	-11.54	-11.53	-11.52	-11.51	-11.5	-11.49	-11.48	-11.47
-11.55	-11.54	-11.53	-11.52	-11.51	-11.5	-11.49	-11.48	-11.47
-11.55	-11.54	-11.53	-11.52	-11.51	-11.5	-11.49	-11.48	-11.47
-11.55	-11.54	-11.53	-11.52	-11.51	-11.5	-11.49	-11.48	-11.47
-11.55	-11.54	-11.53	-11.52	-11.51	-11.5	-11.49	-11.48	-11.47
-11.55	-11.54	-11.53	-11.52	-11.51	-11.5	-11.49	-11.48	-11.47
-11.55	-11.54	-11.53	-11.52	-11.51	-11.5	-11.49	-11.48	-11.47
-11.55	-11.54	-11.53	-11.52	-11.51	-11.5	-11.49	-11.48	-11.47
-11.55	-11.54	-11.53	-11.52	-11.51	-11.5	-11.49	-11.48	-11.47
-11.55	-11.54	-11.53	-11.52	-11.51	-11.5	-11.49	-11.48	-11.47
-11.55	-11.54	-11.53	-11.52	-11.51	-11.5	-11.49	-11.48	-11.47
-11.55	-11.54	-11.53	-11.52	-11.51	-11.5	-11.49	-11.48	-11.47
-11.55	-11.54	-11.53	-11.52	-11.51	-11.5	-11.49	-11.48	-11.47

-11.55	-11.54	-11.53	-11.52	-11.51	-11.5	-11.49	-11.48	-11.47
-11.55	-11.54	-11.53	-11.52	-11.51	-11.5	-11.49	-11.48	-11.47
-11.55	-11.54	-11.53	-11.52	-11.51	-11.5	-11.49	-11.48	-11.47
-11.55	-11.54	-11.53	-11.52	-11.51	-11.5	-11.49	-11.48	-11.47
-11.55	-11.54	-11.53	-11.52	-11.51	-11.5	-11.49	-11.48	-11.47
-11.55	-11.54	-11.53	-11.52	-11.51	-11.5	-11.49	-11.48	-11.47
-11.55	-11.54	-11.53	-11.52	-11.51	-11.5	-11.49	-11.48	-11.47
-11.55	-11.54	-11.53	-11.52	-11.51	-11.5	-11.49	-11.48	-11.47
-11.55	-11.54	-11.53	-11.52	-11.51	-11.5	-11.49	-11.48	-11.47
-11.55	-11.54	-11.53	-11.52	-11.51	-11.5	-11.49	-11.48	-11.47
-11.55	-11.54	-11.53	-11.52	-11.51	-11.5	-11.49	-11.48	-11.47
-11.55	-11.54	-11.53	-11.52	-11.51	-11.5	-11.49	-11.48	-11.47
-11.55	-11.54	-11.53	-11.52	-11.51	-11.5	-11.49	-11.48	-11.47
-11.55	-11.54	-11.53	-11.52	-11.51	-11.5	-11.49	-11.48	-11.47
-11.55	-11.54	-11.53	-11.52	-11.51	-11.5	-11.49	-11.48	-11.47
-11.55	-11.54	-11.53	-11.52	-11.51	-11.5	-11.49	-11.48	-11.47
-11.55	-11.54	-11.53	-11.52	-11.51	-11.5	-11.49	-11.48	-11.47
-11.55	-11.54	-11.53	-11.52	-11.51	-11.5	-11.49	-11.48	-11.47
-11.55	-11.54	-11.53	-11.52	-11.51	-11.5	-11.49	-11.48	-11.47
-11.55	-11.54	-11.53	-11.52	-11.51	-11.5	-11.49	-11.48	-11.47
-11.55	-11.54	-11.53	-11.52	-11.51	-11.5	-11.49	-11.48	-11.47
-11.55	-11.54	-11.53	-11.52	-11.51	-11.5	-11.49	-11.48	-11.47
-11.55	-11.54	-11.53	-11.52	-11.51	-11.5	-11.49	-11.48	-11.47
-11.55	-11.54	-11.53	-11.52	-11.51	-11.5	-11.49	-11.48	-11.47
-11.55	-11.54	-11.53	-11.52	-11.51	-11.5	-11.49	-11.48	-11.47
-11.55	-11.54	-11.53	-11.52	-11.51	-11.5	-11.49	-11.48	-11.47
-11.55	-11.54	-11.53	-11.52	-11.51	-11.5	-11.49	-11.48	-11.47
-11.55	-11.54	-11.53	-11.52	-11.51	-11.5	-11.49	-11.48	-11.47
-11.55	-11.54	-11.53	-11.52	-11.51	-11.5	-11.49	-11.48	-11.47
-11.55	-11.54	-11.53	-11.52	-11.51	-11.5	-11.49	-11.48	-11.47
-11.55	-11.54	-11.53	-11.52	-11.51	-11.5	-11.49	-11.48	-11.47
-11.55	-11.54	-11.53	-11.52	-11.51	-11.5	-11.49	-11.48	-11.47
-11.55	-11.54	-11.53	-11.52	-11.51	-11.5	-11.49	-11.48	-11.47
-11.55	-11.54	-11.53	-11.52	-11.51	-11.5	-11.49	-11.48	-11.47
-11.55	-11.54	-11.53	-11.52	-11.51	-11.5	-11.49	-11.48	-11.47
-11.55	-11.54	-11.53	-11.52	-11.51	-11.5	-11.49	-11.48	-11.47
-11.55	-11.54	-11.53	-11.52	-11.51	-11.5	-11.49	-11.48	-11.47
-11.55	-11.54	-11.53	-11.52	-11.51	-11.5	-11.49	-11.48	-11.47
-11.55	-11.54	-11.53	-11.52	-11.51	-11.5	-11.49	-11.48	-11.47
-11.55	-11.54	-11.53	-11.52	-11.51	-11.5	-11.49	-11.48	-11.47
-11.55	-11.54	-11.53	-11.52	-11.51	-11.5	-11.49	-11.48	-11.47
-11.55	-11.54	-11.53	-11.52	-11.51	-11.5	-11.49	-11.48	-11.47
-11.55	-11.54	-11.53	-11.52	-11.51	-11.5	-11.49	-11.48	-11.47
-11.55	-11.54	-11.53	-11.52	-11.51	-11.5	-11.49	-11.48	-11.47
-11.55	-11.54	-11.53	-11.52	-11.51	-11.5	-11.49	-11.48	-11.47
-11.55	-11.54	-11.53	-11.52	-11.51	-11.5	-11.49	-11.48	-11.47
-11.55	-11.54	-11.53	-11.52	-11.51	-11.5	-11.49	-11.48	-11.47
-11.55	-11.54	-11.53	-11.52	-11.51	-11.5	-11.49	-11.48	-11.47
-11.55	-11.54	-11.53	-11.52	-11.51	-11.5	-11.49	-11.48	-11.47
-11.55	-11.54	-11.53	-11.52	-11.51	-11.5	-11.49	-11.48	-11.47

-11.55	-11.54	-11.53	-11.52	-11.51	-11.5	-11.49	-11.48	-11.47
-11.55	-11.54	-11.53	-11.52	-11.51	-11.5	-11.49	-11.48	-11.47
-11.55	-11.54	-11.53	-11.52	-11.51	-11.5	-11.49	-11.48	-11.47
-11.55	-11.54	-11.53	-11.52	-11.51	-11.5	-11.49	-11.48	-11.47
-11.55	-11.54	-11.53	-11.52	-11.51	-11.5	-11.49	-11.48	-11.47
-11.55	-11.54	-11.53	-11.52	-11.51	-11.5	-11.49	-11.48	-11.47
-11.55	-11.54	-11.53	-11.52	-11.51	-11.5	-11.49	-11.48	-11.47
-11.55	-11.54	-11.53	-11.52	-11.51	-11.5	-11.49	-11.48	-11.47
-11.55	-11.54	-11.53	-11.52	-11.51	-11.5	-11.49	-11.48	-11.47
-11.55	-11.54	-11.53	-11.52	-11.51	-11.5	-11.49	-11.48	-11.47
-11.55	-11.54	-11.53	-11.52	-11.51	-11.5	-11.49	-11.48	-11.47
-11.55	-11.54	-11.53	-11.52	-11.51	-11.5	-11.49	-11.48	-11.47
-11.55	-11.54	-11.53	-11.52	-11.51	-11.5	-11.49	-11.48	-11.47
-11.55	-11.54	-11.53	-11.52	-11.51	-11.5	-11.49	-11.48	-11.47
-11.55	-11.54	-11.53	-11.52	-11.51	-11.5	-11.49	-11.48	-11.47
-11.55	-11.54	-11.53	-11.52	-11.51	-11.5	-11.49	-11.48	-11.47
-11.55	-11.54	-11.53	-11.52	-11.51	-11.5	-11.49	-11.48	-11.47
-11.55	-11.54	-11.53	-11.52	-11.51	-11.5	-11.49	-11.48	-11.47
-11.55	-11.54	-11.53	-11.52	-11.51	-11.5	-11.49	-11.48	-11.47
-11.55	-11.54	-11.53	-11.52	-11.51	-11.5	-11.49	-11.48	-11.47
-11.55	-11.54	-11.53	-11.52	-11.51	-11.5	-11.49	-11.48	-11.47
-11.55	-11.54	-11.53	-11.52	-11.51	-11.5	-11.49	-11.48	-11.47
-11.55	-11.54	-11.53	-11.52	-11.51	-11.5	-11.49	-11.48	-11.47
-11.55	-11.54	-11.53	-11.52	-11.51	-11.5	-11.49	-11.48	-11.47
-11.55	-11.54	-11.53	-11.52	-11.51	-11.5	-11.49	-11.48	-11.47
-11.55	-11.54	-11.53	-11.52	-11.51	-11.5	-11.49	-11.48	-11.47
-11.55	-11.54	-11.53	-11.52	-11.51	-11.5	-11.49	-11.48	-11.47
-11.55	-11.54	-11.53	-11.52	-11.51	-11.5	-11.49	-11.48	-11.47
-11.55	-11.54	-11.53	-11.52	-11.51	-11.5	-11.49	-11.48	-11.47
-11.55	-11.54	-11.53	-11.52	-11.51	-11.5	-11.49	-11.48	-11.47
-11.55	-11.54	-11.53	-11.52	-11.51	-11.5	-11.49	-11.48	-11.47
-11.55	-11.54	-11.53	-11.52	-11.51	-11.5	-11.49	-11.48	-11.47
-11.55	-11.54	-11.53	-11.52	-11.51	-11.5	-11.49	-11.48	-11.47
-11.55	-11.54	-11.53	-11.52	-11.51	-11.5	-11.49	-11.48	-11.47
-11.55	-11.54	-11.53	-11.52	-11.51	-11.5	-11.49	-11.48	-11.47
-11.55	-11.54	-11.53	-11.52	-11.51	-11.5	-11.49	-11.48	-11.47
-11.55	-11.54	-11.53	-11.52	-11.51	-11.5	-11.49	-11.48	-11.47
-11.55	-11.54	-11.53	-11.52	-11.51	-11.5	-11.49	-11.48	-11.47
-11.55	-11.54	-11.53	-11.52	-11.51	-11.5	-11.49	-11.48	-11.47
-11.55	-11.54	-11.53	-11.52	-11.51	-11.5	-11.49	-11.48	-11.47
-11.55	-11.54	-11.53	-11.52	-11.51	-11.5	-11.49	-11.48	-11.47
-11.55	-11.54	-11.53	-11.52	-11.51	-11.5	-11.49	-11.48	-11.47
-11.55	-11.54	-11.53	-11.52	-11.51	-11.5	-11.49	-11.48	-11.47
-11.55	-11.54	-11.53	-11.52	-11.51	-11.5	-11.49	-11.48	-11.47
-11.55	-11.54	-11.53	-11.52	-11.51	-11.5	-11.49	-11.48	-11.47
-11.55	-11.54	-11.53	-11.52	-11.51	-11.5	-11.49	-11.48	-11.47
-11.55	-11.54	-11.53	-11.52	-11.51	-11.5	-11.49	-11.48	-11.47
-11.55	-11.54	-11.53	-11.52	-11.51	-11.5	-11.49	-11.48	-11.47
-11.55	-11.54	-11.53	-11.52	-11.51	-11.5	-11.49	-11.48	-11.47
-11.55	-11.54	-11.53	-11.52	-11.51	-11.5	-11.49	-11.48	-11.47

-11.55	-11.54	-11.53	-11.52	-11.51	-11.5	-11.49	-11.48	-11.47
-11.55	-11.54	-11.53	-11.52	-11.51	-11.5	-11.49	-11.48	-11.47
-11.55	-11.54	-11.53	-11.52	-11.51	-11.5	-11.49	-11.48	-11.47
-11.55	-11.54	-11.53	-11.52	-11.51	-11.5	-11.49	-11.48	-11.47
-11.55	-11.54	-11.53	-11.52	-11.51	-11.5	-11.49	-11.48	-11.47
-11.55	-11.54	-11.53	-11.52	-11.51	-11.5	-11.49	-11.48	-11.47
-11.55	-11.54	-11.53	-11.52	-11.51	-11.5	-11.49	-11.48	-11.47
-11.55	-11.54	-11.53	-11.52	-11.51	-11.5	-11.49	-11.48	-11.47
-11.55	-11.54	-11.53	-11.52	-11.51	-11.5	-11.49	-11.48	-11.47
-11.55	-11.54	-11.53	-11.52	-11.51	-11.5	-11.49	-11.48	-11.47
-11.55	-11.54	-11.53	-11.52	-11.51	-11.5	-11.49	-11.48	-11.47
-11.55	-11.54	-11.53	-11.52	-11.51	-11.5	-11.49	-11.48	-11.47
-11.55	-11.54	-11.53	-11.52	-11.51	-11.5	-11.49	-11.48	-11.47
-11.55	-11.54	-11.53	-11.52	-11.51	-11.5	-11.49	-11.48	-11.47
-11.55	-11.54	-11.53	-11.52	-11.51	-11.5	-11.49	-11.48	-11.47
-11.55	-11.54	-11.53	-11.52	-11.51	-11.5	-11.49	-11.48	-11.47
-11.55	-11.54	-11.53	-11.52	-11.51	-11.5	-11.49	-11.48	-11.47
-11.55	-11.54	-11.53	-11.52	-11.51	-11.5	-11.49	-11.48	-11.47
-11.55	-11.54	-11.53	-11.52	-11.51	-11.5	-11.49	-11.48	-11.47
-11.55	-11.54	-11.53	-11.52	-11.51	-11.5	-11.49	-11.48	-11.47
-11.55	-11.54	-11.53	-11.52	-11.51	-11.5	-11.49	-11.48	-11.47
-11.55	-11.54	-11.53	-11.52	-11.51	-11.5	-11.49	-11.48	-11.47
-11.55	-11.54	-11.53	-11.52	-11.51	-11.5	-11.49	-11.48	-11.47
-11.55	-11.54	-11.53	-11.52	-11.51	-11.5	-11.49	-11.48	-11.47
-11.55	-11.54	-11.53	-11.52	-11.51	-11.5	-11.49	-11.48	-11.47
-11.55	-11.54	-11.53	-11.52	-11.51	-11.5	-11.49	-11.48	-11.47
-11.55	-11.54	-11.53	-11.52	-11.51	-11.5	-11.49	-11.48	-11.47
-11.55	-11.54	-11.53	-11.52	-11.51	-11.5	-11.49	-11.48	-11.47
-11.55	-11.54	-11.53	-11.52	-11.51	-11.5	-11.49	-11.48	-11.47
-11.55	-11.54	-11.53	-11.52	-11.51	-11.5	-11.49	-11.48	-11.47
-11.55	-11.54	-11.53	-11.52	-11.51	-11.5	-11.49	-11.48	-11.47
-11.55	-11.54	-11.53	-11.52	-11.51	-11.5	-11.49	-11.48	-11.47
-11.55	-11.54	-11.53	-11.52	-11.51	-11.5	-11.49	-11.48	-11.47
-11.55	-11.54	-11.53	-11.52	-11.51	-11.5	-11.49	-11.48	-11.47
-11.55	-11.54	-11.53	-11.52	-11.51	-11.5	-11.49	-11.48	-11.47
-11.55	-11.54	-11.53	-11.52	-11.51	-11.5	-11.49	-11.48	-11.47
-11.55	-11.54	-11.53	-11.52	-11.51	-11.5	-11.49	-11.48	-11.47
-11.55	-11.54	-11.53	-11.52	-11.51	-11.5	-11.49	-11.48	-11.47
-11.55	-11.54	-11.53	-11.52	-11.51	-11.5	-11.49	-11.48	-11.47
-11.55	-11.54	-11.53	-11.52	-11.51	-11.5	-11.49	-11.48	-11.47
-11.55	-11.54	-11.53	-11.52	-11.51	-11.5	-11.49	-11.48	-11.47
-11.55	-11.54	-11.53	-11.52	-11.51	-11.5	-11.49	-11.48	-11.47
-11.55	-11.54	-11.53	-11.52	-11.51	-11.5	-11.49	-11.48	-11.47
-11.55	-11.54	-11.53	-11.52	-11.51	-11.5	-11.49	-11.48	-11.47
-11.55	-11.54	-11.53	-11.52	-11.51	-11.5	-11.49	-11.48	-11.47
-11.55	-11.54	-11.53	-11.52	-11.51	-11.5	-11.49	-11.48	-11.47
-11.55	-11.54	-11.53	-11.52	-11.51	-11.5	-11.49	-11.48	-11.47
-11.55	-11.54	-11.53	-11.52	-11.51	-11.5	-11.49	-11.48	-11.47
-11.55	-11.54	-11.53	-11.52	-11.51	-11.5	-11.49	-11.48	-11.47
-11.55	-11.54	-11.53	-11.52	-11.51	-11.5	-11.49	-11.48	-11.47

-11.55	-11.54	-11.53	-11.52	-11.51	-11.5	-11.49	-11.48	-11.47
-11.55	-11.54	-11.53	-11.52	-11.51	-11.5	-11.49	-11.48	-11.47
-11.55	-11.54	-11.53	-11.52	-11.51	-11.5	-11.49	-11.48	-11.47
-11.55	-11.54	-11.53	-11.52	-11.51	-11.5	-11.49	-11.48	-11.47
-11.55	-11.54	-11.53	-11.52	-11.51	-11.5	-11.49	-11.48	-11.47
-11.55	-11.54	-11.53	-11.52	-11.51	-11.5	-11.49	-11.48	-11.47
-11.55	-11.54	-11.53	-11.52	-11.51	-11.5	-11.49	-11.48	-11.47
-11.55	-11.54	-11.53	-11.52	-11.51	-11.5	-11.49	-11.48	-11.47
-11.55	-11.54	-11.53	-11.52	-11.51	-11.5	-11.49	-11.48	-11.47
-11.55	-11.54	-11.53	-11.52	-11.51	-11.5	-11.49	-11.48	-11.47
-11.55	-11.54	-11.53	-11.52	-11.51	-11.5	-11.49	-11.48	-11.47
-11.55	-11.54	-11.53	-11.52	-11.51	-11.5	-11.49	-11.48	-11.47
-11.55	-11.54	-11.53	-11.52	-11.51	-11.5	-11.49	-11.48	-11.47
-11.55	-11.54	-11.53	-11.52	-11.51	-11.5	-11.49	-11.48	-11.47
-11.55	-11.54	-11.53	-11.52	-11.51	-11.5	-11.49	-11.48	-11.47
-11.55	-11.54	-11.53	-11.52	-11.51	-11.5	-11.49	-11.48	-11.47
-11.55	-11.54	-11.53	-11.52	-11.51	-11.5	-11.49	-11.48	-11.47
-11.55	-11.54	-11.53	-11.52	-11.51	-11.5	-11.49	-11.48	-11.47
-11.55	-11.54	-11.53	-11.52	-11.51	-11.5	-11.49	-11.48	-11.47
-11.55	-11.54	-11.53	-11.52	-11.51	-11.5	-11.49	-11.48	-11.47
-11.55	-11.54	-11.53	-11.52	-11.51	-11.5	-11.49	-11.48	-11.47
-11.55	-11.54	-11.53	-11.52	-11.51	-11.5	-11.49	-11.48	-11.47
-11.55	-11.54	-11.53	-11.52	-11.51	-11.5	-11.49	-11.48	-11.47
-11.55	-11.54	-11.53	-11.52	-11.51	-11.5	-11.49	-11.48	-11.47
-11.55	-11.54	-11.53	-11.52	-11.51	-11.5	-11.49	-11.48	-11.47
-11.55	-11.54	-11.53	-11.52	-11.51	-11.5	-11.49	-11.48	-11.47
-11.55	-11.54	-11.53	-11.52	-11.51	-11.5	-11.49	-11.48	-11.47
-11.55	-11.54	-11.53	-11.52	-11.51	-11.5	-11.49	-11.48	-11.47
-11.55	-11.54	-11.53	-11.52	-11.51	-11.5	-11.49	-11.48	-11.47
-11.55	-11.54	-11.53	-11.52	-11.51	-11.5	-11.49	-11.48	-11.47
-11.55	-11.54	-11.53	-11.52	-11.51	-11.5	-11.49	-11.48	-11.47
-11.55	-11.54	-11.53	-11.52	-11.51	-11.5	-11.49	-11.48	-11.47
-11.55	-11.54	-11.53	-11.52	-11.51	-11.5	-11.49	-11.48	-11.47
-11.55	-11.54	-11.53	-11.52	-11.51	-11.5	-11.49	-11.48	-11.47
-11.55	-11.54	-11.53	-11.52	-11.51	-11.5	-11.49	-11.48	-11.47
-11.55	-11.54	-11.53	-11.52	-11.51	-11.5	-11.49	-11.48	-11.47
-11.55	-11.54	-11.53	-11.52	-11.51	-11.5	-11.49	-11.48	-11.47
-11.55	-11.54	-11.53	-11.52	-11.51	-11.5	-11.49	-11.48	-11.47
-11.55	-11.54	-11.53	-11.52	-11.51	-11.5	-11.49	-11.48	-11.47
-11.55	-11.54	-11.53	-11.52	-11.51	-11.5	-11.49	-11.48	-11.47
-11.55	-11.54	-11.53	-11.52	-11.51	-11.5	-11.49	-11.48	-11.47
-11.55	-11.54	-11.53	-11.52	-11.51	-11.5	-11.49	-11.48	-11.47
-11.55	-11.54	-11.53	-11.52	-11.51	-11.5	-11.49	-11.48	-11.47
-11.55	-11.54	-11.53	-11.52	-11.51	-11.5	-11.49	-11.48	-11.47
-11.55	-11.54	-11.53	-11.52	-11.51	-11.5	-11.49	-11.48	-11.47
-11.55	-11.54	-11.53	-11.52	-11.51	-11.5	-11.49	-11.48	-11.47
-11.55	-11.54	-11.53	-11.52	-11.51	-11.5	-11.49	-11.48	-11.47
-11.55	-11.54	-11.53	-11.52	-11.51	-11.5	-11.49	-11.48	-11.47
-11.55	-11.54	-11.53	-11.52	-11.51	-11.5	-11.49	-11.48	-11.47
-11.55	-11.54	-11.53	-11.52	-11.51	-11.5	-11.49	-11.48	-11.47

-11.55	-11.54	-11.53	-11.52	-11.51	-11.5	-11.49	-11.48	-11.47
-11.55	-11.54	-11.53	-11.52	-11.51	-11.5	-11.49	-11.48	-11.47
-11.55	-11.54	-11.53	-11.52	-11.51	-11.5	-11.49	-11.48	-11.47
-11.55	-11.54	-11.53	-11.52	-11.51	-11.5	-11.49	-11.48	-11.47
-11.55	-11.54	-11.53	-11.52	-11.51	-11.5	-11.49	-11.48	-11.47
-11.55	-11.54	-11.53	-11.52	-11.51	-11.5	-11.49	-11.48	-11.47
-11.55	-11.54	-11.53	-11.52	-11.51	-11.5	-11.49	-11.48	-11.47
-11.55	-11.54	-11.53	-11.52	-11.51	-11.5	-11.49	-11.48	-11.47
-11.55	-11.54	-11.53	-11.52	-11.51	-11.5	-11.49	-11.48	-11.47
-11.55	-11.54	-11.53	-11.52	-11.51	-11.5	-11.49	-11.48	-11.47
-11.55	-11.54	-11.53	-11.52	-11.51	-11.5	-11.49	-11.48	-11.47
-11.55	-11.54	-11.53	-11.52	-11.51	-11.5	-11.49	-11.48	-11.47
-11.55	-11.54	-11.53	-11.52	-11.51	-11.5	-11.49	-11.48	-11.47
-11.55	-11.54	-11.53	-11.52	-11.51	-11.5	-11.49	-11.48	-11.47
-11.55	-11.54	-11.53	-11.52	-11.51	-11.5	-11.49	-11.48	-11.47
-11.55	-11.54	-11.53	-11.52	-11.51	-11.5	-11.49	-11.48	-11.47
-11.55	-11.54	-11.53	-11.52	-11.51	-11.5	-11.49	-11.48	-11.47
-11.55	-11.54	-11.53	-11.52	-11.51	-11.5	-11.49	-11.48	-11.47
-11.55	-11.54	-11.53	-11.52	-11.51	-11.5	-11.49	-11.48	-11.47
-11.55	-11.54	-11.53	-11.52	-11.51	-11.5	-11.49	-11.48	-11.47
-11.55	-11.54	-11.53	-11.52	-11.51	-11.5	-11.49	-11.48	-11.47
-11.55	-11.54	-11.53	-11.52	-11.51	-11.5	-11.49	-11.48	-11.47
-11.55	-11.54	-11.53	-11.52	-11.51	-11.5	-11.49	-11.48	-11.47
-11.55	-11.54	-11.53	-11.52	-11.51	-11.5	-11.49	-11.48	-11.47
-11.55	-11.54	-11.53	-11.52	-11.51	-11.5	-11.49	-11.48	-11.47
-11.55	-11.54	-11.53	-11.52	-11.51	-11.5	-11.49	-11.48	-11.47
-11.55	-11.54	-11.53	-11.52	-11.51	-11.5	-11.49	-11.48	-11.47
-11.55	-11.54	-11.53	-11.52	-11.51	-11.5	-11.49	-11.48	-11.47
-11.55	-11.54	-11.53	-11.52	-11.51	-11.5	-11.49	-11.48	-11.47
-11.55	-11.54	-11.53	-11.52	-11.51	-11.5	-11.49	-11.48	-11.47
-11.55	-11.54	-11.53	-11.52	-11.51	-11.5	-11.49	-11.48	-11.47
-11.55	-11.54	-11.53	-11.52	-11.51	-11.5	-11.49	-11.48	-11.47
-11.55	-11.54	-11.53	-11.52	-11.51	-11.5	-11.49	-11.48	-11.47
-11.55	-11.54	-11.53	-11.52	-11.51	-11.5	-11.49	-11.48	-11.47
-11.55	-11.54	-11.53	-11.52	-11.51	-11.5	-11.49	-11.48	-11.47
-11.55	-11.54	-11.53	-11.52	-11.51	-11.5	-11.49	-11.48	-11.47
-11.55	-11.54	-11.53	-11.52	-11.51	-11.5	-11.49	-11.48	-11.47
-11.55	-11.54	-11.53	-11.52	-11.51	-11.5	-11.49	-11.48	-11.47
-11.55	-11.54	-11.53	-11.52	-11.51	-11.5	-11.49	-11.48	-11.47
-11.55	-11.54	-11.53	-11.52	-11.51	-11.5	-11.49	-11.48	-11.47
-11.55	-11.54	-11.53	-11.52	-11.51	-11.5	-11.49	-11.48	-11.47
-11.55	-11.54	-11.53	-11.52	-11.51	-11.5	-11.49	-11.48	-11.47
-11.55	-11.54	-11.53	-11.52	-11.51	-11.5	-11.49	-11.48	-11.47
-11.55	-11.54	-11.53	-11.52	-11.51	-11.5	-11.49	-11.48	-11.47
-11.55	-11.54	-11.53	-11.52	-11.51	-11.5	-11.49	-11.48	-11.47
-11.55	-11.54	-11.53	-11.52	-11.51	-11.5	-11.49	-11.48	-11.47
-11.55	-11.54	-11.53	-11.52	-11.51	-11.5	-11.49	-11.48	-11.47
-11.55	-11.54	-11.53	-11.52	-11.51	-11.5	-11.49	-11.48	-11.47
-11.55	-11.54	-11.53	-11.52	-11.51	-11.5	-11.49	-11.48	-11.47
-11.55	-11.54	-11.53	-11.52	-11.51	-11.5	-11.49	-11.48	-11.47

-11.55	-11.54	-11.53	-11.52	-11.51	-11.5	-11.49	-11.48	-11.47
-11.55	-11.54	-11.53	-11.52	-11.51	-11.5	-11.49	-11.48	-11.47
-11.55	-11.54	-11.53	-11.52	-11.51	-11.5	-11.49	-11.48	-11.47
-11.55	-11.54	-11.53	-11.52	-11.51	-11.5	-11.49	-11.48	-11.47
-11.55	-11.54	-11.53	-11.52	-11.51	-11.5	-11.49	-11.48	-11.47
-11.55	-11.54	-11.53	-11.52	-11.51	-11.5	-11.49	-11.48	-11.47
-11.55	-11.54	-11.53	-11.52	-11.51	-11.5	-11.49	-11.48	-11.47
-11.55	-11.54	-11.53	-11.52	-11.51	-11.5	-11.49	-11.48	-11.47
-11.55	-11.54	-11.53	-11.52	-11.51	-11.5	-11.49	-11.48	-11.47
-11.55	-11.54	-11.53	-11.52	-11.51	-11.5	-11.49	-11.48	-11.47
-11.55	-11.54	-11.53	-11.52	-11.51	-11.5	-11.49	-11.48	-11.47
-11.55	-11.54	-11.53	-11.52	-11.51	-11.5	-11.49	-11.48	-11.47
-11.55	-11.54	-11.53	-11.52	-11.51	-11.5	-11.49	-11.48	-11.47
-11.55	-11.54	-11.53	-11.52	-11.51	-11.5	-11.49	-11.48	-11.47
-11.55	-11.54	-11.53	-11.52	-11.51	-11.5	-11.49	-11.48	-11.47
-11.55	-11.54	-11.53	-11.52	-11.51	-11.5	-11.49	-11.48	-11.47
-11.55	-11.54	-11.53	-11.52	-11.51	-11.5	-11.49	-11.48	-11.47
-11.55	-11.54	-11.53	-11.52	-11.51	-11.5	-11.49	-11.48	-11.47
-11.55	-11.54	-11.53	-11.52	-11.51	-11.5	-11.49	-11.48	-11.47
-11.55	-11.54	-11.53	-11.52	-11.51	-11.5	-11.49	-11.48	-11.47
-11.55	-11.54	-11.53	-11.52	-11.51	-11.5	-11.49	-11.48	-11.47
-11.55	-11.54	-11.53	-11.52	-11.51	-11.5	-11.49	-11.48	-11.47
-11.55	-11.54	-11.53	-11.52	-11.51	-11.5	-11.49	-11.48	-11.47
-11.55	-11.54	-11.53	-11.52	-11.51	-11.5	-11.49	-11.48	-11.47
-11.55	-11.54	-11.53	-11.52	-11.51	-11.5	-11.49	-11.48	-11.47
-11.55	-11.54	-11.53	-11.52	-11.51	-11.5	-11.49	-11.48	-11.47
-11.55	-11.54	-11.53	-11.52	-11.51	-11.5	-11.49	-11.48	-11.47
-11.55	-11.54	-11.53	-11.52	-11.51	-11.5	-11.49	-11.48	-11.47
-11.55	-11.54	-11.53	-11.52	-11.51	-11.5	-11.49	-11.48	-11.47
-11.55	-11.54	-11.53	-11.52	-11.51	-11.5	-11.49	-11.48	-11.47
-11.55	-11.54	-11.53	-11.52	-11.51	-11.5	-11.49	-11.48	-11.47
-11.55	-11.54	-11.53	-11.52	-11.51	-11.5	-11.49	-11.48	-11.47
-11.55	-11.54	-11.53	-11.52	-11.51	-11.5	-11.49	-11.48	-11.47
-11.55	-11.54	-11.53	-11.52	-11.51	-11.5	-11.49	-11.48	-11.47
-11.55	-11.54	-11.53	-11.52	-11.51	-11.5	-11.49	-11.48	-11.47
-11.55	-11.54	-11.53	-11.52	-11.51	-11.5	-11.49	-11.48	-11.47
-11.55	-11.54	-11.53	-11.52	-11.51	-11.5	-11.49	-11.48	-11.47
-11.55	-11.54	-11.53	-11.52	-11.51	-11.5	-11.49	-11.48	-11.47
-11.55	-11.54	-11.53	-11.52	-11.51	-11.5	-11.49	-11.48	-11.47
-11.55	-11.54	-11.53	-11.52	-11.51	-11.5	-11.49	-11.48	-11.47
-11.55	-11.54	-11.53	-11.52	-11.51	-11.5	-11.49	-11.48	-11.47
-11.55	-11.54	-11.53	-11.52	-11.51	-11.5	-11.49	-11.48	-11.47
-11.55	-11.54	-11.53	-11.52	-11.51	-11.5	-11.49	-11.48	-11.47
-11.55	-11.54	-11.53	-11.52	-11.51	-11.5	-11.49	-11.48	-11.47
-11.55	-11.54	-11.53	-11.52	-11.51	-11.5	-11.49	-11.48	-11.47
-11.55	-11.54	-11.53	-11.52	-11.51	-11.5	-11.49	-11.48	-11.47
-11.55	-11.54	-11.53	-11.52	-11.51	-11.5	-11.49	-11.48	-11.47
-11.55	-11.54	-11.53	-11.52	-11.51	-11.5	-11.49	-11.48	-11.47
-11.55	-11.54	-11.53	-11.52	-11.51	-11.5	-11.49	-11.48	-11.47
-11.55	-11.54	-11.53	-11.52	-11.51	-11.5	-11.49	-11.48	-11.47

-11.55	-11.54	-11.53	-11.52	-11.51	-11.5	-11.49	-11.48	-11.47
-11.55	-11.54	-11.53	-11.52	-11.51	-11.5	-11.49	-11.48	-11.47
-11.55	-11.54	-11.53	-11.52	-11.51	-11.5	-11.49	-11.48	-11.47
-11.55	-11.54	-11.53	-11.52	-11.51	-11.5	-11.49	-11.48	-11.47
-11.55	-11.54	-11.53	-11.52	-11.51	-11.5	-11.49	-11.48	-11.47
-11.55	-11.54	-11.53	-11.52	-11.51	-11.5	-11.49	-11.48	-11.47
-11.55	-11.54	-11.53	-11.52	-11.51	-11.5	-11.49	-11.48	-11.47
-11.55	-11.54	-11.53	-11.52	-11.51	-11.5	-11.49	-11.48	-11.47
-11.55	-11.54	-11.53	-11.52	-11.51	-11.5	-11.49	-11.48	-11.47
-11.55	-11.54	-11.53	-11.52	-11.51	-11.5	-11.49	-11.48	-11.47
-11.55	-11.54	-11.53	-11.52	-11.51	-11.5	-11.49	-11.48	-11.47
-11.55	-11.54	-11.53	-11.52	-11.51	-11.5	-11.49	-11.48	-11.47
-11.55	-11.54	-11.53	-11.52	-11.51	-11.5	-11.49	-11.48	-11.47
-11.55	-11.54	-11.53	-11.52	-11.51	-11.5	-11.49	-11.48	-11.47
-11.55	-11.54	-11.53	-11.52	-11.51	-11.5	-11.49	-11.48	-11.47
-11.55	-11.54	-11.53	-11.52	-11.51	-11.5	-11.49	-11.48	-11.47
-11.55	-11.54	-11.53	-11.52	-11.51	-11.5	-11.49	-11.48	-11.47
-11.55	-11.54	-11.53	-11.52	-11.51	-11.5	-11.49	-11.48	-11.47
-11.55	-11.54	-11.53	-11.52	-11.51	-11.5	-11.49	-11.48	-11.47
-11.55	-11.54	-11.53	-11.52	-11.51	-11.5	-11.49	-11.48	-11.47
-11.55	-11.54	-11.53	-11.52	-11.51	-11.5	-11.49	-11.48	-11.47
-11.55	-11.54	-11.53	-11.52	-11.51	-11.5	-11.49	-11.48	-11.47
-11.55	-11.54	-11.53	-11.52	-11.51	-11.5	-11.49	-11.48	-11.47
-11.55	-11.54	-11.53	-11.52	-11.51	-11.5	-11.49	-11.48	-11.47
-11.55	-11.54	-11.53	-11.52	-11.51	-11.5	-11.49	-11.48	-11.47
-11.55	-11.54	-11.53	-11.52	-11.51	-11.5	-11.49	-11.48	-11.47
-11.55	-11.54	-11.53	-11.52	-11.51	-11.5	-11.49	-11.48	-11.47
-11.55	-11.54	-11.53	-11.52	-11.51	-11.5	-11.49	-11.48	-11.47
-11.55	-11.54	-11.53	-11.52	-11.51	-11.5	-11.49	-11.48	-11.47
-11.55	-11.54	-11.53	-11.52	-11.51	-11.5	-11.49	-11.48	-11.47
-11.55	-11.54	-11.53	-11.52	-11.51	-11.5	-11.49	-11.48	-11.47
-11.55	-11.54	-11.53	-11.52	-11.51	-11.5	-11.49	-11.48	-11.47
-11.55	-11.54	-11.53	-11.52	-11.51	-11.5	-11.49	-11.48	-11.47
-11.55	-11.54	-11.53	-11.52	-11.51	-11.5	-11.49	-11.48	-11.47
-11.55	-11.54	-11.53	-11.52	-11.51	-11.5	-11.49	-11.48	-11.47
-11.55	-11.54	-11.53	-11.52	-11.51	-11.5	-11.49	-11.48	-11.47
-11.55	-11.54	-11.53	-11.52	-11.51	-11.5	-11.49	-11.48	-11.47
-11.55	-11.54	-11.53	-11.52	-11.51	-11.5	-11.49	-11.48	-11.47
-11.55	-11.54	-11.53	-11.52	-11.51	-11.5	-11.49	-11.48	-11.47
-11.55	-11.54	-11.53	-11.52	-11.51	-11.5	-11.49	-11.48	-11.47
-11.55	-11.54	-11.53	-11.52	-11.51	-11.5	-11.49	-11.48	-11.47
-11.55	-11.54	-11.53	-11.52	-11.51	-11.5	-11.49	-11.48	-11.47
-11.55	-11.54	-11.53	-11.52	-11.51	-11.5	-11.49	-11.48	-11.47
-11.55	-11.54	-11.53	-11.52	-11.51	-11.5	-11.49	-11.48	-11.47
-11.55	-11.54	-11.53	-11.52	-11.51	-11.5	-11.49	-11.48	-11.47
-11.55	-11.54	-11.53	-11.52	-11.51	-11.5	-11.49	-11.48	-11.47
-11.55	-11.54	-11.53	-11.52	-11.51	-11.5	-11.49	-11.48	-11.47
-11.55	-11.54	-11.53	-11.52	-11.51	-11.5	-11.49	-11.48	-11.47
-11.55	-11.54	-11.53	-11.52	-11.51	-11.5	-11.49	-11.48	-11.47
-11.55	-11.54	-11.53	-11.52	-11.51	-11.5	-11.49	-11.48	-11.47

-11.55	-11.54	-11.53	-11.52	-11.51	-11.5	-11.49	-11.48	-11.47
-11.55	-11.54	-11.53	-11.52	-11.51	-11.5	-11.49	-11.48	-11.47
-11.55	-11.54	-11.53	-11.52	-11.51	-11.5	-11.49	-11.48	-11.47
-11.55	-11.54	-11.53	-11.52	-11.51	-11.5	-11.49	-11.48	-11.47
-11.55	-11.54	-11.53	-11.52	-11.51	-11.5	-11.49	-11.48	-11.47
-11.55	-11.54	-11.53	-11.52	-11.51	-11.5	-11.49	-11.48	-11.47
-11.55	-11.54	-11.53	-11.52	-11.51	-11.5	-11.49	-11.48	-11.47
-11.55	-11.54	-11.53	-11.52	-11.51	-11.5	-11.49	-11.48	-11.47
-11.55	-11.54	-11.53	-11.52	-11.51	-11.5	-11.49	-11.48	-11.47
-11.55	-11.54	-11.53	-11.52	-11.51	-11.5	-11.49	-11.48	-11.47
-11.55	-11.54	-11.53	-11.52	-11.51	-11.5	-11.49	-11.48	-11.47
-11.55	-11.54	-11.53	-11.52	-11.51	-11.5	-11.49	-11.48	-11.47
-11.55	-11.54	-11.53	-11.52	-11.51	-11.5	-11.49	-11.48	-11.47
-11.55	-11.54	-11.53	-11.52	-11.51	-11.5	-11.49	-11.48	-11.47
-11.55	-11.54	-11.53	-11.52	-11.51	-11.5	-11.49	-11.48	-11.47
-11.55	-11.54	-11.53	-11.52	-11.51	-11.5	-11.49	-11.48	-11.47
-11.55	-11.54	-11.53	-11.52	-11.51	-11.5	-11.49	-11.48	-11.47
-11.55	-11.54	-11.53	-11.52	-11.51	-11.5	-11.49	-11.48	-11.47
-11.55	-11.54	-11.53	-11.52	-11.51	-11.5	-11.49	-11.48	-11.47
-11.55	-11.54	-11.53	-11.52	-11.51	-11.5	-11.49	-11.48	-11.47
-11.55	-11.54	-11.53	-11.52	-11.51	-11.5	-11.49	-11.48	-11.47
-11.55	-11.54	-11.53	-11.52	-11.51	-11.5	-11.49	-11.48	-11.47
-11.55	-11.54	-11.53	-11.52	-11.51	-11.5	-11.49	-11.48	-11.47
-11.55	-11.54	-11.53	-11.52	-11.51	-11.5	-11.49	-11.48	-11.47
-11.55	-11.54	-11.53	-11.52	-11.51	-11.5	-11.49	-11.48	-11.47
-11.55	-11.54	-11.53	-11.52	-11.51	-11.5	-11.49	-11.48	-11.47
-11.55	-11.54	-11.53	-11.52	-11.51	-11.5	-11.49	-11.48	-11.47
-11.55	-11.54	-11.53	-11.52	-11.51	-11.5	-11.49	-11.48	-11.47
-11.55	-11.54	-11.53	-11.52	-11.51	-11.5	-11.49	-11.48	-11.47
-11.55	-11.54	-11.53	-11.52	-11.51	-11.5	-11.49	-11.48	-11.47
-11.55	-11.54	-11.53	-11.52	-11.51	-11.5	-11.49	-11.48	-11.47
-11.55	-11.54	-11.53	-11.52	-11.51	-11.5	-11.49	-11.48	-11.47
-11.55	-11.54	-11.53	-11.52	-11.51	-11.5	-11.49	-11.48	-11.47
-11.55	-11.54	-11.53	-11.52	-11.51	-11.5	-11.49	-11.48	-11.47
-11.55	-11.54	-11.53	-11.52	-11.51	-11.5	-11.49	-11.48	-11.47
-11.55	-11.54	-11.53	-11.52	-11.51	-11.5	-11.49	-11.48	-11.47
-11.55	-11.54	-11.53	-11.52	-11.51	-11.5	-11.49	-11.48	-11.47
-11.55	-11.54	-11.53	-11.52	-11.51	-11.5	-11.49	-11.48	-11.47
-11.55	-11.54	-11.53	-11.52	-11.51	-11.5	-11.49	-11.48	-11.47
-11.55	-11.54	-11.53	-11.52	-11.51	-11.5	-11.49	-11.48	-11.47
-11.55	-11.54	-11.53	-11.52	-11.51	-11.5	-11.49	-11.48	-11.47
-11.55	-11.54	-11.53	-11.52	-11.51	-11.5	-11.49	-11.48	-11.47
-11.55	-11.54	-11.53	-11.52	-11.51	-11.5	-11.49	-11.48	-11.47
-11.55	-11.54	-11.53	-11.52	-11.51	-11.5	-11.49	-11.48	-11.47
-11.55	-11.54	-11.53	-11.52	-11.51	-11.5	-11.49	-11.48	-11.47
-11.55	-11.54	-11.53	-11.52	-11.51	-11.5	-11.49	-11.48	-11.47
-11.55	-11.54	-11.53	-11.52	-11.51	-11.5	-11.49	-11.48	-11.47
-11.55	-11.54	-11.53	-11.52	-11.51	-11.5	-11.49	-11.48	-11.47
-11.55	-11.54	-11.53	-11.52	-11.51	-11.5	-11.49	-11.48	-11.47
-11.55	-11.54	-11.53	-11.52	-11.51	-11.5	-11.49	-11.48	-11.47

-11.55	-11.54	-11.53	-11.52	-11.51	-11.5	-11.49	-11.48	-11.47
-11.55	-11.54	-11.53	-11.52	-11.51	-11.5	-11.49	-11.48	-11.47
-11.55	-11.54	-11.53	-11.52	-11.51	-11.5	-11.49	-11.48	-11.47
-11.55	-11.54	-11.53	-11.52	-11.51	-11.5	-11.49	-11.48	-11.47
-11.55	-11.54	-11.53	-11.52	-11.51	-11.5	-11.49	-11.48	-11.47
-11.55	-11.54	-11.53	-11.52	-11.51	-11.5	-11.49	-11.48	-11.47
-11.55	-11.54	-11.53	-11.52	-11.51	-11.5	-11.49	-11.48	-11.47
-11.55	-11.54	-11.53	-11.52	-11.51	-11.5	-11.49	-11.48	-11.47
-11.55	-11.54	-11.53	-11.52	-11.51	-11.5	-11.49	-11.48	-11.47
-11.55	-11.54	-11.53	-11.52	-11.51	-11.5	-11.49	-11.48	-11.47
-11.55	-11.54	-11.53	-11.52	-11.51	-11.5	-11.49	-11.48	-11.47
-11.55	-11.54	-11.53	-11.52	-11.51	-11.5	-11.49	-11.48	-11.47
-11.55	-11.54	-11.53	-11.52	-11.51	-11.5	-11.49	-11.48	-11.47
-11.55	-11.54	-11.53	-11.52	-11.51	-11.5	-11.49	-11.48	-11.47
-11.55	-11.54	-11.53	-11.52	-11.51	-11.5	-11.49	-11.48	-11.47
-11.55	-11.54	-11.53	-11.52	-11.51	-11.5	-11.49	-11.48	-11.47
-11.55	-11.54	-11.53	-11.52	-11.51	-11.5	-11.49	-11.48	-11.47
-11.55	-11.54	-11.53	-11.52	-11.51	-11.5	-11.49	-11.48	-11.47
-11.55	-11.54	-11.53	-11.52	-11.51	-11.5	-11.49	-11.48	-11.47
-11.55	-11.54	-11.53	-11.52	-11.51	-11.5	-11.49	-11.48	-11.47
-11.55	-11.54	-11.53	-11.52	-11.51	-11.5	-11.49	-11.48	-11.47
-11.55	-11.54	-11.53	-11.52	-11.51	-11.5	-11.49	-11.48	-11.47
-11.55	-11.54	-11.53	-11.52	-11.51	-11.5	-11.49	-11.48	-11.47
-11.55	-11.54	-11.53	-11.52	-11.51	-11.5	-11.49	-11.48	-11.47
-11.55	-11.54	-11.53	-11.52	-11.51	-11.5	-11.49	-11.48	-11.47
-11.55	-11.54	-11.53	-11.52	-11.51	-11.5	-11.49	-11.48	-11.47
-11.55	-11.54	-11.53	-11.52	-11.51	-11.5	-11.49	-11.48	-11.47
-11.55	-11.54	-11.53	-11.52	-11.51	-11.5	-11.49	-11.48	-11.47
-11.55	-11.54	-11.53	-11.52	-11.51	-11.5	-11.49	-11.48	-11.47
-11.55	-11.54	-11.53	-11.52	-11.51	-11.5	-11.49	-11.48	-11.47
-11.55	-11.54	-11.53	-11.52	-11.51	-11.5	-11.49	-11.48	-11.47
-11.55	-11.54	-11.53	-11.52	-11.51	-11.5	-11.49	-11.48	-11.47
-11.55	-11.54	-11.53	-11.52	-11.51	-11.5	-11.49	-11.48	-11.47
-11.55	-11.54	-11.53	-11.52	-11.51	-11.5	-11.49	-11.48	-11.47
-11.55	-11.54	-11.53	-11.52	-11.51	-11.5	-11.49	-11.48	-11.47
-11.55	-11.54	-11.53	-11.52	-11.51	-11.5	-11.49	-11.48	-11.47
-11.55	-11.54	-11.53	-11.52	-11.51	-11.5	-11.49	-11.48	-11.47
-11.55	-11.54	-11.53	-11.52	-11.51	-11.5	-11.49	-11.48	-11.47
-11.55	-11.54	-11.53	-11.52	-11.51	-11.5	-11.49	-11.48	-11.47
-11.55	-11.54	-11.53	-11.52	-11.51	-11.5	-11.49	-11.48	-11.47
-11.55	-11.54	-11.53	-11.52	-11.51	-11.5	-11.49	-11.48	-11.47
-11.55	-11.54	-11.53	-11.52	-11.51	-11.5	-11.49	-11.48	-11.47
-11.55	-11.54	-11.53	-11.52	-11.51	-11.5	-11.49	-11.48	-11.47
-11.55	-11.54	-11.53	-11.52	-11.51	-11.5	-11.49	-11.48	-11.47
-11.55	-11.54	-11.53	-11.52	-11.51	-11.5	-11.49	-11.48	-11.47
-11.55	-11.54	-11.53	-11.52	-11.51	-11.5	-11.49	-11.48	-11.47
-11.55	-11.54	-11.53	-11.52	-11.51	-11.5	-11.49	-11.48	-11.47
-11.55	-11.54	-11.53	-11.52	-11.51	-11.5	-11.49	-11.48	-11.47
-11.55	-11.54	-11.53	-11.52	-11.51	-11.5	-11.49	-11.48	-11.47
-11.55	-11.54	-11.53	-11.52	-11.51	-11.5	-11.49	-11.48	-11.47

-11.55	-11.54	-11.53	-11.52	-11.51	-11.5	-11.49	-11.48	-11.47
-11.55	-11.54	-11.53	-11.52	-11.51	-11.5	-11.49	-11.48	-11.47
-11.55	-11.54	-11.53	-11.52	-11.51	-11.5	-11.49	-11.48	-11.47
-11.55	-11.54	-11.53	-11.52	-11.51	-11.5	-11.49	-11.48	-11.47
-11.55	-11.54	-11.53	-11.52	-11.51	-11.5	-11.49	-11.48	-11.47
-11.55	-11.54	-11.53	-11.52	-11.51	-11.5	-11.49	-11.48	-11.47
-11.55	-11.54	-11.53	-11.52	-11.51	-11.5	-11.49	-11.48	-11.47
-11.55	-11.54	-11.53	-11.52	-11.51	-11.5	-11.49	-11.48	-11.47
-11.55	-11.54	-11.53	-11.52	-11.51	-11.5	-11.49	-11.48	-11.47
-11.55	-11.54	-11.53	-11.52	-11.51	-11.5	-11.49	-11.48	-11.47
-11.55	-11.54	-11.53	-11.52	-11.51	-11.5	-11.49	-11.48	-11.47
-11.55	-11.54	-11.53	-11.52	-11.51	-11.5	-11.49	-11.48	-11.47
-11.55	-11.54	-11.53	-11.52	-11.51	-11.5	-11.49	-11.48	-11.47
-11.55	-11.54	-11.53	-11.52	-11.51	-11.5	-11.49	-11.48	-11.47
-11.55	-11.54	-11.53	-11.52	-11.51	-11.5	-11.49	-11.48	-11.47
-11.55	-11.54	-11.53	-11.52	-11.51	-11.5	-11.49	-11.48	-11.47
-11.55	-11.54	-11.53	-11.52	-11.51	-11.5	-11.49	-11.48	-11.47
-11.55	-11.54	-11.53	-11.52	-11.51	-11.5	-11.49	-11.48	-11.47
-11.55	-11.54	-11.53	-11.52	-11.51	-11.5	-11.49	-11.48	-11.47
-11.55	-11.54	-11.53	-11.52	-11.51	-11.5	-11.49	-11.48	-11.47
-11.55	-11.54	-11.53	-11.52	-11.51	-11.5	-11.49	-11.48	-11.47
-11.55	-11.54	-11.53	-11.52	-11.51	-11.5	-11.49	-11.48	-11.47
-11.55	-11.54	-11.53	-11.52	-11.51	-11.5	-11.49	-11.48	-11.47
-11.55	-11.54	-11.53	-11.52	-11.51	-11.5	-11.49	-11.48	-11.47
-11.55	-11.54	-11.53	-11.52	-11.51	-11.5	-11.49	-11.48	-11.47
-11.55	-11.54	-11.53	-11.52	-11.51	-11.5	-11.49	-11.48	-11.47
-11.55	-11.54	-11.53	-11.52	-11.51	-11.5	-11.49	-11.48	-11.47
-11.55	-11.54	-11.53	-11.52	-11.51	-11.5	-11.49	-11.48	-11.47
-11.55	-11.54	-11.53	-11.52	-11.51	-11.5	-11.49	-11.48	-11.47
-11.55	-11.54	-11.53	-11.52	-11.51	-11.5	-11.49	-11.48	-11.47
-11.55	-11.54	-11.53	-11.52	-11.51	-11.5	-11.49	-11.48	-11.47
-11.55	-11.54	-11.53	-11.52	-11.51	-11.5	-11.49	-11.48	-11.47
-11.55	-11.54	-11.53	-11.52	-11.51	-11.5	-11.49	-11.48	-11.47
-11.55	-11.54	-11.53	-11.52	-11.51	-11.5	-11.49	-11.48	-11.47
-11.55	-11.54	-11.53	-11.52	-11.51	-11.5	-11.49	-11.48	-11.47
-11.55	-11.54	-11.53	-11.52	-11.51	-11.5	-11.49	-11.48	-11.47
-11.55	-11.54	-11.53	-11.52	-11.51	-11.5	-11.49	-11.48	-11.47
-11.55	-11.54	-11.53	-11.52	-11.51	-11.5	-11.49	-11.48	-11.47
-11.55	-11.54	-11.53	-11.52	-11.51	-11.5	-11.49	-11.48	-11.47
-11.55	-11.54	-11.53	-11.52	-11.51	-11.5	-11.49	-11.48	-11.47
-11.55	-11.54	-11.53	-11.52	-11.51	-11.5	-11.49	-11.48	-11.47
-11.55	-11.54	-11.53	-11.52	-11.51	-11.5	-11.49	-11.48	-11.47
-11.55	-11.54	-11.53	-11.52	-11.51	-11.5	-11.49	-11.48	-11.47
-11.55	-11.54	-11.53	-11.52	-11.51	-11.5	-11.49	-11.48	-11.47
-11.55	-11.54	-11.53	-11.52	-11.51	-11.5	-11.49	-11.48	-11.47
-11.55	-11.54	-11.53	-11.52	-11.51	-11.5	-11.49	-11.48	-11.47
-11.55	-11.54	-11.53	-11.52	-11.51	-11.5	-11.49	-11.48	-11.47
-11.55	-11.54	-11.53	-11.52	-11.51	-11.5	-11.49	-11.48	-11.47
-11.55	-11.54	-11.53	-11.52	-11.51	-11.5	-11.49	-11.48	-11.47
-11.55	-11.54	-11.53	-11.52	-11.51	-11.5	-11.49	-11.48	-11.47

-11.55	-11.54	-11.53	-11.52	-11.51	-11.5	-11.49	-11.48	-11.47
-11.55	-11.54	-11.53	-11.52	-11.51	-11.5	-11.49	-11.48	-11.47
-11.55	-11.54	-11.53	-11.52	-11.51	-11.5	-11.49	-11.48	-11.47
-11.55	-11.54	-11.53	-11.52	-11.51	-11.5	-11.49	-11.48	-11.47
-11.55	-11.54	-11.53	-11.52	-11.51	-11.5	-11.49	-11.48	-11.47
-11.55	-11.54	-11.53	-11.52	-11.51	-11.5	-11.49	-11.48	-11.47
-11.55	-11.54	-11.53	-11.52	-11.51	-11.5	-11.49	-11.48	-11.47
-11.55	-11.54	-11.53	-11.52	-11.51	-11.5	-11.49	-11.48	-11.47
-11.55	-11.54	-11.53	-11.52	-11.51	-11.5	-11.49	-11.48	-11.47
-11.55	-11.54	-11.53	-11.52	-11.51	-11.5	-11.49	-11.48	-11.47
-11.55	-11.54	-11.53	-11.52	-11.51	-11.5	-11.49	-11.48	-11.47
-11.55	-11.54	-11.53	-11.52	-11.51	-11.5	-11.49	-11.48	-11.47
-11.55	-11.54	-11.53	-11.52	-11.51	-11.5	-11.49	-11.48	-11.47
-11.55	-11.54	-11.53	-11.52	-11.51	-11.5	-11.49	-11.48	-11.47
-11.55	-11.54	-11.53	-11.52	-11.51	-11.5	-11.49	-11.48	-11.47
-11.55	-11.54	-11.53	-11.52	-11.51	-11.5	-11.49	-11.48	-11.47
-11.55	-11.54	-11.53	-11.52	-11.51	-11.5	-11.49	-11.48	-11.47
-11.55	-11.54	-11.53	-11.52	-11.51	-11.5	-11.49	-11.48	-11.47
-11.55	-11.54	-11.53	-11.52	-11.51	-11.5	-11.49	-11.48	-11.47
-11.55	-11.54	-11.53	-11.52	-11.51	-11.5	-11.49	-11.48	-11.47
-11.55	-11.54	-11.53	-11.52	-11.51	-11.5	-11.49	-11.48	-11.47
-11.55	-11.54	-11.53	-11.52	-11.51	-11.5	-11.49	-11.48	-11.47
-11.55	-11.54	-11.53	-11.52	-11.51	-11.5	-11.49	-11.48	-11.47
-11.55	-11.54	-11.53	-11.52	-11.51	-11.5	-11.49	-11.48	-11.47
-11.55	-11.54	-11.53	-11.52	-11.51	-11.5	-11.49	-11.48	-11.47
-11.55	-11.54	-11.53	-11.52	-11.51	-11.5	-11.49	-11.48	-11.47
-11.55	-11.54	-11.53	-11.52	-11.51	-11.5	-11.49	-11.48	-11.47
-11.55	-11.54	-11.53	-11.52	-11.51	-11.5	-11.49	-11.48	-11.47
-11.55	-11.54	-11.53	-11.52	-11.51	-11.5	-11.49	-11.48	-11.47
-11.55	-11.54	-11.53	-11.52	-11.51	-11.5	-11.49	-11.48	-11.47
-11.55	-11.54	-11.53	-11.52	-11.51	-11.5	-11.49	-11.48	-11.47
-11.55	-11.54	-11.53	-11.52	-11.51	-11.5	-11.49	-11.48	-11.47
-11.55	-11.54	-11.53	-11.52	-11.51	-11.5	-11.49	-11.48	-11.47
-11.55	-11.54	-11.53	-11.52	-11.51	-11.5	-11.49	-11.48	-11.47
-11.55	-11.54	-11.53	-11.52	-11.51	-11.5	-11.49	-11.48	-11.47
-11.55	-11.54	-11.53	-11.52	-11.51	-11.5	-11.49	-11.48	-11.47
-11.55	-11.54	-11.53	-11.52	-11.51	-11.5	-11.49	-11.48	-11.47
-11.55	-11.54	-11.53	-11.52	-11.51	-11.5	-11.49	-11.48	-11.47
-11.55	-11.54	-11.53	-11.52	-11.51	-11.5	-11.49	-11.48	-11.47
-11.55	-11.54	-11.53	-11.52	-11.51	-11.5	-11.49	-11.48	-11.47
-11.55	-11.54	-11.53	-11.52	-11.51	-11.5	-11.49	-11.48	-11.47
-11.55	-11.54	-11.53	-11.52	-11.51	-11.5	-11.49	-11.48	-11.47
-11.55	-11.54	-11.53	-11.52	-11.51	-11.5	-11.49	-11.48	-11.47
-11.55	-11.54	-11.53	-11.52	-11.51	-11.5	-11.49	-11.48	-11.47
-11.55	-11.54	-11.53	-11.52	-11.51	-11.5	-11.49	-11.48	-11.47
-11.55	-11.54	-11.53	-11.52	-11.51	-11.5	-11.49	-11.48	-11.47
-11.55	-11.54	-11.53	-11.52	-11.51	-11.5	-11.49	-11.48	-11.47
-11.55	-11.54	-11.53	-11.52	-11.51	-11.5	-11.49	-11.48	-11.47
-11.55	-11.54	-11.53	-11.52	-11.51	-11.5	-11.49	-11.48	-11.47
-11.55	-11.54	-11.53	-11.52	-11.51	-11.5	-11.49	-11.48	-11.47

-11.55	-11.54	-11.53	-11.52	-11.51	-11.5	-11.49	-11.48	-11.47
-11.55	-11.54	-11.53	-11.52	-11.51	-11.5	-11.49	-11.48	-11.47
-11.55	-11.54	-11.53	-11.52	-11.51	-11.5	-11.49	-11.48	-11.47
-11.55	-11.54	-11.53	-11.52	-11.51	-11.5	-11.49	-11.48	-11.47
-11.55	-11.54	-11.53	-11.52	-11.51	-11.5	-11.49	-11.48	-11.47
-11.55	-11.54	-11.53	-11.52	-11.51	-11.5	-11.49	-11.48	-11.47
-11.55	-11.54	-11.53	-11.52	-11.51	-11.5	-11.49	-11.48	-11.47
-11.55	-11.54	-11.53	-11.52	-11.51	-11.5	-11.49	-11.48	-11.47
-11.55	-11.54	-11.53	-11.52	-11.51	-11.5	-11.49	-11.48	-11.47
-11.55	-11.54	-11.53	-11.52	-11.51	-11.5	-11.49	-11.48	-11.47
-11.55	-11.54	-11.53	-11.52	-11.51	-11.5	-11.49	-11.48	-11.47
-11.55	-11.54	-11.53	-11.52	-11.51	-11.5	-11.49	-11.48	-11.47
-11.55	-11.54	-11.53	-11.52	-11.51	-11.5	-11.49	-11.48	-11.47
-11.55	-11.54	-11.53	-11.52	-11.51	-11.5	-11.49	-11.48	-11.47
-11.55	-11.54	-11.53	-11.52	-11.51	-11.5	-11.49	-11.48	-11.47
-11.55	-11.54	-11.53	-11.52	-11.51	-11.5	-11.49	-11.48	-11.47
-11.55	-11.54	-11.53	-11.52	-11.51	-11.5	-11.49	-11.48	-11.47
-11.55	-11.54	-11.53	-11.52	-11.51	-11.5	-11.49	-11.48	-11.47
-11.55	-11.54	-11.53	-11.52	-11.51	-11.5	-11.49	-11.48	-11.47
-11.55	-11.54	-11.53	-11.52	-11.51	-11.5	-11.49	-11.48	-11.47
-11.55	-11.54	-11.53	-11.52	-11.51	-11.5	-11.49	-11.48	-11.47
-11.55	-11.54	-11.53	-11.52	-11.51	-11.5	-11.49	-11.48	-11.47
-11.55	-11.54	-11.53	-11.52	-11.51	-11.5	-11.49	-11.48	-11.47
-11.55	-11.54	-11.53	-11.52	-11.51	-11.5	-11.49	-11.48	-11.47
-11.55	-11.54	-11.53	-11.52	-11.51	-11.5	-11.49	-11.48	-11.47
-11.55	-11.54	-11.53	-11.52	-11.51	-11.5	-11.49	-11.48	-11.47
-11.55	-11.54	-11.53	-11.52	-11.51	-11.5	-11.49	-11.48	-11.47
-11.55	-11.54	-11.53	-11.52	-11.51	-11.5	-11.49	-11.48	-11.47
-11.55	-11.54	-11.53	-11.52	-11.51	-11.5	-11.49	-11.48	-11.47
-11.55	-11.54	-11.53	-11.52	-11.51	-11.5	-11.49	-11.48	-11.47
-11.55	-11.54	-11.53	-11.52	-11.51	-11.5	-11.49	-11.48	-11.47
-11.55	-11.54	-11.53	-11.52	-11.51	-11.5	-11.49	-11.48	-11.47
-11.55	-11.54	-11.53	-11.52	-11.51	-11.5	-11.49	-11.48	-11.47
-11.55	-11.54	-11.53	-11.52	-11.51	-11.5	-11.49	-11.48	-11.47
-11.55	-11.54	-11.53	-11.52	-11.51	-11.5	-11.49	-11.48	-11.47
-11.55	-11.54	-11.53	-11.52	-11.51	-11.5	-11.49	-11.48	-11.47
-11.55	-11.54	-11.53	-11.52	-11.51	-11.5	-11.49	-11.48	-11.47
-11.55	-11.54	-11.53	-11.52	-11.51	-11.5	-11.49	-11.48	-11.47
-11.55	-11.54	-11.53	-11.52	-11.51	-11.5	-11.49	-11.48	-11.47
-11.55	-11.54	-11.53	-11.52	-11.51	-11.5	-11.49	-11.48	-11.47
-11.55	-11.54	-11.53	-11.52	-11.51	-11.5	-11.49	-11.48	-11.47
-11.55	-11.54	-11.53	-11.52	-11.51	-11.5	-11.49	-11.48	-11.47
-11.55	-11.54	-11.53	-11.52	-11.51	-11.5	-11.49	-11.48	-11.47
-11.55	-11.54	-11.53	-11.52	-11.51	-11.5	-11.49	-11.48	-11.47
-11.55	-11.54	-11.53	-11.52	-11.51	-11.5	-11.49	-11.48	-11.47
-11.55	-11.54	-11.53	-11.52	-11.51	-11.5	-11.49	-11.48	-11.47
-11.55	-11.54	-11.53	-11.52	-11.51	-11.5	-11.49	-11.48	-11.47
-11.55	-11.54	-11.53	-11.52	-11.51	-11.5	-11.49	-11.48	-11.47
-11.55	-11.54	-11.53	-11.52	-11.51	-11.5	-11.49	-11.48	-11.47
-11.55	-11.54	-11.53	-11.52	-11.51	-11.5	-11.49	-11.48	-11.47

-11.55	-11.54	-11.53	-11.52	-11.51	-11.5	-11.49	-11.48	-11.47
-11.55	-11.54	-11.53	-11.52	-11.51	-11.5	-11.49	-11.48	-11.47
-11.55	-11.54	-11.53	-11.52	-11.51	-11.5	-11.49	-11.48	-11.47
-11.55	-11.54	-11.53	-11.52	-11.51	-11.5	-11.49	-11.48	-11.47
-11.55	-11.54	-11.53	-11.52	-11.51	-11.5	-11.49	-11.48	-11.47
-11.55	-11.54	-11.53	-11.52	-11.51	-11.5	-11.49	-11.48	-11.47
-11.55	-11.54	-11.53	-11.52	-11.51	-11.5	-11.49	-11.48	-11.47
-11.55	-11.54	-11.53	-11.52	-11.51	-11.5	-11.49	-11.48	-11.47
-11.55	-11.54	-11.53	-11.52	-11.51	-11.5	-11.49	-11.48	-11.47
-11.55	-11.54	-11.53	-11.52	-11.51	-11.5	-11.49	-11.48	-11.47
-11.55	-11.54	-11.53	-11.52	-11.51	-11.5	-11.49	-11.48	-11.47
-11.55	-11.54	-11.53	-11.52	-11.51	-11.5	-11.49	-11.48	-11.47
-11.55	-11.54	-11.53	-11.52	-11.51	-11.5	-11.49	-11.48	-11.47
-11.55	-11.54	-11.53	-11.52	-11.51	-11.5	-11.49	-11.48	-11.47
-11.55	-11.54	-11.53	-11.52	-11.51	-11.5	-11.49	-11.48	-11.47
-11.55	-11.54	-11.53	-11.52	-11.51	-11.5	-11.49	-11.48	-11.47
-11.55	-11.54	-11.53	-11.52	-11.51	-11.5	-11.49	-11.48	-11.47
-11.55	-11.54	-11.53	-11.52	-11.51	-11.5	-11.49	-11.48	-11.47
-11.55	-11.54	-11.53	-11.52	-11.51	-11.5	-11.49	-11.48	-11.47
-11.55	-11.54	-11.53	-11.52	-11.51	-11.5	-11.49	-11.48	-11.47
-11.55	-11.54	-11.53	-11.52	-11.51	-11.5	-11.49	-11.48	-11.47
-11.55	-11.54	-11.53	-11.52	-11.51	-11.5	-11.49	-11.48	-11.47
-11.55	-11.54	-11.53	-11.52	-11.51	-11.5	-11.49	-11.48	-11.47
-11.55	-11.54	-11.53	-11.52	-11.51	-11.5	-11.49	-11.48	-11.47
-11.55	-11.54	-11.53	-11.52	-11.51	-11.5	-11.49	-11.48	-11.47
-11.55	-11.54	-11.53	-11.52	-11.51	-11.5	-11.49	-11.48	-11.47
-11.55	-11.54	-11.53	-11.52	-11.51	-11.5	-11.49	-11.48	-11.47
-11.55	-11.54	-11.53	-11.52	-11.51	-11.5	-11.49	-11.48	-11.47
-11.55	-11.54	-11.53	-11.52	-11.51	-11.5	-11.49	-11.48	-11.47
-11.55	-11.54	-11.53	-11.52	-11.51	-11.5	-11.49	-11.48	-11.47
-11.55	-11.54	-11.53	-11.52	-11.51	-11.5	-11.49	-11.48	-11.47
-11.55	-11.54	-11.53	-11.52	-11.51	-11.5	-11.49	-11.48	-11.47
-11.55	-11.54	-11.53	-11.52	-11.51	-11.5	-11.49	-11.48	-11.47
-11.55	-11.54	-11.53	-11.52	-11.51	-11.5	-11.49	-11.48	-11.47
-11.55	-11.54	-11.53	-11.52	-11.51	-11.5	-11.49	-11.48	-11.47
-11.55	-11.54	-11.53	-11.52	-11.51	-11.5	-11.49	-11.48	-11.47
-11.55	-11.54	-11.53	-11.52	-11.51	-11.5	-11.49	-11.48	-11.47
-11.55	-11.54	-11.53	-11.52	-11.51	-11.5	-11.49	-11.48	-11.47
-11.55	-11.54	-11.53	-11.52	-11.51	-11.5	-11.49	-11.48	-11.47
-11.55	-11.54	-11.53	-11.52	-11.51	-11.5	-11.49	-11.48	-11.47
-11.55	-11.54	-11.53	-11.52	-11.51	-11.5	-11.49	-11.48	-11.47
-11.55	-11.54	-11.53	-11.52	-11.51	-11.5	-11.49	-11.48	-11.47
-11.55	-11.54	-11.53	-11.52	-11.51	-11.5	-11.49	-11.48	-11.47
-11.55	-11.54	-11.53	-11.52	-11.51	-11.5	-11.49	-11.48	-11.47
-11.55	-11.54	-11.53	-11.52	-11.51	-11.5	-11.49	-11.48	-11.47
-11.55	-11.54	-11.53	-11.52	-11.51	-11.5	-11.49	-11.48	-11.47
-11.55	-11.54	-11.53	-11.52	-11.51	-11.5	-11.49	-11.48	-11.47
-11.55	-11.54	-11.53	-11.52	-11.51	-11.5	-11.49	-11.48	-11.47
-11.55	-11.54	-11.53	-11.52	-11.51	-11.5	-11.49	-11.48	-11.47
-11.55	-11.54	-11.53	-11.52	-11.51	-11.5	-11.49	-11.48	-11.47

-11.55	-11.54	-11.53	-11.52	-11.51	-11.5	-11.49	-11.48	-11.47
-11.55	-11.54	-11.53	-11.52	-11.51	-11.5	-11.49	-11.48	-11.47
-11.55	-11.54	-11.53	-11.52	-11.51	-11.5	-11.49	-11.48	-11.47
-11.55	-11.54	-11.53	-11.52	-11.51	-11.5	-11.49	-11.48	-11.47
-11.55	-11.54	-11.53	-11.52	-11.51	-11.5	-11.49	-11.48	-11.47
-11.55	-11.54	-11.53	-11.52	-11.51	-11.5	-11.49	-11.48	-11.47
-11.55	-11.54	-11.53	-11.52	-11.51	-11.5	-11.49	-11.48	-11.47
-11.55	-11.54	-11.53	-11.52	-11.51	-11.5	-11.49	-11.48	-11.47
-11.55	-11.54	-11.53	-11.52	-11.51	-11.5	-11.49	-11.48	-11.47
-11.55	-11.54	-11.53	-11.52	-11.51	-11.5	-11.49	-11.48	-11.47
-11.55	-11.54	-11.53	-11.52	-11.51	-11.5	-11.49	-11.48	-11.47
-11.55	-11.54	-11.53	-11.52	-11.51	-11.5	-11.49	-11.48	-11.47
-11.55	-11.54	-11.53	-11.52	-11.51	-11.5	-11.49	-11.48	-11.47
-11.55	-11.54	-11.53	-11.52	-11.51	-11.5	-11.49	-11.48	-11.47
-11.55	-11.54	-11.53	-11.52	-11.51	-11.5	-11.49	-11.48	-11.47
-11.55	-11.54	-11.53	-11.52	-11.51	-11.5	-11.49	-11.48	-11.47
-11.55	-11.54	-11.53	-11.52	-11.51	-11.5	-11.49	-11.48	-11.47
-11.55	-11.54	-11.53	-11.52	-11.51	-11.5	-11.49	-11.48	-11.47
-11.55	-11.54	-11.53	-11.52	-11.51	-11.5	-11.49	-11.48	-11.47
-11.55	-11.54	-11.53	-11.52	-11.51	-11.5	-11.49	-11.48	-11.47
-11.55	-11.54	-11.53	-11.52	-11.51	-11.5	-11.49	-11.48	-11.47
-11.55	-11.54	-11.53	-11.52	-11.51	-11.5	-11.49	-11.48	-11.47
-11.55	-11.54	-11.53	-11.52	-11.51	-11.5	-11.49	-11.48	-11.47
-11.55	-11.54	-11.53	-11.52	-11.51	-11.5	-11.49	-11.48	-11.47
-11.55	-11.54	-11.53	-11.52	-11.51	-11.5	-11.49	-11.48	-11.47
-11.55	-11.54	-11.53	-11.52	-11.51	-11.5	-11.49	-11.48	-11.47
-11.55	-11.54	-11.53	-11.52	-11.51	-11.5	-11.49	-11.48	-11.47
-11.55	-11.54	-11.53	-11.52	-11.51	-11.5	-11.49	-11.48	-11.47
-11.55	-11.54	-11.53	-11.52	-11.51	-11.5	-11.49	-11.48	-11.47
-11.55	-11.54	-11.53	-11.52	-11.51	-11.5	-11.49	-11.48	-11.47
-11.55	-11.54	-11.53	-11.52	-11.51	-11.5	-11.49	-11.48	-11.47
-11.55	-11.54	-11.53	-11.52	-11.51	-11.5	-11.49	-11.48	-11.47
-11.55	-11.54	-11.53	-11.52	-11.51	-11.5	-11.49	-11.48	-11.47
-11.55	-11.54	-11.53	-11.52	-11.51	-11.5	-11.49	-11.48	-11.47
-11.55	-11.54	-11.53	-11.52	-11.51	-11.5	-11.49	-11.48	-11.47
-11.55	-11.54	-11.53	-11.52	-11.51	-11.5	-11.49	-11.48	-11.47
-11.55	-11.54	-11.53	-11.52	-11.51	-11.5	-11.49	-11.48	-11.47
-11.55	-11.54	-11.53	-11.52	-11.51	-11.5	-11.49	-11.48	-11.47
-11.55	-11.54	-11.53	-11.52	-11.51	-11.5	-11.49	-11.48	-11.47
-11.55	-11.54	-11.53	-11.52	-11.51	-11.5	-11.49	-11.48	-11.47
-11.55	-11.54	-11.53	-11.52	-11.51	-11.5	-11.49	-11.48	-11.47
-11.55	-11.54	-11.53	-11.52	-11.51	-11.5	-11.49	-11.48	-11.47
-11.55	-11.54	-11.53	-11.52	-11.51	-11.5	-11.49	-11.48	-11.47
-11.55	-11.54	-11.53	-11.52	-11.51	-11.5	-11.49	-11.48	-11.47
-11.55	-11.54	-11.53	-11.52	-11.51	-11.5	-11.49	-11.48	-11.47
-11.55	-11.54	-11.53	-11.52	-11.51	-11.5	-11.49	-11.48	-11.47
-11.55	-11.54	-11.53	-11.52	-11.51	-11.5	-11.49	-11.48	-11.47
-11.55	-11.54	-11.53	-11.52	-11.51	-11.5	-11.49	-11.48	-11.47
-11.55	-11.54	-11.53	-11.52	-11.51	-11.5	-11.49	-11.48	-11.47
-11.55	-11.54	-11.53	-11.52	-11.51	-11.5	-11.49	-11.48	-11.47

-11.55	-11.54	-11.53	-11.52	-11.51	-11.5	-11.49	-11.48	-11.47
-11.55	-11.54	-11.53	-11.52	-11.51	-11.5	-11.49	-11.48	-11.47
-11.55	-11.54	-11.53	-11.52	-11.51	-11.5	-11.49	-11.48	-11.47
-11.55	-11.54	-11.53	-11.52	-11.51	-11.5	-11.49	-11.48	-11.47
-11.55	-11.54	-11.53	-11.52	-11.51	-11.5	-11.49	-11.48	-11.47
-11.55	-11.54	-11.53	-11.52	-11.51	-11.5	-11.49	-11.48	-11.47
-11.55	-11.54	-11.53	-11.52	-11.51	-11.5	-11.49	-11.48	-11.47
-11.55	-11.54	-11.53	-11.52	-11.51	-11.5	-11.49	-11.48	-11.47
-11.55	-11.54	-11.53	-11.52	-11.51	-11.5	-11.49	-11.48	-11.47
-11.55	-11.54	-11.53	-11.52	-11.51	-11.5	-11.49	-11.48	-11.47
-11.55	-11.54	-11.53	-11.52	-11.51	-11.5	-11.49	-11.48	-11.47
-11.55	-11.54	-11.53	-11.52	-11.51	-11.5	-11.49	-11.48	-11.47
-11.55	-11.54	-11.53	-11.52	-11.51	-11.5	-11.49	-11.48	-11.47
-11.55	-11.54	-11.53	-11.52	-11.51	-11.5	-11.49	-11.48	-11.47
-11.55	-11.54	-11.53	-11.52	-11.51	-11.5	-11.49	-11.48	-11.47
-11.55	-11.54	-11.53	-11.52	-11.51	-11.5	-11.49	-11.48	-11.47
-11.55	-11.54	-11.53	-11.52	-11.51	-11.5	-11.49	-11.48	-11.47
-11.55	-11.54	-11.53	-11.52	-11.51	-11.5	-11.49	-11.48	-11.47
-11.55	-11.54	-11.53	-11.52	-11.51	-11.5	-11.49	-11.48	-11.47
-11.55	-11.54	-11.53	-11.52	-11.51	-11.5	-11.49	-11.48	-11.47
-11.55	-11.54	-11.53	-11.52	-11.51	-11.5	-11.49	-11.48	-11.47
-11.55	-11.54	-11.53	-11.52	-11.51	-11.5	-11.49	-11.48	-11.47
-11.55	-11.54	-11.53	-11.52	-11.51	-11.5	-11.49	-11.48	-11.47
-11.55	-11.54	-11.53	-11.52	-11.51	-11.5	-11.49	-11.48	-11.47
-11.55	-11.54	-11.53	-11.52	-11.51	-11.5	-11.49	-11.48	-11.47
-11.55	-11.54	-11.53	-11.52	-11.51	-11.5	-11.49	-11.48	-11.47
-11.55	-11.54	-11.53	-11.52	-11.51	-11.5	-11.49	-11.48	-11.47
-11.55	-11.54	-11.53	-11.52	-11.51	-11.5	-11.49	-11.48	-11.47
-11.55	-11.54	-11.53	-11.52	-11.51	-11.5	-11.49	-11.48	-11.47
-11.55	-11.54	-11.53	-11.52	-11.51	-11.5	-11.49	-11.48	-11.47
-11.55	-11.54	-11.53	-11.52	-11.51	-11.5	-11.49	-11.48	-11.47
-11.55	-11.54	-11.53	-11.52	-11.51	-11.5	-11.49	-11.48	-11.47
-11.55	-11.54	-11.53	-11.52	-11.51	-11.5	-11.49	-11.48	-11.47
-11.55	-11.54	-11.53	-11.52	-11.51	-11.5	-11.49	-11.48	-11.47
-11.55	-11.54	-11.53	-11.52	-11.51	-11.5	-11.49	-11.48	-11.47
-11.55	-11.54	-11.53	-11.52	-11.51	-11.5	-11.49	-11.48	-11.47
-11.55	-11.54	-11.53	-11.52	-11.51	-11.5	-11.49	-11.48	-11.47
-11.55	-11.54	-11.53	-11.52	-11.51	-11.5	-11.49	-11.48	-11.47
-11.55	-11.54	-11.53	-11.52	-11.51	-11.5	-11.49	-11.48	-11.47
-11.55	-11.54	-11.53	-11.52	-11.51	-11.5	-11.49	-11.48	-11.47
-11.55	-11.54	-11.53	-11.52	-11.51	-11.5	-11.49	-11.48	-11.47
-11.55	-11.54	-11.53	-11.52	-11.51	-11.5	-11.49	-11.48	-11.47
-11.55	-11.54	-11.53	-11.52	-11.51	-11.5	-11.49	-11.48	-11.47
-11.55	-11.54	-11.53	-11.52	-11.51	-11.5	-11.49	-11.48	-11.47
-11.55	-11.54	-11.53	-11.52	-11.51	-11.5	-11.49	-11.48	-11.47
-11.55	-11.54	-11.53	-11.52	-11.51	-11.5	-11.49	-11.48	-11.47
-11.55	-11.54	-11.53	-11.52	-11.51	-11.5	-11.49	-11.48	-11.47
-11.55	-11.54	-11.53	-11.52	-11.51	-11.5	-11.49	-11.48	-11.47
-11.55	-11.54	-11.53	-11.52	-11.51	-11.5	-11.49	-11.48	-11.47
-11.55	-11.54	-11.53	-11.52	-11.51	-11.5	-11.49	-11.48	-11.47

-11.55	-11.54	-11.53	-11.52	-11.51	-11.5	-11.49	-11.48	-11.47

-11.46	-11.45	-11.44	-11.43	-11.42	-11.41	-11.4	-11.39	-11.38
-11.46	-11.45	-11.44	-11.43	-11.42	-11.41	-11.4	-11.39	-11.38
-11.46	-11.45	-11.44	-11.43	-11.42	-11.41	-11.4	-11.39	-11.38
-11.46	-11.45	-11.44	-11.43	-11.42	-11.41	-11.4	-11.39	-11.38
-11.46	-11.45	-11.44	-11.43	-11.42	-11.41	-11.4	-11.39	-11.38
-11.46	-11.45	-11.44	-11.43	-11.42	-11.41	-11.4	-11.39	-11.38
-11.46	-11.45	-11.44	-11.43	-11.42	-11.41	-11.4	-11.39	-11.38
-11.46	-11.45	-11.44	-11.43	-11.42	-11.41	-11.4	-11.39	-11.38
-11.46	-11.45	-11.44	-11.43	-11.42	-11.41	-11.4	-11.39	-11.38
-11.46	-11.45	-11.44	-11.43	-11.42	-11.41	-11.4	-11.39	-11.38
-11.46	-11.45	-11.44	-11.43	-11.42	-11.41	-11.4	-11.39	-11.38
-11.46	-11.45	-11.44	-11.43	-11.42	-11.41	-11.4	-11.39	-11.38
-11.46	-11.45	-11.44	-11.43	-11.42	-11.41	-11.4	-11.39	-11.38
-11.46	-11.45	-11.44	-11.43	-11.42	-11.41	-11.4	-11.39	-11.38
-11.46	-11.45	-11.44	-11.43	-11.42	-11.41	-11.4	-11.39	-11.38
-11.46	-11.45	-11.44	-11.43	-11.42	-11.41	-11.4	-11.39	-11.38
-11.46	-11.45	-11.44	-11.43	-11.42	-11.41	-11.4	-11.39	-11.38
-11.46	-11.45	-11.44	-11.43	-11.42	-11.41	-11.4	-11.39	-11.38
-11.46	-11.45	-11.44	-11.43	-11.42	-11.41	-11.4	-11.39	-11.38
-11.46	-11.45	-11.44	-11.43	-11.42	-11.41	-11.4	-11.39	-11.38
-11.46	-11.45	-11.44	-11.43	-11.42	-11.41	-11.4	-11.39	-11.38
-11.46	-11.45	-11.44	-11.43	-11.42	-11.41	-11.4	-11.39	-11.38
-11.46	-11.45	-11.44	-11.43	-11.42	-11.41	-11.4	-11.39	-11.38
-11.46	-11.45	-11.44	-11.43	-11.42	-11.41	-11.4	-11.39	-11.38
-11.46	-11.45	-11.44	-11.43	-11.42	-11.41	-11.4	-11.39	-11.38
-11.46	-11.45	-11.44	-11.43	-11.42	-11.41	-11.4	-11.39	-11.38
-11.46	-11.45	-11.44	-11.43	-11.42	-11.41	-11.4	-11.39	-11.38
-11.46	-11.45	-11.44	-11.43	-11.42	-11.41	-11.4	-11.39	-11.38
-11.46	-11.45	-11.44	-11.43	-11.42	-11.41	-11.4	-11.39	-11.38
-11.46	-11.45	-11.44	-11.43	-11.42	-11.41	-11.4	-11.39	-11.38
-11.46	-11.45	-11.44	-11.43	-11.42	-11.41	-11.4	-11.39	-11.38
-11.46	-11.45	-11.44	-11.43	-11.42	-11.41	-11.4	-11.39	-11.38
-11.46	-11.45	-11.44	-11.43	-11.42	-11.41	-11.4	-11.39	-11.38
-11.46	-11.45	-11.44	-11.43	-11.42	-11.41	-11.4	-11.39	-11.38
-11.46	-11.45	-11.44	-11.43	-11.42	-11.41	-11.4	-11.39	-11.38
-11.46	-11.45	-11.44	-11.43	-11.42	-11.41	-11.4	-11.39	-11.38
-11.46	-11.45	-11.44	-11.43	-11.42	-11.41	-11.4	-11.39	-11.38
-11.46	-11.45	-11.44	-11.43	-11.42	-11.41	-11.4	-11.39	-11.38
-11.46	-11.45	-11.44	-11.43	-11.42	-11.41	-11.4	-11.39	-11.38
-11.46	-11.45	-11.44	-11.43	-11.42	-11.41	-11.4	-11.39	-11.38
-11.46	-11.45	-11.44	-11.43	-11.42	-11.41	-11.4	-11.39	-11.38
-11.46	-11.45	-11.44	-11.43	-11.42	-11.41	-11.4	-11.39	-11.38
-11.46	-11.45	-11.44	-11.43	-11.42	-11.41	-11.4	-11.39	-11.38
-11.46	-11.45	-11.44	-11.43	-11.42	-11.41	-11.4	-11.39	-11.38
-11.46	-11.45	-11.44	-11.43	-11.42	-11.41	-11.4	-11.39	-11.38
-11.46	-11.45	-11.44	-11.43	-11.42	-11.41	-11.4	-11.39	-11.38
-11.46	-11.45	-11.44	-11.43	-11.42	-11.41	-11.4	-11.39	-11.38
-11.46	-11.45	-11.44	-11.43	-11.42	-11.41	-11.4	-11.39	-11.38
-11.46	-11.45	-11.44	-11.43	-11.42	-11.41	-11.4	-11.39	-11.38
-11.46	-11.45	-11.44	-11.43	-11.42	-11.41	-11.4	-11.39	-11.38

-11.46	-11.45	-11.44	-11.43	-11.42	-11.41	-11.4	-11.39	-11.38
-11.46	-11.45	-11.44	-11.43	-11.42	-11.41	-11.4	-11.39	-11.38
-11.46	-11.45	-11.44	-11.43	-11.42	-11.41	-11.4	-11.39	-11.38
-11.46	-11.45	-11.44	-11.43	-11.42	-11.41	-11.4	-11.39	-11.38
-11.46	-11.45	-11.44	-11.43	-11.42	-11.41	-11.4	-11.39	-11.38
-11.46	-11.45	-11.44	-11.43	-11.42	-11.41	-11.4	-11.39	-11.38
-11.46	-11.45	-11.44	-11.43	-11.42	-11.41	-11.4	-11.39	-11.38
-11.46	-11.45	-11.44	-11.43	-11.42	-11.41	-11.4	-11.39	-11.38
-11.46	-11.45	-11.44	-11.43	-11.42	-11.41	-11.4	-11.39	-11.38
-11.46	-11.45	-11.44	-11.43	-11.42	-11.41	-11.4	-11.39	-11.38
-11.46	-11.45	-11.44	-11.43	-11.42	-11.41	-11.4	-11.39	-11.38
-11.46	-11.45	-11.44	-11.43	-11.42	-11.41	-11.4	-11.39	-11.38
-11.46	-11.45	-11.44	-11.43	-11.42	-11.41	-11.4	-11.39	-11.38
-11.46	-11.45	-11.44	-11.43	-11.42	-11.41	-11.4	-11.39	-11.38
-11.46	-11.45	-11.44	-11.43	-11.42	-11.41	-11.4	-11.39	-11.38
-11.46	-11.45	-11.44	-11.43	-11.42	-11.41	-11.4	-11.39	-11.38
-11.46	-11.45	-11.44	-11.43	-11.42	-11.41	-11.4	-11.39	-11.38
-11.46	-11.45	-11.44	-11.43	-11.42	-11.41	-11.4	-11.39	-11.38
-11.46	-11.45	-11.44	-11.43	-11.42	-11.41	-11.4	-11.39	-11.38
-11.46	-11.45	-11.44	-11.43	-11.42	-11.41	-11.4	-11.39	-11.38
-11.46	-11.45	-11.44	-11.43	-11.42	-11.41	-11.4	-11.39	-11.38
-11.46	-11.45	-11.44	-11.43	-11.42	-11.41	-11.4	-11.39	-11.38
-11.46	-11.45	-11.44	-11.43	-11.42	-11.41	-11.4	-11.39	-11.38
-11.46	-11.45	-11.44	-11.43	-11.42	-11.41	-11.4	-11.39	-11.38
-11.46	-11.45	-11.44	-11.43	-11.42	-11.41	-11.4	-11.39	-11.38
-11.46	-11.45	-11.44	-11.43	-11.42	-11.41	-11.4	-11.39	-11.38
-11.46	-11.45	-11.44	-11.43	-11.42	-11.41	-11.4	-11.39	-11.38
-11.46	-11.45	-11.44	-11.43	-11.42	-11.41	-11.4	-11.39	-11.38
-11.46	-11.45	-11.44	-11.43	-11.42	-11.41	-11.4	-11.39	-11.38
-11.46	-11.45	-11.44	-11.43	-11.42	-11.41	-11.4	-11.39	-11.38
-11.46	-11.45	-11.44	-11.43	-11.42	-11.41	-11.4	-11.39	-11.38
-11.46	-11.45	-11.44	-11.43	-11.42	-11.41	-11.4	-11.39	-11.38
-11.46	-11.45	-11.44	-11.43	-11.42	-11.41	-11.4	-11.39	-11.38
-11.46	-11.45	-11.44	-11.43	-11.42	-11.41	-11.4	-11.39	-11.38
-11.46	-11.45	-11.44	-11.43	-11.42	-11.41	-11.4	-11.39	-11.38
-11.46	-11.45	-11.44	-11.43	-11.42	-11.41	-11.4	-11.39	-11.38
-11.46	-11.45	-11.44	-11.43	-11.42	-11.41	-11.4	-11.39	-11.38
-11.46	-11.45	-11.44	-11.43	-11.42	-11.41	-11.4	-11.39	-11.38
-11.46	-11.45	-11.44	-11.43	-11.42	-11.41	-11.4	-11.39	-11.38
-11.46	-11.45	-11.44	-11.43	-11.42	-11.41	-11.4	-11.39	-11.38
-11.46	-11.45	-11.44	-11.43	-11.42	-11.41	-11.4	-11.39	-11.38
-11.46	-11.45	-11.44	-11.43	-11.42	-11.41	-11.4	-11.39	-11.38
-11.46	-11.45	-11.44	-11.43	-11.42	-11.41	-11.4	-11.39	-11.38
-11.46	-11.45	-11.44	-11.43	-11.42	-11.41	-11.4	-11.39	-11.38
-11.46	-11.45	-11.44	-11.43	-11.42	-11.41	-11.4	-11.39	-11.38
-11.46	-11.45	-11.44	-11.43	-11.42	-11.41	-11.4	-11.39	-11.38
-11.46	-11.45	-11.44	-11.43	-11.42	-11.41	-11.4	-11.39	-11.38
-11.46	-11.45	-11.44	-11.43	-11.42	-11.41	-11.4	-11.39	-11.38
-11.46	-11.45	-11.44	-11.43	-11.42	-11.41	-11.4	-11.39	-11.38
-11.46	-11.45	-11.44	-11.43	-11.42	-11.41	-11.4	-11.39	-11.38

-11.46	-11.45	-11.44	-11.43	-11.42	-11.41	-11.4	-11.39	-11.38
-11.46	-11.45	-11.44	-11.43	-11.42	-11.41	-11.4	-11.39	-11.38
-11.46	-11.45	-11.44	-11.43	-11.42	-11.41	-11.4	-11.39	-11.38
-11.46	-11.45	-11.44	-11.43	-11.42	-11.41	-11.4	-11.39	-11.38
-11.46	-11.45	-11.44	-11.43	-11.42	-11.41	-11.4	-11.39	-11.38
-11.46	-11.45	-11.44	-11.43	-11.42	-11.41	-11.4	-11.39	-11.38
-11.46	-11.45	-11.44	-11.43	-11.42	-11.41	-11.4	-11.39	-11.38
-11.46	-11.45	-11.44	-11.43	-11.42	-11.41	-11.4	-11.39	-11.38
-11.46	-11.45	-11.44	-11.43	-11.42	-11.41	-11.4	-11.39	-11.38
-11.46	-11.45	-11.44	-11.43	-11.42	-11.41	-11.4	-11.39	-11.38
-11.46	-11.45	-11.44	-11.43	-11.42	-11.41	-11.4	-11.39	-11.38
-11.46	-11.45	-11.44	-11.43	-11.42	-11.41	-11.4	-11.39	-11.38
-11.46	-11.45	-11.44	-11.43	-11.42	-11.41	-11.4	-11.39	-11.38
-11.46	-11.45	-11.44	-11.43	-11.42	-11.41	-11.4	-11.39	-11.38
-11.46	-11.45	-11.44	-11.43	-11.42	-11.41	-11.4	-11.39	-11.38
-11.46	-11.45	-11.44	-11.43	-11.42	-11.41	-11.4	-11.39	-11.38
-11.46	-11.45	-11.44	-11.43	-11.42	-11.41	-11.4	-11.39	-11.38
-11.46	-11.45	-11.44	-11.43	-11.42	-11.41	-11.4	-11.39	-11.38
-11.46	-11.45	-11.44	-11.43	-11.42	-11.41	-11.4	-11.39	-11.38
-11.46	-11.45	-11.44	-11.43	-11.42	-11.41	-11.4	-11.39	-11.38
-11.46	-11.45	-11.44	-11.43	-11.42	-11.41	-11.4	-11.39	-11.38
-11.46	-11.45	-11.44	-11.43	-11.42	-11.41	-11.4	-11.39	-11.38
-11.46	-11.45	-11.44	-11.43	-11.42	-11.41	-11.4	-11.39	-11.38
-11.46	-11.45	-11.44	-11.43	-11.42	-11.41	-11.4	-11.39	-11.38
-11.46	-11.45	-11.44	-11.43	-11.42	-11.41	-11.4	-11.39	-11.38
-11.46	-11.45	-11.44	-11.43	-11.42	-11.41	-11.4	-11.39	-11.38
-11.46	-11.45	-11.44	-11.43	-11.42	-11.41	-11.4	-11.39	-11.38
-11.46	-11.45	-11.44	-11.43	-11.42	-11.41	-11.4	-11.39	-11.38
-11.46	-11.45	-11.44	-11.43	-11.42	-11.41	-11.4	-11.39	-11.38
-11.46	-11.45	-11.44	-11.43	-11.42	-11.41	-11.4	-11.39	-11.38
-11.46	-11.45	-11.44	-11.43	-11.42	-11.41	-11.4	-11.39	-11.38
-11.46	-11.45	-11.44	-11.43	-11.42	-11.41	-11.4	-11.39	-11.38
-11.46	-11.45	-11.44	-11.43	-11.42	-11.41	-11.4	-11.39	-11.38
-11.46	-11.45	-11.44	-11.43	-11.42	-11.41	-11.4	-11.39	-11.38
-11.46	-11.45	-11.44	-11.43	-11.42	-11.41	-11.4	-11.39	-11.38
-11.46	-11.45	-11.44	-11.43	-11.42	-11.41	-11.4	-11.39	-11.38
-11.46	-11.45	-11.44	-11.43	-11.42	-11.41	-11.4	-11.39	-11.38
-11.46	-11.45	-11.44	-11.43	-11.42	-11.41	-11.4	-11.39	-11.38
-11.46	-11.45	-11.44	-11.43	-11.42	-11.41	-11.4	-11.39	-11.38
-11.46	-11.45	-11.44	-11.43	-11.42	-11.41	-11.4	-11.39	-11.38
-11.46	-11.45	-11.44	-11.43	-11.42	-11.41	-11.4	-11.39	-11.38
-11.46	-11.45	-11.44	-11.43	-11.42	-11.41	-11.4	-11.39	-11.38
-11.46	-11.45	-11.44	-11.43	-11.42	-11.41	-11.4	-11.39	-11.38
-11.46	-11.45	-11.44	-11.43	-11.42	-11.41	-11.4	-11.39	-11.38
-11.46	-11.45	-11.44	-11.43	-11.42	-11.41	-11.4	-11.39	-11.38
-11.46	-11.45	-11.44	-11.43	-11.42	-11.41	-11.4	-11.39	-11.38
-11.46	-11.45	-11.44	-11.43	-11.42	-11.41	-11.4	-11.39	-11.38
-11.46	-11.45	-11.44	-11.43	-11.42	-11.41	-11.4	-11.39	-11.38
-11.46	-11.45	-11.44	-11.43	-11.42	-11.41	-11.4	-11.39	-11.38
-11.46	-11.45	-11.44	-11.43	-11.42	-11.41	-11.4	-11.39	-11.38

-11.46	-11.45	-11.44	-11.43	-11.42	-11.41	-11.4	-11.39	-11.38
-11.46	-11.45	-11.44	-11.43	-11.42	-11.41	-11.4	-11.39	-11.38
-11.46	-11.45	-11.44	-11.43	-11.42	-11.41	-11.4	-11.39	-11.38
-11.46	-11.45	-11.44	-11.43	-11.42	-11.41	-11.4	-11.39	-11.38
-11.46	-11.45	-11.44	-11.43	-11.42	-11.41	-11.4	-11.39	-11.38
-11.46	-11.45	-11.44	-11.43	-11.42	-11.41	-11.4	-11.39	-11.38
-11.46	-11.45	-11.44	-11.43	-11.42	-11.41	-11.4	-11.39	-11.38
-11.46	-11.45	-11.44	-11.43	-11.42	-11.41	-11.4	-11.39	-11.38
-11.46	-11.45	-11.44	-11.43	-11.42	-11.41	-11.4	-11.39	-11.38
-11.46	-11.45	-11.44	-11.43	-11.42	-11.41	-11.4	-11.39	-11.38
-11.46	-11.45	-11.44	-11.43	-11.42	-11.41	-11.4	-11.39	-11.38
-11.46	-11.45	-11.44	-11.43	-11.42	-11.41	-11.4	-11.39	-11.38
-11.46	-11.45	-11.44	-11.43	-11.42	-11.41	-11.4	-11.39	-11.38
-11.46	-11.45	-11.44	-11.43	-11.42	-11.41	-11.4	-11.39	-11.38
-11.46	-11.45	-11.44	-11.43	-11.42	-11.41	-11.4	-11.39	-11.38
-11.46	-11.45	-11.44	-11.43	-11.42	-11.41	-11.4	-11.39	-11.38
-11.46	-11.45	-11.44	-11.43	-11.42	-11.41	-11.4	-11.39	-11.38
-11.46	-11.45	-11.44	-11.43	-11.42	-11.41	-11.4	-11.39	-11.38
-11.46	-11.45	-11.44	-11.43	-11.42	-11.41	-11.4	-11.39	-11.38
-11.46	-11.45	-11.44	-11.43	-11.42	-11.41	-11.4	-11.39	-11.38
-11.46	-11.45	-11.44	-11.43	-11.42	-11.41	-11.4	-11.39	-11.38
-11.46	-11.45	-11.44	-11.43	-11.42	-11.41	-11.4	-11.39	-11.38
-11.46	-11.45	-11.44	-11.43	-11.42	-11.41	-11.4	-11.39	-11.38
-11.46	-11.45	-11.44	-11.43	-11.42	-11.41	-11.4	-11.39	-11.38
-11.46	-11.45	-11.44	-11.43	-11.42	-11.41	-11.4	-11.39	-11.38
-11.46	-11.45	-11.44	-11.43	-11.42	-11.41	-11.4	-11.39	-11.38
-11.46	-11.45	-11.44	-11.43	-11.42	-11.41	-11.4	-11.39	-11.38
-11.46	-11.45	-11.44	-11.43	-11.42	-11.41	-11.4	-11.39	-11.38
-11.46	-11.45	-11.44	-11.43	-11.42	-11.41	-11.4	-11.39	-11.38
-11.46	-11.45	-11.44	-11.43	-11.42	-11.41	-11.4	-11.39	-11.38
-11.46	-11.45	-11.44	-11.43	-11.42	-11.41	-11.4	-11.39	-11.38
-11.46	-11.45	-11.44	-11.43	-11.42	-11.41	-11.4	-11.39	-11.38
-11.46	-11.45	-11.44	-11.43	-11.42	-11.41	-11.4	-11.39	-11.38
-11.46	-11.45	-11.44	-11.43	-11.42	-11.41	-11.4	-11.39	-11.38
-11.46	-11.45	-11.44	-11.43	-11.42	-11.41	-11.4	-11.39	-11.38
-11.46	-11.45	-11.44	-11.43	-11.42	-11.41	-11.4	-11.39	-11.38
-11.46	-11.45	-11.44	-11.43	-11.42	-11.41	-11.4	-11.39	-11.38
-11.46	-11.45	-11.44	-11.43	-11.42	-11.41	-11.4	-11.39	-11.38
-11.46	-11.45	-11.44	-11.43	-11.42	-11.41	-11.4	-11.39	-11.38
-11.46	-11.45	-11.44	-11.43	-11.42	-11.41	-11.4	-11.39	-11.38
-11.46	-11.45	-11.44	-11.43	-11.42	-11.41	-11.4	-11.39	-11.38
-11.46	-11.45	-11.44	-11.43	-11.42	-11.41	-11.4	-11.39	-11.38
-11.46	-11.45	-11.44	-11.43	-11.42	-11.41	-11.4	-11.39	-11.38
-11.46	-11.45	-11.44	-11.43	-11.42	-11.41	-11.4	-11.39	-11.38
-11.46	-11.45	-11.44	-11.43	-11.42	-11.41	-11.4	-11.39	-11.38
-11.46	-11.45	-11.44	-11.43	-11.42	-11.41	-11.4	-11.39	-11.38
-11.46	-11.45	-11.44	-11.43	-11.42	-11.41	-11.4	-11.39	-11.38
-11.46	-11.45	-11.44	-11.43	-11.42	-11.41	-11.4	-11.39	-11.38
-11.46	-11.45	-11.44	-11.43	-11.42	-11.41	-11.4	-11.39	-11.38
-11.46	-11.45	-11.44	-11.43	-11.42	-11.41	-11.4	-11.39	-11.38

-11.46	-11.45	-11.44	-11.43	-11.42	-11.41	-11.4	-11.39	-11.38
-11.46	-11.45	-11.44	-11.43	-11.42	-11.41	-11.4	-11.39	-11.38
-11.46	-11.45	-11.44	-11.43	-11.42	-11.41	-11.4	-11.39	-11.38
-11.46	-11.45	-11.44	-11.43	-11.42	-11.41	-11.4	-11.39	-11.38
-11.46	-11.45	-11.44	-11.43	-11.42	-11.41	-11.4	-11.39	-11.38
-11.46	-11.45	-11.44	-11.43	-11.42	-11.41	-11.4	-11.39	-11.38
-11.46	-11.45	-11.44	-11.43	-11.42	-11.41	-11.4	-11.39	-11.38
-11.46	-11.45	-11.44	-11.43	-11.42	-11.41	-11.4	-11.39	-11.38
-11.46	-11.45	-11.44	-11.43	-11.42	-11.41	-11.4	-11.39	-11.38
-11.46	-11.45	-11.44	-11.43	-11.42	-11.41	-11.4	-11.39	-11.38
-11.46	-11.45	-11.44	-11.43	-11.42	-11.41	-11.4	-11.39	-11.38
-11.46	-11.45	-11.44	-11.43	-11.42	-11.41	-11.4	-11.39	-11.38
-11.46	-11.45	-11.44	-11.43	-11.42	-11.41	-11.4	-11.39	-11.38
-11.46	-11.45	-11.44	-11.43	-11.42	-11.41	-11.4	-11.39	-11.38
-11.46	-11.45	-11.44	-11.43	-11.42	-11.41	-11.4	-11.39	-11.38
-11.46	-11.45	-11.44	-11.43	-11.42	-11.41	-11.4	-11.39	-11.38
-11.46	-11.45	-11.44	-11.43	-11.42	-11.41	-11.4	-11.39	-11.38
-11.46	-11.45	-11.44	-11.43	-11.42	-11.41	-11.4	-11.39	-11.38
-11.46	-11.45	-11.44	-11.43	-11.42	-11.41	-11.4	-11.39	-11.38
-11.46	-11.45	-11.44	-11.43	-11.42	-11.41	-11.4	-11.39	-11.38
-11.46	-11.45	-11.44	-11.43	-11.42	-11.41	-11.4	-11.39	-11.38
-11.46	-11.45	-11.44	-11.43	-11.42	-11.41	-11.4	-11.39	-11.38
-11.46	-11.45	-11.44	-11.43	-11.42	-11.41	-11.4	-11.39	-11.38
-11.46	-11.45	-11.44	-11.43	-11.42	-11.41	-11.4	-11.39	-11.38
-11.46	-11.45	-11.44	-11.43	-11.42	-11.41	-11.4	-11.39	-11.38
-11.46	-11.45	-11.44	-11.43	-11.42	-11.41	-11.4	-11.39	-11.38
-11.46	-11.45	-11.44	-11.43	-11.42	-11.41	-11.4	-11.39	-11.38
-11.46	-11.45	-11.44	-11.43	-11.42	-11.41	-11.4	-11.39	-11.38
-11.46	-11.45	-11.44	-11.43	-11.42	-11.41	-11.4	-11.39	-11.38
-11.46	-11.45	-11.44	-11.43	-11.42	-11.41	-11.4	-11.39	-11.38
-11.46	-11.45	-11.44	-11.43	-11.42	-11.41	-11.4	-11.39	-11.38
-11.46	-11.45	-11.44	-11.43	-11.42	-11.41	-11.4	-11.39	-11.38
-11.46	-11.45	-11.44	-11.43	-11.42	-11.41	-11.4	-11.39	-11.38
-11.46	-11.45	-11.44	-11.43	-11.42	-11.41	-11.4	-11.39	-11.38
-11.46	-11.45	-11.44	-11.43	-11.42	-11.41	-11.4	-11.39	-11.38
-11.46	-11.45	-11.44	-11.43	-11.42	-11.41	-11.4	-11.39	-11.38
-11.46	-11.45	-11.44	-11.43	-11.42	-11.41	-11.4	-11.39	-11.38
-11.46	-11.45	-11.44	-11.43	-11.42	-11.41	-11.4	-11.39	-11.38
-11.46	-11.45	-11.44	-11.43	-11.42	-11.41	-11.4	-11.39	-11.38
-11.46	-11.45	-11.44	-11.43	-11.42	-11.41	-11.4	-11.39	-11.38
-11.46	-11.45	-11.44	-11.43	-11.42	-11.41	-11.4	-11.39	-11.38
-11.46	-11.45	-11.44	-11.43	-11.42	-11.41	-11.4	-11.39	-11.38
-11.46	-11.45	-11.44	-11.43	-11.42	-11.41	-11.4	-11.39	-11.38
-11.46	-11.45	-11.44	-11.43	-11.42	-11.41	-11.4	-11.39	-11.38
-11.46	-11.45	-11.44	-11.43	-11.42	-11.41	-11.4	-11.39	-11.38
-11.46	-11.45	-11.44	-11.43	-11.42	-11.41	-11.4	-11.39	-11.38
-11.46	-11.45	-11.44	-11.43	-11.42	-11.41	-11.4	-11.39	-11.38
-11.46	-11.45	-11.44	-11.43	-11.42	-11.41	-11.4	-11.39	-11.38
-11.46	-11.45	-11.44	-11.43	-11.42	-11.41	-11.4	-11.39	-11.38
-11.46	-11.45	-11.44	-11.43	-11.42	-11.41	-11.4	-11.39	-11.38

-11.46	-11.45	-11.44	-11.43	-11.42	-11.41	-11.4	-11.39	-11.38
-11.46	-11.45	-11.44	-11.43	-11.42	-11.41	-11.4	-11.39	-11.38
-11.46	-11.45	-11.44	-11.43	-11.42	-11.41	-11.4	-11.39	-11.38
-11.46	-11.45	-11.44	-11.43	-11.42	-11.41	-11.4	-11.39	-11.38
-11.46	-11.45	-11.44	-11.43	-11.42	-11.41	-11.4	-11.39	-11.38
-11.46	-11.45	-11.44	-11.43	-11.42	-11.41	-11.4	-11.39	-11.38
-11.46	-11.45	-11.44	-11.43	-11.42	-11.41	-11.4	-11.39	-11.38
-11.46	-11.45	-11.44	-11.43	-11.42	-11.41	-11.4	-11.39	-11.38
-11.46	-11.45	-11.44	-11.43	-11.42	-11.41	-11.4	-11.39	-11.38
-11.46	-11.45	-11.44	-11.43	-11.42	-11.41	-11.4	-11.39	-11.38
-11.46	-11.45	-11.44	-11.43	-11.42	-11.41	-11.4	-11.39	-11.38
-11.46	-11.45	-11.44	-11.43	-11.42	-11.41	-11.4	-11.39	-11.38
-11.46	-11.45	-11.44	-11.43	-11.42	-11.41	-11.4	-11.39	-11.38
-11.46	-11.45	-11.44	-11.43	-11.42	-11.41	-11.4	-11.39	-11.38
-11.46	-11.45	-11.44	-11.43	-11.42	-11.41	-11.4	-11.39	-11.38
-11.46	-11.45	-11.44	-11.43	-11.42	-11.41	-11.4	-11.39	-11.38
-11.46	-11.45	-11.44	-11.43	-11.42	-11.41	-11.4	-11.39	-11.38
-11.46	-11.45	-11.44	-11.43	-11.42	-11.41	-11.4	-11.39	-11.38
-11.46	-11.45	-11.44	-11.43	-11.42	-11.41	-11.4	-11.39	-11.38
-11.46	-11.45	-11.44	-11.43	-11.42	-11.41	-11.4	-11.39	-11.38
-11.46	-11.45	-11.44	-11.43	-11.42	-11.41	-11.4	-11.39	-11.38
-11.46	-11.45	-11.44	-11.43	-11.42	-11.41	-11.4	-11.39	-11.38
-11.46	-11.45	-11.44	-11.43	-11.42	-11.41	-11.4	-11.39	-11.38
-11.46	-11.45	-11.44	-11.43	-11.42	-11.41	-11.4	-11.39	-11.38
-11.46	-11.45	-11.44	-11.43	-11.42	-11.41	-11.4	-11.39	-11.38
-11.46	-11.45	-11.44	-11.43	-11.42	-11.41	-11.4	-11.39	-11.38
-11.46	-11.45	-11.44	-11.43	-11.42	-11.41	-11.4	-11.39	-11.38
-11.46	-11.45	-11.44	-11.43	-11.42	-11.41	-11.4	-11.39	-11.38
-11.46	-11.45	-11.44	-11.43	-11.42	-11.41	-11.4	-11.39	-11.38
-11.46	-11.45	-11.44	-11.43	-11.42	-11.41	-11.4	-11.39	-11.38
-11.46	-11.45	-11.44	-11.43	-11.42	-11.41	-11.4	-11.39	-11.38
-11.46	-11.45	-11.44	-11.43	-11.42	-11.41	-11.4	-11.39	-11.38
-11.46	-11.45	-11.44	-11.43	-11.42	-11.41	-11.4	-11.39	-11.38
-11.46	-11.45	-11.44	-11.43	-11.42	-11.41	-11.4	-11.39	-11.38
-11.46	-11.45	-11.44	-11.43	-11.42	-11.41	-11.4	-11.39	-11.38
-11.46	-11.45	-11.44	-11.43	-11.42	-11.41	-11.4	-11.39	-11.38
-11.46	-11.45	-11.44	-11.43	-11.42	-11.41	-11.4	-11.39	-11.38
-11.46	-11.45	-11.44	-11.43	-11.42	-11.41	-11.4	-11.39	-11.38
-11.46	-11.45	-11.44	-11.43	-11.42	-11.41	-11.4	-11.39	-11.38
-11.46	-11.45	-11.44	-11.43	-11.42	-11.41	-11.4	-11.39	-11.38
-11.46	-11.45	-11.44	-11.43	-11.42	-11.41	-11.4	-11.39	-11.38
-11.46	-11.45	-11.44	-11.43	-11.42	-11.41	-11.4	-11.39	-11.38
-11.46	-11.45	-11.44	-11.43	-11.42	-11.41	-11.4	-11.39	-11.38
-11.46	-11.45	-11.44	-11.43	-11.42	-11.41	-11.4	-11.39	-11.38
-11.46	-11.45	-11.44	-11.43	-11.42	-11.41	-11.4	-11.39	-11.38
-11.46	-11.45	-11.44	-11.43	-11.42	-11.41	-11.4	-11.39	-11.38
-11.46	-11.45	-11.44	-11.43	-11.42	-11.41	-11.4	-11.39	-11.38
-11.46	-11.45	-11.44	-11.43	-11.42	-11.41	-11.4	-11.39	-11.38
-11.46	-11.45	-11.44	-11.43	-11.42	-11.41	-11.4	-11.39	-11.38
-11.46	-11.45	-11.44	-11.43	-11.42	-11.41	-11.4	-11.39	-11.38

-11.46	-11.45	-11.44	-11.43	-11.42	-11.41	-11.4	-11.39	-11.38
-11.46	-11.45	-11.44	-11.43	-11.42	-11.41	-11.4	-11.39	-11.38
-11.46	-11.45	-11.44	-11.43	-11.42	-11.41	-11.4	-11.39	-11.38
-11.46	-11.45	-11.44	-11.43	-11.42	-11.41	-11.4	-11.39	-11.38
-11.46	-11.45	-11.44	-11.43	-11.42	-11.41	-11.4	-11.39	-11.38
-11.46	-11.45	-11.44	-11.43	-11.42	-11.41	-11.4	-11.39	-11.38
-11.46	-11.45	-11.44	-11.43	-11.42	-11.41	-11.4	-11.39	-11.38
-11.46	-11.45	-11.44	-11.43	-11.42	-11.41	-11.4	-11.39	-11.38
-11.46	-11.45	-11.44	-11.43	-11.42	-11.41	-11.4	-11.39	-11.38
-11.46	-11.45	-11.44	-11.43	-11.42	-11.41	-11.4	-11.39	-11.38
-11.46	-11.45	-11.44	-11.43	-11.42	-11.41	-11.4	-11.39	-11.38
-11.46	-11.45	-11.44	-11.43	-11.42	-11.41	-11.4	-11.39	-11.38
-11.46	-11.45	-11.44	-11.43	-11.42	-11.41	-11.4	-11.39	-11.38
-11.46	-11.45	-11.44	-11.43	-11.42	-11.41	-11.4	-11.39	-11.38
-11.46	-11.45	-11.44	-11.43	-11.42	-11.41	-11.4	-11.39	-11.38
-11.46	-11.45	-11.44	-11.43	-11.42	-11.41	-11.4	-11.39	-11.38
-11.46	-11.45	-11.44	-11.43	-11.42	-11.41	-11.4	-11.39	-11.38
-11.46	-11.45	-11.44	-11.43	-11.42	-11.41	-11.4	-11.39	-11.38
-11.46	-11.45	-11.44	-11.43	-11.42	-11.41	-11.4	-11.39	-11.38
-11.46	-11.45	-11.44	-11.43	-11.42	-11.41	-11.4	-11.39	-11.38
-11.46	-11.45	-11.44	-11.43	-11.42	-11.41	-11.4	-11.39	-11.38
-11.46	-11.45	-11.44	-11.43	-11.42	-11.41	-11.4	-11.39	-11.38
-11.46	-11.45	-11.44	-11.43	-11.42	-11.41	-11.4	-11.39	-11.38
-11.46	-11.45	-11.44	-11.43	-11.42	-11.41	-11.4	-11.39	-11.38
-11.46	-11.45	-11.44	-11.43	-11.42	-11.41	-11.4	-11.39	-11.38
-11.46	-11.45	-11.44	-11.43	-11.42	-11.41	-11.4	-11.39	-11.38
-11.46	-11.45	-11.44	-11.43	-11.42	-11.41	-11.4	-11.39	-11.38
-11.46	-11.45	-11.44	-11.43	-11.42	-11.41	-11.4	-11.39	-11.38
-11.46	-11.45	-11.44	-11.43	-11.42	-11.41	-11.4	-11.39	-11.38
-11.46	-11.45	-11.44	-11.43	-11.42	-11.41	-11.4	-11.39	-11.38
-11.46	-11.45	-11.44	-11.43	-11.42	-11.41	-11.4	-11.39	-11.38
-11.46	-11.45	-11.44	-11.43	-11.42	-11.41	-11.4	-11.39	-11.38
-11.46	-11.45	-11.44	-11.43	-11.42	-11.41	-11.4	-11.39	-11.38
-11.46	-11.45	-11.44	-11.43	-11.42	-11.41	-11.4	-11.39	-11.38
-11.46	-11.45	-11.44	-11.43	-11.42	-11.41	-11.4	-11.39	-11.38
-11.46	-11.45	-11.44	-11.43	-11.42	-11.41	-11.4	-11.39	-11.38
-11.46	-11.45	-11.44	-11.43	-11.42	-11.41	-11.4	-11.39	-11.38
-11.46	-11.45	-11.44	-11.43	-11.42	-11.41	-11.4	-11.39	-11.38
-11.46	-11.45	-11.44	-11.43	-11.42	-11.41	-11.4	-11.39	-11.38
-11.46	-11.45	-11.44	-11.43	-11.42	-11.41	-11.4	-11.39	-11.38
-11.46	-11.45	-11.44	-11.43	-11.42	-11.41	-11.4	-11.39	-11.38
-11.46	-11.45	-11.44	-11.43	-11.42	-11.41	-11.4	-11.39	-11.38
-11.46	-11.45	-11.44	-11.43	-11.42	-11.41	-11.4	-11.39	-11.38
-11.46	-11.45	-11.44	-11.43	-11.42	-11.41	-11.4	-11.39	-11.38
-11.46	-11.45	-11.44	-11.43	-11.42	-11.41	-11.4	-11.39	-11.38
-11.46	-11.45	-11.44	-11.43	-11.42	-11.41	-11.4	-11.39	-11.38
-11.46	-11.45	-11.44	-11.43	-11.42	-11.41	-11.4	-11.39	-11.38
-11.46	-11.45	-11.44	-11.43	-11.42	-11.41	-11.4	-11.39	-11.38
-11.46	-11.45	-11.44	-11.43	-11.42	-11.41	-11.4	-11.39	-11.38
-11.46	-11.45	-11.44	-11.43	-11.42	-11.41	-11.4	-11.39	-11.38

-11.46	-11.45	-11.44	-11.43	-11.42	-11.41	-11.4	-11.39	-11.38
-11.46	-11.45	-11.44	-11.43	-11.42	-11.41	-11.4	-11.39	-11.38
-11.46	-11.45	-11.44	-11.43	-11.42	-11.41	-11.4	-11.39	-11.38
-11.46	-11.45	-11.44	-11.43	-11.42	-11.41	-11.4	-11.39	-11.38
-11.46	-11.45	-11.44	-11.43	-11.42	-11.41	-11.4	-11.39	-11.38
-11.46	-11.45	-11.44	-11.43	-11.42	-11.41	-11.4	-11.39	-11.38
-11.46	-11.45	-11.44	-11.43	-11.42	-11.41	-11.4	-11.39	-11.38
-11.46	-11.45	-11.44	-11.43	-11.42	-11.41	-11.4	-11.39	-11.38
-11.46	-11.45	-11.44	-11.43	-11.42	-11.41	-11.4	-11.39	-11.38
-11.46	-11.45	-11.44	-11.43	-11.42	-11.41	-11.4	-11.39	-11.38
-11.46	-11.45	-11.44	-11.43	-11.42	-11.41	-11.4	-11.39	-11.38
-11.46	-11.45	-11.44	-11.43	-11.42	-11.41	-11.4	-11.39	-11.38
-11.46	-11.45	-11.44	-11.43	-11.42	-11.41	-11.4	-11.39	-11.38
-11.46	-11.45	-11.44	-11.43	-11.42	-11.41	-11.4	-11.39	-11.38
-11.46	-11.45	-11.44	-11.43	-11.42	-11.41	-11.4	-11.39	-11.38
-11.46	-11.45	-11.44	-11.43	-11.42	-11.41	-11.4	-11.39	-11.38
-11.46	-11.45	-11.44	-11.43	-11.42	-11.41	-11.4	-11.39	-11.38
-11.46	-11.45	-11.44	-11.43	-11.42	-11.41	-11.4	-11.39	-11.38
-11.46	-11.45	-11.44	-11.43	-11.42	-11.41	-11.4	-11.39	-11.38
-11.46	-11.45	-11.44	-11.43	-11.42	-11.41	-11.4	-11.39	-11.38
-11.46	-11.45	-11.44	-11.43	-11.42	-11.41	-11.4	-11.39	-11.38
-11.46	-11.45	-11.44	-11.43	-11.42	-11.41	-11.4	-11.39	-11.38
-11.46	-11.45	-11.44	-11.43	-11.42	-11.41	-11.4	-11.39	-11.38
-11.46	-11.45	-11.44	-11.43	-11.42	-11.41	-11.4	-11.39	-11.38
-11.46	-11.45	-11.44	-11.43	-11.42	-11.41	-11.4	-11.39	-11.38
-11.46	-11.45	-11.44	-11.43	-11.42	-11.41	-11.4	-11.39	-11.38
-11.46	-11.45	-11.44	-11.43	-11.42	-11.41	-11.4	-11.39	-11.38
-11.46	-11.45	-11.44	-11.43	-11.42	-11.41	-11.4	-11.39	-11.38
-11.46	-11.45	-11.44	-11.43	-11.42	-11.41	-11.4	-11.39	-11.38
-11.46	-11.45	-11.44	-11.43	-11.42	-11.41	-11.4	-11.39	-11.38
-11.46	-11.45	-11.44	-11.43	-11.42	-11.41	-11.4	-11.39	-11.38
-11.46	-11.45	-11.44	-11.43	-11.42	-11.41	-11.4	-11.39	-11.38
-11.46	-11.45	-11.44	-11.43	-11.42	-11.41	-11.4	-11.39	-11.38
-11.46	-11.45	-11.44	-11.43	-11.42	-11.41	-11.4	-11.39	-11.38
-11.46	-11.45	-11.44	-11.43	-11.42	-11.41	-11.4	-11.39	-11.38
-11.46	-11.45	-11.44	-11.43	-11.42	-11.41	-11.4	-11.39	-11.38
-11.46	-11.45	-11.44	-11.43	-11.42	-11.41	-11.4	-11.39	-11.38
-11.46	-11.45	-11.44	-11.43	-11.42	-11.41	-11.4	-11.39	-11.38
-11.46	-11.45	-11.44	-11.43	-11.42	-11.41	-11.4	-11.39	-11.38
-11.46	-11.45	-11.44	-11.43	-11.42	-11.41	-11.4	-11.39	-11.38
-11.46	-11.45	-11.44	-11.43	-11.42	-11.41	-11.4	-11.39	-11.38
-11.46	-11.45	-11.44	-11.43	-11.42	-11.41	-11.4	-11.39	-11.38
-11.46	-11.45	-11.44	-11.43	-11.42	-11.41	-11.4	-11.39	-11.38
-11.46	-11.45	-11.44	-11.43	-11.42	-11.41	-11.4	-11.39	-11.38
-11.46	-11.45	-11.44	-11.43	-11.42	-11.41	-11.4	-11.39	-11.38
-11.46	-11.45	-11.44	-11.43	-11.42	-11.41	-11.4	-11.39	-11.38
-11.46	-11.45	-11.44	-11.43	-11.42	-11.41	-11.4	-11.39	-11.38
-11.46	-11.45	-11.44	-11.43	-11.42	-11.41	-11.4	-11.39	-11.38
-11.46	-11.45	-11.44	-11.43	-11.42	-11.41	-11.4	-11.39	-11.38
-11.46	-11.45	-11.44	-11.43	-11.42	-11.41	-11.4	-11.39	-11.38

-11.46	-11.45	-11.44	-11.43	-11.42	-11.41	-11.4	-11.39	-11.38
-11.46	-11.45	-11.44	-11.43	-11.42	-11.41	-11.4	-11.39	-11.38
-11.46	-11.45	-11.44	-11.43	-11.42	-11.41	-11.4	-11.39	-11.38
-11.46	-11.45	-11.44	-11.43	-11.42	-11.41	-11.4	-11.39	-11.38
-11.46	-11.45	-11.44	-11.43	-11.42	-11.41	-11.4	-11.39	-11.38
-11.46	-11.45	-11.44	-11.43	-11.42	-11.41	-11.4	-11.39	-11.38
-11.46	-11.45	-11.44	-11.43	-11.42	-11.41	-11.4	-11.39	-11.38
-11.46	-11.45	-11.44	-11.43	-11.42	-11.41	-11.4	-11.39	-11.38
-11.46	-11.45	-11.44	-11.43	-11.42	-11.41	-11.4	-11.39	-11.38
-11.46	-11.45	-11.44	-11.43	-11.42	-11.41	-11.4	-11.39	-11.38
-11.46	-11.45	-11.44	-11.43	-11.42	-11.41	-11.4	-11.39	-11.38
-11.46	-11.45	-11.44	-11.43	-11.42	-11.41	-11.4	-11.39	-11.38
-11.46	-11.45	-11.44	-11.43	-11.42	-11.41	-11.4	-11.39	-11.38
-11.46	-11.45	-11.44	-11.43	-11.42	-11.41	-11.4	-11.39	-11.38
-11.46	-11.45	-11.44	-11.43	-11.42	-11.41	-11.4	-11.39	-11.38
-11.46	-11.45	-11.44	-11.43	-11.42	-11.41	-11.4	-11.39	-11.38
-11.46	-11.45	-11.44	-11.43	-11.42	-11.41	-11.4	-11.39	-11.38
-11.46	-11.45	-11.44	-11.43	-11.42	-11.41	-11.4	-11.39	-11.38
-11.46	-11.45	-11.44	-11.43	-11.42	-11.41	-11.4	-11.39	-11.38
-11.46	-11.45	-11.44	-11.43	-11.42	-11.41	-11.4	-11.39	-11.38
-11.46	-11.45	-11.44	-11.43	-11.42	-11.41	-11.4	-11.39	-11.38
-11.46	-11.45	-11.44	-11.43	-11.42	-11.41	-11.4	-11.39	-11.38
-11.46	-11.45	-11.44	-11.43	-11.42	-11.41	-11.4	-11.39	-11.38
-11.46	-11.45	-11.44	-11.43	-11.42	-11.41	-11.4	-11.39	-11.38
-11.46	-11.45	-11.44	-11.43	-11.42	-11.41	-11.4	-11.39	-11.38
-11.46	-11.45	-11.44	-11.43	-11.42	-11.41	-11.4	-11.39	-11.38
-11.46	-11.45	-11.44	-11.43	-11.42	-11.41	-11.4	-11.39	-11.38
-11.46	-11.45	-11.44	-11.43	-11.42	-11.41	-11.4	-11.39	-11.38
-11.46	-11.45	-11.44	-11.43	-11.42	-11.41	-11.4	-11.39	-11.38
-11.46	-11.45	-11.44	-11.43	-11.42	-11.41	-11.4	-11.39	-11.38
-11.46	-11.45	-11.44	-11.43	-11.42	-11.41	-11.4	-11.39	-11.38
-11.46	-11.45	-11.44	-11.43	-11.42	-11.41	-11.4	-11.39	-11.38
-11.46	-11.45	-11.44	-11.43	-11.42	-11.41	-11.4	-11.39	-11.38
-11.46	-11.45	-11.44	-11.43	-11.42	-11.41	-11.4	-11.39	-11.38
-11.46	-11.45	-11.44	-11.43	-11.42	-11.41	-11.4	-11.39	-11.38
-11.46	-11.45	-11.44	-11.43	-11.42	-11.41	-11.4	-11.39	-11.38
-11.46	-11.45	-11.44	-11.43	-11.42	-11.41	-11.4	-11.39	-11.38
-11.46	-11.45	-11.44	-11.43	-11.42	-11.41	-11.4	-11.39	-11.38
-11.46	-11.45	-11.44	-11.43	-11.42	-11.41	-11.4	-11.39	-11.38
-11.46	-11.45	-11.44	-11.43	-11.42	-11.41	-11.4	-11.39	-11.38
-11.46	-11.45	-11.44	-11.43	-11.42	-11.41	-11.4	-11.39	-11.38
-11.46	-11.45	-11.44	-11.43	-11.42	-11.41	-11.4	-11.39	-11.38
-11.46	-11.45	-11.44	-11.43	-11.42	-11.41	-11.4	-11.39	-11.38
-11.46	-11.45	-11.44	-11.43	-11.42	-11.41	-11.4	-11.39	-11.38
-11.46	-11.45	-11.44	-11.43	-11.42	-11.41	-11.4	-11.39	-11.38
-11.46	-11.45	-11.44	-11.43	-11.42	-11.41	-11.4	-11.39	-11.38
-11.46	-11.45	-11.44	-11.43	-11.42	-11.41	-11.4	-11.39	-11.38
-11.46	-11.45	-11.44	-11.43	-11.42	-11.41	-11.4	-11.39	-11.38
-11.46	-11.45	-11.44	-11.43	-11.42	-11.41	-11.4	-11.39	-11.38
-11.46	-11.45	-11.44	-11.43	-11.42	-11.41	-11.4	-11.39	-11.38

-11.46	-11.45	-11.44	-11.43	-11.42	-11.41	-11.4	-11.39	-11.38
-11.46	-11.45	-11.44	-11.43	-11.42	-11.41	-11.4	-11.39	-11.38
-11.46	-11.45	-11.44	-11.43	-11.42	-11.41	-11.4	-11.39	-11.38
-11.46	-11.45	-11.44	-11.43	-11.42	-11.41	-11.4	-11.39	-11.38
-11.46	-11.45	-11.44	-11.43	-11.42	-11.41	-11.4	-11.39	-11.38
-11.46	-11.45	-11.44	-11.43	-11.42	-11.41	-11.4	-11.39	-11.38
-11.46	-11.45	-11.44	-11.43	-11.42	-11.41	-11.4	-11.39	-11.38
-11.46	-11.45	-11.44	-11.43	-11.42	-11.41	-11.4	-11.39	-11.38
-11.46	-11.45	-11.44	-11.43	-11.42	-11.41	-11.4	-11.39	-11.38
-11.46	-11.45	-11.44	-11.43	-11.42	-11.41	-11.4	-11.39	-11.38
-11.46	-11.45	-11.44	-11.43	-11.42	-11.41	-11.4	-11.39	-11.38
-11.46	-11.45	-11.44	-11.43	-11.42	-11.41	-11.4	-11.39	-11.38
-11.46	-11.45	-11.44	-11.43	-11.42	-11.41	-11.4	-11.39	-11.38
-11.46	-11.45	-11.44	-11.43	-11.42	-11.41	-11.4	-11.39	-11.38
-11.46	-11.45	-11.44	-11.43	-11.42	-11.41	-11.4	-11.39	-11.38
-11.46	-11.45	-11.44	-11.43	-11.42	-11.41	-11.4	-11.39	-11.38
-11.46	-11.45	-11.44	-11.43	-11.42	-11.41	-11.4	-11.39	-11.38
-11.46	-11.45	-11.44	-11.43	-11.42	-11.41	-11.4	-11.39	-11.38
-11.46	-11.45	-11.44	-11.43	-11.42	-11.41	-11.4	-11.39	-11.38
-11.46	-11.45	-11.44	-11.43	-11.42	-11.41	-11.4	-11.39	-11.38
-11.46	-11.45	-11.44	-11.43	-11.42	-11.41	-11.4	-11.39	-11.38
-11.46	-11.45	-11.44	-11.43	-11.42	-11.41	-11.4	-11.39	-11.38
-11.46	-11.45	-11.44	-11.43	-11.42	-11.41	-11.4	-11.39	-11.38
-11.46	-11.45	-11.44	-11.43	-11.42	-11.41	-11.4	-11.39	-11.38
-11.46	-11.45	-11.44	-11.43	-11.42	-11.41	-11.4	-11.39	-11.38
-11.46	-11.45	-11.44	-11.43	-11.42	-11.41	-11.4	-11.39	-11.38
-11.46	-11.45	-11.44	-11.43	-11.42	-11.41	-11.4	-11.39	-11.38
-11.46	-11.45	-11.44	-11.43	-11.42	-11.41	-11.4	-11.39	-11.38
-11.46	-11.45	-11.44	-11.43	-11.42	-11.41	-11.4	-11.39	-11.38
-11.46	-11.45	-11.44	-11.43	-11.42	-11.41	-11.4	-11.39	-11.38
-11.46	-11.45	-11.44	-11.43	-11.42	-11.41	-11.4	-11.39	-11.38
-11.46	-11.45	-11.44	-11.43	-11.42	-11.41	-11.4	-11.39	-11.38
-11.46	-11.45	-11.44	-11.43	-11.42	-11.41	-11.4	-11.39	-11.38
-11.46	-11.45	-11.44	-11.43	-11.42	-11.41	-11.4	-11.39	-11.38
-11.46	-11.45	-11.44	-11.43	-11.42	-11.41	-11.4	-11.39	-11.38
-11.46	-11.45	-11.44	-11.43	-11.42	-11.41	-11.4	-11.39	-11.38
-11.46	-11.45	-11.44	-11.43	-11.42	-11.41	-11.4	-11.39	-11.38
-11.46	-11.45	-11.44	-11.43	-11.42	-11.41	-11.4	-11.39	-11.38
-11.46	-11.45	-11.44	-11.43	-11.42	-11.41	-11.4	-11.39	-11.38
-11.46	-11.45	-11.44	-11.43	-11.42	-11.41	-11.4	-11.39	-11.38
-11.46	-11.45	-11.44	-11.43	-11.42	-11.41	-11.4	-11.39	-11.38
-11.46	-11.45	-11.44	-11.43	-11.42	-11.41	-11.4	-11.39	-11.38
-11.46	-11.45	-11.44	-11.43	-11.42	-11.41	-11.4	-11.39	-11.38
-11.46	-11.45	-11.44	-11.43	-11.42	-11.41	-11.4	-11.39	-11.38
-11.46	-11.45	-11.44	-11.43	-11.42	-11.41	-11.4	-11.39	-11.38
-11.46	-11.45	-11.44	-11.43	-11.42	-11.41	-11.4	-11.39	-11.38
-11.46	-11.45	-11.44	-11.43	-11.42	-11.41	-11.4	-11.39	-11.38
-11.46	-11.45	-11.44	-11.43	-11.42	-11.41	-11.4	-11.39	-11.38
-11.46	-11.45	-11.44	-11.43	-11.42	-11.41	-11.4	-11.39	-11.38
-11.46	-11.45	-11.44	-11.43	-11.42	-11.41	-11.4	-11.39	-11.38

-11.46	-11.45	-11.44	-11.43	-11.42	-11.41	-11.4	-11.39	-11.38
-11.46	-11.45	-11.44	-11.43	-11.42	-11.41	-11.4	-11.39	-11.38
-11.46	-11.45	-11.44	-11.43	-11.42	-11.41	-11.4	-11.39	-11.38
-11.46	-11.45	-11.44	-11.43	-11.42	-11.41	-11.4	-11.39	-11.38
-11.46	-11.45	-11.44	-11.43	-11.42	-11.41	-11.4	-11.39	-11.38
-11.46	-11.45	-11.44	-11.43	-11.42	-11.41	-11.4	-11.39	-11.38
-11.46	-11.45	-11.44	-11.43	-11.42	-11.41	-11.4	-11.39	-11.38
-11.46	-11.45	-11.44	-11.43	-11.42	-11.41	-11.4	-11.39	-11.38
-11.46	-11.45	-11.44	-11.43	-11.42	-11.41	-11.4	-11.39	-11.38
-11.46	-11.45	-11.44	-11.43	-11.42	-11.41	-11.4	-11.39	-11.38
-11.46	-11.45	-11.44	-11.43	-11.42	-11.41	-11.4	-11.39	-11.38
-11.46	-11.45	-11.44	-11.43	-11.42	-11.41	-11.4	-11.39	-11.38
-11.46	-11.45	-11.44	-11.43	-11.42	-11.41	-11.4	-11.39	-11.38
-11.46	-11.45	-11.44	-11.43	-11.42	-11.41	-11.4	-11.39	-11.38
-11.46	-11.45	-11.44	-11.43	-11.42	-11.41	-11.4	-11.39	-11.38
-11.46	-11.45	-11.44	-11.43	-11.42	-11.41	-11.4	-11.39	-11.38
-11.46	-11.45	-11.44	-11.43	-11.42	-11.41	-11.4	-11.39	-11.38
-11.46	-11.45	-11.44	-11.43	-11.42	-11.41	-11.4	-11.39	-11.38
-11.46	-11.45	-11.44	-11.43	-11.42	-11.41	-11.4	-11.39	-11.38
-11.46	-11.45	-11.44	-11.43	-11.42	-11.41	-11.4	-11.39	-11.38
-11.46	-11.45	-11.44	-11.43	-11.42	-11.41	-11.4	-11.39	-11.38
-11.46	-11.45	-11.44	-11.43	-11.42	-11.41	-11.4	-11.39	-11.38
-11.46	-11.45	-11.44	-11.43	-11.42	-11.41	-11.4	-11.39	-11.38
-11.46	-11.45	-11.44	-11.43	-11.42	-11.41	-11.4	-11.39	-11.38
-11.46	-11.45	-11.44	-11.43	-11.42	-11.41	-11.4	-11.39	-11.38
-11.46	-11.45	-11.44	-11.43	-11.42	-11.41	-11.4	-11.39	-11.38
-11.46	-11.45	-11.44	-11.43	-11.42	-11.41	-11.4	-11.39	-11.38
-11.46	-11.45	-11.44	-11.43	-11.42	-11.41	-11.4	-11.39	-11.38
-11.46	-11.45	-11.44	-11.43	-11.42	-11.41	-11.4	-11.39	-11.38
-11.46	-11.45	-11.44	-11.43	-11.42	-11.41	-11.4	-11.39	-11.38
-11.46	-11.45	-11.44	-11.43	-11.42	-11.41	-11.4	-11.39	-11.38
-11.46	-11.45	-11.44	-11.43	-11.42	-11.41	-11.4	-11.39	-11.38
-11.46	-11.45	-11.44	-11.43	-11.42	-11.41	-11.4	-11.39	-11.38
-11.46	-11.45	-11.44	-11.43	-11.42	-11.41	-11.4	-11.39	-11.38
-11.46	-11.45	-11.44	-11.43	-11.42	-11.41	-11.4	-11.39	-11.38
-11.46	-11.45	-11.44	-11.43	-11.42	-11.41	-11.4	-11.39	-11.38
-11.46	-11.45	-11.44	-11.43	-11.42	-11.41	-11.4	-11.39	-11.38
-11.46	-11.45	-11.44	-11.43	-11.42	-11.41	-11.4	-11.39	-11.38
-11.46	-11.45	-11.44	-11.43	-11.42	-11.41	-11.4	-11.39	-11.38
-11.46	-11.45	-11.44	-11.43	-11.42	-11.41	-11.4	-11.39	-11.38
-11.46	-11.45	-11.44	-11.43	-11.42	-11.41	-11.4	-11.39	-11.38
-11.46	-11.45	-11.44	-11.43	-11.42	-11.41	-11.4	-11.39	-11.38
-11.46	-11.45	-11.44	-11.43	-11.42	-11.41	-11.4	-11.39	-11.38
-11.46	-11.45	-11.44	-11.43	-11.42	-11.41	-11.4	-11.39	-11.38
-11.46	-11.45	-11.44	-11.43	-11.42	-11.41	-11.4	-11.39	-11.38
-11.46	-11.45	-11.44	-11.43	-11.42	-11.41	-11.4	-11.39	-11.38
-11.46	-11.45	-11.44	-11.43	-11.42	-11.41	-11.4	-11.39	-11.38
-11.46	-11.45	-11.44	-11.43	-11.42	-11.41	-11.4	-11.39	-11.38
-11.46	-11.45	-11.44	-11.43	-11.42	-11.41	-11.4	-11.39	-11.38
-11.46	-11.45	-11.44	-11.43	-11.42	-11.41	-11.4	-11.39	-11.38

-11.46	-11.45	-11.44	-11.43	-11.42	-11.41	-11.4	-11.39	-11.38
-11.46	-11.45	-11.44	-11.43	-11.42	-11.41	-11.4	-11.39	-11.38
-11.46	-11.45	-11.44	-11.43	-11.42	-11.41	-11.4	-11.39	-11.38
-11.46	-11.45	-11.44	-11.43	-11.42	-11.41	-11.4	-11.39	-11.38
-11.46	-11.45	-11.44	-11.43	-11.42	-11.41	-11.4	-11.39	-11.38
-11.46	-11.45	-11.44	-11.43	-11.42	-11.41	-11.4	-11.39	-11.38
-11.46	-11.45	-11.44	-11.43	-11.42	-11.41	-11.4	-11.39	-11.38
-11.46	-11.45	-11.44	-11.43	-11.42	-11.41	-11.4	-11.39	-11.38
-11.46	-11.45	-11.44	-11.43	-11.42	-11.41	-11.4	-11.39	-11.38
-11.46	-11.45	-11.44	-11.43	-11.42	-11.41	-11.4	-11.39	-11.38
-11.46	-11.45	-11.44	-11.43	-11.42	-11.41	-11.4	-11.39	-11.38
-11.46	-11.45	-11.44	-11.43	-11.42	-11.41	-11.4	-11.39	-11.38
-11.46	-11.45	-11.44	-11.43	-11.42	-11.41	-11.4	-11.39	-11.38
-11.46	-11.45	-11.44	-11.43	-11.42	-11.41	-11.4	-11.39	-11.38
-11.46	-11.45	-11.44	-11.43	-11.42	-11.41	-11.4	-11.39	-11.38
-11.46	-11.45	-11.44	-11.43	-11.42	-11.41	-11.4	-11.39	-11.38
-11.46	-11.45	-11.44	-11.43	-11.42	-11.41	-11.4	-11.39	-11.38
-11.46	-11.45	-11.44	-11.43	-11.42	-11.41	-11.4	-11.39	-11.38
-11.46	-11.45	-11.44	-11.43	-11.42	-11.41	-11.4	-11.39	-11.38
-11.46	-11.45	-11.44	-11.43	-11.42	-11.41	-11.4	-11.39	-11.38
-11.46	-11.45	-11.44	-11.43	-11.42	-11.41	-11.4	-11.39	-11.38
-11.46	-11.45	-11.44	-11.43	-11.42	-11.41	-11.4	-11.39	-11.38
-11.46	-11.45	-11.44	-11.43	-11.42	-11.41	-11.4	-11.39	-11.38
-11.46	-11.45	-11.44	-11.43	-11.42	-11.41	-11.4	-11.39	-11.38
-11.46	-11.45	-11.44	-11.43	-11.42	-11.41	-11.4	-11.39	-11.38
-11.46	-11.45	-11.44	-11.43	-11.42	-11.41	-11.4	-11.39	-11.38
-11.46	-11.45	-11.44	-11.43	-11.42	-11.41	-11.4	-11.39	-11.38
-11.46	-11.45	-11.44	-11.43	-11.42	-11.41	-11.4	-11.39	-11.38
-11.46	-11.45	-11.44	-11.43	-11.42	-11.41	-11.4	-11.39	-11.38
-11.46	-11.45	-11.44	-11.43	-11.42	-11.41	-11.4	-11.39	-11.38
-11.46	-11.45	-11.44	-11.43	-11.42	-11.41	-11.4	-11.39	-11.38
-11.46	-11.45	-11.44	-11.43	-11.42	-11.41	-11.4	-11.39	-11.38
-11.46	-11.45	-11.44	-11.43	-11.42	-11.41	-11.4	-11.39	-11.38
-11.46	-11.45	-11.44	-11.43	-11.42	-11.41	-11.4	-11.39	-11.38
-11.46	-11.45	-11.44	-11.43	-11.42	-11.41	-11.4	-11.39	-11.38
-11.46	-11.45	-11.44	-11.43	-11.42	-11.41	-11.4	-11.39	-11.38
-11.46	-11.45	-11.44	-11.43	-11.42	-11.41	-11.4	-11.39	-11.38
-11.46	-11.45	-11.44	-11.43	-11.42	-11.41	-11.4	-11.39	-11.38
-11.46	-11.45	-11.44	-11.43	-11.42	-11.41	-11.4	-11.39	-11.38
-11.46	-11.45	-11.44	-11.43	-11.42	-11.41	-11.4	-11.39	-11.38
-11.46	-11.45	-11.44	-11.43	-11.42	-11.41	-11.4	-11.39	-11.38
-11.46	-11.45	-11.44	-11.43	-11.42	-11.41	-11.4	-11.39	-11.38
-11.46	-11.45	-11.44	-11.43	-11.42	-11.41	-11.4	-11.39	-11.38
-11.46	-11.45	-11.44	-11.43	-11.42	-11.41	-11.4	-11.39	-11.38
-11.46	-11.45	-11.44	-11.43	-11.42	-11.41	-11.4	-11.39	-11.38
-11.46	-11.45	-11.44	-11.43	-11.42	-11.41	-11.4	-11.39	-11.38
-11.46	-11.45	-11.44	-11.43	-11.42	-11.41	-11.4	-11.39	-11.38
-11.46	-11.45	-11.44	-11.43	-11.42	-11.41	-11.4	-11.39	-11.38
-11.46	-11.45	-11.44	-11.43	-11.42	-11.41	-11.4	-11.39	-11.38
-11.46	-11.45	-11.44	-11.43	-11.42	-11.41	-11.4	-11.39	-11.38

-11.46	-11.45	-11.44	-11.43	-11.42	-11.41	-11.4	-11.39	-11.38
-11.46	-11.45	-11.44	-11.43	-11.42	-11.41	-11.4	-11.39	-11.38
-11.46	-11.45	-11.44	-11.43	-11.42	-11.41	-11.4	-11.39	-11.38
-11.46	-11.45	-11.44	-11.43	-11.42	-11.41	-11.4	-11.39	-11.38
-11.46	-11.45	-11.44	-11.43	-11.42	-11.41	-11.4	-11.39	-11.38
-11.46	-11.45	-11.44	-11.43	-11.42	-11.41	-11.4	-11.39	-11.38
-11.46	-11.45	-11.44	-11.43	-11.42	-11.41	-11.4	-11.39	-11.38
-11.46	-11.45	-11.44	-11.43	-11.42	-11.41	-11.4	-11.39	-11.38
-11.46	-11.45	-11.44	-11.43	-11.42	-11.41	-11.4	-11.39	-11.38
-11.46	-11.45	-11.44	-11.43	-11.42	-11.41	-11.4	-11.39	-11.38
-11.46	-11.45	-11.44	-11.43	-11.42	-11.41	-11.4	-11.39	-11.38
-11.46	-11.45	-11.44	-11.43	-11.42	-11.41	-11.4	-11.39	-11.38
-11.46	-11.45	-11.44	-11.43	-11.42	-11.41	-11.4	-11.39	-11.38
-11.46	-11.45	-11.44	-11.43	-11.42	-11.41	-11.4	-11.39	-11.38
-11.46	-11.45	-11.44	-11.43	-11.42	-11.41	-11.4	-11.39	-11.38
-11.46	-11.45	-11.44	-11.43	-11.42	-11.41	-11.4	-11.39	-11.38
-11.46	-11.45	-11.44	-11.43	-11.42	-11.41	-11.4	-11.39	-11.38
-11.46	-11.45	-11.44	-11.43	-11.42	-11.41	-11.4	-11.39	-11.38
-11.46	-11.45	-11.44	-11.43	-11.42	-11.41	-11.4	-11.39	-11.38
-11.46	-11.45	-11.44	-11.43	-11.42	-11.41	-11.4	-11.39	-11.38
-11.46	-11.45	-11.44	-11.43	-11.42	-11.41	-11.4	-11.39	-11.38
-11.46	-11.45	-11.44	-11.43	-11.42	-11.41	-11.4	-11.39	-11.38
-11.46	-11.45	-11.44	-11.43	-11.42	-11.41	-11.4	-11.39	-11.38
-11.46	-11.45	-11.44	-11.43	-11.42	-11.41	-11.4	-11.39	-11.38
-11.46	-11.45	-11.44	-11.43	-11.42	-11.41	-11.4	-11.39	-11.38
-11.46	-11.45	-11.44	-11.43	-11.42	-11.41	-11.4	-11.39	-11.38
-11.46	-11.45	-11.44	-11.43	-11.42	-11.41	-11.4	-11.39	-11.38
-11.46	-11.45	-11.44	-11.43	-11.42	-11.41	-11.4	-11.39	-11.38
-11.46	-11.45	-11.44	-11.43	-11.42	-11.41	-11.4	-11.39	-11.38
-11.46	-11.45	-11.44	-11.43	-11.42	-11.41	-11.4	-11.39	-11.38
-11.46	-11.45	-11.44	-11.43	-11.42	-11.41	-11.4	-11.39	-11.38
-11.46	-11.45	-11.44	-11.43	-11.42	-11.41	-11.4	-11.39	-11.38
-11.46	-11.45	-11.44	-11.43	-11.42	-11.41	-11.4	-11.39	-11.38
-11.46	-11.45	-11.44	-11.43	-11.42	-11.41	-11.4	-11.39	-11.38
-11.46	-11.45	-11.44	-11.43	-11.42	-11.41	-11.4	-11.39	-11.38
-11.46	-11.45	-11.44	-11.43	-11.42	-11.41	-11.4	-11.39	-11.38
-11.46	-11.45	-11.44	-11.43	-11.42	-11.41	-11.4	-11.39	-11.38
-11.46	-11.45	-11.44	-11.43	-11.42	-11.41	-11.4	-11.39	-11.38
-11.46	-11.45	-11.44	-11.43	-11.42	-11.41	-11.4	-11.39	-11.38
-11.46	-11.45	-11.44	-11.43	-11.42	-11.41	-11.4	-11.39	-11.38
-11.46	-11.45	-11.44	-11.43	-11.42	-11.41	-11.4	-11.39	-11.38
-11.46	-11.45	-11.44	-11.43	-11.42	-11.41	-11.4	-11.39	-11.38
-11.46	-11.45	-11.44	-11.43	-11.42	-11.41	-11.4	-11.39	-11.38
-11.46	-11.45	-11.44	-11.43	-11.42	-11.41	-11.4	-11.39	-11.38
-11.46	-11.45	-11.44	-11.43	-11.42	-11.41	-11.4	-11.39	-11.38
-11.46	-11.45	-11.44	-11.43	-11.42	-11.41	-11.4	-11.39	-11.38
-11.46	-11.45	-11.44	-11.43	-11.42	-11.41	-11.4	-11.39	-11.38
-11.46	-11.45	-11.44	-11.43	-11.42	-11.41	-11.4	-11.39	-11.38
-11.46	-11.45	-11.44	-11.43	-11.42	-11.41	-11.4	-11.39	-11.38
-11.46	-11.45	-11.44	-11.43	-11.42	-11.41	-11.4	-11.39	-11.38

-11.46	-11.45	-11.44	-11.43	-11.42	-11.41	-11.4	-11.39	-11.38
-11.46	-11.45	-11.44	-11.43	-11.42	-11.41	-11.4	-11.39	-11.38
-11.46	-11.45	-11.44	-11.43	-11.42	-11.41	-11.4	-11.39	-11.38
-11.46	-11.45	-11.44	-11.43	-11.42	-11.41	-11.4	-11.39	-11.38
-11.46	-11.45	-11.44	-11.43	-11.42	-11.41	-11.4	-11.39	-11.38
-11.46	-11.45	-11.44	-11.43	-11.42	-11.41	-11.4	-11.39	-11.38
-11.46	-11.45	-11.44	-11.43	-11.42	-11.41	-11.4	-11.39	-11.38
-11.46	-11.45	-11.44	-11.43	-11.42	-11.41	-11.4	-11.39	-11.38
-11.46	-11.45	-11.44	-11.43	-11.42	-11.41	-11.4	-11.39	-11.38
-11.46	-11.45	-11.44	-11.43	-11.42	-11.41	-11.4	-11.39	-11.38
-11.46	-11.45	-11.44	-11.43	-11.42	-11.41	-11.4	-11.39	-11.38
-11.46	-11.45	-11.44	-11.43	-11.42	-11.41	-11.4	-11.39	-11.38
-11.46	-11.45	-11.44	-11.43	-11.42	-11.41	-11.4	-11.39	-11.38
-11.46	-11.45	-11.44	-11.43	-11.42	-11.41	-11.4	-11.39	-11.38
-11.46	-11.45	-11.44	-11.43	-11.42	-11.41	-11.4	-11.39	-11.38
-11.46	-11.45	-11.44	-11.43	-11.42	-11.41	-11.4	-11.39	-11.38
-11.46	-11.45	-11.44	-11.43	-11.42	-11.41	-11.4	-11.39	-11.38
-11.46	-11.45	-11.44	-11.43	-11.42	-11.41	-11.4	-11.39	-11.38
-11.46	-11.45	-11.44	-11.43	-11.42	-11.41	-11.4	-11.39	-11.38
-11.46	-11.45	-11.44	-11.43	-11.42	-11.41	-11.4	-11.39	-11.38
-11.46	-11.45	-11.44	-11.43	-11.42	-11.41	-11.4	-11.39	-11.38
-11.46	-11.45	-11.44	-11.43	-11.42	-11.41	-11.4	-11.39	-11.38
-11.46	-11.45	-11.44	-11.43	-11.42	-11.41	-11.4	-11.39	-11.38
-11.46	-11.45	-11.44	-11.43	-11.42	-11.41	-11.4	-11.39	-11.38
-11.46	-11.45	-11.44	-11.43	-11.42	-11.41	-11.4	-11.39	-11.38
-11.46	-11.45	-11.44	-11.43	-11.42	-11.41	-11.4	-11.39	-11.38
-11.46	-11.45	-11.44	-11.43	-11.42	-11.41	-11.4	-11.39	-11.38
-11.46	-11.45	-11.44	-11.43	-11.42	-11.41	-11.4	-11.39	-11.38
-11.46	-11.45	-11.44	-11.43	-11.42	-11.41	-11.4	-11.39	-11.38
-11.46	-11.45	-11.44	-11.43	-11.42	-11.41	-11.4	-11.39	-11.38
-11.46	-11.45	-11.44	-11.43	-11.42	-11.41	-11.4	-11.39	-11.38
-11.46	-11.45	-11.44	-11.43	-11.42	-11.41	-11.4	-11.39	-11.38
-11.46	-11.45	-11.44	-11.43	-11.42	-11.41	-11.4	-11.39	-11.38
-11.46	-11.45	-11.44	-11.43	-11.42	-11.41	-11.4	-11.39	-11.38
-11.46	-11.45	-11.44	-11.43	-11.42	-11.41	-11.4	-11.39	-11.38
-11.46	-11.45	-11.44	-11.43	-11.42	-11.41	-11.4	-11.39	-11.38
-11.46	-11.45	-11.44	-11.43	-11.42	-11.41	-11.4	-11.39	-11.38
-11.46	-11.45	-11.44	-11.43	-11.42	-11.41	-11.4	-11.39	-11.38
-11.46	-11.45	-11.44	-11.43	-11.42	-11.41	-11.4	-11.39	-11.38
-11.46	-11.45	-11.44	-11.43	-11.42	-11.41	-11.4	-11.39	-11.38
-11.46	-11.45	-11.44	-11.43	-11.42	-11.41	-11.4	-11.39	-11.38
-11.46	-11.45	-11.44	-11.43	-11.42	-11.41	-11.4	-11.39	-11.38
-11.46	-11.45	-11.44	-11.43	-11.42	-11.41	-11.4	-11.39	-11.38
-11.46	-11.45	-11.44	-11.43	-11.42	-11.41	-11.4	-11.39	-11.38
-11.46	-11.45	-11.44	-11.43	-11.42	-11.41	-11.4	-11.39	-11.38
-11.46	-11.45	-11.44	-11.43	-11.42	-11.41	-11.4	-11.39	-11.38
-11.46	-11.45	-11.44	-11.43	-11.42	-11.41	-11.4	-11.39	-11.38
-11.46	-11.45	-11.44	-11.43	-11.42	-11.41	-11.4	-11.39	-11.38
-11.46	-11.45	-11.44	-11.43	-11.42	-11.41	-11.4	-11.39	-11.38
-11.46	-11.45	-11.44	-11.43	-11.42	-11.41	-11.4	-11.39	-11.38

-11.46	-11.45	-11.44	-11.43	-11.42	-11.41	-11.4	-11.39	-11.38
-11.46	-11.45	-11.44	-11.43	-11.42	-11.41	-11.4	-11.39	-11.38
-11.46	-11.45	-11.44	-11.43	-11.42	-11.41	-11.4	-11.39	-11.38
-11.46	-11.45	-11.44	-11.43	-11.42	-11.41	-11.4	-11.39	-11.38
-11.46	-11.45	-11.44	-11.43	-11.42	-11.41	-11.4	-11.39	-11.38
-11.46	-11.45	-11.44	-11.43	-11.42	-11.41	-11.4	-11.39	-11.38
-11.46	-11.45	-11.44	-11.43	-11.42	-11.41	-11.4	-11.39	-11.38
-11.46	-11.45	-11.44	-11.43	-11.42	-11.41	-11.4	-11.39	-11.38
-11.46	-11.45	-11.44	-11.43	-11.42	-11.41	-11.4	-11.39	-11.38
-11.46	-11.45	-11.44	-11.43	-11.42	-11.41	-11.4	-11.39	-11.38
-11.46	-11.45	-11.44	-11.43	-11.42	-11.41	-11.4	-11.39	-11.38
-11.46	-11.45	-11.44	-11.43	-11.42	-11.41	-11.4	-11.39	-11.38
-11.46	-11.45	-11.44	-11.43	-11.42	-11.41	-11.4	-11.39	-11.38
-11.46	-11.45	-11.44	-11.43	-11.42	-11.41	-11.4	-11.39	-11.38
-11.46	-11.45	-11.44	-11.43	-11.42	-11.41	-11.4	-11.39	-11.38
-11.46	-11.45	-11.44	-11.43	-11.42	-11.41	-11.4	-11.39	-11.38
-11.46	-11.45	-11.44	-11.43	-11.42	-11.41	-11.4	-11.39	-11.38
-11.46	-11.45	-11.44	-11.43	-11.42	-11.41	-11.4	-11.39	-11.38
-11.46	-11.45	-11.44	-11.43	-11.42	-11.41	-11.4	-11.39	-11.38
-11.46	-11.45	-11.44	-11.43	-11.42	-11.41	-11.4	-11.39	-11.38
-11.46	-11.45	-11.44	-11.43	-11.42	-11.41	-11.4	-11.39	-11.38
-11.46	-11.45	-11.44	-11.43	-11.42	-11.41	-11.4	-11.39	-11.38
-11.46	-11.45	-11.44	-11.43	-11.42	-11.41	-11.4	-11.39	-11.38
-11.46	-11.45	-11.44	-11.43	-11.42	-11.41	-11.4	-11.39	-11.38
-11.46	-11.45	-11.44	-11.43	-11.42	-11.41	-11.4	-11.39	-11.38
-11.46	-11.45	-11.44	-11.43	-11.42	-11.41	-11.4	-11.39	-11.38
-11.46	-11.45	-11.44	-11.43	-11.42	-11.41	-11.4	-11.39	-11.38
-11.46	-11.45	-11.44	-11.43	-11.42	-11.41	-11.4	-11.39	-11.38
-11.46	-11.45	-11.44	-11.43	-11.42	-11.41	-11.4	-11.39	-11.38
-11.46	-11.45	-11.44	-11.43	-11.42	-11.41	-11.4	-11.39	-11.38
-11.46	-11.45	-11.44	-11.43	-11.42	-11.41	-11.4	-11.39	-11.38
-11.46	-11.45	-11.44	-11.43	-11.42	-11.41	-11.4	-11.39	-11.38
-11.46	-11.45	-11.44	-11.43	-11.42	-11.41	-11.4	-11.39	-11.38
-11.46	-11.45	-11.44	-11.43	-11.42	-11.41	-11.4	-11.39	-11.38
-11.46	-11.45	-11.44	-11.43	-11.42	-11.41	-11.4	-11.39	-11.38
-11.46	-11.45	-11.44	-11.43	-11.42	-11.41	-11.4	-11.39	-11.38
-11.46	-11.45	-11.44	-11.43	-11.42	-11.41	-11.4	-11.39	-11.38
-11.46	-11.45	-11.44	-11.43	-11.42	-11.41	-11.4	-11.39	-11.38
-11.46	-11.45	-11.44	-11.43	-11.42	-11.41	-11.4	-11.39	-11.38
-11.46	-11.45	-11.44	-11.43	-11.42	-11.41	-11.4	-11.39	-11.38
-11.46	-11.45	-11.44	-11.43	-11.42	-11.41	-11.4	-11.39	-11.38
-11.46	-11.45	-11.44	-11.43	-11.42	-11.41	-11.4	-11.39	-11.38
-11.46	-11.45	-11.44	-11.43	-11.42	-11.41	-11.4	-11.39	-11.38
-11.46	-11.45	-11.44	-11.43	-11.42	-11.41	-11.4	-11.39	-11.38
-11.46	-11.45	-11.44	-11.43	-11.42	-11.41	-11.4	-11.39	-11.38
-11.46	-11.45	-11.44	-11.43	-11.42	-11.41	-11.4	-11.39	-11.38
-11.46	-11.45	-11.44	-11.43	-11.42	-11.41	-11.4	-11.39	-11.38
-11.46	-11.45	-11.44	-11.43	-11.42	-11.41	-11.4	-11.39	-11.38
-11.46	-11.45	-11.44	-11.43	-11.42	-11.41	-11.4	-11.39	-11.38
-11.46	-11.45	-11.44	-11.43	-11.42	-11.41	-11.4	-11.39	-11.38

-11.46	-11.45	-11.44	-11.43	-11.42	-11.41	-11.4	-11.39	-11.38
-11.46	-11.45	-11.44	-11.43	-11.42	-11.41	-11.4	-11.39	-11.38
-11.46	-11.45	-11.44	-11.43	-11.42	-11.41	-11.4	-11.39	-11.38
-11.46	-11.45	-11.44	-11.43	-11.42	-11.41	-11.4	-11.39	-11.38
-11.46	-11.45	-11.44	-11.43	-11.42	-11.41	-11.4	-11.39	-11.38
-11.46	-11.45	-11.44	-11.43	-11.42	-11.41	-11.4	-11.39	-11.38
-11.46	-11.45	-11.44	-11.43	-11.42	-11.41	-11.4	-11.39	-11.38
-11.46	-11.45	-11.44	-11.43	-11.42	-11.41	-11.4	-11.39	-11.38
-11.46	-11.45	-11.44	-11.43	-11.42	-11.41	-11.4	-11.39	-11.38
-11.46	-11.45	-11.44	-11.43	-11.42	-11.41	-11.4	-11.39	-11.38
-11.46	-11.45	-11.44	-11.43	-11.42	-11.41	-11.4	-11.39	-11.38
-11.46	-11.45	-11.44	-11.43	-11.42	-11.41	-11.4	-11.39	-11.38
-11.46	-11.45	-11.44	-11.43	-11.42	-11.41	-11.4	-11.39	-11.38
-11.46	-11.45	-11.44	-11.43	-11.42	-11.41	-11.4	-11.39	-11.38
-11.46	-11.45	-11.44	-11.43	-11.42	-11.41	-11.4	-11.39	-11.38
-11.46	-11.45	-11.44	-11.43	-11.42	-11.41	-11.4	-11.39	-11.38
-11.46	-11.45	-11.44	-11.43	-11.42	-11.41	-11.4	-11.39	-11.38
-11.46	-11.45	-11.44	-11.43	-11.42	-11.41	-11.4	-11.39	-11.38
-11.46	-11.45	-11.44	-11.43	-11.42	-11.41	-11.4	-11.39	-11.38
-11.46	-11.45	-11.44	-11.43	-11.42	-11.41	-11.4	-11.39	-11.38
-11.46	-11.45	-11.44	-11.43	-11.42	-11.41	-11.4	-11.39	-11.38
-11.46	-11.45	-11.44	-11.43	-11.42	-11.41	-11.4	-11.39	-11.38
-11.46	-11.45	-11.44	-11.43	-11.42	-11.41	-11.4	-11.39	-11.38
-11.46	-11.45	-11.44	-11.43	-11.42	-11.41	-11.4	-11.39	-11.38
-11.46	-11.45	-11.44	-11.43	-11.42	-11.41	-11.4	-11.39	-11.38
-11.46	-11.45	-11.44	-11.43	-11.42	-11.41	-11.4	-11.39	-11.38
-11.46	-11.45	-11.44	-11.43	-11.42	-11.41	-11.4	-11.39	-11.38
-11.46	-11.45	-11.44	-11.43	-11.42	-11.41	-11.4	-11.39	-11.38
-11.46	-11.45	-11.44	-11.43	-11.42	-11.41	-11.4	-11.39	-11.38
-11.46	-11.45	-11.44	-11.43	-11.42	-11.41	-11.4	-11.39	-11.38
-11.46	-11.45	-11.44	-11.43	-11.42	-11.41	-11.4	-11.39	-11.38
-11.46	-11.45	-11.44	-11.43	-11.42	-11.41	-11.4	-11.39	-11.38
-11.46	-11.45	-11.44	-11.43	-11.42	-11.41	-11.4	-11.39	-11.38
-11.46	-11.45	-11.44	-11.43	-11.42	-11.41	-11.4	-11.39	-11.38
-11.46	-11.45	-11.44	-11.43	-11.42	-11.41	-11.4	-11.39	-11.38
-11.46	-11.45	-11.44	-11.43	-11.42	-11.41	-11.4	-11.39	-11.38
-11.46	-11.45	-11.44	-11.43	-11.42	-11.41	-11.4	-11.39	-11.38
-11.46	-11.45	-11.44	-11.43	-11.42	-11.41	-11.4	-11.39	-11.38
-11.46	-11.45	-11.44	-11.43	-11.42	-11.41	-11.4	-11.39	-11.38
-11.46	-11.45	-11.44	-11.43	-11.42	-11.41	-11.4	-11.39	-11.38
-11.46	-11.45	-11.44	-11.43	-11.42	-11.41	-11.4	-11.39	-11.38
-11.46	-11.45	-11.44	-11.43	-11.42	-11.41	-11.4	-11.39	-11.38
-11.46	-11.45	-11.44	-11.43	-11.42	-11.41	-11.4	-11.39	-11.38
-11.46	-11.45	-11.44	-11.43	-11.42	-11.41	-11.4	-11.39	-11.38
-11.46	-11.45	-11.44	-11.43	-11.42	-11.41	-11.4	-11.39	-11.38
-11.46	-11.45	-11.44	-11.43	-11.42	-11.41	-11.4	-11.39	-11.38
-11.46	-11.45	-11.44	-11.43	-11.42	-11.41	-11.4	-11.39	-11.38
-11.46	-11.45	-11.44	-11.43	-11.42	-11.41	-11.4	-11.39	-11.38
-11.46	-11.45	-11.44	-11.43	-11.42	-11.41	-11.4	-11.39	-11.38
-11.46	-11.45	-11.44	-11.43	-11.42	-11.41	-11.4	-11.39	-11.38

-11.46	-11.45	-11.44	-11.43	-11.42	-11.41	-11.4	-11.39	-11.38
-11.46	-11.45	-11.44	-11.43	-11.42	-11.41	-11.4	-11.39	-11.38
-11.46	-11.45	-11.44	-11.43	-11.42	-11.41	-11.4	-11.39	-11.38
-11.46	-11.45	-11.44	-11.43	-11.42	-11.41	-11.4	-11.39	-11.38
-11.46	-11.45	-11.44	-11.43	-11.42	-11.41	-11.4	-11.39	-11.38
-11.46	-11.45	-11.44	-11.43	-11.42	-11.41	-11.4	-11.39	-11.38
-11.46	-11.45	-11.44	-11.43	-11.42	-11.41	-11.4	-11.39	-11.38
-11.46	-11.45	-11.44	-11.43	-11.42	-11.41	-11.4	-11.39	-11.38
-11.46	-11.45	-11.44	-11.43	-11.42	-11.41	-11.4	-11.39	-11.38
-11.46	-11.45	-11.44	-11.43	-11.42	-11.41	-11.4	-11.39	-11.38
-11.46	-11.45	-11.44	-11.43	-11.42	-11.41	-11.4	-11.39	-11.38
-11.46	-11.45	-11.44	-11.43	-11.42	-11.41	-11.4	-11.39	-11.38
-11.46	-11.45	-11.44	-11.43	-11.42	-11.41	-11.4	-11.39	-11.38
-11.46	-11.45	-11.44	-11.43	-11.42	-11.41	-11.4	-11.39	-11.38
-11.46	-11.45	-11.44	-11.43	-11.42	-11.41	-11.4	-11.39	-11.38
-11.46	-11.45	-11.44	-11.43	-11.42	-11.41	-11.4	-11.39	-11.38
-11.46	-11.45	-11.44	-11.43	-11.42	-11.41	-11.4	-11.39	-11.38
-11.46	-11.45	-11.44	-11.43	-11.42	-11.41	-11.4	-11.39	-11.38
-11.46	-11.45	-11.44	-11.43	-11.42	-11.41	-11.4	-11.39	-11.38
-11.46	-11.45	-11.44	-11.43	-11.42	-11.41	-11.4	-11.39	-11.38
-11.46	-11.45	-11.44	-11.43	-11.42	-11.41	-11.4	-11.39	-11.38
-11.46	-11.45	-11.44	-11.43	-11.42	-11.41	-11.4	-11.39	-11.38
-11.46	-11.45	-11.44	-11.43	-11.42	-11.41	-11.4	-11.39	-11.38
-11.46	-11.45	-11.44	-11.43	-11.42	-11.41	-11.4	-11.39	-11.38
-11.46	-11.45	-11.44	-11.43	-11.42	-11.41	-11.4	-11.39	-11.38
-11.46	-11.45	-11.44	-11.43	-11.42	-11.41	-11.4	-11.39	-11.38
-11.46	-11.45	-11.44	-11.43	-11.42	-11.41	-11.4	-11.39	-11.38
-11.46	-11.45	-11.44	-11.43	-11.42	-11.41	-11.4	-11.39	-11.38
-11.46	-11.45	-11.44	-11.43	-11.42	-11.41	-11.4	-11.39	-11.38
-11.46	-11.45	-11.44	-11.43	-11.42	-11.41	-11.4	-11.39	-11.38
-11.46	-11.45	-11.44	-11.43	-11.42	-11.41	-11.4	-11.39	-11.38
-11.46	-11.45	-11.44	-11.43	-11.42	-11.41	-11.4	-11.39	-11.38
-11.46	-11.45	-11.44	-11.43	-11.42	-11.41	-11.4	-11.39	-11.38
-11.46	-11.45	-11.44	-11.43	-11.42	-11.41	-11.4	-11.39	-11.38
-11.46	-11.45	-11.44	-11.43	-11.42	-11.41	-11.4	-11.39	-11.38
-11.46	-11.45	-11.44	-11.43	-11.42	-11.41	-11.4	-11.39	-11.38
-11.46	-11.45	-11.44	-11.43	-11.42	-11.41	-11.4	-11.39	-11.38
-11.46	-11.45	-11.44	-11.43	-11.42	-11.41	-11.4	-11.39	-11.38
-11.46	-11.45	-11.44	-11.43	-11.42	-11.41	-11.4	-11.39	-11.38
-11.46	-11.45	-11.44	-11.43	-11.42	-11.41	-11.4	-11.39	-11.38
-11.46	-11.45	-11.44	-11.43	-11.42	-11.41	-11.4	-11.39	-11.38
-11.46	-11.45	-11.44	-11.43	-11.42	-11.41	-11.4	-11.39	-11.38
-11.46	-11.45	-11.44	-11.43	-11.42	-11.41	-11.4	-11.39	-11.38
-11.46	-11.45	-11.44	-11.43	-11.42	-11.41	-11.4	-11.39	-11.38
-11.46	-11.45	-11.44	-11.43	-11.42	-11.41	-11.4	-11.39	-11.38
-11.46	-11.45	-11.44	-11.43	-11.42	-11.41	-11.4	-11.39	-11.38
-11.46	-11.45	-11.44	-11.43	-11.42	-11.41	-11.4	-11.39	-11.38
-11.46	-11.45	-11.44	-11.43	-11.42	-11.41	-11.4	-11.39	-11.38
-11.46	-11.45	-11.44	-11.43	-11.42	-11.41	-11.4	-11.39	-11.38
-11.46	-11.45	-11.44	-11.43	-11.42	-11.41	-11.4	-11.39	-11.38

-11.46	-11.45	-11.44	-11.43	-11.42	-11.41	-11.4	-11.39	-11.38
-11.46	-11.45	-11.44	-11.43	-11.42	-11.41	-11.4	-11.39	-11.38
-11.46	-11.45	-11.44	-11.43	-11.42	-11.41	-11.4	-11.39	-11.38
-11.46	-11.45	-11.44	-11.43	-11.42	-11.41	-11.4	-11.39	-11.38
-11.46	-11.45	-11.44	-11.43	-11.42	-11.41	-11.4	-11.39	-11.38
-11.46	-11.45	-11.44	-11.43	-11.42	-11.41	-11.4	-11.39	-11.38
-11.46	-11.45	-11.44	-11.43	-11.42	-11.41	-11.4	-11.39	-11.38
-11.46	-11.45	-11.44	-11.43	-11.42	-11.41	-11.4	-11.39	-11.38
-11.46	-11.45	-11.44	-11.43	-11.42	-11.41	-11.4	-11.39	-11.38
-11.46	-11.45	-11.44	-11.43	-11.42	-11.41	-11.4	-11.39	-11.38
-11.46	-11.45	-11.44	-11.43	-11.42	-11.41	-11.4	-11.39	-11.38
-11.46	-11.45	-11.44	-11.43	-11.42	-11.41	-11.4	-11.39	-11.38
-11.46	-11.45	-11.44	-11.43	-11.42	-11.41	-11.4	-11.39	-11.38
-11.46	-11.45	-11.44	-11.43	-11.42	-11.41	-11.4	-11.39	-11.38
-11.46	-11.45	-11.44	-11.43	-11.42	-11.41	-11.4	-11.39	-11.38
-11.46	-11.45	-11.44	-11.43	-11.42	-11.41	-11.4	-11.39	-11.38
-11.46	-11.45	-11.44	-11.43	-11.42	-11.41	-11.4	-11.39	-11.38
-11.46	-11.45	-11.44	-11.43	-11.42	-11.41	-11.4	-11.39	-11.38
-11.46	-11.45	-11.44	-11.43	-11.42	-11.41	-11.4	-11.39	-11.38
-11.46	-11.45	-11.44	-11.43	-11.42	-11.41	-11.4	-11.39	-11.38
-11.46	-11.45	-11.44	-11.43	-11.42	-11.41	-11.4	-11.39	-11.38
-11.46	-11.45	-11.44	-11.43	-11.42	-11.41	-11.4	-11.39	-11.38
-11.46	-11.45	-11.44	-11.43	-11.42	-11.41	-11.4	-11.39	-11.38
-11.46	-11.45	-11.44	-11.43	-11.42	-11.41	-11.4	-11.39	-11.38
-11.46	-11.45	-11.44	-11.43	-11.42	-11.41	-11.4	-11.39	-11.38
-11.46	-11.45	-11.44	-11.43	-11.42	-11.41	-11.4	-11.39	-11.38
-11.46	-11.45	-11.44	-11.43	-11.42	-11.41	-11.4	-11.39	-11.38
-11.46	-11.45	-11.44	-11.43	-11.42	-11.41	-11.4	-11.39	-11.38
-11.46	-11.45	-11.44	-11.43	-11.42	-11.41	-11.4	-11.39	-11.38
-11.46	-11.45	-11.44	-11.43	-11.42	-11.41	-11.4	-11.39	-11.38
-11.46	-11.45	-11.44	-11.43	-11.42	-11.41	-11.4	-11.39	-11.38
-11.46	-11.45	-11.44	-11.43	-11.42	-11.41	-11.4	-11.39	-11.38
-11.46	-11.45	-11.44	-11.43	-11.42	-11.41	-11.4	-11.39	-11.38
-11.46	-11.45	-11.44	-11.43	-11.42	-11.41	-11.4	-11.39	-11.38
-11.46	-11.45	-11.44	-11.43	-11.42	-11.41	-11.4	-11.39	-11.38
-11.46	-11.45	-11.44	-11.43	-11.42	-11.41	-11.4	-11.39	-11.38
-11.46	-11.45	-11.44	-11.43	-11.42	-11.41	-11.4	-11.39	-11.38
-11.46	-11.45	-11.44	-11.43	-11.42	-11.41	-11.4	-11.39	-11.38
-11.46	-11.45	-11.44	-11.43	-11.42	-11.41	-11.4	-11.39	-11.38
-11.46	-11.45	-11.44	-11.43	-11.42	-11.41	-11.4	-11.39	-11.38
-11.46	-11.45	-11.44	-11.43	-11.42	-11.41	-11.4	-11.39	-11.38
-11.46	-11.45	-11.44	-11.43	-11.42	-11.41	-11.4	-11.39	-11.38
-11.46	-11.45	-11.44	-11.43	-11.42	-11.41	-11.4	-11.39	-11.38
-11.46	-11.45	-11.44	-11.43	-11.42	-11.41	-11.4	-11.39	-11.38
-11.46	-11.45	-11.44	-11.43	-11.42	-11.41	-11.4	-11.39	-11.38
-11.46	-11.45	-11.44	-11.43	-11.42	-11.41	-11.4	-11.39	-11.38
-11.46	-11.45	-11.44	-11.43	-11.42	-11.41	-11.4	-11.39	-11.38
-11.46	-11.45	-11.44	-11.43	-11.42	-11.41	-11.4	-11.39	-11.38
-11.46	-11.45	-11.44	-11.43	-11.42	-11.41	-11.4	-11.39	-11.38
-11.46	-11.45	-11.44	-11.43	-11.42	-11.41	-11.4	-11.39	-11.38

-11.46	-11.45	-11.44	-11.43	-11.42	-11.41	-11.4	-11.39	-11.38
-11.46	-11.45	-11.44	-11.43	-11.42	-11.41	-11.4	-11.39	-11.38
-11.46	-11.45	-11.44	-11.43	-11.42	-11.41	-11.4	-11.39	-11.38
-11.46	-11.45	-11.44	-11.43	-11.42	-11.41	-11.4	-11.39	-11.38
-11.46	-11.45	-11.44	-11.43	-11.42	-11.41	-11.4	-11.39	-11.38
-11.46	-11.45	-11.44	-11.43	-11.42	-11.41	-11.4	-11.39	-11.38
-11.46	-11.45	-11.44	-11.43	-11.42	-11.41	-11.4	-11.39	-11.38
-11.46	-11.45	-11.44	-11.43	-11.42	-11.41	-11.4	-11.39	-11.38
-11.46	-11.45	-11.44	-11.43	-11.42	-11.41	-11.4	-11.39	-11.38
-11.46	-11.45	-11.44	-11.43	-11.42	-11.41	-11.4	-11.39	-11.38
-11.46	-11.45	-11.44	-11.43	-11.42	-11.41	-11.4	-11.39	-11.38
-11.46	-11.45	-11.44	-11.43	-11.42	-11.41	-11.4	-11.39	-11.38
-11.46	-11.45	-11.44	-11.43	-11.42	-11.41	-11.4	-11.39	-11.38
-11.46	-11.45	-11.44	-11.43	-11.42	-11.41	-11.4	-11.39	-11.38
-11.46	-11.45	-11.44	-11.43	-11.42	-11.41	-11.4	-11.39	-11.38
-11.46	-11.45	-11.44	-11.43	-11.42	-11.41	-11.4	-11.39	-11.38
-11.46	-11.45	-11.44	-11.43	-11.42	-11.41	-11.4	-11.39	-11.38
-11.46	-11.45	-11.44	-11.43	-11.42	-11.41	-11.4	-11.39	-11.38
-11.46	-11.45	-11.44	-11.43	-11.42	-11.41	-11.4	-11.39	-11.38
-11.46	-11.45	-11.44	-11.43	-11.42	-11.41	-11.4	-11.39	-11.38
-11.46	-11.45	-11.44	-11.43	-11.42	-11.41	-11.4	-11.39	-11.38
-11.46	-11.45	-11.44	-11.43	-11.42	-11.41	-11.4	-11.39	-11.38
-11.46	-11.45	-11.44	-11.43	-11.42	-11.41	-11.4	-11.39	-11.38
-11.46	-11.45	-11.44	-11.43	-11.42	-11.41	-11.4	-11.39	-11.38
-11.46	-11.45	-11.44	-11.43	-11.42	-11.41	-11.4	-11.39	-11.38
-11.46	-11.45	-11.44	-11.43	-11.42	-11.41	-11.4	-11.39	-11.38
-11.46	-11.45	-11.44	-11.43	-11.42	-11.41	-11.4	-11.39	-11.38
-11.46	-11.45	-11.44	-11.43	-11.42	-11.41	-11.4	-11.39	-11.38
-11.46	-11.45	-11.44	-11.43	-11.42	-11.41	-11.4	-11.39	-11.38
-11.46	-11.45	-11.44	-11.43	-11.42	-11.41	-11.4	-11.39	-11.38
-11.46	-11.45	-11.44	-11.43	-11.42	-11.41	-11.4	-11.39	-11.38
-11.46	-11.45	-11.44	-11.43	-11.42	-11.41	-11.4	-11.39	-11.38
-11.46	-11.45	-11.44	-11.43	-11.42	-11.41	-11.4	-11.39	-11.38
-11.46	-11.45	-11.44	-11.43	-11.42	-11.41	-11.4	-11.39	-11.38
-11.46	-11.45	-11.44	-11.43	-11.42	-11.41	-11.4	-11.39	-11.38
-11.46	-11.45	-11.44	-11.43	-11.42	-11.41	-11.4	-11.39	-11.38
-11.46	-11.45	-11.44	-11.43	-11.42	-11.41	-11.4	-11.39	-11.38
-11.46	-11.45	-11.44	-11.43	-11.42	-11.41	-11.4	-11.39	-11.38
-11.46	-11.45	-11.44	-11.43	-11.42	-11.41	-11.4	-11.39	-11.38
-11.46	-11.45	-11.44	-11.43	-11.42	-11.41	-11.4	-11.39	-11.38
-11.46	-11.45	-11.44	-11.43	-11.42	-11.41	-11.4	-11.39	-11.38
-11.46	-11.45	-11.44	-11.43	-11.42	-11.41	-11.4	-11.39	-11.38
-11.46	-11.45	-11.44	-11.43	-11.42	-11.41	-11.4	-11.39	-11.38
-11.46	-11.45	-11.44	-11.43	-11.42	-11.41	-11.4	-11.39	-11.38
-11.46	-11.45	-11.44	-11.43	-11.42	-11.41	-11.4	-11.39	-11.38
-11.46	-11.45	-11.44	-11.43	-11.42	-11.41	-11.4	-11.39	-11.38
-11.46	-11.45	-11.44	-11.43	-11.42	-11.41	-11.4	-11.39	-11.38
-11.46	-11.45	-11.44	-11.43	-11.42	-11.41	-11.4	-11.39	-11.38
-11.46	-11.45	-11.44	-11.43	-11.42	-11.41	-11.4	-11.39	-11.38
-11.46	-11.45	-11.44	-11.43	-11.42	-11.41	-11.4	-11.39	-11.38

-11.46	-11.45	-11.44	-11.43	-11.42	-11.41	-11.4	-11.39	-11.38
-11.46	-11.45	-11.44	-11.43	-11.42	-11.41	-11.4	-11.39	-11.38
-11.46	-11.45	-11.44	-11.43	-11.42	-11.41	-11.4	-11.39	-11.38
-11.46	-11.45	-11.44	-11.43	-11.42	-11.41	-11.4	-11.39	-11.38
-11.46	-11.45	-11.44	-11.43	-11.42	-11.41	-11.4	-11.39	-11.38
-11.46	-11.45	-11.44	-11.43	-11.42	-11.41	-11.4	-11.39	-11.38
-11.46	-11.45	-11.44	-11.43	-11.42	-11.41	-11.4	-11.39	-11.38
-11.46	-11.45	-11.44	-11.43	-11.42	-11.41	-11.4	-11.39	-11.38
-11.46	-11.45	-11.44	-11.43	-11.42	-11.41	-11.4	-11.39	-11.38
-11.46	-11.45	-11.44	-11.43	-11.42	-11.41	-11.4	-11.39	-11.38
-11.46	-11.45	-11.44	-11.43	-11.42	-11.41	-11.4	-11.39	-11.38
-11.46	-11.45	-11.44	-11.43	-11.42	-11.41	-11.4	-11.39	-11.38
-11.46	-11.45	-11.44	-11.43	-11.42	-11.41	-11.4	-11.39	-11.38
-11.46	-11.45	-11.44	-11.43	-11.42	-11.41	-11.4	-11.39	-11.38
-11.46	-11.45	-11.44	-11.43	-11.42	-11.41	-11.4	-11.39	-11.38
-11.46	-11.45	-11.44	-11.43	-11.42	-11.41	-11.4	-11.39	-11.38
-11.46	-11.45	-11.44	-11.43	-11.42	-11.41	-11.4	-11.39	-11.38
-11.46	-11.45	-11.44	-11.43	-11.42	-11.41	-11.4	-11.39	-11.38
-11.46	-11.45	-11.44	-11.43	-11.42	-11.41	-11.4	-11.39	-11.38
-11.46	-11.45	-11.44	-11.43	-11.42	-11.41	-11.4	-11.39	-11.38
-11.46	-11.45	-11.44	-11.43	-11.42	-11.41	-11.4	-11.39	-11.38
-11.46	-11.45	-11.44	-11.43	-11.42	-11.41	-11.4	-11.39	-11.38
-11.46	-11.45	-11.44	-11.43	-11.42	-11.41	-11.4	-11.39	-11.38
-11.46	-11.45	-11.44	-11.43	-11.42	-11.41	-11.4	-11.39	-11.38
-11.46	-11.45	-11.44	-11.43	-11.42	-11.41	-11.4	-11.39	-11.38
-11.46	-11.45	-11.44	-11.43	-11.42	-11.41	-11.4	-11.39	-11.38
-11.46	-11.45	-11.44	-11.43	-11.42	-11.41	-11.4	-11.39	-11.38
-11.46	-11.45	-11.44	-11.43	-11.42	-11.41	-11.4	-11.39	-11.38
-11.46	-11.45	-11.44	-11.43	-11.42	-11.41	-11.4	-11.39	-11.38
-11.46	-11.45	-11.44	-11.43	-11.42	-11.41	-11.4	-11.39	-11.38
-11.46	-11.45	-11.44	-11.43	-11.42	-11.41	-11.4	-11.39	-11.38
-11.46	-11.45	-11.44	-11.43	-11.42	-11.41	-11.4	-11.39	-11.38
-11.46	-11.45	-11.44	-11.43	-11.42	-11.41	-11.4	-11.39	-11.38
-11.46	-11.45	-11.44	-11.43	-11.42	-11.41	-11.4	-11.39	-11.38
-11.46	-11.45	-11.44	-11.43	-11.42	-11.41	-11.4	-11.39	-11.38
-11.46	-11.45	-11.44	-11.43	-11.42	-11.41	-11.4	-11.39	-11.38
-11.46	-11.45	-11.44	-11.43	-11.42	-11.41	-11.4	-11.39	-11.38
-11.46	-11.45	-11.44	-11.43	-11.42	-11.41	-11.4	-11.39	-11.38
-11.46	-11.45	-11.44	-11.43	-11.42	-11.41	-11.4	-11.39	-11.38
-11.46	-11.45	-11.44	-11.43	-11.42	-11.41	-11.4	-11.39	-11.38
-11.46	-11.45	-11.44	-11.43	-11.42	-11.41	-11.4	-11.39	-11.38
-11.46	-11.45	-11.44	-11.43	-11.42	-11.41	-11.4	-11.39	-11.38
-11.46	-11.45	-11.44	-11.43	-11.42	-11.41	-11.4	-11.39	-11.38
-11.46	-11.45	-11.44	-11.43	-11.42	-11.41	-11.4	-11.39	-11.38
-11.46	-11.45	-11.44	-11.43	-11.42	-11.41	-11.4	-11.39	-11.38
-11.46	-11.45	-11.44	-11.43	-11.42	-11.41	-11.4	-11.39	-11.38
-11.46	-11.45	-11.44	-11.43	-11.42	-11.41	-11.4	-11.39	-11.38
-11.46	-11.45	-11.44	-11.43	-11.42	-11.41	-11.4	-11.39	-11.38
-11.46	-11.45	-11.44	-11.43	-11.42	-11.41	-11.4	-11.39	-11.38
-11.46	-11.45	-11.44	-11.43	-11.42	-11.41	-11.4	-11.39	-11.38

-11.46	-11.45	-11.44	-11.43	-11.42	-11.41	-11.4	-11.39	-11.38
-11.46	-11.45	-11.44	-11.43	-11.42	-11.41	-11.4	-11.39	-11.38
-11.46	-11.45	-11.44	-11.43	-11.42	-11.41	-11.4	-11.39	-11.38
-11.46	-11.45	-11.44	-11.43	-11.42	-11.41	-11.4	-11.39	-11.38
-11.46	-11.45	-11.44	-11.43	-11.42	-11.41	-11.4	-11.39	-11.38
-11.46	-11.45	-11.44	-11.43	-11.42	-11.41	-11.4	-11.39	-11.38
-11.46	-11.45	-11.44	-11.43	-11.42	-11.41	-11.4	-11.39	-11.38
-11.46	-11.45	-11.44	-11.43	-11.42	-11.41	-11.4	-11.39	-11.38
-11.46	-11.45	-11.44	-11.43	-11.42	-11.41	-11.4	-11.39	-11.38
-11.46	-11.45	-11.44	-11.43	-11.42	-11.41	-11.4	-11.39	-11.38
-11.46	-11.45	-11.44	-11.43	-11.42	-11.41	-11.4	-11.39	-11.38
-11.46	-11.45	-11.44	-11.43	-11.42	-11.41	-11.4	-11.39	-11.38
-11.46	-11.45	-11.44	-11.43	-11.42	-11.41	-11.4	-11.39	-11.38
-11.46	-11.45	-11.44	-11.43	-11.42	-11.41	-11.4	-11.39	-11.38
-11.46	-11.45	-11.44	-11.43	-11.42	-11.41	-11.4	-11.39	-11.38
-11.46	-11.45	-11.44	-11.43	-11.42	-11.41	-11.4	-11.39	-11.38
-11.46	-11.45	-11.44	-11.43	-11.42	-11.41	-11.4	-11.39	-11.38
-11.46	-11.45	-11.44	-11.43	-11.42	-11.41	-11.4	-11.39	-11.38
-11.46	-11.45	-11.44	-11.43	-11.42	-11.41	-11.4	-11.39	-11.38
-11.46	-11.45	-11.44	-11.43	-11.42	-11.41	-11.4	-11.39	-11.38
-11.46	-11.45	-11.44	-11.43	-11.42	-11.41	-11.4	-11.39	-11.38
-11.46	-11.45	-11.44	-11.43	-11.42	-11.41	-11.4	-11.39	-11.38
-11.46	-11.45	-11.44	-11.43	-11.42	-11.41	-11.4	-11.39	-11.38
-11.46	-11.45	-11.44	-11.43	-11.42	-11.41	-11.4	-11.39	-11.38
-11.46	-11.45	-11.44	-11.43	-11.42	-11.41	-11.4	-11.39	-11.38
-11.46	-11.45	-11.44	-11.43	-11.42	-11.41	-11.4	-11.39	-11.38
-11.46	-11.45	-11.44	-11.43	-11.42	-11.41	-11.4	-11.39	-11.38
-11.46	-11.45	-11.44	-11.43	-11.42	-11.41	-11.4	-11.39	-11.38
-11.46	-11.45	-11.44	-11.43	-11.42	-11.41	-11.4	-11.39	-11.38
-11.46	-11.45	-11.44	-11.43	-11.42	-11.41	-11.4	-11.39	-11.38
-11.46	-11.45	-11.44	-11.43	-11.42	-11.41	-11.4	-11.39	-11.38
-11.46	-11.45	-11.44	-11.43	-11.42	-11.41	-11.4	-11.39	-11.38
-11.46	-11.45	-11.44	-11.43	-11.42	-11.41	-11.4	-11.39	-11.38
-11.46	-11.45	-11.44	-11.43	-11.42	-11.41	-11.4	-11.39	-11.38
-11.46	-11.45	-11.44	-11.43	-11.42	-11.41	-11.4	-11.39	-11.38
-11.46	-11.45	-11.44	-11.43	-11.42	-11.41	-11.4	-11.39	-11.38
-11.46	-11.45	-11.44	-11.43	-11.42	-11.41	-11.4	-11.39	-11.38
-11.46	-11.45	-11.44	-11.43	-11.42	-11.41	-11.4	-11.39	-11.38
-11.46	-11.45	-11.44	-11.43	-11.42	-11.41	-11.4	-11.39	-11.38
-11.46	-11.45	-11.44	-11.43	-11.42	-11.41	-11.4	-11.39	-11.38
-11.46	-11.45	-11.44	-11.43	-11.42	-11.41	-11.4	-11.39	-11.38
-11.46	-11.45	-11.44	-11.43	-11.42	-11.41	-11.4	-11.39	-11.38
-11.46	-11.45	-11.44	-11.43	-11.42	-11.41	-11.4	-11.39	-11.38
-11.46	-11.45	-11.44	-11.43	-11.42	-11.41	-11.4	-11.39	-11.38
-11.46	-11.45	-11.44	-11.43	-11.42	-11.41	-11.4	-11.39	-11.38
-11.46	-11.45	-11.44	-11.43	-11.42	-11.41	-11.4	-11.39	-11.38
-11.46	-11.45	-11.44	-11.43	-11.42	-11.41	-11.4	-11.39	-11.38
-11.46	-11.45	-11.44	-11.43	-11.42	-11.41	-11.4	-11.39	-11.38
-11.46	-11.45	-11.44	-11.43	-11.42	-11.41	-11.4	-11.39	-11.38
-11.46	-11.45	-11.44	-11.43	-11.42	-11.41	-11.4	-11.39	-11.38

-11.46	-11.45	-11.44	-11.43	-11.42	-11.41	-11.4	-11.39	-11.38
-11.46	-11.45	-11.44	-11.43	-11.42	-11.41	-11.4	-11.39	-11.38
-11.46	-11.45	-11.44	-11.43	-11.42	-11.41	-11.4	-11.39	-11.38
-11.46	-11.45	-11.44	-11.43	-11.42	-11.41	-11.4	-11.39	-11.38
-11.46	-11.45	-11.44	-11.43	-11.42	-11.41	-11.4	-11.39	-11.38
-11.46	-11.45	-11.44	-11.43	-11.42	-11.41	-11.4	-11.39	-11.38
-11.46	-11.45	-11.44	-11.43	-11.42	-11.41	-11.4	-11.39	-11.38
-11.46	-11.45	-11.44	-11.43	-11.42	-11.41	-11.4	-11.39	-11.38
-11.46	-11.45	-11.44	-11.43	-11.42	-11.41	-11.4	-11.39	-11.38
-11.46	-11.45	-11.44	-11.43	-11.42	-11.41	-11.4	-11.39	-11.38
-11.46	-11.45	-11.44	-11.43	-11.42	-11.41	-11.4	-11.39	-11.38
-11.46	-11.45	-11.44	-11.43	-11.42	-11.41	-11.4	-11.39	-11.38
-11.46	-11.45	-11.44	-11.43	-11.42	-11.41	-11.4	-11.39	-11.38
-11.46	-11.45	-11.44	-11.43	-11.42	-11.41	-11.4	-11.39	-11.38
-11.46	-11.45	-11.44	-11.43	-11.42	-11.41	-11.4	-11.39	-11.38
-11.46	-11.45	-11.44	-11.43	-11.42	-11.41	-11.4	-11.39	-11.38
-11.46	-11.45	-11.44	-11.43	-11.42	-11.41	-11.4	-11.39	-11.38
-11.46	-11.45	-11.44	-11.43	-11.42	-11.41	-11.4	-11.39	-11.38
-11.46	-11.45	-11.44	-11.43	-11.42	-11.41	-11.4	-11.39	-11.38
-11.46	-11.45	-11.44	-11.43	-11.42	-11.41	-11.4	-11.39	-11.38
-11.46	-11.45	-11.44	-11.43	-11.42	-11.41	-11.4	-11.39	-11.38
-11.46	-11.45	-11.44	-11.43	-11.42	-11.41	-11.4	-11.39	-11.38
-11.46	-11.45	-11.44	-11.43	-11.42	-11.41	-11.4	-11.39	-11.38
-11.46	-11.45	-11.44	-11.43	-11.42	-11.41	-11.4	-11.39	-11.38
-11.46	-11.45	-11.44	-11.43	-11.42	-11.41	-11.4	-11.39	-11.38
-11.46	-11.45	-11.44	-11.43	-11.42	-11.41	-11.4	-11.39	-11.38
-11.46	-11.45	-11.44	-11.43	-11.42	-11.41	-11.4	-11.39	-11.38
-11.46	-11.45	-11.44	-11.43	-11.42	-11.41	-11.4	-11.39	-11.38
-11.46	-11.45	-11.44	-11.43	-11.42	-11.41	-11.4	-11.39	-11.38
-11.46	-11.45	-11.44	-11.43	-11.42	-11.41	-11.4	-11.39	-11.38
-11.46	-11.45	-11.44	-11.43	-11.42	-11.41	-11.4	-11.39	-11.38
-11.46	-11.45	-11.44	-11.43	-11.42	-11.41	-11.4	-11.39	-11.38
-11.46	-11.45	-11.44	-11.43	-11.42	-11.41	-11.4	-11.39	-11.38
-11.46	-11.45	-11.44	-11.43	-11.42	-11.41	-11.4	-11.39	-11.38
-11.46	-11.45	-11.44	-11.43	-11.42	-11.41	-11.4	-11.39	-11.38
-11.46	-11.45	-11.44	-11.43	-11.42	-11.41	-11.4	-11.39	-11.38
-11.46	-11.45	-11.44	-11.43	-11.42	-11.41	-11.4	-11.39	-11.38
-11.46	-11.45	-11.44	-11.43	-11.42	-11.41	-11.4	-11.39	-11.38
-11.46	-11.45	-11.44	-11.43	-11.42	-11.41	-11.4	-11.39	-11.38
-11.46	-11.45	-11.44	-11.43	-11.42	-11.41	-11.4	-11.39	-11.38
-11.46	-11.45	-11.44	-11.43	-11.42	-11.41	-11.4	-11.39	-11.38
-11.46	-11.45	-11.44	-11.43	-11.42	-11.41	-11.4	-11.39	-11.38
-11.46	-11.45	-11.44	-11.43	-11.42	-11.41	-11.4	-11.39	-11.38
-11.46	-11.45	-11.44	-11.43	-11.42	-11.41	-11.4	-11.39	-11.38
-11.46	-11.45	-11.44	-11.43	-11.42	-11.41	-11.4	-11.39	-11.38
-11.46	-11.45	-11.44	-11.43	-11.42	-11.41	-11.4	-11.39	-11.38
-11.46	-11.45	-11.44	-11.43	-11.42	-11.41	-11.4	-11.39	-11.38
-11.46	-11.45	-11.44	-11.43	-11.42	-11.41	-11.4	-11.39	-11.38
-11.46	-11.45	-11.44	-11.43	-11.42	-11.41	-11.4	-11.39	-11.38
-11.46	-11.45	-11.44	-11.43	-11.42	-11.41	-11.4	-11.39	-11.38

-11.46	-11.45	-11.44	-11.43	-11.42	-11.41	-11.4	-11.39	-11.38
-11.46	-11.45	-11.44	-11.43	-11.42	-11.41	-11.4	-11.39	-11.38
-11.46	-11.45	-11.44	-11.43	-11.42	-11.41	-11.4	-11.39	-11.38
-11.46	-11.45	-11.44	-11.43	-11.42	-11.41	-11.4	-11.39	-11.38
-11.46	-11.45	-11.44	-11.43	-11.42	-11.41	-11.4	-11.39	-11.38
-11.46	-11.45	-11.44	-11.43	-11.42	-11.41	-11.4	-11.39	-11.38
-11.46	-11.45	-11.44	-11.43	-11.42	-11.41	-11.4	-11.39	-11.38
-11.46	-11.45	-11.44	-11.43	-11.42	-11.41	-11.4	-11.39	-11.38
-11.46	-11.45	-11.44	-11.43	-11.42	-11.41	-11.4	-11.39	-11.38
-11.46	-11.45	-11.44	-11.43	-11.42	-11.41	-11.4	-11.39	-11.38
-11.46	-11.45	-11.44	-11.43	-11.42	-11.41	-11.4	-11.39	-11.38
-11.46	-11.45	-11.44	-11.43	-11.42	-11.41	-11.4	-11.39	-11.38
-11.46	-11.45	-11.44	-11.43	-11.42	-11.41	-11.4	-11.39	-11.38
-11.46	-11.45	-11.44	-11.43	-11.42	-11.41	-11.4	-11.39	-11.38
-11.46	-11.45	-11.44	-11.43	-11.42	-11.41	-11.4	-11.39	-11.38
-11.46	-11.45	-11.44	-11.43	-11.42	-11.41	-11.4	-11.39	-11.38
-11.46	-11.45	-11.44	-11.43	-11.42	-11.41	-11.4	-11.39	-11.38
-11.46	-11.45	-11.44	-11.43	-11.42	-11.41	-11.4	-11.39	-11.38
-11.46	-11.45	-11.44	-11.43	-11.42	-11.41	-11.4	-11.39	-11.38
-11.46	-11.45	-11.44	-11.43	-11.42	-11.41	-11.4	-11.39	-11.38
-11.46	-11.45	-11.44	-11.43	-11.42	-11.41	-11.4	-11.39	-11.38
-11.46	-11.45	-11.44	-11.43	-11.42	-11.41	-11.4	-11.39	-11.38
-11.46	-11.45	-11.44	-11.43	-11.42	-11.41	-11.4	-11.39	-11.38
-11.46	-11.45	-11.44	-11.43	-11.42	-11.41	-11.4	-11.39	-11.38
-11.46	-11.45	-11.44	-11.43	-11.42	-11.41	-11.4	-11.39	-11.38
-11.46	-11.45	-11.44	-11.43	-11.42	-11.41	-11.4	-11.39	-11.38
-11.46	-11.45	-11.44	-11.43	-11.42	-11.41	-11.4	-11.39	-11.38
-11.46	-11.45	-11.44	-11.43	-11.42	-11.41	-11.4	-11.39	-11.38
-11.46	-11.45	-11.44	-11.43	-11.42	-11.41	-11.4	-11.39	-11.38
-11.46	-11.45	-11.44	-11.43	-11.42	-11.41	-11.4	-11.39	-11.38
-11.46	-11.45	-11.44	-11.43	-11.42	-11.41	-11.4	-11.39	-11.38
-11.46	-11.45	-11.44	-11.43	-11.42	-11.41	-11.4	-11.39	-11.38
-11.46	-11.45	-11.44	-11.43	-11.42	-11.41	-11.4	-11.39	-11.38
-11.46	-11.45	-11.44	-11.43	-11.42	-11.41	-11.4	-11.39	-11.38
-11.46	-11.45	-11.44	-11.43	-11.42	-11.41	-11.4	-11.39	-11.38
-11.46	-11.45	-11.44	-11.43	-11.42	-11.41	-11.4	-11.39	-11.38
-11.46	-11.45	-11.44	-11.43	-11.42	-11.41	-11.4	-11.39	-11.38
-11.46	-11.45	-11.44	-11.43	-11.42	-11.41	-11.4	-11.39	-11.38
-11.46	-11.45	-11.44	-11.43	-11.42	-11.41	-11.4	-11.39	-11.38
-11.46	-11.45	-11.44	-11.43	-11.42	-11.41	-11.4	-11.39	-11.38
-11.46	-11.45	-11.44	-11.43	-11.42	-11.41	-11.4	-11.39	-11.38
-11.46	-11.45	-11.44	-11.43	-11.42	-11.41	-11.4	-11.39	-11.38
-11.46	-11.45	-11.44	-11.43	-11.42	-11.41	-11.4	-11.39	-11.38
-11.46	-11.45	-11.44	-11.43	-11.42	-11.41	-11.4	-11.39	-11.38
-11.46	-11.45	-11.44	-11.43	-11.42	-11.41	-11.4	-11.39	-11.38
-11.46	-11.45	-11.44	-11.43	-11.42	-11.41	-11.4	-11.39	-11.38
-11.46	-11.45	-11.44	-11.43	-11.42	-11.41	-11.4	-11.39	-11.38
-11.46	-11.45	-11.44	-11.43	-11.42	-11.41	-11.4	-11.39	-11.38
-11.46	-11.45	-11.44	-11.43	-11.42	-11.41	-11.4	-11.39	-11.38
-11.46	-11.45	-11.44	-11.43	-11.42	-11.41	-11.4	-11.39	-11.38

-11.46	-11.45	-11.44	-11.43	-11.42	-11.41	-11.4	-11.39	-11.38
-11.46	-11.45	-11.44	-11.43	-11.42	-11.41	-11.4	-11.39	-11.38
-11.46	-11.45	-11.44	-11.43	-11.42	-11.41	-11.4	-11.39	-11.38
-11.46	-11.45	-11.44	-11.43	-11.42	-11.41	-11.4	-11.39	-11.38
-11.46	-11.45	-11.44	-11.43	-11.42	-11.41	-11.4	-11.39	-11.38
-11.46	-11.45	-11.44	-11.43	-11.42	-11.41	-11.4	-11.39	-11.38
-11.46	-11.45	-11.44	-11.43	-11.42	-11.41	-11.4	-11.39	-11.38
-11.46	-11.45	-11.44	-11.43	-11.42	-11.41	-11.4	-11.39	-11.38
-11.46	-11.45	-11.44	-11.43	-11.42	-11.41	-11.4	-11.39	-11.38
-11.46	-11.45	-11.44	-11.43	-11.42	-11.41	-11.4	-11.39	-11.38
-11.46	-11.45	-11.44	-11.43	-11.42	-11.41	-11.4	-11.39	-11.38
-11.46	-11.45	-11.44	-11.43	-11.42	-11.41	-11.4	-11.39	-11.38
-11.46	-11.45	-11.44	-11.43	-11.42	-11.41	-11.4	-11.39	-11.38
-11.46	-11.45	-11.44	-11.43	-11.42	-11.41	-11.4	-11.39	-11.38
-11.46	-11.45	-11.44	-11.43	-11.42	-11.41	-11.4	-11.39	-11.38
-11.46	-11.45	-11.44	-11.43	-11.42	-11.41	-11.4	-11.39	-11.38
-11.46	-11.45	-11.44	-11.43	-11.42	-11.41	-11.4	-11.39	-11.38
-11.46	-11.45	-11.44	-11.43	-11.42	-11.41	-11.4	-11.39	-11.38
-11.46	-11.45	-11.44	-11.43	-11.42	-11.41	-11.4	-11.39	-11.38
-11.46	-11.45	-11.44	-11.43	-11.42	-11.41	-11.4	-11.39	-11.38
-11.46	-11.45	-11.44	-11.43	-11.42	-11.41	-11.4	-11.39	-11.38
-11.46	-11.45	-11.44	-11.43	-11.42	-11.41	-11.4	-11.39	-11.38
-11.46	-11.45	-11.44	-11.43	-11.42	-11.41	-11.4	-11.39	-11.38
-11.46	-11.45	-11.44	-11.43	-11.42	-11.41	-11.4	-11.39	-11.38
-11.46	-11.45	-11.44	-11.43	-11.42	-11.41	-11.4	-11.39	-11.38
-11.46	-11.45	-11.44	-11.43	-11.42	-11.41	-11.4	-11.39	-11.38
-11.46	-11.45	-11.44	-11.43	-11.42	-11.41	-11.4	-11.39	-11.38
-11.46	-11.45	-11.44	-11.43	-11.42	-11.41	-11.4	-11.39	-11.38
-11.46	-11.45	-11.44	-11.43	-11.42	-11.41	-11.4	-11.39	-11.38
-11.46	-11.45	-11.44	-11.43	-11.42	-11.41	-11.4	-11.39	-11.38
-11.46	-11.45	-11.44	-11.43	-11.42	-11.41	-11.4	-11.39	-11.38
-11.46	-11.45	-11.44	-11.43	-11.42	-11.41	-11.4	-11.39	-11.38
-11.46	-11.45	-11.44	-11.43	-11.42	-11.41	-11.4	-11.39	-11.38
-11.46	-11.45	-11.44	-11.43	-11.42	-11.41	-11.4	-11.39	-11.38
-11.46	-11.45	-11.44	-11.43	-11.42	-11.41	-11.4	-11.39	-11.38
-11.46	-11.45	-11.44	-11.43	-11.42	-11.41	-11.4	-11.39	-11.38
-11.46	-11.45	-11.44	-11.43	-11.42	-11.41	-11.4	-11.39	-11.38
-11.46	-11.45	-11.44	-11.43	-11.42	-11.41	-11.4	-11.39	-11.38
-11.46	-11.45	-11.44	-11.43	-11.42	-11.41	-11.4	-11.39	-11.38
-11.46	-11.45	-11.44	-11.43	-11.42	-11.41	-11.4	-11.39	-11.38
-11.46	-11.45	-11.44	-11.43	-11.42	-11.41	-11.4	-11.39	-11.38
-11.46	-11.45	-11.44	-11.43	-11.42	-11.41	-11.4	-11.39	-11.38
-11.46	-11.45	-11.44	-11.43	-11.42	-11.41	-11.4	-11.39	-11.38
-11.46	-11.45	-11.44	-11.43	-11.42	-11.41	-11.4	-11.39	-11.38
-11.46	-11.45	-11.44	-11.43	-11.42	-11.41	-11.4	-11.39	-11.38
-11.46	-11.45	-11.44	-11.43	-11.42	-11.41	-11.4	-11.39	-11.38
-11.46	-11.45	-11.44	-11.43	-11.42	-11.41	-11.4	-11.39	-11.38
-11.46	-11.45	-11.44	-11.43	-11.42	-11.41	-11.4	-11.39	-11.38
-11.46	-11.45	-11.44	-11.43	-11.42	-11.41	-11.4	-11.39	-11.38
-11.46	-11.45	-11.44	-11.43	-11.42	-11.41	-11.4	-11.39	-11.38

-11.46	-11.45	-11.44	-11.43	-11.42	-11.41	-11.4	-11.39	-11.38
-11.46	-11.45	-11.44	-11.43	-11.42	-11.41	-11.4	-11.39	-11.38
-11.46	-11.45	-11.44	-11.43	-11.42	-11.41	-11.4	-11.39	-11.38
-11.46	-11.45	-11.44	-11.43	-11.42	-11.41	-11.4	-11.39	-11.38
-11.46	-11.45	-11.44	-11.43	-11.42	-11.41	-11.4	-11.39	-11.38
-11.46	-11.45	-11.44	-11.43	-11.42	-11.41	-11.4	-11.39	-11.38
-11.46	-11.45	-11.44	-11.43	-11.42	-11.41	-11.4	-11.39	-11.38
-11.46	-11.45	-11.44	-11.43	-11.42	-11.41	-11.4	-11.39	-11.38
-11.46	-11.45	-11.44	-11.43	-11.42	-11.41	-11.4	-11.39	-11.38
-11.46	-11.45	-11.44	-11.43	-11.42	-11.41	-11.4	-11.39	-11.38
-11.46	-11.45	-11.44	-11.43	-11.42	-11.41	-11.4	-11.39	-11.38
-11.46	-11.45	-11.44	-11.43	-11.42	-11.41	-11.4	-11.39	-11.38
-11.46	-11.45	-11.44	-11.43	-11.42	-11.41	-11.4	-11.39	-11.38
-11.46	-11.45	-11.44	-11.43	-11.42	-11.41	-11.4	-11.39	-11.38
-11.46	-11.45	-11.44	-11.43	-11.42	-11.41	-11.4	-11.39	-11.38
-11.46	-11.45	-11.44	-11.43	-11.42	-11.41	-11.4	-11.39	-11.38
-11.46	-11.45	-11.44	-11.43	-11.42	-11.41	-11.4	-11.39	-11.38
-11.46	-11.45	-11.44	-11.43	-11.42	-11.41	-11.4	-11.39	-11.38
-11.46	-11.45	-11.44	-11.43	-11.42	-11.41	-11.4	-11.39	-11.38
-11.46	-11.45	-11.44	-11.43	-11.42	-11.41	-11.4	-11.39	-11.38
-11.46	-11.45	-11.44	-11.43	-11.42	-11.41	-11.4	-11.39	-11.38
-11.46	-11.45	-11.44	-11.43	-11.42	-11.41	-11.4	-11.39	-11.38
-11.46	-11.45	-11.44	-11.43	-11.42	-11.41	-11.4	-11.39	-11.38
-11.46	-11.45	-11.44	-11.43	-11.42	-11.41	-11.4	-11.39	-11.38
-11.46	-11.45	-11.44	-11.43	-11.42	-11.41	-11.4	-11.39	-11.38
-11.46	-11.45	-11.44	-11.43	-11.42	-11.41	-11.4	-11.39	-11.38
-11.46	-11.45	-11.44	-11.43	-11.42	-11.41	-11.4	-11.39	-11.38
-11.46	-11.45	-11.44	-11.43	-11.42	-11.41	-11.4	-11.39	-11.38
-11.46	-11.45	-11.44	-11.43	-11.42	-11.41	-11.4	-11.39	-11.38
-11.46	-11.45	-11.44	-11.43	-11.42	-11.41	-11.4	-11.39	-11.38
-11.46	-11.45	-11.44	-11.43	-11.42	-11.41	-11.4	-11.39	-11.38
-11.46	-11.45	-11.44	-11.43	-11.42	-11.41	-11.4	-11.39	-11.38
-11.46	-11.45	-11.44	-11.43	-11.42	-11.41	-11.4	-11.39	-11.38
-11.46	-11.45	-11.44	-11.43	-11.42	-11.41	-11.4	-11.39	-11.38
-11.46	-11.45	-11.44	-11.43	-11.42	-11.41	-11.4	-11.39	-11.38
-11.46	-11.45	-11.44	-11.43	-11.42	-11.41	-11.4	-11.39	-11.38
-11.46	-11.45	-11.44	-11.43	-11.42	-11.41	-11.4	-11.39	-11.38
-11.46	-11.45	-11.44	-11.43	-11.42	-11.41	-11.4	-11.39	-11.38
-11.46	-11.45	-11.44	-11.43	-11.42	-11.41	-11.4	-11.39	-11.38
-11.46	-11.45	-11.44	-11.43	-11.42	-11.41	-11.4	-11.39	-11.38
-11.46	-11.45	-11.44	-11.43	-11.42	-11.41	-11.4	-11.39	-11.38
-11.46	-11.45	-11.44	-11.43	-11.42	-11.41	-11.4	-11.39	-11.38
-11.46	-11.45	-11.44	-11.43	-11.42	-11.41	-11.4	-11.39	-11.38
-11.46	-11.45	-11.44	-11.43	-11.42	-11.41	-11.4	-11.39	-11.38
-11.46	-11.45	-11.44	-11.43	-11.42	-11.41	-11.4	-11.39	-11.38
-11.46	-11.45	-11.44	-11.43	-11.42	-11.41	-11.4	-11.39	-11.38
-11.46	-11.45	-11.44	-11.43	-11.42	-11.41	-11.4	-11.39	-11.38
-11.46	-11.45	-11.44	-11.43	-11.42	-11.41	-11.4	-11.39	-11.38
-11.46	-11.45	-11.44	-11.43	-11.42	-11.41	-11.4	-11.39	-11.38
-11.46	-11.45	-11.44	-11.43	-11.42	-11.41	-11.4	-11.39	-11.38

-11.46	-11.45	-11.44	-11.43	-11.42	-11.41	-11.4	-11.39	-11.38
-11.46	-11.45	-11.44	-11.43	-11.42	-11.41	-11.4	-11.39	-11.38
-11.46	-11.45	-11.44	-11.43	-11.42	-11.41	-11.4	-11.39	-11.38
-11.46	-11.45	-11.44	-11.43	-11.42	-11.41	-11.4	-11.39	-11.38
-11.46	-11.45	-11.44	-11.43	-11.42	-11.41	-11.4	-11.39	-11.38
-11.46	-11.45	-11.44	-11.43	-11.42	-11.41	-11.4	-11.39	-11.38
-11.46	-11.45	-11.44	-11.43	-11.42	-11.41	-11.4	-11.39	-11.38
-11.46	-11.45	-11.44	-11.43	-11.42	-11.41	-11.4	-11.39	-11.38
-11.46	-11.45	-11.44	-11.43	-11.42	-11.41	-11.4	-11.39	-11.38
-11.46	-11.45	-11.44	-11.43	-11.42	-11.41	-11.4	-11.39	-11.38
-11.46	-11.45	-11.44	-11.43	-11.42	-11.41	-11.4	-11.39	-11.38
-11.46	-11.45	-11.44	-11.43	-11.42	-11.41	-11.4	-11.39	-11.38
-11.46	-11.45	-11.44	-11.43	-11.42	-11.41	-11.4	-11.39	-11.38
-11.46	-11.45	-11.44	-11.43	-11.42	-11.41	-11.4	-11.39	-11.38
-11.46	-11.45	-11.44	-11.43	-11.42	-11.41	-11.4	-11.39	-11.38
-11.46	-11.45	-11.44	-11.43	-11.42	-11.41	-11.4	-11.39	-11.38
-11.46	-11.45	-11.44	-11.43	-11.42	-11.41	-11.4	-11.39	-11.38
-11.46	-11.45	-11.44	-11.43	-11.42	-11.41	-11.4	-11.39	-11.38
-11.46	-11.45	-11.44	-11.43	-11.42	-11.41	-11.4	-11.39	-11.38
-11.46	-11.45	-11.44	-11.43	-11.42	-11.41	-11.4	-11.39	-11.38
-11.46	-11.45	-11.44	-11.43	-11.42	-11.41	-11.4	-11.39	-11.38
-11.46	-11.45	-11.44	-11.43	-11.42	-11.41	-11.4	-11.39	-11.38
-11.46	-11.45	-11.44	-11.43	-11.42	-11.41	-11.4	-11.39	-11.38
-11.46	-11.45	-11.44	-11.43	-11.42	-11.41	-11.4	-11.39	-11.38
-11.46	-11.45	-11.44	-11.43	-11.42	-11.41	-11.4	-11.39	-11.38
-11.46	-11.45	-11.44	-11.43	-11.42	-11.41	-11.4	-11.39	-11.38
-11.46	-11.45	-11.44	-11.43	-11.42	-11.41	-11.4	-11.39	-11.38
-11.46	-11.45	-11.44	-11.43	-11.42	-11.41	-11.4	-11.39	-11.38
-11.46	-11.45	-11.44	-11.43	-11.42	-11.41	-11.4	-11.39	-11.38
-11.46	-11.45	-11.44	-11.43	-11.42	-11.41	-11.4	-11.39	-11.38
-11.46	-11.45	-11.44	-11.43	-11.42	-11.41	-11.4	-11.39	-11.38
-11.46	-11.45	-11.44	-11.43	-11.42	-11.41	-11.4	-11.39	-11.38
-11.46	-11.45	-11.44	-11.43	-11.42	-11.41	-11.4	-11.39	-11.38
-11.46	-11.45	-11.44	-11.43	-11.42	-11.41	-11.4	-11.39	-11.38
-11.46	-11.45	-11.44	-11.43	-11.42	-11.41	-11.4	-11.39	-11.38
-11.46	-11.45	-11.44	-11.43	-11.42	-11.41	-11.4	-11.39	-11.38
-11.46	-11.45	-11.44	-11.43	-11.42	-11.41	-11.4	-11.39	-11.38
-11.46	-11.45	-11.44	-11.43	-11.42	-11.41	-11.4	-11.39	-11.38
-11.46	-11.45	-11.44	-11.43	-11.42	-11.41	-11.4	-11.39	-11.38
-11.46	-11.45	-11.44	-11.43	-11.42	-11.41	-11.4	-11.39	-11.38
-11.46	-11.45	-11.44	-11.43	-11.42	-11.41	-11.4	-11.39	-11.38
-11.46	-11.45	-11.44	-11.43	-11.42	-11.41	-11.4	-11.39	-11.38
-11.46	-11.45	-11.44	-11.43	-11.42	-11.41	-11.4	-11.39	-11.38
-11.46	-11.45	-11.44	-11.43	-11.42	-11.41	-11.4	-11.39	-11.38
-11.46	-11.45	-11.44	-11.43	-11.42	-11.41	-11.4	-11.39	-11.38
-11.46	-11.45	-11.44	-11.43	-11.42	-11.41	-11.4	-11.39	-11.38
-11.46	-11.45	-11.44	-11.43	-11.42	-11.41	-11.4	-11.39	-11.38
-11.46	-11.45	-11.44	-11.43	-11.42	-11.41	-11.4	-11.39	-11.38
-11.46	-11.45	-11.44	-11.43	-11.42	-11.41	-11.4	-11.39	-11.38
-11.46	-11.45	-11.44	-11.43	-11.42	-11.41	-11.4	-11.39	-11.38

-11.46	-11.45	-11.44	-11.43	-11.42	-11.41	-11.4	-11.39	-11.38
-11.46	-11.45	-11.44	-11.43	-11.42	-11.41	-11.4	-11.39	-11.38
-11.46	-11.45	-11.44	-11.43	-11.42	-11.41	-11.4	-11.39	-11.38
-11.46	-11.45	-11.44	-11.43	-11.42	-11.41	-11.4	-11.39	-11.38
-11.46	-11.45	-11.44	-11.43	-11.42	-11.41	-11.4	-11.39	-11.38
-11.46	-11.45	-11.44	-11.43	-11.42	-11.41	-11.4	-11.39	-11.38
-11.46	-11.45	-11.44	-11.43	-11.42	-11.41	-11.4	-11.39	-11.38
-11.46	-11.45	-11.44	-11.43	-11.42	-11.41	-11.4	-11.39	-11.38
-11.46	-11.45	-11.44	-11.43	-11.42	-11.41	-11.4	-11.39	-11.38
-11.46	-11.45	-11.44	-11.43	-11.42	-11.41	-11.4	-11.39	-11.38
-11.46	-11.45	-11.44	-11.43	-11.42	-11.41	-11.4	-11.39	-11.38
-11.46	-11.45	-11.44	-11.43	-11.42	-11.41	-11.4	-11.39	-11.38
-11.46	-11.45	-11.44	-11.43	-11.42	-11.41	-11.4	-11.39	-11.38
-11.46	-11.45	-11.44	-11.43	-11.42	-11.41	-11.4	-11.39	-11.38
-11.46	-11.45	-11.44	-11.43	-11.42	-11.41	-11.4	-11.39	-11.38
-11.46	-11.45	-11.44	-11.43	-11.42	-11.41	-11.4	-11.39	-11.38
-11.46	-11.45	-11.44	-11.43	-11.42	-11.41	-11.4	-11.39	-11.38
-11.46	-11.45	-11.44	-11.43	-11.42	-11.41	-11.4	-11.39	-11.38
-11.46	-11.45	-11.44	-11.43	-11.42	-11.41	-11.4	-11.39	-11.38
-11.46	-11.45	-11.44	-11.43	-11.42	-11.41	-11.4	-11.39	-11.38
-11.46	-11.45	-11.44	-11.43	-11.42	-11.41	-11.4	-11.39	-11.38
-11.46	-11.45	-11.44	-11.43	-11.42	-11.41	-11.4	-11.39	-11.38
-11.46	-11.45	-11.44	-11.43	-11.42	-11.41	-11.4	-11.39	-11.38
-11.46	-11.45	-11.44	-11.43	-11.42	-11.41	-11.4	-11.39	-11.38
-11.46	-11.45	-11.44	-11.43	-11.42	-11.41	-11.4	-11.39	-11.38
-11.46	-11.45	-11.44	-11.43	-11.42	-11.41	-11.4	-11.39	-11.38
-11.46	-11.45	-11.44	-11.43	-11.42	-11.41	-11.4	-11.39	-11.38
-11.46	-11.45	-11.44	-11.43	-11.42	-11.41	-11.4	-11.39	-11.38
-11.46	-11.45	-11.44	-11.43	-11.42	-11.41	-11.4	-11.39	-11.38
-11.46	-11.45	-11.44	-11.43	-11.42	-11.41	-11.4	-11.39	-11.38
-11.46	-11.45	-11.44	-11.43	-11.42	-11.41	-11.4	-11.39	-11.38
-11.46	-11.45	-11.44	-11.43	-11.42	-11.41	-11.4	-11.39	-11.38
-11.46	-11.45	-11.44	-11.43	-11.42	-11.41	-11.4	-11.39	-11.38
-11.46	-11.45	-11.44	-11.43	-11.42	-11.41	-11.4	-11.39	-11.38
-11.46	-11.45	-11.44	-11.43	-11.42	-11.41	-11.4	-11.39	-11.38
-11.46	-11.45	-11.44	-11.43	-11.42	-11.41	-11.4	-11.39	-11.38
-11.46	-11.45	-11.44	-11.43	-11.42	-11.41	-11.4	-11.39	-11.38
-11.46	-11.45	-11.44	-11.43	-11.42	-11.41	-11.4	-11.39	-11.38
-11.46	-11.45	-11.44	-11.43	-11.42	-11.41	-11.4	-11.39	-11.38
-11.46	-11.45	-11.44	-11.43	-11.42	-11.41	-11.4	-11.39	-11.38
-11.46	-11.45	-11.44	-11.43	-11.42	-11.41	-11.4	-11.39	-11.38
-11.46	-11.45	-11.44	-11.43	-11.42	-11.41	-11.4	-11.39	-11.38
-11.46	-11.45	-11.44	-11.43	-11.42	-11.41	-11.4	-11.39	-11.38
-11.46	-11.45	-11.44	-11.43	-11.42	-11.41	-11.4	-11.39	-11.38
-11.46	-11.45	-11.44	-11.43	-11.42	-11.41	-11.4	-11.39	-11.38
-11.46	-11.45	-11.44	-11.43	-11.42	-11.41	-11.4	-11.39	-11.38
-11.46	-11.45	-11.44	-11.43	-11.42	-11.41	-11.4	-11.39	-11.38
-11.46	-11.45	-11.44	-11.43	-11.42	-11.41	-11.4	-11.39	-11.38
-11.46	-11.45	-11.44	-11.43	-11.42	-11.41	-11.4	-11.39	-11.38
-11.46	-11.45	-11.44	-11.43	-11.42	-11.41	-11.4	-11.39	-11.38

-11.46	-11.45	-11.44	-11.43	-11.42	-11.41	-11.4	-11.39	-11.38
-11.46	-11.45	-11.44	-11.43	-11.42	-11.41	-11.4	-11.39	-11.38
-11.46	-11.45	-11.44	-11.43	-11.42	-11.41	-11.4	-11.39	-11.38
-11.46	-11.45	-11.44	-11.43	-11.42	-11.41	-11.4	-11.39	-11.38
-11.46	-11.45	-11.44	-11.43	-11.42	-11.41	-11.4	-11.39	-11.38
-11.46	-11.45	-11.44	-11.43	-11.42	-11.41	-11.4	-11.39	-11.38
-11.46	-11.45	-11.44	-11.43	-11.42	-11.41	-11.4	-11.39	-11.38
-11.46	-11.45	-11.44	-11.43	-11.42	-11.41	-11.4	-11.39	-11.38
-11.46	-11.45	-11.44	-11.43	-11.42	-11.41	-11.4	-11.39	-11.38
-11.46	-11.45	-11.44	-11.43	-11.42	-11.41	-11.4	-11.39	-11.38
-11.46	-11.45	-11.44	-11.43	-11.42	-11.41	-11.4	-11.39	-11.38
-11.46	-11.45	-11.44	-11.43	-11.42	-11.41	-11.4	-11.39	-11.38
-11.46	-11.45	-11.44	-11.43	-11.42	-11.41	-11.4	-11.39	-11.38
-11.46	-11.45	-11.44	-11.43	-11.42	-11.41	-11.4	-11.39	-11.38
-11.46	-11.45	-11.44	-11.43	-11.42	-11.41	-11.4	-11.39	-11.38
-11.46	-11.45	-11.44	-11.43	-11.42	-11.41	-11.4	-11.39	-11.38
-11.46	-11.45	-11.44	-11.43	-11.42	-11.41	-11.4	-11.39	-11.38
-11.46	-11.45	-11.44	-11.43	-11.42	-11.41	-11.4	-11.39	-11.38
-11.46	-11.45	-11.44	-11.43	-11.42	-11.41	-11.4	-11.39	-11.38
-11.46	-11.45	-11.44	-11.43	-11.42	-11.41	-11.4	-11.39	-11.38
-11.46	-11.45	-11.44	-11.43	-11.42	-11.41	-11.4	-11.39	-11.38
-11.46	-11.45	-11.44	-11.43	-11.42	-11.41	-11.4	-11.39	-11.38
-11.46	-11.45	-11.44	-11.43	-11.42	-11.41	-11.4	-11.39	-11.38
-11.46	-11.45	-11.44	-11.43	-11.42	-11.41	-11.4	-11.39	-11.38
-11.46	-11.45	-11.44	-11.43	-11.42	-11.41	-11.4	-11.39	-11.38
-11.46	-11.45	-11.44	-11.43	-11.42	-11.41	-11.4	-11.39	-11.38
-11.46	-11.45	-11.44	-11.43	-11.42	-11.41	-11.4	-11.39	-11.38
-11.46	-11.45	-11.44	-11.43	-11.42	-11.41	-11.4	-11.39	-11.38
-11.46	-11.45	-11.44	-11.43	-11.42	-11.41	-11.4	-11.39	-11.38
-11.46	-11.45	-11.44	-11.43	-11.42	-11.41	-11.4	-11.39	-11.38
-11.46	-11.45	-11.44	-11.43	-11.42	-11.41	-11.4	-11.39	-11.38
-11.46	-11.45	-11.44	-11.43	-11.42	-11.41	-11.4	-11.39	-11.38
-11.46	-11.45	-11.44	-11.43	-11.42	-11.41	-11.4	-11.39	-11.38
-11.46	-11.45	-11.44	-11.43	-11.42	-11.41	-11.4	-11.39	-11.38
-11.46	-11.45	-11.44	-11.43	-11.42	-11.41	-11.4	-11.39	-11.38
-11.46	-11.45	-11.44	-11.43	-11.42	-11.41	-11.4	-11.39	-11.38
-11.46	-11.45	-11.44	-11.43	-11.42	-11.41	-11.4	-11.39	-11.38
-11.46	-11.45	-11.44	-11.43	-11.42	-11.41	-11.4	-11.39	-11.38
-11.46	-11.45	-11.44	-11.43	-11.42	-11.41	-11.4	-11.39	-11.38
-11.46	-11.45	-11.44	-11.43	-11.42	-11.41	-11.4	-11.39	-11.38
-11.46	-11.45	-11.44	-11.43	-11.42	-11.41	-11.4	-11.39	-11.38
-11.46	-11.45	-11.44	-11.43	-11.42	-11.41	-11.4	-11.39	-11.38
-11.46	-11.45	-11.44	-11.43	-11.42	-11.41	-11.4	-11.39	-11.38
-11.46	-11.45	-11.44	-11.43	-11.42	-11.41	-11.4	-11.39	-11.38
-11.46	-11.45	-11.44	-11.43	-11.42	-11.41	-11.4	-11.39	-11.38
-11.46	-11.45	-11.44	-11.43	-11.42	-11.41	-11.4	-11.39	-11.38
-11.46	-11.45	-11.44	-11.43	-11.42	-11.41	-11.4	-11.39	-11.38
-11.46	-11.45	-11.44	-11.43	-11.42	-11.41	-11.4	-11.39	-11.38
-11.46	-11.45	-11.44	-11.43	-11.42	-11.41	-11.4	-11.39	-11.38
-11.46	-11.45	-11.44	-11.43	-11.42	-11.41	-11.4	-11.39	-11.38

-11.46	-11.45	-11.44	-11.43	-11.42	-11.41	-11.4	-11.39	-11.38
-11.46	-11.45	-11.44	-11.43	-11.42	-11.41	-11.4	-11.39	-11.38
-11.46	-11.45	-11.44	-11.43	-11.42	-11.41	-11.4	-11.39	-11.38
-11.46	-11.45	-11.44	-11.43	-11.42	-11.41	-11.4	-11.39	-11.38
-11.46	-11.45	-11.44	-11.43	-11.42	-11.41	-11.4	-11.39	-11.38
-11.46	-11.45	-11.44	-11.43	-11.42	-11.41	-11.4	-11.39	-11.38
-11.46	-11.45	-11.44	-11.43	-11.42	-11.41	-11.4	-11.39	-11.38
-11.46	-11.45	-11.44	-11.43	-11.42	-11.41	-11.4	-11.39	-11.38
-11.46	-11.45	-11.44	-11.43	-11.42	-11.41	-11.4	-11.39	-11.38
-11.46	-11.45	-11.44	-11.43	-11.42	-11.41	-11.4	-11.39	-11.38
-11.46	-11.45	-11.44	-11.43	-11.42	-11.41	-11.4	-11.39	-11.38
-11.46	-11.45	-11.44	-11.43	-11.42	-11.41	-11.4	-11.39	-11.38
-11.46	-11.45	-11.44	-11.43	-11.42	-11.41	-11.4	-11.39	-11.38
-11.46	-11.45	-11.44	-11.43	-11.42	-11.41	-11.4	-11.39	-11.38
-11.46	-11.45	-11.44	-11.43	-11.42	-11.41	-11.4	-11.39	-11.38
-11.46	-11.45	-11.44	-11.43	-11.42	-11.41	-11.4	-11.39	-11.38
-11.46	-11.45	-11.44	-11.43	-11.42	-11.41	-11.4	-11.39	-11.38
-11.46	-11.45	-11.44	-11.43	-11.42	-11.41	-11.4	-11.39	-11.38
-11.46	-11.45	-11.44	-11.43	-11.42	-11.41	-11.4	-11.39	-11.38
-11.46	-11.45	-11.44	-11.43	-11.42	-11.41	-11.4	-11.39	-11.38
-11.46	-11.45	-11.44	-11.43	-11.42	-11.41	-11.4	-11.39	-11.38
-11.46	-11.45	-11.44	-11.43	-11.42	-11.41	-11.4	-11.39	-11.38
-11.46	-11.45	-11.44	-11.43	-11.42	-11.41	-11.4	-11.39	-11.38
-11.46	-11.45	-11.44	-11.43	-11.42	-11.41	-11.4	-11.39	-11.38
-11.46	-11.45	-11.44	-11.43	-11.42	-11.41	-11.4	-11.39	-11.38
-11.46	-11.45	-11.44	-11.43	-11.42	-11.41	-11.4	-11.39	-11.38
-11.46	-11.45	-11.44	-11.43	-11.42	-11.41	-11.4	-11.39	-11.38
-11.46	-11.45	-11.44	-11.43	-11.42	-11.41	-11.4	-11.39	-11.38
-11.46	-11.45	-11.44	-11.43	-11.42	-11.41	-11.4	-11.39	-11.38
-11.46	-11.45	-11.44	-11.43	-11.42	-11.41	-11.4	-11.39	-11.38
-11.46	-11.45	-11.44	-11.43	-11.42	-11.41	-11.4	-11.39	-11.38
-11.46	-11.45	-11.44	-11.43	-11.42	-11.41	-11.4	-11.39	-11.38
-11.46	-11.45	-11.44	-11.43	-11.42	-11.41	-11.4	-11.39	-11.38
-11.46	-11.45	-11.44	-11.43	-11.42	-11.41	-11.4	-11.39	-11.38
-11.46	-11.45	-11.44	-11.43	-11.42	-11.41	-11.4	-11.39	-11.38
-11.46	-11.45	-11.44	-11.43	-11.42	-11.41	-11.4	-11.39	-11.38
-11.46	-11.45	-11.44	-11.43	-11.42	-11.41	-11.4	-11.39	-11.38
-11.46	-11.45	-11.44	-11.43	-11.42	-11.41	-11.4	-11.39	-11.38
-11.46	-11.45	-11.44	-11.43	-11.42	-11.41	-11.4	-11.39	-11.38
-11.46	-11.45	-11.44	-11.43	-11.42	-11.41	-11.4	-11.39	-11.38
-11.46	-11.45	-11.44	-11.43	-11.42	-11.41	-11.4	-11.39	-11.38
-11.46	-11.45	-11.44	-11.43	-11.42	-11.41	-11.4	-11.39	-11.38
-11.46	-11.45	-11.44	-11.43	-11.42	-11.41	-11.4	-11.39	-11.38
-11.46	-11.45	-11.44	-11.43	-11.42	-11.41	-11.4	-11.39	-11.38
-11.46	-11.45	-11.44	-11.43	-11.42	-11.41	-11.4	-11.39	-11.38
-11.46	-11.45	-11.44	-11.43	-11.42	-11.41	-11.4	-11.39	-11.38
-11.46	-11.45	-11.44	-11.43	-11.42	-11.41	-11.4	-11.39	-11.38
-11.46	-11.45	-11.44	-11.43	-11.42	-11.41	-11.4	-11.39	-11.38
-11.46	-11.45	-11.44	-11.43	-11.42	-11.41	-11.4	-11.39	-11.38
-11.46	-11.45	-11.44	-11.43	-11.42	-11.41	-11.4	-11.39	-11.38

-11.46	-11.45	-11.44	-11.43	-11.42	-11.41	-11.4	-11.39	-11.38
-11.46	-11.45	-11.44	-11.43	-11.42	-11.41	-11.4	-11.39	-11.38
-11.46	-11.45	-11.44	-11.43	-11.42	-11.41	-11.4	-11.39	-11.38
-11.46	-11.45	-11.44	-11.43	-11.42	-11.41	-11.4	-11.39	-11.38
-11.46	-11.45	-11.44	-11.43	-11.42	-11.41	-11.4	-11.39	-11.38
-11.46	-11.45	-11.44	-11.43	-11.42	-11.41	-11.4	-11.39	-11.38
-11.46	-11.45	-11.44	-11.43	-11.42	-11.41	-11.4	-11.39	-11.38
-11.46	-11.45	-11.44	-11.43	-11.42	-11.41	-11.4	-11.39	-11.38
-11.46	-11.45	-11.44	-11.43	-11.42	-11.41	-11.4	-11.39	-11.38
-11.46	-11.45	-11.44	-11.43	-11.42	-11.41	-11.4	-11.39	-11.38
-11.46	-11.45	-11.44	-11.43	-11.42	-11.41	-11.4	-11.39	-11.38
-11.46	-11.45	-11.44	-11.43	-11.42	-11.41	-11.4	-11.39	-11.38
-11.46	-11.45	-11.44	-11.43	-11.42	-11.41	-11.4	-11.39	-11.38
-11.46	-11.45	-11.44	-11.43	-11.42	-11.41	-11.4	-11.39	-11.38
-11.46	-11.45	-11.44	-11.43	-11.42	-11.41	-11.4	-11.39	-11.38
-11.46	-11.45	-11.44	-11.43	-11.42	-11.41	-11.4	-11.39	-11.38
-11.46	-11.45	-11.44	-11.43	-11.42	-11.41	-11.4	-11.39	-11.38
-11.46	-11.45	-11.44	-11.43	-11.42	-11.41	-11.4	-11.39	-11.38
-11.46	-11.45	-11.44	-11.43	-11.42	-11.41	-11.4	-11.39	-11.38
-11.46	-11.45	-11.44	-11.43	-11.42	-11.41	-11.4	-11.39	-11.38
-11.46	-11.45	-11.44	-11.43	-11.42	-11.41	-11.4	-11.39	-11.38
-11.46	-11.45	-11.44	-11.43	-11.42	-11.41	-11.4	-11.39	-11.38
-11.46	-11.45	-11.44	-11.43	-11.42	-11.41	-11.4	-11.39	-11.38
-11.46	-11.45	-11.44	-11.43	-11.42	-11.41	-11.4	-11.39	-11.38
-11.46	-11.45	-11.44	-11.43	-11.42	-11.41	-11.4	-11.39	-11.38
-11.46	-11.45	-11.44	-11.43	-11.42	-11.41	-11.4	-11.39	-11.38
-11.46	-11.45	-11.44	-11.43	-11.42	-11.41	-11.4	-11.39	-11.38
-11.46	-11.45	-11.44	-11.43	-11.42	-11.41	-11.4	-11.39	-11.38
-11.46	-11.45	-11.44	-11.43	-11.42	-11.41	-11.4	-11.39	-11.38
-11.46	-11.45	-11.44	-11.43	-11.42	-11.41	-11.4	-11.39	-11.38
-11.46	-11.45	-11.44	-11.43	-11.42	-11.41	-11.4	-11.39	-11.38
-11.46	-11.45	-11.44	-11.43	-11.42	-11.41	-11.4	-11.39	-11.38
-11.46	-11.45	-11.44	-11.43	-11.42	-11.41	-11.4	-11.39	-11.38
-11.46	-11.45	-11.44	-11.43	-11.42	-11.41	-11.4	-11.39	-11.38
-11.46	-11.45	-11.44	-11.43	-11.42	-11.41	-11.4	-11.39	-11.38
-11.46	-11.45	-11.44	-11.43	-11.42	-11.41	-11.4	-11.39	-11.38
-11.46	-11.45	-11.44	-11.43	-11.42	-11.41	-11.4	-11.39	-11.38
-11.46	-11.45	-11.44	-11.43	-11.42	-11.41	-11.4	-11.39	-11.38
-11.46	-11.45	-11.44	-11.43	-11.42	-11.41	-11.4	-11.39	-11.38
-11.46	-11.45	-11.44	-11.43	-11.42	-11.41	-11.4	-11.39	-11.38
-11.46	-11.45	-11.44	-11.43	-11.42	-11.41	-11.4	-11.39	-11.38
-11.46	-11.45	-11.44	-11.43	-11.42	-11.41	-11.4	-11.39	-11.38
-11.46	-11.45	-11.44	-11.43	-11.42	-11.41	-11.4	-11.39	-11.38
-11.46	-11.45	-11.44	-11.43	-11.42	-11.41	-11.4	-11.39	-11.38
-11.46	-11.45	-11.44	-11.43	-11.42	-11.41	-11.4	-11.39	-11.38
-11.46	-11.45	-11.44	-11.43	-11.42	-11.41	-11.4	-11.39	-11.38
-11.46	-11.45	-11.44	-11.43	-11.42	-11.41	-11.4	-11.39	-11.38
-11.46	-11.45	-11.44	-11.43	-11.42	-11.41	-11.4	-11.39	-11.38
-11.46	-11.45	-11.44	-11.43	-11.42	-11.41	-11.4	-11.39	-11.38
-11.46	-11.45	-11.44	-11.43	-11.42	-11.41	-11.4	-11.39	-11.38

-11.46	-11.45	-11.44	-11.43	-11.42	-11.41	-11.4	-11.39	-11.38
-11.46	-11.45	-11.44	-11.43	-11.42	-11.41	-11.4	-11.39	-11.38
-11.46	-11.45	-11.44	-11.43	-11.42	-11.41	-11.4	-11.39	-11.38
-11.46	-11.45	-11.44	-11.43	-11.42	-11.41	-11.4	-11.39	-11.38
-11.46	-11.45	-11.44	-11.43	-11.42	-11.41	-11.4	-11.39	-11.38
-11.46	-11.45	-11.44	-11.43	-11.42	-11.41	-11.4	-11.39	-11.38
-11.46	-11.45	-11.44	-11.43	-11.42	-11.41	-11.4	-11.39	-11.38
-11.46	-11.45	-11.44	-11.43	-11.42	-11.41	-11.4	-11.39	-11.38
-11.46	-11.45	-11.44	-11.43	-11.42	-11.41	-11.4	-11.39	-11.38
-11.46	-11.45	-11.44	-11.43	-11.42	-11.41	-11.4	-11.39	-11.38
-11.46	-11.45	-11.44	-11.43	-11.42	-11.41	-11.4	-11.39	-11.38
-11.46	-11.45	-11.44	-11.43	-11.42	-11.41	-11.4	-11.39	-11.38
-11.46	-11.45	-11.44	-11.43	-11.42	-11.41	-11.4	-11.39	-11.38
-11.46	-11.45	-11.44	-11.43	-11.42	-11.41	-11.4	-11.39	-11.38
-11.46	-11.45	-11.44	-11.43	-11.42	-11.41	-11.4	-11.39	-11.38
-11.46	-11.45	-11.44	-11.43	-11.42	-11.41	-11.4	-11.39	-11.38
-11.46	-11.45	-11.44	-11.43	-11.42	-11.41	-11.4	-11.39	-11.38
-11.46	-11.45	-11.44	-11.43	-11.42	-11.41	-11.4	-11.39	-11.38
-11.46	-11.45	-11.44	-11.43	-11.42	-11.41	-11.4	-11.39	-11.38
-11.46	-11.45	-11.44	-11.43	-11.42	-11.41	-11.4	-11.39	-11.38
-11.46	-11.45	-11.44	-11.43	-11.42	-11.41	-11.4	-11.39	-11.38
-11.46	-11.45	-11.44	-11.43	-11.42	-11.41	-11.4	-11.39	-11.38
-11.46	-11.45	-11.44	-11.43	-11.42	-11.41	-11.4	-11.39	-11.38
-11.46	-11.45	-11.44	-11.43	-11.42	-11.41	-11.4	-11.39	-11.38
-11.46	-11.45	-11.44	-11.43	-11.42	-11.41	-11.4	-11.39	-11.38
-11.46	-11.45	-11.44	-11.43	-11.42	-11.41	-11.4	-11.39	-11.38
-11.46	-11.45	-11.44	-11.43	-11.42	-11.41	-11.4	-11.39	-11.38
-11.46	-11.45	-11.44	-11.43	-11.42	-11.41	-11.4	-11.39	-11.38
-11.46	-11.45	-11.44	-11.43	-11.42	-11.41	-11.4	-11.39	-11.38
-11.46	-11.45	-11.44	-11.43	-11.42	-11.41	-11.4	-11.39	-11.38
-11.46	-11.45	-11.44	-11.43	-11.42	-11.41	-11.4	-11.39	-11.38
-11.46	-11.45	-11.44	-11.43	-11.42	-11.41	-11.4	-11.39	-11.38
-11.46	-11.45	-11.44	-11.43	-11.42	-11.41	-11.4	-11.39	-11.38
-11.46	-11.45	-11.44	-11.43	-11.42	-11.41	-11.4	-11.39	-11.38
-11.46	-11.45	-11.44	-11.43	-11.42	-11.41	-11.4	-11.39	-11.38
-11.46	-11.45	-11.44	-11.43	-11.42	-11.41	-11.4	-11.39	-11.38
-11.46	-11.45	-11.44	-11.43	-11.42	-11.41	-11.4	-11.39	-11.38
-11.46	-11.45	-11.44	-11.43	-11.42	-11.41	-11.4	-11.39	-11.38
-11.46	-11.45	-11.44	-11.43	-11.42	-11.41	-11.4	-11.39	-11.38
-11.46	-11.45	-11.44	-11.43	-11.42	-11.41	-11.4	-11.39	-11.38
-11.46	-11.45	-11.44	-11.43	-11.42	-11.41	-11.4	-11.39	-11.38
-11.46	-11.45	-11.44	-11.43	-11.42	-11.41	-11.4	-11.39	-11.38
-11.46	-11.45	-11.44	-11.43	-11.42	-11.41	-11.4	-11.39	-11.38
-11.46	-11.45	-11.44	-11.43	-11.42	-11.41	-11.4	-11.39	-11.38
-11.46	-11.45	-11.44	-11.43	-11.42	-11.41	-11.4	-11.39	-11.38
-11.46	-11.45	-11.44	-11.43	-11.42	-11.41	-11.4	-11.39	-11.38
-11.46	-11.45	-11.44	-11.43	-11.42	-11.41	-11.4	-11.39	-11.38
-11.46	-11.45	-11.44	-11.43	-11.42	-11.41	-11.4	-11.39	-11.38
-11.46	-11.45	-11.44	-11.43	-11.42	-11.41	-11.4	-11.39	-11.38
-11.46	-11.45	-11.44	-11.43	-11.42	-11.41	-11.4	-11.39	-11.38

-11.46	-11.45	-11.44	-11.43	-11.42	-11.41	-11.4	-11.39	-11.38
-11.46	-11.45	-11.44	-11.43	-11.42	-11.41	-11.4	-11.39	-11.38
-11.46	-11.45	-11.44	-11.43	-11.42	-11.41	-11.4	-11.39	-11.38
-11.46	-11.45	-11.44	-11.43	-11.42	-11.41	-11.4	-11.39	-11.38
-11.46	-11.45	-11.44	-11.43	-11.42	-11.41	-11.4	-11.39	-11.38
-11.46	-11.45	-11.44	-11.43	-11.42	-11.41	-11.4	-11.39	-11.38
-11.46	-11.45	-11.44	-11.43	-11.42	-11.41	-11.4	-11.39	-11.38
-11.46	-11.45	-11.44	-11.43	-11.42	-11.41	-11.4	-11.39	-11.38
-11.46	-11.45	-11.44	-11.43	-11.42	-11.41	-11.4	-11.39	-11.38
-11.46	-11.45	-11.44	-11.43	-11.42	-11.41	-11.4	-11.39	-11.38
-11.46	-11.45	-11.44	-11.43	-11.42	-11.41	-11.4	-11.39	-11.38
-11.46	-11.45	-11.44	-11.43	-11.42	-11.41	-11.4	-11.39	-11.38
-11.46	-11.45	-11.44	-11.43	-11.42	-11.41	-11.4	-11.39	-11.38
-11.46	-11.45	-11.44	-11.43	-11.42	-11.41	-11.4	-11.39	-11.38
-11.46	-11.45	-11.44	-11.43	-11.42	-11.41	-11.4	-11.39	-11.38
-11.46	-11.45	-11.44	-11.43	-11.42	-11.41	-11.4	-11.39	-11.38
-11.46	-11.45	-11.44	-11.43	-11.42	-11.41	-11.4	-11.39	-11.38
-11.46	-11.45	-11.44	-11.43	-11.42	-11.41	-11.4	-11.39	-11.38
-11.46	-11.45	-11.44	-11.43	-11.42	-11.41	-11.4	-11.39	-11.38
-11.46	-11.45	-11.44	-11.43	-11.42	-11.41	-11.4	-11.39	-11.38
-11.46	-11.45	-11.44	-11.43	-11.42	-11.41	-11.4	-11.39	-11.38
-11.46	-11.45	-11.44	-11.43	-11.42	-11.41	-11.4	-11.39	-11.38
-11.46	-11.45	-11.44	-11.43	-11.42	-11.41	-11.4	-11.39	-11.38
-11.46	-11.45	-11.44	-11.43	-11.42	-11.41	-11.4	-11.39	-11.38
-11.46	-11.45	-11.44	-11.43	-11.42	-11.41	-11.4	-11.39	-11.38
-11.46	-11.45	-11.44	-11.43	-11.42	-11.41	-11.4	-11.39	-11.38
-11.46	-11.45	-11.44	-11.43	-11.42	-11.41	-11.4	-11.39	-11.38
-11.46	-11.45	-11.44	-11.43	-11.42	-11.41	-11.4	-11.39	-11.38
-11.46	-11.45	-11.44	-11.43	-11.42	-11.41	-11.4	-11.39	-11.38
-11.46	-11.45	-11.44	-11.43	-11.42	-11.41	-11.4	-11.39	-11.38
-11.46	-11.45	-11.44	-11.43	-11.42	-11.41	-11.4	-11.39	-11.38
-11.46	-11.45	-11.44	-11.43	-11.42	-11.41	-11.4	-11.39	-11.38
-11.46	-11.45	-11.44	-11.43	-11.42	-11.41	-11.4	-11.39	-11.38
-11.46	-11.45	-11.44	-11.43	-11.42	-11.41	-11.4	-11.39	-11.38
-11.46	-11.45	-11.44	-11.43	-11.42	-11.41	-11.4	-11.39	-11.38
-11.46	-11.45	-11.44	-11.43	-11.42	-11.41	-11.4	-11.39	-11.38
-11.46	-11.45	-11.44	-11.43	-11.42	-11.41	-11.4	-11.39	-11.38
-11.46	-11.45	-11.44	-11.43	-11.42	-11.41	-11.4	-11.39	-11.38
-11.46	-11.45	-11.44	-11.43	-11.42	-11.41	-11.4	-11.39	-11.38
-11.46	-11.45	-11.44	-11.43	-11.42	-11.41	-11.4	-11.39	-11.38
-11.46	-11.45	-11.44	-11.43	-11.42	-11.41	-11.4	-11.39	-11.38
-11.46	-11.45	-11.44	-11.43	-11.42	-11.41	-11.4	-11.39	-11.38
-11.46	-11.45	-11.44	-11.43	-11.42	-11.41	-11.4	-11.39	-11.38
-11.46	-11.45	-11.44	-11.43	-11.42	-11.41	-11.4	-11.39	-11.38
-11.46	-11.45	-11.44	-11.43	-11.42	-11.41	-11.4	-11.39	-11.38
-11.46	-11.45	-11.44	-11.43	-11.42	-11.41	-11.4	-11.39	-11.38
-11.46	-11.45	-11.44	-11.43	-11.42	-11.41	-11.4	-11.39	-11.38
-11.46	-11.45	-11.44	-11.43	-11.42	-11.41	-11.4	-11.39	-11.38
-11.46	-11.45	-11.44	-11.43	-11.42	-11.41	-11.4	-11.39	-11.38
-11.46	-11.45	-11.44	-11.43	-11.42	-11.41	-11.4	-11.39	-11.38

-11.46	-11.45	-11.44	-11.43	-11.42	-11.41	-11.4	-11.39	-11.38
-11.46	-11.45	-11.44	-11.43	-11.42	-11.41	-11.4	-11.39	-11.38
-11.46	-11.45	-11.44	-11.43	-11.42	-11.41	-11.4	-11.39	-11.38
-11.46	-11.45	-11.44	-11.43	-11.42	-11.41	-11.4	-11.39	-11.38
-11.46	-11.45	-11.44	-11.43	-11.42	-11.41	-11.4	-11.39	-11.38
-11.46	-11.45	-11.44	-11.43	-11.42	-11.41	-11.4	-11.39	-11.38
-11.46	-11.45	-11.44	-11.43	-11.42	-11.41	-11.4	-11.39	-11.38
-11.46	-11.45	-11.44	-11.43	-11.42	-11.41	-11.4	-11.39	-11.38
-11.46	-11.45	-11.44	-11.43	-11.42	-11.41	-11.4	-11.39	-11.38
-11.46	-11.45	-11.44	-11.43	-11.42	-11.41	-11.4	-11.39	-11.38
-11.46	-11.45	-11.44	-11.43	-11.42	-11.41	-11.4	-11.39	-11.38
-11.46	-11.45	-11.44	-11.43	-11.42	-11.41	-11.4	-11.39	-11.38
-11.46	-11.45	-11.44	-11.43	-11.42	-11.41	-11.4	-11.39	-11.38
-11.46	-11.45	-11.44	-11.43	-11.42	-11.41	-11.4	-11.39	-11.38
-11.46	-11.45	-11.44	-11.43	-11.42	-11.41	-11.4	-11.39	-11.38
-11.46	-11.45	-11.44	-11.43	-11.42	-11.41	-11.4	-11.39	-11.38
-11.46	-11.45	-11.44	-11.43	-11.42	-11.41	-11.4	-11.39	-11.38
-11.46	-11.45	-11.44	-11.43	-11.42	-11.41	-11.4	-11.39	-11.38
-11.46	-11.45	-11.44	-11.43	-11.42	-11.41	-11.4	-11.39	-11.38
-11.46	-11.45	-11.44	-11.43	-11.42	-11.41	-11.4	-11.39	-11.38
-11.46	-11.45	-11.44	-11.43	-11.42	-11.41	-11.4	-11.39	-11.38
-11.46	-11.45	-11.44	-11.43	-11.42	-11.41	-11.4	-11.39	-11.38
-11.46	-11.45	-11.44	-11.43	-11.42	-11.41	-11.4	-11.39	-11.38
-11.46	-11.45	-11.44	-11.43	-11.42	-11.41	-11.4	-11.39	-11.38
-11.46	-11.45	-11.44	-11.43	-11.42	-11.41	-11.4	-11.39	-11.38
-11.46	-11.45	-11.44	-11.43	-11.42	-11.41	-11.4	-11.39	-11.38
-11.46	-11.45	-11.44	-11.43	-11.42	-11.41	-11.4	-11.39	-11.38
-11.46	-11.45	-11.44	-11.43	-11.42	-11.41	-11.4	-11.39	-11.38
-11.46	-11.45	-11.44	-11.43	-11.42	-11.41	-11.4	-11.39	-11.38
-11.46	-11.45	-11.44	-11.43	-11.42	-11.41	-11.4	-11.39	-11.38
-11.46	-11.45	-11.44	-11.43	-11.42	-11.41	-11.4	-11.39	-11.38
-11.46	-11.45	-11.44	-11.43	-11.42	-11.41	-11.4	-11.39	-11.38
-11.46	-11.45	-11.44	-11.43	-11.42	-11.41	-11.4	-11.39	-11.38
-11.46	-11.45	-11.44	-11.43	-11.42	-11.41	-11.4	-11.39	-11.38
-11.46	-11.45	-11.44	-11.43	-11.42	-11.41	-11.4	-11.39	-11.38
-11.46	-11.45	-11.44	-11.43	-11.42	-11.41	-11.4	-11.39	-11.38
-11.46	-11.45	-11.44	-11.43	-11.42	-11.41	-11.4	-11.39	-11.38
-11.46	-11.45	-11.44	-11.43	-11.42	-11.41	-11.4	-11.39	-11.38
-11.46	-11.45	-11.44	-11.43	-11.42	-11.41	-11.4	-11.39	-11.38
-11.46	-11.45	-11.44	-11.43	-11.42	-11.41	-11.4	-11.39	-11.38
-11.46	-11.45	-11.44	-11.43	-11.42	-11.41	-11.4	-11.39	-11.38
-11.46	-11.45	-11.44	-11.43	-11.42	-11.41	-11.4	-11.39	-11.38
-11.46	-11.45	-11.44	-11.43	-11.42	-11.41	-11.4	-11.39	-11.38
-11.46	-11.45	-11.44	-11.43	-11.42	-11.41	-11.4	-11.39	-11.38
-11.46	-11.45	-11.44	-11.43	-11.42	-11.41	-11.4	-11.39	-11.38
-11.46	-11.45	-11.44	-11.43	-11.42	-11.41	-11.4	-11.39	-11.38
-11.46	-11.45	-11.44	-11.43	-11.42	-11.41	-11.4	-11.39	-11.38
-11.46	-11.45	-11.44	-11.43	-11.42	-11.41	-11.4	-11.39	-11.38
-11.46	-11.45	-11.44	-11.43	-11.42	-11.41	-11.4	-11.39	-11.38
-11.46	-11.45	-11.44	-11.43	-11.42	-11.41	-11.4	-11.39	-11.38

-11.46	-11.45	-11.44	-11.43	-11.42	-11.41	-11.4	-11.39	-11.38
-11.46	-11.45	-11.44	-11.43	-11.42	-11.41	-11.4	-11.39	-11.38
-11.46	-11.45	-11.44	-11.43	-11.42	-11.41	-11.4	-11.39	-11.38
-11.46	-11.45	-11.44	-11.43	-11.42	-11.41	-11.4	-11.39	-11.38
-11.46	-11.45	-11.44	-11.43	-11.42	-11.41	-11.4	-11.39	-11.38
-11.46	-11.45	-11.44	-11.43	-11.42	-11.41	-11.4	-11.39	-11.38
-11.46	-11.45	-11.44	-11.43	-11.42	-11.41	-11.4	-11.39	-11.38
-11.46	-11.45	-11.44	-11.43	-11.42	-11.41	-11.4	-11.39	-11.38
-11.46	-11.45	-11.44	-11.43	-11.42	-11.41	-11.4	-11.39	-11.38
-11.46	-11.45	-11.44	-11.43	-11.42	-11.41	-11.4	-11.39	-11.38
-11.46	-11.45	-11.44	-11.43	-11.42	-11.41	-11.4	-11.39	-11.38
-11.46	-11.45	-11.44	-11.43	-11.42	-11.41	-11.4	-11.39	-11.38
-11.46	-11.45	-11.44	-11.43	-11.42	-11.41	-11.4	-11.39	-11.38
-11.46	-11.45	-11.44	-11.43	-11.42	-11.41	-11.4	-11.39	-11.38
-11.46	-11.45	-11.44	-11.43	-11.42	-11.41	-11.4	-11.39	-11.38
-11.46	-11.45	-11.44	-11.43	-11.42	-11.41	-11.4	-11.39	-11.38
-11.46	-11.45	-11.44	-11.43	-11.42	-11.41	-11.4	-11.39	-11.38
-11.46	-11.45	-11.44	-11.43	-11.42	-11.41	-11.4	-11.39	-11.38
-11.46	-11.45	-11.44	-11.43	-11.42	-11.41	-11.4	-11.39	-11.38
-11.46	-11.45	-11.44	-11.43	-11.42	-11.41	-11.4	-11.39	-11.38
-11.46	-11.45	-11.44	-11.43	-11.42	-11.41	-11.4	-11.39	-11.38
-11.46	-11.45	-11.44	-11.43	-11.42	-11.41	-11.4	-11.39	-11.38
-11.46	-11.45	-11.44	-11.43	-11.42	-11.41	-11.4	-11.39	-11.38
-11.46	-11.45	-11.44	-11.43	-11.42	-11.41	-11.4	-11.39	-11.38
-11.46	-11.45	-11.44	-11.43	-11.42	-11.41	-11.4	-11.39	-11.38
-11.46	-11.45	-11.44	-11.43	-11.42	-11.41	-11.4	-11.39	-11.38
-11.46	-11.45	-11.44	-11.43	-11.42	-11.41	-11.4	-11.39	-11.38
-11.46	-11.45	-11.44	-11.43	-11.42	-11.41	-11.4	-11.39	-11.38
-11.46	-11.45	-11.44	-11.43	-11.42	-11.41	-11.4	-11.39	-11.38
-11.46	-11.45	-11.44	-11.43	-11.42	-11.41	-11.4	-11.39	-11.38
-11.46	-11.45	-11.44	-11.43	-11.42	-11.41	-11.4	-11.39	-11.38
-11.46	-11.45	-11.44	-11.43	-11.42	-11.41	-11.4	-11.39	-11.38
-11.46	-11.45	-11.44	-11.43	-11.42	-11.41	-11.4	-11.39	-11.38
-11.46	-11.45	-11.44	-11.43	-11.42	-11.41	-11.4	-11.39	-11.38
-11.46	-11.45	-11.44	-11.43	-11.42	-11.41	-11.4	-11.39	-11.38
-11.46	-11.45	-11.44	-11.43	-11.42	-11.41	-11.4	-11.39	-11.38
-11.46	-11.45	-11.44	-11.43	-11.42	-11.41	-11.4	-11.39	-11.38
-11.46	-11.45	-11.44	-11.43	-11.42	-11.41	-11.4	-11.39	-11.38
-11.46	-11.45	-11.44	-11.43	-11.42	-11.41	-11.4	-11.39	-11.38
-11.46	-11.45	-11.44	-11.43	-11.42	-11.41	-11.4	-11.39	-11.38
-11.46	-11.45	-11.44	-11.43	-11.42	-11.41	-11.4	-11.39	-11.38
-11.46	-11.45	-11.44	-11.43	-11.42	-11.41	-11.4	-11.39	-11.38
-11.46	-11.45	-11.44	-11.43	-11.42	-11.41	-11.4	-11.39	-11.38
-11.46	-11.45	-11.44	-11.43	-11.42	-11.41	-11.4	-11.39	-11.38
-11.46	-11.45	-11.44	-11.43	-11.42	-11.41	-11.4	-11.39	-11.38
-11.46	-11.45	-11.44	-11.43	-11.42	-11.41	-11.4	-11.39	-11.38
-11.46	-11.45	-11.44	-11.43	-11.42	-11.41	-11.4	-11.39	-11.38
-11.46	-11.45	-11.44	-11.43	-11.42	-11.41	-11.4	-11.39	-11.38
-11.46	-11.45	-11.44	-11.43	-11.42	-11.41	-11.4	-11.39	-11.38
-11.46	-11.45	-11.44	-11.43	-11.42	-11.41	-11.4	-11.39	-11.38

-11.46	-11.45	-11.44	-11.43	-11.42	-11.41	-11.4	-11.39	-11.38
-11.46	-11.45	-11.44	-11.43	-11.42	-11.41	-11.4	-11.39	-11.38
-11.46	-11.45	-11.44	-11.43	-11.42	-11.41	-11.4	-11.39	-11.38
-11.46	-11.45	-11.44	-11.43	-11.42	-11.41	-11.4	-11.39	-11.38
-11.46	-11.45	-11.44	-11.43	-11.42	-11.41	-11.4	-11.39	-11.38
-11.46	-11.45	-11.44	-11.43	-11.42	-11.41	-11.4	-11.39	-11.38
-11.46	-11.45	-11.44	-11.43	-11.42	-11.41	-11.4	-11.39	-11.38
-11.46	-11.45	-11.44	-11.43	-11.42	-11.41	-11.4	-11.39	-11.38
-11.46	-11.45	-11.44	-11.43	-11.42	-11.41	-11.4	-11.39	-11.38
-11.46	-11.45	-11.44	-11.43	-11.42	-11.41	-11.4	-11.39	-11.38
-11.46	-11.45	-11.44	-11.43	-11.42	-11.41	-11.4	-11.39	-11.38
-11.46	-11.45	-11.44	-11.43	-11.42	-11.41	-11.4	-11.39	-11.38
-11.46	-11.45	-11.44	-11.43	-11.42	-11.41	-11.4	-11.39	-11.38
-11.46	-11.45	-11.44	-11.43	-11.42	-11.41	-11.4	-11.39	-11.38
-11.46	-11.45	-11.44	-11.43	-11.42	-11.41	-11.4	-11.39	-11.38
-11.46	-11.45	-11.44	-11.43	-11.42	-11.41	-11.4	-11.39	-11.38
-11.46	-11.45	-11.44	-11.43	-11.42	-11.41	-11.4	-11.39	-11.38
-11.46	-11.45	-11.44	-11.43	-11.42	-11.41	-11.4	-11.39	-11.38
-11.46	-11.45	-11.44	-11.43	-11.42	-11.41	-11.4	-11.39	-11.38
-11.46	-11.45	-11.44	-11.43	-11.42	-11.41	-11.4	-11.39	-11.38
-11.46	-11.45	-11.44	-11.43	-11.42	-11.41	-11.4	-11.39	-11.38
-11.46	-11.45	-11.44	-11.43	-11.42	-11.41	-11.4	-11.39	-11.38
-11.46	-11.45	-11.44	-11.43	-11.42	-11.41	-11.4	-11.39	-11.38
-11.46	-11.45	-11.44	-11.43	-11.42	-11.41	-11.4	-11.39	-11.38
-11.46	-11.45	-11.44	-11.43	-11.42	-11.41	-11.4	-11.39	-11.38
-11.46	-11.45	-11.44	-11.43	-11.42	-11.41	-11.4	-11.39	-11.38
-11.46	-11.45	-11.44	-11.43	-11.42	-11.41	-11.4	-11.39	-11.38
-11.46	-11.45	-11.44	-11.43	-11.42	-11.41	-11.4	-11.39	-11.38
-11.46	-11.45	-11.44	-11.43	-11.42	-11.41	-11.4	-11.39	-11.38
-11.46	-11.45	-11.44	-11.43	-11.42	-11.41	-11.4	-11.39	-11.38
-11.46	-11.45	-11.44	-11.43	-11.42	-11.41	-11.4	-11.39	-11.38
-11.46	-11.45	-11.44	-11.43	-11.42	-11.41	-11.4	-11.39	-11.38
-11.46	-11.45	-11.44	-11.43	-11.42	-11.41	-11.4	-11.39	-11.38
-11.46	-11.45	-11.44	-11.43	-11.42	-11.41	-11.4	-11.39	-11.38
-11.46	-11.45	-11.44	-11.43	-11.42	-11.41	-11.4	-11.39	-11.38
-11.46	-11.45	-11.44	-11.43	-11.42	-11.41	-11.4	-11.39	-11.38
-11.46	-11.45	-11.44	-11.43	-11.42	-11.41	-11.4	-11.39	-11.38
-11.46	-11.45	-11.44	-11.43	-11.42	-11.41	-11.4	-11.39	-11.38
-11.46	-11.45	-11.44	-11.43	-11.42	-11.41	-11.4	-11.39	-11.38
-11.46	-11.45	-11.44	-11.43	-11.42	-11.41	-11.4	-11.39	-11.38
-11.46	-11.45	-11.44	-11.43	-11.42	-11.41	-11.4	-11.39	-11.38
-11.46	-11.45	-11.44	-11.43	-11.42	-11.41	-11.4	-11.39	-11.38
-11.46	-11.45	-11.44	-11.43	-11.42	-11.41	-11.4	-11.39	-11.38
-11.46	-11.45	-11.44	-11.43	-11.42	-11.41	-11.4	-11.39	-11.38
-11.46	-11.45	-11.44	-11.43	-11.42	-11.41	-11.4	-11.39	-11.38
-11.46	-11.45	-11.44	-11.43	-11.42	-11.41	-11.4	-11.39	-11.38
-11.46	-11.45	-11.44	-11.43	-11.42	-11.41	-11.4	-11.39	-11.38
-11.46	-11.45	-11.44	-11.43	-11.42	-11.41	-11.4	-11.39	-11.38
-11.46	-11.45	-11.44	-11.43	-11.42	-11.41	-11.4	-11.39	-11.38
-11.46	-11.45	-11.44	-11.43	-11.42	-11.41	-11.4	-11.39	-11.38

-11.46	-11.45	-11.44	-11.43	-11.42	-11.41	-11.4	-11.39	-11.38
-11.46	-11.45	-11.44	-11.43	-11.42	-11.41	-11.4	-11.39	-11.38
-11.46	-11.45	-11.44	-11.43	-11.42	-11.41	-11.4	-11.39	-11.38
-11.46	-11.45	-11.44	-11.43	-11.42	-11.41	-11.4	-11.39	-11.38
-11.46	-11.45	-11.44	-11.43	-11.42	-11.41	-11.4	-11.39	-11.38
-11.46	-11.45	-11.44	-11.43	-11.42	-11.41	-11.4	-11.39	-11.38
-11.46	-11.45	-11.44	-11.43	-11.42	-11.41	-11.4	-11.39	-11.38
-11.46	-11.45	-11.44	-11.43	-11.42	-11.41	-11.4	-11.39	-11.38
-11.46	-11.45	-11.44	-11.43	-11.42	-11.41	-11.4	-11.39	-11.38
-11.46	-11.45	-11.44	-11.43	-11.42	-11.41	-11.4	-11.39	-11.38
-11.46	-11.45	-11.44	-11.43	-11.42	-11.41	-11.4	-11.39	-11.38
-11.46	-11.45	-11.44	-11.43	-11.42	-11.41	-11.4	-11.39	-11.38
-11.46	-11.45	-11.44	-11.43	-11.42	-11.41	-11.4	-11.39	-11.38
-11.46	-11.45	-11.44	-11.43	-11.42	-11.41	-11.4	-11.39	-11.38
-11.46	-11.45	-11.44	-11.43	-11.42	-11.41	-11.4	-11.39	-11.38
-11.46	-11.45	-11.44	-11.43	-11.42	-11.41	-11.4	-11.39	-11.38
-11.46	-11.45	-11.44	-11.43	-11.42	-11.41	-11.4	-11.39	-11.38
-11.46	-11.45	-11.44	-11.43	-11.42	-11.41	-11.4	-11.39	-11.38
-11.46	-11.45	-11.44	-11.43	-11.42	-11.41	-11.4	-11.39	-11.38
-11.46	-11.45	-11.44	-11.43	-11.42	-11.41	-11.4	-11.39	-11.38
-11.46	-11.45	-11.44	-11.43	-11.42	-11.41	-11.4	-11.39	-11.38
-11.46	-11.45	-11.44	-11.43	-11.42	-11.41	-11.4	-11.39	-11.38
-11.46	-11.45	-11.44	-11.43	-11.42	-11.41	-11.4	-11.39	-11.38
-11.46	-11.45	-11.44	-11.43	-11.42	-11.41	-11.4	-11.39	-11.38
-11.46	-11.45	-11.44	-11.43	-11.42	-11.41	-11.4	-11.39	-11.38
-11.46	-11.45	-11.44	-11.43	-11.42	-11.41	-11.4	-11.39	-11.38
-11.46	-11.45	-11.44	-11.43	-11.42	-11.41	-11.4	-11.39	-11.38
-11.46	-11.45	-11.44	-11.43	-11.42	-11.41	-11.4	-11.39	-11.38
-11.46	-11.45	-11.44	-11.43	-11.42	-11.41	-11.4	-11.39	-11.38
-11.46	-11.45	-11.44	-11.43	-11.42	-11.41	-11.4	-11.39	-11.38
-11.46	-11.45	-11.44	-11.43	-11.42	-11.41	-11.4	-11.39	-11.38
-11.46	-11.45	-11.44	-11.43	-11.42	-11.41	-11.4	-11.39	-11.38
-11.46	-11.45	-11.44	-11.43	-11.42	-11.41	-11.4	-11.39	-11.38
-11.46	-11.45	-11.44	-11.43	-11.42	-11.41	-11.4	-11.39	-11.38
-11.46	-11.45	-11.44	-11.43	-11.42	-11.41	-11.4	-11.39	-11.38
-11.46	-11.45	-11.44	-11.43	-11.42	-11.41	-11.4	-11.39	-11.38
-11.46	-11.45	-11.44	-11.43	-11.42	-11.41	-11.4	-11.39	-11.38
-11.46	-11.45	-11.44	-11.43	-11.42	-11.41	-11.4	-11.39	-11.38
-11.46	-11.45	-11.44	-11.43	-11.42	-11.41	-11.4	-11.39	-11.38
-11.46	-11.45	-11.44	-11.43	-11.42	-11.41	-11.4	-11.39	-11.38
-11.46	-11.45	-11.44	-11.43	-11.42	-11.41	-11.4	-11.39	-11.38
-11.46	-11.45	-11.44	-11.43	-11.42	-11.41	-11.4	-11.39	-11.38
-11.46	-11.45	-11.44	-11.43	-11.42	-11.41	-11.4	-11.39	-11.38
-11.46	-11.45	-11.44	-11.43	-11.42	-11.41	-11.4	-11.39	-11.38
-11.46	-11.45	-11.44	-11.43	-11.42	-11.41	-11.4	-11.39	-11.38
-11.46	-11.45	-11.44	-11.43	-11.42	-11.41	-11.4	-11.39	-11.38
-11.46	-11.45	-11.44	-11.43	-11.42	-11.41	-11.4	-11.39	-11.38
-11.46	-11.45	-11.44	-11.43	-11.42	-11.41	-11.4	-11.39	-11.38
-11.46	-11.45	-11.44	-11.43	-11.42	-11.41	-11.4	-11.39	-11.38
-11.46	-11.45	-11.44	-11.43	-11.42	-11.41	-11.4	-11.39	-11.38

-11.46	-11.45	-11.44	-11.43	-11.42	-11.41	-11.4	-11.39	-11.38
-11.46	-11.45	-11.44	-11.43	-11.42	-11.41	-11.4	-11.39	-11.38
-11.46	-11.45	-11.44	-11.43	-11.42	-11.41	-11.4	-11.39	-11.38
-11.46	-11.45	-11.44	-11.43	-11.42	-11.41	-11.4	-11.39	-11.38
-11.46	-11.45	-11.44	-11.43	-11.42	-11.41	-11.4	-11.39	-11.38
-11.46	-11.45	-11.44	-11.43	-11.42	-11.41	-11.4	-11.39	-11.38
-11.46	-11.45	-11.44	-11.43	-11.42	-11.41	-11.4	-11.39	-11.38
-11.46	-11.45	-11.44	-11.43	-11.42	-11.41	-11.4	-11.39	-11.38
-11.46	-11.45	-11.44	-11.43	-11.42	-11.41	-11.4	-11.39	-11.38
-11.46	-11.45	-11.44	-11.43	-11.42	-11.41	-11.4	-11.39	-11.38
-11.46	-11.45	-11.44	-11.43	-11.42	-11.41	-11.4	-11.39	-11.38
-11.46	-11.45	-11.44	-11.43	-11.42	-11.41	-11.4	-11.39	-11.38
-11.46	-11.45	-11.44	-11.43	-11.42	-11.41	-11.4	-11.39	-11.38
-11.46	-11.45	-11.44	-11.43	-11.42	-11.41	-11.4	-11.39	-11.38
-11.46	-11.45	-11.44	-11.43	-11.42	-11.41	-11.4	-11.39	-11.38
-11.46	-11.45	-11.44	-11.43	-11.42	-11.41	-11.4	-11.39	-11.38
-11.46	-11.45	-11.44	-11.43	-11.42	-11.41	-11.4	-11.39	-11.38
-11.46	-11.45	-11.44	-11.43	-11.42	-11.41	-11.4	-11.39	-11.38
-11.46	-11.45	-11.44	-11.43	-11.42	-11.41	-11.4	-11.39	-11.38
-11.46	-11.45	-11.44	-11.43	-11.42	-11.41	-11.4	-11.39	-11.38
-11.46	-11.45	-11.44	-11.43	-11.42	-11.41	-11.4	-11.39	-11.38
-11.46	-11.45	-11.44	-11.43	-11.42	-11.41	-11.4	-11.39	-11.38
-11.46	-11.45	-11.44	-11.43	-11.42	-11.41	-11.4	-11.39	-11.38
-11.46	-11.45	-11.44	-11.43	-11.42	-11.41	-11.4	-11.39	-11.38
-11.46	-11.45	-11.44	-11.43	-11.42	-11.41	-11.4	-11.39	-11.38
-11.46	-11.45	-11.44	-11.43	-11.42	-11.41	-11.4	-11.39	-11.38
-11.46	-11.45	-11.44	-11.43	-11.42	-11.41	-11.4	-11.39	-11.38
-11.46	-11.45	-11.44	-11.43	-11.42	-11.41	-11.4	-11.39	-11.38
-11.46	-11.45	-11.44	-11.43	-11.42	-11.41	-11.4	-11.39	-11.38
-11.46	-11.45	-11.44	-11.43	-11.42	-11.41	-11.4	-11.39	-11.38
-11.46	-11.45	-11.44	-11.43	-11.42	-11.41	-11.4	-11.39	-11.38
-11.46	-11.45	-11.44	-11.43	-11.42	-11.41	-11.4	-11.39	-11.38
-11.46	-11.45	-11.44	-11.43	-11.42	-11.41	-11.4	-11.39	-11.38
-11.46	-11.45	-11.44	-11.43	-11.42	-11.41	-11.4	-11.39	-11.38
-11.46	-11.45	-11.44	-11.43	-11.42	-11.41	-11.4	-11.39	-11.38
-11.46	-11.45	-11.44	-11.43	-11.42	-11.41	-11.4	-11.39	-11.38
-11.46	-11.45	-11.44	-11.43	-11.42	-11.41	-11.4	-11.39	-11.38
-11.46	-11.45	-11.44	-11.43	-11.42	-11.41	-11.4	-11.39	-11.38
-11.46	-11.45	-11.44	-11.43	-11.42	-11.41	-11.4	-11.39	-11.38
-11.46	-11.45	-11.44	-11.43	-11.42	-11.41	-11.4	-11.39	-11.38
-11.46	-11.45	-11.44	-11.43	-11.42	-11.41	-11.4	-11.39	-11.38
-11.46	-11.45	-11.44	-11.43	-11.42	-11.41	-11.4	-11.39	-11.38
-11.46	-11.45	-11.44	-11.43	-11.42	-11.41	-11.4	-11.39	-11.38
-11.46	-11.45	-11.44	-11.43	-11.42	-11.41	-11.4	-11.39	-11.38
-11.46	-11.45	-11.44	-11.43	-11.42	-11.41	-11.4	-11.39	-11.38
-11.46	-11.45	-11.44	-11.43	-11.42	-11.41	-11.4	-11.39	-11.38
-11.46	-11.45	-11.44	-11.43	-11.42	-11.41	-11.4	-11.39	-11.38
-11.46	-11.45	-11.44	-11.43	-11.42	-11.41	-11.4	-11.39	-11.38
-11.46	-11.45	-11.44	-11.43	-11.42	-11.41	-11.4	-11.39	-11.38
-11.46	-11.45	-11.44	-11.43	-11.42	-11.41	-11.4	-11.39	-11.38

-11.46	-11.45	-11.44	-11.43	-11.42	-11.41	-11.4	-11.39	-11.38
-11.46	-11.45	-11.44	-11.43	-11.42	-11.41	-11.4	-11.39	-11.38
-11.46	-11.45	-11.44	-11.43	-11.42	-11.41	-11.4	-11.39	-11.38
-11.46	-11.45	-11.44	-11.43	-11.42	-11.41	-11.4	-11.39	-11.38
-11.46	-11.45	-11.44	-11.43	-11.42	-11.41	-11.4	-11.39	-11.38
-11.46	-11.45	-11.44	-11.43	-11.42	-11.41	-11.4	-11.39	-11.38
-11.46	-11.45	-11.44	-11.43	-11.42	-11.41	-11.4	-11.39	-11.38
-11.46	-11.45	-11.44	-11.43	-11.42	-11.41	-11.4	-11.39	-11.38
-11.46	-11.45	-11.44	-11.43	-11.42	-11.41	-11.4	-11.39	-11.38
-11.46	-11.45	-11.44	-11.43	-11.42	-11.41	-11.4	-11.39	-11.38
-11.46	-11.45	-11.44	-11.43	-11.42	-11.41	-11.4	-11.39	-11.38
-11.46	-11.45	-11.44	-11.43	-11.42	-11.41	-11.4	-11.39	-11.38
-11.46	-11.45	-11.44	-11.43	-11.42	-11.41	-11.4	-11.39	-11.38
-11.46	-11.45	-11.44	-11.43	-11.42	-11.41	-11.4	-11.39	-11.38
-11.46	-11.45	-11.44	-11.43	-11.42	-11.41	-11.4	-11.39	-11.38
-11.46	-11.45	-11.44	-11.43	-11.42	-11.41	-11.4	-11.39	-11.38
-11.46	-11.45	-11.44	-11.43	-11.42	-11.41	-11.4	-11.39	-11.38
-11.46	-11.45	-11.44	-11.43	-11.42	-11.41	-11.4	-11.39	-11.38
-11.46	-11.45	-11.44	-11.43	-11.42	-11.41	-11.4	-11.39	-11.38
-11.46	-11.45	-11.44	-11.43	-11.42	-11.41	-11.4	-11.39	-11.38
-11.46	-11.45	-11.44	-11.43	-11.42	-11.41	-11.4	-11.39	-11.38
-11.46	-11.45	-11.44	-11.43	-11.42	-11.41	-11.4	-11.39	-11.38
-11.46	-11.45	-11.44	-11.43	-11.42	-11.41	-11.4	-11.39	-11.38
-11.46	-11.45	-11.44	-11.43	-11.42	-11.41	-11.4	-11.39	-11.38
-11.46	-11.45	-11.44	-11.43	-11.42	-11.41	-11.4	-11.39	-11.38
-11.46	-11.45	-11.44	-11.43	-11.42	-11.41	-11.4	-11.39	-11.38
-11.46	-11.45	-11.44	-11.43	-11.42	-11.41	-11.4	-11.39	-11.38
-11.46	-11.45	-11.44	-11.43	-11.42	-11.41	-11.4	-11.39	-11.38
-11.46	-11.45	-11.44	-11.43	-11.42	-11.41	-11.4	-11.39	-11.38
-11.46	-11.45	-11.44	-11.43	-11.42	-11.41	-11.4	-11.39	-11.38
-11.46	-11.45	-11.44	-11.43	-11.42	-11.41	-11.4	-11.39	-11.38
-11.46	-11.45	-11.44	-11.43	-11.42	-11.41	-11.4	-11.39	-11.38
-11.46	-11.45	-11.44	-11.43	-11.42	-11.41	-11.4	-11.39	-11.38
-11.46	-11.45	-11.44	-11.43	-11.42	-11.41	-11.4	-11.39	-11.38
-11.46	-11.45	-11.44	-11.43	-11.42	-11.41	-11.4	-11.39	-11.38
-11.46	-11.45	-11.44	-11.43	-11.42	-11.41	-11.4	-11.39	-11.38
-11.46	-11.45	-11.44	-11.43	-11.42	-11.41	-11.4	-11.39	-11.38
-11.46	-11.45	-11.44	-11.43	-11.42	-11.41	-11.4	-11.39	-11.38
-11.46	-11.45	-11.44	-11.43	-11.42	-11.41	-11.4	-11.39	-11.38
-11.46	-11.45	-11.44	-11.43	-11.42	-11.41	-11.4	-11.39	-11.38
-11.46	-11.45	-11.44	-11.43	-11.42	-11.41	-11.4	-11.39	-11.38
-11.46	-11.45	-11.44	-11.43	-11.42	-11.41	-11.4	-11.39	-11.38
-11.46	-11.45	-11.44	-11.43	-11.42	-11.41	-11.4	-11.39	-11.38
-11.46	-11.45	-11.44	-11.43	-11.42	-11.41	-11.4	-11.39	-11.38
-11.46	-11.45	-11.44	-11.43	-11.42	-11.41	-11.4	-11.39	-11.38
-11.46	-11.45	-11.44	-11.43	-11.42	-11.41	-11.4	-11.39	-11.38
-11.46	-11.45	-11.44	-11.43	-11.42	-11.41	-11.4	-11.39	-11.38
-11.46	-11.45	-11.44	-11.43	-11.42	-11.41	-11.4	-11.39	-11.38
-11.46	-11.45	-11.44	-11.43	-11.42	-11.41	-11.4	-11.39	-11.38
-11.46	-11.45	-11.44	-11.43	-11.42	-11.41	-11.4	-11.39	-11.38

-11.46	-11.45	-11.44	-11.43	-11.42	-11.41	-11.4	-11.39	-11.38
-11.46	-11.45	-11.44	-11.43	-11.42	-11.41	-11.4	-11.39	-11.38
-11.46	-11.45	-11.44	-11.43	-11.42	-11.41	-11.4	-11.39	-11.38
-11.46	-11.45	-11.44	-11.43	-11.42	-11.41	-11.4	-11.39	-11.38
-11.46	-11.45	-11.44	-11.43	-11.42	-11.41	-11.4	-11.39	-11.38
-11.46	-11.45	-11.44	-11.43	-11.42	-11.41	-11.4	-11.39	-11.38
-11.46	-11.45	-11.44	-11.43	-11.42	-11.41	-11.4	-11.39	-11.38
-11.46	-11.45	-11.44	-11.43	-11.42	-11.41	-11.4	-11.39	-11.38
-11.46	-11.45	-11.44	-11.43	-11.42	-11.41	-11.4	-11.39	-11.38
-11.46	-11.45	-11.44	-11.43	-11.42	-11.41	-11.4	-11.39	-11.38
-11.46	-11.45	-11.44	-11.43	-11.42	-11.41	-11.4	-11.39	-11.38
-11.46	-11.45	-11.44	-11.43	-11.42	-11.41	-11.4	-11.39	-11.38
-11.46	-11.45	-11.44	-11.43	-11.42	-11.41	-11.4	-11.39	-11.38
-11.46	-11.45	-11.44	-11.43	-11.42	-11.41	-11.4	-11.39	-11.38
-11.46	-11.45	-11.44	-11.43	-11.42	-11.41	-11.4	-11.39	-11.38
-11.46	-11.45	-11.44	-11.43	-11.42	-11.41	-11.4	-11.39	-11.38
-11.46	-11.45	-11.44	-11.43	-11.42	-11.41	-11.4	-11.39	-11.38
-11.46	-11.45	-11.44	-11.43	-11.42	-11.41	-11.4	-11.39	-11.38
-11.46	-11.45	-11.44	-11.43	-11.42	-11.41	-11.4	-11.39	-11.38
-11.46	-11.45	-11.44	-11.43	-11.42	-11.41	-11.4	-11.39	-11.38
-11.46	-11.45	-11.44	-11.43	-11.42	-11.41	-11.4	-11.39	-11.38
-11.46	-11.45	-11.44	-11.43	-11.42	-11.41	-11.4	-11.39	-11.38
-11.46	-11.45	-11.44	-11.43	-11.42	-11.41	-11.4	-11.39	-11.38
-11.46	-11.45	-11.44	-11.43	-11.42	-11.41	-11.4	-11.39	-11.38
-11.46	-11.45	-11.44	-11.43	-11.42	-11.41	-11.4	-11.39	-11.38
-11.46	-11.45	-11.44	-11.43	-11.42	-11.41	-11.4	-11.39	-11.38
-11.46	-11.45	-11.44	-11.43	-11.42	-11.41	-11.4	-11.39	-11.38
-11.46	-11.45	-11.44	-11.43	-11.42	-11.41	-11.4	-11.39	-11.38
-11.46	-11.45	-11.44	-11.43	-11.42	-11.41	-11.4	-11.39	-11.38
-11.46	-11.45	-11.44	-11.43	-11.42	-11.41	-11.4	-11.39	-11.38
-11.46	-11.45	-11.44	-11.43	-11.42	-11.41	-11.4	-11.39	-11.38
-11.46	-11.45	-11.44	-11.43	-11.42	-11.41	-11.4	-11.39	-11.38
-11.46	-11.45	-11.44	-11.43	-11.42	-11.41	-11.4	-11.39	-11.38
-11.46	-11.45	-11.44	-11.43	-11.42	-11.41	-11.4	-11.39	-11.38
-11.46	-11.45	-11.44	-11.43	-11.42	-11.41	-11.4	-11.39	-11.38
-11.46	-11.45	-11.44	-11.43	-11.42	-11.41	-11.4	-11.39	-11.38
-11.46	-11.45	-11.44	-11.43	-11.42	-11.41	-11.4	-11.39	-11.38
-11.46	-11.45	-11.44	-11.43	-11.42	-11.41	-11.4	-11.39	-11.38
-11.46	-11.45	-11.44	-11.43	-11.42	-11.41	-11.4	-11.39	-11.38
-11.46	-11.45	-11.44	-11.43	-11.42	-11.41	-11.4	-11.39	-11.38
-11.46	-11.45	-11.44	-11.43	-11.42	-11.41	-11.4	-11.39	-11.38
-11.46	-11.45	-11.44	-11.43	-11.42	-11.41	-11.4	-11.39	-11.38
-11.46	-11.45	-11.44	-11.43	-11.42	-11.41	-11.4	-11.39	-11.38
-11.46	-11.45	-11.44	-11.43	-11.42	-11.41	-11.4	-11.39	-11.38
-11.46	-11.45	-11.44	-11.43	-11.42	-11.41	-11.4	-11.39	-11.38
-11.46	-11.45	-11.44	-11.43	-11.42	-11.41	-11.4	-11.39	-11.38
-11.46	-11.45	-11.44	-11.43	-11.42	-11.41	-11.4	-11.39	-11.38
-11.46	-11.45	-11.44	-11.43	-11.42	-11.41	-11.4	-11.39	-11.38
-11.46	-11.45	-11.44	-11.43	-11.42	-11.41	-11.4	-11.39	-11.38
-11.46	-11.45	-11.44	-11.43	-11.42	-11.41	-11.4	-11.39	-11.38

-11.46	-11.45	-11.44	-11.43	-11.42	-11.41	-11.4	-11.39	-11.38
-11.46	-11.45	-11.44	-11.43	-11.42	-11.41	-11.4	-11.39	-11.38
-11.46	-11.45	-11.44	-11.43	-11.42	-11.41	-11.4	-11.39	-11.38
-11.46	-11.45	-11.44	-11.43	-11.42	-11.41	-11.4	-11.39	-11.38
-11.46	-11.45	-11.44	-11.43	-11.42	-11.41	-11.4	-11.39	-11.38
-11.46	-11.45	-11.44	-11.43	-11.42	-11.41	-11.4	-11.39	-11.38
-11.46	-11.45	-11.44	-11.43	-11.42	-11.41	-11.4	-11.39	-11.38
-11.46	-11.45	-11.44	-11.43	-11.42	-11.41	-11.4	-11.39	-11.38
-11.46	-11.45	-11.44	-11.43	-11.42	-11.41	-11.4	-11.39	-11.38
-11.46	-11.45	-11.44	-11.43	-11.42	-11.41	-11.4	-11.39	-11.38
-11.46	-11.45	-11.44	-11.43	-11.42	-11.41	-11.4	-11.39	-11.38
-11.46	-11.45	-11.44	-11.43	-11.42	-11.41	-11.4	-11.39	-11.38
-11.46	-11.45	-11.44	-11.43	-11.42	-11.41	-11.4	-11.39	-11.38
-11.46	-11.45	-11.44	-11.43	-11.42	-11.41	-11.4	-11.39	-11.38
-11.46	-11.45	-11.44	-11.43	-11.42	-11.41	-11.4	-11.39	-11.38
-11.46	-11.45	-11.44	-11.43	-11.42	-11.41	-11.4	-11.39	-11.38
-11.46	-11.45	-11.44	-11.43	-11.42	-11.41	-11.4	-11.39	-11.38
-11.46	-11.45	-11.44	-11.43	-11.42	-11.41	-11.4	-11.39	-11.38
-11.46	-11.45	-11.44	-11.43	-11.42	-11.41	-11.4	-11.39	-11.38
-11.46	-11.45	-11.44	-11.43	-11.42	-11.41	-11.4	-11.39	-11.38
-11.46	-11.45	-11.44	-11.43	-11.42	-11.41	-11.4	-11.39	-11.38
-11.46	-11.45	-11.44	-11.43	-11.42	-11.41	-11.4	-11.39	-11.38
-11.46	-11.45	-11.44	-11.43	-11.42	-11.41	-11.4	-11.39	-11.38
-11.46	-11.45	-11.44	-11.43	-11.42	-11.41	-11.4	-11.39	-11.38
-11.46	-11.45	-11.44	-11.43	-11.42	-11.41	-11.4	-11.39	-11.38
-11.46	-11.45	-11.44	-11.43	-11.42	-11.41	-11.4	-11.39	-11.38
-11.46	-11.45	-11.44	-11.43	-11.42	-11.41	-11.4	-11.39	-11.38
-11.46	-11.45	-11.44	-11.43	-11.42	-11.41	-11.4	-11.39	-11.38
-11.46	-11.45	-11.44	-11.43	-11.42	-11.41	-11.4	-11.39	-11.38
-11.46	-11.45	-11.44	-11.43	-11.42	-11.41	-11.4	-11.39	-11.38
-11.46	-11.45	-11.44	-11.43	-11.42	-11.41	-11.4	-11.39	-11.38
-11.46	-11.45	-11.44	-11.43	-11.42	-11.41	-11.4	-11.39	-11.38
-11.46	-11.45	-11.44	-11.43	-11.42	-11.41	-11.4	-11.39	-11.38
-11.46	-11.45	-11.44	-11.43	-11.42	-11.41	-11.4	-11.39	-11.38
-11.46	-11.45	-11.44	-11.43	-11.42	-11.41	-11.4	-11.39	-11.38
-11.46	-11.45	-11.44	-11.43	-11.42	-11.41	-11.4	-11.39	-11.38
-11.46	-11.45	-11.44	-11.43	-11.42	-11.41	-11.4	-11.39	-11.38
-11.46	-11.45	-11.44	-11.43	-11.42	-11.41	-11.4	-11.39	-11.38
-11.46	-11.45	-11.44	-11.43	-11.42	-11.41	-11.4	-11.39	-11.38
-11.46	-11.45	-11.44	-11.43	-11.42	-11.41	-11.4	-11.39	-11.38
-11.46	-11.45	-11.44	-11.43	-11.42	-11.41	-11.4	-11.39	-11.38
-11.46	-11.45	-11.44	-11.43	-11.42	-11.41	-11.4	-11.39	-11.38
-11.46	-11.45	-11.44	-11.43	-11.42	-11.41	-11.4	-11.39	-11.38
-11.46	-11.45	-11.44	-11.43	-11.42	-11.41	-11.4	-11.39	-11.38
-11.46	-11.45	-11.44	-11.43	-11.42	-11.41	-11.4	-11.39	-11.38
-11.46	-11.45	-11.44	-11.43	-11.42	-11.41	-11.4	-11.39	-11.38
-11.46	-11.45	-11.44	-11.43	-11.42	-11.41	-11.4	-11.39	-11.38
-11.46	-11.45	-11.44	-11.43	-11.42	-11.41	-11.4	-11.39	-11.38
-11.46	-11.45	-11.44	-11.43	-11.42	-11.41	-11.4	-11.39	-11.38
-11.46	-11.45	-11.44	-11.43	-11.42	-11.41	-11.4	-11.39	-11.38

-11.46	-11.45	-11.44	-11.43	-11.42	-11.41	-11.4	-11.39	-11.38
-11.46	-11.45	-11.44	-11.43	-11.42	-11.41	-11.4	-11.39	-11.38
-11.46	-11.45	-11.44	-11.43	-11.42	-11.41	-11.4	-11.39	-11.38
-11.46	-11.45	-11.44	-11.43	-11.42	-11.41	-11.4	-11.39	-11.38
-11.46	-11.45	-11.44	-11.43	-11.42	-11.41	-11.4	-11.39	-11.38
-11.46	-11.45	-11.44	-11.43	-11.42	-11.41	-11.4	-11.39	-11.38
-11.46	-11.45	-11.44	-11.43	-11.42	-11.41	-11.4	-11.39	-11.38
-11.46	-11.45	-11.44	-11.43	-11.42	-11.41	-11.4	-11.39	-11.38
-11.46	-11.45	-11.44	-11.43	-11.42	-11.41	-11.4	-11.39	-11.38
-11.46	-11.45	-11.44	-11.43	-11.42	-11.41	-11.4	-11.39	-11.38
-11.46	-11.45	-11.44	-11.43	-11.42	-11.41	-11.4	-11.39	-11.38
-11.46	-11.45	-11.44	-11.43	-11.42	-11.41	-11.4	-11.39	-11.38
-11.46	-11.45	-11.44	-11.43	-11.42	-11.41	-11.4	-11.39	-11.38
-11.46	-11.45	-11.44	-11.43	-11.42	-11.41	-11.4	-11.39	-11.38
-11.46	-11.45	-11.44	-11.43	-11.42	-11.41	-11.4	-11.39	-11.38
-11.46	-11.45	-11.44	-11.43	-11.42	-11.41	-11.4	-11.39	-11.38
-11.46	-11.45	-11.44	-11.43	-11.42	-11.41	-11.4	-11.39	-11.38
-11.46	-11.45	-11.44	-11.43	-11.42	-11.41	-11.4	-11.39	-11.38
-11.46	-11.45	-11.44	-11.43	-11.42	-11.41	-11.4	-11.39	-11.38
-11.46	-11.45	-11.44	-11.43	-11.42	-11.41	-11.4	-11.39	-11.38
-11.46	-11.45	-11.44	-11.43	-11.42	-11.41	-11.4	-11.39	-11.38
-11.46	-11.45	-11.44	-11.43	-11.42	-11.41	-11.4	-11.39	-11.38
-11.46	-11.45	-11.44	-11.43	-11.42	-11.41	-11.4	-11.39	-11.38
-11.46	-11.45	-11.44	-11.43	-11.42	-11.41	-11.4	-11.39	-11.38
-11.46	-11.45	-11.44	-11.43	-11.42	-11.41	-11.4	-11.39	-11.38
-11.46	-11.45	-11.44	-11.43	-11.42	-11.41	-11.4	-11.39	-11.38
-11.46	-11.45	-11.44	-11.43	-11.42	-11.41	-11.4	-11.39	-11.38
-11.46	-11.45	-11.44	-11.43	-11.42	-11.41	-11.4	-11.39	-11.38
-11.46	-11.45	-11.44	-11.43	-11.42	-11.41	-11.4	-11.39	-11.38
-11.46	-11.45	-11.44	-11.43	-11.42	-11.41	-11.4	-11.39	-11.38
-11.46	-11.45	-11.44	-11.43	-11.42	-11.41	-11.4	-11.39	-11.38
-11.46	-11.45	-11.44	-11.43	-11.42	-11.41	-11.4	-11.39	-11.38
-11.46	-11.45	-11.44	-11.43	-11.42	-11.41	-11.4	-11.39	-11.38
-11.46	-11.45	-11.44	-11.43	-11.42	-11.41	-11.4	-11.39	-11.38
-11.46	-11.45	-11.44	-11.43	-11.42	-11.41	-11.4	-11.39	-11.38
-11.46	-11.45	-11.44	-11.43	-11.42	-11.41	-11.4	-11.39	-11.38
-11.46	-11.45	-11.44	-11.43	-11.42	-11.41	-11.4	-11.39	-11.38
-11.46	-11.45	-11.44	-11.43	-11.42	-11.41	-11.4	-11.39	-11.38
-11.46	-11.45	-11.44	-11.43	-11.42	-11.41	-11.4	-11.39	-11.38
-11.46	-11.45	-11.44	-11.43	-11.42	-11.41	-11.4	-11.39	-11.38
-11.46	-11.45	-11.44	-11.43	-11.42	-11.41	-11.4	-11.39	-11.38
-11.46	-11.45	-11.44	-11.43	-11.42	-11.41	-11.4	-11.39	-11.38
-11.46	-11.45	-11.44	-11.43	-11.42	-11.41	-11.4	-11.39	-11.38
-11.46	-11.45	-11.44	-11.43	-11.42	-11.41	-11.4	-11.39	-11.38
-11.46	-11.45	-11.44	-11.43	-11.42	-11.41	-11.4	-11.39	-11.38
-11.46	-11.45	-11.44	-11.43	-11.42	-11.41	-11.4	-11.39	-11.38
-11.46	-11.45	-11.44	-11.43	-11.42	-11.41	-11.4	-11.39	-11.38
-11.46	-11.45	-11.44	-11.43	-11.42	-11.41	-11.4	-11.39	-11.38
-11.46	-11.45	-11.44	-11.43	-11.42	-11.41	-11.4	-11.39	-11.38
-11.46	-11.45	-11.44	-11.43	-11.42	-11.41	-11.4	-11.39	-11.38

-11.46	-11.45	-11.44	-11.43	-11.42	-11.41	-11.4	-11.39	-11.38
-11.46	-11.45	-11.44	-11.43	-11.42	-11.41	-11.4	-11.39	-11.38
-11.46	-11.45	-11.44	-11.43	-11.42	-11.41	-11.4	-11.39	-11.38
-11.46	-11.45	-11.44	-11.43	-11.42	-11.41	-11.4	-11.39	-11.38
-11.46	-11.45	-11.44	-11.43	-11.42	-11.41	-11.4	-11.39	-11.38
-11.46	-11.45	-11.44	-11.43	-11.42	-11.41	-11.4	-11.39	-11.38
-11.46	-11.45	-11.44	-11.43	-11.42	-11.41	-11.4	-11.39	-11.38
-11.46	-11.45	-11.44	-11.43	-11.42	-11.41	-11.4	-11.39	-11.38
-11.46	-11.45	-11.44	-11.43	-11.42	-11.41	-11.4	-11.39	-11.38
-11.46	-11.45	-11.44	-11.43	-11.42	-11.41	-11.4	-11.39	-11.38
-11.46	-11.45	-11.44	-11.43	-11.42	-11.41	-11.4	-11.39	-11.38
-11.46	-11.45	-11.44	-11.43	-11.42	-11.41	-11.4	-11.39	-11.38
-11.46	-11.45	-11.44	-11.43	-11.42	-11.41	-11.4	-11.39	-11.38
-11.46	-11.45	-11.44	-11.43	-11.42	-11.41	-11.4	-11.39	-11.38
-11.46	-11.45	-11.44	-11.43	-11.42	-11.41	-11.4	-11.39	-11.38
-11.46	-11.45	-11.44	-11.43	-11.42	-11.41	-11.4	-11.39	-11.38
-11.46	-11.45	-11.44	-11.43	-11.42	-11.41	-11.4	-11.39	-11.38
-11.46	-11.45	-11.44	-11.43	-11.42	-11.41	-11.4	-11.39	-11.38
-11.46	-11.45	-11.44	-11.43	-11.42	-11.41	-11.4	-11.39	-11.38
-11.46	-11.45	-11.44	-11.43	-11.42	-11.41	-11.4	-11.39	-11.38
-11.46	-11.45	-11.44	-11.43	-11.42	-11.41	-11.4	-11.39	-11.38
-11.46	-11.45	-11.44	-11.43	-11.42	-11.41	-11.4	-11.39	-11.38
-11.46	-11.45	-11.44	-11.43	-11.42	-11.41	-11.4	-11.39	-11.38
-11.46	-11.45	-11.44	-11.43	-11.42	-11.41	-11.4	-11.39	-11.38
-11.46	-11.45	-11.44	-11.43	-11.42	-11.41	-11.4	-11.39	-11.38
-11.46	-11.45	-11.44	-11.43	-11.42	-11.41	-11.4	-11.39	-11.38
-11.46	-11.45	-11.44	-11.43	-11.42	-11.41	-11.4	-11.39	-11.38
-11.46	-11.45	-11.44	-11.43	-11.42	-11.41	-11.4	-11.39	-11.38
-11.46	-11.45	-11.44	-11.43	-11.42	-11.41	-11.4	-11.39	-11.38
-11.46	-11.45	-11.44	-11.43	-11.42	-11.41	-11.4	-11.39	-11.38
-11.46	-11.45	-11.44	-11.43	-11.42	-11.41	-11.4	-11.39	-11.38
-11.46	-11.45	-11.44	-11.43	-11.42	-11.41	-11.4	-11.39	-11.38
-11.46	-11.45	-11.44	-11.43	-11.42	-11.41	-11.4	-11.39	-11.38
-11.46	-11.45	-11.44	-11.43	-11.42	-11.41	-11.4	-11.39	-11.38
-11.46	-11.45	-11.44	-11.43	-11.42	-11.41	-11.4	-11.39	-11.38
-11.46	-11.45	-11.44	-11.43	-11.42	-11.41	-11.4	-11.39	-11.38
-11.46	-11.45	-11.44	-11.43	-11.42	-11.41	-11.4	-11.39	-11.38
-11.46	-11.45	-11.44	-11.43	-11.42	-11.41	-11.4	-11.39	-11.38
-11.46	-11.45	-11.44	-11.43	-11.42	-11.41	-11.4	-11.39	-11.38
-11.46	-11.45	-11.44	-11.43	-11.42	-11.41	-11.4	-11.39	-11.38
-11.46	-11.45	-11.44	-11.43	-11.42	-11.41	-11.4	-11.39	-11.38
-11.46	-11.45	-11.44	-11.43	-11.42	-11.41	-11.4	-11.39	-11.38
-11.46	-11.45	-11.44	-11.43	-11.42	-11.41	-11.4	-11.39	-11.38
-11.46	-11.45	-11.44	-11.43	-11.42	-11.41	-11.4	-11.39	-11.38
-11.46	-11.45	-11.44	-11.43	-11.42	-11.41	-11.4	-11.39	-11.38
-11.46	-11.45	-11.44	-11.43	-11.42	-11.41	-11.4	-11.39	-11.38
-11.46	-11.45	-11.44	-11.43	-11.42	-11.41	-11.4	-11.39	-11.38
-11.46	-11.45	-11.44	-11.43	-11.42	-11.41	-11.4	-11.39	-11.38
-11.46	-11.45	-11.44	-11.43	-11.42	-11.41	-11.4	-11.39	-11.38
-11.46	-11.45	-11.44	-11.43	-11.42	-11.41	-11.4	-11.39	-11.38

-11.46	-11.45	-11.44	-11.43	-11.42	-11.41	-11.4	-11.39	-11.38
-11.46	-11.45	-11.44	-11.43	-11.42	-11.41	-11.4	-11.39	-11.38
-11.46	-11.45	-11.44	-11.43	-11.42	-11.41	-11.4	-11.39	-11.38
-11.46	-11.45	-11.44	-11.43	-11.42	-11.41	-11.4	-11.39	-11.38
-11.46	-11.45	-11.44	-11.43	-11.42	-11.41	-11.4	-11.39	-11.38
-11.46	-11.45	-11.44	-11.43	-11.42	-11.41	-11.4	-11.39	-11.38
-11.46	-11.45	-11.44	-11.43	-11.42	-11.41	-11.4	-11.39	-11.38
-11.46	-11.45	-11.44	-11.43	-11.42	-11.41	-11.4	-11.39	-11.38
-11.46	-11.45	-11.44	-11.43	-11.42	-11.41	-11.4	-11.39	-11.38
-11.46	-11.45	-11.44	-11.43	-11.42	-11.41	-11.4	-11.39	-11.38
-11.46	-11.45	-11.44	-11.43	-11.42	-11.41	-11.4	-11.39	-11.38
-11.46	-11.45	-11.44	-11.43	-11.42	-11.41	-11.4	-11.39	-11.38
-11.46	-11.45	-11.44	-11.43	-11.42	-11.41	-11.4	-11.39	-11.38
-11.46	-11.45	-11.44	-11.43	-11.42	-11.41	-11.4	-11.39	-11.38
-11.46	-11.45	-11.44	-11.43	-11.42	-11.41	-11.4	-11.39	-11.38
-11.46	-11.45	-11.44	-11.43	-11.42	-11.41	-11.4	-11.39	-11.38
-11.46	-11.45	-11.44	-11.43	-11.42	-11.41	-11.4	-11.39	-11.38
-11.46	-11.45	-11.44	-11.43	-11.42	-11.41	-11.4	-11.39	-11.38
-11.46	-11.45	-11.44	-11.43	-11.42	-11.41	-11.4	-11.39	-11.38
-11.46	-11.45	-11.44	-11.43	-11.42	-11.41	-11.4	-11.39	-11.38
-11.46	-11.45	-11.44	-11.43	-11.42	-11.41	-11.4	-11.39	-11.38
-11.46	-11.45	-11.44	-11.43	-11.42	-11.41	-11.4	-11.39	-11.38
-11.46	-11.45	-11.44	-11.43	-11.42	-11.41	-11.4	-11.39	-11.38
-11.46	-11.45	-11.44	-11.43	-11.42	-11.41	-11.4	-11.39	-11.38
-11.46	-11.45	-11.44	-11.43	-11.42	-11.41	-11.4	-11.39	-11.38
-11.46	-11.45	-11.44	-11.43	-11.42	-11.41	-11.4	-11.39	-11.38
-11.46	-11.45	-11.44	-11.43	-11.42	-11.41	-11.4	-11.39	-11.38
-11.46	-11.45	-11.44	-11.43	-11.42	-11.41	-11.4	-11.39	-11.38
-11.46	-11.45	-11.44	-11.43	-11.42	-11.41	-11.4	-11.39	-11.38
-11.46	-11.45	-11.44	-11.43	-11.42	-11.41	-11.4	-11.39	-11.38
-11.46	-11.45	-11.44	-11.43	-11.42	-11.41	-11.4	-11.39	-11.38
-11.46	-11.45	-11.44	-11.43	-11.42	-11.41	-11.4	-11.39	-11.38
-11.46	-11.45	-11.44	-11.43	-11.42	-11.41	-11.4	-11.39	-11.38
-11.46	-11.45	-11.44	-11.43	-11.42	-11.41	-11.4	-11.39	-11.38
-11.46	-11.45	-11.44	-11.43	-11.42	-11.41	-11.4	-11.39	-11.38
-11.46	-11.45	-11.44	-11.43	-11.42	-11.41	-11.4	-11.39	-11.38
-11.46	-11.45	-11.44	-11.43	-11.42	-11.41	-11.4	-11.39	-11.38
-11.46	-11.45	-11.44	-11.43	-11.42	-11.41	-11.4	-11.39	-11.38
-11.46	-11.45	-11.44	-11.43	-11.42	-11.41	-11.4	-11.39	-11.38
-11.46	-11.45	-11.44	-11.43	-11.42	-11.41	-11.4	-11.39	-11.38
-11.46	-11.45	-11.44	-11.43	-11.42	-11.41	-11.4	-11.39	-11.38
-11.46	-11.45	-11.44	-11.43	-11.42	-11.41	-11.4	-11.39	-11.38
-11.46	-11.45	-11.44	-11.43	-11.42	-11.41	-11.4	-11.39	-11.38
-11.46	-11.45	-11.44	-11.43	-11.42	-11.41	-11.4	-11.39	-11.38
-11.46	-11.45	-11.44	-11.43	-11.42	-11.41	-11.4	-11.39	-11.38
-11.46	-11.45	-11.44	-11.43	-11.42	-11.41	-11.4	-11.39	-11.38
-11.46	-11.45	-11.44	-11.43	-11.42	-11.41	-11.4	-11.39	-11.38
-11.46	-11.45	-11.44	-11.43	-11.42	-11.41	-11.4	-11.39	-11.38
-11.46	-11.45	-11.44	-11.43	-11.42	-11.41	-11.4	-11.39	-11.38
-11.46	-11.45	-11.44	-11.43	-11.42	-11.41	-11.4	-11.39	-11.38

-11.46	-11.45	-11.44	-11.43	-11.42	-11.41	-11.4	-11.39	-11.38
-11.46	-11.45	-11.44	-11.43	-11.42	-11.41	-11.4	-11.39	-11.38
-11.46	-11.45	-11.44	-11.43	-11.42	-11.41	-11.4	-11.39	-11.38
-11.46	-11.45	-11.44	-11.43	-11.42	-11.41	-11.4	-11.39	-11.38
-11.46	-11.45	-11.44	-11.43	-11.42	-11.41	-11.4	-11.39	-11.38
-11.46	-11.45	-11.44	-11.43	-11.42	-11.41	-11.4	-11.39	-11.38
-11.46	-11.45	-11.44	-11.43	-11.42	-11.41	-11.4	-11.39	-11.38
-11.46	-11.45	-11.44	-11.43	-11.42	-11.41	-11.4	-11.39	-11.38
-11.46	-11.45	-11.44	-11.43	-11.42	-11.41	-11.4	-11.39	-11.38
-11.46	-11.45	-11.44	-11.43	-11.42	-11.41	-11.4	-11.39	-11.38
-11.46	-11.45	-11.44	-11.43	-11.42	-11.41	-11.4	-11.39	-11.38
-11.46	-11.45	-11.44	-11.43	-11.42	-11.41	-11.4	-11.39	-11.38
-11.46	-11.45	-11.44	-11.43	-11.42	-11.41	-11.4	-11.39	-11.38
-11.46	-11.45	-11.44	-11.43	-11.42	-11.41	-11.4	-11.39	-11.38
-11.46	-11.45	-11.44	-11.43	-11.42	-11.41	-11.4	-11.39	-11.38
-11.46	-11.45	-11.44	-11.43	-11.42	-11.41	-11.4	-11.39	-11.38
-11.46	-11.45	-11.44	-11.43	-11.42	-11.41	-11.4	-11.39	-11.38
-11.46	-11.45	-11.44	-11.43	-11.42	-11.41	-11.4	-11.39	-11.38
-11.46	-11.45	-11.44	-11.43	-11.42	-11.41	-11.4	-11.39	-11.38
-11.46	-11.45	-11.44	-11.43	-11.42	-11.41	-11.4	-11.39	-11.38
-11.46	-11.45	-11.44	-11.43	-11.42	-11.41	-11.4	-11.39	-11.38
-11.46	-11.45	-11.44	-11.43	-11.42	-11.41	-11.4	-11.39	-11.38
-11.46	-11.45	-11.44	-11.43	-11.42	-11.41	-11.4	-11.39	-11.38
-11.46	-11.45	-11.44	-11.43	-11.42	-11.41	-11.4	-11.39	-11.38
-11.46	-11.45	-11.44	-11.43	-11.42	-11.41	-11.4	-11.39	-11.38
-11.46	-11.45	-11.44	-11.43	-11.42	-11.41	-11.4	-11.39	-11.38
-11.46	-11.45	-11.44	-11.43	-11.42	-11.41	-11.4	-11.39	-11.38
-11.46	-11.45	-11.44	-11.43	-11.42	-11.41	-11.4	-11.39	-11.38
-11.46	-11.45	-11.44	-11.43	-11.42	-11.41	-11.4	-11.39	-11.38
-11.46	-11.45	-11.44	-11.43	-11.42	-11.41	-11.4	-11.39	-11.38
-11.46	-11.45	-11.44	-11.43	-11.42	-11.41	-11.4	-11.39	-11.38
-11.46	-11.45	-11.44	-11.43	-11.42	-11.41	-11.4	-11.39	-11.38
-11.46	-11.45	-11.44	-11.43	-11.42	-11.41	-11.4	-11.39	-11.38
-11.46	-11.45	-11.44	-11.43	-11.42	-11.41	-11.4	-11.39	-11.38
-11.46	-11.45	-11.44	-11.43	-11.42	-11.41	-11.4	-11.39	-11.38
-11.46	-11.45	-11.44	-11.43	-11.42	-11.41	-11.4	-11.39	-11.38
-11.46	-11.45	-11.44	-11.43	-11.42	-11.41	-11.4	-11.39	-11.38
-11.46	-11.45	-11.44	-11.43	-11.42	-11.41	-11.4	-11.39	-11.38
-11.46	-11.45	-11.44	-11.43	-11.42	-11.41	-11.4	-11.39	-11.38
-11.46	-11.45	-11.44	-11.43	-11.42	-11.41	-11.4	-11.39	-11.38
-11.46	-11.45	-11.44	-11.43	-11.42	-11.41	-11.4	-11.39	-11.38
-11.46	-11.45	-11.44	-11.43	-11.42	-11.41	-11.4	-11.39	-11.38
-11.46	-11.45	-11.44	-11.43	-11.42	-11.41	-11.4	-11.39	-11.38
-11.46	-11.45	-11.44	-11.43	-11.42	-11.41	-11.4	-11.39	-11.38
-11.46	-11.45	-11.44	-11.43	-11.42	-11.41	-11.4	-11.39	-11.38
-11.46	-11.45	-11.44	-11.43	-11.42	-11.41	-11.4	-11.39	-11.38
-11.46	-11.45	-11.44	-11.43	-11.42	-11.41	-11.4	-11.39	-11.38
-11.46	-11.45	-11.44	-11.43	-11.42	-11.41	-11.4	-11.39	-11.38
-11.46	-11.45	-11.44	-11.43	-11.42	-11.41	-11.4	-11.39	-11.38
-11.46	-11.45	-11.44	-11.43	-11.42	-11.41	-11.4	-11.39	-11.38

-11.46	-11.45	-11.44	-11.43	-11.42	-11.41	-11.4	-11.39	-11.38
-11.46	-11.45	-11.44	-11.43	-11.42	-11.41	-11.4	-11.39	-11.38
-11.46	-11.45	-11.44	-11.43	-11.42	-11.41	-11.4	-11.39	-11.38
-11.46	-11.45	-11.44	-11.43	-11.42	-11.41	-11.4	-11.39	-11.38
-11.46	-11.45	-11.44	-11.43	-11.42	-11.41	-11.4	-11.39	-11.38
-11.46	-11.45	-11.44	-11.43	-11.42	-11.41	-11.4	-11.39	-11.38
-11.46	-11.45	-11.44	-11.43	-11.42	-11.41	-11.4	-11.39	-11.38
-11.46	-11.45	-11.44	-11.43	-11.42	-11.41	-11.4	-11.39	-11.38
-11.46	-11.45	-11.44	-11.43	-11.42	-11.41	-11.4	-11.39	-11.38
-11.46	-11.45	-11.44	-11.43	-11.42	-11.41	-11.4	-11.39	-11.38
-11.46	-11.45	-11.44	-11.43	-11.42	-11.41	-11.4	-11.39	-11.38
-11.46	-11.45	-11.44	-11.43	-11.42	-11.41	-11.4	-11.39	-11.38
-11.46	-11.45	-11.44	-11.43	-11.42	-11.41	-11.4	-11.39	-11.38
-11.46	-11.45	-11.44	-11.43	-11.42	-11.41	-11.4	-11.39	-11.38
-11.46	-11.45	-11.44	-11.43	-11.42	-11.41	-11.4	-11.39	-11.38
-11.46	-11.45	-11.44	-11.43	-11.42	-11.41	-11.4	-11.39	-11.38
-11.46	-11.45	-11.44	-11.43	-11.42	-11.41	-11.4	-11.39	-11.38
-11.46	-11.45	-11.44	-11.43	-11.42	-11.41	-11.4	-11.39	-11.38
-11.46	-11.45	-11.44	-11.43	-11.42	-11.41	-11.4	-11.39	-11.38
-11.46	-11.45	-11.44	-11.43	-11.42	-11.41	-11.4	-11.39	-11.38
-11.46	-11.45	-11.44	-11.43	-11.42	-11.41	-11.4	-11.39	-11.38
-11.46	-11.45	-11.44	-11.43	-11.42	-11.41	-11.4	-11.39	-11.38
-11.46	-11.45	-11.44	-11.43	-11.42	-11.41	-11.4	-11.39	-11.38
-11.46	-11.45	-11.44	-11.43	-11.42	-11.41	-11.4	-11.39	-11.38
-11.46	-11.45	-11.44	-11.43	-11.42	-11.41	-11.4	-11.39	-11.38
-11.46	-11.45	-11.44	-11.43	-11.42	-11.41	-11.4	-11.39	-11.38
-11.46	-11.45	-11.44	-11.43	-11.42	-11.41	-11.4	-11.39	-11.38
-11.46	-11.45	-11.44	-11.43	-11.42	-11.41	-11.4	-11.39	-11.38
-11.46	-11.45	-11.44	-11.43	-11.42	-11.41	-11.4	-11.39	-11.38
-11.46	-11.45	-11.44	-11.43	-11.42	-11.41	-11.4	-11.39	-11.38
-11.46	-11.45	-11.44	-11.43	-11.42	-11.41	-11.4	-11.39	-11.38
-11.46	-11.45	-11.44	-11.43	-11.42	-11.41	-11.4	-11.39	-11.38
-11.46	-11.45	-11.44	-11.43	-11.42	-11.41	-11.4	-11.39	-11.38
-11.46	-11.45	-11.44	-11.43	-11.42	-11.41	-11.4	-11.39	-11.38
-11.46	-11.45	-11.44	-11.43	-11.42	-11.41	-11.4	-11.39	-11.38
-11.46	-11.45	-11.44	-11.43	-11.42	-11.41	-11.4	-11.39	-11.38
-11.46	-11.45	-11.44	-11.43	-11.42	-11.41	-11.4	-11.39	-11.38
-11.46	-11.45	-11.44	-11.43	-11.42	-11.41	-11.4	-11.39	-11.38
-11.46	-11.45	-11.44	-11.43	-11.42	-11.41	-11.4	-11.39	-11.38
-11.46	-11.45	-11.44	-11.43	-11.42	-11.41	-11.4	-11.39	-11.38
-11.46	-11.45	-11.44	-11.43	-11.42	-11.41	-11.4	-11.39	-11.38
-11.46	-11.45	-11.44	-11.43	-11.42	-11.41	-11.4	-11.39	-11.38
-11.46	-11.45	-11.44	-11.43	-11.42	-11.41	-11.4	-11.39	-11.38
-11.46	-11.45	-11.44	-11.43	-11.42	-11.41	-11.4	-11.39	-11.38
-11.46	-11.45	-11.44	-11.43	-11.42	-11.41	-11.4	-11.39	-11.38
-11.46	-11.45	-11.44	-11.43	-11.42	-11.41	-11.4	-11.39	-11.38
-11.46	-11.45	-11.44	-11.43	-11.42	-11.41	-11.4	-11.39	-11.38
-11.46	-11.45	-11.44	-11.43	-11.42	-11.41	-11.4	-11.39	-11.38
-11.46	-11.45	-11.44	-11.43	-11.42	-11.41	-11.4	-11.39	-11.38
-11.46	-11.45	-11.44	-11.43	-11.42	-11.41	-11.4	-11.39	-11.38

-11.46	-11.45	-11.44	-11.43	-11.42	-11.41	-11.4	-11.39	-11.38
-11.46	-11.45	-11.44	-11.43	-11.42	-11.41	-11.4	-11.39	-11.38
-11.46	-11.45	-11.44	-11.43	-11.42	-11.41	-11.4	-11.39	-11.38
-11.46	-11.45	-11.44	-11.43	-11.42	-11.41	-11.4	-11.39	-11.38
-11.46	-11.45	-11.44	-11.43	-11.42	-11.41	-11.4	-11.39	-11.38
-11.46	-11.45	-11.44	-11.43	-11.42	-11.41	-11.4	-11.39	-11.38
-11.46	-11.45	-11.44	-11.43	-11.42	-11.41	-11.4	-11.39	-11.38
-11.46	-11.45	-11.44	-11.43	-11.42	-11.41	-11.4	-11.39	-11.38
-11.46	-11.45	-11.44	-11.43	-11.42	-11.41	-11.4	-11.39	-11.38
-11.46	-11.45	-11.44	-11.43	-11.42	-11.41	-11.4	-11.39	-11.38
-11.46	-11.45	-11.44	-11.43	-11.42	-11.41	-11.4	-11.39	-11.38
-11.46	-11.45	-11.44	-11.43	-11.42	-11.41	-11.4	-11.39	-11.38
-11.46	-11.45	-11.44	-11.43	-11.42	-11.41	-11.4	-11.39	-11.38
-11.46	-11.45	-11.44	-11.43	-11.42	-11.41	-11.4	-11.39	-11.38
-11.46	-11.45	-11.44	-11.43	-11.42	-11.41	-11.4	-11.39	-11.38
-11.46	-11.45	-11.44	-11.43	-11.42	-11.41	-11.4	-11.39	-11.38
-11.46	-11.45	-11.44	-11.43	-11.42	-11.41	-11.4	-11.39	-11.38
-11.46	-11.45	-11.44	-11.43	-11.42	-11.41	-11.4	-11.39	-11.38
-11.46	-11.45	-11.44	-11.43	-11.42	-11.41	-11.4	-11.39	-11.38
-11.46	-11.45	-11.44	-11.43	-11.42	-11.41	-11.4	-11.39	-11.38
-11.46	-11.45	-11.44	-11.43	-11.42	-11.41	-11.4	-11.39	-11.38
-11.46	-11.45	-11.44	-11.43	-11.42	-11.41	-11.4	-11.39	-11.38
-11.46	-11.45	-11.44	-11.43	-11.42	-11.41	-11.4	-11.39	-11.38
-11.46	-11.45	-11.44	-11.43	-11.42	-11.41	-11.4	-11.39	-11.38
-11.46	-11.45	-11.44	-11.43	-11.42	-11.41	-11.4	-11.39	-11.38
-11.46	-11.45	-11.44	-11.43	-11.42	-11.41	-11.4	-11.39	-11.38
-11.46	-11.45	-11.44	-11.43	-11.42	-11.41	-11.4	-11.39	-11.38
-11.46	-11.45	-11.44	-11.43	-11.42	-11.41	-11.4	-11.39	-11.38
-11.46	-11.45	-11.44	-11.43	-11.42	-11.41	-11.4	-11.39	-11.38
-11.46	-11.45	-11.44	-11.43	-11.42	-11.41	-11.4	-11.39	-11.38
-11.46	-11.45	-11.44	-11.43	-11.42	-11.41	-11.4	-11.39	-11.38
-11.46	-11.45	-11.44	-11.43	-11.42	-11.41	-11.4	-11.39	-11.38
-11.46	-11.45	-11.44	-11.43	-11.42	-11.41	-11.4	-11.39	-11.38
-11.46	-11.45	-11.44	-11.43	-11.42	-11.41	-11.4	-11.39	-11.38
-11.46	-11.45	-11.44	-11.43	-11.42	-11.41	-11.4	-11.39	-11.38
-11.46	-11.45	-11.44	-11.43	-11.42	-11.41	-11.4	-11.39	-11.38
-11.46	-11.45	-11.44	-11.43	-11.42	-11.41	-11.4	-11.39	-11.38
-11.46	-11.45	-11.44	-11.43	-11.42	-11.41	-11.4	-11.39	-11.38
-11.46	-11.45	-11.44	-11.43	-11.42	-11.41	-11.4	-11.39	-11.38
-11.46	-11.45	-11.44	-11.43	-11.42	-11.41	-11.4	-11.39	-11.38
-11.46	-11.45	-11.44	-11.43	-11.42	-11.41	-11.4	-11.39	-11.38
-11.46	-11.45	-11.44	-11.43	-11.42	-11.41	-11.4	-11.39	-11.38
-11.46	-11.45	-11.44	-11.43	-11.42	-11.41	-11.4	-11.39	-11.38
-11.46	-11.45	-11.44	-11.43	-11.42	-11.41	-11.4	-11.39	-11.38
-11.46	-11.45	-11.44	-11.43	-11.42	-11.41	-11.4	-11.39	-11.38
-11.46	-11.45	-11.44	-11.43	-11.42	-11.41	-11.4	-11.39	-11.38
-11.46	-11.45	-11.44	-11.43	-11.42	-11.41	-11.4	-11.39	-11.38
-11.46	-11.45	-11.44	-11.43	-11.42	-11.41	-11.4	-11.39	-11.38
-11.46	-11.45	-11.44	-11.43	-11.42	-11.41	-11.4	-11.39	-11.38
-11.46	-11.45	-11.44	-11.43	-11.42	-11.41	-11.4	-11.39	-11.38

-11.46	-11.45	-11.44	-11.43	-11.42	-11.41	-11.4	-11.39	-11.38
-11.46	-11.45	-11.44	-11.43	-11.42	-11.41	-11.4	-11.39	-11.38
-11.46	-11.45	-11.44	-11.43	-11.42	-11.41	-11.4	-11.39	-11.38
-11.46	-11.45	-11.44	-11.43	-11.42	-11.41	-11.4	-11.39	-11.38
-11.46	-11.45	-11.44	-11.43	-11.42	-11.41	-11.4	-11.39	-11.38
-11.46	-11.45	-11.44	-11.43	-11.42	-11.41	-11.4	-11.39	-11.38
-11.46	-11.45	-11.44	-11.43	-11.42	-11.41	-11.4	-11.39	-11.38
-11.46	-11.45	-11.44	-11.43	-11.42	-11.41	-11.4	-11.39	-11.38
-11.46	-11.45	-11.44	-11.43	-11.42	-11.41	-11.4	-11.39	-11.38
-11.46	-11.45	-11.44	-11.43	-11.42	-11.41	-11.4	-11.39	-11.38
-11.46	-11.45	-11.44	-11.43	-11.42	-11.41	-11.4	-11.39	-11.38
-11.46	-11.45	-11.44	-11.43	-11.42	-11.41	-11.4	-11.39	-11.38
-11.46	-11.45	-11.44	-11.43	-11.42	-11.41	-11.4	-11.39	-11.38
-11.46	-11.45	-11.44	-11.43	-11.42	-11.41	-11.4	-11.39	-11.38
-11.46	-11.45	-11.44	-11.43	-11.42	-11.41	-11.4	-11.39	-11.38
-11.46	-11.45	-11.44	-11.43	-11.42	-11.41	-11.4	-11.39	-11.38
-11.46	-11.45	-11.44	-11.43	-11.42	-11.41	-11.4	-11.39	-11.38
-11.46	-11.45	-11.44	-11.43	-11.42	-11.41	-11.4	-11.39	-11.38
-11.46	-11.45	-11.44	-11.43	-11.42	-11.41	-11.4	-11.39	-11.38
-11.46	-11.45	-11.44	-11.43	-11.42	-11.41	-11.4	-11.39	-11.38
-11.46	-11.45	-11.44	-11.43	-11.42	-11.41	-11.4	-11.39	-11.38
-11.46	-11.45	-11.44	-11.43	-11.42	-11.41	-11.4	-11.39	-11.38
-11.46	-11.45	-11.44	-11.43	-11.42	-11.41	-11.4	-11.39	-11.38
-11.46	-11.45	-11.44	-11.43	-11.42	-11.41	-11.4	-11.39	-11.38
-11.46	-11.45	-11.44	-11.43	-11.42	-11.41	-11.4	-11.39	-11.38
-11.46	-11.45	-11.44	-11.43	-11.42	-11.41	-11.4	-11.39	-11.38
-11.46	-11.45	-11.44	-11.43	-11.42	-11.41	-11.4	-11.39	-11.38
-11.46	-11.45	-11.44	-11.43	-11.42	-11.41	-11.4	-11.39	-11.38
-11.46	-11.45	-11.44	-11.43	-11.42	-11.41	-11.4	-11.39	-11.38
-11.46	-11.45	-11.44	-11.43	-11.42	-11.41	-11.4	-11.39	-11.38
-11.46	-11.45	-11.44	-11.43	-11.42	-11.41	-11.4	-11.39	-11.38
-11.46	-11.45	-11.44	-11.43	-11.42	-11.41	-11.4	-11.39	-11.38
-11.46	-11.45	-11.44	-11.43	-11.42	-11.41	-11.4	-11.39	-11.38
-11.46	-11.45	-11.44	-11.43	-11.42	-11.41	-11.4	-11.39	-11.38
-11.46	-11.45	-11.44	-11.43	-11.42	-11.41	-11.4	-11.39	-11.38
-11.46	-11.45	-11.44	-11.43	-11.42	-11.41	-11.4	-11.39	-11.38
-11.46	-11.45	-11.44	-11.43	-11.42	-11.41	-11.4	-11.39	-11.38
-11.46	-11.45	-11.44	-11.43	-11.42	-11.41	-11.4	-11.39	-11.38
-11.46	-11.45	-11.44	-11.43	-11.42	-11.41	-11.4	-11.39	-11.38
-11.46	-11.45	-11.44	-11.43	-11.42	-11.41	-11.4	-11.39	-11.38
-11.46	-11.45	-11.44	-11.43	-11.42	-11.41	-11.4	-11.39	-11.38
-11.46	-11.45	-11.44	-11.43	-11.42	-11.41	-11.4	-11.39	-11.38
-11.46	-11.45	-11.44	-11.43	-11.42	-11.41	-11.4	-11.39	-11.38
-11.46	-11.45	-11.44	-11.43	-11.42	-11.41	-11.4	-11.39	-11.38
-11.46	-11.45	-11.44	-11.43	-11.42	-11.41	-11.4	-11.39	-11.38
-11.46	-11.45	-11.44	-11.43	-11.42	-11.41	-11.4	-11.39	-11.38
-11.46	-11.45	-11.44	-11.43	-11.42	-11.41	-11.4	-11.39	-11.38
-11.46	-11.45	-11.44	-11.43	-11.42	-11.41	-11.4	-11.39	-11.38
-11.46	-11.45	-11.44	-11.43	-11.42	-11.41	-11.4	-11.39	-11.38
-11.46	-11.45	-11.44	-11.43	-11.42	-11.41	-11.4	-11.39	-11.38

-11.46	-11.45	-11.44	-11.43	-11.42	-11.41	-11.4	-11.39	-11.38
-11.46	-11.45	-11.44	-11.43	-11.42	-11.41	-11.4	-11.39	-11.38
-11.46	-11.45	-11.44	-11.43	-11.42	-11.41	-11.4	-11.39	-11.38
-11.46	-11.45	-11.44	-11.43	-11.42	-11.41	-11.4	-11.39	-11.38
-11.46	-11.45	-11.44	-11.43	-11.42	-11.41	-11.4	-11.39	-11.38
-11.46	-11.45	-11.44	-11.43	-11.42	-11.41	-11.4	-11.39	-11.38
-11.46	-11.45	-11.44	-11.43	-11.42	-11.41	-11.4	-11.39	-11.38
-11.46	-11.45	-11.44	-11.43	-11.42	-11.41	-11.4	-11.39	-11.38
-11.46	-11.45	-11.44	-11.43	-11.42	-11.41	-11.4	-11.39	-11.38
-11.46	-11.45	-11.44	-11.43	-11.42	-11.41	-11.4	-11.39	-11.38
-11.46	-11.45	-11.44	-11.43	-11.42	-11.41	-11.4	-11.39	-11.38
-11.46	-11.45	-11.44	-11.43	-11.42	-11.41	-11.4	-11.39	-11.38
-11.46	-11.45	-11.44	-11.43	-11.42	-11.41	-11.4	-11.39	-11.38
-11.46	-11.45	-11.44	-11.43	-11.42	-11.41	-11.4	-11.39	-11.38
-11.46	-11.45	-11.44	-11.43	-11.42	-11.41	-11.4	-11.39	-11.38
-11.46	-11.45	-11.44	-11.43	-11.42	-11.41	-11.4	-11.39	-11.38
-11.46	-11.45	-11.44	-11.43	-11.42	-11.41	-11.4	-11.39	-11.38
-11.46	-11.45	-11.44	-11.43	-11.42	-11.41	-11.4	-11.39	-11.38
-11.46	-11.45	-11.44	-11.43	-11.42	-11.41	-11.4	-11.39	-11.38
-11.46	-11.45	-11.44	-11.43	-11.42	-11.41	-11.4	-11.39	-11.38
-11.46	-11.45	-11.44	-11.43	-11.42	-11.41	-11.4	-11.39	-11.38
-11.46	-11.45	-11.44	-11.43	-11.42	-11.41	-11.4	-11.39	-11.38
-11.46	-11.45	-11.44	-11.43	-11.42	-11.41	-11.4	-11.39	-11.38
-11.46	-11.45	-11.44	-11.43	-11.42	-11.41	-11.4	-11.39	-11.38
-11.46	-11.45	-11.44	-11.43	-11.42	-11.41	-11.4	-11.39	-11.38
-11.46	-11.45	-11.44	-11.43	-11.42	-11.41	-11.4	-11.39	-11.38
-11.46	-11.45	-11.44	-11.43	-11.42	-11.41	-11.4	-11.39	-11.38
-11.46	-11.45	-11.44	-11.43	-11.42	-11.41	-11.4	-11.39	-11.38
-11.46	-11.45	-11.44	-11.43	-11.42	-11.41	-11.4	-11.39	-11.38
-11.46	-11.45	-11.44	-11.43	-11.42	-11.41	-11.4	-11.39	-11.38
-11.46	-11.45	-11.44	-11.43	-11.42	-11.41	-11.4	-11.39	-11.38
-11.46	-11.45	-11.44	-11.43	-11.42	-11.41	-11.4	-11.39	-11.38
-11.46	-11.45	-11.44	-11.43	-11.42	-11.41	-11.4	-11.39	-11.38
-11.46	-11.45	-11.44	-11.43	-11.42	-11.41	-11.4	-11.39	-11.38
-11.46	-11.45	-11.44	-11.43	-11.42	-11.41	-11.4	-11.39	-11.38
-11.46	-11.45	-11.44	-11.43	-11.42	-11.41	-11.4	-11.39	-11.38
-11.46	-11.45	-11.44	-11.43	-11.42	-11.41	-11.4	-11.39	-11.38
-11.46	-11.45	-11.44	-11.43	-11.42	-11.41	-11.4	-11.39	-11.38
-11.46	-11.45	-11.44	-11.43	-11.42	-11.41	-11.4	-11.39	-11.38
-11.46	-11.45	-11.44	-11.43	-11.42	-11.41	-11.4	-11.39	-11.38
-11.46	-11.45	-11.44	-11.43	-11.42	-11.41	-11.4	-11.39	-11.38
-11.46	-11.45	-11.44	-11.43	-11.42	-11.41	-11.4	-11.39	-11.38
-11.46	-11.45	-11.44	-11.43	-11.42	-11.41	-11.4	-11.39	-11.38
-11.46	-11.45	-11.44	-11.43	-11.42	-11.41	-11.4	-11.39	-11.38
-11.46	-11.45	-11.44	-11.43	-11.42	-11.41	-11.4	-11.39	-11.38
-11.46	-11.45	-11.44	-11.43	-11.42	-11.41	-11.4	-11.39	-11.38
-11.46	-11.45	-11.44	-11.43	-11.42	-11.41	-11.4	-11.39	-11.38
-11.46	-11.45	-11.44	-11.43	-11.42	-11.41	-11.4	-11.39	-11.38
-11.46	-11.45	-11.44	-11.43	-11.42	-11.41	-11.4	-11.39	-11.38
-11.46	-11.45	-11.44	-11.43	-11.42	-11.41	-11.4	-11.39	-11.38

-11.46	-11.45	-11.44	-11.43	-11.42	-11.41	-11.4	-11.39	-11.38

-11.37	-11.36	-11.35	-11.34	-11.33	-11.32	-11.31	-11.3	-11.29
-11.37	-11.36	-11.35	-11.34	-11.33	-11.32	-11.31	-11.3	-11.29
-11.37	-11.36	-11.35	-11.34	-11.33	-11.32	-11.31	-11.3	-11.29
-11.37	-11.36	-11.35	-11.34	-11.33	-11.32	-11.31	-11.3	-11.29
-11.37	-11.36	-11.35	-11.34	-11.33	-11.32	-11.31	-11.3	-11.29
-11.37	-11.36	-11.35	-11.34	-11.33	-11.32	-11.31	-11.3	-11.29
-11.37	-11.36	-11.35	-11.34	-11.33	-11.32	-11.31	-11.3	-11.29
-11.37	-11.36	-11.35	-11.34	-11.33	-11.32	-11.31	-11.3	-11.29
-11.37	-11.36	-11.35	-11.34	-11.33	-11.32	-11.31	-11.3	-11.29
-11.37	-11.36	-11.35	-11.34	-11.33	-11.32	-11.31	-11.3	-11.29
-11.37	-11.36	-11.35	-11.34	-11.33	-11.32	-11.31	-11.3	-11.29
-11.37	-11.36	-11.35	-11.34	-11.33	-11.32	-11.31	-11.3	-11.29
-11.37	-11.36	-11.35	-11.34	-11.33	-11.32	-11.31	-11.3	-11.29
-11.37	-11.36	-11.35	-11.34	-11.33	-11.32	-11.31	-11.3	-11.29
-11.37	-11.36	-11.35	-11.34	-11.33	-11.32	-11.31	-11.3	-11.29
-11.37	-11.36	-11.35	-11.34	-11.33	-11.32	-11.31	-11.3	-11.29
-11.37	-11.36	-11.35	-11.34	-11.33	-11.32	-11.31	-11.3	-11.29
-11.37	-11.36	-11.35	-11.34	-11.33	-11.32	-11.31	-11.3	-11.29
-11.37	-11.36	-11.35	-11.34	-11.33	-11.32	-11.31	-11.3	-11.29
-11.37	-11.36	-11.35	-11.34	-11.33	-11.32	-11.31	-11.3	-11.29
-11.37	-11.36	-11.35	-11.34	-11.33	-11.32	-11.31	-11.3	-11.29
-11.37	-11.36	-11.35	-11.34	-11.33	-11.32	-11.31	-11.3	-11.29
-11.37	-11.36	-11.35	-11.34	-11.33	-11.32	-11.31	-11.3	-11.29
-11.37	-11.36	-11.35	-11.34	-11.33	-11.32	-11.31	-11.3	-11.29
-11.37	-11.36	-11.35	-11.34	-11.33	-11.32	-11.31	-11.3	-11.29
-11.37	-11.36	-11.35	-11.34	-11.33	-11.32	-11.31	-11.3	-11.29
-11.37	-11.36	-11.35	-11.34	-11.33	-11.32	-11.31	-11.3	-11.29
-11.37	-11.36	-11.35	-11.34	-11.33	-11.32	-11.31	-11.3	-11.29
-11.37	-11.36	-11.35	-11.34	-11.33	-11.32	-11.31	-11.3	-11.29
-11.37	-11.36	-11.35	-11.34	-11.33	-11.32	-11.31	-11.3	-11.29
-11.37	-11.36	-11.35	-11.34	-11.33	-11.32	-11.31	-11.3	-11.29
-11.37	-11.36	-11.35	-11.34	-11.33	-11.32	-11.31	-11.3	-11.29
-11.37	-11.36	-11.35	-11.34	-11.33	-11.32	-11.31	-11.3	-11.29
-11.37	-11.36	-11.35	-11.34	-11.33	-11.32	-11.31	-11.3	-11.29
-11.37	-11.36	-11.35	-11.34	-11.33	-11.32	-11.31	-11.3	-11.29
-11.37	-11.36	-11.35	-11.34	-11.33	-11.32	-11.31	-11.3	-11.29
-11.37	-11.36	-11.35	-11.34	-11.33	-11.32	-11.31	-11.3	-11.29
-11.37	-11.36	-11.35	-11.34	-11.33	-11.32	-11.31	-11.3	-11.29
-11.37	-11.36	-11.35	-11.34	-11.33	-11.32	-11.31	-11.3	-11.29
-11.37	-11.36	-11.35	-11.34	-11.33	-11.32	-11.31	-11.3	-11.29
-11.37	-11.36	-11.35	-11.34	-11.33	-11.32	-11.31	-11.3	-11.29
-11.37	-11.36	-11.35	-11.34	-11.33	-11.32	-11.31	-11.3	-11.29
-11.37	-11.36	-11.35	-11.34	-11.33	-11.32	-11.31	-11.3	-11.29
-11.37	-11.36	-11.35	-11.34	-11.33	-11.32	-11.31	-11.3	-11.29
-11.37	-11.36	-11.35	-11.34	-11.33	-11.32	-11.31	-11.3	-11.29
-11.37	-11.36	-11.35	-11.34	-11.33	-11.32	-11.31	-11.3	-11.29
-11.37	-11.36	-11.35	-11.34	-11.33	-11.32	-11.31	-11.3	-11.29
-11.37	-11.36	-11.35	-11.34	-11.33	-11.32	-11.31	-11.3	-11.29
-11.37	-11.36	-11.35	-11.34	-11.33	-11.32	-11.31	-11.3	-11.29
-11.37	-11.36	-11.35	-11.34	-11.33	-11.32	-11.31	-11.3	-11.29

-11.37	-11.36	-11.35	-11.34	-11.33	-11.32	-11.31	-11.3	-11.29
-11.37	-11.36	-11.35	-11.34	-11.33	-11.32	-11.31	-11.3	-11.29
-11.37	-11.36	-11.35	-11.34	-11.33	-11.32	-11.31	-11.3	-11.29
-11.37	-11.36	-11.35	-11.34	-11.33	-11.32	-11.31	-11.3	-11.29
-11.37	-11.36	-11.35	-11.34	-11.33	-11.32	-11.31	-11.3	-11.29
-11.37	-11.36	-11.35	-11.34	-11.33	-11.32	-11.31	-11.3	-11.29
-11.37	-11.36	-11.35	-11.34	-11.33	-11.32	-11.31	-11.3	-11.29
-11.37	-11.36	-11.35	-11.34	-11.33	-11.32	-11.31	-11.3	-11.29
-11.37	-11.36	-11.35	-11.34	-11.33	-11.32	-11.31	-11.3	-11.29
-11.37	-11.36	-11.35	-11.34	-11.33	-11.32	-11.31	-11.3	-11.29
-11.37	-11.36	-11.35	-11.34	-11.33	-11.32	-11.31	-11.3	-11.29
-11.37	-11.36	-11.35	-11.34	-11.33	-11.32	-11.31	-11.3	-11.29
-11.37	-11.36	-11.35	-11.34	-11.33	-11.32	-11.31	-11.3	-11.29
-11.37	-11.36	-11.35	-11.34	-11.33	-11.32	-11.31	-11.3	-11.29
-11.37	-11.36	-11.35	-11.34	-11.33	-11.32	-11.31	-11.3	-11.29
-11.37	-11.36	-11.35	-11.34	-11.33	-11.32	-11.31	-11.3	-11.29
-11.37	-11.36	-11.35	-11.34	-11.33	-11.32	-11.31	-11.3	-11.29
-11.37	-11.36	-11.35	-11.34	-11.33	-11.32	-11.31	-11.3	-11.29
-11.37	-11.36	-11.35	-11.34	-11.33	-11.32	-11.31	-11.3	-11.29
-11.37	-11.36	-11.35	-11.34	-11.33	-11.32	-11.31	-11.3	-11.29
-11.37	-11.36	-11.35	-11.34	-11.33	-11.32	-11.31	-11.3	-11.29
-11.37	-11.36	-11.35	-11.34	-11.33	-11.32	-11.31	-11.3	-11.29
-11.37	-11.36	-11.35	-11.34	-11.33	-11.32	-11.31	-11.3	-11.29
-11.37	-11.36	-11.35	-11.34	-11.33	-11.32	-11.31	-11.3	-11.29
-11.37	-11.36	-11.35	-11.34	-11.33	-11.32	-11.31	-11.3	-11.29
-11.37	-11.36	-11.35	-11.34	-11.33	-11.32	-11.31	-11.3	-11.29
-11.37	-11.36	-11.35	-11.34	-11.33	-11.32	-11.31	-11.3	-11.29
-11.37	-11.36	-11.35	-11.34	-11.33	-11.32	-11.31	-11.3	-11.29
-11.37	-11.36	-11.35	-11.34	-11.33	-11.32	-11.31	-11.3	-11.29
-11.37	-11.36	-11.35	-11.34	-11.33	-11.32	-11.31	-11.3	-11.29
-11.37	-11.36	-11.35	-11.34	-11.33	-11.32	-11.31	-11.3	-11.29
-11.37	-11.36	-11.35	-11.34	-11.33	-11.32	-11.31	-11.3	-11.29
-11.37	-11.36	-11.35	-11.34	-11.33	-11.32	-11.31	-11.3	-11.29
-11.37	-11.36	-11.35	-11.34	-11.33	-11.32	-11.31	-11.3	-11.29
-11.37	-11.36	-11.35	-11.34	-11.33	-11.32	-11.31	-11.3	-11.29
-11.37	-11.36	-11.35	-11.34	-11.33	-11.32	-11.31	-11.3	-11.29
-11.37	-11.36	-11.35	-11.34	-11.33	-11.32	-11.31	-11.3	-11.29
-11.37	-11.36	-11.35	-11.34	-11.33	-11.32	-11.31	-11.3	-11.29
-11.37	-11.36	-11.35	-11.34	-11.33	-11.32	-11.31	-11.3	-11.29
-11.37	-11.36	-11.35	-11.34	-11.33	-11.32	-11.31	-11.3	-11.29
-11.37	-11.36	-11.35	-11.34	-11.33	-11.32	-11.31	-11.3	-11.29
-11.37	-11.36	-11.35	-11.34	-11.33	-11.32	-11.31	-11.3	-11.29
-11.37	-11.36	-11.35	-11.34	-11.33	-11.32	-11.31	-11.3	-11.29
-11.37	-11.36	-11.35	-11.34	-11.33	-11.32	-11.31	-11.3	-11.29
-11.37	-11.36	-11.35	-11.34	-11.33	-11.32	-11.31	-11.3	-11.29
-11.37	-11.36	-11.35	-11.34	-11.33	-11.32	-11.31	-11.3	-11.29
-11.37	-11.36	-11.35	-11.34	-11.33	-11.32	-11.31	-11.3	-11.29
-11.37	-11.36	-11.35	-11.34	-11.33	-11.32	-11.31	-11.3	-11.29
-11.37	-11.36	-11.35	-11.34	-11.33	-11.32	-11.31	-11.3	-11.29
-11.37	-11.36	-11.35	-11.34	-11.33	-11.32	-11.31	-11.3	-11.29

-11.37	-11.36	-11.35	-11.34	-11.33	-11.32	-11.31	-11.3	-11.29
-11.37	-11.36	-11.35	-11.34	-11.33	-11.32	-11.31	-11.3	-11.29
-11.37	-11.36	-11.35	-11.34	-11.33	-11.32	-11.31	-11.3	-11.29
-11.37	-11.36	-11.35	-11.34	-11.33	-11.32	-11.31	-11.3	-11.29
-11.37	-11.36	-11.35	-11.34	-11.33	-11.32	-11.31	-11.3	-11.29
-11.37	-11.36	-11.35	-11.34	-11.33	-11.32	-11.31	-11.3	-11.29
-11.37	-11.36	-11.35	-11.34	-11.33	-11.32	-11.31	-11.3	-11.29
-11.37	-11.36	-11.35	-11.34	-11.33	-11.32	-11.31	-11.3	-11.29
-11.37	-11.36	-11.35	-11.34	-11.33	-11.32	-11.31	-11.3	-11.29
-11.37	-11.36	-11.35	-11.34	-11.33	-11.32	-11.31	-11.3	-11.29
-11.37	-11.36	-11.35	-11.34	-11.33	-11.32	-11.31	-11.3	-11.29
-11.37	-11.36	-11.35	-11.34	-11.33	-11.32	-11.31	-11.3	-11.29
-11.37	-11.36	-11.35	-11.34	-11.33	-11.32	-11.31	-11.3	-11.29
-11.37	-11.36	-11.35	-11.34	-11.33	-11.32	-11.31	-11.3	-11.29
-11.37	-11.36	-11.35	-11.34	-11.33	-11.32	-11.31	-11.3	-11.29
-11.37	-11.36	-11.35	-11.34	-11.33	-11.32	-11.31	-11.3	-11.29
-11.37	-11.36	-11.35	-11.34	-11.33	-11.32	-11.31	-11.3	-11.29
-11.37	-11.36	-11.35	-11.34	-11.33	-11.32	-11.31	-11.3	-11.29
-11.37	-11.36	-11.35	-11.34	-11.33	-11.32	-11.31	-11.3	-11.29
-11.37	-11.36	-11.35	-11.34	-11.33	-11.32	-11.31	-11.3	-11.29
-11.37	-11.36	-11.35	-11.34	-11.33	-11.32	-11.31	-11.3	-11.29
-11.37	-11.36	-11.35	-11.34	-11.33	-11.32	-11.31	-11.3	-11.29
-11.37	-11.36	-11.35	-11.34	-11.33	-11.32	-11.31	-11.3	-11.29
-11.37	-11.36	-11.35	-11.34	-11.33	-11.32	-11.31	-11.3	-11.29
-11.37	-11.36	-11.35	-11.34	-11.33	-11.32	-11.31	-11.3	-11.29
-11.37	-11.36	-11.35	-11.34	-11.33	-11.32	-11.31	-11.3	-11.29
-11.37	-11.36	-11.35	-11.34	-11.33	-11.32	-11.31	-11.3	-11.29
-11.37	-11.36	-11.35	-11.34	-11.33	-11.32	-11.31	-11.3	-11.29
-11.37	-11.36	-11.35	-11.34	-11.33	-11.32	-11.31	-11.3	-11.29
-11.37	-11.36	-11.35	-11.34	-11.33	-11.32	-11.31	-11.3	-11.29
-11.37	-11.36	-11.35	-11.34	-11.33	-11.32	-11.31	-11.3	-11.29
-11.37	-11.36	-11.35	-11.34	-11.33	-11.32	-11.31	-11.3	-11.29
-11.37	-11.36	-11.35	-11.34	-11.33	-11.32	-11.31	-11.3	-11.29
-11.37	-11.36	-11.35	-11.34	-11.33	-11.32	-11.31	-11.3	-11.29
-11.37	-11.36	-11.35	-11.34	-11.33	-11.32	-11.31	-11.3	-11.29
-11.37	-11.36	-11.35	-11.34	-11.33	-11.32	-11.31	-11.3	-11.29
-11.37	-11.36	-11.35	-11.34	-11.33	-11.32	-11.31	-11.3	-11.29
-11.37	-11.36	-11.35	-11.34	-11.33	-11.32	-11.31	-11.3	-11.29
-11.37	-11.36	-11.35	-11.34	-11.33	-11.32	-11.31	-11.3	-11.29
-11.37	-11.36	-11.35	-11.34	-11.33	-11.32	-11.31	-11.3	-11.29
-11.37	-11.36	-11.35	-11.34	-11.33	-11.32	-11.31	-11.3	-11.29
-11.37	-11.36	-11.35	-11.34	-11.33	-11.32	-11.31	-11.3	-11.29
-11.37	-11.36	-11.35	-11.34	-11.33	-11.32	-11.31	-11.3	-11.29
-11.37	-11.36	-11.35	-11.34	-11.33	-11.32	-11.31	-11.3	-11.29
-11.37	-11.36	-11.35	-11.34	-11.33	-11.32	-11.31	-11.3	-11.29
-11.37	-11.36	-11.35	-11.34	-11.33	-11.32	-11.31	-11.3	-11.29
-11.37	-11.36	-11.35	-11.34	-11.33	-11.32	-11.31	-11.3	-11.29
-11.37	-11.36	-11.35	-11.34	-11.33	-11.32	-11.31	-11.3	-11.29
-11.37	-11.36	-11.35	-11.34	-11.33	-11.32	-11.31	-11.3	-11.29
-11.37	-11.36	-11.35	-11.34	-11.33	-11.32	-11.31	-11.3	-11.29

-11.37	-11.36	-11.35	-11.34	-11.33	-11.32	-11.31	-11.3	-11.29
-11.37	-11.36	-11.35	-11.34	-11.33	-11.32	-11.31	-11.3	-11.29
-11.37	-11.36	-11.35	-11.34	-11.33	-11.32	-11.31	-11.3	-11.29
-11.37	-11.36	-11.35	-11.34	-11.33	-11.32	-11.31	-11.3	-11.29
-11.37	-11.36	-11.35	-11.34	-11.33	-11.32	-11.31	-11.3	-11.29
-11.37	-11.36	-11.35	-11.34	-11.33	-11.32	-11.31	-11.3	-11.29
-11.37	-11.36	-11.35	-11.34	-11.33	-11.32	-11.31	-11.3	-11.29
-11.37	-11.36	-11.35	-11.34	-11.33	-11.32	-11.31	-11.3	-11.29
-11.37	-11.36	-11.35	-11.34	-11.33	-11.32	-11.31	-11.3	-11.29
-11.37	-11.36	-11.35	-11.34	-11.33	-11.32	-11.31	-11.3	-11.29
-11.37	-11.36	-11.35	-11.34	-11.33	-11.32	-11.31	-11.3	-11.29
-11.37	-11.36	-11.35	-11.34	-11.33	-11.32	-11.31	-11.3	-11.29
-11.37	-11.36	-11.35	-11.34	-11.33	-11.32	-11.31	-11.3	-11.29
-11.37	-11.36	-11.35	-11.34	-11.33	-11.32	-11.31	-11.3	-11.29
-11.37	-11.36	-11.35	-11.34	-11.33	-11.32	-11.31	-11.3	-11.29
-11.37	-11.36	-11.35	-11.34	-11.33	-11.32	-11.31	-11.3	-11.29
-11.37	-11.36	-11.35	-11.34	-11.33	-11.32	-11.31	-11.3	-11.29
-11.37	-11.36	-11.35	-11.34	-11.33	-11.32	-11.31	-11.3	-11.29
-11.37	-11.36	-11.35	-11.34	-11.33	-11.32	-11.31	-11.3	-11.29
-11.37	-11.36	-11.35	-11.34	-11.33	-11.32	-11.31	-11.3	-11.29
-11.37	-11.36	-11.35	-11.34	-11.33	-11.32	-11.31	-11.3	-11.29
-11.37	-11.36	-11.35	-11.34	-11.33	-11.32	-11.31	-11.3	-11.29
-11.37	-11.36	-11.35	-11.34	-11.33	-11.32	-11.31	-11.3	-11.29
-11.37	-11.36	-11.35	-11.34	-11.33	-11.32	-11.31	-11.3	-11.29
-11.37	-11.36	-11.35	-11.34	-11.33	-11.32	-11.31	-11.3	-11.29
-11.37	-11.36	-11.35	-11.34	-11.33	-11.32	-11.31	-11.3	-11.29
-11.37	-11.36	-11.35	-11.34	-11.33	-11.32	-11.31	-11.3	-11.29
-11.37	-11.36	-11.35	-11.34	-11.33	-11.32	-11.31	-11.3	-11.29
-11.37	-11.36	-11.35	-11.34	-11.33	-11.32	-11.31	-11.3	-11.29
-11.37	-11.36	-11.35	-11.34	-11.33	-11.32	-11.31	-11.3	-11.29
-11.37	-11.36	-11.35	-11.34	-11.33	-11.32	-11.31	-11.3	-11.29
-11.37	-11.36	-11.35	-11.34	-11.33	-11.32	-11.31	-11.3	-11.29
-11.37	-11.36	-11.35	-11.34	-11.33	-11.32	-11.31	-11.3	-11.29
-11.37	-11.36	-11.35	-11.34	-11.33	-11.32	-11.31	-11.3	-11.29
-11.37	-11.36	-11.35	-11.34	-11.33	-11.32	-11.31	-11.3	-11.29
-11.37	-11.36	-11.35	-11.34	-11.33	-11.32	-11.31	-11.3	-11.29
-11.37	-11.36	-11.35	-11.34	-11.33	-11.32	-11.31	-11.3	-11.29
-11.37	-11.36	-11.35	-11.34	-11.33	-11.32	-11.31	-11.3	-11.29
-11.37	-11.36	-11.35	-11.34	-11.33	-11.32	-11.31	-11.3	-11.29
-11.37	-11.36	-11.35	-11.34	-11.33	-11.32	-11.31	-11.3	-11.29
-11.37	-11.36	-11.35	-11.34	-11.33	-11.32	-11.31	-11.3	-11.29
-11.37	-11.36	-11.35	-11.34	-11.33	-11.32	-11.31	-11.3	-11.29
-11.37	-11.36	-11.35	-11.34	-11.33	-11.32	-11.31	-11.3	-11.29
-11.37	-11.36	-11.35	-11.34	-11.33	-11.32	-11.31	-11.3	-11.29
-11.37	-11.36	-11.35	-11.34	-11.33	-11.32	-11.31	-11.3	-11.29
-11.37	-11.36	-11.35	-11.34	-11.33	-11.32	-11.31	-11.3	-11.29
-11.37	-11.36	-11.35	-11.34	-11.33	-11.32	-11.31	-11.3	-11.29
-11.37	-11.36	-11.35	-11.34	-11.33	-11.32	-11.31	-11.3	-11.29
-11.37	-11.36	-11.35	-11.34	-11.33	-11.32	-11.31	-11.3	-11.29
-11.37	-11.36	-11.35	-11.34	-11.33	-11.32	-11.31	-11.3	-11.29

-11.37	-11.36	-11.35	-11.34	-11.33	-11.32	-11.31	-11.3	-11.29
-11.37	-11.36	-11.35	-11.34	-11.33	-11.32	-11.31	-11.3	-11.29
-11.37	-11.36	-11.35	-11.34	-11.33	-11.32	-11.31	-11.3	-11.29
-11.37	-11.36	-11.35	-11.34	-11.33	-11.32	-11.31	-11.3	-11.29
-11.37	-11.36	-11.35	-11.34	-11.33	-11.32	-11.31	-11.3	-11.29
-11.37	-11.36	-11.35	-11.34	-11.33	-11.32	-11.31	-11.3	-11.29
-11.37	-11.36	-11.35	-11.34	-11.33	-11.32	-11.31	-11.3	-11.29
-11.37	-11.36	-11.35	-11.34	-11.33	-11.32	-11.31	-11.3	-11.29
-11.37	-11.36	-11.35	-11.34	-11.33	-11.32	-11.31	-11.3	-11.29
-11.37	-11.36	-11.35	-11.34	-11.33	-11.32	-11.31	-11.3	-11.29
-11.37	-11.36	-11.35	-11.34	-11.33	-11.32	-11.31	-11.3	-11.29
-11.37	-11.36	-11.35	-11.34	-11.33	-11.32	-11.31	-11.3	-11.29
-11.37	-11.36	-11.35	-11.34	-11.33	-11.32	-11.31	-11.3	-11.29
-11.37	-11.36	-11.35	-11.34	-11.33	-11.32	-11.31	-11.3	-11.29
-11.37	-11.36	-11.35	-11.34	-11.33	-11.32	-11.31	-11.3	-11.29
-11.37	-11.36	-11.35	-11.34	-11.33	-11.32	-11.31	-11.3	-11.29
-11.37	-11.36	-11.35	-11.34	-11.33	-11.32	-11.31	-11.3	-11.29
-11.37	-11.36	-11.35	-11.34	-11.33	-11.32	-11.31	-11.3	-11.29
-11.37	-11.36	-11.35	-11.34	-11.33	-11.32	-11.31	-11.3	-11.29
-11.37	-11.36	-11.35	-11.34	-11.33	-11.32	-11.31	-11.3	-11.29
-11.37	-11.36	-11.35	-11.34	-11.33	-11.32	-11.31	-11.3	-11.29
-11.37	-11.36	-11.35	-11.34	-11.33	-11.32	-11.31	-11.3	-11.29
-11.37	-11.36	-11.35	-11.34	-11.33	-11.32	-11.31	-11.3	-11.29
-11.37	-11.36	-11.35	-11.34	-11.33	-11.32	-11.31	-11.3	-11.29
-11.37	-11.36	-11.35	-11.34	-11.33	-11.32	-11.31	-11.3	-11.29
-11.37	-11.36	-11.35	-11.34	-11.33	-11.32	-11.31	-11.3	-11.29
-11.37	-11.36	-11.35	-11.34	-11.33	-11.32	-11.31	-11.3	-11.29
-11.37	-11.36	-11.35	-11.34	-11.33	-11.32	-11.31	-11.3	-11.29
-11.37	-11.36	-11.35	-11.34	-11.33	-11.32	-11.31	-11.3	-11.29
-11.37	-11.36	-11.35	-11.34	-11.33	-11.32	-11.31	-11.3	-11.29
-11.37	-11.36	-11.35	-11.34	-11.33	-11.32	-11.31	-11.3	-11.29
-11.37	-11.36	-11.35	-11.34	-11.33	-11.32	-11.31	-11.3	-11.29
-11.37	-11.36	-11.35	-11.34	-11.33	-11.32	-11.31	-11.3	-11.29
-11.37	-11.36	-11.35	-11.34	-11.33	-11.32	-11.31	-11.3	-11.29
-11.37	-11.36	-11.35	-11.34	-11.33	-11.32	-11.31	-11.3	-11.29
-11.37	-11.36	-11.35	-11.34	-11.33	-11.32	-11.31	-11.3	-11.29
-11.37	-11.36	-11.35	-11.34	-11.33	-11.32	-11.31	-11.3	-11.29
-11.37	-11.36	-11.35	-11.34	-11.33	-11.32	-11.31	-11.3	-11.29
-11.37	-11.36	-11.35	-11.34	-11.33	-11.32	-11.31	-11.3	-11.29
-11.37	-11.36	-11.35	-11.34	-11.33	-11.32	-11.31	-11.3	-11.29
-11.37	-11.36	-11.35	-11.34	-11.33	-11.32	-11.31	-11.3	-11.29
-11.37	-11.36	-11.35	-11.34	-11.33	-11.32	-11.31	-11.3	-11.29
-11.37	-11.36	-11.35	-11.34	-11.33	-11.32	-11.31	-11.3	-11.29
-11.37	-11.36	-11.35	-11.34	-11.33	-11.32	-11.31	-11.3	-11.29
-11.37	-11.36	-11.35	-11.34	-11.33	-11.32	-11.31	-11.3	-11.29
-11.37	-11.36	-11.35	-11.34	-11.33	-11.32	-11.31	-11.3	-11.29
-11.37	-11.36	-11.35	-11.34	-11.33	-11.32	-11.31	-11.3	-11.29
-11.37	-11.36	-11.35	-11.34	-11.33	-11.32	-11.31	-11.3	-11.29
-11.37	-11.36	-11.35	-11.34	-11.33	-11.32	-11.31	-11.3	-11.29
-11.37	-11.36	-11.35	-11.34	-11.33	-11.32	-11.31	-11.3	-11.29

-11.37	-11.36	-11.35	-11.34	-11.33	-11.32	-11.31	-11.3	-11.29
-11.37	-11.36	-11.35	-11.34	-11.33	-11.32	-11.31	-11.3	-11.29
-11.37	-11.36	-11.35	-11.34	-11.33	-11.32	-11.31	-11.3	-11.29
-11.37	-11.36	-11.35	-11.34	-11.33	-11.32	-11.31	-11.3	-11.29
-11.37	-11.36	-11.35	-11.34	-11.33	-11.32	-11.31	-11.3	-11.29
-11.37	-11.36	-11.35	-11.34	-11.33	-11.32	-11.31	-11.3	-11.29
-11.37	-11.36	-11.35	-11.34	-11.33	-11.32	-11.31	-11.3	-11.29
-11.37	-11.36	-11.35	-11.34	-11.33	-11.32	-11.31	-11.3	-11.29
-11.37	-11.36	-11.35	-11.34	-11.33	-11.32	-11.31	-11.3	-11.29
-11.37	-11.36	-11.35	-11.34	-11.33	-11.32	-11.31	-11.3	-11.29
-11.37	-11.36	-11.35	-11.34	-11.33	-11.32	-11.31	-11.3	-11.29
-11.37	-11.36	-11.35	-11.34	-11.33	-11.32	-11.31	-11.3	-11.29
-11.37	-11.36	-11.35	-11.34	-11.33	-11.32	-11.31	-11.3	-11.29
-11.37	-11.36	-11.35	-11.34	-11.33	-11.32	-11.31	-11.3	-11.29
-11.37	-11.36	-11.35	-11.34	-11.33	-11.32	-11.31	-11.3	-11.29
-11.37	-11.36	-11.35	-11.34	-11.33	-11.32	-11.31	-11.3	-11.29
-11.37	-11.36	-11.35	-11.34	-11.33	-11.32	-11.31	-11.3	-11.29
-11.37	-11.36	-11.35	-11.34	-11.33	-11.32	-11.31	-11.3	-11.29
-11.37	-11.36	-11.35	-11.34	-11.33	-11.32	-11.31	-11.3	-11.29
-11.37	-11.36	-11.35	-11.34	-11.33	-11.32	-11.31	-11.3	-11.29
-11.37	-11.36	-11.35	-11.34	-11.33	-11.32	-11.31	-11.3	-11.29
-11.37	-11.36	-11.35	-11.34	-11.33	-11.32	-11.31	-11.3	-11.29
-11.37	-11.36	-11.35	-11.34	-11.33	-11.32	-11.31	-11.3	-11.29
-11.37	-11.36	-11.35	-11.34	-11.33	-11.32	-11.31	-11.3	-11.29
-11.37	-11.36	-11.35	-11.34	-11.33	-11.32	-11.31	-11.3	-11.29
-11.37	-11.36	-11.35	-11.34	-11.33	-11.32	-11.31	-11.3	-11.29
-11.37	-11.36	-11.35	-11.34	-11.33	-11.32	-11.31	-11.3	-11.29
-11.37	-11.36	-11.35	-11.34	-11.33	-11.32	-11.31	-11.3	-11.29
-11.37	-11.36	-11.35	-11.34	-11.33	-11.32	-11.31	-11.3	-11.29
-11.37	-11.36	-11.35	-11.34	-11.33	-11.32	-11.31	-11.3	-11.29
-11.37	-11.36	-11.35	-11.34	-11.33	-11.32	-11.31	-11.3	-11.29
-11.37	-11.36	-11.35	-11.34	-11.33	-11.32	-11.31	-11.3	-11.29
-11.37	-11.36	-11.35	-11.34	-11.33	-11.32	-11.31	-11.3	-11.29
-11.37	-11.36	-11.35	-11.34	-11.33	-11.32	-11.31	-11.3	-11.29
-11.37	-11.36	-11.35	-11.34	-11.33	-11.32	-11.31	-11.3	-11.29
-11.37	-11.36	-11.35	-11.34	-11.33	-11.32	-11.31	-11.3	-11.29
-11.37	-11.36	-11.35	-11.34	-11.33	-11.32	-11.31	-11.3	-11.29
-11.37	-11.36	-11.35	-11.34	-11.33	-11.32	-11.31	-11.3	-11.29
-11.37	-11.36	-11.35	-11.34	-11.33	-11.32	-11.31	-11.3	-11.29
-11.37	-11.36	-11.35	-11.34	-11.33	-11.32	-11.31	-11.3	-11.29
-11.37	-11.36	-11.35	-11.34	-11.33	-11.32	-11.31	-11.3	-11.29
-11.37	-11.36	-11.35	-11.34	-11.33	-11.32	-11.31	-11.3	-11.29
-11.37	-11.36	-11.35	-11.34	-11.33	-11.32	-11.31	-11.3	-11.29
-11.37	-11.36	-11.35	-11.34	-11.33	-11.32	-11.31	-11.3	-11.29
-11.37	-11.36	-11.35	-11.34	-11.33	-11.32	-11.31	-11.3	-11.29
-11.37	-11.36	-11.35	-11.34	-11.33	-11.32	-11.31	-11.3	-11.29
-11.37	-11.36	-11.35	-11.34	-11.33	-11.32	-11.31	-11.3	-11.29
-11.37	-11.36	-11.35	-11.34	-11.33	-11.32	-11.31	-11.3	-11.29
-11.37	-11.36	-11.35	-11.34	-11.33	-11.32	-11.31	-11.3	-11.29
-11.37	-11.36	-11.35	-11.34	-11.33	-11.32	-11.31	-11.3	-11.29

-11.37	-11.36	-11.35	-11.34	-11.33	-11.32	-11.31	-11.3	-11.29
-11.37	-11.36	-11.35	-11.34	-11.33	-11.32	-11.31	-11.3	-11.29
-11.37	-11.36	-11.35	-11.34	-11.33	-11.32	-11.31	-11.3	-11.29
-11.37	-11.36	-11.35	-11.34	-11.33	-11.32	-11.31	-11.3	-11.29
-11.37	-11.36	-11.35	-11.34	-11.33	-11.32	-11.31	-11.3	-11.29
-11.37	-11.36	-11.35	-11.34	-11.33	-11.32	-11.31	-11.3	-11.29
-11.37	-11.36	-11.35	-11.34	-11.33	-11.32	-11.31	-11.3	-11.29
-11.37	-11.36	-11.35	-11.34	-11.33	-11.32	-11.31	-11.3	-11.29
-11.37	-11.36	-11.35	-11.34	-11.33	-11.32	-11.31	-11.3	-11.29
-11.37	-11.36	-11.35	-11.34	-11.33	-11.32	-11.31	-11.3	-11.29
-11.37	-11.36	-11.35	-11.34	-11.33	-11.32	-11.31	-11.3	-11.29
-11.37	-11.36	-11.35	-11.34	-11.33	-11.32	-11.31	-11.3	-11.29
-11.37	-11.36	-11.35	-11.34	-11.33	-11.32	-11.31	-11.3	-11.29
-11.37	-11.36	-11.35	-11.34	-11.33	-11.32	-11.31	-11.3	-11.29
-11.37	-11.36	-11.35	-11.34	-11.33	-11.32	-11.31	-11.3	-11.29
-11.37	-11.36	-11.35	-11.34	-11.33	-11.32	-11.31	-11.3	-11.29
-11.37	-11.36	-11.35	-11.34	-11.33	-11.32	-11.31	-11.3	-11.29
-11.37	-11.36	-11.35	-11.34	-11.33	-11.32	-11.31	-11.3	-11.29
-11.37	-11.36	-11.35	-11.34	-11.33	-11.32	-11.31	-11.3	-11.29
-11.37	-11.36	-11.35	-11.34	-11.33	-11.32	-11.31	-11.3	-11.29
-11.37	-11.36	-11.35	-11.34	-11.33	-11.32	-11.31	-11.3	-11.29
-11.37	-11.36	-11.35	-11.34	-11.33	-11.32	-11.31	-11.3	-11.29
-11.37	-11.36	-11.35	-11.34	-11.33	-11.32	-11.31	-11.3	-11.29
-11.37	-11.36	-11.35	-11.34	-11.33	-11.32	-11.31	-11.3	-11.29
-11.37	-11.36	-11.35	-11.34	-11.33	-11.32	-11.31	-11.3	-11.29
-11.37	-11.36	-11.35	-11.34	-11.33	-11.32	-11.31	-11.3	-11.29
-11.37	-11.36	-11.35	-11.34	-11.33	-11.32	-11.31	-11.3	-11.29
-11.37	-11.36	-11.35	-11.34	-11.33	-11.32	-11.31	-11.3	-11.29
-11.37	-11.36	-11.35	-11.34	-11.33	-11.32	-11.31	-11.3	-11.29
-11.37	-11.36	-11.35	-11.34	-11.33	-11.32	-11.31	-11.3	-11.29
-11.37	-11.36	-11.35	-11.34	-11.33	-11.32	-11.31	-11.3	-11.29
-11.37	-11.36	-11.35	-11.34	-11.33	-11.32	-11.31	-11.3	-11.29
-11.37	-11.36	-11.35	-11.34	-11.33	-11.32	-11.31	-11.3	-11.29
-11.37	-11.36	-11.35	-11.34	-11.33	-11.32	-11.31	-11.3	-11.29
-11.37	-11.36	-11.35	-11.34	-11.33	-11.32	-11.31	-11.3	-11.29
-11.37	-11.36	-11.35	-11.34	-11.33	-11.32	-11.31	-11.3	-11.29
-11.37	-11.36	-11.35	-11.34	-11.33	-11.32	-11.31	-11.3	-11.29
-11.37	-11.36	-11.35	-11.34	-11.33	-11.32	-11.31	-11.3	-11.29
-11.37	-11.36	-11.35	-11.34	-11.33	-11.32	-11.31	-11.3	-11.29
-11.37	-11.36	-11.35	-11.34	-11.33	-11.32	-11.31	-11.3	-11.29
-11.37	-11.36	-11.35	-11.34	-11.33	-11.32	-11.31	-11.3	-11.29
-11.37	-11.36	-11.35	-11.34	-11.33	-11.32	-11.31	-11.3	-11.29
-11.37	-11.36	-11.35	-11.34	-11.33	-11.32	-11.31	-11.3	-11.29
-11.37	-11.36	-11.35	-11.34	-11.33	-11.32	-11.31	-11.3	-11.29
-11.37	-11.36	-11.35	-11.34	-11.33	-11.32	-11.31	-11.3	-11.29
-11.37	-11.36	-11.35	-11.34	-11.33	-11.32	-11.31	-11.3	-11.29
-11.37	-11.36	-11.35	-11.34	-11.33	-11.32	-11.31	-11.3	-11.29
-11.37	-11.36	-11.35	-11.34	-11.33	-11.32	-11.31	-11.3	-11.29
-11.37	-11.36	-11.35	-11.34	-11.33	-11.32	-11.31	-11.3	-11.29
-11.37	-11.36	-11.35	-11.34	-11.33	-11.32	-11.31	-11.3	-11.29

-11.37	-11.36	-11.35	-11.34	-11.33	-11.32	-11.31	-11.3	-11.29
-11.37	-11.36	-11.35	-11.34	-11.33	-11.32	-11.31	-11.3	-11.29
-11.37	-11.36	-11.35	-11.34	-11.33	-11.32	-11.31	-11.3	-11.29
-11.37	-11.36	-11.35	-11.34	-11.33	-11.32	-11.31	-11.3	-11.29
-11.37	-11.36	-11.35	-11.34	-11.33	-11.32	-11.31	-11.3	-11.29
-11.37	-11.36	-11.35	-11.34	-11.33	-11.32	-11.31	-11.3	-11.29
-11.37	-11.36	-11.35	-11.34	-11.33	-11.32	-11.31	-11.3	-11.29
-11.37	-11.36	-11.35	-11.34	-11.33	-11.32	-11.31	-11.3	-11.29
-11.37	-11.36	-11.35	-11.34	-11.33	-11.32	-11.31	-11.3	-11.29
-11.37	-11.36	-11.35	-11.34	-11.33	-11.32	-11.31	-11.3	-11.29
-11.37	-11.36	-11.35	-11.34	-11.33	-11.32	-11.31	-11.3	-11.29
-11.37	-11.36	-11.35	-11.34	-11.33	-11.32	-11.31	-11.3	-11.29
-11.37	-11.36	-11.35	-11.34	-11.33	-11.32	-11.31	-11.3	-11.29
-11.37	-11.36	-11.35	-11.34	-11.33	-11.32	-11.31	-11.3	-11.29
-11.37	-11.36	-11.35	-11.34	-11.33	-11.32	-11.31	-11.3	-11.29
-11.37	-11.36	-11.35	-11.34	-11.33	-11.32	-11.31	-11.3	-11.29
-11.37	-11.36	-11.35	-11.34	-11.33	-11.32	-11.31	-11.3	-11.29
-11.37	-11.36	-11.35	-11.34	-11.33	-11.32	-11.31	-11.3	-11.29
-11.37	-11.36	-11.35	-11.34	-11.33	-11.32	-11.31	-11.3	-11.29
-11.37	-11.36	-11.35	-11.34	-11.33	-11.32	-11.31	-11.3	-11.29
-11.37	-11.36	-11.35	-11.34	-11.33	-11.32	-11.31	-11.3	-11.29
-11.37	-11.36	-11.35	-11.34	-11.33	-11.32	-11.31	-11.3	-11.29
-11.37	-11.36	-11.35	-11.34	-11.33	-11.32	-11.31	-11.3	-11.29
-11.37	-11.36	-11.35	-11.34	-11.33	-11.32	-11.31	-11.3	-11.29
-11.37	-11.36	-11.35	-11.34	-11.33	-11.32	-11.31	-11.3	-11.29
-11.37	-11.36	-11.35	-11.34	-11.33	-11.32	-11.31	-11.3	-11.29
-11.37	-11.36	-11.35	-11.34	-11.33	-11.32	-11.31	-11.3	-11.29
-11.37	-11.36	-11.35	-11.34	-11.33	-11.32	-11.31	-11.3	-11.29
-11.37	-11.36	-11.35	-11.34	-11.33	-11.32	-11.31	-11.3	-11.29
-11.37	-11.36	-11.35	-11.34	-11.33	-11.32	-11.31	-11.3	-11.29
-11.37	-11.36	-11.35	-11.34	-11.33	-11.32	-11.31	-11.3	-11.29
-11.37	-11.36	-11.35	-11.34	-11.33	-11.32	-11.31	-11.3	-11.29
-11.37	-11.36	-11.35	-11.34	-11.33	-11.32	-11.31	-11.3	-11.29
-11.37	-11.36	-11.35	-11.34	-11.33	-11.32	-11.31	-11.3	-11.29
-11.37	-11.36	-11.35	-11.34	-11.33	-11.32	-11.31	-11.3	-11.29
-11.37	-11.36	-11.35	-11.34	-11.33	-11.32	-11.31	-11.3	-11.29
-11.37	-11.36	-11.35	-11.34	-11.33	-11.32	-11.31	-11.3	-11.29
-11.37	-11.36	-11.35	-11.34	-11.33	-11.32	-11.31	-11.3	-11.29
-11.37	-11.36	-11.35	-11.34	-11.33	-11.32	-11.31	-11.3	-11.29
-11.37	-11.36	-11.35	-11.34	-11.33	-11.32	-11.31	-11.3	-11.29
-11.37	-11.36	-11.35	-11.34	-11.33	-11.32	-11.31	-11.3	-11.29
-11.37	-11.36	-11.35	-11.34	-11.33	-11.32	-11.31	-11.3	-11.29
-11.37	-11.36	-11.35	-11.34	-11.33	-11.32	-11.31	-11.3	-11.29
-11.37	-11.36	-11.35	-11.34	-11.33	-11.32	-11.31	-11.3	-11.29
-11.37	-11.36	-11.35	-11.34	-11.33	-11.32	-11.31	-11.3	-11.29
-11.37	-11.36	-11.35	-11.34	-11.33	-11.32	-11.31	-11.3	-11.29
-11.37	-11.36	-11.35	-11.34	-11.33	-11.32	-11.31	-11.3	-11.29
-11.37	-11.36	-11.35	-11.34	-11.33	-11.32	-11.31	-11.3	-11.29
-11.37	-11.36	-11.35	-11.34	-11.33	-11.32	-11.31	-11.3	-11.29
-11.37	-11.36	-11.35	-11.34	-11.33	-11.32	-11.31	-11.3	-11.29

-11.37	-11.36	-11.35	-11.34	-11.33	-11.32	-11.31	-11.3	-11.29
-11.37	-11.36	-11.35	-11.34	-11.33	-11.32	-11.31	-11.3	-11.29
-11.37	-11.36	-11.35	-11.34	-11.33	-11.32	-11.31	-11.3	-11.29
-11.37	-11.36	-11.35	-11.34	-11.33	-11.32	-11.31	-11.3	-11.29
-11.37	-11.36	-11.35	-11.34	-11.33	-11.32	-11.31	-11.3	-11.29
-11.37	-11.36	-11.35	-11.34	-11.33	-11.32	-11.31	-11.3	-11.29
-11.37	-11.36	-11.35	-11.34	-11.33	-11.32	-11.31	-11.3	-11.29
-11.37	-11.36	-11.35	-11.34	-11.33	-11.32	-11.31	-11.3	-11.29
-11.37	-11.36	-11.35	-11.34	-11.33	-11.32	-11.31	-11.3	-11.29
-11.37	-11.36	-11.35	-11.34	-11.33	-11.32	-11.31	-11.3	-11.29
-11.37	-11.36	-11.35	-11.34	-11.33	-11.32	-11.31	-11.3	-11.29
-11.37	-11.36	-11.35	-11.34	-11.33	-11.32	-11.31	-11.3	-11.29
-11.37	-11.36	-11.35	-11.34	-11.33	-11.32	-11.31	-11.3	-11.29
-11.37	-11.36	-11.35	-11.34	-11.33	-11.32	-11.31	-11.3	-11.29
-11.37	-11.36	-11.35	-11.34	-11.33	-11.32	-11.31	-11.3	-11.29
-11.37	-11.36	-11.35	-11.34	-11.33	-11.32	-11.31	-11.3	-11.29
-11.37	-11.36	-11.35	-11.34	-11.33	-11.32	-11.31	-11.3	-11.29
-11.37	-11.36	-11.35	-11.34	-11.33	-11.32	-11.31	-11.3	-11.29
-11.37	-11.36	-11.35	-11.34	-11.33	-11.32	-11.31	-11.3	-11.29
-11.37	-11.36	-11.35	-11.34	-11.33	-11.32	-11.31	-11.3	-11.29
-11.37	-11.36	-11.35	-11.34	-11.33	-11.32	-11.31	-11.3	-11.29
-11.37	-11.36	-11.35	-11.34	-11.33	-11.32	-11.31	-11.3	-11.29
-11.37	-11.36	-11.35	-11.34	-11.33	-11.32	-11.31	-11.3	-11.29
-11.37	-11.36	-11.35	-11.34	-11.33	-11.32	-11.31	-11.3	-11.29
-11.37	-11.36	-11.35	-11.34	-11.33	-11.32	-11.31	-11.3	-11.29
-11.37	-11.36	-11.35	-11.34	-11.33	-11.32	-11.31	-11.3	-11.29
-11.37	-11.36	-11.35	-11.34	-11.33	-11.32	-11.31	-11.3	-11.29
-11.37	-11.36	-11.35	-11.34	-11.33	-11.32	-11.31	-11.3	-11.29
-11.37	-11.36	-11.35	-11.34	-11.33	-11.32	-11.31	-11.3	-11.29
-11.37	-11.36	-11.35	-11.34	-11.33	-11.32	-11.31	-11.3	-11.29
-11.37	-11.36	-11.35	-11.34	-11.33	-11.32	-11.31	-11.3	-11.29
-11.37	-11.36	-11.35	-11.34	-11.33	-11.32	-11.31	-11.3	-11.29
-11.37	-11.36	-11.35	-11.34	-11.33	-11.32	-11.31	-11.3	-11.29
-11.37	-11.36	-11.35	-11.34	-11.33	-11.32	-11.31	-11.3	-11.29
-11.37	-11.36	-11.35	-11.34	-11.33	-11.32	-11.31	-11.3	-11.29
-11.37	-11.36	-11.35	-11.34	-11.33	-11.32	-11.31	-11.3	-11.29
-11.37	-11.36	-11.35	-11.34	-11.33	-11.32	-11.31	-11.3	-11.29
-11.37	-11.36	-11.35	-11.34	-11.33	-11.32	-11.31	-11.3	-11.29
-11.37	-11.36	-11.35	-11.34	-11.33	-11.32	-11.31	-11.3	-11.29
-11.37	-11.36	-11.35	-11.34	-11.33	-11.32	-11.31	-11.3	-11.29
-11.37	-11.36	-11.35	-11.34	-11.33	-11.32	-11.31	-11.3	-11.29
-11.37	-11.36	-11.35	-11.34	-11.33	-11.32	-11.31	-11.3	-11.29
-11.37	-11.36	-11.35	-11.34	-11.33	-11.32	-11.31	-11.3	-11.29
-11.37	-11.36	-11.35	-11.34	-11.33	-11.32	-11.31	-11.3	-11.29
-11.37	-11.36	-11.35	-11.34	-11.33	-11.32	-11.31	-11.3	-11.29
-11.37	-11.36	-11.35	-11.34	-11.33	-11.32	-11.31	-11.3	-11.29
-11.37	-11.36	-11.35	-11.34	-11.33	-11.32	-11.31	-11.3	-11.29
-11.37	-11.36	-11.35	-11.34	-11.33	-11.32	-11.31	-11.3	-11.29
-11.37	-11.36	-11.35	-11.34	-11.33	-11.32	-11.31	-11.3	-11.29
-11.37	-11.36	-11.35	-11.34	-11.33	-11.32	-11.31	-11.3	-11.29

-11.37	-11.36	-11.35	-11.34	-11.33	-11.32	-11.31	-11.3	-11.29
-11.37	-11.36	-11.35	-11.34	-11.33	-11.32	-11.31	-11.3	-11.29
-11.37	-11.36	-11.35	-11.34	-11.33	-11.32	-11.31	-11.3	-11.29
-11.37	-11.36	-11.35	-11.34	-11.33	-11.32	-11.31	-11.3	-11.29
-11.37	-11.36	-11.35	-11.34	-11.33	-11.32	-11.31	-11.3	-11.29
-11.37	-11.36	-11.35	-11.34	-11.33	-11.32	-11.31	-11.3	-11.29
-11.37	-11.36	-11.35	-11.34	-11.33	-11.32	-11.31	-11.3	-11.29
-11.37	-11.36	-11.35	-11.34	-11.33	-11.32	-11.31	-11.3	-11.29
-11.37	-11.36	-11.35	-11.34	-11.33	-11.32	-11.31	-11.3	-11.29
-11.37	-11.36	-11.35	-11.34	-11.33	-11.32	-11.31	-11.3	-11.29
-11.37	-11.36	-11.35	-11.34	-11.33	-11.32	-11.31	-11.3	-11.29
-11.37	-11.36	-11.35	-11.34	-11.33	-11.32	-11.31	-11.3	-11.29
-11.37	-11.36	-11.35	-11.34	-11.33	-11.32	-11.31	-11.3	-11.29
-11.37	-11.36	-11.35	-11.34	-11.33	-11.32	-11.31	-11.3	-11.29
-11.37	-11.36	-11.35	-11.34	-11.33	-11.32	-11.31	-11.3	-11.29
-11.37	-11.36	-11.35	-11.34	-11.33	-11.32	-11.31	-11.3	-11.29
-11.37	-11.36	-11.35	-11.34	-11.33	-11.32	-11.31	-11.3	-11.29
-11.37	-11.36	-11.35	-11.34	-11.33	-11.32	-11.31	-11.3	-11.29
-11.37	-11.36	-11.35	-11.34	-11.33	-11.32	-11.31	-11.3	-11.29
-11.37	-11.36	-11.35	-11.34	-11.33	-11.32	-11.31	-11.3	-11.29
-11.37	-11.36	-11.35	-11.34	-11.33	-11.32	-11.31	-11.3	-11.29
-11.37	-11.36	-11.35	-11.34	-11.33	-11.32	-11.31	-11.3	-11.29
-11.37	-11.36	-11.35	-11.34	-11.33	-11.32	-11.31	-11.3	-11.29
-11.37	-11.36	-11.35	-11.34	-11.33	-11.32	-11.31	-11.3	-11.29
-11.37	-11.36	-11.35	-11.34	-11.33	-11.32	-11.31	-11.3	-11.29
-11.37	-11.36	-11.35	-11.34	-11.33	-11.32	-11.31	-11.3	-11.29
-11.37	-11.36	-11.35	-11.34	-11.33	-11.32	-11.31	-11.3	-11.29
-11.37	-11.36	-11.35	-11.34	-11.33	-11.32	-11.31	-11.3	-11.29
-11.37	-11.36	-11.35	-11.34	-11.33	-11.32	-11.31	-11.3	-11.29
-11.37	-11.36	-11.35	-11.34	-11.33	-11.32	-11.31	-11.3	-11.29
-11.37	-11.36	-11.35	-11.34	-11.33	-11.32	-11.31	-11.3	-11.29
-11.37	-11.36	-11.35	-11.34	-11.33	-11.32	-11.31	-11.3	-11.29
-11.37	-11.36	-11.35	-11.34	-11.33	-11.32	-11.31	-11.3	-11.29
-11.37	-11.36	-11.35	-11.34	-11.33	-11.32	-11.31	-11.3	-11.29
-11.37	-11.36	-11.35	-11.34	-11.33	-11.32	-11.31	-11.3	-11.29
-11.37	-11.36	-11.35	-11.34	-11.33	-11.32	-11.31	-11.3	-11.29
-11.37	-11.36	-11.35	-11.34	-11.33	-11.32	-11.31	-11.3	-11.29
-11.37	-11.36	-11.35	-11.34	-11.33	-11.32	-11.31	-11.3	-11.29
-11.37	-11.36	-11.35	-11.34	-11.33	-11.32	-11.31	-11.3	-11.29
-11.37	-11.36	-11.35	-11.34	-11.33	-11.32	-11.31	-11.3	-11.29
-11.37	-11.36	-11.35	-11.34	-11.33	-11.32	-11.31	-11.3	-11.29
-11.37	-11.36	-11.35	-11.34	-11.33	-11.32	-11.31	-11.3	-11.29
-11.37	-11.36	-11.35	-11.34	-11.33	-11.32	-11.31	-11.3	-11.29
-11.37	-11.36	-11.35	-11.34	-11.33	-11.32	-11.31	-11.3	-11.29
-11.37	-11.36	-11.35	-11.34	-11.33	-11.32	-11.31	-11.3	-11.29
-11.37	-11.36	-11.35	-11.34	-11.33	-11.32	-11.31	-11.3	-11.29
-11.37	-11.36	-11.35	-11.34	-11.33	-11.32	-11.31	-11.3	-11.29
-11.37	-11.36	-11.35	-11.34	-11.33	-11.32	-11.31	-11.3	-11.29
-11.37	-11.36	-11.35	-11.34	-11.33	-11.32	-11.31	-11.3	-11.29
-11.37	-11.36	-11.35	-11.34	-11.33	-11.32	-11.31	-11.3	-11.29

-11.37	-11.36	-11.35	-11.34	-11.33	-11.32	-11.31	-11.3	-11.29
-11.37	-11.36	-11.35	-11.34	-11.33	-11.32	-11.31	-11.3	-11.29
-11.37	-11.36	-11.35	-11.34	-11.33	-11.32	-11.31	-11.3	-11.29
-11.37	-11.36	-11.35	-11.34	-11.33	-11.32	-11.31	-11.3	-11.29
-11.37	-11.36	-11.35	-11.34	-11.33	-11.32	-11.31	-11.3	-11.29
-11.37	-11.36	-11.35	-11.34	-11.33	-11.32	-11.31	-11.3	-11.29
-11.37	-11.36	-11.35	-11.34	-11.33	-11.32	-11.31	-11.3	-11.29
-11.37	-11.36	-11.35	-11.34	-11.33	-11.32	-11.31	-11.3	-11.29
-11.37	-11.36	-11.35	-11.34	-11.33	-11.32	-11.31	-11.3	-11.29
-11.37	-11.36	-11.35	-11.34	-11.33	-11.32	-11.31	-11.3	-11.29
-11.37	-11.36	-11.35	-11.34	-11.33	-11.32	-11.31	-11.3	-11.29
-11.37	-11.36	-11.35	-11.34	-11.33	-11.32	-11.31	-11.3	-11.29
-11.37	-11.36	-11.35	-11.34	-11.33	-11.32	-11.31	-11.3	-11.29
-11.37	-11.36	-11.35	-11.34	-11.33	-11.32	-11.31	-11.3	-11.29
-11.37	-11.36	-11.35	-11.34	-11.33	-11.32	-11.31	-11.3	-11.29
-11.37	-11.36	-11.35	-11.34	-11.33	-11.32	-11.31	-11.3	-11.29
-11.37	-11.36	-11.35	-11.34	-11.33	-11.32	-11.31	-11.3	-11.29
-11.37	-11.36	-11.35	-11.34	-11.33	-11.32	-11.31	-11.3	-11.29
-11.37	-11.36	-11.35	-11.34	-11.33	-11.32	-11.31	-11.3	-11.29
-11.37	-11.36	-11.35	-11.34	-11.33	-11.32	-11.31	-11.3	-11.29
-11.37	-11.36	-11.35	-11.34	-11.33	-11.32	-11.31	-11.3	-11.29
-11.37	-11.36	-11.35	-11.34	-11.33	-11.32	-11.31	-11.3	-11.29
-11.37	-11.36	-11.35	-11.34	-11.33	-11.32	-11.31	-11.3	-11.29
-11.37	-11.36	-11.35	-11.34	-11.33	-11.32	-11.31	-11.3	-11.29
-11.37	-11.36	-11.35	-11.34	-11.33	-11.32	-11.31	-11.3	-11.29
-11.37	-11.36	-11.35	-11.34	-11.33	-11.32	-11.31	-11.3	-11.29
-11.37	-11.36	-11.35	-11.34	-11.33	-11.32	-11.31	-11.3	-11.29
-11.37	-11.36	-11.35	-11.34	-11.33	-11.32	-11.31	-11.3	-11.29
-11.37	-11.36	-11.35	-11.34	-11.33	-11.32	-11.31	-11.3	-11.29
-11.37	-11.36	-11.35	-11.34	-11.33	-11.32	-11.31	-11.3	-11.29
-11.37	-11.36	-11.35	-11.34	-11.33	-11.32	-11.31	-11.3	-11.29
-11.37	-11.36	-11.35	-11.34	-11.33	-11.32	-11.31	-11.3	-11.29
-11.37	-11.36	-11.35	-11.34	-11.33	-11.32	-11.31	-11.3	-11.29
-11.37	-11.36	-11.35	-11.34	-11.33	-11.32	-11.31	-11.3	-11.29
-11.37	-11.36	-11.35	-11.34	-11.33	-11.32	-11.31	-11.3	-11.29
-11.37	-11.36	-11.35	-11.34	-11.33	-11.32	-11.31	-11.3	-11.29
-11.37	-11.36	-11.35	-11.34	-11.33	-11.32	-11.31	-11.3	-11.29
-11.37	-11.36	-11.35	-11.34	-11.33	-11.32	-11.31	-11.3	-11.29
-11.37	-11.36	-11.35	-11.34	-11.33	-11.32	-11.31	-11.3	-11.29
-11.37	-11.36	-11.35	-11.34	-11.33	-11.32	-11.31	-11.3	-11.29
-11.37	-11.36	-11.35	-11.34	-11.33	-11.32	-11.31	-11.3	-11.29
-11.37	-11.36	-11.35	-11.34	-11.33	-11.32	-11.31	-11.3	-11.29
-11.37	-11.36	-11.35	-11.34	-11.33	-11.32	-11.31	-11.3	-11.29
-11.37	-11.36	-11.35	-11.34	-11.33	-11.32	-11.31	-11.3	-11.29
-11.37	-11.36	-11.35	-11.34	-11.33	-11.32	-11.31	-11.3	-11.29
-11.37	-11.36	-11.35	-11.34	-11.33	-11.32	-11.31	-11.3	-11.29
-11.37	-11.36	-11.35	-11.34	-11.33	-11.32	-11.31	-11.3	-11.29
-11.37	-11.36	-11.35	-11.34	-11.33	-11.32	-11.31	-11.3	-11.29
-11.37	-11.36	-11.35	-11.34	-11.33	-11.32	-11.31	-11.3	-11.29
-11.37	-11.36	-11.35	-11.34	-11.33	-11.32	-11.31	-11.3	-11.29

-11.37	-11.36	-11.35	-11.34	-11.33	-11.32	-11.31	-11.3	-11.29
-11.37	-11.36	-11.35	-11.34	-11.33	-11.32	-11.31	-11.3	-11.29
-11.37	-11.36	-11.35	-11.34	-11.33	-11.32	-11.31	-11.3	-11.29
-11.37	-11.36	-11.35	-11.34	-11.33	-11.32	-11.31	-11.3	-11.29
-11.37	-11.36	-11.35	-11.34	-11.33	-11.32	-11.31	-11.3	-11.29
-11.37	-11.36	-11.35	-11.34	-11.33	-11.32	-11.31	-11.3	-11.29
-11.37	-11.36	-11.35	-11.34	-11.33	-11.32	-11.31	-11.3	-11.29
-11.37	-11.36	-11.35	-11.34	-11.33	-11.32	-11.31	-11.3	-11.29
-11.37	-11.36	-11.35	-11.34	-11.33	-11.32	-11.31	-11.3	-11.29
-11.37	-11.36	-11.35	-11.34	-11.33	-11.32	-11.31	-11.3	-11.29
-11.37	-11.36	-11.35	-11.34	-11.33	-11.32	-11.31	-11.3	-11.29
-11.37	-11.36	-11.35	-11.34	-11.33	-11.32	-11.31	-11.3	-11.29
-11.37	-11.36	-11.35	-11.34	-11.33	-11.32	-11.31	-11.3	-11.29
-11.37	-11.36	-11.35	-11.34	-11.33	-11.32	-11.31	-11.3	-11.29
-11.37	-11.36	-11.35	-11.34	-11.33	-11.32	-11.31	-11.3	-11.29
-11.37	-11.36	-11.35	-11.34	-11.33	-11.32	-11.31	-11.3	-11.29
-11.37	-11.36	-11.35	-11.34	-11.33	-11.32	-11.31	-11.3	-11.29
-11.37	-11.36	-11.35	-11.34	-11.33	-11.32	-11.31	-11.3	-11.29
-11.37	-11.36	-11.35	-11.34	-11.33	-11.32	-11.31	-11.3	-11.29
-11.37	-11.36	-11.35	-11.34	-11.33	-11.32	-11.31	-11.3	-11.29
-11.37	-11.36	-11.35	-11.34	-11.33	-11.32	-11.31	-11.3	-11.29
-11.37	-11.36	-11.35	-11.34	-11.33	-11.32	-11.31	-11.3	-11.29
-11.37	-11.36	-11.35	-11.34	-11.33	-11.32	-11.31	-11.3	-11.29
-11.37	-11.36	-11.35	-11.34	-11.33	-11.32	-11.31	-11.3	-11.29
-11.37	-11.36	-11.35	-11.34	-11.33	-11.32	-11.31	-11.3	-11.29
-11.37	-11.36	-11.35	-11.34	-11.33	-11.32	-11.31	-11.3	-11.29
-11.37	-11.36	-11.35	-11.34	-11.33	-11.32	-11.31	-11.3	-11.29
-11.37	-11.36	-11.35	-11.34	-11.33	-11.32	-11.31	-11.3	-11.29
-11.37	-11.36	-11.35	-11.34	-11.33	-11.32	-11.31	-11.3	-11.29
-11.37	-11.36	-11.35	-11.34	-11.33	-11.32	-11.31	-11.3	-11.29
-11.37	-11.36	-11.35	-11.34	-11.33	-11.32	-11.31	-11.3	-11.29
-11.37	-11.36	-11.35	-11.34	-11.33	-11.32	-11.31	-11.3	-11.29
-11.37	-11.36	-11.35	-11.34	-11.33	-11.32	-11.31	-11.3	-11.29
-11.37	-11.36	-11.35	-11.34	-11.33	-11.32	-11.31	-11.3	-11.29
-11.37	-11.36	-11.35	-11.34	-11.33	-11.32	-11.31	-11.3	-11.29
-11.37	-11.36	-11.35	-11.34	-11.33	-11.32	-11.31	-11.3	-11.29
-11.37	-11.36	-11.35	-11.34	-11.33	-11.32	-11.31	-11.3	-11.29
-11.37	-11.36	-11.35	-11.34	-11.33	-11.32	-11.31	-11.3	-11.29
-11.37	-11.36	-11.35	-11.34	-11.33	-11.32	-11.31	-11.3	-11.29
-11.37	-11.36	-11.35	-11.34	-11.33	-11.32	-11.31	-11.3	-11.29
-11.37	-11.36	-11.35	-11.34	-11.33	-11.32	-11.31	-11.3	-11.29
-11.37	-11.36	-11.35	-11.34	-11.33	-11.32	-11.31	-11.3	-11.29
-11.37	-11.36	-11.35	-11.34	-11.33	-11.32	-11.31	-11.3	-11.29
-11.37	-11.36	-11.35	-11.34	-11.33	-11.32	-11.31	-11.3	-11.29
-11.37	-11.36	-11.35	-11.34	-11.33	-11.32	-11.31	-11.3	-11.29
-11.37	-11.36	-11.35	-11.34	-11.33	-11.32	-11.31	-11.3	-11.29
-11.37	-11.36	-11.35	-11.34	-11.33	-11.32	-11.31	-11.3	-11.29
-11.37	-11.36	-11.35	-11.34	-11.33	-11.32	-11.31	-11.3	-11.29
-11.37	-11.36	-11.35	-11.34	-11.33	-11.32	-11.31	-11.3	-11.29
-11.37	-11.36	-11.35	-11.34	-11.33	-11.32	-11.31	-11.3	-11.29

-11.37	-11.36	-11.35	-11.34	-11.33	-11.32	-11.31	-11.3	-11.29
-11.37	-11.36	-11.35	-11.34	-11.33	-11.32	-11.31	-11.3	-11.29
-11.37	-11.36	-11.35	-11.34	-11.33	-11.32	-11.31	-11.3	-11.29
-11.37	-11.36	-11.35	-11.34	-11.33	-11.32	-11.31	-11.3	-11.29
-11.37	-11.36	-11.35	-11.34	-11.33	-11.32	-11.31	-11.3	-11.29
-11.37	-11.36	-11.35	-11.34	-11.33	-11.32	-11.31	-11.3	-11.29
-11.37	-11.36	-11.35	-11.34	-11.33	-11.32	-11.31	-11.3	-11.29
-11.37	-11.36	-11.35	-11.34	-11.33	-11.32	-11.31	-11.3	-11.29
-11.37	-11.36	-11.35	-11.34	-11.33	-11.32	-11.31	-11.3	-11.29
-11.37	-11.36	-11.35	-11.34	-11.33	-11.32	-11.31	-11.3	-11.29
-11.37	-11.36	-11.35	-11.34	-11.33	-11.32	-11.31	-11.3	-11.29
-11.37	-11.36	-11.35	-11.34	-11.33	-11.32	-11.31	-11.3	-11.29
-11.37	-11.36	-11.35	-11.34	-11.33	-11.32	-11.31	-11.3	-11.29
-11.37	-11.36	-11.35	-11.34	-11.33	-11.32	-11.31	-11.3	-11.29
-11.37	-11.36	-11.35	-11.34	-11.33	-11.32	-11.31	-11.3	-11.29
-11.37	-11.36	-11.35	-11.34	-11.33	-11.32	-11.31	-11.3	-11.29
-11.37	-11.36	-11.35	-11.34	-11.33	-11.32	-11.31	-11.3	-11.29
-11.37	-11.36	-11.35	-11.34	-11.33	-11.32	-11.31	-11.3	-11.29
-11.37	-11.36	-11.35	-11.34	-11.33	-11.32	-11.31	-11.3	-11.29
-11.37	-11.36	-11.35	-11.34	-11.33	-11.32	-11.31	-11.3	-11.29
-11.37	-11.36	-11.35	-11.34	-11.33	-11.32	-11.31	-11.3	-11.29
-11.37	-11.36	-11.35	-11.34	-11.33	-11.32	-11.31	-11.3	-11.29
-11.37	-11.36	-11.35	-11.34	-11.33	-11.32	-11.31	-11.3	-11.29
-11.37	-11.36	-11.35	-11.34	-11.33	-11.32	-11.31	-11.3	-11.29
-11.37	-11.36	-11.35	-11.34	-11.33	-11.32	-11.31	-11.3	-11.29
-11.37	-11.36	-11.35	-11.34	-11.33	-11.32	-11.31	-11.3	-11.29
-11.37	-11.36	-11.35	-11.34	-11.33	-11.32	-11.31	-11.3	-11.29
-11.37	-11.36	-11.35	-11.34	-11.33	-11.32	-11.31	-11.3	-11.29
-11.37	-11.36	-11.35	-11.34	-11.33	-11.32	-11.31	-11.3	-11.29
-11.37	-11.36	-11.35	-11.34	-11.33	-11.32	-11.31	-11.3	-11.29
-11.37	-11.36	-11.35	-11.34	-11.33	-11.32	-11.31	-11.3	-11.29
-11.37	-11.36	-11.35	-11.34	-11.33	-11.32	-11.31	-11.3	-11.29
-11.37	-11.36	-11.35	-11.34	-11.33	-11.32	-11.31	-11.3	-11.29
-11.37	-11.36	-11.35	-11.34	-11.33	-11.32	-11.31	-11.3	-11.29
-11.37	-11.36	-11.35	-11.34	-11.33	-11.32	-11.31	-11.3	-11.29
-11.37	-11.36	-11.35	-11.34	-11.33	-11.32	-11.31	-11.3	-11.29
-11.37	-11.36	-11.35	-11.34	-11.33	-11.32	-11.31	-11.3	-11.29
-11.37	-11.36	-11.35	-11.34	-11.33	-11.32	-11.31	-11.3	-11.29
-11.37	-11.36	-11.35	-11.34	-11.33	-11.32	-11.31	-11.3	-11.29
-11.37	-11.36	-11.35	-11.34	-11.33	-11.32	-11.31	-11.3	-11.29
-11.37	-11.36	-11.35	-11.34	-11.33	-11.32	-11.31	-11.3	-11.29
-11.37	-11.36	-11.35	-11.34	-11.33	-11.32	-11.31	-11.3	-11.29
-11.37	-11.36	-11.35	-11.34	-11.33	-11.32	-11.31	-11.3	-11.29
-11.37	-11.36	-11.35	-11.34	-11.33	-11.32	-11.31	-11.3	-11.29
-11.37	-11.36	-11.35	-11.34	-11.33	-11.32	-11.31	-11.3	-11.29
-11.37	-11.36	-11.35	-11.34	-11.33	-11.32	-11.31	-11.3	-11.29
-11.37	-11.36	-11.35	-11.34	-11.33	-11.32	-11.31	-11.3	-11.29
-11.37	-11.36	-11.35	-11.34	-11.33	-11.32	-11.31	-11.3	-11.29
-11.37	-11.36	-11.35	-11.34	-11.33	-11.32	-11.31	-11.3	-11.29
-11.37	-11.36	-11.35	-11.34	-11.33	-11.32	-11.31	-11.3	-11.29

-11.37	-11.36	-11.35	-11.34	-11.33	-11.32	-11.31	-11.3	-11.29
-11.37	-11.36	-11.35	-11.34	-11.33	-11.32	-11.31	-11.3	-11.29
-11.37	-11.36	-11.35	-11.34	-11.33	-11.32	-11.31	-11.3	-11.29
-11.37	-11.36	-11.35	-11.34	-11.33	-11.32	-11.31	-11.3	-11.29
-11.37	-11.36	-11.35	-11.34	-11.33	-11.32	-11.31	-11.3	-11.29
-11.37	-11.36	-11.35	-11.34	-11.33	-11.32	-11.31	-11.3	-11.29
-11.37	-11.36	-11.35	-11.34	-11.33	-11.32	-11.31	-11.3	-11.29
-11.37	-11.36	-11.35	-11.34	-11.33	-11.32	-11.31	-11.3	-11.29
-11.37	-11.36	-11.35	-11.34	-11.33	-11.32	-11.31	-11.3	-11.29
-11.37	-11.36	-11.35	-11.34	-11.33	-11.32	-11.31	-11.3	-11.29
-11.37	-11.36	-11.35	-11.34	-11.33	-11.32	-11.31	-11.3	-11.29
-11.37	-11.36	-11.35	-11.34	-11.33	-11.32	-11.31	-11.3	-11.29
-11.37	-11.36	-11.35	-11.34	-11.33	-11.32	-11.31	-11.3	-11.29
-11.37	-11.36	-11.35	-11.34	-11.33	-11.32	-11.31	-11.3	-11.29
-11.37	-11.36	-11.35	-11.34	-11.33	-11.32	-11.31	-11.3	-11.29
-11.37	-11.36	-11.35	-11.34	-11.33	-11.32	-11.31	-11.3	-11.29
-11.37	-11.36	-11.35	-11.34	-11.33	-11.32	-11.31	-11.3	-11.29
-11.37	-11.36	-11.35	-11.34	-11.33	-11.32	-11.31	-11.3	-11.29
-11.37	-11.36	-11.35	-11.34	-11.33	-11.32	-11.31	-11.3	-11.29
-11.37	-11.36	-11.35	-11.34	-11.33	-11.32	-11.31	-11.3	-11.29
-11.37	-11.36	-11.35	-11.34	-11.33	-11.32	-11.31	-11.3	-11.29
-11.37	-11.36	-11.35	-11.34	-11.33	-11.32	-11.31	-11.3	-11.29
-11.37	-11.36	-11.35	-11.34	-11.33	-11.32	-11.31	-11.3	-11.29
-11.37	-11.36	-11.35	-11.34	-11.33	-11.32	-11.31	-11.3	-11.29
-11.37	-11.36	-11.35	-11.34	-11.33	-11.32	-11.31	-11.3	-11.29
-11.37	-11.36	-11.35	-11.34	-11.33	-11.32	-11.31	-11.3	-11.29
-11.37	-11.36	-11.35	-11.34	-11.33	-11.32	-11.31	-11.3	-11.29
-11.37	-11.36	-11.35	-11.34	-11.33	-11.32	-11.31	-11.3	-11.29
-11.37	-11.36	-11.35	-11.34	-11.33	-11.32	-11.31	-11.3	-11.29
-11.37	-11.36	-11.35	-11.34	-11.33	-11.32	-11.31	-11.3	-11.29
-11.37	-11.36	-11.35	-11.34	-11.33	-11.32	-11.31	-11.3	-11.29
-11.37	-11.36	-11.35	-11.34	-11.33	-11.32	-11.31	-11.3	-11.29
-11.37	-11.36	-11.35	-11.34	-11.33	-11.32	-11.31	-11.3	-11.29
-11.37	-11.36	-11.35	-11.34	-11.33	-11.32	-11.31	-11.3	-11.29
-11.37	-11.36	-11.35	-11.34	-11.33	-11.32	-11.31	-11.3	-11.29
-11.37	-11.36	-11.35	-11.34	-11.33	-11.32	-11.31	-11.3	-11.29
-11.37	-11.36	-11.35	-11.34	-11.33	-11.32	-11.31	-11.3	-11.29
-11.37	-11.36	-11.35	-11.34	-11.33	-11.32	-11.31	-11.3	-11.29
-11.37	-11.36	-11.35	-11.34	-11.33	-11.32	-11.31	-11.3	-11.29
-11.37	-11.36	-11.35	-11.34	-11.33	-11.32	-11.31	-11.3	-11.29
-11.37	-11.36	-11.35	-11.34	-11.33	-11.32	-11.31	-11.3	-11.29
-11.37	-11.36	-11.35	-11.34	-11.33	-11.32	-11.31	-11.3	-11.29
-11.37	-11.36	-11.35	-11.34	-11.33	-11.32	-11.31	-11.3	-11.29
-11.37	-11.36	-11.35	-11.34	-11.33	-11.32	-11.31	-11.3	-11.29
-11.37	-11.36	-11.35	-11.34	-11.33	-11.32	-11.31	-11.3	-11.29
-11.37	-11.36	-11.35	-11.34	-11.33	-11.32	-11.31	-11.3	-11.29
-11.37	-11.36	-11.35	-11.34	-11.33	-11.32	-11.31	-11.3	-11.29
-11.37	-11.36	-11.35	-11.34	-11.33	-11.32	-11.31	-11.3	-11.29
-11.37	-11.36	-11.35	-11.34	-11.33	-11.32	-11.31	-11.3	-11.29
-11.37	-11.36	-11.35	-11.34	-11.33	-11.32	-11.31	-11.3	-11.29

-11.37	-11.36	-11.35	-11.34	-11.33	-11.32	-11.31	-11.3	-11.29
-11.37	-11.36	-11.35	-11.34	-11.33	-11.32	-11.31	-11.3	-11.29
-11.37	-11.36	-11.35	-11.34	-11.33	-11.32	-11.31	-11.3	-11.29
-11.37	-11.36	-11.35	-11.34	-11.33	-11.32	-11.31	-11.3	-11.29
-11.37	-11.36	-11.35	-11.34	-11.33	-11.32	-11.31	-11.3	-11.29
-11.37	-11.36	-11.35	-11.34	-11.33	-11.32	-11.31	-11.3	-11.29
-11.37	-11.36	-11.35	-11.34	-11.33	-11.32	-11.31	-11.3	-11.29
-11.37	-11.36	-11.35	-11.34	-11.33	-11.32	-11.31	-11.3	-11.29
-11.37	-11.36	-11.35	-11.34	-11.33	-11.32	-11.31	-11.3	-11.29
-11.37	-11.36	-11.35	-11.34	-11.33	-11.32	-11.31	-11.3	-11.29
-11.37	-11.36	-11.35	-11.34	-11.33	-11.32	-11.31	-11.3	-11.29
-11.37	-11.36	-11.35	-11.34	-11.33	-11.32	-11.31	-11.3	-11.29
-11.37	-11.36	-11.35	-11.34	-11.33	-11.32	-11.31	-11.3	-11.29
-11.37	-11.36	-11.35	-11.34	-11.33	-11.32	-11.31	-11.3	-11.29
-11.37	-11.36	-11.35	-11.34	-11.33	-11.32	-11.31	-11.3	-11.29
-11.37	-11.36	-11.35	-11.34	-11.33	-11.32	-11.31	-11.3	-11.29
-11.37	-11.36	-11.35	-11.34	-11.33	-11.32	-11.31	-11.3	-11.29
-11.37	-11.36	-11.35	-11.34	-11.33	-11.32	-11.31	-11.3	-11.29
-11.37	-11.36	-11.35	-11.34	-11.33	-11.32	-11.31	-11.3	-11.29
-11.37	-11.36	-11.35	-11.34	-11.33	-11.32	-11.31	-11.3	-11.29
-11.37	-11.36	-11.35	-11.34	-11.33	-11.32	-11.31	-11.3	-11.29
-11.37	-11.36	-11.35	-11.34	-11.33	-11.32	-11.31	-11.3	-11.29
-11.37	-11.36	-11.35	-11.34	-11.33	-11.32	-11.31	-11.3	-11.29
-11.37	-11.36	-11.35	-11.34	-11.33	-11.32	-11.31	-11.3	-11.29
-11.37	-11.36	-11.35	-11.34	-11.33	-11.32	-11.31	-11.3	-11.29
-11.37	-11.36	-11.35	-11.34	-11.33	-11.32	-11.31	-11.3	-11.29
-11.37	-11.36	-11.35	-11.34	-11.33	-11.32	-11.31	-11.3	-11.29
-11.37	-11.36	-11.35	-11.34	-11.33	-11.32	-11.31	-11.3	-11.29
-11.37	-11.36	-11.35	-11.34	-11.33	-11.32	-11.31	-11.3	-11.29
-11.37	-11.36	-11.35	-11.34	-11.33	-11.32	-11.31	-11.3	-11.29
-11.37	-11.36	-11.35	-11.34	-11.33	-11.32	-11.31	-11.3	-11.29
-11.37	-11.36	-11.35	-11.34	-11.33	-11.32	-11.31	-11.3	-11.29
-11.37	-11.36	-11.35	-11.34	-11.33	-11.32	-11.31	-11.3	-11.29
-11.37	-11.36	-11.35	-11.34	-11.33	-11.32	-11.31	-11.3	-11.29
-11.37	-11.36	-11.35	-11.34	-11.33	-11.32	-11.31	-11.3	-11.29
-11.37	-11.36	-11.35	-11.34	-11.33	-11.32	-11.31	-11.3	-11.29
-11.37	-11.36	-11.35	-11.34	-11.33	-11.32	-11.31	-11.3	-11.29
-11.37	-11.36	-11.35	-11.34	-11.33	-11.32	-11.31	-11.3	-11.29
-11.37	-11.36	-11.35	-11.34	-11.33	-11.32	-11.31	-11.3	-11.29
-11.37	-11.36	-11.35	-11.34	-11.33	-11.32	-11.31	-11.3	-11.29
-11.37	-11.36	-11.35	-11.34	-11.33	-11.32	-11.31	-11.3	-11.29
-11.37	-11.36	-11.35	-11.34	-11.33	-11.32	-11.31	-11.3	-11.29
-11.37	-11.36	-11.35	-11.34	-11.33	-11.32	-11.31	-11.3	-11.29
-11.37	-11.36	-11.35	-11.34	-11.33	-11.32	-11.31	-11.3	-11.29
-11.37	-11.36	-11.35	-11.34	-11.33	-11.32	-11.31	-11.3	-11.29
-11.37	-11.36	-11.35	-11.34	-11.33	-11.32	-11.31	-11.3	-11.29
-11.37	-11.36	-11.35	-11.34	-11.33	-11.32	-11.31	-11.3	-11.29
-11.37	-11.36	-11.35	-11.34	-11.33	-11.32	-11.31	-11.3	-11.29
-11.37	-11.36	-11.35	-11.34	-11.33	-11.32	-11.31	-11.3	-11.29
-11.37	-11.36	-11.35	-11.34	-11.33	-11.32	-11.31	-11.3	-11.29

-11.37	-11.36	-11.35	-11.34	-11.33	-11.32	-11.31	-11.3	-11.29
-11.37	-11.36	-11.35	-11.34	-11.33	-11.32	-11.31	-11.3	-11.29
-11.37	-11.36	-11.35	-11.34	-11.33	-11.32	-11.31	-11.3	-11.29
-11.37	-11.36	-11.35	-11.34	-11.33	-11.32	-11.31	-11.3	-11.29
-11.37	-11.36	-11.35	-11.34	-11.33	-11.32	-11.31	-11.3	-11.29
-11.37	-11.36	-11.35	-11.34	-11.33	-11.32	-11.31	-11.3	-11.29
-11.37	-11.36	-11.35	-11.34	-11.33	-11.32	-11.31	-11.3	-11.29
-11.37	-11.36	-11.35	-11.34	-11.33	-11.32	-11.31	-11.3	-11.29
-11.37	-11.36	-11.35	-11.34	-11.33	-11.32	-11.31	-11.3	-11.29
-11.37	-11.36	-11.35	-11.34	-11.33	-11.32	-11.31	-11.3	-11.29
-11.37	-11.36	-11.35	-11.34	-11.33	-11.32	-11.31	-11.3	-11.29
-11.37	-11.36	-11.35	-11.34	-11.33	-11.32	-11.31	-11.3	-11.29
-11.37	-11.36	-11.35	-11.34	-11.33	-11.32	-11.31	-11.3	-11.29
-11.37	-11.36	-11.35	-11.34	-11.33	-11.32	-11.31	-11.3	-11.29
-11.37	-11.36	-11.35	-11.34	-11.33	-11.32	-11.31	-11.3	-11.29
-11.37	-11.36	-11.35	-11.34	-11.33	-11.32	-11.31	-11.3	-11.29
-11.37	-11.36	-11.35	-11.34	-11.33	-11.32	-11.31	-11.3	-11.29
-11.37	-11.36	-11.35	-11.34	-11.33	-11.32	-11.31	-11.3	-11.29
-11.37	-11.36	-11.35	-11.34	-11.33	-11.32	-11.31	-11.3	-11.29
-11.37	-11.36	-11.35	-11.34	-11.33	-11.32	-11.31	-11.3	-11.29
-11.37	-11.36	-11.35	-11.34	-11.33	-11.32	-11.31	-11.3	-11.29
-11.37	-11.36	-11.35	-11.34	-11.33	-11.32	-11.31	-11.3	-11.29
-11.37	-11.36	-11.35	-11.34	-11.33	-11.32	-11.31	-11.3	-11.29
-11.37	-11.36	-11.35	-11.34	-11.33	-11.32	-11.31	-11.3	-11.29
-11.37	-11.36	-11.35	-11.34	-11.33	-11.32	-11.31	-11.3	-11.29
-11.37	-11.36	-11.35	-11.34	-11.33	-11.32	-11.31	-11.3	-11.29
-11.37	-11.36	-11.35	-11.34	-11.33	-11.32	-11.31	-11.3	-11.29
-11.37	-11.36	-11.35	-11.34	-11.33	-11.32	-11.31	-11.3	-11.29
-11.37	-11.36	-11.35	-11.34	-11.33	-11.32	-11.31	-11.3	-11.29
-11.37	-11.36	-11.35	-11.34	-11.33	-11.32	-11.31	-11.3	-11.29
-11.37	-11.36	-11.35	-11.34	-11.33	-11.32	-11.31	-11.3	-11.29
-11.37	-11.36	-11.35	-11.34	-11.33	-11.32	-11.31	-11.3	-11.29
-11.37	-11.36	-11.35	-11.34	-11.33	-11.32	-11.31	-11.3	-11.29
-11.37	-11.36	-11.35	-11.34	-11.33	-11.32	-11.31	-11.3	-11.29
-11.37	-11.36	-11.35	-11.34	-11.33	-11.32	-11.31	-11.3	-11.29
-11.37	-11.36	-11.35	-11.34	-11.33	-11.32	-11.31	-11.3	-11.29
-11.37	-11.36	-11.35	-11.34	-11.33	-11.32	-11.31	-11.3	-11.29
-11.37	-11.36	-11.35	-11.34	-11.33	-11.32	-11.31	-11.3	-11.29
-11.37	-11.36	-11.35	-11.34	-11.33	-11.32	-11.31	-11.3	-11.29
-11.37	-11.36	-11.35	-11.34	-11.33	-11.32	-11.31	-11.3	-11.29
-11.37	-11.36	-11.35	-11.34	-11.33	-11.32	-11.31	-11.3	-11.29
-11.37	-11.36	-11.35	-11.34	-11.33	-11.32	-11.31	-11.3	-11.29
-11.37	-11.36	-11.35	-11.34	-11.33	-11.32	-11.31	-11.3	-11.29
-11.37	-11.36	-11.35	-11.34	-11.33	-11.32	-11.31	-11.3	-11.29
-11.37	-11.36	-11.35	-11.34	-11.33	-11.32	-11.31	-11.3	-11.29
-11.37	-11.36	-11.35	-11.34	-11.33	-11.32	-11.31	-11.3	-11.29
-11.37	-11.36	-11.35	-11.34	-11.33	-11.32	-11.31	-11.3	-11.29
-11.37	-11.36	-11.35	-11.34	-11.33	-11.32	-11.31	-11.3	-11.29
-11.37	-11.36	-11.35	-11.34	-11.33	-11.32	-11.31	-11.3	-11.29
-11.37	-11.36	-11.35	-11.34	-11.33	-11.32	-11.31	-11.3	-11.29

-11.37	-11.36	-11.35	-11.34	-11.33	-11.32	-11.31	-11.3	-11.29
-11.37	-11.36	-11.35	-11.34	-11.33	-11.32	-11.31	-11.3	-11.29
-11.37	-11.36	-11.35	-11.34	-11.33	-11.32	-11.31	-11.3	-11.29
-11.37	-11.36	-11.35	-11.34	-11.33	-11.32	-11.31	-11.3	-11.29
-11.37	-11.36	-11.35	-11.34	-11.33	-11.32	-11.31	-11.3	-11.29
-11.37	-11.36	-11.35	-11.34	-11.33	-11.32	-11.31	-11.3	-11.29
-11.37	-11.36	-11.35	-11.34	-11.33	-11.32	-11.31	-11.3	-11.29
-11.37	-11.36	-11.35	-11.34	-11.33	-11.32	-11.31	-11.3	-11.29
-11.37	-11.36	-11.35	-11.34	-11.33	-11.32	-11.31	-11.3	-11.29
-11.37	-11.36	-11.35	-11.34	-11.33	-11.32	-11.31	-11.3	-11.29
-11.37	-11.36	-11.35	-11.34	-11.33	-11.32	-11.31	-11.3	-11.29
-11.37	-11.36	-11.35	-11.34	-11.33	-11.32	-11.31	-11.3	-11.29
-11.37	-11.36	-11.35	-11.34	-11.33	-11.32	-11.31	-11.3	-11.29
-11.37	-11.36	-11.35	-11.34	-11.33	-11.32	-11.31	-11.3	-11.29
-11.37	-11.36	-11.35	-11.34	-11.33	-11.32	-11.31	-11.3	-11.29
-11.37	-11.36	-11.35	-11.34	-11.33	-11.32	-11.31	-11.3	-11.29
-11.37	-11.36	-11.35	-11.34	-11.33	-11.32	-11.31	-11.3	-11.29
-11.37	-11.36	-11.35	-11.34	-11.33	-11.32	-11.31	-11.3	-11.29
-11.37	-11.36	-11.35	-11.34	-11.33	-11.32	-11.31	-11.3	-11.29
-11.37	-11.36	-11.35	-11.34	-11.33	-11.32	-11.31	-11.3	-11.29
-11.37	-11.36	-11.35	-11.34	-11.33	-11.32	-11.31	-11.3	-11.29
-11.37	-11.36	-11.35	-11.34	-11.33	-11.32	-11.31	-11.3	-11.29
-11.37	-11.36	-11.35	-11.34	-11.33	-11.32	-11.31	-11.3	-11.29
-11.37	-11.36	-11.35	-11.34	-11.33	-11.32	-11.31	-11.3	-11.29
-11.37	-11.36	-11.35	-11.34	-11.33	-11.32	-11.31	-11.3	-11.29
-11.37	-11.36	-11.35	-11.34	-11.33	-11.32	-11.31	-11.3	-11.29
-11.37	-11.36	-11.35	-11.34	-11.33	-11.32	-11.31	-11.3	-11.29
-11.37	-11.36	-11.35	-11.34	-11.33	-11.32	-11.31	-11.3	-11.29
-11.37	-11.36	-11.35	-11.34	-11.33	-11.32	-11.31	-11.3	-11.29
-11.37	-11.36	-11.35	-11.34	-11.33	-11.32	-11.31	-11.3	-11.29
-11.37	-11.36	-11.35	-11.34	-11.33	-11.32	-11.31	-11.3	-11.29
-11.37	-11.36	-11.35	-11.34	-11.33	-11.32	-11.31	-11.3	-11.29
-11.37	-11.36	-11.35	-11.34	-11.33	-11.32	-11.31	-11.3	-11.29
-11.37	-11.36	-11.35	-11.34	-11.33	-11.32	-11.31	-11.3	-11.29
-11.37	-11.36	-11.35	-11.34	-11.33	-11.32	-11.31	-11.3	-11.29
-11.37	-11.36	-11.35	-11.34	-11.33	-11.32	-11.31	-11.3	-11.29
-11.37	-11.36	-11.35	-11.34	-11.33	-11.32	-11.31	-11.3	-11.29
-11.37	-11.36	-11.35	-11.34	-11.33	-11.32	-11.31	-11.3	-11.29
-11.37	-11.36	-11.35	-11.34	-11.33	-11.32	-11.31	-11.3	-11.29
-11.37	-11.36	-11.35	-11.34	-11.33	-11.32	-11.31	-11.3	-11.29
-11.37	-11.36	-11.35	-11.34	-11.33	-11.32	-11.31	-11.3	-11.29
-11.37	-11.36	-11.35	-11.34	-11.33	-11.32	-11.31	-11.3	-11.29
-11.37	-11.36	-11.35	-11.34	-11.33	-11.32	-11.31	-11.3	-11.29
-11.37	-11.36	-11.35	-11.34	-11.33	-11.32	-11.31	-11.3	-11.29
-11.37	-11.36	-11.35	-11.34	-11.33	-11.32	-11.31	-11.3	-11.29
-11.37	-11.36	-11.35	-11.34	-11.33	-11.32	-11.31	-11.3	-11.29
-11.37	-11.36	-11.35	-11.34	-11.33	-11.32	-11.31	-11.3	-11.29
-11.37	-11.36	-11.35	-11.34	-11.33	-11.32	-11.31	-11.3	-11.29
-11.37	-11.36	-11.35	-11.34	-11.33	-11.32	-11.31	-11.3	-11.29
-11.37	-11.36	-11.35	-11.34	-11.33	-11.32	-11.31	-11.3	-11.29

-11.37	-11.36	-11.35	-11.34	-11.33	-11.32	-11.31	-11.3	-11.29
-11.37	-11.36	-11.35	-11.34	-11.33	-11.32	-11.31	-11.3	-11.29
-11.37	-11.36	-11.35	-11.34	-11.33	-11.32	-11.31	-11.3	-11.29
-11.37	-11.36	-11.35	-11.34	-11.33	-11.32	-11.31	-11.3	-11.29
-11.37	-11.36	-11.35	-11.34	-11.33	-11.32	-11.31	-11.3	-11.29
-11.37	-11.36	-11.35	-11.34	-11.33	-11.32	-11.31	-11.3	-11.29
-11.37	-11.36	-11.35	-11.34	-11.33	-11.32	-11.31	-11.3	-11.29
-11.37	-11.36	-11.35	-11.34	-11.33	-11.32	-11.31	-11.3	-11.29
-11.37	-11.36	-11.35	-11.34	-11.33	-11.32	-11.31	-11.3	-11.29
-11.37	-11.36	-11.35	-11.34	-11.33	-11.32	-11.31	-11.3	-11.29
-11.37	-11.36	-11.35	-11.34	-11.33	-11.32	-11.31	-11.3	-11.29
-11.37	-11.36	-11.35	-11.34	-11.33	-11.32	-11.31	-11.3	-11.29
-11.37	-11.36	-11.35	-11.34	-11.33	-11.32	-11.31	-11.3	-11.29
-11.37	-11.36	-11.35	-11.34	-11.33	-11.32	-11.31	-11.3	-11.29
-11.37	-11.36	-11.35	-11.34	-11.33	-11.32	-11.31	-11.3	-11.29
-11.37	-11.36	-11.35	-11.34	-11.33	-11.32	-11.31	-11.3	-11.29
-11.37	-11.36	-11.35	-11.34	-11.33	-11.32	-11.31	-11.3	-11.29
-11.37	-11.36	-11.35	-11.34	-11.33	-11.32	-11.31	-11.3	-11.29
-11.37	-11.36	-11.35	-11.34	-11.33	-11.32	-11.31	-11.3	-11.29
-11.37	-11.36	-11.35	-11.34	-11.33	-11.32	-11.31	-11.3	-11.29
-11.37	-11.36	-11.35	-11.34	-11.33	-11.32	-11.31	-11.3	-11.29
-11.37	-11.36	-11.35	-11.34	-11.33	-11.32	-11.31	-11.3	-11.29
-11.37	-11.36	-11.35	-11.34	-11.33	-11.32	-11.31	-11.3	-11.29
-11.37	-11.36	-11.35	-11.34	-11.33	-11.32	-11.31	-11.3	-11.29
-11.37	-11.36	-11.35	-11.34	-11.33	-11.32	-11.31	-11.3	-11.29
-11.37	-11.36	-11.35	-11.34	-11.33	-11.32	-11.31	-11.3	-11.29
-11.37	-11.36	-11.35	-11.34	-11.33	-11.32	-11.31	-11.3	-11.29
-11.37	-11.36	-11.35	-11.34	-11.33	-11.32	-11.31	-11.3	-11.29
-11.37	-11.36	-11.35	-11.34	-11.33	-11.32	-11.31	-11.3	-11.29
-11.37	-11.36	-11.35	-11.34	-11.33	-11.32	-11.31	-11.3	-11.29
-11.37	-11.36	-11.35	-11.34	-11.33	-11.32	-11.31	-11.3	-11.29
-11.37	-11.36	-11.35	-11.34	-11.33	-11.32	-11.31	-11.3	-11.29
-11.37	-11.36	-11.35	-11.34	-11.33	-11.32	-11.31	-11.3	-11.29
-11.37	-11.36	-11.35	-11.34	-11.33	-11.32	-11.31	-11.3	-11.29
-11.37	-11.36	-11.35	-11.34	-11.33	-11.32	-11.31	-11.3	-11.29
-11.37	-11.36	-11.35	-11.34	-11.33	-11.32	-11.31	-11.3	-11.29
-11.37	-11.36	-11.35	-11.34	-11.33	-11.32	-11.31	-11.3	-11.29
-11.37	-11.36	-11.35	-11.34	-11.33	-11.32	-11.31	-11.3	-11.29
-11.37	-11.36	-11.35	-11.34	-11.33	-11.32	-11.31	-11.3	-11.29
-11.37	-11.36	-11.35	-11.34	-11.33	-11.32	-11.31	-11.3	-11.29
-11.37	-11.36	-11.35	-11.34	-11.33	-11.32	-11.31	-11.3	-11.29
-11.37	-11.36	-11.35	-11.34	-11.33	-11.32	-11.31	-11.3	-11.29
-11.37	-11.36	-11.35	-11.34	-11.33	-11.32	-11.31	-11.3	-11.29
-11.37	-11.36	-11.35	-11.34	-11.33	-11.32	-11.31	-11.3	-11.29
-11.37	-11.36	-11.35	-11.34	-11.33	-11.32	-11.31	-11.3	-11.29
-11.37	-11.36	-11.35	-11.34	-11.33	-11.32	-11.31	-11.3	-11.29
-11.37	-11.36	-11.35	-11.34	-11.33	-11.32	-11.31	-11.3	-11.29
-11.37	-11.36	-11.35	-11.34	-11.33	-11.32	-11.31	-11.3	-11.29
-11.37	-11.36	-11.35	-11.34	-11.33	-11.32	-11.31	-11.3	-11.29
-11.37	-11.36	-11.35	-11.34	-11.33	-11.32	-11.31	-11.3	-11.29

-11.37	-11.36	-11.35	-11.34	-11.33	-11.32	-11.31	-11.3	-11.29
-11.37	-11.36	-11.35	-11.34	-11.33	-11.32	-11.31	-11.3	-11.29
-11.37	-11.36	-11.35	-11.34	-11.33	-11.32	-11.31	-11.3	-11.29
-11.37	-11.36	-11.35	-11.34	-11.33	-11.32	-11.31	-11.3	-11.29
-11.37	-11.36	-11.35	-11.34	-11.33	-11.32	-11.31	-11.3	-11.29
-11.37	-11.36	-11.35	-11.34	-11.33	-11.32	-11.31	-11.3	-11.29
-11.37	-11.36	-11.35	-11.34	-11.33	-11.32	-11.31	-11.3	-11.29
-11.37	-11.36	-11.35	-11.34	-11.33	-11.32	-11.31	-11.3	-11.29
-11.37	-11.36	-11.35	-11.34	-11.33	-11.32	-11.31	-11.3	-11.29
-11.37	-11.36	-11.35	-11.34	-11.33	-11.32	-11.31	-11.3	-11.29
-11.37	-11.36	-11.35	-11.34	-11.33	-11.32	-11.31	-11.3	-11.29
-11.37	-11.36	-11.35	-11.34	-11.33	-11.32	-11.31	-11.3	-11.29
-11.37	-11.36	-11.35	-11.34	-11.33	-11.32	-11.31	-11.3	-11.29
-11.37	-11.36	-11.35	-11.34	-11.33	-11.32	-11.31	-11.3	-11.29
-11.37	-11.36	-11.35	-11.34	-11.33	-11.32	-11.31	-11.3	-11.29
-11.37	-11.36	-11.35	-11.34	-11.33	-11.32	-11.31	-11.3	-11.29
-11.37	-11.36	-11.35	-11.34	-11.33	-11.32	-11.31	-11.3	-11.29
-11.37	-11.36	-11.35	-11.34	-11.33	-11.32	-11.31	-11.3	-11.29
-11.37	-11.36	-11.35	-11.34	-11.33	-11.32	-11.31	-11.3	-11.29
-11.37	-11.36	-11.35	-11.34	-11.33	-11.32	-11.31	-11.3	-11.29
-11.37	-11.36	-11.35	-11.34	-11.33	-11.32	-11.31	-11.3	-11.29
-11.37	-11.36	-11.35	-11.34	-11.33	-11.32	-11.31	-11.3	-11.29
-11.37	-11.36	-11.35	-11.34	-11.33	-11.32	-11.31	-11.3	-11.29
-11.37	-11.36	-11.35	-11.34	-11.33	-11.32	-11.31	-11.3	-11.29
-11.37	-11.36	-11.35	-11.34	-11.33	-11.32	-11.31	-11.3	-11.29
-11.37	-11.36	-11.35	-11.34	-11.33	-11.32	-11.31	-11.3	-11.29
-11.37	-11.36	-11.35	-11.34	-11.33	-11.32	-11.31	-11.3	-11.29
-11.37	-11.36	-11.35	-11.34	-11.33	-11.32	-11.31	-11.3	-11.29
-11.37	-11.36	-11.35	-11.34	-11.33	-11.32	-11.31	-11.3	-11.29
-11.37	-11.36	-11.35	-11.34	-11.33	-11.32	-11.31	-11.3	-11.29
-11.37	-11.36	-11.35	-11.34	-11.33	-11.32	-11.31	-11.3	-11.29
-11.37	-11.36	-11.35	-11.34	-11.33	-11.32	-11.31	-11.3	-11.29
-11.37	-11.36	-11.35	-11.34	-11.33	-11.32	-11.31	-11.3	-11.29
-11.37	-11.36	-11.35	-11.34	-11.33	-11.32	-11.31	-11.3	-11.29
-11.37	-11.36	-11.35	-11.34	-11.33	-11.32	-11.31	-11.3	-11.29
-11.37	-11.36	-11.35	-11.34	-11.33	-11.32	-11.31	-11.3	-11.29
-11.37	-11.36	-11.35	-11.34	-11.33	-11.32	-11.31	-11.3	-11.29
-11.37	-11.36	-11.35	-11.34	-11.33	-11.32	-11.31	-11.3	-11.29
-11.37	-11.36	-11.35	-11.34	-11.33	-11.32	-11.31	-11.3	-11.29
-11.37	-11.36	-11.35	-11.34	-11.33	-11.32	-11.31	-11.3	-11.29
-11.37	-11.36	-11.35	-11.34	-11.33	-11.32	-11.31	-11.3	-11.29
-11.37	-11.36	-11.35	-11.34	-11.33	-11.32	-11.31	-11.3	-11.29
-11.37	-11.36	-11.35	-11.34	-11.33	-11.32	-11.31	-11.3	-11.29
-11.37	-11.36	-11.35	-11.34	-11.33	-11.32	-11.31	-11.3	-11.29
-11.37	-11.36	-11.35	-11.34	-11.33	-11.32	-11.31	-11.3	-11.29
-11.37	-11.36	-11.35	-11.34	-11.33	-11.32	-11.31	-11.3	-11.29
-11.37	-11.36	-11.35	-11.34	-11.33	-11.32	-11.31	-11.3	-11.29
-11.37	-11.36	-11.35	-11.34	-11.33	-11.32	-11.31	-11.3	-11.29
-11.37	-11.36	-11.35	-11.34	-11.33	-11.32	-11.31	-11.3	-11.29
-11.37	-11.36	-11.35	-11.34	-11.33	-11.32	-11.31	-11.3	-11.29

-11.37	-11.36	-11.35	-11.34	-11.33	-11.32	-11.31	-11.3	-11.29
-11.37	-11.36	-11.35	-11.34	-11.33	-11.32	-11.31	-11.3	-11.29
-11.37	-11.36	-11.35	-11.34	-11.33	-11.32	-11.31	-11.3	-11.29
-11.37	-11.36	-11.35	-11.34	-11.33	-11.32	-11.31	-11.3	-11.29
-11.37	-11.36	-11.35	-11.34	-11.33	-11.32	-11.31	-11.3	-11.29
-11.37	-11.36	-11.35	-11.34	-11.33	-11.32	-11.31	-11.3	-11.29
-11.37	-11.36	-11.35	-11.34	-11.33	-11.32	-11.31	-11.3	-11.29
-11.37	-11.36	-11.35	-11.34	-11.33	-11.32	-11.31	-11.3	-11.29
-11.37	-11.36	-11.35	-11.34	-11.33	-11.32	-11.31	-11.3	-11.29
-11.37	-11.36	-11.35	-11.34	-11.33	-11.32	-11.31	-11.3	-11.29
-11.37	-11.36	-11.35	-11.34	-11.33	-11.32	-11.31	-11.3	-11.29
-11.37	-11.36	-11.35	-11.34	-11.33	-11.32	-11.31	-11.3	-11.29
-11.37	-11.36	-11.35	-11.34	-11.33	-11.32	-11.31	-11.3	-11.29
-11.37	-11.36	-11.35	-11.34	-11.33	-11.32	-11.31	-11.3	-11.29
-11.37	-11.36	-11.35	-11.34	-11.33	-11.32	-11.31	-11.3	-11.29
-11.37	-11.36	-11.35	-11.34	-11.33	-11.32	-11.31	-11.3	-11.29
-11.37	-11.36	-11.35	-11.34	-11.33	-11.32	-11.31	-11.3	-11.29
-11.37	-11.36	-11.35	-11.34	-11.33	-11.32	-11.31	-11.3	-11.29
-11.37	-11.36	-11.35	-11.34	-11.33	-11.32	-11.31	-11.3	-11.29
-11.37	-11.36	-11.35	-11.34	-11.33	-11.32	-11.31	-11.3	-11.29
-11.37	-11.36	-11.35	-11.34	-11.33	-11.32	-11.31	-11.3	-11.29
-11.37	-11.36	-11.35	-11.34	-11.33	-11.32	-11.31	-11.3	-11.29
-11.37	-11.36	-11.35	-11.34	-11.33	-11.32	-11.31	-11.3	-11.29
-11.37	-11.36	-11.35	-11.34	-11.33	-11.32	-11.31	-11.3	-11.29
-11.37	-11.36	-11.35	-11.34	-11.33	-11.32	-11.31	-11.3	-11.29
-11.37	-11.36	-11.35	-11.34	-11.33	-11.32	-11.31	-11.3	-11.29
-11.37	-11.36	-11.35	-11.34	-11.33	-11.32	-11.31	-11.3	-11.29
-11.37	-11.36	-11.35	-11.34	-11.33	-11.32	-11.31	-11.3	-11.29
-11.37	-11.36	-11.35	-11.34	-11.33	-11.32	-11.31	-11.3	-11.29
-11.37	-11.36	-11.35	-11.34	-11.33	-11.32	-11.31	-11.3	-11.29
-11.37	-11.36	-11.35	-11.34	-11.33	-11.32	-11.31	-11.3	-11.29
-11.37	-11.36	-11.35	-11.34	-11.33	-11.32	-11.31	-11.3	-11.29
-11.37	-11.36	-11.35	-11.34	-11.33	-11.32	-11.31	-11.3	-11.29
-11.37	-11.36	-11.35	-11.34	-11.33	-11.32	-11.31	-11.3	-11.29
-11.37	-11.36	-11.35	-11.34	-11.33	-11.32	-11.31	-11.3	-11.29
-11.37	-11.36	-11.35	-11.34	-11.33	-11.32	-11.31	-11.3	-11.29
-11.37	-11.36	-11.35	-11.34	-11.33	-11.32	-11.31	-11.3	-11.29
-11.37	-11.36	-11.35	-11.34	-11.33	-11.32	-11.31	-11.3	-11.29
-11.37	-11.36	-11.35	-11.34	-11.33	-11.32	-11.31	-11.3	-11.29
-11.37	-11.36	-11.35	-11.34	-11.33	-11.32	-11.31	-11.3	-11.29
-11.37	-11.36	-11.35	-11.34	-11.33	-11.32	-11.31	-11.3	-11.29
-11.37	-11.36	-11.35	-11.34	-11.33	-11.32	-11.31	-11.3	-11.29
-11.37	-11.36	-11.35	-11.34	-11.33	-11.32	-11.31	-11.3	-11.29
-11.37	-11.36	-11.35	-11.34	-11.33	-11.32	-11.31	-11.3	-11.29
-11.37	-11.36	-11.35	-11.34	-11.33	-11.32	-11.31	-11.3	-11.29
-11.37	-11.36	-11.35	-11.34	-11.33	-11.32	-11.31	-11.3	-11.29
-11.37	-11.36	-11.35	-11.34	-11.33	-11.32	-11.31	-11.3	-11.29
-11.37	-11.36	-11.35	-11.34	-11.33	-11.32	-11.31	-11.3	-11.29
-11.37	-11.36	-11.35	-11.34	-11.33	-11.32	-11.31	-11.3	-11.29
-11.37	-11.36	-11.35	-11.34	-11.33	-11.32	-11.31	-11.3	-11.29

-11.37	-11.36	-11.35	-11.34	-11.33	-11.32	-11.31	-11.3	-11.29
-11.37	-11.36	-11.35	-11.34	-11.33	-11.32	-11.31	-11.3	-11.29
-11.37	-11.36	-11.35	-11.34	-11.33	-11.32	-11.31	-11.3	-11.29
-11.37	-11.36	-11.35	-11.34	-11.33	-11.32	-11.31	-11.3	-11.29
-11.37	-11.36	-11.35	-11.34	-11.33	-11.32	-11.31	-11.3	-11.29
-11.37	-11.36	-11.35	-11.34	-11.33	-11.32	-11.31	-11.3	-11.29
-11.37	-11.36	-11.35	-11.34	-11.33	-11.32	-11.31	-11.3	-11.29
-11.37	-11.36	-11.35	-11.34	-11.33	-11.32	-11.31	-11.3	-11.29
-11.37	-11.36	-11.35	-11.34	-11.33	-11.32	-11.31	-11.3	-11.29
-11.37	-11.36	-11.35	-11.34	-11.33	-11.32	-11.31	-11.3	-11.29
-11.37	-11.36	-11.35	-11.34	-11.33	-11.32	-11.31	-11.3	-11.29
-11.37	-11.36	-11.35	-11.34	-11.33	-11.32	-11.31	-11.3	-11.29
-11.37	-11.36	-11.35	-11.34	-11.33	-11.32	-11.31	-11.3	-11.29
-11.37	-11.36	-11.35	-11.34	-11.33	-11.32	-11.31	-11.3	-11.29
-11.37	-11.36	-11.35	-11.34	-11.33	-11.32	-11.31	-11.3	-11.29
-11.37	-11.36	-11.35	-11.34	-11.33	-11.32	-11.31	-11.3	-11.29
-11.37	-11.36	-11.35	-11.34	-11.33	-11.32	-11.31	-11.3	-11.29
-11.37	-11.36	-11.35	-11.34	-11.33	-11.32	-11.31	-11.3	-11.29
-11.37	-11.36	-11.35	-11.34	-11.33	-11.32	-11.31	-11.3	-11.29
-11.37	-11.36	-11.35	-11.34	-11.33	-11.32	-11.31	-11.3	-11.29
-11.37	-11.36	-11.35	-11.34	-11.33	-11.32	-11.31	-11.3	-11.29
-11.37	-11.36	-11.35	-11.34	-11.33	-11.32	-11.31	-11.3	-11.29
-11.37	-11.36	-11.35	-11.34	-11.33	-11.32	-11.31	-11.3	-11.29
-11.37	-11.36	-11.35	-11.34	-11.33	-11.32	-11.31	-11.3	-11.29
-11.37	-11.36	-11.35	-11.34	-11.33	-11.32	-11.31	-11.3	-11.29
-11.37	-11.36	-11.35	-11.34	-11.33	-11.32	-11.31	-11.3	-11.29
-11.37	-11.36	-11.35	-11.34	-11.33	-11.32	-11.31	-11.3	-11.29
-11.37	-11.36	-11.35	-11.34	-11.33	-11.32	-11.31	-11.3	-11.29
-11.37	-11.36	-11.35	-11.34	-11.33	-11.32	-11.31	-11.3	-11.29
-11.37	-11.36	-11.35	-11.34	-11.33	-11.32	-11.31	-11.3	-11.29
-11.37	-11.36	-11.35	-11.34	-11.33	-11.32	-11.31	-11.3	-11.29
-11.37	-11.36	-11.35	-11.34	-11.33	-11.32	-11.31	-11.3	-11.29
-11.37	-11.36	-11.35	-11.34	-11.33	-11.32	-11.31	-11.3	-11.29
-11.37	-11.36	-11.35	-11.34	-11.33	-11.32	-11.31	-11.3	-11.29
-11.37	-11.36	-11.35	-11.34	-11.33	-11.32	-11.31	-11.3	-11.29
-11.37	-11.36	-11.35	-11.34	-11.33	-11.32	-11.31	-11.3	-11.29
-11.37	-11.36	-11.35	-11.34	-11.33	-11.32	-11.31	-11.3	-11.29
-11.37	-11.36	-11.35	-11.34	-11.33	-11.32	-11.31	-11.3	-11.29
-11.37	-11.36	-11.35	-11.34	-11.33	-11.32	-11.31	-11.3	-11.29
-11.37	-11.36	-11.35	-11.34	-11.33	-11.32	-11.31	-11.3	-11.29
-11.37	-11.36	-11.35	-11.34	-11.33	-11.32	-11.31	-11.3	-11.29
-11.37	-11.36	-11.35	-11.34	-11.33	-11.32	-11.31	-11.3	-11.29
-11.37	-11.36	-11.35	-11.34	-11.33	-11.32	-11.31	-11.3	-11.29
-11.37	-11.36	-11.35	-11.34	-11.33	-11.32	-11.31	-11.3	-11.29
-11.37	-11.36	-11.35	-11.34	-11.33	-11.32	-11.31	-11.3	-11.29
-11.37	-11.36	-11.35	-11.34	-11.33	-11.32	-11.31	-11.3	-11.29
-11.37	-11.36	-11.35	-11.34	-11.33	-11.32	-11.31	-11.3	-11.29
-11.37	-11.36	-11.35	-11.34	-11.33	-11.32	-11.31	-11.3	-11.29
-11.37	-11.36	-11.35	-11.34	-11.33	-11.32	-11.31	-11.3	-11.29
-11.37	-11.36	-11.35	-11.34	-11.33	-11.32	-11.31	-11.3	-11.29

-11.37	-11.36	-11.35	-11.34	-11.33	-11.32	-11.31	-11.3	-11.29
-11.37	-11.36	-11.35	-11.34	-11.33	-11.32	-11.31	-11.3	-11.29
-11.37	-11.36	-11.35	-11.34	-11.33	-11.32	-11.31	-11.3	-11.29
-11.37	-11.36	-11.35	-11.34	-11.33	-11.32	-11.31	-11.3	-11.29
-11.37	-11.36	-11.35	-11.34	-11.33	-11.32	-11.31	-11.3	-11.29
-11.37	-11.36	-11.35	-11.34	-11.33	-11.32	-11.31	-11.3	-11.29
-11.37	-11.36	-11.35	-11.34	-11.33	-11.32	-11.31	-11.3	-11.29
-11.37	-11.36	-11.35	-11.34	-11.33	-11.32	-11.31	-11.3	-11.29
-11.37	-11.36	-11.35	-11.34	-11.33	-11.32	-11.31	-11.3	-11.29
-11.37	-11.36	-11.35	-11.34	-11.33	-11.32	-11.31	-11.3	-11.29
-11.37	-11.36	-11.35	-11.34	-11.33	-11.32	-11.31	-11.3	-11.29
-11.37	-11.36	-11.35	-11.34	-11.33	-11.32	-11.31	-11.3	-11.29
-11.37	-11.36	-11.35	-11.34	-11.33	-11.32	-11.31	-11.3	-11.29
-11.37	-11.36	-11.35	-11.34	-11.33	-11.32	-11.31	-11.3	-11.29
-11.37	-11.36	-11.35	-11.34	-11.33	-11.32	-11.31	-11.3	-11.29
-11.37	-11.36	-11.35	-11.34	-11.33	-11.32	-11.31	-11.3	-11.29
-11.37	-11.36	-11.35	-11.34	-11.33	-11.32	-11.31	-11.3	-11.29
-11.37	-11.36	-11.35	-11.34	-11.33	-11.32	-11.31	-11.3	-11.29
-11.37	-11.36	-11.35	-11.34	-11.33	-11.32	-11.31	-11.3	-11.29
-11.37	-11.36	-11.35	-11.34	-11.33	-11.32	-11.31	-11.3	-11.29
-11.37	-11.36	-11.35	-11.34	-11.33	-11.32	-11.31	-11.3	-11.29
-11.37	-11.36	-11.35	-11.34	-11.33	-11.32	-11.31	-11.3	-11.29
-11.37	-11.36	-11.35	-11.34	-11.33	-11.32	-11.31	-11.3	-11.29
-11.37	-11.36	-11.35	-11.34	-11.33	-11.32	-11.31	-11.3	-11.29
-11.37	-11.36	-11.35	-11.34	-11.33	-11.32	-11.31	-11.3	-11.29
-11.37	-11.36	-11.35	-11.34	-11.33	-11.32	-11.31	-11.3	-11.29
-11.37	-11.36	-11.35	-11.34	-11.33	-11.32	-11.31	-11.3	-11.29
-11.37	-11.36	-11.35	-11.34	-11.33	-11.32	-11.31	-11.3	-11.29
-11.37	-11.36	-11.35	-11.34	-11.33	-11.32	-11.31	-11.3	-11.29
-11.37	-11.36	-11.35	-11.34	-11.33	-11.32	-11.31	-11.3	-11.29
-11.37	-11.36	-11.35	-11.34	-11.33	-11.32	-11.31	-11.3	-11.29
-11.37	-11.36	-11.35	-11.34	-11.33	-11.32	-11.31	-11.3	-11.29
-11.37	-11.36	-11.35	-11.34	-11.33	-11.32	-11.31	-11.3	-11.29
-11.37	-11.36	-11.35	-11.34	-11.33	-11.32	-11.31	-11.3	-11.29
-11.37	-11.36	-11.35	-11.34	-11.33	-11.32	-11.31	-11.3	-11.29
-11.37	-11.36	-11.35	-11.34	-11.33	-11.32	-11.31	-11.3	-11.29
-11.37	-11.36	-11.35	-11.34	-11.33	-11.32	-11.31	-11.3	-11.29
-11.37	-11.36	-11.35	-11.34	-11.33	-11.32	-11.31	-11.3	-11.29
-11.37	-11.36	-11.35	-11.34	-11.33	-11.32	-11.31	-11.3	-11.29
-11.37	-11.36	-11.35	-11.34	-11.33	-11.32	-11.31	-11.3	-11.29
-11.37	-11.36	-11.35	-11.34	-11.33	-11.32	-11.31	-11.3	-11.29
-11.37	-11.36	-11.35	-11.34	-11.33	-11.32	-11.31	-11.3	-11.29
-11.37	-11.36	-11.35	-11.34	-11.33	-11.32	-11.31	-11.3	-11.29
-11.37	-11.36	-11.35	-11.34	-11.33	-11.32	-11.31	-11.3	-11.29
-11.37	-11.36	-11.35	-11.34	-11.33	-11.32	-11.31	-11.3	-11.29
-11.37	-11.36	-11.35	-11.34	-11.33	-11.32	-11.31	-11.3	-11.29
-11.37	-11.36	-11.35	-11.34	-11.33	-11.32	-11.31	-11.3	-11.29
-11.37	-11.36	-11.35	-11.34	-11.33	-11.32	-11.31	-11.3	-11.29
-11.37	-11.36	-11.35	-11.34	-11.33	-11.32	-11.31	-11.3	-11.29
-11.37	-11.36	-11.35	-11.34	-11.33	-11.32	-11.31	-11.3	-11.29

-11.37	-11.36	-11.35	-11.34	-11.33	-11.32	-11.31	-11.3	-11.29
-11.37	-11.36	-11.35	-11.34	-11.33	-11.32	-11.31	-11.3	-11.29
-11.37	-11.36	-11.35	-11.34	-11.33	-11.32	-11.31	-11.3	-11.29
-11.37	-11.36	-11.35	-11.34	-11.33	-11.32	-11.31	-11.3	-11.29
-11.37	-11.36	-11.35	-11.34	-11.33	-11.32	-11.31	-11.3	-11.29
-11.37	-11.36	-11.35	-11.34	-11.33	-11.32	-11.31	-11.3	-11.29
-11.37	-11.36	-11.35	-11.34	-11.33	-11.32	-11.31	-11.3	-11.29
-11.37	-11.36	-11.35	-11.34	-11.33	-11.32	-11.31	-11.3	-11.29
-11.37	-11.36	-11.35	-11.34	-11.33	-11.32	-11.31	-11.3	-11.29
-11.37	-11.36	-11.35	-11.34	-11.33	-11.32	-11.31	-11.3	-11.29
-11.37	-11.36	-11.35	-11.34	-11.33	-11.32	-11.31	-11.3	-11.29
-11.37	-11.36	-11.35	-11.34	-11.33	-11.32	-11.31	-11.3	-11.29
-11.37	-11.36	-11.35	-11.34	-11.33	-11.32	-11.31	-11.3	-11.29
-11.37	-11.36	-11.35	-11.34	-11.33	-11.32	-11.31	-11.3	-11.29
-11.37	-11.36	-11.35	-11.34	-11.33	-11.32	-11.31	-11.3	-11.29
-11.37	-11.36	-11.35	-11.34	-11.33	-11.32	-11.31	-11.3	-11.29
-11.37	-11.36	-11.35	-11.34	-11.33	-11.32	-11.31	-11.3	-11.29
-11.37	-11.36	-11.35	-11.34	-11.33	-11.32	-11.31	-11.3	-11.29
-11.37	-11.36	-11.35	-11.34	-11.33	-11.32	-11.31	-11.3	-11.29
-11.37	-11.36	-11.35	-11.34	-11.33	-11.32	-11.31	-11.3	-11.29
-11.37	-11.36	-11.35	-11.34	-11.33	-11.32	-11.31	-11.3	-11.29
-11.37	-11.36	-11.35	-11.34	-11.33	-11.32	-11.31	-11.3	-11.29
-11.37	-11.36	-11.35	-11.34	-11.33	-11.32	-11.31	-11.3	-11.29
-11.37	-11.36	-11.35	-11.34	-11.33	-11.32	-11.31	-11.3	-11.29
-11.37	-11.36	-11.35	-11.34	-11.33	-11.32	-11.31	-11.3	-11.29
-11.37	-11.36	-11.35	-11.34	-11.33	-11.32	-11.31	-11.3	-11.29
-11.37	-11.36	-11.35	-11.34	-11.33	-11.32	-11.31	-11.3	-11.29
-11.37	-11.36	-11.35	-11.34	-11.33	-11.32	-11.31	-11.3	-11.29
-11.37	-11.36	-11.35	-11.34	-11.33	-11.32	-11.31	-11.3	-11.29
-11.37	-11.36	-11.35	-11.34	-11.33	-11.32	-11.31	-11.3	-11.29
-11.37	-11.36	-11.35	-11.34	-11.33	-11.32	-11.31	-11.3	-11.29
-11.37	-11.36	-11.35	-11.34	-11.33	-11.32	-11.31	-11.3	-11.29
-11.37	-11.36	-11.35	-11.34	-11.33	-11.32	-11.31	-11.3	-11.29
-11.37	-11.36	-11.35	-11.34	-11.33	-11.32	-11.31	-11.3	-11.29
-11.37	-11.36	-11.35	-11.34	-11.33	-11.32	-11.31	-11.3	-11.29
-11.37	-11.36	-11.35	-11.34	-11.33	-11.32	-11.31	-11.3	-11.29
-11.37	-11.36	-11.35	-11.34	-11.33	-11.32	-11.31	-11.3	-11.29
-11.37	-11.36	-11.35	-11.34	-11.33	-11.32	-11.31	-11.3	-11.29
-11.37	-11.36	-11.35	-11.34	-11.33	-11.32	-11.31	-11.3	-11.29
-11.37	-11.36	-11.35	-11.34	-11.33	-11.32	-11.31	-11.3	-11.29
-11.37	-11.36	-11.35	-11.34	-11.33	-11.32	-11.31	-11.3	-11.29
-11.37	-11.36	-11.35	-11.34	-11.33	-11.32	-11.31	-11.3	-11.29
-11.37	-11.36	-11.35	-11.34	-11.33	-11.32	-11.31	-11.3	-11.29
-11.37	-11.36	-11.35	-11.34	-11.33	-11.32	-11.31	-11.3	-11.29
-11.37	-11.36	-11.35	-11.34	-11.33	-11.32	-11.31	-11.3	-11.29
-11.37	-11.36	-11.35	-11.34	-11.33	-11.32	-11.31	-11.3	-11.29
-11.37	-11.36	-11.35	-11.34	-11.33	-11.32	-11.31	-11.3	-11.29
-11.37	-11.36	-11.35	-11.34	-11.33	-11.32	-11.31	-11.3	-11.29
-11.37	-11.36	-11.35	-11.34	-11.33	-11.32	-11.31	-11.3	-11.29
-11.37	-11.36	-11.35	-11.34	-11.33	-11.32	-11.31	-11.3	-11.29

-11.37	-11.36	-11.35	-11.34	-11.33	-11.32	-11.31	-11.3	-11.29
-11.37	-11.36	-11.35	-11.34	-11.33	-11.32	-11.31	-11.3	-11.29
-11.37	-11.36	-11.35	-11.34	-11.33	-11.32	-11.31	-11.3	-11.29
-11.37	-11.36	-11.35	-11.34	-11.33	-11.32	-11.31	-11.3	-11.29
-11.37	-11.36	-11.35	-11.34	-11.33	-11.32	-11.31	-11.3	-11.29
-11.37	-11.36	-11.35	-11.34	-11.33	-11.32	-11.31	-11.3	-11.29
-11.37	-11.36	-11.35	-11.34	-11.33	-11.32	-11.31	-11.3	-11.29
-11.37	-11.36	-11.35	-11.34	-11.33	-11.32	-11.31	-11.3	-11.29
-11.37	-11.36	-11.35	-11.34	-11.33	-11.32	-11.31	-11.3	-11.29
-11.37	-11.36	-11.35	-11.34	-11.33	-11.32	-11.31	-11.3	-11.29
-11.37	-11.36	-11.35	-11.34	-11.33	-11.32	-11.31	-11.3	-11.29
-11.37	-11.36	-11.35	-11.34	-11.33	-11.32	-11.31	-11.3	-11.29
-11.37	-11.36	-11.35	-11.34	-11.33	-11.32	-11.31	-11.3	-11.29
-11.37	-11.36	-11.35	-11.34	-11.33	-11.32	-11.31	-11.3	-11.29
-11.37	-11.36	-11.35	-11.34	-11.33	-11.32	-11.31	-11.3	-11.29
-11.37	-11.36	-11.35	-11.34	-11.33	-11.32	-11.31	-11.3	-11.29
-11.37	-11.36	-11.35	-11.34	-11.33	-11.32	-11.31	-11.3	-11.29
-11.37	-11.36	-11.35	-11.34	-11.33	-11.32	-11.31	-11.3	-11.29
-11.37	-11.36	-11.35	-11.34	-11.33	-11.32	-11.31	-11.3	-11.29
-11.37	-11.36	-11.35	-11.34	-11.33	-11.32	-11.31	-11.3	-11.29
-11.37	-11.36	-11.35	-11.34	-11.33	-11.32	-11.31	-11.3	-11.29
-11.37	-11.36	-11.35	-11.34	-11.33	-11.32	-11.31	-11.3	-11.29
-11.37	-11.36	-11.35	-11.34	-11.33	-11.32	-11.31	-11.3	-11.29
-11.37	-11.36	-11.35	-11.34	-11.33	-11.32	-11.31	-11.3	-11.29
-11.37	-11.36	-11.35	-11.34	-11.33	-11.32	-11.31	-11.3	-11.29
-11.37	-11.36	-11.35	-11.34	-11.33	-11.32	-11.31	-11.3	-11.29
-11.37	-11.36	-11.35	-11.34	-11.33	-11.32	-11.31	-11.3	-11.29
-11.37	-11.36	-11.35	-11.34	-11.33	-11.32	-11.31	-11.3	-11.29
-11.37	-11.36	-11.35	-11.34	-11.33	-11.32	-11.31	-11.3	-11.29
-11.37	-11.36	-11.35	-11.34	-11.33	-11.32	-11.31	-11.3	-11.29
-11.37	-11.36	-11.35	-11.34	-11.33	-11.32	-11.31	-11.3	-11.29
-11.37	-11.36	-11.35	-11.34	-11.33	-11.32	-11.31	-11.3	-11.29
-11.37	-11.36	-11.35	-11.34	-11.33	-11.32	-11.31	-11.3	-11.29
-11.37	-11.36	-11.35	-11.34	-11.33	-11.32	-11.31	-11.3	-11.29
-11.37	-11.36	-11.35	-11.34	-11.33	-11.32	-11.31	-11.3	-11.29
-11.37	-11.36	-11.35	-11.34	-11.33	-11.32	-11.31	-11.3	-11.29
-11.37	-11.36	-11.35	-11.34	-11.33	-11.32	-11.31	-11.3	-11.29
-11.37	-11.36	-11.35	-11.34	-11.33	-11.32	-11.31	-11.3	-11.29
-11.37	-11.36	-11.35	-11.34	-11.33	-11.32	-11.31	-11.3	-11.29
-11.37	-11.36	-11.35	-11.34	-11.33	-11.32	-11.31	-11.3	-11.29
-11.37	-11.36	-11.35	-11.34	-11.33	-11.32	-11.31	-11.3	-11.29
-11.37	-11.36	-11.35	-11.34	-11.33	-11.32	-11.31	-11.3	-11.29
-11.37	-11.36	-11.35	-11.34	-11.33	-11.32	-11.31	-11.3	-11.29
-11.37	-11.36	-11.35	-11.34	-11.33	-11.32	-11.31	-11.3	-11.29
-11.37	-11.36	-11.35	-11.34	-11.33	-11.32	-11.31	-11.3	-11.29
-11.37	-11.36	-11.35	-11.34	-11.33	-11.32	-11.31	-11.3	-11.29
-11.37	-11.36	-11.35	-11.34	-11.33	-11.32	-11.31	-11.3	-11.29
-11.37	-11.36	-11.35	-11.34	-11.33	-11.32	-11.31	-11.3	-11.29
-11.37	-11.36	-11.35	-11.34	-11.33	-11.32	-11.31	-11.3	-11.29
-11.37	-11.36	-11.35	-11.34	-11.33	-11.32	-11.31	-11.3	-11.29

-11.37	-11.36	-11.35	-11.34	-11.33	-11.32	-11.31	-11.3	-11.29
-11.37	-11.36	-11.35	-11.34	-11.33	-11.32	-11.31	-11.3	-11.29
-11.37	-11.36	-11.35	-11.34	-11.33	-11.32	-11.31	-11.3	-11.29
-11.37	-11.36	-11.35	-11.34	-11.33	-11.32	-11.31	-11.3	-11.29
-11.37	-11.36	-11.35	-11.34	-11.33	-11.32	-11.31	-11.3	-11.29
-11.37	-11.36	-11.35	-11.34	-11.33	-11.32	-11.31	-11.3	-11.29
-11.37	-11.36	-11.35	-11.34	-11.33	-11.32	-11.31	-11.3	-11.29
-11.37	-11.36	-11.35	-11.34	-11.33	-11.32	-11.31	-11.3	-11.29
-11.37	-11.36	-11.35	-11.34	-11.33	-11.32	-11.31	-11.3	-11.29
-11.37	-11.36	-11.35	-11.34	-11.33	-11.32	-11.31	-11.3	-11.29
-11.37	-11.36	-11.35	-11.34	-11.33	-11.32	-11.31	-11.3	-11.29
-11.37	-11.36	-11.35	-11.34	-11.33	-11.32	-11.31	-11.3	-11.29
-11.37	-11.36	-11.35	-11.34	-11.33	-11.32	-11.31	-11.3	-11.29
-11.37	-11.36	-11.35	-11.34	-11.33	-11.32	-11.31	-11.3	-11.29
-11.37	-11.36	-11.35	-11.34	-11.33	-11.32	-11.31	-11.3	-11.29
-11.37	-11.36	-11.35	-11.34	-11.33	-11.32	-11.31	-11.3	-11.29
-11.37	-11.36	-11.35	-11.34	-11.33	-11.32	-11.31	-11.3	-11.29
-11.37	-11.36	-11.35	-11.34	-11.33	-11.32	-11.31	-11.3	-11.29
-11.37	-11.36	-11.35	-11.34	-11.33	-11.32	-11.31	-11.3	-11.29
-11.37	-11.36	-11.35	-11.34	-11.33	-11.32	-11.31	-11.3	-11.29
-11.37	-11.36	-11.35	-11.34	-11.33	-11.32	-11.31	-11.3	-11.29
-11.37	-11.36	-11.35	-11.34	-11.33	-11.32	-11.31	-11.3	-11.29
-11.37	-11.36	-11.35	-11.34	-11.33	-11.32	-11.31	-11.3	-11.29
-11.37	-11.36	-11.35	-11.34	-11.33	-11.32	-11.31	-11.3	-11.29
-11.37	-11.36	-11.35	-11.34	-11.33	-11.32	-11.31	-11.3	-11.29
-11.37	-11.36	-11.35	-11.34	-11.33	-11.32	-11.31	-11.3	-11.29
-11.37	-11.36	-11.35	-11.34	-11.33	-11.32	-11.31	-11.3	-11.29
-11.37	-11.36	-11.35	-11.34	-11.33	-11.32	-11.31	-11.3	-11.29
-11.37	-11.36	-11.35	-11.34	-11.33	-11.32	-11.31	-11.3	-11.29
-11.37	-11.36	-11.35	-11.34	-11.33	-11.32	-11.31	-11.3	-11.29
-11.37	-11.36	-11.35	-11.34	-11.33	-11.32	-11.31	-11.3	-11.29
-11.37	-11.36	-11.35	-11.34	-11.33	-11.32	-11.31	-11.3	-11.29
-11.37	-11.36	-11.35	-11.34	-11.33	-11.32	-11.31	-11.3	-11.29
-11.37	-11.36	-11.35	-11.34	-11.33	-11.32	-11.31	-11.3	-11.29
-11.37	-11.36	-11.35	-11.34	-11.33	-11.32	-11.31	-11.3	-11.29
-11.37	-11.36	-11.35	-11.34	-11.33	-11.32	-11.31	-11.3	-11.29
-11.37	-11.36	-11.35	-11.34	-11.33	-11.32	-11.31	-11.3	-11.29
-11.37	-11.36	-11.35	-11.34	-11.33	-11.32	-11.31	-11.3	-11.29
-11.37	-11.36	-11.35	-11.34	-11.33	-11.32	-11.31	-11.3	-11.29
-11.37	-11.36	-11.35	-11.34	-11.33	-11.32	-11.31	-11.3	-11.29
-11.37	-11.36	-11.35	-11.34	-11.33	-11.32	-11.31	-11.3	-11.29
-11.37	-11.36	-11.35	-11.34	-11.33	-11.32	-11.31	-11.3	-11.29
-11.37	-11.36	-11.35	-11.34	-11.33	-11.32	-11.31	-11.3	-11.29
-11.37	-11.36	-11.35	-11.34	-11.33	-11.32	-11.31	-11.3	-11.29
-11.37	-11.36	-11.35	-11.34	-11.33	-11.32	-11.31	-11.3	-11.29
-11.37	-11.36	-11.35	-11.34	-11.33	-11.32	-11.31	-11.3	-11.29
-11.37	-11.36	-11.35	-11.34	-11.33	-11.32	-11.31	-11.3	-11.29
-11.37	-11.36	-11.35	-11.34	-11.33	-11.32	-11.31	-11.3	-11.29
-11.37	-11.36	-11.35	-11.34	-11.33	-11.32	-11.31	-11.3	-11.29
-11.37	-11.36	-11.35	-11.34	-11.33	-11.32	-11.31	-11.3	-11.29

-11.37	-11.36	-11.35	-11.34	-11.33	-11.32	-11.31	-11.3	-11.29
-11.37	-11.36	-11.35	-11.34	-11.33	-11.32	-11.31	-11.3	-11.29
-11.37	-11.36	-11.35	-11.34	-11.33	-11.32	-11.31	-11.3	-11.29
-11.37	-11.36	-11.35	-11.34	-11.33	-11.32	-11.31	-11.3	-11.29
-11.37	-11.36	-11.35	-11.34	-11.33	-11.32	-11.31	-11.3	-11.29
-11.37	-11.36	-11.35	-11.34	-11.33	-11.32	-11.31	-11.3	-11.29
-11.37	-11.36	-11.35	-11.34	-11.33	-11.32	-11.31	-11.3	-11.29
-11.37	-11.36	-11.35	-11.34	-11.33	-11.32	-11.31	-11.3	-11.29
-11.37	-11.36	-11.35	-11.34	-11.33	-11.32	-11.31	-11.3	-11.29
-11.37	-11.36	-11.35	-11.34	-11.33	-11.32	-11.31	-11.3	-11.29
-11.37	-11.36	-11.35	-11.34	-11.33	-11.32	-11.31	-11.3	-11.29
-11.37	-11.36	-11.35	-11.34	-11.33	-11.32	-11.31	-11.3	-11.29
-11.37	-11.36	-11.35	-11.34	-11.33	-11.32	-11.31	-11.3	-11.29
-11.37	-11.36	-11.35	-11.34	-11.33	-11.32	-11.31	-11.3	-11.29
-11.37	-11.36	-11.35	-11.34	-11.33	-11.32	-11.31	-11.3	-11.29
-11.37	-11.36	-11.35	-11.34	-11.33	-11.32	-11.31	-11.3	-11.29
-11.37	-11.36	-11.35	-11.34	-11.33	-11.32	-11.31	-11.3	-11.29
-11.37	-11.36	-11.35	-11.34	-11.33	-11.32	-11.31	-11.3	-11.29
-11.37	-11.36	-11.35	-11.34	-11.33	-11.32	-11.31	-11.3	-11.29
-11.37	-11.36	-11.35	-11.34	-11.33	-11.32	-11.31	-11.3	-11.29
-11.37	-11.36	-11.35	-11.34	-11.33	-11.32	-11.31	-11.3	-11.29
-11.37	-11.36	-11.35	-11.34	-11.33	-11.32	-11.31	-11.3	-11.29
-11.37	-11.36	-11.35	-11.34	-11.33	-11.32	-11.31	-11.3	-11.29
-11.37	-11.36	-11.35	-11.34	-11.33	-11.32	-11.31	-11.3	-11.29
-11.37	-11.36	-11.35	-11.34	-11.33	-11.32	-11.31	-11.3	-11.29
-11.37	-11.36	-11.35	-11.34	-11.33	-11.32	-11.31	-11.3	-11.29
-11.37	-11.36	-11.35	-11.34	-11.33	-11.32	-11.31	-11.3	-11.29
-11.37	-11.36	-11.35	-11.34	-11.33	-11.32	-11.31	-11.3	-11.29
-11.37	-11.36	-11.35	-11.34	-11.33	-11.32	-11.31	-11.3	-11.29
-11.37	-11.36	-11.35	-11.34	-11.33	-11.32	-11.31	-11.3	-11.29
-11.37	-11.36	-11.35	-11.34	-11.33	-11.32	-11.31	-11.3	-11.29
-11.37	-11.36	-11.35	-11.34	-11.33	-11.32	-11.31	-11.3	-11.29
-11.37	-11.36	-11.35	-11.34	-11.33	-11.32	-11.31	-11.3	-11.29
-11.37	-11.36	-11.35	-11.34	-11.33	-11.32	-11.31	-11.3	-11.29
-11.37	-11.36	-11.35	-11.34	-11.33	-11.32	-11.31	-11.3	-11.29
-11.37	-11.36	-11.35	-11.34	-11.33	-11.32	-11.31	-11.3	-11.29
-11.37	-11.36	-11.35	-11.34	-11.33	-11.32	-11.31	-11.3	-11.29
-11.37	-11.36	-11.35	-11.34	-11.33	-11.32	-11.31	-11.3	-11.29
-11.37	-11.36	-11.35	-11.34	-11.33	-11.32	-11.31	-11.3	-11.29
-11.37	-11.36	-11.35	-11.34	-11.33	-11.32	-11.31	-11.3	-11.29
-11.37	-11.36	-11.35	-11.34	-11.33	-11.32	-11.31	-11.3	-11.29
-11.37	-11.36	-11.35	-11.34	-11.33	-11.32	-11.31	-11.3	-11.29
-11.37	-11.36	-11.35	-11.34	-11.33	-11.32	-11.31	-11.3	-11.29
-11.37	-11.36	-11.35	-11.34	-11.33	-11.32	-11.31	-11.3	-11.29
-11.37	-11.36	-11.35	-11.34	-11.33	-11.32	-11.31	-11.3	-11.29
-11.37	-11.36	-11.35	-11.34	-11.33	-11.32	-11.31	-11.3	-11.29
-11.37	-11.36	-11.35	-11.34	-11.33	-11.32	-11.31	-11.3	-11.29
-11.37	-11.36	-11.35	-11.34	-11.33	-11.32	-11.31	-11.3	-11.29
-11.37	-11.36	-11.35	-11.34	-11.33	-11.32	-11.31	-11.3	-11.29
-11.37	-11.36	-11.35	-11.34	-11.33	-11.32	-11.31	-11.3	-11.29

-11.37	-11.36	-11.35	-11.34	-11.33	-11.32	-11.31	-11.3	-11.29
-11.37	-11.36	-11.35	-11.34	-11.33	-11.32	-11.31	-11.3	-11.29
-11.37	-11.36	-11.35	-11.34	-11.33	-11.32	-11.31	-11.3	-11.29
-11.37	-11.36	-11.35	-11.34	-11.33	-11.32	-11.31	-11.3	-11.29
-11.37	-11.36	-11.35	-11.34	-11.33	-11.32	-11.31	-11.3	-11.29
-11.37	-11.36	-11.35	-11.34	-11.33	-11.32	-11.31	-11.3	-11.29
-11.37	-11.36	-11.35	-11.34	-11.33	-11.32	-11.31	-11.3	-11.29
-11.37	-11.36	-11.35	-11.34	-11.33	-11.32	-11.31	-11.3	-11.29
-11.37	-11.36	-11.35	-11.34	-11.33	-11.32	-11.31	-11.3	-11.29
-11.37	-11.36	-11.35	-11.34	-11.33	-11.32	-11.31	-11.3	-11.29
-11.37	-11.36	-11.35	-11.34	-11.33	-11.32	-11.31	-11.3	-11.29
-11.37	-11.36	-11.35	-11.34	-11.33	-11.32	-11.31	-11.3	-11.29
-11.37	-11.36	-11.35	-11.34	-11.33	-11.32	-11.31	-11.3	-11.29
-11.37	-11.36	-11.35	-11.34	-11.33	-11.32	-11.31	-11.3	-11.29
-11.37	-11.36	-11.35	-11.34	-11.33	-11.32	-11.31	-11.3	-11.29
-11.37	-11.36	-11.35	-11.34	-11.33	-11.32	-11.31	-11.3	-11.29
-11.37	-11.36	-11.35	-11.34	-11.33	-11.32	-11.31	-11.3	-11.29
-11.37	-11.36	-11.35	-11.34	-11.33	-11.32	-11.31	-11.3	-11.29
-11.37	-11.36	-11.35	-11.34	-11.33	-11.32	-11.31	-11.3	-11.29
-11.37	-11.36	-11.35	-11.34	-11.33	-11.32	-11.31	-11.3	-11.29
-11.37	-11.36	-11.35	-11.34	-11.33	-11.32	-11.31	-11.3	-11.29
-11.37	-11.36	-11.35	-11.34	-11.33	-11.32	-11.31	-11.3	-11.29
-11.37	-11.36	-11.35	-11.34	-11.33	-11.32	-11.31	-11.3	-11.29
-11.37	-11.36	-11.35	-11.34	-11.33	-11.32	-11.31	-11.3	-11.29
-11.37	-11.36	-11.35	-11.34	-11.33	-11.32	-11.31	-11.3	-11.29
-11.37	-11.36	-11.35	-11.34	-11.33	-11.32	-11.31	-11.3	-11.29
-11.37	-11.36	-11.35	-11.34	-11.33	-11.32	-11.31	-11.3	-11.29
-11.37	-11.36	-11.35	-11.34	-11.33	-11.32	-11.31	-11.3	-11.29
-11.37	-11.36	-11.35	-11.34	-11.33	-11.32	-11.31	-11.3	-11.29
-11.37	-11.36	-11.35	-11.34	-11.33	-11.32	-11.31	-11.3	-11.29
-11.37	-11.36	-11.35	-11.34	-11.33	-11.32	-11.31	-11.3	-11.29
-11.37	-11.36	-11.35	-11.34	-11.33	-11.32	-11.31	-11.3	-11.29
-11.37	-11.36	-11.35	-11.34	-11.33	-11.32	-11.31	-11.3	-11.29
-11.37	-11.36	-11.35	-11.34	-11.33	-11.32	-11.31	-11.3	-11.29
-11.37	-11.36	-11.35	-11.34	-11.33	-11.32	-11.31	-11.3	-11.29
-11.37	-11.36	-11.35	-11.34	-11.33	-11.32	-11.31	-11.3	-11.29
-11.37	-11.36	-11.35	-11.34	-11.33	-11.32	-11.31	-11.3	-11.29
-11.37	-11.36	-11.35	-11.34	-11.33	-11.32	-11.31	-11.3	-11.29
-11.37	-11.36	-11.35	-11.34	-11.33	-11.32	-11.31	-11.3	-11.29
-11.37	-11.36	-11.35	-11.34	-11.33	-11.32	-11.31	-11.3	-11.29
-11.37	-11.36	-11.35	-11.34	-11.33	-11.32	-11.31	-11.3	-11.29
-11.37	-11.36	-11.35	-11.34	-11.33	-11.32	-11.31	-11.3	-11.29
-11.37	-11.36	-11.35	-11.34	-11.33	-11.32	-11.31	-11.3	-11.29
-11.37	-11.36	-11.35	-11.34	-11.33	-11.32	-11.31	-11.3	-11.29
-11.37	-11.36	-11.35	-11.34	-11.33	-11.32	-11.31	-11.3	-11.29
-11.37	-11.36	-11.35	-11.34	-11.33	-11.32	-11.31	-11.3	-11.29
-11.37	-11.36	-11.35	-11.34	-11.33	-11.32	-11.31	-11.3	-11.29
-11.37	-11.36	-11.35	-11.34	-11.33	-11.32	-11.31	-11.3	-11.29
-11.37	-11.36	-11.35	-11.34	-11.33	-11.32	-11.31	-11.3	-11.29
-11.37	-11.36	-11.35	-11.34	-11.33	-11.32	-11.31	-11.3	-11.29

-11.37	-11.36	-11.35	-11.34	-11.33	-11.32	-11.31	-11.3	-11.29
-11.37	-11.36	-11.35	-11.34	-11.33	-11.32	-11.31	-11.3	-11.29
-11.37	-11.36	-11.35	-11.34	-11.33	-11.32	-11.31	-11.3	-11.29
-11.37	-11.36	-11.35	-11.34	-11.33	-11.32	-11.31	-11.3	-11.29
-11.37	-11.36	-11.35	-11.34	-11.33	-11.32	-11.31	-11.3	-11.29
-11.37	-11.36	-11.35	-11.34	-11.33	-11.32	-11.31	-11.3	-11.29
-11.37	-11.36	-11.35	-11.34	-11.33	-11.32	-11.31	-11.3	-11.29
-11.37	-11.36	-11.35	-11.34	-11.33	-11.32	-11.31	-11.3	-11.29
-11.37	-11.36	-11.35	-11.34	-11.33	-11.32	-11.31	-11.3	-11.29
-11.37	-11.36	-11.35	-11.34	-11.33	-11.32	-11.31	-11.3	-11.29
-11.37	-11.36	-11.35	-11.34	-11.33	-11.32	-11.31	-11.3	-11.29
-11.37	-11.36	-11.35	-11.34	-11.33	-11.32	-11.31	-11.3	-11.29
-11.37	-11.36	-11.35	-11.34	-11.33	-11.32	-11.31	-11.3	-11.29
-11.37	-11.36	-11.35	-11.34	-11.33	-11.32	-11.31	-11.3	-11.29
-11.37	-11.36	-11.35	-11.34	-11.33	-11.32	-11.31	-11.3	-11.29
-11.37	-11.36	-11.35	-11.34	-11.33	-11.32	-11.31	-11.3	-11.29
-11.37	-11.36	-11.35	-11.34	-11.33	-11.32	-11.31	-11.3	-11.29
-11.37	-11.36	-11.35	-11.34	-11.33	-11.32	-11.31	-11.3	-11.29
-11.37	-11.36	-11.35	-11.34	-11.33	-11.32	-11.31	-11.3	-11.29
-11.37	-11.36	-11.35	-11.34	-11.33	-11.32	-11.31	-11.3	-11.29
-11.37	-11.36	-11.35	-11.34	-11.33	-11.32	-11.31	-11.3	-11.29
-11.37	-11.36	-11.35	-11.34	-11.33	-11.32	-11.31	-11.3	-11.29
-11.37	-11.36	-11.35	-11.34	-11.33	-11.32	-11.31	-11.3	-11.29
-11.37	-11.36	-11.35	-11.34	-11.33	-11.32	-11.31	-11.3	-11.29
-11.37	-11.36	-11.35	-11.34	-11.33	-11.32	-11.31	-11.3	-11.29
-11.37	-11.36	-11.35	-11.34	-11.33	-11.32	-11.31	-11.3	-11.29
-11.37	-11.36	-11.35	-11.34	-11.33	-11.32	-11.31	-11.3	-11.29
-11.37	-11.36	-11.35	-11.34	-11.33	-11.32	-11.31	-11.3	-11.29
-11.37	-11.36	-11.35	-11.34	-11.33	-11.32	-11.31	-11.3	-11.29
-11.37	-11.36	-11.35	-11.34	-11.33	-11.32	-11.31	-11.3	-11.29
-11.37	-11.36	-11.35	-11.34	-11.33	-11.32	-11.31	-11.3	-11.29
-11.37	-11.36	-11.35	-11.34	-11.33	-11.32	-11.31	-11.3	-11.29
-11.37	-11.36	-11.35	-11.34	-11.33	-11.32	-11.31	-11.3	-11.29
-11.37	-11.36	-11.35	-11.34	-11.33	-11.32	-11.31	-11.3	-11.29
-11.37	-11.36	-11.35	-11.34	-11.33	-11.32	-11.31	-11.3	-11.29
-11.37	-11.36	-11.35	-11.34	-11.33	-11.32	-11.31	-11.3	-11.29
-11.37	-11.36	-11.35	-11.34	-11.33	-11.32	-11.31	-11.3	-11.29
-11.37	-11.36	-11.35	-11.34	-11.33	-11.32	-11.31	-11.3	-11.29
-11.37	-11.36	-11.35	-11.34	-11.33	-11.32	-11.31	-11.3	-11.29
-11.37	-11.36	-11.35	-11.34	-11.33	-11.32	-11.31	-11.3	-11.29
-11.37	-11.36	-11.35	-11.34	-11.33	-11.32	-11.31	-11.3	-11.29
-11.37	-11.36	-11.35	-11.34	-11.33	-11.32	-11.31	-11.3	-11.29
-11.37	-11.36	-11.35	-11.34	-11.33	-11.32	-11.31	-11.3	-11.29
-11.37	-11.36	-11.35	-11.34	-11.33	-11.32	-11.31	-11.3	-11.29
-11.37	-11.36	-11.35	-11.34	-11.33	-11.32	-11.31	-11.3	-11.29
-11.37	-11.36	-11.35	-11.34	-11.33	-11.32	-11.31	-11.3	-11.29
-11.37	-11.36	-11.35	-11.34	-11.33	-11.32	-11.31	-11.3	-11.29
-11.37	-11.36	-11.35	-11.34	-11.33	-11.32	-11.31	-11.3	-11.29
-11.37	-11.36	-11.35	-11.34	-11.33	-11.32	-11.31	-11.3	-11.29
-11.37	-11.36	-11.35	-11.34	-11.33	-11.32	-11.31	-11.3	-11.29

-11.37	-11.36	-11.35	-11.34	-11.33	-11.32	-11.31	-11.3	-11.29
-11.37	-11.36	-11.35	-11.34	-11.33	-11.32	-11.31	-11.3	-11.29
-11.37	-11.36	-11.35	-11.34	-11.33	-11.32	-11.31	-11.3	-11.29
-11.37	-11.36	-11.35	-11.34	-11.33	-11.32	-11.31	-11.3	-11.29
-11.37	-11.36	-11.35	-11.34	-11.33	-11.32	-11.31	-11.3	-11.29
-11.37	-11.36	-11.35	-11.34	-11.33	-11.32	-11.31	-11.3	-11.29
-11.37	-11.36	-11.35	-11.34	-11.33	-11.32	-11.31	-11.3	-11.29
-11.37	-11.36	-11.35	-11.34	-11.33	-11.32	-11.31	-11.3	-11.29
-11.37	-11.36	-11.35	-11.34	-11.33	-11.32	-11.31	-11.3	-11.29
-11.37	-11.36	-11.35	-11.34	-11.33	-11.32	-11.31	-11.3	-11.29
-11.37	-11.36	-11.35	-11.34	-11.33	-11.32	-11.31	-11.3	-11.29
-11.37	-11.36	-11.35	-11.34	-11.33	-11.32	-11.31	-11.3	-11.29
-11.37	-11.36	-11.35	-11.34	-11.33	-11.32	-11.31	-11.3	-11.29
-11.37	-11.36	-11.35	-11.34	-11.33	-11.32	-11.31	-11.3	-11.29
-11.37	-11.36	-11.35	-11.34	-11.33	-11.32	-11.31	-11.3	-11.29
-11.37	-11.36	-11.35	-11.34	-11.33	-11.32	-11.31	-11.3	-11.29
-11.37	-11.36	-11.35	-11.34	-11.33	-11.32	-11.31	-11.3	-11.29
-11.37	-11.36	-11.35	-11.34	-11.33	-11.32	-11.31	-11.3	-11.29
-11.37	-11.36	-11.35	-11.34	-11.33	-11.32	-11.31	-11.3	-11.29
-11.37	-11.36	-11.35	-11.34	-11.33	-11.32	-11.31	-11.3	-11.29
-11.37	-11.36	-11.35	-11.34	-11.33	-11.32	-11.31	-11.3	-11.29
-11.37	-11.36	-11.35	-11.34	-11.33	-11.32	-11.31	-11.3	-11.29
-11.37	-11.36	-11.35	-11.34	-11.33	-11.32	-11.31	-11.3	-11.29
-11.37	-11.36	-11.35	-11.34	-11.33	-11.32	-11.31	-11.3	-11.29
-11.37	-11.36	-11.35	-11.34	-11.33	-11.32	-11.31	-11.3	-11.29
-11.37	-11.36	-11.35	-11.34	-11.33	-11.32	-11.31	-11.3	-11.29
-11.37	-11.36	-11.35	-11.34	-11.33	-11.32	-11.31	-11.3	-11.29
-11.37	-11.36	-11.35	-11.34	-11.33	-11.32	-11.31	-11.3	-11.29
-11.37	-11.36	-11.35	-11.34	-11.33	-11.32	-11.31	-11.3	-11.29
-11.37	-11.36	-11.35	-11.34	-11.33	-11.32	-11.31	-11.3	-11.29
-11.37	-11.36	-11.35	-11.34	-11.33	-11.32	-11.31	-11.3	-11.29
-11.37	-11.36	-11.35	-11.34	-11.33	-11.32	-11.31	-11.3	-11.29
-11.37	-11.36	-11.35	-11.34	-11.33	-11.32	-11.31	-11.3	-11.29
-11.37	-11.36	-11.35	-11.34	-11.33	-11.32	-11.31	-11.3	-11.29
-11.37	-11.36	-11.35	-11.34	-11.33	-11.32	-11.31	-11.3	-11.29
-11.37	-11.36	-11.35	-11.34	-11.33	-11.32	-11.31	-11.3	-11.29
-11.37	-11.36	-11.35	-11.34	-11.33	-11.32	-11.31	-11.3	-11.29
-11.37	-11.36	-11.35	-11.34	-11.33	-11.32	-11.31	-11.3	-11.29
-11.37	-11.36	-11.35	-11.34	-11.33	-11.32	-11.31	-11.3	-11.29
-11.37	-11.36	-11.35	-11.34	-11.33	-11.32	-11.31	-11.3	-11.29
-11.37	-11.36	-11.35	-11.34	-11.33	-11.32	-11.31	-11.3	-11.29
-11.37	-11.36	-11.35	-11.34	-11.33	-11.32	-11.31	-11.3	-11.29
-11.37	-11.36	-11.35	-11.34	-11.33	-11.32	-11.31	-11.3	-11.29
-11.37	-11.36	-11.35	-11.34	-11.33	-11.32	-11.31	-11.3	-11.29
-11.37	-11.36	-11.35	-11.34	-11.33	-11.32	-11.31	-11.3	-11.29
-11.37	-11.36	-11.35	-11.34	-11.33	-11.32	-11.31	-11.3	-11.29
-11.37	-11.36	-11.35	-11.34	-11.33	-11.32	-11.31	-11.3	-11.29
-11.37	-11.36	-11.35	-11.34	-11.33	-11.32	-11.31	-11.3	-11.29
-11.37	-11.36	-11.35	-11.34	-11.33	-11.32	-11.31	-11.3	-11.29
-11.37	-11.36	-11.35	-11.34	-11.33	-11.32	-11.31	-11.3	-11.29

-11.37	-11.36	-11.35	-11.34	-11.33	-11.32	-11.31	-11.3	-11.29
-11.37	-11.36	-11.35	-11.34	-11.33	-11.32	-11.31	-11.3	-11.29
-11.37	-11.36	-11.35	-11.34	-11.33	-11.32	-11.31	-11.3	-11.29
-11.37	-11.36	-11.35	-11.34	-11.33	-11.32	-11.31	-11.3	-11.29
-11.37	-11.36	-11.35	-11.34	-11.33	-11.32	-11.31	-11.3	-11.29
-11.37	-11.36	-11.35	-11.34	-11.33	-11.32	-11.31	-11.3	-11.29
-11.37	-11.36	-11.35	-11.34	-11.33	-11.32	-11.31	-11.3	-11.29
-11.37	-11.36	-11.35	-11.34	-11.33	-11.32	-11.31	-11.3	-11.29
-11.37	-11.36	-11.35	-11.34	-11.33	-11.32	-11.31	-11.3	-11.29
-11.37	-11.36	-11.35	-11.34	-11.33	-11.32	-11.31	-11.3	-11.29
-11.37	-11.36	-11.35	-11.34	-11.33	-11.32	-11.31	-11.3	-11.29
-11.37	-11.36	-11.35	-11.34	-11.33	-11.32	-11.31	-11.3	-11.29
-11.37	-11.36	-11.35	-11.34	-11.33	-11.32	-11.31	-11.3	-11.29
-11.37	-11.36	-11.35	-11.34	-11.33	-11.32	-11.31	-11.3	-11.29
-11.37	-11.36	-11.35	-11.34	-11.33	-11.32	-11.31	-11.3	-11.29
-11.37	-11.36	-11.35	-11.34	-11.33	-11.32	-11.31	-11.3	-11.29
-11.37	-11.36	-11.35	-11.34	-11.33	-11.32	-11.31	-11.3	-11.29
-11.37	-11.36	-11.35	-11.34	-11.33	-11.32	-11.31	-11.3	-11.29
-11.37	-11.36	-11.35	-11.34	-11.33	-11.32	-11.31	-11.3	-11.29
-11.37	-11.36	-11.35	-11.34	-11.33	-11.32	-11.31	-11.3	-11.29
-11.37	-11.36	-11.35	-11.34	-11.33	-11.32	-11.31	-11.3	-11.29
-11.37	-11.36	-11.35	-11.34	-11.33	-11.32	-11.31	-11.3	-11.29
-11.37	-11.36	-11.35	-11.34	-11.33	-11.32	-11.31	-11.3	-11.29
-11.37	-11.36	-11.35	-11.34	-11.33	-11.32	-11.31	-11.3	-11.29
-11.37	-11.36	-11.35	-11.34	-11.33	-11.32	-11.31	-11.3	-11.29
-11.37	-11.36	-11.35	-11.34	-11.33	-11.32	-11.31	-11.3	-11.29
-11.37	-11.36	-11.35	-11.34	-11.33	-11.32	-11.31	-11.3	-11.29
-11.37	-11.36	-11.35	-11.34	-11.33	-11.32	-11.31	-11.3	-11.29
-11.37	-11.36	-11.35	-11.34	-11.33	-11.32	-11.31	-11.3	-11.29
-11.37	-11.36	-11.35	-11.34	-11.33	-11.32	-11.31	-11.3	-11.29
-11.37	-11.36	-11.35	-11.34	-11.33	-11.32	-11.31	-11.3	-11.29
-11.37	-11.36	-11.35	-11.34	-11.33	-11.32	-11.31	-11.3	-11.29
-11.37	-11.36	-11.35	-11.34	-11.33	-11.32	-11.31	-11.3	-11.29
-11.37	-11.36	-11.35	-11.34	-11.33	-11.32	-11.31	-11.3	-11.29
-11.37	-11.36	-11.35	-11.34	-11.33	-11.32	-11.31	-11.3	-11.29
-11.37	-11.36	-11.35	-11.34	-11.33	-11.32	-11.31	-11.3	-11.29
-11.37	-11.36	-11.35	-11.34	-11.33	-11.32	-11.31	-11.3	-11.29
-11.37	-11.36	-11.35	-11.34	-11.33	-11.32	-11.31	-11.3	-11.29
-11.37	-11.36	-11.35	-11.34	-11.33	-11.32	-11.31	-11.3	-11.29
-11.37	-11.36	-11.35	-11.34	-11.33	-11.32	-11.31	-11.3	-11.29
-11.37	-11.36	-11.35	-11.34	-11.33	-11.32	-11.31	-11.3	-11.29
-11.37	-11.36	-11.35	-11.34	-11.33	-11.32	-11.31	-11.3	-11.29
-11.37	-11.36	-11.35	-11.34	-11.33	-11.32	-11.31	-11.3	-11.29
-11.37	-11.36	-11.35	-11.34	-11.33	-11.32	-11.31	-11.3	-11.29
-11.37	-11.36	-11.35	-11.34	-11.33	-11.32	-11.31	-11.3	-11.29
-11.37	-11.36	-11.35	-11.34	-11.33	-11.32	-11.31	-11.3	-11.29
-11.37	-11.36	-11.35	-11.34	-11.33	-11.32	-11.31	-11.3	-11.29
-11.37	-11.36	-11.35	-11.34	-11.33	-11.32	-11.31	-11.3	-11.29
-11.37	-11.36	-11.35	-11.34	-11.33	-11.32	-11.31	-11.3	-11.29
-11.37	-11.36	-11.35	-11.34	-11.33	-11.32	-11.31	-11.3	-11.29

-11.37	-11.36	-11.35	-11.34	-11.33	-11.32	-11.31	-11.3	-11.29
-11.37	-11.36	-11.35	-11.34	-11.33	-11.32	-11.31	-11.3	-11.29
-11.37	-11.36	-11.35	-11.34	-11.33	-11.32	-11.31	-11.3	-11.29
-11.37	-11.36	-11.35	-11.34	-11.33	-11.32	-11.31	-11.3	-11.29
-11.37	-11.36	-11.35	-11.34	-11.33	-11.32	-11.31	-11.3	-11.29
-11.37	-11.36	-11.35	-11.34	-11.33	-11.32	-11.31	-11.3	-11.29
-11.37	-11.36	-11.35	-11.34	-11.33	-11.32	-11.31	-11.3	-11.29
-11.37	-11.36	-11.35	-11.34	-11.33	-11.32	-11.31	-11.3	-11.29
-11.37	-11.36	-11.35	-11.34	-11.33	-11.32	-11.31	-11.3	-11.29
-11.37	-11.36	-11.35	-11.34	-11.33	-11.32	-11.31	-11.3	-11.29
-11.37	-11.36	-11.35	-11.34	-11.33	-11.32	-11.31	-11.3	-11.29
-11.37	-11.36	-11.35	-11.34	-11.33	-11.32	-11.31	-11.3	-11.29
-11.37	-11.36	-11.35	-11.34	-11.33	-11.32	-11.31	-11.3	-11.29
-11.37	-11.36	-11.35	-11.34	-11.33	-11.32	-11.31	-11.3	-11.29
-11.37	-11.36	-11.35	-11.34	-11.33	-11.32	-11.31	-11.3	-11.29
-11.37	-11.36	-11.35	-11.34	-11.33	-11.32	-11.31	-11.3	-11.29
-11.37	-11.36	-11.35	-11.34	-11.33	-11.32	-11.31	-11.3	-11.29
-11.37	-11.36	-11.35	-11.34	-11.33	-11.32	-11.31	-11.3	-11.29
-11.37	-11.36	-11.35	-11.34	-11.33	-11.32	-11.31	-11.3	-11.29
-11.37	-11.36	-11.35	-11.34	-11.33	-11.32	-11.31	-11.3	-11.29
-11.37	-11.36	-11.35	-11.34	-11.33	-11.32	-11.31	-11.3	-11.29
-11.37	-11.36	-11.35	-11.34	-11.33	-11.32	-11.31	-11.3	-11.29
-11.37	-11.36	-11.35	-11.34	-11.33	-11.32	-11.31	-11.3	-11.29
-11.37	-11.36	-11.35	-11.34	-11.33	-11.32	-11.31	-11.3	-11.29
-11.37	-11.36	-11.35	-11.34	-11.33	-11.32	-11.31	-11.3	-11.29
-11.37	-11.36	-11.35	-11.34	-11.33	-11.32	-11.31	-11.3	-11.29
-11.37	-11.36	-11.35	-11.34	-11.33	-11.32	-11.31	-11.3	-11.29
-11.37	-11.36	-11.35	-11.34	-11.33	-11.32	-11.31	-11.3	-11.29
-11.37	-11.36	-11.35	-11.34	-11.33	-11.32	-11.31	-11.3	-11.29
-11.37	-11.36	-11.35	-11.34	-11.33	-11.32	-11.31	-11.3	-11.29
-11.37	-11.36	-11.35	-11.34	-11.33	-11.32	-11.31	-11.3	-11.29
-11.37	-11.36	-11.35	-11.34	-11.33	-11.32	-11.31	-11.3	-11.29
-11.37	-11.36	-11.35	-11.34	-11.33	-11.32	-11.31	-11.3	-11.29
-11.37	-11.36	-11.35	-11.34	-11.33	-11.32	-11.31	-11.3	-11.29
-11.37	-11.36	-11.35	-11.34	-11.33	-11.32	-11.31	-11.3	-11.29
-11.37	-11.36	-11.35	-11.34	-11.33	-11.32	-11.31	-11.3	-11.29
-11.37	-11.36	-11.35	-11.34	-11.33	-11.32	-11.31	-11.3	-11.29
-11.37	-11.36	-11.35	-11.34	-11.33	-11.32	-11.31	-11.3	-11.29
-11.37	-11.36	-11.35	-11.34	-11.33	-11.32	-11.31	-11.3	-11.29
-11.37	-11.36	-11.35	-11.34	-11.33	-11.32	-11.31	-11.3	-11.29
-11.37	-11.36	-11.35	-11.34	-11.33	-11.32	-11.31	-11.3	-11.29
-11.37	-11.36	-11.35	-11.34	-11.33	-11.32	-11.31	-11.3	-11.29
-11.37	-11.36	-11.35	-11.34	-11.33	-11.32	-11.31	-11.3	-11.29
-11.37	-11.36	-11.35	-11.34	-11.33	-11.32	-11.31	-11.3	-11.29
-11.37	-11.36	-11.35	-11.34	-11.33	-11.32	-11.31	-11.3	-11.29
-11.37	-11.36	-11.35	-11.34	-11.33	-11.32	-11.31	-11.3	-11.29
-11.37	-11.36	-11.35	-11.34	-11.33	-11.32	-11.31	-11.3	-11.29
-11.37	-11.36	-11.35	-11.34	-11.33	-11.32	-11.31	-11.3	-11.29
-11.37	-11.36	-11.35	-11.34	-11.33	-11.32	-11.31	-11.3	-11.29
-11.37	-11.36	-11.35	-11.34	-11.33	-11.32	-11.31	-11.3	-11.29

-11.37	-11.36	-11.35	-11.34	-11.33	-11.32	-11.31	-11.3	-11.29
-11.37	-11.36	-11.35	-11.34	-11.33	-11.32	-11.31	-11.3	-11.29
-11.37	-11.36	-11.35	-11.34	-11.33	-11.32	-11.31	-11.3	-11.29
-11.37	-11.36	-11.35	-11.34	-11.33	-11.32	-11.31	-11.3	-11.29
-11.37	-11.36	-11.35	-11.34	-11.33	-11.32	-11.31	-11.3	-11.29
-11.37	-11.36	-11.35	-11.34	-11.33	-11.32	-11.31	-11.3	-11.29
-11.37	-11.36	-11.35	-11.34	-11.33	-11.32	-11.31	-11.3	-11.29
-11.37	-11.36	-11.35	-11.34	-11.33	-11.32	-11.31	-11.3	-11.29
-11.37	-11.36	-11.35	-11.34	-11.33	-11.32	-11.31	-11.3	-11.29
-11.37	-11.36	-11.35	-11.34	-11.33	-11.32	-11.31	-11.3	-11.29
-11.37	-11.36	-11.35	-11.34	-11.33	-11.32	-11.31	-11.3	-11.29
-11.37	-11.36	-11.35	-11.34	-11.33	-11.32	-11.31	-11.3	-11.29
-11.37	-11.36	-11.35	-11.34	-11.33	-11.32	-11.31	-11.3	-11.29
-11.37	-11.36	-11.35	-11.34	-11.33	-11.32	-11.31	-11.3	-11.29
-11.37	-11.36	-11.35	-11.34	-11.33	-11.32	-11.31	-11.3	-11.29
-11.37	-11.36	-11.35	-11.34	-11.33	-11.32	-11.31	-11.3	-11.29
-11.37	-11.36	-11.35	-11.34	-11.33	-11.32	-11.31	-11.3	-11.29
-11.37	-11.36	-11.35	-11.34	-11.33	-11.32	-11.31	-11.3	-11.29
-11.37	-11.36	-11.35	-11.34	-11.33	-11.32	-11.31	-11.3	-11.29
-11.37	-11.36	-11.35	-11.34	-11.33	-11.32	-11.31	-11.3	-11.29
-11.37	-11.36	-11.35	-11.34	-11.33	-11.32	-11.31	-11.3	-11.29
-11.37	-11.36	-11.35	-11.34	-11.33	-11.32	-11.31	-11.3	-11.29
-11.37	-11.36	-11.35	-11.34	-11.33	-11.32	-11.31	-11.3	-11.29
-11.37	-11.36	-11.35	-11.34	-11.33	-11.32	-11.31	-11.3	-11.29
-11.37	-11.36	-11.35	-11.34	-11.33	-11.32	-11.31	-11.3	-11.29
-11.37	-11.36	-11.35	-11.34	-11.33	-11.32	-11.31	-11.3	-11.29
-11.37	-11.36	-11.35	-11.34	-11.33	-11.32	-11.31	-11.3	-11.29
-11.37	-11.36	-11.35	-11.34	-11.33	-11.32	-11.31	-11.3	-11.29
-11.37	-11.36	-11.35	-11.34	-11.33	-11.32	-11.31	-11.3	-11.29
-11.37	-11.36	-11.35	-11.34	-11.33	-11.32	-11.31	-11.3	-11.29
-11.37	-11.36	-11.35	-11.34	-11.33	-11.32	-11.31	-11.3	-11.29
-11.37	-11.36	-11.35	-11.34	-11.33	-11.32	-11.31	-11.3	-11.29
-11.37	-11.36	-11.35	-11.34	-11.33	-11.32	-11.31	-11.3	-11.29
-11.37	-11.36	-11.35	-11.34	-11.33	-11.32	-11.31	-11.3	-11.29
-11.37	-11.36	-11.35	-11.34	-11.33	-11.32	-11.31	-11.3	-11.29
-11.37	-11.36	-11.35	-11.34	-11.33	-11.32	-11.31	-11.3	-11.29
-11.37	-11.36	-11.35	-11.34	-11.33	-11.32	-11.31	-11.3	-11.29
-11.37	-11.36	-11.35	-11.34	-11.33	-11.32	-11.31	-11.3	-11.29
-11.37	-11.36	-11.35	-11.34	-11.33	-11.32	-11.31	-11.3	-11.29
-11.37	-11.36	-11.35	-11.34	-11.33	-11.32	-11.31	-11.3	-11.29
-11.37	-11.36	-11.35	-11.34	-11.33	-11.32	-11.31	-11.3	-11.29
-11.37	-11.36	-11.35	-11.34	-11.33	-11.32	-11.31	-11.3	-11.29
-11.37	-11.36	-11.35	-11.34	-11.33	-11.32	-11.31	-11.3	-11.29
-11.37	-11.36	-11.35	-11.34	-11.33	-11.32	-11.31	-11.3	-11.29
-11.37	-11.36	-11.35	-11.34	-11.33	-11.32	-11.31	-11.3	-11.29
-11.37	-11.36	-11.35	-11.34	-11.33	-11.32	-11.31	-11.3	-11.29
-11.37	-11.36	-11.35	-11.34	-11.33	-11.32	-11.31	-11.3	-11.29
-11.37	-11.36	-11.35	-11.34	-11.33	-11.32	-11.31	-11.3	-11.29
-11.37	-11.36	-11.35	-11.34	-11.33	-11.32	-11.31	-11.3	-11.29
-11.37	-11.36	-11.35	-11.34	-11.33	-11.32	-11.31	-11.3	-11.29

-11.37	-11.36	-11.35	-11.34	-11.33	-11.32	-11.31	-11.3	-11.29
-11.37	-11.36	-11.35	-11.34	-11.33	-11.32	-11.31	-11.3	-11.29
-11.37	-11.36	-11.35	-11.34	-11.33	-11.32	-11.31	-11.3	-11.29
-11.37	-11.36	-11.35	-11.34	-11.33	-11.32	-11.31	-11.3	-11.29
-11.37	-11.36	-11.35	-11.34	-11.33	-11.32	-11.31	-11.3	-11.29
-11.37	-11.36	-11.35	-11.34	-11.33	-11.32	-11.31	-11.3	-11.29
-11.37	-11.36	-11.35	-11.34	-11.33	-11.32	-11.31	-11.3	-11.29
-11.37	-11.36	-11.35	-11.34	-11.33	-11.32	-11.31	-11.3	-11.29
-11.37	-11.36	-11.35	-11.34	-11.33	-11.32	-11.31	-11.3	-11.29
-11.37	-11.36	-11.35	-11.34	-11.33	-11.32	-11.31	-11.3	-11.29
-11.37	-11.36	-11.35	-11.34	-11.33	-11.32	-11.31	-11.3	-11.29
-11.37	-11.36	-11.35	-11.34	-11.33	-11.32	-11.31	-11.3	-11.29
-11.37	-11.36	-11.35	-11.34	-11.33	-11.32	-11.31	-11.3	-11.29
-11.37	-11.36	-11.35	-11.34	-11.33	-11.32	-11.31	-11.3	-11.29
-11.37	-11.36	-11.35	-11.34	-11.33	-11.32	-11.31	-11.3	-11.29
-11.37	-11.36	-11.35	-11.34	-11.33	-11.32	-11.31	-11.3	-11.29
-11.37	-11.36	-11.35	-11.34	-11.33	-11.32	-11.31	-11.3	-11.29
-11.37	-11.36	-11.35	-11.34	-11.33	-11.32	-11.31	-11.3	-11.29
-11.37	-11.36	-11.35	-11.34	-11.33	-11.32	-11.31	-11.3	-11.29
-11.37	-11.36	-11.35	-11.34	-11.33	-11.32	-11.31	-11.3	-11.29
-11.37	-11.36	-11.35	-11.34	-11.33	-11.32	-11.31	-11.3	-11.29
-11.37	-11.36	-11.35	-11.34	-11.33	-11.32	-11.31	-11.3	-11.29
-11.37	-11.36	-11.35	-11.34	-11.33	-11.32	-11.31	-11.3	-11.29
-11.37	-11.36	-11.35	-11.34	-11.33	-11.32	-11.31	-11.3	-11.29
-11.37	-11.36	-11.35	-11.34	-11.33	-11.32	-11.31	-11.3	-11.29
-11.37	-11.36	-11.35	-11.34	-11.33	-11.32	-11.31	-11.3	-11.29
-11.37	-11.36	-11.35	-11.34	-11.33	-11.32	-11.31	-11.3	-11.29
-11.37	-11.36	-11.35	-11.34	-11.33	-11.32	-11.31	-11.3	-11.29
-11.37	-11.36	-11.35	-11.34	-11.33	-11.32	-11.31	-11.3	-11.29
-11.37	-11.36	-11.35	-11.34	-11.33	-11.32	-11.31	-11.3	-11.29
-11.37	-11.36	-11.35	-11.34	-11.33	-11.32	-11.31	-11.3	-11.29
-11.37	-11.36	-11.35	-11.34	-11.33	-11.32	-11.31	-11.3	-11.29
-11.37	-11.36	-11.35	-11.34	-11.33	-11.32	-11.31	-11.3	-11.29
-11.37	-11.36	-11.35	-11.34	-11.33	-11.32	-11.31	-11.3	-11.29
-11.37	-11.36	-11.35	-11.34	-11.33	-11.32	-11.31	-11.3	-11.29
-11.37	-11.36	-11.35	-11.34	-11.33	-11.32	-11.31	-11.3	-11.29
-11.37	-11.36	-11.35	-11.34	-11.33	-11.32	-11.31	-11.3	-11.29
-11.37	-11.36	-11.35	-11.34	-11.33	-11.32	-11.31	-11.3	-11.29
-11.37	-11.36	-11.35	-11.34	-11.33	-11.32	-11.31	-11.3	-11.29
-11.37	-11.36	-11.35	-11.34	-11.33	-11.32	-11.31	-11.3	-11.29
-11.37	-11.36	-11.35	-11.34	-11.33	-11.32	-11.31	-11.3	-11.29
-11.37	-11.36	-11.35	-11.34	-11.33	-11.32	-11.31	-11.3	-11.29
-11.37	-11.36	-11.35	-11.34	-11.33	-11.32	-11.31	-11.3	-11.29
-11.37	-11.36	-11.35	-11.34	-11.33	-11.32	-11.31	-11.3	-11.29
-11.37	-11.36	-11.35	-11.34	-11.33	-11.32	-11.31	-11.3	-11.29
-11.37	-11.36	-11.35	-11.34	-11.33	-11.32	-11.31	-11.3	-11.29
-11.37	-11.36	-11.35	-11.34	-11.33	-11.32	-11.31	-11.3	-11.29
-11.37	-11.36	-11.35	-11.34	-11.33	-11.32	-11.31	-11.3	-11.29
-11.37	-11.36	-11.35	-11.34	-11.33	-11.32	-11.31	-11.3	-11.29
-11.37	-11.36	-11.35	-11.34	-11.33	-11.32	-11.31	-11.3	-11.29

-11.37	-11.36	-11.35	-11.34	-11.33	-11.32	-11.31	-11.3	-11.29
-11.37	-11.36	-11.35	-11.34	-11.33	-11.32	-11.31	-11.3	-11.29
-11.37	-11.36	-11.35	-11.34	-11.33	-11.32	-11.31	-11.3	-11.29
-11.37	-11.36	-11.35	-11.34	-11.33	-11.32	-11.31	-11.3	-11.29
-11.37	-11.36	-11.35	-11.34	-11.33	-11.32	-11.31	-11.3	-11.29
-11.37	-11.36	-11.35	-11.34	-11.33	-11.32	-11.31	-11.3	-11.29
-11.37	-11.36	-11.35	-11.34	-11.33	-11.32	-11.31	-11.3	-11.29
-11.37	-11.36	-11.35	-11.34	-11.33	-11.32	-11.31	-11.3	-11.29
-11.37	-11.36	-11.35	-11.34	-11.33	-11.32	-11.31	-11.3	-11.29
-11.37	-11.36	-11.35	-11.34	-11.33	-11.32	-11.31	-11.3	-11.29
-11.37	-11.36	-11.35	-11.34	-11.33	-11.32	-11.31	-11.3	-11.29
-11.37	-11.36	-11.35	-11.34	-11.33	-11.32	-11.31	-11.3	-11.29
-11.37	-11.36	-11.35	-11.34	-11.33	-11.32	-11.31	-11.3	-11.29
-11.37	-11.36	-11.35	-11.34	-11.33	-11.32	-11.31	-11.3	-11.29
-11.37	-11.36	-11.35	-11.34	-11.33	-11.32	-11.31	-11.3	-11.29
-11.37	-11.36	-11.35	-11.34	-11.33	-11.32	-11.31	-11.3	-11.29
-11.37	-11.36	-11.35	-11.34	-11.33	-11.32	-11.31	-11.3	-11.29
-11.37	-11.36	-11.35	-11.34	-11.33	-11.32	-11.31	-11.3	-11.29
-11.37	-11.36	-11.35	-11.34	-11.33	-11.32	-11.31	-11.3	-11.29
-11.37	-11.36	-11.35	-11.34	-11.33	-11.32	-11.31	-11.3	-11.29
-11.37	-11.36	-11.35	-11.34	-11.33	-11.32	-11.31	-11.3	-11.29
-11.37	-11.36	-11.35	-11.34	-11.33	-11.32	-11.31	-11.3	-11.29
-11.37	-11.36	-11.35	-11.34	-11.33	-11.32	-11.31	-11.3	-11.29
-11.37	-11.36	-11.35	-11.34	-11.33	-11.32	-11.31	-11.3	-11.29
-11.37	-11.36	-11.35	-11.34	-11.33	-11.32	-11.31	-11.3	-11.29
-11.37	-11.36	-11.35	-11.34	-11.33	-11.32	-11.31	-11.3	-11.29
-11.37	-11.36	-11.35	-11.34	-11.33	-11.32	-11.31	-11.3	-11.29
-11.37	-11.36	-11.35	-11.34	-11.33	-11.32	-11.31	-11.3	-11.29
-11.37	-11.36	-11.35	-11.34	-11.33	-11.32	-11.31	-11.3	-11.29
-11.37	-11.36	-11.35	-11.34	-11.33	-11.32	-11.31	-11.3	-11.29
-11.37	-11.36	-11.35	-11.34	-11.33	-11.32	-11.31	-11.3	-11.29
-11.37	-11.36	-11.35	-11.34	-11.33	-11.32	-11.31	-11.3	-11.29
-11.37	-11.36	-11.35	-11.34	-11.33	-11.32	-11.31	-11.3	-11.29
-11.37	-11.36	-11.35	-11.34	-11.33	-11.32	-11.31	-11.3	-11.29
-11.37	-11.36	-11.35	-11.34	-11.33	-11.32	-11.31	-11.3	-11.29
-11.37	-11.36	-11.35	-11.34	-11.33	-11.32	-11.31	-11.3	-11.29
-11.37	-11.36	-11.35	-11.34	-11.33	-11.32	-11.31	-11.3	-11.29
-11.37	-11.36	-11.35	-11.34	-11.33	-11.32	-11.31	-11.3	-11.29
-11.37	-11.36	-11.35	-11.34	-11.33	-11.32	-11.31	-11.3	-11.29
-11.37	-11.36	-11.35	-11.34	-11.33	-11.32	-11.31	-11.3	-11.29
-11.37	-11.36	-11.35	-11.34	-11.33	-11.32	-11.31	-11.3	-11.29
-11.37	-11.36	-11.35	-11.34	-11.33	-11.32	-11.31	-11.3	-11.29
-11.37	-11.36	-11.35	-11.34	-11.33	-11.32	-11.31	-11.3	-11.29
-11.37	-11.36	-11.35	-11.34	-11.33	-11.32	-11.31	-11.3	-11.29
-11.37	-11.36	-11.35	-11.34	-11.33	-11.32	-11.31	-11.3	-11.29
-11.37	-11.36	-11.35	-11.34	-11.33	-11.32	-11.31	-11.3	-11.29
-11.37	-11.36	-11.35	-11.34	-11.33	-11.32	-11.31	-11.3	-11.29
-11.37	-11.36	-11.35	-11.34	-11.33	-11.32	-11.31	-11.3	-11.29
-11.37	-11.36	-11.35	-11.34	-11.33	-11.32	-11.31	-11.3	-11.29
-11.37	-11.36	-11.35	-11.34	-11.33	-11.32	-11.31	-11.3	-11.29

-11.37	-11.36	-11.35	-11.34	-11.33	-11.32	-11.31	-11.3	-11.29
-11.37	-11.36	-11.35	-11.34	-11.33	-11.32	-11.31	-11.3	-11.29
-11.37	-11.36	-11.35	-11.34	-11.33	-11.32	-11.31	-11.3	-11.29
-11.37	-11.36	-11.35	-11.34	-11.33	-11.32	-11.31	-11.3	-11.29
-11.37	-11.36	-11.35	-11.34	-11.33	-11.32	-11.31	-11.3	-11.29
-11.37	-11.36	-11.35	-11.34	-11.33	-11.32	-11.31	-11.3	-11.29
-11.37	-11.36	-11.35	-11.34	-11.33	-11.32	-11.31	-11.3	-11.29
-11.37	-11.36	-11.35	-11.34	-11.33	-11.32	-11.31	-11.3	-11.29
-11.37	-11.36	-11.35	-11.34	-11.33	-11.32	-11.31	-11.3	-11.29
-11.37	-11.36	-11.35	-11.34	-11.33	-11.32	-11.31	-11.3	-11.29
-11.37	-11.36	-11.35	-11.34	-11.33	-11.32	-11.31	-11.3	-11.29
-11.37	-11.36	-11.35	-11.34	-11.33	-11.32	-11.31	-11.3	-11.29
-11.37	-11.36	-11.35	-11.34	-11.33	-11.32	-11.31	-11.3	-11.29
-11.37	-11.36	-11.35	-11.34	-11.33	-11.32	-11.31	-11.3	-11.29
-11.37	-11.36	-11.35	-11.34	-11.33	-11.32	-11.31	-11.3	-11.29
-11.37	-11.36	-11.35	-11.34	-11.33	-11.32	-11.31	-11.3	-11.29
-11.37	-11.36	-11.35	-11.34	-11.33	-11.32	-11.31	-11.3	-11.29
-11.37	-11.36	-11.35	-11.34	-11.33	-11.32	-11.31	-11.3	-11.29
-11.37	-11.36	-11.35	-11.34	-11.33	-11.32	-11.31	-11.3	-11.29
-11.37	-11.36	-11.35	-11.34	-11.33	-11.32	-11.31	-11.3	-11.29
-11.37	-11.36	-11.35	-11.34	-11.33	-11.32	-11.31	-11.3	-11.29
-11.37	-11.36	-11.35	-11.34	-11.33	-11.32	-11.31	-11.3	-11.29
-11.37	-11.36	-11.35	-11.34	-11.33	-11.32	-11.31	-11.3	-11.29
-11.37	-11.36	-11.35	-11.34	-11.33	-11.32	-11.31	-11.3	-11.29
-11.37	-11.36	-11.35	-11.34	-11.33	-11.32	-11.31	-11.3	-11.29
-11.37	-11.36	-11.35	-11.34	-11.33	-11.32	-11.31	-11.3	-11.29
-11.37	-11.36	-11.35	-11.34	-11.33	-11.32	-11.31	-11.3	-11.29
-11.37	-11.36	-11.35	-11.34	-11.33	-11.32	-11.31	-11.3	-11.29
-11.37	-11.36	-11.35	-11.34	-11.33	-11.32	-11.31	-11.3	-11.29
-11.37	-11.36	-11.35	-11.34	-11.33	-11.32	-11.31	-11.3	-11.29
-11.37	-11.36	-11.35	-11.34	-11.33	-11.32	-11.31	-11.3	-11.29
-11.37	-11.36	-11.35	-11.34	-11.33	-11.32	-11.31	-11.3	-11.29
-11.37	-11.36	-11.35	-11.34	-11.33	-11.32	-11.31	-11.3	-11.29
-11.37	-11.36	-11.35	-11.34	-11.33	-11.32	-11.31	-11.3	-11.29
-11.37	-11.36	-11.35	-11.34	-11.33	-11.32	-11.31	-11.3	-11.29
-11.37	-11.36	-11.35	-11.34	-11.33	-11.32	-11.31	-11.3	-11.29
-11.37	-11.36	-11.35	-11.34	-11.33	-11.32	-11.31	-11.3	-11.29
-11.37	-11.36	-11.35	-11.34	-11.33	-11.32	-11.31	-11.3	-11.29
-11.37	-11.36	-11.35	-11.34	-11.33	-11.32	-11.31	-11.3	-11.29
-11.37	-11.36	-11.35	-11.34	-11.33	-11.32	-11.31	-11.3	-11.29
-11.37	-11.36	-11.35	-11.34	-11.33	-11.32	-11.31	-11.3	-11.29
-11.37	-11.36	-11.35	-11.34	-11.33	-11.32	-11.31	-11.3	-11.29
-11.37	-11.36	-11.35	-11.34	-11.33	-11.32	-11.31	-11.3	-11.29
-11.37	-11.36	-11.35	-11.34	-11.33	-11.32	-11.31	-11.3	-11.29
-11.37	-11.36	-11.35	-11.34	-11.33	-11.32	-11.31	-11.3	-11.29
-11.37	-11.36	-11.35	-11.34	-11.33	-11.32	-11.31	-11.3	-11.29
-11.37	-11.36	-11.35	-11.34	-11.33	-11.32	-11.31	-11.3	-11.29
-11.37	-11.36	-11.35	-11.34	-11.33	-11.32	-11.31	-11.3	-11.29
-11.37	-11.36	-11.35	-11.34	-11.33	-11.32	-11.31	-11.3	-11.29
-11.37	-11.36	-11.35	-11.34	-11.33	-11.32	-11.31	-11.3	-11.29

-11.37	-11.36	-11.35	-11.34	-11.33	-11.32	-11.31	-11.3	-11.29
-11.37	-11.36	-11.35	-11.34	-11.33	-11.32	-11.31	-11.3	-11.29
-11.37	-11.36	-11.35	-11.34	-11.33	-11.32	-11.31	-11.3	-11.29
-11.37	-11.36	-11.35	-11.34	-11.33	-11.32	-11.31	-11.3	-11.29
-11.37	-11.36	-11.35	-11.34	-11.33	-11.32	-11.31	-11.3	-11.29
-11.37	-11.36	-11.35	-11.34	-11.33	-11.32	-11.31	-11.3	-11.29
-11.37	-11.36	-11.35	-11.34	-11.33	-11.32	-11.31	-11.3	-11.29
-11.37	-11.36	-11.35	-11.34	-11.33	-11.32	-11.31	-11.3	-11.29
-11.37	-11.36	-11.35	-11.34	-11.33	-11.32	-11.31	-11.3	-11.29
-11.37	-11.36	-11.35	-11.34	-11.33	-11.32	-11.31	-11.3	-11.29
-11.37	-11.36	-11.35	-11.34	-11.33	-11.32	-11.31	-11.3	-11.29
-11.37	-11.36	-11.35	-11.34	-11.33	-11.32	-11.31	-11.3	-11.29
-11.37	-11.36	-11.35	-11.34	-11.33	-11.32	-11.31	-11.3	-11.29
-11.37	-11.36	-11.35	-11.34	-11.33	-11.32	-11.31	-11.3	-11.29
-11.37	-11.36	-11.35	-11.34	-11.33	-11.32	-11.31	-11.3	-11.29
-11.37	-11.36	-11.35	-11.34	-11.33	-11.32	-11.31	-11.3	-11.29
-11.37	-11.36	-11.35	-11.34	-11.33	-11.32	-11.31	-11.3	-11.29
-11.37	-11.36	-11.35	-11.34	-11.33	-11.32	-11.31	-11.3	-11.29
-11.37	-11.36	-11.35	-11.34	-11.33	-11.32	-11.31	-11.3	-11.29
-11.37	-11.36	-11.35	-11.34	-11.33	-11.32	-11.31	-11.3	-11.29
-11.37	-11.36	-11.35	-11.34	-11.33	-11.32	-11.31	-11.3	-11.29
-11.37	-11.36	-11.35	-11.34	-11.33	-11.32	-11.31	-11.3	-11.29
-11.37	-11.36	-11.35	-11.34	-11.33	-11.32	-11.31	-11.3	-11.29
-11.37	-11.36	-11.35	-11.34	-11.33	-11.32	-11.31	-11.3	-11.29
-11.37	-11.36	-11.35	-11.34	-11.33	-11.32	-11.31	-11.3	-11.29
-11.37	-11.36	-11.35	-11.34	-11.33	-11.32	-11.31	-11.3	-11.29
-11.37	-11.36	-11.35	-11.34	-11.33	-11.32	-11.31	-11.3	-11.29
-11.37	-11.36	-11.35	-11.34	-11.33	-11.32	-11.31	-11.3	-11.29
-11.37	-11.36	-11.35	-11.34	-11.33	-11.32	-11.31	-11.3	-11.29
-11.37	-11.36	-11.35	-11.34	-11.33	-11.32	-11.31	-11.3	-11.29
-11.37	-11.36	-11.35	-11.34	-11.33	-11.32	-11.31	-11.3	-11.29
-11.37	-11.36	-11.35	-11.34	-11.33	-11.32	-11.31	-11.3	-11.29
-11.37	-11.36	-11.35	-11.34	-11.33	-11.32	-11.31	-11.3	-11.29
-11.37	-11.36	-11.35	-11.34	-11.33	-11.32	-11.31	-11.3	-11.29
-11.37	-11.36	-11.35	-11.34	-11.33	-11.32	-11.31	-11.3	-11.29
-11.37	-11.36	-11.35	-11.34	-11.33	-11.32	-11.31	-11.3	-11.29
-11.37	-11.36	-11.35	-11.34	-11.33	-11.32	-11.31	-11.3	-11.29
-11.37	-11.36	-11.35	-11.34	-11.33	-11.32	-11.31	-11.3	-11.29
-11.37	-11.36	-11.35	-11.34	-11.33	-11.32	-11.31	-11.3	-11.29
-11.37	-11.36	-11.35	-11.34	-11.33	-11.32	-11.31	-11.3	-11.29
-11.37	-11.36	-11.35	-11.34	-11.33	-11.32	-11.31	-11.3	-11.29
-11.37	-11.36	-11.35	-11.34	-11.33	-11.32	-11.31	-11.3	-11.29
-11.37	-11.36	-11.35	-11.34	-11.33	-11.32	-11.31	-11.3	-11.29
-11.37	-11.36	-11.35	-11.34	-11.33	-11.32	-11.31	-11.3	-11.29
-11.37	-11.36	-11.35	-11.34	-11.33	-11.32	-11.31	-11.3	-11.29
-11.37	-11.36	-11.35	-11.34	-11.33	-11.32	-11.31	-11.3	-11.29
-11.37	-11.36	-11.35	-11.34	-11.33	-11.32	-11.31	-11.3	-11.29
-11.37	-11.36	-11.35	-11.34	-11.33	-11.32	-11.31	-11.3	-11.29
-11.37	-11.36	-11.35	-11.34	-11.33	-11.32	-11.31	-11.3	-11.29
-11.37	-11.36	-11.35	-11.34	-11.33	-11.32	-11.31	-11.3	-11.29

-11.37	-11.36	-11.35	-11.34	-11.33	-11.32	-11.31	-11.3	-11.29
-11.37	-11.36	-11.35	-11.34	-11.33	-11.32	-11.31	-11.3	-11.29
-11.37	-11.36	-11.35	-11.34	-11.33	-11.32	-11.31	-11.3	-11.29
-11.37	-11.36	-11.35	-11.34	-11.33	-11.32	-11.31	-11.3	-11.29
-11.37	-11.36	-11.35	-11.34	-11.33	-11.32	-11.31	-11.3	-11.29
-11.37	-11.36	-11.35	-11.34	-11.33	-11.32	-11.31	-11.3	-11.29
-11.37	-11.36	-11.35	-11.34	-11.33	-11.32	-11.31	-11.3	-11.29
-11.37	-11.36	-11.35	-11.34	-11.33	-11.32	-11.31	-11.3	-11.29
-11.37	-11.36	-11.35	-11.34	-11.33	-11.32	-11.31	-11.3	-11.29
-11.37	-11.36	-11.35	-11.34	-11.33	-11.32	-11.31	-11.3	-11.29
-11.37	-11.36	-11.35	-11.34	-11.33	-11.32	-11.31	-11.3	-11.29
-11.37	-11.36	-11.35	-11.34	-11.33	-11.32	-11.31	-11.3	-11.29
-11.37	-11.36	-11.35	-11.34	-11.33	-11.32	-11.31	-11.3	-11.29
-11.37	-11.36	-11.35	-11.34	-11.33	-11.32	-11.31	-11.3	-11.29
-11.37	-11.36	-11.35	-11.34	-11.33	-11.32	-11.31	-11.3	-11.29
-11.37	-11.36	-11.35	-11.34	-11.33	-11.32	-11.31	-11.3	-11.29
-11.37	-11.36	-11.35	-11.34	-11.33	-11.32	-11.31	-11.3	-11.29
-11.37	-11.36	-11.35	-11.34	-11.33	-11.32	-11.31	-11.3	-11.29
-11.37	-11.36	-11.35	-11.34	-11.33	-11.32	-11.31	-11.3	-11.29
-11.37	-11.36	-11.35	-11.34	-11.33	-11.32	-11.31	-11.3	-11.29
-11.37	-11.36	-11.35	-11.34	-11.33	-11.32	-11.31	-11.3	-11.29
-11.37	-11.36	-11.35	-11.34	-11.33	-11.32	-11.31	-11.3	-11.29
-11.37	-11.36	-11.35	-11.34	-11.33	-11.32	-11.31	-11.3	-11.29
-11.37	-11.36	-11.35	-11.34	-11.33	-11.32	-11.31	-11.3	-11.29
-11.37	-11.36	-11.35	-11.34	-11.33	-11.32	-11.31	-11.3	-11.29
-11.37	-11.36	-11.35	-11.34	-11.33	-11.32	-11.31	-11.3	-11.29
-11.37	-11.36	-11.35	-11.34	-11.33	-11.32	-11.31	-11.3	-11.29
-11.37	-11.36	-11.35	-11.34	-11.33	-11.32	-11.31	-11.3	-11.29
-11.37	-11.36	-11.35	-11.34	-11.33	-11.32	-11.31	-11.3	-11.29
-11.37	-11.36	-11.35	-11.34	-11.33	-11.32	-11.31	-11.3	-11.29
-11.37	-11.36	-11.35	-11.34	-11.33	-11.32	-11.31	-11.3	-11.29
-11.37	-11.36	-11.35	-11.34	-11.33	-11.32	-11.31	-11.3	-11.29
-11.37	-11.36	-11.35	-11.34	-11.33	-11.32	-11.31	-11.3	-11.29
-11.37	-11.36	-11.35	-11.34	-11.33	-11.32	-11.31	-11.3	-11.29
-11.37	-11.36	-11.35	-11.34	-11.33	-11.32	-11.31	-11.3	-11.29
-11.37	-11.36	-11.35	-11.34	-11.33	-11.32	-11.31	-11.3	-11.29
-11.37	-11.36	-11.35	-11.34	-11.33	-11.32	-11.31	-11.3	-11.29
-11.37	-11.36	-11.35	-11.34	-11.33	-11.32	-11.31	-11.3	-11.29
-11.37	-11.36	-11.35	-11.34	-11.33	-11.32	-11.31	-11.3	-11.29
-11.37	-11.36	-11.35	-11.34	-11.33	-11.32	-11.31	-11.3	-11.29
-11.37	-11.36	-11.35	-11.34	-11.33	-11.32	-11.31	-11.3	-11.29
-11.37	-11.36	-11.35	-11.34	-11.33	-11.32	-11.31	-11.3	-11.29
-11.37	-11.36	-11.35	-11.34	-11.33	-11.32	-11.31	-11.3	-11.29
-11.37	-11.36	-11.35	-11.34	-11.33	-11.32	-11.31	-11.3	-11.29
-11.37	-11.36	-11.35	-11.34	-11.33	-11.32	-11.31	-11.3	-11.29
-11.37	-11.36	-11.35	-11.34	-11.33	-11.32	-11.31	-11.3	-11.29
-11.37	-11.36	-11.35	-11.34	-11.33	-11.32	-11.31	-11.3	-11.29
-11.37	-11.36	-11.35	-11.34	-11.33	-11.32	-11.31	-11.3	-11.29
-11.37	-11.36	-11.35	-11.34	-11.33	-11.32	-11.31	-11.3	-11.29
-11.37	-11.36	-11.35	-11.34	-11.33	-11.32	-11.31	-11.3	-11.29

-11.37	-11.36	-11.35	-11.34	-11.33	-11.32	-11.31	-11.3	-11.29
-11.37	-11.36	-11.35	-11.34	-11.33	-11.32	-11.31	-11.3	-11.29
-11.37	-11.36	-11.35	-11.34	-11.33	-11.32	-11.31	-11.3	-11.29
-11.37	-11.36	-11.35	-11.34	-11.33	-11.32	-11.31	-11.3	-11.29
-11.37	-11.36	-11.35	-11.34	-11.33	-11.32	-11.31	-11.3	-11.29
-11.37	-11.36	-11.35	-11.34	-11.33	-11.32	-11.31	-11.3	-11.29
-11.37	-11.36	-11.35	-11.34	-11.33	-11.32	-11.31	-11.3	-11.29
-11.37	-11.36	-11.35	-11.34	-11.33	-11.32	-11.31	-11.3	-11.29
-11.37	-11.36	-11.35	-11.34	-11.33	-11.32	-11.31	-11.3	-11.29
-11.37	-11.36	-11.35	-11.34	-11.33	-11.32	-11.31	-11.3	-11.29
-11.37	-11.36	-11.35	-11.34	-11.33	-11.32	-11.31	-11.3	-11.29
-11.37	-11.36	-11.35	-11.34	-11.33	-11.32	-11.31	-11.3	-11.29
-11.37	-11.36	-11.35	-11.34	-11.33	-11.32	-11.31	-11.3	-11.29
-11.37	-11.36	-11.35	-11.34	-11.33	-11.32	-11.31	-11.3	-11.29
-11.37	-11.36	-11.35	-11.34	-11.33	-11.32	-11.31	-11.3	-11.29
-11.37	-11.36	-11.35	-11.34	-11.33	-11.32	-11.31	-11.3	-11.29
-11.37	-11.36	-11.35	-11.34	-11.33	-11.32	-11.31	-11.3	-11.29
-11.37	-11.36	-11.35	-11.34	-11.33	-11.32	-11.31	-11.3	-11.29
-11.37	-11.36	-11.35	-11.34	-11.33	-11.32	-11.31	-11.3	-11.29
-11.37	-11.36	-11.35	-11.34	-11.33	-11.32	-11.31	-11.3	-11.29
-11.37	-11.36	-11.35	-11.34	-11.33	-11.32	-11.31	-11.3	-11.29
-11.37	-11.36	-11.35	-11.34	-11.33	-11.32	-11.31	-11.3	-11.29
-11.37	-11.36	-11.35	-11.34	-11.33	-11.32	-11.31	-11.3	-11.29
-11.37	-11.36	-11.35	-11.34	-11.33	-11.32	-11.31	-11.3	-11.29
-11.37	-11.36	-11.35	-11.34	-11.33	-11.32	-11.31	-11.3	-11.29
-11.37	-11.36	-11.35	-11.34	-11.33	-11.32	-11.31	-11.3	-11.29
-11.37	-11.36	-11.35	-11.34	-11.33	-11.32	-11.31	-11.3	-11.29
-11.37	-11.36	-11.35	-11.34	-11.33	-11.32	-11.31	-11.3	-11.29
-11.37	-11.36	-11.35	-11.34	-11.33	-11.32	-11.31	-11.3	-11.29
-11.37	-11.36	-11.35	-11.34	-11.33	-11.32	-11.31	-11.3	-11.29
-11.37	-11.36	-11.35	-11.34	-11.33	-11.32	-11.31	-11.3	-11.29
-11.37	-11.36	-11.35	-11.34	-11.33	-11.32	-11.31	-11.3	-11.29
-11.37	-11.36	-11.35	-11.34	-11.33	-11.32	-11.31	-11.3	-11.29
-11.37	-11.36	-11.35	-11.34	-11.33	-11.32	-11.31	-11.3	-11.29
-11.37	-11.36	-11.35	-11.34	-11.33	-11.32	-11.31	-11.3	-11.29
-11.37	-11.36	-11.35	-11.34	-11.33	-11.32	-11.31	-11.3	-11.29
-11.37	-11.36	-11.35	-11.34	-11.33	-11.32	-11.31	-11.3	-11.29
-11.37	-11.36	-11.35	-11.34	-11.33	-11.32	-11.31	-11.3	-11.29
-11.37	-11.36	-11.35	-11.34	-11.33	-11.32	-11.31	-11.3	-11.29
-11.37	-11.36	-11.35	-11.34	-11.33	-11.32	-11.31	-11.3	-11.29
-11.37	-11.36	-11.35	-11.34	-11.33	-11.32	-11.31	-11.3	-11.29
-11.37	-11.36	-11.35	-11.34	-11.33	-11.32	-11.31	-11.3	-11.29
-11.37	-11.36	-11.35	-11.34	-11.33	-11.32	-11.31	-11.3	-11.29
-11.37	-11.36	-11.35	-11.34	-11.33	-11.32	-11.31	-11.3	-11.29
-11.37	-11.36	-11.35	-11.34	-11.33	-11.32	-11.31	-11.3	-11.29
-11.37	-11.36	-11.35	-11.34	-11.33	-11.32	-11.31	-11.3	-11.29
-11.37	-11.36	-11.35	-11.34	-11.33	-11.32	-11.31	-11.3	-11.29
-11.37	-11.36	-11.35	-11.34	-11.33	-11.32	-11.31	-11.3	-11.29
-11.37	-11.36	-11.35	-11.34	-11.33	-11.32	-11.31	-11.3	-11.29
-11.37	-11.36	-11.35	-11.34	-11.33	-11.32	-11.31	-11.3	-11.29

-11.37	-11.36	-11.35	-11.34	-11.33	-11.32	-11.31	-11.3	-11.29
-11.37	-11.36	-11.35	-11.34	-11.33	-11.32	-11.31	-11.3	-11.29
-11.37	-11.36	-11.35	-11.34	-11.33	-11.32	-11.31	-11.3	-11.29
-11.37	-11.36	-11.35	-11.34	-11.33	-11.32	-11.31	-11.3	-11.29
-11.37	-11.36	-11.35	-11.34	-11.33	-11.32	-11.31	-11.3	-11.29
-11.37	-11.36	-11.35	-11.34	-11.33	-11.32	-11.31	-11.3	-11.29
-11.37	-11.36	-11.35	-11.34	-11.33	-11.32	-11.31	-11.3	-11.29
-11.37	-11.36	-11.35	-11.34	-11.33	-11.32	-11.31	-11.3	-11.29
-11.37	-11.36	-11.35	-11.34	-11.33	-11.32	-11.31	-11.3	-11.29
-11.37	-11.36	-11.35	-11.34	-11.33	-11.32	-11.31	-11.3	-11.29
-11.37	-11.36	-11.35	-11.34	-11.33	-11.32	-11.31	-11.3	-11.29
-11.37	-11.36	-11.35	-11.34	-11.33	-11.32	-11.31	-11.3	-11.29
-11.37	-11.36	-11.35	-11.34	-11.33	-11.32	-11.31	-11.3	-11.29
-11.37	-11.36	-11.35	-11.34	-11.33	-11.32	-11.31	-11.3	-11.29
-11.37	-11.36	-11.35	-11.34	-11.33	-11.32	-11.31	-11.3	-11.29
-11.37	-11.36	-11.35	-11.34	-11.33	-11.32	-11.31	-11.3	-11.29
-11.37	-11.36	-11.35	-11.34	-11.33	-11.32	-11.31	-11.3	-11.29
-11.37	-11.36	-11.35	-11.34	-11.33	-11.32	-11.31	-11.3	-11.29
-11.37	-11.36	-11.35	-11.34	-11.33	-11.32	-11.31	-11.3	-11.29
-11.37	-11.36	-11.35	-11.34	-11.33	-11.32	-11.31	-11.3	-11.29
-11.37	-11.36	-11.35	-11.34	-11.33	-11.32	-11.31	-11.3	-11.29
-11.37	-11.36	-11.35	-11.34	-11.33	-11.32	-11.31	-11.3	-11.29
-11.37	-11.36	-11.35	-11.34	-11.33	-11.32	-11.31	-11.3	-11.29
-11.37	-11.36	-11.35	-11.34	-11.33	-11.32	-11.31	-11.3	-11.29
-11.37	-11.36	-11.35	-11.34	-11.33	-11.32	-11.31	-11.3	-11.29
-11.37	-11.36	-11.35	-11.34	-11.33	-11.32	-11.31	-11.3	-11.29
-11.37	-11.36	-11.35	-11.34	-11.33	-11.32	-11.31	-11.3	-11.29
-11.37	-11.36	-11.35	-11.34	-11.33	-11.32	-11.31	-11.3	-11.29
-11.37	-11.36	-11.35	-11.34	-11.33	-11.32	-11.31	-11.3	-11.29
-11.37	-11.36	-11.35	-11.34	-11.33	-11.32	-11.31	-11.3	-11.29
-11.37	-11.36	-11.35	-11.34	-11.33	-11.32	-11.31	-11.3	-11.29
-11.37	-11.36	-11.35	-11.34	-11.33	-11.32	-11.31	-11.3	-11.29
-11.37	-11.36	-11.35	-11.34	-11.33	-11.32	-11.31	-11.3	-11.29
-11.37	-11.36	-11.35	-11.34	-11.33	-11.32	-11.31	-11.3	-11.29
-11.37	-11.36	-11.35	-11.34	-11.33	-11.32	-11.31	-11.3	-11.29
-11.37	-11.36	-11.35	-11.34	-11.33	-11.32	-11.31	-11.3	-11.29
-11.37	-11.36	-11.35	-11.34	-11.33	-11.32	-11.31	-11.3	-11.29
-11.37	-11.36	-11.35	-11.34	-11.33	-11.32	-11.31	-11.3	-11.29
-11.37	-11.36	-11.35	-11.34	-11.33	-11.32	-11.31	-11.3	-11.29
-11.37	-11.36	-11.35	-11.34	-11.33	-11.32	-11.31	-11.3	-11.29
-11.37	-11.36	-11.35	-11.34	-11.33	-11.32	-11.31	-11.3	-11.29
-11.37	-11.36	-11.35	-11.34	-11.33	-11.32	-11.31	-11.3	-11.29
-11.37	-11.36	-11.35	-11.34	-11.33	-11.32	-11.31	-11.3	-11.29
-11.37	-11.36	-11.35	-11.34	-11.33	-11.32	-11.31	-11.3	-11.29
-11.37	-11.36	-11.35	-11.34	-11.33	-11.32	-11.31	-11.3	-11.29
-11.37	-11.36	-11.35	-11.34	-11.33	-11.32	-11.31	-11.3	-11.29
-11.37	-11.36	-11.35	-11.34	-11.33	-11.32	-11.31	-11.3	-11.29
-11.37	-11.36	-11.35	-11.34	-11.33	-11.32	-11.31	-11.3	-11.29
-11.37	-11.36	-11.35	-11.34	-11.33	-11.32	-11.31	-11.3	-11.29
-11.37	-11.36	-11.35	-11.34	-11.33	-11.32	-11.31	-11.3	-11.29

-11.37	-11.36	-11.35	-11.34	-11.33	-11.32	-11.31	-11.3	-11.29
-11.37	-11.36	-11.35	-11.34	-11.33	-11.32	-11.31	-11.3	-11.29
-11.37	-11.36	-11.35	-11.34	-11.33	-11.32	-11.31	-11.3	-11.29
-11.37	-11.36	-11.35	-11.34	-11.33	-11.32	-11.31	-11.3	-11.29
-11.37	-11.36	-11.35	-11.34	-11.33	-11.32	-11.31	-11.3	-11.29
-11.37	-11.36	-11.35	-11.34	-11.33	-11.32	-11.31	-11.3	-11.29
-11.37	-11.36	-11.35	-11.34	-11.33	-11.32	-11.31	-11.3	-11.29
-11.37	-11.36	-11.35	-11.34	-11.33	-11.32	-11.31	-11.3	-11.29
-11.37	-11.36	-11.35	-11.34	-11.33	-11.32	-11.31	-11.3	-11.29
-11.37	-11.36	-11.35	-11.34	-11.33	-11.32	-11.31	-11.3	-11.29
-11.37	-11.36	-11.35	-11.34	-11.33	-11.32	-11.31	-11.3	-11.29
-11.37	-11.36	-11.35	-11.34	-11.33	-11.32	-11.31	-11.3	-11.29
-11.37	-11.36	-11.35	-11.34	-11.33	-11.32	-11.31	-11.3	-11.29
-11.37	-11.36	-11.35	-11.34	-11.33	-11.32	-11.31	-11.3	-11.29
-11.37	-11.36	-11.35	-11.34	-11.33	-11.32	-11.31	-11.3	-11.29
-11.37	-11.36	-11.35	-11.34	-11.33	-11.32	-11.31	-11.3	-11.29
-11.37	-11.36	-11.35	-11.34	-11.33	-11.32	-11.31	-11.3	-11.29
-11.37	-11.36	-11.35	-11.34	-11.33	-11.32	-11.31	-11.3	-11.29
-11.37	-11.36	-11.35	-11.34	-11.33	-11.32	-11.31	-11.3	-11.29
-11.37	-11.36	-11.35	-11.34	-11.33	-11.32	-11.31	-11.3	-11.29
-11.37	-11.36	-11.35	-11.34	-11.33	-11.32	-11.31	-11.3	-11.29
-11.37	-11.36	-11.35	-11.34	-11.33	-11.32	-11.31	-11.3	-11.29
-11.37	-11.36	-11.35	-11.34	-11.33	-11.32	-11.31	-11.3	-11.29
-11.37	-11.36	-11.35	-11.34	-11.33	-11.32	-11.31	-11.3	-11.29
-11.37	-11.36	-11.35	-11.34	-11.33	-11.32	-11.31	-11.3	-11.29
-11.37	-11.36	-11.35	-11.34	-11.33	-11.32	-11.31	-11.3	-11.29
-11.37	-11.36	-11.35	-11.34	-11.33	-11.32	-11.31	-11.3	-11.29
-11.37	-11.36	-11.35	-11.34	-11.33	-11.32	-11.31	-11.3	-11.29
-11.37	-11.36	-11.35	-11.34	-11.33	-11.32	-11.31	-11.3	-11.29
-11.37	-11.36	-11.35	-11.34	-11.33	-11.32	-11.31	-11.3	-11.29
-11.37	-11.36	-11.35	-11.34	-11.33	-11.32	-11.31	-11.3	-11.29
-11.37	-11.36	-11.35	-11.34	-11.33	-11.32	-11.31	-11.3	-11.29
-11.37	-11.36	-11.35	-11.34	-11.33	-11.32	-11.31	-11.3	-11.29
-11.37	-11.36	-11.35	-11.34	-11.33	-11.32	-11.31	-11.3	-11.29
-11.37	-11.36	-11.35	-11.34	-11.33	-11.32	-11.31	-11.3	-11.29
-11.37	-11.36	-11.35	-11.34	-11.33	-11.32	-11.31	-11.3	-11.29
-11.37	-11.36	-11.35	-11.34	-11.33	-11.32	-11.31	-11.3	-11.29
-11.37	-11.36	-11.35	-11.34	-11.33	-11.32	-11.31	-11.3	-11.29
-11.37	-11.36	-11.35	-11.34	-11.33	-11.32	-11.31	-11.3	-11.29
-11.37	-11.36	-11.35	-11.34	-11.33	-11.32	-11.31	-11.3	-11.29
-11.37	-11.36	-11.35	-11.34	-11.33	-11.32	-11.31	-11.3	-11.29
-11.37	-11.36	-11.35	-11.34	-11.33	-11.32	-11.31	-11.3	-11.29
-11.37	-11.36	-11.35	-11.34	-11.33	-11.32	-11.31	-11.3	-11.29
-11.37	-11.36	-11.35	-11.34	-11.33	-11.32	-11.31	-11.3	-11.29
-11.37	-11.36	-11.35	-11.34	-11.33	-11.32	-11.31	-11.3	-11.29
-11.37	-11.36	-11.35	-11.34	-11.33	-11.32	-11.31	-11.3	-11.29
-11.37	-11.36	-11.35	-11.34	-11.33	-11.32	-11.31	-11.3	-11.29
-11.37	-11.36	-11.35	-11.34	-11.33	-11.32	-11.31	-11.3	-11.29
-11.37	-11.36	-11.35	-11.34	-11.33	-11.32	-11.31	-11.3	-11.29
-11.37	-11.36	-11.35	-11.34	-11.33	-11.32	-11.31	-11.3	-11.29

-11.37	-11.36	-11.35	-11.34	-11.33	-11.32	-11.31	-11.3	-11.29
-11.37	-11.36	-11.35	-11.34	-11.33	-11.32	-11.31	-11.3	-11.29
-11.37	-11.36	-11.35	-11.34	-11.33	-11.32	-11.31	-11.3	-11.29
-11.37	-11.36	-11.35	-11.34	-11.33	-11.32	-11.31	-11.3	-11.29
-11.37	-11.36	-11.35	-11.34	-11.33	-11.32	-11.31	-11.3	-11.29
-11.37	-11.36	-11.35	-11.34	-11.33	-11.32	-11.31	-11.3	-11.29
-11.37	-11.36	-11.35	-11.34	-11.33	-11.32	-11.31	-11.3	-11.29
-11.37	-11.36	-11.35	-11.34	-11.33	-11.32	-11.31	-11.3	-11.29
-11.37	-11.36	-11.35	-11.34	-11.33	-11.32	-11.31	-11.3	-11.29
-11.37	-11.36	-11.35	-11.34	-11.33	-11.32	-11.31	-11.3	-11.29
-11.37	-11.36	-11.35	-11.34	-11.33	-11.32	-11.31	-11.3	-11.29
-11.37	-11.36	-11.35	-11.34	-11.33	-11.32	-11.31	-11.3	-11.29
-11.37	-11.36	-11.35	-11.34	-11.33	-11.32	-11.31	-11.3	-11.29
-11.37	-11.36	-11.35	-11.34	-11.33	-11.32	-11.31	-11.3	-11.29
-11.37	-11.36	-11.35	-11.34	-11.33	-11.32	-11.31	-11.3	-11.29
-11.37	-11.36	-11.35	-11.34	-11.33	-11.32	-11.31	-11.3	-11.29
-11.37	-11.36	-11.35	-11.34	-11.33	-11.32	-11.31	-11.3	-11.29
-11.37	-11.36	-11.35	-11.34	-11.33	-11.32	-11.31	-11.3	-11.29
-11.37	-11.36	-11.35	-11.34	-11.33	-11.32	-11.31	-11.3	-11.29
-11.37	-11.36	-11.35	-11.34	-11.33	-11.32	-11.31	-11.3	-11.29
-11.37	-11.36	-11.35	-11.34	-11.33	-11.32	-11.31	-11.3	-11.29
-11.37	-11.36	-11.35	-11.34	-11.33	-11.32	-11.31	-11.3	-11.29
-11.37	-11.36	-11.35	-11.34	-11.33	-11.32	-11.31	-11.3	-11.29
-11.37	-11.36	-11.35	-11.34	-11.33	-11.32	-11.31	-11.3	-11.29
-11.37	-11.36	-11.35	-11.34	-11.33	-11.32	-11.31	-11.3	-11.29
-11.37	-11.36	-11.35	-11.34	-11.33	-11.32	-11.31	-11.3	-11.29
-11.37	-11.36	-11.35	-11.34	-11.33	-11.32	-11.31	-11.3	-11.29
-11.37	-11.36	-11.35	-11.34	-11.33	-11.32	-11.31	-11.3	-11.29
-11.37	-11.36	-11.35	-11.34	-11.33	-11.32	-11.31	-11.3	-11.29
-11.37	-11.36	-11.35	-11.34	-11.33	-11.32	-11.31	-11.3	-11.29
-11.37	-11.36	-11.35	-11.34	-11.33	-11.32	-11.31	-11.3	-11.29
-11.37	-11.36	-11.35	-11.34	-11.33	-11.32	-11.31	-11.3	-11.29
-11.37	-11.36	-11.35	-11.34	-11.33	-11.32	-11.31	-11.3	-11.29
-11.37	-11.36	-11.35	-11.34	-11.33	-11.32	-11.31	-11.3	-11.29
-11.37	-11.36	-11.35	-11.34	-11.33	-11.32	-11.31	-11.3	-11.29
-11.37	-11.36	-11.35	-11.34	-11.33	-11.32	-11.31	-11.3	-11.29
-11.37	-11.36	-11.35	-11.34	-11.33	-11.32	-11.31	-11.3	-11.29
-11.37	-11.36	-11.35	-11.34	-11.33	-11.32	-11.31	-11.3	-11.29
-11.37	-11.36	-11.35	-11.34	-11.33	-11.32	-11.31	-11.3	-11.29
-11.37	-11.36	-11.35	-11.34	-11.33	-11.32	-11.31	-11.3	-11.29
-11.37	-11.36	-11.35	-11.34	-11.33	-11.32	-11.31	-11.3	-11.29
-11.37	-11.36	-11.35	-11.34	-11.33	-11.32	-11.31	-11.3	-11.29
-11.37	-11.36	-11.35	-11.34	-11.33	-11.32	-11.31	-11.3	-11.29
-11.37	-11.36	-11.35	-11.34	-11.33	-11.32	-11.31	-11.3	-11.29
-11.37	-11.36	-11.35	-11.34	-11.33	-11.32	-11.31	-11.3	-11.29
-11.37	-11.36	-11.35	-11.34	-11.33	-11.32	-11.31	-11.3	-11.29
-11.37	-11.36	-11.35	-11.34	-11.33	-11.32	-11.31	-11.3	-11.29
-11.37	-11.36	-11.35	-11.34	-11.33	-11.32	-11.31	-11.3	-11.29
-11.37	-11.36	-11.35	-11.34	-11.33	-11.32	-11.31	-11.3	-11.29
-11.37	-11.36	-11.35	-11.34	-11.33	-11.32	-11.31	-11.3	-11.29

-11.37	-11.36	-11.35	-11.34	-11.33	-11.32	-11.31	-11.3	-11.29
-11.37	-11.36	-11.35	-11.34	-11.33	-11.32	-11.31	-11.3	-11.29
-11.37	-11.36	-11.35	-11.34	-11.33	-11.32	-11.31	-11.3	-11.29
-11.37	-11.36	-11.35	-11.34	-11.33	-11.32	-11.31	-11.3	-11.29
-11.37	-11.36	-11.35	-11.34	-11.33	-11.32	-11.31	-11.3	-11.29
-11.37	-11.36	-11.35	-11.34	-11.33	-11.32	-11.31	-11.3	-11.29
-11.37	-11.36	-11.35	-11.34	-11.33	-11.32	-11.31	-11.3	-11.29
-11.37	-11.36	-11.35	-11.34	-11.33	-11.32	-11.31	-11.3	-11.29
-11.37	-11.36	-11.35	-11.34	-11.33	-11.32	-11.31	-11.3	-11.29
-11.37	-11.36	-11.35	-11.34	-11.33	-11.32	-11.31	-11.3	-11.29
-11.37	-11.36	-11.35	-11.34	-11.33	-11.32	-11.31	-11.3	-11.29
-11.37	-11.36	-11.35	-11.34	-11.33	-11.32	-11.31	-11.3	-11.29
-11.37	-11.36	-11.35	-11.34	-11.33	-11.32	-11.31	-11.3	-11.29
-11.37	-11.36	-11.35	-11.34	-11.33	-11.32	-11.31	-11.3	-11.29
-11.37	-11.36	-11.35	-11.34	-11.33	-11.32	-11.31	-11.3	-11.29
-11.37	-11.36	-11.35	-11.34	-11.33	-11.32	-11.31	-11.3	-11.29
-11.37	-11.36	-11.35	-11.34	-11.33	-11.32	-11.31	-11.3	-11.29
-11.37	-11.36	-11.35	-11.34	-11.33	-11.32	-11.31	-11.3	-11.29
-11.37	-11.36	-11.35	-11.34	-11.33	-11.32	-11.31	-11.3	-11.29
-11.37	-11.36	-11.35	-11.34	-11.33	-11.32	-11.31	-11.3	-11.29
-11.37	-11.36	-11.35	-11.34	-11.33	-11.32	-11.31	-11.3	-11.29
-11.37	-11.36	-11.35	-11.34	-11.33	-11.32	-11.31	-11.3	-11.29
-11.37	-11.36	-11.35	-11.34	-11.33	-11.32	-11.31	-11.3	-11.29
-11.37	-11.36	-11.35	-11.34	-11.33	-11.32	-11.31	-11.3	-11.29
-11.37	-11.36	-11.35	-11.34	-11.33	-11.32	-11.31	-11.3	-11.29
-11.37	-11.36	-11.35	-11.34	-11.33	-11.32	-11.31	-11.3	-11.29
-11.37	-11.36	-11.35	-11.34	-11.33	-11.32	-11.31	-11.3	-11.29
-11.37	-11.36	-11.35	-11.34	-11.33	-11.32	-11.31	-11.3	-11.29
-11.37	-11.36	-11.35	-11.34	-11.33	-11.32	-11.31	-11.3	-11.29
-11.37	-11.36	-11.35	-11.34	-11.33	-11.32	-11.31	-11.3	-11.29
-11.37	-11.36	-11.35	-11.34	-11.33	-11.32	-11.31	-11.3	-11.29
-11.37	-11.36	-11.35	-11.34	-11.33	-11.32	-11.31	-11.3	-11.29
-11.37	-11.36	-11.35	-11.34	-11.33	-11.32	-11.31	-11.3	-11.29
-11.37	-11.36	-11.35	-11.34	-11.33	-11.32	-11.31	-11.3	-11.29
-11.37	-11.36	-11.35	-11.34	-11.33	-11.32	-11.31	-11.3	-11.29
-11.37	-11.36	-11.35	-11.34	-11.33	-11.32	-11.31	-11.3	-11.29
-11.37	-11.36	-11.35	-11.34	-11.33	-11.32	-11.31	-11.3	-11.29
-11.37	-11.36	-11.35	-11.34	-11.33	-11.32	-11.31	-11.3	-11.29
-11.37	-11.36	-11.35	-11.34	-11.33	-11.32	-11.31	-11.3	-11.29
-11.37	-11.36	-11.35	-11.34	-11.33	-11.32	-11.31	-11.3	-11.29
-11.37	-11.36	-11.35	-11.34	-11.33	-11.32	-11.31	-11.3	-11.29
-11.37	-11.36	-11.35	-11.34	-11.33	-11.32	-11.31	-11.3	-11.29
-11.37	-11.36	-11.35	-11.34	-11.33	-11.32	-11.31	-11.3	-11.29
-11.37	-11.36	-11.35	-11.34	-11.33	-11.32	-11.31	-11.3	-11.29
-11.37	-11.36	-11.35	-11.34	-11.33	-11.32	-11.31	-11.3	-11.29
-11.37	-11.36	-11.35	-11.34	-11.33	-11.32	-11.31	-11.3	-11.29
-11.37	-11.36	-11.35	-11.34	-11.33	-11.32	-11.31	-11.3	-11.29
-11.37	-11.36	-11.35	-11.34	-11.33	-11.32	-11.31	-11.3	-11.29
-11.37	-11.36	-11.35	-11.34	-11.33	-11.32	-11.31	-11.3	-11.29
-11.37	-11.36	-11.35	-11.34	-11.33	-11.32	-11.31	-11.3	-11.29

-11.37	-11.36	-11.35	-11.34	-11.33	-11.32	-11.31	-11.3	-11.29
-11.37	-11.36	-11.35	-11.34	-11.33	-11.32	-11.31	-11.3	-11.29
-11.37	-11.36	-11.35	-11.34	-11.33	-11.32	-11.31	-11.3	-11.29
-11.37	-11.36	-11.35	-11.34	-11.33	-11.32	-11.31	-11.3	-11.29
-11.37	-11.36	-11.35	-11.34	-11.33	-11.32	-11.31	-11.3	-11.29
-11.37	-11.36	-11.35	-11.34	-11.33	-11.32	-11.31	-11.3	-11.29
-11.37	-11.36	-11.35	-11.34	-11.33	-11.32	-11.31	-11.3	-11.29
-11.37	-11.36	-11.35	-11.34	-11.33	-11.32	-11.31	-11.3	-11.29
-11.37	-11.36	-11.35	-11.34	-11.33	-11.32	-11.31	-11.3	-11.29
-11.37	-11.36	-11.35	-11.34	-11.33	-11.32	-11.31	-11.3	-11.29
-11.37	-11.36	-11.35	-11.34	-11.33	-11.32	-11.31	-11.3	-11.29
-11.37	-11.36	-11.35	-11.34	-11.33	-11.32	-11.31	-11.3	-11.29
-11.37	-11.36	-11.35	-11.34	-11.33	-11.32	-11.31	-11.3	-11.29
-11.37	-11.36	-11.35	-11.34	-11.33	-11.32	-11.31	-11.3	-11.29
-11.37	-11.36	-11.35	-11.34	-11.33	-11.32	-11.31	-11.3	-11.29
-11.37	-11.36	-11.35	-11.34	-11.33	-11.32	-11.31	-11.3	-11.29
-11.37	-11.36	-11.35	-11.34	-11.33	-11.32	-11.31	-11.3	-11.29
-11.37	-11.36	-11.35	-11.34	-11.33	-11.32	-11.31	-11.3	-11.29
-11.37	-11.36	-11.35	-11.34	-11.33	-11.32	-11.31	-11.3	-11.29
-11.37	-11.36	-11.35	-11.34	-11.33	-11.32	-11.31	-11.3	-11.29
-11.37	-11.36	-11.35	-11.34	-11.33	-11.32	-11.31	-11.3	-11.29
-11.37	-11.36	-11.35	-11.34	-11.33	-11.32	-11.31	-11.3	-11.29
-11.37	-11.36	-11.35	-11.34	-11.33	-11.32	-11.31	-11.3	-11.29
-11.37	-11.36	-11.35	-11.34	-11.33	-11.32	-11.31	-11.3	-11.29
-11.37	-11.36	-11.35	-11.34	-11.33	-11.32	-11.31	-11.3	-11.29
-11.37	-11.36	-11.35	-11.34	-11.33	-11.32	-11.31	-11.3	-11.29
-11.37	-11.36	-11.35	-11.34	-11.33	-11.32	-11.31	-11.3	-11.29
-11.37	-11.36	-11.35	-11.34	-11.33	-11.32	-11.31	-11.3	-11.29
-11.37	-11.36	-11.35	-11.34	-11.33	-11.32	-11.31	-11.3	-11.29
-11.37	-11.36	-11.35	-11.34	-11.33	-11.32	-11.31	-11.3	-11.29
-11.37	-11.36	-11.35	-11.34	-11.33	-11.32	-11.31	-11.3	-11.29
-11.37	-11.36	-11.35	-11.34	-11.33	-11.32	-11.31	-11.3	-11.29
-11.37	-11.36	-11.35	-11.34	-11.33	-11.32	-11.31	-11.3	-11.29
-11.37	-11.36	-11.35	-11.34	-11.33	-11.32	-11.31	-11.3	-11.29
-11.37	-11.36	-11.35	-11.34	-11.33	-11.32	-11.31	-11.3	-11.29
-11.37	-11.36	-11.35	-11.34	-11.33	-11.32	-11.31	-11.3	-11.29
-11.37	-11.36	-11.35	-11.34	-11.33	-11.32	-11.31	-11.3	-11.29
-11.37	-11.36	-11.35	-11.34	-11.33	-11.32	-11.31	-11.3	-11.29
-11.37	-11.36	-11.35	-11.34	-11.33	-11.32	-11.31	-11.3	-11.29
-11.37	-11.36	-11.35	-11.34	-11.33	-11.32	-11.31	-11.3	-11.29
-11.37	-11.36	-11.35	-11.34	-11.33	-11.32	-11.31	-11.3	-11.29
-11.37	-11.36	-11.35	-11.34	-11.33	-11.32	-11.31	-11.3	-11.29
-11.37	-11.36	-11.35	-11.34	-11.33	-11.32	-11.31	-11.3	-11.29
-11.37	-11.36	-11.35	-11.34	-11.33	-11.32	-11.31	-11.3	-11.29
-11.37	-11.36	-11.35	-11.34	-11.33	-11.32	-11.31	-11.3	-11.29
-11.37	-11.36	-11.35	-11.34	-11.33	-11.32	-11.31	-11.3	-11.29
-11.37	-11.36	-11.35	-11.34	-11.33	-11.32	-11.31	-11.3	-11.29
-11.37	-11.36	-11.35	-11.34	-11.33	-11.32	-11.31	-11.3	-11.29
-11.37	-11.36	-11.35	-11.34	-11.33	-11.32	-11.31	-11.3	-11.29
-11.37	-11.36	-11.35	-11.34	-11.33	-11.32	-11.31	-11.3	-11.29

-11.37	-11.36	-11.35	-11.34	-11.33	-11.32	-11.31	-11.3	-11.29
-11.37	-11.36	-11.35	-11.34	-11.33	-11.32	-11.31	-11.3	-11.29
-11.37	-11.36	-11.35	-11.34	-11.33	-11.32	-11.31	-11.3	-11.29
-11.37	-11.36	-11.35	-11.34	-11.33	-11.32	-11.31	-11.3	-11.29
-11.37	-11.36	-11.35	-11.34	-11.33	-11.32	-11.31	-11.3	-11.29
-11.37	-11.36	-11.35	-11.34	-11.33	-11.32	-11.31	-11.3	-11.29
-11.37	-11.36	-11.35	-11.34	-11.33	-11.32	-11.31	-11.3	-11.29
-11.37	-11.36	-11.35	-11.34	-11.33	-11.32	-11.31	-11.3	-11.29
-11.37	-11.36	-11.35	-11.34	-11.33	-11.32	-11.31	-11.3	-11.29
-11.37	-11.36	-11.35	-11.34	-11.33	-11.32	-11.31	-11.3	-11.29
-11.37	-11.36	-11.35	-11.34	-11.33	-11.32	-11.31	-11.3	-11.29
-11.37	-11.36	-11.35	-11.34	-11.33	-11.32	-11.31	-11.3	-11.29
-11.37	-11.36	-11.35	-11.34	-11.33	-11.32	-11.31	-11.3	-11.29
-11.37	-11.36	-11.35	-11.34	-11.33	-11.32	-11.31	-11.3	-11.29
-11.37	-11.36	-11.35	-11.34	-11.33	-11.32	-11.31	-11.3	-11.29
-11.37	-11.36	-11.35	-11.34	-11.33	-11.32	-11.31	-11.3	-11.29
-11.37	-11.36	-11.35	-11.34	-11.33	-11.32	-11.31	-11.3	-11.29
-11.37	-11.36	-11.35	-11.34	-11.33	-11.32	-11.31	-11.3	-11.29
-11.37	-11.36	-11.35	-11.34	-11.33	-11.32	-11.31	-11.3	-11.29
-11.37	-11.36	-11.35	-11.34	-11.33	-11.32	-11.31	-11.3	-11.29
-11.37	-11.36	-11.35	-11.34	-11.33	-11.32	-11.31	-11.3	-11.29
-11.37	-11.36	-11.35	-11.34	-11.33	-11.32	-11.31	-11.3	-11.29
-11.37	-11.36	-11.35	-11.34	-11.33	-11.32	-11.31	-11.3	-11.29
-11.37	-11.36	-11.35	-11.34	-11.33	-11.32	-11.31	-11.3	-11.29
-11.37	-11.36	-11.35	-11.34	-11.33	-11.32	-11.31	-11.3	-11.29
-11.37	-11.36	-11.35	-11.34	-11.33	-11.32	-11.31	-11.3	-11.29
-11.37	-11.36	-11.35	-11.34	-11.33	-11.32	-11.31	-11.3	-11.29
-11.37	-11.36	-11.35	-11.34	-11.33	-11.32	-11.31	-11.3	-11.29
-11.37	-11.36	-11.35	-11.34	-11.33	-11.32	-11.31	-11.3	-11.29
-11.37	-11.36	-11.35	-11.34	-11.33	-11.32	-11.31	-11.3	-11.29
-11.37	-11.36	-11.35	-11.34	-11.33	-11.32	-11.31	-11.3	-11.29
-11.37	-11.36	-11.35	-11.34	-11.33	-11.32	-11.31	-11.3	-11.29
-11.37	-11.36	-11.35	-11.34	-11.33	-11.32	-11.31	-11.3	-11.29
-11.37	-11.36	-11.35	-11.34	-11.33	-11.32	-11.31	-11.3	-11.29
-11.37	-11.36	-11.35	-11.34	-11.33	-11.32	-11.31	-11.3	-11.29
-11.37	-11.36	-11.35	-11.34	-11.33	-11.32	-11.31	-11.3	-11.29
-11.37	-11.36	-11.35	-11.34	-11.33	-11.32	-11.31	-11.3	-11.29
-11.37	-11.36	-11.35	-11.34	-11.33	-11.32	-11.31	-11.3	-11.29
-11.37	-11.36	-11.35	-11.34	-11.33	-11.32	-11.31	-11.3	-11.29
-11.37	-11.36	-11.35	-11.34	-11.33	-11.32	-11.31	-11.3	-11.29
-11.37	-11.36	-11.35	-11.34	-11.33	-11.32	-11.31	-11.3	-11.29
-11.37	-11.36	-11.35	-11.34	-11.33	-11.32	-11.31	-11.3	-11.29
-11.37	-11.36	-11.35	-11.34	-11.33	-11.32	-11.31	-11.3	-11.29
-11.37	-11.36	-11.35	-11.34	-11.33	-11.32	-11.31	-11.3	-11.29
-11.37	-11.36	-11.35	-11.34	-11.33	-11.32	-11.31	-11.3	-11.29
-11.37	-11.36	-11.35	-11.34	-11.33	-11.32	-11.31	-11.3	-11.29
-11.37	-11.36	-11.35	-11.34	-11.33	-11.32	-11.31	-11.3	-11.29
-11.37	-11.36	-11.35	-11.34	-11.33	-11.32	-11.31	-11.3	-11.29
-11.37	-11.36	-11.35	-11.34	-11.33	-11.32	-11.31	-11.3	-11.29
-11.37	-11.36	-11.35	-11.34	-11.33	-11.32	-11.31	-11.3	-11.29

-11.37	-11.36	-11.35	-11.34	-11.33	-11.32	-11.31	-11.3	-11.29
-11.37	-11.36	-11.35	-11.34	-11.33	-11.32	-11.31	-11.3	-11.29
-11.37	-11.36	-11.35	-11.34	-11.33	-11.32	-11.31	-11.3	-11.29
-11.37	-11.36	-11.35	-11.34	-11.33	-11.32	-11.31	-11.3	-11.29
-11.37	-11.36	-11.35	-11.34	-11.33	-11.32	-11.31	-11.3	-11.29
-11.37	-11.36	-11.35	-11.34	-11.33	-11.32	-11.31	-11.3	-11.29
-11.37	-11.36	-11.35	-11.34	-11.33	-11.32	-11.31	-11.3	-11.29
-11.37	-11.36	-11.35	-11.34	-11.33	-11.32	-11.31	-11.3	-11.29
-11.37	-11.36	-11.35	-11.34	-11.33	-11.32	-11.31	-11.3	-11.29
-11.37	-11.36	-11.35	-11.34	-11.33	-11.32	-11.31	-11.3	-11.29
-11.37	-11.36	-11.35	-11.34	-11.33	-11.32	-11.31	-11.3	-11.29
-11.37	-11.36	-11.35	-11.34	-11.33	-11.32	-11.31	-11.3	-11.29
-11.37	-11.36	-11.35	-11.34	-11.33	-11.32	-11.31	-11.3	-11.29
-11.37	-11.36	-11.35	-11.34	-11.33	-11.32	-11.31	-11.3	-11.29
-11.37	-11.36	-11.35	-11.34	-11.33	-11.32	-11.31	-11.3	-11.29
-11.37	-11.36	-11.35	-11.34	-11.33	-11.32	-11.31	-11.3	-11.29
-11.37	-11.36	-11.35	-11.34	-11.33	-11.32	-11.31	-11.3	-11.29
-11.37	-11.36	-11.35	-11.34	-11.33	-11.32	-11.31	-11.3	-11.29
-11.37	-11.36	-11.35	-11.34	-11.33	-11.32	-11.31	-11.3	-11.29
-11.37	-11.36	-11.35	-11.34	-11.33	-11.32	-11.31	-11.3	-11.29
-11.37	-11.36	-11.35	-11.34	-11.33	-11.32	-11.31	-11.3	-11.29
-11.37	-11.36	-11.35	-11.34	-11.33	-11.32	-11.31	-11.3	-11.29
-11.37	-11.36	-11.35	-11.34	-11.33	-11.32	-11.31	-11.3	-11.29
-11.37	-11.36	-11.35	-11.34	-11.33	-11.32	-11.31	-11.3	-11.29
-11.37	-11.36	-11.35	-11.34	-11.33	-11.32	-11.31	-11.3	-11.29
-11.37	-11.36	-11.35	-11.34	-11.33	-11.32	-11.31	-11.3	-11.29
-11.37	-11.36	-11.35	-11.34	-11.33	-11.32	-11.31	-11.3	-11.29
-11.37	-11.36	-11.35	-11.34	-11.33	-11.32	-11.31	-11.3	-11.29
-11.37	-11.36	-11.35	-11.34	-11.33	-11.32	-11.31	-11.3	-11.29
-11.37	-11.36	-11.35	-11.34	-11.33	-11.32	-11.31	-11.3	-11.29
-11.37	-11.36	-11.35	-11.34	-11.33	-11.32	-11.31	-11.3	-11.29
-11.37	-11.36	-11.35	-11.34	-11.33	-11.32	-11.31	-11.3	-11.29
-11.37	-11.36	-11.35	-11.34	-11.33	-11.32	-11.31	-11.3	-11.29
-11.37	-11.36	-11.35	-11.34	-11.33	-11.32	-11.31	-11.3	-11.29
-11.37	-11.36	-11.35	-11.34	-11.33	-11.32	-11.31	-11.3	-11.29
-11.37	-11.36	-11.35	-11.34	-11.33	-11.32	-11.31	-11.3	-11.29
-11.37	-11.36	-11.35	-11.34	-11.33	-11.32	-11.31	-11.3	-11.29
-11.37	-11.36	-11.35	-11.34	-11.33	-11.32	-11.31	-11.3	-11.29
-11.37	-11.36	-11.35	-11.34	-11.33	-11.32	-11.31	-11.3	-11.29
-11.37	-11.36	-11.35	-11.34	-11.33	-11.32	-11.31	-11.3	-11.29
-11.37	-11.36	-11.35	-11.34	-11.33	-11.32	-11.31	-11.3	-11.29
-11.37	-11.36	-11.35	-11.34	-11.33	-11.32	-11.31	-11.3	-11.29
-11.37	-11.36	-11.35	-11.34	-11.33	-11.32	-11.31	-11.3	-11.29
-11.37	-11.36	-11.35	-11.34	-11.33	-11.32	-11.31	-11.3	-11.29
-11.37	-11.36	-11.35	-11.34	-11.33	-11.32	-11.31	-11.3	-11.29
-11.37	-11.36	-11.35	-11.34	-11.33	-11.32	-11.31	-11.3	-11.29
-11.37	-11.36	-11.35	-11.34	-11.33	-11.32	-11.31	-11.3	-11.29
-11.37	-11.36	-11.35	-11.34	-11.33	-11.32	-11.31	-11.3	-11.29
-11.37	-11.36	-11.35	-11.34	-11.33	-11.32	-11.31	-11.3	-11.29
-11.37	-11.36	-11.35	-11.34	-11.33	-11.32	-11.31	-11.3	-11.29

-11.37	-11.36	-11.35	-11.34	-11.33	-11.32	-11.31	-11.3	-11.29
-11.37	-11.36	-11.35	-11.34	-11.33	-11.32	-11.31	-11.3	-11.29
-11.37	-11.36	-11.35	-11.34	-11.33	-11.32	-11.31	-11.3	-11.29
-11.37	-11.36	-11.35	-11.34	-11.33	-11.32	-11.31	-11.3	-11.29
-11.37	-11.36	-11.35	-11.34	-11.33	-11.32	-11.31	-11.3	-11.29
-11.37	-11.36	-11.35	-11.34	-11.33	-11.32	-11.31	-11.3	-11.29
-11.37	-11.36	-11.35	-11.34	-11.33	-11.32	-11.31	-11.3	-11.29
-11.37	-11.36	-11.35	-11.34	-11.33	-11.32	-11.31	-11.3	-11.29
-11.37	-11.36	-11.35	-11.34	-11.33	-11.32	-11.31	-11.3	-11.29
-11.37	-11.36	-11.35	-11.34	-11.33	-11.32	-11.31	-11.3	-11.29
-11.37	-11.36	-11.35	-11.34	-11.33	-11.32	-11.31	-11.3	-11.29
-11.37	-11.36	-11.35	-11.34	-11.33	-11.32	-11.31	-11.3	-11.29
-11.37	-11.36	-11.35	-11.34	-11.33	-11.32	-11.31	-11.3	-11.29
-11.37	-11.36	-11.35	-11.34	-11.33	-11.32	-11.31	-11.3	-11.29
-11.37	-11.36	-11.35	-11.34	-11.33	-11.32	-11.31	-11.3	-11.29
-11.37	-11.36	-11.35	-11.34	-11.33	-11.32	-11.31	-11.3	-11.29
-11.37	-11.36	-11.35	-11.34	-11.33	-11.32	-11.31	-11.3	-11.29
-11.37	-11.36	-11.35	-11.34	-11.33	-11.32	-11.31	-11.3	-11.29
-11.37	-11.36	-11.35	-11.34	-11.33	-11.32	-11.31	-11.3	-11.29
-11.37	-11.36	-11.35	-11.34	-11.33	-11.32	-11.31	-11.3	-11.29
-11.37	-11.36	-11.35	-11.34	-11.33	-11.32	-11.31	-11.3	-11.29
-11.37	-11.36	-11.35	-11.34	-11.33	-11.32	-11.31	-11.3	-11.29
-11.37	-11.36	-11.35	-11.34	-11.33	-11.32	-11.31	-11.3	-11.29
-11.37	-11.36	-11.35	-11.34	-11.33	-11.32	-11.31	-11.3	-11.29
-11.37	-11.36	-11.35	-11.34	-11.33	-11.32	-11.31	-11.3	-11.29
-11.37	-11.36	-11.35	-11.34	-11.33	-11.32	-11.31	-11.3	-11.29
-11.37	-11.36	-11.35	-11.34	-11.33	-11.32	-11.31	-11.3	-11.29
-11.37	-11.36	-11.35	-11.34	-11.33	-11.32	-11.31	-11.3	-11.29
-11.37	-11.36	-11.35	-11.34	-11.33	-11.32	-11.31	-11.3	-11.29
-11.37	-11.36	-11.35	-11.34	-11.33	-11.32	-11.31	-11.3	-11.29
-11.37	-11.36	-11.35	-11.34	-11.33	-11.32	-11.31	-11.3	-11.29
-11.37	-11.36	-11.35	-11.34	-11.33	-11.32	-11.31	-11.3	-11.29
-11.37	-11.36	-11.35	-11.34	-11.33	-11.32	-11.31	-11.3	-11.29
-11.37	-11.36	-11.35	-11.34	-11.33	-11.32	-11.31	-11.3	-11.29
-11.37	-11.36	-11.35	-11.34	-11.33	-11.32	-11.31	-11.3	-11.29
-11.37	-11.36	-11.35	-11.34	-11.33	-11.32	-11.31	-11.3	-11.29
-11.37	-11.36	-11.35	-11.34	-11.33	-11.32	-11.31	-11.3	-11.29
-11.37	-11.36	-11.35	-11.34	-11.33	-11.32	-11.31	-11.3	-11.29
-11.37	-11.36	-11.35	-11.34	-11.33	-11.32	-11.31	-11.3	-11.29
-11.37	-11.36	-11.35	-11.34	-11.33	-11.32	-11.31	-11.3	-11.29
-11.37	-11.36	-11.35	-11.34	-11.33	-11.32	-11.31	-11.3	-11.29
-11.37	-11.36	-11.35	-11.34	-11.33	-11.32	-11.31	-11.3	-11.29
-11.37	-11.36	-11.35	-11.34	-11.33	-11.32	-11.31	-11.3	-11.29
-11.37	-11.36	-11.35	-11.34	-11.33	-11.32	-11.31	-11.3	-11.29
-11.37	-11.36	-11.35	-11.34	-11.33	-11.32	-11.31	-11.3	-11.29
-11.37	-11.36	-11.35	-11.34	-11.33	-11.32	-11.31	-11.3	-11.29
-11.37	-11.36	-11.35	-11.34	-11.33	-11.32	-11.31	-11.3	-11.29
-11.37	-11.36	-11.35	-11.34	-11.33	-11.32	-11.31	-11.3	-11.29
-11.37	-11.36	-11.35	-11.34	-11.33	-11.32	-11.31	-11.3	-11.29
-11.37	-11.36	-11.35	-11.34	-11.33	-11.32	-11.31	-11.3	-11.29

-11.37	-11.36	-11.35	-11.34	-11.33	-11.32	-11.31	-11.3	-11.29
-11.37	-11.36	-11.35	-11.34	-11.33	-11.32	-11.31	-11.3	-11.29
-11.37	-11.36	-11.35	-11.34	-11.33	-11.32	-11.31	-11.3	-11.29
-11.37	-11.36	-11.35	-11.34	-11.33	-11.32	-11.31	-11.3	-11.29
-11.37	-11.36	-11.35	-11.34	-11.33	-11.32	-11.31	-11.3	-11.29
-11.37	-11.36	-11.35	-11.34	-11.33	-11.32	-11.31	-11.3	-11.29
-11.37	-11.36	-11.35	-11.34	-11.33	-11.32	-11.31	-11.3	-11.29
-11.37	-11.36	-11.35	-11.34	-11.33	-11.32	-11.31	-11.3	-11.29
-11.37	-11.36	-11.35	-11.34	-11.33	-11.32	-11.31	-11.3	-11.29
-11.37	-11.36	-11.35	-11.34	-11.33	-11.32	-11.31	-11.3	-11.29
-11.37	-11.36	-11.35	-11.34	-11.33	-11.32	-11.31	-11.3	-11.29
-11.37	-11.36	-11.35	-11.34	-11.33	-11.32	-11.31	-11.3	-11.29
-11.37	-11.36	-11.35	-11.34	-11.33	-11.32	-11.31	-11.3	-11.29
-11.37	-11.36	-11.35	-11.34	-11.33	-11.32	-11.31	-11.3	-11.29
-11.37	-11.36	-11.35	-11.34	-11.33	-11.32	-11.31	-11.3	-11.29
-11.37	-11.36	-11.35	-11.34	-11.33	-11.32	-11.31	-11.3	-11.29
-11.37	-11.36	-11.35	-11.34	-11.33	-11.32	-11.31	-11.3	-11.29
-11.37	-11.36	-11.35	-11.34	-11.33	-11.32	-11.31	-11.3	-11.29
-11.37	-11.36	-11.35	-11.34	-11.33	-11.32	-11.31	-11.3	-11.29
-11.37	-11.36	-11.35	-11.34	-11.33	-11.32	-11.31	-11.3	-11.29
-11.37	-11.36	-11.35	-11.34	-11.33	-11.32	-11.31	-11.3	-11.29
-11.37	-11.36	-11.35	-11.34	-11.33	-11.32	-11.31	-11.3	-11.29
-11.37	-11.36	-11.35	-11.34	-11.33	-11.32	-11.31	-11.3	-11.29
-11.37	-11.36	-11.35	-11.34	-11.33	-11.32	-11.31	-11.3	-11.29
-11.37	-11.36	-11.35	-11.34	-11.33	-11.32	-11.31	-11.3	-11.29
-11.37	-11.36	-11.35	-11.34	-11.33	-11.32	-11.31	-11.3	-11.29
-11.37	-11.36	-11.35	-11.34	-11.33	-11.32	-11.31	-11.3	-11.29
-11.37	-11.36	-11.35	-11.34	-11.33	-11.32	-11.31	-11.3	-11.29
-11.37	-11.36	-11.35	-11.34	-11.33	-11.32	-11.31	-11.3	-11.29
-11.37	-11.36	-11.35	-11.34	-11.33	-11.32	-11.31	-11.3	-11.29
-11.37	-11.36	-11.35	-11.34	-11.33	-11.32	-11.31	-11.3	-11.29
-11.37	-11.36	-11.35	-11.34	-11.33	-11.32	-11.31	-11.3	-11.29
-11.37	-11.36	-11.35	-11.34	-11.33	-11.32	-11.31	-11.3	-11.29
-11.37	-11.36	-11.35	-11.34	-11.33	-11.32	-11.31	-11.3	-11.29
-11.37	-11.36	-11.35	-11.34	-11.33	-11.32	-11.31	-11.3	-11.29
-11.37	-11.36	-11.35	-11.34	-11.33	-11.32	-11.31	-11.3	-11.29
-11.37	-11.36	-11.35	-11.34	-11.33	-11.32	-11.31	-11.3	-11.29
-11.37	-11.36	-11.35	-11.34	-11.33	-11.32	-11.31	-11.3	-11.29
-11.37	-11.36	-11.35	-11.34	-11.33	-11.32	-11.31	-11.3	-11.29
-11.37	-11.36	-11.35	-11.34	-11.33	-11.32	-11.31	-11.3	-11.29
-11.37	-11.36	-11.35	-11.34	-11.33	-11.32	-11.31	-11.3	-11.29
-11.37	-11.36	-11.35	-11.34	-11.33	-11.32	-11.31	-11.3	-11.29
-11.37	-11.36	-11.35	-11.34	-11.33	-11.32	-11.31	-11.3	-11.29
-11.37	-11.36	-11.35	-11.34	-11.33	-11.32	-11.31	-11.3	-11.29
-11.37	-11.36	-11.35	-11.34	-11.33	-11.32	-11.31	-11.3	-11.29
-11.37	-11.36	-11.35	-11.34	-11.33	-11.32	-11.31	-11.3	-11.29
-11.37	-11.36	-11.35	-11.34	-11.33	-11.32	-11.31	-11.3	-11.29
-11.37	-11.36	-11.35	-11.34	-11.33	-11.32	-11.31	-11.3	-11.29
-11.37	-11.36	-11.35	-11.34	-11.33	-11.32	-11.31	-11.3	-11.29
-11.37	-11.36	-11.35	-11.34	-11.33	-11.32	-11.31	-11.3	-11.29

-11.37	-11.36	-11.35	-11.34	-11.33	-11.32	-11.31	-11.3	-11.29
-11.37	-11.36	-11.35	-11.34	-11.33	-11.32	-11.31	-11.3	-11.29
-11.37	-11.36	-11.35	-11.34	-11.33	-11.32	-11.31	-11.3	-11.29
-11.37	-11.36	-11.35	-11.34	-11.33	-11.32	-11.31	-11.3	-11.29
-11.37	-11.36	-11.35	-11.34	-11.33	-11.32	-11.31	-11.3	-11.29
-11.37	-11.36	-11.35	-11.34	-11.33	-11.32	-11.31	-11.3	-11.29
-11.37	-11.36	-11.35	-11.34	-11.33	-11.32	-11.31	-11.3	-11.29
-11.37	-11.36	-11.35	-11.34	-11.33	-11.32	-11.31	-11.3	-11.29
-11.37	-11.36	-11.35	-11.34	-11.33	-11.32	-11.31	-11.3	-11.29
-11.37	-11.36	-11.35	-11.34	-11.33	-11.32	-11.31	-11.3	-11.29
-11.37	-11.36	-11.35	-11.34	-11.33	-11.32	-11.31	-11.3	-11.29
-11.37	-11.36	-11.35	-11.34	-11.33	-11.32	-11.31	-11.3	-11.29
-11.37	-11.36	-11.35	-11.34	-11.33	-11.32	-11.31	-11.3	-11.29
-11.37	-11.36	-11.35	-11.34	-11.33	-11.32	-11.31	-11.3	-11.29
-11.37	-11.36	-11.35	-11.34	-11.33	-11.32	-11.31	-11.3	-11.29
-11.37	-11.36	-11.35	-11.34	-11.33	-11.32	-11.31	-11.3	-11.29
-11.37	-11.36	-11.35	-11.34	-11.33	-11.32	-11.31	-11.3	-11.29
-11.37	-11.36	-11.35	-11.34	-11.33	-11.32	-11.31	-11.3	-11.29
-11.37	-11.36	-11.35	-11.34	-11.33	-11.32	-11.31	-11.3	-11.29
-11.37	-11.36	-11.35	-11.34	-11.33	-11.32	-11.31	-11.3	-11.29
-11.37	-11.36	-11.35	-11.34	-11.33	-11.32	-11.31	-11.3	-11.29
-11.37	-11.36	-11.35	-11.34	-11.33	-11.32	-11.31	-11.3	-11.29
-11.37	-11.36	-11.35	-11.34	-11.33	-11.32	-11.31	-11.3	-11.29
-11.37	-11.36	-11.35	-11.34	-11.33	-11.32	-11.31	-11.3	-11.29
-11.37	-11.36	-11.35	-11.34	-11.33	-11.32	-11.31	-11.3	-11.29
-11.37	-11.36	-11.35	-11.34	-11.33	-11.32	-11.31	-11.3	-11.29
-11.37	-11.36	-11.35	-11.34	-11.33	-11.32	-11.31	-11.3	-11.29
-11.37	-11.36	-11.35	-11.34	-11.33	-11.32	-11.31	-11.3	-11.29
-11.37	-11.36	-11.35	-11.34	-11.33	-11.32	-11.31	-11.3	-11.29
-11.37	-11.36	-11.35	-11.34	-11.33	-11.32	-11.31	-11.3	-11.29
-11.37	-11.36	-11.35	-11.34	-11.33	-11.32	-11.31	-11.3	-11.29
-11.37	-11.36	-11.35	-11.34	-11.33	-11.32	-11.31	-11.3	-11.29
-11.37	-11.36	-11.35	-11.34	-11.33	-11.32	-11.31	-11.3	-11.29
-11.37	-11.36	-11.35	-11.34	-11.33	-11.32	-11.31	-11.3	-11.29
-11.37	-11.36	-11.35	-11.34	-11.33	-11.32	-11.31	-11.3	-11.29
-11.37	-11.36	-11.35	-11.34	-11.33	-11.32	-11.31	-11.3	-11.29
-11.37	-11.36	-11.35	-11.34	-11.33	-11.32	-11.31	-11.3	-11.29
-11.37	-11.36	-11.35	-11.34	-11.33	-11.32	-11.31	-11.3	-11.29
-11.37	-11.36	-11.35	-11.34	-11.33	-11.32	-11.31	-11.3	-11.29
-11.37	-11.36	-11.35	-11.34	-11.33	-11.32	-11.31	-11.3	-11.29
-11.37	-11.36	-11.35	-11.34	-11.33	-11.32	-11.31	-11.3	-11.29
-11.37	-11.36	-11.35	-11.34	-11.33	-11.32	-11.31	-11.3	-11.29
-11.37	-11.36	-11.35	-11.34	-11.33	-11.32	-11.31	-11.3	-11.29
-11.37	-11.36	-11.35	-11.34	-11.33	-11.32	-11.31	-11.3	-11.29
-11.37	-11.36	-11.35	-11.34	-11.33	-11.32	-11.31	-11.3	-11.29
-11.37	-11.36	-11.35	-11.34	-11.33	-11.32	-11.31	-11.3	-11.29
-11.37	-11.36	-11.35	-11.34	-11.33	-11.32	-11.31	-11.3	-11.29
-11.37	-11.36	-11.35	-11.34	-11.33	-11.32	-11.31	-11.3	-11.29
-11.37	-11.36	-11.35	-11.34	-11.33	-11.32	-11.31	-11.3	-11.29
-11.37	-11.36	-11.35	-11.34	-11.33	-11.32	-11.31	-11.3	-11.29

-11.37 -11.36 -11.35 -11.34 -11.33 -11.32 -11.31 -11.3 -11.29

-11.28	-11.27	-11.26	-11.25	-11.24	-11.23	-11.22	-11.21	-11.2
-11.28	-11.27	-11.26	-11.25	-11.24	-11.23	-11.22	-11.21	-11.2
-11.28	-11.27	-11.26	-11.25	-11.24	-11.23	-11.22	-11.21	-11.2
-11.28	-11.27	-11.26	-11.25	-11.24	-11.23	-11.22	-11.21	-11.2
-11.28	-11.27	-11.26	-11.25	-11.24	-11.23	-11.22	-11.21	-11.2
-11.28	-11.27	-11.26	-11.25	-11.24	-11.23	-11.22	-11.21	-11.2
-11.28	-11.27	-11.26	-11.25	-11.24	-11.23	-11.22	-11.21	-11.2
-11.28	-11.27	-11.26	-11.25	-11.24	-11.23	-11.22	-11.21	-11.2
-11.28	-11.27	-11.26	-11.25	-11.24	-11.23	-11.22	-11.21	-11.2
-11.28	-11.27	-11.26	-11.25	-11.24	-11.23	-11.22	-11.21	-11.2
-11.28	-11.27	-11.26	-11.25	-11.24	-11.23	-11.22	-11.21	-11.2
-11.28	-11.27	-11.26	-11.25	-11.24	-11.23	-11.22	-11.21	-11.2
-11.28	-11.27	-11.26	-11.25	-11.24	-11.23	-11.22	-11.21	-11.2
-11.28	-11.27	-11.26	-11.25	-11.24	-11.23	-11.22	-11.21	-11.2
-11.28	-11.27	-11.26	-11.25	-11.24	-11.23	-11.22	-11.21	-11.2
-11.28	-11.27	-11.26	-11.25	-11.24	-11.23	-11.22	-11.21	-11.2
-11.28	-11.27	-11.26	-11.25	-11.24	-11.23	-11.22	-11.21	-11.2
-11.28	-11.27	-11.26	-11.25	-11.24	-11.23	-11.22	-11.21	-11.2
-11.28	-11.27	-11.26	-11.25	-11.24	-11.23	-11.22	-11.21	-11.2
-11.28	-11.27	-11.26	-11.25	-11.24	-11.23	-11.22	-11.21	-11.2
-11.28	-11.27	-11.26	-11.25	-11.24	-11.23	-11.22	-11.21	-11.2
-11.28	-11.27	-11.26	-11.25	-11.24	-11.23	-11.22	-11.21	-11.2
-11.28	-11.27	-11.26	-11.25	-11.24	-11.23	-11.22	-11.21	-11.2
-11.28	-11.27	-11.26	-11.25	-11.24	-11.23	-11.22	-11.21	-11.2
-11.28	-11.27	-11.26	-11.25	-11.24	-11.23	-11.22	-11.21	-11.2
-11.28	-11.27	-11.26	-11.25	-11.24	-11.23	-11.22	-11.21	-11.2
-11.28	-11.27	-11.26	-11.25	-11.24	-11.23	-11.22	-11.21	-11.2
-11.28	-11.27	-11.26	-11.25	-11.24	-11.23	-11.22	-11.21	-11.2
-11.28	-11.27	-11.26	-11.25	-11.24	-11.23	-11.22	-11.21	-11.2
-11.28	-11.27	-11.26	-11.25	-11.24	-11.23	-11.22	-11.21	-11.2
-11.28	-11.27	-11.26	-11.25	-11.24	-11.23	-11.22	-11.21	-11.2
-11.28	-11.27	-11.26	-11.25	-11.24	-11.23	-11.22	-11.21	-11.2
-11.28	-11.27	-11.26	-11.25	-11.24	-11.23	-11.22	-11.21	-11.2
-11.28	-11.27	-11.26	-11.25	-11.24	-11.23	-11.22	-11.21	-11.2
-11.28	-11.27	-11.26	-11.25	-11.24	-11.23	-11.22	-11.21	-11.2
-11.28	-11.27	-11.26	-11.25	-11.24	-11.23	-11.22	-11.21	-11.2
-11.28	-11.27	-11.26	-11.25	-11.24	-11.23	-11.22	-11.21	-11.2
-11.28	-11.27	-11.26	-11.25	-11.24	-11.23	-11.22	-11.21	-11.2
-11.28	-11.27	-11.26	-11.25	-11.24	-11.23	-11.22	-11.21	-11.2
-11.28	-11.27	-11.26	-11.25	-11.24	-11.23	-11.22	-11.21	-11.2
-11.28	-11.27	-11.26	-11.25	-11.24	-11.23	-11.22	-11.21	-11.2
-11.28	-11.27	-11.26	-11.25	-11.24	-11.23	-11.22	-11.21	-11.2
-11.28	-11.27	-11.26	-11.25	-11.24	-11.23	-11.22	-11.21	-11.2
-11.28	-11.27	-11.26	-11.25	-11.24	-11.23	-11.22	-11.21	-11.2
-11.28	-11.27	-11.26	-11.25	-11.24	-11.23	-11.22	-11.21	-11.2
-11.28	-11.27	-11.26	-11.25	-11.24	-11.23	-11.22	-11.21	-11.2
-11.28	-11.27	-11.26	-11.25	-11.24	-11.23	-11.22	-11.21	-11.2
-11.28	-11.27	-11.26	-11.25	-11.24	-11.23	-11.22	-11.21	-11.2
-11.28	-11.27	-11.26	-11.25	-11.24	-11.23	-11.22	-11.21	-11.2
-11.28	-11.27	-11.26	-11.25	-11.24	-11.23	-11.22	-11.21	-11.2

-11.28	-11.27	-11.26	-11.25	-11.24	-11.23	-11.22	-11.21	-11.2
-11.28	-11.27	-11.26	-11.25	-11.24	-11.23	-11.22	-11.21	-11.2
-11.28	-11.27	-11.26	-11.25	-11.24	-11.23	-11.22	-11.21	-11.2
-11.28	-11.27	-11.26	-11.25	-11.24	-11.23	-11.22	-11.21	-11.2
-11.28	-11.27	-11.26	-11.25	-11.24	-11.23	-11.22	-11.21	-11.2
-11.28	-11.27	-11.26	-11.25	-11.24	-11.23	-11.22	-11.21	-11.2
-11.28	-11.27	-11.26	-11.25	-11.24	-11.23	-11.22	-11.21	-11.2
-11.28	-11.27	-11.26	-11.25	-11.24	-11.23	-11.22	-11.21	-11.2
-11.28	-11.27	-11.26	-11.25	-11.24	-11.23	-11.22	-11.21	-11.2
-11.28	-11.27	-11.26	-11.25	-11.24	-11.23	-11.22	-11.21	-11.2
-11.28	-11.27	-11.26	-11.25	-11.24	-11.23	-11.22	-11.21	-11.2
-11.28	-11.27	-11.26	-11.25	-11.24	-11.23	-11.22	-11.21	-11.2
-11.28	-11.27	-11.26	-11.25	-11.24	-11.23	-11.22	-11.21	-11.2
-11.28	-11.27	-11.26	-11.25	-11.24	-11.23	-11.22	-11.21	-11.2
-11.28	-11.27	-11.26	-11.25	-11.24	-11.23	-11.22	-11.21	-11.2
-11.28	-11.27	-11.26	-11.25	-11.24	-11.23	-11.22	-11.21	-11.2
-11.28	-11.27	-11.26	-11.25	-11.24	-11.23	-11.22	-11.21	-11.2
-11.28	-11.27	-11.26	-11.25	-11.24	-11.23	-11.22	-11.21	-11.2
-11.28	-11.27	-11.26	-11.25	-11.24	-11.23	-11.22	-11.21	-11.2
-11.28	-11.27	-11.26	-11.25	-11.24	-11.23	-11.22	-11.21	-11.2
-11.28	-11.27	-11.26	-11.25	-11.24	-11.23	-11.22	-11.21	-11.2
-11.28	-11.27	-11.26	-11.25	-11.24	-11.23	-11.22	-11.21	-11.2
-11.28	-11.27	-11.26	-11.25	-11.24	-11.23	-11.22	-11.21	-11.2
-11.28	-11.27	-11.26	-11.25	-11.24	-11.23	-11.22	-11.21	-11.2
-11.28	-11.27	-11.26	-11.25	-11.24	-11.23	-11.22	-11.21	-11.2
-11.28	-11.27	-11.26	-11.25	-11.24	-11.23	-11.22	-11.21	-11.2
-11.28	-11.27	-11.26	-11.25	-11.24	-11.23	-11.22	-11.21	-11.2
-11.28	-11.27	-11.26	-11.25	-11.24	-11.23	-11.22	-11.21	-11.2
-11.28	-11.27	-11.26	-11.25	-11.24	-11.23	-11.22	-11.21	-11.2
-11.28	-11.27	-11.26	-11.25	-11.24	-11.23	-11.22	-11.21	-11.2
-11.28	-11.27	-11.26	-11.25	-11.24	-11.23	-11.22	-11.21	-11.2
-11.28	-11.27	-11.26	-11.25	-11.24	-11.23	-11.22	-11.21	-11.2
-11.28	-11.27	-11.26	-11.25	-11.24	-11.23	-11.22	-11.21	-11.2
-11.28	-11.27	-11.26	-11.25	-11.24	-11.23	-11.22	-11.21	-11.2
-11.28	-11.27	-11.26	-11.25	-11.24	-11.23	-11.22	-11.21	-11.2
-11.28	-11.27	-11.26	-11.25	-11.24	-11.23	-11.22	-11.21	-11.2
-11.28	-11.27	-11.26	-11.25	-11.24	-11.23	-11.22	-11.21	-11.2
-11.28	-11.27	-11.26	-11.25	-11.24	-11.23	-11.22	-11.21	-11.2
-11.28	-11.27	-11.26	-11.25	-11.24	-11.23	-11.22	-11.21	-11.2
-11.28	-11.27	-11.26	-11.25	-11.24	-11.23	-11.22	-11.21	-11.2
-11.28	-11.27	-11.26	-11.25	-11.24	-11.23	-11.22	-11.21	-11.2
-11.28	-11.27	-11.26	-11.25	-11.24	-11.23	-11.22	-11.21	-11.2
-11.28	-11.27	-11.26	-11.25	-11.24	-11.23	-11.22	-11.21	-11.2
-11.28	-11.27	-11.26	-11.25	-11.24	-11.23	-11.22	-11.21	-11.2
-11.28	-11.27	-11.26	-11.25	-11.24	-11.23	-11.22	-11.21	-11.2
-11.28	-11.27	-11.26	-11.25	-11.24	-11.23	-11.22	-11.21	-11.2
-11.28	-11.27	-11.26	-11.25	-11.24	-11.23	-11.22	-11.21	-11.2
-11.28	-11.27	-11.26	-11.25	-11.24	-11.23	-11.22	-11.21	-11.2
-11.28	-11.27	-11.26	-11.25	-11.24	-11.23	-11.22	-11.21	-11.2
-11.28	-11.27	-11.26	-11.25	-11.24	-11.23	-11.22	-11.21	-11.2

-11.28	-11.27	-11.26	-11.25	-11.24	-11.23	-11.22	-11.21	-11.2
-11.28	-11.27	-11.26	-11.25	-11.24	-11.23	-11.22	-11.21	-11.2
-11.28	-11.27	-11.26	-11.25	-11.24	-11.23	-11.22	-11.21	-11.2
-11.28	-11.27	-11.26	-11.25	-11.24	-11.23	-11.22	-11.21	-11.2
-11.28	-11.27	-11.26	-11.25	-11.24	-11.23	-11.22	-11.21	-11.2
-11.28	-11.27	-11.26	-11.25	-11.24	-11.23	-11.22	-11.21	-11.2
-11.28	-11.27	-11.26	-11.25	-11.24	-11.23	-11.22	-11.21	-11.2
-11.28	-11.27	-11.26	-11.25	-11.24	-11.23	-11.22	-11.21	-11.2
-11.28	-11.27	-11.26	-11.25	-11.24	-11.23	-11.22	-11.21	-11.2
-11.28	-11.27	-11.26	-11.25	-11.24	-11.23	-11.22	-11.21	-11.2
-11.28	-11.27	-11.26	-11.25	-11.24	-11.23	-11.22	-11.21	-11.2
-11.28	-11.27	-11.26	-11.25	-11.24	-11.23	-11.22	-11.21	-11.2
-11.28	-11.27	-11.26	-11.25	-11.24	-11.23	-11.22	-11.21	-11.2
-11.28	-11.27	-11.26	-11.25	-11.24	-11.23	-11.22	-11.21	-11.2
-11.28	-11.27	-11.26	-11.25	-11.24	-11.23	-11.22	-11.21	-11.2
-11.28	-11.27	-11.26	-11.25	-11.24	-11.23	-11.22	-11.21	-11.2
-11.28	-11.27	-11.26	-11.25	-11.24	-11.23	-11.22	-11.21	-11.2
-11.28	-11.27	-11.26	-11.25	-11.24	-11.23	-11.22	-11.21	-11.2
-11.28	-11.27	-11.26	-11.25	-11.24	-11.23	-11.22	-11.21	-11.2
-11.28	-11.27	-11.26	-11.25	-11.24	-11.23	-11.22	-11.21	-11.2
-11.28	-11.27	-11.26	-11.25	-11.24	-11.23	-11.22	-11.21	-11.2
-11.28	-11.27	-11.26	-11.25	-11.24	-11.23	-11.22	-11.21	-11.2
-11.28	-11.27	-11.26	-11.25	-11.24	-11.23	-11.22	-11.21	-11.2
-11.28	-11.27	-11.26	-11.25	-11.24	-11.23	-11.22	-11.21	-11.2
-11.28	-11.27	-11.26	-11.25	-11.24	-11.23	-11.22	-11.21	-11.2
-11.28	-11.27	-11.26	-11.25	-11.24	-11.23	-11.22	-11.21	-11.2
-11.28	-11.27	-11.26	-11.25	-11.24	-11.23	-11.22	-11.21	-11.2
-11.28	-11.27	-11.26	-11.25	-11.24	-11.23	-11.22	-11.21	-11.2
-11.28	-11.27	-11.26	-11.25	-11.24	-11.23	-11.22	-11.21	-11.2
-11.28	-11.27	-11.26	-11.25	-11.24	-11.23	-11.22	-11.21	-11.2
-11.28	-11.27	-11.26	-11.25	-11.24	-11.23	-11.22	-11.21	-11.2
-11.28	-11.27	-11.26	-11.25	-11.24	-11.23	-11.22	-11.21	-11.2
-11.28	-11.27	-11.26	-11.25	-11.24	-11.23	-11.22	-11.21	-11.2
-11.28	-11.27	-11.26	-11.25	-11.24	-11.23	-11.22	-11.21	-11.2
-11.28	-11.27	-11.26	-11.25	-11.24	-11.23	-11.22	-11.21	-11.2
-11.28	-11.27	-11.26	-11.25	-11.24	-11.23	-11.22	-11.21	-11.2
-11.28	-11.27	-11.26	-11.25	-11.24	-11.23	-11.22	-11.21	-11.2
-11.28	-11.27	-11.26	-11.25	-11.24	-11.23	-11.22	-11.21	-11.2
-11.28	-11.27	-11.26	-11.25	-11.24	-11.23	-11.22	-11.21	-11.2
-11.28	-11.27	-11.26	-11.25	-11.24	-11.23	-11.22	-11.21	-11.2
-11.28	-11.27	-11.26	-11.25	-11.24	-11.23	-11.22	-11.21	-11.2
-11.28	-11.27	-11.26	-11.25	-11.24	-11.23	-11.22	-11.21	-11.2
-11.28	-11.27	-11.26	-11.25	-11.24	-11.23	-11.22	-11.21	-11.2
-11.28	-11.27	-11.26	-11.25	-11.24	-11.23	-11.22	-11.21	-11.2
-11.28	-11.27	-11.26	-11.25	-11.24	-11.23	-11.22	-11.21	-11.2
-11.28	-11.27	-11.26	-11.25	-11.24	-11.23	-11.22	-11.21	-11.2
-11.28	-11.27	-11.26	-11.25	-11.24	-11.23	-11.22	-11.21	-11.2
-11.28	-11.27	-11.26	-11.25	-11.24	-11.23	-11.22	-11.21	-11.2
-11.28	-11.27	-11.26	-11.25	-11.24	-11.23	-11.22	-11.21	-11.2
-11.28	-11.27	-11.26	-11.25	-11.24	-11.23	-11.22	-11.21	-11.2

-11.28	-11.27	-11.26	-11.25	-11.24	-11.23	-11.22	-11.21	-11.2
-11.28	-11.27	-11.26	-11.25	-11.24	-11.23	-11.22	-11.21	-11.2
-11.28	-11.27	-11.26	-11.25	-11.24	-11.23	-11.22	-11.21	-11.2
-11.28	-11.27	-11.26	-11.25	-11.24	-11.23	-11.22	-11.21	-11.2
-11.28	-11.27	-11.26	-11.25	-11.24	-11.23	-11.22	-11.21	-11.2
-11.28	-11.27	-11.26	-11.25	-11.24	-11.23	-11.22	-11.21	-11.2
-11.28	-11.27	-11.26	-11.25	-11.24	-11.23	-11.22	-11.21	-11.2
-11.28	-11.27	-11.26	-11.25	-11.24	-11.23	-11.22	-11.21	-11.2
-11.28	-11.27	-11.26	-11.25	-11.24	-11.23	-11.22	-11.21	-11.2
-11.28	-11.27	-11.26	-11.25	-11.24	-11.23	-11.22	-11.21	-11.2
-11.28	-11.27	-11.26	-11.25	-11.24	-11.23	-11.22	-11.21	-11.2
-11.28	-11.27	-11.26	-11.25	-11.24	-11.23	-11.22	-11.21	-11.2
-11.28	-11.27	-11.26	-11.25	-11.24	-11.23	-11.22	-11.21	-11.2
-11.28	-11.27	-11.26	-11.25	-11.24	-11.23	-11.22	-11.21	-11.2
-11.28	-11.27	-11.26	-11.25	-11.24	-11.23	-11.22	-11.21	-11.2
-11.28	-11.27	-11.26	-11.25	-11.24	-11.23	-11.22	-11.21	-11.2
-11.28	-11.27	-11.26	-11.25	-11.24	-11.23	-11.22	-11.21	-11.2
-11.28	-11.27	-11.26	-11.25	-11.24	-11.23	-11.22	-11.21	-11.2
-11.28	-11.27	-11.26	-11.25	-11.24	-11.23	-11.22	-11.21	-11.2
-11.28	-11.27	-11.26	-11.25	-11.24	-11.23	-11.22	-11.21	-11.2
-11.28	-11.27	-11.26	-11.25	-11.24	-11.23	-11.22	-11.21	-11.2
-11.28	-11.27	-11.26	-11.25	-11.24	-11.23	-11.22	-11.21	-11.2
-11.28	-11.27	-11.26	-11.25	-11.24	-11.23	-11.22	-11.21	-11.2
-11.28	-11.27	-11.26	-11.25	-11.24	-11.23	-11.22	-11.21	-11.2
-11.28	-11.27	-11.26	-11.25	-11.24	-11.23	-11.22	-11.21	-11.2
-11.28	-11.27	-11.26	-11.25	-11.24	-11.23	-11.22	-11.21	-11.2
-11.28	-11.27	-11.26	-11.25	-11.24	-11.23	-11.22	-11.21	-11.2
-11.28	-11.27	-11.26	-11.25	-11.24	-11.23	-11.22	-11.21	-11.2
-11.28	-11.27	-11.26	-11.25	-11.24	-11.23	-11.22	-11.21	-11.2
-11.28	-11.27	-11.26	-11.25	-11.24	-11.23	-11.22	-11.21	-11.2
-11.28	-11.27	-11.26	-11.25	-11.24	-11.23	-11.22	-11.21	-11.2
-11.28	-11.27	-11.26	-11.25	-11.24	-11.23	-11.22	-11.21	-11.2
-11.28	-11.27	-11.26	-11.25	-11.24	-11.23	-11.22	-11.21	-11.2
-11.28	-11.27	-11.26	-11.25	-11.24	-11.23	-11.22	-11.21	-11.2
-11.28	-11.27	-11.26	-11.25	-11.24	-11.23	-11.22	-11.21	-11.2
-11.28	-11.27	-11.26	-11.25	-11.24	-11.23	-11.22	-11.21	-11.2
-11.28	-11.27	-11.26	-11.25	-11.24	-11.23	-11.22	-11.21	-11.2
-11.28	-11.27	-11.26	-11.25	-11.24	-11.23	-11.22	-11.21	-11.2
-11.28	-11.27	-11.26	-11.25	-11.24	-11.23	-11.22	-11.21	-11.2
-11.28	-11.27	-11.26	-11.25	-11.24	-11.23	-11.22	-11.21	-11.2
-11.28	-11.27	-11.26	-11.25	-11.24	-11.23	-11.22	-11.21	-11.2
-11.28	-11.27	-11.26	-11.25	-11.24	-11.23	-11.22	-11.21	-11.2
-11.28	-11.27	-11.26	-11.25	-11.24	-11.23	-11.22	-11.21	-11.2
-11.28	-11.27	-11.26	-11.25	-11.24	-11.23	-11.22	-11.21	-11.2
-11.28	-11.27	-11.26	-11.25	-11.24	-11.23	-11.22	-11.21	-11.2
-11.28	-11.27	-11.26	-11.25	-11.24	-11.23	-11.22	-11.21	-11.2
-11.28	-11.27	-11.26	-11.25	-11.24	-11.23	-11.22	-11.21	-11.2
-11.28	-11.27	-11.26	-11.25	-11.24	-11.23	-11.22	-11.21	-11.2
-11.28	-11.27	-11.26	-11.25	-11.24	-11.23	-11.22	-11.21	-11.2
-11.28	-11.27	-11.26	-11.25	-11.24	-11.23	-11.22	-11.21	-11.2

-11.28	-11.27	-11.26	-11.25	-11.24	-11.23	-11.22	-11.21	-11.2
-11.28	-11.27	-11.26	-11.25	-11.24	-11.23	-11.22	-11.21	-11.2
-11.28	-11.27	-11.26	-11.25	-11.24	-11.23	-11.22	-11.21	-11.2
-11.28	-11.27	-11.26	-11.25	-11.24	-11.23	-11.22	-11.21	-11.2
-11.28	-11.27	-11.26	-11.25	-11.24	-11.23	-11.22	-11.21	-11.2
-11.28	-11.27	-11.26	-11.25	-11.24	-11.23	-11.22	-11.21	-11.2
-11.28	-11.27	-11.26	-11.25	-11.24	-11.23	-11.22	-11.21	-11.2
-11.28	-11.27	-11.26	-11.25	-11.24	-11.23	-11.22	-11.21	-11.2
-11.28	-11.27	-11.26	-11.25	-11.24	-11.23	-11.22	-11.21	-11.2
-11.28	-11.27	-11.26	-11.25	-11.24	-11.23	-11.22	-11.21	-11.2
-11.28	-11.27	-11.26	-11.25	-11.24	-11.23	-11.22	-11.21	-11.2
-11.28	-11.27	-11.26	-11.25	-11.24	-11.23	-11.22	-11.21	-11.2
-11.28	-11.27	-11.26	-11.25	-11.24	-11.23	-11.22	-11.21	-11.2
-11.28	-11.27	-11.26	-11.25	-11.24	-11.23	-11.22	-11.21	-11.2
-11.28	-11.27	-11.26	-11.25	-11.24	-11.23	-11.22	-11.21	-11.2
-11.28	-11.27	-11.26	-11.25	-11.24	-11.23	-11.22	-11.21	-11.2
-11.28	-11.27	-11.26	-11.25	-11.24	-11.23	-11.22	-11.21	-11.2
-11.28	-11.27	-11.26	-11.25	-11.24	-11.23	-11.22	-11.21	-11.2
-11.28	-11.27	-11.26	-11.25	-11.24	-11.23	-11.22	-11.21	-11.2
-11.28	-11.27	-11.26	-11.25	-11.24	-11.23	-11.22	-11.21	-11.2
-11.28	-11.27	-11.26	-11.25	-11.24	-11.23	-11.22	-11.21	-11.2
-11.28	-11.27	-11.26	-11.25	-11.24	-11.23	-11.22	-11.21	-11.2
-11.28	-11.27	-11.26	-11.25	-11.24	-11.23	-11.22	-11.21	-11.2
-11.28	-11.27	-11.26	-11.25	-11.24	-11.23	-11.22	-11.21	-11.2
-11.28	-11.27	-11.26	-11.25	-11.24	-11.23	-11.22	-11.21	-11.2
-11.28	-11.27	-11.26	-11.25	-11.24	-11.23	-11.22	-11.21	-11.2
-11.28	-11.27	-11.26	-11.25	-11.24	-11.23	-11.22	-11.21	-11.2
-11.28	-11.27	-11.26	-11.25	-11.24	-11.23	-11.22	-11.21	-11.2
-11.28	-11.27	-11.26	-11.25	-11.24	-11.23	-11.22	-11.21	-11.2
-11.28	-11.27	-11.26	-11.25	-11.24	-11.23	-11.22	-11.21	-11.2
-11.28	-11.27	-11.26	-11.25	-11.24	-11.23	-11.22	-11.21	-11.2
-11.28	-11.27	-11.26	-11.25	-11.24	-11.23	-11.22	-11.21	-11.2
-11.28	-11.27	-11.26	-11.25	-11.24	-11.23	-11.22	-11.21	-11.2
-11.28	-11.27	-11.26	-11.25	-11.24	-11.23	-11.22	-11.21	-11.2
-11.28	-11.27	-11.26	-11.25	-11.24	-11.23	-11.22	-11.21	-11.2
-11.28	-11.27	-11.26	-11.25	-11.24	-11.23	-11.22	-11.21	-11.2
-11.28	-11.27	-11.26	-11.25	-11.24	-11.23	-11.22	-11.21	-11.2
-11.28	-11.27	-11.26	-11.25	-11.24	-11.23	-11.22	-11.21	-11.2
-11.28	-11.27	-11.26	-11.25	-11.24	-11.23	-11.22	-11.21	-11.2
-11.28	-11.27	-11.26	-11.25	-11.24	-11.23	-11.22	-11.21	-11.2
-11.28	-11.27	-11.26	-11.25	-11.24	-11.23	-11.22	-11.21	-11.2
-11.28	-11.27	-11.26	-11.25	-11.24	-11.23	-11.22	-11.21	-11.2
-11.28	-11.27	-11.26	-11.25	-11.24	-11.23	-11.22	-11.21	-11.2
-11.28	-11.27	-11.26	-11.25	-11.24	-11.23	-11.22	-11.21	-11.2
-11.28	-11.27	-11.26	-11.25	-11.24	-11.23	-11.22	-11.21	-11.2
-11.28	-11.27	-11.26	-11.25	-11.24	-11.23	-11.22	-11.21	-11.2
-11.28	-11.27	-11.26	-11.25	-11.24	-11.23	-11.22	-11.21	-11.2
-11.28	-11.27	-11.26	-11.25	-11.24	-11.23	-11.22	-11.21	-11.2
-11.28	-11.27	-11.26	-11.25	-11.24	-11.23	-11.22	-11.21	-11.2
-11.28	-11.27	-11.26	-11.25	-11.24	-11.23	-11.22	-11.21	-11.2

-11.28	-11.27	-11.26	-11.25	-11.24	-11.23	-11.22	-11.21	-11.2
-11.28	-11.27	-11.26	-11.25	-11.24	-11.23	-11.22	-11.21	-11.2
-11.28	-11.27	-11.26	-11.25	-11.24	-11.23	-11.22	-11.21	-11.2
-11.28	-11.27	-11.26	-11.25	-11.24	-11.23	-11.22	-11.21	-11.2
-11.28	-11.27	-11.26	-11.25	-11.24	-11.23	-11.22	-11.21	-11.2
-11.28	-11.27	-11.26	-11.25	-11.24	-11.23	-11.22	-11.21	-11.2
-11.28	-11.27	-11.26	-11.25	-11.24	-11.23	-11.22	-11.21	-11.2
-11.28	-11.27	-11.26	-11.25	-11.24	-11.23	-11.22	-11.21	-11.2
-11.28	-11.27	-11.26	-11.25	-11.24	-11.23	-11.22	-11.21	-11.2
-11.28	-11.27	-11.26	-11.25	-11.24	-11.23	-11.22	-11.21	-11.2
-11.28	-11.27	-11.26	-11.25	-11.24	-11.23	-11.22	-11.21	-11.2
-11.28	-11.27	-11.26	-11.25	-11.24	-11.23	-11.22	-11.21	-11.2
-11.28	-11.27	-11.26	-11.25	-11.24	-11.23	-11.22	-11.21	-11.2
-11.28	-11.27	-11.26	-11.25	-11.24	-11.23	-11.22	-11.21	-11.2
-11.28	-11.27	-11.26	-11.25	-11.24	-11.23	-11.22	-11.21	-11.2
-11.28	-11.27	-11.26	-11.25	-11.24	-11.23	-11.22	-11.21	-11.2
-11.28	-11.27	-11.26	-11.25	-11.24	-11.23	-11.22	-11.21	-11.2
-11.28	-11.27	-11.26	-11.25	-11.24	-11.23	-11.22	-11.21	-11.2
-11.28	-11.27	-11.26	-11.25	-11.24	-11.23	-11.22	-11.21	-11.2
-11.28	-11.27	-11.26	-11.25	-11.24	-11.23	-11.22	-11.21	-11.2
-11.28	-11.27	-11.26	-11.25	-11.24	-11.23	-11.22	-11.21	-11.2
-11.28	-11.27	-11.26	-11.25	-11.24	-11.23	-11.22	-11.21	-11.2
-11.28	-11.27	-11.26	-11.25	-11.24	-11.23	-11.22	-11.21	-11.2
-11.28	-11.27	-11.26	-11.25	-11.24	-11.23	-11.22	-11.21	-11.2
-11.28	-11.27	-11.26	-11.25	-11.24	-11.23	-11.22	-11.21	-11.2
-11.28	-11.27	-11.26	-11.25	-11.24	-11.23	-11.22	-11.21	-11.2
-11.28	-11.27	-11.26	-11.25	-11.24	-11.23	-11.22	-11.21	-11.2
-11.28	-11.27	-11.26	-11.25	-11.24	-11.23	-11.22	-11.21	-11.2
-11.28	-11.27	-11.26	-11.25	-11.24	-11.23	-11.22	-11.21	-11.2
-11.28	-11.27	-11.26	-11.25	-11.24	-11.23	-11.22	-11.21	-11.2
-11.28	-11.27	-11.26	-11.25	-11.24	-11.23	-11.22	-11.21	-11.2
-11.28	-11.27	-11.26	-11.25	-11.24	-11.23	-11.22	-11.21	-11.2
-11.28	-11.27	-11.26	-11.25	-11.24	-11.23	-11.22	-11.21	-11.2
-11.28	-11.27	-11.26	-11.25	-11.24	-11.23	-11.22	-11.21	-11.2
-11.28	-11.27	-11.26	-11.25	-11.24	-11.23	-11.22	-11.21	-11.2
-11.28	-11.27	-11.26	-11.25	-11.24	-11.23	-11.22	-11.21	-11.2
-11.28	-11.27	-11.26	-11.25	-11.24	-11.23	-11.22	-11.21	-11.2
-11.28	-11.27	-11.26	-11.25	-11.24	-11.23	-11.22	-11.21	-11.2
-11.28	-11.27	-11.26	-11.25	-11.24	-11.23	-11.22	-11.21	-11.2
-11.28	-11.27	-11.26	-11.25	-11.24	-11.23	-11.22	-11.21	-11.2
-11.28	-11.27	-11.26	-11.25	-11.24	-11.23	-11.22	-11.21	-11.2
-11.28	-11.27	-11.26	-11.25	-11.24	-11.23	-11.22	-11.21	-11.2
-11.28	-11.27	-11.26	-11.25	-11.24	-11.23	-11.22	-11.21	-11.2
-11.28	-11.27	-11.26	-11.25	-11.24	-11.23	-11.22	-11.21	-11.2
-11.28	-11.27	-11.26	-11.25	-11.24	-11.23	-11.22	-11.21	-11.2
-11.28	-11.27	-11.26	-11.25	-11.24	-11.23	-11.22	-11.21	-11.2
-11.28	-11.27	-11.26	-11.25	-11.24	-11.23	-11.22	-11.21	-11.2
-11.28	-11.27	-11.26	-11.25	-11.24	-11.23	-11.22	-11.21	-11.2
-11.28	-11.27	-11.26	-11.25	-11.24	-11.23	-11.22	-11.21	-11.2
-11.28	-11.27	-11.26	-11.25	-11.24	-11.23	-11.22	-11.21	-11.2

-11.28	-11.27	-11.26	-11.25	-11.24	-11.23	-11.22	-11.21	-11.2
-11.28	-11.27	-11.26	-11.25	-11.24	-11.23	-11.22	-11.21	-11.2
-11.28	-11.27	-11.26	-11.25	-11.24	-11.23	-11.22	-11.21	-11.2
-11.28	-11.27	-11.26	-11.25	-11.24	-11.23	-11.22	-11.21	-11.2
-11.28	-11.27	-11.26	-11.25	-11.24	-11.23	-11.22	-11.21	-11.2
-11.28	-11.27	-11.26	-11.25	-11.24	-11.23	-11.22	-11.21	-11.2
-11.28	-11.27	-11.26	-11.25	-11.24	-11.23	-11.22	-11.21	-11.2
-11.28	-11.27	-11.26	-11.25	-11.24	-11.23	-11.22	-11.21	-11.2
-11.28	-11.27	-11.26	-11.25	-11.24	-11.23	-11.22	-11.21	-11.2
-11.28	-11.27	-11.26	-11.25	-11.24	-11.23	-11.22	-11.21	-11.2
-11.28	-11.27	-11.26	-11.25	-11.24	-11.23	-11.22	-11.21	-11.2
-11.28	-11.27	-11.26	-11.25	-11.24	-11.23	-11.22	-11.21	-11.2
-11.28	-11.27	-11.26	-11.25	-11.24	-11.23	-11.22	-11.21	-11.2
-11.28	-11.27	-11.26	-11.25	-11.24	-11.23	-11.22	-11.21	-11.2
-11.28	-11.27	-11.26	-11.25	-11.24	-11.23	-11.22	-11.21	-11.2
-11.28	-11.27	-11.26	-11.25	-11.24	-11.23	-11.22	-11.21	-11.2
-11.28	-11.27	-11.26	-11.25	-11.24	-11.23	-11.22	-11.21	-11.2
-11.28	-11.27	-11.26	-11.25	-11.24	-11.23	-11.22	-11.21	-11.2
-11.28	-11.27	-11.26	-11.25	-11.24	-11.23	-11.22	-11.21	-11.2
-11.28	-11.27	-11.26	-11.25	-11.24	-11.23	-11.22	-11.21	-11.2
-11.28	-11.27	-11.26	-11.25	-11.24	-11.23	-11.22	-11.21	-11.2
-11.28	-11.27	-11.26	-11.25	-11.24	-11.23	-11.22	-11.21	-11.2
-11.28	-11.27	-11.26	-11.25	-11.24	-11.23	-11.22	-11.21	-11.2
-11.28	-11.27	-11.26	-11.25	-11.24	-11.23	-11.22	-11.21	-11.2
-11.28	-11.27	-11.26	-11.25	-11.24	-11.23	-11.22	-11.21	-11.2
-11.28	-11.27	-11.26	-11.25	-11.24	-11.23	-11.22	-11.21	-11.2
-11.28	-11.27	-11.26	-11.25	-11.24	-11.23	-11.22	-11.21	-11.2
-11.28	-11.27	-11.26	-11.25	-11.24	-11.23	-11.22	-11.21	-11.2
-11.28	-11.27	-11.26	-11.25	-11.24	-11.23	-11.22	-11.21	-11.2
-11.28	-11.27	-11.26	-11.25	-11.24	-11.23	-11.22	-11.21	-11.2
-11.28	-11.27	-11.26	-11.25	-11.24	-11.23	-11.22	-11.21	-11.2
-11.28	-11.27	-11.26	-11.25	-11.24	-11.23	-11.22	-11.21	-11.2
-11.28	-11.27	-11.26	-11.25	-11.24	-11.23	-11.22	-11.21	-11.2
-11.28	-11.27	-11.26	-11.25	-11.24	-11.23	-11.22	-11.21	-11.2
-11.28	-11.27	-11.26	-11.25	-11.24	-11.23	-11.22	-11.21	-11.2
-11.28	-11.27	-11.26	-11.25	-11.24	-11.23	-11.22	-11.21	-11.2
-11.28	-11.27	-11.26	-11.25	-11.24	-11.23	-11.22	-11.21	-11.2
-11.28	-11.27	-11.26	-11.25	-11.24	-11.23	-11.22	-11.21	-11.2
-11.28	-11.27	-11.26	-11.25	-11.24	-11.23	-11.22	-11.21	-11.2
-11.28	-11.27	-11.26	-11.25	-11.24	-11.23	-11.22	-11.21	-11.2
-11.28	-11.27	-11.26	-11.25	-11.24	-11.23	-11.22	-11.21	-11.2
-11.28	-11.27	-11.26	-11.25	-11.24	-11.23	-11.22	-11.21	-11.2
-11.28	-11.27	-11.26	-11.25	-11.24	-11.23	-11.22	-11.21	-11.2
-11.28	-11.27	-11.26	-11.25	-11.24	-11.23	-11.22	-11.21	-11.2
-11.28	-11.27	-11.26	-11.25	-11.24	-11.23	-11.22	-11.21	-11.2
-11.28	-11.27	-11.26	-11.25	-11.24	-11.23	-11.22	-11.21	-11.2
-11.28	-11.27	-11.26	-11.25	-11.24	-11.23	-11.22	-11.21	-11.2
-11.28	-11.27	-11.26	-11.25	-11.24	-11.23	-11.22	-11.21	-11.2
-11.28	-11.27	-11.26	-11.25	-11.24	-11.23	-11.22	-11.21	-11.2
-11.28	-11.27	-11.26	-11.25	-11.24	-11.23	-11.22	-11.21	-11.2

-11.28	-11.27	-11.26	-11.25	-11.24	-11.23	-11.22	-11.21	-11.2
-11.28	-11.27	-11.26	-11.25	-11.24	-11.23	-11.22	-11.21	-11.2
-11.28	-11.27	-11.26	-11.25	-11.24	-11.23	-11.22	-11.21	-11.2
-11.28	-11.27	-11.26	-11.25	-11.24	-11.23	-11.22	-11.21	-11.2
-11.28	-11.27	-11.26	-11.25	-11.24	-11.23	-11.22	-11.21	-11.2
-11.28	-11.27	-11.26	-11.25	-11.24	-11.23	-11.22	-11.21	-11.2
-11.28	-11.27	-11.26	-11.25	-11.24	-11.23	-11.22	-11.21	-11.2
-11.28	-11.27	-11.26	-11.25	-11.24	-11.23	-11.22	-11.21	-11.2
-11.28	-11.27	-11.26	-11.25	-11.24	-11.23	-11.22	-11.21	-11.2
-11.28	-11.27	-11.26	-11.25	-11.24	-11.23	-11.22	-11.21	-11.2
-11.28	-11.27	-11.26	-11.25	-11.24	-11.23	-11.22	-11.21	-11.2
-11.28	-11.27	-11.26	-11.25	-11.24	-11.23	-11.22	-11.21	-11.2
-11.28	-11.27	-11.26	-11.25	-11.24	-11.23	-11.22	-11.21	-11.2
-11.28	-11.27	-11.26	-11.25	-11.24	-11.23	-11.22	-11.21	-11.2
-11.28	-11.27	-11.26	-11.25	-11.24	-11.23	-11.22	-11.21	-11.2
-11.28	-11.27	-11.26	-11.25	-11.24	-11.23	-11.22	-11.21	-11.2
-11.28	-11.27	-11.26	-11.25	-11.24	-11.23	-11.22	-11.21	-11.2
-11.28	-11.27	-11.26	-11.25	-11.24	-11.23	-11.22	-11.21	-11.2
-11.28	-11.27	-11.26	-11.25	-11.24	-11.23	-11.22	-11.21	-11.2
-11.28	-11.27	-11.26	-11.25	-11.24	-11.23	-11.22	-11.21	-11.2
-11.28	-11.27	-11.26	-11.25	-11.24	-11.23	-11.22	-11.21	-11.2
-11.28	-11.27	-11.26	-11.25	-11.24	-11.23	-11.22	-11.21	-11.2
-11.28	-11.27	-11.26	-11.25	-11.24	-11.23	-11.22	-11.21	-11.2
-11.28	-11.27	-11.26	-11.25	-11.24	-11.23	-11.22	-11.21	-11.2
-11.28	-11.27	-11.26	-11.25	-11.24	-11.23	-11.22	-11.21	-11.2
-11.28	-11.27	-11.26	-11.25	-11.24	-11.23	-11.22	-11.21	-11.2
-11.28	-11.27	-11.26	-11.25	-11.24	-11.23	-11.22	-11.21	-11.2
-11.28	-11.27	-11.26	-11.25	-11.24	-11.23	-11.22	-11.21	-11.2
-11.28	-11.27	-11.26	-11.25	-11.24	-11.23	-11.22	-11.21	-11.2
-11.28	-11.27	-11.26	-11.25	-11.24	-11.23	-11.22	-11.21	-11.2
-11.28	-11.27	-11.26	-11.25	-11.24	-11.23	-11.22	-11.21	-11.2
-11.28	-11.27	-11.26	-11.25	-11.24	-11.23	-11.22	-11.21	-11.2
-11.28	-11.27	-11.26	-11.25	-11.24	-11.23	-11.22	-11.21	-11.2
-11.28	-11.27	-11.26	-11.25	-11.24	-11.23	-11.22	-11.21	-11.2
-11.28	-11.27	-11.26	-11.25	-11.24	-11.23	-11.22	-11.21	-11.2
-11.28	-11.27	-11.26	-11.25	-11.24	-11.23	-11.22	-11.21	-11.2
-11.28	-11.27	-11.26	-11.25	-11.24	-11.23	-11.22	-11.21	-11.2
-11.28	-11.27	-11.26	-11.25	-11.24	-11.23	-11.22	-11.21	-11.2
-11.28	-11.27	-11.26	-11.25	-11.24	-11.23	-11.22	-11.21	-11.2
-11.28	-11.27	-11.26	-11.25	-11.24	-11.23	-11.22	-11.21	-11.2
-11.28	-11.27	-11.26	-11.25	-11.24	-11.23	-11.22	-11.21	-11.2
-11.28	-11.27	-11.26	-11.25	-11.24	-11.23	-11.22	-11.21	-11.2
-11.28	-11.27	-11.26	-11.25	-11.24	-11.23	-11.22	-11.21	-11.2
-11.28	-11.27	-11.26	-11.25	-11.24	-11.23	-11.22	-11.21	-11.2
-11.28	-11.27	-11.26	-11.25	-11.24	-11.23	-11.22	-11.21	-11.2
-11.28	-11.27	-11.26	-11.25	-11.24	-11.23	-11.22	-11.21	-11.2
-11.28	-11.27	-11.26	-11.25	-11.24	-11.23	-11.22	-11.21	-11.2
-11.28	-11.27	-11.26	-11.25	-11.24	-11.23	-11.22	-11.21	-11.2
-11.28	-11.27	-11.26	-11.25	-11.24	-11.23	-11.22	-11.21	-11.2
-11.28	-11.27	-11.26	-11.25	-11.24	-11.23	-11.22	-11.21	-11.2

-11.28	-11.27	-11.26	-11.25	-11.24	-11.23	-11.22	-11.21	-11.2
-11.28	-11.27	-11.26	-11.25	-11.24	-11.23	-11.22	-11.21	-11.2
-11.28	-11.27	-11.26	-11.25	-11.24	-11.23	-11.22	-11.21	-11.2
-11.28	-11.27	-11.26	-11.25	-11.24	-11.23	-11.22	-11.21	-11.2
-11.28	-11.27	-11.26	-11.25	-11.24	-11.23	-11.22	-11.21	-11.2
-11.28	-11.27	-11.26	-11.25	-11.24	-11.23	-11.22	-11.21	-11.2
-11.28	-11.27	-11.26	-11.25	-11.24	-11.23	-11.22	-11.21	-11.2
-11.28	-11.27	-11.26	-11.25	-11.24	-11.23	-11.22	-11.21	-11.2
-11.28	-11.27	-11.26	-11.25	-11.24	-11.23	-11.22	-11.21	-11.2
-11.28	-11.27	-11.26	-11.25	-11.24	-11.23	-11.22	-11.21	-11.2
-11.28	-11.27	-11.26	-11.25	-11.24	-11.23	-11.22	-11.21	-11.2
-11.28	-11.27	-11.26	-11.25	-11.24	-11.23	-11.22	-11.21	-11.2
-11.28	-11.27	-11.26	-11.25	-11.24	-11.23	-11.22	-11.21	-11.2
-11.28	-11.27	-11.26	-11.25	-11.24	-11.23	-11.22	-11.21	-11.2
-11.28	-11.27	-11.26	-11.25	-11.24	-11.23	-11.22	-11.21	-11.2
-11.28	-11.27	-11.26	-11.25	-11.24	-11.23	-11.22	-11.21	-11.2
-11.28	-11.27	-11.26	-11.25	-11.24	-11.23	-11.22	-11.21	-11.2
-11.28	-11.27	-11.26	-11.25	-11.24	-11.23	-11.22	-11.21	-11.2
-11.28	-11.27	-11.26	-11.25	-11.24	-11.23	-11.22	-11.21	-11.2
-11.28	-11.27	-11.26	-11.25	-11.24	-11.23	-11.22	-11.21	-11.2
-11.28	-11.27	-11.26	-11.25	-11.24	-11.23	-11.22	-11.21	-11.2
-11.28	-11.27	-11.26	-11.25	-11.24	-11.23	-11.22	-11.21	-11.2
-11.28	-11.27	-11.26	-11.25	-11.24	-11.23	-11.22	-11.21	-11.2
-11.28	-11.27	-11.26	-11.25	-11.24	-11.23	-11.22	-11.21	-11.2
-11.28	-11.27	-11.26	-11.25	-11.24	-11.23	-11.22	-11.21	-11.2
-11.28	-11.27	-11.26	-11.25	-11.24	-11.23	-11.22	-11.21	-11.2
-11.28	-11.27	-11.26	-11.25	-11.24	-11.23	-11.22	-11.21	-11.2
-11.28	-11.27	-11.26	-11.25	-11.24	-11.23	-11.22	-11.21	-11.2
-11.28	-11.27	-11.26	-11.25	-11.24	-11.23	-11.22	-11.21	-11.2
-11.28	-11.27	-11.26	-11.25	-11.24	-11.23	-11.22	-11.21	-11.2
-11.28	-11.27	-11.26	-11.25	-11.24	-11.23	-11.22	-11.21	-11.2
-11.28	-11.27	-11.26	-11.25	-11.24	-11.23	-11.22	-11.21	-11.2
-11.28	-11.27	-11.26	-11.25	-11.24	-11.23	-11.22	-11.21	-11.2
-11.28	-11.27	-11.26	-11.25	-11.24	-11.23	-11.22	-11.21	-11.2
-11.28	-11.27	-11.26	-11.25	-11.24	-11.23	-11.22	-11.21	-11.2
-11.28	-11.27	-11.26	-11.25	-11.24	-11.23	-11.22	-11.21	-11.2
-11.28	-11.27	-11.26	-11.25	-11.24	-11.23	-11.22	-11.21	-11.2
-11.28	-11.27	-11.26	-11.25	-11.24	-11.23	-11.22	-11.21	-11.2
-11.28	-11.27	-11.26	-11.25	-11.24	-11.23	-11.22	-11.21	-11.2
-11.28	-11.27	-11.26	-11.25	-11.24	-11.23	-11.22	-11.21	-11.2
-11.28	-11.27	-11.26	-11.25	-11.24	-11.23	-11.22	-11.21	-11.2
-11.28	-11.27	-11.26	-11.25	-11.24	-11.23	-11.22	-11.21	-11.2
-11.28	-11.27	-11.26	-11.25	-11.24	-11.23	-11.22	-11.21	-11.2
-11.28	-11.27	-11.26	-11.25	-11.24	-11.23	-11.22	-11.21	-11.2
-11.28	-11.27	-11.26	-11.25	-11.24	-11.23	-11.22	-11.21	-11.2
-11.28	-11.27	-11.26	-11.25	-11.24	-11.23	-11.22	-11.21	-11.2
-11.28	-11.27	-11.26	-11.25	-11.24	-11.23	-11.22	-11.21	-11.2
-11.28	-11.27	-11.26	-11.25	-11.24	-11.23	-11.22	-11.21	-11.2
-11.28	-11.27	-11.26	-11.25	-11.24	-11.23	-11.22	-11.21	-11.2
-11.28	-11.27	-11.26	-11.25	-11.24	-11.23	-11.22	-11.21	-11.2

-11.28	-11.27	-11.26	-11.25	-11.24	-11.23	-11.22	-11.21	-11.2
-11.28	-11.27	-11.26	-11.25	-11.24	-11.23	-11.22	-11.21	-11.2
-11.28	-11.27	-11.26	-11.25	-11.24	-11.23	-11.22	-11.21	-11.2
-11.28	-11.27	-11.26	-11.25	-11.24	-11.23	-11.22	-11.21	-11.2
-11.28	-11.27	-11.26	-11.25	-11.24	-11.23	-11.22	-11.21	-11.2
-11.28	-11.27	-11.26	-11.25	-11.24	-11.23	-11.22	-11.21	-11.2
-11.28	-11.27	-11.26	-11.25	-11.24	-11.23	-11.22	-11.21	-11.2
-11.28	-11.27	-11.26	-11.25	-11.24	-11.23	-11.22	-11.21	-11.2
-11.28	-11.27	-11.26	-11.25	-11.24	-11.23	-11.22	-11.21	-11.2
-11.28	-11.27	-11.26	-11.25	-11.24	-11.23	-11.22	-11.21	-11.2
-11.28	-11.27	-11.26	-11.25	-11.24	-11.23	-11.22	-11.21	-11.2
-11.28	-11.27	-11.26	-11.25	-11.24	-11.23	-11.22	-11.21	-11.2
-11.28	-11.27	-11.26	-11.25	-11.24	-11.23	-11.22	-11.21	-11.2
-11.28	-11.27	-11.26	-11.25	-11.24	-11.23	-11.22	-11.21	-11.2
-11.28	-11.27	-11.26	-11.25	-11.24	-11.23	-11.22	-11.21	-11.2
-11.28	-11.27	-11.26	-11.25	-11.24	-11.23	-11.22	-11.21	-11.2
-11.28	-11.27	-11.26	-11.25	-11.24	-11.23	-11.22	-11.21	-11.2
-11.28	-11.27	-11.26	-11.25	-11.24	-11.23	-11.22	-11.21	-11.2
-11.28	-11.27	-11.26	-11.25	-11.24	-11.23	-11.22	-11.21	-11.2
-11.28	-11.27	-11.26	-11.25	-11.24	-11.23	-11.22	-11.21	-11.2
-11.28	-11.27	-11.26	-11.25	-11.24	-11.23	-11.22	-11.21	-11.2
-11.28	-11.27	-11.26	-11.25	-11.24	-11.23	-11.22	-11.21	-11.2
-11.28	-11.27	-11.26	-11.25	-11.24	-11.23	-11.22	-11.21	-11.2
-11.28	-11.27	-11.26	-11.25	-11.24	-11.23	-11.22	-11.21	-11.2
-11.28	-11.27	-11.26	-11.25	-11.24	-11.23	-11.22	-11.21	-11.2
-11.28	-11.27	-11.26	-11.25	-11.24	-11.23	-11.22	-11.21	-11.2
-11.28	-11.27	-11.26	-11.25	-11.24	-11.23	-11.22	-11.21	-11.2
-11.28	-11.27	-11.26	-11.25	-11.24	-11.23	-11.22	-11.21	-11.2
-11.28	-11.27	-11.26	-11.25	-11.24	-11.23	-11.22	-11.21	-11.2
-11.28	-11.27	-11.26	-11.25	-11.24	-11.23	-11.22	-11.21	-11.2
-11.28	-11.27	-11.26	-11.25	-11.24	-11.23	-11.22	-11.21	-11.2
-11.28	-11.27	-11.26	-11.25	-11.24	-11.23	-11.22	-11.21	-11.2
-11.28	-11.27	-11.26	-11.25	-11.24	-11.23	-11.22	-11.21	-11.2
-11.28	-11.27	-11.26	-11.25	-11.24	-11.23	-11.22	-11.21	-11.2
-11.28	-11.27	-11.26	-11.25	-11.24	-11.23	-11.22	-11.21	-11.2
-11.28	-11.27	-11.26	-11.25	-11.24	-11.23	-11.22	-11.21	-11.2
-11.28	-11.27	-11.26	-11.25	-11.24	-11.23	-11.22	-11.21	-11.2
-11.28	-11.27	-11.26	-11.25	-11.24	-11.23	-11.22	-11.21	-11.2
-11.28	-11.27	-11.26	-11.25	-11.24	-11.23	-11.22	-11.21	-11.2
-11.28	-11.27	-11.26	-11.25	-11.24	-11.23	-11.22	-11.21	-11.2
-11.28	-11.27	-11.26	-11.25	-11.24	-11.23	-11.22	-11.21	-11.2
-11.28	-11.27	-11.26	-11.25	-11.24	-11.23	-11.22	-11.21	-11.2
-11.28	-11.27	-11.26	-11.25	-11.24	-11.23	-11.22	-11.21	-11.2
-11.28	-11.27	-11.26	-11.25	-11.24	-11.23	-11.22	-11.21	-11.2
-11.28	-11.27	-11.26	-11.25	-11.24	-11.23	-11.22	-11.21	-11.2
-11.28	-11.27	-11.26	-11.25	-11.24	-11.23	-11.22	-11.21	-11.2
-11.28	-11.27	-11.26	-11.25	-11.24	-11.23	-11.22	-11.21	-11.2
-11.28	-11.27	-11.26	-11.25	-11.24	-11.23	-11.22	-11.21	-11.2
-11.28	-11.27	-11.26	-11.25	-11.24	-11.23	-11.22	-11.21	-11.2
-11.28	-11.27	-11.26	-11.25	-11.24	-11.23	-11.22	-11.21	-11.2

-11.28	-11.27	-11.26	-11.25	-11.24	-11.23	-11.22	-11.21	-11.2
-11.28	-11.27	-11.26	-11.25	-11.24	-11.23	-11.22	-11.21	-11.2
-11.28	-11.27	-11.26	-11.25	-11.24	-11.23	-11.22	-11.21	-11.2
-11.28	-11.27	-11.26	-11.25	-11.24	-11.23	-11.22	-11.21	-11.2
-11.28	-11.27	-11.26	-11.25	-11.24	-11.23	-11.22	-11.21	-11.2
-11.28	-11.27	-11.26	-11.25	-11.24	-11.23	-11.22	-11.21	-11.2
-11.28	-11.27	-11.26	-11.25	-11.24	-11.23	-11.22	-11.21	-11.2
-11.28	-11.27	-11.26	-11.25	-11.24	-11.23	-11.22	-11.21	-11.2
-11.28	-11.27	-11.26	-11.25	-11.24	-11.23	-11.22	-11.21	-11.2
-11.28	-11.27	-11.26	-11.25	-11.24	-11.23	-11.22	-11.21	-11.2
-11.28	-11.27	-11.26	-11.25	-11.24	-11.23	-11.22	-11.21	-11.2
-11.28	-11.27	-11.26	-11.25	-11.24	-11.23	-11.22	-11.21	-11.2
-11.28	-11.27	-11.26	-11.25	-11.24	-11.23	-11.22	-11.21	-11.2
-11.28	-11.27	-11.26	-11.25	-11.24	-11.23	-11.22	-11.21	-11.2
-11.28	-11.27	-11.26	-11.25	-11.24	-11.23	-11.22	-11.21	-11.2
-11.28	-11.27	-11.26	-11.25	-11.24	-11.23	-11.22	-11.21	-11.2
-11.28	-11.27	-11.26	-11.25	-11.24	-11.23	-11.22	-11.21	-11.2
-11.28	-11.27	-11.26	-11.25	-11.24	-11.23	-11.22	-11.21	-11.2
-11.28	-11.27	-11.26	-11.25	-11.24	-11.23	-11.22	-11.21	-11.2
-11.28	-11.27	-11.26	-11.25	-11.24	-11.23	-11.22	-11.21	-11.2
-11.28	-11.27	-11.26	-11.25	-11.24	-11.23	-11.22	-11.21	-11.2
-11.28	-11.27	-11.26	-11.25	-11.24	-11.23	-11.22	-11.21	-11.2
-11.28	-11.27	-11.26	-11.25	-11.24	-11.23	-11.22	-11.21	-11.2
-11.28	-11.27	-11.26	-11.25	-11.24	-11.23	-11.22	-11.21	-11.2
-11.28	-11.27	-11.26	-11.25	-11.24	-11.23	-11.22	-11.21	-11.2
-11.28	-11.27	-11.26	-11.25	-11.24	-11.23	-11.22	-11.21	-11.2
-11.28	-11.27	-11.26	-11.25	-11.24	-11.23	-11.22	-11.21	-11.2
-11.28	-11.27	-11.26	-11.25	-11.24	-11.23	-11.22	-11.21	-11.2
-11.28	-11.27	-11.26	-11.25	-11.24	-11.23	-11.22	-11.21	-11.2
-11.28	-11.27	-11.26	-11.25	-11.24	-11.23	-11.22	-11.21	-11.2
-11.28	-11.27	-11.26	-11.25	-11.24	-11.23	-11.22	-11.21	-11.2
-11.28	-11.27	-11.26	-11.25	-11.24	-11.23	-11.22	-11.21	-11.2
-11.28	-11.27	-11.26	-11.25	-11.24	-11.23	-11.22	-11.21	-11.2
-11.28	-11.27	-11.26	-11.25	-11.24	-11.23	-11.22	-11.21	-11.2
-11.28	-11.27	-11.26	-11.25	-11.24	-11.23	-11.22	-11.21	-11.2
-11.28	-11.27	-11.26	-11.25	-11.24	-11.23	-11.22	-11.21	-11.2
-11.28	-11.27	-11.26	-11.25	-11.24	-11.23	-11.22	-11.21	-11.2
-11.28	-11.27	-11.26	-11.25	-11.24	-11.23	-11.22	-11.21	-11.2
-11.28	-11.27	-11.26	-11.25	-11.24	-11.23	-11.22	-11.21	-11.2
-11.28	-11.27	-11.26	-11.25	-11.24	-11.23	-11.22	-11.21	-11.2
-11.28	-11.27	-11.26	-11.25	-11.24	-11.23	-11.22	-11.21	-11.2
-11.28	-11.27	-11.26	-11.25	-11.24	-11.23	-11.22	-11.21	-11.2
-11.28	-11.27	-11.26	-11.25	-11.24	-11.23	-11.22	-11.21	-11.2
-11.28	-11.27	-11.26	-11.25	-11.24	-11.23	-11.22	-11.21	-11.2
-11.28	-11.27	-11.26	-11.25	-11.24	-11.23	-11.22	-11.21	-11.2
-11.28	-11.27	-11.26	-11.25	-11.24	-11.23	-11.22	-11.21	-11.2
-11.28	-11.27	-11.26	-11.25	-11.24	-11.23	-11.22	-11.21	-11.2
-11.28	-11.27	-11.26	-11.25	-11.24	-11.23	-11.22	-11.21	-11.2
-11.28	-11.27	-11.26	-11.25	-11.24	-11.23	-11.22	-11.21	-11.2
-11.28	-11.27	-11.26	-11.25	-11.24	-11.23	-11.22	-11.21	-11.2

-11.28	-11.27	-11.26	-11.25	-11.24	-11.23	-11.22	-11.21	-11.2
-11.28	-11.27	-11.26	-11.25	-11.24	-11.23	-11.22	-11.21	-11.2
-11.28	-11.27	-11.26	-11.25	-11.24	-11.23	-11.22	-11.21	-11.2
-11.28	-11.27	-11.26	-11.25	-11.24	-11.23	-11.22	-11.21	-11.2
-11.28	-11.27	-11.26	-11.25	-11.24	-11.23	-11.22	-11.21	-11.2
-11.28	-11.27	-11.26	-11.25	-11.24	-11.23	-11.22	-11.21	-11.2
-11.28	-11.27	-11.26	-11.25	-11.24	-11.23	-11.22	-11.21	-11.2
-11.28	-11.27	-11.26	-11.25	-11.24	-11.23	-11.22	-11.21	-11.2
-11.28	-11.27	-11.26	-11.25	-11.24	-11.23	-11.22	-11.21	-11.2
-11.28	-11.27	-11.26	-11.25	-11.24	-11.23	-11.22	-11.21	-11.2
-11.28	-11.27	-11.26	-11.25	-11.24	-11.23	-11.22	-11.21	-11.2
-11.28	-11.27	-11.26	-11.25	-11.24	-11.23	-11.22	-11.21	-11.2
-11.28	-11.27	-11.26	-11.25	-11.24	-11.23	-11.22	-11.21	-11.2
-11.28	-11.27	-11.26	-11.25	-11.24	-11.23	-11.22	-11.21	-11.2
-11.28	-11.27	-11.26	-11.25	-11.24	-11.23	-11.22	-11.21	-11.2
-11.28	-11.27	-11.26	-11.25	-11.24	-11.23	-11.22	-11.21	-11.2
-11.28	-11.27	-11.26	-11.25	-11.24	-11.23	-11.22	-11.21	-11.2
-11.28	-11.27	-11.26	-11.25	-11.24	-11.23	-11.22	-11.21	-11.2
-11.28	-11.27	-11.26	-11.25	-11.24	-11.23	-11.22	-11.21	-11.2
-11.28	-11.27	-11.26	-11.25	-11.24	-11.23	-11.22	-11.21	-11.2
-11.28	-11.27	-11.26	-11.25	-11.24	-11.23	-11.22	-11.21	-11.2
-11.28	-11.27	-11.26	-11.25	-11.24	-11.23	-11.22	-11.21	-11.2
-11.28	-11.27	-11.26	-11.25	-11.24	-11.23	-11.22	-11.21	-11.2
-11.28	-11.27	-11.26	-11.25	-11.24	-11.23	-11.22	-11.21	-11.2
-11.28	-11.27	-11.26	-11.25	-11.24	-11.23	-11.22	-11.21	-11.2
-11.28	-11.27	-11.26	-11.25	-11.24	-11.23	-11.22	-11.21	-11.2
-11.28	-11.27	-11.26	-11.25	-11.24	-11.23	-11.22	-11.21	-11.2
-11.28	-11.27	-11.26	-11.25	-11.24	-11.23	-11.22	-11.21	-11.2
-11.28	-11.27	-11.26	-11.25	-11.24	-11.23	-11.22	-11.21	-11.2
-11.28	-11.27	-11.26	-11.25	-11.24	-11.23	-11.22	-11.21	-11.2
-11.28	-11.27	-11.26	-11.25	-11.24	-11.23	-11.22	-11.21	-11.2
-11.28	-11.27	-11.26	-11.25	-11.24	-11.23	-11.22	-11.21	-11.2
-11.28	-11.27	-11.26	-11.25	-11.24	-11.23	-11.22	-11.21	-11.2
-11.28	-11.27	-11.26	-11.25	-11.24	-11.23	-11.22	-11.21	-11.2
-11.28	-11.27	-11.26	-11.25	-11.24	-11.23	-11.22	-11.21	-11.2
-11.28	-11.27	-11.26	-11.25	-11.24	-11.23	-11.22	-11.21	-11.2
-11.28	-11.27	-11.26	-11.25	-11.24	-11.23	-11.22	-11.21	-11.2
-11.28	-11.27	-11.26	-11.25	-11.24	-11.23	-11.22	-11.21	-11.2
-11.28	-11.27	-11.26	-11.25	-11.24	-11.23	-11.22	-11.21	-11.2
-11.28	-11.27	-11.26	-11.25	-11.24	-11.23	-11.22	-11.21	-11.2
-11.28	-11.27	-11.26	-11.25	-11.24	-11.23	-11.22	-11.21	-11.2
-11.28	-11.27	-11.26	-11.25	-11.24	-11.23	-11.22	-11.21	-11.2
-11.28	-11.27	-11.26	-11.25	-11.24	-11.23	-11.22	-11.21	-11.2
-11.28	-11.27	-11.26	-11.25	-11.24	-11.23	-11.22	-11.21	-11.2
-11.28	-11.27	-11.26	-11.25	-11.24	-11.23	-11.22	-11.21	-11.2
-11.28	-11.27	-11.26	-11.25	-11.24	-11.23	-11.22	-11.21	-11.2
-11.28	-11.27	-11.26	-11.25	-11.24	-11.23	-11.22	-11.21	-11.2
-11.28	-11.27	-11.26	-11.25	-11.24	-11.23	-11.22	-11.21	-11.2
-11.28	-11.27	-11.26	-11.25	-11.24	-11.23	-11.22	-11.21	-11.2
-11.28	-11.27	-11.26	-11.25	-11.24	-11.23	-11.22	-11.21	-11.2

-11.28	-11.27	-11.26	-11.25	-11.24	-11.23	-11.22	-11.21	-11.2
-11.28	-11.27	-11.26	-11.25	-11.24	-11.23	-11.22	-11.21	-11.2
-11.28	-11.27	-11.26	-11.25	-11.24	-11.23	-11.22	-11.21	-11.2
-11.28	-11.27	-11.26	-11.25	-11.24	-11.23	-11.22	-11.21	-11.2
-11.28	-11.27	-11.26	-11.25	-11.24	-11.23	-11.22	-11.21	-11.2
-11.28	-11.27	-11.26	-11.25	-11.24	-11.23	-11.22	-11.21	-11.2
-11.28	-11.27	-11.26	-11.25	-11.24	-11.23	-11.22	-11.21	-11.2
-11.28	-11.27	-11.26	-11.25	-11.24	-11.23	-11.22	-11.21	-11.2
-11.28	-11.27	-11.26	-11.25	-11.24	-11.23	-11.22	-11.21	-11.2
-11.28	-11.27	-11.26	-11.25	-11.24	-11.23	-11.22	-11.21	-11.2
-11.28	-11.27	-11.26	-11.25	-11.24	-11.23	-11.22	-11.21	-11.2
-11.28	-11.27	-11.26	-11.25	-11.24	-11.23	-11.22	-11.21	-11.2
-11.28	-11.27	-11.26	-11.25	-11.24	-11.23	-11.22	-11.21	-11.2
-11.28	-11.27	-11.26	-11.25	-11.24	-11.23	-11.22	-11.21	-11.2
-11.28	-11.27	-11.26	-11.25	-11.24	-11.23	-11.22	-11.21	-11.2
-11.28	-11.27	-11.26	-11.25	-11.24	-11.23	-11.22	-11.21	-11.2
-11.28	-11.27	-11.26	-11.25	-11.24	-11.23	-11.22	-11.21	-11.2
-11.28	-11.27	-11.26	-11.25	-11.24	-11.23	-11.22	-11.21	-11.2
-11.28	-11.27	-11.26	-11.25	-11.24	-11.23	-11.22	-11.21	-11.2
-11.28	-11.27	-11.26	-11.25	-11.24	-11.23	-11.22	-11.21	-11.2
-11.28	-11.27	-11.26	-11.25	-11.24	-11.23	-11.22	-11.21	-11.2
-11.28	-11.27	-11.26	-11.25	-11.24	-11.23	-11.22	-11.21	-11.2
-11.28	-11.27	-11.26	-11.25	-11.24	-11.23	-11.22	-11.21	-11.2
-11.28	-11.27	-11.26	-11.25	-11.24	-11.23	-11.22	-11.21	-11.2
-11.28	-11.27	-11.26	-11.25	-11.24	-11.23	-11.22	-11.21	-11.2
-11.28	-11.27	-11.26	-11.25	-11.24	-11.23	-11.22	-11.21	-11.2
-11.28	-11.27	-11.26	-11.25	-11.24	-11.23	-11.22	-11.21	-11.2
-11.28	-11.27	-11.26	-11.25	-11.24	-11.23	-11.22	-11.21	-11.2
-11.28	-11.27	-11.26	-11.25	-11.24	-11.23	-11.22	-11.21	-11.2
-11.28	-11.27	-11.26	-11.25	-11.24	-11.23	-11.22	-11.21	-11.2
-11.28	-11.27	-11.26	-11.25	-11.24	-11.23	-11.22	-11.21	-11.2
-11.28	-11.27	-11.26	-11.25	-11.24	-11.23	-11.22	-11.21	-11.2
-11.28	-11.27	-11.26	-11.25	-11.24	-11.23	-11.22	-11.21	-11.2
-11.28	-11.27	-11.26	-11.25	-11.24	-11.23	-11.22	-11.21	-11.2
-11.28	-11.27	-11.26	-11.25	-11.24	-11.23	-11.22	-11.21	-11.2
-11.28	-11.27	-11.26	-11.25	-11.24	-11.23	-11.22	-11.21	-11.2
-11.28	-11.27	-11.26	-11.25	-11.24	-11.23	-11.22	-11.21	-11.2
-11.28	-11.27	-11.26	-11.25	-11.24	-11.23	-11.22	-11.21	-11.2
-11.28	-11.27	-11.26	-11.25	-11.24	-11.23	-11.22	-11.21	-11.2
-11.28	-11.27	-11.26	-11.25	-11.24	-11.23	-11.22	-11.21	-11.2
-11.28	-11.27	-11.26	-11.25	-11.24	-11.23	-11.22	-11.21	-11.2
-11.28	-11.27	-11.26	-11.25	-11.24	-11.23	-11.22	-11.21	-11.2
-11.28	-11.27	-11.26	-11.25	-11.24	-11.23	-11.22	-11.21	-11.2
-11.28	-11.27	-11.26	-11.25	-11.24	-11.23	-11.22	-11.21	-11.2
-11.28	-11.27	-11.26	-11.25	-11.24	-11.23	-11.22	-11.21	-11.2
-11.28	-11.27	-11.26	-11.25	-11.24	-11.23	-11.22	-11.21	-11.2
-11.28	-11.27	-11.26	-11.25	-11.24	-11.23	-11.22	-11.21	-11.2
-11.28	-11.27	-11.26	-11.25	-11.24	-11.23	-11.22	-11.21	-11.2
-11.28	-11.27	-11.26	-11.25	-11.24	-11.23	-11.22	-11.21	-11.2
-11.28	-11.27	-11.26	-11.25	-11.24	-11.23	-11.22	-11.21	-11.2

-11.28	-11.27	-11.26	-11.25	-11.24	-11.23	-11.22	-11.21	-11.2
-11.28	-11.27	-11.26	-11.25	-11.24	-11.23	-11.22	-11.21	-11.2
-11.28	-11.27	-11.26	-11.25	-11.24	-11.23	-11.22	-11.21	-11.2
-11.28	-11.27	-11.26	-11.25	-11.24	-11.23	-11.22	-11.21	-11.2
-11.28	-11.27	-11.26	-11.25	-11.24	-11.23	-11.22	-11.21	-11.2
-11.28	-11.27	-11.26	-11.25	-11.24	-11.23	-11.22	-11.21	-11.2
-11.28	-11.27	-11.26	-11.25	-11.24	-11.23	-11.22	-11.21	-11.2
-11.28	-11.27	-11.26	-11.25	-11.24	-11.23	-11.22	-11.21	-11.2
-11.28	-11.27	-11.26	-11.25	-11.24	-11.23	-11.22	-11.21	-11.2
-11.28	-11.27	-11.26	-11.25	-11.24	-11.23	-11.22	-11.21	-11.2
-11.28	-11.27	-11.26	-11.25	-11.24	-11.23	-11.22	-11.21	-11.2
-11.28	-11.27	-11.26	-11.25	-11.24	-11.23	-11.22	-11.21	-11.2
-11.28	-11.27	-11.26	-11.25	-11.24	-11.23	-11.22	-11.21	-11.2
-11.28	-11.27	-11.26	-11.25	-11.24	-11.23	-11.22	-11.21	-11.2
-11.28	-11.27	-11.26	-11.25	-11.24	-11.23	-11.22	-11.21	-11.2
-11.28	-11.27	-11.26	-11.25	-11.24	-11.23	-11.22	-11.21	-11.2
-11.28	-11.27	-11.26	-11.25	-11.24	-11.23	-11.22	-11.21	-11.2
-11.28	-11.27	-11.26	-11.25	-11.24	-11.23	-11.22	-11.21	-11.2
-11.28	-11.27	-11.26	-11.25	-11.24	-11.23	-11.22	-11.21	-11.2
-11.28	-11.27	-11.26	-11.25	-11.24	-11.23	-11.22	-11.21	-11.2
-11.28	-11.27	-11.26	-11.25	-11.24	-11.23	-11.22	-11.21	-11.2
-11.28	-11.27	-11.26	-11.25	-11.24	-11.23	-11.22	-11.21	-11.2
-11.28	-11.27	-11.26	-11.25	-11.24	-11.23	-11.22	-11.21	-11.2
-11.28	-11.27	-11.26	-11.25	-11.24	-11.23	-11.22	-11.21	-11.2
-11.28	-11.27	-11.26	-11.25	-11.24	-11.23	-11.22	-11.21	-11.2
-11.28	-11.27	-11.26	-11.25	-11.24	-11.23	-11.22	-11.21	-11.2
-11.28	-11.27	-11.26	-11.25	-11.24	-11.23	-11.22	-11.21	-11.2
-11.28	-11.27	-11.26	-11.25	-11.24	-11.23	-11.22	-11.21	-11.2
-11.28	-11.27	-11.26	-11.25	-11.24	-11.23	-11.22	-11.21	-11.2
-11.28	-11.27	-11.26	-11.25	-11.24	-11.23	-11.22	-11.21	-11.2
-11.28	-11.27	-11.26	-11.25	-11.24	-11.23	-11.22	-11.21	-11.2
-11.28	-11.27	-11.26	-11.25	-11.24	-11.23	-11.22	-11.21	-11.2
-11.28	-11.27	-11.26	-11.25	-11.24	-11.23	-11.22	-11.21	-11.2
-11.28	-11.27	-11.26	-11.25	-11.24	-11.23	-11.22	-11.21	-11.2
-11.28	-11.27	-11.26	-11.25	-11.24	-11.23	-11.22	-11.21	-11.2
-11.28	-11.27	-11.26	-11.25	-11.24	-11.23	-11.22	-11.21	-11.2
-11.28	-11.27	-11.26	-11.25	-11.24	-11.23	-11.22	-11.21	-11.2
-11.28	-11.27	-11.26	-11.25	-11.24	-11.23	-11.22	-11.21	-11.2
-11.28	-11.27	-11.26	-11.25	-11.24	-11.23	-11.22	-11.21	-11.2
-11.28	-11.27	-11.26	-11.25	-11.24	-11.23	-11.22	-11.21	-11.2
-11.28	-11.27	-11.26	-11.25	-11.24	-11.23	-11.22	-11.21	-11.2
-11.28	-11.27	-11.26	-11.25	-11.24	-11.23	-11.22	-11.21	-11.2
-11.28	-11.27	-11.26	-11.25	-11.24	-11.23	-11.22	-11.21	-11.2
-11.28	-11.27	-11.26	-11.25	-11.24	-11.23	-11.22	-11.21	-11.2
-11.28	-11.27	-11.26	-11.25	-11.24	-11.23	-11.22	-11.21	-11.2
-11.28	-11.27	-11.26	-11.25	-11.24	-11.23	-11.22	-11.21	-11.2
-11.28	-11.27	-11.26	-11.25	-11.24	-11.23	-11.22	-11.21	-11.2
-11.28	-11.27	-11.26	-11.25	-11.24	-11.23	-11.22	-11.21	-11.2
-11.28	-11.27	-11.26	-11.25	-11.24	-11.23	-11.22	-11.21	-11.2
-11.28	-11.27	-11.26	-11.25	-11.24	-11.23	-11.22	-11.21	-11.2

-11.28	-11.27	-11.26	-11.25	-11.24	-11.23	-11.22	-11.21	-11.2
-11.28	-11.27	-11.26	-11.25	-11.24	-11.23	-11.22	-11.21	-11.2
-11.28	-11.27	-11.26	-11.25	-11.24	-11.23	-11.22	-11.21	-11.2
-11.28	-11.27	-11.26	-11.25	-11.24	-11.23	-11.22	-11.21	-11.2
-11.28	-11.27	-11.26	-11.25	-11.24	-11.23	-11.22	-11.21	-11.2
-11.28	-11.27	-11.26	-11.25	-11.24	-11.23	-11.22	-11.21	-11.2
-11.28	-11.27	-11.26	-11.25	-11.24	-11.23	-11.22	-11.21	-11.2
-11.28	-11.27	-11.26	-11.25	-11.24	-11.23	-11.22	-11.21	-11.2
-11.28	-11.27	-11.26	-11.25	-11.24	-11.23	-11.22	-11.21	-11.2
-11.28	-11.27	-11.26	-11.25	-11.24	-11.23	-11.22	-11.21	-11.2
-11.28	-11.27	-11.26	-11.25	-11.24	-11.23	-11.22	-11.21	-11.2
-11.28	-11.27	-11.26	-11.25	-11.24	-11.23	-11.22	-11.21	-11.2
-11.28	-11.27	-11.26	-11.25	-11.24	-11.23	-11.22	-11.21	-11.2
-11.28	-11.27	-11.26	-11.25	-11.24	-11.23	-11.22	-11.21	-11.2
-11.28	-11.27	-11.26	-11.25	-11.24	-11.23	-11.22	-11.21	-11.2
-11.28	-11.27	-11.26	-11.25	-11.24	-11.23	-11.22	-11.21	-11.2
-11.28	-11.27	-11.26	-11.25	-11.24	-11.23	-11.22	-11.21	-11.2
-11.28	-11.27	-11.26	-11.25	-11.24	-11.23	-11.22	-11.21	-11.2
-11.28	-11.27	-11.26	-11.25	-11.24	-11.23	-11.22	-11.21	-11.2
-11.28	-11.27	-11.26	-11.25	-11.24	-11.23	-11.22	-11.21	-11.2
-11.28	-11.27	-11.26	-11.25	-11.24	-11.23	-11.22	-11.21	-11.2
-11.28	-11.27	-11.26	-11.25	-11.24	-11.23	-11.22	-11.21	-11.2
-11.28	-11.27	-11.26	-11.25	-11.24	-11.23	-11.22	-11.21	-11.2
-11.28	-11.27	-11.26	-11.25	-11.24	-11.23	-11.22	-11.21	-11.2
-11.28	-11.27	-11.26	-11.25	-11.24	-11.23	-11.22	-11.21	-11.2
-11.28	-11.27	-11.26	-11.25	-11.24	-11.23	-11.22	-11.21	-11.2
-11.28	-11.27	-11.26	-11.25	-11.24	-11.23	-11.22	-11.21	-11.2
-11.28	-11.27	-11.26	-11.25	-11.24	-11.23	-11.22	-11.21	-11.2
-11.28	-11.27	-11.26	-11.25	-11.24	-11.23	-11.22	-11.21	-11.2
-11.28	-11.27	-11.26	-11.25	-11.24	-11.23	-11.22	-11.21	-11.2
-11.28	-11.27	-11.26	-11.25	-11.24	-11.23	-11.22	-11.21	-11.2
-11.28	-11.27	-11.26	-11.25	-11.24	-11.23	-11.22	-11.21	-11.2
-11.28	-11.27	-11.26	-11.25	-11.24	-11.23	-11.22	-11.21	-11.2
-11.28	-11.27	-11.26	-11.25	-11.24	-11.23	-11.22	-11.21	-11.2
-11.28	-11.27	-11.26	-11.25	-11.24	-11.23	-11.22	-11.21	-11.2
-11.28	-11.27	-11.26	-11.25	-11.24	-11.23	-11.22	-11.21	-11.2
-11.28	-11.27	-11.26	-11.25	-11.24	-11.23	-11.22	-11.21	-11.2
-11.28	-11.27	-11.26	-11.25	-11.24	-11.23	-11.22	-11.21	-11.2
-11.28	-11.27	-11.26	-11.25	-11.24	-11.23	-11.22	-11.21	-11.2
-11.28	-11.27	-11.26	-11.25	-11.24	-11.23	-11.22	-11.21	-11.2
-11.28	-11.27	-11.26	-11.25	-11.24	-11.23	-11.22	-11.21	-11.2
-11.28	-11.27	-11.26	-11.25	-11.24	-11.23	-11.22	-11.21	-11.2
-11.28	-11.27	-11.26	-11.25	-11.24	-11.23	-11.22	-11.21	-11.2
-11.28	-11.27	-11.26	-11.25	-11.24	-11.23	-11.22	-11.21	-11.2
-11.28	-11.27	-11.26	-11.25	-11.24	-11.23	-11.22	-11.21	-11.2
-11.28	-11.27	-11.26	-11.25	-11.24	-11.23	-11.22	-11.21	-11.2
-11.28	-11.27	-11.26	-11.25	-11.24	-11.23	-11.22	-11.21	-11.2
-11.28	-11.27	-11.26	-11.25	-11.24	-11.23	-11.22	-11.21	-11.2
-11.28	-11.27	-11.26	-11.25	-11.24	-11.23	-11.22	-11.21	-11.2
-11.28	-11.27	-11.26	-11.25	-11.24	-11.23	-11.22	-11.21	-11.2

-11.28	-11.27	-11.26	-11.25	-11.24	-11.23	-11.22	-11.21	-11.2
-11.28	-11.27	-11.26	-11.25	-11.24	-11.23	-11.22	-11.21	-11.2
-11.28	-11.27	-11.26	-11.25	-11.24	-11.23	-11.22	-11.21	-11.2
-11.28	-11.27	-11.26	-11.25	-11.24	-11.23	-11.22	-11.21	-11.2
-11.28	-11.27	-11.26	-11.25	-11.24	-11.23	-11.22	-11.21	-11.2
-11.28	-11.27	-11.26	-11.25	-11.24	-11.23	-11.22	-11.21	-11.2
-11.28	-11.27	-11.26	-11.25	-11.24	-11.23	-11.22	-11.21	-11.2
-11.28	-11.27	-11.26	-11.25	-11.24	-11.23	-11.22	-11.21	-11.2
-11.28	-11.27	-11.26	-11.25	-11.24	-11.23	-11.22	-11.21	-11.2
-11.28	-11.27	-11.26	-11.25	-11.24	-11.23	-11.22	-11.21	-11.2
-11.28	-11.27	-11.26	-11.25	-11.24	-11.23	-11.22	-11.21	-11.2
-11.28	-11.27	-11.26	-11.25	-11.24	-11.23	-11.22	-11.21	-11.2
-11.28	-11.27	-11.26	-11.25	-11.24	-11.23	-11.22	-11.21	-11.2
-11.28	-11.27	-11.26	-11.25	-11.24	-11.23	-11.22	-11.21	-11.2
-11.28	-11.27	-11.26	-11.25	-11.24	-11.23	-11.22	-11.21	-11.2
-11.28	-11.27	-11.26	-11.25	-11.24	-11.23	-11.22	-11.21	-11.2
-11.28	-11.27	-11.26	-11.25	-11.24	-11.23	-11.22	-11.21	-11.2
-11.28	-11.27	-11.26	-11.25	-11.24	-11.23	-11.22	-11.21	-11.2
-11.28	-11.27	-11.26	-11.25	-11.24	-11.23	-11.22	-11.21	-11.2
-11.28	-11.27	-11.26	-11.25	-11.24	-11.23	-11.22	-11.21	-11.2
-11.28	-11.27	-11.26	-11.25	-11.24	-11.23	-11.22	-11.21	-11.2
-11.28	-11.27	-11.26	-11.25	-11.24	-11.23	-11.22	-11.21	-11.2
-11.28	-11.27	-11.26	-11.25	-11.24	-11.23	-11.22	-11.21	-11.2
-11.28	-11.27	-11.26	-11.25	-11.24	-11.23	-11.22	-11.21	-11.2
-11.28	-11.27	-11.26	-11.25	-11.24	-11.23	-11.22	-11.21	-11.2
-11.28	-11.27	-11.26	-11.25	-11.24	-11.23	-11.22	-11.21	-11.2
-11.28	-11.27	-11.26	-11.25	-11.24	-11.23	-11.22	-11.21	-11.2
-11.28	-11.27	-11.26	-11.25	-11.24	-11.23	-11.22	-11.21	-11.2
-11.28	-11.27	-11.26	-11.25	-11.24	-11.23	-11.22	-11.21	-11.2
-11.28	-11.27	-11.26	-11.25	-11.24	-11.23	-11.22	-11.21	-11.2
-11.28	-11.27	-11.26	-11.25	-11.24	-11.23	-11.22	-11.21	-11.2
-11.28	-11.27	-11.26	-11.25	-11.24	-11.23	-11.22	-11.21	-11.2
-11.28	-11.27	-11.26	-11.25	-11.24	-11.23	-11.22	-11.21	-11.2
-11.28	-11.27	-11.26	-11.25	-11.24	-11.23	-11.22	-11.21	-11.2
-11.28	-11.27	-11.26	-11.25	-11.24	-11.23	-11.22	-11.21	-11.2
-11.28	-11.27	-11.26	-11.25	-11.24	-11.23	-11.22	-11.21	-11.2
-11.28	-11.27	-11.26	-11.25	-11.24	-11.23	-11.22	-11.21	-11.2
-11.28	-11.27	-11.26	-11.25	-11.24	-11.23	-11.22	-11.21	-11.2
-11.28	-11.27	-11.26	-11.25	-11.24	-11.23	-11.22	-11.21	-11.2
-11.28	-11.27	-11.26	-11.25	-11.24	-11.23	-11.22	-11.21	-11.2
-11.28	-11.27	-11.26	-11.25	-11.24	-11.23	-11.22	-11.21	-11.2
-11.28	-11.27	-11.26	-11.25	-11.24	-11.23	-11.22	-11.21	-11.2
-11.28	-11.27	-11.26	-11.25	-11.24	-11.23	-11.22	-11.21	-11.2
-11.28	-11.27	-11.26	-11.25	-11.24	-11.23	-11.22	-11.21	-11.2
-11.28	-11.27	-11.26	-11.25	-11.24	-11.23	-11.22	-11.21	-11.2
-11.28	-11.27	-11.26	-11.25	-11.24	-11.23	-11.22	-11.21	-11.2
-11.28	-11.27	-11.26	-11.25	-11.24	-11.23	-11.22	-11.21	-11.2
-11.28	-11.27	-11.26	-11.25	-11.24	-11.23	-11.22	-11.21	-11.2
-11.28	-11.27	-11.26	-11.25	-11.24	-11.23	-11.22	-11.21	-11.2
-11.28	-11.27	-11.26	-11.25	-11.24	-11.23	-11.22	-11.21	-11.2

-11.28	-11.27	-11.26	-11.25	-11.24	-11.23	-11.22	-11.21	-11.2
-11.28	-11.27	-11.26	-11.25	-11.24	-11.23	-11.22	-11.21	-11.2
-11.28	-11.27	-11.26	-11.25	-11.24	-11.23	-11.22	-11.21	-11.2
-11.28	-11.27	-11.26	-11.25	-11.24	-11.23	-11.22	-11.21	-11.2
-11.28	-11.27	-11.26	-11.25	-11.24	-11.23	-11.22	-11.21	-11.2
-11.28	-11.27	-11.26	-11.25	-11.24	-11.23	-11.22	-11.21	-11.2
-11.28	-11.27	-11.26	-11.25	-11.24	-11.23	-11.22	-11.21	-11.2
-11.28	-11.27	-11.26	-11.25	-11.24	-11.23	-11.22	-11.21	-11.2
-11.28	-11.27	-11.26	-11.25	-11.24	-11.23	-11.22	-11.21	-11.2
-11.28	-11.27	-11.26	-11.25	-11.24	-11.23	-11.22	-11.21	-11.2
-11.28	-11.27	-11.26	-11.25	-11.24	-11.23	-11.22	-11.21	-11.2
-11.28	-11.27	-11.26	-11.25	-11.24	-11.23	-11.22	-11.21	-11.2
-11.28	-11.27	-11.26	-11.25	-11.24	-11.23	-11.22	-11.21	-11.2
-11.28	-11.27	-11.26	-11.25	-11.24	-11.23	-11.22	-11.21	-11.2
-11.28	-11.27	-11.26	-11.25	-11.24	-11.23	-11.22	-11.21	-11.2
-11.28	-11.27	-11.26	-11.25	-11.24	-11.23	-11.22	-11.21	-11.2
-11.28	-11.27	-11.26	-11.25	-11.24	-11.23	-11.22	-11.21	-11.2
-11.28	-11.27	-11.26	-11.25	-11.24	-11.23	-11.22	-11.21	-11.2
-11.28	-11.27	-11.26	-11.25	-11.24	-11.23	-11.22	-11.21	-11.2
-11.28	-11.27	-11.26	-11.25	-11.24	-11.23	-11.22	-11.21	-11.2
-11.28	-11.27	-11.26	-11.25	-11.24	-11.23	-11.22	-11.21	-11.2
-11.28	-11.27	-11.26	-11.25	-11.24	-11.23	-11.22	-11.21	-11.2
-11.28	-11.27	-11.26	-11.25	-11.24	-11.23	-11.22	-11.21	-11.2
-11.28	-11.27	-11.26	-11.25	-11.24	-11.23	-11.22	-11.21	-11.2
-11.28	-11.27	-11.26	-11.25	-11.24	-11.23	-11.22	-11.21	-11.2
-11.28	-11.27	-11.26	-11.25	-11.24	-11.23	-11.22	-11.21	-11.2
-11.28	-11.27	-11.26	-11.25	-11.24	-11.23	-11.22	-11.21	-11.2
-11.28	-11.27	-11.26	-11.25	-11.24	-11.23	-11.22	-11.21	-11.2
-11.28	-11.27	-11.26	-11.25	-11.24	-11.23	-11.22	-11.21	-11.2
-11.28	-11.27	-11.26	-11.25	-11.24	-11.23	-11.22	-11.21	-11.2
-11.28	-11.27	-11.26	-11.25	-11.24	-11.23	-11.22	-11.21	-11.2
-11.28	-11.27	-11.26	-11.25	-11.24	-11.23	-11.22	-11.21	-11.2
-11.28	-11.27	-11.26	-11.25	-11.24	-11.23	-11.22	-11.21	-11.2
-11.28	-11.27	-11.26	-11.25	-11.24	-11.23	-11.22	-11.21	-11.2
-11.28	-11.27	-11.26	-11.25	-11.24	-11.23	-11.22	-11.21	-11.2
-11.28	-11.27	-11.26	-11.25	-11.24	-11.23	-11.22	-11.21	-11.2
-11.28	-11.27	-11.26	-11.25	-11.24	-11.23	-11.22	-11.21	-11.2
-11.28	-11.27	-11.26	-11.25	-11.24	-11.23	-11.22	-11.21	-11.2
-11.28	-11.27	-11.26	-11.25	-11.24	-11.23	-11.22	-11.21	-11.2
-11.28	-11.27	-11.26	-11.25	-11.24	-11.23	-11.22	-11.21	-11.2
-11.28	-11.27	-11.26	-11.25	-11.24	-11.23	-11.22	-11.21	-11.2
-11.28	-11.27	-11.26	-11.25	-11.24	-11.23	-11.22	-11.21	-11.2
-11.28	-11.27	-11.26	-11.25	-11.24	-11.23	-11.22	-11.21	-11.2
-11.28	-11.27	-11.26	-11.25	-11.24	-11.23	-11.22	-11.21	-11.2
-11.28	-11.27	-11.26	-11.25	-11.24	-11.23	-11.22	-11.21	-11.2
-11.28	-11.27	-11.26	-11.25	-11.24	-11.23	-11.22	-11.21	-11.2
-11.28	-11.27	-11.26	-11.25	-11.24	-11.23	-11.22	-11.21	-11.2
-11.28	-11.27	-11.26	-11.25	-11.24	-11.23	-11.22	-11.21	-11.2
-11.28	-11.27	-11.26	-11.25	-11.24	-11.23	-11.22	-11.21	-11.2
-11.28	-11.27	-11.26	-11.25	-11.24	-11.23	-11.22	-11.21	-11.2

-11.28	-11.27	-11.26	-11.25	-11.24	-11.23	-11.22	-11.21	-11.2
-11.28	-11.27	-11.26	-11.25	-11.24	-11.23	-11.22	-11.21	-11.2
-11.28	-11.27	-11.26	-11.25	-11.24	-11.23	-11.22	-11.21	-11.2
-11.28	-11.27	-11.26	-11.25	-11.24	-11.23	-11.22	-11.21	-11.2
-11.28	-11.27	-11.26	-11.25	-11.24	-11.23	-11.22	-11.21	-11.2
-11.28	-11.27	-11.26	-11.25	-11.24	-11.23	-11.22	-11.21	-11.2
-11.28	-11.27	-11.26	-11.25	-11.24	-11.23	-11.22	-11.21	-11.2
-11.28	-11.27	-11.26	-11.25	-11.24	-11.23	-11.22	-11.21	-11.2
-11.28	-11.27	-11.26	-11.25	-11.24	-11.23	-11.22	-11.21	-11.2
-11.28	-11.27	-11.26	-11.25	-11.24	-11.23	-11.22	-11.21	-11.2
-11.28	-11.27	-11.26	-11.25	-11.24	-11.23	-11.22	-11.21	-11.2
-11.28	-11.27	-11.26	-11.25	-11.24	-11.23	-11.22	-11.21	-11.2
-11.28	-11.27	-11.26	-11.25	-11.24	-11.23	-11.22	-11.21	-11.2
-11.28	-11.27	-11.26	-11.25	-11.24	-11.23	-11.22	-11.21	-11.2
-11.28	-11.27	-11.26	-11.25	-11.24	-11.23	-11.22	-11.21	-11.2
-11.28	-11.27	-11.26	-11.25	-11.24	-11.23	-11.22	-11.21	-11.2
-11.28	-11.27	-11.26	-11.25	-11.24	-11.23	-11.22	-11.21	-11.2
-11.28	-11.27	-11.26	-11.25	-11.24	-11.23	-11.22	-11.21	-11.2
-11.28	-11.27	-11.26	-11.25	-11.24	-11.23	-11.22	-11.21	-11.2
-11.28	-11.27	-11.26	-11.25	-11.24	-11.23	-11.22	-11.21	-11.2
-11.28	-11.27	-11.26	-11.25	-11.24	-11.23	-11.22	-11.21	-11.2
-11.28	-11.27	-11.26	-11.25	-11.24	-11.23	-11.22	-11.21	-11.2
-11.28	-11.27	-11.26	-11.25	-11.24	-11.23	-11.22	-11.21	-11.2
-11.28	-11.27	-11.26	-11.25	-11.24	-11.23	-11.22	-11.21	-11.2
-11.28	-11.27	-11.26	-11.25	-11.24	-11.23	-11.22	-11.21	-11.2
-11.28	-11.27	-11.26	-11.25	-11.24	-11.23	-11.22	-11.21	-11.2
-11.28	-11.27	-11.26	-11.25	-11.24	-11.23	-11.22	-11.21	-11.2
-11.28	-11.27	-11.26	-11.25	-11.24	-11.23	-11.22	-11.21	-11.2
-11.28	-11.27	-11.26	-11.25	-11.24	-11.23	-11.22	-11.21	-11.2
-11.28	-11.27	-11.26	-11.25	-11.24	-11.23	-11.22	-11.21	-11.2
-11.28	-11.27	-11.26	-11.25	-11.24	-11.23	-11.22	-11.21	-11.2
-11.28	-11.27	-11.26	-11.25	-11.24	-11.23	-11.22	-11.21	-11.2
-11.28	-11.27	-11.26	-11.25	-11.24	-11.23	-11.22	-11.21	-11.2
-11.28	-11.27	-11.26	-11.25	-11.24	-11.23	-11.22	-11.21	-11.2
-11.28	-11.27	-11.26	-11.25	-11.24	-11.23	-11.22	-11.21	-11.2
-11.28	-11.27	-11.26	-11.25	-11.24	-11.23	-11.22	-11.21	-11.2
-11.28	-11.27	-11.26	-11.25	-11.24	-11.23	-11.22	-11.21	-11.2
-11.28	-11.27	-11.26	-11.25	-11.24	-11.23	-11.22	-11.21	-11.2
-11.28	-11.27	-11.26	-11.25	-11.24	-11.23	-11.22	-11.21	-11.2
-11.28	-11.27	-11.26	-11.25	-11.24	-11.23	-11.22	-11.21	-11.2
-11.28	-11.27	-11.26	-11.25	-11.24	-11.23	-11.22	-11.21	-11.2
-11.28	-11.27	-11.26	-11.25	-11.24	-11.23	-11.22	-11.21	-11.2
-11.28	-11.27	-11.26	-11.25	-11.24	-11.23	-11.22	-11.21	-11.2
-11.28	-11.27	-11.26	-11.25	-11.24	-11.23	-11.22	-11.21	-11.2
-11.28	-11.27	-11.26	-11.25	-11.24	-11.23	-11.22	-11.21	-11.2
-11.28	-11.27	-11.26	-11.25	-11.24	-11.23	-11.22	-11.21	-11.2
-11.28	-11.27	-11.26	-11.25	-11.24	-11.23	-11.22	-11.21	-11.2
-11.28	-11.27	-11.26	-11.25	-11.24	-11.23	-11.22	-11.21	-11.2
-11.28	-11.27	-11.26	-11.25	-11.24	-11.23	-11.22	-11.21	-11.2
-11.28	-11.27	-11.26	-11.25	-11.24	-11.23	-11.22	-11.21	-11.2

-11.28	-11.27	-11.26	-11.25	-11.24	-11.23	-11.22	-11.21	-11.2
-11.28	-11.27	-11.26	-11.25	-11.24	-11.23	-11.22	-11.21	-11.2
-11.28	-11.27	-11.26	-11.25	-11.24	-11.23	-11.22	-11.21	-11.2
-11.28	-11.27	-11.26	-11.25	-11.24	-11.23	-11.22	-11.21	-11.2
-11.28	-11.27	-11.26	-11.25	-11.24	-11.23	-11.22	-11.21	-11.2
-11.28	-11.27	-11.26	-11.25	-11.24	-11.23	-11.22	-11.21	-11.2
-11.28	-11.27	-11.26	-11.25	-11.24	-11.23	-11.22	-11.21	-11.2
-11.28	-11.27	-11.26	-11.25	-11.24	-11.23	-11.22	-11.21	-11.2
-11.28	-11.27	-11.26	-11.25	-11.24	-11.23	-11.22	-11.21	-11.2
-11.28	-11.27	-11.26	-11.25	-11.24	-11.23	-11.22	-11.21	-11.2
-11.28	-11.27	-11.26	-11.25	-11.24	-11.23	-11.22	-11.21	-11.2
-11.28	-11.27	-11.26	-11.25	-11.24	-11.23	-11.22	-11.21	-11.2
-11.28	-11.27	-11.26	-11.25	-11.24	-11.23	-11.22	-11.21	-11.2
-11.28	-11.27	-11.26	-11.25	-11.24	-11.23	-11.22	-11.21	-11.2
-11.28	-11.27	-11.26	-11.25	-11.24	-11.23	-11.22	-11.21	-11.2
-11.28	-11.27	-11.26	-11.25	-11.24	-11.23	-11.22	-11.21	-11.2
-11.28	-11.27	-11.26	-11.25	-11.24	-11.23	-11.22	-11.21	-11.2
-11.28	-11.27	-11.26	-11.25	-11.24	-11.23	-11.22	-11.21	-11.2
-11.28	-11.27	-11.26	-11.25	-11.24	-11.23	-11.22	-11.21	-11.2
-11.28	-11.27	-11.26	-11.25	-11.24	-11.23	-11.22	-11.21	-11.2
-11.28	-11.27	-11.26	-11.25	-11.24	-11.23	-11.22	-11.21	-11.2
-11.28	-11.27	-11.26	-11.25	-11.24	-11.23	-11.22	-11.21	-11.2
-11.28	-11.27	-11.26	-11.25	-11.24	-11.23	-11.22	-11.21	-11.2
-11.28	-11.27	-11.26	-11.25	-11.24	-11.23	-11.22	-11.21	-11.2
-11.28	-11.27	-11.26	-11.25	-11.24	-11.23	-11.22	-11.21	-11.2
-11.28	-11.27	-11.26	-11.25	-11.24	-11.23	-11.22	-11.21	-11.2
-11.28	-11.27	-11.26	-11.25	-11.24	-11.23	-11.22	-11.21	-11.2
-11.28	-11.27	-11.26	-11.25	-11.24	-11.23	-11.22	-11.21	-11.2
-11.28	-11.27	-11.26	-11.25	-11.24	-11.23	-11.22	-11.21	-11.2
-11.28	-11.27	-11.26	-11.25	-11.24	-11.23	-11.22	-11.21	-11.2
-11.28	-11.27	-11.26	-11.25	-11.24	-11.23	-11.22	-11.21	-11.2
-11.28	-11.27	-11.26	-11.25	-11.24	-11.23	-11.22	-11.21	-11.2
-11.28	-11.27	-11.26	-11.25	-11.24	-11.23	-11.22	-11.21	-11.2
-11.28	-11.27	-11.26	-11.25	-11.24	-11.23	-11.22	-11.21	-11.2
-11.28	-11.27	-11.26	-11.25	-11.24	-11.23	-11.22	-11.21	-11.2
-11.28	-11.27	-11.26	-11.25	-11.24	-11.23	-11.22	-11.21	-11.2
-11.28	-11.27	-11.26	-11.25	-11.24	-11.23	-11.22	-11.21	-11.2
-11.28	-11.27	-11.26	-11.25	-11.24	-11.23	-11.22	-11.21	-11.2
-11.28	-11.27	-11.26	-11.25	-11.24	-11.23	-11.22	-11.21	-11.2
-11.28	-11.27	-11.26	-11.25	-11.24	-11.23	-11.22	-11.21	-11.2
-11.28	-11.27	-11.26	-11.25	-11.24	-11.23	-11.22	-11.21	-11.2
-11.28	-11.27	-11.26	-11.25	-11.24	-11.23	-11.22	-11.21	-11.2
-11.28	-11.27	-11.26	-11.25	-11.24	-11.23	-11.22	-11.21	-11.2
-11.28	-11.27	-11.26	-11.25	-11.24	-11.23	-11.22	-11.21	-11.2
-11.28	-11.27	-11.26	-11.25	-11.24	-11.23	-11.22	-11.21	-11.2
-11.28	-11.27	-11.26	-11.25	-11.24	-11.23	-11.22	-11.21	-11.2
-11.28	-11.27	-11.26	-11.25	-11.24	-11.23	-11.22	-11.21	-11.2
-11.28	-11.27	-11.26	-11.25	-11.24	-11.23	-11.22	-11.21	-11.2
-11.28	-11.27	-11.26	-11.25	-11.24	-11.23	-11.22	-11.21	-11.2
-11.28	-11.27	-11.26	-11.25	-11.24	-11.23	-11.22	-11.21	-11.2

-11.28	-11.27	-11.26	-11.25	-11.24	-11.23	-11.22	-11.21	-11.2
-11.28	-11.27	-11.26	-11.25	-11.24	-11.23	-11.22	-11.21	-11.2
-11.28	-11.27	-11.26	-11.25	-11.24	-11.23	-11.22	-11.21	-11.2
-11.28	-11.27	-11.26	-11.25	-11.24	-11.23	-11.22	-11.21	-11.2
-11.28	-11.27	-11.26	-11.25	-11.24	-11.23	-11.22	-11.21	-11.2
-11.28	-11.27	-11.26	-11.25	-11.24	-11.23	-11.22	-11.21	-11.2
-11.28	-11.27	-11.26	-11.25	-11.24	-11.23	-11.22	-11.21	-11.2
-11.28	-11.27	-11.26	-11.25	-11.24	-11.23	-11.22	-11.21	-11.2
-11.28	-11.27	-11.26	-11.25	-11.24	-11.23	-11.22	-11.21	-11.2
-11.28	-11.27	-11.26	-11.25	-11.24	-11.23	-11.22	-11.21	-11.2
-11.28	-11.27	-11.26	-11.25	-11.24	-11.23	-11.22	-11.21	-11.2
-11.28	-11.27	-11.26	-11.25	-11.24	-11.23	-11.22	-11.21	-11.2
-11.28	-11.27	-11.26	-11.25	-11.24	-11.23	-11.22	-11.21	-11.2
-11.28	-11.27	-11.26	-11.25	-11.24	-11.23	-11.22	-11.21	-11.2
-11.28	-11.27	-11.26	-11.25	-11.24	-11.23	-11.22	-11.21	-11.2
-11.28	-11.27	-11.26	-11.25	-11.24	-11.23	-11.22	-11.21	-11.2
-11.28	-11.27	-11.26	-11.25	-11.24	-11.23	-11.22	-11.21	-11.2
-11.28	-11.27	-11.26	-11.25	-11.24	-11.23	-11.22	-11.21	-11.2
-11.28	-11.27	-11.26	-11.25	-11.24	-11.23	-11.22	-11.21	-11.2
-11.28	-11.27	-11.26	-11.25	-11.24	-11.23	-11.22	-11.21	-11.2
-11.28	-11.27	-11.26	-11.25	-11.24	-11.23	-11.22	-11.21	-11.2
-11.28	-11.27	-11.26	-11.25	-11.24	-11.23	-11.22	-11.21	-11.2
-11.28	-11.27	-11.26	-11.25	-11.24	-11.23	-11.22	-11.21	-11.2
-11.28	-11.27	-11.26	-11.25	-11.24	-11.23	-11.22	-11.21	-11.2
-11.28	-11.27	-11.26	-11.25	-11.24	-11.23	-11.22	-11.21	-11.2
-11.28	-11.27	-11.26	-11.25	-11.24	-11.23	-11.22	-11.21	-11.2
-11.28	-11.27	-11.26	-11.25	-11.24	-11.23	-11.22	-11.21	-11.2
-11.28	-11.27	-11.26	-11.25	-11.24	-11.23	-11.22	-11.21	-11.2
-11.28	-11.27	-11.26	-11.25	-11.24	-11.23	-11.22	-11.21	-11.2
-11.28	-11.27	-11.26	-11.25	-11.24	-11.23	-11.22	-11.21	-11.2
-11.28	-11.27	-11.26	-11.25	-11.24	-11.23	-11.22	-11.21	-11.2
-11.28	-11.27	-11.26	-11.25	-11.24	-11.23	-11.22	-11.21	-11.2
-11.28	-11.27	-11.26	-11.25	-11.24	-11.23	-11.22	-11.21	-11.2
-11.28	-11.27	-11.26	-11.25	-11.24	-11.23	-11.22	-11.21	-11.2
-11.28	-11.27	-11.26	-11.25	-11.24	-11.23	-11.22	-11.21	-11.2
-11.28	-11.27	-11.26	-11.25	-11.24	-11.23	-11.22	-11.21	-11.2
-11.28	-11.27	-11.26	-11.25	-11.24	-11.23	-11.22	-11.21	-11.2
-11.28	-11.27	-11.26	-11.25	-11.24	-11.23	-11.22	-11.21	-11.2
-11.28	-11.27	-11.26	-11.25	-11.24	-11.23	-11.22	-11.21	-11.2
-11.28	-11.27	-11.26	-11.25	-11.24	-11.23	-11.22	-11.21	-11.2
-11.28	-11.27	-11.26	-11.25	-11.24	-11.23	-11.22	-11.21	-11.2
-11.28	-11.27	-11.26	-11.25	-11.24	-11.23	-11.22	-11.21	-11.2
-11.28	-11.27	-11.26	-11.25	-11.24	-11.23	-11.22	-11.21	-11.2
-11.28	-11.27	-11.26	-11.25	-11.24	-11.23	-11.22	-11.21	-11.2
-11.28	-11.27	-11.26	-11.25	-11.24	-11.23	-11.22	-11.21	-11.2
-11.28	-11.27	-11.26	-11.25	-11.24	-11.23	-11.22	-11.21	-11.2
-11.28	-11.27	-11.26	-11.25	-11.24	-11.23	-11.22	-11.21	-11.2
-11.28	-11.27	-11.26	-11.25	-11.24	-11.23	-11.22	-11.21	-11.2
-11.28	-11.27	-11.26	-11.25	-11.24	-11.23	-11.22	-11.21	-11.2
-11.28	-11.27	-11.26	-11.25	-11.24	-11.23	-11.22	-11.21	-11.2

-11.28	-11.27	-11.26	-11.25	-11.24	-11.23	-11.22	-11.21	-11.2
-11.28	-11.27	-11.26	-11.25	-11.24	-11.23	-11.22	-11.21	-11.2
-11.28	-11.27	-11.26	-11.25	-11.24	-11.23	-11.22	-11.21	-11.2
-11.28	-11.27	-11.26	-11.25	-11.24	-11.23	-11.22	-11.21	-11.2
-11.28	-11.27	-11.26	-11.25	-11.24	-11.23	-11.22	-11.21	-11.2
-11.28	-11.27	-11.26	-11.25	-11.24	-11.23	-11.22	-11.21	-11.2
-11.28	-11.27	-11.26	-11.25	-11.24	-11.23	-11.22	-11.21	-11.2
-11.28	-11.27	-11.26	-11.25	-11.24	-11.23	-11.22	-11.21	-11.2
-11.28	-11.27	-11.26	-11.25	-11.24	-11.23	-11.22	-11.21	-11.2
-11.28	-11.27	-11.26	-11.25	-11.24	-11.23	-11.22	-11.21	-11.2
-11.28	-11.27	-11.26	-11.25	-11.24	-11.23	-11.22	-11.21	-11.2
-11.28	-11.27	-11.26	-11.25	-11.24	-11.23	-11.22	-11.21	-11.2
-11.28	-11.27	-11.26	-11.25	-11.24	-11.23	-11.22	-11.21	-11.2
-11.28	-11.27	-11.26	-11.25	-11.24	-11.23	-11.22	-11.21	-11.2
-11.28	-11.27	-11.26	-11.25	-11.24	-11.23	-11.22	-11.21	-11.2
-11.28	-11.27	-11.26	-11.25	-11.24	-11.23	-11.22	-11.21	-11.2
-11.28	-11.27	-11.26	-11.25	-11.24	-11.23	-11.22	-11.21	-11.2
-11.28	-11.27	-11.26	-11.25	-11.24	-11.23	-11.22	-11.21	-11.2
-11.28	-11.27	-11.26	-11.25	-11.24	-11.23	-11.22	-11.21	-11.2
-11.28	-11.27	-11.26	-11.25	-11.24	-11.23	-11.22	-11.21	-11.2
-11.28	-11.27	-11.26	-11.25	-11.24	-11.23	-11.22	-11.21	-11.2
-11.28	-11.27	-11.26	-11.25	-11.24	-11.23	-11.22	-11.21	-11.2
-11.28	-11.27	-11.26	-11.25	-11.24	-11.23	-11.22	-11.21	-11.2
-11.28	-11.27	-11.26	-11.25	-11.24	-11.23	-11.22	-11.21	-11.2
-11.28	-11.27	-11.26	-11.25	-11.24	-11.23	-11.22	-11.21	-11.2
-11.28	-11.27	-11.26	-11.25	-11.24	-11.23	-11.22	-11.21	-11.2
-11.28	-11.27	-11.26	-11.25	-11.24	-11.23	-11.22	-11.21	-11.2
-11.28	-11.27	-11.26	-11.25	-11.24	-11.23	-11.22	-11.21	-11.2
-11.28	-11.27	-11.26	-11.25	-11.24	-11.23	-11.22	-11.21	-11.2
-11.28	-11.27	-11.26	-11.25	-11.24	-11.23	-11.22	-11.21	-11.2
-11.28	-11.27	-11.26	-11.25	-11.24	-11.23	-11.22	-11.21	-11.2
-11.28	-11.27	-11.26	-11.25	-11.24	-11.23	-11.22	-11.21	-11.2
-11.28	-11.27	-11.26	-11.25	-11.24	-11.23	-11.22	-11.21	-11.2
-11.28	-11.27	-11.26	-11.25	-11.24	-11.23	-11.22	-11.21	-11.2
-11.28	-11.27	-11.26	-11.25	-11.24	-11.23	-11.22	-11.21	-11.2
-11.28	-11.27	-11.26	-11.25	-11.24	-11.23	-11.22	-11.21	-11.2
-11.28	-11.27	-11.26	-11.25	-11.24	-11.23	-11.22	-11.21	-11.2
-11.28	-11.27	-11.26	-11.25	-11.24	-11.23	-11.22	-11.21	-11.2
-11.28	-11.27	-11.26	-11.25	-11.24	-11.23	-11.22	-11.21	-11.2
-11.28	-11.27	-11.26	-11.25	-11.24	-11.23	-11.22	-11.21	-11.2
-11.28	-11.27	-11.26	-11.25	-11.24	-11.23	-11.22	-11.21	-11.2
-11.28	-11.27	-11.26	-11.25	-11.24	-11.23	-11.22	-11.21	-11.2
-11.28	-11.27	-11.26	-11.25	-11.24	-11.23	-11.22	-11.21	-11.2
-11.28	-11.27	-11.26	-11.25	-11.24	-11.23	-11.22	-11.21	-11.2
-11.28	-11.27	-11.26	-11.25	-11.24	-11.23	-11.22	-11.21	-11.2
-11.28	-11.27	-11.26	-11.25	-11.24	-11.23	-11.22	-11.21	-11.2
-11.28	-11.27	-11.26	-11.25	-11.24	-11.23	-11.22	-11.21	-11.2
-11.28	-11.27	-11.26	-11.25	-11.24	-11.23	-11.22	-11.21	-11.2
-11.28	-11.27	-11.26	-11.25	-11.24	-11.23	-11.22	-11.21	-11.2
-11.28	-11.27	-11.26	-11.25	-11.24	-11.23	-11.22	-11.21	-11.2

-11.28	-11.27	-11.26	-11.25	-11.24	-11.23	-11.22	-11.21	-11.2
-11.28	-11.27	-11.26	-11.25	-11.24	-11.23	-11.22	-11.21	-11.2
-11.28	-11.27	-11.26	-11.25	-11.24	-11.23	-11.22	-11.21	-11.2
-11.28	-11.27	-11.26	-11.25	-11.24	-11.23	-11.22	-11.21	-11.2
-11.28	-11.27	-11.26	-11.25	-11.24	-11.23	-11.22	-11.21	-11.2
-11.28	-11.27	-11.26	-11.25	-11.24	-11.23	-11.22	-11.21	-11.2
-11.28	-11.27	-11.26	-11.25	-11.24	-11.23	-11.22	-11.21	-11.2
-11.28	-11.27	-11.26	-11.25	-11.24	-11.23	-11.22	-11.21	-11.2
-11.28	-11.27	-11.26	-11.25	-11.24	-11.23	-11.22	-11.21	-11.2
-11.28	-11.27	-11.26	-11.25	-11.24	-11.23	-11.22	-11.21	-11.2
-11.28	-11.27	-11.26	-11.25	-11.24	-11.23	-11.22	-11.21	-11.2
-11.28	-11.27	-11.26	-11.25	-11.24	-11.23	-11.22	-11.21	-11.2
-11.28	-11.27	-11.26	-11.25	-11.24	-11.23	-11.22	-11.21	-11.2
-11.28	-11.27	-11.26	-11.25	-11.24	-11.23	-11.22	-11.21	-11.2
-11.28	-11.27	-11.26	-11.25	-11.24	-11.23	-11.22	-11.21	-11.2
-11.28	-11.27	-11.26	-11.25	-11.24	-11.23	-11.22	-11.21	-11.2
-11.28	-11.27	-11.26	-11.25	-11.24	-11.23	-11.22	-11.21	-11.2
-11.28	-11.27	-11.26	-11.25	-11.24	-11.23	-11.22	-11.21	-11.2
-11.28	-11.27	-11.26	-11.25	-11.24	-11.23	-11.22	-11.21	-11.2
-11.28	-11.27	-11.26	-11.25	-11.24	-11.23	-11.22	-11.21	-11.2
-11.28	-11.27	-11.26	-11.25	-11.24	-11.23	-11.22	-11.21	-11.2
-11.28	-11.27	-11.26	-11.25	-11.24	-11.23	-11.22	-11.21	-11.2
-11.28	-11.27	-11.26	-11.25	-11.24	-11.23	-11.22	-11.21	-11.2
-11.28	-11.27	-11.26	-11.25	-11.24	-11.23	-11.22	-11.21	-11.2
-11.28	-11.27	-11.26	-11.25	-11.24	-11.23	-11.22	-11.21	-11.2
-11.28	-11.27	-11.26	-11.25	-11.24	-11.23	-11.22	-11.21	-11.2
-11.28	-11.27	-11.26	-11.25	-11.24	-11.23	-11.22	-11.21	-11.2
-11.28	-11.27	-11.26	-11.25	-11.24	-11.23	-11.22	-11.21	-11.2
-11.28	-11.27	-11.26	-11.25	-11.24	-11.23	-11.22	-11.21	-11.2
-11.28	-11.27	-11.26	-11.25	-11.24	-11.23	-11.22	-11.21	-11.2
-11.28	-11.27	-11.26	-11.25	-11.24	-11.23	-11.22	-11.21	-11.2
-11.28	-11.27	-11.26	-11.25	-11.24	-11.23	-11.22	-11.21	-11.2
-11.28	-11.27	-11.26	-11.25	-11.24	-11.23	-11.22	-11.21	-11.2
-11.28	-11.27	-11.26	-11.25	-11.24	-11.23	-11.22	-11.21	-11.2
-11.28	-11.27	-11.26	-11.25	-11.24	-11.23	-11.22	-11.21	-11.2
-11.28	-11.27	-11.26	-11.25	-11.24	-11.23	-11.22	-11.21	-11.2
-11.28	-11.27	-11.26	-11.25	-11.24	-11.23	-11.22	-11.21	-11.2
-11.28	-11.27	-11.26	-11.25	-11.24	-11.23	-11.22	-11.21	-11.2
-11.28	-11.27	-11.26	-11.25	-11.24	-11.23	-11.22	-11.21	-11.2
-11.28	-11.27	-11.26	-11.25	-11.24	-11.23	-11.22	-11.21	-11.2
-11.28	-11.27	-11.26	-11.25	-11.24	-11.23	-11.22	-11.21	-11.2
-11.28	-11.27	-11.26	-11.25	-11.24	-11.23	-11.22	-11.21	-11.2
-11.28	-11.27	-11.26	-11.25	-11.24	-11.23	-11.22	-11.21	-11.2
-11.28	-11.27	-11.26	-11.25	-11.24	-11.23	-11.22	-11.21	-11.2
-11.28	-11.27	-11.26	-11.25	-11.24	-11.23	-11.22	-11.21	-11.2
-11.28	-11.27	-11.26	-11.25	-11.24	-11.23	-11.22	-11.21	-11.2
-11.28	-11.27	-11.26	-11.25	-11.24	-11.23	-11.22	-11.21	-11.2
-11.28	-11.27	-11.26	-11.25	-11.24	-11.23	-11.22	-11.21	-11.2
-11.28	-11.27	-11.26	-11.25	-11.24	-11.23	-11.22	-11.21	-11.2
-11.28	-11.27	-11.26	-11.25	-11.24	-11.23	-11.22	-11.21	-11.2

-11.28	-11.27	-11.26	-11.25	-11.24	-11.23	-11.22	-11.21	-11.2
-11.28	-11.27	-11.26	-11.25	-11.24	-11.23	-11.22	-11.21	-11.2
-11.28	-11.27	-11.26	-11.25	-11.24	-11.23	-11.22	-11.21	-11.2
-11.28	-11.27	-11.26	-11.25	-11.24	-11.23	-11.22	-11.21	-11.2
-11.28	-11.27	-11.26	-11.25	-11.24	-11.23	-11.22	-11.21	-11.2
-11.28	-11.27	-11.26	-11.25	-11.24	-11.23	-11.22	-11.21	-11.2
-11.28	-11.27	-11.26	-11.25	-11.24	-11.23	-11.22	-11.21	-11.2
-11.28	-11.27	-11.26	-11.25	-11.24	-11.23	-11.22	-11.21	-11.2
-11.28	-11.27	-11.26	-11.25	-11.24	-11.23	-11.22	-11.21	-11.2
-11.28	-11.27	-11.26	-11.25	-11.24	-11.23	-11.22	-11.21	-11.2
-11.28	-11.27	-11.26	-11.25	-11.24	-11.23	-11.22	-11.21	-11.2
-11.28	-11.27	-11.26	-11.25	-11.24	-11.23	-11.22	-11.21	-11.2
-11.28	-11.27	-11.26	-11.25	-11.24	-11.23	-11.22	-11.21	-11.2
-11.28	-11.27	-11.26	-11.25	-11.24	-11.23	-11.22	-11.21	-11.2
-11.28	-11.27	-11.26	-11.25	-11.24	-11.23	-11.22	-11.21	-11.2
-11.28	-11.27	-11.26	-11.25	-11.24	-11.23	-11.22	-11.21	-11.2
-11.28	-11.27	-11.26	-11.25	-11.24	-11.23	-11.22	-11.21	-11.2
-11.28	-11.27	-11.26	-11.25	-11.24	-11.23	-11.22	-11.21	-11.2
-11.28	-11.27	-11.26	-11.25	-11.24	-11.23	-11.22	-11.21	-11.2
-11.28	-11.27	-11.26	-11.25	-11.24	-11.23	-11.22	-11.21	-11.2
-11.28	-11.27	-11.26	-11.25	-11.24	-11.23	-11.22	-11.21	-11.2
-11.28	-11.27	-11.26	-11.25	-11.24	-11.23	-11.22	-11.21	-11.2
-11.28	-11.27	-11.26	-11.25	-11.24	-11.23	-11.22	-11.21	-11.2
-11.28	-11.27	-11.26	-11.25	-11.24	-11.23	-11.22	-11.21	-11.2
-11.28	-11.27	-11.26	-11.25	-11.24	-11.23	-11.22	-11.21	-11.2
-11.28	-11.27	-11.26	-11.25	-11.24	-11.23	-11.22	-11.21	-11.2
-11.28	-11.27	-11.26	-11.25	-11.24	-11.23	-11.22	-11.21	-11.2
-11.28	-11.27	-11.26	-11.25	-11.24	-11.23	-11.22	-11.21	-11.2
-11.28	-11.27	-11.26	-11.25	-11.24	-11.23	-11.22	-11.21	-11.2
-11.28	-11.27	-11.26	-11.25	-11.24	-11.23	-11.22	-11.21	-11.2
-11.28	-11.27	-11.26	-11.25	-11.24	-11.23	-11.22	-11.21	-11.2
-11.28	-11.27	-11.26	-11.25	-11.24	-11.23	-11.22	-11.21	-11.2
-11.28	-11.27	-11.26	-11.25	-11.24	-11.23	-11.22	-11.21	-11.2
-11.28	-11.27	-11.26	-11.25	-11.24	-11.23	-11.22	-11.21	-11.2
-11.28	-11.27	-11.26	-11.25	-11.24	-11.23	-11.22	-11.21	-11.2
-11.28	-11.27	-11.26	-11.25	-11.24	-11.23	-11.22	-11.21	-11.2
-11.28	-11.27	-11.26	-11.25	-11.24	-11.23	-11.22	-11.21	-11.2
-11.28	-11.27	-11.26	-11.25	-11.24	-11.23	-11.22	-11.21	-11.2
-11.28	-11.27	-11.26	-11.25	-11.24	-11.23	-11.22	-11.21	-11.2
-11.28	-11.27	-11.26	-11.25	-11.24	-11.23	-11.22	-11.21	-11.2
-11.28	-11.27	-11.26	-11.25	-11.24	-11.23	-11.22	-11.21	-11.2
-11.28	-11.27	-11.26	-11.25	-11.24	-11.23	-11.22	-11.21	-11.2
-11.28	-11.27	-11.26	-11.25	-11.24	-11.23	-11.22	-11.21	-11.2
-11.28	-11.27	-11.26	-11.25	-11.24	-11.23	-11.22	-11.21	-11.2
-11.28	-11.27	-11.26	-11.25	-11.24	-11.23	-11.22	-11.21	-11.2
-11.28	-11.27	-11.26	-11.25	-11.24	-11.23	-11.22	-11.21	-11.2
-11.28	-11.27	-11.26	-11.25	-11.24	-11.23	-11.22	-11.21	-11.2
-11.28	-11.27	-11.26	-11.25	-11.24	-11.23	-11.22	-11.21	-11.2
-11.28	-11.27	-11.26	-11.25	-11.24	-11.23	-11.22	-11.21	-11.2
-11.28	-11.27	-11.26	-11.25	-11.24	-11.23	-11.22	-11.21	-11.2

-11.28	-11.27	-11.26	-11.25	-11.24	-11.23	-11.22	-11.21	-11.2
-11.28	-11.27	-11.26	-11.25	-11.24	-11.23	-11.22	-11.21	-11.2
-11.28	-11.27	-11.26	-11.25	-11.24	-11.23	-11.22	-11.21	-11.2
-11.28	-11.27	-11.26	-11.25	-11.24	-11.23	-11.22	-11.21	-11.2
-11.28	-11.27	-11.26	-11.25	-11.24	-11.23	-11.22	-11.21	-11.2
-11.28	-11.27	-11.26	-11.25	-11.24	-11.23	-11.22	-11.21	-11.2
-11.28	-11.27	-11.26	-11.25	-11.24	-11.23	-11.22	-11.21	-11.2
-11.28	-11.27	-11.26	-11.25	-11.24	-11.23	-11.22	-11.21	-11.2
-11.28	-11.27	-11.26	-11.25	-11.24	-11.23	-11.22	-11.21	-11.2
-11.28	-11.27	-11.26	-11.25	-11.24	-11.23	-11.22	-11.21	-11.2
-11.28	-11.27	-11.26	-11.25	-11.24	-11.23	-11.22	-11.21	-11.2
-11.28	-11.27	-11.26	-11.25	-11.24	-11.23	-11.22	-11.21	-11.2
-11.28	-11.27	-11.26	-11.25	-11.24	-11.23	-11.22	-11.21	-11.2
-11.28	-11.27	-11.26	-11.25	-11.24	-11.23	-11.22	-11.21	-11.2
-11.28	-11.27	-11.26	-11.25	-11.24	-11.23	-11.22	-11.21	-11.2
-11.28	-11.27	-11.26	-11.25	-11.24	-11.23	-11.22	-11.21	-11.2
-11.28	-11.27	-11.26	-11.25	-11.24	-11.23	-11.22	-11.21	-11.2
-11.28	-11.27	-11.26	-11.25	-11.24	-11.23	-11.22	-11.21	-11.2
-11.28	-11.27	-11.26	-11.25	-11.24	-11.23	-11.22	-11.21	-11.2
-11.28	-11.27	-11.26	-11.25	-11.24	-11.23	-11.22	-11.21	-11.2
-11.28	-11.27	-11.26	-11.25	-11.24	-11.23	-11.22	-11.21	-11.2
-11.28	-11.27	-11.26	-11.25	-11.24	-11.23	-11.22	-11.21	-11.2
-11.28	-11.27	-11.26	-11.25	-11.24	-11.23	-11.22	-11.21	-11.2
-11.28	-11.27	-11.26	-11.25	-11.24	-11.23	-11.22	-11.21	-11.2
-11.28	-11.27	-11.26	-11.25	-11.24	-11.23	-11.22	-11.21	-11.2
-11.28	-11.27	-11.26	-11.25	-11.24	-11.23	-11.22	-11.21	-11.2
-11.28	-11.27	-11.26	-11.25	-11.24	-11.23	-11.22	-11.21	-11.2
-11.28	-11.27	-11.26	-11.25	-11.24	-11.23	-11.22	-11.21	-11.2
-11.28	-11.27	-11.26	-11.25	-11.24	-11.23	-11.22	-11.21	-11.2
-11.28	-11.27	-11.26	-11.25	-11.24	-11.23	-11.22	-11.21	-11.2
-11.28	-11.27	-11.26	-11.25	-11.24	-11.23	-11.22	-11.21	-11.2
-11.28	-11.27	-11.26	-11.25	-11.24	-11.23	-11.22	-11.21	-11.2
-11.28	-11.27	-11.26	-11.25	-11.24	-11.23	-11.22	-11.21	-11.2
-11.28	-11.27	-11.26	-11.25	-11.24	-11.23	-11.22	-11.21	-11.2
-11.28	-11.27	-11.26	-11.25	-11.24	-11.23	-11.22	-11.21	-11.2
-11.28	-11.27	-11.26	-11.25	-11.24	-11.23	-11.22	-11.21	-11.2
-11.28	-11.27	-11.26	-11.25	-11.24	-11.23	-11.22	-11.21	-11.2
-11.28	-11.27	-11.26	-11.25	-11.24	-11.23	-11.22	-11.21	-11.2
-11.28	-11.27	-11.26	-11.25	-11.24	-11.23	-11.22	-11.21	-11.2
-11.28	-11.27	-11.26	-11.25	-11.24	-11.23	-11.22	-11.21	-11.2
-11.28	-11.27	-11.26	-11.25	-11.24	-11.23	-11.22	-11.21	-11.2
-11.28	-11.27	-11.26	-11.25	-11.24	-11.23	-11.22	-11.21	-11.2
-11.28	-11.27	-11.26	-11.25	-11.24	-11.23	-11.22	-11.21	-11.2
-11.28	-11.27	-11.26	-11.25	-11.24	-11.23	-11.22	-11.21	-11.2
-11.28	-11.27	-11.26	-11.25	-11.24	-11.23	-11.22	-11.21	-11.2
-11.28	-11.27	-11.26	-11.25	-11.24	-11.23	-11.22	-11.21	-11.2
-11.28	-11.27	-11.26	-11.25	-11.24	-11.23	-11.22	-11.21	-11.2
-11.28	-11.27	-11.26	-11.25	-11.24	-11.23	-11.22	-11.21	-11.2
-11.28	-11.27	-11.26	-11.25	-11.24	-11.23	-11.22	-11.21	-11.2
-11.28	-11.27	-11.26	-11.25	-11.24	-11.23	-11.22	-11.21	-11.2

-11.28	-11.27	-11.26	-11.25	-11.24	-11.23	-11.22	-11.21	-11.2
-11.28	-11.27	-11.26	-11.25	-11.24	-11.23	-11.22	-11.21	-11.2
-11.28	-11.27	-11.26	-11.25	-11.24	-11.23	-11.22	-11.21	-11.2
-11.28	-11.27	-11.26	-11.25	-11.24	-11.23	-11.22	-11.21	-11.2
-11.28	-11.27	-11.26	-11.25	-11.24	-11.23	-11.22	-11.21	-11.2
-11.28	-11.27	-11.26	-11.25	-11.24	-11.23	-11.22	-11.21	-11.2
-11.28	-11.27	-11.26	-11.25	-11.24	-11.23	-11.22	-11.21	-11.2
-11.28	-11.27	-11.26	-11.25	-11.24	-11.23	-11.22	-11.21	-11.2
-11.28	-11.27	-11.26	-11.25	-11.24	-11.23	-11.22	-11.21	-11.2
-11.28	-11.27	-11.26	-11.25	-11.24	-11.23	-11.22	-11.21	-11.2
-11.28	-11.27	-11.26	-11.25	-11.24	-11.23	-11.22	-11.21	-11.2
-11.28	-11.27	-11.26	-11.25	-11.24	-11.23	-11.22	-11.21	-11.2
-11.28	-11.27	-11.26	-11.25	-11.24	-11.23	-11.22	-11.21	-11.2
-11.28	-11.27	-11.26	-11.25	-11.24	-11.23	-11.22	-11.21	-11.2
-11.28	-11.27	-11.26	-11.25	-11.24	-11.23	-11.22	-11.21	-11.2
-11.28	-11.27	-11.26	-11.25	-11.24	-11.23	-11.22	-11.21	-11.2
-11.28	-11.27	-11.26	-11.25	-11.24	-11.23	-11.22	-11.21	-11.2
-11.28	-11.27	-11.26	-11.25	-11.24	-11.23	-11.22	-11.21	-11.2
-11.28	-11.27	-11.26	-11.25	-11.24	-11.23	-11.22	-11.21	-11.2
-11.28	-11.27	-11.26	-11.25	-11.24	-11.23	-11.22	-11.21	-11.2
-11.28	-11.27	-11.26	-11.25	-11.24	-11.23	-11.22	-11.21	-11.2
-11.28	-11.27	-11.26	-11.25	-11.24	-11.23	-11.22	-11.21	-11.2
-11.28	-11.27	-11.26	-11.25	-11.24	-11.23	-11.22	-11.21	-11.2
-11.28	-11.27	-11.26	-11.25	-11.24	-11.23	-11.22	-11.21	-11.2
-11.28	-11.27	-11.26	-11.25	-11.24	-11.23	-11.22	-11.21	-11.2
-11.28	-11.27	-11.26	-11.25	-11.24	-11.23	-11.22	-11.21	-11.2
-11.28	-11.27	-11.26	-11.25	-11.24	-11.23	-11.22	-11.21	-11.2
-11.28	-11.27	-11.26	-11.25	-11.24	-11.23	-11.22	-11.21	-11.2
-11.28	-11.27	-11.26	-11.25	-11.24	-11.23	-11.22	-11.21	-11.2
-11.28	-11.27	-11.26	-11.25	-11.24	-11.23	-11.22	-11.21	-11.2
-11.28	-11.27	-11.26	-11.25	-11.24	-11.23	-11.22	-11.21	-11.2
-11.28	-11.27	-11.26	-11.25	-11.24	-11.23	-11.22	-11.21	-11.2
-11.28	-11.27	-11.26	-11.25	-11.24	-11.23	-11.22	-11.21	-11.2
-11.28	-11.27	-11.26	-11.25	-11.24	-11.23	-11.22	-11.21	-11.2
-11.28	-11.27	-11.26	-11.25	-11.24	-11.23	-11.22	-11.21	-11.2
-11.28	-11.27	-11.26	-11.25	-11.24	-11.23	-11.22	-11.21	-11.2
-11.28	-11.27	-11.26	-11.25	-11.24	-11.23	-11.22	-11.21	-11.2
-11.28	-11.27	-11.26	-11.25	-11.24	-11.23	-11.22	-11.21	-11.2
-11.28	-11.27	-11.26	-11.25	-11.24	-11.23	-11.22	-11.21	-11.2
-11.28	-11.27	-11.26	-11.25	-11.24	-11.23	-11.22	-11.21	-11.2
-11.28	-11.27	-11.26	-11.25	-11.24	-11.23	-11.22	-11.21	-11.2
-11.28	-11.27	-11.26	-11.25	-11.24	-11.23	-11.22	-11.21	-11.2
-11.28	-11.27	-11.26	-11.25	-11.24	-11.23	-11.22	-11.21	-11.2
-11.28	-11.27	-11.26	-11.25	-11.24	-11.23	-11.22	-11.21	-11.2
-11.28	-11.27	-11.26	-11.25	-11.24	-11.23	-11.22	-11.21	-11.2
-11.28	-11.27	-11.26	-11.25	-11.24	-11.23	-11.22	-11.21	-11.2
-11.28	-11.27	-11.26	-11.25	-11.24	-11.23	-11.22	-11.21	-11.2
-11.28	-11.27	-11.26	-11.25	-11.24	-11.23	-11.22	-11.21	-11.2
-11.28	-11.27	-11.26	-11.25	-11.24	-11.23	-11.22	-11.21	-11.2
-11.28	-11.27	-11.26	-11.25	-11.24	-11.23	-11.22	-11.21	-11.2

-11.28	-11.27	-11.26	-11.25	-11.24	-11.23	-11.22	-11.21	-11.2
-11.28	-11.27	-11.26	-11.25	-11.24	-11.23	-11.22	-11.21	-11.2
-11.28	-11.27	-11.26	-11.25	-11.24	-11.23	-11.22	-11.21	-11.2
-11.28	-11.27	-11.26	-11.25	-11.24	-11.23	-11.22	-11.21	-11.2
-11.28	-11.27	-11.26	-11.25	-11.24	-11.23	-11.22	-11.21	-11.2
-11.28	-11.27	-11.26	-11.25	-11.24	-11.23	-11.22	-11.21	-11.2
-11.28	-11.27	-11.26	-11.25	-11.24	-11.23	-11.22	-11.21	-11.2
-11.28	-11.27	-11.26	-11.25	-11.24	-11.23	-11.22	-11.21	-11.2
-11.28	-11.27	-11.26	-11.25	-11.24	-11.23	-11.22	-11.21	-11.2
-11.28	-11.27	-11.26	-11.25	-11.24	-11.23	-11.22	-11.21	-11.2
-11.28	-11.27	-11.26	-11.25	-11.24	-11.23	-11.22	-11.21	-11.2
-11.28	-11.27	-11.26	-11.25	-11.24	-11.23	-11.22	-11.21	-11.2
-11.28	-11.27	-11.26	-11.25	-11.24	-11.23	-11.22	-11.21	-11.2
-11.28	-11.27	-11.26	-11.25	-11.24	-11.23	-11.22	-11.21	-11.2
-11.28	-11.27	-11.26	-11.25	-11.24	-11.23	-11.22	-11.21	-11.2
-11.28	-11.27	-11.26	-11.25	-11.24	-11.23	-11.22	-11.21	-11.2
-11.28	-11.27	-11.26	-11.25	-11.24	-11.23	-11.22	-11.21	-11.2
-11.28	-11.27	-11.26	-11.25	-11.24	-11.23	-11.22	-11.21	-11.2
-11.28	-11.27	-11.26	-11.25	-11.24	-11.23	-11.22	-11.21	-11.2
-11.28	-11.27	-11.26	-11.25	-11.24	-11.23	-11.22	-11.21	-11.2
-11.28	-11.27	-11.26	-11.25	-11.24	-11.23	-11.22	-11.21	-11.2
-11.28	-11.27	-11.26	-11.25	-11.24	-11.23	-11.22	-11.21	-11.2
-11.28	-11.27	-11.26	-11.25	-11.24	-11.23	-11.22	-11.21	-11.2
-11.28	-11.27	-11.26	-11.25	-11.24	-11.23	-11.22	-11.21	-11.2
-11.28	-11.27	-11.26	-11.25	-11.24	-11.23	-11.22	-11.21	-11.2
-11.28	-11.27	-11.26	-11.25	-11.24	-11.23	-11.22	-11.21	-11.2
-11.28	-11.27	-11.26	-11.25	-11.24	-11.23	-11.22	-11.21	-11.2
-11.28	-11.27	-11.26	-11.25	-11.24	-11.23	-11.22	-11.21	-11.2
-11.28	-11.27	-11.26	-11.25	-11.24	-11.23	-11.22	-11.21	-11.2
-11.28	-11.27	-11.26	-11.25	-11.24	-11.23	-11.22	-11.21	-11.2
-11.28	-11.27	-11.26	-11.25	-11.24	-11.23	-11.22	-11.21	-11.2
-11.28	-11.27	-11.26	-11.25	-11.24	-11.23	-11.22	-11.21	-11.2
-11.28	-11.27	-11.26	-11.25	-11.24	-11.23	-11.22	-11.21	-11.2
-11.28	-11.27	-11.26	-11.25	-11.24	-11.23	-11.22	-11.21	-11.2
-11.28	-11.27	-11.26	-11.25	-11.24	-11.23	-11.22	-11.21	-11.2
-11.28	-11.27	-11.26	-11.25	-11.24	-11.23	-11.22	-11.21	-11.2
-11.28	-11.27	-11.26	-11.25	-11.24	-11.23	-11.22	-11.21	-11.2
-11.28	-11.27	-11.26	-11.25	-11.24	-11.23	-11.22	-11.21	-11.2
-11.28	-11.27	-11.26	-11.25	-11.24	-11.23	-11.22	-11.21	-11.2
-11.28	-11.27	-11.26	-11.25	-11.24	-11.23	-11.22	-11.21	-11.2
-11.28	-11.27	-11.26	-11.25	-11.24	-11.23	-11.22	-11.21	-11.2
-11.28	-11.27	-11.26	-11.25	-11.24	-11.23	-11.22	-11.21	-11.2
-11.28	-11.27	-11.26	-11.25	-11.24	-11.23	-11.22	-11.21	-11.2
-11.28	-11.27	-11.26	-11.25	-11.24	-11.23	-11.22	-11.21	-11.2
-11.28	-11.27	-11.26	-11.25	-11.24	-11.23	-11.22	-11.21	-11.2
-11.28	-11.27	-11.26	-11.25	-11.24	-11.23	-11.22	-11.21	-11.2
-11.28	-11.27	-11.26	-11.25	-11.24	-11.23	-11.22	-11.21	-11.2
-11.28	-11.27	-11.26	-11.25	-11.24	-11.23	-11.22	-11.21	-11.2
-11.28	-11.27	-11.26	-11.25	-11.24	-11.23	-11.22	-11.21	-11.2
-11.28	-11.27	-11.26	-11.25	-11.24	-11.23	-11.22	-11.21	-11.2

-11.28	-11.27	-11.26	-11.25	-11.24	-11.23	-11.22	-11.21	-11.2
-11.28	-11.27	-11.26	-11.25	-11.24	-11.23	-11.22	-11.21	-11.2
-11.28	-11.27	-11.26	-11.25	-11.24	-11.23	-11.22	-11.21	-11.2
-11.28	-11.27	-11.26	-11.25	-11.24	-11.23	-11.22	-11.21	-11.2
-11.28	-11.27	-11.26	-11.25	-11.24	-11.23	-11.22	-11.21	-11.2
-11.28	-11.27	-11.26	-11.25	-11.24	-11.23	-11.22	-11.21	-11.2
-11.28	-11.27	-11.26	-11.25	-11.24	-11.23	-11.22	-11.21	-11.2
-11.28	-11.27	-11.26	-11.25	-11.24	-11.23	-11.22	-11.21	-11.2
-11.28	-11.27	-11.26	-11.25	-11.24	-11.23	-11.22	-11.21	-11.2
-11.28	-11.27	-11.26	-11.25	-11.24	-11.23	-11.22	-11.21	-11.2
-11.28	-11.27	-11.26	-11.25	-11.24	-11.23	-11.22	-11.21	-11.2
-11.28	-11.27	-11.26	-11.25	-11.24	-11.23	-11.22	-11.21	-11.2
-11.28	-11.27	-11.26	-11.25	-11.24	-11.23	-11.22	-11.21	-11.2
-11.28	-11.27	-11.26	-11.25	-11.24	-11.23	-11.22	-11.21	-11.2
-11.28	-11.27	-11.26	-11.25	-11.24	-11.23	-11.22	-11.21	-11.2
-11.28	-11.27	-11.26	-11.25	-11.24	-11.23	-11.22	-11.21	-11.2
-11.28	-11.27	-11.26	-11.25	-11.24	-11.23	-11.22	-11.21	-11.2
-11.28	-11.27	-11.26	-11.25	-11.24	-11.23	-11.22	-11.21	-11.2
-11.28	-11.27	-11.26	-11.25	-11.24	-11.23	-11.22	-11.21	-11.2
-11.28	-11.27	-11.26	-11.25	-11.24	-11.23	-11.22	-11.21	-11.2
-11.28	-11.27	-11.26	-11.25	-11.24	-11.23	-11.22	-11.21	-11.2
-11.28	-11.27	-11.26	-11.25	-11.24	-11.23	-11.22	-11.21	-11.2
-11.28	-11.27	-11.26	-11.25	-11.24	-11.23	-11.22	-11.21	-11.2
-11.28	-11.27	-11.26	-11.25	-11.24	-11.23	-11.22	-11.21	-11.2
-11.28	-11.27	-11.26	-11.25	-11.24	-11.23	-11.22	-11.21	-11.2
-11.28	-11.27	-11.26	-11.25	-11.24	-11.23	-11.22	-11.21	-11.2
-11.28	-11.27	-11.26	-11.25	-11.24	-11.23	-11.22	-11.21	-11.2
-11.28	-11.27	-11.26	-11.25	-11.24	-11.23	-11.22	-11.21	-11.2
-11.28	-11.27	-11.26	-11.25	-11.24	-11.23	-11.22	-11.21	-11.2
-11.28	-11.27	-11.26	-11.25	-11.24	-11.23	-11.22	-11.21	-11.2
-11.28	-11.27	-11.26	-11.25	-11.24	-11.23	-11.22	-11.21	-11.2
-11.28	-11.27	-11.26	-11.25	-11.24	-11.23	-11.22	-11.21	-11.2
-11.28	-11.27	-11.26	-11.25	-11.24	-11.23	-11.22	-11.21	-11.2
-11.28	-11.27	-11.26	-11.25	-11.24	-11.23	-11.22	-11.21	-11.2
-11.28	-11.27	-11.26	-11.25	-11.24	-11.23	-11.22	-11.21	-11.2
-11.28	-11.27	-11.26	-11.25	-11.24	-11.23	-11.22	-11.21	-11.2
-11.28	-11.27	-11.26	-11.25	-11.24	-11.23	-11.22	-11.21	-11.2
-11.28	-11.27	-11.26	-11.25	-11.24	-11.23	-11.22	-11.21	-11.2
-11.28	-11.27	-11.26	-11.25	-11.24	-11.23	-11.22	-11.21	-11.2
-11.28	-11.27	-11.26	-11.25	-11.24	-11.23	-11.22	-11.21	-11.2
-11.28	-11.27	-11.26	-11.25	-11.24	-11.23	-11.22	-11.21	-11.2
-11.28	-11.27	-11.26	-11.25	-11.24	-11.23	-11.22	-11.21	-11.2
-11.28	-11.27	-11.26	-11.25	-11.24	-11.23	-11.22	-11.21	-11.2
-11.28	-11.27	-11.26	-11.25	-11.24	-11.23	-11.22	-11.21	-11.2
-11.28	-11.27	-11.26	-11.25	-11.24	-11.23	-11.22	-11.21	-11.2
-11.28	-11.27	-11.26	-11.25	-11.24	-11.23	-11.22	-11.21	-11.2
-11.28	-11.27	-11.26	-11.25	-11.24	-11.23	-11.22	-11.21	-11.2
-11.28	-11.27	-11.26	-11.25	-11.24	-11.23	-11.22	-11.21	-11.2
-11.28	-11.27	-11.26	-11.25	-11.24	-11.23	-11.22	-11.21	-11.2
-11.28	-11.27	-11.26	-11.25	-11.24	-11.23	-11.22	-11.21	-11.2

-11.28	-11.27	-11.26	-11.25	-11.24	-11.23	-11.22	-11.21	-11.2
-11.28	-11.27	-11.26	-11.25	-11.24	-11.23	-11.22	-11.21	-11.2
-11.28	-11.27	-11.26	-11.25	-11.24	-11.23	-11.22	-11.21	-11.2
-11.28	-11.27	-11.26	-11.25	-11.24	-11.23	-11.22	-11.21	-11.2
-11.28	-11.27	-11.26	-11.25	-11.24	-11.23	-11.22	-11.21	-11.2
-11.28	-11.27	-11.26	-11.25	-11.24	-11.23	-11.22	-11.21	-11.2
-11.28	-11.27	-11.26	-11.25	-11.24	-11.23	-11.22	-11.21	-11.2
-11.28	-11.27	-11.26	-11.25	-11.24	-11.23	-11.22	-11.21	-11.2
-11.28	-11.27	-11.26	-11.25	-11.24	-11.23	-11.22	-11.21	-11.2
-11.28	-11.27	-11.26	-11.25	-11.24	-11.23	-11.22	-11.21	-11.2
-11.28	-11.27	-11.26	-11.25	-11.24	-11.23	-11.22	-11.21	-11.2
-11.28	-11.27	-11.26	-11.25	-11.24	-11.23	-11.22	-11.21	-11.2
-11.28	-11.27	-11.26	-11.25	-11.24	-11.23	-11.22	-11.21	-11.2
-11.28	-11.27	-11.26	-11.25	-11.24	-11.23	-11.22	-11.21	-11.2
-11.28	-11.27	-11.26	-11.25	-11.24	-11.23	-11.22	-11.21	-11.2
-11.28	-11.27	-11.26	-11.25	-11.24	-11.23	-11.22	-11.21	-11.2
-11.28	-11.27	-11.26	-11.25	-11.24	-11.23	-11.22	-11.21	-11.2
-11.28	-11.27	-11.26	-11.25	-11.24	-11.23	-11.22	-11.21	-11.2
-11.28	-11.27	-11.26	-11.25	-11.24	-11.23	-11.22	-11.21	-11.2
-11.28	-11.27	-11.26	-11.25	-11.24	-11.23	-11.22	-11.21	-11.2
-11.28	-11.27	-11.26	-11.25	-11.24	-11.23	-11.22	-11.21	-11.2
-11.28	-11.27	-11.26	-11.25	-11.24	-11.23	-11.22	-11.21	-11.2
-11.28	-11.27	-11.26	-11.25	-11.24	-11.23	-11.22	-11.21	-11.2
-11.28	-11.27	-11.26	-11.25	-11.24	-11.23	-11.22	-11.21	-11.2
-11.28	-11.27	-11.26	-11.25	-11.24	-11.23	-11.22	-11.21	-11.2
-11.28	-11.27	-11.26	-11.25	-11.24	-11.23	-11.22	-11.21	-11.2
-11.28	-11.27	-11.26	-11.25	-11.24	-11.23	-11.22	-11.21	-11.2
-11.28	-11.27	-11.26	-11.25	-11.24	-11.23	-11.22	-11.21	-11.2
-11.28	-11.27	-11.26	-11.25	-11.24	-11.23	-11.22	-11.21	-11.2
-11.28	-11.27	-11.26	-11.25	-11.24	-11.23	-11.22	-11.21	-11.2
-11.28	-11.27	-11.26	-11.25	-11.24	-11.23	-11.22	-11.21	-11.2
-11.28	-11.27	-11.26	-11.25	-11.24	-11.23	-11.22	-11.21	-11.2
-11.28	-11.27	-11.26	-11.25	-11.24	-11.23	-11.22	-11.21	-11.2
-11.28	-11.27	-11.26	-11.25	-11.24	-11.23	-11.22	-11.21	-11.2
-11.28	-11.27	-11.26	-11.25	-11.24	-11.23	-11.22	-11.21	-11.2
-11.28	-11.27	-11.26	-11.25	-11.24	-11.23	-11.22	-11.21	-11.2
-11.28	-11.27	-11.26	-11.25	-11.24	-11.23	-11.22	-11.21	-11.2
-11.28	-11.27	-11.26	-11.25	-11.24	-11.23	-11.22	-11.21	-11.2
-11.28	-11.27	-11.26	-11.25	-11.24	-11.23	-11.22	-11.21	-11.2
-11.28	-11.27	-11.26	-11.25	-11.24	-11.23	-11.22	-11.21	-11.2
-11.28	-11.27	-11.26	-11.25	-11.24	-11.23	-11.22	-11.21	-11.2
-11.28	-11.27	-11.26	-11.25	-11.24	-11.23	-11.22	-11.21	-11.2
-11.28	-11.27	-11.26	-11.25	-11.24	-11.23	-11.22	-11.21	-11.2
-11.28	-11.27	-11.26	-11.25	-11.24	-11.23	-11.22	-11.21	-11.2
-11.28	-11.27	-11.26	-11.25	-11.24	-11.23	-11.22	-11.21	-11.2
-11.28	-11.27	-11.26	-11.25	-11.24	-11.23	-11.22	-11.21	-11.2
-11.28	-11.27	-11.26	-11.25	-11.24	-11.23	-11.22	-11.21	-11.2
-11.28	-11.27	-11.26	-11.25	-11.24	-11.23	-11.22	-11.21	-11.2
-11.28	-11.27	-11.26	-11.25	-11.24	-11.23	-11.22	-11.21	-11.2
-11.28	-11.27	-11.26	-11.25	-11.24	-11.23	-11.22	-11.21	-11.2

-11.28	-11.27	-11.26	-11.25	-11.24	-11.23	-11.22	-11.21	-11.2
-11.28	-11.27	-11.26	-11.25	-11.24	-11.23	-11.22	-11.21	-11.2
-11.28	-11.27	-11.26	-11.25	-11.24	-11.23	-11.22	-11.21	-11.2
-11.28	-11.27	-11.26	-11.25	-11.24	-11.23	-11.22	-11.21	-11.2
-11.28	-11.27	-11.26	-11.25	-11.24	-11.23	-11.22	-11.21	-11.2
-11.28	-11.27	-11.26	-11.25	-11.24	-11.23	-11.22	-11.21	-11.2
-11.28	-11.27	-11.26	-11.25	-11.24	-11.23	-11.22	-11.21	-11.2
-11.28	-11.27	-11.26	-11.25	-11.24	-11.23	-11.22	-11.21	-11.2
-11.28	-11.27	-11.26	-11.25	-11.24	-11.23	-11.22	-11.21	-11.2
-11.28	-11.27	-11.26	-11.25	-11.24	-11.23	-11.22	-11.21	-11.2
-11.28	-11.27	-11.26	-11.25	-11.24	-11.23	-11.22	-11.21	-11.2
-11.28	-11.27	-11.26	-11.25	-11.24	-11.23	-11.22	-11.21	-11.2
-11.28	-11.27	-11.26	-11.25	-11.24	-11.23	-11.22	-11.21	-11.2
-11.28	-11.27	-11.26	-11.25	-11.24	-11.23	-11.22	-11.21	-11.2
-11.28	-11.27	-11.26	-11.25	-11.24	-11.23	-11.22	-11.21	-11.2
-11.28	-11.27	-11.26	-11.25	-11.24	-11.23	-11.22	-11.21	-11.2
-11.28	-11.27	-11.26	-11.25	-11.24	-11.23	-11.22	-11.21	-11.2
-11.28	-11.27	-11.26	-11.25	-11.24	-11.23	-11.22	-11.21	-11.2
-11.28	-11.27	-11.26	-11.25	-11.24	-11.23	-11.22	-11.21	-11.2
-11.28	-11.27	-11.26	-11.25	-11.24	-11.23	-11.22	-11.21	-11.2
-11.28	-11.27	-11.26	-11.25	-11.24	-11.23	-11.22	-11.21	-11.2
-11.28	-11.27	-11.26	-11.25	-11.24	-11.23	-11.22	-11.21	-11.2
-11.28	-11.27	-11.26	-11.25	-11.24	-11.23	-11.22	-11.21	-11.2
-11.28	-11.27	-11.26	-11.25	-11.24	-11.23	-11.22	-11.21	-11.2
-11.28	-11.27	-11.26	-11.25	-11.24	-11.23	-11.22	-11.21	-11.2
-11.28	-11.27	-11.26	-11.25	-11.24	-11.23	-11.22	-11.21	-11.2
-11.28	-11.27	-11.26	-11.25	-11.24	-11.23	-11.22	-11.21	-11.2
-11.28	-11.27	-11.26	-11.25	-11.24	-11.23	-11.22	-11.21	-11.2
-11.28	-11.27	-11.26	-11.25	-11.24	-11.23	-11.22	-11.21	-11.2
-11.28	-11.27	-11.26	-11.25	-11.24	-11.23	-11.22	-11.21	-11.2
-11.28	-11.27	-11.26	-11.25	-11.24	-11.23	-11.22	-11.21	-11.2
-11.28	-11.27	-11.26	-11.25	-11.24	-11.23	-11.22	-11.21	-11.2
-11.28	-11.27	-11.26	-11.25	-11.24	-11.23	-11.22	-11.21	-11.2
-11.28	-11.27	-11.26	-11.25	-11.24	-11.23	-11.22	-11.21	-11.2
-11.28	-11.27	-11.26	-11.25	-11.24	-11.23	-11.22	-11.21	-11.2
-11.28	-11.27	-11.26	-11.25	-11.24	-11.23	-11.22	-11.21	-11.2
-11.28	-11.27	-11.26	-11.25	-11.24	-11.23	-11.22	-11.21	-11.2
-11.28	-11.27	-11.26	-11.25	-11.24	-11.23	-11.22	-11.21	-11.2
-11.28	-11.27	-11.26	-11.25	-11.24	-11.23	-11.22	-11.21	-11.2
-11.28	-11.27	-11.26	-11.25	-11.24	-11.23	-11.22	-11.21	-11.2
-11.28	-11.27	-11.26	-11.25	-11.24	-11.23	-11.22	-11.21	-11.2
-11.28	-11.27	-11.26	-11.25	-11.24	-11.23	-11.22	-11.21	-11.2
-11.28	-11.27	-11.26	-11.25	-11.24	-11.23	-11.22	-11.21	-11.2
-11.28	-11.27	-11.26	-11.25	-11.24	-11.23	-11.22	-11.21	-11.2
-11.28	-11.27	-11.26	-11.25	-11.24	-11.23	-11.22	-11.21	-11.2
-11.28	-11.27	-11.26	-11.25	-11.24	-11.23	-11.22	-11.21	-11.2
-11.28	-11.27	-11.26	-11.25	-11.24	-11.23	-11.22	-11.21	-11.2
-11.28	-11.27	-11.26	-11.25	-11.24	-11.23	-11.22	-11.21	-11.2
-11.28	-11.27	-11.26	-11.25	-11.24	-11.23	-11.22	-11.21	-11.2
-11.28	-11.27	-11.26	-11.25	-11.24	-11.23	-11.22	-11.21	-11.2

-11.28	-11.27	-11.26	-11.25	-11.24	-11.23	-11.22	-11.21	-11.2
-11.28	-11.27	-11.26	-11.25	-11.24	-11.23	-11.22	-11.21	-11.2
-11.28	-11.27	-11.26	-11.25	-11.24	-11.23	-11.22	-11.21	-11.2
-11.28	-11.27	-11.26	-11.25	-11.24	-11.23	-11.22	-11.21	-11.2
-11.28	-11.27	-11.26	-11.25	-11.24	-11.23	-11.22	-11.21	-11.2
-11.28	-11.27	-11.26	-11.25	-11.24	-11.23	-11.22	-11.21	-11.2
-11.28	-11.27	-11.26	-11.25	-11.24	-11.23	-11.22	-11.21	-11.2
-11.28	-11.27	-11.26	-11.25	-11.24	-11.23	-11.22	-11.21	-11.2
-11.28	-11.27	-11.26	-11.25	-11.24	-11.23	-11.22	-11.21	-11.2
-11.28	-11.27	-11.26	-11.25	-11.24	-11.23	-11.22	-11.21	-11.2
-11.28	-11.27	-11.26	-11.25	-11.24	-11.23	-11.22	-11.21	-11.2
-11.28	-11.27	-11.26	-11.25	-11.24	-11.23	-11.22	-11.21	-11.2
-11.28	-11.27	-11.26	-11.25	-11.24	-11.23	-11.22	-11.21	-11.2
-11.28	-11.27	-11.26	-11.25	-11.24	-11.23	-11.22	-11.21	-11.2
-11.28	-11.27	-11.26	-11.25	-11.24	-11.23	-11.22	-11.21	-11.2
-11.28	-11.27	-11.26	-11.25	-11.24	-11.23	-11.22	-11.21	-11.2
-11.28	-11.27	-11.26	-11.25	-11.24	-11.23	-11.22	-11.21	-11.2
-11.28	-11.27	-11.26	-11.25	-11.24	-11.23	-11.22	-11.21	-11.2
-11.28	-11.27	-11.26	-11.25	-11.24	-11.23	-11.22	-11.21	-11.2
-11.28	-11.27	-11.26	-11.25	-11.24	-11.23	-11.22	-11.21	-11.2
-11.28	-11.27	-11.26	-11.25	-11.24	-11.23	-11.22	-11.21	-11.2
-11.28	-11.27	-11.26	-11.25	-11.24	-11.23	-11.22	-11.21	-11.2
-11.28	-11.27	-11.26	-11.25	-11.24	-11.23	-11.22	-11.21	-11.2
-11.28	-11.27	-11.26	-11.25	-11.24	-11.23	-11.22	-11.21	-11.2
-11.28	-11.27	-11.26	-11.25	-11.24	-11.23	-11.22	-11.21	-11.2
-11.28	-11.27	-11.26	-11.25	-11.24	-11.23	-11.22	-11.21	-11.2
-11.28	-11.27	-11.26	-11.25	-11.24	-11.23	-11.22	-11.21	-11.2
-11.28	-11.27	-11.26	-11.25	-11.24	-11.23	-11.22	-11.21	-11.2
-11.28	-11.27	-11.26	-11.25	-11.24	-11.23	-11.22	-11.21	-11.2
-11.28	-11.27	-11.26	-11.25	-11.24	-11.23	-11.22	-11.21	-11.2
-11.28	-11.27	-11.26	-11.25	-11.24	-11.23	-11.22	-11.21	-11.2
-11.28	-11.27	-11.26	-11.25	-11.24	-11.23	-11.22	-11.21	-11.2
-11.28	-11.27	-11.26	-11.25	-11.24	-11.23	-11.22	-11.21	-11.2
-11.28	-11.27	-11.26	-11.25	-11.24	-11.23	-11.22	-11.21	-11.2
-11.28	-11.27	-11.26	-11.25	-11.24	-11.23	-11.22	-11.21	-11.2
-11.28	-11.27	-11.26	-11.25	-11.24	-11.23	-11.22	-11.21	-11.2
-11.28	-11.27	-11.26	-11.25	-11.24	-11.23	-11.22	-11.21	-11.2
-11.28	-11.27	-11.26	-11.25	-11.24	-11.23	-11.22	-11.21	-11.2
-11.28	-11.27	-11.26	-11.25	-11.24	-11.23	-11.22	-11.21	-11.2
-11.28	-11.27	-11.26	-11.25	-11.24	-11.23	-11.22	-11.21	-11.2
-11.28	-11.27	-11.26	-11.25	-11.24	-11.23	-11.22	-11.21	-11.2
-11.28	-11.27	-11.26	-11.25	-11.24	-11.23	-11.22	-11.21	-11.2
-11.28	-11.27	-11.26	-11.25	-11.24	-11.23	-11.22	-11.21	-11.2
-11.28	-11.27	-11.26	-11.25	-11.24	-11.23	-11.22	-11.21	-11.2
-11.28	-11.27	-11.26	-11.25	-11.24	-11.23	-11.22	-11.21	-11.2
-11.28	-11.27	-11.26	-11.25	-11.24	-11.23	-11.22	-11.21	-11.2
-11.28	-11.27	-11.26	-11.25	-11.24	-11.23	-11.22	-11.21	-11.2
-11.28	-11.27	-11.26	-11.25	-11.24	-11.23	-11.22	-11.21	-11.2
-11.28	-11.27	-11.26	-11.25	-11.24	-11.23	-11.22	-11.21	-11.2
-11.28	-11.27	-11.26	-11.25	-11.24	-11.23	-11.22	-11.21	-11.2

-11.28	-11.27	-11.26	-11.25	-11.24	-11.23	-11.22	-11.21	-11.2
-11.28	-11.27	-11.26	-11.25	-11.24	-11.23	-11.22	-11.21	-11.2
-11.28	-11.27	-11.26	-11.25	-11.24	-11.23	-11.22	-11.21	-11.2
-11.28	-11.27	-11.26	-11.25	-11.24	-11.23	-11.22	-11.21	-11.2
-11.28	-11.27	-11.26	-11.25	-11.24	-11.23	-11.22	-11.21	-11.2
-11.28	-11.27	-11.26	-11.25	-11.24	-11.23	-11.22	-11.21	-11.2
-11.28	-11.27	-11.26	-11.25	-11.24	-11.23	-11.22	-11.21	-11.2
-11.28	-11.27	-11.26	-11.25	-11.24	-11.23	-11.22	-11.21	-11.2
-11.28	-11.27	-11.26	-11.25	-11.24	-11.23	-11.22	-11.21	-11.2
-11.28	-11.27	-11.26	-11.25	-11.24	-11.23	-11.22	-11.21	-11.2
-11.28	-11.27	-11.26	-11.25	-11.24	-11.23	-11.22	-11.21	-11.2
-11.28	-11.27	-11.26	-11.25	-11.24	-11.23	-11.22	-11.21	-11.2
-11.28	-11.27	-11.26	-11.25	-11.24	-11.23	-11.22	-11.21	-11.2
-11.28	-11.27	-11.26	-11.25	-11.24	-11.23	-11.22	-11.21	-11.2
-11.28	-11.27	-11.26	-11.25	-11.24	-11.23	-11.22	-11.21	-11.2
-11.28	-11.27	-11.26	-11.25	-11.24	-11.23	-11.22	-11.21	-11.2
-11.28	-11.27	-11.26	-11.25	-11.24	-11.23	-11.22	-11.21	-11.2
-11.28	-11.27	-11.26	-11.25	-11.24	-11.23	-11.22	-11.21	-11.2
-11.28	-11.27	-11.26	-11.25	-11.24	-11.23	-11.22	-11.21	-11.2
-11.28	-11.27	-11.26	-11.25	-11.24	-11.23	-11.22	-11.21	-11.2
-11.28	-11.27	-11.26	-11.25	-11.24	-11.23	-11.22	-11.21	-11.2
-11.28	-11.27	-11.26	-11.25	-11.24	-11.23	-11.22	-11.21	-11.2
-11.28	-11.27	-11.26	-11.25	-11.24	-11.23	-11.22	-11.21	-11.2
-11.28	-11.27	-11.26	-11.25	-11.24	-11.23	-11.22	-11.21	-11.2
-11.28	-11.27	-11.26	-11.25	-11.24	-11.23	-11.22	-11.21	-11.2
-11.28	-11.27	-11.26	-11.25	-11.24	-11.23	-11.22	-11.21	-11.2
-11.28	-11.27	-11.26	-11.25	-11.24	-11.23	-11.22	-11.21	-11.2
-11.28	-11.27	-11.26	-11.25	-11.24	-11.23	-11.22	-11.21	-11.2
-11.28	-11.27	-11.26	-11.25	-11.24	-11.23	-11.22	-11.21	-11.2
-11.28	-11.27	-11.26	-11.25	-11.24	-11.23	-11.22	-11.21	-11.2
-11.28	-11.27	-11.26	-11.25	-11.24	-11.23	-11.22	-11.21	-11.2
-11.28	-11.27	-11.26	-11.25	-11.24	-11.23	-11.22	-11.21	-11.2
-11.28	-11.27	-11.26	-11.25	-11.24	-11.23	-11.22	-11.21	-11.2
-11.28	-11.27	-11.26	-11.25	-11.24	-11.23	-11.22	-11.21	-11.2
-11.28	-11.27	-11.26	-11.25	-11.24	-11.23	-11.22	-11.21	-11.2
-11.28	-11.27	-11.26	-11.25	-11.24	-11.23	-11.22	-11.21	-11.2
-11.28	-11.27	-11.26	-11.25	-11.24	-11.23	-11.22	-11.21	-11.2
-11.28	-11.27	-11.26	-11.25	-11.24	-11.23	-11.22	-11.21	-11.2
-11.28	-11.27	-11.26	-11.25	-11.24	-11.23	-11.22	-11.21	-11.2
-11.28	-11.27	-11.26	-11.25	-11.24	-11.23	-11.22	-11.21	-11.2
-11.28	-11.27	-11.26	-11.25	-11.24	-11.23	-11.22	-11.21	-11.2
-11.28	-11.27	-11.26	-11.25	-11.24	-11.23	-11.22	-11.21	-11.2
-11.28	-11.27	-11.26	-11.25	-11.24	-11.23	-11.22	-11.21	-11.2
-11.28	-11.27	-11.26	-11.25	-11.24	-11.23	-11.22	-11.21	-11.2
-11.28	-11.27	-11.26	-11.25	-11.24	-11.23	-11.22	-11.21	-11.2
-11.28	-11.27	-11.26	-11.25	-11.24	-11.23	-11.22	-11.21	-11.2
-11.28	-11.27	-11.26	-11.25	-11.24	-11.23	-11.22	-11.21	-11.2
-11.28	-11.27	-11.26	-11.25	-11.24	-11.23	-11.22	-11.21	-11.2
-11.28	-11.27	-11.26	-11.25	-11.24	-11.23	-11.22	-11.21	-11.2
-11.28	-11.27	-11.26	-11.25	-11.24	-11.23	-11.22	-11.21	-11.2

-11.28	-11.27	-11.26	-11.25	-11.24	-11.23	-11.22	-11.21	-11.2
-11.28	-11.27	-11.26	-11.25	-11.24	-11.23	-11.22	-11.21	-11.2
-11.28	-11.27	-11.26	-11.25	-11.24	-11.23	-11.22	-11.21	-11.2
-11.28	-11.27	-11.26	-11.25	-11.24	-11.23	-11.22	-11.21	-11.2
-11.28	-11.27	-11.26	-11.25	-11.24	-11.23	-11.22	-11.21	-11.2
-11.28	-11.27	-11.26	-11.25	-11.24	-11.23	-11.22	-11.21	-11.2
-11.28	-11.27	-11.26	-11.25	-11.24	-11.23	-11.22	-11.21	-11.2
-11.28	-11.27	-11.26	-11.25	-11.24	-11.23	-11.22	-11.21	-11.2
-11.28	-11.27	-11.26	-11.25	-11.24	-11.23	-11.22	-11.21	-11.2
-11.28	-11.27	-11.26	-11.25	-11.24	-11.23	-11.22	-11.21	-11.2
-11.28	-11.27	-11.26	-11.25	-11.24	-11.23	-11.22	-11.21	-11.2
-11.28	-11.27	-11.26	-11.25	-11.24	-11.23	-11.22	-11.21	-11.2
-11.28	-11.27	-11.26	-11.25	-11.24	-11.23	-11.22	-11.21	-11.2
-11.28	-11.27	-11.26	-11.25	-11.24	-11.23	-11.22	-11.21	-11.2
-11.28	-11.27	-11.26	-11.25	-11.24	-11.23	-11.22	-11.21	-11.2
-11.28	-11.27	-11.26	-11.25	-11.24	-11.23	-11.22	-11.21	-11.2
-11.28	-11.27	-11.26	-11.25	-11.24	-11.23	-11.22	-11.21	-11.2
-11.28	-11.27	-11.26	-11.25	-11.24	-11.23	-11.22	-11.21	-11.2
-11.28	-11.27	-11.26	-11.25	-11.24	-11.23	-11.22	-11.21	-11.2
-11.28	-11.27	-11.26	-11.25	-11.24	-11.23	-11.22	-11.21	-11.2
-11.28	-11.27	-11.26	-11.25	-11.24	-11.23	-11.22	-11.21	-11.2
-11.28	-11.27	-11.26	-11.25	-11.24	-11.23	-11.22	-11.21	-11.2
-11.28	-11.27	-11.26	-11.25	-11.24	-11.23	-11.22	-11.21	-11.2
-11.28	-11.27	-11.26	-11.25	-11.24	-11.23	-11.22	-11.21	-11.2
-11.28	-11.27	-11.26	-11.25	-11.24	-11.23	-11.22	-11.21	-11.2
-11.28	-11.27	-11.26	-11.25	-11.24	-11.23	-11.22	-11.21	-11.2
-11.28	-11.27	-11.26	-11.25	-11.24	-11.23	-11.22	-11.21	-11.2
-11.28	-11.27	-11.26	-11.25	-11.24	-11.23	-11.22	-11.21	-11.2
-11.28	-11.27	-11.26	-11.25	-11.24	-11.23	-11.22	-11.21	-11.2
-11.28	-11.27	-11.26	-11.25	-11.24	-11.23	-11.22	-11.21	-11.2
-11.28	-11.27	-11.26	-11.25	-11.24	-11.23	-11.22	-11.21	-11.2
-11.28	-11.27	-11.26	-11.25	-11.24	-11.23	-11.22	-11.21	-11.2
-11.28	-11.27	-11.26	-11.25	-11.24	-11.23	-11.22	-11.21	-11.2
-11.28	-11.27	-11.26	-11.25	-11.24	-11.23	-11.22	-11.21	-11.2
-11.28	-11.27	-11.26	-11.25	-11.24	-11.23	-11.22	-11.21	-11.2
-11.28	-11.27	-11.26	-11.25	-11.24	-11.23	-11.22	-11.21	-11.2
-11.28	-11.27	-11.26	-11.25	-11.24	-11.23	-11.22	-11.21	-11.2
-11.28	-11.27	-11.26	-11.25	-11.24	-11.23	-11.22	-11.21	-11.2
-11.28	-11.27	-11.26	-11.25	-11.24	-11.23	-11.22	-11.21	-11.2
-11.28	-11.27	-11.26	-11.25	-11.24	-11.23	-11.22	-11.21	-11.2
-11.28	-11.27	-11.26	-11.25	-11.24	-11.23	-11.22	-11.21	-11.2
-11.28	-11.27	-11.26	-11.25	-11.24	-11.23	-11.22	-11.21	-11.2
-11.28	-11.27	-11.26	-11.25	-11.24	-11.23	-11.22	-11.21	-11.2
-11.28	-11.27	-11.26	-11.25	-11.24	-11.23	-11.22	-11.21	-11.2
-11.28	-11.27	-11.26	-11.25	-11.24	-11.23	-11.22	-11.21	-11.2
-11.28	-11.27	-11.26	-11.25	-11.24	-11.23	-11.22	-11.21	-11.2
-11.28	-11.27	-11.26	-11.25	-11.24	-11.23	-11.22	-11.21	-11.2
-11.28	-11.27	-11.26	-11.25	-11.24	-11.23	-11.22	-11.21	-11.2
-11.28	-11.27	-11.26	-11.25	-11.24	-11.23	-11.22	-11.21	-11.2
-11.28	-11.27	-11.26	-11.25	-11.24	-11.23	-11.22	-11.21	-11.2

-11.28	-11.27	-11.26	-11.25	-11.24	-11.23	-11.22	-11.21	-11.2
-11.28	-11.27	-11.26	-11.25	-11.24	-11.23	-11.22	-11.21	-11.2
-11.28	-11.27	-11.26	-11.25	-11.24	-11.23	-11.22	-11.21	-11.2
-11.28	-11.27	-11.26	-11.25	-11.24	-11.23	-11.22	-11.21	-11.2
-11.28	-11.27	-11.26	-11.25	-11.24	-11.23	-11.22	-11.21	-11.2
-11.28	-11.27	-11.26	-11.25	-11.24	-11.23	-11.22	-11.21	-11.2
-11.28	-11.27	-11.26	-11.25	-11.24	-11.23	-11.22	-11.21	-11.2
-11.28	-11.27	-11.26	-11.25	-11.24	-11.23	-11.22	-11.21	-11.2
-11.28	-11.27	-11.26	-11.25	-11.24	-11.23	-11.22	-11.21	-11.2
-11.28	-11.27	-11.26	-11.25	-11.24	-11.23	-11.22	-11.21	-11.2
-11.28	-11.27	-11.26	-11.25	-11.24	-11.23	-11.22	-11.21	-11.2
-11.28	-11.27	-11.26	-11.25	-11.24	-11.23	-11.22	-11.21	-11.2
-11.28	-11.27	-11.26	-11.25	-11.24	-11.23	-11.22	-11.21	-11.2
-11.28	-11.27	-11.26	-11.25	-11.24	-11.23	-11.22	-11.21	-11.2
-11.28	-11.27	-11.26	-11.25	-11.24	-11.23	-11.22	-11.21	-11.2
-11.28	-11.27	-11.26	-11.25	-11.24	-11.23	-11.22	-11.21	-11.2
-11.28	-11.27	-11.26	-11.25	-11.24	-11.23	-11.22	-11.21	-11.2
-11.28	-11.27	-11.26	-11.25	-11.24	-11.23	-11.22	-11.21	-11.2
-11.28	-11.27	-11.26	-11.25	-11.24	-11.23	-11.22	-11.21	-11.2
-11.28	-11.27	-11.26	-11.25	-11.24	-11.23	-11.22	-11.21	-11.2
-11.28	-11.27	-11.26	-11.25	-11.24	-11.23	-11.22	-11.21	-11.2
-11.28	-11.27	-11.26	-11.25	-11.24	-11.23	-11.22	-11.21	-11.2
-11.28	-11.27	-11.26	-11.25	-11.24	-11.23	-11.22	-11.21	-11.2
-11.28	-11.27	-11.26	-11.25	-11.24	-11.23	-11.22	-11.21	-11.2
-11.28	-11.27	-11.26	-11.25	-11.24	-11.23	-11.22	-11.21	-11.2
-11.28	-11.27	-11.26	-11.25	-11.24	-11.23	-11.22	-11.21	-11.2
-11.28	-11.27	-11.26	-11.25	-11.24	-11.23	-11.22	-11.21	-11.2
-11.28	-11.27	-11.26	-11.25	-11.24	-11.23	-11.22	-11.21	-11.2
-11.28	-11.27	-11.26	-11.25	-11.24	-11.23	-11.22	-11.21	-11.2
-11.28	-11.27	-11.26	-11.25	-11.24	-11.23	-11.22	-11.21	-11.2
-11.28	-11.27	-11.26	-11.25	-11.24	-11.23	-11.22	-11.21	-11.2
-11.28	-11.27	-11.26	-11.25	-11.24	-11.23	-11.22	-11.21	-11.2
-11.28	-11.27	-11.26	-11.25	-11.24	-11.23	-11.22	-11.21	-11.2
-11.28	-11.27	-11.26	-11.25	-11.24	-11.23	-11.22	-11.21	-11.2
-11.28	-11.27	-11.26	-11.25	-11.24	-11.23	-11.22	-11.21	-11.2
-11.28	-11.27	-11.26	-11.25	-11.24	-11.23	-11.22	-11.21	-11.2
-11.28	-11.27	-11.26	-11.25	-11.24	-11.23	-11.22	-11.21	-11.2
-11.28	-11.27	-11.26	-11.25	-11.24	-11.23	-11.22	-11.21	-11.2
-11.28	-11.27	-11.26	-11.25	-11.24	-11.23	-11.22	-11.21	-11.2
-11.28	-11.27	-11.26	-11.25	-11.24	-11.23	-11.22	-11.21	-11.2
-11.28	-11.27	-11.26	-11.25	-11.24	-11.23	-11.22	-11.21	-11.2
-11.28	-11.27	-11.26	-11.25	-11.24	-11.23	-11.22	-11.21	-11.2
-11.28	-11.27	-11.26	-11.25	-11.24	-11.23	-11.22	-11.21	-11.2
-11.28	-11.27	-11.26	-11.25	-11.24	-11.23	-11.22	-11.21	-11.2
-11.28	-11.27	-11.26	-11.25	-11.24	-11.23	-11.22	-11.21	-11.2
-11.28	-11.27	-11.26	-11.25	-11.24	-11.23	-11.22	-11.21	-11.2
-11.28	-11.27	-11.26	-11.25	-11.24	-11.23	-11.22	-11.21	-11.2
-11.28	-11.27	-11.26	-11.25	-11.24	-11.23	-11.22	-11.21	-11.2
-11.28	-11.27	-11.26	-11.25	-11.24	-11.23	-11.22	-11.21	-11.2
-11.28	-11.27	-11.26	-11.25	-11.24	-11.23	-11.22	-11.21	-11.2

-11.28	-11.27	-11.26	-11.25	-11.24	-11.23	-11.22	-11.21	-11.2
-11.28	-11.27	-11.26	-11.25	-11.24	-11.23	-11.22	-11.21	-11.2
-11.28	-11.27	-11.26	-11.25	-11.24	-11.23	-11.22	-11.21	-11.2
-11.28	-11.27	-11.26	-11.25	-11.24	-11.23	-11.22	-11.21	-11.2
-11.28	-11.27	-11.26	-11.25	-11.24	-11.23	-11.22	-11.21	-11.2
-11.28	-11.27	-11.26	-11.25	-11.24	-11.23	-11.22	-11.21	-11.2
-11.28	-11.27	-11.26	-11.25	-11.24	-11.23	-11.22	-11.21	-11.2
-11.28	-11.27	-11.26	-11.25	-11.24	-11.23	-11.22	-11.21	-11.2
-11.28	-11.27	-11.26	-11.25	-11.24	-11.23	-11.22	-11.21	-11.2
-11.28	-11.27	-11.26	-11.25	-11.24	-11.23	-11.22	-11.21	-11.2
-11.28	-11.27	-11.26	-11.25	-11.24	-11.23	-11.22	-11.21	-11.2
-11.28	-11.27	-11.26	-11.25	-11.24	-11.23	-11.22	-11.21	-11.2
-11.28	-11.27	-11.26	-11.25	-11.24	-11.23	-11.22	-11.21	-11.2
-11.28	-11.27	-11.26	-11.25	-11.24	-11.23	-11.22	-11.21	-11.2
-11.28	-11.27	-11.26	-11.25	-11.24	-11.23	-11.22	-11.21	-11.2
-11.28	-11.27	-11.26	-11.25	-11.24	-11.23	-11.22	-11.21	-11.2
-11.28	-11.27	-11.26	-11.25	-11.24	-11.23	-11.22	-11.21	-11.2
-11.28	-11.27	-11.26	-11.25	-11.24	-11.23	-11.22	-11.21	-11.2
-11.28	-11.27	-11.26	-11.25	-11.24	-11.23	-11.22	-11.21	-11.2
-11.28	-11.27	-11.26	-11.25	-11.24	-11.23	-11.22	-11.21	-11.2
-11.28	-11.27	-11.26	-11.25	-11.24	-11.23	-11.22	-11.21	-11.2
-11.28	-11.27	-11.26	-11.25	-11.24	-11.23	-11.22	-11.21	-11.2
-11.28	-11.27	-11.26	-11.25	-11.24	-11.23	-11.22	-11.21	-11.2
-11.28	-11.27	-11.26	-11.25	-11.24	-11.23	-11.22	-11.21	-11.2
-11.28	-11.27	-11.26	-11.25	-11.24	-11.23	-11.22	-11.21	-11.2
-11.28	-11.27	-11.26	-11.25	-11.24	-11.23	-11.22	-11.21	-11.2
-11.28	-11.27	-11.26	-11.25	-11.24	-11.23	-11.22	-11.21	-11.2
-11.28	-11.27	-11.26	-11.25	-11.24	-11.23	-11.22	-11.21	-11.2
-11.28	-11.27	-11.26	-11.25	-11.24	-11.23	-11.22	-11.21	-11.2
-11.28	-11.27	-11.26	-11.25	-11.24	-11.23	-11.22	-11.21	-11.2
-11.28	-11.27	-11.26	-11.25	-11.24	-11.23	-11.22	-11.21	-11.2
-11.28	-11.27	-11.26	-11.25	-11.24	-11.23	-11.22	-11.21	-11.2
-11.28	-11.27	-11.26	-11.25	-11.24	-11.23	-11.22	-11.21	-11.2
-11.28	-11.27	-11.26	-11.25	-11.24	-11.23	-11.22	-11.21	-11.2
-11.28	-11.27	-11.26	-11.25	-11.24	-11.23	-11.22	-11.21	-11.2
-11.28	-11.27	-11.26	-11.25	-11.24	-11.23	-11.22	-11.21	-11.2
-11.28	-11.27	-11.26	-11.25	-11.24	-11.23	-11.22	-11.21	-11.2
-11.28	-11.27	-11.26	-11.25	-11.24	-11.23	-11.22	-11.21	-11.2
-11.28	-11.27	-11.26	-11.25	-11.24	-11.23	-11.22	-11.21	-11.2
-11.28	-11.27	-11.26	-11.25	-11.24	-11.23	-11.22	-11.21	-11.2
-11.28	-11.27	-11.26	-11.25	-11.24	-11.23	-11.22	-11.21	-11.2
-11.28	-11.27	-11.26	-11.25	-11.24	-11.23	-11.22	-11.21	-11.2
-11.28	-11.27	-11.26	-11.25	-11.24	-11.23	-11.22	-11.21	-11.2
-11.28	-11.27	-11.26	-11.25	-11.24	-11.23	-11.22	-11.21	-11.2
-11.28	-11.27	-11.26	-11.25	-11.24	-11.23	-11.22	-11.21	-11.2
-11.28	-11.27	-11.26	-11.25	-11.24	-11.23	-11.22	-11.21	-11.2
-11.28	-11.27	-11.26	-11.25	-11.24	-11.23	-11.22	-11.21	-11.2
-11.28	-11.27	-11.26	-11.25	-11.24	-11.23	-11.22	-11.21	-11.2
-11.28	-11.27	-11.26	-11.25	-11.24	-11.23	-11.22	-11.21	-11.2
-11.28	-11.27	-11.26	-11.25	-11.24	-11.23	-11.22	-11.21	-11.2

-11.28	-11.27	-11.26	-11.25	-11.24	-11.23	-11.22	-11.21	-11.2
-11.28	-11.27	-11.26	-11.25	-11.24	-11.23	-11.22	-11.21	-11.2
-11.28	-11.27	-11.26	-11.25	-11.24	-11.23	-11.22	-11.21	-11.2
-11.28	-11.27	-11.26	-11.25	-11.24	-11.23	-11.22	-11.21	-11.2
-11.28	-11.27	-11.26	-11.25	-11.24	-11.23	-11.22	-11.21	-11.2
-11.28	-11.27	-11.26	-11.25	-11.24	-11.23	-11.22	-11.21	-11.2
-11.28	-11.27	-11.26	-11.25	-11.24	-11.23	-11.22	-11.21	-11.2
-11.28	-11.27	-11.26	-11.25	-11.24	-11.23	-11.22	-11.21	-11.2
-11.28	-11.27	-11.26	-11.25	-11.24	-11.23	-11.22	-11.21	-11.2
-11.28	-11.27	-11.26	-11.25	-11.24	-11.23	-11.22	-11.21	-11.2
-11.28	-11.27	-11.26	-11.25	-11.24	-11.23	-11.22	-11.21	-11.2
-11.28	-11.27	-11.26	-11.25	-11.24	-11.23	-11.22	-11.21	-11.2
-11.28	-11.27	-11.26	-11.25	-11.24	-11.23	-11.22	-11.21	-11.2
-11.28	-11.27	-11.26	-11.25	-11.24	-11.23	-11.22	-11.21	-11.2
-11.28	-11.27	-11.26	-11.25	-11.24	-11.23	-11.22	-11.21	-11.2
-11.28	-11.27	-11.26	-11.25	-11.24	-11.23	-11.22	-11.21	-11.2
-11.28	-11.27	-11.26	-11.25	-11.24	-11.23	-11.22	-11.21	-11.2
-11.28	-11.27	-11.26	-11.25	-11.24	-11.23	-11.22	-11.21	-11.2
-11.28	-11.27	-11.26	-11.25	-11.24	-11.23	-11.22	-11.21	-11.2
-11.28	-11.27	-11.26	-11.25	-11.24	-11.23	-11.22	-11.21	-11.2
-11.28	-11.27	-11.26	-11.25	-11.24	-11.23	-11.22	-11.21	-11.2
-11.28	-11.27	-11.26	-11.25	-11.24	-11.23	-11.22	-11.21	-11.2
-11.28	-11.27	-11.26	-11.25	-11.24	-11.23	-11.22	-11.21	-11.2
-11.28	-11.27	-11.26	-11.25	-11.24	-11.23	-11.22	-11.21	-11.2
-11.28	-11.27	-11.26	-11.25	-11.24	-11.23	-11.22	-11.21	-11.2
-11.28	-11.27	-11.26	-11.25	-11.24	-11.23	-11.22	-11.21	-11.2
-11.28	-11.27	-11.26	-11.25	-11.24	-11.23	-11.22	-11.21	-11.2
-11.28	-11.27	-11.26	-11.25	-11.24	-11.23	-11.22	-11.21	-11.2
-11.28	-11.27	-11.26	-11.25	-11.24	-11.23	-11.22	-11.21	-11.2
-11.28	-11.27	-11.26	-11.25	-11.24	-11.23	-11.22	-11.21	-11.2
-11.28	-11.27	-11.26	-11.25	-11.24	-11.23	-11.22	-11.21	-11.2
-11.28	-11.27	-11.26	-11.25	-11.24	-11.23	-11.22	-11.21	-11.2
-11.28	-11.27	-11.26	-11.25	-11.24	-11.23	-11.22	-11.21	-11.2
-11.28	-11.27	-11.26	-11.25	-11.24	-11.23	-11.22	-11.21	-11.2
-11.28	-11.27	-11.26	-11.25	-11.24	-11.23	-11.22	-11.21	-11.2
-11.28	-11.27	-11.26	-11.25	-11.24	-11.23	-11.22	-11.21	-11.2
-11.28	-11.27	-11.26	-11.25	-11.24	-11.23	-11.22	-11.21	-11.2
-11.28	-11.27	-11.26	-11.25	-11.24	-11.23	-11.22	-11.21	-11.2
-11.28	-11.27	-11.26	-11.25	-11.24	-11.23	-11.22	-11.21	-11.2
-11.28	-11.27	-11.26	-11.25	-11.24	-11.23	-11.22	-11.21	-11.2
-11.28	-11.27	-11.26	-11.25	-11.24	-11.23	-11.22	-11.21	-11.2
-11.28	-11.27	-11.26	-11.25	-11.24	-11.23	-11.22	-11.21	-11.2
-11.28	-11.27	-11.26	-11.25	-11.24	-11.23	-11.22	-11.21	-11.2
-11.28	-11.27	-11.26	-11.25	-11.24	-11.23	-11.22	-11.21	-11.2
-11.28	-11.27	-11.26	-11.25	-11.24	-11.23	-11.22	-11.21	-11.2
-11.28	-11.27	-11.26	-11.25	-11.24	-11.23	-11.22	-11.21	-11.2
-11.28	-11.27	-11.26	-11.25	-11.24	-11.23	-11.22	-11.21	-11.2
-11.28	-11.27	-11.26	-11.25	-11.24	-11.23	-11.22	-11.21	-11.2
-11.28	-11.27	-11.26	-11.25	-11.24	-11.23	-11.22	-11.21	-11.2
-11.28	-11.27	-11.26	-11.25	-11.24	-11.23	-11.22	-11.21	-11.2

-11.28	-11.27	-11.26	-11.25	-11.24	-11.23	-11.22	-11.21	-11.2
-11.28	-11.27	-11.26	-11.25	-11.24	-11.23	-11.22	-11.21	-11.2
-11.28	-11.27	-11.26	-11.25	-11.24	-11.23	-11.22	-11.21	-11.2
-11.28	-11.27	-11.26	-11.25	-11.24	-11.23	-11.22	-11.21	-11.2
-11.28	-11.27	-11.26	-11.25	-11.24	-11.23	-11.22	-11.21	-11.2
-11.28	-11.27	-11.26	-11.25	-11.24	-11.23	-11.22	-11.21	-11.2
-11.28	-11.27	-11.26	-11.25	-11.24	-11.23	-11.22	-11.21	-11.2
-11.28	-11.27	-11.26	-11.25	-11.24	-11.23	-11.22	-11.21	-11.2
-11.28	-11.27	-11.26	-11.25	-11.24	-11.23	-11.22	-11.21	-11.2
-11.28	-11.27	-11.26	-11.25	-11.24	-11.23	-11.22	-11.21	-11.2
-11.28	-11.27	-11.26	-11.25	-11.24	-11.23	-11.22	-11.21	-11.2
-11.28	-11.27	-11.26	-11.25	-11.24	-11.23	-11.22	-11.21	-11.2
-11.28	-11.27	-11.26	-11.25	-11.24	-11.23	-11.22	-11.21	-11.2
-11.28	-11.27	-11.26	-11.25	-11.24	-11.23	-11.22	-11.21	-11.2
-11.28	-11.27	-11.26	-11.25	-11.24	-11.23	-11.22	-11.21	-11.2
-11.28	-11.27	-11.26	-11.25	-11.24	-11.23	-11.22	-11.21	-11.2
-11.28	-11.27	-11.26	-11.25	-11.24	-11.23	-11.22	-11.21	-11.2
-11.28	-11.27	-11.26	-11.25	-11.24	-11.23	-11.22	-11.21	-11.2
-11.28	-11.27	-11.26	-11.25	-11.24	-11.23	-11.22	-11.21	-11.2
-11.28	-11.27	-11.26	-11.25	-11.24	-11.23	-11.22	-11.21	-11.2
-11.28	-11.27	-11.26	-11.25	-11.24	-11.23	-11.22	-11.21	-11.2
-11.28	-11.27	-11.26	-11.25	-11.24	-11.23	-11.22	-11.21	-11.2
-11.28	-11.27	-11.26	-11.25	-11.24	-11.23	-11.22	-11.21	-11.2
-11.28	-11.27	-11.26	-11.25	-11.24	-11.23	-11.22	-11.21	-11.2
-11.28	-11.27	-11.26	-11.25	-11.24	-11.23	-11.22	-11.21	-11.2
-11.28	-11.27	-11.26	-11.25	-11.24	-11.23	-11.22	-11.21	-11.2
-11.28	-11.27	-11.26	-11.25	-11.24	-11.23	-11.22	-11.21	-11.2
-11.28	-11.27	-11.26	-11.25	-11.24	-11.23	-11.22	-11.21	-11.2
-11.28	-11.27	-11.26	-11.25	-11.24	-11.23	-11.22	-11.21	-11.2
-11.28	-11.27	-11.26	-11.25	-11.24	-11.23	-11.22	-11.21	-11.2
-11.28	-11.27	-11.26	-11.25	-11.24	-11.23	-11.22	-11.21	-11.2
-11.28	-11.27	-11.26	-11.25	-11.24	-11.23	-11.22	-11.21	-11.2
-11.28	-11.27	-11.26	-11.25	-11.24	-11.23	-11.22	-11.21	-11.2
-11.28	-11.27	-11.26	-11.25	-11.24	-11.23	-11.22	-11.21	-11.2
-11.28	-11.27	-11.26	-11.25	-11.24	-11.23	-11.22	-11.21	-11.2
-11.28	-11.27	-11.26	-11.25	-11.24	-11.23	-11.22	-11.21	-11.2
-11.28	-11.27	-11.26	-11.25	-11.24	-11.23	-11.22	-11.21	-11.2
-11.28	-11.27	-11.26	-11.25	-11.24	-11.23	-11.22	-11.21	-11.2
-11.28	-11.27	-11.26	-11.25	-11.24	-11.23	-11.22	-11.21	-11.2
-11.28	-11.27	-11.26	-11.25	-11.24	-11.23	-11.22	-11.21	-11.2
-11.28	-11.27	-11.26	-11.25	-11.24	-11.23	-11.22	-11.21	-11.2
-11.28	-11.27	-11.26	-11.25	-11.24	-11.23	-11.22	-11.21	-11.2
-11.28	-11.27	-11.26	-11.25	-11.24	-11.23	-11.22	-11.21	-11.2
-11.28	-11.27	-11.26	-11.25	-11.24	-11.23	-11.22	-11.21	-11.2
-11.28	-11.27	-11.26	-11.25	-11.24	-11.23	-11.22	-11.21	-11.2
-11.28	-11.27	-11.26	-11.25	-11.24	-11.23	-11.22	-11.21	-11.2
-11.28	-11.27	-11.26	-11.25	-11.24	-11.23	-11.22	-11.21	-11.2
-11.28	-11.27	-11.26	-11.25	-11.24	-11.23	-11.22	-11.21	-11.2
-11.28	-11.27	-11.26	-11.25	-11.24	-11.23	-11.22	-11.21	-11.2
-11.28	-11.27	-11.26	-11.25	-11.24	-11.23	-11.22	-11.21	-11.2

-11.28	-11.27	-11.26	-11.25	-11.24	-11.23	-11.22	-11.21	-11.2
-11.28	-11.27	-11.26	-11.25	-11.24	-11.23	-11.22	-11.21	-11.2
-11.28	-11.27	-11.26	-11.25	-11.24	-11.23	-11.22	-11.21	-11.2
-11.28	-11.27	-11.26	-11.25	-11.24	-11.23	-11.22	-11.21	-11.2
-11.28	-11.27	-11.26	-11.25	-11.24	-11.23	-11.22	-11.21	-11.2
-11.28	-11.27	-11.26	-11.25	-11.24	-11.23	-11.22	-11.21	-11.2
-11.28	-11.27	-11.26	-11.25	-11.24	-11.23	-11.22	-11.21	-11.2
-11.28	-11.27	-11.26	-11.25	-11.24	-11.23	-11.22	-11.21	-11.2
-11.28	-11.27	-11.26	-11.25	-11.24	-11.23	-11.22	-11.21	-11.2
-11.28	-11.27	-11.26	-11.25	-11.24	-11.23	-11.22	-11.21	-11.2
-11.28	-11.27	-11.26	-11.25	-11.24	-11.23	-11.22	-11.21	-11.2
-11.28	-11.27	-11.26	-11.25	-11.24	-11.23	-11.22	-11.21	-11.2
-11.28	-11.27	-11.26	-11.25	-11.24	-11.23	-11.22	-11.21	-11.2
-11.28	-11.27	-11.26	-11.25	-11.24	-11.23	-11.22	-11.21	-11.2
-11.28	-11.27	-11.26	-11.25	-11.24	-11.23	-11.22	-11.21	-11.2
-11.28	-11.27	-11.26	-11.25	-11.24	-11.23	-11.22	-11.21	-11.2
-11.28	-11.27	-11.26	-11.25	-11.24	-11.23	-11.22	-11.21	-11.2
-11.28	-11.27	-11.26	-11.25	-11.24	-11.23	-11.22	-11.21	-11.2
-11.28	-11.27	-11.26	-11.25	-11.24	-11.23	-11.22	-11.21	-11.2
-11.28	-11.27	-11.26	-11.25	-11.24	-11.23	-11.22	-11.21	-11.2
-11.28	-11.27	-11.26	-11.25	-11.24	-11.23	-11.22	-11.21	-11.2
-11.28	-11.27	-11.26	-11.25	-11.24	-11.23	-11.22	-11.21	-11.2
-11.28	-11.27	-11.26	-11.25	-11.24	-11.23	-11.22	-11.21	-11.2
-11.28	-11.27	-11.26	-11.25	-11.24	-11.23	-11.22	-11.21	-11.2
-11.28	-11.27	-11.26	-11.25	-11.24	-11.23	-11.22	-11.21	-11.2
-11.28	-11.27	-11.26	-11.25	-11.24	-11.23	-11.22	-11.21	-11.2
-11.28	-11.27	-11.26	-11.25	-11.24	-11.23	-11.22	-11.21	-11.2
-11.28	-11.27	-11.26	-11.25	-11.24	-11.23	-11.22	-11.21	-11.2
-11.28	-11.27	-11.26	-11.25	-11.24	-11.23	-11.22	-11.21	-11.2
-11.28	-11.27	-11.26	-11.25	-11.24	-11.23	-11.22	-11.21	-11.2
-11.28	-11.27	-11.26	-11.25	-11.24	-11.23	-11.22	-11.21	-11.2
-11.28	-11.27	-11.26	-11.25	-11.24	-11.23	-11.22	-11.21	-11.2
-11.28	-11.27	-11.26	-11.25	-11.24	-11.23	-11.22	-11.21	-11.2
-11.28	-11.27	-11.26	-11.25	-11.24	-11.23	-11.22	-11.21	-11.2
-11.28	-11.27	-11.26	-11.25	-11.24	-11.23	-11.22	-11.21	-11.2
-11.28	-11.27	-11.26	-11.25	-11.24	-11.23	-11.22	-11.21	-11.2
-11.28	-11.27	-11.26	-11.25	-11.24	-11.23	-11.22	-11.21	-11.2
-11.28	-11.27	-11.26	-11.25	-11.24	-11.23	-11.22	-11.21	-11.2
-11.28	-11.27	-11.26	-11.25	-11.24	-11.23	-11.22	-11.21	-11.2
-11.28	-11.27	-11.26	-11.25	-11.24	-11.23	-11.22	-11.21	-11.2
-11.28	-11.27	-11.26	-11.25	-11.24	-11.23	-11.22	-11.21	-11.2
-11.28	-11.27	-11.26	-11.25	-11.24	-11.23	-11.22	-11.21	-11.2
-11.28	-11.27	-11.26	-11.25	-11.24	-11.23	-11.22	-11.21	-11.2
-11.28	-11.27	-11.26	-11.25	-11.24	-11.23	-11.22	-11.21	-11.2
-11.28	-11.27	-11.26	-11.25	-11.24	-11.23	-11.22	-11.21	-11.2
-11.28	-11.27	-11.26	-11.25	-11.24	-11.23	-11.22	-11.21	-11.2
-11.28	-11.27	-11.26	-11.25	-11.24	-11.23	-11.22	-11.21	-11.2
-11.28	-11.27	-11.26	-11.25	-11.24	-11.23	-11.22	-11.21	-11.2
-11.28	-11.27	-11.26	-11.25	-11.24	-11.23	-11.22	-11.21	-11.2
-11.28	-11.27	-11.26	-11.25	-11.24	-11.23	-11.22	-11.21	-11.2

-11.28	-11.27	-11.26	-11.25	-11.24	-11.23	-11.22	-11.21	-11.2
-11.28	-11.27	-11.26	-11.25	-11.24	-11.23	-11.22	-11.21	-11.2
-11.28	-11.27	-11.26	-11.25	-11.24	-11.23	-11.22	-11.21	-11.2
-11.28	-11.27	-11.26	-11.25	-11.24	-11.23	-11.22	-11.21	-11.2
-11.28	-11.27	-11.26	-11.25	-11.24	-11.23	-11.22	-11.21	-11.2
-11.28	-11.27	-11.26	-11.25	-11.24	-11.23	-11.22	-11.21	-11.2
-11.28	-11.27	-11.26	-11.25	-11.24	-11.23	-11.22	-11.21	-11.2
-11.28	-11.27	-11.26	-11.25	-11.24	-11.23	-11.22	-11.21	-11.2
-11.28	-11.27	-11.26	-11.25	-11.24	-11.23	-11.22	-11.21	-11.2
-11.28	-11.27	-11.26	-11.25	-11.24	-11.23	-11.22	-11.21	-11.2
-11.28	-11.27	-11.26	-11.25	-11.24	-11.23	-11.22	-11.21	-11.2
-11.28	-11.27	-11.26	-11.25	-11.24	-11.23	-11.22	-11.21	-11.2
-11.28	-11.27	-11.26	-11.25	-11.24	-11.23	-11.22	-11.21	-11.2
-11.28	-11.27	-11.26	-11.25	-11.24	-11.23	-11.22	-11.21	-11.2
-11.28	-11.27	-11.26	-11.25	-11.24	-11.23	-11.22	-11.21	-11.2
-11.28	-11.27	-11.26	-11.25	-11.24	-11.23	-11.22	-11.21	-11.2
-11.28	-11.27	-11.26	-11.25	-11.24	-11.23	-11.22	-11.21	-11.2
-11.28	-11.27	-11.26	-11.25	-11.24	-11.23	-11.22	-11.21	-11.2
-11.28	-11.27	-11.26	-11.25	-11.24	-11.23	-11.22	-11.21	-11.2
-11.28	-11.27	-11.26	-11.25	-11.24	-11.23	-11.22	-11.21	-11.2
-11.28	-11.27	-11.26	-11.25	-11.24	-11.23	-11.22	-11.21	-11.2
-11.28	-11.27	-11.26	-11.25	-11.24	-11.23	-11.22	-11.21	-11.2
-11.28	-11.27	-11.26	-11.25	-11.24	-11.23	-11.22	-11.21	-11.2
-11.28	-11.27	-11.26	-11.25	-11.24	-11.23	-11.22	-11.21	-11.2
-11.28	-11.27	-11.26	-11.25	-11.24	-11.23	-11.22	-11.21	-11.2
-11.28	-11.27	-11.26	-11.25	-11.24	-11.23	-11.22	-11.21	-11.2
-11.28	-11.27	-11.26	-11.25	-11.24	-11.23	-11.22	-11.21	-11.2
-11.28	-11.27	-11.26	-11.25	-11.24	-11.23	-11.22	-11.21	-11.2
-11.28	-11.27	-11.26	-11.25	-11.24	-11.23	-11.22	-11.21	-11.2
-11.28	-11.27	-11.26	-11.25	-11.24	-11.23	-11.22	-11.21	-11.2
-11.28	-11.27	-11.26	-11.25	-11.24	-11.23	-11.22	-11.21	-11.2
-11.28	-11.27	-11.26	-11.25	-11.24	-11.23	-11.22	-11.21	-11.2
-11.28	-11.27	-11.26	-11.25	-11.24	-11.23	-11.22	-11.21	-11.2
-11.28	-11.27	-11.26	-11.25	-11.24	-11.23	-11.22	-11.21	-11.2
-11.28	-11.27	-11.26	-11.25	-11.24	-11.23	-11.22	-11.21	-11.2
-11.28	-11.27	-11.26	-11.25	-11.24	-11.23	-11.22	-11.21	-11.2
-11.28	-11.27	-11.26	-11.25	-11.24	-11.23	-11.22	-11.21	-11.2
-11.28	-11.27	-11.26	-11.25	-11.24	-11.23	-11.22	-11.21	-11.2
-11.28	-11.27	-11.26	-11.25	-11.24	-11.23	-11.22	-11.21	-11.2
-11.28	-11.27	-11.26	-11.25	-11.24	-11.23	-11.22	-11.21	-11.2
-11.28	-11.27	-11.26	-11.25	-11.24	-11.23	-11.22	-11.21	-11.2
-11.28	-11.27	-11.26	-11.25	-11.24	-11.23	-11.22	-11.21	-11.2
-11.28	-11.27	-11.26	-11.25	-11.24	-11.23	-11.22	-11.21	-11.2
-11.28	-11.27	-11.26	-11.25	-11.24	-11.23	-11.22	-11.21	-11.2
-11.28	-11.27	-11.26	-11.25	-11.24	-11.23	-11.22	-11.21	-11.2
-11.28	-11.27	-11.26	-11.25	-11.24	-11.23	-11.22	-11.21	-11.2
-11.28	-11.27	-11.26	-11.25	-11.24	-11.23	-11.22	-11.21	-11.2
-11.28	-11.27	-11.26	-11.25	-11.24	-11.23	-11.22	-11.21	-11.2
-11.28	-11.27	-11.26	-11.25	-11.24	-11.23	-11.22	-11.21	-11.2
-11.28	-11.27	-11.26	-11.25	-11.24	-11.23	-11.22	-11.21	-11.2

-11.28	-11.27	-11.26	-11.25	-11.24	-11.23	-11.22	-11.21	-11.2
-11.28	-11.27	-11.26	-11.25	-11.24	-11.23	-11.22	-11.21	-11.2
-11.28	-11.27	-11.26	-11.25	-11.24	-11.23	-11.22	-11.21	-11.2
-11.28	-11.27	-11.26	-11.25	-11.24	-11.23	-11.22	-11.21	-11.2
-11.28	-11.27	-11.26	-11.25	-11.24	-11.23	-11.22	-11.21	-11.2
-11.28	-11.27	-11.26	-11.25	-11.24	-11.23	-11.22	-11.21	-11.2
-11.28	-11.27	-11.26	-11.25	-11.24	-11.23	-11.22	-11.21	-11.2
-11.28	-11.27	-11.26	-11.25	-11.24	-11.23	-11.22	-11.21	-11.2
-11.28	-11.27	-11.26	-11.25	-11.24	-11.23	-11.22	-11.21	-11.2
-11.28	-11.27	-11.26	-11.25	-11.24	-11.23	-11.22	-11.21	-11.2
-11.28	-11.27	-11.26	-11.25	-11.24	-11.23	-11.22	-11.21	-11.2
-11.28	-11.27	-11.26	-11.25	-11.24	-11.23	-11.22	-11.21	-11.2
-11.28	-11.27	-11.26	-11.25	-11.24	-11.23	-11.22	-11.21	-11.2
-11.28	-11.27	-11.26	-11.25	-11.24	-11.23	-11.22	-11.21	-11.2
-11.28	-11.27	-11.26	-11.25	-11.24	-11.23	-11.22	-11.21	-11.2
-11.28	-11.27	-11.26	-11.25	-11.24	-11.23	-11.22	-11.21	-11.2
-11.28	-11.27	-11.26	-11.25	-11.24	-11.23	-11.22	-11.21	-11.2
-11.28	-11.27	-11.26	-11.25	-11.24	-11.23	-11.22	-11.21	-11.2
-11.28	-11.27	-11.26	-11.25	-11.24	-11.23	-11.22	-11.21	-11.2
-11.28	-11.27	-11.26	-11.25	-11.24	-11.23	-11.22	-11.21	-11.2
-11.28	-11.27	-11.26	-11.25	-11.24	-11.23	-11.22	-11.21	-11.2
-11.28	-11.27	-11.26	-11.25	-11.24	-11.23	-11.22	-11.21	-11.2
-11.28	-11.27	-11.26	-11.25	-11.24	-11.23	-11.22	-11.21	-11.2
-11.28	-11.27	-11.26	-11.25	-11.24	-11.23	-11.22	-11.21	-11.2
-11.28	-11.27	-11.26	-11.25	-11.24	-11.23	-11.22	-11.21	-11.2
-11.28	-11.27	-11.26	-11.25	-11.24	-11.23	-11.22	-11.21	-11.2
-11.28	-11.27	-11.26	-11.25	-11.24	-11.23	-11.22	-11.21	-11.2
-11.28	-11.27	-11.26	-11.25	-11.24	-11.23	-11.22	-11.21	-11.2
-11.28	-11.27	-11.26	-11.25	-11.24	-11.23	-11.22	-11.21	-11.2
-11.28	-11.27	-11.26	-11.25	-11.24	-11.23	-11.22	-11.21	-11.2
-11.28	-11.27	-11.26	-11.25	-11.24	-11.23	-11.22	-11.21	-11.2
-11.28	-11.27	-11.26	-11.25	-11.24	-11.23	-11.22	-11.21	-11.2
-11.28	-11.27	-11.26	-11.25	-11.24	-11.23	-11.22	-11.21	-11.2
-11.28	-11.27	-11.26	-11.25	-11.24	-11.23	-11.22	-11.21	-11.2
-11.28	-11.27	-11.26	-11.25	-11.24	-11.23	-11.22	-11.21	-11.2
-11.28	-11.27	-11.26	-11.25	-11.24	-11.23	-11.22	-11.21	-11.2
-11.28	-11.27	-11.26	-11.25	-11.24	-11.23	-11.22	-11.21	-11.2
-11.28	-11.27	-11.26	-11.25	-11.24	-11.23	-11.22	-11.21	-11.2
-11.28	-11.27	-11.26	-11.25	-11.24	-11.23	-11.22	-11.21	-11.2
-11.28	-11.27	-11.26	-11.25	-11.24	-11.23	-11.22	-11.21	-11.2
-11.28	-11.27	-11.26	-11.25	-11.24	-11.23	-11.22	-11.21	-11.2
-11.28	-11.27	-11.26	-11.25	-11.24	-11.23	-11.22	-11.21	-11.2
-11.28	-11.27	-11.26	-11.25	-11.24	-11.23	-11.22	-11.21	-11.2
-11.28	-11.27	-11.26	-11.25	-11.24	-11.23	-11.22	-11.21	-11.2
-11.28	-11.27	-11.26	-11.25	-11.24	-11.23	-11.22	-11.21	-11.2
-11.28	-11.27	-11.26	-11.25	-11.24	-11.23	-11.22	-11.21	-11.2
-11.28	-11.27	-11.26	-11.25	-11.24	-11.23	-11.22	-11.21	-11.2
-11.28	-11.27	-11.26	-11.25	-11.24	-11.23	-11.22	-11.21	-11.2
-11.28	-11.27	-11.26	-11.25	-11.24	-11.23	-11.22	-11.21	-11.2
-11.28	-11.27	-11.26	-11.25	-11.24	-11.23	-11.22	-11.21	-11.2

-11.28	-11.27	-11.26	-11.25	-11.24	-11.23	-11.22	-11.21	-11.2
-11.28	-11.27	-11.26	-11.25	-11.24	-11.23	-11.22	-11.21	-11.2
-11.28	-11.27	-11.26	-11.25	-11.24	-11.23	-11.22	-11.21	-11.2
-11.28	-11.27	-11.26	-11.25	-11.24	-11.23	-11.22	-11.21	-11.2
-11.28	-11.27	-11.26	-11.25	-11.24	-11.23	-11.22	-11.21	-11.2
-11.28	-11.27	-11.26	-11.25	-11.24	-11.23	-11.22	-11.21	-11.2
-11.28	-11.27	-11.26	-11.25	-11.24	-11.23	-11.22	-11.21	-11.2
-11.28	-11.27	-11.26	-11.25	-11.24	-11.23	-11.22	-11.21	-11.2
-11.28	-11.27	-11.26	-11.25	-11.24	-11.23	-11.22	-11.21	-11.2
-11.28	-11.27	-11.26	-11.25	-11.24	-11.23	-11.22	-11.21	-11.2
-11.28	-11.27	-11.26	-11.25	-11.24	-11.23	-11.22	-11.21	-11.2
-11.28	-11.27	-11.26	-11.25	-11.24	-11.23	-11.22	-11.21	-11.2
-11.28	-11.27	-11.26	-11.25	-11.24	-11.23	-11.22	-11.21	-11.2
-11.28	-11.27	-11.26	-11.25	-11.24	-11.23	-11.22	-11.21	-11.2
-11.28	-11.27	-11.26	-11.25	-11.24	-11.23	-11.22	-11.21	-11.2
-11.28	-11.27	-11.26	-11.25	-11.24	-11.23	-11.22	-11.21	-11.2
-11.28	-11.27	-11.26	-11.25	-11.24	-11.23	-11.22	-11.21	-11.2
-11.28	-11.27	-11.26	-11.25	-11.24	-11.23	-11.22	-11.21	-11.2
-11.28	-11.27	-11.26	-11.25	-11.24	-11.23	-11.22	-11.21	-11.2
-11.28	-11.27	-11.26	-11.25	-11.24	-11.23	-11.22	-11.21	-11.2
-11.28	-11.27	-11.26	-11.25	-11.24	-11.23	-11.22	-11.21	-11.2
-11.28	-11.27	-11.26	-11.25	-11.24	-11.23	-11.22	-11.21	-11.2
-11.28	-11.27	-11.26	-11.25	-11.24	-11.23	-11.22	-11.21	-11.2
-11.28	-11.27	-11.26	-11.25	-11.24	-11.23	-11.22	-11.21	-11.2
-11.28	-11.27	-11.26	-11.25	-11.24	-11.23	-11.22	-11.21	-11.2
-11.28	-11.27	-11.26	-11.25	-11.24	-11.23	-11.22	-11.21	-11.2
-11.28	-11.27	-11.26	-11.25	-11.24	-11.23	-11.22	-11.21	-11.2
-11.28	-11.27	-11.26	-11.25	-11.24	-11.23	-11.22	-11.21	-11.2
-11.28	-11.27	-11.26	-11.25	-11.24	-11.23	-11.22	-11.21	-11.2
-11.28	-11.27	-11.26	-11.25	-11.24	-11.23	-11.22	-11.21	-11.2
-11.28	-11.27	-11.26	-11.25	-11.24	-11.23	-11.22	-11.21	-11.2
-11.28	-11.27	-11.26	-11.25	-11.24	-11.23	-11.22	-11.21	-11.2
-11.28	-11.27	-11.26	-11.25	-11.24	-11.23	-11.22	-11.21	-11.2
-11.28	-11.27	-11.26	-11.25	-11.24	-11.23	-11.22	-11.21	-11.2
-11.28	-11.27	-11.26	-11.25	-11.24	-11.23	-11.22	-11.21	-11.2
-11.28	-11.27	-11.26	-11.25	-11.24	-11.23	-11.22	-11.21	-11.2
-11.28	-11.27	-11.26	-11.25	-11.24	-11.23	-11.22	-11.21	-11.2
-11.28	-11.27	-11.26	-11.25	-11.24	-11.23	-11.22	-11.21	-11.2
-11.28	-11.27	-11.26	-11.25	-11.24	-11.23	-11.22	-11.21	-11.2
-11.28	-11.27	-11.26	-11.25	-11.24	-11.23	-11.22	-11.21	-11.2
-11.28	-11.27	-11.26	-11.25	-11.24	-11.23	-11.22	-11.21	-11.2
-11.28	-11.27	-11.26	-11.25	-11.24	-11.23	-11.22	-11.21	-11.2
-11.28	-11.27	-11.26	-11.25	-11.24	-11.23	-11.22	-11.21	-11.2
-11.28	-11.27	-11.26	-11.25	-11.24	-11.23	-11.22	-11.21	-11.2
-11.28	-11.27	-11.26	-11.25	-11.24	-11.23	-11.22	-11.21	-11.2
-11.28	-11.27	-11.26	-11.25	-11.24	-11.23	-11.22	-11.21	-11.2
-11.28	-11.27	-11.26	-11.25	-11.24	-11.23	-11.22	-11.21	-11.2
-11.28	-11.27	-11.26	-11.25	-11.24	-11.23	-11.22	-11.21	-11.2
-11.28	-11.27	-11.26	-11.25	-11.24	-11.23	-11.22	-11.21	-11.2
-11.28	-11.27	-11.26	-11.25	-11.24	-11.23	-11.22	-11.21	-11.2

-11.28	-11.27	-11.26	-11.25	-11.24	-11.23	-11.22	-11.21	-11.2
-11.28	-11.27	-11.26	-11.25	-11.24	-11.23	-11.22	-11.21	-11.2
-11.28	-11.27	-11.26	-11.25	-11.24	-11.23	-11.22	-11.21	-11.2
-11.28	-11.27	-11.26	-11.25	-11.24	-11.23	-11.22	-11.21	-11.2
-11.28	-11.27	-11.26	-11.25	-11.24	-11.23	-11.22	-11.21	-11.2
-11.28	-11.27	-11.26	-11.25	-11.24	-11.23	-11.22	-11.21	-11.2
-11.28	-11.27	-11.26	-11.25	-11.24	-11.23	-11.22	-11.21	-11.2
-11.28	-11.27	-11.26	-11.25	-11.24	-11.23	-11.22	-11.21	-11.2
-11.28	-11.27	-11.26	-11.25	-11.24	-11.23	-11.22	-11.21	-11.2
-11.28	-11.27	-11.26	-11.25	-11.24	-11.23	-11.22	-11.21	-11.2
-11.28	-11.27	-11.26	-11.25	-11.24	-11.23	-11.22	-11.21	-11.2
-11.28	-11.27	-11.26	-11.25	-11.24	-11.23	-11.22	-11.21	-11.2
-11.28	-11.27	-11.26	-11.25	-11.24	-11.23	-11.22	-11.21	-11.2
-11.28	-11.27	-11.26	-11.25	-11.24	-11.23	-11.22	-11.21	-11.2
-11.28	-11.27	-11.26	-11.25	-11.24	-11.23	-11.22	-11.21	-11.2
-11.28	-11.27	-11.26	-11.25	-11.24	-11.23	-11.22	-11.21	-11.2
-11.28	-11.27	-11.26	-11.25	-11.24	-11.23	-11.22	-11.21	-11.2
-11.28	-11.27	-11.26	-11.25	-11.24	-11.23	-11.22	-11.21	-11.2
-11.28	-11.27	-11.26	-11.25	-11.24	-11.23	-11.22	-11.21	-11.2
-11.28	-11.27	-11.26	-11.25	-11.24	-11.23	-11.22	-11.21	-11.2
-11.28	-11.27	-11.26	-11.25	-11.24	-11.23	-11.22	-11.21	-11.2
-11.28	-11.27	-11.26	-11.25	-11.24	-11.23	-11.22	-11.21	-11.2
-11.28	-11.27	-11.26	-11.25	-11.24	-11.23	-11.22	-11.21	-11.2
-11.28	-11.27	-11.26	-11.25	-11.24	-11.23	-11.22	-11.21	-11.2
-11.28	-11.27	-11.26	-11.25	-11.24	-11.23	-11.22	-11.21	-11.2
-11.28	-11.27	-11.26	-11.25	-11.24	-11.23	-11.22	-11.21	-11.2
-11.28	-11.27	-11.26	-11.25	-11.24	-11.23	-11.22	-11.21	-11.2
-11.28	-11.27	-11.26	-11.25	-11.24	-11.23	-11.22	-11.21	-11.2
-11.28	-11.27	-11.26	-11.25	-11.24	-11.23	-11.22	-11.21	-11.2
-11.28	-11.27	-11.26	-11.25	-11.24	-11.23	-11.22	-11.21	-11.2
-11.28	-11.27	-11.26	-11.25	-11.24	-11.23	-11.22	-11.21	-11.2
-11.28	-11.27	-11.26	-11.25	-11.24	-11.23	-11.22	-11.21	-11.2
-11.28	-11.27	-11.26	-11.25	-11.24	-11.23	-11.22	-11.21	-11.2
-11.28	-11.27	-11.26	-11.25	-11.24	-11.23	-11.22	-11.21	-11.2
-11.28	-11.27	-11.26	-11.25	-11.24	-11.23	-11.22	-11.21	-11.2
-11.28	-11.27	-11.26	-11.25	-11.24	-11.23	-11.22	-11.21	-11.2
-11.28	-11.27	-11.26	-11.25	-11.24	-11.23	-11.22	-11.21	-11.2
-11.28	-11.27	-11.26	-11.25	-11.24	-11.23	-11.22	-11.21	-11.2
-11.28	-11.27	-11.26	-11.25	-11.24	-11.23	-11.22	-11.21	-11.2
-11.28	-11.27	-11.26	-11.25	-11.24	-11.23	-11.22	-11.21	-11.2
-11.28	-11.27	-11.26	-11.25	-11.24	-11.23	-11.22	-11.21	-11.2
-11.28	-11.27	-11.26	-11.25	-11.24	-11.23	-11.22	-11.21	-11.2
-11.28	-11.27	-11.26	-11.25	-11.24	-11.23	-11.22	-11.21	-11.2
-11.28	-11.27	-11.26	-11.25	-11.24	-11.23	-11.22	-11.21	-11.2
-11.28	-11.27	-11.26	-11.25	-11.24	-11.23	-11.22	-11.21	-11.2
-11.28	-11.27	-11.26	-11.25	-11.24	-11.23	-11.22	-11.21	-11.2
-11.28	-11.27	-11.26	-11.25	-11.24	-11.23	-11.22	-11.21	-11.2
-11.28	-11.27	-11.26	-11.25	-11.24	-11.23	-11.22	-11.21	-11.2
-11.28	-11.27	-11.26	-11.25	-11.24	-11.23	-11.22	-11.21	-11.2
-11.28	-11.27	-11.26	-11.25	-11.24	-11.23	-11.22	-11.21	-11.2

-11.28	-11.27	-11.26	-11.25	-11.24	-11.23	-11.22	-11.21	-11.2
-11.28	-11.27	-11.26	-11.25	-11.24	-11.23	-11.22	-11.21	-11.2
-11.28	-11.27	-11.26	-11.25	-11.24	-11.23	-11.22	-11.21	-11.2
-11.28	-11.27	-11.26	-11.25	-11.24	-11.23	-11.22	-11.21	-11.2
-11.28	-11.27	-11.26	-11.25	-11.24	-11.23	-11.22	-11.21	-11.2
-11.28	-11.27	-11.26	-11.25	-11.24	-11.23	-11.22	-11.21	-11.2
-11.28	-11.27	-11.26	-11.25	-11.24	-11.23	-11.22	-11.21	-11.2
-11.28	-11.27	-11.26	-11.25	-11.24	-11.23	-11.22	-11.21	-11.2
-11.28	-11.27	-11.26	-11.25	-11.24	-11.23	-11.22	-11.21	-11.2
-11.28	-11.27	-11.26	-11.25	-11.24	-11.23	-11.22	-11.21	-11.2
-11.28	-11.27	-11.26	-11.25	-11.24	-11.23	-11.22	-11.21	-11.2
-11.28	-11.27	-11.26	-11.25	-11.24	-11.23	-11.22	-11.21	-11.2
-11.28	-11.27	-11.26	-11.25	-11.24	-11.23	-11.22	-11.21	-11.2
-11.28	-11.27	-11.26	-11.25	-11.24	-11.23	-11.22	-11.21	-11.2
-11.28	-11.27	-11.26	-11.25	-11.24	-11.23	-11.22	-11.21	-11.2
-11.28	-11.27	-11.26	-11.25	-11.24	-11.23	-11.22	-11.21	-11.2
-11.28	-11.27	-11.26	-11.25	-11.24	-11.23	-11.22	-11.21	-11.2
-11.28	-11.27	-11.26	-11.25	-11.24	-11.23	-11.22	-11.21	-11.2
-11.28	-11.27	-11.26	-11.25	-11.24	-11.23	-11.22	-11.21	-11.2
-11.28	-11.27	-11.26	-11.25	-11.24	-11.23	-11.22	-11.21	-11.2
-11.28	-11.27	-11.26	-11.25	-11.24	-11.23	-11.22	-11.21	-11.2
-11.28	-11.27	-11.26	-11.25	-11.24	-11.23	-11.22	-11.21	-11.2
-11.28	-11.27	-11.26	-11.25	-11.24	-11.23	-11.22	-11.21	-11.2
-11.28	-11.27	-11.26	-11.25	-11.24	-11.23	-11.22	-11.21	-11.2
-11.28	-11.27	-11.26	-11.25	-11.24	-11.23	-11.22	-11.21	-11.2
-11.28	-11.27	-11.26	-11.25	-11.24	-11.23	-11.22	-11.21	-11.2
-11.28	-11.27	-11.26	-11.25	-11.24	-11.23	-11.22	-11.21	-11.2
-11.28	-11.27	-11.26	-11.25	-11.24	-11.23	-11.22	-11.21	-11.2
-11.28	-11.27	-11.26	-11.25	-11.24	-11.23	-11.22	-11.21	-11.2
-11.28	-11.27	-11.26	-11.25	-11.24	-11.23	-11.22	-11.21	-11.2
-11.28	-11.27	-11.26	-11.25	-11.24	-11.23	-11.22	-11.21	-11.2
-11.28	-11.27	-11.26	-11.25	-11.24	-11.23	-11.22	-11.21	-11.2
-11.28	-11.27	-11.26	-11.25	-11.24	-11.23	-11.22	-11.21	-11.2
-11.28	-11.27	-11.26	-11.25	-11.24	-11.23	-11.22	-11.21	-11.2
-11.28	-11.27	-11.26	-11.25	-11.24	-11.23	-11.22	-11.21	-11.2
-11.28	-11.27	-11.26	-11.25	-11.24	-11.23	-11.22	-11.21	-11.2
-11.28	-11.27	-11.26	-11.25	-11.24	-11.23	-11.22	-11.21	-11.2
-11.28	-11.27	-11.26	-11.25	-11.24	-11.23	-11.22	-11.21	-11.2
-11.28	-11.27	-11.26	-11.25	-11.24	-11.23	-11.22	-11.21	-11.2
-11.28	-11.27	-11.26	-11.25	-11.24	-11.23	-11.22	-11.21	-11.2
-11.28	-11.27	-11.26	-11.25	-11.24	-11.23	-11.22	-11.21	-11.2
-11.28	-11.27	-11.26	-11.25	-11.24	-11.23	-11.22	-11.21	-11.2
-11.28	-11.27	-11.26	-11.25	-11.24	-11.23	-11.22	-11.21	-11.2
-11.28	-11.27	-11.26	-11.25	-11.24	-11.23	-11.22	-11.21	-11.2
-11.28	-11.27	-11.26	-11.25	-11.24	-11.23	-11.22	-11.21	-11.2
-11.28	-11.27	-11.26	-11.25	-11.24	-11.23	-11.22	-11.21	-11.2
-11.28	-11.27	-11.26	-11.25	-11.24	-11.23	-11.22	-11.21	-11.2
-11.28	-11.27	-11.26	-11.25	-11.24	-11.23	-11.22	-11.21	-11.2
-11.28	-11.27	-11.26	-11.25	-11.24	-11.23	-11.22	-11.21	-11.2
-11.28	-11.27	-11.26	-11.25	-11.24	-11.23	-11.22	-11.21	-11.2

-11.28	-11.27	-11.26	-11.25	-11.24	-11.23	-11.22	-11.21	-11.2
-11.28	-11.27	-11.26	-11.25	-11.24	-11.23	-11.22	-11.21	-11.2
-11.28	-11.27	-11.26	-11.25	-11.24	-11.23	-11.22	-11.21	-11.2
-11.28	-11.27	-11.26	-11.25	-11.24	-11.23	-11.22	-11.21	-11.2
-11.28	-11.27	-11.26	-11.25	-11.24	-11.23	-11.22	-11.21	-11.2
-11.28	-11.27	-11.26	-11.25	-11.24	-11.23	-11.22	-11.21	-11.2
-11.28	-11.27	-11.26	-11.25	-11.24	-11.23	-11.22	-11.21	-11.2
-11.28	-11.27	-11.26	-11.25	-11.24	-11.23	-11.22	-11.21	-11.2
-11.28	-11.27	-11.26	-11.25	-11.24	-11.23	-11.22	-11.21	-11.2
-11.28	-11.27	-11.26	-11.25	-11.24	-11.23	-11.22	-11.21	-11.2
-11.28	-11.27	-11.26	-11.25	-11.24	-11.23	-11.22	-11.21	-11.2
-11.28	-11.27	-11.26	-11.25	-11.24	-11.23	-11.22	-11.21	-11.2
-11.28	-11.27	-11.26	-11.25	-11.24	-11.23	-11.22	-11.21	-11.2
-11.28	-11.27	-11.26	-11.25	-11.24	-11.23	-11.22	-11.21	-11.2
-11.28	-11.27	-11.26	-11.25	-11.24	-11.23	-11.22	-11.21	-11.2
-11.28	-11.27	-11.26	-11.25	-11.24	-11.23	-11.22	-11.21	-11.2
-11.28	-11.27	-11.26	-11.25	-11.24	-11.23	-11.22	-11.21	-11.2
-11.28	-11.27	-11.26	-11.25	-11.24	-11.23	-11.22	-11.21	-11.2
-11.28	-11.27	-11.26	-11.25	-11.24	-11.23	-11.22	-11.21	-11.2
-11.28	-11.27	-11.26	-11.25	-11.24	-11.23	-11.22	-11.21	-11.2
-11.28	-11.27	-11.26	-11.25	-11.24	-11.23	-11.22	-11.21	-11.2
-11.28	-11.27	-11.26	-11.25	-11.24	-11.23	-11.22	-11.21	-11.2
-11.28	-11.27	-11.26	-11.25	-11.24	-11.23	-11.22	-11.21	-11.2
-11.28	-11.27	-11.26	-11.25	-11.24	-11.23	-11.22	-11.21	-11.2
-11.28	-11.27	-11.26	-11.25	-11.24	-11.23	-11.22	-11.21	-11.2
-11.28	-11.27	-11.26	-11.25	-11.24	-11.23	-11.22	-11.21	-11.2
-11.28	-11.27	-11.26	-11.25	-11.24	-11.23	-11.22	-11.21	-11.2
-11.28	-11.27	-11.26	-11.25	-11.24	-11.23	-11.22	-11.21	-11.2
-11.28	-11.27	-11.26	-11.25	-11.24	-11.23	-11.22	-11.21	-11.2
-11.28	-11.27	-11.26	-11.25	-11.24	-11.23	-11.22	-11.21	-11.2
-11.28	-11.27	-11.26	-11.25	-11.24	-11.23	-11.22	-11.21	-11.2
-11.28	-11.27	-11.26	-11.25	-11.24	-11.23	-11.22	-11.21	-11.2
-11.28	-11.27	-11.26	-11.25	-11.24	-11.23	-11.22	-11.21	-11.2
-11.28	-11.27	-11.26	-11.25	-11.24	-11.23	-11.22	-11.21	-11.2
-11.28	-11.27	-11.26	-11.25	-11.24	-11.23	-11.22	-11.21	-11.2
-11.28	-11.27	-11.26	-11.25	-11.24	-11.23	-11.22	-11.21	-11.2
-11.28	-11.27	-11.26	-11.25	-11.24	-11.23	-11.22	-11.21	-11.2
-11.28	-11.27	-11.26	-11.25	-11.24	-11.23	-11.22	-11.21	-11.2
-11.28	-11.27	-11.26	-11.25	-11.24	-11.23	-11.22	-11.21	-11.2
-11.28	-11.27	-11.26	-11.25	-11.24	-11.23	-11.22	-11.21	-11.2
-11.28	-11.27	-11.26	-11.25	-11.24	-11.23	-11.22	-11.21	-11.2
-11.28	-11.27	-11.26	-11.25	-11.24	-11.23	-11.22	-11.21	-11.2
-11.28	-11.27	-11.26	-11.25	-11.24	-11.23	-11.22	-11.21	-11.2
-11.28	-11.27	-11.26	-11.25	-11.24	-11.23	-11.22	-11.21	-11.2
-11.28	-11.27	-11.26	-11.25	-11.24	-11.23	-11.22	-11.21	-11.2
-11.28	-11.27	-11.26	-11.25	-11.24	-11.23	-11.22	-11.21	-11.2
-11.28	-11.27	-11.26	-11.25	-11.24	-11.23	-11.22	-11.21	-11.2
-11.28	-11.27	-11.26	-11.25	-11.24	-11.23	-11.22	-11.21	-11.2
-11.28	-11.27	-11.26	-11.25	-11.24	-11.23	-11.22	-11.21	-11.2
-11.28	-11.27	-11.26	-11.25	-11.24	-11.23	-11.22	-11.21	-11.2

-11.28	-11.27	-11.26	-11.25	-11.24	-11.23	-11.22	-11.21	-11.2
-11.28	-11.27	-11.26	-11.25	-11.24	-11.23	-11.22	-11.21	-11.2
-11.28	-11.27	-11.26	-11.25	-11.24	-11.23	-11.22	-11.21	-11.2
-11.28	-11.27	-11.26	-11.25	-11.24	-11.23	-11.22	-11.21	-11.2
-11.28	-11.27	-11.26	-11.25	-11.24	-11.23	-11.22	-11.21	-11.2
-11.28	-11.27	-11.26	-11.25	-11.24	-11.23	-11.22	-11.21	-11.2
-11.28	-11.27	-11.26	-11.25	-11.24	-11.23	-11.22	-11.21	-11.2
-11.28	-11.27	-11.26	-11.25	-11.24	-11.23	-11.22	-11.21	-11.2
-11.28	-11.27	-11.26	-11.25	-11.24	-11.23	-11.22	-11.21	-11.2
-11.28	-11.27	-11.26	-11.25	-11.24	-11.23	-11.22	-11.21	-11.2
-11.28	-11.27	-11.26	-11.25	-11.24	-11.23	-11.22	-11.21	-11.2
-11.28	-11.27	-11.26	-11.25	-11.24	-11.23	-11.22	-11.21	-11.2
-11.28	-11.27	-11.26	-11.25	-11.24	-11.23	-11.22	-11.21	-11.2
-11.28	-11.27	-11.26	-11.25	-11.24	-11.23	-11.22	-11.21	-11.2
-11.28	-11.27	-11.26	-11.25	-11.24	-11.23	-11.22	-11.21	-11.2
-11.28	-11.27	-11.26	-11.25	-11.24	-11.23	-11.22	-11.21	-11.2
-11.28	-11.27	-11.26	-11.25	-11.24	-11.23	-11.22	-11.21	-11.2
-11.28	-11.27	-11.26	-11.25	-11.24	-11.23	-11.22	-11.21	-11.2
-11.28	-11.27	-11.26	-11.25	-11.24	-11.23	-11.22	-11.21	-11.2
-11.28	-11.27	-11.26	-11.25	-11.24	-11.23	-11.22	-11.21	-11.2
-11.28	-11.27	-11.26	-11.25	-11.24	-11.23	-11.22	-11.21	-11.2
-11.28	-11.27	-11.26	-11.25	-11.24	-11.23	-11.22	-11.21	-11.2
-11.28	-11.27	-11.26	-11.25	-11.24	-11.23	-11.22	-11.21	-11.2
-11.28	-11.27	-11.26	-11.25	-11.24	-11.23	-11.22	-11.21	-11.2
-11.28	-11.27	-11.26	-11.25	-11.24	-11.23	-11.22	-11.21	-11.2
-11.28	-11.27	-11.26	-11.25	-11.24	-11.23	-11.22	-11.21	-11.2
-11.28	-11.27	-11.26	-11.25	-11.24	-11.23	-11.22	-11.21	-11.2
-11.28	-11.27	-11.26	-11.25	-11.24	-11.23	-11.22	-11.21	-11.2
-11.28	-11.27	-11.26	-11.25	-11.24	-11.23	-11.22	-11.21	-11.2
-11.28	-11.27	-11.26	-11.25	-11.24	-11.23	-11.22	-11.21	-11.2
-11.28	-11.27	-11.26	-11.25	-11.24	-11.23	-11.22	-11.21	-11.2
-11.28	-11.27	-11.26	-11.25	-11.24	-11.23	-11.22	-11.21	-11.2
-11.28	-11.27	-11.26	-11.25	-11.24	-11.23	-11.22	-11.21	-11.2
-11.28	-11.27	-11.26	-11.25	-11.24	-11.23	-11.22	-11.21	-11.2
-11.28	-11.27	-11.26	-11.25	-11.24	-11.23	-11.22	-11.21	-11.2
-11.28	-11.27	-11.26	-11.25	-11.24	-11.23	-11.22	-11.21	-11.2
-11.28	-11.27	-11.26	-11.25	-11.24	-11.23	-11.22	-11.21	-11.2
-11.28	-11.27	-11.26	-11.25	-11.24	-11.23	-11.22	-11.21	-11.2
-11.28	-11.27	-11.26	-11.25	-11.24	-11.23	-11.22	-11.21	-11.2
-11.28	-11.27	-11.26	-11.25	-11.24	-11.23	-11.22	-11.21	-11.2
-11.28	-11.27	-11.26	-11.25	-11.24	-11.23	-11.22	-11.21	-11.2
-11.28	-11.27	-11.26	-11.25	-11.24	-11.23	-11.22	-11.21	-11.2
-11.28	-11.27	-11.26	-11.25	-11.24	-11.23	-11.22	-11.21	-11.2
-11.28	-11.27	-11.26	-11.25	-11.24	-11.23	-11.22	-11.21	-11.2
-11.28	-11.27	-11.26	-11.25	-11.24	-11.23	-11.22	-11.21	-11.2
-11.28	-11.27	-11.26	-11.25	-11.24	-11.23	-11.22	-11.21	-11.2
-11.28	-11.27	-11.26	-11.25	-11.24	-11.23	-11.22	-11.21	-11.2
-11.28	-11.27	-11.26	-11.25	-11.24	-11.23	-11.22	-11.21	-11.2
-11.28	-11.27	-11.26	-11.25	-11.24	-11.23	-11.22	-11.21	-11.2
-11.28	-11.27	-11.26	-11.25	-11.24	-11.23	-11.22	-11.21	-11.2

-11.28	-11.27	-11.26	-11.25	-11.24	-11.23	-11.22	-11.21	-11.2
-11.28	-11.27	-11.26	-11.25	-11.24	-11.23	-11.22	-11.21	-11.2
-11.28	-11.27	-11.26	-11.25	-11.24	-11.23	-11.22	-11.21	-11.2
-11.28	-11.27	-11.26	-11.25	-11.24	-11.23	-11.22	-11.21	-11.2
-11.28	-11.27	-11.26	-11.25	-11.24	-11.23	-11.22	-11.21	-11.2
-11.28	-11.27	-11.26	-11.25	-11.24	-11.23	-11.22	-11.21	-11.2
-11.28	-11.27	-11.26	-11.25	-11.24	-11.23	-11.22	-11.21	-11.2
-11.28	-11.27	-11.26	-11.25	-11.24	-11.23	-11.22	-11.21	-11.2
-11.28	-11.27	-11.26	-11.25	-11.24	-11.23	-11.22	-11.21	-11.2
-11.28	-11.27	-11.26	-11.25	-11.24	-11.23	-11.22	-11.21	-11.2
-11.28	-11.27	-11.26	-11.25	-11.24	-11.23	-11.22	-11.21	-11.2
-11.28	-11.27	-11.26	-11.25	-11.24	-11.23	-11.22	-11.21	-11.2
-11.28	-11.27	-11.26	-11.25	-11.24	-11.23	-11.22	-11.21	-11.2
-11.28	-11.27	-11.26	-11.25	-11.24	-11.23	-11.22	-11.21	-11.2
-11.28	-11.27	-11.26	-11.25	-11.24	-11.23	-11.22	-11.21	-11.2
-11.28	-11.27	-11.26	-11.25	-11.24	-11.23	-11.22	-11.21	-11.2
-11.28	-11.27	-11.26	-11.25	-11.24	-11.23	-11.22	-11.21	-11.2
-11.28	-11.27	-11.26	-11.25	-11.24	-11.23	-11.22	-11.21	-11.2
-11.28	-11.27	-11.26	-11.25	-11.24	-11.23	-11.22	-11.21	-11.2
-11.28	-11.27	-11.26	-11.25	-11.24	-11.23	-11.22	-11.21	-11.2
-11.28	-11.27	-11.26	-11.25	-11.24	-11.23	-11.22	-11.21	-11.2
-11.28	-11.27	-11.26	-11.25	-11.24	-11.23	-11.22	-11.21	-11.2
-11.28	-11.27	-11.26	-11.25	-11.24	-11.23	-11.22	-11.21	-11.2
-11.28	-11.27	-11.26	-11.25	-11.24	-11.23	-11.22	-11.21	-11.2
-11.28	-11.27	-11.26	-11.25	-11.24	-11.23	-11.22	-11.21	-11.2
-11.28	-11.27	-11.26	-11.25	-11.24	-11.23	-11.22	-11.21	-11.2
-11.28	-11.27	-11.26	-11.25	-11.24	-11.23	-11.22	-11.21	-11.2
-11.28	-11.27	-11.26	-11.25	-11.24	-11.23	-11.22	-11.21	-11.2
-11.28	-11.27	-11.26	-11.25	-11.24	-11.23	-11.22	-11.21	-11.2
-11.28	-11.27	-11.26	-11.25	-11.24	-11.23	-11.22	-11.21	-11.2
-11.28	-11.27	-11.26	-11.25	-11.24	-11.23	-11.22	-11.21	-11.2
-11.28	-11.27	-11.26	-11.25	-11.24	-11.23	-11.22	-11.21	-11.2
-11.28	-11.27	-11.26	-11.25	-11.24	-11.23	-11.22	-11.21	-11.2
-11.28	-11.27	-11.26	-11.25	-11.24	-11.23	-11.22	-11.21	-11.2
-11.28	-11.27	-11.26	-11.25	-11.24	-11.23	-11.22	-11.21	-11.2
-11.28	-11.27	-11.26	-11.25	-11.24	-11.23	-11.22	-11.21	-11.2
-11.28	-11.27	-11.26	-11.25	-11.24	-11.23	-11.22	-11.21	-11.2
-11.28	-11.27	-11.26	-11.25	-11.24	-11.23	-11.22	-11.21	-11.2
-11.28	-11.27	-11.26	-11.25	-11.24	-11.23	-11.22	-11.21	-11.2
-11.28	-11.27	-11.26	-11.25	-11.24	-11.23	-11.22	-11.21	-11.2
-11.28	-11.27	-11.26	-11.25	-11.24	-11.23	-11.22	-11.21	-11.2
-11.28	-11.27	-11.26	-11.25	-11.24	-11.23	-11.22	-11.21	-11.2
-11.28	-11.27	-11.26	-11.25	-11.24	-11.23	-11.22	-11.21	-11.2
-11.28	-11.27	-11.26	-11.25	-11.24	-11.23	-11.22	-11.21	-11.2
-11.28	-11.27	-11.26	-11.25	-11.24	-11.23	-11.22	-11.21	-11.2
-11.28	-11.27	-11.26	-11.25	-11.24	-11.23	-11.22	-11.21	-11.2
-11.28	-11.27	-11.26	-11.25	-11.24	-11.23	-11.22	-11.21	-11.2
-11.28	-11.27	-11.26	-11.25	-11.24	-11.23	-11.22	-11.21	-11.2
-11.28	-11.27	-11.26	-11.25	-11.24	-11.23	-11.22	-11.21	-11.2
-11.28	-11.27	-11.26	-11.25	-11.24	-11.23	-11.22	-11.21	-11.2

-11.28	-11.27	-11.26	-11.25	-11.24	-11.23	-11.22	-11.21	-11.2
-11.28	-11.27	-11.26	-11.25	-11.24	-11.23	-11.22	-11.21	-11.2
-11.28	-11.27	-11.26	-11.25	-11.24	-11.23	-11.22	-11.21	-11.2
-11.28	-11.27	-11.26	-11.25	-11.24	-11.23	-11.22	-11.21	-11.2
-11.28	-11.27	-11.26	-11.25	-11.24	-11.23	-11.22	-11.21	-11.2
-11.28	-11.27	-11.26	-11.25	-11.24	-11.23	-11.22	-11.21	-11.2
-11.28	-11.27	-11.26	-11.25	-11.24	-11.23	-11.22	-11.21	-11.2
-11.28	-11.27	-11.26	-11.25	-11.24	-11.23	-11.22	-11.21	-11.2
-11.28	-11.27	-11.26	-11.25	-11.24	-11.23	-11.22	-11.21	-11.2
-11.28	-11.27	-11.26	-11.25	-11.24	-11.23	-11.22	-11.21	-11.2
-11.28	-11.27	-11.26	-11.25	-11.24	-11.23	-11.22	-11.21	-11.2
-11.28	-11.27	-11.26	-11.25	-11.24	-11.23	-11.22	-11.21	-11.2
-11.28	-11.27	-11.26	-11.25	-11.24	-11.23	-11.22	-11.21	-11.2
-11.28	-11.27	-11.26	-11.25	-11.24	-11.23	-11.22	-11.21	-11.2
-11.28	-11.27	-11.26	-11.25	-11.24	-11.23	-11.22	-11.21	-11.2
-11.28	-11.27	-11.26	-11.25	-11.24	-11.23	-11.22	-11.21	-11.2
-11.28	-11.27	-11.26	-11.25	-11.24	-11.23	-11.22	-11.21	-11.2
-11.28	-11.27	-11.26	-11.25	-11.24	-11.23	-11.22	-11.21	-11.2
-11.28	-11.27	-11.26	-11.25	-11.24	-11.23	-11.22	-11.21	-11.2
-11.28	-11.27	-11.26	-11.25	-11.24	-11.23	-11.22	-11.21	-11.2
-11.28	-11.27	-11.26	-11.25	-11.24	-11.23	-11.22	-11.21	-11.2
-11.28	-11.27	-11.26	-11.25	-11.24	-11.23	-11.22	-11.21	-11.2
-11.28	-11.27	-11.26	-11.25	-11.24	-11.23	-11.22	-11.21	-11.2
-11.28	-11.27	-11.26	-11.25	-11.24	-11.23	-11.22	-11.21	-11.2
-11.28	-11.27	-11.26	-11.25	-11.24	-11.23	-11.22	-11.21	-11.2
-11.28	-11.27	-11.26	-11.25	-11.24	-11.23	-11.22	-11.21	-11.2
-11.28	-11.27	-11.26	-11.25	-11.24	-11.23	-11.22	-11.21	-11.2
-11.28	-11.27	-11.26	-11.25	-11.24	-11.23	-11.22	-11.21	-11.2
-11.28	-11.27	-11.26	-11.25	-11.24	-11.23	-11.22	-11.21	-11.2
-11.28	-11.27	-11.26	-11.25	-11.24	-11.23	-11.22	-11.21	-11.2
-11.28	-11.27	-11.26	-11.25	-11.24	-11.23	-11.22	-11.21	-11.2
-11.28	-11.27	-11.26	-11.25	-11.24	-11.23	-11.22	-11.21	-11.2
-11.28	-11.27	-11.26	-11.25	-11.24	-11.23	-11.22	-11.21	-11.2
-11.28	-11.27	-11.26	-11.25	-11.24	-11.23	-11.22	-11.21	-11.2
-11.28	-11.27	-11.26	-11.25	-11.24	-11.23	-11.22	-11.21	-11.2
-11.28	-11.27	-11.26	-11.25	-11.24	-11.23	-11.22	-11.21	-11.2
-11.28	-11.27	-11.26	-11.25	-11.24	-11.23	-11.22	-11.21	-11.2
-11.28	-11.27	-11.26	-11.25	-11.24	-11.23	-11.22	-11.21	-11.2
-11.28	-11.27	-11.26	-11.25	-11.24	-11.23	-11.22	-11.21	-11.2
-11.28	-11.27	-11.26	-11.25	-11.24	-11.23	-11.22	-11.21	-11.2
-11.28	-11.27	-11.26	-11.25	-11.24	-11.23	-11.22	-11.21	-11.2
-11.28	-11.27	-11.26	-11.25	-11.24	-11.23	-11.22	-11.21	-11.2
-11.28	-11.27	-11.26	-11.25	-11.24	-11.23	-11.22	-11.21	-11.2
-11.28	-11.27	-11.26	-11.25	-11.24	-11.23	-11.22	-11.21	-11.2
-11.28	-11.27	-11.26	-11.25	-11.24	-11.23	-11.22	-11.21	-11.2
-11.28	-11.27	-11.26	-11.25	-11.24	-11.23	-11.22	-11.21	-11.2
-11.28	-11.27	-11.26	-11.25	-11.24	-11.23	-11.22	-11.21	-11.2
-11.28	-11.27	-11.26	-11.25	-11.24	-11.23	-11.22	-11.21	-11.2
-11.28	-11.27	-11.26	-11.25	-11.24	-11.23	-11.22	-11.21	-11.2
-11.28	-11.27	-11.26	-11.25	-11.24	-11.23	-11.22	-11.21	-11.2

-11.28	-11.27	-11.26	-11.25	-11.24	-11.23	-11.22	-11.21	-11.2
-11.28	-11.27	-11.26	-11.25	-11.24	-11.23	-11.22	-11.21	-11.2
-11.28	-11.27	-11.26	-11.25	-11.24	-11.23	-11.22	-11.21	-11.2
-11.28	-11.27	-11.26	-11.25	-11.24	-11.23	-11.22	-11.21	-11.2
-11.28	-11.27	-11.26	-11.25	-11.24	-11.23	-11.22	-11.21	-11.2
-11.28	-11.27	-11.26	-11.25	-11.24	-11.23	-11.22	-11.21	-11.2
-11.28	-11.27	-11.26	-11.25	-11.24	-11.23	-11.22	-11.21	-11.2
-11.28	-11.27	-11.26	-11.25	-11.24	-11.23	-11.22	-11.21	-11.2
-11.28	-11.27	-11.26	-11.25	-11.24	-11.23	-11.22	-11.21	-11.2
-11.28	-11.27	-11.26	-11.25	-11.24	-11.23	-11.22	-11.21	-11.2
-11.28	-11.27	-11.26	-11.25	-11.24	-11.23	-11.22	-11.21	-11.2
-11.28	-11.27	-11.26	-11.25	-11.24	-11.23	-11.22	-11.21	-11.2
-11.28	-11.27	-11.26	-11.25	-11.24	-11.23	-11.22	-11.21	-11.2
-11.28	-11.27	-11.26	-11.25	-11.24	-11.23	-11.22	-11.21	-11.2
-11.28	-11.27	-11.26	-11.25	-11.24	-11.23	-11.22	-11.21	-11.2
-11.28	-11.27	-11.26	-11.25	-11.24	-11.23	-11.22	-11.21	-11.2
-11.28	-11.27	-11.26	-11.25	-11.24	-11.23	-11.22	-11.21	-11.2
-11.28	-11.27	-11.26	-11.25	-11.24	-11.23	-11.22	-11.21	-11.2
-11.28	-11.27	-11.26	-11.25	-11.24	-11.23	-11.22	-11.21	-11.2
-11.28	-11.27	-11.26	-11.25	-11.24	-11.23	-11.22	-11.21	-11.2
-11.28	-11.27	-11.26	-11.25	-11.24	-11.23	-11.22	-11.21	-11.2
-11.28	-11.27	-11.26	-11.25	-11.24	-11.23	-11.22	-11.21	-11.2
-11.28	-11.27	-11.26	-11.25	-11.24	-11.23	-11.22	-11.21	-11.2
-11.28	-11.27	-11.26	-11.25	-11.24	-11.23	-11.22	-11.21	-11.2
-11.28	-11.27	-11.26	-11.25	-11.24	-11.23	-11.22	-11.21	-11.2
-11.28	-11.27	-11.26	-11.25	-11.24	-11.23	-11.22	-11.21	-11.2
-11.28	-11.27	-11.26	-11.25	-11.24	-11.23	-11.22	-11.21	-11.2
-11.28	-11.27	-11.26	-11.25	-11.24	-11.23	-11.22	-11.21	-11.2
-11.28	-11.27	-11.26	-11.25	-11.24	-11.23	-11.22	-11.21	-11.2
-11.28	-11.27	-11.26	-11.25	-11.24	-11.23	-11.22	-11.21	-11.2
-11.28	-11.27	-11.26	-11.25	-11.24	-11.23	-11.22	-11.21	-11.2
-11.28	-11.27	-11.26	-11.25	-11.24	-11.23	-11.22	-11.21	-11.2
-11.28	-11.27	-11.26	-11.25	-11.24	-11.23	-11.22	-11.21	-11.2
-11.28	-11.27	-11.26	-11.25	-11.24	-11.23	-11.22	-11.21	-11.2
-11.28	-11.27	-11.26	-11.25	-11.24	-11.23	-11.22	-11.21	-11.2
-11.28	-11.27	-11.26	-11.25	-11.24	-11.23	-11.22	-11.21	-11.2
-11.28	-11.27	-11.26	-11.25	-11.24	-11.23	-11.22	-11.21	-11.2
-11.28	-11.27	-11.26	-11.25	-11.24	-11.23	-11.22	-11.21	-11.2
-11.28	-11.27	-11.26	-11.25	-11.24	-11.23	-11.22	-11.21	-11.2
-11.28	-11.27	-11.26	-11.25	-11.24	-11.23	-11.22	-11.21	-11.2
-11.28	-11.27	-11.26	-11.25	-11.24	-11.23	-11.22	-11.21	-11.2
-11.28	-11.27	-11.26	-11.25	-11.24	-11.23	-11.22	-11.21	-11.2
-11.28	-11.27	-11.26	-11.25	-11.24	-11.23	-11.22	-11.21	-11.2
-11.28	-11.27	-11.26	-11.25	-11.24	-11.23	-11.22	-11.21	-11.2
-11.28	-11.27	-11.26	-11.25	-11.24	-11.23	-11.22	-11.21	-11.2
-11.28	-11.27	-11.26	-11.25	-11.24	-11.23	-11.22	-11.21	-11.2
-11.28	-11.27	-11.26	-11.25	-11.24	-11.23	-11.22	-11.21	-11.2
-11.28	-11.27	-11.26	-11.25	-11.24	-11.23	-11.22	-11.21	-11.2
-11.28	-11.27	-11.26	-11.25	-11.24	-11.23	-11.22	-11.21	-11.2
-11.28	-11.27	-11.26	-11.25	-11.24	-11.23	-11.22	-11.21	-11.2

-11.28	-11.27	-11.26	-11.25	-11.24	-11.23	-11.22	-11.21	-11.2
-11.28	-11.27	-11.26	-11.25	-11.24	-11.23	-11.22	-11.21	-11.2
-11.28	-11.27	-11.26	-11.25	-11.24	-11.23	-11.22	-11.21	-11.2
-11.28	-11.27	-11.26	-11.25	-11.24	-11.23	-11.22	-11.21	-11.2
-11.28	-11.27	-11.26	-11.25	-11.24	-11.23	-11.22	-11.21	-11.2
-11.28	-11.27	-11.26	-11.25	-11.24	-11.23	-11.22	-11.21	-11.2
-11.28	-11.27	-11.26	-11.25	-11.24	-11.23	-11.22	-11.21	-11.2
-11.28	-11.27	-11.26	-11.25	-11.24	-11.23	-11.22	-11.21	-11.2
-11.28	-11.27	-11.26	-11.25	-11.24	-11.23	-11.22	-11.21	-11.2
-11.28	-11.27	-11.26	-11.25	-11.24	-11.23	-11.22	-11.21	-11.2
-11.28	-11.27	-11.26	-11.25	-11.24	-11.23	-11.22	-11.21	-11.2
-11.28	-11.27	-11.26	-11.25	-11.24	-11.23	-11.22	-11.21	-11.2
-11.28	-11.27	-11.26	-11.25	-11.24	-11.23	-11.22	-11.21	-11.2
-11.28	-11.27	-11.26	-11.25	-11.24	-11.23	-11.22	-11.21	-11.2
-11.28	-11.27	-11.26	-11.25	-11.24	-11.23	-11.22	-11.21	-11.2
-11.28	-11.27	-11.26	-11.25	-11.24	-11.23	-11.22	-11.21	-11.2
-11.28	-11.27	-11.26	-11.25	-11.24	-11.23	-11.22	-11.21	-11.2
-11.28	-11.27	-11.26	-11.25	-11.24	-11.23	-11.22	-11.21	-11.2
-11.28	-11.27	-11.26	-11.25	-11.24	-11.23	-11.22	-11.21	-11.2
-11.28	-11.27	-11.26	-11.25	-11.24	-11.23	-11.22	-11.21	-11.2
-11.28	-11.27	-11.26	-11.25	-11.24	-11.23	-11.22	-11.21	-11.2
-11.28	-11.27	-11.26	-11.25	-11.24	-11.23	-11.22	-11.21	-11.2
-11.28	-11.27	-11.26	-11.25	-11.24	-11.23	-11.22	-11.21	-11.2
-11.28	-11.27	-11.26	-11.25	-11.24	-11.23	-11.22	-11.21	-11.2
-11.28	-11.27	-11.26	-11.25	-11.24	-11.23	-11.22	-11.21	-11.2
-11.28	-11.27	-11.26	-11.25	-11.24	-11.23	-11.22	-11.21	-11.2
-11.28	-11.27	-11.26	-11.25	-11.24	-11.23	-11.22	-11.21	-11.2
-11.28	-11.27	-11.26	-11.25	-11.24	-11.23	-11.22	-11.21	-11.2
-11.28	-11.27	-11.26	-11.25	-11.24	-11.23	-11.22	-11.21	-11.2
-11.28	-11.27	-11.26	-11.25	-11.24	-11.23	-11.22	-11.21	-11.2
-11.28	-11.27	-11.26	-11.25	-11.24	-11.23	-11.22	-11.21	-11.2
-11.28	-11.27	-11.26	-11.25	-11.24	-11.23	-11.22	-11.21	-11.2
-11.28	-11.27	-11.26	-11.25	-11.24	-11.23	-11.22	-11.21	-11.2
-11.28	-11.27	-11.26	-11.25	-11.24	-11.23	-11.22	-11.21	-11.2
-11.28	-11.27	-11.26	-11.25	-11.24	-11.23	-11.22	-11.21	-11.2
-11.28	-11.27	-11.26	-11.25	-11.24	-11.23	-11.22	-11.21	-11.2
-11.28	-11.27	-11.26	-11.25	-11.24	-11.23	-11.22	-11.21	-11.2
-11.28	-11.27	-11.26	-11.25	-11.24	-11.23	-11.22	-11.21	-11.2
-11.28	-11.27	-11.26	-11.25	-11.24	-11.23	-11.22	-11.21	-11.2
-11.28	-11.27	-11.26	-11.25	-11.24	-11.23	-11.22	-11.21	-11.2
-11.28	-11.27	-11.26	-11.25	-11.24	-11.23	-11.22	-11.21	-11.2
-11.28	-11.27	-11.26	-11.25	-11.24	-11.23	-11.22	-11.21	-11.2
-11.28	-11.27	-11.26	-11.25	-11.24	-11.23	-11.22	-11.21	-11.2
-11.28	-11.27	-11.26	-11.25	-11.24	-11.23	-11.22	-11.21	-11.2
-11.28	-11.27	-11.26	-11.25	-11.24	-11.23	-11.22	-11.21	-11.2
-11.28	-11.27	-11.26	-11.25	-11.24	-11.23	-11.22	-11.21	-11.2
-11.28	-11.27	-11.26	-11.25	-11.24	-11.23	-11.22	-11.21	-11.2
-11.28	-11.27	-11.26	-11.25	-11.24	-11.23	-11.22	-11.21	-11.2
-11.28	-11.27	-11.26	-11.25	-11.24	-11.23	-11.22	-11.21	-11.2
-11.28	-11.27	-11.26	-11.25	-11.24	-11.23	-11.22	-11.21	-11.2

-11.28	-11.27	-11.26	-11.25	-11.24	-11.23	-11.22	-11.21	-11.2

-11.19	-11.18	-11.17	-11.16	-11.15	-11.14	-11.13	-11.12	-11.11
-11.19	-11.18	-11.17	-11.16	-11.15	-11.14	-11.13	-11.12	-11.11
-11.19	-11.18	-11.17	-11.16	-11.15	-11.14	-11.13	-11.12	-11.11
-11.19	-11.18	-11.17	-11.16	-11.15	-11.14	-11.13	-11.12	-11.11
-11.19	-11.18	-11.17	-11.16	-11.15	-11.14	-11.13	-11.12	-11.11
-11.19	-11.18	-11.17	-11.16	-11.15	-11.14	-11.13	-11.12	-11.11
-11.19	-11.18	-11.17	-11.16	-11.15	-11.14	-11.13	-11.12	-11.11
-11.19	-11.18	-11.17	-11.16	-11.15	-11.14	-11.13	-11.12	-11.11
-11.19	-11.18	-11.17	-11.16	-11.15	-11.14	-11.13	-11.12	-11.11
-11.19	-11.18	-11.17	-11.16	-11.15	-11.14	-11.13	-11.12	-11.11
-11.19	-11.18	-11.17	-11.16	-11.15	-11.14	-11.13	-11.12	-11.11
-11.19	-11.18	-11.17	-11.16	-11.15	-11.14	-11.13	-11.12	-11.11
-11.19	-11.18	-11.17	-11.16	-11.15	-11.14	-11.13	-11.12	-11.11
-11.19	-11.18	-11.17	-11.16	-11.15	-11.14	-11.13	-11.12	-11.11
-11.19	-11.18	-11.17	-11.16	-11.15	-11.14	-11.13	-11.12	-11.11
-11.19	-11.18	-11.17	-11.16	-11.15	-11.14	-11.13	-11.12	-11.11
-11.19	-11.18	-11.17	-11.16	-11.15	-11.14	-11.13	-11.12	-11.11
-11.19	-11.18	-11.17	-11.16	-11.15	-11.14	-11.13	-11.12	-11.11
-11.19	-11.18	-11.17	-11.16	-11.15	-11.14	-11.13	-11.12	-11.11
-11.19	-11.18	-11.17	-11.16	-11.15	-11.14	-11.13	-11.12	-11.11
-11.19	-11.18	-11.17	-11.16	-11.15	-11.14	-11.13	-11.12	-11.11
-11.19	-11.18	-11.17	-11.16	-11.15	-11.14	-11.13	-11.12	-11.11
-11.19	-11.18	-11.17	-11.16	-11.15	-11.14	-11.13	-11.12	-11.11
-11.19	-11.18	-11.17	-11.16	-11.15	-11.14	-11.13	-11.12	-11.11
-11.19	-11.18	-11.17	-11.16	-11.15	-11.14	-11.13	-11.12	-11.11
-11.19	-11.18	-11.17	-11.16	-11.15	-11.14	-11.13	-11.12	-11.11
-11.19	-11.18	-11.17	-11.16	-11.15	-11.14	-11.13	-11.12	-11.11
-11.19	-11.18	-11.17	-11.16	-11.15	-11.14	-11.13	-11.12	-11.11
-11.19	-11.18	-11.17	-11.16	-11.15	-11.14	-11.13	-11.12	-11.11
-11.19	-11.18	-11.17	-11.16	-11.15	-11.14	-11.13	-11.12	-11.11
-11.19	-11.18	-11.17	-11.16	-11.15	-11.14	-11.13	-11.12	-11.11
-11.19	-11.18	-11.17	-11.16	-11.15	-11.14	-11.13	-11.12	-11.11
-11.19	-11.18	-11.17	-11.16	-11.15	-11.14	-11.13	-11.12	-11.11
-11.19	-11.18	-11.17	-11.16	-11.15	-11.14	-11.13	-11.12	-11.11
-11.19	-11.18	-11.17	-11.16	-11.15	-11.14	-11.13	-11.12	-11.11
-11.19	-11.18	-11.17	-11.16	-11.15	-11.14	-11.13	-11.12	-11.11
-11.19	-11.18	-11.17	-11.16	-11.15	-11.14	-11.13	-11.12	-11.11
-11.19	-11.18	-11.17	-11.16	-11.15	-11.14	-11.13	-11.12	-11.11
-11.19	-11.18	-11.17	-11.16	-11.15	-11.14	-11.13	-11.12	-11.11
-11.19	-11.18	-11.17	-11.16	-11.15	-11.14	-11.13	-11.12	-11.11
-11.19	-11.18	-11.17	-11.16	-11.15	-11.14	-11.13	-11.12	-11.11
-11.19	-11.18	-11.17	-11.16	-11.15	-11.14	-11.13	-11.12	-11.11
-11.19	-11.18	-11.17	-11.16	-11.15	-11.14	-11.13	-11.12	-11.11
-11.19	-11.18	-11.17	-11.16	-11.15	-11.14	-11.13	-11.12	-11.11
-11.19	-11.18	-11.17	-11.16	-11.15	-11.14	-11.13	-11.12	-11.11
-11.19	-11.18	-11.17	-11.16	-11.15	-11.14	-11.13	-11.12	-11.11
-11.19	-11.18	-11.17	-11.16	-11.15	-11.14	-11.13	-11.12	-11.11
-11.19	-11.18	-11.17	-11.16	-11.15	-11.14	-11.13	-11.12	-11.11
-11.19	-11.18	-11.17	-11.16	-11.15	-11.14	-11.13	-11.12	-11.11
-11.19	-11.18	-11.17	-11.16	-11.15	-11.14	-11.13	-11.12	-11.11

-11.19	-11.18	-11.17	-11.16	-11.15	-11.14	-11.13	-11.12	-11.11
-11.19	-11.18	-11.17	-11.16	-11.15	-11.14	-11.13	-11.12	-11.11
-11.19	-11.18	-11.17	-11.16	-11.15	-11.14	-11.13	-11.12	-11.11
-11.19	-11.18	-11.17	-11.16	-11.15	-11.14	-11.13	-11.12	-11.11
-11.19	-11.18	-11.17	-11.16	-11.15	-11.14	-11.13	-11.12	-11.11
-11.19	-11.18	-11.17	-11.16	-11.15	-11.14	-11.13	-11.12	-11.11
-11.19	-11.18	-11.17	-11.16	-11.15	-11.14	-11.13	-11.12	-11.11
-11.19	-11.18	-11.17	-11.16	-11.15	-11.14	-11.13	-11.12	-11.11
-11.19	-11.18	-11.17	-11.16	-11.15	-11.14	-11.13	-11.12	-11.11
-11.19	-11.18	-11.17	-11.16	-11.15	-11.14	-11.13	-11.12	-11.11
-11.19	-11.18	-11.17	-11.16	-11.15	-11.14	-11.13	-11.12	-11.11
-11.19	-11.18	-11.17	-11.16	-11.15	-11.14	-11.13	-11.12	-11.11
-11.19	-11.18	-11.17	-11.16	-11.15	-11.14	-11.13	-11.12	-11.11
-11.19	-11.18	-11.17	-11.16	-11.15	-11.14	-11.13	-11.12	-11.11
-11.19	-11.18	-11.17	-11.16	-11.15	-11.14	-11.13	-11.12	-11.11
-11.19	-11.18	-11.17	-11.16	-11.15	-11.14	-11.13	-11.12	-11.11
-11.19	-11.18	-11.17	-11.16	-11.15	-11.14	-11.13	-11.12	-11.11
-11.19	-11.18	-11.17	-11.16	-11.15	-11.14	-11.13	-11.12	-11.11
-11.19	-11.18	-11.17	-11.16	-11.15	-11.14	-11.13	-11.12	-11.11
-11.19	-11.18	-11.17	-11.16	-11.15	-11.14	-11.13	-11.12	-11.11
-11.19	-11.18	-11.17	-11.16	-11.15	-11.14	-11.13	-11.12	-11.11
-11.19	-11.18	-11.17	-11.16	-11.15	-11.14	-11.13	-11.12	-11.11
-11.19	-11.18	-11.17	-11.16	-11.15	-11.14	-11.13	-11.12	-11.11
-11.19	-11.18	-11.17	-11.16	-11.15	-11.14	-11.13	-11.12	-11.11
-11.19	-11.18	-11.17	-11.16	-11.15	-11.14	-11.13	-11.12	-11.11
-11.19	-11.18	-11.17	-11.16	-11.15	-11.14	-11.13	-11.12	-11.11
-11.19	-11.18	-11.17	-11.16	-11.15	-11.14	-11.13	-11.12	-11.11
-11.19	-11.18	-11.17	-11.16	-11.15	-11.14	-11.13	-11.12	-11.11
-11.19	-11.18	-11.17	-11.16	-11.15	-11.14	-11.13	-11.12	-11.11
-11.19	-11.18	-11.17	-11.16	-11.15	-11.14	-11.13	-11.12	-11.11
-11.19	-11.18	-11.17	-11.16	-11.15	-11.14	-11.13	-11.12	-11.11
-11.19	-11.18	-11.17	-11.16	-11.15	-11.14	-11.13	-11.12	-11.11
-11.19	-11.18	-11.17	-11.16	-11.15	-11.14	-11.13	-11.12	-11.11
-11.19	-11.18	-11.17	-11.16	-11.15	-11.14	-11.13	-11.12	-11.11
-11.19	-11.18	-11.17	-11.16	-11.15	-11.14	-11.13	-11.12	-11.11
-11.19	-11.18	-11.17	-11.16	-11.15	-11.14	-11.13	-11.12	-11.11
-11.19	-11.18	-11.17	-11.16	-11.15	-11.14	-11.13	-11.12	-11.11
-11.19	-11.18	-11.17	-11.16	-11.15	-11.14	-11.13	-11.12	-11.11
-11.19	-11.18	-11.17	-11.16	-11.15	-11.14	-11.13	-11.12	-11.11
-11.19	-11.18	-11.17	-11.16	-11.15	-11.14	-11.13	-11.12	-11.11
-11.19	-11.18	-11.17	-11.16	-11.15	-11.14	-11.13	-11.12	-11.11
-11.19	-11.18	-11.17	-11.16	-11.15	-11.14	-11.13	-11.12	-11.11
-11.19	-11.18	-11.17	-11.16	-11.15	-11.14	-11.13	-11.12	-11.11
-11.19	-11.18	-11.17	-11.16	-11.15	-11.14	-11.13	-11.12	-11.11
-11.19	-11.18	-11.17	-11.16	-11.15	-11.14	-11.13	-11.12	-11.11
-11.19	-11.18	-11.17	-11.16	-11.15	-11.14	-11.13	-11.12	-11.11
-11.19	-11.18	-11.17	-11.16	-11.15	-11.14	-11.13	-11.12	-11.11
-11.19	-11.18	-11.17	-11.16	-11.15	-11.14	-11.13	-11.12	-11.11
-11.19	-11.18	-11.17	-11.16	-11.15	-11.14	-11.13	-11.12	-11.11
-11.19	-11.18	-11.17	-11.16	-11.15	-11.14	-11.13	-11.12	-11.11

-11.19	-11.18	-11.17	-11.16	-11.15	-11.14	-11.13	-11.12	-11.11
-11.19	-11.18	-11.17	-11.16	-11.15	-11.14	-11.13	-11.12	-11.11
-11.19	-11.18	-11.17	-11.16	-11.15	-11.14	-11.13	-11.12	-11.11
-11.19	-11.18	-11.17	-11.16	-11.15	-11.14	-11.13	-11.12	-11.11
-11.19	-11.18	-11.17	-11.16	-11.15	-11.14	-11.13	-11.12	-11.11
-11.19	-11.18	-11.17	-11.16	-11.15	-11.14	-11.13	-11.12	-11.11
-11.19	-11.18	-11.17	-11.16	-11.15	-11.14	-11.13	-11.12	-11.11
-11.19	-11.18	-11.17	-11.16	-11.15	-11.14	-11.13	-11.12	-11.11
-11.19	-11.18	-11.17	-11.16	-11.15	-11.14	-11.13	-11.12	-11.11
-11.19	-11.18	-11.17	-11.16	-11.15	-11.14	-11.13	-11.12	-11.11
-11.19	-11.18	-11.17	-11.16	-11.15	-11.14	-11.13	-11.12	-11.11
-11.19	-11.18	-11.17	-11.16	-11.15	-11.14	-11.13	-11.12	-11.11
-11.19	-11.18	-11.17	-11.16	-11.15	-11.14	-11.13	-11.12	-11.11
-11.19	-11.18	-11.17	-11.16	-11.15	-11.14	-11.13	-11.12	-11.11
-11.19	-11.18	-11.17	-11.16	-11.15	-11.14	-11.13	-11.12	-11.11
-11.19	-11.18	-11.17	-11.16	-11.15	-11.14	-11.13	-11.12	-11.11
-11.19	-11.18	-11.17	-11.16	-11.15	-11.14	-11.13	-11.12	-11.11
-11.19	-11.18	-11.17	-11.16	-11.15	-11.14	-11.13	-11.12	-11.11
-11.19	-11.18	-11.17	-11.16	-11.15	-11.14	-11.13	-11.12	-11.11
-11.19	-11.18	-11.17	-11.16	-11.15	-11.14	-11.13	-11.12	-11.11
-11.19	-11.18	-11.17	-11.16	-11.15	-11.14	-11.13	-11.12	-11.11
-11.19	-11.18	-11.17	-11.16	-11.15	-11.14	-11.13	-11.12	-11.11
-11.19	-11.18	-11.17	-11.16	-11.15	-11.14	-11.13	-11.12	-11.11
-11.19	-11.18	-11.17	-11.16	-11.15	-11.14	-11.13	-11.12	-11.11
-11.19	-11.18	-11.17	-11.16	-11.15	-11.14	-11.13	-11.12	-11.11
-11.19	-11.18	-11.17	-11.16	-11.15	-11.14	-11.13	-11.12	-11.11
-11.19	-11.18	-11.17	-11.16	-11.15	-11.14	-11.13	-11.12	-11.11
-11.19	-11.18	-11.17	-11.16	-11.15	-11.14	-11.13	-11.12	-11.11
-11.19	-11.18	-11.17	-11.16	-11.15	-11.14	-11.13	-11.12	-11.11
-11.19	-11.18	-11.17	-11.16	-11.15	-11.14	-11.13	-11.12	-11.11
-11.19	-11.18	-11.17	-11.16	-11.15	-11.14	-11.13	-11.12	-11.11
-11.19	-11.18	-11.17	-11.16	-11.15	-11.14	-11.13	-11.12	-11.11
-11.19	-11.18	-11.17	-11.16	-11.15	-11.14	-11.13	-11.12	-11.11
-11.19	-11.18	-11.17	-11.16	-11.15	-11.14	-11.13	-11.12	-11.11
-11.19	-11.18	-11.17	-11.16	-11.15	-11.14	-11.13	-11.12	-11.11
-11.19	-11.18	-11.17	-11.16	-11.15	-11.14	-11.13	-11.12	-11.11
-11.19	-11.18	-11.17	-11.16	-11.15	-11.14	-11.13	-11.12	-11.11
-11.19	-11.18	-11.17	-11.16	-11.15	-11.14	-11.13	-11.12	-11.11
-11.19	-11.18	-11.17	-11.16	-11.15	-11.14	-11.13	-11.12	-11.11
-11.19	-11.18	-11.17	-11.16	-11.15	-11.14	-11.13	-11.12	-11.11
-11.19	-11.18	-11.17	-11.16	-11.15	-11.14	-11.13	-11.12	-11.11
-11.19	-11.18	-11.17	-11.16	-11.15	-11.14	-11.13	-11.12	-11.11
-11.19	-11.18	-11.17	-11.16	-11.15	-11.14	-11.13	-11.12	-11.11
-11.19	-11.18	-11.17	-11.16	-11.15	-11.14	-11.13	-11.12	-11.11
-11.19	-11.18	-11.17	-11.16	-11.15	-11.14	-11.13	-11.12	-11.11
-11.19	-11.18	-11.17	-11.16	-11.15	-11.14	-11.13	-11.12	-11.11
-11.19	-11.18	-11.17	-11.16	-11.15	-11.14	-11.13	-11.12	-11.11
-11.19	-11.18	-11.17	-11.16	-11.15	-11.14	-11.13	-11.12	-11.11
-11.19	-11.18	-11.17	-11.16	-11.15	-11.14	-11.13	-11.12	-11.11
-11.19	-11.18	-11.17	-11.16	-11.15	-11.14	-11.13	-11.12	-11.11

-11.19	-11.18	-11.17	-11.16	-11.15	-11.14	-11.13	-11.12	-11.11
-11.19	-11.18	-11.17	-11.16	-11.15	-11.14	-11.13	-11.12	-11.11
-11.19	-11.18	-11.17	-11.16	-11.15	-11.14	-11.13	-11.12	-11.11
-11.19	-11.18	-11.17	-11.16	-11.15	-11.14	-11.13	-11.12	-11.11
-11.19	-11.18	-11.17	-11.16	-11.15	-11.14	-11.13	-11.12	-11.11
-11.19	-11.18	-11.17	-11.16	-11.15	-11.14	-11.13	-11.12	-11.11
-11.19	-11.18	-11.17	-11.16	-11.15	-11.14	-11.13	-11.12	-11.11
-11.19	-11.18	-11.17	-11.16	-11.15	-11.14	-11.13	-11.12	-11.11
-11.19	-11.18	-11.17	-11.16	-11.15	-11.14	-11.13	-11.12	-11.11
-11.19	-11.18	-11.17	-11.16	-11.15	-11.14	-11.13	-11.12	-11.11
-11.19	-11.18	-11.17	-11.16	-11.15	-11.14	-11.13	-11.12	-11.11
-11.19	-11.18	-11.17	-11.16	-11.15	-11.14	-11.13	-11.12	-11.11
-11.19	-11.18	-11.17	-11.16	-11.15	-11.14	-11.13	-11.12	-11.11
-11.19	-11.18	-11.17	-11.16	-11.15	-11.14	-11.13	-11.12	-11.11
-11.19	-11.18	-11.17	-11.16	-11.15	-11.14	-11.13	-11.12	-11.11
-11.19	-11.18	-11.17	-11.16	-11.15	-11.14	-11.13	-11.12	-11.11
-11.19	-11.18	-11.17	-11.16	-11.15	-11.14	-11.13	-11.12	-11.11
-11.19	-11.18	-11.17	-11.16	-11.15	-11.14	-11.13	-11.12	-11.11
-11.19	-11.18	-11.17	-11.16	-11.15	-11.14	-11.13	-11.12	-11.11
-11.19	-11.18	-11.17	-11.16	-11.15	-11.14	-11.13	-11.12	-11.11
-11.19	-11.18	-11.17	-11.16	-11.15	-11.14	-11.13	-11.12	-11.11
-11.19	-11.18	-11.17	-11.16	-11.15	-11.14	-11.13	-11.12	-11.11
-11.19	-11.18	-11.17	-11.16	-11.15	-11.14	-11.13	-11.12	-11.11
-11.19	-11.18	-11.17	-11.16	-11.15	-11.14	-11.13	-11.12	-11.11
-11.19	-11.18	-11.17	-11.16	-11.15	-11.14	-11.13	-11.12	-11.11
-11.19	-11.18	-11.17	-11.16	-11.15	-11.14	-11.13	-11.12	-11.11
-11.19	-11.18	-11.17	-11.16	-11.15	-11.14	-11.13	-11.12	-11.11
-11.19	-11.18	-11.17	-11.16	-11.15	-11.14	-11.13	-11.12	-11.11
-11.19	-11.18	-11.17	-11.16	-11.15	-11.14	-11.13	-11.12	-11.11
-11.19	-11.18	-11.17	-11.16	-11.15	-11.14	-11.13	-11.12	-11.11
-11.19	-11.18	-11.17	-11.16	-11.15	-11.14	-11.13	-11.12	-11.11
-11.19	-11.18	-11.17	-11.16	-11.15	-11.14	-11.13	-11.12	-11.11
-11.19	-11.18	-11.17	-11.16	-11.15	-11.14	-11.13	-11.12	-11.11
-11.19	-11.18	-11.17	-11.16	-11.15	-11.14	-11.13	-11.12	-11.11
-11.19	-11.18	-11.17	-11.16	-11.15	-11.14	-11.13	-11.12	-11.11
-11.19	-11.18	-11.17	-11.16	-11.15	-11.14	-11.13	-11.12	-11.11
-11.19	-11.18	-11.17	-11.16	-11.15	-11.14	-11.13	-11.12	-11.11
-11.19	-11.18	-11.17	-11.16	-11.15	-11.14	-11.13	-11.12	-11.11
-11.19	-11.18	-11.17	-11.16	-11.15	-11.14	-11.13	-11.12	-11.11
-11.19	-11.18	-11.17	-11.16	-11.15	-11.14	-11.13	-11.12	-11.11
-11.19	-11.18	-11.17	-11.16	-11.15	-11.14	-11.13	-11.12	-11.11
-11.19	-11.18	-11.17	-11.16	-11.15	-11.14	-11.13	-11.12	-11.11
-11.19	-11.18	-11.17	-11.16	-11.15	-11.14	-11.13	-11.12	-11.11
-11.19	-11.18	-11.17	-11.16	-11.15	-11.14	-11.13	-11.12	-11.11
-11.19	-11.18	-11.17	-11.16	-11.15	-11.14	-11.13	-11.12	-11.11
-11.19	-11.18	-11.17	-11.16	-11.15	-11.14	-11.13	-11.12	-11.11
-11.19	-11.18	-11.17	-11.16	-11.15	-11.14	-11.13	-11.12	-11.11
-11.19	-11.18	-11.17	-11.16	-11.15	-11.14	-11.13	-11.12	-11.11
-11.19	-11.18	-11.17	-11.16	-11.15	-11.14	-11.13	-11.12	-11.11
-11.19	-11.18	-11.17	-11.16	-11.15	-11.14	-11.13	-11.12	-11.11

-11.19	-11.18	-11.17	-11.16	-11.15	-11.14	-11.13	-11.12	-11.11
-11.19	-11.18	-11.17	-11.16	-11.15	-11.14	-11.13	-11.12	-11.11
-11.19	-11.18	-11.17	-11.16	-11.15	-11.14	-11.13	-11.12	-11.11
-11.19	-11.18	-11.17	-11.16	-11.15	-11.14	-11.13	-11.12	-11.11
-11.19	-11.18	-11.17	-11.16	-11.15	-11.14	-11.13	-11.12	-11.11
-11.19	-11.18	-11.17	-11.16	-11.15	-11.14	-11.13	-11.12	-11.11
-11.19	-11.18	-11.17	-11.16	-11.15	-11.14	-11.13	-11.12	-11.11
-11.19	-11.18	-11.17	-11.16	-11.15	-11.14	-11.13	-11.12	-11.11
-11.19	-11.18	-11.17	-11.16	-11.15	-11.14	-11.13	-11.12	-11.11
-11.19	-11.18	-11.17	-11.16	-11.15	-11.14	-11.13	-11.12	-11.11
-11.19	-11.18	-11.17	-11.16	-11.15	-11.14	-11.13	-11.12	-11.11
-11.19	-11.18	-11.17	-11.16	-11.15	-11.14	-11.13	-11.12	-11.11
-11.19	-11.18	-11.17	-11.16	-11.15	-11.14	-11.13	-11.12	-11.11
-11.19	-11.18	-11.17	-11.16	-11.15	-11.14	-11.13	-11.12	-11.11
-11.19	-11.18	-11.17	-11.16	-11.15	-11.14	-11.13	-11.12	-11.11
-11.19	-11.18	-11.17	-11.16	-11.15	-11.14	-11.13	-11.12	-11.11
-11.19	-11.18	-11.17	-11.16	-11.15	-11.14	-11.13	-11.12	-11.11
-11.19	-11.18	-11.17	-11.16	-11.15	-11.14	-11.13	-11.12	-11.11
-11.19	-11.18	-11.17	-11.16	-11.15	-11.14	-11.13	-11.12	-11.11
-11.19	-11.18	-11.17	-11.16	-11.15	-11.14	-11.13	-11.12	-11.11
-11.19	-11.18	-11.17	-11.16	-11.15	-11.14	-11.13	-11.12	-11.11
-11.19	-11.18	-11.17	-11.16	-11.15	-11.14	-11.13	-11.12	-11.11
-11.19	-11.18	-11.17	-11.16	-11.15	-11.14	-11.13	-11.12	-11.11
-11.19	-11.18	-11.17	-11.16	-11.15	-11.14	-11.13	-11.12	-11.11
-11.19	-11.18	-11.17	-11.16	-11.15	-11.14	-11.13	-11.12	-11.11
-11.19	-11.18	-11.17	-11.16	-11.15	-11.14	-11.13	-11.12	-11.11
-11.19	-11.18	-11.17	-11.16	-11.15	-11.14	-11.13	-11.12	-11.11
-11.19	-11.18	-11.17	-11.16	-11.15	-11.14	-11.13	-11.12	-11.11
-11.19	-11.18	-11.17	-11.16	-11.15	-11.14	-11.13	-11.12	-11.11
-11.19	-11.18	-11.17	-11.16	-11.15	-11.14	-11.13	-11.12	-11.11
-11.19	-11.18	-11.17	-11.16	-11.15	-11.14	-11.13	-11.12	-11.11
-11.19	-11.18	-11.17	-11.16	-11.15	-11.14	-11.13	-11.12	-11.11
-11.19	-11.18	-11.17	-11.16	-11.15	-11.14	-11.13	-11.12	-11.11
-11.19	-11.18	-11.17	-11.16	-11.15	-11.14	-11.13	-11.12	-11.11
-11.19	-11.18	-11.17	-11.16	-11.15	-11.14	-11.13	-11.12	-11.11
-11.19	-11.18	-11.17	-11.16	-11.15	-11.14	-11.13	-11.12	-11.11
-11.19	-11.18	-11.17	-11.16	-11.15	-11.14	-11.13	-11.12	-11.11
-11.19	-11.18	-11.17	-11.16	-11.15	-11.14	-11.13	-11.12	-11.11
-11.19	-11.18	-11.17	-11.16	-11.15	-11.14	-11.13	-11.12	-11.11
-11.19	-11.18	-11.17	-11.16	-11.15	-11.14	-11.13	-11.12	-11.11
-11.19	-11.18	-11.17	-11.16	-11.15	-11.14	-11.13	-11.12	-11.11
-11.19	-11.18	-11.17	-11.16	-11.15	-11.14	-11.13	-11.12	-11.11
-11.19	-11.18	-11.17	-11.16	-11.15	-11.14	-11.13	-11.12	-11.11
-11.19	-11.18	-11.17	-11.16	-11.15	-11.14	-11.13	-11.12	-11.11
-11.19	-11.18	-11.17	-11.16	-11.15	-11.14	-11.13	-11.12	-11.11
-11.19	-11.18	-11.17	-11.16	-11.15	-11.14	-11.13	-11.12	-11.11
-11.19	-11.18	-11.17	-11.16	-11.15	-11.14	-11.13	-11.12	-11.11
-11.19	-11.18	-11.17	-11.16	-11.15	-11.14	-11.13	-11.12	-11.11
-11.19	-11.18	-11.17	-11.16	-11.15	-11.14	-11.13	-11.12	-11.11
-11.19	-11.18	-11.17	-11.16	-11.15	-11.14	-11.13	-11.12	-11.11

-11.19	-11.18	-11.17	-11.16	-11.15	-11.14	-11.13	-11.12	-11.11
-11.19	-11.18	-11.17	-11.16	-11.15	-11.14	-11.13	-11.12	-11.11
-11.19	-11.18	-11.17	-11.16	-11.15	-11.14	-11.13	-11.12	-11.11
-11.19	-11.18	-11.17	-11.16	-11.15	-11.14	-11.13	-11.12	-11.11
-11.19	-11.18	-11.17	-11.16	-11.15	-11.14	-11.13	-11.12	-11.11
-11.19	-11.18	-11.17	-11.16	-11.15	-11.14	-11.13	-11.12	-11.11
-11.19	-11.18	-11.17	-11.16	-11.15	-11.14	-11.13	-11.12	-11.11
-11.19	-11.18	-11.17	-11.16	-11.15	-11.14	-11.13	-11.12	-11.11
-11.19	-11.18	-11.17	-11.16	-11.15	-11.14	-11.13	-11.12	-11.11
-11.19	-11.18	-11.17	-11.16	-11.15	-11.14	-11.13	-11.12	-11.11
-11.19	-11.18	-11.17	-11.16	-11.15	-11.14	-11.13	-11.12	-11.11
-11.19	-11.18	-11.17	-11.16	-11.15	-11.14	-11.13	-11.12	-11.11
-11.19	-11.18	-11.17	-11.16	-11.15	-11.14	-11.13	-11.12	-11.11
-11.19	-11.18	-11.17	-11.16	-11.15	-11.14	-11.13	-11.12	-11.11
-11.19	-11.18	-11.17	-11.16	-11.15	-11.14	-11.13	-11.12	-11.11
-11.19	-11.18	-11.17	-11.16	-11.15	-11.14	-11.13	-11.12	-11.11
-11.19	-11.18	-11.17	-11.16	-11.15	-11.14	-11.13	-11.12	-11.11
-11.19	-11.18	-11.17	-11.16	-11.15	-11.14	-11.13	-11.12	-11.11
-11.19	-11.18	-11.17	-11.16	-11.15	-11.14	-11.13	-11.12	-11.11
-11.19	-11.18	-11.17	-11.16	-11.15	-11.14	-11.13	-11.12	-11.11
-11.19	-11.18	-11.17	-11.16	-11.15	-11.14	-11.13	-11.12	-11.11
-11.19	-11.18	-11.17	-11.16	-11.15	-11.14	-11.13	-11.12	-11.11
-11.19	-11.18	-11.17	-11.16	-11.15	-11.14	-11.13	-11.12	-11.11
-11.19	-11.18	-11.17	-11.16	-11.15	-11.14	-11.13	-11.12	-11.11
-11.19	-11.18	-11.17	-11.16	-11.15	-11.14	-11.13	-11.12	-11.11
-11.19	-11.18	-11.17	-11.16	-11.15	-11.14	-11.13	-11.12	-11.11
-11.19	-11.18	-11.17	-11.16	-11.15	-11.14	-11.13	-11.12	-11.11
-11.19	-11.18	-11.17	-11.16	-11.15	-11.14	-11.13	-11.12	-11.11
-11.19	-11.18	-11.17	-11.16	-11.15	-11.14	-11.13	-11.12	-11.11
-11.19	-11.18	-11.17	-11.16	-11.15	-11.14	-11.13	-11.12	-11.11
-11.19	-11.18	-11.17	-11.16	-11.15	-11.14	-11.13	-11.12	-11.11
-11.19	-11.18	-11.17	-11.16	-11.15	-11.14	-11.13	-11.12	-11.11
-11.19	-11.18	-11.17	-11.16	-11.15	-11.14	-11.13	-11.12	-11.11
-11.19	-11.18	-11.17	-11.16	-11.15	-11.14	-11.13	-11.12	-11.11
-11.19	-11.18	-11.17	-11.16	-11.15	-11.14	-11.13	-11.12	-11.11
-11.19	-11.18	-11.17	-11.16	-11.15	-11.14	-11.13	-11.12	-11.11
-11.19	-11.18	-11.17	-11.16	-11.15	-11.14	-11.13	-11.12	-11.11
-11.19	-11.18	-11.17	-11.16	-11.15	-11.14	-11.13	-11.12	-11.11
-11.19	-11.18	-11.17	-11.16	-11.15	-11.14	-11.13	-11.12	-11.11
-11.19	-11.18	-11.17	-11.16	-11.15	-11.14	-11.13	-11.12	-11.11
-11.19	-11.18	-11.17	-11.16	-11.15	-11.14	-11.13	-11.12	-11.11
-11.19	-11.18	-11.17	-11.16	-11.15	-11.14	-11.13	-11.12	-11.11
-11.19	-11.18	-11.17	-11.16	-11.15	-11.14	-11.13	-11.12	-11.11
-11.19	-11.18	-11.17	-11.16	-11.15	-11.14	-11.13	-11.12	-11.11
-11.19	-11.18	-11.17	-11.16	-11.15	-11.14	-11.13	-11.12	-11.11
-11.19	-11.18	-11.17	-11.16	-11.15	-11.14	-11.13	-11.12	-11.11
-11.19	-11.18	-11.17	-11.16	-11.15	-11.14	-11.13	-11.12	-11.11
-11.19	-11.18	-11.17	-11.16	-11.15	-11.14	-11.13	-11.12	-11.11
-11.19	-11.18	-11.17	-11.16	-11.15	-11.14	-11.13	-11.12	-11.11
-11.19	-11.18	-11.17	-11.16	-11.15	-11.14	-11.13	-11.12	-11.11

-11.19	-11.18	-11.17	-11.16	-11.15	-11.14	-11.13	-11.12	-11.11
-11.19	-11.18	-11.17	-11.16	-11.15	-11.14	-11.13	-11.12	-11.11
-11.19	-11.18	-11.17	-11.16	-11.15	-11.14	-11.13	-11.12	-11.11
-11.19	-11.18	-11.17	-11.16	-11.15	-11.14	-11.13	-11.12	-11.11
-11.19	-11.18	-11.17	-11.16	-11.15	-11.14	-11.13	-11.12	-11.11
-11.19	-11.18	-11.17	-11.16	-11.15	-11.14	-11.13	-11.12	-11.11
-11.19	-11.18	-11.17	-11.16	-11.15	-11.14	-11.13	-11.12	-11.11
-11.19	-11.18	-11.17	-11.16	-11.15	-11.14	-11.13	-11.12	-11.11
-11.19	-11.18	-11.17	-11.16	-11.15	-11.14	-11.13	-11.12	-11.11
-11.19	-11.18	-11.17	-11.16	-11.15	-11.14	-11.13	-11.12	-11.11
-11.19	-11.18	-11.17	-11.16	-11.15	-11.14	-11.13	-11.12	-11.11
-11.19	-11.18	-11.17	-11.16	-11.15	-11.14	-11.13	-11.12	-11.11
-11.19	-11.18	-11.17	-11.16	-11.15	-11.14	-11.13	-11.12	-11.11
-11.19	-11.18	-11.17	-11.16	-11.15	-11.14	-11.13	-11.12	-11.11
-11.19	-11.18	-11.17	-11.16	-11.15	-11.14	-11.13	-11.12	-11.11
-11.19	-11.18	-11.17	-11.16	-11.15	-11.14	-11.13	-11.12	-11.11
-11.19	-11.18	-11.17	-11.16	-11.15	-11.14	-11.13	-11.12	-11.11
-11.19	-11.18	-11.17	-11.16	-11.15	-11.14	-11.13	-11.12	-11.11
-11.19	-11.18	-11.17	-11.16	-11.15	-11.14	-11.13	-11.12	-11.11
-11.19	-11.18	-11.17	-11.16	-11.15	-11.14	-11.13	-11.12	-11.11
-11.19	-11.18	-11.17	-11.16	-11.15	-11.14	-11.13	-11.12	-11.11
-11.19	-11.18	-11.17	-11.16	-11.15	-11.14	-11.13	-11.12	-11.11
-11.19	-11.18	-11.17	-11.16	-11.15	-11.14	-11.13	-11.12	-11.11
-11.19	-11.18	-11.17	-11.16	-11.15	-11.14	-11.13	-11.12	-11.11
-11.19	-11.18	-11.17	-11.16	-11.15	-11.14	-11.13	-11.12	-11.11
-11.19	-11.18	-11.17	-11.16	-11.15	-11.14	-11.13	-11.12	-11.11
-11.19	-11.18	-11.17	-11.16	-11.15	-11.14	-11.13	-11.12	-11.11
-11.19	-11.18	-11.17	-11.16	-11.15	-11.14	-11.13	-11.12	-11.11
-11.19	-11.18	-11.17	-11.16	-11.15	-11.14	-11.13	-11.12	-11.11
-11.19	-11.18	-11.17	-11.16	-11.15	-11.14	-11.13	-11.12	-11.11
-11.19	-11.18	-11.17	-11.16	-11.15	-11.14	-11.13	-11.12	-11.11
-11.19	-11.18	-11.17	-11.16	-11.15	-11.14	-11.13	-11.12	-11.11
-11.19	-11.18	-11.17	-11.16	-11.15	-11.14	-11.13	-11.12	-11.11
-11.19	-11.18	-11.17	-11.16	-11.15	-11.14	-11.13	-11.12	-11.11
-11.19	-11.18	-11.17	-11.16	-11.15	-11.14	-11.13	-11.12	-11.11
-11.19	-11.18	-11.17	-11.16	-11.15	-11.14	-11.13	-11.12	-11.11
-11.19	-11.18	-11.17	-11.16	-11.15	-11.14	-11.13	-11.12	-11.11
-11.19	-11.18	-11.17	-11.16	-11.15	-11.14	-11.13	-11.12	-11.11
-11.19	-11.18	-11.17	-11.16	-11.15	-11.14	-11.13	-11.12	-11.11
-11.19	-11.18	-11.17	-11.16	-11.15	-11.14	-11.13	-11.12	-11.11
-11.19	-11.18	-11.17	-11.16	-11.15	-11.14	-11.13	-11.12	-11.11
-11.19	-11.18	-11.17	-11.16	-11.15	-11.14	-11.13	-11.12	-11.11
-11.19	-11.18	-11.17	-11.16	-11.15	-11.14	-11.13	-11.12	-11.11
-11.19	-11.18	-11.17	-11.16	-11.15	-11.14	-11.13	-11.12	-11.11
-11.19	-11.18	-11.17	-11.16	-11.15	-11.14	-11.13	-11.12	-11.11
-11.19	-11.18	-11.17	-11.16	-11.15	-11.14	-11.13	-11.12	-11.11
-11.19	-11.18	-11.17	-11.16	-11.15	-11.14	-11.13	-11.12	-11.11
-11.19	-11.18	-11.17	-11.16	-11.15	-11.14	-11.13	-11.12	-11.11
-11.19	-11.18	-11.17	-11.16	-11.15	-11.14	-11.13	-11.12	-11.11
-11.19	-11.18	-11.17	-11.16	-11.15	-11.14	-11.13	-11.12	-11.11

-11.19	-11.18	-11.17	-11.16	-11.15	-11.14	-11.13	-11.12	-11.11
-11.19	-11.18	-11.17	-11.16	-11.15	-11.14	-11.13	-11.12	-11.11
-11.19	-11.18	-11.17	-11.16	-11.15	-11.14	-11.13	-11.12	-11.11
-11.19	-11.18	-11.17	-11.16	-11.15	-11.14	-11.13	-11.12	-11.11
-11.19	-11.18	-11.17	-11.16	-11.15	-11.14	-11.13	-11.12	-11.11
-11.19	-11.18	-11.17	-11.16	-11.15	-11.14	-11.13	-11.12	-11.11
-11.19	-11.18	-11.17	-11.16	-11.15	-11.14	-11.13	-11.12	-11.11
-11.19	-11.18	-11.17	-11.16	-11.15	-11.14	-11.13	-11.12	-11.11
-11.19	-11.18	-11.17	-11.16	-11.15	-11.14	-11.13	-11.12	-11.11
-11.19	-11.18	-11.17	-11.16	-11.15	-11.14	-11.13	-11.12	-11.11
-11.19	-11.18	-11.17	-11.16	-11.15	-11.14	-11.13	-11.12	-11.11
-11.19	-11.18	-11.17	-11.16	-11.15	-11.14	-11.13	-11.12	-11.11
-11.19	-11.18	-11.17	-11.16	-11.15	-11.14	-11.13	-11.12	-11.11
-11.19	-11.18	-11.17	-11.16	-11.15	-11.14	-11.13	-11.12	-11.11
-11.19	-11.18	-11.17	-11.16	-11.15	-11.14	-11.13	-11.12	-11.11
-11.19	-11.18	-11.17	-11.16	-11.15	-11.14	-11.13	-11.12	-11.11
-11.19	-11.18	-11.17	-11.16	-11.15	-11.14	-11.13	-11.12	-11.11
-11.19	-11.18	-11.17	-11.16	-11.15	-11.14	-11.13	-11.12	-11.11
-11.19	-11.18	-11.17	-11.16	-11.15	-11.14	-11.13	-11.12	-11.11
-11.19	-11.18	-11.17	-11.16	-11.15	-11.14	-11.13	-11.12	-11.11
-11.19	-11.18	-11.17	-11.16	-11.15	-11.14	-11.13	-11.12	-11.11
-11.19	-11.18	-11.17	-11.16	-11.15	-11.14	-11.13	-11.12	-11.11
-11.19	-11.18	-11.17	-11.16	-11.15	-11.14	-11.13	-11.12	-11.11
-11.19	-11.18	-11.17	-11.16	-11.15	-11.14	-11.13	-11.12	-11.11
-11.19	-11.18	-11.17	-11.16	-11.15	-11.14	-11.13	-11.12	-11.11
-11.19	-11.18	-11.17	-11.16	-11.15	-11.14	-11.13	-11.12	-11.11
-11.19	-11.18	-11.17	-11.16	-11.15	-11.14	-11.13	-11.12	-11.11
-11.19	-11.18	-11.17	-11.16	-11.15	-11.14	-11.13	-11.12	-11.11
-11.19	-11.18	-11.17	-11.16	-11.15	-11.14	-11.13	-11.12	-11.11
-11.19	-11.18	-11.17	-11.16	-11.15	-11.14	-11.13	-11.12	-11.11
-11.19	-11.18	-11.17	-11.16	-11.15	-11.14	-11.13	-11.12	-11.11
-11.19	-11.18	-11.17	-11.16	-11.15	-11.14	-11.13	-11.12	-11.11
-11.19	-11.18	-11.17	-11.16	-11.15	-11.14	-11.13	-11.12	-11.11
-11.19	-11.18	-11.17	-11.16	-11.15	-11.14	-11.13	-11.12	-11.11
-11.19	-11.18	-11.17	-11.16	-11.15	-11.14	-11.13	-11.12	-11.11
-11.19	-11.18	-11.17	-11.16	-11.15	-11.14	-11.13	-11.12	-11.11
-11.19	-11.18	-11.17	-11.16	-11.15	-11.14	-11.13	-11.12	-11.11
-11.19	-11.18	-11.17	-11.16	-11.15	-11.14	-11.13	-11.12	-11.11
-11.19	-11.18	-11.17	-11.16	-11.15	-11.14	-11.13	-11.12	-11.11
-11.19	-11.18	-11.17	-11.16	-11.15	-11.14	-11.13	-11.12	-11.11
-11.19	-11.18	-11.17	-11.16	-11.15	-11.14	-11.13	-11.12	-11.11
-11.19	-11.18	-11.17	-11.16	-11.15	-11.14	-11.13	-11.12	-11.11
-11.19	-11.18	-11.17	-11.16	-11.15	-11.14	-11.13	-11.12	-11.11
-11.19	-11.18	-11.17	-11.16	-11.15	-11.14	-11.13	-11.12	-11.11
-11.19	-11.18	-11.17	-11.16	-11.15	-11.14	-11.13	-11.12	-11.11
-11.19	-11.18	-11.17	-11.16	-11.15	-11.14	-11.13	-11.12	-11.11
-11.19	-11.18	-11.17	-11.16	-11.15	-11.14	-11.13	-11.12	-11.11
-11.19	-11.18	-11.17	-11.16	-11.15	-11.14	-11.13	-11.12	-11.11
-11.19	-11.18	-11.17	-11.16	-11.15	-11.14	-11.13	-11.12	-11.11
-11.19	-11.18	-11.17	-11.16	-11.15	-11.14	-11.13	-11.12	-11.11

-11.19	-11.18	-11.17	-11.16	-11.15	-11.14	-11.13	-11.12	-11.11
-11.19	-11.18	-11.17	-11.16	-11.15	-11.14	-11.13	-11.12	-11.11
-11.19	-11.18	-11.17	-11.16	-11.15	-11.14	-11.13	-11.12	-11.11
-11.19	-11.18	-11.17	-11.16	-11.15	-11.14	-11.13	-11.12	-11.11
-11.19	-11.18	-11.17	-11.16	-11.15	-11.14	-11.13	-11.12	-11.11
-11.19	-11.18	-11.17	-11.16	-11.15	-11.14	-11.13	-11.12	-11.11
-11.19	-11.18	-11.17	-11.16	-11.15	-11.14	-11.13	-11.12	-11.11
-11.19	-11.18	-11.17	-11.16	-11.15	-11.14	-11.13	-11.12	-11.11
-11.19	-11.18	-11.17	-11.16	-11.15	-11.14	-11.13	-11.12	-11.11
-11.19	-11.18	-11.17	-11.16	-11.15	-11.14	-11.13	-11.12	-11.11
-11.19	-11.18	-11.17	-11.16	-11.15	-11.14	-11.13	-11.12	-11.11
-11.19	-11.18	-11.17	-11.16	-11.15	-11.14	-11.13	-11.12	-11.11
-11.19	-11.18	-11.17	-11.16	-11.15	-11.14	-11.13	-11.12	-11.11
-11.19	-11.18	-11.17	-11.16	-11.15	-11.14	-11.13	-11.12	-11.11
-11.19	-11.18	-11.17	-11.16	-11.15	-11.14	-11.13	-11.12	-11.11
-11.19	-11.18	-11.17	-11.16	-11.15	-11.14	-11.13	-11.12	-11.11
-11.19	-11.18	-11.17	-11.16	-11.15	-11.14	-11.13	-11.12	-11.11
-11.19	-11.18	-11.17	-11.16	-11.15	-11.14	-11.13	-11.12	-11.11
-11.19	-11.18	-11.17	-11.16	-11.15	-11.14	-11.13	-11.12	-11.11
-11.19	-11.18	-11.17	-11.16	-11.15	-11.14	-11.13	-11.12	-11.11
-11.19	-11.18	-11.17	-11.16	-11.15	-11.14	-11.13	-11.12	-11.11
-11.19	-11.18	-11.17	-11.16	-11.15	-11.14	-11.13	-11.12	-11.11
-11.19	-11.18	-11.17	-11.16	-11.15	-11.14	-11.13	-11.12	-11.11
-11.19	-11.18	-11.17	-11.16	-11.15	-11.14	-11.13	-11.12	-11.11
-11.19	-11.18	-11.17	-11.16	-11.15	-11.14	-11.13	-11.12	-11.11
-11.19	-11.18	-11.17	-11.16	-11.15	-11.14	-11.13	-11.12	-11.11
-11.19	-11.18	-11.17	-11.16	-11.15	-11.14	-11.13	-11.12	-11.11
-11.19	-11.18	-11.17	-11.16	-11.15	-11.14	-11.13	-11.12	-11.11
-11.19	-11.18	-11.17	-11.16	-11.15	-11.14	-11.13	-11.12	-11.11
-11.19	-11.18	-11.17	-11.16	-11.15	-11.14	-11.13	-11.12	-11.11
-11.19	-11.18	-11.17	-11.16	-11.15	-11.14	-11.13	-11.12	-11.11
-11.19	-11.18	-11.17	-11.16	-11.15	-11.14	-11.13	-11.12	-11.11
-11.19	-11.18	-11.17	-11.16	-11.15	-11.14	-11.13	-11.12	-11.11
-11.19	-11.18	-11.17	-11.16	-11.15	-11.14	-11.13	-11.12	-11.11
-11.19	-11.18	-11.17	-11.16	-11.15	-11.14	-11.13	-11.12	-11.11
-11.19	-11.18	-11.17	-11.16	-11.15	-11.14	-11.13	-11.12	-11.11
-11.19	-11.18	-11.17	-11.16	-11.15	-11.14	-11.13	-11.12	-11.11
-11.19	-11.18	-11.17	-11.16	-11.15	-11.14	-11.13	-11.12	-11.11
-11.19	-11.18	-11.17	-11.16	-11.15	-11.14	-11.13	-11.12	-11.11
-11.19	-11.18	-11.17	-11.16	-11.15	-11.14	-11.13	-11.12	-11.11
-11.19	-11.18	-11.17	-11.16	-11.15	-11.14	-11.13	-11.12	-11.11
-11.19	-11.18	-11.17	-11.16	-11.15	-11.14	-11.13	-11.12	-11.11
-11.19	-11.18	-11.17	-11.16	-11.15	-11.14	-11.13	-11.12	-11.11
-11.19	-11.18	-11.17	-11.16	-11.15	-11.14	-11.13	-11.12	-11.11
-11.19	-11.18	-11.17	-11.16	-11.15	-11.14	-11.13	-11.12	-11.11
-11.19	-11.18	-11.17	-11.16	-11.15	-11.14	-11.13	-11.12	-11.11
-11.19	-11.18	-11.17	-11.16	-11.15	-11.14	-11.13	-11.12	-11.11
-11.19	-11.18	-11.17	-11.16	-11.15	-11.14	-11.13	-11.12	-11.11
-11.19	-11.18	-11.17	-11.16	-11.15	-11.14	-11.13	-11.12	-11.11
-11.19	-11.18	-11.17	-11.16	-11.15	-11.14	-11.13	-11.12	-11.11

-11.19	-11.18	-11.17	-11.16	-11.15	-11.14	-11.13	-11.12	-11.11
-11.19	-11.18	-11.17	-11.16	-11.15	-11.14	-11.13	-11.12	-11.11
-11.19	-11.18	-11.17	-11.16	-11.15	-11.14	-11.13	-11.12	-11.11
-11.19	-11.18	-11.17	-11.16	-11.15	-11.14	-11.13	-11.12	-11.11
-11.19	-11.18	-11.17	-11.16	-11.15	-11.14	-11.13	-11.12	-11.11
-11.19	-11.18	-11.17	-11.16	-11.15	-11.14	-11.13	-11.12	-11.11
-11.19	-11.18	-11.17	-11.16	-11.15	-11.14	-11.13	-11.12	-11.11
-11.19	-11.18	-11.17	-11.16	-11.15	-11.14	-11.13	-11.12	-11.11
-11.19	-11.18	-11.17	-11.16	-11.15	-11.14	-11.13	-11.12	-11.11
-11.19	-11.18	-11.17	-11.16	-11.15	-11.14	-11.13	-11.12	-11.11
-11.19	-11.18	-11.17	-11.16	-11.15	-11.14	-11.13	-11.12	-11.11
-11.19	-11.18	-11.17	-11.16	-11.15	-11.14	-11.13	-11.12	-11.11
-11.19	-11.18	-11.17	-11.16	-11.15	-11.14	-11.13	-11.12	-11.11
-11.19	-11.18	-11.17	-11.16	-11.15	-11.14	-11.13	-11.12	-11.11
-11.19	-11.18	-11.17	-11.16	-11.15	-11.14	-11.13	-11.12	-11.11
-11.19	-11.18	-11.17	-11.16	-11.15	-11.14	-11.13	-11.12	-11.11
-11.19	-11.18	-11.17	-11.16	-11.15	-11.14	-11.13	-11.12	-11.11
-11.19	-11.18	-11.17	-11.16	-11.15	-11.14	-11.13	-11.12	-11.11
-11.19	-11.18	-11.17	-11.16	-11.15	-11.14	-11.13	-11.12	-11.11
-11.19	-11.18	-11.17	-11.16	-11.15	-11.14	-11.13	-11.12	-11.11
-11.19	-11.18	-11.17	-11.16	-11.15	-11.14	-11.13	-11.12	-11.11
-11.19	-11.18	-11.17	-11.16	-11.15	-11.14	-11.13	-11.12	-11.11
-11.19	-11.18	-11.17	-11.16	-11.15	-11.14	-11.13	-11.12	-11.11
-11.19	-11.18	-11.17	-11.16	-11.15	-11.14	-11.13	-11.12	-11.11
-11.19	-11.18	-11.17	-11.16	-11.15	-11.14	-11.13	-11.12	-11.11
-11.19	-11.18	-11.17	-11.16	-11.15	-11.14	-11.13	-11.12	-11.11
-11.19	-11.18	-11.17	-11.16	-11.15	-11.14	-11.13	-11.12	-11.11
-11.19	-11.18	-11.17	-11.16	-11.15	-11.14	-11.13	-11.12	-11.11
-11.19	-11.18	-11.17	-11.16	-11.15	-11.14	-11.13	-11.12	-11.11
-11.19	-11.18	-11.17	-11.16	-11.15	-11.14	-11.13	-11.12	-11.11
-11.19	-11.18	-11.17	-11.16	-11.15	-11.14	-11.13	-11.12	-11.11
-11.19	-11.18	-11.17	-11.16	-11.15	-11.14	-11.13	-11.12	-11.11
-11.19	-11.18	-11.17	-11.16	-11.15	-11.14	-11.13	-11.12	-11.11
-11.19	-11.18	-11.17	-11.16	-11.15	-11.14	-11.13	-11.12	-11.11
-11.19	-11.18	-11.17	-11.16	-11.15	-11.14	-11.13	-11.12	-11.11
-11.19	-11.18	-11.17	-11.16	-11.15	-11.14	-11.13	-11.12	-11.11
-11.19	-11.18	-11.17	-11.16	-11.15	-11.14	-11.13	-11.12	-11.11
-11.19	-11.18	-11.17	-11.16	-11.15	-11.14	-11.13	-11.12	-11.11
-11.19	-11.18	-11.17	-11.16	-11.15	-11.14	-11.13	-11.12	-11.11
-11.19	-11.18	-11.17	-11.16	-11.15	-11.14	-11.13	-11.12	-11.11
-11.19	-11.18	-11.17	-11.16	-11.15	-11.14	-11.13	-11.12	-11.11
-11.19	-11.18	-11.17	-11.16	-11.15	-11.14	-11.13	-11.12	-11.11
-11.19	-11.18	-11.17	-11.16	-11.15	-11.14	-11.13	-11.12	-11.11
-11.19	-11.18	-11.17	-11.16	-11.15	-11.14	-11.13	-11.12	-11.11
-11.19	-11.18	-11.17	-11.16	-11.15	-11.14	-11.13	-11.12	-11.11
-11.19	-11.18	-11.17	-11.16	-11.15	-11.14	-11.13	-11.12	-11.11
-11.19	-11.18	-11.17	-11.16	-11.15	-11.14	-11.13	-11.12	-11.11
-11.19	-11.18	-11.17	-11.16	-11.15	-11.14	-11.13	-11.12	-11.11
-11.19	-11.18	-11.17	-11.16	-11.15	-11.14	-11.13	-11.12	-11.11
-11.19	-11.18	-11.17	-11.16	-11.15	-11.14	-11.13	-11.12	-11.11

-11.19	-11.18	-11.17	-11.16	-11.15	-11.14	-11.13	-11.12	-11.11
-11.19	-11.18	-11.17	-11.16	-11.15	-11.14	-11.13	-11.12	-11.11
-11.19	-11.18	-11.17	-11.16	-11.15	-11.14	-11.13	-11.12	-11.11
-11.19	-11.18	-11.17	-11.16	-11.15	-11.14	-11.13	-11.12	-11.11
-11.19	-11.18	-11.17	-11.16	-11.15	-11.14	-11.13	-11.12	-11.11
-11.19	-11.18	-11.17	-11.16	-11.15	-11.14	-11.13	-11.12	-11.11
-11.19	-11.18	-11.17	-11.16	-11.15	-11.14	-11.13	-11.12	-11.11
-11.19	-11.18	-11.17	-11.16	-11.15	-11.14	-11.13	-11.12	-11.11
-11.19	-11.18	-11.17	-11.16	-11.15	-11.14	-11.13	-11.12	-11.11
-11.19	-11.18	-11.17	-11.16	-11.15	-11.14	-11.13	-11.12	-11.11
-11.19	-11.18	-11.17	-11.16	-11.15	-11.14	-11.13	-11.12	-11.11
-11.19	-11.18	-11.17	-11.16	-11.15	-11.14	-11.13	-11.12	-11.11
-11.19	-11.18	-11.17	-11.16	-11.15	-11.14	-11.13	-11.12	-11.11
-11.19	-11.18	-11.17	-11.16	-11.15	-11.14	-11.13	-11.12	-11.11
-11.19	-11.18	-11.17	-11.16	-11.15	-11.14	-11.13	-11.12	-11.11
-11.19	-11.18	-11.17	-11.16	-11.15	-11.14	-11.13	-11.12	-11.11
-11.19	-11.18	-11.17	-11.16	-11.15	-11.14	-11.13	-11.12	-11.11
-11.19	-11.18	-11.17	-11.16	-11.15	-11.14	-11.13	-11.12	-11.11
-11.19	-11.18	-11.17	-11.16	-11.15	-11.14	-11.13	-11.12	-11.11
-11.19	-11.18	-11.17	-11.16	-11.15	-11.14	-11.13	-11.12	-11.11
-11.19	-11.18	-11.17	-11.16	-11.15	-11.14	-11.13	-11.12	-11.11
-11.19	-11.18	-11.17	-11.16	-11.15	-11.14	-11.13	-11.12	-11.11
-11.19	-11.18	-11.17	-11.16	-11.15	-11.14	-11.13	-11.12	-11.11
-11.19	-11.18	-11.17	-11.16	-11.15	-11.14	-11.13	-11.12	-11.11
-11.19	-11.18	-11.17	-11.16	-11.15	-11.14	-11.13	-11.12	-11.11
-11.19	-11.18	-11.17	-11.16	-11.15	-11.14	-11.13	-11.12	-11.11
-11.19	-11.18	-11.17	-11.16	-11.15	-11.14	-11.13	-11.12	-11.11
-11.19	-11.18	-11.17	-11.16	-11.15	-11.14	-11.13	-11.12	-11.11
-11.19	-11.18	-11.17	-11.16	-11.15	-11.14	-11.13	-11.12	-11.11
-11.19	-11.18	-11.17	-11.16	-11.15	-11.14	-11.13	-11.12	-11.11
-11.19	-11.18	-11.17	-11.16	-11.15	-11.14	-11.13	-11.12	-11.11
-11.19	-11.18	-11.17	-11.16	-11.15	-11.14	-11.13	-11.12	-11.11
-11.19	-11.18	-11.17	-11.16	-11.15	-11.14	-11.13	-11.12	-11.11
-11.19	-11.18	-11.17	-11.16	-11.15	-11.14	-11.13	-11.12	-11.11
-11.19	-11.18	-11.17	-11.16	-11.15	-11.14	-11.13	-11.12	-11.11
-11.19	-11.18	-11.17	-11.16	-11.15	-11.14	-11.13	-11.12	-11.11
-11.19	-11.18	-11.17	-11.16	-11.15	-11.14	-11.13	-11.12	-11.11
-11.19	-11.18	-11.17	-11.16	-11.15	-11.14	-11.13	-11.12	-11.11
-11.19	-11.18	-11.17	-11.16	-11.15	-11.14	-11.13	-11.12	-11.11
-11.19	-11.18	-11.17	-11.16	-11.15	-11.14	-11.13	-11.12	-11.11
-11.19	-11.18	-11.17	-11.16	-11.15	-11.14	-11.13	-11.12	-11.11
-11.19	-11.18	-11.17	-11.16	-11.15	-11.14	-11.13	-11.12	-11.11
-11.19	-11.18	-11.17	-11.16	-11.15	-11.14	-11.13	-11.12	-11.11
-11.19	-11.18	-11.17	-11.16	-11.15	-11.14	-11.13	-11.12	-11.11
-11.19	-11.18	-11.17	-11.16	-11.15	-11.14	-11.13	-11.12	-11.11
-11.19	-11.18	-11.17	-11.16	-11.15	-11.14	-11.13	-11.12	-11.11
-11.19	-11.18	-11.17	-11.16	-11.15	-11.14	-11.13	-11.12	-11.11
-11.19	-11.18	-11.17	-11.16	-11.15	-11.14	-11.13	-11.12	-11.11
-11.19	-11.18	-11.17	-11.16	-11.15	-11.14	-11.13	-11.12	-11.11
-11.19	-11.18	-11.17	-11.16	-11.15	-11.14	-11.13	-11.12	-11.11

-11.19	-11.18	-11.17	-11.16	-11.15	-11.14	-11.13	-11.12	-11.11
-11.19	-11.18	-11.17	-11.16	-11.15	-11.14	-11.13	-11.12	-11.11
-11.19	-11.18	-11.17	-11.16	-11.15	-11.14	-11.13	-11.12	-11.11
-11.19	-11.18	-11.17	-11.16	-11.15	-11.14	-11.13	-11.12	-11.11
-11.19	-11.18	-11.17	-11.16	-11.15	-11.14	-11.13	-11.12	-11.11
-11.19	-11.18	-11.17	-11.16	-11.15	-11.14	-11.13	-11.12	-11.11
-11.19	-11.18	-11.17	-11.16	-11.15	-11.14	-11.13	-11.12	-11.11
-11.19	-11.18	-11.17	-11.16	-11.15	-11.14	-11.13	-11.12	-11.11
-11.19	-11.18	-11.17	-11.16	-11.15	-11.14	-11.13	-11.12	-11.11
-11.19	-11.18	-11.17	-11.16	-11.15	-11.14	-11.13	-11.12	-11.11
-11.19	-11.18	-11.17	-11.16	-11.15	-11.14	-11.13	-11.12	-11.11
-11.19	-11.18	-11.17	-11.16	-11.15	-11.14	-11.13	-11.12	-11.11
-11.19	-11.18	-11.17	-11.16	-11.15	-11.14	-11.13	-11.12	-11.11
-11.19	-11.18	-11.17	-11.16	-11.15	-11.14	-11.13	-11.12	-11.11
-11.19	-11.18	-11.17	-11.16	-11.15	-11.14	-11.13	-11.12	-11.11
-11.19	-11.18	-11.17	-11.16	-11.15	-11.14	-11.13	-11.12	-11.11
-11.19	-11.18	-11.17	-11.16	-11.15	-11.14	-11.13	-11.12	-11.11
-11.19	-11.18	-11.17	-11.16	-11.15	-11.14	-11.13	-11.12	-11.11
-11.19	-11.18	-11.17	-11.16	-11.15	-11.14	-11.13	-11.12	-11.11
-11.19	-11.18	-11.17	-11.16	-11.15	-11.14	-11.13	-11.12	-11.11
-11.19	-11.18	-11.17	-11.16	-11.15	-11.14	-11.13	-11.12	-11.11
-11.19	-11.18	-11.17	-11.16	-11.15	-11.14	-11.13	-11.12	-11.11
-11.19	-11.18	-11.17	-11.16	-11.15	-11.14	-11.13	-11.12	-11.11
-11.19	-11.18	-11.17	-11.16	-11.15	-11.14	-11.13	-11.12	-11.11
-11.19	-11.18	-11.17	-11.16	-11.15	-11.14	-11.13	-11.12	-11.11
-11.19	-11.18	-11.17	-11.16	-11.15	-11.14	-11.13	-11.12	-11.11
-11.19	-11.18	-11.17	-11.16	-11.15	-11.14	-11.13	-11.12	-11.11
-11.19	-11.18	-11.17	-11.16	-11.15	-11.14	-11.13	-11.12	-11.11
-11.19	-11.18	-11.17	-11.16	-11.15	-11.14	-11.13	-11.12	-11.11
-11.19	-11.18	-11.17	-11.16	-11.15	-11.14	-11.13	-11.12	-11.11
-11.19	-11.18	-11.17	-11.16	-11.15	-11.14	-11.13	-11.12	-11.11
-11.19	-11.18	-11.17	-11.16	-11.15	-11.14	-11.13	-11.12	-11.11
-11.19	-11.18	-11.17	-11.16	-11.15	-11.14	-11.13	-11.12	-11.11
-11.19	-11.18	-11.17	-11.16	-11.15	-11.14	-11.13	-11.12	-11.11
-11.19	-11.18	-11.17	-11.16	-11.15	-11.14	-11.13	-11.12	-11.11
-11.19	-11.18	-11.17	-11.16	-11.15	-11.14	-11.13	-11.12	-11.11
-11.19	-11.18	-11.17	-11.16	-11.15	-11.14	-11.13	-11.12	-11.11
-11.19	-11.18	-11.17	-11.16	-11.15	-11.14	-11.13	-11.12	-11.11
-11.19	-11.18	-11.17	-11.16	-11.15	-11.14	-11.13	-11.12	-11.11
-11.19	-11.18	-11.17	-11.16	-11.15	-11.14	-11.13	-11.12	-11.11
-11.19	-11.18	-11.17	-11.16	-11.15	-11.14	-11.13	-11.12	-11.11
-11.19	-11.18	-11.17	-11.16	-11.15	-11.14	-11.13	-11.12	-11.11
-11.19	-11.18	-11.17	-11.16	-11.15	-11.14	-11.13	-11.12	-11.11
-11.19	-11.18	-11.17	-11.16	-11.15	-11.14	-11.13	-11.12	-11.11
-11.19	-11.18	-11.17	-11.16	-11.15	-11.14	-11.13	-11.12	-11.11
-11.19	-11.18	-11.17	-11.16	-11.15	-11.14	-11.13	-11.12	-11.11
-11.19	-11.18	-11.17	-11.16	-11.15	-11.14	-11.13	-11.12	-11.11
-11.19	-11.18	-11.17	-11.16	-11.15	-11.14	-11.13	-11.12	-11.11
-11.19	-11.18	-11.17	-11.16	-11.15	-11.14	-11.13	-11.12	-11.11
-11.19	-11.18	-11.17	-11.16	-11.15	-11.14	-11.13	-11.12	-11.11

-11.19	-11.18	-11.17	-11.16	-11.15	-11.14	-11.13	-11.12	-11.11
-11.19	-11.18	-11.17	-11.16	-11.15	-11.14	-11.13	-11.12	-11.11
-11.19	-11.18	-11.17	-11.16	-11.15	-11.14	-11.13	-11.12	-11.11
-11.19	-11.18	-11.17	-11.16	-11.15	-11.14	-11.13	-11.12	-11.11
-11.19	-11.18	-11.17	-11.16	-11.15	-11.14	-11.13	-11.12	-11.11
-11.19	-11.18	-11.17	-11.16	-11.15	-11.14	-11.13	-11.12	-11.11
-11.19	-11.18	-11.17	-11.16	-11.15	-11.14	-11.13	-11.12	-11.11
-11.19	-11.18	-11.17	-11.16	-11.15	-11.14	-11.13	-11.12	-11.11
-11.19	-11.18	-11.17	-11.16	-11.15	-11.14	-11.13	-11.12	-11.11
-11.19	-11.18	-11.17	-11.16	-11.15	-11.14	-11.13	-11.12	-11.11
-11.19	-11.18	-11.17	-11.16	-11.15	-11.14	-11.13	-11.12	-11.11
-11.19	-11.18	-11.17	-11.16	-11.15	-11.14	-11.13	-11.12	-11.11
-11.19	-11.18	-11.17	-11.16	-11.15	-11.14	-11.13	-11.12	-11.11
-11.19	-11.18	-11.17	-11.16	-11.15	-11.14	-11.13	-11.12	-11.11
-11.19	-11.18	-11.17	-11.16	-11.15	-11.14	-11.13	-11.12	-11.11
-11.19	-11.18	-11.17	-11.16	-11.15	-11.14	-11.13	-11.12	-11.11
-11.19	-11.18	-11.17	-11.16	-11.15	-11.14	-11.13	-11.12	-11.11
-11.19	-11.18	-11.17	-11.16	-11.15	-11.14	-11.13	-11.12	-11.11
-11.19	-11.18	-11.17	-11.16	-11.15	-11.14	-11.13	-11.12	-11.11
-11.19	-11.18	-11.17	-11.16	-11.15	-11.14	-11.13	-11.12	-11.11
-11.19	-11.18	-11.17	-11.16	-11.15	-11.14	-11.13	-11.12	-11.11
-11.19	-11.18	-11.17	-11.16	-11.15	-11.14	-11.13	-11.12	-11.11
-11.19	-11.18	-11.17	-11.16	-11.15	-11.14	-11.13	-11.12	-11.11
-11.19	-11.18	-11.17	-11.16	-11.15	-11.14	-11.13	-11.12	-11.11
-11.19	-11.18	-11.17	-11.16	-11.15	-11.14	-11.13	-11.12	-11.11
-11.19	-11.18	-11.17	-11.16	-11.15	-11.14	-11.13	-11.12	-11.11
-11.19	-11.18	-11.17	-11.16	-11.15	-11.14	-11.13	-11.12	-11.11
-11.19	-11.18	-11.17	-11.16	-11.15	-11.14	-11.13	-11.12	-11.11
-11.19	-11.18	-11.17	-11.16	-11.15	-11.14	-11.13	-11.12	-11.11
-11.19	-11.18	-11.17	-11.16	-11.15	-11.14	-11.13	-11.12	-11.11
-11.19	-11.18	-11.17	-11.16	-11.15	-11.14	-11.13	-11.12	-11.11
-11.19	-11.18	-11.17	-11.16	-11.15	-11.14	-11.13	-11.12	-11.11
-11.19	-11.18	-11.17	-11.16	-11.15	-11.14	-11.13	-11.12	-11.11
-11.19	-11.18	-11.17	-11.16	-11.15	-11.14	-11.13	-11.12	-11.11
-11.19	-11.18	-11.17	-11.16	-11.15	-11.14	-11.13	-11.12	-11.11
-11.19	-11.18	-11.17	-11.16	-11.15	-11.14	-11.13	-11.12	-11.11
-11.19	-11.18	-11.17	-11.16	-11.15	-11.14	-11.13	-11.12	-11.11
-11.19	-11.18	-11.17	-11.16	-11.15	-11.14	-11.13	-11.12	-11.11
-11.19	-11.18	-11.17	-11.16	-11.15	-11.14	-11.13	-11.12	-11.11
-11.19	-11.18	-11.17	-11.16	-11.15	-11.14	-11.13	-11.12	-11.11
-11.19	-11.18	-11.17	-11.16	-11.15	-11.14	-11.13	-11.12	-11.11
-11.19	-11.18	-11.17	-11.16	-11.15	-11.14	-11.13	-11.12	-11.11
-11.19	-11.18	-11.17	-11.16	-11.15	-11.14	-11.13	-11.12	-11.11
-11.19	-11.18	-11.17	-11.16	-11.15	-11.14	-11.13	-11.12	-11.11
-11.19	-11.18	-11.17	-11.16	-11.15	-11.14	-11.13	-11.12	-11.11
-11.19	-11.18	-11.17	-11.16	-11.15	-11.14	-11.13	-11.12	-11.11
-11.19	-11.18	-11.17	-11.16	-11.15	-11.14	-11.13	-11.12	-11.11
-11.19	-11.18	-11.17	-11.16	-11.15	-11.14	-11.13	-11.12	-11.11
-11.19	-11.18	-11.17	-11.16	-11.15	-11.14	-11.13	-11.12	-11.11
-11.19	-11.18	-11.17	-11.16	-11.15	-11.14	-11.13	-11.12	-11.11

-11.19	-11.18	-11.17	-11.16	-11.15	-11.14	-11.13	-11.12	-11.11
-11.19	-11.18	-11.17	-11.16	-11.15	-11.14	-11.13	-11.12	-11.11
-11.19	-11.18	-11.17	-11.16	-11.15	-11.14	-11.13	-11.12	-11.11
-11.19	-11.18	-11.17	-11.16	-11.15	-11.14	-11.13	-11.12	-11.11
-11.19	-11.18	-11.17	-11.16	-11.15	-11.14	-11.13	-11.12	-11.11
-11.19	-11.18	-11.17	-11.16	-11.15	-11.14	-11.13	-11.12	-11.11
-11.19	-11.18	-11.17	-11.16	-11.15	-11.14	-11.13	-11.12	-11.11
-11.19	-11.18	-11.17	-11.16	-11.15	-11.14	-11.13	-11.12	-11.11
-11.19	-11.18	-11.17	-11.16	-11.15	-11.14	-11.13	-11.12	-11.11
-11.19	-11.18	-11.17	-11.16	-11.15	-11.14	-11.13	-11.12	-11.11
-11.19	-11.18	-11.17	-11.16	-11.15	-11.14	-11.13	-11.12	-11.11
-11.19	-11.18	-11.17	-11.16	-11.15	-11.14	-11.13	-11.12	-11.11
-11.19	-11.18	-11.17	-11.16	-11.15	-11.14	-11.13	-11.12	-11.11
-11.19	-11.18	-11.17	-11.16	-11.15	-11.14	-11.13	-11.12	-11.11
-11.19	-11.18	-11.17	-11.16	-11.15	-11.14	-11.13	-11.12	-11.11
-11.19	-11.18	-11.17	-11.16	-11.15	-11.14	-11.13	-11.12	-11.11
-11.19	-11.18	-11.17	-11.16	-11.15	-11.14	-11.13	-11.12	-11.11
-11.19	-11.18	-11.17	-11.16	-11.15	-11.14	-11.13	-11.12	-11.11
-11.19	-11.18	-11.17	-11.16	-11.15	-11.14	-11.13	-11.12	-11.11
-11.19	-11.18	-11.17	-11.16	-11.15	-11.14	-11.13	-11.12	-11.11
-11.19	-11.18	-11.17	-11.16	-11.15	-11.14	-11.13	-11.12	-11.11
-11.19	-11.18	-11.17	-11.16	-11.15	-11.14	-11.13	-11.12	-11.11
-11.19	-11.18	-11.17	-11.16	-11.15	-11.14	-11.13	-11.12	-11.11
-11.19	-11.18	-11.17	-11.16	-11.15	-11.14	-11.13	-11.12	-11.11
-11.19	-11.18	-11.17	-11.16	-11.15	-11.14	-11.13	-11.12	-11.11
-11.19	-11.18	-11.17	-11.16	-11.15	-11.14	-11.13	-11.12	-11.11
-11.19	-11.18	-11.17	-11.16	-11.15	-11.14	-11.13	-11.12	-11.11
-11.19	-11.18	-11.17	-11.16	-11.15	-11.14	-11.13	-11.12	-11.11
-11.19	-11.18	-11.17	-11.16	-11.15	-11.14	-11.13	-11.12	-11.11
-11.19	-11.18	-11.17	-11.16	-11.15	-11.14	-11.13	-11.12	-11.11
-11.19	-11.18	-11.17	-11.16	-11.15	-11.14	-11.13	-11.12	-11.11
-11.19	-11.18	-11.17	-11.16	-11.15	-11.14	-11.13	-11.12	-11.11
-11.19	-11.18	-11.17	-11.16	-11.15	-11.14	-11.13	-11.12	-11.11
-11.19	-11.18	-11.17	-11.16	-11.15	-11.14	-11.13	-11.12	-11.11
-11.19	-11.18	-11.17	-11.16	-11.15	-11.14	-11.13	-11.12	-11.11
-11.19	-11.18	-11.17	-11.16	-11.15	-11.14	-11.13	-11.12	-11.11
-11.19	-11.18	-11.17	-11.16	-11.15	-11.14	-11.13	-11.12	-11.11
-11.19	-11.18	-11.17	-11.16	-11.15	-11.14	-11.13	-11.12	-11.11
-11.19	-11.18	-11.17	-11.16	-11.15	-11.14	-11.13	-11.12	-11.11
-11.19	-11.18	-11.17	-11.16	-11.15	-11.14	-11.13	-11.12	-11.11
-11.19	-11.18	-11.17	-11.16	-11.15	-11.14	-11.13	-11.12	-11.11
-11.19	-11.18	-11.17	-11.16	-11.15	-11.14	-11.13	-11.12	-11.11
-11.19	-11.18	-11.17	-11.16	-11.15	-11.14	-11.13	-11.12	-11.11
-11.19	-11.18	-11.17	-11.16	-11.15	-11.14	-11.13	-11.12	-11.11
-11.19	-11.18	-11.17	-11.16	-11.15	-11.14	-11.13	-11.12	-11.11
-11.19	-11.18	-11.17	-11.16	-11.15	-11.14	-11.13	-11.12	-11.11
-11.19	-11.18	-11.17	-11.16	-11.15	-11.14	-11.13	-11.12	-11.11
-11.19	-11.18	-11.17	-11.16	-11.15	-11.14	-11.13	-11.12	-11.11
-11.19	-11.18	-11.17	-11.16	-11.15	-11.14	-11.13	-11.12	-11.11
-11.19	-11.18	-11.17	-11.16	-11.15	-11.14	-11.13	-11.12	-11.11

-11.19	-11.18	-11.17	-11.16	-11.15	-11.14	-11.13	-11.12	-11.11
-11.19	-11.18	-11.17	-11.16	-11.15	-11.14	-11.13	-11.12	-11.11
-11.19	-11.18	-11.17	-11.16	-11.15	-11.14	-11.13	-11.12	-11.11
-11.19	-11.18	-11.17	-11.16	-11.15	-11.14	-11.13	-11.12	-11.11
-11.19	-11.18	-11.17	-11.16	-11.15	-11.14	-11.13	-11.12	-11.11
-11.19	-11.18	-11.17	-11.16	-11.15	-11.14	-11.13	-11.12	-11.11
-11.19	-11.18	-11.17	-11.16	-11.15	-11.14	-11.13	-11.12	-11.11
-11.19	-11.18	-11.17	-11.16	-11.15	-11.14	-11.13	-11.12	-11.11
-11.19	-11.18	-11.17	-11.16	-11.15	-11.14	-11.13	-11.12	-11.11
-11.19	-11.18	-11.17	-11.16	-11.15	-11.14	-11.13	-11.12	-11.11
-11.19	-11.18	-11.17	-11.16	-11.15	-11.14	-11.13	-11.12	-11.11
-11.19	-11.18	-11.17	-11.16	-11.15	-11.14	-11.13	-11.12	-11.11
-11.19	-11.18	-11.17	-11.16	-11.15	-11.14	-11.13	-11.12	-11.11
-11.19	-11.18	-11.17	-11.16	-11.15	-11.14	-11.13	-11.12	-11.11
-11.19	-11.18	-11.17	-11.16	-11.15	-11.14	-11.13	-11.12	-11.11
-11.19	-11.18	-11.17	-11.16	-11.15	-11.14	-11.13	-11.12	-11.11
-11.19	-11.18	-11.17	-11.16	-11.15	-11.14	-11.13	-11.12	-11.11
-11.19	-11.18	-11.17	-11.16	-11.15	-11.14	-11.13	-11.12	-11.11
-11.19	-11.18	-11.17	-11.16	-11.15	-11.14	-11.13	-11.12	-11.11
-11.19	-11.18	-11.17	-11.16	-11.15	-11.14	-11.13	-11.12	-11.11
-11.19	-11.18	-11.17	-11.16	-11.15	-11.14	-11.13	-11.12	-11.11
-11.19	-11.18	-11.17	-11.16	-11.15	-11.14	-11.13	-11.12	-11.11
-11.19	-11.18	-11.17	-11.16	-11.15	-11.14	-11.13	-11.12	-11.11
-11.19	-11.18	-11.17	-11.16	-11.15	-11.14	-11.13	-11.12	-11.11
-11.19	-11.18	-11.17	-11.16	-11.15	-11.14	-11.13	-11.12	-11.11
-11.19	-11.18	-11.17	-11.16	-11.15	-11.14	-11.13	-11.12	-11.11
-11.19	-11.18	-11.17	-11.16	-11.15	-11.14	-11.13	-11.12	-11.11
-11.19	-11.18	-11.17	-11.16	-11.15	-11.14	-11.13	-11.12	-11.11
-11.19	-11.18	-11.17	-11.16	-11.15	-11.14	-11.13	-11.12	-11.11
-11.19	-11.18	-11.17	-11.16	-11.15	-11.14	-11.13	-11.12	-11.11
-11.19	-11.18	-11.17	-11.16	-11.15	-11.14	-11.13	-11.12	-11.11
-11.19	-11.18	-11.17	-11.16	-11.15	-11.14	-11.13	-11.12	-11.11
-11.19	-11.18	-11.17	-11.16	-11.15	-11.14	-11.13	-11.12	-11.11
-11.19	-11.18	-11.17	-11.16	-11.15	-11.14	-11.13	-11.12	-11.11
-11.19	-11.18	-11.17	-11.16	-11.15	-11.14	-11.13	-11.12	-11.11
-11.19	-11.18	-11.17	-11.16	-11.15	-11.14	-11.13	-11.12	-11.11
-11.19	-11.18	-11.17	-11.16	-11.15	-11.14	-11.13	-11.12	-11.11
-11.19	-11.18	-11.17	-11.16	-11.15	-11.14	-11.13	-11.12	-11.11
-11.19	-11.18	-11.17	-11.16	-11.15	-11.14	-11.13	-11.12	-11.11
-11.19	-11.18	-11.17	-11.16	-11.15	-11.14	-11.13	-11.12	-11.11
-11.19	-11.18	-11.17	-11.16	-11.15	-11.14	-11.13	-11.12	-11.11
-11.19	-11.18	-11.17	-11.16	-11.15	-11.14	-11.13	-11.12	-11.11
-11.19	-11.18	-11.17	-11.16	-11.15	-11.14	-11.13	-11.12	-11.11
-11.19	-11.18	-11.17	-11.16	-11.15	-11.14	-11.13	-11.12	-11.11
-11.19	-11.18	-11.17	-11.16	-11.15	-11.14	-11.13	-11.12	-11.11
-11.19	-11.18	-11.17	-11.16	-11.15	-11.14	-11.13	-11.12	-11.11
-11.19	-11.18	-11.17	-11.16	-11.15	-11.14	-11.13	-11.12	-11.11
-11.19	-11.18	-11.17	-11.16	-11.15	-11.14	-11.13	-11.12	-11.11
-11.19	-11.18	-11.17	-11.16	-11.15	-11.14	-11.13	-11.12	-11.11
-11.19	-11.18	-11.17	-11.16	-11.15	-11.14	-11.13	-11.12	-11.11

-11.19	-11.18	-11.17	-11.16	-11.15	-11.14	-11.13	-11.12	-11.11
-11.19	-11.18	-11.17	-11.16	-11.15	-11.14	-11.13	-11.12	-11.11
-11.19	-11.18	-11.17	-11.16	-11.15	-11.14	-11.13	-11.12	-11.11
-11.19	-11.18	-11.17	-11.16	-11.15	-11.14	-11.13	-11.12	-11.11
-11.19	-11.18	-11.17	-11.16	-11.15	-11.14	-11.13	-11.12	-11.11
-11.19	-11.18	-11.17	-11.16	-11.15	-11.14	-11.13	-11.12	-11.11
-11.19	-11.18	-11.17	-11.16	-11.15	-11.14	-11.13	-11.12	-11.11
-11.19	-11.18	-11.17	-11.16	-11.15	-11.14	-11.13	-11.12	-11.11
-11.19	-11.18	-11.17	-11.16	-11.15	-11.14	-11.13	-11.12	-11.11
-11.19	-11.18	-11.17	-11.16	-11.15	-11.14	-11.13	-11.12	-11.11
-11.19	-11.18	-11.17	-11.16	-11.15	-11.14	-11.13	-11.12	-11.11
-11.19	-11.18	-11.17	-11.16	-11.15	-11.14	-11.13	-11.12	-11.11
-11.19	-11.18	-11.17	-11.16	-11.15	-11.14	-11.13	-11.12	-11.11
-11.19	-11.18	-11.17	-11.16	-11.15	-11.14	-11.13	-11.12	-11.11
-11.19	-11.18	-11.17	-11.16	-11.15	-11.14	-11.13	-11.12	-11.11
-11.19	-11.18	-11.17	-11.16	-11.15	-11.14	-11.13	-11.12	-11.11
-11.19	-11.18	-11.17	-11.16	-11.15	-11.14	-11.13	-11.12	-11.11
-11.19	-11.18	-11.17	-11.16	-11.15	-11.14	-11.13	-11.12	-11.11
-11.19	-11.18	-11.17	-11.16	-11.15	-11.14	-11.13	-11.12	-11.11
-11.19	-11.18	-11.17	-11.16	-11.15	-11.14	-11.13	-11.12	-11.11
-11.19	-11.18	-11.17	-11.16	-11.15	-11.14	-11.13	-11.12	-11.11
-11.19	-11.18	-11.17	-11.16	-11.15	-11.14	-11.13	-11.12	-11.11
-11.19	-11.18	-11.17	-11.16	-11.15	-11.14	-11.13	-11.12	-11.11
-11.19	-11.18	-11.17	-11.16	-11.15	-11.14	-11.13	-11.12	-11.11
-11.19	-11.18	-11.17	-11.16	-11.15	-11.14	-11.13	-11.12	-11.11
-11.19	-11.18	-11.17	-11.16	-11.15	-11.14	-11.13	-11.12	-11.11
-11.19	-11.18	-11.17	-11.16	-11.15	-11.14	-11.13	-11.12	-11.11
-11.19	-11.18	-11.17	-11.16	-11.15	-11.14	-11.13	-11.12	-11.11
-11.19	-11.18	-11.17	-11.16	-11.15	-11.14	-11.13	-11.12	-11.11
-11.19	-11.18	-11.17	-11.16	-11.15	-11.14	-11.13	-11.12	-11.11
-11.19	-11.18	-11.17	-11.16	-11.15	-11.14	-11.13	-11.12	-11.11
-11.19	-11.18	-11.17	-11.16	-11.15	-11.14	-11.13	-11.12	-11.11
-11.19	-11.18	-11.17	-11.16	-11.15	-11.14	-11.13	-11.12	-11.11
-11.19	-11.18	-11.17	-11.16	-11.15	-11.14	-11.13	-11.12	-11.11
-11.19	-11.18	-11.17	-11.16	-11.15	-11.14	-11.13	-11.12	-11.11
-11.19	-11.18	-11.17	-11.16	-11.15	-11.14	-11.13	-11.12	-11.11
-11.19	-11.18	-11.17	-11.16	-11.15	-11.14	-11.13	-11.12	-11.11
-11.19	-11.18	-11.17	-11.16	-11.15	-11.14	-11.13	-11.12	-11.11
-11.19	-11.18	-11.17	-11.16	-11.15	-11.14	-11.13	-11.12	-11.11
-11.19	-11.18	-11.17	-11.16	-11.15	-11.14	-11.13	-11.12	-11.11
-11.19	-11.18	-11.17	-11.16	-11.15	-11.14	-11.13	-11.12	-11.11
-11.19	-11.18	-11.17	-11.16	-11.15	-11.14	-11.13	-11.12	-11.11
-11.19	-11.18	-11.17	-11.16	-11.15	-11.14	-11.13	-11.12	-11.11
-11.19	-11.18	-11.17	-11.16	-11.15	-11.14	-11.13	-11.12	-11.11
-11.19	-11.18	-11.17	-11.16	-11.15	-11.14	-11.13	-11.12	-11.11
-11.19	-11.18	-11.17	-11.16	-11.15	-11.14	-11.13	-11.12	-11.11
-11.19	-11.18	-11.17	-11.16	-11.15	-11.14	-11.13	-11.12	-11.11
-11.19	-11.18	-11.17	-11.16	-11.15	-11.14	-11.13	-11.12	-11.11
-11.19	-11.18	-11.17	-11.16	-11.15	-11.14	-11.13	-11.12	-11.11
-11.19	-11.18	-11.17	-11.16	-11.15	-11.14	-11.13	-11.12	-11.11

-11.19	-11.18	-11.17	-11.16	-11.15	-11.14	-11.13	-11.12	-11.11
-11.19	-11.18	-11.17	-11.16	-11.15	-11.14	-11.13	-11.12	-11.11
-11.19	-11.18	-11.17	-11.16	-11.15	-11.14	-11.13	-11.12	-11.11
-11.19	-11.18	-11.17	-11.16	-11.15	-11.14	-11.13	-11.12	-11.11
-11.19	-11.18	-11.17	-11.16	-11.15	-11.14	-11.13	-11.12	-11.11
-11.19	-11.18	-11.17	-11.16	-11.15	-11.14	-11.13	-11.12	-11.11
-11.19	-11.18	-11.17	-11.16	-11.15	-11.14	-11.13	-11.12	-11.11
-11.19	-11.18	-11.17	-11.16	-11.15	-11.14	-11.13	-11.12	-11.11
-11.19	-11.18	-11.17	-11.16	-11.15	-11.14	-11.13	-11.12	-11.11
-11.19	-11.18	-11.17	-11.16	-11.15	-11.14	-11.13	-11.12	-11.11
-11.19	-11.18	-11.17	-11.16	-11.15	-11.14	-11.13	-11.12	-11.11
-11.19	-11.18	-11.17	-11.16	-11.15	-11.14	-11.13	-11.12	-11.11
-11.19	-11.18	-11.17	-11.16	-11.15	-11.14	-11.13	-11.12	-11.11
-11.19	-11.18	-11.17	-11.16	-11.15	-11.14	-11.13	-11.12	-11.11
-11.19	-11.18	-11.17	-11.16	-11.15	-11.14	-11.13	-11.12	-11.11
-11.19	-11.18	-11.17	-11.16	-11.15	-11.14	-11.13	-11.12	-11.11
-11.19	-11.18	-11.17	-11.16	-11.15	-11.14	-11.13	-11.12	-11.11
-11.19	-11.18	-11.17	-11.16	-11.15	-11.14	-11.13	-11.12	-11.11
-11.19	-11.18	-11.17	-11.16	-11.15	-11.14	-11.13	-11.12	-11.11
-11.19	-11.18	-11.17	-11.16	-11.15	-11.14	-11.13	-11.12	-11.11
-11.19	-11.18	-11.17	-11.16	-11.15	-11.14	-11.13	-11.12	-11.11
-11.19	-11.18	-11.17	-11.16	-11.15	-11.14	-11.13	-11.12	-11.11
-11.19	-11.18	-11.17	-11.16	-11.15	-11.14	-11.13	-11.12	-11.11
-11.19	-11.18	-11.17	-11.16	-11.15	-11.14	-11.13	-11.12	-11.11
-11.19	-11.18	-11.17	-11.16	-11.15	-11.14	-11.13	-11.12	-11.11
-11.19	-11.18	-11.17	-11.16	-11.15	-11.14	-11.13	-11.12	-11.11
-11.19	-11.18	-11.17	-11.16	-11.15	-11.14	-11.13	-11.12	-11.11
-11.19	-11.18	-11.17	-11.16	-11.15	-11.14	-11.13	-11.12	-11.11
-11.19	-11.18	-11.17	-11.16	-11.15	-11.14	-11.13	-11.12	-11.11
-11.19	-11.18	-11.17	-11.16	-11.15	-11.14	-11.13	-11.12	-11.11
-11.19	-11.18	-11.17	-11.16	-11.15	-11.14	-11.13	-11.12	-11.11
-11.19	-11.18	-11.17	-11.16	-11.15	-11.14	-11.13	-11.12	-11.11
-11.19	-11.18	-11.17	-11.16	-11.15	-11.14	-11.13	-11.12	-11.11
-11.19	-11.18	-11.17	-11.16	-11.15	-11.14	-11.13	-11.12	-11.11
-11.19	-11.18	-11.17	-11.16	-11.15	-11.14	-11.13	-11.12	-11.11
-11.19	-11.18	-11.17	-11.16	-11.15	-11.14	-11.13	-11.12	-11.11
-11.19	-11.18	-11.17	-11.16	-11.15	-11.14	-11.13	-11.12	-11.11
-11.19	-11.18	-11.17	-11.16	-11.15	-11.14	-11.13	-11.12	-11.11
-11.19	-11.18	-11.17	-11.16	-11.15	-11.14	-11.13	-11.12	-11.11
-11.19	-11.18	-11.17	-11.16	-11.15	-11.14	-11.13	-11.12	-11.11
-11.19	-11.18	-11.17	-11.16	-11.15	-11.14	-11.13	-11.12	-11.11
-11.19	-11.18	-11.17	-11.16	-11.15	-11.14	-11.13	-11.12	-11.11
-11.19	-11.18	-11.17	-11.16	-11.15	-11.14	-11.13	-11.12	-11.11
-11.19	-11.18	-11.17	-11.16	-11.15	-11.14	-11.13	-11.12	-11.11
-11.19	-11.18	-11.17	-11.16	-11.15	-11.14	-11.13	-11.12	-11.11
-11.19	-11.18	-11.17	-11.16	-11.15	-11.14	-11.13	-11.12	-11.11
-11.19	-11.18	-11.17	-11.16	-11.15	-11.14	-11.13	-11.12	-11.11
-11.19	-11.18	-11.17	-11.16	-11.15	-11.14	-11.13	-11.12	-11.11
-11.19	-11.18	-11.17	-11.16	-11.15	-11.14	-11.13	-11.12	-11.11
-11.19	-11.18	-11.17	-11.16	-11.15	-11.14	-11.13	-11.12	-11.11

-11.19	-11.18	-11.17	-11.16	-11.15	-11.14	-11.13	-11.12	-11.11
-11.19	-11.18	-11.17	-11.16	-11.15	-11.14	-11.13	-11.12	-11.11
-11.19	-11.18	-11.17	-11.16	-11.15	-11.14	-11.13	-11.12	-11.11
-11.19	-11.18	-11.17	-11.16	-11.15	-11.14	-11.13	-11.12	-11.11
-11.19	-11.18	-11.17	-11.16	-11.15	-11.14	-11.13	-11.12	-11.11
-11.19	-11.18	-11.17	-11.16	-11.15	-11.14	-11.13	-11.12	-11.11
-11.19	-11.18	-11.17	-11.16	-11.15	-11.14	-11.13	-11.12	-11.11
-11.19	-11.18	-11.17	-11.16	-11.15	-11.14	-11.13	-11.12	-11.11
-11.19	-11.18	-11.17	-11.16	-11.15	-11.14	-11.13	-11.12	-11.11
-11.19	-11.18	-11.17	-11.16	-11.15	-11.14	-11.13	-11.12	-11.11
-11.19	-11.18	-11.17	-11.16	-11.15	-11.14	-11.13	-11.12	-11.11
-11.19	-11.18	-11.17	-11.16	-11.15	-11.14	-11.13	-11.12	-11.11
-11.19	-11.18	-11.17	-11.16	-11.15	-11.14	-11.13	-11.12	-11.11
-11.19	-11.18	-11.17	-11.16	-11.15	-11.14	-11.13	-11.12	-11.11
-11.19	-11.18	-11.17	-11.16	-11.15	-11.14	-11.13	-11.12	-11.11
-11.19	-11.18	-11.17	-11.16	-11.15	-11.14	-11.13	-11.12	-11.11
-11.19	-11.18	-11.17	-11.16	-11.15	-11.14	-11.13	-11.12	-11.11
-11.19	-11.18	-11.17	-11.16	-11.15	-11.14	-11.13	-11.12	-11.11
-11.19	-11.18	-11.17	-11.16	-11.15	-11.14	-11.13	-11.12	-11.11
-11.19	-11.18	-11.17	-11.16	-11.15	-11.14	-11.13	-11.12	-11.11
-11.19	-11.18	-11.17	-11.16	-11.15	-11.14	-11.13	-11.12	-11.11
-11.19	-11.18	-11.17	-11.16	-11.15	-11.14	-11.13	-11.12	-11.11
-11.19	-11.18	-11.17	-11.16	-11.15	-11.14	-11.13	-11.12	-11.11
-11.19	-11.18	-11.17	-11.16	-11.15	-11.14	-11.13	-11.12	-11.11
-11.19	-11.18	-11.17	-11.16	-11.15	-11.14	-11.13	-11.12	-11.11
-11.19	-11.18	-11.17	-11.16	-11.15	-11.14	-11.13	-11.12	-11.11
-11.19	-11.18	-11.17	-11.16	-11.15	-11.14	-11.13	-11.12	-11.11
-11.19	-11.18	-11.17	-11.16	-11.15	-11.14	-11.13	-11.12	-11.11
-11.19	-11.18	-11.17	-11.16	-11.15	-11.14	-11.13	-11.12	-11.11
-11.19	-11.18	-11.17	-11.16	-11.15	-11.14	-11.13	-11.12	-11.11
-11.19	-11.18	-11.17	-11.16	-11.15	-11.14	-11.13	-11.12	-11.11
-11.19	-11.18	-11.17	-11.16	-11.15	-11.14	-11.13	-11.12	-11.11
-11.19	-11.18	-11.17	-11.16	-11.15	-11.14	-11.13	-11.12	-11.11
-11.19	-11.18	-11.17	-11.16	-11.15	-11.14	-11.13	-11.12	-11.11
-11.19	-11.18	-11.17	-11.16	-11.15	-11.14	-11.13	-11.12	-11.11
-11.19	-11.18	-11.17	-11.16	-11.15	-11.14	-11.13	-11.12	-11.11
-11.19	-11.18	-11.17	-11.16	-11.15	-11.14	-11.13	-11.12	-11.11
-11.19	-11.18	-11.17	-11.16	-11.15	-11.14	-11.13	-11.12	-11.11
-11.19	-11.18	-11.17	-11.16	-11.15	-11.14	-11.13	-11.12	-11.11
-11.19	-11.18	-11.17	-11.16	-11.15	-11.14	-11.13	-11.12	-11.11
-11.19	-11.18	-11.17	-11.16	-11.15	-11.14	-11.13	-11.12	-11.11
-11.19	-11.18	-11.17	-11.16	-11.15	-11.14	-11.13	-11.12	-11.11
-11.19	-11.18	-11.17	-11.16	-11.15	-11.14	-11.13	-11.12	-11.11
-11.19	-11.18	-11.17	-11.16	-11.15	-11.14	-11.13	-11.12	-11.11
-11.19	-11.18	-11.17	-11.16	-11.15	-11.14	-11.13	-11.12	-11.11
-11.19	-11.18	-11.17	-11.16	-11.15	-11.14	-11.13	-11.12	-11.11
-11.19	-11.18	-11.17	-11.16	-11.15	-11.14	-11.13	-11.12	-11.11
-11.19	-11.18	-11.17	-11.16	-11.15	-11.14	-11.13	-11.12	-11.11
-11.19	-11.18	-11.17	-11.16	-11.15	-11.14	-11.13	-11.12	-11.11
-11.19	-11.18	-11.17	-11.16	-11.15	-11.14	-11.13	-11.12	-11.11

-11.19	-11.18	-11.17	-11.16	-11.15	-11.14	-11.13	-11.12	-11.11
-11.19	-11.18	-11.17	-11.16	-11.15	-11.14	-11.13	-11.12	-11.11
-11.19	-11.18	-11.17	-11.16	-11.15	-11.14	-11.13	-11.12	-11.11
-11.19	-11.18	-11.17	-11.16	-11.15	-11.14	-11.13	-11.12	-11.11
-11.19	-11.18	-11.17	-11.16	-11.15	-11.14	-11.13	-11.12	-11.11
-11.19	-11.18	-11.17	-11.16	-11.15	-11.14	-11.13	-11.12	-11.11
-11.19	-11.18	-11.17	-11.16	-11.15	-11.14	-11.13	-11.12	-11.11
-11.19	-11.18	-11.17	-11.16	-11.15	-11.14	-11.13	-11.12	-11.11
-11.19	-11.18	-11.17	-11.16	-11.15	-11.14	-11.13	-11.12	-11.11
-11.19	-11.18	-11.17	-11.16	-11.15	-11.14	-11.13	-11.12	-11.11
-11.19	-11.18	-11.17	-11.16	-11.15	-11.14	-11.13	-11.12	-11.11
-11.19	-11.18	-11.17	-11.16	-11.15	-11.14	-11.13	-11.12	-11.11
-11.19	-11.18	-11.17	-11.16	-11.15	-11.14	-11.13	-11.12	-11.11
-11.19	-11.18	-11.17	-11.16	-11.15	-11.14	-11.13	-11.12	-11.11
-11.19	-11.18	-11.17	-11.16	-11.15	-11.14	-11.13	-11.12	-11.11
-11.19	-11.18	-11.17	-11.16	-11.15	-11.14	-11.13	-11.12	-11.11
-11.19	-11.18	-11.17	-11.16	-11.15	-11.14	-11.13	-11.12	-11.11
-11.19	-11.18	-11.17	-11.16	-11.15	-11.14	-11.13	-11.12	-11.11
-11.19	-11.18	-11.17	-11.16	-11.15	-11.14	-11.13	-11.12	-11.11
-11.19	-11.18	-11.17	-11.16	-11.15	-11.14	-11.13	-11.12	-11.11
-11.19	-11.18	-11.17	-11.16	-11.15	-11.14	-11.13	-11.12	-11.11
-11.19	-11.18	-11.17	-11.16	-11.15	-11.14	-11.13	-11.12	-11.11
-11.19	-11.18	-11.17	-11.16	-11.15	-11.14	-11.13	-11.12	-11.11
-11.19	-11.18	-11.17	-11.16	-11.15	-11.14	-11.13	-11.12	-11.11
-11.19	-11.18	-11.17	-11.16	-11.15	-11.14	-11.13	-11.12	-11.11
-11.19	-11.18	-11.17	-11.16	-11.15	-11.14	-11.13	-11.12	-11.11
-11.19	-11.18	-11.17	-11.16	-11.15	-11.14	-11.13	-11.12	-11.11
-11.19	-11.18	-11.17	-11.16	-11.15	-11.14	-11.13	-11.12	-11.11
-11.19	-11.18	-11.17	-11.16	-11.15	-11.14	-11.13	-11.12	-11.11
-11.19	-11.18	-11.17	-11.16	-11.15	-11.14	-11.13	-11.12	-11.11
-11.19	-11.18	-11.17	-11.16	-11.15	-11.14	-11.13	-11.12	-11.11
-11.19	-11.18	-11.17	-11.16	-11.15	-11.14	-11.13	-11.12	-11.11
-11.19	-11.18	-11.17	-11.16	-11.15	-11.14	-11.13	-11.12	-11.11
-11.19	-11.18	-11.17	-11.16	-11.15	-11.14	-11.13	-11.12	-11.11
-11.19	-11.18	-11.17	-11.16	-11.15	-11.14	-11.13	-11.12	-11.11
-11.19	-11.18	-11.17	-11.16	-11.15	-11.14	-11.13	-11.12	-11.11
-11.19	-11.18	-11.17	-11.16	-11.15	-11.14	-11.13	-11.12	-11.11
-11.19	-11.18	-11.17	-11.16	-11.15	-11.14	-11.13	-11.12	-11.11
-11.19	-11.18	-11.17	-11.16	-11.15	-11.14	-11.13	-11.12	-11.11
-11.19	-11.18	-11.17	-11.16	-11.15	-11.14	-11.13	-11.12	-11.11
-11.19	-11.18	-11.17	-11.16	-11.15	-11.14	-11.13	-11.12	-11.11
-11.19	-11.18	-11.17	-11.16	-11.15	-11.14	-11.13	-11.12	-11.11
-11.19	-11.18	-11.17	-11.16	-11.15	-11.14	-11.13	-11.12	-11.11
-11.19	-11.18	-11.17	-11.16	-11.15	-11.14	-11.13	-11.12	-11.11
-11.19	-11.18	-11.17	-11.16	-11.15	-11.14	-11.13	-11.12	-11.11
-11.19	-11.18	-11.17	-11.16	-11.15	-11.14	-11.13	-11.12	-11.11
-11.19	-11.18	-11.17	-11.16	-11.15	-11.14	-11.13	-11.12	-11.11
-11.19	-11.18	-11.17	-11.16	-11.15	-11.14	-11.13	-11.12	-11.11
-11.19	-11.18	-11.17	-11.16	-11.15	-11.14	-11.13	-11.12	-11.11
-11.19	-11.18	-11.17	-11.16	-11.15	-11.14	-11.13	-11.12	-11.11

-11.19	-11.18	-11.17	-11.16	-11.15	-11.14	-11.13	-11.12	-11.11
-11.19	-11.18	-11.17	-11.16	-11.15	-11.14	-11.13	-11.12	-11.11
-11.19	-11.18	-11.17	-11.16	-11.15	-11.14	-11.13	-11.12	-11.11
-11.19	-11.18	-11.17	-11.16	-11.15	-11.14	-11.13	-11.12	-11.11
-11.19	-11.18	-11.17	-11.16	-11.15	-11.14	-11.13	-11.12	-11.11
-11.19	-11.18	-11.17	-11.16	-11.15	-11.14	-11.13	-11.12	-11.11
-11.19	-11.18	-11.17	-11.16	-11.15	-11.14	-11.13	-11.12	-11.11
-11.19	-11.18	-11.17	-11.16	-11.15	-11.14	-11.13	-11.12	-11.11
-11.19	-11.18	-11.17	-11.16	-11.15	-11.14	-11.13	-11.12	-11.11
-11.19	-11.18	-11.17	-11.16	-11.15	-11.14	-11.13	-11.12	-11.11
-11.19	-11.18	-11.17	-11.16	-11.15	-11.14	-11.13	-11.12	-11.11
-11.19	-11.18	-11.17	-11.16	-11.15	-11.14	-11.13	-11.12	-11.11
-11.19	-11.18	-11.17	-11.16	-11.15	-11.14	-11.13	-11.12	-11.11
-11.19	-11.18	-11.17	-11.16	-11.15	-11.14	-11.13	-11.12	-11.11
-11.19	-11.18	-11.17	-11.16	-11.15	-11.14	-11.13	-11.12	-11.11
-11.19	-11.18	-11.17	-11.16	-11.15	-11.14	-11.13	-11.12	-11.11
-11.19	-11.18	-11.17	-11.16	-11.15	-11.14	-11.13	-11.12	-11.11
-11.19	-11.18	-11.17	-11.16	-11.15	-11.14	-11.13	-11.12	-11.11
-11.19	-11.18	-11.17	-11.16	-11.15	-11.14	-11.13	-11.12	-11.11
-11.19	-11.18	-11.17	-11.16	-11.15	-11.14	-11.13	-11.12	-11.11
-11.19	-11.18	-11.17	-11.16	-11.15	-11.14	-11.13	-11.12	-11.11
-11.19	-11.18	-11.17	-11.16	-11.15	-11.14	-11.13	-11.12	-11.11
-11.19	-11.18	-11.17	-11.16	-11.15	-11.14	-11.13	-11.12	-11.11
-11.19	-11.18	-11.17	-11.16	-11.15	-11.14	-11.13	-11.12	-11.11
-11.19	-11.18	-11.17	-11.16	-11.15	-11.14	-11.13	-11.12	-11.11
-11.19	-11.18	-11.17	-11.16	-11.15	-11.14	-11.13	-11.12	-11.11
-11.19	-11.18	-11.17	-11.16	-11.15	-11.14	-11.13	-11.12	-11.11
-11.19	-11.18	-11.17	-11.16	-11.15	-11.14	-11.13	-11.12	-11.11
-11.19	-11.18	-11.17	-11.16	-11.15	-11.14	-11.13	-11.12	-11.11
-11.19	-11.18	-11.17	-11.16	-11.15	-11.14	-11.13	-11.12	-11.11
-11.19	-11.18	-11.17	-11.16	-11.15	-11.14	-11.13	-11.12	-11.11
-11.19	-11.18	-11.17	-11.16	-11.15	-11.14	-11.13	-11.12	-11.11
-11.19	-11.18	-11.17	-11.16	-11.15	-11.14	-11.13	-11.12	-11.11
-11.19	-11.18	-11.17	-11.16	-11.15	-11.14	-11.13	-11.12	-11.11
-11.19	-11.18	-11.17	-11.16	-11.15	-11.14	-11.13	-11.12	-11.11
-11.19	-11.18	-11.17	-11.16	-11.15	-11.14	-11.13	-11.12	-11.11
-11.19	-11.18	-11.17	-11.16	-11.15	-11.14	-11.13	-11.12	-11.11
-11.19	-11.18	-11.17	-11.16	-11.15	-11.14	-11.13	-11.12	-11.11
-11.19	-11.18	-11.17	-11.16	-11.15	-11.14	-11.13	-11.12	-11.11
-11.19	-11.18	-11.17	-11.16	-11.15	-11.14	-11.13	-11.12	-11.11
-11.19	-11.18	-11.17	-11.16	-11.15	-11.14	-11.13	-11.12	-11.11
-11.19	-11.18	-11.17	-11.16	-11.15	-11.14	-11.13	-11.12	-11.11
-11.19	-11.18	-11.17	-11.16	-11.15	-11.14	-11.13	-11.12	-11.11
-11.19	-11.18	-11.17	-11.16	-11.15	-11.14	-11.13	-11.12	-11.11
-11.19	-11.18	-11.17	-11.16	-11.15	-11.14	-11.13	-11.12	-11.11
-11.19	-11.18	-11.17	-11.16	-11.15	-11.14	-11.13	-11.12	-11.11
-11.19	-11.18	-11.17	-11.16	-11.15	-11.14	-11.13	-11.12	-11.11
-11.19	-11.18	-11.17	-11.16	-11.15	-11.14	-11.13	-11.12	-11.11
-11.19	-11.18	-11.17	-11.16	-11.15	-11.14	-11.13	-11.12	-11.11
-11.19	-11.18	-11.17	-11.16	-11.15	-11.14	-11.13	-11.12	-11.11

-11.19	-11.18	-11.17	-11.16	-11.15	-11.14	-11.13	-11.12	-11.11
-11.19	-11.18	-11.17	-11.16	-11.15	-11.14	-11.13	-11.12	-11.11
-11.19	-11.18	-11.17	-11.16	-11.15	-11.14	-11.13	-11.12	-11.11
-11.19	-11.18	-11.17	-11.16	-11.15	-11.14	-11.13	-11.12	-11.11
-11.19	-11.18	-11.17	-11.16	-11.15	-11.14	-11.13	-11.12	-11.11
-11.19	-11.18	-11.17	-11.16	-11.15	-11.14	-11.13	-11.12	-11.11
-11.19	-11.18	-11.17	-11.16	-11.15	-11.14	-11.13	-11.12	-11.11
-11.19	-11.18	-11.17	-11.16	-11.15	-11.14	-11.13	-11.12	-11.11
-11.19	-11.18	-11.17	-11.16	-11.15	-11.14	-11.13	-11.12	-11.11
-11.19	-11.18	-11.17	-11.16	-11.15	-11.14	-11.13	-11.12	-11.11
-11.19	-11.18	-11.17	-11.16	-11.15	-11.14	-11.13	-11.12	-11.11
-11.19	-11.18	-11.17	-11.16	-11.15	-11.14	-11.13	-11.12	-11.11
-11.19	-11.18	-11.17	-11.16	-11.15	-11.14	-11.13	-11.12	-11.11
-11.19	-11.18	-11.17	-11.16	-11.15	-11.14	-11.13	-11.12	-11.11
-11.19	-11.18	-11.17	-11.16	-11.15	-11.14	-11.13	-11.12	-11.11
-11.19	-11.18	-11.17	-11.16	-11.15	-11.14	-11.13	-11.12	-11.11
-11.19	-11.18	-11.17	-11.16	-11.15	-11.14	-11.13	-11.12	-11.11
-11.19	-11.18	-11.17	-11.16	-11.15	-11.14	-11.13	-11.12	-11.11
-11.19	-11.18	-11.17	-11.16	-11.15	-11.14	-11.13	-11.12	-11.11
-11.19	-11.18	-11.17	-11.16	-11.15	-11.14	-11.13	-11.12	-11.11
-11.19	-11.18	-11.17	-11.16	-11.15	-11.14	-11.13	-11.12	-11.11
-11.19	-11.18	-11.17	-11.16	-11.15	-11.14	-11.13	-11.12	-11.11
-11.19	-11.18	-11.17	-11.16	-11.15	-11.14	-11.13	-11.12	-11.11
-11.19	-11.18	-11.17	-11.16	-11.15	-11.14	-11.13	-11.12	-11.11
-11.19	-11.18	-11.17	-11.16	-11.15	-11.14	-11.13	-11.12	-11.11
-11.19	-11.18	-11.17	-11.16	-11.15	-11.14	-11.13	-11.12	-11.11
-11.19	-11.18	-11.17	-11.16	-11.15	-11.14	-11.13	-11.12	-11.11
-11.19	-11.18	-11.17	-11.16	-11.15	-11.14	-11.13	-11.12	-11.11
-11.19	-11.18	-11.17	-11.16	-11.15	-11.14	-11.13	-11.12	-11.11
-11.19	-11.18	-11.17	-11.16	-11.15	-11.14	-11.13	-11.12	-11.11
-11.19	-11.18	-11.17	-11.16	-11.15	-11.14	-11.13	-11.12	-11.11
-11.19	-11.18	-11.17	-11.16	-11.15	-11.14	-11.13	-11.12	-11.11
-11.19	-11.18	-11.17	-11.16	-11.15	-11.14	-11.13	-11.12	-11.11
-11.19	-11.18	-11.17	-11.16	-11.15	-11.14	-11.13	-11.12	-11.11
-11.19	-11.18	-11.17	-11.16	-11.15	-11.14	-11.13	-11.12	-11.11
-11.19	-11.18	-11.17	-11.16	-11.15	-11.14	-11.13	-11.12	-11.11
-11.19	-11.18	-11.17	-11.16	-11.15	-11.14	-11.13	-11.12	-11.11
-11.19	-11.18	-11.17	-11.16	-11.15	-11.14	-11.13	-11.12	-11.11
-11.19	-11.18	-11.17	-11.16	-11.15	-11.14	-11.13	-11.12	-11.11
-11.19	-11.18	-11.17	-11.16	-11.15	-11.14	-11.13	-11.12	-11.11
-11.19	-11.18	-11.17	-11.16	-11.15	-11.14	-11.13	-11.12	-11.11
-11.19	-11.18	-11.17	-11.16	-11.15	-11.14	-11.13	-11.12	-11.11
-11.19	-11.18	-11.17	-11.16	-11.15	-11.14	-11.13	-11.12	-11.11
-11.19	-11.18	-11.17	-11.16	-11.15	-11.14	-11.13	-11.12	-11.11
-11.19	-11.18	-11.17	-11.16	-11.15	-11.14	-11.13	-11.12	-11.11
-11.19	-11.18	-11.17	-11.16	-11.15	-11.14	-11.13	-11.12	-11.11
-11.19	-11.18	-11.17	-11.16	-11.15	-11.14	-11.13	-11.12	-11.11
-11.19	-11.18	-11.17	-11.16	-11.15	-11.14	-11.13	-11.12	-11.11
-11.19	-11.18	-11.17	-11.16	-11.15	-11.14	-11.13	-11.12	-11.11
-11.19	-11.18	-11.17	-11.16	-11.15	-11.14	-11.13	-11.12	-11.11

-11.19	-11.18	-11.17	-11.16	-11.15	-11.14	-11.13	-11.12	-11.11
-11.19	-11.18	-11.17	-11.16	-11.15	-11.14	-11.13	-11.12	-11.11
-11.19	-11.18	-11.17	-11.16	-11.15	-11.14	-11.13	-11.12	-11.11
-11.19	-11.18	-11.17	-11.16	-11.15	-11.14	-11.13	-11.12	-11.11
-11.19	-11.18	-11.17	-11.16	-11.15	-11.14	-11.13	-11.12	-11.11
-11.19	-11.18	-11.17	-11.16	-11.15	-11.14	-11.13	-11.12	-11.11
-11.19	-11.18	-11.17	-11.16	-11.15	-11.14	-11.13	-11.12	-11.11
-11.19	-11.18	-11.17	-11.16	-11.15	-11.14	-11.13	-11.12	-11.11
-11.19	-11.18	-11.17	-11.16	-11.15	-11.14	-11.13	-11.12	-11.11
-11.19	-11.18	-11.17	-11.16	-11.15	-11.14	-11.13	-11.12	-11.11
-11.19	-11.18	-11.17	-11.16	-11.15	-11.14	-11.13	-11.12	-11.11
-11.19	-11.18	-11.17	-11.16	-11.15	-11.14	-11.13	-11.12	-11.11
-11.19	-11.18	-11.17	-11.16	-11.15	-11.14	-11.13	-11.12	-11.11
-11.19	-11.18	-11.17	-11.16	-11.15	-11.14	-11.13	-11.12	-11.11
-11.19	-11.18	-11.17	-11.16	-11.15	-11.14	-11.13	-11.12	-11.11
-11.19	-11.18	-11.17	-11.16	-11.15	-11.14	-11.13	-11.12	-11.11
-11.19	-11.18	-11.17	-11.16	-11.15	-11.14	-11.13	-11.12	-11.11
-11.19	-11.18	-11.17	-11.16	-11.15	-11.14	-11.13	-11.12	-11.11
-11.19	-11.18	-11.17	-11.16	-11.15	-11.14	-11.13	-11.12	-11.11
-11.19	-11.18	-11.17	-11.16	-11.15	-11.14	-11.13	-11.12	-11.11
-11.19	-11.18	-11.17	-11.16	-11.15	-11.14	-11.13	-11.12	-11.11
-11.19	-11.18	-11.17	-11.16	-11.15	-11.14	-11.13	-11.12	-11.11
-11.19	-11.18	-11.17	-11.16	-11.15	-11.14	-11.13	-11.12	-11.11
-11.19	-11.18	-11.17	-11.16	-11.15	-11.14	-11.13	-11.12	-11.11
-11.19	-11.18	-11.17	-11.16	-11.15	-11.14	-11.13	-11.12	-11.11
-11.19	-11.18	-11.17	-11.16	-11.15	-11.14	-11.13	-11.12	-11.11
-11.19	-11.18	-11.17	-11.16	-11.15	-11.14	-11.13	-11.12	-11.11
-11.19	-11.18	-11.17	-11.16	-11.15	-11.14	-11.13	-11.12	-11.11
-11.19	-11.18	-11.17	-11.16	-11.15	-11.14	-11.13	-11.12	-11.11
-11.19	-11.18	-11.17	-11.16	-11.15	-11.14	-11.13	-11.12	-11.11
-11.19	-11.18	-11.17	-11.16	-11.15	-11.14	-11.13	-11.12	-11.11
-11.19	-11.18	-11.17	-11.16	-11.15	-11.14	-11.13	-11.12	-11.11
-11.19	-11.18	-11.17	-11.16	-11.15	-11.14	-11.13	-11.12	-11.11
-11.19	-11.18	-11.17	-11.16	-11.15	-11.14	-11.13	-11.12	-11.11
-11.19	-11.18	-11.17	-11.16	-11.15	-11.14	-11.13	-11.12	-11.11
-11.19	-11.18	-11.17	-11.16	-11.15	-11.14	-11.13	-11.12	-11.11
-11.19	-11.18	-11.17	-11.16	-11.15	-11.14	-11.13	-11.12	-11.11
-11.19	-11.18	-11.17	-11.16	-11.15	-11.14	-11.13	-11.12	-11.11
-11.19	-11.18	-11.17	-11.16	-11.15	-11.14	-11.13	-11.12	-11.11
-11.19	-11.18	-11.17	-11.16	-11.15	-11.14	-11.13	-11.12	-11.11
-11.19	-11.18	-11.17	-11.16	-11.15	-11.14	-11.13	-11.12	-11.11
-11.19	-11.18	-11.17	-11.16	-11.15	-11.14	-11.13	-11.12	-11.11
-11.19	-11.18	-11.17	-11.16	-11.15	-11.14	-11.13	-11.12	-11.11
-11.19	-11.18	-11.17	-11.16	-11.15	-11.14	-11.13	-11.12	-11.11
-11.19	-11.18	-11.17	-11.16	-11.15	-11.14	-11.13	-11.12	-11.11
-11.19	-11.18	-11.17	-11.16	-11.15	-11.14	-11.13	-11.12	-11.11
-11.19	-11.18	-11.17	-11.16	-11.15	-11.14	-11.13	-11.12	-11.11
-11.19	-11.18	-11.17	-11.16	-11.15	-11.14	-11.13	-11.12	-11.11
-11.19	-11.18	-11.17	-11.16	-11.15	-11.14	-11.13	-11.12	-11.11
-11.19	-11.18	-11.17	-11.16	-11.15	-11.14	-11.13	-11.12	-11.11

-11.19	-11.18	-11.17	-11.16	-11.15	-11.14	-11.13	-11.12	-11.11
-11.19	-11.18	-11.17	-11.16	-11.15	-11.14	-11.13	-11.12	-11.11
-11.19	-11.18	-11.17	-11.16	-11.15	-11.14	-11.13	-11.12	-11.11
-11.19	-11.18	-11.17	-11.16	-11.15	-11.14	-11.13	-11.12	-11.11
-11.19	-11.18	-11.17	-11.16	-11.15	-11.14	-11.13	-11.12	-11.11
-11.19	-11.18	-11.17	-11.16	-11.15	-11.14	-11.13	-11.12	-11.11
-11.19	-11.18	-11.17	-11.16	-11.15	-11.14	-11.13	-11.12	-11.11
-11.19	-11.18	-11.17	-11.16	-11.15	-11.14	-11.13	-11.12	-11.11
-11.19	-11.18	-11.17	-11.16	-11.15	-11.14	-11.13	-11.12	-11.11
-11.19	-11.18	-11.17	-11.16	-11.15	-11.14	-11.13	-11.12	-11.11
-11.19	-11.18	-11.17	-11.16	-11.15	-11.14	-11.13	-11.12	-11.11
-11.19	-11.18	-11.17	-11.16	-11.15	-11.14	-11.13	-11.12	-11.11
-11.19	-11.18	-11.17	-11.16	-11.15	-11.14	-11.13	-11.12	-11.11
-11.19	-11.18	-11.17	-11.16	-11.15	-11.14	-11.13	-11.12	-11.11
-11.19	-11.18	-11.17	-11.16	-11.15	-11.14	-11.13	-11.12	-11.11
-11.19	-11.18	-11.17	-11.16	-11.15	-11.14	-11.13	-11.12	-11.11
-11.19	-11.18	-11.17	-11.16	-11.15	-11.14	-11.13	-11.12	-11.11
-11.19	-11.18	-11.17	-11.16	-11.15	-11.14	-11.13	-11.12	-11.11
-11.19	-11.18	-11.17	-11.16	-11.15	-11.14	-11.13	-11.12	-11.11
-11.19	-11.18	-11.17	-11.16	-11.15	-11.14	-11.13	-11.12	-11.11
-11.19	-11.18	-11.17	-11.16	-11.15	-11.14	-11.13	-11.12	-11.11
-11.19	-11.18	-11.17	-11.16	-11.15	-11.14	-11.13	-11.12	-11.11
-11.19	-11.18	-11.17	-11.16	-11.15	-11.14	-11.13	-11.12	-11.11
-11.19	-11.18	-11.17	-11.16	-11.15	-11.14	-11.13	-11.12	-11.11
-11.19	-11.18	-11.17	-11.16	-11.15	-11.14	-11.13	-11.12	-11.11
-11.19	-11.18	-11.17	-11.16	-11.15	-11.14	-11.13	-11.12	-11.11
-11.19	-11.18	-11.17	-11.16	-11.15	-11.14	-11.13	-11.12	-11.11
-11.19	-11.18	-11.17	-11.16	-11.15	-11.14	-11.13	-11.12	-11.11
-11.19	-11.18	-11.17	-11.16	-11.15	-11.14	-11.13	-11.12	-11.11
-11.19	-11.18	-11.17	-11.16	-11.15	-11.14	-11.13	-11.12	-11.11
-11.19	-11.18	-11.17	-11.16	-11.15	-11.14	-11.13	-11.12	-11.11
-11.19	-11.18	-11.17	-11.16	-11.15	-11.14	-11.13	-11.12	-11.11
-11.19	-11.18	-11.17	-11.16	-11.15	-11.14	-11.13	-11.12	-11.11
-11.19	-11.18	-11.17	-11.16	-11.15	-11.14	-11.13	-11.12	-11.11
-11.19	-11.18	-11.17	-11.16	-11.15	-11.14	-11.13	-11.12	-11.11
-11.19	-11.18	-11.17	-11.16	-11.15	-11.14	-11.13	-11.12	-11.11
-11.19	-11.18	-11.17	-11.16	-11.15	-11.14	-11.13	-11.12	-11.11
-11.19	-11.18	-11.17	-11.16	-11.15	-11.14	-11.13	-11.12	-11.11
-11.19	-11.18	-11.17	-11.16	-11.15	-11.14	-11.13	-11.12	-11.11
-11.19	-11.18	-11.17	-11.16	-11.15	-11.14	-11.13	-11.12	-11.11
-11.19	-11.18	-11.17	-11.16	-11.15	-11.14	-11.13	-11.12	-11.11
-11.19	-11.18	-11.17	-11.16	-11.15	-11.14	-11.13	-11.12	-11.11
-11.19	-11.18	-11.17	-11.16	-11.15	-11.14	-11.13	-11.12	-11.11
-11.19	-11.18	-11.17	-11.16	-11.15	-11.14	-11.13	-11.12	-11.11
-11.19	-11.18	-11.17	-11.16	-11.15	-11.14	-11.13	-11.12	-11.11
-11.19	-11.18	-11.17	-11.16	-11.15	-11.14	-11.13	-11.12	-11.11
-11.19	-11.18	-11.17	-11.16	-11.15	-11.14	-11.13	-11.12	-11.11
-11.19	-11.18	-11.17	-11.16	-11.15	-11.14	-11.13	-11.12	-11.11
-11.19	-11.18	-11.17	-11.16	-11.15	-11.14	-11.13	-11.12	-11.11
-11.19	-11.18	-11.17	-11.16	-11.15	-11.14	-11.13	-11.12	-11.11

-11.19	-11.18	-11.17	-11.16	-11.15	-11.14	-11.13	-11.12	-11.11
-11.19	-11.18	-11.17	-11.16	-11.15	-11.14	-11.13	-11.12	-11.11
-11.19	-11.18	-11.17	-11.16	-11.15	-11.14	-11.13	-11.12	-11.11
-11.19	-11.18	-11.17	-11.16	-11.15	-11.14	-11.13	-11.12	-11.11
-11.19	-11.18	-11.17	-11.16	-11.15	-11.14	-11.13	-11.12	-11.11
-11.19	-11.18	-11.17	-11.16	-11.15	-11.14	-11.13	-11.12	-11.11
-11.19	-11.18	-11.17	-11.16	-11.15	-11.14	-11.13	-11.12	-11.11
-11.19	-11.18	-11.17	-11.16	-11.15	-11.14	-11.13	-11.12	-11.11
-11.19	-11.18	-11.17	-11.16	-11.15	-11.14	-11.13	-11.12	-11.11
-11.19	-11.18	-11.17	-11.16	-11.15	-11.14	-11.13	-11.12	-11.11
-11.19	-11.18	-11.17	-11.16	-11.15	-11.14	-11.13	-11.12	-11.11
-11.19	-11.18	-11.17	-11.16	-11.15	-11.14	-11.13	-11.12	-11.11
-11.19	-11.18	-11.17	-11.16	-11.15	-11.14	-11.13	-11.12	-11.11
-11.19	-11.18	-11.17	-11.16	-11.15	-11.14	-11.13	-11.12	-11.11
-11.19	-11.18	-11.17	-11.16	-11.15	-11.14	-11.13	-11.12	-11.11
-11.19	-11.18	-11.17	-11.16	-11.15	-11.14	-11.13	-11.12	-11.11
-11.19	-11.18	-11.17	-11.16	-11.15	-11.14	-11.13	-11.12	-11.11
-11.19	-11.18	-11.17	-11.16	-11.15	-11.14	-11.13	-11.12	-11.11
-11.19	-11.18	-11.17	-11.16	-11.15	-11.14	-11.13	-11.12	-11.11
-11.19	-11.18	-11.17	-11.16	-11.15	-11.14	-11.13	-11.12	-11.11
-11.19	-11.18	-11.17	-11.16	-11.15	-11.14	-11.13	-11.12	-11.11
-11.19	-11.18	-11.17	-11.16	-11.15	-11.14	-11.13	-11.12	-11.11
-11.19	-11.18	-11.17	-11.16	-11.15	-11.14	-11.13	-11.12	-11.11
-11.19	-11.18	-11.17	-11.16	-11.15	-11.14	-11.13	-11.12	-11.11
-11.19	-11.18	-11.17	-11.16	-11.15	-11.14	-11.13	-11.12	-11.11
-11.19	-11.18	-11.17	-11.16	-11.15	-11.14	-11.13	-11.12	-11.11
-11.19	-11.18	-11.17	-11.16	-11.15	-11.14	-11.13	-11.12	-11.11
-11.19	-11.18	-11.17	-11.16	-11.15	-11.14	-11.13	-11.12	-11.11
-11.19	-11.18	-11.17	-11.16	-11.15	-11.14	-11.13	-11.12	-11.11
-11.19	-11.18	-11.17	-11.16	-11.15	-11.14	-11.13	-11.12	-11.11
-11.19	-11.18	-11.17	-11.16	-11.15	-11.14	-11.13	-11.12	-11.11
-11.19	-11.18	-11.17	-11.16	-11.15	-11.14	-11.13	-11.12	-11.11
-11.19	-11.18	-11.17	-11.16	-11.15	-11.14	-11.13	-11.12	-11.11
-11.19	-11.18	-11.17	-11.16	-11.15	-11.14	-11.13	-11.12	-11.11
-11.19	-11.18	-11.17	-11.16	-11.15	-11.14	-11.13	-11.12	-11.11
-11.19	-11.18	-11.17	-11.16	-11.15	-11.14	-11.13	-11.12	-11.11
-11.19	-11.18	-11.17	-11.16	-11.15	-11.14	-11.13	-11.12	-11.11
-11.19	-11.18	-11.17	-11.16	-11.15	-11.14	-11.13	-11.12	-11.11
-11.19	-11.18	-11.17	-11.16	-11.15	-11.14	-11.13	-11.12	-11.11
-11.19	-11.18	-11.17	-11.16	-11.15	-11.14	-11.13	-11.12	-11.11
-11.19	-11.18	-11.17	-11.16	-11.15	-11.14	-11.13	-11.12	-11.11
-11.19	-11.18	-11.17	-11.16	-11.15	-11.14	-11.13	-11.12	-11.11
-11.19	-11.18	-11.17	-11.16	-11.15	-11.14	-11.13	-11.12	-11.11
-11.19	-11.18	-11.17	-11.16	-11.15	-11.14	-11.13	-11.12	-11.11
-11.19	-11.18	-11.17	-11.16	-11.15	-11.14	-11.13	-11.12	-11.11
-11.19	-11.18	-11.17	-11.16	-11.15	-11.14	-11.13	-11.12	-11.11
-11.19	-11.18	-11.17	-11.16	-11.15	-11.14	-11.13	-11.12	-11.11
-11.19	-11.18	-11.17	-11.16	-11.15	-11.14	-11.13	-11.12	-11.11
-11.19	-11.18	-11.17	-11.16	-11.15	-11.14	-11.13	-11.12	-11.11
-11.19	-11.18	-11.17	-11.16	-11.15	-11.14	-11.13	-11.12	-11.11

-11.19	-11.18	-11.17	-11.16	-11.15	-11.14	-11.13	-11.12	-11.11
-11.19	-11.18	-11.17	-11.16	-11.15	-11.14	-11.13	-11.12	-11.11
-11.19	-11.18	-11.17	-11.16	-11.15	-11.14	-11.13	-11.12	-11.11
-11.19	-11.18	-11.17	-11.16	-11.15	-11.14	-11.13	-11.12	-11.11
-11.19	-11.18	-11.17	-11.16	-11.15	-11.14	-11.13	-11.12	-11.11
-11.19	-11.18	-11.17	-11.16	-11.15	-11.14	-11.13	-11.12	-11.11
-11.19	-11.18	-11.17	-11.16	-11.15	-11.14	-11.13	-11.12	-11.11
-11.19	-11.18	-11.17	-11.16	-11.15	-11.14	-11.13	-11.12	-11.11
-11.19	-11.18	-11.17	-11.16	-11.15	-11.14	-11.13	-11.12	-11.11
-11.19	-11.18	-11.17	-11.16	-11.15	-11.14	-11.13	-11.12	-11.11
-11.19	-11.18	-11.17	-11.16	-11.15	-11.14	-11.13	-11.12	-11.11
-11.19	-11.18	-11.17	-11.16	-11.15	-11.14	-11.13	-11.12	-11.11
-11.19	-11.18	-11.17	-11.16	-11.15	-11.14	-11.13	-11.12	-11.11
-11.19	-11.18	-11.17	-11.16	-11.15	-11.14	-11.13	-11.12	-11.11
-11.19	-11.18	-11.17	-11.16	-11.15	-11.14	-11.13	-11.12	-11.11
-11.19	-11.18	-11.17	-11.16	-11.15	-11.14	-11.13	-11.12	-11.11
-11.19	-11.18	-11.17	-11.16	-11.15	-11.14	-11.13	-11.12	-11.11
-11.19	-11.18	-11.17	-11.16	-11.15	-11.14	-11.13	-11.12	-11.11
-11.19	-11.18	-11.17	-11.16	-11.15	-11.14	-11.13	-11.12	-11.11
-11.19	-11.18	-11.17	-11.16	-11.15	-11.14	-11.13	-11.12	-11.11
-11.19	-11.18	-11.17	-11.16	-11.15	-11.14	-11.13	-11.12	-11.11
-11.19	-11.18	-11.17	-11.16	-11.15	-11.14	-11.13	-11.12	-11.11
-11.19	-11.18	-11.17	-11.16	-11.15	-11.14	-11.13	-11.12	-11.11
-11.19	-11.18	-11.17	-11.16	-11.15	-11.14	-11.13	-11.12	-11.11
-11.19	-11.18	-11.17	-11.16	-11.15	-11.14	-11.13	-11.12	-11.11
-11.19	-11.18	-11.17	-11.16	-11.15	-11.14	-11.13	-11.12	-11.11
-11.19	-11.18	-11.17	-11.16	-11.15	-11.14	-11.13	-11.12	-11.11
-11.19	-11.18	-11.17	-11.16	-11.15	-11.14	-11.13	-11.12	-11.11
-11.19	-11.18	-11.17	-11.16	-11.15	-11.14	-11.13	-11.12	-11.11
-11.19	-11.18	-11.17	-11.16	-11.15	-11.14	-11.13	-11.12	-11.11
-11.19	-11.18	-11.17	-11.16	-11.15	-11.14	-11.13	-11.12	-11.11
-11.19	-11.18	-11.17	-11.16	-11.15	-11.14	-11.13	-11.12	-11.11
-11.19	-11.18	-11.17	-11.16	-11.15	-11.14	-11.13	-11.12	-11.11
-11.19	-11.18	-11.17	-11.16	-11.15	-11.14	-11.13	-11.12	-11.11
-11.19	-11.18	-11.17	-11.16	-11.15	-11.14	-11.13	-11.12	-11.11
-11.19	-11.18	-11.17	-11.16	-11.15	-11.14	-11.13	-11.12	-11.11
-11.19	-11.18	-11.17	-11.16	-11.15	-11.14	-11.13	-11.12	-11.11
-11.19	-11.18	-11.17	-11.16	-11.15	-11.14	-11.13	-11.12	-11.11
-11.19	-11.18	-11.17	-11.16	-11.15	-11.14	-11.13	-11.12	-11.11
-11.19	-11.18	-11.17	-11.16	-11.15	-11.14	-11.13	-11.12	-11.11
-11.19	-11.18	-11.17	-11.16	-11.15	-11.14	-11.13	-11.12	-11.11
-11.19	-11.18	-11.17	-11.16	-11.15	-11.14	-11.13	-11.12	-11.11
-11.19	-11.18	-11.17	-11.16	-11.15	-11.14	-11.13	-11.12	-11.11
-11.19	-11.18	-11.17	-11.16	-11.15	-11.14	-11.13	-11.12	-11.11
-11.19	-11.18	-11.17	-11.16	-11.15	-11.14	-11.13	-11.12	-11.11
-11.19	-11.18	-11.17	-11.16	-11.15	-11.14	-11.13	-11.12	-11.11
-11.19	-11.18	-11.17	-11.16	-11.15	-11.14	-11.13	-11.12	-11.11
-11.19	-11.18	-11.17	-11.16	-11.15	-11.14	-11.13	-11.12	-11.11
-11.19	-11.18	-11.17	-11.16	-11.15	-11.14	-11.13	-11.12	-11.11
-11.19	-11.18	-11.17	-11.16	-11.15	-11.14	-11.13	-11.12	-11.11

-11.19	-11.18	-11.17	-11.16	-11.15	-11.14	-11.13	-11.12	-11.11
-11.19	-11.18	-11.17	-11.16	-11.15	-11.14	-11.13	-11.12	-11.11
-11.19	-11.18	-11.17	-11.16	-11.15	-11.14	-11.13	-11.12	-11.11
-11.19	-11.18	-11.17	-11.16	-11.15	-11.14	-11.13	-11.12	-11.11
-11.19	-11.18	-11.17	-11.16	-11.15	-11.14	-11.13	-11.12	-11.11
-11.19	-11.18	-11.17	-11.16	-11.15	-11.14	-11.13	-11.12	-11.11
-11.19	-11.18	-11.17	-11.16	-11.15	-11.14	-11.13	-11.12	-11.11
-11.19	-11.18	-11.17	-11.16	-11.15	-11.14	-11.13	-11.12	-11.11
-11.19	-11.18	-11.17	-11.16	-11.15	-11.14	-11.13	-11.12	-11.11
-11.19	-11.18	-11.17	-11.16	-11.15	-11.14	-11.13	-11.12	-11.11
-11.19	-11.18	-11.17	-11.16	-11.15	-11.14	-11.13	-11.12	-11.11
-11.19	-11.18	-11.17	-11.16	-11.15	-11.14	-11.13	-11.12	-11.11
-11.19	-11.18	-11.17	-11.16	-11.15	-11.14	-11.13	-11.12	-11.11
-11.19	-11.18	-11.17	-11.16	-11.15	-11.14	-11.13	-11.12	-11.11
-11.19	-11.18	-11.17	-11.16	-11.15	-11.14	-11.13	-11.12	-11.11
-11.19	-11.18	-11.17	-11.16	-11.15	-11.14	-11.13	-11.12	-11.11
-11.19	-11.18	-11.17	-11.16	-11.15	-11.14	-11.13	-11.12	-11.11
-11.19	-11.18	-11.17	-11.16	-11.15	-11.14	-11.13	-11.12	-11.11
-11.19	-11.18	-11.17	-11.16	-11.15	-11.14	-11.13	-11.12	-11.11
-11.19	-11.18	-11.17	-11.16	-11.15	-11.14	-11.13	-11.12	-11.11
-11.19	-11.18	-11.17	-11.16	-11.15	-11.14	-11.13	-11.12	-11.11
-11.19	-11.18	-11.17	-11.16	-11.15	-11.14	-11.13	-11.12	-11.11
-11.19	-11.18	-11.17	-11.16	-11.15	-11.14	-11.13	-11.12	-11.11
-11.19	-11.18	-11.17	-11.16	-11.15	-11.14	-11.13	-11.12	-11.11
-11.19	-11.18	-11.17	-11.16	-11.15	-11.14	-11.13	-11.12	-11.11
-11.19	-11.18	-11.17	-11.16	-11.15	-11.14	-11.13	-11.12	-11.11
-11.19	-11.18	-11.17	-11.16	-11.15	-11.14	-11.13	-11.12	-11.11
-11.19	-11.18	-11.17	-11.16	-11.15	-11.14	-11.13	-11.12	-11.11
-11.19	-11.18	-11.17	-11.16	-11.15	-11.14	-11.13	-11.12	-11.11
-11.19	-11.18	-11.17	-11.16	-11.15	-11.14	-11.13	-11.12	-11.11
-11.19	-11.18	-11.17	-11.16	-11.15	-11.14	-11.13	-11.12	-11.11
-11.19	-11.18	-11.17	-11.16	-11.15	-11.14	-11.13	-11.12	-11.11
-11.19	-11.18	-11.17	-11.16	-11.15	-11.14	-11.13	-11.12	-11.11
-11.19	-11.18	-11.17	-11.16	-11.15	-11.14	-11.13	-11.12	-11.11
-11.19	-11.18	-11.17	-11.16	-11.15	-11.14	-11.13	-11.12	-11.11
-11.19	-11.18	-11.17	-11.16	-11.15	-11.14	-11.13	-11.12	-11.11
-11.19	-11.18	-11.17	-11.16	-11.15	-11.14	-11.13	-11.12	-11.11
-11.19	-11.18	-11.17	-11.16	-11.15	-11.14	-11.13	-11.12	-11.11
-11.19	-11.18	-11.17	-11.16	-11.15	-11.14	-11.13	-11.12	-11.11
-11.19	-11.18	-11.17	-11.16	-11.15	-11.14	-11.13	-11.12	-11.11
-11.19	-11.18	-11.17	-11.16	-11.15	-11.14	-11.13	-11.12	-11.11
-11.19	-11.18	-11.17	-11.16	-11.15	-11.14	-11.13	-11.12	-11.11
-11.19	-11.18	-11.17	-11.16	-11.15	-11.14	-11.13	-11.12	-11.11
-11.19	-11.18	-11.17	-11.16	-11.15	-11.14	-11.13	-11.12	-11.11
-11.19	-11.18	-11.17	-11.16	-11.15	-11.14	-11.13	-11.12	-11.11
-11.19	-11.18	-11.17	-11.16	-11.15	-11.14	-11.13	-11.12	-11.11
-11.19	-11.18	-11.17	-11.16	-11.15	-11.14	-11.13	-11.12	-11.11
-11.19	-11.18	-11.17	-11.16	-11.15	-11.14	-11.13	-11.12	-11.11
-11.19	-11.18	-11.17	-11.16	-11.15	-11.14	-11.13	-11.12	-11.11
-11.19	-11.18	-11.17	-11.16	-11.15	-11.14	-11.13	-11.12	-11.11

-11.19	-11.18	-11.17	-11.16	-11.15	-11.14	-11.13	-11.12	-11.11
-11.19	-11.18	-11.17	-11.16	-11.15	-11.14	-11.13	-11.12	-11.11
-11.19	-11.18	-11.17	-11.16	-11.15	-11.14	-11.13	-11.12	-11.11
-11.19	-11.18	-11.17	-11.16	-11.15	-11.14	-11.13	-11.12	-11.11
-11.19	-11.18	-11.17	-11.16	-11.15	-11.14	-11.13	-11.12	-11.11
-11.19	-11.18	-11.17	-11.16	-11.15	-11.14	-11.13	-11.12	-11.11
-11.19	-11.18	-11.17	-11.16	-11.15	-11.14	-11.13	-11.12	-11.11
-11.19	-11.18	-11.17	-11.16	-11.15	-11.14	-11.13	-11.12	-11.11
-11.19	-11.18	-11.17	-11.16	-11.15	-11.14	-11.13	-11.12	-11.11
-11.19	-11.18	-11.17	-11.16	-11.15	-11.14	-11.13	-11.12	-11.11
-11.19	-11.18	-11.17	-11.16	-11.15	-11.14	-11.13	-11.12	-11.11
-11.19	-11.18	-11.17	-11.16	-11.15	-11.14	-11.13	-11.12	-11.11
-11.19	-11.18	-11.17	-11.16	-11.15	-11.14	-11.13	-11.12	-11.11
-11.19	-11.18	-11.17	-11.16	-11.15	-11.14	-11.13	-11.12	-11.11
-11.19	-11.18	-11.17	-11.16	-11.15	-11.14	-11.13	-11.12	-11.11
-11.19	-11.18	-11.17	-11.16	-11.15	-11.14	-11.13	-11.12	-11.11
-11.19	-11.18	-11.17	-11.16	-11.15	-11.14	-11.13	-11.12	-11.11
-11.19	-11.18	-11.17	-11.16	-11.15	-11.14	-11.13	-11.12	-11.11
-11.19	-11.18	-11.17	-11.16	-11.15	-11.14	-11.13	-11.12	-11.11
-11.19	-11.18	-11.17	-11.16	-11.15	-11.14	-11.13	-11.12	-11.11
-11.19	-11.18	-11.17	-11.16	-11.15	-11.14	-11.13	-11.12	-11.11
-11.19	-11.18	-11.17	-11.16	-11.15	-11.14	-11.13	-11.12	-11.11
-11.19	-11.18	-11.17	-11.16	-11.15	-11.14	-11.13	-11.12	-11.11
-11.19	-11.18	-11.17	-11.16	-11.15	-11.14	-11.13	-11.12	-11.11
-11.19	-11.18	-11.17	-11.16	-11.15	-11.14	-11.13	-11.12	-11.11
-11.19	-11.18	-11.17	-11.16	-11.15	-11.14	-11.13	-11.12	-11.11
-11.19	-11.18	-11.17	-11.16	-11.15	-11.14	-11.13	-11.12	-11.11
-11.19	-11.18	-11.17	-11.16	-11.15	-11.14	-11.13	-11.12	-11.11
-11.19	-11.18	-11.17	-11.16	-11.15	-11.14	-11.13	-11.12	-11.11
-11.19	-11.18	-11.17	-11.16	-11.15	-11.14	-11.13	-11.12	-11.11
-11.19	-11.18	-11.17	-11.16	-11.15	-11.14	-11.13	-11.12	-11.11
-11.19	-11.18	-11.17	-11.16	-11.15	-11.14	-11.13	-11.12	-11.11
-11.19	-11.18	-11.17	-11.16	-11.15	-11.14	-11.13	-11.12	-11.11
-11.19	-11.18	-11.17	-11.16	-11.15	-11.14	-11.13	-11.12	-11.11
-11.19	-11.18	-11.17	-11.16	-11.15	-11.14	-11.13	-11.12	-11.11
-11.19	-11.18	-11.17	-11.16	-11.15	-11.14	-11.13	-11.12	-11.11
-11.19	-11.18	-11.17	-11.16	-11.15	-11.14	-11.13	-11.12	-11.11
-11.19	-11.18	-11.17	-11.16	-11.15	-11.14	-11.13	-11.12	-11.11
-11.19	-11.18	-11.17	-11.16	-11.15	-11.14	-11.13	-11.12	-11.11
-11.19	-11.18	-11.17	-11.16	-11.15	-11.14	-11.13	-11.12	-11.11
-11.19	-11.18	-11.17	-11.16	-11.15	-11.14	-11.13	-11.12	-11.11
-11.19	-11.18	-11.17	-11.16	-11.15	-11.14	-11.13	-11.12	-11.11
-11.19	-11.18	-11.17	-11.16	-11.15	-11.14	-11.13	-11.12	-11.11
-11.19	-11.18	-11.17	-11.16	-11.15	-11.14	-11.13	-11.12	-11.11
-11.19	-11.18	-11.17	-11.16	-11.15	-11.14	-11.13	-11.12	-11.11
-11.19	-11.18	-11.17	-11.16	-11.15	-11.14	-11.13	-11.12	-11.11
-11.19	-11.18	-11.17	-11.16	-11.15	-11.14	-11.13	-11.12	-11.11
-11.19	-11.18	-11.17	-11.16	-11.15	-11.14	-11.13	-11.12	-11.11
-11.19	-11.18	-11.17	-11.16	-11.15	-11.14	-11.13	-11.12	-11.11
-11.19	-11.18	-11.17	-11.16	-11.15	-11.14	-11.13	-11.12	-11.11

-11.19	-11.18	-11.17	-11.16	-11.15	-11.14	-11.13	-11.12	-11.11
-11.19	-11.18	-11.17	-11.16	-11.15	-11.14	-11.13	-11.12	-11.11
-11.19	-11.18	-11.17	-11.16	-11.15	-11.14	-11.13	-11.12	-11.11
-11.19	-11.18	-11.17	-11.16	-11.15	-11.14	-11.13	-11.12	-11.11
-11.19	-11.18	-11.17	-11.16	-11.15	-11.14	-11.13	-11.12	-11.11
-11.19	-11.18	-11.17	-11.16	-11.15	-11.14	-11.13	-11.12	-11.11
-11.19	-11.18	-11.17	-11.16	-11.15	-11.14	-11.13	-11.12	-11.11
-11.19	-11.18	-11.17	-11.16	-11.15	-11.14	-11.13	-11.12	-11.11
-11.19	-11.18	-11.17	-11.16	-11.15	-11.14	-11.13	-11.12	-11.11
-11.19	-11.18	-11.17	-11.16	-11.15	-11.14	-11.13	-11.12	-11.11
-11.19	-11.18	-11.17	-11.16	-11.15	-11.14	-11.13	-11.12	-11.11
-11.19	-11.18	-11.17	-11.16	-11.15	-11.14	-11.13	-11.12	-11.11
-11.19	-11.18	-11.17	-11.16	-11.15	-11.14	-11.13	-11.12	-11.11
-11.19	-11.18	-11.17	-11.16	-11.15	-11.14	-11.13	-11.12	-11.11
-11.19	-11.18	-11.17	-11.16	-11.15	-11.14	-11.13	-11.12	-11.11
-11.19	-11.18	-11.17	-11.16	-11.15	-11.14	-11.13	-11.12	-11.11
-11.19	-11.18	-11.17	-11.16	-11.15	-11.14	-11.13	-11.12	-11.11
-11.19	-11.18	-11.17	-11.16	-11.15	-11.14	-11.13	-11.12	-11.11
-11.19	-11.18	-11.17	-11.16	-11.15	-11.14	-11.13	-11.12	-11.11
-11.19	-11.18	-11.17	-11.16	-11.15	-11.14	-11.13	-11.12	-11.11
-11.19	-11.18	-11.17	-11.16	-11.15	-11.14	-11.13	-11.12	-11.11
-11.19	-11.18	-11.17	-11.16	-11.15	-11.14	-11.13	-11.12	-11.11
-11.19	-11.18	-11.17	-11.16	-11.15	-11.14	-11.13	-11.12	-11.11
-11.19	-11.18	-11.17	-11.16	-11.15	-11.14	-11.13	-11.12	-11.11
-11.19	-11.18	-11.17	-11.16	-11.15	-11.14	-11.13	-11.12	-11.11
-11.19	-11.18	-11.17	-11.16	-11.15	-11.14	-11.13	-11.12	-11.11
-11.19	-11.18	-11.17	-11.16	-11.15	-11.14	-11.13	-11.12	-11.11
-11.19	-11.18	-11.17	-11.16	-11.15	-11.14	-11.13	-11.12	-11.11
-11.19	-11.18	-11.17	-11.16	-11.15	-11.14	-11.13	-11.12	-11.11
-11.19	-11.18	-11.17	-11.16	-11.15	-11.14	-11.13	-11.12	-11.11
-11.19	-11.18	-11.17	-11.16	-11.15	-11.14	-11.13	-11.12	-11.11
-11.19	-11.18	-11.17	-11.16	-11.15	-11.14	-11.13	-11.12	-11.11
-11.19	-11.18	-11.17	-11.16	-11.15	-11.14	-11.13	-11.12	-11.11
-11.19	-11.18	-11.17	-11.16	-11.15	-11.14	-11.13	-11.12	-11.11
-11.19	-11.18	-11.17	-11.16	-11.15	-11.14	-11.13	-11.12	-11.11
-11.19	-11.18	-11.17	-11.16	-11.15	-11.14	-11.13	-11.12	-11.11
-11.19	-11.18	-11.17	-11.16	-11.15	-11.14	-11.13	-11.12	-11.11
-11.19	-11.18	-11.17	-11.16	-11.15	-11.14	-11.13	-11.12	-11.11
-11.19	-11.18	-11.17	-11.16	-11.15	-11.14	-11.13	-11.12	-11.11
-11.19	-11.18	-11.17	-11.16	-11.15	-11.14	-11.13	-11.12	-11.11
-11.19	-11.18	-11.17	-11.16	-11.15	-11.14	-11.13	-11.12	-11.11
-11.19	-11.18	-11.17	-11.16	-11.15	-11.14	-11.13	-11.12	-11.11
-11.19	-11.18	-11.17	-11.16	-11.15	-11.14	-11.13	-11.12	-11.11
-11.19	-11.18	-11.17	-11.16	-11.15	-11.14	-11.13	-11.12	-11.11
-11.19	-11.18	-11.17	-11.16	-11.15	-11.14	-11.13	-11.12	-11.11
-11.19	-11.18	-11.17	-11.16	-11.15	-11.14	-11.13	-11.12	-11.11
-11.19	-11.18	-11.17	-11.16	-11.15	-11.14	-11.13	-11.12	-11.11
-11.19	-11.18	-11.17	-11.16	-11.15	-11.14	-11.13	-11.12	-11.11
-11.19	-11.18	-11.17	-11.16	-11.15	-11.14	-11.13	-11.12	-11.11
-11.19	-11.18	-11.17	-11.16	-11.15	-11.14	-11.13	-11.12	-11.11

-11.19	-11.18	-11.17	-11.16	-11.15	-11.14	-11.13	-11.12	-11.11
-11.19	-11.18	-11.17	-11.16	-11.15	-11.14	-11.13	-11.12	-11.11
-11.19	-11.18	-11.17	-11.16	-11.15	-11.14	-11.13	-11.12	-11.11
-11.19	-11.18	-11.17	-11.16	-11.15	-11.14	-11.13	-11.12	-11.11
-11.19	-11.18	-11.17	-11.16	-11.15	-11.14	-11.13	-11.12	-11.11
-11.19	-11.18	-11.17	-11.16	-11.15	-11.14	-11.13	-11.12	-11.11
-11.19	-11.18	-11.17	-11.16	-11.15	-11.14	-11.13	-11.12	-11.11
-11.19	-11.18	-11.17	-11.16	-11.15	-11.14	-11.13	-11.12	-11.11
-11.19	-11.18	-11.17	-11.16	-11.15	-11.14	-11.13	-11.12	-11.11
-11.19	-11.18	-11.17	-11.16	-11.15	-11.14	-11.13	-11.12	-11.11
-11.19	-11.18	-11.17	-11.16	-11.15	-11.14	-11.13	-11.12	-11.11
-11.19	-11.18	-11.17	-11.16	-11.15	-11.14	-11.13	-11.12	-11.11
-11.19	-11.18	-11.17	-11.16	-11.15	-11.14	-11.13	-11.12	-11.11
-11.19	-11.18	-11.17	-11.16	-11.15	-11.14	-11.13	-11.12	-11.11
-11.19	-11.18	-11.17	-11.16	-11.15	-11.14	-11.13	-11.12	-11.11
-11.19	-11.18	-11.17	-11.16	-11.15	-11.14	-11.13	-11.12	-11.11
-11.19	-11.18	-11.17	-11.16	-11.15	-11.14	-11.13	-11.12	-11.11
-11.19	-11.18	-11.17	-11.16	-11.15	-11.14	-11.13	-11.12	-11.11
-11.19	-11.18	-11.17	-11.16	-11.15	-11.14	-11.13	-11.12	-11.11
-11.19	-11.18	-11.17	-11.16	-11.15	-11.14	-11.13	-11.12	-11.11
-11.19	-11.18	-11.17	-11.16	-11.15	-11.14	-11.13	-11.12	-11.11
-11.19	-11.18	-11.17	-11.16	-11.15	-11.14	-11.13	-11.12	-11.11
-11.19	-11.18	-11.17	-11.16	-11.15	-11.14	-11.13	-11.12	-11.11
-11.19	-11.18	-11.17	-11.16	-11.15	-11.14	-11.13	-11.12	-11.11
-11.19	-11.18	-11.17	-11.16	-11.15	-11.14	-11.13	-11.12	-11.11
-11.19	-11.18	-11.17	-11.16	-11.15	-11.14	-11.13	-11.12	-11.11
-11.19	-11.18	-11.17	-11.16	-11.15	-11.14	-11.13	-11.12	-11.11
-11.19	-11.18	-11.17	-11.16	-11.15	-11.14	-11.13	-11.12	-11.11
-11.19	-11.18	-11.17	-11.16	-11.15	-11.14	-11.13	-11.12	-11.11
-11.19	-11.18	-11.17	-11.16	-11.15	-11.14	-11.13	-11.12	-11.11
-11.19	-11.18	-11.17	-11.16	-11.15	-11.14	-11.13	-11.12	-11.11
-11.19	-11.18	-11.17	-11.16	-11.15	-11.14	-11.13	-11.12	-11.11
-11.19	-11.18	-11.17	-11.16	-11.15	-11.14	-11.13	-11.12	-11.11
-11.19	-11.18	-11.17	-11.16	-11.15	-11.14	-11.13	-11.12	-11.11
-11.19	-11.18	-11.17	-11.16	-11.15	-11.14	-11.13	-11.12	-11.11
-11.19	-11.18	-11.17	-11.16	-11.15	-11.14	-11.13	-11.12	-11.11
-11.19	-11.18	-11.17	-11.16	-11.15	-11.14	-11.13	-11.12	-11.11
-11.19	-11.18	-11.17	-11.16	-11.15	-11.14	-11.13	-11.12	-11.11
-11.19	-11.18	-11.17	-11.16	-11.15	-11.14	-11.13	-11.12	-11.11
-11.19	-11.18	-11.17	-11.16	-11.15	-11.14	-11.13	-11.12	-11.11
-11.19	-11.18	-11.17	-11.16	-11.15	-11.14	-11.13	-11.12	-11.11
-11.19	-11.18	-11.17	-11.16	-11.15	-11.14	-11.13	-11.12	-11.11
-11.19	-11.18	-11.17	-11.16	-11.15	-11.14	-11.13	-11.12	-11.11
-11.19	-11.18	-11.17	-11.16	-11.15	-11.14	-11.13	-11.12	-11.11
-11.19	-11.18	-11.17	-11.16	-11.15	-11.14	-11.13	-11.12	-11.11
-11.19	-11.18	-11.17	-11.16	-11.15	-11.14	-11.13	-11.12	-11.11
-11.19	-11.18	-11.17	-11.16	-11.15	-11.14	-11.13	-11.12	-11.11
-11.19	-11.18	-11.17	-11.16	-11.15	-11.14	-11.13	-11.12	-11.11
-11.19	-11.18	-11.17	-11.16	-11.15	-11.14	-11.13	-11.12	-11.11
-11.19	-11.18	-11.17	-11.16	-11.15	-11.14	-11.13	-11.12	-11.11

-11.19	-11.18	-11.17	-11.16	-11.15	-11.14	-11.13	-11.12	-11.11
-11.19	-11.18	-11.17	-11.16	-11.15	-11.14	-11.13	-11.12	-11.11
-11.19	-11.18	-11.17	-11.16	-11.15	-11.14	-11.13	-11.12	-11.11
-11.19	-11.18	-11.17	-11.16	-11.15	-11.14	-11.13	-11.12	-11.11
-11.19	-11.18	-11.17	-11.16	-11.15	-11.14	-11.13	-11.12	-11.11
-11.19	-11.18	-11.17	-11.16	-11.15	-11.14	-11.13	-11.12	-11.11
-11.19	-11.18	-11.17	-11.16	-11.15	-11.14	-11.13	-11.12	-11.11
-11.19	-11.18	-11.17	-11.16	-11.15	-11.14	-11.13	-11.12	-11.11
-11.19	-11.18	-11.17	-11.16	-11.15	-11.14	-11.13	-11.12	-11.11
-11.19	-11.18	-11.17	-11.16	-11.15	-11.14	-11.13	-11.12	-11.11
-11.19	-11.18	-11.17	-11.16	-11.15	-11.14	-11.13	-11.12	-11.11
-11.19	-11.18	-11.17	-11.16	-11.15	-11.14	-11.13	-11.12	-11.11
-11.19	-11.18	-11.17	-11.16	-11.15	-11.14	-11.13	-11.12	-11.11
-11.19	-11.18	-11.17	-11.16	-11.15	-11.14	-11.13	-11.12	-11.11
-11.19	-11.18	-11.17	-11.16	-11.15	-11.14	-11.13	-11.12	-11.11
-11.19	-11.18	-11.17	-11.16	-11.15	-11.14	-11.13	-11.12	-11.11
-11.19	-11.18	-11.17	-11.16	-11.15	-11.14	-11.13	-11.12	-11.11
-11.19	-11.18	-11.17	-11.16	-11.15	-11.14	-11.13	-11.12	-11.11
-11.19	-11.18	-11.17	-11.16	-11.15	-11.14	-11.13	-11.12	-11.11
-11.19	-11.18	-11.17	-11.16	-11.15	-11.14	-11.13	-11.12	-11.11
-11.19	-11.18	-11.17	-11.16	-11.15	-11.14	-11.13	-11.12	-11.11
-11.19	-11.18	-11.17	-11.16	-11.15	-11.14	-11.13	-11.12	-11.11
-11.19	-11.18	-11.17	-11.16	-11.15	-11.14	-11.13	-11.12	-11.11
-11.19	-11.18	-11.17	-11.16	-11.15	-11.14	-11.13	-11.12	-11.11
-11.19	-11.18	-11.17	-11.16	-11.15	-11.14	-11.13	-11.12	-11.11
-11.19	-11.18	-11.17	-11.16	-11.15	-11.14	-11.13	-11.12	-11.11
-11.19	-11.18	-11.17	-11.16	-11.15	-11.14	-11.13	-11.12	-11.11
-11.19	-11.18	-11.17	-11.16	-11.15	-11.14	-11.13	-11.12	-11.11
-11.19	-11.18	-11.17	-11.16	-11.15	-11.14	-11.13	-11.12	-11.11
-11.19	-11.18	-11.17	-11.16	-11.15	-11.14	-11.13	-11.12	-11.11
-11.19	-11.18	-11.17	-11.16	-11.15	-11.14	-11.13	-11.12	-11.11
-11.19	-11.18	-11.17	-11.16	-11.15	-11.14	-11.13	-11.12	-11.11
-11.19	-11.18	-11.17	-11.16	-11.15	-11.14	-11.13	-11.12	-11.11
-11.19	-11.18	-11.17	-11.16	-11.15	-11.14	-11.13	-11.12	-11.11
-11.19	-11.18	-11.17	-11.16	-11.15	-11.14	-11.13	-11.12	-11.11
-11.19	-11.18	-11.17	-11.16	-11.15	-11.14	-11.13	-11.12	-11.11
-11.19	-11.18	-11.17	-11.16	-11.15	-11.14	-11.13	-11.12	-11.11
-11.19	-11.18	-11.17	-11.16	-11.15	-11.14	-11.13	-11.12	-11.11
-11.19	-11.18	-11.17	-11.16	-11.15	-11.14	-11.13	-11.12	-11.11
-11.19	-11.18	-11.17	-11.16	-11.15	-11.14	-11.13	-11.12	-11.11
-11.19	-11.18	-11.17	-11.16	-11.15	-11.14	-11.13	-11.12	-11.11
-11.19	-11.18	-11.17	-11.16	-11.15	-11.14	-11.13	-11.12	-11.11
-11.19	-11.18	-11.17	-11.16	-11.15	-11.14	-11.13	-11.12	-11.11
-11.19	-11.18	-11.17	-11.16	-11.15	-11.14	-11.13	-11.12	-11.11
-11.19	-11.18	-11.17	-11.16	-11.15	-11.14	-11.13	-11.12	-11.11
-11.19	-11.18	-11.17	-11.16	-11.15	-11.14	-11.13	-11.12	-11.11
-11.19	-11.18	-11.17	-11.16	-11.15	-11.14	-11.13	-11.12	-11.11
-11.19	-11.18	-11.17	-11.16	-11.15	-11.14	-11.13	-11.12	-11.11
-11.19	-11.18	-11.17	-11.16	-11.15	-11.14	-11.13	-11.12	-11.11
-11.19	-11.18	-11.17	-11.16	-11.15	-11.14	-11.13	-11.12	-11.11

-11.19	-11.18	-11.17	-11.16	-11.15	-11.14	-11.13	-11.12	-11.11
-11.19	-11.18	-11.17	-11.16	-11.15	-11.14	-11.13	-11.12	-11.11
-11.19	-11.18	-11.17	-11.16	-11.15	-11.14	-11.13	-11.12	-11.11
-11.19	-11.18	-11.17	-11.16	-11.15	-11.14	-11.13	-11.12	-11.11
-11.19	-11.18	-11.17	-11.16	-11.15	-11.14	-11.13	-11.12	-11.11
-11.19	-11.18	-11.17	-11.16	-11.15	-11.14	-11.13	-11.12	-11.11
-11.19	-11.18	-11.17	-11.16	-11.15	-11.14	-11.13	-11.12	-11.11
-11.19	-11.18	-11.17	-11.16	-11.15	-11.14	-11.13	-11.12	-11.11
-11.19	-11.18	-11.17	-11.16	-11.15	-11.14	-11.13	-11.12	-11.11
-11.19	-11.18	-11.17	-11.16	-11.15	-11.14	-11.13	-11.12	-11.11
-11.19	-11.18	-11.17	-11.16	-11.15	-11.14	-11.13	-11.12	-11.11
-11.19	-11.18	-11.17	-11.16	-11.15	-11.14	-11.13	-11.12	-11.11
-11.19	-11.18	-11.17	-11.16	-11.15	-11.14	-11.13	-11.12	-11.11
-11.19	-11.18	-11.17	-11.16	-11.15	-11.14	-11.13	-11.12	-11.11
-11.19	-11.18	-11.17	-11.16	-11.15	-11.14	-11.13	-11.12	-11.11
-11.19	-11.18	-11.17	-11.16	-11.15	-11.14	-11.13	-11.12	-11.11
-11.19	-11.18	-11.17	-11.16	-11.15	-11.14	-11.13	-11.12	-11.11
-11.19	-11.18	-11.17	-11.16	-11.15	-11.14	-11.13	-11.12	-11.11
-11.19	-11.18	-11.17	-11.16	-11.15	-11.14	-11.13	-11.12	-11.11
-11.19	-11.18	-11.17	-11.16	-11.15	-11.14	-11.13	-11.12	-11.11
-11.19	-11.18	-11.17	-11.16	-11.15	-11.14	-11.13	-11.12	-11.11
-11.19	-11.18	-11.17	-11.16	-11.15	-11.14	-11.13	-11.12	-11.11
-11.19	-11.18	-11.17	-11.16	-11.15	-11.14	-11.13	-11.12	-11.11
-11.19	-11.18	-11.17	-11.16	-11.15	-11.14	-11.13	-11.12	-11.11
-11.19	-11.18	-11.17	-11.16	-11.15	-11.14	-11.13	-11.12	-11.11
-11.19	-11.18	-11.17	-11.16	-11.15	-11.14	-11.13	-11.12	-11.11
-11.19	-11.18	-11.17	-11.16	-11.15	-11.14	-11.13	-11.12	-11.11
-11.19	-11.18	-11.17	-11.16	-11.15	-11.14	-11.13	-11.12	-11.11
-11.19	-11.18	-11.17	-11.16	-11.15	-11.14	-11.13	-11.12	-11.11
-11.19	-11.18	-11.17	-11.16	-11.15	-11.14	-11.13	-11.12	-11.11
-11.19	-11.18	-11.17	-11.16	-11.15	-11.14	-11.13	-11.12	-11.11
-11.19	-11.18	-11.17	-11.16	-11.15	-11.14	-11.13	-11.12	-11.11
-11.19	-11.18	-11.17	-11.16	-11.15	-11.14	-11.13	-11.12	-11.11
-11.19	-11.18	-11.17	-11.16	-11.15	-11.14	-11.13	-11.12	-11.11
-11.19	-11.18	-11.17	-11.16	-11.15	-11.14	-11.13	-11.12	-11.11
-11.19	-11.18	-11.17	-11.16	-11.15	-11.14	-11.13	-11.12	-11.11
-11.19	-11.18	-11.17	-11.16	-11.15	-11.14	-11.13	-11.12	-11.11
-11.19	-11.18	-11.17	-11.16	-11.15	-11.14	-11.13	-11.12	-11.11
-11.19	-11.18	-11.17	-11.16	-11.15	-11.14	-11.13	-11.12	-11.11
-11.19	-11.18	-11.17	-11.16	-11.15	-11.14	-11.13	-11.12	-11.11
-11.19	-11.18	-11.17	-11.16	-11.15	-11.14	-11.13	-11.12	-11.11
-11.19	-11.18	-11.17	-11.16	-11.15	-11.14	-11.13	-11.12	-11.11
-11.19	-11.18	-11.17	-11.16	-11.15	-11.14	-11.13	-11.12	-11.11
-11.19	-11.18	-11.17	-11.16	-11.15	-11.14	-11.13	-11.12	-11.11
-11.19	-11.18	-11.17	-11.16	-11.15	-11.14	-11.13	-11.12	-11.11
-11.19	-11.18	-11.17	-11.16	-11.15	-11.14	-11.13	-11.12	-11.11
-11.19	-11.18	-11.17	-11.16	-11.15	-11.14	-11.13	-11.12	-11.11
-11.19	-11.18	-11.17	-11.16	-11.15	-11.14	-11.13	-11.12	-11.11
-11.19	-11.18	-11.17	-11.16	-11.15	-11.14	-11.13	-11.12	-11.11
-11.19	-11.18	-11.17	-11.16	-11.15	-11.14	-11.13	-11.12	-11.11

-11.19	-11.18	-11.17	-11.16	-11.15	-11.14	-11.13	-11.12	-11.11
-11.19	-11.18	-11.17	-11.16	-11.15	-11.14	-11.13	-11.12	-11.11
-11.19	-11.18	-11.17	-11.16	-11.15	-11.14	-11.13	-11.12	-11.11
-11.19	-11.18	-11.17	-11.16	-11.15	-11.14	-11.13	-11.12	-11.11
-11.19	-11.18	-11.17	-11.16	-11.15	-11.14	-11.13	-11.12	-11.11
-11.19	-11.18	-11.17	-11.16	-11.15	-11.14	-11.13	-11.12	-11.11
-11.19	-11.18	-11.17	-11.16	-11.15	-11.14	-11.13	-11.12	-11.11
-11.19	-11.18	-11.17	-11.16	-11.15	-11.14	-11.13	-11.12	-11.11
-11.19	-11.18	-11.17	-11.16	-11.15	-11.14	-11.13	-11.12	-11.11
-11.19	-11.18	-11.17	-11.16	-11.15	-11.14	-11.13	-11.12	-11.11
-11.19	-11.18	-11.17	-11.16	-11.15	-11.14	-11.13	-11.12	-11.11
-11.19	-11.18	-11.17	-11.16	-11.15	-11.14	-11.13	-11.12	-11.11
-11.19	-11.18	-11.17	-11.16	-11.15	-11.14	-11.13	-11.12	-11.11
-11.19	-11.18	-11.17	-11.16	-11.15	-11.14	-11.13	-11.12	-11.11
-11.19	-11.18	-11.17	-11.16	-11.15	-11.14	-11.13	-11.12	-11.11
-11.19	-11.18	-11.17	-11.16	-11.15	-11.14	-11.13	-11.12	-11.11
-11.19	-11.18	-11.17	-11.16	-11.15	-11.14	-11.13	-11.12	-11.11
-11.19	-11.18	-11.17	-11.16	-11.15	-11.14	-11.13	-11.12	-11.11
-11.19	-11.18	-11.17	-11.16	-11.15	-11.14	-11.13	-11.12	-11.11
-11.19	-11.18	-11.17	-11.16	-11.15	-11.14	-11.13	-11.12	-11.11
-11.19	-11.18	-11.17	-11.16	-11.15	-11.14	-11.13	-11.12	-11.11
-11.19	-11.18	-11.17	-11.16	-11.15	-11.14	-11.13	-11.12	-11.11
-11.19	-11.18	-11.17	-11.16	-11.15	-11.14	-11.13	-11.12	-11.11
-11.19	-11.18	-11.17	-11.16	-11.15	-11.14	-11.13	-11.12	-11.11
-11.19	-11.18	-11.17	-11.16	-11.15	-11.14	-11.13	-11.12	-11.11
-11.19	-11.18	-11.17	-11.16	-11.15	-11.14	-11.13	-11.12	-11.11
-11.19	-11.18	-11.17	-11.16	-11.15	-11.14	-11.13	-11.12	-11.11
-11.19	-11.18	-11.17	-11.16	-11.15	-11.14	-11.13	-11.12	-11.11
-11.19	-11.18	-11.17	-11.16	-11.15	-11.14	-11.13	-11.12	-11.11
-11.19	-11.18	-11.17	-11.16	-11.15	-11.14	-11.13	-11.12	-11.11
-11.19	-11.18	-11.17	-11.16	-11.15	-11.14	-11.13	-11.12	-11.11
-11.19	-11.18	-11.17	-11.16	-11.15	-11.14	-11.13	-11.12	-11.11
-11.19	-11.18	-11.17	-11.16	-11.15	-11.14	-11.13	-11.12	-11.11
-11.19	-11.18	-11.17	-11.16	-11.15	-11.14	-11.13	-11.12	-11.11
-11.19	-11.18	-11.17	-11.16	-11.15	-11.14	-11.13	-11.12	-11.11
-11.19	-11.18	-11.17	-11.16	-11.15	-11.14	-11.13	-11.12	-11.11
-11.19	-11.18	-11.17	-11.16	-11.15	-11.14	-11.13	-11.12	-11.11
-11.19	-11.18	-11.17	-11.16	-11.15	-11.14	-11.13	-11.12	-11.11
-11.19	-11.18	-11.17	-11.16	-11.15	-11.14	-11.13	-11.12	-11.11
-11.19	-11.18	-11.17	-11.16	-11.15	-11.14	-11.13	-11.12	-11.11
-11.19	-11.18	-11.17	-11.16	-11.15	-11.14	-11.13	-11.12	-11.11
-11.19	-11.18	-11.17	-11.16	-11.15	-11.14	-11.13	-11.12	-11.11
-11.19	-11.18	-11.17	-11.16	-11.15	-11.14	-11.13	-11.12	-11.11
-11.19	-11.18	-11.17	-11.16	-11.15	-11.14	-11.13	-11.12	-11.11
-11.19	-11.18	-11.17	-11.16	-11.15	-11.14	-11.13	-11.12	-11.11
-11.19	-11.18	-11.17	-11.16	-11.15	-11.14	-11.13	-11.12	-11.11
-11.19	-11.18	-11.17	-11.16	-11.15	-11.14	-11.13	-11.12	-11.11
-11.19	-11.18	-11.17	-11.16	-11.15	-11.14	-11.13	-11.12	-11.11
-11.19	-11.18	-11.17	-11.16	-11.15	-11.14	-11.13	-11.12	-11.11
-11.19	-11.18	-11.17	-11.16	-11.15	-11.14	-11.13	-11.12	-11.11

-11.19	-11.18	-11.17	-11.16	-11.15	-11.14	-11.13	-11.12	-11.11
-11.19	-11.18	-11.17	-11.16	-11.15	-11.14	-11.13	-11.12	-11.11
-11.19	-11.18	-11.17	-11.16	-11.15	-11.14	-11.13	-11.12	-11.11
-11.19	-11.18	-11.17	-11.16	-11.15	-11.14	-11.13	-11.12	-11.11
-11.19	-11.18	-11.17	-11.16	-11.15	-11.14	-11.13	-11.12	-11.11
-11.19	-11.18	-11.17	-11.16	-11.15	-11.14	-11.13	-11.12	-11.11
-11.19	-11.18	-11.17	-11.16	-11.15	-11.14	-11.13	-11.12	-11.11
-11.19	-11.18	-11.17	-11.16	-11.15	-11.14	-11.13	-11.12	-11.11
-11.19	-11.18	-11.17	-11.16	-11.15	-11.14	-11.13	-11.12	-11.11
-11.19	-11.18	-11.17	-11.16	-11.15	-11.14	-11.13	-11.12	-11.11
-11.19	-11.18	-11.17	-11.16	-11.15	-11.14	-11.13	-11.12	-11.11
-11.19	-11.18	-11.17	-11.16	-11.15	-11.14	-11.13	-11.12	-11.11
-11.19	-11.18	-11.17	-11.16	-11.15	-11.14	-11.13	-11.12	-11.11
-11.19	-11.18	-11.17	-11.16	-11.15	-11.14	-11.13	-11.12	-11.11
-11.19	-11.18	-11.17	-11.16	-11.15	-11.14	-11.13	-11.12	-11.11
-11.19	-11.18	-11.17	-11.16	-11.15	-11.14	-11.13	-11.12	-11.11
-11.19	-11.18	-11.17	-11.16	-11.15	-11.14	-11.13	-11.12	-11.11
-11.19	-11.18	-11.17	-11.16	-11.15	-11.14	-11.13	-11.12	-11.11
-11.19	-11.18	-11.17	-11.16	-11.15	-11.14	-11.13	-11.12	-11.11
-11.19	-11.18	-11.17	-11.16	-11.15	-11.14	-11.13	-11.12	-11.11
-11.19	-11.18	-11.17	-11.16	-11.15	-11.14	-11.13	-11.12	-11.11
-11.19	-11.18	-11.17	-11.16	-11.15	-11.14	-11.13	-11.12	-11.11
-11.19	-11.18	-11.17	-11.16	-11.15	-11.14	-11.13	-11.12	-11.11
-11.19	-11.18	-11.17	-11.16	-11.15	-11.14	-11.13	-11.12	-11.11
-11.19	-11.18	-11.17	-11.16	-11.15	-11.14	-11.13	-11.12	-11.11
-11.19	-11.18	-11.17	-11.16	-11.15	-11.14	-11.13	-11.12	-11.11
-11.19	-11.18	-11.17	-11.16	-11.15	-11.14	-11.13	-11.12	-11.11
-11.19	-11.18	-11.17	-11.16	-11.15	-11.14	-11.13	-11.12	-11.11
-11.19	-11.18	-11.17	-11.16	-11.15	-11.14	-11.13	-11.12	-11.11
-11.19	-11.18	-11.17	-11.16	-11.15	-11.14	-11.13	-11.12	-11.11
-11.19	-11.18	-11.17	-11.16	-11.15	-11.14	-11.13	-11.12	-11.11
-11.19	-11.18	-11.17	-11.16	-11.15	-11.14	-11.13	-11.12	-11.11
-11.19	-11.18	-11.17	-11.16	-11.15	-11.14	-11.13	-11.12	-11.11
-11.19	-11.18	-11.17	-11.16	-11.15	-11.14	-11.13	-11.12	-11.11
-11.19	-11.18	-11.17	-11.16	-11.15	-11.14	-11.13	-11.12	-11.11
-11.19	-11.18	-11.17	-11.16	-11.15	-11.14	-11.13	-11.12	-11.11
-11.19	-11.18	-11.17	-11.16	-11.15	-11.14	-11.13	-11.12	-11.11
-11.19	-11.18	-11.17	-11.16	-11.15	-11.14	-11.13	-11.12	-11.11
-11.19	-11.18	-11.17	-11.16	-11.15	-11.14	-11.13	-11.12	-11.11
-11.19	-11.18	-11.17	-11.16	-11.15	-11.14	-11.13	-11.12	-11.11
-11.19	-11.18	-11.17	-11.16	-11.15	-11.14	-11.13	-11.12	-11.11
-11.19	-11.18	-11.17	-11.16	-11.15	-11.14	-11.13	-11.12	-11.11
-11.19	-11.18	-11.17	-11.16	-11.15	-11.14	-11.13	-11.12	-11.11
-11.19	-11.18	-11.17	-11.16	-11.15	-11.14	-11.13	-11.12	-11.11
-11.19	-11.18	-11.17	-11.16	-11.15	-11.14	-11.13	-11.12	-11.11
-11.19	-11.18	-11.17	-11.16	-11.15	-11.14	-11.13	-11.12	-11.11
-11.19	-11.18	-11.17	-11.16	-11.15	-11.14	-11.13	-11.12	-11.11
-11.19	-11.18	-11.17	-11.16	-11.15	-11.14	-11.13	-11.12	-11.11
-11.19	-11.18	-11.17	-11.16	-11.15	-11.14	-11.13	-11.12	-11.11
-11.19	-11.18	-11.17	-11.16	-11.15	-11.14	-11.13	-11.12	-11.11

-11.19	-11.18	-11.17	-11.16	-11.15	-11.14	-11.13	-11.12	-11.11
-11.19	-11.18	-11.17	-11.16	-11.15	-11.14	-11.13	-11.12	-11.11
-11.19	-11.18	-11.17	-11.16	-11.15	-11.14	-11.13	-11.12	-11.11
-11.19	-11.18	-11.17	-11.16	-11.15	-11.14	-11.13	-11.12	-11.11
-11.19	-11.18	-11.17	-11.16	-11.15	-11.14	-11.13	-11.12	-11.11
-11.19	-11.18	-11.17	-11.16	-11.15	-11.14	-11.13	-11.12	-11.11
-11.19	-11.18	-11.17	-11.16	-11.15	-11.14	-11.13	-11.12	-11.11
-11.19	-11.18	-11.17	-11.16	-11.15	-11.14	-11.13	-11.12	-11.11
-11.19	-11.18	-11.17	-11.16	-11.15	-11.14	-11.13	-11.12	-11.11
-11.19	-11.18	-11.17	-11.16	-11.15	-11.14	-11.13	-11.12	-11.11
-11.19	-11.18	-11.17	-11.16	-11.15	-11.14	-11.13	-11.12	-11.11
-11.19	-11.18	-11.17	-11.16	-11.15	-11.14	-11.13	-11.12	-11.11
-11.19	-11.18	-11.17	-11.16	-11.15	-11.14	-11.13	-11.12	-11.11
-11.19	-11.18	-11.17	-11.16	-11.15	-11.14	-11.13	-11.12	-11.11
-11.19	-11.18	-11.17	-11.16	-11.15	-11.14	-11.13	-11.12	-11.11
-11.19	-11.18	-11.17	-11.16	-11.15	-11.14	-11.13	-11.12	-11.11
-11.19	-11.18	-11.17	-11.16	-11.15	-11.14	-11.13	-11.12	-11.11
-11.19	-11.18	-11.17	-11.16	-11.15	-11.14	-11.13	-11.12	-11.11
-11.19	-11.18	-11.17	-11.16	-11.15	-11.14	-11.13	-11.12	-11.11
-11.19	-11.18	-11.17	-11.16	-11.15	-11.14	-11.13	-11.12	-11.11
-11.19	-11.18	-11.17	-11.16	-11.15	-11.14	-11.13	-11.12	-11.11
-11.19	-11.18	-11.17	-11.16	-11.15	-11.14	-11.13	-11.12	-11.11
-11.19	-11.18	-11.17	-11.16	-11.15	-11.14	-11.13	-11.12	-11.11
-11.19	-11.18	-11.17	-11.16	-11.15	-11.14	-11.13	-11.12	-11.11
-11.19	-11.18	-11.17	-11.16	-11.15	-11.14	-11.13	-11.12	-11.11
-11.19	-11.18	-11.17	-11.16	-11.15	-11.14	-11.13	-11.12	-11.11
-11.19	-11.18	-11.17	-11.16	-11.15	-11.14	-11.13	-11.12	-11.11
-11.19	-11.18	-11.17	-11.16	-11.15	-11.14	-11.13	-11.12	-11.11
-11.19	-11.18	-11.17	-11.16	-11.15	-11.14	-11.13	-11.12	-11.11
-11.19	-11.18	-11.17	-11.16	-11.15	-11.14	-11.13	-11.12	-11.11
-11.19	-11.18	-11.17	-11.16	-11.15	-11.14	-11.13	-11.12	-11.11
-11.19	-11.18	-11.17	-11.16	-11.15	-11.14	-11.13	-11.12	-11.11
-11.19	-11.18	-11.17	-11.16	-11.15	-11.14	-11.13	-11.12	-11.11
-11.19	-11.18	-11.17	-11.16	-11.15	-11.14	-11.13	-11.12	-11.11
-11.19	-11.18	-11.17	-11.16	-11.15	-11.14	-11.13	-11.12	-11.11
-11.19	-11.18	-11.17	-11.16	-11.15	-11.14	-11.13	-11.12	-11.11
-11.19	-11.18	-11.17	-11.16	-11.15	-11.14	-11.13	-11.12	-11.11
-11.19	-11.18	-11.17	-11.16	-11.15	-11.14	-11.13	-11.12	-11.11
-11.19	-11.18	-11.17	-11.16	-11.15	-11.14	-11.13	-11.12	-11.11
-11.19	-11.18	-11.17	-11.16	-11.15	-11.14	-11.13	-11.12	-11.11
-11.19	-11.18	-11.17	-11.16	-11.15	-11.14	-11.13	-11.12	-11.11
-11.19	-11.18	-11.17	-11.16	-11.15	-11.14	-11.13	-11.12	-11.11
-11.19	-11.18	-11.17	-11.16	-11.15	-11.14	-11.13	-11.12	-11.11
-11.19	-11.18	-11.17	-11.16	-11.15	-11.14	-11.13	-11.12	-11.11
-11.19	-11.18	-11.17	-11.16	-11.15	-11.14	-11.13	-11.12	-11.11
-11.19	-11.18	-11.17	-11.16	-11.15	-11.14	-11.13	-11.12	-11.11
-11.19	-11.18	-11.17	-11.16	-11.15	-11.14	-11.13	-11.12	-11.11
-11.19	-11.18	-11.17	-11.16	-11.15	-11.14	-11.13	-11.12	-11.11
-11.19	-11.18	-11.17	-11.16	-11.15	-11.14	-11.13	-11.12	-11.11
-11.19	-11.18	-11.17	-11.16	-11.15	-11.14	-11.13	-11.12	-11.11

-11.19	-11.18	-11.17	-11.16	-11.15	-11.14	-11.13	-11.12	-11.11
-11.19	-11.18	-11.17	-11.16	-11.15	-11.14	-11.13	-11.12	-11.11
-11.19	-11.18	-11.17	-11.16	-11.15	-11.14	-11.13	-11.12	-11.11
-11.19	-11.18	-11.17	-11.16	-11.15	-11.14	-11.13	-11.12	-11.11
-11.19	-11.18	-11.17	-11.16	-11.15	-11.14	-11.13	-11.12	-11.11
-11.19	-11.18	-11.17	-11.16	-11.15	-11.14	-11.13	-11.12	-11.11
-11.19	-11.18	-11.17	-11.16	-11.15	-11.14	-11.13	-11.12	-11.11
-11.19	-11.18	-11.17	-11.16	-11.15	-11.14	-11.13	-11.12	-11.11
-11.19	-11.18	-11.17	-11.16	-11.15	-11.14	-11.13	-11.12	-11.11
-11.19	-11.18	-11.17	-11.16	-11.15	-11.14	-11.13	-11.12	-11.11
-11.19	-11.18	-11.17	-11.16	-11.15	-11.14	-11.13	-11.12	-11.11
-11.19	-11.18	-11.17	-11.16	-11.15	-11.14	-11.13	-11.12	-11.11
-11.19	-11.18	-11.17	-11.16	-11.15	-11.14	-11.13	-11.12	-11.11
-11.19	-11.18	-11.17	-11.16	-11.15	-11.14	-11.13	-11.12	-11.11
-11.19	-11.18	-11.17	-11.16	-11.15	-11.14	-11.13	-11.12	-11.11
-11.19	-11.18	-11.17	-11.16	-11.15	-11.14	-11.13	-11.12	-11.11
-11.19	-11.18	-11.17	-11.16	-11.15	-11.14	-11.13	-11.12	-11.11
-11.19	-11.18	-11.17	-11.16	-11.15	-11.14	-11.13	-11.12	-11.11
-11.19	-11.18	-11.17	-11.16	-11.15	-11.14	-11.13	-11.12	-11.11
-11.19	-11.18	-11.17	-11.16	-11.15	-11.14	-11.13	-11.12	-11.11
-11.19	-11.18	-11.17	-11.16	-11.15	-11.14	-11.13	-11.12	-11.11
-11.19	-11.18	-11.17	-11.16	-11.15	-11.14	-11.13	-11.12	-11.11
-11.19	-11.18	-11.17	-11.16	-11.15	-11.14	-11.13	-11.12	-11.11
-11.19	-11.18	-11.17	-11.16	-11.15	-11.14	-11.13	-11.12	-11.11
-11.19	-11.18	-11.17	-11.16	-11.15	-11.14	-11.13	-11.12	-11.11
-11.19	-11.18	-11.17	-11.16	-11.15	-11.14	-11.13	-11.12	-11.11
-11.19	-11.18	-11.17	-11.16	-11.15	-11.14	-11.13	-11.12	-11.11
-11.19	-11.18	-11.17	-11.16	-11.15	-11.14	-11.13	-11.12	-11.11
-11.19	-11.18	-11.17	-11.16	-11.15	-11.14	-11.13	-11.12	-11.11
-11.19	-11.18	-11.17	-11.16	-11.15	-11.14	-11.13	-11.12	-11.11
-11.19	-11.18	-11.17	-11.16	-11.15	-11.14	-11.13	-11.12	-11.11
-11.19	-11.18	-11.17	-11.16	-11.15	-11.14	-11.13	-11.12	-11.11
-11.19	-11.18	-11.17	-11.16	-11.15	-11.14	-11.13	-11.12	-11.11
-11.19	-11.18	-11.17	-11.16	-11.15	-11.14	-11.13	-11.12	-11.11
-11.19	-11.18	-11.17	-11.16	-11.15	-11.14	-11.13	-11.12	-11.11
-11.19	-11.18	-11.17	-11.16	-11.15	-11.14	-11.13	-11.12	-11.11
-11.19	-11.18	-11.17	-11.16	-11.15	-11.14	-11.13	-11.12	-11.11
-11.19	-11.18	-11.17	-11.16	-11.15	-11.14	-11.13	-11.12	-11.11
-11.19	-11.18	-11.17	-11.16	-11.15	-11.14	-11.13	-11.12	-11.11
-11.19	-11.18	-11.17	-11.16	-11.15	-11.14	-11.13	-11.12	-11.11
-11.19	-11.18	-11.17	-11.16	-11.15	-11.14	-11.13	-11.12	-11.11
-11.19	-11.18	-11.17	-11.16	-11.15	-11.14	-11.13	-11.12	-11.11
-11.19	-11.18	-11.17	-11.16	-11.15	-11.14	-11.13	-11.12	-11.11
-11.19	-11.18	-11.17	-11.16	-11.15	-11.14	-11.13	-11.12	-11.11
-11.19	-11.18	-11.17	-11.16	-11.15	-11.14	-11.13	-11.12	-11.11
-11.19	-11.18	-11.17	-11.16	-11.15	-11.14	-11.13	-11.12	-11.11
-11.19	-11.18	-11.17	-11.16	-11.15	-11.14	-11.13	-11.12	-11.11
-11.19	-11.18	-11.17	-11.16	-11.15	-11.14	-11.13	-11.12	-11.11
-11.19	-11.18	-11.17	-11.16	-11.15	-11.14	-11.13	-11.12	-11.11
-11.19	-11.18	-11.17	-11.16	-11.15	-11.14	-11.13	-11.12	-11.11

-11.19	-11.18	-11.17	-11.16	-11.15	-11.14	-11.13	-11.12	-11.11
-11.19	-11.18	-11.17	-11.16	-11.15	-11.14	-11.13	-11.12	-11.11
-11.19	-11.18	-11.17	-11.16	-11.15	-11.14	-11.13	-11.12	-11.11
-11.19	-11.18	-11.17	-11.16	-11.15	-11.14	-11.13	-11.12	-11.11
-11.19	-11.18	-11.17	-11.16	-11.15	-11.14	-11.13	-11.12	-11.11
-11.19	-11.18	-11.17	-11.16	-11.15	-11.14	-11.13	-11.12	-11.11
-11.19	-11.18	-11.17	-11.16	-11.15	-11.14	-11.13	-11.12	-11.11
-11.19	-11.18	-11.17	-11.16	-11.15	-11.14	-11.13	-11.12	-11.11
-11.19	-11.18	-11.17	-11.16	-11.15	-11.14	-11.13	-11.12	-11.11
-11.19	-11.18	-11.17	-11.16	-11.15	-11.14	-11.13	-11.12	-11.11
-11.19	-11.18	-11.17	-11.16	-11.15	-11.14	-11.13	-11.12	-11.11
-11.19	-11.18	-11.17	-11.16	-11.15	-11.14	-11.13	-11.12	-11.11
-11.19	-11.18	-11.17	-11.16	-11.15	-11.14	-11.13	-11.12	-11.11
-11.19	-11.18	-11.17	-11.16	-11.15	-11.14	-11.13	-11.12	-11.11
-11.19	-11.18	-11.17	-11.16	-11.15	-11.14	-11.13	-11.12	-11.11
-11.19	-11.18	-11.17	-11.16	-11.15	-11.14	-11.13	-11.12	-11.11
-11.19	-11.18	-11.17	-11.16	-11.15	-11.14	-11.13	-11.12	-11.11
-11.19	-11.18	-11.17	-11.16	-11.15	-11.14	-11.13	-11.12	-11.11
-11.19	-11.18	-11.17	-11.16	-11.15	-11.14	-11.13	-11.12	-11.11
-11.19	-11.18	-11.17	-11.16	-11.15	-11.14	-11.13	-11.12	-11.11
-11.19	-11.18	-11.17	-11.16	-11.15	-11.14	-11.13	-11.12	-11.11
-11.19	-11.18	-11.17	-11.16	-11.15	-11.14	-11.13	-11.12	-11.11
-11.19	-11.18	-11.17	-11.16	-11.15	-11.14	-11.13	-11.12	-11.11
-11.19	-11.18	-11.17	-11.16	-11.15	-11.14	-11.13	-11.12	-11.11
-11.19	-11.18	-11.17	-11.16	-11.15	-11.14	-11.13	-11.12	-11.11
-11.19	-11.18	-11.17	-11.16	-11.15	-11.14	-11.13	-11.12	-11.11
-11.19	-11.18	-11.17	-11.16	-11.15	-11.14	-11.13	-11.12	-11.11
-11.19	-11.18	-11.17	-11.16	-11.15	-11.14	-11.13	-11.12	-11.11
-11.19	-11.18	-11.17	-11.16	-11.15	-11.14	-11.13	-11.12	-11.11
-11.19	-11.18	-11.17	-11.16	-11.15	-11.14	-11.13	-11.12	-11.11
-11.19	-11.18	-11.17	-11.16	-11.15	-11.14	-11.13	-11.12	-11.11
-11.19	-11.18	-11.17	-11.16	-11.15	-11.14	-11.13	-11.12	-11.11
-11.19	-11.18	-11.17	-11.16	-11.15	-11.14	-11.13	-11.12	-11.11
-11.19	-11.18	-11.17	-11.16	-11.15	-11.14	-11.13	-11.12	-11.11
-11.19	-11.18	-11.17	-11.16	-11.15	-11.14	-11.13	-11.12	-11.11
-11.19	-11.18	-11.17	-11.16	-11.15	-11.14	-11.13	-11.12	-11.11
-11.19	-11.18	-11.17	-11.16	-11.15	-11.14	-11.13	-11.12	-11.11
-11.19	-11.18	-11.17	-11.16	-11.15	-11.14	-11.13	-11.12	-11.11
-11.19	-11.18	-11.17	-11.16	-11.15	-11.14	-11.13	-11.12	-11.11
-11.19	-11.18	-11.17	-11.16	-11.15	-11.14	-11.13	-11.12	-11.11
-11.19	-11.18	-11.17	-11.16	-11.15	-11.14	-11.13	-11.12	-11.11
-11.19	-11.18	-11.17	-11.16	-11.15	-11.14	-11.13	-11.12	-11.11
-11.19	-11.18	-11.17	-11.16	-11.15	-11.14	-11.13	-11.12	-11.11
-11.19	-11.18	-11.17	-11.16	-11.15	-11.14	-11.13	-11.12	-11.11
-11.19	-11.18	-11.17	-11.16	-11.15	-11.14	-11.13	-11.12	-11.11
-11.19	-11.18	-11.17	-11.16	-11.15	-11.14	-11.13	-11.12	-11.11
-11.19	-11.18	-11.17	-11.16	-11.15	-11.14	-11.13	-11.12	-11.11
-11.19	-11.18	-11.17	-11.16	-11.15	-11.14	-11.13	-11.12	-11.11
-11.19	-11.18	-11.17	-11.16	-11.15	-11.14	-11.13	-11.12	-11.11
-11.19	-11.18	-11.17	-11.16	-11.15	-11.14	-11.13	-11.12	-11.11

-11.19	-11.18	-11.17	-11.16	-11.15	-11.14	-11.13	-11.12	-11.11
-11.19	-11.18	-11.17	-11.16	-11.15	-11.14	-11.13	-11.12	-11.11
-11.19	-11.18	-11.17	-11.16	-11.15	-11.14	-11.13	-11.12	-11.11
-11.19	-11.18	-11.17	-11.16	-11.15	-11.14	-11.13	-11.12	-11.11
-11.19	-11.18	-11.17	-11.16	-11.15	-11.14	-11.13	-11.12	-11.11
-11.19	-11.18	-11.17	-11.16	-11.15	-11.14	-11.13	-11.12	-11.11
-11.19	-11.18	-11.17	-11.16	-11.15	-11.14	-11.13	-11.12	-11.11
-11.19	-11.18	-11.17	-11.16	-11.15	-11.14	-11.13	-11.12	-11.11
-11.19	-11.18	-11.17	-11.16	-11.15	-11.14	-11.13	-11.12	-11.11
-11.19	-11.18	-11.17	-11.16	-11.15	-11.14	-11.13	-11.12	-11.11
-11.19	-11.18	-11.17	-11.16	-11.15	-11.14	-11.13	-11.12	-11.11
-11.19	-11.18	-11.17	-11.16	-11.15	-11.14	-11.13	-11.12	-11.11
-11.19	-11.18	-11.17	-11.16	-11.15	-11.14	-11.13	-11.12	-11.11
-11.19	-11.18	-11.17	-11.16	-11.15	-11.14	-11.13	-11.12	-11.11
-11.19	-11.18	-11.17	-11.16	-11.15	-11.14	-11.13	-11.12	-11.11
-11.19	-11.18	-11.17	-11.16	-11.15	-11.14	-11.13	-11.12	-11.11
-11.19	-11.18	-11.17	-11.16	-11.15	-11.14	-11.13	-11.12	-11.11
-11.19	-11.18	-11.17	-11.16	-11.15	-11.14	-11.13	-11.12	-11.11
-11.19	-11.18	-11.17	-11.16	-11.15	-11.14	-11.13	-11.12	-11.11
-11.19	-11.18	-11.17	-11.16	-11.15	-11.14	-11.13	-11.12	-11.11
-11.19	-11.18	-11.17	-11.16	-11.15	-11.14	-11.13	-11.12	-11.11
-11.19	-11.18	-11.17	-11.16	-11.15	-11.14	-11.13	-11.12	-11.11
-11.19	-11.18	-11.17	-11.16	-11.15	-11.14	-11.13	-11.12	-11.11
-11.19	-11.18	-11.17	-11.16	-11.15	-11.14	-11.13	-11.12	-11.11
-11.19	-11.18	-11.17	-11.16	-11.15	-11.14	-11.13	-11.12	-11.11
-11.19	-11.18	-11.17	-11.16	-11.15	-11.14	-11.13	-11.12	-11.11
-11.19	-11.18	-11.17	-11.16	-11.15	-11.14	-11.13	-11.12	-11.11
-11.19	-11.18	-11.17	-11.16	-11.15	-11.14	-11.13	-11.12	-11.11
-11.19	-11.18	-11.17	-11.16	-11.15	-11.14	-11.13	-11.12	-11.11
-11.19	-11.18	-11.17	-11.16	-11.15	-11.14	-11.13	-11.12	-11.11
-11.19	-11.18	-11.17	-11.16	-11.15	-11.14	-11.13	-11.12	-11.11
-11.19	-11.18	-11.17	-11.16	-11.15	-11.14	-11.13	-11.12	-11.11
-11.19	-11.18	-11.17	-11.16	-11.15	-11.14	-11.13	-11.12	-11.11
-11.19	-11.18	-11.17	-11.16	-11.15	-11.14	-11.13	-11.12	-11.11
-11.19	-11.18	-11.17	-11.16	-11.15	-11.14	-11.13	-11.12	-11.11
-11.19	-11.18	-11.17	-11.16	-11.15	-11.14	-11.13	-11.12	-11.11
-11.19	-11.18	-11.17	-11.16	-11.15	-11.14	-11.13	-11.12	-11.11
-11.19	-11.18	-11.17	-11.16	-11.15	-11.14	-11.13	-11.12	-11.11
-11.19	-11.18	-11.17	-11.16	-11.15	-11.14	-11.13	-11.12	-11.11
-11.19	-11.18	-11.17	-11.16	-11.15	-11.14	-11.13	-11.12	-11.11
-11.19	-11.18	-11.17	-11.16	-11.15	-11.14	-11.13	-11.12	-11.11
-11.19	-11.18	-11.17	-11.16	-11.15	-11.14	-11.13	-11.12	-11.11
-11.19	-11.18	-11.17	-11.16	-11.15	-11.14	-11.13	-11.12	-11.11
-11.19	-11.18	-11.17	-11.16	-11.15	-11.14	-11.13	-11.12	-11.11
-11.19	-11.18	-11.17	-11.16	-11.15	-11.14	-11.13	-11.12	-11.11
-11.19	-11.18	-11.17	-11.16	-11.15	-11.14	-11.13	-11.12	-11.11
-11.19	-11.18	-11.17	-11.16	-11.15	-11.14	-11.13	-11.12	-11.11
-11.19	-11.18	-11.17	-11.16	-11.15	-11.14	-11.13	-11.12	-11.11
-11.19	-11.18	-11.17	-11.16	-11.15	-11.14	-11.13	-11.12	-11.11
-11.19	-11.18	-11.17	-11.16	-11.15	-11.14	-11.13	-11.12	-11.11

-11.19	-11.18	-11.17	-11.16	-11.15	-11.14	-11.13	-11.12	-11.11
-11.19	-11.18	-11.17	-11.16	-11.15	-11.14	-11.13	-11.12	-11.11
-11.19	-11.18	-11.17	-11.16	-11.15	-11.14	-11.13	-11.12	-11.11
-11.19	-11.18	-11.17	-11.16	-11.15	-11.14	-11.13	-11.12	-11.11
-11.19	-11.18	-11.17	-11.16	-11.15	-11.14	-11.13	-11.12	-11.11
-11.19	-11.18	-11.17	-11.16	-11.15	-11.14	-11.13	-11.12	-11.11
-11.19	-11.18	-11.17	-11.16	-11.15	-11.14	-11.13	-11.12	-11.11
-11.19	-11.18	-11.17	-11.16	-11.15	-11.14	-11.13	-11.12	-11.11
-11.19	-11.18	-11.17	-11.16	-11.15	-11.14	-11.13	-11.12	-11.11
-11.19	-11.18	-11.17	-11.16	-11.15	-11.14	-11.13	-11.12	-11.11
-11.19	-11.18	-11.17	-11.16	-11.15	-11.14	-11.13	-11.12	-11.11
-11.19	-11.18	-11.17	-11.16	-11.15	-11.14	-11.13	-11.12	-11.11
-11.19	-11.18	-11.17	-11.16	-11.15	-11.14	-11.13	-11.12	-11.11
-11.19	-11.18	-11.17	-11.16	-11.15	-11.14	-11.13	-11.12	-11.11
-11.19	-11.18	-11.17	-11.16	-11.15	-11.14	-11.13	-11.12	-11.11
-11.19	-11.18	-11.17	-11.16	-11.15	-11.14	-11.13	-11.12	-11.11
-11.19	-11.18	-11.17	-11.16	-11.15	-11.14	-11.13	-11.12	-11.11
-11.19	-11.18	-11.17	-11.16	-11.15	-11.14	-11.13	-11.12	-11.11
-11.19	-11.18	-11.17	-11.16	-11.15	-11.14	-11.13	-11.12	-11.11
-11.19	-11.18	-11.17	-11.16	-11.15	-11.14	-11.13	-11.12	-11.11
-11.19	-11.18	-11.17	-11.16	-11.15	-11.14	-11.13	-11.12	-11.11
-11.19	-11.18	-11.17	-11.16	-11.15	-11.14	-11.13	-11.12	-11.11
-11.19	-11.18	-11.17	-11.16	-11.15	-11.14	-11.13	-11.12	-11.11
-11.19	-11.18	-11.17	-11.16	-11.15	-11.14	-11.13	-11.12	-11.11
-11.19	-11.18	-11.17	-11.16	-11.15	-11.14	-11.13	-11.12	-11.11
-11.19	-11.18	-11.17	-11.16	-11.15	-11.14	-11.13	-11.12	-11.11
-11.19	-11.18	-11.17	-11.16	-11.15	-11.14	-11.13	-11.12	-11.11
-11.19	-11.18	-11.17	-11.16	-11.15	-11.14	-11.13	-11.12	-11.11
-11.19	-11.18	-11.17	-11.16	-11.15	-11.14	-11.13	-11.12	-11.11
-11.19	-11.18	-11.17	-11.16	-11.15	-11.14	-11.13	-11.12	-11.11
-11.19	-11.18	-11.17	-11.16	-11.15	-11.14	-11.13	-11.12	-11.11
-11.19	-11.18	-11.17	-11.16	-11.15	-11.14	-11.13	-11.12	-11.11
-11.19	-11.18	-11.17	-11.16	-11.15	-11.14	-11.13	-11.12	-11.11
-11.19	-11.18	-11.17	-11.16	-11.15	-11.14	-11.13	-11.12	-11.11
-11.19	-11.18	-11.17	-11.16	-11.15	-11.14	-11.13	-11.12	-11.11
-11.19	-11.18	-11.17	-11.16	-11.15	-11.14	-11.13	-11.12	-11.11
-11.19	-11.18	-11.17	-11.16	-11.15	-11.14	-11.13	-11.12	-11.11
-11.19	-11.18	-11.17	-11.16	-11.15	-11.14	-11.13	-11.12	-11.11
-11.19	-11.18	-11.17	-11.16	-11.15	-11.14	-11.13	-11.12	-11.11
-11.19	-11.18	-11.17	-11.16	-11.15	-11.14	-11.13	-11.12	-11.11
-11.19	-11.18	-11.17	-11.16	-11.15	-11.14	-11.13	-11.12	-11.11
-11.19	-11.18	-11.17	-11.16	-11.15	-11.14	-11.13	-11.12	-11.11
-11.19	-11.18	-11.17	-11.16	-11.15	-11.14	-11.13	-11.12	-11.11
-11.19	-11.18	-11.17	-11.16	-11.15	-11.14	-11.13	-11.12	-11.11
-11.19	-11.18	-11.17	-11.16	-11.15	-11.14	-11.13	-11.12	-11.11
-11.19	-11.18	-11.17	-11.16	-11.15	-11.14	-11.13	-11.12	-11.11
-11.19	-11.18	-11.17	-11.16	-11.15	-11.14	-11.13	-11.12	-11.11
-11.19	-11.18	-11.17	-11.16	-11.15	-11.14	-11.13	-11.12	-11.11
-11.19	-11.18	-11.17	-11.16	-11.15	-11.14	-11.13	-11.12	-11.11
-11.19	-11.18	-11.17	-11.16	-11.15	-11.14	-11.13	-11.12	-11.11

-11.19	-11.18	-11.17	-11.16	-11.15	-11.14	-11.13	-11.12	-11.11
-11.19	-11.18	-11.17	-11.16	-11.15	-11.14	-11.13	-11.12	-11.11
-11.19	-11.18	-11.17	-11.16	-11.15	-11.14	-11.13	-11.12	-11.11
-11.19	-11.18	-11.17	-11.16	-11.15	-11.14	-11.13	-11.12	-11.11
-11.19	-11.18	-11.17	-11.16	-11.15	-11.14	-11.13	-11.12	-11.11
-11.19	-11.18	-11.17	-11.16	-11.15	-11.14	-11.13	-11.12	-11.11
-11.19	-11.18	-11.17	-11.16	-11.15	-11.14	-11.13	-11.12	-11.11
-11.19	-11.18	-11.17	-11.16	-11.15	-11.14	-11.13	-11.12	-11.11
-11.19	-11.18	-11.17	-11.16	-11.15	-11.14	-11.13	-11.12	-11.11
-11.19	-11.18	-11.17	-11.16	-11.15	-11.14	-11.13	-11.12	-11.11
-11.19	-11.18	-11.17	-11.16	-11.15	-11.14	-11.13	-11.12	-11.11
-11.19	-11.18	-11.17	-11.16	-11.15	-11.14	-11.13	-11.12	-11.11
-11.19	-11.18	-11.17	-11.16	-11.15	-11.14	-11.13	-11.12	-11.11
-11.19	-11.18	-11.17	-11.16	-11.15	-11.14	-11.13	-11.12	-11.11
-11.19	-11.18	-11.17	-11.16	-11.15	-11.14	-11.13	-11.12	-11.11
-11.19	-11.18	-11.17	-11.16	-11.15	-11.14	-11.13	-11.12	-11.11
-11.19	-11.18	-11.17	-11.16	-11.15	-11.14	-11.13	-11.12	-11.11
-11.19	-11.18	-11.17	-11.16	-11.15	-11.14	-11.13	-11.12	-11.11
-11.19	-11.18	-11.17	-11.16	-11.15	-11.14	-11.13	-11.12	-11.11
-11.19	-11.18	-11.17	-11.16	-11.15	-11.14	-11.13	-11.12	-11.11
-11.19	-11.18	-11.17	-11.16	-11.15	-11.14	-11.13	-11.12	-11.11
-11.19	-11.18	-11.17	-11.16	-11.15	-11.14	-11.13	-11.12	-11.11
-11.19	-11.18	-11.17	-11.16	-11.15	-11.14	-11.13	-11.12	-11.11
-11.19	-11.18	-11.17	-11.16	-11.15	-11.14	-11.13	-11.12	-11.11
-11.19	-11.18	-11.17	-11.16	-11.15	-11.14	-11.13	-11.12	-11.11
-11.19	-11.18	-11.17	-11.16	-11.15	-11.14	-11.13	-11.12	-11.11
-11.19	-11.18	-11.17	-11.16	-11.15	-11.14	-11.13	-11.12	-11.11
-11.19	-11.18	-11.17	-11.16	-11.15	-11.14	-11.13	-11.12	-11.11
-11.19	-11.18	-11.17	-11.16	-11.15	-11.14	-11.13	-11.12	-11.11
-11.19	-11.18	-11.17	-11.16	-11.15	-11.14	-11.13	-11.12	-11.11
-11.19	-11.18	-11.17	-11.16	-11.15	-11.14	-11.13	-11.12	-11.11
-11.19	-11.18	-11.17	-11.16	-11.15	-11.14	-11.13	-11.12	-11.11
-11.19	-11.18	-11.17	-11.16	-11.15	-11.14	-11.13	-11.12	-11.11
-11.19	-11.18	-11.17	-11.16	-11.15	-11.14	-11.13	-11.12	-11.11
-11.19	-11.18	-11.17	-11.16	-11.15	-11.14	-11.13	-11.12	-11.11
-11.19	-11.18	-11.17	-11.16	-11.15	-11.14	-11.13	-11.12	-11.11
-11.19	-11.18	-11.17	-11.16	-11.15	-11.14	-11.13	-11.12	-11.11
-11.19	-11.18	-11.17	-11.16	-11.15	-11.14	-11.13	-11.12	-11.11
-11.19	-11.18	-11.17	-11.16	-11.15	-11.14	-11.13	-11.12	-11.11
-11.19	-11.18	-11.17	-11.16	-11.15	-11.14	-11.13	-11.12	-11.11
-11.19	-11.18	-11.17	-11.16	-11.15	-11.14	-11.13	-11.12	-11.11
-11.19	-11.18	-11.17	-11.16	-11.15	-11.14	-11.13	-11.12	-11.11
-11.19	-11.18	-11.17	-11.16	-11.15	-11.14	-11.13	-11.12	-11.11
-11.19	-11.18	-11.17	-11.16	-11.15	-11.14	-11.13	-11.12	-11.11
-11.19	-11.18	-11.17	-11.16	-11.15	-11.14	-11.13	-11.12	-11.11
-11.19	-11.18	-11.17	-11.16	-11.15	-11.14	-11.13	-11.12	-11.11
-11.19	-11.18	-11.17	-11.16	-11.15	-11.14	-11.13	-11.12	-11.11
-11.19	-11.18	-11.17	-11.16	-11.15	-11.14	-11.13	-11.12	-11.11
-11.19	-11.18	-11.17	-11.16	-11.15	-11.14	-11.13	-11.12	-11.11
-11.19	-11.18	-11.17	-11.16	-11.15	-11.14	-11.13	-11.12	-11.11

-11.19	-11.18	-11.17	-11.16	-11.15	-11.14	-11.13	-11.12	-11.11
-11.19	-11.18	-11.17	-11.16	-11.15	-11.14	-11.13	-11.12	-11.11
-11.19	-11.18	-11.17	-11.16	-11.15	-11.14	-11.13	-11.12	-11.11
-11.19	-11.18	-11.17	-11.16	-11.15	-11.14	-11.13	-11.12	-11.11
-11.19	-11.18	-11.17	-11.16	-11.15	-11.14	-11.13	-11.12	-11.11
-11.19	-11.18	-11.17	-11.16	-11.15	-11.14	-11.13	-11.12	-11.11
-11.19	-11.18	-11.17	-11.16	-11.15	-11.14	-11.13	-11.12	-11.11
-11.19	-11.18	-11.17	-11.16	-11.15	-11.14	-11.13	-11.12	-11.11
-11.19	-11.18	-11.17	-11.16	-11.15	-11.14	-11.13	-11.12	-11.11
-11.19	-11.18	-11.17	-11.16	-11.15	-11.14	-11.13	-11.12	-11.11
-11.19	-11.18	-11.17	-11.16	-11.15	-11.14	-11.13	-11.12	-11.11
-11.19	-11.18	-11.17	-11.16	-11.15	-11.14	-11.13	-11.12	-11.11
-11.19	-11.18	-11.17	-11.16	-11.15	-11.14	-11.13	-11.12	-11.11
-11.19	-11.18	-11.17	-11.16	-11.15	-11.14	-11.13	-11.12	-11.11
-11.19	-11.18	-11.17	-11.16	-11.15	-11.14	-11.13	-11.12	-11.11
-11.19	-11.18	-11.17	-11.16	-11.15	-11.14	-11.13	-11.12	-11.11
-11.19	-11.18	-11.17	-11.16	-11.15	-11.14	-11.13	-11.12	-11.11
-11.19	-11.18	-11.17	-11.16	-11.15	-11.14	-11.13	-11.12	-11.11
-11.19	-11.18	-11.17	-11.16	-11.15	-11.14	-11.13	-11.12	-11.11
-11.19	-11.18	-11.17	-11.16	-11.15	-11.14	-11.13	-11.12	-11.11
-11.19	-11.18	-11.17	-11.16	-11.15	-11.14	-11.13	-11.12	-11.11
-11.19	-11.18	-11.17	-11.16	-11.15	-11.14	-11.13	-11.12	-11.11
-11.19	-11.18	-11.17	-11.16	-11.15	-11.14	-11.13	-11.12	-11.11
-11.19	-11.18	-11.17	-11.16	-11.15	-11.14	-11.13	-11.12	-11.11
-11.19	-11.18	-11.17	-11.16	-11.15	-11.14	-11.13	-11.12	-11.11
-11.19	-11.18	-11.17	-11.16	-11.15	-11.14	-11.13	-11.12	-11.11
-11.19	-11.18	-11.17	-11.16	-11.15	-11.14	-11.13	-11.12	-11.11
-11.19	-11.18	-11.17	-11.16	-11.15	-11.14	-11.13	-11.12	-11.11
-11.19	-11.18	-11.17	-11.16	-11.15	-11.14	-11.13	-11.12	-11.11
-11.19	-11.18	-11.17	-11.16	-11.15	-11.14	-11.13	-11.12	-11.11
-11.19	-11.18	-11.17	-11.16	-11.15	-11.14	-11.13	-11.12	-11.11
-11.19	-11.18	-11.17	-11.16	-11.15	-11.14	-11.13	-11.12	-11.11
-11.19	-11.18	-11.17	-11.16	-11.15	-11.14	-11.13	-11.12	-11.11
-11.19	-11.18	-11.17	-11.16	-11.15	-11.14	-11.13	-11.12	-11.11
-11.19	-11.18	-11.17	-11.16	-11.15	-11.14	-11.13	-11.12	-11.11
-11.19	-11.18	-11.17	-11.16	-11.15	-11.14	-11.13	-11.12	-11.11
-11.19	-11.18	-11.17	-11.16	-11.15	-11.14	-11.13	-11.12	-11.11
-11.19	-11.18	-11.17	-11.16	-11.15	-11.14	-11.13	-11.12	-11.11
-11.19	-11.18	-11.17	-11.16	-11.15	-11.14	-11.13	-11.12	-11.11
-11.19	-11.18	-11.17	-11.16	-11.15	-11.14	-11.13	-11.12	-11.11
-11.19	-11.18	-11.17	-11.16	-11.15	-11.14	-11.13	-11.12	-11.11
-11.19	-11.18	-11.17	-11.16	-11.15	-11.14	-11.13	-11.12	-11.11
-11.19	-11.18	-11.17	-11.16	-11.15	-11.14	-11.13	-11.12	-11.11
-11.19	-11.18	-11.17	-11.16	-11.15	-11.14	-11.13	-11.12	-11.11
-11.19	-11.18	-11.17	-11.16	-11.15	-11.14	-11.13	-11.12	-11.11
-11.19	-11.18	-11.17	-11.16	-11.15	-11.14	-11.13	-11.12	-11.11
-11.19	-11.18	-11.17	-11.16	-11.15	-11.14	-11.13	-11.12	-11.11
-11.19	-11.18	-11.17	-11.16	-11.15	-11.14	-11.13	-11.12	-11.11
-11.19	-11.18	-11.17	-11.16	-11.15	-11.14	-11.13	-11.12	-11.11
-11.19	-11.18	-11.17	-11.16	-11.15	-11.14	-11.13	-11.12	-11.11

-11.19	-11.18	-11.17	-11.16	-11.15	-11.14	-11.13	-11.12	-11.11
-11.19	-11.18	-11.17	-11.16	-11.15	-11.14	-11.13	-11.12	-11.11
-11.19	-11.18	-11.17	-11.16	-11.15	-11.14	-11.13	-11.12	-11.11
-11.19	-11.18	-11.17	-11.16	-11.15	-11.14	-11.13	-11.12	-11.11
-11.19	-11.18	-11.17	-11.16	-11.15	-11.14	-11.13	-11.12	-11.11
-11.19	-11.18	-11.17	-11.16	-11.15	-11.14	-11.13	-11.12	-11.11
-11.19	-11.18	-11.17	-11.16	-11.15	-11.14	-11.13	-11.12	-11.11
-11.19	-11.18	-11.17	-11.16	-11.15	-11.14	-11.13	-11.12	-11.11
-11.19	-11.18	-11.17	-11.16	-11.15	-11.14	-11.13	-11.12	-11.11
-11.19	-11.18	-11.17	-11.16	-11.15	-11.14	-11.13	-11.12	-11.11
-11.19	-11.18	-11.17	-11.16	-11.15	-11.14	-11.13	-11.12	-11.11
-11.19	-11.18	-11.17	-11.16	-11.15	-11.14	-11.13	-11.12	-11.11
-11.19	-11.18	-11.17	-11.16	-11.15	-11.14	-11.13	-11.12	-11.11
-11.19	-11.18	-11.17	-11.16	-11.15	-11.14	-11.13	-11.12	-11.11
-11.19	-11.18	-11.17	-11.16	-11.15	-11.14	-11.13	-11.12	-11.11
-11.19	-11.18	-11.17	-11.16	-11.15	-11.14	-11.13	-11.12	-11.11
-11.19	-11.18	-11.17	-11.16	-11.15	-11.14	-11.13	-11.12	-11.11
-11.19	-11.18	-11.17	-11.16	-11.15	-11.14	-11.13	-11.12	-11.11
-11.19	-11.18	-11.17	-11.16	-11.15	-11.14	-11.13	-11.12	-11.11
-11.19	-11.18	-11.17	-11.16	-11.15	-11.14	-11.13	-11.12	-11.11
-11.19	-11.18	-11.17	-11.16	-11.15	-11.14	-11.13	-11.12	-11.11
-11.19	-11.18	-11.17	-11.16	-11.15	-11.14	-11.13	-11.12	-11.11
-11.19	-11.18	-11.17	-11.16	-11.15	-11.14	-11.13	-11.12	-11.11
-11.19	-11.18	-11.17	-11.16	-11.15	-11.14	-11.13	-11.12	-11.11
-11.19	-11.18	-11.17	-11.16	-11.15	-11.14	-11.13	-11.12	-11.11
-11.19	-11.18	-11.17	-11.16	-11.15	-11.14	-11.13	-11.12	-11.11
-11.19	-11.18	-11.17	-11.16	-11.15	-11.14	-11.13	-11.12	-11.11
-11.19	-11.18	-11.17	-11.16	-11.15	-11.14	-11.13	-11.12	-11.11
-11.19	-11.18	-11.17	-11.16	-11.15	-11.14	-11.13	-11.12	-11.11
-11.19	-11.18	-11.17	-11.16	-11.15	-11.14	-11.13	-11.12	-11.11
-11.19	-11.18	-11.17	-11.16	-11.15	-11.14	-11.13	-11.12	-11.11
-11.19	-11.18	-11.17	-11.16	-11.15	-11.14	-11.13	-11.12	-11.11
-11.19	-11.18	-11.17	-11.16	-11.15	-11.14	-11.13	-11.12	-11.11
-11.19	-11.18	-11.17	-11.16	-11.15	-11.14	-11.13	-11.12	-11.11
-11.19	-11.18	-11.17	-11.16	-11.15	-11.14	-11.13	-11.12	-11.11
-11.19	-11.18	-11.17	-11.16	-11.15	-11.14	-11.13	-11.12	-11.11
-11.19	-11.18	-11.17	-11.16	-11.15	-11.14	-11.13	-11.12	-11.11
-11.19	-11.18	-11.17	-11.16	-11.15	-11.14	-11.13	-11.12	-11.11
-11.19	-11.18	-11.17	-11.16	-11.15	-11.14	-11.13	-11.12	-11.11
-11.19	-11.18	-11.17	-11.16	-11.15	-11.14	-11.13	-11.12	-11.11
-11.19	-11.18	-11.17	-11.16	-11.15	-11.14	-11.13	-11.12	-11.11
-11.19	-11.18	-11.17	-11.16	-11.15	-11.14	-11.13	-11.12	-11.11
-11.19	-11.18	-11.17	-11.16	-11.15	-11.14	-11.13	-11.12	-11.11
-11.19	-11.18	-11.17	-11.16	-11.15	-11.14	-11.13	-11.12	-11.11
-11.19	-11.18	-11.17	-11.16	-11.15	-11.14	-11.13	-11.12	-11.11
-11.19	-11.18	-11.17	-11.16	-11.15	-11.14	-11.13	-11.12	-11.11
-11.19	-11.18	-11.17	-11.16	-11.15	-11.14	-11.13	-11.12	-11.11
-11.19	-11.18	-11.17	-11.16	-11.15	-11.14	-11.13	-11.12	-11.11
-11.19	-11.18	-11.17	-11.16	-11.15	-11.14	-11.13	-11.12	-11.11
-11.19	-11.18	-11.17	-11.16	-11.15	-11.14	-11.13	-11.12	-11.11

-11.19	-11.18	-11.17	-11.16	-11.15	-11.14	-11.13	-11.12	-11.11
-11.19	-11.18	-11.17	-11.16	-11.15	-11.14	-11.13	-11.12	-11.11
-11.19	-11.18	-11.17	-11.16	-11.15	-11.14	-11.13	-11.12	-11.11
-11.19	-11.18	-11.17	-11.16	-11.15	-11.14	-11.13	-11.12	-11.11
-11.19	-11.18	-11.17	-11.16	-11.15	-11.14	-11.13	-11.12	-11.11
-11.19	-11.18	-11.17	-11.16	-11.15	-11.14	-11.13	-11.12	-11.11
-11.19	-11.18	-11.17	-11.16	-11.15	-11.14	-11.13	-11.12	-11.11
-11.19	-11.18	-11.17	-11.16	-11.15	-11.14	-11.13	-11.12	-11.11
-11.19	-11.18	-11.17	-11.16	-11.15	-11.14	-11.13	-11.12	-11.11
-11.19	-11.18	-11.17	-11.16	-11.15	-11.14	-11.13	-11.12	-11.11
-11.19	-11.18	-11.17	-11.16	-11.15	-11.14	-11.13	-11.12	-11.11
-11.19	-11.18	-11.17	-11.16	-11.15	-11.14	-11.13	-11.12	-11.11
-11.19	-11.18	-11.17	-11.16	-11.15	-11.14	-11.13	-11.12	-11.11
-11.19	-11.18	-11.17	-11.16	-11.15	-11.14	-11.13	-11.12	-11.11
-11.19	-11.18	-11.17	-11.16	-11.15	-11.14	-11.13	-11.12	-11.11
-11.19	-11.18	-11.17	-11.16	-11.15	-11.14	-11.13	-11.12	-11.11
-11.19	-11.18	-11.17	-11.16	-11.15	-11.14	-11.13	-11.12	-11.11
-11.19	-11.18	-11.17	-11.16	-11.15	-11.14	-11.13	-11.12	-11.11
-11.19	-11.18	-11.17	-11.16	-11.15	-11.14	-11.13	-11.12	-11.11
-11.19	-11.18	-11.17	-11.16	-11.15	-11.14	-11.13	-11.12	-11.11
-11.19	-11.18	-11.17	-11.16	-11.15	-11.14	-11.13	-11.12	-11.11
-11.19	-11.18	-11.17	-11.16	-11.15	-11.14	-11.13	-11.12	-11.11
-11.19	-11.18	-11.17	-11.16	-11.15	-11.14	-11.13	-11.12	-11.11
-11.19	-11.18	-11.17	-11.16	-11.15	-11.14	-11.13	-11.12	-11.11
-11.19	-11.18	-11.17	-11.16	-11.15	-11.14	-11.13	-11.12	-11.11
-11.19	-11.18	-11.17	-11.16	-11.15	-11.14	-11.13	-11.12	-11.11
-11.19	-11.18	-11.17	-11.16	-11.15	-11.14	-11.13	-11.12	-11.11
-11.19	-11.18	-11.17	-11.16	-11.15	-11.14	-11.13	-11.12	-11.11
-11.19	-11.18	-11.17	-11.16	-11.15	-11.14	-11.13	-11.12	-11.11
-11.19	-11.18	-11.17	-11.16	-11.15	-11.14	-11.13	-11.12	-11.11
-11.19	-11.18	-11.17	-11.16	-11.15	-11.14	-11.13	-11.12	-11.11
-11.19	-11.18	-11.17	-11.16	-11.15	-11.14	-11.13	-11.12	-11.11
-11.19	-11.18	-11.17	-11.16	-11.15	-11.14	-11.13	-11.12	-11.11
-11.19	-11.18	-11.17	-11.16	-11.15	-11.14	-11.13	-11.12	-11.11
-11.19	-11.18	-11.17	-11.16	-11.15	-11.14	-11.13	-11.12	-11.11
-11.19	-11.18	-11.17	-11.16	-11.15	-11.14	-11.13	-11.12	-11.11
-11.19	-11.18	-11.17	-11.16	-11.15	-11.14	-11.13	-11.12	-11.11
-11.19	-11.18	-11.17	-11.16	-11.15	-11.14	-11.13	-11.12	-11.11
-11.19	-11.18	-11.17	-11.16	-11.15	-11.14	-11.13	-11.12	-11.11
-11.19	-11.18	-11.17	-11.16	-11.15	-11.14	-11.13	-11.12	-11.11
-11.19	-11.18	-11.17	-11.16	-11.15	-11.14	-11.13	-11.12	-11.11
-11.19	-11.18	-11.17	-11.16	-11.15	-11.14	-11.13	-11.12	-11.11
-11.19	-11.18	-11.17	-11.16	-11.15	-11.14	-11.13	-11.12	-11.11
-11.19	-11.18	-11.17	-11.16	-11.15	-11.14	-11.13	-11.12	-11.11
-11.19	-11.18	-11.17	-11.16	-11.15	-11.14	-11.13	-11.12	-11.11
-11.19	-11.18	-11.17	-11.16	-11.15	-11.14	-11.13	-11.12	-11.11
-11.19	-11.18	-11.17	-11.16	-11.15	-11.14	-11.13	-11.12	-11.11
-11.19	-11.18	-11.17	-11.16	-11.15	-11.14	-11.13	-11.12	-11.11
-11.19	-11.18	-11.17	-11.16	-11.15	-11.14	-11.13	-11.12	-11.11
-11.19	-11.18	-11.17	-11.16	-11.15	-11.14	-11.13	-11.12	-11.11

-11.19	-11.18	-11.17	-11.16	-11.15	-11.14	-11.13	-11.12	-11.11
-11.19	-11.18	-11.17	-11.16	-11.15	-11.14	-11.13	-11.12	-11.11
-11.19	-11.18	-11.17	-11.16	-11.15	-11.14	-11.13	-11.12	-11.11
-11.19	-11.18	-11.17	-11.16	-11.15	-11.14	-11.13	-11.12	-11.11
-11.19	-11.18	-11.17	-11.16	-11.15	-11.14	-11.13	-11.12	-11.11
-11.19	-11.18	-11.17	-11.16	-11.15	-11.14	-11.13	-11.12	-11.11
-11.19	-11.18	-11.17	-11.16	-11.15	-11.14	-11.13	-11.12	-11.11
-11.19	-11.18	-11.17	-11.16	-11.15	-11.14	-11.13	-11.12	-11.11
-11.19	-11.18	-11.17	-11.16	-11.15	-11.14	-11.13	-11.12	-11.11
-11.19	-11.18	-11.17	-11.16	-11.15	-11.14	-11.13	-11.12	-11.11
-11.19	-11.18	-11.17	-11.16	-11.15	-11.14	-11.13	-11.12	-11.11
-11.19	-11.18	-11.17	-11.16	-11.15	-11.14	-11.13	-11.12	-11.11
-11.19	-11.18	-11.17	-11.16	-11.15	-11.14	-11.13	-11.12	-11.11
-11.19	-11.18	-11.17	-11.16	-11.15	-11.14	-11.13	-11.12	-11.11
-11.19	-11.18	-11.17	-11.16	-11.15	-11.14	-11.13	-11.12	-11.11
-11.19	-11.18	-11.17	-11.16	-11.15	-11.14	-11.13	-11.12	-11.11
-11.19	-11.18	-11.17	-11.16	-11.15	-11.14	-11.13	-11.12	-11.11
-11.19	-11.18	-11.17	-11.16	-11.15	-11.14	-11.13	-11.12	-11.11
-11.19	-11.18	-11.17	-11.16	-11.15	-11.14	-11.13	-11.12	-11.11
-11.19	-11.18	-11.17	-11.16	-11.15	-11.14	-11.13	-11.12	-11.11
-11.19	-11.18	-11.17	-11.16	-11.15	-11.14	-11.13	-11.12	-11.11
-11.19	-11.18	-11.17	-11.16	-11.15	-11.14	-11.13	-11.12	-11.11
-11.19	-11.18	-11.17	-11.16	-11.15	-11.14	-11.13	-11.12	-11.11
-11.19	-11.18	-11.17	-11.16	-11.15	-11.14	-11.13	-11.12	-11.11
-11.19	-11.18	-11.17	-11.16	-11.15	-11.14	-11.13	-11.12	-11.11
-11.19	-11.18	-11.17	-11.16	-11.15	-11.14	-11.13	-11.12	-11.11
-11.19	-11.18	-11.17	-11.16	-11.15	-11.14	-11.13	-11.12	-11.11
-11.19	-11.18	-11.17	-11.16	-11.15	-11.14	-11.13	-11.12	-11.11
-11.19	-11.18	-11.17	-11.16	-11.15	-11.14	-11.13	-11.12	-11.11
-11.19	-11.18	-11.17	-11.16	-11.15	-11.14	-11.13	-11.12	-11.11
-11.19	-11.18	-11.17	-11.16	-11.15	-11.14	-11.13	-11.12	-11.11
-11.19	-11.18	-11.17	-11.16	-11.15	-11.14	-11.13	-11.12	-11.11
-11.19	-11.18	-11.17	-11.16	-11.15	-11.14	-11.13	-11.12	-11.11
-11.19	-11.18	-11.17	-11.16	-11.15	-11.14	-11.13	-11.12	-11.11
-11.19	-11.18	-11.17	-11.16	-11.15	-11.14	-11.13	-11.12	-11.11
-11.19	-11.18	-11.17	-11.16	-11.15	-11.14	-11.13	-11.12	-11.11
-11.19	-11.18	-11.17	-11.16	-11.15	-11.14	-11.13	-11.12	-11.11
-11.19	-11.18	-11.17	-11.16	-11.15	-11.14	-11.13	-11.12	-11.11
-11.19	-11.18	-11.17	-11.16	-11.15	-11.14	-11.13	-11.12	-11.11
-11.19	-11.18	-11.17	-11.16	-11.15	-11.14	-11.13	-11.12	-11.11
-11.19	-11.18	-11.17	-11.16	-11.15	-11.14	-11.13	-11.12	-11.11
-11.19	-11.18	-11.17	-11.16	-11.15	-11.14	-11.13	-11.12	-11.11
-11.19	-11.18	-11.17	-11.16	-11.15	-11.14	-11.13	-11.12	-11.11
-11.19	-11.18	-11.17	-11.16	-11.15	-11.14	-11.13	-11.12	-11.11
-11.19	-11.18	-11.17	-11.16	-11.15	-11.14	-11.13	-11.12	-11.11
-11.19	-11.18	-11.17	-11.16	-11.15	-11.14	-11.13	-11.12	-11.11
-11.19	-11.18	-11.17	-11.16	-11.15	-11.14	-11.13	-11.12	-11.11
-11.19	-11.18	-11.17	-11.16	-11.15	-11.14	-11.13	-11.12	-11.11
-11.19	-11.18	-11.17	-11.16	-11.15	-11.14	-11.13	-11.12	-11.11
-11.19	-11.18	-11.17	-11.16	-11.15	-11.14	-11.13	-11.12	-11.11

-11.19	-11.18	-11.17	-11.16	-11.15	-11.14	-11.13	-11.12	-11.11
-11.19	-11.18	-11.17	-11.16	-11.15	-11.14	-11.13	-11.12	-11.11
-11.19	-11.18	-11.17	-11.16	-11.15	-11.14	-11.13	-11.12	-11.11
-11.19	-11.18	-11.17	-11.16	-11.15	-11.14	-11.13	-11.12	-11.11
-11.19	-11.18	-11.17	-11.16	-11.15	-11.14	-11.13	-11.12	-11.11
-11.19	-11.18	-11.17	-11.16	-11.15	-11.14	-11.13	-11.12	-11.11
-11.19	-11.18	-11.17	-11.16	-11.15	-11.14	-11.13	-11.12	-11.11
-11.19	-11.18	-11.17	-11.16	-11.15	-11.14	-11.13	-11.12	-11.11
-11.19	-11.18	-11.17	-11.16	-11.15	-11.14	-11.13	-11.12	-11.11
-11.19	-11.18	-11.17	-11.16	-11.15	-11.14	-11.13	-11.12	-11.11
-11.19	-11.18	-11.17	-11.16	-11.15	-11.14	-11.13	-11.12	-11.11
-11.19	-11.18	-11.17	-11.16	-11.15	-11.14	-11.13	-11.12	-11.11
-11.19	-11.18	-11.17	-11.16	-11.15	-11.14	-11.13	-11.12	-11.11
-11.19	-11.18	-11.17	-11.16	-11.15	-11.14	-11.13	-11.12	-11.11
-11.19	-11.18	-11.17	-11.16	-11.15	-11.14	-11.13	-11.12	-11.11
-11.19	-11.18	-11.17	-11.16	-11.15	-11.14	-11.13	-11.12	-11.11
-11.19	-11.18	-11.17	-11.16	-11.15	-11.14	-11.13	-11.12	-11.11
-11.19	-11.18	-11.17	-11.16	-11.15	-11.14	-11.13	-11.12	-11.11
-11.19	-11.18	-11.17	-11.16	-11.15	-11.14	-11.13	-11.12	-11.11
-11.19	-11.18	-11.17	-11.16	-11.15	-11.14	-11.13	-11.12	-11.11
-11.19	-11.18	-11.17	-11.16	-11.15	-11.14	-11.13	-11.12	-11.11
-11.19	-11.18	-11.17	-11.16	-11.15	-11.14	-11.13	-11.12	-11.11
-11.19	-11.18	-11.17	-11.16	-11.15	-11.14	-11.13	-11.12	-11.11
-11.19	-11.18	-11.17	-11.16	-11.15	-11.14	-11.13	-11.12	-11.11
-11.19	-11.18	-11.17	-11.16	-11.15	-11.14	-11.13	-11.12	-11.11
-11.19	-11.18	-11.17	-11.16	-11.15	-11.14	-11.13	-11.12	-11.11
-11.19	-11.18	-11.17	-11.16	-11.15	-11.14	-11.13	-11.12	-11.11
-11.19	-11.18	-11.17	-11.16	-11.15	-11.14	-11.13	-11.12	-11.11
-11.19	-11.18	-11.17	-11.16	-11.15	-11.14	-11.13	-11.12	-11.11
-11.19	-11.18	-11.17	-11.16	-11.15	-11.14	-11.13	-11.12	-11.11
-11.19	-11.18	-11.17	-11.16	-11.15	-11.14	-11.13	-11.12	-11.11
-11.19	-11.18	-11.17	-11.16	-11.15	-11.14	-11.13	-11.12	-11.11
-11.19	-11.18	-11.17	-11.16	-11.15	-11.14	-11.13	-11.12	-11.11
-11.19	-11.18	-11.17	-11.16	-11.15	-11.14	-11.13	-11.12	-11.11
-11.19	-11.18	-11.17	-11.16	-11.15	-11.14	-11.13	-11.12	-11.11
-11.19	-11.18	-11.17	-11.16	-11.15	-11.14	-11.13	-11.12	-11.11
-11.19	-11.18	-11.17	-11.16	-11.15	-11.14	-11.13	-11.12	-11.11
-11.19	-11.18	-11.17	-11.16	-11.15	-11.14	-11.13	-11.12	-11.11
-11.19	-11.18	-11.17	-11.16	-11.15	-11.14	-11.13	-11.12	-11.11
-11.19	-11.18	-11.17	-11.16	-11.15	-11.14	-11.13	-11.12	-11.11
-11.19	-11.18	-11.17	-11.16	-11.15	-11.14	-11.13	-11.12	-11.11
-11.19	-11.18	-11.17	-11.16	-11.15	-11.14	-11.13	-11.12	-11.11
-11.19	-11.18	-11.17	-11.16	-11.15	-11.14	-11.13	-11.12	-11.11
-11.19	-11.18	-11.17	-11.16	-11.15	-11.14	-11.13	-11.12	-11.11
-11.19	-11.18	-11.17	-11.16	-11.15	-11.14	-11.13	-11.12	-11.11
-11.19	-11.18	-11.17	-11.16	-11.15	-11.14	-11.13	-11.12	-11.11
-11.19	-11.18	-11.17	-11.16	-11.15	-11.14	-11.13	-11.12	-11.11
-11.19	-11.18	-11.17	-11.16	-11.15	-11.14	-11.13	-11.12	-11.11
-11.19	-11.18	-11.17	-11.16	-11.15	-11.14	-11.13	-11.12	-11.11
-11.19	-11.18	-11.17	-11.16	-11.15	-11.14	-11.13	-11.12	-11.11

-11.19	-11.18	-11.17	-11.16	-11.15	-11.14	-11.13	-11.12	-11.11
-11.19	-11.18	-11.17	-11.16	-11.15	-11.14	-11.13	-11.12	-11.11
-11.19	-11.18	-11.17	-11.16	-11.15	-11.14	-11.13	-11.12	-11.11
-11.19	-11.18	-11.17	-11.16	-11.15	-11.14	-11.13	-11.12	-11.11
-11.19	-11.18	-11.17	-11.16	-11.15	-11.14	-11.13	-11.12	-11.11
-11.19	-11.18	-11.17	-11.16	-11.15	-11.14	-11.13	-11.12	-11.11
-11.19	-11.18	-11.17	-11.16	-11.15	-11.14	-11.13	-11.12	-11.11
-11.19	-11.18	-11.17	-11.16	-11.15	-11.14	-11.13	-11.12	-11.11
-11.19	-11.18	-11.17	-11.16	-11.15	-11.14	-11.13	-11.12	-11.11
-11.19	-11.18	-11.17	-11.16	-11.15	-11.14	-11.13	-11.12	-11.11
-11.19	-11.18	-11.17	-11.16	-11.15	-11.14	-11.13	-11.12	-11.11
-11.19	-11.18	-11.17	-11.16	-11.15	-11.14	-11.13	-11.12	-11.11
-11.19	-11.18	-11.17	-11.16	-11.15	-11.14	-11.13	-11.12	-11.11
-11.19	-11.18	-11.17	-11.16	-11.15	-11.14	-11.13	-11.12	-11.11
-11.19	-11.18	-11.17	-11.16	-11.15	-11.14	-11.13	-11.12	-11.11
-11.19	-11.18	-11.17	-11.16	-11.15	-11.14	-11.13	-11.12	-11.11
-11.19	-11.18	-11.17	-11.16	-11.15	-11.14	-11.13	-11.12	-11.11
-11.19	-11.18	-11.17	-11.16	-11.15	-11.14	-11.13	-11.12	-11.11
-11.19	-11.18	-11.17	-11.16	-11.15	-11.14	-11.13	-11.12	-11.11
-11.19	-11.18	-11.17	-11.16	-11.15	-11.14	-11.13	-11.12	-11.11
-11.19	-11.18	-11.17	-11.16	-11.15	-11.14	-11.13	-11.12	-11.11
-11.19	-11.18	-11.17	-11.16	-11.15	-11.14	-11.13	-11.12	-11.11
-11.19	-11.18	-11.17	-11.16	-11.15	-11.14	-11.13	-11.12	-11.11
-11.19	-11.18	-11.17	-11.16	-11.15	-11.14	-11.13	-11.12	-11.11
-11.19	-11.18	-11.17	-11.16	-11.15	-11.14	-11.13	-11.12	-11.11
-11.19	-11.18	-11.17	-11.16	-11.15	-11.14	-11.13	-11.12	-11.11
-11.19	-11.18	-11.17	-11.16	-11.15	-11.14	-11.13	-11.12	-11.11
-11.19	-11.18	-11.17	-11.16	-11.15	-11.14	-11.13	-11.12	-11.11
-11.19	-11.18	-11.17	-11.16	-11.15	-11.14	-11.13	-11.12	-11.11
-11.19	-11.18	-11.17	-11.16	-11.15	-11.14	-11.13	-11.12	-11.11
-11.19	-11.18	-11.17	-11.16	-11.15	-11.14	-11.13	-11.12	-11.11
-11.19	-11.18	-11.17	-11.16	-11.15	-11.14	-11.13	-11.12	-11.11
-11.19	-11.18	-11.17	-11.16	-11.15	-11.14	-11.13	-11.12	-11.11
-11.19	-11.18	-11.17	-11.16	-11.15	-11.14	-11.13	-11.12	-11.11
-11.19	-11.18	-11.17	-11.16	-11.15	-11.14	-11.13	-11.12	-11.11
-11.19	-11.18	-11.17	-11.16	-11.15	-11.14	-11.13	-11.12	-11.11
-11.19	-11.18	-11.17	-11.16	-11.15	-11.14	-11.13	-11.12	-11.11
-11.19	-11.18	-11.17	-11.16	-11.15	-11.14	-11.13	-11.12	-11.11
-11.19	-11.18	-11.17	-11.16	-11.15	-11.14	-11.13	-11.12	-11.11
-11.19	-11.18	-11.17	-11.16	-11.15	-11.14	-11.13	-11.12	-11.11
-11.19	-11.18	-11.17	-11.16	-11.15	-11.14	-11.13	-11.12	-11.11
-11.19	-11.18	-11.17	-11.16	-11.15	-11.14	-11.13	-11.12	-11.11
-11.19	-11.18	-11.17	-11.16	-11.15	-11.14	-11.13	-11.12	-11.11
-11.19	-11.18	-11.17	-11.16	-11.15	-11.14	-11.13	-11.12	-11.11
-11.19	-11.18	-11.17	-11.16	-11.15	-11.14	-11.13	-11.12	-11.11
-11.19	-11.18	-11.17	-11.16	-11.15	-11.14	-11.13	-11.12	-11.11
-11.19	-11.18	-11.17	-11.16	-11.15	-11.14	-11.13	-11.12	-11.11
-11.19	-11.18	-11.17	-11.16	-11.15	-11.14	-11.13	-11.12	-11.11
-11.19	-11.18	-11.17	-11.16	-11.15	-11.14	-11.13	-11.12	-11.11
-11.19	-11.18	-11.17	-11.16	-11.15	-11.14	-11.13	-11.12	-11.11

-11.19	-11.18	-11.17	-11.16	-11.15	-11.14	-11.13	-11.12	-11.11
-11.19	-11.18	-11.17	-11.16	-11.15	-11.14	-11.13	-11.12	-11.11
-11.19	-11.18	-11.17	-11.16	-11.15	-11.14	-11.13	-11.12	-11.11
-11.19	-11.18	-11.17	-11.16	-11.15	-11.14	-11.13	-11.12	-11.11
-11.19	-11.18	-11.17	-11.16	-11.15	-11.14	-11.13	-11.12	-11.11
-11.19	-11.18	-11.17	-11.16	-11.15	-11.14	-11.13	-11.12	-11.11
-11.19	-11.18	-11.17	-11.16	-11.15	-11.14	-11.13	-11.12	-11.11
-11.19	-11.18	-11.17	-11.16	-11.15	-11.14	-11.13	-11.12	-11.11
-11.19	-11.18	-11.17	-11.16	-11.15	-11.14	-11.13	-11.12	-11.11
-11.19	-11.18	-11.17	-11.16	-11.15	-11.14	-11.13	-11.12	-11.11
-11.19	-11.18	-11.17	-11.16	-11.15	-11.14	-11.13	-11.12	-11.11
-11.19	-11.18	-11.17	-11.16	-11.15	-11.14	-11.13	-11.12	-11.11
-11.19	-11.18	-11.17	-11.16	-11.15	-11.14	-11.13	-11.12	-11.11
-11.19	-11.18	-11.17	-11.16	-11.15	-11.14	-11.13	-11.12	-11.11
-11.19	-11.18	-11.17	-11.16	-11.15	-11.14	-11.13	-11.12	-11.11
-11.19	-11.18	-11.17	-11.16	-11.15	-11.14	-11.13	-11.12	-11.11
-11.19	-11.18	-11.17	-11.16	-11.15	-11.14	-11.13	-11.12	-11.11
-11.19	-11.18	-11.17	-11.16	-11.15	-11.14	-11.13	-11.12	-11.11
-11.19	-11.18	-11.17	-11.16	-11.15	-11.14	-11.13	-11.12	-11.11
-11.19	-11.18	-11.17	-11.16	-11.15	-11.14	-11.13	-11.12	-11.11
-11.19	-11.18	-11.17	-11.16	-11.15	-11.14	-11.13	-11.12	-11.11
-11.19	-11.18	-11.17	-11.16	-11.15	-11.14	-11.13	-11.12	-11.11
-11.19	-11.18	-11.17	-11.16	-11.15	-11.14	-11.13	-11.12	-11.11
-11.19	-11.18	-11.17	-11.16	-11.15	-11.14	-11.13	-11.12	-11.11
-11.19	-11.18	-11.17	-11.16	-11.15	-11.14	-11.13	-11.12	-11.11
-11.19	-11.18	-11.17	-11.16	-11.15	-11.14	-11.13	-11.12	-11.11
-11.19	-11.18	-11.17	-11.16	-11.15	-11.14	-11.13	-11.12	-11.11
-11.19	-11.18	-11.17	-11.16	-11.15	-11.14	-11.13	-11.12	-11.11
-11.19	-11.18	-11.17	-11.16	-11.15	-11.14	-11.13	-11.12	-11.11
-11.19	-11.18	-11.17	-11.16	-11.15	-11.14	-11.13	-11.12	-11.11
-11.19	-11.18	-11.17	-11.16	-11.15	-11.14	-11.13	-11.12	-11.11
-11.19	-11.18	-11.17	-11.16	-11.15	-11.14	-11.13	-11.12	-11.11
-11.19	-11.18	-11.17	-11.16	-11.15	-11.14	-11.13	-11.12	-11.11
-11.19	-11.18	-11.17	-11.16	-11.15	-11.14	-11.13	-11.12	-11.11
-11.19	-11.18	-11.17	-11.16	-11.15	-11.14	-11.13	-11.12	-11.11
-11.19	-11.18	-11.17	-11.16	-11.15	-11.14	-11.13	-11.12	-11.11
-11.19	-11.18	-11.17	-11.16	-11.15	-11.14	-11.13	-11.12	-11.11
-11.19	-11.18	-11.17	-11.16	-11.15	-11.14	-11.13	-11.12	-11.11
-11.19	-11.18	-11.17	-11.16	-11.15	-11.14	-11.13	-11.12	-11.11
-11.19	-11.18	-11.17	-11.16	-11.15	-11.14	-11.13	-11.12	-11.11
-11.19	-11.18	-11.17	-11.16	-11.15	-11.14	-11.13	-11.12	-11.11
-11.19	-11.18	-11.17	-11.16	-11.15	-11.14	-11.13	-11.12	-11.11
-11.19	-11.18	-11.17	-11.16	-11.15	-11.14	-11.13	-11.12	-11.11
-11.19	-11.18	-11.17	-11.16	-11.15	-11.14	-11.13	-11.12	-11.11
-11.19	-11.18	-11.17	-11.16	-11.15	-11.14	-11.13	-11.12	-11.11
-11.19	-11.18	-11.17	-11.16	-11.15	-11.14	-11.13	-11.12	-11.11
-11.19	-11.18	-11.17	-11.16	-11.15	-11.14	-11.13	-11.12	-11.11
-11.19	-11.18	-11.17	-11.16	-11.15	-11.14	-11.13	-11.12	-11.11
-11.19	-11.18	-11.17	-11.16	-11.15	-11.14	-11.13	-11.12	-11.11
-11.19	-11.18	-11.17	-11.16	-11.15	-11.14	-11.13	-11.12	-11.11

-11.19	-11.18	-11.17	-11.16	-11.15	-11.14	-11.13	-11.12	-11.11
-11.19	-11.18	-11.17	-11.16	-11.15	-11.14	-11.13	-11.12	-11.11
-11.19	-11.18	-11.17	-11.16	-11.15	-11.14	-11.13	-11.12	-11.11
-11.19	-11.18	-11.17	-11.16	-11.15	-11.14	-11.13	-11.12	-11.11
-11.19	-11.18	-11.17	-11.16	-11.15	-11.14	-11.13	-11.12	-11.11
-11.19	-11.18	-11.17	-11.16	-11.15	-11.14	-11.13	-11.12	-11.11
-11.19	-11.18	-11.17	-11.16	-11.15	-11.14	-11.13	-11.12	-11.11
-11.19	-11.18	-11.17	-11.16	-11.15	-11.14	-11.13	-11.12	-11.11
-11.19	-11.18	-11.17	-11.16	-11.15	-11.14	-11.13	-11.12	-11.11
-11.19	-11.18	-11.17	-11.16	-11.15	-11.14	-11.13	-11.12	-11.11
-11.19	-11.18	-11.17	-11.16	-11.15	-11.14	-11.13	-11.12	-11.11
-11.19	-11.18	-11.17	-11.16	-11.15	-11.14	-11.13	-11.12	-11.11
-11.19	-11.18	-11.17	-11.16	-11.15	-11.14	-11.13	-11.12	-11.11
-11.19	-11.18	-11.17	-11.16	-11.15	-11.14	-11.13	-11.12	-11.11
-11.19	-11.18	-11.17	-11.16	-11.15	-11.14	-11.13	-11.12	-11.11
-11.19	-11.18	-11.17	-11.16	-11.15	-11.14	-11.13	-11.12	-11.11
-11.19	-11.18	-11.17	-11.16	-11.15	-11.14	-11.13	-11.12	-11.11
-11.19	-11.18	-11.17	-11.16	-11.15	-11.14	-11.13	-11.12	-11.11
-11.19	-11.18	-11.17	-11.16	-11.15	-11.14	-11.13	-11.12	-11.11
-11.19	-11.18	-11.17	-11.16	-11.15	-11.14	-11.13	-11.12	-11.11
-11.19	-11.18	-11.17	-11.16	-11.15	-11.14	-11.13	-11.12	-11.11
-11.19	-11.18	-11.17	-11.16	-11.15	-11.14	-11.13	-11.12	-11.11
-11.19	-11.18	-11.17	-11.16	-11.15	-11.14	-11.13	-11.12	-11.11
-11.19	-11.18	-11.17	-11.16	-11.15	-11.14	-11.13	-11.12	-11.11
-11.19	-11.18	-11.17	-11.16	-11.15	-11.14	-11.13	-11.12	-11.11
-11.19	-11.18	-11.17	-11.16	-11.15	-11.14	-11.13	-11.12	-11.11
-11.19	-11.18	-11.17	-11.16	-11.15	-11.14	-11.13	-11.12	-11.11
-11.19	-11.18	-11.17	-11.16	-11.15	-11.14	-11.13	-11.12	-11.11
-11.19	-11.18	-11.17	-11.16	-11.15	-11.14	-11.13	-11.12	-11.11
-11.19	-11.18	-11.17	-11.16	-11.15	-11.14	-11.13	-11.12	-11.11
-11.19	-11.18	-11.17	-11.16	-11.15	-11.14	-11.13	-11.12	-11.11
-11.19	-11.18	-11.17	-11.16	-11.15	-11.14	-11.13	-11.12	-11.11
-11.19	-11.18	-11.17	-11.16	-11.15	-11.14	-11.13	-11.12	-11.11
-11.19	-11.18	-11.17	-11.16	-11.15	-11.14	-11.13	-11.12	-11.11
-11.19	-11.18	-11.17	-11.16	-11.15	-11.14	-11.13	-11.12	-11.11
-11.19	-11.18	-11.17	-11.16	-11.15	-11.14	-11.13	-11.12	-11.11
-11.19	-11.18	-11.17	-11.16	-11.15	-11.14	-11.13	-11.12	-11.11
-11.19	-11.18	-11.17	-11.16	-11.15	-11.14	-11.13	-11.12	-11.11
-11.19	-11.18	-11.17	-11.16	-11.15	-11.14	-11.13	-11.12	-11.11
-11.19	-11.18	-11.17	-11.16	-11.15	-11.14	-11.13	-11.12	-11.11
-11.19	-11.18	-11.17	-11.16	-11.15	-11.14	-11.13	-11.12	-11.11
-11.19	-11.18	-11.17	-11.16	-11.15	-11.14	-11.13	-11.12	-11.11
-11.19	-11.18	-11.17	-11.16	-11.15	-11.14	-11.13	-11.12	-11.11
-11.19	-11.18	-11.17	-11.16	-11.15	-11.14	-11.13	-11.12	-11.11
-11.19	-11.18	-11.17	-11.16	-11.15	-11.14	-11.13	-11.12	-11.11
-11.19	-11.18	-11.17	-11.16	-11.15	-11.14	-11.13	-11.12	-11.11
-11.19	-11.18	-11.17	-11.16	-11.15	-11.14	-11.13	-11.12	-11.11
-11.19	-11.18	-11.17	-11.16	-11.15	-11.14	-11.13	-11.12	-11.11
-11.19	-11.18	-11.17	-11.16	-11.15	-11.14	-11.13	-11.12	-11.11
-11.19	-11.18	-11.17	-11.16	-11.15	-11.14	-11.13	-11.12	-11.11

-11.19	-11.18	-11.17	-11.16	-11.15	-11.14	-11.13	-11.12	-11.11
-11.19	-11.18	-11.17	-11.16	-11.15	-11.14	-11.13	-11.12	-11.11
-11.19	-11.18	-11.17	-11.16	-11.15	-11.14	-11.13	-11.12	-11.11
-11.19	-11.18	-11.17	-11.16	-11.15	-11.14	-11.13	-11.12	-11.11
-11.19	-11.18	-11.17	-11.16	-11.15	-11.14	-11.13	-11.12	-11.11
-11.19	-11.18	-11.17	-11.16	-11.15	-11.14	-11.13	-11.12	-11.11
-11.19	-11.18	-11.17	-11.16	-11.15	-11.14	-11.13	-11.12	-11.11
-11.19	-11.18	-11.17	-11.16	-11.15	-11.14	-11.13	-11.12	-11.11
-11.19	-11.18	-11.17	-11.16	-11.15	-11.14	-11.13	-11.12	-11.11
-11.19	-11.18	-11.17	-11.16	-11.15	-11.14	-11.13	-11.12	-11.11
-11.19	-11.18	-11.17	-11.16	-11.15	-11.14	-11.13	-11.12	-11.11
-11.19	-11.18	-11.17	-11.16	-11.15	-11.14	-11.13	-11.12	-11.11
-11.19	-11.18	-11.17	-11.16	-11.15	-11.14	-11.13	-11.12	-11.11
-11.19	-11.18	-11.17	-11.16	-11.15	-11.14	-11.13	-11.12	-11.11
-11.19	-11.18	-11.17	-11.16	-11.15	-11.14	-11.13	-11.12	-11.11
-11.19	-11.18	-11.17	-11.16	-11.15	-11.14	-11.13	-11.12	-11.11
-11.19	-11.18	-11.17	-11.16	-11.15	-11.14	-11.13	-11.12	-11.11
-11.19	-11.18	-11.17	-11.16	-11.15	-11.14	-11.13	-11.12	-11.11
-11.19	-11.18	-11.17	-11.16	-11.15	-11.14	-11.13	-11.12	-11.11
-11.19	-11.18	-11.17	-11.16	-11.15	-11.14	-11.13	-11.12	-11.11
-11.19	-11.18	-11.17	-11.16	-11.15	-11.14	-11.13	-11.12	-11.11
-11.19	-11.18	-11.17	-11.16	-11.15	-11.14	-11.13	-11.12	-11.11
-11.19	-11.18	-11.17	-11.16	-11.15	-11.14	-11.13	-11.12	-11.11
-11.19	-11.18	-11.17	-11.16	-11.15	-11.14	-11.13	-11.12	-11.11
-11.19	-11.18	-11.17	-11.16	-11.15	-11.14	-11.13	-11.12	-11.11
-11.19	-11.18	-11.17	-11.16	-11.15	-11.14	-11.13	-11.12	-11.11
-11.19	-11.18	-11.17	-11.16	-11.15	-11.14	-11.13	-11.12	-11.11
-11.19	-11.18	-11.17	-11.16	-11.15	-11.14	-11.13	-11.12	-11.11
-11.19	-11.18	-11.17	-11.16	-11.15	-11.14	-11.13	-11.12	-11.11
-11.19	-11.18	-11.17	-11.16	-11.15	-11.14	-11.13	-11.12	-11.11
-11.19	-11.18	-11.17	-11.16	-11.15	-11.14	-11.13	-11.12	-11.11
-11.19	-11.18	-11.17	-11.16	-11.15	-11.14	-11.13	-11.12	-11.11
-11.19	-11.18	-11.17	-11.16	-11.15	-11.14	-11.13	-11.12	-11.11
-11.19	-11.18	-11.17	-11.16	-11.15	-11.14	-11.13	-11.12	-11.11
-11.19	-11.18	-11.17	-11.16	-11.15	-11.14	-11.13	-11.12	-11.11
-11.19	-11.18	-11.17	-11.16	-11.15	-11.14	-11.13	-11.12	-11.11
-11.19	-11.18	-11.17	-11.16	-11.15	-11.14	-11.13	-11.12	-11.11
-11.19	-11.18	-11.17	-11.16	-11.15	-11.14	-11.13	-11.12	-11.11
-11.19	-11.18	-11.17	-11.16	-11.15	-11.14	-11.13	-11.12	-11.11
-11.19	-11.18	-11.17	-11.16	-11.15	-11.14	-11.13	-11.12	-11.11
-11.19	-11.18	-11.17	-11.16	-11.15	-11.14	-11.13	-11.12	-11.11
-11.19	-11.18	-11.17	-11.16	-11.15	-11.14	-11.13	-11.12	-11.11
-11.19	-11.18	-11.17	-11.16	-11.15	-11.14	-11.13	-11.12	-11.11
-11.19	-11.18	-11.17	-11.16	-11.15	-11.14	-11.13	-11.12	-11.11
-11.19	-11.18	-11.17	-11.16	-11.15	-11.14	-11.13	-11.12	-11.11
-11.19	-11.18	-11.17	-11.16	-11.15	-11.14	-11.13	-11.12	-11.11
-11.19	-11.18	-11.17	-11.16	-11.15	-11.14	-11.13	-11.12	-11.11
-11.19	-11.18	-11.17	-11.16	-11.15	-11.14	-11.13	-11.12	-11.11
-11.19	-11.18	-11.17	-11.16	-11.15	-11.14	-11.13	-11.12	-11.11
-11.19	-11.18	-11.17	-11.16	-11.15	-11.14	-11.13	-11.12	-11.11

-11.19	-11.18	-11.17	-11.16	-11.15	-11.14	-11.13	-11.12	-11.11

-11.1	-11.09	-11.08	-11.07	-11.06	-11.05	-11.04	-11.03	-11.02
-11.1	-11.09	-11.08	-11.07	-11.06	-11.05	-11.04	-11.03	-11.02
-11.1	-11.09	-11.08	-11.07	-11.06	-11.05	-11.04	-11.03	-11.02
-11.1	-11.09	-11.08	-11.07	-11.06	-11.05	-11.04	-11.03	-11.02
-11.1	-11.09	-11.08	-11.07	-11.06	-11.05	-11.04	-11.03	-11.02
-11.1	-11.09	-11.08	-11.07	-11.06	-11.05	-11.04	-11.03	-11.02
-11.1	-11.09	-11.08	-11.07	-11.06	-11.05	-11.04	-11.03	-11.02
-11.1	-11.09	-11.08	-11.07	-11.06	-11.05	-11.04	-11.03	-11.02
-11.1	-11.09	-11.08	-11.07	-11.06	-11.05	-11.04	-11.03	-11.02
-11.1	-11.09	-11.08	-11.07	-11.06	-11.05	-11.04	-11.03	-11.02
-11.1	-11.09	-11.08	-11.07	-11.06	-11.05	-11.04	-11.03	-11.02
-11.1	-11.09	-11.08	-11.07	-11.06	-11.05	-11.04	-11.03	-11.02
-11.1	-11.09	-11.08	-11.07	-11.06	-11.05	-11.04	-11.03	-11.02
-11.1	-11.09	-11.08	-11.07	-11.06	-11.05	-11.04	-11.03	-11.02
-11.1	-11.09	-11.08	-11.07	-11.06	-11.05	-11.04	-11.03	-11.02
-11.1	-11.09	-11.08	-11.07	-11.06	-11.05	-11.04	-11.03	-11.02
-11.1	-11.09	-11.08	-11.07	-11.06	-11.05	-11.04	-11.03	-11.02
-11.1	-11.09	-11.08	-11.07	-11.06	-11.05	-11.04	-11.03	-11.02
-11.1	-11.09	-11.08	-11.07	-11.06	-11.05	-11.04	-11.03	-11.02
-11.1	-11.09	-11.08	-11.07	-11.06	-11.05	-11.04	-11.03	-11.02
-11.1	-11.09	-11.08	-11.07	-11.06	-11.05	-11.04	-11.03	-11.02
-11.1	-11.09	-11.08	-11.07	-11.06	-11.05	-11.04	-11.03	-11.02
-11.1	-11.09	-11.08	-11.07	-11.06	-11.05	-11.04	-11.03	-11.02
-11.1	-11.09	-11.08	-11.07	-11.06	-11.05	-11.04	-11.03	-11.02
-11.1	-11.09	-11.08	-11.07	-11.06	-11.05	-11.04	-11.03	-11.02
-11.1	-11.09	-11.08	-11.07	-11.06	-11.05	-11.04	-11.03	-11.02
-11.1	-11.09	-11.08	-11.07	-11.06	-11.05	-11.04	-11.03	-11.02
-11.1	-11.09	-11.08	-11.07	-11.06	-11.05	-11.04	-11.03	-11.02
-11.1	-11.09	-11.08	-11.07	-11.06	-11.05	-11.04	-11.03	-11.02
-11.1	-11.09	-11.08	-11.07	-11.06	-11.05	-11.04	-11.03	-11.02
-11.1	-11.09	-11.08	-11.07	-11.06	-11.05	-11.04	-11.03	-11.02
-11.1	-11.09	-11.08	-11.07	-11.06	-11.05	-11.04	-11.03	-11.02
-11.1	-11.09	-11.08	-11.07	-11.06	-11.05	-11.04	-11.03	-11.02
-11.1	-11.09	-11.08	-11.07	-11.06	-11.05	-11.04	-11.03	-11.02
-11.1	-11.09	-11.08	-11.07	-11.06	-11.05	-11.04	-11.03	-11.02
-11.1	-11.09	-11.08	-11.07	-11.06	-11.05	-11.04	-11.03	-11.02
-11.1	-11.09	-11.08	-11.07	-11.06	-11.05	-11.04	-11.03	-11.02
-11.1	-11.09	-11.08	-11.07	-11.06	-11.05	-11.04	-11.03	-11.02
-11.1	-11.09	-11.08	-11.07	-11.06	-11.05	-11.04	-11.03	-11.02
-11.1	-11.09	-11.08	-11.07	-11.06	-11.05	-11.04	-11.03	-11.02
-11.1	-11.09	-11.08	-11.07	-11.06	-11.05	-11.04	-11.03	-11.02
-11.1	-11.09	-11.08	-11.07	-11.06	-11.05	-11.04	-11.03	-11.02
-11.1	-11.09	-11.08	-11.07	-11.06	-11.05	-11.04	-11.03	-11.02
-11.1	-11.09	-11.08	-11.07	-11.06	-11.05	-11.04	-11.03	-11.02
-11.1	-11.09	-11.08	-11.07	-11.06	-11.05	-11.04	-11.03	-11.02
-11.1	-11.09	-11.08	-11.07	-11.06	-11.05	-11.04	-11.03	-11.02
-11.1	-11.09	-11.08	-11.07	-11.06	-11.05	-11.04	-11.03	-11.02
-11.1	-11.09	-11.08	-11.07	-11.06	-11.05	-11.04	-11.03	-11.02
-11.1	-11.09	-11.08	-11.07	-11.06	-11.05	-11.04	-11.03	-11.02
-11.1	-11.09	-11.08	-11.07	-11.06	-11.05	-11.04	-11.03	-11.02

-11.1	-11.09	-11.08	-11.07	-11.06	-11.05	-11.04	-11.03	-11.02
-11.1	-11.09	-11.08	-11.07	-11.06	-11.05	-11.04	-11.03	-11.02
-11.1	-11.09	-11.08	-11.07	-11.06	-11.05	-11.04	-11.03	-11.02
-11.1	-11.09	-11.08	-11.07	-11.06	-11.05	-11.04	-11.03	-11.02
-11.1	-11.09	-11.08	-11.07	-11.06	-11.05	-11.04	-11.03	-11.02
-11.1	-11.09	-11.08	-11.07	-11.06	-11.05	-11.04	-11.03	-11.02
-11.1	-11.09	-11.08	-11.07	-11.06	-11.05	-11.04	-11.03	-11.02
[truncated: 439,860 more chars]
