# Supplementary material for: Artefact-free topography based scleral-asymmetry
Source: PLoS One. 2019 Jul 26;14(7):e0219789. doi: 10.1371/journal.pone.0219789 (PMC6660081; doi:10.1371/journal.pone.0219789)
Supplement: S1 Table — (ZIP) [file Y.pdf › Y.pdf]

-12	-12	-12	-12	-12	-12	-12	-12	-12
-11.99	-11.99	-11.99	-11.99	-11.99	-11.99	-11.99	-11.99	-11.99
-11.98	-11.98	-11.98	-11.98	-11.98	-11.98	-11.98	-11.98	-11.98
-11.97	-11.97	-11.97	-11.97	-11.97	-11.97	-11.97	-11.97	-11.97
-11.96	-11.96	-11.96	-11.96	-11.96	-11.96	-11.96	-11.96	-11.96
-11.95	-11.95	-11.95	-11.95	-11.95	-11.95	-11.95	-11.95	-11.95
-11.94	-11.94	-11.94	-11.94	-11.94	-11.94	-11.94	-11.94	-11.94
-11.93	-11.93	-11.93	-11.93	-11.93	-11.93	-11.93	-11.93	-11.93
-11.92	-11.92	-11.92	-11.92	-11.92	-11.92	-11.92	-11.92	-11.92
-11.91	-11.91	-11.91	-11.91	-11.91	-11.91	-11.91	-11.91	-11.91
-11.9	-11.9	-11.9	-11.9	-11.9	-11.9	-11.9	-11.9	-11.9
-11.89	-11.89	-11.89	-11.89	-11.89	-11.89	-11.89	-11.89	-11.89
-11.88	-11.88	-11.88	-11.88	-11.88	-11.88	-11.88	-11.88	-11.88
-11.87	-11.87	-11.87	-11.87	-11.87	-11.87	-11.87	-11.87	-11.87
-11.86	-11.86	-11.86	-11.86	-11.86	-11.86	-11.86	-11.86	-11.86
-11.85	-11.85	-11.85	-11.85	-11.85	-11.85	-11.85	-11.85	-11.85
-11.84	-11.84	-11.84	-11.84	-11.84	-11.84	-11.84	-11.84	-11.84
-11.83	-11.83	-11.83	-11.83	-11.83	-11.83	-11.83	-11.83	-11.83
-11.82	-11.82	-11.82	-11.82	-11.82	-11.82	-11.82	-11.82	-11.82
-11.81	-11.81	-11.81	-11.81	-11.81	-11.81	-11.81	-11.81	-11.81
-11.8	-11.8	-11.8	-11.8	-11.8	-11.8	-11.8	-11.8	-11.8
-11.79	-11.79	-11.79	-11.79	-11.79	-11.79	-11.79	-11.79	-11.79
-11.78	-11.78	-11.78	-11.78	-11.78	-11.78	-11.78	-11.78	-11.78
-11.77	-11.77	-11.77	-11.77	-11.77	-11.77	-11.77	-11.77	-11.77
-11.76	-11.76	-11.76	-11.76	-11.76	-11.76	-11.76	-11.76	-11.76
-11.75	-11.75	-11.75	-11.75	-11.75	-11.75	-11.75	-11.75	-11.75
-11.74	-11.74	-11.74	-11.74	-11.74	-11.74	-11.74	-11.74	-11.74
-11.73	-11.73	-11.73	-11.73	-11.73	-11.73	-11.73	-11.73	-11.73
-11.72	-11.72	-11.72	-11.72	-11.72	-11.72	-11.72	-11.72	-11.72
-11.71	-11.71	-11.71	-11.71	-11.71	-11.71	-11.71	-11.71	-11.71
-11.7	-11.7	-11.7	-11.7	-11.7	-11.7	-11.7	-11.7	-11.7
-11.69	-11.69	-11.69	-11.69	-11.69	-11.69	-11.69	-11.69	-11.69
-11.68	-11.68	-11.68	-11.68	-11.68	-11.68	-11.68	-11.68	-11.68
-11.67	-11.67	-11.67	-11.67	-11.67	-11.67	-11.67	-11.67	-11.67
-11.66	-11.66	-11.66	-11.66	-11.66	-11.66	-11.66	-11.66	-11.66
-11.65	-11.65	-11.65	-11.65	-11.65	-11.65	-11.65	-11.65	-11.65
-11.64	-11.64	-11.64	-11.64	-11.64	-11.64	-11.64	-11.64	-11.64
-11.63	-11.63	-11.63	-11.63	-11.63	-11.63	-11.63	-11.63	-11.63
-11.62	-11.62	-11.62	-11.62	-11.62	-11.62	-11.62	-11.62	-11.62
-11.61	-11.61	-11.61	-11.61	-11.61	-11.61	-11.61	-11.61	-11.61
-11.6	-11.6	-11.6	-11.6	-11.6	-11.6	-11.6	-11.6	-11.6
-11.59	-11.59	-11.59	-11.59	-11.59	-11.59	-11.59	-11.59	-11.59
-11.58	-11.58	-11.58	-11.58	-11.58	-11.58	-11.58	-11.58	-11.58
-11.57	-11.57	-11.57	-11.57	-11.57	-11.57	-11.57	-11.57	-11.57
-11.56	-11.56	-11.56	-11.56	-11.56	-11.56	-11.56	-11.56	-11.56
-11.55	-11.55	-11.55	-11.55	-11.55	-11.55	-11.55	-11.55	-11.55
-11.54	-11.54	-11.54	-11.54	-11.54	-11.54	-11.54	-11.54	-11.54
-11.53	-11.53	-11.53	-11.53	-11.53	-11.53	-11.53	-11.53	-11.53
-11.52	-11.52	-11.52	-11.52	-11.52	-11.52	-11.52	-11.52	-11.52
-11.51	-11.51	-11.51	-11.51	-11.51	-11.51	-11.51	-11.51	-11.51

-11.5	-11.5	-11.5	-11.5	-11.5	-11.5	-11.5	-11.5	-11.5
-11.49	-11.49	-11.49	-11.49	-11.49	-11.49	-11.49	-11.49	-11.49
-11.48	-11.48	-11.48	-11.48	-11.48	-11.48	-11.48	-11.48	-11.48
-11.47	-11.47	-11.47	-11.47	-11.47	-11.47	-11.47	-11.47	-11.47
-11.46	-11.46	-11.46	-11.46	-11.46	-11.46	-11.46	-11.46	-11.46
-11.45	-11.45	-11.45	-11.45	-11.45	-11.45	-11.45	-11.45	-11.45
-11.44	-11.44	-11.44	-11.44	-11.44	-11.44	-11.44	-11.44	-11.44
-11.43	-11.43	-11.43	-11.43	-11.43	-11.43	-11.43	-11.43	-11.43
-11.42	-11.42	-11.42	-11.42	-11.42	-11.42	-11.42	-11.42	-11.42
-11.41	-11.41	-11.41	-11.41	-11.41	-11.41	-11.41	-11.41	-11.41
-11.4	-11.4	-11.4	-11.4	-11.4	-11.4	-11.4	-11.4	-11.4
-11.39	-11.39	-11.39	-11.39	-11.39	-11.39	-11.39	-11.39	-11.39
-11.38	-11.38	-11.38	-11.38	-11.38	-11.38	-11.38	-11.38	-11.38
-11.37	-11.37	-11.37	-11.37	-11.37	-11.37	-11.37	-11.37	-11.37
-11.36	-11.36	-11.36	-11.36	-11.36	-11.36	-11.36	-11.36	-11.36
-11.35	-11.35	-11.35	-11.35	-11.35	-11.35	-11.35	-11.35	-11.35
-11.34	-11.34	-11.34	-11.34	-11.34	-11.34	-11.34	-11.34	-11.34
-11.33	-11.33	-11.33	-11.33	-11.33	-11.33	-11.33	-11.33	-11.33
-11.32	-11.32	-11.32	-11.32	-11.32	-11.32	-11.32	-11.32	-11.32
-11.31	-11.31	-11.31	-11.31	-11.31	-11.31	-11.31	-11.31	-11.31
-11.3	-11.3	-11.3	-11.3	-11.3	-11.3	-11.3	-11.3	-11.3
-11.29	-11.29	-11.29	-11.29	-11.29	-11.29	-11.29	-11.29	-11.29
-11.28	-11.28	-11.28	-11.28	-11.28	-11.28	-11.28	-11.28	-11.28
-11.27	-11.27	-11.27	-11.27	-11.27	-11.27	-11.27	-11.27	-11.27
-11.26	-11.26	-11.26	-11.26	-11.26	-11.26	-11.26	-11.26	-11.26
-11.25	-11.25	-11.25	-11.25	-11.25	-11.25	-11.25	-11.25	-11.25
-11.24	-11.24	-11.24	-11.24	-11.24	-11.24	-11.24	-11.24	-11.24
-11.23	-11.23	-11.23	-11.23	-11.23	-11.23	-11.23	-11.23	-11.23
-11.22	-11.22	-11.22	-11.22	-11.22	-11.22	-11.22	-11.22	-11.22
-11.21	-11.21	-11.21	-11.21	-11.21	-11.21	-11.21	-11.21	-11.21
-11.2	-11.2	-11.2	-11.2	-11.2	-11.2	-11.2	-11.2	-11.2
-11.19	-11.19	-11.19	-11.19	-11.19	-11.19	-11.19	-11.19	-11.19
-11.18	-11.18	-11.18	-11.18	-11.18	-11.18	-11.18	-11.18	-11.18
-11.17	-11.17	-11.17	-11.17	-11.17	-11.17	-11.17	-11.17	-11.17
-11.16	-11.16	-11.16	-11.16	-11.16	-11.16	-11.16	-11.16	-11.16
-11.15	-11.15	-11.15	-11.15	-11.15	-11.15	-11.15	-11.15	-11.15
-11.14	-11.14	-11.14	-11.14	-11.14	-11.14	-11.14	-11.14	-11.14
-11.13	-11.13	-11.13	-11.13	-11.13	-11.13	-11.13	-11.13	-11.13
-11.12	-11.12	-11.12	-11.12	-11.12	-11.12	-11.12	-11.12	-11.12
-11.11	-11.11	-11.11	-11.11	-11.11	-11.11	-11.11	-11.11	-11.11
-11.1	-11.1	-11.1	-11.1	-11.1	-11.1	-11.1	-11.1	-11.1
-11.09	-11.09	-11.09	-11.09	-11.09	-11.09	-11.09	-11.09	-11.09
-11.08	-11.08	-11.08	-11.08	-11.08	-11.08	-11.08	-11.08	-11.08
-11.07	-11.07	-11.07	-11.07	-11.07	-11.07	-11.07	-11.07	-11.07
-11.06	-11.06	-11.06	-11.06	-11.06	-11.06	-11.06	-11.06	-11.06
-11.05	-11.05	-11.05	-11.05	-11.05	-11.05	-11.05	-11.05	-11.05
-11.04	-11.04	-11.04	-11.04	-11.04	-11.04	-11.04	-11.04	-11.04
-11.03	-11.03	-11.03	-11.03	-11.03	-11.03	-11.03	-11.03	-11.03
-11.02	-11.02	-11.02	-11.02	-11.02	-11.02	-11.02	-11.02	-11.02
-11.01	-11.01	-11.01	-11.01	-11.01	-11.01	-11.01	-11.01	-11.01

-11	-11	-11	-11	-11	-11	-11	-11	-11
-10.99	-10.99	-10.99	-10.99	-10.99	-10.99	-10.99	-10.99	-10.99
-10.98	-10.98	-10.98	-10.98	-10.98	-10.98	-10.98	-10.98	-10.98
-10.97	-10.97	-10.97	-10.97	-10.97	-10.97	-10.97	-10.97	-10.97
-10.96	-10.96	-10.96	-10.96	-10.96	-10.96	-10.96	-10.96	-10.96
-10.95	-10.95	-10.95	-10.95	-10.95	-10.95	-10.95	-10.95	-10.95
-10.94	-10.94	-10.94	-10.94	-10.94	-10.94	-10.94	-10.94	-10.94
-10.93	-10.93	-10.93	-10.93	-10.93	-10.93	-10.93	-10.93	-10.93
-10.92	-10.92	-10.92	-10.92	-10.92	-10.92	-10.92	-10.92	-10.92
-10.91	-10.91	-10.91	-10.91	-10.91	-10.91	-10.91	-10.91	-10.91
-10.9	-10.9	-10.9	-10.9	-10.9	-10.9	-10.9	-10.9	-10.9
-10.89	-10.89	-10.89	-10.89	-10.89	-10.89	-10.89	-10.89	-10.89
-10.88	-10.88	-10.88	-10.88	-10.88	-10.88	-10.88	-10.88	-10.88
-10.87	-10.87	-10.87	-10.87	-10.87	-10.87	-10.87	-10.87	-10.87
-10.86	-10.86	-10.86	-10.86	-10.86	-10.86	-10.86	-10.86	-10.86
-10.85	-10.85	-10.85	-10.85	-10.85	-10.85	-10.85	-10.85	-10.85
-10.84	-10.84	-10.84	-10.84	-10.84	-10.84	-10.84	-10.84	-10.84
-10.83	-10.83	-10.83	-10.83	-10.83	-10.83	-10.83	-10.83	-10.83
-10.82	-10.82	-10.82	-10.82	-10.82	-10.82	-10.82	-10.82	-10.82
-10.81	-10.81	-10.81	-10.81	-10.81	-10.81	-10.81	-10.81	-10.81
-10.8	-10.8	-10.8	-10.8	-10.8	-10.8	-10.8	-10.8	-10.8
-10.79	-10.79	-10.79	-10.79	-10.79	-10.79	-10.79	-10.79	-10.79
-10.78	-10.78	-10.78	-10.78	-10.78	-10.78	-10.78	-10.78	-10.78
-10.77	-10.77	-10.77	-10.77	-10.77	-10.77	-10.77	-10.77	-10.77
-10.76	-10.76	-10.76	-10.76	-10.76	-10.76	-10.76	-10.76	-10.76
-10.75	-10.75	-10.75	-10.75	-10.75	-10.75	-10.75	-10.75	-10.75
-10.74	-10.74	-10.74	-10.74	-10.74	-10.74	-10.74	-10.74	-10.74
-10.73	-10.73	-10.73	-10.73	-10.73	-10.73	-10.73	-10.73	-10.73
-10.72	-10.72	-10.72	-10.72	-10.72	-10.72	-10.72	-10.72	-10.72
-10.71	-10.71	-10.71	-10.71	-10.71	-10.71	-10.71	-10.71	-10.71
-10.7	-10.7	-10.7	-10.7	-10.7	-10.7	-10.7	-10.7	-10.7
-10.69	-10.69	-10.69	-10.69	-10.69	-10.69	-10.69	-10.69	-10.69
-10.68	-10.68	-10.68	-10.68	-10.68	-10.68	-10.68	-10.68	-10.68
-10.67	-10.67	-10.67	-10.67	-10.67	-10.67	-10.67	-10.67	-10.67
-10.66	-10.66	-10.66	-10.66	-10.66	-10.66	-10.66	-10.66	-10.66
-10.65	-10.65	-10.65	-10.65	-10.65	-10.65	-10.65	-10.65	-10.65
-10.64	-10.64	-10.64	-10.64	-10.64	-10.64	-10.64	-10.64	-10.64
-10.63	-10.63	-10.63	-10.63	-10.63	-10.63	-10.63	-10.63	-10.63
-10.62	-10.62	-10.62	-10.62	-10.62	-10.62	-10.62	-10.62	-10.62
-10.61	-10.61	-10.61	-10.61	-10.61	-10.61	-10.61	-10.61	-10.61
-10.6	-10.6	-10.6	-10.6	-10.6	-10.6	-10.6	-10.6	-10.6
-10.59	-10.59	-10.59	-10.59	-10.59	-10.59	-10.59	-10.59	-10.59
-10.58	-10.58	-10.58	-10.58	-10.58	-10.58	-10.58	-10.58	-10.58
-10.57	-10.57	-10.57	-10.57	-10.57	-10.57	-10.57	-10.57	-10.57
-10.56	-10.56	-10.56	-10.56	-10.56	-10.56	-10.56	-10.56	-10.56
-10.55	-10.55	-10.55	-10.55	-10.55	-10.55	-10.55	-10.55	-10.55
-10.54	-10.54	-10.54	-10.54	-10.54	-10.54	-10.54	-10.54	-10.54
-10.53	-10.53	-10.53	-10.53	-10.53	-10.53	-10.53	-10.53	-10.53
-10.52	-10.52	-10.52	-10.52	-10.52	-10.52	-10.52	-10.52	-10.52
-10.51	-10.51	-10.51	-10.51	-10.51	-10.51	-10.51	-10.51	-10.51

-10.5	-10.5	-10.5	-10.5	-10.5	-10.5	-10.5	-10.5	-10.5
-10.49	-10.49	-10.49	-10.49	-10.49	-10.49	-10.49	-10.49	-10.49
-10.48	-10.48	-10.48	-10.48	-10.48	-10.48	-10.48	-10.48	-10.48
-10.47	-10.47	-10.47	-10.47	-10.47	-10.47	-10.47	-10.47	-10.47
-10.46	-10.46	-10.46	-10.46	-10.46	-10.46	-10.46	-10.46	-10.46
-10.45	-10.45	-10.45	-10.45	-10.45	-10.45	-10.45	-10.45	-10.45
-10.44	-10.44	-10.44	-10.44	-10.44	-10.44	-10.44	-10.44	-10.44
-10.43	-10.43	-10.43	-10.43	-10.43	-10.43	-10.43	-10.43	-10.43
-10.42	-10.42	-10.42	-10.42	-10.42	-10.42	-10.42	-10.42	-10.42
-10.41	-10.41	-10.41	-10.41	-10.41	-10.41	-10.41	-10.41	-10.41
-10.4	-10.4	-10.4	-10.4	-10.4	-10.4	-10.4	-10.4	-10.4
-10.39	-10.39	-10.39	-10.39	-10.39	-10.39	-10.39	-10.39	-10.39
-10.38	-10.38	-10.38	-10.38	-10.38	-10.38	-10.38	-10.38	-10.38
-10.37	-10.37	-10.37	-10.37	-10.37	-10.37	-10.37	-10.37	-10.37
-10.36	-10.36	-10.36	-10.36	-10.36	-10.36	-10.36	-10.36	-10.36
-10.35	-10.35	-10.35	-10.35	-10.35	-10.35	-10.35	-10.35	-10.35
-10.34	-10.34	-10.34	-10.34	-10.34	-10.34	-10.34	-10.34	-10.34
-10.33	-10.33	-10.33	-10.33	-10.33	-10.33	-10.33	-10.33	-10.33
-10.32	-10.32	-10.32	-10.32	-10.32	-10.32	-10.32	-10.32	-10.32
-10.31	-10.31	-10.31	-10.31	-10.31	-10.31	-10.31	-10.31	-10.31
-10.3	-10.3	-10.3	-10.3	-10.3	-10.3	-10.3	-10.3	-10.3
-10.29	-10.29	-10.29	-10.29	-10.29	-10.29	-10.29	-10.29	-10.29
-10.28	-10.28	-10.28	-10.28	-10.28	-10.28	-10.28	-10.28	-10.28
-10.27	-10.27	-10.27	-10.27	-10.27	-10.27	-10.27	-10.27	-10.27
-10.26	-10.26	-10.26	-10.26	-10.26	-10.26	-10.26	-10.26	-10.26
-10.25	-10.25	-10.25	-10.25	-10.25	-10.25	-10.25	-10.25	-10.25
-10.24	-10.24	-10.24	-10.24	-10.24	-10.24	-10.24	-10.24	-10.24
-10.23	-10.23	-10.23	-10.23	-10.23	-10.23	-10.23	-10.23	-10.23
-10.22	-10.22	-10.22	-10.22	-10.22	-10.22	-10.22	-10.22	-10.22
-10.21	-10.21	-10.21	-10.21	-10.21	-10.21	-10.21	-10.21	-10.21
-10.2	-10.2	-10.2	-10.2	-10.2	-10.2	-10.2	-10.2	-10.2
-10.19	-10.19	-10.19	-10.19	-10.19	-10.19	-10.19	-10.19	-10.19
-10.18	-10.18	-10.18	-10.18	-10.18	-10.18	-10.18	-10.18	-10.18
-10.17	-10.17	-10.17	-10.17	-10.17	-10.17	-10.17	-10.17	-10.17
-10.16	-10.16	-10.16	-10.16	-10.16	-10.16	-10.16	-10.16	-10.16
-10.15	-10.15	-10.15	-10.15	-10.15	-10.15	-10.15	-10.15	-10.15
-10.14	-10.14	-10.14	-10.14	-10.14	-10.14	-10.14	-10.14	-10.14
-10.13	-10.13	-10.13	-10.13	-10.13	-10.13	-10.13	-10.13	-10.13
-10.12	-10.12	-10.12	-10.12	-10.12	-10.12	-10.12	-10.12	-10.12
-10.11	-10.11	-10.11	-10.11	-10.11	-10.11	-10.11	-10.11	-10.11
-10.1	-10.1	-10.1	-10.1	-10.1	-10.1	-10.1	-10.1	-10.1
-10.09	-10.09	-10.09	-10.09	-10.09	-10.09	-10.09	-10.09	-10.09
-10.08	-10.08	-10.08	-10.08	-10.08	-10.08	-10.08	-10.08	-10.08
-10.07	-10.07	-10.07	-10.07	-10.07	-10.07	-10.07	-10.07	-10.07
-10.06	-10.06	-10.06	-10.06	-10.06	-10.06	-10.06	-10.06	-10.06
-10.05	-10.05	-10.05	-10.05	-10.05	-10.05	-10.05	-10.05	-10.05
-10.04	-10.04	-10.04	-10.04	-10.04	-10.04	-10.04	-10.04	-10.04
-10.03	-10.03	-10.03	-10.03	-10.03	-10.03	-10.03	-10.03	-10.03
-10.02	-10.02	-10.02	-10.02	-10.02	-10.02	-10.02	-10.02	-10.02
-10.01	-10.01	-10.01	-10.01	-10.01	-10.01	-10.01	-10.01	-10.01

-10	-10	-10	-10	-10	-10	-10	-10	-10
-9.99	-9.99	-9.99	-9.99	-9.99	-9.99	-9.99	-9.99	-9.99
-9.98	-9.98	-9.98	-9.98	-9.98	-9.98	-9.98	-9.98	-9.98
-9.97	-9.97	-9.97	-9.97	-9.97	-9.97	-9.97	-9.97	-9.97
-9.96	-9.96	-9.96	-9.96	-9.96	-9.96	-9.96	-9.96	-9.96
-9.95	-9.95	-9.95	-9.95	-9.95	-9.95	-9.95	-9.95	-9.95
-9.94	-9.94	-9.94	-9.94	-9.94	-9.94	-9.94	-9.94	-9.94
-9.93	-9.93	-9.93	-9.93	-9.93	-9.93	-9.93	-9.93	-9.93
-9.92	-9.92	-9.92	-9.92	-9.92	-9.92	-9.92	-9.92	-9.92
-9.91	-9.91	-9.91	-9.91	-9.91	-9.91	-9.91	-9.91	-9.91
-9.9	-9.9	-9.9	-9.9	-9.9	-9.9	-9.9	-9.9	-9.9
-9.89	-9.89	-9.89	-9.89	-9.89	-9.89	-9.89	-9.89	-9.89
-9.88	-9.88	-9.88	-9.88	-9.88	-9.88	-9.88	-9.88	-9.88
-9.87	-9.87	-9.87	-9.87	-9.87	-9.87	-9.87	-9.87	-9.87
-9.86	-9.86	-9.86	-9.86	-9.86	-9.86	-9.86	-9.86	-9.86
-9.85	-9.85	-9.85	-9.85	-9.85	-9.85	-9.85	-9.85	-9.85
-9.84	-9.84	-9.84	-9.84	-9.84	-9.84	-9.84	-9.84	-9.84
-9.83	-9.83	-9.83	-9.83	-9.83	-9.83	-9.83	-9.83	-9.83
-9.82	-9.82	-9.82	-9.82	-9.82	-9.82	-9.82	-9.82	-9.82
-9.81	-9.81	-9.81	-9.81	-9.81	-9.81	-9.81	-9.81	-9.81
-9.8	-9.8	-9.8	-9.8	-9.8	-9.8	-9.8	-9.8	-9.8
-9.79	-9.79	-9.79	-9.79	-9.79	-9.79	-9.79	-9.79	-9.79
-9.78	-9.78	-9.78	-9.78	-9.78	-9.78	-9.78	-9.78	-9.78
-9.77	-9.77	-9.77	-9.77	-9.77	-9.77	-9.77	-9.77	-9.77
-9.76	-9.76	-9.76	-9.76	-9.76	-9.76	-9.76	-9.76	-9.76
-9.75	-9.75	-9.75	-9.75	-9.75	-9.75	-9.75	-9.75	-9.75
-9.74	-9.74	-9.74	-9.74	-9.74	-9.74	-9.74	-9.74	-9.74
-9.73	-9.73	-9.73	-9.73	-9.73	-9.73	-9.73	-9.73	-9.73
-9.72	-9.72	-9.72	-9.72	-9.72	-9.72	-9.72	-9.72	-9.72
-9.71	-9.71	-9.71	-9.71	-9.71	-9.71	-9.71	-9.71	-9.71
-9.7	-9.7	-9.7	-9.7	-9.7	-9.7	-9.7	-9.7	-9.7
-9.69	-9.69	-9.69	-9.69	-9.69	-9.69	-9.69	-9.69	-9.69
-9.68	-9.68	-9.68	-9.68	-9.68	-9.68	-9.68	-9.68	-9.68
-9.67	-9.67	-9.67	-9.67	-9.67	-9.67	-9.67	-9.67	-9.67
-9.66	-9.66	-9.66	-9.66	-9.66	-9.66	-9.66	-9.66	-9.66
-9.65	-9.65	-9.65	-9.65	-9.65	-9.65	-9.65	-9.65	-9.65
-9.64	-9.64	-9.64	-9.64	-9.64	-9.64	-9.64	-9.64	-9.64
-9.63	-9.63	-9.63	-9.63	-9.63	-9.63	-9.63	-9.63	-9.63
-9.62	-9.62	-9.62	-9.62	-9.62	-9.62	-9.62	-9.62	-9.62
-9.61	-9.61	-9.61	-9.61	-9.61	-9.61	-9.61	-9.61	-9.61
-9.6	-9.6	-9.6	-9.6	-9.6	-9.6	-9.6	-9.6	-9.6
-9.59	-9.59	-9.59	-9.59	-9.59	-9.59	-9.59	-9.59	-9.59
-9.58	-9.58	-9.58	-9.58	-9.58	-9.58	-9.58	-9.58	-9.58
-9.57	-9.57	-9.57	-9.57	-9.57	-9.57	-9.57	-9.57	-9.57
-9.56	-9.56	-9.56	-9.56	-9.56	-9.56	-9.56	-9.56	-9.56
-9.55	-9.55	-9.55	-9.55	-9.55	-9.55	-9.55	-9.55	-9.55
-9.54	-9.54	-9.54	-9.54	-9.54	-9.54	-9.54	-9.54	-9.54
-9.53	-9.53	-9.53	-9.53	-9.53	-9.53	-9.53	-9.53	-9.53
-9.52	-9.52	-9.52	-9.52	-9.52	-9.52	-9.52	-9.52	-9.52
-9.51	-9.51	-9.51	-9.51	-9.51	-9.51	-9.51	-9.51	-9.51

-9.5	-9.5	-9.5	-9.5	-9.5	-9.5	-9.5	-9.5	-9.5
-9.49	-9.49	-9.49	-9.49	-9.49	-9.49	-9.49	-9.49	-9.49
-9.48	-9.48	-9.48	-9.48	-9.48	-9.48	-9.48	-9.48	-9.48
-9.47	-9.47	-9.47	-9.47	-9.47	-9.47	-9.47	-9.47	-9.47
-9.46	-9.46	-9.46	-9.46	-9.46	-9.46	-9.46	-9.46	-9.46
-9.45	-9.45	-9.45	-9.45	-9.45	-9.45	-9.45	-9.45	-9.45
-9.44	-9.44	-9.44	-9.44	-9.44	-9.44	-9.44	-9.44	-9.44
-9.43	-9.43	-9.43	-9.43	-9.43	-9.43	-9.43	-9.43	-9.43
-9.42	-9.42	-9.42	-9.42	-9.42	-9.42	-9.42	-9.42	-9.42
-9.41	-9.41	-9.41	-9.41	-9.41	-9.41	-9.41	-9.41	-9.41
-9.4	-9.4	-9.4	-9.4	-9.4	-9.4	-9.4	-9.4	-9.4
-9.39	-9.39	-9.39	-9.39	-9.39	-9.39	-9.39	-9.39	-9.39
-9.38	-9.38	-9.38	-9.38	-9.38	-9.38	-9.38	-9.38	-9.38
-9.37	-9.37	-9.37	-9.37	-9.37	-9.37	-9.37	-9.37	-9.37
-9.36	-9.36	-9.36	-9.36	-9.36	-9.36	-9.36	-9.36	-9.36
-9.35	-9.35	-9.35	-9.35	-9.35	-9.35	-9.35	-9.35	-9.35
-9.34	-9.34	-9.34	-9.34	-9.34	-9.34	-9.34	-9.34	-9.34
-9.33	-9.33	-9.33	-9.33	-9.33	-9.33	-9.33	-9.33	-9.33
-9.32	-9.32	-9.32	-9.32	-9.32	-9.32	-9.32	-9.32	-9.32
-9.31	-9.31	-9.31	-9.31	-9.31	-9.31	-9.31	-9.31	-9.31
-9.3	-9.3	-9.3	-9.3	-9.3	-9.3	-9.3	-9.3	-9.3
-9.29	-9.29	-9.29	-9.29	-9.29	-9.29	-9.29	-9.29	-9.29
-9.28	-9.28	-9.28	-9.28	-9.28	-9.28	-9.28	-9.28	-9.28
-9.27	-9.27	-9.27	-9.27	-9.27	-9.27	-9.27	-9.27	-9.27
-9.26	-9.26	-9.26	-9.26	-9.26	-9.26	-9.26	-9.26	-9.26
-9.25	-9.25	-9.25	-9.25	-9.25	-9.25	-9.25	-9.25	-9.25
-9.24	-9.24	-9.24	-9.24	-9.24	-9.24	-9.24	-9.24	-9.24
-9.23	-9.23	-9.23	-9.23	-9.23	-9.23	-9.23	-9.23	-9.23
-9.22	-9.22	-9.22	-9.22	-9.22	-9.22	-9.22	-9.22	-9.22
-9.21	-9.21	-9.21	-9.21	-9.21	-9.21	-9.21	-9.21	-9.21
-9.2	-9.2	-9.2	-9.2	-9.2	-9.2	-9.2	-9.2	-9.2
-9.19	-9.19	-9.19	-9.19	-9.19	-9.19	-9.19	-9.19	-9.19
-9.18	-9.18	-9.18	-9.18	-9.18	-9.18	-9.18	-9.18	-9.18
-9.17	-9.17	-9.17	-9.17	-9.17	-9.17	-9.17	-9.17	-9.17
-9.16	-9.16	-9.16	-9.16	-9.16	-9.16	-9.16	-9.16	-9.16
-9.15	-9.15	-9.15	-9.15	-9.15	-9.15	-9.15	-9.15	-9.15
-9.14	-9.14	-9.14	-9.14	-9.14	-9.14	-9.14	-9.14	-9.14
-9.13	-9.13	-9.13	-9.13	-9.13	-9.13	-9.13	-9.13	-9.13
-9.12	-9.12	-9.12	-9.12	-9.12	-9.12	-9.12	-9.12	-9.12
-9.11	-9.11	-9.11	-9.11	-9.11	-9.11	-9.11	-9.11	-9.11
-9.1	-9.1	-9.1	-9.1	-9.1	-9.1	-9.1	-9.1	-9.1
-9.09	-9.09	-9.09	-9.09	-9.09	-9.09	-9.09	-9.09	-9.09
-9.08	-9.08	-9.08	-9.08	-9.08	-9.08	-9.08	-9.08	-9.08
-9.07	-9.07	-9.07	-9.07	-9.07	-9.07	-9.07	-9.07	-9.07
-9.06	-9.06	-9.06	-9.06	-9.06	-9.06	-9.06	-9.06	-9.06
-9.05	-9.05	-9.05	-9.05	-9.05	-9.05	-9.05	-9.05	-9.05
-9.04	-9.04	-9.04	-9.04	-9.04	-9.04	-9.04	-9.04	-9.04
-9.03	-9.03	-9.03	-9.03	-9.03	-9.03	-9.03	-9.03	-9.03
-9.02	-9.02	-9.02	-9.02	-9.02	-9.02	-9.02	-9.02	-9.02
-9.01	-9.01	-9.01	-9.01	-9.01	-9.01	-9.01	-9.01	-9.01

-9	-9	-9	-9	-9	-9	-9	-9	-9
-8.99	-8.99	-8.99	-8.99	-8.99	-8.99	-8.99	-8.99	-8.99
-8.98	-8.98	-8.98	-8.98	-8.98	-8.98	-8.98	-8.98	-8.98
-8.97	-8.97	-8.97	-8.97	-8.97	-8.97	-8.97	-8.97	-8.97
-8.96	-8.96	-8.96	-8.96	-8.96	-8.96	-8.96	-8.96	-8.96
-8.95	-8.95	-8.95	-8.95	-8.95	-8.95	-8.95	-8.95	-8.95
-8.94	-8.94	-8.94	-8.94	-8.94	-8.94	-8.94	-8.94	-8.94
-8.93	-8.93	-8.93	-8.93	-8.93	-8.93	-8.93	-8.93	-8.93
-8.92	-8.92	-8.92	-8.92	-8.92	-8.92	-8.92	-8.92	-8.92
-8.91	-8.91	-8.91	-8.91	-8.91	-8.91	-8.91	-8.91	-8.91
-8.9	-8.9	-8.9	-8.9	-8.9	-8.9	-8.9	-8.9	-8.9
-8.89	-8.89	-8.89	-8.89	-8.89	-8.89	-8.89	-8.89	-8.89
-8.88	-8.88	-8.88	-8.88	-8.88	-8.88	-8.88	-8.88	-8.88
-8.87	-8.87	-8.87	-8.87	-8.87	-8.87	-8.87	-8.87	-8.87
-8.86	-8.86	-8.86	-8.86	-8.86	-8.86	-8.86	-8.86	-8.86
-8.85	-8.85	-8.85	-8.85	-8.85	-8.85	-8.85	-8.85	-8.85
-8.84	-8.84	-8.84	-8.84	-8.84	-8.84	-8.84	-8.84	-8.84
-8.83	-8.83	-8.83	-8.83	-8.83	-8.83	-8.83	-8.83	-8.83
-8.82	-8.82	-8.82	-8.82	-8.82	-8.82	-8.82	-8.82	-8.82
-8.81	-8.81	-8.81	-8.81	-8.81	-8.81	-8.81	-8.81	-8.81
-8.8	-8.8	-8.8	-8.8	-8.8	-8.8	-8.8	-8.8	-8.8
-8.79	-8.79	-8.79	-8.79	-8.79	-8.79	-8.79	-8.79	-8.79
-8.78	-8.78	-8.78	-8.78	-8.78	-8.78	-8.78	-8.78	-8.78
-8.77	-8.77	-8.77	-8.77	-8.77	-8.77	-8.77	-8.77	-8.77
-8.76	-8.76	-8.76	-8.76	-8.76	-8.76	-8.76	-8.76	-8.76
-8.75	-8.75	-8.75	-8.75	-8.75	-8.75	-8.75	-8.75	-8.75
-8.74	-8.74	-8.74	-8.74	-8.74	-8.74	-8.74	-8.74	-8.74
-8.73	-8.73	-8.73	-8.73	-8.73	-8.73	-8.73	-8.73	-8.73
-8.72	-8.72	-8.72	-8.72	-8.72	-8.72	-8.72	-8.72	-8.72
-8.71	-8.71	-8.71	-8.71	-8.71	-8.71	-8.71	-8.71	-8.71
-8.7	-8.7	-8.7	-8.7	-8.7	-8.7	-8.7	-8.7	-8.7
-8.69	-8.69	-8.69	-8.69	-8.69	-8.69	-8.69	-8.69	-8.69
-8.68	-8.68	-8.68	-8.68	-8.68	-8.68	-8.68	-8.68	-8.68
-8.67	-8.67	-8.67	-8.67	-8.67	-8.67	-8.67	-8.67	-8.67
-8.66	-8.66	-8.66	-8.66	-8.66	-8.66	-8.66	-8.66	-8.66
-8.65	-8.65	-8.65	-8.65	-8.65	-8.65	-8.65	-8.65	-8.65
-8.64	-8.64	-8.64	-8.64	-8.64	-8.64	-8.64	-8.64	-8.64
-8.63	-8.63	-8.63	-8.63	-8.63	-8.63	-8.63	-8.63	-8.63
-8.62	-8.62	-8.62	-8.62	-8.62	-8.62	-8.62	-8.62	-8.62
-8.61	-8.61	-8.61	-8.61	-8.61	-8.61	-8.61	-8.61	-8.61
-8.6	-8.6	-8.6	-8.6	-8.6	-8.6	-8.6	-8.6	-8.6
-8.59	-8.59	-8.59	-8.59	-8.59	-8.59	-8.59	-8.59	-8.59
-8.58	-8.58	-8.58	-8.58	-8.58	-8.58	-8.58	-8.58	-8.58
-8.57	-8.57	-8.57	-8.57	-8.57	-8.57	-8.57	-8.57	-8.57
-8.56	-8.56	-8.56	-8.56	-8.56	-8.56	-8.56	-8.56	-8.56
-8.55	-8.55	-8.55	-8.55	-8.55	-8.55	-8.55	-8.55	-8.55
-8.54	-8.54	-8.54	-8.54	-8.54	-8.54	-8.54	-8.54	-8.54
-8.53	-8.53	-8.53	-8.53	-8.53	-8.53	-8.53	-8.53	-8.53
-8.52	-8.52	-8.52	-8.52	-8.52	-8.52	-8.52	-8.52	-8.52
-8.51	-8.51	-8.51	-8.51	-8.51	-8.51	-8.51	-8.51	-8.51

-8.5	-8.5	-8.5	-8.5	-8.5	-8.5	-8.5	-8.5	-8.5
-8.49	-8.49	-8.49	-8.49	-8.49	-8.49	-8.49	-8.49	-8.49
-8.48	-8.48	-8.48	-8.48	-8.48	-8.48	-8.48	-8.48	-8.48
-8.47	-8.47	-8.47	-8.47	-8.47	-8.47	-8.47	-8.47	-8.47
-8.46	-8.46	-8.46	-8.46	-8.46	-8.46	-8.46	-8.46	-8.46
-8.45	-8.45	-8.45	-8.45	-8.45	-8.45	-8.45	-8.45	-8.45
-8.44	-8.44	-8.44	-8.44	-8.44	-8.44	-8.44	-8.44	-8.44
-8.43	-8.43	-8.43	-8.43	-8.43	-8.43	-8.43	-8.43	-8.43
-8.42	-8.42	-8.42	-8.42	-8.42	-8.42	-8.42	-8.42	-8.42
-8.41	-8.41	-8.41	-8.41	-8.41	-8.41	-8.41	-8.41	-8.41
-8.4	-8.4	-8.4	-8.4	-8.4	-8.4	-8.4	-8.4	-8.4
-8.39	-8.39	-8.39	-8.39	-8.39	-8.39	-8.39	-8.39	-8.39
-8.38	-8.38	-8.38	-8.38	-8.38	-8.38	-8.38	-8.38	-8.38
-8.37	-8.37	-8.37	-8.37	-8.37	-8.37	-8.37	-8.37	-8.37
-8.36	-8.36	-8.36	-8.36	-8.36	-8.36	-8.36	-8.36	-8.36
-8.35	-8.35	-8.35	-8.35	-8.35	-8.35	-8.35	-8.35	-8.35
-8.34	-8.34	-8.34	-8.34	-8.34	-8.34	-8.34	-8.34	-8.34
-8.33	-8.33	-8.33	-8.33	-8.33	-8.33	-8.33	-8.33	-8.33
-8.32	-8.32	-8.32	-8.32	-8.32	-8.32	-8.32	-8.32	-8.32
-8.31	-8.31	-8.31	-8.31	-8.31	-8.31	-8.31	-8.31	-8.31
-8.3	-8.3	-8.3	-8.3	-8.3	-8.3	-8.3	-8.3	-8.3
-8.29	-8.29	-8.29	-8.29	-8.29	-8.29	-8.29	-8.29	-8.29
-8.28	-8.28	-8.28	-8.28	-8.28	-8.28	-8.28	-8.28	-8.28
-8.27	-8.27	-8.27	-8.27	-8.27	-8.27	-8.27	-8.27	-8.27
-8.26	-8.26	-8.26	-8.26	-8.26	-8.26	-8.26	-8.26	-8.26
-8.25	-8.25	-8.25	-8.25	-8.25	-8.25	-8.25	-8.25	-8.25
-8.24	-8.24	-8.24	-8.24	-8.24	-8.24	-8.24	-8.24	-8.24
-8.23	-8.23	-8.23	-8.23	-8.23	-8.23	-8.23	-8.23	-8.23
-8.22	-8.22	-8.22	-8.22	-8.22	-8.22	-8.22	-8.22	-8.22
-8.21	-8.21	-8.21	-8.21	-8.21	-8.21	-8.21	-8.21	-8.21
-8.2	-8.2	-8.2	-8.2	-8.2	-8.2	-8.2	-8.2	-8.2
-8.19	-8.19	-8.19	-8.19	-8.19	-8.19	-8.19	-8.19	-8.19
-8.18	-8.18	-8.18	-8.18	-8.18	-8.18	-8.18	-8.18	-8.18
-8.17	-8.17	-8.17	-8.17	-8.17	-8.17	-8.17	-8.17	-8.17
-8.16	-8.16	-8.16	-8.16	-8.16	-8.16	-8.16	-8.16	-8.16
-8.15	-8.15	-8.15	-8.15	-8.15	-8.15	-8.15	-8.15	-8.15
-8.14	-8.14	-8.14	-8.14	-8.14	-8.14	-8.14	-8.14	-8.14
-8.13	-8.13	-8.13	-8.13	-8.13	-8.13	-8.13	-8.13	-8.13
-8.12	-8.12	-8.12	-8.12	-8.12	-8.12	-8.12	-8.12	-8.12
-8.11	-8.11	-8.11	-8.11	-8.11	-8.11	-8.11	-8.11	-8.11
-8.1	-8.1	-8.1	-8.1	-8.1	-8.1	-8.1	-8.1	-8.1
-8.09	-8.09	-8.09	-8.09	-8.09	-8.09	-8.09	-8.09	-8.09
-8.08	-8.08	-8.08	-8.08	-8.08	-8.08	-8.08	-8.08	-8.08
-8.07	-8.07	-8.07	-8.07	-8.07	-8.07	-8.07	-8.07	-8.07
-8.06	-8.06	-8.06	-8.06	-8.06	-8.06	-8.06	-8.06	-8.06
-8.05	-8.05	-8.05	-8.05	-8.05	-8.05	-8.05	-8.05	-8.05
-8.04	-8.04	-8.04	-8.04	-8.04	-8.04	-8.04	-8.04	-8.04
-8.03	-8.03	-8.03	-8.03	-8.03	-8.03	-8.03	-8.03	-8.03
-8.02	-8.02	-8.02	-8.02	-8.02	-8.02	-8.02	-8.02	-8.02
-8.01	-8.01	-8.01	-8.01	-8.01	-8.01	-8.01	-8.01	-8.01

-8	-8	-8	-8	-8	-8	-8	-8	-8
-7.99	-7.99	-7.99	-7.99	-7.99	-7.99	-7.99	-7.99	-7.99
-7.98	-7.98	-7.98	-7.98	-7.98	-7.98	-7.98	-7.98	-7.98
-7.97	-7.97	-7.97	-7.97	-7.97	-7.97	-7.97	-7.97	-7.97
-7.96	-7.96	-7.96	-7.96	-7.96	-7.96	-7.96	-7.96	-7.96
-7.95	-7.95	-7.95	-7.95	-7.95	-7.95	-7.95	-7.95	-7.95
-7.94	-7.94	-7.94	-7.94	-7.94	-7.94	-7.94	-7.94	-7.94
-7.93	-7.93	-7.93	-7.93	-7.93	-7.93	-7.93	-7.93	-7.93
-7.92	-7.92	-7.92	-7.92	-7.92	-7.92	-7.92	-7.92	-7.92
-7.91	-7.91	-7.91	-7.91	-7.91	-7.91	-7.91	-7.91	-7.91
-7.9	-7.9	-7.9	-7.9	-7.9	-7.9	-7.9	-7.9	-7.9
-7.89	-7.89	-7.89	-7.89	-7.89	-7.89	-7.89	-7.89	-7.89
-7.88	-7.88	-7.88	-7.88	-7.88	-7.88	-7.88	-7.88	-7.88
-7.87	-7.87	-7.87	-7.87	-7.87	-7.87	-7.87	-7.87	-7.87
-7.86	-7.86	-7.86	-7.86	-7.86	-7.86	-7.86	-7.86	-7.86
-7.85	-7.85	-7.85	-7.85	-7.85	-7.85	-7.85	-7.85	-7.85
-7.84	-7.84	-7.84	-7.84	-7.84	-7.84	-7.84	-7.84	-7.84
-7.83	-7.83	-7.83	-7.83	-7.83	-7.83	-7.83	-7.83	-7.83
-7.82	-7.82	-7.82	-7.82	-7.82	-7.82	-7.82	-7.82	-7.82
-7.81	-7.81	-7.81	-7.81	-7.81	-7.81	-7.81	-7.81	-7.81
-7.8	-7.8	-7.8	-7.8	-7.8	-7.8	-7.8	-7.8	-7.8
-7.79	-7.79	-7.79	-7.79	-7.79	-7.79	-7.79	-7.79	-7.79
-7.78	-7.78	-7.78	-7.78	-7.78	-7.78	-7.78	-7.78	-7.78
-7.77	-7.77	-7.77	-7.77	-7.77	-7.77	-7.77	-7.77	-7.77
-7.76	-7.76	-7.76	-7.76	-7.76	-7.76	-7.76	-7.76	-7.76
-7.75	-7.75	-7.75	-7.75	-7.75	-7.75	-7.75	-7.75	-7.75
-7.74	-7.74	-7.74	-7.74	-7.74	-7.74	-7.74	-7.74	-7.74
-7.73	-7.73	-7.73	-7.73	-7.73	-7.73	-7.73	-7.73	-7.73
-7.72	-7.72	-7.72	-7.72	-7.72	-7.72	-7.72	-7.72	-7.72
-7.71	-7.71	-7.71	-7.71	-7.71	-7.71	-7.71	-7.71	-7.71
-7.7	-7.7	-7.7	-7.7	-7.7	-7.7	-7.7	-7.7	-7.7
-7.69	-7.69	-7.69	-7.69	-7.69	-7.69	-7.69	-7.69	-7.69
-7.68	-7.68	-7.68	-7.68	-7.68	-7.68	-7.68	-7.68	-7.68
-7.67	-7.67	-7.67	-7.67	-7.67	-7.67	-7.67	-7.67	-7.67
-7.66	-7.66	-7.66	-7.66	-7.66	-7.66	-7.66	-7.66	-7.66
-7.65	-7.65	-7.65	-7.65	-7.65	-7.65	-7.65	-7.65	-7.65
-7.64	-7.64	-7.64	-7.64	-7.64	-7.64	-7.64	-7.64	-7.64
-7.63	-7.63	-7.63	-7.63	-7.63	-7.63	-7.63	-7.63	-7.63
-7.62	-7.62	-7.62	-7.62	-7.62	-7.62	-7.62	-7.62	-7.62
-7.61	-7.61	-7.61	-7.61	-7.61	-7.61	-7.61	-7.61	-7.61
-7.6	-7.6	-7.6	-7.6	-7.6	-7.6	-7.6	-7.6	-7.6
-7.59	-7.59	-7.59	-7.59	-7.59	-7.59	-7.59	-7.59	-7.59
-7.58	-7.58	-7.58	-7.58	-7.58	-7.58	-7.58	-7.58	-7.58
-7.57	-7.57	-7.57	-7.57	-7.57	-7.57	-7.57	-7.57	-7.57
-7.56	-7.56	-7.56	-7.56	-7.56	-7.56	-7.56	-7.56	-7.56
-7.55	-7.55	-7.55	-7.55	-7.55	-7.55	-7.55	-7.55	-7.55
-7.54	-7.54	-7.54	-7.54	-7.54	-7.54	-7.54	-7.54	-7.54
-7.53	-7.53	-7.53	-7.53	-7.53	-7.53	-7.53	-7.53	-7.53
-7.52	-7.52	-7.52	-7.52	-7.52	-7.52	-7.52	-7.52	-7.52
-7.51	-7.51	-7.51	-7.51	-7.51	-7.51	-7.51	-7.51	-7.51

-7.5	-7.5	-7.5	-7.5	-7.5	-7.5	-7.5	-7.5	-7.5
-7.49	-7.49	-7.49	-7.49	-7.49	-7.49	-7.49	-7.49	-7.49
-7.48	-7.48	-7.48	-7.48	-7.48	-7.48	-7.48	-7.48	-7.48
-7.47	-7.47	-7.47	-7.47	-7.47	-7.47	-7.47	-7.47	-7.47
-7.46	-7.46	-7.46	-7.46	-7.46	-7.46	-7.46	-7.46	-7.46
-7.45	-7.45	-7.45	-7.45	-7.45	-7.45	-7.45	-7.45	-7.45
-7.44	-7.44	-7.44	-7.44	-7.44	-7.44	-7.44	-7.44	-7.44
-7.43	-7.43	-7.43	-7.43	-7.43	-7.43	-7.43	-7.43	-7.43
-7.42	-7.42	-7.42	-7.42	-7.42	-7.42	-7.42	-7.42	-7.42
-7.41	-7.41	-7.41	-7.41	-7.41	-7.41	-7.41	-7.41	-7.41
-7.4	-7.4	-7.4	-7.4	-7.4	-7.4	-7.4	-7.4	-7.4
-7.39	-7.39	-7.39	-7.39	-7.39	-7.39	-7.39	-7.39	-7.39
-7.38	-7.38	-7.38	-7.38	-7.38	-7.38	-7.38	-7.38	-7.38
-7.37	-7.37	-7.37	-7.37	-7.37	-7.37	-7.37	-7.37	-7.37
-7.36	-7.36	-7.36	-7.36	-7.36	-7.36	-7.36	-7.36	-7.36
-7.35	-7.35	-7.35	-7.35	-7.35	-7.35	-7.35	-7.35	-7.35
-7.34	-7.34	-7.34	-7.34	-7.34	-7.34	-7.34	-7.34	-7.34
-7.33	-7.33	-7.33	-7.33	-7.33	-7.33	-7.33	-7.33	-7.33
-7.32	-7.32	-7.32	-7.32	-7.32	-7.32	-7.32	-7.32	-7.32
-7.31	-7.31	-7.31	-7.31	-7.31	-7.31	-7.31	-7.31	-7.31
-7.3	-7.3	-7.3	-7.3	-7.3	-7.3	-7.3	-7.3	-7.3
-7.29	-7.29	-7.29	-7.29	-7.29	-7.29	-7.29	-7.29	-7.29
-7.28	-7.28	-7.28	-7.28	-7.28	-7.28	-7.28	-7.28	-7.28
-7.27	-7.27	-7.27	-7.27	-7.27	-7.27	-7.27	-7.27	-7.27
-7.26	-7.26	-7.26	-7.26	-7.26	-7.26	-7.26	-7.26	-7.26
-7.25	-7.25	-7.25	-7.25	-7.25	-7.25	-7.25	-7.25	-7.25
-7.24	-7.24	-7.24	-7.24	-7.24	-7.24	-7.24	-7.24	-7.24
-7.23	-7.23	-7.23	-7.23	-7.23	-7.23	-7.23	-7.23	-7.23
-7.22	-7.22	-7.22	-7.22	-7.22	-7.22	-7.22	-7.22	-7.22
-7.21	-7.21	-7.21	-7.21	-7.21	-7.21	-7.21	-7.21	-7.21
-7.2	-7.2	-7.2	-7.2	-7.2	-7.2	-7.2	-7.2	-7.2
-7.19	-7.19	-7.19	-7.19	-7.19	-7.19	-7.19	-7.19	-7.19
-7.18	-7.18	-7.18	-7.18	-7.18	-7.18	-7.18	-7.18	-7.18
-7.17	-7.17	-7.17	-7.17	-7.17	-7.17	-7.17	-7.17	-7.17
-7.16	-7.16	-7.16	-7.16	-7.16	-7.16	-7.16	-7.16	-7.16
-7.15	-7.15	-7.15	-7.15	-7.15	-7.15	-7.15	-7.15	-7.15
-7.14	-7.14	-7.14	-7.14	-7.14	-7.14	-7.14	-7.14	-7.14
-7.13	-7.13	-7.13	-7.13	-7.13	-7.13	-7.13	-7.13	-7.13
-7.12	-7.12	-7.12	-7.12	-7.12	-7.12	-7.12	-7.12	-7.12
-7.11	-7.11	-7.11	-7.11	-7.11	-7.11	-7.11	-7.11	-7.11
-7.1	-7.1	-7.1	-7.1	-7.1	-7.1	-7.1	-7.1	-7.1
-7.09	-7.09	-7.09	-7.09	-7.09	-7.09	-7.09	-7.09	-7.09
-7.08	-7.08	-7.08	-7.08	-7.08	-7.08	-7.08	-7.08	-7.08
-7.07	-7.07	-7.07	-7.07	-7.07	-7.07	-7.07	-7.07	-7.07
-7.06	-7.06	-7.06	-7.06	-7.06	-7.06	-7.06	-7.06	-7.06
-7.05	-7.05	-7.05	-7.05	-7.05	-7.05	-7.05	-7.05	-7.05
-7.04	-7.04	-7.04	-7.04	-7.04	-7.04	-7.04	-7.04	-7.04
-7.03	-7.03	-7.03	-7.03	-7.03	-7.03	-7.03	-7.03	-7.03
-7.02	-7.02	-7.02	-7.02	-7.02	-7.02	-7.02	-7.02	-7.02
-7.01	-7.01	-7.01	-7.01	-7.01	-7.01	-7.01	-7.01	-7.01

-7	-7	-7	-7	-7	-7	-7	-7	-7
-6.99	-6.99	-6.99	-6.99	-6.99	-6.99	-6.99	-6.99	-6.99
-6.98	-6.98	-6.98	-6.98	-6.98	-6.98	-6.98	-6.98	-6.98
-6.97	-6.97	-6.97	-6.97	-6.97	-6.97	-6.97	-6.97	-6.97
-6.96	-6.96	-6.96	-6.96	-6.96	-6.96	-6.96	-6.96	-6.96
-6.95	-6.95	-6.95	-6.95	-6.95	-6.95	-6.95	-6.95	-6.95
-6.94	-6.94	-6.94	-6.94	-6.94	-6.94	-6.94	-6.94	-6.94
-6.93	-6.93	-6.93	-6.93	-6.93	-6.93	-6.93	-6.93	-6.93
-6.92	-6.92	-6.92	-6.92	-6.92	-6.92	-6.92	-6.92	-6.92
-6.91	-6.91	-6.91	-6.91	-6.91	-6.91	-6.91	-6.91	-6.91
-6.9	-6.9	-6.9	-6.9	-6.9	-6.9	-6.9	-6.9	-6.9
-6.89	-6.89	-6.89	-6.89	-6.89	-6.89	-6.89	-6.89	-6.89
-6.88	-6.88	-6.88	-6.88	-6.88	-6.88	-6.88	-6.88	-6.88
-6.87	-6.87	-6.87	-6.87	-6.87	-6.87	-6.87	-6.87	-6.87
-6.86	-6.86	-6.86	-6.86	-6.86	-6.86	-6.86	-6.86	-6.86
-6.85	-6.85	-6.85	-6.85	-6.85	-6.85	-6.85	-6.85	-6.85
-6.84	-6.84	-6.84	-6.84	-6.84	-6.84	-6.84	-6.84	-6.84
-6.83	-6.83	-6.83	-6.83	-6.83	-6.83	-6.83	-6.83	-6.83
-6.82	-6.82	-6.82	-6.82	-6.82	-6.82	-6.82	-6.82	-6.82
-6.81	-6.81	-6.81	-6.81	-6.81	-6.81	-6.81	-6.81	-6.81
-6.8	-6.8	-6.8	-6.8	-6.8	-6.8	-6.8	-6.8	-6.8
-6.79	-6.79	-6.79	-6.79	-6.79	-6.79	-6.79	-6.79	-6.79
-6.78	-6.78	-6.78	-6.78	-6.78	-6.78	-6.78	-6.78	-6.78
-6.77	-6.77	-6.77	-6.77	-6.77	-6.77	-6.77	-6.77	-6.77
-6.76	-6.76	-6.76	-6.76	-6.76	-6.76	-6.76	-6.76	-6.76
-6.75	-6.75	-6.75	-6.75	-6.75	-6.75	-6.75	-6.75	-6.75
-6.74	-6.74	-6.74	-6.74	-6.74	-6.74	-6.74	-6.74	-6.74
-6.73	-6.73	-6.73	-6.73	-6.73	-6.73	-6.73	-6.73	-6.73
-6.72	-6.72	-6.72	-6.72	-6.72	-6.72	-6.72	-6.72	-6.72
-6.71	-6.71	-6.71	-6.71	-6.71	-6.71	-6.71	-6.71	-6.71
-6.7	-6.7	-6.7	-6.7	-6.7	-6.7	-6.7	-6.7	-6.7
-6.69	-6.69	-6.69	-6.69	-6.69	-6.69	-6.69	-6.69	-6.69
-6.68	-6.68	-6.68	-6.68	-6.68	-6.68	-6.68	-6.68	-6.68
-6.67	-6.67	-6.67	-6.67	-6.67	-6.67	-6.67	-6.67	-6.67
-6.66	-6.66	-6.66	-6.66	-6.66	-6.66	-6.66	-6.66	-6.66
-6.65	-6.65	-6.65	-6.65	-6.65	-6.65	-6.65	-6.65	-6.65
-6.64	-6.64	-6.64	-6.64	-6.64	-6.64	-6.64	-6.64	-6.64
-6.63	-6.63	-6.63	-6.63	-6.63	-6.63	-6.63	-6.63	-6.63
-6.62	-6.62	-6.62	-6.62	-6.62	-6.62	-6.62	-6.62	-6.62
-6.61	-6.61	-6.61	-6.61	-6.61	-6.61	-6.61	-6.61	-6.61
-6.6	-6.6	-6.6	-6.6	-6.6	-6.6	-6.6	-6.6	-6.6
-6.59	-6.59	-6.59	-6.59	-6.59	-6.59	-6.59	-6.59	-6.59
-6.58	-6.58	-6.58	-6.58	-6.58	-6.58	-6.58	-6.58	-6.58
-6.57	-6.57	-6.57	-6.57	-6.57	-6.57	-6.57	-6.57	-6.57
-6.56	-6.56	-6.56	-6.56	-6.56	-6.56	-6.56	-6.56	-6.56
-6.55	-6.55	-6.55	-6.55	-6.55	-6.55	-6.55	-6.55	-6.55
-6.54	-6.54	-6.54	-6.54	-6.54	-6.54	-6.54	-6.54	-6.54
-6.53	-6.53	-6.53	-6.53	-6.53	-6.53	-6.53	-6.53	-6.53
-6.52	-6.52	-6.52	-6.52	-6.52	-6.52	-6.52	-6.52	-6.52
-6.51	-6.51	-6.51	-6.51	-6.51	-6.51	-6.51	-6.51	-6.51

-6.5	-6.5	-6.5	-6.5	-6.5	-6.5	-6.5	-6.5	-6.5
-6.49	-6.49	-6.49	-6.49	-6.49	-6.49	-6.49	-6.49	-6.49
-6.48	-6.48	-6.48	-6.48	-6.48	-6.48	-6.48	-6.48	-6.48
-6.47	-6.47	-6.47	-6.47	-6.47	-6.47	-6.47	-6.47	-6.47
-6.46	-6.46	-6.46	-6.46	-6.46	-6.46	-6.46	-6.46	-6.46
-6.45	-6.45	-6.45	-6.45	-6.45	-6.45	-6.45	-6.45	-6.45
-6.44	-6.44	-6.44	-6.44	-6.44	-6.44	-6.44	-6.44	-6.44
-6.43	-6.43	-6.43	-6.43	-6.43	-6.43	-6.43	-6.43	-6.43
-6.42	-6.42	-6.42	-6.42	-6.42	-6.42	-6.42	-6.42	-6.42
-6.41	-6.41	-6.41	-6.41	-6.41	-6.41	-6.41	-6.41	-6.41
-6.4	-6.4	-6.4	-6.4	-6.4	-6.4	-6.4	-6.4	-6.4
-6.39	-6.39	-6.39	-6.39	-6.39	-6.39	-6.39	-6.39	-6.39
-6.38	-6.38	-6.38	-6.38	-6.38	-6.38	-6.38	-6.38	-6.38
-6.37	-6.37	-6.37	-6.37	-6.37	-6.37	-6.37	-6.37	-6.37
-6.36	-6.36	-6.36	-6.36	-6.36	-6.36	-6.36	-6.36	-6.36
-6.35	-6.35	-6.35	-6.35	-6.35	-6.35	-6.35	-6.35	-6.35
-6.34	-6.34	-6.34	-6.34	-6.34	-6.34	-6.34	-6.34	-6.34
-6.33	-6.33	-6.33	-6.33	-6.33	-6.33	-6.33	-6.33	-6.33
-6.32	-6.32	-6.32	-6.32	-6.32	-6.32	-6.32	-6.32	-6.32
-6.31	-6.31	-6.31	-6.31	-6.31	-6.31	-6.31	-6.31	-6.31
-6.3	-6.3	-6.3	-6.3	-6.3	-6.3	-6.3	-6.3	-6.3
-6.29	-6.29	-6.29	-6.29	-6.29	-6.29	-6.29	-6.29	-6.29
-6.28	-6.28	-6.28	-6.28	-6.28	-6.28	-6.28	-6.28	-6.28
-6.27	-6.27	-6.27	-6.27	-6.27	-6.27	-6.27	-6.27	-6.27
-6.26	-6.26	-6.26	-6.26	-6.26	-6.26	-6.26	-6.26	-6.26
-6.25	-6.25	-6.25	-6.25	-6.25	-6.25	-6.25	-6.25	-6.25
-6.24	-6.24	-6.24	-6.24	-6.24	-6.24	-6.24	-6.24	-6.24
-6.23	-6.23	-6.23	-6.23	-6.23	-6.23	-6.23	-6.23	-6.23
-6.22	-6.22	-6.22	-6.22	-6.22	-6.22	-6.22	-6.22	-6.22
-6.21	-6.21	-6.21	-6.21	-6.21	-6.21	-6.21	-6.21	-6.21
-6.2	-6.2	-6.2	-6.2	-6.2	-6.2	-6.2	-6.2	-6.2
-6.19	-6.19	-6.19	-6.19	-6.19	-6.19	-6.19	-6.19	-6.19
-6.18	-6.18	-6.18	-6.18	-6.18	-6.18	-6.18	-6.18	-6.18
-6.17	-6.17	-6.17	-6.17	-6.17	-6.17	-6.17	-6.17	-6.17
-6.16	-6.16	-6.16	-6.16	-6.16	-6.16	-6.16	-6.16	-6.16
-6.15	-6.15	-6.15	-6.15	-6.15	-6.15	-6.15	-6.15	-6.15
-6.14	-6.14	-6.14	-6.14	-6.14	-6.14	-6.14	-6.14	-6.14
-6.13	-6.13	-6.13	-6.13	-6.13	-6.13	-6.13	-6.13	-6.13
-6.12	-6.12	-6.12	-6.12	-6.12	-6.12	-6.12	-6.12	-6.12
-6.11	-6.11	-6.11	-6.11	-6.11	-6.11	-6.11	-6.11	-6.11
-6.1	-6.1	-6.1	-6.1	-6.1	-6.1	-6.1	-6.1	-6.1
-6.09	-6.09	-6.09	-6.09	-6.09	-6.09	-6.09	-6.09	-6.09
-6.08	-6.08	-6.08	-6.08	-6.08	-6.08	-6.08	-6.08	-6.08
-6.07	-6.07	-6.07	-6.07	-6.07	-6.07	-6.07	-6.07	-6.07
-6.06	-6.06	-6.06	-6.06	-6.06	-6.06	-6.06	-6.06	-6.06
-6.05	-6.05	-6.05	-6.05	-6.05	-6.05	-6.05	-6.05	-6.05
-6.04	-6.04	-6.04	-6.04	-6.04	-6.04	-6.04	-6.04	-6.04
-6.03	-6.03	-6.03	-6.03	-6.03	-6.03	-6.03	-6.03	-6.03
-6.02	-6.02	-6.02	-6.02	-6.02	-6.02	-6.02	-6.02	-6.02
-6.01	-6.01	-6.01	-6.01	-6.01	-6.01	-6.01	-6.01	-6.01

-6	-6	-6	-6	-6	-6	-6	-6	-6
-5.99	-5.99	-5.99	-5.99	-5.99	-5.99	-5.99	-5.99	-5.99
-5.98	-5.98	-5.98	-5.98	-5.98	-5.98	-5.98	-5.98	-5.98
-5.97	-5.97	-5.97	-5.97	-5.97	-5.97	-5.97	-5.97	-5.97
-5.96	-5.96	-5.96	-5.96	-5.96	-5.96	-5.96	-5.96	-5.96
-5.95	-5.95	-5.95	-5.95	-5.95	-5.95	-5.95	-5.95	-5.95
-5.94	-5.94	-5.94	-5.94	-5.94	-5.94	-5.94	-5.94	-5.94
-5.93	-5.93	-5.93	-5.93	-5.93	-5.93	-5.93	-5.93	-5.93
-5.92	-5.92	-5.92	-5.92	-5.92	-5.92	-5.92	-5.92	-5.92
-5.91	-5.91	-5.91	-5.91	-5.91	-5.91	-5.91	-5.91	-5.91
-5.9	-5.9	-5.9	-5.9	-5.9	-5.9	-5.9	-5.9	-5.9
-5.89	-5.89	-5.89	-5.89	-5.89	-5.89	-5.89	-5.89	-5.89
-5.88	-5.88	-5.88	-5.88	-5.88	-5.88	-5.88	-5.88	-5.88
-5.87	-5.87	-5.87	-5.87	-5.87	-5.87	-5.87	-5.87	-5.87
-5.86	-5.86	-5.86	-5.86	-5.86	-5.86	-5.86	-5.86	-5.86
-5.85	-5.85	-5.85	-5.85	-5.85	-5.85	-5.85	-5.85	-5.85
-5.84	-5.84	-5.84	-5.84	-5.84	-5.84	-5.84	-5.84	-5.84
-5.83	-5.83	-5.83	-5.83	-5.83	-5.83	-5.83	-5.83	-5.83
-5.82	-5.82	-5.82	-5.82	-5.82	-5.82	-5.82	-5.82	-5.82
-5.81	-5.81	-5.81	-5.81	-5.81	-5.81	-5.81	-5.81	-5.81
-5.8	-5.8	-5.8	-5.8	-5.8	-5.8	-5.8	-5.8	-5.8
-5.79	-5.79	-5.79	-5.79	-5.79	-5.79	-5.79	-5.79	-5.79
-5.78	-5.78	-5.78	-5.78	-5.78	-5.78	-5.78	-5.78	-5.78
-5.77	-5.77	-5.77	-5.77	-5.77	-5.77	-5.77	-5.77	-5.77
-5.76	-5.76	-5.76	-5.76	-5.76	-5.76	-5.76	-5.76	-5.76
-5.75	-5.75	-5.75	-5.75	-5.75	-5.75	-5.75	-5.75	-5.75
-5.74	-5.74	-5.74	-5.74	-5.74	-5.74	-5.74	-5.74	-5.74
-5.73	-5.73	-5.73	-5.73	-5.73	-5.73	-5.73	-5.73	-5.73
-5.72	-5.72	-5.72	-5.72	-5.72	-5.72	-5.72	-5.72	-5.72
-5.71	-5.71	-5.71	-5.71	-5.71	-5.71	-5.71	-5.71	-5.71
-5.7	-5.7	-5.7	-5.7	-5.7	-5.7	-5.7	-5.7	-5.7
-5.69	-5.69	-5.69	-5.69	-5.69	-5.69	-5.69	-5.69	-5.69
-5.68	-5.68	-5.68	-5.68	-5.68	-5.68	-5.68	-5.68	-5.68
-5.67	-5.67	-5.67	-5.67	-5.67	-5.67	-5.67	-5.67	-5.67
-5.66	-5.66	-5.66	-5.66	-5.66	-5.66	-5.66	-5.66	-5.66
-5.65	-5.65	-5.65	-5.65	-5.65	-5.65	-5.65	-5.65	-5.65
-5.64	-5.64	-5.64	-5.64	-5.64	-5.64	-5.64	-5.64	-5.64
-5.63	-5.63	-5.63	-5.63	-5.63	-5.63	-5.63	-5.63	-5.63
-5.62	-5.62	-5.62	-5.62	-5.62	-5.62	-5.62	-5.62	-5.62
-5.61	-5.61	-5.61	-5.61	-5.61	-5.61	-5.61	-5.61	-5.61
-5.6	-5.6	-5.6	-5.6	-5.6	-5.6	-5.6	-5.6	-5.6
-5.59	-5.59	-5.59	-5.59	-5.59	-5.59	-5.59	-5.59	-5.59
-5.58	-5.58	-5.58	-5.58	-5.58	-5.58	-5.58	-5.58	-5.58
-5.57	-5.57	-5.57	-5.57	-5.57	-5.57	-5.57	-5.57	-5.57
-5.56	-5.56	-5.56	-5.56	-5.56	-5.56	-5.56	-5.56	-5.56
-5.55	-5.55	-5.55	-5.55	-5.55	-5.55	-5.55	-5.55	-5.55
-5.54	-5.54	-5.54	-5.54	-5.54	-5.54	-5.54	-5.54	-5.54
-5.53	-5.53	-5.53	-5.53	-5.53	-5.53	-5.53	-5.53	-5.53
-5.52	-5.52	-5.52	-5.52	-5.52	-5.52	-5.52	-5.52	-5.52
-5.51	-5.51	-5.51	-5.51	-5.51	-5.51	-5.51	-5.51	-5.51

-5.5	-5.5	-5.5	-5.5	-5.5	-5.5	-5.5	-5.5	-5.5
-5.49	-5.49	-5.49	-5.49	-5.49	-5.49	-5.49	-5.49	-5.49
-5.48	-5.48	-5.48	-5.48	-5.48	-5.48	-5.48	-5.48	-5.48
-5.47	-5.47	-5.47	-5.47	-5.47	-5.47	-5.47	-5.47	-5.47
-5.46	-5.46	-5.46	-5.46	-5.46	-5.46	-5.46	-5.46	-5.46
-5.45	-5.45	-5.45	-5.45	-5.45	-5.45	-5.45	-5.45	-5.45
-5.44	-5.44	-5.44	-5.44	-5.44	-5.44	-5.44	-5.44	-5.44
-5.43	-5.43	-5.43	-5.43	-5.43	-5.43	-5.43	-5.43	-5.43
-5.42	-5.42	-5.42	-5.42	-5.42	-5.42	-5.42	-5.42	-5.42
-5.41	-5.41	-5.41	-5.41	-5.41	-5.41	-5.41	-5.41	-5.41
-5.4	-5.4	-5.4	-5.4	-5.4	-5.4	-5.4	-5.4	-5.4
-5.39	-5.39	-5.39	-5.39	-5.39	-5.39	-5.39	-5.39	-5.39
-5.38	-5.38	-5.38	-5.38	-5.38	-5.38	-5.38	-5.38	-5.38
-5.37	-5.37	-5.37	-5.37	-5.37	-5.37	-5.37	-5.37	-5.37
-5.36	-5.36	-5.36	-5.36	-5.36	-5.36	-5.36	-5.36	-5.36
-5.35	-5.35	-5.35	-5.35	-5.35	-5.35	-5.35	-5.35	-5.35
-5.34	-5.34	-5.34	-5.34	-5.34	-5.34	-5.34	-5.34	-5.34
-5.33	-5.33	-5.33	-5.33	-5.33	-5.33	-5.33	-5.33	-5.33
-5.32	-5.32	-5.32	-5.32	-5.32	-5.32	-5.32	-5.32	-5.32
-5.31	-5.31	-5.31	-5.31	-5.31	-5.31	-5.31	-5.31	-5.31
-5.3	-5.3	-5.3	-5.3	-5.3	-5.3	-5.3	-5.3	-5.3
-5.29	-5.29	-5.29	-5.29	-5.29	-5.29	-5.29	-5.29	-5.29
-5.28	-5.28	-5.28	-5.28	-5.28	-5.28	-5.28	-5.28	-5.28
-5.27	-5.27	-5.27	-5.27	-5.27	-5.27	-5.27	-5.27	-5.27
-5.26	-5.26	-5.26	-5.26	-5.26	-5.26	-5.26	-5.26	-5.26
-5.25	-5.25	-5.25	-5.25	-5.25	-5.25	-5.25	-5.25	-5.25
-5.24	-5.24	-5.24	-5.24	-5.24	-5.24	-5.24	-5.24	-5.24
-5.23	-5.23	-5.23	-5.23	-5.23	-5.23	-5.23	-5.23	-5.23
-5.22	-5.22	-5.22	-5.22	-5.22	-5.22	-5.22	-5.22	-5.22
-5.21	-5.21	-5.21	-5.21	-5.21	-5.21	-5.21	-5.21	-5.21
-5.2	-5.2	-5.2	-5.2	-5.2	-5.2	-5.2	-5.2	-5.2
-5.19	-5.19	-5.19	-5.19	-5.19	-5.19	-5.19	-5.19	-5.19
-5.18	-5.18	-5.18	-5.18	-5.18	-5.18	-5.18	-5.18	-5.18
-5.17	-5.17	-5.17	-5.17	-5.17	-5.17	-5.17	-5.17	-5.17
-5.16	-5.16	-5.16	-5.16	-5.16	-5.16	-5.16	-5.16	-5.16
-5.15	-5.15	-5.15	-5.15	-5.15	-5.15	-5.15	-5.15	-5.15
-5.14	-5.14	-5.14	-5.14	-5.14	-5.14	-5.14	-5.14	-5.14
-5.13	-5.13	-5.13	-5.13	-5.13	-5.13	-5.13	-5.13	-5.13
-5.12	-5.12	-5.12	-5.12	-5.12	-5.12	-5.12	-5.12	-5.12
-5.11	-5.11	-5.11	-5.11	-5.11	-5.11	-5.11	-5.11	-5.11
-5.1	-5.1	-5.1	-5.1	-5.1	-5.1	-5.1	-5.1	-5.1
-5.09	-5.09	-5.09	-5.09	-5.09	-5.09	-5.09	-5.09	-5.09
-5.08	-5.08	-5.08	-5.08	-5.08	-5.08	-5.08	-5.08	-5.08
-5.07	-5.07	-5.07	-5.07	-5.07	-5.07	-5.07	-5.07	-5.07
-5.06	-5.06	-5.06	-5.06	-5.06	-5.06	-5.06	-5.06	-5.06
-5.05	-5.05	-5.05	-5.05	-5.05	-5.05	-5.05	-5.05	-5.05
-5.04	-5.04	-5.04	-5.04	-5.04	-5.04	-5.04	-5.04	-5.04
-5.03	-5.03	-5.03	-5.03	-5.03	-5.03	-5.03	-5.03	-5.03
-5.02	-5.02	-5.02	-5.02	-5.02	-5.02	-5.02	-5.02	-5.02
-5.01	-5.01	-5.01	-5.01	-5.01	-5.01	-5.01	-5.01	-5.01

-5	-5	-5	-5	-5	-5	-5	-5	-5
-4.99	-4.99	-4.99	-4.99	-4.99	-4.99	-4.99	-4.99	-4.99
-4.98	-4.98	-4.98	-4.98	-4.98	-4.98	-4.98	-4.98	-4.98
-4.97	-4.97	-4.97	-4.97	-4.97	-4.97	-4.97	-4.97	-4.97
-4.96	-4.96	-4.96	-4.96	-4.96	-4.96	-4.96	-4.96	-4.96
-4.95	-4.95	-4.95	-4.95	-4.95	-4.95	-4.95	-4.95	-4.95
-4.94	-4.94	-4.94	-4.94	-4.94	-4.94	-4.94	-4.94	-4.94
-4.93	-4.93	-4.93	-4.93	-4.93	-4.93	-4.93	-4.93	-4.93
-4.92	-4.92	-4.92	-4.92	-4.92	-4.92	-4.92	-4.92	-4.92
-4.91	-4.91	-4.91	-4.91	-4.91	-4.91	-4.91	-4.91	-4.91
-4.9	-4.9	-4.9	-4.9	-4.9	-4.9	-4.9	-4.9	-4.9
-4.89	-4.89	-4.89	-4.89	-4.89	-4.89	-4.89	-4.89	-4.89
-4.88	-4.88	-4.88	-4.88	-4.88	-4.88	-4.88	-4.88	-4.88
-4.87	-4.87	-4.87	-4.87	-4.87	-4.87	-4.87	-4.87	-4.87
-4.86	-4.86	-4.86	-4.86	-4.86	-4.86	-4.86	-4.86	-4.86
-4.85	-4.85	-4.85	-4.85	-4.85	-4.85	-4.85	-4.85	-4.85
-4.84	-4.84	-4.84	-4.84	-4.84	-4.84	-4.84	-4.84	-4.84
-4.83	-4.83	-4.83	-4.83	-4.83	-4.83	-4.83	-4.83	-4.83
-4.82	-4.82	-4.82	-4.82	-4.82	-4.82	-4.82	-4.82	-4.82
-4.81	-4.81	-4.81	-4.81	-4.81	-4.81	-4.81	-4.81	-4.81
-4.8	-4.8	-4.8	-4.8	-4.8	-4.8	-4.8	-4.8	-4.8
-4.79	-4.79	-4.79	-4.79	-4.79	-4.79	-4.79	-4.79	-4.79
-4.78	-4.78	-4.78	-4.78	-4.78	-4.78	-4.78	-4.78	-4.78
-4.77	-4.77	-4.77	-4.77	-4.77	-4.77	-4.77	-4.77	-4.77
-4.76	-4.76	-4.76	-4.76	-4.76	-4.76	-4.76	-4.76	-4.76
-4.75	-4.75	-4.75	-4.75	-4.75	-4.75	-4.75	-4.75	-4.75
-4.74	-4.74	-4.74	-4.74	-4.74	-4.74	-4.74	-4.74	-4.74
-4.73	-4.73	-4.73	-4.73	-4.73	-4.73	-4.73	-4.73	-4.73
-4.72	-4.72	-4.72	-4.72	-4.72	-4.72	-4.72	-4.72	-4.72
-4.71	-4.71	-4.71	-4.71	-4.71	-4.71	-4.71	-4.71	-4.71
-4.7	-4.7	-4.7	-4.7	-4.7	-4.7	-4.7	-4.7	-4.7
-4.69	-4.69	-4.69	-4.69	-4.69	-4.69	-4.69	-4.69	-4.69
-4.68	-4.68	-4.68	-4.68	-4.68	-4.68	-4.68	-4.68	-4.68
-4.67	-4.67	-4.67	-4.67	-4.67	-4.67	-4.67	-4.67	-4.67
-4.66	-4.66	-4.66	-4.66	-4.66	-4.66	-4.66	-4.66	-4.66
-4.65	-4.65	-4.65	-4.65	-4.65	-4.65	-4.65	-4.65	-4.65
-4.64	-4.64	-4.64	-4.64	-4.64	-4.64	-4.64	-4.64	-4.64
-4.63	-4.63	-4.63	-4.63	-4.63	-4.63	-4.63	-4.63	-4.63
-4.62	-4.62	-4.62	-4.62	-4.62	-4.62	-4.62	-4.62	-4.62
-4.61	-4.61	-4.61	-4.61	-4.61	-4.61	-4.61	-4.61	-4.61
-4.6	-4.6	-4.6	-4.6	-4.6	-4.6	-4.6	-4.6	-4.6
-4.59	-4.59	-4.59	-4.59	-4.59	-4.59	-4.59	-4.59	-4.59
-4.58	-4.58	-4.58	-4.58	-4.58	-4.58	-4.58	-4.58	-4.58
-4.57	-4.57	-4.57	-4.57	-4.57	-4.57	-4.57	-4.57	-4.57
-4.56	-4.56	-4.56	-4.56	-4.56	-4.56	-4.56	-4.56	-4.56
-4.55	-4.55	-4.55	-4.55	-4.55	-4.55	-4.55	-4.55	-4.55
-4.54	-4.54	-4.54	-4.54	-4.54	-4.54	-4.54	-4.54	-4.54
-4.53	-4.53	-4.53	-4.53	-4.53	-4.53	-4.53	-4.53	-4.53
-4.52	-4.52	-4.52	-4.52	-4.52	-4.52	-4.52	-4.52	-4.52
-4.51	-4.51	-4.51	-4.51	-4.51	-4.51	-4.51	-4.51	-4.51

-4.5	-4.5	-4.5	-4.5	-4.5	-4.5	-4.5	-4.5	-4.5
-4.49	-4.49	-4.49	-4.49	-4.49	-4.49	-4.49	-4.49	-4.49
-4.48	-4.48	-4.48	-4.48	-4.48	-4.48	-4.48	-4.48	-4.48
-4.47	-4.47	-4.47	-4.47	-4.47	-4.47	-4.47	-4.47	-4.47
-4.46	-4.46	-4.46	-4.46	-4.46	-4.46	-4.46	-4.46	-4.46
-4.45	-4.45	-4.45	-4.45	-4.45	-4.45	-4.45	-4.45	-4.45
-4.44	-4.44	-4.44	-4.44	-4.44	-4.44	-4.44	-4.44	-4.44
-4.43	-4.43	-4.43	-4.43	-4.43	-4.43	-4.43	-4.43	-4.43
-4.42	-4.42	-4.42	-4.42	-4.42	-4.42	-4.42	-4.42	-4.42
-4.41	-4.41	-4.41	-4.41	-4.41	-4.41	-4.41	-4.41	-4.41
-4.4	-4.4	-4.4	-4.4	-4.4	-4.4	-4.4	-4.4	-4.4
-4.39	-4.39	-4.39	-4.39	-4.39	-4.39	-4.39	-4.39	-4.39
-4.38	-4.38	-4.38	-4.38	-4.38	-4.38	-4.38	-4.38	-4.38
-4.37	-4.37	-4.37	-4.37	-4.37	-4.37	-4.37	-4.37	-4.37
-4.36	-4.36	-4.36	-4.36	-4.36	-4.36	-4.36	-4.36	-4.36
-4.35	-4.35	-4.35	-4.35	-4.35	-4.35	-4.35	-4.35	-4.35
-4.34	-4.34	-4.34	-4.34	-4.34	-4.34	-4.34	-4.34	-4.34
-4.33	-4.33	-4.33	-4.33	-4.33	-4.33	-4.33	-4.33	-4.33
-4.32	-4.32	-4.32	-4.32	-4.32	-4.32	-4.32	-4.32	-4.32
-4.31	-4.31	-4.31	-4.31	-4.31	-4.31	-4.31	-4.31	-4.31
-4.3	-4.3	-4.3	-4.3	-4.3	-4.3	-4.3	-4.3	-4.3
-4.29	-4.29	-4.29	-4.29	-4.29	-4.29	-4.29	-4.29	-4.29
-4.28	-4.28	-4.28	-4.28	-4.28	-4.28	-4.28	-4.28	-4.28
-4.27	-4.27	-4.27	-4.27	-4.27	-4.27	-4.27	-4.27	-4.27
-4.26	-4.26	-4.26	-4.26	-4.26	-4.26	-4.26	-4.26	-4.26
-4.25	-4.25	-4.25	-4.25	-4.25	-4.25	-4.25	-4.25	-4.25
-4.24	-4.24	-4.24	-4.24	-4.24	-4.24	-4.24	-4.24	-4.24
-4.23	-4.23	-4.23	-4.23	-4.23	-4.23	-4.23	-4.23	-4.23
-4.22	-4.22	-4.22	-4.22	-4.22	-4.22	-4.22	-4.22	-4.22
-4.21	-4.21	-4.21	-4.21	-4.21	-4.21	-4.21	-4.21	-4.21
-4.2	-4.2	-4.2	-4.2	-4.2	-4.2	-4.2	-4.2	-4.2
-4.19	-4.19	-4.19	-4.19	-4.19	-4.19	-4.19	-4.19	-4.19
-4.18	-4.18	-4.18	-4.18	-4.18	-4.18	-4.18	-4.18	-4.18
-4.17	-4.17	-4.17	-4.17	-4.17	-4.17	-4.17	-4.17	-4.17
-4.16	-4.16	-4.16	-4.16	-4.16	-4.16	-4.16	-4.16	-4.16
-4.15	-4.15	-4.15	-4.15	-4.15	-4.15	-4.15	-4.15	-4.15
-4.14	-4.14	-4.14	-4.14	-4.14	-4.14	-4.14	-4.14	-4.14
-4.13	-4.13	-4.13	-4.13	-4.13	-4.13	-4.13	-4.13	-4.13
-4.12	-4.12	-4.12	-4.12	-4.12	-4.12	-4.12	-4.12	-4.12
-4.11	-4.11	-4.11	-4.11	-4.11	-4.11	-4.11	-4.11	-4.11
-4.1	-4.1	-4.1	-4.1	-4.1	-4.1	-4.1	-4.1	-4.1
-4.09	-4.09	-4.09	-4.09	-4.09	-4.09	-4.09	-4.09	-4.09
-4.08	-4.08	-4.08	-4.08	-4.08	-4.08	-4.08	-4.08	-4.08
-4.07	-4.07	-4.07	-4.07	-4.07	-4.07	-4.07	-4.07	-4.07
-4.06	-4.06	-4.06	-4.06	-4.06	-4.06	-4.06	-4.06	-4.06
-4.05	-4.05	-4.05	-4.05	-4.05	-4.05	-4.05	-4.05	-4.05
-4.04	-4.04	-4.04	-4.04	-4.04	-4.04	-4.04	-4.04	-4.04
-4.03	-4.03	-4.03	-4.03	-4.03	-4.03	-4.03	-4.03	-4.03
-4.02	-4.02	-4.02	-4.02	-4.02	-4.02	-4.02	-4.02	-4.02
-4.01	-4.01	-4.01	-4.01	-4.01	-4.01	-4.01	-4.01	-4.01

-4	-4	-4	-4	-4	-4	-4	-4	-4
-3.99	-3.99	-3.99	-3.99	-3.99	-3.99	-3.99	-3.99	-3.99
-3.98	-3.98	-3.98	-3.98	-3.98	-3.98	-3.98	-3.98	-3.98
-3.97	-3.97	-3.97	-3.97	-3.97	-3.97	-3.97	-3.97	-3.97
-3.96	-3.96	-3.96	-3.96	-3.96	-3.96	-3.96	-3.96	-3.96
-3.95	-3.95	-3.95	-3.95	-3.95	-3.95	-3.95	-3.95	-3.95
-3.94	-3.94	-3.94	-3.94	-3.94	-3.94	-3.94	-3.94	-3.94
-3.93	-3.93	-3.93	-3.93	-3.93	-3.93	-3.93	-3.93	-3.93
-3.92	-3.92	-3.92	-3.92	-3.92	-3.92	-3.92	-3.92	-3.92
-3.91	-3.91	-3.91	-3.91	-3.91	-3.91	-3.91	-3.91	-3.91
-3.9	-3.9	-3.9	-3.9	-3.9	-3.9	-3.9	-3.9	-3.9
-3.89	-3.89	-3.89	-3.89	-3.89	-3.89	-3.89	-3.89	-3.89
-3.88	-3.88	-3.88	-3.88	-3.88	-3.88	-3.88	-3.88	-3.88
-3.87	-3.87	-3.87	-3.87	-3.87	-3.87	-3.87	-3.87	-3.87
-3.86	-3.86	-3.86	-3.86	-3.86	-3.86	-3.86	-3.86	-3.86
-3.85	-3.85	-3.85	-3.85	-3.85	-3.85	-3.85	-3.85	-3.85
-3.84	-3.84	-3.84	-3.84	-3.84	-3.84	-3.84	-3.84	-3.84
-3.83	-3.83	-3.83	-3.83	-3.83	-3.83	-3.83	-3.83	-3.83
-3.82	-3.82	-3.82	-3.82	-3.82	-3.82	-3.82	-3.82	-3.82
-3.81	-3.81	-3.81	-3.81	-3.81	-3.81	-3.81	-3.81	-3.81
-3.8	-3.8	-3.8	-3.8	-3.8	-3.8	-3.8	-3.8	-3.8
-3.79	-3.79	-3.79	-3.79	-3.79	-3.79	-3.79	-3.79	-3.79
-3.78	-3.78	-3.78	-3.78	-3.78	-3.78	-3.78	-3.78	-3.78
-3.77	-3.77	-3.77	-3.77	-3.77	-3.77	-3.77	-3.77	-3.77
-3.76	-3.76	-3.76	-3.76	-3.76	-3.76	-3.76	-3.76	-3.76
-3.75	-3.75	-3.75	-3.75	-3.75	-3.75	-3.75	-3.75	-3.75
-3.74	-3.74	-3.74	-3.74	-3.74	-3.74	-3.74	-3.74	-3.74
-3.73	-3.73	-3.73	-3.73	-3.73	-3.73	-3.73	-3.73	-3.73
-3.72	-3.72	-3.72	-3.72	-3.72	-3.72	-3.72	-3.72	-3.72
-3.71	-3.71	-3.71	-3.71	-3.71	-3.71	-3.71	-3.71	-3.71
-3.7	-3.7	-3.7	-3.7	-3.7	-3.7	-3.7	-3.7	-3.7
-3.69	-3.69	-3.69	-3.69	-3.69	-3.69	-3.69	-3.69	-3.69
-3.68	-3.68	-3.68	-3.68	-3.68	-3.68	-3.68	-3.68	-3.68
-3.67	-3.67	-3.67	-3.67	-3.67	-3.67	-3.67	-3.67	-3.67
-3.66	-3.66	-3.66	-3.66	-3.66	-3.66	-3.66	-3.66	-3.66
-3.65	-3.65	-3.65	-3.65	-3.65	-3.65	-3.65	-3.65	-3.65
-3.64	-3.64	-3.64	-3.64	-3.64	-3.64	-3.64	-3.64	-3.64
-3.63	-3.63	-3.63	-3.63	-3.63	-3.63	-3.63	-3.63	-3.63
-3.62	-3.62	-3.62	-3.62	-3.62	-3.62	-3.62	-3.62	-3.62
-3.61	-3.61	-3.61	-3.61	-3.61	-3.61	-3.61	-3.61	-3.61
-3.6	-3.6	-3.6	-3.6	-3.6	-3.6	-3.6	-3.6	-3.6
-3.59	-3.59	-3.59	-3.59	-3.59	-3.59	-3.59	-3.59	-3.59
-3.58	-3.58	-3.58	-3.58	-3.58	-3.58	-3.58	-3.58	-3.58
-3.57	-3.57	-3.57	-3.57	-3.57	-3.57	-3.57	-3.57	-3.57
-3.56	-3.56	-3.56	-3.56	-3.56	-3.56	-3.56	-3.56	-3.56
-3.55	-3.55	-3.55	-3.55	-3.55	-3.55	-3.55	-3.55	-3.55
-3.54	-3.54	-3.54	-3.54	-3.54	-3.54	-3.54	-3.54	-3.54
-3.53	-3.53	-3.53	-3.53	-3.53	-3.53	-3.53	-3.53	-3.53
-3.52	-3.52	-3.52	-3.52	-3.52	-3.52	-3.52	-3.52	-3.52
-3.51	-3.51	-3.51	-3.51	-3.51	-3.51	-3.51	-3.51	-3.51

-3.5	-3.5	-3.5	-3.5	-3.5	-3.5	-3.5	-3.5	-3.5
-3.49	-3.49	-3.49	-3.49	-3.49	-3.49	-3.49	-3.49	-3.49
-3.48	-3.48	-3.48	-3.48	-3.48	-3.48	-3.48	-3.48	-3.48
-3.47	-3.47	-3.47	-3.47	-3.47	-3.47	-3.47	-3.47	-3.47
-3.46	-3.46	-3.46	-3.46	-3.46	-3.46	-3.46	-3.46	-3.46
-3.45	-3.45	-3.45	-3.45	-3.45	-3.45	-3.45	-3.45	-3.45
-3.44	-3.44	-3.44	-3.44	-3.44	-3.44	-3.44	-3.44	-3.44
-3.43	-3.43	-3.43	-3.43	-3.43	-3.43	-3.43	-3.43	-3.43
-3.42	-3.42	-3.42	-3.42	-3.42	-3.42	-3.42	-3.42	-3.42
-3.41	-3.41	-3.41	-3.41	-3.41	-3.41	-3.41	-3.41	-3.41
-3.4	-3.4	-3.4	-3.4	-3.4	-3.4	-3.4	-3.4	-3.4
-3.39	-3.39	-3.39	-3.39	-3.39	-3.39	-3.39	-3.39	-3.39
-3.38	-3.38	-3.38	-3.38	-3.38	-3.38	-3.38	-3.38	-3.38
-3.37	-3.37	-3.37	-3.37	-3.37	-3.37	-3.37	-3.37	-3.37
-3.36	-3.36	-3.36	-3.36	-3.36	-3.36	-3.36	-3.36	-3.36
-3.35	-3.35	-3.35	-3.35	-3.35	-3.35	-3.35	-3.35	-3.35
-3.34	-3.34	-3.34	-3.34	-3.34	-3.34	-3.34	-3.34	-3.34
-3.33	-3.33	-3.33	-3.33	-3.33	-3.33	-3.33	-3.33	-3.33
-3.32	-3.32	-3.32	-3.32	-3.32	-3.32	-3.32	-3.32	-3.32
-3.31	-3.31	-3.31	-3.31	-3.31	-3.31	-3.31	-3.31	-3.31
-3.3	-3.3	-3.3	-3.3	-3.3	-3.3	-3.3	-3.3	-3.3
-3.29	-3.29	-3.29	-3.29	-3.29	-3.29	-3.29	-3.29	-3.29
-3.28	-3.28	-3.28	-3.28	-3.28	-3.28	-3.28	-3.28	-3.28
-3.27	-3.27	-3.27	-3.27	-3.27	-3.27	-3.27	-3.27	-3.27
-3.26	-3.26	-3.26	-3.26	-3.26	-3.26	-3.26	-3.26	-3.26
-3.25	-3.25	-3.25	-3.25	-3.25	-3.25	-3.25	-3.25	-3.25
-3.24	-3.24	-3.24	-3.24	-3.24	-3.24	-3.24	-3.24	-3.24
-3.23	-3.23	-3.23	-3.23	-3.23	-3.23	-3.23	-3.23	-3.23
-3.22	-3.22	-3.22	-3.22	-3.22	-3.22	-3.22	-3.22	-3.22
-3.21	-3.21	-3.21	-3.21	-3.21	-3.21	-3.21	-3.21	-3.21
-3.2	-3.2	-3.2	-3.2	-3.2	-3.2	-3.2	-3.2	-3.2
-3.19	-3.19	-3.19	-3.19	-3.19	-3.19	-3.19	-3.19	-3.19
-3.18	-3.18	-3.18	-3.18	-3.18	-3.18	-3.18	-3.18	-3.18
-3.17	-3.17	-3.17	-3.17	-3.17	-3.17	-3.17	-3.17	-3.17
-3.16	-3.16	-3.16	-3.16	-3.16	-3.16	-3.16	-3.16	-3.16
-3.15	-3.15	-3.15	-3.15	-3.15	-3.15	-3.15	-3.15	-3.15
-3.14	-3.14	-3.14	-3.14	-3.14	-3.14	-3.14	-3.14	-3.14
-3.13	-3.13	-3.13	-3.13	-3.13	-3.13	-3.13	-3.13	-3.13
-3.12	-3.12	-3.12	-3.12	-3.12	-3.12	-3.12	-3.12	-3.12
-3.11	-3.11	-3.11	-3.11	-3.11	-3.11	-3.11	-3.11	-3.11
-3.1	-3.1	-3.1	-3.1	-3.1	-3.1	-3.1	-3.1	-3.1
-3.09	-3.09	-3.09	-3.09	-3.09	-3.09	-3.09	-3.09	-3.09
-3.08	-3.08	-3.08	-3.08	-3.08	-3.08	-3.08	-3.08	-3.08
-3.07	-3.07	-3.07	-3.07	-3.07	-3.07	-3.07	-3.07	-3.07
-3.06	-3.06	-3.06	-3.06	-3.06	-3.06	-3.06	-3.06	-3.06
-3.05	-3.05	-3.05	-3.05	-3.05	-3.05	-3.05	-3.05	-3.05
-3.04	-3.04	-3.04	-3.04	-3.04	-3.04	-3.04	-3.04	-3.04
-3.03	-3.03	-3.03	-3.03	-3.03	-3.03	-3.03	-3.03	-3.03
-3.02	-3.02	-3.02	-3.02	-3.02	-3.02	-3.02	-3.02	-3.02
-3.01	-3.01	-3.01	-3.01	-3.01	-3.01	-3.01	-3.01	-3.01

-3	-3	-3	-3	-3	-3	-3	-3	-3
-2.99	-2.99	-2.99	-2.99	-2.99	-2.99	-2.99	-2.99	-2.99
-2.98	-2.98	-2.98	-2.98	-2.98	-2.98	-2.98	-2.98	-2.98
-2.97	-2.97	-2.97	-2.97	-2.97	-2.97	-2.97	-2.97	-2.97
-2.96	-2.96	-2.96	-2.96	-2.96	-2.96	-2.96	-2.96	-2.96
-2.95	-2.95	-2.95	-2.95	-2.95	-2.95	-2.95	-2.95	-2.95
-2.94	-2.94	-2.94	-2.94	-2.94	-2.94	-2.94	-2.94	-2.94
-2.93	-2.93	-2.93	-2.93	-2.93	-2.93	-2.93	-2.93	-2.93
-2.92	-2.92	-2.92	-2.92	-2.92	-2.92	-2.92	-2.92	-2.92
-2.91	-2.91	-2.91	-2.91	-2.91	-2.91	-2.91	-2.91	-2.91
-2.9	-2.9	-2.9	-2.9	-2.9	-2.9	-2.9	-2.9	-2.9
-2.89	-2.89	-2.89	-2.89	-2.89	-2.89	-2.89	-2.89	-2.89
-2.88	-2.88	-2.88	-2.88	-2.88	-2.88	-2.88	-2.88	-2.88
-2.87	-2.87	-2.87	-2.87	-2.87	-2.87	-2.87	-2.87	-2.87
-2.86	-2.86	-2.86	-2.86	-2.86	-2.86	-2.86	-2.86	-2.86
-2.85	-2.85	-2.85	-2.85	-2.85	-2.85	-2.85	-2.85	-2.85
-2.84	-2.84	-2.84	-2.84	-2.84	-2.84	-2.84	-2.84	-2.84
-2.83	-2.83	-2.83	-2.83	-2.83	-2.83	-2.83	-2.83	-2.83
-2.82	-2.82	-2.82	-2.82	-2.82	-2.82	-2.82	-2.82	-2.82
-2.81	-2.81	-2.81	-2.81	-2.81	-2.81	-2.81	-2.81	-2.81
-2.8	-2.8	-2.8	-2.8	-2.8	-2.8	-2.8	-2.8	-2.8
-2.79	-2.79	-2.79	-2.79	-2.79	-2.79	-2.79	-2.79	-2.79
-2.78	-2.78	-2.78	-2.78	-2.78	-2.78	-2.78	-2.78	-2.78
-2.77	-2.77	-2.77	-2.77	-2.77	-2.77	-2.77	-2.77	-2.77
-2.76	-2.76	-2.76	-2.76	-2.76	-2.76	-2.76	-2.76	-2.76
-2.75	-2.75	-2.75	-2.75	-2.75	-2.75	-2.75	-2.75	-2.75
-2.74	-2.74	-2.74	-2.74	-2.74	-2.74	-2.74	-2.74	-2.74
-2.73	-2.73	-2.73	-2.73	-2.73	-2.73	-2.73	-2.73	-2.73
-2.72	-2.72	-2.72	-2.72	-2.72	-2.72	-2.72	-2.72	-2.72
-2.71	-2.71	-2.71	-2.71	-2.71	-2.71	-2.71	-2.71	-2.71
-2.7	-2.7	-2.7	-2.7	-2.7	-2.7	-2.7	-2.7	-2.7
-2.69	-2.69	-2.69	-2.69	-2.69	-2.69	-2.69	-2.69	-2.69
-2.68	-2.68	-2.68	-2.68	-2.68	-2.68	-2.68	-2.68	-2.68
-2.67	-2.67	-2.67	-2.67	-2.67	-2.67	-2.67	-2.67	-2.67
-2.66	-2.66	-2.66	-2.66	-2.66	-2.66	-2.66	-2.66	-2.66
-2.65	-2.65	-2.65	-2.65	-2.65	-2.65	-2.65	-2.65	-2.65
-2.64	-2.64	-2.64	-2.64	-2.64	-2.64	-2.64	-2.64	-2.64
-2.63	-2.63	-2.63	-2.63	-2.63	-2.63	-2.63	-2.63	-2.63
-2.62	-2.62	-2.62	-2.62	-2.62	-2.62	-2.62	-2.62	-2.62
-2.61	-2.61	-2.61	-2.61	-2.61	-2.61	-2.61	-2.61	-2.61
-2.6	-2.6	-2.6	-2.6	-2.6	-2.6	-2.6	-2.6	-2.6
-2.59	-2.59	-2.59	-2.59	-2.59	-2.59	-2.59	-2.59	-2.59
-2.58	-2.58	-2.58	-2.58	-2.58	-2.58	-2.58	-2.58	-2.58
-2.57	-2.57	-2.57	-2.57	-2.57	-2.57	-2.57	-2.57	-2.57
-2.56	-2.56	-2.56	-2.56	-2.56	-2.56	-2.56	-2.56	-2.56
-2.55	-2.55	-2.55	-2.55	-2.55	-2.55	-2.55	-2.55	-2.55
-2.54	-2.54	-2.54	-2.54	-2.54	-2.54	-2.54	-2.54	-2.54
-2.53	-2.53	-2.53	-2.53	-2.53	-2.53	-2.53	-2.53	-2.53
-2.52	-2.52	-2.52	-2.52	-2.52	-2.52	-2.52	-2.52	-2.52
-2.51	-2.51	-2.51	-2.51	-2.51	-2.51	-2.51	-2.51	-2.51

-2.5	-2.5	-2.5	-2.5	-2.5	-2.5	-2.5	-2.5	-2.5
-2.49	-2.49	-2.49	-2.49	-2.49	-2.49	-2.49	-2.49	-2.49
-2.48	-2.48	-2.48	-2.48	-2.48	-2.48	-2.48	-2.48	-2.48
-2.47	-2.47	-2.47	-2.47	-2.47	-2.47	-2.47	-2.47	-2.47
-2.46	-2.46	-2.46	-2.46	-2.46	-2.46	-2.46	-2.46	-2.46
-2.45	-2.45	-2.45	-2.45	-2.45	-2.45	-2.45	-2.45	-2.45
-2.44	-2.44	-2.44	-2.44	-2.44	-2.44	-2.44	-2.44	-2.44
-2.43	-2.43	-2.43	-2.43	-2.43	-2.43	-2.43	-2.43	-2.43
-2.42	-2.42	-2.42	-2.42	-2.42	-2.42	-2.42	-2.42	-2.42
-2.41	-2.41	-2.41	-2.41	-2.41	-2.41	-2.41	-2.41	-2.41
-2.4	-2.4	-2.4	-2.4	-2.4	-2.4	-2.4	-2.4	-2.4
-2.39	-2.39	-2.39	-2.39	-2.39	-2.39	-2.39	-2.39	-2.39
-2.38	-2.38	-2.38	-2.38	-2.38	-2.38	-2.38	-2.38	-2.38
-2.37	-2.37	-2.37	-2.37	-2.37	-2.37	-2.37	-2.37	-2.37
-2.36	-2.36	-2.36	-2.36	-2.36	-2.36	-2.36	-2.36	-2.36
-2.35	-2.35	-2.35	-2.35	-2.35	-2.35	-2.35	-2.35	-2.35
-2.34	-2.34	-2.34	-2.34	-2.34	-2.34	-2.34	-2.34	-2.34
-2.33	-2.33	-2.33	-2.33	-2.33	-2.33	-2.33	-2.33	-2.33
-2.32	-2.32	-2.32	-2.32	-2.32	-2.32	-2.32	-2.32	-2.32
-2.31	-2.31	-2.31	-2.31	-2.31	-2.31	-2.31	-2.31	-2.31
-2.3	-2.3	-2.3	-2.3	-2.3	-2.3	-2.3	-2.3	-2.3
-2.29	-2.29	-2.29	-2.29	-2.29	-2.29	-2.29	-2.29	-2.29
-2.28	-2.28	-2.28	-2.28	-2.28	-2.28	-2.28	-2.28	-2.28
-2.27	-2.27	-2.27	-2.27	-2.27	-2.27	-2.27	-2.27	-2.27
-2.26	-2.26	-2.26	-2.26	-2.26	-2.26	-2.26	-2.26	-2.26
-2.25	-2.25	-2.25	-2.25	-2.25	-2.25	-2.25	-2.25	-2.25
-2.24	-2.24	-2.24	-2.24	-2.24	-2.24	-2.24	-2.24	-2.24
-2.23	-2.23	-2.23	-2.23	-2.23	-2.23	-2.23	-2.23	-2.23
-2.22	-2.22	-2.22	-2.22	-2.22	-2.22	-2.22	-2.22	-2.22
-2.21	-2.21	-2.21	-2.21	-2.21	-2.21	-2.21	-2.21	-2.21
-2.2	-2.2	-2.2	-2.2	-2.2	-2.2	-2.2	-2.2	-2.2
-2.19	-2.19	-2.19	-2.19	-2.19	-2.19	-2.19	-2.19	-2.19
-2.18	-2.18	-2.18	-2.18	-2.18	-2.18	-2.18	-2.18	-2.18
-2.17	-2.17	-2.17	-2.17	-2.17	-2.17	-2.17	-2.17	-2.17
-2.16	-2.16	-2.16	-2.16	-2.16	-2.16	-2.16	-2.16	-2.16
-2.15	-2.15	-2.15	-2.15	-2.15	-2.15	-2.15	-2.15	-2.15
-2.14	-2.14	-2.14	-2.14	-2.14	-2.14	-2.14	-2.14	-2.14
-2.13	-2.13	-2.13	-2.13	-2.13	-2.13	-2.13	-2.13	-2.13
-2.12	-2.12	-2.12	-2.12	-2.12	-2.12	-2.12	-2.12	-2.12
-2.11	-2.11	-2.11	-2.11	-2.11	-2.11	-2.11	-2.11	-2.11
-2.1	-2.1	-2.1	-2.1	-2.1	-2.1	-2.1	-2.1	-2.1
-2.09	-2.09	-2.09	-2.09	-2.09	-2.09	-2.09	-2.09	-2.09
-2.08	-2.08	-2.08	-2.08	-2.08	-2.08	-2.08	-2.08	-2.08
-2.07	-2.07	-2.07	-2.07	-2.07	-2.07	-2.07	-2.07	-2.07
-2.06	-2.06	-2.06	-2.06	-2.06	-2.06	-2.06	-2.06	-2.06
-2.05	-2.05	-2.05	-2.05	-2.05	-2.05	-2.05	-2.05	-2.05
-2.04	-2.04	-2.04	-2.04	-2.04	-2.04	-2.04	-2.04	-2.04
-2.03	-2.03	-2.03	-2.03	-2.03	-2.03	-2.03	-2.03	-2.03
-2.02	-2.02	-2.02	-2.02	-2.02	-2.02	-2.02	-2.02	-2.02
-2.01	-2.01	-2.01	-2.01	-2.01	-2.01	-2.01	-2.01	-2.01

-2	-2	-2	-2	-2	-2	-2	-2	-2
-1.99	-1.99	-1.99	-1.99	-1.99	-1.99	-1.99	-1.99	-1.99
-1.98	-1.98	-1.98	-1.98	-1.98	-1.98	-1.98	-1.98	-1.98
-1.97	-1.97	-1.97	-1.97	-1.97	-1.97	-1.97	-1.97	-1.97
-1.96	-1.96	-1.96	-1.96	-1.96	-1.96	-1.96	-1.96	-1.96
-1.95	-1.95	-1.95	-1.95	-1.95	-1.95	-1.95	-1.95	-1.95
-1.94	-1.94	-1.94	-1.94	-1.94	-1.94	-1.94	-1.94	-1.94
-1.93	-1.93	-1.93	-1.93	-1.93	-1.93	-1.93	-1.93	-1.93
-1.92	-1.92	-1.92	-1.92	-1.92	-1.92	-1.92	-1.92	-1.92
-1.91	-1.91	-1.91	-1.91	-1.91	-1.91	-1.91	-1.91	-1.91
-1.9	-1.9	-1.9	-1.9	-1.9	-1.9	-1.9	-1.9	-1.9
-1.89	-1.89	-1.89	-1.89	-1.89	-1.89	-1.89	-1.89	-1.89
-1.88	-1.88	-1.88	-1.88	-1.88	-1.88	-1.88	-1.88	-1.88
-1.87	-1.87	-1.87	-1.87	-1.87	-1.87	-1.87	-1.87	-1.87
-1.86	-1.86	-1.86	-1.86	-1.86	-1.86	-1.86	-1.86	-1.86
-1.85	-1.85	-1.85	-1.85	-1.85	-1.85	-1.85	-1.85	-1.85
-1.84	-1.84	-1.84	-1.84	-1.84	-1.84	-1.84	-1.84	-1.84
-1.83	-1.83	-1.83	-1.83	-1.83	-1.83	-1.83	-1.83	-1.83
-1.82	-1.82	-1.82	-1.82	-1.82	-1.82	-1.82	-1.82	-1.82
-1.81	-1.81	-1.81	-1.81	-1.81	-1.81	-1.81	-1.81	-1.81
-1.8	-1.8	-1.8	-1.8	-1.8	-1.8	-1.8	-1.8	-1.8
-1.79	-1.79	-1.79	-1.79	-1.79	-1.79	-1.79	-1.79	-1.79
-1.78	-1.78	-1.78	-1.78	-1.78	-1.78	-1.78	-1.78	-1.78
-1.77	-1.77	-1.77	-1.77	-1.77	-1.77	-1.77	-1.77	-1.77
-1.76	-1.76	-1.76	-1.76	-1.76	-1.76	-1.76	-1.76	-1.76
-1.75	-1.75	-1.75	-1.75	-1.75	-1.75	-1.75	-1.75	-1.75
-1.74	-1.74	-1.74	-1.74	-1.74	-1.74	-1.74	-1.74	-1.74
-1.73	-1.73	-1.73	-1.73	-1.73	-1.73	-1.73	-1.73	-1.73
-1.72	-1.72	-1.72	-1.72	-1.72	-1.72	-1.72	-1.72	-1.72
-1.71	-1.71	-1.71	-1.71	-1.71	-1.71	-1.71	-1.71	-1.71
-1.7	-1.7	-1.7	-1.7	-1.7	-1.7	-1.7	-1.7	-1.7
-1.69	-1.69	-1.69	-1.69	-1.69	-1.69	-1.69	-1.69	-1.69
-1.68	-1.68	-1.68	-1.68	-1.68	-1.68	-1.68	-1.68	-1.68
-1.67	-1.67	-1.67	-1.67	-1.67	-1.67	-1.67	-1.67	-1.67
-1.66	-1.66	-1.66	-1.66	-1.66	-1.66	-1.66	-1.66	-1.66
-1.65	-1.65	-1.65	-1.65	-1.65	-1.65	-1.65	-1.65	-1.65
-1.64	-1.64	-1.64	-1.64	-1.64	-1.64	-1.64	-1.64	-1.64
-1.63	-1.63	-1.63	-1.63	-1.63	-1.63	-1.63	-1.63	-1.63
-1.62	-1.62	-1.62	-1.62	-1.62	-1.62	-1.62	-1.62	-1.62
-1.61	-1.61	-1.61	-1.61	-1.61	-1.61	-1.61	-1.61	-1.61
-1.6	-1.6	-1.6	-1.6	-1.6	-1.6	-1.6	-1.6	-1.6
-1.59	-1.59	-1.59	-1.59	-1.59	-1.59	-1.59	-1.59	-1.59
-1.58	-1.58	-1.58	-1.58	-1.58	-1.58	-1.58	-1.58	-1.58
-1.57	-1.57	-1.57	-1.57	-1.57	-1.57	-1.57	-1.57	-1.57
-1.56	-1.56	-1.56	-1.56	-1.56	-1.56	-1.56	-1.56	-1.56
-1.55	-1.55	-1.55	-1.55	-1.55	-1.55	-1.55	-1.55	-1.55
-1.54	-1.54	-1.54	-1.54	-1.54	-1.54	-1.54	-1.54	-1.54
-1.53	-1.53	-1.53	-1.53	-1.53	-1.53	-1.53	-1.53	-1.53
-1.52	-1.52	-1.52	-1.52	-1.52	-1.52	-1.52	-1.52	-1.52
-1.51	-1.51	-1.51	-1.51	-1.51	-1.51	-1.51	-1.51	-1.51

-1.5	-1.5	-1.5	-1.5	-1.5	-1.5	-1.5	-1.5	-1.5
-1.49	-1.49	-1.49	-1.49	-1.49	-1.49	-1.49	-1.49	-1.49
-1.48	-1.48	-1.48	-1.48	-1.48	-1.48	-1.48	-1.48	-1.48
-1.47	-1.47	-1.47	-1.47	-1.47	-1.47	-1.47	-1.47	-1.47
-1.46	-1.46	-1.46	-1.46	-1.46	-1.46	-1.46	-1.46	-1.46
-1.45	-1.45	-1.45	-1.45	-1.45	-1.45	-1.45	-1.45	-1.45
-1.44	-1.44	-1.44	-1.44	-1.44	-1.44	-1.44	-1.44	-1.44
-1.43	-1.43	-1.43	-1.43	-1.43	-1.43	-1.43	-1.43	-1.43
-1.42	-1.42	-1.42	-1.42	-1.42	-1.42	-1.42	-1.42	-1.42
-1.41	-1.41	-1.41	-1.41	-1.41	-1.41	-1.41	-1.41	-1.41
-1.4	-1.4	-1.4	-1.4	-1.4	-1.4	-1.4	-1.4	-1.4
-1.39	-1.39	-1.39	-1.39	-1.39	-1.39	-1.39	-1.39	-1.39
-1.38	-1.38	-1.38	-1.38	-1.38	-1.38	-1.38	-1.38	-1.38
-1.37	-1.37	-1.37	-1.37	-1.37	-1.37	-1.37	-1.37	-1.37
-1.36	-1.36	-1.36	-1.36	-1.36	-1.36	-1.36	-1.36	-1.36
-1.35	-1.35	-1.35	-1.35	-1.35	-1.35	-1.35	-1.35	-1.35
-1.34	-1.34	-1.34	-1.34	-1.34	-1.34	-1.34	-1.34	-1.34
-1.33	-1.33	-1.33	-1.33	-1.33	-1.33	-1.33	-1.33	-1.33
-1.32	-1.32	-1.32	-1.32	-1.32	-1.32	-1.32	-1.32	-1.32
-1.31	-1.31	-1.31	-1.31	-1.31	-1.31	-1.31	-1.31	-1.31
-1.3	-1.3	-1.3	-1.3	-1.3	-1.3	-1.3	-1.3	-1.3
-1.29	-1.29	-1.29	-1.29	-1.29	-1.29	-1.29	-1.29	-1.29
-1.28	-1.28	-1.28	-1.28	-1.28	-1.28	-1.28	-1.28	-1.28
-1.27	-1.27	-1.27	-1.27	-1.27	-1.27	-1.27	-1.27	-1.27
-1.26	-1.26	-1.26	-1.26	-1.26	-1.26	-1.26	-1.26	-1.26
-1.25	-1.25	-1.25	-1.25	-1.25	-1.25	-1.25	-1.25	-1.25
-1.24	-1.24	-1.24	-1.24	-1.24	-1.24	-1.24	-1.24	-1.24
-1.23	-1.23	-1.23	-1.23	-1.23	-1.23	-1.23	-1.23	-1.23
-1.22	-1.22	-1.22	-1.22	-1.22	-1.22	-1.22	-1.22	-1.22
-1.21	-1.21	-1.21	-1.21	-1.21	-1.21	-1.21	-1.21	-1.21
-1.2	-1.2	-1.2	-1.2	-1.2	-1.2	-1.2	-1.2	-1.2
-1.19	-1.19	-1.19	-1.19	-1.19	-1.19	-1.19	-1.19	-1.19
-1.18	-1.18	-1.18	-1.18	-1.18	-1.18	-1.18	-1.18	-1.18
-1.17	-1.17	-1.17	-1.17	-1.17	-1.17	-1.17	-1.17	-1.17
-1.16	-1.16	-1.16	-1.16	-1.16	-1.16	-1.16	-1.16	-1.16
-1.15	-1.15	-1.15	-1.15	-1.15	-1.15	-1.15	-1.15	-1.15
-1.14	-1.14	-1.14	-1.14	-1.14	-1.14	-1.14	-1.14	-1.14
-1.13	-1.13	-1.13	-1.13	-1.13	-1.13	-1.13	-1.13	-1.13
-1.12	-1.12	-1.12	-1.12	-1.12	-1.12	-1.12	-1.12	-1.12
-1.11	-1.11	-1.11	-1.11	-1.11	-1.11	-1.11	-1.11	-1.11
-1.1	-1.1	-1.1	-1.1	-1.1	-1.1	-1.1	-1.1	-1.1
-1.09	-1.09	-1.09	-1.09	-1.09	-1.09	-1.09	-1.09	-1.09
-1.08	-1.08	-1.08	-1.08	-1.08	-1.08	-1.08	-1.08	-1.08
-1.07	-1.07	-1.07	-1.07	-1.07	-1.07	-1.07	-1.07	-1.07
-1.06	-1.06	-1.06	-1.06	-1.06	-1.06	-1.06	-1.06	-1.06
-1.05	-1.05	-1.05	-1.05	-1.05	-1.05	-1.05	-1.05	-1.05
-1.04	-1.04	-1.04	-1.04	-1.04	-1.04	-1.04	-1.04	-1.04
-1.03	-1.03	-1.03	-1.03	-1.03	-1.03	-1.03	-1.03	-1.03
-1.02	-1.02	-1.02	-1.02	-1.02	-1.02	-1.02	-1.02	-1.02
-1.01	-1.01	-1.01	-1.01	-1.01	-1.01	-1.01	-1.01	-1.01

-1	-1	-1	-1	-1	-1	-1	-1	-1
-0.99	-0.99	-0.99	-0.99	-0.99	-0.99	-0.99	-0.99	-0.99
-0.98	-0.98	-0.98	-0.98	-0.98	-0.98	-0.98	-0.98	-0.98
-0.97	-0.97	-0.97	-0.97	-0.97	-0.97	-0.97	-0.97	-0.97
-0.96	-0.96	-0.96	-0.96	-0.96	-0.96	-0.96	-0.96	-0.96
-0.95	-0.95	-0.95	-0.95	-0.95	-0.95	-0.95	-0.95	-0.95
-0.94	-0.94	-0.94	-0.94	-0.94	-0.94	-0.94	-0.94	-0.94
-0.93	-0.93	-0.93	-0.93	-0.93	-0.93	-0.93	-0.93	-0.93
-0.92	-0.92	-0.92	-0.92	-0.92	-0.92	-0.92	-0.92	-0.92
-0.91	-0.91	-0.91	-0.91	-0.91	-0.91	-0.91	-0.91	-0.91
-0.9	-0.9	-0.9	-0.9	-0.9	-0.9	-0.9	-0.9	-0.9
-0.89	-0.89	-0.89	-0.89	-0.89	-0.89	-0.89	-0.89	-0.89
-0.88	-0.88	-0.88	-0.88	-0.88	-0.88	-0.88	-0.88	-0.88
-0.87	-0.87	-0.87	-0.87	-0.87	-0.87	-0.87	-0.87	-0.87
-0.86	-0.86	-0.86	-0.86	-0.86	-0.86	-0.86	-0.86	-0.86
-0.85	-0.85	-0.85	-0.85	-0.85	-0.85	-0.85	-0.85	-0.85
-0.84	-0.84	-0.84	-0.84	-0.84	-0.84	-0.84	-0.84	-0.84
-0.83	-0.83	-0.83	-0.83	-0.83	-0.83	-0.83	-0.83	-0.83
-0.82	-0.82	-0.82	-0.82	-0.82	-0.82	-0.82	-0.82	-0.82
-0.81	-0.81	-0.81	-0.81	-0.81	-0.81	-0.81	-0.81	-0.81
-0.8	-0.8	-0.8	-0.8	-0.8	-0.8	-0.8	-0.8	-0.8
-0.79	-0.79	-0.79	-0.79	-0.79	-0.79	-0.79	-0.79	-0.79
-0.78	-0.78	-0.78	-0.78	-0.78	-0.78	-0.78	-0.78	-0.78
-0.77	-0.77	-0.77	-0.77	-0.77	-0.77	-0.77	-0.77	-0.77
-0.76	-0.76	-0.76	-0.76	-0.76	-0.76	-0.76	-0.76	-0.76
-0.75	-0.75	-0.75	-0.75	-0.75	-0.75	-0.75	-0.75	-0.75
-0.74	-0.74	-0.74	-0.74	-0.74	-0.74	-0.74	-0.74	-0.74
-0.73	-0.73	-0.73	-0.73	-0.73	-0.73	-0.73	-0.73	-0.73
-0.72	-0.72	-0.72	-0.72	-0.72	-0.72	-0.72	-0.72	-0.72
-0.71	-0.71	-0.71	-0.71	-0.71	-0.71	-0.71	-0.71	-0.71
-0.7	-0.7	-0.7	-0.7	-0.7	-0.7	-0.7	-0.7	-0.7
-0.69	-0.69	-0.69	-0.69	-0.69	-0.69	-0.69	-0.69	-0.69
-0.68	-0.68	-0.68	-0.68	-0.68	-0.68	-0.68	-0.68	-0.68
-0.67	-0.67	-0.67	-0.67	-0.67	-0.67	-0.67	-0.67	-0.67
-0.66	-0.66	-0.66	-0.66	-0.66	-0.66	-0.66	-0.66	-0.66
-0.65	-0.65	-0.65	-0.65	-0.65	-0.65	-0.65	-0.65	-0.65
-0.64	-0.64	-0.64	-0.64	-0.64	-0.64	-0.64	-0.64	-0.64
-0.63	-0.63	-0.63	-0.63	-0.63	-0.63	-0.63	-0.63	-0.63
-0.62	-0.62	-0.62	-0.62	-0.62	-0.62	-0.62	-0.62	-0.62
-0.61	-0.61	-0.61	-0.61	-0.61	-0.61	-0.61	-0.61	-0.61
-0.6	-0.6	-0.6	-0.6	-0.6	-0.6	-0.6	-0.6	-0.6
-0.59	-0.59	-0.59	-0.59	-0.59	-0.59	-0.59	-0.59	-0.59
-0.58	-0.58	-0.58	-0.58	-0.58	-0.58	-0.58	-0.58	-0.58
-0.57	-0.57	-0.57	-0.57	-0.57	-0.57	-0.57	-0.57	-0.57
-0.56	-0.56	-0.56	-0.56	-0.56	-0.56	-0.56	-0.56	-0.56
-0.55	-0.55	-0.55	-0.55	-0.55	-0.55	-0.55	-0.55	-0.55
-0.54	-0.54	-0.54	-0.54	-0.54	-0.54	-0.54	-0.54	-0.54
-0.53	-0.53	-0.53	-0.53	-0.53	-0.53	-0.53	-0.53	-0.53
-0.52	-0.52	-0.52	-0.52	-0.52	-0.52	-0.52	-0.52	-0.52
-0.51	-0.51	-0.51	-0.51	-0.51	-0.51	-0.51	-0.51	-0.51

-0.5	-0.5	-0.5	-0.5	-0.5	-0.5	-0.5	-0.5	-0.5
-0.49	-0.49	-0.49	-0.49	-0.49	-0.49	-0.49	-0.49	-0.49
-0.48	-0.48	-0.48	-0.48	-0.48	-0.48	-0.48	-0.48	-0.48
-0.47	-0.47	-0.47	-0.47	-0.47	-0.47	-0.47	-0.47	-0.47
-0.46	-0.46	-0.46	-0.46	-0.46	-0.46	-0.46	-0.46	-0.46
-0.45	-0.45	-0.45	-0.45	-0.45	-0.45	-0.45	-0.45	-0.45
-0.44	-0.44	-0.44	-0.44	-0.44	-0.44	-0.44	-0.44	-0.44
-0.43	-0.43	-0.43	-0.43	-0.43	-0.43	-0.43	-0.43	-0.43
-0.42	-0.42	-0.42	-0.42	-0.42	-0.42	-0.42	-0.42	-0.42
-0.41	-0.41	-0.41	-0.41	-0.41	-0.41	-0.41	-0.41	-0.41
-0.4	-0.4	-0.4	-0.4	-0.4	-0.4	-0.4	-0.4	-0.4
-0.39	-0.39	-0.39	-0.39	-0.39	-0.39	-0.39	-0.39	-0.39
-0.38	-0.38	-0.38	-0.38	-0.38	-0.38	-0.38	-0.38	-0.38
-0.37	-0.37	-0.37	-0.37	-0.37	-0.37	-0.37	-0.37	-0.37
-0.36	-0.36	-0.36	-0.36	-0.36	-0.36	-0.36	-0.36	-0.36
-0.35	-0.35	-0.35	-0.35	-0.35	-0.35	-0.35	-0.35	-0.35
-0.34	-0.34	-0.34	-0.34	-0.34	-0.34	-0.34	-0.34	-0.34
-0.33	-0.33	-0.33	-0.33	-0.33	-0.33	-0.33	-0.33	-0.33
-0.32	-0.32	-0.32	-0.32	-0.32	-0.32	-0.32	-0.32	-0.32
-0.31	-0.31	-0.31	-0.31	-0.31	-0.31	-0.31	-0.31	-0.31
-0.3	-0.3	-0.3	-0.3	-0.3	-0.3	-0.3	-0.3	-0.3
-0.29	-0.29	-0.29	-0.29	-0.29	-0.29	-0.29	-0.29	-0.29
-0.28	-0.28	-0.28	-0.28	-0.28	-0.28	-0.28	-0.28	-0.28
-0.27	-0.27	-0.27	-0.27	-0.27	-0.27	-0.27	-0.27	-0.27
-0.26	-0.26	-0.26	-0.26	-0.26	-0.26	-0.26	-0.26	-0.26
-0.25	-0.25	-0.25	-0.25	-0.25	-0.25	-0.25	-0.25	-0.25
-0.24	-0.24	-0.24	-0.24	-0.24	-0.24	-0.24	-0.24	-0.24
-0.23	-0.23	-0.23	-0.23	-0.23	-0.23	-0.23	-0.23	-0.23
-0.22	-0.22	-0.22	-0.22	-0.22	-0.22	-0.22	-0.22	-0.22
-0.21	-0.21	-0.21	-0.21	-0.21	-0.21	-0.21	-0.21	-0.21
-0.2	-0.2	-0.2	-0.2	-0.2	-0.2	-0.2	-0.2	-0.2
-0.19	-0.19	-0.19	-0.19	-0.19	-0.19	-0.19	-0.19	-0.19
-0.18	-0.18	-0.18	-0.18	-0.18	-0.18	-0.18	-0.18	-0.18
-0.17	-0.17	-0.17	-0.17	-0.17	-0.17	-0.17	-0.17	-0.17
-0.16	-0.16	-0.16	-0.16	-0.16	-0.16	-0.16	-0.16	-0.16
-0.15	-0.15	-0.15	-0.15	-0.15	-0.15	-0.15	-0.15	-0.15
-0.14	-0.14	-0.14	-0.14	-0.14	-0.14	-0.14	-0.14	-0.14
-0.13	-0.13	-0.13	-0.13	-0.13	-0.13	-0.13	-0.13	-0.13
-0.12	-0.12	-0.12	-0.12	-0.12	-0.12	-0.12	-0.12	-0.12
-0.11	-0.11	-0.11	-0.11	-0.11	-0.11	-0.11	-0.11	-0.11
-0.1	-0.1	-0.1	-0.1	-0.1	-0.1	-0.1	-0.1	-0.1
-0.09	-0.09	-0.09	-0.09	-0.09	-0.09	-0.09	-0.09	-0.09
-0.08	-0.08	-0.08	-0.08	-0.08	-0.08	-0.08	-0.08	-0.08
-0.07	-0.07	-0.07	-0.07	-0.07	-0.07	-0.07	-0.07	-0.07
-0.06	-0.06	-0.06	-0.06	-0.06	-0.06	-0.06	-0.06	-0.06
-0.05	-0.05	-0.05	-0.05	-0.05	-0.05	-0.05	-0.05	-0.05
-0.04	-0.04	-0.04	-0.04	-0.04	-0.04	-0.04	-0.04	-0.04
-0.03	-0.03	-0.03	-0.03	-0.03	-0.03	-0.03	-0.03	-0.03
-0.02	-0.02	-0.02	-0.02	-0.02	-0.02	-0.02	-0.02	-0.02
-0.01	-0.01	-0.01	-0.01	-0.01	-0.01	-0.01	-0.01	-0.01

0	0	0	0	0	0	0	0	0
0.01	0.01	0.01	0.01	0.01	0.01	0.01	0.01	0.01
0.02	0.02	0.02	0.02	0.02	0.02	0.02	0.02	0.02
0.03	0.03	0.03	0.03	0.03	0.03	0.03	0.03	0.03
0.04	0.04	0.04	0.04	0.04	0.04	0.04	0.04	0.04
0.05	0.05	0.05	0.05	0.05	0.05	0.05	0.05	0.05
0.06	0.06	0.06	0.06	0.06	0.06	0.06	0.06	0.06
0.07	0.07	0.07	0.07	0.07	0.07	0.07	0.07	0.07
0.08	0.08	0.08	0.08	0.08	0.08	0.08	0.08	0.08
0.09	0.09	0.09	0.09	0.09	0.09	0.09	0.09	0.09
0.1	0.1	0.1	0.1	0.1	0.1	0.1	0.1	0.1
0.11	0.11	0.11	0.11	0.11	0.11	0.11	0.11	0.11
0.12	0.12	0.12	0.12	0.12	0.12	0.12	0.12	0.12
0.13	0.13	0.13	0.13	0.13	0.13	0.13	0.13	0.13
0.14	0.14	0.14	0.14	0.14	0.14	0.14	0.14	0.14
0.15	0.15	0.15	0.15	0.15	0.15	0.15	0.15	0.15
0.16	0.16	0.16	0.16	0.16	0.16	0.16	0.16	0.16
0.17	0.17	0.17	0.17	0.17	0.17	0.17	0.17	0.17
0.18	0.18	0.18	0.18	0.18	0.18	0.18	0.18	0.18
0.19	0.19	0.19	0.19	0.19	0.19	0.19	0.19	0.19
0.2	0.2	0.2	0.2	0.2	0.2	0.2	0.2	0.2
0.21	0.21	0.21	0.21	0.21	0.21	0.21	0.21	0.21
0.22	0.22	0.22	0.22	0.22	0.22	0.22	0.22	0.22
0.23	0.23	0.23	0.23	0.23	0.23	0.23	0.23	0.23
0.24	0.24	0.24	0.24	0.24	0.24	0.24	0.24	0.24
0.25	0.25	0.25	0.25	0.25	0.25	0.25	0.25	0.25
0.26	0.26	0.26	0.26	0.26	0.26	0.26	0.26	0.26
0.27	0.27	0.27	0.27	0.27	0.27	0.27	0.27	0.27
0.28	0.28	0.28	0.28	0.28	0.28	0.28	0.28	0.28
0.29	0.29	0.29	0.29	0.29	0.29	0.29	0.29	0.29
0.3	0.3	0.3	0.3	0.3	0.3	0.3	0.3	0.3
0.31	0.31	0.31	0.31	0.31	0.31	0.31	0.31	0.31
0.32	0.32	0.32	0.32	0.32	0.32	0.32	0.32	0.32
0.33	0.33	0.33	0.33	0.33	0.33	0.33	0.33	0.33
0.34	0.34	0.34	0.34	0.34	0.34	0.34	0.34	0.34
0.35	0.35	0.35	0.35	0.35	0.35	0.35	0.35	0.35
0.36	0.36	0.36	0.36	0.36	0.36	0.36	0.36	0.36
0.37	0.37	0.37	0.37	0.37	0.37	0.37	0.37	0.37
0.38	0.38	0.38	0.38	0.38	0.38	0.38	0.38	0.38
0.39	0.39	0.39	0.39	0.39	0.39	0.39	0.39	0.39
0.4	0.4	0.4	0.4	0.4	0.4	0.4	0.4	0.4
0.41	0.41	0.41	0.41	0.41	0.41	0.41	0.41	0.41
0.42	0.42	0.42	0.42	0.42	0.42	0.42	0.42	0.42
0.43	0.43	0.43	0.43	0.43	0.43	0.43	0.43	0.43
0.44	0.44	0.44	0.44	0.44	0.44	0.44	0.44	0.44
0.45	0.45	0.45	0.45	0.45	0.45	0.45	0.45	0.45
0.46	0.46	0.46	0.46	0.46	0.46	0.46	0.46	0.46
0.47	0.47	0.47	0.47	0.47	0.47	0.47	0.47	0.47
0.48	0.48	0.48	0.48	0.48	0.48	0.48	0.48	0.48
0.49	0.49	0.49	0.49	0.49	0.49	0.49	0.49	0.49

0.5	0.5	0.5	0.5	0.5	0.5	0.5	0.5	0.5
0.51	0.51	0.51	0.51	0.51	0.51	0.51	0.51	0.51
0.52	0.52	0.52	0.52	0.52	0.52	0.52	0.52	0.52
0.53	0.53	0.53	0.53	0.53	0.53	0.53	0.53	0.53
0.54	0.54	0.54	0.54	0.54	0.54	0.54	0.54	0.54
0.55	0.55	0.55	0.55	0.55	0.55	0.55	0.55	0.55
0.56	0.56	0.56	0.56	0.56	0.56	0.56	0.56	0.56
0.57	0.57	0.57	0.57	0.57	0.57	0.57	0.57	0.57
0.58	0.58	0.58	0.58	0.58	0.58	0.58	0.58	0.58
0.59	0.59	0.59	0.59	0.59	0.59	0.59	0.59	0.59
0.6	0.6	0.6	0.6	0.6	0.6	0.6	0.6	0.6
0.61	0.61	0.61	0.61	0.61	0.61	0.61	0.61	0.61
0.62	0.62	0.62	0.62	0.62	0.62	0.62	0.62	0.62
0.63	0.63	0.63	0.63	0.63	0.63	0.63	0.63	0.63
0.64	0.64	0.64	0.64	0.64	0.64	0.64	0.64	0.64
0.65	0.65	0.65	0.65	0.65	0.65	0.65	0.65	0.65
0.66	0.66	0.66	0.66	0.66	0.66	0.66	0.66	0.66
0.67	0.67	0.67	0.67	0.67	0.67	0.67	0.67	0.67
0.68	0.68	0.68	0.68	0.68	0.68	0.68	0.68	0.68
0.69	0.69	0.69	0.69	0.69	0.69	0.69	0.69	0.69
0.7	0.7	0.7	0.7	0.7	0.7	0.7	0.7	0.7
0.71	0.71	0.71	0.71	0.71	0.71	0.71	0.71	0.71
0.72	0.72	0.72	0.72	0.72	0.72	0.72	0.72	0.72
0.73	0.73	0.73	0.73	0.73	0.73	0.73	0.73	0.73
0.74	0.74	0.74	0.74	0.74	0.74	0.74	0.74	0.74
0.75	0.75	0.75	0.75	0.75	0.75	0.75	0.75	0.75
0.76	0.76	0.76	0.76	0.76	0.76	0.76	0.76	0.76
0.77	0.77	0.77	0.77	0.77	0.77	0.77	0.77	0.77
0.78	0.78	0.78	0.78	0.78	0.78	0.78	0.78	0.78
0.79	0.79	0.79	0.79	0.79	0.79	0.79	0.79	0.79
0.8	0.8	0.8	0.8	0.8	0.8	0.8	0.8	0.8
0.81	0.81	0.81	0.81	0.81	0.81	0.81	0.81	0.81
0.82	0.82	0.82	0.82	0.82	0.82	0.82	0.82	0.82
0.83	0.83	0.83	0.83	0.83	0.83	0.83	0.83	0.83
0.84	0.84	0.84	0.84	0.84	0.84	0.84	0.84	0.84
0.85	0.85	0.85	0.85	0.85	0.85	0.85	0.85	0.85
0.86	0.86	0.86	0.86	0.86	0.86	0.86	0.86	0.86
0.87	0.87	0.87	0.87	0.87	0.87	0.87	0.87	0.87
0.88	0.88	0.88	0.88	0.88	0.88	0.88	0.88	0.88
0.89	0.89	0.89	0.89	0.89	0.89	0.89	0.89	0.89
0.9	0.9	0.9	0.9	0.9	0.9	0.9	0.9	0.9
0.91	0.91	0.91	0.91	0.91	0.91	0.91	0.91	0.91
0.92	0.92	0.92	0.92	0.92	0.92	0.92	0.92	0.92
0.93	0.93	0.93	0.93	0.93	0.93	0.93	0.93	0.93
0.94	0.94	0.94	0.94	0.94	0.94	0.94	0.94	0.94
0.95	0.95	0.95	0.95	0.95	0.95	0.95	0.95	0.95
0.96	0.96	0.96	0.96	0.96	0.96	0.96	0.96	0.96
0.97	0.97	0.97	0.97	0.97	0.97	0.97	0.97	0.97
0.98	0.98	0.98	0.98	0.98	0.98	0.98	0.98	0.98
0.99	0.99	0.99	0.99	0.99	0.99	0.99	0.99	0.99

1	1	1	1	1	1	1	1	1
1.01	1.01	1.01	1.01	1.01	1.01	1.01	1.01	1.01
1.02	1.02	1.02	1.02	1.02	1.02	1.02	1.02	1.02
1.03	1.03	1.03	1.03	1.03	1.03	1.03	1.03	1.03
1.04	1.04	1.04	1.04	1.04	1.04	1.04	1.04	1.04
1.05	1.05	1.05	1.05	1.05	1.05	1.05	1.05	1.05
1.06	1.06	1.06	1.06	1.06	1.06	1.06	1.06	1.06
1.07	1.07	1.07	1.07	1.07	1.07	1.07	1.07	1.07
1.08	1.08	1.08	1.08	1.08	1.08	1.08	1.08	1.08
1.09	1.09	1.09	1.09	1.09	1.09	1.09	1.09	1.09
1.1	1.1	1.1	1.1	1.1	1.1	1.1	1.1	1.1
1.11	1.11	1.11	1.11	1.11	1.11	1.11	1.11	1.11
1.12	1.12	1.12	1.12	1.12	1.12	1.12	1.12	1.12
1.13	1.13	1.13	1.13	1.13	1.13	1.13	1.13	1.13
1.14	1.14	1.14	1.14	1.14	1.14	1.14	1.14	1.14
1.15	1.15	1.15	1.15	1.15	1.15	1.15	1.15	1.15
1.16	1.16	1.16	1.16	1.16	1.16	1.16	1.16	1.16
1.17	1.17	1.17	1.17	1.17	1.17	1.17	1.17	1.17
1.18	1.18	1.18	1.18	1.18	1.18	1.18	1.18	1.18
1.19	1.19	1.19	1.19	1.19	1.19	1.19	1.19	1.19
1.2	1.2	1.2	1.2	1.2	1.2	1.2	1.2	1.2
1.21	1.21	1.21	1.21	1.21	1.21	1.21	1.21	1.21
1.22	1.22	1.22	1.22	1.22	1.22	1.22	1.22	1.22
1.23	1.23	1.23	1.23	1.23	1.23	1.23	1.23	1.23
1.24	1.24	1.24	1.24	1.24	1.24	1.24	1.24	1.24
1.25	1.25	1.25	1.25	1.25	1.25	1.25	1.25	1.25
1.26	1.26	1.26	1.26	1.26	1.26	1.26	1.26	1.26
1.27	1.27	1.27	1.27	1.27	1.27	1.27	1.27	1.27
1.28	1.28	1.28	1.28	1.28	1.28	1.28	1.28	1.28
1.29	1.29	1.29	1.29	1.29	1.29	1.29	1.29	1.29
1.3	1.3	1.3	1.3	1.3	1.3	1.3	1.3	1.3
1.31	1.31	1.31	1.31	1.31	1.31	1.31	1.31	1.31
1.32	1.32	1.32	1.32	1.32	1.32	1.32	1.32	1.32
1.33	1.33	1.33	1.33	1.33	1.33	1.33	1.33	1.33
1.34	1.34	1.34	1.34	1.34	1.34	1.34	1.34	1.34
1.35	1.35	1.35	1.35	1.35	1.35	1.35	1.35	1.35
1.36	1.36	1.36	1.36	1.36	1.36	1.36	1.36	1.36
1.37	1.37	1.37	1.37	1.37	1.37	1.37	1.37	1.37
1.38	1.38	1.38	1.38	1.38	1.38	1.38	1.38	1.38
1.39	1.39	1.39	1.39	1.39	1.39	1.39	1.39	1.39
1.4	1.4	1.4	1.4	1.4	1.4	1.4	1.4	1.4
1.41	1.41	1.41	1.41	1.41	1.41	1.41	1.41	1.41
1.42	1.42	1.42	1.42	1.42	1.42	1.42	1.42	1.42
1.43	1.43	1.43	1.43	1.43	1.43	1.43	1.43	1.43
1.44	1.44	1.44	1.44	1.44	1.44	1.44	1.44	1.44
1.45	1.45	1.45	1.45	1.45	1.45	1.45	1.45	1.45
1.46	1.46	1.46	1.46	1.46	1.46	1.46	1.46	1.46
1.47	1.47	1.47	1.47	1.47	1.47	1.47	1.47	1.47
1.48	1.48	1.48	1.48	1.48	1.48	1.48	1.48	1.48
1.49	1.49	1.49	1.49	1.49	1.49	1.49	1.49	1.49

1.5	1.5	1.5	1.5	1.5	1.5	1.5	1.5	1.5
1.51	1.51	1.51	1.51	1.51	1.51	1.51	1.51	1.51
1.52	1.52	1.52	1.52	1.52	1.52	1.52	1.52	1.52
1.53	1.53	1.53	1.53	1.53	1.53	1.53	1.53	1.53
1.54	1.54	1.54	1.54	1.54	1.54	1.54	1.54	1.54
1.55	1.55	1.55	1.55	1.55	1.55	1.55	1.55	1.55
1.56	1.56	1.56	1.56	1.56	1.56	1.56	1.56	1.56
1.57	1.57	1.57	1.57	1.57	1.57	1.57	1.57	1.57
1.58	1.58	1.58	1.58	1.58	1.58	1.58	1.58	1.58
1.59	1.59	1.59	1.59	1.59	1.59	1.59	1.59	1.59
1.6	1.6	1.6	1.6	1.6	1.6	1.6	1.6	1.6
1.61	1.61	1.61	1.61	1.61	1.61	1.61	1.61	1.61
1.62	1.62	1.62	1.62	1.62	1.62	1.62	1.62	1.62
1.63	1.63	1.63	1.63	1.63	1.63	1.63	1.63	1.63
1.64	1.64	1.64	1.64	1.64	1.64	1.64	1.64	1.64
1.65	1.65	1.65	1.65	1.65	1.65	1.65	1.65	1.65
1.66	1.66	1.66	1.66	1.66	1.66	1.66	1.66	1.66
1.67	1.67	1.67	1.67	1.67	1.67	1.67	1.67	1.67
1.68	1.68	1.68	1.68	1.68	1.68	1.68	1.68	1.68
1.69	1.69	1.69	1.69	1.69	1.69	1.69	1.69	1.69
1.7	1.7	1.7	1.7	1.7	1.7	1.7	1.7	1.7
1.71	1.71	1.71	1.71	1.71	1.71	1.71	1.71	1.71
1.72	1.72	1.72	1.72	1.72	1.72	1.72	1.72	1.72
1.73	1.73	1.73	1.73	1.73	1.73	1.73	1.73	1.73
1.74	1.74	1.74	1.74	1.74	1.74	1.74	1.74	1.74
1.75	1.75	1.75	1.75	1.75	1.75	1.75	1.75	1.75
1.76	1.76	1.76	1.76	1.76	1.76	1.76	1.76	1.76
1.77	1.77	1.77	1.77	1.77	1.77	1.77	1.77	1.77
1.78	1.78	1.78	1.78	1.78	1.78	1.78	1.78	1.78
1.79	1.79	1.79	1.79	1.79	1.79	1.79	1.79	1.79
1.8	1.8	1.8	1.8	1.8	1.8	1.8	1.8	1.8
1.81	1.81	1.81	1.81	1.81	1.81	1.81	1.81	1.81
1.82	1.82	1.82	1.82	1.82	1.82	1.82	1.82	1.82
1.83	1.83	1.83	1.83	1.83	1.83	1.83	1.83	1.83
1.84	1.84	1.84	1.84	1.84	1.84	1.84	1.84	1.84
1.85	1.85	1.85	1.85	1.85	1.85	1.85	1.85	1.85
1.86	1.86	1.86	1.86	1.86	1.86	1.86	1.86	1.86
1.87	1.87	1.87	1.87	1.87	1.87	1.87	1.87	1.87
1.88	1.88	1.88	1.88	1.88	1.88	1.88	1.88	1.88
1.89	1.89	1.89	1.89	1.89	1.89	1.89	1.89	1.89
1.9	1.9	1.9	1.9	1.9	1.9	1.9	1.9	1.9
1.91	1.91	1.91	1.91	1.91	1.91	1.91	1.91	1.91
1.92	1.92	1.92	1.92	1.92	1.92	1.92	1.92	1.92
1.93	1.93	1.93	1.93	1.93	1.93	1.93	1.93	1.93
1.94	1.94	1.94	1.94	1.94	1.94	1.94	1.94	1.94
1.95	1.95	1.95	1.95	1.95	1.95	1.95	1.95	1.95
1.96	1.96	1.96	1.96	1.96	1.96	1.96	1.96	1.96
1.97	1.97	1.97	1.97	1.97	1.97	1.97	1.97	1.97
1.98	1.98	1.98	1.98	1.98	1.98	1.98	1.98	1.98
1.99	1.99	1.99	1.99	1.99	1.99	1.99	1.99	1.99

2	2	2	2	2	2	2	2	2
2.01	2.01	2.01	2.01	2.01	2.01	2.01	2.01	2.01
2.02	2.02	2.02	2.02	2.02	2.02	2.02	2.02	2.02
2.03	2.03	2.03	2.03	2.03	2.03	2.03	2.03	2.03
2.04	2.04	2.04	2.04	2.04	2.04	2.04	2.04	2.04
2.05	2.05	2.05	2.05	2.05	2.05	2.05	2.05	2.05
2.06	2.06	2.06	2.06	2.06	2.06	2.06	2.06	2.06
2.07	2.07	2.07	2.07	2.07	2.07	2.07	2.07	2.07
2.08	2.08	2.08	2.08	2.08	2.08	2.08	2.08	2.08
2.09	2.09	2.09	2.09	2.09	2.09	2.09	2.09	2.09
2.1	2.1	2.1	2.1	2.1	2.1	2.1	2.1	2.1
2.11	2.11	2.11	2.11	2.11	2.11	2.11	2.11	2.11
2.12	2.12	2.12	2.12	2.12	2.12	2.12	2.12	2.12
2.13	2.13	2.13	2.13	2.13	2.13	2.13	2.13	2.13
2.14	2.14	2.14	2.14	2.14	2.14	2.14	2.14	2.14
2.15	2.15	2.15	2.15	2.15	2.15	2.15	2.15	2.15
2.16	2.16	2.16	2.16	2.16	2.16	2.16	2.16	2.16
2.17	2.17	2.17	2.17	2.17	2.17	2.17	2.17	2.17
2.18	2.18	2.18	2.18	2.18	2.18	2.18	2.18	2.18
2.19	2.19	2.19	2.19	2.19	2.19	2.19	2.19	2.19
2.2	2.2	2.2	2.2	2.2	2.2	2.2	2.2	2.2
2.21	2.21	2.21	2.21	2.21	2.21	2.21	2.21	2.21
2.22	2.22	2.22	2.22	2.22	2.22	2.22	2.22	2.22
2.23	2.23	2.23	2.23	2.23	2.23	2.23	2.23	2.23
2.24	2.24	2.24	2.24	2.24	2.24	2.24	2.24	2.24
2.25	2.25	2.25	2.25	2.25	2.25	2.25	2.25	2.25
2.26	2.26	2.26	2.26	2.26	2.26	2.26	2.26	2.26
2.27	2.27	2.27	2.27	2.27	2.27	2.27	2.27	2.27
2.28	2.28	2.28	2.28	2.28	2.28	2.28	2.28	2.28
2.29	2.29	2.29	2.29	2.29	2.29	2.29	2.29	2.29
2.3	2.3	2.3	2.3	2.3	2.3	2.3	2.3	2.3
2.31	2.31	2.31	2.31	2.31	2.31	2.31	2.31	2.31
2.32	2.32	2.32	2.32	2.32	2.32	2.32	2.32	2.32
2.33	2.33	2.33	2.33	2.33	2.33	2.33	2.33	2.33
2.34	2.34	2.34	2.34	2.34	2.34	2.34	2.34	2.34
2.35	2.35	2.35	2.35	2.35	2.35	2.35	2.35	2.35
2.36	2.36	2.36	2.36	2.36	2.36	2.36	2.36	2.36
2.37	2.37	2.37	2.37	2.37	2.37	2.37	2.37	2.37
2.38	2.38	2.38	2.38	2.38	2.38	2.38	2.38	2.38
2.39	2.39	2.39	2.39	2.39	2.39	2.39	2.39	2.39
2.4	2.4	2.4	2.4	2.4	2.4	2.4	2.4	2.4
2.41	2.41	2.41	2.41	2.41	2.41	2.41	2.41	2.41
2.42	2.42	2.42	2.42	2.42	2.42	2.42	2.42	2.42
2.43	2.43	2.43	2.43	2.43	2.43	2.43	2.43	2.43
2.44	2.44	2.44	2.44	2.44	2.44	2.44	2.44	2.44
2.45	2.45	2.45	2.45	2.45	2.45	2.45	2.45	2.45
2.46	2.46	2.46	2.46	2.46	2.46	2.46	2.46	2.46
2.47	2.47	2.47	2.47	2.47	2.47	2.47	2.47	2.47
2.48	2.48	2.48	2.48	2.48	2.48	2.48	2.48	2.48
2.49	2.49	2.49	2.49	2.49	2.49	2.49	2.49	2.49

2.5	2.5	2.5	2.5	2.5	2.5	2.5	2.5	2.5
2.51	2.51	2.51	2.51	2.51	2.51	2.51	2.51	2.51
2.52	2.52	2.52	2.52	2.52	2.52	2.52	2.52	2.52
2.53	2.53	2.53	2.53	2.53	2.53	2.53	2.53	2.53
2.54	2.54	2.54	2.54	2.54	2.54	2.54	2.54	2.54
2.55	2.55	2.55	2.55	2.55	2.55	2.55	2.55	2.55
2.56	2.56	2.56	2.56	2.56	2.56	2.56	2.56	2.56
2.57	2.57	2.57	2.57	2.57	2.57	2.57	2.57	2.57
2.58	2.58	2.58	2.58	2.58	2.58	2.58	2.58	2.58
2.59	2.59	2.59	2.59	2.59	2.59	2.59	2.59	2.59
2.6	2.6	2.6	2.6	2.6	2.6	2.6	2.6	2.6
2.61	2.61	2.61	2.61	2.61	2.61	2.61	2.61	2.61
2.62	2.62	2.62	2.62	2.62	2.62	2.62	2.62	2.62
2.63	2.63	2.63	2.63	2.63	2.63	2.63	2.63	2.63
2.64	2.64	2.64	2.64	2.64	2.64	2.64	2.64	2.64
2.65	2.65	2.65	2.65	2.65	2.65	2.65	2.65	2.65
2.66	2.66	2.66	2.66	2.66	2.66	2.66	2.66	2.66
2.67	2.67	2.67	2.67	2.67	2.67	2.67	2.67	2.67
2.68	2.68	2.68	2.68	2.68	2.68	2.68	2.68	2.68
2.69	2.69	2.69	2.69	2.69	2.69	2.69	2.69	2.69
2.7	2.7	2.7	2.7	2.7	2.7	2.7	2.7	2.7
2.71	2.71	2.71	2.71	2.71	2.71	2.71	2.71	2.71
2.72	2.72	2.72	2.72	2.72	2.72	2.72	2.72	2.72
2.73	2.73	2.73	2.73	2.73	2.73	2.73	2.73	2.73
2.74	2.74	2.74	2.74	2.74	2.74	2.74	2.74	2.74
2.75	2.75	2.75	2.75	2.75	2.75	2.75	2.75	2.75
2.76	2.76	2.76	2.76	2.76	2.76	2.76	2.76	2.76
2.77	2.77	2.77	2.77	2.77	2.77	2.77	2.77	2.77
2.78	2.78	2.78	2.78	2.78	2.78	2.78	2.78	2.78
2.79	2.79	2.79	2.79	2.79	2.79	2.79	2.79	2.79
2.8	2.8	2.8	2.8	2.8	2.8	2.8	2.8	2.8
2.81	2.81	2.81	2.81	2.81	2.81	2.81	2.81	2.81
2.82	2.82	2.82	2.82	2.82	2.82	2.82	2.82	2.82
2.83	2.83	2.83	2.83	2.83	2.83	2.83	2.83	2.83
2.84	2.84	2.84	2.84	2.84	2.84	2.84	2.84	2.84
2.85	2.85	2.85	2.85	2.85	2.85	2.85	2.85	2.85
2.86	2.86	2.86	2.86	2.86	2.86	2.86	2.86	2.86
2.87	2.87	2.87	2.87	2.87	2.87	2.87	2.87	2.87
2.88	2.88	2.88	2.88	2.88	2.88	2.88	2.88	2.88
2.89	2.89	2.89	2.89	2.89	2.89	2.89	2.89	2.89
2.9	2.9	2.9	2.9	2.9	2.9	2.9	2.9	2.9
2.91	2.91	2.91	2.91	2.91	2.91	2.91	2.91	2.91
2.92	2.92	2.92	2.92	2.92	2.92	2.92	2.92	2.92
2.93	2.93	2.93	2.93	2.93	2.93	2.93	2.93	2.93
2.94	2.94	2.94	2.94	2.94	2.94	2.94	2.94	2.94
2.95	2.95	2.95	2.95	2.95	2.95	2.95	2.95	2.95
2.96	2.96	2.96	2.96	2.96	2.96	2.96	2.96	2.96
2.97	2.97	2.97	2.97	2.97	2.97	2.97	2.97	2.97
2.98	2.98	2.98	2.98	2.98	2.98	2.98	2.98	2.98
2.99	2.99	2.99	2.99	2.99	2.99	2.99	2.99	2.99

3	3	3	3	3	3	3	3	3
3.01	3.01	3.01	3.01	3.01	3.01	3.01	3.01	3.01
3.02	3.02	3.02	3.02	3.02	3.02	3.02	3.02	3.02
3.03	3.03	3.03	3.03	3.03	3.03	3.03	3.03	3.03
3.04	3.04	3.04	3.04	3.04	3.04	3.04	3.04	3.04
3.05	3.05	3.05	3.05	3.05	3.05	3.05	3.05	3.05
3.06	3.06	3.06	3.06	3.06	3.06	3.06	3.06	3.06
3.07	3.07	3.07	3.07	3.07	3.07	3.07	3.07	3.07
3.08	3.08	3.08	3.08	3.08	3.08	3.08	3.08	3.08
3.09	3.09	3.09	3.09	3.09	3.09	3.09	3.09	3.09
3.1	3.1	3.1	3.1	3.1	3.1	3.1	3.1	3.1
3.11	3.11	3.11	3.11	3.11	3.11	3.11	3.11	3.11
3.12	3.12	3.12	3.12	3.12	3.12	3.12	3.12	3.12
3.13	3.13	3.13	3.13	3.13	3.13	3.13	3.13	3.13
3.14	3.14	3.14	3.14	3.14	3.14	3.14	3.14	3.14
3.15	3.15	3.15	3.15	3.15	3.15	3.15	3.15	3.15
3.16	3.16	3.16	3.16	3.16	3.16	3.16	3.16	3.16
3.17	3.17	3.17	3.17	3.17	3.17	3.17	3.17	3.17
3.18	3.18	3.18	3.18	3.18	3.18	3.18	3.18	3.18
3.19	3.19	3.19	3.19	3.19	3.19	3.19	3.19	3.19
3.2	3.2	3.2	3.2	3.2	3.2	3.2	3.2	3.2
3.21	3.21	3.21	3.21	3.21	3.21	3.21	3.21	3.21
3.22	3.22	3.22	3.22	3.22	3.22	3.22	3.22	3.22
3.23	3.23	3.23	3.23	3.23	3.23	3.23	3.23	3.23
3.24	3.24	3.24	3.24	3.24	3.24	3.24	3.24	3.24
3.25	3.25	3.25	3.25	3.25	3.25	3.25	3.25	3.25
3.26	3.26	3.26	3.26	3.26	3.26	3.26	3.26	3.26
3.27	3.27	3.27	3.27	3.27	3.27	3.27	3.27	3.27
3.28	3.28	3.28	3.28	3.28	3.28	3.28	3.28	3.28
3.29	3.29	3.29	3.29	3.29	3.29	3.29	3.29	3.29
3.3	3.3	3.3	3.3	3.3	3.3	3.3	3.3	3.3
3.31	3.31	3.31	3.31	3.31	3.31	3.31	3.31	3.31
3.32	3.32	3.32	3.32	3.32	3.32	3.32	3.32	3.32
3.33	3.33	3.33	3.33	3.33	3.33	3.33	3.33	3.33
3.34	3.34	3.34	3.34	3.34	3.34	3.34	3.34	3.34
3.35	3.35	3.35	3.35	3.35	3.35	3.35	3.35	3.35
3.36	3.36	3.36	3.36	3.36	3.36	3.36	3.36	3.36
3.37	3.37	3.37	3.37	3.37	3.37	3.37	3.37	3.37
3.38	3.38	3.38	3.38	3.38	3.38	3.38	3.38	3.38
3.39	3.39	3.39	3.39	3.39	3.39	3.39	3.39	3.39
3.4	3.4	3.4	3.4	3.4	3.4	3.4	3.4	3.4
3.41	3.41	3.41	3.41	3.41	3.41	3.41	3.41	3.41
3.42	3.42	3.42	3.42	3.42	3.42	3.42	3.42	3.42
3.43	3.43	3.43	3.43	3.43	3.43	3.43	3.43	3.43
3.44	3.44	3.44	3.44	3.44	3.44	3.44	3.44	3.44
3.45	3.45	3.45	3.45	3.45	3.45	3.45	3.45	3.45
3.46	3.46	3.46	3.46	3.46	3.46	3.46	3.46	3.46
3.47	3.47	3.47	3.47	3.47	3.47	3.47	3.47	3.47
3.48	3.48	3.48	3.48	3.48	3.48	3.48	3.48	3.48
3.49	3.49	3.49	3.49	3.49	3.49	3.49	3.49	3.49

3.5	3.5	3.5	3.5	3.5	3.5	3.5	3.5	3.5
3.51	3.51	3.51	3.51	3.51	3.51	3.51	3.51	3.51
3.52	3.52	3.52	3.52	3.52	3.52	3.52	3.52	3.52
3.53	3.53	3.53	3.53	3.53	3.53	3.53	3.53	3.53
3.54	3.54	3.54	3.54	3.54	3.54	3.54	3.54	3.54
3.55	3.55	3.55	3.55	3.55	3.55	3.55	3.55	3.55
3.56	3.56	3.56	3.56	3.56	3.56	3.56	3.56	3.56
3.57	3.57	3.57	3.57	3.57	3.57	3.57	3.57	3.57
3.58	3.58	3.58	3.58	3.58	3.58	3.58	3.58	3.58
3.59	3.59	3.59	3.59	3.59	3.59	3.59	3.59	3.59
3.6	3.6	3.6	3.6	3.6	3.6	3.6	3.6	3.6
3.61	3.61	3.61	3.61	3.61	3.61	3.61	3.61	3.61
3.62	3.62	3.62	3.62	3.62	3.62	3.62	3.62	3.62
3.63	3.63	3.63	3.63	3.63	3.63	3.63	3.63	3.63
3.64	3.64	3.64	3.64	3.64	3.64	3.64	3.64	3.64
3.65	3.65	3.65	3.65	3.65	3.65	3.65	3.65	3.65
3.66	3.66	3.66	3.66	3.66	3.66	3.66	3.66	3.66
3.67	3.67	3.67	3.67	3.67	3.67	3.67	3.67	3.67
3.68	3.68	3.68	3.68	3.68	3.68	3.68	3.68	3.68
3.69	3.69	3.69	3.69	3.69	3.69	3.69	3.69	3.69
3.7	3.7	3.7	3.7	3.7	3.7	3.7	3.7	3.7
3.71	3.71	3.71	3.71	3.71	3.71	3.71	3.71	3.71
3.72	3.72	3.72	3.72	3.72	3.72	3.72	3.72	3.72
3.73	3.73	3.73	3.73	3.73	3.73	3.73	3.73	3.73
3.74	3.74	3.74	3.74	3.74	3.74	3.74	3.74	3.74
3.75	3.75	3.75	3.75	3.75	3.75	3.75	3.75	3.75
3.76	3.76	3.76	3.76	3.76	3.76	3.76	3.76	3.76
3.77	3.77	3.77	3.77	3.77	3.77	3.77	3.77	3.77
3.78	3.78	3.78	3.78	3.78	3.78	3.78	3.78	3.78
3.79	3.79	3.79	3.79	3.79	3.79	3.79	3.79	3.79
3.8	3.8	3.8	3.8	3.8	3.8	3.8	3.8	3.8
3.81	3.81	3.81	3.81	3.81	3.81	3.81	3.81	3.81
3.82	3.82	3.82	3.82	3.82	3.82	3.82	3.82	3.82
3.83	3.83	3.83	3.83	3.83	3.83	3.83	3.83	3.83
3.84	3.84	3.84	3.84	3.84	3.84	3.84	3.84	3.84
3.85	3.85	3.85	3.85	3.85	3.85	3.85	3.85	3.85
3.86	3.86	3.86	3.86	3.86	3.86	3.86	3.86	3.86
3.87	3.87	3.87	3.87	3.87	3.87	3.87	3.87	3.87
3.88	3.88	3.88	3.88	3.88	3.88	3.88	3.88	3.88
3.89	3.89	3.89	3.89	3.89	3.89	3.89	3.89	3.89
3.9	3.9	3.9	3.9	3.9	3.9	3.9	3.9	3.9
3.91	3.91	3.91	3.91	3.91	3.91	3.91	3.91	3.91
3.92	3.92	3.92	3.92	3.92	3.92	3.92	3.92	3.92
3.93	3.93	3.93	3.93	3.93	3.93	3.93	3.93	3.93
3.94	3.94	3.94	3.94	3.94	3.94	3.94	3.94	3.94
3.95	3.95	3.95	3.95	3.95	3.95	3.95	3.95	3.95
3.96	3.96	3.96	3.96	3.96	3.96	3.96	3.96	3.96
3.97	3.97	3.97	3.97	3.97	3.97	3.97	3.97	3.97
3.98	3.98	3.98	3.98	3.98	3.98	3.98	3.98	3.98
3.99	3.99	3.99	3.99	3.99	3.99	3.99	3.99	3.99

4	4	4	4	4	4	4	4	4
4.01	4.01	4.01	4.01	4.01	4.01	4.01	4.01	4.01
4.02	4.02	4.02	4.02	4.02	4.02	4.02	4.02	4.02
4.03	4.03	4.03	4.03	4.03	4.03	4.03	4.03	4.03
4.04	4.04	4.04	4.04	4.04	4.04	4.04	4.04	4.04
4.05	4.05	4.05	4.05	4.05	4.05	4.05	4.05	4.05
4.06	4.06	4.06	4.06	4.06	4.06	4.06	4.06	4.06
4.07	4.07	4.07	4.07	4.07	4.07	4.07	4.07	4.07
4.08	4.08	4.08	4.08	4.08	4.08	4.08	4.08	4.08
4.09	4.09	4.09	4.09	4.09	4.09	4.09	4.09	4.09
4.1	4.1	4.1	4.1	4.1	4.1	4.1	4.1	4.1
4.11	4.11	4.11	4.11	4.11	4.11	4.11	4.11	4.11
4.12	4.12	4.12	4.12	4.12	4.12	4.12	4.12	4.12
4.13	4.13	4.13	4.13	4.13	4.13	4.13	4.13	4.13
4.14	4.14	4.14	4.14	4.14	4.14	4.14	4.14	4.14
4.15	4.15	4.15	4.15	4.15	4.15	4.15	4.15	4.15
4.16	4.16	4.16	4.16	4.16	4.16	4.16	4.16	4.16
4.17	4.17	4.17	4.17	4.17	4.17	4.17	4.17	4.17
4.18	4.18	4.18	4.18	4.18	4.18	4.18	4.18	4.18
4.19	4.19	4.19	4.19	4.19	4.19	4.19	4.19	4.19
4.2	4.2	4.2	4.2	4.2	4.2	4.2	4.2	4.2
4.21	4.21	4.21	4.21	4.21	4.21	4.21	4.21	4.21
4.22	4.22	4.22	4.22	4.22	4.22	4.22	4.22	4.22
4.23	4.23	4.23	4.23	4.23	4.23	4.23	4.23	4.23
4.24	4.24	4.24	4.24	4.24	4.24	4.24	4.24	4.24
4.25	4.25	4.25	4.25	4.25	4.25	4.25	4.25	4.25
4.26	4.26	4.26	4.26	4.26	4.26	4.26	4.26	4.26
4.27	4.27	4.27	4.27	4.27	4.27	4.27	4.27	4.27
4.28	4.28	4.28	4.28	4.28	4.28	4.28	4.28	4.28
4.29	4.29	4.29	4.29	4.29	4.29	4.29	4.29	4.29
4.3	4.3	4.3	4.3	4.3	4.3	4.3	4.3	4.3
4.31	4.31	4.31	4.31	4.31	4.31	4.31	4.31	4.31
4.32	4.32	4.32	4.32	4.32	4.32	4.32	4.32	4.32
4.33	4.33	4.33	4.33	4.33	4.33	4.33	4.33	4.33
4.34	4.34	4.34	4.34	4.34	4.34	4.34	4.34	4.34
4.35	4.35	4.35	4.35	4.35	4.35	4.35	4.35	4.35
4.36	4.36	4.36	4.36	4.36	4.36	4.36	4.36	4.36
4.37	4.37	4.37	4.37	4.37	4.37	4.37	4.37	4.37
4.38	4.38	4.38	4.38	4.38	4.38	4.38	4.38	4.38
4.39	4.39	4.39	4.39	4.39	4.39	4.39	4.39	4.39
4.4	4.4	4.4	4.4	4.4	4.4	4.4	4.4	4.4
4.41	4.41	4.41	4.41	4.41	4.41	4.41	4.41	4.41
4.42	4.42	4.42	4.42	4.42	4.42	4.42	4.42	4.42
4.43	4.43	4.43	4.43	4.43	4.43	4.43	4.43	4.43
4.44	4.44	4.44	4.44	4.44	4.44	4.44	4.44	4.44
4.45	4.45	4.45	4.45	4.45	4.45	4.45	4.45	4.45
4.46	4.46	4.46	4.46	4.46	4.46	4.46	4.46	4.46
4.47	4.47	4.47	4.47	4.47	4.47	4.47	4.47	4.47
4.48	4.48	4.48	4.48	4.48	4.48	4.48	4.48	4.48
4.49	4.49	4.49	4.49	4.49	4.49	4.49	4.49	4.49

4.5	4.5	4.5	4.5	4.5	4.5	4.5	4.5	4.5
4.51	4.51	4.51	4.51	4.51	4.51	4.51	4.51	4.51
4.52	4.52	4.52	4.52	4.52	4.52	4.52	4.52	4.52
4.53	4.53	4.53	4.53	4.53	4.53	4.53	4.53	4.53
4.54	4.54	4.54	4.54	4.54	4.54	4.54	4.54	4.54
4.55	4.55	4.55	4.55	4.55	4.55	4.55	4.55	4.55
4.56	4.56	4.56	4.56	4.56	4.56	4.56	4.56	4.56
4.57	4.57	4.57	4.57	4.57	4.57	4.57	4.57	4.57
4.58	4.58	4.58	4.58	4.58	4.58	4.58	4.58	4.58
4.59	4.59	4.59	4.59	4.59	4.59	4.59	4.59	4.59
4.6	4.6	4.6	4.6	4.6	4.6	4.6	4.6	4.6
4.61	4.61	4.61	4.61	4.61	4.61	4.61	4.61	4.61
4.62	4.62	4.62	4.62	4.62	4.62	4.62	4.62	4.62
4.63	4.63	4.63	4.63	4.63	4.63	4.63	4.63	4.63
4.64	4.64	4.64	4.64	4.64	4.64	4.64	4.64	4.64
4.65	4.65	4.65	4.65	4.65	4.65	4.65	4.65	4.65
4.66	4.66	4.66	4.66	4.66	4.66	4.66	4.66	4.66
4.67	4.67	4.67	4.67	4.67	4.67	4.67	4.67	4.67
4.68	4.68	4.68	4.68	4.68	4.68	4.68	4.68	4.68
4.69	4.69	4.69	4.69	4.69	4.69	4.69	4.69	4.69
4.7	4.7	4.7	4.7	4.7	4.7	4.7	4.7	4.7
4.71	4.71	4.71	4.71	4.71	4.71	4.71	4.71	4.71
4.72	4.72	4.72	4.72	4.72	4.72	4.72	4.72	4.72
4.73	4.73	4.73	4.73	4.73	4.73	4.73	4.73	4.73
4.74	4.74	4.74	4.74	4.74	4.74	4.74	4.74	4.74
4.75	4.75	4.75	4.75	4.75	4.75	4.75	4.75	4.75
4.76	4.76	4.76	4.76	4.76	4.76	4.76	4.76	4.76
4.77	4.77	4.77	4.77	4.77	4.77	4.77	4.77	4.77
4.78	4.78	4.78	4.78	4.78	4.78	4.78	4.78	4.78
4.79	4.79	4.79	4.79	4.79	4.79	4.79	4.79	4.79
4.8	4.8	4.8	4.8	4.8	4.8	4.8	4.8	4.8
4.81	4.81	4.81	4.81	4.81	4.81	4.81	4.81	4.81
4.82	4.82	4.82	4.82	4.82	4.82	4.82	4.82	4.82
4.83	4.83	4.83	4.83	4.83	4.83	4.83	4.83	4.83
4.84	4.84	4.84	4.84	4.84	4.84	4.84	4.84	4.84
4.85	4.85	4.85	4.85	4.85	4.85	4.85	4.85	4.85
4.86	4.86	4.86	4.86	4.86	4.86	4.86	4.86	4.86
4.87	4.87	4.87	4.87	4.87	4.87	4.87	4.87	4.87
4.88	4.88	4.88	4.88	4.88	4.88	4.88	4.88	4.88
4.89	4.89	4.89	4.89	4.89	4.89	4.89	4.89	4.89
4.9	4.9	4.9	4.9	4.9	4.9	4.9	4.9	4.9
4.91	4.91	4.91	4.91	4.91	4.91	4.91	4.91	4.91
4.92	4.92	4.92	4.92	4.92	4.92	4.92	4.92	4.92
4.93	4.93	4.93	4.93	4.93	4.93	4.93	4.93	4.93
4.94	4.94	4.94	4.94	4.94	4.94	4.94	4.94	4.94
4.95	4.95	4.95	4.95	4.95	4.95	4.95	4.95	4.95
4.96	4.96	4.96	4.96	4.96	4.96	4.96	4.96	4.96
4.97	4.97	4.97	4.97	4.97	4.97	4.97	4.97	4.97
4.98	4.98	4.98	4.98	4.98	4.98	4.98	4.98	4.98
4.99	4.99	4.99	4.99	4.99	4.99	4.99	4.99	4.99

5	5	5	5	5	5	5	5	5
5.01	5.01	5.01	5.01	5.01	5.01	5.01	5.01	5.01
5.02	5.02	5.02	5.02	5.02	5.02	5.02	5.02	5.02
5.03	5.03	5.03	5.03	5.03	5.03	5.03	5.03	5.03
5.04	5.04	5.04	5.04	5.04	5.04	5.04	5.04	5.04
5.05	5.05	5.05	5.05	5.05	5.05	5.05	5.05	5.05
5.06	5.06	5.06	5.06	5.06	5.06	5.06	5.06	5.06
5.07	5.07	5.07	5.07	5.07	5.07	5.07	5.07	5.07
5.08	5.08	5.08	5.08	5.08	5.08	5.08	5.08	5.08
5.09	5.09	5.09	5.09	5.09	5.09	5.09	5.09	5.09
5.1	5.1	5.1	5.1	5.1	5.1	5.1	5.1	5.1
5.11	5.11	5.11	5.11	5.11	5.11	5.11	5.11	5.11
5.12	5.12	5.12	5.12	5.12	5.12	5.12	5.12	5.12
5.13	5.13	5.13	5.13	5.13	5.13	5.13	5.13	5.13
5.14	5.14	5.14	5.14	5.14	5.14	5.14	5.14	5.14
5.15	5.15	5.15	5.15	5.15	5.15	5.15	5.15	5.15
5.16	5.16	5.16	5.16	5.16	5.16	5.16	5.16	5.16
5.17	5.17	5.17	5.17	5.17	5.17	5.17	5.17	5.17
5.18	5.18	5.18	5.18	5.18	5.18	5.18	5.18	5.18
5.19	5.19	5.19	5.19	5.19	5.19	5.19	5.19	5.19
5.2	5.2	5.2	5.2	5.2	5.2	5.2	5.2	5.2
5.21	5.21	5.21	5.21	5.21	5.21	5.21	5.21	5.21
5.22	5.22	5.22	5.22	5.22	5.22	5.22	5.22	5.22
5.23	5.23	5.23	5.23	5.23	5.23	5.23	5.23	5.23
5.24	5.24	5.24	5.24	5.24	5.24	5.24	5.24	5.24
5.25	5.25	5.25	5.25	5.25	5.25	5.25	5.25	5.25
5.26	5.26	5.26	5.26	5.26	5.26	5.26	5.26	5.26
5.27	5.27	5.27	5.27	5.27	5.27	5.27	5.27	5.27
5.28	5.28	5.28	5.28	5.28	5.28	5.28	5.28	5.28
5.29	5.29	5.29	5.29	5.29	5.29	5.29	5.29	5.29
5.3	5.3	5.3	5.3	5.3	5.3	5.3	5.3	5.3
5.31	5.31	5.31	5.31	5.31	5.31	5.31	5.31	5.31
5.32	5.32	5.32	5.32	5.32	5.32	5.32	5.32	5.32
5.33	5.33	5.33	5.33	5.33	5.33	5.33	5.33	5.33
5.34	5.34	5.34	5.34	5.34	5.34	5.34	5.34	5.34
5.35	5.35	5.35	5.35	5.35	5.35	5.35	5.35	5.35
5.36	5.36	5.36	5.36	5.36	5.36	5.36	5.36	5.36
5.37	5.37	5.37	5.37	5.37	5.37	5.37	5.37	5.37
5.38	5.38	5.38	5.38	5.38	5.38	5.38	5.38	5.38
5.39	5.39	5.39	5.39	5.39	5.39	5.39	5.39	5.39
5.4	5.4	5.4	5.4	5.4	5.4	5.4	5.4	5.4
5.41	5.41	5.41	5.41	5.41	5.41	5.41	5.41	5.41
5.42	5.42	5.42	5.42	5.42	5.42	5.42	5.42	5.42
5.43	5.43	5.43	5.43	5.43	5.43	5.43	5.43	5.43
5.44	5.44	5.44	5.44	5.44	5.44	5.44	5.44	5.44
5.45	5.45	5.45	5.45	5.45	5.45	5.45	5.45	5.45
5.46	5.46	5.46	5.46	5.46	5.46	5.46	5.46	5.46
5.47	5.47	5.47	5.47	5.47	5.47	5.47	5.47	5.47
5.48	5.48	5.48	5.48	5.48	5.48	5.48	5.48	5.48
5.49	5.49	5.49	5.49	5.49	5.49	5.49	5.49	5.49

5.5	5.5	5.5	5.5	5.5	5.5	5.5	5.5	5.5
5.51	5.51	5.51	5.51	5.51	5.51	5.51	5.51	5.51
5.52	5.52	5.52	5.52	5.52	5.52	5.52	5.52	5.52
5.53	5.53	5.53	5.53	5.53	5.53	5.53	5.53	5.53
5.54	5.54	5.54	5.54	5.54	5.54	5.54	5.54	5.54
5.55	5.55	5.55	5.55	5.55	5.55	5.55	5.55	5.55
5.56	5.56	5.56	5.56	5.56	5.56	5.56	5.56	5.56
5.57	5.57	5.57	5.57	5.57	5.57	5.57	5.57	5.57
5.58	5.58	5.58	5.58	5.58	5.58	5.58	5.58	5.58
5.59	5.59	5.59	5.59	5.59	5.59	5.59	5.59	5.59
5.6	5.6	5.6	5.6	5.6	5.6	5.6	5.6	5.6
5.61	5.61	5.61	5.61	5.61	5.61	5.61	5.61	5.61
5.62	5.62	5.62	5.62	5.62	5.62	5.62	5.62	5.62
5.63	5.63	5.63	5.63	5.63	5.63	5.63	5.63	5.63
5.64	5.64	5.64	5.64	5.64	5.64	5.64	5.64	5.64
5.65	5.65	5.65	5.65	5.65	5.65	5.65	5.65	5.65
5.66	5.66	5.66	5.66	5.66	5.66	5.66	5.66	5.66
5.67	5.67	5.67	5.67	5.67	5.67	5.67	5.67	5.67
5.68	5.68	5.68	5.68	5.68	5.68	5.68	5.68	5.68
5.69	5.69	5.69	5.69	5.69	5.69	5.69	5.69	5.69
5.7	5.7	5.7	5.7	5.7	5.7	5.7	5.7	5.7
5.71	5.71	5.71	5.71	5.71	5.71	5.71	5.71	5.71
5.72	5.72	5.72	5.72	5.72	5.72	5.72	5.72	5.72
5.73	5.73	5.73	5.73	5.73	5.73	5.73	5.73	5.73
5.74	5.74	5.74	5.74	5.74	5.74	5.74	5.74	5.74
5.75	5.75	5.75	5.75	5.75	5.75	5.75	5.75	5.75
5.76	5.76	5.76	5.76	5.76	5.76	5.76	5.76	5.76
5.77	5.77	5.77	5.77	5.77	5.77	5.77	5.77	5.77
5.78	5.78	5.78	5.78	5.78	5.78	5.78	5.78	5.78
5.79	5.79	5.79	5.79	5.79	5.79	5.79	5.79	5.79
5.8	5.8	5.8	5.8	5.8	5.8	5.8	5.8	5.8
5.81	5.81	5.81	5.81	5.81	5.81	5.81	5.81	5.81
5.82	5.82	5.82	5.82	5.82	5.82	5.82	5.82	5.82
5.83	5.83	5.83	5.83	5.83	5.83	5.83	5.83	5.83
5.84	5.84	5.84	5.84	5.84	5.84	5.84	5.84	5.84
5.85	5.85	5.85	5.85	5.85	5.85	5.85	5.85	5.85
5.86	5.86	5.86	5.86	5.86	5.86	5.86	5.86	5.86
5.87	5.87	5.87	5.87	5.87	5.87	5.87	5.87	5.87
5.88	5.88	5.88	5.88	5.88	5.88	5.88	5.88	5.88
5.89	5.89	5.89	5.89	5.89	5.89	5.89	5.89	5.89
5.9	5.9	5.9	5.9	5.9	5.9	5.9	5.9	5.9
5.91	5.91	5.91	5.91	5.91	5.91	5.91	5.91	5.91
5.92	5.92	5.92	5.92	5.92	5.92	5.92	5.92	5.92
5.93	5.93	5.93	5.93	5.93	5.93	5.93	5.93	5.93
5.94	5.94	5.94	5.94	5.94	5.94	5.94	5.94	5.94
5.95	5.95	5.95	5.95	5.95	5.95	5.95	5.95	5.95
5.96	5.96	5.96	5.96	5.96	5.96	5.96	5.96	5.96
5.97	5.97	5.97	5.97	5.97	5.97	5.97	5.97	5.97
5.98	5.98	5.98	5.98	5.98	5.98	5.98	5.98	5.98
5.99	5.99	5.99	5.99	5.99	5.99	5.99	5.99	5.99

6	6	6	6	6	6	6	6	6
6.01	6.01	6.01	6.01	6.01	6.01	6.01	6.01	6.01
6.02	6.02	6.02	6.02	6.02	6.02	6.02	6.02	6.02
6.03	6.03	6.03	6.03	6.03	6.03	6.03	6.03	6.03
6.04	6.04	6.04	6.04	6.04	6.04	6.04	6.04	6.04
6.05	6.05	6.05	6.05	6.05	6.05	6.05	6.05	6.05
6.06	6.06	6.06	6.06	6.06	6.06	6.06	6.06	6.06
6.07	6.07	6.07	6.07	6.07	6.07	6.07	6.07	6.07
6.08	6.08	6.08	6.08	6.08	6.08	6.08	6.08	6.08
6.09	6.09	6.09	6.09	6.09	6.09	6.09	6.09	6.09
6.1	6.1	6.1	6.1	6.1	6.1	6.1	6.1	6.1
6.11	6.11	6.11	6.11	6.11	6.11	6.11	6.11	6.11
6.12	6.12	6.12	6.12	6.12	6.12	6.12	6.12	6.12
6.13	6.13	6.13	6.13	6.13	6.13	6.13	6.13	6.13
6.14	6.14	6.14	6.14	6.14	6.14	6.14	6.14	6.14
6.15	6.15	6.15	6.15	6.15	6.15	6.15	6.15	6.15
6.16	6.16	6.16	6.16	6.16	6.16	6.16	6.16	6.16
6.17	6.17	6.17	6.17	6.17	6.17	6.17	6.17	6.17
6.18	6.18	6.18	6.18	6.18	6.18	6.18	6.18	6.18
6.19	6.19	6.19	6.19	6.19	6.19	6.19	6.19	6.19
6.2	6.2	6.2	6.2	6.2	6.2	6.2	6.2	6.2
6.21	6.21	6.21	6.21	6.21	6.21	6.21	6.21	6.21
6.22	6.22	6.22	6.22	6.22	6.22	6.22	6.22	6.22
6.23	6.23	6.23	6.23	6.23	6.23	6.23	6.23	6.23
6.24	6.24	6.24	6.24	6.24	6.24	6.24	6.24	6.24
6.25	6.25	6.25	6.25	6.25	6.25	6.25	6.25	6.25
6.26	6.26	6.26	6.26	6.26	6.26	6.26	6.26	6.26
6.27	6.27	6.27	6.27	6.27	6.27	6.27	6.27	6.27
6.28	6.28	6.28	6.28	6.28	6.28	6.28	6.28	6.28
6.29	6.29	6.29	6.29	6.29	6.29	6.29	6.29	6.29
6.3	6.3	6.3	6.3	6.3	6.3	6.3	6.3	6.3
6.31	6.31	6.31	6.31	6.31	6.31	6.31	6.31	6.31
6.32	6.32	6.32	6.32	6.32	6.32	6.32	6.32	6.32
6.33	6.33	6.33	6.33	6.33	6.33	6.33	6.33	6.33
6.34	6.34	6.34	6.34	6.34	6.34	6.34	6.34	6.34
6.35	6.35	6.35	6.35	6.35	6.35	6.35	6.35	6.35
6.36	6.36	6.36	6.36	6.36	6.36	6.36	6.36	6.36
6.37	6.37	6.37	6.37	6.37	6.37	6.37	6.37	6.37
6.38	6.38	6.38	6.38	6.38	6.38	6.38	6.38	6.38
6.39	6.39	6.39	6.39	6.39	6.39	6.39	6.39	6.39
6.4	6.4	6.4	6.4	6.4	6.4	6.4	6.4	6.4
6.41	6.41	6.41	6.41	6.41	6.41	6.41	6.41	6.41
6.42	6.42	6.42	6.42	6.42	6.42	6.42	6.42	6.42
6.43	6.43	6.43	6.43	6.43	6.43	6.43	6.43	6.43
6.44	6.44	6.44	6.44	6.44	6.44	6.44	6.44	6.44
6.45	6.45	6.45	6.45	6.45	6.45	6.45	6.45	6.45
6.46	6.46	6.46	6.46	6.46	6.46	6.46	6.46	6.46
6.47	6.47	6.47	6.47	6.47	6.47	6.47	6.47	6.47
6.48	6.48	6.48	6.48	6.48	6.48	6.48	6.48	6.48
6.49	6.49	6.49	6.49	6.49	6.49	6.49	6.49	6.49

6.5	6.5	6.5	6.5	6.5	6.5	6.5	6.5	6.5
6.51	6.51	6.51	6.51	6.51	6.51	6.51	6.51	6.51
6.52	6.52	6.52	6.52	6.52	6.52	6.52	6.52	6.52
6.53	6.53	6.53	6.53	6.53	6.53	6.53	6.53	6.53
6.54	6.54	6.54	6.54	6.54	6.54	6.54	6.54	6.54
6.55	6.55	6.55	6.55	6.55	6.55	6.55	6.55	6.55
6.56	6.56	6.56	6.56	6.56	6.56	6.56	6.56	6.56
6.57	6.57	6.57	6.57	6.57	6.57	6.57	6.57	6.57
6.58	6.58	6.58	6.58	6.58	6.58	6.58	6.58	6.58
6.59	6.59	6.59	6.59	6.59	6.59	6.59	6.59	6.59
6.6	6.6	6.6	6.6	6.6	6.6	6.6	6.6	6.6
6.61	6.61	6.61	6.61	6.61	6.61	6.61	6.61	6.61
6.62	6.62	6.62	6.62	6.62	6.62	6.62	6.62	6.62
6.63	6.63	6.63	6.63	6.63	6.63	6.63	6.63	6.63
6.64	6.64	6.64	6.64	6.64	6.64	6.64	6.64	6.64
6.65	6.65	6.65	6.65	6.65	6.65	6.65	6.65	6.65
6.66	6.66	6.66	6.66	6.66	6.66	6.66	6.66	6.66
6.67	6.67	6.67	6.67	6.67	6.67	6.67	6.67	6.67
6.68	6.68	6.68	6.68	6.68	6.68	6.68	6.68	6.68
6.69	6.69	6.69	6.69	6.69	6.69	6.69	6.69	6.69
6.7	6.7	6.7	6.7	6.7	6.7	6.7	6.7	6.7
6.71	6.71	6.71	6.71	6.71	6.71	6.71	6.71	6.71
6.72	6.72	6.72	6.72	6.72	6.72	6.72	6.72	6.72
6.73	6.73	6.73	6.73	6.73	6.73	6.73	6.73	6.73
6.74	6.74	6.74	6.74	6.74	6.74	6.74	6.74	6.74
6.75	6.75	6.75	6.75	6.75	6.75	6.75	6.75	6.75
6.76	6.76	6.76	6.76	6.76	6.76	6.76	6.76	6.76
6.77	6.77	6.77	6.77	6.77	6.77	6.77	6.77	6.77
6.78	6.78	6.78	6.78	6.78	6.78	6.78	6.78	6.78
6.79	6.79	6.79	6.79	6.79	6.79	6.79	6.79	6.79
6.8	6.8	6.8	6.8	6.8	6.8	6.8	6.8	6.8
6.81	6.81	6.81	6.81	6.81	6.81	6.81	6.81	6.81
6.82	6.82	6.82	6.82	6.82	6.82	6.82	6.82	6.82
6.83	6.83	6.83	6.83	6.83	6.83	6.83	6.83	6.83
6.84	6.84	6.84	6.84	6.84	6.84	6.84	6.84	6.84
6.85	6.85	6.85	6.85	6.85	6.85	6.85	6.85	6.85
6.86	6.86	6.86	6.86	6.86	6.86	6.86	6.86	6.86
6.87	6.87	6.87	6.87	6.87	6.87	6.87	6.87	6.87
6.88	6.88	6.88	6.88	6.88	6.88	6.88	6.88	6.88
6.89	6.89	6.89	6.89	6.89	6.89	6.89	6.89	6.89
6.9	6.9	6.9	6.9	6.9	6.9	6.9	6.9	6.9
6.91	6.91	6.91	6.91	6.91	6.91	6.91	6.91	6.91
6.92	6.92	6.92	6.92	6.92	6.92	6.92	6.92	6.92
6.93	6.93	6.93	6.93	6.93	6.93	6.93	6.93	6.93
6.94	6.94	6.94	6.94	6.94	6.94	6.94	6.94	6.94
6.95	6.95	6.95	6.95	6.95	6.95	6.95	6.95	6.95
6.96	6.96	6.96	6.96	6.96	6.96	6.96	6.96	6.96
6.97	6.97	6.97	6.97	6.97	6.97	6.97	6.97	6.97
6.98	6.98	6.98	6.98	6.98	6.98	6.98	6.98	6.98
6.99	6.99	6.99	6.99	6.99	6.99	6.99	6.99	6.99

7	7	7	7	7	7	7	7	7
7.01	7.01	7.01	7.01	7.01	7.01	7.01	7.01	7.01
7.02	7.02	7.02	7.02	7.02	7.02	7.02	7.02	7.02
7.03	7.03	7.03	7.03	7.03	7.03	7.03	7.03	7.03
7.04	7.04	7.04	7.04	7.04	7.04	7.04	7.04	7.04
7.05	7.05	7.05	7.05	7.05	7.05	7.05	7.05	7.05
7.06	7.06	7.06	7.06	7.06	7.06	7.06	7.06	7.06
7.07	7.07	7.07	7.07	7.07	7.07	7.07	7.07	7.07
7.08	7.08	7.08	7.08	7.08	7.08	7.08	7.08	7.08
7.09	7.09	7.09	7.09	7.09	7.09	7.09	7.09	7.09
7.1	7.1	7.1	7.1	7.1	7.1	7.1	7.1	7.1
7.11	7.11	7.11	7.11	7.11	7.11	7.11	7.11	7.11
7.12	7.12	7.12	7.12	7.12	7.12	7.12	7.12	7.12
7.13	7.13	7.13	7.13	7.13	7.13	7.13	7.13	7.13
7.14	7.14	7.14	7.14	7.14	7.14	7.14	7.14	7.14
7.15	7.15	7.15	7.15	7.15	7.15	7.15	7.15	7.15
7.16	7.16	7.16	7.16	7.16	7.16	7.16	7.16	7.16
7.17	7.17	7.17	7.17	7.17	7.17	7.17	7.17	7.17
7.18	7.18	7.18	7.18	7.18	7.18	7.18	7.18	7.18
7.19	7.19	7.19	7.19	7.19	7.19	7.19	7.19	7.19
7.2	7.2	7.2	7.2	7.2	7.2	7.2	7.2	7.2
7.21	7.21	7.21	7.21	7.21	7.21	7.21	7.21	7.21
7.22	7.22	7.22	7.22	7.22	7.22	7.22	7.22	7.22
7.23	7.23	7.23	7.23	7.23	7.23	7.23	7.23	7.23
7.24	7.24	7.24	7.24	7.24	7.24	7.24	7.24	7.24
7.25	7.25	7.25	7.25	7.25	7.25	7.25	7.25	7.25
7.26	7.26	7.26	7.26	7.26	7.26	7.26	7.26	7.26
7.27	7.27	7.27	7.27	7.27	7.27	7.27	7.27	7.27
7.28	7.28	7.28	7.28	7.28	7.28	7.28	7.28	7.28
7.29	7.29	7.29	7.29	7.29	7.29	7.29	7.29	7.29
7.3	7.3	7.3	7.3	7.3	7.3	7.3	7.3	7.3
7.31	7.31	7.31	7.31	7.31	7.31	7.31	7.31	7.31
7.32	7.32	7.32	7.32	7.32	7.32	7.32	7.32	7.32
7.33	7.33	7.33	7.33	7.33	7.33	7.33	7.33	7.33
7.34	7.34	7.34	7.34	7.34	7.34	7.34	7.34	7.34
7.35	7.35	7.35	7.35	7.35	7.35	7.35	7.35	7.35
7.36	7.36	7.36	7.36	7.36	7.36	7.36	7.36	7.36
7.37	7.37	7.37	7.37	7.37	7.37	7.37	7.37	7.37
7.38	7.38	7.38	7.38	7.38	7.38	7.38	7.38	7.38
7.39	7.39	7.39	7.39	7.39	7.39	7.39	7.39	7.39
7.4	7.4	7.4	7.4	7.4	7.4	7.4	7.4	7.4
7.41	7.41	7.41	7.41	7.41	7.41	7.41	7.41	7.41
7.42	7.42	7.42	7.42	7.42	7.42	7.42	7.42	7.42
7.43	7.43	7.43	7.43	7.43	7.43	7.43	7.43	7.43
7.44	7.44	7.44	7.44	7.44	7.44	7.44	7.44	7.44
7.45	7.45	7.45	7.45	7.45	7.45	7.45	7.45	7.45
7.46	7.46	7.46	7.46	7.46	7.46	7.46	7.46	7.46
7.47	7.47	7.47	7.47	7.47	7.47	7.47	7.47	7.47
7.48	7.48	7.48	7.48	7.48	7.48	7.48	7.48	7.48
7.49	7.49	7.49	7.49	7.49	7.49	7.49	7.49	7.49

7.5	7.5	7.5	7.5	7.5	7.5	7.5	7.5	7.5
7.51	7.51	7.51	7.51	7.51	7.51	7.51	7.51	7.51
7.52	7.52	7.52	7.52	7.52	7.52	7.52	7.52	7.52
7.53	7.53	7.53	7.53	7.53	7.53	7.53	7.53	7.53
7.54	7.54	7.54	7.54	7.54	7.54	7.54	7.54	7.54
7.55	7.55	7.55	7.55	7.55	7.55	7.55	7.55	7.55
7.56	7.56	7.56	7.56	7.56	7.56	7.56	7.56	7.56
7.57	7.57	7.57	7.57	7.57	7.57	7.57	7.57	7.57
7.58	7.58	7.58	7.58	7.58	7.58	7.58	7.58	7.58
7.59	7.59	7.59	7.59	7.59	7.59	7.59	7.59	7.59
7.6	7.6	7.6	7.6	7.6	7.6	7.6	7.6	7.6
7.61	7.61	7.61	7.61	7.61	7.61	7.61	7.61	7.61
7.62	7.62	7.62	7.62	7.62	7.62	7.62	7.62	7.62
7.63	7.63	7.63	7.63	7.63	7.63	7.63	7.63	7.63
7.64	7.64	7.64	7.64	7.64	7.64	7.64	7.64	7.64
7.65	7.65	7.65	7.65	7.65	7.65	7.65	7.65	7.65
7.66	7.66	7.66	7.66	7.66	7.66	7.66	7.66	7.66
7.67	7.67	7.67	7.67	7.67	7.67	7.67	7.67	7.67
7.68	7.68	7.68	7.68	7.68	7.68	7.68	7.68	7.68
7.69	7.69	7.69	7.69	7.69	7.69	7.69	7.69	7.69
7.7	7.7	7.7	7.7	7.7	7.7	7.7	7.7	7.7
7.71	7.71	7.71	7.71	7.71	7.71	7.71	7.71	7.71
7.72	7.72	7.72	7.72	7.72	7.72	7.72	7.72	7.72
7.73	7.73	7.73	7.73	7.73	7.73	7.73	7.73	7.73
7.74	7.74	7.74	7.74	7.74	7.74	7.74	7.74	7.74
7.75	7.75	7.75	7.75	7.75	7.75	7.75	7.75	7.75
7.76	7.76	7.76	7.76	7.76	7.76	7.76	7.76	7.76
7.77	7.77	7.77	7.77	7.77	7.77	7.77	7.77	7.77
7.78	7.78	7.78	7.78	7.78	7.78	7.78	7.78	7.78
7.79	7.79	7.79	7.79	7.79	7.79	7.79	7.79	7.79
7.8	7.8	7.8	7.8	7.8	7.8	7.8	7.8	7.8
7.81	7.81	7.81	7.81	7.81	7.81	7.81	7.81	7.81
7.82	7.82	7.82	7.82	7.82	7.82	7.82	7.82	7.82
7.83	7.83	7.83	7.83	7.83	7.83	7.83	7.83	7.83
7.84	7.84	7.84	7.84	7.84	7.84	7.84	7.84	7.84
7.85	7.85	7.85	7.85	7.85	7.85	7.85	7.85	7.85
7.86	7.86	7.86	7.86	7.86	7.86	7.86	7.86	7.86
7.87	7.87	7.87	7.87	7.87	7.87	7.87	7.87	7.87
7.88	7.88	7.88	7.88	7.88	7.88	7.88	7.88	7.88
7.89	7.89	7.89	7.89	7.89	7.89	7.89	7.89	7.89
7.9	7.9	7.9	7.9	7.9	7.9	7.9	7.9	7.9
7.91	7.91	7.91	7.91	7.91	7.91	7.91	7.91	7.91
7.92	7.92	7.92	7.92	7.92	7.92	7.92	7.92	7.92
7.93	7.93	7.93	7.93	7.93	7.93	7.93	7.93	7.93
7.94	7.94	7.94	7.94	7.94	7.94	7.94	7.94	7.94
7.95	7.95	7.95	7.95	7.95	7.95	7.95	7.95	7.95
7.96	7.96	7.96	7.96	7.96	7.96	7.96	7.96	7.96
7.97	7.97	7.97	7.97	7.97	7.97	7.97	7.97	7.97
7.98	7.98	7.98	7.98	7.98	7.98	7.98	7.98	7.98
7.99	7.99	7.99	7.99	7.99	7.99	7.99	7.99	7.99

8	8	8	8	8	8	8	8	8
8.01	8.01	8.01	8.01	8.01	8.01	8.01	8.01	8.01
8.02	8.02	8.02	8.02	8.02	8.02	8.02	8.02	8.02
8.03	8.03	8.03	8.03	8.03	8.03	8.03	8.03	8.03
8.04	8.04	8.04	8.04	8.04	8.04	8.04	8.04	8.04
8.05	8.05	8.05	8.05	8.05	8.05	8.05	8.05	8.05
8.06	8.06	8.06	8.06	8.06	8.06	8.06	8.06	8.06
8.07	8.07	8.07	8.07	8.07	8.07	8.07	8.07	8.07
8.08	8.08	8.08	8.08	8.08	8.08	8.08	8.08	8.08
8.09	8.09	8.09	8.09	8.09	8.09	8.09	8.09	8.09
8.1	8.1	8.1	8.1	8.1	8.1	8.1	8.1	8.1
8.11	8.11	8.11	8.11	8.11	8.11	8.11	8.11	8.11
8.12	8.12	8.12	8.12	8.12	8.12	8.12	8.12	8.12
8.13	8.13	8.13	8.13	8.13	8.13	8.13	8.13	8.13
8.14	8.14	8.14	8.14	8.14	8.14	8.14	8.14	8.14
8.15	8.15	8.15	8.15	8.15	8.15	8.15	8.15	8.15
8.16	8.16	8.16	8.16	8.16	8.16	8.16	8.16	8.16
8.17	8.17	8.17	8.17	8.17	8.17	8.17	8.17	8.17
8.18	8.18	8.18	8.18	8.18	8.18	8.18	8.18	8.18
8.19	8.19	8.19	8.19	8.19	8.19	8.19	8.19	8.19
8.2	8.2	8.2	8.2	8.2	8.2	8.2	8.2	8.2
8.21	8.21	8.21	8.21	8.21	8.21	8.21	8.21	8.21
8.22	8.22	8.22	8.22	8.22	8.22	8.22	8.22	8.22
8.23	8.23	8.23	8.23	8.23	8.23	8.23	8.23	8.23
8.24	8.24	8.24	8.24	8.24	8.24	8.24	8.24	8.24
8.25	8.25	8.25	8.25	8.25	8.25	8.25	8.25	8.25
8.26	8.26	8.26	8.26	8.26	8.26	8.26	8.26	8.26
8.27	8.27	8.27	8.27	8.27	8.27	8.27	8.27	8.27
8.28	8.28	8.28	8.28	8.28	8.28	8.28	8.28	8.28
8.29	8.29	8.29	8.29	8.29	8.29	8.29	8.29	8.29
8.3	8.3	8.3	8.3	8.3	8.3	8.3	8.3	8.3
8.31	8.31	8.31	8.31	8.31	8.31	8.31	8.31	8.31
8.32	8.32	8.32	8.32	8.32	8.32	8.32	8.32	8.32
8.33	8.33	8.33	8.33	8.33	8.33	8.33	8.33	8.33
8.34	8.34	8.34	8.34	8.34	8.34	8.34	8.34	8.34
8.35	8.35	8.35	8.35	8.35	8.35	8.35	8.35	8.35
8.36	8.36	8.36	8.36	8.36	8.36	8.36	8.36	8.36
8.37	8.37	8.37	8.37	8.37	8.37	8.37	8.37	8.37
8.38	8.38	8.38	8.38	8.38	8.38	8.38	8.38	8.38
8.39	8.39	8.39	8.39	8.39	8.39	8.39	8.39	8.39
8.4	8.4	8.4	8.4	8.4	8.4	8.4	8.4	8.4
8.41	8.41	8.41	8.41	8.41	8.41	8.41	8.41	8.41
8.42	8.42	8.42	8.42	8.42	8.42	8.42	8.42	8.42
8.43	8.43	8.43	8.43	8.43	8.43	8.43	8.43	8.43
8.44	8.44	8.44	8.44	8.44	8.44	8.44	8.44	8.44
8.45	8.45	8.45	8.45	8.45	8.45	8.45	8.45	8.45
8.46	8.46	8.46	8.46	8.46	8.46	8.46	8.46	8.46
8.47	8.47	8.47	8.47	8.47	8.47	8.47	8.47	8.47
8.48	8.48	8.48	8.48	8.48	8.48	8.48	8.48	8.48
8.49	8.49	8.49	8.49	8.49	8.49	8.49	8.49	8.49

8.5	8.5	8.5	8.5	8.5	8.5	8.5	8.5	8.5
8.51	8.51	8.51	8.51	8.51	8.51	8.51	8.51	8.51
8.52	8.52	8.52	8.52	8.52	8.52	8.52	8.52	8.52
8.53	8.53	8.53	8.53	8.53	8.53	8.53	8.53	8.53
8.54	8.54	8.54	8.54	8.54	8.54	8.54	8.54	8.54
8.55	8.55	8.55	8.55	8.55	8.55	8.55	8.55	8.55
8.56	8.56	8.56	8.56	8.56	8.56	8.56	8.56	8.56
8.57	8.57	8.57	8.57	8.57	8.57	8.57	8.57	8.57
8.58	8.58	8.58	8.58	8.58	8.58	8.58	8.58	8.58
8.59	8.59	8.59	8.59	8.59	8.59	8.59	8.59	8.59
8.6	8.6	8.6	8.6	8.6	8.6	8.6	8.6	8.6
8.61	8.61	8.61	8.61	8.61	8.61	8.61	8.61	8.61
8.62	8.62	8.62	8.62	8.62	8.62	8.62	8.62	8.62
8.63	8.63	8.63	8.63	8.63	8.63	8.63	8.63	8.63
8.64	8.64	8.64	8.64	8.64	8.64	8.64	8.64	8.64
8.65	8.65	8.65	8.65	8.65	8.65	8.65	8.65	8.65
8.66	8.66	8.66	8.66	8.66	8.66	8.66	8.66	8.66
8.67	8.67	8.67	8.67	8.67	8.67	8.67	8.67	8.67
8.68	8.68	8.68	8.68	8.68	8.68	8.68	8.68	8.68
8.69	8.69	8.69	8.69	8.69	8.69	8.69	8.69	8.69
8.7	8.7	8.7	8.7	8.7	8.7	8.7	8.7	8.7
8.71	8.71	8.71	8.71	8.71	8.71	8.71	8.71	8.71
8.72	8.72	8.72	8.72	8.72	8.72	8.72	8.72	8.72
8.73	8.73	8.73	8.73	8.73	8.73	8.73	8.73	8.73
8.74	8.74	8.74	8.74	8.74	8.74	8.74	8.74	8.74
8.75	8.75	8.75	8.75	8.75	8.75	8.75	8.75	8.75
8.76	8.76	8.76	8.76	8.76	8.76	8.76	8.76	8.76
8.77	8.77	8.77	8.77	8.77	8.77	8.77	8.77	8.77
8.78	8.78	8.78	8.78	8.78	8.78	8.78	8.78	8.78
8.79	8.79	8.79	8.79	8.79	8.79	8.79	8.79	8.79
8.8	8.8	8.8	8.8	8.8	8.8	8.8	8.8	8.8
8.81	8.81	8.81	8.81	8.81	8.81	8.81	8.81	8.81
8.82	8.82	8.82	8.82	8.82	8.82	8.82	8.82	8.82
8.83	8.83	8.83	8.83	8.83	8.83	8.83	8.83	8.83
8.84	8.84	8.84	8.84	8.84	8.84	8.84	8.84	8.84
8.85	8.85	8.85	8.85	8.85	8.85	8.85	8.85	8.85
8.86	8.86	8.86	8.86	8.86	8.86	8.86	8.86	8.86
8.87	8.87	8.87	8.87	8.87	8.87	8.87	8.87	8.87
8.88	8.88	8.88	8.88	8.88	8.88	8.88	8.88	8.88
8.89	8.89	8.89	8.89	8.89	8.89	8.89	8.89	8.89
8.9	8.9	8.9	8.9	8.9	8.9	8.9	8.9	8.9
8.91	8.91	8.91	8.91	8.91	8.91	8.91	8.91	8.91
8.92	8.92	8.92	8.92	8.92	8.92	8.92	8.92	8.92
8.93	8.93	8.93	8.93	8.93	8.93	8.93	8.93	8.93
8.94	8.94	8.94	8.94	8.94	8.94	8.94	8.94	8.94
8.95	8.95	8.95	8.95	8.95	8.95	8.95	8.95	8.95
8.96	8.96	8.96	8.96	8.96	8.96	8.96	8.96	8.96
8.97	8.97	8.97	8.97	8.97	8.97	8.97	8.97	8.97
8.98	8.98	8.98	8.98	8.98	8.98	8.98	8.98	8.98
8.99	8.99	8.99	8.99	8.99	8.99	8.99	8.99	8.99

9	9	9	9	9	9	9	9	9
9.01	9.01	9.01	9.01	9.01	9.01	9.01	9.01	9.01
9.02	9.02	9.02	9.02	9.02	9.02	9.02	9.02	9.02
9.03	9.03	9.03	9.03	9.03	9.03	9.03	9.03	9.03
9.04	9.04	9.04	9.04	9.04	9.04	9.04	9.04	9.04
9.05	9.05	9.05	9.05	9.05	9.05	9.05	9.05	9.05
9.06	9.06	9.06	9.06	9.06	9.06	9.06	9.06	9.06
9.07	9.07	9.07	9.07	9.07	9.07	9.07	9.07	9.07
9.08	9.08	9.08	9.08	9.08	9.08	9.08	9.08	9.08
9.09	9.09	9.09	9.09	9.09	9.09	9.09	9.09	9.09
9.1	9.1	9.1	9.1	9.1	9.1	9.1	9.1	9.1
9.11	9.11	9.11	9.11	9.11	9.11	9.11	9.11	9.11
9.12	9.12	9.12	9.12	9.12	9.12	9.12	9.12	9.12
9.13	9.13	9.13	9.13	9.13	9.13	9.13	9.13	9.13
9.14	9.14	9.14	9.14	9.14	9.14	9.14	9.14	9.14
9.15	9.15	9.15	9.15	9.15	9.15	9.15	9.15	9.15
9.16	9.16	9.16	9.16	9.16	9.16	9.16	9.16	9.16
9.17	9.17	9.17	9.17	9.17	9.17	9.17	9.17	9.17
9.18	9.18	9.18	9.18	9.18	9.18	9.18	9.18	9.18
9.19	9.19	9.19	9.19	9.19	9.19	9.19	9.19	9.19
9.2	9.2	9.2	9.2	9.2	9.2	9.2	9.2	9.2
9.21	9.21	9.21	9.21	9.21	9.21	9.21	9.21	9.21
9.22	9.22	9.22	9.22	9.22	9.22	9.22	9.22	9.22
9.23	9.23	9.23	9.23	9.23	9.23	9.23	9.23	9.23
9.24	9.24	9.24	9.24	9.24	9.24	9.24	9.24	9.24
9.25	9.25	9.25	9.25	9.25	9.25	9.25	9.25	9.25
9.26	9.26	9.26	9.26	9.26	9.26	9.26	9.26	9.26
9.27	9.27	9.27	9.27	9.27	9.27	9.27	9.27	9.27
9.28	9.28	9.28	9.28	9.28	9.28	9.28	9.28	9.28
9.29	9.29	9.29	9.29	9.29	9.29	9.29	9.29	9.29
9.3	9.3	9.3	9.3	9.3	9.3	9.3	9.3	9.3
9.31	9.31	9.31	9.31	9.31	9.31	9.31	9.31	9.31
9.32	9.32	9.32	9.32	9.32	9.32	9.32	9.32	9.32
9.33	9.33	9.33	9.33	9.33	9.33	9.33	9.33	9.33
9.34	9.34	9.34	9.34	9.34	9.34	9.34	9.34	9.34
9.35	9.35	9.35	9.35	9.35	9.35	9.35	9.35	9.35
9.36	9.36	9.36	9.36	9.36	9.36	9.36	9.36	9.36
9.37	9.37	9.37	9.37	9.37	9.37	9.37	9.37	9.37
9.38	9.38	9.38	9.38	9.38	9.38	9.38	9.38	9.38
9.39	9.39	9.39	9.39	9.39	9.39	9.39	9.39	9.39
9.4	9.4	9.4	9.4	9.4	9.4	9.4	9.4	9.4
9.41	9.41	9.41	9.41	9.41	9.41	9.41	9.41	9.41
9.42	9.42	9.42	9.42	9.42	9.42	9.42	9.42	9.42
9.43	9.43	9.43	9.43	9.43	9.43	9.43	9.43	9.43
9.44	9.44	9.44	9.44	9.44	9.44	9.44	9.44	9.44
9.45	9.45	9.45	9.45	9.45	9.45	9.45	9.45	9.45
9.46	9.46	9.46	9.46	9.46	9.46	9.46	9.46	9.46
9.47	9.47	9.47	9.47	9.47	9.47	9.47	9.47	9.47
9.48	9.48	9.48	9.48	9.48	9.48	9.48	9.48	9.48
9.49	9.49	9.49	9.49	9.49	9.49	9.49	9.49	9.49

9.5	9.5	9.5	9.5	9.5	9.5	9.5	9.5	9.5
9.51	9.51	9.51	9.51	9.51	9.51	9.51	9.51	9.51
9.52	9.52	9.52	9.52	9.52	9.52	9.52	9.52	9.52
9.53	9.53	9.53	9.53	9.53	9.53	9.53	9.53	9.53
9.54	9.54	9.54	9.54	9.54	9.54	9.54	9.54	9.54
9.55	9.55	9.55	9.55	9.55	9.55	9.55	9.55	9.55
9.56	9.56	9.56	9.56	9.56	9.56	9.56	9.56	9.56
9.57	9.57	9.57	9.57	9.57	9.57	9.57	9.57	9.57
9.58	9.58	9.58	9.58	9.58	9.58	9.58	9.58	9.58
9.59	9.59	9.59	9.59	9.59	9.59	9.59	9.59	9.59
9.6	9.6	9.6	9.6	9.6	9.6	9.6	9.6	9.6
9.61	9.61	9.61	9.61	9.61	9.61	9.61	9.61	9.61
9.62	9.62	9.62	9.62	9.62	9.62	9.62	9.62	9.62
9.63	9.63	9.63	9.63	9.63	9.63	9.63	9.63	9.63
9.64	9.64	9.64	9.64	9.64	9.64	9.64	9.64	9.64
9.65	9.65	9.65	9.65	9.65	9.65	9.65	9.65	9.65
9.66	9.66	9.66	9.66	9.66	9.66	9.66	9.66	9.66
9.67	9.67	9.67	9.67	9.67	9.67	9.67	9.67	9.67
9.68	9.68	9.68	9.68	9.68	9.68	9.68	9.68	9.68
9.69	9.69	9.69	9.69	9.69	9.69	9.69	9.69	9.69
9.7	9.7	9.7	9.7	9.7	9.7	9.7	9.7	9.7
9.71	9.71	9.71	9.71	9.71	9.71	9.71	9.71	9.71
9.72	9.72	9.72	9.72	9.72	9.72	9.72	9.72	9.72
9.73	9.73	9.73	9.73	9.73	9.73	9.73	9.73	9.73
9.74	9.74	9.74	9.74	9.74	9.74	9.74	9.74	9.74
9.75	9.75	9.75	9.75	9.75	9.75	9.75	9.75	9.75
9.76	9.76	9.76	9.76	9.76	9.76	9.76	9.76	9.76
9.77	9.77	9.77	9.77	9.77	9.77	9.77	9.77	9.77
9.78	9.78	9.78	9.78	9.78	9.78	9.78	9.78	9.78
9.79	9.79	9.79	9.79	9.79	9.79	9.79	9.79	9.79
9.8	9.8	9.8	9.8	9.8	9.8	9.8	9.8	9.8
9.81	9.81	9.81	9.81	9.81	9.81	9.81	9.81	9.81
9.82	9.82	9.82	9.82	9.82	9.82	9.82	9.82	9.82
9.83	9.83	9.83	9.83	9.83	9.83	9.83	9.83	9.83
9.84	9.84	9.84	9.84	9.84	9.84	9.84	9.84	9.84
9.85	9.85	9.85	9.85	9.85	9.85	9.85	9.85	9.85
9.86	9.86	9.86	9.86	9.86	9.86	9.86	9.86	9.86
9.87	9.87	9.87	9.87	9.87	9.87	9.87	9.87	9.87
9.88	9.88	9.88	9.88	9.88	9.88	9.88	9.88	9.88
9.89	9.89	9.89	9.89	9.89	9.89	9.89	9.89	9.89
9.9	9.9	9.9	9.9	9.9	9.9	9.9	9.9	9.9
9.91	9.91	9.91	9.91	9.91	9.91	9.91	9.91	9.91
9.92	9.92	9.92	9.92	9.92	9.92	9.92	9.92	9.92
9.93	9.93	9.93	9.93	9.93	9.93	9.93	9.93	9.93
9.94	9.94	9.94	9.94	9.94	9.94	9.94	9.94	9.94
9.95	9.95	9.95	9.95	9.95	9.95	9.95	9.95	9.95
9.96	9.96	9.96	9.96	9.96	9.96	9.96	9.96	9.96
9.97	9.97	9.97	9.97	9.97	9.97	9.97	9.97	9.97
9.98	9.98	9.98	9.98	9.98	9.98	9.98	9.98	9.98
9.99	9.99	9.99	9.99	9.99	9.99	9.99	9.99	9.99

10	10	10	10	10	10	10	10	10
10.01	10.01	10.01	10.01	10.01	10.01	10.01	10.01	10.01
10.02	10.02	10.02	10.02	10.02	10.02	10.02	10.02	10.02
10.03	10.03	10.03	10.03	10.03	10.03	10.03	10.03	10.03
10.04	10.04	10.04	10.04	10.04	10.04	10.04	10.04	10.04
10.05	10.05	10.05	10.05	10.05	10.05	10.05	10.05	10.05
10.06	10.06	10.06	10.06	10.06	10.06	10.06	10.06	10.06
10.07	10.07	10.07	10.07	10.07	10.07	10.07	10.07	10.07
10.08	10.08	10.08	10.08	10.08	10.08	10.08	10.08	10.08
10.09	10.09	10.09	10.09	10.09	10.09	10.09	10.09	10.09
10.1	10.1	10.1	10.1	10.1	10.1	10.1	10.1	10.1
10.11	10.11	10.11	10.11	10.11	10.11	10.11	10.11	10.11
10.12	10.12	10.12	10.12	10.12	10.12	10.12	10.12	10.12
10.13	10.13	10.13	10.13	10.13	10.13	10.13	10.13	10.13
10.14	10.14	10.14	10.14	10.14	10.14	10.14	10.14	10.14
10.15	10.15	10.15	10.15	10.15	10.15	10.15	10.15	10.15
10.16	10.16	10.16	10.16	10.16	10.16	10.16	10.16	10.16
10.17	10.17	10.17	10.17	10.17	10.17	10.17	10.17	10.17
10.18	10.18	10.18	10.18	10.18	10.18	10.18	10.18	10.18
10.19	10.19	10.19	10.19	10.19	10.19	10.19	10.19	10.19
10.2	10.2	10.2	10.2	10.2	10.2	10.2	10.2	10.2
10.21	10.21	10.21	10.21	10.21	10.21	10.21	10.21	10.21
10.22	10.22	10.22	10.22	10.22	10.22	10.22	10.22	10.22
10.23	10.23	10.23	10.23	10.23	10.23	10.23	10.23	10.23
10.24	10.24	10.24	10.24	10.24	10.24	10.24	10.24	10.24
10.25	10.25	10.25	10.25	10.25	10.25	10.25	10.25	10.25
10.26	10.26	10.26	10.26	10.26	10.26	10.26	10.26	10.26
10.27	10.27	10.27	10.27	10.27	10.27	10.27	10.27	10.27
10.28	10.28	10.28	10.28	10.28	10.28	10.28	10.28	10.28
10.29	10.29	10.29	10.29	10.29	10.29	10.29	10.29	10.29
10.3	10.3	10.3	10.3	10.3	10.3	10.3	10.3	10.3
10.31	10.31	10.31	10.31	10.31	10.31	10.31	10.31	10.31
10.32	10.32	10.32	10.32	10.32	10.32	10.32	10.32	10.32
10.33	10.33	10.33	10.33	10.33	10.33	10.33	10.33	10.33
10.34	10.34	10.34	10.34	10.34	10.34	10.34	10.34	10.34
10.35	10.35	10.35	10.35	10.35	10.35	10.35	10.35	10.35
10.36	10.36	10.36	10.36	10.36	10.36	10.36	10.36	10.36
10.37	10.37	10.37	10.37	10.37	10.37	10.37	10.37	10.37
10.38	10.38	10.38	10.38	10.38	10.38	10.38	10.38	10.38
10.39	10.39	10.39	10.39	10.39	10.39	10.39	10.39	10.39
10.4	10.4	10.4	10.4	10.4	10.4	10.4	10.4	10.4
10.41	10.41	10.41	10.41	10.41	10.41	10.41	10.41	10.41
10.42	10.42	10.42	10.42	10.42	10.42	10.42	10.42	10.42
10.43	10.43	10.43	10.43	10.43	10.43	10.43	10.43	10.43
10.44	10.44	10.44	10.44	10.44	10.44	10.44	10.44	10.44
10.45	10.45	10.45	10.45	10.45	10.45	10.45	10.45	10.45
10.46	10.46	10.46	10.46	10.46	10.46	10.46	10.46	10.46
10.47	10.47	10.47	10.47	10.47	10.47	10.47	10.47	10.47
10.48	10.48	10.48	10.48	10.48	10.48	10.48	10.48	10.48
10.49	10.49	10.49	10.49	10.49	10.49	10.49	10.49	10.49

10.5	10.5	10.5	10.5	10.5	10.5	10.5	10.5	10.5
10.51	10.51	10.51	10.51	10.51	10.51	10.51	10.51	10.51
10.52	10.52	10.52	10.52	10.52	10.52	10.52	10.52	10.52
10.53	10.53	10.53	10.53	10.53	10.53	10.53	10.53	10.53
10.54	10.54	10.54	10.54	10.54	10.54	10.54	10.54	10.54
10.55	10.55	10.55	10.55	10.55	10.55	10.55	10.55	10.55
10.56	10.56	10.56	10.56	10.56	10.56	10.56	10.56	10.56
10.57	10.57	10.57	10.57	10.57	10.57	10.57	10.57	10.57
10.58	10.58	10.58	10.58	10.58	10.58	10.58	10.58	10.58
10.59	10.59	10.59	10.59	10.59	10.59	10.59	10.59	10.59
10.6	10.6	10.6	10.6	10.6	10.6	10.6	10.6	10.6
10.61	10.61	10.61	10.61	10.61	10.61	10.61	10.61	10.61
10.62	10.62	10.62	10.62	10.62	10.62	10.62	10.62	10.62
10.63	10.63	10.63	10.63	10.63	10.63	10.63	10.63	10.63
10.64	10.64	10.64	10.64	10.64	10.64	10.64	10.64	10.64
10.65	10.65	10.65	10.65	10.65	10.65	10.65	10.65	10.65
10.66	10.66	10.66	10.66	10.66	10.66	10.66	10.66	10.66
10.67	10.67	10.67	10.67	10.67	10.67	10.67	10.67	10.67
10.68	10.68	10.68	10.68	10.68	10.68	10.68	10.68	10.68
10.69	10.69	10.69	10.69	10.69	10.69	10.69	10.69	10.69
10.7	10.7	10.7	10.7	10.7	10.7	10.7	10.7	10.7
10.71	10.71	10.71	10.71	10.71	10.71	10.71	10.71	10.71
10.72	10.72	10.72	10.72	10.72	10.72	10.72	10.72	10.72
10.73	10.73	10.73	10.73	10.73	10.73	10.73	10.73	10.73
10.74	10.74	10.74	10.74	10.74	10.74	10.74	10.74	10.74
10.75	10.75	10.75	10.75	10.75	10.75	10.75	10.75	10.75
10.76	10.76	10.76	10.76	10.76	10.76	10.76	10.76	10.76
10.77	10.77	10.77	10.77	10.77	10.77	10.77	10.77	10.77
10.78	10.78	10.78	10.78	10.78	10.78	10.78	10.78	10.78
10.79	10.79	10.79	10.79	10.79	10.79	10.79	10.79	10.79
10.8	10.8	10.8	10.8	10.8	10.8	10.8	10.8	10.8
10.81	10.81	10.81	10.81	10.81	10.81	10.81	10.81	10.81
10.82	10.82	10.82	10.82	10.82	10.82	10.82	10.82	10.82
10.83	10.83	10.83	10.83	10.83	10.83	10.83	10.83	10.83
10.84	10.84	10.84	10.84	10.84	10.84	10.84	10.84	10.84
10.85	10.85	10.85	10.85	10.85	10.85	10.85	10.85	10.85
10.86	10.86	10.86	10.86	10.86	10.86	10.86	10.86	10.86
10.87	10.87	10.87	10.87	10.87	10.87	10.87	10.87	10.87
10.88	10.88	10.88	10.88	10.88	10.88	10.88	10.88	10.88
10.89	10.89	10.89	10.89	10.89	10.89	10.89	10.89	10.89
10.9	10.9	10.9	10.9	10.9	10.9	10.9	10.9	10.9
10.91	10.91	10.91	10.91	10.91	10.91	10.91	10.91	10.91
10.92	10.92	10.92	10.92	10.92	10.92	10.92	10.92	10.92
10.93	10.93	10.93	10.93	10.93	10.93	10.93	10.93	10.93
10.94	10.94	10.94	10.94	10.94	10.94	10.94	10.94	10.94
10.95	10.95	10.95	10.95	10.95	10.95	10.95	10.95	10.95
10.96	10.96	10.96	10.96	10.96	10.96	10.96	10.96	10.96
10.97	10.97	10.97	10.97	10.97	10.97	10.97	10.97	10.97
10.98	10.98	10.98	10.98	10.98	10.98	10.98	10.98	10.98
10.99	10.99	10.99	10.99	10.99	10.99	10.99	10.99	10.99

11	11	11	11	11	11	11	11	11
11.01	11.01	11.01	11.01	11.01	11.01	11.01	11.01	11.01
11.02	11.02	11.02	11.02	11.02	11.02	11.02	11.02	11.02
11.03	11.03	11.03	11.03	11.03	11.03	11.03	11.03	11.03
11.04	11.04	11.04	11.04	11.04	11.04	11.04	11.04	11.04
11.05	11.05	11.05	11.05	11.05	11.05	11.05	11.05	11.05
11.06	11.06	11.06	11.06	11.06	11.06	11.06	11.06	11.06
11.07	11.07	11.07	11.07	11.07	11.07	11.07	11.07	11.07
11.08	11.08	11.08	11.08	11.08	11.08	11.08	11.08	11.08
11.09	11.09	11.09	11.09	11.09	11.09	11.09	11.09	11.09
11.1	11.1	11.1	11.1	11.1	11.1	11.1	11.1	11.1
11.11	11.11	11.11	11.11	11.11	11.11	11.11	11.11	11.11
11.12	11.12	11.12	11.12	11.12	11.12	11.12	11.12	11.12
11.13	11.13	11.13	11.13	11.13	11.13	11.13	11.13	11.13
11.14	11.14	11.14	11.14	11.14	11.14	11.14	11.14	11.14
11.15	11.15	11.15	11.15	11.15	11.15	11.15	11.15	11.15
11.16	11.16	11.16	11.16	11.16	11.16	11.16	11.16	11.16
11.17	11.17	11.17	11.17	11.17	11.17	11.17	11.17	11.17
11.18	11.18	11.18	11.18	11.18	11.18	11.18	11.18	11.18
11.19	11.19	11.19	11.19	11.19	11.19	11.19	11.19	11.19
11.2	11.2	11.2	11.2	11.2	11.2	11.2	11.2	11.2
11.21	11.21	11.21	11.21	11.21	11.21	11.21	11.21	11.21
11.22	11.22	11.22	11.22	11.22	11.22	11.22	11.22	11.22
11.23	11.23	11.23	11.23	11.23	11.23	11.23	11.23	11.23
11.24	11.24	11.24	11.24	11.24	11.24	11.24	11.24	11.24
11.25	11.25	11.25	11.25	11.25	11.25	11.25	11.25	11.25
11.26	11.26	11.26	11.26	11.26	11.26	11.26	11.26	11.26
11.27	11.27	11.27	11.27	11.27	11.27	11.27	11.27	11.27
11.28	11.28	11.28	11.28	11.28	11.28	11.28	11.28	11.28
11.29	11.29	11.29	11.29	11.29	11.29	11.29	11.29	11.29
11.3	11.3	11.3	11.3	11.3	11.3	11.3	11.3	11.3
11.31	11.31	11.31	11.31	11.31	11.31	11.31	11.31	11.31
11.32	11.32	11.32	11.32	11.32	11.32	11.32	11.32	11.32
11.33	11.33	11.33	11.33	11.33	11.33	11.33	11.33	11.33
11.34	11.34	11.34	11.34	11.34	11.34	11.34	11.34	11.34
11.35	11.35	11.35	11.35	11.35	11.35	11.35	11.35	11.35
11.36	11.36	11.36	11.36	11.36	11.36	11.36	11.36	11.36
11.37	11.37	11.37	11.37	11.37	11.37	11.37	11.37	11.37
11.38	11.38	11.38	11.38	11.38	11.38	11.38	11.38	11.38
11.39	11.39	11.39	11.39	11.39	11.39	11.39	11.39	11.39
11.4	11.4	11.4	11.4	11.4	11.4	11.4	11.4	11.4
11.41	11.41	11.41	11.41	11.41	11.41	11.41	11.41	11.41
11.42	11.42	11.42	11.42	11.42	11.42	11.42	11.42	11.42
11.43	11.43	11.43	11.43	11.43	11.43	11.43	11.43	11.43
11.44	11.44	11.44	11.44	11.44	11.44	11.44	11.44	11.44
11.45	11.45	11.45	11.45	11.45	11.45	11.45	11.45	11.45
11.46	11.46	11.46	11.46	11.46	11.46	11.46	11.46	11.46
11.47	11.47	11.47	11.47	11.47	11.47	11.47	11.47	11.47
11.48	11.48	11.48	11.48	11.48	11.48	11.48	11.48	11.48
11.49	11.49	11.49	11.49	11.49	11.49	11.49	11.49	11.49

11.5	11.5	11.5	11.5	11.5	11.5	11.5	11.5	11.5
11.51	11.51	11.51	11.51	11.51	11.51	11.51	11.51	11.51
11.52	11.52	11.52	11.52	11.52	11.52	11.52	11.52	11.52
11.53	11.53	11.53	11.53	11.53	11.53	11.53	11.53	11.53
11.54	11.54	11.54	11.54	11.54	11.54	11.54	11.54	11.54
11.55	11.55	11.55	11.55	11.55	11.55	11.55	11.55	11.55
11.56	11.56	11.56	11.56	11.56	11.56	11.56	11.56	11.56
11.57	11.57	11.57	11.57	11.57	11.57	11.57	11.57	11.57
11.58	11.58	11.58	11.58	11.58	11.58	11.58	11.58	11.58
11.59	11.59	11.59	11.59	11.59	11.59	11.59	11.59	11.59
11.6	11.6	11.6	11.6	11.6	11.6	11.6	11.6	11.6
11.61	11.61	11.61	11.61	11.61	11.61	11.61	11.61	11.61
11.62	11.62	11.62	11.62	11.62	11.62	11.62	11.62	11.62
11.63	11.63	11.63	11.63	11.63	11.63	11.63	11.63	11.63
11.64	11.64	11.64	11.64	11.64	11.64	11.64	11.64	11.64
11.65	11.65	11.65	11.65	11.65	11.65	11.65	11.65	11.65
11.66	11.66	11.66	11.66	11.66	11.66	11.66	11.66	11.66
11.67	11.67	11.67	11.67	11.67	11.67	11.67	11.67	11.67
11.68	11.68	11.68	11.68	11.68	11.68	11.68	11.68	11.68
11.69	11.69	11.69	11.69	11.69	11.69	11.69	11.69	11.69
11.7	11.7	11.7	11.7	11.7	11.7	11.7	11.7	11.7
11.71	11.71	11.71	11.71	11.71	11.71	11.71	11.71	11.71
11.72	11.72	11.72	11.72	11.72	11.72	11.72	11.72	11.72
11.73	11.73	11.73	11.73	11.73	11.73	11.73	11.73	11.73
11.74	11.74	11.74	11.74	11.74	11.74	11.74	11.74	11.74
11.75	11.75	11.75	11.75	11.75	11.75	11.75	11.75	11.75
11.76	11.76	11.76	11.76	11.76	11.76	11.76	11.76	11.76
11.77	11.77	11.77	11.77	11.77	11.77	11.77	11.77	11.77
11.78	11.78	11.78	11.78	11.78	11.78	11.78	11.78	11.78
11.79	11.79	11.79	11.79	11.79	11.79	11.79	11.79	11.79
11.8	11.8	11.8	11.8	11.8	11.8	11.8	11.8	11.8
11.81	11.81	11.81	11.81	11.81	11.81	11.81	11.81	11.81
11.82	11.82	11.82	11.82	11.82	11.82	11.82	11.82	11.82
11.83	11.83	11.83	11.83	11.83	11.83	11.83	11.83	11.83
11.84	11.84	11.84	11.84	11.84	11.84	11.84	11.84	11.84
11.85	11.85	11.85	11.85	11.85	11.85	11.85	11.85	11.85
11.86	11.86	11.86	11.86	11.86	11.86	11.86	11.86	11.86
11.87	11.87	11.87	11.87	11.87	11.87	11.87	11.87	11.87
11.88	11.88	11.88	11.88	11.88	11.88	11.88	11.88	11.88
11.89	11.89	11.89	11.89	11.89	11.89	11.89	11.89	11.89
11.9	11.9	11.9	11.9	11.9	11.9	11.9	11.9	11.9
11.91	11.91	11.91	11.91	11.91	11.91	11.91	11.91	11.91
11.92	11.92	11.92	11.92	11.92	11.92	11.92	11.92	11.92
11.93	11.93	11.93	11.93	11.93	11.93	11.93	11.93	11.93
11.94	11.94	11.94	11.94	11.94	11.94	11.94	11.94	11.94
11.95	11.95	11.95	11.95	11.95	11.95	11.95	11.95	11.95
11.96	11.96	11.96	11.96	11.96	11.96	11.96	11.96	11.96
11.97	11.97	11.97	11.97	11.97	11.97	11.97	11.97	11.97
11.98	11.98	11.98	11.98	11.98	11.98	11.98	11.98	11.98
11.99	11.99	11.99	11.99	11.99	11.99	11.99	11.99	11.99

12	12	12	12	12	12	12	12	12

-12	-12	-12	-12	-12	-12	-12	-12	-12
-11.99	-11.99	-11.99	-11.99	-11.99	-11.99	-11.99	-11.99	-11.99
-11.98	-11.98	-11.98	-11.98	-11.98	-11.98	-11.98	-11.98	-11.98
-11.97	-11.97	-11.97	-11.97	-11.97	-11.97	-11.97	-11.97	-11.97
-11.96	-11.96	-11.96	-11.96	-11.96	-11.96	-11.96	-11.96	-11.96
-11.95	-11.95	-11.95	-11.95	-11.95	-11.95	-11.95	-11.95	-11.95
-11.94	-11.94	-11.94	-11.94	-11.94	-11.94	-11.94	-11.94	-11.94
-11.93	-11.93	-11.93	-11.93	-11.93	-11.93	-11.93	-11.93	-11.93
-11.92	-11.92	-11.92	-11.92	-11.92	-11.92	-11.92	-11.92	-11.92
-11.91	-11.91	-11.91	-11.91	-11.91	-11.91	-11.91	-11.91	-11.91
-11.9	-11.9	-11.9	-11.9	-11.9	-11.9	-11.9	-11.9	-11.9
-11.89	-11.89	-11.89	-11.89	-11.89	-11.89	-11.89	-11.89	-11.89
-11.88	-11.88	-11.88	-11.88	-11.88	-11.88	-11.88	-11.88	-11.88
-11.87	-11.87	-11.87	-11.87	-11.87	-11.87	-11.87	-11.87	-11.87
-11.86	-11.86	-11.86	-11.86	-11.86	-11.86	-11.86	-11.86	-11.86
-11.85	-11.85	-11.85	-11.85	-11.85	-11.85	-11.85	-11.85	-11.85
-11.84	-11.84	-11.84	-11.84	-11.84	-11.84	-11.84	-11.84	-11.84
-11.83	-11.83	-11.83	-11.83	-11.83	-11.83	-11.83	-11.83	-11.83
-11.82	-11.82	-11.82	-11.82	-11.82	-11.82	-11.82	-11.82	-11.82
-11.81	-11.81	-11.81	-11.81	-11.81	-11.81	-11.81	-11.81	-11.81
-11.8	-11.8	-11.8	-11.8	-11.8	-11.8	-11.8	-11.8	-11.8
-11.79	-11.79	-11.79	-11.79	-11.79	-11.79	-11.79	-11.79	-11.79
-11.78	-11.78	-11.78	-11.78	-11.78	-11.78	-11.78	-11.78	-11.78
-11.77	-11.77	-11.77	-11.77	-11.77	-11.77	-11.77	-11.77	-11.77
-11.76	-11.76	-11.76	-11.76	-11.76	-11.76	-11.76	-11.76	-11.76
-11.75	-11.75	-11.75	-11.75	-11.75	-11.75	-11.75	-11.75	-11.75
-11.74	-11.74	-11.74	-11.74	-11.74	-11.74	-11.74	-11.74	-11.74
-11.73	-11.73	-11.73	-11.73	-11.73	-11.73	-11.73	-11.73	-11.73
-11.72	-11.72	-11.72	-11.72	-11.72	-11.72	-11.72	-11.72	-11.72
-11.71	-11.71	-11.71	-11.71	-11.71	-11.71	-11.71	-11.71	-11.71
-11.7	-11.7	-11.7	-11.7	-11.7	-11.7	-11.7	-11.7	-11.7
-11.69	-11.69	-11.69	-11.69	-11.69	-11.69	-11.69	-11.69	-11.69
-11.68	-11.68	-11.68	-11.68	-11.68	-11.68	-11.68	-11.68	-11.68
-11.67	-11.67	-11.67	-11.67	-11.67	-11.67	-11.67	-11.67	-11.67
-11.66	-11.66	-11.66	-11.66	-11.66	-11.66	-11.66	-11.66	-11.66
-11.65	-11.65	-11.65	-11.65	-11.65	-11.65	-11.65	-11.65	-11.65
-11.64	-11.64	-11.64	-11.64	-11.64	-11.64	-11.64	-11.64	-11.64
-11.63	-11.63	-11.63	-11.63	-11.63	-11.63	-11.63	-11.63	-11.63
-11.62	-11.62	-11.62	-11.62	-11.62	-11.62	-11.62	-11.62	-11.62
-11.61	-11.61	-11.61	-11.61	-11.61	-11.61	-11.61	-11.61	-11.61
-11.6	-11.6	-11.6	-11.6	-11.6	-11.6	-11.6	-11.6	-11.6
-11.59	-11.59	-11.59	-11.59	-11.59	-11.59	-11.59	-11.59	-11.59
-11.58	-11.58	-11.58	-11.58	-11.58	-11.58	-11.58	-11.58	-11.58
-11.57	-11.57	-11.57	-11.57	-11.57	-11.57	-11.57	-11.57	-11.57
-11.56	-11.56	-11.56	-11.56	-11.56	-11.56	-11.56	-11.56	-11.56
-11.55	-11.55	-11.55	-11.55	-11.55	-11.55	-11.55	-11.55	-11.55
-11.54	-11.54	-11.54	-11.54	-11.54	-11.54	-11.54	-11.54	-11.54
-11.53	-11.53	-11.53	-11.53	-11.53	-11.53	-11.53	-11.53	-11.53
-11.52	-11.52	-11.52	-11.52	-11.52	-11.52	-11.52	-11.52	-11.52
-11.51	-11.51	-11.51	-11.51	-11.51	-11.51	-11.51	-11.51	-11.51

-11.5	-11.5	-11.5	-11.5	-11.5	-11.5	-11.5	-11.5	-11.5
-11.49	-11.49	-11.49	-11.49	-11.49	-11.49	-11.49	-11.49	-11.49
-11.48	-11.48	-11.48	-11.48	-11.48	-11.48	-11.48	-11.48	-11.48
-11.47	-11.47	-11.47	-11.47	-11.47	-11.47	-11.47	-11.47	-11.47
-11.46	-11.46	-11.46	-11.46	-11.46	-11.46	-11.46	-11.46	-11.46
-11.45	-11.45	-11.45	-11.45	-11.45	-11.45	-11.45	-11.45	-11.45
-11.44	-11.44	-11.44	-11.44	-11.44	-11.44	-11.44	-11.44	-11.44
-11.43	-11.43	-11.43	-11.43	-11.43	-11.43	-11.43	-11.43	-11.43
-11.42	-11.42	-11.42	-11.42	-11.42	-11.42	-11.42	-11.42	-11.42
-11.41	-11.41	-11.41	-11.41	-11.41	-11.41	-11.41	-11.41	-11.41
-11.4	-11.4	-11.4	-11.4	-11.4	-11.4	-11.4	-11.4	-11.4
-11.39	-11.39	-11.39	-11.39	-11.39	-11.39	-11.39	-11.39	-11.39
-11.38	-11.38	-11.38	-11.38	-11.38	-11.38	-11.38	-11.38	-11.38
-11.37	-11.37	-11.37	-11.37	-11.37	-11.37	-11.37	-11.37	-11.37
-11.36	-11.36	-11.36	-11.36	-11.36	-11.36	-11.36	-11.36	-11.36
-11.35	-11.35	-11.35	-11.35	-11.35	-11.35	-11.35	-11.35	-11.35
-11.34	-11.34	-11.34	-11.34	-11.34	-11.34	-11.34	-11.34	-11.34
-11.33	-11.33	-11.33	-11.33	-11.33	-11.33	-11.33	-11.33	-11.33
-11.32	-11.32	-11.32	-11.32	-11.32	-11.32	-11.32	-11.32	-11.32
-11.31	-11.31	-11.31	-11.31	-11.31	-11.31	-11.31	-11.31	-11.31
-11.3	-11.3	-11.3	-11.3	-11.3	-11.3	-11.3	-11.3	-11.3
-11.29	-11.29	-11.29	-11.29	-11.29	-11.29	-11.29	-11.29	-11.29
-11.28	-11.28	-11.28	-11.28	-11.28	-11.28	-11.28	-11.28	-11.28
-11.27	-11.27	-11.27	-11.27	-11.27	-11.27	-11.27	-11.27	-11.27
-11.26	-11.26	-11.26	-11.26	-11.26	-11.26	-11.26	-11.26	-11.26
-11.25	-11.25	-11.25	-11.25	-11.25	-11.25	-11.25	-11.25	-11.25
-11.24	-11.24	-11.24	-11.24	-11.24	-11.24	-11.24	-11.24	-11.24
-11.23	-11.23	-11.23	-11.23	-11.23	-11.23	-11.23	-11.23	-11.23
-11.22	-11.22	-11.22	-11.22	-11.22	-11.22	-11.22	-11.22	-11.22
-11.21	-11.21	-11.21	-11.21	-11.21	-11.21	-11.21	-11.21	-11.21
-11.2	-11.2	-11.2	-11.2	-11.2	-11.2	-11.2	-11.2	-11.2
-11.19	-11.19	-11.19	-11.19	-11.19	-11.19	-11.19	-11.19	-11.19
-11.18	-11.18	-11.18	-11.18	-11.18	-11.18	-11.18	-11.18	-11.18
-11.17	-11.17	-11.17	-11.17	-11.17	-11.17	-11.17	-11.17	-11.17
-11.16	-11.16	-11.16	-11.16	-11.16	-11.16	-11.16	-11.16	-11.16
-11.15	-11.15	-11.15	-11.15	-11.15	-11.15	-11.15	-11.15	-11.15
-11.14	-11.14	-11.14	-11.14	-11.14	-11.14	-11.14	-11.14	-11.14
-11.13	-11.13	-11.13	-11.13	-11.13	-11.13	-11.13	-11.13	-11.13
-11.12	-11.12	-11.12	-11.12	-11.12	-11.12	-11.12	-11.12	-11.12
-11.11	-11.11	-11.11	-11.11	-11.11	-11.11	-11.11	-11.11	-11.11
-11.1	-11.1	-11.1	-11.1	-11.1	-11.1	-11.1	-11.1	-11.1
-11.09	-11.09	-11.09	-11.09	-11.09	-11.09	-11.09	-11.09	-11.09
-11.08	-11.08	-11.08	-11.08	-11.08	-11.08	-11.08	-11.08	-11.08
-11.07	-11.07	-11.07	-11.07	-11.07	-11.07	-11.07	-11.07	-11.07
-11.06	-11.06	-11.06	-11.06	-11.06	-11.06	-11.06	-11.06	-11.06
-11.05	-11.05	-11.05	-11.05	-11.05	-11.05	-11.05	-11.05	-11.05
-11.04	-11.04	-11.04	-11.04	-11.04	-11.04	-11.04	-11.04	-11.04
-11.03	-11.03	-11.03	-11.03	-11.03	-11.03	-11.03	-11.03	-11.03
-11.02	-11.02	-11.02	-11.02	-11.02	-11.02	-11.02	-11.02	-11.02
-11.01	-11.01	-11.01	-11.01	-11.01	-11.01	-11.01	-11.01	-11.01

-11	-11	-11	-11	-11	-11	-11	-11	-11
-10.99	-10.99	-10.99	-10.99	-10.99	-10.99	-10.99	-10.99	-10.99
-10.98	-10.98	-10.98	-10.98	-10.98	-10.98	-10.98	-10.98	-10.98
-10.97	-10.97	-10.97	-10.97	-10.97	-10.97	-10.97	-10.97	-10.97
-10.96	-10.96	-10.96	-10.96	-10.96	-10.96	-10.96	-10.96	-10.96
-10.95	-10.95	-10.95	-10.95	-10.95	-10.95	-10.95	-10.95	-10.95
-10.94	-10.94	-10.94	-10.94	-10.94	-10.94	-10.94	-10.94	-10.94
-10.93	-10.93	-10.93	-10.93	-10.93	-10.93	-10.93	-10.93	-10.93
-10.92	-10.92	-10.92	-10.92	-10.92	-10.92	-10.92	-10.92	-10.92
-10.91	-10.91	-10.91	-10.91	-10.91	-10.91	-10.91	-10.91	-10.91
-10.9	-10.9	-10.9	-10.9	-10.9	-10.9	-10.9	-10.9	-10.9
-10.89	-10.89	-10.89	-10.89	-10.89	-10.89	-10.89	-10.89	-10.89
-10.88	-10.88	-10.88	-10.88	-10.88	-10.88	-10.88	-10.88	-10.88
-10.87	-10.87	-10.87	-10.87	-10.87	-10.87	-10.87	-10.87	-10.87
-10.86	-10.86	-10.86	-10.86	-10.86	-10.86	-10.86	-10.86	-10.86
-10.85	-10.85	-10.85	-10.85	-10.85	-10.85	-10.85	-10.85	-10.85
-10.84	-10.84	-10.84	-10.84	-10.84	-10.84	-10.84	-10.84	-10.84
-10.83	-10.83	-10.83	-10.83	-10.83	-10.83	-10.83	-10.83	-10.83
-10.82	-10.82	-10.82	-10.82	-10.82	-10.82	-10.82	-10.82	-10.82
-10.81	-10.81	-10.81	-10.81	-10.81	-10.81	-10.81	-10.81	-10.81
-10.8	-10.8	-10.8	-10.8	-10.8	-10.8	-10.8	-10.8	-10.8
-10.79	-10.79	-10.79	-10.79	-10.79	-10.79	-10.79	-10.79	-10.79
-10.78	-10.78	-10.78	-10.78	-10.78	-10.78	-10.78	-10.78	-10.78
-10.77	-10.77	-10.77	-10.77	-10.77	-10.77	-10.77	-10.77	-10.77
-10.76	-10.76	-10.76	-10.76	-10.76	-10.76	-10.76	-10.76	-10.76
-10.75	-10.75	-10.75	-10.75	-10.75	-10.75	-10.75	-10.75	-10.75
-10.74	-10.74	-10.74	-10.74	-10.74	-10.74	-10.74	-10.74	-10.74
-10.73	-10.73	-10.73	-10.73	-10.73	-10.73	-10.73	-10.73	-10.73
-10.72	-10.72	-10.72	-10.72	-10.72	-10.72	-10.72	-10.72	-10.72
-10.71	-10.71	-10.71	-10.71	-10.71	-10.71	-10.71	-10.71	-10.71
-10.7	-10.7	-10.7	-10.7	-10.7	-10.7	-10.7	-10.7	-10.7
-10.69	-10.69	-10.69	-10.69	-10.69	-10.69	-10.69	-10.69	-10.69
-10.68	-10.68	-10.68	-10.68	-10.68	-10.68	-10.68	-10.68	-10.68
-10.67	-10.67	-10.67	-10.67	-10.67	-10.67	-10.67	-10.67	-10.67
-10.66	-10.66	-10.66	-10.66	-10.66	-10.66	-10.66	-10.66	-10.66
-10.65	-10.65	-10.65	-10.65	-10.65	-10.65	-10.65	-10.65	-10.65
-10.64	-10.64	-10.64	-10.64	-10.64	-10.64	-10.64	-10.64	-10.64
-10.63	-10.63	-10.63	-10.63	-10.63	-10.63	-10.63	-10.63	-10.63
-10.62	-10.62	-10.62	-10.62	-10.62	-10.62	-10.62	-10.62	-10.62
-10.61	-10.61	-10.61	-10.61	-10.61	-10.61	-10.61	-10.61	-10.61
-10.6	-10.6	-10.6	-10.6	-10.6	-10.6	-10.6	-10.6	-10.6
-10.59	-10.59	-10.59	-10.59	-10.59	-10.59	-10.59	-10.59	-10.59
-10.58	-10.58	-10.58	-10.58	-10.58	-10.58	-10.58	-10.58	-10.58
-10.57	-10.57	-10.57	-10.57	-10.57	-10.57	-10.57	-10.57	-10.57
-10.56	-10.56	-10.56	-10.56	-10.56	-10.56	-10.56	-10.56	-10.56
-10.55	-10.55	-10.55	-10.55	-10.55	-10.55	-10.55	-10.55	-10.55
-10.54	-10.54	-10.54	-10.54	-10.54	-10.54	-10.54	-10.54	-10.54
-10.53	-10.53	-10.53	-10.53	-10.53	-10.53	-10.53	-10.53	-10.53
-10.52	-10.52	-10.52	-10.52	-10.52	-10.52	-10.52	-10.52	-10.52
-10.51	-10.51	-10.51	-10.51	-10.51	-10.51	-10.51	-10.51	-10.51

-10.5	-10.5	-10.5	-10.5	-10.5	-10.5	-10.5	-10.5	-10.5
-10.49	-10.49	-10.49	-10.49	-10.49	-10.49	-10.49	-10.49	-10.49
-10.48	-10.48	-10.48	-10.48	-10.48	-10.48	-10.48	-10.48	-10.48
-10.47	-10.47	-10.47	-10.47	-10.47	-10.47	-10.47	-10.47	-10.47
-10.46	-10.46	-10.46	-10.46	-10.46	-10.46	-10.46	-10.46	-10.46
-10.45	-10.45	-10.45	-10.45	-10.45	-10.45	-10.45	-10.45	-10.45
-10.44	-10.44	-10.44	-10.44	-10.44	-10.44	-10.44	-10.44	-10.44
-10.43	-10.43	-10.43	-10.43	-10.43	-10.43	-10.43	-10.43	-10.43
-10.42	-10.42	-10.42	-10.42	-10.42	-10.42	-10.42	-10.42	-10.42
-10.41	-10.41	-10.41	-10.41	-10.41	-10.41	-10.41	-10.41	-10.41
-10.4	-10.4	-10.4	-10.4	-10.4	-10.4	-10.4	-10.4	-10.4
-10.39	-10.39	-10.39	-10.39	-10.39	-10.39	-10.39	-10.39	-10.39
-10.38	-10.38	-10.38	-10.38	-10.38	-10.38	-10.38	-10.38	-10.38
-10.37	-10.37	-10.37	-10.37	-10.37	-10.37	-10.37	-10.37	-10.37
-10.36	-10.36	-10.36	-10.36	-10.36	-10.36	-10.36	-10.36	-10.36
-10.35	-10.35	-10.35	-10.35	-10.35	-10.35	-10.35	-10.35	-10.35
-10.34	-10.34	-10.34	-10.34	-10.34	-10.34	-10.34	-10.34	-10.34
-10.33	-10.33	-10.33	-10.33	-10.33	-10.33	-10.33	-10.33	-10.33
-10.32	-10.32	-10.32	-10.32	-10.32	-10.32	-10.32	-10.32	-10.32
-10.31	-10.31	-10.31	-10.31	-10.31	-10.31	-10.31	-10.31	-10.31
-10.3	-10.3	-10.3	-10.3	-10.3	-10.3	-10.3	-10.3	-10.3
-10.29	-10.29	-10.29	-10.29	-10.29	-10.29	-10.29	-10.29	-10.29
-10.28	-10.28	-10.28	-10.28	-10.28	-10.28	-10.28	-10.28	-10.28
-10.27	-10.27	-10.27	-10.27	-10.27	-10.27	-10.27	-10.27	-10.27
-10.26	-10.26	-10.26	-10.26	-10.26	-10.26	-10.26	-10.26	-10.26
-10.25	-10.25	-10.25	-10.25	-10.25	-10.25	-10.25	-10.25	-10.25
-10.24	-10.24	-10.24	-10.24	-10.24	-10.24	-10.24	-10.24	-10.24
-10.23	-10.23	-10.23	-10.23	-10.23	-10.23	-10.23	-10.23	-10.23
-10.22	-10.22	-10.22	-10.22	-10.22	-10.22	-10.22	-10.22	-10.22
-10.21	-10.21	-10.21	-10.21	-10.21	-10.21	-10.21	-10.21	-10.21
-10.2	-10.2	-10.2	-10.2	-10.2	-10.2	-10.2	-10.2	-10.2
-10.19	-10.19	-10.19	-10.19	-10.19	-10.19	-10.19	-10.19	-10.19
-10.18	-10.18	-10.18	-10.18	-10.18	-10.18	-10.18	-10.18	-10.18
-10.17	-10.17	-10.17	-10.17	-10.17	-10.17	-10.17	-10.17	-10.17
-10.16	-10.16	-10.16	-10.16	-10.16	-10.16	-10.16	-10.16	-10.16
-10.15	-10.15	-10.15	-10.15	-10.15	-10.15	-10.15	-10.15	-10.15
-10.14	-10.14	-10.14	-10.14	-10.14	-10.14	-10.14	-10.14	-10.14
-10.13	-10.13	-10.13	-10.13	-10.13	-10.13	-10.13	-10.13	-10.13
-10.12	-10.12	-10.12	-10.12	-10.12	-10.12	-10.12	-10.12	-10.12
-10.11	-10.11	-10.11	-10.11	-10.11	-10.11	-10.11	-10.11	-10.11
-10.1	-10.1	-10.1	-10.1	-10.1	-10.1	-10.1	-10.1	-10.1
-10.09	-10.09	-10.09	-10.09	-10.09	-10.09	-10.09	-10.09	-10.09
-10.08	-10.08	-10.08	-10.08	-10.08	-10.08	-10.08	-10.08	-10.08
-10.07	-10.07	-10.07	-10.07	-10.07	-10.07	-10.07	-10.07	-10.07
-10.06	-10.06	-10.06	-10.06	-10.06	-10.06	-10.06	-10.06	-10.06
-10.05	-10.05	-10.05	-10.05	-10.05	-10.05	-10.05	-10.05	-10.05
-10.04	-10.04	-10.04	-10.04	-10.04	-10.04	-10.04	-10.04	-10.04
-10.03	-10.03	-10.03	-10.03	-10.03	-10.03	-10.03	-10.03	-10.03
-10.02	-10.02	-10.02	-10.02	-10.02	-10.02	-10.02	-10.02	-10.02
-10.01	-10.01	-10.01	-10.01	-10.01	-10.01	-10.01	-10.01	-10.01

-10	-10	-10	-10	-10	-10	-10	-10	-10
-9.99	-9.99	-9.99	-9.99	-9.99	-9.99	-9.99	-9.99	-9.99
-9.98	-9.98	-9.98	-9.98	-9.98	-9.98	-9.98	-9.98	-9.98
-9.97	-9.97	-9.97	-9.97	-9.97	-9.97	-9.97	-9.97	-9.97
-9.96	-9.96	-9.96	-9.96	-9.96	-9.96	-9.96	-9.96	-9.96
-9.95	-9.95	-9.95	-9.95	-9.95	-9.95	-9.95	-9.95	-9.95
-9.94	-9.94	-9.94	-9.94	-9.94	-9.94	-9.94	-9.94	-9.94
-9.93	-9.93	-9.93	-9.93	-9.93	-9.93	-9.93	-9.93	-9.93
-9.92	-9.92	-9.92	-9.92	-9.92	-9.92	-9.92	-9.92	-9.92
-9.91	-9.91	-9.91	-9.91	-9.91	-9.91	-9.91	-9.91	-9.91
-9.9	-9.9	-9.9	-9.9	-9.9	-9.9	-9.9	-9.9	-9.9
-9.89	-9.89	-9.89	-9.89	-9.89	-9.89	-9.89	-9.89	-9.89
-9.88	-9.88	-9.88	-9.88	-9.88	-9.88	-9.88	-9.88	-9.88
-9.87	-9.87	-9.87	-9.87	-9.87	-9.87	-9.87	-9.87	-9.87
-9.86	-9.86	-9.86	-9.86	-9.86	-9.86	-9.86	-9.86	-9.86
-9.85	-9.85	-9.85	-9.85	-9.85	-9.85	-9.85	-9.85	-9.85
-9.84	-9.84	-9.84	-9.84	-9.84	-9.84	-9.84	-9.84	-9.84
-9.83	-9.83	-9.83	-9.83	-9.83	-9.83	-9.83	-9.83	-9.83
-9.82	-9.82	-9.82	-9.82	-9.82	-9.82	-9.82	-9.82	-9.82
-9.81	-9.81	-9.81	-9.81	-9.81	-9.81	-9.81	-9.81	-9.81
-9.8	-9.8	-9.8	-9.8	-9.8	-9.8	-9.8	-9.8	-9.8
-9.79	-9.79	-9.79	-9.79	-9.79	-9.79	-9.79	-9.79	-9.79
-9.78	-9.78	-9.78	-9.78	-9.78	-9.78	-9.78	-9.78	-9.78
-9.77	-9.77	-9.77	-9.77	-9.77	-9.77	-9.77	-9.77	-9.77
-9.76	-9.76	-9.76	-9.76	-9.76	-9.76	-9.76	-9.76	-9.76
-9.75	-9.75	-9.75	-9.75	-9.75	-9.75	-9.75	-9.75	-9.75
-9.74	-9.74	-9.74	-9.74	-9.74	-9.74	-9.74	-9.74	-9.74
-9.73	-9.73	-9.73	-9.73	-9.73	-9.73	-9.73	-9.73	-9.73
-9.72	-9.72	-9.72	-9.72	-9.72	-9.72	-9.72	-9.72	-9.72
-9.71	-9.71	-9.71	-9.71	-9.71	-9.71	-9.71	-9.71	-9.71
-9.7	-9.7	-9.7	-9.7	-9.7	-9.7	-9.7	-9.7	-9.7
-9.69	-9.69	-9.69	-9.69	-9.69	-9.69	-9.69	-9.69	-9.69
-9.68	-9.68	-9.68	-9.68	-9.68	-9.68	-9.68	-9.68	-9.68
-9.67	-9.67	-9.67	-9.67	-9.67	-9.67	-9.67	-9.67	-9.67
-9.66	-9.66	-9.66	-9.66	-9.66	-9.66	-9.66	-9.66	-9.66
-9.65	-9.65	-9.65	-9.65	-9.65	-9.65	-9.65	-9.65	-9.65
-9.64	-9.64	-9.64	-9.64	-9.64	-9.64	-9.64	-9.64	-9.64
-9.63	-9.63	-9.63	-9.63	-9.63	-9.63	-9.63	-9.63	-9.63
-9.62	-9.62	-9.62	-9.62	-9.62	-9.62	-9.62	-9.62	-9.62
-9.61	-9.61	-9.61	-9.61	-9.61	-9.61	-9.61	-9.61	-9.61
-9.6	-9.6	-9.6	-9.6	-9.6	-9.6	-9.6	-9.6	-9.6
-9.59	-9.59	-9.59	-9.59	-9.59	-9.59	-9.59	-9.59	-9.59
-9.58	-9.58	-9.58	-9.58	-9.58	-9.58	-9.58	-9.58	-9.58
-9.57	-9.57	-9.57	-9.57	-9.57	-9.57	-9.57	-9.57	-9.57
-9.56	-9.56	-9.56	-9.56	-9.56	-9.56	-9.56	-9.56	-9.56
-9.55	-9.55	-9.55	-9.55	-9.55	-9.55	-9.55	-9.55	-9.55
-9.54	-9.54	-9.54	-9.54	-9.54	-9.54	-9.54	-9.54	-9.54
-9.53	-9.53	-9.53	-9.53	-9.53	-9.53	-9.53	-9.53	-9.53
-9.52	-9.52	-9.52	-9.52	-9.52	-9.52	-9.52	-9.52	-9.52
-9.51	-9.51	-9.51	-9.51	-9.51	-9.51	-9.51	-9.51	-9.51

-9.5	-9.5	-9.5	-9.5	-9.5	-9.5	-9.5	-9.5	-9.5
-9.49	-9.49	-9.49	-9.49	-9.49	-9.49	-9.49	-9.49	-9.49
-9.48	-9.48	-9.48	-9.48	-9.48	-9.48	-9.48	-9.48	-9.48
-9.47	-9.47	-9.47	-9.47	-9.47	-9.47	-9.47	-9.47	-9.47
-9.46	-9.46	-9.46	-9.46	-9.46	-9.46	-9.46	-9.46	-9.46
-9.45	-9.45	-9.45	-9.45	-9.45	-9.45	-9.45	-9.45	-9.45
-9.44	-9.44	-9.44	-9.44	-9.44	-9.44	-9.44	-9.44	-9.44
-9.43	-9.43	-9.43	-9.43	-9.43	-9.43	-9.43	-9.43	-9.43
-9.42	-9.42	-9.42	-9.42	-9.42	-9.42	-9.42	-9.42	-9.42
-9.41	-9.41	-9.41	-9.41	-9.41	-9.41	-9.41	-9.41	-9.41
-9.4	-9.4	-9.4	-9.4	-9.4	-9.4	-9.4	-9.4	-9.4
-9.39	-9.39	-9.39	-9.39	-9.39	-9.39	-9.39	-9.39	-9.39
-9.38	-9.38	-9.38	-9.38	-9.38	-9.38	-9.38	-9.38	-9.38
-9.37	-9.37	-9.37	-9.37	-9.37	-9.37	-9.37	-9.37	-9.37
-9.36	-9.36	-9.36	-9.36	-9.36	-9.36	-9.36	-9.36	-9.36
-9.35	-9.35	-9.35	-9.35	-9.35	-9.35	-9.35	-9.35	-9.35
-9.34	-9.34	-9.34	-9.34	-9.34	-9.34	-9.34	-9.34	-9.34
-9.33	-9.33	-9.33	-9.33	-9.33	-9.33	-9.33	-9.33	-9.33
-9.32	-9.32	-9.32	-9.32	-9.32	-9.32	-9.32	-9.32	-9.32
-9.31	-9.31	-9.31	-9.31	-9.31	-9.31	-9.31	-9.31	-9.31
-9.3	-9.3	-9.3	-9.3	-9.3	-9.3	-9.3	-9.3	-9.3
-9.29	-9.29	-9.29	-9.29	-9.29	-9.29	-9.29	-9.29	-9.29
-9.28	-9.28	-9.28	-9.28	-9.28	-9.28	-9.28	-9.28	-9.28
-9.27	-9.27	-9.27	-9.27	-9.27	-9.27	-9.27	-9.27	-9.27
-9.26	-9.26	-9.26	-9.26	-9.26	-9.26	-9.26	-9.26	-9.26
-9.25	-9.25	-9.25	-9.25	-9.25	-9.25	-9.25	-9.25	-9.25
-9.24	-9.24	-9.24	-9.24	-9.24	-9.24	-9.24	-9.24	-9.24
-9.23	-9.23	-9.23	-9.23	-9.23	-9.23	-9.23	-9.23	-9.23
-9.22	-9.22	-9.22	-9.22	-9.22	-9.22	-9.22	-9.22	-9.22
-9.21	-9.21	-9.21	-9.21	-9.21	-9.21	-9.21	-9.21	-9.21
-9.2	-9.2	-9.2	-9.2	-9.2	-9.2	-9.2	-9.2	-9.2
-9.19	-9.19	-9.19	-9.19	-9.19	-9.19	-9.19	-9.19	-9.19
-9.18	-9.18	-9.18	-9.18	-9.18	-9.18	-9.18	-9.18	-9.18
-9.17	-9.17	-9.17	-9.17	-9.17	-9.17	-9.17	-9.17	-9.17
-9.16	-9.16	-9.16	-9.16	-9.16	-9.16	-9.16	-9.16	-9.16
-9.15	-9.15	-9.15	-9.15	-9.15	-9.15	-9.15	-9.15	-9.15
-9.14	-9.14	-9.14	-9.14	-9.14	-9.14	-9.14	-9.14	-9.14
-9.13	-9.13	-9.13	-9.13	-9.13	-9.13	-9.13	-9.13	-9.13
-9.12	-9.12	-9.12	-9.12	-9.12	-9.12	-9.12	-9.12	-9.12
-9.11	-9.11	-9.11	-9.11	-9.11	-9.11	-9.11	-9.11	-9.11
-9.1	-9.1	-9.1	-9.1	-9.1	-9.1	-9.1	-9.1	-9.1
-9.09	-9.09	-9.09	-9.09	-9.09	-9.09	-9.09	-9.09	-9.09
-9.08	-9.08	-9.08	-9.08	-9.08	-9.08	-9.08	-9.08	-9.08
-9.07	-9.07	-9.07	-9.07	-9.07	-9.07	-9.07	-9.07	-9.07
-9.06	-9.06	-9.06	-9.06	-9.06	-9.06	-9.06	-9.06	-9.06
-9.05	-9.05	-9.05	-9.05	-9.05	-9.05	-9.05	-9.05	-9.05
-9.04	-9.04	-9.04	-9.04	-9.04	-9.04	-9.04	-9.04	-9.04
-9.03	-9.03	-9.03	-9.03	-9.03	-9.03	-9.03	-9.03	-9.03
-9.02	-9.02	-9.02	-9.02	-9.02	-9.02	-9.02	-9.02	-9.02
-9.01	-9.01	-9.01	-9.01	-9.01	-9.01	-9.01	-9.01	-9.01

-9	-9	-9	-9	-9	-9	-9	-9	-9
-8.99	-8.99	-8.99	-8.99	-8.99	-8.99	-8.99	-8.99	-8.99
-8.98	-8.98	-8.98	-8.98	-8.98	-8.98	-8.98	-8.98	-8.98
-8.97	-8.97	-8.97	-8.97	-8.97	-8.97	-8.97	-8.97	-8.97
-8.96	-8.96	-8.96	-8.96	-8.96	-8.96	-8.96	-8.96	-8.96
-8.95	-8.95	-8.95	-8.95	-8.95	-8.95	-8.95	-8.95	-8.95
-8.94	-8.94	-8.94	-8.94	-8.94	-8.94	-8.94	-8.94	-8.94
-8.93	-8.93	-8.93	-8.93	-8.93	-8.93	-8.93	-8.93	-8.93
-8.92	-8.92	-8.92	-8.92	-8.92	-8.92	-8.92	-8.92	-8.92
-8.91	-8.91	-8.91	-8.91	-8.91	-8.91	-8.91	-8.91	-8.91
-8.9	-8.9	-8.9	-8.9	-8.9	-8.9	-8.9	-8.9	-8.9
-8.89	-8.89	-8.89	-8.89	-8.89	-8.89	-8.89	-8.89	-8.89
-8.88	-8.88	-8.88	-8.88	-8.88	-8.88	-8.88	-8.88	-8.88
-8.87	-8.87	-8.87	-8.87	-8.87	-8.87	-8.87	-8.87	-8.87
-8.86	-8.86	-8.86	-8.86	-8.86	-8.86	-8.86	-8.86	-8.86
-8.85	-8.85	-8.85	-8.85	-8.85	-8.85	-8.85	-8.85	-8.85
-8.84	-8.84	-8.84	-8.84	-8.84	-8.84	-8.84	-8.84	-8.84
-8.83	-8.83	-8.83	-8.83	-8.83	-8.83	-8.83	-8.83	-8.83
-8.82	-8.82	-8.82	-8.82	-8.82	-8.82	-8.82	-8.82	-8.82
-8.81	-8.81	-8.81	-8.81	-8.81	-8.81	-8.81	-8.81	-8.81
-8.8	-8.8	-8.8	-8.8	-8.8	-8.8	-8.8	-8.8	-8.8
-8.79	-8.79	-8.79	-8.79	-8.79	-8.79	-8.79	-8.79	-8.79
-8.78	-8.78	-8.78	-8.78	-8.78	-8.78	-8.78	-8.78	-8.78
-8.77	-8.77	-8.77	-8.77	-8.77	-8.77	-8.77	-8.77	-8.77
-8.76	-8.76	-8.76	-8.76	-8.76	-8.76	-8.76	-8.76	-8.76
-8.75	-8.75	-8.75	-8.75	-8.75	-8.75	-8.75	-8.75	-8.75
-8.74	-8.74	-8.74	-8.74	-8.74	-8.74	-8.74	-8.74	-8.74
-8.73	-8.73	-8.73	-8.73	-8.73	-8.73	-8.73	-8.73	-8.73
-8.72	-8.72	-8.72	-8.72	-8.72	-8.72	-8.72	-8.72	-8.72
-8.71	-8.71	-8.71	-8.71	-8.71	-8.71	-8.71	-8.71	-8.71
-8.7	-8.7	-8.7	-8.7	-8.7	-8.7	-8.7	-8.7	-8.7
-8.69	-8.69	-8.69	-8.69	-8.69	-8.69	-8.69	-8.69	-8.69
-8.68	-8.68	-8.68	-8.68	-8.68	-8.68	-8.68	-8.68	-8.68
-8.67	-8.67	-8.67	-8.67	-8.67	-8.67	-8.67	-8.67	-8.67
-8.66	-8.66	-8.66	-8.66	-8.66	-8.66	-8.66	-8.66	-8.66
-8.65	-8.65	-8.65	-8.65	-8.65	-8.65	-8.65	-8.65	-8.65
-8.64	-8.64	-8.64	-8.64	-8.64	-8.64	-8.64	-8.64	-8.64
-8.63	-8.63	-8.63	-8.63	-8.63	-8.63	-8.63	-8.63	-8.63
-8.62	-8.62	-8.62	-8.62	-8.62	-8.62	-8.62	-8.62	-8.62
-8.61	-8.61	-8.61	-8.61	-8.61	-8.61	-8.61	-8.61	-8.61
-8.6	-8.6	-8.6	-8.6	-8.6	-8.6	-8.6	-8.6	-8.6
-8.59	-8.59	-8.59	-8.59	-8.59	-8.59	-8.59	-8.59	-8.59
-8.58	-8.58	-8.58	-8.58	-8.58	-8.58	-8.58	-8.58	-8.58
-8.57	-8.57	-8.57	-8.57	-8.57	-8.57	-8.57	-8.57	-8.57
-8.56	-8.56	-8.56	-8.56	-8.56	-8.56	-8.56	-8.56	-8.56
-8.55	-8.55	-8.55	-8.55	-8.55	-8.55	-8.55	-8.55	-8.55
-8.54	-8.54	-8.54	-8.54	-8.54	-8.54	-8.54	-8.54	-8.54
-8.53	-8.53	-8.53	-8.53	-8.53	-8.53	-8.53	-8.53	-8.53
-8.52	-8.52	-8.52	-8.52	-8.52	-8.52	-8.52	-8.52	-8.52
-8.51	-8.51	-8.51	-8.51	-8.51	-8.51	-8.51	-8.51	-8.51

-8.5	-8.5	-8.5	-8.5	-8.5	-8.5	-8.5	-8.5	-8.5
-8.49	-8.49	-8.49	-8.49	-8.49	-8.49	-8.49	-8.49	-8.49
-8.48	-8.48	-8.48	-8.48	-8.48	-8.48	-8.48	-8.48	-8.48
-8.47	-8.47	-8.47	-8.47	-8.47	-8.47	-8.47	-8.47	-8.47
-8.46	-8.46	-8.46	-8.46	-8.46	-8.46	-8.46	-8.46	-8.46
-8.45	-8.45	-8.45	-8.45	-8.45	-8.45	-8.45	-8.45	-8.45
-8.44	-8.44	-8.44	-8.44	-8.44	-8.44	-8.44	-8.44	-8.44
-8.43	-8.43	-8.43	-8.43	-8.43	-8.43	-8.43	-8.43	-8.43
-8.42	-8.42	-8.42	-8.42	-8.42	-8.42	-8.42	-8.42	-8.42
-8.41	-8.41	-8.41	-8.41	-8.41	-8.41	-8.41	-8.41	-8.41
-8.4	-8.4	-8.4	-8.4	-8.4	-8.4	-8.4	-8.4	-8.4
-8.39	-8.39	-8.39	-8.39	-8.39	-8.39	-8.39	-8.39	-8.39
-8.38	-8.38	-8.38	-8.38	-8.38	-8.38	-8.38	-8.38	-8.38
-8.37	-8.37	-8.37	-8.37	-8.37	-8.37	-8.37	-8.37	-8.37
-8.36	-8.36	-8.36	-8.36	-8.36	-8.36	-8.36	-8.36	-8.36
-8.35	-8.35	-8.35	-8.35	-8.35	-8.35	-8.35	-8.35	-8.35
-8.34	-8.34	-8.34	-8.34	-8.34	-8.34	-8.34	-8.34	-8.34
-8.33	-8.33	-8.33	-8.33	-8.33	-8.33	-8.33	-8.33	-8.33
-8.32	-8.32	-8.32	-8.32	-8.32	-8.32	-8.32	-8.32	-8.32
-8.31	-8.31	-8.31	-8.31	-8.31	-8.31	-8.31	-8.31	-8.31
-8.3	-8.3	-8.3	-8.3	-8.3	-8.3	-8.3	-8.3	-8.3
-8.29	-8.29	-8.29	-8.29	-8.29	-8.29	-8.29	-8.29	-8.29
-8.28	-8.28	-8.28	-8.28	-8.28	-8.28	-8.28	-8.28	-8.28
-8.27	-8.27	-8.27	-8.27	-8.27	-8.27	-8.27	-8.27	-8.27
-8.26	-8.26	-8.26	-8.26	-8.26	-8.26	-8.26	-8.26	-8.26
-8.25	-8.25	-8.25	-8.25	-8.25	-8.25	-8.25	-8.25	-8.25
-8.24	-8.24	-8.24	-8.24	-8.24	-8.24	-8.24	-8.24	-8.24
-8.23	-8.23	-8.23	-8.23	-8.23	-8.23	-8.23	-8.23	-8.23
-8.22	-8.22	-8.22	-8.22	-8.22	-8.22	-8.22	-8.22	-8.22
-8.21	-8.21	-8.21	-8.21	-8.21	-8.21	-8.21	-8.21	-8.21
-8.2	-8.2	-8.2	-8.2	-8.2	-8.2	-8.2	-8.2	-8.2
-8.19	-8.19	-8.19	-8.19	-8.19	-8.19	-8.19	-8.19	-8.19
-8.18	-8.18	-8.18	-8.18	-8.18	-8.18	-8.18	-8.18	-8.18
-8.17	-8.17	-8.17	-8.17	-8.17	-8.17	-8.17	-8.17	-8.17
-8.16	-8.16	-8.16	-8.16	-8.16	-8.16	-8.16	-8.16	-8.16
-8.15	-8.15	-8.15	-8.15	-8.15	-8.15	-8.15	-8.15	-8.15
-8.14	-8.14	-8.14	-8.14	-8.14	-8.14	-8.14	-8.14	-8.14
-8.13	-8.13	-8.13	-8.13	-8.13	-8.13	-8.13	-8.13	-8.13
-8.12	-8.12	-8.12	-8.12	-8.12	-8.12	-8.12	-8.12	-8.12
-8.11	-8.11	-8.11	-8.11	-8.11	-8.11	-8.11	-8.11	-8.11
-8.1	-8.1	-8.1	-8.1	-8.1	-8.1	-8.1	-8.1	-8.1
-8.09	-8.09	-8.09	-8.09	-8.09	-8.09	-8.09	-8.09	-8.09
-8.08	-8.08	-8.08	-8.08	-8.08	-8.08	-8.08	-8.08	-8.08
-8.07	-8.07	-8.07	-8.07	-8.07	-8.07	-8.07	-8.07	-8.07
-8.06	-8.06	-8.06	-8.06	-8.06	-8.06	-8.06	-8.06	-8.06
-8.05	-8.05	-8.05	-8.05	-8.05	-8.05	-8.05	-8.05	-8.05
-8.04	-8.04	-8.04	-8.04	-8.04	-8.04	-8.04	-8.04	-8.04
-8.03	-8.03	-8.03	-8.03	-8.03	-8.03	-8.03	-8.03	-8.03
-8.02	-8.02	-8.02	-8.02	-8.02	-8.02	-8.02	-8.02	-8.02
-8.01	-8.01	-8.01	-8.01	-8.01	-8.01	-8.01	-8.01	-8.01

-8	-8	-8	-8	-8	-8	-8	-8	-8
-7.99	-7.99	-7.99	-7.99	-7.99	-7.99	-7.99	-7.99	-7.99
-7.98	-7.98	-7.98	-7.98	-7.98	-7.98	-7.98	-7.98	-7.98
-7.97	-7.97	-7.97	-7.97	-7.97	-7.97	-7.97	-7.97	-7.97
-7.96	-7.96	-7.96	-7.96	-7.96	-7.96	-7.96	-7.96	-7.96
-7.95	-7.95	-7.95	-7.95	-7.95	-7.95	-7.95	-7.95	-7.95
-7.94	-7.94	-7.94	-7.94	-7.94	-7.94	-7.94	-7.94	-7.94
-7.93	-7.93	-7.93	-7.93	-7.93	-7.93	-7.93	-7.93	-7.93
-7.92	-7.92	-7.92	-7.92	-7.92	-7.92	-7.92	-7.92	-7.92
-7.91	-7.91	-7.91	-7.91	-7.91	-7.91	-7.91	-7.91	-7.91
-7.9	-7.9	-7.9	-7.9	-7.9	-7.9	-7.9	-7.9	-7.9
-7.89	-7.89	-7.89	-7.89	-7.89	-7.89	-7.89	-7.89	-7.89
-7.88	-7.88	-7.88	-7.88	-7.88	-7.88	-7.88	-7.88	-7.88
-7.87	-7.87	-7.87	-7.87	-7.87	-7.87	-7.87	-7.87	-7.87
-7.86	-7.86	-7.86	-7.86	-7.86	-7.86	-7.86	-7.86	-7.86
-7.85	-7.85	-7.85	-7.85	-7.85	-7.85	-7.85	-7.85	-7.85
-7.84	-7.84	-7.84	-7.84	-7.84	-7.84	-7.84	-7.84	-7.84
-7.83	-7.83	-7.83	-7.83	-7.83	-7.83	-7.83	-7.83	-7.83
-7.82	-7.82	-7.82	-7.82	-7.82	-7.82	-7.82	-7.82	-7.82
-7.81	-7.81	-7.81	-7.81	-7.81	-7.81	-7.81	-7.81	-7.81
-7.8	-7.8	-7.8	-7.8	-7.8	-7.8	-7.8	-7.8	-7.8
-7.79	-7.79	-7.79	-7.79	-7.79	-7.79	-7.79	-7.79	-7.79
-7.78	-7.78	-7.78	-7.78	-7.78	-7.78	-7.78	-7.78	-7.78
-7.77	-7.77	-7.77	-7.77	-7.77	-7.77	-7.77	-7.77	-7.77
-7.76	-7.76	-7.76	-7.76	-7.76	-7.76	-7.76	-7.76	-7.76
-7.75	-7.75	-7.75	-7.75	-7.75	-7.75	-7.75	-7.75	-7.75
-7.74	-7.74	-7.74	-7.74	-7.74	-7.74	-7.74	-7.74	-7.74
-7.73	-7.73	-7.73	-7.73	-7.73	-7.73	-7.73	-7.73	-7.73
-7.72	-7.72	-7.72	-7.72	-7.72	-7.72	-7.72	-7.72	-7.72
-7.71	-7.71	-7.71	-7.71	-7.71	-7.71	-7.71	-7.71	-7.71
-7.7	-7.7	-7.7	-7.7	-7.7	-7.7	-7.7	-7.7	-7.7
-7.69	-7.69	-7.69	-7.69	-7.69	-7.69	-7.69	-7.69	-7.69
-7.68	-7.68	-7.68	-7.68	-7.68	-7.68	-7.68	-7.68	-7.68
-7.67	-7.67	-7.67	-7.67	-7.67	-7.67	-7.67	-7.67	-7.67
-7.66	-7.66	-7.66	-7.66	-7.66	-7.66	-7.66	-7.66	-7.66
-7.65	-7.65	-7.65	-7.65	-7.65	-7.65	-7.65	-7.65	-7.65
-7.64	-7.64	-7.64	-7.64	-7.64	-7.64	-7.64	-7.64	-7.64
-7.63	-7.63	-7.63	-7.63	-7.63	-7.63	-7.63	-7.63	-7.63
-7.62	-7.62	-7.62	-7.62	-7.62	-7.62	-7.62	-7.62	-7.62
-7.61	-7.61	-7.61	-7.61	-7.61	-7.61	-7.61	-7.61	-7.61
-7.6	-7.6	-7.6	-7.6	-7.6	-7.6	-7.6	-7.6	-7.6
-7.59	-7.59	-7.59	-7.59	-7.59	-7.59	-7.59	-7.59	-7.59
-7.58	-7.58	-7.58	-7.58	-7.58	-7.58	-7.58	-7.58	-7.58
-7.57	-7.57	-7.57	-7.57	-7.57	-7.57	-7.57	-7.57	-7.57
-7.56	-7.56	-7.56	-7.56	-7.56	-7.56	-7.56	-7.56	-7.56
-7.55	-7.55	-7.55	-7.55	-7.55	-7.55	-7.55	-7.55	-7.55
-7.54	-7.54	-7.54	-7.54	-7.54	-7.54	-7.54	-7.54	-7.54
-7.53	-7.53	-7.53	-7.53	-7.53	-7.53	-7.53	-7.53	-7.53
-7.52	-7.52	-7.52	-7.52	-7.52	-7.52	-7.52	-7.52	-7.52
-7.51	-7.51	-7.51	-7.51	-7.51	-7.51	-7.51	-7.51	-7.51

-7.5	-7.5	-7.5	-7.5	-7.5	-7.5	-7.5	-7.5	-7.5
-7.49	-7.49	-7.49	-7.49	-7.49	-7.49	-7.49	-7.49	-7.49
-7.48	-7.48	-7.48	-7.48	-7.48	-7.48	-7.48	-7.48	-7.48
-7.47	-7.47	-7.47	-7.47	-7.47	-7.47	-7.47	-7.47	-7.47
-7.46	-7.46	-7.46	-7.46	-7.46	-7.46	-7.46	-7.46	-7.46
-7.45	-7.45	-7.45	-7.45	-7.45	-7.45	-7.45	-7.45	-7.45
-7.44	-7.44	-7.44	-7.44	-7.44	-7.44	-7.44	-7.44	-7.44
-7.43	-7.43	-7.43	-7.43	-7.43	-7.43	-7.43	-7.43	-7.43
-7.42	-7.42	-7.42	-7.42	-7.42	-7.42	-7.42	-7.42	-7.42
-7.41	-7.41	-7.41	-7.41	-7.41	-7.41	-7.41	-7.41	-7.41
-7.4	-7.4	-7.4	-7.4	-7.4	-7.4	-7.4	-7.4	-7.4
-7.39	-7.39	-7.39	-7.39	-7.39	-7.39	-7.39	-7.39	-7.39
-7.38	-7.38	-7.38	-7.38	-7.38	-7.38	-7.38	-7.38	-7.38
-7.37	-7.37	-7.37	-7.37	-7.37	-7.37	-7.37	-7.37	-7.37
-7.36	-7.36	-7.36	-7.36	-7.36	-7.36	-7.36	-7.36	-7.36
-7.35	-7.35	-7.35	-7.35	-7.35	-7.35	-7.35	-7.35	-7.35
-7.34	-7.34	-7.34	-7.34	-7.34	-7.34	-7.34	-7.34	-7.34
-7.33	-7.33	-7.33	-7.33	-7.33	-7.33	-7.33	-7.33	-7.33
-7.32	-7.32	-7.32	-7.32	-7.32	-7.32	-7.32	-7.32	-7.32
-7.31	-7.31	-7.31	-7.31	-7.31	-7.31	-7.31	-7.31	-7.31
-7.3	-7.3	-7.3	-7.3	-7.3	-7.3	-7.3	-7.3	-7.3
-7.29	-7.29	-7.29	-7.29	-7.29	-7.29	-7.29	-7.29	-7.29
-7.28	-7.28	-7.28	-7.28	-7.28	-7.28	-7.28	-7.28	-7.28
-7.27	-7.27	-7.27	-7.27	-7.27	-7.27	-7.27	-7.27	-7.27
-7.26	-7.26	-7.26	-7.26	-7.26	-7.26	-7.26	-7.26	-7.26
-7.25	-7.25	-7.25	-7.25	-7.25	-7.25	-7.25	-7.25	-7.25
-7.24	-7.24	-7.24	-7.24	-7.24	-7.24	-7.24	-7.24	-7.24
-7.23	-7.23	-7.23	-7.23	-7.23	-7.23	-7.23	-7.23	-7.23
-7.22	-7.22	-7.22	-7.22	-7.22	-7.22	-7.22	-7.22	-7.22
-7.21	-7.21	-7.21	-7.21	-7.21	-7.21	-7.21	-7.21	-7.21
-7.2	-7.2	-7.2	-7.2	-7.2	-7.2	-7.2	-7.2	-7.2
-7.19	-7.19	-7.19	-7.19	-7.19	-7.19	-7.19	-7.19	-7.19
-7.18	-7.18	-7.18	-7.18	-7.18	-7.18	-7.18	-7.18	-7.18
-7.17	-7.17	-7.17	-7.17	-7.17	-7.17	-7.17	-7.17	-7.17
-7.16	-7.16	-7.16	-7.16	-7.16	-7.16	-7.16	-7.16	-7.16
-7.15	-7.15	-7.15	-7.15	-7.15	-7.15	-7.15	-7.15	-7.15
-7.14	-7.14	-7.14	-7.14	-7.14	-7.14	-7.14	-7.14	-7.14
-7.13	-7.13	-7.13	-7.13	-7.13	-7.13	-7.13	-7.13	-7.13
-7.12	-7.12	-7.12	-7.12	-7.12	-7.12	-7.12	-7.12	-7.12
-7.11	-7.11	-7.11	-7.11	-7.11	-7.11	-7.11	-7.11	-7.11
-7.1	-7.1	-7.1	-7.1	-7.1	-7.1	-7.1	-7.1	-7.1
-7.09	-7.09	-7.09	-7.09	-7.09	-7.09	-7.09	-7.09	-7.09
-7.08	-7.08	-7.08	-7.08	-7.08	-7.08	-7.08	-7.08	-7.08
-7.07	-7.07	-7.07	-7.07	-7.07	-7.07	-7.07	-7.07	-7.07
-7.06	-7.06	-7.06	-7.06	-7.06	-7.06	-7.06	-7.06	-7.06
-7.05	-7.05	-7.05	-7.05	-7.05	-7.05	-7.05	-7.05	-7.05
-7.04	-7.04	-7.04	-7.04	-7.04	-7.04	-7.04	-7.04	-7.04
-7.03	-7.03	-7.03	-7.03	-7.03	-7.03	-7.03	-7.03	-7.03
-7.02	-7.02	-7.02	-7.02	-7.02	-7.02	-7.02	-7.02	-7.02
-7.01	-7.01	-7.01	-7.01	-7.01	-7.01	-7.01	-7.01	-7.01

-7	-7	-7	-7	-7	-7	-7	-7	-7
-6.99	-6.99	-6.99	-6.99	-6.99	-6.99	-6.99	-6.99	-6.99
-6.98	-6.98	-6.98	-6.98	-6.98	-6.98	-6.98	-6.98	-6.98
-6.97	-6.97	-6.97	-6.97	-6.97	-6.97	-6.97	-6.97	-6.97
-6.96	-6.96	-6.96	-6.96	-6.96	-6.96	-6.96	-6.96	-6.96
-6.95	-6.95	-6.95	-6.95	-6.95	-6.95	-6.95	-6.95	-6.95
-6.94	-6.94	-6.94	-6.94	-6.94	-6.94	-6.94	-6.94	-6.94
-6.93	-6.93	-6.93	-6.93	-6.93	-6.93	-6.93	-6.93	-6.93
-6.92	-6.92	-6.92	-6.92	-6.92	-6.92	-6.92	-6.92	-6.92
-6.91	-6.91	-6.91	-6.91	-6.91	-6.91	-6.91	-6.91	-6.91
-6.9	-6.9	-6.9	-6.9	-6.9	-6.9	-6.9	-6.9	-6.9
-6.89	-6.89	-6.89	-6.89	-6.89	-6.89	-6.89	-6.89	-6.89
-6.88	-6.88	-6.88	-6.88	-6.88	-6.88	-6.88	-6.88	-6.88
-6.87	-6.87	-6.87	-6.87	-6.87	-6.87	-6.87	-6.87	-6.87
-6.86	-6.86	-6.86	-6.86	-6.86	-6.86	-6.86	-6.86	-6.86
-6.85	-6.85	-6.85	-6.85	-6.85	-6.85	-6.85	-6.85	-6.85
-6.84	-6.84	-6.84	-6.84	-6.84	-6.84	-6.84	-6.84	-6.84
-6.83	-6.83	-6.83	-6.83	-6.83	-6.83	-6.83	-6.83	-6.83
-6.82	-6.82	-6.82	-6.82	-6.82	-6.82	-6.82	-6.82	-6.82
-6.81	-6.81	-6.81	-6.81	-6.81	-6.81	-6.81	-6.81	-6.81
-6.8	-6.8	-6.8	-6.8	-6.8	-6.8	-6.8	-6.8	-6.8
-6.79	-6.79	-6.79	-6.79	-6.79	-6.79	-6.79	-6.79	-6.79
-6.78	-6.78	-6.78	-6.78	-6.78	-6.78	-6.78	-6.78	-6.78
-6.77	-6.77	-6.77	-6.77	-6.77	-6.77	-6.77	-6.77	-6.77
-6.76	-6.76	-6.76	-6.76	-6.76	-6.76	-6.76	-6.76	-6.76
-6.75	-6.75	-6.75	-6.75	-6.75	-6.75	-6.75	-6.75	-6.75
-6.74	-6.74	-6.74	-6.74	-6.74	-6.74	-6.74	-6.74	-6.74
-6.73	-6.73	-6.73	-6.73	-6.73	-6.73	-6.73	-6.73	-6.73
-6.72	-6.72	-6.72	-6.72	-6.72	-6.72	-6.72	-6.72	-6.72
-6.71	-6.71	-6.71	-6.71	-6.71	-6.71	-6.71	-6.71	-6.71
-6.7	-6.7	-6.7	-6.7	-6.7	-6.7	-6.7	-6.7	-6.7
-6.69	-6.69	-6.69	-6.69	-6.69	-6.69	-6.69	-6.69	-6.69
-6.68	-6.68	-6.68	-6.68	-6.68	-6.68	-6.68	-6.68	-6.68
-6.67	-6.67	-6.67	-6.67	-6.67	-6.67	-6.67	-6.67	-6.67
-6.66	-6.66	-6.66	-6.66	-6.66	-6.66	-6.66	-6.66	-6.66
-6.65	-6.65	-6.65	-6.65	-6.65	-6.65	-6.65	-6.65	-6.65
-6.64	-6.64	-6.64	-6.64	-6.64	-6.64	-6.64	-6.64	-6.64
-6.63	-6.63	-6.63	-6.63	-6.63	-6.63	-6.63	-6.63	-6.63
-6.62	-6.62	-6.62	-6.62	-6.62	-6.62	-6.62	-6.62	-6.62
-6.61	-6.61	-6.61	-6.61	-6.61	-6.61	-6.61	-6.61	-6.61
-6.6	-6.6	-6.6	-6.6	-6.6	-6.6	-6.6	-6.6	-6.6
-6.59	-6.59	-6.59	-6.59	-6.59	-6.59	-6.59	-6.59	-6.59
-6.58	-6.58	-6.58	-6.58	-6.58	-6.58	-6.58	-6.58	-6.58
-6.57	-6.57	-6.57	-6.57	-6.57	-6.57	-6.57	-6.57	-6.57
-6.56	-6.56	-6.56	-6.56	-6.56	-6.56	-6.56	-6.56	-6.56
-6.55	-6.55	-6.55	-6.55	-6.55	-6.55	-6.55	-6.55	-6.55
-6.54	-6.54	-6.54	-6.54	-6.54	-6.54	-6.54	-6.54	-6.54
-6.53	-6.53	-6.53	-6.53	-6.53	-6.53	-6.53	-6.53	-6.53
-6.52	-6.52	-6.52	-6.52	-6.52	-6.52	-6.52	-6.52	-6.52
-6.51	-6.51	-6.51	-6.51	-6.51	-6.51	-6.51	-6.51	-6.51

-6.5	-6.5	-6.5	-6.5	-6.5	-6.5	-6.5	-6.5	-6.5
-6.49	-6.49	-6.49	-6.49	-6.49	-6.49	-6.49	-6.49	-6.49
-6.48	-6.48	-6.48	-6.48	-6.48	-6.48	-6.48	-6.48	-6.48
-6.47	-6.47	-6.47	-6.47	-6.47	-6.47	-6.47	-6.47	-6.47
-6.46	-6.46	-6.46	-6.46	-6.46	-6.46	-6.46	-6.46	-6.46
-6.45	-6.45	-6.45	-6.45	-6.45	-6.45	-6.45	-6.45	-6.45
-6.44	-6.44	-6.44	-6.44	-6.44	-6.44	-6.44	-6.44	-6.44
-6.43	-6.43	-6.43	-6.43	-6.43	-6.43	-6.43	-6.43	-6.43
-6.42	-6.42	-6.42	-6.42	-6.42	-6.42	-6.42	-6.42	-6.42
-6.41	-6.41	-6.41	-6.41	-6.41	-6.41	-6.41	-6.41	-6.41
-6.4	-6.4	-6.4	-6.4	-6.4	-6.4	-6.4	-6.4	-6.4
-6.39	-6.39	-6.39	-6.39	-6.39	-6.39	-6.39	-6.39	-6.39
-6.38	-6.38	-6.38	-6.38	-6.38	-6.38	-6.38	-6.38	-6.38
-6.37	-6.37	-6.37	-6.37	-6.37	-6.37	-6.37	-6.37	-6.37
-6.36	-6.36	-6.36	-6.36	-6.36	-6.36	-6.36	-6.36	-6.36
-6.35	-6.35	-6.35	-6.35	-6.35	-6.35	-6.35	-6.35	-6.35
-6.34	-6.34	-6.34	-6.34	-6.34	-6.34	-6.34	-6.34	-6.34
-6.33	-6.33	-6.33	-6.33	-6.33	-6.33	-6.33	-6.33	-6.33
-6.32	-6.32	-6.32	-6.32	-6.32	-6.32	-6.32	-6.32	-6.32
-6.31	-6.31	-6.31	-6.31	-6.31	-6.31	-6.31	-6.31	-6.31
-6.3	-6.3	-6.3	-6.3	-6.3	-6.3	-6.3	-6.3	-6.3
-6.29	-6.29	-6.29	-6.29	-6.29	-6.29	-6.29	-6.29	-6.29
-6.28	-6.28	-6.28	-6.28	-6.28	-6.28	-6.28	-6.28	-6.28
-6.27	-6.27	-6.27	-6.27	-6.27	-6.27	-6.27	-6.27	-6.27
-6.26	-6.26	-6.26	-6.26	-6.26	-6.26	-6.26	-6.26	-6.26
-6.25	-6.25	-6.25	-6.25	-6.25	-6.25	-6.25	-6.25	-6.25
-6.24	-6.24	-6.24	-6.24	-6.24	-6.24	-6.24	-6.24	-6.24
-6.23	-6.23	-6.23	-6.23	-6.23	-6.23	-6.23	-6.23	-6.23
-6.22	-6.22	-6.22	-6.22	-6.22	-6.22	-6.22	-6.22	-6.22
-6.21	-6.21	-6.21	-6.21	-6.21	-6.21	-6.21	-6.21	-6.21
-6.2	-6.2	-6.2	-6.2	-6.2	-6.2	-6.2	-6.2	-6.2
-6.19	-6.19	-6.19	-6.19	-6.19	-6.19	-6.19	-6.19	-6.19
-6.18	-6.18	-6.18	-6.18	-6.18	-6.18	-6.18	-6.18	-6.18
-6.17	-6.17	-6.17	-6.17	-6.17	-6.17	-6.17	-6.17	-6.17
-6.16	-6.16	-6.16	-6.16	-6.16	-6.16	-6.16	-6.16	-6.16
-6.15	-6.15	-6.15	-6.15	-6.15	-6.15	-6.15	-6.15	-6.15
-6.14	-6.14	-6.14	-6.14	-6.14	-6.14	-6.14	-6.14	-6.14
-6.13	-6.13	-6.13	-6.13	-6.13	-6.13	-6.13	-6.13	-6.13
-6.12	-6.12	-6.12	-6.12	-6.12	-6.12	-6.12	-6.12	-6.12
-6.11	-6.11	-6.11	-6.11	-6.11	-6.11	-6.11	-6.11	-6.11
-6.1	-6.1	-6.1	-6.1	-6.1	-6.1	-6.1	-6.1	-6.1
-6.09	-6.09	-6.09	-6.09	-6.09	-6.09	-6.09	-6.09	-6.09
-6.08	-6.08	-6.08	-6.08	-6.08	-6.08	-6.08	-6.08	-6.08
-6.07	-6.07	-6.07	-6.07	-6.07	-6.07	-6.07	-6.07	-6.07
-6.06	-6.06	-6.06	-6.06	-6.06	-6.06	-6.06	-6.06	-6.06
-6.05	-6.05	-6.05	-6.05	-6.05	-6.05	-6.05	-6.05	-6.05
-6.04	-6.04	-6.04	-6.04	-6.04	-6.04	-6.04	-6.04	-6.04
-6.03	-6.03	-6.03	-6.03	-6.03	-6.03	-6.03	-6.03	-6.03
-6.02	-6.02	-6.02	-6.02	-6.02	-6.02	-6.02	-6.02	-6.02
-6.01	-6.01	-6.01	-6.01	-6.01	-6.01	-6.01	-6.01	-6.01

-6	-6	-6	-6	-6	-6	-6	-6	-6
-5.99	-5.99	-5.99	-5.99	-5.99	-5.99	-5.99	-5.99	-5.99
-5.98	-5.98	-5.98	-5.98	-5.98	-5.98	-5.98	-5.98	-5.98
-5.97	-5.97	-5.97	-5.97	-5.97	-5.97	-5.97	-5.97	-5.97
-5.96	-5.96	-5.96	-5.96	-5.96	-5.96	-5.96	-5.96	-5.96
-5.95	-5.95	-5.95	-5.95	-5.95	-5.95	-5.95	-5.95	-5.95
-5.94	-5.94	-5.94	-5.94	-5.94	-5.94	-5.94	-5.94	-5.94
-5.93	-5.93	-5.93	-5.93	-5.93	-5.93	-5.93	-5.93	-5.93
-5.92	-5.92	-5.92	-5.92	-5.92	-5.92	-5.92	-5.92	-5.92
-5.91	-5.91	-5.91	-5.91	-5.91	-5.91	-5.91	-5.91	-5.91
-5.9	-5.9	-5.9	-5.9	-5.9	-5.9	-5.9	-5.9	-5.9
-5.89	-5.89	-5.89	-5.89	-5.89	-5.89	-5.89	-5.89	-5.89
-5.88	-5.88	-5.88	-5.88	-5.88	-5.88	-5.88	-5.88	-5.88
-5.87	-5.87	-5.87	-5.87	-5.87	-5.87	-5.87	-5.87	-5.87
-5.86	-5.86	-5.86	-5.86	-5.86	-5.86	-5.86	-5.86	-5.86
-5.85	-5.85	-5.85	-5.85	-5.85	-5.85	-5.85	-5.85	-5.85
-5.84	-5.84	-5.84	-5.84	-5.84	-5.84	-5.84	-5.84	-5.84
-5.83	-5.83	-5.83	-5.83	-5.83	-5.83	-5.83	-5.83	-5.83
-5.82	-5.82	-5.82	-5.82	-5.82	-5.82	-5.82	-5.82	-5.82
-5.81	-5.81	-5.81	-5.81	-5.81	-5.81	-5.81	-5.81	-5.81
-5.8	-5.8	-5.8	-5.8	-5.8	-5.8	-5.8	-5.8	-5.8
-5.79	-5.79	-5.79	-5.79	-5.79	-5.79	-5.79	-5.79	-5.79
-5.78	-5.78	-5.78	-5.78	-5.78	-5.78	-5.78	-5.78	-5.78
-5.77	-5.77	-5.77	-5.77	-5.77	-5.77	-5.77	-5.77	-5.77
-5.76	-5.76	-5.76	-5.76	-5.76	-5.76	-5.76	-5.76	-5.76
-5.75	-5.75	-5.75	-5.75	-5.75	-5.75	-5.75	-5.75	-5.75
-5.74	-5.74	-5.74	-5.74	-5.74	-5.74	-5.74	-5.74	-5.74
-5.73	-5.73	-5.73	-5.73	-5.73	-5.73	-5.73	-5.73	-5.73
-5.72	-5.72	-5.72	-5.72	-5.72	-5.72	-5.72	-5.72	-5.72
-5.71	-5.71	-5.71	-5.71	-5.71	-5.71	-5.71	-5.71	-5.71
-5.7	-5.7	-5.7	-5.7	-5.7	-5.7	-5.7	-5.7	-5.7
-5.69	-5.69	-5.69	-5.69	-5.69	-5.69	-5.69	-5.69	-5.69
-5.68	-5.68	-5.68	-5.68	-5.68	-5.68	-5.68	-5.68	-5.68
-5.67	-5.67	-5.67	-5.67	-5.67	-5.67	-5.67	-5.67	-5.67
-5.66	-5.66	-5.66	-5.66	-5.66	-5.66	-5.66	-5.66	-5.66
-5.65	-5.65	-5.65	-5.65	-5.65	-5.65	-5.65	-5.65	-5.65
-5.64	-5.64	-5.64	-5.64	-5.64	-5.64	-5.64	-5.64	-5.64
-5.63	-5.63	-5.63	-5.63	-5.63	-5.63	-5.63	-5.63	-5.63
-5.62	-5.62	-5.62	-5.62	-5.62	-5.62	-5.62	-5.62	-5.62
-5.61	-5.61	-5.61	-5.61	-5.61	-5.61	-5.61	-5.61	-5.61
-5.6	-5.6	-5.6	-5.6	-5.6	-5.6	-5.6	-5.6	-5.6
-5.59	-5.59	-5.59	-5.59	-5.59	-5.59	-5.59	-5.59	-5.59
-5.58	-5.58	-5.58	-5.58	-5.58	-5.58	-5.58	-5.58	-5.58
-5.57	-5.57	-5.57	-5.57	-5.57	-5.57	-5.57	-5.57	-5.57
-5.56	-5.56	-5.56	-5.56	-5.56	-5.56	-5.56	-5.56	-5.56
-5.55	-5.55	-5.55	-5.55	-5.55	-5.55	-5.55	-5.55	-5.55
-5.54	-5.54	-5.54	-5.54	-5.54	-5.54	-5.54	-5.54	-5.54
-5.53	-5.53	-5.53	-5.53	-5.53	-5.53	-5.53	-5.53	-5.53
-5.52	-5.52	-5.52	-5.52	-5.52	-5.52	-5.52	-5.52	-5.52
-5.51	-5.51	-5.51	-5.51	-5.51	-5.51	-5.51	-5.51	-5.51

-5.5	-5.5	-5.5	-5.5	-5.5	-5.5	-5.5	-5.5	-5.5
-5.49	-5.49	-5.49	-5.49	-5.49	-5.49	-5.49	-5.49	-5.49
-5.48	-5.48	-5.48	-5.48	-5.48	-5.48	-5.48	-5.48	-5.48
-5.47	-5.47	-5.47	-5.47	-5.47	-5.47	-5.47	-5.47	-5.47
-5.46	-5.46	-5.46	-5.46	-5.46	-5.46	-5.46	-5.46	-5.46
-5.45	-5.45	-5.45	-5.45	-5.45	-5.45	-5.45	-5.45	-5.45
-5.44	-5.44	-5.44	-5.44	-5.44	-5.44	-5.44	-5.44	-5.44
-5.43	-5.43	-5.43	-5.43	-5.43	-5.43	-5.43	-5.43	-5.43
-5.42	-5.42	-5.42	-5.42	-5.42	-5.42	-5.42	-5.42	-5.42
-5.41	-5.41	-5.41	-5.41	-5.41	-5.41	-5.41	-5.41	-5.41
-5.4	-5.4	-5.4	-5.4	-5.4	-5.4	-5.4	-5.4	-5.4
-5.39	-5.39	-5.39	-5.39	-5.39	-5.39	-5.39	-5.39	-5.39
-5.38	-5.38	-5.38	-5.38	-5.38	-5.38	-5.38	-5.38	-5.38
-5.37	-5.37	-5.37	-5.37	-5.37	-5.37	-5.37	-5.37	-5.37
-5.36	-5.36	-5.36	-5.36	-5.36	-5.36	-5.36	-5.36	-5.36
-5.35	-5.35	-5.35	-5.35	-5.35	-5.35	-5.35	-5.35	-5.35
-5.34	-5.34	-5.34	-5.34	-5.34	-5.34	-5.34	-5.34	-5.34
-5.33	-5.33	-5.33	-5.33	-5.33	-5.33	-5.33	-5.33	-5.33
-5.32	-5.32	-5.32	-5.32	-5.32	-5.32	-5.32	-5.32	-5.32
-5.31	-5.31	-5.31	-5.31	-5.31	-5.31	-5.31	-5.31	-5.31
-5.3	-5.3	-5.3	-5.3	-5.3	-5.3	-5.3	-5.3	-5.3
-5.29	-5.29	-5.29	-5.29	-5.29	-5.29	-5.29	-5.29	-5.29
-5.28	-5.28	-5.28	-5.28	-5.28	-5.28	-5.28	-5.28	-5.28
-5.27	-5.27	-5.27	-5.27	-5.27	-5.27	-5.27	-5.27	-5.27
-5.26	-5.26	-5.26	-5.26	-5.26	-5.26	-5.26	-5.26	-5.26
-5.25	-5.25	-5.25	-5.25	-5.25	-5.25	-5.25	-5.25	-5.25
-5.24	-5.24	-5.24	-5.24	-5.24	-5.24	-5.24	-5.24	-5.24
-5.23	-5.23	-5.23	-5.23	-5.23	-5.23	-5.23	-5.23	-5.23
-5.22	-5.22	-5.22	-5.22	-5.22	-5.22	-5.22	-5.22	-5.22
-5.21	-5.21	-5.21	-5.21	-5.21	-5.21	-5.21	-5.21	-5.21
-5.2	-5.2	-5.2	-5.2	-5.2	-5.2	-5.2	-5.2	-5.2
-5.19	-5.19	-5.19	-5.19	-5.19	-5.19	-5.19	-5.19	-5.19
-5.18	-5.18	-5.18	-5.18	-5.18	-5.18	-5.18	-5.18	-5.18
-5.17	-5.17	-5.17	-5.17	-5.17	-5.17	-5.17	-5.17	-5.17
-5.16	-5.16	-5.16	-5.16	-5.16	-5.16	-5.16	-5.16	-5.16
-5.15	-5.15	-5.15	-5.15	-5.15	-5.15	-5.15	-5.15	-5.15
-5.14	-5.14	-5.14	-5.14	-5.14	-5.14	-5.14	-5.14	-5.14
-5.13	-5.13	-5.13	-5.13	-5.13	-5.13	-5.13	-5.13	-5.13
-5.12	-5.12	-5.12	-5.12	-5.12	-5.12	-5.12	-5.12	-5.12
-5.11	-5.11	-5.11	-5.11	-5.11	-5.11	-5.11	-5.11	-5.11
-5.1	-5.1	-5.1	-5.1	-5.1	-5.1	-5.1	-5.1	-5.1
-5.09	-5.09	-5.09	-5.09	-5.09	-5.09	-5.09	-5.09	-5.09
-5.08	-5.08	-5.08	-5.08	-5.08	-5.08	-5.08	-5.08	-5.08
-5.07	-5.07	-5.07	-5.07	-5.07	-5.07	-5.07	-5.07	-5.07
-5.06	-5.06	-5.06	-5.06	-5.06	-5.06	-5.06	-5.06	-5.06
-5.05	-5.05	-5.05	-5.05	-5.05	-5.05	-5.05	-5.05	-5.05
-5.04	-5.04	-5.04	-5.04	-5.04	-5.04	-5.04	-5.04	-5.04
-5.03	-5.03	-5.03	-5.03	-5.03	-5.03	-5.03	-5.03	-5.03
-5.02	-5.02	-5.02	-5.02	-5.02	-5.02	-5.02	-5.02	-5.02
-5.01	-5.01	-5.01	-5.01	-5.01	-5.01	-5.01	-5.01	-5.01

-5	-5	-5	-5	-5	-5	-5	-5	-5
-4.99	-4.99	-4.99	-4.99	-4.99	-4.99	-4.99	-4.99	-4.99
-4.98	-4.98	-4.98	-4.98	-4.98	-4.98	-4.98	-4.98	-4.98
-4.97	-4.97	-4.97	-4.97	-4.97	-4.97	-4.97	-4.97	-4.97
-4.96	-4.96	-4.96	-4.96	-4.96	-4.96	-4.96	-4.96	-4.96
-4.95	-4.95	-4.95	-4.95	-4.95	-4.95	-4.95	-4.95	-4.95
-4.94	-4.94	-4.94	-4.94	-4.94	-4.94	-4.94	-4.94	-4.94
-4.93	-4.93	-4.93	-4.93	-4.93	-4.93	-4.93	-4.93	-4.93
-4.92	-4.92	-4.92	-4.92	-4.92	-4.92	-4.92	-4.92	-4.92
-4.91	-4.91	-4.91	-4.91	-4.91	-4.91	-4.91	-4.91	-4.91
-4.9	-4.9	-4.9	-4.9	-4.9	-4.9	-4.9	-4.9	-4.9
-4.89	-4.89	-4.89	-4.89	-4.89	-4.89	-4.89	-4.89	-4.89
-4.88	-4.88	-4.88	-4.88	-4.88	-4.88	-4.88	-4.88	-4.88
-4.87	-4.87	-4.87	-4.87	-4.87	-4.87	-4.87	-4.87	-4.87
-4.86	-4.86	-4.86	-4.86	-4.86	-4.86	-4.86	-4.86	-4.86
-4.85	-4.85	-4.85	-4.85	-4.85	-4.85	-4.85	-4.85	-4.85
-4.84	-4.84	-4.84	-4.84	-4.84	-4.84	-4.84	-4.84	-4.84
-4.83	-4.83	-4.83	-4.83	-4.83	-4.83	-4.83	-4.83	-4.83
-4.82	-4.82	-4.82	-4.82	-4.82	-4.82	-4.82	-4.82	-4.82
-4.81	-4.81	-4.81	-4.81	-4.81	-4.81	-4.81	-4.81	-4.81
-4.8	-4.8	-4.8	-4.8	-4.8	-4.8	-4.8	-4.8	-4.8
-4.79	-4.79	-4.79	-4.79	-4.79	-4.79	-4.79	-4.79	-4.79
-4.78	-4.78	-4.78	-4.78	-4.78	-4.78	-4.78	-4.78	-4.78
-4.77	-4.77	-4.77	-4.77	-4.77	-4.77	-4.77	-4.77	-4.77
-4.76	-4.76	-4.76	-4.76	-4.76	-4.76	-4.76	-4.76	-4.76
-4.75	-4.75	-4.75	-4.75	-4.75	-4.75	-4.75	-4.75	-4.75
-4.74	-4.74	-4.74	-4.74	-4.74	-4.74	-4.74	-4.74	-4.74
-4.73	-4.73	-4.73	-4.73	-4.73	-4.73	-4.73	-4.73	-4.73
-4.72	-4.72	-4.72	-4.72	-4.72	-4.72	-4.72	-4.72	-4.72
-4.71	-4.71	-4.71	-4.71	-4.71	-4.71	-4.71	-4.71	-4.71
-4.7	-4.7	-4.7	-4.7	-4.7	-4.7	-4.7	-4.7	-4.7
-4.69	-4.69	-4.69	-4.69	-4.69	-4.69	-4.69	-4.69	-4.69
-4.68	-4.68	-4.68	-4.68	-4.68	-4.68	-4.68	-4.68	-4.68
-4.67	-4.67	-4.67	-4.67	-4.67	-4.67	-4.67	-4.67	-4.67
-4.66	-4.66	-4.66	-4.66	-4.66	-4.66	-4.66	-4.66	-4.66
-4.65	-4.65	-4.65	-4.65	-4.65	-4.65	-4.65	-4.65	-4.65
-4.64	-4.64	-4.64	-4.64	-4.64	-4.64	-4.64	-4.64	-4.64
-4.63	-4.63	-4.63	-4.63	-4.63	-4.63	-4.63	-4.63	-4.63
-4.62	-4.62	-4.62	-4.62	-4.62	-4.62	-4.62	-4.62	-4.62
-4.61	-4.61	-4.61	-4.61	-4.61	-4.61	-4.61	-4.61	-4.61
-4.6	-4.6	-4.6	-4.6	-4.6	-4.6	-4.6	-4.6	-4.6
-4.59	-4.59	-4.59	-4.59	-4.59	-4.59	-4.59	-4.59	-4.59
-4.58	-4.58	-4.58	-4.58	-4.58	-4.58	-4.58	-4.58	-4.58
-4.57	-4.57	-4.57	-4.57	-4.57	-4.57	-4.57	-4.57	-4.57
-4.56	-4.56	-4.56	-4.56	-4.56	-4.56	-4.56	-4.56	-4.56
-4.55	-4.55	-4.55	-4.55	-4.55	-4.55	-4.55	-4.55	-4.55
-4.54	-4.54	-4.54	-4.54	-4.54	-4.54	-4.54	-4.54	-4.54
-4.53	-4.53	-4.53	-4.53	-4.53	-4.53	-4.53	-4.53	-4.53
-4.52	-4.52	-4.52	-4.52	-4.52	-4.52	-4.52	-4.52	-4.52
-4.51	-4.51	-4.51	-4.51	-4.51	-4.51	-4.51	-4.51	-4.51

-4.5	-4.5	-4.5	-4.5	-4.5	-4.5	-4.5	-4.5	-4.5
-4.49	-4.49	-4.49	-4.49	-4.49	-4.49	-4.49	-4.49	-4.49
-4.48	-4.48	-4.48	-4.48	-4.48	-4.48	-4.48	-4.48	-4.48
-4.47	-4.47	-4.47	-4.47	-4.47	-4.47	-4.47	-4.47	-4.47
-4.46	-4.46	-4.46	-4.46	-4.46	-4.46	-4.46	-4.46	-4.46
-4.45	-4.45	-4.45	-4.45	-4.45	-4.45	-4.45	-4.45	-4.45
-4.44	-4.44	-4.44	-4.44	-4.44	-4.44	-4.44	-4.44	-4.44
-4.43	-4.43	-4.43	-4.43	-4.43	-4.43	-4.43	-4.43	-4.43
-4.42	-4.42	-4.42	-4.42	-4.42	-4.42	-4.42	-4.42	-4.42
-4.41	-4.41	-4.41	-4.41	-4.41	-4.41	-4.41	-4.41	-4.41
-4.4	-4.4	-4.4	-4.4	-4.4	-4.4	-4.4	-4.4	-4.4
-4.39	-4.39	-4.39	-4.39	-4.39	-4.39	-4.39	-4.39	-4.39
-4.38	-4.38	-4.38	-4.38	-4.38	-4.38	-4.38	-4.38	-4.38
-4.37	-4.37	-4.37	-4.37	-4.37	-4.37	-4.37	-4.37	-4.37
-4.36	-4.36	-4.36	-4.36	-4.36	-4.36	-4.36	-4.36	-4.36
-4.35	-4.35	-4.35	-4.35	-4.35	-4.35	-4.35	-4.35	-4.35
-4.34	-4.34	-4.34	-4.34	-4.34	-4.34	-4.34	-4.34	-4.34
-4.33	-4.33	-4.33	-4.33	-4.33	-4.33	-4.33	-4.33	-4.33
-4.32	-4.32	-4.32	-4.32	-4.32	-4.32	-4.32	-4.32	-4.32
-4.31	-4.31	-4.31	-4.31	-4.31	-4.31	-4.31	-4.31	-4.31
-4.3	-4.3	-4.3	-4.3	-4.3	-4.3	-4.3	-4.3	-4.3
-4.29	-4.29	-4.29	-4.29	-4.29	-4.29	-4.29	-4.29	-4.29
-4.28	-4.28	-4.28	-4.28	-4.28	-4.28	-4.28	-4.28	-4.28
-4.27	-4.27	-4.27	-4.27	-4.27	-4.27	-4.27	-4.27	-4.27
-4.26	-4.26	-4.26	-4.26	-4.26	-4.26	-4.26	-4.26	-4.26
-4.25	-4.25	-4.25	-4.25	-4.25	-4.25	-4.25	-4.25	-4.25
-4.24	-4.24	-4.24	-4.24	-4.24	-4.24	-4.24	-4.24	-4.24
-4.23	-4.23	-4.23	-4.23	-4.23	-4.23	-4.23	-4.23	-4.23
-4.22	-4.22	-4.22	-4.22	-4.22	-4.22	-4.22	-4.22	-4.22
-4.21	-4.21	-4.21	-4.21	-4.21	-4.21	-4.21	-4.21	-4.21
-4.2	-4.2	-4.2	-4.2	-4.2	-4.2	-4.2	-4.2	-4.2
-4.19	-4.19	-4.19	-4.19	-4.19	-4.19	-4.19	-4.19	-4.19
-4.18	-4.18	-4.18	-4.18	-4.18	-4.18	-4.18	-4.18	-4.18
-4.17	-4.17	-4.17	-4.17	-4.17	-4.17	-4.17	-4.17	-4.17
-4.16	-4.16	-4.16	-4.16	-4.16	-4.16	-4.16	-4.16	-4.16
-4.15	-4.15	-4.15	-4.15	-4.15	-4.15	-4.15	-4.15	-4.15
-4.14	-4.14	-4.14	-4.14	-4.14	-4.14	-4.14	-4.14	-4.14
-4.13	-4.13	-4.13	-4.13	-4.13	-4.13	-4.13	-4.13	-4.13
-4.12	-4.12	-4.12	-4.12	-4.12	-4.12	-4.12	-4.12	-4.12
-4.11	-4.11	-4.11	-4.11	-4.11	-4.11	-4.11	-4.11	-4.11
-4.1	-4.1	-4.1	-4.1	-4.1	-4.1	-4.1	-4.1	-4.1
-4.09	-4.09	-4.09	-4.09	-4.09	-4.09	-4.09	-4.09	-4.09
-4.08	-4.08	-4.08	-4.08	-4.08	-4.08	-4.08	-4.08	-4.08
-4.07	-4.07	-4.07	-4.07	-4.07	-4.07	-4.07	-4.07	-4.07
-4.06	-4.06	-4.06	-4.06	-4.06	-4.06	-4.06	-4.06	-4.06
-4.05	-4.05	-4.05	-4.05	-4.05	-4.05	-4.05	-4.05	-4.05
-4.04	-4.04	-4.04	-4.04	-4.04	-4.04	-4.04	-4.04	-4.04
-4.03	-4.03	-4.03	-4.03	-4.03	-4.03	-4.03	-4.03	-4.03
-4.02	-4.02	-4.02	-4.02	-4.02	-4.02	-4.02	-4.02	-4.02
-4.01	-4.01	-4.01	-4.01	-4.01	-4.01	-4.01	-4.01	-4.01

-4	-4	-4	-4	-4	-4	-4	-4	-4
-3.99	-3.99	-3.99	-3.99	-3.99	-3.99	-3.99	-3.99	-3.99
-3.98	-3.98	-3.98	-3.98	-3.98	-3.98	-3.98	-3.98	-3.98
-3.97	-3.97	-3.97	-3.97	-3.97	-3.97	-3.97	-3.97	-3.97
-3.96	-3.96	-3.96	-3.96	-3.96	-3.96	-3.96	-3.96	-3.96
-3.95	-3.95	-3.95	-3.95	-3.95	-3.95	-3.95	-3.95	-3.95
-3.94	-3.94	-3.94	-3.94	-3.94	-3.94	-3.94	-3.94	-3.94
-3.93	-3.93	-3.93	-3.93	-3.93	-3.93	-3.93	-3.93	-3.93
-3.92	-3.92	-3.92	-3.92	-3.92	-3.92	-3.92	-3.92	-3.92
-3.91	-3.91	-3.91	-3.91	-3.91	-3.91	-3.91	-3.91	-3.91
-3.9	-3.9	-3.9	-3.9	-3.9	-3.9	-3.9	-3.9	-3.9
-3.89	-3.89	-3.89	-3.89	-3.89	-3.89	-3.89	-3.89	-3.89
-3.88	-3.88	-3.88	-3.88	-3.88	-3.88	-3.88	-3.88	-3.88
-3.87	-3.87	-3.87	-3.87	-3.87	-3.87	-3.87	-3.87	-3.87
-3.86	-3.86	-3.86	-3.86	-3.86	-3.86	-3.86	-3.86	-3.86
-3.85	-3.85	-3.85	-3.85	-3.85	-3.85	-3.85	-3.85	-3.85
-3.84	-3.84	-3.84	-3.84	-3.84	-3.84	-3.84	-3.84	-3.84
-3.83	-3.83	-3.83	-3.83	-3.83	-3.83	-3.83	-3.83	-3.83
-3.82	-3.82	-3.82	-3.82	-3.82	-3.82	-3.82	-3.82	-3.82
-3.81	-3.81	-3.81	-3.81	-3.81	-3.81	-3.81	-3.81	-3.81
-3.8	-3.8	-3.8	-3.8	-3.8	-3.8	-3.8	-3.8	-3.8
-3.79	-3.79	-3.79	-3.79	-3.79	-3.79	-3.79	-3.79	-3.79
-3.78	-3.78	-3.78	-3.78	-3.78	-3.78	-3.78	-3.78	-3.78
-3.77	-3.77	-3.77	-3.77	-3.77	-3.77	-3.77	-3.77	-3.77
-3.76	-3.76	-3.76	-3.76	-3.76	-3.76	-3.76	-3.76	-3.76
-3.75	-3.75	-3.75	-3.75	-3.75	-3.75	-3.75	-3.75	-3.75
-3.74	-3.74	-3.74	-3.74	-3.74	-3.74	-3.74	-3.74	-3.74
-3.73	-3.73	-3.73	-3.73	-3.73	-3.73	-3.73	-3.73	-3.73
-3.72	-3.72	-3.72	-3.72	-3.72	-3.72	-3.72	-3.72	-3.72
-3.71	-3.71	-3.71	-3.71	-3.71	-3.71	-3.71	-3.71	-3.71
-3.7	-3.7	-3.7	-3.7	-3.7	-3.7	-3.7	-3.7	-3.7
-3.69	-3.69	-3.69	-3.69	-3.69	-3.69	-3.69	-3.69	-3.69
-3.68	-3.68	-3.68	-3.68	-3.68	-3.68	-3.68	-3.68	-3.68
-3.67	-3.67	-3.67	-3.67	-3.67	-3.67	-3.67	-3.67	-3.67
-3.66	-3.66	-3.66	-3.66	-3.66	-3.66	-3.66	-3.66	-3.66
-3.65	-3.65	-3.65	-3.65	-3.65	-3.65	-3.65	-3.65	-3.65
-3.64	-3.64	-3.64	-3.64	-3.64	-3.64	-3.64	-3.64	-3.64
-3.63	-3.63	-3.63	-3.63	-3.63	-3.63	-3.63	-3.63	-3.63
-3.62	-3.62	-3.62	-3.62	-3.62	-3.62	-3.62	-3.62	-3.62
-3.61	-3.61	-3.61	-3.61	-3.61	-3.61	-3.61	-3.61	-3.61
-3.6	-3.6	-3.6	-3.6	-3.6	-3.6	-3.6	-3.6	-3.6
-3.59	-3.59	-3.59	-3.59	-3.59	-3.59	-3.59	-3.59	-3.59
-3.58	-3.58	-3.58	-3.58	-3.58	-3.58	-3.58	-3.58	-3.58
-3.57	-3.57	-3.57	-3.57	-3.57	-3.57	-3.57	-3.57	-3.57
-3.56	-3.56	-3.56	-3.56	-3.56	-3.56	-3.56	-3.56	-3.56
-3.55	-3.55	-3.55	-3.55	-3.55	-3.55	-3.55	-3.55	-3.55
-3.54	-3.54	-3.54	-3.54	-3.54	-3.54	-3.54	-3.54	-3.54
-3.53	-3.53	-3.53	-3.53	-3.53	-3.53	-3.53	-3.53	-3.53
-3.52	-3.52	-3.52	-3.52	-3.52	-3.52	-3.52	-3.52	-3.52
-3.51	-3.51	-3.51	-3.51	-3.51	-3.51	-3.51	-3.51	-3.51

-3.5	-3.5	-3.5	-3.5	-3.5	-3.5	-3.5	-3.5	-3.5
-3.49	-3.49	-3.49	-3.49	-3.49	-3.49	-3.49	-3.49	-3.49
-3.48	-3.48	-3.48	-3.48	-3.48	-3.48	-3.48	-3.48	-3.48
-3.47	-3.47	-3.47	-3.47	-3.47	-3.47	-3.47	-3.47	-3.47
-3.46	-3.46	-3.46	-3.46	-3.46	-3.46	-3.46	-3.46	-3.46
-3.45	-3.45	-3.45	-3.45	-3.45	-3.45	-3.45	-3.45	-3.45
-3.44	-3.44	-3.44	-3.44	-3.44	-3.44	-3.44	-3.44	-3.44
-3.43	-3.43	-3.43	-3.43	-3.43	-3.43	-3.43	-3.43	-3.43
-3.42	-3.42	-3.42	-3.42	-3.42	-3.42	-3.42	-3.42	-3.42
-3.41	-3.41	-3.41	-3.41	-3.41	-3.41	-3.41	-3.41	-3.41
-3.4	-3.4	-3.4	-3.4	-3.4	-3.4	-3.4	-3.4	-3.4
-3.39	-3.39	-3.39	-3.39	-3.39	-3.39	-3.39	-3.39	-3.39
-3.38	-3.38	-3.38	-3.38	-3.38	-3.38	-3.38	-3.38	-3.38
-3.37	-3.37	-3.37	-3.37	-3.37	-3.37	-3.37	-3.37	-3.37
-3.36	-3.36	-3.36	-3.36	-3.36	-3.36	-3.36	-3.36	-3.36
-3.35	-3.35	-3.35	-3.35	-3.35	-3.35	-3.35	-3.35	-3.35
-3.34	-3.34	-3.34	-3.34	-3.34	-3.34	-3.34	-3.34	-3.34
-3.33	-3.33	-3.33	-3.33	-3.33	-3.33	-3.33	-3.33	-3.33
-3.32	-3.32	-3.32	-3.32	-3.32	-3.32	-3.32	-3.32	-3.32
-3.31	-3.31	-3.31	-3.31	-3.31	-3.31	-3.31	-3.31	-3.31
-3.3	-3.3	-3.3	-3.3	-3.3	-3.3	-3.3	-3.3	-3.3
-3.29	-3.29	-3.29	-3.29	-3.29	-3.29	-3.29	-3.29	-3.29
-3.28	-3.28	-3.28	-3.28	-3.28	-3.28	-3.28	-3.28	-3.28
-3.27	-3.27	-3.27	-3.27	-3.27	-3.27	-3.27	-3.27	-3.27
-3.26	-3.26	-3.26	-3.26	-3.26	-3.26	-3.26	-3.26	-3.26
-3.25	-3.25	-3.25	-3.25	-3.25	-3.25	-3.25	-3.25	-3.25
-3.24	-3.24	-3.24	-3.24	-3.24	-3.24	-3.24	-3.24	-3.24
-3.23	-3.23	-3.23	-3.23	-3.23	-3.23	-3.23	-3.23	-3.23
-3.22	-3.22	-3.22	-3.22	-3.22	-3.22	-3.22	-3.22	-3.22
-3.21	-3.21	-3.21	-3.21	-3.21	-3.21	-3.21	-3.21	-3.21
-3.2	-3.2	-3.2	-3.2	-3.2	-3.2	-3.2	-3.2	-3.2
-3.19	-3.19	-3.19	-3.19	-3.19	-3.19	-3.19	-3.19	-3.19
-3.18	-3.18	-3.18	-3.18	-3.18	-3.18	-3.18	-3.18	-3.18
-3.17	-3.17	-3.17	-3.17	-3.17	-3.17	-3.17	-3.17	-3.17
-3.16	-3.16	-3.16	-3.16	-3.16	-3.16	-3.16	-3.16	-3.16
-3.15	-3.15	-3.15	-3.15	-3.15	-3.15	-3.15	-3.15	-3.15
-3.14	-3.14	-3.14	-3.14	-3.14	-3.14	-3.14	-3.14	-3.14
-3.13	-3.13	-3.13	-3.13	-3.13	-3.13	-3.13	-3.13	-3.13
-3.12	-3.12	-3.12	-3.12	-3.12	-3.12	-3.12	-3.12	-3.12
-3.11	-3.11	-3.11	-3.11	-3.11	-3.11	-3.11	-3.11	-3.11
-3.1	-3.1	-3.1	-3.1	-3.1	-3.1	-3.1	-3.1	-3.1
-3.09	-3.09	-3.09	-3.09	-3.09	-3.09	-3.09	-3.09	-3.09
-3.08	-3.08	-3.08	-3.08	-3.08	-3.08	-3.08	-3.08	-3.08
-3.07	-3.07	-3.07	-3.07	-3.07	-3.07	-3.07	-3.07	-3.07
-3.06	-3.06	-3.06	-3.06	-3.06	-3.06	-3.06	-3.06	-3.06
-3.05	-3.05	-3.05	-3.05	-3.05	-3.05	-3.05	-3.05	-3.05
-3.04	-3.04	-3.04	-3.04	-3.04	-3.04	-3.04	-3.04	-3.04
-3.03	-3.03	-3.03	-3.03	-3.03	-3.03	-3.03	-3.03	-3.03
-3.02	-3.02	-3.02	-3.02	-3.02	-3.02	-3.02	-3.02	-3.02
-3.01	-3.01	-3.01	-3.01	-3.01	-3.01	-3.01	-3.01	-3.01

-3	-3	-3	-3	-3	-3	-3	-3	-3
-2.99	-2.99	-2.99	-2.99	-2.99	-2.99	-2.99	-2.99	-2.99
-2.98	-2.98	-2.98	-2.98	-2.98	-2.98	-2.98	-2.98	-2.98
-2.97	-2.97	-2.97	-2.97	-2.97	-2.97	-2.97	-2.97	-2.97
-2.96	-2.96	-2.96	-2.96	-2.96	-2.96	-2.96	-2.96	-2.96
-2.95	-2.95	-2.95	-2.95	-2.95	-2.95	-2.95	-2.95	-2.95
-2.94	-2.94	-2.94	-2.94	-2.94	-2.94	-2.94	-2.94	-2.94
-2.93	-2.93	-2.93	-2.93	-2.93	-2.93	-2.93	-2.93	-2.93
-2.92	-2.92	-2.92	-2.92	-2.92	-2.92	-2.92	-2.92	-2.92
-2.91	-2.91	-2.91	-2.91	-2.91	-2.91	-2.91	-2.91	-2.91
-2.9	-2.9	-2.9	-2.9	-2.9	-2.9	-2.9	-2.9	-2.9
-2.89	-2.89	-2.89	-2.89	-2.89	-2.89	-2.89	-2.89	-2.89
-2.88	-2.88	-2.88	-2.88	-2.88	-2.88	-2.88	-2.88	-2.88
-2.87	-2.87	-2.87	-2.87	-2.87	-2.87	-2.87	-2.87	-2.87
-2.86	-2.86	-2.86	-2.86	-2.86	-2.86	-2.86	-2.86	-2.86
-2.85	-2.85	-2.85	-2.85	-2.85	-2.85	-2.85	-2.85	-2.85
-2.84	-2.84	-2.84	-2.84	-2.84	-2.84	-2.84	-2.84	-2.84
-2.83	-2.83	-2.83	-2.83	-2.83	-2.83	-2.83	-2.83	-2.83
-2.82	-2.82	-2.82	-2.82	-2.82	-2.82	-2.82	-2.82	-2.82
-2.81	-2.81	-2.81	-2.81	-2.81	-2.81	-2.81	-2.81	-2.81
-2.8	-2.8	-2.8	-2.8	-2.8	-2.8	-2.8	-2.8	-2.8
-2.79	-2.79	-2.79	-2.79	-2.79	-2.79	-2.79	-2.79	-2.79
-2.78	-2.78	-2.78	-2.78	-2.78	-2.78	-2.78	-2.78	-2.78
-2.77	-2.77	-2.77	-2.77	-2.77	-2.77	-2.77	-2.77	-2.77
-2.76	-2.76	-2.76	-2.76	-2.76	-2.76	-2.76	-2.76	-2.76
-2.75	-2.75	-2.75	-2.75	-2.75	-2.75	-2.75	-2.75	-2.75
-2.74	-2.74	-2.74	-2.74	-2.74	-2.74	-2.74	-2.74	-2.74
-2.73	-2.73	-2.73	-2.73	-2.73	-2.73	-2.73	-2.73	-2.73
-2.72	-2.72	-2.72	-2.72	-2.72	-2.72	-2.72	-2.72	-2.72
-2.71	-2.71	-2.71	-2.71	-2.71	-2.71	-2.71	-2.71	-2.71
-2.7	-2.7	-2.7	-2.7	-2.7	-2.7	-2.7	-2.7	-2.7
-2.69	-2.69	-2.69	-2.69	-2.69	-2.69	-2.69	-2.69	-2.69
-2.68	-2.68	-2.68	-2.68	-2.68	-2.68	-2.68	-2.68	-2.68
-2.67	-2.67	-2.67	-2.67	-2.67	-2.67	-2.67	-2.67	-2.67
-2.66	-2.66	-2.66	-2.66	-2.66	-2.66	-2.66	-2.66	-2.66
-2.65	-2.65	-2.65	-2.65	-2.65	-2.65	-2.65	-2.65	-2.65
-2.64	-2.64	-2.64	-2.64	-2.64	-2.64	-2.64	-2.64	-2.64
-2.63	-2.63	-2.63	-2.63	-2.63	-2.63	-2.63	-2.63	-2.63
-2.62	-2.62	-2.62	-2.62	-2.62	-2.62	-2.62	-2.62	-2.62
-2.61	-2.61	-2.61	-2.61	-2.61	-2.61	-2.61	-2.61	-2.61
-2.6	-2.6	-2.6	-2.6	-2.6	-2.6	-2.6	-2.6	-2.6
-2.59	-2.59	-2.59	-2.59	-2.59	-2.59	-2.59	-2.59	-2.59
-2.58	-2.58	-2.58	-2.58	-2.58	-2.58	-2.58	-2.58	-2.58
-2.57	-2.57	-2.57	-2.57	-2.57	-2.57	-2.57	-2.57	-2.57
-2.56	-2.56	-2.56	-2.56	-2.56	-2.56	-2.56	-2.56	-2.56
-2.55	-2.55	-2.55	-2.55	-2.55	-2.55	-2.55	-2.55	-2.55
-2.54	-2.54	-2.54	-2.54	-2.54	-2.54	-2.54	-2.54	-2.54
-2.53	-2.53	-2.53	-2.53	-2.53	-2.53	-2.53	-2.53	-2.53
-2.52	-2.52	-2.52	-2.52	-2.52	-2.52	-2.52	-2.52	-2.52
-2.51	-2.51	-2.51	-2.51	-2.51	-2.51	-2.51	-2.51	-2.51

-2.5	-2.5	-2.5	-2.5	-2.5	-2.5	-2.5	-2.5	-2.5
-2.49	-2.49	-2.49	-2.49	-2.49	-2.49	-2.49	-2.49	-2.49
-2.48	-2.48	-2.48	-2.48	-2.48	-2.48	-2.48	-2.48	-2.48
-2.47	-2.47	-2.47	-2.47	-2.47	-2.47	-2.47	-2.47	-2.47
-2.46	-2.46	-2.46	-2.46	-2.46	-2.46	-2.46	-2.46	-2.46
-2.45	-2.45	-2.45	-2.45	-2.45	-2.45	-2.45	-2.45	-2.45
-2.44	-2.44	-2.44	-2.44	-2.44	-2.44	-2.44	-2.44	-2.44
-2.43	-2.43	-2.43	-2.43	-2.43	-2.43	-2.43	-2.43	-2.43
-2.42	-2.42	-2.42	-2.42	-2.42	-2.42	-2.42	-2.42	-2.42
-2.41	-2.41	-2.41	-2.41	-2.41	-2.41	-2.41	-2.41	-2.41
-2.4	-2.4	-2.4	-2.4	-2.4	-2.4	-2.4	-2.4	-2.4
-2.39	-2.39	-2.39	-2.39	-2.39	-2.39	-2.39	-2.39	-2.39
-2.38	-2.38	-2.38	-2.38	-2.38	-2.38	-2.38	-2.38	-2.38
-2.37	-2.37	-2.37	-2.37	-2.37	-2.37	-2.37	-2.37	-2.37
-2.36	-2.36	-2.36	-2.36	-2.36	-2.36	-2.36	-2.36	-2.36
-2.35	-2.35	-2.35	-2.35	-2.35	-2.35	-2.35	-2.35	-2.35
-2.34	-2.34	-2.34	-2.34	-2.34	-2.34	-2.34	-2.34	-2.34
-2.33	-2.33	-2.33	-2.33	-2.33	-2.33	-2.33	-2.33	-2.33
-2.32	-2.32	-2.32	-2.32	-2.32	-2.32	-2.32	-2.32	-2.32
-2.31	-2.31	-2.31	-2.31	-2.31	-2.31	-2.31	-2.31	-2.31
-2.3	-2.3	-2.3	-2.3	-2.3	-2.3	-2.3	-2.3	-2.3
-2.29	-2.29	-2.29	-2.29	-2.29	-2.29	-2.29	-2.29	-2.29
-2.28	-2.28	-2.28	-2.28	-2.28	-2.28	-2.28	-2.28	-2.28
-2.27	-2.27	-2.27	-2.27	-2.27	-2.27	-2.27	-2.27	-2.27
-2.26	-2.26	-2.26	-2.26	-2.26	-2.26	-2.26	-2.26	-2.26
-2.25	-2.25	-2.25	-2.25	-2.25	-2.25	-2.25	-2.25	-2.25
-2.24	-2.24	-2.24	-2.24	-2.24	-2.24	-2.24	-2.24	-2.24
-2.23	-2.23	-2.23	-2.23	-2.23	-2.23	-2.23	-2.23	-2.23
-2.22	-2.22	-2.22	-2.22	-2.22	-2.22	-2.22	-2.22	-2.22
-2.21	-2.21	-2.21	-2.21	-2.21	-2.21	-2.21	-2.21	-2.21
-2.2	-2.2	-2.2	-2.2	-2.2	-2.2	-2.2	-2.2	-2.2
-2.19	-2.19	-2.19	-2.19	-2.19	-2.19	-2.19	-2.19	-2.19
-2.18	-2.18	-2.18	-2.18	-2.18	-2.18	-2.18	-2.18	-2.18
-2.17	-2.17	-2.17	-2.17	-2.17	-2.17	-2.17	-2.17	-2.17
-2.16	-2.16	-2.16	-2.16	-2.16	-2.16	-2.16	-2.16	-2.16
-2.15	-2.15	-2.15	-2.15	-2.15	-2.15	-2.15	-2.15	-2.15
-2.14	-2.14	-2.14	-2.14	-2.14	-2.14	-2.14	-2.14	-2.14
-2.13	-2.13	-2.13	-2.13	-2.13	-2.13	-2.13	-2.13	-2.13
-2.12	-2.12	-2.12	-2.12	-2.12	-2.12	-2.12	-2.12	-2.12
-2.11	-2.11	-2.11	-2.11	-2.11	-2.11	-2.11	-2.11	-2.11
-2.1	-2.1	-2.1	-2.1	-2.1	-2.1	-2.1	-2.1	-2.1
-2.09	-2.09	-2.09	-2.09	-2.09	-2.09	-2.09	-2.09	-2.09
-2.08	-2.08	-2.08	-2.08	-2.08	-2.08	-2.08	-2.08	-2.08
-2.07	-2.07	-2.07	-2.07	-2.07	-2.07	-2.07	-2.07	-2.07
-2.06	-2.06	-2.06	-2.06	-2.06	-2.06	-2.06	-2.06	-2.06
-2.05	-2.05	-2.05	-2.05	-2.05	-2.05	-2.05	-2.05	-2.05
-2.04	-2.04	-2.04	-2.04	-2.04	-2.04	-2.04	-2.04	-2.04
-2.03	-2.03	-2.03	-2.03	-2.03	-2.03	-2.03	-2.03	-2.03
-2.02	-2.02	-2.02	-2.02	-2.02	-2.02	-2.02	-2.02	-2.02
-2.01	-2.01	-2.01	-2.01	-2.01	-2.01	-2.01	-2.01	-2.01

-2	-2	-2	-2	-2	-2	-2	-2	-2
-1.99	-1.99	-1.99	-1.99	-1.99	-1.99	-1.99	-1.99	-1.99
-1.98	-1.98	-1.98	-1.98	-1.98	-1.98	-1.98	-1.98	-1.98
-1.97	-1.97	-1.97	-1.97	-1.97	-1.97	-1.97	-1.97	-1.97
-1.96	-1.96	-1.96	-1.96	-1.96	-1.96	-1.96	-1.96	-1.96
-1.95	-1.95	-1.95	-1.95	-1.95	-1.95	-1.95	-1.95	-1.95
-1.94	-1.94	-1.94	-1.94	-1.94	-1.94	-1.94	-1.94	-1.94
-1.93	-1.93	-1.93	-1.93	-1.93	-1.93	-1.93	-1.93	-1.93
-1.92	-1.92	-1.92	-1.92	-1.92	-1.92	-1.92	-1.92	-1.92
-1.91	-1.91	-1.91	-1.91	-1.91	-1.91	-1.91	-1.91	-1.91
-1.9	-1.9	-1.9	-1.9	-1.9	-1.9	-1.9	-1.9	-1.9
-1.89	-1.89	-1.89	-1.89	-1.89	-1.89	-1.89	-1.89	-1.89
-1.88	-1.88	-1.88	-1.88	-1.88	-1.88	-1.88	-1.88	-1.88
-1.87	-1.87	-1.87	-1.87	-1.87	-1.87	-1.87	-1.87	-1.87
-1.86	-1.86	-1.86	-1.86	-1.86	-1.86	-1.86	-1.86	-1.86
-1.85	-1.85	-1.85	-1.85	-1.85	-1.85	-1.85	-1.85	-1.85
-1.84	-1.84	-1.84	-1.84	-1.84	-1.84	-1.84	-1.84	-1.84
-1.83	-1.83	-1.83	-1.83	-1.83	-1.83	-1.83	-1.83	-1.83
-1.82	-1.82	-1.82	-1.82	-1.82	-1.82	-1.82	-1.82	-1.82
-1.81	-1.81	-1.81	-1.81	-1.81	-1.81	-1.81	-1.81	-1.81
-1.8	-1.8	-1.8	-1.8	-1.8	-1.8	-1.8	-1.8	-1.8
-1.79	-1.79	-1.79	-1.79	-1.79	-1.79	-1.79	-1.79	-1.79
-1.78	-1.78	-1.78	-1.78	-1.78	-1.78	-1.78	-1.78	-1.78
-1.77	-1.77	-1.77	-1.77	-1.77	-1.77	-1.77	-1.77	-1.77
-1.76	-1.76	-1.76	-1.76	-1.76	-1.76	-1.76	-1.76	-1.76
-1.75	-1.75	-1.75	-1.75	-1.75	-1.75	-1.75	-1.75	-1.75
-1.74	-1.74	-1.74	-1.74	-1.74	-1.74	-1.74	-1.74	-1.74
-1.73	-1.73	-1.73	-1.73	-1.73	-1.73	-1.73	-1.73	-1.73
-1.72	-1.72	-1.72	-1.72	-1.72	-1.72	-1.72	-1.72	-1.72
-1.71	-1.71	-1.71	-1.71	-1.71	-1.71	-1.71	-1.71	-1.71
-1.7	-1.7	-1.7	-1.7	-1.7	-1.7	-1.7	-1.7	-1.7
-1.69	-1.69	-1.69	-1.69	-1.69	-1.69	-1.69	-1.69	-1.69
-1.68	-1.68	-1.68	-1.68	-1.68	-1.68	-1.68	-1.68	-1.68
-1.67	-1.67	-1.67	-1.67	-1.67	-1.67	-1.67	-1.67	-1.67
-1.66	-1.66	-1.66	-1.66	-1.66	-1.66	-1.66	-1.66	-1.66
-1.65	-1.65	-1.65	-1.65	-1.65	-1.65	-1.65	-1.65	-1.65
-1.64	-1.64	-1.64	-1.64	-1.64	-1.64	-1.64	-1.64	-1.64
-1.63	-1.63	-1.63	-1.63	-1.63	-1.63	-1.63	-1.63	-1.63
-1.62	-1.62	-1.62	-1.62	-1.62	-1.62	-1.62	-1.62	-1.62
-1.61	-1.61	-1.61	-1.61	-1.61	-1.61	-1.61	-1.61	-1.61
-1.6	-1.6	-1.6	-1.6	-1.6	-1.6	-1.6	-1.6	-1.6
-1.59	-1.59	-1.59	-1.59	-1.59	-1.59	-1.59	-1.59	-1.59
-1.58	-1.58	-1.58	-1.58	-1.58	-1.58	-1.58	-1.58	-1.58
-1.57	-1.57	-1.57	-1.57	-1.57	-1.57	-1.57	-1.57	-1.57
-1.56	-1.56	-1.56	-1.56	-1.56	-1.56	-1.56	-1.56	-1.56
-1.55	-1.55	-1.55	-1.55	-1.55	-1.55	-1.55	-1.55	-1.55
-1.54	-1.54	-1.54	-1.54	-1.54	-1.54	-1.54	-1.54	-1.54
-1.53	-1.53	-1.53	-1.53	-1.53	-1.53	-1.53	-1.53	-1.53
-1.52	-1.52	-1.52	-1.52	-1.52	-1.52	-1.52	-1.52	-1.52
-1.51	-1.51	-1.51	-1.51	-1.51	-1.51	-1.51	-1.51	-1.51

-1.5	-1.5	-1.5	-1.5	-1.5	-1.5	-1.5	-1.5	-1.5
-1.49	-1.49	-1.49	-1.49	-1.49	-1.49	-1.49	-1.49	-1.49
-1.48	-1.48	-1.48	-1.48	-1.48	-1.48	-1.48	-1.48	-1.48
-1.47	-1.47	-1.47	-1.47	-1.47	-1.47	-1.47	-1.47	-1.47
-1.46	-1.46	-1.46	-1.46	-1.46	-1.46	-1.46	-1.46	-1.46
-1.45	-1.45	-1.45	-1.45	-1.45	-1.45	-1.45	-1.45	-1.45
-1.44	-1.44	-1.44	-1.44	-1.44	-1.44	-1.44	-1.44	-1.44
-1.43	-1.43	-1.43	-1.43	-1.43	-1.43	-1.43	-1.43	-1.43
-1.42	-1.42	-1.42	-1.42	-1.42	-1.42	-1.42	-1.42	-1.42
-1.41	-1.41	-1.41	-1.41	-1.41	-1.41	-1.41	-1.41	-1.41
-1.4	-1.4	-1.4	-1.4	-1.4	-1.4	-1.4	-1.4	-1.4
-1.39	-1.39	-1.39	-1.39	-1.39	-1.39	-1.39	-1.39	-1.39
-1.38	-1.38	-1.38	-1.38	-1.38	-1.38	-1.38	-1.38	-1.38
-1.37	-1.37	-1.37	-1.37	-1.37	-1.37	-1.37	-1.37	-1.37
-1.36	-1.36	-1.36	-1.36	-1.36	-1.36	-1.36	-1.36	-1.36
-1.35	-1.35	-1.35	-1.35	-1.35	-1.35	-1.35	-1.35	-1.35
-1.34	-1.34	-1.34	-1.34	-1.34	-1.34	-1.34	-1.34	-1.34
-1.33	-1.33	-1.33	-1.33	-1.33	-1.33	-1.33	-1.33	-1.33
-1.32	-1.32	-1.32	-1.32	-1.32	-1.32	-1.32	-1.32	-1.32
-1.31	-1.31	-1.31	-1.31	-1.31	-1.31	-1.31	-1.31	-1.31
-1.3	-1.3	-1.3	-1.3	-1.3	-1.3	-1.3	-1.3	-1.3
-1.29	-1.29	-1.29	-1.29	-1.29	-1.29	-1.29	-1.29	-1.29
-1.28	-1.28	-1.28	-1.28	-1.28	-1.28	-1.28	-1.28	-1.28
-1.27	-1.27	-1.27	-1.27	-1.27	-1.27	-1.27	-1.27	-1.27
-1.26	-1.26	-1.26	-1.26	-1.26	-1.26	-1.26	-1.26	-1.26
-1.25	-1.25	-1.25	-1.25	-1.25	-1.25	-1.25	-1.25	-1.25
-1.24	-1.24	-1.24	-1.24	-1.24	-1.24	-1.24	-1.24	-1.24
-1.23	-1.23	-1.23	-1.23	-1.23	-1.23	-1.23	-1.23	-1.23
-1.22	-1.22	-1.22	-1.22	-1.22	-1.22	-1.22	-1.22	-1.22
-1.21	-1.21	-1.21	-1.21	-1.21	-1.21	-1.21	-1.21	-1.21
-1.2	-1.2	-1.2	-1.2	-1.2	-1.2	-1.2	-1.2	-1.2
-1.19	-1.19	-1.19	-1.19	-1.19	-1.19	-1.19	-1.19	-1.19
-1.18	-1.18	-1.18	-1.18	-1.18	-1.18	-1.18	-1.18	-1.18
-1.17	-1.17	-1.17	-1.17	-1.17	-1.17	-1.17	-1.17	-1.17
-1.16	-1.16	-1.16	-1.16	-1.16	-1.16	-1.16	-1.16	-1.16
-1.15	-1.15	-1.15	-1.15	-1.15	-1.15	-1.15	-1.15	-1.15
-1.14	-1.14	-1.14	-1.14	-1.14	-1.14	-1.14	-1.14	-1.14
-1.13	-1.13	-1.13	-1.13	-1.13	-1.13	-1.13	-1.13	-1.13
-1.12	-1.12	-1.12	-1.12	-1.12	-1.12	-1.12	-1.12	-1.12
-1.11	-1.11	-1.11	-1.11	-1.11	-1.11	-1.11	-1.11	-1.11
-1.1	-1.1	-1.1	-1.1	-1.1	-1.1	-1.1	-1.1	-1.1
-1.09	-1.09	-1.09	-1.09	-1.09	-1.09	-1.09	-1.09	-1.09
-1.08	-1.08	-1.08	-1.08	-1.08	-1.08	-1.08	-1.08	-1.08
-1.07	-1.07	-1.07	-1.07	-1.07	-1.07	-1.07	-1.07	-1.07
-1.06	-1.06	-1.06	-1.06	-1.06	-1.06	-1.06	-1.06	-1.06
-1.05	-1.05	-1.05	-1.05	-1.05	-1.05	-1.05	-1.05	-1.05
-1.04	-1.04	-1.04	-1.04	-1.04	-1.04	-1.04	-1.04	-1.04
-1.03	-1.03	-1.03	-1.03	-1.03	-1.03	-1.03	-1.03	-1.03
-1.02	-1.02	-1.02	-1.02	-1.02	-1.02	-1.02	-1.02	-1.02
-1.01	-1.01	-1.01	-1.01	-1.01	-1.01	-1.01	-1.01	-1.01

-1	-1	-1	-1	-1	-1	-1	-1	-1
-0.99	-0.99	-0.99	-0.99	-0.99	-0.99	-0.99	-0.99	-0.99
-0.98	-0.98	-0.98	-0.98	-0.98	-0.98	-0.98	-0.98	-0.98
-0.97	-0.97	-0.97	-0.97	-0.97	-0.97	-0.97	-0.97	-0.97
-0.96	-0.96	-0.96	-0.96	-0.96	-0.96	-0.96	-0.96	-0.96
-0.95	-0.95	-0.95	-0.95	-0.95	-0.95	-0.95	-0.95	-0.95
-0.94	-0.94	-0.94	-0.94	-0.94	-0.94	-0.94	-0.94	-0.94
-0.93	-0.93	-0.93	-0.93	-0.93	-0.93	-0.93	-0.93	-0.93
-0.92	-0.92	-0.92	-0.92	-0.92	-0.92	-0.92	-0.92	-0.92
-0.91	-0.91	-0.91	-0.91	-0.91	-0.91	-0.91	-0.91	-0.91
-0.9	-0.9	-0.9	-0.9	-0.9	-0.9	-0.9	-0.9	-0.9
-0.89	-0.89	-0.89	-0.89	-0.89	-0.89	-0.89	-0.89	-0.89
-0.88	-0.88	-0.88	-0.88	-0.88	-0.88	-0.88	-0.88	-0.88
-0.87	-0.87	-0.87	-0.87	-0.87	-0.87	-0.87	-0.87	-0.87
-0.86	-0.86	-0.86	-0.86	-0.86	-0.86	-0.86	-0.86	-0.86
-0.85	-0.85	-0.85	-0.85	-0.85	-0.85	-0.85	-0.85	-0.85
-0.84	-0.84	-0.84	-0.84	-0.84	-0.84	-0.84	-0.84	-0.84
-0.83	-0.83	-0.83	-0.83	-0.83	-0.83	-0.83	-0.83	-0.83
-0.82	-0.82	-0.82	-0.82	-0.82	-0.82	-0.82	-0.82	-0.82
-0.81	-0.81	-0.81	-0.81	-0.81	-0.81	-0.81	-0.81	-0.81
-0.8	-0.8	-0.8	-0.8	-0.8	-0.8	-0.8	-0.8	-0.8
-0.79	-0.79	-0.79	-0.79	-0.79	-0.79	-0.79	-0.79	-0.79
-0.78	-0.78	-0.78	-0.78	-0.78	-0.78	-0.78	-0.78	-0.78
-0.77	-0.77	-0.77	-0.77	-0.77	-0.77	-0.77	-0.77	-0.77
-0.76	-0.76	-0.76	-0.76	-0.76	-0.76	-0.76	-0.76	-0.76
-0.75	-0.75	-0.75	-0.75	-0.75	-0.75	-0.75	-0.75	-0.75
-0.74	-0.74	-0.74	-0.74	-0.74	-0.74	-0.74	-0.74	-0.74
-0.73	-0.73	-0.73	-0.73	-0.73	-0.73	-0.73	-0.73	-0.73
-0.72	-0.72	-0.72	-0.72	-0.72	-0.72	-0.72	-0.72	-0.72
-0.71	-0.71	-0.71	-0.71	-0.71	-0.71	-0.71	-0.71	-0.71
-0.7	-0.7	-0.7	-0.7	-0.7	-0.7	-0.7	-0.7	-0.7
-0.69	-0.69	-0.69	-0.69	-0.69	-0.69	-0.69	-0.69	-0.69
-0.68	-0.68	-0.68	-0.68	-0.68	-0.68	-0.68	-0.68	-0.68
-0.67	-0.67	-0.67	-0.67	-0.67	-0.67	-0.67	-0.67	-0.67
-0.66	-0.66	-0.66	-0.66	-0.66	-0.66	-0.66	-0.66	-0.66
-0.65	-0.65	-0.65	-0.65	-0.65	-0.65	-0.65	-0.65	-0.65
-0.64	-0.64	-0.64	-0.64	-0.64	-0.64	-0.64	-0.64	-0.64
-0.63	-0.63	-0.63	-0.63	-0.63	-0.63	-0.63	-0.63	-0.63
-0.62	-0.62	-0.62	-0.62	-0.62	-0.62	-0.62	-0.62	-0.62
-0.61	-0.61	-0.61	-0.61	-0.61	-0.61	-0.61	-0.61	-0.61
-0.6	-0.6	-0.6	-0.6	-0.6	-0.6	-0.6	-0.6	-0.6
-0.59	-0.59	-0.59	-0.59	-0.59	-0.59	-0.59	-0.59	-0.59
-0.58	-0.58	-0.58	-0.58	-0.58	-0.58	-0.58	-0.58	-0.58
-0.57	-0.57	-0.57	-0.57	-0.57	-0.57	-0.57	-0.57	-0.57
-0.56	-0.56	-0.56	-0.56	-0.56	-0.56	-0.56	-0.56	-0.56
-0.55	-0.55	-0.55	-0.55	-0.55	-0.55	-0.55	-0.55	-0.55
-0.54	-0.54	-0.54	-0.54	-0.54	-0.54	-0.54	-0.54	-0.54
-0.53	-0.53	-0.53	-0.53	-0.53	-0.53	-0.53	-0.53	-0.53
-0.52	-0.52	-0.52	-0.52	-0.52	-0.52	-0.52	-0.52	-0.52
-0.51	-0.51	-0.51	-0.51	-0.51	-0.51	-0.51	-0.51	-0.51

-0.5	-0.5	-0.5	-0.5	-0.5	-0.5	-0.5	-0.5	-0.5
-0.49	-0.49	-0.49	-0.49	-0.49	-0.49	-0.49	-0.49	-0.49
-0.48	-0.48	-0.48	-0.48	-0.48	-0.48	-0.48	-0.48	-0.48
-0.47	-0.47	-0.47	-0.47	-0.47	-0.47	-0.47	-0.47	-0.47
-0.46	-0.46	-0.46	-0.46	-0.46	-0.46	-0.46	-0.46	-0.46
-0.45	-0.45	-0.45	-0.45	-0.45	-0.45	-0.45	-0.45	-0.45
-0.44	-0.44	-0.44	-0.44	-0.44	-0.44	-0.44	-0.44	-0.44
-0.43	-0.43	-0.43	-0.43	-0.43	-0.43	-0.43	-0.43	-0.43
-0.42	-0.42	-0.42	-0.42	-0.42	-0.42	-0.42	-0.42	-0.42
-0.41	-0.41	-0.41	-0.41	-0.41	-0.41	-0.41	-0.41	-0.41
-0.4	-0.4	-0.4	-0.4	-0.4	-0.4	-0.4	-0.4	-0.4
-0.39	-0.39	-0.39	-0.39	-0.39	-0.39	-0.39	-0.39	-0.39
-0.38	-0.38	-0.38	-0.38	-0.38	-0.38	-0.38	-0.38	-0.38
-0.37	-0.37	-0.37	-0.37	-0.37	-0.37	-0.37	-0.37	-0.37
-0.36	-0.36	-0.36	-0.36	-0.36	-0.36	-0.36	-0.36	-0.36
-0.35	-0.35	-0.35	-0.35	-0.35	-0.35	-0.35	-0.35	-0.35
-0.34	-0.34	-0.34	-0.34	-0.34	-0.34	-0.34	-0.34	-0.34
-0.33	-0.33	-0.33	-0.33	-0.33	-0.33	-0.33	-0.33	-0.33
-0.32	-0.32	-0.32	-0.32	-0.32	-0.32	-0.32	-0.32	-0.32
-0.31	-0.31	-0.31	-0.31	-0.31	-0.31	-0.31	-0.31	-0.31
-0.3	-0.3	-0.3	-0.3	-0.3	-0.3	-0.3	-0.3	-0.3
-0.29	-0.29	-0.29	-0.29	-0.29	-0.29	-0.29	-0.29	-0.29
-0.28	-0.28	-0.28	-0.28	-0.28	-0.28	-0.28	-0.28	-0.28
-0.27	-0.27	-0.27	-0.27	-0.27	-0.27	-0.27	-0.27	-0.27
-0.26	-0.26	-0.26	-0.26	-0.26	-0.26	-0.26	-0.26	-0.26
-0.25	-0.25	-0.25	-0.25	-0.25	-0.25	-0.25	-0.25	-0.25
-0.24	-0.24	-0.24	-0.24	-0.24	-0.24	-0.24	-0.24	-0.24
-0.23	-0.23	-0.23	-0.23	-0.23	-0.23	-0.23	-0.23	-0.23
-0.22	-0.22	-0.22	-0.22	-0.22	-0.22	-0.22	-0.22	-0.22
-0.21	-0.21	-0.21	-0.21	-0.21	-0.21	-0.21	-0.21	-0.21
-0.2	-0.2	-0.2	-0.2	-0.2	-0.2	-0.2	-0.2	-0.2
-0.19	-0.19	-0.19	-0.19	-0.19	-0.19	-0.19	-0.19	-0.19
-0.18	-0.18	-0.18	-0.18	-0.18	-0.18	-0.18	-0.18	-0.18
-0.17	-0.17	-0.17	-0.17	-0.17	-0.17	-0.17	-0.17	-0.17
-0.16	-0.16	-0.16	-0.16	-0.16	-0.16	-0.16	-0.16	-0.16
-0.15	-0.15	-0.15	-0.15	-0.15	-0.15	-0.15	-0.15	-0.15
-0.14	-0.14	-0.14	-0.14	-0.14	-0.14	-0.14	-0.14	-0.14
-0.13	-0.13	-0.13	-0.13	-0.13	-0.13	-0.13	-0.13	-0.13
-0.12	-0.12	-0.12	-0.12	-0.12	-0.12	-0.12	-0.12	-0.12
-0.11	-0.11	-0.11	-0.11	-0.11	-0.11	-0.11	-0.11	-0.11
-0.1	-0.1	-0.1	-0.1	-0.1	-0.1	-0.1	-0.1	-0.1
-0.09	-0.09	-0.09	-0.09	-0.09	-0.09	-0.09	-0.09	-0.09
-0.08	-0.08	-0.08	-0.08	-0.08	-0.08	-0.08	-0.08	-0.08
-0.07	-0.07	-0.07	-0.07	-0.07	-0.07	-0.07	-0.07	-0.07
-0.06	-0.06	-0.06	-0.06	-0.06	-0.06	-0.06	-0.06	-0.06
-0.05	-0.05	-0.05	-0.05	-0.05	-0.05	-0.05	-0.05	-0.05
-0.04	-0.04	-0.04	-0.04	-0.04	-0.04	-0.04	-0.04	-0.04
-0.03	-0.03	-0.03	-0.03	-0.03	-0.03	-0.03	-0.03	-0.03
-0.02	-0.02	-0.02	-0.02	-0.02	-0.02	-0.02	-0.02	-0.02
-0.01	-0.01	-0.01	-0.01	-0.01	-0.01	-0.01	-0.01	-0.01

0	0	0	0	0	0	0	0	0
0.01	0.01	0.01	0.01	0.01	0.01	0.01	0.01	0.01
0.02	0.02	0.02	0.02	0.02	0.02	0.02	0.02	0.02
0.03	0.03	0.03	0.03	0.03	0.03	0.03	0.03	0.03
0.04	0.04	0.04	0.04	0.04	0.04	0.04	0.04	0.04
0.05	0.05	0.05	0.05	0.05	0.05	0.05	0.05	0.05
0.06	0.06	0.06	0.06	0.06	0.06	0.06	0.06	0.06
0.07	0.07	0.07	0.07	0.07	0.07	0.07	0.07	0.07
0.08	0.08	0.08	0.08	0.08	0.08	0.08	0.08	0.08
0.09	0.09	0.09	0.09	0.09	0.09	0.09	0.09	0.09
0.1	0.1	0.1	0.1	0.1	0.1	0.1	0.1	0.1
0.11	0.11	0.11	0.11	0.11	0.11	0.11	0.11	0.11
0.12	0.12	0.12	0.12	0.12	0.12	0.12	0.12	0.12
0.13	0.13	0.13	0.13	0.13	0.13	0.13	0.13	0.13
0.14	0.14	0.14	0.14	0.14	0.14	0.14	0.14	0.14
0.15	0.15	0.15	0.15	0.15	0.15	0.15	0.15	0.15
0.16	0.16	0.16	0.16	0.16	0.16	0.16	0.16	0.16
0.17	0.17	0.17	0.17	0.17	0.17	0.17	0.17	0.17
0.18	0.18	0.18	0.18	0.18	0.18	0.18	0.18	0.18
0.19	0.19	0.19	0.19	0.19	0.19	0.19	0.19	0.19
0.2	0.2	0.2	0.2	0.2	0.2	0.2	0.2	0.2
0.21	0.21	0.21	0.21	0.21	0.21	0.21	0.21	0.21
0.22	0.22	0.22	0.22	0.22	0.22	0.22	0.22	0.22
0.23	0.23	0.23	0.23	0.23	0.23	0.23	0.23	0.23
0.24	0.24	0.24	0.24	0.24	0.24	0.24	0.24	0.24
0.25	0.25	0.25	0.25	0.25	0.25	0.25	0.25	0.25
0.26	0.26	0.26	0.26	0.26	0.26	0.26	0.26	0.26
0.27	0.27	0.27	0.27	0.27	0.27	0.27	0.27	0.27
0.28	0.28	0.28	0.28	0.28	0.28	0.28	0.28	0.28
0.29	0.29	0.29	0.29	0.29	0.29	0.29	0.29	0.29
0.3	0.3	0.3	0.3	0.3	0.3	0.3	0.3	0.3
0.31	0.31	0.31	0.31	0.31	0.31	0.31	0.31	0.31
0.32	0.32	0.32	0.32	0.32	0.32	0.32	0.32	0.32
0.33	0.33	0.33	0.33	0.33	0.33	0.33	0.33	0.33
0.34	0.34	0.34	0.34	0.34	0.34	0.34	0.34	0.34
0.35	0.35	0.35	0.35	0.35	0.35	0.35	0.35	0.35
0.36	0.36	0.36	0.36	0.36	0.36	0.36	0.36	0.36
0.37	0.37	0.37	0.37	0.37	0.37	0.37	0.37	0.37
0.38	0.38	0.38	0.38	0.38	0.38	0.38	0.38	0.38
0.39	0.39	0.39	0.39	0.39	0.39	0.39	0.39	0.39
0.4	0.4	0.4	0.4	0.4	0.4	0.4	0.4	0.4
0.41	0.41	0.41	0.41	0.41	0.41	0.41	0.41	0.41
0.42	0.42	0.42	0.42	0.42	0.42	0.42	0.42	0.42
0.43	0.43	0.43	0.43	0.43	0.43	0.43	0.43	0.43
0.44	0.44	0.44	0.44	0.44	0.44	0.44	0.44	0.44
0.45	0.45	0.45	0.45	0.45	0.45	0.45	0.45	0.45
0.46	0.46	0.46	0.46	0.46	0.46	0.46	0.46	0.46
0.47	0.47	0.47	0.47	0.47	0.47	0.47	0.47	0.47
0.48	0.48	0.48	0.48	0.48	0.48	0.48	0.48	0.48
0.49	0.49	0.49	0.49	0.49	0.49	0.49	0.49	0.49

0.5	0.5	0.5	0.5	0.5	0.5	0.5	0.5	0.5
0.51	0.51	0.51	0.51	0.51	0.51	0.51	0.51	0.51
0.52	0.52	0.52	0.52	0.52	0.52	0.52	0.52	0.52
0.53	0.53	0.53	0.53	0.53	0.53	0.53	0.53	0.53
0.54	0.54	0.54	0.54	0.54	0.54	0.54	0.54	0.54
0.55	0.55	0.55	0.55	0.55	0.55	0.55	0.55	0.55
0.56	0.56	0.56	0.56	0.56	0.56	0.56	0.56	0.56
0.57	0.57	0.57	0.57	0.57	0.57	0.57	0.57	0.57
0.58	0.58	0.58	0.58	0.58	0.58	0.58	0.58	0.58
0.59	0.59	0.59	0.59	0.59	0.59	0.59	0.59	0.59
0.6	0.6	0.6	0.6	0.6	0.6	0.6	0.6	0.6
0.61	0.61	0.61	0.61	0.61	0.61	0.61	0.61	0.61
0.62	0.62	0.62	0.62	0.62	0.62	0.62	0.62	0.62
0.63	0.63	0.63	0.63	0.63	0.63	0.63	0.63	0.63
0.64	0.64	0.64	0.64	0.64	0.64	0.64	0.64	0.64
0.65	0.65	0.65	0.65	0.65	0.65	0.65	0.65	0.65
0.66	0.66	0.66	0.66	0.66	0.66	0.66	0.66	0.66
0.67	0.67	0.67	0.67	0.67	0.67	0.67	0.67	0.67
0.68	0.68	0.68	0.68	0.68	0.68	0.68	0.68	0.68
0.69	0.69	0.69	0.69	0.69	0.69	0.69	0.69	0.69
0.7	0.7	0.7	0.7	0.7	0.7	0.7	0.7	0.7
0.71	0.71	0.71	0.71	0.71	0.71	0.71	0.71	0.71
0.72	0.72	0.72	0.72	0.72	0.72	0.72	0.72	0.72
0.73	0.73	0.73	0.73	0.73	0.73	0.73	0.73	0.73
0.74	0.74	0.74	0.74	0.74	0.74	0.74	0.74	0.74
0.75	0.75	0.75	0.75	0.75	0.75	0.75	0.75	0.75
0.76	0.76	0.76	0.76	0.76	0.76	0.76	0.76	0.76
0.77	0.77	0.77	0.77	0.77	0.77	0.77	0.77	0.77
0.78	0.78	0.78	0.78	0.78	0.78	0.78	0.78	0.78
0.79	0.79	0.79	0.79	0.79	0.79	0.79	0.79	0.79
0.8	0.8	0.8	0.8	0.8	0.8	0.8	0.8	0.8
0.81	0.81	0.81	0.81	0.81	0.81	0.81	0.81	0.81
0.82	0.82	0.82	0.82	0.82	0.82	0.82	0.82	0.82
0.83	0.83	0.83	0.83	0.83	0.83	0.83	0.83	0.83
0.84	0.84	0.84	0.84	0.84	0.84	0.84	0.84	0.84
0.85	0.85	0.85	0.85	0.85	0.85	0.85	0.85	0.85
0.86	0.86	0.86	0.86	0.86	0.86	0.86	0.86	0.86
0.87	0.87	0.87	0.87	0.87	0.87	0.87	0.87	0.87
0.88	0.88	0.88	0.88	0.88	0.88	0.88	0.88	0.88
0.89	0.89	0.89	0.89	0.89	0.89	0.89	0.89	0.89
0.9	0.9	0.9	0.9	0.9	0.9	0.9	0.9	0.9
0.91	0.91	0.91	0.91	0.91	0.91	0.91	0.91	0.91
0.92	0.92	0.92	0.92	0.92	0.92	0.92	0.92	0.92
0.93	0.93	0.93	0.93	0.93	0.93	0.93	0.93	0.93
0.94	0.94	0.94	0.94	0.94	0.94	0.94	0.94	0.94
0.95	0.95	0.95	0.95	0.95	0.95	0.95	0.95	0.95
0.96	0.96	0.96	0.96	0.96	0.96	0.96	0.96	0.96
0.97	0.97	0.97	0.97	0.97	0.97	0.97	0.97	0.97
0.98	0.98	0.98	0.98	0.98	0.98	0.98	0.98	0.98
0.99	0.99	0.99	0.99	0.99	0.99	0.99	0.99	0.99

1	1	1	1	1	1	1	1	1
1.01	1.01	1.01	1.01	1.01	1.01	1.01	1.01	1.01
1.02	1.02	1.02	1.02	1.02	1.02	1.02	1.02	1.02
1.03	1.03	1.03	1.03	1.03	1.03	1.03	1.03	1.03
1.04	1.04	1.04	1.04	1.04	1.04	1.04	1.04	1.04
1.05	1.05	1.05	1.05	1.05	1.05	1.05	1.05	1.05
1.06	1.06	1.06	1.06	1.06	1.06	1.06	1.06	1.06
1.07	1.07	1.07	1.07	1.07	1.07	1.07	1.07	1.07
1.08	1.08	1.08	1.08	1.08	1.08	1.08	1.08	1.08
1.09	1.09	1.09	1.09	1.09	1.09	1.09	1.09	1.09
1.1	1.1	1.1	1.1	1.1	1.1	1.1	1.1	1.1
1.11	1.11	1.11	1.11	1.11	1.11	1.11	1.11	1.11
1.12	1.12	1.12	1.12	1.12	1.12	1.12	1.12	1.12
1.13	1.13	1.13	1.13	1.13	1.13	1.13	1.13	1.13
1.14	1.14	1.14	1.14	1.14	1.14	1.14	1.14	1.14
1.15	1.15	1.15	1.15	1.15	1.15	1.15	1.15	1.15
1.16	1.16	1.16	1.16	1.16	1.16	1.16	1.16	1.16
1.17	1.17	1.17	1.17	1.17	1.17	1.17	1.17	1.17
1.18	1.18	1.18	1.18	1.18	1.18	1.18	1.18	1.18
1.19	1.19	1.19	1.19	1.19	1.19	1.19	1.19	1.19
1.2	1.2	1.2	1.2	1.2	1.2	1.2	1.2	1.2
1.21	1.21	1.21	1.21	1.21	1.21	1.21	1.21	1.21
1.22	1.22	1.22	1.22	1.22	1.22	1.22	1.22	1.22
1.23	1.23	1.23	1.23	1.23	1.23	1.23	1.23	1.23
1.24	1.24	1.24	1.24	1.24	1.24	1.24	1.24	1.24
1.25	1.25	1.25	1.25	1.25	1.25	1.25	1.25	1.25
1.26	1.26	1.26	1.26	1.26	1.26	1.26	1.26	1.26
1.27	1.27	1.27	1.27	1.27	1.27	1.27	1.27	1.27
1.28	1.28	1.28	1.28	1.28	1.28	1.28	1.28	1.28
1.29	1.29	1.29	1.29	1.29	1.29	1.29	1.29	1.29
1.3	1.3	1.3	1.3	1.3	1.3	1.3	1.3	1.3
1.31	1.31	1.31	1.31	1.31	1.31	1.31	1.31	1.31
1.32	1.32	1.32	1.32	1.32	1.32	1.32	1.32	1.32
1.33	1.33	1.33	1.33	1.33	1.33	1.33	1.33	1.33
1.34	1.34	1.34	1.34	1.34	1.34	1.34	1.34	1.34
1.35	1.35	1.35	1.35	1.35	1.35	1.35	1.35	1.35
1.36	1.36	1.36	1.36	1.36	1.36	1.36	1.36	1.36
1.37	1.37	1.37	1.37	1.37	1.37	1.37	1.37	1.37
1.38	1.38	1.38	1.38	1.38	1.38	1.38	1.38	1.38
1.39	1.39	1.39	1.39	1.39	1.39	1.39	1.39	1.39
1.4	1.4	1.4	1.4	1.4	1.4	1.4	1.4	1.4
1.41	1.41	1.41	1.41	1.41	1.41	1.41	1.41	1.41
1.42	1.42	1.42	1.42	1.42	1.42	1.42	1.42	1.42
1.43	1.43	1.43	1.43	1.43	1.43	1.43	1.43	1.43
1.44	1.44	1.44	1.44	1.44	1.44	1.44	1.44	1.44
1.45	1.45	1.45	1.45	1.45	1.45	1.45	1.45	1.45
1.46	1.46	1.46	1.46	1.46	1.46	1.46	1.46	1.46
1.47	1.47	1.47	1.47	1.47	1.47	1.47	1.47	1.47
1.48	1.48	1.48	1.48	1.48	1.48	1.48	1.48	1.48
1.49	1.49	1.49	1.49	1.49	1.49	1.49	1.49	1.49

1.5	1.5	1.5	1.5	1.5	1.5	1.5	1.5	1.5
1.51	1.51	1.51	1.51	1.51	1.51	1.51	1.51	1.51
1.52	1.52	1.52	1.52	1.52	1.52	1.52	1.52	1.52
1.53	1.53	1.53	1.53	1.53	1.53	1.53	1.53	1.53
1.54	1.54	1.54	1.54	1.54	1.54	1.54	1.54	1.54
1.55	1.55	1.55	1.55	1.55	1.55	1.55	1.55	1.55
1.56	1.56	1.56	1.56	1.56	1.56	1.56	1.56	1.56
1.57	1.57	1.57	1.57	1.57	1.57	1.57	1.57	1.57
1.58	1.58	1.58	1.58	1.58	1.58	1.58	1.58	1.58
1.59	1.59	1.59	1.59	1.59	1.59	1.59	1.59	1.59
1.6	1.6	1.6	1.6	1.6	1.6	1.6	1.6	1.6
1.61	1.61	1.61	1.61	1.61	1.61	1.61	1.61	1.61
1.62	1.62	1.62	1.62	1.62	1.62	1.62	1.62	1.62
1.63	1.63	1.63	1.63	1.63	1.63	1.63	1.63	1.63
1.64	1.64	1.64	1.64	1.64	1.64	1.64	1.64	1.64
1.65	1.65	1.65	1.65	1.65	1.65	1.65	1.65	1.65
1.66	1.66	1.66	1.66	1.66	1.66	1.66	1.66	1.66
1.67	1.67	1.67	1.67	1.67	1.67	1.67	1.67	1.67
1.68	1.68	1.68	1.68	1.68	1.68	1.68	1.68	1.68
1.69	1.69	1.69	1.69	1.69	1.69	1.69	1.69	1.69
1.7	1.7	1.7	1.7	1.7	1.7	1.7	1.7	1.7
1.71	1.71	1.71	1.71	1.71	1.71	1.71	1.71	1.71
1.72	1.72	1.72	1.72	1.72	1.72	1.72	1.72	1.72
1.73	1.73	1.73	1.73	1.73	1.73	1.73	1.73	1.73
1.74	1.74	1.74	1.74	1.74	1.74	1.74	1.74	1.74
1.75	1.75	1.75	1.75	1.75	1.75	1.75	1.75	1.75
1.76	1.76	1.76	1.76	1.76	1.76	1.76	1.76	1.76
1.77	1.77	1.77	1.77	1.77	1.77	1.77	1.77	1.77
1.78	1.78	1.78	1.78	1.78	1.78	1.78	1.78	1.78
1.79	1.79	1.79	1.79	1.79	1.79	1.79	1.79	1.79
1.8	1.8	1.8	1.8	1.8	1.8	1.8	1.8	1.8
1.81	1.81	1.81	1.81	1.81	1.81	1.81	1.81	1.81
1.82	1.82	1.82	1.82	1.82	1.82	1.82	1.82	1.82
1.83	1.83	1.83	1.83	1.83	1.83	1.83	1.83	1.83
1.84	1.84	1.84	1.84	1.84	1.84	1.84	1.84	1.84
1.85	1.85	1.85	1.85	1.85	1.85	1.85	1.85	1.85
1.86	1.86	1.86	1.86	1.86	1.86	1.86	1.86	1.86
1.87	1.87	1.87	1.87	1.87	1.87	1.87	1.87	1.87
1.88	1.88	1.88	1.88	1.88	1.88	1.88	1.88	1.88
1.89	1.89	1.89	1.89	1.89	1.89	1.89	1.89	1.89
1.9	1.9	1.9	1.9	1.9	1.9	1.9	1.9	1.9
1.91	1.91	1.91	1.91	1.91	1.91	1.91	1.91	1.91
1.92	1.92	1.92	1.92	1.92	1.92	1.92	1.92	1.92
1.93	1.93	1.93	1.93	1.93	1.93	1.93	1.93	1.93
1.94	1.94	1.94	1.94	1.94	1.94	1.94	1.94	1.94
1.95	1.95	1.95	1.95	1.95	1.95	1.95	1.95	1.95
1.96	1.96	1.96	1.96	1.96	1.96	1.96	1.96	1.96
1.97	1.97	1.97	1.97	1.97	1.97	1.97	1.97	1.97
1.98	1.98	1.98	1.98	1.98	1.98	1.98	1.98	1.98
1.99	1.99	1.99	1.99	1.99	1.99	1.99	1.99	1.99

2	2	2	2	2	2	2	2	2
2.01	2.01	2.01	2.01	2.01	2.01	2.01	2.01	2.01
2.02	2.02	2.02	2.02	2.02	2.02	2.02	2.02	2.02
2.03	2.03	2.03	2.03	2.03	2.03	2.03	2.03	2.03
2.04	2.04	2.04	2.04	2.04	2.04	2.04	2.04	2.04
2.05	2.05	2.05	2.05	2.05	2.05	2.05	2.05	2.05
2.06	2.06	2.06	2.06	2.06	2.06	2.06	2.06	2.06
2.07	2.07	2.07	2.07	2.07	2.07	2.07	2.07	2.07
2.08	2.08	2.08	2.08	2.08	2.08	2.08	2.08	2.08
2.09	2.09	2.09	2.09	2.09	2.09	2.09	2.09	2.09
2.1	2.1	2.1	2.1	2.1	2.1	2.1	2.1	2.1
2.11	2.11	2.11	2.11	2.11	2.11	2.11	2.11	2.11
2.12	2.12	2.12	2.12	2.12	2.12	2.12	2.12	2.12
2.13	2.13	2.13	2.13	2.13	2.13	2.13	2.13	2.13
2.14	2.14	2.14	2.14	2.14	2.14	2.14	2.14	2.14
2.15	2.15	2.15	2.15	2.15	2.15	2.15	2.15	2.15
2.16	2.16	2.16	2.16	2.16	2.16	2.16	2.16	2.16
2.17	2.17	2.17	2.17	2.17	2.17	2.17	2.17	2.17
2.18	2.18	2.18	2.18	2.18	2.18	2.18	2.18	2.18
2.19	2.19	2.19	2.19	2.19	2.19	2.19	2.19	2.19
2.2	2.2	2.2	2.2	2.2	2.2	2.2	2.2	2.2
2.21	2.21	2.21	2.21	2.21	2.21	2.21	2.21	2.21
2.22	2.22	2.22	2.22	2.22	2.22	2.22	2.22	2.22
2.23	2.23	2.23	2.23	2.23	2.23	2.23	2.23	2.23
2.24	2.24	2.24	2.24	2.24	2.24	2.24	2.24	2.24
2.25	2.25	2.25	2.25	2.25	2.25	2.25	2.25	2.25
2.26	2.26	2.26	2.26	2.26	2.26	2.26	2.26	2.26
2.27	2.27	2.27	2.27	2.27	2.27	2.27	2.27	2.27
2.28	2.28	2.28	2.28	2.28	2.28	2.28	2.28	2.28
2.29	2.29	2.29	2.29	2.29	2.29	2.29	2.29	2.29
2.3	2.3	2.3	2.3	2.3	2.3	2.3	2.3	2.3
2.31	2.31	2.31	2.31	2.31	2.31	2.31	2.31	2.31
2.32	2.32	2.32	2.32	2.32	2.32	2.32	2.32	2.32
2.33	2.33	2.33	2.33	2.33	2.33	2.33	2.33	2.33
2.34	2.34	2.34	2.34	2.34	2.34	2.34	2.34	2.34
2.35	2.35	2.35	2.35	2.35	2.35	2.35	2.35	2.35
2.36	2.36	2.36	2.36	2.36	2.36	2.36	2.36	2.36
2.37	2.37	2.37	2.37	2.37	2.37	2.37	2.37	2.37
2.38	2.38	2.38	2.38	2.38	2.38	2.38	2.38	2.38
2.39	2.39	2.39	2.39	2.39	2.39	2.39	2.39	2.39
2.4	2.4	2.4	2.4	2.4	2.4	2.4	2.4	2.4
2.41	2.41	2.41	2.41	2.41	2.41	2.41	2.41	2.41
2.42	2.42	2.42	2.42	2.42	2.42	2.42	2.42	2.42
2.43	2.43	2.43	2.43	2.43	2.43	2.43	2.43	2.43
2.44	2.44	2.44	2.44	2.44	2.44	2.44	2.44	2.44
2.45	2.45	2.45	2.45	2.45	2.45	2.45	2.45	2.45
2.46	2.46	2.46	2.46	2.46	2.46	2.46	2.46	2.46
2.47	2.47	2.47	2.47	2.47	2.47	2.47	2.47	2.47
2.48	2.48	2.48	2.48	2.48	2.48	2.48	2.48	2.48
2.49	2.49	2.49	2.49	2.49	2.49	2.49	2.49	2.49

2.5	2.5	2.5	2.5	2.5	2.5	2.5	2.5	2.5
2.51	2.51	2.51	2.51	2.51	2.51	2.51	2.51	2.51
2.52	2.52	2.52	2.52	2.52	2.52	2.52	2.52	2.52
2.53	2.53	2.53	2.53	2.53	2.53	2.53	2.53	2.53
2.54	2.54	2.54	2.54	2.54	2.54	2.54	2.54	2.54
2.55	2.55	2.55	2.55	2.55	2.55	2.55	2.55	2.55
2.56	2.56	2.56	2.56	2.56	2.56	2.56	2.56	2.56
2.57	2.57	2.57	2.57	2.57	2.57	2.57	2.57	2.57
2.58	2.58	2.58	2.58	2.58	2.58	2.58	2.58	2.58
2.59	2.59	2.59	2.59	2.59	2.59	2.59	2.59	2.59
2.6	2.6	2.6	2.6	2.6	2.6	2.6	2.6	2.6
2.61	2.61	2.61	2.61	2.61	2.61	2.61	2.61	2.61
2.62	2.62	2.62	2.62	2.62	2.62	2.62	2.62	2.62
2.63	2.63	2.63	2.63	2.63	2.63	2.63	2.63	2.63
2.64	2.64	2.64	2.64	2.64	2.64	2.64	2.64	2.64
2.65	2.65	2.65	2.65	2.65	2.65	2.65	2.65	2.65
2.66	2.66	2.66	2.66	2.66	2.66	2.66	2.66	2.66
2.67	2.67	2.67	2.67	2.67	2.67	2.67	2.67	2.67
2.68	2.68	2.68	2.68	2.68	2.68	2.68	2.68	2.68
2.69	2.69	2.69	2.69	2.69	2.69	2.69	2.69	2.69
2.7	2.7	2.7	2.7	2.7	2.7	2.7	2.7	2.7
2.71	2.71	2.71	2.71	2.71	2.71	2.71	2.71	2.71
2.72	2.72	2.72	2.72	2.72	2.72	2.72	2.72	2.72
2.73	2.73	2.73	2.73	2.73	2.73	2.73	2.73	2.73
2.74	2.74	2.74	2.74	2.74	2.74	2.74	2.74	2.74
2.75	2.75	2.75	2.75	2.75	2.75	2.75	2.75	2.75
2.76	2.76	2.76	2.76	2.76	2.76	2.76	2.76	2.76
2.77	2.77	2.77	2.77	2.77	2.77	2.77	2.77	2.77
2.78	2.78	2.78	2.78	2.78	2.78	2.78	2.78	2.78
2.79	2.79	2.79	2.79	2.79	2.79	2.79	2.79	2.79
2.8	2.8	2.8	2.8	2.8	2.8	2.8	2.8	2.8
2.81	2.81	2.81	2.81	2.81	2.81	2.81	2.81	2.81
2.82	2.82	2.82	2.82	2.82	2.82	2.82	2.82	2.82
2.83	2.83	2.83	2.83	2.83	2.83	2.83	2.83	2.83
2.84	2.84	2.84	2.84	2.84	2.84	2.84	2.84	2.84
2.85	2.85	2.85	2.85	2.85	2.85	2.85	2.85	2.85
2.86	2.86	2.86	2.86	2.86	2.86	2.86	2.86	2.86
2.87	2.87	2.87	2.87	2.87	2.87	2.87	2.87	2.87
2.88	2.88	2.88	2.88	2.88	2.88	2.88	2.88	2.88
2.89	2.89	2.89	2.89	2.89	2.89	2.89	2.89	2.89
2.9	2.9	2.9	2.9	2.9	2.9	2.9	2.9	2.9
2.91	2.91	2.91	2.91	2.91	2.91	2.91	2.91	2.91
2.92	2.92	2.92	2.92	2.92	2.92	2.92	2.92	2.92
2.93	2.93	2.93	2.93	2.93	2.93	2.93	2.93	2.93
2.94	2.94	2.94	2.94	2.94	2.94	2.94	2.94	2.94
2.95	2.95	2.95	2.95	2.95	2.95	2.95	2.95	2.95
2.96	2.96	2.96	2.96	2.96	2.96	2.96	2.96	2.96
2.97	2.97	2.97	2.97	2.97	2.97	2.97	2.97	2.97
2.98	2.98	2.98	2.98	2.98	2.98	2.98	2.98	2.98
2.99	2.99	2.99	2.99	2.99	2.99	2.99	2.99	2.99

3	3	3	3	3	3	3	3	3
3.01	3.01	3.01	3.01	3.01	3.01	3.01	3.01	3.01
3.02	3.02	3.02	3.02	3.02	3.02	3.02	3.02	3.02
3.03	3.03	3.03	3.03	3.03	3.03	3.03	3.03	3.03
3.04	3.04	3.04	3.04	3.04	3.04	3.04	3.04	3.04
3.05	3.05	3.05	3.05	3.05	3.05	3.05	3.05	3.05
3.06	3.06	3.06	3.06	3.06	3.06	3.06	3.06	3.06
3.07	3.07	3.07	3.07	3.07	3.07	3.07	3.07	3.07
3.08	3.08	3.08	3.08	3.08	3.08	3.08	3.08	3.08
3.09	3.09	3.09	3.09	3.09	3.09	3.09	3.09	3.09
3.1	3.1	3.1	3.1	3.1	3.1	3.1	3.1	3.1
3.11	3.11	3.11	3.11	3.11	3.11	3.11	3.11	3.11
3.12	3.12	3.12	3.12	3.12	3.12	3.12	3.12	3.12
3.13	3.13	3.13	3.13	3.13	3.13	3.13	3.13	3.13
3.14	3.14	3.14	3.14	3.14	3.14	3.14	3.14	3.14
3.15	3.15	3.15	3.15	3.15	3.15	3.15	3.15	3.15
3.16	3.16	3.16	3.16	3.16	3.16	3.16	3.16	3.16
3.17	3.17	3.17	3.17	3.17	3.17	3.17	3.17	3.17
3.18	3.18	3.18	3.18	3.18	3.18	3.18	3.18	3.18
3.19	3.19	3.19	3.19	3.19	3.19	3.19	3.19	3.19
3.2	3.2	3.2	3.2	3.2	3.2	3.2	3.2	3.2
3.21	3.21	3.21	3.21	3.21	3.21	3.21	3.21	3.21
3.22	3.22	3.22	3.22	3.22	3.22	3.22	3.22	3.22
3.23	3.23	3.23	3.23	3.23	3.23	3.23	3.23	3.23
3.24	3.24	3.24	3.24	3.24	3.24	3.24	3.24	3.24
3.25	3.25	3.25	3.25	3.25	3.25	3.25	3.25	3.25
3.26	3.26	3.26	3.26	3.26	3.26	3.26	3.26	3.26
3.27	3.27	3.27	3.27	3.27	3.27	3.27	3.27	3.27
3.28	3.28	3.28	3.28	3.28	3.28	3.28	3.28	3.28
3.29	3.29	3.29	3.29	3.29	3.29	3.29	3.29	3.29
3.3	3.3	3.3	3.3	3.3	3.3	3.3	3.3	3.3
3.31	3.31	3.31	3.31	3.31	3.31	3.31	3.31	3.31
3.32	3.32	3.32	3.32	3.32	3.32	3.32	3.32	3.32
3.33	3.33	3.33	3.33	3.33	3.33	3.33	3.33	3.33
3.34	3.34	3.34	3.34	3.34	3.34	3.34	3.34	3.34
3.35	3.35	3.35	3.35	3.35	3.35	3.35	3.35	3.35
3.36	3.36	3.36	3.36	3.36	3.36	3.36	3.36	3.36
3.37	3.37	3.37	3.37	3.37	3.37	3.37	3.37	3.37
3.38	3.38	3.38	3.38	3.38	3.38	3.38	3.38	3.38
3.39	3.39	3.39	3.39	3.39	3.39	3.39	3.39	3.39
3.4	3.4	3.4	3.4	3.4	3.4	3.4	3.4	3.4
3.41	3.41	3.41	3.41	3.41	3.41	3.41	3.41	3.41
3.42	3.42	3.42	3.42	3.42	3.42	3.42	3.42	3.42
3.43	3.43	3.43	3.43	3.43	3.43	3.43	3.43	3.43
3.44	3.44	3.44	3.44	3.44	3.44	3.44	3.44	3.44
3.45	3.45	3.45	3.45	3.45	3.45	3.45	3.45	3.45
3.46	3.46	3.46	3.46	3.46	3.46	3.46	3.46	3.46
3.47	3.47	3.47	3.47	3.47	3.47	3.47	3.47	3.47
3.48	3.48	3.48	3.48	3.48	3.48	3.48	3.48	3.48
3.49	3.49	3.49	3.49	3.49	3.49	3.49	3.49	3.49

3.5	3.5	3.5	3.5	3.5	3.5	3.5	3.5	3.5
3.51	3.51	3.51	3.51	3.51	3.51	3.51	3.51	3.51
3.52	3.52	3.52	3.52	3.52	3.52	3.52	3.52	3.52
3.53	3.53	3.53	3.53	3.53	3.53	3.53	3.53	3.53
3.54	3.54	3.54	3.54	3.54	3.54	3.54	3.54	3.54
3.55	3.55	3.55	3.55	3.55	3.55	3.55	3.55	3.55
3.56	3.56	3.56	3.56	3.56	3.56	3.56	3.56	3.56
3.57	3.57	3.57	3.57	3.57	3.57	3.57	3.57	3.57
3.58	3.58	3.58	3.58	3.58	3.58	3.58	3.58	3.58
3.59	3.59	3.59	3.59	3.59	3.59	3.59	3.59	3.59
3.6	3.6	3.6	3.6	3.6	3.6	3.6	3.6	3.6
3.61	3.61	3.61	3.61	3.61	3.61	3.61	3.61	3.61
3.62	3.62	3.62	3.62	3.62	3.62	3.62	3.62	3.62
3.63	3.63	3.63	3.63	3.63	3.63	3.63	3.63	3.63
3.64	3.64	3.64	3.64	3.64	3.64	3.64	3.64	3.64
3.65	3.65	3.65	3.65	3.65	3.65	3.65	3.65	3.65
3.66	3.66	3.66	3.66	3.66	3.66	3.66	3.66	3.66
3.67	3.67	3.67	3.67	3.67	3.67	3.67	3.67	3.67
3.68	3.68	3.68	3.68	3.68	3.68	3.68	3.68	3.68
3.69	3.69	3.69	3.69	3.69	3.69	3.69	3.69	3.69
3.7	3.7	3.7	3.7	3.7	3.7	3.7	3.7	3.7
3.71	3.71	3.71	3.71	3.71	3.71	3.71	3.71	3.71
3.72	3.72	3.72	3.72	3.72	3.72	3.72	3.72	3.72
3.73	3.73	3.73	3.73	3.73	3.73	3.73	3.73	3.73
3.74	3.74	3.74	3.74	3.74	3.74	3.74	3.74	3.74
3.75	3.75	3.75	3.75	3.75	3.75	3.75	3.75	3.75
3.76	3.76	3.76	3.76	3.76	3.76	3.76	3.76	3.76
3.77	3.77	3.77	3.77	3.77	3.77	3.77	3.77	3.77
3.78	3.78	3.78	3.78	3.78	3.78	3.78	3.78	3.78
3.79	3.79	3.79	3.79	3.79	3.79	3.79	3.79	3.79
3.8	3.8	3.8	3.8	3.8	3.8	3.8	3.8	3.8
3.81	3.81	3.81	3.81	3.81	3.81	3.81	3.81	3.81
3.82	3.82	3.82	3.82	3.82	3.82	3.82	3.82	3.82
3.83	3.83	3.83	3.83	3.83	3.83	3.83	3.83	3.83
3.84	3.84	3.84	3.84	3.84	3.84	3.84	3.84	3.84
3.85	3.85	3.85	3.85	3.85	3.85	3.85	3.85	3.85
3.86	3.86	3.86	3.86	3.86	3.86	3.86	3.86	3.86
3.87	3.87	3.87	3.87	3.87	3.87	3.87	3.87	3.87
3.88	3.88	3.88	3.88	3.88	3.88	3.88	3.88	3.88
3.89	3.89	3.89	3.89	3.89	3.89	3.89	3.89	3.89
3.9	3.9	3.9	3.9	3.9	3.9	3.9	3.9	3.9
3.91	3.91	3.91	3.91	3.91	3.91	3.91	3.91	3.91
3.92	3.92	3.92	3.92	3.92	3.92	3.92	3.92	3.92
3.93	3.93	3.93	3.93	3.93	3.93	3.93	3.93	3.93
3.94	3.94	3.94	3.94	3.94	3.94	3.94	3.94	3.94
3.95	3.95	3.95	3.95	3.95	3.95	3.95	3.95	3.95
3.96	3.96	3.96	3.96	3.96	3.96	3.96	3.96	3.96
3.97	3.97	3.97	3.97	3.97	3.97	3.97	3.97	3.97
3.98	3.98	3.98	3.98	3.98	3.98	3.98	3.98	3.98
3.99	3.99	3.99	3.99	3.99	3.99	3.99	3.99	3.99

4	4	4	4	4	4	4	4	4
4.01	4.01	4.01	4.01	4.01	4.01	4.01	4.01	4.01
4.02	4.02	4.02	4.02	4.02	4.02	4.02	4.02	4.02
4.03	4.03	4.03	4.03	4.03	4.03	4.03	4.03	4.03
4.04	4.04	4.04	4.04	4.04	4.04	4.04	4.04	4.04
4.05	4.05	4.05	4.05	4.05	4.05	4.05	4.05	4.05
4.06	4.06	4.06	4.06	4.06	4.06	4.06	4.06	4.06
4.07	4.07	4.07	4.07	4.07	4.07	4.07	4.07	4.07
4.08	4.08	4.08	4.08	4.08	4.08	4.08	4.08	4.08
4.09	4.09	4.09	4.09	4.09	4.09	4.09	4.09	4.09
4.1	4.1	4.1	4.1	4.1	4.1	4.1	4.1	4.1
4.11	4.11	4.11	4.11	4.11	4.11	4.11	4.11	4.11
4.12	4.12	4.12	4.12	4.12	4.12	4.12	4.12	4.12
4.13	4.13	4.13	4.13	4.13	4.13	4.13	4.13	4.13
4.14	4.14	4.14	4.14	4.14	4.14	4.14	4.14	4.14
4.15	4.15	4.15	4.15	4.15	4.15	4.15	4.15	4.15
4.16	4.16	4.16	4.16	4.16	4.16	4.16	4.16	4.16
4.17	4.17	4.17	4.17	4.17	4.17	4.17	4.17	4.17
4.18	4.18	4.18	4.18	4.18	4.18	4.18	4.18	4.18
4.19	4.19	4.19	4.19	4.19	4.19	4.19	4.19	4.19
4.2	4.2	4.2	4.2	4.2	4.2	4.2	4.2	4.2
4.21	4.21	4.21	4.21	4.21	4.21	4.21	4.21	4.21
4.22	4.22	4.22	4.22	4.22	4.22	4.22	4.22	4.22
4.23	4.23	4.23	4.23	4.23	4.23	4.23	4.23	4.23
4.24	4.24	4.24	4.24	4.24	4.24	4.24	4.24	4.24
4.25	4.25	4.25	4.25	4.25	4.25	4.25	4.25	4.25
4.26	4.26	4.26	4.26	4.26	4.26	4.26	4.26	4.26
4.27	4.27	4.27	4.27	4.27	4.27	4.27	4.27	4.27
4.28	4.28	4.28	4.28	4.28	4.28	4.28	4.28	4.28
4.29	4.29	4.29	4.29	4.29	4.29	4.29	4.29	4.29
4.3	4.3	4.3	4.3	4.3	4.3	4.3	4.3	4.3
4.31	4.31	4.31	4.31	4.31	4.31	4.31	4.31	4.31
4.32	4.32	4.32	4.32	4.32	4.32	4.32	4.32	4.32
4.33	4.33	4.33	4.33	4.33	4.33	4.33	4.33	4.33
4.34	4.34	4.34	4.34	4.34	4.34	4.34	4.34	4.34
4.35	4.35	4.35	4.35	4.35	4.35	4.35	4.35	4.35
4.36	4.36	4.36	4.36	4.36	4.36	4.36	4.36	4.36
4.37	4.37	4.37	4.37	4.37	4.37	4.37	4.37	4.37
4.38	4.38	4.38	4.38	4.38	4.38	4.38	4.38	4.38
4.39	4.39	4.39	4.39	4.39	4.39	4.39	4.39	4.39
4.4	4.4	4.4	4.4	4.4	4.4	4.4	4.4	4.4
4.41	4.41	4.41	4.41	4.41	4.41	4.41	4.41	4.41
4.42	4.42	4.42	4.42	4.42	4.42	4.42	4.42	4.42
4.43	4.43	4.43	4.43	4.43	4.43	4.43	4.43	4.43
4.44	4.44	4.44	4.44	4.44	4.44	4.44	4.44	4.44
4.45	4.45	4.45	4.45	4.45	4.45	4.45	4.45	4.45
4.46	4.46	4.46	4.46	4.46	4.46	4.46	4.46	4.46
4.47	4.47	4.47	4.47	4.47	4.47	4.47	4.47	4.47
4.48	4.48	4.48	4.48	4.48	4.48	4.48	4.48	4.48
4.49	4.49	4.49	4.49	4.49	4.49	4.49	4.49	4.49

4.5	4.5	4.5	4.5	4.5	4.5	4.5	4.5	4.5
4.51	4.51	4.51	4.51	4.51	4.51	4.51	4.51	4.51
4.52	4.52	4.52	4.52	4.52	4.52	4.52	4.52	4.52
4.53	4.53	4.53	4.53	4.53	4.53	4.53	4.53	4.53
4.54	4.54	4.54	4.54	4.54	4.54	4.54	4.54	4.54
4.55	4.55	4.55	4.55	4.55	4.55	4.55	4.55	4.55
4.56	4.56	4.56	4.56	4.56	4.56	4.56	4.56	4.56
4.57	4.57	4.57	4.57	4.57	4.57	4.57	4.57	4.57
4.58	4.58	4.58	4.58	4.58	4.58	4.58	4.58	4.58
4.59	4.59	4.59	4.59	4.59	4.59	4.59	4.59	4.59
4.6	4.6	4.6	4.6	4.6	4.6	4.6	4.6	4.6
4.61	4.61	4.61	4.61	4.61	4.61	4.61	4.61	4.61
4.62	4.62	4.62	4.62	4.62	4.62	4.62	4.62	4.62
4.63	4.63	4.63	4.63	4.63	4.63	4.63	4.63	4.63
4.64	4.64	4.64	4.64	4.64	4.64	4.64	4.64	4.64
4.65	4.65	4.65	4.65	4.65	4.65	4.65	4.65	4.65
4.66	4.66	4.66	4.66	4.66	4.66	4.66	4.66	4.66
4.67	4.67	4.67	4.67	4.67	4.67	4.67	4.67	4.67
4.68	4.68	4.68	4.68	4.68	4.68	4.68	4.68	4.68
4.69	4.69	4.69	4.69	4.69	4.69	4.69	4.69	4.69
4.7	4.7	4.7	4.7	4.7	4.7	4.7	4.7	4.7
4.71	4.71	4.71	4.71	4.71	4.71	4.71	4.71	4.71
4.72	4.72	4.72	4.72	4.72	4.72	4.72	4.72	4.72
4.73	4.73	4.73	4.73	4.73	4.73	4.73	4.73	4.73
4.74	4.74	4.74	4.74	4.74	4.74	4.74	4.74	4.74
4.75	4.75	4.75	4.75	4.75	4.75	4.75	4.75	4.75
4.76	4.76	4.76	4.76	4.76	4.76	4.76	4.76	4.76
4.77	4.77	4.77	4.77	4.77	4.77	4.77	4.77	4.77
4.78	4.78	4.78	4.78	4.78	4.78	4.78	4.78	4.78
4.79	4.79	4.79	4.79	4.79	4.79	4.79	4.79	4.79
4.8	4.8	4.8	4.8	4.8	4.8	4.8	4.8	4.8
4.81	4.81	4.81	4.81	4.81	4.81	4.81	4.81	4.81
4.82	4.82	4.82	4.82	4.82	4.82	4.82	4.82	4.82
4.83	4.83	4.83	4.83	4.83	4.83	4.83	4.83	4.83
4.84	4.84	4.84	4.84	4.84	4.84	4.84	4.84	4.84
4.85	4.85	4.85	4.85	4.85	4.85	4.85	4.85	4.85
4.86	4.86	4.86	4.86	4.86	4.86	4.86	4.86	4.86
4.87	4.87	4.87	4.87	4.87	4.87	4.87	4.87	4.87
4.88	4.88	4.88	4.88	4.88	4.88	4.88	4.88	4.88
4.89	4.89	4.89	4.89	4.89	4.89	4.89	4.89	4.89
4.9	4.9	4.9	4.9	4.9	4.9	4.9	4.9	4.9
4.91	4.91	4.91	4.91	4.91	4.91	4.91	4.91	4.91
4.92	4.92	4.92	4.92	4.92	4.92	4.92	4.92	4.92
4.93	4.93	4.93	4.93	4.93	4.93	4.93	4.93	4.93
4.94	4.94	4.94	4.94	4.94	4.94	4.94	4.94	4.94
4.95	4.95	4.95	4.95	4.95	4.95	4.95	4.95	4.95
4.96	4.96	4.96	4.96	4.96	4.96	4.96	4.96	4.96
4.97	4.97	4.97	4.97	4.97	4.97	4.97	4.97	4.97
4.98	4.98	4.98	4.98	4.98	4.98	4.98	4.98	4.98
4.99	4.99	4.99	4.99	4.99	4.99	4.99	4.99	4.99

5	5	5	5	5	5	5	5	5
5.01	5.01	5.01	5.01	5.01	5.01	5.01	5.01	5.01
5.02	5.02	5.02	5.02	5.02	5.02	5.02	5.02	5.02
5.03	5.03	5.03	5.03	5.03	5.03	5.03	5.03	5.03
5.04	5.04	5.04	5.04	5.04	5.04	5.04	5.04	5.04
5.05	5.05	5.05	5.05	5.05	5.05	5.05	5.05	5.05
5.06	5.06	5.06	5.06	5.06	5.06	5.06	5.06	5.06
5.07	5.07	5.07	5.07	5.07	5.07	5.07	5.07	5.07
5.08	5.08	5.08	5.08	5.08	5.08	5.08	5.08	5.08
5.09	5.09	5.09	5.09	5.09	5.09	5.09	5.09	5.09
5.1	5.1	5.1	5.1	5.1	5.1	5.1	5.1	5.1
5.11	5.11	5.11	5.11	5.11	5.11	5.11	5.11	5.11
5.12	5.12	5.12	5.12	5.12	5.12	5.12	5.12	5.12
5.13	5.13	5.13	5.13	5.13	5.13	5.13	5.13	5.13
5.14	5.14	5.14	5.14	5.14	5.14	5.14	5.14	5.14
5.15	5.15	5.15	5.15	5.15	5.15	5.15	5.15	5.15
5.16	5.16	5.16	5.16	5.16	5.16	5.16	5.16	5.16
5.17	5.17	5.17	5.17	5.17	5.17	5.17	5.17	5.17
5.18	5.18	5.18	5.18	5.18	5.18	5.18	5.18	5.18
5.19	5.19	5.19	5.19	5.19	5.19	5.19	5.19	5.19
5.2	5.2	5.2	5.2	5.2	5.2	5.2	5.2	5.2
5.21	5.21	5.21	5.21	5.21	5.21	5.21	5.21	5.21
5.22	5.22	5.22	5.22	5.22	5.22	5.22	5.22	5.22
5.23	5.23	5.23	5.23	5.23	5.23	5.23	5.23	5.23
5.24	5.24	5.24	5.24	5.24	5.24	5.24	5.24	5.24
5.25	5.25	5.25	5.25	5.25	5.25	5.25	5.25	5.25
5.26	5.26	5.26	5.26	5.26	5.26	5.26	5.26	5.26
5.27	5.27	5.27	5.27	5.27	5.27	5.27	5.27	5.27
5.28	5.28	5.28	5.28	5.28	5.28	5.28	5.28	5.28
5.29	5.29	5.29	5.29	5.29	5.29	5.29	5.29	5.29
5.3	5.3	5.3	5.3	5.3	5.3	5.3	5.3	5.3
5.31	5.31	5.31	5.31	5.31	5.31	5.31	5.31	5.31
5.32	5.32	5.32	5.32	5.32	5.32	5.32	5.32	5.32
5.33	5.33	5.33	5.33	5.33	5.33	5.33	5.33	5.33
5.34	5.34	5.34	5.34	5.34	5.34	5.34	5.34	5.34
5.35	5.35	5.35	5.35	5.35	5.35	5.35	5.35	5.35
5.36	5.36	5.36	5.36	5.36	5.36	5.36	5.36	5.36
5.37	5.37	5.37	5.37	5.37	5.37	5.37	5.37	5.37
5.38	5.38	5.38	5.38	5.38	5.38	5.38	5.38	5.38
5.39	5.39	5.39	5.39	5.39	5.39	5.39	5.39	5.39
5.4	5.4	5.4	5.4	5.4	5.4	5.4	5.4	5.4
5.41	5.41	5.41	5.41	5.41	5.41	5.41	5.41	5.41
5.42	5.42	5.42	5.42	5.42	5.42	5.42	5.42	5.42
5.43	5.43	5.43	5.43	5.43	5.43	5.43	5.43	5.43
5.44	5.44	5.44	5.44	5.44	5.44	5.44	5.44	5.44
5.45	5.45	5.45	5.45	5.45	5.45	5.45	5.45	5.45
5.46	5.46	5.46	5.46	5.46	5.46	5.46	5.46	5.46
5.47	5.47	5.47	5.47	5.47	5.47	5.47	5.47	5.47
5.48	5.48	5.48	5.48	5.48	5.48	5.48	5.48	5.48
5.49	5.49	5.49	5.49	5.49	5.49	5.49	5.49	5.49

5.5	5.5	5.5	5.5	5.5	5.5	5.5	5.5	5.5
5.51	5.51	5.51	5.51	5.51	5.51	5.51	5.51	5.51
5.52	5.52	5.52	5.52	5.52	5.52	5.52	5.52	5.52
5.53	5.53	5.53	5.53	5.53	5.53	5.53	5.53	5.53
5.54	5.54	5.54	5.54	5.54	5.54	5.54	5.54	5.54
5.55	5.55	5.55	5.55	5.55	5.55	5.55	5.55	5.55
5.56	5.56	5.56	5.56	5.56	5.56	5.56	5.56	5.56
5.57	5.57	5.57	5.57	5.57	5.57	5.57	5.57	5.57
5.58	5.58	5.58	5.58	5.58	5.58	5.58	5.58	5.58
5.59	5.59	5.59	5.59	5.59	5.59	5.59	5.59	5.59
5.6	5.6	5.6	5.6	5.6	5.6	5.6	5.6	5.6
5.61	5.61	5.61	5.61	5.61	5.61	5.61	5.61	5.61
5.62	5.62	5.62	5.62	5.62	5.62	5.62	5.62	5.62
5.63	5.63	5.63	5.63	5.63	5.63	5.63	5.63	5.63
5.64	5.64	5.64	5.64	5.64	5.64	5.64	5.64	5.64
5.65	5.65	5.65	5.65	5.65	5.65	5.65	5.65	5.65
5.66	5.66	5.66	5.66	5.66	5.66	5.66	5.66	5.66
5.67	5.67	5.67	5.67	5.67	5.67	5.67	5.67	5.67
5.68	5.68	5.68	5.68	5.68	5.68	5.68	5.68	5.68
5.69	5.69	5.69	5.69	5.69	5.69	5.69	5.69	5.69
5.7	5.7	5.7	5.7	5.7	5.7	5.7	5.7	5.7
5.71	5.71	5.71	5.71	5.71	5.71	5.71	5.71	5.71
5.72	5.72	5.72	5.72	5.72	5.72	5.72	5.72	5.72
5.73	5.73	5.73	5.73	5.73	5.73	5.73	5.73	5.73
5.74	5.74	5.74	5.74	5.74	5.74	5.74	5.74	5.74
5.75	5.75	5.75	5.75	5.75	5.75	5.75	5.75	5.75
5.76	5.76	5.76	5.76	5.76	5.76	5.76	5.76	5.76
5.77	5.77	5.77	5.77	5.77	5.77	5.77	5.77	5.77
5.78	5.78	5.78	5.78	5.78	5.78	5.78	5.78	5.78
5.79	5.79	5.79	5.79	5.79	5.79	5.79	5.79	5.79
5.8	5.8	5.8	5.8	5.8	5.8	5.8	5.8	5.8
5.81	5.81	5.81	5.81	5.81	5.81	5.81	5.81	5.81
5.82	5.82	5.82	5.82	5.82	5.82	5.82	5.82	5.82
5.83	5.83	5.83	5.83	5.83	5.83	5.83	5.83	5.83
5.84	5.84	5.84	5.84	5.84	5.84	5.84	5.84	5.84
5.85	5.85	5.85	5.85	5.85	5.85	5.85	5.85	5.85
5.86	5.86	5.86	5.86	5.86	5.86	5.86	5.86	5.86
5.87	5.87	5.87	5.87	5.87	5.87	5.87	5.87	5.87
5.88	5.88	5.88	5.88	5.88	5.88	5.88	5.88	5.88
5.89	5.89	5.89	5.89	5.89	5.89	5.89	5.89	5.89
5.9	5.9	5.9	5.9	5.9	5.9	5.9	5.9	5.9
5.91	5.91	5.91	5.91	5.91	5.91	5.91	5.91	5.91
5.92	5.92	5.92	5.92	5.92	5.92	5.92	5.92	5.92
5.93	5.93	5.93	5.93	5.93	5.93	5.93	5.93	5.93
5.94	5.94	5.94	5.94	5.94	5.94	5.94	5.94	5.94
5.95	5.95	5.95	5.95	5.95	5.95	5.95	5.95	5.95
5.96	5.96	5.96	5.96	5.96	5.96	5.96	5.96	5.96
5.97	5.97	5.97	5.97	5.97	5.97	5.97	5.97	5.97
5.98	5.98	5.98	5.98	5.98	5.98	5.98	5.98	5.98
5.99	5.99	5.99	5.99	5.99	5.99	5.99	5.99	5.99

6	6	6	6	6	6	6	6	6
6.01	6.01	6.01	6.01	6.01	6.01	6.01	6.01	6.01
6.02	6.02	6.02	6.02	6.02	6.02	6.02	6.02	6.02
6.03	6.03	6.03	6.03	6.03	6.03	6.03	6.03	6.03
6.04	6.04	6.04	6.04	6.04	6.04	6.04	6.04	6.04
6.05	6.05	6.05	6.05	6.05	6.05	6.05	6.05	6.05
6.06	6.06	6.06	6.06	6.06	6.06	6.06	6.06	6.06
6.07	6.07	6.07	6.07	6.07	6.07	6.07	6.07	6.07
6.08	6.08	6.08	6.08	6.08	6.08	6.08	6.08	6.08
6.09	6.09	6.09	6.09	6.09	6.09	6.09	6.09	6.09
6.1	6.1	6.1	6.1	6.1	6.1	6.1	6.1	6.1
6.11	6.11	6.11	6.11	6.11	6.11	6.11	6.11	6.11
6.12	6.12	6.12	6.12	6.12	6.12	6.12	6.12	6.12
6.13	6.13	6.13	6.13	6.13	6.13	6.13	6.13	6.13
6.14	6.14	6.14	6.14	6.14	6.14	6.14	6.14	6.14
6.15	6.15	6.15	6.15	6.15	6.15	6.15	6.15	6.15
6.16	6.16	6.16	6.16	6.16	6.16	6.16	6.16	6.16
6.17	6.17	6.17	6.17	6.17	6.17	6.17	6.17	6.17
6.18	6.18	6.18	6.18	6.18	6.18	6.18	6.18	6.18
6.19	6.19	6.19	6.19	6.19	6.19	6.19	6.19	6.19
6.2	6.2	6.2	6.2	6.2	6.2	6.2	6.2	6.2
6.21	6.21	6.21	6.21	6.21	6.21	6.21	6.21	6.21
6.22	6.22	6.22	6.22	6.22	6.22	6.22	6.22	6.22
6.23	6.23	6.23	6.23	6.23	6.23	6.23	6.23	6.23
6.24	6.24	6.24	6.24	6.24	6.24	6.24	6.24	6.24
6.25	6.25	6.25	6.25	6.25	6.25	6.25	6.25	6.25
6.26	6.26	6.26	6.26	6.26	6.26	6.26	6.26	6.26
6.27	6.27	6.27	6.27	6.27	6.27	6.27	6.27	6.27
6.28	6.28	6.28	6.28	6.28	6.28	6.28	6.28	6.28
6.29	6.29	6.29	6.29	6.29	6.29	6.29	6.29	6.29
6.3	6.3	6.3	6.3	6.3	6.3	6.3	6.3	6.3
6.31	6.31	6.31	6.31	6.31	6.31	6.31	6.31	6.31
6.32	6.32	6.32	6.32	6.32	6.32	6.32	6.32	6.32
6.33	6.33	6.33	6.33	6.33	6.33	6.33	6.33	6.33
6.34	6.34	6.34	6.34	6.34	6.34	6.34	6.34	6.34
6.35	6.35	6.35	6.35	6.35	6.35	6.35	6.35	6.35
6.36	6.36	6.36	6.36	6.36	6.36	6.36	6.36	6.36
6.37	6.37	6.37	6.37	6.37	6.37	6.37	6.37	6.37
6.38	6.38	6.38	6.38	6.38	6.38	6.38	6.38	6.38
6.39	6.39	6.39	6.39	6.39	6.39	6.39	6.39	6.39
6.4	6.4	6.4	6.4	6.4	6.4	6.4	6.4	6.4
6.41	6.41	6.41	6.41	6.41	6.41	6.41	6.41	6.41
6.42	6.42	6.42	6.42	6.42	6.42	6.42	6.42	6.42
6.43	6.43	6.43	6.43	6.43	6.43	6.43	6.43	6.43
6.44	6.44	6.44	6.44	6.44	6.44	6.44	6.44	6.44
6.45	6.45	6.45	6.45	6.45	6.45	6.45	6.45	6.45
6.46	6.46	6.46	6.46	6.46	6.46	6.46	6.46	6.46
6.47	6.47	6.47	6.47	6.47	6.47	6.47	6.47	6.47
6.48	6.48	6.48	6.48	6.48	6.48	6.48	6.48	6.48
6.49	6.49	6.49	6.49	6.49	6.49	6.49	6.49	6.49

6.5	6.5	6.5	6.5	6.5	6.5	6.5	6.5	6.5
6.51	6.51	6.51	6.51	6.51	6.51	6.51	6.51	6.51
6.52	6.52	6.52	6.52	6.52	6.52	6.52	6.52	6.52
6.53	6.53	6.53	6.53	6.53	6.53	6.53	6.53	6.53
6.54	6.54	6.54	6.54	6.54	6.54	6.54	6.54	6.54
6.55	6.55	6.55	6.55	6.55	6.55	6.55	6.55	6.55
6.56	6.56	6.56	6.56	6.56	6.56	6.56	6.56	6.56
6.57	6.57	6.57	6.57	6.57	6.57	6.57	6.57	6.57
6.58	6.58	6.58	6.58	6.58	6.58	6.58	6.58	6.58
6.59	6.59	6.59	6.59	6.59	6.59	6.59	6.59	6.59
6.6	6.6	6.6	6.6	6.6	6.6	6.6	6.6	6.6
6.61	6.61	6.61	6.61	6.61	6.61	6.61	6.61	6.61
6.62	6.62	6.62	6.62	6.62	6.62	6.62	6.62	6.62
6.63	6.63	6.63	6.63	6.63	6.63	6.63	6.63	6.63
6.64	6.64	6.64	6.64	6.64	6.64	6.64	6.64	6.64
6.65	6.65	6.65	6.65	6.65	6.65	6.65	6.65	6.65
6.66	6.66	6.66	6.66	6.66	6.66	6.66	6.66	6.66
6.67	6.67	6.67	6.67	6.67	6.67	6.67	6.67	6.67
6.68	6.68	6.68	6.68	6.68	6.68	6.68	6.68	6.68
6.69	6.69	6.69	6.69	6.69	6.69	6.69	6.69	6.69
6.7	6.7	6.7	6.7	6.7	6.7	6.7	6.7	6.7
6.71	6.71	6.71	6.71	6.71	6.71	6.71	6.71	6.71
6.72	6.72	6.72	6.72	6.72	6.72	6.72	6.72	6.72
6.73	6.73	6.73	6.73	6.73	6.73	6.73	6.73	6.73
6.74	6.74	6.74	6.74	6.74	6.74	6.74	6.74	6.74
6.75	6.75	6.75	6.75	6.75	6.75	6.75	6.75	6.75
6.76	6.76	6.76	6.76	6.76	6.76	6.76	6.76	6.76
6.77	6.77	6.77	6.77	6.77	6.77	6.77	6.77	6.77
6.78	6.78	6.78	6.78	6.78	6.78	6.78	6.78	6.78
6.79	6.79	6.79	6.79	6.79	6.79	6.79	6.79	6.79
6.8	6.8	6.8	6.8	6.8	6.8	6.8	6.8	6.8
6.81	6.81	6.81	6.81	6.81	6.81	6.81	6.81	6.81
6.82	6.82	6.82	6.82	6.82	6.82	6.82	6.82	6.82
6.83	6.83	6.83	6.83	6.83	6.83	6.83	6.83	6.83
6.84	6.84	6.84	6.84	6.84	6.84	6.84	6.84	6.84
6.85	6.85	6.85	6.85	6.85	6.85	6.85	6.85	6.85
6.86	6.86	6.86	6.86	6.86	6.86	6.86	6.86	6.86
6.87	6.87	6.87	6.87	6.87	6.87	6.87	6.87	6.87
6.88	6.88	6.88	6.88	6.88	6.88	6.88	6.88	6.88
6.89	6.89	6.89	6.89	6.89	6.89	6.89	6.89	6.89
6.9	6.9	6.9	6.9	6.9	6.9	6.9	6.9	6.9
6.91	6.91	6.91	6.91	6.91	6.91	6.91	6.91	6.91
6.92	6.92	6.92	6.92	6.92	6.92	6.92	6.92	6.92
6.93	6.93	6.93	6.93	6.93	6.93	6.93	6.93	6.93
6.94	6.94	6.94	6.94	6.94	6.94	6.94	6.94	6.94
6.95	6.95	6.95	6.95	6.95	6.95	6.95	6.95	6.95
6.96	6.96	6.96	6.96	6.96	6.96	6.96	6.96	6.96
6.97	6.97	6.97	6.97	6.97	6.97	6.97	6.97	6.97
6.98	6.98	6.98	6.98	6.98	6.98	6.98	6.98	6.98
6.99	6.99	6.99	6.99	6.99	6.99	6.99	6.99	6.99

7	7	7	7	7	7	7	7	7
7.01	7.01	7.01	7.01	7.01	7.01	7.01	7.01	7.01
7.02	7.02	7.02	7.02	7.02	7.02	7.02	7.02	7.02
7.03	7.03	7.03	7.03	7.03	7.03	7.03	7.03	7.03
7.04	7.04	7.04	7.04	7.04	7.04	7.04	7.04	7.04
7.05	7.05	7.05	7.05	7.05	7.05	7.05	7.05	7.05
7.06	7.06	7.06	7.06	7.06	7.06	7.06	7.06	7.06
7.07	7.07	7.07	7.07	7.07	7.07	7.07	7.07	7.07
7.08	7.08	7.08	7.08	7.08	7.08	7.08	7.08	7.08
7.09	7.09	7.09	7.09	7.09	7.09	7.09	7.09	7.09
7.1	7.1	7.1	7.1	7.1	7.1	7.1	7.1	7.1
7.11	7.11	7.11	7.11	7.11	7.11	7.11	7.11	7.11
7.12	7.12	7.12	7.12	7.12	7.12	7.12	7.12	7.12
7.13	7.13	7.13	7.13	7.13	7.13	7.13	7.13	7.13
7.14	7.14	7.14	7.14	7.14	7.14	7.14	7.14	7.14
7.15	7.15	7.15	7.15	7.15	7.15	7.15	7.15	7.15
7.16	7.16	7.16	7.16	7.16	7.16	7.16	7.16	7.16
7.17	7.17	7.17	7.17	7.17	7.17	7.17	7.17	7.17
7.18	7.18	7.18	7.18	7.18	7.18	7.18	7.18	7.18
7.19	7.19	7.19	7.19	7.19	7.19	7.19	7.19	7.19
7.2	7.2	7.2	7.2	7.2	7.2	7.2	7.2	7.2
7.21	7.21	7.21	7.21	7.21	7.21	7.21	7.21	7.21
7.22	7.22	7.22	7.22	7.22	7.22	7.22	7.22	7.22
7.23	7.23	7.23	7.23	7.23	7.23	7.23	7.23	7.23
7.24	7.24	7.24	7.24	7.24	7.24	7.24	7.24	7.24
7.25	7.25	7.25	7.25	7.25	7.25	7.25	7.25	7.25
7.26	7.26	7.26	7.26	7.26	7.26	7.26	7.26	7.26
7.27	7.27	7.27	7.27	7.27	7.27	7.27	7.27	7.27
7.28	7.28	7.28	7.28	7.28	7.28	7.28	7.28	7.28
7.29	7.29	7.29	7.29	7.29	7.29	7.29	7.29	7.29
7.3	7.3	7.3	7.3	7.3	7.3	7.3	7.3	7.3
7.31	7.31	7.31	7.31	7.31	7.31	7.31	7.31	7.31
7.32	7.32	7.32	7.32	7.32	7.32	7.32	7.32	7.32
7.33	7.33	7.33	7.33	7.33	7.33	7.33	7.33	7.33
7.34	7.34	7.34	7.34	7.34	7.34	7.34	7.34	7.34
7.35	7.35	7.35	7.35	7.35	7.35	7.35	7.35	7.35
7.36	7.36	7.36	7.36	7.36	7.36	7.36	7.36	7.36
7.37	7.37	7.37	7.37	7.37	7.37	7.37	7.37	7.37
7.38	7.38	7.38	7.38	7.38	7.38	7.38	7.38	7.38
7.39	7.39	7.39	7.39	7.39	7.39	7.39	7.39	7.39
7.4	7.4	7.4	7.4	7.4	7.4	7.4	7.4	7.4
7.41	7.41	7.41	7.41	7.41	7.41	7.41	7.41	7.41
7.42	7.42	7.42	7.42	7.42	7.42	7.42	7.42	7.42
7.43	7.43	7.43	7.43	7.43	7.43	7.43	7.43	7.43
7.44	7.44	7.44	7.44	7.44	7.44	7.44	7.44	7.44
7.45	7.45	7.45	7.45	7.45	7.45	7.45	7.45	7.45
7.46	7.46	7.46	7.46	7.46	7.46	7.46	7.46	7.46
7.47	7.47	7.47	7.47	7.47	7.47	7.47	7.47	7.47
7.48	7.48	7.48	7.48	7.48	7.48	7.48	7.48	7.48
7.49	7.49	7.49	7.49	7.49	7.49	7.49	7.49	7.49

7.5	7.5	7.5	7.5	7.5	7.5	7.5	7.5	7.5
7.51	7.51	7.51	7.51	7.51	7.51	7.51	7.51	7.51
7.52	7.52	7.52	7.52	7.52	7.52	7.52	7.52	7.52
7.53	7.53	7.53	7.53	7.53	7.53	7.53	7.53	7.53
7.54	7.54	7.54	7.54	7.54	7.54	7.54	7.54	7.54
7.55	7.55	7.55	7.55	7.55	7.55	7.55	7.55	7.55
7.56	7.56	7.56	7.56	7.56	7.56	7.56	7.56	7.56
7.57	7.57	7.57	7.57	7.57	7.57	7.57	7.57	7.57
7.58	7.58	7.58	7.58	7.58	7.58	7.58	7.58	7.58
7.59	7.59	7.59	7.59	7.59	7.59	7.59	7.59	7.59
7.6	7.6	7.6	7.6	7.6	7.6	7.6	7.6	7.6
7.61	7.61	7.61	7.61	7.61	7.61	7.61	7.61	7.61
7.62	7.62	7.62	7.62	7.62	7.62	7.62	7.62	7.62
7.63	7.63	7.63	7.63	7.63	7.63	7.63	7.63	7.63
7.64	7.64	7.64	7.64	7.64	7.64	7.64	7.64	7.64
7.65	7.65	7.65	7.65	7.65	7.65	7.65	7.65	7.65
7.66	7.66	7.66	7.66	7.66	7.66	7.66	7.66	7.66
7.67	7.67	7.67	7.67	7.67	7.67	7.67	7.67	7.67
7.68	7.68	7.68	7.68	7.68	7.68	7.68	7.68	7.68
7.69	7.69	7.69	7.69	7.69	7.69	7.69	7.69	7.69
7.7	7.7	7.7	7.7	7.7	7.7	7.7	7.7	7.7
7.71	7.71	7.71	7.71	7.71	7.71	7.71	7.71	7.71
7.72	7.72	7.72	7.72	7.72	7.72	7.72	7.72	7.72
7.73	7.73	7.73	7.73	7.73	7.73	7.73	7.73	7.73
7.74	7.74	7.74	7.74	7.74	7.74	7.74	7.74	7.74
7.75	7.75	7.75	7.75	7.75	7.75	7.75	7.75	7.75
7.76	7.76	7.76	7.76	7.76	7.76	7.76	7.76	7.76
7.77	7.77	7.77	7.77	7.77	7.77	7.77	7.77	7.77
7.78	7.78	7.78	7.78	7.78	7.78	7.78	7.78	7.78
7.79	7.79	7.79	7.79	7.79	7.79	7.79	7.79	7.79
7.8	7.8	7.8	7.8	7.8	7.8	7.8	7.8	7.8
7.81	7.81	7.81	7.81	7.81	7.81	7.81	7.81	7.81
7.82	7.82	7.82	7.82	7.82	7.82	7.82	7.82	7.82
7.83	7.83	7.83	7.83	7.83	7.83	7.83	7.83	7.83
7.84	7.84	7.84	7.84	7.84	7.84	7.84	7.84	7.84
7.85	7.85	7.85	7.85	7.85	7.85	7.85	7.85	7.85
7.86	7.86	7.86	7.86	7.86	7.86	7.86	7.86	7.86
7.87	7.87	7.87	7.87	7.87	7.87	7.87	7.87	7.87
7.88	7.88	7.88	7.88	7.88	7.88	7.88	7.88	7.88
7.89	7.89	7.89	7.89	7.89	7.89	7.89	7.89	7.89
7.9	7.9	7.9	7.9	7.9	7.9	7.9	7.9	7.9
7.91	7.91	7.91	7.91	7.91	7.91	7.91	7.91	7.91
7.92	7.92	7.92	7.92	7.92	7.92	7.92	7.92	7.92
7.93	7.93	7.93	7.93	7.93	7.93	7.93	7.93	7.93
7.94	7.94	7.94	7.94	7.94	7.94	7.94	7.94	7.94
7.95	7.95	7.95	7.95	7.95	7.95	7.95	7.95	7.95
7.96	7.96	7.96	7.96	7.96	7.96	7.96	7.96	7.96
7.97	7.97	7.97	7.97	7.97	7.97	7.97	7.97	7.97
7.98	7.98	7.98	7.98	7.98	7.98	7.98	7.98	7.98
7.99	7.99	7.99	7.99	7.99	7.99	7.99	7.99	7.99

8	8	8	8	8	8	8	8	8
8.01	8.01	8.01	8.01	8.01	8.01	8.01	8.01	8.01
8.02	8.02	8.02	8.02	8.02	8.02	8.02	8.02	8.02
8.03	8.03	8.03	8.03	8.03	8.03	8.03	8.03	8.03
8.04	8.04	8.04	8.04	8.04	8.04	8.04	8.04	8.04
8.05	8.05	8.05	8.05	8.05	8.05	8.05	8.05	8.05
8.06	8.06	8.06	8.06	8.06	8.06	8.06	8.06	8.06
8.07	8.07	8.07	8.07	8.07	8.07	8.07	8.07	8.07
8.08	8.08	8.08	8.08	8.08	8.08	8.08	8.08	8.08
8.09	8.09	8.09	8.09	8.09	8.09	8.09	8.09	8.09
8.1	8.1	8.1	8.1	8.1	8.1	8.1	8.1	8.1
8.11	8.11	8.11	8.11	8.11	8.11	8.11	8.11	8.11
8.12	8.12	8.12	8.12	8.12	8.12	8.12	8.12	8.12
8.13	8.13	8.13	8.13	8.13	8.13	8.13	8.13	8.13
8.14	8.14	8.14	8.14	8.14	8.14	8.14	8.14	8.14
8.15	8.15	8.15	8.15	8.15	8.15	8.15	8.15	8.15
8.16	8.16	8.16	8.16	8.16	8.16	8.16	8.16	8.16
8.17	8.17	8.17	8.17	8.17	8.17	8.17	8.17	8.17
8.18	8.18	8.18	8.18	8.18	8.18	8.18	8.18	8.18
8.19	8.19	8.19	8.19	8.19	8.19	8.19	8.19	8.19
8.2	8.2	8.2	8.2	8.2	8.2	8.2	8.2	8.2
8.21	8.21	8.21	8.21	8.21	8.21	8.21	8.21	8.21
8.22	8.22	8.22	8.22	8.22	8.22	8.22	8.22	8.22
8.23	8.23	8.23	8.23	8.23	8.23	8.23	8.23	8.23
8.24	8.24	8.24	8.24	8.24	8.24	8.24	8.24	8.24
8.25	8.25	8.25	8.25	8.25	8.25	8.25	8.25	8.25
8.26	8.26	8.26	8.26	8.26	8.26	8.26	8.26	8.26
8.27	8.27	8.27	8.27	8.27	8.27	8.27	8.27	8.27
8.28	8.28	8.28	8.28	8.28	8.28	8.28	8.28	8.28
8.29	8.29	8.29	8.29	8.29	8.29	8.29	8.29	8.29
8.3	8.3	8.3	8.3	8.3	8.3	8.3	8.3	8.3
8.31	8.31	8.31	8.31	8.31	8.31	8.31	8.31	8.31
8.32	8.32	8.32	8.32	8.32	8.32	8.32	8.32	8.32
8.33	8.33	8.33	8.33	8.33	8.33	8.33	8.33	8.33
8.34	8.34	8.34	8.34	8.34	8.34	8.34	8.34	8.34
8.35	8.35	8.35	8.35	8.35	8.35	8.35	8.35	8.35
8.36	8.36	8.36	8.36	8.36	8.36	8.36	8.36	8.36
8.37	8.37	8.37	8.37	8.37	8.37	8.37	8.37	8.37
8.38	8.38	8.38	8.38	8.38	8.38	8.38	8.38	8.38
8.39	8.39	8.39	8.39	8.39	8.39	8.39	8.39	8.39
8.4	8.4	8.4	8.4	8.4	8.4	8.4	8.4	8.4
8.41	8.41	8.41	8.41	8.41	8.41	8.41	8.41	8.41
8.42	8.42	8.42	8.42	8.42	8.42	8.42	8.42	8.42
8.43	8.43	8.43	8.43	8.43	8.43	8.43	8.43	8.43
8.44	8.44	8.44	8.44	8.44	8.44	8.44	8.44	8.44
8.45	8.45	8.45	8.45	8.45	8.45	8.45	8.45	8.45
8.46	8.46	8.46	8.46	8.46	8.46	8.46	8.46	8.46
8.47	8.47	8.47	8.47	8.47	8.47	8.47	8.47	8.47
8.48	8.48	8.48	8.48	8.48	8.48	8.48	8.48	8.48
8.49	8.49	8.49	8.49	8.49	8.49	8.49	8.49	8.49

8.5	8.5	8.5	8.5	8.5	8.5	8.5	8.5	8.5
8.51	8.51	8.51	8.51	8.51	8.51	8.51	8.51	8.51
8.52	8.52	8.52	8.52	8.52	8.52	8.52	8.52	8.52
8.53	8.53	8.53	8.53	8.53	8.53	8.53	8.53	8.53
8.54	8.54	8.54	8.54	8.54	8.54	8.54	8.54	8.54
8.55	8.55	8.55	8.55	8.55	8.55	8.55	8.55	8.55
8.56	8.56	8.56	8.56	8.56	8.56	8.56	8.56	8.56
8.57	8.57	8.57	8.57	8.57	8.57	8.57	8.57	8.57
8.58	8.58	8.58	8.58	8.58	8.58	8.58	8.58	8.58
8.59	8.59	8.59	8.59	8.59	8.59	8.59	8.59	8.59
8.6	8.6	8.6	8.6	8.6	8.6	8.6	8.6	8.6
8.61	8.61	8.61	8.61	8.61	8.61	8.61	8.61	8.61
8.62	8.62	8.62	8.62	8.62	8.62	8.62	8.62	8.62
8.63	8.63	8.63	8.63	8.63	8.63	8.63	8.63	8.63
8.64	8.64	8.64	8.64	8.64	8.64	8.64	8.64	8.64
8.65	8.65	8.65	8.65	8.65	8.65	8.65	8.65	8.65
8.66	8.66	8.66	8.66	8.66	8.66	8.66	8.66	8.66
8.67	8.67	8.67	8.67	8.67	8.67	8.67	8.67	8.67
8.68	8.68	8.68	8.68	8.68	8.68	8.68	8.68	8.68
8.69	8.69	8.69	8.69	8.69	8.69	8.69	8.69	8.69
8.7	8.7	8.7	8.7	8.7	8.7	8.7	8.7	8.7
8.71	8.71	8.71	8.71	8.71	8.71	8.71	8.71	8.71
8.72	8.72	8.72	8.72	8.72	8.72	8.72	8.72	8.72
8.73	8.73	8.73	8.73	8.73	8.73	8.73	8.73	8.73
8.74	8.74	8.74	8.74	8.74	8.74	8.74	8.74	8.74
8.75	8.75	8.75	8.75	8.75	8.75	8.75	8.75	8.75
8.76	8.76	8.76	8.76	8.76	8.76	8.76	8.76	8.76
8.77	8.77	8.77	8.77	8.77	8.77	8.77	8.77	8.77
8.78	8.78	8.78	8.78	8.78	8.78	8.78	8.78	8.78
8.79	8.79	8.79	8.79	8.79	8.79	8.79	8.79	8.79
8.8	8.8	8.8	8.8	8.8	8.8	8.8	8.8	8.8
8.81	8.81	8.81	8.81	8.81	8.81	8.81	8.81	8.81
8.82	8.82	8.82	8.82	8.82	8.82	8.82	8.82	8.82
8.83	8.83	8.83	8.83	8.83	8.83	8.83	8.83	8.83
8.84	8.84	8.84	8.84	8.84	8.84	8.84	8.84	8.84
8.85	8.85	8.85	8.85	8.85	8.85	8.85	8.85	8.85
8.86	8.86	8.86	8.86	8.86	8.86	8.86	8.86	8.86
8.87	8.87	8.87	8.87	8.87	8.87	8.87	8.87	8.87
8.88	8.88	8.88	8.88	8.88	8.88	8.88	8.88	8.88
8.89	8.89	8.89	8.89	8.89	8.89	8.89	8.89	8.89
8.9	8.9	8.9	8.9	8.9	8.9	8.9	8.9	8.9
8.91	8.91	8.91	8.91	8.91	8.91	8.91	8.91	8.91
8.92	8.92	8.92	8.92	8.92	8.92	8.92	8.92	8.92
8.93	8.93	8.93	8.93	8.93	8.93	8.93	8.93	8.93
8.94	8.94	8.94	8.94	8.94	8.94	8.94	8.94	8.94
8.95	8.95	8.95	8.95	8.95	8.95	8.95	8.95	8.95
8.96	8.96	8.96	8.96	8.96	8.96	8.96	8.96	8.96
8.97	8.97	8.97	8.97	8.97	8.97	8.97	8.97	8.97
8.98	8.98	8.98	8.98	8.98	8.98	8.98	8.98	8.98
8.99	8.99	8.99	8.99	8.99	8.99	8.99	8.99	8.99

9	9	9	9	9	9	9	9	9
9.01	9.01	9.01	9.01	9.01	9.01	9.01	9.01	9.01
9.02	9.02	9.02	9.02	9.02	9.02	9.02	9.02	9.02
9.03	9.03	9.03	9.03	9.03	9.03	9.03	9.03	9.03
9.04	9.04	9.04	9.04	9.04	9.04	9.04	9.04	9.04
9.05	9.05	9.05	9.05	9.05	9.05	9.05	9.05	9.05
9.06	9.06	9.06	9.06	9.06	9.06	9.06	9.06	9.06
9.07	9.07	9.07	9.07	9.07	9.07	9.07	9.07	9.07
9.08	9.08	9.08	9.08	9.08	9.08	9.08	9.08	9.08
9.09	9.09	9.09	9.09	9.09	9.09	9.09	9.09	9.09
9.1	9.1	9.1	9.1	9.1	9.1	9.1	9.1	9.1
9.11	9.11	9.11	9.11	9.11	9.11	9.11	9.11	9.11
9.12	9.12	9.12	9.12	9.12	9.12	9.12	9.12	9.12
9.13	9.13	9.13	9.13	9.13	9.13	9.13	9.13	9.13
9.14	9.14	9.14	9.14	9.14	9.14	9.14	9.14	9.14
9.15	9.15	9.15	9.15	9.15	9.15	9.15	9.15	9.15
9.16	9.16	9.16	9.16	9.16	9.16	9.16	9.16	9.16
9.17	9.17	9.17	9.17	9.17	9.17	9.17	9.17	9.17
9.18	9.18	9.18	9.18	9.18	9.18	9.18	9.18	9.18
9.19	9.19	9.19	9.19	9.19	9.19	9.19	9.19	9.19
9.2	9.2	9.2	9.2	9.2	9.2	9.2	9.2	9.2
9.21	9.21	9.21	9.21	9.21	9.21	9.21	9.21	9.21
9.22	9.22	9.22	9.22	9.22	9.22	9.22	9.22	9.22
9.23	9.23	9.23	9.23	9.23	9.23	9.23	9.23	9.23
9.24	9.24	9.24	9.24	9.24	9.24	9.24	9.24	9.24
9.25	9.25	9.25	9.25	9.25	9.25	9.25	9.25	9.25
9.26	9.26	9.26	9.26	9.26	9.26	9.26	9.26	9.26
9.27	9.27	9.27	9.27	9.27	9.27	9.27	9.27	9.27
9.28	9.28	9.28	9.28	9.28	9.28	9.28	9.28	9.28
9.29	9.29	9.29	9.29	9.29	9.29	9.29	9.29	9.29
9.3	9.3	9.3	9.3	9.3	9.3	9.3	9.3	9.3
9.31	9.31	9.31	9.31	9.31	9.31	9.31	9.31	9.31
9.32	9.32	9.32	9.32	9.32	9.32	9.32	9.32	9.32
9.33	9.33	9.33	9.33	9.33	9.33	9.33	9.33	9.33
9.34	9.34	9.34	9.34	9.34	9.34	9.34	9.34	9.34
9.35	9.35	9.35	9.35	9.35	9.35	9.35	9.35	9.35
9.36	9.36	9.36	9.36	9.36	9.36	9.36	9.36	9.36
9.37	9.37	9.37	9.37	9.37	9.37	9.37	9.37	9.37
9.38	9.38	9.38	9.38	9.38	9.38	9.38	9.38	9.38
9.39	9.39	9.39	9.39	9.39	9.39	9.39	9.39	9.39
9.4	9.4	9.4	9.4	9.4	9.4	9.4	9.4	9.4
9.41	9.41	9.41	9.41	9.41	9.41	9.41	9.41	9.41
9.42	9.42	9.42	9.42	9.42	9.42	9.42	9.42	9.42
9.43	9.43	9.43	9.43	9.43	9.43	9.43	9.43	9.43
9.44	9.44	9.44	9.44	9.44	9.44	9.44	9.44	9.44
9.45	9.45	9.45	9.45	9.45	9.45	9.45	9.45	9.45
9.46	9.46	9.46	9.46	9.46	9.46	9.46	9.46	9.46
9.47	9.47	9.47	9.47	9.47	9.47	9.47	9.47	9.47
9.48	9.48	9.48	9.48	9.48	9.48	9.48	9.48	9.48
9.49	9.49	9.49	9.49	9.49	9.49	9.49	9.49	9.49

9.5	9.5	9.5	9.5	9.5	9.5	9.5	9.5	9.5
9.51	9.51	9.51	9.51	9.51	9.51	9.51	9.51	9.51
9.52	9.52	9.52	9.52	9.52	9.52	9.52	9.52	9.52
9.53	9.53	9.53	9.53	9.53	9.53	9.53	9.53	9.53
9.54	9.54	9.54	9.54	9.54	9.54	9.54	9.54	9.54
9.55	9.55	9.55	9.55	9.55	9.55	9.55	9.55	9.55
9.56	9.56	9.56	9.56	9.56	9.56	9.56	9.56	9.56
9.57	9.57	9.57	9.57	9.57	9.57	9.57	9.57	9.57
9.58	9.58	9.58	9.58	9.58	9.58	9.58	9.58	9.58
9.59	9.59	9.59	9.59	9.59	9.59	9.59	9.59	9.59
9.6	9.6	9.6	9.6	9.6	9.6	9.6	9.6	9.6
9.61	9.61	9.61	9.61	9.61	9.61	9.61	9.61	9.61
9.62	9.62	9.62	9.62	9.62	9.62	9.62	9.62	9.62
9.63	9.63	9.63	9.63	9.63	9.63	9.63	9.63	9.63
9.64	9.64	9.64	9.64	9.64	9.64	9.64	9.64	9.64
9.65	9.65	9.65	9.65	9.65	9.65	9.65	9.65	9.65
9.66	9.66	9.66	9.66	9.66	9.66	9.66	9.66	9.66
9.67	9.67	9.67	9.67	9.67	9.67	9.67	9.67	9.67
9.68	9.68	9.68	9.68	9.68	9.68	9.68	9.68	9.68
9.69	9.69	9.69	9.69	9.69	9.69	9.69	9.69	9.69
9.7	9.7	9.7	9.7	9.7	9.7	9.7	9.7	9.7
9.71	9.71	9.71	9.71	9.71	9.71	9.71	9.71	9.71
9.72	9.72	9.72	9.72	9.72	9.72	9.72	9.72	9.72
9.73	9.73	9.73	9.73	9.73	9.73	9.73	9.73	9.73
9.74	9.74	9.74	9.74	9.74	9.74	9.74	9.74	9.74
9.75	9.75	9.75	9.75	9.75	9.75	9.75	9.75	9.75
9.76	9.76	9.76	9.76	9.76	9.76	9.76	9.76	9.76
9.77	9.77	9.77	9.77	9.77	9.77	9.77	9.77	9.77
9.78	9.78	9.78	9.78	9.78	9.78	9.78	9.78	9.78
9.79	9.79	9.79	9.79	9.79	9.79	9.79	9.79	9.79
9.8	9.8	9.8	9.8	9.8	9.8	9.8	9.8	9.8
9.81	9.81	9.81	9.81	9.81	9.81	9.81	9.81	9.81
9.82	9.82	9.82	9.82	9.82	9.82	9.82	9.82	9.82
9.83	9.83	9.83	9.83	9.83	9.83	9.83	9.83	9.83
9.84	9.84	9.84	9.84	9.84	9.84	9.84	9.84	9.84
9.85	9.85	9.85	9.85	9.85	9.85	9.85	9.85	9.85
9.86	9.86	9.86	9.86	9.86	9.86	9.86	9.86	9.86
9.87	9.87	9.87	9.87	9.87	9.87	9.87	9.87	9.87
9.88	9.88	9.88	9.88	9.88	9.88	9.88	9.88	9.88
9.89	9.89	9.89	9.89	9.89	9.89	9.89	9.89	9.89
9.9	9.9	9.9	9.9	9.9	9.9	9.9	9.9	9.9
9.91	9.91	9.91	9.91	9.91	9.91	9.91	9.91	9.91
9.92	9.92	9.92	9.92	9.92	9.92	9.92	9.92	9.92
9.93	9.93	9.93	9.93	9.93	9.93	9.93	9.93	9.93
9.94	9.94	9.94	9.94	9.94	9.94	9.94	9.94	9.94
9.95	9.95	9.95	9.95	9.95	9.95	9.95	9.95	9.95
9.96	9.96	9.96	9.96	9.96	9.96	9.96	9.96	9.96
9.97	9.97	9.97	9.97	9.97	9.97	9.97	9.97	9.97
9.98	9.98	9.98	9.98	9.98	9.98	9.98	9.98	9.98
9.99	9.99	9.99	9.99	9.99	9.99	9.99	9.99	9.99

10	10	10	10	10	10	10	10	10
10.01	10.01	10.01	10.01	10.01	10.01	10.01	10.01	10.01
10.02	10.02	10.02	10.02	10.02	10.02	10.02	10.02	10.02
10.03	10.03	10.03	10.03	10.03	10.03	10.03	10.03	10.03
10.04	10.04	10.04	10.04	10.04	10.04	10.04	10.04	10.04
10.05	10.05	10.05	10.05	10.05	10.05	10.05	10.05	10.05
10.06	10.06	10.06	10.06	10.06	10.06	10.06	10.06	10.06
10.07	10.07	10.07	10.07	10.07	10.07	10.07	10.07	10.07
10.08	10.08	10.08	10.08	10.08	10.08	10.08	10.08	10.08
10.09	10.09	10.09	10.09	10.09	10.09	10.09	10.09	10.09
10.1	10.1	10.1	10.1	10.1	10.1	10.1	10.1	10.1
10.11	10.11	10.11	10.11	10.11	10.11	10.11	10.11	10.11
10.12	10.12	10.12	10.12	10.12	10.12	10.12	10.12	10.12
10.13	10.13	10.13	10.13	10.13	10.13	10.13	10.13	10.13
10.14	10.14	10.14	10.14	10.14	10.14	10.14	10.14	10.14
10.15	10.15	10.15	10.15	10.15	10.15	10.15	10.15	10.15
10.16	10.16	10.16	10.16	10.16	10.16	10.16	10.16	10.16
10.17	10.17	10.17	10.17	10.17	10.17	10.17	10.17	10.17
10.18	10.18	10.18	10.18	10.18	10.18	10.18	10.18	10.18
10.19	10.19	10.19	10.19	10.19	10.19	10.19	10.19	10.19
10.2	10.2	10.2	10.2	10.2	10.2	10.2	10.2	10.2
10.21	10.21	10.21	10.21	10.21	10.21	10.21	10.21	10.21
10.22	10.22	10.22	10.22	10.22	10.22	10.22	10.22	10.22
10.23	10.23	10.23	10.23	10.23	10.23	10.23	10.23	10.23
10.24	10.24	10.24	10.24	10.24	10.24	10.24	10.24	10.24
10.25	10.25	10.25	10.25	10.25	10.25	10.25	10.25	10.25
10.26	10.26	10.26	10.26	10.26	10.26	10.26	10.26	10.26
10.27	10.27	10.27	10.27	10.27	10.27	10.27	10.27	10.27
10.28	10.28	10.28	10.28	10.28	10.28	10.28	10.28	10.28
10.29	10.29	10.29	10.29	10.29	10.29	10.29	10.29	10.29
10.3	10.3	10.3	10.3	10.3	10.3	10.3	10.3	10.3
10.31	10.31	10.31	10.31	10.31	10.31	10.31	10.31	10.31
10.32	10.32	10.32	10.32	10.32	10.32	10.32	10.32	10.32
10.33	10.33	10.33	10.33	10.33	10.33	10.33	10.33	10.33
10.34	10.34	10.34	10.34	10.34	10.34	10.34	10.34	10.34
10.35	10.35	10.35	10.35	10.35	10.35	10.35	10.35	10.35
10.36	10.36	10.36	10.36	10.36	10.36	10.36	10.36	10.36
10.37	10.37	10.37	10.37	10.37	10.37	10.37	10.37	10.37
10.38	10.38	10.38	10.38	10.38	10.38	10.38	10.38	10.38
10.39	10.39	10.39	10.39	10.39	10.39	10.39	10.39	10.39
10.4	10.4	10.4	10.4	10.4	10.4	10.4	10.4	10.4
10.41	10.41	10.41	10.41	10.41	10.41	10.41	10.41	10.41
10.42	10.42	10.42	10.42	10.42	10.42	10.42	10.42	10.42
10.43	10.43	10.43	10.43	10.43	10.43	10.43	10.43	10.43
10.44	10.44	10.44	10.44	10.44	10.44	10.44	10.44	10.44
10.45	10.45	10.45	10.45	10.45	10.45	10.45	10.45	10.45
10.46	10.46	10.46	10.46	10.46	10.46	10.46	10.46	10.46
10.47	10.47	10.47	10.47	10.47	10.47	10.47	10.47	10.47
10.48	10.48	10.48	10.48	10.48	10.48	10.48	10.48	10.48
10.49	10.49	10.49	10.49	10.49	10.49	10.49	10.49	10.49

10.5	10.5	10.5	10.5	10.5	10.5	10.5	10.5	10.5
10.51	10.51	10.51	10.51	10.51	10.51	10.51	10.51	10.51
10.52	10.52	10.52	10.52	10.52	10.52	10.52	10.52	10.52
10.53	10.53	10.53	10.53	10.53	10.53	10.53	10.53	10.53
10.54	10.54	10.54	10.54	10.54	10.54	10.54	10.54	10.54
10.55	10.55	10.55	10.55	10.55	10.55	10.55	10.55	10.55
10.56	10.56	10.56	10.56	10.56	10.56	10.56	10.56	10.56
10.57	10.57	10.57	10.57	10.57	10.57	10.57	10.57	10.57
10.58	10.58	10.58	10.58	10.58	10.58	10.58	10.58	10.58
10.59	10.59	10.59	10.59	10.59	10.59	10.59	10.59	10.59
10.6	10.6	10.6	10.6	10.6	10.6	10.6	10.6	10.6
10.61	10.61	10.61	10.61	10.61	10.61	10.61	10.61	10.61
10.62	10.62	10.62	10.62	10.62	10.62	10.62	10.62	10.62
10.63	10.63	10.63	10.63	10.63	10.63	10.63	10.63	10.63
10.64	10.64	10.64	10.64	10.64	10.64	10.64	10.64	10.64
10.65	10.65	10.65	10.65	10.65	10.65	10.65	10.65	10.65
10.66	10.66	10.66	10.66	10.66	10.66	10.66	10.66	10.66
10.67	10.67	10.67	10.67	10.67	10.67	10.67	10.67	10.67
10.68	10.68	10.68	10.68	10.68	10.68	10.68	10.68	10.68
10.69	10.69	10.69	10.69	10.69	10.69	10.69	10.69	10.69
10.7	10.7	10.7	10.7	10.7	10.7	10.7	10.7	10.7
10.71	10.71	10.71	10.71	10.71	10.71	10.71	10.71	10.71
10.72	10.72	10.72	10.72	10.72	10.72	10.72	10.72	10.72
10.73	10.73	10.73	10.73	10.73	10.73	10.73	10.73	10.73
10.74	10.74	10.74	10.74	10.74	10.74	10.74	10.74	10.74
10.75	10.75	10.75	10.75	10.75	10.75	10.75	10.75	10.75
10.76	10.76	10.76	10.76	10.76	10.76	10.76	10.76	10.76
10.77	10.77	10.77	10.77	10.77	10.77	10.77	10.77	10.77
10.78	10.78	10.78	10.78	10.78	10.78	10.78	10.78	10.78
10.79	10.79	10.79	10.79	10.79	10.79	10.79	10.79	10.79
10.8	10.8	10.8	10.8	10.8	10.8	10.8	10.8	10.8
10.81	10.81	10.81	10.81	10.81	10.81	10.81	10.81	10.81
10.82	10.82	10.82	10.82	10.82	10.82	10.82	10.82	10.82
10.83	10.83	10.83	10.83	10.83	10.83	10.83	10.83	10.83
10.84	10.84	10.84	10.84	10.84	10.84	10.84	10.84	10.84
10.85	10.85	10.85	10.85	10.85	10.85	10.85	10.85	10.85
10.86	10.86	10.86	10.86	10.86	10.86	10.86	10.86	10.86
10.87	10.87	10.87	10.87	10.87	10.87	10.87	10.87	10.87
10.88	10.88	10.88	10.88	10.88	10.88	10.88	10.88	10.88
10.89	10.89	10.89	10.89	10.89	10.89	10.89	10.89	10.89
10.9	10.9	10.9	10.9	10.9	10.9	10.9	10.9	10.9
10.91	10.91	10.91	10.91	10.91	10.91	10.91	10.91	10.91
10.92	10.92	10.92	10.92	10.92	10.92	10.92	10.92	10.92
10.93	10.93	10.93	10.93	10.93	10.93	10.93	10.93	10.93
10.94	10.94	10.94	10.94	10.94	10.94	10.94	10.94	10.94
10.95	10.95	10.95	10.95	10.95	10.95	10.95	10.95	10.95
10.96	10.96	10.96	10.96	10.96	10.96	10.96	10.96	10.96
10.97	10.97	10.97	10.97	10.97	10.97	10.97	10.97	10.97
10.98	10.98	10.98	10.98	10.98	10.98	10.98	10.98	10.98
10.99	10.99	10.99	10.99	10.99	10.99	10.99	10.99	10.99

11	11	11	11	11	11	11	11	11
11.01	11.01	11.01	11.01	11.01	11.01	11.01	11.01	11.01
11.02	11.02	11.02	11.02	11.02	11.02	11.02	11.02	11.02
11.03	11.03	11.03	11.03	11.03	11.03	11.03	11.03	11.03
11.04	11.04	11.04	11.04	11.04	11.04	11.04	11.04	11.04
11.05	11.05	11.05	11.05	11.05	11.05	11.05	11.05	11.05
11.06	11.06	11.06	11.06	11.06	11.06	11.06	11.06	11.06
11.07	11.07	11.07	11.07	11.07	11.07	11.07	11.07	11.07
11.08	11.08	11.08	11.08	11.08	11.08	11.08	11.08	11.08
11.09	11.09	11.09	11.09	11.09	11.09	11.09	11.09	11.09
11.1	11.1	11.1	11.1	11.1	11.1	11.1	11.1	11.1
11.11	11.11	11.11	11.11	11.11	11.11	11.11	11.11	11.11
11.12	11.12	11.12	11.12	11.12	11.12	11.12	11.12	11.12
11.13	11.13	11.13	11.13	11.13	11.13	11.13	11.13	11.13
11.14	11.14	11.14	11.14	11.14	11.14	11.14	11.14	11.14
11.15	11.15	11.15	11.15	11.15	11.15	11.15	11.15	11.15
11.16	11.16	11.16	11.16	11.16	11.16	11.16	11.16	11.16
11.17	11.17	11.17	11.17	11.17	11.17	11.17	11.17	11.17
11.18	11.18	11.18	11.18	11.18	11.18	11.18	11.18	11.18
11.19	11.19	11.19	11.19	11.19	11.19	11.19	11.19	11.19
11.2	11.2	11.2	11.2	11.2	11.2	11.2	11.2	11.2
11.21	11.21	11.21	11.21	11.21	11.21	11.21	11.21	11.21
11.22	11.22	11.22	11.22	11.22	11.22	11.22	11.22	11.22
11.23	11.23	11.23	11.23	11.23	11.23	11.23	11.23	11.23
11.24	11.24	11.24	11.24	11.24	11.24	11.24	11.24	11.24
11.25	11.25	11.25	11.25	11.25	11.25	11.25	11.25	11.25
11.26	11.26	11.26	11.26	11.26	11.26	11.26	11.26	11.26
11.27	11.27	11.27	11.27	11.27	11.27	11.27	11.27	11.27
11.28	11.28	11.28	11.28	11.28	11.28	11.28	11.28	11.28
11.29	11.29	11.29	11.29	11.29	11.29	11.29	11.29	11.29
11.3	11.3	11.3	11.3	11.3	11.3	11.3	11.3	11.3
11.31	11.31	11.31	11.31	11.31	11.31	11.31	11.31	11.31
11.32	11.32	11.32	11.32	11.32	11.32	11.32	11.32	11.32
11.33	11.33	11.33	11.33	11.33	11.33	11.33	11.33	11.33
11.34	11.34	11.34	11.34	11.34	11.34	11.34	11.34	11.34
11.35	11.35	11.35	11.35	11.35	11.35	11.35	11.35	11.35
11.36	11.36	11.36	11.36	11.36	11.36	11.36	11.36	11.36
11.37	11.37	11.37	11.37	11.37	11.37	11.37	11.37	11.37
11.38	11.38	11.38	11.38	11.38	11.38	11.38	11.38	11.38
11.39	11.39	11.39	11.39	11.39	11.39	11.39	11.39	11.39
11.4	11.4	11.4	11.4	11.4	11.4	11.4	11.4	11.4
11.41	11.41	11.41	11.41	11.41	11.41	11.41	11.41	11.41
11.42	11.42	11.42	11.42	11.42	11.42	11.42	11.42	11.42
11.43	11.43	11.43	11.43	11.43	11.43	11.43	11.43	11.43
11.44	11.44	11.44	11.44	11.44	11.44	11.44	11.44	11.44
11.45	11.45	11.45	11.45	11.45	11.45	11.45	11.45	11.45
11.46	11.46	11.46	11.46	11.46	11.46	11.46	11.46	11.46
11.47	11.47	11.47	11.47	11.47	11.47	11.47	11.47	11.47
11.48	11.48	11.48	11.48	11.48	11.48	11.48	11.48	11.48
11.49	11.49	11.49	11.49	11.49	11.49	11.49	11.49	11.49

11.5	11.5	11.5	11.5	11.5	11.5	11.5	11.5	11.5
11.51	11.51	11.51	11.51	11.51	11.51	11.51	11.51	11.51
11.52	11.52	11.52	11.52	11.52	11.52	11.52	11.52	11.52
11.53	11.53	11.53	11.53	11.53	11.53	11.53	11.53	11.53
11.54	11.54	11.54	11.54	11.54	11.54	11.54	11.54	11.54
11.55	11.55	11.55	11.55	11.55	11.55	11.55	11.55	11.55
11.56	11.56	11.56	11.56	11.56	11.56	11.56	11.56	11.56
11.57	11.57	11.57	11.57	11.57	11.57	11.57	11.57	11.57
11.58	11.58	11.58	11.58	11.58	11.58	11.58	11.58	11.58
11.59	11.59	11.59	11.59	11.59	11.59	11.59	11.59	11.59
11.6	11.6	11.6	11.6	11.6	11.6	11.6	11.6	11.6
11.61	11.61	11.61	11.61	11.61	11.61	11.61	11.61	11.61
11.62	11.62	11.62	11.62	11.62	11.62	11.62	11.62	11.62
11.63	11.63	11.63	11.63	11.63	11.63	11.63	11.63	11.63
11.64	11.64	11.64	11.64	11.64	11.64	11.64	11.64	11.64
11.65	11.65	11.65	11.65	11.65	11.65	11.65	11.65	11.65
11.66	11.66	11.66	11.66	11.66	11.66	11.66	11.66	11.66
11.67	11.67	11.67	11.67	11.67	11.67	11.67	11.67	11.67
11.68	11.68	11.68	11.68	11.68	11.68	11.68	11.68	11.68
11.69	11.69	11.69	11.69	11.69	11.69	11.69	11.69	11.69
11.7	11.7	11.7	11.7	11.7	11.7	11.7	11.7	11.7
11.71	11.71	11.71	11.71	11.71	11.71	11.71	11.71	11.71
11.72	11.72	11.72	11.72	11.72	11.72	11.72	11.72	11.72
11.73	11.73	11.73	11.73	11.73	11.73	11.73	11.73	11.73
11.74	11.74	11.74	11.74	11.74	11.74	11.74	11.74	11.74
11.75	11.75	11.75	11.75	11.75	11.75	11.75	11.75	11.75
11.76	11.76	11.76	11.76	11.76	11.76	11.76	11.76	11.76
11.77	11.77	11.77	11.77	11.77	11.77	11.77	11.77	11.77
11.78	11.78	11.78	11.78	11.78	11.78	11.78	11.78	11.78
11.79	11.79	11.79	11.79	11.79	11.79	11.79	11.79	11.79
11.8	11.8	11.8	11.8	11.8	11.8	11.8	11.8	11.8
11.81	11.81	11.81	11.81	11.81	11.81	11.81	11.81	11.81
11.82	11.82	11.82	11.82	11.82	11.82	11.82	11.82	11.82
11.83	11.83	11.83	11.83	11.83	11.83	11.83	11.83	11.83
11.84	11.84	11.84	11.84	11.84	11.84	11.84	11.84	11.84
11.85	11.85	11.85	11.85	11.85	11.85	11.85	11.85	11.85
11.86	11.86	11.86	11.86	11.86	11.86	11.86	11.86	11.86
11.87	11.87	11.87	11.87	11.87	11.87	11.87	11.87	11.87
11.88	11.88	11.88	11.88	11.88	11.88	11.88	11.88	11.88
11.89	11.89	11.89	11.89	11.89	11.89	11.89	11.89	11.89
11.9	11.9	11.9	11.9	11.9	11.9	11.9	11.9	11.9
11.91	11.91	11.91	11.91	11.91	11.91	11.91	11.91	11.91
11.92	11.92	11.92	11.92	11.92	11.92	11.92	11.92	11.92
11.93	11.93	11.93	11.93	11.93	11.93	11.93	11.93	11.93
11.94	11.94	11.94	11.94	11.94	11.94	11.94	11.94	11.94
11.95	11.95	11.95	11.95	11.95	11.95	11.95	11.95	11.95
11.96	11.96	11.96	11.96	11.96	11.96	11.96	11.96	11.96
11.97	11.97	11.97	11.97	11.97	11.97	11.97	11.97	11.97
11.98	11.98	11.98	11.98	11.98	11.98	11.98	11.98	11.98
11.99	11.99	11.99	11.99	11.99	11.99	11.99	11.99	11.99

12	12	12	12	12	12	12	12	12

-12	-12	-12	-12	-12	-12	-12	-12	-12
-11.99	-11.99	-11.99	-11.99	-11.99	-11.99	-11.99	-11.99	-11.99
-11.98	-11.98	-11.98	-11.98	-11.98	-11.98	-11.98	-11.98	-11.98
-11.97	-11.97	-11.97	-11.97	-11.97	-11.97	-11.97	-11.97	-11.97
-11.96	-11.96	-11.96	-11.96	-11.96	-11.96	-11.96	-11.96	-11.96
-11.95	-11.95	-11.95	-11.95	-11.95	-11.95	-11.95	-11.95	-11.95
-11.94	-11.94	-11.94	-11.94	-11.94	-11.94	-11.94	-11.94	-11.94
-11.93	-11.93	-11.93	-11.93	-11.93	-11.93	-11.93	-11.93	-11.93
-11.92	-11.92	-11.92	-11.92	-11.92	-11.92	-11.92	-11.92	-11.92
-11.91	-11.91	-11.91	-11.91	-11.91	-11.91	-11.91	-11.91	-11.91
-11.9	-11.9	-11.9	-11.9	-11.9	-11.9	-11.9	-11.9	-11.9
-11.89	-11.89	-11.89	-11.89	-11.89	-11.89	-11.89	-11.89	-11.89
-11.88	-11.88	-11.88	-11.88	-11.88	-11.88	-11.88	-11.88	-11.88
-11.87	-11.87	-11.87	-11.87	-11.87	-11.87	-11.87	-11.87	-11.87
-11.86	-11.86	-11.86	-11.86	-11.86	-11.86	-11.86	-11.86	-11.86
-11.85	-11.85	-11.85	-11.85	-11.85	-11.85	-11.85	-11.85	-11.85
-11.84	-11.84	-11.84	-11.84	-11.84	-11.84	-11.84	-11.84	-11.84
-11.83	-11.83	-11.83	-11.83	-11.83	-11.83	-11.83	-11.83	-11.83
-11.82	-11.82	-11.82	-11.82	-11.82	-11.82	-11.82	-11.82	-11.82
-11.81	-11.81	-11.81	-11.81	-11.81	-11.81	-11.81	-11.81	-11.81
-11.8	-11.8	-11.8	-11.8	-11.8	-11.8	-11.8	-11.8	-11.8
-11.79	-11.79	-11.79	-11.79	-11.79	-11.79	-11.79	-11.79	-11.79
-11.78	-11.78	-11.78	-11.78	-11.78	-11.78	-11.78	-11.78	-11.78
-11.77	-11.77	-11.77	-11.77	-11.77	-11.77	-11.77	-11.77	-11.77
-11.76	-11.76	-11.76	-11.76	-11.76	-11.76	-11.76	-11.76	-11.76
-11.75	-11.75	-11.75	-11.75	-11.75	-11.75	-11.75	-11.75	-11.75
-11.74	-11.74	-11.74	-11.74	-11.74	-11.74	-11.74	-11.74	-11.74
-11.73	-11.73	-11.73	-11.73	-11.73	-11.73	-11.73	-11.73	-11.73
-11.72	-11.72	-11.72	-11.72	-11.72	-11.72	-11.72	-11.72	-11.72
-11.71	-11.71	-11.71	-11.71	-11.71	-11.71	-11.71	-11.71	-11.71
-11.7	-11.7	-11.7	-11.7	-11.7	-11.7	-11.7	-11.7	-11.7
-11.69	-11.69	-11.69	-11.69	-11.69	-11.69	-11.69	-11.69	-11.69
-11.68	-11.68	-11.68	-11.68	-11.68	-11.68	-11.68	-11.68	-11.68
-11.67	-11.67	-11.67	-11.67	-11.67	-11.67	-11.67	-11.67	-11.67
-11.66	-11.66	-11.66	-11.66	-11.66	-11.66	-11.66	-11.66	-11.66
-11.65	-11.65	-11.65	-11.65	-11.65	-11.65	-11.65	-11.65	-11.65
-11.64	-11.64	-11.64	-11.64	-11.64	-11.64	-11.64	-11.64	-11.64
-11.63	-11.63	-11.63	-11.63	-11.63	-11.63	-11.63	-11.63	-11.63
-11.62	-11.62	-11.62	-11.62	-11.62	-11.62	-11.62	-11.62	-11.62
-11.61	-11.61	-11.61	-11.61	-11.61	-11.61	-11.61	-11.61	-11.61
-11.6	-11.6	-11.6	-11.6	-11.6	-11.6	-11.6	-11.6	-11.6
-11.59	-11.59	-11.59	-11.59	-11.59	-11.59	-11.59	-11.59	-11.59
-11.58	-11.58	-11.58	-11.58	-11.58	-11.58	-11.58	-11.58	-11.58
-11.57	-11.57	-11.57	-11.57	-11.57	-11.57	-11.57	-11.57	-11.57
-11.56	-11.56	-11.56	-11.56	-11.56	-11.56	-11.56	-11.56	-11.56
-11.55	-11.55	-11.55	-11.55	-11.55	-11.55	-11.55	-11.55	-11.55
-11.54	-11.54	-11.54	-11.54	-11.54	-11.54	-11.54	-11.54	-11.54
-11.53	-11.53	-11.53	-11.53	-11.53	-11.53	-11.53	-11.53	-11.53
-11.52	-11.52	-11.52	-11.52	-11.52	-11.52	-11.52	-11.52	-11.52
-11.51	-11.51	-11.51	-11.51	-11.51	-11.51	-11.51	-11.51	-11.51

-11.5	-11.5	-11.5	-11.5	-11.5	-11.5	-11.5	-11.5	-11.5
-11.49	-11.49	-11.49	-11.49	-11.49	-11.49	-11.49	-11.49	-11.49
-11.48	-11.48	-11.48	-11.48	-11.48	-11.48	-11.48	-11.48	-11.48
-11.47	-11.47	-11.47	-11.47	-11.47	-11.47	-11.47	-11.47	-11.47
-11.46	-11.46	-11.46	-11.46	-11.46	-11.46	-11.46	-11.46	-11.46
-11.45	-11.45	-11.45	-11.45	-11.45	-11.45	-11.45	-11.45	-11.45
-11.44	-11.44	-11.44	-11.44	-11.44	-11.44	-11.44	-11.44	-11.44
-11.43	-11.43	-11.43	-11.43	-11.43	-11.43	-11.43	-11.43	-11.43
-11.42	-11.42	-11.42	-11.42	-11.42	-11.42	-11.42	-11.42	-11.42
-11.41	-11.41	-11.41	-11.41	-11.41	-11.41	-11.41	-11.41	-11.41
-11.4	-11.4	-11.4	-11.4	-11.4	-11.4	-11.4	-11.4	-11.4
-11.39	-11.39	-11.39	-11.39	-11.39	-11.39	-11.39	-11.39	-11.39
-11.38	-11.38	-11.38	-11.38	-11.38	-11.38	-11.38	-11.38	-11.38
-11.37	-11.37	-11.37	-11.37	-11.37	-11.37	-11.37	-11.37	-11.37
-11.36	-11.36	-11.36	-11.36	-11.36	-11.36	-11.36	-11.36	-11.36
-11.35	-11.35	-11.35	-11.35	-11.35	-11.35	-11.35	-11.35	-11.35
-11.34	-11.34	-11.34	-11.34	-11.34	-11.34	-11.34	-11.34	-11.34
-11.33	-11.33	-11.33	-11.33	-11.33	-11.33	-11.33	-11.33	-11.33
-11.32	-11.32	-11.32	-11.32	-11.32	-11.32	-11.32	-11.32	-11.32
-11.31	-11.31	-11.31	-11.31	-11.31	-11.31	-11.31	-11.31	-11.31
-11.3	-11.3	-11.3	-11.3	-11.3	-11.3	-11.3	-11.3	-11.3
-11.29	-11.29	-11.29	-11.29	-11.29	-11.29	-11.29	-11.29	-11.29
-11.28	-11.28	-11.28	-11.28	-11.28	-11.28	-11.28	-11.28	-11.28
-11.27	-11.27	-11.27	-11.27	-11.27	-11.27	-11.27	-11.27	-11.27
-11.26	-11.26	-11.26	-11.26	-11.26	-11.26	-11.26	-11.26	-11.26
-11.25	-11.25	-11.25	-11.25	-11.25	-11.25	-11.25	-11.25	-11.25
-11.24	-11.24	-11.24	-11.24	-11.24	-11.24	-11.24	-11.24	-11.24
-11.23	-11.23	-11.23	-11.23	-11.23	-11.23	-11.23	-11.23	-11.23
-11.22	-11.22	-11.22	-11.22	-11.22	-11.22	-11.22	-11.22	-11.22
-11.21	-11.21	-11.21	-11.21	-11.21	-11.21	-11.21	-11.21	-11.21
-11.2	-11.2	-11.2	-11.2	-11.2	-11.2	-11.2	-11.2	-11.2
-11.19	-11.19	-11.19	-11.19	-11.19	-11.19	-11.19	-11.19	-11.19
-11.18	-11.18	-11.18	-11.18	-11.18	-11.18	-11.18	-11.18	-11.18
-11.17	-11.17	-11.17	-11.17	-11.17	-11.17	-11.17	-11.17	-11.17
-11.16	-11.16	-11.16	-11.16	-11.16	-11.16	-11.16	-11.16	-11.16
-11.15	-11.15	-11.15	-11.15	-11.15	-11.15	-11.15	-11.15	-11.15
-11.14	-11.14	-11.14	-11.14	-11.14	-11.14	-11.14	-11.14	-11.14
-11.13	-11.13	-11.13	-11.13	-11.13	-11.13	-11.13	-11.13	-11.13
-11.12	-11.12	-11.12	-11.12	-11.12	-11.12	-11.12	-11.12	-11.12
-11.11	-11.11	-11.11	-11.11	-11.11	-11.11	-11.11	-11.11	-11.11
-11.1	-11.1	-11.1	-11.1	-11.1	-11.1	-11.1	-11.1	-11.1
-11.09	-11.09	-11.09	-11.09	-11.09	-11.09	-11.09	-11.09	-11.09
-11.08	-11.08	-11.08	-11.08	-11.08	-11.08	-11.08	-11.08	-11.08
-11.07	-11.07	-11.07	-11.07	-11.07	-11.07	-11.07	-11.07	-11.07
-11.06	-11.06	-11.06	-11.06	-11.06	-11.06	-11.06	-11.06	-11.06
-11.05	-11.05	-11.05	-11.05	-11.05	-11.05	-11.05	-11.05	-11.05
-11.04	-11.04	-11.04	-11.04	-11.04	-11.04	-11.04	-11.04	-11.04
-11.03	-11.03	-11.03	-11.03	-11.03	-11.03	-11.03	-11.03	-11.03
-11.02	-11.02	-11.02	-11.02	-11.02	-11.02	-11.02	-11.02	-11.02
-11.01	-11.01	-11.01	-11.01	-11.01	-11.01	-11.01	-11.01	-11.01

-11	-11	-11	-11	-11	-11	-11	-11	-11
-10.99	-10.99	-10.99	-10.99	-10.99	-10.99	-10.99	-10.99	-10.99
-10.98	-10.98	-10.98	-10.98	-10.98	-10.98	-10.98	-10.98	-10.98
-10.97	-10.97	-10.97	-10.97	-10.97	-10.97	-10.97	-10.97	-10.97
-10.96	-10.96	-10.96	-10.96	-10.96	-10.96	-10.96	-10.96	-10.96
-10.95	-10.95	-10.95	-10.95	-10.95	-10.95	-10.95	-10.95	-10.95
-10.94	-10.94	-10.94	-10.94	-10.94	-10.94	-10.94	-10.94	-10.94
-10.93	-10.93	-10.93	-10.93	-10.93	-10.93	-10.93	-10.93	-10.93
-10.92	-10.92	-10.92	-10.92	-10.92	-10.92	-10.92	-10.92	-10.92
-10.91	-10.91	-10.91	-10.91	-10.91	-10.91	-10.91	-10.91	-10.91
-10.9	-10.9	-10.9	-10.9	-10.9	-10.9	-10.9	-10.9	-10.9
-10.89	-10.89	-10.89	-10.89	-10.89	-10.89	-10.89	-10.89	-10.89
-10.88	-10.88	-10.88	-10.88	-10.88	-10.88	-10.88	-10.88	-10.88
-10.87	-10.87	-10.87	-10.87	-10.87	-10.87	-10.87	-10.87	-10.87
-10.86	-10.86	-10.86	-10.86	-10.86	-10.86	-10.86	-10.86	-10.86
-10.85	-10.85	-10.85	-10.85	-10.85	-10.85	-10.85	-10.85	-10.85
-10.84	-10.84	-10.84	-10.84	-10.84	-10.84	-10.84	-10.84	-10.84
-10.83	-10.83	-10.83	-10.83	-10.83	-10.83	-10.83	-10.83	-10.83
-10.82	-10.82	-10.82	-10.82	-10.82	-10.82	-10.82	-10.82	-10.82
-10.81	-10.81	-10.81	-10.81	-10.81	-10.81	-10.81	-10.81	-10.81
-10.8	-10.8	-10.8	-10.8	-10.8	-10.8	-10.8	-10.8	-10.8
-10.79	-10.79	-10.79	-10.79	-10.79	-10.79	-10.79	-10.79	-10.79
-10.78	-10.78	-10.78	-10.78	-10.78	-10.78	-10.78	-10.78	-10.78
-10.77	-10.77	-10.77	-10.77	-10.77	-10.77	-10.77	-10.77	-10.77
-10.76	-10.76	-10.76	-10.76	-10.76	-10.76	-10.76	-10.76	-10.76
-10.75	-10.75	-10.75	-10.75	-10.75	-10.75	-10.75	-10.75	-10.75
-10.74	-10.74	-10.74	-10.74	-10.74	-10.74	-10.74	-10.74	-10.74
-10.73	-10.73	-10.73	-10.73	-10.73	-10.73	-10.73	-10.73	-10.73
-10.72	-10.72	-10.72	-10.72	-10.72	-10.72	-10.72	-10.72	-10.72
-10.71	-10.71	-10.71	-10.71	-10.71	-10.71	-10.71	-10.71	-10.71
-10.7	-10.7	-10.7	-10.7	-10.7	-10.7	-10.7	-10.7	-10.7
-10.69	-10.69	-10.69	-10.69	-10.69	-10.69	-10.69	-10.69	-10.69
-10.68	-10.68	-10.68	-10.68	-10.68	-10.68	-10.68	-10.68	-10.68
-10.67	-10.67	-10.67	-10.67	-10.67	-10.67	-10.67	-10.67	-10.67
-10.66	-10.66	-10.66	-10.66	-10.66	-10.66	-10.66	-10.66	-10.66
-10.65	-10.65	-10.65	-10.65	-10.65	-10.65	-10.65	-10.65	-10.65
-10.64	-10.64	-10.64	-10.64	-10.64	-10.64	-10.64	-10.64	-10.64
-10.63	-10.63	-10.63	-10.63	-10.63	-10.63	-10.63	-10.63	-10.63
-10.62	-10.62	-10.62	-10.62	-10.62	-10.62	-10.62	-10.62	-10.62
-10.61	-10.61	-10.61	-10.61	-10.61	-10.61	-10.61	-10.61	-10.61
-10.6	-10.6	-10.6	-10.6	-10.6	-10.6	-10.6	-10.6	-10.6
-10.59	-10.59	-10.59	-10.59	-10.59	-10.59	-10.59	-10.59	-10.59
-10.58	-10.58	-10.58	-10.58	-10.58	-10.58	-10.58	-10.58	-10.58
-10.57	-10.57	-10.57	-10.57	-10.57	-10.57	-10.57	-10.57	-10.57
-10.56	-10.56	-10.56	-10.56	-10.56	-10.56	-10.56	-10.56	-10.56
-10.55	-10.55	-10.55	-10.55	-10.55	-10.55	-10.55	-10.55	-10.55
-10.54	-10.54	-10.54	-10.54	-10.54	-10.54	-10.54	-10.54	-10.54
-10.53	-10.53	-10.53	-10.53	-10.53	-10.53	-10.53	-10.53	-10.53
-10.52	-10.52	-10.52	-10.52	-10.52	-10.52	-10.52	-10.52	-10.52
-10.51	-10.51	-10.51	-10.51	-10.51	-10.51	-10.51	-10.51	-10.51

-10.5	-10.5	-10.5	-10.5	-10.5	-10.5	-10.5	-10.5	-10.5
-10.49	-10.49	-10.49	-10.49	-10.49	-10.49	-10.49	-10.49	-10.49
-10.48	-10.48	-10.48	-10.48	-10.48	-10.48	-10.48	-10.48	-10.48
-10.47	-10.47	-10.47	-10.47	-10.47	-10.47	-10.47	-10.47	-10.47
-10.46	-10.46	-10.46	-10.46	-10.46	-10.46	-10.46	-10.46	-10.46
-10.45	-10.45	-10.45	-10.45	-10.45	-10.45	-10.45	-10.45	-10.45
-10.44	-10.44	-10.44	-10.44	-10.44	-10.44	-10.44	-10.44	-10.44
-10.43	-10.43	-10.43	-10.43	-10.43	-10.43	-10.43	-10.43	-10.43
-10.42	-10.42	-10.42	-10.42	-10.42	-10.42	-10.42	-10.42	-10.42
-10.41	-10.41	-10.41	-10.41	-10.41	-10.41	-10.41	-10.41	-10.41
-10.4	-10.4	-10.4	-10.4	-10.4	-10.4	-10.4	-10.4	-10.4
-10.39	-10.39	-10.39	-10.39	-10.39	-10.39	-10.39	-10.39	-10.39
-10.38	-10.38	-10.38	-10.38	-10.38	-10.38	-10.38	-10.38	-10.38
-10.37	-10.37	-10.37	-10.37	-10.37	-10.37	-10.37	-10.37	-10.37
-10.36	-10.36	-10.36	-10.36	-10.36	-10.36	-10.36	-10.36	-10.36
-10.35	-10.35	-10.35	-10.35	-10.35	-10.35	-10.35	-10.35	-10.35
-10.34	-10.34	-10.34	-10.34	-10.34	-10.34	-10.34	-10.34	-10.34
-10.33	-10.33	-10.33	-10.33	-10.33	-10.33	-10.33	-10.33	-10.33
-10.32	-10.32	-10.32	-10.32	-10.32	-10.32	-10.32	-10.32	-10.32
-10.31	-10.31	-10.31	-10.31	-10.31	-10.31	-10.31	-10.31	-10.31
-10.3	-10.3	-10.3	-10.3	-10.3	-10.3	-10.3	-10.3	-10.3
-10.29	-10.29	-10.29	-10.29	-10.29	-10.29	-10.29	-10.29	-10.29
-10.28	-10.28	-10.28	-10.28	-10.28	-10.28	-10.28	-10.28	-10.28
-10.27	-10.27	-10.27	-10.27	-10.27	-10.27	-10.27	-10.27	-10.27
-10.26	-10.26	-10.26	-10.26	-10.26	-10.26	-10.26	-10.26	-10.26
-10.25	-10.25	-10.25	-10.25	-10.25	-10.25	-10.25	-10.25	-10.25
-10.24	-10.24	-10.24	-10.24	-10.24	-10.24	-10.24	-10.24	-10.24
-10.23	-10.23	-10.23	-10.23	-10.23	-10.23	-10.23	-10.23	-10.23
-10.22	-10.22	-10.22	-10.22	-10.22	-10.22	-10.22	-10.22	-10.22
-10.21	-10.21	-10.21	-10.21	-10.21	-10.21	-10.21	-10.21	-10.21
-10.2	-10.2	-10.2	-10.2	-10.2	-10.2	-10.2	-10.2	-10.2
-10.19	-10.19	-10.19	-10.19	-10.19	-10.19	-10.19	-10.19	-10.19
-10.18	-10.18	-10.18	-10.18	-10.18	-10.18	-10.18	-10.18	-10.18
-10.17	-10.17	-10.17	-10.17	-10.17	-10.17	-10.17	-10.17	-10.17
-10.16	-10.16	-10.16	-10.16	-10.16	-10.16	-10.16	-10.16	-10.16
-10.15	-10.15	-10.15	-10.15	-10.15	-10.15	-10.15	-10.15	-10.15
-10.14	-10.14	-10.14	-10.14	-10.14	-10.14	-10.14	-10.14	-10.14
-10.13	-10.13	-10.13	-10.13	-10.13	-10.13	-10.13	-10.13	-10.13
-10.12	-10.12	-10.12	-10.12	-10.12	-10.12	-10.12	-10.12	-10.12
-10.11	-10.11	-10.11	-10.11	-10.11	-10.11	-10.11	-10.11	-10.11
-10.1	-10.1	-10.1	-10.1	-10.1	-10.1	-10.1	-10.1	-10.1
-10.09	-10.09	-10.09	-10.09	-10.09	-10.09	-10.09	-10.09	-10.09
-10.08	-10.08	-10.08	-10.08	-10.08	-10.08	-10.08	-10.08	-10.08
-10.07	-10.07	-10.07	-10.07	-10.07	-10.07	-10.07	-10.07	-10.07
-10.06	-10.06	-10.06	-10.06	-10.06	-10.06	-10.06	-10.06	-10.06
-10.05	-10.05	-10.05	-10.05	-10.05	-10.05	-10.05	-10.05	-10.05
-10.04	-10.04	-10.04	-10.04	-10.04	-10.04	-10.04	-10.04	-10.04
-10.03	-10.03	-10.03	-10.03	-10.03	-10.03	-10.03	-10.03	-10.03
-10.02	-10.02	-10.02	-10.02	-10.02	-10.02	-10.02	-10.02	-10.02
-10.01	-10.01	-10.01	-10.01	-10.01	-10.01	-10.01	-10.01	-10.01

-10	-10	-10	-10	-10	-10	-10	-10	-10
-9.99	-9.99	-9.99	-9.99	-9.99	-9.99	-9.99	-9.99	-9.99
-9.98	-9.98	-9.98	-9.98	-9.98	-9.98	-9.98	-9.98	-9.98
-9.97	-9.97	-9.97	-9.97	-9.97	-9.97	-9.97	-9.97	-9.97
-9.96	-9.96	-9.96	-9.96	-9.96	-9.96	-9.96	-9.96	-9.96
-9.95	-9.95	-9.95	-9.95	-9.95	-9.95	-9.95	-9.95	-9.95
-9.94	-9.94	-9.94	-9.94	-9.94	-9.94	-9.94	-9.94	-9.94
-9.93	-9.93	-9.93	-9.93	-9.93	-9.93	-9.93	-9.93	-9.93
-9.92	-9.92	-9.92	-9.92	-9.92	-9.92	-9.92	-9.92	-9.92
-9.91	-9.91	-9.91	-9.91	-9.91	-9.91	-9.91	-9.91	-9.91
-9.9	-9.9	-9.9	-9.9	-9.9	-9.9	-9.9	-9.9	-9.9
-9.89	-9.89	-9.89	-9.89	-9.89	-9.89	-9.89	-9.89	-9.89
-9.88	-9.88	-9.88	-9.88	-9.88	-9.88	-9.88	-9.88	-9.88
-9.87	-9.87	-9.87	-9.87	-9.87	-9.87	-9.87	-9.87	-9.87
-9.86	-9.86	-9.86	-9.86	-9.86	-9.86	-9.86	-9.86	-9.86
-9.85	-9.85	-9.85	-9.85	-9.85	-9.85	-9.85	-9.85	-9.85
-9.84	-9.84	-9.84	-9.84	-9.84	-9.84	-9.84	-9.84	-9.84
-9.83	-9.83	-9.83	-9.83	-9.83	-9.83	-9.83	-9.83	-9.83
-9.82	-9.82	-9.82	-9.82	-9.82	-9.82	-9.82	-9.82	-9.82
-9.81	-9.81	-9.81	-9.81	-9.81	-9.81	-9.81	-9.81	-9.81
-9.8	-9.8	-9.8	-9.8	-9.8	-9.8	-9.8	-9.8	-9.8
-9.79	-9.79	-9.79	-9.79	-9.79	-9.79	-9.79	-9.79	-9.79
-9.78	-9.78	-9.78	-9.78	-9.78	-9.78	-9.78	-9.78	-9.78
-9.77	-9.77	-9.77	-9.77	-9.77	-9.77	-9.77	-9.77	-9.77
-9.76	-9.76	-9.76	-9.76	-9.76	-9.76	-9.76	-9.76	-9.76
-9.75	-9.75	-9.75	-9.75	-9.75	-9.75	-9.75	-9.75	-9.75
-9.74	-9.74	-9.74	-9.74	-9.74	-9.74	-9.74	-9.74	-9.74
-9.73	-9.73	-9.73	-9.73	-9.73	-9.73	-9.73	-9.73	-9.73
-9.72	-9.72	-9.72	-9.72	-9.72	-9.72	-9.72	-9.72	-9.72
-9.71	-9.71	-9.71	-9.71	-9.71	-9.71	-9.71	-9.71	-9.71
-9.7	-9.7	-9.7	-9.7	-9.7	-9.7	-9.7	-9.7	-9.7
-9.69	-9.69	-9.69	-9.69	-9.69	-9.69	-9.69	-9.69	-9.69
-9.68	-9.68	-9.68	-9.68	-9.68	-9.68	-9.68	-9.68	-9.68
-9.67	-9.67	-9.67	-9.67	-9.67	-9.67	-9.67	-9.67	-9.67
-9.66	-9.66	-9.66	-9.66	-9.66	-9.66	-9.66	-9.66	-9.66
-9.65	-9.65	-9.65	-9.65	-9.65	-9.65	-9.65	-9.65	-9.65
-9.64	-9.64	-9.64	-9.64	-9.64	-9.64	-9.64	-9.64	-9.64
-9.63	-9.63	-9.63	-9.63	-9.63	-9.63	-9.63	-9.63	-9.63
-9.62	-9.62	-9.62	-9.62	-9.62	-9.62	-9.62	-9.62	-9.62
-9.61	-9.61	-9.61	-9.61	-9.61	-9.61	-9.61	-9.61	-9.61
-9.6	-9.6	-9.6	-9.6	-9.6	-9.6	-9.6	-9.6	-9.6
-9.59	-9.59	-9.59	-9.59	-9.59	-9.59	-9.59	-9.59	-9.59
-9.58	-9.58	-9.58	-9.58	-9.58	-9.58	-9.58	-9.58	-9.58
-9.57	-9.57	-9.57	-9.57	-9.57	-9.57	-9.57	-9.57	-9.57
-9.56	-9.56	-9.56	-9.56	-9.56	-9.56	-9.56	-9.56	-9.56
-9.55	-9.55	-9.55	-9.55	-9.55	-9.55	-9.55	-9.55	-9.55
-9.54	-9.54	-9.54	-9.54	-9.54	-9.54	-9.54	-9.54	-9.54
-9.53	-9.53	-9.53	-9.53	-9.53	-9.53	-9.53	-9.53	-9.53
-9.52	-9.52	-9.52	-9.52	-9.52	-9.52	-9.52	-9.52	-9.52
-9.51	-9.51	-9.51	-9.51	-9.51	-9.51	-9.51	-9.51	-9.51

-9.5	-9.5	-9.5	-9.5	-9.5	-9.5	-9.5	-9.5	-9.5
-9.49	-9.49	-9.49	-9.49	-9.49	-9.49	-9.49	-9.49	-9.49
-9.48	-9.48	-9.48	-9.48	-9.48	-9.48	-9.48	-9.48	-9.48
-9.47	-9.47	-9.47	-9.47	-9.47	-9.47	-9.47	-9.47	-9.47
-9.46	-9.46	-9.46	-9.46	-9.46	-9.46	-9.46	-9.46	-9.46
-9.45	-9.45	-9.45	-9.45	-9.45	-9.45	-9.45	-9.45	-9.45
-9.44	-9.44	-9.44	-9.44	-9.44	-9.44	-9.44	-9.44	-9.44
-9.43	-9.43	-9.43	-9.43	-9.43	-9.43	-9.43	-9.43	-9.43
-9.42	-9.42	-9.42	-9.42	-9.42	-9.42	-9.42	-9.42	-9.42
-9.41	-9.41	-9.41	-9.41	-9.41	-9.41	-9.41	-9.41	-9.41
-9.4	-9.4	-9.4	-9.4	-9.4	-9.4	-9.4	-9.4	-9.4
-9.39	-9.39	-9.39	-9.39	-9.39	-9.39	-9.39	-9.39	-9.39
-9.38	-9.38	-9.38	-9.38	-9.38	-9.38	-9.38	-9.38	-9.38
-9.37	-9.37	-9.37	-9.37	-9.37	-9.37	-9.37	-9.37	-9.37
-9.36	-9.36	-9.36	-9.36	-9.36	-9.36	-9.36	-9.36	-9.36
-9.35	-9.35	-9.35	-9.35	-9.35	-9.35	-9.35	-9.35	-9.35
-9.34	-9.34	-9.34	-9.34	-9.34	-9.34	-9.34	-9.34	-9.34
-9.33	-9.33	-9.33	-9.33	-9.33	-9.33	-9.33	-9.33	-9.33
-9.32	-9.32	-9.32	-9.32	-9.32	-9.32	-9.32	-9.32	-9.32
-9.31	-9.31	-9.31	-9.31	-9.31	-9.31	-9.31	-9.31	-9.31
-9.3	-9.3	-9.3	-9.3	-9.3	-9.3	-9.3	-9.3	-9.3
-9.29	-9.29	-9.29	-9.29	-9.29	-9.29	-9.29	-9.29	-9.29
-9.28	-9.28	-9.28	-9.28	-9.28	-9.28	-9.28	-9.28	-9.28
-9.27	-9.27	-9.27	-9.27	-9.27	-9.27	-9.27	-9.27	-9.27
-9.26	-9.26	-9.26	-9.26	-9.26	-9.26	-9.26	-9.26	-9.26
-9.25	-9.25	-9.25	-9.25	-9.25	-9.25	-9.25	-9.25	-9.25
-9.24	-9.24	-9.24	-9.24	-9.24	-9.24	-9.24	-9.24	-9.24
-9.23	-9.23	-9.23	-9.23	-9.23	-9.23	-9.23	-9.23	-9.23
-9.22	-9.22	-9.22	-9.22	-9.22	-9.22	-9.22	-9.22	-9.22
-9.21	-9.21	-9.21	-9.21	-9.21	-9.21	-9.21	-9.21	-9.21
-9.2	-9.2	-9.2	-9.2	-9.2	-9.2	-9.2	-9.2	-9.2
-9.19	-9.19	-9.19	-9.19	-9.19	-9.19	-9.19	-9.19	-9.19
-9.18	-9.18	-9.18	-9.18	-9.18	-9.18	-9.18	-9.18	-9.18
-9.17	-9.17	-9.17	-9.17	-9.17	-9.17	-9.17	-9.17	-9.17
-9.16	-9.16	-9.16	-9.16	-9.16	-9.16	-9.16	-9.16	-9.16
-9.15	-9.15	-9.15	-9.15	-9.15	-9.15	-9.15	-9.15	-9.15
-9.14	-9.14	-9.14	-9.14	-9.14	-9.14	-9.14	-9.14	-9.14
-9.13	-9.13	-9.13	-9.13	-9.13	-9.13	-9.13	-9.13	-9.13
-9.12	-9.12	-9.12	-9.12	-9.12	-9.12	-9.12	-9.12	-9.12
-9.11	-9.11	-9.11	-9.11	-9.11	-9.11	-9.11	-9.11	-9.11
-9.1	-9.1	-9.1	-9.1	-9.1	-9.1	-9.1	-9.1	-9.1
-9.09	-9.09	-9.09	-9.09	-9.09	-9.09	-9.09	-9.09	-9.09
-9.08	-9.08	-9.08	-9.08	-9.08	-9.08	-9.08	-9.08	-9.08
-9.07	-9.07	-9.07	-9.07	-9.07	-9.07	-9.07	-9.07	-9.07
-9.06	-9.06	-9.06	-9.06	-9.06	-9.06	-9.06	-9.06	-9.06
-9.05	-9.05	-9.05	-9.05	-9.05	-9.05	-9.05	-9.05	-9.05
-9.04	-9.04	-9.04	-9.04	-9.04	-9.04	-9.04	-9.04	-9.04
-9.03	-9.03	-9.03	-9.03	-9.03	-9.03	-9.03	-9.03	-9.03
-9.02	-9.02	-9.02	-9.02	-9.02	-9.02	-9.02	-9.02	-9.02
-9.01	-9.01	-9.01	-9.01	-9.01	-9.01	-9.01	-9.01	-9.01

-9	-9	-9	-9	-9	-9	-9	-9	-9
-8.99	-8.99	-8.99	-8.99	-8.99	-8.99	-8.99	-8.99	-8.99
-8.98	-8.98	-8.98	-8.98	-8.98	-8.98	-8.98	-8.98	-8.98
-8.97	-8.97	-8.97	-8.97	-8.97	-8.97	-8.97	-8.97	-8.97
-8.96	-8.96	-8.96	-8.96	-8.96	-8.96	-8.96	-8.96	-8.96
-8.95	-8.95	-8.95	-8.95	-8.95	-8.95	-8.95	-8.95	-8.95
-8.94	-8.94	-8.94	-8.94	-8.94	-8.94	-8.94	-8.94	-8.94
-8.93	-8.93	-8.93	-8.93	-8.93	-8.93	-8.93	-8.93	-8.93
-8.92	-8.92	-8.92	-8.92	-8.92	-8.92	-8.92	-8.92	-8.92
-8.91	-8.91	-8.91	-8.91	-8.91	-8.91	-8.91	-8.91	-8.91
-8.9	-8.9	-8.9	-8.9	-8.9	-8.9	-8.9	-8.9	-8.9
-8.89	-8.89	-8.89	-8.89	-8.89	-8.89	-8.89	-8.89	-8.89
-8.88	-8.88	-8.88	-8.88	-8.88	-8.88	-8.88	-8.88	-8.88
-8.87	-8.87	-8.87	-8.87	-8.87	-8.87	-8.87	-8.87	-8.87
-8.86	-8.86	-8.86	-8.86	-8.86	-8.86	-8.86	-8.86	-8.86
-8.85	-8.85	-8.85	-8.85	-8.85	-8.85	-8.85	-8.85	-8.85
-8.84	-8.84	-8.84	-8.84	-8.84	-8.84	-8.84	-8.84	-8.84
-8.83	-8.83	-8.83	-8.83	-8.83	-8.83	-8.83	-8.83	-8.83
-8.82	-8.82	-8.82	-8.82	-8.82	-8.82	-8.82	-8.82	-8.82
-8.81	-8.81	-8.81	-8.81	-8.81	-8.81	-8.81	-8.81	-8.81
-8.8	-8.8	-8.8	-8.8	-8.8	-8.8	-8.8	-8.8	-8.8
-8.79	-8.79	-8.79	-8.79	-8.79	-8.79	-8.79	-8.79	-8.79
-8.78	-8.78	-8.78	-8.78	-8.78	-8.78	-8.78	-8.78	-8.78
-8.77	-8.77	-8.77	-8.77	-8.77	-8.77	-8.77	-8.77	-8.77
-8.76	-8.76	-8.76	-8.76	-8.76	-8.76	-8.76	-8.76	-8.76
-8.75	-8.75	-8.75	-8.75	-8.75	-8.75	-8.75	-8.75	-8.75
-8.74	-8.74	-8.74	-8.74	-8.74	-8.74	-8.74	-8.74	-8.74
-8.73	-8.73	-8.73	-8.73	-8.73	-8.73	-8.73	-8.73	-8.73
-8.72	-8.72	-8.72	-8.72	-8.72	-8.72	-8.72	-8.72	-8.72
-8.71	-8.71	-8.71	-8.71	-8.71	-8.71	-8.71	-8.71	-8.71
-8.7	-8.7	-8.7	-8.7	-8.7	-8.7	-8.7	-8.7	-8.7
-8.69	-8.69	-8.69	-8.69	-8.69	-8.69	-8.69	-8.69	-8.69
-8.68	-8.68	-8.68	-8.68	-8.68	-8.68	-8.68	-8.68	-8.68
-8.67	-8.67	-8.67	-8.67	-8.67	-8.67	-8.67	-8.67	-8.67
-8.66	-8.66	-8.66	-8.66	-8.66	-8.66	-8.66	-8.66	-8.66
-8.65	-8.65	-8.65	-8.65	-8.65	-8.65	-8.65	-8.65	-8.65
-8.64	-8.64	-8.64	-8.64	-8.64	-8.64	-8.64	-8.64	-8.64
-8.63	-8.63	-8.63	-8.63	-8.63	-8.63	-8.63	-8.63	-8.63
-8.62	-8.62	-8.62	-8.62	-8.62	-8.62	-8.62	-8.62	-8.62
-8.61	-8.61	-8.61	-8.61	-8.61	-8.61	-8.61	-8.61	-8.61
-8.6	-8.6	-8.6	-8.6	-8.6	-8.6	-8.6	-8.6	-8.6
-8.59	-8.59	-8.59	-8.59	-8.59	-8.59	-8.59	-8.59	-8.59
-8.58	-8.58	-8.58	-8.58	-8.58	-8.58	-8.58	-8.58	-8.58
-8.57	-8.57	-8.57	-8.57	-8.57	-8.57	-8.57	-8.57	-8.57
-8.56	-8.56	-8.56	-8.56	-8.56	-8.56	-8.56	-8.56	-8.56
-8.55	-8.55	-8.55	-8.55	-8.55	-8.55	-8.55	-8.55	-8.55
-8.54	-8.54	-8.54	-8.54	-8.54	-8.54	-8.54	-8.54	-8.54
-8.53	-8.53	-8.53	-8.53	-8.53	-8.53	-8.53	-8.53	-8.53
-8.52	-8.52	-8.52	-8.52	-8.52	-8.52	-8.52	-8.52	-8.52
-8.51	-8.51	-8.51	-8.51	-8.51	-8.51	-8.51	-8.51	-8.51

-8.5	-8.5	-8.5	-8.5	-8.5	-8.5	-8.5	-8.5	-8.5
-8.49	-8.49	-8.49	-8.49	-8.49	-8.49	-8.49	-8.49	-8.49
-8.48	-8.48	-8.48	-8.48	-8.48	-8.48	-8.48	-8.48	-8.48
-8.47	-8.47	-8.47	-8.47	-8.47	-8.47	-8.47	-8.47	-8.47
-8.46	-8.46	-8.46	-8.46	-8.46	-8.46	-8.46	-8.46	-8.46
-8.45	-8.45	-8.45	-8.45	-8.45	-8.45	-8.45	-8.45	-8.45
-8.44	-8.44	-8.44	-8.44	-8.44	-8.44	-8.44	-8.44	-8.44
-8.43	-8.43	-8.43	-8.43	-8.43	-8.43	-8.43	-8.43	-8.43
-8.42	-8.42	-8.42	-8.42	-8.42	-8.42	-8.42	-8.42	-8.42
-8.41	-8.41	-8.41	-8.41	-8.41	-8.41	-8.41	-8.41	-8.41
-8.4	-8.4	-8.4	-8.4	-8.4	-8.4	-8.4	-8.4	-8.4
-8.39	-8.39	-8.39	-8.39	-8.39	-8.39	-8.39	-8.39	-8.39
-8.38	-8.38	-8.38	-8.38	-8.38	-8.38	-8.38	-8.38	-8.38
-8.37	-8.37	-8.37	-8.37	-8.37	-8.37	-8.37	-8.37	-8.37
-8.36	-8.36	-8.36	-8.36	-8.36	-8.36	-8.36	-8.36	-8.36
-8.35	-8.35	-8.35	-8.35	-8.35	-8.35	-8.35	-8.35	-8.35
-8.34	-8.34	-8.34	-8.34	-8.34	-8.34	-8.34	-8.34	-8.34
-8.33	-8.33	-8.33	-8.33	-8.33	-8.33	-8.33	-8.33	-8.33
-8.32	-8.32	-8.32	-8.32	-8.32	-8.32	-8.32	-8.32	-8.32
-8.31	-8.31	-8.31	-8.31	-8.31	-8.31	-8.31	-8.31	-8.31
-8.3	-8.3	-8.3	-8.3	-8.3	-8.3	-8.3	-8.3	-8.3
-8.29	-8.29	-8.29	-8.29	-8.29	-8.29	-8.29	-8.29	-8.29
-8.28	-8.28	-8.28	-8.28	-8.28	-8.28	-8.28	-8.28	-8.28
-8.27	-8.27	-8.27	-8.27	-8.27	-8.27	-8.27	-8.27	-8.27
-8.26	-8.26	-8.26	-8.26	-8.26	-8.26	-8.26	-8.26	-8.26
-8.25	-8.25	-8.25	-8.25	-8.25	-8.25	-8.25	-8.25	-8.25
-8.24	-8.24	-8.24	-8.24	-8.24	-8.24	-8.24	-8.24	-8.24
-8.23	-8.23	-8.23	-8.23	-8.23	-8.23	-8.23	-8.23	-8.23
-8.22	-8.22	-8.22	-8.22	-8.22	-8.22	-8.22	-8.22	-8.22
-8.21	-8.21	-8.21	-8.21	-8.21	-8.21	-8.21	-8.21	-8.21
-8.2	-8.2	-8.2	-8.2	-8.2	-8.2	-8.2	-8.2	-8.2
-8.19	-8.19	-8.19	-8.19	-8.19	-8.19	-8.19	-8.19	-8.19
-8.18	-8.18	-8.18	-8.18	-8.18	-8.18	-8.18	-8.18	-8.18
-8.17	-8.17	-8.17	-8.17	-8.17	-8.17	-8.17	-8.17	-8.17
-8.16	-8.16	-8.16	-8.16	-8.16	-8.16	-8.16	-8.16	-8.16
-8.15	-8.15	-8.15	-8.15	-8.15	-8.15	-8.15	-8.15	-8.15
-8.14	-8.14	-8.14	-8.14	-8.14	-8.14	-8.14	-8.14	-8.14
-8.13	-8.13	-8.13	-8.13	-8.13	-8.13	-8.13	-8.13	-8.13
-8.12	-8.12	-8.12	-8.12	-8.12	-8.12	-8.12	-8.12	-8.12
-8.11	-8.11	-8.11	-8.11	-8.11	-8.11	-8.11	-8.11	-8.11
-8.1	-8.1	-8.1	-8.1	-8.1	-8.1	-8.1	-8.1	-8.1
-8.09	-8.09	-8.09	-8.09	-8.09	-8.09	-8.09	-8.09	-8.09
-8.08	-8.08	-8.08	-8.08	-8.08	-8.08	-8.08	-8.08	-8.08
-8.07	-8.07	-8.07	-8.07	-8.07	-8.07	-8.07	-8.07	-8.07
-8.06	-8.06	-8.06	-8.06	-8.06	-8.06	-8.06	-8.06	-8.06
-8.05	-8.05	-8.05	-8.05	-8.05	-8.05	-8.05	-8.05	-8.05
-8.04	-8.04	-8.04	-8.04	-8.04	-8.04	-8.04	-8.04	-8.04
-8.03	-8.03	-8.03	-8.03	-8.03	-8.03	-8.03	-8.03	-8.03
-8.02	-8.02	-8.02	-8.02	-8.02	-8.02	-8.02	-8.02	-8.02
-8.01	-8.01	-8.01	-8.01	-8.01	-8.01	-8.01	-8.01	-8.01

-8	-8	-8	-8	-8	-8	-8	-8	-8
-7.99	-7.99	-7.99	-7.99	-7.99	-7.99	-7.99	-7.99	-7.99
-7.98	-7.98	-7.98	-7.98	-7.98	-7.98	-7.98	-7.98	-7.98
-7.97	-7.97	-7.97	-7.97	-7.97	-7.97	-7.97	-7.97	-7.97
-7.96	-7.96	-7.96	-7.96	-7.96	-7.96	-7.96	-7.96	-7.96
-7.95	-7.95	-7.95	-7.95	-7.95	-7.95	-7.95	-7.95	-7.95
-7.94	-7.94	-7.94	-7.94	-7.94	-7.94	-7.94	-7.94	-7.94
-7.93	-7.93	-7.93	-7.93	-7.93	-7.93	-7.93	-7.93	-7.93
-7.92	-7.92	-7.92	-7.92	-7.92	-7.92	-7.92	-7.92	-7.92
-7.91	-7.91	-7.91	-7.91	-7.91	-7.91	-7.91	-7.91	-7.91
-7.9	-7.9	-7.9	-7.9	-7.9	-7.9	-7.9	-7.9	-7.9
-7.89	-7.89	-7.89	-7.89	-7.89	-7.89	-7.89	-7.89	-7.89
-7.88	-7.88	-7.88	-7.88	-7.88	-7.88	-7.88	-7.88	-7.88
-7.87	-7.87	-7.87	-7.87	-7.87	-7.87	-7.87	-7.87	-7.87
-7.86	-7.86	-7.86	-7.86	-7.86	-7.86	-7.86	-7.86	-7.86
-7.85	-7.85	-7.85	-7.85	-7.85	-7.85	-7.85	-7.85	-7.85
-7.84	-7.84	-7.84	-7.84	-7.84	-7.84	-7.84	-7.84	-7.84
-7.83	-7.83	-7.83	-7.83	-7.83	-7.83	-7.83	-7.83	-7.83
-7.82	-7.82	-7.82	-7.82	-7.82	-7.82	-7.82	-7.82	-7.82
-7.81	-7.81	-7.81	-7.81	-7.81	-7.81	-7.81	-7.81	-7.81
-7.8	-7.8	-7.8	-7.8	-7.8	-7.8	-7.8	-7.8	-7.8
-7.79	-7.79	-7.79	-7.79	-7.79	-7.79	-7.79	-7.79	-7.79
-7.78	-7.78	-7.78	-7.78	-7.78	-7.78	-7.78	-7.78	-7.78
-7.77	-7.77	-7.77	-7.77	-7.77	-7.77	-7.77	-7.77	-7.77
-7.76	-7.76	-7.76	-7.76	-7.76	-7.76	-7.76	-7.76	-7.76
-7.75	-7.75	-7.75	-7.75	-7.75	-7.75	-7.75	-7.75	-7.75
-7.74	-7.74	-7.74	-7.74	-7.74	-7.74	-7.74	-7.74	-7.74
-7.73	-7.73	-7.73	-7.73	-7.73	-7.73	-7.73	-7.73	-7.73
-7.72	-7.72	-7.72	-7.72	-7.72	-7.72	-7.72	-7.72	-7.72
-7.71	-7.71	-7.71	-7.71	-7.71	-7.71	-7.71	-7.71	-7.71
-7.7	-7.7	-7.7	-7.7	-7.7	-7.7	-7.7	-7.7	-7.7
-7.69	-7.69	-7.69	-7.69	-7.69	-7.69	-7.69	-7.69	-7.69
-7.68	-7.68	-7.68	-7.68	-7.68	-7.68	-7.68	-7.68	-7.68
-7.67	-7.67	-7.67	-7.67	-7.67	-7.67	-7.67	-7.67	-7.67
-7.66	-7.66	-7.66	-7.66	-7.66	-7.66	-7.66	-7.66	-7.66
-7.65	-7.65	-7.65	-7.65	-7.65	-7.65	-7.65	-7.65	-7.65
-7.64	-7.64	-7.64	-7.64	-7.64	-7.64	-7.64	-7.64	-7.64
-7.63	-7.63	-7.63	-7.63	-7.63	-7.63	-7.63	-7.63	-7.63
-7.62	-7.62	-7.62	-7.62	-7.62	-7.62	-7.62	-7.62	-7.62
-7.61	-7.61	-7.61	-7.61	-7.61	-7.61	-7.61	-7.61	-7.61
-7.6	-7.6	-7.6	-7.6	-7.6	-7.6	-7.6	-7.6	-7.6
-7.59	-7.59	-7.59	-7.59	-7.59	-7.59	-7.59	-7.59	-7.59
-7.58	-7.58	-7.58	-7.58	-7.58	-7.58	-7.58	-7.58	-7.58
-7.57	-7.57	-7.57	-7.57	-7.57	-7.57	-7.57	-7.57	-7.57
-7.56	-7.56	-7.56	-7.56	-7.56	-7.56	-7.56	-7.56	-7.56
-7.55	-7.55	-7.55	-7.55	-7.55	-7.55	-7.55	-7.55	-7.55
-7.54	-7.54	-7.54	-7.54	-7.54	-7.54	-7.54	-7.54	-7.54
-7.53	-7.53	-7.53	-7.53	-7.53	-7.53	-7.53	-7.53	-7.53
-7.52	-7.52	-7.52	-7.52	-7.52	-7.52	-7.52	-7.52	-7.52
-7.51	-7.51	-7.51	-7.51	-7.51	-7.51	-7.51	-7.51	-7.51

-7.5	-7.5	-7.5	-7.5	-7.5	-7.5	-7.5	-7.5	-7.5
-7.49	-7.49	-7.49	-7.49	-7.49	-7.49	-7.49	-7.49	-7.49
-7.48	-7.48	-7.48	-7.48	-7.48	-7.48	-7.48	-7.48	-7.48
-7.47	-7.47	-7.47	-7.47	-7.47	-7.47	-7.47	-7.47	-7.47
-7.46	-7.46	-7.46	-7.46	-7.46	-7.46	-7.46	-7.46	-7.46
-7.45	-7.45	-7.45	-7.45	-7.45	-7.45	-7.45	-7.45	-7.45
-7.44	-7.44	-7.44	-7.44	-7.44	-7.44	-7.44	-7.44	-7.44
-7.43	-7.43	-7.43	-7.43	-7.43	-7.43	-7.43	-7.43	-7.43
-7.42	-7.42	-7.42	-7.42	-7.42	-7.42	-7.42	-7.42	-7.42
-7.41	-7.41	-7.41	-7.41	-7.41	-7.41	-7.41	-7.41	-7.41
-7.4	-7.4	-7.4	-7.4	-7.4	-7.4	-7.4	-7.4	-7.4
-7.39	-7.39	-7.39	-7.39	-7.39	-7.39	-7.39	-7.39	-7.39
-7.38	-7.38	-7.38	-7.38	-7.38	-7.38	-7.38	-7.38	-7.38
-7.37	-7.37	-7.37	-7.37	-7.37	-7.37	-7.37	-7.37	-7.37
-7.36	-7.36	-7.36	-7.36	-7.36	-7.36	-7.36	-7.36	-7.36
-7.35	-7.35	-7.35	-7.35	-7.35	-7.35	-7.35	-7.35	-7.35
-7.34	-7.34	-7.34	-7.34	-7.34	-7.34	-7.34	-7.34	-7.34
-7.33	-7.33	-7.33	-7.33	-7.33	-7.33	-7.33	-7.33	-7.33
-7.32	-7.32	-7.32	-7.32	-7.32	-7.32	-7.32	-7.32	-7.32
-7.31	-7.31	-7.31	-7.31	-7.31	-7.31	-7.31	-7.31	-7.31
-7.3	-7.3	-7.3	-7.3	-7.3	-7.3	-7.3	-7.3	-7.3
-7.29	-7.29	-7.29	-7.29	-7.29	-7.29	-7.29	-7.29	-7.29
-7.28	-7.28	-7.28	-7.28	-7.28	-7.28	-7.28	-7.28	-7.28
-7.27	-7.27	-7.27	-7.27	-7.27	-7.27	-7.27	-7.27	-7.27
-7.26	-7.26	-7.26	-7.26	-7.26	-7.26	-7.26	-7.26	-7.26
-7.25	-7.25	-7.25	-7.25	-7.25	-7.25	-7.25	-7.25	-7.25
-7.24	-7.24	-7.24	-7.24	-7.24	-7.24	-7.24	-7.24	-7.24
-7.23	-7.23	-7.23	-7.23	-7.23	-7.23	-7.23	-7.23	-7.23
-7.22	-7.22	-7.22	-7.22	-7.22	-7.22	-7.22	-7.22	-7.22
-7.21	-7.21	-7.21	-7.21	-7.21	-7.21	-7.21	-7.21	-7.21
-7.2	-7.2	-7.2	-7.2	-7.2	-7.2	-7.2	-7.2	-7.2
-7.19	-7.19	-7.19	-7.19	-7.19	-7.19	-7.19	-7.19	-7.19
-7.18	-7.18	-7.18	-7.18	-7.18	-7.18	-7.18	-7.18	-7.18
-7.17	-7.17	-7.17	-7.17	-7.17	-7.17	-7.17	-7.17	-7.17
-7.16	-7.16	-7.16	-7.16	-7.16	-7.16	-7.16	-7.16	-7.16
-7.15	-7.15	-7.15	-7.15	-7.15	-7.15	-7.15	-7.15	-7.15
-7.14	-7.14	-7.14	-7.14	-7.14	-7.14	-7.14	-7.14	-7.14
-7.13	-7.13	-7.13	-7.13	-7.13	-7.13	-7.13	-7.13	-7.13
-7.12	-7.12	-7.12	-7.12	-7.12	-7.12	-7.12	-7.12	-7.12
-7.11	-7.11	-7.11	-7.11	-7.11	-7.11	-7.11	-7.11	-7.11
-7.1	-7.1	-7.1	-7.1	-7.1	-7.1	-7.1	-7.1	-7.1
-7.09	-7.09	-7.09	-7.09	-7.09	-7.09	-7.09	-7.09	-7.09
-7.08	-7.08	-7.08	-7.08	-7.08	-7.08	-7.08	-7.08	-7.08
-7.07	-7.07	-7.07	-7.07	-7.07	-7.07	-7.07	-7.07	-7.07
-7.06	-7.06	-7.06	-7.06	-7.06	-7.06	-7.06	-7.06	-7.06
-7.05	-7.05	-7.05	-7.05	-7.05	-7.05	-7.05	-7.05	-7.05
-7.04	-7.04	-7.04	-7.04	-7.04	-7.04	-7.04	-7.04	-7.04
-7.03	-7.03	-7.03	-7.03	-7.03	-7.03	-7.03	-7.03	-7.03
-7.02	-7.02	-7.02	-7.02	-7.02	-7.02	-7.02	-7.02	-7.02
-7.01	-7.01	-7.01	-7.01	-7.01	-7.01	-7.01	-7.01	-7.01

-7	-7	-7	-7	-7	-7	-7	-7	-7
-6.99	-6.99	-6.99	-6.99	-6.99	-6.99	-6.99	-6.99	-6.99
-6.98	-6.98	-6.98	-6.98	-6.98	-6.98	-6.98	-6.98	-6.98
-6.97	-6.97	-6.97	-6.97	-6.97	-6.97	-6.97	-6.97	-6.97
-6.96	-6.96	-6.96	-6.96	-6.96	-6.96	-6.96	-6.96	-6.96
-6.95	-6.95	-6.95	-6.95	-6.95	-6.95	-6.95	-6.95	-6.95
-6.94	-6.94	-6.94	-6.94	-6.94	-6.94	-6.94	-6.94	-6.94
-6.93	-6.93	-6.93	-6.93	-6.93	-6.93	-6.93	-6.93	-6.93
-6.92	-6.92	-6.92	-6.92	-6.92	-6.92	-6.92	-6.92	-6.92
-6.91	-6.91	-6.91	-6.91	-6.91	-6.91	-6.91	-6.91	-6.91
-6.9	-6.9	-6.9	-6.9	-6.9	-6.9	-6.9	-6.9	-6.9
-6.89	-6.89	-6.89	-6.89	-6.89	-6.89	-6.89	-6.89	-6.89
-6.88	-6.88	-6.88	-6.88	-6.88	-6.88	-6.88	-6.88	-6.88
-6.87	-6.87	-6.87	-6.87	-6.87	-6.87	-6.87	-6.87	-6.87
-6.86	-6.86	-6.86	-6.86	-6.86	-6.86	-6.86	-6.86	-6.86
-6.85	-6.85	-6.85	-6.85	-6.85	-6.85	-6.85	-6.85	-6.85
-6.84	-6.84	-6.84	-6.84	-6.84	-6.84	-6.84	-6.84	-6.84
-6.83	-6.83	-6.83	-6.83	-6.83	-6.83	-6.83	-6.83	-6.83
-6.82	-6.82	-6.82	-6.82	-6.82	-6.82	-6.82	-6.82	-6.82
-6.81	-6.81	-6.81	-6.81	-6.81	-6.81	-6.81	-6.81	-6.81
-6.8	-6.8	-6.8	-6.8	-6.8	-6.8	-6.8	-6.8	-6.8
-6.79	-6.79	-6.79	-6.79	-6.79	-6.79	-6.79	-6.79	-6.79
-6.78	-6.78	-6.78	-6.78	-6.78	-6.78	-6.78	-6.78	-6.78
-6.77	-6.77	-6.77	-6.77	-6.77	-6.77	-6.77	-6.77	-6.77
-6.76	-6.76	-6.76	-6.76	-6.76	-6.76	-6.76	-6.76	-6.76
-6.75	-6.75	-6.75	-6.75	-6.75	-6.75	-6.75	-6.75	-6.75
-6.74	-6.74	-6.74	-6.74	-6.74	-6.74	-6.74	-6.74	-6.74
-6.73	-6.73	-6.73	-6.73	-6.73	-6.73	-6.73	-6.73	-6.73
-6.72	-6.72	-6.72	-6.72	-6.72	-6.72	-6.72	-6.72	-6.72
-6.71	-6.71	-6.71	-6.71	-6.71	-6.71	-6.71	-6.71	-6.71
-6.7	-6.7	-6.7	-6.7	-6.7	-6.7	-6.7	-6.7	-6.7
-6.69	-6.69	-6.69	-6.69	-6.69	-6.69	-6.69	-6.69	-6.69
-6.68	-6.68	-6.68	-6.68	-6.68	-6.68	-6.68	-6.68	-6.68
-6.67	-6.67	-6.67	-6.67	-6.67	-6.67	-6.67	-6.67	-6.67
-6.66	-6.66	-6.66	-6.66	-6.66	-6.66	-6.66	-6.66	-6.66
-6.65	-6.65	-6.65	-6.65	-6.65	-6.65	-6.65	-6.65	-6.65
-6.64	-6.64	-6.64	-6.64	-6.64	-6.64	-6.64	-6.64	-6.64
-6.63	-6.63	-6.63	-6.63	-6.63	-6.63	-6.63	-6.63	-6.63
-6.62	-6.62	-6.62	-6.62	-6.62	-6.62	-6.62	-6.62	-6.62
-6.61	-6.61	-6.61	-6.61	-6.61	-6.61	-6.61	-6.61	-6.61
-6.6	-6.6	-6.6	-6.6	-6.6	-6.6	-6.6	-6.6	-6.6
-6.59	-6.59	-6.59	-6.59	-6.59	-6.59	-6.59	-6.59	-6.59
-6.58	-6.58	-6.58	-6.58	-6.58	-6.58	-6.58	-6.58	-6.58
-6.57	-6.57	-6.57	-6.57	-6.57	-6.57	-6.57	-6.57	-6.57
-6.56	-6.56	-6.56	-6.56	-6.56	-6.56	-6.56	-6.56	-6.56
-6.55	-6.55	-6.55	-6.55	-6.55	-6.55	-6.55	-6.55	-6.55
-6.54	-6.54	-6.54	-6.54	-6.54	-6.54	-6.54	-6.54	-6.54
-6.53	-6.53	-6.53	-6.53	-6.53	-6.53	-6.53	-6.53	-6.53
-6.52	-6.52	-6.52	-6.52	-6.52	-6.52	-6.52	-6.52	-6.52
-6.51	-6.51	-6.51	-6.51	-6.51	-6.51	-6.51	-6.51	-6.51

-6.5	-6.5	-6.5	-6.5	-6.5	-6.5	-6.5	-6.5	-6.5
-6.49	-6.49	-6.49	-6.49	-6.49	-6.49	-6.49	-6.49	-6.49
-6.48	-6.48	-6.48	-6.48	-6.48	-6.48	-6.48	-6.48	-6.48
-6.47	-6.47	-6.47	-6.47	-6.47	-6.47	-6.47	-6.47	-6.47
-6.46	-6.46	-6.46	-6.46	-6.46	-6.46	-6.46	-6.46	-6.46
-6.45	-6.45	-6.45	-6.45	-6.45	-6.45	-6.45	-6.45	-6.45
-6.44	-6.44	-6.44	-6.44	-6.44	-6.44	-6.44	-6.44	-6.44
-6.43	-6.43	-6.43	-6.43	-6.43	-6.43	-6.43	-6.43	-6.43
-6.42	-6.42	-6.42	-6.42	-6.42	-6.42	-6.42	-6.42	-6.42
-6.41	-6.41	-6.41	-6.41	-6.41	-6.41	-6.41	-6.41	-6.41
-6.4	-6.4	-6.4	-6.4	-6.4	-6.4	-6.4	-6.4	-6.4
-6.39	-6.39	-6.39	-6.39	-6.39	-6.39	-6.39	-6.39	-6.39
-6.38	-6.38	-6.38	-6.38	-6.38	-6.38	-6.38	-6.38	-6.38
-6.37	-6.37	-6.37	-6.37	-6.37	-6.37	-6.37	-6.37	-6.37
-6.36	-6.36	-6.36	-6.36	-6.36	-6.36	-6.36	-6.36	-6.36
-6.35	-6.35	-6.35	-6.35	-6.35	-6.35	-6.35	-6.35	-6.35
-6.34	-6.34	-6.34	-6.34	-6.34	-6.34	-6.34	-6.34	-6.34
-6.33	-6.33	-6.33	-6.33	-6.33	-6.33	-6.33	-6.33	-6.33
-6.32	-6.32	-6.32	-6.32	-6.32	-6.32	-6.32	-6.32	-6.32
-6.31	-6.31	-6.31	-6.31	-6.31	-6.31	-6.31	-6.31	-6.31
-6.3	-6.3	-6.3	-6.3	-6.3	-6.3	-6.3	-6.3	-6.3
-6.29	-6.29	-6.29	-6.29	-6.29	-6.29	-6.29	-6.29	-6.29
-6.28	-6.28	-6.28	-6.28	-6.28	-6.28	-6.28	-6.28	-6.28
-6.27	-6.27	-6.27	-6.27	-6.27	-6.27	-6.27	-6.27	-6.27
-6.26	-6.26	-6.26	-6.26	-6.26	-6.26	-6.26	-6.26	-6.26
-6.25	-6.25	-6.25	-6.25	-6.25	-6.25	-6.25	-6.25	-6.25
-6.24	-6.24	-6.24	-6.24	-6.24	-6.24	-6.24	-6.24	-6.24
-6.23	-6.23	-6.23	-6.23	-6.23	-6.23	-6.23	-6.23	-6.23
-6.22	-6.22	-6.22	-6.22	-6.22	-6.22	-6.22	-6.22	-6.22
-6.21	-6.21	-6.21	-6.21	-6.21	-6.21	-6.21	-6.21	-6.21
-6.2	-6.2	-6.2	-6.2	-6.2	-6.2	-6.2	-6.2	-6.2
-6.19	-6.19	-6.19	-6.19	-6.19	-6.19	-6.19	-6.19	-6.19
-6.18	-6.18	-6.18	-6.18	-6.18	-6.18	-6.18	-6.18	-6.18
-6.17	-6.17	-6.17	-6.17	-6.17	-6.17	-6.17	-6.17	-6.17
-6.16	-6.16	-6.16	-6.16	-6.16	-6.16	-6.16	-6.16	-6.16
-6.15	-6.15	-6.15	-6.15	-6.15	-6.15	-6.15	-6.15	-6.15
-6.14	-6.14	-6.14	-6.14	-6.14	-6.14	-6.14	-6.14	-6.14
-6.13	-6.13	-6.13	-6.13	-6.13	-6.13	-6.13	-6.13	-6.13
-6.12	-6.12	-6.12	-6.12	-6.12	-6.12	-6.12	-6.12	-6.12
-6.11	-6.11	-6.11	-6.11	-6.11	-6.11	-6.11	-6.11	-6.11
-6.1	-6.1	-6.1	-6.1	-6.1	-6.1	-6.1	-6.1	-6.1
-6.09	-6.09	-6.09	-6.09	-6.09	-6.09	-6.09	-6.09	-6.09
-6.08	-6.08	-6.08	-6.08	-6.08	-6.08	-6.08	-6.08	-6.08
-6.07	-6.07	-6.07	-6.07	-6.07	-6.07	-6.07	-6.07	-6.07
-6.06	-6.06	-6.06	-6.06	-6.06	-6.06	-6.06	-6.06	-6.06
-6.05	-6.05	-6.05	-6.05	-6.05	-6.05	-6.05	-6.05	-6.05
-6.04	-6.04	-6.04	-6.04	-6.04	-6.04	-6.04	-6.04	-6.04
-6.03	-6.03	-6.03	-6.03	-6.03	-6.03	-6.03	-6.03	-6.03
-6.02	-6.02	-6.02	-6.02	-6.02	-6.02	-6.02	-6.02	-6.02
-6.01	-6.01	-6.01	-6.01	-6.01	-6.01	-6.01	-6.01	-6.01

-6	-6	-6	-6	-6	-6	-6	-6	-6
-5.99	-5.99	-5.99	-5.99	-5.99	-5.99	-5.99	-5.99	-5.99
-5.98	-5.98	-5.98	-5.98	-5.98	-5.98	-5.98	-5.98	-5.98
-5.97	-5.97	-5.97	-5.97	-5.97	-5.97	-5.97	-5.97	-5.97
-5.96	-5.96	-5.96	-5.96	-5.96	-5.96	-5.96	-5.96	-5.96
-5.95	-5.95	-5.95	-5.95	-5.95	-5.95	-5.95	-5.95	-5.95
-5.94	-5.94	-5.94	-5.94	-5.94	-5.94	-5.94	-5.94	-5.94
-5.93	-5.93	-5.93	-5.93	-5.93	-5.93	-5.93	-5.93	-5.93
-5.92	-5.92	-5.92	-5.92	-5.92	-5.92	-5.92	-5.92	-5.92
-5.91	-5.91	-5.91	-5.91	-5.91	-5.91	-5.91	-5.91	-5.91
-5.9	-5.9	-5.9	-5.9	-5.9	-5.9	-5.9	-5.9	-5.9
-5.89	-5.89	-5.89	-5.89	-5.89	-5.89	-5.89	-5.89	-5.89
-5.88	-5.88	-5.88	-5.88	-5.88	-5.88	-5.88	-5.88	-5.88
-5.87	-5.87	-5.87	-5.87	-5.87	-5.87	-5.87	-5.87	-5.87
-5.86	-5.86	-5.86	-5.86	-5.86	-5.86	-5.86	-5.86	-5.86
-5.85	-5.85	-5.85	-5.85	-5.85	-5.85	-5.85	-5.85	-5.85
-5.84	-5.84	-5.84	-5.84	-5.84	-5.84	-5.84	-5.84	-5.84
-5.83	-5.83	-5.83	-5.83	-5.83	-5.83	-5.83	-5.83	-5.83
-5.82	-5.82	-5.82	-5.82	-5.82	-5.82	-5.82	-5.82	-5.82
-5.81	-5.81	-5.81	-5.81	-5.81	-5.81	-5.81	-5.81	-5.81
-5.8	-5.8	-5.8	-5.8	-5.8	-5.8	-5.8	-5.8	-5.8
-5.79	-5.79	-5.79	-5.79	-5.79	-5.79	-5.79	-5.79	-5.79
-5.78	-5.78	-5.78	-5.78	-5.78	-5.78	-5.78	-5.78	-5.78
-5.77	-5.77	-5.77	-5.77	-5.77	-5.77	-5.77	-5.77	-5.77
-5.76	-5.76	-5.76	-5.76	-5.76	-5.76	-5.76	-5.76	-5.76
-5.75	-5.75	-5.75	-5.75	-5.75	-5.75	-5.75	-5.75	-5.75
-5.74	-5.74	-5.74	-5.74	-5.74	-5.74	-5.74	-5.74	-5.74
-5.73	-5.73	-5.73	-5.73	-5.73	-5.73	-5.73	-5.73	-5.73
-5.72	-5.72	-5.72	-5.72	-5.72	-5.72	-5.72	-5.72	-5.72
-5.71	-5.71	-5.71	-5.71	-5.71	-5.71	-5.71	-5.71	-5.71
-5.7	-5.7	-5.7	-5.7	-5.7	-5.7	-5.7	-5.7	-5.7
-5.69	-5.69	-5.69	-5.69	-5.69	-5.69	-5.69	-5.69	-5.69
-5.68	-5.68	-5.68	-5.68	-5.68	-5.68	-5.68	-5.68	-5.68
-5.67	-5.67	-5.67	-5.67	-5.67	-5.67	-5.67	-5.67	-5.67
-5.66	-5.66	-5.66	-5.66	-5.66	-5.66	-5.66	-5.66	-5.66
-5.65	-5.65	-5.65	-5.65	-5.65	-5.65	-5.65	-5.65	-5.65
-5.64	-5.64	-5.64	-5.64	-5.64	-5.64	-5.64	-5.64	-5.64
-5.63	-5.63	-5.63	-5.63	-5.63	-5.63	-5.63	-5.63	-5.63
-5.62	-5.62	-5.62	-5.62	-5.62	-5.62	-5.62	-5.62	-5.62
-5.61	-5.61	-5.61	-5.61	-5.61	-5.61	-5.61	-5.61	-5.61
-5.6	-5.6	-5.6	-5.6	-5.6	-5.6	-5.6	-5.6	-5.6
-5.59	-5.59	-5.59	-5.59	-5.59	-5.59	-5.59	-5.59	-5.59
-5.58	-5.58	-5.58	-5.58	-5.58	-5.58	-5.58	-5.58	-5.58
-5.57	-5.57	-5.57	-5.57	-5.57	-5.57	-5.57	-5.57	-5.57
-5.56	-5.56	-5.56	-5.56	-5.56	-5.56	-5.56	-5.56	-5.56
-5.55	-5.55	-5.55	-5.55	-5.55	-5.55	-5.55	-5.55	-5.55
-5.54	-5.54	-5.54	-5.54	-5.54	-5.54	-5.54	-5.54	-5.54
-5.53	-5.53	-5.53	-5.53	-5.53	-5.53	-5.53	-5.53	-5.53
-5.52	-5.52	-5.52	-5.52	-5.52	-5.52	-5.52	-5.52	-5.52
-5.51	-5.51	-5.51	-5.51	-5.51	-5.51	-5.51	-5.51	-5.51

-5.5	-5.5	-5.5	-5.5	-5.5	-5.5	-5.5	-5.5	-5.5
-5.49	-5.49	-5.49	-5.49	-5.49	-5.49	-5.49	-5.49	-5.49
-5.48	-5.48	-5.48	-5.48	-5.48	-5.48	-5.48	-5.48	-5.48
-5.47	-5.47	-5.47	-5.47	-5.47	-5.47	-5.47	-5.47	-5.47
-5.46	-5.46	-5.46	-5.46	-5.46	-5.46	-5.46	-5.46	-5.46
-5.45	-5.45	-5.45	-5.45	-5.45	-5.45	-5.45	-5.45	-5.45
-5.44	-5.44	-5.44	-5.44	-5.44	-5.44	-5.44	-5.44	-5.44
-5.43	-5.43	-5.43	-5.43	-5.43	-5.43	-5.43	-5.43	-5.43
-5.42	-5.42	-5.42	-5.42	-5.42	-5.42	-5.42	-5.42	-5.42
-5.41	-5.41	-5.41	-5.41	-5.41	-5.41	-5.41	-5.41	-5.41
-5.4	-5.4	-5.4	-5.4	-5.4	-5.4	-5.4	-5.4	-5.4
-5.39	-5.39	-5.39	-5.39	-5.39	-5.39	-5.39	-5.39	-5.39
-5.38	-5.38	-5.38	-5.38	-5.38	-5.38	-5.38	-5.38	-5.38
-5.37	-5.37	-5.37	-5.37	-5.37	-5.37	-5.37	-5.37	-5.37
-5.36	-5.36	-5.36	-5.36	-5.36	-5.36	-5.36	-5.36	-5.36
-5.35	-5.35	-5.35	-5.35	-5.35	-5.35	-5.35	-5.35	-5.35
-5.34	-5.34	-5.34	-5.34	-5.34	-5.34	-5.34	-5.34	-5.34
-5.33	-5.33	-5.33	-5.33	-5.33	-5.33	-5.33	-5.33	-5.33
-5.32	-5.32	-5.32	-5.32	-5.32	-5.32	-5.32	-5.32	-5.32
-5.31	-5.31	-5.31	-5.31	-5.31	-5.31	-5.31	-5.31	-5.31
-5.3	-5.3	-5.3	-5.3	-5.3	-5.3	-5.3	-5.3	-5.3
-5.29	-5.29	-5.29	-5.29	-5.29	-5.29	-5.29	-5.29	-5.29
-5.28	-5.28	-5.28	-5.28	-5.28	-5.28	-5.28	-5.28	-5.28
-5.27	-5.27	-5.27	-5.27	-5.27	-5.27	-5.27	-5.27	-5.27
-5.26	-5.26	-5.26	-5.26	-5.26	-5.26	-5.26	-5.26	-5.26
-5.25	-5.25	-5.25	-5.25	-5.25	-5.25	-5.25	-5.25	-5.25
-5.24	-5.24	-5.24	-5.24	-5.24	-5.24	-5.24	-5.24	-5.24
-5.23	-5.23	-5.23	-5.23	-5.23	-5.23	-5.23	-5.23	-5.23
-5.22	-5.22	-5.22	-5.22	-5.22	-5.22	-5.22	-5.22	-5.22
-5.21	-5.21	-5.21	-5.21	-5.21	-5.21	-5.21	-5.21	-5.21
-5.2	-5.2	-5.2	-5.2	-5.2	-5.2	-5.2	-5.2	-5.2
-5.19	-5.19	-5.19	-5.19	-5.19	-5.19	-5.19	-5.19	-5.19
-5.18	-5.18	-5.18	-5.18	-5.18	-5.18	-5.18	-5.18	-5.18
-5.17	-5.17	-5.17	-5.17	-5.17	-5.17	-5.17	-5.17	-5.17
-5.16	-5.16	-5.16	-5.16	-5.16	-5.16	-5.16	-5.16	-5.16
-5.15	-5.15	-5.15	-5.15	-5.15	-5.15	-5.15	-5.15	-5.15
-5.14	-5.14	-5.14	-5.14	-5.14	-5.14	-5.14	-5.14	-5.14
-5.13	-5.13	-5.13	-5.13	-5.13	-5.13	-5.13	-5.13	-5.13
-5.12	-5.12	-5.12	-5.12	-5.12	-5.12	-5.12	-5.12	-5.12
-5.11	-5.11	-5.11	-5.11	-5.11	-5.11	-5.11	-5.11	-5.11
-5.1	-5.1	-5.1	-5.1	-5.1	-5.1	-5.1	-5.1	-5.1
-5.09	-5.09	-5.09	-5.09	-5.09	-5.09	-5.09	-5.09	-5.09
-5.08	-5.08	-5.08	-5.08	-5.08	-5.08	-5.08	-5.08	-5.08
-5.07	-5.07	-5.07	-5.07	-5.07	-5.07	-5.07	-5.07	-5.07
-5.06	-5.06	-5.06	-5.06	-5.06	-5.06	-5.06	-5.06	-5.06
-5.05	-5.05	-5.05	-5.05	-5.05	-5.05	-5.05	-5.05	-5.05
-5.04	-5.04	-5.04	-5.04	-5.04	-5.04	-5.04	-5.04	-5.04
-5.03	-5.03	-5.03	-5.03	-5.03	-5.03	-5.03	-5.03	-5.03
-5.02	-5.02	-5.02	-5.02	-5.02	-5.02	-5.02	-5.02	-5.02
-5.01	-5.01	-5.01	-5.01	-5.01	-5.01	-5.01	-5.01	-5.01

-5	-5	-5	-5	-5	-5	-5	-5	-5
-4.99	-4.99	-4.99	-4.99	-4.99	-4.99	-4.99	-4.99	-4.99
-4.98	-4.98	-4.98	-4.98	-4.98	-4.98	-4.98	-4.98	-4.98
-4.97	-4.97	-4.97	-4.97	-4.97	-4.97	-4.97	-4.97	-4.97
-4.96	-4.96	-4.96	-4.96	-4.96	-4.96	-4.96	-4.96	-4.96
-4.95	-4.95	-4.95	-4.95	-4.95	-4.95	-4.95	-4.95	-4.95
-4.94	-4.94	-4.94	-4.94	-4.94	-4.94	-4.94	-4.94	-4.94
-4.93	-4.93	-4.93	-4.93	-4.93	-4.93	-4.93	-4.93	-4.93
-4.92	-4.92	-4.92	-4.92	-4.92	-4.92	-4.92	-4.92	-4.92
-4.91	-4.91	-4.91	-4.91	-4.91	-4.91	-4.91	-4.91	-4.91
-4.9	-4.9	-4.9	-4.9	-4.9	-4.9	-4.9	-4.9	-4.9
-4.89	-4.89	-4.89	-4.89	-4.89	-4.89	-4.89	-4.89	-4.89
-4.88	-4.88	-4.88	-4.88	-4.88	-4.88	-4.88	-4.88	-4.88
-4.87	-4.87	-4.87	-4.87	-4.87	-4.87	-4.87	-4.87	-4.87
-4.86	-4.86	-4.86	-4.86	-4.86	-4.86	-4.86	-4.86	-4.86
-4.85	-4.85	-4.85	-4.85	-4.85	-4.85	-4.85	-4.85	-4.85
-4.84	-4.84	-4.84	-4.84	-4.84	-4.84	-4.84	-4.84	-4.84
-4.83	-4.83	-4.83	-4.83	-4.83	-4.83	-4.83	-4.83	-4.83
-4.82	-4.82	-4.82	-4.82	-4.82	-4.82	-4.82	-4.82	-4.82
-4.81	-4.81	-4.81	-4.81	-4.81	-4.81	-4.81	-4.81	-4.81
-4.8	-4.8	-4.8	-4.8	-4.8	-4.8	-4.8	-4.8	-4.8
-4.79	-4.79	-4.79	-4.79	-4.79	-4.79	-4.79	-4.79	-4.79
-4.78	-4.78	-4.78	-4.78	-4.78	-4.78	-4.78	-4.78	-4.78
-4.77	-4.77	-4.77	-4.77	-4.77	-4.77	-4.77	-4.77	-4.77
-4.76	-4.76	-4.76	-4.76	-4.76	-4.76	-4.76	-4.76	-4.76
-4.75	-4.75	-4.75	-4.75	-4.75	-4.75	-4.75	-4.75	-4.75
-4.74	-4.74	-4.74	-4.74	-4.74	-4.74	-4.74	-4.74	-4.74
-4.73	-4.73	-4.73	-4.73	-4.73	-4.73	-4.73	-4.73	-4.73
-4.72	-4.72	-4.72	-4.72	-4.72	-4.72	-4.72	-4.72	-4.72
-4.71	-4.71	-4.71	-4.71	-4.71	-4.71	-4.71	-4.71	-4.71
-4.7	-4.7	-4.7	-4.7	-4.7	-4.7	-4.7	-4.7	-4.7
-4.69	-4.69	-4.69	-4.69	-4.69	-4.69	-4.69	-4.69	-4.69
-4.68	-4.68	-4.68	-4.68	-4.68	-4.68	-4.68	-4.68	-4.68
-4.67	-4.67	-4.67	-4.67	-4.67	-4.67	-4.67	-4.67	-4.67
-4.66	-4.66	-4.66	-4.66	-4.66	-4.66	-4.66	-4.66	-4.66
-4.65	-4.65	-4.65	-4.65	-4.65	-4.65	-4.65	-4.65	-4.65
-4.64	-4.64	-4.64	-4.64	-4.64	-4.64	-4.64	-4.64	-4.64
-4.63	-4.63	-4.63	-4.63	-4.63	-4.63	-4.63	-4.63	-4.63
-4.62	-4.62	-4.62	-4.62	-4.62	-4.62	-4.62	-4.62	-4.62
-4.61	-4.61	-4.61	-4.61	-4.61	-4.61	-4.61	-4.61	-4.61
-4.6	-4.6	-4.6	-4.6	-4.6	-4.6	-4.6	-4.6	-4.6
-4.59	-4.59	-4.59	-4.59	-4.59	-4.59	-4.59	-4.59	-4.59
-4.58	-4.58	-4.58	-4.58	-4.58	-4.58	-4.58	-4.58	-4.58
-4.57	-4.57	-4.57	-4.57	-4.57	-4.57	-4.57	-4.57	-4.57
-4.56	-4.56	-4.56	-4.56	-4.56	-4.56	-4.56	-4.56	-4.56
-4.55	-4.55	-4.55	-4.55	-4.55	-4.55	-4.55	-4.55	-4.55
-4.54	-4.54	-4.54	-4.54	-4.54	-4.54	-4.54	-4.54	-4.54
-4.53	-4.53	-4.53	-4.53	-4.53	-4.53	-4.53	-4.53	-4.53
-4.52	-4.52	-4.52	-4.52	-4.52	-4.52	-4.52	-4.52	-4.52
-4.51	-4.51	-4.51	-4.51	-4.51	-4.51	-4.51	-4.51	-4.51

-4.5	-4.5	-4.5	-4.5	-4.5	-4.5	-4.5	-4.5	-4.5
-4.49	-4.49	-4.49	-4.49	-4.49	-4.49	-4.49	-4.49	-4.49
-4.48	-4.48	-4.48	-4.48	-4.48	-4.48	-4.48	-4.48	-4.48
-4.47	-4.47	-4.47	-4.47	-4.47	-4.47	-4.47	-4.47	-4.47
-4.46	-4.46	-4.46	-4.46	-4.46	-4.46	-4.46	-4.46	-4.46
-4.45	-4.45	-4.45	-4.45	-4.45	-4.45	-4.45	-4.45	-4.45
-4.44	-4.44	-4.44	-4.44	-4.44	-4.44	-4.44	-4.44	-4.44
-4.43	-4.43	-4.43	-4.43	-4.43	-4.43	-4.43	-4.43	-4.43
-4.42	-4.42	-4.42	-4.42	-4.42	-4.42	-4.42	-4.42	-4.42
-4.41	-4.41	-4.41	-4.41	-4.41	-4.41	-4.41	-4.41	-4.41
-4.4	-4.4	-4.4	-4.4	-4.4	-4.4	-4.4	-4.4	-4.4
-4.39	-4.39	-4.39	-4.39	-4.39	-4.39	-4.39	-4.39	-4.39
-4.38	-4.38	-4.38	-4.38	-4.38	-4.38	-4.38	-4.38	-4.38
-4.37	-4.37	-4.37	-4.37	-4.37	-4.37	-4.37	-4.37	-4.37
-4.36	-4.36	-4.36	-4.36	-4.36	-4.36	-4.36	-4.36	-4.36
-4.35	-4.35	-4.35	-4.35	-4.35	-4.35	-4.35	-4.35	-4.35
-4.34	-4.34	-4.34	-4.34	-4.34	-4.34	-4.34	-4.34	-4.34
-4.33	-4.33	-4.33	-4.33	-4.33	-4.33	-4.33	-4.33	-4.33
-4.32	-4.32	-4.32	-4.32	-4.32	-4.32	-4.32	-4.32	-4.32
-4.31	-4.31	-4.31	-4.31	-4.31	-4.31	-4.31	-4.31	-4.31
-4.3	-4.3	-4.3	-4.3	-4.3	-4.3	-4.3	-4.3	-4.3
-4.29	-4.29	-4.29	-4.29	-4.29	-4.29	-4.29	-4.29	-4.29
-4.28	-4.28	-4.28	-4.28	-4.28	-4.28	-4.28	-4.28	-4.28
-4.27	-4.27	-4.27	-4.27	-4.27	-4.27	-4.27	-4.27	-4.27
-4.26	-4.26	-4.26	-4.26	-4.26	-4.26	-4.26	-4.26	-4.26
-4.25	-4.25	-4.25	-4.25	-4.25	-4.25	-4.25	-4.25	-4.25
-4.24	-4.24	-4.24	-4.24	-4.24	-4.24	-4.24	-4.24	-4.24
-4.23	-4.23	-4.23	-4.23	-4.23	-4.23	-4.23	-4.23	-4.23
-4.22	-4.22	-4.22	-4.22	-4.22	-4.22	-4.22	-4.22	-4.22
-4.21	-4.21	-4.21	-4.21	-4.21	-4.21	-4.21	-4.21	-4.21
-4.2	-4.2	-4.2	-4.2	-4.2	-4.2	-4.2	-4.2	-4.2
-4.19	-4.19	-4.19	-4.19	-4.19	-4.19	-4.19	-4.19	-4.19
-4.18	-4.18	-4.18	-4.18	-4.18	-4.18	-4.18	-4.18	-4.18
-4.17	-4.17	-4.17	-4.17	-4.17	-4.17	-4.17	-4.17	-4.17
-4.16	-4.16	-4.16	-4.16	-4.16	-4.16	-4.16	-4.16	-4.16
-4.15	-4.15	-4.15	-4.15	-4.15	-4.15	-4.15	-4.15	-4.15
-4.14	-4.14	-4.14	-4.14	-4.14	-4.14	-4.14	-4.14	-4.14
-4.13	-4.13	-4.13	-4.13	-4.13	-4.13	-4.13	-4.13	-4.13
-4.12	-4.12	-4.12	-4.12	-4.12	-4.12	-4.12	-4.12	-4.12
-4.11	-4.11	-4.11	-4.11	-4.11	-4.11	-4.11	-4.11	-4.11
-4.1	-4.1	-4.1	-4.1	-4.1	-4.1	-4.1	-4.1	-4.1
-4.09	-4.09	-4.09	-4.09	-4.09	-4.09	-4.09	-4.09	-4.09
-4.08	-4.08	-4.08	-4.08	-4.08	-4.08	-4.08	-4.08	-4.08
-4.07	-4.07	-4.07	-4.07	-4.07	-4.07	-4.07	-4.07	-4.07
-4.06	-4.06	-4.06	-4.06	-4.06	-4.06	-4.06	-4.06	-4.06
-4.05	-4.05	-4.05	-4.05	-4.05	-4.05	-4.05	-4.05	-4.05
-4.04	-4.04	-4.04	-4.04	-4.04	-4.04	-4.04	-4.04	-4.04
-4.03	-4.03	-4.03	-4.03	-4.03	-4.03	-4.03	-4.03	-4.03
-4.02	-4.02	-4.02	-4.02	-4.02	-4.02	-4.02	-4.02	-4.02
-4.01	-4.01	-4.01	-4.01	-4.01	-4.01	-4.01	-4.01	-4.01

-4	-4	-4	-4	-4	-4	-4	-4	-4
-3.99	-3.99	-3.99	-3.99	-3.99	-3.99	-3.99	-3.99	-3.99
-3.98	-3.98	-3.98	-3.98	-3.98	-3.98	-3.98	-3.98	-3.98
-3.97	-3.97	-3.97	-3.97	-3.97	-3.97	-3.97	-3.97	-3.97
-3.96	-3.96	-3.96	-3.96	-3.96	-3.96	-3.96	-3.96	-3.96
-3.95	-3.95	-3.95	-3.95	-3.95	-3.95	-3.95	-3.95	-3.95
-3.94	-3.94	-3.94	-3.94	-3.94	-3.94	-3.94	-3.94	-3.94
-3.93	-3.93	-3.93	-3.93	-3.93	-3.93	-3.93	-3.93	-3.93
-3.92	-3.92	-3.92	-3.92	-3.92	-3.92	-3.92	-3.92	-3.92
-3.91	-3.91	-3.91	-3.91	-3.91	-3.91	-3.91	-3.91	-3.91
-3.9	-3.9	-3.9	-3.9	-3.9	-3.9	-3.9	-3.9	-3.9
-3.89	-3.89	-3.89	-3.89	-3.89	-3.89	-3.89	-3.89	-3.89
-3.88	-3.88	-3.88	-3.88	-3.88	-3.88	-3.88	-3.88	-3.88
-3.87	-3.87	-3.87	-3.87	-3.87	-3.87	-3.87	-3.87	-3.87
-3.86	-3.86	-3.86	-3.86	-3.86	-3.86	-3.86	-3.86	-3.86
-3.85	-3.85	-3.85	-3.85	-3.85	-3.85	-3.85	-3.85	-3.85
-3.84	-3.84	-3.84	-3.84	-3.84	-3.84	-3.84	-3.84	-3.84
-3.83	-3.83	-3.83	-3.83	-3.83	-3.83	-3.83	-3.83	-3.83
-3.82	-3.82	-3.82	-3.82	-3.82	-3.82	-3.82	-3.82	-3.82
-3.81	-3.81	-3.81	-3.81	-3.81	-3.81	-3.81	-3.81	-3.81
-3.8	-3.8	-3.8	-3.8	-3.8	-3.8	-3.8	-3.8	-3.8
-3.79	-3.79	-3.79	-3.79	-3.79	-3.79	-3.79	-3.79	-3.79
-3.78	-3.78	-3.78	-3.78	-3.78	-3.78	-3.78	-3.78	-3.78
-3.77	-3.77	-3.77	-3.77	-3.77	-3.77	-3.77	-3.77	-3.77
-3.76	-3.76	-3.76	-3.76	-3.76	-3.76	-3.76	-3.76	-3.76
-3.75	-3.75	-3.75	-3.75	-3.75	-3.75	-3.75	-3.75	-3.75
-3.74	-3.74	-3.74	-3.74	-3.74	-3.74	-3.74	-3.74	-3.74
-3.73	-3.73	-3.73	-3.73	-3.73	-3.73	-3.73	-3.73	-3.73
-3.72	-3.72	-3.72	-3.72	-3.72	-3.72	-3.72	-3.72	-3.72
-3.71	-3.71	-3.71	-3.71	-3.71	-3.71	-3.71	-3.71	-3.71
-3.7	-3.7	-3.7	-3.7	-3.7	-3.7	-3.7	-3.7	-3.7
-3.69	-3.69	-3.69	-3.69	-3.69	-3.69	-3.69	-3.69	-3.69
-3.68	-3.68	-3.68	-3.68	-3.68	-3.68	-3.68	-3.68	-3.68
-3.67	-3.67	-3.67	-3.67	-3.67	-3.67	-3.67	-3.67	-3.67
-3.66	-3.66	-3.66	-3.66	-3.66	-3.66	-3.66	-3.66	-3.66
-3.65	-3.65	-3.65	-3.65	-3.65	-3.65	-3.65	-3.65	-3.65
-3.64	-3.64	-3.64	-3.64	-3.64	-3.64	-3.64	-3.64	-3.64
-3.63	-3.63	-3.63	-3.63	-3.63	-3.63	-3.63	-3.63	-3.63
-3.62	-3.62	-3.62	-3.62	-3.62	-3.62	-3.62	-3.62	-3.62
-3.61	-3.61	-3.61	-3.61	-3.61	-3.61	-3.61	-3.61	-3.61
-3.6	-3.6	-3.6	-3.6	-3.6	-3.6	-3.6	-3.6	-3.6
-3.59	-3.59	-3.59	-3.59	-3.59	-3.59	-3.59	-3.59	-3.59
-3.58	-3.58	-3.58	-3.58	-3.58	-3.58	-3.58	-3.58	-3.58
-3.57	-3.57	-3.57	-3.57	-3.57	-3.57	-3.57	-3.57	-3.57
-3.56	-3.56	-3.56	-3.56	-3.56	-3.56	-3.56	-3.56	-3.56
-3.55	-3.55	-3.55	-3.55	-3.55	-3.55	-3.55	-3.55	-3.55
-3.54	-3.54	-3.54	-3.54	-3.54	-3.54	-3.54	-3.54	-3.54
-3.53	-3.53	-3.53	-3.53	-3.53	-3.53	-3.53	-3.53	-3.53
-3.52	-3.52	-3.52	-3.52	-3.52	-3.52	-3.52	-3.52	-3.52
-3.51	-3.51	-3.51	-3.51	-3.51	-3.51	-3.51	-3.51	-3.51

-3.5	-3.5	-3.5	-3.5	-3.5	-3.5	-3.5	-3.5	-3.5
-3.49	-3.49	-3.49	-3.49	-3.49	-3.49	-3.49	-3.49	-3.49
-3.48	-3.48	-3.48	-3.48	-3.48	-3.48	-3.48	-3.48	-3.48
-3.47	-3.47	-3.47	-3.47	-3.47	-3.47	-3.47	-3.47	-3.47
-3.46	-3.46	-3.46	-3.46	-3.46	-3.46	-3.46	-3.46	-3.46
-3.45	-3.45	-3.45	-3.45	-3.45	-3.45	-3.45	-3.45	-3.45
-3.44	-3.44	-3.44	-3.44	-3.44	-3.44	-3.44	-3.44	-3.44
-3.43	-3.43	-3.43	-3.43	-3.43	-3.43	-3.43	-3.43	-3.43
-3.42	-3.42	-3.42	-3.42	-3.42	-3.42	-3.42	-3.42	-3.42
-3.41	-3.41	-3.41	-3.41	-3.41	-3.41	-3.41	-3.41	-3.41
-3.4	-3.4	-3.4	-3.4	-3.4	-3.4	-3.4	-3.4	-3.4
-3.39	-3.39	-3.39	-3.39	-3.39	-3.39	-3.39	-3.39	-3.39
-3.38	-3.38	-3.38	-3.38	-3.38	-3.38	-3.38	-3.38	-3.38
-3.37	-3.37	-3.37	-3.37	-3.37	-3.37	-3.37	-3.37	-3.37
-3.36	-3.36	-3.36	-3.36	-3.36	-3.36	-3.36	-3.36	-3.36
-3.35	-3.35	-3.35	-3.35	-3.35	-3.35	-3.35	-3.35	-3.35
-3.34	-3.34	-3.34	-3.34	-3.34	-3.34	-3.34	-3.34	-3.34
-3.33	-3.33	-3.33	-3.33	-3.33	-3.33	-3.33	-3.33	-3.33
-3.32	-3.32	-3.32	-3.32	-3.32	-3.32	-3.32	-3.32	-3.32
-3.31	-3.31	-3.31	-3.31	-3.31	-3.31	-3.31	-3.31	-3.31
-3.3	-3.3	-3.3	-3.3	-3.3	-3.3	-3.3	-3.3	-3.3
-3.29	-3.29	-3.29	-3.29	-3.29	-3.29	-3.29	-3.29	-3.29
-3.28	-3.28	-3.28	-3.28	-3.28	-3.28	-3.28	-3.28	-3.28
-3.27	-3.27	-3.27	-3.27	-3.27	-3.27	-3.27	-3.27	-3.27
-3.26	-3.26	-3.26	-3.26	-3.26	-3.26	-3.26	-3.26	-3.26
-3.25	-3.25	-3.25	-3.25	-3.25	-3.25	-3.25	-3.25	-3.25
-3.24	-3.24	-3.24	-3.24	-3.24	-3.24	-3.24	-3.24	-3.24
-3.23	-3.23	-3.23	-3.23	-3.23	-3.23	-3.23	-3.23	-3.23
-3.22	-3.22	-3.22	-3.22	-3.22	-3.22	-3.22	-3.22	-3.22
-3.21	-3.21	-3.21	-3.21	-3.21	-3.21	-3.21	-3.21	-3.21
-3.2	-3.2	-3.2	-3.2	-3.2	-3.2	-3.2	-3.2	-3.2
-3.19	-3.19	-3.19	-3.19	-3.19	-3.19	-3.19	-3.19	-3.19
-3.18	-3.18	-3.18	-3.18	-3.18	-3.18	-3.18	-3.18	-3.18
-3.17	-3.17	-3.17	-3.17	-3.17	-3.17	-3.17	-3.17	-3.17
-3.16	-3.16	-3.16	-3.16	-3.16	-3.16	-3.16	-3.16	-3.16
-3.15	-3.15	-3.15	-3.15	-3.15	-3.15	-3.15	-3.15	-3.15
-3.14	-3.14	-3.14	-3.14	-3.14	-3.14	-3.14	-3.14	-3.14
-3.13	-3.13	-3.13	-3.13	-3.13	-3.13	-3.13	-3.13	-3.13
-3.12	-3.12	-3.12	-3.12	-3.12	-3.12	-3.12	-3.12	-3.12
-3.11	-3.11	-3.11	-3.11	-3.11	-3.11	-3.11	-3.11	-3.11
-3.1	-3.1	-3.1	-3.1	-3.1	-3.1	-3.1	-3.1	-3.1
-3.09	-3.09	-3.09	-3.09	-3.09	-3.09	-3.09	-3.09	-3.09
-3.08	-3.08	-3.08	-3.08	-3.08	-3.08	-3.08	-3.08	-3.08
-3.07	-3.07	-3.07	-3.07	-3.07	-3.07	-3.07	-3.07	-3.07
-3.06	-3.06	-3.06	-3.06	-3.06	-3.06	-3.06	-3.06	-3.06
-3.05	-3.05	-3.05	-3.05	-3.05	-3.05	-3.05	-3.05	-3.05
-3.04	-3.04	-3.04	-3.04	-3.04	-3.04	-3.04	-3.04	-3.04
-3.03	-3.03	-3.03	-3.03	-3.03	-3.03	-3.03	-3.03	-3.03
-3.02	-3.02	-3.02	-3.02	-3.02	-3.02	-3.02	-3.02	-3.02
-3.01	-3.01	-3.01	-3.01	-3.01	-3.01	-3.01	-3.01	-3.01

-3	-3	-3	-3	-3	-3	-3	-3	-3
-2.99	-2.99	-2.99	-2.99	-2.99	-2.99	-2.99	-2.99	-2.99
-2.98	-2.98	-2.98	-2.98	-2.98	-2.98	-2.98	-2.98	-2.98
-2.97	-2.97	-2.97	-2.97	-2.97	-2.97	-2.97	-2.97	-2.97
-2.96	-2.96	-2.96	-2.96	-2.96	-2.96	-2.96	-2.96	-2.96
-2.95	-2.95	-2.95	-2.95	-2.95	-2.95	-2.95	-2.95	-2.95
-2.94	-2.94	-2.94	-2.94	-2.94	-2.94	-2.94	-2.94	-2.94
-2.93	-2.93	-2.93	-2.93	-2.93	-2.93	-2.93	-2.93	-2.93
-2.92	-2.92	-2.92	-2.92	-2.92	-2.92	-2.92	-2.92	-2.92
-2.91	-2.91	-2.91	-2.91	-2.91	-2.91	-2.91	-2.91	-2.91
-2.9	-2.9	-2.9	-2.9	-2.9	-2.9	-2.9	-2.9	-2.9
-2.89	-2.89	-2.89	-2.89	-2.89	-2.89	-2.89	-2.89	-2.89
-2.88	-2.88	-2.88	-2.88	-2.88	-2.88	-2.88	-2.88	-2.88
-2.87	-2.87	-2.87	-2.87	-2.87	-2.87	-2.87	-2.87	-2.87
-2.86	-2.86	-2.86	-2.86	-2.86	-2.86	-2.86	-2.86	-2.86
-2.85	-2.85	-2.85	-2.85	-2.85	-2.85	-2.85	-2.85	-2.85
-2.84	-2.84	-2.84	-2.84	-2.84	-2.84	-2.84	-2.84	-2.84
-2.83	-2.83	-2.83	-2.83	-2.83	-2.83	-2.83	-2.83	-2.83
-2.82	-2.82	-2.82	-2.82	-2.82	-2.82	-2.82	-2.82	-2.82
-2.81	-2.81	-2.81	-2.81	-2.81	-2.81	-2.81	-2.81	-2.81
-2.8	-2.8	-2.8	-2.8	-2.8	-2.8	-2.8	-2.8	-2.8
-2.79	-2.79	-2.79	-2.79	-2.79	-2.79	-2.79	-2.79	-2.79
-2.78	-2.78	-2.78	-2.78	-2.78	-2.78	-2.78	-2.78	-2.78
-2.77	-2.77	-2.77	-2.77	-2.77	-2.77	-2.77	-2.77	-2.77
-2.76	-2.76	-2.76	-2.76	-2.76	-2.76	-2.76	-2.76	-2.76
-2.75	-2.75	-2.75	-2.75	-2.75	-2.75	-2.75	-2.75	-2.75
-2.74	-2.74	-2.74	-2.74	-2.74	-2.74	-2.74	-2.74	-2.74
-2.73	-2.73	-2.73	-2.73	-2.73	-2.73	-2.73	-2.73	-2.73
-2.72	-2.72	-2.72	-2.72	-2.72	-2.72	-2.72	-2.72	-2.72
-2.71	-2.71	-2.71	-2.71	-2.71	-2.71	-2.71	-2.71	-2.71
-2.7	-2.7	-2.7	-2.7	-2.7	-2.7	-2.7	-2.7	-2.7
-2.69	-2.69	-2.69	-2.69	-2.69	-2.69	-2.69	-2.69	-2.69
-2.68	-2.68	-2.68	-2.68	-2.68	-2.68	-2.68	-2.68	-2.68
-2.67	-2.67	-2.67	-2.67	-2.67	-2.67	-2.67	-2.67	-2.67
-2.66	-2.66	-2.66	-2.66	-2.66	-2.66	-2.66	-2.66	-2.66
-2.65	-2.65	-2.65	-2.65	-2.65	-2.65	-2.65	-2.65	-2.65
-2.64	-2.64	-2.64	-2.64	-2.64	-2.64	-2.64	-2.64	-2.64
-2.63	-2.63	-2.63	-2.63	-2.63	-2.63	-2.63	-2.63	-2.63
-2.62	-2.62	-2.62	-2.62	-2.62	-2.62	-2.62	-2.62	-2.62
-2.61	-2.61	-2.61	-2.61	-2.61	-2.61	-2.61	-2.61	-2.61
-2.6	-2.6	-2.6	-2.6	-2.6	-2.6	-2.6	-2.6	-2.6
-2.59	-2.59	-2.59	-2.59	-2.59	-2.59	-2.59	-2.59	-2.59
-2.58	-2.58	-2.58	-2.58	-2.58	-2.58	-2.58	-2.58	-2.58
-2.57	-2.57	-2.57	-2.57	-2.57	-2.57	-2.57	-2.57	-2.57
-2.56	-2.56	-2.56	-2.56	-2.56	-2.56	-2.56	-2.56	-2.56
-2.55	-2.55	-2.55	-2.55	-2.55	-2.55	-2.55	-2.55	-2.55
-2.54	-2.54	-2.54	-2.54	-2.54	-2.54	-2.54	-2.54	-2.54
-2.53	-2.53	-2.53	-2.53	-2.53	-2.53	-2.53	-2.53	-2.53
-2.52	-2.52	-2.52	-2.52	-2.52	-2.52	-2.52	-2.52	-2.52
-2.51	-2.51	-2.51	-2.51	-2.51	-2.51	-2.51	-2.51	-2.51

-2.5	-2.5	-2.5	-2.5	-2.5	-2.5	-2.5	-2.5	-2.5
-2.49	-2.49	-2.49	-2.49	-2.49	-2.49	-2.49	-2.49	-2.49
-2.48	-2.48	-2.48	-2.48	-2.48	-2.48	-2.48	-2.48	-2.48
-2.47	-2.47	-2.47	-2.47	-2.47	-2.47	-2.47	-2.47	-2.47
-2.46	-2.46	-2.46	-2.46	-2.46	-2.46	-2.46	-2.46	-2.46
-2.45	-2.45	-2.45	-2.45	-2.45	-2.45	-2.45	-2.45	-2.45
-2.44	-2.44	-2.44	-2.44	-2.44	-2.44	-2.44	-2.44	-2.44
-2.43	-2.43	-2.43	-2.43	-2.43	-2.43	-2.43	-2.43	-2.43
-2.42	-2.42	-2.42	-2.42	-2.42	-2.42	-2.42	-2.42	-2.42
-2.41	-2.41	-2.41	-2.41	-2.41	-2.41	-2.41	-2.41	-2.41
-2.4	-2.4	-2.4	-2.4	-2.4	-2.4	-2.4	-2.4	-2.4
-2.39	-2.39	-2.39	-2.39	-2.39	-2.39	-2.39	-2.39	-2.39
-2.38	-2.38	-2.38	-2.38	-2.38	-2.38	-2.38	-2.38	-2.38
-2.37	-2.37	-2.37	-2.37	-2.37	-2.37	-2.37	-2.37	-2.37
-2.36	-2.36	-2.36	-2.36	-2.36	-2.36	-2.36	-2.36	-2.36
-2.35	-2.35	-2.35	-2.35	-2.35	-2.35	-2.35	-2.35	-2.35
-2.34	-2.34	-2.34	-2.34	-2.34	-2.34	-2.34	-2.34	-2.34
-2.33	-2.33	-2.33	-2.33	-2.33	-2.33	-2.33	-2.33	-2.33
-2.32	-2.32	-2.32	-2.32	-2.32	-2.32	-2.32	-2.32	-2.32
-2.31	-2.31	-2.31	-2.31	-2.31	-2.31	-2.31	-2.31	-2.31
-2.3	-2.3	-2.3	-2.3	-2.3	-2.3	-2.3	-2.3	-2.3
-2.29	-2.29	-2.29	-2.29	-2.29	-2.29	-2.29	-2.29	-2.29
-2.28	-2.28	-2.28	-2.28	-2.28	-2.28	-2.28	-2.28	-2.28
-2.27	-2.27	-2.27	-2.27	-2.27	-2.27	-2.27	-2.27	-2.27
-2.26	-2.26	-2.26	-2.26	-2.26	-2.26	-2.26	-2.26	-2.26
-2.25	-2.25	-2.25	-2.25	-2.25	-2.25	-2.25	-2.25	-2.25
-2.24	-2.24	-2.24	-2.24	-2.24	-2.24	-2.24	-2.24	-2.24
-2.23	-2.23	-2.23	-2.23	-2.23	-2.23	-2.23	-2.23	-2.23
-2.22	-2.22	-2.22	-2.22	-2.22	-2.22	-2.22	-2.22	-2.22
-2.21	-2.21	-2.21	-2.21	-2.21	-2.21	-2.21	-2.21	-2.21
-2.2	-2.2	-2.2	-2.2	-2.2	-2.2	-2.2	-2.2	-2.2
-2.19	-2.19	-2.19	-2.19	-2.19	-2.19	-2.19	-2.19	-2.19
-2.18	-2.18	-2.18	-2.18	-2.18	-2.18	-2.18	-2.18	-2.18
-2.17	-2.17	-2.17	-2.17	-2.17	-2.17	-2.17	-2.17	-2.17
-2.16	-2.16	-2.16	-2.16	-2.16	-2.16	-2.16	-2.16	-2.16
-2.15	-2.15	-2.15	-2.15	-2.15	-2.15	-2.15	-2.15	-2.15
-2.14	-2.14	-2.14	-2.14	-2.14	-2.14	-2.14	-2.14	-2.14
-2.13	-2.13	-2.13	-2.13	-2.13	-2.13	-2.13	-2.13	-2.13
-2.12	-2.12	-2.12	-2.12	-2.12	-2.12	-2.12	-2.12	-2.12
-2.11	-2.11	-2.11	-2.11	-2.11	-2.11	-2.11	-2.11	-2.11
-2.1	-2.1	-2.1	-2.1	-2.1	-2.1	-2.1	-2.1	-2.1
-2.09	-2.09	-2.09	-2.09	-2.09	-2.09	-2.09	-2.09	-2.09
-2.08	-2.08	-2.08	-2.08	-2.08	-2.08	-2.08	-2.08	-2.08
-2.07	-2.07	-2.07	-2.07	-2.07	-2.07	-2.07	-2.07	-2.07
-2.06	-2.06	-2.06	-2.06	-2.06	-2.06	-2.06	-2.06	-2.06
-2.05	-2.05	-2.05	-2.05	-2.05	-2.05	-2.05	-2.05	-2.05
-2.04	-2.04	-2.04	-2.04	-2.04	-2.04	-2.04	-2.04	-2.04
-2.03	-2.03	-2.03	-2.03	-2.03	-2.03	-2.03	-2.03	-2.03
-2.02	-2.02	-2.02	-2.02	-2.02	-2.02	-2.02	-2.02	-2.02
-2.01	-2.01	-2.01	-2.01	-2.01	-2.01	-2.01	-2.01	-2.01

-2	-2	-2	-2	-2	-2	-2	-2	-2
-1.99	-1.99	-1.99	-1.99	-1.99	-1.99	-1.99	-1.99	-1.99
-1.98	-1.98	-1.98	-1.98	-1.98	-1.98	-1.98	-1.98	-1.98
-1.97	-1.97	-1.97	-1.97	-1.97	-1.97	-1.97	-1.97	-1.97
-1.96	-1.96	-1.96	-1.96	-1.96	-1.96	-1.96	-1.96	-1.96
-1.95	-1.95	-1.95	-1.95	-1.95	-1.95	-1.95	-1.95	-1.95
-1.94	-1.94	-1.94	-1.94	-1.94	-1.94	-1.94	-1.94	-1.94
-1.93	-1.93	-1.93	-1.93	-1.93	-1.93	-1.93	-1.93	-1.93
-1.92	-1.92	-1.92	-1.92	-1.92	-1.92	-1.92	-1.92	-1.92
-1.91	-1.91	-1.91	-1.91	-1.91	-1.91	-1.91	-1.91	-1.91
-1.9	-1.9	-1.9	-1.9	-1.9	-1.9	-1.9	-1.9	-1.9
-1.89	-1.89	-1.89	-1.89	-1.89	-1.89	-1.89	-1.89	-1.89
-1.88	-1.88	-1.88	-1.88	-1.88	-1.88	-1.88	-1.88	-1.88
-1.87	-1.87	-1.87	-1.87	-1.87	-1.87	-1.87	-1.87	-1.87
-1.86	-1.86	-1.86	-1.86	-1.86	-1.86	-1.86	-1.86	-1.86
-1.85	-1.85	-1.85	-1.85	-1.85	-1.85	-1.85	-1.85	-1.85
-1.84	-1.84	-1.84	-1.84	-1.84	-1.84	-1.84	-1.84	-1.84
-1.83	-1.83	-1.83	-1.83	-1.83	-1.83	-1.83	-1.83	-1.83
-1.82	-1.82	-1.82	-1.82	-1.82	-1.82	-1.82	-1.82	-1.82
-1.81	-1.81	-1.81	-1.81	-1.81	-1.81	-1.81	-1.81	-1.81
-1.8	-1.8	-1.8	-1.8	-1.8	-1.8	-1.8	-1.8	-1.8
-1.79	-1.79	-1.79	-1.79	-1.79	-1.79	-1.79	-1.79	-1.79
-1.78	-1.78	-1.78	-1.78	-1.78	-1.78	-1.78	-1.78	-1.78
-1.77	-1.77	-1.77	-1.77	-1.77	-1.77	-1.77	-1.77	-1.77
-1.76	-1.76	-1.76	-1.76	-1.76	-1.76	-1.76	-1.76	-1.76
-1.75	-1.75	-1.75	-1.75	-1.75	-1.75	-1.75	-1.75	-1.75
-1.74	-1.74	-1.74	-1.74	-1.74	-1.74	-1.74	-1.74	-1.74
-1.73	-1.73	-1.73	-1.73	-1.73	-1.73	-1.73	-1.73	-1.73
-1.72	-1.72	-1.72	-1.72	-1.72	-1.72	-1.72	-1.72	-1.72
-1.71	-1.71	-1.71	-1.71	-1.71	-1.71	-1.71	-1.71	-1.71
-1.7	-1.7	-1.7	-1.7	-1.7	-1.7	-1.7	-1.7	-1.7
-1.69	-1.69	-1.69	-1.69	-1.69	-1.69	-1.69	-1.69	-1.69
-1.68	-1.68	-1.68	-1.68	-1.68	-1.68	-1.68	-1.68	-1.68
-1.67	-1.67	-1.67	-1.67	-1.67	-1.67	-1.67	-1.67	-1.67
-1.66	-1.66	-1.66	-1.66	-1.66	-1.66	-1.66	-1.66	-1.66
-1.65	-1.65	-1.65	-1.65	-1.65	-1.65	-1.65	-1.65	-1.65
-1.64	-1.64	-1.64	-1.64	-1.64	-1.64	-1.64	-1.64	-1.64
-1.63	-1.63	-1.63	-1.63	-1.63	-1.63	-1.63	-1.63	-1.63
-1.62	-1.62	-1.62	-1.62	-1.62	-1.62	-1.62	-1.62	-1.62
-1.61	-1.61	-1.61	-1.61	-1.61	-1.61	-1.61	-1.61	-1.61
-1.6	-1.6	-1.6	-1.6	-1.6	-1.6	-1.6	-1.6	-1.6
-1.59	-1.59	-1.59	-1.59	-1.59	-1.59	-1.59	-1.59	-1.59
-1.58	-1.58	-1.58	-1.58	-1.58	-1.58	-1.58	-1.58	-1.58
-1.57	-1.57	-1.57	-1.57	-1.57	-1.57	-1.57	-1.57	-1.57
-1.56	-1.56	-1.56	-1.56	-1.56	-1.56	-1.56	-1.56	-1.56
-1.55	-1.55	-1.55	-1.55	-1.55	-1.55	-1.55	-1.55	-1.55
-1.54	-1.54	-1.54	-1.54	-1.54	-1.54	-1.54	-1.54	-1.54
-1.53	-1.53	-1.53	-1.53	-1.53	-1.53	-1.53	-1.53	-1.53
-1.52	-1.52	-1.52	-1.52	-1.52	-1.52	-1.52	-1.52	-1.52
-1.51	-1.51	-1.51	-1.51	-1.51	-1.51	-1.51	-1.51	-1.51

-1.5	-1.5	-1.5	-1.5	-1.5	-1.5	-1.5	-1.5	-1.5
-1.49	-1.49	-1.49	-1.49	-1.49	-1.49	-1.49	-1.49	-1.49
-1.48	-1.48	-1.48	-1.48	-1.48	-1.48	-1.48	-1.48	-1.48
-1.47	-1.47	-1.47	-1.47	-1.47	-1.47	-1.47	-1.47	-1.47
-1.46	-1.46	-1.46	-1.46	-1.46	-1.46	-1.46	-1.46	-1.46
-1.45	-1.45	-1.45	-1.45	-1.45	-1.45	-1.45	-1.45	-1.45
-1.44	-1.44	-1.44	-1.44	-1.44	-1.44	-1.44	-1.44	-1.44
-1.43	-1.43	-1.43	-1.43	-1.43	-1.43	-1.43	-1.43	-1.43
-1.42	-1.42	-1.42	-1.42	-1.42	-1.42	-1.42	-1.42	-1.42
-1.41	-1.41	-1.41	-1.41	-1.41	-1.41	-1.41	-1.41	-1.41
-1.4	-1.4	-1.4	-1.4	-1.4	-1.4	-1.4	-1.4	-1.4
-1.39	-1.39	-1.39	-1.39	-1.39	-1.39	-1.39	-1.39	-1.39
-1.38	-1.38	-1.38	-1.38	-1.38	-1.38	-1.38	-1.38	-1.38
-1.37	-1.37	-1.37	-1.37	-1.37	-1.37	-1.37	-1.37	-1.37
-1.36	-1.36	-1.36	-1.36	-1.36	-1.36	-1.36	-1.36	-1.36
-1.35	-1.35	-1.35	-1.35	-1.35	-1.35	-1.35	-1.35	-1.35
-1.34	-1.34	-1.34	-1.34	-1.34	-1.34	-1.34	-1.34	-1.34
-1.33	-1.33	-1.33	-1.33	-1.33	-1.33	-1.33	-1.33	-1.33
-1.32	-1.32	-1.32	-1.32	-1.32	-1.32	-1.32	-1.32	-1.32
-1.31	-1.31	-1.31	-1.31	-1.31	-1.31	-1.31	-1.31	-1.31
-1.3	-1.3	-1.3	-1.3	-1.3	-1.3	-1.3	-1.3	-1.3
-1.29	-1.29	-1.29	-1.29	-1.29	-1.29	-1.29	-1.29	-1.29
-1.28	-1.28	-1.28	-1.28	-1.28	-1.28	-1.28	-1.28	-1.28
-1.27	-1.27	-1.27	-1.27	-1.27	-1.27	-1.27	-1.27	-1.27
-1.26	-1.26	-1.26	-1.26	-1.26	-1.26	-1.26	-1.26	-1.26
-1.25	-1.25	-1.25	-1.25	-1.25	-1.25	-1.25	-1.25	-1.25
-1.24	-1.24	-1.24	-1.24	-1.24	-1.24	-1.24	-1.24	-1.24
-1.23	-1.23	-1.23	-1.23	-1.23	-1.23	-1.23	-1.23	-1.23
-1.22	-1.22	-1.22	-1.22	-1.22	-1.22	-1.22	-1.22	-1.22
-1.21	-1.21	-1.21	-1.21	-1.21	-1.21	-1.21	-1.21	-1.21
-1.2	-1.2	-1.2	-1.2	-1.2	-1.2	-1.2	-1.2	-1.2
-1.19	-1.19	-1.19	-1.19	-1.19	-1.19	-1.19	-1.19	-1.19
-1.18	-1.18	-1.18	-1.18	-1.18	-1.18	-1.18	-1.18	-1.18
-1.17	-1.17	-1.17	-1.17	-1.17	-1.17	-1.17	-1.17	-1.17
-1.16	-1.16	-1.16	-1.16	-1.16	-1.16	-1.16	-1.16	-1.16
-1.15	-1.15	-1.15	-1.15	-1.15	-1.15	-1.15	-1.15	-1.15
-1.14	-1.14	-1.14	-1.14	-1.14	-1.14	-1.14	-1.14	-1.14
-1.13	-1.13	-1.13	-1.13	-1.13	-1.13	-1.13	-1.13	-1.13
-1.12	-1.12	-1.12	-1.12	-1.12	-1.12	-1.12	-1.12	-1.12
-1.11	-1.11	-1.11	-1.11	-1.11	-1.11	-1.11	-1.11	-1.11
-1.1	-1.1	-1.1	-1.1	-1.1	-1.1	-1.1	-1.1	-1.1
-1.09	-1.09	-1.09	-1.09	-1.09	-1.09	-1.09	-1.09	-1.09
-1.08	-1.08	-1.08	-1.08	-1.08	-1.08	-1.08	-1.08	-1.08
-1.07	-1.07	-1.07	-1.07	-1.07	-1.07	-1.07	-1.07	-1.07
-1.06	-1.06	-1.06	-1.06	-1.06	-1.06	-1.06	-1.06	-1.06
-1.05	-1.05	-1.05	-1.05	-1.05	-1.05	-1.05	-1.05	-1.05
-1.04	-1.04	-1.04	-1.04	-1.04	-1.04	-1.04	-1.04	-1.04
-1.03	-1.03	-1.03	-1.03	-1.03	-1.03	-1.03	-1.03	-1.03
-1.02	-1.02	-1.02	-1.02	-1.02	-1.02	-1.02	-1.02	-1.02
-1.01	-1.01	-1.01	-1.01	-1.01	-1.01	-1.01	-1.01	-1.01

-1	-1	-1	-1	-1	-1	-1	-1	-1
-0.99	-0.99	-0.99	-0.99	-0.99	-0.99	-0.99	-0.99	-0.99
-0.98	-0.98	-0.98	-0.98	-0.98	-0.98	-0.98	-0.98	-0.98
-0.97	-0.97	-0.97	-0.97	-0.97	-0.97	-0.97	-0.97	-0.97
-0.96	-0.96	-0.96	-0.96	-0.96	-0.96	-0.96	-0.96	-0.96
-0.95	-0.95	-0.95	-0.95	-0.95	-0.95	-0.95	-0.95	-0.95
-0.94	-0.94	-0.94	-0.94	-0.94	-0.94	-0.94	-0.94	-0.94
-0.93	-0.93	-0.93	-0.93	-0.93	-0.93	-0.93	-0.93	-0.93
-0.92	-0.92	-0.92	-0.92	-0.92	-0.92	-0.92	-0.92	-0.92
-0.91	-0.91	-0.91	-0.91	-0.91	-0.91	-0.91	-0.91	-0.91
-0.9	-0.9	-0.9	-0.9	-0.9	-0.9	-0.9	-0.9	-0.9
-0.89	-0.89	-0.89	-0.89	-0.89	-0.89	-0.89	-0.89	-0.89
-0.88	-0.88	-0.88	-0.88	-0.88	-0.88	-0.88	-0.88	-0.88
-0.87	-0.87	-0.87	-0.87	-0.87	-0.87	-0.87	-0.87	-0.87
-0.86	-0.86	-0.86	-0.86	-0.86	-0.86	-0.86	-0.86	-0.86
-0.85	-0.85	-0.85	-0.85	-0.85	-0.85	-0.85	-0.85	-0.85
-0.84	-0.84	-0.84	-0.84	-0.84	-0.84	-0.84	-0.84	-0.84
-0.83	-0.83	-0.83	-0.83	-0.83	-0.83	-0.83	-0.83	-0.83
-0.82	-0.82	-0.82	-0.82	-0.82	-0.82	-0.82	-0.82	-0.82
-0.81	-0.81	-0.81	-0.81	-0.81	-0.81	-0.81	-0.81	-0.81
-0.8	-0.8	-0.8	-0.8	-0.8	-0.8	-0.8	-0.8	-0.8
-0.79	-0.79	-0.79	-0.79	-0.79	-0.79	-0.79	-0.79	-0.79
-0.78	-0.78	-0.78	-0.78	-0.78	-0.78	-0.78	-0.78	-0.78
-0.77	-0.77	-0.77	-0.77	-0.77	-0.77	-0.77	-0.77	-0.77
-0.76	-0.76	-0.76	-0.76	-0.76	-0.76	-0.76	-0.76	-0.76
-0.75	-0.75	-0.75	-0.75	-0.75	-0.75	-0.75	-0.75	-0.75
-0.74	-0.74	-0.74	-0.74	-0.74	-0.74	-0.74	-0.74	-0.74
-0.73	-0.73	-0.73	-0.73	-0.73	-0.73	-0.73	-0.73	-0.73
-0.72	-0.72	-0.72	-0.72	-0.72	-0.72	-0.72	-0.72	-0.72
-0.71	-0.71	-0.71	-0.71	-0.71	-0.71	-0.71	-0.71	-0.71
-0.7	-0.7	-0.7	-0.7	-0.7	-0.7	-0.7	-0.7	-0.7
-0.69	-0.69	-0.69	-0.69	-0.69	-0.69	-0.69	-0.69	-0.69
-0.68	-0.68	-0.68	-0.68	-0.68	-0.68	-0.68	-0.68	-0.68
-0.67	-0.67	-0.67	-0.67	-0.67	-0.67	-0.67	-0.67	-0.67
-0.66	-0.66	-0.66	-0.66	-0.66	-0.66	-0.66	-0.66	-0.66
-0.65	-0.65	-0.65	-0.65	-0.65	-0.65	-0.65	-0.65	-0.65
-0.64	-0.64	-0.64	-0.64	-0.64	-0.64	-0.64	-0.64	-0.64
-0.63	-0.63	-0.63	-0.63	-0.63	-0.63	-0.63	-0.63	-0.63
-0.62	-0.62	-0.62	-0.62	-0.62	-0.62	-0.62	-0.62	-0.62
-0.61	-0.61	-0.61	-0.61	-0.61	-0.61	-0.61	-0.61	-0.61
-0.6	-0.6	-0.6	-0.6	-0.6	-0.6	-0.6	-0.6	-0.6
-0.59	-0.59	-0.59	-0.59	-0.59	-0.59	-0.59	-0.59	-0.59
-0.58	-0.58	-0.58	-0.58	-0.58	-0.58	-0.58	-0.58	-0.58
-0.57	-0.57	-0.57	-0.57	-0.57	-0.57	-0.57	-0.57	-0.57
-0.56	-0.56	-0.56	-0.56	-0.56	-0.56	-0.56	-0.56	-0.56
-0.55	-0.55	-0.55	-0.55	-0.55	-0.55	-0.55	-0.55	-0.55
-0.54	-0.54	-0.54	-0.54	-0.54	-0.54	-0.54	-0.54	-0.54
-0.53	-0.53	-0.53	-0.53	-0.53	-0.53	-0.53	-0.53	-0.53
-0.52	-0.52	-0.52	-0.52	-0.52	-0.52	-0.52	-0.52	-0.52
-0.51	-0.51	-0.51	-0.51	-0.51	-0.51	-0.51	-0.51	-0.51

-0.5	-0.5	-0.5	-0.5	-0.5	-0.5	-0.5	-0.5	-0.5
-0.49	-0.49	-0.49	-0.49	-0.49	-0.49	-0.49	-0.49	-0.49
-0.48	-0.48	-0.48	-0.48	-0.48	-0.48	-0.48	-0.48	-0.48
-0.47	-0.47	-0.47	-0.47	-0.47	-0.47	-0.47	-0.47	-0.47
-0.46	-0.46	-0.46	-0.46	-0.46	-0.46	-0.46	-0.46	-0.46
-0.45	-0.45	-0.45	-0.45	-0.45	-0.45	-0.45	-0.45	-0.45
-0.44	-0.44	-0.44	-0.44	-0.44	-0.44	-0.44	-0.44	-0.44
-0.43	-0.43	-0.43	-0.43	-0.43	-0.43	-0.43	-0.43	-0.43
-0.42	-0.42	-0.42	-0.42	-0.42	-0.42	-0.42	-0.42	-0.42
-0.41	-0.41	-0.41	-0.41	-0.41	-0.41	-0.41	-0.41	-0.41
-0.4	-0.4	-0.4	-0.4	-0.4	-0.4	-0.4	-0.4	-0.4
-0.39	-0.39	-0.39	-0.39	-0.39	-0.39	-0.39	-0.39	-0.39
-0.38	-0.38	-0.38	-0.38	-0.38	-0.38	-0.38	-0.38	-0.38
-0.37	-0.37	-0.37	-0.37	-0.37	-0.37	-0.37	-0.37	-0.37
-0.36	-0.36	-0.36	-0.36	-0.36	-0.36	-0.36	-0.36	-0.36
-0.35	-0.35	-0.35	-0.35	-0.35	-0.35	-0.35	-0.35	-0.35
-0.34	-0.34	-0.34	-0.34	-0.34	-0.34	-0.34	-0.34	-0.34
-0.33	-0.33	-0.33	-0.33	-0.33	-0.33	-0.33	-0.33	-0.33
-0.32	-0.32	-0.32	-0.32	-0.32	-0.32	-0.32	-0.32	-0.32
-0.31	-0.31	-0.31	-0.31	-0.31	-0.31	-0.31	-0.31	-0.31
-0.3	-0.3	-0.3	-0.3	-0.3	-0.3	-0.3	-0.3	-0.3
-0.29	-0.29	-0.29	-0.29	-0.29	-0.29	-0.29	-0.29	-0.29
-0.28	-0.28	-0.28	-0.28	-0.28	-0.28	-0.28	-0.28	-0.28
-0.27	-0.27	-0.27	-0.27	-0.27	-0.27	-0.27	-0.27	-0.27
-0.26	-0.26	-0.26	-0.26	-0.26	-0.26	-0.26	-0.26	-0.26
-0.25	-0.25	-0.25	-0.25	-0.25	-0.25	-0.25	-0.25	-0.25
-0.24	-0.24	-0.24	-0.24	-0.24	-0.24	-0.24	-0.24	-0.24
-0.23	-0.23	-0.23	-0.23	-0.23	-0.23	-0.23	-0.23	-0.23
-0.22	-0.22	-0.22	-0.22	-0.22	-0.22	-0.22	-0.22	-0.22
-0.21	-0.21	-0.21	-0.21	-0.21	-0.21	-0.21	-0.21	-0.21
-0.2	-0.2	-0.2	-0.2	-0.2	-0.2	-0.2	-0.2	-0.2
-0.19	-0.19	-0.19	-0.19	-0.19	-0.19	-0.19	-0.19	-0.19
-0.18	-0.18	-0.18	-0.18	-0.18	-0.18	-0.18	-0.18	-0.18
-0.17	-0.17	-0.17	-0.17	-0.17	-0.17	-0.17	-0.17	-0.17
-0.16	-0.16	-0.16	-0.16	-0.16	-0.16	-0.16	-0.16	-0.16
-0.15	-0.15	-0.15	-0.15	-0.15	-0.15	-0.15	-0.15	-0.15
-0.14	-0.14	-0.14	-0.14	-0.14	-0.14	-0.14	-0.14	-0.14
-0.13	-0.13	-0.13	-0.13	-0.13	-0.13	-0.13	-0.13	-0.13
-0.12	-0.12	-0.12	-0.12	-0.12	-0.12	-0.12	-0.12	-0.12
-0.11	-0.11	-0.11	-0.11	-0.11	-0.11	-0.11	-0.11	-0.11
-0.1	-0.1	-0.1	-0.1	-0.1	-0.1	-0.1	-0.1	-0.1
-0.09	-0.09	-0.09	-0.09	-0.09	-0.09	-0.09	-0.09	-0.09
-0.08	-0.08	-0.08	-0.08	-0.08	-0.08	-0.08	-0.08	-0.08
-0.07	-0.07	-0.07	-0.07	-0.07	-0.07	-0.07	-0.07	-0.07
-0.06	-0.06	-0.06	-0.06	-0.06	-0.06	-0.06	-0.06	-0.06
-0.05	-0.05	-0.05	-0.05	-0.05	-0.05	-0.05	-0.05	-0.05
-0.04	-0.04	-0.04	-0.04	-0.04	-0.04	-0.04	-0.04	-0.04
-0.03	-0.03	-0.03	-0.03	-0.03	-0.03	-0.03	-0.03	-0.03
-0.02	-0.02	-0.02	-0.02	-0.02	-0.02	-0.02	-0.02	-0.02
-0.01	-0.01	-0.01	-0.01	-0.01	-0.01	-0.01	-0.01	-0.01

0	0	0	0	0	0	0	0	0
0.01	0.01	0.01	0.01	0.01	0.01	0.01	0.01	0.01
0.02	0.02	0.02	0.02	0.02	0.02	0.02	0.02	0.02
0.03	0.03	0.03	0.03	0.03	0.03	0.03	0.03	0.03
0.04	0.04	0.04	0.04	0.04	0.04	0.04	0.04	0.04
0.05	0.05	0.05	0.05	0.05	0.05	0.05	0.05	0.05
0.06	0.06	0.06	0.06	0.06	0.06	0.06	0.06	0.06
0.07	0.07	0.07	0.07	0.07	0.07	0.07	0.07	0.07
0.08	0.08	0.08	0.08	0.08	0.08	0.08	0.08	0.08
0.09	0.09	0.09	0.09	0.09	0.09	0.09	0.09	0.09
0.1	0.1	0.1	0.1	0.1	0.1	0.1	0.1	0.1
0.11	0.11	0.11	0.11	0.11	0.11	0.11	0.11	0.11
0.12	0.12	0.12	0.12	0.12	0.12	0.12	0.12	0.12
0.13	0.13	0.13	0.13	0.13	0.13	0.13	0.13	0.13
0.14	0.14	0.14	0.14	0.14	0.14	0.14	0.14	0.14
0.15	0.15	0.15	0.15	0.15	0.15	0.15	0.15	0.15
0.16	0.16	0.16	0.16	0.16	0.16	0.16	0.16	0.16
0.17	0.17	0.17	0.17	0.17	0.17	0.17	0.17	0.17
0.18	0.18	0.18	0.18	0.18	0.18	0.18	0.18	0.18
0.19	0.19	0.19	0.19	0.19	0.19	0.19	0.19	0.19
0.2	0.2	0.2	0.2	0.2	0.2	0.2	0.2	0.2
0.21	0.21	0.21	0.21	0.21	0.21	0.21	0.21	0.21
0.22	0.22	0.22	0.22	0.22	0.22	0.22	0.22	0.22
0.23	0.23	0.23	0.23	0.23	0.23	0.23	0.23	0.23
0.24	0.24	0.24	0.24	0.24	0.24	0.24	0.24	0.24
0.25	0.25	0.25	0.25	0.25	0.25	0.25	0.25	0.25
0.26	0.26	0.26	0.26	0.26	0.26	0.26	0.26	0.26
0.27	0.27	0.27	0.27	0.27	0.27	0.27	0.27	0.27
0.28	0.28	0.28	0.28	0.28	0.28	0.28	0.28	0.28
0.29	0.29	0.29	0.29	0.29	0.29	0.29	0.29	0.29
0.3	0.3	0.3	0.3	0.3	0.3	0.3	0.3	0.3
0.31	0.31	0.31	0.31	0.31	0.31	0.31	0.31	0.31
0.32	0.32	0.32	0.32	0.32	0.32	0.32	0.32	0.32
0.33	0.33	0.33	0.33	0.33	0.33	0.33	0.33	0.33
0.34	0.34	0.34	0.34	0.34	0.34	0.34	0.34	0.34
0.35	0.35	0.35	0.35	0.35	0.35	0.35	0.35	0.35
0.36	0.36	0.36	0.36	0.36	0.36	0.36	0.36	0.36
0.37	0.37	0.37	0.37	0.37	0.37	0.37	0.37	0.37
0.38	0.38	0.38	0.38	0.38	0.38	0.38	0.38	0.38
0.39	0.39	0.39	0.39	0.39	0.39	0.39	0.39	0.39
0.4	0.4	0.4	0.4	0.4	0.4	0.4	0.4	0.4
0.41	0.41	0.41	0.41	0.41	0.41	0.41	0.41	0.41
0.42	0.42	0.42	0.42	0.42	0.42	0.42	0.42	0.42
0.43	0.43	0.43	0.43	0.43	0.43	0.43	0.43	0.43
0.44	0.44	0.44	0.44	0.44	0.44	0.44	0.44	0.44
0.45	0.45	0.45	0.45	0.45	0.45	0.45	0.45	0.45
0.46	0.46	0.46	0.46	0.46	0.46	0.46	0.46	0.46
0.47	0.47	0.47	0.47	0.47	0.47	0.47	0.47	0.47
0.48	0.48	0.48	0.48	0.48	0.48	0.48	0.48	0.48
0.49	0.49	0.49	0.49	0.49	0.49	0.49	0.49	0.49

0.5	0.5	0.5	0.5	0.5	0.5	0.5	0.5	0.5
0.51	0.51	0.51	0.51	0.51	0.51	0.51	0.51	0.51
0.52	0.52	0.52	0.52	0.52	0.52	0.52	0.52	0.52
0.53	0.53	0.53	0.53	0.53	0.53	0.53	0.53	0.53
0.54	0.54	0.54	0.54	0.54	0.54	0.54	0.54	0.54
0.55	0.55	0.55	0.55	0.55	0.55	0.55	0.55	0.55
0.56	0.56	0.56	0.56	0.56	0.56	0.56	0.56	0.56
0.57	0.57	0.57	0.57	0.57	0.57	0.57	0.57	0.57
0.58	0.58	0.58	0.58	0.58	0.58	0.58	0.58	0.58
0.59	0.59	0.59	0.59	0.59	0.59	0.59	0.59	0.59
0.6	0.6	0.6	0.6	0.6	0.6	0.6	0.6	0.6
0.61	0.61	0.61	0.61	0.61	0.61	0.61	0.61	0.61
0.62	0.62	0.62	0.62	0.62	0.62	0.62	0.62	0.62
0.63	0.63	0.63	0.63	0.63	0.63	0.63	0.63	0.63
0.64	0.64	0.64	0.64	0.64	0.64	0.64	0.64	0.64
0.65	0.65	0.65	0.65	0.65	0.65	0.65	0.65	0.65
0.66	0.66	0.66	0.66	0.66	0.66	0.66	0.66	0.66
0.67	0.67	0.67	0.67	0.67	0.67	0.67	0.67	0.67
0.68	0.68	0.68	0.68	0.68	0.68	0.68	0.68	0.68
0.69	0.69	0.69	0.69	0.69	0.69	0.69	0.69	0.69
0.7	0.7	0.7	0.7	0.7	0.7	0.7	0.7	0.7
0.71	0.71	0.71	0.71	0.71	0.71	0.71	0.71	0.71
0.72	0.72	0.72	0.72	0.72	0.72	0.72	0.72	0.72
0.73	0.73	0.73	0.73	0.73	0.73	0.73	0.73	0.73
0.74	0.74	0.74	0.74	0.74	0.74	0.74	0.74	0.74
0.75	0.75	0.75	0.75	0.75	0.75	0.75	0.75	0.75
0.76	0.76	0.76	0.76	0.76	0.76	0.76	0.76	0.76
0.77	0.77	0.77	0.77	0.77	0.77	0.77	0.77	0.77
0.78	0.78	0.78	0.78	0.78	0.78	0.78	0.78	0.78
0.79	0.79	0.79	0.79	0.79	0.79	0.79	0.79	0.79
0.8	0.8	0.8	0.8	0.8	0.8	0.8	0.8	0.8
0.81	0.81	0.81	0.81	0.81	0.81	0.81	0.81	0.81
0.82	0.82	0.82	0.82	0.82	0.82	0.82	0.82	0.82
0.83	0.83	0.83	0.83	0.83	0.83	0.83	0.83	0.83
0.84	0.84	0.84	0.84	0.84	0.84	0.84	0.84	0.84
0.85	0.85	0.85	0.85	0.85	0.85	0.85	0.85	0.85
0.86	0.86	0.86	0.86	0.86	0.86	0.86	0.86	0.86
0.87	0.87	0.87	0.87	0.87	0.87	0.87	0.87	0.87
0.88	0.88	0.88	0.88	0.88	0.88	0.88	0.88	0.88
0.89	0.89	0.89	0.89	0.89	0.89	0.89	0.89	0.89
0.9	0.9	0.9	0.9	0.9	0.9	0.9	0.9	0.9
0.91	0.91	0.91	0.91	0.91	0.91	0.91	0.91	0.91
0.92	0.92	0.92	0.92	0.92	0.92	0.92	0.92	0.92
0.93	0.93	0.93	0.93	0.93	0.93	0.93	0.93	0.93
0.94	0.94	0.94	0.94	0.94	0.94	0.94	0.94	0.94
0.95	0.95	0.95	0.95	0.95	0.95	0.95	0.95	0.95
0.96	0.96	0.96	0.96	0.96	0.96	0.96	0.96	0.96
0.97	0.97	0.97	0.97	0.97	0.97	0.97	0.97	0.97
0.98	0.98	0.98	0.98	0.98	0.98	0.98	0.98	0.98
0.99	0.99	0.99	0.99	0.99	0.99	0.99	0.99	0.99

1	1	1	1	1	1	1	1	1
1.01	1.01	1.01	1.01	1.01	1.01	1.01	1.01	1.01
1.02	1.02	1.02	1.02	1.02	1.02	1.02	1.02	1.02
1.03	1.03	1.03	1.03	1.03	1.03	1.03	1.03	1.03
1.04	1.04	1.04	1.04	1.04	1.04	1.04	1.04	1.04
1.05	1.05	1.05	1.05	1.05	1.05	1.05	1.05	1.05
1.06	1.06	1.06	1.06	1.06	1.06	1.06	1.06	1.06
1.07	1.07	1.07	1.07	1.07	1.07	1.07	1.07	1.07
1.08	1.08	1.08	1.08	1.08	1.08	1.08	1.08	1.08
1.09	1.09	1.09	1.09	1.09	1.09	1.09	1.09	1.09
1.1	1.1	1.1	1.1	1.1	1.1	1.1	1.1	1.1
1.11	1.11	1.11	1.11	1.11	1.11	1.11	1.11	1.11
1.12	1.12	1.12	1.12	1.12	1.12	1.12	1.12	1.12
1.13	1.13	1.13	1.13	1.13	1.13	1.13	1.13	1.13
1.14	1.14	1.14	1.14	1.14	1.14	1.14	1.14	1.14
1.15	1.15	1.15	1.15	1.15	1.15	1.15	1.15	1.15
1.16	1.16	1.16	1.16	1.16	1.16	1.16	1.16	1.16
1.17	1.17	1.17	1.17	1.17	1.17	1.17	1.17	1.17
1.18	1.18	1.18	1.18	1.18	1.18	1.18	1.18	1.18
1.19	1.19	1.19	1.19	1.19	1.19	1.19	1.19	1.19
1.2	1.2	1.2	1.2	1.2	1.2	1.2	1.2	1.2
1.21	1.21	1.21	1.21	1.21	1.21	1.21	1.21	1.21
1.22	1.22	1.22	1.22	1.22	1.22	1.22	1.22	1.22
1.23	1.23	1.23	1.23	1.23	1.23	1.23	1.23	1.23
1.24	1.24	1.24	1.24	1.24	1.24	1.24	1.24	1.24
1.25	1.25	1.25	1.25	1.25	1.25	1.25	1.25	1.25
1.26	1.26	1.26	1.26	1.26	1.26	1.26	1.26	1.26
1.27	1.27	1.27	1.27	1.27	1.27	1.27	1.27	1.27
1.28	1.28	1.28	1.28	1.28	1.28	1.28	1.28	1.28
1.29	1.29	1.29	1.29	1.29	1.29	1.29	1.29	1.29
1.3	1.3	1.3	1.3	1.3	1.3	1.3	1.3	1.3
1.31	1.31	1.31	1.31	1.31	1.31	1.31	1.31	1.31
1.32	1.32	1.32	1.32	1.32	1.32	1.32	1.32	1.32
1.33	1.33	1.33	1.33	1.33	1.33	1.33	1.33	1.33
1.34	1.34	1.34	1.34	1.34	1.34	1.34	1.34	1.34
1.35	1.35	1.35	1.35	1.35	1.35	1.35	1.35	1.35
1.36	1.36	1.36	1.36	1.36	1.36	1.36	1.36	1.36
1.37	1.37	1.37	1.37	1.37	1.37	1.37	1.37	1.37
1.38	1.38	1.38	1.38	1.38	1.38	1.38	1.38	1.38
1.39	1.39	1.39	1.39	1.39	1.39	1.39	1.39	1.39
1.4	1.4	1.4	1.4	1.4	1.4	1.4	1.4	1.4
1.41	1.41	1.41	1.41	1.41	1.41	1.41	1.41	1.41
1.42	1.42	1.42	1.42	1.42	1.42	1.42	1.42	1.42
1.43	1.43	1.43	1.43	1.43	1.43	1.43	1.43	1.43
1.44	1.44	1.44	1.44	1.44	1.44	1.44	1.44	1.44
1.45	1.45	1.45	1.45	1.45	1.45	1.45	1.45	1.45
1.46	1.46	1.46	1.46	1.46	1.46	1.46	1.46	1.46
1.47	1.47	1.47	1.47	1.47	1.47	1.47	1.47	1.47
1.48	1.48	1.48	1.48	1.48	1.48	1.48	1.48	1.48
1.49	1.49	1.49	1.49	1.49	1.49	1.49	1.49	1.49

1.5	1.5	1.5	1.5	1.5	1.5	1.5	1.5	1.5
1.51	1.51	1.51	1.51	1.51	1.51	1.51	1.51	1.51
1.52	1.52	1.52	1.52	1.52	1.52	1.52	1.52	1.52
1.53	1.53	1.53	1.53	1.53	1.53	1.53	1.53	1.53
1.54	1.54	1.54	1.54	1.54	1.54	1.54	1.54	1.54
1.55	1.55	1.55	1.55	1.55	1.55	1.55	1.55	1.55
1.56	1.56	1.56	1.56	1.56	1.56	1.56	1.56	1.56
1.57	1.57	1.57	1.57	1.57	1.57	1.57	1.57	1.57
1.58	1.58	1.58	1.58	1.58	1.58	1.58	1.58	1.58
1.59	1.59	1.59	1.59	1.59	1.59	1.59	1.59	1.59
1.6	1.6	1.6	1.6	1.6	1.6	1.6	1.6	1.6
1.61	1.61	1.61	1.61	1.61	1.61	1.61	1.61	1.61
1.62	1.62	1.62	1.62	1.62	1.62	1.62	1.62	1.62
1.63	1.63	1.63	1.63	1.63	1.63	1.63	1.63	1.63
1.64	1.64	1.64	1.64	1.64	1.64	1.64	1.64	1.64
1.65	1.65	1.65	1.65	1.65	1.65	1.65	1.65	1.65
1.66	1.66	1.66	1.66	1.66	1.66	1.66	1.66	1.66
1.67	1.67	1.67	1.67	1.67	1.67	1.67	1.67	1.67
1.68	1.68	1.68	1.68	1.68	1.68	1.68	1.68	1.68
1.69	1.69	1.69	1.69	1.69	1.69	1.69	1.69	1.69
1.7	1.7	1.7	1.7	1.7	1.7	1.7	1.7	1.7
1.71	1.71	1.71	1.71	1.71	1.71	1.71	1.71	1.71
1.72	1.72	1.72	1.72	1.72	1.72	1.72	1.72	1.72
1.73	1.73	1.73	1.73	1.73	1.73	1.73	1.73	1.73
1.74	1.74	1.74	1.74	1.74	1.74	1.74	1.74	1.74
1.75	1.75	1.75	1.75	1.75	1.75	1.75	1.75	1.75
1.76	1.76	1.76	1.76	1.76	1.76	1.76	1.76	1.76
1.77	1.77	1.77	1.77	1.77	1.77	1.77	1.77	1.77
1.78	1.78	1.78	1.78	1.78	1.78	1.78	1.78	1.78
1.79	1.79	1.79	1.79	1.79	1.79	1.79	1.79	1.79
1.8	1.8	1.8	1.8	1.8	1.8	1.8	1.8	1.8
1.81	1.81	1.81	1.81	1.81	1.81	1.81	1.81	1.81
1.82	1.82	1.82	1.82	1.82	1.82	1.82	1.82	1.82
1.83	1.83	1.83	1.83	1.83	1.83	1.83	1.83	1.83
1.84	1.84	1.84	1.84	1.84	1.84	1.84	1.84	1.84
1.85	1.85	1.85	1.85	1.85	1.85	1.85	1.85	1.85
1.86	1.86	1.86	1.86	1.86	1.86	1.86	1.86	1.86
1.87	1.87	1.87	1.87	1.87	1.87	1.87	1.87	1.87
1.88	1.88	1.88	1.88	1.88	1.88	1.88	1.88	1.88
1.89	1.89	1.89	1.89	1.89	1.89	1.89	1.89	1.89
1.9	1.9	1.9	1.9	1.9	1.9	1.9	1.9	1.9
1.91	1.91	1.91	1.91	1.91	1.91	1.91	1.91	1.91
1.92	1.92	1.92	1.92	1.92	1.92	1.92	1.92	1.92
1.93	1.93	1.93	1.93	1.93	1.93	1.93	1.93	1.93
1.94	1.94	1.94	1.94	1.94	1.94	1.94	1.94	1.94
1.95	1.95	1.95	1.95	1.95	1.95	1.95	1.95	1.95
1.96	1.96	1.96	1.96	1.96	1.96	1.96	1.96	1.96
1.97	1.97	1.97	1.97	1.97	1.97	1.97	1.97	1.97
1.98	1.98	1.98	1.98	1.98	1.98	1.98	1.98	1.98
1.99	1.99	1.99	1.99	1.99	1.99	1.99	1.99	1.99

2	2	2	2	2	2	2	2	2
2.01	2.01	2.01	2.01	2.01	2.01	2.01	2.01	2.01
2.02	2.02	2.02	2.02	2.02	2.02	2.02	2.02	2.02
2.03	2.03	2.03	2.03	2.03	2.03	2.03	2.03	2.03
2.04	2.04	2.04	2.04	2.04	2.04	2.04	2.04	2.04
2.05	2.05	2.05	2.05	2.05	2.05	2.05	2.05	2.05
2.06	2.06	2.06	2.06	2.06	2.06	2.06	2.06	2.06
2.07	2.07	2.07	2.07	2.07	2.07	2.07	2.07	2.07
2.08	2.08	2.08	2.08	2.08	2.08	2.08	2.08	2.08
2.09	2.09	2.09	2.09	2.09	2.09	2.09	2.09	2.09
2.1	2.1	2.1	2.1	2.1	2.1	2.1	2.1	2.1
2.11	2.11	2.11	2.11	2.11	2.11	2.11	2.11	2.11
2.12	2.12	2.12	2.12	2.12	2.12	2.12	2.12	2.12
2.13	2.13	2.13	2.13	2.13	2.13	2.13	2.13	2.13
2.14	2.14	2.14	2.14	2.14	2.14	2.14	2.14	2.14
2.15	2.15	2.15	2.15	2.15	2.15	2.15	2.15	2.15
2.16	2.16	2.16	2.16	2.16	2.16	2.16	2.16	2.16
2.17	2.17	2.17	2.17	2.17	2.17	2.17	2.17	2.17
2.18	2.18	2.18	2.18	2.18	2.18	2.18	2.18	2.18
2.19	2.19	2.19	2.19	2.19	2.19	2.19	2.19	2.19
2.2	2.2	2.2	2.2	2.2	2.2	2.2	2.2	2.2
2.21	2.21	2.21	2.21	2.21	2.21	2.21	2.21	2.21
2.22	2.22	2.22	2.22	2.22	2.22	2.22	2.22	2.22
2.23	2.23	2.23	2.23	2.23	2.23	2.23	2.23	2.23
2.24	2.24	2.24	2.24	2.24	2.24	2.24	2.24	2.24
2.25	2.25	2.25	2.25	2.25	2.25	2.25	2.25	2.25
2.26	2.26	2.26	2.26	2.26	2.26	2.26	2.26	2.26
2.27	2.27	2.27	2.27	2.27	2.27	2.27	2.27	2.27
2.28	2.28	2.28	2.28	2.28	2.28	2.28	2.28	2.28
2.29	2.29	2.29	2.29	2.29	2.29	2.29	2.29	2.29
2.3	2.3	2.3	2.3	2.3	2.3	2.3	2.3	2.3
2.31	2.31	2.31	2.31	2.31	2.31	2.31	2.31	2.31
2.32	2.32	2.32	2.32	2.32	2.32	2.32	2.32	2.32
2.33	2.33	2.33	2.33	2.33	2.33	2.33	2.33	2.33
2.34	2.34	2.34	2.34	2.34	2.34	2.34	2.34	2.34
2.35	2.35	2.35	2.35	2.35	2.35	2.35	2.35	2.35
2.36	2.36	2.36	2.36	2.36	2.36	2.36	2.36	2.36
2.37	2.37	2.37	2.37	2.37	2.37	2.37	2.37	2.37
2.38	2.38	2.38	2.38	2.38	2.38	2.38	2.38	2.38
2.39	2.39	2.39	2.39	2.39	2.39	2.39	2.39	2.39
2.4	2.4	2.4	2.4	2.4	2.4	2.4	2.4	2.4
2.41	2.41	2.41	2.41	2.41	2.41	2.41	2.41	2.41
2.42	2.42	2.42	2.42	2.42	2.42	2.42	2.42	2.42
2.43	2.43	2.43	2.43	2.43	2.43	2.43	2.43	2.43
2.44	2.44	2.44	2.44	2.44	2.44	2.44	2.44	2.44
2.45	2.45	2.45	2.45	2.45	2.45	2.45	2.45	2.45
2.46	2.46	2.46	2.46	2.46	2.46	2.46	2.46	2.46
2.47	2.47	2.47	2.47	2.47	2.47	2.47	2.47	2.47
2.48	2.48	2.48	2.48	2.48	2.48	2.48	2.48	2.48
2.49	2.49	2.49	2.49	2.49	2.49	2.49	2.49	2.49

2.5	2.5	2.5	2.5	2.5	2.5	2.5	2.5	2.5
2.51	2.51	2.51	2.51	2.51	2.51	2.51	2.51	2.51
2.52	2.52	2.52	2.52	2.52	2.52	2.52	2.52	2.52
2.53	2.53	2.53	2.53	2.53	2.53	2.53	2.53	2.53
2.54	2.54	2.54	2.54	2.54	2.54	2.54	2.54	2.54
2.55	2.55	2.55	2.55	2.55	2.55	2.55	2.55	2.55
2.56	2.56	2.56	2.56	2.56	2.56	2.56	2.56	2.56
2.57	2.57	2.57	2.57	2.57	2.57	2.57	2.57	2.57
2.58	2.58	2.58	2.58	2.58	2.58	2.58	2.58	2.58
2.59	2.59	2.59	2.59	2.59	2.59	2.59	2.59	2.59
2.6	2.6	2.6	2.6	2.6	2.6	2.6	2.6	2.6
2.61	2.61	2.61	2.61	2.61	2.61	2.61	2.61	2.61
2.62	2.62	2.62	2.62	2.62	2.62	2.62	2.62	2.62
2.63	2.63	2.63	2.63	2.63	2.63	2.63	2.63	2.63
2.64	2.64	2.64	2.64	2.64	2.64	2.64	2.64	2.64
2.65	2.65	2.65	2.65	2.65	2.65	2.65	2.65	2.65
2.66	2.66	2.66	2.66	2.66	2.66	2.66	2.66	2.66
2.67	2.67	2.67	2.67	2.67	2.67	2.67	2.67	2.67
2.68	2.68	2.68	2.68	2.68	2.68	2.68	2.68	2.68
2.69	2.69	2.69	2.69	2.69	2.69	2.69	2.69	2.69
2.7	2.7	2.7	2.7	2.7	2.7	2.7	2.7	2.7
2.71	2.71	2.71	2.71	2.71	2.71	2.71	2.71	2.71
2.72	2.72	2.72	2.72	2.72	2.72	2.72	2.72	2.72
2.73	2.73	2.73	2.73	2.73	2.73	2.73	2.73	2.73
2.74	2.74	2.74	2.74	2.74	2.74	2.74	2.74	2.74
2.75	2.75	2.75	2.75	2.75	2.75	2.75	2.75	2.75
2.76	2.76	2.76	2.76	2.76	2.76	2.76	2.76	2.76
2.77	2.77	2.77	2.77	2.77	2.77	2.77	2.77	2.77
2.78	2.78	2.78	2.78	2.78	2.78	2.78	2.78	2.78
2.79	2.79	2.79	2.79	2.79	2.79	2.79	2.79	2.79
2.8	2.8	2.8	2.8	2.8	2.8	2.8	2.8	2.8
2.81	2.81	2.81	2.81	2.81	2.81	2.81	2.81	2.81
2.82	2.82	2.82	2.82	2.82	2.82	2.82	2.82	2.82
2.83	2.83	2.83	2.83	2.83	2.83	2.83	2.83	2.83
2.84	2.84	2.84	2.84	2.84	2.84	2.84	2.84	2.84
2.85	2.85	2.85	2.85	2.85	2.85	2.85	2.85	2.85
2.86	2.86	2.86	2.86	2.86	2.86	2.86	2.86	2.86
2.87	2.87	2.87	2.87	2.87	2.87	2.87	2.87	2.87
2.88	2.88	2.88	2.88	2.88	2.88	2.88	2.88	2.88
2.89	2.89	2.89	2.89	2.89	2.89	2.89	2.89	2.89
2.9	2.9	2.9	2.9	2.9	2.9	2.9	2.9	2.9
2.91	2.91	2.91	2.91	2.91	2.91	2.91	2.91	2.91
2.92	2.92	2.92	2.92	2.92	2.92	2.92	2.92	2.92
2.93	2.93	2.93	2.93	2.93	2.93	2.93	2.93	2.93
2.94	2.94	2.94	2.94	2.94	2.94	2.94	2.94	2.94
2.95	2.95	2.95	2.95	2.95	2.95	2.95	2.95	2.95
2.96	2.96	2.96	2.96	2.96	2.96	2.96	2.96	2.96
2.97	2.97	2.97	2.97	2.97	2.97	2.97	2.97	2.97
2.98	2.98	2.98	2.98	2.98	2.98	2.98	2.98	2.98
2.99	2.99	2.99	2.99	2.99	2.99	2.99	2.99	2.99

3	3	3	3	3	3	3	3	3
3.01	3.01	3.01	3.01	3.01	3.01	3.01	3.01	3.01
3.02	3.02	3.02	3.02	3.02	3.02	3.02	3.02	3.02
3.03	3.03	3.03	3.03	3.03	3.03	3.03	3.03	3.03
3.04	3.04	3.04	3.04	3.04	3.04	3.04	3.04	3.04
3.05	3.05	3.05	3.05	3.05	3.05	3.05	3.05	3.05
3.06	3.06	3.06	3.06	3.06	3.06	3.06	3.06	3.06
3.07	3.07	3.07	3.07	3.07	3.07	3.07	3.07	3.07
3.08	3.08	3.08	3.08	3.08	3.08	3.08	3.08	3.08
3.09	3.09	3.09	3.09	3.09	3.09	3.09	3.09	3.09
3.1	3.1	3.1	3.1	3.1	3.1	3.1	3.1	3.1
3.11	3.11	3.11	3.11	3.11	3.11	3.11	3.11	3.11
3.12	3.12	3.12	3.12	3.12	3.12	3.12	3.12	3.12
3.13	3.13	3.13	3.13	3.13	3.13	3.13	3.13	3.13
3.14	3.14	3.14	3.14	3.14	3.14	3.14	3.14	3.14
3.15	3.15	3.15	3.15	3.15	3.15	3.15	3.15	3.15
3.16	3.16	3.16	3.16	3.16	3.16	3.16	3.16	3.16
3.17	3.17	3.17	3.17	3.17	3.17	3.17	3.17	3.17
3.18	3.18	3.18	3.18	3.18	3.18	3.18	3.18	3.18
3.19	3.19	3.19	3.19	3.19	3.19	3.19	3.19	3.19
3.2	3.2	3.2	3.2	3.2	3.2	3.2	3.2	3.2
3.21	3.21	3.21	3.21	3.21	3.21	3.21	3.21	3.21
3.22	3.22	3.22	3.22	3.22	3.22	3.22	3.22	3.22
3.23	3.23	3.23	3.23	3.23	3.23	3.23	3.23	3.23
3.24	3.24	3.24	3.24	3.24	3.24	3.24	3.24	3.24
3.25	3.25	3.25	3.25	3.25	3.25	3.25	3.25	3.25
3.26	3.26	3.26	3.26	3.26	3.26	3.26	3.26	3.26
3.27	3.27	3.27	3.27	3.27	3.27	3.27	3.27	3.27
3.28	3.28	3.28	3.28	3.28	3.28	3.28	3.28	3.28
3.29	3.29	3.29	3.29	3.29	3.29	3.29	3.29	3.29
3.3	3.3	3.3	3.3	3.3	3.3	3.3	3.3	3.3
3.31	3.31	3.31	3.31	3.31	3.31	3.31	3.31	3.31
3.32	3.32	3.32	3.32	3.32	3.32	3.32	3.32	3.32
3.33	3.33	3.33	3.33	3.33	3.33	3.33	3.33	3.33
3.34	3.34	3.34	3.34	3.34	3.34	3.34	3.34	3.34
3.35	3.35	3.35	3.35	3.35	3.35	3.35	3.35	3.35
3.36	3.36	3.36	3.36	3.36	3.36	3.36	3.36	3.36
3.37	3.37	3.37	3.37	3.37	3.37	3.37	3.37	3.37
3.38	3.38	3.38	3.38	3.38	3.38	3.38	3.38	3.38
3.39	3.39	3.39	3.39	3.39	3.39	3.39	3.39	3.39
3.4	3.4	3.4	3.4	3.4	3.4	3.4	3.4	3.4
3.41	3.41	3.41	3.41	3.41	3.41	3.41	3.41	3.41
3.42	3.42	3.42	3.42	3.42	3.42	3.42	3.42	3.42
3.43	3.43	3.43	3.43	3.43	3.43	3.43	3.43	3.43
3.44	3.44	3.44	3.44	3.44	3.44	3.44	3.44	3.44
3.45	3.45	3.45	3.45	3.45	3.45	3.45	3.45	3.45
3.46	3.46	3.46	3.46	3.46	3.46	3.46	3.46	3.46
3.47	3.47	3.47	3.47	3.47	3.47	3.47	3.47	3.47
3.48	3.48	3.48	3.48	3.48	3.48	3.48	3.48	3.48
3.49	3.49	3.49	3.49	3.49	3.49	3.49	3.49	3.49

3.5	3.5	3.5	3.5	3.5	3.5	3.5	3.5	3.5
3.51	3.51	3.51	3.51	3.51	3.51	3.51	3.51	3.51
3.52	3.52	3.52	3.52	3.52	3.52	3.52	3.52	3.52
3.53	3.53	3.53	3.53	3.53	3.53	3.53	3.53	3.53
3.54	3.54	3.54	3.54	3.54	3.54	3.54	3.54	3.54
3.55	3.55	3.55	3.55	3.55	3.55	3.55	3.55	3.55
3.56	3.56	3.56	3.56	3.56	3.56	3.56	3.56	3.56
3.57	3.57	3.57	3.57	3.57	3.57	3.57	3.57	3.57
3.58	3.58	3.58	3.58	3.58	3.58	3.58	3.58	3.58
3.59	3.59	3.59	3.59	3.59	3.59	3.59	3.59	3.59
3.6	3.6	3.6	3.6	3.6	3.6	3.6	3.6	3.6
3.61	3.61	3.61	3.61	3.61	3.61	3.61	3.61	3.61
3.62	3.62	3.62	3.62	3.62	3.62	3.62	3.62	3.62
3.63	3.63	3.63	3.63	3.63	3.63	3.63	3.63	3.63
3.64	3.64	3.64	3.64	3.64	3.64	3.64	3.64	3.64
3.65	3.65	3.65	3.65	3.65	3.65	3.65	3.65	3.65
3.66	3.66	3.66	3.66	3.66	3.66	3.66	3.66	3.66
3.67	3.67	3.67	3.67	3.67	3.67	3.67	3.67	3.67
3.68	3.68	3.68	3.68	3.68	3.68	3.68	3.68	3.68
3.69	3.69	3.69	3.69	3.69	3.69	3.69	3.69	3.69
3.7	3.7	3.7	3.7	3.7	3.7	3.7	3.7	3.7
3.71	3.71	3.71	3.71	3.71	3.71	3.71	3.71	3.71
3.72	3.72	3.72	3.72	3.72	3.72	3.72	3.72	3.72
3.73	3.73	3.73	3.73	3.73	3.73	3.73	3.73	3.73
3.74	3.74	3.74	3.74	3.74	3.74	3.74	3.74	3.74
3.75	3.75	3.75	3.75	3.75	3.75	3.75	3.75	3.75
3.76	3.76	3.76	3.76	3.76	3.76	3.76	3.76	3.76
3.77	3.77	3.77	3.77	3.77	3.77	3.77	3.77	3.77
3.78	3.78	3.78	3.78	3.78	3.78	3.78	3.78	3.78
3.79	3.79	3.79	3.79	3.79	3.79	3.79	3.79	3.79
3.8	3.8	3.8	3.8	3.8	3.8	3.8	3.8	3.8
3.81	3.81	3.81	3.81	3.81	3.81	3.81	3.81	3.81
3.82	3.82	3.82	3.82	3.82	3.82	3.82	3.82	3.82
3.83	3.83	3.83	3.83	3.83	3.83	3.83	3.83	3.83
3.84	3.84	3.84	3.84	3.84	3.84	3.84	3.84	3.84
3.85	3.85	3.85	3.85	3.85	3.85	3.85	3.85	3.85
3.86	3.86	3.86	3.86	3.86	3.86	3.86	3.86	3.86
3.87	3.87	3.87	3.87	3.87	3.87	3.87	3.87	3.87
3.88	3.88	3.88	3.88	3.88	3.88	3.88	3.88	3.88
3.89	3.89	3.89	3.89	3.89	3.89	3.89	3.89	3.89
3.9	3.9	3.9	3.9	3.9	3.9	3.9	3.9	3.9
3.91	3.91	3.91	3.91	3.91	3.91	3.91	3.91	3.91
3.92	3.92	3.92	3.92	3.92	3.92	3.92	3.92	3.92
3.93	3.93	3.93	3.93	3.93	3.93	3.93	3.93	3.93
3.94	3.94	3.94	3.94	3.94	3.94	3.94	3.94	3.94
3.95	3.95	3.95	3.95	3.95	3.95	3.95	3.95	3.95
3.96	3.96	3.96	3.96	3.96	3.96	3.96	3.96	3.96
3.97	3.97	3.97	3.97	3.97	3.97	3.97	3.97	3.97
3.98	3.98	3.98	3.98	3.98	3.98	3.98	3.98	3.98
3.99	3.99	3.99	3.99	3.99	3.99	3.99	3.99	3.99

4	4	4	4	4	4	4	4	4
4.01	4.01	4.01	4.01	4.01	4.01	4.01	4.01	4.01
4.02	4.02	4.02	4.02	4.02	4.02	4.02	4.02	4.02
4.03	4.03	4.03	4.03	4.03	4.03	4.03	4.03	4.03
4.04	4.04	4.04	4.04	4.04	4.04	4.04	4.04	4.04
4.05	4.05	4.05	4.05	4.05	4.05	4.05	4.05	4.05
4.06	4.06	4.06	4.06	4.06	4.06	4.06	4.06	4.06
4.07	4.07	4.07	4.07	4.07	4.07	4.07	4.07	4.07
4.08	4.08	4.08	4.08	4.08	4.08	4.08	4.08	4.08
4.09	4.09	4.09	4.09	4.09	4.09	4.09	4.09	4.09
4.1	4.1	4.1	4.1	4.1	4.1	4.1	4.1	4.1
4.11	4.11	4.11	4.11	4.11	4.11	4.11	4.11	4.11
4.12	4.12	4.12	4.12	4.12	4.12	4.12	4.12	4.12
4.13	4.13	4.13	4.13	4.13	4.13	4.13	4.13	4.13
4.14	4.14	4.14	4.14	4.14	4.14	4.14	4.14	4.14
4.15	4.15	4.15	4.15	4.15	4.15	4.15	4.15	4.15
4.16	4.16	4.16	4.16	4.16	4.16	4.16	4.16	4.16
4.17	4.17	4.17	4.17	4.17	4.17	4.17	4.17	4.17
4.18	4.18	4.18	4.18	4.18	4.18	4.18	4.18	4.18
4.19	4.19	4.19	4.19	4.19	4.19	4.19	4.19	4.19
4.2	4.2	4.2	4.2	4.2	4.2	4.2	4.2	4.2
4.21	4.21	4.21	4.21	4.21	4.21	4.21	4.21	4.21
4.22	4.22	4.22	4.22	4.22	4.22	4.22	4.22	4.22
4.23	4.23	4.23	4.23	4.23	4.23	4.23	4.23	4.23
4.24	4.24	4.24	4.24	4.24	4.24	4.24	4.24	4.24
4.25	4.25	4.25	4.25	4.25	4.25	4.25	4.25	4.25
4.26	4.26	4.26	4.26	4.26	4.26	4.26	4.26	4.26
4.27	4.27	4.27	4.27	4.27	4.27	4.27	4.27	4.27
4.28	4.28	4.28	4.28	4.28	4.28	4.28	4.28	4.28
4.29	4.29	4.29	4.29	4.29	4.29	4.29	4.29	4.29
4.3	4.3	4.3	4.3	4.3	4.3	4.3	4.3	4.3
4.31	4.31	4.31	4.31	4.31	4.31	4.31	4.31	4.31
4.32	4.32	4.32	4.32	4.32	4.32	4.32	4.32	4.32
4.33	4.33	4.33	4.33	4.33	4.33	4.33	4.33	4.33
4.34	4.34	4.34	4.34	4.34	4.34	4.34	4.34	4.34
4.35	4.35	4.35	4.35	4.35	4.35	4.35	4.35	4.35
4.36	4.36	4.36	4.36	4.36	4.36	4.36	4.36	4.36
4.37	4.37	4.37	4.37	4.37	4.37	4.37	4.37	4.37
4.38	4.38	4.38	4.38	4.38	4.38	4.38	4.38	4.38
4.39	4.39	4.39	4.39	4.39	4.39	4.39	4.39	4.39
4.4	4.4	4.4	4.4	4.4	4.4	4.4	4.4	4.4
4.41	4.41	4.41	4.41	4.41	4.41	4.41	4.41	4.41
4.42	4.42	4.42	4.42	4.42	4.42	4.42	4.42	4.42
4.43	4.43	4.43	4.43	4.43	4.43	4.43	4.43	4.43
4.44	4.44	4.44	4.44	4.44	4.44	4.44	4.44	4.44
4.45	4.45	4.45	4.45	4.45	4.45	4.45	4.45	4.45
4.46	4.46	4.46	4.46	4.46	4.46	4.46	4.46	4.46
4.47	4.47	4.47	4.47	4.47	4.47	4.47	4.47	4.47
4.48	4.48	4.48	4.48	4.48	4.48	4.48	4.48	4.48
4.49	4.49	4.49	4.49	4.49	4.49	4.49	4.49	4.49

4.5	4.5	4.5	4.5	4.5	4.5	4.5	4.5	4.5
4.51	4.51	4.51	4.51	4.51	4.51	4.51	4.51	4.51
4.52	4.52	4.52	4.52	4.52	4.52	4.52	4.52	4.52
4.53	4.53	4.53	4.53	4.53	4.53	4.53	4.53	4.53
4.54	4.54	4.54	4.54	4.54	4.54	4.54	4.54	4.54
4.55	4.55	4.55	4.55	4.55	4.55	4.55	4.55	4.55
4.56	4.56	4.56	4.56	4.56	4.56	4.56	4.56	4.56
4.57	4.57	4.57	4.57	4.57	4.57	4.57	4.57	4.57
4.58	4.58	4.58	4.58	4.58	4.58	4.58	4.58	4.58
4.59	4.59	4.59	4.59	4.59	4.59	4.59	4.59	4.59
4.6	4.6	4.6	4.6	4.6	4.6	4.6	4.6	4.6
4.61	4.61	4.61	4.61	4.61	4.61	4.61	4.61	4.61
4.62	4.62	4.62	4.62	4.62	4.62	4.62	4.62	4.62
4.63	4.63	4.63	4.63	4.63	4.63	4.63	4.63	4.63
4.64	4.64	4.64	4.64	4.64	4.64	4.64	4.64	4.64
4.65	4.65	4.65	4.65	4.65	4.65	4.65	4.65	4.65
4.66	4.66	4.66	4.66	4.66	4.66	4.66	4.66	4.66
4.67	4.67	4.67	4.67	4.67	4.67	4.67	4.67	4.67
4.68	4.68	4.68	4.68	4.68	4.68	4.68	4.68	4.68
4.69	4.69	4.69	4.69	4.69	4.69	4.69	4.69	4.69
4.7	4.7	4.7	4.7	4.7	4.7	4.7	4.7	4.7
4.71	4.71	4.71	4.71	4.71	4.71	4.71	4.71	4.71
4.72	4.72	4.72	4.72	4.72	4.72	4.72	4.72	4.72
4.73	4.73	4.73	4.73	4.73	4.73	4.73	4.73	4.73
4.74	4.74	4.74	4.74	4.74	4.74	4.74	4.74	4.74
4.75	4.75	4.75	4.75	4.75	4.75	4.75	4.75	4.75
4.76	4.76	4.76	4.76	4.76	4.76	4.76	4.76	4.76
4.77	4.77	4.77	4.77	4.77	4.77	4.77	4.77	4.77
4.78	4.78	4.78	4.78	4.78	4.78	4.78	4.78	4.78
4.79	4.79	4.79	4.79	4.79	4.79	4.79	4.79	4.79
4.8	4.8	4.8	4.8	4.8	4.8	4.8	4.8	4.8
4.81	4.81	4.81	4.81	4.81	4.81	4.81	4.81	4.81
4.82	4.82	4.82	4.82	4.82	4.82	4.82	4.82	4.82
4.83	4.83	4.83	4.83	4.83	4.83	4.83	4.83	4.83
4.84	4.84	4.84	4.84	4.84	4.84	4.84	4.84	4.84
4.85	4.85	4.85	4.85	4.85	4.85	4.85	4.85	4.85
4.86	4.86	4.86	4.86	4.86	4.86	4.86	4.86	4.86
4.87	4.87	4.87	4.87	4.87	4.87	4.87	4.87	4.87
4.88	4.88	4.88	4.88	4.88	4.88	4.88	4.88	4.88
4.89	4.89	4.89	4.89	4.89	4.89	4.89	4.89	4.89
4.9	4.9	4.9	4.9	4.9	4.9	4.9	4.9	4.9
4.91	4.91	4.91	4.91	4.91	4.91	4.91	4.91	4.91
4.92	4.92	4.92	4.92	4.92	4.92	4.92	4.92	4.92
4.93	4.93	4.93	4.93	4.93	4.93	4.93	4.93	4.93
4.94	4.94	4.94	4.94	4.94	4.94	4.94	4.94	4.94
4.95	4.95	4.95	4.95	4.95	4.95	4.95	4.95	4.95
4.96	4.96	4.96	4.96	4.96	4.96	4.96	4.96	4.96
4.97	4.97	4.97	4.97	4.97	4.97	4.97	4.97	4.97
4.98	4.98	4.98	4.98	4.98	4.98	4.98	4.98	4.98
4.99	4.99	4.99	4.99	4.99	4.99	4.99	4.99	4.99

5	5	5	5	5	5	5	5	5
5.01	5.01	5.01	5.01	5.01	5.01	5.01	5.01	5.01
5.02	5.02	5.02	5.02	5.02	5.02	5.02	5.02	5.02
5.03	5.03	5.03	5.03	5.03	5.03	5.03	5.03	5.03
5.04	5.04	5.04	5.04	5.04	5.04	5.04	5.04	5.04
5.05	5.05	5.05	5.05	5.05	5.05	5.05	5.05	5.05
5.06	5.06	5.06	5.06	5.06	5.06	5.06	5.06	5.06
5.07	5.07	5.07	5.07	5.07	5.07	5.07	5.07	5.07
5.08	5.08	5.08	5.08	5.08	5.08	5.08	5.08	5.08
5.09	5.09	5.09	5.09	5.09	5.09	5.09	5.09	5.09
5.1	5.1	5.1	5.1	5.1	5.1	5.1	5.1	5.1
5.11	5.11	5.11	5.11	5.11	5.11	5.11	5.11	5.11
5.12	5.12	5.12	5.12	5.12	5.12	5.12	5.12	5.12
5.13	5.13	5.13	5.13	5.13	5.13	5.13	5.13	5.13
5.14	5.14	5.14	5.14	5.14	5.14	5.14	5.14	5.14
5.15	5.15	5.15	5.15	5.15	5.15	5.15	5.15	5.15
5.16	5.16	5.16	5.16	5.16	5.16	5.16	5.16	5.16
5.17	5.17	5.17	5.17	5.17	5.17	5.17	5.17	5.17
5.18	5.18	5.18	5.18	5.18	5.18	5.18	5.18	5.18
5.19	5.19	5.19	5.19	5.19	5.19	5.19	5.19	5.19
5.2	5.2	5.2	5.2	5.2	5.2	5.2	5.2	5.2
5.21	5.21	5.21	5.21	5.21	5.21	5.21	5.21	5.21
5.22	5.22	5.22	5.22	5.22	5.22	5.22	5.22	5.22
5.23	5.23	5.23	5.23	5.23	5.23	5.23	5.23	5.23
5.24	5.24	5.24	5.24	5.24	5.24	5.24	5.24	5.24
5.25	5.25	5.25	5.25	5.25	5.25	5.25	5.25	5.25
5.26	5.26	5.26	5.26	5.26	5.26	5.26	5.26	5.26
5.27	5.27	5.27	5.27	5.27	5.27	5.27	5.27	5.27
5.28	5.28	5.28	5.28	5.28	5.28	5.28	5.28	5.28
5.29	5.29	5.29	5.29	5.29	5.29	5.29	5.29	5.29
5.3	5.3	5.3	5.3	5.3	5.3	5.3	5.3	5.3
5.31	5.31	5.31	5.31	5.31	5.31	5.31	5.31	5.31
5.32	5.32	5.32	5.32	5.32	5.32	5.32	5.32	5.32
5.33	5.33	5.33	5.33	5.33	5.33	5.33	5.33	5.33
5.34	5.34	5.34	5.34	5.34	5.34	5.34	5.34	5.34
5.35	5.35	5.35	5.35	5.35	5.35	5.35	5.35	5.35
5.36	5.36	5.36	5.36	5.36	5.36	5.36	5.36	5.36
5.37	5.37	5.37	5.37	5.37	5.37	5.37	5.37	5.37
5.38	5.38	5.38	5.38	5.38	5.38	5.38	5.38	5.38
5.39	5.39	5.39	5.39	5.39	5.39	5.39	5.39	5.39
5.4	5.4	5.4	5.4	5.4	5.4	5.4	5.4	5.4
5.41	5.41	5.41	5.41	5.41	5.41	5.41	5.41	5.41
5.42	5.42	5.42	5.42	5.42	5.42	5.42	5.42	5.42
5.43	5.43	5.43	5.43	5.43	5.43	5.43	5.43	5.43
5.44	5.44	5.44	5.44	5.44	5.44	5.44	5.44	5.44
5.45	5.45	5.45	5.45	5.45	5.45	5.45	5.45	5.45
5.46	5.46	5.46	5.46	5.46	5.46	5.46	5.46	5.46
5.47	5.47	5.47	5.47	5.47	5.47	5.47	5.47	5.47
5.48	5.48	5.48	5.48	5.48	5.48	5.48	5.48	5.48
5.49	5.49	5.49	5.49	5.49	5.49	5.49	5.49	5.49

5.5	5.5	5.5	5.5	5.5	5.5	5.5	5.5	5.5
5.51	5.51	5.51	5.51	5.51	5.51	5.51	5.51	5.51
5.52	5.52	5.52	5.52	5.52	5.52	5.52	5.52	5.52
5.53	5.53	5.53	5.53	5.53	5.53	5.53	5.53	5.53
5.54	5.54	5.54	5.54	5.54	5.54	5.54	5.54	5.54
5.55	5.55	5.55	5.55	5.55	5.55	5.55	5.55	5.55
5.56	5.56	5.56	5.56	5.56	5.56	5.56	5.56	5.56
5.57	5.57	5.57	5.57	5.57	5.57	5.57	5.57	5.57
5.58	5.58	5.58	5.58	5.58	5.58	5.58	5.58	5.58
5.59	5.59	5.59	5.59	5.59	5.59	5.59	5.59	5.59
5.6	5.6	5.6	5.6	5.6	5.6	5.6	5.6	5.6
5.61	5.61	5.61	5.61	5.61	5.61	5.61	5.61	5.61
5.62	5.62	5.62	5.62	5.62	5.62	5.62	5.62	5.62
5.63	5.63	5.63	5.63	5.63	5.63	5.63	5.63	5.63
5.64	5.64	5.64	5.64	5.64	5.64	5.64	5.64	5.64
5.65	5.65	5.65	5.65	5.65	5.65	5.65	5.65	5.65
5.66	5.66	5.66	5.66	5.66	5.66	5.66	5.66	5.66
5.67	5.67	5.67	5.67	5.67	5.67	5.67	5.67	5.67
5.68	5.68	5.68	5.68	5.68	5.68	5.68	5.68	5.68
5.69	5.69	5.69	5.69	5.69	5.69	5.69	5.69	5.69
5.7	5.7	5.7	5.7	5.7	5.7	5.7	5.7	5.7
5.71	5.71	5.71	5.71	5.71	5.71	5.71	5.71	5.71
5.72	5.72	5.72	5.72	5.72	5.72	5.72	5.72	5.72
5.73	5.73	5.73	5.73	5.73	5.73	5.73	5.73	5.73
5.74	5.74	5.74	5.74	5.74	5.74	5.74	5.74	5.74
5.75	5.75	5.75	5.75	5.75	5.75	5.75	5.75	5.75
5.76	5.76	5.76	5.76	5.76	5.76	5.76	5.76	5.76
5.77	5.77	5.77	5.77	5.77	5.77	5.77	5.77	5.77
5.78	5.78	5.78	5.78	5.78	5.78	5.78	5.78	5.78
5.79	5.79	5.79	5.79	5.79	5.79	5.79	5.79	5.79
5.8	5.8	5.8	5.8	5.8	5.8	5.8	5.8	5.8
5.81	5.81	5.81	5.81	5.81	5.81	5.81	5.81	5.81
5.82	5.82	5.82	5.82	5.82	5.82	5.82	5.82	5.82
5.83	5.83	5.83	5.83	5.83	5.83	5.83	5.83	5.83
5.84	5.84	5.84	5.84	5.84	5.84	5.84	5.84	5.84
5.85	5.85	5.85	5.85	5.85	5.85	5.85	5.85	5.85
5.86	5.86	5.86	5.86	5.86	5.86	5.86	5.86	5.86
5.87	5.87	5.87	5.87	5.87	5.87	5.87	5.87	5.87
5.88	5.88	5.88	5.88	5.88	5.88	5.88	5.88	5.88
5.89	5.89	5.89	5.89	5.89	5.89	5.89	5.89	5.89
5.9	5.9	5.9	5.9	5.9	5.9	5.9	5.9	5.9
5.91	5.91	5.91	5.91	5.91	5.91	5.91	5.91	5.91
5.92	5.92	5.92	5.92	5.92	5.92	5.92	5.92	5.92
5.93	5.93	5.93	5.93	5.93	5.93	5.93	5.93	5.93
5.94	5.94	5.94	5.94	5.94	5.94	5.94	5.94	5.94
5.95	5.95	5.95	5.95	5.95	5.95	5.95	5.95	5.95
5.96	5.96	5.96	5.96	5.96	5.96	5.96	5.96	5.96
5.97	5.97	5.97	5.97	5.97	5.97	5.97	5.97	5.97
5.98	5.98	5.98	5.98	5.98	5.98	5.98	5.98	5.98
5.99	5.99	5.99	5.99	5.99	5.99	5.99	5.99	5.99

6	6	6	6	6	6	6	6	6
6.01	6.01	6.01	6.01	6.01	6.01	6.01	6.01	6.01
6.02	6.02	6.02	6.02	6.02	6.02	6.02	6.02	6.02
6.03	6.03	6.03	6.03	6.03	6.03	6.03	6.03	6.03
6.04	6.04	6.04	6.04	6.04	6.04	6.04	6.04	6.04
6.05	6.05	6.05	6.05	6.05	6.05	6.05	6.05	6.05
6.06	6.06	6.06	6.06	6.06	6.06	6.06	6.06	6.06
6.07	6.07	6.07	6.07	6.07	6.07	6.07	6.07	6.07
6.08	6.08	6.08	6.08	6.08	6.08	6.08	6.08	6.08
6.09	6.09	6.09	6.09	6.09	6.09	6.09	6.09	6.09
6.1	6.1	6.1	6.1	6.1	6.1	6.1	6.1	6.1
6.11	6.11	6.11	6.11	6.11	6.11	6.11	6.11	6.11
6.12	6.12	6.12	6.12	6.12	6.12	6.12	6.12	6.12
6.13	6.13	6.13	6.13	6.13	6.13	6.13	6.13	6.13
6.14	6.14	6.14	6.14	6.14	6.14	6.14	6.14	6.14
6.15	6.15	6.15	6.15	6.15	6.15	6.15	6.15	6.15
6.16	6.16	6.16	6.16	6.16	6.16	6.16	6.16	6.16
6.17	6.17	6.17	6.17	6.17	6.17	6.17	6.17	6.17
6.18	6.18	6.18	6.18	6.18	6.18	6.18	6.18	6.18
6.19	6.19	6.19	6.19	6.19	6.19	6.19	6.19	6.19
6.2	6.2	6.2	6.2	6.2	6.2	6.2	6.2	6.2
6.21	6.21	6.21	6.21	6.21	6.21	6.21	6.21	6.21
6.22	6.22	6.22	6.22	6.22	6.22	6.22	6.22	6.22
6.23	6.23	6.23	6.23	6.23	6.23	6.23	6.23	6.23
6.24	6.24	6.24	6.24	6.24	6.24	6.24	6.24	6.24
6.25	6.25	6.25	6.25	6.25	6.25	6.25	6.25	6.25
6.26	6.26	6.26	6.26	6.26	6.26	6.26	6.26	6.26
6.27	6.27	6.27	6.27	6.27	6.27	6.27	6.27	6.27
6.28	6.28	6.28	6.28	6.28	6.28	6.28	6.28	6.28
6.29	6.29	6.29	6.29	6.29	6.29	6.29	6.29	6.29
6.3	6.3	6.3	6.3	6.3	6.3	6.3	6.3	6.3
6.31	6.31	6.31	6.31	6.31	6.31	6.31	6.31	6.31
6.32	6.32	6.32	6.32	6.32	6.32	6.32	6.32	6.32
6.33	6.33	6.33	6.33	6.33	6.33	6.33	6.33	6.33
6.34	6.34	6.34	6.34	6.34	6.34	6.34	6.34	6.34
6.35	6.35	6.35	6.35	6.35	6.35	6.35	6.35	6.35
6.36	6.36	6.36	6.36	6.36	6.36	6.36	6.36	6.36
6.37	6.37	6.37	6.37	6.37	6.37	6.37	6.37	6.37
6.38	6.38	6.38	6.38	6.38	6.38	6.38	6.38	6.38
6.39	6.39	6.39	6.39	6.39	6.39	6.39	6.39	6.39
6.4	6.4	6.4	6.4	6.4	6.4	6.4	6.4	6.4
6.41	6.41	6.41	6.41	6.41	6.41	6.41	6.41	6.41
6.42	6.42	6.42	6.42	6.42	6.42	6.42	6.42	6.42
6.43	6.43	6.43	6.43	6.43	6.43	6.43	6.43	6.43
6.44	6.44	6.44	6.44	6.44	6.44	6.44	6.44	6.44
6.45	6.45	6.45	6.45	6.45	6.45	6.45	6.45	6.45
6.46	6.46	6.46	6.46	6.46	6.46	6.46	6.46	6.46
6.47	6.47	6.47	6.47	6.47	6.47	6.47	6.47	6.47
6.48	6.48	6.48	6.48	6.48	6.48	6.48	6.48	6.48
6.49	6.49	6.49	6.49	6.49	6.49	6.49	6.49	6.49

6.5	6.5	6.5	6.5	6.5	6.5	6.5	6.5	6.5
6.51	6.51	6.51	6.51	6.51	6.51	6.51	6.51	6.51
6.52	6.52	6.52	6.52	6.52	6.52	6.52	6.52	6.52
6.53	6.53	6.53	6.53	6.53	6.53	6.53	6.53	6.53
6.54	6.54	6.54	6.54	6.54	6.54	6.54	6.54	6.54
6.55	6.55	6.55	6.55	6.55	6.55	6.55	6.55	6.55
6.56	6.56	6.56	6.56	6.56	6.56	6.56	6.56	6.56
6.57	6.57	6.57	6.57	6.57	6.57	6.57	6.57	6.57
6.58	6.58	6.58	6.58	6.58	6.58	6.58	6.58	6.58
6.59	6.59	6.59	6.59	6.59	6.59	6.59	6.59	6.59
6.6	6.6	6.6	6.6	6.6	6.6	6.6	6.6	6.6
6.61	6.61	6.61	6.61	6.61	6.61	6.61	6.61	6.61
6.62	6.62	6.62	6.62	6.62	6.62	6.62	6.62	6.62
6.63	6.63	6.63	6.63	6.63	6.63	6.63	6.63	6.63
6.64	6.64	6.64	6.64	6.64	6.64	6.64	6.64	6.64
6.65	6.65	6.65	6.65	6.65	6.65	6.65	6.65	6.65
6.66	6.66	6.66	6.66	6.66	6.66	6.66	6.66	6.66
6.67	6.67	6.67	6.67	6.67	6.67	6.67	6.67	6.67
6.68	6.68	6.68	6.68	6.68	6.68	6.68	6.68	6.68
6.69	6.69	6.69	6.69	6.69	6.69	6.69	6.69	6.69
6.7	6.7	6.7	6.7	6.7	6.7	6.7	6.7	6.7
6.71	6.71	6.71	6.71	6.71	6.71	6.71	6.71	6.71
6.72	6.72	6.72	6.72	6.72	6.72	6.72	6.72	6.72
6.73	6.73	6.73	6.73	6.73	6.73	6.73	6.73	6.73
6.74	6.74	6.74	6.74	6.74	6.74	6.74	6.74	6.74
6.75	6.75	6.75	6.75	6.75	6.75	6.75	6.75	6.75
6.76	6.76	6.76	6.76	6.76	6.76	6.76	6.76	6.76
6.77	6.77	6.77	6.77	6.77	6.77	6.77	6.77	6.77
6.78	6.78	6.78	6.78	6.78	6.78	6.78	6.78	6.78
6.79	6.79	6.79	6.79	6.79	6.79	6.79	6.79	6.79
6.8	6.8	6.8	6.8	6.8	6.8	6.8	6.8	6.8
6.81	6.81	6.81	6.81	6.81	6.81	6.81	6.81	6.81
6.82	6.82	6.82	6.82	6.82	6.82	6.82	6.82	6.82
6.83	6.83	6.83	6.83	6.83	6.83	6.83	6.83	6.83
6.84	6.84	6.84	6.84	6.84	6.84	6.84	6.84	6.84
6.85	6.85	6.85	6.85	6.85	6.85	6.85	6.85	6.85
6.86	6.86	6.86	6.86	6.86	6.86	6.86	6.86	6.86
6.87	6.87	6.87	6.87	6.87	6.87	6.87	6.87	6.87
6.88	6.88	6.88	6.88	6.88	6.88	6.88	6.88	6.88
6.89	6.89	6.89	6.89	6.89	6.89	6.89	6.89	6.89
6.9	6.9	6.9	6.9	6.9	6.9	6.9	6.9	6.9
6.91	6.91	6.91	6.91	6.91	6.91	6.91	6.91	6.91
6.92	6.92	6.92	6.92	6.92	6.92	6.92	6.92	6.92
6.93	6.93	6.93	6.93	6.93	6.93	6.93	6.93	6.93
6.94	6.94	6.94	6.94	6.94	6.94	6.94	6.94	6.94
6.95	6.95	6.95	6.95	6.95	6.95	6.95	6.95	6.95
6.96	6.96	6.96	6.96	6.96	6.96	6.96	6.96	6.96
6.97	6.97	6.97	6.97	6.97	6.97	6.97	6.97	6.97
6.98	6.98	6.98	6.98	6.98	6.98	6.98	6.98	6.98
6.99	6.99	6.99	6.99	6.99	6.99	6.99	6.99	6.99

7	7	7	7	7	7	7	7	7
7.01	7.01	7.01	7.01	7.01	7.01	7.01	7.01	7.01
7.02	7.02	7.02	7.02	7.02	7.02	7.02	7.02	7.02
7.03	7.03	7.03	7.03	7.03	7.03	7.03	7.03	7.03
7.04	7.04	7.04	7.04	7.04	7.04	7.04	7.04	7.04
7.05	7.05	7.05	7.05	7.05	7.05	7.05	7.05	7.05
7.06	7.06	7.06	7.06	7.06	7.06	7.06	7.06	7.06
7.07	7.07	7.07	7.07	7.07	7.07	7.07	7.07	7.07
7.08	7.08	7.08	7.08	7.08	7.08	7.08	7.08	7.08
7.09	7.09	7.09	7.09	7.09	7.09	7.09	7.09	7.09
7.1	7.1	7.1	7.1	7.1	7.1	7.1	7.1	7.1
7.11	7.11	7.11	7.11	7.11	7.11	7.11	7.11	7.11
7.12	7.12	7.12	7.12	7.12	7.12	7.12	7.12	7.12
7.13	7.13	7.13	7.13	7.13	7.13	7.13	7.13	7.13
7.14	7.14	7.14	7.14	7.14	7.14	7.14	7.14	7.14
7.15	7.15	7.15	7.15	7.15	7.15	7.15	7.15	7.15
7.16	7.16	7.16	7.16	7.16	7.16	7.16	7.16	7.16
7.17	7.17	7.17	7.17	7.17	7.17	7.17	7.17	7.17
7.18	7.18	7.18	7.18	7.18	7.18	7.18	7.18	7.18
7.19	7.19	7.19	7.19	7.19	7.19	7.19	7.19	7.19
7.2	7.2	7.2	7.2	7.2	7.2	7.2	7.2	7.2
7.21	7.21	7.21	7.21	7.21	7.21	7.21	7.21	7.21
7.22	7.22	7.22	7.22	7.22	7.22	7.22	7.22	7.22
7.23	7.23	7.23	7.23	7.23	7.23	7.23	7.23	7.23
7.24	7.24	7.24	7.24	7.24	7.24	7.24	7.24	7.24
7.25	7.25	7.25	7.25	7.25	7.25	7.25	7.25	7.25
7.26	7.26	7.26	7.26	7.26	7.26	7.26	7.26	7.26
7.27	7.27	7.27	7.27	7.27	7.27	7.27	7.27	7.27
7.28	7.28	7.28	7.28	7.28	7.28	7.28	7.28	7.28
7.29	7.29	7.29	7.29	7.29	7.29	7.29	7.29	7.29
7.3	7.3	7.3	7.3	7.3	7.3	7.3	7.3	7.3
7.31	7.31	7.31	7.31	7.31	7.31	7.31	7.31	7.31
7.32	7.32	7.32	7.32	7.32	7.32	7.32	7.32	7.32
7.33	7.33	7.33	7.33	7.33	7.33	7.33	7.33	7.33
7.34	7.34	7.34	7.34	7.34	7.34	7.34	7.34	7.34
7.35	7.35	7.35	7.35	7.35	7.35	7.35	7.35	7.35
7.36	7.36	7.36	7.36	7.36	7.36	7.36	7.36	7.36
7.37	7.37	7.37	7.37	7.37	7.37	7.37	7.37	7.37
7.38	7.38	7.38	7.38	7.38	7.38	7.38	7.38	7.38
7.39	7.39	7.39	7.39	7.39	7.39	7.39	7.39	7.39
7.4	7.4	7.4	7.4	7.4	7.4	7.4	7.4	7.4
7.41	7.41	7.41	7.41	7.41	7.41	7.41	7.41	7.41
7.42	7.42	7.42	7.42	7.42	7.42	7.42	7.42	7.42
7.43	7.43	7.43	7.43	7.43	7.43	7.43	7.43	7.43
7.44	7.44	7.44	7.44	7.44	7.44	7.44	7.44	7.44
7.45	7.45	7.45	7.45	7.45	7.45	7.45	7.45	7.45
7.46	7.46	7.46	7.46	7.46	7.46	7.46	7.46	7.46
7.47	7.47	7.47	7.47	7.47	7.47	7.47	7.47	7.47
7.48	7.48	7.48	7.48	7.48	7.48	7.48	7.48	7.48
7.49	7.49	7.49	7.49	7.49	7.49	7.49	7.49	7.49

7.5	7.5	7.5	7.5	7.5	7.5	7.5	7.5	7.5
7.51	7.51	7.51	7.51	7.51	7.51	7.51	7.51	7.51
7.52	7.52	7.52	7.52	7.52	7.52	7.52	7.52	7.52
7.53	7.53	7.53	7.53	7.53	7.53	7.53	7.53	7.53
7.54	7.54	7.54	7.54	7.54	7.54	7.54	7.54	7.54
7.55	7.55	7.55	7.55	7.55	7.55	7.55	7.55	7.55
7.56	7.56	7.56	7.56	7.56	7.56	7.56	7.56	7.56
7.57	7.57	7.57	7.57	7.57	7.57	7.57	7.57	7.57
7.58	7.58	7.58	7.58	7.58	7.58	7.58	7.58	7.58
7.59	7.59	7.59	7.59	7.59	7.59	7.59	7.59	7.59
7.6	7.6	7.6	7.6	7.6	7.6	7.6	7.6	7.6
7.61	7.61	7.61	7.61	7.61	7.61	7.61	7.61	7.61
7.62	7.62	7.62	7.62	7.62	7.62	7.62	7.62	7.62
7.63	7.63	7.63	7.63	7.63	7.63	7.63	7.63	7.63
7.64	7.64	7.64	7.64	7.64	7.64	7.64	7.64	7.64
7.65	7.65	7.65	7.65	7.65	7.65	7.65	7.65	7.65
7.66	7.66	7.66	7.66	7.66	7.66	7.66	7.66	7.66
7.67	7.67	7.67	7.67	7.67	7.67	7.67	7.67	7.67
7.68	7.68	7.68	7.68	7.68	7.68	7.68	7.68	7.68
7.69	7.69	7.69	7.69	7.69	7.69	7.69	7.69	7.69
7.7	7.7	7.7	7.7	7.7	7.7	7.7	7.7	7.7
7.71	7.71	7.71	7.71	7.71	7.71	7.71	7.71	7.71
7.72	7.72	7.72	7.72	7.72	7.72	7.72	7.72	7.72
7.73	7.73	7.73	7.73	7.73	7.73	7.73	7.73	7.73
7.74	7.74	7.74	7.74	7.74	7.74	7.74	7.74	7.74
7.75	7.75	7.75	7.75	7.75	7.75	7.75	7.75	7.75
7.76	7.76	7.76	7.76	7.76	7.76	7.76	7.76	7.76
7.77	7.77	7.77	7.77	7.77	7.77	7.77	7.77	7.77
7.78	7.78	7.78	7.78	7.78	7.78	7.78	7.78	7.78
7.79	7.79	7.79	7.79	7.79	7.79	7.79	7.79	7.79
7.8	7.8	7.8	7.8	7.8	7.8	7.8	7.8	7.8
7.81	7.81	7.81	7.81	7.81	7.81	7.81	7.81	7.81
7.82	7.82	7.82	7.82	7.82	7.82	7.82	7.82	7.82
7.83	7.83	7.83	7.83	7.83	7.83	7.83	7.83	7.83
7.84	7.84	7.84	7.84	7.84	7.84	7.84	7.84	7.84
7.85	7.85	7.85	7.85	7.85	7.85	7.85	7.85	7.85
7.86	7.86	7.86	7.86	7.86	7.86	7.86	7.86	7.86
7.87	7.87	7.87	7.87	7.87	7.87	7.87	7.87	7.87
7.88	7.88	7.88	7.88	7.88	7.88	7.88	7.88	7.88
7.89	7.89	7.89	7.89	7.89	7.89	7.89	7.89	7.89
7.9	7.9	7.9	7.9	7.9	7.9	7.9	7.9	7.9
7.91	7.91	7.91	7.91	7.91	7.91	7.91	7.91	7.91
7.92	7.92	7.92	7.92	7.92	7.92	7.92	7.92	7.92
7.93	7.93	7.93	7.93	7.93	7.93	7.93	7.93	7.93
7.94	7.94	7.94	7.94	7.94	7.94	7.94	7.94	7.94
7.95	7.95	7.95	7.95	7.95	7.95	7.95	7.95	7.95
7.96	7.96	7.96	7.96	7.96	7.96	7.96	7.96	7.96
7.97	7.97	7.97	7.97	7.97	7.97	7.97	7.97	7.97
7.98	7.98	7.98	7.98	7.98	7.98	7.98	7.98	7.98
7.99	7.99	7.99	7.99	7.99	7.99	7.99	7.99	7.99

8	8	8	8	8	8	8	8	8
8.01	8.01	8.01	8.01	8.01	8.01	8.01	8.01	8.01
8.02	8.02	8.02	8.02	8.02	8.02	8.02	8.02	8.02
8.03	8.03	8.03	8.03	8.03	8.03	8.03	8.03	8.03
8.04	8.04	8.04	8.04	8.04	8.04	8.04	8.04	8.04
8.05	8.05	8.05	8.05	8.05	8.05	8.05	8.05	8.05
8.06	8.06	8.06	8.06	8.06	8.06	8.06	8.06	8.06
8.07	8.07	8.07	8.07	8.07	8.07	8.07	8.07	8.07
8.08	8.08	8.08	8.08	8.08	8.08	8.08	8.08	8.08
8.09	8.09	8.09	8.09	8.09	8.09	8.09	8.09	8.09
8.1	8.1	8.1	8.1	8.1	8.1	8.1	8.1	8.1
8.11	8.11	8.11	8.11	8.11	8.11	8.11	8.11	8.11
8.12	8.12	8.12	8.12	8.12	8.12	8.12	8.12	8.12
8.13	8.13	8.13	8.13	8.13	8.13	8.13	8.13	8.13
8.14	8.14	8.14	8.14	8.14	8.14	8.14	8.14	8.14
8.15	8.15	8.15	8.15	8.15	8.15	8.15	8.15	8.15
8.16	8.16	8.16	8.16	8.16	8.16	8.16	8.16	8.16
8.17	8.17	8.17	8.17	8.17	8.17	8.17	8.17	8.17
8.18	8.18	8.18	8.18	8.18	8.18	8.18	8.18	8.18
8.19	8.19	8.19	8.19	8.19	8.19	8.19	8.19	8.19
8.2	8.2	8.2	8.2	8.2	8.2	8.2	8.2	8.2
8.21	8.21	8.21	8.21	8.21	8.21	8.21	8.21	8.21
8.22	8.22	8.22	8.22	8.22	8.22	8.22	8.22	8.22
8.23	8.23	8.23	8.23	8.23	8.23	8.23	8.23	8.23
8.24	8.24	8.24	8.24	8.24	8.24	8.24	8.24	8.24
8.25	8.25	8.25	8.25	8.25	8.25	8.25	8.25	8.25
8.26	8.26	8.26	8.26	8.26	8.26	8.26	8.26	8.26
8.27	8.27	8.27	8.27	8.27	8.27	8.27	8.27	8.27
8.28	8.28	8.28	8.28	8.28	8.28	8.28	8.28	8.28
8.29	8.29	8.29	8.29	8.29	8.29	8.29	8.29	8.29
8.3	8.3	8.3	8.3	8.3	8.3	8.3	8.3	8.3
8.31	8.31	8.31	8.31	8.31	8.31	8.31	8.31	8.31
8.32	8.32	8.32	8.32	8.32	8.32	8.32	8.32	8.32
8.33	8.33	8.33	8.33	8.33	8.33	8.33	8.33	8.33
8.34	8.34	8.34	8.34	8.34	8.34	8.34	8.34	8.34
8.35	8.35	8.35	8.35	8.35	8.35	8.35	8.35	8.35
8.36	8.36	8.36	8.36	8.36	8.36	8.36	8.36	8.36
8.37	8.37	8.37	8.37	8.37	8.37	8.37	8.37	8.37
8.38	8.38	8.38	8.38	8.38	8.38	8.38	8.38	8.38
8.39	8.39	8.39	8.39	8.39	8.39	8.39	8.39	8.39
8.4	8.4	8.4	8.4	8.4	8.4	8.4	8.4	8.4
8.41	8.41	8.41	8.41	8.41	8.41	8.41	8.41	8.41
8.42	8.42	8.42	8.42	8.42	8.42	8.42	8.42	8.42
8.43	8.43	8.43	8.43	8.43	8.43	8.43	8.43	8.43
8.44	8.44	8.44	8.44	8.44	8.44	8.44	8.44	8.44
8.45	8.45	8.45	8.45	8.45	8.45	8.45	8.45	8.45
8.46	8.46	8.46	8.46	8.46	8.46	8.46	8.46	8.46
8.47	8.47	8.47	8.47	8.47	8.47	8.47	8.47	8.47
8.48	8.48	8.48	8.48	8.48	8.48	8.48	8.48	8.48
8.49	8.49	8.49	8.49	8.49	8.49	8.49	8.49	8.49

8.5	8.5	8.5	8.5	8.5	8.5	8.5	8.5	8.5
8.51	8.51	8.51	8.51	8.51	8.51	8.51	8.51	8.51
8.52	8.52	8.52	8.52	8.52	8.52	8.52	8.52	8.52
8.53	8.53	8.53	8.53	8.53	8.53	8.53	8.53	8.53
8.54	8.54	8.54	8.54	8.54	8.54	8.54	8.54	8.54
8.55	8.55	8.55	8.55	8.55	8.55	8.55	8.55	8.55
8.56	8.56	8.56	8.56	8.56	8.56	8.56	8.56	8.56
8.57	8.57	8.57	8.57	8.57	8.57	8.57	8.57	8.57
8.58	8.58	8.58	8.58	8.58	8.58	8.58	8.58	8.58
8.59	8.59	8.59	8.59	8.59	8.59	8.59	8.59	8.59
8.6	8.6	8.6	8.6	8.6	8.6	8.6	8.6	8.6
8.61	8.61	8.61	8.61	8.61	8.61	8.61	8.61	8.61
8.62	8.62	8.62	8.62	8.62	8.62	8.62	8.62	8.62
8.63	8.63	8.63	8.63	8.63	8.63	8.63	8.63	8.63
8.64	8.64	8.64	8.64	8.64	8.64	8.64	8.64	8.64
8.65	8.65	8.65	8.65	8.65	8.65	8.65	8.65	8.65
8.66	8.66	8.66	8.66	8.66	8.66	8.66	8.66	8.66
8.67	8.67	8.67	8.67	8.67	8.67	8.67	8.67	8.67
8.68	8.68	8.68	8.68	8.68	8.68	8.68	8.68	8.68
8.69	8.69	8.69	8.69	8.69	8.69	8.69	8.69	8.69
8.7	8.7	8.7	8.7	8.7	8.7	8.7	8.7	8.7
8.71	8.71	8.71	8.71	8.71	8.71	8.71	8.71	8.71
8.72	8.72	8.72	8.72	8.72	8.72	8.72	8.72	8.72
8.73	8.73	8.73	8.73	8.73	8.73	8.73	8.73	8.73
8.74	8.74	8.74	8.74	8.74	8.74	8.74	8.74	8.74
8.75	8.75	8.75	8.75	8.75	8.75	8.75	8.75	8.75
8.76	8.76	8.76	8.76	8.76	8.76	8.76	8.76	8.76
8.77	8.77	8.77	8.77	8.77	8.77	8.77	8.77	8.77
8.78	8.78	8.78	8.78	8.78	8.78	8.78	8.78	8.78
8.79	8.79	8.79	8.79	8.79	8.79	8.79	8.79	8.79
8.8	8.8	8.8	8.8	8.8	8.8	8.8	8.8	8.8
8.81	8.81	8.81	8.81	8.81	8.81	8.81	8.81	8.81
8.82	8.82	8.82	8.82	8.82	8.82	8.82	8.82	8.82
8.83	8.83	8.83	8.83	8.83	8.83	8.83	8.83	8.83
8.84	8.84	8.84	8.84	8.84	8.84	8.84	8.84	8.84
8.85	8.85	8.85	8.85	8.85	8.85	8.85	8.85	8.85
8.86	8.86	8.86	8.86	8.86	8.86	8.86	8.86	8.86
8.87	8.87	8.87	8.87	8.87	8.87	8.87	8.87	8.87
8.88	8.88	8.88	8.88	8.88	8.88	8.88	8.88	8.88
8.89	8.89	8.89	8.89	8.89	8.89	8.89	8.89	8.89
8.9	8.9	8.9	8.9	8.9	8.9	8.9	8.9	8.9
8.91	8.91	8.91	8.91	8.91	8.91	8.91	8.91	8.91
8.92	8.92	8.92	8.92	8.92	8.92	8.92	8.92	8.92
8.93	8.93	8.93	8.93	8.93	8.93	8.93	8.93	8.93
8.94	8.94	8.94	8.94	8.94	8.94	8.94	8.94	8.94
8.95	8.95	8.95	8.95	8.95	8.95	8.95	8.95	8.95
8.96	8.96	8.96	8.96	8.96	8.96	8.96	8.96	8.96
8.97	8.97	8.97	8.97	8.97	8.97	8.97	8.97	8.97
8.98	8.98	8.98	8.98	8.98	8.98	8.98	8.98	8.98
8.99	8.99	8.99	8.99	8.99	8.99	8.99	8.99	8.99

9	9	9	9	9	9	9	9	9
9.01	9.01	9.01	9.01	9.01	9.01	9.01	9.01	9.01
9.02	9.02	9.02	9.02	9.02	9.02	9.02	9.02	9.02
9.03	9.03	9.03	9.03	9.03	9.03	9.03	9.03	9.03
9.04	9.04	9.04	9.04	9.04	9.04	9.04	9.04	9.04
9.05	9.05	9.05	9.05	9.05	9.05	9.05	9.05	9.05
9.06	9.06	9.06	9.06	9.06	9.06	9.06	9.06	9.06
9.07	9.07	9.07	9.07	9.07	9.07	9.07	9.07	9.07
9.08	9.08	9.08	9.08	9.08	9.08	9.08	9.08	9.08
9.09	9.09	9.09	9.09	9.09	9.09	9.09	9.09	9.09
9.1	9.1	9.1	9.1	9.1	9.1	9.1	9.1	9.1
9.11	9.11	9.11	9.11	9.11	9.11	9.11	9.11	9.11
9.12	9.12	9.12	9.12	9.12	9.12	9.12	9.12	9.12
9.13	9.13	9.13	9.13	9.13	9.13	9.13	9.13	9.13
9.14	9.14	9.14	9.14	9.14	9.14	9.14	9.14	9.14
9.15	9.15	9.15	9.15	9.15	9.15	9.15	9.15	9.15
9.16	9.16	9.16	9.16	9.16	9.16	9.16	9.16	9.16
9.17	9.17	9.17	9.17	9.17	9.17	9.17	9.17	9.17
9.18	9.18	9.18	9.18	9.18	9.18	9.18	9.18	9.18
9.19	9.19	9.19	9.19	9.19	9.19	9.19	9.19	9.19
9.2	9.2	9.2	9.2	9.2	9.2	9.2	9.2	9.2
9.21	9.21	9.21	9.21	9.21	9.21	9.21	9.21	9.21
9.22	9.22	9.22	9.22	9.22	9.22	9.22	9.22	9.22
9.23	9.23	9.23	9.23	9.23	9.23	9.23	9.23	9.23
9.24	9.24	9.24	9.24	9.24	9.24	9.24	9.24	9.24
9.25	9.25	9.25	9.25	9.25	9.25	9.25	9.25	9.25
9.26	9.26	9.26	9.26	9.26	9.26	9.26	9.26	9.26
9.27	9.27	9.27	9.27	9.27	9.27	9.27	9.27	9.27
9.28	9.28	9.28	9.28	9.28	9.28	9.28	9.28	9.28
9.29	9.29	9.29	9.29	9.29	9.29	9.29	9.29	9.29
9.3	9.3	9.3	9.3	9.3	9.3	9.3	9.3	9.3
9.31	9.31	9.31	9.31	9.31	9.31	9.31	9.31	9.31
9.32	9.32	9.32	9.32	9.32	9.32	9.32	9.32	9.32
9.33	9.33	9.33	9.33	9.33	9.33	9.33	9.33	9.33
9.34	9.34	9.34	9.34	9.34	9.34	9.34	9.34	9.34
9.35	9.35	9.35	9.35	9.35	9.35	9.35	9.35	9.35
9.36	9.36	9.36	9.36	9.36	9.36	9.36	9.36	9.36
9.37	9.37	9.37	9.37	9.37	9.37	9.37	9.37	9.37
9.38	9.38	9.38	9.38	9.38	9.38	9.38	9.38	9.38
9.39	9.39	9.39	9.39	9.39	9.39	9.39	9.39	9.39
9.4	9.4	9.4	9.4	9.4	9.4	9.4	9.4	9.4
9.41	9.41	9.41	9.41	9.41	9.41	9.41	9.41	9.41
9.42	9.42	9.42	9.42	9.42	9.42	9.42	9.42	9.42
9.43	9.43	9.43	9.43	9.43	9.43	9.43	9.43	9.43
9.44	9.44	9.44	9.44	9.44	9.44	9.44	9.44	9.44
9.45	9.45	9.45	9.45	9.45	9.45	9.45	9.45	9.45
9.46	9.46	9.46	9.46	9.46	9.46	9.46	9.46	9.46
9.47	9.47	9.47	9.47	9.47	9.47	9.47	9.47	9.47
9.48	9.48	9.48	9.48	9.48	9.48	9.48	9.48	9.48
9.49	9.49	9.49	9.49	9.49	9.49	9.49	9.49	9.49

9.5	9.5	9.5	9.5	9.5	9.5	9.5	9.5	9.5
9.51	9.51	9.51	9.51	9.51	9.51	9.51	9.51	9.51
9.52	9.52	9.52	9.52	9.52	9.52	9.52	9.52	9.52
9.53	9.53	9.53	9.53	9.53	9.53	9.53	9.53	9.53
9.54	9.54	9.54	9.54	9.54	9.54	9.54	9.54	9.54
9.55	9.55	9.55	9.55	9.55	9.55	9.55	9.55	9.55
9.56	9.56	9.56	9.56	9.56	9.56	9.56	9.56	9.56
9.57	9.57	9.57	9.57	9.57	9.57	9.57	9.57	9.57
9.58	9.58	9.58	9.58	9.58	9.58	9.58	9.58	9.58
9.59	9.59	9.59	9.59	9.59	9.59	9.59	9.59	9.59
9.6	9.6	9.6	9.6	9.6	9.6	9.6	9.6	9.6
9.61	9.61	9.61	9.61	9.61	9.61	9.61	9.61	9.61
9.62	9.62	9.62	9.62	9.62	9.62	9.62	9.62	9.62
9.63	9.63	9.63	9.63	9.63	9.63	9.63	9.63	9.63
9.64	9.64	9.64	9.64	9.64	9.64	9.64	9.64	9.64
9.65	9.65	9.65	9.65	9.65	9.65	9.65	9.65	9.65
9.66	9.66	9.66	9.66	9.66	9.66	9.66	9.66	9.66
9.67	9.67	9.67	9.67	9.67	9.67	9.67	9.67	9.67
9.68	9.68	9.68	9.68	9.68	9.68	9.68	9.68	9.68
9.69	9.69	9.69	9.69	9.69	9.69	9.69	9.69	9.69
9.7	9.7	9.7	9.7	9.7	9.7	9.7	9.7	9.7
9.71	9.71	9.71	9.71	9.71	9.71	9.71	9.71	9.71
9.72	9.72	9.72	9.72	9.72	9.72	9.72	9.72	9.72
9.73	9.73	9.73	9.73	9.73	9.73	9.73	9.73	9.73
9.74	9.74	9.74	9.74	9.74	9.74	9.74	9.74	9.74
9.75	9.75	9.75	9.75	9.75	9.75	9.75	9.75	9.75
9.76	9.76	9.76	9.76	9.76	9.76	9.76	9.76	9.76
9.77	9.77	9.77	9.77	9.77	9.77	9.77	9.77	9.77
9.78	9.78	9.78	9.78	9.78	9.78	9.78	9.78	9.78
9.79	9.79	9.79	9.79	9.79	9.79	9.79	9.79	9.79
9.8	9.8	9.8	9.8	9.8	9.8	9.8	9.8	9.8
9.81	9.81	9.81	9.81	9.81	9.81	9.81	9.81	9.81
9.82	9.82	9.82	9.82	9.82	9.82	9.82	9.82	9.82
9.83	9.83	9.83	9.83	9.83	9.83	9.83	9.83	9.83
9.84	9.84	9.84	9.84	9.84	9.84	9.84	9.84	9.84
9.85	9.85	9.85	9.85	9.85	9.85	9.85	9.85	9.85
9.86	9.86	9.86	9.86	9.86	9.86	9.86	9.86	9.86
9.87	9.87	9.87	9.87	9.87	9.87	9.87	9.87	9.87
9.88	9.88	9.88	9.88	9.88	9.88	9.88	9.88	9.88
9.89	9.89	9.89	9.89	9.89	9.89	9.89	9.89	9.89
9.9	9.9	9.9	9.9	9.9	9.9	9.9	9.9	9.9
9.91	9.91	9.91	9.91	9.91	9.91	9.91	9.91	9.91
9.92	9.92	9.92	9.92	9.92	9.92	9.92	9.92	9.92
9.93	9.93	9.93	9.93	9.93	9.93	9.93	9.93	9.93
9.94	9.94	9.94	9.94	9.94	9.94	9.94	9.94	9.94
9.95	9.95	9.95	9.95	9.95	9.95	9.95	9.95	9.95
9.96	9.96	9.96	9.96	9.96	9.96	9.96	9.96	9.96
9.97	9.97	9.97	9.97	9.97	9.97	9.97	9.97	9.97
9.98	9.98	9.98	9.98	9.98	9.98	9.98	9.98	9.98
9.99	9.99	9.99	9.99	9.99	9.99	9.99	9.99	9.99

10	10	10	10	10	10	10	10	10
10.01	10.01	10.01	10.01	10.01	10.01	10.01	10.01	10.01
10.02	10.02	10.02	10.02	10.02	10.02	10.02	10.02	10.02
10.03	10.03	10.03	10.03	10.03	10.03	10.03	10.03	10.03
10.04	10.04	10.04	10.04	10.04	10.04	10.04	10.04	10.04
10.05	10.05	10.05	10.05	10.05	10.05	10.05	10.05	10.05
10.06	10.06	10.06	10.06	10.06	10.06	10.06	10.06	10.06
10.07	10.07	10.07	10.07	10.07	10.07	10.07	10.07	10.07
10.08	10.08	10.08	10.08	10.08	10.08	10.08	10.08	10.08
10.09	10.09	10.09	10.09	10.09	10.09	10.09	10.09	10.09
10.1	10.1	10.1	10.1	10.1	10.1	10.1	10.1	10.1
10.11	10.11	10.11	10.11	10.11	10.11	10.11	10.11	10.11
10.12	10.12	10.12	10.12	10.12	10.12	10.12	10.12	10.12
10.13	10.13	10.13	10.13	10.13	10.13	10.13	10.13	10.13
10.14	10.14	10.14	10.14	10.14	10.14	10.14	10.14	10.14
10.15	10.15	10.15	10.15	10.15	10.15	10.15	10.15	10.15
10.16	10.16	10.16	10.16	10.16	10.16	10.16	10.16	10.16
10.17	10.17	10.17	10.17	10.17	10.17	10.17	10.17	10.17
10.18	10.18	10.18	10.18	10.18	10.18	10.18	10.18	10.18
10.19	10.19	10.19	10.19	10.19	10.19	10.19	10.19	10.19
10.2	10.2	10.2	10.2	10.2	10.2	10.2	10.2	10.2
10.21	10.21	10.21	10.21	10.21	10.21	10.21	10.21	10.21
10.22	10.22	10.22	10.22	10.22	10.22	10.22	10.22	10.22
10.23	10.23	10.23	10.23	10.23	10.23	10.23	10.23	10.23
10.24	10.24	10.24	10.24	10.24	10.24	10.24	10.24	10.24
10.25	10.25	10.25	10.25	10.25	10.25	10.25	10.25	10.25
10.26	10.26	10.26	10.26	10.26	10.26	10.26	10.26	10.26
10.27	10.27	10.27	10.27	10.27	10.27	10.27	10.27	10.27
10.28	10.28	10.28	10.28	10.28	10.28	10.28	10.28	10.28
10.29	10.29	10.29	10.29	10.29	10.29	10.29	10.29	10.29
10.3	10.3	10.3	10.3	10.3	10.3	10.3	10.3	10.3
10.31	10.31	10.31	10.31	10.31	10.31	10.31	10.31	10.31
10.32	10.32	10.32	10.32	10.32	10.32	10.32	10.32	10.32
10.33	10.33	10.33	10.33	10.33	10.33	10.33	10.33	10.33
10.34	10.34	10.34	10.34	10.34	10.34	10.34	10.34	10.34
10.35	10.35	10.35	10.35	10.35	10.35	10.35	10.35	10.35
10.36	10.36	10.36	10.36	10.36	10.36	10.36	10.36	10.36
10.37	10.37	10.37	10.37	10.37	10.37	10.37	10.37	10.37
10.38	10.38	10.38	10.38	10.38	10.38	10.38	10.38	10.38
10.39	10.39	10.39	10.39	10.39	10.39	10.39	10.39	10.39
10.4	10.4	10.4	10.4	10.4	10.4	10.4	10.4	10.4
10.41	10.41	10.41	10.41	10.41	10.41	10.41	10.41	10.41
10.42	10.42	10.42	10.42	10.42	10.42	10.42	10.42	10.42
10.43	10.43	10.43	10.43	10.43	10.43	10.43	10.43	10.43
10.44	10.44	10.44	10.44	10.44	10.44	10.44	10.44	10.44
10.45	10.45	10.45	10.45	10.45	10.45	10.45	10.45	10.45
10.46	10.46	10.46	10.46	10.46	10.46	10.46	10.46	10.46
10.47	10.47	10.47	10.47	10.47	10.47	10.47	10.47	10.47
10.48	10.48	10.48	10.48	10.48	10.48	10.48	10.48	10.48
10.49	10.49	10.49	10.49	10.49	10.49	10.49	10.49	10.49

10.5	10.5	10.5	10.5	10.5	10.5	10.5	10.5	10.5
10.51	10.51	10.51	10.51	10.51	10.51	10.51	10.51	10.51
10.52	10.52	10.52	10.52	10.52	10.52	10.52	10.52	10.52
10.53	10.53	10.53	10.53	10.53	10.53	10.53	10.53	10.53
10.54	10.54	10.54	10.54	10.54	10.54	10.54	10.54	10.54
10.55	10.55	10.55	10.55	10.55	10.55	10.55	10.55	10.55
10.56	10.56	10.56	10.56	10.56	10.56	10.56	10.56	10.56
10.57	10.57	10.57	10.57	10.57	10.57	10.57	10.57	10.57
10.58	10.58	10.58	10.58	10.58	10.58	10.58	10.58	10.58
10.59	10.59	10.59	10.59	10.59	10.59	10.59	10.59	10.59
10.6	10.6	10.6	10.6	10.6	10.6	10.6	10.6	10.6
10.61	10.61	10.61	10.61	10.61	10.61	10.61	10.61	10.61
10.62	10.62	10.62	10.62	10.62	10.62	10.62	10.62	10.62
10.63	10.63	10.63	10.63	10.63	10.63	10.63	10.63	10.63
10.64	10.64	10.64	10.64	10.64	10.64	10.64	10.64	10.64
10.65	10.65	10.65	10.65	10.65	10.65	10.65	10.65	10.65
10.66	10.66	10.66	10.66	10.66	10.66	10.66	10.66	10.66
10.67	10.67	10.67	10.67	10.67	10.67	10.67	10.67	10.67
10.68	10.68	10.68	10.68	10.68	10.68	10.68	10.68	10.68
10.69	10.69	10.69	10.69	10.69	10.69	10.69	10.69	10.69
10.7	10.7	10.7	10.7	10.7	10.7	10.7	10.7	10.7
10.71	10.71	10.71	10.71	10.71	10.71	10.71	10.71	10.71
10.72	10.72	10.72	10.72	10.72	10.72	10.72	10.72	10.72
10.73	10.73	10.73	10.73	10.73	10.73	10.73	10.73	10.73
10.74	10.74	10.74	10.74	10.74	10.74	10.74	10.74	10.74
10.75	10.75	10.75	10.75	10.75	10.75	10.75	10.75	10.75
10.76	10.76	10.76	10.76	10.76	10.76	10.76	10.76	10.76
10.77	10.77	10.77	10.77	10.77	10.77	10.77	10.77	10.77
10.78	10.78	10.78	10.78	10.78	10.78	10.78	10.78	10.78
10.79	10.79	10.79	10.79	10.79	10.79	10.79	10.79	10.79
10.8	10.8	10.8	10.8	10.8	10.8	10.8	10.8	10.8
10.81	10.81	10.81	10.81	10.81	10.81	10.81	10.81	10.81
10.82	10.82	10.82	10.82	10.82	10.82	10.82	10.82	10.82
10.83	10.83	10.83	10.83	10.83	10.83	10.83	10.83	10.83
10.84	10.84	10.84	10.84	10.84	10.84	10.84	10.84	10.84
10.85	10.85	10.85	10.85	10.85	10.85	10.85	10.85	10.85
10.86	10.86	10.86	10.86	10.86	10.86	10.86	10.86	10.86
10.87	10.87	10.87	10.87	10.87	10.87	10.87	10.87	10.87
10.88	10.88	10.88	10.88	10.88	10.88	10.88	10.88	10.88
10.89	10.89	10.89	10.89	10.89	10.89	10.89	10.89	10.89
10.9	10.9	10.9	10.9	10.9	10.9	10.9	10.9	10.9
10.91	10.91	10.91	10.91	10.91	10.91	10.91	10.91	10.91
10.92	10.92	10.92	10.92	10.92	10.92	10.92	10.92	10.92
10.93	10.93	10.93	10.93	10.93	10.93	10.93	10.93	10.93
10.94	10.94	10.94	10.94	10.94	10.94	10.94	10.94	10.94
10.95	10.95	10.95	10.95	10.95	10.95	10.95	10.95	10.95
10.96	10.96	10.96	10.96	10.96	10.96	10.96	10.96	10.96
10.97	10.97	10.97	10.97	10.97	10.97	10.97	10.97	10.97
10.98	10.98	10.98	10.98	10.98	10.98	10.98	10.98	10.98
10.99	10.99	10.99	10.99	10.99	10.99	10.99	10.99	10.99

11	11	11	11	11	11	11	11	11
11.01	11.01	11.01	11.01	11.01	11.01	11.01	11.01	11.01
11.02	11.02	11.02	11.02	11.02	11.02	11.02	11.02	11.02
11.03	11.03	11.03	11.03	11.03	11.03	11.03	11.03	11.03
11.04	11.04	11.04	11.04	11.04	11.04	11.04	11.04	11.04
11.05	11.05	11.05	11.05	11.05	11.05	11.05	11.05	11.05
11.06	11.06	11.06	11.06	11.06	11.06	11.06	11.06	11.06
11.07	11.07	11.07	11.07	11.07	11.07	11.07	11.07	11.07
11.08	11.08	11.08	11.08	11.08	11.08	11.08	11.08	11.08
11.09	11.09	11.09	11.09	11.09	11.09	11.09	11.09	11.09
11.1	11.1	11.1	11.1	11.1	11.1	11.1	11.1	11.1
11.11	11.11	11.11	11.11	11.11	11.11	11.11	11.11	11.11
11.12	11.12	11.12	11.12	11.12	11.12	11.12	11.12	11.12
11.13	11.13	11.13	11.13	11.13	11.13	11.13	11.13	11.13
11.14	11.14	11.14	11.14	11.14	11.14	11.14	11.14	11.14
11.15	11.15	11.15	11.15	11.15	11.15	11.15	11.15	11.15
11.16	11.16	11.16	11.16	11.16	11.16	11.16	11.16	11.16
11.17	11.17	11.17	11.17	11.17	11.17	11.17	11.17	11.17
11.18	11.18	11.18	11.18	11.18	11.18	11.18	11.18	11.18
11.19	11.19	11.19	11.19	11.19	11.19	11.19	11.19	11.19
11.2	11.2	11.2	11.2	11.2	11.2	11.2	11.2	11.2
11.21	11.21	11.21	11.21	11.21	11.21	11.21	11.21	11.21
11.22	11.22	11.22	11.22	11.22	11.22	11.22	11.22	11.22
11.23	11.23	11.23	11.23	11.23	11.23	11.23	11.23	11.23
11.24	11.24	11.24	11.24	11.24	11.24	11.24	11.24	11.24
11.25	11.25	11.25	11.25	11.25	11.25	11.25	11.25	11.25
11.26	11.26	11.26	11.26	11.26	11.26	11.26	11.26	11.26
11.27	11.27	11.27	11.27	11.27	11.27	11.27	11.27	11.27
11.28	11.28	11.28	11.28	11.28	11.28	11.28	11.28	11.28
11.29	11.29	11.29	11.29	11.29	11.29	11.29	11.29	11.29
11.3	11.3	11.3	11.3	11.3	11.3	11.3	11.3	11.3
11.31	11.31	11.31	11.31	11.31	11.31	11.31	11.31	11.31
11.32	11.32	11.32	11.32	11.32	11.32	11.32	11.32	11.32
11.33	11.33	11.33	11.33	11.33	11.33	11.33	11.33	11.33
11.34	11.34	11.34	11.34	11.34	11.34	11.34	11.34	11.34
11.35	11.35	11.35	11.35	11.35	11.35	11.35	11.35	11.35
11.36	11.36	11.36	11.36	11.36	11.36	11.36	11.36	11.36
11.37	11.37	11.37	11.37	11.37	11.37	11.37	11.37	11.37
11.38	11.38	11.38	11.38	11.38	11.38	11.38	11.38	11.38
11.39	11.39	11.39	11.39	11.39	11.39	11.39	11.39	11.39
11.4	11.4	11.4	11.4	11.4	11.4	11.4	11.4	11.4
11.41	11.41	11.41	11.41	11.41	11.41	11.41	11.41	11.41
11.42	11.42	11.42	11.42	11.42	11.42	11.42	11.42	11.42
11.43	11.43	11.43	11.43	11.43	11.43	11.43	11.43	11.43
11.44	11.44	11.44	11.44	11.44	11.44	11.44	11.44	11.44
11.45	11.45	11.45	11.45	11.45	11.45	11.45	11.45	11.45
11.46	11.46	11.46	11.46	11.46	11.46	11.46	11.46	11.46
11.47	11.47	11.47	11.47	11.47	11.47	11.47	11.47	11.47
11.48	11.48	11.48	11.48	11.48	11.48	11.48	11.48	11.48
11.49	11.49	11.49	11.49	11.49	11.49	11.49	11.49	11.49

11.5	11.5	11.5	11.5	11.5	11.5	11.5	11.5	11.5
11.51	11.51	11.51	11.51	11.51	11.51	11.51	11.51	11.51
11.52	11.52	11.52	11.52	11.52	11.52	11.52	11.52	11.52
11.53	11.53	11.53	11.53	11.53	11.53	11.53	11.53	11.53
11.54	11.54	11.54	11.54	11.54	11.54	11.54	11.54	11.54
11.55	11.55	11.55	11.55	11.55	11.55	11.55	11.55	11.55
11.56	11.56	11.56	11.56	11.56	11.56	11.56	11.56	11.56
11.57	11.57	11.57	11.57	11.57	11.57	11.57	11.57	11.57
11.58	11.58	11.58	11.58	11.58	11.58	11.58	11.58	11.58
11.59	11.59	11.59	11.59	11.59	11.59	11.59	11.59	11.59
11.6	11.6	11.6	11.6	11.6	11.6	11.6	11.6	11.6
11.61	11.61	11.61	11.61	11.61	11.61	11.61	11.61	11.61
11.62	11.62	11.62	11.62	11.62	11.62	11.62	11.62	11.62
11.63	11.63	11.63	11.63	11.63	11.63	11.63	11.63	11.63
11.64	11.64	11.64	11.64	11.64	11.64	11.64	11.64	11.64
11.65	11.65	11.65	11.65	11.65	11.65	11.65	11.65	11.65
11.66	11.66	11.66	11.66	11.66	11.66	11.66	11.66	11.66
11.67	11.67	11.67	11.67	11.67	11.67	11.67	11.67	11.67
11.68	11.68	11.68	11.68	11.68	11.68	11.68	11.68	11.68
11.69	11.69	11.69	11.69	11.69	11.69	11.69	11.69	11.69
11.7	11.7	11.7	11.7	11.7	11.7	11.7	11.7	11.7
11.71	11.71	11.71	11.71	11.71	11.71	11.71	11.71	11.71
11.72	11.72	11.72	11.72	11.72	11.72	11.72	11.72	11.72
11.73	11.73	11.73	11.73	11.73	11.73	11.73	11.73	11.73
11.74	11.74	11.74	11.74	11.74	11.74	11.74	11.74	11.74
11.75	11.75	11.75	11.75	11.75	11.75	11.75	11.75	11.75
11.76	11.76	11.76	11.76	11.76	11.76	11.76	11.76	11.76
11.77	11.77	11.77	11.77	11.77	11.77	11.77	11.77	11.77
11.78	11.78	11.78	11.78	11.78	11.78	11.78	11.78	11.78
11.79	11.79	11.79	11.79	11.79	11.79	11.79	11.79	11.79
11.8	11.8	11.8	11.8	11.8	11.8	11.8	11.8	11.8
11.81	11.81	11.81	11.81	11.81	11.81	11.81	11.81	11.81
11.82	11.82	11.82	11.82	11.82	11.82	11.82	11.82	11.82
11.83	11.83	11.83	11.83	11.83	11.83	11.83	11.83	11.83
11.84	11.84	11.84	11.84	11.84	11.84	11.84	11.84	11.84
11.85	11.85	11.85	11.85	11.85	11.85	11.85	11.85	11.85
11.86	11.86	11.86	11.86	11.86	11.86	11.86	11.86	11.86
11.87	11.87	11.87	11.87	11.87	11.87	11.87	11.87	11.87
11.88	11.88	11.88	11.88	11.88	11.88	11.88	11.88	11.88
11.89	11.89	11.89	11.89	11.89	11.89	11.89	11.89	11.89
11.9	11.9	11.9	11.9	11.9	11.9	11.9	11.9	11.9
11.91	11.91	11.91	11.91	11.91	11.91	11.91	11.91	11.91
11.92	11.92	11.92	11.92	11.92	11.92	11.92	11.92	11.92
11.93	11.93	11.93	11.93	11.93	11.93	11.93	11.93	11.93
11.94	11.94	11.94	11.94	11.94	11.94	11.94	11.94	11.94
11.95	11.95	11.95	11.95	11.95	11.95	11.95	11.95	11.95
11.96	11.96	11.96	11.96	11.96	11.96	11.96	11.96	11.96
11.97	11.97	11.97	11.97	11.97	11.97	11.97	11.97	11.97
11.98	11.98	11.98	11.98	11.98	11.98	11.98	11.98	11.98
11.99	11.99	11.99	11.99	11.99	11.99	11.99	11.99	11.99

12	12	12	12	12	12	12	12	12

-12	-12	-12	-12	-12	-12	-12	-12	-12
-11.99	-11.99	-11.99	-11.99	-11.99	-11.99	-11.99	-11.99	-11.99
-11.98	-11.98	-11.98	-11.98	-11.98	-11.98	-11.98	-11.98	-11.98
-11.97	-11.97	-11.97	-11.97	-11.97	-11.97	-11.97	-11.97	-11.97
-11.96	-11.96	-11.96	-11.96	-11.96	-11.96	-11.96	-11.96	-11.96
-11.95	-11.95	-11.95	-11.95	-11.95	-11.95	-11.95	-11.95	-11.95
-11.94	-11.94	-11.94	-11.94	-11.94	-11.94	-11.94	-11.94	-11.94
-11.93	-11.93	-11.93	-11.93	-11.93	-11.93	-11.93	-11.93	-11.93
-11.92	-11.92	-11.92	-11.92	-11.92	-11.92	-11.92	-11.92	-11.92
-11.91	-11.91	-11.91	-11.91	-11.91	-11.91	-11.91	-11.91	-11.91
-11.9	-11.9	-11.9	-11.9	-11.9	-11.9	-11.9	-11.9	-11.9
-11.89	-11.89	-11.89	-11.89	-11.89	-11.89	-11.89	-11.89	-11.89
-11.88	-11.88	-11.88	-11.88	-11.88	-11.88	-11.88	-11.88	-11.88
-11.87	-11.87	-11.87	-11.87	-11.87	-11.87	-11.87	-11.87	-11.87
-11.86	-11.86	-11.86	-11.86	-11.86	-11.86	-11.86	-11.86	-11.86
-11.85	-11.85	-11.85	-11.85	-11.85	-11.85	-11.85	-11.85	-11.85
-11.84	-11.84	-11.84	-11.84	-11.84	-11.84	-11.84	-11.84	-11.84
-11.83	-11.83	-11.83	-11.83	-11.83	-11.83	-11.83	-11.83	-11.83
-11.82	-11.82	-11.82	-11.82	-11.82	-11.82	-11.82	-11.82	-11.82
-11.81	-11.81	-11.81	-11.81	-11.81	-11.81	-11.81	-11.81	-11.81
-11.8	-11.8	-11.8	-11.8	-11.8	-11.8	-11.8	-11.8	-11.8
-11.79	-11.79	-11.79	-11.79	-11.79	-11.79	-11.79	-11.79	-11.79
-11.78	-11.78	-11.78	-11.78	-11.78	-11.78	-11.78	-11.78	-11.78
-11.77	-11.77	-11.77	-11.77	-11.77	-11.77	-11.77	-11.77	-11.77
-11.76	-11.76	-11.76	-11.76	-11.76	-11.76	-11.76	-11.76	-11.76
-11.75	-11.75	-11.75	-11.75	-11.75	-11.75	-11.75	-11.75	-11.75
-11.74	-11.74	-11.74	-11.74	-11.74	-11.74	-11.74	-11.74	-11.74
-11.73	-11.73	-11.73	-11.73	-11.73	-11.73	-11.73	-11.73	-11.73
-11.72	-11.72	-11.72	-11.72	-11.72	-11.72	-11.72	-11.72	-11.72
-11.71	-11.71	-11.71	-11.71	-11.71	-11.71	-11.71	-11.71	-11.71
-11.7	-11.7	-11.7	-11.7	-11.7	-11.7	-11.7	-11.7	-11.7
-11.69	-11.69	-11.69	-11.69	-11.69	-11.69	-11.69	-11.69	-11.69
-11.68	-11.68	-11.68	-11.68	-11.68	-11.68	-11.68	-11.68	-11.68
-11.67	-11.67	-11.67	-11.67	-11.67	-11.67	-11.67	-11.67	-11.67
-11.66	-11.66	-11.66	-11.66	-11.66	-11.66	-11.66	-11.66	-11.66
-11.65	-11.65	-11.65	-11.65	-11.65	-11.65	-11.65	-11.65	-11.65
-11.64	-11.64	-11.64	-11.64	-11.64	-11.64	-11.64	-11.64	-11.64
-11.63	-11.63	-11.63	-11.63	-11.63	-11.63	-11.63	-11.63	-11.63
-11.62	-11.62	-11.62	-11.62	-11.62	-11.62	-11.62	-11.62	-11.62
-11.61	-11.61	-11.61	-11.61	-11.61	-11.61	-11.61	-11.61	-11.61
-11.6	-11.6	-11.6	-11.6	-11.6	-11.6	-11.6	-11.6	-11.6
-11.59	-11.59	-11.59	-11.59	-11.59	-11.59	-11.59	-11.59	-11.59
-11.58	-11.58	-11.58	-11.58	-11.58	-11.58	-11.58	-11.58	-11.58
-11.57	-11.57	-11.57	-11.57	-11.57	-11.57	-11.57	-11.57	-11.57
-11.56	-11.56	-11.56	-11.56	-11.56	-11.56	-11.56	-11.56	-11.56
-11.55	-11.55	-11.55	-11.55	-11.55	-11.55	-11.55	-11.55	-11.55
-11.54	-11.54	-11.54	-11.54	-11.54	-11.54	-11.54	-11.54	-11.54
-11.53	-11.53	-11.53	-11.53	-11.53	-11.53	-11.53	-11.53	-11.53
-11.52	-11.52	-11.52	-11.52	-11.52	-11.52	-11.52	-11.52	-11.52
-11.51	-11.51	-11.51	-11.51	-11.51	-11.51	-11.51	-11.51	-11.51

-11.5	-11.5	-11.5	-11.5	-11.5	-11.5	-11.5	-11.5	-11.5
-11.49	-11.49	-11.49	-11.49	-11.49	-11.49	-11.49	-11.49	-11.49
-11.48	-11.48	-11.48	-11.48	-11.48	-11.48	-11.48	-11.48	-11.48
-11.47	-11.47	-11.47	-11.47	-11.47	-11.47	-11.47	-11.47	-11.47
-11.46	-11.46	-11.46	-11.46	-11.46	-11.46	-11.46	-11.46	-11.46
-11.45	-11.45	-11.45	-11.45	-11.45	-11.45	-11.45	-11.45	-11.45
-11.44	-11.44	-11.44	-11.44	-11.44	-11.44	-11.44	-11.44	-11.44
-11.43	-11.43	-11.43	-11.43	-11.43	-11.43	-11.43	-11.43	-11.43
-11.42	-11.42	-11.42	-11.42	-11.42	-11.42	-11.42	-11.42	-11.42
-11.41	-11.41	-11.41	-11.41	-11.41	-11.41	-11.41	-11.41	-11.41
-11.4	-11.4	-11.4	-11.4	-11.4	-11.4	-11.4	-11.4	-11.4
-11.39	-11.39	-11.39	-11.39	-11.39	-11.39	-11.39	-11.39	-11.39
-11.38	-11.38	-11.38	-11.38	-11.38	-11.38	-11.38	-11.38	-11.38
-11.37	-11.37	-11.37	-11.37	-11.37	-11.37	-11.37	-11.37	-11.37
-11.36	-11.36	-11.36	-11.36	-11.36	-11.36	-11.36	-11.36	-11.36
-11.35	-11.35	-11.35	-11.35	-11.35	-11.35	-11.35	-11.35	-11.35
-11.34	-11.34	-11.34	-11.34	-11.34	-11.34	-11.34	-11.34	-11.34
-11.33	-11.33	-11.33	-11.33	-11.33	-11.33	-11.33	-11.33	-11.33
-11.32	-11.32	-11.32	-11.32	-11.32	-11.32	-11.32	-11.32	-11.32
-11.31	-11.31	-11.31	-11.31	-11.31	-11.31	-11.31	-11.31	-11.31
-11.3	-11.3	-11.3	-11.3	-11.3	-11.3	-11.3	-11.3	-11.3
-11.29	-11.29	-11.29	-11.29	-11.29	-11.29	-11.29	-11.29	-11.29
-11.28	-11.28	-11.28	-11.28	-11.28	-11.28	-11.28	-11.28	-11.28
-11.27	-11.27	-11.27	-11.27	-11.27	-11.27	-11.27	-11.27	-11.27
-11.26	-11.26	-11.26	-11.26	-11.26	-11.26	-11.26	-11.26	-11.26
-11.25	-11.25	-11.25	-11.25	-11.25	-11.25	-11.25	-11.25	-11.25
-11.24	-11.24	-11.24	-11.24	-11.24	-11.24	-11.24	-11.24	-11.24
-11.23	-11.23	-11.23	-11.23	-11.23	-11.23	-11.23	-11.23	-11.23
-11.22	-11.22	-11.22	-11.22	-11.22	-11.22	-11.22	-11.22	-11.22
-11.21	-11.21	-11.21	-11.21	-11.21	-11.21	-11.21	-11.21	-11.21
-11.2	-11.2	-11.2	-11.2	-11.2	-11.2	-11.2	-11.2	-11.2
-11.19	-11.19	-11.19	-11.19	-11.19	-11.19	-11.19	-11.19	-11.19
-11.18	-11.18	-11.18	-11.18	-11.18	-11.18	-11.18	-11.18	-11.18
-11.17	-11.17	-11.17	-11.17	-11.17	-11.17	-11.17	-11.17	-11.17
-11.16	-11.16	-11.16	-11.16	-11.16	-11.16	-11.16	-11.16	-11.16
-11.15	-11.15	-11.15	-11.15	-11.15	-11.15	-11.15	-11.15	-11.15
-11.14	-11.14	-11.14	-11.14	-11.14	-11.14	-11.14	-11.14	-11.14
-11.13	-11.13	-11.13	-11.13	-11.13	-11.13	-11.13	-11.13	-11.13
-11.12	-11.12	-11.12	-11.12	-11.12	-11.12	-11.12	-11.12	-11.12
-11.11	-11.11	-11.11	-11.11	-11.11	-11.11	-11.11	-11.11	-11.11
-11.1	-11.1	-11.1	-11.1	-11.1	-11.1	-11.1	-11.1	-11.1
-11.09	-11.09	-11.09	-11.09	-11.09	-11.09	-11.09	-11.09	-11.09
-11.08	-11.08	-11.08	-11.08	-11.08	-11.08	-11.08	-11.08	-11.08
-11.07	-11.07	-11.07	-11.07	-11.07	-11.07	-11.07	-11.07	-11.07
-11.06	-11.06	-11.06	-11.06	-11.06	-11.06	-11.06	-11.06	-11.06
-11.05	-11.05	-11.05	-11.05	-11.05	-11.05	-11.05	-11.05	-11.05
-11.04	-11.04	-11.04	-11.04	-11.04	-11.04	-11.04	-11.04	-11.04
-11.03	-11.03	-11.03	-11.03	-11.03	-11.03	-11.03	-11.03	-11.03
-11.02	-11.02	-11.02	-11.02	-11.02	-11.02	-11.02	-11.02	-11.02
-11.01	-11.01	-11.01	-11.01	-11.01	-11.01	-11.01	-11.01	-11.01

-11	-11	-11	-11	-11	-11	-11	-11	-11
-10.99	-10.99	-10.99	-10.99	-10.99	-10.99	-10.99	-10.99	-10.99
-10.98	-10.98	-10.98	-10.98	-10.98	-10.98	-10.98	-10.98	-10.98
-10.97	-10.97	-10.97	-10.97	-10.97	-10.97	-10.97	-10.97	-10.97
-10.96	-10.96	-10.96	-10.96	-10.96	-10.96	-10.96	-10.96	-10.96
-10.95	-10.95	-10.95	-10.95	-10.95	-10.95	-10.95	-10.95	-10.95
-10.94	-10.94	-10.94	-10.94	-10.94	-10.94	-10.94	-10.94	-10.94
-10.93	-10.93	-10.93	-10.93	-10.93	-10.93	-10.93	-10.93	-10.93
-10.92	-10.92	-10.92	-10.92	-10.92	-10.92	-10.92	-10.92	-10.92
-10.91	-10.91	-10.91	-10.91	-10.91	-10.91	-10.91	-10.91	-10.91
-10.9	-10.9	-10.9	-10.9	-10.9	-10.9	-10.9	-10.9	-10.9
-10.89	-10.89	-10.89	-10.89	-10.89	-10.89	-10.89	-10.89	-10.89
-10.88	-10.88	-10.88	-10.88	-10.88	-10.88	-10.88	-10.88	-10.88
-10.87	-10.87	-10.87	-10.87	-10.87	-10.87	-10.87	-10.87	-10.87
-10.86	-10.86	-10.86	-10.86	-10.86	-10.86	-10.86	-10.86	-10.86
-10.85	-10.85	-10.85	-10.85	-10.85	-10.85	-10.85	-10.85	-10.85
-10.84	-10.84	-10.84	-10.84	-10.84	-10.84	-10.84	-10.84	-10.84
-10.83	-10.83	-10.83	-10.83	-10.83	-10.83	-10.83	-10.83	-10.83
-10.82	-10.82	-10.82	-10.82	-10.82	-10.82	-10.82	-10.82	-10.82
-10.81	-10.81	-10.81	-10.81	-10.81	-10.81	-10.81	-10.81	-10.81
-10.8	-10.8	-10.8	-10.8	-10.8	-10.8	-10.8	-10.8	-10.8
-10.79	-10.79	-10.79	-10.79	-10.79	-10.79	-10.79	-10.79	-10.79
-10.78	-10.78	-10.78	-10.78	-10.78	-10.78	-10.78	-10.78	-10.78
-10.77	-10.77	-10.77	-10.77	-10.77	-10.77	-10.77	-10.77	-10.77
-10.76	-10.76	-10.76	-10.76	-10.76	-10.76	-10.76	-10.76	-10.76
-10.75	-10.75	-10.75	-10.75	-10.75	-10.75	-10.75	-10.75	-10.75
-10.74	-10.74	-10.74	-10.74	-10.74	-10.74	-10.74	-10.74	-10.74
-10.73	-10.73	-10.73	-10.73	-10.73	-10.73	-10.73	-10.73	-10.73
-10.72	-10.72	-10.72	-10.72	-10.72	-10.72	-10.72	-10.72	-10.72
-10.71	-10.71	-10.71	-10.71	-10.71	-10.71	-10.71	-10.71	-10.71
-10.7	-10.7	-10.7	-10.7	-10.7	-10.7	-10.7	-10.7	-10.7
-10.69	-10.69	-10.69	-10.69	-10.69	-10.69	-10.69	-10.69	-10.69
-10.68	-10.68	-10.68	-10.68	-10.68	-10.68	-10.68	-10.68	-10.68
-10.67	-10.67	-10.67	-10.67	-10.67	-10.67	-10.67	-10.67	-10.67
-10.66	-10.66	-10.66	-10.66	-10.66	-10.66	-10.66	-10.66	-10.66
-10.65	-10.65	-10.65	-10.65	-10.65	-10.65	-10.65	-10.65	-10.65
-10.64	-10.64	-10.64	-10.64	-10.64	-10.64	-10.64	-10.64	-10.64
-10.63	-10.63	-10.63	-10.63	-10.63	-10.63	-10.63	-10.63	-10.63
-10.62	-10.62	-10.62	-10.62	-10.62	-10.62	-10.62	-10.62	-10.62
-10.61	-10.61	-10.61	-10.61	-10.61	-10.61	-10.61	-10.61	-10.61
-10.6	-10.6	-10.6	-10.6	-10.6	-10.6	-10.6	-10.6	-10.6
-10.59	-10.59	-10.59	-10.59	-10.59	-10.59	-10.59	-10.59	-10.59
-10.58	-10.58	-10.58	-10.58	-10.58	-10.58	-10.58	-10.58	-10.58
-10.57	-10.57	-10.57	-10.57	-10.57	-10.57	-10.57	-10.57	-10.57
-10.56	-10.56	-10.56	-10.56	-10.56	-10.56	-10.56	-10.56	-10.56
-10.55	-10.55	-10.55	-10.55	-10.55	-10.55	-10.55	-10.55	-10.55
-10.54	-10.54	-10.54	-10.54	-10.54	-10.54	-10.54	-10.54	-10.54
-10.53	-10.53	-10.53	-10.53	-10.53	-10.53	-10.53	-10.53	-10.53
-10.52	-10.52	-10.52	-10.52	-10.52	-10.52	-10.52	-10.52	-10.52
-10.51	-10.51	-10.51	-10.51	-10.51	-10.51	-10.51	-10.51	-10.51

-10.5	-10.5	-10.5	-10.5	-10.5	-10.5	-10.5	-10.5	-10.5
-10.49	-10.49	-10.49	-10.49	-10.49	-10.49	-10.49	-10.49	-10.49
-10.48	-10.48	-10.48	-10.48	-10.48	-10.48	-10.48	-10.48	-10.48
-10.47	-10.47	-10.47	-10.47	-10.47	-10.47	-10.47	-10.47	-10.47
-10.46	-10.46	-10.46	-10.46	-10.46	-10.46	-10.46	-10.46	-10.46
-10.45	-10.45	-10.45	-10.45	-10.45	-10.45	-10.45	-10.45	-10.45
-10.44	-10.44	-10.44	-10.44	-10.44	-10.44	-10.44	-10.44	-10.44
-10.43	-10.43	-10.43	-10.43	-10.43	-10.43	-10.43	-10.43	-10.43
-10.42	-10.42	-10.42	-10.42	-10.42	-10.42	-10.42	-10.42	-10.42
-10.41	-10.41	-10.41	-10.41	-10.41	-10.41	-10.41	-10.41	-10.41
-10.4	-10.4	-10.4	-10.4	-10.4	-10.4	-10.4	-10.4	-10.4
-10.39	-10.39	-10.39	-10.39	-10.39	-10.39	-10.39	-10.39	-10.39
-10.38	-10.38	-10.38	-10.38	-10.38	-10.38	-10.38	-10.38	-10.38
-10.37	-10.37	-10.37	-10.37	-10.37	-10.37	-10.37	-10.37	-10.37
-10.36	-10.36	-10.36	-10.36	-10.36	-10.36	-10.36	-10.36	-10.36
-10.35	-10.35	-10.35	-10.35	-10.35	-10.35	-10.35	-10.35	-10.35
-10.34	-10.34	-10.34	-10.34	-10.34	-10.34	-10.34	-10.34	-10.34
-10.33	-10.33	-10.33	-10.33	-10.33	-10.33	-10.33	-10.33	-10.33
-10.32	-10.32	-10.32	-10.32	-10.32	-10.32	-10.32	-10.32	-10.32
-10.31	-10.31	-10.31	-10.31	-10.31	-10.31	-10.31	-10.31	-10.31
-10.3	-10.3	-10.3	-10.3	-10.3	-10.3	-10.3	-10.3	-10.3
-10.29	-10.29	-10.29	-10.29	-10.29	-10.29	-10.29	-10.29	-10.29
-10.28	-10.28	-10.28	-10.28	-10.28	-10.28	-10.28	-10.28	-10.28
-10.27	-10.27	-10.27	-10.27	-10.27	-10.27	-10.27	-10.27	-10.27
-10.26	-10.26	-10.26	-10.26	-10.26	-10.26	-10.26	-10.26	-10.26
-10.25	-10.25	-10.25	-10.25	-10.25	-10.25	-10.25	-10.25	-10.25
-10.24	-10.24	-10.24	-10.24	-10.24	-10.24	-10.24	-10.24	-10.24
-10.23	-10.23	-10.23	-10.23	-10.23	-10.23	-10.23	-10.23	-10.23
-10.22	-10.22	-10.22	-10.22	-10.22	-10.22	-10.22	-10.22	-10.22
-10.21	-10.21	-10.21	-10.21	-10.21	-10.21	-10.21	-10.21	-10.21
-10.2	-10.2	-10.2	-10.2	-10.2	-10.2	-10.2	-10.2	-10.2
-10.19	-10.19	-10.19	-10.19	-10.19	-10.19	-10.19	-10.19	-10.19
-10.18	-10.18	-10.18	-10.18	-10.18	-10.18	-10.18	-10.18	-10.18
-10.17	-10.17	-10.17	-10.17	-10.17	-10.17	-10.17	-10.17	-10.17
-10.16	-10.16	-10.16	-10.16	-10.16	-10.16	-10.16	-10.16	-10.16
-10.15	-10.15	-10.15	-10.15	-10.15	-10.15	-10.15	-10.15	-10.15
-10.14	-10.14	-10.14	-10.14	-10.14	-10.14	-10.14	-10.14	-10.14
-10.13	-10.13	-10.13	-10.13	-10.13	-10.13	-10.13	-10.13	-10.13
-10.12	-10.12	-10.12	-10.12	-10.12	-10.12	-10.12	-10.12	-10.12
-10.11	-10.11	-10.11	-10.11	-10.11	-10.11	-10.11	-10.11	-10.11
-10.1	-10.1	-10.1	-10.1	-10.1	-10.1	-10.1	-10.1	-10.1
-10.09	-10.09	-10.09	-10.09	-10.09	-10.09	-10.09	-10.09	-10.09
-10.08	-10.08	-10.08	-10.08	-10.08	-10.08	-10.08	-10.08	-10.08
-10.07	-10.07	-10.07	-10.07	-10.07	-10.07	-10.07	-10.07	-10.07
-10.06	-10.06	-10.06	-10.06	-10.06	-10.06	-10.06	-10.06	-10.06
-10.05	-10.05	-10.05	-10.05	-10.05	-10.05	-10.05	-10.05	-10.05
-10.04	-10.04	-10.04	-10.04	-10.04	-10.04	-10.04	-10.04	-10.04
-10.03	-10.03	-10.03	-10.03	-10.03	-10.03	-10.03	-10.03	-10.03
-10.02	-10.02	-10.02	-10.02	-10.02	-10.02	-10.02	-10.02	-10.02
-10.01	-10.01	-10.01	-10.01	-10.01	-10.01	-10.01	-10.01	-10.01

-10	-10	-10	-10	-10	-10	-10	-10	-10
-9.99	-9.99	-9.99	-9.99	-9.99	-9.99	-9.99	-9.99	-9.99
-9.98	-9.98	-9.98	-9.98	-9.98	-9.98	-9.98	-9.98	-9.98
-9.97	-9.97	-9.97	-9.97	-9.97	-9.97	-9.97	-9.97	-9.97
-9.96	-9.96	-9.96	-9.96	-9.96	-9.96	-9.96	-9.96	-9.96
-9.95	-9.95	-9.95	-9.95	-9.95	-9.95	-9.95	-9.95	-9.95
-9.94	-9.94	-9.94	-9.94	-9.94	-9.94	-9.94	-9.94	-9.94
-9.93	-9.93	-9.93	-9.93	-9.93	-9.93	-9.93	-9.93	-9.93
-9.92	-9.92	-9.92	-9.92	-9.92	-9.92	-9.92	-9.92	-9.92
-9.91	-9.91	-9.91	-9.91	-9.91	-9.91	-9.91	-9.91	-9.91
-9.9	-9.9	-9.9	-9.9	-9.9	-9.9	-9.9	-9.9	-9.9
-9.89	-9.89	-9.89	-9.89	-9.89	-9.89	-9.89	-9.89	-9.89
-9.88	-9.88	-9.88	-9.88	-9.88	-9.88	-9.88	-9.88	-9.88
-9.87	-9.87	-9.87	-9.87	-9.87	-9.87	-9.87	-9.87	-9.87
-9.86	-9.86	-9.86	-9.86	-9.86	-9.86	-9.86	-9.86	-9.86
-9.85	-9.85	-9.85	-9.85	-9.85	-9.85	-9.85	-9.85	-9.85
-9.84	-9.84	-9.84	-9.84	-9.84	-9.84	-9.84	-9.84	-9.84
-9.83	-9.83	-9.83	-9.83	-9.83	-9.83	-9.83	-9.83	-9.83
-9.82	-9.82	-9.82	-9.82	-9.82	-9.82	-9.82	-9.82	-9.82
-9.81	-9.81	-9.81	-9.81	-9.81	-9.81	-9.81	-9.81	-9.81
-9.8	-9.8	-9.8	-9.8	-9.8	-9.8	-9.8	-9.8	-9.8
-9.79	-9.79	-9.79	-9.79	-9.79	-9.79	-9.79	-9.79	-9.79
-9.78	-9.78	-9.78	-9.78	-9.78	-9.78	-9.78	-9.78	-9.78
-9.77	-9.77	-9.77	-9.77	-9.77	-9.77	-9.77	-9.77	-9.77
-9.76	-9.76	-9.76	-9.76	-9.76	-9.76	-9.76	-9.76	-9.76
-9.75	-9.75	-9.75	-9.75	-9.75	-9.75	-9.75	-9.75	-9.75
-9.74	-9.74	-9.74	-9.74	-9.74	-9.74	-9.74	-9.74	-9.74
-9.73	-9.73	-9.73	-9.73	-9.73	-9.73	-9.73	-9.73	-9.73
-9.72	-9.72	-9.72	-9.72	-9.72	-9.72	-9.72	-9.72	-9.72
-9.71	-9.71	-9.71	-9.71	-9.71	-9.71	-9.71	-9.71	-9.71
-9.7	-9.7	-9.7	-9.7	-9.7	-9.7	-9.7	-9.7	-9.7
-9.69	-9.69	-9.69	-9.69	-9.69	-9.69	-9.69	-9.69	-9.69
-9.68	-9.68	-9.68	-9.68	-9.68	-9.68	-9.68	-9.68	-9.68
-9.67	-9.67	-9.67	-9.67	-9.67	-9.67	-9.67	-9.67	-9.67
-9.66	-9.66	-9.66	-9.66	-9.66	-9.66	-9.66	-9.66	-9.66
-9.65	-9.65	-9.65	-9.65	-9.65	-9.65	-9.65	-9.65	-9.65
-9.64	-9.64	-9.64	-9.64	-9.64	-9.64	-9.64	-9.64	-9.64
-9.63	-9.63	-9.63	-9.63	-9.63	-9.63	-9.63	-9.63	-9.63
-9.62	-9.62	-9.62	-9.62	-9.62	-9.62	-9.62	-9.62	-9.62
-9.61	-9.61	-9.61	-9.61	-9.61	-9.61	-9.61	-9.61	-9.61
-9.6	-9.6	-9.6	-9.6	-9.6	-9.6	-9.6	-9.6	-9.6
-9.59	-9.59	-9.59	-9.59	-9.59	-9.59	-9.59	-9.59	-9.59
-9.58	-9.58	-9.58	-9.58	-9.58	-9.58	-9.58	-9.58	-9.58
-9.57	-9.57	-9.57	-9.57	-9.57	-9.57	-9.57	-9.57	-9.57
-9.56	-9.56	-9.56	-9.56	-9.56	-9.56	-9.56	-9.56	-9.56
-9.55	-9.55	-9.55	-9.55	-9.55	-9.55	-9.55	-9.55	-9.55
-9.54	-9.54	-9.54	-9.54	-9.54	-9.54	-9.54	-9.54	-9.54
-9.53	-9.53	-9.53	-9.53	-9.53	-9.53	-9.53	-9.53	-9.53
-9.52	-9.52	-9.52	-9.52	-9.52	-9.52	-9.52	-9.52	-9.52
-9.51	-9.51	-9.51	-9.51	-9.51	-9.51	-9.51	-9.51	-9.51

-9.5	-9.5	-9.5	-9.5	-9.5	-9.5	-9.5	-9.5	-9.5
-9.49	-9.49	-9.49	-9.49	-9.49	-9.49	-9.49	-9.49	-9.49
-9.48	-9.48	-9.48	-9.48	-9.48	-9.48	-9.48	-9.48	-9.48
-9.47	-9.47	-9.47	-9.47	-9.47	-9.47	-9.47	-9.47	-9.47
-9.46	-9.46	-9.46	-9.46	-9.46	-9.46	-9.46	-9.46	-9.46
-9.45	-9.45	-9.45	-9.45	-9.45	-9.45	-9.45	-9.45	-9.45
-9.44	-9.44	-9.44	-9.44	-9.44	-9.44	-9.44	-9.44	-9.44
-9.43	-9.43	-9.43	-9.43	-9.43	-9.43	-9.43	-9.43	-9.43
-9.42	-9.42	-9.42	-9.42	-9.42	-9.42	-9.42	-9.42	-9.42
-9.41	-9.41	-9.41	-9.41	-9.41	-9.41	-9.41	-9.41	-9.41
-9.4	-9.4	-9.4	-9.4	-9.4	-9.4	-9.4	-9.4	-9.4
-9.39	-9.39	-9.39	-9.39	-9.39	-9.39	-9.39	-9.39	-9.39
-9.38	-9.38	-9.38	-9.38	-9.38	-9.38	-9.38	-9.38	-9.38
-9.37	-9.37	-9.37	-9.37	-9.37	-9.37	-9.37	-9.37	-9.37
-9.36	-9.36	-9.36	-9.36	-9.36	-9.36	-9.36	-9.36	-9.36
-9.35	-9.35	-9.35	-9.35	-9.35	-9.35	-9.35	-9.35	-9.35
-9.34	-9.34	-9.34	-9.34	-9.34	-9.34	-9.34	-9.34	-9.34
-9.33	-9.33	-9.33	-9.33	-9.33	-9.33	-9.33	-9.33	-9.33
-9.32	-9.32	-9.32	-9.32	-9.32	-9.32	-9.32	-9.32	-9.32
-9.31	-9.31	-9.31	-9.31	-9.31	-9.31	-9.31	-9.31	-9.31
-9.3	-9.3	-9.3	-9.3	-9.3	-9.3	-9.3	-9.3	-9.3
-9.29	-9.29	-9.29	-9.29	-9.29	-9.29	-9.29	-9.29	-9.29
-9.28	-9.28	-9.28	-9.28	-9.28	-9.28	-9.28	-9.28	-9.28
-9.27	-9.27	-9.27	-9.27	-9.27	-9.27	-9.27	-9.27	-9.27
-9.26	-9.26	-9.26	-9.26	-9.26	-9.26	-9.26	-9.26	-9.26
-9.25	-9.25	-9.25	-9.25	-9.25	-9.25	-9.25	-9.25	-9.25
-9.24	-9.24	-9.24	-9.24	-9.24	-9.24	-9.24	-9.24	-9.24
-9.23	-9.23	-9.23	-9.23	-9.23	-9.23	-9.23	-9.23	-9.23
-9.22	-9.22	-9.22	-9.22	-9.22	-9.22	-9.22	-9.22	-9.22
-9.21	-9.21	-9.21	-9.21	-9.21	-9.21	-9.21	-9.21	-9.21
-9.2	-9.2	-9.2	-9.2	-9.2	-9.2	-9.2	-9.2	-9.2
-9.19	-9.19	-9.19	-9.19	-9.19	-9.19	-9.19	-9.19	-9.19
-9.18	-9.18	-9.18	-9.18	-9.18	-9.18	-9.18	-9.18	-9.18
-9.17	-9.17	-9.17	-9.17	-9.17	-9.17	-9.17	-9.17	-9.17
-9.16	-9.16	-9.16	-9.16	-9.16	-9.16	-9.16	-9.16	-9.16
-9.15	-9.15	-9.15	-9.15	-9.15	-9.15	-9.15	-9.15	-9.15
-9.14	-9.14	-9.14	-9.14	-9.14	-9.14	-9.14	-9.14	-9.14
-9.13	-9.13	-9.13	-9.13	-9.13	-9.13	-9.13	-9.13	-9.13
-9.12	-9.12	-9.12	-9.12	-9.12	-9.12	-9.12	-9.12	-9.12
-9.11	-9.11	-9.11	-9.11	-9.11	-9.11	-9.11	-9.11	-9.11
-9.1	-9.1	-9.1	-9.1	-9.1	-9.1	-9.1	-9.1	-9.1
-9.09	-9.09	-9.09	-9.09	-9.09	-9.09	-9.09	-9.09	-9.09
-9.08	-9.08	-9.08	-9.08	-9.08	-9.08	-9.08	-9.08	-9.08
-9.07	-9.07	-9.07	-9.07	-9.07	-9.07	-9.07	-9.07	-9.07
-9.06	-9.06	-9.06	-9.06	-9.06	-9.06	-9.06	-9.06	-9.06
-9.05	-9.05	-9.05	-9.05	-9.05	-9.05	-9.05	-9.05	-9.05
-9.04	-9.04	-9.04	-9.04	-9.04	-9.04	-9.04	-9.04	-9.04
-9.03	-9.03	-9.03	-9.03	-9.03	-9.03	-9.03	-9.03	-9.03
-9.02	-9.02	-9.02	-9.02	-9.02	-9.02	-9.02	-9.02	-9.02
-9.01	-9.01	-9.01	-9.01	-9.01	-9.01	-9.01	-9.01	-9.01

-9	-9	-9	-9	-9	-9	-9	-9	-9
-8.99	-8.99	-8.99	-8.99	-8.99	-8.99	-8.99	-8.99	-8.99
-8.98	-8.98	-8.98	-8.98	-8.98	-8.98	-8.98	-8.98	-8.98
-8.97	-8.97	-8.97	-8.97	-8.97	-8.97	-8.97	-8.97	-8.97
-8.96	-8.96	-8.96	-8.96	-8.96	-8.96	-8.96	-8.96	-8.96
-8.95	-8.95	-8.95	-8.95	-8.95	-8.95	-8.95	-8.95	-8.95
-8.94	-8.94	-8.94	-8.94	-8.94	-8.94	-8.94	-8.94	-8.94
-8.93	-8.93	-8.93	-8.93	-8.93	-8.93	-8.93	-8.93	-8.93
-8.92	-8.92	-8.92	-8.92	-8.92	-8.92	-8.92	-8.92	-8.92
-8.91	-8.91	-8.91	-8.91	-8.91	-8.91	-8.91	-8.91	-8.91
-8.9	-8.9	-8.9	-8.9	-8.9	-8.9	-8.9	-8.9	-8.9
-8.89	-8.89	-8.89	-8.89	-8.89	-8.89	-8.89	-8.89	-8.89
-8.88	-8.88	-8.88	-8.88	-8.88	-8.88	-8.88	-8.88	-8.88
-8.87	-8.87	-8.87	-8.87	-8.87	-8.87	-8.87	-8.87	-8.87
-8.86	-8.86	-8.86	-8.86	-8.86	-8.86	-8.86	-8.86	-8.86
-8.85	-8.85	-8.85	-8.85	-8.85	-8.85	-8.85	-8.85	-8.85
-8.84	-8.84	-8.84	-8.84	-8.84	-8.84	-8.84	-8.84	-8.84
-8.83	-8.83	-8.83	-8.83	-8.83	-8.83	-8.83	-8.83	-8.83
-8.82	-8.82	-8.82	-8.82	-8.82	-8.82	-8.82	-8.82	-8.82
-8.81	-8.81	-8.81	-8.81	-8.81	-8.81	-8.81	-8.81	-8.81
-8.8	-8.8	-8.8	-8.8	-8.8	-8.8	-8.8	-8.8	-8.8
-8.79	-8.79	-8.79	-8.79	-8.79	-8.79	-8.79	-8.79	-8.79
-8.78	-8.78	-8.78	-8.78	-8.78	-8.78	-8.78	-8.78	-8.78
-8.77	-8.77	-8.77	-8.77	-8.77	-8.77	-8.77	-8.77	-8.77
-8.76	-8.76	-8.76	-8.76	-8.76	-8.76	-8.76	-8.76	-8.76
-8.75	-8.75	-8.75	-8.75	-8.75	-8.75	-8.75	-8.75	-8.75
-8.74	-8.74	-8.74	-8.74	-8.74	-8.74	-8.74	-8.74	-8.74
-8.73	-8.73	-8.73	-8.73	-8.73	-8.73	-8.73	-8.73	-8.73
-8.72	-8.72	-8.72	-8.72	-8.72	-8.72	-8.72	-8.72	-8.72
-8.71	-8.71	-8.71	-8.71	-8.71	-8.71	-8.71	-8.71	-8.71
-8.7	-8.7	-8.7	-8.7	-8.7	-8.7	-8.7	-8.7	-8.7
-8.69	-8.69	-8.69	-8.69	-8.69	-8.69	-8.69	-8.69	-8.69
-8.68	-8.68	-8.68	-8.68	-8.68	-8.68	-8.68	-8.68	-8.68
-8.67	-8.67	-8.67	-8.67	-8.67	-8.67	-8.67	-8.67	-8.67
-8.66	-8.66	-8.66	-8.66	-8.66	-8.66	-8.66	-8.66	-8.66
-8.65	-8.65	-8.65	-8.65	-8.65	-8.65	-8.65	-8.65	-8.65
-8.64	-8.64	-8.64	-8.64	-8.64	-8.64	-8.64	-8.64	-8.64
-8.63	-8.63	-8.63	-8.63	-8.63	-8.63	-8.63	-8.63	-8.63
-8.62	-8.62	-8.62	-8.62	-8.62	-8.62	-8.62	-8.62	-8.62
-8.61	-8.61	-8.61	-8.61	-8.61	-8.61	-8.61	-8.61	-8.61
-8.6	-8.6	-8.6	-8.6	-8.6	-8.6	-8.6	-8.6	-8.6
-8.59	-8.59	-8.59	-8.59	-8.59	-8.59	-8.59	-8.59	-8.59
-8.58	-8.58	-8.58	-8.58	-8.58	-8.58	-8.58	-8.58	-8.58
-8.57	-8.57	-8.57	-8.57	-8.57	-8.57	-8.57	-8.57	-8.57
-8.56	-8.56	-8.56	-8.56	-8.56	-8.56	-8.56	-8.56	-8.56
-8.55	-8.55	-8.55	-8.55	-8.55	-8.55	-8.55	-8.55	-8.55
-8.54	-8.54	-8.54	-8.54	-8.54	-8.54	-8.54	-8.54	-8.54
-8.53	-8.53	-8.53	-8.53	-8.53	-8.53	-8.53	-8.53	-8.53
-8.52	-8.52	-8.52	-8.52	-8.52	-8.52	-8.52	-8.52	-8.52
-8.51	-8.51	-8.51	-8.51	-8.51	-8.51	-8.51	-8.51	-8.51

-8.5	-8.5	-8.5	-8.5	-8.5	-8.5	-8.5	-8.5	-8.5
-8.49	-8.49	-8.49	-8.49	-8.49	-8.49	-8.49	-8.49	-8.49
-8.48	-8.48	-8.48	-8.48	-8.48	-8.48	-8.48	-8.48	-8.48
-8.47	-8.47	-8.47	-8.47	-8.47	-8.47	-8.47	-8.47	-8.47
-8.46	-8.46	-8.46	-8.46	-8.46	-8.46	-8.46	-8.46	-8.46
-8.45	-8.45	-8.45	-8.45	-8.45	-8.45	-8.45	-8.45	-8.45
-8.44	-8.44	-8.44	-8.44	-8.44	-8.44	-8.44	-8.44	-8.44
-8.43	-8.43	-8.43	-8.43	-8.43	-8.43	-8.43	-8.43	-8.43
-8.42	-8.42	-8.42	-8.42	-8.42	-8.42	-8.42	-8.42	-8.42
-8.41	-8.41	-8.41	-8.41	-8.41	-8.41	-8.41	-8.41	-8.41
-8.4	-8.4	-8.4	-8.4	-8.4	-8.4	-8.4	-8.4	-8.4
-8.39	-8.39	-8.39	-8.39	-8.39	-8.39	-8.39	-8.39	-8.39
-8.38	-8.38	-8.38	-8.38	-8.38	-8.38	-8.38	-8.38	-8.38
-8.37	-8.37	-8.37	-8.37	-8.37	-8.37	-8.37	-8.37	-8.37
-8.36	-8.36	-8.36	-8.36	-8.36	-8.36	-8.36	-8.36	-8.36
-8.35	-8.35	-8.35	-8.35	-8.35	-8.35	-8.35	-8.35	-8.35
-8.34	-8.34	-8.34	-8.34	-8.34	-8.34	-8.34	-8.34	-8.34
-8.33	-8.33	-8.33	-8.33	-8.33	-8.33	-8.33	-8.33	-8.33
-8.32	-8.32	-8.32	-8.32	-8.32	-8.32	-8.32	-8.32	-8.32
-8.31	-8.31	-8.31	-8.31	-8.31	-8.31	-8.31	-8.31	-8.31
-8.3	-8.3	-8.3	-8.3	-8.3	-8.3	-8.3	-8.3	-8.3
-8.29	-8.29	-8.29	-8.29	-8.29	-8.29	-8.29	-8.29	-8.29
-8.28	-8.28	-8.28	-8.28	-8.28	-8.28	-8.28	-8.28	-8.28
-8.27	-8.27	-8.27	-8.27	-8.27	-8.27	-8.27	-8.27	-8.27
-8.26	-8.26	-8.26	-8.26	-8.26	-8.26	-8.26	-8.26	-8.26
-8.25	-8.25	-8.25	-8.25	-8.25	-8.25	-8.25	-8.25	-8.25
-8.24	-8.24	-8.24	-8.24	-8.24	-8.24	-8.24	-8.24	-8.24
-8.23	-8.23	-8.23	-8.23	-8.23	-8.23	-8.23	-8.23	-8.23
-8.22	-8.22	-8.22	-8.22	-8.22	-8.22	-8.22	-8.22	-8.22
-8.21	-8.21	-8.21	-8.21	-8.21	-8.21	-8.21	-8.21	-8.21
-8.2	-8.2	-8.2	-8.2	-8.2	-8.2	-8.2	-8.2	-8.2
-8.19	-8.19	-8.19	-8.19	-8.19	-8.19	-8.19	-8.19	-8.19
-8.18	-8.18	-8.18	-8.18	-8.18	-8.18	-8.18	-8.18	-8.18
-8.17	-8.17	-8.17	-8.17	-8.17	-8.17	-8.17	-8.17	-8.17
-8.16	-8.16	-8.16	-8.16	-8.16	-8.16	-8.16	-8.16	-8.16
-8.15	-8.15	-8.15	-8.15	-8.15	-8.15	-8.15	-8.15	-8.15
-8.14	-8.14	-8.14	-8.14	-8.14	-8.14	-8.14	-8.14	-8.14
-8.13	-8.13	-8.13	-8.13	-8.13	-8.13	-8.13	-8.13	-8.13
-8.12	-8.12	-8.12	-8.12	-8.12	-8.12	-8.12	-8.12	-8.12
-8.11	-8.11	-8.11	-8.11	-8.11	-8.11	-8.11	-8.11	-8.11
-8.1	-8.1	-8.1	-8.1	-8.1	-8.1	-8.1	-8.1	-8.1
-8.09	-8.09	-8.09	-8.09	-8.09	-8.09	-8.09	-8.09	-8.09
-8.08	-8.08	-8.08	-8.08	-8.08	-8.08	-8.08	-8.08	-8.08
-8.07	-8.07	-8.07	-8.07	-8.07	-8.07	-8.07	-8.07	-8.07
-8.06	-8.06	-8.06	-8.06	-8.06	-8.06	-8.06	-8.06	-8.06
-8.05	-8.05	-8.05	-8.05	-8.05	-8.05	-8.05	-8.05	-8.05
-8.04	-8.04	-8.04	-8.04	-8.04	-8.04	-8.04	-8.04	-8.04
-8.03	-8.03	-8.03	-8.03	-8.03	-8.03	-8.03	-8.03	-8.03
-8.02	-8.02	-8.02	-8.02	-8.02	-8.02	-8.02	-8.02	-8.02
-8.01	-8.01	-8.01	-8.01	-8.01	-8.01	-8.01	-8.01	-8.01

-8	-8	-8	-8	-8	-8	-8	-8	-8
-7.99	-7.99	-7.99	-7.99	-7.99	-7.99	-7.99	-7.99	-7.99
-7.98	-7.98	-7.98	-7.98	-7.98	-7.98	-7.98	-7.98	-7.98
-7.97	-7.97	-7.97	-7.97	-7.97	-7.97	-7.97	-7.97	-7.97
-7.96	-7.96	-7.96	-7.96	-7.96	-7.96	-7.96	-7.96	-7.96
-7.95	-7.95	-7.95	-7.95	-7.95	-7.95	-7.95	-7.95	-7.95
-7.94	-7.94	-7.94	-7.94	-7.94	-7.94	-7.94	-7.94	-7.94
-7.93	-7.93	-7.93	-7.93	-7.93	-7.93	-7.93	-7.93	-7.93
-7.92	-7.92	-7.92	-7.92	-7.92	-7.92	-7.92	-7.92	-7.92
-7.91	-7.91	-7.91	-7.91	-7.91	-7.91	-7.91	-7.91	-7.91
-7.9	-7.9	-7.9	-7.9	-7.9	-7.9	-7.9	-7.9	-7.9
-7.89	-7.89	-7.89	-7.89	-7.89	-7.89	-7.89	-7.89	-7.89
-7.88	-7.88	-7.88	-7.88	-7.88	-7.88	-7.88	-7.88	-7.88
-7.87	-7.87	-7.87	-7.87	-7.87	-7.87	-7.87	-7.87	-7.87
-7.86	-7.86	-7.86	-7.86	-7.86	-7.86	-7.86	-7.86	-7.86
-7.85	-7.85	-7.85	-7.85	-7.85	-7.85	-7.85	-7.85	-7.85
-7.84	-7.84	-7.84	-7.84	-7.84	-7.84	-7.84	-7.84	-7.84
-7.83	-7.83	-7.83	-7.83	-7.83	-7.83	-7.83	-7.83	-7.83
-7.82	-7.82	-7.82	-7.82	-7.82	-7.82	-7.82	-7.82	-7.82
-7.81	-7.81	-7.81	-7.81	-7.81	-7.81	-7.81	-7.81	-7.81
-7.8	-7.8	-7.8	-7.8	-7.8	-7.8	-7.8	-7.8	-7.8
-7.79	-7.79	-7.79	-7.79	-7.79	-7.79	-7.79	-7.79	-7.79
-7.78	-7.78	-7.78	-7.78	-7.78	-7.78	-7.78	-7.78	-7.78
-7.77	-7.77	-7.77	-7.77	-7.77	-7.77	-7.77	-7.77	-7.77
-7.76	-7.76	-7.76	-7.76	-7.76	-7.76	-7.76	-7.76	-7.76
-7.75	-7.75	-7.75	-7.75	-7.75	-7.75	-7.75	-7.75	-7.75
-7.74	-7.74	-7.74	-7.74	-7.74	-7.74	-7.74	-7.74	-7.74
-7.73	-7.73	-7.73	-7.73	-7.73	-7.73	-7.73	-7.73	-7.73
-7.72	-7.72	-7.72	-7.72	-7.72	-7.72	-7.72	-7.72	-7.72
-7.71	-7.71	-7.71	-7.71	-7.71	-7.71	-7.71	-7.71	-7.71
-7.7	-7.7	-7.7	-7.7	-7.7	-7.7	-7.7	-7.7	-7.7
-7.69	-7.69	-7.69	-7.69	-7.69	-7.69	-7.69	-7.69	-7.69
-7.68	-7.68	-7.68	-7.68	-7.68	-7.68	-7.68	-7.68	-7.68
-7.67	-7.67	-7.67	-7.67	-7.67	-7.67	-7.67	-7.67	-7.67
-7.66	-7.66	-7.66	-7.66	-7.66	-7.66	-7.66	-7.66	-7.66
-7.65	-7.65	-7.65	-7.65	-7.65	-7.65	-7.65	-7.65	-7.65
-7.64	-7.64	-7.64	-7.64	-7.64	-7.64	-7.64	-7.64	-7.64
-7.63	-7.63	-7.63	-7.63	-7.63	-7.63	-7.63	-7.63	-7.63
-7.62	-7.62	-7.62	-7.62	-7.62	-7.62	-7.62	-7.62	-7.62
-7.61	-7.61	-7.61	-7.61	-7.61	-7.61	-7.61	-7.61	-7.61
-7.6	-7.6	-7.6	-7.6	-7.6	-7.6	-7.6	-7.6	-7.6
-7.59	-7.59	-7.59	-7.59	-7.59	-7.59	-7.59	-7.59	-7.59
-7.58	-7.58	-7.58	-7.58	-7.58	-7.58	-7.58	-7.58	-7.58
-7.57	-7.57	-7.57	-7.57	-7.57	-7.57	-7.57	-7.57	-7.57
-7.56	-7.56	-7.56	-7.56	-7.56	-7.56	-7.56	-7.56	-7.56
-7.55	-7.55	-7.55	-7.55	-7.55	-7.55	-7.55	-7.55	-7.55
-7.54	-7.54	-7.54	-7.54	-7.54	-7.54	-7.54	-7.54	-7.54
-7.53	-7.53	-7.53	-7.53	-7.53	-7.53	-7.53	-7.53	-7.53
-7.52	-7.52	-7.52	-7.52	-7.52	-7.52	-7.52	-7.52	-7.52
-7.51	-7.51	-7.51	-7.51	-7.51	-7.51	-7.51	-7.51	-7.51

-7.5	-7.5	-7.5	-7.5	-7.5	-7.5	-7.5	-7.5	-7.5
-7.49	-7.49	-7.49	-7.49	-7.49	-7.49	-7.49	-7.49	-7.49
-7.48	-7.48	-7.48	-7.48	-7.48	-7.48	-7.48	-7.48	-7.48
-7.47	-7.47	-7.47	-7.47	-7.47	-7.47	-7.47	-7.47	-7.47
-7.46	-7.46	-7.46	-7.46	-7.46	-7.46	-7.46	-7.46	-7.46
-7.45	-7.45	-7.45	-7.45	-7.45	-7.45	-7.45	-7.45	-7.45
-7.44	-7.44	-7.44	-7.44	-7.44	-7.44	-7.44	-7.44	-7.44
-7.43	-7.43	-7.43	-7.43	-7.43	-7.43	-7.43	-7.43	-7.43
-7.42	-7.42	-7.42	-7.42	-7.42	-7.42	-7.42	-7.42	-7.42
-7.41	-7.41	-7.41	-7.41	-7.41	-7.41	-7.41	-7.41	-7.41
-7.4	-7.4	-7.4	-7.4	-7.4	-7.4	-7.4	-7.4	-7.4
-7.39	-7.39	-7.39	-7.39	-7.39	-7.39	-7.39	-7.39	-7.39
-7.38	-7.38	-7.38	-7.38	-7.38	-7.38	-7.38	-7.38	-7.38
-7.37	-7.37	-7.37	-7.37	-7.37	-7.37	-7.37	-7.37	-7.37
-7.36	-7.36	-7.36	-7.36	-7.36	-7.36	-7.36	-7.36	-7.36
-7.35	-7.35	-7.35	-7.35	-7.35	-7.35	-7.35	-7.35	-7.35
-7.34	-7.34	-7.34	-7.34	-7.34	-7.34	-7.34	-7.34	-7.34
-7.33	-7.33	-7.33	-7.33	-7.33	-7.33	-7.33	-7.33	-7.33
-7.32	-7.32	-7.32	-7.32	-7.32	-7.32	-7.32	-7.32	-7.32
-7.31	-7.31	-7.31	-7.31	-7.31	-7.31	-7.31	-7.31	-7.31
-7.3	-7.3	-7.3	-7.3	-7.3	-7.3	-7.3	-7.3	-7.3
-7.29	-7.29	-7.29	-7.29	-7.29	-7.29	-7.29	-7.29	-7.29
-7.28	-7.28	-7.28	-7.28	-7.28	-7.28	-7.28	-7.28	-7.28
-7.27	-7.27	-7.27	-7.27	-7.27	-7.27	-7.27	-7.27	-7.27
-7.26	-7.26	-7.26	-7.26	-7.26	-7.26	-7.26	-7.26	-7.26
-7.25	-7.25	-7.25	-7.25	-7.25	-7.25	-7.25	-7.25	-7.25
-7.24	-7.24	-7.24	-7.24	-7.24	-7.24	-7.24	-7.24	-7.24
-7.23	-7.23	-7.23	-7.23	-7.23	-7.23	-7.23	-7.23	-7.23
-7.22	-7.22	-7.22	-7.22	-7.22	-7.22	-7.22	-7.22	-7.22
-7.21	-7.21	-7.21	-7.21	-7.21	-7.21	-7.21	-7.21	-7.21
-7.2	-7.2	-7.2	-7.2	-7.2	-7.2	-7.2	-7.2	-7.2
-7.19	-7.19	-7.19	-7.19	-7.19	-7.19	-7.19	-7.19	-7.19
-7.18	-7.18	-7.18	-7.18	-7.18	-7.18	-7.18	-7.18	-7.18
-7.17	-7.17	-7.17	-7.17	-7.17	-7.17	-7.17	-7.17	-7.17
-7.16	-7.16	-7.16	-7.16	-7.16	-7.16	-7.16	-7.16	-7.16
-7.15	-7.15	-7.15	-7.15	-7.15	-7.15	-7.15	-7.15	-7.15
-7.14	-7.14	-7.14	-7.14	-7.14	-7.14	-7.14	-7.14	-7.14
-7.13	-7.13	-7.13	-7.13	-7.13	-7.13	-7.13	-7.13	-7.13
-7.12	-7.12	-7.12	-7.12	-7.12	-7.12	-7.12	-7.12	-7.12
-7.11	-7.11	-7.11	-7.11	-7.11	-7.11	-7.11	-7.11	-7.11
-7.1	-7.1	-7.1	-7.1	-7.1	-7.1	-7.1	-7.1	-7.1
-7.09	-7.09	-7.09	-7.09	-7.09	-7.09	-7.09	-7.09	-7.09
-7.08	-7.08	-7.08	-7.08	-7.08	-7.08	-7.08	-7.08	-7.08
-7.07	-7.07	-7.07	-7.07	-7.07	-7.07	-7.07	-7.07	-7.07
-7.06	-7.06	-7.06	-7.06	-7.06	-7.06	-7.06	-7.06	-7.06
-7.05	-7.05	-7.05	-7.05	-7.05	-7.05	-7.05	-7.05	-7.05
-7.04	-7.04	-7.04	-7.04	-7.04	-7.04	-7.04	-7.04	-7.04
-7.03	-7.03	-7.03	-7.03	-7.03	-7.03	-7.03	-7.03	-7.03
-7.02	-7.02	-7.02	-7.02	-7.02	-7.02	-7.02	-7.02	-7.02
-7.01	-7.01	-7.01	-7.01	-7.01	-7.01	-7.01	-7.01	-7.01

-7	-7	-7	-7	-7	-7	-7	-7	-7
-6.99	-6.99	-6.99	-6.99	-6.99	-6.99	-6.99	-6.99	-6.99
-6.98	-6.98	-6.98	-6.98	-6.98	-6.98	-6.98	-6.98	-6.98
-6.97	-6.97	-6.97	-6.97	-6.97	-6.97	-6.97	-6.97	-6.97
-6.96	-6.96	-6.96	-6.96	-6.96	-6.96	-6.96	-6.96	-6.96
-6.95	-6.95	-6.95	-6.95	-6.95	-6.95	-6.95	-6.95	-6.95
-6.94	-6.94	-6.94	-6.94	-6.94	-6.94	-6.94	-6.94	-6.94
-6.93	-6.93	-6.93	-6.93	-6.93	-6.93	-6.93	-6.93	-6.93
-6.92	-6.92	-6.92	-6.92	-6.92	-6.92	-6.92	-6.92	-6.92
-6.91	-6.91	-6.91	-6.91	-6.91	-6.91	-6.91	-6.91	-6.91
-6.9	-6.9	-6.9	-6.9	-6.9	-6.9	-6.9	-6.9	-6.9
-6.89	-6.89	-6.89	-6.89	-6.89	-6.89	-6.89	-6.89	-6.89
-6.88	-6.88	-6.88	-6.88	-6.88	-6.88	-6.88	-6.88	-6.88
-6.87	-6.87	-6.87	-6.87	-6.87	-6.87	-6.87	-6.87	-6.87
-6.86	-6.86	-6.86	-6.86	-6.86	-6.86	-6.86	-6.86	-6.86
-6.85	-6.85	-6.85	-6.85	-6.85	-6.85	-6.85	-6.85	-6.85
-6.84	-6.84	-6.84	-6.84	-6.84	-6.84	-6.84	-6.84	-6.84
-6.83	-6.83	-6.83	-6.83	-6.83	-6.83	-6.83	-6.83	-6.83
-6.82	-6.82	-6.82	-6.82	-6.82	-6.82	-6.82	-6.82	-6.82
-6.81	-6.81	-6.81	-6.81	-6.81	-6.81	-6.81	-6.81	-6.81
-6.8	-6.8	-6.8	-6.8	-6.8	-6.8	-6.8	-6.8	-6.8
-6.79	-6.79	-6.79	-6.79	-6.79	-6.79	-6.79	-6.79	-6.79
-6.78	-6.78	-6.78	-6.78	-6.78	-6.78	-6.78	-6.78	-6.78
-6.77	-6.77	-6.77	-6.77	-6.77	-6.77	-6.77	-6.77	-6.77
-6.76	-6.76	-6.76	-6.76	-6.76	-6.76	-6.76	-6.76	-6.76
-6.75	-6.75	-6.75	-6.75	-6.75	-6.75	-6.75	-6.75	-6.75
-6.74	-6.74	-6.74	-6.74	-6.74	-6.74	-6.74	-6.74	-6.74
-6.73	-6.73	-6.73	-6.73	-6.73	-6.73	-6.73	-6.73	-6.73
-6.72	-6.72	-6.72	-6.72	-6.72	-6.72	-6.72	-6.72	-6.72
-6.71	-6.71	-6.71	-6.71	-6.71	-6.71	-6.71	-6.71	-6.71
-6.7	-6.7	-6.7	-6.7	-6.7	-6.7	-6.7	-6.7	-6.7
-6.69	-6.69	-6.69	-6.69	-6.69	-6.69	-6.69	-6.69	-6.69
-6.68	-6.68	-6.68	-6.68	-6.68	-6.68	-6.68	-6.68	-6.68
-6.67	-6.67	-6.67	-6.67	-6.67	-6.67	-6.67	-6.67	-6.67
-6.66	-6.66	-6.66	-6.66	-6.66	-6.66	-6.66	-6.66	-6.66
-6.65	-6.65	-6.65	-6.65	-6.65	-6.65	-6.65	-6.65	-6.65
-6.64	-6.64	-6.64	-6.64	-6.64	-6.64	-6.64	-6.64	-6.64
-6.63	-6.63	-6.63	-6.63	-6.63	-6.63	-6.63	-6.63	-6.63
-6.62	-6.62	-6.62	-6.62	-6.62	-6.62	-6.62	-6.62	-6.62
-6.61	-6.61	-6.61	-6.61	-6.61	-6.61	-6.61	-6.61	-6.61
-6.6	-6.6	-6.6	-6.6	-6.6	-6.6	-6.6	-6.6	-6.6
-6.59	-6.59	-6.59	-6.59	-6.59	-6.59	-6.59	-6.59	-6.59
-6.58	-6.58	-6.58	-6.58	-6.58	-6.58	-6.58	-6.58	-6.58
-6.57	-6.57	-6.57	-6.57	-6.57	-6.57	-6.57	-6.57	-6.57
-6.56	-6.56	-6.56	-6.56	-6.56	-6.56	-6.56	-6.56	-6.56
-6.55	-6.55	-6.55	-6.55	-6.55	-6.55	-6.55	-6.55	-6.55
-6.54	-6.54	-6.54	-6.54	-6.54	-6.54	-6.54	-6.54	-6.54
-6.53	-6.53	-6.53	-6.53	-6.53	-6.53	-6.53	-6.53	-6.53
-6.52	-6.52	-6.52	-6.52	-6.52	-6.52	-6.52	-6.52	-6.52
-6.51	-6.51	-6.51	-6.51	-6.51	-6.51	-6.51	-6.51	-6.51

-6.5	-6.5	-6.5	-6.5	-6.5	-6.5	-6.5	-6.5	-6.5
-6.49	-6.49	-6.49	-6.49	-6.49	-6.49	-6.49	-6.49	-6.49
-6.48	-6.48	-6.48	-6.48	-6.48	-6.48	-6.48	-6.48	-6.48
-6.47	-6.47	-6.47	-6.47	-6.47	-6.47	-6.47	-6.47	-6.47
-6.46	-6.46	-6.46	-6.46	-6.46	-6.46	-6.46	-6.46	-6.46
-6.45	-6.45	-6.45	-6.45	-6.45	-6.45	-6.45	-6.45	-6.45
-6.44	-6.44	-6.44	-6.44	-6.44	-6.44	-6.44	-6.44	-6.44
-6.43	-6.43	-6.43	-6.43	-6.43	-6.43	-6.43	-6.43	-6.43
-6.42	-6.42	-6.42	-6.42	-6.42	-6.42	-6.42	-6.42	-6.42
-6.41	-6.41	-6.41	-6.41	-6.41	-6.41	-6.41	-6.41	-6.41
-6.4	-6.4	-6.4	-6.4	-6.4	-6.4	-6.4	-6.4	-6.4
-6.39	-6.39	-6.39	-6.39	-6.39	-6.39	-6.39	-6.39	-6.39
-6.38	-6.38	-6.38	-6.38	-6.38	-6.38	-6.38	-6.38	-6.38
-6.37	-6.37	-6.37	-6.37	-6.37	-6.37	-6.37	-6.37	-6.37
-6.36	-6.36	-6.36	-6.36	-6.36	-6.36	-6.36	-6.36	-6.36
-6.35	-6.35	-6.35	-6.35	-6.35	-6.35	-6.35	-6.35	-6.35
-6.34	-6.34	-6.34	-6.34	-6.34	-6.34	-6.34	-6.34	-6.34
-6.33	-6.33	-6.33	-6.33	-6.33	-6.33	-6.33	-6.33	-6.33
-6.32	-6.32	-6.32	-6.32	-6.32	-6.32	-6.32	-6.32	-6.32
-6.31	-6.31	-6.31	-6.31	-6.31	-6.31	-6.31	-6.31	-6.31
-6.3	-6.3	-6.3	-6.3	-6.3	-6.3	-6.3	-6.3	-6.3
-6.29	-6.29	-6.29	-6.29	-6.29	-6.29	-6.29	-6.29	-6.29
-6.28	-6.28	-6.28	-6.28	-6.28	-6.28	-6.28	-6.28	-6.28
-6.27	-6.27	-6.27	-6.27	-6.27	-6.27	-6.27	-6.27	-6.27
-6.26	-6.26	-6.26	-6.26	-6.26	-6.26	-6.26	-6.26	-6.26
-6.25	-6.25	-6.25	-6.25	-6.25	-6.25	-6.25	-6.25	-6.25
-6.24	-6.24	-6.24	-6.24	-6.24	-6.24	-6.24	-6.24	-6.24
-6.23	-6.23	-6.23	-6.23	-6.23	-6.23	-6.23	-6.23	-6.23
-6.22	-6.22	-6.22	-6.22	-6.22	-6.22	-6.22	-6.22	-6.22
-6.21	-6.21	-6.21	-6.21	-6.21	-6.21	-6.21	-6.21	-6.21
-6.2	-6.2	-6.2	-6.2	-6.2	-6.2	-6.2	-6.2	-6.2
-6.19	-6.19	-6.19	-6.19	-6.19	-6.19	-6.19	-6.19	-6.19
-6.18	-6.18	-6.18	-6.18	-6.18	-6.18	-6.18	-6.18	-6.18
-6.17	-6.17	-6.17	-6.17	-6.17	-6.17	-6.17	-6.17	-6.17
-6.16	-6.16	-6.16	-6.16	-6.16	-6.16	-6.16	-6.16	-6.16
-6.15	-6.15	-6.15	-6.15	-6.15	-6.15	-6.15	-6.15	-6.15
-6.14	-6.14	-6.14	-6.14	-6.14	-6.14	-6.14	-6.14	-6.14
-6.13	-6.13	-6.13	-6.13	-6.13	-6.13	-6.13	-6.13	-6.13
-6.12	-6.12	-6.12	-6.12	-6.12	-6.12	-6.12	-6.12	-6.12
-6.11	-6.11	-6.11	-6.11	-6.11	-6.11	-6.11	-6.11	-6.11
-6.1	-6.1	-6.1	-6.1	-6.1	-6.1	-6.1	-6.1	-6.1
-6.09	-6.09	-6.09	-6.09	-6.09	-6.09	-6.09	-6.09	-6.09
-6.08	-6.08	-6.08	-6.08	-6.08	-6.08	-6.08	-6.08	-6.08
-6.07	-6.07	-6.07	-6.07	-6.07	-6.07	-6.07	-6.07	-6.07
-6.06	-6.06	-6.06	-6.06	-6.06	-6.06	-6.06	-6.06	-6.06
-6.05	-6.05	-6.05	-6.05	-6.05	-6.05	-6.05	-6.05	-6.05
-6.04	-6.04	-6.04	-6.04	-6.04	-6.04	-6.04	-6.04	-6.04
-6.03	-6.03	-6.03	-6.03	-6.03	-6.03	-6.03	-6.03	-6.03
-6.02	-6.02	-6.02	-6.02	-6.02	-6.02	-6.02	-6.02	-6.02
-6.01	-6.01	-6.01	-6.01	-6.01	-6.01	-6.01	-6.01	-6.01

-6	-6	-6	-6	-6	-6	-6	-6	-6
-5.99	-5.99	-5.99	-5.99	-5.99	-5.99	-5.99	-5.99	-5.99
-5.98	-5.98	-5.98	-5.98	-5.98	-5.98	-5.98	-5.98	-5.98
-5.97	-5.97	-5.97	-5.97	-5.97	-5.97	-5.97	-5.97	-5.97
-5.96	-5.96	-5.96	-5.96	-5.96	-5.96	-5.96	-5.96	-5.96
-5.95	-5.95	-5.95	-5.95	-5.95	-5.95	-5.95	-5.95	-5.95
-5.94	-5.94	-5.94	-5.94	-5.94	-5.94	-5.94	-5.94	-5.94
-5.93	-5.93	-5.93	-5.93	-5.93	-5.93	-5.93	-5.93	-5.93
-5.92	-5.92	-5.92	-5.92	-5.92	-5.92	-5.92	-5.92	-5.92
-5.91	-5.91	-5.91	-5.91	-5.91	-5.91	-5.91	-5.91	-5.91
-5.9	-5.9	-5.9	-5.9	-5.9	-5.9	-5.9	-5.9	-5.9
-5.89	-5.89	-5.89	-5.89	-5.89	-5.89	-5.89	-5.89	-5.89
-5.88	-5.88	-5.88	-5.88	-5.88	-5.88	-5.88	-5.88	-5.88
-5.87	-5.87	-5.87	-5.87	-5.87	-5.87	-5.87	-5.87	-5.87
-5.86	-5.86	-5.86	-5.86	-5.86	-5.86	-5.86	-5.86	-5.86
-5.85	-5.85	-5.85	-5.85	-5.85	-5.85	-5.85	-5.85	-5.85
-5.84	-5.84	-5.84	-5.84	-5.84	-5.84	-5.84	-5.84	-5.84
-5.83	-5.83	-5.83	-5.83	-5.83	-5.83	-5.83	-5.83	-5.83
-5.82	-5.82	-5.82	-5.82	-5.82	-5.82	-5.82	-5.82	-5.82
-5.81	-5.81	-5.81	-5.81	-5.81	-5.81	-5.81	-5.81	-5.81
-5.8	-5.8	-5.8	-5.8	-5.8	-5.8	-5.8	-5.8	-5.8
-5.79	-5.79	-5.79	-5.79	-5.79	-5.79	-5.79	-5.79	-5.79
-5.78	-5.78	-5.78	-5.78	-5.78	-5.78	-5.78	-5.78	-5.78
-5.77	-5.77	-5.77	-5.77	-5.77	-5.77	-5.77	-5.77	-5.77
-5.76	-5.76	-5.76	-5.76	-5.76	-5.76	-5.76	-5.76	-5.76
-5.75	-5.75	-5.75	-5.75	-5.75	-5.75	-5.75	-5.75	-5.75
-5.74	-5.74	-5.74	-5.74	-5.74	-5.74	-5.74	-5.74	-5.74
-5.73	-5.73	-5.73	-5.73	-5.73	-5.73	-5.73	-5.73	-5.73
-5.72	-5.72	-5.72	-5.72	-5.72	-5.72	-5.72	-5.72	-5.72
-5.71	-5.71	-5.71	-5.71	-5.71	-5.71	-5.71	-5.71	-5.71
-5.7	-5.7	-5.7	-5.7	-5.7	-5.7	-5.7	-5.7	-5.7
-5.69	-5.69	-5.69	-5.69	-5.69	-5.69	-5.69	-5.69	-5.69
-5.68	-5.68	-5.68	-5.68	-5.68	-5.68	-5.68	-5.68	-5.68
-5.67	-5.67	-5.67	-5.67	-5.67	-5.67	-5.67	-5.67	-5.67
-5.66	-5.66	-5.66	-5.66	-5.66	-5.66	-5.66	-5.66	-5.66
-5.65	-5.65	-5.65	-5.65	-5.65	-5.65	-5.65	-5.65	-5.65
-5.64	-5.64	-5.64	-5.64	-5.64	-5.64	-5.64	-5.64	-5.64
-5.63	-5.63	-5.63	-5.63	-5.63	-5.63	-5.63	-5.63	-5.63
-5.62	-5.62	-5.62	-5.62	-5.62	-5.62	-5.62	-5.62	-5.62
-5.61	-5.61	-5.61	-5.61	-5.61	-5.61	-5.61	-5.61	-5.61
-5.6	-5.6	-5.6	-5.6	-5.6	-5.6	-5.6	-5.6	-5.6
-5.59	-5.59	-5.59	-5.59	-5.59	-5.59	-5.59	-5.59	-5.59
-5.58	-5.58	-5.58	-5.58	-5.58	-5.58	-5.58	-5.58	-5.58
-5.57	-5.57	-5.57	-5.57	-5.57	-5.57	-5.57	-5.57	-5.57
-5.56	-5.56	-5.56	-5.56	-5.56	-5.56	-5.56	-5.56	-5.56
-5.55	-5.55	-5.55	-5.55	-5.55	-5.55	-5.55	-5.55	-5.55
-5.54	-5.54	-5.54	-5.54	-5.54	-5.54	-5.54	-5.54	-5.54
-5.53	-5.53	-5.53	-5.53	-5.53	-5.53	-5.53	-5.53	-5.53
-5.52	-5.52	-5.52	-5.52	-5.52	-5.52	-5.52	-5.52	-5.52
-5.51	-5.51	-5.51	-5.51	-5.51	-5.51	-5.51	-5.51	-5.51

-5.5	-5.5	-5.5	-5.5	-5.5	-5.5	-5.5	-5.5	-5.5
-5.49	-5.49	-5.49	-5.49	-5.49	-5.49	-5.49	-5.49	-5.49
-5.48	-5.48	-5.48	-5.48	-5.48	-5.48	-5.48	-5.48	-5.48
-5.47	-5.47	-5.47	-5.47	-5.47	-5.47	-5.47	-5.47	-5.47
-5.46	-5.46	-5.46	-5.46	-5.46	-5.46	-5.46	-5.46	-5.46
-5.45	-5.45	-5.45	-5.45	-5.45	-5.45	-5.45	-5.45	-5.45
-5.44	-5.44	-5.44	-5.44	-5.44	-5.44	-5.44	-5.44	-5.44
-5.43	-5.43	-5.43	-5.43	-5.43	-5.43	-5.43	-5.43	-5.43
-5.42	-5.42	-5.42	-5.42	-5.42	-5.42	-5.42	-5.42	-5.42
-5.41	-5.41	-5.41	-5.41	-5.41	-5.41	-5.41	-5.41	-5.41
-5.4	-5.4	-5.4	-5.4	-5.4	-5.4	-5.4	-5.4	-5.4
-5.39	-5.39	-5.39	-5.39	-5.39	-5.39	-5.39	-5.39	-5.39
-5.38	-5.38	-5.38	-5.38	-5.38	-5.38	-5.38	-5.38	-5.38
-5.37	-5.37	-5.37	-5.37	-5.37	-5.37	-5.37	-5.37	-5.37
-5.36	-5.36	-5.36	-5.36	-5.36	-5.36	-5.36	-5.36	-5.36
-5.35	-5.35	-5.35	-5.35	-5.35	-5.35	-5.35	-5.35	-5.35
-5.34	-5.34	-5.34	-5.34	-5.34	-5.34	-5.34	-5.34	-5.34
-5.33	-5.33	-5.33	-5.33	-5.33	-5.33	-5.33	-5.33	-5.33
-5.32	-5.32	-5.32	-5.32	-5.32	-5.32	-5.32	-5.32	-5.32
-5.31	-5.31	-5.31	-5.31	-5.31	-5.31	-5.31	-5.31	-5.31
-5.3	-5.3	-5.3	-5.3	-5.3	-5.3	-5.3	-5.3	-5.3
-5.29	-5.29	-5.29	-5.29	-5.29	-5.29	-5.29	-5.29	-5.29
-5.28	-5.28	-5.28	-5.28	-5.28	-5.28	-5.28	-5.28	-5.28
-5.27	-5.27	-5.27	-5.27	-5.27	-5.27	-5.27	-5.27	-5.27
-5.26	-5.26	-5.26	-5.26	-5.26	-5.26	-5.26	-5.26	-5.26
-5.25	-5.25	-5.25	-5.25	-5.25	-5.25	-5.25	-5.25	-5.25
-5.24	-5.24	-5.24	-5.24	-5.24	-5.24	-5.24	-5.24	-5.24
-5.23	-5.23	-5.23	-5.23	-5.23	-5.23	-5.23	-5.23	-5.23
-5.22	-5.22	-5.22	-5.22	-5.22	-5.22	-5.22	-5.22	-5.22
-5.21	-5.21	-5.21	-5.21	-5.21	-5.21	-5.21	-5.21	-5.21
-5.2	-5.2	-5.2	-5.2	-5.2	-5.2	-5.2	-5.2	-5.2
-5.19	-5.19	-5.19	-5.19	-5.19	-5.19	-5.19	-5.19	-5.19
-5.18	-5.18	-5.18	-5.18	-5.18	-5.18	-5.18	-5.18	-5.18
-5.17	-5.17	-5.17	-5.17	-5.17	-5.17	-5.17	-5.17	-5.17
-5.16	-5.16	-5.16	-5.16	-5.16	-5.16	-5.16	-5.16	-5.16
-5.15	-5.15	-5.15	-5.15	-5.15	-5.15	-5.15	-5.15	-5.15
-5.14	-5.14	-5.14	-5.14	-5.14	-5.14	-5.14	-5.14	-5.14
-5.13	-5.13	-5.13	-5.13	-5.13	-5.13	-5.13	-5.13	-5.13
-5.12	-5.12	-5.12	-5.12	-5.12	-5.12	-5.12	-5.12	-5.12
-5.11	-5.11	-5.11	-5.11	-5.11	-5.11	-5.11	-5.11	-5.11
-5.1	-5.1	-5.1	-5.1	-5.1	-5.1	-5.1	-5.1	-5.1
-5.09	-5.09	-5.09	-5.09	-5.09	-5.09	-5.09	-5.09	-5.09
-5.08	-5.08	-5.08	-5.08	-5.08	-5.08	-5.08	-5.08	-5.08
-5.07	-5.07	-5.07	-5.07	-5.07	-5.07	-5.07	-5.07	-5.07
-5.06	-5.06	-5.06	-5.06	-5.06	-5.06	-5.06	-5.06	-5.06
-5.05	-5.05	-5.05	-5.05	-5.05	-5.05	-5.05	-5.05	-5.05
-5.04	-5.04	-5.04	-5.04	-5.04	-5.04	-5.04	-5.04	-5.04
-5.03	-5.03	-5.03	-5.03	-5.03	-5.03	-5.03	-5.03	-5.03
-5.02	-5.02	-5.02	-5.02	-5.02	-5.02	-5.02	-5.02	-5.02
-5.01	-5.01	-5.01	-5.01	-5.01	-5.01	-5.01	-5.01	-5.01

-5	-5	-5	-5	-5	-5	-5	-5	-5
-4.99	-4.99	-4.99	-4.99	-4.99	-4.99	-4.99	-4.99	-4.99
-4.98	-4.98	-4.98	-4.98	-4.98	-4.98	-4.98	-4.98	-4.98
-4.97	-4.97	-4.97	-4.97	-4.97	-4.97	-4.97	-4.97	-4.97
-4.96	-4.96	-4.96	-4.96	-4.96	-4.96	-4.96	-4.96	-4.96
-4.95	-4.95	-4.95	-4.95	-4.95	-4.95	-4.95	-4.95	-4.95
-4.94	-4.94	-4.94	-4.94	-4.94	-4.94	-4.94	-4.94	-4.94
-4.93	-4.93	-4.93	-4.93	-4.93	-4.93	-4.93	-4.93	-4.93
-4.92	-4.92	-4.92	-4.92	-4.92	-4.92	-4.92	-4.92	-4.92
-4.91	-4.91	-4.91	-4.91	-4.91	-4.91	-4.91	-4.91	-4.91
-4.9	-4.9	-4.9	-4.9	-4.9	-4.9	-4.9	-4.9	-4.9
-4.89	-4.89	-4.89	-4.89	-4.89	-4.89	-4.89	-4.89	-4.89
-4.88	-4.88	-4.88	-4.88	-4.88	-4.88	-4.88	-4.88	-4.88
-4.87	-4.87	-4.87	-4.87	-4.87	-4.87	-4.87	-4.87	-4.87
-4.86	-4.86	-4.86	-4.86	-4.86	-4.86	-4.86	-4.86	-4.86
-4.85	-4.85	-4.85	-4.85	-4.85	-4.85	-4.85	-4.85	-4.85
-4.84	-4.84	-4.84	-4.84	-4.84	-4.84	-4.84	-4.84	-4.84
-4.83	-4.83	-4.83	-4.83	-4.83	-4.83	-4.83	-4.83	-4.83
-4.82	-4.82	-4.82	-4.82	-4.82	-4.82	-4.82	-4.82	-4.82
-4.81	-4.81	-4.81	-4.81	-4.81	-4.81	-4.81	-4.81	-4.81
-4.8	-4.8	-4.8	-4.8	-4.8	-4.8	-4.8	-4.8	-4.8
-4.79	-4.79	-4.79	-4.79	-4.79	-4.79	-4.79	-4.79	-4.79
-4.78	-4.78	-4.78	-4.78	-4.78	-4.78	-4.78	-4.78	-4.78
-4.77	-4.77	-4.77	-4.77	-4.77	-4.77	-4.77	-4.77	-4.77
-4.76	-4.76	-4.76	-4.76	-4.76	-4.76	-4.76	-4.76	-4.76
-4.75	-4.75	-4.75	-4.75	-4.75	-4.75	-4.75	-4.75	-4.75
-4.74	-4.74	-4.74	-4.74	-4.74	-4.74	-4.74	-4.74	-4.74
-4.73	-4.73	-4.73	-4.73	-4.73	-4.73	-4.73	-4.73	-4.73
-4.72	-4.72	-4.72	-4.72	-4.72	-4.72	-4.72	-4.72	-4.72
-4.71	-4.71	-4.71	-4.71	-4.71	-4.71	-4.71	-4.71	-4.71
-4.7	-4.7	-4.7	-4.7	-4.7	-4.7	-4.7	-4.7	-4.7
-4.69	-4.69	-4.69	-4.69	-4.69	-4.69	-4.69	-4.69	-4.69
-4.68	-4.68	-4.68	-4.68	-4.68	-4.68	-4.68	-4.68	-4.68
-4.67	-4.67	-4.67	-4.67	-4.67	-4.67	-4.67	-4.67	-4.67
-4.66	-4.66	-4.66	-4.66	-4.66	-4.66	-4.66	-4.66	-4.66
-4.65	-4.65	-4.65	-4.65	-4.65	-4.65	-4.65	-4.65	-4.65
-4.64	-4.64	-4.64	-4.64	-4.64	-4.64	-4.64	-4.64	-4.64
-4.63	-4.63	-4.63	-4.63	-4.63	-4.63	-4.63	-4.63	-4.63
-4.62	-4.62	-4.62	-4.62	-4.62	-4.62	-4.62	-4.62	-4.62
-4.61	-4.61	-4.61	-4.61	-4.61	-4.61	-4.61	-4.61	-4.61
-4.6	-4.6	-4.6	-4.6	-4.6	-4.6	-4.6	-4.6	-4.6
-4.59	-4.59	-4.59	-4.59	-4.59	-4.59	-4.59	-4.59	-4.59
-4.58	-4.58	-4.58	-4.58	-4.58	-4.58	-4.58	-4.58	-4.58
-4.57	-4.57	-4.57	-4.57	-4.57	-4.57	-4.57	-4.57	-4.57
-4.56	-4.56	-4.56	-4.56	-4.56	-4.56	-4.56	-4.56	-4.56
-4.55	-4.55	-4.55	-4.55	-4.55	-4.55	-4.55	-4.55	-4.55
-4.54	-4.54	-4.54	-4.54	-4.54	-4.54	-4.54	-4.54	-4.54
-4.53	-4.53	-4.53	-4.53	-4.53	-4.53	-4.53	-4.53	-4.53
-4.52	-4.52	-4.52	-4.52	-4.52	-4.52	-4.52	-4.52	-4.52
-4.51	-4.51	-4.51	-4.51	-4.51	-4.51	-4.51	-4.51	-4.51

-4.5	-4.5	-4.5	-4.5	-4.5	-4.5	-4.5	-4.5	-4.5
-4.49	-4.49	-4.49	-4.49	-4.49	-4.49	-4.49	-4.49	-4.49
-4.48	-4.48	-4.48	-4.48	-4.48	-4.48	-4.48	-4.48	-4.48
-4.47	-4.47	-4.47	-4.47	-4.47	-4.47	-4.47	-4.47	-4.47
-4.46	-4.46	-4.46	-4.46	-4.46	-4.46	-4.46	-4.46	-4.46
-4.45	-4.45	-4.45	-4.45	-4.45	-4.45	-4.45	-4.45	-4.45
-4.44	-4.44	-4.44	-4.44	-4.44	-4.44	-4.44	-4.44	-4.44
-4.43	-4.43	-4.43	-4.43	-4.43	-4.43	-4.43	-4.43	-4.43
-4.42	-4.42	-4.42	-4.42	-4.42	-4.42	-4.42	-4.42	-4.42
-4.41	-4.41	-4.41	-4.41	-4.41	-4.41	-4.41	-4.41	-4.41
-4.4	-4.4	-4.4	-4.4	-4.4	-4.4	-4.4	-4.4	-4.4
-4.39	-4.39	-4.39	-4.39	-4.39	-4.39	-4.39	-4.39	-4.39
-4.38	-4.38	-4.38	-4.38	-4.38	-4.38	-4.38	-4.38	-4.38
-4.37	-4.37	-4.37	-4.37	-4.37	-4.37	-4.37	-4.37	-4.37
-4.36	-4.36	-4.36	-4.36	-4.36	-4.36	-4.36	-4.36	-4.36
-4.35	-4.35	-4.35	-4.35	-4.35	-4.35	-4.35	-4.35	-4.35
-4.34	-4.34	-4.34	-4.34	-4.34	-4.34	-4.34	-4.34	-4.34
-4.33	-4.33	-4.33	-4.33	-4.33	-4.33	-4.33	-4.33	-4.33
-4.32	-4.32	-4.32	-4.32	-4.32	-4.32	-4.32	-4.32	-4.32
-4.31	-4.31	-4.31	-4.31	-4.31	-4.31	-4.31	-4.31	-4.31
-4.3	-4.3	-4.3	-4.3	-4.3	-4.3	-4.3	-4.3	-4.3
-4.29	-4.29	-4.29	-4.29	-4.29	-4.29	-4.29	-4.29	-4.29
-4.28	-4.28	-4.28	-4.28	-4.28	-4.28	-4.28	-4.28	-4.28
-4.27	-4.27	-4.27	-4.27	-4.27	-4.27	-4.27	-4.27	-4.27
-4.26	-4.26	-4.26	-4.26	-4.26	-4.26	-4.26	-4.26	-4.26
-4.25	-4.25	-4.25	-4.25	-4.25	-4.25	-4.25	-4.25	-4.25
-4.24	-4.24	-4.24	-4.24	-4.24	-4.24	-4.24	-4.24	-4.24
-4.23	-4.23	-4.23	-4.23	-4.23	-4.23	-4.23	-4.23	-4.23
-4.22	-4.22	-4.22	-4.22	-4.22	-4.22	-4.22	-4.22	-4.22
-4.21	-4.21	-4.21	-4.21	-4.21	-4.21	-4.21	-4.21	-4.21
-4.2	-4.2	-4.2	-4.2	-4.2	-4.2	-4.2	-4.2	-4.2
-4.19	-4.19	-4.19	-4.19	-4.19	-4.19	-4.19	-4.19	-4.19
-4.18	-4.18	-4.18	-4.18	-4.18	-4.18	-4.18	-4.18	-4.18
-4.17	-4.17	-4.17	-4.17	-4.17	-4.17	-4.17	-4.17	-4.17
-4.16	-4.16	-4.16	-4.16	-4.16	-4.16	-4.16	-4.16	-4.16
-4.15	-4.15	-4.15	-4.15	-4.15	-4.15	-4.15	-4.15	-4.15
-4.14	-4.14	-4.14	-4.14	-4.14	-4.14	-4.14	-4.14	-4.14
-4.13	-4.13	-4.13	-4.13	-4.13	-4.13	-4.13	-4.13	-4.13
-4.12	-4.12	-4.12	-4.12	-4.12	-4.12	-4.12	-4.12	-4.12
-4.11	-4.11	-4.11	-4.11	-4.11	-4.11	-4.11	-4.11	-4.11
-4.1	-4.1	-4.1	-4.1	-4.1	-4.1	-4.1	-4.1	-4.1
-4.09	-4.09	-4.09	-4.09	-4.09	-4.09	-4.09	-4.09	-4.09
-4.08	-4.08	-4.08	-4.08	-4.08	-4.08	-4.08	-4.08	-4.08
-4.07	-4.07	-4.07	-4.07	-4.07	-4.07	-4.07	-4.07	-4.07
-4.06	-4.06	-4.06	-4.06	-4.06	-4.06	-4.06	-4.06	-4.06
-4.05	-4.05	-4.05	-4.05	-4.05	-4.05	-4.05	-4.05	-4.05
-4.04	-4.04	-4.04	-4.04	-4.04	-4.04	-4.04	-4.04	-4.04
-4.03	-4.03	-4.03	-4.03	-4.03	-4.03	-4.03	-4.03	-4.03
-4.02	-4.02	-4.02	-4.02	-4.02	-4.02	-4.02	-4.02	-4.02
-4.01	-4.01	-4.01	-4.01	-4.01	-4.01	-4.01	-4.01	-4.01

-4	-4	-4	-4	-4	-4	-4	-4	-4
-3.99	-3.99	-3.99	-3.99	-3.99	-3.99	-3.99	-3.99	-3.99
-3.98	-3.98	-3.98	-3.98	-3.98	-3.98	-3.98	-3.98	-3.98
-3.97	-3.97	-3.97	-3.97	-3.97	-3.97	-3.97	-3.97	-3.97
-3.96	-3.96	-3.96	-3.96	-3.96	-3.96	-3.96	-3.96	-3.96
-3.95	-3.95	-3.95	-3.95	-3.95	-3.95	-3.95	-3.95	-3.95
-3.94	-3.94	-3.94	-3.94	-3.94	-3.94	-3.94	-3.94	-3.94
-3.93	-3.93	-3.93	-3.93	-3.93	-3.93	-3.93	-3.93	-3.93
-3.92	-3.92	-3.92	-3.92	-3.92	-3.92	-3.92	-3.92	-3.92
-3.91	-3.91	-3.91	-3.91	-3.91	-3.91	-3.91	-3.91	-3.91
-3.9	-3.9	-3.9	-3.9	-3.9	-3.9	-3.9	-3.9	-3.9
-3.89	-3.89	-3.89	-3.89	-3.89	-3.89	-3.89	-3.89	-3.89
-3.88	-3.88	-3.88	-3.88	-3.88	-3.88	-3.88	-3.88	-3.88
-3.87	-3.87	-3.87	-3.87	-3.87	-3.87	-3.87	-3.87	-3.87
-3.86	-3.86	-3.86	-3.86	-3.86	-3.86	-3.86	-3.86	-3.86
-3.85	-3.85	-3.85	-3.85	-3.85	-3.85	-3.85	-3.85	-3.85
-3.84	-3.84	-3.84	-3.84	-3.84	-3.84	-3.84	-3.84	-3.84
-3.83	-3.83	-3.83	-3.83	-3.83	-3.83	-3.83	-3.83	-3.83
-3.82	-3.82	-3.82	-3.82	-3.82	-3.82	-3.82	-3.82	-3.82
-3.81	-3.81	-3.81	-3.81	-3.81	-3.81	-3.81	-3.81	-3.81
-3.8	-3.8	-3.8	-3.8	-3.8	-3.8	-3.8	-3.8	-3.8
-3.79	-3.79	-3.79	-3.79	-3.79	-3.79	-3.79	-3.79	-3.79
-3.78	-3.78	-3.78	-3.78	-3.78	-3.78	-3.78	-3.78	-3.78
-3.77	-3.77	-3.77	-3.77	-3.77	-3.77	-3.77	-3.77	-3.77
-3.76	-3.76	-3.76	-3.76	-3.76	-3.76	-3.76	-3.76	-3.76
-3.75	-3.75	-3.75	-3.75	-3.75	-3.75	-3.75	-3.75	-3.75
-3.74	-3.74	-3.74	-3.74	-3.74	-3.74	-3.74	-3.74	-3.74
-3.73	-3.73	-3.73	-3.73	-3.73	-3.73	-3.73	-3.73	-3.73
-3.72	-3.72	-3.72	-3.72	-3.72	-3.72	-3.72	-3.72	-3.72
-3.71	-3.71	-3.71	-3.71	-3.71	-3.71	-3.71	-3.71	-3.71
-3.7	-3.7	-3.7	-3.7	-3.7	-3.7	-3.7	-3.7	-3.7
-3.69	-3.69	-3.69	-3.69	-3.69	-3.69	-3.69	-3.69	-3.69
-3.68	-3.68	-3.68	-3.68	-3.68	-3.68	-3.68	-3.68	-3.68
-3.67	-3.67	-3.67	-3.67	-3.67	-3.67	-3.67	-3.67	-3.67
-3.66	-3.66	-3.66	-3.66	-3.66	-3.66	-3.66	-3.66	-3.66
-3.65	-3.65	-3.65	-3.65	-3.65	-3.65	-3.65	-3.65	-3.65
-3.64	-3.64	-3.64	-3.64	-3.64	-3.64	-3.64	-3.64	-3.64
-3.63	-3.63	-3.63	-3.63	-3.63	-3.63	-3.63	-3.63	-3.63
-3.62	-3.62	-3.62	-3.62	-3.62	-3.62	-3.62	-3.62	-3.62
-3.61	-3.61	-3.61	-3.61	-3.61	-3.61	-3.61	-3.61	-3.61
-3.6	-3.6	-3.6	-3.6	-3.6	-3.6	-3.6	-3.6	-3.6
-3.59	-3.59	-3.59	-3.59	-3.59	-3.59	-3.59	-3.59	-3.59
-3.58	-3.58	-3.58	-3.58	-3.58	-3.58	-3.58	-3.58	-3.58
-3.57	-3.57	-3.57	-3.57	-3.57	-3.57	-3.57	-3.57	-3.57
-3.56	-3.56	-3.56	-3.56	-3.56	-3.56	-3.56	-3.56	-3.56
-3.55	-3.55	-3.55	-3.55	-3.55	-3.55	-3.55	-3.55	-3.55
-3.54	-3.54	-3.54	-3.54	-3.54	-3.54	-3.54	-3.54	-3.54
-3.53	-3.53	-3.53	-3.53	-3.53	-3.53	-3.53	-3.53	-3.53
-3.52	-3.52	-3.52	-3.52	-3.52	-3.52	-3.52	-3.52	-3.52
-3.51	-3.51	-3.51	-3.51	-3.51	-3.51	-3.51	-3.51	-3.51

-3.5	-3.5	-3.5	-3.5	-3.5	-3.5	-3.5	-3.5	-3.5
-3.49	-3.49	-3.49	-3.49	-3.49	-3.49	-3.49	-3.49	-3.49
-3.48	-3.48	-3.48	-3.48	-3.48	-3.48	-3.48	-3.48	-3.48
-3.47	-3.47	-3.47	-3.47	-3.47	-3.47	-3.47	-3.47	-3.47
-3.46	-3.46	-3.46	-3.46	-3.46	-3.46	-3.46	-3.46	-3.46
-3.45	-3.45	-3.45	-3.45	-3.45	-3.45	-3.45	-3.45	-3.45
-3.44	-3.44	-3.44	-3.44	-3.44	-3.44	-3.44	-3.44	-3.44
-3.43	-3.43	-3.43	-3.43	-3.43	-3.43	-3.43	-3.43	-3.43
-3.42	-3.42	-3.42	-3.42	-3.42	-3.42	-3.42	-3.42	-3.42
-3.41	-3.41	-3.41	-3.41	-3.41	-3.41	-3.41	-3.41	-3.41
-3.4	-3.4	-3.4	-3.4	-3.4	-3.4	-3.4	-3.4	-3.4
-3.39	-3.39	-3.39	-3.39	-3.39	-3.39	-3.39	-3.39	-3.39
-3.38	-3.38	-3.38	-3.38	-3.38	-3.38	-3.38	-3.38	-3.38
-3.37	-3.37	-3.37	-3.37	-3.37	-3.37	-3.37	-3.37	-3.37
-3.36	-3.36	-3.36	-3.36	-3.36	-3.36	-3.36	-3.36	-3.36
-3.35	-3.35	-3.35	-3.35	-3.35	-3.35	-3.35	-3.35	-3.35
-3.34	-3.34	-3.34	-3.34	-3.34	-3.34	-3.34	-3.34	-3.34
-3.33	-3.33	-3.33	-3.33	-3.33	-3.33	-3.33	-3.33	-3.33
-3.32	-3.32	-3.32	-3.32	-3.32	-3.32	-3.32	-3.32	-3.32
-3.31	-3.31	-3.31	-3.31	-3.31	-3.31	-3.31	-3.31	-3.31
-3.3	-3.3	-3.3	-3.3	-3.3	-3.3	-3.3	-3.3	-3.3
-3.29	-3.29	-3.29	-3.29	-3.29	-3.29	-3.29	-3.29	-3.29
-3.28	-3.28	-3.28	-3.28	-3.28	-3.28	-3.28	-3.28	-3.28
-3.27	-3.27	-3.27	-3.27	-3.27	-3.27	-3.27	-3.27	-3.27
-3.26	-3.26	-3.26	-3.26	-3.26	-3.26	-3.26	-3.26	-3.26
-3.25	-3.25	-3.25	-3.25	-3.25	-3.25	-3.25	-3.25	-3.25
-3.24	-3.24	-3.24	-3.24	-3.24	-3.24	-3.24	-3.24	-3.24
-3.23	-3.23	-3.23	-3.23	-3.23	-3.23	-3.23	-3.23	-3.23
-3.22	-3.22	-3.22	-3.22	-3.22	-3.22	-3.22	-3.22	-3.22
-3.21	-3.21	-3.21	-3.21	-3.21	-3.21	-3.21	-3.21	-3.21
-3.2	-3.2	-3.2	-3.2	-3.2	-3.2	-3.2	-3.2	-3.2
-3.19	-3.19	-3.19	-3.19	-3.19	-3.19	-3.19	-3.19	-3.19
-3.18	-3.18	-3.18	-3.18	-3.18	-3.18	-3.18	-3.18	-3.18
-3.17	-3.17	-3.17	-3.17	-3.17	-3.17	-3.17	-3.17	-3.17
-3.16	-3.16	-3.16	-3.16	-3.16	-3.16	-3.16	-3.16	-3.16
-3.15	-3.15	-3.15	-3.15	-3.15	-3.15	-3.15	-3.15	-3.15
-3.14	-3.14	-3.14	-3.14	-3.14	-3.14	-3.14	-3.14	-3.14
-3.13	-3.13	-3.13	-3.13	-3.13	-3.13	-3.13	-3.13	-3.13
-3.12	-3.12	-3.12	-3.12	-3.12	-3.12	-3.12	-3.12	-3.12
-3.11	-3.11	-3.11	-3.11	-3.11	-3.11	-3.11	-3.11	-3.11
-3.1	-3.1	-3.1	-3.1	-3.1	-3.1	-3.1	-3.1	-3.1
-3.09	-3.09	-3.09	-3.09	-3.09	-3.09	-3.09	-3.09	-3.09
-3.08	-3.08	-3.08	-3.08	-3.08	-3.08	-3.08	-3.08	-3.08
-3.07	-3.07	-3.07	-3.07	-3.07	-3.07	-3.07	-3.07	-3.07
-3.06	-3.06	-3.06	-3.06	-3.06	-3.06	-3.06	-3.06	-3.06
-3.05	-3.05	-3.05	-3.05	-3.05	-3.05	-3.05	-3.05	-3.05
-3.04	-3.04	-3.04	-3.04	-3.04	-3.04	-3.04	-3.04	-3.04
-3.03	-3.03	-3.03	-3.03	-3.03	-3.03	-3.03	-3.03	-3.03
-3.02	-3.02	-3.02	-3.02	-3.02	-3.02	-3.02	-3.02	-3.02
-3.01	-3.01	-3.01	-3.01	-3.01	-3.01	-3.01	-3.01	-3.01

-3	-3	-3	-3	-3	-3	-3	-3	-3
-2.99	-2.99	-2.99	-2.99	-2.99	-2.99	-2.99	-2.99	-2.99
-2.98	-2.98	-2.98	-2.98	-2.98	-2.98	-2.98	-2.98	-2.98
-2.97	-2.97	-2.97	-2.97	-2.97	-2.97	-2.97	-2.97	-2.97
-2.96	-2.96	-2.96	-2.96	-2.96	-2.96	-2.96	-2.96	-2.96
-2.95	-2.95	-2.95	-2.95	-2.95	-2.95	-2.95	-2.95	-2.95
-2.94	-2.94	-2.94	-2.94	-2.94	-2.94	-2.94	-2.94	-2.94
-2.93	-2.93	-2.93	-2.93	-2.93	-2.93	-2.93	-2.93	-2.93
-2.92	-2.92	-2.92	-2.92	-2.92	-2.92	-2.92	-2.92	-2.92
-2.91	-2.91	-2.91	-2.91	-2.91	-2.91	-2.91	-2.91	-2.91
-2.9	-2.9	-2.9	-2.9	-2.9	-2.9	-2.9	-2.9	-2.9
-2.89	-2.89	-2.89	-2.89	-2.89	-2.89	-2.89	-2.89	-2.89
-2.88	-2.88	-2.88	-2.88	-2.88	-2.88	-2.88	-2.88	-2.88
-2.87	-2.87	-2.87	-2.87	-2.87	-2.87	-2.87	-2.87	-2.87
-2.86	-2.86	-2.86	-2.86	-2.86	-2.86	-2.86	-2.86	-2.86
-2.85	-2.85	-2.85	-2.85	-2.85	-2.85	-2.85	-2.85	-2.85
-2.84	-2.84	-2.84	-2.84	-2.84	-2.84	-2.84	-2.84	-2.84
-2.83	-2.83	-2.83	-2.83	-2.83	-2.83	-2.83	-2.83	-2.83
-2.82	-2.82	-2.82	-2.82	-2.82	-2.82	-2.82	-2.82	-2.82
-2.81	-2.81	-2.81	-2.81	-2.81	-2.81	-2.81	-2.81	-2.81
-2.8	-2.8	-2.8	-2.8	-2.8	-2.8	-2.8	-2.8	-2.8
-2.79	-2.79	-2.79	-2.79	-2.79	-2.79	-2.79	-2.79	-2.79
-2.78	-2.78	-2.78	-2.78	-2.78	-2.78	-2.78	-2.78	-2.78
-2.77	-2.77	-2.77	-2.77	-2.77	-2.77	-2.77	-2.77	-2.77
-2.76	-2.76	-2.76	-2.76	-2.76	-2.76	-2.76	-2.76	-2.76
-2.75	-2.75	-2.75	-2.75	-2.75	-2.75	-2.75	-2.75	-2.75
-2.74	-2.74	-2.74	-2.74	-2.74	-2.74	-2.74	-2.74	-2.74
-2.73	-2.73	-2.73	-2.73	-2.73	-2.73	-2.73	-2.73	-2.73
-2.72	-2.72	-2.72	-2.72	-2.72	-2.72	-2.72	-2.72	-2.72
-2.71	-2.71	-2.71	-2.71	-2.71	-2.71	-2.71	-2.71	-2.71
-2.7	-2.7	-2.7	-2.7	-2.7	-2.7	-2.7	-2.7	-2.7
-2.69	-2.69	-2.69	-2.69	-2.69	-2.69	-2.69	-2.69	-2.69
-2.68	-2.68	-2.68	-2.68	-2.68	-2.68	-2.68	-2.68	-2.68
-2.67	-2.67	-2.67	-2.67	-2.67	-2.67	-2.67	-2.67	-2.67
-2.66	-2.66	-2.66	-2.66	-2.66	-2.66	-2.66	-2.66	-2.66
-2.65	-2.65	-2.65	-2.65	-2.65	-2.65	-2.65	-2.65	-2.65
-2.64	-2.64	-2.64	-2.64	-2.64	-2.64	-2.64	-2.64	-2.64
-2.63	-2.63	-2.63	-2.63	-2.63	-2.63	-2.63	-2.63	-2.63
-2.62	-2.62	-2.62	-2.62	-2.62	-2.62	-2.62	-2.62	-2.62
-2.61	-2.61	-2.61	-2.61	-2.61	-2.61	-2.61	-2.61	-2.61
-2.6	-2.6	-2.6	-2.6	-2.6	-2.6	-2.6	-2.6	-2.6
-2.59	-2.59	-2.59	-2.59	-2.59	-2.59	-2.59	-2.59	-2.59
-2.58	-2.58	-2.58	-2.58	-2.58	-2.58	-2.58	-2.58	-2.58
-2.57	-2.57	-2.57	-2.57	-2.57	-2.57	-2.57	-2.57	-2.57
-2.56	-2.56	-2.56	-2.56	-2.56	-2.56	-2.56	-2.56	-2.56
-2.55	-2.55	-2.55	-2.55	-2.55	-2.55	-2.55	-2.55	-2.55
-2.54	-2.54	-2.54	-2.54	-2.54	-2.54	-2.54	-2.54	-2.54
-2.53	-2.53	-2.53	-2.53	-2.53	-2.53	-2.53	-2.53	-2.53
-2.52	-2.52	-2.52	-2.52	-2.52	-2.52	-2.52	-2.52	-2.52
-2.51	-2.51	-2.51	-2.51	-2.51	-2.51	-2.51	-2.51	-2.51

-2.5	-2.5	-2.5	-2.5	-2.5	-2.5	-2.5	-2.5	-2.5
-2.49	-2.49	-2.49	-2.49	-2.49	-2.49	-2.49	-2.49	-2.49
-2.48	-2.48	-2.48	-2.48	-2.48	-2.48	-2.48	-2.48	-2.48
-2.47	-2.47	-2.47	-2.47	-2.47	-2.47	-2.47	-2.47	-2.47
-2.46	-2.46	-2.46	-2.46	-2.46	-2.46	-2.46	-2.46	-2.46
-2.45	-2.45	-2.45	-2.45	-2.45	-2.45	-2.45	-2.45	-2.45
-2.44	-2.44	-2.44	-2.44	-2.44	-2.44	-2.44	-2.44	-2.44
-2.43	-2.43	-2.43	-2.43	-2.43	-2.43	-2.43	-2.43	-2.43
-2.42	-2.42	-2.42	-2.42	-2.42	-2.42	-2.42	-2.42	-2.42
-2.41	-2.41	-2.41	-2.41	-2.41	-2.41	-2.41	-2.41	-2.41
-2.4	-2.4	-2.4	-2.4	-2.4	-2.4	-2.4	-2.4	-2.4
-2.39	-2.39	-2.39	-2.39	-2.39	-2.39	-2.39	-2.39	-2.39
-2.38	-2.38	-2.38	-2.38	-2.38	-2.38	-2.38	-2.38	-2.38
-2.37	-2.37	-2.37	-2.37	-2.37	-2.37	-2.37	-2.37	-2.37
-2.36	-2.36	-2.36	-2.36	-2.36	-2.36	-2.36	-2.36	-2.36
-2.35	-2.35	-2.35	-2.35	-2.35	-2.35	-2.35	-2.35	-2.35
-2.34	-2.34	-2.34	-2.34	-2.34	-2.34	-2.34	-2.34	-2.34
-2.33	-2.33	-2.33	-2.33	-2.33	-2.33	-2.33	-2.33	-2.33
-2.32	-2.32	-2.32	-2.32	-2.32	-2.32	-2.32	-2.32	-2.32
-2.31	-2.31	-2.31	-2.31	-2.31	-2.31	-2.31	-2.31	-2.31
-2.3	-2.3	-2.3	-2.3	-2.3	-2.3	-2.3	-2.3	-2.3
-2.29	-2.29	-2.29	-2.29	-2.29	-2.29	-2.29	-2.29	-2.29
-2.28	-2.28	-2.28	-2.28	-2.28	-2.28	-2.28	-2.28	-2.28
-2.27	-2.27	-2.27	-2.27	-2.27	-2.27	-2.27	-2.27	-2.27
-2.26	-2.26	-2.26	-2.26	-2.26	-2.26	-2.26	-2.26	-2.26
-2.25	-2.25	-2.25	-2.25	-2.25	-2.25	-2.25	-2.25	-2.25
-2.24	-2.24	-2.24	-2.24	-2.24	-2.24	-2.24	-2.24	-2.24
-2.23	-2.23	-2.23	-2.23	-2.23	-2.23	-2.23	-2.23	-2.23
-2.22	-2.22	-2.22	-2.22	-2.22	-2.22	-2.22	-2.22	-2.22
-2.21	-2.21	-2.21	-2.21	-2.21	-2.21	-2.21	-2.21	-2.21
-2.2	-2.2	-2.2	-2.2	-2.2	-2.2	-2.2	-2.2	-2.2
-2.19	-2.19	-2.19	-2.19	-2.19	-2.19	-2.19	-2.19	-2.19
-2.18	-2.18	-2.18	-2.18	-2.18	-2.18	-2.18	-2.18	-2.18
-2.17	-2.17	-2.17	-2.17	-2.17	-2.17	-2.17	-2.17	-2.17
-2.16	-2.16	-2.16	-2.16	-2.16	-2.16	-2.16	-2.16	-2.16
-2.15	-2.15	-2.15	-2.15	-2.15	-2.15	-2.15	-2.15	-2.15
-2.14	-2.14	-2.14	-2.14	-2.14	-2.14	-2.14	-2.14	-2.14
-2.13	-2.13	-2.13	-2.13	-2.13	-2.13	-2.13	-2.13	-2.13
-2.12	-2.12	-2.12	-2.12	-2.12	-2.12	-2.12	-2.12	-2.12
-2.11	-2.11	-2.11	-2.11	-2.11	-2.11	-2.11	-2.11	-2.11
-2.1	-2.1	-2.1	-2.1	-2.1	-2.1	-2.1	-2.1	-2.1
-2.09	-2.09	-2.09	-2.09	-2.09	-2.09	-2.09	-2.09	-2.09
-2.08	-2.08	-2.08	-2.08	-2.08	-2.08	-2.08	-2.08	-2.08
-2.07	-2.07	-2.07	-2.07	-2.07	-2.07	-2.07	-2.07	-2.07
-2.06	-2.06	-2.06	-2.06	-2.06	-2.06	-2.06	-2.06	-2.06
-2.05	-2.05	-2.05	-2.05	-2.05	-2.05	-2.05	-2.05	-2.05
-2.04	-2.04	-2.04	-2.04	-2.04	-2.04	-2.04	-2.04	-2.04
-2.03	-2.03	-2.03	-2.03	-2.03	-2.03	-2.03	-2.03	-2.03
-2.02	-2.02	-2.02	-2.02	-2.02	-2.02	-2.02	-2.02	-2.02
-2.01	-2.01	-2.01	-2.01	-2.01	-2.01	-2.01	-2.01	-2.01

-2	-2	-2	-2	-2	-2	-2	-2	-2
-1.99	-1.99	-1.99	-1.99	-1.99	-1.99	-1.99	-1.99	-1.99
-1.98	-1.98	-1.98	-1.98	-1.98	-1.98	-1.98	-1.98	-1.98
-1.97	-1.97	-1.97	-1.97	-1.97	-1.97	-1.97	-1.97	-1.97
-1.96	-1.96	-1.96	-1.96	-1.96	-1.96	-1.96	-1.96	-1.96
-1.95	-1.95	-1.95	-1.95	-1.95	-1.95	-1.95	-1.95	-1.95
-1.94	-1.94	-1.94	-1.94	-1.94	-1.94	-1.94	-1.94	-1.94
-1.93	-1.93	-1.93	-1.93	-1.93	-1.93	-1.93	-1.93	-1.93
-1.92	-1.92	-1.92	-1.92	-1.92	-1.92	-1.92	-1.92	-1.92
-1.91	-1.91	-1.91	-1.91	-1.91	-1.91	-1.91	-1.91	-1.91
-1.9	-1.9	-1.9	-1.9	-1.9	-1.9	-1.9	-1.9	-1.9
-1.89	-1.89	-1.89	-1.89	-1.89	-1.89	-1.89	-1.89	-1.89
-1.88	-1.88	-1.88	-1.88	-1.88	-1.88	-1.88	-1.88	-1.88
-1.87	-1.87	-1.87	-1.87	-1.87	-1.87	-1.87	-1.87	-1.87
-1.86	-1.86	-1.86	-1.86	-1.86	-1.86	-1.86	-1.86	-1.86
-1.85	-1.85	-1.85	-1.85	-1.85	-1.85	-1.85	-1.85	-1.85
-1.84	-1.84	-1.84	-1.84	-1.84	-1.84	-1.84	-1.84	-1.84
-1.83	-1.83	-1.83	-1.83	-1.83	-1.83	-1.83	-1.83	-1.83
-1.82	-1.82	-1.82	-1.82	-1.82	-1.82	-1.82	-1.82	-1.82
-1.81	-1.81	-1.81	-1.81	-1.81	-1.81	-1.81	-1.81	-1.81
-1.8	-1.8	-1.8	-1.8	-1.8	-1.8	-1.8	-1.8	-1.8
-1.79	-1.79	-1.79	-1.79	-1.79	-1.79	-1.79	-1.79	-1.79
-1.78	-1.78	-1.78	-1.78	-1.78	-1.78	-1.78	-1.78	-1.78
-1.77	-1.77	-1.77	-1.77	-1.77	-1.77	-1.77	-1.77	-1.77
-1.76	-1.76	-1.76	-1.76	-1.76	-1.76	-1.76	-1.76	-1.76
-1.75	-1.75	-1.75	-1.75	-1.75	-1.75	-1.75	-1.75	-1.75
-1.74	-1.74	-1.74	-1.74	-1.74	-1.74	-1.74	-1.74	-1.74
-1.73	-1.73	-1.73	-1.73	-1.73	-1.73	-1.73	-1.73	-1.73
-1.72	-1.72	-1.72	-1.72	-1.72	-1.72	-1.72	-1.72	-1.72
-1.71	-1.71	-1.71	-1.71	-1.71	-1.71	-1.71	-1.71	-1.71
-1.7	-1.7	-1.7	-1.7	-1.7	-1.7	-1.7	-1.7	-1.7
-1.69	-1.69	-1.69	-1.69	-1.69	-1.69	-1.69	-1.69	-1.69
-1.68	-1.68	-1.68	-1.68	-1.68	-1.68	-1.68	-1.68	-1.68
-1.67	-1.67	-1.67	-1.67	-1.67	-1.67	-1.67	-1.67	-1.67
-1.66	-1.66	-1.66	-1.66	-1.66	-1.66	-1.66	-1.66	-1.66
-1.65	-1.65	-1.65	-1.65	-1.65	-1.65	-1.65	-1.65	-1.65
-1.64	-1.64	-1.64	-1.64	-1.64	-1.64	-1.64	-1.64	-1.64
-1.63	-1.63	-1.63	-1.63	-1.63	-1.63	-1.63	-1.63	-1.63
-1.62	-1.62	-1.62	-1.62	-1.62	-1.62	-1.62	-1.62	-1.62
-1.61	-1.61	-1.61	-1.61	-1.61	-1.61	-1.61	-1.61	-1.61
-1.6	-1.6	-1.6	-1.6	-1.6	-1.6	-1.6	-1.6	-1.6
-1.59	-1.59	-1.59	-1.59	-1.59	-1.59	-1.59	-1.59	-1.59
-1.58	-1.58	-1.58	-1.58	-1.58	-1.58	-1.58	-1.58	-1.58
-1.57	-1.57	-1.57	-1.57	-1.57	-1.57	-1.57	-1.57	-1.57
-1.56	-1.56	-1.56	-1.56	-1.56	-1.56	-1.56	-1.56	-1.56
-1.55	-1.55	-1.55	-1.55	-1.55	-1.55	-1.55	-1.55	-1.55
-1.54	-1.54	-1.54	-1.54	-1.54	-1.54	-1.54	-1.54	-1.54
-1.53	-1.53	-1.53	-1.53	-1.53	-1.53	-1.53	-1.53	-1.53
-1.52	-1.52	-1.52	-1.52	-1.52	-1.52	-1.52	-1.52	-1.52
-1.51	-1.51	-1.51	-1.51	-1.51	-1.51	-1.51	-1.51	-1.51

-1.5	-1.5	-1.5	-1.5	-1.5	-1.5	-1.5	-1.5	-1.5
-1.49	-1.49	-1.49	-1.49	-1.49	-1.49	-1.49	-1.49	-1.49
-1.48	-1.48	-1.48	-1.48	-1.48	-1.48	-1.48	-1.48	-1.48
-1.47	-1.47	-1.47	-1.47	-1.47	-1.47	-1.47	-1.47	-1.47
-1.46	-1.46	-1.46	-1.46	-1.46	-1.46	-1.46	-1.46	-1.46
-1.45	-1.45	-1.45	-1.45	-1.45	-1.45	-1.45	-1.45	-1.45
-1.44	-1.44	-1.44	-1.44	-1.44	-1.44	-1.44	-1.44	-1.44
-1.43	-1.43	-1.43	-1.43	-1.43	-1.43	-1.43	-1.43	-1.43
-1.42	-1.42	-1.42	-1.42	-1.42	-1.42	-1.42	-1.42	-1.42
-1.41	-1.41	-1.41	-1.41	-1.41	-1.41	-1.41	-1.41	-1.41
-1.4	-1.4	-1.4	-1.4	-1.4	-1.4	-1.4	-1.4	-1.4
-1.39	-1.39	-1.39	-1.39	-1.39	-1.39	-1.39	-1.39	-1.39
-1.38	-1.38	-1.38	-1.38	-1.38	-1.38	-1.38	-1.38	-1.38
-1.37	-1.37	-1.37	-1.37	-1.37	-1.37	-1.37	-1.37	-1.37
-1.36	-1.36	-1.36	-1.36	-1.36	-1.36	-1.36	-1.36	-1.36
-1.35	-1.35	-1.35	-1.35	-1.35	-1.35	-1.35	-1.35	-1.35
-1.34	-1.34	-1.34	-1.34	-1.34	-1.34	-1.34	-1.34	-1.34
-1.33	-1.33	-1.33	-1.33	-1.33	-1.33	-1.33	-1.33	-1.33
-1.32	-1.32	-1.32	-1.32	-1.32	-1.32	-1.32	-1.32	-1.32
-1.31	-1.31	-1.31	-1.31	-1.31	-1.31	-1.31	-1.31	-1.31
-1.3	-1.3	-1.3	-1.3	-1.3	-1.3	-1.3	-1.3	-1.3
-1.29	-1.29	-1.29	-1.29	-1.29	-1.29	-1.29	-1.29	-1.29
-1.28	-1.28	-1.28	-1.28	-1.28	-1.28	-1.28	-1.28	-1.28
-1.27	-1.27	-1.27	-1.27	-1.27	-1.27	-1.27	-1.27	-1.27
-1.26	-1.26	-1.26	-1.26	-1.26	-1.26	-1.26	-1.26	-1.26
-1.25	-1.25	-1.25	-1.25	-1.25	-1.25	-1.25	-1.25	-1.25
-1.24	-1.24	-1.24	-1.24	-1.24	-1.24	-1.24	-1.24	-1.24
-1.23	-1.23	-1.23	-1.23	-1.23	-1.23	-1.23	-1.23	-1.23
-1.22	-1.22	-1.22	-1.22	-1.22	-1.22	-1.22	-1.22	-1.22
-1.21	-1.21	-1.21	-1.21	-1.21	-1.21	-1.21	-1.21	-1.21
-1.2	-1.2	-1.2	-1.2	-1.2	-1.2	-1.2	-1.2	-1.2
-1.19	-1.19	-1.19	-1.19	-1.19	-1.19	-1.19	-1.19	-1.19
-1.18	-1.18	-1.18	-1.18	-1.18	-1.18	-1.18	-1.18	-1.18
-1.17	-1.17	-1.17	-1.17	-1.17	-1.17	-1.17	-1.17	-1.17
-1.16	-1.16	-1.16	-1.16	-1.16	-1.16	-1.16	-1.16	-1.16
-1.15	-1.15	-1.15	-1.15	-1.15	-1.15	-1.15	-1.15	-1.15
-1.14	-1.14	-1.14	-1.14	-1.14	-1.14	-1.14	-1.14	-1.14
-1.13	-1.13	-1.13	-1.13	-1.13	-1.13	-1.13	-1.13	-1.13
-1.12	-1.12	-1.12	-1.12	-1.12	-1.12	-1.12	-1.12	-1.12
-1.11	-1.11	-1.11	-1.11	-1.11	-1.11	-1.11	-1.11	-1.11
-1.1	-1.1	-1.1	-1.1	-1.1	-1.1	-1.1	-1.1	-1.1
-1.09	-1.09	-1.09	-1.09	-1.09	-1.09	-1.09	-1.09	-1.09
-1.08	-1.08	-1.08	-1.08	-1.08	-1.08	-1.08	-1.08	-1.08
-1.07	-1.07	-1.07	-1.07	-1.07	-1.07	-1.07	-1.07	-1.07
-1.06	-1.06	-1.06	-1.06	-1.06	-1.06	-1.06	-1.06	-1.06
-1.05	-1.05	-1.05	-1.05	-1.05	-1.05	-1.05	-1.05	-1.05
-1.04	-1.04	-1.04	-1.04	-1.04	-1.04	-1.04	-1.04	-1.04
-1.03	-1.03	-1.03	-1.03	-1.03	-1.03	-1.03	-1.03	-1.03
-1.02	-1.02	-1.02	-1.02	-1.02	-1.02	-1.02	-1.02	-1.02
-1.01	-1.01	-1.01	-1.01	-1.01	-1.01	-1.01	-1.01	-1.01

-1	-1	-1	-1	-1	-1	-1	-1	-1
-0.99	-0.99	-0.99	-0.99	-0.99	-0.99	-0.99	-0.99	-0.99
-0.98	-0.98	-0.98	-0.98	-0.98	-0.98	-0.98	-0.98	-0.98
-0.97	-0.97	-0.97	-0.97	-0.97	-0.97	-0.97	-0.97	-0.97
-0.96	-0.96	-0.96	-0.96	-0.96	-0.96	-0.96	-0.96	-0.96
-0.95	-0.95	-0.95	-0.95	-0.95	-0.95	-0.95	-0.95	-0.95
-0.94	-0.94	-0.94	-0.94	-0.94	-0.94	-0.94	-0.94	-0.94
-0.93	-0.93	-0.93	-0.93	-0.93	-0.93	-0.93	-0.93	-0.93
-0.92	-0.92	-0.92	-0.92	-0.92	-0.92	-0.92	-0.92	-0.92
-0.91	-0.91	-0.91	-0.91	-0.91	-0.91	-0.91	-0.91	-0.91
-0.9	-0.9	-0.9	-0.9	-0.9	-0.9	-0.9	-0.9	-0.9
-0.89	-0.89	-0.89	-0.89	-0.89	-0.89	-0.89	-0.89	-0.89
-0.88	-0.88	-0.88	-0.88	-0.88	-0.88	-0.88	-0.88	-0.88
-0.87	-0.87	-0.87	-0.87	-0.87	-0.87	-0.87	-0.87	-0.87
-0.86	-0.86	-0.86	-0.86	-0.86	-0.86	-0.86	-0.86	-0.86
-0.85	-0.85	-0.85	-0.85	-0.85	-0.85	-0.85	-0.85	-0.85
-0.84	-0.84	-0.84	-0.84	-0.84	-0.84	-0.84	-0.84	-0.84
-0.83	-0.83	-0.83	-0.83	-0.83	-0.83	-0.83	-0.83	-0.83
-0.82	-0.82	-0.82	-0.82	-0.82	-0.82	-0.82	-0.82	-0.82
-0.81	-0.81	-0.81	-0.81	-0.81	-0.81	-0.81	-0.81	-0.81
-0.8	-0.8	-0.8	-0.8	-0.8	-0.8	-0.8	-0.8	-0.8
-0.79	-0.79	-0.79	-0.79	-0.79	-0.79	-0.79	-0.79	-0.79
-0.78	-0.78	-0.78	-0.78	-0.78	-0.78	-0.78	-0.78	-0.78
-0.77	-0.77	-0.77	-0.77	-0.77	-0.77	-0.77	-0.77	-0.77
-0.76	-0.76	-0.76	-0.76	-0.76	-0.76	-0.76	-0.76	-0.76
-0.75	-0.75	-0.75	-0.75	-0.75	-0.75	-0.75	-0.75	-0.75
-0.74	-0.74	-0.74	-0.74	-0.74	-0.74	-0.74	-0.74	-0.74
-0.73	-0.73	-0.73	-0.73	-0.73	-0.73	-0.73	-0.73	-0.73
-0.72	-0.72	-0.72	-0.72	-0.72	-0.72	-0.72	-0.72	-0.72
-0.71	-0.71	-0.71	-0.71	-0.71	-0.71	-0.71	-0.71	-0.71
-0.7	-0.7	-0.7	-0.7	-0.7	-0.7	-0.7	-0.7	-0.7
-0.69	-0.69	-0.69	-0.69	-0.69	-0.69	-0.69	-0.69	-0.69
-0.68	-0.68	-0.68	-0.68	-0.68	-0.68	-0.68	-0.68	-0.68
-0.67	-0.67	-0.67	-0.67	-0.67	-0.67	-0.67	-0.67	-0.67
-0.66	-0.66	-0.66	-0.66	-0.66	-0.66	-0.66	-0.66	-0.66
-0.65	-0.65	-0.65	-0.65	-0.65	-0.65	-0.65	-0.65	-0.65
-0.64	-0.64	-0.64	-0.64	-0.64	-0.64	-0.64	-0.64	-0.64
-0.63	-0.63	-0.63	-0.63	-0.63	-0.63	-0.63	-0.63	-0.63
-0.62	-0.62	-0.62	-0.62	-0.62	-0.62	-0.62	-0.62	-0.62
-0.61	-0.61	-0.61	-0.61	-0.61	-0.61	-0.61	-0.61	-0.61
-0.6	-0.6	-0.6	-0.6	-0.6	-0.6	-0.6	-0.6	-0.6
-0.59	-0.59	-0.59	-0.59	-0.59	-0.59	-0.59	-0.59	-0.59
-0.58	-0.58	-0.58	-0.58	-0.58	-0.58	-0.58	-0.58	-0.58
-0.57	-0.57	-0.57	-0.57	-0.57	-0.57	-0.57	-0.57	-0.57
-0.56	-0.56	-0.56	-0.56	-0.56	-0.56	-0.56	-0.56	-0.56
-0.55	-0.55	-0.55	-0.55	-0.55	-0.55	-0.55	-0.55	-0.55
-0.54	-0.54	-0.54	-0.54	-0.54	-0.54	-0.54	-0.54	-0.54
-0.53	-0.53	-0.53	-0.53	-0.53	-0.53	-0.53	-0.53	-0.53
-0.52	-0.52	-0.52	-0.52	-0.52	-0.52	-0.52	-0.52	-0.52
-0.51	-0.51	-0.51	-0.51	-0.51	-0.51	-0.51	-0.51	-0.51

-0.5	-0.5	-0.5	-0.5	-0.5	-0.5	-0.5	-0.5	-0.5
-0.49	-0.49	-0.49	-0.49	-0.49	-0.49	-0.49	-0.49	-0.49
-0.48	-0.48	-0.48	-0.48	-0.48	-0.48	-0.48	-0.48	-0.48
-0.47	-0.47	-0.47	-0.47	-0.47	-0.47	-0.47	-0.47	-0.47
-0.46	-0.46	-0.46	-0.46	-0.46	-0.46	-0.46	-0.46	-0.46
-0.45	-0.45	-0.45	-0.45	-0.45	-0.45	-0.45	-0.45	-0.45
-0.44	-0.44	-0.44	-0.44	-0.44	-0.44	-0.44	-0.44	-0.44
-0.43	-0.43	-0.43	-0.43	-0.43	-0.43	-0.43	-0.43	-0.43
-0.42	-0.42	-0.42	-0.42	-0.42	-0.42	-0.42	-0.42	-0.42
-0.41	-0.41	-0.41	-0.41	-0.41	-0.41	-0.41	-0.41	-0.41
-0.4	-0.4	-0.4	-0.4	-0.4	-0.4	-0.4	-0.4	-0.4
-0.39	-0.39	-0.39	-0.39	-0.39	-0.39	-0.39	-0.39	-0.39
-0.38	-0.38	-0.38	-0.38	-0.38	-0.38	-0.38	-0.38	-0.38
-0.37	-0.37	-0.37	-0.37	-0.37	-0.37	-0.37	-0.37	-0.37
-0.36	-0.36	-0.36	-0.36	-0.36	-0.36	-0.36	-0.36	-0.36
-0.35	-0.35	-0.35	-0.35	-0.35	-0.35	-0.35	-0.35	-0.35
-0.34	-0.34	-0.34	-0.34	-0.34	-0.34	-0.34	-0.34	-0.34
-0.33	-0.33	-0.33	-0.33	-0.33	-0.33	-0.33	-0.33	-0.33
-0.32	-0.32	-0.32	-0.32	-0.32	-0.32	-0.32	-0.32	-0.32
-0.31	-0.31	-0.31	-0.31	-0.31	-0.31	-0.31	-0.31	-0.31
-0.3	-0.3	-0.3	-0.3	-0.3	-0.3	-0.3	-0.3	-0.3
-0.29	-0.29	-0.29	-0.29	-0.29	-0.29	-0.29	-0.29	-0.29
-0.28	-0.28	-0.28	-0.28	-0.28	-0.28	-0.28	-0.28	-0.28
-0.27	-0.27	-0.27	-0.27	-0.27	-0.27	-0.27	-0.27	-0.27
-0.26	-0.26	-0.26	-0.26	-0.26	-0.26	-0.26	-0.26	-0.26
-0.25	-0.25	-0.25	-0.25	-0.25	-0.25	-0.25	-0.25	-0.25
-0.24	-0.24	-0.24	-0.24	-0.24	-0.24	-0.24	-0.24	-0.24
-0.23	-0.23	-0.23	-0.23	-0.23	-0.23	-0.23	-0.23	-0.23
-0.22	-0.22	-0.22	-0.22	-0.22	-0.22	-0.22	-0.22	-0.22
-0.21	-0.21	-0.21	-0.21	-0.21	-0.21	-0.21	-0.21	-0.21
-0.2	-0.2	-0.2	-0.2	-0.2	-0.2	-0.2	-0.2	-0.2
-0.19	-0.19	-0.19	-0.19	-0.19	-0.19	-0.19	-0.19	-0.19
-0.18	-0.18	-0.18	-0.18	-0.18	-0.18	-0.18	-0.18	-0.18
-0.17	-0.17	-0.17	-0.17	-0.17	-0.17	-0.17	-0.17	-0.17
-0.16	-0.16	-0.16	-0.16	-0.16	-0.16	-0.16	-0.16	-0.16
-0.15	-0.15	-0.15	-0.15	-0.15	-0.15	-0.15	-0.15	-0.15
-0.14	-0.14	-0.14	-0.14	-0.14	-0.14	-0.14	-0.14	-0.14
-0.13	-0.13	-0.13	-0.13	-0.13	-0.13	-0.13	-0.13	-0.13
-0.12	-0.12	-0.12	-0.12	-0.12	-0.12	-0.12	-0.12	-0.12
-0.11	-0.11	-0.11	-0.11	-0.11	-0.11	-0.11	-0.11	-0.11
-0.1	-0.1	-0.1	-0.1	-0.1	-0.1	-0.1	-0.1	-0.1
-0.09	-0.09	-0.09	-0.09	-0.09	-0.09	-0.09	-0.09	-0.09
-0.08	-0.08	-0.08	-0.08	-0.08	-0.08	-0.08	-0.08	-0.08
-0.07	-0.07	-0.07	-0.07	-0.07	-0.07	-0.07	-0.07	-0.07
-0.06	-0.06	-0.06	-0.06	-0.06	-0.06	-0.06	-0.06	-0.06
-0.05	-0.05	-0.05	-0.05	-0.05	-0.05	-0.05	-0.05	-0.05
-0.04	-0.04	-0.04	-0.04	-0.04	-0.04	-0.04	-0.04	-0.04
-0.03	-0.03	-0.03	-0.03	-0.03	-0.03	-0.03	-0.03	-0.03
-0.02	-0.02	-0.02	-0.02	-0.02	-0.02	-0.02	-0.02	-0.02
-0.01	-0.01	-0.01	-0.01	-0.01	-0.01	-0.01	-0.01	-0.01

0	0	0	0	0	0	0	0	0
0.01	0.01	0.01	0.01	0.01	0.01	0.01	0.01	0.01
0.02	0.02	0.02	0.02	0.02	0.02	0.02	0.02	0.02
0.03	0.03	0.03	0.03	0.03	0.03	0.03	0.03	0.03
0.04	0.04	0.04	0.04	0.04	0.04	0.04	0.04	0.04
0.05	0.05	0.05	0.05	0.05	0.05	0.05	0.05	0.05
0.06	0.06	0.06	0.06	0.06	0.06	0.06	0.06	0.06
0.07	0.07	0.07	0.07	0.07	0.07	0.07	0.07	0.07
0.08	0.08	0.08	0.08	0.08	0.08	0.08	0.08	0.08
0.09	0.09	0.09	0.09	0.09	0.09	0.09	0.09	0.09
0.1	0.1	0.1	0.1	0.1	0.1	0.1	0.1	0.1
0.11	0.11	0.11	0.11	0.11	0.11	0.11	0.11	0.11
0.12	0.12	0.12	0.12	0.12	0.12	0.12	0.12	0.12
0.13	0.13	0.13	0.13	0.13	0.13	0.13	0.13	0.13
0.14	0.14	0.14	0.14	0.14	0.14	0.14	0.14	0.14
0.15	0.15	0.15	0.15	0.15	0.15	0.15	0.15	0.15
0.16	0.16	0.16	0.16	0.16	0.16	0.16	0.16	0.16
0.17	0.17	0.17	0.17	0.17	0.17	0.17	0.17	0.17
0.18	0.18	0.18	0.18	0.18	0.18	0.18	0.18	0.18
0.19	0.19	0.19	0.19	0.19	0.19	0.19	0.19	0.19
0.2	0.2	0.2	0.2	0.2	0.2	0.2	0.2	0.2
0.21	0.21	0.21	0.21	0.21	0.21	0.21	0.21	0.21
0.22	0.22	0.22	0.22	0.22	0.22	0.22	0.22	0.22
0.23	0.23	0.23	0.23	0.23	0.23	0.23	0.23	0.23
0.24	0.24	0.24	0.24	0.24	0.24	0.24	0.24	0.24
0.25	0.25	0.25	0.25	0.25	0.25	0.25	0.25	0.25
0.26	0.26	0.26	0.26	0.26	0.26	0.26	0.26	0.26
0.27	0.27	0.27	0.27	0.27	0.27	0.27	0.27	0.27
0.28	0.28	0.28	0.28	0.28	0.28	0.28	0.28	0.28
0.29	0.29	0.29	0.29	0.29	0.29	0.29	0.29	0.29
0.3	0.3	0.3	0.3	0.3	0.3	0.3	0.3	0.3
0.31	0.31	0.31	0.31	0.31	0.31	0.31	0.31	0.31
0.32	0.32	0.32	0.32	0.32	0.32	0.32	0.32	0.32
0.33	0.33	0.33	0.33	0.33	0.33	0.33	0.33	0.33
0.34	0.34	0.34	0.34	0.34	0.34	0.34	0.34	0.34
0.35	0.35	0.35	0.35	0.35	0.35	0.35	0.35	0.35
0.36	0.36	0.36	0.36	0.36	0.36	0.36	0.36	0.36
0.37	0.37	0.37	0.37	0.37	0.37	0.37	0.37	0.37
0.38	0.38	0.38	0.38	0.38	0.38	0.38	0.38	0.38
0.39	0.39	0.39	0.39	0.39	0.39	0.39	0.39	0.39
0.4	0.4	0.4	0.4	0.4	0.4	0.4	0.4	0.4
0.41	0.41	0.41	0.41	0.41	0.41	0.41	0.41	0.41
0.42	0.42	0.42	0.42	0.42	0.42	0.42	0.42	0.42
0.43	0.43	0.43	0.43	0.43	0.43	0.43	0.43	0.43
0.44	0.44	0.44	0.44	0.44	0.44	0.44	0.44	0.44
0.45	0.45	0.45	0.45	0.45	0.45	0.45	0.45	0.45
0.46	0.46	0.46	0.46	0.46	0.46	0.46	0.46	0.46
0.47	0.47	0.47	0.47	0.47	0.47	0.47	0.47	0.47
0.48	0.48	0.48	0.48	0.48	0.48	0.48	0.48	0.48
0.49	0.49	0.49	0.49	0.49	0.49	0.49	0.49	0.49

0.5	0.5	0.5	0.5	0.5	0.5	0.5	0.5	0.5
0.51	0.51	0.51	0.51	0.51	0.51	0.51	0.51	0.51
0.52	0.52	0.52	0.52	0.52	0.52	0.52	0.52	0.52
0.53	0.53	0.53	0.53	0.53	0.53	0.53	0.53	0.53
0.54	0.54	0.54	0.54	0.54	0.54	0.54	0.54	0.54
0.55	0.55	0.55	0.55	0.55	0.55	0.55	0.55	0.55
0.56	0.56	0.56	0.56	0.56	0.56	0.56	0.56	0.56
0.57	0.57	0.57	0.57	0.57	0.57	0.57	0.57	0.57
0.58	0.58	0.58	0.58	0.58	0.58	0.58	0.58	0.58
0.59	0.59	0.59	0.59	0.59	0.59	0.59	0.59	0.59
0.6	0.6	0.6	0.6	0.6	0.6	0.6	0.6	0.6
0.61	0.61	0.61	0.61	0.61	0.61	0.61	0.61	0.61
0.62	0.62	0.62	0.62	0.62	0.62	0.62	0.62	0.62
0.63	0.63	0.63	0.63	0.63	0.63	0.63	0.63	0.63
0.64	0.64	0.64	0.64	0.64	0.64	0.64	0.64	0.64
0.65	0.65	0.65	0.65	0.65	0.65	0.65	0.65	0.65
0.66	0.66	0.66	0.66	0.66	0.66	0.66	0.66	0.66
0.67	0.67	0.67	0.67	0.67	0.67	0.67	0.67	0.67
0.68	0.68	0.68	0.68	0.68	0.68	0.68	0.68	0.68
0.69	0.69	0.69	0.69	0.69	0.69	0.69	0.69	0.69
0.7	0.7	0.7	0.7	0.7	0.7	0.7	0.7	0.7
0.71	0.71	0.71	0.71	0.71	0.71	0.71	0.71	0.71
0.72	0.72	0.72	0.72	0.72	0.72	0.72	0.72	0.72
0.73	0.73	0.73	0.73	0.73	0.73	0.73	0.73	0.73
0.74	0.74	0.74	0.74	0.74	0.74	0.74	0.74	0.74
0.75	0.75	0.75	0.75	0.75	0.75	0.75	0.75	0.75
0.76	0.76	0.76	0.76	0.76	0.76	0.76	0.76	0.76
0.77	0.77	0.77	0.77	0.77	0.77	0.77	0.77	0.77
0.78	0.78	0.78	0.78	0.78	0.78	0.78	0.78	0.78
0.79	0.79	0.79	0.79	0.79	0.79	0.79	0.79	0.79
0.8	0.8	0.8	0.8	0.8	0.8	0.8	0.8	0.8
0.81	0.81	0.81	0.81	0.81	0.81	0.81	0.81	0.81
0.82	0.82	0.82	0.82	0.82	0.82	0.82	0.82	0.82
0.83	0.83	0.83	0.83	0.83	0.83	0.83	0.83	0.83
0.84	0.84	0.84	0.84	0.84	0.84	0.84	0.84	0.84
0.85	0.85	0.85	0.85	0.85	0.85	0.85	0.85	0.85
0.86	0.86	0.86	0.86	0.86	0.86	0.86	0.86	0.86
0.87	0.87	0.87	0.87	0.87	0.87	0.87	0.87	0.87
0.88	0.88	0.88	0.88	0.88	0.88	0.88	0.88	0.88
0.89	0.89	0.89	0.89	0.89	0.89	0.89	0.89	0.89
0.9	0.9	0.9	0.9	0.9	0.9	0.9	0.9	0.9
0.91	0.91	0.91	0.91	0.91	0.91	0.91	0.91	0.91
0.92	0.92	0.92	0.92	0.92	0.92	0.92	0.92	0.92
0.93	0.93	0.93	0.93	0.93	0.93	0.93	0.93	0.93
0.94	0.94	0.94	0.94	0.94	0.94	0.94	0.94	0.94
0.95	0.95	0.95	0.95	0.95	0.95	0.95	0.95	0.95
0.96	0.96	0.96	0.96	0.96	0.96	0.96	0.96	0.96
0.97	0.97	0.97	0.97	0.97	0.97	0.97	0.97	0.97
0.98	0.98	0.98	0.98	0.98	0.98	0.98	0.98	0.98
0.99	0.99	0.99	0.99	0.99	0.99	0.99	0.99	0.99

1	1	1	1	1	1	1	1	1
1.01	1.01	1.01	1.01	1.01	1.01	1.01	1.01	1.01
1.02	1.02	1.02	1.02	1.02	1.02	1.02	1.02	1.02
1.03	1.03	1.03	1.03	1.03	1.03	1.03	1.03	1.03
1.04	1.04	1.04	1.04	1.04	1.04	1.04	1.04	1.04
1.05	1.05	1.05	1.05	1.05	1.05	1.05	1.05	1.05
1.06	1.06	1.06	1.06	1.06	1.06	1.06	1.06	1.06
1.07	1.07	1.07	1.07	1.07	1.07	1.07	1.07	1.07
1.08	1.08	1.08	1.08	1.08	1.08	1.08	1.08	1.08
1.09	1.09	1.09	1.09	1.09	1.09	1.09	1.09	1.09
1.1	1.1	1.1	1.1	1.1	1.1	1.1	1.1	1.1
1.11	1.11	1.11	1.11	1.11	1.11	1.11	1.11	1.11
1.12	1.12	1.12	1.12	1.12	1.12	1.12	1.12	1.12
1.13	1.13	1.13	1.13	1.13	1.13	1.13	1.13	1.13
1.14	1.14	1.14	1.14	1.14	1.14	1.14	1.14	1.14
1.15	1.15	1.15	1.15	1.15	1.15	1.15	1.15	1.15
1.16	1.16	1.16	1.16	1.16	1.16	1.16	1.16	1.16
1.17	1.17	1.17	1.17	1.17	1.17	1.17	1.17	1.17
1.18	1.18	1.18	1.18	1.18	1.18	1.18	1.18	1.18
1.19	1.19	1.19	1.19	1.19	1.19	1.19	1.19	1.19
1.2	1.2	1.2	1.2	1.2	1.2	1.2	1.2	1.2
1.21	1.21	1.21	1.21	1.21	1.21	1.21	1.21	1.21
1.22	1.22	1.22	1.22	1.22	1.22	1.22	1.22	1.22
1.23	1.23	1.23	1.23	1.23	1.23	1.23	1.23	1.23
1.24	1.24	1.24	1.24	1.24	1.24	1.24	1.24	1.24
1.25	1.25	1.25	1.25	1.25	1.25	1.25	1.25	1.25
1.26	1.26	1.26	1.26	1.26	1.26	1.26	1.26	1.26
1.27	1.27	1.27	1.27	1.27	1.27	1.27	1.27	1.27
1.28	1.28	1.28	1.28	1.28	1.28	1.28	1.28	1.28
1.29	1.29	1.29	1.29	1.29	1.29	1.29	1.29	1.29
1.3	1.3	1.3	1.3	1.3	1.3	1.3	1.3	1.3
1.31	1.31	1.31	1.31	1.31	1.31	1.31	1.31	1.31
1.32	1.32	1.32	1.32	1.32	1.32	1.32	1.32	1.32
1.33	1.33	1.33	1.33	1.33	1.33	1.33	1.33	1.33
1.34	1.34	1.34	1.34	1.34	1.34	1.34	1.34	1.34
1.35	1.35	1.35	1.35	1.35	1.35	1.35	1.35	1.35
1.36	1.36	1.36	1.36	1.36	1.36	1.36	1.36	1.36
1.37	1.37	1.37	1.37	1.37	1.37	1.37	1.37	1.37
1.38	1.38	1.38	1.38	1.38	1.38	1.38	1.38	1.38
1.39	1.39	1.39	1.39	1.39	1.39	1.39	1.39	1.39
1.4	1.4	1.4	1.4	1.4	1.4	1.4	1.4	1.4
1.41	1.41	1.41	1.41	1.41	1.41	1.41	1.41	1.41
1.42	1.42	1.42	1.42	1.42	1.42	1.42	1.42	1.42
1.43	1.43	1.43	1.43	1.43	1.43	1.43	1.43	1.43
1.44	1.44	1.44	1.44	1.44	1.44	1.44	1.44	1.44
1.45	1.45	1.45	1.45	1.45	1.45	1.45	1.45	1.45
1.46	1.46	1.46	1.46	1.46	1.46	1.46	1.46	1.46
1.47	1.47	1.47	1.47	1.47	1.47	1.47	1.47	1.47
1.48	1.48	1.48	1.48	1.48	1.48	1.48	1.48	1.48
1.49	1.49	1.49	1.49	1.49	1.49	1.49	1.49	1.49

1.5	1.5	1.5	1.5	1.5	1.5	1.5	1.5	1.5
1.51	1.51	1.51	1.51	1.51	1.51	1.51	1.51	1.51
1.52	1.52	1.52	1.52	1.52	1.52	1.52	1.52	1.52
1.53	1.53	1.53	1.53	1.53	1.53	1.53	1.53	1.53
1.54	1.54	1.54	1.54	1.54	1.54	1.54	1.54	1.54
1.55	1.55	1.55	1.55	1.55	1.55	1.55	1.55	1.55
1.56	1.56	1.56	1.56	1.56	1.56	1.56	1.56	1.56
1.57	1.57	1.57	1.57	1.57	1.57	1.57	1.57	1.57
1.58	1.58	1.58	1.58	1.58	1.58	1.58	1.58	1.58
1.59	1.59	1.59	1.59	1.59	1.59	1.59	1.59	1.59
1.6	1.6	1.6	1.6	1.6	1.6	1.6	1.6	1.6
1.61	1.61	1.61	1.61	1.61	1.61	1.61	1.61	1.61
1.62	1.62	1.62	1.62	1.62	1.62	1.62	1.62	1.62
1.63	1.63	1.63	1.63	1.63	1.63	1.63	1.63	1.63
1.64	1.64	1.64	1.64	1.64	1.64	1.64	1.64	1.64
1.65	1.65	1.65	1.65	1.65	1.65	1.65	1.65	1.65
1.66	1.66	1.66	1.66	1.66	1.66	1.66	1.66	1.66
1.67	1.67	1.67	1.67	1.67	1.67	1.67	1.67	1.67
1.68	1.68	1.68	1.68	1.68	1.68	1.68	1.68	1.68
1.69	1.69	1.69	1.69	1.69	1.69	1.69	1.69	1.69
1.7	1.7	1.7	1.7	1.7	1.7	1.7	1.7	1.7
1.71	1.71	1.71	1.71	1.71	1.71	1.71	1.71	1.71
1.72	1.72	1.72	1.72	1.72	1.72	1.72	1.72	1.72
1.73	1.73	1.73	1.73	1.73	1.73	1.73	1.73	1.73
1.74	1.74	1.74	1.74	1.74	1.74	1.74	1.74	1.74
1.75	1.75	1.75	1.75	1.75	1.75	1.75	1.75	1.75
1.76	1.76	1.76	1.76	1.76	1.76	1.76	1.76	1.76
1.77	1.77	1.77	1.77	1.77	1.77	1.77	1.77	1.77
1.78	1.78	1.78	1.78	1.78	1.78	1.78	1.78	1.78
1.79	1.79	1.79	1.79	1.79	1.79	1.79	1.79	1.79
1.8	1.8	1.8	1.8	1.8	1.8	1.8	1.8	1.8
1.81	1.81	1.81	1.81	1.81	1.81	1.81	1.81	1.81
1.82	1.82	1.82	1.82	1.82	1.82	1.82	1.82	1.82
1.83	1.83	1.83	1.83	1.83	1.83	1.83	1.83	1.83
1.84	1.84	1.84	1.84	1.84	1.84	1.84	1.84	1.84
1.85	1.85	1.85	1.85	1.85	1.85	1.85	1.85	1.85
1.86	1.86	1.86	1.86	1.86	1.86	1.86	1.86	1.86
1.87	1.87	1.87	1.87	1.87	1.87	1.87	1.87	1.87
1.88	1.88	1.88	1.88	1.88	1.88	1.88	1.88	1.88
1.89	1.89	1.89	1.89	1.89	1.89	1.89	1.89	1.89
1.9	1.9	1.9	1.9	1.9	1.9	1.9	1.9	1.9
1.91	1.91	1.91	1.91	1.91	1.91	1.91	1.91	1.91
1.92	1.92	1.92	1.92	1.92	1.92	1.92	1.92	1.92
1.93	1.93	1.93	1.93	1.93	1.93	1.93	1.93	1.93
1.94	1.94	1.94	1.94	1.94	1.94	1.94	1.94	1.94
1.95	1.95	1.95	1.95	1.95	1.95	1.95	1.95	1.95
1.96	1.96	1.96	1.96	1.96	1.96	1.96	1.96	1.96
1.97	1.97	1.97	1.97	1.97	1.97	1.97	1.97	1.97
1.98	1.98	1.98	1.98	1.98	1.98	1.98	1.98	1.98
1.99	1.99	1.99	1.99	1.99	1.99	1.99	1.99	1.99

2	2	2	2	2	2	2	2	2
2.01	2.01	2.01	2.01	2.01	2.01	2.01	2.01	2.01
2.02	2.02	2.02	2.02	2.02	2.02	2.02	2.02	2.02
2.03	2.03	2.03	2.03	2.03	2.03	2.03	2.03	2.03
2.04	2.04	2.04	2.04	2.04	2.04	2.04	2.04	2.04
2.05	2.05	2.05	2.05	2.05	2.05	2.05	2.05	2.05
2.06	2.06	2.06	2.06	2.06	2.06	2.06	2.06	2.06
2.07	2.07	2.07	2.07	2.07	2.07	2.07	2.07	2.07
2.08	2.08	2.08	2.08	2.08	2.08	2.08	2.08	2.08
2.09	2.09	2.09	2.09	2.09	2.09	2.09	2.09	2.09
2.1	2.1	2.1	2.1	2.1	2.1	2.1	2.1	2.1
2.11	2.11	2.11	2.11	2.11	2.11	2.11	2.11	2.11
2.12	2.12	2.12	2.12	2.12	2.12	2.12	2.12	2.12
2.13	2.13	2.13	2.13	2.13	2.13	2.13	2.13	2.13
2.14	2.14	2.14	2.14	2.14	2.14	2.14	2.14	2.14
2.15	2.15	2.15	2.15	2.15	2.15	2.15	2.15	2.15
2.16	2.16	2.16	2.16	2.16	2.16	2.16	2.16	2.16
2.17	2.17	2.17	2.17	2.17	2.17	2.17	2.17	2.17
2.18	2.18	2.18	2.18	2.18	2.18	2.18	2.18	2.18
2.19	2.19	2.19	2.19	2.19	2.19	2.19	2.19	2.19
2.2	2.2	2.2	2.2	2.2	2.2	2.2	2.2	2.2
2.21	2.21	2.21	2.21	2.21	2.21	2.21	2.21	2.21
2.22	2.22	2.22	2.22	2.22	2.22	2.22	2.22	2.22
2.23	2.23	2.23	2.23	2.23	2.23	2.23	2.23	2.23
2.24	2.24	2.24	2.24	2.24	2.24	2.24	2.24	2.24
2.25	2.25	2.25	2.25	2.25	2.25	2.25	2.25	2.25
2.26	2.26	2.26	2.26	2.26	2.26	2.26	2.26	2.26
2.27	2.27	2.27	2.27	2.27	2.27	2.27	2.27	2.27
2.28	2.28	2.28	2.28	2.28	2.28	2.28	2.28	2.28
2.29	2.29	2.29	2.29	2.29	2.29	2.29	2.29	2.29
2.3	2.3	2.3	2.3	2.3	2.3	2.3	2.3	2.3
2.31	2.31	2.31	2.31	2.31	2.31	2.31	2.31	2.31
2.32	2.32	2.32	2.32	2.32	2.32	2.32	2.32	2.32
2.33	2.33	2.33	2.33	2.33	2.33	2.33	2.33	2.33
2.34	2.34	2.34	2.34	2.34	2.34	2.34	2.34	2.34
2.35	2.35	2.35	2.35	2.35	2.35	2.35	2.35	2.35
2.36	2.36	2.36	2.36	2.36	2.36	2.36	2.36	2.36
2.37	2.37	2.37	2.37	2.37	2.37	2.37	2.37	2.37
2.38	2.38	2.38	2.38	2.38	2.38	2.38	2.38	2.38
2.39	2.39	2.39	2.39	2.39	2.39	2.39	2.39	2.39
2.4	2.4	2.4	2.4	2.4	2.4	2.4	2.4	2.4
2.41	2.41	2.41	2.41	2.41	2.41	2.41	2.41	2.41
2.42	2.42	2.42	2.42	2.42	2.42	2.42	2.42	2.42
2.43	2.43	2.43	2.43	2.43	2.43	2.43	2.43	2.43
2.44	2.44	2.44	2.44	2.44	2.44	2.44	2.44	2.44
2.45	2.45	2.45	2.45	2.45	2.45	2.45	2.45	2.45
2.46	2.46	2.46	2.46	2.46	2.46	2.46	2.46	2.46
2.47	2.47	2.47	2.47	2.47	2.47	2.47	2.47	2.47
2.48	2.48	2.48	2.48	2.48	2.48	2.48	2.48	2.48
2.49	2.49	2.49	2.49	2.49	2.49	2.49	2.49	2.49

2.5	2.5	2.5	2.5	2.5	2.5	2.5	2.5	2.5
2.51	2.51	2.51	2.51	2.51	2.51	2.51	2.51	2.51
2.52	2.52	2.52	2.52	2.52	2.52	2.52	2.52	2.52
2.53	2.53	2.53	2.53	2.53	2.53	2.53	2.53	2.53
2.54	2.54	2.54	2.54	2.54	2.54	2.54	2.54	2.54
2.55	2.55	2.55	2.55	2.55	2.55	2.55	2.55	2.55
2.56	2.56	2.56	2.56	2.56	2.56	2.56	2.56	2.56
2.57	2.57	2.57	2.57	2.57	2.57	2.57	2.57	2.57
2.58	2.58	2.58	2.58	2.58	2.58	2.58	2.58	2.58
2.59	2.59	2.59	2.59	2.59	2.59	2.59	2.59	2.59
2.6	2.6	2.6	2.6	2.6	2.6	2.6	2.6	2.6
2.61	2.61	2.61	2.61	2.61	2.61	2.61	2.61	2.61
2.62	2.62	2.62	2.62	2.62	2.62	2.62	2.62	2.62
2.63	2.63	2.63	2.63	2.63	2.63	2.63	2.63	2.63
2.64	2.64	2.64	2.64	2.64	2.64	2.64	2.64	2.64
2.65	2.65	2.65	2.65	2.65	2.65	2.65	2.65	2.65
2.66	2.66	2.66	2.66	2.66	2.66	2.66	2.66	2.66
2.67	2.67	2.67	2.67	2.67	2.67	2.67	2.67	2.67
2.68	2.68	2.68	2.68	2.68	2.68	2.68	2.68	2.68
2.69	2.69	2.69	2.69	2.69	2.69	2.69	2.69	2.69
2.7	2.7	2.7	2.7	2.7	2.7	2.7	2.7	2.7
2.71	2.71	2.71	2.71	2.71	2.71	2.71	2.71	2.71
2.72	2.72	2.72	2.72	2.72	2.72	2.72	2.72	2.72
2.73	2.73	2.73	2.73	2.73	2.73	2.73	2.73	2.73
2.74	2.74	2.74	2.74	2.74	2.74	2.74	2.74	2.74
2.75	2.75	2.75	2.75	2.75	2.75	2.75	2.75	2.75
2.76	2.76	2.76	2.76	2.76	2.76	2.76	2.76	2.76
2.77	2.77	2.77	2.77	2.77	2.77	2.77	2.77	2.77
2.78	2.78	2.78	2.78	2.78	2.78	2.78	2.78	2.78
2.79	2.79	2.79	2.79	2.79	2.79	2.79	2.79	2.79
2.8	2.8	2.8	2.8	2.8	2.8	2.8	2.8	2.8
2.81	2.81	2.81	2.81	2.81	2.81	2.81	2.81	2.81
2.82	2.82	2.82	2.82	2.82	2.82	2.82	2.82	2.82
2.83	2.83	2.83	2.83	2.83	2.83	2.83	2.83	2.83
2.84	2.84	2.84	2.84	2.84	2.84	2.84	2.84	2.84
2.85	2.85	2.85	2.85	2.85	2.85	2.85	2.85	2.85
2.86	2.86	2.86	2.86	2.86	2.86	2.86	2.86	2.86
2.87	2.87	2.87	2.87	2.87	2.87	2.87	2.87	2.87
2.88	2.88	2.88	2.88	2.88	2.88	2.88	2.88	2.88
2.89	2.89	2.89	2.89	2.89	2.89	2.89	2.89	2.89
2.9	2.9	2.9	2.9	2.9	2.9	2.9	2.9	2.9
2.91	2.91	2.91	2.91	2.91	2.91	2.91	2.91	2.91
2.92	2.92	2.92	2.92	2.92	2.92	2.92	2.92	2.92
2.93	2.93	2.93	2.93	2.93	2.93	2.93	2.93	2.93
2.94	2.94	2.94	2.94	2.94	2.94	2.94	2.94	2.94
2.95	2.95	2.95	2.95	2.95	2.95	2.95	2.95	2.95
2.96	2.96	2.96	2.96	2.96	2.96	2.96	2.96	2.96
2.97	2.97	2.97	2.97	2.97	2.97	2.97	2.97	2.97
2.98	2.98	2.98	2.98	2.98	2.98	2.98	2.98	2.98
2.99	2.99	2.99	2.99	2.99	2.99	2.99	2.99	2.99

3	3	3	3	3	3	3	3	3
3.01	3.01	3.01	3.01	3.01	3.01	3.01	3.01	3.01
3.02	3.02	3.02	3.02	3.02	3.02	3.02	3.02	3.02
3.03	3.03	3.03	3.03	3.03	3.03	3.03	3.03	3.03
3.04	3.04	3.04	3.04	3.04	3.04	3.04	3.04	3.04
3.05	3.05	3.05	3.05	3.05	3.05	3.05	3.05	3.05
3.06	3.06	3.06	3.06	3.06	3.06	3.06	3.06	3.06
3.07	3.07	3.07	3.07	3.07	3.07	3.07	3.07	3.07
3.08	3.08	3.08	3.08	3.08	3.08	3.08	3.08	3.08
3.09	3.09	3.09	3.09	3.09	3.09	3.09	3.09	3.09
3.1	3.1	3.1	3.1	3.1	3.1	3.1	3.1	3.1
3.11	3.11	3.11	3.11	3.11	3.11	3.11	3.11	3.11
3.12	3.12	3.12	3.12	3.12	3.12	3.12	3.12	3.12
3.13	3.13	3.13	3.13	3.13	3.13	3.13	3.13	3.13
3.14	3.14	3.14	3.14	3.14	3.14	3.14	3.14	3.14
3.15	3.15	3.15	3.15	3.15	3.15	3.15	3.15	3.15
3.16	3.16	3.16	3.16	3.16	3.16	3.16	3.16	3.16
3.17	3.17	3.17	3.17	3.17	3.17	3.17	3.17	3.17
3.18	3.18	3.18	3.18	3.18	3.18	3.18	3.18	3.18
3.19	3.19	3.19	3.19	3.19	3.19	3.19	3.19	3.19
3.2	3.2	3.2	3.2	3.2	3.2	3.2	3.2	3.2
3.21	3.21	3.21	3.21	3.21	3.21	3.21	3.21	3.21
3.22	3.22	3.22	3.22	3.22	3.22	3.22	3.22	3.22
3.23	3.23	3.23	3.23	3.23	3.23	3.23	3.23	3.23
3.24	3.24	3.24	3.24	3.24	3.24	3.24	3.24	3.24
3.25	3.25	3.25	3.25	3.25	3.25	3.25	3.25	3.25
3.26	3.26	3.26	3.26	3.26	3.26	3.26	3.26	3.26
3.27	3.27	3.27	3.27	3.27	3.27	3.27	3.27	3.27
3.28	3.28	3.28	3.28	3.28	3.28	3.28	3.28	3.28
3.29	3.29	3.29	3.29	3.29	3.29	3.29	3.29	3.29
3.3	3.3	3.3	3.3	3.3	3.3	3.3	3.3	3.3
3.31	3.31	3.31	3.31	3.31	3.31	3.31	3.31	3.31
3.32	3.32	3.32	3.32	3.32	3.32	3.32	3.32	3.32
3.33	3.33	3.33	3.33	3.33	3.33	3.33	3.33	3.33
3.34	3.34	3.34	3.34	3.34	3.34	3.34	3.34	3.34
3.35	3.35	3.35	3.35	3.35	3.35	3.35	3.35	3.35
3.36	3.36	3.36	3.36	3.36	3.36	3.36	3.36	3.36
3.37	3.37	3.37	3.37	3.37	3.37	3.37	3.37	3.37
3.38	3.38	3.38	3.38	3.38	3.38	3.38	3.38	3.38
3.39	3.39	3.39	3.39	3.39	3.39	3.39	3.39	3.39
3.4	3.4	3.4	3.4	3.4	3.4	3.4	3.4	3.4
3.41	3.41	3.41	3.41	3.41	3.41	3.41	3.41	3.41
3.42	3.42	3.42	3.42	3.42	3.42	3.42	3.42	3.42
3.43	3.43	3.43	3.43	3.43	3.43	3.43	3.43	3.43
3.44	3.44	3.44	3.44	3.44	3.44	3.44	3.44	3.44
3.45	3.45	3.45	3.45	3.45	3.45	3.45	3.45	3.45
3.46	3.46	3.46	3.46	3.46	3.46	3.46	3.46	3.46
3.47	3.47	3.47	3.47	3.47	3.47	3.47	3.47	3.47
3.48	3.48	3.48	3.48	3.48	3.48	3.48	3.48	3.48
3.49	3.49	3.49	3.49	3.49	3.49	3.49	3.49	3.49

3.5	3.5	3.5	3.5	3.5	3.5	3.5	3.5	3.5
3.51	3.51	3.51	3.51	3.51	3.51	3.51	3.51	3.51
3.52	3.52	3.52	3.52	3.52	3.52	3.52	3.52	3.52
3.53	3.53	3.53	3.53	3.53	3.53	3.53	3.53	3.53
3.54	3.54	3.54	3.54	3.54	3.54	3.54	3.54	3.54
3.55	3.55	3.55	3.55	3.55	3.55	3.55	3.55	3.55
3.56	3.56	3.56	3.56	3.56	3.56	3.56	3.56	3.56
3.57	3.57	3.57	3.57	3.57	3.57	3.57	3.57	3.57
3.58	3.58	3.58	3.58	3.58	3.58	3.58	3.58	3.58
3.59	3.59	3.59	3.59	3.59	3.59	3.59	3.59	3.59
3.6	3.6	3.6	3.6	3.6	3.6	3.6	3.6	3.6
3.61	3.61	3.61	3.61	3.61	3.61	3.61	3.61	3.61
3.62	3.62	3.62	3.62	3.62	3.62	3.62	3.62	3.62
3.63	3.63	3.63	3.63	3.63	3.63	3.63	3.63	3.63
3.64	3.64	3.64	3.64	3.64	3.64	3.64	3.64	3.64
3.65	3.65	3.65	3.65	3.65	3.65	3.65	3.65	3.65
3.66	3.66	3.66	3.66	3.66	3.66	3.66	3.66	3.66
3.67	3.67	3.67	3.67	3.67	3.67	3.67	3.67	3.67
3.68	3.68	3.68	3.68	3.68	3.68	3.68	3.68	3.68
3.69	3.69	3.69	3.69	3.69	3.69	3.69	3.69	3.69
3.7	3.7	3.7	3.7	3.7	3.7	3.7	3.7	3.7
3.71	3.71	3.71	3.71	3.71	3.71	3.71	3.71	3.71
3.72	3.72	3.72	3.72	3.72	3.72	3.72	3.72	3.72
3.73	3.73	3.73	3.73	3.73	3.73	3.73	3.73	3.73
3.74	3.74	3.74	3.74	3.74	3.74	3.74	3.74	3.74
3.75	3.75	3.75	3.75	3.75	3.75	3.75	3.75	3.75
3.76	3.76	3.76	3.76	3.76	3.76	3.76	3.76	3.76
3.77	3.77	3.77	3.77	3.77	3.77	3.77	3.77	3.77
3.78	3.78	3.78	3.78	3.78	3.78	3.78	3.78	3.78
3.79	3.79	3.79	3.79	3.79	3.79	3.79	3.79	3.79
3.8	3.8	3.8	3.8	3.8	3.8	3.8	3.8	3.8
3.81	3.81	3.81	3.81	3.81	3.81	3.81	3.81	3.81
3.82	3.82	3.82	3.82	3.82	3.82	3.82	3.82	3.82
3.83	3.83	3.83	3.83	3.83	3.83	3.83	3.83	3.83
3.84	3.84	3.84	3.84	3.84	3.84	3.84	3.84	3.84
3.85	3.85	3.85	3.85	3.85	3.85	3.85	3.85	3.85
3.86	3.86	3.86	3.86	3.86	3.86	3.86	3.86	3.86
3.87	3.87	3.87	3.87	3.87	3.87	3.87	3.87	3.87
3.88	3.88	3.88	3.88	3.88	3.88	3.88	3.88	3.88
3.89	3.89	3.89	3.89	3.89	3.89	3.89	3.89	3.89
3.9	3.9	3.9	3.9	3.9	3.9	3.9	3.9	3.9
3.91	3.91	3.91	3.91	3.91	3.91	3.91	3.91	3.91
3.92	3.92	3.92	3.92	3.92	3.92	3.92	3.92	3.92
3.93	3.93	3.93	3.93	3.93	3.93	3.93	3.93	3.93
3.94	3.94	3.94	3.94	3.94	3.94	3.94	3.94	3.94
3.95	3.95	3.95	3.95	3.95	3.95	3.95	3.95	3.95
3.96	3.96	3.96	3.96	3.96	3.96	3.96	3.96	3.96
3.97	3.97	3.97	3.97	3.97	3.97	3.97	3.97	3.97
3.98	3.98	3.98	3.98	3.98	3.98	3.98	3.98	3.98
3.99	3.99	3.99	3.99	3.99	3.99	3.99	3.99	3.99

4	4	4	4	4	4	4	4	4
4.01	4.01	4.01	4.01	4.01	4.01	4.01	4.01	4.01
4.02	4.02	4.02	4.02	4.02	4.02	4.02	4.02	4.02
4.03	4.03	4.03	4.03	4.03	4.03	4.03	4.03	4.03
4.04	4.04	4.04	4.04	4.04	4.04	4.04	4.04	4.04
4.05	4.05	4.05	4.05	4.05	4.05	4.05	4.05	4.05
4.06	4.06	4.06	4.06	4.06	4.06	4.06	4.06	4.06
4.07	4.07	4.07	4.07	4.07	4.07	4.07	4.07	4.07
4.08	4.08	4.08	4.08	4.08	4.08	4.08	4.08	4.08
4.09	4.09	4.09	4.09	4.09	4.09	4.09	4.09	4.09
4.1	4.1	4.1	4.1	4.1	4.1	4.1	4.1	4.1
4.11	4.11	4.11	4.11	4.11	4.11	4.11	4.11	4.11
4.12	4.12	4.12	4.12	4.12	4.12	4.12	4.12	4.12
4.13	4.13	4.13	4.13	4.13	4.13	4.13	4.13	4.13
4.14	4.14	4.14	4.14	4.14	4.14	4.14	4.14	4.14
4.15	4.15	4.15	4.15	4.15	4.15	4.15	4.15	4.15
4.16	4.16	4.16	4.16	4.16	4.16	4.16	4.16	4.16
4.17	4.17	4.17	4.17	4.17	4.17	4.17	4.17	4.17
4.18	4.18	4.18	4.18	4.18	4.18	4.18	4.18	4.18
4.19	4.19	4.19	4.19	4.19	4.19	4.19	4.19	4.19
4.2	4.2	4.2	4.2	4.2	4.2	4.2	4.2	4.2
4.21	4.21	4.21	4.21	4.21	4.21	4.21	4.21	4.21
4.22	4.22	4.22	4.22	4.22	4.22	4.22	4.22	4.22
4.23	4.23	4.23	4.23	4.23	4.23	4.23	4.23	4.23
4.24	4.24	4.24	4.24	4.24	4.24	4.24	4.24	4.24
4.25	4.25	4.25	4.25	4.25	4.25	4.25	4.25	4.25
4.26	4.26	4.26	4.26	4.26	4.26	4.26	4.26	4.26
4.27	4.27	4.27	4.27	4.27	4.27	4.27	4.27	4.27
4.28	4.28	4.28	4.28	4.28	4.28	4.28	4.28	4.28
4.29	4.29	4.29	4.29	4.29	4.29	4.29	4.29	4.29
4.3	4.3	4.3	4.3	4.3	4.3	4.3	4.3	4.3
4.31	4.31	4.31	4.31	4.31	4.31	4.31	4.31	4.31
4.32	4.32	4.32	4.32	4.32	4.32	4.32	4.32	4.32
4.33	4.33	4.33	4.33	4.33	4.33	4.33	4.33	4.33
4.34	4.34	4.34	4.34	4.34	4.34	4.34	4.34	4.34
4.35	4.35	4.35	4.35	4.35	4.35	4.35	4.35	4.35
4.36	4.36	4.36	4.36	4.36	4.36	4.36	4.36	4.36
4.37	4.37	4.37	4.37	4.37	4.37	4.37	4.37	4.37
4.38	4.38	4.38	4.38	4.38	4.38	4.38	4.38	4.38
4.39	4.39	4.39	4.39	4.39	4.39	4.39	4.39	4.39
4.4	4.4	4.4	4.4	4.4	4.4	4.4	4.4	4.4
4.41	4.41	4.41	4.41	4.41	4.41	4.41	4.41	4.41
4.42	4.42	4.42	4.42	4.42	4.42	4.42	4.42	4.42
4.43	4.43	4.43	4.43	4.43	4.43	4.43	4.43	4.43
4.44	4.44	4.44	4.44	4.44	4.44	4.44	4.44	4.44
4.45	4.45	4.45	4.45	4.45	4.45	4.45	4.45	4.45
4.46	4.46	4.46	4.46	4.46	4.46	4.46	4.46	4.46
4.47	4.47	4.47	4.47	4.47	4.47	4.47	4.47	4.47
4.48	4.48	4.48	4.48	4.48	4.48	4.48	4.48	4.48
4.49	4.49	4.49	4.49	4.49	4.49	4.49	4.49	4.49

4.5	4.5	4.5	4.5	4.5	4.5	4.5	4.5	4.5
4.51	4.51	4.51	4.51	4.51	4.51	4.51	4.51	4.51
4.52	4.52	4.52	4.52	4.52	4.52	4.52	4.52	4.52
4.53	4.53	4.53	4.53	4.53	4.53	4.53	4.53	4.53
4.54	4.54	4.54	4.54	4.54	4.54	4.54	4.54	4.54
4.55	4.55	4.55	4.55	4.55	4.55	4.55	4.55	4.55
4.56	4.56	4.56	4.56	4.56	4.56	4.56	4.56	4.56
4.57	4.57	4.57	4.57	4.57	4.57	4.57	4.57	4.57
4.58	4.58	4.58	4.58	4.58	4.58	4.58	4.58	4.58
4.59	4.59	4.59	4.59	4.59	4.59	4.59	4.59	4.59
4.6	4.6	4.6	4.6	4.6	4.6	4.6	4.6	4.6
4.61	4.61	4.61	4.61	4.61	4.61	4.61	4.61	4.61
4.62	4.62	4.62	4.62	4.62	4.62	4.62	4.62	4.62
4.63	4.63	4.63	4.63	4.63	4.63	4.63	4.63	4.63
4.64	4.64	4.64	4.64	4.64	4.64	4.64	4.64	4.64
4.65	4.65	4.65	4.65	4.65	4.65	4.65	4.65	4.65
4.66	4.66	4.66	4.66	4.66	4.66	4.66	4.66	4.66
4.67	4.67	4.67	4.67	4.67	4.67	4.67	4.67	4.67
4.68	4.68	4.68	4.68	4.68	4.68	4.68	4.68	4.68
4.69	4.69	4.69	4.69	4.69	4.69	4.69	4.69	4.69
4.7	4.7	4.7	4.7	4.7	4.7	4.7	4.7	4.7
4.71	4.71	4.71	4.71	4.71	4.71	4.71	4.71	4.71
4.72	4.72	4.72	4.72	4.72	4.72	4.72	4.72	4.72
4.73	4.73	4.73	4.73	4.73	4.73	4.73	4.73	4.73
4.74	4.74	4.74	4.74	4.74	4.74	4.74	4.74	4.74
4.75	4.75	4.75	4.75	4.75	4.75	4.75	4.75	4.75
4.76	4.76	4.76	4.76	4.76	4.76	4.76	4.76	4.76
4.77	4.77	4.77	4.77	4.77	4.77	4.77	4.77	4.77
4.78	4.78	4.78	4.78	4.78	4.78	4.78	4.78	4.78
4.79	4.79	4.79	4.79	4.79	4.79	4.79	4.79	4.79
4.8	4.8	4.8	4.8	4.8	4.8	4.8	4.8	4.8
4.81	4.81	4.81	4.81	4.81	4.81	4.81	4.81	4.81
4.82	4.82	4.82	4.82	4.82	4.82	4.82	4.82	4.82
4.83	4.83	4.83	4.83	4.83	4.83	4.83	4.83	4.83
4.84	4.84	4.84	4.84	4.84	4.84	4.84	4.84	4.84
4.85	4.85	4.85	4.85	4.85	4.85	4.85	4.85	4.85
4.86	4.86	4.86	4.86	4.86	4.86	4.86	4.86	4.86
4.87	4.87	4.87	4.87	4.87	4.87	4.87	4.87	4.87
4.88	4.88	4.88	4.88	4.88	4.88	4.88	4.88	4.88
4.89	4.89	4.89	4.89	4.89	4.89	4.89	4.89	4.89
4.9	4.9	4.9	4.9	4.9	4.9	4.9	4.9	4.9
4.91	4.91	4.91	4.91	4.91	4.91	4.91	4.91	4.91
4.92	4.92	4.92	4.92	4.92	4.92	4.92	4.92	4.92
4.93	4.93	4.93	4.93	4.93	4.93	4.93	4.93	4.93
4.94	4.94	4.94	4.94	4.94	4.94	4.94	4.94	4.94
4.95	4.95	4.95	4.95	4.95	4.95	4.95	4.95	4.95
4.96	4.96	4.96	4.96	4.96	4.96	4.96	4.96	4.96
4.97	4.97	4.97	4.97	4.97	4.97	4.97	4.97	4.97
4.98	4.98	4.98	4.98	4.98	4.98	4.98	4.98	4.98
4.99	4.99	4.99	4.99	4.99	4.99	4.99	4.99	4.99

5	5	5	5	5	5	5	5	5
5.01	5.01	5.01	5.01	5.01	5.01	5.01	5.01	5.01
5.02	5.02	5.02	5.02	5.02	5.02	5.02	5.02	5.02
5.03	5.03	5.03	5.03	5.03	5.03	5.03	5.03	5.03
5.04	5.04	5.04	5.04	5.04	5.04	5.04	5.04	5.04
5.05	5.05	5.05	5.05	5.05	5.05	5.05	5.05	5.05
5.06	5.06	5.06	5.06	5.06	5.06	5.06	5.06	5.06
5.07	5.07	5.07	5.07	5.07	5.07	5.07	5.07	5.07
5.08	5.08	5.08	5.08	5.08	5.08	5.08	5.08	5.08
5.09	5.09	5.09	5.09	5.09	5.09	5.09	5.09	5.09
5.1	5.1	5.1	5.1	5.1	5.1	5.1	5.1	5.1
5.11	5.11	5.11	5.11	5.11	5.11	5.11	5.11	5.11
5.12	5.12	5.12	5.12	5.12	5.12	5.12	5.12	5.12
5.13	5.13	5.13	5.13	5.13	5.13	5.13	5.13	5.13
5.14	5.14	5.14	5.14	5.14	5.14	5.14	5.14	5.14
5.15	5.15	5.15	5.15	5.15	5.15	5.15	5.15	5.15
5.16	5.16	5.16	5.16	5.16	5.16	5.16	5.16	5.16
5.17	5.17	5.17	5.17	5.17	5.17	5.17	5.17	5.17
5.18	5.18	5.18	5.18	5.18	5.18	5.18	5.18	5.18
5.19	5.19	5.19	5.19	5.19	5.19	5.19	5.19	5.19
5.2	5.2	5.2	5.2	5.2	5.2	5.2	5.2	5.2
5.21	5.21	5.21	5.21	5.21	5.21	5.21	5.21	5.21
5.22	5.22	5.22	5.22	5.22	5.22	5.22	5.22	5.22
5.23	5.23	5.23	5.23	5.23	5.23	5.23	5.23	5.23
5.24	5.24	5.24	5.24	5.24	5.24	5.24	5.24	5.24
5.25	5.25	5.25	5.25	5.25	5.25	5.25	5.25	5.25
5.26	5.26	5.26	5.26	5.26	5.26	5.26	5.26	5.26
5.27	5.27	5.27	5.27	5.27	5.27	5.27	5.27	5.27
5.28	5.28	5.28	5.28	5.28	5.28	5.28	5.28	5.28
5.29	5.29	5.29	5.29	5.29	5.29	5.29	5.29	5.29
5.3	5.3	5.3	5.3	5.3	5.3	5.3	5.3	5.3
5.31	5.31	5.31	5.31	5.31	5.31	5.31	5.31	5.31
5.32	5.32	5.32	5.32	5.32	5.32	5.32	5.32	5.32
5.33	5.33	5.33	5.33	5.33	5.33	5.33	5.33	5.33
5.34	5.34	5.34	5.34	5.34	5.34	5.34	5.34	5.34
5.35	5.35	5.35	5.35	5.35	5.35	5.35	5.35	5.35
5.36	5.36	5.36	5.36	5.36	5.36	5.36	5.36	5.36
5.37	5.37	5.37	5.37	5.37	5.37	5.37	5.37	5.37
5.38	5.38	5.38	5.38	5.38	5.38	5.38	5.38	5.38
5.39	5.39	5.39	5.39	5.39	5.39	5.39	5.39	5.39
5.4	5.4	5.4	5.4	5.4	5.4	5.4	5.4	5.4
5.41	5.41	5.41	5.41	5.41	5.41	5.41	5.41	5.41
5.42	5.42	5.42	5.42	5.42	5.42	5.42	5.42	5.42
5.43	5.43	5.43	5.43	5.43	5.43	5.43	5.43	5.43
5.44	5.44	5.44	5.44	5.44	5.44	5.44	5.44	5.44
5.45	5.45	5.45	5.45	5.45	5.45	5.45	5.45	5.45
5.46	5.46	5.46	5.46	5.46	5.46	5.46	5.46	5.46
5.47	5.47	5.47	5.47	5.47	5.47	5.47	5.47	5.47
5.48	5.48	5.48	5.48	5.48	5.48	5.48	5.48	5.48
5.49	5.49	5.49	5.49	5.49	5.49	5.49	5.49	5.49

5.5	5.5	5.5	5.5	5.5	5.5	5.5	5.5	5.5
5.51	5.51	5.51	5.51	5.51	5.51	5.51	5.51	5.51
5.52	5.52	5.52	5.52	5.52	5.52	5.52	5.52	5.52
5.53	5.53	5.53	5.53	5.53	5.53	5.53	5.53	5.53
5.54	5.54	5.54	5.54	5.54	5.54	5.54	5.54	5.54
5.55	5.55	5.55	5.55	5.55	5.55	5.55	5.55	5.55
5.56	5.56	5.56	5.56	5.56	5.56	5.56	5.56	5.56
5.57	5.57	5.57	5.57	5.57	5.57	5.57	5.57	5.57
5.58	5.58	5.58	5.58	5.58	5.58	5.58	5.58	5.58
5.59	5.59	5.59	5.59	5.59	5.59	5.59	5.59	5.59
5.6	5.6	5.6	5.6	5.6	5.6	5.6	5.6	5.6
5.61	5.61	5.61	5.61	5.61	5.61	5.61	5.61	5.61
5.62	5.62	5.62	5.62	5.62	5.62	5.62	5.62	5.62
5.63	5.63	5.63	5.63	5.63	5.63	5.63	5.63	5.63
5.64	5.64	5.64	5.64	5.64	5.64	5.64	5.64	5.64
5.65	5.65	5.65	5.65	5.65	5.65	5.65	5.65	5.65
5.66	5.66	5.66	5.66	5.66	5.66	5.66	5.66	5.66
5.67	5.67	5.67	5.67	5.67	5.67	5.67	5.67	5.67
5.68	5.68	5.68	5.68	5.68	5.68	5.68	5.68	5.68
5.69	5.69	5.69	5.69	5.69	5.69	5.69	5.69	5.69
5.7	5.7	5.7	5.7	5.7	5.7	5.7	5.7	5.7
5.71	5.71	5.71	5.71	5.71	5.71	5.71	5.71	5.71
5.72	5.72	5.72	5.72	5.72	5.72	5.72	5.72	5.72
5.73	5.73	5.73	5.73	5.73	5.73	5.73	5.73	5.73
5.74	5.74	5.74	5.74	5.74	5.74	5.74	5.74	5.74
5.75	5.75	5.75	5.75	5.75	5.75	5.75	5.75	5.75
5.76	5.76	5.76	5.76	5.76	5.76	5.76	5.76	5.76
5.77	5.77	5.77	5.77	5.77	5.77	5.77	5.77	5.77
5.78	5.78	5.78	5.78	5.78	5.78	5.78	5.78	5.78
5.79	5.79	5.79	5.79	5.79	5.79	5.79	5.79	5.79
5.8	5.8	5.8	5.8	5.8	5.8	5.8	5.8	5.8
5.81	5.81	5.81	5.81	5.81	5.81	5.81	5.81	5.81
5.82	5.82	5.82	5.82	5.82	5.82	5.82	5.82	5.82
5.83	5.83	5.83	5.83	5.83	5.83	5.83	5.83	5.83
5.84	5.84	5.84	5.84	5.84	5.84	5.84	5.84	5.84
5.85	5.85	5.85	5.85	5.85	5.85	5.85	5.85	5.85
5.86	5.86	5.86	5.86	5.86	5.86	5.86	5.86	5.86
5.87	5.87	5.87	5.87	5.87	5.87	5.87	5.87	5.87
5.88	5.88	5.88	5.88	5.88	5.88	5.88	5.88	5.88
5.89	5.89	5.89	5.89	5.89	5.89	5.89	5.89	5.89
5.9	5.9	5.9	5.9	5.9	5.9	5.9	5.9	5.9
5.91	5.91	5.91	5.91	5.91	5.91	5.91	5.91	5.91
5.92	5.92	5.92	5.92	5.92	5.92	5.92	5.92	5.92
5.93	5.93	5.93	5.93	5.93	5.93	5.93	5.93	5.93
5.94	5.94	5.94	5.94	5.94	5.94	5.94	5.94	5.94
5.95	5.95	5.95	5.95	5.95	5.95	5.95	5.95	5.95
5.96	5.96	5.96	5.96	5.96	5.96	5.96	5.96	5.96
5.97	5.97	5.97	5.97	5.97	5.97	5.97	5.97	5.97
5.98	5.98	5.98	5.98	5.98	5.98	5.98	5.98	5.98
5.99	5.99	5.99	5.99	5.99	5.99	5.99	5.99	5.99

6	6	6	6	6	6	6	6	6
6.01	6.01	6.01	6.01	6.01	6.01	6.01	6.01	6.01
6.02	6.02	6.02	6.02	6.02	6.02	6.02	6.02	6.02
6.03	6.03	6.03	6.03	6.03	6.03	6.03	6.03	6.03
6.04	6.04	6.04	6.04	6.04	6.04	6.04	6.04	6.04
6.05	6.05	6.05	6.05	6.05	6.05	6.05	6.05	6.05
6.06	6.06	6.06	6.06	6.06	6.06	6.06	6.06	6.06
6.07	6.07	6.07	6.07	6.07	6.07	6.07	6.07	6.07
6.08	6.08	6.08	6.08	6.08	6.08	6.08	6.08	6.08
6.09	6.09	6.09	6.09	6.09	6.09	6.09	6.09	6.09
6.1	6.1	6.1	6.1	6.1	6.1	6.1	6.1	6.1
6.11	6.11	6.11	6.11	6.11	6.11	6.11	6.11	6.11
6.12	6.12	6.12	6.12	6.12	6.12	6.12	6.12	6.12
6.13	6.13	6.13	6.13	6.13	6.13	6.13	6.13	6.13
6.14	6.14	6.14	6.14	6.14	6.14	6.14	6.14	6.14
6.15	6.15	6.15	6.15	6.15	6.15	6.15	6.15	6.15
6.16	6.16	6.16	6.16	6.16	6.16	6.16	6.16	6.16
6.17	6.17	6.17	6.17	6.17	6.17	6.17	6.17	6.17
6.18	6.18	6.18	6.18	6.18	6.18	6.18	6.18	6.18
6.19	6.19	6.19	6.19	6.19	6.19	6.19	6.19	6.19
6.2	6.2	6.2	6.2	6.2	6.2	6.2	6.2	6.2
6.21	6.21	6.21	6.21	6.21	6.21	6.21	6.21	6.21
6.22	6.22	6.22	6.22	6.22	6.22	6.22	6.22	6.22
6.23	6.23	6.23	6.23	6.23	6.23	6.23	6.23	6.23
6.24	6.24	6.24	6.24	6.24	6.24	6.24	6.24	6.24
6.25	6.25	6.25	6.25	6.25	6.25	6.25	6.25	6.25
6.26	6.26	6.26	6.26	6.26	6.26	6.26	6.26	6.26
6.27	6.27	6.27	6.27	6.27	6.27	6.27	6.27	6.27
6.28	6.28	6.28	6.28	6.28	6.28	6.28	6.28	6.28
6.29	6.29	6.29	6.29	6.29	6.29	6.29	6.29	6.29
6.3	6.3	6.3	6.3	6.3	6.3	6.3	6.3	6.3
6.31	6.31	6.31	6.31	6.31	6.31	6.31	6.31	6.31
6.32	6.32	6.32	6.32	6.32	6.32	6.32	6.32	6.32
6.33	6.33	6.33	6.33	6.33	6.33	6.33	6.33	6.33
6.34	6.34	6.34	6.34	6.34	6.34	6.34	6.34	6.34
6.35	6.35	6.35	6.35	6.35	6.35	6.35	6.35	6.35
6.36	6.36	6.36	6.36	6.36	6.36	6.36	6.36	6.36
6.37	6.37	6.37	6.37	6.37	6.37	6.37	6.37	6.37
6.38	6.38	6.38	6.38	6.38	6.38	6.38	6.38	6.38
6.39	6.39	6.39	6.39	6.39	6.39	6.39	6.39	6.39
6.4	6.4	6.4	6.4	6.4	6.4	6.4	6.4	6.4
6.41	6.41	6.41	6.41	6.41	6.41	6.41	6.41	6.41
6.42	6.42	6.42	6.42	6.42	6.42	6.42	6.42	6.42
6.43	6.43	6.43	6.43	6.43	6.43	6.43	6.43	6.43
6.44	6.44	6.44	6.44	6.44	6.44	6.44	6.44	6.44
6.45	6.45	6.45	6.45	6.45	6.45	6.45	6.45	6.45
6.46	6.46	6.46	6.46	6.46	6.46	6.46	6.46	6.46
6.47	6.47	6.47	6.47	6.47	6.47	6.47	6.47	6.47
6.48	6.48	6.48	6.48	6.48	6.48	6.48	6.48	6.48
6.49	6.49	6.49	6.49	6.49	6.49	6.49	6.49	6.49

6.5	6.5	6.5	6.5	6.5	6.5	6.5	6.5	6.5
6.51	6.51	6.51	6.51	6.51	6.51	6.51	6.51	6.51
6.52	6.52	6.52	6.52	6.52	6.52	6.52	6.52	6.52
6.53	6.53	6.53	6.53	6.53	6.53	6.53	6.53	6.53
6.54	6.54	6.54	6.54	6.54	6.54	6.54	6.54	6.54
6.55	6.55	6.55	6.55	6.55	6.55	6.55	6.55	6.55
6.56	6.56	6.56	6.56	6.56	6.56	6.56	6.56	6.56
6.57	6.57	6.57	6.57	6.57	6.57	6.57	6.57	6.57
6.58	6.58	6.58	6.58	6.58	6.58	6.58	6.58	6.58
6.59	6.59	6.59	6.59	6.59	6.59	6.59	6.59	6.59
6.6	6.6	6.6	6.6	6.6	6.6	6.6	6.6	6.6
6.61	6.61	6.61	6.61	6.61	6.61	6.61	6.61	6.61
6.62	6.62	6.62	6.62	6.62	6.62	6.62	6.62	6.62
6.63	6.63	6.63	6.63	6.63	6.63	6.63	6.63	6.63
6.64	6.64	6.64	6.64	6.64	6.64	6.64	6.64	6.64
6.65	6.65	6.65	6.65	6.65	6.65	6.65	6.65	6.65
6.66	6.66	6.66	6.66	6.66	6.66	6.66	6.66	6.66
6.67	6.67	6.67	6.67	6.67	6.67	6.67	6.67	6.67
6.68	6.68	6.68	6.68	6.68	6.68	6.68	6.68	6.68
6.69	6.69	6.69	6.69	6.69	6.69	6.69	6.69	6.69
6.7	6.7	6.7	6.7	6.7	6.7	6.7	6.7	6.7
6.71	6.71	6.71	6.71	6.71	6.71	6.71	6.71	6.71
6.72	6.72	6.72	6.72	6.72	6.72	6.72	6.72	6.72
6.73	6.73	6.73	6.73	6.73	6.73	6.73	6.73	6.73
6.74	6.74	6.74	6.74	6.74	6.74	6.74	6.74	6.74
6.75	6.75	6.75	6.75	6.75	6.75	6.75	6.75	6.75
6.76	6.76	6.76	6.76	6.76	6.76	6.76	6.76	6.76
6.77	6.77	6.77	6.77	6.77	6.77	6.77	6.77	6.77
6.78	6.78	6.78	6.78	6.78	6.78	6.78	6.78	6.78
6.79	6.79	6.79	6.79	6.79	6.79	6.79	6.79	6.79
6.8	6.8	6.8	6.8	6.8	6.8	6.8	6.8	6.8
6.81	6.81	6.81	6.81	6.81	6.81	6.81	6.81	6.81
6.82	6.82	6.82	6.82	6.82	6.82	6.82	6.82	6.82
6.83	6.83	6.83	6.83	6.83	6.83	6.83	6.83	6.83
6.84	6.84	6.84	6.84	6.84	6.84	6.84	6.84	6.84
6.85	6.85	6.85	6.85	6.85	6.85	6.85	6.85	6.85
6.86	6.86	6.86	6.86	6.86	6.86	6.86	6.86	6.86
6.87	6.87	6.87	6.87	6.87	6.87	6.87	6.87	6.87
6.88	6.88	6.88	6.88	6.88	6.88	6.88	6.88	6.88
6.89	6.89	6.89	6.89	6.89	6.89	6.89	6.89	6.89
6.9	6.9	6.9	6.9	6.9	6.9	6.9	6.9	6.9
6.91	6.91	6.91	6.91	6.91	6.91	6.91	6.91	6.91
6.92	6.92	6.92	6.92	6.92	6.92	6.92	6.92	6.92
6.93	6.93	6.93	6.93	6.93	6.93	6.93	6.93	6.93
6.94	6.94	6.94	6.94	6.94	6.94	6.94	6.94	6.94
6.95	6.95	6.95	6.95	6.95	6.95	6.95	6.95	6.95
6.96	6.96	6.96	6.96	6.96	6.96	6.96	6.96	6.96
6.97	6.97	6.97	6.97	6.97	6.97	6.97	6.97	6.97
6.98	6.98	6.98	6.98	6.98	6.98	6.98	6.98	6.98
6.99	6.99	6.99	6.99	6.99	6.99	6.99	6.99	6.99

7	7	7	7	7	7	7	7	7
7.01	7.01	7.01	7.01	7.01	7.01	7.01	7.01	7.01
7.02	7.02	7.02	7.02	7.02	7.02	7.02	7.02	7.02
7.03	7.03	7.03	7.03	7.03	7.03	7.03	7.03	7.03
7.04	7.04	7.04	7.04	7.04	7.04	7.04	7.04	7.04
7.05	7.05	7.05	7.05	7.05	7.05	7.05	7.05	7.05
7.06	7.06	7.06	7.06	7.06	7.06	7.06	7.06	7.06
7.07	7.07	7.07	7.07	7.07	7.07	7.07	7.07	7.07
7.08	7.08	7.08	7.08	7.08	7.08	7.08	7.08	7.08
7.09	7.09	7.09	7.09	7.09	7.09	7.09	7.09	7.09
7.1	7.1	7.1	7.1	7.1	7.1	7.1	7.1	7.1
7.11	7.11	7.11	7.11	7.11	7.11	7.11	7.11	7.11
7.12	7.12	7.12	7.12	7.12	7.12	7.12	7.12	7.12
7.13	7.13	7.13	7.13	7.13	7.13	7.13	7.13	7.13
7.14	7.14	7.14	7.14	7.14	7.14	7.14	7.14	7.14
7.15	7.15	7.15	7.15	7.15	7.15	7.15	7.15	7.15
7.16	7.16	7.16	7.16	7.16	7.16	7.16	7.16	7.16
7.17	7.17	7.17	7.17	7.17	7.17	7.17	7.17	7.17
7.18	7.18	7.18	7.18	7.18	7.18	7.18	7.18	7.18
7.19	7.19	7.19	7.19	7.19	7.19	7.19	7.19	7.19
7.2	7.2	7.2	7.2	7.2	7.2	7.2	7.2	7.2
7.21	7.21	7.21	7.21	7.21	7.21	7.21	7.21	7.21
7.22	7.22	7.22	7.22	7.22	7.22	7.22	7.22	7.22
7.23	7.23	7.23	7.23	7.23	7.23	7.23	7.23	7.23
7.24	7.24	7.24	7.24	7.24	7.24	7.24	7.24	7.24
7.25	7.25	7.25	7.25	7.25	7.25	7.25	7.25	7.25
7.26	7.26	7.26	7.26	7.26	7.26	7.26	7.26	7.26
7.27	7.27	7.27	7.27	7.27	7.27	7.27	7.27	7.27
7.28	7.28	7.28	7.28	7.28	7.28	7.28	7.28	7.28
7.29	7.29	7.29	7.29	7.29	7.29	7.29	7.29	7.29
7.3	7.3	7.3	7.3	7.3	7.3	7.3	7.3	7.3
7.31	7.31	7.31	7.31	7.31	7.31	7.31	7.31	7.31
7.32	7.32	7.32	7.32	7.32	7.32	7.32	7.32	7.32
7.33	7.33	7.33	7.33	7.33	7.33	7.33	7.33	7.33
7.34	7.34	7.34	7.34	7.34	7.34	7.34	7.34	7.34
7.35	7.35	7.35	7.35	7.35	7.35	7.35	7.35	7.35
7.36	7.36	7.36	7.36	7.36	7.36	7.36	7.36	7.36
7.37	7.37	7.37	7.37	7.37	7.37	7.37	7.37	7.37
7.38	7.38	7.38	7.38	7.38	7.38	7.38	7.38	7.38
7.39	7.39	7.39	7.39	7.39	7.39	7.39	7.39	7.39
7.4	7.4	7.4	7.4	7.4	7.4	7.4	7.4	7.4
7.41	7.41	7.41	7.41	7.41	7.41	7.41	7.41	7.41
7.42	7.42	7.42	7.42	7.42	7.42	7.42	7.42	7.42
7.43	7.43	7.43	7.43	7.43	7.43	7.43	7.43	7.43
7.44	7.44	7.44	7.44	7.44	7.44	7.44	7.44	7.44
7.45	7.45	7.45	7.45	7.45	7.45	7.45	7.45	7.45
7.46	7.46	7.46	7.46	7.46	7.46	7.46	7.46	7.46
7.47	7.47	7.47	7.47	7.47	7.47	7.47	7.47	7.47
7.48	7.48	7.48	7.48	7.48	7.48	7.48	7.48	7.48
7.49	7.49	7.49	7.49	7.49	7.49	7.49	7.49	7.49

7.5	7.5	7.5	7.5	7.5	7.5	7.5	7.5	7.5
7.51	7.51	7.51	7.51	7.51	7.51	7.51	7.51	7.51
7.52	7.52	7.52	7.52	7.52	7.52	7.52	7.52	7.52
7.53	7.53	7.53	7.53	7.53	7.53	7.53	7.53	7.53
7.54	7.54	7.54	7.54	7.54	7.54	7.54	7.54	7.54
7.55	7.55	7.55	7.55	7.55	7.55	7.55	7.55	7.55
7.56	7.56	7.56	7.56	7.56	7.56	7.56	7.56	7.56
7.57	7.57	7.57	7.57	7.57	7.57	7.57	7.57	7.57
7.58	7.58	7.58	7.58	7.58	7.58	7.58	7.58	7.58
7.59	7.59	7.59	7.59	7.59	7.59	7.59	7.59	7.59
7.6	7.6	7.6	7.6	7.6	7.6	7.6	7.6	7.6
7.61	7.61	7.61	7.61	7.61	7.61	7.61	7.61	7.61
7.62	7.62	7.62	7.62	7.62	7.62	7.62	7.62	7.62
7.63	7.63	7.63	7.63	7.63	7.63	7.63	7.63	7.63
7.64	7.64	7.64	7.64	7.64	7.64	7.64	7.64	7.64
7.65	7.65	7.65	7.65	7.65	7.65	7.65	7.65	7.65
7.66	7.66	7.66	7.66	7.66	7.66	7.66	7.66	7.66
7.67	7.67	7.67	7.67	7.67	7.67	7.67	7.67	7.67
7.68	7.68	7.68	7.68	7.68	7.68	7.68	7.68	7.68
7.69	7.69	7.69	7.69	7.69	7.69	7.69	7.69	7.69
7.7	7.7	7.7	7.7	7.7	7.7	7.7	7.7	7.7
7.71	7.71	7.71	7.71	7.71	7.71	7.71	7.71	7.71
7.72	7.72	7.72	7.72	7.72	7.72	7.72	7.72	7.72
7.73	7.73	7.73	7.73	7.73	7.73	7.73	7.73	7.73
7.74	7.74	7.74	7.74	7.74	7.74	7.74	7.74	7.74
7.75	7.75	7.75	7.75	7.75	7.75	7.75	7.75	7.75
7.76	7.76	7.76	7.76	7.76	7.76	7.76	7.76	7.76
7.77	7.77	7.77	7.77	7.77	7.77	7.77	7.77	7.77
7.78	7.78	7.78	7.78	7.78	7.78	7.78	7.78	7.78
7.79	7.79	7.79	7.79	7.79	7.79	7.79	7.79	7.79
7.8	7.8	7.8	7.8	7.8	7.8	7.8	7.8	7.8
7.81	7.81	7.81	7.81	7.81	7.81	7.81	7.81	7.81
7.82	7.82	7.82	7.82	7.82	7.82	7.82	7.82	7.82
7.83	7.83	7.83	7.83	7.83	7.83	7.83	7.83	7.83
7.84	7.84	7.84	7.84	7.84	7.84	7.84	7.84	7.84
7.85	7.85	7.85	7.85	7.85	7.85	7.85	7.85	7.85
7.86	7.86	7.86	7.86	7.86	7.86	7.86	7.86	7.86
7.87	7.87	7.87	7.87	7.87	7.87	7.87	7.87	7.87
7.88	7.88	7.88	7.88	7.88	7.88	7.88	7.88	7.88
7.89	7.89	7.89	7.89	7.89	7.89	7.89	7.89	7.89
7.9	7.9	7.9	7.9	7.9	7.9	7.9	7.9	7.9
7.91	7.91	7.91	7.91	7.91	7.91	7.91	7.91	7.91
7.92	7.92	7.92	7.92	7.92	7.92	7.92	7.92	7.92
7.93	7.93	7.93	7.93	7.93	7.93	7.93	7.93	7.93
7.94	7.94	7.94	7.94	7.94	7.94	7.94	7.94	7.94
7.95	7.95	7.95	7.95	7.95	7.95	7.95	7.95	7.95
7.96	7.96	7.96	7.96	7.96	7.96	7.96	7.96	7.96
7.97	7.97	7.97	7.97	7.97	7.97	7.97	7.97	7.97
7.98	7.98	7.98	7.98	7.98	7.98	7.98	7.98	7.98
7.99	7.99	7.99	7.99	7.99	7.99	7.99	7.99	7.99

8	8	8	8	8	8	8	8	8
8.01	8.01	8.01	8.01	8.01	8.01	8.01	8.01	8.01
8.02	8.02	8.02	8.02	8.02	8.02	8.02	8.02	8.02
8.03	8.03	8.03	8.03	8.03	8.03	8.03	8.03	8.03
8.04	8.04	8.04	8.04	8.04	8.04	8.04	8.04	8.04
8.05	8.05	8.05	8.05	8.05	8.05	8.05	8.05	8.05
8.06	8.06	8.06	8.06	8.06	8.06	8.06	8.06	8.06
8.07	8.07	8.07	8.07	8.07	8.07	8.07	8.07	8.07
8.08	8.08	8.08	8.08	8.08	8.08	8.08	8.08	8.08
8.09	8.09	8.09	8.09	8.09	8.09	8.09	8.09	8.09
8.1	8.1	8.1	8.1	8.1	8.1	8.1	8.1	8.1
8.11	8.11	8.11	8.11	8.11	8.11	8.11	8.11	8.11
8.12	8.12	8.12	8.12	8.12	8.12	8.12	8.12	8.12
8.13	8.13	8.13	8.13	8.13	8.13	8.13	8.13	8.13
8.14	8.14	8.14	8.14	8.14	8.14	8.14	8.14	8.14
8.15	8.15	8.15	8.15	8.15	8.15	8.15	8.15	8.15
8.16	8.16	8.16	8.16	8.16	8.16	8.16	8.16	8.16
8.17	8.17	8.17	8.17	8.17	8.17	8.17	8.17	8.17
8.18	8.18	8.18	8.18	8.18	8.18	8.18	8.18	8.18
8.19	8.19	8.19	8.19	8.19	8.19	8.19	8.19	8.19
8.2	8.2	8.2	8.2	8.2	8.2	8.2	8.2	8.2
8.21	8.21	8.21	8.21	8.21	8.21	8.21	8.21	8.21
8.22	8.22	8.22	8.22	8.22	8.22	8.22	8.22	8.22
8.23	8.23	8.23	8.23	8.23	8.23	8.23	8.23	8.23
8.24	8.24	8.24	8.24	8.24	8.24	8.24	8.24	8.24
8.25	8.25	8.25	8.25	8.25	8.25	8.25	8.25	8.25
8.26	8.26	8.26	8.26	8.26	8.26	8.26	8.26	8.26
8.27	8.27	8.27	8.27	8.27	8.27	8.27	8.27	8.27
8.28	8.28	8.28	8.28	8.28	8.28	8.28	8.28	8.28
8.29	8.29	8.29	8.29	8.29	8.29	8.29	8.29	8.29
8.3	8.3	8.3	8.3	8.3	8.3	8.3	8.3	8.3
8.31	8.31	8.31	8.31	8.31	8.31	8.31	8.31	8.31
8.32	8.32	8.32	8.32	8.32	8.32	8.32	8.32	8.32
8.33	8.33	8.33	8.33	8.33	8.33	8.33	8.33	8.33
8.34	8.34	8.34	8.34	8.34	8.34	8.34	8.34	8.34
8.35	8.35	8.35	8.35	8.35	8.35	8.35	8.35	8.35
8.36	8.36	8.36	8.36	8.36	8.36	8.36	8.36	8.36
8.37	8.37	8.37	8.37	8.37	8.37	8.37	8.37	8.37
8.38	8.38	8.38	8.38	8.38	8.38	8.38	8.38	8.38
8.39	8.39	8.39	8.39	8.39	8.39	8.39	8.39	8.39
8.4	8.4	8.4	8.4	8.4	8.4	8.4	8.4	8.4
8.41	8.41	8.41	8.41	8.41	8.41	8.41	8.41	8.41
8.42	8.42	8.42	8.42	8.42	8.42	8.42	8.42	8.42
8.43	8.43	8.43	8.43	8.43	8.43	8.43	8.43	8.43
8.44	8.44	8.44	8.44	8.44	8.44	8.44	8.44	8.44
8.45	8.45	8.45	8.45	8.45	8.45	8.45	8.45	8.45
8.46	8.46	8.46	8.46	8.46	8.46	8.46	8.46	8.46
8.47	8.47	8.47	8.47	8.47	8.47	8.47	8.47	8.47
8.48	8.48	8.48	8.48	8.48	8.48	8.48	8.48	8.48
8.49	8.49	8.49	8.49	8.49	8.49	8.49	8.49	8.49

8.5	8.5	8.5	8.5	8.5	8.5	8.5	8.5	8.5
8.51	8.51	8.51	8.51	8.51	8.51	8.51	8.51	8.51
8.52	8.52	8.52	8.52	8.52	8.52	8.52	8.52	8.52
8.53	8.53	8.53	8.53	8.53	8.53	8.53	8.53	8.53
8.54	8.54	8.54	8.54	8.54	8.54	8.54	8.54	8.54
8.55	8.55	8.55	8.55	8.55	8.55	8.55	8.55	8.55
8.56	8.56	8.56	8.56	8.56	8.56	8.56	8.56	8.56
8.57	8.57	8.57	8.57	8.57	8.57	8.57	8.57	8.57
8.58	8.58	8.58	8.58	8.58	8.58	8.58	8.58	8.58
8.59	8.59	8.59	8.59	8.59	8.59	8.59	8.59	8.59
8.6	8.6	8.6	8.6	8.6	8.6	8.6	8.6	8.6
8.61	8.61	8.61	8.61	8.61	8.61	8.61	8.61	8.61
8.62	8.62	8.62	8.62	8.62	8.62	8.62	8.62	8.62
8.63	8.63	8.63	8.63	8.63	8.63	8.63	8.63	8.63
8.64	8.64	8.64	8.64	8.64	8.64	8.64	8.64	8.64
8.65	8.65	8.65	8.65	8.65	8.65	8.65	8.65	8.65
8.66	8.66	8.66	8.66	8.66	8.66	8.66	8.66	8.66
8.67	8.67	8.67	8.67	8.67	8.67	8.67	8.67	8.67
8.68	8.68	8.68	8.68	8.68	8.68	8.68	8.68	8.68
8.69	8.69	8.69	8.69	8.69	8.69	8.69	8.69	8.69
8.7	8.7	8.7	8.7	8.7	8.7	8.7	8.7	8.7
8.71	8.71	8.71	8.71	8.71	8.71	8.71	8.71	8.71
8.72	8.72	8.72	8.72	8.72	8.72	8.72	8.72	8.72
8.73	8.73	8.73	8.73	8.73	8.73	8.73	8.73	8.73
8.74	8.74	8.74	8.74	8.74	8.74	8.74	8.74	8.74
8.75	8.75	8.75	8.75	8.75	8.75	8.75	8.75	8.75
8.76	8.76	8.76	8.76	8.76	8.76	8.76	8.76	8.76
8.77	8.77	8.77	8.77	8.77	8.77	8.77	8.77	8.77
8.78	8.78	8.78	8.78	8.78	8.78	8.78	8.78	8.78
8.79	8.79	8.79	8.79	8.79	8.79	8.79	8.79	8.79
8.8	8.8	8.8	8.8	8.8	8.8	8.8	8.8	8.8
8.81	8.81	8.81	8.81	8.81	8.81	8.81	8.81	8.81
8.82	8.82	8.82	8.82	8.82	8.82	8.82	8.82	8.82
8.83	8.83	8.83	8.83	8.83	8.83	8.83	8.83	8.83
8.84	8.84	8.84	8.84	8.84	8.84	8.84	8.84	8.84
8.85	8.85	8.85	8.85	8.85	8.85	8.85	8.85	8.85
8.86	8.86	8.86	8.86	8.86	8.86	8.86	8.86	8.86
8.87	8.87	8.87	8.87	8.87	8.87	8.87	8.87	8.87
8.88	8.88	8.88	8.88	8.88	8.88	8.88	8.88	8.88
8.89	8.89	8.89	8.89	8.89	8.89	8.89	8.89	8.89
8.9	8.9	8.9	8.9	8.9	8.9	8.9	8.9	8.9
8.91	8.91	8.91	8.91	8.91	8.91	8.91	8.91	8.91
8.92	8.92	8.92	8.92	8.92	8.92	8.92	8.92	8.92
8.93	8.93	8.93	8.93	8.93	8.93	8.93	8.93	8.93
8.94	8.94	8.94	8.94	8.94	8.94	8.94	8.94	8.94
8.95	8.95	8.95	8.95	8.95	8.95	8.95	8.95	8.95
8.96	8.96	8.96	8.96	8.96	8.96	8.96	8.96	8.96
8.97	8.97	8.97	8.97	8.97	8.97	8.97	8.97	8.97
8.98	8.98	8.98	8.98	8.98	8.98	8.98	8.98	8.98
8.99	8.99	8.99	8.99	8.99	8.99	8.99	8.99	8.99

9	9	9	9	9	9	9	9	9
9.01	9.01	9.01	9.01	9.01	9.01	9.01	9.01	9.01
9.02	9.02	9.02	9.02	9.02	9.02	9.02	9.02	9.02
9.03	9.03	9.03	9.03	9.03	9.03	9.03	9.03	9.03
9.04	9.04	9.04	9.04	9.04	9.04	9.04	9.04	9.04
9.05	9.05	9.05	9.05	9.05	9.05	9.05	9.05	9.05
9.06	9.06	9.06	9.06	9.06	9.06	9.06	9.06	9.06
9.07	9.07	9.07	9.07	9.07	9.07	9.07	9.07	9.07
9.08	9.08	9.08	9.08	9.08	9.08	9.08	9.08	9.08
9.09	9.09	9.09	9.09	9.09	9.09	9.09	9.09	9.09
9.1	9.1	9.1	9.1	9.1	9.1	9.1	9.1	9.1
9.11	9.11	9.11	9.11	9.11	9.11	9.11	9.11	9.11
9.12	9.12	9.12	9.12	9.12	9.12	9.12	9.12	9.12
9.13	9.13	9.13	9.13	9.13	9.13	9.13	9.13	9.13
9.14	9.14	9.14	9.14	9.14	9.14	9.14	9.14	9.14
9.15	9.15	9.15	9.15	9.15	9.15	9.15	9.15	9.15
9.16	9.16	9.16	9.16	9.16	9.16	9.16	9.16	9.16
9.17	9.17	9.17	9.17	9.17	9.17	9.17	9.17	9.17
9.18	9.18	9.18	9.18	9.18	9.18	9.18	9.18	9.18
9.19	9.19	9.19	9.19	9.19	9.19	9.19	9.19	9.19
9.2	9.2	9.2	9.2	9.2	9.2	9.2	9.2	9.2
9.21	9.21	9.21	9.21	9.21	9.21	9.21	9.21	9.21
9.22	9.22	9.22	9.22	9.22	9.22	9.22	9.22	9.22
9.23	9.23	9.23	9.23	9.23	9.23	9.23	9.23	9.23
9.24	9.24	9.24	9.24	9.24	9.24	9.24	9.24	9.24
9.25	9.25	9.25	9.25	9.25	9.25	9.25	9.25	9.25
9.26	9.26	9.26	9.26	9.26	9.26	9.26	9.26	9.26
9.27	9.27	9.27	9.27	9.27	9.27	9.27	9.27	9.27
9.28	9.28	9.28	9.28	9.28	9.28	9.28	9.28	9.28
9.29	9.29	9.29	9.29	9.29	9.29	9.29	9.29	9.29
9.3	9.3	9.3	9.3	9.3	9.3	9.3	9.3	9.3
9.31	9.31	9.31	9.31	9.31	9.31	9.31	9.31	9.31
9.32	9.32	9.32	9.32	9.32	9.32	9.32	9.32	9.32
9.33	9.33	9.33	9.33	9.33	9.33	9.33	9.33	9.33
9.34	9.34	9.34	9.34	9.34	9.34	9.34	9.34	9.34
9.35	9.35	9.35	9.35	9.35	9.35	9.35	9.35	9.35
9.36	9.36	9.36	9.36	9.36	9.36	9.36	9.36	9.36
9.37	9.37	9.37	9.37	9.37	9.37	9.37	9.37	9.37
9.38	9.38	9.38	9.38	9.38	9.38	9.38	9.38	9.38
9.39	9.39	9.39	9.39	9.39	9.39	9.39	9.39	9.39
9.4	9.4	9.4	9.4	9.4	9.4	9.4	9.4	9.4
9.41	9.41	9.41	9.41	9.41	9.41	9.41	9.41	9.41
9.42	9.42	9.42	9.42	9.42	9.42	9.42	9.42	9.42
9.43	9.43	9.43	9.43	9.43	9.43	9.43	9.43	9.43
9.44	9.44	9.44	9.44	9.44	9.44	9.44	9.44	9.44
9.45	9.45	9.45	9.45	9.45	9.45	9.45	9.45	9.45
9.46	9.46	9.46	9.46	9.46	9.46	9.46	9.46	9.46
9.47	9.47	9.47	9.47	9.47	9.47	9.47	9.47	9.47
9.48	9.48	9.48	9.48	9.48	9.48	9.48	9.48	9.48
9.49	9.49	9.49	9.49	9.49	9.49	9.49	9.49	9.49

9.5	9.5	9.5	9.5	9.5	9.5	9.5	9.5	9.5
9.51	9.51	9.51	9.51	9.51	9.51	9.51	9.51	9.51
9.52	9.52	9.52	9.52	9.52	9.52	9.52	9.52	9.52
9.53	9.53	9.53	9.53	9.53	9.53	9.53	9.53	9.53
9.54	9.54	9.54	9.54	9.54	9.54	9.54	9.54	9.54
9.55	9.55	9.55	9.55	9.55	9.55	9.55	9.55	9.55
9.56	9.56	9.56	9.56	9.56	9.56	9.56	9.56	9.56
9.57	9.57	9.57	9.57	9.57	9.57	9.57	9.57	9.57
9.58	9.58	9.58	9.58	9.58	9.58	9.58	9.58	9.58
9.59	9.59	9.59	9.59	9.59	9.59	9.59	9.59	9.59
9.6	9.6	9.6	9.6	9.6	9.6	9.6	9.6	9.6
9.61	9.61	9.61	9.61	9.61	9.61	9.61	9.61	9.61
9.62	9.62	9.62	9.62	9.62	9.62	9.62	9.62	9.62
9.63	9.63	9.63	9.63	9.63	9.63	9.63	9.63	9.63
9.64	9.64	9.64	9.64	9.64	9.64	9.64	9.64	9.64
9.65	9.65	9.65	9.65	9.65	9.65	9.65	9.65	9.65
9.66	9.66	9.66	9.66	9.66	9.66	9.66	9.66	9.66
9.67	9.67	9.67	9.67	9.67	9.67	9.67	9.67	9.67
9.68	9.68	9.68	9.68	9.68	9.68	9.68	9.68	9.68
9.69	9.69	9.69	9.69	9.69	9.69	9.69	9.69	9.69
9.7	9.7	9.7	9.7	9.7	9.7	9.7	9.7	9.7
9.71	9.71	9.71	9.71	9.71	9.71	9.71	9.71	9.71
9.72	9.72	9.72	9.72	9.72	9.72	9.72	9.72	9.72
9.73	9.73	9.73	9.73	9.73	9.73	9.73	9.73	9.73
9.74	9.74	9.74	9.74	9.74	9.74	9.74	9.74	9.74
9.75	9.75	9.75	9.75	9.75	9.75	9.75	9.75	9.75
9.76	9.76	9.76	9.76	9.76	9.76	9.76	9.76	9.76
9.77	9.77	9.77	9.77	9.77	9.77	9.77	9.77	9.77
9.78	9.78	9.78	9.78	9.78	9.78	9.78	9.78	9.78
9.79	9.79	9.79	9.79	9.79	9.79	9.79	9.79	9.79
9.8	9.8	9.8	9.8	9.8	9.8	9.8	9.8	9.8
9.81	9.81	9.81	9.81	9.81	9.81	9.81	9.81	9.81
9.82	9.82	9.82	9.82	9.82	9.82	9.82	9.82	9.82
9.83	9.83	9.83	9.83	9.83	9.83	9.83	9.83	9.83
9.84	9.84	9.84	9.84	9.84	9.84	9.84	9.84	9.84
9.85	9.85	9.85	9.85	9.85	9.85	9.85	9.85	9.85
9.86	9.86	9.86	9.86	9.86	9.86	9.86	9.86	9.86
9.87	9.87	9.87	9.87	9.87	9.87	9.87	9.87	9.87
9.88	9.88	9.88	9.88	9.88	9.88	9.88	9.88	9.88
9.89	9.89	9.89	9.89	9.89	9.89	9.89	9.89	9.89
9.9	9.9	9.9	9.9	9.9	9.9	9.9	9.9	9.9
9.91	9.91	9.91	9.91	9.91	9.91	9.91	9.91	9.91
9.92	9.92	9.92	9.92	9.92	9.92	9.92	9.92	9.92
9.93	9.93	9.93	9.93	9.93	9.93	9.93	9.93	9.93
9.94	9.94	9.94	9.94	9.94	9.94	9.94	9.94	9.94
9.95	9.95	9.95	9.95	9.95	9.95	9.95	9.95	9.95
9.96	9.96	9.96	9.96	9.96	9.96	9.96	9.96	9.96
9.97	9.97	9.97	9.97	9.97	9.97	9.97	9.97	9.97
9.98	9.98	9.98	9.98	9.98	9.98	9.98	9.98	9.98
9.99	9.99	9.99	9.99	9.99	9.99	9.99	9.99	9.99

10	10	10	10	10	10	10	10	10
10.01	10.01	10.01	10.01	10.01	10.01	10.01	10.01	10.01
10.02	10.02	10.02	10.02	10.02	10.02	10.02	10.02	10.02
10.03	10.03	10.03	10.03	10.03	10.03	10.03	10.03	10.03
10.04	10.04	10.04	10.04	10.04	10.04	10.04	10.04	10.04
10.05	10.05	10.05	10.05	10.05	10.05	10.05	10.05	10.05
10.06	10.06	10.06	10.06	10.06	10.06	10.06	10.06	10.06
10.07	10.07	10.07	10.07	10.07	10.07	10.07	10.07	10.07
10.08	10.08	10.08	10.08	10.08	10.08	10.08	10.08	10.08
10.09	10.09	10.09	10.09	10.09	10.09	10.09	10.09	10.09
10.1	10.1	10.1	10.1	10.1	10.1	10.1	10.1	10.1
10.11	10.11	10.11	10.11	10.11	10.11	10.11	10.11	10.11
10.12	10.12	10.12	10.12	10.12	10.12	10.12	10.12	10.12
10.13	10.13	10.13	10.13	10.13	10.13	10.13	10.13	10.13
10.14	10.14	10.14	10.14	10.14	10.14	10.14	10.14	10.14
10.15	10.15	10.15	10.15	10.15	10.15	10.15	10.15	10.15
10.16	10.16	10.16	10.16	10.16	10.16	10.16	10.16	10.16
10.17	10.17	10.17	10.17	10.17	10.17	10.17	10.17	10.17
10.18	10.18	10.18	10.18	10.18	10.18	10.18	10.18	10.18
10.19	10.19	10.19	10.19	10.19	10.19	10.19	10.19	10.19
10.2	10.2	10.2	10.2	10.2	10.2	10.2	10.2	10.2
10.21	10.21	10.21	10.21	10.21	10.21	10.21	10.21	10.21
10.22	10.22	10.22	10.22	10.22	10.22	10.22	10.22	10.22
10.23	10.23	10.23	10.23	10.23	10.23	10.23	10.23	10.23
10.24	10.24	10.24	10.24	10.24	10.24	10.24	10.24	10.24
10.25	10.25	10.25	10.25	10.25	10.25	10.25	10.25	10.25
10.26	10.26	10.26	10.26	10.26	10.26	10.26	10.26	10.26
10.27	10.27	10.27	10.27	10.27	10.27	10.27	10.27	10.27
10.28	10.28	10.28	10.28	10.28	10.28	10.28	10.28	10.28
10.29	10.29	10.29	10.29	10.29	10.29	10.29	10.29	10.29
10.3	10.3	10.3	10.3	10.3	10.3	10.3	10.3	10.3
10.31	10.31	10.31	10.31	10.31	10.31	10.31	10.31	10.31
10.32	10.32	10.32	10.32	10.32	10.32	10.32	10.32	10.32
10.33	10.33	10.33	10.33	10.33	10.33	10.33	10.33	10.33
10.34	10.34	10.34	10.34	10.34	10.34	10.34	10.34	10.34
10.35	10.35	10.35	10.35	10.35	10.35	10.35	10.35	10.35
10.36	10.36	10.36	10.36	10.36	10.36	10.36	10.36	10.36
10.37	10.37	10.37	10.37	10.37	10.37	10.37	10.37	10.37
10.38	10.38	10.38	10.38	10.38	10.38	10.38	10.38	10.38
10.39	10.39	10.39	10.39	10.39	10.39	10.39	10.39	10.39
10.4	10.4	10.4	10.4	10.4	10.4	10.4	10.4	10.4
10.41	10.41	10.41	10.41	10.41	10.41	10.41	10.41	10.41
10.42	10.42	10.42	10.42	10.42	10.42	10.42	10.42	10.42
10.43	10.43	10.43	10.43	10.43	10.43	10.43	10.43	10.43
10.44	10.44	10.44	10.44	10.44	10.44	10.44	10.44	10.44
10.45	10.45	10.45	10.45	10.45	10.45	10.45	10.45	10.45
10.46	10.46	10.46	10.46	10.46	10.46	10.46	10.46	10.46
10.47	10.47	10.47	10.47	10.47	10.47	10.47	10.47	10.47
10.48	10.48	10.48	10.48	10.48	10.48	10.48	10.48	10.48
10.49	10.49	10.49	10.49	10.49	10.49	10.49	10.49	10.49

10.5	10.5	10.5	10.5	10.5	10.5	10.5	10.5	10.5
10.51	10.51	10.51	10.51	10.51	10.51	10.51	10.51	10.51
10.52	10.52	10.52	10.52	10.52	10.52	10.52	10.52	10.52
10.53	10.53	10.53	10.53	10.53	10.53	10.53	10.53	10.53
10.54	10.54	10.54	10.54	10.54	10.54	10.54	10.54	10.54
10.55	10.55	10.55	10.55	10.55	10.55	10.55	10.55	10.55
10.56	10.56	10.56	10.56	10.56	10.56	10.56	10.56	10.56
10.57	10.57	10.57	10.57	10.57	10.57	10.57	10.57	10.57
10.58	10.58	10.58	10.58	10.58	10.58	10.58	10.58	10.58
10.59	10.59	10.59	10.59	10.59	10.59	10.59	10.59	10.59
10.6	10.6	10.6	10.6	10.6	10.6	10.6	10.6	10.6
10.61	10.61	10.61	10.61	10.61	10.61	10.61	10.61	10.61
10.62	10.62	10.62	10.62	10.62	10.62	10.62	10.62	10.62
10.63	10.63	10.63	10.63	10.63	10.63	10.63	10.63	10.63
10.64	10.64	10.64	10.64	10.64	10.64	10.64	10.64	10.64
10.65	10.65	10.65	10.65	10.65	10.65	10.65	10.65	10.65
10.66	10.66	10.66	10.66	10.66	10.66	10.66	10.66	10.66
10.67	10.67	10.67	10.67	10.67	10.67	10.67	10.67	10.67
10.68	10.68	10.68	10.68	10.68	10.68	10.68	10.68	10.68
10.69	10.69	10.69	10.69	10.69	10.69	10.69	10.69	10.69
10.7	10.7	10.7	10.7	10.7	10.7	10.7	10.7	10.7
10.71	10.71	10.71	10.71	10.71	10.71	10.71	10.71	10.71
10.72	10.72	10.72	10.72	10.72	10.72	10.72	10.72	10.72
10.73	10.73	10.73	10.73	10.73	10.73	10.73	10.73	10.73
10.74	10.74	10.74	10.74	10.74	10.74	10.74	10.74	10.74
10.75	10.75	10.75	10.75	10.75	10.75	10.75	10.75	10.75
10.76	10.76	10.76	10.76	10.76	10.76	10.76	10.76	10.76
10.77	10.77	10.77	10.77	10.77	10.77	10.77	10.77	10.77
10.78	10.78	10.78	10.78	10.78	10.78	10.78	10.78	10.78
10.79	10.79	10.79	10.79	10.79	10.79	10.79	10.79	10.79
10.8	10.8	10.8	10.8	10.8	10.8	10.8	10.8	10.8
10.81	10.81	10.81	10.81	10.81	10.81	10.81	10.81	10.81
10.82	10.82	10.82	10.82	10.82	10.82	10.82	10.82	10.82
10.83	10.83	10.83	10.83	10.83	10.83	10.83	10.83	10.83
10.84	10.84	10.84	10.84	10.84	10.84	10.84	10.84	10.84
10.85	10.85	10.85	10.85	10.85	10.85	10.85	10.85	10.85
10.86	10.86	10.86	10.86	10.86	10.86	10.86	10.86	10.86
10.87	10.87	10.87	10.87	10.87	10.87	10.87	10.87	10.87
10.88	10.88	10.88	10.88	10.88	10.88	10.88	10.88	10.88
10.89	10.89	10.89	10.89	10.89	10.89	10.89	10.89	10.89
10.9	10.9	10.9	10.9	10.9	10.9	10.9	10.9	10.9
10.91	10.91	10.91	10.91	10.91	10.91	10.91	10.91	10.91
10.92	10.92	10.92	10.92	10.92	10.92	10.92	10.92	10.92
10.93	10.93	10.93	10.93	10.93	10.93	10.93	10.93	10.93
10.94	10.94	10.94	10.94	10.94	10.94	10.94	10.94	10.94
10.95	10.95	10.95	10.95	10.95	10.95	10.95	10.95	10.95
10.96	10.96	10.96	10.96	10.96	10.96	10.96	10.96	10.96
10.97	10.97	10.97	10.97	10.97	10.97	10.97	10.97	10.97
10.98	10.98	10.98	10.98	10.98	10.98	10.98	10.98	10.98
10.99	10.99	10.99	10.99	10.99	10.99	10.99	10.99	10.99

11	11	11	11	11	11	11	11	11
11.01	11.01	11.01	11.01	11.01	11.01	11.01	11.01	11.01
11.02	11.02	11.02	11.02	11.02	11.02	11.02	11.02	11.02
11.03	11.03	11.03	11.03	11.03	11.03	11.03	11.03	11.03
11.04	11.04	11.04	11.04	11.04	11.04	11.04	11.04	11.04
11.05	11.05	11.05	11.05	11.05	11.05	11.05	11.05	11.05
11.06	11.06	11.06	11.06	11.06	11.06	11.06	11.06	11.06
11.07	11.07	11.07	11.07	11.07	11.07	11.07	11.07	11.07
11.08	11.08	11.08	11.08	11.08	11.08	11.08	11.08	11.08
11.09	11.09	11.09	11.09	11.09	11.09	11.09	11.09	11.09
11.1	11.1	11.1	11.1	11.1	11.1	11.1	11.1	11.1
11.11	11.11	11.11	11.11	11.11	11.11	11.11	11.11	11.11
11.12	11.12	11.12	11.12	11.12	11.12	11.12	11.12	11.12
11.13	11.13	11.13	11.13	11.13	11.13	11.13	11.13	11.13
11.14	11.14	11.14	11.14	11.14	11.14	11.14	11.14	11.14
11.15	11.15	11.15	11.15	11.15	11.15	11.15	11.15	11.15
11.16	11.16	11.16	11.16	11.16	11.16	11.16	11.16	11.16
11.17	11.17	11.17	11.17	11.17	11.17	11.17	11.17	11.17
11.18	11.18	11.18	11.18	11.18	11.18	11.18	11.18	11.18
11.19	11.19	11.19	11.19	11.19	11.19	11.19	11.19	11.19
11.2	11.2	11.2	11.2	11.2	11.2	11.2	11.2	11.2
11.21	11.21	11.21	11.21	11.21	11.21	11.21	11.21	11.21
11.22	11.22	11.22	11.22	11.22	11.22	11.22	11.22	11.22
11.23	11.23	11.23	11.23	11.23	11.23	11.23	11.23	11.23
11.24	11.24	11.24	11.24	11.24	11.24	11.24	11.24	11.24
11.25	11.25	11.25	11.25	11.25	11.25	11.25	11.25	11.25
11.26	11.26	11.26	11.26	11.26	11.26	11.26	11.26	11.26
11.27	11.27	11.27	11.27	11.27	11.27	11.27	11.27	11.27
11.28	11.28	11.28	11.28	11.28	11.28	11.28	11.28	11.28
11.29	11.29	11.29	11.29	11.29	11.29	11.29	11.29	11.29
11.3	11.3	11.3	11.3	11.3	11.3	11.3	11.3	11.3
11.31	11.31	11.31	11.31	11.31	11.31	11.31	11.31	11.31
11.32	11.32	11.32	11.32	11.32	11.32	11.32	11.32	11.32
11.33	11.33	11.33	11.33	11.33	11.33	11.33	11.33	11.33
11.34	11.34	11.34	11.34	11.34	11.34	11.34	11.34	11.34
11.35	11.35	11.35	11.35	11.35	11.35	11.35	11.35	11.35
11.36	11.36	11.36	11.36	11.36	11.36	11.36	11.36	11.36
11.37	11.37	11.37	11.37	11.37	11.37	11.37	11.37	11.37
11.38	11.38	11.38	11.38	11.38	11.38	11.38	11.38	11.38
11.39	11.39	11.39	11.39	11.39	11.39	11.39	11.39	11.39
11.4	11.4	11.4	11.4	11.4	11.4	11.4	11.4	11.4
11.41	11.41	11.41	11.41	11.41	11.41	11.41	11.41	11.41
11.42	11.42	11.42	11.42	11.42	11.42	11.42	11.42	11.42
11.43	11.43	11.43	11.43	11.43	11.43	11.43	11.43	11.43
11.44	11.44	11.44	11.44	11.44	11.44	11.44	11.44	11.44
11.45	11.45	11.45	11.45	11.45	11.45	11.45	11.45	11.45
11.46	11.46	11.46	11.46	11.46	11.46	11.46	11.46	11.46
11.47	11.47	11.47	11.47	11.47	11.47	11.47	11.47	11.47
11.48	11.48	11.48	11.48	11.48	11.48	11.48	11.48	11.48
11.49	11.49	11.49	11.49	11.49	11.49	11.49	11.49	11.49

11.5	11.5	11.5	11.5	11.5	11.5	11.5	11.5	11.5
11.51	11.51	11.51	11.51	11.51	11.51	11.51	11.51	11.51
11.52	11.52	11.52	11.52	11.52	11.52	11.52	11.52	11.52
11.53	11.53	11.53	11.53	11.53	11.53	11.53	11.53	11.53
11.54	11.54	11.54	11.54	11.54	11.54	11.54	11.54	11.54
11.55	11.55	11.55	11.55	11.55	11.55	11.55	11.55	11.55
11.56	11.56	11.56	11.56	11.56	11.56	11.56	11.56	11.56
11.57	11.57	11.57	11.57	11.57	11.57	11.57	11.57	11.57
11.58	11.58	11.58	11.58	11.58	11.58	11.58	11.58	11.58
11.59	11.59	11.59	11.59	11.59	11.59	11.59	11.59	11.59
11.6	11.6	11.6	11.6	11.6	11.6	11.6	11.6	11.6
11.61	11.61	11.61	11.61	11.61	11.61	11.61	11.61	11.61
11.62	11.62	11.62	11.62	11.62	11.62	11.62	11.62	11.62
11.63	11.63	11.63	11.63	11.63	11.63	11.63	11.63	11.63
11.64	11.64	11.64	11.64	11.64	11.64	11.64	11.64	11.64
11.65	11.65	11.65	11.65	11.65	11.65	11.65	11.65	11.65
11.66	11.66	11.66	11.66	11.66	11.66	11.66	11.66	11.66
11.67	11.67	11.67	11.67	11.67	11.67	11.67	11.67	11.67
11.68	11.68	11.68	11.68	11.68	11.68	11.68	11.68	11.68
11.69	11.69	11.69	11.69	11.69	11.69	11.69	11.69	11.69
11.7	11.7	11.7	11.7	11.7	11.7	11.7	11.7	11.7
11.71	11.71	11.71	11.71	11.71	11.71	11.71	11.71	11.71
11.72	11.72	11.72	11.72	11.72	11.72	11.72	11.72	11.72
11.73	11.73	11.73	11.73	11.73	11.73	11.73	11.73	11.73
11.74	11.74	11.74	11.74	11.74	11.74	11.74	11.74	11.74
11.75	11.75	11.75	11.75	11.75	11.75	11.75	11.75	11.75
11.76	11.76	11.76	11.76	11.76	11.76	11.76	11.76	11.76
11.77	11.77	11.77	11.77	11.77	11.77	11.77	11.77	11.77
11.78	11.78	11.78	11.78	11.78	11.78	11.78	11.78	11.78
11.79	11.79	11.79	11.79	11.79	11.79	11.79	11.79	11.79
11.8	11.8	11.8	11.8	11.8	11.8	11.8	11.8	11.8
11.81	11.81	11.81	11.81	11.81	11.81	11.81	11.81	11.81
11.82	11.82	11.82	11.82	11.82	11.82	11.82	11.82	11.82
11.83	11.83	11.83	11.83	11.83	11.83	11.83	11.83	11.83
11.84	11.84	11.84	11.84	11.84	11.84	11.84	11.84	11.84
11.85	11.85	11.85	11.85	11.85	11.85	11.85	11.85	11.85
11.86	11.86	11.86	11.86	11.86	11.86	11.86	11.86	11.86
11.87	11.87	11.87	11.87	11.87	11.87	11.87	11.87	11.87
11.88	11.88	11.88	11.88	11.88	11.88	11.88	11.88	11.88
11.89	11.89	11.89	11.89	11.89	11.89	11.89	11.89	11.89
11.9	11.9	11.9	11.9	11.9	11.9	11.9	11.9	11.9
11.91	11.91	11.91	11.91	11.91	11.91	11.91	11.91	11.91
11.92	11.92	11.92	11.92	11.92	11.92	11.92	11.92	11.92
11.93	11.93	11.93	11.93	11.93	11.93	11.93	11.93	11.93
11.94	11.94	11.94	11.94	11.94	11.94	11.94	11.94	11.94
11.95	11.95	11.95	11.95	11.95	11.95	11.95	11.95	11.95
11.96	11.96	11.96	11.96	11.96	11.96	11.96	11.96	11.96
11.97	11.97	11.97	11.97	11.97	11.97	11.97	11.97	11.97
11.98	11.98	11.98	11.98	11.98	11.98	11.98	11.98	11.98
11.99	11.99	11.99	11.99	11.99	11.99	11.99	11.99	11.99

12	12	12	12	12	12	12	12	12

-12	-12	-12	-12	-12	-12	-12	-12	-12
-11.99	-11.99	-11.99	-11.99	-11.99	-11.99	-11.99	-11.99	-11.99
-11.98	-11.98	-11.98	-11.98	-11.98	-11.98	-11.98	-11.98	-11.98
-11.97	-11.97	-11.97	-11.97	-11.97	-11.97	-11.97	-11.97	-11.97
-11.96	-11.96	-11.96	-11.96	-11.96	-11.96	-11.96	-11.96	-11.96
-11.95	-11.95	-11.95	-11.95	-11.95	-11.95	-11.95	-11.95	-11.95
-11.94	-11.94	-11.94	-11.94	-11.94	-11.94	-11.94	-11.94	-11.94
-11.93	-11.93	-11.93	-11.93	-11.93	-11.93	-11.93	-11.93	-11.93
-11.92	-11.92	-11.92	-11.92	-11.92	-11.92	-11.92	-11.92	-11.92
-11.91	-11.91	-11.91	-11.91	-11.91	-11.91	-11.91	-11.91	-11.91
-11.9	-11.9	-11.9	-11.9	-11.9	-11.9	-11.9	-11.9	-11.9
-11.89	-11.89	-11.89	-11.89	-11.89	-11.89	-11.89	-11.89	-11.89
-11.88	-11.88	-11.88	-11.88	-11.88	-11.88	-11.88	-11.88	-11.88
-11.87	-11.87	-11.87	-11.87	-11.87	-11.87	-11.87	-11.87	-11.87
-11.86	-11.86	-11.86	-11.86	-11.86	-11.86	-11.86	-11.86	-11.86
-11.85	-11.85	-11.85	-11.85	-11.85	-11.85	-11.85	-11.85	-11.85
-11.84	-11.84	-11.84	-11.84	-11.84	-11.84	-11.84	-11.84	-11.84
-11.83	-11.83	-11.83	-11.83	-11.83	-11.83	-11.83	-11.83	-11.83
-11.82	-11.82	-11.82	-11.82	-11.82	-11.82	-11.82	-11.82	-11.82
-11.81	-11.81	-11.81	-11.81	-11.81	-11.81	-11.81	-11.81	-11.81
-11.8	-11.8	-11.8	-11.8	-11.8	-11.8	-11.8	-11.8	-11.8
-11.79	-11.79	-11.79	-11.79	-11.79	-11.79	-11.79	-11.79	-11.79
-11.78	-11.78	-11.78	-11.78	-11.78	-11.78	-11.78	-11.78	-11.78
-11.77	-11.77	-11.77	-11.77	-11.77	-11.77	-11.77	-11.77	-11.77
-11.76	-11.76	-11.76	-11.76	-11.76	-11.76	-11.76	-11.76	-11.76
-11.75	-11.75	-11.75	-11.75	-11.75	-11.75	-11.75	-11.75	-11.75
-11.74	-11.74	-11.74	-11.74	-11.74	-11.74	-11.74	-11.74	-11.74
-11.73	-11.73	-11.73	-11.73	-11.73	-11.73	-11.73	-11.73	-11.73
-11.72	-11.72	-11.72	-11.72	-11.72	-11.72	-11.72	-11.72	-11.72
-11.71	-11.71	-11.71	-11.71	-11.71	-11.71	-11.71	-11.71	-11.71
-11.7	-11.7	-11.7	-11.7	-11.7	-11.7	-11.7	-11.7	-11.7
-11.69	-11.69	-11.69	-11.69	-11.69	-11.69	-11.69	-11.69	-11.69
-11.68	-11.68	-11.68	-11.68	-11.68	-11.68	-11.68	-11.68	-11.68
-11.67	-11.67	-11.67	-11.67	-11.67	-11.67	-11.67	-11.67	-11.67
-11.66	-11.66	-11.66	-11.66	-11.66	-11.66	-11.66	-11.66	-11.66
-11.65	-11.65	-11.65	-11.65	-11.65	-11.65	-11.65	-11.65	-11.65
-11.64	-11.64	-11.64	-11.64	-11.64	-11.64	-11.64	-11.64	-11.64
-11.63	-11.63	-11.63	-11.63	-11.63	-11.63	-11.63	-11.63	-11.63
-11.62	-11.62	-11.62	-11.62	-11.62	-11.62	-11.62	-11.62	-11.62
-11.61	-11.61	-11.61	-11.61	-11.61	-11.61	-11.61	-11.61	-11.61
-11.6	-11.6	-11.6	-11.6	-11.6	-11.6	-11.6	-11.6	-11.6
-11.59	-11.59	-11.59	-11.59	-11.59	-11.59	-11.59	-11.59	-11.59
-11.58	-11.58	-11.58	-11.58	-11.58	-11.58	-11.58	-11.58	-11.58
-11.57	-11.57	-11.57	-11.57	-11.57	-11.57	-11.57	-11.57	-11.57
-11.56	-11.56	-11.56	-11.56	-11.56	-11.56	-11.56	-11.56	-11.56
-11.55	-11.55	-11.55	-11.55	-11.55	-11.55	-11.55	-11.55	-11.55
-11.54	-11.54	-11.54	-11.54	-11.54	-11.54	-11.54	-11.54	-11.54
-11.53	-11.53	-11.53	-11.53	-11.53	-11.53	-11.53	-11.53	-11.53
-11.52	-11.52	-11.52	-11.52	-11.52	-11.52	-11.52	-11.52	-11.52
-11.51	-11.51	-11.51	-11.51	-11.51	-11.51	-11.51	-11.51	-11.51

-11.5	-11.5	-11.5	-11.5	-11.5	-11.5	-11.5	-11.5	-11.5
-11.49	-11.49	-11.49	-11.49	-11.49	-11.49	-11.49	-11.49	-11.49
-11.48	-11.48	-11.48	-11.48	-11.48	-11.48	-11.48	-11.48	-11.48
-11.47	-11.47	-11.47	-11.47	-11.47	-11.47	-11.47	-11.47	-11.47
-11.46	-11.46	-11.46	-11.46	-11.46	-11.46	-11.46	-11.46	-11.46
-11.45	-11.45	-11.45	-11.45	-11.45	-11.45	-11.45	-11.45	-11.45
-11.44	-11.44	-11.44	-11.44	-11.44	-11.44	-11.44	-11.44	-11.44
-11.43	-11.43	-11.43	-11.43	-11.43	-11.43	-11.43	-11.43	-11.43
-11.42	-11.42	-11.42	-11.42	-11.42	-11.42	-11.42	-11.42	-11.42
-11.41	-11.41	-11.41	-11.41	-11.41	-11.41	-11.41	-11.41	-11.41
-11.4	-11.4	-11.4	-11.4	-11.4	-11.4	-11.4	-11.4	-11.4
-11.39	-11.39	-11.39	-11.39	-11.39	-11.39	-11.39	-11.39	-11.39
-11.38	-11.38	-11.38	-11.38	-11.38	-11.38	-11.38	-11.38	-11.38
-11.37	-11.37	-11.37	-11.37	-11.37	-11.37	-11.37	-11.37	-11.37
-11.36	-11.36	-11.36	-11.36	-11.36	-11.36	-11.36	-11.36	-11.36
-11.35	-11.35	-11.35	-11.35	-11.35	-11.35	-11.35	-11.35	-11.35
-11.34	-11.34	-11.34	-11.34	-11.34	-11.34	-11.34	-11.34	-11.34
-11.33	-11.33	-11.33	-11.33	-11.33	-11.33	-11.33	-11.33	-11.33
-11.32	-11.32	-11.32	-11.32	-11.32	-11.32	-11.32	-11.32	-11.32
-11.31	-11.31	-11.31	-11.31	-11.31	-11.31	-11.31	-11.31	-11.31
-11.3	-11.3	-11.3	-11.3	-11.3	-11.3	-11.3	-11.3	-11.3
-11.29	-11.29	-11.29	-11.29	-11.29	-11.29	-11.29	-11.29	-11.29
-11.28	-11.28	-11.28	-11.28	-11.28	-11.28	-11.28	-11.28	-11.28
-11.27	-11.27	-11.27	-11.27	-11.27	-11.27	-11.27	-11.27	-11.27
-11.26	-11.26	-11.26	-11.26	-11.26	-11.26	-11.26	-11.26	-11.26
-11.25	-11.25	-11.25	-11.25	-11.25	-11.25	-11.25	-11.25	-11.25
-11.24	-11.24	-11.24	-11.24	-11.24	-11.24	-11.24	-11.24	-11.24
-11.23	-11.23	-11.23	-11.23	-11.23	-11.23	-11.23	-11.23	-11.23
-11.22	-11.22	-11.22	-11.22	-11.22	-11.22	-11.22	-11.22	-11.22
-11.21	-11.21	-11.21	-11.21	-11.21	-11.21	-11.21	-11.21	-11.21
-11.2	-11.2	-11.2	-11.2	-11.2	-11.2	-11.2	-11.2	-11.2
-11.19	-11.19	-11.19	-11.19	-11.19	-11.19	-11.19	-11.19	-11.19
-11.18	-11.18	-11.18	-11.18	-11.18	-11.18	-11.18	-11.18	-11.18
-11.17	-11.17	-11.17	-11.17	-11.17	-11.17	-11.17	-11.17	-11.17
-11.16	-11.16	-11.16	-11.16	-11.16	-11.16	-11.16	-11.16	-11.16
-11.15	-11.15	-11.15	-11.15	-11.15	-11.15	-11.15	-11.15	-11.15
-11.14	-11.14	-11.14	-11.14	-11.14	-11.14	-11.14	-11.14	-11.14
-11.13	-11.13	-11.13	-11.13	-11.13	-11.13	-11.13	-11.13	-11.13
-11.12	-11.12	-11.12	-11.12	-11.12	-11.12	-11.12	-11.12	-11.12
-11.11	-11.11	-11.11	-11.11	-11.11	-11.11	-11.11	-11.11	-11.11
-11.1	-11.1	-11.1	-11.1	-11.1	-11.1	-11.1	-11.1	-11.1
-11.09	-11.09	-11.09	-11.09	-11.09	-11.09	-11.09	-11.09	-11.09
-11.08	-11.08	-11.08	-11.08	-11.08	-11.08	-11.08	-11.08	-11.08
-11.07	-11.07	-11.07	-11.07	-11.07	-11.07	-11.07	-11.07	-11.07
-11.06	-11.06	-11.06	-11.06	-11.06	-11.06	-11.06	-11.06	-11.06
-11.05	-11.05	-11.05	-11.05	-11.05	-11.05	-11.05	-11.05	-11.05
-11.04	-11.04	-11.04	-11.04	-11.04	-11.04	-11.04	-11.04	-11.04
-11.03	-11.03	-11.03	-11.03	-11.03	-11.03	-11.03	-11.03	-11.03
-11.02	-11.02	-11.02	-11.02	-11.02	-11.02	-11.02	-11.02	-11.02
-11.01	-11.01	-11.01	-11.01	-11.01	-11.01	-11.01	-11.01	-11.01

-11	-11	-11	-11	-11	-11	-11	-11	-11
-10.99	-10.99	-10.99	-10.99	-10.99	-10.99	-10.99	-10.99	-10.99
-10.98	-10.98	-10.98	-10.98	-10.98	-10.98	-10.98	-10.98	-10.98
-10.97	-10.97	-10.97	-10.97	-10.97	-10.97	-10.97	-10.97	-10.97
-10.96	-10.96	-10.96	-10.96	-10.96	-10.96	-10.96	-10.96	-10.96
-10.95	-10.95	-10.95	-10.95	-10.95	-10.95	-10.95	-10.95	-10.95
-10.94	-10.94	-10.94	-10.94	-10.94	-10.94	-10.94	-10.94	-10.94
-10.93	-10.93	-10.93	-10.93	-10.93	-10.93	-10.93	-10.93	-10.93
-10.92	-10.92	-10.92	-10.92	-10.92	-10.92	-10.92	-10.92	-10.92
-10.91	-10.91	-10.91	-10.91	-10.91	-10.91	-10.91	-10.91	-10.91
-10.9	-10.9	-10.9	-10.9	-10.9	-10.9	-10.9	-10.9	-10.9
-10.89	-10.89	-10.89	-10.89	-10.89	-10.89	-10.89	-10.89	-10.89
-10.88	-10.88	-10.88	-10.88	-10.88	-10.88	-10.88	-10.88	-10.88
-10.87	-10.87	-10.87	-10.87	-10.87	-10.87	-10.87	-10.87	-10.87
-10.86	-10.86	-10.86	-10.86	-10.86	-10.86	-10.86	-10.86	-10.86
-10.85	-10.85	-10.85	-10.85	-10.85	-10.85	-10.85	-10.85	-10.85
-10.84	-10.84	-10.84	-10.84	-10.84	-10.84	-10.84	-10.84	-10.84
-10.83	-10.83	-10.83	-10.83	-10.83	-10.83	-10.83	-10.83	-10.83
-10.82	-10.82	-10.82	-10.82	-10.82	-10.82	-10.82	-10.82	-10.82
-10.81	-10.81	-10.81	-10.81	-10.81	-10.81	-10.81	-10.81	-10.81
-10.8	-10.8	-10.8	-10.8	-10.8	-10.8	-10.8	-10.8	-10.8
-10.79	-10.79	-10.79	-10.79	-10.79	-10.79	-10.79	-10.79	-10.79
-10.78	-10.78	-10.78	-10.78	-10.78	-10.78	-10.78	-10.78	-10.78
-10.77	-10.77	-10.77	-10.77	-10.77	-10.77	-10.77	-10.77	-10.77
-10.76	-10.76	-10.76	-10.76	-10.76	-10.76	-10.76	-10.76	-10.76
-10.75	-10.75	-10.75	-10.75	-10.75	-10.75	-10.75	-10.75	-10.75
-10.74	-10.74	-10.74	-10.74	-10.74	-10.74	-10.74	-10.74	-10.74
-10.73	-10.73	-10.73	-10.73	-10.73	-10.73	-10.73	-10.73	-10.73
-10.72	-10.72	-10.72	-10.72	-10.72	-10.72	-10.72	-10.72	-10.72
-10.71	-10.71	-10.71	-10.71	-10.71	-10.71	-10.71	-10.71	-10.71
-10.7	-10.7	-10.7	-10.7	-10.7	-10.7	-10.7	-10.7	-10.7
-10.69	-10.69	-10.69	-10.69	-10.69	-10.69	-10.69	-10.69	-10.69
-10.68	-10.68	-10.68	-10.68	-10.68	-10.68	-10.68	-10.68	-10.68
-10.67	-10.67	-10.67	-10.67	-10.67	-10.67	-10.67	-10.67	-10.67
-10.66	-10.66	-10.66	-10.66	-10.66	-10.66	-10.66	-10.66	-10.66
-10.65	-10.65	-10.65	-10.65	-10.65	-10.65	-10.65	-10.65	-10.65
-10.64	-10.64	-10.64	-10.64	-10.64	-10.64	-10.64	-10.64	-10.64
-10.63	-10.63	-10.63	-10.63	-10.63	-10.63	-10.63	-10.63	-10.63
-10.62	-10.62	-10.62	-10.62	-10.62	-10.62	-10.62	-10.62	-10.62
-10.61	-10.61	-10.61	-10.61	-10.61	-10.61	-10.61	-10.61	-10.61
-10.6	-10.6	-10.6	-10.6	-10.6	-10.6	-10.6	-10.6	-10.6
-10.59	-10.59	-10.59	-10.59	-10.59	-10.59	-10.59	-10.59	-10.59
-10.58	-10.58	-10.58	-10.58	-10.58	-10.58	-10.58	-10.58	-10.58
-10.57	-10.57	-10.57	-10.57	-10.57	-10.57	-10.57	-10.57	-10.57
-10.56	-10.56	-10.56	-10.56	-10.56	-10.56	-10.56	-10.56	-10.56
-10.55	-10.55	-10.55	-10.55	-10.55	-10.55	-10.55	-10.55	-10.55
-10.54	-10.54	-10.54	-10.54	-10.54	-10.54	-10.54	-10.54	-10.54
-10.53	-10.53	-10.53	-10.53	-10.53	-10.53	-10.53	-10.53	-10.53
-10.52	-10.52	-10.52	-10.52	-10.52	-10.52	-10.52	-10.52	-10.52
-10.51	-10.51	-10.51	-10.51	-10.51	-10.51	-10.51	-10.51	-10.51

-10.5	-10.5	-10.5	-10.5	-10.5	-10.5	-10.5	-10.5	-10.5
-10.49	-10.49	-10.49	-10.49	-10.49	-10.49	-10.49	-10.49	-10.49
-10.48	-10.48	-10.48	-10.48	-10.48	-10.48	-10.48	-10.48	-10.48
-10.47	-10.47	-10.47	-10.47	-10.47	-10.47	-10.47	-10.47	-10.47
-10.46	-10.46	-10.46	-10.46	-10.46	-10.46	-10.46	-10.46	-10.46
-10.45	-10.45	-10.45	-10.45	-10.45	-10.45	-10.45	-10.45	-10.45
-10.44	-10.44	-10.44	-10.44	-10.44	-10.44	-10.44	-10.44	-10.44
-10.43	-10.43	-10.43	-10.43	-10.43	-10.43	-10.43	-10.43	-10.43
-10.42	-10.42	-10.42	-10.42	-10.42	-10.42	-10.42	-10.42	-10.42
-10.41	-10.41	-10.41	-10.41	-10.41	-10.41	-10.41	-10.41	-10.41
-10.4	-10.4	-10.4	-10.4	-10.4	-10.4	-10.4	-10.4	-10.4
-10.39	-10.39	-10.39	-10.39	-10.39	-10.39	-10.39	-10.39	-10.39
-10.38	-10.38	-10.38	-10.38	-10.38	-10.38	-10.38	-10.38	-10.38
-10.37	-10.37	-10.37	-10.37	-10.37	-10.37	-10.37	-10.37	-10.37
-10.36	-10.36	-10.36	-10.36	-10.36	-10.36	-10.36	-10.36	-10.36
-10.35	-10.35	-10.35	-10.35	-10.35	-10.35	-10.35	-10.35	-10.35
-10.34	-10.34	-10.34	-10.34	-10.34	-10.34	-10.34	-10.34	-10.34
-10.33	-10.33	-10.33	-10.33	-10.33	-10.33	-10.33	-10.33	-10.33
-10.32	-10.32	-10.32	-10.32	-10.32	-10.32	-10.32	-10.32	-10.32
-10.31	-10.31	-10.31	-10.31	-10.31	-10.31	-10.31	-10.31	-10.31
-10.3	-10.3	-10.3	-10.3	-10.3	-10.3	-10.3	-10.3	-10.3
-10.29	-10.29	-10.29	-10.29	-10.29	-10.29	-10.29	-10.29	-10.29
-10.28	-10.28	-10.28	-10.28	-10.28	-10.28	-10.28	-10.28	-10.28
-10.27	-10.27	-10.27	-10.27	-10.27	-10.27	-10.27	-10.27	-10.27
-10.26	-10.26	-10.26	-10.26	-10.26	-10.26	-10.26	-10.26	-10.26
-10.25	-10.25	-10.25	-10.25	-10.25	-10.25	-10.25	-10.25	-10.25
-10.24	-10.24	-10.24	-10.24	-10.24	-10.24	-10.24	-10.24	-10.24
-10.23	-10.23	-10.23	-10.23	-10.23	-10.23	-10.23	-10.23	-10.23
-10.22	-10.22	-10.22	-10.22	-10.22	-10.22	-10.22	-10.22	-10.22
-10.21	-10.21	-10.21	-10.21	-10.21	-10.21	-10.21	-10.21	-10.21
-10.2	-10.2	-10.2	-10.2	-10.2	-10.2	-10.2	-10.2	-10.2
-10.19	-10.19	-10.19	-10.19	-10.19	-10.19	-10.19	-10.19	-10.19
-10.18	-10.18	-10.18	-10.18	-10.18	-10.18	-10.18	-10.18	-10.18
-10.17	-10.17	-10.17	-10.17	-10.17	-10.17	-10.17	-10.17	-10.17
-10.16	-10.16	-10.16	-10.16	-10.16	-10.16	-10.16	-10.16	-10.16
-10.15	-10.15	-10.15	-10.15	-10.15	-10.15	-10.15	-10.15	-10.15
-10.14	-10.14	-10.14	-10.14	-10.14	-10.14	-10.14	-10.14	-10.14
-10.13	-10.13	-10.13	-10.13	-10.13	-10.13	-10.13	-10.13	-10.13
-10.12	-10.12	-10.12	-10.12	-10.12	-10.12	-10.12	-10.12	-10.12
-10.11	-10.11	-10.11	-10.11	-10.11	-10.11	-10.11	-10.11	-10.11
-10.1	-10.1	-10.1	-10.1	-10.1	-10.1	-10.1	-10.1	-10.1
-10.09	-10.09	-10.09	-10.09	-10.09	-10.09	-10.09	-10.09	-10.09
-10.08	-10.08	-10.08	-10.08	-10.08	-10.08	-10.08	-10.08	-10.08
-10.07	-10.07	-10.07	-10.07	-10.07	-10.07	-10.07	-10.07	-10.07
-10.06	-10.06	-10.06	-10.06	-10.06	-10.06	-10.06	-10.06	-10.06
-10.05	-10.05	-10.05	-10.05	-10.05	-10.05	-10.05	-10.05	-10.05
-10.04	-10.04	-10.04	-10.04	-10.04	-10.04	-10.04	-10.04	-10.04
-10.03	-10.03	-10.03	-10.03	-10.03	-10.03	-10.03	-10.03	-10.03
-10.02	-10.02	-10.02	-10.02	-10.02	-10.02	-10.02	-10.02	-10.02
-10.01	-10.01	-10.01	-10.01	-10.01	-10.01	-10.01	-10.01	-10.01

-10	-10	-10	-10	-10	-10	-10	-10	-10
-9.99	-9.99	-9.99	-9.99	-9.99	-9.99	-9.99	-9.99	-9.99
-9.98	-9.98	-9.98	-9.98	-9.98	-9.98	-9.98	-9.98	-9.98
-9.97	-9.97	-9.97	-9.97	-9.97	-9.97	-9.97	-9.97	-9.97
-9.96	-9.96	-9.96	-9.96	-9.96	-9.96	-9.96	-9.96	-9.96
-9.95	-9.95	-9.95	-9.95	-9.95	-9.95	-9.95	-9.95	-9.95
-9.94	-9.94	-9.94	-9.94	-9.94	-9.94	-9.94	-9.94	-9.94
-9.93	-9.93	-9.93	-9.93	-9.93	-9.93	-9.93	-9.93	-9.93
-9.92	-9.92	-9.92	-9.92	-9.92	-9.92	-9.92	-9.92	-9.92
-9.91	-9.91	-9.91	-9.91	-9.91	-9.91	-9.91	-9.91	-9.91
-9.9	-9.9	-9.9	-9.9	-9.9	-9.9	-9.9	-9.9	-9.9
-9.89	-9.89	-9.89	-9.89	-9.89	-9.89	-9.89	-9.89	-9.89
-9.88	-9.88	-9.88	-9.88	-9.88	-9.88	-9.88	-9.88	-9.88
-9.87	-9.87	-9.87	-9.87	-9.87	-9.87	-9.87	-9.87	-9.87
-9.86	-9.86	-9.86	-9.86	-9.86	-9.86	-9.86	-9.86	-9.86
-9.85	-9.85	-9.85	-9.85	-9.85	-9.85	-9.85	-9.85	-9.85
-9.84	-9.84	-9.84	-9.84	-9.84	-9.84	-9.84	-9.84	-9.84
-9.83	-9.83	-9.83	-9.83	-9.83	-9.83	-9.83	-9.83	-9.83
-9.82	-9.82	-9.82	-9.82	-9.82	-9.82	-9.82	-9.82	-9.82
-9.81	-9.81	-9.81	-9.81	-9.81	-9.81	-9.81	-9.81	-9.81
-9.8	-9.8	-9.8	-9.8	-9.8	-9.8	-9.8	-9.8	-9.8
-9.79	-9.79	-9.79	-9.79	-9.79	-9.79	-9.79	-9.79	-9.79
-9.78	-9.78	-9.78	-9.78	-9.78	-9.78	-9.78	-9.78	-9.78
-9.77	-9.77	-9.77	-9.77	-9.77	-9.77	-9.77	-9.77	-9.77
-9.76	-9.76	-9.76	-9.76	-9.76	-9.76	-9.76	-9.76	-9.76
-9.75	-9.75	-9.75	-9.75	-9.75	-9.75	-9.75	-9.75	-9.75
-9.74	-9.74	-9.74	-9.74	-9.74	-9.74	-9.74	-9.74	-9.74
-9.73	-9.73	-9.73	-9.73	-9.73	-9.73	-9.73	-9.73	-9.73
-9.72	-9.72	-9.72	-9.72	-9.72	-9.72	-9.72	-9.72	-9.72
-9.71	-9.71	-9.71	-9.71	-9.71	-9.71	-9.71	-9.71	-9.71
-9.7	-9.7	-9.7	-9.7	-9.7	-9.7	-9.7	-9.7	-9.7
-9.69	-9.69	-9.69	-9.69	-9.69	-9.69	-9.69	-9.69	-9.69
-9.68	-9.68	-9.68	-9.68	-9.68	-9.68	-9.68	-9.68	-9.68
-9.67	-9.67	-9.67	-9.67	-9.67	-9.67	-9.67	-9.67	-9.67
-9.66	-9.66	-9.66	-9.66	-9.66	-9.66	-9.66	-9.66	-9.66
-9.65	-9.65	-9.65	-9.65	-9.65	-9.65	-9.65	-9.65	-9.65
-9.64	-9.64	-9.64	-9.64	-9.64	-9.64	-9.64	-9.64	-9.64
-9.63	-9.63	-9.63	-9.63	-9.63	-9.63	-9.63	-9.63	-9.63
-9.62	-9.62	-9.62	-9.62	-9.62	-9.62	-9.62	-9.62	-9.62
-9.61	-9.61	-9.61	-9.61	-9.61	-9.61	-9.61	-9.61	-9.61
-9.6	-9.6	-9.6	-9.6	-9.6	-9.6	-9.6	-9.6	-9.6
-9.59	-9.59	-9.59	-9.59	-9.59	-9.59	-9.59	-9.59	-9.59
-9.58	-9.58	-9.58	-9.58	-9.58	-9.58	-9.58	-9.58	-9.58
-9.57	-9.57	-9.57	-9.57	-9.57	-9.57	-9.57	-9.57	-9.57
-9.56	-9.56	-9.56	-9.56	-9.56	-9.56	-9.56	-9.56	-9.56
-9.55	-9.55	-9.55	-9.55	-9.55	-9.55	-9.55	-9.55	-9.55
-9.54	-9.54	-9.54	-9.54	-9.54	-9.54	-9.54	-9.54	-9.54
-9.53	-9.53	-9.53	-9.53	-9.53	-9.53	-9.53	-9.53	-9.53
-9.52	-9.52	-9.52	-9.52	-9.52	-9.52	-9.52	-9.52	-9.52
-9.51	-9.51	-9.51	-9.51	-9.51	-9.51	-9.51	-9.51	-9.51

-9.5	-9.5	-9.5	-9.5	-9.5	-9.5	-9.5	-9.5	-9.5
-9.49	-9.49	-9.49	-9.49	-9.49	-9.49	-9.49	-9.49	-9.49
-9.48	-9.48	-9.48	-9.48	-9.48	-9.48	-9.48	-9.48	-9.48
-9.47	-9.47	-9.47	-9.47	-9.47	-9.47	-9.47	-9.47	-9.47
-9.46	-9.46	-9.46	-9.46	-9.46	-9.46	-9.46	-9.46	-9.46
-9.45	-9.45	-9.45	-9.45	-9.45	-9.45	-9.45	-9.45	-9.45
-9.44	-9.44	-9.44	-9.44	-9.44	-9.44	-9.44	-9.44	-9.44
-9.43	-9.43	-9.43	-9.43	-9.43	-9.43	-9.43	-9.43	-9.43
-9.42	-9.42	-9.42	-9.42	-9.42	-9.42	-9.42	-9.42	-9.42
-9.41	-9.41	-9.41	-9.41	-9.41	-9.41	-9.41	-9.41	-9.41
-9.4	-9.4	-9.4	-9.4	-9.4	-9.4	-9.4	-9.4	-9.4
-9.39	-9.39	-9.39	-9.39	-9.39	-9.39	-9.39	-9.39	-9.39
-9.38	-9.38	-9.38	-9.38	-9.38	-9.38	-9.38	-9.38	-9.38
-9.37	-9.37	-9.37	-9.37	-9.37	-9.37	-9.37	-9.37	-9.37
-9.36	-9.36	-9.36	-9.36	-9.36	-9.36	-9.36	-9.36	-9.36
-9.35	-9.35	-9.35	-9.35	-9.35	-9.35	-9.35	-9.35	-9.35
-9.34	-9.34	-9.34	-9.34	-9.34	-9.34	-9.34	-9.34	-9.34
-9.33	-9.33	-9.33	-9.33	-9.33	-9.33	-9.33	-9.33	-9.33
-9.32	-9.32	-9.32	-9.32	-9.32	-9.32	-9.32	-9.32	-9.32
-9.31	-9.31	-9.31	-9.31	-9.31	-9.31	-9.31	-9.31	-9.31
-9.3	-9.3	-9.3	-9.3	-9.3	-9.3	-9.3	-9.3	-9.3
-9.29	-9.29	-9.29	-9.29	-9.29	-9.29	-9.29	-9.29	-9.29
-9.28	-9.28	-9.28	-9.28	-9.28	-9.28	-9.28	-9.28	-9.28
-9.27	-9.27	-9.27	-9.27	-9.27	-9.27	-9.27	-9.27	-9.27
-9.26	-9.26	-9.26	-9.26	-9.26	-9.26	-9.26	-9.26	-9.26
-9.25	-9.25	-9.25	-9.25	-9.25	-9.25	-9.25	-9.25	-9.25
-9.24	-9.24	-9.24	-9.24	-9.24	-9.24	-9.24	-9.24	-9.24
-9.23	-9.23	-9.23	-9.23	-9.23	-9.23	-9.23	-9.23	-9.23
-9.22	-9.22	-9.22	-9.22	-9.22	-9.22	-9.22	-9.22	-9.22
-9.21	-9.21	-9.21	-9.21	-9.21	-9.21	-9.21	-9.21	-9.21
-9.2	-9.2	-9.2	-9.2	-9.2	-9.2	-9.2	-9.2	-9.2
-9.19	-9.19	-9.19	-9.19	-9.19	-9.19	-9.19	-9.19	-9.19
-9.18	-9.18	-9.18	-9.18	-9.18	-9.18	-9.18	-9.18	-9.18
-9.17	-9.17	-9.17	-9.17	-9.17	-9.17	-9.17	-9.17	-9.17
-9.16	-9.16	-9.16	-9.16	-9.16	-9.16	-9.16	-9.16	-9.16
-9.15	-9.15	-9.15	-9.15	-9.15	-9.15	-9.15	-9.15	-9.15
-9.14	-9.14	-9.14	-9.14	-9.14	-9.14	-9.14	-9.14	-9.14
-9.13	-9.13	-9.13	-9.13	-9.13	-9.13	-9.13	-9.13	-9.13
-9.12	-9.12	-9.12	-9.12	-9.12	-9.12	-9.12	-9.12	-9.12
-9.11	-9.11	-9.11	-9.11	-9.11	-9.11	-9.11	-9.11	-9.11
-9.1	-9.1	-9.1	-9.1	-9.1	-9.1	-9.1	-9.1	-9.1
-9.09	-9.09	-9.09	-9.09	-9.09	-9.09	-9.09	-9.09	-9.09
-9.08	-9.08	-9.08	-9.08	-9.08	-9.08	-9.08	-9.08	-9.08
-9.07	-9.07	-9.07	-9.07	-9.07	-9.07	-9.07	-9.07	-9.07
-9.06	-9.06	-9.06	-9.06	-9.06	-9.06	-9.06	-9.06	-9.06
-9.05	-9.05	-9.05	-9.05	-9.05	-9.05	-9.05	-9.05	-9.05
-9.04	-9.04	-9.04	-9.04	-9.04	-9.04	-9.04	-9.04	-9.04
-9.03	-9.03	-9.03	-9.03	-9.03	-9.03	-9.03	-9.03	-9.03
-9.02	-9.02	-9.02	-9.02	-9.02	-9.02	-9.02	-9.02	-9.02
-9.01	-9.01	-9.01	-9.01	-9.01	-9.01	-9.01	-9.01	-9.01

-9	-9	-9	-9	-9	-9	-9	-9	-9
-8.99	-8.99	-8.99	-8.99	-8.99	-8.99	-8.99	-8.99	-8.99
-8.98	-8.98	-8.98	-8.98	-8.98	-8.98	-8.98	-8.98	-8.98
-8.97	-8.97	-8.97	-8.97	-8.97	-8.97	-8.97	-8.97	-8.97
-8.96	-8.96	-8.96	-8.96	-8.96	-8.96	-8.96	-8.96	-8.96
-8.95	-8.95	-8.95	-8.95	-8.95	-8.95	-8.95	-8.95	-8.95
-8.94	-8.94	-8.94	-8.94	-8.94	-8.94	-8.94	-8.94	-8.94
-8.93	-8.93	-8.93	-8.93	-8.93	-8.93	-8.93	-8.93	-8.93
-8.92	-8.92	-8.92	-8.92	-8.92	-8.92	-8.92	-8.92	-8.92
-8.91	-8.91	-8.91	-8.91	-8.91	-8.91	-8.91	-8.91	-8.91
-8.9	-8.9	-8.9	-8.9	-8.9	-8.9	-8.9	-8.9	-8.9
-8.89	-8.89	-8.89	-8.89	-8.89	-8.89	-8.89	-8.89	-8.89
-8.88	-8.88	-8.88	-8.88	-8.88	-8.88	-8.88	-8.88	-8.88
-8.87	-8.87	-8.87	-8.87	-8.87	-8.87	-8.87	-8.87	-8.87
-8.86	-8.86	-8.86	-8.86	-8.86	-8.86	-8.86	-8.86	-8.86
-8.85	-8.85	-8.85	-8.85	-8.85	-8.85	-8.85	-8.85	-8.85
-8.84	-8.84	-8.84	-8.84	-8.84	-8.84	-8.84	-8.84	-8.84
-8.83	-8.83	-8.83	-8.83	-8.83	-8.83	-8.83	-8.83	-8.83
-8.82	-8.82	-8.82	-8.82	-8.82	-8.82	-8.82	-8.82	-8.82
-8.81	-8.81	-8.81	-8.81	-8.81	-8.81	-8.81	-8.81	-8.81
-8.8	-8.8	-8.8	-8.8	-8.8	-8.8	-8.8	-8.8	-8.8
-8.79	-8.79	-8.79	-8.79	-8.79	-8.79	-8.79	-8.79	-8.79
-8.78	-8.78	-8.78	-8.78	-8.78	-8.78	-8.78	-8.78	-8.78
-8.77	-8.77	-8.77	-8.77	-8.77	-8.77	-8.77	-8.77	-8.77
-8.76	-8.76	-8.76	-8.76	-8.76	-8.76	-8.76	-8.76	-8.76
-8.75	-8.75	-8.75	-8.75	-8.75	-8.75	-8.75	-8.75	-8.75
-8.74	-8.74	-8.74	-8.74	-8.74	-8.74	-8.74	-8.74	-8.74
-8.73	-8.73	-8.73	-8.73	-8.73	-8.73	-8.73	-8.73	-8.73
-8.72	-8.72	-8.72	-8.72	-8.72	-8.72	-8.72	-8.72	-8.72
-8.71	-8.71	-8.71	-8.71	-8.71	-8.71	-8.71	-8.71	-8.71
-8.7	-8.7	-8.7	-8.7	-8.7	-8.7	-8.7	-8.7	-8.7
-8.69	-8.69	-8.69	-8.69	-8.69	-8.69	-8.69	-8.69	-8.69
-8.68	-8.68	-8.68	-8.68	-8.68	-8.68	-8.68	-8.68	-8.68
-8.67	-8.67	-8.67	-8.67	-8.67	-8.67	-8.67	-8.67	-8.67
-8.66	-8.66	-8.66	-8.66	-8.66	-8.66	-8.66	-8.66	-8.66
-8.65	-8.65	-8.65	-8.65	-8.65	-8.65	-8.65	-8.65	-8.65
-8.64	-8.64	-8.64	-8.64	-8.64	-8.64	-8.64	-8.64	-8.64
-8.63	-8.63	-8.63	-8.63	-8.63	-8.63	-8.63	-8.63	-8.63
-8.62	-8.62	-8.62	-8.62	-8.62	-8.62	-8.62	-8.62	-8.62
-8.61	-8.61	-8.61	-8.61	-8.61	-8.61	-8.61	-8.61	-8.61
-8.6	-8.6	-8.6	-8.6	-8.6	-8.6	-8.6	-8.6	-8.6
-8.59	-8.59	-8.59	-8.59	-8.59	-8.59	-8.59	-8.59	-8.59
-8.58	-8.58	-8.58	-8.58	-8.58	-8.58	-8.58	-8.58	-8.58
-8.57	-8.57	-8.57	-8.57	-8.57	-8.57	-8.57	-8.57	-8.57
-8.56	-8.56	-8.56	-8.56	-8.56	-8.56	-8.56	-8.56	-8.56
-8.55	-8.55	-8.55	-8.55	-8.55	-8.55	-8.55	-8.55	-8.55
-8.54	-8.54	-8.54	-8.54	-8.54	-8.54	-8.54	-8.54	-8.54
-8.53	-8.53	-8.53	-8.53	-8.53	-8.53	-8.53	-8.53	-8.53
-8.52	-8.52	-8.52	-8.52	-8.52	-8.52	-8.52	-8.52	-8.52
-8.51	-8.51	-8.51	-8.51	-8.51	-8.51	-8.51	-8.51	-8.51

-8.5	-8.5	-8.5	-8.5	-8.5	-8.5	-8.5	-8.5	-8.5
-8.49	-8.49	-8.49	-8.49	-8.49	-8.49	-8.49	-8.49	-8.49
-8.48	-8.48	-8.48	-8.48	-8.48	-8.48	-8.48	-8.48	-8.48
-8.47	-8.47	-8.47	-8.47	-8.47	-8.47	-8.47	-8.47	-8.47
-8.46	-8.46	-8.46	-8.46	-8.46	-8.46	-8.46	-8.46	-8.46
-8.45	-8.45	-8.45	-8.45	-8.45	-8.45	-8.45	-8.45	-8.45
-8.44	-8.44	-8.44	-8.44	-8.44	-8.44	-8.44	-8.44	-8.44
-8.43	-8.43	-8.43	-8.43	-8.43	-8.43	-8.43	-8.43	-8.43
-8.42	-8.42	-8.42	-8.42	-8.42	-8.42	-8.42	-8.42	-8.42
-8.41	-8.41	-8.41	-8.41	-8.41	-8.41	-8.41	-8.41	-8.41
-8.4	-8.4	-8.4	-8.4	-8.4	-8.4	-8.4	-8.4	-8.4
-8.39	-8.39	-8.39	-8.39	-8.39	-8.39	-8.39	-8.39	-8.39
-8.38	-8.38	-8.38	-8.38	-8.38	-8.38	-8.38	-8.38	-8.38
-8.37	-8.37	-8.37	-8.37	-8.37	-8.37	-8.37	-8.37	-8.37
-8.36	-8.36	-8.36	-8.36	-8.36	-8.36	-8.36	-8.36	-8.36
-8.35	-8.35	-8.35	-8.35	-8.35	-8.35	-8.35	-8.35	-8.35
-8.34	-8.34	-8.34	-8.34	-8.34	-8.34	-8.34	-8.34	-8.34
-8.33	-8.33	-8.33	-8.33	-8.33	-8.33	-8.33	-8.33	-8.33
-8.32	-8.32	-8.32	-8.32	-8.32	-8.32	-8.32	-8.32	-8.32
-8.31	-8.31	-8.31	-8.31	-8.31	-8.31	-8.31	-8.31	-8.31
-8.3	-8.3	-8.3	-8.3	-8.3	-8.3	-8.3	-8.3	-8.3
-8.29	-8.29	-8.29	-8.29	-8.29	-8.29	-8.29	-8.29	-8.29
-8.28	-8.28	-8.28	-8.28	-8.28	-8.28	-8.28	-8.28	-8.28
-8.27	-8.27	-8.27	-8.27	-8.27	-8.27	-8.27	-8.27	-8.27
-8.26	-8.26	-8.26	-8.26	-8.26	-8.26	-8.26	-8.26	-8.26
-8.25	-8.25	-8.25	-8.25	-8.25	-8.25	-8.25	-8.25	-8.25
-8.24	-8.24	-8.24	-8.24	-8.24	-8.24	-8.24	-8.24	-8.24
-8.23	-8.23	-8.23	-8.23	-8.23	-8.23	-8.23	-8.23	-8.23
-8.22	-8.22	-8.22	-8.22	-8.22	-8.22	-8.22	-8.22	-8.22
-8.21	-8.21	-8.21	-8.21	-8.21	-8.21	-8.21	-8.21	-8.21
-8.2	-8.2	-8.2	-8.2	-8.2	-8.2	-8.2	-8.2	-8.2
-8.19	-8.19	-8.19	-8.19	-8.19	-8.19	-8.19	-8.19	-8.19
-8.18	-8.18	-8.18	-8.18	-8.18	-8.18	-8.18	-8.18	-8.18
-8.17	-8.17	-8.17	-8.17	-8.17	-8.17	-8.17	-8.17	-8.17
-8.16	-8.16	-8.16	-8.16	-8.16	-8.16	-8.16	-8.16	-8.16
-8.15	-8.15	-8.15	-8.15	-8.15	-8.15	-8.15	-8.15	-8.15
-8.14	-8.14	-8.14	-8.14	-8.14	-8.14	-8.14	-8.14	-8.14
-8.13	-8.13	-8.13	-8.13	-8.13	-8.13	-8.13	-8.13	-8.13
-8.12	-8.12	-8.12	-8.12	-8.12	-8.12	-8.12	-8.12	-8.12
-8.11	-8.11	-8.11	-8.11	-8.11	-8.11	-8.11	-8.11	-8.11
-8.1	-8.1	-8.1	-8.1	-8.1	-8.1	-8.1	-8.1	-8.1
-8.09	-8.09	-8.09	-8.09	-8.09	-8.09	-8.09	-8.09	-8.09
-8.08	-8.08	-8.08	-8.08	-8.08	-8.08	-8.08	-8.08	-8.08
-8.07	-8.07	-8.07	-8.07	-8.07	-8.07	-8.07	-8.07	-8.07
-8.06	-8.06	-8.06	-8.06	-8.06	-8.06	-8.06	-8.06	-8.06
-8.05	-8.05	-8.05	-8.05	-8.05	-8.05	-8.05	-8.05	-8.05
-8.04	-8.04	-8.04	-8.04	-8.04	-8.04	-8.04	-8.04	-8.04
-8.03	-8.03	-8.03	-8.03	-8.03	-8.03	-8.03	-8.03	-8.03
-8.02	-8.02	-8.02	-8.02	-8.02	-8.02	-8.02	-8.02	-8.02
-8.01	-8.01	-8.01	-8.01	-8.01	-8.01	-8.01	-8.01	-8.01

-8	-8	-8	-8	-8	-8	-8	-8	-8
-7.99	-7.99	-7.99	-7.99	-7.99	-7.99	-7.99	-7.99	-7.99
-7.98	-7.98	-7.98	-7.98	-7.98	-7.98	-7.98	-7.98	-7.98
-7.97	-7.97	-7.97	-7.97	-7.97	-7.97	-7.97	-7.97	-7.97
-7.96	-7.96	-7.96	-7.96	-7.96	-7.96	-7.96	-7.96	-7.96
-7.95	-7.95	-7.95	-7.95	-7.95	-7.95	-7.95	-7.95	-7.95
-7.94	-7.94	-7.94	-7.94	-7.94	-7.94	-7.94	-7.94	-7.94
-7.93	-7.93	-7.93	-7.93	-7.93	-7.93	-7.93	-7.93	-7.93
-7.92	-7.92	-7.92	-7.92	-7.92	-7.92	-7.92	-7.92	-7.92
-7.91	-7.91	-7.91	-7.91	-7.91	-7.91	-7.91	-7.91	-7.91
-7.9	-7.9	-7.9	-7.9	-7.9	-7.9	-7.9	-7.9	-7.9
-7.89	-7.89	-7.89	-7.89	-7.89	-7.89	-7.89	-7.89	-7.89
-7.88	-7.88	-7.88	-7.88	-7.88	-7.88	-7.88	-7.88	-7.88
-7.87	-7.87	-7.87	-7.87	-7.87	-7.87	-7.87	-7.87	-7.87
-7.86	-7.86	-7.86	-7.86	-7.86	-7.86	-7.86	-7.86	-7.86
-7.85	-7.85	-7.85	-7.85	-7.85	-7.85	-7.85	-7.85	-7.85
-7.84	-7.84	-7.84	-7.84	-7.84	-7.84	-7.84	-7.84	-7.84
-7.83	-7.83	-7.83	-7.83	-7.83	-7.83	-7.83	-7.83	-7.83
-7.82	-7.82	-7.82	-7.82	-7.82	-7.82	-7.82	-7.82	-7.82
-7.81	-7.81	-7.81	-7.81	-7.81	-7.81	-7.81	-7.81	-7.81
-7.8	-7.8	-7.8	-7.8	-7.8	-7.8	-7.8	-7.8	-7.8
-7.79	-7.79	-7.79	-7.79	-7.79	-7.79	-7.79	-7.79	-7.79
-7.78	-7.78	-7.78	-7.78	-7.78	-7.78	-7.78	-7.78	-7.78
-7.77	-7.77	-7.77	-7.77	-7.77	-7.77	-7.77	-7.77	-7.77
-7.76	-7.76	-7.76	-7.76	-7.76	-7.76	-7.76	-7.76	-7.76
-7.75	-7.75	-7.75	-7.75	-7.75	-7.75	-7.75	-7.75	-7.75
-7.74	-7.74	-7.74	-7.74	-7.74	-7.74	-7.74	-7.74	-7.74
-7.73	-7.73	-7.73	-7.73	-7.73	-7.73	-7.73	-7.73	-7.73
-7.72	-7.72	-7.72	-7.72	-7.72	-7.72	-7.72	-7.72	-7.72
-7.71	-7.71	-7.71	-7.71	-7.71	-7.71	-7.71	-7.71	-7.71
-7.7	-7.7	-7.7	-7.7	-7.7	-7.7	-7.7	-7.7	-7.7
-7.69	-7.69	-7.69	-7.69	-7.69	-7.69	-7.69	-7.69	-7.69
-7.68	-7.68	-7.68	-7.68	-7.68	-7.68	-7.68	-7.68	-7.68
-7.67	-7.67	-7.67	-7.67	-7.67	-7.67	-7.67	-7.67	-7.67
-7.66	-7.66	-7.66	-7.66	-7.66	-7.66	-7.66	-7.66	-7.66
-7.65	-7.65	-7.65	-7.65	-7.65	-7.65	-7.65	-7.65	-7.65
-7.64	-7.64	-7.64	-7.64	-7.64	-7.64	-7.64	-7.64	-7.64
-7.63	-7.63	-7.63	-7.63	-7.63	-7.63	-7.63	-7.63	-7.63
-7.62	-7.62	-7.62	-7.62	-7.62	-7.62	-7.62	-7.62	-7.62
-7.61	-7.61	-7.61	-7.61	-7.61	-7.61	-7.61	-7.61	-7.61
-7.6	-7.6	-7.6	-7.6	-7.6	-7.6	-7.6	-7.6	-7.6
-7.59	-7.59	-7.59	-7.59	-7.59	-7.59	-7.59	-7.59	-7.59
-7.58	-7.58	-7.58	-7.58	-7.58	-7.58	-7.58	-7.58	-7.58
-7.57	-7.57	-7.57	-7.57	-7.57	-7.57	-7.57	-7.57	-7.57
-7.56	-7.56	-7.56	-7.56	-7.56	-7.56	-7.56	-7.56	-7.56
-7.55	-7.55	-7.55	-7.55	-7.55	-7.55	-7.55	-7.55	-7.55
-7.54	-7.54	-7.54	-7.54	-7.54	-7.54	-7.54	-7.54	-7.54
-7.53	-7.53	-7.53	-7.53	-7.53	-7.53	-7.53	-7.53	-7.53
-7.52	-7.52	-7.52	-7.52	-7.52	-7.52	-7.52	-7.52	-7.52
-7.51	-7.51	-7.51	-7.51	-7.51	-7.51	-7.51	-7.51	-7.51

-7.5	-7.5	-7.5	-7.5	-7.5	-7.5	-7.5	-7.5	-7.5
-7.49	-7.49	-7.49	-7.49	-7.49	-7.49	-7.49	-7.49	-7.49
-7.48	-7.48	-7.48	-7.48	-7.48	-7.48	-7.48	-7.48	-7.48
-7.47	-7.47	-7.47	-7.47	-7.47	-7.47	-7.47	-7.47	-7.47
-7.46	-7.46	-7.46	-7.46	-7.46	-7.46	-7.46	-7.46	-7.46
-7.45	-7.45	-7.45	-7.45	-7.45	-7.45	-7.45	-7.45	-7.45
-7.44	-7.44	-7.44	-7.44	-7.44	-7.44	-7.44	-7.44	-7.44
-7.43	-7.43	-7.43	-7.43	-7.43	-7.43	-7.43	-7.43	-7.43
-7.42	-7.42	-7.42	-7.42	-7.42	-7.42	-7.42	-7.42	-7.42
-7.41	-7.41	-7.41	-7.41	-7.41	-7.41	-7.41	-7.41	-7.41
-7.4	-7.4	-7.4	-7.4	-7.4	-7.4	-7.4	-7.4	-7.4
-7.39	-7.39	-7.39	-7.39	-7.39	-7.39	-7.39	-7.39	-7.39
-7.38	-7.38	-7.38	-7.38	-7.38	-7.38	-7.38	-7.38	-7.38
-7.37	-7.37	-7.37	-7.37	-7.37	-7.37	-7.37	-7.37	-7.37
-7.36	-7.36	-7.36	-7.36	-7.36	-7.36	-7.36	-7.36	-7.36
-7.35	-7.35	-7.35	-7.35	-7.35	-7.35	-7.35	-7.35	-7.35
-7.34	-7.34	-7.34	-7.34	-7.34	-7.34	-7.34	-7.34	-7.34
-7.33	-7.33	-7.33	-7.33	-7.33	-7.33	-7.33	-7.33	-7.33
-7.32	-7.32	-7.32	-7.32	-7.32	-7.32	-7.32	-7.32	-7.32
-7.31	-7.31	-7.31	-7.31	-7.31	-7.31	-7.31	-7.31	-7.31
-7.3	-7.3	-7.3	-7.3	-7.3	-7.3	-7.3	-7.3	-7.3
-7.29	-7.29	-7.29	-7.29	-7.29	-7.29	-7.29	-7.29	-7.29
-7.28	-7.28	-7.28	-7.28	-7.28	-7.28	-7.28	-7.28	-7.28
-7.27	-7.27	-7.27	-7.27	-7.27	-7.27	-7.27	-7.27	-7.27
-7.26	-7.26	-7.26	-7.26	-7.26	-7.26	-7.26	-7.26	-7.26
-7.25	-7.25	-7.25	-7.25	-7.25	-7.25	-7.25	-7.25	-7.25
-7.24	-7.24	-7.24	-7.24	-7.24	-7.24	-7.24	-7.24	-7.24
-7.23	-7.23	-7.23	-7.23	-7.23	-7.23	-7.23	-7.23	-7.23
-7.22	-7.22	-7.22	-7.22	-7.22	-7.22	-7.22	-7.22	-7.22
-7.21	-7.21	-7.21	-7.21	-7.21	-7.21	-7.21	-7.21	-7.21
-7.2	-7.2	-7.2	-7.2	-7.2	-7.2	-7.2	-7.2	-7.2
-7.19	-7.19	-7.19	-7.19	-7.19	-7.19	-7.19	-7.19	-7.19
-7.18	-7.18	-7.18	-7.18	-7.18	-7.18	-7.18	-7.18	-7.18
-7.17	-7.17	-7.17	-7.17	-7.17	-7.17	-7.17	-7.17	-7.17
-7.16	-7.16	-7.16	-7.16	-7.16	-7.16	-7.16	-7.16	-7.16
-7.15	-7.15	-7.15	-7.15	-7.15	-7.15	-7.15	-7.15	-7.15
-7.14	-7.14	-7.14	-7.14	-7.14	-7.14	-7.14	-7.14	-7.14
-7.13	-7.13	-7.13	-7.13	-7.13	-7.13	-7.13	-7.13	-7.13
-7.12	-7.12	-7.12	-7.12	-7.12	-7.12	-7.12	-7.12	-7.12
-7.11	-7.11	-7.11	-7.11	-7.11	-7.11	-7.11	-7.11	-7.11
-7.1	-7.1	-7.1	-7.1	-7.1	-7.1	-7.1	-7.1	-7.1
-7.09	-7.09	-7.09	-7.09	-7.09	-7.09	-7.09	-7.09	-7.09
-7.08	-7.08	-7.08	-7.08	-7.08	-7.08	-7.08	-7.08	-7.08
-7.07	-7.07	-7.07	-7.07	-7.07	-7.07	-7.07	-7.07	-7.07
-7.06	-7.06	-7.06	-7.06	-7.06	-7.06	-7.06	-7.06	-7.06
-7.05	-7.05	-7.05	-7.05	-7.05	-7.05	-7.05	-7.05	-7.05
-7.04	-7.04	-7.04	-7.04	-7.04	-7.04	-7.04	-7.04	-7.04
-7.03	-7.03	-7.03	-7.03	-7.03	-7.03	-7.03	-7.03	-7.03
-7.02	-7.02	-7.02	-7.02	-7.02	-7.02	-7.02	-7.02	-7.02
-7.01	-7.01	-7.01	-7.01	-7.01	-7.01	-7.01	-7.01	-7.01

-7	-7	-7	-7	-7	-7	-7	-7	-7
-6.99	-6.99	-6.99	-6.99	-6.99	-6.99	-6.99	-6.99	-6.99
-6.98	-6.98	-6.98	-6.98	-6.98	-6.98	-6.98	-6.98	-6.98
-6.97	-6.97	-6.97	-6.97	-6.97	-6.97	-6.97	-6.97	-6.97
-6.96	-6.96	-6.96	-6.96	-6.96	-6.96	-6.96	-6.96	-6.96
-6.95	-6.95	-6.95	-6.95	-6.95	-6.95	-6.95	-6.95	-6.95
-6.94	-6.94	-6.94	-6.94	-6.94	-6.94	-6.94	-6.94	-6.94
-6.93	-6.93	-6.93	-6.93	-6.93	-6.93	-6.93	-6.93	-6.93
-6.92	-6.92	-6.92	-6.92	-6.92	-6.92	-6.92	-6.92	-6.92
-6.91	-6.91	-6.91	-6.91	-6.91	-6.91	-6.91	-6.91	-6.91
-6.9	-6.9	-6.9	-6.9	-6.9	-6.9	-6.9	-6.9	-6.9
-6.89	-6.89	-6.89	-6.89	-6.89	-6.89	-6.89	-6.89	-6.89
-6.88	-6.88	-6.88	-6.88	-6.88	-6.88	-6.88	-6.88	-6.88
-6.87	-6.87	-6.87	-6.87	-6.87	-6.87	-6.87	-6.87	-6.87
-6.86	-6.86	-6.86	-6.86	-6.86	-6.86	-6.86	-6.86	-6.86
-6.85	-6.85	-6.85	-6.85	-6.85	-6.85	-6.85	-6.85	-6.85
-6.84	-6.84	-6.84	-6.84	-6.84	-6.84	-6.84	-6.84	-6.84
-6.83	-6.83	-6.83	-6.83	-6.83	-6.83	-6.83	-6.83	-6.83
-6.82	-6.82	-6.82	-6.82	-6.82	-6.82	-6.82	-6.82	-6.82
-6.81	-6.81	-6.81	-6.81	-6.81	-6.81	-6.81	-6.81	-6.81
-6.8	-6.8	-6.8	-6.8	-6.8	-6.8	-6.8	-6.8	-6.8
-6.79	-6.79	-6.79	-6.79	-6.79	-6.79	-6.79	-6.79	-6.79
-6.78	-6.78	-6.78	-6.78	-6.78	-6.78	-6.78	-6.78	-6.78
-6.77	-6.77	-6.77	-6.77	-6.77	-6.77	-6.77	-6.77	-6.77
-6.76	-6.76	-6.76	-6.76	-6.76	-6.76	-6.76	-6.76	-6.76
-6.75	-6.75	-6.75	-6.75	-6.75	-6.75	-6.75	-6.75	-6.75
-6.74	-6.74	-6.74	-6.74	-6.74	-6.74	-6.74	-6.74	-6.74
-6.73	-6.73	-6.73	-6.73	-6.73	-6.73	-6.73	-6.73	-6.73
-6.72	-6.72	-6.72	-6.72	-6.72	-6.72	-6.72	-6.72	-6.72
-6.71	-6.71	-6.71	-6.71	-6.71	-6.71	-6.71	-6.71	-6.71
-6.7	-6.7	-6.7	-6.7	-6.7	-6.7	-6.7	-6.7	-6.7
-6.69	-6.69	-6.69	-6.69	-6.69	-6.69	-6.69	-6.69	-6.69
-6.68	-6.68	-6.68	-6.68	-6.68	-6.68	-6.68	-6.68	-6.68
-6.67	-6.67	-6.67	-6.67	-6.67	-6.67	-6.67	-6.67	-6.67
-6.66	-6.66	-6.66	-6.66	-6.66	-6.66	-6.66	-6.66	-6.66
-6.65	-6.65	-6.65	-6.65	-6.65	-6.65	-6.65	-6.65	-6.65
-6.64	-6.64	-6.64	-6.64	-6.64	-6.64	-6.64	-6.64	-6.64
-6.63	-6.63	-6.63	-6.63	-6.63	-6.63	-6.63	-6.63	-6.63
-6.62	-6.62	-6.62	-6.62	-6.62	-6.62	-6.62	-6.62	-6.62
-6.61	-6.61	-6.61	-6.61	-6.61	-6.61	-6.61	-6.61	-6.61
-6.6	-6.6	-6.6	-6.6	-6.6	-6.6	-6.6	-6.6	-6.6
-6.59	-6.59	-6.59	-6.59	-6.59	-6.59	-6.59	-6.59	-6.59
-6.58	-6.58	-6.58	-6.58	-6.58	-6.58	-6.58	-6.58	-6.58
-6.57	-6.57	-6.57	-6.57	-6.57	-6.57	-6.57	-6.57	-6.57
-6.56	-6.56	-6.56	-6.56	-6.56	-6.56	-6.56	-6.56	-6.56
-6.55	-6.55	-6.55	-6.55	-6.55	-6.55	-6.55	-6.55	-6.55
-6.54	-6.54	-6.54	-6.54	-6.54	-6.54	-6.54	-6.54	-6.54
-6.53	-6.53	-6.53	-6.53	-6.53	-6.53	-6.53	-6.53	-6.53
-6.52	-6.52	-6.52	-6.52	-6.52	-6.52	-6.52	-6.52	-6.52
-6.51	-6.51	-6.51	-6.51	-6.51	-6.51	-6.51	-6.51	-6.51

-6.5	-6.5	-6.5	-6.5	-6.5	-6.5	-6.5	-6.5	-6.5
-6.49	-6.49	-6.49	-6.49	-6.49	-6.49	-6.49	-6.49	-6.49
-6.48	-6.48	-6.48	-6.48	-6.48	-6.48	-6.48	-6.48	-6.48
-6.47	-6.47	-6.47	-6.47	-6.47	-6.47	-6.47	-6.47	-6.47
-6.46	-6.46	-6.46	-6.46	-6.46	-6.46	-6.46	-6.46	-6.46
-6.45	-6.45	-6.45	-6.45	-6.45	-6.45	-6.45	-6.45	-6.45
-6.44	-6.44	-6.44	-6.44	-6.44	-6.44	-6.44	-6.44	-6.44
-6.43	-6.43	-6.43	-6.43	-6.43	-6.43	-6.43	-6.43	-6.43
-6.42	-6.42	-6.42	-6.42	-6.42	-6.42	-6.42	-6.42	-6.42
-6.41	-6.41	-6.41	-6.41	-6.41	-6.41	-6.41	-6.41	-6.41
-6.4	-6.4	-6.4	-6.4	-6.4	-6.4	-6.4	-6.4	-6.4
-6.39	-6.39	-6.39	-6.39	-6.39	-6.39	-6.39	-6.39	-6.39
-6.38	-6.38	-6.38	-6.38	-6.38	-6.38	-6.38	-6.38	-6.38
-6.37	-6.37	-6.37	-6.37	-6.37	-6.37	-6.37	-6.37	-6.37
-6.36	-6.36	-6.36	-6.36	-6.36	-6.36	-6.36	-6.36	-6.36
-6.35	-6.35	-6.35	-6.35	-6.35	-6.35	-6.35	-6.35	-6.35
-6.34	-6.34	-6.34	-6.34	-6.34	-6.34	-6.34	-6.34	-6.34
-6.33	-6.33	-6.33	-6.33	-6.33	-6.33	-6.33	-6.33	-6.33
-6.32	-6.32	-6.32	-6.32	-6.32	-6.32	-6.32	-6.32	-6.32
-6.31	-6.31	-6.31	-6.31	-6.31	-6.31	-6.31	-6.31	-6.31
-6.3	-6.3	-6.3	-6.3	-6.3	-6.3	-6.3	-6.3	-6.3
-6.29	-6.29	-6.29	-6.29	-6.29	-6.29	-6.29	-6.29	-6.29
-6.28	-6.28	-6.28	-6.28	-6.28	-6.28	-6.28	-6.28	-6.28
-6.27	-6.27	-6.27	-6.27	-6.27	-6.27	-6.27	-6.27	-6.27
-6.26	-6.26	-6.26	-6.26	-6.26	-6.26	-6.26	-6.26	-6.26
-6.25	-6.25	-6.25	-6.25	-6.25	-6.25	-6.25	-6.25	-6.25
-6.24	-6.24	-6.24	-6.24	-6.24	-6.24	-6.24	-6.24	-6.24
-6.23	-6.23	-6.23	-6.23	-6.23	-6.23	-6.23	-6.23	-6.23
-6.22	-6.22	-6.22	-6.22	-6.22	-6.22	-6.22	-6.22	-6.22
-6.21	-6.21	-6.21	-6.21	-6.21	-6.21	-6.21	-6.21	-6.21
-6.2	-6.2	-6.2	-6.2	-6.2	-6.2	-6.2	-6.2	-6.2
-6.19	-6.19	-6.19	-6.19	-6.19	-6.19	-6.19	-6.19	-6.19
-6.18	-6.18	-6.18	-6.18	-6.18	-6.18	-6.18	-6.18	-6.18
-6.17	-6.17	-6.17	-6.17	-6.17	-6.17	-6.17	-6.17	-6.17
-6.16	-6.16	-6.16	-6.16	-6.16	-6.16	-6.16	-6.16	-6.16
-6.15	-6.15	-6.15	-6.15	-6.15	-6.15	-6.15	-6.15	-6.15
-6.14	-6.14	-6.14	-6.14	-6.14	-6.14	-6.14	-6.14	-6.14
-6.13	-6.13	-6.13	-6.13	-6.13	-6.13	-6.13	-6.13	-6.13
-6.12	-6.12	-6.12	-6.12	-6.12	-6.12	-6.12	-6.12	-6.12
-6.11	-6.11	-6.11	-6.11	-6.11	-6.11	-6.11	-6.11	-6.11
-6.1	-6.1	-6.1	-6.1	-6.1	-6.1	-6.1	-6.1	-6.1
-6.09	-6.09	-6.09	-6.09	-6.09	-6.09	-6.09	-6.09	-6.09
-6.08	-6.08	-6.08	-6.08	-6.08	-6.08	-6.08	-6.08	-6.08
-6.07	-6.07	-6.07	-6.07	-6.07	-6.07	-6.07	-6.07	-6.07
-6.06	-6.06	-6.06	-6.06	-6.06	-6.06	-6.06	-6.06	-6.06
-6.05	-6.05	-6.05	-6.05	-6.05	-6.05	-6.05	-6.05	-6.05
-6.04	-6.04	-6.04	-6.04	-6.04	-6.04	-6.04	-6.04	-6.04
-6.03	-6.03	-6.03	-6.03	-6.03	-6.03	-6.03	-6.03	-6.03
-6.02	-6.02	-6.02	-6.02	-6.02	-6.02	-6.02	-6.02	-6.02
-6.01	-6.01	-6.01	-6.01	-6.01	-6.01	-6.01	-6.01	-6.01

-6	-6	-6	-6	-6	-6	-6	-6	-6
-5.99	-5.99	-5.99	-5.99	-5.99	-5.99	-5.99	-5.99	-5.99
-5.98	-5.98	-5.98	-5.98	-5.98	-5.98	-5.98	-5.98	-5.98
-5.97	-5.97	-5.97	-5.97	-5.97	-5.97	-5.97	-5.97	-5.97
-5.96	-5.96	-5.96	-5.96	-5.96	-5.96	-5.96	-5.96	-5.96
-5.95	-5.95	-5.95	-5.95	-5.95	-5.95	-5.95	-5.95	-5.95
-5.94	-5.94	-5.94	-5.94	-5.94	-5.94	-5.94	-5.94	-5.94
-5.93	-5.93	-5.93	-5.93	-5.93	-5.93	-5.93	-5.93	-5.93
-5.92	-5.92	-5.92	-5.92	-5.92	-5.92	-5.92	-5.92	-5.92
-5.91	-5.91	-5.91	-5.91	-5.91	-5.91	-5.91	-5.91	-5.91
-5.9	-5.9	-5.9	-5.9	-5.9	-5.9	-5.9	-5.9	-5.9
-5.89	-5.89	-5.89	-5.89	-5.89	-5.89	-5.89	-5.89	-5.89
-5.88	-5.88	-5.88	-5.88	-5.88	-5.88	-5.88	-5.88	-5.88
-5.87	-5.87	-5.87	-5.87	-5.87	-5.87	-5.87	-5.87	-5.87
-5.86	-5.86	-5.86	-5.86	-5.86	-5.86	-5.86	-5.86	-5.86
-5.85	-5.85	-5.85	-5.85	-5.85	-5.85	-5.85	-5.85	-5.85
-5.84	-5.84	-5.84	-5.84	-5.84	-5.84	-5.84	-5.84	-5.84
-5.83	-5.83	-5.83	-5.83	-5.83	-5.83	-5.83	-5.83	-5.83
-5.82	-5.82	-5.82	-5.82	-5.82	-5.82	-5.82	-5.82	-5.82
-5.81	-5.81	-5.81	-5.81	-5.81	-5.81	-5.81	-5.81	-5.81
-5.8	-5.8	-5.8	-5.8	-5.8	-5.8	-5.8	-5.8	-5.8
-5.79	-5.79	-5.79	-5.79	-5.79	-5.79	-5.79	-5.79	-5.79
-5.78	-5.78	-5.78	-5.78	-5.78	-5.78	-5.78	-5.78	-5.78
-5.77	-5.77	-5.77	-5.77	-5.77	-5.77	-5.77	-5.77	-5.77
-5.76	-5.76	-5.76	-5.76	-5.76	-5.76	-5.76	-5.76	-5.76
-5.75	-5.75	-5.75	-5.75	-5.75	-5.75	-5.75	-5.75	-5.75
-5.74	-5.74	-5.74	-5.74	-5.74	-5.74	-5.74	-5.74	-5.74
-5.73	-5.73	-5.73	-5.73	-5.73	-5.73	-5.73	-5.73	-5.73
-5.72	-5.72	-5.72	-5.72	-5.72	-5.72	-5.72	-5.72	-5.72
-5.71	-5.71	-5.71	-5.71	-5.71	-5.71	-5.71	-5.71	-5.71
-5.7	-5.7	-5.7	-5.7	-5.7	-5.7	-5.7	-5.7	-5.7
-5.69	-5.69	-5.69	-5.69	-5.69	-5.69	-5.69	-5.69	-5.69
-5.68	-5.68	-5.68	-5.68	-5.68	-5.68	-5.68	-5.68	-5.68
-5.67	-5.67	-5.67	-5.67	-5.67	-5.67	-5.67	-5.67	-5.67
-5.66	-5.66	-5.66	-5.66	-5.66	-5.66	-5.66	-5.66	-5.66
-5.65	-5.65	-5.65	-5.65	-5.65	-5.65	-5.65	-5.65	-5.65
-5.64	-5.64	-5.64	-5.64	-5.64	-5.64	-5.64	-5.64	-5.64
-5.63	-5.63	-5.63	-5.63	-5.63	-5.63	-5.63	-5.63	-5.63
-5.62	-5.62	-5.62	-5.62	-5.62	-5.62	-5.62	-5.62	-5.62
-5.61	-5.61	-5.61	-5.61	-5.61	-5.61	-5.61	-5.61	-5.61
-5.6	-5.6	-5.6	-5.6	-5.6	-5.6	-5.6	-5.6	-5.6
-5.59	-5.59	-5.59	-5.59	-5.59	-5.59	-5.59	-5.59	-5.59
-5.58	-5.58	-5.58	-5.58	-5.58	-5.58	-5.58	-5.58	-5.58
-5.57	-5.57	-5.57	-5.57	-5.57	-5.57	-5.57	-5.57	-5.57
-5.56	-5.56	-5.56	-5.56	-5.56	-5.56	-5.56	-5.56	-5.56
-5.55	-5.55	-5.55	-5.55	-5.55	-5.55	-5.55	-5.55	-5.55
-5.54	-5.54	-5.54	-5.54	-5.54	-5.54	-5.54	-5.54	-5.54
-5.53	-5.53	-5.53	-5.53	-5.53	-5.53	-5.53	-5.53	-5.53
-5.52	-5.52	-5.52	-5.52	-5.52	-5.52	-5.52	-5.52	-5.52
-5.51	-5.51	-5.51	-5.51	-5.51	-5.51	-5.51	-5.51	-5.51

-5.5	-5.5	-5.5	-5.5	-5.5	-5.5	-5.5	-5.5	-5.5
-5.49	-5.49	-5.49	-5.49	-5.49	-5.49	-5.49	-5.49	-5.49
-5.48	-5.48	-5.48	-5.48	-5.48	-5.48	-5.48	-5.48	-5.48
-5.47	-5.47	-5.47	-5.47	-5.47	-5.47	-5.47	-5.47	-5.47
-5.46	-5.46	-5.46	-5.46	-5.46	-5.46	-5.46	-5.46	-5.46
-5.45	-5.45	-5.45	-5.45	-5.45	-5.45	-5.45	-5.45	-5.45
-5.44	-5.44	-5.44	-5.44	-5.44	-5.44	-5.44	-5.44	-5.44
-5.43	-5.43	-5.43	-5.43	-5.43	-5.43	-5.43	-5.43	-5.43
-5.42	-5.42	-5.42	-5.42	-5.42	-5.42	-5.42	-5.42	-5.42
-5.41	-5.41	-5.41	-5.41	-5.41	-5.41	-5.41	-5.41	-5.41
-5.4	-5.4	-5.4	-5.4	-5.4	-5.4	-5.4	-5.4	-5.4
-5.39	-5.39	-5.39	-5.39	-5.39	-5.39	-5.39	-5.39	-5.39
-5.38	-5.38	-5.38	-5.38	-5.38	-5.38	-5.38	-5.38	-5.38
-5.37	-5.37	-5.37	-5.37	-5.37	-5.37	-5.37	-5.37	-5.37
-5.36	-5.36	-5.36	-5.36	-5.36	-5.36	-5.36	-5.36	-5.36
-5.35	-5.35	-5.35	-5.35	-5.35	-5.35	-5.35	-5.35	-5.35
-5.34	-5.34	-5.34	-5.34	-5.34	-5.34	-5.34	-5.34	-5.34
-5.33	-5.33	-5.33	-5.33	-5.33	-5.33	-5.33	-5.33	-5.33
-5.32	-5.32	-5.32	-5.32	-5.32	-5.32	-5.32	-5.32	-5.32
-5.31	-5.31	-5.31	-5.31	-5.31	-5.31	-5.31	-5.31	-5.31
-5.3	-5.3	-5.3	-5.3	-5.3	-5.3	-5.3	-5.3	-5.3
-5.29	-5.29	-5.29	-5.29	-5.29	-5.29	-5.29	-5.29	-5.29
-5.28	-5.28	-5.28	-5.28	-5.28	-5.28	-5.28	-5.28	-5.28
-5.27	-5.27	-5.27	-5.27	-5.27	-5.27	-5.27	-5.27	-5.27
-5.26	-5.26	-5.26	-5.26	-5.26	-5.26	-5.26	-5.26	-5.26
-5.25	-5.25	-5.25	-5.25	-5.25	-5.25	-5.25	-5.25	-5.25
-5.24	-5.24	-5.24	-5.24	-5.24	-5.24	-5.24	-5.24	-5.24
-5.23	-5.23	-5.23	-5.23	-5.23	-5.23	-5.23	-5.23	-5.23
-5.22	-5.22	-5.22	-5.22	-5.22	-5.22	-5.22	-5.22	-5.22
-5.21	-5.21	-5.21	-5.21	-5.21	-5.21	-5.21	-5.21	-5.21
-5.2	-5.2	-5.2	-5.2	-5.2	-5.2	-5.2	-5.2	-5.2
-5.19	-5.19	-5.19	-5.19	-5.19	-5.19	-5.19	-5.19	-5.19
-5.18	-5.18	-5.18	-5.18	-5.18	-5.18	-5.18	-5.18	-5.18
-5.17	-5.17	-5.17	-5.17	-5.17	-5.17	-5.17	-5.17	-5.17
-5.16	-5.16	-5.16	-5.16	-5.16	-5.16	-5.16	-5.16	-5.16
-5.15	-5.15	-5.15	-5.15	-5.15	-5.15	-5.15	-5.15	-5.15
-5.14	-5.14	-5.14	-5.14	-5.14	-5.14	-5.14	-5.14	-5.14
-5.13	-5.13	-5.13	-5.13	-5.13	-5.13	-5.13	-5.13	-5.13
-5.12	-5.12	-5.12	-5.12	-5.12	-5.12	-5.12	-5.12	-5.12
-5.11	-5.11	-5.11	-5.11	-5.11	-5.11	-5.11	-5.11	-5.11
-5.1	-5.1	-5.1	-5.1	-5.1	-5.1	-5.1	-5.1	-5.1
-5.09	-5.09	-5.09	-5.09	-5.09	-5.09	-5.09	-5.09	-5.09
-5.08	-5.08	-5.08	-5.08	-5.08	-5.08	-5.08	-5.08	-5.08
-5.07	-5.07	-5.07	-5.07	-5.07	-5.07	-5.07	-5.07	-5.07
-5.06	-5.06	-5.06	-5.06	-5.06	-5.06	-5.06	-5.06	-5.06
-5.05	-5.05	-5.05	-5.05	-5.05	-5.05	-5.05	-5.05	-5.05
-5.04	-5.04	-5.04	-5.04	-5.04	-5.04	-5.04	-5.04	-5.04
-5.03	-5.03	-5.03	-5.03	-5.03	-5.03	-5.03	-5.03	-5.03
-5.02	-5.02	-5.02	-5.02	-5.02	-5.02	-5.02	-5.02	-5.02
-5.01	-5.01	-5.01	-5.01	-5.01	-5.01	-5.01	-5.01	-5.01

-5	-5	-5	-5	-5	-5	-5	-5	-5
-4.99	-4.99	-4.99	-4.99	-4.99	-4.99	-4.99	-4.99	-4.99
-4.98	-4.98	-4.98	-4.98	-4.98	-4.98	-4.98	-4.98	-4.98
-4.97	-4.97	-4.97	-4.97	-4.97	-4.97	-4.97	-4.97	-4.97
-4.96	-4.96	-4.96	-4.96	-4.96	-4.96	-4.96	-4.96	-4.96
-4.95	-4.95	-4.95	-4.95	-4.95	-4.95	-4.95	-4.95	-4.95
-4.94	-4.94	-4.94	-4.94	-4.94	-4.94	-4.94	-4.94	-4.94
-4.93	-4.93	-4.93	-4.93	-4.93	-4.93	-4.93	-4.93	-4.93
-4.92	-4.92	-4.92	-4.92	-4.92	-4.92	-4.92	-4.92	-4.92
-4.91	-4.91	-4.91	-4.91	-4.91	-4.91	-4.91	-4.91	-4.91
-4.9	-4.9	-4.9	-4.9	-4.9	-4.9	-4.9	-4.9	-4.9
-4.89	-4.89	-4.89	-4.89	-4.89	-4.89	-4.89	-4.89	-4.89
-4.88	-4.88	-4.88	-4.88	-4.88	-4.88	-4.88	-4.88	-4.88
-4.87	-4.87	-4.87	-4.87	-4.87	-4.87	-4.87	-4.87	-4.87
-4.86	-4.86	-4.86	-4.86	-4.86	-4.86	-4.86	-4.86	-4.86
-4.85	-4.85	-4.85	-4.85	-4.85	-4.85	-4.85	-4.85	-4.85
-4.84	-4.84	-4.84	-4.84	-4.84	-4.84	-4.84	-4.84	-4.84
-4.83	-4.83	-4.83	-4.83	-4.83	-4.83	-4.83	-4.83	-4.83
-4.82	-4.82	-4.82	-4.82	-4.82	-4.82	-4.82	-4.82	-4.82
-4.81	-4.81	-4.81	-4.81	-4.81	-4.81	-4.81	-4.81	-4.81
-4.8	-4.8	-4.8	-4.8	-4.8	-4.8	-4.8	-4.8	-4.8
-4.79	-4.79	-4.79	-4.79	-4.79	-4.79	-4.79	-4.79	-4.79
-4.78	-4.78	-4.78	-4.78	-4.78	-4.78	-4.78	-4.78	-4.78
-4.77	-4.77	-4.77	-4.77	-4.77	-4.77	-4.77	-4.77	-4.77
-4.76	-4.76	-4.76	-4.76	-4.76	-4.76	-4.76	-4.76	-4.76
-4.75	-4.75	-4.75	-4.75	-4.75	-4.75	-4.75	-4.75	-4.75
-4.74	-4.74	-4.74	-4.74	-4.74	-4.74	-4.74	-4.74	-4.74
-4.73	-4.73	-4.73	-4.73	-4.73	-4.73	-4.73	-4.73	-4.73
-4.72	-4.72	-4.72	-4.72	-4.72	-4.72	-4.72	-4.72	-4.72
-4.71	-4.71	-4.71	-4.71	-4.71	-4.71	-4.71	-4.71	-4.71
-4.7	-4.7	-4.7	-4.7	-4.7	-4.7	-4.7	-4.7	-4.7
-4.69	-4.69	-4.69	-4.69	-4.69	-4.69	-4.69	-4.69	-4.69
-4.68	-4.68	-4.68	-4.68	-4.68	-4.68	-4.68	-4.68	-4.68
-4.67	-4.67	-4.67	-4.67	-4.67	-4.67	-4.67	-4.67	-4.67
-4.66	-4.66	-4.66	-4.66	-4.66	-4.66	-4.66	-4.66	-4.66
-4.65	-4.65	-4.65	-4.65	-4.65	-4.65	-4.65	-4.65	-4.65
-4.64	-4.64	-4.64	-4.64	-4.64	-4.64	-4.64	-4.64	-4.64
-4.63	-4.63	-4.63	-4.63	-4.63	-4.63	-4.63	-4.63	-4.63
-4.62	-4.62	-4.62	-4.62	-4.62	-4.62	-4.62	-4.62	-4.62
-4.61	-4.61	-4.61	-4.61	-4.61	-4.61	-4.61	-4.61	-4.61
-4.6	-4.6	-4.6	-4.6	-4.6	-4.6	-4.6	-4.6	-4.6
-4.59	-4.59	-4.59	-4.59	-4.59	-4.59	-4.59	-4.59	-4.59
-4.58	-4.58	-4.58	-4.58	-4.58	-4.58	-4.58	-4.58	-4.58
-4.57	-4.57	-4.57	-4.57	-4.57	-4.57	-4.57	-4.57	-4.57
-4.56	-4.56	-4.56	-4.56	-4.56	-4.56	-4.56	-4.56	-4.56
-4.55	-4.55	-4.55	-4.55	-4.55	-4.55	-4.55	-4.55	-4.55
-4.54	-4.54	-4.54	-4.54	-4.54	-4.54	-4.54	-4.54	-4.54
-4.53	-4.53	-4.53	-4.53	-4.53	-4.53	-4.53	-4.53	-4.53
-4.52	-4.52	-4.52	-4.52	-4.52	-4.52	-4.52	-4.52	-4.52
-4.51	-4.51	-4.51	-4.51	-4.51	-4.51	-4.51	-4.51	-4.51

-4.5	-4.5	-4.5	-4.5	-4.5	-4.5	-4.5	-4.5	-4.5
-4.49	-4.49	-4.49	-4.49	-4.49	-4.49	-4.49	-4.49	-4.49
-4.48	-4.48	-4.48	-4.48	-4.48	-4.48	-4.48	-4.48	-4.48
-4.47	-4.47	-4.47	-4.47	-4.47	-4.47	-4.47	-4.47	-4.47
-4.46	-4.46	-4.46	-4.46	-4.46	-4.46	-4.46	-4.46	-4.46
-4.45	-4.45	-4.45	-4.45	-4.45	-4.45	-4.45	-4.45	-4.45
-4.44	-4.44	-4.44	-4.44	-4.44	-4.44	-4.44	-4.44	-4.44
-4.43	-4.43	-4.43	-4.43	-4.43	-4.43	-4.43	-4.43	-4.43
-4.42	-4.42	-4.42	-4.42	-4.42	-4.42	-4.42	-4.42	-4.42
-4.41	-4.41	-4.41	-4.41	-4.41	-4.41	-4.41	-4.41	-4.41
-4.4	-4.4	-4.4	-4.4	-4.4	-4.4	-4.4	-4.4	-4.4
-4.39	-4.39	-4.39	-4.39	-4.39	-4.39	-4.39	-4.39	-4.39
-4.38	-4.38	-4.38	-4.38	-4.38	-4.38	-4.38	-4.38	-4.38
-4.37	-4.37	-4.37	-4.37	-4.37	-4.37	-4.37	-4.37	-4.37
-4.36	-4.36	-4.36	-4.36	-4.36	-4.36	-4.36	-4.36	-4.36
-4.35	-4.35	-4.35	-4.35	-4.35	-4.35	-4.35	-4.35	-4.35
-4.34	-4.34	-4.34	-4.34	-4.34	-4.34	-4.34	-4.34	-4.34
-4.33	-4.33	-4.33	-4.33	-4.33	-4.33	-4.33	-4.33	-4.33
-4.32	-4.32	-4.32	-4.32	-4.32	-4.32	-4.32	-4.32	-4.32
-4.31	-4.31	-4.31	-4.31	-4.31	-4.31	-4.31	-4.31	-4.31
-4.3	-4.3	-4.3	-4.3	-4.3	-4.3	-4.3	-4.3	-4.3
-4.29	-4.29	-4.29	-4.29	-4.29	-4.29	-4.29	-4.29	-4.29
-4.28	-4.28	-4.28	-4.28	-4.28	-4.28	-4.28	-4.28	-4.28
-4.27	-4.27	-4.27	-4.27	-4.27	-4.27	-4.27	-4.27	-4.27
-4.26	-4.26	-4.26	-4.26	-4.26	-4.26	-4.26	-4.26	-4.26
-4.25	-4.25	-4.25	-4.25	-4.25	-4.25	-4.25	-4.25	-4.25
-4.24	-4.24	-4.24	-4.24	-4.24	-4.24	-4.24	-4.24	-4.24
-4.23	-4.23	-4.23	-4.23	-4.23	-4.23	-4.23	-4.23	-4.23
-4.22	-4.22	-4.22	-4.22	-4.22	-4.22	-4.22	-4.22	-4.22
-4.21	-4.21	-4.21	-4.21	-4.21	-4.21	-4.21	-4.21	-4.21
-4.2	-4.2	-4.2	-4.2	-4.2	-4.2	-4.2	-4.2	-4.2
-4.19	-4.19	-4.19	-4.19	-4.19	-4.19	-4.19	-4.19	-4.19
-4.18	-4.18	-4.18	-4.18	-4.18	-4.18	-4.18	-4.18	-4.18
-4.17	-4.17	-4.17	-4.17	-4.17	-4.17	-4.17	-4.17	-4.17
-4.16	-4.16	-4.16	-4.16	-4.16	-4.16	-4.16	-4.16	-4.16
-4.15	-4.15	-4.15	-4.15	-4.15	-4.15	-4.15	-4.15	-4.15
-4.14	-4.14	-4.14	-4.14	-4.14	-4.14	-4.14	-4.14	-4.14
-4.13	-4.13	-4.13	-4.13	-4.13	-4.13	-4.13	-4.13	-4.13
-4.12	-4.12	-4.12	-4.12	-4.12	-4.12	-4.12	-4.12	-4.12
-4.11	-4.11	-4.11	-4.11	-4.11	-4.11	-4.11	-4.11	-4.11
-4.1	-4.1	-4.1	-4.1	-4.1	-4.1	-4.1	-4.1	-4.1
-4.09	-4.09	-4.09	-4.09	-4.09	-4.09	-4.09	-4.09	-4.09
-4.08	-4.08	-4.08	-4.08	-4.08	-4.08	-4.08	-4.08	-4.08
-4.07	-4.07	-4.07	-4.07	-4.07	-4.07	-4.07	-4.07	-4.07
-4.06	-4.06	-4.06	-4.06	-4.06	-4.06	-4.06	-4.06	-4.06
-4.05	-4.05	-4.05	-4.05	-4.05	-4.05	-4.05	-4.05	-4.05
-4.04	-4.04	-4.04	-4.04	-4.04	-4.04	-4.04	-4.04	-4.04
-4.03	-4.03	-4.03	-4.03	-4.03	-4.03	-4.03	-4.03	-4.03
-4.02	-4.02	-4.02	-4.02	-4.02	-4.02	-4.02	-4.02	-4.02
-4.01	-4.01	-4.01	-4.01	-4.01	-4.01	-4.01	-4.01	-4.01

-4	-4	-4	-4	-4	-4	-4	-4	-4
-3.99	-3.99	-3.99	-3.99	-3.99	-3.99	-3.99	-3.99	-3.99
-3.98	-3.98	-3.98	-3.98	-3.98	-3.98	-3.98	-3.98	-3.98
-3.97	-3.97	-3.97	-3.97	-3.97	-3.97	-3.97	-3.97	-3.97
-3.96	-3.96	-3.96	-3.96	-3.96	-3.96	-3.96	-3.96	-3.96
-3.95	-3.95	-3.95	-3.95	-3.95	-3.95	-3.95	-3.95	-3.95
-3.94	-3.94	-3.94	-3.94	-3.94	-3.94	-3.94	-3.94	-3.94
-3.93	-3.93	-3.93	-3.93	-3.93	-3.93	-3.93	-3.93	-3.93
-3.92	-3.92	-3.92	-3.92	-3.92	-3.92	-3.92	-3.92	-3.92
-3.91	-3.91	-3.91	-3.91	-3.91	-3.91	-3.91	-3.91	-3.91
-3.9	-3.9	-3.9	-3.9	-3.9	-3.9	-3.9	-3.9	-3.9
-3.89	-3.89	-3.89	-3.89	-3.89	-3.89	-3.89	-3.89	-3.89
-3.88	-3.88	-3.88	-3.88	-3.88	-3.88	-3.88	-3.88	-3.88
-3.87	-3.87	-3.87	-3.87	-3.87	-3.87	-3.87	-3.87	-3.87
-3.86	-3.86	-3.86	-3.86	-3.86	-3.86	-3.86	-3.86	-3.86
-3.85	-3.85	-3.85	-3.85	-3.85	-3.85	-3.85	-3.85	-3.85
-3.84	-3.84	-3.84	-3.84	-3.84	-3.84	-3.84	-3.84	-3.84
-3.83	-3.83	-3.83	-3.83	-3.83	-3.83	-3.83	-3.83	-3.83
-3.82	-3.82	-3.82	-3.82	-3.82	-3.82	-3.82	-3.82	-3.82
-3.81	-3.81	-3.81	-3.81	-3.81	-3.81	-3.81	-3.81	-3.81
-3.8	-3.8	-3.8	-3.8	-3.8	-3.8	-3.8	-3.8	-3.8
-3.79	-3.79	-3.79	-3.79	-3.79	-3.79	-3.79	-3.79	-3.79
-3.78	-3.78	-3.78	-3.78	-3.78	-3.78	-3.78	-3.78	-3.78
-3.77	-3.77	-3.77	-3.77	-3.77	-3.77	-3.77	-3.77	-3.77
-3.76	-3.76	-3.76	-3.76	-3.76	-3.76	-3.76	-3.76	-3.76
-3.75	-3.75	-3.75	-3.75	-3.75	-3.75	-3.75	-3.75	-3.75
-3.74	-3.74	-3.74	-3.74	-3.74	-3.74	-3.74	-3.74	-3.74
-3.73	-3.73	-3.73	-3.73	-3.73	-3.73	-3.73	-3.73	-3.73
-3.72	-3.72	-3.72	-3.72	-3.72	-3.72	-3.72	-3.72	-3.72
-3.71	-3.71	-3.71	-3.71	-3.71	-3.71	-3.71	-3.71	-3.71
-3.7	-3.7	-3.7	-3.7	-3.7	-3.7	-3.7	-3.7	-3.7
-3.69	-3.69	-3.69	-3.69	-3.69	-3.69	-3.69	-3.69	-3.69
-3.68	-3.68	-3.68	-3.68	-3.68	-3.68	-3.68	-3.68	-3.68
-3.67	-3.67	-3.67	-3.67	-3.67	-3.67	-3.67	-3.67	-3.67
-3.66	-3.66	-3.66	-3.66	-3.66	-3.66	-3.66	-3.66	-3.66
-3.65	-3.65	-3.65	-3.65	-3.65	-3.65	-3.65	-3.65	-3.65
-3.64	-3.64	-3.64	-3.64	-3.64	-3.64	-3.64	-3.64	-3.64
-3.63	-3.63	-3.63	-3.63	-3.63	-3.63	-3.63	-3.63	-3.63
-3.62	-3.62	-3.62	-3.62	-3.62	-3.62	-3.62	-3.62	-3.62
-3.61	-3.61	-3.61	-3.61	-3.61	-3.61	-3.61	-3.61	-3.61
-3.6	-3.6	-3.6	-3.6	-3.6	-3.6	-3.6	-3.6	-3.6
-3.59	-3.59	-3.59	-3.59	-3.59	-3.59	-3.59	-3.59	-3.59
-3.58	-3.58	-3.58	-3.58	-3.58	-3.58	-3.58	-3.58	-3.58
-3.57	-3.57	-3.57	-3.57	-3.57	-3.57	-3.57	-3.57	-3.57
-3.56	-3.56	-3.56	-3.56	-3.56	-3.56	-3.56	-3.56	-3.56
-3.55	-3.55	-3.55	-3.55	-3.55	-3.55	-3.55	-3.55	-3.55
-3.54	-3.54	-3.54	-3.54	-3.54	-3.54	-3.54	-3.54	-3.54
-3.53	-3.53	-3.53	-3.53	-3.53	-3.53	-3.53	-3.53	-3.53
-3.52	-3.52	-3.52	-3.52	-3.52	-3.52	-3.52	-3.52	-3.52
-3.51	-3.51	-3.51	-3.51	-3.51	-3.51	-3.51	-3.51	-3.51

-3.5	-3.5	-3.5	-3.5	-3.5	-3.5	-3.5	-3.5	-3.5
-3.49	-3.49	-3.49	-3.49	-3.49	-3.49	-3.49	-3.49	-3.49
-3.48	-3.48	-3.48	-3.48	-3.48	-3.48	-3.48	-3.48	-3.48
-3.47	-3.47	-3.47	-3.47	-3.47	-3.47	-3.47	-3.47	-3.47
-3.46	-3.46	-3.46	-3.46	-3.46	-3.46	-3.46	-3.46	-3.46
-3.45	-3.45	-3.45	-3.45	-3.45	-3.45	-3.45	-3.45	-3.45
-3.44	-3.44	-3.44	-3.44	-3.44	-3.44	-3.44	-3.44	-3.44
-3.43	-3.43	-3.43	-3.43	-3.43	-3.43	-3.43	-3.43	-3.43
-3.42	-3.42	-3.42	-3.42	-3.42	-3.42	-3.42	-3.42	-3.42
-3.41	-3.41	-3.41	-3.41	-3.41	-3.41	-3.41	-3.41	-3.41
-3.4	-3.4	-3.4	-3.4	-3.4	-3.4	-3.4	-3.4	-3.4
-3.39	-3.39	-3.39	-3.39	-3.39	-3.39	-3.39	-3.39	-3.39
-3.38	-3.38	-3.38	-3.38	-3.38	-3.38	-3.38	-3.38	-3.38
-3.37	-3.37	-3.37	-3.37	-3.37	-3.37	-3.37	-3.37	-3.37
-3.36	-3.36	-3.36	-3.36	-3.36	-3.36	-3.36	-3.36	-3.36
-3.35	-3.35	-3.35	-3.35	-3.35	-3.35	-3.35	-3.35	-3.35
-3.34	-3.34	-3.34	-3.34	-3.34	-3.34	-3.34	-3.34	-3.34
-3.33	-3.33	-3.33	-3.33	-3.33	-3.33	-3.33	-3.33	-3.33
-3.32	-3.32	-3.32	-3.32	-3.32	-3.32	-3.32	-3.32	-3.32
-3.31	-3.31	-3.31	-3.31	-3.31	-3.31	-3.31	-3.31	-3.31
-3.3	-3.3	-3.3	-3.3	-3.3	-3.3	-3.3	-3.3	-3.3
-3.29	-3.29	-3.29	-3.29	-3.29	-3.29	-3.29	-3.29	-3.29
-3.28	-3.28	-3.28	-3.28	-3.28	-3.28	-3.28	-3.28	-3.28
-3.27	-3.27	-3.27	-3.27	-3.27	-3.27	-3.27	-3.27	-3.27
-3.26	-3.26	-3.26	-3.26	-3.26	-3.26	-3.26	-3.26	-3.26
-3.25	-3.25	-3.25	-3.25	-3.25	-3.25	-3.25	-3.25	-3.25
-3.24	-3.24	-3.24	-3.24	-3.24	-3.24	-3.24	-3.24	-3.24
-3.23	-3.23	-3.23	-3.23	-3.23	-3.23	-3.23	-3.23	-3.23
-3.22	-3.22	-3.22	-3.22	-3.22	-3.22	-3.22	-3.22	-3.22
-3.21	-3.21	-3.21	-3.21	-3.21	-3.21	-3.21	-3.21	-3.21
-3.2	-3.2	-3.2	-3.2	-3.2	-3.2	-3.2	-3.2	-3.2
-3.19	-3.19	-3.19	-3.19	-3.19	-3.19	-3.19	-3.19	-3.19
-3.18	-3.18	-3.18	-3.18	-3.18	-3.18	-3.18	-3.18	-3.18
-3.17	-3.17	-3.17	-3.17	-3.17	-3.17	-3.17	-3.17	-3.17
-3.16	-3.16	-3.16	-3.16	-3.16	-3.16	-3.16	-3.16	-3.16
-3.15	-3.15	-3.15	-3.15	-3.15	-3.15	-3.15	-3.15	-3.15
-3.14	-3.14	-3.14	-3.14	-3.14	-3.14	-3.14	-3.14	-3.14
-3.13	-3.13	-3.13	-3.13	-3.13	-3.13	-3.13	-3.13	-3.13
-3.12	-3.12	-3.12	-3.12	-3.12	-3.12	-3.12	-3.12	-3.12
-3.11	-3.11	-3.11	-3.11	-3.11	-3.11	-3.11	-3.11	-3.11
-3.1	-3.1	-3.1	-3.1	-3.1	-3.1	-3.1	-3.1	-3.1
-3.09	-3.09	-3.09	-3.09	-3.09	-3.09	-3.09	-3.09	-3.09
-3.08	-3.08	-3.08	-3.08	-3.08	-3.08	-3.08	-3.08	-3.08
-3.07	-3.07	-3.07	-3.07	-3.07	-3.07	-3.07	-3.07	-3.07
-3.06	-3.06	-3.06	-3.06	-3.06	-3.06	-3.06	-3.06	-3.06
-3.05	-3.05	-3.05	-3.05	-3.05	-3.05	-3.05	-3.05	-3.05
-3.04	-3.04	-3.04	-3.04	-3.04	-3.04	-3.04	-3.04	-3.04
-3.03	-3.03	-3.03	-3.03	-3.03	-3.03	-3.03	-3.03	-3.03
-3.02	-3.02	-3.02	-3.02	-3.02	-3.02	-3.02	-3.02	-3.02
-3.01	-3.01	-3.01	-3.01	-3.01	-3.01	-3.01	-3.01	-3.01

-3	-3	-3	-3	-3	-3	-3	-3	-3
-2.99	-2.99	-2.99	-2.99	-2.99	-2.99	-2.99	-2.99	-2.99
-2.98	-2.98	-2.98	-2.98	-2.98	-2.98	-2.98	-2.98	-2.98
-2.97	-2.97	-2.97	-2.97	-2.97	-2.97	-2.97	-2.97	-2.97
-2.96	-2.96	-2.96	-2.96	-2.96	-2.96	-2.96	-2.96	-2.96
-2.95	-2.95	-2.95	-2.95	-2.95	-2.95	-2.95	-2.95	-2.95
-2.94	-2.94	-2.94	-2.94	-2.94	-2.94	-2.94	-2.94	-2.94
-2.93	-2.93	-2.93	-2.93	-2.93	-2.93	-2.93	-2.93	-2.93
-2.92	-2.92	-2.92	-2.92	-2.92	-2.92	-2.92	-2.92	-2.92
-2.91	-2.91	-2.91	-2.91	-2.91	-2.91	-2.91	-2.91	-2.91
-2.9	-2.9	-2.9	-2.9	-2.9	-2.9	-2.9	-2.9	-2.9
-2.89	-2.89	-2.89	-2.89	-2.89	-2.89	-2.89	-2.89	-2.89
-2.88	-2.88	-2.88	-2.88	-2.88	-2.88	-2.88	-2.88	-2.88
-2.87	-2.87	-2.87	-2.87	-2.87	-2.87	-2.87	-2.87	-2.87
-2.86	-2.86	-2.86	-2.86	-2.86	-2.86	-2.86	-2.86	-2.86
-2.85	-2.85	-2.85	-2.85	-2.85	-2.85	-2.85	-2.85	-2.85
-2.84	-2.84	-2.84	-2.84	-2.84	-2.84	-2.84	-2.84	-2.84
-2.83	-2.83	-2.83	-2.83	-2.83	-2.83	-2.83	-2.83	-2.83
-2.82	-2.82	-2.82	-2.82	-2.82	-2.82	-2.82	-2.82	-2.82
-2.81	-2.81	-2.81	-2.81	-2.81	-2.81	-2.81	-2.81	-2.81
-2.8	-2.8	-2.8	-2.8	-2.8	-2.8	-2.8	-2.8	-2.8
-2.79	-2.79	-2.79	-2.79	-2.79	-2.79	-2.79	-2.79	-2.79
-2.78	-2.78	-2.78	-2.78	-2.78	-2.78	-2.78	-2.78	-2.78
-2.77	-2.77	-2.77	-2.77	-2.77	-2.77	-2.77	-2.77	-2.77
-2.76	-2.76	-2.76	-2.76	-2.76	-2.76	-2.76	-2.76	-2.76
-2.75	-2.75	-2.75	-2.75	-2.75	-2.75	-2.75	-2.75	-2.75
-2.74	-2.74	-2.74	-2.74	-2.74	-2.74	-2.74	-2.74	-2.74
-2.73	-2.73	-2.73	-2.73	-2.73	-2.73	-2.73	-2.73	-2.73
-2.72	-2.72	-2.72	-2.72	-2.72	-2.72	-2.72	-2.72	-2.72
-2.71	-2.71	-2.71	-2.71	-2.71	-2.71	-2.71	-2.71	-2.71
-2.7	-2.7	-2.7	-2.7	-2.7	-2.7	-2.7	-2.7	-2.7
-2.69	-2.69	-2.69	-2.69	-2.69	-2.69	-2.69	-2.69	-2.69
-2.68	-2.68	-2.68	-2.68	-2.68	-2.68	-2.68	-2.68	-2.68
-2.67	-2.67	-2.67	-2.67	-2.67	-2.67	-2.67	-2.67	-2.67
-2.66	-2.66	-2.66	-2.66	-2.66	-2.66	-2.66	-2.66	-2.66
-2.65	-2.65	-2.65	-2.65	-2.65	-2.65	-2.65	-2.65	-2.65
-2.64	-2.64	-2.64	-2.64	-2.64	-2.64	-2.64	-2.64	-2.64
-2.63	-2.63	-2.63	-2.63	-2.63	-2.63	-2.63	-2.63	-2.63
-2.62	-2.62	-2.62	-2.62	-2.62	-2.62	-2.62	-2.62	-2.62
-2.61	-2.61	-2.61	-2.61	-2.61	-2.61	-2.61	-2.61	-2.61
-2.6	-2.6	-2.6	-2.6	-2.6	-2.6	-2.6	-2.6	-2.6
-2.59	-2.59	-2.59	-2.59	-2.59	-2.59	-2.59	-2.59	-2.59
-2.58	-2.58	-2.58	-2.58	-2.58	-2.58	-2.58	-2.58	-2.58
-2.57	-2.57	-2.57	-2.57	-2.57	-2.57	-2.57	-2.57	-2.57
-2.56	-2.56	-2.56	-2.56	-2.56	-2.56	-2.56	-2.56	-2.56
-2.55	-2.55	-2.55	-2.55	-2.55	-2.55	-2.55	-2.55	-2.55
-2.54	-2.54	-2.54	-2.54	-2.54	-2.54	-2.54	-2.54	-2.54
-2.53	-2.53	-2.53	-2.53	-2.53	-2.53	-2.53	-2.53	-2.53
-2.52	-2.52	-2.52	-2.52	-2.52	-2.52	-2.52	-2.52	-2.52
-2.51	-2.51	-2.51	-2.51	-2.51	-2.51	-2.51	-2.51	-2.51

-2.5	-2.5	-2.5	-2.5	-2.5	-2.5	-2.5	-2.5	-2.5
-2.49	-2.49	-2.49	-2.49	-2.49	-2.49	-2.49	-2.49	-2.49
-2.48	-2.48	-2.48	-2.48	-2.48	-2.48	-2.48	-2.48	-2.48
-2.47	-2.47	-2.47	-2.47	-2.47	-2.47	-2.47	-2.47	-2.47
-2.46	-2.46	-2.46	-2.46	-2.46	-2.46	-2.46	-2.46	-2.46
-2.45	-2.45	-2.45	-2.45	-2.45	-2.45	-2.45	-2.45	-2.45
-2.44	-2.44	-2.44	-2.44	-2.44	-2.44	-2.44	-2.44	-2.44
-2.43	-2.43	-2.43	-2.43	-2.43	-2.43	-2.43	-2.43	-2.43
-2.42	-2.42	-2.42	-2.42	-2.42	-2.42	-2.42	-2.42	-2.42
-2.41	-2.41	-2.41	-2.41	-2.41	-2.41	-2.41	-2.41	-2.41
-2.4	-2.4	-2.4	-2.4	-2.4	-2.4	-2.4	-2.4	-2.4
-2.39	-2.39	-2.39	-2.39	-2.39	-2.39	-2.39	-2.39	-2.39
-2.38	-2.38	-2.38	-2.38	-2.38	-2.38	-2.38	-2.38	-2.38
-2.37	-2.37	-2.37	-2.37	-2.37	-2.37	-2.37	-2.37	-2.37
-2.36	-2.36	-2.36	-2.36	-2.36	-2.36	-2.36	-2.36	-2.36
-2.35	-2.35	-2.35	-2.35	-2.35	-2.35	-2.35	-2.35	-2.35
-2.34	-2.34	-2.34	-2.34	-2.34	-2.34	-2.34	-2.34	-2.34
-2.33	-2.33	-2.33	-2.33	-2.33	-2.33	-2.33	-2.33	-2.33
-2.32	-2.32	-2.32	-2.32	-2.32	-2.32	-2.32	-2.32	-2.32
-2.31	-2.31	-2.31	-2.31	-2.31	-2.31	-2.31	-2.31	-2.31
-2.3	-2.3	-2.3	-2.3	-2.3	-2.3	-2.3	-2.3	-2.3
-2.29	-2.29	-2.29	-2.29	-2.29	-2.29	-2.29	-2.29	-2.29
-2.28	-2.28	-2.28	-2.28	-2.28	-2.28	-2.28	-2.28	-2.28
-2.27	-2.27	-2.27	-2.27	-2.27	-2.27	-2.27	-2.27	-2.27
-2.26	-2.26	-2.26	-2.26	-2.26	-2.26	-2.26	-2.26	-2.26
-2.25	-2.25	-2.25	-2.25	-2.25	-2.25	-2.25	-2.25	-2.25
-2.24	-2.24	-2.24	-2.24	-2.24	-2.24	-2.24	-2.24	-2.24
-2.23	-2.23	-2.23	-2.23	-2.23	-2.23	-2.23	-2.23	-2.23
-2.22	-2.22	-2.22	-2.22	-2.22	-2.22	-2.22	-2.22	-2.22
-2.21	-2.21	-2.21	-2.21	-2.21	-2.21	-2.21	-2.21	-2.21
-2.2	-2.2	-2.2	-2.2	-2.2	-2.2	-2.2	-2.2	-2.2
-2.19	-2.19	-2.19	-2.19	-2.19	-2.19	-2.19	-2.19	-2.19
-2.18	-2.18	-2.18	-2.18	-2.18	-2.18	-2.18	-2.18	-2.18
-2.17	-2.17	-2.17	-2.17	-2.17	-2.17	-2.17	-2.17	-2.17
-2.16	-2.16	-2.16	-2.16	-2.16	-2.16	-2.16	-2.16	-2.16
-2.15	-2.15	-2.15	-2.15	-2.15	-2.15	-2.15	-2.15	-2.15
-2.14	-2.14	-2.14	-2.14	-2.14	-2.14	-2.14	-2.14	-2.14
-2.13	-2.13	-2.13	-2.13	-2.13	-2.13	-2.13	-2.13	-2.13
-2.12	-2.12	-2.12	-2.12	-2.12	-2.12	-2.12	-2.12	-2.12
-2.11	-2.11	-2.11	-2.11	-2.11	-2.11	-2.11	-2.11	-2.11
-2.1	-2.1	-2.1	-2.1	-2.1	-2.1	-2.1	-2.1	-2.1
-2.09	-2.09	-2.09	-2.09	-2.09	-2.09	-2.09	-2.09	-2.09
-2.08	-2.08	-2.08	-2.08	-2.08	-2.08	-2.08	-2.08	-2.08
-2.07	-2.07	-2.07	-2.07	-2.07	-2.07	-2.07	-2.07	-2.07
-2.06	-2.06	-2.06	-2.06	-2.06	-2.06	-2.06	-2.06	-2.06
-2.05	-2.05	-2.05	-2.05	-2.05	-2.05	-2.05	-2.05	-2.05
-2.04	-2.04	-2.04	-2.04	-2.04	-2.04	-2.04	-2.04	-2.04
-2.03	-2.03	-2.03	-2.03	-2.03	-2.03	-2.03	-2.03	-2.03
-2.02	-2.02	-2.02	-2.02	-2.02	-2.02	-2.02	-2.02	-2.02
-2.01	-2.01	-2.01	-2.01	-2.01	-2.01	-2.01	-2.01	-2.01

-2	-2	-2	-2	-2	-2	-2	-2	-2
-1.99	-1.99	-1.99	-1.99	-1.99	-1.99	-1.99	-1.99	-1.99
-1.98	-1.98	-1.98	-1.98	-1.98	-1.98	-1.98	-1.98	-1.98
-1.97	-1.97	-1.97	-1.97	-1.97	-1.97	-1.97	-1.97	-1.97
-1.96	-1.96	-1.96	-1.96	-1.96	-1.96	-1.96	-1.96	-1.96
-1.95	-1.95	-1.95	-1.95	-1.95	-1.95	-1.95	-1.95	-1.95
-1.94	-1.94	-1.94	-1.94	-1.94	-1.94	-1.94	-1.94	-1.94
-1.93	-1.93	-1.93	-1.93	-1.93	-1.93	-1.93	-1.93	-1.93
-1.92	-1.92	-1.92	-1.92	-1.92	-1.92	-1.92	-1.92	-1.92
-1.91	-1.91	-1.91	-1.91	-1.91	-1.91	-1.91	-1.91	-1.91
-1.9	-1.9	-1.9	-1.9	-1.9	-1.9	-1.9	-1.9	-1.9
-1.89	-1.89	-1.89	-1.89	-1.89	-1.89	-1.89	-1.89	-1.89
-1.88	-1.88	-1.88	-1.88	-1.88	-1.88	-1.88	-1.88	-1.88
-1.87	-1.87	-1.87	-1.87	-1.87	-1.87	-1.87	-1.87	-1.87
-1.86	-1.86	-1.86	-1.86	-1.86	-1.86	-1.86	-1.86	-1.86
-1.85	-1.85	-1.85	-1.85	-1.85	-1.85	-1.85	-1.85	-1.85
-1.84	-1.84	-1.84	-1.84	-1.84	-1.84	-1.84	-1.84	-1.84
-1.83	-1.83	-1.83	-1.83	-1.83	-1.83	-1.83	-1.83	-1.83
-1.82	-1.82	-1.82	-1.82	-1.82	-1.82	-1.82	-1.82	-1.82
-1.81	-1.81	-1.81	-1.81	-1.81	-1.81	-1.81	-1.81	-1.81
-1.8	-1.8	-1.8	-1.8	-1.8	-1.8	-1.8	-1.8	-1.8
-1.79	-1.79	-1.79	-1.79	-1.79	-1.79	-1.79	-1.79	-1.79
-1.78	-1.78	-1.78	-1.78	-1.78	-1.78	-1.78	-1.78	-1.78
-1.77	-1.77	-1.77	-1.77	-1.77	-1.77	-1.77	-1.77	-1.77
-1.76	-1.76	-1.76	-1.76	-1.76	-1.76	-1.76	-1.76	-1.76
-1.75	-1.75	-1.75	-1.75	-1.75	-1.75	-1.75	-1.75	-1.75
-1.74	-1.74	-1.74	-1.74	-1.74	-1.74	-1.74	-1.74	-1.74
-1.73	-1.73	-1.73	-1.73	-1.73	-1.73	-1.73	-1.73	-1.73
-1.72	-1.72	-1.72	-1.72	-1.72	-1.72	-1.72	-1.72	-1.72
-1.71	-1.71	-1.71	-1.71	-1.71	-1.71	-1.71	-1.71	-1.71
-1.7	-1.7	-1.7	-1.7	-1.7	-1.7	-1.7	-1.7	-1.7
-1.69	-1.69	-1.69	-1.69	-1.69	-1.69	-1.69	-1.69	-1.69
-1.68	-1.68	-1.68	-1.68	-1.68	-1.68	-1.68	-1.68	-1.68
-1.67	-1.67	-1.67	-1.67	-1.67	-1.67	-1.67	-1.67	-1.67
-1.66	-1.66	-1.66	-1.66	-1.66	-1.66	-1.66	-1.66	-1.66
-1.65	-1.65	-1.65	-1.65	-1.65	-1.65	-1.65	-1.65	-1.65
-1.64	-1.64	-1.64	-1.64	-1.64	-1.64	-1.64	-1.64	-1.64
-1.63	-1.63	-1.63	-1.63	-1.63	-1.63	-1.63	-1.63	-1.63
-1.62	-1.62	-1.62	-1.62	-1.62	-1.62	-1.62	-1.62	-1.62
-1.61	-1.61	-1.61	-1.61	-1.61	-1.61	-1.61	-1.61	-1.61
-1.6	-1.6	-1.6	-1.6	-1.6	-1.6	-1.6	-1.6	-1.6
-1.59	-1.59	-1.59	-1.59	-1.59	-1.59	-1.59	-1.59	-1.59
-1.58	-1.58	-1.58	-1.58	-1.58	-1.58	-1.58	-1.58	-1.58
-1.57	-1.57	-1.57	-1.57	-1.57	-1.57	-1.57	-1.57	-1.57
-1.56	-1.56	-1.56	-1.56	-1.56	-1.56	-1.56	-1.56	-1.56
-1.55	-1.55	-1.55	-1.55	-1.55	-1.55	-1.55	-1.55	-1.55
-1.54	-1.54	-1.54	-1.54	-1.54	-1.54	-1.54	-1.54	-1.54
-1.53	-1.53	-1.53	-1.53	-1.53	-1.53	-1.53	-1.53	-1.53
-1.52	-1.52	-1.52	-1.52	-1.52	-1.52	-1.52	-1.52	-1.52
-1.51	-1.51	-1.51	-1.51	-1.51	-1.51	-1.51	-1.51	-1.51

-1.5	-1.5	-1.5	-1.5	-1.5	-1.5	-1.5	-1.5	-1.5
-1.49	-1.49	-1.49	-1.49	-1.49	-1.49	-1.49	-1.49	-1.49
-1.48	-1.48	-1.48	-1.48	-1.48	-1.48	-1.48	-1.48	-1.48
-1.47	-1.47	-1.47	-1.47	-1.47	-1.47	-1.47	-1.47	-1.47
-1.46	-1.46	-1.46	-1.46	-1.46	-1.46	-1.46	-1.46	-1.46
-1.45	-1.45	-1.45	-1.45	-1.45	-1.45	-1.45	-1.45	-1.45
-1.44	-1.44	-1.44	-1.44	-1.44	-1.44	-1.44	-1.44	-1.44
-1.43	-1.43	-1.43	-1.43	-1.43	-1.43	-1.43	-1.43	-1.43
-1.42	-1.42	-1.42	-1.42	-1.42	-1.42	-1.42	-1.42	-1.42
-1.41	-1.41	-1.41	-1.41	-1.41	-1.41	-1.41	-1.41	-1.41
-1.4	-1.4	-1.4	-1.4	-1.4	-1.4	-1.4	-1.4	-1.4
-1.39	-1.39	-1.39	-1.39	-1.39	-1.39	-1.39	-1.39	-1.39
-1.38	-1.38	-1.38	-1.38	-1.38	-1.38	-1.38	-1.38	-1.38
-1.37	-1.37	-1.37	-1.37	-1.37	-1.37	-1.37	-1.37	-1.37
-1.36	-1.36	-1.36	-1.36	-1.36	-1.36	-1.36	-1.36	-1.36
-1.35	-1.35	-1.35	-1.35	-1.35	-1.35	-1.35	-1.35	-1.35
-1.34	-1.34	-1.34	-1.34	-1.34	-1.34	-1.34	-1.34	-1.34
-1.33	-1.33	-1.33	-1.33	-1.33	-1.33	-1.33	-1.33	-1.33
-1.32	-1.32	-1.32	-1.32	-1.32	-1.32	-1.32	-1.32	-1.32
-1.31	-1.31	-1.31	-1.31	-1.31	-1.31	-1.31	-1.31	-1.31
-1.3	-1.3	-1.3	-1.3	-1.3	-1.3	-1.3	-1.3	-1.3
-1.29	-1.29	-1.29	-1.29	-1.29	-1.29	-1.29	-1.29	-1.29
-1.28	-1.28	-1.28	-1.28	-1.28	-1.28	-1.28	-1.28	-1.28
-1.27	-1.27	-1.27	-1.27	-1.27	-1.27	-1.27	-1.27	-1.27
-1.26	-1.26	-1.26	-1.26	-1.26	-1.26	-1.26	-1.26	-1.26
-1.25	-1.25	-1.25	-1.25	-1.25	-1.25	-1.25	-1.25	-1.25
-1.24	-1.24	-1.24	-1.24	-1.24	-1.24	-1.24	-1.24	-1.24
-1.23	-1.23	-1.23	-1.23	-1.23	-1.23	-1.23	-1.23	-1.23
-1.22	-1.22	-1.22	-1.22	-1.22	-1.22	-1.22	-1.22	-1.22
-1.21	-1.21	-1.21	-1.21	-1.21	-1.21	-1.21	-1.21	-1.21
-1.2	-1.2	-1.2	-1.2	-1.2	-1.2	-1.2	-1.2	-1.2
-1.19	-1.19	-1.19	-1.19	-1.19	-1.19	-1.19	-1.19	-1.19
-1.18	-1.18	-1.18	-1.18	-1.18	-1.18	-1.18	-1.18	-1.18
-1.17	-1.17	-1.17	-1.17	-1.17	-1.17	-1.17	-1.17	-1.17
-1.16	-1.16	-1.16	-1.16	-1.16	-1.16	-1.16	-1.16	-1.16
-1.15	-1.15	-1.15	-1.15	-1.15	-1.15	-1.15	-1.15	-1.15
-1.14	-1.14	-1.14	-1.14	-1.14	-1.14	-1.14	-1.14	-1.14
-1.13	-1.13	-1.13	-1.13	-1.13	-1.13	-1.13	-1.13	-1.13
-1.12	-1.12	-1.12	-1.12	-1.12	-1.12	-1.12	-1.12	-1.12
-1.11	-1.11	-1.11	-1.11	-1.11	-1.11	-1.11	-1.11	-1.11
-1.1	-1.1	-1.1	-1.1	-1.1	-1.1	-1.1	-1.1	-1.1
-1.09	-1.09	-1.09	-1.09	-1.09	-1.09	-1.09	-1.09	-1.09
-1.08	-1.08	-1.08	-1.08	-1.08	-1.08	-1.08	-1.08	-1.08
-1.07	-1.07	-1.07	-1.07	-1.07	-1.07	-1.07	-1.07	-1.07
-1.06	-1.06	-1.06	-1.06	-1.06	-1.06	-1.06	-1.06	-1.06
-1.05	-1.05	-1.05	-1.05	-1.05	-1.05	-1.05	-1.05	-1.05
-1.04	-1.04	-1.04	-1.04	-1.04	-1.04	-1.04	-1.04	-1.04
-1.03	-1.03	-1.03	-1.03	-1.03	-1.03	-1.03	-1.03	-1.03
-1.02	-1.02	-1.02	-1.02	-1.02	-1.02	-1.02	-1.02	-1.02
-1.01	-1.01	-1.01	-1.01	-1.01	-1.01	-1.01	-1.01	-1.01

-1	-1	-1	-1	-1	-1	-1	-1	-1
-0.99	-0.99	-0.99	-0.99	-0.99	-0.99	-0.99	-0.99	-0.99
-0.98	-0.98	-0.98	-0.98	-0.98	-0.98	-0.98	-0.98	-0.98
-0.97	-0.97	-0.97	-0.97	-0.97	-0.97	-0.97	-0.97	-0.97
-0.96	-0.96	-0.96	-0.96	-0.96	-0.96	-0.96	-0.96	-0.96
-0.95	-0.95	-0.95	-0.95	-0.95	-0.95	-0.95	-0.95	-0.95
-0.94	-0.94	-0.94	-0.94	-0.94	-0.94	-0.94	-0.94	-0.94
-0.93	-0.93	-0.93	-0.93	-0.93	-0.93	-0.93	-0.93	-0.93
-0.92	-0.92	-0.92	-0.92	-0.92	-0.92	-0.92	-0.92	-0.92
-0.91	-0.91	-0.91	-0.91	-0.91	-0.91	-0.91	-0.91	-0.91
-0.9	-0.9	-0.9	-0.9	-0.9	-0.9	-0.9	-0.9	-0.9
-0.89	-0.89	-0.89	-0.89	-0.89	-0.89	-0.89	-0.89	-0.89
-0.88	-0.88	-0.88	-0.88	-0.88	-0.88	-0.88	-0.88	-0.88
-0.87	-0.87	-0.87	-0.87	-0.87	-0.87	-0.87	-0.87	-0.87
-0.86	-0.86	-0.86	-0.86	-0.86	-0.86	-0.86	-0.86	-0.86
-0.85	-0.85	-0.85	-0.85	-0.85	-0.85	-0.85	-0.85	-0.85
-0.84	-0.84	-0.84	-0.84	-0.84	-0.84	-0.84	-0.84	-0.84
-0.83	-0.83	-0.83	-0.83	-0.83	-0.83	-0.83	-0.83	-0.83
-0.82	-0.82	-0.82	-0.82	-0.82	-0.82	-0.82	-0.82	-0.82
-0.81	-0.81	-0.81	-0.81	-0.81	-0.81	-0.81	-0.81	-0.81
-0.8	-0.8	-0.8	-0.8	-0.8	-0.8	-0.8	-0.8	-0.8
-0.79	-0.79	-0.79	-0.79	-0.79	-0.79	-0.79	-0.79	-0.79
-0.78	-0.78	-0.78	-0.78	-0.78	-0.78	-0.78	-0.78	-0.78
-0.77	-0.77	-0.77	-0.77	-0.77	-0.77	-0.77	-0.77	-0.77
-0.76	-0.76	-0.76	-0.76	-0.76	-0.76	-0.76	-0.76	-0.76
-0.75	-0.75	-0.75	-0.75	-0.75	-0.75	-0.75	-0.75	-0.75
-0.74	-0.74	-0.74	-0.74	-0.74	-0.74	-0.74	-0.74	-0.74
-0.73	-0.73	-0.73	-0.73	-0.73	-0.73	-0.73	-0.73	-0.73
-0.72	-0.72	-0.72	-0.72	-0.72	-0.72	-0.72	-0.72	-0.72
-0.71	-0.71	-0.71	-0.71	-0.71	-0.71	-0.71	-0.71	-0.71
-0.7	-0.7	-0.7	-0.7	-0.7	-0.7	-0.7	-0.7	-0.7
-0.69	-0.69	-0.69	-0.69	-0.69	-0.69	-0.69	-0.69	-0.69
-0.68	-0.68	-0.68	-0.68	-0.68	-0.68	-0.68	-0.68	-0.68
-0.67	-0.67	-0.67	-0.67	-0.67	-0.67	-0.67	-0.67	-0.67
-0.66	-0.66	-0.66	-0.66	-0.66	-0.66	-0.66	-0.66	-0.66
-0.65	-0.65	-0.65	-0.65	-0.65	-0.65	-0.65	-0.65	-0.65
-0.64	-0.64	-0.64	-0.64	-0.64	-0.64	-0.64	-0.64	-0.64
-0.63	-0.63	-0.63	-0.63	-0.63	-0.63	-0.63	-0.63	-0.63
-0.62	-0.62	-0.62	-0.62	-0.62	-0.62	-0.62	-0.62	-0.62
-0.61	-0.61	-0.61	-0.61	-0.61	-0.61	-0.61	-0.61	-0.61
-0.6	-0.6	-0.6	-0.6	-0.6	-0.6	-0.6	-0.6	-0.6
-0.59	-0.59	-0.59	-0.59	-0.59	-0.59	-0.59	-0.59	-0.59
-0.58	-0.58	-0.58	-0.58	-0.58	-0.58	-0.58	-0.58	-0.58
-0.57	-0.57	-0.57	-0.57	-0.57	-0.57	-0.57	-0.57	-0.57
-0.56	-0.56	-0.56	-0.56	-0.56	-0.56	-0.56	-0.56	-0.56
-0.55	-0.55	-0.55	-0.55	-0.55	-0.55	-0.55	-0.55	-0.55
-0.54	-0.54	-0.54	-0.54	-0.54	-0.54	-0.54	-0.54	-0.54
-0.53	-0.53	-0.53	-0.53	-0.53	-0.53	-0.53	-0.53	-0.53
-0.52	-0.52	-0.52	-0.52	-0.52	-0.52	-0.52	-0.52	-0.52
-0.51	-0.51	-0.51	-0.51	-0.51	-0.51	-0.51	-0.51	-0.51

-0.5	-0.5	-0.5	-0.5	-0.5	-0.5	-0.5	-0.5	-0.5
-0.49	-0.49	-0.49	-0.49	-0.49	-0.49	-0.49	-0.49	-0.49
-0.48	-0.48	-0.48	-0.48	-0.48	-0.48	-0.48	-0.48	-0.48
-0.47	-0.47	-0.47	-0.47	-0.47	-0.47	-0.47	-0.47	-0.47
-0.46	-0.46	-0.46	-0.46	-0.46	-0.46	-0.46	-0.46	-0.46
-0.45	-0.45	-0.45	-0.45	-0.45	-0.45	-0.45	-0.45	-0.45
-0.44	-0.44	-0.44	-0.44	-0.44	-0.44	-0.44	-0.44	-0.44
-0.43	-0.43	-0.43	-0.43	-0.43	-0.43	-0.43	-0.43	-0.43
-0.42	-0.42	-0.42	-0.42	-0.42	-0.42	-0.42	-0.42	-0.42
-0.41	-0.41	-0.41	-0.41	-0.41	-0.41	-0.41	-0.41	-0.41
-0.4	-0.4	-0.4	-0.4	-0.4	-0.4	-0.4	-0.4	-0.4
-0.39	-0.39	-0.39	-0.39	-0.39	-0.39	-0.39	-0.39	-0.39
-0.38	-0.38	-0.38	-0.38	-0.38	-0.38	-0.38	-0.38	-0.38
-0.37	-0.37	-0.37	-0.37	-0.37	-0.37	-0.37	-0.37	-0.37
-0.36	-0.36	-0.36	-0.36	-0.36	-0.36	-0.36	-0.36	-0.36
-0.35	-0.35	-0.35	-0.35	-0.35	-0.35	-0.35	-0.35	-0.35
-0.34	-0.34	-0.34	-0.34	-0.34	-0.34	-0.34	-0.34	-0.34
-0.33	-0.33	-0.33	-0.33	-0.33	-0.33	-0.33	-0.33	-0.33
-0.32	-0.32	-0.32	-0.32	-0.32	-0.32	-0.32	-0.32	-0.32
-0.31	-0.31	-0.31	-0.31	-0.31	-0.31	-0.31	-0.31	-0.31
-0.3	-0.3	-0.3	-0.3	-0.3	-0.3	-0.3	-0.3	-0.3
-0.29	-0.29	-0.29	-0.29	-0.29	-0.29	-0.29	-0.29	-0.29
-0.28	-0.28	-0.28	-0.28	-0.28	-0.28	-0.28	-0.28	-0.28
-0.27	-0.27	-0.27	-0.27	-0.27	-0.27	-0.27	-0.27	-0.27
-0.26	-0.26	-0.26	-0.26	-0.26	-0.26	-0.26	-0.26	-0.26
-0.25	-0.25	-0.25	-0.25	-0.25	-0.25	-0.25	-0.25	-0.25
-0.24	-0.24	-0.24	-0.24	-0.24	-0.24	-0.24	-0.24	-0.24
-0.23	-0.23	-0.23	-0.23	-0.23	-0.23	-0.23	-0.23	-0.23
-0.22	-0.22	-0.22	-0.22	-0.22	-0.22	-0.22	-0.22	-0.22
-0.21	-0.21	-0.21	-0.21	-0.21	-0.21	-0.21	-0.21	-0.21
-0.2	-0.2	-0.2	-0.2	-0.2	-0.2	-0.2	-0.2	-0.2
-0.19	-0.19	-0.19	-0.19	-0.19	-0.19	-0.19	-0.19	-0.19
-0.18	-0.18	-0.18	-0.18	-0.18	-0.18	-0.18	-0.18	-0.18
-0.17	-0.17	-0.17	-0.17	-0.17	-0.17	-0.17	-0.17	-0.17
-0.16	-0.16	-0.16	-0.16	-0.16	-0.16	-0.16	-0.16	-0.16
-0.15	-0.15	-0.15	-0.15	-0.15	-0.15	-0.15	-0.15	-0.15
-0.14	-0.14	-0.14	-0.14	-0.14	-0.14	-0.14	-0.14	-0.14
-0.13	-0.13	-0.13	-0.13	-0.13	-0.13	-0.13	-0.13	-0.13
-0.12	-0.12	-0.12	-0.12	-0.12	-0.12	-0.12	-0.12	-0.12
-0.11	-0.11	-0.11	-0.11	-0.11	-0.11	-0.11	-0.11	-0.11
-0.1	-0.1	-0.1	-0.1	-0.1	-0.1	-0.1	-0.1	-0.1
-0.09	-0.09	-0.09	-0.09	-0.09	-0.09	-0.09	-0.09	-0.09
-0.08	-0.08	-0.08	-0.08	-0.08	-0.08	-0.08	-0.08	-0.08
-0.07	-0.07	-0.07	-0.07	-0.07	-0.07	-0.07	-0.07	-0.07
-0.06	-0.06	-0.06	-0.06	-0.06	-0.06	-0.06	-0.06	-0.06
-0.05	-0.05	-0.05	-0.05	-0.05	-0.05	-0.05	-0.05	-0.05
-0.04	-0.04	-0.04	-0.04	-0.04	-0.04	-0.04	-0.04	-0.04
-0.03	-0.03	-0.03	-0.03	-0.03	-0.03	-0.03	-0.03	-0.03
-0.02	-0.02	-0.02	-0.02	-0.02	-0.02	-0.02	-0.02	-0.02
-0.01	-0.01	-0.01	-0.01	-0.01	-0.01	-0.01	-0.01	-0.01

0	0	0	0	0	0	0	0	0
0.01	0.01	0.01	0.01	0.01	0.01	0.01	0.01	0.01
0.02	0.02	0.02	0.02	0.02	0.02	0.02	0.02	0.02
0.03	0.03	0.03	0.03	0.03	0.03	0.03	0.03	0.03
0.04	0.04	0.04	0.04	0.04	0.04	0.04	0.04	0.04
0.05	0.05	0.05	0.05	0.05	0.05	0.05	0.05	0.05
0.06	0.06	0.06	0.06	0.06	0.06	0.06	0.06	0.06
0.07	0.07	0.07	0.07	0.07	0.07	0.07	0.07	0.07
0.08	0.08	0.08	0.08	0.08	0.08	0.08	0.08	0.08
0.09	0.09	0.09	0.09	0.09	0.09	0.09	0.09	0.09
0.1	0.1	0.1	0.1	0.1	0.1	0.1	0.1	0.1
0.11	0.11	0.11	0.11	0.11	0.11	0.11	0.11	0.11
0.12	0.12	0.12	0.12	0.12	0.12	0.12	0.12	0.12
0.13	0.13	0.13	0.13	0.13	0.13	0.13	0.13	0.13
0.14	0.14	0.14	0.14	0.14	0.14	0.14	0.14	0.14
0.15	0.15	0.15	0.15	0.15	0.15	0.15	0.15	0.15
0.16	0.16	0.16	0.16	0.16	0.16	0.16	0.16	0.16
0.17	0.17	0.17	0.17	0.17	0.17	0.17	0.17	0.17
0.18	0.18	0.18	0.18	0.18	0.18	0.18	0.18	0.18
0.19	0.19	0.19	0.19	0.19	0.19	0.19	0.19	0.19
0.2	0.2	0.2	0.2	0.2	0.2	0.2	0.2	0.2
0.21	0.21	0.21	0.21	0.21	0.21	0.21	0.21	0.21
0.22	0.22	0.22	0.22	0.22	0.22	0.22	0.22	0.22
0.23	0.23	0.23	0.23	0.23	0.23	0.23	0.23	0.23
0.24	0.24	0.24	0.24	0.24	0.24	0.24	0.24	0.24
0.25	0.25	0.25	0.25	0.25	0.25	0.25	0.25	0.25
0.26	0.26	0.26	0.26	0.26	0.26	0.26	0.26	0.26
0.27	0.27	0.27	0.27	0.27	0.27	0.27	0.27	0.27
0.28	0.28	0.28	0.28	0.28	0.28	0.28	0.28	0.28
0.29	0.29	0.29	0.29	0.29	0.29	0.29	0.29	0.29
0.3	0.3	0.3	0.3	0.3	0.3	0.3	0.3	0.3
0.31	0.31	0.31	0.31	0.31	0.31	0.31	0.31	0.31
0.32	0.32	0.32	0.32	0.32	0.32	0.32	0.32	0.32
0.33	0.33	0.33	0.33	0.33	0.33	0.33	0.33	0.33
0.34	0.34	0.34	0.34	0.34	0.34	0.34	0.34	0.34
0.35	0.35	0.35	0.35	0.35	0.35	0.35	0.35	0.35
0.36	0.36	0.36	0.36	0.36	0.36	0.36	0.36	0.36
0.37	0.37	0.37	0.37	0.37	0.37	0.37	0.37	0.37
0.38	0.38	0.38	0.38	0.38	0.38	0.38	0.38	0.38
0.39	0.39	0.39	0.39	0.39	0.39	0.39	0.39	0.39
0.4	0.4	0.4	0.4	0.4	0.4	0.4	0.4	0.4
0.41	0.41	0.41	0.41	0.41	0.41	0.41	0.41	0.41
0.42	0.42	0.42	0.42	0.42	0.42	0.42	0.42	0.42
0.43	0.43	0.43	0.43	0.43	0.43	0.43	0.43	0.43
0.44	0.44	0.44	0.44	0.44	0.44	0.44	0.44	0.44
0.45	0.45	0.45	0.45	0.45	0.45	0.45	0.45	0.45
0.46	0.46	0.46	0.46	0.46	0.46	0.46	0.46	0.46
0.47	0.47	0.47	0.47	0.47	0.47	0.47	0.47	0.47
0.48	0.48	0.48	0.48	0.48	0.48	0.48	0.48	0.48
0.49	0.49	0.49	0.49	0.49	0.49	0.49	0.49	0.49

0.5	0.5	0.5	0.5	0.5	0.5	0.5	0.5	0.5
0.51	0.51	0.51	0.51	0.51	0.51	0.51	0.51	0.51
0.52	0.52	0.52	0.52	0.52	0.52	0.52	0.52	0.52
0.53	0.53	0.53	0.53	0.53	0.53	0.53	0.53	0.53
0.54	0.54	0.54	0.54	0.54	0.54	0.54	0.54	0.54
0.55	0.55	0.55	0.55	0.55	0.55	0.55	0.55	0.55
0.56	0.56	0.56	0.56	0.56	0.56	0.56	0.56	0.56
0.57	0.57	0.57	0.57	0.57	0.57	0.57	0.57	0.57
0.58	0.58	0.58	0.58	0.58	0.58	0.58	0.58	0.58
0.59	0.59	0.59	0.59	0.59	0.59	0.59	0.59	0.59
0.6	0.6	0.6	0.6	0.6	0.6	0.6	0.6	0.6
0.61	0.61	0.61	0.61	0.61	0.61	0.61	0.61	0.61
0.62	0.62	0.62	0.62	0.62	0.62	0.62	0.62	0.62
0.63	0.63	0.63	0.63	0.63	0.63	0.63	0.63	0.63
0.64	0.64	0.64	0.64	0.64	0.64	0.64	0.64	0.64
0.65	0.65	0.65	0.65	0.65	0.65	0.65	0.65	0.65
0.66	0.66	0.66	0.66	0.66	0.66	0.66	0.66	0.66
0.67	0.67	0.67	0.67	0.67	0.67	0.67	0.67	0.67
0.68	0.68	0.68	0.68	0.68	0.68	0.68	0.68	0.68
0.69	0.69	0.69	0.69	0.69	0.69	0.69	0.69	0.69
0.7	0.7	0.7	0.7	0.7	0.7	0.7	0.7	0.7
0.71	0.71	0.71	0.71	0.71	0.71	0.71	0.71	0.71
0.72	0.72	0.72	0.72	0.72	0.72	0.72	0.72	0.72
0.73	0.73	0.73	0.73	0.73	0.73	0.73	0.73	0.73
0.74	0.74	0.74	0.74	0.74	0.74	0.74	0.74	0.74
0.75	0.75	0.75	0.75	0.75	0.75	0.75	0.75	0.75
0.76	0.76	0.76	0.76	0.76	0.76	0.76	0.76	0.76
0.77	0.77	0.77	0.77	0.77	0.77	0.77	0.77	0.77
0.78	0.78	0.78	0.78	0.78	0.78	0.78	0.78	0.78
0.79	0.79	0.79	0.79	0.79	0.79	0.79	0.79	0.79
0.8	0.8	0.8	0.8	0.8	0.8	0.8	0.8	0.8
0.81	0.81	0.81	0.81	0.81	0.81	0.81	0.81	0.81
0.82	0.82	0.82	0.82	0.82	0.82	0.82	0.82	0.82
0.83	0.83	0.83	0.83	0.83	0.83	0.83	0.83	0.83
0.84	0.84	0.84	0.84	0.84	0.84	0.84	0.84	0.84
0.85	0.85	0.85	0.85	0.85	0.85	0.85	0.85	0.85
0.86	0.86	0.86	0.86	0.86	0.86	0.86	0.86	0.86
0.87	0.87	0.87	0.87	0.87	0.87	0.87	0.87	0.87
0.88	0.88	0.88	0.88	0.88	0.88	0.88	0.88	0.88
0.89	0.89	0.89	0.89	0.89	0.89	0.89	0.89	0.89
0.9	0.9	0.9	0.9	0.9	0.9	0.9	0.9	0.9
0.91	0.91	0.91	0.91	0.91	0.91	0.91	0.91	0.91
0.92	0.92	0.92	0.92	0.92	0.92	0.92	0.92	0.92
0.93	0.93	0.93	0.93	0.93	0.93	0.93	0.93	0.93
0.94	0.94	0.94	0.94	0.94	0.94	0.94	0.94	0.94
0.95	0.95	0.95	0.95	0.95	0.95	0.95	0.95	0.95
0.96	0.96	0.96	0.96	0.96	0.96	0.96	0.96	0.96
0.97	0.97	0.97	0.97	0.97	0.97	0.97	0.97	0.97
0.98	0.98	0.98	0.98	0.98	0.98	0.98	0.98	0.98
0.99	0.99	0.99	0.99	0.99	0.99	0.99	0.99	0.99

1	1	1	1	1	1	1	1	1
1.01	1.01	1.01	1.01	1.01	1.01	1.01	1.01	1.01
1.02	1.02	1.02	1.02	1.02	1.02	1.02	1.02	1.02
1.03	1.03	1.03	1.03	1.03	1.03	1.03	1.03	1.03
1.04	1.04	1.04	1.04	1.04	1.04	1.04	1.04	1.04
1.05	1.05	1.05	1.05	1.05	1.05	1.05	1.05	1.05
1.06	1.06	1.06	1.06	1.06	1.06	1.06	1.06	1.06
1.07	1.07	1.07	1.07	1.07	1.07	1.07	1.07	1.07
1.08	1.08	1.08	1.08	1.08	1.08	1.08	1.08	1.08
1.09	1.09	1.09	1.09	1.09	1.09	1.09	1.09	1.09
1.1	1.1	1.1	1.1	1.1	1.1	1.1	1.1	1.1
1.11	1.11	1.11	1.11	1.11	1.11	1.11	1.11	1.11
1.12	1.12	1.12	1.12	1.12	1.12	1.12	1.12	1.12
1.13	1.13	1.13	1.13	1.13	1.13	1.13	1.13	1.13
1.14	1.14	1.14	1.14	1.14	1.14	1.14	1.14	1.14
1.15	1.15	1.15	1.15	1.15	1.15	1.15	1.15	1.15
1.16	1.16	1.16	1.16	1.16	1.16	1.16	1.16	1.16
1.17	1.17	1.17	1.17	1.17	1.17	1.17	1.17	1.17
1.18	1.18	1.18	1.18	1.18	1.18	1.18	1.18	1.18
1.19	1.19	1.19	1.19	1.19	1.19	1.19	1.19	1.19
1.2	1.2	1.2	1.2	1.2	1.2	1.2	1.2	1.2
1.21	1.21	1.21	1.21	1.21	1.21	1.21	1.21	1.21
1.22	1.22	1.22	1.22	1.22	1.22	1.22	1.22	1.22
1.23	1.23	1.23	1.23	1.23	1.23	1.23	1.23	1.23
1.24	1.24	1.24	1.24	1.24	1.24	1.24	1.24	1.24
1.25	1.25	1.25	1.25	1.25	1.25	1.25	1.25	1.25
1.26	1.26	1.26	1.26	1.26	1.26	1.26	1.26	1.26
1.27	1.27	1.27	1.27	1.27	1.27	1.27	1.27	1.27
1.28	1.28	1.28	1.28	1.28	1.28	1.28	1.28	1.28
1.29	1.29	1.29	1.29	1.29	1.29	1.29	1.29	1.29
1.3	1.3	1.3	1.3	1.3	1.3	1.3	1.3	1.3
1.31	1.31	1.31	1.31	1.31	1.31	1.31	1.31	1.31
1.32	1.32	1.32	1.32	1.32	1.32	1.32	1.32	1.32
1.33	1.33	1.33	1.33	1.33	1.33	1.33	1.33	1.33
1.34	1.34	1.34	1.34	1.34	1.34	1.34	1.34	1.34
1.35	1.35	1.35	1.35	1.35	1.35	1.35	1.35	1.35
1.36	1.36	1.36	1.36	1.36	1.36	1.36	1.36	1.36
1.37	1.37	1.37	1.37	1.37	1.37	1.37	1.37	1.37
1.38	1.38	1.38	1.38	1.38	1.38	1.38	1.38	1.38
1.39	1.39	1.39	1.39	1.39	1.39	1.39	1.39	1.39
1.4	1.4	1.4	1.4	1.4	1.4	1.4	1.4	1.4
1.41	1.41	1.41	1.41	1.41	1.41	1.41	1.41	1.41
1.42	1.42	1.42	1.42	1.42	1.42	1.42	1.42	1.42
1.43	1.43	1.43	1.43	1.43	1.43	1.43	1.43	1.43
1.44	1.44	1.44	1.44	1.44	1.44	1.44	1.44	1.44
1.45	1.45	1.45	1.45	1.45	1.45	1.45	1.45	1.45
1.46	1.46	1.46	1.46	1.46	1.46	1.46	1.46	1.46
1.47	1.47	1.47	1.47	1.47	1.47	1.47	1.47	1.47
1.48	1.48	1.48	1.48	1.48	1.48	1.48	1.48	1.48
1.49	1.49	1.49	1.49	1.49	1.49	1.49	1.49	1.49

1.5	1.5	1.5	1.5	1.5	1.5	1.5	1.5	1.5
1.51	1.51	1.51	1.51	1.51	1.51	1.51	1.51	1.51
1.52	1.52	1.52	1.52	1.52	1.52	1.52	1.52	1.52
1.53	1.53	1.53	1.53	1.53	1.53	1.53	1.53	1.53
1.54	1.54	1.54	1.54	1.54	1.54	1.54	1.54	1.54
1.55	1.55	1.55	1.55	1.55	1.55	1.55	1.55	1.55
1.56	1.56	1.56	1.56	1.56	1.56	1.56	1.56	1.56
1.57	1.57	1.57	1.57	1.57	1.57	1.57	1.57	1.57
1.58	1.58	1.58	1.58	1.58	1.58	1.58	1.58	1.58
1.59	1.59	1.59	1.59	1.59	1.59	1.59	1.59	1.59
1.6	1.6	1.6	1.6	1.6	1.6	1.6	1.6	1.6
1.61	1.61	1.61	1.61	1.61	1.61	1.61	1.61	1.61
1.62	1.62	1.62	1.62	1.62	1.62	1.62	1.62	1.62
1.63	1.63	1.63	1.63	1.63	1.63	1.63	1.63	1.63
1.64	1.64	1.64	1.64	1.64	1.64	1.64	1.64	1.64
1.65	1.65	1.65	1.65	1.65	1.65	1.65	1.65	1.65
1.66	1.66	1.66	1.66	1.66	1.66	1.66	1.66	1.66
1.67	1.67	1.67	1.67	1.67	1.67	1.67	1.67	1.67
1.68	1.68	1.68	1.68	1.68	1.68	1.68	1.68	1.68
1.69	1.69	1.69	1.69	1.69	1.69	1.69	1.69	1.69
1.7	1.7	1.7	1.7	1.7	1.7	1.7	1.7	1.7
1.71	1.71	1.71	1.71	1.71	1.71	1.71	1.71	1.71
1.72	1.72	1.72	1.72	1.72	1.72	1.72	1.72	1.72
1.73	1.73	1.73	1.73	1.73	1.73	1.73	1.73	1.73
1.74	1.74	1.74	1.74	1.74	1.74	1.74	1.74	1.74
1.75	1.75	1.75	1.75	1.75	1.75	1.75	1.75	1.75
1.76	1.76	1.76	1.76	1.76	1.76	1.76	1.76	1.76
1.77	1.77	1.77	1.77	1.77	1.77	1.77	1.77	1.77
1.78	1.78	1.78	1.78	1.78	1.78	1.78	1.78	1.78
1.79	1.79	1.79	1.79	1.79	1.79	1.79	1.79	1.79
1.8	1.8	1.8	1.8	1.8	1.8	1.8	1.8	1.8
1.81	1.81	1.81	1.81	1.81	1.81	1.81	1.81	1.81
1.82	1.82	1.82	1.82	1.82	1.82	1.82	1.82	1.82
1.83	1.83	1.83	1.83	1.83	1.83	1.83	1.83	1.83
1.84	1.84	1.84	1.84	1.84	1.84	1.84	1.84	1.84
1.85	1.85	1.85	1.85	1.85	1.85	1.85	1.85	1.85
1.86	1.86	1.86	1.86	1.86	1.86	1.86	1.86	1.86
1.87	1.87	1.87	1.87	1.87	1.87	1.87	1.87	1.87
1.88	1.88	1.88	1.88	1.88	1.88	1.88	1.88	1.88
1.89	1.89	1.89	1.89	1.89	1.89	1.89	1.89	1.89
1.9	1.9	1.9	1.9	1.9	1.9	1.9	1.9	1.9
1.91	1.91	1.91	1.91	1.91	1.91	1.91	1.91	1.91
1.92	1.92	1.92	1.92	1.92	1.92	1.92	1.92	1.92
1.93	1.93	1.93	1.93	1.93	1.93	1.93	1.93	1.93
1.94	1.94	1.94	1.94	1.94	1.94	1.94	1.94	1.94
1.95	1.95	1.95	1.95	1.95	1.95	1.95	1.95	1.95
1.96	1.96	1.96	1.96	1.96	1.96	1.96	1.96	1.96
1.97	1.97	1.97	1.97	1.97	1.97	1.97	1.97	1.97
1.98	1.98	1.98	1.98	1.98	1.98	1.98	1.98	1.98
1.99	1.99	1.99	1.99	1.99	1.99	1.99	1.99	1.99

2	2	2	2	2	2	2	2	2
2.01	2.01	2.01	2.01	2.01	2.01	2.01	2.01	2.01
2.02	2.02	2.02	2.02	2.02	2.02	2.02	2.02	2.02
2.03	2.03	2.03	2.03	2.03	2.03	2.03	2.03	2.03
2.04	2.04	2.04	2.04	2.04	2.04	2.04	2.04	2.04
2.05	2.05	2.05	2.05	2.05	2.05	2.05	2.05	2.05
2.06	2.06	2.06	2.06	2.06	2.06	2.06	2.06	2.06
2.07	2.07	2.07	2.07	2.07	2.07	2.07	2.07	2.07
2.08	2.08	2.08	2.08	2.08	2.08	2.08	2.08	2.08
2.09	2.09	2.09	2.09	2.09	2.09	2.09	2.09	2.09
2.1	2.1	2.1	2.1	2.1	2.1	2.1	2.1	2.1
2.11	2.11	2.11	2.11	2.11	2.11	2.11	2.11	2.11
2.12	2.12	2.12	2.12	2.12	2.12	2.12	2.12	2.12
2.13	2.13	2.13	2.13	2.13	2.13	2.13	2.13	2.13
2.14	2.14	2.14	2.14	2.14	2.14	2.14	2.14	2.14
2.15	2.15	2.15	2.15	2.15	2.15	2.15	2.15	2.15
2.16	2.16	2.16	2.16	2.16	2.16	2.16	2.16	2.16
2.17	2.17	2.17	2.17	2.17	2.17	2.17	2.17	2.17
2.18	2.18	2.18	2.18	2.18	2.18	2.18	2.18	2.18
2.19	2.19	2.19	2.19	2.19	2.19	2.19	2.19	2.19
2.2	2.2	2.2	2.2	2.2	2.2	2.2	2.2	2.2
2.21	2.21	2.21	2.21	2.21	2.21	2.21	2.21	2.21
2.22	2.22	2.22	2.22	2.22	2.22	2.22	2.22	2.22
2.23	2.23	2.23	2.23	2.23	2.23	2.23	2.23	2.23
2.24	2.24	2.24	2.24	2.24	2.24	2.24	2.24	2.24
2.25	2.25	2.25	2.25	2.25	2.25	2.25	2.25	2.25
2.26	2.26	2.26	2.26	2.26	2.26	2.26	2.26	2.26
2.27	2.27	2.27	2.27	2.27	2.27	2.27	2.27	2.27
2.28	2.28	2.28	2.28	2.28	2.28	2.28	2.28	2.28
2.29	2.29	2.29	2.29	2.29	2.29	2.29	2.29	2.29
2.3	2.3	2.3	2.3	2.3	2.3	2.3	2.3	2.3
2.31	2.31	2.31	2.31	2.31	2.31	2.31	2.31	2.31
2.32	2.32	2.32	2.32	2.32	2.32	2.32	2.32	2.32
2.33	2.33	2.33	2.33	2.33	2.33	2.33	2.33	2.33
2.34	2.34	2.34	2.34	2.34	2.34	2.34	2.34	2.34
2.35	2.35	2.35	2.35	2.35	2.35	2.35	2.35	2.35
2.36	2.36	2.36	2.36	2.36	2.36	2.36	2.36	2.36
2.37	2.37	2.37	2.37	2.37	2.37	2.37	2.37	2.37
2.38	2.38	2.38	2.38	2.38	2.38	2.38	2.38	2.38
2.39	2.39	2.39	2.39	2.39	2.39	2.39	2.39	2.39
2.4	2.4	2.4	2.4	2.4	2.4	2.4	2.4	2.4
2.41	2.41	2.41	2.41	2.41	2.41	2.41	2.41	2.41
2.42	2.42	2.42	2.42	2.42	2.42	2.42	2.42	2.42
2.43	2.43	2.43	2.43	2.43	2.43	2.43	2.43	2.43
2.44	2.44	2.44	2.44	2.44	2.44	2.44	2.44	2.44
2.45	2.45	2.45	2.45	2.45	2.45	2.45	2.45	2.45
2.46	2.46	2.46	2.46	2.46	2.46	2.46	2.46	2.46
2.47	2.47	2.47	2.47	2.47	2.47	2.47	2.47	2.47
2.48	2.48	2.48	2.48	2.48	2.48	2.48	2.48	2.48
2.49	2.49	2.49	2.49	2.49	2.49	2.49	2.49	2.49

2.5	2.5	2.5	2.5	2.5	2.5	2.5	2.5	2.5
2.51	2.51	2.51	2.51	2.51	2.51	2.51	2.51	2.51
2.52	2.52	2.52	2.52	2.52	2.52	2.52	2.52	2.52
2.53	2.53	2.53	2.53	2.53	2.53	2.53	2.53	2.53
2.54	2.54	2.54	2.54	2.54	2.54	2.54	2.54	2.54
2.55	2.55	2.55	2.55	2.55	2.55	2.55	2.55	2.55
2.56	2.56	2.56	2.56	2.56	2.56	2.56	2.56	2.56
2.57	2.57	2.57	2.57	2.57	2.57	2.57	2.57	2.57
2.58	2.58	2.58	2.58	2.58	2.58	2.58	2.58	2.58
2.59	2.59	2.59	2.59	2.59	2.59	2.59	2.59	2.59
2.6	2.6	2.6	2.6	2.6	2.6	2.6	2.6	2.6
2.61	2.61	2.61	2.61	2.61	2.61	2.61	2.61	2.61
2.62	2.62	2.62	2.62	2.62	2.62	2.62	2.62	2.62
2.63	2.63	2.63	2.63	2.63	2.63	2.63	2.63	2.63
2.64	2.64	2.64	2.64	2.64	2.64	2.64	2.64	2.64
2.65	2.65	2.65	2.65	2.65	2.65	2.65	2.65	2.65
2.66	2.66	2.66	2.66	2.66	2.66	2.66	2.66	2.66
2.67	2.67	2.67	2.67	2.67	2.67	2.67	2.67	2.67
2.68	2.68	2.68	2.68	2.68	2.68	2.68	2.68	2.68
2.69	2.69	2.69	2.69	2.69	2.69	2.69	2.69	2.69
2.7	2.7	2.7	2.7	2.7	2.7	2.7	2.7	2.7
2.71	2.71	2.71	2.71	2.71	2.71	2.71	2.71	2.71
2.72	2.72	2.72	2.72	2.72	2.72	2.72	2.72	2.72
2.73	2.73	2.73	2.73	2.73	2.73	2.73	2.73	2.73
2.74	2.74	2.74	2.74	2.74	2.74	2.74	2.74	2.74
2.75	2.75	2.75	2.75	2.75	2.75	2.75	2.75	2.75
2.76	2.76	2.76	2.76	2.76	2.76	2.76	2.76	2.76
2.77	2.77	2.77	2.77	2.77	2.77	2.77	2.77	2.77
2.78	2.78	2.78	2.78	2.78	2.78	2.78	2.78	2.78
2.79	2.79	2.79	2.79	2.79	2.79	2.79	2.79	2.79
2.8	2.8	2.8	2.8	2.8	2.8	2.8	2.8	2.8
2.81	2.81	2.81	2.81	2.81	2.81	2.81	2.81	2.81
2.82	2.82	2.82	2.82	2.82	2.82	2.82	2.82	2.82
2.83	2.83	2.83	2.83	2.83	2.83	2.83	2.83	2.83
2.84	2.84	2.84	2.84	2.84	2.84	2.84	2.84	2.84
2.85	2.85	2.85	2.85	2.85	2.85	2.85	2.85	2.85
2.86	2.86	2.86	2.86	2.86	2.86	2.86	2.86	2.86
2.87	2.87	2.87	2.87	2.87	2.87	2.87	2.87	2.87
2.88	2.88	2.88	2.88	2.88	2.88	2.88	2.88	2.88
2.89	2.89	2.89	2.89	2.89	2.89	2.89	2.89	2.89
2.9	2.9	2.9	2.9	2.9	2.9	2.9	2.9	2.9
2.91	2.91	2.91	2.91	2.91	2.91	2.91	2.91	2.91
2.92	2.92	2.92	2.92	2.92	2.92	2.92	2.92	2.92
2.93	2.93	2.93	2.93	2.93	2.93	2.93	2.93	2.93
2.94	2.94	2.94	2.94	2.94	2.94	2.94	2.94	2.94
2.95	2.95	2.95	2.95	2.95	2.95	2.95	2.95	2.95
2.96	2.96	2.96	2.96	2.96	2.96	2.96	2.96	2.96
2.97	2.97	2.97	2.97	2.97	2.97	2.97	2.97	2.97
2.98	2.98	2.98	2.98	2.98	2.98	2.98	2.98	2.98
2.99	2.99	2.99	2.99	2.99	2.99	2.99	2.99	2.99

3	3	3	3	3	3	3	3	3
3.01	3.01	3.01	3.01	3.01	3.01	3.01	3.01	3.01
3.02	3.02	3.02	3.02	3.02	3.02	3.02	3.02	3.02
3.03	3.03	3.03	3.03	3.03	3.03	3.03	3.03	3.03
3.04	3.04	3.04	3.04	3.04	3.04	3.04	3.04	3.04
3.05	3.05	3.05	3.05	3.05	3.05	3.05	3.05	3.05
3.06	3.06	3.06	3.06	3.06	3.06	3.06	3.06	3.06
3.07	3.07	3.07	3.07	3.07	3.07	3.07	3.07	3.07
3.08	3.08	3.08	3.08	3.08	3.08	3.08	3.08	3.08
3.09	3.09	3.09	3.09	3.09	3.09	3.09	3.09	3.09
3.1	3.1	3.1	3.1	3.1	3.1	3.1	3.1	3.1
3.11	3.11	3.11	3.11	3.11	3.11	3.11	3.11	3.11
3.12	3.12	3.12	3.12	3.12	3.12	3.12	3.12	3.12
3.13	3.13	3.13	3.13	3.13	3.13	3.13	3.13	3.13
3.14	3.14	3.14	3.14	3.14	3.14	3.14	3.14	3.14
3.15	3.15	3.15	3.15	3.15	3.15	3.15	3.15	3.15
3.16	3.16	3.16	3.16	3.16	3.16	3.16	3.16	3.16
3.17	3.17	3.17	3.17	3.17	3.17	3.17	3.17	3.17
3.18	3.18	3.18	3.18	3.18	3.18	3.18	3.18	3.18
3.19	3.19	3.19	3.19	3.19	3.19	3.19	3.19	3.19
3.2	3.2	3.2	3.2	3.2	3.2	3.2	3.2	3.2
3.21	3.21	3.21	3.21	3.21	3.21	3.21	3.21	3.21
3.22	3.22	3.22	3.22	3.22	3.22	3.22	3.22	3.22
3.23	3.23	3.23	3.23	3.23	3.23	3.23	3.23	3.23
3.24	3.24	3.24	3.24	3.24	3.24	3.24	3.24	3.24
3.25	3.25	3.25	3.25	3.25	3.25	3.25	3.25	3.25
3.26	3.26	3.26	3.26	3.26	3.26	3.26	3.26	3.26
3.27	3.27	3.27	3.27	3.27	3.27	3.27	3.27	3.27
3.28	3.28	3.28	3.28	3.28	3.28	3.28	3.28	3.28
3.29	3.29	3.29	3.29	3.29	3.29	3.29	3.29	3.29
3.3	3.3	3.3	3.3	3.3	3.3	3.3	3.3	3.3
3.31	3.31	3.31	3.31	3.31	3.31	3.31	3.31	3.31
3.32	3.32	3.32	3.32	3.32	3.32	3.32	3.32	3.32
3.33	3.33	3.33	3.33	3.33	3.33	3.33	3.33	3.33
3.34	3.34	3.34	3.34	3.34	3.34	3.34	3.34	3.34
3.35	3.35	3.35	3.35	3.35	3.35	3.35	3.35	3.35
3.36	3.36	3.36	3.36	3.36	3.36	3.36	3.36	3.36
3.37	3.37	3.37	3.37	3.37	3.37	3.37	3.37	3.37
3.38	3.38	3.38	3.38	3.38	3.38	3.38	3.38	3.38
3.39	3.39	3.39	3.39	3.39	3.39	3.39	3.39	3.39
3.4	3.4	3.4	3.4	3.4	3.4	3.4	3.4	3.4
3.41	3.41	3.41	3.41	3.41	3.41	3.41	3.41	3.41
3.42	3.42	3.42	3.42	3.42	3.42	3.42	3.42	3.42
3.43	3.43	3.43	3.43	3.43	3.43	3.43	3.43	3.43
3.44	3.44	3.44	3.44	3.44	3.44	3.44	3.44	3.44
3.45	3.45	3.45	3.45	3.45	3.45	3.45	3.45	3.45
3.46	3.46	3.46	3.46	3.46	3.46	3.46	3.46	3.46
3.47	3.47	3.47	3.47	3.47	3.47	3.47	3.47	3.47
3.48	3.48	3.48	3.48	3.48	3.48	3.48	3.48	3.48
3.49	3.49	3.49	3.49	3.49	3.49	3.49	3.49	3.49

3.5	3.5	3.5	3.5	3.5	3.5	3.5	3.5	3.5
3.51	3.51	3.51	3.51	3.51	3.51	3.51	3.51	3.51
3.52	3.52	3.52	3.52	3.52	3.52	3.52	3.52	3.52
3.53	3.53	3.53	3.53	3.53	3.53	3.53	3.53	3.53
3.54	3.54	3.54	3.54	3.54	3.54	3.54	3.54	3.54
3.55	3.55	3.55	3.55	3.55	3.55	3.55	3.55	3.55
3.56	3.56	3.56	3.56	3.56	3.56	3.56	3.56	3.56
3.57	3.57	3.57	3.57	3.57	3.57	3.57	3.57	3.57
3.58	3.58	3.58	3.58	3.58	3.58	3.58	3.58	3.58
3.59	3.59	3.59	3.59	3.59	3.59	3.59	3.59	3.59
3.6	3.6	3.6	3.6	3.6	3.6	3.6	3.6	3.6
3.61	3.61	3.61	3.61	3.61	3.61	3.61	3.61	3.61
3.62	3.62	3.62	3.62	3.62	3.62	3.62	3.62	3.62
3.63	3.63	3.63	3.63	3.63	3.63	3.63	3.63	3.63
3.64	3.64	3.64	3.64	3.64	3.64	3.64	3.64	3.64
3.65	3.65	3.65	3.65	3.65	3.65	3.65	3.65	3.65
3.66	3.66	3.66	3.66	3.66	3.66	3.66	3.66	3.66
3.67	3.67	3.67	3.67	3.67	3.67	3.67	3.67	3.67
3.68	3.68	3.68	3.68	3.68	3.68	3.68	3.68	3.68
3.69	3.69	3.69	3.69	3.69	3.69	3.69	3.69	3.69
3.7	3.7	3.7	3.7	3.7	3.7	3.7	3.7	3.7
3.71	3.71	3.71	3.71	3.71	3.71	3.71	3.71	3.71
3.72	3.72	3.72	3.72	3.72	3.72	3.72	3.72	3.72
3.73	3.73	3.73	3.73	3.73	3.73	3.73	3.73	3.73
3.74	3.74	3.74	3.74	3.74	3.74	3.74	3.74	3.74
3.75	3.75	3.75	3.75	3.75	3.75	3.75	3.75	3.75
3.76	3.76	3.76	3.76	3.76	3.76	3.76	3.76	3.76
3.77	3.77	3.77	3.77	3.77	3.77	3.77	3.77	3.77
3.78	3.78	3.78	3.78	3.78	3.78	3.78	3.78	3.78
3.79	3.79	3.79	3.79	3.79	3.79	3.79	3.79	3.79
3.8	3.8	3.8	3.8	3.8	3.8	3.8	3.8	3.8
3.81	3.81	3.81	3.81	3.81	3.81	3.81	3.81	3.81
3.82	3.82	3.82	3.82	3.82	3.82	3.82	3.82	3.82
3.83	3.83	3.83	3.83	3.83	3.83	3.83	3.83	3.83
3.84	3.84	3.84	3.84	3.84	3.84	3.84	3.84	3.84
3.85	3.85	3.85	3.85	3.85	3.85	3.85	3.85	3.85
3.86	3.86	3.86	3.86	3.86	3.86	3.86	3.86	3.86
3.87	3.87	3.87	3.87	3.87	3.87	3.87	3.87	3.87
3.88	3.88	3.88	3.88	3.88	3.88	3.88	3.88	3.88
3.89	3.89	3.89	3.89	3.89	3.89	3.89	3.89	3.89
3.9	3.9	3.9	3.9	3.9	3.9	3.9	3.9	3.9
3.91	3.91	3.91	3.91	3.91	3.91	3.91	3.91	3.91
3.92	3.92	3.92	3.92	3.92	3.92	3.92	3.92	3.92
3.93	3.93	3.93	3.93	3.93	3.93	3.93	3.93	3.93
3.94	3.94	3.94	3.94	3.94	3.94	3.94	3.94	3.94
3.95	3.95	3.95	3.95	3.95	3.95	3.95	3.95	3.95
3.96	3.96	3.96	3.96	3.96	3.96	3.96	3.96	3.96
3.97	3.97	3.97	3.97	3.97	3.97	3.97	3.97	3.97
3.98	3.98	3.98	3.98	3.98	3.98	3.98	3.98	3.98
3.99	3.99	3.99	3.99	3.99	3.99	3.99	3.99	3.99

4	4	4	4	4	4	4	4	4
4.01	4.01	4.01	4.01	4.01	4.01	4.01	4.01	4.01
4.02	4.02	4.02	4.02	4.02	4.02	4.02	4.02	4.02
4.03	4.03	4.03	4.03	4.03	4.03	4.03	4.03	4.03
4.04	4.04	4.04	4.04	4.04	4.04	4.04	4.04	4.04
4.05	4.05	4.05	4.05	4.05	4.05	4.05	4.05	4.05
4.06	4.06	4.06	4.06	4.06	4.06	4.06	4.06	4.06
4.07	4.07	4.07	4.07	4.07	4.07	4.07	4.07	4.07
4.08	4.08	4.08	4.08	4.08	4.08	4.08	4.08	4.08
4.09	4.09	4.09	4.09	4.09	4.09	4.09	4.09	4.09
4.1	4.1	4.1	4.1	4.1	4.1	4.1	4.1	4.1
4.11	4.11	4.11	4.11	4.11	4.11	4.11	4.11	4.11
4.12	4.12	4.12	4.12	4.12	4.12	4.12	4.12	4.12
4.13	4.13	4.13	4.13	4.13	4.13	4.13	4.13	4.13
4.14	4.14	4.14	4.14	4.14	4.14	4.14	4.14	4.14
4.15	4.15	4.15	4.15	4.15	4.15	4.15	4.15	4.15
4.16	4.16	4.16	4.16	4.16	4.16	4.16	4.16	4.16
4.17	4.17	4.17	4.17	4.17	4.17	4.17	4.17	4.17
4.18	4.18	4.18	4.18	4.18	4.18	4.18	4.18	4.18
4.19	4.19	4.19	4.19	4.19	4.19	4.19	4.19	4.19
4.2	4.2	4.2	4.2	4.2	4.2	4.2	4.2	4.2
4.21	4.21	4.21	4.21	4.21	4.21	4.21	4.21	4.21
4.22	4.22	4.22	4.22	4.22	4.22	4.22	4.22	4.22
4.23	4.23	4.23	4.23	4.23	4.23	4.23	4.23	4.23
4.24	4.24	4.24	4.24	4.24	4.24	4.24	4.24	4.24
4.25	4.25	4.25	4.25	4.25	4.25	4.25	4.25	4.25
4.26	4.26	4.26	4.26	4.26	4.26	4.26	4.26	4.26
4.27	4.27	4.27	4.27	4.27	4.27	4.27	4.27	4.27
4.28	4.28	4.28	4.28	4.28	4.28	4.28	4.28	4.28
4.29	4.29	4.29	4.29	4.29	4.29	4.29	4.29	4.29
4.3	4.3	4.3	4.3	4.3	4.3	4.3	4.3	4.3
4.31	4.31	4.31	4.31	4.31	4.31	4.31	4.31	4.31
4.32	4.32	4.32	4.32	4.32	4.32	4.32	4.32	4.32
4.33	4.33	4.33	4.33	4.33	4.33	4.33	4.33	4.33
4.34	4.34	4.34	4.34	4.34	4.34	4.34	4.34	4.34
4.35	4.35	4.35	4.35	4.35	4.35	4.35	4.35	4.35
4.36	4.36	4.36	4.36	4.36	4.36	4.36	4.36	4.36
4.37	4.37	4.37	4.37	4.37	4.37	4.37	4.37	4.37
4.38	4.38	4.38	4.38	4.38	4.38	4.38	4.38	4.38
4.39	4.39	4.39	4.39	4.39	4.39	4.39	4.39	4.39
4.4	4.4	4.4	4.4	4.4	4.4	4.4	4.4	4.4
4.41	4.41	4.41	4.41	4.41	4.41	4.41	4.41	4.41
4.42	4.42	4.42	4.42	4.42	4.42	4.42	4.42	4.42
4.43	4.43	4.43	4.43	4.43	4.43	4.43	4.43	4.43
4.44	4.44	4.44	4.44	4.44	4.44	4.44	4.44	4.44
4.45	4.45	4.45	4.45	4.45	4.45	4.45	4.45	4.45
4.46	4.46	4.46	4.46	4.46	4.46	4.46	4.46	4.46
4.47	4.47	4.47	4.47	4.47	4.47	4.47	4.47	4.47
4.48	4.48	4.48	4.48	4.48	4.48	4.48	4.48	4.48
4.49	4.49	4.49	4.49	4.49	4.49	4.49	4.49	4.49

4.5	4.5	4.5	4.5	4.5	4.5	4.5	4.5	4.5
4.51	4.51	4.51	4.51	4.51	4.51	4.51	4.51	4.51
4.52	4.52	4.52	4.52	4.52	4.52	4.52	4.52	4.52
4.53	4.53	4.53	4.53	4.53	4.53	4.53	4.53	4.53
4.54	4.54	4.54	4.54	4.54	4.54	4.54	4.54	4.54
4.55	4.55	4.55	4.55	4.55	4.55	4.55	4.55	4.55
4.56	4.56	4.56	4.56	4.56	4.56	4.56	4.56	4.56
4.57	4.57	4.57	4.57	4.57	4.57	4.57	4.57	4.57
4.58	4.58	4.58	4.58	4.58	4.58	4.58	4.58	4.58
4.59	4.59	4.59	4.59	4.59	4.59	4.59	4.59	4.59
4.6	4.6	4.6	4.6	4.6	4.6	4.6	4.6	4.6
4.61	4.61	4.61	4.61	4.61	4.61	4.61	4.61	4.61
4.62	4.62	4.62	4.62	4.62	4.62	4.62	4.62	4.62
4.63	4.63	4.63	4.63	4.63	4.63	4.63	4.63	4.63
4.64	4.64	4.64	4.64	4.64	4.64	4.64	4.64	4.64
4.65	4.65	4.65	4.65	4.65	4.65	4.65	4.65	4.65
4.66	4.66	4.66	4.66	4.66	4.66	4.66	4.66	4.66
4.67	4.67	4.67	4.67	4.67	4.67	4.67	4.67	4.67
4.68	4.68	4.68	4.68	4.68	4.68	4.68	4.68	4.68
4.69	4.69	4.69	4.69	4.69	4.69	4.69	4.69	4.69
4.7	4.7	4.7	4.7	4.7	4.7	4.7	4.7	4.7
4.71	4.71	4.71	4.71	4.71	4.71	4.71	4.71	4.71
4.72	4.72	4.72	4.72	4.72	4.72	4.72	4.72	4.72
4.73	4.73	4.73	4.73	4.73	4.73	4.73	4.73	4.73
4.74	4.74	4.74	4.74	4.74	4.74	4.74	4.74	4.74
4.75	4.75	4.75	4.75	4.75	4.75	4.75	4.75	4.75
4.76	4.76	4.76	4.76	4.76	4.76	4.76	4.76	4.76
4.77	4.77	4.77	4.77	4.77	4.77	4.77	4.77	4.77
4.78	4.78	4.78	4.78	4.78	4.78	4.78	4.78	4.78
4.79	4.79	4.79	4.79	4.79	4.79	4.79	4.79	4.79
4.8	4.8	4.8	4.8	4.8	4.8	4.8	4.8	4.8
4.81	4.81	4.81	4.81	4.81	4.81	4.81	4.81	4.81
4.82	4.82	4.82	4.82	4.82	4.82	4.82	4.82	4.82
4.83	4.83	4.83	4.83	4.83	4.83	4.83	4.83	4.83
4.84	4.84	4.84	4.84	4.84	4.84	4.84	4.84	4.84
4.85	4.85	4.85	4.85	4.85	4.85	4.85	4.85	4.85
4.86	4.86	4.86	4.86	4.86	4.86	4.86	4.86	4.86
4.87	4.87	4.87	4.87	4.87	4.87	4.87	4.87	4.87
4.88	4.88	4.88	4.88	4.88	4.88	4.88	4.88	4.88
4.89	4.89	4.89	4.89	4.89	4.89	4.89	4.89	4.89
4.9	4.9	4.9	4.9	4.9	4.9	4.9	4.9	4.9
4.91	4.91	4.91	4.91	4.91	4.91	4.91	4.91	4.91
4.92	4.92	4.92	4.92	4.92	4.92	4.92	4.92	4.92
4.93	4.93	4.93	4.93	4.93	4.93	4.93	4.93	4.93
4.94	4.94	4.94	4.94	4.94	4.94	4.94	4.94	4.94
4.95	4.95	4.95	4.95	4.95	4.95	4.95	4.95	4.95
4.96	4.96	4.96	4.96	4.96	4.96	4.96	4.96	4.96
4.97	4.97	4.97	4.97	4.97	4.97	4.97	4.97	4.97
4.98	4.98	4.98	4.98	4.98	4.98	4.98	4.98	4.98
4.99	4.99	4.99	4.99	4.99	4.99	4.99	4.99	4.99

5	5	5	5	5	5	5	5	5
5.01	5.01	5.01	5.01	5.01	5.01	5.01	5.01	5.01
5.02	5.02	5.02	5.02	5.02	5.02	5.02	5.02	5.02
5.03	5.03	5.03	5.03	5.03	5.03	5.03	5.03	5.03
5.04	5.04	5.04	5.04	5.04	5.04	5.04	5.04	5.04
5.05	5.05	5.05	5.05	5.05	5.05	5.05	5.05	5.05
5.06	5.06	5.06	5.06	5.06	5.06	5.06	5.06	5.06
5.07	5.07	5.07	5.07	5.07	5.07	5.07	5.07	5.07
5.08	5.08	5.08	5.08	5.08	5.08	5.08	5.08	5.08
5.09	5.09	5.09	5.09	5.09	5.09	5.09	5.09	5.09
5.1	5.1	5.1	5.1	5.1	5.1	5.1	5.1	5.1
5.11	5.11	5.11	5.11	5.11	5.11	5.11	5.11	5.11
5.12	5.12	5.12	5.12	5.12	5.12	5.12	5.12	5.12
5.13	5.13	5.13	5.13	5.13	5.13	5.13	5.13	5.13
5.14	5.14	5.14	5.14	5.14	5.14	5.14	5.14	5.14
5.15	5.15	5.15	5.15	5.15	5.15	5.15	5.15	5.15
5.16	5.16	5.16	5.16	5.16	5.16	5.16	5.16	5.16
5.17	5.17	5.17	5.17	5.17	5.17	5.17	5.17	5.17
5.18	5.18	5.18	5.18	5.18	5.18	5.18	5.18	5.18
5.19	5.19	5.19	5.19	5.19	5.19	5.19	5.19	5.19
5.2	5.2	5.2	5.2	5.2	5.2	5.2	5.2	5.2
5.21	5.21	5.21	5.21	5.21	5.21	5.21	5.21	5.21
5.22	5.22	5.22	5.22	5.22	5.22	5.22	5.22	5.22
5.23	5.23	5.23	5.23	5.23	5.23	5.23	5.23	5.23
5.24	5.24	5.24	5.24	5.24	5.24	5.24	5.24	5.24
5.25	5.25	5.25	5.25	5.25	5.25	5.25	5.25	5.25
5.26	5.26	5.26	5.26	5.26	5.26	5.26	5.26	5.26
5.27	5.27	5.27	5.27	5.27	5.27	5.27	5.27	5.27
5.28	5.28	5.28	5.28	5.28	5.28	5.28	5.28	5.28
5.29	5.29	5.29	5.29	5.29	5.29	5.29	5.29	5.29
5.3	5.3	5.3	5.3	5.3	5.3	5.3	5.3	5.3
5.31	5.31	5.31	5.31	5.31	5.31	5.31	5.31	5.31
5.32	5.32	5.32	5.32	5.32	5.32	5.32	5.32	5.32
5.33	5.33	5.33	5.33	5.33	5.33	5.33	5.33	5.33
5.34	5.34	5.34	5.34	5.34	5.34	5.34	5.34	5.34
5.35	5.35	5.35	5.35	5.35	5.35	5.35	5.35	5.35
5.36	5.36	5.36	5.36	5.36	5.36	5.36	5.36	5.36
5.37	5.37	5.37	5.37	5.37	5.37	5.37	5.37	5.37
5.38	5.38	5.38	5.38	5.38	5.38	5.38	5.38	5.38
5.39	5.39	5.39	5.39	5.39	5.39	5.39	5.39	5.39
5.4	5.4	5.4	5.4	5.4	5.4	5.4	5.4	5.4
5.41	5.41	5.41	5.41	5.41	5.41	5.41	5.41	5.41
5.42	5.42	5.42	5.42	5.42	5.42	5.42	5.42	5.42
5.43	5.43	5.43	5.43	5.43	5.43	5.43	5.43	5.43
5.44	5.44	5.44	5.44	5.44	5.44	5.44	5.44	5.44
5.45	5.45	5.45	5.45	5.45	5.45	5.45	5.45	5.45
5.46	5.46	5.46	5.46	5.46	5.46	5.46	5.46	5.46
5.47	5.47	5.47	5.47	5.47	5.47	5.47	5.47	5.47
5.48	5.48	5.48	5.48	5.48	5.48	5.48	5.48	5.48
5.49	5.49	5.49	5.49	5.49	5.49	5.49	5.49	5.49

5.5	5.5	5.5	5.5	5.5	5.5	5.5	5.5	5.5
5.51	5.51	5.51	5.51	5.51	5.51	5.51	5.51	5.51
5.52	5.52	5.52	5.52	5.52	5.52	5.52	5.52	5.52
5.53	5.53	5.53	5.53	5.53	5.53	5.53	5.53	5.53
5.54	5.54	5.54	5.54	5.54	5.54	5.54	5.54	5.54
5.55	5.55	5.55	5.55	5.55	5.55	5.55	5.55	5.55
5.56	5.56	5.56	5.56	5.56	5.56	5.56	5.56	5.56
5.57	5.57	5.57	5.57	5.57	5.57	5.57	5.57	5.57
5.58	5.58	5.58	5.58	5.58	5.58	5.58	5.58	5.58
5.59	5.59	5.59	5.59	5.59	5.59	5.59	5.59	5.59
5.6	5.6	5.6	5.6	5.6	5.6	5.6	5.6	5.6
5.61	5.61	5.61	5.61	5.61	5.61	5.61	5.61	5.61
5.62	5.62	5.62	5.62	5.62	5.62	5.62	5.62	5.62
5.63	5.63	5.63	5.63	5.63	5.63	5.63	5.63	5.63
5.64	5.64	5.64	5.64	5.64	5.64	5.64	5.64	5.64
5.65	5.65	5.65	5.65	5.65	5.65	5.65	5.65	5.65
5.66	5.66	5.66	5.66	5.66	5.66	5.66	5.66	5.66
5.67	5.67	5.67	5.67	5.67	5.67	5.67	5.67	5.67
5.68	5.68	5.68	5.68	5.68	5.68	5.68	5.68	5.68
5.69	5.69	5.69	5.69	5.69	5.69	5.69	5.69	5.69
5.7	5.7	5.7	5.7	5.7	5.7	5.7	5.7	5.7
5.71	5.71	5.71	5.71	5.71	5.71	5.71	5.71	5.71
5.72	5.72	5.72	5.72	5.72	5.72	5.72	5.72	5.72
5.73	5.73	5.73	5.73	5.73	5.73	5.73	5.73	5.73
5.74	5.74	5.74	5.74	5.74	5.74	5.74	5.74	5.74
5.75	5.75	5.75	5.75	5.75	5.75	5.75	5.75	5.75
5.76	5.76	5.76	5.76	5.76	5.76	5.76	5.76	5.76
5.77	5.77	5.77	5.77	5.77	5.77	5.77	5.77	5.77
5.78	5.78	5.78	5.78	5.78	5.78	5.78	5.78	5.78
5.79	5.79	5.79	5.79	5.79	5.79	5.79	5.79	5.79
5.8	5.8	5.8	5.8	5.8	5.8	5.8	5.8	5.8
5.81	5.81	5.81	5.81	5.81	5.81	5.81	5.81	5.81
5.82	5.82	5.82	5.82	5.82	5.82	5.82	5.82	5.82
5.83	5.83	5.83	5.83	5.83	5.83	5.83	5.83	5.83
5.84	5.84	5.84	5.84	5.84	5.84	5.84	5.84	5.84
5.85	5.85	5.85	5.85	5.85	5.85	5.85	5.85	5.85
5.86	5.86	5.86	5.86	5.86	5.86	5.86	5.86	5.86
5.87	5.87	5.87	5.87	5.87	5.87	5.87	5.87	5.87
5.88	5.88	5.88	5.88	5.88	5.88	5.88	5.88	5.88
5.89	5.89	5.89	5.89	5.89	5.89	5.89	5.89	5.89
5.9	5.9	5.9	5.9	5.9	5.9	5.9	5.9	5.9
5.91	5.91	5.91	5.91	5.91	5.91	5.91	5.91	5.91
5.92	5.92	5.92	5.92	5.92	5.92	5.92	5.92	5.92
5.93	5.93	5.93	5.93	5.93	5.93	5.93	5.93	5.93
5.94	5.94	5.94	5.94	5.94	5.94	5.94	5.94	5.94
5.95	5.95	5.95	5.95	5.95	5.95	5.95	5.95	5.95
5.96	5.96	5.96	5.96	5.96	5.96	5.96	5.96	5.96
5.97	5.97	5.97	5.97	5.97	5.97	5.97	5.97	5.97
5.98	5.98	5.98	5.98	5.98	5.98	5.98	5.98	5.98
5.99	5.99	5.99	5.99	5.99	5.99	5.99	5.99	5.99

6	6	6	6	6	6	6	6	6
6.01	6.01	6.01	6.01	6.01	6.01	6.01	6.01	6.01
6.02	6.02	6.02	6.02	6.02	6.02	6.02	6.02	6.02
6.03	6.03	6.03	6.03	6.03	6.03	6.03	6.03	6.03
6.04	6.04	6.04	6.04	6.04	6.04	6.04	6.04	6.04
6.05	6.05	6.05	6.05	6.05	6.05	6.05	6.05	6.05
6.06	6.06	6.06	6.06	6.06	6.06	6.06	6.06	6.06
6.07	6.07	6.07	6.07	6.07	6.07	6.07	6.07	6.07
6.08	6.08	6.08	6.08	6.08	6.08	6.08	6.08	6.08
6.09	6.09	6.09	6.09	6.09	6.09	6.09	6.09	6.09
6.1	6.1	6.1	6.1	6.1	6.1	6.1	6.1	6.1
6.11	6.11	6.11	6.11	6.11	6.11	6.11	6.11	6.11
6.12	6.12	6.12	6.12	6.12	6.12	6.12	6.12	6.12
6.13	6.13	6.13	6.13	6.13	6.13	6.13	6.13	6.13
6.14	6.14	6.14	6.14	6.14	6.14	6.14	6.14	6.14
6.15	6.15	6.15	6.15	6.15	6.15	6.15	6.15	6.15
6.16	6.16	6.16	6.16	6.16	6.16	6.16	6.16	6.16
6.17	6.17	6.17	6.17	6.17	6.17	6.17	6.17	6.17
6.18	6.18	6.18	6.18	6.18	6.18	6.18	6.18	6.18
6.19	6.19	6.19	6.19	6.19	6.19	6.19	6.19	6.19
6.2	6.2	6.2	6.2	6.2	6.2	6.2	6.2	6.2
6.21	6.21	6.21	6.21	6.21	6.21	6.21	6.21	6.21
6.22	6.22	6.22	6.22	6.22	6.22	6.22	6.22	6.22
6.23	6.23	6.23	6.23	6.23	6.23	6.23	6.23	6.23
6.24	6.24	6.24	6.24	6.24	6.24	6.24	6.24	6.24
6.25	6.25	6.25	6.25	6.25	6.25	6.25	6.25	6.25
6.26	6.26	6.26	6.26	6.26	6.26	6.26	6.26	6.26
6.27	6.27	6.27	6.27	6.27	6.27	6.27	6.27	6.27
6.28	6.28	6.28	6.28	6.28	6.28	6.28	6.28	6.28
6.29	6.29	6.29	6.29	6.29	6.29	6.29	6.29	6.29
6.3	6.3	6.3	6.3	6.3	6.3	6.3	6.3	6.3
6.31	6.31	6.31	6.31	6.31	6.31	6.31	6.31	6.31
6.32	6.32	6.32	6.32	6.32	6.32	6.32	6.32	6.32
6.33	6.33	6.33	6.33	6.33	6.33	6.33	6.33	6.33
6.34	6.34	6.34	6.34	6.34	6.34	6.34	6.34	6.34
6.35	6.35	6.35	6.35	6.35	6.35	6.35	6.35	6.35
6.36	6.36	6.36	6.36	6.36	6.36	6.36	6.36	6.36
6.37	6.37	6.37	6.37	6.37	6.37	6.37	6.37	6.37
6.38	6.38	6.38	6.38	6.38	6.38	6.38	6.38	6.38
6.39	6.39	6.39	6.39	6.39	6.39	6.39	6.39	6.39
6.4	6.4	6.4	6.4	6.4	6.4	6.4	6.4	6.4
6.41	6.41	6.41	6.41	6.41	6.41	6.41	6.41	6.41
6.42	6.42	6.42	6.42	6.42	6.42	6.42	6.42	6.42
6.43	6.43	6.43	6.43	6.43	6.43	6.43	6.43	6.43
6.44	6.44	6.44	6.44	6.44	6.44	6.44	6.44	6.44
6.45	6.45	6.45	6.45	6.45	6.45	6.45	6.45	6.45
6.46	6.46	6.46	6.46	6.46	6.46	6.46	6.46	6.46
6.47	6.47	6.47	6.47	6.47	6.47	6.47	6.47	6.47
6.48	6.48	6.48	6.48	6.48	6.48	6.48	6.48	6.48
6.49	6.49	6.49	6.49	6.49	6.49	6.49	6.49	6.49

6.5	6.5	6.5	6.5	6.5	6.5	6.5	6.5	6.5
6.51	6.51	6.51	6.51	6.51	6.51	6.51	6.51	6.51
6.52	6.52	6.52	6.52	6.52	6.52	6.52	6.52	6.52
6.53	6.53	6.53	6.53	6.53	6.53	6.53	6.53	6.53
6.54	6.54	6.54	6.54	6.54	6.54	6.54	6.54	6.54
6.55	6.55	6.55	6.55	6.55	6.55	6.55	6.55	6.55
6.56	6.56	6.56	6.56	6.56	6.56	6.56	6.56	6.56
6.57	6.57	6.57	6.57	6.57	6.57	6.57	6.57	6.57
6.58	6.58	6.58	6.58	6.58	6.58	6.58	6.58	6.58
6.59	6.59	6.59	6.59	6.59	6.59	6.59	6.59	6.59
6.6	6.6	6.6	6.6	6.6	6.6	6.6	6.6	6.6
6.61	6.61	6.61	6.61	6.61	6.61	6.61	6.61	6.61
6.62	6.62	6.62	6.62	6.62	6.62	6.62	6.62	6.62
6.63	6.63	6.63	6.63	6.63	6.63	6.63	6.63	6.63
6.64	6.64	6.64	6.64	6.64	6.64	6.64	6.64	6.64
6.65	6.65	6.65	6.65	6.65	6.65	6.65	6.65	6.65
6.66	6.66	6.66	6.66	6.66	6.66	6.66	6.66	6.66
6.67	6.67	6.67	6.67	6.67	6.67	6.67	6.67	6.67
6.68	6.68	6.68	6.68	6.68	6.68	6.68	6.68	6.68
6.69	6.69	6.69	6.69	6.69	6.69	6.69	6.69	6.69
6.7	6.7	6.7	6.7	6.7	6.7	6.7	6.7	6.7
6.71	6.71	6.71	6.71	6.71	6.71	6.71	6.71	6.71
6.72	6.72	6.72	6.72	6.72	6.72	6.72	6.72	6.72
6.73	6.73	6.73	6.73	6.73	6.73	6.73	6.73	6.73
6.74	6.74	6.74	6.74	6.74	6.74	6.74	6.74	6.74
6.75	6.75	6.75	6.75	6.75	6.75	6.75	6.75	6.75
6.76	6.76	6.76	6.76	6.76	6.76	6.76	6.76	6.76
6.77	6.77	6.77	6.77	6.77	6.77	6.77	6.77	6.77
6.78	6.78	6.78	6.78	6.78	6.78	6.78	6.78	6.78
6.79	6.79	6.79	6.79	6.79	6.79	6.79	6.79	6.79
6.8	6.8	6.8	6.8	6.8	6.8	6.8	6.8	6.8
6.81	6.81	6.81	6.81	6.81	6.81	6.81	6.81	6.81
6.82	6.82	6.82	6.82	6.82	6.82	6.82	6.82	6.82
6.83	6.83	6.83	6.83	6.83	6.83	6.83	6.83	6.83
6.84	6.84	6.84	6.84	6.84	6.84	6.84	6.84	6.84
6.85	6.85	6.85	6.85	6.85	6.85	6.85	6.85	6.85
6.86	6.86	6.86	6.86	6.86	6.86	6.86	6.86	6.86
6.87	6.87	6.87	6.87	6.87	6.87	6.87	6.87	6.87
6.88	6.88	6.88	6.88	6.88	6.88	6.88	6.88	6.88
6.89	6.89	6.89	6.89	6.89	6.89	6.89	6.89	6.89
6.9	6.9	6.9	6.9	6.9	6.9	6.9	6.9	6.9
6.91	6.91	6.91	6.91	6.91	6.91	6.91	6.91	6.91
6.92	6.92	6.92	6.92	6.92	6.92	6.92	6.92	6.92
6.93	6.93	6.93	6.93	6.93	6.93	6.93	6.93	6.93
6.94	6.94	6.94	6.94	6.94	6.94	6.94	6.94	6.94
6.95	6.95	6.95	6.95	6.95	6.95	6.95	6.95	6.95
6.96	6.96	6.96	6.96	6.96	6.96	6.96	6.96	6.96
6.97	6.97	6.97	6.97	6.97	6.97	6.97	6.97	6.97
6.98	6.98	6.98	6.98	6.98	6.98	6.98	6.98	6.98
6.99	6.99	6.99	6.99	6.99	6.99	6.99	6.99	6.99

7	7	7	7	7	7	7	7	7
7.01	7.01	7.01	7.01	7.01	7.01	7.01	7.01	7.01
7.02	7.02	7.02	7.02	7.02	7.02	7.02	7.02	7.02
7.03	7.03	7.03	7.03	7.03	7.03	7.03	7.03	7.03
7.04	7.04	7.04	7.04	7.04	7.04	7.04	7.04	7.04
7.05	7.05	7.05	7.05	7.05	7.05	7.05	7.05	7.05
7.06	7.06	7.06	7.06	7.06	7.06	7.06	7.06	7.06
7.07	7.07	7.07	7.07	7.07	7.07	7.07	7.07	7.07
7.08	7.08	7.08	7.08	7.08	7.08	7.08	7.08	7.08
7.09	7.09	7.09	7.09	7.09	7.09	7.09	7.09	7.09
7.1	7.1	7.1	7.1	7.1	7.1	7.1	7.1	7.1
7.11	7.11	7.11	7.11	7.11	7.11	7.11	7.11	7.11
7.12	7.12	7.12	7.12	7.12	7.12	7.12	7.12	7.12
7.13	7.13	7.13	7.13	7.13	7.13	7.13	7.13	7.13
7.14	7.14	7.14	7.14	7.14	7.14	7.14	7.14	7.14
7.15	7.15	7.15	7.15	7.15	7.15	7.15	7.15	7.15
7.16	7.16	7.16	7.16	7.16	7.16	7.16	7.16	7.16
7.17	7.17	7.17	7.17	7.17	7.17	7.17	7.17	7.17
7.18	7.18	7.18	7.18	7.18	7.18	7.18	7.18	7.18
7.19	7.19	7.19	7.19	7.19	7.19	7.19	7.19	7.19
7.2	7.2	7.2	7.2	7.2	7.2	7.2	7.2	7.2
7.21	7.21	7.21	7.21	7.21	7.21	7.21	7.21	7.21
7.22	7.22	7.22	7.22	7.22	7.22	7.22	7.22	7.22
7.23	7.23	7.23	7.23	7.23	7.23	7.23	7.23	7.23
7.24	7.24	7.24	7.24	7.24	7.24	7.24	7.24	7.24
7.25	7.25	7.25	7.25	7.25	7.25	7.25	7.25	7.25
7.26	7.26	7.26	7.26	7.26	7.26	7.26	7.26	7.26
7.27	7.27	7.27	7.27	7.27	7.27	7.27	7.27	7.27
7.28	7.28	7.28	7.28	7.28	7.28	7.28	7.28	7.28
7.29	7.29	7.29	7.29	7.29	7.29	7.29	7.29	7.29
7.3	7.3	7.3	7.3	7.3	7.3	7.3	7.3	7.3
7.31	7.31	7.31	7.31	7.31	7.31	7.31	7.31	7.31
7.32	7.32	7.32	7.32	7.32	7.32	7.32	7.32	7.32
7.33	7.33	7.33	7.33	7.33	7.33	7.33	7.33	7.33
7.34	7.34	7.34	7.34	7.34	7.34	7.34	7.34	7.34
7.35	7.35	7.35	7.35	7.35	7.35	7.35	7.35	7.35
7.36	7.36	7.36	7.36	7.36	7.36	7.36	7.36	7.36
7.37	7.37	7.37	7.37	7.37	7.37	7.37	7.37	7.37
7.38	7.38	7.38	7.38	7.38	7.38	7.38	7.38	7.38
7.39	7.39	7.39	7.39	7.39	7.39	7.39	7.39	7.39
7.4	7.4	7.4	7.4	7.4	7.4	7.4	7.4	7.4
7.41	7.41	7.41	7.41	7.41	7.41	7.41	7.41	7.41
7.42	7.42	7.42	7.42	7.42	7.42	7.42	7.42	7.42
7.43	7.43	7.43	7.43	7.43	7.43	7.43	7.43	7.43
7.44	7.44	7.44	7.44	7.44	7.44	7.44	7.44	7.44
7.45	7.45	7.45	7.45	7.45	7.45	7.45	7.45	7.45
7.46	7.46	7.46	7.46	7.46	7.46	7.46	7.46	7.46
7.47	7.47	7.47	7.47	7.47	7.47	7.47	7.47	7.47
7.48	7.48	7.48	7.48	7.48	7.48	7.48	7.48	7.48
7.49	7.49	7.49	7.49	7.49	7.49	7.49	7.49	7.49

7.5	7.5	7.5	7.5	7.5	7.5	7.5	7.5	7.5
7.51	7.51	7.51	7.51	7.51	7.51	7.51	7.51	7.51
7.52	7.52	7.52	7.52	7.52	7.52	7.52	7.52	7.52
7.53	7.53	7.53	7.53	7.53	7.53	7.53	7.53	7.53
7.54	7.54	7.54	7.54	7.54	7.54	7.54	7.54	7.54
7.55	7.55	7.55	7.55	7.55	7.55	7.55	7.55	7.55
7.56	7.56	7.56	7.56	7.56	7.56	7.56	7.56	7.56
7.57	7.57	7.57	7.57	7.57	7.57	7.57	7.57	7.57
7.58	7.58	7.58	7.58	7.58	7.58	7.58	7.58	7.58
7.59	7.59	7.59	7.59	7.59	7.59	7.59	7.59	7.59
7.6	7.6	7.6	7.6	7.6	7.6	7.6	7.6	7.6
7.61	7.61	7.61	7.61	7.61	7.61	7.61	7.61	7.61
7.62	7.62	7.62	7.62	7.62	7.62	7.62	7.62	7.62
7.63	7.63	7.63	7.63	7.63	7.63	7.63	7.63	7.63
7.64	7.64	7.64	7.64	7.64	7.64	7.64	7.64	7.64
7.65	7.65	7.65	7.65	7.65	7.65	7.65	7.65	7.65
7.66	7.66	7.66	7.66	7.66	7.66	7.66	7.66	7.66
7.67	7.67	7.67	7.67	7.67	7.67	7.67	7.67	7.67
7.68	7.68	7.68	7.68	7.68	7.68	7.68	7.68	7.68
7.69	7.69	7.69	7.69	7.69	7.69	7.69	7.69	7.69
7.7	7.7	7.7	7.7	7.7	7.7	7.7	7.7	7.7
7.71	7.71	7.71	7.71	7.71	7.71	7.71	7.71	7.71
7.72	7.72	7.72	7.72	7.72	7.72	7.72	7.72	7.72
7.73	7.73	7.73	7.73	7.73	7.73	7.73	7.73	7.73
7.74	7.74	7.74	7.74	7.74	7.74	7.74	7.74	7.74
7.75	7.75	7.75	7.75	7.75	7.75	7.75	7.75	7.75
7.76	7.76	7.76	7.76	7.76	7.76	7.76	7.76	7.76
7.77	7.77	7.77	7.77	7.77	7.77	7.77	7.77	7.77
7.78	7.78	7.78	7.78	7.78	7.78	7.78	7.78	7.78
7.79	7.79	7.79	7.79	7.79	7.79	7.79	7.79	7.79
7.8	7.8	7.8	7.8	7.8	7.8	7.8	7.8	7.8
7.81	7.81	7.81	7.81	7.81	7.81	7.81	7.81	7.81
7.82	7.82	7.82	7.82	7.82	7.82	7.82	7.82	7.82
7.83	7.83	7.83	7.83	7.83	7.83	7.83	7.83	7.83
7.84	7.84	7.84	7.84	7.84	7.84	7.84	7.84	7.84
7.85	7.85	7.85	7.85	7.85	7.85	7.85	7.85	7.85
7.86	7.86	7.86	7.86	7.86	7.86	7.86	7.86	7.86
7.87	7.87	7.87	7.87	7.87	7.87	7.87	7.87	7.87
7.88	7.88	7.88	7.88	7.88	7.88	7.88	7.88	7.88
7.89	7.89	7.89	7.89	7.89	7.89	7.89	7.89	7.89
7.9	7.9	7.9	7.9	7.9	7.9	7.9	7.9	7.9
7.91	7.91	7.91	7.91	7.91	7.91	7.91	7.91	7.91
7.92	7.92	7.92	7.92	7.92	7.92	7.92	7.92	7.92
7.93	7.93	7.93	7.93	7.93	7.93	7.93	7.93	7.93
7.94	7.94	7.94	7.94	7.94	7.94	7.94	7.94	7.94
7.95	7.95	7.95	7.95	7.95	7.95	7.95	7.95	7.95
7.96	7.96	7.96	7.96	7.96	7.96	7.96	7.96	7.96
7.97	7.97	7.97	7.97	7.97	7.97	7.97	7.97	7.97
7.98	7.98	7.98	7.98	7.98	7.98	7.98	7.98	7.98
7.99	7.99	7.99	7.99	7.99	7.99	7.99	7.99	7.99

8	8	8	8	8	8	8	8	8
8.01	8.01	8.01	8.01	8.01	8.01	8.01	8.01	8.01
8.02	8.02	8.02	8.02	8.02	8.02	8.02	8.02	8.02
8.03	8.03	8.03	8.03	8.03	8.03	8.03	8.03	8.03
8.04	8.04	8.04	8.04	8.04	8.04	8.04	8.04	8.04
8.05	8.05	8.05	8.05	8.05	8.05	8.05	8.05	8.05
8.06	8.06	8.06	8.06	8.06	8.06	8.06	8.06	8.06
8.07	8.07	8.07	8.07	8.07	8.07	8.07	8.07	8.07
8.08	8.08	8.08	8.08	8.08	8.08	8.08	8.08	8.08
8.09	8.09	8.09	8.09	8.09	8.09	8.09	8.09	8.09
8.1	8.1	8.1	8.1	8.1	8.1	8.1	8.1	8.1
8.11	8.11	8.11	8.11	8.11	8.11	8.11	8.11	8.11
8.12	8.12	8.12	8.12	8.12	8.12	8.12	8.12	8.12
8.13	8.13	8.13	8.13	8.13	8.13	8.13	8.13	8.13
8.14	8.14	8.14	8.14	8.14	8.14	8.14	8.14	8.14
8.15	8.15	8.15	8.15	8.15	8.15	8.15	8.15	8.15
8.16	8.16	8.16	8.16	8.16	8.16	8.16	8.16	8.16
8.17	8.17	8.17	8.17	8.17	8.17	8.17	8.17	8.17
8.18	8.18	8.18	8.18	8.18	8.18	8.18	8.18	8.18
8.19	8.19	8.19	8.19	8.19	8.19	8.19	8.19	8.19
8.2	8.2	8.2	8.2	8.2	8.2	8.2	8.2	8.2
8.21	8.21	8.21	8.21	8.21	8.21	8.21	8.21	8.21
8.22	8.22	8.22	8.22	8.22	8.22	8.22	8.22	8.22
8.23	8.23	8.23	8.23	8.23	8.23	8.23	8.23	8.23
8.24	8.24	8.24	8.24	8.24	8.24	8.24	8.24	8.24
8.25	8.25	8.25	8.25	8.25	8.25	8.25	8.25	8.25
8.26	8.26	8.26	8.26	8.26	8.26	8.26	8.26	8.26
8.27	8.27	8.27	8.27	8.27	8.27	8.27	8.27	8.27
8.28	8.28	8.28	8.28	8.28	8.28	8.28	8.28	8.28
8.29	8.29	8.29	8.29	8.29	8.29	8.29	8.29	8.29
8.3	8.3	8.3	8.3	8.3	8.3	8.3	8.3	8.3
8.31	8.31	8.31	8.31	8.31	8.31	8.31	8.31	8.31
8.32	8.32	8.32	8.32	8.32	8.32	8.32	8.32	8.32
8.33	8.33	8.33	8.33	8.33	8.33	8.33	8.33	8.33
8.34	8.34	8.34	8.34	8.34	8.34	8.34	8.34	8.34
8.35	8.35	8.35	8.35	8.35	8.35	8.35	8.35	8.35
8.36	8.36	8.36	8.36	8.36	8.36	8.36	8.36	8.36
8.37	8.37	8.37	8.37	8.37	8.37	8.37	8.37	8.37
8.38	8.38	8.38	8.38	8.38	8.38	8.38	8.38	8.38
8.39	8.39	8.39	8.39	8.39	8.39	8.39	8.39	8.39
8.4	8.4	8.4	8.4	8.4	8.4	8.4	8.4	8.4
8.41	8.41	8.41	8.41	8.41	8.41	8.41	8.41	8.41
8.42	8.42	8.42	8.42	8.42	8.42	8.42	8.42	8.42
8.43	8.43	8.43	8.43	8.43	8.43	8.43	8.43	8.43
8.44	8.44	8.44	8.44	8.44	8.44	8.44	8.44	8.44
8.45	8.45	8.45	8.45	8.45	8.45	8.45	8.45	8.45
8.46	8.46	8.46	8.46	8.46	8.46	8.46	8.46	8.46
8.47	8.47	8.47	8.47	8.47	8.47	8.47	8.47	8.47
8.48	8.48	8.48	8.48	8.48	8.48	8.48	8.48	8.48
8.49	8.49	8.49	8.49	8.49	8.49	8.49	8.49	8.49

8.5	8.5	8.5	8.5	8.5	8.5	8.5	8.5	8.5
8.51	8.51	8.51	8.51	8.51	8.51	8.51	8.51	8.51
8.52	8.52	8.52	8.52	8.52	8.52	8.52	8.52	8.52
8.53	8.53	8.53	8.53	8.53	8.53	8.53	8.53	8.53
8.54	8.54	8.54	8.54	8.54	8.54	8.54	8.54	8.54
8.55	8.55	8.55	8.55	8.55	8.55	8.55	8.55	8.55
8.56	8.56	8.56	8.56	8.56	8.56	8.56	8.56	8.56
8.57	8.57	8.57	8.57	8.57	8.57	8.57	8.57	8.57
8.58	8.58	8.58	8.58	8.58	8.58	8.58	8.58	8.58
8.59	8.59	8.59	8.59	8.59	8.59	8.59	8.59	8.59
8.6	8.6	8.6	8.6	8.6	8.6	8.6	8.6	8.6
8.61	8.61	8.61	8.61	8.61	8.61	8.61	8.61	8.61
8.62	8.62	8.62	8.62	8.62	8.62	8.62	8.62	8.62
8.63	8.63	8.63	8.63	8.63	8.63	8.63	8.63	8.63
8.64	8.64	8.64	8.64	8.64	8.64	8.64	8.64	8.64
8.65	8.65	8.65	8.65	8.65	8.65	8.65	8.65	8.65
8.66	8.66	8.66	8.66	8.66	8.66	8.66	8.66	8.66
8.67	8.67	8.67	8.67	8.67	8.67	8.67	8.67	8.67
8.68	8.68	8.68	8.68	8.68	8.68	8.68	8.68	8.68
8.69	8.69	8.69	8.69	8.69	8.69	8.69	8.69	8.69
8.7	8.7	8.7	8.7	8.7	8.7	8.7	8.7	8.7
8.71	8.71	8.71	8.71	8.71	8.71	8.71	8.71	8.71
8.72	8.72	8.72	8.72	8.72	8.72	8.72	8.72	8.72
8.73	8.73	8.73	8.73	8.73	8.73	8.73	8.73	8.73
8.74	8.74	8.74	8.74	8.74	8.74	8.74	8.74	8.74
8.75	8.75	8.75	8.75	8.75	8.75	8.75	8.75	8.75
8.76	8.76	8.76	8.76	8.76	8.76	8.76	8.76	8.76
8.77	8.77	8.77	8.77	8.77	8.77	8.77	8.77	8.77
8.78	8.78	8.78	8.78	8.78	8.78	8.78	8.78	8.78
8.79	8.79	8.79	8.79	8.79	8.79	8.79	8.79	8.79
8.8	8.8	8.8	8.8	8.8	8.8	8.8	8.8	8.8
8.81	8.81	8.81	8.81	8.81	8.81	8.81	8.81	8.81
8.82	8.82	8.82	8.82	8.82	8.82	8.82	8.82	8.82
8.83	8.83	8.83	8.83	8.83	8.83	8.83	8.83	8.83
8.84	8.84	8.84	8.84	8.84	8.84	8.84	8.84	8.84
8.85	8.85	8.85	8.85	8.85	8.85	8.85	8.85	8.85
8.86	8.86	8.86	8.86	8.86	8.86	8.86	8.86	8.86
8.87	8.87	8.87	8.87	8.87	8.87	8.87	8.87	8.87
8.88	8.88	8.88	8.88	8.88	8.88	8.88	8.88	8.88
8.89	8.89	8.89	8.89	8.89	8.89	8.89	8.89	8.89
8.9	8.9	8.9	8.9	8.9	8.9	8.9	8.9	8.9
8.91	8.91	8.91	8.91	8.91	8.91	8.91	8.91	8.91
8.92	8.92	8.92	8.92	8.92	8.92	8.92	8.92	8.92
8.93	8.93	8.93	8.93	8.93	8.93	8.93	8.93	8.93
8.94	8.94	8.94	8.94	8.94	8.94	8.94	8.94	8.94
8.95	8.95	8.95	8.95	8.95	8.95	8.95	8.95	8.95
8.96	8.96	8.96	8.96	8.96	8.96	8.96	8.96	8.96
8.97	8.97	8.97	8.97	8.97	8.97	8.97	8.97	8.97
8.98	8.98	8.98	8.98	8.98	8.98	8.98	8.98	8.98
8.99	8.99	8.99	8.99	8.99	8.99	8.99	8.99	8.99

9	9	9	9	9	9	9	9	9
9.01	9.01	9.01	9.01	9.01	9.01	9.01	9.01	9.01
9.02	9.02	9.02	9.02	9.02	9.02	9.02	9.02	9.02
9.03	9.03	9.03	9.03	9.03	9.03	9.03	9.03	9.03
9.04	9.04	9.04	9.04	9.04	9.04	9.04	9.04	9.04
9.05	9.05	9.05	9.05	9.05	9.05	9.05	9.05	9.05
9.06	9.06	9.06	9.06	9.06	9.06	9.06	9.06	9.06
9.07	9.07	9.07	9.07	9.07	9.07	9.07	9.07	9.07
9.08	9.08	9.08	9.08	9.08	9.08	9.08	9.08	9.08
9.09	9.09	9.09	9.09	9.09	9.09	9.09	9.09	9.09
9.1	9.1	9.1	9.1	9.1	9.1	9.1	9.1	9.1
9.11	9.11	9.11	9.11	9.11	9.11	9.11	9.11	9.11
9.12	9.12	9.12	9.12	9.12	9.12	9.12	9.12	9.12
9.13	9.13	9.13	9.13	9.13	9.13	9.13	9.13	9.13
9.14	9.14	9.14	9.14	9.14	9.14	9.14	9.14	9.14
9.15	9.15	9.15	9.15	9.15	9.15	9.15	9.15	9.15
9.16	9.16	9.16	9.16	9.16	9.16	9.16	9.16	9.16
9.17	9.17	9.17	9.17	9.17	9.17	9.17	9.17	9.17
9.18	9.18	9.18	9.18	9.18	9.18	9.18	9.18	9.18
9.19	9.19	9.19	9.19	9.19	9.19	9.19	9.19	9.19
9.2	9.2	9.2	9.2	9.2	9.2	9.2	9.2	9.2
9.21	9.21	9.21	9.21	9.21	9.21	9.21	9.21	9.21
9.22	9.22	9.22	9.22	9.22	9.22	9.22	9.22	9.22
9.23	9.23	9.23	9.23	9.23	9.23	9.23	9.23	9.23
9.24	9.24	9.24	9.24	9.24	9.24	9.24	9.24	9.24
9.25	9.25	9.25	9.25	9.25	9.25	9.25	9.25	9.25
9.26	9.26	9.26	9.26	9.26	9.26	9.26	9.26	9.26
9.27	9.27	9.27	9.27	9.27	9.27	9.27	9.27	9.27
9.28	9.28	9.28	9.28	9.28	9.28	9.28	9.28	9.28
9.29	9.29	9.29	9.29	9.29	9.29	9.29	9.29	9.29
9.3	9.3	9.3	9.3	9.3	9.3	9.3	9.3	9.3
9.31	9.31	9.31	9.31	9.31	9.31	9.31	9.31	9.31
9.32	9.32	9.32	9.32	9.32	9.32	9.32	9.32	9.32
9.33	9.33	9.33	9.33	9.33	9.33	9.33	9.33	9.33
9.34	9.34	9.34	9.34	9.34	9.34	9.34	9.34	9.34
9.35	9.35	9.35	9.35	9.35	9.35	9.35	9.35	9.35
9.36	9.36	9.36	9.36	9.36	9.36	9.36	9.36	9.36
9.37	9.37	9.37	9.37	9.37	9.37	9.37	9.37	9.37
9.38	9.38	9.38	9.38	9.38	9.38	9.38	9.38	9.38
9.39	9.39	9.39	9.39	9.39	9.39	9.39	9.39	9.39
9.4	9.4	9.4	9.4	9.4	9.4	9.4	9.4	9.4
9.41	9.41	9.41	9.41	9.41	9.41	9.41	9.41	9.41
9.42	9.42	9.42	9.42	9.42	9.42	9.42	9.42	9.42
9.43	9.43	9.43	9.43	9.43	9.43	9.43	9.43	9.43
9.44	9.44	9.44	9.44	9.44	9.44	9.44	9.44	9.44
9.45	9.45	9.45	9.45	9.45	9.45	9.45	9.45	9.45
9.46	9.46	9.46	9.46	9.46	9.46	9.46	9.46	9.46
9.47	9.47	9.47	9.47	9.47	9.47	9.47	9.47	9.47
9.48	9.48	9.48	9.48	9.48	9.48	9.48	9.48	9.48
9.49	9.49	9.49	9.49	9.49	9.49	9.49	9.49	9.49

9.5	9.5	9.5	9.5	9.5	9.5	9.5	9.5	9.5
9.51	9.51	9.51	9.51	9.51	9.51	9.51	9.51	9.51
9.52	9.52	9.52	9.52	9.52	9.52	9.52	9.52	9.52
9.53	9.53	9.53	9.53	9.53	9.53	9.53	9.53	9.53
9.54	9.54	9.54	9.54	9.54	9.54	9.54	9.54	9.54
9.55	9.55	9.55	9.55	9.55	9.55	9.55	9.55	9.55
9.56	9.56	9.56	9.56	9.56	9.56	9.56	9.56	9.56
9.57	9.57	9.57	9.57	9.57	9.57	9.57	9.57	9.57
9.58	9.58	9.58	9.58	9.58	9.58	9.58	9.58	9.58
9.59	9.59	9.59	9.59	9.59	9.59	9.59	9.59	9.59
9.6	9.6	9.6	9.6	9.6	9.6	9.6	9.6	9.6
9.61	9.61	9.61	9.61	9.61	9.61	9.61	9.61	9.61
9.62	9.62	9.62	9.62	9.62	9.62	9.62	9.62	9.62
9.63	9.63	9.63	9.63	9.63	9.63	9.63	9.63	9.63
9.64	9.64	9.64	9.64	9.64	9.64	9.64	9.64	9.64
9.65	9.65	9.65	9.65	9.65	9.65	9.65	9.65	9.65
9.66	9.66	9.66	9.66	9.66	9.66	9.66	9.66	9.66
9.67	9.67	9.67	9.67	9.67	9.67	9.67	9.67	9.67
9.68	9.68	9.68	9.68	9.68	9.68	9.68	9.68	9.68
9.69	9.69	9.69	9.69	9.69	9.69	9.69	9.69	9.69
9.7	9.7	9.7	9.7	9.7	9.7	9.7	9.7	9.7
9.71	9.71	9.71	9.71	9.71	9.71	9.71	9.71	9.71
9.72	9.72	9.72	9.72	9.72	9.72	9.72	9.72	9.72
9.73	9.73	9.73	9.73	9.73	9.73	9.73	9.73	9.73
9.74	9.74	9.74	9.74	9.74	9.74	9.74	9.74	9.74
9.75	9.75	9.75	9.75	9.75	9.75	9.75	9.75	9.75
9.76	9.76	9.76	9.76	9.76	9.76	9.76	9.76	9.76
9.77	9.77	9.77	9.77	9.77	9.77	9.77	9.77	9.77
9.78	9.78	9.78	9.78	9.78	9.78	9.78	9.78	9.78
9.79	9.79	9.79	9.79	9.79	9.79	9.79	9.79	9.79
9.8	9.8	9.8	9.8	9.8	9.8	9.8	9.8	9.8
9.81	9.81	9.81	9.81	9.81	9.81	9.81	9.81	9.81
9.82	9.82	9.82	9.82	9.82	9.82	9.82	9.82	9.82
9.83	9.83	9.83	9.83	9.83	9.83	9.83	9.83	9.83
9.84	9.84	9.84	9.84	9.84	9.84	9.84	9.84	9.84
9.85	9.85	9.85	9.85	9.85	9.85	9.85	9.85	9.85
9.86	9.86	9.86	9.86	9.86	9.86	9.86	9.86	9.86
9.87	9.87	9.87	9.87	9.87	9.87	9.87	9.87	9.87
9.88	9.88	9.88	9.88	9.88	9.88	9.88	9.88	9.88
9.89	9.89	9.89	9.89	9.89	9.89	9.89	9.89	9.89
9.9	9.9	9.9	9.9	9.9	9.9	9.9	9.9	9.9
9.91	9.91	9.91	9.91	9.91	9.91	9.91	9.91	9.91
9.92	9.92	9.92	9.92	9.92	9.92	9.92	9.92	9.92
9.93	9.93	9.93	9.93	9.93	9.93	9.93	9.93	9.93
9.94	9.94	9.94	9.94	9.94	9.94	9.94	9.94	9.94
9.95	9.95	9.95	9.95	9.95	9.95	9.95	9.95	9.95
9.96	9.96	9.96	9.96	9.96	9.96	9.96	9.96	9.96
9.97	9.97	9.97	9.97	9.97	9.97	9.97	9.97	9.97
9.98	9.98	9.98	9.98	9.98	9.98	9.98	9.98	9.98
9.99	9.99	9.99	9.99	9.99	9.99	9.99	9.99	9.99

10	10	10	10	10	10	10	10	10
10.01	10.01	10.01	10.01	10.01	10.01	10.01	10.01	10.01
10.02	10.02	10.02	10.02	10.02	10.02	10.02	10.02	10.02
10.03	10.03	10.03	10.03	10.03	10.03	10.03	10.03	10.03
10.04	10.04	10.04	10.04	10.04	10.04	10.04	10.04	10.04
10.05	10.05	10.05	10.05	10.05	10.05	10.05	10.05	10.05
10.06	10.06	10.06	10.06	10.06	10.06	10.06	10.06	10.06
10.07	10.07	10.07	10.07	10.07	10.07	10.07	10.07	10.07
10.08	10.08	10.08	10.08	10.08	10.08	10.08	10.08	10.08
10.09	10.09	10.09	10.09	10.09	10.09	10.09	10.09	10.09
10.1	10.1	10.1	10.1	10.1	10.1	10.1	10.1	10.1
10.11	10.11	10.11	10.11	10.11	10.11	10.11	10.11	10.11
10.12	10.12	10.12	10.12	10.12	10.12	10.12	10.12	10.12
10.13	10.13	10.13	10.13	10.13	10.13	10.13	10.13	10.13
10.14	10.14	10.14	10.14	10.14	10.14	10.14	10.14	10.14
10.15	10.15	10.15	10.15	10.15	10.15	10.15	10.15	10.15
10.16	10.16	10.16	10.16	10.16	10.16	10.16	10.16	10.16
10.17	10.17	10.17	10.17	10.17	10.17	10.17	10.17	10.17
10.18	10.18	10.18	10.18	10.18	10.18	10.18	10.18	10.18
10.19	10.19	10.19	10.19	10.19	10.19	10.19	10.19	10.19
10.2	10.2	10.2	10.2	10.2	10.2	10.2	10.2	10.2
10.21	10.21	10.21	10.21	10.21	10.21	10.21	10.21	10.21
10.22	10.22	10.22	10.22	10.22	10.22	10.22	10.22	10.22
10.23	10.23	10.23	10.23	10.23	10.23	10.23	10.23	10.23
10.24	10.24	10.24	10.24	10.24	10.24	10.24	10.24	10.24
10.25	10.25	10.25	10.25	10.25	10.25	10.25	10.25	10.25
10.26	10.26	10.26	10.26	10.26	10.26	10.26	10.26	10.26
10.27	10.27	10.27	10.27	10.27	10.27	10.27	10.27	10.27
10.28	10.28	10.28	10.28	10.28	10.28	10.28	10.28	10.28
10.29	10.29	10.29	10.29	10.29	10.29	10.29	10.29	10.29
10.3	10.3	10.3	10.3	10.3	10.3	10.3	10.3	10.3
10.31	10.31	10.31	10.31	10.31	10.31	10.31	10.31	10.31
10.32	10.32	10.32	10.32	10.32	10.32	10.32	10.32	10.32
10.33	10.33	10.33	10.33	10.33	10.33	10.33	10.33	10.33
10.34	10.34	10.34	10.34	10.34	10.34	10.34	10.34	10.34
10.35	10.35	10.35	10.35	10.35	10.35	10.35	10.35	10.35
10.36	10.36	10.36	10.36	10.36	10.36	10.36	10.36	10.36
10.37	10.37	10.37	10.37	10.37	10.37	10.37	10.37	10.37
10.38	10.38	10.38	10.38	10.38	10.38	10.38	10.38	10.38
10.39	10.39	10.39	10.39	10.39	10.39	10.39	10.39	10.39
10.4	10.4	10.4	10.4	10.4	10.4	10.4	10.4	10.4
10.41	10.41	10.41	10.41	10.41	10.41	10.41	10.41	10.41
10.42	10.42	10.42	10.42	10.42	10.42	10.42	10.42	10.42
10.43	10.43	10.43	10.43	10.43	10.43	10.43	10.43	10.43
10.44	10.44	10.44	10.44	10.44	10.44	10.44	10.44	10.44
10.45	10.45	10.45	10.45	10.45	10.45	10.45	10.45	10.45
10.46	10.46	10.46	10.46	10.46	10.46	10.46	10.46	10.46
10.47	10.47	10.47	10.47	10.47	10.47	10.47	10.47	10.47
10.48	10.48	10.48	10.48	10.48	10.48	10.48	10.48	10.48
10.49	10.49	10.49	10.49	10.49	10.49	10.49	10.49	10.49

10.5	10.5	10.5	10.5	10.5	10.5	10.5	10.5	10.5
10.51	10.51	10.51	10.51	10.51	10.51	10.51	10.51	10.51
10.52	10.52	10.52	10.52	10.52	10.52	10.52	10.52	10.52
10.53	10.53	10.53	10.53	10.53	10.53	10.53	10.53	10.53
10.54	10.54	10.54	10.54	10.54	10.54	10.54	10.54	10.54
10.55	10.55	10.55	10.55	10.55	10.55	10.55	10.55	10.55
10.56	10.56	10.56	10.56	10.56	10.56	10.56	10.56	10.56
10.57	10.57	10.57	10.57	10.57	10.57	10.57	10.57	10.57
10.58	10.58	10.58	10.58	10.58	10.58	10.58	10.58	10.58
10.59	10.59	10.59	10.59	10.59	10.59	10.59	10.59	10.59
10.6	10.6	10.6	10.6	10.6	10.6	10.6	10.6	10.6
10.61	10.61	10.61	10.61	10.61	10.61	10.61	10.61	10.61
10.62	10.62	10.62	10.62	10.62	10.62	10.62	10.62	10.62
10.63	10.63	10.63	10.63	10.63	10.63	10.63	10.63	10.63
10.64	10.64	10.64	10.64	10.64	10.64	10.64	10.64	10.64
10.65	10.65	10.65	10.65	10.65	10.65	10.65	10.65	10.65
10.66	10.66	10.66	10.66	10.66	10.66	10.66	10.66	10.66
10.67	10.67	10.67	10.67	10.67	10.67	10.67	10.67	10.67
10.68	10.68	10.68	10.68	10.68	10.68	10.68	10.68	10.68
10.69	10.69	10.69	10.69	10.69	10.69	10.69	10.69	10.69
10.7	10.7	10.7	10.7	10.7	10.7	10.7	10.7	10.7
10.71	10.71	10.71	10.71	10.71	10.71	10.71	10.71	10.71
10.72	10.72	10.72	10.72	10.72	10.72	10.72	10.72	10.72
10.73	10.73	10.73	10.73	10.73	10.73	10.73	10.73	10.73
10.74	10.74	10.74	10.74	10.74	10.74	10.74	10.74	10.74
10.75	10.75	10.75	10.75	10.75	10.75	10.75	10.75	10.75
10.76	10.76	10.76	10.76	10.76	10.76	10.76	10.76	10.76
10.77	10.77	10.77	10.77	10.77	10.77	10.77	10.77	10.77
10.78	10.78	10.78	10.78	10.78	10.78	10.78	10.78	10.78
10.79	10.79	10.79	10.79	10.79	10.79	10.79	10.79	10.79
10.8	10.8	10.8	10.8	10.8	10.8	10.8	10.8	10.8
10.81	10.81	10.81	10.81	10.81	10.81	10.81	10.81	10.81
10.82	10.82	10.82	10.82	10.82	10.82	10.82	10.82	10.82
10.83	10.83	10.83	10.83	10.83	10.83	10.83	10.83	10.83
10.84	10.84	10.84	10.84	10.84	10.84	10.84	10.84	10.84
10.85	10.85	10.85	10.85	10.85	10.85	10.85	10.85	10.85
10.86	10.86	10.86	10.86	10.86	10.86	10.86	10.86	10.86
10.87	10.87	10.87	10.87	10.87	10.87	10.87	10.87	10.87
10.88	10.88	10.88	10.88	10.88	10.88	10.88	10.88	10.88
10.89	10.89	10.89	10.89	10.89	10.89	10.89	10.89	10.89
10.9	10.9	10.9	10.9	10.9	10.9	10.9	10.9	10.9
10.91	10.91	10.91	10.91	10.91	10.91	10.91	10.91	10.91
10.92	10.92	10.92	10.92	10.92	10.92	10.92	10.92	10.92
10.93	10.93	10.93	10.93	10.93	10.93	10.93	10.93	10.93
10.94	10.94	10.94	10.94	10.94	10.94	10.94	10.94	10.94
10.95	10.95	10.95	10.95	10.95	10.95	10.95	10.95	10.95
10.96	10.96	10.96	10.96	10.96	10.96	10.96	10.96	10.96
10.97	10.97	10.97	10.97	10.97	10.97	10.97	10.97	10.97
10.98	10.98	10.98	10.98	10.98	10.98	10.98	10.98	10.98
10.99	10.99	10.99	10.99	10.99	10.99	10.99	10.99	10.99

11	11	11	11	11	11	11	11	11
11.01	11.01	11.01	11.01	11.01	11.01	11.01	11.01	11.01
11.02	11.02	11.02	11.02	11.02	11.02	11.02	11.02	11.02
11.03	11.03	11.03	11.03	11.03	11.03	11.03	11.03	11.03
11.04	11.04	11.04	11.04	11.04	11.04	11.04	11.04	11.04
11.05	11.05	11.05	11.05	11.05	11.05	11.05	11.05	11.05
11.06	11.06	11.06	11.06	11.06	11.06	11.06	11.06	11.06
11.07	11.07	11.07	11.07	11.07	11.07	11.07	11.07	11.07
11.08	11.08	11.08	11.08	11.08	11.08	11.08	11.08	11.08
11.09	11.09	11.09	11.09	11.09	11.09	11.09	11.09	11.09
11.1	11.1	11.1	11.1	11.1	11.1	11.1	11.1	11.1
11.11	11.11	11.11	11.11	11.11	11.11	11.11	11.11	11.11
11.12	11.12	11.12	11.12	11.12	11.12	11.12	11.12	11.12
11.13	11.13	11.13	11.13	11.13	11.13	11.13	11.13	11.13
11.14	11.14	11.14	11.14	11.14	11.14	11.14	11.14	11.14
11.15	11.15	11.15	11.15	11.15	11.15	11.15	11.15	11.15
11.16	11.16	11.16	11.16	11.16	11.16	11.16	11.16	11.16
11.17	11.17	11.17	11.17	11.17	11.17	11.17	11.17	11.17
11.18	11.18	11.18	11.18	11.18	11.18	11.18	11.18	11.18
11.19	11.19	11.19	11.19	11.19	11.19	11.19	11.19	11.19
11.2	11.2	11.2	11.2	11.2	11.2	11.2	11.2	11.2
11.21	11.21	11.21	11.21	11.21	11.21	11.21	11.21	11.21
11.22	11.22	11.22	11.22	11.22	11.22	11.22	11.22	11.22
11.23	11.23	11.23	11.23	11.23	11.23	11.23	11.23	11.23
11.24	11.24	11.24	11.24	11.24	11.24	11.24	11.24	11.24
11.25	11.25	11.25	11.25	11.25	11.25	11.25	11.25	11.25
11.26	11.26	11.26	11.26	11.26	11.26	11.26	11.26	11.26
11.27	11.27	11.27	11.27	11.27	11.27	11.27	11.27	11.27
11.28	11.28	11.28	11.28	11.28	11.28	11.28	11.28	11.28
11.29	11.29	11.29	11.29	11.29	11.29	11.29	11.29	11.29
11.3	11.3	11.3	11.3	11.3	11.3	11.3	11.3	11.3
11.31	11.31	11.31	11.31	11.31	11.31	11.31	11.31	11.31
11.32	11.32	11.32	11.32	11.32	11.32	11.32	11.32	11.32
11.33	11.33	11.33	11.33	11.33	11.33	11.33	11.33	11.33
11.34	11.34	11.34	11.34	11.34	11.34	11.34	11.34	11.34
11.35	11.35	11.35	11.35	11.35	11.35	11.35	11.35	11.35
11.36	11.36	11.36	11.36	11.36	11.36	11.36	11.36	11.36
11.37	11.37	11.37	11.37	11.37	11.37	11.37	11.37	11.37
11.38	11.38	11.38	11.38	11.38	11.38	11.38	11.38	11.38
11.39	11.39	11.39	11.39	11.39	11.39	11.39	11.39	11.39
11.4	11.4	11.4	11.4	11.4	11.4	11.4	11.4	11.4
11.41	11.41	11.41	11.41	11.41	11.41	11.41	11.41	11.41
11.42	11.42	11.42	11.42	11.42	11.42	11.42	11.42	11.42
11.43	11.43	11.43	11.43	11.43	11.43	11.43	11.43	11.43
11.44	11.44	11.44	11.44	11.44	11.44	11.44	11.44	11.44
11.45	11.45	11.45	11.45	11.45	11.45	11.45	11.45	11.45
11.46	11.46	11.46	11.46	11.46	11.46	11.46	11.46	11.46
11.47	11.47	11.47	11.47	11.47	11.47	11.47	11.47	11.47
11.48	11.48	11.48	11.48	11.48	11.48	11.48	11.48	11.48
11.49	11.49	11.49	11.49	11.49	11.49	11.49	11.49	11.49

11.5	11.5	11.5	11.5	11.5	11.5	11.5	11.5	11.5
11.51	11.51	11.51	11.51	11.51	11.51	11.51	11.51	11.51
11.52	11.52	11.52	11.52	11.52	11.52	11.52	11.52	11.52
11.53	11.53	11.53	11.53	11.53	11.53	11.53	11.53	11.53
11.54	11.54	11.54	11.54	11.54	11.54	11.54	11.54	11.54
11.55	11.55	11.55	11.55	11.55	11.55	11.55	11.55	11.55
11.56	11.56	11.56	11.56	11.56	11.56	11.56	11.56	11.56
11.57	11.57	11.57	11.57	11.57	11.57	11.57	11.57	11.57
11.58	11.58	11.58	11.58	11.58	11.58	11.58	11.58	11.58
11.59	11.59	11.59	11.59	11.59	11.59	11.59	11.59	11.59
11.6	11.6	11.6	11.6	11.6	11.6	11.6	11.6	11.6
11.61	11.61	11.61	11.61	11.61	11.61	11.61	11.61	11.61
11.62	11.62	11.62	11.62	11.62	11.62	11.62	11.62	11.62
11.63	11.63	11.63	11.63	11.63	11.63	11.63	11.63	11.63
11.64	11.64	11.64	11.64	11.64	11.64	11.64	11.64	11.64
11.65	11.65	11.65	11.65	11.65	11.65	11.65	11.65	11.65
11.66	11.66	11.66	11.66	11.66	11.66	11.66	11.66	11.66
11.67	11.67	11.67	11.67	11.67	11.67	11.67	11.67	11.67
11.68	11.68	11.68	11.68	11.68	11.68	11.68	11.68	11.68
11.69	11.69	11.69	11.69	11.69	11.69	11.69	11.69	11.69
11.7	11.7	11.7	11.7	11.7	11.7	11.7	11.7	11.7
11.71	11.71	11.71	11.71	11.71	11.71	11.71	11.71	11.71
11.72	11.72	11.72	11.72	11.72	11.72	11.72	11.72	11.72
11.73	11.73	11.73	11.73	11.73	11.73	11.73	11.73	11.73
11.74	11.74	11.74	11.74	11.74	11.74	11.74	11.74	11.74
11.75	11.75	11.75	11.75	11.75	11.75	11.75	11.75	11.75
11.76	11.76	11.76	11.76	11.76	11.76	11.76	11.76	11.76
11.77	11.77	11.77	11.77	11.77	11.77	11.77	11.77	11.77
11.78	11.78	11.78	11.78	11.78	11.78	11.78	11.78	11.78
11.79	11.79	11.79	11.79	11.79	11.79	11.79	11.79	11.79
11.8	11.8	11.8	11.8	11.8	11.8	11.8	11.8	11.8
11.81	11.81	11.81	11.81	11.81	11.81	11.81	11.81	11.81
11.82	11.82	11.82	11.82	11.82	11.82	11.82	11.82	11.82
11.83	11.83	11.83	11.83	11.83	11.83	11.83	11.83	11.83
11.84	11.84	11.84	11.84	11.84	11.84	11.84	11.84	11.84
11.85	11.85	11.85	11.85	11.85	11.85	11.85	11.85	11.85
11.86	11.86	11.86	11.86	11.86	11.86	11.86	11.86	11.86
11.87	11.87	11.87	11.87	11.87	11.87	11.87	11.87	11.87
11.88	11.88	11.88	11.88	11.88	11.88	11.88	11.88	11.88
11.89	11.89	11.89	11.89	11.89	11.89	11.89	11.89	11.89
11.9	11.9	11.9	11.9	11.9	11.9	11.9	11.9	11.9
11.91	11.91	11.91	11.91	11.91	11.91	11.91	11.91	11.91
11.92	11.92	11.92	11.92	11.92	11.92	11.92	11.92	11.92
11.93	11.93	11.93	11.93	11.93	11.93	11.93	11.93	11.93
11.94	11.94	11.94	11.94	11.94	11.94	11.94	11.94	11.94
11.95	11.95	11.95	11.95	11.95	11.95	11.95	11.95	11.95
11.96	11.96	11.96	11.96	11.96	11.96	11.96	11.96	11.96
11.97	11.97	11.97	11.97	11.97	11.97	11.97	11.97	11.97
11.98	11.98	11.98	11.98	11.98	11.98	11.98	11.98	11.98
11.99	11.99	11.99	11.99	11.99	11.99	11.99	11.99	11.99

12	12	12	12	12	12	12	12	12

-12	-12	-12	-12	-12	-12	-12	-12	-12
-11.99	-11.99	-11.99	-11.99	-11.99	-11.99	-11.99	-11.99	-11.99
-11.98	-11.98	-11.98	-11.98	-11.98	-11.98	-11.98	-11.98	-11.98
-11.97	-11.97	-11.97	-11.97	-11.97	-11.97	-11.97	-11.97	-11.97
-11.96	-11.96	-11.96	-11.96	-11.96	-11.96	-11.96	-11.96	-11.96
-11.95	-11.95	-11.95	-11.95	-11.95	-11.95	-11.95	-11.95	-11.95
-11.94	-11.94	-11.94	-11.94	-11.94	-11.94	-11.94	-11.94	-11.94
-11.93	-11.93	-11.93	-11.93	-11.93	-11.93	-11.93	-11.93	-11.93
-11.92	-11.92	-11.92	-11.92	-11.92	-11.92	-11.92	-11.92	-11.92
-11.91	-11.91	-11.91	-11.91	-11.91	-11.91	-11.91	-11.91	-11.91
-11.9	-11.9	-11.9	-11.9	-11.9	-11.9	-11.9	-11.9	-11.9
-11.89	-11.89	-11.89	-11.89	-11.89	-11.89	-11.89	-11.89	-11.89
-11.88	-11.88	-11.88	-11.88	-11.88	-11.88	-11.88	-11.88	-11.88
-11.87	-11.87	-11.87	-11.87	-11.87	-11.87	-11.87	-11.87	-11.87
-11.86	-11.86	-11.86	-11.86	-11.86	-11.86	-11.86	-11.86	-11.86
-11.85	-11.85	-11.85	-11.85	-11.85	-11.85	-11.85	-11.85	-11.85
-11.84	-11.84	-11.84	-11.84	-11.84	-11.84	-11.84	-11.84	-11.84
-11.83	-11.83	-11.83	-11.83	-11.83	-11.83	-11.83	-11.83	-11.83
-11.82	-11.82	-11.82	-11.82	-11.82	-11.82	-11.82	-11.82	-11.82
-11.81	-11.81	-11.81	-11.81	-11.81	-11.81	-11.81	-11.81	-11.81
-11.8	-11.8	-11.8	-11.8	-11.8	-11.8	-11.8	-11.8	-11.8
-11.79	-11.79	-11.79	-11.79	-11.79	-11.79	-11.79	-11.79	-11.79
-11.78	-11.78	-11.78	-11.78	-11.78	-11.78	-11.78	-11.78	-11.78
-11.77	-11.77	-11.77	-11.77	-11.77	-11.77	-11.77	-11.77	-11.77
-11.76	-11.76	-11.76	-11.76	-11.76	-11.76	-11.76	-11.76	-11.76
-11.75	-11.75	-11.75	-11.75	-11.75	-11.75	-11.75	-11.75	-11.75
-11.74	-11.74	-11.74	-11.74	-11.74	-11.74	-11.74	-11.74	-11.74
-11.73	-11.73	-11.73	-11.73	-11.73	-11.73	-11.73	-11.73	-11.73
-11.72	-11.72	-11.72	-11.72	-11.72	-11.72	-11.72	-11.72	-11.72
-11.71	-11.71	-11.71	-11.71	-11.71	-11.71	-11.71	-11.71	-11.71
-11.7	-11.7	-11.7	-11.7	-11.7	-11.7	-11.7	-11.7	-11.7
-11.69	-11.69	-11.69	-11.69	-11.69	-11.69	-11.69	-11.69	-11.69
-11.68	-11.68	-11.68	-11.68	-11.68	-11.68	-11.68	-11.68	-11.68
-11.67	-11.67	-11.67	-11.67	-11.67	-11.67	-11.67	-11.67	-11.67
-11.66	-11.66	-11.66	-11.66	-11.66	-11.66	-11.66	-11.66	-11.66
-11.65	-11.65	-11.65	-11.65	-11.65	-11.65	-11.65	-11.65	-11.65
-11.64	-11.64	-11.64	-11.64	-11.64	-11.64	-11.64	-11.64	-11.64
-11.63	-11.63	-11.63	-11.63	-11.63	-11.63	-11.63	-11.63	-11.63
-11.62	-11.62	-11.62	-11.62	-11.62	-11.62	-11.62	-11.62	-11.62
-11.61	-11.61	-11.61	-11.61	-11.61	-11.61	-11.61	-11.61	-11.61
-11.6	-11.6	-11.6	-11.6	-11.6	-11.6	-11.6	-11.6	-11.6
-11.59	-11.59	-11.59	-11.59	-11.59	-11.59	-11.59	-11.59	-11.59
-11.58	-11.58	-11.58	-11.58	-11.58	-11.58	-11.58	-11.58	-11.58
-11.57	-11.57	-11.57	-11.57	-11.57	-11.57	-11.57	-11.57	-11.57
-11.56	-11.56	-11.56	-11.56	-11.56	-11.56	-11.56	-11.56	-11.56
-11.55	-11.55	-11.55	-11.55	-11.55	-11.55	-11.55	-11.55	-11.55
-11.54	-11.54	-11.54	-11.54	-11.54	-11.54	-11.54	-11.54	-11.54
-11.53	-11.53	-11.53	-11.53	-11.53	-11.53	-11.53	-11.53	-11.53
-11.52	-11.52	-11.52	-11.52	-11.52	-11.52	-11.52	-11.52	-11.52
-11.51	-11.51	-11.51	-11.51	-11.51	-11.51	-11.51	-11.51	-11.51

-11.5	-11.5	-11.5	-11.5	-11.5	-11.5	-11.5	-11.5	-11.5
-11.49	-11.49	-11.49	-11.49	-11.49	-11.49	-11.49	-11.49	-11.49
-11.48	-11.48	-11.48	-11.48	-11.48	-11.48	-11.48	-11.48	-11.48
-11.47	-11.47	-11.47	-11.47	-11.47	-11.47	-11.47	-11.47	-11.47
-11.46	-11.46	-11.46	-11.46	-11.46	-11.46	-11.46	-11.46	-11.46
-11.45	-11.45	-11.45	-11.45	-11.45	-11.45	-11.45	-11.45	-11.45
-11.44	-11.44	-11.44	-11.44	-11.44	-11.44	-11.44	-11.44	-11.44
-11.43	-11.43	-11.43	-11.43	-11.43	-11.43	-11.43	-11.43	-11.43
-11.42	-11.42	-11.42	-11.42	-11.42	-11.42	-11.42	-11.42	-11.42
-11.41	-11.41	-11.41	-11.41	-11.41	-11.41	-11.41	-11.41	-11.41
-11.4	-11.4	-11.4	-11.4	-11.4	-11.4	-11.4	-11.4	-11.4
-11.39	-11.39	-11.39	-11.39	-11.39	-11.39	-11.39	-11.39	-11.39
-11.38	-11.38	-11.38	-11.38	-11.38	-11.38	-11.38	-11.38	-11.38
-11.37	-11.37	-11.37	-11.37	-11.37	-11.37	-11.37	-11.37	-11.37
-11.36	-11.36	-11.36	-11.36	-11.36	-11.36	-11.36	-11.36	-11.36
-11.35	-11.35	-11.35	-11.35	-11.35	-11.35	-11.35	-11.35	-11.35
-11.34	-11.34	-11.34	-11.34	-11.34	-11.34	-11.34	-11.34	-11.34
-11.33	-11.33	-11.33	-11.33	-11.33	-11.33	-11.33	-11.33	-11.33
-11.32	-11.32	-11.32	-11.32	-11.32	-11.32	-11.32	-11.32	-11.32
-11.31	-11.31	-11.31	-11.31	-11.31	-11.31	-11.31	-11.31	-11.31
-11.3	-11.3	-11.3	-11.3	-11.3	-11.3	-11.3	-11.3	-11.3
-11.29	-11.29	-11.29	-11.29	-11.29	-11.29	-11.29	-11.29	-11.29
-11.28	-11.28	-11.28	-11.28	-11.28	-11.28	-11.28	-11.28	-11.28
-11.27	-11.27	-11.27	-11.27	-11.27	-11.27	-11.27	-11.27	-11.27
-11.26	-11.26	-11.26	-11.26	-11.26	-11.26	-11.26	-11.26	-11.26
-11.25	-11.25	-11.25	-11.25	-11.25	-11.25	-11.25	-11.25	-11.25
-11.24	-11.24	-11.24	-11.24	-11.24	-11.24	-11.24	-11.24	-11.24
-11.23	-11.23	-11.23	-11.23	-11.23	-11.23	-11.23	-11.23	-11.23
-11.22	-11.22	-11.22	-11.22	-11.22	-11.22	-11.22	-11.22	-11.22
-11.21	-11.21	-11.21	-11.21	-11.21	-11.21	-11.21	-11.21	-11.21
-11.2	-11.2	-11.2	-11.2	-11.2	-11.2	-11.2	-11.2	-11.2
-11.19	-11.19	-11.19	-11.19	-11.19	-11.19	-11.19	-11.19	-11.19
-11.18	-11.18	-11.18	-11.18	-11.18	-11.18	-11.18	-11.18	-11.18
-11.17	-11.17	-11.17	-11.17	-11.17	-11.17	-11.17	-11.17	-11.17
-11.16	-11.16	-11.16	-11.16	-11.16	-11.16	-11.16	-11.16	-11.16
-11.15	-11.15	-11.15	-11.15	-11.15	-11.15	-11.15	-11.15	-11.15
-11.14	-11.14	-11.14	-11.14	-11.14	-11.14	-11.14	-11.14	-11.14
-11.13	-11.13	-11.13	-11.13	-11.13	-11.13	-11.13	-11.13	-11.13
-11.12	-11.12	-11.12	-11.12	-11.12	-11.12	-11.12	-11.12	-11.12
-11.11	-11.11	-11.11	-11.11	-11.11	-11.11	-11.11	-11.11	-11.11
-11.1	-11.1	-11.1	-11.1	-11.1	-11.1	-11.1	-11.1	-11.1
-11.09	-11.09	-11.09	-11.09	-11.09	-11.09	-11.09	-11.09	-11.09
-11.08	-11.08	-11.08	-11.08	-11.08	-11.08	-11.08	-11.08	-11.08
-11.07	-11.07	-11.07	-11.07	-11.07	-11.07	-11.07	-11.07	-11.07
-11.06	-11.06	-11.06	-11.06	-11.06	-11.06	-11.06	-11.06	-11.06
-11.05	-11.05	-11.05	-11.05	-11.05	-11.05	-11.05	-11.05	-11.05
-11.04	-11.04	-11.04	-11.04	-11.04	-11.04	-11.04	-11.04	-11.04
-11.03	-11.03	-11.03	-11.03	-11.03	-11.03	-11.03	-11.03	-11.03
-11.02	-11.02	-11.02	-11.02	-11.02	-11.02	-11.02	-11.02	-11.02
-11.01	-11.01	-11.01	-11.01	-11.01	-11.01	-11.01	-11.01	-11.01

-11	-11	-11	-11	-11	-11	-11	-11	-11
-10.99	-10.99	-10.99	-10.99	-10.99	-10.99	-10.99	-10.99	-10.99
-10.98	-10.98	-10.98	-10.98	-10.98	-10.98	-10.98	-10.98	-10.98
-10.97	-10.97	-10.97	-10.97	-10.97	-10.97	-10.97	-10.97	-10.97
-10.96	-10.96	-10.96	-10.96	-10.96	-10.96	-10.96	-10.96	-10.96
-10.95	-10.95	-10.95	-10.95	-10.95	-10.95	-10.95	-10.95	-10.95
-10.94	-10.94	-10.94	-10.94	-10.94	-10.94	-10.94	-10.94	-10.94
-10.93	-10.93	-10.93	-10.93	-10.93	-10.93	-10.93	-10.93	-10.93
-10.92	-10.92	-10.92	-10.92	-10.92	-10.92	-10.92	-10.92	-10.92
-10.91	-10.91	-10.91	-10.91	-10.91	-10.91	-10.91	-10.91	-10.91
-10.9	-10.9	-10.9	-10.9	-10.9	-10.9	-10.9	-10.9	-10.9
-10.89	-10.89	-10.89	-10.89	-10.89	-10.89	-10.89	-10.89	-10.89
-10.88	-10.88	-10.88	-10.88	-10.88	-10.88	-10.88	-10.88	-10.88
-10.87	-10.87	-10.87	-10.87	-10.87	-10.87	-10.87	-10.87	-10.87
-10.86	-10.86	-10.86	-10.86	-10.86	-10.86	-10.86	-10.86	-10.86
-10.85	-10.85	-10.85	-10.85	-10.85	-10.85	-10.85	-10.85	-10.85
-10.84	-10.84	-10.84	-10.84	-10.84	-10.84	-10.84	-10.84	-10.84
-10.83	-10.83	-10.83	-10.83	-10.83	-10.83	-10.83	-10.83	-10.83
-10.82	-10.82	-10.82	-10.82	-10.82	-10.82	-10.82	-10.82	-10.82
-10.81	-10.81	-10.81	-10.81	-10.81	-10.81	-10.81	-10.81	-10.81
-10.8	-10.8	-10.8	-10.8	-10.8	-10.8	-10.8	-10.8	-10.8
-10.79	-10.79	-10.79	-10.79	-10.79	-10.79	-10.79	-10.79	-10.79
-10.78	-10.78	-10.78	-10.78	-10.78	-10.78	-10.78	-10.78	-10.78
-10.77	-10.77	-10.77	-10.77	-10.77	-10.77	-10.77	-10.77	-10.77
-10.76	-10.76	-10.76	-10.76	-10.76	-10.76	-10.76	-10.76	-10.76
-10.75	-10.75	-10.75	-10.75	-10.75	-10.75	-10.75	-10.75	-10.75
-10.74	-10.74	-10.74	-10.74	-10.74	-10.74	-10.74	-10.74	-10.74
-10.73	-10.73	-10.73	-10.73	-10.73	-10.73	-10.73	-10.73	-10.73
-10.72	-10.72	-10.72	-10.72	-10.72	-10.72	-10.72	-10.72	-10.72
-10.71	-10.71	-10.71	-10.71	-10.71	-10.71	-10.71	-10.71	-10.71
-10.7	-10.7	-10.7	-10.7	-10.7	-10.7	-10.7	-10.7	-10.7
-10.69	-10.69	-10.69	-10.69	-10.69	-10.69	-10.69	-10.69	-10.69
-10.68	-10.68	-10.68	-10.68	-10.68	-10.68	-10.68	-10.68	-10.68
-10.67	-10.67	-10.67	-10.67	-10.67	-10.67	-10.67	-10.67	-10.67
-10.66	-10.66	-10.66	-10.66	-10.66	-10.66	-10.66	-10.66	-10.66
-10.65	-10.65	-10.65	-10.65	-10.65	-10.65	-10.65	-10.65	-10.65
-10.64	-10.64	-10.64	-10.64	-10.64	-10.64	-10.64	-10.64	-10.64
-10.63	-10.63	-10.63	-10.63	-10.63	-10.63	-10.63	-10.63	-10.63
-10.62	-10.62	-10.62	-10.62	-10.62	-10.62	-10.62	-10.62	-10.62
-10.61	-10.61	-10.61	-10.61	-10.61	-10.61	-10.61	-10.61	-10.61
-10.6	-10.6	-10.6	-10.6	-10.6	-10.6	-10.6	-10.6	-10.6
-10.59	-10.59	-10.59	-10.59	-10.59	-10.59	-10.59	-10.59	-10.59
-10.58	-10.58	-10.58	-10.58	-10.58	-10.58	-10.58	-10.58	-10.58
-10.57	-10.57	-10.57	-10.57	-10.57	-10.57	-10.57	-10.57	-10.57
-10.56	-10.56	-10.56	-10.56	-10.56	-10.56	-10.56	-10.56	-10.56
-10.55	-10.55	-10.55	-10.55	-10.55	-10.55	-10.55	-10.55	-10.55
-10.54	-10.54	-10.54	-10.54	-10.54	-10.54	-10.54	-10.54	-10.54
-10.53	-10.53	-10.53	-10.53	-10.53	-10.53	-10.53	-10.53	-10.53
-10.52	-10.52	-10.52	-10.52	-10.52	-10.52	-10.52	-10.52	-10.52
-10.51	-10.51	-10.51	-10.51	-10.51	-10.51	-10.51	-10.51	-10.51

-10.5	-10.5	-10.5	-10.5	-10.5	-10.5	-10.5	-10.5	-10.5
-10.49	-10.49	-10.49	-10.49	-10.49	-10.49	-10.49	-10.49	-10.49
-10.48	-10.48	-10.48	-10.48	-10.48	-10.48	-10.48	-10.48	-10.48
-10.47	-10.47	-10.47	-10.47	-10.47	-10.47	-10.47	-10.47	-10.47
-10.46	-10.46	-10.46	-10.46	-10.46	-10.46	-10.46	-10.46	-10.46
-10.45	-10.45	-10.45	-10.45	-10.45	-10.45	-10.45	-10.45	-10.45
-10.44	-10.44	-10.44	-10.44	-10.44	-10.44	-10.44	-10.44	-10.44
-10.43	-10.43	-10.43	-10.43	-10.43	-10.43	-10.43	-10.43	-10.43
-10.42	-10.42	-10.42	-10.42	-10.42	-10.42	-10.42	-10.42	-10.42
-10.41	-10.41	-10.41	-10.41	-10.41	-10.41	-10.41	-10.41	-10.41
-10.4	-10.4	-10.4	-10.4	-10.4	-10.4	-10.4	-10.4	-10.4
-10.39	-10.39	-10.39	-10.39	-10.39	-10.39	-10.39	-10.39	-10.39
-10.38	-10.38	-10.38	-10.38	-10.38	-10.38	-10.38	-10.38	-10.38
-10.37	-10.37	-10.37	-10.37	-10.37	-10.37	-10.37	-10.37	-10.37
-10.36	-10.36	-10.36	-10.36	-10.36	-10.36	-10.36	-10.36	-10.36
-10.35	-10.35	-10.35	-10.35	-10.35	-10.35	-10.35	-10.35	-10.35
-10.34	-10.34	-10.34	-10.34	-10.34	-10.34	-10.34	-10.34	-10.34
-10.33	-10.33	-10.33	-10.33	-10.33	-10.33	-10.33	-10.33	-10.33
-10.32	-10.32	-10.32	-10.32	-10.32	-10.32	-10.32	-10.32	-10.32
-10.31	-10.31	-10.31	-10.31	-10.31	-10.31	-10.31	-10.31	-10.31
-10.3	-10.3	-10.3	-10.3	-10.3	-10.3	-10.3	-10.3	-10.3
-10.29	-10.29	-10.29	-10.29	-10.29	-10.29	-10.29	-10.29	-10.29
-10.28	-10.28	-10.28	-10.28	-10.28	-10.28	-10.28	-10.28	-10.28
-10.27	-10.27	-10.27	-10.27	-10.27	-10.27	-10.27	-10.27	-10.27
-10.26	-10.26	-10.26	-10.26	-10.26	-10.26	-10.26	-10.26	-10.26
-10.25	-10.25	-10.25	-10.25	-10.25	-10.25	-10.25	-10.25	-10.25
-10.24	-10.24	-10.24	-10.24	-10.24	-10.24	-10.24	-10.24	-10.24
-10.23	-10.23	-10.23	-10.23	-10.23	-10.23	-10.23	-10.23	-10.23
-10.22	-10.22	-10.22	-10.22	-10.22	-10.22	-10.22	-10.22	-10.22
-10.21	-10.21	-10.21	-10.21	-10.21	-10.21	-10.21	-10.21	-10.21
-10.2	-10.2	-10.2	-10.2	-10.2	-10.2	-10.2	-10.2	-10.2
-10.19	-10.19	-10.19	-10.19	-10.19	-10.19	-10.19	-10.19	-10.19
-10.18	-10.18	-10.18	-10.18	-10.18	-10.18	-10.18	-10.18	-10.18
-10.17	-10.17	-10.17	-10.17	-10.17	-10.17	-10.17	-10.17	-10.17
-10.16	-10.16	-10.16	-10.16	-10.16	-10.16	-10.16	-10.16	-10.16
-10.15	-10.15	-10.15	-10.15	-10.15	-10.15	-10.15	-10.15	-10.15
-10.14	-10.14	-10.14	-10.14	-10.14	-10.14	-10.14	-10.14	-10.14
-10.13	-10.13	-10.13	-10.13	-10.13	-10.13	-10.13	-10.13	-10.13
-10.12	-10.12	-10.12	-10.12	-10.12	-10.12	-10.12	-10.12	-10.12
-10.11	-10.11	-10.11	-10.11	-10.11	-10.11	-10.11	-10.11	-10.11
-10.1	-10.1	-10.1	-10.1	-10.1	-10.1	-10.1	-10.1	-10.1
-10.09	-10.09	-10.09	-10.09	-10.09	-10.09	-10.09	-10.09	-10.09
-10.08	-10.08	-10.08	-10.08	-10.08	-10.08	-10.08	-10.08	-10.08
-10.07	-10.07	-10.07	-10.07	-10.07	-10.07	-10.07	-10.07	-10.07
-10.06	-10.06	-10.06	-10.06	-10.06	-10.06	-10.06	-10.06	-10.06
-10.05	-10.05	-10.05	-10.05	-10.05	-10.05	-10.05	-10.05	-10.05
-10.04	-10.04	-10.04	-10.04	-10.04	-10.04	-10.04	-10.04	-10.04
-10.03	-10.03	-10.03	-10.03	-10.03	-10.03	-10.03	-10.03	-10.03
-10.02	-10.02	-10.02	-10.02	-10.02	-10.02	-10.02	-10.02	-10.02
-10.01	-10.01	-10.01	-10.01	-10.01	-10.01	-10.01	-10.01	-10.01

-10	-10	-10	-10	-10	-10	-10	-10	-10
-9.99	-9.99	-9.99	-9.99	-9.99	-9.99	-9.99	-9.99	-9.99
-9.98	-9.98	-9.98	-9.98	-9.98	-9.98	-9.98	-9.98	-9.98
-9.97	-9.97	-9.97	-9.97	-9.97	-9.97	-9.97	-9.97	-9.97
-9.96	-9.96	-9.96	-9.96	-9.96	-9.96	-9.96	-9.96	-9.96
-9.95	-9.95	-9.95	-9.95	-9.95	-9.95	-9.95	-9.95	-9.95
-9.94	-9.94	-9.94	-9.94	-9.94	-9.94	-9.94	-9.94	-9.94
-9.93	-9.93	-9.93	-9.93	-9.93	-9.93	-9.93	-9.93	-9.93
-9.92	-9.92	-9.92	-9.92	-9.92	-9.92	-9.92	-9.92	-9.92
-9.91	-9.91	-9.91	-9.91	-9.91	-9.91	-9.91	-9.91	-9.91
-9.9	-9.9	-9.9	-9.9	-9.9	-9.9	-9.9	-9.9	-9.9
-9.89	-9.89	-9.89	-9.89	-9.89	-9.89	-9.89	-9.89	-9.89
-9.88	-9.88	-9.88	-9.88	-9.88	-9.88	-9.88	-9.88	-9.88
-9.87	-9.87	-9.87	-9.87	-9.87	-9.87	-9.87	-9.87	-9.87
-9.86	-9.86	-9.86	-9.86	-9.86	-9.86	-9.86	-9.86	-9.86
-9.85	-9.85	-9.85	-9.85	-9.85	-9.85	-9.85	-9.85	-9.85
-9.84	-9.84	-9.84	-9.84	-9.84	-9.84	-9.84	-9.84	-9.84
-9.83	-9.83	-9.83	-9.83	-9.83	-9.83	-9.83	-9.83	-9.83
-9.82	-9.82	-9.82	-9.82	-9.82	-9.82	-9.82	-9.82	-9.82
-9.81	-9.81	-9.81	-9.81	-9.81	-9.81	-9.81	-9.81	-9.81
-9.8	-9.8	-9.8	-9.8	-9.8	-9.8	-9.8	-9.8	-9.8
-9.79	-9.79	-9.79	-9.79	-9.79	-9.79	-9.79	-9.79	-9.79
-9.78	-9.78	-9.78	-9.78	-9.78	-9.78	-9.78	-9.78	-9.78
-9.77	-9.77	-9.77	-9.77	-9.77	-9.77	-9.77	-9.77	-9.77
-9.76	-9.76	-9.76	-9.76	-9.76	-9.76	-9.76	-9.76	-9.76
-9.75	-9.75	-9.75	-9.75	-9.75	-9.75	-9.75	-9.75	-9.75
-9.74	-9.74	-9.74	-9.74	-9.74	-9.74	-9.74	-9.74	-9.74
-9.73	-9.73	-9.73	-9.73	-9.73	-9.73	-9.73	-9.73	-9.73
-9.72	-9.72	-9.72	-9.72	-9.72	-9.72	-9.72	-9.72	-9.72
-9.71	-9.71	-9.71	-9.71	-9.71	-9.71	-9.71	-9.71	-9.71
-9.7	-9.7	-9.7	-9.7	-9.7	-9.7	-9.7	-9.7	-9.7
-9.69	-9.69	-9.69	-9.69	-9.69	-9.69	-9.69	-9.69	-9.69
-9.68	-9.68	-9.68	-9.68	-9.68	-9.68	-9.68	-9.68	-9.68
-9.67	-9.67	-9.67	-9.67	-9.67	-9.67	-9.67	-9.67	-9.67
-9.66	-9.66	-9.66	-9.66	-9.66	-9.66	-9.66	-9.66	-9.66
-9.65	-9.65	-9.65	-9.65	-9.65	-9.65	-9.65	-9.65	-9.65
-9.64	-9.64	-9.64	-9.64	-9.64	-9.64	-9.64	-9.64	-9.64
-9.63	-9.63	-9.63	-9.63	-9.63	-9.63	-9.63	-9.63	-9.63
-9.62	-9.62	-9.62	-9.62	-9.62	-9.62	-9.62	-9.62	-9.62
-9.61	-9.61	-9.61	-9.61	-9.61	-9.61	-9.61	-9.61	-9.61
-9.6	-9.6	-9.6	-9.6	-9.6	-9.6	-9.6	-9.6	-9.6
-9.59	-9.59	-9.59	-9.59	-9.59	-9.59	-9.59	-9.59	-9.59
-9.58	-9.58	-9.58	-9.58	-9.58	-9.58	-9.58	-9.58	-9.58
-9.57	-9.57	-9.57	-9.57	-9.57	-9.57	-9.57	-9.57	-9.57
-9.56	-9.56	-9.56	-9.56	-9.56	-9.56	-9.56	-9.56	-9.56
-9.55	-9.55	-9.55	-9.55	-9.55	-9.55	-9.55	-9.55	-9.55
-9.54	-9.54	-9.54	-9.54	-9.54	-9.54	-9.54	-9.54	-9.54
-9.53	-9.53	-9.53	-9.53	-9.53	-9.53	-9.53	-9.53	-9.53
-9.52	-9.52	-9.52	-9.52	-9.52	-9.52	-9.52	-9.52	-9.52
-9.51	-9.51	-9.51	-9.51	-9.51	-9.51	-9.51	-9.51	-9.51

-9.5	-9.5	-9.5	-9.5	-9.5	-9.5	-9.5	-9.5	-9.5
-9.49	-9.49	-9.49	-9.49	-9.49	-9.49	-9.49	-9.49	-9.49
-9.48	-9.48	-9.48	-9.48	-9.48	-9.48	-9.48	-9.48	-9.48
-9.47	-9.47	-9.47	-9.47	-9.47	-9.47	-9.47	-9.47	-9.47
-9.46	-9.46	-9.46	-9.46	-9.46	-9.46	-9.46	-9.46	-9.46
-9.45	-9.45	-9.45	-9.45	-9.45	-9.45	-9.45	-9.45	-9.45
-9.44	-9.44	-9.44	-9.44	-9.44	-9.44	-9.44	-9.44	-9.44
-9.43	-9.43	-9.43	-9.43	-9.43	-9.43	-9.43	-9.43	-9.43
-9.42	-9.42	-9.42	-9.42	-9.42	-9.42	-9.42	-9.42	-9.42
-9.41	-9.41	-9.41	-9.41	-9.41	-9.41	-9.41	-9.41	-9.41
-9.4	-9.4	-9.4	-9.4	-9.4	-9.4	-9.4	-9.4	-9.4
-9.39	-9.39	-9.39	-9.39	-9.39	-9.39	-9.39	-9.39	-9.39
-9.38	-9.38	-9.38	-9.38	-9.38	-9.38	-9.38	-9.38	-9.38
-9.37	-9.37	-9.37	-9.37	-9.37	-9.37	-9.37	-9.37	-9.37
-9.36	-9.36	-9.36	-9.36	-9.36	-9.36	-9.36	-9.36	-9.36
-9.35	-9.35	-9.35	-9.35	-9.35	-9.35	-9.35	-9.35	-9.35
-9.34	-9.34	-9.34	-9.34	-9.34	-9.34	-9.34	-9.34	-9.34
-9.33	-9.33	-9.33	-9.33	-9.33	-9.33	-9.33	-9.33	-9.33
-9.32	-9.32	-9.32	-9.32	-9.32	-9.32	-9.32	-9.32	-9.32
-9.31	-9.31	-9.31	-9.31	-9.31	-9.31	-9.31	-9.31	-9.31
-9.3	-9.3	-9.3	-9.3	-9.3	-9.3	-9.3	-9.3	-9.3
-9.29	-9.29	-9.29	-9.29	-9.29	-9.29	-9.29	-9.29	-9.29
-9.28	-9.28	-9.28	-9.28	-9.28	-9.28	-9.28	-9.28	-9.28
-9.27	-9.27	-9.27	-9.27	-9.27	-9.27	-9.27	-9.27	-9.27
-9.26	-9.26	-9.26	-9.26	-9.26	-9.26	-9.26	-9.26	-9.26
-9.25	-9.25	-9.25	-9.25	-9.25	-9.25	-9.25	-9.25	-9.25
-9.24	-9.24	-9.24	-9.24	-9.24	-9.24	-9.24	-9.24	-9.24
-9.23	-9.23	-9.23	-9.23	-9.23	-9.23	-9.23	-9.23	-9.23
-9.22	-9.22	-9.22	-9.22	-9.22	-9.22	-9.22	-9.22	-9.22
-9.21	-9.21	-9.21	-9.21	-9.21	-9.21	-9.21	-9.21	-9.21
-9.2	-9.2	-9.2	-9.2	-9.2	-9.2	-9.2	-9.2	-9.2
-9.19	-9.19	-9.19	-9.19	-9.19	-9.19	-9.19	-9.19	-9.19
-9.18	-9.18	-9.18	-9.18	-9.18	-9.18	-9.18	-9.18	-9.18
-9.17	-9.17	-9.17	-9.17	-9.17	-9.17	-9.17	-9.17	-9.17
-9.16	-9.16	-9.16	-9.16	-9.16	-9.16	-9.16	-9.16	-9.16
-9.15	-9.15	-9.15	-9.15	-9.15	-9.15	-9.15	-9.15	-9.15
-9.14	-9.14	-9.14	-9.14	-9.14	-9.14	-9.14	-9.14	-9.14
-9.13	-9.13	-9.13	-9.13	-9.13	-9.13	-9.13	-9.13	-9.13
-9.12	-9.12	-9.12	-9.12	-9.12	-9.12	-9.12	-9.12	-9.12
-9.11	-9.11	-9.11	-9.11	-9.11	-9.11	-9.11	-9.11	-9.11
-9.1	-9.1	-9.1	-9.1	-9.1	-9.1	-9.1	-9.1	-9.1
-9.09	-9.09	-9.09	-9.09	-9.09	-9.09	-9.09	-9.09	-9.09
-9.08	-9.08	-9.08	-9.08	-9.08	-9.08	-9.08	-9.08	-9.08
-9.07	-9.07	-9.07	-9.07	-9.07	-9.07	-9.07	-9.07	-9.07
-9.06	-9.06	-9.06	-9.06	-9.06	-9.06	-9.06	-9.06	-9.06
-9.05	-9.05	-9.05	-9.05	-9.05	-9.05	-9.05	-9.05	-9.05
-9.04	-9.04	-9.04	-9.04	-9.04	-9.04	-9.04	-9.04	-9.04
-9.03	-9.03	-9.03	-9.03	-9.03	-9.03	-9.03	-9.03	-9.03
-9.02	-9.02	-9.02	-9.02	-9.02	-9.02	-9.02	-9.02	-9.02
-9.01	-9.01	-9.01	-9.01	-9.01	-9.01	-9.01	-9.01	-9.01

-9	-9	-9	-9	-9	-9	-9	-9	-9
-8.99	-8.99	-8.99	-8.99	-8.99	-8.99	-8.99	-8.99	-8.99
-8.98	-8.98	-8.98	-8.98	-8.98	-8.98	-8.98	-8.98	-8.98
-8.97	-8.97	-8.97	-8.97	-8.97	-8.97	-8.97	-8.97	-8.97
-8.96	-8.96	-8.96	-8.96	-8.96	-8.96	-8.96	-8.96	-8.96
-8.95	-8.95	-8.95	-8.95	-8.95	-8.95	-8.95	-8.95	-8.95
-8.94	-8.94	-8.94	-8.94	-8.94	-8.94	-8.94	-8.94	-8.94
-8.93	-8.93	-8.93	-8.93	-8.93	-8.93	-8.93	-8.93	-8.93
-8.92	-8.92	-8.92	-8.92	-8.92	-8.92	-8.92	-8.92	-8.92
-8.91	-8.91	-8.91	-8.91	-8.91	-8.91	-8.91	-8.91	-8.91
-8.9	-8.9	-8.9	-8.9	-8.9	-8.9	-8.9	-8.9	-8.9
-8.89	-8.89	-8.89	-8.89	-8.89	-8.89	-8.89	-8.89	-8.89
-8.88	-8.88	-8.88	-8.88	-8.88	-8.88	-8.88	-8.88	-8.88
-8.87	-8.87	-8.87	-8.87	-8.87	-8.87	-8.87	-8.87	-8.87
-8.86	-8.86	-8.86	-8.86	-8.86	-8.86	-8.86	-8.86	-8.86
-8.85	-8.85	-8.85	-8.85	-8.85	-8.85	-8.85	-8.85	-8.85
-8.84	-8.84	-8.84	-8.84	-8.84	-8.84	-8.84	-8.84	-8.84
-8.83	-8.83	-8.83	-8.83	-8.83	-8.83	-8.83	-8.83	-8.83
-8.82	-8.82	-8.82	-8.82	-8.82	-8.82	-8.82	-8.82	-8.82
-8.81	-8.81	-8.81	-8.81	-8.81	-8.81	-8.81	-8.81	-8.81
-8.8	-8.8	-8.8	-8.8	-8.8	-8.8	-8.8	-8.8	-8.8
-8.79	-8.79	-8.79	-8.79	-8.79	-8.79	-8.79	-8.79	-8.79
-8.78	-8.78	-8.78	-8.78	-8.78	-8.78	-8.78	-8.78	-8.78
-8.77	-8.77	-8.77	-8.77	-8.77	-8.77	-8.77	-8.77	-8.77
-8.76	-8.76	-8.76	-8.76	-8.76	-8.76	-8.76	-8.76	-8.76
-8.75	-8.75	-8.75	-8.75	-8.75	-8.75	-8.75	-8.75	-8.75
-8.74	-8.74	-8.74	-8.74	-8.74	-8.74	-8.74	-8.74	-8.74
-8.73	-8.73	-8.73	-8.73	-8.73	-8.73	-8.73	-8.73	-8.73
-8.72	-8.72	-8.72	-8.72	-8.72	-8.72	-8.72	-8.72	-8.72
-8.71	-8.71	-8.71	-8.71	-8.71	-8.71	-8.71	-8.71	-8.71
-8.7	-8.7	-8.7	-8.7	-8.7	-8.7	-8.7	-8.7	-8.7
-8.69	-8.69	-8.69	-8.69	-8.69	-8.69	-8.69	-8.69	-8.69
-8.68	-8.68	-8.68	-8.68	-8.68	-8.68	-8.68	-8.68	-8.68
-8.67	-8.67	-8.67	-8.67	-8.67	-8.67	-8.67	-8.67	-8.67
-8.66	-8.66	-8.66	-8.66	-8.66	-8.66	-8.66	-8.66	-8.66
-8.65	-8.65	-8.65	-8.65	-8.65	-8.65	-8.65	-8.65	-8.65
-8.64	-8.64	-8.64	-8.64	-8.64	-8.64	-8.64	-8.64	-8.64
-8.63	-8.63	-8.63	-8.63	-8.63	-8.63	-8.63	-8.63	-8.63
-8.62	-8.62	-8.62	-8.62	-8.62	-8.62	-8.62	-8.62	-8.62
-8.61	-8.61	-8.61	-8.61	-8.61	-8.61	-8.61	-8.61	-8.61
-8.6	-8.6	-8.6	-8.6	-8.6	-8.6	-8.6	-8.6	-8.6
-8.59	-8.59	-8.59	-8.59	-8.59	-8.59	-8.59	-8.59	-8.59
-8.58	-8.58	-8.58	-8.58	-8.58	-8.58	-8.58	-8.58	-8.58
-8.57	-8.57	-8.57	-8.57	-8.57	-8.57	-8.57	-8.57	-8.57
-8.56	-8.56	-8.56	-8.56	-8.56	-8.56	-8.56	-8.56	-8.56
-8.55	-8.55	-8.55	-8.55	-8.55	-8.55	-8.55	-8.55	-8.55
-8.54	-8.54	-8.54	-8.54	-8.54	-8.54	-8.54	-8.54	-8.54
-8.53	-8.53	-8.53	-8.53	-8.53	-8.53	-8.53	-8.53	-8.53
-8.52	-8.52	-8.52	-8.52	-8.52	-8.52	-8.52	-8.52	-8.52
-8.51	-8.51	-8.51	-8.51	-8.51	-8.51	-8.51	-8.51	-8.51

-8.5	-8.5	-8.5	-8.5	-8.5	-8.5	-8.5	-8.5	-8.5
-8.49	-8.49	-8.49	-8.49	-8.49	-8.49	-8.49	-8.49	-8.49
-8.48	-8.48	-8.48	-8.48	-8.48	-8.48	-8.48	-8.48	-8.48
-8.47	-8.47	-8.47	-8.47	-8.47	-8.47	-8.47	-8.47	-8.47
-8.46	-8.46	-8.46	-8.46	-8.46	-8.46	-8.46	-8.46	-8.46
-8.45	-8.45	-8.45	-8.45	-8.45	-8.45	-8.45	-8.45	-8.45
-8.44	-8.44	-8.44	-8.44	-8.44	-8.44	-8.44	-8.44	-8.44
-8.43	-8.43	-8.43	-8.43	-8.43	-8.43	-8.43	-8.43	-8.43
-8.42	-8.42	-8.42	-8.42	-8.42	-8.42	-8.42	-8.42	-8.42
-8.41	-8.41	-8.41	-8.41	-8.41	-8.41	-8.41	-8.41	-8.41
-8.4	-8.4	-8.4	-8.4	-8.4	-8.4	-8.4	-8.4	-8.4
-8.39	-8.39	-8.39	-8.39	-8.39	-8.39	-8.39	-8.39	-8.39
-8.38	-8.38	-8.38	-8.38	-8.38	-8.38	-8.38	-8.38	-8.38
-8.37	-8.37	-8.37	-8.37	-8.37	-8.37	-8.37	-8.37	-8.37
-8.36	-8.36	-8.36	-8.36	-8.36	-8.36	-8.36	-8.36	-8.36
-8.35	-8.35	-8.35	-8.35	-8.35	-8.35	-8.35	-8.35	-8.35
-8.34	-8.34	-8.34	-8.34	-8.34	-8.34	-8.34	-8.34	-8.34
-8.33	-8.33	-8.33	-8.33	-8.33	-8.33	-8.33	-8.33	-8.33
-8.32	-8.32	-8.32	-8.32	-8.32	-8.32	-8.32	-8.32	-8.32
-8.31	-8.31	-8.31	-8.31	-8.31	-8.31	-8.31	-8.31	-8.31
-8.3	-8.3	-8.3	-8.3	-8.3	-8.3	-8.3	-8.3	-8.3
-8.29	-8.29	-8.29	-8.29	-8.29	-8.29	-8.29	-8.29	-8.29
-8.28	-8.28	-8.28	-8.28	-8.28	-8.28	-8.28	-8.28	-8.28
-8.27	-8.27	-8.27	-8.27	-8.27	-8.27	-8.27	-8.27	-8.27
-8.26	-8.26	-8.26	-8.26	-8.26	-8.26	-8.26	-8.26	-8.26
-8.25	-8.25	-8.25	-8.25	-8.25	-8.25	-8.25	-8.25	-8.25
-8.24	-8.24	-8.24	-8.24	-8.24	-8.24	-8.24	-8.24	-8.24
-8.23	-8.23	-8.23	-8.23	-8.23	-8.23	-8.23	-8.23	-8.23
-8.22	-8.22	-8.22	-8.22	-8.22	-8.22	-8.22	-8.22	-8.22
-8.21	-8.21	-8.21	-8.21	-8.21	-8.21	-8.21	-8.21	-8.21
-8.2	-8.2	-8.2	-8.2	-8.2	-8.2	-8.2	-8.2	-8.2
-8.19	-8.19	-8.19	-8.19	-8.19	-8.19	-8.19	-8.19	-8.19
-8.18	-8.18	-8.18	-8.18	-8.18	-8.18	-8.18	-8.18	-8.18
-8.17	-8.17	-8.17	-8.17	-8.17	-8.17	-8.17	-8.17	-8.17
-8.16	-8.16	-8.16	-8.16	-8.16	-8.16	-8.16	-8.16	-8.16
-8.15	-8.15	-8.15	-8.15	-8.15	-8.15	-8.15	-8.15	-8.15
-8.14	-8.14	-8.14	-8.14	-8.14	-8.14	-8.14	-8.14	-8.14
-8.13	-8.13	-8.13	-8.13	-8.13	-8.13	-8.13	-8.13	-8.13
-8.12	-8.12	-8.12	-8.12	-8.12	-8.12	-8.12	-8.12	-8.12
-8.11	-8.11	-8.11	-8.11	-8.11	-8.11	-8.11	-8.11	-8.11
-8.1	-8.1	-8.1	-8.1	-8.1	-8.1	-8.1	-8.1	-8.1
-8.09	-8.09	-8.09	-8.09	-8.09	-8.09	-8.09	-8.09	-8.09
-8.08	-8.08	-8.08	-8.08	-8.08	-8.08	-8.08	-8.08	-8.08
-8.07	-8.07	-8.07	-8.07	-8.07	-8.07	-8.07	-8.07	-8.07
-8.06	-8.06	-8.06	-8.06	-8.06	-8.06	-8.06	-8.06	-8.06
-8.05	-8.05	-8.05	-8.05	-8.05	-8.05	-8.05	-8.05	-8.05
-8.04	-8.04	-8.04	-8.04	-8.04	-8.04	-8.04	-8.04	-8.04
-8.03	-8.03	-8.03	-8.03	-8.03	-8.03	-8.03	-8.03	-8.03
-8.02	-8.02	-8.02	-8.02	-8.02	-8.02	-8.02	-8.02	-8.02
-8.01	-8.01	-8.01	-8.01	-8.01	-8.01	-8.01	-8.01	-8.01

-8	-8	-8	-8	-8	-8	-8	-8	-8
-7.99	-7.99	-7.99	-7.99	-7.99	-7.99	-7.99	-7.99	-7.99
-7.98	-7.98	-7.98	-7.98	-7.98	-7.98	-7.98	-7.98	-7.98
-7.97	-7.97	-7.97	-7.97	-7.97	-7.97	-7.97	-7.97	-7.97
-7.96	-7.96	-7.96	-7.96	-7.96	-7.96	-7.96	-7.96	-7.96
-7.95	-7.95	-7.95	-7.95	-7.95	-7.95	-7.95	-7.95	-7.95
-7.94	-7.94	-7.94	-7.94	-7.94	-7.94	-7.94	-7.94	-7.94
-7.93	-7.93	-7.93	-7.93	-7.93	-7.93	-7.93	-7.93	-7.93
-7.92	-7.92	-7.92	-7.92	-7.92	-7.92	-7.92	-7.92	-7.92
-7.91	-7.91	-7.91	-7.91	-7.91	-7.91	-7.91	-7.91	-7.91
-7.9	-7.9	-7.9	-7.9	-7.9	-7.9	-7.9	-7.9	-7.9
-7.89	-7.89	-7.89	-7.89	-7.89	-7.89	-7.89	-7.89	-7.89
-7.88	-7.88	-7.88	-7.88	-7.88	-7.88	-7.88	-7.88	-7.88
-7.87	-7.87	-7.87	-7.87	-7.87	-7.87	-7.87	-7.87	-7.87
-7.86	-7.86	-7.86	-7.86	-7.86	-7.86	-7.86	-7.86	-7.86
-7.85	-7.85	-7.85	-7.85	-7.85	-7.85	-7.85	-7.85	-7.85
-7.84	-7.84	-7.84	-7.84	-7.84	-7.84	-7.84	-7.84	-7.84
-7.83	-7.83	-7.83	-7.83	-7.83	-7.83	-7.83	-7.83	-7.83
-7.82	-7.82	-7.82	-7.82	-7.82	-7.82	-7.82	-7.82	-7.82
-7.81	-7.81	-7.81	-7.81	-7.81	-7.81	-7.81	-7.81	-7.81
-7.8	-7.8	-7.8	-7.8	-7.8	-7.8	-7.8	-7.8	-7.8
-7.79	-7.79	-7.79	-7.79	-7.79	-7.79	-7.79	-7.79	-7.79
-7.78	-7.78	-7.78	-7.78	-7.78	-7.78	-7.78	-7.78	-7.78
-7.77	-7.77	-7.77	-7.77	-7.77	-7.77	-7.77	-7.77	-7.77
-7.76	-7.76	-7.76	-7.76	-7.76	-7.76	-7.76	-7.76	-7.76
-7.75	-7.75	-7.75	-7.75	-7.75	-7.75	-7.75	-7.75	-7.75
-7.74	-7.74	-7.74	-7.74	-7.74	-7.74	-7.74	-7.74	-7.74
-7.73	-7.73	-7.73	-7.73	-7.73	-7.73	-7.73	-7.73	-7.73
-7.72	-7.72	-7.72	-7.72	-7.72	-7.72	-7.72	-7.72	-7.72
-7.71	-7.71	-7.71	-7.71	-7.71	-7.71	-7.71	-7.71	-7.71
-7.7	-7.7	-7.7	-7.7	-7.7	-7.7	-7.7	-7.7	-7.7
-7.69	-7.69	-7.69	-7.69	-7.69	-7.69	-7.69	-7.69	-7.69
-7.68	-7.68	-7.68	-7.68	-7.68	-7.68	-7.68	-7.68	-7.68
-7.67	-7.67	-7.67	-7.67	-7.67	-7.67	-7.67	-7.67	-7.67
-7.66	-7.66	-7.66	-7.66	-7.66	-7.66	-7.66	-7.66	-7.66
-7.65	-7.65	-7.65	-7.65	-7.65	-7.65	-7.65	-7.65	-7.65
-7.64	-7.64	-7.64	-7.64	-7.64	-7.64	-7.64	-7.64	-7.64
-7.63	-7.63	-7.63	-7.63	-7.63	-7.63	-7.63	-7.63	-7.63
-7.62	-7.62	-7.62	-7.62	-7.62	-7.62	-7.62	-7.62	-7.62
-7.61	-7.61	-7.61	-7.61	-7.61	-7.61	-7.61	-7.61	-7.61
-7.6	-7.6	-7.6	-7.6	-7.6	-7.6	-7.6	-7.6	-7.6
-7.59	-7.59	-7.59	-7.59	-7.59	-7.59	-7.59	-7.59	-7.59
-7.58	-7.58	-7.58	-7.58	-7.58	-7.58	-7.58	-7.58	-7.58
-7.57	-7.57	-7.57	-7.57	-7.57	-7.57	-7.57	-7.57	-7.57
-7.56	-7.56	-7.56	-7.56	-7.56	-7.56	-7.56	-7.56	-7.56
-7.55	-7.55	-7.55	-7.55	-7.55	-7.55	-7.55	-7.55	-7.55
-7.54	-7.54	-7.54	-7.54	-7.54	-7.54	-7.54	-7.54	-7.54
-7.53	-7.53	-7.53	-7.53	-7.53	-7.53	-7.53	-7.53	-7.53
-7.52	-7.52	-7.52	-7.52	-7.52	-7.52	-7.52	-7.52	-7.52
-7.51	-7.51	-7.51	-7.51	-7.51	-7.51	-7.51	-7.51	-7.51

-7.5	-7.5	-7.5	-7.5	-7.5	-7.5	-7.5	-7.5	-7.5
-7.49	-7.49	-7.49	-7.49	-7.49	-7.49	-7.49	-7.49	-7.49
-7.48	-7.48	-7.48	-7.48	-7.48	-7.48	-7.48	-7.48	-7.48
-7.47	-7.47	-7.47	-7.47	-7.47	-7.47	-7.47	-7.47	-7.47
-7.46	-7.46	-7.46	-7.46	-7.46	-7.46	-7.46	-7.46	-7.46
-7.45	-7.45	-7.45	-7.45	-7.45	-7.45	-7.45	-7.45	-7.45
-7.44	-7.44	-7.44	-7.44	-7.44	-7.44	-7.44	-7.44	-7.44
-7.43	-7.43	-7.43	-7.43	-7.43	-7.43	-7.43	-7.43	-7.43
-7.42	-7.42	-7.42	-7.42	-7.42	-7.42	-7.42	-7.42	-7.42
-7.41	-7.41	-7.41	-7.41	-7.41	-7.41	-7.41	-7.41	-7.41
-7.4	-7.4	-7.4	-7.4	-7.4	-7.4	-7.4	-7.4	-7.4
-7.39	-7.39	-7.39	-7.39	-7.39	-7.39	-7.39	-7.39	-7.39
-7.38	-7.38	-7.38	-7.38	-7.38	-7.38	-7.38	-7.38	-7.38
-7.37	-7.37	-7.37	-7.37	-7.37	-7.37	-7.37	-7.37	-7.37
-7.36	-7.36	-7.36	-7.36	-7.36	-7.36	-7.36	-7.36	-7.36
-7.35	-7.35	-7.35	-7.35	-7.35	-7.35	-7.35	-7.35	-7.35
-7.34	-7.34	-7.34	-7.34	-7.34	-7.34	-7.34	-7.34	-7.34
-7.33	-7.33	-7.33	-7.33	-7.33	-7.33	-7.33	-7.33	-7.33
-7.32	-7.32	-7.32	-7.32	-7.32	-7.32	-7.32	-7.32	-7.32
-7.31	-7.31	-7.31	-7.31	-7.31	-7.31	-7.31	-7.31	-7.31
-7.3	-7.3	-7.3	-7.3	-7.3	-7.3	-7.3	-7.3	-7.3
-7.29	-7.29	-7.29	-7.29	-7.29	-7.29	-7.29	-7.29	-7.29
-7.28	-7.28	-7.28	-7.28	-7.28	-7.28	-7.28	-7.28	-7.28
-7.27	-7.27	-7.27	-7.27	-7.27	-7.27	-7.27	-7.27	-7.27
-7.26	-7.26	-7.26	-7.26	-7.26	-7.26	-7.26	-7.26	-7.26
-7.25	-7.25	-7.25	-7.25	-7.25	-7.25	-7.25	-7.25	-7.25
-7.24	-7.24	-7.24	-7.24	-7.24	-7.24	-7.24	-7.24	-7.24
-7.23	-7.23	-7.23	-7.23	-7.23	-7.23	-7.23	-7.23	-7.23
-7.22	-7.22	-7.22	-7.22	-7.22	-7.22	-7.22	-7.22	-7.22
-7.21	-7.21	-7.21	-7.21	-7.21	-7.21	-7.21	-7.21	-7.21
-7.2	-7.2	-7.2	-7.2	-7.2	-7.2	-7.2	-7.2	-7.2
-7.19	-7.19	-7.19	-7.19	-7.19	-7.19	-7.19	-7.19	-7.19
-7.18	-7.18	-7.18	-7.18	-7.18	-7.18	-7.18	-7.18	-7.18
-7.17	-7.17	-7.17	-7.17	-7.17	-7.17	-7.17	-7.17	-7.17
-7.16	-7.16	-7.16	-7.16	-7.16	-7.16	-7.16	-7.16	-7.16
-7.15	-7.15	-7.15	-7.15	-7.15	-7.15	-7.15	-7.15	-7.15
-7.14	-7.14	-7.14	-7.14	-7.14	-7.14	-7.14	-7.14	-7.14
-7.13	-7.13	-7.13	-7.13	-7.13	-7.13	-7.13	-7.13	-7.13
-7.12	-7.12	-7.12	-7.12	-7.12	-7.12	-7.12	-7.12	-7.12
-7.11	-7.11	-7.11	-7.11	-7.11	-7.11	-7.11	-7.11	-7.11
-7.1	-7.1	-7.1	-7.1	-7.1	-7.1	-7.1	-7.1	-7.1
-7.09	-7.09	-7.09	-7.09	-7.09	-7.09	-7.09	-7.09	-7.09
-7.08	-7.08	-7.08	-7.08	-7.08	-7.08	-7.08	-7.08	-7.08
-7.07	-7.07	-7.07	-7.07	-7.07	-7.07	-7.07	-7.07	-7.07
-7.06	-7.06	-7.06	-7.06	-7.06	-7.06	-7.06	-7.06	-7.06
-7.05	-7.05	-7.05	-7.05	-7.05	-7.05	-7.05	-7.05	-7.05
-7.04	-7.04	-7.04	-7.04	-7.04	-7.04	-7.04	-7.04	-7.04
-7.03	-7.03	-7.03	-7.03	-7.03	-7.03	-7.03	-7.03	-7.03
-7.02	-7.02	-7.02	-7.02	-7.02	-7.02	-7.02	-7.02	-7.02
-7.01	-7.01	-7.01	-7.01	-7.01	-7.01	-7.01	-7.01	-7.01

-7	-7	-7	-7	-7	-7	-7	-7	-7
-6.99	-6.99	-6.99	-6.99	-6.99	-6.99	-6.99	-6.99	-6.99
-6.98	-6.98	-6.98	-6.98	-6.98	-6.98	-6.98	-6.98	-6.98
-6.97	-6.97	-6.97	-6.97	-6.97	-6.97	-6.97	-6.97	-6.97
-6.96	-6.96	-6.96	-6.96	-6.96	-6.96	-6.96	-6.96	-6.96
-6.95	-6.95	-6.95	-6.95	-6.95	-6.95	-6.95	-6.95	-6.95
-6.94	-6.94	-6.94	-6.94	-6.94	-6.94	-6.94	-6.94	-6.94
-6.93	-6.93	-6.93	-6.93	-6.93	-6.93	-6.93	-6.93	-6.93
-6.92	-6.92	-6.92	-6.92	-6.92	-6.92	-6.92	-6.92	-6.92
-6.91	-6.91	-6.91	-6.91	-6.91	-6.91	-6.91	-6.91	-6.91
-6.9	-6.9	-6.9	-6.9	-6.9	-6.9	-6.9	-6.9	-6.9
-6.89	-6.89	-6.89	-6.89	-6.89	-6.89	-6.89	-6.89	-6.89
-6.88	-6.88	-6.88	-6.88	-6.88	-6.88	-6.88	-6.88	-6.88
-6.87	-6.87	-6.87	-6.87	-6.87	-6.87	-6.87	-6.87	-6.87
-6.86	-6.86	-6.86	-6.86	-6.86	-6.86	-6.86	-6.86	-6.86
-6.85	-6.85	-6.85	-6.85	-6.85	-6.85	-6.85	-6.85	-6.85
-6.84	-6.84	-6.84	-6.84	-6.84	-6.84	-6.84	-6.84	-6.84
-6.83	-6.83	-6.83	-6.83	-6.83	-6.83	-6.83	-6.83	-6.83
-6.82	-6.82	-6.82	-6.82	-6.82	-6.82	-6.82	-6.82	-6.82
-6.81	-6.81	-6.81	-6.81	-6.81	-6.81	-6.81	-6.81	-6.81
-6.8	-6.8	-6.8	-6.8	-6.8	-6.8	-6.8	-6.8	-6.8
-6.79	-6.79	-6.79	-6.79	-6.79	-6.79	-6.79	-6.79	-6.79
-6.78	-6.78	-6.78	-6.78	-6.78	-6.78	-6.78	-6.78	-6.78
-6.77	-6.77	-6.77	-6.77	-6.77	-6.77	-6.77	-6.77	-6.77
-6.76	-6.76	-6.76	-6.76	-6.76	-6.76	-6.76	-6.76	-6.76
-6.75	-6.75	-6.75	-6.75	-6.75	-6.75	-6.75	-6.75	-6.75
-6.74	-6.74	-6.74	-6.74	-6.74	-6.74	-6.74	-6.74	-6.74
-6.73	-6.73	-6.73	-6.73	-6.73	-6.73	-6.73	-6.73	-6.73
-6.72	-6.72	-6.72	-6.72	-6.72	-6.72	-6.72	-6.72	-6.72
-6.71	-6.71	-6.71	-6.71	-6.71	-6.71	-6.71	-6.71	-6.71
-6.7	-6.7	-6.7	-6.7	-6.7	-6.7	-6.7	-6.7	-6.7
-6.69	-6.69	-6.69	-6.69	-6.69	-6.69	-6.69	-6.69	-6.69
-6.68	-6.68	-6.68	-6.68	-6.68	-6.68	-6.68	-6.68	-6.68
-6.67	-6.67	-6.67	-6.67	-6.67	-6.67	-6.67	-6.67	-6.67
-6.66	-6.66	-6.66	-6.66	-6.66	-6.66	-6.66	-6.66	-6.66
-6.65	-6.65	-6.65	-6.65	-6.65	-6.65	-6.65	-6.65	-6.65
-6.64	-6.64	-6.64	-6.64	-6.64	-6.64	-6.64	-6.64	-6.64
-6.63	-6.63	-6.63	-6.63	-6.63	-6.63	-6.63	-6.63	-6.63
-6.62	-6.62	-6.62	-6.62	-6.62	-6.62	-6.62	-6.62	-6.62
-6.61	-6.61	-6.61	-6.61	-6.61	-6.61	-6.61	-6.61	-6.61
-6.6	-6.6	-6.6	-6.6	-6.6	-6.6	-6.6	-6.6	-6.6
-6.59	-6.59	-6.59	-6.59	-6.59	-6.59	-6.59	-6.59	-6.59
-6.58	-6.58	-6.58	-6.58	-6.58	-6.58	-6.58	-6.58	-6.58
-6.57	-6.57	-6.57	-6.57	-6.57	-6.57	-6.57	-6.57	-6.57
-6.56	-6.56	-6.56	-6.56	-6.56	-6.56	-6.56	-6.56	-6.56
-6.55	-6.55	-6.55	-6.55	-6.55	-6.55	-6.55	-6.55	-6.55
-6.54	-6.54	-6.54	-6.54	-6.54	-6.54	-6.54	-6.54	-6.54
-6.53	-6.53	-6.53	-6.53	-6.53	-6.53	-6.53	-6.53	-6.53
-6.52	-6.52	-6.52	-6.52	-6.52	-6.52	-6.52	-6.52	-6.52
-6.51	-6.51	-6.51	-6.51	-6.51	-6.51	-6.51	-6.51	-6.51

-6.5	-6.5	-6.5	-6.5	-6.5	-6.5	-6.5	-6.5	-6.5
-6.49	-6.49	-6.49	-6.49	-6.49	-6.49	-6.49	-6.49	-6.49
-6.48	-6.48	-6.48	-6.48	-6.48	-6.48	-6.48	-6.48	-6.48
-6.47	-6.47	-6.47	-6.47	-6.47	-6.47	-6.47	-6.47	-6.47
-6.46	-6.46	-6.46	-6.46	-6.46	-6.46	-6.46	-6.46	-6.46
-6.45	-6.45	-6.45	-6.45	-6.45	-6.45	-6.45	-6.45	-6.45
-6.44	-6.44	-6.44	-6.44	-6.44	-6.44	-6.44	-6.44	-6.44
-6.43	-6.43	-6.43	-6.43	-6.43	-6.43	-6.43	-6.43	-6.43
-6.42	-6.42	-6.42	-6.42	-6.42	-6.42	-6.42	-6.42	-6.42
-6.41	-6.41	-6.41	-6.41	-6.41	-6.41	-6.41	-6.41	-6.41
-6.4	-6.4	-6.4	-6.4	-6.4	-6.4	-6.4	-6.4	-6.4
-6.39	-6.39	-6.39	-6.39	-6.39	-6.39	-6.39	-6.39	-6.39
-6.38	-6.38	-6.38	-6.38	-6.38	-6.38	-6.38	-6.38	-6.38
-6.37	-6.37	-6.37	-6.37	-6.37	-6.37	-6.37	-6.37	-6.37
-6.36	-6.36	-6.36	-6.36	-6.36	-6.36	-6.36	-6.36	-6.36
-6.35	-6.35	-6.35	-6.35	-6.35	-6.35	-6.35	-6.35	-6.35
-6.34	-6.34	-6.34	-6.34	-6.34	-6.34	-6.34	-6.34	-6.34
-6.33	-6.33	-6.33	-6.33	-6.33	-6.33	-6.33	-6.33	-6.33
-6.32	-6.32	-6.32	-6.32	-6.32	-6.32	-6.32	-6.32	-6.32
-6.31	-6.31	-6.31	-6.31	-6.31	-6.31	-6.31	-6.31	-6.31
-6.3	-6.3	-6.3	-6.3	-6.3	-6.3	-6.3	-6.3	-6.3
-6.29	-6.29	-6.29	-6.29	-6.29	-6.29	-6.29	-6.29	-6.29
-6.28	-6.28	-6.28	-6.28	-6.28	-6.28	-6.28	-6.28	-6.28
-6.27	-6.27	-6.27	-6.27	-6.27	-6.27	-6.27	-6.27	-6.27
-6.26	-6.26	-6.26	-6.26	-6.26	-6.26	-6.26	-6.26	-6.26
-6.25	-6.25	-6.25	-6.25	-6.25	-6.25	-6.25	-6.25	-6.25
-6.24	-6.24	-6.24	-6.24	-6.24	-6.24	-6.24	-6.24	-6.24
-6.23	-6.23	-6.23	-6.23	-6.23	-6.23	-6.23	-6.23	-6.23
-6.22	-6.22	-6.22	-6.22	-6.22	-6.22	-6.22	-6.22	-6.22
-6.21	-6.21	-6.21	-6.21	-6.21	-6.21	-6.21	-6.21	-6.21
-6.2	-6.2	-6.2	-6.2	-6.2	-6.2	-6.2	-6.2	-6.2
-6.19	-6.19	-6.19	-6.19	-6.19	-6.19	-6.19	-6.19	-6.19
-6.18	-6.18	-6.18	-6.18	-6.18	-6.18	-6.18	-6.18	-6.18
-6.17	-6.17	-6.17	-6.17	-6.17	-6.17	-6.17	-6.17	-6.17
-6.16	-6.16	-6.16	-6.16	-6.16	-6.16	-6.16	-6.16	-6.16
-6.15	-6.15	-6.15	-6.15	-6.15	-6.15	-6.15	-6.15	-6.15
-6.14	-6.14	-6.14	-6.14	-6.14	-6.14	-6.14	-6.14	-6.14
-6.13	-6.13	-6.13	-6.13	-6.13	-6.13	-6.13	-6.13	-6.13
-6.12	-6.12	-6.12	-6.12	-6.12	-6.12	-6.12	-6.12	-6.12
-6.11	-6.11	-6.11	-6.11	-6.11	-6.11	-6.11	-6.11	-6.11
-6.1	-6.1	-6.1	-6.1	-6.1	-6.1	-6.1	-6.1	-6.1
-6.09	-6.09	-6.09	-6.09	-6.09	-6.09	-6.09	-6.09	-6.09
-6.08	-6.08	-6.08	-6.08	-6.08	-6.08	-6.08	-6.08	-6.08
-6.07	-6.07	-6.07	-6.07	-6.07	-6.07	-6.07	-6.07	-6.07
-6.06	-6.06	-6.06	-6.06	-6.06	-6.06	-6.06	-6.06	-6.06
-6.05	-6.05	-6.05	-6.05	-6.05	-6.05	-6.05	-6.05	-6.05
-6.04	-6.04	-6.04	-6.04	-6.04	-6.04	-6.04	-6.04	-6.04
-6.03	-6.03	-6.03	-6.03	-6.03	-6.03	-6.03	-6.03	-6.03
-6.02	-6.02	-6.02	-6.02	-6.02	-6.02	-6.02	-6.02	-6.02
-6.01	-6.01	-6.01	-6.01	-6.01	-6.01	-6.01	-6.01	-6.01

-6	-6	-6	-6	-6	-6	-6	-6	-6
-5.99	-5.99	-5.99	-5.99	-5.99	-5.99	-5.99	-5.99	-5.99
-5.98	-5.98	-5.98	-5.98	-5.98	-5.98	-5.98	-5.98	-5.98
-5.97	-5.97	-5.97	-5.97	-5.97	-5.97	-5.97	-5.97	-5.97
-5.96	-5.96	-5.96	-5.96	-5.96	-5.96	-5.96	-5.96	-5.96
-5.95	-5.95	-5.95	-5.95	-5.95	-5.95	-5.95	-5.95	-5.95
-5.94	-5.94	-5.94	-5.94	-5.94	-5.94	-5.94	-5.94	-5.94
-5.93	-5.93	-5.93	-5.93	-5.93	-5.93	-5.93	-5.93	-5.93
-5.92	-5.92	-5.92	-5.92	-5.92	-5.92	-5.92	-5.92	-5.92
-5.91	-5.91	-5.91	-5.91	-5.91	-5.91	-5.91	-5.91	-5.91
-5.9	-5.9	-5.9	-5.9	-5.9	-5.9	-5.9	-5.9	-5.9
-5.89	-5.89	-5.89	-5.89	-5.89	-5.89	-5.89	-5.89	-5.89
-5.88	-5.88	-5.88	-5.88	-5.88	-5.88	-5.88	-5.88	-5.88
-5.87	-5.87	-5.87	-5.87	-5.87	-5.87	-5.87	-5.87	-5.87
-5.86	-5.86	-5.86	-5.86	-5.86	-5.86	-5.86	-5.86	-5.86
-5.85	-5.85	-5.85	-5.85	-5.85	-5.85	-5.85	-5.85	-5.85
-5.84	-5.84	-5.84	-5.84	-5.84	-5.84	-5.84	-5.84	-5.84
-5.83	-5.83	-5.83	-5.83	-5.83	-5.83	-5.83	-5.83	-5.83
-5.82	-5.82	-5.82	-5.82	-5.82	-5.82	-5.82	-5.82	-5.82
-5.81	-5.81	-5.81	-5.81	-5.81	-5.81	-5.81	-5.81	-5.81
-5.8	-5.8	-5.8	-5.8	-5.8	-5.8	-5.8	-5.8	-5.8
-5.79	-5.79	-5.79	-5.79	-5.79	-5.79	-5.79	-5.79	-5.79
-5.78	-5.78	-5.78	-5.78	-5.78	-5.78	-5.78	-5.78	-5.78
-5.77	-5.77	-5.77	-5.77	-5.77	-5.77	-5.77	-5.77	-5.77
-5.76	-5.76	-5.76	-5.76	-5.76	-5.76	-5.76	-5.76	-5.76
-5.75	-5.75	-5.75	-5.75	-5.75	-5.75	-5.75	-5.75	-5.75
-5.74	-5.74	-5.74	-5.74	-5.74	-5.74	-5.74	-5.74	-5.74
-5.73	-5.73	-5.73	-5.73	-5.73	-5.73	-5.73	-5.73	-5.73
-5.72	-5.72	-5.72	-5.72	-5.72	-5.72	-5.72	-5.72	-5.72
-5.71	-5.71	-5.71	-5.71	-5.71	-5.71	-5.71	-5.71	-5.71
-5.7	-5.7	-5.7	-5.7	-5.7	-5.7	-5.7	-5.7	-5.7
-5.69	-5.69	-5.69	-5.69	-5.69	-5.69	-5.69	-5.69	-5.69
-5.68	-5.68	-5.68	-5.68	-5.68	-5.68	-5.68	-5.68	-5.68
-5.67	-5.67	-5.67	-5.67	-5.67	-5.67	-5.67	-5.67	-5.67
-5.66	-5.66	-5.66	-5.66	-5.66	-5.66	-5.66	-5.66	-5.66
-5.65	-5.65	-5.65	-5.65	-5.65	-5.65	-5.65	-5.65	-5.65
-5.64	-5.64	-5.64	-5.64	-5.64	-5.64	-5.64	-5.64	-5.64
-5.63	-5.63	-5.63	-5.63	-5.63	-5.63	-5.63	-5.63	-5.63
-5.62	-5.62	-5.62	-5.62	-5.62	-5.62	-5.62	-5.62	-5.62
-5.61	-5.61	-5.61	-5.61	-5.61	-5.61	-5.61	-5.61	-5.61
-5.6	-5.6	-5.6	-5.6	-5.6	-5.6	-5.6	-5.6	-5.6
-5.59	-5.59	-5.59	-5.59	-5.59	-5.59	-5.59	-5.59	-5.59
-5.58	-5.58	-5.58	-5.58	-5.58	-5.58	-5.58	-5.58	-5.58
-5.57	-5.57	-5.57	-5.57	-5.57	-5.57	-5.57	-5.57	-5.57
-5.56	-5.56	-5.56	-5.56	-5.56	-5.56	-5.56	-5.56	-5.56
-5.55	-5.55	-5.55	-5.55	-5.55	-5.55	-5.55	-5.55	-5.55
-5.54	-5.54	-5.54	-5.54	-5.54	-5.54	-5.54	-5.54	-5.54
-5.53	-5.53	-5.53	-5.53	-5.53	-5.53	-5.53	-5.53	-5.53
-5.52	-5.52	-5.52	-5.52	-5.52	-5.52	-5.52	-5.52	-5.52
-5.51	-5.51	-5.51	-5.51	-5.51	-5.51	-5.51	-5.51	-5.51

-5.5	-5.5	-5.5	-5.5	-5.5	-5.5	-5.5	-5.5	-5.5
-5.49	-5.49	-5.49	-5.49	-5.49	-5.49	-5.49	-5.49	-5.49
-5.48	-5.48	-5.48	-5.48	-5.48	-5.48	-5.48	-5.48	-5.48
-5.47	-5.47	-5.47	-5.47	-5.47	-5.47	-5.47	-5.47	-5.47
-5.46	-5.46	-5.46	-5.46	-5.46	-5.46	-5.46	-5.46	-5.46
-5.45	-5.45	-5.45	-5.45	-5.45	-5.45	-5.45	-5.45	-5.45
-5.44	-5.44	-5.44	-5.44	-5.44	-5.44	-5.44	-5.44	-5.44
-5.43	-5.43	-5.43	-5.43	-5.43	-5.43	-5.43	-5.43	-5.43
-5.42	-5.42	-5.42	-5.42	-5.42	-5.42	-5.42	-5.42	-5.42
-5.41	-5.41	-5.41	-5.41	-5.41	-5.41	-5.41	-5.41	-5.41
-5.4	-5.4	-5.4	-5.4	-5.4	-5.4	-5.4	-5.4	-5.4
-5.39	-5.39	-5.39	-5.39	-5.39	-5.39	-5.39	-5.39	-5.39
-5.38	-5.38	-5.38	-5.38	-5.38	-5.38	-5.38	-5.38	-5.38
-5.37	-5.37	-5.37	-5.37	-5.37	-5.37	-5.37	-5.37	-5.37
-5.36	-5.36	-5.36	-5.36	-5.36	-5.36	-5.36	-5.36	-5.36
-5.35	-5.35	-5.35	-5.35	-5.35	-5.35	-5.35	-5.35	-5.35
-5.34	-5.34	-5.34	-5.34	-5.34	-5.34	-5.34	-5.34	-5.34
-5.33	-5.33	-5.33	-5.33	-5.33	-5.33	-5.33	-5.33	-5.33
-5.32	-5.32	-5.32	-5.32	-5.32	-5.32	-5.32	-5.32	-5.32
-5.31	-5.31	-5.31	-5.31	-5.31	-5.31	-5.31	-5.31	-5.31
-5.3	-5.3	-5.3	-5.3	-5.3	-5.3	-5.3	-5.3	-5.3
-5.29	-5.29	-5.29	-5.29	-5.29	-5.29	-5.29	-5.29	-5.29
-5.28	-5.28	-5.28	-5.28	-5.28	-5.28	-5.28	-5.28	-5.28
-5.27	-5.27	-5.27	-5.27	-5.27	-5.27	-5.27	-5.27	-5.27
-5.26	-5.26	-5.26	-5.26	-5.26	-5.26	-5.26	-5.26	-5.26
-5.25	-5.25	-5.25	-5.25	-5.25	-5.25	-5.25	-5.25	-5.25
-5.24	-5.24	-5.24	-5.24	-5.24	-5.24	-5.24	-5.24	-5.24
-5.23	-5.23	-5.23	-5.23	-5.23	-5.23	-5.23	-5.23	-5.23
-5.22	-5.22	-5.22	-5.22	-5.22	-5.22	-5.22	-5.22	-5.22
-5.21	-5.21	-5.21	-5.21	-5.21	-5.21	-5.21	-5.21	-5.21
-5.2	-5.2	-5.2	-5.2	-5.2	-5.2	-5.2	-5.2	-5.2
-5.19	-5.19	-5.19	-5.19	-5.19	-5.19	-5.19	-5.19	-5.19
-5.18	-5.18	-5.18	-5.18	-5.18	-5.18	-5.18	-5.18	-5.18
-5.17	-5.17	-5.17	-5.17	-5.17	-5.17	-5.17	-5.17	-5.17
-5.16	-5.16	-5.16	-5.16	-5.16	-5.16	-5.16	-5.16	-5.16
-5.15	-5.15	-5.15	-5.15	-5.15	-5.15	-5.15	-5.15	-5.15
-5.14	-5.14	-5.14	-5.14	-5.14	-5.14	-5.14	-5.14	-5.14
-5.13	-5.13	-5.13	-5.13	-5.13	-5.13	-5.13	-5.13	-5.13
-5.12	-5.12	-5.12	-5.12	-5.12	-5.12	-5.12	-5.12	-5.12
-5.11	-5.11	-5.11	-5.11	-5.11	-5.11	-5.11	-5.11	-5.11
-5.1	-5.1	-5.1	-5.1	-5.1	-5.1	-5.1	-5.1	-5.1
-5.09	-5.09	-5.09	-5.09	-5.09	-5.09	-5.09	-5.09	-5.09
-5.08	-5.08	-5.08	-5.08	-5.08	-5.08	-5.08	-5.08	-5.08
-5.07	-5.07	-5.07	-5.07	-5.07	-5.07	-5.07	-5.07	-5.07
-5.06	-5.06	-5.06	-5.06	-5.06	-5.06	-5.06	-5.06	-5.06
-5.05	-5.05	-5.05	-5.05	-5.05	-5.05	-5.05	-5.05	-5.05
-5.04	-5.04	-5.04	-5.04	-5.04	-5.04	-5.04	-5.04	-5.04
-5.03	-5.03	-5.03	-5.03	-5.03	-5.03	-5.03	-5.03	-5.03
-5.02	-5.02	-5.02	-5.02	-5.02	-5.02	-5.02	-5.02	-5.02
-5.01	-5.01	-5.01	-5.01	-5.01	-5.01	-5.01	-5.01	-5.01

-5	-5	-5	-5	-5	-5	-5	-5	-5
-4.99	-4.99	-4.99	-4.99	-4.99	-4.99	-4.99	-4.99	-4.99
-4.98	-4.98	-4.98	-4.98	-4.98	-4.98	-4.98	-4.98	-4.98
-4.97	-4.97	-4.97	-4.97	-4.97	-4.97	-4.97	-4.97	-4.97
-4.96	-4.96	-4.96	-4.96	-4.96	-4.96	-4.96	-4.96	-4.96
-4.95	-4.95	-4.95	-4.95	-4.95	-4.95	-4.95	-4.95	-4.95
-4.94	-4.94	-4.94	-4.94	-4.94	-4.94	-4.94	-4.94	-4.94
-4.93	-4.93	-4.93	-4.93	-4.93	-4.93	-4.93	-4.93	-4.93
-4.92	-4.92	-4.92	-4.92	-4.92	-4.92	-4.92	-4.92	-4.92
-4.91	-4.91	-4.91	-4.91	-4.91	-4.91	-4.91	-4.91	-4.91
-4.9	-4.9	-4.9	-4.9	-4.9	-4.9	-4.9	-4.9	-4.9
-4.89	-4.89	-4.89	-4.89	-4.89	-4.89	-4.89	-4.89	-4.89
-4.88	-4.88	-4.88	-4.88	-4.88	-4.88	-4.88	-4.88	-4.88
-4.87	-4.87	-4.87	-4.87	-4.87	-4.87	-4.87	-4.87	-4.87
-4.86	-4.86	-4.86	-4.86	-4.86	-4.86	-4.86	-4.86	-4.86
-4.85	-4.85	-4.85	-4.85	-4.85	-4.85	-4.85	-4.85	-4.85
-4.84	-4.84	-4.84	-4.84	-4.84	-4.84	-4.84	-4.84	-4.84
-4.83	-4.83	-4.83	-4.83	-4.83	-4.83	-4.83	-4.83	-4.83
-4.82	-4.82	-4.82	-4.82	-4.82	-4.82	-4.82	-4.82	-4.82
-4.81	-4.81	-4.81	-4.81	-4.81	-4.81	-4.81	-4.81	-4.81
-4.8	-4.8	-4.8	-4.8	-4.8	-4.8	-4.8	-4.8	-4.8
-4.79	-4.79	-4.79	-4.79	-4.79	-4.79	-4.79	-4.79	-4.79
-4.78	-4.78	-4.78	-4.78	-4.78	-4.78	-4.78	-4.78	-4.78
-4.77	-4.77	-4.77	-4.77	-4.77	-4.77	-4.77	-4.77	-4.77
-4.76	-4.76	-4.76	-4.76	-4.76	-4.76	-4.76	-4.76	-4.76
-4.75	-4.75	-4.75	-4.75	-4.75	-4.75	-4.75	-4.75	-4.75
-4.74	-4.74	-4.74	-4.74	-4.74	-4.74	-4.74	-4.74	-4.74
-4.73	-4.73	-4.73	-4.73	-4.73	-4.73	-4.73	-4.73	-4.73
-4.72	-4.72	-4.72	-4.72	-4.72	-4.72	-4.72	-4.72	-4.72
-4.71	-4.71	-4.71	-4.71	-4.71	-4.71	-4.71	-4.71	-4.71
-4.7	-4.7	-4.7	-4.7	-4.7	-4.7	-4.7	-4.7	-4.7
-4.69	-4.69	-4.69	-4.69	-4.69	-4.69	-4.69	-4.69	-4.69
-4.68	-4.68	-4.68	-4.68	-4.68	-4.68	-4.68	-4.68	-4.68
-4.67	-4.67	-4.67	-4.67	-4.67	-4.67	-4.67	-4.67	-4.67
-4.66	-4.66	-4.66	-4.66	-4.66	-4.66	-4.66	-4.66	-4.66
-4.65	-4.65	-4.65	-4.65	-4.65	-4.65	-4.65	-4.65	-4.65
-4.64	-4.64	-4.64	-4.64	-4.64	-4.64	-4.64	-4.64	-4.64
-4.63	-4.63	-4.63	-4.63	-4.63	-4.63	-4.63	-4.63	-4.63
-4.62	-4.62	-4.62	-4.62	-4.62	-4.62	-4.62	-4.62	-4.62
-4.61	-4.61	-4.61	-4.61	-4.61	-4.61	-4.61	-4.61	-4.61
-4.6	-4.6	-4.6	-4.6	-4.6	-4.6	-4.6	-4.6	-4.6
-4.59	-4.59	-4.59	-4.59	-4.59	-4.59	-4.59	-4.59	-4.59
-4.58	-4.58	-4.58	-4.58	-4.58	-4.58	-4.58	-4.58	-4.58
-4.57	-4.57	-4.57	-4.57	-4.57	-4.57	-4.57	-4.57	-4.57
-4.56	-4.56	-4.56	-4.56	-4.56	-4.56	-4.56	-4.56	-4.56
-4.55	-4.55	-4.55	-4.55	-4.55	-4.55	-4.55	-4.55	-4.55
-4.54	-4.54	-4.54	-4.54	-4.54	-4.54	-4.54	-4.54	-4.54
-4.53	-4.53	-4.53	-4.53	-4.53	-4.53	-4.53	-4.53	-4.53
-4.52	-4.52	-4.52	-4.52	-4.52	-4.52	-4.52	-4.52	-4.52
-4.51	-4.51	-4.51	-4.51	-4.51	-4.51	-4.51	-4.51	-4.51

-4.5	-4.5	-4.5	-4.5	-4.5	-4.5	-4.5	-4.5	-4.5
-4.49	-4.49	-4.49	-4.49	-4.49	-4.49	-4.49	-4.49	-4.49
-4.48	-4.48	-4.48	-4.48	-4.48	-4.48	-4.48	-4.48	-4.48
-4.47	-4.47	-4.47	-4.47	-4.47	-4.47	-4.47	-4.47	-4.47
-4.46	-4.46	-4.46	-4.46	-4.46	-4.46	-4.46	-4.46	-4.46
-4.45	-4.45	-4.45	-4.45	-4.45	-4.45	-4.45	-4.45	-4.45
-4.44	-4.44	-4.44	-4.44	-4.44	-4.44	-4.44	-4.44	-4.44
-4.43	-4.43	-4.43	-4.43	-4.43	-4.43	-4.43	-4.43	-4.43
-4.42	-4.42	-4.42	-4.42	-4.42	-4.42	-4.42	-4.42	-4.42
-4.41	-4.41	-4.41	-4.41	-4.41	-4.41	-4.41	-4.41	-4.41
-4.4	-4.4	-4.4	-4.4	-4.4	-4.4	-4.4	-4.4	-4.4
-4.39	-4.39	-4.39	-4.39	-4.39	-4.39	-4.39	-4.39	-4.39
-4.38	-4.38	-4.38	-4.38	-4.38	-4.38	-4.38	-4.38	-4.38
-4.37	-4.37	-4.37	-4.37	-4.37	-4.37	-4.37	-4.37	-4.37
-4.36	-4.36	-4.36	-4.36	-4.36	-4.36	-4.36	-4.36	-4.36
-4.35	-4.35	-4.35	-4.35	-4.35	-4.35	-4.35	-4.35	-4.35
-4.34	-4.34	-4.34	-4.34	-4.34	-4.34	-4.34	-4.34	-4.34
-4.33	-4.33	-4.33	-4.33	-4.33	-4.33	-4.33	-4.33	-4.33
-4.32	-4.32	-4.32	-4.32	-4.32	-4.32	-4.32	-4.32	-4.32
-4.31	-4.31	-4.31	-4.31	-4.31	-4.31	-4.31	-4.31	-4.31
-4.3	-4.3	-4.3	-4.3	-4.3	-4.3	-4.3	-4.3	-4.3
-4.29	-4.29	-4.29	-4.29	-4.29	-4.29	-4.29	-4.29	-4.29
-4.28	-4.28	-4.28	-4.28	-4.28	-4.28	-4.28	-4.28	-4.28
-4.27	-4.27	-4.27	-4.27	-4.27	-4.27	-4.27	-4.27	-4.27
-4.26	-4.26	-4.26	-4.26	-4.26	-4.26	-4.26	-4.26	-4.26
-4.25	-4.25	-4.25	-4.25	-4.25	-4.25	-4.25	-4.25	-4.25
-4.24	-4.24	-4.24	-4.24	-4.24	-4.24	-4.24	-4.24	-4.24
-4.23	-4.23	-4.23	-4.23	-4.23	-4.23	-4.23	-4.23	-4.23
-4.22	-4.22	-4.22	-4.22	-4.22	-4.22	-4.22	-4.22	-4.22
-4.21	-4.21	-4.21	-4.21	-4.21	-4.21	-4.21	-4.21	-4.21
-4.2	-4.2	-4.2	-4.2	-4.2	-4.2	-4.2	-4.2	-4.2
-4.19	-4.19	-4.19	-4.19	-4.19	-4.19	-4.19	-4.19	-4.19
-4.18	-4.18	-4.18	-4.18	-4.18	-4.18	-4.18	-4.18	-4.18
-4.17	-4.17	-4.17	-4.17	-4.17	-4.17	-4.17	-4.17	-4.17
-4.16	-4.16	-4.16	-4.16	-4.16	-4.16	-4.16	-4.16	-4.16
-4.15	-4.15	-4.15	-4.15	-4.15	-4.15	-4.15	-4.15	-4.15
-4.14	-4.14	-4.14	-4.14	-4.14	-4.14	-4.14	-4.14	-4.14
-4.13	-4.13	-4.13	-4.13	-4.13	-4.13	-4.13	-4.13	-4.13
-4.12	-4.12	-4.12	-4.12	-4.12	-4.12	-4.12	-4.12	-4.12
-4.11	-4.11	-4.11	-4.11	-4.11	-4.11	-4.11	-4.11	-4.11
-4.1	-4.1	-4.1	-4.1	-4.1	-4.1	-4.1	-4.1	-4.1
-4.09	-4.09	-4.09	-4.09	-4.09	-4.09	-4.09	-4.09	-4.09
-4.08	-4.08	-4.08	-4.08	-4.08	-4.08	-4.08	-4.08	-4.08
-4.07	-4.07	-4.07	-4.07	-4.07	-4.07	-4.07	-4.07	-4.07
-4.06	-4.06	-4.06	-4.06	-4.06	-4.06	-4.06	-4.06	-4.06
-4.05	-4.05	-4.05	-4.05	-4.05	-4.05	-4.05	-4.05	-4.05
-4.04	-4.04	-4.04	-4.04	-4.04	-4.04	-4.04	-4.04	-4.04
-4.03	-4.03	-4.03	-4.03	-4.03	-4.03	-4.03	-4.03	-4.03
-4.02	-4.02	-4.02	-4.02	-4.02	-4.02	-4.02	-4.02	-4.02
-4.01	-4.01	-4.01	-4.01	-4.01	-4.01	-4.01	-4.01	-4.01

-4	-4	-4	-4	-4	-4	-4	-4	-4
-3.99	-3.99	-3.99	-3.99	-3.99	-3.99	-3.99	-3.99	-3.99
-3.98	-3.98	-3.98	-3.98	-3.98	-3.98	-3.98	-3.98	-3.98
-3.97	-3.97	-3.97	-3.97	-3.97	-3.97	-3.97	-3.97	-3.97
-3.96	-3.96	-3.96	-3.96	-3.96	-3.96	-3.96	-3.96	-3.96
-3.95	-3.95	-3.95	-3.95	-3.95	-3.95	-3.95	-3.95	-3.95
-3.94	-3.94	-3.94	-3.94	-3.94	-3.94	-3.94	-3.94	-3.94
-3.93	-3.93	-3.93	-3.93	-3.93	-3.93	-3.93	-3.93	-3.93
-3.92	-3.92	-3.92	-3.92	-3.92	-3.92	-3.92	-3.92	-3.92
-3.91	-3.91	-3.91	-3.91	-3.91	-3.91	-3.91	-3.91	-3.91
-3.9	-3.9	-3.9	-3.9	-3.9	-3.9	-3.9	-3.9	-3.9
-3.89	-3.89	-3.89	-3.89	-3.89	-3.89	-3.89	-3.89	-3.89
-3.88	-3.88	-3.88	-3.88	-3.88	-3.88	-3.88	-3.88	-3.88
-3.87	-3.87	-3.87	-3.87	-3.87	-3.87	-3.87	-3.87	-3.87
-3.86	-3.86	-3.86	-3.86	-3.86	-3.86	-3.86	-3.86	-3.86
-3.85	-3.85	-3.85	-3.85	-3.85	-3.85	-3.85	-3.85	-3.85
-3.84	-3.84	-3.84	-3.84	-3.84	-3.84	-3.84	-3.84	-3.84
-3.83	-3.83	-3.83	-3.83	-3.83	-3.83	-3.83	-3.83	-3.83
-3.82	-3.82	-3.82	-3.82	-3.82	-3.82	-3.82	-3.82	-3.82
-3.81	-3.81	-3.81	-3.81	-3.81	-3.81	-3.81	-3.81	-3.81
-3.8	-3.8	-3.8	-3.8	-3.8	-3.8	-3.8	-3.8	-3.8
-3.79	-3.79	-3.79	-3.79	-3.79	-3.79	-3.79	-3.79	-3.79
-3.78	-3.78	-3.78	-3.78	-3.78	-3.78	-3.78	-3.78	-3.78
-3.77	-3.77	-3.77	-3.77	-3.77	-3.77	-3.77	-3.77	-3.77
-3.76	-3.76	-3.76	-3.76	-3.76	-3.76	-3.76	-3.76	-3.76
-3.75	-3.75	-3.75	-3.75	-3.75	-3.75	-3.75	-3.75	-3.75
-3.74	-3.74	-3.74	-3.74	-3.74	-3.74	-3.74	-3.74	-3.74
-3.73	-3.73	-3.73	-3.73	-3.73	-3.73	-3.73	-3.73	-3.73
-3.72	-3.72	-3.72	-3.72	-3.72	-3.72	-3.72	-3.72	-3.72
-3.71	-3.71	-3.71	-3.71	-3.71	-3.71	-3.71	-3.71	-3.71
-3.7	-3.7	-3.7	-3.7	-3.7	-3.7	-3.7	-3.7	-3.7
-3.69	-3.69	-3.69	-3.69	-3.69	-3.69	-3.69	-3.69	-3.69
-3.68	-3.68	-3.68	-3.68	-3.68	-3.68	-3.68	-3.68	-3.68
-3.67	-3.67	-3.67	-3.67	-3.67	-3.67	-3.67	-3.67	-3.67
-3.66	-3.66	-3.66	-3.66	-3.66	-3.66	-3.66	-3.66	-3.66
-3.65	-3.65	-3.65	-3.65	-3.65	-3.65	-3.65	-3.65	-3.65
-3.64	-3.64	-3.64	-3.64	-3.64	-3.64	-3.64	-3.64	-3.64
-3.63	-3.63	-3.63	-3.63	-3.63	-3.63	-3.63	-3.63	-3.63
-3.62	-3.62	-3.62	-3.62	-3.62	-3.62	-3.62	-3.62	-3.62
-3.61	-3.61	-3.61	-3.61	-3.61	-3.61	-3.61	-3.61	-3.61
-3.6	-3.6	-3.6	-3.6	-3.6	-3.6	-3.6	-3.6	-3.6
-3.59	-3.59	-3.59	-3.59	-3.59	-3.59	-3.59	-3.59	-3.59
-3.58	-3.58	-3.58	-3.58	-3.58	-3.58	-3.58	-3.58	-3.58
-3.57	-3.57	-3.57	-3.57	-3.57	-3.57	-3.57	-3.57	-3.57
-3.56	-3.56	-3.56	-3.56	-3.56	-3.56	-3.56	-3.56	-3.56
-3.55	-3.55	-3.55	-3.55	-3.55	-3.55	-3.55	-3.55	-3.55
-3.54	-3.54	-3.54	-3.54	-3.54	-3.54	-3.54	-3.54	-3.54
-3.53	-3.53	-3.53	-3.53	-3.53	-3.53	-3.53	-3.53	-3.53
-3.52	-3.52	-3.52	-3.52	-3.52	-3.52	-3.52	-3.52	-3.52
-3.51	-3.51	-3.51	-3.51	-3.51	-3.51	-3.51	-3.51	-3.51

-3.5	-3.5	-3.5	-3.5	-3.5	-3.5	-3.5	-3.5	-3.5
-3.49	-3.49	-3.49	-3.49	-3.49	-3.49	-3.49	-3.49	-3.49
-3.48	-3.48	-3.48	-3.48	-3.48	-3.48	-3.48	-3.48	-3.48
-3.47	-3.47	-3.47	-3.47	-3.47	-3.47	-3.47	-3.47	-3.47
-3.46	-3.46	-3.46	-3.46	-3.46	-3.46	-3.46	-3.46	-3.46
-3.45	-3.45	-3.45	-3.45	-3.45	-3.45	-3.45	-3.45	-3.45
-3.44	-3.44	-3.44	-3.44	-3.44	-3.44	-3.44	-3.44	-3.44
-3.43	-3.43	-3.43	-3.43	-3.43	-3.43	-3.43	-3.43	-3.43
-3.42	-3.42	-3.42	-3.42	-3.42	-3.42	-3.42	-3.42	-3.42
-3.41	-3.41	-3.41	-3.41	-3.41	-3.41	-3.41	-3.41	-3.41
-3.4	-3.4	-3.4	-3.4	-3.4	-3.4	-3.4	-3.4	-3.4
-3.39	-3.39	-3.39	-3.39	-3.39	-3.39	-3.39	-3.39	-3.39
-3.38	-3.38	-3.38	-3.38	-3.38	-3.38	-3.38	-3.38	-3.38
-3.37	-3.37	-3.37	-3.37	-3.37	-3.37	-3.37	-3.37	-3.37
-3.36	-3.36	-3.36	-3.36	-3.36	-3.36	-3.36	-3.36	-3.36
-3.35	-3.35	-3.35	-3.35	-3.35	-3.35	-3.35	-3.35	-3.35
-3.34	-3.34	-3.34	-3.34	-3.34	-3.34	-3.34	-3.34	-3.34
-3.33	-3.33	-3.33	-3.33	-3.33	-3.33	-3.33	-3.33	-3.33
-3.32	-3.32	-3.32	-3.32	-3.32	-3.32	-3.32	-3.32	-3.32
-3.31	-3.31	-3.31	-3.31	-3.31	-3.31	-3.31	-3.31	-3.31
-3.3	-3.3	-3.3	-3.3	-3.3	-3.3	-3.3	-3.3	-3.3
-3.29	-3.29	-3.29	-3.29	-3.29	-3.29	-3.29	-3.29	-3.29
-3.28	-3.28	-3.28	-3.28	-3.28	-3.28	-3.28	-3.28	-3.28
-3.27	-3.27	-3.27	-3.27	-3.27	-3.27	-3.27	-3.27	-3.27
-3.26	-3.26	-3.26	-3.26	-3.26	-3.26	-3.26	-3.26	-3.26
-3.25	-3.25	-3.25	-3.25	-3.25	-3.25	-3.25	-3.25	-3.25
-3.24	-3.24	-3.24	-3.24	-3.24	-3.24	-3.24	-3.24	-3.24
-3.23	-3.23	-3.23	-3.23	-3.23	-3.23	-3.23	-3.23	-3.23
-3.22	-3.22	-3.22	-3.22	-3.22	-3.22	-3.22	-3.22	-3.22
-3.21	-3.21	-3.21	-3.21	-3.21	-3.21	-3.21	-3.21	-3.21
-3.2	-3.2	-3.2	-3.2	-3.2	-3.2	-3.2	-3.2	-3.2
-3.19	-3.19	-3.19	-3.19	-3.19	-3.19	-3.19	-3.19	-3.19
-3.18	-3.18	-3.18	-3.18	-3.18	-3.18	-3.18	-3.18	-3.18
-3.17	-3.17	-3.17	-3.17	-3.17	-3.17	-3.17	-3.17	-3.17
-3.16	-3.16	-3.16	-3.16	-3.16	-3.16	-3.16	-3.16	-3.16
-3.15	-3.15	-3.15	-3.15	-3.15	-3.15	-3.15	-3.15	-3.15
-3.14	-3.14	-3.14	-3.14	-3.14	-3.14	-3.14	-3.14	-3.14
-3.13	-3.13	-3.13	-3.13	-3.13	-3.13	-3.13	-3.13	-3.13
-3.12	-3.12	-3.12	-3.12	-3.12	-3.12	-3.12	-3.12	-3.12
-3.11	-3.11	-3.11	-3.11	-3.11	-3.11	-3.11	-3.11	-3.11
-3.1	-3.1	-3.1	-3.1	-3.1	-3.1	-3.1	-3.1	-3.1
-3.09	-3.09	-3.09	-3.09	-3.09	-3.09	-3.09	-3.09	-3.09
-3.08	-3.08	-3.08	-3.08	-3.08	-3.08	-3.08	-3.08	-3.08
-3.07	-3.07	-3.07	-3.07	-3.07	-3.07	-3.07	-3.07	-3.07
-3.06	-3.06	-3.06	-3.06	-3.06	-3.06	-3.06	-3.06	-3.06
-3.05	-3.05	-3.05	-3.05	-3.05	-3.05	-3.05	-3.05	-3.05
-3.04	-3.04	-3.04	-3.04	-3.04	-3.04	-3.04	-3.04	-3.04
-3.03	-3.03	-3.03	-3.03	-3.03	-3.03	-3.03	-3.03	-3.03
-3.02	-3.02	-3.02	-3.02	-3.02	-3.02	-3.02	-3.02	-3.02
-3.01	-3.01	-3.01	-3.01	-3.01	-3.01	-3.01	-3.01	-3.01

-3	-3	-3	-3	-3	-3	-3	-3	-3
-2.99	-2.99	-2.99	-2.99	-2.99	-2.99	-2.99	-2.99	-2.99
-2.98	-2.98	-2.98	-2.98	-2.98	-2.98	-2.98	-2.98	-2.98
-2.97	-2.97	-2.97	-2.97	-2.97	-2.97	-2.97	-2.97	-2.97
-2.96	-2.96	-2.96	-2.96	-2.96	-2.96	-2.96	-2.96	-2.96
-2.95	-2.95	-2.95	-2.95	-2.95	-2.95	-2.95	-2.95	-2.95
-2.94	-2.94	-2.94	-2.94	-2.94	-2.94	-2.94	-2.94	-2.94
-2.93	-2.93	-2.93	-2.93	-2.93	-2.93	-2.93	-2.93	-2.93
-2.92	-2.92	-2.92	-2.92	-2.92	-2.92	-2.92	-2.92	-2.92
-2.91	-2.91	-2.91	-2.91	-2.91	-2.91	-2.91	-2.91	-2.91
-2.9	-2.9	-2.9	-2.9	-2.9	-2.9	-2.9	-2.9	-2.9
-2.89	-2.89	-2.89	-2.89	-2.89	-2.89	-2.89	-2.89	-2.89
-2.88	-2.88	-2.88	-2.88	-2.88	-2.88	-2.88	-2.88	-2.88
-2.87	-2.87	-2.87	-2.87	-2.87	-2.87	-2.87	-2.87	-2.87
-2.86	-2.86	-2.86	-2.86	-2.86	-2.86	-2.86	-2.86	-2.86
-2.85	-2.85	-2.85	-2.85	-2.85	-2.85	-2.85	-2.85	-2.85
-2.84	-2.84	-2.84	-2.84	-2.84	-2.84	-2.84	-2.84	-2.84
-2.83	-2.83	-2.83	-2.83	-2.83	-2.83	-2.83	-2.83	-2.83
-2.82	-2.82	-2.82	-2.82	-2.82	-2.82	-2.82	-2.82	-2.82
-2.81	-2.81	-2.81	-2.81	-2.81	-2.81	-2.81	-2.81	-2.81
-2.8	-2.8	-2.8	-2.8	-2.8	-2.8	-2.8	-2.8	-2.8
-2.79	-2.79	-2.79	-2.79	-2.79	-2.79	-2.79	-2.79	-2.79
-2.78	-2.78	-2.78	-2.78	-2.78	-2.78	-2.78	-2.78	-2.78
-2.77	-2.77	-2.77	-2.77	-2.77	-2.77	-2.77	-2.77	-2.77
-2.76	-2.76	-2.76	-2.76	-2.76	-2.76	-2.76	-2.76	-2.76
-2.75	-2.75	-2.75	-2.75	-2.75	-2.75	-2.75	-2.75	-2.75
-2.74	-2.74	-2.74	-2.74	-2.74	-2.74	-2.74	-2.74	-2.74
-2.73	-2.73	-2.73	-2.73	-2.73	-2.73	-2.73	-2.73	-2.73
-2.72	-2.72	-2.72	-2.72	-2.72	-2.72	-2.72	-2.72	-2.72
-2.71	-2.71	-2.71	-2.71	-2.71	-2.71	-2.71	-2.71	-2.71
-2.7	-2.7	-2.7	-2.7	-2.7	-2.7	-2.7	-2.7	-2.7
-2.69	-2.69	-2.69	-2.69	-2.69	-2.69	-2.69	-2.69	-2.69
-2.68	-2.68	-2.68	-2.68	-2.68	-2.68	-2.68	-2.68	-2.68
-2.67	-2.67	-2.67	-2.67	-2.67	-2.67	-2.67	-2.67	-2.67
-2.66	-2.66	-2.66	-2.66	-2.66	-2.66	-2.66	-2.66	-2.66
-2.65	-2.65	-2.65	-2.65	-2.65	-2.65	-2.65	-2.65	-2.65
-2.64	-2.64	-2.64	-2.64	-2.64	-2.64	-2.64	-2.64	-2.64
-2.63	-2.63	-2.63	-2.63	-2.63	-2.63	-2.63	-2.63	-2.63
-2.62	-2.62	-2.62	-2.62	-2.62	-2.62	-2.62	-2.62	-2.62
-2.61	-2.61	-2.61	-2.61	-2.61	-2.61	-2.61	-2.61	-2.61
-2.6	-2.6	-2.6	-2.6	-2.6	-2.6	-2.6	-2.6	-2.6
-2.59	-2.59	-2.59	-2.59	-2.59	-2.59	-2.59	-2.59	-2.59
-2.58	-2.58	-2.58	-2.58	-2.58	-2.58	-2.58	-2.58	-2.58
-2.57	-2.57	-2.57	-2.57	-2.57	-2.57	-2.57	-2.57	-2.57
-2.56	-2.56	-2.56	-2.56	-2.56	-2.56	-2.56	-2.56	-2.56
-2.55	-2.55	-2.55	-2.55	-2.55	-2.55	-2.55	-2.55	-2.55
-2.54	-2.54	-2.54	-2.54	-2.54	-2.54	-2.54	-2.54	-2.54
-2.53	-2.53	-2.53	-2.53	-2.53	-2.53	-2.53	-2.53	-2.53
-2.52	-2.52	-2.52	-2.52	-2.52	-2.52	-2.52	-2.52	-2.52
-2.51	-2.51	-2.51	-2.51	-2.51	-2.51	-2.51	-2.51	-2.51

-2.5	-2.5	-2.5	-2.5	-2.5	-2.5	-2.5	-2.5	-2.5
-2.49	-2.49	-2.49	-2.49	-2.49	-2.49	-2.49	-2.49	-2.49
-2.48	-2.48	-2.48	-2.48	-2.48	-2.48	-2.48	-2.48	-2.48
-2.47	-2.47	-2.47	-2.47	-2.47	-2.47	-2.47	-2.47	-2.47
-2.46	-2.46	-2.46	-2.46	-2.46	-2.46	-2.46	-2.46	-2.46
-2.45	-2.45	-2.45	-2.45	-2.45	-2.45	-2.45	-2.45	-2.45
-2.44	-2.44	-2.44	-2.44	-2.44	-2.44	-2.44	-2.44	-2.44
-2.43	-2.43	-2.43	-2.43	-2.43	-2.43	-2.43	-2.43	-2.43
-2.42	-2.42	-2.42	-2.42	-2.42	-2.42	-2.42	-2.42	-2.42
-2.41	-2.41	-2.41	-2.41	-2.41	-2.41	-2.41	-2.41	-2.41
-2.4	-2.4	-2.4	-2.4	-2.4	-2.4	-2.4	-2.4	-2.4
-2.39	-2.39	-2.39	-2.39	-2.39	-2.39	-2.39	-2.39	-2.39
-2.38	-2.38	-2.38	-2.38	-2.38	-2.38	-2.38	-2.38	-2.38
-2.37	-2.37	-2.37	-2.37	-2.37	-2.37	-2.37	-2.37	-2.37
-2.36	-2.36	-2.36	-2.36	-2.36	-2.36	-2.36	-2.36	-2.36
-2.35	-2.35	-2.35	-2.35	-2.35	-2.35	-2.35	-2.35	-2.35
-2.34	-2.34	-2.34	-2.34	-2.34	-2.34	-2.34	-2.34	-2.34
-2.33	-2.33	-2.33	-2.33	-2.33	-2.33	-2.33	-2.33	-2.33
-2.32	-2.32	-2.32	-2.32	-2.32	-2.32	-2.32	-2.32	-2.32
-2.31	-2.31	-2.31	-2.31	-2.31	-2.31	-2.31	-2.31	-2.31
-2.3	-2.3	-2.3	-2.3	-2.3	-2.3	-2.3	-2.3	-2.3
-2.29	-2.29	-2.29	-2.29	-2.29	-2.29	-2.29	-2.29	-2.29
-2.28	-2.28	-2.28	-2.28	-2.28	-2.28	-2.28	-2.28	-2.28
-2.27	-2.27	-2.27	-2.27	-2.27	-2.27	-2.27	-2.27	-2.27
-2.26	-2.26	-2.26	-2.26	-2.26	-2.26	-2.26	-2.26	-2.26
-2.25	-2.25	-2.25	-2.25	-2.25	-2.25	-2.25	-2.25	-2.25
-2.24	-2.24	-2.24	-2.24	-2.24	-2.24	-2.24	-2.24	-2.24
-2.23	-2.23	-2.23	-2.23	-2.23	-2.23	-2.23	-2.23	-2.23
-2.22	-2.22	-2.22	-2.22	-2.22	-2.22	-2.22	-2.22	-2.22
-2.21	-2.21	-2.21	-2.21	-2.21	-2.21	-2.21	-2.21	-2.21
-2.2	-2.2	-2.2	-2.2	-2.2	-2.2	-2.2	-2.2	-2.2
-2.19	-2.19	-2.19	-2.19	-2.19	-2.19	-2.19	-2.19	-2.19
-2.18	-2.18	-2.18	-2.18	-2.18	-2.18	-2.18	-2.18	-2.18
-2.17	-2.17	-2.17	-2.17	-2.17	-2.17	-2.17	-2.17	-2.17
-2.16	-2.16	-2.16	-2.16	-2.16	-2.16	-2.16	-2.16	-2.16
-2.15	-2.15	-2.15	-2.15	-2.15	-2.15	-2.15	-2.15	-2.15
-2.14	-2.14	-2.14	-2.14	-2.14	-2.14	-2.14	-2.14	-2.14
-2.13	-2.13	-2.13	-2.13	-2.13	-2.13	-2.13	-2.13	-2.13
-2.12	-2.12	-2.12	-2.12	-2.12	-2.12	-2.12	-2.12	-2.12
-2.11	-2.11	-2.11	-2.11	-2.11	-2.11	-2.11	-2.11	-2.11
-2.1	-2.1	-2.1	-2.1	-2.1	-2.1	-2.1	-2.1	-2.1
-2.09	-2.09	-2.09	-2.09	-2.09	-2.09	-2.09	-2.09	-2.09
-2.08	-2.08	-2.08	-2.08	-2.08	-2.08	-2.08	-2.08	-2.08
-2.07	-2.07	-2.07	-2.07	-2.07	-2.07	-2.07	-2.07	-2.07
-2.06	-2.06	-2.06	-2.06	-2.06	-2.06	-2.06	-2.06	-2.06
-2.05	-2.05	-2.05	-2.05	-2.05	-2.05	-2.05	-2.05	-2.05
-2.04	-2.04	-2.04	-2.04	-2.04	-2.04	-2.04	-2.04	-2.04
-2.03	-2.03	-2.03	-2.03	-2.03	-2.03	-2.03	-2.03	-2.03
-2.02	-2.02	-2.02	-2.02	-2.02	-2.02	-2.02	-2.02	-2.02
-2.01	-2.01	-2.01	-2.01	-2.01	-2.01	-2.01	-2.01	-2.01

-2	-2	-2	-2	-2	-2	-2	-2	-2
-1.99	-1.99	-1.99	-1.99	-1.99	-1.99	-1.99	-1.99	-1.99
-1.98	-1.98	-1.98	-1.98	-1.98	-1.98	-1.98	-1.98	-1.98
-1.97	-1.97	-1.97	-1.97	-1.97	-1.97	-1.97	-1.97	-1.97
-1.96	-1.96	-1.96	-1.96	-1.96	-1.96	-1.96	-1.96	-1.96
-1.95	-1.95	-1.95	-1.95	-1.95	-1.95	-1.95	-1.95	-1.95
-1.94	-1.94	-1.94	-1.94	-1.94	-1.94	-1.94	-1.94	-1.94
-1.93	-1.93	-1.93	-1.93	-1.93	-1.93	-1.93	-1.93	-1.93
-1.92	-1.92	-1.92	-1.92	-1.92	-1.92	-1.92	-1.92	-1.92
-1.91	-1.91	-1.91	-1.91	-1.91	-1.91	-1.91	-1.91	-1.91
-1.9	-1.9	-1.9	-1.9	-1.9	-1.9	-1.9	-1.9	-1.9
-1.89	-1.89	-1.89	-1.89	-1.89	-1.89	-1.89	-1.89	-1.89
-1.88	-1.88	-1.88	-1.88	-1.88	-1.88	-1.88	-1.88	-1.88
-1.87	-1.87	-1.87	-1.87	-1.87	-1.87	-1.87	-1.87	-1.87
-1.86	-1.86	-1.86	-1.86	-1.86	-1.86	-1.86	-1.86	-1.86
-1.85	-1.85	-1.85	-1.85	-1.85	-1.85	-1.85	-1.85	-1.85
-1.84	-1.84	-1.84	-1.84	-1.84	-1.84	-1.84	-1.84	-1.84
-1.83	-1.83	-1.83	-1.83	-1.83	-1.83	-1.83	-1.83	-1.83
-1.82	-1.82	-1.82	-1.82	-1.82	-1.82	-1.82	-1.82	-1.82
-1.81	-1.81	-1.81	-1.81	-1.81	-1.81	-1.81	-1.81	-1.81
-1.8	-1.8	-1.8	-1.8	-1.8	-1.8	-1.8	-1.8	-1.8
-1.79	-1.79	-1.79	-1.79	-1.79	-1.79	-1.79	-1.79	-1.79
-1.78	-1.78	-1.78	-1.78	-1.78	-1.78	-1.78	-1.78	-1.78
-1.77	-1.77	-1.77	-1.77	-1.77	-1.77	-1.77	-1.77	-1.77
-1.76	-1.76	-1.76	-1.76	-1.76	-1.76	-1.76	-1.76	-1.76
-1.75	-1.75	-1.75	-1.75	-1.75	-1.75	-1.75	-1.75	-1.75
-1.74	-1.74	-1.74	-1.74	-1.74	-1.74	-1.74	-1.74	-1.74
-1.73	-1.73	-1.73	-1.73	-1.73	-1.73	-1.73	-1.73	-1.73
-1.72	-1.72	-1.72	-1.72	-1.72	-1.72	-1.72	-1.72	-1.72
-1.71	-1.71	-1.71	-1.71	-1.71	-1.71	-1.71	-1.71	-1.71
-1.7	-1.7	-1.7	-1.7	-1.7	-1.7	-1.7	-1.7	-1.7
-1.69	-1.69	-1.69	-1.69	-1.69	-1.69	-1.69	-1.69	-1.69
-1.68	-1.68	-1.68	-1.68	-1.68	-1.68	-1.68	-1.68	-1.68
-1.67	-1.67	-1.67	-1.67	-1.67	-1.67	-1.67	-1.67	-1.67
-1.66	-1.66	-1.66	-1.66	-1.66	-1.66	-1.66	-1.66	-1.66
-1.65	-1.65	-1.65	-1.65	-1.65	-1.65	-1.65	-1.65	-1.65
-1.64	-1.64	-1.64	-1.64	-1.64	-1.64	-1.64	-1.64	-1.64
-1.63	-1.63	-1.63	-1.63	-1.63	-1.63	-1.63	-1.63	-1.63
-1.62	-1.62	-1.62	-1.62	-1.62	-1.62	-1.62	-1.62	-1.62
-1.61	-1.61	-1.61	-1.61	-1.61	-1.61	-1.61	-1.61	-1.61
-1.6	-1.6	-1.6	-1.6	-1.6	-1.6	-1.6	-1.6	-1.6
-1.59	-1.59	-1.59	-1.59	-1.59	-1.59	-1.59	-1.59	-1.59
-1.58	-1.58	-1.58	-1.58	-1.58	-1.58	-1.58	-1.58	-1.58
-1.57	-1.57	-1.57	-1.57	-1.57	-1.57	-1.57	-1.57	-1.57
-1.56	-1.56	-1.56	-1.56	-1.56	-1.56	-1.56	-1.56	-1.56
-1.55	-1.55	-1.55	-1.55	-1.55	-1.55	-1.55	-1.55	-1.55
-1.54	-1.54	-1.54	-1.54	-1.54	-1.54	-1.54	-1.54	-1.54
-1.53	-1.53	-1.53	-1.53	-1.53	-1.53	-1.53	-1.53	-1.53
-1.52	-1.52	-1.52	-1.52	-1.52	-1.52	-1.52	-1.52	-1.52
-1.51	-1.51	-1.51	-1.51	-1.51	-1.51	-1.51	-1.51	-1.51

-1.5	-1.5	-1.5	-1.5	-1.5	-1.5	-1.5	-1.5	-1.5
-1.49	-1.49	-1.49	-1.49	-1.49	-1.49	-1.49	-1.49	-1.49
-1.48	-1.48	-1.48	-1.48	-1.48	-1.48	-1.48	-1.48	-1.48
-1.47	-1.47	-1.47	-1.47	-1.47	-1.47	-1.47	-1.47	-1.47
-1.46	-1.46	-1.46	-1.46	-1.46	-1.46	-1.46	-1.46	-1.46
-1.45	-1.45	-1.45	-1.45	-1.45	-1.45	-1.45	-1.45	-1.45
-1.44	-1.44	-1.44	-1.44	-1.44	-1.44	-1.44	-1.44	-1.44
-1.43	-1.43	-1.43	-1.43	-1.43	-1.43	-1.43	-1.43	-1.43
-1.42	-1.42	-1.42	-1.42	-1.42	-1.42	-1.42	-1.42	-1.42
-1.41	-1.41	-1.41	-1.41	-1.41	-1.41	-1.41	-1.41	-1.41
-1.4	-1.4	-1.4	-1.4	-1.4	-1.4	-1.4	-1.4	-1.4
-1.39	-1.39	-1.39	-1.39	-1.39	-1.39	-1.39	-1.39	-1.39
-1.38	-1.38	-1.38	-1.38	-1.38	-1.38	-1.38	-1.38	-1.38
-1.37	-1.37	-1.37	-1.37	-1.37	-1.37	-1.37	-1.37	-1.37
-1.36	-1.36	-1.36	-1.36	-1.36	-1.36	-1.36	-1.36	-1.36
-1.35	-1.35	-1.35	-1.35	-1.35	-1.35	-1.35	-1.35	-1.35
-1.34	-1.34	-1.34	-1.34	-1.34	-1.34	-1.34	-1.34	-1.34
-1.33	-1.33	-1.33	-1.33	-1.33	-1.33	-1.33	-1.33	-1.33
-1.32	-1.32	-1.32	-1.32	-1.32	-1.32	-1.32	-1.32	-1.32
-1.31	-1.31	-1.31	-1.31	-1.31	-1.31	-1.31	-1.31	-1.31
-1.3	-1.3	-1.3	-1.3	-1.3	-1.3	-1.3	-1.3	-1.3
-1.29	-1.29	-1.29	-1.29	-1.29	-1.29	-1.29	-1.29	-1.29
-1.28	-1.28	-1.28	-1.28	-1.28	-1.28	-1.28	-1.28	-1.28
-1.27	-1.27	-1.27	-1.27	-1.27	-1.27	-1.27	-1.27	-1.27
-1.26	-1.26	-1.26	-1.26	-1.26	-1.26	-1.26	-1.26	-1.26
-1.25	-1.25	-1.25	-1.25	-1.25	-1.25	-1.25	-1.25	-1.25
-1.24	-1.24	-1.24	-1.24	-1.24	-1.24	-1.24	-1.24	-1.24
-1.23	-1.23	-1.23	-1.23	-1.23	-1.23	-1.23	-1.23	-1.23
-1.22	-1.22	-1.22	-1.22	-1.22	-1.22	-1.22	-1.22	-1.22
-1.21	-1.21	-1.21	-1.21	-1.21	-1.21	-1.21	-1.21	-1.21
-1.2	-1.2	-1.2	-1.2	-1.2	-1.2	-1.2	-1.2	-1.2
-1.19	-1.19	-1.19	-1.19	-1.19	-1.19	-1.19	-1.19	-1.19
-1.18	-1.18	-1.18	-1.18	-1.18	-1.18	-1.18	-1.18	-1.18
-1.17	-1.17	-1.17	-1.17	-1.17	-1.17	-1.17	-1.17	-1.17
-1.16	-1.16	-1.16	-1.16	-1.16	-1.16	-1.16	-1.16	-1.16
-1.15	-1.15	-1.15	-1.15	-1.15	-1.15	-1.15	-1.15	-1.15
-1.14	-1.14	-1.14	-1.14	-1.14	-1.14	-1.14	-1.14	-1.14
-1.13	-1.13	-1.13	-1.13	-1.13	-1.13	-1.13	-1.13	-1.13
-1.12	-1.12	-1.12	-1.12	-1.12	-1.12	-1.12	-1.12	-1.12
-1.11	-1.11	-1.11	-1.11	-1.11	-1.11	-1.11	-1.11	-1.11
-1.1	-1.1	-1.1	-1.1	-1.1	-1.1	-1.1	-1.1	-1.1
-1.09	-1.09	-1.09	-1.09	-1.09	-1.09	-1.09	-1.09	-1.09
-1.08	-1.08	-1.08	-1.08	-1.08	-1.08	-1.08	-1.08	-1.08
-1.07	-1.07	-1.07	-1.07	-1.07	-1.07	-1.07	-1.07	-1.07
-1.06	-1.06	-1.06	-1.06	-1.06	-1.06	-1.06	-1.06	-1.06
-1.05	-1.05	-1.05	-1.05	-1.05	-1.05	-1.05	-1.05	-1.05
-1.04	-1.04	-1.04	-1.04	-1.04	-1.04	-1.04	-1.04	-1.04
-1.03	-1.03	-1.03	-1.03	-1.03	-1.03	-1.03	-1.03	-1.03
-1.02	-1.02	-1.02	-1.02	-1.02	-1.02	-1.02	-1.02	-1.02
-1.01	-1.01	-1.01	-1.01	-1.01	-1.01	-1.01	-1.01	-1.01

-1	-1	-1	-1	-1	-1	-1	-1	-1
-0.99	-0.99	-0.99	-0.99	-0.99	-0.99	-0.99	-0.99	-0.99
-0.98	-0.98	-0.98	-0.98	-0.98	-0.98	-0.98	-0.98	-0.98
-0.97	-0.97	-0.97	-0.97	-0.97	-0.97	-0.97	-0.97	-0.97
-0.96	-0.96	-0.96	-0.96	-0.96	-0.96	-0.96	-0.96	-0.96
-0.95	-0.95	-0.95	-0.95	-0.95	-0.95	-0.95	-0.95	-0.95
-0.94	-0.94	-0.94	-0.94	-0.94	-0.94	-0.94	-0.94	-0.94
-0.93	-0.93	-0.93	-0.93	-0.93	-0.93	-0.93	-0.93	-0.93
-0.92	-0.92	-0.92	-0.92	-0.92	-0.92	-0.92	-0.92	-0.92
-0.91	-0.91	-0.91	-0.91	-0.91	-0.91	-0.91	-0.91	-0.91
-0.9	-0.9	-0.9	-0.9	-0.9	-0.9	-0.9	-0.9	-0.9
-0.89	-0.89	-0.89	-0.89	-0.89	-0.89	-0.89	-0.89	-0.89
-0.88	-0.88	-0.88	-0.88	-0.88	-0.88	-0.88	-0.88	-0.88
-0.87	-0.87	-0.87	-0.87	-0.87	-0.87	-0.87	-0.87	-0.87
-0.86	-0.86	-0.86	-0.86	-0.86	-0.86	-0.86	-0.86	-0.86
-0.85	-0.85	-0.85	-0.85	-0.85	-0.85	-0.85	-0.85	-0.85
-0.84	-0.84	-0.84	-0.84	-0.84	-0.84	-0.84	-0.84	-0.84
-0.83	-0.83	-0.83	-0.83	-0.83	-0.83	-0.83	-0.83	-0.83
-0.82	-0.82	-0.82	-0.82	-0.82	-0.82	-0.82	-0.82	-0.82
-0.81	-0.81	-0.81	-0.81	-0.81	-0.81	-0.81	-0.81	-0.81
-0.8	-0.8	-0.8	-0.8	-0.8	-0.8	-0.8	-0.8	-0.8
-0.79	-0.79	-0.79	-0.79	-0.79	-0.79	-0.79	-0.79	-0.79
-0.78	-0.78	-0.78	-0.78	-0.78	-0.78	-0.78	-0.78	-0.78
-0.77	-0.77	-0.77	-0.77	-0.77	-0.77	-0.77	-0.77	-0.77
-0.76	-0.76	-0.76	-0.76	-0.76	-0.76	-0.76	-0.76	-0.76
-0.75	-0.75	-0.75	-0.75	-0.75	-0.75	-0.75	-0.75	-0.75
-0.74	-0.74	-0.74	-0.74	-0.74	-0.74	-0.74	-0.74	-0.74
-0.73	-0.73	-0.73	-0.73	-0.73	-0.73	-0.73	-0.73	-0.73
-0.72	-0.72	-0.72	-0.72	-0.72	-0.72	-0.72	-0.72	-0.72
-0.71	-0.71	-0.71	-0.71	-0.71	-0.71	-0.71	-0.71	-0.71
-0.7	-0.7	-0.7	-0.7	-0.7	-0.7	-0.7	-0.7	-0.7
-0.69	-0.69	-0.69	-0.69	-0.69	-0.69	-0.69	-0.69	-0.69
-0.68	-0.68	-0.68	-0.68	-0.68	-0.68	-0.68	-0.68	-0.68
-0.67	-0.67	-0.67	-0.67	-0.67	-0.67	-0.67	-0.67	-0.67
-0.66	-0.66	-0.66	-0.66	-0.66	-0.66	-0.66	-0.66	-0.66
-0.65	-0.65	-0.65	-0.65	-0.65	-0.65	-0.65	-0.65	-0.65
-0.64	-0.64	-0.64	-0.64	-0.64	-0.64	-0.64	-0.64	-0.64
-0.63	-0.63	-0.63	-0.63	-0.63	-0.63	-0.63	-0.63	-0.63
-0.62	-0.62	-0.62	-0.62	-0.62	-0.62	-0.62	-0.62	-0.62
-0.61	-0.61	-0.61	-0.61	-0.61	-0.61	-0.61	-0.61	-0.61
-0.6	-0.6	-0.6	-0.6	-0.6	-0.6	-0.6	-0.6	-0.6
-0.59	-0.59	-0.59	-0.59	-0.59	-0.59	-0.59	-0.59	-0.59
-0.58	-0.58	-0.58	-0.58	-0.58	-0.58	-0.58	-0.58	-0.58
-0.57	-0.57	-0.57	-0.57	-0.57	-0.57	-0.57	-0.57	-0.57
-0.56	-0.56	-0.56	-0.56	-0.56	-0.56	-0.56	-0.56	-0.56
-0.55	-0.55	-0.55	-0.55	-0.55	-0.55	-0.55	-0.55	-0.55
-0.54	-0.54	-0.54	-0.54	-0.54	-0.54	-0.54	-0.54	-0.54
-0.53	-0.53	-0.53	-0.53	-0.53	-0.53	-0.53	-0.53	-0.53
-0.52	-0.52	-0.52	-0.52	-0.52	-0.52	-0.52	-0.52	-0.52
-0.51	-0.51	-0.51	-0.51	-0.51	-0.51	-0.51	-0.51	-0.51

-0.5	-0.5	-0.5	-0.5	-0.5	-0.5	-0.5	-0.5	-0.5
-0.49	-0.49	-0.49	-0.49	-0.49	-0.49	-0.49	-0.49	-0.49
-0.48	-0.48	-0.48	-0.48	-0.48	-0.48	-0.48	-0.48	-0.48
-0.47	-0.47	-0.47	-0.47	-0.47	-0.47	-0.47	-0.47	-0.47
-0.46	-0.46	-0.46	-0.46	-0.46	-0.46	-0.46	-0.46	-0.46
-0.45	-0.45	-0.45	-0.45	-0.45	-0.45	-0.45	-0.45	-0.45
-0.44	-0.44	-0.44	-0.44	-0.44	-0.44	-0.44	-0.44	-0.44
-0.43	-0.43	-0.43	-0.43	-0.43	-0.43	-0.43	-0.43	-0.43
-0.42	-0.42	-0.42	-0.42	-0.42	-0.42	-0.42	-0.42	-0.42
-0.41	-0.41	-0.41	-0.41	-0.41	-0.41	-0.41	-0.41	-0.41
-0.4	-0.4	-0.4	-0.4	-0.4	-0.4	-0.4	-0.4	-0.4
-0.39	-0.39	-0.39	-0.39	-0.39	-0.39	-0.39	-0.39	-0.39
-0.38	-0.38	-0.38	-0.38	-0.38	-0.38	-0.38	-0.38	-0.38
-0.37	-0.37	-0.37	-0.37	-0.37	-0.37	-0.37	-0.37	-0.37
-0.36	-0.36	-0.36	-0.36	-0.36	-0.36	-0.36	-0.36	-0.36
-0.35	-0.35	-0.35	-0.35	-0.35	-0.35	-0.35	-0.35	-0.35
-0.34	-0.34	-0.34	-0.34	-0.34	-0.34	-0.34	-0.34	-0.34
-0.33	-0.33	-0.33	-0.33	-0.33	-0.33	-0.33	-0.33	-0.33
-0.32	-0.32	-0.32	-0.32	-0.32	-0.32	-0.32	-0.32	-0.32
-0.31	-0.31	-0.31	-0.31	-0.31	-0.31	-0.31	-0.31	-0.31
-0.3	-0.3	-0.3	-0.3	-0.3	-0.3	-0.3	-0.3	-0.3
-0.29	-0.29	-0.29	-0.29	-0.29	-0.29	-0.29	-0.29	-0.29
-0.28	-0.28	-0.28	-0.28	-0.28	-0.28	-0.28	-0.28	-0.28
-0.27	-0.27	-0.27	-0.27	-0.27	-0.27	-0.27	-0.27	-0.27
-0.26	-0.26	-0.26	-0.26	-0.26	-0.26	-0.26	-0.26	-0.26
-0.25	-0.25	-0.25	-0.25	-0.25	-0.25	-0.25	-0.25	-0.25
-0.24	-0.24	-0.24	-0.24	-0.24	-0.24	-0.24	-0.24	-0.24
-0.23	-0.23	-0.23	-0.23	-0.23	-0.23	-0.23	-0.23	-0.23
-0.22	-0.22	-0.22	-0.22	-0.22	-0.22	-0.22	-0.22	-0.22
-0.21	-0.21	-0.21	-0.21	-0.21	-0.21	-0.21	-0.21	-0.21
-0.2	-0.2	-0.2	-0.2	-0.2	-0.2	-0.2	-0.2	-0.2
-0.19	-0.19	-0.19	-0.19	-0.19	-0.19	-0.19	-0.19	-0.19
-0.18	-0.18	-0.18	-0.18	-0.18	-0.18	-0.18	-0.18	-0.18
-0.17	-0.17	-0.17	-0.17	-0.17	-0.17	-0.17	-0.17	-0.17
-0.16	-0.16	-0.16	-0.16	-0.16	-0.16	-0.16	-0.16	-0.16
-0.15	-0.15	-0.15	-0.15	-0.15	-0.15	-0.15	-0.15	-0.15
-0.14	-0.14	-0.14	-0.14	-0.14	-0.14	-0.14	-0.14	-0.14
-0.13	-0.13	-0.13	-0.13	-0.13	-0.13	-0.13	-0.13	-0.13
-0.12	-0.12	-0.12	-0.12	-0.12	-0.12	-0.12	-0.12	-0.12
-0.11	-0.11	-0.11	-0.11	-0.11	-0.11	-0.11	-0.11	-0.11
-0.1	-0.1	-0.1	-0.1	-0.1	-0.1	-0.1	-0.1	-0.1
-0.09	-0.09	-0.09	-0.09	-0.09	-0.09	-0.09	-0.09	-0.09
-0.08	-0.08	-0.08	-0.08	-0.08	-0.08	-0.08	-0.08	-0.08
-0.07	-0.07	-0.07	-0.07	-0.07	-0.07	-0.07	-0.07	-0.07
-0.06	-0.06	-0.06	-0.06	-0.06	-0.06	-0.06	-0.06	-0.06
-0.05	-0.05	-0.05	-0.05	-0.05	-0.05	-0.05	-0.05	-0.05
-0.04	-0.04	-0.04	-0.04	-0.04	-0.04	-0.04	-0.04	-0.04
-0.03	-0.03	-0.03	-0.03	-0.03	-0.03	-0.03	-0.03	-0.03
-0.02	-0.02	-0.02	-0.02	-0.02	-0.02	-0.02	-0.02	-0.02
-0.01	-0.01	-0.01	-0.01	-0.01	-0.01	-0.01	-0.01	-0.01

0	0	0	0	0	0	0	0	0
0.01	0.01	0.01	0.01	0.01	0.01	0.01	0.01	0.01
0.02	0.02	0.02	0.02	0.02	0.02	0.02	0.02	0.02
0.03	0.03	0.03	0.03	0.03	0.03	0.03	0.03	0.03
0.04	0.04	0.04	0.04	0.04	0.04	0.04	0.04	0.04
0.05	0.05	0.05	0.05	0.05	0.05	0.05	0.05	0.05
0.06	0.06	0.06	0.06	0.06	0.06	0.06	0.06	0.06
0.07	0.07	0.07	0.07	0.07	0.07	0.07	0.07	0.07
0.08	0.08	0.08	0.08	0.08	0.08	0.08	0.08	0.08
0.09	0.09	0.09	0.09	0.09	0.09	0.09	0.09	0.09
0.1	0.1	0.1	0.1	0.1	0.1	0.1	0.1	0.1
0.11	0.11	0.11	0.11	0.11	0.11	0.11	0.11	0.11
0.12	0.12	0.12	0.12	0.12	0.12	0.12	0.12	0.12
0.13	0.13	0.13	0.13	0.13	0.13	0.13	0.13	0.13
0.14	0.14	0.14	0.14	0.14	0.14	0.14	0.14	0.14
0.15	0.15	0.15	0.15	0.15	0.15	0.15	0.15	0.15
0.16	0.16	0.16	0.16	0.16	0.16	0.16	0.16	0.16
0.17	0.17	0.17	0.17	0.17	0.17	0.17	0.17	0.17
0.18	0.18	0.18	0.18	0.18	0.18	0.18	0.18	0.18
0.19	0.19	0.19	0.19	0.19	0.19	0.19	0.19	0.19
0.2	0.2	0.2	0.2	0.2	0.2	0.2	0.2	0.2
0.21	0.21	0.21	0.21	0.21	0.21	0.21	0.21	0.21
0.22	0.22	0.22	0.22	0.22	0.22	0.22	0.22	0.22
0.23	0.23	0.23	0.23	0.23	0.23	0.23	0.23	0.23
0.24	0.24	0.24	0.24	0.24	0.24	0.24	0.24	0.24
0.25	0.25	0.25	0.25	0.25	0.25	0.25	0.25	0.25
0.26	0.26	0.26	0.26	0.26	0.26	0.26	0.26	0.26
0.27	0.27	0.27	0.27	0.27	0.27	0.27	0.27	0.27
0.28	0.28	0.28	0.28	0.28	0.28	0.28	0.28	0.28
0.29	0.29	0.29	0.29	0.29	0.29	0.29	0.29	0.29
0.3	0.3	0.3	0.3	0.3	0.3	0.3	0.3	0.3
0.31	0.31	0.31	0.31	0.31	0.31	0.31	0.31	0.31
0.32	0.32	0.32	0.32	0.32	0.32	0.32	0.32	0.32
0.33	0.33	0.33	0.33	0.33	0.33	0.33	0.33	0.33
0.34	0.34	0.34	0.34	0.34	0.34	0.34	0.34	0.34
0.35	0.35	0.35	0.35	0.35	0.35	0.35	0.35	0.35
0.36	0.36	0.36	0.36	0.36	0.36	0.36	0.36	0.36
0.37	0.37	0.37	0.37	0.37	0.37	0.37	0.37	0.37
0.38	0.38	0.38	0.38	0.38	0.38	0.38	0.38	0.38
0.39	0.39	0.39	0.39	0.39	0.39	0.39	0.39	0.39
0.4	0.4	0.4	0.4	0.4	0.4	0.4	0.4	0.4
0.41	0.41	0.41	0.41	0.41	0.41	0.41	0.41	0.41
0.42	0.42	0.42	0.42	0.42	0.42	0.42	0.42	0.42
0.43	0.43	0.43	0.43	0.43	0.43	0.43	0.43	0.43
0.44	0.44	0.44	0.44	0.44	0.44	0.44	0.44	0.44
0.45	0.45	0.45	0.45	0.45	0.45	0.45	0.45	0.45
0.46	0.46	0.46	0.46	0.46	0.46	0.46	0.46	0.46
0.47	0.47	0.47	0.47	0.47	0.47	0.47	0.47	0.47
0.48	0.48	0.48	0.48	0.48	0.48	0.48	0.48	0.48
0.49	0.49	0.49	0.49	0.49	0.49	0.49	0.49	0.49

0.5	0.5	0.5	0.5	0.5	0.5	0.5	0.5	0.5
0.51	0.51	0.51	0.51	0.51	0.51	0.51	0.51	0.51
0.52	0.52	0.52	0.52	0.52	0.52	0.52	0.52	0.52
0.53	0.53	0.53	0.53	0.53	0.53	0.53	0.53	0.53
0.54	0.54	0.54	0.54	0.54	0.54	0.54	0.54	0.54
0.55	0.55	0.55	0.55	0.55	0.55	0.55	0.55	0.55
0.56	0.56	0.56	0.56	0.56	0.56	0.56	0.56	0.56
0.57	0.57	0.57	0.57	0.57	0.57	0.57	0.57	0.57
0.58	0.58	0.58	0.58	0.58	0.58	0.58	0.58	0.58
0.59	0.59	0.59	0.59	0.59	0.59	0.59	0.59	0.59
0.6	0.6	0.6	0.6	0.6	0.6	0.6	0.6	0.6
0.61	0.61	0.61	0.61	0.61	0.61	0.61	0.61	0.61
0.62	0.62	0.62	0.62	0.62	0.62	0.62	0.62	0.62
0.63	0.63	0.63	0.63	0.63	0.63	0.63	0.63	0.63
0.64	0.64	0.64	0.64	0.64	0.64	0.64	0.64	0.64
0.65	0.65	0.65	0.65	0.65	0.65	0.65	0.65	0.65
0.66	0.66	0.66	0.66	0.66	0.66	0.66	0.66	0.66
0.67	0.67	0.67	0.67	0.67	0.67	0.67	0.67	0.67
0.68	0.68	0.68	0.68	0.68	0.68	0.68	0.68	0.68
0.69	0.69	0.69	0.69	0.69	0.69	0.69	0.69	0.69
0.7	0.7	0.7	0.7	0.7	0.7	0.7	0.7	0.7
0.71	0.71	0.71	0.71	0.71	0.71	0.71	0.71	0.71
0.72	0.72	0.72	0.72	0.72	0.72	0.72	0.72	0.72
0.73	0.73	0.73	0.73	0.73	0.73	0.73	0.73	0.73
0.74	0.74	0.74	0.74	0.74	0.74	0.74	0.74	0.74
0.75	0.75	0.75	0.75	0.75	0.75	0.75	0.75	0.75
0.76	0.76	0.76	0.76	0.76	0.76	0.76	0.76	0.76
0.77	0.77	0.77	0.77	0.77	0.77	0.77	0.77	0.77
0.78	0.78	0.78	0.78	0.78	0.78	0.78	0.78	0.78
0.79	0.79	0.79	0.79	0.79	0.79	0.79	0.79	0.79
0.8	0.8	0.8	0.8	0.8	0.8	0.8	0.8	0.8
0.81	0.81	0.81	0.81	0.81	0.81	0.81	0.81	0.81
0.82	0.82	0.82	0.82	0.82	0.82	0.82	0.82	0.82
0.83	0.83	0.83	0.83	0.83	0.83	0.83	0.83	0.83
0.84	0.84	0.84	0.84	0.84	0.84	0.84	0.84	0.84
0.85	0.85	0.85	0.85	0.85	0.85	0.85	0.85	0.85
0.86	0.86	0.86	0.86	0.86	0.86	0.86	0.86	0.86
0.87	0.87	0.87	0.87	0.87	0.87	0.87	0.87	0.87
0.88	0.88	0.88	0.88	0.88	0.88	0.88	0.88	0.88
0.89	0.89	0.89	0.89	0.89	0.89	0.89	0.89	0.89
0.9	0.9	0.9	0.9	0.9	0.9	0.9	0.9	0.9
0.91	0.91	0.91	0.91	0.91	0.91	0.91	0.91	0.91
0.92	0.92	0.92	0.92	0.92	0.92	0.92	0.92	0.92
0.93	0.93	0.93	0.93	0.93	0.93	0.93	0.93	0.93
0.94	0.94	0.94	0.94	0.94	0.94	0.94	0.94	0.94
0.95	0.95	0.95	0.95	0.95	0.95	0.95	0.95	0.95
0.96	0.96	0.96	0.96	0.96	0.96	0.96	0.96	0.96
0.97	0.97	0.97	0.97	0.97	0.97	0.97	0.97	0.97
0.98	0.98	0.98	0.98	0.98	0.98	0.98	0.98	0.98
0.99	0.99	0.99	0.99	0.99	0.99	0.99	0.99	0.99

1	1	1	1	1	1	1	1	1
1.01	1.01	1.01	1.01	1.01	1.01	1.01	1.01	1.01
1.02	1.02	1.02	1.02	1.02	1.02	1.02	1.02	1.02
1.03	1.03	1.03	1.03	1.03	1.03	1.03	1.03	1.03
1.04	1.04	1.04	1.04	1.04	1.04	1.04	1.04	1.04
1.05	1.05	1.05	1.05	1.05	1.05	1.05	1.05	1.05
1.06	1.06	1.06	1.06	1.06	1.06	1.06	1.06	1.06
1.07	1.07	1.07	1.07	1.07	1.07	1.07	1.07	1.07
1.08	1.08	1.08	1.08	1.08	1.08	1.08	1.08	1.08
1.09	1.09	1.09	1.09	1.09	1.09	1.09	1.09	1.09
1.1	1.1	1.1	1.1	1.1	1.1	1.1	1.1	1.1
1.11	1.11	1.11	1.11	1.11	1.11	1.11	1.11	1.11
1.12	1.12	1.12	1.12	1.12	1.12	1.12	1.12	1.12
1.13	1.13	1.13	1.13	1.13	1.13	1.13	1.13	1.13
1.14	1.14	1.14	1.14	1.14	1.14	1.14	1.14	1.14
1.15	1.15	1.15	1.15	1.15	1.15	1.15	1.15	1.15
1.16	1.16	1.16	1.16	1.16	1.16	1.16	1.16	1.16
1.17	1.17	1.17	1.17	1.17	1.17	1.17	1.17	1.17
1.18	1.18	1.18	1.18	1.18	1.18	1.18	1.18	1.18
1.19	1.19	1.19	1.19	1.19	1.19	1.19	1.19	1.19
1.2	1.2	1.2	1.2	1.2	1.2	1.2	1.2	1.2
1.21	1.21	1.21	1.21	1.21	1.21	1.21	1.21	1.21
1.22	1.22	1.22	1.22	1.22	1.22	1.22	1.22	1.22
1.23	1.23	1.23	1.23	1.23	1.23	1.23	1.23	1.23
1.24	1.24	1.24	1.24	1.24	1.24	1.24	1.24	1.24
1.25	1.25	1.25	1.25	1.25	1.25	1.25	1.25	1.25
1.26	1.26	1.26	1.26	1.26	1.26	1.26	1.26	1.26
1.27	1.27	1.27	1.27	1.27	1.27	1.27	1.27	1.27
1.28	1.28	1.28	1.28	1.28	1.28	1.28	1.28	1.28
1.29	1.29	1.29	1.29	1.29	1.29	1.29	1.29	1.29
1.3	1.3	1.3	1.3	1.3	1.3	1.3	1.3	1.3
1.31	1.31	1.31	1.31	1.31	1.31	1.31	1.31	1.31
1.32	1.32	1.32	1.32	1.32	1.32	1.32	1.32	1.32
1.33	1.33	1.33	1.33	1.33	1.33	1.33	1.33	1.33
1.34	1.34	1.34	1.34	1.34	1.34	1.34	1.34	1.34
1.35	1.35	1.35	1.35	1.35	1.35	1.35	1.35	1.35
1.36	1.36	1.36	1.36	1.36	1.36	1.36	1.36	1.36
1.37	1.37	1.37	1.37	1.37	1.37	1.37	1.37	1.37
1.38	1.38	1.38	1.38	1.38	1.38	1.38	1.38	1.38
1.39	1.39	1.39	1.39	1.39	1.39	1.39	1.39	1.39
1.4	1.4	1.4	1.4	1.4	1.4	1.4	1.4	1.4
1.41	1.41	1.41	1.41	1.41	1.41	1.41	1.41	1.41
1.42	1.42	1.42	1.42	1.42	1.42	1.42	1.42	1.42
1.43	1.43	1.43	1.43	1.43	1.43	1.43	1.43	1.43
1.44	1.44	1.44	1.44	1.44	1.44	1.44	1.44	1.44
1.45	1.45	1.45	1.45	1.45	1.45	1.45	1.45	1.45
1.46	1.46	1.46	1.46	1.46	1.46	1.46	1.46	1.46
1.47	1.47	1.47	1.47	1.47	1.47	1.47	1.47	1.47
1.48	1.48	1.48	1.48	1.48	1.48	1.48	1.48	1.48
1.49	1.49	1.49	1.49	1.49	1.49	1.49	1.49	1.49

1.5	1.5	1.5	1.5	1.5	1.5	1.5	1.5	1.5
1.51	1.51	1.51	1.51	1.51	1.51	1.51	1.51	1.51
1.52	1.52	1.52	1.52	1.52	1.52	1.52	1.52	1.52
1.53	1.53	1.53	1.53	1.53	1.53	1.53	1.53	1.53
1.54	1.54	1.54	1.54	1.54	1.54	1.54	1.54	1.54
1.55	1.55	1.55	1.55	1.55	1.55	1.55	1.55	1.55
1.56	1.56	1.56	1.56	1.56	1.56	1.56	1.56	1.56
1.57	1.57	1.57	1.57	1.57	1.57	1.57	1.57	1.57
1.58	1.58	1.58	1.58	1.58	1.58	1.58	1.58	1.58
1.59	1.59	1.59	1.59	1.59	1.59	1.59	1.59	1.59
1.6	1.6	1.6	1.6	1.6	1.6	1.6	1.6	1.6
1.61	1.61	1.61	1.61	1.61	1.61	1.61	1.61	1.61
1.62	1.62	1.62	1.62	1.62	1.62	1.62	1.62	1.62
1.63	1.63	1.63	1.63	1.63	1.63	1.63	1.63	1.63
1.64	1.64	1.64	1.64	1.64	1.64	1.64	1.64	1.64
1.65	1.65	1.65	1.65	1.65	1.65	1.65	1.65	1.65
1.66	1.66	1.66	1.66	1.66	1.66	1.66	1.66	1.66
1.67	1.67	1.67	1.67	1.67	1.67	1.67	1.67	1.67
1.68	1.68	1.68	1.68	1.68	1.68	1.68	1.68	1.68
1.69	1.69	1.69	1.69	1.69	1.69	1.69	1.69	1.69
1.7	1.7	1.7	1.7	1.7	1.7	1.7	1.7	1.7
1.71	1.71	1.71	1.71	1.71	1.71	1.71	1.71	1.71
1.72	1.72	1.72	1.72	1.72	1.72	1.72	1.72	1.72
1.73	1.73	1.73	1.73	1.73	1.73	1.73	1.73	1.73
1.74	1.74	1.74	1.74	1.74	1.74	1.74	1.74	1.74
1.75	1.75	1.75	1.75	1.75	1.75	1.75	1.75	1.75
1.76	1.76	1.76	1.76	1.76	1.76	1.76	1.76	1.76
1.77	1.77	1.77	1.77	1.77	1.77	1.77	1.77	1.77
1.78	1.78	1.78	1.78	1.78	1.78	1.78	1.78	1.78
1.79	1.79	1.79	1.79	1.79	1.79	1.79	1.79	1.79
1.8	1.8	1.8	1.8	1.8	1.8	1.8	1.8	1.8
1.81	1.81	1.81	1.81	1.81	1.81	1.81	1.81	1.81
1.82	1.82	1.82	1.82	1.82	1.82	1.82	1.82	1.82
1.83	1.83	1.83	1.83	1.83	1.83	1.83	1.83	1.83
1.84	1.84	1.84	1.84	1.84	1.84	1.84	1.84	1.84
1.85	1.85	1.85	1.85	1.85	1.85	1.85	1.85	1.85
1.86	1.86	1.86	1.86	1.86	1.86	1.86	1.86	1.86
1.87	1.87	1.87	1.87	1.87	1.87	1.87	1.87	1.87
1.88	1.88	1.88	1.88	1.88	1.88	1.88	1.88	1.88
1.89	1.89	1.89	1.89	1.89	1.89	1.89	1.89	1.89
1.9	1.9	1.9	1.9	1.9	1.9	1.9	1.9	1.9
1.91	1.91	1.91	1.91	1.91	1.91	1.91	1.91	1.91
1.92	1.92	1.92	1.92	1.92	1.92	1.92	1.92	1.92
1.93	1.93	1.93	1.93	1.93	1.93	1.93	1.93	1.93
1.94	1.94	1.94	1.94	1.94	1.94	1.94	1.94	1.94
1.95	1.95	1.95	1.95	1.95	1.95	1.95	1.95	1.95
1.96	1.96	1.96	1.96	1.96	1.96	1.96	1.96	1.96
1.97	1.97	1.97	1.97	1.97	1.97	1.97	1.97	1.97
1.98	1.98	1.98	1.98	1.98	1.98	1.98	1.98	1.98
1.99	1.99	1.99	1.99	1.99	1.99	1.99	1.99	1.99

2	2	2	2	2	2	2	2	2
2.01	2.01	2.01	2.01	2.01	2.01	2.01	2.01	2.01
2.02	2.02	2.02	2.02	2.02	2.02	2.02	2.02	2.02
2.03	2.03	2.03	2.03	2.03	2.03	2.03	2.03	2.03
2.04	2.04	2.04	2.04	2.04	2.04	2.04	2.04	2.04
2.05	2.05	2.05	2.05	2.05	2.05	2.05	2.05	2.05
2.06	2.06	2.06	2.06	2.06	2.06	2.06	2.06	2.06
2.07	2.07	2.07	2.07	2.07	2.07	2.07	2.07	2.07
2.08	2.08	2.08	2.08	2.08	2.08	2.08	2.08	2.08
2.09	2.09	2.09	2.09	2.09	2.09	2.09	2.09	2.09
2.1	2.1	2.1	2.1	2.1	2.1	2.1	2.1	2.1
2.11	2.11	2.11	2.11	2.11	2.11	2.11	2.11	2.11
2.12	2.12	2.12	2.12	2.12	2.12	2.12	2.12	2.12
2.13	2.13	2.13	2.13	2.13	2.13	2.13	2.13	2.13
2.14	2.14	2.14	2.14	2.14	2.14	2.14	2.14	2.14
2.15	2.15	2.15	2.15	2.15	2.15	2.15	2.15	2.15
2.16	2.16	2.16	2.16	2.16	2.16	2.16	2.16	2.16
2.17	2.17	2.17	2.17	2.17	2.17	2.17	2.17	2.17
2.18	2.18	2.18	2.18	2.18	2.18	2.18	2.18	2.18
2.19	2.19	2.19	2.19	2.19	2.19	2.19	2.19	2.19
2.2	2.2	2.2	2.2	2.2	2.2	2.2	2.2	2.2
2.21	2.21	2.21	2.21	2.21	2.21	2.21	2.21	2.21
2.22	2.22	2.22	2.22	2.22	2.22	2.22	2.22	2.22
2.23	2.23	2.23	2.23	2.23	2.23	2.23	2.23	2.23
2.24	2.24	2.24	2.24	2.24	2.24	2.24	2.24	2.24
2.25	2.25	2.25	2.25	2.25	2.25	2.25	2.25	2.25
2.26	2.26	2.26	2.26	2.26	2.26	2.26	2.26	2.26
2.27	2.27	2.27	2.27	2.27	2.27	2.27	2.27	2.27
2.28	2.28	2.28	2.28	2.28	2.28	2.28	2.28	2.28
2.29	2.29	2.29	2.29	2.29	2.29	2.29	2.29	2.29
2.3	2.3	2.3	2.3	2.3	2.3	2.3	2.3	2.3
2.31	2.31	2.31	2.31	2.31	2.31	2.31	2.31	2.31
2.32	2.32	2.32	2.32	2.32	2.32	2.32	2.32	2.32
2.33	2.33	2.33	2.33	2.33	2.33	2.33	2.33	2.33
2.34	2.34	2.34	2.34	2.34	2.34	2.34	2.34	2.34
2.35	2.35	2.35	2.35	2.35	2.35	2.35	2.35	2.35
2.36	2.36	2.36	2.36	2.36	2.36	2.36	2.36	2.36
2.37	2.37	2.37	2.37	2.37	2.37	2.37	2.37	2.37
2.38	2.38	2.38	2.38	2.38	2.38	2.38	2.38	2.38
2.39	2.39	2.39	2.39	2.39	2.39	2.39	2.39	2.39
2.4	2.4	2.4	2.4	2.4	2.4	2.4	2.4	2.4
2.41	2.41	2.41	2.41	2.41	2.41	2.41	2.41	2.41
2.42	2.42	2.42	2.42	2.42	2.42	2.42	2.42	2.42
2.43	2.43	2.43	2.43	2.43	2.43	2.43	2.43	2.43
2.44	2.44	2.44	2.44	2.44	2.44	2.44	2.44	2.44
2.45	2.45	2.45	2.45	2.45	2.45	2.45	2.45	2.45
2.46	2.46	2.46	2.46	2.46	2.46	2.46	2.46	2.46
2.47	2.47	2.47	2.47	2.47	2.47	2.47	2.47	2.47
2.48	2.48	2.48	2.48	2.48	2.48	2.48	2.48	2.48
2.49	2.49	2.49	2.49	2.49	2.49	2.49	2.49	2.49

2.5	2.5	2.5	2.5	2.5	2.5	2.5	2.5	2.5
2.51	2.51	2.51	2.51	2.51	2.51	2.51	2.51	2.51
2.52	2.52	2.52	2.52	2.52	2.52	2.52	2.52	2.52
2.53	2.53	2.53	2.53	2.53	2.53	2.53	2.53	2.53
2.54	2.54	2.54	2.54	2.54	2.54	2.54	2.54	2.54
2.55	2.55	2.55	2.55	2.55	2.55	2.55	2.55	2.55
2.56	2.56	2.56	2.56	2.56	2.56	2.56	2.56	2.56
2.57	2.57	2.57	2.57	2.57	2.57	2.57	2.57	2.57
2.58	2.58	2.58	2.58	2.58	2.58	2.58	2.58	2.58
2.59	2.59	2.59	2.59	2.59	2.59	2.59	2.59	2.59
2.6	2.6	2.6	2.6	2.6	2.6	2.6	2.6	2.6
2.61	2.61	2.61	2.61	2.61	2.61	2.61	2.61	2.61
2.62	2.62	2.62	2.62	2.62	2.62	2.62	2.62	2.62
2.63	2.63	2.63	2.63	2.63	2.63	2.63	2.63	2.63
2.64	2.64	2.64	2.64	2.64	2.64	2.64	2.64	2.64
2.65	2.65	2.65	2.65	2.65	2.65	2.65	2.65	2.65
2.66	2.66	2.66	2.66	2.66	2.66	2.66	2.66	2.66
2.67	2.67	2.67	2.67	2.67	2.67	2.67	2.67	2.67
2.68	2.68	2.68	2.68	2.68	2.68	2.68	2.68	2.68
2.69	2.69	2.69	2.69	2.69	2.69	2.69	2.69	2.69
2.7	2.7	2.7	2.7	2.7	2.7	2.7	2.7	2.7
2.71	2.71	2.71	2.71	2.71	2.71	2.71	2.71	2.71
2.72	2.72	2.72	2.72	2.72	2.72	2.72	2.72	2.72
2.73	2.73	2.73	2.73	2.73	2.73	2.73	2.73	2.73
2.74	2.74	2.74	2.74	2.74	2.74	2.74	2.74	2.74
2.75	2.75	2.75	2.75	2.75	2.75	2.75	2.75	2.75
2.76	2.76	2.76	2.76	2.76	2.76	2.76	2.76	2.76
2.77	2.77	2.77	2.77	2.77	2.77	2.77	2.77	2.77
2.78	2.78	2.78	2.78	2.78	2.78	2.78	2.78	2.78
2.79	2.79	2.79	2.79	2.79	2.79	2.79	2.79	2.79
2.8	2.8	2.8	2.8	2.8	2.8	2.8	2.8	2.8
2.81	2.81	2.81	2.81	2.81	2.81	2.81	2.81	2.81
2.82	2.82	2.82	2.82	2.82	2.82	2.82	2.82	2.82
2.83	2.83	2.83	2.83	2.83	2.83	2.83	2.83	2.83
2.84	2.84	2.84	2.84	2.84	2.84	2.84	2.84	2.84
2.85	2.85	2.85	2.85	2.85	2.85	2.85	2.85	2.85
2.86	2.86	2.86	2.86	2.86	2.86	2.86	2.86	2.86
2.87	2.87	2.87	2.87	2.87	2.87	2.87	2.87	2.87
2.88	2.88	2.88	2.88	2.88	2.88	2.88	2.88	2.88
2.89	2.89	2.89	2.89	2.89	2.89	2.89	2.89	2.89
2.9	2.9	2.9	2.9	2.9	2.9	2.9	2.9	2.9
2.91	2.91	2.91	2.91	2.91	2.91	2.91	2.91	2.91
2.92	2.92	2.92	2.92	2.92	2.92	2.92	2.92	2.92
2.93	2.93	2.93	2.93	2.93	2.93	2.93	2.93	2.93
2.94	2.94	2.94	2.94	2.94	2.94	2.94	2.94	2.94
2.95	2.95	2.95	2.95	2.95	2.95	2.95	2.95	2.95
2.96	2.96	2.96	2.96	2.96	2.96	2.96	2.96	2.96
2.97	2.97	2.97	2.97	2.97	2.97	2.97	2.97	2.97
2.98	2.98	2.98	2.98	2.98	2.98	2.98	2.98	2.98
2.99	2.99	2.99	2.99	2.99	2.99	2.99	2.99	2.99

3	3	3	3	3	3	3	3	3
3.01	3.01	3.01	3.01	3.01	3.01	3.01	3.01	3.01
3.02	3.02	3.02	3.02	3.02	3.02	3.02	3.02	3.02
3.03	3.03	3.03	3.03	3.03	3.03	3.03	3.03	3.03
3.04	3.04	3.04	3.04	3.04	3.04	3.04	3.04	3.04
3.05	3.05	3.05	3.05	3.05	3.05	3.05	3.05	3.05
3.06	3.06	3.06	3.06	3.06	3.06	3.06	3.06	3.06
3.07	3.07	3.07	3.07	3.07	3.07	3.07	3.07	3.07
3.08	3.08	3.08	3.08	3.08	3.08	3.08	3.08	3.08
3.09	3.09	3.09	3.09	3.09	3.09	3.09	3.09	3.09
3.1	3.1	3.1	3.1	3.1	3.1	3.1	3.1	3.1
3.11	3.11	3.11	3.11	3.11	3.11	3.11	3.11	3.11
3.12	3.12	3.12	3.12	3.12	3.12	3.12	3.12	3.12
3.13	3.13	3.13	3.13	3.13	3.13	3.13	3.13	3.13
3.14	3.14	3.14	3.14	3.14	3.14	3.14	3.14	3.14
3.15	3.15	3.15	3.15	3.15	3.15	3.15	3.15	3.15
3.16	3.16	3.16	3.16	3.16	3.16	3.16	3.16	3.16
3.17	3.17	3.17	3.17	3.17	3.17	3.17	3.17	3.17
3.18	3.18	3.18	3.18	3.18	3.18	3.18	3.18	3.18
3.19	3.19	3.19	3.19	3.19	3.19	3.19	3.19	3.19
3.2	3.2	3.2	3.2	3.2	3.2	3.2	3.2	3.2
3.21	3.21	3.21	3.21	3.21	3.21	3.21	3.21	3.21
3.22	3.22	3.22	3.22	3.22	3.22	3.22	3.22	3.22
3.23	3.23	3.23	3.23	3.23	3.23	3.23	3.23	3.23
3.24	3.24	3.24	3.24	3.24	3.24	3.24	3.24	3.24
3.25	3.25	3.25	3.25	3.25	3.25	3.25	3.25	3.25
3.26	3.26	3.26	3.26	3.26	3.26	3.26	3.26	3.26
3.27	3.27	3.27	3.27	3.27	3.27	3.27	3.27	3.27
3.28	3.28	3.28	3.28	3.28	3.28	3.28	3.28	3.28
3.29	3.29	3.29	3.29	3.29	3.29	3.29	3.29	3.29
3.3	3.3	3.3	3.3	3.3	3.3	3.3	3.3	3.3
3.31	3.31	3.31	3.31	3.31	3.31	3.31	3.31	3.31
3.32	3.32	3.32	3.32	3.32	3.32	3.32	3.32	3.32
3.33	3.33	3.33	3.33	3.33	3.33	3.33	3.33	3.33
3.34	3.34	3.34	3.34	3.34	3.34	3.34	3.34	3.34
3.35	3.35	3.35	3.35	3.35	3.35	3.35	3.35	3.35
3.36	3.36	3.36	3.36	3.36	3.36	3.36	3.36	3.36
3.37	3.37	3.37	3.37	3.37	3.37	3.37	3.37	3.37
3.38	3.38	3.38	3.38	3.38	3.38	3.38	3.38	3.38
3.39	3.39	3.39	3.39	3.39	3.39	3.39	3.39	3.39
3.4	3.4	3.4	3.4	3.4	3.4	3.4	3.4	3.4
3.41	3.41	3.41	3.41	3.41	3.41	3.41	3.41	3.41
3.42	3.42	3.42	3.42	3.42	3.42	3.42	3.42	3.42
3.43	3.43	3.43	3.43	3.43	3.43	3.43	3.43	3.43
3.44	3.44	3.44	3.44	3.44	3.44	3.44	3.44	3.44
3.45	3.45	3.45	3.45	3.45	3.45	3.45	3.45	3.45
3.46	3.46	3.46	3.46	3.46	3.46	3.46	3.46	3.46
3.47	3.47	3.47	3.47	3.47	3.47	3.47	3.47	3.47
3.48	3.48	3.48	3.48	3.48	3.48	3.48	3.48	3.48
3.49	3.49	3.49	3.49	3.49	3.49	3.49	3.49	3.49

3.5	3.5	3.5	3.5	3.5	3.5	3.5	3.5	3.5
3.51	3.51	3.51	3.51	3.51	3.51	3.51	3.51	3.51
3.52	3.52	3.52	3.52	3.52	3.52	3.52	3.52	3.52
3.53	3.53	3.53	3.53	3.53	3.53	3.53	3.53	3.53
3.54	3.54	3.54	3.54	3.54	3.54	3.54	3.54	3.54
3.55	3.55	3.55	3.55	3.55	3.55	3.55	3.55	3.55
3.56	3.56	3.56	3.56	3.56	3.56	3.56	3.56	3.56
3.57	3.57	3.57	3.57	3.57	3.57	3.57	3.57	3.57
3.58	3.58	3.58	3.58	3.58	3.58	3.58	3.58	3.58
3.59	3.59	3.59	3.59	3.59	3.59	3.59	3.59	3.59
3.6	3.6	3.6	3.6	3.6	3.6	3.6	3.6	3.6
3.61	3.61	3.61	3.61	3.61	3.61	3.61	3.61	3.61
3.62	3.62	3.62	3.62	3.62	3.62	3.62	3.62	3.62
3.63	3.63	3.63	3.63	3.63	3.63	3.63	3.63	3.63
3.64	3.64	3.64	3.64	3.64	3.64	3.64	3.64	3.64
3.65	3.65	3.65	3.65	3.65	3.65	3.65	3.65	3.65
3.66	3.66	3.66	3.66	3.66	3.66	3.66	3.66	3.66
3.67	3.67	3.67	3.67	3.67	3.67	3.67	3.67	3.67
3.68	3.68	3.68	3.68	3.68	3.68	3.68	3.68	3.68
3.69	3.69	3.69	3.69	3.69	3.69	3.69	3.69	3.69
3.7	3.7	3.7	3.7	3.7	3.7	3.7	3.7	3.7
3.71	3.71	3.71	3.71	3.71	3.71	3.71	3.71	3.71
3.72	3.72	3.72	3.72	3.72	3.72	3.72	3.72	3.72
3.73	3.73	3.73	3.73	3.73	3.73	3.73	3.73	3.73
3.74	3.74	3.74	3.74	3.74	3.74	3.74	3.74	3.74
3.75	3.75	3.75	3.75	3.75	3.75	3.75	3.75	3.75
3.76	3.76	3.76	3.76	3.76	3.76	3.76	3.76	3.76
3.77	3.77	3.77	3.77	3.77	3.77	3.77	3.77	3.77
3.78	3.78	3.78	3.78	3.78	3.78	3.78	3.78	3.78
3.79	3.79	3.79	3.79	3.79	3.79	3.79	3.79	3.79
3.8	3.8	3.8	3.8	3.8	3.8	3.8	3.8	3.8
3.81	3.81	3.81	3.81	3.81	3.81	3.81	3.81	3.81
3.82	3.82	3.82	3.82	3.82	3.82	3.82	3.82	3.82
3.83	3.83	3.83	3.83	3.83	3.83	3.83	3.83	3.83
3.84	3.84	3.84	3.84	3.84	3.84	3.84	3.84	3.84
3.85	3.85	3.85	3.85	3.85	3.85	3.85	3.85	3.85
3.86	3.86	3.86	3.86	3.86	3.86	3.86	3.86	3.86
3.87	3.87	3.87	3.87	3.87	3.87	3.87	3.87	3.87
3.88	3.88	3.88	3.88	3.88	3.88	3.88	3.88	3.88
3.89	3.89	3.89	3.89	3.89	3.89	3.89	3.89	3.89
3.9	3.9	3.9	3.9	3.9	3.9	3.9	3.9	3.9
3.91	3.91	3.91	3.91	3.91	3.91	3.91	3.91	3.91
3.92	3.92	3.92	3.92	3.92	3.92	3.92	3.92	3.92
3.93	3.93	3.93	3.93	3.93	3.93	3.93	3.93	3.93
3.94	3.94	3.94	3.94	3.94	3.94	3.94	3.94	3.94
3.95	3.95	3.95	3.95	3.95	3.95	3.95	3.95	3.95
3.96	3.96	3.96	3.96	3.96	3.96	3.96	3.96	3.96
3.97	3.97	3.97	3.97	3.97	3.97	3.97	3.97	3.97
3.98	3.98	3.98	3.98	3.98	3.98	3.98	3.98	3.98
3.99	3.99	3.99	3.99	3.99	3.99	3.99	3.99	3.99

4	4	4	4	4	4	4	4	4
4.01	4.01	4.01	4.01	4.01	4.01	4.01	4.01	4.01
4.02	4.02	4.02	4.02	4.02	4.02	4.02	4.02	4.02
4.03	4.03	4.03	4.03	4.03	4.03	4.03	4.03	4.03
4.04	4.04	4.04	4.04	4.04	4.04	4.04	4.04	4.04
4.05	4.05	4.05	4.05	4.05	4.05	4.05	4.05	4.05
4.06	4.06	4.06	4.06	4.06	4.06	4.06	4.06	4.06
4.07	4.07	4.07	4.07	4.07	4.07	4.07	4.07	4.07
4.08	4.08	4.08	4.08	4.08	4.08	4.08	4.08	4.08
4.09	4.09	4.09	4.09	4.09	4.09	4.09	4.09	4.09
4.1	4.1	4.1	4.1	4.1	4.1	4.1	4.1	4.1
4.11	4.11	4.11	4.11	4.11	4.11	4.11	4.11	4.11
4.12	4.12	4.12	4.12	4.12	4.12	4.12	4.12	4.12
4.13	4.13	4.13	4.13	4.13	4.13	4.13	4.13	4.13
4.14	4.14	4.14	4.14	4.14	4.14	4.14	4.14	4.14
4.15	4.15	4.15	4.15	4.15	4.15	4.15	4.15	4.15
4.16	4.16	4.16	4.16	4.16	4.16	4.16	4.16	4.16
4.17	4.17	4.17	4.17	4.17	4.17	4.17	4.17	4.17
4.18	4.18	4.18	4.18	4.18	4.18	4.18	4.18	4.18
4.19	4.19	4.19	4.19	4.19	4.19	4.19	4.19	4.19
4.2	4.2	4.2	4.2	4.2	4.2	4.2	4.2	4.2
4.21	4.21	4.21	4.21	4.21	4.21	4.21	4.21	4.21
4.22	4.22	4.22	4.22	4.22	4.22	4.22	4.22	4.22
4.23	4.23	4.23	4.23	4.23	4.23	4.23	4.23	4.23
4.24	4.24	4.24	4.24	4.24	4.24	4.24	4.24	4.24
4.25	4.25	4.25	4.25	4.25	4.25	4.25	4.25	4.25
4.26	4.26	4.26	4.26	4.26	4.26	4.26	4.26	4.26
4.27	4.27	4.27	4.27	4.27	4.27	4.27	4.27	4.27
4.28	4.28	4.28	4.28	4.28	4.28	4.28	4.28	4.28
4.29	4.29	4.29	4.29	4.29	4.29	4.29	4.29	4.29
4.3	4.3	4.3	4.3	4.3	4.3	4.3	4.3	4.3
4.31	4.31	4.31	4.31	4.31	4.31	4.31	4.31	4.31
4.32	4.32	4.32	4.32	4.32	4.32	4.32	4.32	4.32
4.33	4.33	4.33	4.33	4.33	4.33	4.33	4.33	4.33
4.34	4.34	4.34	4.34	4.34	4.34	4.34	4.34	4.34
4.35	4.35	4.35	4.35	4.35	4.35	4.35	4.35	4.35
4.36	4.36	4.36	4.36	4.36	4.36	4.36	4.36	4.36
4.37	4.37	4.37	4.37	4.37	4.37	4.37	4.37	4.37
4.38	4.38	4.38	4.38	4.38	4.38	4.38	4.38	4.38
4.39	4.39	4.39	4.39	4.39	4.39	4.39	4.39	4.39
4.4	4.4	4.4	4.4	4.4	4.4	4.4	4.4	4.4
4.41	4.41	4.41	4.41	4.41	4.41	4.41	4.41	4.41
4.42	4.42	4.42	4.42	4.42	4.42	4.42	4.42	4.42
4.43	4.43	4.43	4.43	4.43	4.43	4.43	4.43	4.43
4.44	4.44	4.44	4.44	4.44	4.44	4.44	4.44	4.44
4.45	4.45	4.45	4.45	4.45	4.45	4.45	4.45	4.45
4.46	4.46	4.46	4.46	4.46	4.46	4.46	4.46	4.46
4.47	4.47	4.47	4.47	4.47	4.47	4.47	4.47	4.47
4.48	4.48	4.48	4.48	4.48	4.48	4.48	4.48	4.48
4.49	4.49	4.49	4.49	4.49	4.49	4.49	4.49	4.49

4.5	4.5	4.5	4.5	4.5	4.5	4.5	4.5	4.5
4.51	4.51	4.51	4.51	4.51	4.51	4.51	4.51	4.51
4.52	4.52	4.52	4.52	4.52	4.52	4.52	4.52	4.52
4.53	4.53	4.53	4.53	4.53	4.53	4.53	4.53	4.53
4.54	4.54	4.54	4.54	4.54	4.54	4.54	4.54	4.54
4.55	4.55	4.55	4.55	4.55	4.55	4.55	4.55	4.55
4.56	4.56	4.56	4.56	4.56	4.56	4.56	4.56	4.56
4.57	4.57	4.57	4.57	4.57	4.57	4.57	4.57	4.57
4.58	4.58	4.58	4.58	4.58	4.58	4.58	4.58	4.58
4.59	4.59	4.59	4.59	4.59	4.59	4.59	4.59	4.59
4.6	4.6	4.6	4.6	4.6	4.6	4.6	4.6	4.6
4.61	4.61	4.61	4.61	4.61	4.61	4.61	4.61	4.61
4.62	4.62	4.62	4.62	4.62	4.62	4.62	4.62	4.62
4.63	4.63	4.63	4.63	4.63	4.63	4.63	4.63	4.63
4.64	4.64	4.64	4.64	4.64	4.64	4.64	4.64	4.64
4.65	4.65	4.65	4.65	4.65	4.65	4.65	4.65	4.65
4.66	4.66	4.66	4.66	4.66	4.66	4.66	4.66	4.66
4.67	4.67	4.67	4.67	4.67	4.67	4.67	4.67	4.67
4.68	4.68	4.68	4.68	4.68	4.68	4.68	4.68	4.68
4.69	4.69	4.69	4.69	4.69	4.69	4.69	4.69	4.69
4.7	4.7	4.7	4.7	4.7	4.7	4.7	4.7	4.7
4.71	4.71	4.71	4.71	4.71	4.71	4.71	4.71	4.71
4.72	4.72	4.72	4.72	4.72	4.72	4.72	4.72	4.72
4.73	4.73	4.73	4.73	4.73	4.73	4.73	4.73	4.73
4.74	4.74	4.74	4.74	4.74	4.74	4.74	4.74	4.74
4.75	4.75	4.75	4.75	4.75	4.75	4.75	4.75	4.75
4.76	4.76	4.76	4.76	4.76	4.76	4.76	4.76	4.76
4.77	4.77	4.77	4.77	4.77	4.77	4.77	4.77	4.77
4.78	4.78	4.78	4.78	4.78	4.78	4.78	4.78	4.78
4.79	4.79	4.79	4.79	4.79	4.79	4.79	4.79	4.79
4.8	4.8	4.8	4.8	4.8	4.8	4.8	4.8	4.8
4.81	4.81	4.81	4.81	4.81	4.81	4.81	4.81	4.81
4.82	4.82	4.82	4.82	4.82	4.82	4.82	4.82	4.82
4.83	4.83	4.83	4.83	4.83	4.83	4.83	4.83	4.83
4.84	4.84	4.84	4.84	4.84	4.84	4.84	4.84	4.84
4.85	4.85	4.85	4.85	4.85	4.85	4.85	4.85	4.85
4.86	4.86	4.86	4.86	4.86	4.86	4.86	4.86	4.86
4.87	4.87	4.87	4.87	4.87	4.87	4.87	4.87	4.87
4.88	4.88	4.88	4.88	4.88	4.88	4.88	4.88	4.88
4.89	4.89	4.89	4.89	4.89	4.89	4.89	4.89	4.89
4.9	4.9	4.9	4.9	4.9	4.9	4.9	4.9	4.9
4.91	4.91	4.91	4.91	4.91	4.91	4.91	4.91	4.91
4.92	4.92	4.92	4.92	4.92	4.92	4.92	4.92	4.92
4.93	4.93	4.93	4.93	4.93	4.93	4.93	4.93	4.93
4.94	4.94	4.94	4.94	4.94	4.94	4.94	4.94	4.94
4.95	4.95	4.95	4.95	4.95	4.95	4.95	4.95	4.95
4.96	4.96	4.96	4.96	4.96	4.96	4.96	4.96	4.96
4.97	4.97	4.97	4.97	4.97	4.97	4.97	4.97	4.97
4.98	4.98	4.98	4.98	4.98	4.98	4.98	4.98	4.98
4.99	4.99	4.99	4.99	4.99	4.99	4.99	4.99	4.99

5	5	5	5	5	5	5	5	5
5.01	5.01	5.01	5.01	5.01	5.01	5.01	5.01	5.01
5.02	5.02	5.02	5.02	5.02	5.02	5.02	5.02	5.02
5.03	5.03	5.03	5.03	5.03	5.03	5.03	5.03	5.03
5.04	5.04	5.04	5.04	5.04	5.04	5.04	5.04	5.04
5.05	5.05	5.05	5.05	5.05	5.05	5.05	5.05	5.05
5.06	5.06	5.06	5.06	5.06	5.06	5.06	5.06	5.06
5.07	5.07	5.07	5.07	5.07	5.07	5.07	5.07	5.07
5.08	5.08	5.08	5.08	5.08	5.08	5.08	5.08	5.08
5.09	5.09	5.09	5.09	5.09	5.09	5.09	5.09	5.09
5.1	5.1	5.1	5.1	5.1	5.1	5.1	5.1	5.1
5.11	5.11	5.11	5.11	5.11	5.11	5.11	5.11	5.11
5.12	5.12	5.12	5.12	5.12	5.12	5.12	5.12	5.12
5.13	5.13	5.13	5.13	5.13	5.13	5.13	5.13	5.13
5.14	5.14	5.14	5.14	5.14	5.14	5.14	5.14	5.14
5.15	5.15	5.15	5.15	5.15	5.15	5.15	5.15	5.15
5.16	5.16	5.16	5.16	5.16	5.16	5.16	5.16	5.16
5.17	5.17	5.17	5.17	5.17	5.17	5.17	5.17	5.17
5.18	5.18	5.18	5.18	5.18	5.18	5.18	5.18	5.18
5.19	5.19	5.19	5.19	5.19	5.19	5.19	5.19	5.19
5.2	5.2	5.2	5.2	5.2	5.2	5.2	5.2	5.2
5.21	5.21	5.21	5.21	5.21	5.21	5.21	5.21	5.21
5.22	5.22	5.22	5.22	5.22	5.22	5.22	5.22	5.22
5.23	5.23	5.23	5.23	5.23	5.23	5.23	5.23	5.23
5.24	5.24	5.24	5.24	5.24	5.24	5.24	5.24	5.24
5.25	5.25	5.25	5.25	5.25	5.25	5.25	5.25	5.25
5.26	5.26	5.26	5.26	5.26	5.26	5.26	5.26	5.26
5.27	5.27	5.27	5.27	5.27	5.27	5.27	5.27	5.27
5.28	5.28	5.28	5.28	5.28	5.28	5.28	5.28	5.28
5.29	5.29	5.29	5.29	5.29	5.29	5.29	5.29	5.29
5.3	5.3	5.3	5.3	5.3	5.3	5.3	5.3	5.3
5.31	5.31	5.31	5.31	5.31	5.31	5.31	5.31	5.31
5.32	5.32	5.32	5.32	5.32	5.32	5.32	5.32	5.32
5.33	5.33	5.33	5.33	5.33	5.33	5.33	5.33	5.33
5.34	5.34	5.34	5.34	5.34	5.34	5.34	5.34	5.34
5.35	5.35	5.35	5.35	5.35	5.35	5.35	5.35	5.35
5.36	5.36	5.36	5.36	5.36	5.36	5.36	5.36	5.36
5.37	5.37	5.37	5.37	5.37	5.37	5.37	5.37	5.37
5.38	5.38	5.38	5.38	5.38	5.38	5.38	5.38	5.38
5.39	5.39	5.39	5.39	5.39	5.39	5.39	5.39	5.39
5.4	5.4	5.4	5.4	5.4	5.4	5.4	5.4	5.4
5.41	5.41	5.41	5.41	5.41	5.41	5.41	5.41	5.41
5.42	5.42	5.42	5.42	5.42	5.42	5.42	5.42	5.42
5.43	5.43	5.43	5.43	5.43	5.43	5.43	5.43	5.43
5.44	5.44	5.44	5.44	5.44	5.44	5.44	5.44	5.44
5.45	5.45	5.45	5.45	5.45	5.45	5.45	5.45	5.45
5.46	5.46	5.46	5.46	5.46	5.46	5.46	5.46	5.46
5.47	5.47	5.47	5.47	5.47	5.47	5.47	5.47	5.47
5.48	5.48	5.48	5.48	5.48	5.48	5.48	5.48	5.48
5.49	5.49	5.49	5.49	5.49	5.49	5.49	5.49	5.49

5.5	5.5	5.5	5.5	5.5	5.5	5.5	5.5	5.5
5.51	5.51	5.51	5.51	5.51	5.51	5.51	5.51	5.51
5.52	5.52	5.52	5.52	5.52	5.52	5.52	5.52	5.52
5.53	5.53	5.53	5.53	5.53	5.53	5.53	5.53	5.53
5.54	5.54	5.54	5.54	5.54	5.54	5.54	5.54	5.54
5.55	5.55	5.55	5.55	5.55	5.55	5.55	5.55	5.55
5.56	5.56	5.56	5.56	5.56	5.56	5.56	5.56	5.56
5.57	5.57	5.57	5.57	5.57	5.57	5.57	5.57	5.57
5.58	5.58	5.58	5.58	5.58	5.58	5.58	5.58	5.58
5.59	5.59	5.59	5.59	5.59	5.59	5.59	5.59	5.59
5.6	5.6	5.6	5.6	5.6	5.6	5.6	5.6	5.6
5.61	5.61	5.61	5.61	5.61	5.61	5.61	5.61	5.61
5.62	5.62	5.62	5.62	5.62	5.62	5.62	5.62	5.62
5.63	5.63	5.63	5.63	5.63	5.63	5.63	5.63	5.63
5.64	5.64	5.64	5.64	5.64	5.64	5.64	5.64	5.64
5.65	5.65	5.65	5.65	5.65	5.65	5.65	5.65	5.65
5.66	5.66	5.66	5.66	5.66	5.66	5.66	5.66	5.66
5.67	5.67	5.67	5.67	5.67	5.67	5.67	5.67	5.67
5.68	5.68	5.68	5.68	5.68	5.68	5.68	5.68	5.68
5.69	5.69	5.69	5.69	5.69	5.69	5.69	5.69	5.69
5.7	5.7	5.7	5.7	5.7	5.7	5.7	5.7	5.7
5.71	5.71	5.71	5.71	5.71	5.71	5.71	5.71	5.71
5.72	5.72	5.72	5.72	5.72	5.72	5.72	5.72	5.72
5.73	5.73	5.73	5.73	5.73	5.73	5.73	5.73	5.73
5.74	5.74	5.74	5.74	5.74	5.74	5.74	5.74	5.74
5.75	5.75	5.75	5.75	5.75	5.75	5.75	5.75	5.75
5.76	5.76	5.76	5.76	5.76	5.76	5.76	5.76	5.76
5.77	5.77	5.77	5.77	5.77	5.77	5.77	5.77	5.77
5.78	5.78	5.78	5.78	5.78	5.78	5.78	5.78	5.78
5.79	5.79	5.79	5.79	5.79	5.79	5.79	5.79	5.79
5.8	5.8	5.8	5.8	5.8	5.8	5.8	5.8	5.8
5.81	5.81	5.81	5.81	5.81	5.81	5.81	5.81	5.81
5.82	5.82	5.82	5.82	5.82	5.82	5.82	5.82	5.82
5.83	5.83	5.83	5.83	5.83	5.83	5.83	5.83	5.83
5.84	5.84	5.84	5.84	5.84	5.84	5.84	5.84	5.84
5.85	5.85	5.85	5.85	5.85	5.85	5.85	5.85	5.85
5.86	5.86	5.86	5.86	5.86	5.86	5.86	5.86	5.86
5.87	5.87	5.87	5.87	5.87	5.87	5.87	5.87	5.87
5.88	5.88	5.88	5.88	5.88	5.88	5.88	5.88	5.88
5.89	5.89	5.89	5.89	5.89	5.89	5.89	5.89	5.89
5.9	5.9	5.9	5.9	5.9	5.9	5.9	5.9	5.9
5.91	5.91	5.91	5.91	5.91	5.91	5.91	5.91	5.91
5.92	5.92	5.92	5.92	5.92	5.92	5.92	5.92	5.92
5.93	5.93	5.93	5.93	5.93	5.93	5.93	5.93	5.93
5.94	5.94	5.94	5.94	5.94	5.94	5.94	5.94	5.94
5.95	5.95	5.95	5.95	5.95	5.95	5.95	5.95	5.95
5.96	5.96	5.96	5.96	5.96	5.96	5.96	5.96	5.96
5.97	5.97	5.97	5.97	5.97	5.97	5.97	5.97	5.97
5.98	5.98	5.98	5.98	5.98	5.98	5.98	5.98	5.98
5.99	5.99	5.99	5.99	5.99	5.99	5.99	5.99	5.99

6	6	6	6	6	6	6	6	6
6.01	6.01	6.01	6.01	6.01	6.01	6.01	6.01	6.01
6.02	6.02	6.02	6.02	6.02	6.02	6.02	6.02	6.02
6.03	6.03	6.03	6.03	6.03	6.03	6.03	6.03	6.03
6.04	6.04	6.04	6.04	6.04	6.04	6.04	6.04	6.04
6.05	6.05	6.05	6.05	6.05	6.05	6.05	6.05	6.05
6.06	6.06	6.06	6.06	6.06	6.06	6.06	6.06	6.06
6.07	6.07	6.07	6.07	6.07	6.07	6.07	6.07	6.07
6.08	6.08	6.08	6.08	6.08	6.08	6.08	6.08	6.08
6.09	6.09	6.09	6.09	6.09	6.09	6.09	6.09	6.09
6.1	6.1	6.1	6.1	6.1	6.1	6.1	6.1	6.1
6.11	6.11	6.11	6.11	6.11	6.11	6.11	6.11	6.11
6.12	6.12	6.12	6.12	6.12	6.12	6.12	6.12	6.12
6.13	6.13	6.13	6.13	6.13	6.13	6.13	6.13	6.13
6.14	6.14	6.14	6.14	6.14	6.14	6.14	6.14	6.14
6.15	6.15	6.15	6.15	6.15	6.15	6.15	6.15	6.15
6.16	6.16	6.16	6.16	6.16	6.16	6.16	6.16	6.16
6.17	6.17	6.17	6.17	6.17	6.17	6.17	6.17	6.17
6.18	6.18	6.18	6.18	6.18	6.18	6.18	6.18	6.18
6.19	6.19	6.19	6.19	6.19	6.19	6.19	6.19	6.19
6.2	6.2	6.2	6.2	6.2	6.2	6.2	6.2	6.2
6.21	6.21	6.21	6.21	6.21	6.21	6.21	6.21	6.21
6.22	6.22	6.22	6.22	6.22	6.22	6.22	6.22	6.22
6.23	6.23	6.23	6.23	6.23	6.23	6.23	6.23	6.23
6.24	6.24	6.24	6.24	6.24	6.24	6.24	6.24	6.24
6.25	6.25	6.25	6.25	6.25	6.25	6.25	6.25	6.25
6.26	6.26	6.26	6.26	6.26	6.26	6.26	6.26	6.26
6.27	6.27	6.27	6.27	6.27	6.27	6.27	6.27	6.27
6.28	6.28	6.28	6.28	6.28	6.28	6.28	6.28	6.28
6.29	6.29	6.29	6.29	6.29	6.29	6.29	6.29	6.29
6.3	6.3	6.3	6.3	6.3	6.3	6.3	6.3	6.3
6.31	6.31	6.31	6.31	6.31	6.31	6.31	6.31	6.31
6.32	6.32	6.32	6.32	6.32	6.32	6.32	6.32	6.32
6.33	6.33	6.33	6.33	6.33	6.33	6.33	6.33	6.33
6.34	6.34	6.34	6.34	6.34	6.34	6.34	6.34	6.34
6.35	6.35	6.35	6.35	6.35	6.35	6.35	6.35	6.35
6.36	6.36	6.36	6.36	6.36	6.36	6.36	6.36	6.36
6.37	6.37	6.37	6.37	6.37	6.37	6.37	6.37	6.37
6.38	6.38	6.38	6.38	6.38	6.38	6.38	6.38	6.38
6.39	6.39	6.39	6.39	6.39	6.39	6.39	6.39	6.39
6.4	6.4	6.4	6.4	6.4	6.4	6.4	6.4	6.4
6.41	6.41	6.41	6.41	6.41	6.41	6.41	6.41	6.41
6.42	6.42	6.42	6.42	6.42	6.42	6.42	6.42	6.42
6.43	6.43	6.43	6.43	6.43	6.43	6.43	6.43	6.43
6.44	6.44	6.44	6.44	6.44	6.44	6.44	6.44	6.44
6.45	6.45	6.45	6.45	6.45	6.45	6.45	6.45	6.45
6.46	6.46	6.46	6.46	6.46	6.46	6.46	6.46	6.46
6.47	6.47	6.47	6.47	6.47	6.47	6.47	6.47	6.47
6.48	6.48	6.48	6.48	6.48	6.48	6.48	6.48	6.48
6.49	6.49	6.49	6.49	6.49	6.49	6.49	6.49	6.49

6.5	6.5	6.5	6.5	6.5	6.5	6.5	6.5	6.5
6.51	6.51	6.51	6.51	6.51	6.51	6.51	6.51	6.51
6.52	6.52	6.52	6.52	6.52	6.52	6.52	6.52	6.52
6.53	6.53	6.53	6.53	6.53	6.53	6.53	6.53	6.53
6.54	6.54	6.54	6.54	6.54	6.54	6.54	6.54	6.54
6.55	6.55	6.55	6.55	6.55	6.55	6.55	6.55	6.55
6.56	6.56	6.56	6.56	6.56	6.56	6.56	6.56	6.56
6.57	6.57	6.57	6.57	6.57	6.57	6.57	6.57	6.57
6.58	6.58	6.58	6.58	6.58	6.58	6.58	6.58	6.58
6.59	6.59	6.59	6.59	6.59	6.59	6.59	6.59	6.59
6.6	6.6	6.6	6.6	6.6	6.6	6.6	6.6	6.6
6.61	6.61	6.61	6.61	6.61	6.61	6.61	6.61	6.61
6.62	6.62	6.62	6.62	6.62	6.62	6.62	6.62	6.62
6.63	6.63	6.63	6.63	6.63	6.63	6.63	6.63	6.63
6.64	6.64	6.64	6.64	6.64	6.64	6.64	6.64	6.64
6.65	6.65	6.65	6.65	6.65	6.65	6.65	6.65	6.65
6.66	6.66	6.66	6.66	6.66	6.66	6.66	6.66	6.66
6.67	6.67	6.67	6.67	6.67	6.67	6.67	6.67	6.67
6.68	6.68	6.68	6.68	6.68	6.68	6.68	6.68	6.68
6.69	6.69	6.69	6.69	6.69	6.69	6.69	6.69	6.69
6.7	6.7	6.7	6.7	6.7	6.7	6.7	6.7	6.7
6.71	6.71	6.71	6.71	6.71	6.71	6.71	6.71	6.71
6.72	6.72	6.72	6.72	6.72	6.72	6.72	6.72	6.72
6.73	6.73	6.73	6.73	6.73	6.73	6.73	6.73	6.73
6.74	6.74	6.74	6.74	6.74	6.74	6.74	6.74	6.74
6.75	6.75	6.75	6.75	6.75	6.75	6.75	6.75	6.75
6.76	6.76	6.76	6.76	6.76	6.76	6.76	6.76	6.76
6.77	6.77	6.77	6.77	6.77	6.77	6.77	6.77	6.77
6.78	6.78	6.78	6.78	6.78	6.78	6.78	6.78	6.78
6.79	6.79	6.79	6.79	6.79	6.79	6.79	6.79	6.79
6.8	6.8	6.8	6.8	6.8	6.8	6.8	6.8	6.8
6.81	6.81	6.81	6.81	6.81	6.81	6.81	6.81	6.81
6.82	6.82	6.82	6.82	6.82	6.82	6.82	6.82	6.82
6.83	6.83	6.83	6.83	6.83	6.83	6.83	6.83	6.83
6.84	6.84	6.84	6.84	6.84	6.84	6.84	6.84	6.84
6.85	6.85	6.85	6.85	6.85	6.85	6.85	6.85	6.85
6.86	6.86	6.86	6.86	6.86	6.86	6.86	6.86	6.86
6.87	6.87	6.87	6.87	6.87	6.87	6.87	6.87	6.87
6.88	6.88	6.88	6.88	6.88	6.88	6.88	6.88	6.88
6.89	6.89	6.89	6.89	6.89	6.89	6.89	6.89	6.89
6.9	6.9	6.9	6.9	6.9	6.9	6.9	6.9	6.9
6.91	6.91	6.91	6.91	6.91	6.91	6.91	6.91	6.91
6.92	6.92	6.92	6.92	6.92	6.92	6.92	6.92	6.92
6.93	6.93	6.93	6.93	6.93	6.93	6.93	6.93	6.93
6.94	6.94	6.94	6.94	6.94	6.94	6.94	6.94	6.94
6.95	6.95	6.95	6.95	6.95	6.95	6.95	6.95	6.95
6.96	6.96	6.96	6.96	6.96	6.96	6.96	6.96	6.96
6.97	6.97	6.97	6.97	6.97	6.97	6.97	6.97	6.97
6.98	6.98	6.98	6.98	6.98	6.98	6.98	6.98	6.98
6.99	6.99	6.99	6.99	6.99	6.99	6.99	6.99	6.99

7	7	7	7	7	7	7	7	7
7.01	7.01	7.01	7.01	7.01	7.01	7.01	7.01	7.01
7.02	7.02	7.02	7.02	7.02	7.02	7.02	7.02	7.02
7.03	7.03	7.03	7.03	7.03	7.03	7.03	7.03	7.03
7.04	7.04	7.04	7.04	7.04	7.04	7.04	7.04	7.04
7.05	7.05	7.05	7.05	7.05	7.05	7.05	7.05	7.05
7.06	7.06	7.06	7.06	7.06	7.06	7.06	7.06	7.06
7.07	7.07	7.07	7.07	7.07	7.07	7.07	7.07	7.07
7.08	7.08	7.08	7.08	7.08	7.08	7.08	7.08	7.08
7.09	7.09	7.09	7.09	7.09	7.09	7.09	7.09	7.09
7.1	7.1	7.1	7.1	7.1	7.1	7.1	7.1	7.1
7.11	7.11	7.11	7.11	7.11	7.11	7.11	7.11	7.11
7.12	7.12	7.12	7.12	7.12	7.12	7.12	7.12	7.12
7.13	7.13	7.13	7.13	7.13	7.13	7.13	7.13	7.13
7.14	7.14	7.14	7.14	7.14	7.14	7.14	7.14	7.14
7.15	7.15	7.15	7.15	7.15	7.15	7.15	7.15	7.15
7.16	7.16	7.16	7.16	7.16	7.16	7.16	7.16	7.16
7.17	7.17	7.17	7.17	7.17	7.17	7.17	7.17	7.17
7.18	7.18	7.18	7.18	7.18	7.18	7.18	7.18	7.18
7.19	7.19	7.19	7.19	7.19	7.19	7.19	7.19	7.19
7.2	7.2	7.2	7.2	7.2	7.2	7.2	7.2	7.2
7.21	7.21	7.21	7.21	7.21	7.21	7.21	7.21	7.21
7.22	7.22	7.22	7.22	7.22	7.22	7.22	7.22	7.22
7.23	7.23	7.23	7.23	7.23	7.23	7.23	7.23	7.23
7.24	7.24	7.24	7.24	7.24	7.24	7.24	7.24	7.24
7.25	7.25	7.25	7.25	7.25	7.25	7.25	7.25	7.25
7.26	7.26	7.26	7.26	7.26	7.26	7.26	7.26	7.26
7.27	7.27	7.27	7.27	7.27	7.27	7.27	7.27	7.27
7.28	7.28	7.28	7.28	7.28	7.28	7.28	7.28	7.28
7.29	7.29	7.29	7.29	7.29	7.29	7.29	7.29	7.29
7.3	7.3	7.3	7.3	7.3	7.3	7.3	7.3	7.3
7.31	7.31	7.31	7.31	7.31	7.31	7.31	7.31	7.31
7.32	7.32	7.32	7.32	7.32	7.32	7.32	7.32	7.32
7.33	7.33	7.33	7.33	7.33	7.33	7.33	7.33	7.33
7.34	7.34	7.34	7.34	7.34	7.34	7.34	7.34	7.34
7.35	7.35	7.35	7.35	7.35	7.35	7.35	7.35	7.35
7.36	7.36	7.36	7.36	7.36	7.36	7.36	7.36	7.36
7.37	7.37	7.37	7.37	7.37	7.37	7.37	7.37	7.37
7.38	7.38	7.38	7.38	7.38	7.38	7.38	7.38	7.38
7.39	7.39	7.39	7.39	7.39	7.39	7.39	7.39	7.39
7.4	7.4	7.4	7.4	7.4	7.4	7.4	7.4	7.4
7.41	7.41	7.41	7.41	7.41	7.41	7.41	7.41	7.41
7.42	7.42	7.42	7.42	7.42	7.42	7.42	7.42	7.42
7.43	7.43	7.43	7.43	7.43	7.43	7.43	7.43	7.43
7.44	7.44	7.44	7.44	7.44	7.44	7.44	7.44	7.44
7.45	7.45	7.45	7.45	7.45	7.45	7.45	7.45	7.45
7.46	7.46	7.46	7.46	7.46	7.46	7.46	7.46	7.46
7.47	7.47	7.47	7.47	7.47	7.47	7.47	7.47	7.47
7.48	7.48	7.48	7.48	7.48	7.48	7.48	7.48	7.48
7.49	7.49	7.49	7.49	7.49	7.49	7.49	7.49	7.49

7.5	7.5	7.5	7.5	7.5	7.5	7.5	7.5	7.5
7.51	7.51	7.51	7.51	7.51	7.51	7.51	7.51	7.51
7.52	7.52	7.52	7.52	7.52	7.52	7.52	7.52	7.52
7.53	7.53	7.53	7.53	7.53	7.53	7.53	7.53	7.53
7.54	7.54	7.54	7.54	7.54	7.54	7.54	7.54	7.54
7.55	7.55	7.55	7.55	7.55	7.55	7.55	7.55	7.55
7.56	7.56	7.56	7.56	7.56	7.56	7.56	7.56	7.56
7.57	7.57	7.57	7.57	7.57	7.57	7.57	7.57	7.57
7.58	7.58	7.58	7.58	7.58	7.58	7.58	7.58	7.58
7.59	7.59	7.59	7.59	7.59	7.59	7.59	7.59	7.59
7.6	7.6	7.6	7.6	7.6	7.6	7.6	7.6	7.6
7.61	7.61	7.61	7.61	7.61	7.61	7.61	7.61	7.61
7.62	7.62	7.62	7.62	7.62	7.62	7.62	7.62	7.62
7.63	7.63	7.63	7.63	7.63	7.63	7.63	7.63	7.63
7.64	7.64	7.64	7.64	7.64	7.64	7.64	7.64	7.64
7.65	7.65	7.65	7.65	7.65	7.65	7.65	7.65	7.65
7.66	7.66	7.66	7.66	7.66	7.66	7.66	7.66	7.66
7.67	7.67	7.67	7.67	7.67	7.67	7.67	7.67	7.67
7.68	7.68	7.68	7.68	7.68	7.68	7.68	7.68	7.68
7.69	7.69	7.69	7.69	7.69	7.69	7.69	7.69	7.69
7.7	7.7	7.7	7.7	7.7	7.7	7.7	7.7	7.7
7.71	7.71	7.71	7.71	7.71	7.71	7.71	7.71	7.71
7.72	7.72	7.72	7.72	7.72	7.72	7.72	7.72	7.72
7.73	7.73	7.73	7.73	7.73	7.73	7.73	7.73	7.73
7.74	7.74	7.74	7.74	7.74	7.74	7.74	7.74	7.74
7.75	7.75	7.75	7.75	7.75	7.75	7.75	7.75	7.75
7.76	7.76	7.76	7.76	7.76	7.76	7.76	7.76	7.76
7.77	7.77	7.77	7.77	7.77	7.77	7.77	7.77	7.77
7.78	7.78	7.78	7.78	7.78	7.78	7.78	7.78	7.78
7.79	7.79	7.79	7.79	7.79	7.79	7.79	7.79	7.79
7.8	7.8	7.8	7.8	7.8	7.8	7.8	7.8	7.8
7.81	7.81	7.81	7.81	7.81	7.81	7.81	7.81	7.81
7.82	7.82	7.82	7.82	7.82	7.82	7.82	7.82	7.82
7.83	7.83	7.83	7.83	7.83	7.83	7.83	7.83	7.83
7.84	7.84	7.84	7.84	7.84	7.84	7.84	7.84	7.84
7.85	7.85	7.85	7.85	7.85	7.85	7.85	7.85	7.85
7.86	7.86	7.86	7.86	7.86	7.86	7.86	7.86	7.86
7.87	7.87	7.87	7.87	7.87	7.87	7.87	7.87	7.87
7.88	7.88	7.88	7.88	7.88	7.88	7.88	7.88	7.88
7.89	7.89	7.89	7.89	7.89	7.89	7.89	7.89	7.89
7.9	7.9	7.9	7.9	7.9	7.9	7.9	7.9	7.9
7.91	7.91	7.91	7.91	7.91	7.91	7.91	7.91	7.91
7.92	7.92	7.92	7.92	7.92	7.92	7.92	7.92	7.92
7.93	7.93	7.93	7.93	7.93	7.93	7.93	7.93	7.93
7.94	7.94	7.94	7.94	7.94	7.94	7.94	7.94	7.94
7.95	7.95	7.95	7.95	7.95	7.95	7.95	7.95	7.95
7.96	7.96	7.96	7.96	7.96	7.96	7.96	7.96	7.96
7.97	7.97	7.97	7.97	7.97	7.97	7.97	7.97	7.97
7.98	7.98	7.98	7.98	7.98	7.98	7.98	7.98	7.98
7.99	7.99	7.99	7.99	7.99	7.99	7.99	7.99	7.99

8	8	8	8	8	8	8	8	8
8.01	8.01	8.01	8.01	8.01	8.01	8.01	8.01	8.01
8.02	8.02	8.02	8.02	8.02	8.02	8.02	8.02	8.02
8.03	8.03	8.03	8.03	8.03	8.03	8.03	8.03	8.03
8.04	8.04	8.04	8.04	8.04	8.04	8.04	8.04	8.04
8.05	8.05	8.05	8.05	8.05	8.05	8.05	8.05	8.05
8.06	8.06	8.06	8.06	8.06	8.06	8.06	8.06	8.06
8.07	8.07	8.07	8.07	8.07	8.07	8.07	8.07	8.07
8.08	8.08	8.08	8.08	8.08	8.08	8.08	8.08	8.08
8.09	8.09	8.09	8.09	8.09	8.09	8.09	8.09	8.09
8.1	8.1	8.1	8.1	8.1	8.1	8.1	8.1	8.1
8.11	8.11	8.11	8.11	8.11	8.11	8.11	8.11	8.11
8.12	8.12	8.12	8.12	8.12	8.12	8.12	8.12	8.12
8.13	8.13	8.13	8.13	8.13	8.13	8.13	8.13	8.13
8.14	8.14	8.14	8.14	8.14	8.14	8.14	8.14	8.14
8.15	8.15	8.15	8.15	8.15	8.15	8.15	8.15	8.15
8.16	8.16	8.16	8.16	8.16	8.16	8.16	8.16	8.16
8.17	8.17	8.17	8.17	8.17	8.17	8.17	8.17	8.17
8.18	8.18	8.18	8.18	8.18	8.18	8.18	8.18	8.18
8.19	8.19	8.19	8.19	8.19	8.19	8.19	8.19	8.19
8.2	8.2	8.2	8.2	8.2	8.2	8.2	8.2	8.2
8.21	8.21	8.21	8.21	8.21	8.21	8.21	8.21	8.21
8.22	8.22	8.22	8.22	8.22	8.22	8.22	8.22	8.22
8.23	8.23	8.23	8.23	8.23	8.23	8.23	8.23	8.23
8.24	8.24	8.24	8.24	8.24	8.24	8.24	8.24	8.24
8.25	8.25	8.25	8.25	8.25	8.25	8.25	8.25	8.25
8.26	8.26	8.26	8.26	8.26	8.26	8.26	8.26	8.26
8.27	8.27	8.27	8.27	8.27	8.27	8.27	8.27	8.27
8.28	8.28	8.28	8.28	8.28	8.28	8.28	8.28	8.28
8.29	8.29	8.29	8.29	8.29	8.29	8.29	8.29	8.29
8.3	8.3	8.3	8.3	8.3	8.3	8.3	8.3	8.3
8.31	8.31	8.31	8.31	8.31	8.31	8.31	8.31	8.31
8.32	8.32	8.32	8.32	8.32	8.32	8.32	8.32	8.32
8.33	8.33	8.33	8.33	8.33	8.33	8.33	8.33	8.33
8.34	8.34	8.34	8.34	8.34	8.34	8.34	8.34	8.34
8.35	8.35	8.35	8.35	8.35	8.35	8.35	8.35	8.35
8.36	8.36	8.36	8.36	8.36	8.36	8.36	8.36	8.36
8.37	8.37	8.37	8.37	8.37	8.37	8.37	8.37	8.37
8.38	8.38	8.38	8.38	8.38	8.38	8.38	8.38	8.38
8.39	8.39	8.39	8.39	8.39	8.39	8.39	8.39	8.39
8.4	8.4	8.4	8.4	8.4	8.4	8.4	8.4	8.4
8.41	8.41	8.41	8.41	8.41	8.41	8.41	8.41	8.41
8.42	8.42	8.42	8.42	8.42	8.42	8.42	8.42	8.42
8.43	8.43	8.43	8.43	8.43	8.43	8.43	8.43	8.43
8.44	8.44	8.44	8.44	8.44	8.44	8.44	8.44	8.44
8.45	8.45	8.45	8.45	8.45	8.45	8.45	8.45	8.45
8.46	8.46	8.46	8.46	8.46	8.46	8.46	8.46	8.46
8.47	8.47	8.47	8.47	8.47	8.47	8.47	8.47	8.47
8.48	8.48	8.48	8.48	8.48	8.48	8.48	8.48	8.48
8.49	8.49	8.49	8.49	8.49	8.49	8.49	8.49	8.49

8.5	8.5	8.5	8.5	8.5	8.5	8.5	8.5	8.5
8.51	8.51	8.51	8.51	8.51	8.51	8.51	8.51	8.51
8.52	8.52	8.52	8.52	8.52	8.52	8.52	8.52	8.52
8.53	8.53	8.53	8.53	8.53	8.53	8.53	8.53	8.53
8.54	8.54	8.54	8.54	8.54	8.54	8.54	8.54	8.54
8.55	8.55	8.55	8.55	8.55	8.55	8.55	8.55	8.55
8.56	8.56	8.56	8.56	8.56	8.56	8.56	8.56	8.56
8.57	8.57	8.57	8.57	8.57	8.57	8.57	8.57	8.57
8.58	8.58	8.58	8.58	8.58	8.58	8.58	8.58	8.58
8.59	8.59	8.59	8.59	8.59	8.59	8.59	8.59	8.59
8.6	8.6	8.6	8.6	8.6	8.6	8.6	8.6	8.6
8.61	8.61	8.61	8.61	8.61	8.61	8.61	8.61	8.61
8.62	8.62	8.62	8.62	8.62	8.62	8.62	8.62	8.62
8.63	8.63	8.63	8.63	8.63	8.63	8.63	8.63	8.63
8.64	8.64	8.64	8.64	8.64	8.64	8.64	8.64	8.64
8.65	8.65	8.65	8.65	8.65	8.65	8.65	8.65	8.65
8.66	8.66	8.66	8.66	8.66	8.66	8.66	8.66	8.66
8.67	8.67	8.67	8.67	8.67	8.67	8.67	8.67	8.67
8.68	8.68	8.68	8.68	8.68	8.68	8.68	8.68	8.68
8.69	8.69	8.69	8.69	8.69	8.69	8.69	8.69	8.69
8.7	8.7	8.7	8.7	8.7	8.7	8.7	8.7	8.7
8.71	8.71	8.71	8.71	8.71	8.71	8.71	8.71	8.71
8.72	8.72	8.72	8.72	8.72	8.72	8.72	8.72	8.72
8.73	8.73	8.73	8.73	8.73	8.73	8.73	8.73	8.73
8.74	8.74	8.74	8.74	8.74	8.74	8.74	8.74	8.74
8.75	8.75	8.75	8.75	8.75	8.75	8.75	8.75	8.75
8.76	8.76	8.76	8.76	8.76	8.76	8.76	8.76	8.76
8.77	8.77	8.77	8.77	8.77	8.77	8.77	8.77	8.77
8.78	8.78	8.78	8.78	8.78	8.78	8.78	8.78	8.78
8.79	8.79	8.79	8.79	8.79	8.79	8.79	8.79	8.79
8.8	8.8	8.8	8.8	8.8	8.8	8.8	8.8	8.8
8.81	8.81	8.81	8.81	8.81	8.81	8.81	8.81	8.81
8.82	8.82	8.82	8.82	8.82	8.82	8.82	8.82	8.82
8.83	8.83	8.83	8.83	8.83	8.83	8.83	8.83	8.83
8.84	8.84	8.84	8.84	8.84	8.84	8.84	8.84	8.84
8.85	8.85	8.85	8.85	8.85	8.85	8.85	8.85	8.85
8.86	8.86	8.86	8.86	8.86	8.86	8.86	8.86	8.86
8.87	8.87	8.87	8.87	8.87	8.87	8.87	8.87	8.87
8.88	8.88	8.88	8.88	8.88	8.88	8.88	8.88	8.88
8.89	8.89	8.89	8.89	8.89	8.89	8.89	8.89	8.89
8.9	8.9	8.9	8.9	8.9	8.9	8.9	8.9	8.9
8.91	8.91	8.91	8.91	8.91	8.91	8.91	8.91	8.91
8.92	8.92	8.92	8.92	8.92	8.92	8.92	8.92	8.92
8.93	8.93	8.93	8.93	8.93	8.93	8.93	8.93	8.93
8.94	8.94	8.94	8.94	8.94	8.94	8.94	8.94	8.94
8.95	8.95	8.95	8.95	8.95	8.95	8.95	8.95	8.95
8.96	8.96	8.96	8.96	8.96	8.96	8.96	8.96	8.96
8.97	8.97	8.97	8.97	8.97	8.97	8.97	8.97	8.97
8.98	8.98	8.98	8.98	8.98	8.98	8.98	8.98	8.98
8.99	8.99	8.99	8.99	8.99	8.99	8.99	8.99	8.99

9	9	9	9	9	9	9	9	9
9.01	9.01	9.01	9.01	9.01	9.01	9.01	9.01	9.01
9.02	9.02	9.02	9.02	9.02	9.02	9.02	9.02	9.02
9.03	9.03	9.03	9.03	9.03	9.03	9.03	9.03	9.03
9.04	9.04	9.04	9.04	9.04	9.04	9.04	9.04	9.04
9.05	9.05	9.05	9.05	9.05	9.05	9.05	9.05	9.05
9.06	9.06	9.06	9.06	9.06	9.06	9.06	9.06	9.06
9.07	9.07	9.07	9.07	9.07	9.07	9.07	9.07	9.07
9.08	9.08	9.08	9.08	9.08	9.08	9.08	9.08	9.08
9.09	9.09	9.09	9.09	9.09	9.09	9.09	9.09	9.09
9.1	9.1	9.1	9.1	9.1	9.1	9.1	9.1	9.1
9.11	9.11	9.11	9.11	9.11	9.11	9.11	9.11	9.11
9.12	9.12	9.12	9.12	9.12	9.12	9.12	9.12	9.12
9.13	9.13	9.13	9.13	9.13	9.13	9.13	9.13	9.13
9.14	9.14	9.14	9.14	9.14	9.14	9.14	9.14	9.14
9.15	9.15	9.15	9.15	9.15	9.15	9.15	9.15	9.15
9.16	9.16	9.16	9.16	9.16	9.16	9.16	9.16	9.16
9.17	9.17	9.17	9.17	9.17	9.17	9.17	9.17	9.17
9.18	9.18	9.18	9.18	9.18	9.18	9.18	9.18	9.18
9.19	9.19	9.19	9.19	9.19	9.19	9.19	9.19	9.19
9.2	9.2	9.2	9.2	9.2	9.2	9.2	9.2	9.2
9.21	9.21	9.21	9.21	9.21	9.21	9.21	9.21	9.21
9.22	9.22	9.22	9.22	9.22	9.22	9.22	9.22	9.22
9.23	9.23	9.23	9.23	9.23	9.23	9.23	9.23	9.23
9.24	9.24	9.24	9.24	9.24	9.24	9.24	9.24	9.24
9.25	9.25	9.25	9.25	9.25	9.25	9.25	9.25	9.25
9.26	9.26	9.26	9.26	9.26	9.26	9.26	9.26	9.26
9.27	9.27	9.27	9.27	9.27	9.27	9.27	9.27	9.27
9.28	9.28	9.28	9.28	9.28	9.28	9.28	9.28	9.28
9.29	9.29	9.29	9.29	9.29	9.29	9.29	9.29	9.29
9.3	9.3	9.3	9.3	9.3	9.3	9.3	9.3	9.3
9.31	9.31	9.31	9.31	9.31	9.31	9.31	9.31	9.31
9.32	9.32	9.32	9.32	9.32	9.32	9.32	9.32	9.32
9.33	9.33	9.33	9.33	9.33	9.33	9.33	9.33	9.33
9.34	9.34	9.34	9.34	9.34	9.34	9.34	9.34	9.34
9.35	9.35	9.35	9.35	9.35	9.35	9.35	9.35	9.35
9.36	9.36	9.36	9.36	9.36	9.36	9.36	9.36	9.36
9.37	9.37	9.37	9.37	9.37	9.37	9.37	9.37	9.37
9.38	9.38	9.38	9.38	9.38	9.38	9.38	9.38	9.38
9.39	9.39	9.39	9.39	9.39	9.39	9.39	9.39	9.39
9.4	9.4	9.4	9.4	9.4	9.4	9.4	9.4	9.4
9.41	9.41	9.41	9.41	9.41	9.41	9.41	9.41	9.41
9.42	9.42	9.42	9.42	9.42	9.42	9.42	9.42	9.42
9.43	9.43	9.43	9.43	9.43	9.43	9.43	9.43	9.43
9.44	9.44	9.44	9.44	9.44	9.44	9.44	9.44	9.44
9.45	9.45	9.45	9.45	9.45	9.45	9.45	9.45	9.45
9.46	9.46	9.46	9.46	9.46	9.46	9.46	9.46	9.46
9.47	9.47	9.47	9.47	9.47	9.47	9.47	9.47	9.47
9.48	9.48	9.48	9.48	9.48	9.48	9.48	9.48	9.48
9.49	9.49	9.49	9.49	9.49	9.49	9.49	9.49	9.49

9.5	9.5	9.5	9.5	9.5	9.5	9.5	9.5	9.5
9.51	9.51	9.51	9.51	9.51	9.51	9.51	9.51	9.51
9.52	9.52	9.52	9.52	9.52	9.52	9.52	9.52	9.52
9.53	9.53	9.53	9.53	9.53	9.53	9.53	9.53	9.53
9.54	9.54	9.54	9.54	9.54	9.54	9.54	9.54	9.54
9.55	9.55	9.55	9.55	9.55	9.55	9.55	9.55	9.55
9.56	9.56	9.56	9.56	9.56	9.56	9.56	9.56	9.56
9.57	9.57	9.57	9.57	9.57	9.57	9.57	9.57	9.57
9.58	9.58	9.58	9.58	9.58	9.58	9.58	9.58	9.58
9.59	9.59	9.59	9.59	9.59	9.59	9.59	9.59	9.59
9.6	9.6	9.6	9.6	9.6	9.6	9.6	9.6	9.6
9.61	9.61	9.61	9.61	9.61	9.61	9.61	9.61	9.61
9.62	9.62	9.62	9.62	9.62	9.62	9.62	9.62	9.62
9.63	9.63	9.63	9.63	9.63	9.63	9.63	9.63	9.63
9.64	9.64	9.64	9.64	9.64	9.64	9.64	9.64	9.64
9.65	9.65	9.65	9.65	9.65	9.65	9.65	9.65	9.65
9.66	9.66	9.66	9.66	9.66	9.66	9.66	9.66	9.66
9.67	9.67	9.67	9.67	9.67	9.67	9.67	9.67	9.67
9.68	9.68	9.68	9.68	9.68	9.68	9.68	9.68	9.68
9.69	9.69	9.69	9.69	9.69	9.69	9.69	9.69	9.69
9.7	9.7	9.7	9.7	9.7	9.7	9.7	9.7	9.7
9.71	9.71	9.71	9.71	9.71	9.71	9.71	9.71	9.71
9.72	9.72	9.72	9.72	9.72	9.72	9.72	9.72	9.72
9.73	9.73	9.73	9.73	9.73	9.73	9.73	9.73	9.73
9.74	9.74	9.74	9.74	9.74	9.74	9.74	9.74	9.74
9.75	9.75	9.75	9.75	9.75	9.75	9.75	9.75	9.75
9.76	9.76	9.76	9.76	9.76	9.76	9.76	9.76	9.76
9.77	9.77	9.77	9.77	9.77	9.77	9.77	9.77	9.77
9.78	9.78	9.78	9.78	9.78	9.78	9.78	9.78	9.78
9.79	9.79	9.79	9.79	9.79	9.79	9.79	9.79	9.79
9.8	9.8	9.8	9.8	9.8	9.8	9.8	9.8	9.8
9.81	9.81	9.81	9.81	9.81	9.81	9.81	9.81	9.81
9.82	9.82	9.82	9.82	9.82	9.82	9.82	9.82	9.82
9.83	9.83	9.83	9.83	9.83	9.83	9.83	9.83	9.83
9.84	9.84	9.84	9.84	9.84	9.84	9.84	9.84	9.84
9.85	9.85	9.85	9.85	9.85	9.85	9.85	9.85	9.85
9.86	9.86	9.86	9.86	9.86	9.86	9.86	9.86	9.86
9.87	9.87	9.87	9.87	9.87	9.87	9.87	9.87	9.87
9.88	9.88	9.88	9.88	9.88	9.88	9.88	9.88	9.88
9.89	9.89	9.89	9.89	9.89	9.89	9.89	9.89	9.89
9.9	9.9	9.9	9.9	9.9	9.9	9.9	9.9	9.9
9.91	9.91	9.91	9.91	9.91	9.91	9.91	9.91	9.91
9.92	9.92	9.92	9.92	9.92	9.92	9.92	9.92	9.92
9.93	9.93	9.93	9.93	9.93	9.93	9.93	9.93	9.93
9.94	9.94	9.94	9.94	9.94	9.94	9.94	9.94	9.94
9.95	9.95	9.95	9.95	9.95	9.95	9.95	9.95	9.95
9.96	9.96	9.96	9.96	9.96	9.96	9.96	9.96	9.96
9.97	9.97	9.97	9.97	9.97	9.97	9.97	9.97	9.97
9.98	9.98	9.98	9.98	9.98	9.98	9.98	9.98	9.98
9.99	9.99	9.99	9.99	9.99	9.99	9.99	9.99	9.99

10	10	10	10	10	10	10	10	10
10.01	10.01	10.01	10.01	10.01	10.01	10.01	10.01	10.01
10.02	10.02	10.02	10.02	10.02	10.02	10.02	10.02	10.02
10.03	10.03	10.03	10.03	10.03	10.03	10.03	10.03	10.03
10.04	10.04	10.04	10.04	10.04	10.04	10.04	10.04	10.04
10.05	10.05	10.05	10.05	10.05	10.05	10.05	10.05	10.05
10.06	10.06	10.06	10.06	10.06	10.06	10.06	10.06	10.06
10.07	10.07	10.07	10.07	10.07	10.07	10.07	10.07	10.07
10.08	10.08	10.08	10.08	10.08	10.08	10.08	10.08	10.08
10.09	10.09	10.09	10.09	10.09	10.09	10.09	10.09	10.09
10.1	10.1	10.1	10.1	10.1	10.1	10.1	10.1	10.1
10.11	10.11	10.11	10.11	10.11	10.11	10.11	10.11	10.11
10.12	10.12	10.12	10.12	10.12	10.12	10.12	10.12	10.12
10.13	10.13	10.13	10.13	10.13	10.13	10.13	10.13	10.13
10.14	10.14	10.14	10.14	10.14	10.14	10.14	10.14	10.14
10.15	10.15	10.15	10.15	10.15	10.15	10.15	10.15	10.15
10.16	10.16	10.16	10.16	10.16	10.16	10.16	10.16	10.16
10.17	10.17	10.17	10.17	10.17	10.17	10.17	10.17	10.17
10.18	10.18	10.18	10.18	10.18	10.18	10.18	10.18	10.18
10.19	10.19	10.19	10.19	10.19	10.19	10.19	10.19	10.19
10.2	10.2	10.2	10.2	10.2	10.2	10.2	10.2	10.2
10.21	10.21	10.21	10.21	10.21	10.21	10.21	10.21	10.21
10.22	10.22	10.22	10.22	10.22	10.22	10.22	10.22	10.22
10.23	10.23	10.23	10.23	10.23	10.23	10.23	10.23	10.23
10.24	10.24	10.24	10.24	10.24	10.24	10.24	10.24	10.24
10.25	10.25	10.25	10.25	10.25	10.25	10.25	10.25	10.25
10.26	10.26	10.26	10.26	10.26	10.26	10.26	10.26	10.26
10.27	10.27	10.27	10.27	10.27	10.27	10.27	10.27	10.27
10.28	10.28	10.28	10.28	10.28	10.28	10.28	10.28	10.28
10.29	10.29	10.29	10.29	10.29	10.29	10.29	10.29	10.29
10.3	10.3	10.3	10.3	10.3	10.3	10.3	10.3	10.3
10.31	10.31	10.31	10.31	10.31	10.31	10.31	10.31	10.31
10.32	10.32	10.32	10.32	10.32	10.32	10.32	10.32	10.32
10.33	10.33	10.33	10.33	10.33	10.33	10.33	10.33	10.33
10.34	10.34	10.34	10.34	10.34	10.34	10.34	10.34	10.34
10.35	10.35	10.35	10.35	10.35	10.35	10.35	10.35	10.35
10.36	10.36	10.36	10.36	10.36	10.36	10.36	10.36	10.36
10.37	10.37	10.37	10.37	10.37	10.37	10.37	10.37	10.37
10.38	10.38	10.38	10.38	10.38	10.38	10.38	10.38	10.38
10.39	10.39	10.39	10.39	10.39	10.39	10.39	10.39	10.39
10.4	10.4	10.4	10.4	10.4	10.4	10.4	10.4	10.4
10.41	10.41	10.41	10.41	10.41	10.41	10.41	10.41	10.41
10.42	10.42	10.42	10.42	10.42	10.42	10.42	10.42	10.42
10.43	10.43	10.43	10.43	10.43	10.43	10.43	10.43	10.43
10.44	10.44	10.44	10.44	10.44	10.44	10.44	10.44	10.44
10.45	10.45	10.45	10.45	10.45	10.45	10.45	10.45	10.45
10.46	10.46	10.46	10.46	10.46	10.46	10.46	10.46	10.46
10.47	10.47	10.47	10.47	10.47	10.47	10.47	10.47	10.47
10.48	10.48	10.48	10.48	10.48	10.48	10.48	10.48	10.48
10.49	10.49	10.49	10.49	10.49	10.49	10.49	10.49	10.49

10.5	10.5	10.5	10.5	10.5	10.5	10.5	10.5	10.5
10.51	10.51	10.51	10.51	10.51	10.51	10.51	10.51	10.51
10.52	10.52	10.52	10.52	10.52	10.52	10.52	10.52	10.52
10.53	10.53	10.53	10.53	10.53	10.53	10.53	10.53	10.53
10.54	10.54	10.54	10.54	10.54	10.54	10.54	10.54	10.54
10.55	10.55	10.55	10.55	10.55	10.55	10.55	10.55	10.55
10.56	10.56	10.56	10.56	10.56	10.56	10.56	10.56	10.56
10.57	10.57	10.57	10.57	10.57	10.57	10.57	10.57	10.57
10.58	10.58	10.58	10.58	10.58	10.58	10.58	10.58	10.58
10.59	10.59	10.59	10.59	10.59	10.59	10.59	10.59	10.59
10.6	10.6	10.6	10.6	10.6	10.6	10.6	10.6	10.6
10.61	10.61	10.61	10.61	10.61	10.61	10.61	10.61	10.61
10.62	10.62	10.62	10.62	10.62	10.62	10.62	10.62	10.62
10.63	10.63	10.63	10.63	10.63	10.63	10.63	10.63	10.63
10.64	10.64	10.64	10.64	10.64	10.64	10.64	10.64	10.64
10.65	10.65	10.65	10.65	10.65	10.65	10.65	10.65	10.65
10.66	10.66	10.66	10.66	10.66	10.66	10.66	10.66	10.66
10.67	10.67	10.67	10.67	10.67	10.67	10.67	10.67	10.67
10.68	10.68	10.68	10.68	10.68	10.68	10.68	10.68	10.68
10.69	10.69	10.69	10.69	10.69	10.69	10.69	10.69	10.69
10.7	10.7	10.7	10.7	10.7	10.7	10.7	10.7	10.7
10.71	10.71	10.71	10.71	10.71	10.71	10.71	10.71	10.71
10.72	10.72	10.72	10.72	10.72	10.72	10.72	10.72	10.72
10.73	10.73	10.73	10.73	10.73	10.73	10.73	10.73	10.73
10.74	10.74	10.74	10.74	10.74	10.74	10.74	10.74	10.74
10.75	10.75	10.75	10.75	10.75	10.75	10.75	10.75	10.75
10.76	10.76	10.76	10.76	10.76	10.76	10.76	10.76	10.76
10.77	10.77	10.77	10.77	10.77	10.77	10.77	10.77	10.77
10.78	10.78	10.78	10.78	10.78	10.78	10.78	10.78	10.78
10.79	10.79	10.79	10.79	10.79	10.79	10.79	10.79	10.79
10.8	10.8	10.8	10.8	10.8	10.8	10.8	10.8	10.8
10.81	10.81	10.81	10.81	10.81	10.81	10.81	10.81	10.81
10.82	10.82	10.82	10.82	10.82	10.82	10.82	10.82	10.82
10.83	10.83	10.83	10.83	10.83	10.83	10.83	10.83	10.83
10.84	10.84	10.84	10.84	10.84	10.84	10.84	10.84	10.84
10.85	10.85	10.85	10.85	10.85	10.85	10.85	10.85	10.85
10.86	10.86	10.86	10.86	10.86	10.86	10.86	10.86	10.86
10.87	10.87	10.87	10.87	10.87	10.87	10.87	10.87	10.87
10.88	10.88	10.88	10.88	10.88	10.88	10.88	10.88	10.88
10.89	10.89	10.89	10.89	10.89	10.89	10.89	10.89	10.89
10.9	10.9	10.9	10.9	10.9	10.9	10.9	10.9	10.9
10.91	10.91	10.91	10.91	10.91	10.91	10.91	10.91	10.91
10.92	10.92	10.92	10.92	10.92	10.92	10.92	10.92	10.92
10.93	10.93	10.93	10.93	10.93	10.93	10.93	10.93	10.93
10.94	10.94	10.94	10.94	10.94	10.94	10.94	10.94	10.94
10.95	10.95	10.95	10.95	10.95	10.95	10.95	10.95	10.95
10.96	10.96	10.96	10.96	10.96	10.96	10.96	10.96	10.96
10.97	10.97	10.97	10.97	10.97	10.97	10.97	10.97	10.97
10.98	10.98	10.98	10.98	10.98	10.98	10.98	10.98	10.98
10.99	10.99	10.99	10.99	10.99	10.99	10.99	10.99	10.99

11	11	11	11	11	11	11	11	11
11.01	11.01	11.01	11.01	11.01	11.01	11.01	11.01	11.01
11.02	11.02	11.02	11.02	11.02	11.02	11.02	11.02	11.02
11.03	11.03	11.03	11.03	11.03	11.03	11.03	11.03	11.03
11.04	11.04	11.04	11.04	11.04	11.04	11.04	11.04	11.04
11.05	11.05	11.05	11.05	11.05	11.05	11.05	11.05	11.05
11.06	11.06	11.06	11.06	11.06	11.06	11.06	11.06	11.06
11.07	11.07	11.07	11.07	11.07	11.07	11.07	11.07	11.07
11.08	11.08	11.08	11.08	11.08	11.08	11.08	11.08	11.08
11.09	11.09	11.09	11.09	11.09	11.09	11.09	11.09	11.09
11.1	11.1	11.1	11.1	11.1	11.1	11.1	11.1	11.1
11.11	11.11	11.11	11.11	11.11	11.11	11.11	11.11	11.11
11.12	11.12	11.12	11.12	11.12	11.12	11.12	11.12	11.12
11.13	11.13	11.13	11.13	11.13	11.13	11.13	11.13	11.13
11.14	11.14	11.14	11.14	11.14	11.14	11.14	11.14	11.14
11.15	11.15	11.15	11.15	11.15	11.15	11.15	11.15	11.15
11.16	11.16	11.16	11.16	11.16	11.16	11.16	11.16	11.16
11.17	11.17	11.17	11.17	11.17	11.17	11.17	11.17	11.17
11.18	11.18	11.18	11.18	11.18	11.18	11.18	11.18	11.18
11.19	11.19	11.19	11.19	11.19	11.19	11.19	11.19	11.19
11.2	11.2	11.2	11.2	11.2	11.2	11.2	11.2	11.2
11.21	11.21	11.21	11.21	11.21	11.21	11.21	11.21	11.21
11.22	11.22	11.22	11.22	11.22	11.22	11.22	11.22	11.22
11.23	11.23	11.23	11.23	11.23	11.23	11.23	11.23	11.23
11.24	11.24	11.24	11.24	11.24	11.24	11.24	11.24	11.24
11.25	11.25	11.25	11.25	11.25	11.25	11.25	11.25	11.25
11.26	11.26	11.26	11.26	11.26	11.26	11.26	11.26	11.26
11.27	11.27	11.27	11.27	11.27	11.27	11.27	11.27	11.27
11.28	11.28	11.28	11.28	11.28	11.28	11.28	11.28	11.28
11.29	11.29	11.29	11.29	11.29	11.29	11.29	11.29	11.29
11.3	11.3	11.3	11.3	11.3	11.3	11.3	11.3	11.3
11.31	11.31	11.31	11.31	11.31	11.31	11.31	11.31	11.31
11.32	11.32	11.32	11.32	11.32	11.32	11.32	11.32	11.32
11.33	11.33	11.33	11.33	11.33	11.33	11.33	11.33	11.33
11.34	11.34	11.34	11.34	11.34	11.34	11.34	11.34	11.34
11.35	11.35	11.35	11.35	11.35	11.35	11.35	11.35	11.35
11.36	11.36	11.36	11.36	11.36	11.36	11.36	11.36	11.36
11.37	11.37	11.37	11.37	11.37	11.37	11.37	11.37	11.37
11.38	11.38	11.38	11.38	11.38	11.38	11.38	11.38	11.38
11.39	11.39	11.39	11.39	11.39	11.39	11.39	11.39	11.39
11.4	11.4	11.4	11.4	11.4	11.4	11.4	11.4	11.4
11.41	11.41	11.41	11.41	11.41	11.41	11.41	11.41	11.41
11.42	11.42	11.42	11.42	11.42	11.42	11.42	11.42	11.42
11.43	11.43	11.43	11.43	11.43	11.43	11.43	11.43	11.43
11.44	11.44	11.44	11.44	11.44	11.44	11.44	11.44	11.44
11.45	11.45	11.45	11.45	11.45	11.45	11.45	11.45	11.45
11.46	11.46	11.46	11.46	11.46	11.46	11.46	11.46	11.46
11.47	11.47	11.47	11.47	11.47	11.47	11.47	11.47	11.47
11.48	11.48	11.48	11.48	11.48	11.48	11.48	11.48	11.48
11.49	11.49	11.49	11.49	11.49	11.49	11.49	11.49	11.49

11.5	11.5	11.5	11.5	11.5	11.5	11.5	11.5	11.5
11.51	11.51	11.51	11.51	11.51	11.51	11.51	11.51	11.51
11.52	11.52	11.52	11.52	11.52	11.52	11.52	11.52	11.52
11.53	11.53	11.53	11.53	11.53	11.53	11.53	11.53	11.53
11.54	11.54	11.54	11.54	11.54	11.54	11.54	11.54	11.54
11.55	11.55	11.55	11.55	11.55	11.55	11.55	11.55	11.55
11.56	11.56	11.56	11.56	11.56	11.56	11.56	11.56	11.56
11.57	11.57	11.57	11.57	11.57	11.57	11.57	11.57	11.57
11.58	11.58	11.58	11.58	11.58	11.58	11.58	11.58	11.58
11.59	11.59	11.59	11.59	11.59	11.59	11.59	11.59	11.59
11.6	11.6	11.6	11.6	11.6	11.6	11.6	11.6	11.6
11.61	11.61	11.61	11.61	11.61	11.61	11.61	11.61	11.61
11.62	11.62	11.62	11.62	11.62	11.62	11.62	11.62	11.62
11.63	11.63	11.63	11.63	11.63	11.63	11.63	11.63	11.63
11.64	11.64	11.64	11.64	11.64	11.64	11.64	11.64	11.64
11.65	11.65	11.65	11.65	11.65	11.65	11.65	11.65	11.65
11.66	11.66	11.66	11.66	11.66	11.66	11.66	11.66	11.66
11.67	11.67	11.67	11.67	11.67	11.67	11.67	11.67	11.67
11.68	11.68	11.68	11.68	11.68	11.68	11.68	11.68	11.68
11.69	11.69	11.69	11.69	11.69	11.69	11.69	11.69	11.69
11.7	11.7	11.7	11.7	11.7	11.7	11.7	11.7	11.7
11.71	11.71	11.71	11.71	11.71	11.71	11.71	11.71	11.71
11.72	11.72	11.72	11.72	11.72	11.72	11.72	11.72	11.72
11.73	11.73	11.73	11.73	11.73	11.73	11.73	11.73	11.73
11.74	11.74	11.74	11.74	11.74	11.74	11.74	11.74	11.74
11.75	11.75	11.75	11.75	11.75	11.75	11.75	11.75	11.75
11.76	11.76	11.76	11.76	11.76	11.76	11.76	11.76	11.76
11.77	11.77	11.77	11.77	11.77	11.77	11.77	11.77	11.77
11.78	11.78	11.78	11.78	11.78	11.78	11.78	11.78	11.78
11.79	11.79	11.79	11.79	11.79	11.79	11.79	11.79	11.79
11.8	11.8	11.8	11.8	11.8	11.8	11.8	11.8	11.8
11.81	11.81	11.81	11.81	11.81	11.81	11.81	11.81	11.81
11.82	11.82	11.82	11.82	11.82	11.82	11.82	11.82	11.82
11.83	11.83	11.83	11.83	11.83	11.83	11.83	11.83	11.83
11.84	11.84	11.84	11.84	11.84	11.84	11.84	11.84	11.84
11.85	11.85	11.85	11.85	11.85	11.85	11.85	11.85	11.85
11.86	11.86	11.86	11.86	11.86	11.86	11.86	11.86	11.86
11.87	11.87	11.87	11.87	11.87	11.87	11.87	11.87	11.87
11.88	11.88	11.88	11.88	11.88	11.88	11.88	11.88	11.88
11.89	11.89	11.89	11.89	11.89	11.89	11.89	11.89	11.89
11.9	11.9	11.9	11.9	11.9	11.9	11.9	11.9	11.9
11.91	11.91	11.91	11.91	11.91	11.91	11.91	11.91	11.91
11.92	11.92	11.92	11.92	11.92	11.92	11.92	11.92	11.92
11.93	11.93	11.93	11.93	11.93	11.93	11.93	11.93	11.93
11.94	11.94	11.94	11.94	11.94	11.94	11.94	11.94	11.94
11.95	11.95	11.95	11.95	11.95	11.95	11.95	11.95	11.95
11.96	11.96	11.96	11.96	11.96	11.96	11.96	11.96	11.96
11.97	11.97	11.97	11.97	11.97	11.97	11.97	11.97	11.97
11.98	11.98	11.98	11.98	11.98	11.98	11.98	11.98	11.98
11.99	11.99	11.99	11.99	11.99	11.99	11.99	11.99	11.99

12	12	12	12	12	12	12	12	12

-12	-12	-12	-12	-12	-12	-12	-12	-12
-11.99	-11.99	-11.99	-11.99	-11.99	-11.99	-11.99	-11.99	-11.99
-11.98	-11.98	-11.98	-11.98	-11.98	-11.98	-11.98	-11.98	-11.98
-11.97	-11.97	-11.97	-11.97	-11.97	-11.97	-11.97	-11.97	-11.97
-11.96	-11.96	-11.96	-11.96	-11.96	-11.96	-11.96	-11.96	-11.96
-11.95	-11.95	-11.95	-11.95	-11.95	-11.95	-11.95	-11.95	-11.95
-11.94	-11.94	-11.94	-11.94	-11.94	-11.94	-11.94	-11.94	-11.94
-11.93	-11.93	-11.93	-11.93	-11.93	-11.93	-11.93	-11.93	-11.93
-11.92	-11.92	-11.92	-11.92	-11.92	-11.92	-11.92	-11.92	-11.92
-11.91	-11.91	-11.91	-11.91	-11.91	-11.91	-11.91	-11.91	-11.91
-11.9	-11.9	-11.9	-11.9	-11.9	-11.9	-11.9	-11.9	-11.9
-11.89	-11.89	-11.89	-11.89	-11.89	-11.89	-11.89	-11.89	-11.89
-11.88	-11.88	-11.88	-11.88	-11.88	-11.88	-11.88	-11.88	-11.88
-11.87	-11.87	-11.87	-11.87	-11.87	-11.87	-11.87	-11.87	-11.87
-11.86	-11.86	-11.86	-11.86	-11.86	-11.86	-11.86	-11.86	-11.86
-11.85	-11.85	-11.85	-11.85	-11.85	-11.85	-11.85	-11.85	-11.85
-11.84	-11.84	-11.84	-11.84	-11.84	-11.84	-11.84	-11.84	-11.84
-11.83	-11.83	-11.83	-11.83	-11.83	-11.83	-11.83	-11.83	-11.83
-11.82	-11.82	-11.82	-11.82	-11.82	-11.82	-11.82	-11.82	-11.82
-11.81	-11.81	-11.81	-11.81	-11.81	-11.81	-11.81	-11.81	-11.81
-11.8	-11.8	-11.8	-11.8	-11.8	-11.8	-11.8	-11.8	-11.8
-11.79	-11.79	-11.79	-11.79	-11.79	-11.79	-11.79	-11.79	-11.79
-11.78	-11.78	-11.78	-11.78	-11.78	-11.78	-11.78	-11.78	-11.78
-11.77	-11.77	-11.77	-11.77	-11.77	-11.77	-11.77	-11.77	-11.77
-11.76	-11.76	-11.76	-11.76	-11.76	-11.76	-11.76	-11.76	-11.76
-11.75	-11.75	-11.75	-11.75	-11.75	-11.75	-11.75	-11.75	-11.75
-11.74	-11.74	-11.74	-11.74	-11.74	-11.74	-11.74	-11.74	-11.74
-11.73	-11.73	-11.73	-11.73	-11.73	-11.73	-11.73	-11.73	-11.73
-11.72	-11.72	-11.72	-11.72	-11.72	-11.72	-11.72	-11.72	-11.72
-11.71	-11.71	-11.71	-11.71	-11.71	-11.71	-11.71	-11.71	-11.71
-11.7	-11.7	-11.7	-11.7	-11.7	-11.7	-11.7	-11.7	-11.7
-11.69	-11.69	-11.69	-11.69	-11.69	-11.69	-11.69	-11.69	-11.69
-11.68	-11.68	-11.68	-11.68	-11.68	-11.68	-11.68	-11.68	-11.68
-11.67	-11.67	-11.67	-11.67	-11.67	-11.67	-11.67	-11.67	-11.67
-11.66	-11.66	-11.66	-11.66	-11.66	-11.66	-11.66	-11.66	-11.66
-11.65	-11.65	-11.65	-11.65	-11.65	-11.65	-11.65	-11.65	-11.65
-11.64	-11.64	-11.64	-11.64	-11.64	-11.64	-11.64	-11.64	-11.64
-11.63	-11.63	-11.63	-11.63	-11.63	-11.63	-11.63	-11.63	-11.63
-11.62	-11.62	-11.62	-11.62	-11.62	-11.62	-11.62	-11.62	-11.62
-11.61	-11.61	-11.61	-11.61	-11.61	-11.61	-11.61	-11.61	-11.61
-11.6	-11.6	-11.6	-11.6	-11.6	-11.6	-11.6	-11.6	-11.6
-11.59	-11.59	-11.59	-11.59	-11.59	-11.59	-11.59	-11.59	-11.59
-11.58	-11.58	-11.58	-11.58	-11.58	-11.58	-11.58	-11.58	-11.58
-11.57	-11.57	-11.57	-11.57	-11.57	-11.57	-11.57	-11.57	-11.57
-11.56	-11.56	-11.56	-11.56	-11.56	-11.56	-11.56	-11.56	-11.56
-11.55	-11.55	-11.55	-11.55	-11.55	-11.55	-11.55	-11.55	-11.55
-11.54	-11.54	-11.54	-11.54	-11.54	-11.54	-11.54	-11.54	-11.54
-11.53	-11.53	-11.53	-11.53	-11.53	-11.53	-11.53	-11.53	-11.53
-11.52	-11.52	-11.52	-11.52	-11.52	-11.52	-11.52	-11.52	-11.52
-11.51	-11.51	-11.51	-11.51	-11.51	-11.51	-11.51	-11.51	-11.51

-11.5	-11.5	-11.5	-11.5	-11.5	-11.5	-11.5	-11.5	-11.5
-11.49	-11.49	-11.49	-11.49	-11.49	-11.49	-11.49	-11.49	-11.49
-11.48	-11.48	-11.48	-11.48	-11.48	-11.48	-11.48	-11.48	-11.48
-11.47	-11.47	-11.47	-11.47	-11.47	-11.47	-11.47	-11.47	-11.47
-11.46	-11.46	-11.46	-11.46	-11.46	-11.46	-11.46	-11.46	-11.46
-11.45	-11.45	-11.45	-11.45	-11.45	-11.45	-11.45	-11.45	-11.45
-11.44	-11.44	-11.44	-11.44	-11.44	-11.44	-11.44	-11.44	-11.44
-11.43	-11.43	-11.43	-11.43	-11.43	-11.43	-11.43	-11.43	-11.43
-11.42	-11.42	-11.42	-11.42	-11.42	-11.42	-11.42	-11.42	-11.42
-11.41	-11.41	-11.41	-11.41	-11.41	-11.41	-11.41	-11.41	-11.41
-11.4	-11.4	-11.4	-11.4	-11.4	-11.4	-11.4	-11.4	-11.4
-11.39	-11.39	-11.39	-11.39	-11.39	-11.39	-11.39	-11.39	-11.39
-11.38	-11.38	-11.38	-11.38	-11.38	-11.38	-11.38	-11.38	-11.38
-11.37	-11.37	-11.37	-11.37	-11.37	-11.37	-11.37	-11.37	-11.37
-11.36	-11.36	-11.36	-11.36	-11.36	-11.36	-11.36	-11.36	-11.36
-11.35	-11.35	-11.35	-11.35	-11.35	-11.35	-11.35	-11.35	-11.35
-11.34	-11.34	-11.34	-11.34	-11.34	-11.34	-11.34	-11.34	-11.34
-11.33	-11.33	-11.33	-11.33	-11.33	-11.33	-11.33	-11.33	-11.33
-11.32	-11.32	-11.32	-11.32	-11.32	-11.32	-11.32	-11.32	-11.32
-11.31	-11.31	-11.31	-11.31	-11.31	-11.31	-11.31	-11.31	-11.31
-11.3	-11.3	-11.3	-11.3	-11.3	-11.3	-11.3	-11.3	-11.3
-11.29	-11.29	-11.29	-11.29	-11.29	-11.29	-11.29	-11.29	-11.29
-11.28	-11.28	-11.28	-11.28	-11.28	-11.28	-11.28	-11.28	-11.28
-11.27	-11.27	-11.27	-11.27	-11.27	-11.27	-11.27	-11.27	-11.27
-11.26	-11.26	-11.26	-11.26	-11.26	-11.26	-11.26	-11.26	-11.26
-11.25	-11.25	-11.25	-11.25	-11.25	-11.25	-11.25	-11.25	-11.25
-11.24	-11.24	-11.24	-11.24	-11.24	-11.24	-11.24	-11.24	-11.24
-11.23	-11.23	-11.23	-11.23	-11.23	-11.23	-11.23	-11.23	-11.23
-11.22	-11.22	-11.22	-11.22	-11.22	-11.22	-11.22	-11.22	-11.22
-11.21	-11.21	-11.21	-11.21	-11.21	-11.21	-11.21	-11.21	-11.21
-11.2	-11.2	-11.2	-11.2	-11.2	-11.2	-11.2	-11.2	-11.2
-11.19	-11.19	-11.19	-11.19	-11.19	-11.19	-11.19	-11.19	-11.19
-11.18	-11.18	-11.18	-11.18	-11.18	-11.18	-11.18	-11.18	-11.18
-11.17	-11.17	-11.17	-11.17	-11.17	-11.17	-11.17	-11.17	-11.17
-11.16	-11.16	-11.16	-11.16	-11.16	-11.16	-11.16	-11.16	-11.16
-11.15	-11.15	-11.15	-11.15	-11.15	-11.15	-11.15	-11.15	-11.15
-11.14	-11.14	-11.14	-11.14	-11.14	-11.14	-11.14	-11.14	-11.14
-11.13	-11.13	-11.13	-11.13	-11.13	-11.13	-11.13	-11.13	-11.13
-11.12	-11.12	-11.12	-11.12	-11.12	-11.12	-11.12	-11.12	-11.12
-11.11	-11.11	-11.11	-11.11	-11.11	-11.11	-11.11	-11.11	-11.11
-11.1	-11.1	-11.1	-11.1	-11.1	-11.1	-11.1	-11.1	-11.1
-11.09	-11.09	-11.09	-11.09	-11.09	-11.09	-11.09	-11.09	-11.09
-11.08	-11.08	-11.08	-11.08	-11.08	-11.08	-11.08	-11.08	-11.08
-11.07	-11.07	-11.07	-11.07	-11.07	-11.07	-11.07	-11.07	-11.07
-11.06	-11.06	-11.06	-11.06	-11.06	-11.06	-11.06	-11.06	-11.06
-11.05	-11.05	-11.05	-11.05	-11.05	-11.05	-11.05	-11.05	-11.05
-11.04	-11.04	-11.04	-11.04	-11.04	-11.04	-11.04	-11.04	-11.04
-11.03	-11.03	-11.03	-11.03	-11.03	-11.03	-11.03	-11.03	-11.03
-11.02	-11.02	-11.02	-11.02	-11.02	-11.02	-11.02	-11.02	-11.02
-11.01	-11.01	-11.01	-11.01	-11.01	-11.01	-11.01	-11.01	-11.01

-11	-11	-11	-11	-11	-11	-11	-11	-11
-10.99	-10.99	-10.99	-10.99	-10.99	-10.99	-10.99	-10.99	-10.99
-10.98	-10.98	-10.98	-10.98	-10.98	-10.98	-10.98	-10.98	-10.98
-10.97	-10.97	-10.97	-10.97	-10.97	-10.97	-10.97	-10.97	-10.97
-10.96	-10.96	-10.96	-10.96	-10.96	-10.96	-10.96	-10.96	-10.96
-10.95	-10.95	-10.95	-10.95	-10.95	-10.95	-10.95	-10.95	-10.95
-10.94	-10.94	-10.94	-10.94	-10.94	-10.94	-10.94	-10.94	-10.94
-10.93	-10.93	-10.93	-10.93	-10.93	-10.93	-10.93	-10.93	-10.93
-10.92	-10.92	-10.92	-10.92	-10.92	-10.92	-10.92	-10.92	-10.92
-10.91	-10.91	-10.91	-10.91	-10.91	-10.91	-10.91	-10.91	-10.91
-10.9	-10.9	-10.9	-10.9	-10.9	-10.9	-10.9	-10.9	-10.9
-10.89	-10.89	-10.89	-10.89	-10.89	-10.89	-10.89	-10.89	-10.89
-10.88	-10.88	-10.88	-10.88	-10.88	-10.88	-10.88	-10.88	-10.88
-10.87	-10.87	-10.87	-10.87	-10.87	-10.87	-10.87	-10.87	-10.87
-10.86	-10.86	-10.86	-10.86	-10.86	-10.86	-10.86	-10.86	-10.86
-10.85	-10.85	-10.85	-10.85	-10.85	-10.85	-10.85	-10.85	-10.85
-10.84	-10.84	-10.84	-10.84	-10.84	-10.84	-10.84	-10.84	-10.84
-10.83	-10.83	-10.83	-10.83	-10.83	-10.83	-10.83	-10.83	-10.83
-10.82	-10.82	-10.82	-10.82	-10.82	-10.82	-10.82	-10.82	-10.82
-10.81	-10.81	-10.81	-10.81	-10.81	-10.81	-10.81	-10.81	-10.81
-10.8	-10.8	-10.8	-10.8	-10.8	-10.8	-10.8	-10.8	-10.8
-10.79	-10.79	-10.79	-10.79	-10.79	-10.79	-10.79	-10.79	-10.79
-10.78	-10.78	-10.78	-10.78	-10.78	-10.78	-10.78	-10.78	-10.78
-10.77	-10.77	-10.77	-10.77	-10.77	-10.77	-10.77	-10.77	-10.77
-10.76	-10.76	-10.76	-10.76	-10.76	-10.76	-10.76	-10.76	-10.76
-10.75	-10.75	-10.75	-10.75	-10.75	-10.75	-10.75	-10.75	-10.75
-10.74	-10.74	-10.74	-10.74	-10.74	-10.74	-10.74	-10.74	-10.74
-10.73	-10.73	-10.73	-10.73	-10.73	-10.73	-10.73	-10.73	-10.73
-10.72	-10.72	-10.72	-10.72	-10.72	-10.72	-10.72	-10.72	-10.72
-10.71	-10.71	-10.71	-10.71	-10.71	-10.71	-10.71	-10.71	-10.71
-10.7	-10.7	-10.7	-10.7	-10.7	-10.7	-10.7	-10.7	-10.7
-10.69	-10.69	-10.69	-10.69	-10.69	-10.69	-10.69	-10.69	-10.69
-10.68	-10.68	-10.68	-10.68	-10.68	-10.68	-10.68	-10.68	-10.68
-10.67	-10.67	-10.67	-10.67	-10.67	-10.67	-10.67	-10.67	-10.67
-10.66	-10.66	-10.66	-10.66	-10.66	-10.66	-10.66	-10.66	-10.66
-10.65	-10.65	-10.65	-10.65	-10.65	-10.65	-10.65	-10.65	-10.65
-10.64	-10.64	-10.64	-10.64	-10.64	-10.64	-10.64	-10.64	-10.64
-10.63	-10.63	-10.63	-10.63	-10.63	-10.63	-10.63	-10.63	-10.63
-10.62	-10.62	-10.62	-10.62	-10.62	-10.62	-10.62	-10.62	-10.62
-10.61	-10.61	-10.61	-10.61	-10.61	-10.61	-10.61	-10.61	-10.61
-10.6	-10.6	-10.6	-10.6	-10.6	-10.6	-10.6	-10.6	-10.6
-10.59	-10.59	-10.59	-10.59	-10.59	-10.59	-10.59	-10.59	-10.59
-10.58	-10.58	-10.58	-10.58	-10.58	-10.58	-10.58	-10.58	-10.58
-10.57	-10.57	-10.57	-10.57	-10.57	-10.57	-10.57	-10.57	-10.57
-10.56	-10.56	-10.56	-10.56	-10.56	-10.56	-10.56	-10.56	-10.56
-10.55	-10.55	-10.55	-10.55	-10.55	-10.55	-10.55	-10.55	-10.55
-10.54	-10.54	-10.54	-10.54	-10.54	-10.54	-10.54	-10.54	-10.54
-10.53	-10.53	-10.53	-10.53	-10.53	-10.53	-10.53	-10.53	-10.53
-10.52	-10.52	-10.52	-10.52	-10.52	-10.52	-10.52	-10.52	-10.52
-10.51	-10.51	-10.51	-10.51	-10.51	-10.51	-10.51	-10.51	-10.51

-10.5	-10.5	-10.5	-10.5	-10.5	-10.5	-10.5	-10.5	-10.5
-10.49	-10.49	-10.49	-10.49	-10.49	-10.49	-10.49	-10.49	-10.49
-10.48	-10.48	-10.48	-10.48	-10.48	-10.48	-10.48	-10.48	-10.48
-10.47	-10.47	-10.47	-10.47	-10.47	-10.47	-10.47	-10.47	-10.47
-10.46	-10.46	-10.46	-10.46	-10.46	-10.46	-10.46	-10.46	-10.46
-10.45	-10.45	-10.45	-10.45	-10.45	-10.45	-10.45	-10.45	-10.45
-10.44	-10.44	-10.44	-10.44	-10.44	-10.44	-10.44	-10.44	-10.44
-10.43	-10.43	-10.43	-10.43	-10.43	-10.43	-10.43	-10.43	-10.43
-10.42	-10.42	-10.42	-10.42	-10.42	-10.42	-10.42	-10.42	-10.42
-10.41	-10.41	-10.41	-10.41	-10.41	-10.41	-10.41	-10.41	-10.41
-10.4	-10.4	-10.4	-10.4	-10.4	-10.4	-10.4	-10.4	-10.4
-10.39	-10.39	-10.39	-10.39	-10.39	-10.39	-10.39	-10.39	-10.39
-10.38	-10.38	-10.38	-10.38	-10.38	-10.38	-10.38	-10.38	-10.38
-10.37	-10.37	-10.37	-10.37	-10.37	-10.37	-10.37	-10.37	-10.37
-10.36	-10.36	-10.36	-10.36	-10.36	-10.36	-10.36	-10.36	-10.36
-10.35	-10.35	-10.35	-10.35	-10.35	-10.35	-10.35	-10.35	-10.35
-10.34	-10.34	-10.34	-10.34	-10.34	-10.34	-10.34	-10.34	-10.34
-10.33	-10.33	-10.33	-10.33	-10.33	-10.33	-10.33	-10.33	-10.33
-10.32	-10.32	-10.32	-10.32	-10.32	-10.32	-10.32	-10.32	-10.32
-10.31	-10.31	-10.31	-10.31	-10.31	-10.31	-10.31	-10.31	-10.31
-10.3	-10.3	-10.3	-10.3	-10.3	-10.3	-10.3	-10.3	-10.3
-10.29	-10.29	-10.29	-10.29	-10.29	-10.29	-10.29	-10.29	-10.29
-10.28	-10.28	-10.28	-10.28	-10.28	-10.28	-10.28	-10.28	-10.28
-10.27	-10.27	-10.27	-10.27	-10.27	-10.27	-10.27	-10.27	-10.27
-10.26	-10.26	-10.26	-10.26	-10.26	-10.26	-10.26	-10.26	-10.26
-10.25	-10.25	-10.25	-10.25	-10.25	-10.25	-10.25	-10.25	-10.25
-10.24	-10.24	-10.24	-10.24	-10.24	-10.24	-10.24	-10.24	-10.24
-10.23	-10.23	-10.23	-10.23	-10.23	-10.23	-10.23	-10.23	-10.23
-10.22	-10.22	-10.22	-10.22	-10.22	-10.22	-10.22	-10.22	-10.22
-10.21	-10.21	-10.21	-10.21	-10.21	-10.21	-10.21	-10.21	-10.21
-10.2	-10.2	-10.2	-10.2	-10.2	-10.2	-10.2	-10.2	-10.2
-10.19	-10.19	-10.19	-10.19	-10.19	-10.19	-10.19	-10.19	-10.19
-10.18	-10.18	-10.18	-10.18	-10.18	-10.18	-10.18	-10.18	-10.18
-10.17	-10.17	-10.17	-10.17	-10.17	-10.17	-10.17	-10.17	-10.17
-10.16	-10.16	-10.16	-10.16	-10.16	-10.16	-10.16	-10.16	-10.16
-10.15	-10.15	-10.15	-10.15	-10.15	-10.15	-10.15	-10.15	-10.15
-10.14	-10.14	-10.14	-10.14	-10.14	-10.14	-10.14	-10.14	-10.14
-10.13	-10.13	-10.13	-10.13	-10.13	-10.13	-10.13	-10.13	-10.13
-10.12	-10.12	-10.12	-10.12	-10.12	-10.12	-10.12	-10.12	-10.12
-10.11	-10.11	-10.11	-10.11	-10.11	-10.11	-10.11	-10.11	-10.11
-10.1	-10.1	-10.1	-10.1	-10.1	-10.1	-10.1	-10.1	-10.1
-10.09	-10.09	-10.09	-10.09	-10.09	-10.09	-10.09	-10.09	-10.09
-10.08	-10.08	-10.08	-10.08	-10.08	-10.08	-10.08	-10.08	-10.08
-10.07	-10.07	-10.07	-10.07	-10.07	-10.07	-10.07	-10.07	-10.07
-10.06	-10.06	-10.06	-10.06	-10.06	-10.06	-10.06	-10.06	-10.06
-10.05	-10.05	-10.05	-10.05	-10.05	-10.05	-10.05	-10.05	-10.05
-10.04	-10.04	-10.04	-10.04	-10.04	-10.04	-10.04	-10.04	-10.04
-10.03	-10.03	-10.03	-10.03	-10.03	-10.03	-10.03	-10.03	-10.03
-10.02	-10.02	-10.02	-10.02	-10.02	-10.02	-10.02	-10.02	-10.02
-10.01	-10.01	-10.01	-10.01	-10.01	-10.01	-10.01	-10.01	-10.01

-10	-10	-10	-10	-10	-10	-10	-10	-10
-9.99	-9.99	-9.99	-9.99	-9.99	-9.99	-9.99	-9.99	-9.99
-9.98	-9.98	-9.98	-9.98	-9.98	-9.98	-9.98	-9.98	-9.98
-9.97	-9.97	-9.97	-9.97	-9.97	-9.97	-9.97	-9.97	-9.97
-9.96	-9.96	-9.96	-9.96	-9.96	-9.96	-9.96	-9.96	-9.96
-9.95	-9.95	-9.95	-9.95	-9.95	-9.95	-9.95	-9.95	-9.95
-9.94	-9.94	-9.94	-9.94	-9.94	-9.94	-9.94	-9.94	-9.94
-9.93	-9.93	-9.93	-9.93	-9.93	-9.93	-9.93	-9.93	-9.93
-9.92	-9.92	-9.92	-9.92	-9.92	-9.92	-9.92	-9.92	-9.92
-9.91	-9.91	-9.91	-9.91	-9.91	-9.91	-9.91	-9.91	-9.91
-9.9	-9.9	-9.9	-9.9	-9.9	-9.9	-9.9	-9.9	-9.9
-9.89	-9.89	-9.89	-9.89	-9.89	-9.89	-9.89	-9.89	-9.89
-9.88	-9.88	-9.88	-9.88	-9.88	-9.88	-9.88	-9.88	-9.88
-9.87	-9.87	-9.87	-9.87	-9.87	-9.87	-9.87	-9.87	-9.87
-9.86	-9.86	-9.86	-9.86	-9.86	-9.86	-9.86	-9.86	-9.86
-9.85	-9.85	-9.85	-9.85	-9.85	-9.85	-9.85	-9.85	-9.85
-9.84	-9.84	-9.84	-9.84	-9.84	-9.84	-9.84	-9.84	-9.84
-9.83	-9.83	-9.83	-9.83	-9.83	-9.83	-9.83	-9.83	-9.83
-9.82	-9.82	-9.82	-9.82	-9.82	-9.82	-9.82	-9.82	-9.82
-9.81	-9.81	-9.81	-9.81	-9.81	-9.81	-9.81	-9.81	-9.81
-9.8	-9.8	-9.8	-9.8	-9.8	-9.8	-9.8	-9.8	-9.8
-9.79	-9.79	-9.79	-9.79	-9.79	-9.79	-9.79	-9.79	-9.79
-9.78	-9.78	-9.78	-9.78	-9.78	-9.78	-9.78	-9.78	-9.78
-9.77	-9.77	-9.77	-9.77	-9.77	-9.77	-9.77	-9.77	-9.77
-9.76	-9.76	-9.76	-9.76	-9.76	-9.76	-9.76	-9.76	-9.76
-9.75	-9.75	-9.75	-9.75	-9.75	-9.75	-9.75	-9.75	-9.75
-9.74	-9.74	-9.74	-9.74	-9.74	-9.74	-9.74	-9.74	-9.74
-9.73	-9.73	-9.73	-9.73	-9.73	-9.73	-9.73	-9.73	-9.73
-9.72	-9.72	-9.72	-9.72	-9.72	-9.72	-9.72	-9.72	-9.72
-9.71	-9.71	-9.71	-9.71	-9.71	-9.71	-9.71	-9.71	-9.71
-9.7	-9.7	-9.7	-9.7	-9.7	-9.7	-9.7	-9.7	-9.7
-9.69	-9.69	-9.69	-9.69	-9.69	-9.69	-9.69	-9.69	-9.69
-9.68	-9.68	-9.68	-9.68	-9.68	-9.68	-9.68	-9.68	-9.68
-9.67	-9.67	-9.67	-9.67	-9.67	-9.67	-9.67	-9.67	-9.67
-9.66	-9.66	-9.66	-9.66	-9.66	-9.66	-9.66	-9.66	-9.66
-9.65	-9.65	-9.65	-9.65	-9.65	-9.65	-9.65	-9.65	-9.65
-9.64	-9.64	-9.64	-9.64	-9.64	-9.64	-9.64	-9.64	-9.64
-9.63	-9.63	-9.63	-9.63	-9.63	-9.63	-9.63	-9.63	-9.63
-9.62	-9.62	-9.62	-9.62	-9.62	-9.62	-9.62	-9.62	-9.62
-9.61	-9.61	-9.61	-9.61	-9.61	-9.61	-9.61	-9.61	-9.61
-9.6	-9.6	-9.6	-9.6	-9.6	-9.6	-9.6	-9.6	-9.6
-9.59	-9.59	-9.59	-9.59	-9.59	-9.59	-9.59	-9.59	-9.59
-9.58	-9.58	-9.58	-9.58	-9.58	-9.58	-9.58	-9.58	-9.58
-9.57	-9.57	-9.57	-9.57	-9.57	-9.57	-9.57	-9.57	-9.57
-9.56	-9.56	-9.56	-9.56	-9.56	-9.56	-9.56	-9.56	-9.56
-9.55	-9.55	-9.55	-9.55	-9.55	-9.55	-9.55	-9.55	-9.55
-9.54	-9.54	-9.54	-9.54	-9.54	-9.54	-9.54	-9.54	-9.54
-9.53	-9.53	-9.53	-9.53	-9.53	-9.53	-9.53	-9.53	-9.53
-9.52	-9.52	-9.52	-9.52	-9.52	-9.52	-9.52	-9.52	-9.52
-9.51	-9.51	-9.51	-9.51	-9.51	-9.51	-9.51	-9.51	-9.51

-9.5	-9.5	-9.5	-9.5	-9.5	-9.5	-9.5	-9.5	-9.5
-9.49	-9.49	-9.49	-9.49	-9.49	-9.49	-9.49	-9.49	-9.49
-9.48	-9.48	-9.48	-9.48	-9.48	-9.48	-9.48	-9.48	-9.48
-9.47	-9.47	-9.47	-9.47	-9.47	-9.47	-9.47	-9.47	-9.47
-9.46	-9.46	-9.46	-9.46	-9.46	-9.46	-9.46	-9.46	-9.46
-9.45	-9.45	-9.45	-9.45	-9.45	-9.45	-9.45	-9.45	-9.45
-9.44	-9.44	-9.44	-9.44	-9.44	-9.44	-9.44	-9.44	-9.44
-9.43	-9.43	-9.43	-9.43	-9.43	-9.43	-9.43	-9.43	-9.43
-9.42	-9.42	-9.42	-9.42	-9.42	-9.42	-9.42	-9.42	-9.42
-9.41	-9.41	-9.41	-9.41	-9.41	-9.41	-9.41	-9.41	-9.41
-9.4	-9.4	-9.4	-9.4	-9.4	-9.4	-9.4	-9.4	-9.4
-9.39	-9.39	-9.39	-9.39	-9.39	-9.39	-9.39	-9.39	-9.39
-9.38	-9.38	-9.38	-9.38	-9.38	-9.38	-9.38	-9.38	-9.38
-9.37	-9.37	-9.37	-9.37	-9.37	-9.37	-9.37	-9.37	-9.37
-9.36	-9.36	-9.36	-9.36	-9.36	-9.36	-9.36	-9.36	-9.36
-9.35	-9.35	-9.35	-9.35	-9.35	-9.35	-9.35	-9.35	-9.35
-9.34	-9.34	-9.34	-9.34	-9.34	-9.34	-9.34	-9.34	-9.34
-9.33	-9.33	-9.33	-9.33	-9.33	-9.33	-9.33	-9.33	-9.33
-9.32	-9.32	-9.32	-9.32	-9.32	-9.32	-9.32	-9.32	-9.32
-9.31	-9.31	-9.31	-9.31	-9.31	-9.31	-9.31	-9.31	-9.31
-9.3	-9.3	-9.3	-9.3	-9.3	-9.3	-9.3	-9.3	-9.3
-9.29	-9.29	-9.29	-9.29	-9.29	-9.29	-9.29	-9.29	-9.29
-9.28	-9.28	-9.28	-9.28	-9.28	-9.28	-9.28	-9.28	-9.28
-9.27	-9.27	-9.27	-9.27	-9.27	-9.27	-9.27	-9.27	-9.27
-9.26	-9.26	-9.26	-9.26	-9.26	-9.26	-9.26	-9.26	-9.26
-9.25	-9.25	-9.25	-9.25	-9.25	-9.25	-9.25	-9.25	-9.25
-9.24	-9.24	-9.24	-9.24	-9.24	-9.24	-9.24	-9.24	-9.24
-9.23	-9.23	-9.23	-9.23	-9.23	-9.23	-9.23	-9.23	-9.23
-9.22	-9.22	-9.22	-9.22	-9.22	-9.22	-9.22	-9.22	-9.22
-9.21	-9.21	-9.21	-9.21	-9.21	-9.21	-9.21	-9.21	-9.21
-9.2	-9.2	-9.2	-9.2	-9.2	-9.2	-9.2	-9.2	-9.2
-9.19	-9.19	-9.19	-9.19	-9.19	-9.19	-9.19	-9.19	-9.19
-9.18	-9.18	-9.18	-9.18	-9.18	-9.18	-9.18	-9.18	-9.18
-9.17	-9.17	-9.17	-9.17	-9.17	-9.17	-9.17	-9.17	-9.17
-9.16	-9.16	-9.16	-9.16	-9.16	-9.16	-9.16	-9.16	-9.16
-9.15	-9.15	-9.15	-9.15	-9.15	-9.15	-9.15	-9.15	-9.15
-9.14	-9.14	-9.14	-9.14	-9.14	-9.14	-9.14	-9.14	-9.14
-9.13	-9.13	-9.13	-9.13	-9.13	-9.13	-9.13	-9.13	-9.13
-9.12	-9.12	-9.12	-9.12	-9.12	-9.12	-9.12	-9.12	-9.12
-9.11	-9.11	-9.11	-9.11	-9.11	-9.11	-9.11	-9.11	-9.11
-9.1	-9.1	-9.1	-9.1	-9.1	-9.1	-9.1	-9.1	-9.1
-9.09	-9.09	-9.09	-9.09	-9.09	-9.09	-9.09	-9.09	-9.09
-9.08	-9.08	-9.08	-9.08	-9.08	-9.08	-9.08	-9.08	-9.08
-9.07	-9.07	-9.07	-9.07	-9.07	-9.07	-9.07	-9.07	-9.07
-9.06	-9.06	-9.06	-9.06	-9.06	-9.06	-9.06	-9.06	-9.06
-9.05	-9.05	-9.05	-9.05	-9.05	-9.05	-9.05	-9.05	-9.05
-9.04	-9.04	-9.04	-9.04	-9.04	-9.04	-9.04	-9.04	-9.04
-9.03	-9.03	-9.03	-9.03	-9.03	-9.03	-9.03	-9.03	-9.03
-9.02	-9.02	-9.02	-9.02	-9.02	-9.02	-9.02	-9.02	-9.02
-9.01	-9.01	-9.01	-9.01	-9.01	-9.01	-9.01	-9.01	-9.01

-9	-9	-9	-9	-9	-9	-9	-9	-9
-8.99	-8.99	-8.99	-8.99	-8.99	-8.99	-8.99	-8.99	-8.99
-8.98	-8.98	-8.98	-8.98	-8.98	-8.98	-8.98	-8.98	-8.98
-8.97	-8.97	-8.97	-8.97	-8.97	-8.97	-8.97	-8.97	-8.97
-8.96	-8.96	-8.96	-8.96	-8.96	-8.96	-8.96	-8.96	-8.96
-8.95	-8.95	-8.95	-8.95	-8.95	-8.95	-8.95	-8.95	-8.95
-8.94	-8.94	-8.94	-8.94	-8.94	-8.94	-8.94	-8.94	-8.94
-8.93	-8.93	-8.93	-8.93	-8.93	-8.93	-8.93	-8.93	-8.93
-8.92	-8.92	-8.92	-8.92	-8.92	-8.92	-8.92	-8.92	-8.92
-8.91	-8.91	-8.91	-8.91	-8.91	-8.91	-8.91	-8.91	-8.91
-8.9	-8.9	-8.9	-8.9	-8.9	-8.9	-8.9	-8.9	-8.9
-8.89	-8.89	-8.89	-8.89	-8.89	-8.89	-8.89	-8.89	-8.89
-8.88	-8.88	-8.88	-8.88	-8.88	-8.88	-8.88	-8.88	-8.88
-8.87	-8.87	-8.87	-8.87	-8.87	-8.87	-8.87	-8.87	-8.87
-8.86	-8.86	-8.86	-8.86	-8.86	-8.86	-8.86	-8.86	-8.86
-8.85	-8.85	-8.85	-8.85	-8.85	-8.85	-8.85	-8.85	-8.85
-8.84	-8.84	-8.84	-8.84	-8.84	-8.84	-8.84	-8.84	-8.84
-8.83	-8.83	-8.83	-8.83	-8.83	-8.83	-8.83	-8.83	-8.83
-8.82	-8.82	-8.82	-8.82	-8.82	-8.82	-8.82	-8.82	-8.82
-8.81	-8.81	-8.81	-8.81	-8.81	-8.81	-8.81	-8.81	-8.81
-8.8	-8.8	-8.8	-8.8	-8.8	-8.8	-8.8	-8.8	-8.8
-8.79	-8.79	-8.79	-8.79	-8.79	-8.79	-8.79	-8.79	-8.79
-8.78	-8.78	-8.78	-8.78	-8.78	-8.78	-8.78	-8.78	-8.78
-8.77	-8.77	-8.77	-8.77	-8.77	-8.77	-8.77	-8.77	-8.77
-8.76	-8.76	-8.76	-8.76	-8.76	-8.76	-8.76	-8.76	-8.76
-8.75	-8.75	-8.75	-8.75	-8.75	-8.75	-8.75	-8.75	-8.75
-8.74	-8.74	-8.74	-8.74	-8.74	-8.74	-8.74	-8.74	-8.74
-8.73	-8.73	-8.73	-8.73	-8.73	-8.73	-8.73	-8.73	-8.73
-8.72	-8.72	-8.72	-8.72	-8.72	-8.72	-8.72	-8.72	-8.72
-8.71	-8.71	-8.71	-8.71	-8.71	-8.71	-8.71	-8.71	-8.71
-8.7	-8.7	-8.7	-8.7	-8.7	-8.7	-8.7	-8.7	-8.7
-8.69	-8.69	-8.69	-8.69	-8.69	-8.69	-8.69	-8.69	-8.69
-8.68	-8.68	-8.68	-8.68	-8.68	-8.68	-8.68	-8.68	-8.68
-8.67	-8.67	-8.67	-8.67	-8.67	-8.67	-8.67	-8.67	-8.67
-8.66	-8.66	-8.66	-8.66	-8.66	-8.66	-8.66	-8.66	-8.66
-8.65	-8.65	-8.65	-8.65	-8.65	-8.65	-8.65	-8.65	-8.65
-8.64	-8.64	-8.64	-8.64	-8.64	-8.64	-8.64	-8.64	-8.64
-8.63	-8.63	-8.63	-8.63	-8.63	-8.63	-8.63	-8.63	-8.63
-8.62	-8.62	-8.62	-8.62	-8.62	-8.62	-8.62	-8.62	-8.62
-8.61	-8.61	-8.61	-8.61	-8.61	-8.61	-8.61	-8.61	-8.61
-8.6	-8.6	-8.6	-8.6	-8.6	-8.6	-8.6	-8.6	-8.6
-8.59	-8.59	-8.59	-8.59	-8.59	-8.59	-8.59	-8.59	-8.59
-8.58	-8.58	-8.58	-8.58	-8.58	-8.58	-8.58	-8.58	-8.58
-8.57	-8.57	-8.57	-8.57	-8.57	-8.57	-8.57	-8.57	-8.57
-8.56	-8.56	-8.56	-8.56	-8.56	-8.56	-8.56	-8.56	-8.56
-8.55	-8.55	-8.55	-8.55	-8.55	-8.55	-8.55	-8.55	-8.55
-8.54	-8.54	-8.54	-8.54	-8.54	-8.54	-8.54	-8.54	-8.54
-8.53	-8.53	-8.53	-8.53	-8.53	-8.53	-8.53	-8.53	-8.53
-8.52	-8.52	-8.52	-8.52	-8.52	-8.52	-8.52	-8.52	-8.52
-8.51	-8.51	-8.51	-8.51	-8.51	-8.51	-8.51	-8.51	-8.51

-8.5	-8.5	-8.5	-8.5	-8.5	-8.5	-8.5	-8.5	-8.5
-8.49	-8.49	-8.49	-8.49	-8.49	-8.49	-8.49	-8.49	-8.49
-8.48	-8.48	-8.48	-8.48	-8.48	-8.48	-8.48	-8.48	-8.48
-8.47	-8.47	-8.47	-8.47	-8.47	-8.47	-8.47	-8.47	-8.47
-8.46	-8.46	-8.46	-8.46	-8.46	-8.46	-8.46	-8.46	-8.46
-8.45	-8.45	-8.45	-8.45	-8.45	-8.45	-8.45	-8.45	-8.45
-8.44	-8.44	-8.44	-8.44	-8.44	-8.44	-8.44	-8.44	-8.44
-8.43	-8.43	-8.43	-8.43	-8.43	-8.43	-8.43	-8.43	-8.43
-8.42	-8.42	-8.42	-8.42	-8.42	-8.42	-8.42	-8.42	-8.42
-8.41	-8.41	-8.41	-8.41	-8.41	-8.41	-8.41	-8.41	-8.41
-8.4	-8.4	-8.4	-8.4	-8.4	-8.4	-8.4	-8.4	-8.4
-8.39	-8.39	-8.39	-8.39	-8.39	-8.39	-8.39	-8.39	-8.39
-8.38	-8.38	-8.38	-8.38	-8.38	-8.38	-8.38	-8.38	-8.38
-8.37	-8.37	-8.37	-8.37	-8.37	-8.37	-8.37	-8.37	-8.37
-8.36	-8.36	-8.36	-8.36	-8.36	-8.36	-8.36	-8.36	-8.36
-8.35	-8.35	-8.35	-8.35	-8.35	-8.35	-8.35	-8.35	-8.35
-8.34	-8.34	-8.34	-8.34	-8.34	-8.34	-8.34	-8.34	-8.34
-8.33	-8.33	-8.33	-8.33	-8.33	-8.33	-8.33	-8.33	-8.33
-8.32	-8.32	-8.32	-8.32	-8.32	-8.32	-8.32	-8.32	-8.32
-8.31	-8.31	-8.31	-8.31	-8.31	-8.31	-8.31	-8.31	-8.31
-8.3	-8.3	-8.3	-8.3	-8.3	-8.3	-8.3	-8.3	-8.3
-8.29	-8.29	-8.29	-8.29	-8.29	-8.29	-8.29	-8.29	-8.29
-8.28	-8.28	-8.28	-8.28	-8.28	-8.28	-8.28	-8.28	-8.28
-8.27	-8.27	-8.27	-8.27	-8.27	-8.27	-8.27	-8.27	-8.27
-8.26	-8.26	-8.26	-8.26	-8.26	-8.26	-8.26	-8.26	-8.26
-8.25	-8.25	-8.25	-8.25	-8.25	-8.25	-8.25	-8.25	-8.25
-8.24	-8.24	-8.24	-8.24	-8.24	-8.24	-8.24	-8.24	-8.24
-8.23	-8.23	-8.23	-8.23	-8.23	-8.23	-8.23	-8.23	-8.23
-8.22	-8.22	-8.22	-8.22	-8.22	-8.22	-8.22	-8.22	-8.22
-8.21	-8.21	-8.21	-8.21	-8.21	-8.21	-8.21	-8.21	-8.21
-8.2	-8.2	-8.2	-8.2	-8.2	-8.2	-8.2	-8.2	-8.2
-8.19	-8.19	-8.19	-8.19	-8.19	-8.19	-8.19	-8.19	-8.19
-8.18	-8.18	-8.18	-8.18	-8.18	-8.18	-8.18	-8.18	-8.18
-8.17	-8.17	-8.17	-8.17	-8.17	-8.17	-8.17	-8.17	-8.17
-8.16	-8.16	-8.16	-8.16	-8.16	-8.16	-8.16	-8.16	-8.16
-8.15	-8.15	-8.15	-8.15	-8.15	-8.15	-8.15	-8.15	-8.15
-8.14	-8.14	-8.14	-8.14	-8.14	-8.14	-8.14	-8.14	-8.14
-8.13	-8.13	-8.13	-8.13	-8.13	-8.13	-8.13	-8.13	-8.13
-8.12	-8.12	-8.12	-8.12	-8.12	-8.12	-8.12	-8.12	-8.12
-8.11	-8.11	-8.11	-8.11	-8.11	-8.11	-8.11	-8.11	-8.11
-8.1	-8.1	-8.1	-8.1	-8.1	-8.1	-8.1	-8.1	-8.1
-8.09	-8.09	-8.09	-8.09	-8.09	-8.09	-8.09	-8.09	-8.09
-8.08	-8.08	-8.08	-8.08	-8.08	-8.08	-8.08	-8.08	-8.08
-8.07	-8.07	-8.07	-8.07	-8.07	-8.07	-8.07	-8.07	-8.07
-8.06	-8.06	-8.06	-8.06	-8.06	-8.06	-8.06	-8.06	-8.06
-8.05	-8.05	-8.05	-8.05	-8.05	-8.05	-8.05	-8.05	-8.05
-8.04	-8.04	-8.04	-8.04	-8.04	-8.04	-8.04	-8.04	-8.04
-8.03	-8.03	-8.03	-8.03	-8.03	-8.03	-8.03	-8.03	-8.03
-8.02	-8.02	-8.02	-8.02	-8.02	-8.02	-8.02	-8.02	-8.02
-8.01	-8.01	-8.01	-8.01	-8.01	-8.01	-8.01	-8.01	-8.01

-8	-8	-8	-8	-8	-8	-8	-8	-8
-7.99	-7.99	-7.99	-7.99	-7.99	-7.99	-7.99	-7.99	-7.99
-7.98	-7.98	-7.98	-7.98	-7.98	-7.98	-7.98	-7.98	-7.98
-7.97	-7.97	-7.97	-7.97	-7.97	-7.97	-7.97	-7.97	-7.97
-7.96	-7.96	-7.96	-7.96	-7.96	-7.96	-7.96	-7.96	-7.96
-7.95	-7.95	-7.95	-7.95	-7.95	-7.95	-7.95	-7.95	-7.95
-7.94	-7.94	-7.94	-7.94	-7.94	-7.94	-7.94	-7.94	-7.94
-7.93	-7.93	-7.93	-7.93	-7.93	-7.93	-7.93	-7.93	-7.93
-7.92	-7.92	-7.92	-7.92	-7.92	-7.92	-7.92	-7.92	-7.92
-7.91	-7.91	-7.91	-7.91	-7.91	-7.91	-7.91	-7.91	-7.91
-7.9	-7.9	-7.9	-7.9	-7.9	-7.9	-7.9	-7.9	-7.9
-7.89	-7.89	-7.89	-7.89	-7.89	-7.89	-7.89	-7.89	-7.89
-7.88	-7.88	-7.88	-7.88	-7.88	-7.88	-7.88	-7.88	-7.88
-7.87	-7.87	-7.87	-7.87	-7.87	-7.87	-7.87	-7.87	-7.87
-7.86	-7.86	-7.86	-7.86	-7.86	-7.86	-7.86	-7.86	-7.86
-7.85	-7.85	-7.85	-7.85	-7.85	-7.85	-7.85	-7.85	-7.85
-7.84	-7.84	-7.84	-7.84	-7.84	-7.84	-7.84	-7.84	-7.84
-7.83	-7.83	-7.83	-7.83	-7.83	-7.83	-7.83	-7.83	-7.83
-7.82	-7.82	-7.82	-7.82	-7.82	-7.82	-7.82	-7.82	-7.82
-7.81	-7.81	-7.81	-7.81	-7.81	-7.81	-7.81	-7.81	-7.81
-7.8	-7.8	-7.8	-7.8	-7.8	-7.8	-7.8	-7.8	-7.8
-7.79	-7.79	-7.79	-7.79	-7.79	-7.79	-7.79	-7.79	-7.79
-7.78	-7.78	-7.78	-7.78	-7.78	-7.78	-7.78	-7.78	-7.78
-7.77	-7.77	-7.77	-7.77	-7.77	-7.77	-7.77	-7.77	-7.77
-7.76	-7.76	-7.76	-7.76	-7.76	-7.76	-7.76	-7.76	-7.76
-7.75	-7.75	-7.75	-7.75	-7.75	-7.75	-7.75	-7.75	-7.75
-7.74	-7.74	-7.74	-7.74	-7.74	-7.74	-7.74	-7.74	-7.74
-7.73	-7.73	-7.73	-7.73	-7.73	-7.73	-7.73	-7.73	-7.73
-7.72	-7.72	-7.72	-7.72	-7.72	-7.72	-7.72	-7.72	-7.72
-7.71	-7.71	-7.71	-7.71	-7.71	-7.71	-7.71	-7.71	-7.71
-7.7	-7.7	-7.7	-7.7	-7.7	-7.7	-7.7	-7.7	-7.7
-7.69	-7.69	-7.69	-7.69	-7.69	-7.69	-7.69	-7.69	-7.69
-7.68	-7.68	-7.68	-7.68	-7.68	-7.68	-7.68	-7.68	-7.68
-7.67	-7.67	-7.67	-7.67	-7.67	-7.67	-7.67	-7.67	-7.67
-7.66	-7.66	-7.66	-7.66	-7.66	-7.66	-7.66	-7.66	-7.66
-7.65	-7.65	-7.65	-7.65	-7.65	-7.65	-7.65	-7.65	-7.65
-7.64	-7.64	-7.64	-7.64	-7.64	-7.64	-7.64	-7.64	-7.64
-7.63	-7.63	-7.63	-7.63	-7.63	-7.63	-7.63	-7.63	-7.63
-7.62	-7.62	-7.62	-7.62	-7.62	-7.62	-7.62	-7.62	-7.62
-7.61	-7.61	-7.61	-7.61	-7.61	-7.61	-7.61	-7.61	-7.61
-7.6	-7.6	-7.6	-7.6	-7.6	-7.6	-7.6	-7.6	-7.6
-7.59	-7.59	-7.59	-7.59	-7.59	-7.59	-7.59	-7.59	-7.59
-7.58	-7.58	-7.58	-7.58	-7.58	-7.58	-7.58	-7.58	-7.58
-7.57	-7.57	-7.57	-7.57	-7.57	-7.57	-7.57	-7.57	-7.57
-7.56	-7.56	-7.56	-7.56	-7.56	-7.56	-7.56	-7.56	-7.56
-7.55	-7.55	-7.55	-7.55	-7.55	-7.55	-7.55	-7.55	-7.55
-7.54	-7.54	-7.54	-7.54	-7.54	-7.54	-7.54	-7.54	-7.54
-7.53	-7.53	-7.53	-7.53	-7.53	-7.53	-7.53	-7.53	-7.53
-7.52	-7.52	-7.52	-7.52	-7.52	-7.52	-7.52	-7.52	-7.52
-7.51	-7.51	-7.51	-7.51	-7.51	-7.51	-7.51	-7.51	-7.51

-7.5	-7.5	-7.5	-7.5	-7.5	-7.5	-7.5	-7.5	-7.5
-7.49	-7.49	-7.49	-7.49	-7.49	-7.49	-7.49	-7.49	-7.49
-7.48	-7.48	-7.48	-7.48	-7.48	-7.48	-7.48	-7.48	-7.48
-7.47	-7.47	-7.47	-7.47	-7.47	-7.47	-7.47	-7.47	-7.47
-7.46	-7.46	-7.46	-7.46	-7.46	-7.46	-7.46	-7.46	-7.46
-7.45	-7.45	-7.45	-7.45	-7.45	-7.45	-7.45	-7.45	-7.45
-7.44	-7.44	-7.44	-7.44	-7.44	-7.44	-7.44	-7.44	-7.44
-7.43	-7.43	-7.43	-7.43	-7.43	-7.43	-7.43	-7.43	-7.43
-7.42	-7.42	-7.42	-7.42	-7.42	-7.42	-7.42	-7.42	-7.42
-7.41	-7.41	-7.41	-7.41	-7.41	-7.41	-7.41	-7.41	-7.41
-7.4	-7.4	-7.4	-7.4	-7.4	-7.4	-7.4	-7.4	-7.4
-7.39	-7.39	-7.39	-7.39	-7.39	-7.39	-7.39	-7.39	-7.39
-7.38	-7.38	-7.38	-7.38	-7.38	-7.38	-7.38	-7.38	-7.38
-7.37	-7.37	-7.37	-7.37	-7.37	-7.37	-7.37	-7.37	-7.37
-7.36	-7.36	-7.36	-7.36	-7.36	-7.36	-7.36	-7.36	-7.36
-7.35	-7.35	-7.35	-7.35	-7.35	-7.35	-7.35	-7.35	-7.35
-7.34	-7.34	-7.34	-7.34	-7.34	-7.34	-7.34	-7.34	-7.34
-7.33	-7.33	-7.33	-7.33	-7.33	-7.33	-7.33	-7.33	-7.33
-7.32	-7.32	-7.32	-7.32	-7.32	-7.32	-7.32	-7.32	-7.32
-7.31	-7.31	-7.31	-7.31	-7.31	-7.31	-7.31	-7.31	-7.31
-7.3	-7.3	-7.3	-7.3	-7.3	-7.3	-7.3	-7.3	-7.3
-7.29	-7.29	-7.29	-7.29	-7.29	-7.29	-7.29	-7.29	-7.29
-7.28	-7.28	-7.28	-7.28	-7.28	-7.28	-7.28	-7.28	-7.28
-7.27	-7.27	-7.27	-7.27	-7.27	-7.27	-7.27	-7.27	-7.27
-7.26	-7.26	-7.26	-7.26	-7.26	-7.26	-7.26	-7.26	-7.26
-7.25	-7.25	-7.25	-7.25	-7.25	-7.25	-7.25	-7.25	-7.25
-7.24	-7.24	-7.24	-7.24	-7.24	-7.24	-7.24	-7.24	-7.24
-7.23	-7.23	-7.23	-7.23	-7.23	-7.23	-7.23	-7.23	-7.23
-7.22	-7.22	-7.22	-7.22	-7.22	-7.22	-7.22	-7.22	-7.22
-7.21	-7.21	-7.21	-7.21	-7.21	-7.21	-7.21	-7.21	-7.21
-7.2	-7.2	-7.2	-7.2	-7.2	-7.2	-7.2	-7.2	-7.2
-7.19	-7.19	-7.19	-7.19	-7.19	-7.19	-7.19	-7.19	-7.19
-7.18	-7.18	-7.18	-7.18	-7.18	-7.18	-7.18	-7.18	-7.18
-7.17	-7.17	-7.17	-7.17	-7.17	-7.17	-7.17	-7.17	-7.17
-7.16	-7.16	-7.16	-7.16	-7.16	-7.16	-7.16	-7.16	-7.16
-7.15	-7.15	-7.15	-7.15	-7.15	-7.15	-7.15	-7.15	-7.15
-7.14	-7.14	-7.14	-7.14	-7.14	-7.14	-7.14	-7.14	-7.14
-7.13	-7.13	-7.13	-7.13	-7.13	-7.13	-7.13	-7.13	-7.13
-7.12	-7.12	-7.12	-7.12	-7.12	-7.12	-7.12	-7.12	-7.12
-7.11	-7.11	-7.11	-7.11	-7.11	-7.11	-7.11	-7.11	-7.11
-7.1	-7.1	-7.1	-7.1	-7.1	-7.1	-7.1	-7.1	-7.1
-7.09	-7.09	-7.09	-7.09	-7.09	-7.09	-7.09	-7.09	-7.09
-7.08	-7.08	-7.08	-7.08	-7.08	-7.08	-7.08	-7.08	-7.08
-7.07	-7.07	-7.07	-7.07	-7.07	-7.07	-7.07	-7.07	-7.07
-7.06	-7.06	-7.06	-7.06	-7.06	-7.06	-7.06	-7.06	-7.06
-7.05	-7.05	-7.05	-7.05	-7.05	-7.05	-7.05	-7.05	-7.05
-7.04	-7.04	-7.04	-7.04	-7.04	-7.04	-7.04	-7.04	-7.04
-7.03	-7.03	-7.03	-7.03	-7.03	-7.03	-7.03	-7.03	-7.03
-7.02	-7.02	-7.02	-7.02	-7.02	-7.02	-7.02	-7.02	-7.02
-7.01	-7.01	-7.01	-7.01	-7.01	-7.01	-7.01	-7.01	-7.01

-7	-7	-7	-7	-7	-7	-7	-7	-7
-6.99	-6.99	-6.99	-6.99	-6.99	-6.99	-6.99	-6.99	-6.99
-6.98	-6.98	-6.98	-6.98	-6.98	-6.98	-6.98	-6.98	-6.98
-6.97	-6.97	-6.97	-6.97	-6.97	-6.97	-6.97	-6.97	-6.97
-6.96	-6.96	-6.96	-6.96	-6.96	-6.96	-6.96	-6.96	-6.96
-6.95	-6.95	-6.95	-6.95	-6.95	-6.95	-6.95	-6.95	-6.95
-6.94	-6.94	-6.94	-6.94	-6.94	-6.94	-6.94	-6.94	-6.94
-6.93	-6.93	-6.93	-6.93	-6.93	-6.93	-6.93	-6.93	-6.93
-6.92	-6.92	-6.92	-6.92	-6.92	-6.92	-6.92	-6.92	-6.92
-6.91	-6.91	-6.91	-6.91	-6.91	-6.91	-6.91	-6.91	-6.91
-6.9	-6.9	-6.9	-6.9	-6.9	-6.9	-6.9	-6.9	-6.9
-6.89	-6.89	-6.89	-6.89	-6.89	-6.89	-6.89	-6.89	-6.89
-6.88	-6.88	-6.88	-6.88	-6.88	-6.88	-6.88	-6.88	-6.88
-6.87	-6.87	-6.87	-6.87	-6.87	-6.87	-6.87	-6.87	-6.87
-6.86	-6.86	-6.86	-6.86	-6.86	-6.86	-6.86	-6.86	-6.86
-6.85	-6.85	-6.85	-6.85	-6.85	-6.85	-6.85	-6.85	-6.85
-6.84	-6.84	-6.84	-6.84	-6.84	-6.84	-6.84	-6.84	-6.84
-6.83	-6.83	-6.83	-6.83	-6.83	-6.83	-6.83	-6.83	-6.83
-6.82	-6.82	-6.82	-6.82	-6.82	-6.82	-6.82	-6.82	-6.82
-6.81	-6.81	-6.81	-6.81	-6.81	-6.81	-6.81	-6.81	-6.81
-6.8	-6.8	-6.8	-6.8	-6.8	-6.8	-6.8	-6.8	-6.8
-6.79	-6.79	-6.79	-6.79	-6.79	-6.79	-6.79	-6.79	-6.79
-6.78	-6.78	-6.78	-6.78	-6.78	-6.78	-6.78	-6.78	-6.78
-6.77	-6.77	-6.77	-6.77	-6.77	-6.77	-6.77	-6.77	-6.77
-6.76	-6.76	-6.76	-6.76	-6.76	-6.76	-6.76	-6.76	-6.76
-6.75	-6.75	-6.75	-6.75	-6.75	-6.75	-6.75	-6.75	-6.75
-6.74	-6.74	-6.74	-6.74	-6.74	-6.74	-6.74	-6.74	-6.74
-6.73	-6.73	-6.73	-6.73	-6.73	-6.73	-6.73	-6.73	-6.73
-6.72	-6.72	-6.72	-6.72	-6.72	-6.72	-6.72	-6.72	-6.72
-6.71	-6.71	-6.71	-6.71	-6.71	-6.71	-6.71	-6.71	-6.71
-6.7	-6.7	-6.7	-6.7	-6.7	-6.7	-6.7	-6.7	-6.7
-6.69	-6.69	-6.69	-6.69	-6.69	-6.69	-6.69	-6.69	-6.69
-6.68	-6.68	-6.68	-6.68	-6.68	-6.68	-6.68	-6.68	-6.68
-6.67	-6.67	-6.67	-6.67	-6.67	-6.67	-6.67	-6.67	-6.67
-6.66	-6.66	-6.66	-6.66	-6.66	-6.66	-6.66	-6.66	-6.66
-6.65	-6.65	-6.65	-6.65	-6.65	-6.65	-6.65	-6.65	-6.65
-6.64	-6.64	-6.64	-6.64	-6.64	-6.64	-6.64	-6.64	-6.64
-6.63	-6.63	-6.63	-6.63	-6.63	-6.63	-6.63	-6.63	-6.63
-6.62	-6.62	-6.62	-6.62	-6.62	-6.62	-6.62	-6.62	-6.62
-6.61	-6.61	-6.61	-6.61	-6.61	-6.61	-6.61	-6.61	-6.61
-6.6	-6.6	-6.6	-6.6	-6.6	-6.6	-6.6	-6.6	-6.6
-6.59	-6.59	-6.59	-6.59	-6.59	-6.59	-6.59	-6.59	-6.59
-6.58	-6.58	-6.58	-6.58	-6.58	-6.58	-6.58	-6.58	-6.58
-6.57	-6.57	-6.57	-6.57	-6.57	-6.57	-6.57	-6.57	-6.57
-6.56	-6.56	-6.56	-6.56	-6.56	-6.56	-6.56	-6.56	-6.56
-6.55	-6.55	-6.55	-6.55	-6.55	-6.55	-6.55	-6.55	-6.55
-6.54	-6.54	-6.54	-6.54	-6.54	-6.54	-6.54	-6.54	-6.54
-6.53	-6.53	-6.53	-6.53	-6.53	-6.53	-6.53	-6.53	-6.53
-6.52	-6.52	-6.52	-6.52	-6.52	-6.52	-6.52	-6.52	-6.52
-6.51	-6.51	-6.51	-6.51	-6.51	-6.51	-6.51	-6.51	-6.51

-6.5	-6.5	-6.5	-6.5	-6.5	-6.5	-6.5	-6.5	-6.5
-6.49	-6.49	-6.49	-6.49	-6.49	-6.49	-6.49	-6.49	-6.49
-6.48	-6.48	-6.48	-6.48	-6.48	-6.48	-6.48	-6.48	-6.48
-6.47	-6.47	-6.47	-6.47	-6.47	-6.47	-6.47	-6.47	-6.47
-6.46	-6.46	-6.46	-6.46	-6.46	-6.46	-6.46	-6.46	-6.46
-6.45	-6.45	-6.45	-6.45	-6.45	-6.45	-6.45	-6.45	-6.45
-6.44	-6.44	-6.44	-6.44	-6.44	-6.44	-6.44	-6.44	-6.44
-6.43	-6.43	-6.43	-6.43	-6.43	-6.43	-6.43	-6.43	-6.43
-6.42	-6.42	-6.42	-6.42	-6.42	-6.42	-6.42	-6.42	-6.42
-6.41	-6.41	-6.41	-6.41	-6.41	-6.41	-6.41	-6.41	-6.41
-6.4	-6.4	-6.4	-6.4	-6.4	-6.4	-6.4	-6.4	-6.4
-6.39	-6.39	-6.39	-6.39	-6.39	-6.39	-6.39	-6.39	-6.39
-6.38	-6.38	-6.38	-6.38	-6.38	-6.38	-6.38	-6.38	-6.38
-6.37	-6.37	-6.37	-6.37	-6.37	-6.37	-6.37	-6.37	-6.37
-6.36	-6.36	-6.36	-6.36	-6.36	-6.36	-6.36	-6.36	-6.36
-6.35	-6.35	-6.35	-6.35	-6.35	-6.35	-6.35	-6.35	-6.35
-6.34	-6.34	-6.34	-6.34	-6.34	-6.34	-6.34	-6.34	-6.34
-6.33	-6.33	-6.33	-6.33	-6.33	-6.33	-6.33	-6.33	-6.33
-6.32	-6.32	-6.32	-6.32	-6.32	-6.32	-6.32	-6.32	-6.32
-6.31	-6.31	-6.31	-6.31	-6.31	-6.31	-6.31	-6.31	-6.31
-6.3	-6.3	-6.3	-6.3	-6.3	-6.3	-6.3	-6.3	-6.3
-6.29	-6.29	-6.29	-6.29	-6.29	-6.29	-6.29	-6.29	-6.29
-6.28	-6.28	-6.28	-6.28	-6.28	-6.28	-6.28	-6.28	-6.28
-6.27	-6.27	-6.27	-6.27	-6.27	-6.27	-6.27	-6.27	-6.27
-6.26	-6.26	-6.26	-6.26	-6.26	-6.26	-6.26	-6.26	-6.26
-6.25	-6.25	-6.25	-6.25	-6.25	-6.25	-6.25	-6.25	-6.25
-6.24	-6.24	-6.24	-6.24	-6.24	-6.24	-6.24	-6.24	-6.24
-6.23	-6.23	-6.23	-6.23	-6.23	-6.23	-6.23	-6.23	-6.23
-6.22	-6.22	-6.22	-6.22	-6.22	-6.22	-6.22	-6.22	-6.22
-6.21	-6.21	-6.21	-6.21	-6.21	-6.21	-6.21	-6.21	-6.21
-6.2	-6.2	-6.2	-6.2	-6.2	-6.2	-6.2	-6.2	-6.2
-6.19	-6.19	-6.19	-6.19	-6.19	-6.19	-6.19	-6.19	-6.19
-6.18	-6.18	-6.18	-6.18	-6.18	-6.18	-6.18	-6.18	-6.18
-6.17	-6.17	-6.17	-6.17	-6.17	-6.17	-6.17	-6.17	-6.17
-6.16	-6.16	-6.16	-6.16	-6.16	-6.16	-6.16	-6.16	-6.16
-6.15	-6.15	-6.15	-6.15	-6.15	-6.15	-6.15	-6.15	-6.15
-6.14	-6.14	-6.14	-6.14	-6.14	-6.14	-6.14	-6.14	-6.14
-6.13	-6.13	-6.13	-6.13	-6.13	-6.13	-6.13	-6.13	-6.13
-6.12	-6.12	-6.12	-6.12	-6.12	-6.12	-6.12	-6.12	-6.12
-6.11	-6.11	-6.11	-6.11	-6.11	-6.11	-6.11	-6.11	-6.11
-6.1	-6.1	-6.1	-6.1	-6.1	-6.1	-6.1	-6.1	-6.1
-6.09	-6.09	-6.09	-6.09	-6.09	-6.09	-6.09	-6.09	-6.09
-6.08	-6.08	-6.08	-6.08	-6.08	-6.08	-6.08	-6.08	-6.08
-6.07	-6.07	-6.07	-6.07	-6.07	-6.07	-6.07	-6.07	-6.07
-6.06	-6.06	-6.06	-6.06	-6.06	-6.06	-6.06	-6.06	-6.06
-6.05	-6.05	-6.05	-6.05	-6.05	-6.05	-6.05	-6.05	-6.05
-6.04	-6.04	-6.04	-6.04	-6.04	-6.04	-6.04	-6.04	-6.04
-6.03	-6.03	-6.03	-6.03	-6.03	-6.03	-6.03	-6.03	-6.03
-6.02	-6.02	-6.02	-6.02	-6.02	-6.02	-6.02	-6.02	-6.02
-6.01	-6.01	-6.01	-6.01	-6.01	-6.01	-6.01	-6.01	-6.01

-6	-6	-6	-6	-6	-6	-6	-6	-6
-5.99	-5.99	-5.99	-5.99	-5.99	-5.99	-5.99	-5.99	-5.99
-5.98	-5.98	-5.98	-5.98	-5.98	-5.98	-5.98	-5.98	-5.98
-5.97	-5.97	-5.97	-5.97	-5.97	-5.97	-5.97	-5.97	-5.97
-5.96	-5.96	-5.96	-5.96	-5.96	-5.96	-5.96	-5.96	-5.96
-5.95	-5.95	-5.95	-5.95	-5.95	-5.95	-5.95	-5.95	-5.95
-5.94	-5.94	-5.94	-5.94	-5.94	-5.94	-5.94	-5.94	-5.94
-5.93	-5.93	-5.93	-5.93	-5.93	-5.93	-5.93	-5.93	-5.93
-5.92	-5.92	-5.92	-5.92	-5.92	-5.92	-5.92	-5.92	-5.92
-5.91	-5.91	-5.91	-5.91	-5.91	-5.91	-5.91	-5.91	-5.91
-5.9	-5.9	-5.9	-5.9	-5.9	-5.9	-5.9	-5.9	-5.9
-5.89	-5.89	-5.89	-5.89	-5.89	-5.89	-5.89	-5.89	-5.89
-5.88	-5.88	-5.88	-5.88	-5.88	-5.88	-5.88	-5.88	-5.88
-5.87	-5.87	-5.87	-5.87	-5.87	-5.87	-5.87	-5.87	-5.87
-5.86	-5.86	-5.86	-5.86	-5.86	-5.86	-5.86	-5.86	-5.86
-5.85	-5.85	-5.85	-5.85	-5.85	-5.85	-5.85	-5.85	-5.85
-5.84	-5.84	-5.84	-5.84	-5.84	-5.84	-5.84	-5.84	-5.84
-5.83	-5.83	-5.83	-5.83	-5.83	-5.83	-5.83	-5.83	-5.83
-5.82	-5.82	-5.82	-5.82	-5.82	-5.82	-5.82	-5.82	-5.82
-5.81	-5.81	-5.81	-5.81	-5.81	-5.81	-5.81	-5.81	-5.81
-5.8	-5.8	-5.8	-5.8	-5.8	-5.8	-5.8	-5.8	-5.8
-5.79	-5.79	-5.79	-5.79	-5.79	-5.79	-5.79	-5.79	-5.79
-5.78	-5.78	-5.78	-5.78	-5.78	-5.78	-5.78	-5.78	-5.78
-5.77	-5.77	-5.77	-5.77	-5.77	-5.77	-5.77	-5.77	-5.77
-5.76	-5.76	-5.76	-5.76	-5.76	-5.76	-5.76	-5.76	-5.76
-5.75	-5.75	-5.75	-5.75	-5.75	-5.75	-5.75	-5.75	-5.75
-5.74	-5.74	-5.74	-5.74	-5.74	-5.74	-5.74	-5.74	-5.74
-5.73	-5.73	-5.73	-5.73	-5.73	-5.73	-5.73	-5.73	-5.73
-5.72	-5.72	-5.72	-5.72	-5.72	-5.72	-5.72	-5.72	-5.72
-5.71	-5.71	-5.71	-5.71	-5.71	-5.71	-5.71	-5.71	-5.71
-5.7	-5.7	-5.7	-5.7	-5.7	-5.7	-5.7	-5.7	-5.7
-5.69	-5.69	-5.69	-5.69	-5.69	-5.69	-5.69	-5.69	-5.69
-5.68	-5.68	-5.68	-5.68	-5.68	-5.68	-5.68	-5.68	-5.68
-5.67	-5.67	-5.67	-5.67	-5.67	-5.67	-5.67	-5.67	-5.67
-5.66	-5.66	-5.66	-5.66	-5.66	-5.66	-5.66	-5.66	-5.66
-5.65	-5.65	-5.65	-5.65	-5.65	-5.65	-5.65	-5.65	-5.65
-5.64	-5.64	-5.64	-5.64	-5.64	-5.64	-5.64	-5.64	-5.64
-5.63	-5.63	-5.63	-5.63	-5.63	-5.63	-5.63	-5.63	-5.63
-5.62	-5.62	-5.62	-5.62	-5.62	-5.62	-5.62	-5.62	-5.62
-5.61	-5.61	-5.61	-5.61	-5.61	-5.61	-5.61	-5.61	-5.61
-5.6	-5.6	-5.6	-5.6	-5.6	-5.6	-5.6	-5.6	-5.6
-5.59	-5.59	-5.59	-5.59	-5.59	-5.59	-5.59	-5.59	-5.59
-5.58	-5.58	-5.58	-5.58	-5.58	-5.58	-5.58	-5.58	-5.58
-5.57	-5.57	-5.57	-5.57	-5.57	-5.57	-5.57	-5.57	-5.57
-5.56	-5.56	-5.56	-5.56	-5.56	-5.56	-5.56	-5.56	-5.56
-5.55	-5.55	-5.55	-5.55	-5.55	-5.55	-5.55	-5.55	-5.55
-5.54	-5.54	-5.54	-5.54	-5.54	-5.54	-5.54	-5.54	-5.54
-5.53	-5.53	-5.53	-5.53	-5.53	-5.53	-5.53	-5.53	-5.53
-5.52	-5.52	-5.52	-5.52	-5.52	-5.52	-5.52	-5.52	-5.52
-5.51	-5.51	-5.51	-5.51	-5.51	-5.51	-5.51	-5.51	-5.51

-5.5	-5.5	-5.5	-5.5	-5.5	-5.5	-5.5	-5.5	-5.5
-5.49	-5.49	-5.49	-5.49	-5.49	-5.49	-5.49	-5.49	-5.49
-5.48	-5.48	-5.48	-5.48	-5.48	-5.48	-5.48	-5.48	-5.48
-5.47	-5.47	-5.47	-5.47	-5.47	-5.47	-5.47	-5.47	-5.47
-5.46	-5.46	-5.46	-5.46	-5.46	-5.46	-5.46	-5.46	-5.46
-5.45	-5.45	-5.45	-5.45	-5.45	-5.45	-5.45	-5.45	-5.45
-5.44	-5.44	-5.44	-5.44	-5.44	-5.44	-5.44	-5.44	-5.44
-5.43	-5.43	-5.43	-5.43	-5.43	-5.43	-5.43	-5.43	-5.43
-5.42	-5.42	-5.42	-5.42	-5.42	-5.42	-5.42	-5.42	-5.42
-5.41	-5.41	-5.41	-5.41	-5.41	-5.41	-5.41	-5.41	-5.41
-5.4	-5.4	-5.4	-5.4	-5.4	-5.4	-5.4	-5.4	-5.4
-5.39	-5.39	-5.39	-5.39	-5.39	-5.39	-5.39	-5.39	-5.39
-5.38	-5.38	-5.38	-5.38	-5.38	-5.38	-5.38	-5.38	-5.38
-5.37	-5.37	-5.37	-5.37	-5.37	-5.37	-5.37	-5.37	-5.37
-5.36	-5.36	-5.36	-5.36	-5.36	-5.36	-5.36	-5.36	-5.36
-5.35	-5.35	-5.35	-5.35	-5.35	-5.35	-5.35	-5.35	-5.35
-5.34	-5.34	-5.34	-5.34	-5.34	-5.34	-5.34	-5.34	-5.34
-5.33	-5.33	-5.33	-5.33	-5.33	-5.33	-5.33	-5.33	-5.33
-5.32	-5.32	-5.32	-5.32	-5.32	-5.32	-5.32	-5.32	-5.32
-5.31	-5.31	-5.31	-5.31	-5.31	-5.31	-5.31	-5.31	-5.31
-5.3	-5.3	-5.3	-5.3	-5.3	-5.3	-5.3	-5.3	-5.3
-5.29	-5.29	-5.29	-5.29	-5.29	-5.29	-5.29	-5.29	-5.29
-5.28	-5.28	-5.28	-5.28	-5.28	-5.28	-5.28	-5.28	-5.28
-5.27	-5.27	-5.27	-5.27	-5.27	-5.27	-5.27	-5.27	-5.27
-5.26	-5.26	-5.26	-5.26	-5.26	-5.26	-5.26	-5.26	-5.26
-5.25	-5.25	-5.25	-5.25	-5.25	-5.25	-5.25	-5.25	-5.25
-5.24	-5.24	-5.24	-5.24	-5.24	-5.24	-5.24	-5.24	-5.24
-5.23	-5.23	-5.23	-5.23	-5.23	-5.23	-5.23	-5.23	-5.23
-5.22	-5.22	-5.22	-5.22	-5.22	-5.22	-5.22	-5.22	-5.22
-5.21	-5.21	-5.21	-5.21	-5.21	-5.21	-5.21	-5.21	-5.21
-5.2	-5.2	-5.2	-5.2	-5.2	-5.2	-5.2	-5.2	-5.2
-5.19	-5.19	-5.19	-5.19	-5.19	-5.19	-5.19	-5.19	-5.19
-5.18	-5.18	-5.18	-5.18	-5.18	-5.18	-5.18	-5.18	-5.18
-5.17	-5.17	-5.17	-5.17	-5.17	-5.17	-5.17	-5.17	-5.17
-5.16	-5.16	-5.16	-5.16	-5.16	-5.16	-5.16	-5.16	-5.16
-5.15	-5.15	-5.15	-5.15	-5.15	-5.15	-5.15	-5.15	-5.15
-5.14	-5.14	-5.14	-5.14	-5.14	-5.14	-5.14	-5.14	-5.14
-5.13	-5.13	-5.13	-5.13	-5.13	-5.13	-5.13	-5.13	-5.13
-5.12	-5.12	-5.12	-5.12	-5.12	-5.12	-5.12	-5.12	-5.12
-5.11	-5.11	-5.11	-5.11	-5.11	-5.11	-5.11	-5.11	-5.11
-5.1	-5.1	-5.1	-5.1	-5.1	-5.1	-5.1	-5.1	-5.1
-5.09	-5.09	-5.09	-5.09	-5.09	-5.09	-5.09	-5.09	-5.09
-5.08	-5.08	-5.08	-5.08	-5.08	-5.08	-5.08	-5.08	-5.08
-5.07	-5.07	-5.07	-5.07	-5.07	-5.07	-5.07	-5.07	-5.07
-5.06	-5.06	-5.06	-5.06	-5.06	-5.06	-5.06	-5.06	-5.06
-5.05	-5.05	-5.05	-5.05	-5.05	-5.05	-5.05	-5.05	-5.05
-5.04	-5.04	-5.04	-5.04	-5.04	-5.04	-5.04	-5.04	-5.04
-5.03	-5.03	-5.03	-5.03	-5.03	-5.03	-5.03	-5.03	-5.03
-5.02	-5.02	-5.02	-5.02	-5.02	-5.02	-5.02	-5.02	-5.02
-5.01	-5.01	-5.01	-5.01	-5.01	-5.01	-5.01	-5.01	-5.01

-5	-5	-5	-5	-5	-5	-5	-5	-5
-4.99	-4.99	-4.99	-4.99	-4.99	-4.99	-4.99	-4.99	-4.99
-4.98	-4.98	-4.98	-4.98	-4.98	-4.98	-4.98	-4.98	-4.98
-4.97	-4.97	-4.97	-4.97	-4.97	-4.97	-4.97	-4.97	-4.97
-4.96	-4.96	-4.96	-4.96	-4.96	-4.96	-4.96	-4.96	-4.96
-4.95	-4.95	-4.95	-4.95	-4.95	-4.95	-4.95	-4.95	-4.95
-4.94	-4.94	-4.94	-4.94	-4.94	-4.94	-4.94	-4.94	-4.94
-4.93	-4.93	-4.93	-4.93	-4.93	-4.93	-4.93	-4.93	-4.93
-4.92	-4.92	-4.92	-4.92	-4.92	-4.92	-4.92	-4.92	-4.92
-4.91	-4.91	-4.91	-4.91	-4.91	-4.91	-4.91	-4.91	-4.91
-4.9	-4.9	-4.9	-4.9	-4.9	-4.9	-4.9	-4.9	-4.9
-4.89	-4.89	-4.89	-4.89	-4.89	-4.89	-4.89	-4.89	-4.89
-4.88	-4.88	-4.88	-4.88	-4.88	-4.88	-4.88	-4.88	-4.88
-4.87	-4.87	-4.87	-4.87	-4.87	-4.87	-4.87	-4.87	-4.87
-4.86	-4.86	-4.86	-4.86	-4.86	-4.86	-4.86	-4.86	-4.86
-4.85	-4.85	-4.85	-4.85	-4.85	-4.85	-4.85	-4.85	-4.85
-4.84	-4.84	-4.84	-4.84	-4.84	-4.84	-4.84	-4.84	-4.84
-4.83	-4.83	-4.83	-4.83	-4.83	-4.83	-4.83	-4.83	-4.83
-4.82	-4.82	-4.82	-4.82	-4.82	-4.82	-4.82	-4.82	-4.82
-4.81	-4.81	-4.81	-4.81	-4.81	-4.81	-4.81	-4.81	-4.81
-4.8	-4.8	-4.8	-4.8	-4.8	-4.8	-4.8	-4.8	-4.8
-4.79	-4.79	-4.79	-4.79	-4.79	-4.79	-4.79	-4.79	-4.79
-4.78	-4.78	-4.78	-4.78	-4.78	-4.78	-4.78	-4.78	-4.78
-4.77	-4.77	-4.77	-4.77	-4.77	-4.77	-4.77	-4.77	-4.77
-4.76	-4.76	-4.76	-4.76	-4.76	-4.76	-4.76	-4.76	-4.76
-4.75	-4.75	-4.75	-4.75	-4.75	-4.75	-4.75	-4.75	-4.75
-4.74	-4.74	-4.74	-4.74	-4.74	-4.74	-4.74	-4.74	-4.74
-4.73	-4.73	-4.73	-4.73	-4.73	-4.73	-4.73	-4.73	-4.73
-4.72	-4.72	-4.72	-4.72	-4.72	-4.72	-4.72	-4.72	-4.72
-4.71	-4.71	-4.71	-4.71	-4.71	-4.71	-4.71	-4.71	-4.71
-4.7	-4.7	-4.7	-4.7	-4.7	-4.7	-4.7	-4.7	-4.7
-4.69	-4.69	-4.69	-4.69	-4.69	-4.69	-4.69	-4.69	-4.69
-4.68	-4.68	-4.68	-4.68	-4.68	-4.68	-4.68	-4.68	-4.68
-4.67	-4.67	-4.67	-4.67	-4.67	-4.67	-4.67	-4.67	-4.67
-4.66	-4.66	-4.66	-4.66	-4.66	-4.66	-4.66	-4.66	-4.66
-4.65	-4.65	-4.65	-4.65	-4.65	-4.65	-4.65	-4.65	-4.65
-4.64	-4.64	-4.64	-4.64	-4.64	-4.64	-4.64	-4.64	-4.64
-4.63	-4.63	-4.63	-4.63	-4.63	-4.63	-4.63	-4.63	-4.63
-4.62	-4.62	-4.62	-4.62	-4.62	-4.62	-4.62	-4.62	-4.62
-4.61	-4.61	-4.61	-4.61	-4.61	-4.61	-4.61	-4.61	-4.61
-4.6	-4.6	-4.6	-4.6	-4.6	-4.6	-4.6	-4.6	-4.6
-4.59	-4.59	-4.59	-4.59	-4.59	-4.59	-4.59	-4.59	-4.59
-4.58	-4.58	-4.58	-4.58	-4.58	-4.58	-4.58	-4.58	-4.58
-4.57	-4.57	-4.57	-4.57	-4.57	-4.57	-4.57	-4.57	-4.57
-4.56	-4.56	-4.56	-4.56	-4.56	-4.56	-4.56	-4.56	-4.56
-4.55	-4.55	-4.55	-4.55	-4.55	-4.55	-4.55	-4.55	-4.55
-4.54	-4.54	-4.54	-4.54	-4.54	-4.54	-4.54	-4.54	-4.54
-4.53	-4.53	-4.53	-4.53	-4.53	-4.53	-4.53	-4.53	-4.53
-4.52	-4.52	-4.52	-4.52	-4.52	-4.52	-4.52	-4.52	-4.52
-4.51	-4.51	-4.51	-4.51	-4.51	-4.51	-4.51	-4.51	-4.51

-4.5	-4.5	-4.5	-4.5	-4.5	-4.5	-4.5	-4.5	-4.5
-4.49	-4.49	-4.49	-4.49	-4.49	-4.49	-4.49	-4.49	-4.49
-4.48	-4.48	-4.48	-4.48	-4.48	-4.48	-4.48	-4.48	-4.48
-4.47	-4.47	-4.47	-4.47	-4.47	-4.47	-4.47	-4.47	-4.47
-4.46	-4.46	-4.46	-4.46	-4.46	-4.46	-4.46	-4.46	-4.46
-4.45	-4.45	-4.45	-4.45	-4.45	-4.45	-4.45	-4.45	-4.45
-4.44	-4.44	-4.44	-4.44	-4.44	-4.44	-4.44	-4.44	-4.44
-4.43	-4.43	-4.43	-4.43	-4.43	-4.43	-4.43	-4.43	-4.43
-4.42	-4.42	-4.42	-4.42	-4.42	-4.42	-4.42	-4.42	-4.42
-4.41	-4.41	-4.41	-4.41	-4.41	-4.41	-4.41	-4.41	-4.41
-4.4	-4.4	-4.4	-4.4	-4.4	-4.4	-4.4	-4.4	-4.4
-4.39	-4.39	-4.39	-4.39	-4.39	-4.39	-4.39	-4.39	-4.39
-4.38	-4.38	-4.38	-4.38	-4.38	-4.38	-4.38	-4.38	-4.38
-4.37	-4.37	-4.37	-4.37	-4.37	-4.37	-4.37	-4.37	-4.37
-4.36	-4.36	-4.36	-4.36	-4.36	-4.36	-4.36	-4.36	-4.36
-4.35	-4.35	-4.35	-4.35	-4.35	-4.35	-4.35	-4.35	-4.35
-4.34	-4.34	-4.34	-4.34	-4.34	-4.34	-4.34	-4.34	-4.34
-4.33	-4.33	-4.33	-4.33	-4.33	-4.33	-4.33	-4.33	-4.33
-4.32	-4.32	-4.32	-4.32	-4.32	-4.32	-4.32	-4.32	-4.32
-4.31	-4.31	-4.31	-4.31	-4.31	-4.31	-4.31	-4.31	-4.31
-4.3	-4.3	-4.3	-4.3	-4.3	-4.3	-4.3	-4.3	-4.3
-4.29	-4.29	-4.29	-4.29	-4.29	-4.29	-4.29	-4.29	-4.29
-4.28	-4.28	-4.28	-4.28	-4.28	-4.28	-4.28	-4.28	-4.28
-4.27	-4.27	-4.27	-4.27	-4.27	-4.27	-4.27	-4.27	-4.27
-4.26	-4.26	-4.26	-4.26	-4.26	-4.26	-4.26	-4.26	-4.26
-4.25	-4.25	-4.25	-4.25	-4.25	-4.25	-4.25	-4.25	-4.25
-4.24	-4.24	-4.24	-4.24	-4.24	-4.24	-4.24	-4.24	-4.24
-4.23	-4.23	-4.23	-4.23	-4.23	-4.23	-4.23	-4.23	-4.23
-4.22	-4.22	-4.22	-4.22	-4.22	-4.22	-4.22	-4.22	-4.22
-4.21	-4.21	-4.21	-4.21	-4.21	-4.21	-4.21	-4.21	-4.21
-4.2	-4.2	-4.2	-4.2	-4.2	-4.2	-4.2	-4.2	-4.2
-4.19	-4.19	-4.19	-4.19	-4.19	-4.19	-4.19	-4.19	-4.19
-4.18	-4.18	-4.18	-4.18	-4.18	-4.18	-4.18	-4.18	-4.18
-4.17	-4.17	-4.17	-4.17	-4.17	-4.17	-4.17	-4.17	-4.17
-4.16	-4.16	-4.16	-4.16	-4.16	-4.16	-4.16	-4.16	-4.16
-4.15	-4.15	-4.15	-4.15	-4.15	-4.15	-4.15	-4.15	-4.15
-4.14	-4.14	-4.14	-4.14	-4.14	-4.14	-4.14	-4.14	-4.14
-4.13	-4.13	-4.13	-4.13	-4.13	-4.13	-4.13	-4.13	-4.13
-4.12	-4.12	-4.12	-4.12	-4.12	-4.12	-4.12	-4.12	-4.12
-4.11	-4.11	-4.11	-4.11	-4.11	-4.11	-4.11	-4.11	-4.11
-4.1	-4.1	-4.1	-4.1	-4.1	-4.1	-4.1	-4.1	-4.1
-4.09	-4.09	-4.09	-4.09	-4.09	-4.09	-4.09	-4.09	-4.09
-4.08	-4.08	-4.08	-4.08	-4.08	-4.08	-4.08	-4.08	-4.08
-4.07	-4.07	-4.07	-4.07	-4.07	-4.07	-4.07	-4.07	-4.07
-4.06	-4.06	-4.06	-4.06	-4.06	-4.06	-4.06	-4.06	-4.06
-4.05	-4.05	-4.05	-4.05	-4.05	-4.05	-4.05	-4.05	-4.05
-4.04	-4.04	-4.04	-4.04	-4.04	-4.04	-4.04	-4.04	-4.04
-4.03	-4.03	-4.03	-4.03	-4.03	-4.03	-4.03	-4.03	-4.03
-4.02	-4.02	-4.02	-4.02	-4.02	-4.02	-4.02	-4.02	-4.02
-4.01	-4.01	-4.01	-4.01	-4.01	-4.01	-4.01	-4.01	-4.01

-4	-4	-4	-4	-4	-4	-4	-4	-4
-3.99	-3.99	-3.99	-3.99	-3.99	-3.99	-3.99	-3.99	-3.99
-3.98	-3.98	-3.98	-3.98	-3.98	-3.98	-3.98	-3.98	-3.98
-3.97	-3.97	-3.97	-3.97	-3.97	-3.97	-3.97	-3.97	-3.97
-3.96	-3.96	-3.96	-3.96	-3.96	-3.96	-3.96	-3.96	-3.96
-3.95	-3.95	-3.95	-3.95	-3.95	-3.95	-3.95	-3.95	-3.95
-3.94	-3.94	-3.94	-3.94	-3.94	-3.94	-3.94	-3.94	-3.94
-3.93	-3.93	-3.93	-3.93	-3.93	-3.93	-3.93	-3.93	-3.93
-3.92	-3.92	-3.92	-3.92	-3.92	-3.92	-3.92	-3.92	-3.92
-3.91	-3.91	-3.91	-3.91	-3.91	-3.91	-3.91	-3.91	-3.91
-3.9	-3.9	-3.9	-3.9	-3.9	-3.9	-3.9	-3.9	-3.9
-3.89	-3.89	-3.89	-3.89	-3.89	-3.89	-3.89	-3.89	-3.89
-3.88	-3.88	-3.88	-3.88	-3.88	-3.88	-3.88	-3.88	-3.88
-3.87	-3.87	-3.87	-3.87	-3.87	-3.87	-3.87	-3.87	-3.87
-3.86	-3.86	-3.86	-3.86	-3.86	-3.86	-3.86	-3.86	-3.86
-3.85	-3.85	-3.85	-3.85	-3.85	-3.85	-3.85	-3.85	-3.85
-3.84	-3.84	-3.84	-3.84	-3.84	-3.84	-3.84	-3.84	-3.84
-3.83	-3.83	-3.83	-3.83	-3.83	-3.83	-3.83	-3.83	-3.83
-3.82	-3.82	-3.82	-3.82	-3.82	-3.82	-3.82	-3.82	-3.82
-3.81	-3.81	-3.81	-3.81	-3.81	-3.81	-3.81	-3.81	-3.81
-3.8	-3.8	-3.8	-3.8	-3.8	-3.8	-3.8	-3.8	-3.8
-3.79	-3.79	-3.79	-3.79	-3.79	-3.79	-3.79	-3.79	-3.79
-3.78	-3.78	-3.78	-3.78	-3.78	-3.78	-3.78	-3.78	-3.78
-3.77	-3.77	-3.77	-3.77	-3.77	-3.77	-3.77	-3.77	-3.77
-3.76	-3.76	-3.76	-3.76	-3.76	-3.76	-3.76	-3.76	-3.76
-3.75	-3.75	-3.75	-3.75	-3.75	-3.75	-3.75	-3.75	-3.75
-3.74	-3.74	-3.74	-3.74	-3.74	-3.74	-3.74	-3.74	-3.74
-3.73	-3.73	-3.73	-3.73	-3.73	-3.73	-3.73	-3.73	-3.73
-3.72	-3.72	-3.72	-3.72	-3.72	-3.72	-3.72	-3.72	-3.72
-3.71	-3.71	-3.71	-3.71	-3.71	-3.71	-3.71	-3.71	-3.71
-3.7	-3.7	-3.7	-3.7	-3.7	-3.7	-3.7	-3.7	-3.7
-3.69	-3.69	-3.69	-3.69	-3.69	-3.69	-3.69	-3.69	-3.69
-3.68	-3.68	-3.68	-3.68	-3.68	-3.68	-3.68	-3.68	-3.68
-3.67	-3.67	-3.67	-3.67	-3.67	-3.67	-3.67	-3.67	-3.67
-3.66	-3.66	-3.66	-3.66	-3.66	-3.66	-3.66	-3.66	-3.66
-3.65	-3.65	-3.65	-3.65	-3.65	-3.65	-3.65	-3.65	-3.65
-3.64	-3.64	-3.64	-3.64	-3.64	-3.64	-3.64	-3.64	-3.64
-3.63	-3.63	-3.63	-3.63	-3.63	-3.63	-3.63	-3.63	-3.63
-3.62	-3.62	-3.62	-3.62	-3.62	-3.62	-3.62	-3.62	-3.62
-3.61	-3.61	-3.61	-3.61	-3.61	-3.61	-3.61	-3.61	-3.61
-3.6	-3.6	-3.6	-3.6	-3.6	-3.6	-3.6	-3.6	-3.6
-3.59	-3.59	-3.59	-3.59	-3.59	-3.59	-3.59	-3.59	-3.59
-3.58	-3.58	-3.58	-3.58	-3.58	-3.58	-3.58	-3.58	-3.58
-3.57	-3.57	-3.57	-3.57	-3.57	-3.57	-3.57	-3.57	-3.57
-3.56	-3.56	-3.56	-3.56	-3.56	-3.56	-3.56	-3.56	-3.56
-3.55	-3.55	-3.55	-3.55	-3.55	-3.55	-3.55	-3.55	-3.55
-3.54	-3.54	-3.54	-3.54	-3.54	-3.54	-3.54	-3.54	-3.54
-3.53	-3.53	-3.53	-3.53	-3.53	-3.53	-3.53	-3.53	-3.53
-3.52	-3.52	-3.52	-3.52	-3.52	-3.52	-3.52	-3.52	-3.52
-3.51	-3.51	-3.51	-3.51	-3.51	-3.51	-3.51	-3.51	-3.51

-3.5	-3.5	-3.5	-3.5	-3.5	-3.5	-3.5	-3.5	-3.5
-3.49	-3.49	-3.49	-3.49	-3.49	-3.49	-3.49	-3.49	-3.49
-3.48	-3.48	-3.48	-3.48	-3.48	-3.48	-3.48	-3.48	-3.48
-3.47	-3.47	-3.47	-3.47	-3.47	-3.47	-3.47	-3.47	-3.47
-3.46	-3.46	-3.46	-3.46	-3.46	-3.46	-3.46	-3.46	-3.46
-3.45	-3.45	-3.45	-3.45	-3.45	-3.45	-3.45	-3.45	-3.45
-3.44	-3.44	-3.44	-3.44	-3.44	-3.44	-3.44	-3.44	-3.44
-3.43	-3.43	-3.43	-3.43	-3.43	-3.43	-3.43	-3.43	-3.43
-3.42	-3.42	-3.42	-3.42	-3.42	-3.42	-3.42	-3.42	-3.42
-3.41	-3.41	-3.41	-3.41	-3.41	-3.41	-3.41	-3.41	-3.41
-3.4	-3.4	-3.4	-3.4	-3.4	-3.4	-3.4	-3.4	-3.4
-3.39	-3.39	-3.39	-3.39	-3.39	-3.39	-3.39	-3.39	-3.39
-3.38	-3.38	-3.38	-3.38	-3.38	-3.38	-3.38	-3.38	-3.38
-3.37	-3.37	-3.37	-3.37	-3.37	-3.37	-3.37	-3.37	-3.37
-3.36	-3.36	-3.36	-3.36	-3.36	-3.36	-3.36	-3.36	-3.36
-3.35	-3.35	-3.35	-3.35	-3.35	-3.35	-3.35	-3.35	-3.35
-3.34	-3.34	-3.34	-3.34	-3.34	-3.34	-3.34	-3.34	-3.34
-3.33	-3.33	-3.33	-3.33	-3.33	-3.33	-3.33	-3.33	-3.33
-3.32	-3.32	-3.32	-3.32	-3.32	-3.32	-3.32	-3.32	-3.32
-3.31	-3.31	-3.31	-3.31	-3.31	-3.31	-3.31	-3.31	-3.31
-3.3	-3.3	-3.3	-3.3	-3.3	-3.3	-3.3	-3.3	-3.3
-3.29	-3.29	-3.29	-3.29	-3.29	-3.29	-3.29	-3.29	-3.29
-3.28	-3.28	-3.28	-3.28	-3.28	-3.28	-3.28	-3.28	-3.28
-3.27	-3.27	-3.27	-3.27	-3.27	-3.27	-3.27	-3.27	-3.27
-3.26	-3.26	-3.26	-3.26	-3.26	-3.26	-3.26	-3.26	-3.26
-3.25	-3.25	-3.25	-3.25	-3.25	-3.25	-3.25	-3.25	-3.25
-3.24	-3.24	-3.24	-3.24	-3.24	-3.24	-3.24	-3.24	-3.24
-3.23	-3.23	-3.23	-3.23	-3.23	-3.23	-3.23	-3.23	-3.23
-3.22	-3.22	-3.22	-3.22	-3.22	-3.22	-3.22	-3.22	-3.22
-3.21	-3.21	-3.21	-3.21	-3.21	-3.21	-3.21	-3.21	-3.21
-3.2	-3.2	-3.2	-3.2	-3.2	-3.2	-3.2	-3.2	-3.2
-3.19	-3.19	-3.19	-3.19	-3.19	-3.19	-3.19	-3.19	-3.19
-3.18	-3.18	-3.18	-3.18	-3.18	-3.18	-3.18	-3.18	-3.18
-3.17	-3.17	-3.17	-3.17	-3.17	-3.17	-3.17	-3.17	-3.17
-3.16	-3.16	-3.16	-3.16	-3.16	-3.16	-3.16	-3.16	-3.16
-3.15	-3.15	-3.15	-3.15	-3.15	-3.15	-3.15	-3.15	-3.15
-3.14	-3.14	-3.14	-3.14	-3.14	-3.14	-3.14	-3.14	-3.14
-3.13	-3.13	-3.13	-3.13	-3.13	-3.13	-3.13	-3.13	-3.13
-3.12	-3.12	-3.12	-3.12	-3.12	-3.12	-3.12	-3.12	-3.12
-3.11	-3.11	-3.11	-3.11	-3.11	-3.11	-3.11	-3.11	-3.11
-3.1	-3.1	-3.1	-3.1	-3.1	-3.1	-3.1	-3.1	-3.1
-3.09	-3.09	-3.09	-3.09	-3.09	-3.09	-3.09	-3.09	-3.09
-3.08	-3.08	-3.08	-3.08	-3.08	-3.08	-3.08	-3.08	-3.08
-3.07	-3.07	-3.07	-3.07	-3.07	-3.07	-3.07	-3.07	-3.07
-3.06	-3.06	-3.06	-3.06	-3.06	-3.06	-3.06	-3.06	-3.06
-3.05	-3.05	-3.05	-3.05	-3.05	-3.05	-3.05	-3.05	-3.05
-3.04	-3.04	-3.04	-3.04	-3.04	-3.04	-3.04	-3.04	-3.04
-3.03	-3.03	-3.03	-3.03	-3.03	-3.03	-3.03	-3.03	-3.03
-3.02	-3.02	-3.02	-3.02	-3.02	-3.02	-3.02	-3.02	-3.02
-3.01	-3.01	-3.01	-3.01	-3.01	-3.01	-3.01	-3.01	-3.01

-3	-3	-3	-3	-3	-3	-3	-3	-3
-2.99	-2.99	-2.99	-2.99	-2.99	-2.99	-2.99	-2.99	-2.99
-2.98	-2.98	-2.98	-2.98	-2.98	-2.98	-2.98	-2.98	-2.98
-2.97	-2.97	-2.97	-2.97	-2.97	-2.97	-2.97	-2.97	-2.97
-2.96	-2.96	-2.96	-2.96	-2.96	-2.96	-2.96	-2.96	-2.96
-2.95	-2.95	-2.95	-2.95	-2.95	-2.95	-2.95	-2.95	-2.95
-2.94	-2.94	-2.94	-2.94	-2.94	-2.94	-2.94	-2.94	-2.94
-2.93	-2.93	-2.93	-2.93	-2.93	-2.93	-2.93	-2.93	-2.93
-2.92	-2.92	-2.92	-2.92	-2.92	-2.92	-2.92	-2.92	-2.92
-2.91	-2.91	-2.91	-2.91	-2.91	-2.91	-2.91	-2.91	-2.91
-2.9	-2.9	-2.9	-2.9	-2.9	-2.9	-2.9	-2.9	-2.9
-2.89	-2.89	-2.89	-2.89	-2.89	-2.89	-2.89	-2.89	-2.89
-2.88	-2.88	-2.88	-2.88	-2.88	-2.88	-2.88	-2.88	-2.88
-2.87	-2.87	-2.87	-2.87	-2.87	-2.87	-2.87	-2.87	-2.87
-2.86	-2.86	-2.86	-2.86	-2.86	-2.86	-2.86	-2.86	-2.86
-2.85	-2.85	-2.85	-2.85	-2.85	-2.85	-2.85	-2.85	-2.85
-2.84	-2.84	-2.84	-2.84	-2.84	-2.84	-2.84	-2.84	-2.84
-2.83	-2.83	-2.83	-2.83	-2.83	-2.83	-2.83	-2.83	-2.83
-2.82	-2.82	-2.82	-2.82	-2.82	-2.82	-2.82	-2.82	-2.82
-2.81	-2.81	-2.81	-2.81	-2.81	-2.81	-2.81	-2.81	-2.81
-2.8	-2.8	-2.8	-2.8	-2.8	-2.8	-2.8	-2.8	-2.8
-2.79	-2.79	-2.79	-2.79	-2.79	-2.79	-2.79	-2.79	-2.79
-2.78	-2.78	-2.78	-2.78	-2.78	-2.78	-2.78	-2.78	-2.78
-2.77	-2.77	-2.77	-2.77	-2.77	-2.77	-2.77	-2.77	-2.77
-2.76	-2.76	-2.76	-2.76	-2.76	-2.76	-2.76	-2.76	-2.76
-2.75	-2.75	-2.75	-2.75	-2.75	-2.75	-2.75	-2.75	-2.75
-2.74	-2.74	-2.74	-2.74	-2.74	-2.74	-2.74	-2.74	-2.74
-2.73	-2.73	-2.73	-2.73	-2.73	-2.73	-2.73	-2.73	-2.73
-2.72	-2.72	-2.72	-2.72	-2.72	-2.72	-2.72	-2.72	-2.72
-2.71	-2.71	-2.71	-2.71	-2.71	-2.71	-2.71	-2.71	-2.71
-2.7	-2.7	-2.7	-2.7	-2.7	-2.7	-2.7	-2.7	-2.7
-2.69	-2.69	-2.69	-2.69	-2.69	-2.69	-2.69	-2.69	-2.69
-2.68	-2.68	-2.68	-2.68	-2.68	-2.68	-2.68	-2.68	-2.68
-2.67	-2.67	-2.67	-2.67	-2.67	-2.67	-2.67	-2.67	-2.67
-2.66	-2.66	-2.66	-2.66	-2.66	-2.66	-2.66	-2.66	-2.66
-2.65	-2.65	-2.65	-2.65	-2.65	-2.65	-2.65	-2.65	-2.65
-2.64	-2.64	-2.64	-2.64	-2.64	-2.64	-2.64	-2.64	-2.64
-2.63	-2.63	-2.63	-2.63	-2.63	-2.63	-2.63	-2.63	-2.63
-2.62	-2.62	-2.62	-2.62	-2.62	-2.62	-2.62	-2.62	-2.62
-2.61	-2.61	-2.61	-2.61	-2.61	-2.61	-2.61	-2.61	-2.61
-2.6	-2.6	-2.6	-2.6	-2.6	-2.6	-2.6	-2.6	-2.6
-2.59	-2.59	-2.59	-2.59	-2.59	-2.59	-2.59	-2.59	-2.59
-2.58	-2.58	-2.58	-2.58	-2.58	-2.58	-2.58	-2.58	-2.58
-2.57	-2.57	-2.57	-2.57	-2.57	-2.57	-2.57	-2.57	-2.57
-2.56	-2.56	-2.56	-2.56	-2.56	-2.56	-2.56	-2.56	-2.56
-2.55	-2.55	-2.55	-2.55	-2.55	-2.55	-2.55	-2.55	-2.55
-2.54	-2.54	-2.54	-2.54	-2.54	-2.54	-2.54	-2.54	-2.54
-2.53	-2.53	-2.53	-2.53	-2.53	-2.53	-2.53	-2.53	-2.53
-2.52	-2.52	-2.52	-2.52	-2.52	-2.52	-2.52	-2.52	-2.52
-2.51	-2.51	-2.51	-2.51	-2.51	-2.51	-2.51	-2.51	-2.51

-2.5	-2.5	-2.5	-2.5	-2.5	-2.5	-2.5	-2.5	-2.5
-2.49	-2.49	-2.49	-2.49	-2.49	-2.49	-2.49	-2.49	-2.49
-2.48	-2.48	-2.48	-2.48	-2.48	-2.48	-2.48	-2.48	-2.48
-2.47	-2.47	-2.47	-2.47	-2.47	-2.47	-2.47	-2.47	-2.47
-2.46	-2.46	-2.46	-2.46	-2.46	-2.46	-2.46	-2.46	-2.46
-2.45	-2.45	-2.45	-2.45	-2.45	-2.45	-2.45	-2.45	-2.45
-2.44	-2.44	-2.44	-2.44	-2.44	-2.44	-2.44	-2.44	-2.44
-2.43	-2.43	-2.43	-2.43	-2.43	-2.43	-2.43	-2.43	-2.43
-2.42	-2.42	-2.42	-2.42	-2.42	-2.42	-2.42	-2.42	-2.42
-2.41	-2.41	-2.41	-2.41	-2.41	-2.41	-2.41	-2.41	-2.41
-2.4	-2.4	-2.4	-2.4	-2.4	-2.4	-2.4	-2.4	-2.4
-2.39	-2.39	-2.39	-2.39	-2.39	-2.39	-2.39	-2.39	-2.39
-2.38	-2.38	-2.38	-2.38	-2.38	-2.38	-2.38	-2.38	-2.38
-2.37	-2.37	-2.37	-2.37	-2.37	-2.37	-2.37	-2.37	-2.37
-2.36	-2.36	-2.36	-2.36	-2.36	-2.36	-2.36	-2.36	-2.36
-2.35	-2.35	-2.35	-2.35	-2.35	-2.35	-2.35	-2.35	-2.35
-2.34	-2.34	-2.34	-2.34	-2.34	-2.34	-2.34	-2.34	-2.34
-2.33	-2.33	-2.33	-2.33	-2.33	-2.33	-2.33	-2.33	-2.33
-2.32	-2.32	-2.32	-2.32	-2.32	-2.32	-2.32	-2.32	-2.32
-2.31	-2.31	-2.31	-2.31	-2.31	-2.31	-2.31	-2.31	-2.31
-2.3	-2.3	-2.3	-2.3	-2.3	-2.3	-2.3	-2.3	-2.3
-2.29	-2.29	-2.29	-2.29	-2.29	-2.29	-2.29	-2.29	-2.29
-2.28	-2.28	-2.28	-2.28	-2.28	-2.28	-2.28	-2.28	-2.28
-2.27	-2.27	-2.27	-2.27	-2.27	-2.27	-2.27	-2.27	-2.27
-2.26	-2.26	-2.26	-2.26	-2.26	-2.26	-2.26	-2.26	-2.26
-2.25	-2.25	-2.25	-2.25	-2.25	-2.25	-2.25	-2.25	-2.25
-2.24	-2.24	-2.24	-2.24	-2.24	-2.24	-2.24	-2.24	-2.24
-2.23	-2.23	-2.23	-2.23	-2.23	-2.23	-2.23	-2.23	-2.23
-2.22	-2.22	-2.22	-2.22	-2.22	-2.22	-2.22	-2.22	-2.22
-2.21	-2.21	-2.21	-2.21	-2.21	-2.21	-2.21	-2.21	-2.21
-2.2	-2.2	-2.2	-2.2	-2.2	-2.2	-2.2	-2.2	-2.2
-2.19	-2.19	-2.19	-2.19	-2.19	-2.19	-2.19	-2.19	-2.19
-2.18	-2.18	-2.18	-2.18	-2.18	-2.18	-2.18	-2.18	-2.18
-2.17	-2.17	-2.17	-2.17	-2.17	-2.17	-2.17	-2.17	-2.17
-2.16	-2.16	-2.16	-2.16	-2.16	-2.16	-2.16	-2.16	-2.16
-2.15	-2.15	-2.15	-2.15	-2.15	-2.15	-2.15	-2.15	-2.15
-2.14	-2.14	-2.14	-2.14	-2.14	-2.14	-2.14	-2.14	-2.14
-2.13	-2.13	-2.13	-2.13	-2.13	-2.13	-2.13	-2.13	-2.13
-2.12	-2.12	-2.12	-2.12	-2.12	-2.12	-2.12	-2.12	-2.12
-2.11	-2.11	-2.11	-2.11	-2.11	-2.11	-2.11	-2.11	-2.11
-2.1	-2.1	-2.1	-2.1	-2.1	-2.1	-2.1	-2.1	-2.1
-2.09	-2.09	-2.09	-2.09	-2.09	-2.09	-2.09	-2.09	-2.09
-2.08	-2.08	-2.08	-2.08	-2.08	-2.08	-2.08	-2.08	-2.08
-2.07	-2.07	-2.07	-2.07	-2.07	-2.07	-2.07	-2.07	-2.07
-2.06	-2.06	-2.06	-2.06	-2.06	-2.06	-2.06	-2.06	-2.06
-2.05	-2.05	-2.05	-2.05	-2.05	-2.05	-2.05	-2.05	-2.05
-2.04	-2.04	-2.04	-2.04	-2.04	-2.04	-2.04	-2.04	-2.04
-2.03	-2.03	-2.03	-2.03	-2.03	-2.03	-2.03	-2.03	-2.03
-2.02	-2.02	-2.02	-2.02	-2.02	-2.02	-2.02	-2.02	-2.02
-2.01	-2.01	-2.01	-2.01	-2.01	-2.01	-2.01	-2.01	-2.01

-2	-2	-2	-2	-2	-2	-2	-2	-2
-1.99	-1.99	-1.99	-1.99	-1.99	-1.99	-1.99	-1.99	-1.99
-1.98	-1.98	-1.98	-1.98	-1.98	-1.98	-1.98	-1.98	-1.98
-1.97	-1.97	-1.97	-1.97	-1.97	-1.97	-1.97	-1.97	-1.97
-1.96	-1.96	-1.96	-1.96	-1.96	-1.96	-1.96	-1.96	-1.96
-1.95	-1.95	-1.95	-1.95	-1.95	-1.95	-1.95	-1.95	-1.95
-1.94	-1.94	-1.94	-1.94	-1.94	-1.94	-1.94	-1.94	-1.94
-1.93	-1.93	-1.93	-1.93	-1.93	-1.93	-1.93	-1.93	-1.93
-1.92	-1.92	-1.92	-1.92	-1.92	-1.92	-1.92	-1.92	-1.92
-1.91	-1.91	-1.91	-1.91	-1.91	-1.91	-1.91	-1.91	-1.91
-1.9	-1.9	-1.9	-1.9	-1.9	-1.9	-1.9	-1.9	-1.9
-1.89	-1.89	-1.89	-1.89	-1.89	-1.89	-1.89	-1.89	-1.89
-1.88	-1.88	-1.88	-1.88	-1.88	-1.88	-1.88	-1.88	-1.88
-1.87	-1.87	-1.87	-1.87	-1.87	-1.87	-1.87	-1.87	-1.87
-1.86	-1.86	-1.86	-1.86	-1.86	-1.86	-1.86	-1.86	-1.86
-1.85	-1.85	-1.85	-1.85	-1.85	-1.85	-1.85	-1.85	-1.85
-1.84	-1.84	-1.84	-1.84	-1.84	-1.84	-1.84	-1.84	-1.84
-1.83	-1.83	-1.83	-1.83	-1.83	-1.83	-1.83	-1.83	-1.83
-1.82	-1.82	-1.82	-1.82	-1.82	-1.82	-1.82	-1.82	-1.82
-1.81	-1.81	-1.81	-1.81	-1.81	-1.81	-1.81	-1.81	-1.81
-1.8	-1.8	-1.8	-1.8	-1.8	-1.8	-1.8	-1.8	-1.8
-1.79	-1.79	-1.79	-1.79	-1.79	-1.79	-1.79	-1.79	-1.79
-1.78	-1.78	-1.78	-1.78	-1.78	-1.78	-1.78	-1.78	-1.78
-1.77	-1.77	-1.77	-1.77	-1.77	-1.77	-1.77	-1.77	-1.77
-1.76	-1.76	-1.76	-1.76	-1.76	-1.76	-1.76	-1.76	-1.76
-1.75	-1.75	-1.75	-1.75	-1.75	-1.75	-1.75	-1.75	-1.75
-1.74	-1.74	-1.74	-1.74	-1.74	-1.74	-1.74	-1.74	-1.74
-1.73	-1.73	-1.73	-1.73	-1.73	-1.73	-1.73	-1.73	-1.73
-1.72	-1.72	-1.72	-1.72	-1.72	-1.72	-1.72	-1.72	-1.72
-1.71	-1.71	-1.71	-1.71	-1.71	-1.71	-1.71	-1.71	-1.71
-1.7	-1.7	-1.7	-1.7	-1.7	-1.7	-1.7	-1.7	-1.7
-1.69	-1.69	-1.69	-1.69	-1.69	-1.69	-1.69	-1.69	-1.69
-1.68	-1.68	-1.68	-1.68	-1.68	-1.68	-1.68	-1.68	-1.68
-1.67	-1.67	-1.67	-1.67	-1.67	-1.67	-1.67	-1.67	-1.67
-1.66	-1.66	-1.66	-1.66	-1.66	-1.66	-1.66	-1.66	-1.66
-1.65	-1.65	-1.65	-1.65	-1.65	-1.65	-1.65	-1.65	-1.65
-1.64	-1.64	-1.64	-1.64	-1.64	-1.64	-1.64	-1.64	-1.64
-1.63	-1.63	-1.63	-1.63	-1.63	-1.63	-1.63	-1.63	-1.63
-1.62	-1.62	-1.62	-1.62	-1.62	-1.62	-1.62	-1.62	-1.62
-1.61	-1.61	-1.61	-1.61	-1.61	-1.61	-1.61	-1.61	-1.61
-1.6	-1.6	-1.6	-1.6	-1.6	-1.6	-1.6	-1.6	-1.6
-1.59	-1.59	-1.59	-1.59	-1.59	-1.59	-1.59	-1.59	-1.59
-1.58	-1.58	-1.58	-1.58	-1.58	-1.58	-1.58	-1.58	-1.58
-1.57	-1.57	-1.57	-1.57	-1.57	-1.57	-1.57	-1.57	-1.57
-1.56	-1.56	-1.56	-1.56	-1.56	-1.56	-1.56	-1.56	-1.56
-1.55	-1.55	-1.55	-1.55	-1.55	-1.55	-1.55	-1.55	-1.55
-1.54	-1.54	-1.54	-1.54	-1.54	-1.54	-1.54	-1.54	-1.54
-1.53	-1.53	-1.53	-1.53	-1.53	-1.53	-1.53	-1.53	-1.53
-1.52	-1.52	-1.52	-1.52	-1.52	-1.52	-1.52	-1.52	-1.52
-1.51	-1.51	-1.51	-1.51	-1.51	-1.51	-1.51	-1.51	-1.51

-1.5	-1.5	-1.5	-1.5	-1.5	-1.5	-1.5	-1.5	-1.5
-1.49	-1.49	-1.49	-1.49	-1.49	-1.49	-1.49	-1.49	-1.49
-1.48	-1.48	-1.48	-1.48	-1.48	-1.48	-1.48	-1.48	-1.48
-1.47	-1.47	-1.47	-1.47	-1.47	-1.47	-1.47	-1.47	-1.47
-1.46	-1.46	-1.46	-1.46	-1.46	-1.46	-1.46	-1.46	-1.46
-1.45	-1.45	-1.45	-1.45	-1.45	-1.45	-1.45	-1.45	-1.45
-1.44	-1.44	-1.44	-1.44	-1.44	-1.44	-1.44	-1.44	-1.44
-1.43	-1.43	-1.43	-1.43	-1.43	-1.43	-1.43	-1.43	-1.43
-1.42	-1.42	-1.42	-1.42	-1.42	-1.42	-1.42	-1.42	-1.42
-1.41	-1.41	-1.41	-1.41	-1.41	-1.41	-1.41	-1.41	-1.41
-1.4	-1.4	-1.4	-1.4	-1.4	-1.4	-1.4	-1.4	-1.4
-1.39	-1.39	-1.39	-1.39	-1.39	-1.39	-1.39	-1.39	-1.39
-1.38	-1.38	-1.38	-1.38	-1.38	-1.38	-1.38	-1.38	-1.38
-1.37	-1.37	-1.37	-1.37	-1.37	-1.37	-1.37	-1.37	-1.37
-1.36	-1.36	-1.36	-1.36	-1.36	-1.36	-1.36	-1.36	-1.36
-1.35	-1.35	-1.35	-1.35	-1.35	-1.35	-1.35	-1.35	-1.35
-1.34	-1.34	-1.34	-1.34	-1.34	-1.34	-1.34	-1.34	-1.34
-1.33	-1.33	-1.33	-1.33	-1.33	-1.33	-1.33	-1.33	-1.33
-1.32	-1.32	-1.32	-1.32	-1.32	-1.32	-1.32	-1.32	-1.32
-1.31	-1.31	-1.31	-1.31	-1.31	-1.31	-1.31	-1.31	-1.31
-1.3	-1.3	-1.3	-1.3	-1.3	-1.3	-1.3	-1.3	-1.3
-1.29	-1.29	-1.29	-1.29	-1.29	-1.29	-1.29	-1.29	-1.29
-1.28	-1.28	-1.28	-1.28	-1.28	-1.28	-1.28	-1.28	-1.28
-1.27	-1.27	-1.27	-1.27	-1.27	-1.27	-1.27	-1.27	-1.27
-1.26	-1.26	-1.26	-1.26	-1.26	-1.26	-1.26	-1.26	-1.26
-1.25	-1.25	-1.25	-1.25	-1.25	-1.25	-1.25	-1.25	-1.25
-1.24	-1.24	-1.24	-1.24	-1.24	-1.24	-1.24	-1.24	-1.24
-1.23	-1.23	-1.23	-1.23	-1.23	-1.23	-1.23	-1.23	-1.23
-1.22	-1.22	-1.22	-1.22	-1.22	-1.22	-1.22	-1.22	-1.22
-1.21	-1.21	-1.21	-1.21	-1.21	-1.21	-1.21	-1.21	-1.21
-1.2	-1.2	-1.2	-1.2	-1.2	-1.2	-1.2	-1.2	-1.2
-1.19	-1.19	-1.19	-1.19	-1.19	-1.19	-1.19	-1.19	-1.19
-1.18	-1.18	-1.18	-1.18	-1.18	-1.18	-1.18	-1.18	-1.18
-1.17	-1.17	-1.17	-1.17	-1.17	-1.17	-1.17	-1.17	-1.17
-1.16	-1.16	-1.16	-1.16	-1.16	-1.16	-1.16	-1.16	-1.16
-1.15	-1.15	-1.15	-1.15	-1.15	-1.15	-1.15	-1.15	-1.15
-1.14	-1.14	-1.14	-1.14	-1.14	-1.14	-1.14	-1.14	-1.14
-1.13	-1.13	-1.13	-1.13	-1.13	-1.13	-1.13	-1.13	-1.13
-1.12	-1.12	-1.12	-1.12	-1.12	-1.12	-1.12	-1.12	-1.12
-1.11	-1.11	-1.11	-1.11	-1.11	-1.11	-1.11	-1.11	-1.11
-1.1	-1.1	-1.1	-1.1	-1.1	-1.1	-1.1	-1.1	-1.1
-1.09	-1.09	-1.09	-1.09	-1.09	-1.09	-1.09	-1.09	-1.09
-1.08	-1.08	-1.08	-1.08	-1.08	-1.08	-1.08	-1.08	-1.08
-1.07	-1.07	-1.07	-1.07	-1.07	-1.07	-1.07	-1.07	-1.07
-1.06	-1.06	-1.06	-1.06	-1.06	-1.06	-1.06	-1.06	-1.06
-1.05	-1.05	-1.05	-1.05	-1.05	-1.05	-1.05	-1.05	-1.05
-1.04	-1.04	-1.04	-1.04	-1.04	-1.04	-1.04	-1.04	-1.04
-1.03	-1.03	-1.03	-1.03	-1.03	-1.03	-1.03	-1.03	-1.03
-1.02	-1.02	-1.02	-1.02	-1.02	-1.02	-1.02	-1.02	-1.02
-1.01	-1.01	-1.01	-1.01	-1.01	-1.01	-1.01	-1.01	-1.01

-1	-1	-1	-1	-1	-1	-1	-1	-1
-0.99	-0.99	-0.99	-0.99	-0.99	-0.99	-0.99	-0.99	-0.99
-0.98	-0.98	-0.98	-0.98	-0.98	-0.98	-0.98	-0.98	-0.98
-0.97	-0.97	-0.97	-0.97	-0.97	-0.97	-0.97	-0.97	-0.97
-0.96	-0.96	-0.96	-0.96	-0.96	-0.96	-0.96	-0.96	-0.96
-0.95	-0.95	-0.95	-0.95	-0.95	-0.95	-0.95	-0.95	-0.95
-0.94	-0.94	-0.94	-0.94	-0.94	-0.94	-0.94	-0.94	-0.94
-0.93	-0.93	-0.93	-0.93	-0.93	-0.93	-0.93	-0.93	-0.93
-0.92	-0.92	-0.92	-0.92	-0.92	-0.92	-0.92	-0.92	-0.92
-0.91	-0.91	-0.91	-0.91	-0.91	-0.91	-0.91	-0.91	-0.91
-0.9	-0.9	-0.9	-0.9	-0.9	-0.9	-0.9	-0.9	-0.9
-0.89	-0.89	-0.89	-0.89	-0.89	-0.89	-0.89	-0.89	-0.89
-0.88	-0.88	-0.88	-0.88	-0.88	-0.88	-0.88	-0.88	-0.88
-0.87	-0.87	-0.87	-0.87	-0.87	-0.87	-0.87	-0.87	-0.87
-0.86	-0.86	-0.86	-0.86	-0.86	-0.86	-0.86	-0.86	-0.86
-0.85	-0.85	-0.85	-0.85	-0.85	-0.85	-0.85	-0.85	-0.85
-0.84	-0.84	-0.84	-0.84	-0.84	-0.84	-0.84	-0.84	-0.84
-0.83	-0.83	-0.83	-0.83	-0.83	-0.83	-0.83	-0.83	-0.83
-0.82	-0.82	-0.82	-0.82	-0.82	-0.82	-0.82	-0.82	-0.82
-0.81	-0.81	-0.81	-0.81	-0.81	-0.81	-0.81	-0.81	-0.81
-0.8	-0.8	-0.8	-0.8	-0.8	-0.8	-0.8	-0.8	-0.8
-0.79	-0.79	-0.79	-0.79	-0.79	-0.79	-0.79	-0.79	-0.79
-0.78	-0.78	-0.78	-0.78	-0.78	-0.78	-0.78	-0.78	-0.78
-0.77	-0.77	-0.77	-0.77	-0.77	-0.77	-0.77	-0.77	-0.77
-0.76	-0.76	-0.76	-0.76	-0.76	-0.76	-0.76	-0.76	-0.76
-0.75	-0.75	-0.75	-0.75	-0.75	-0.75	-0.75	-0.75	-0.75
-0.74	-0.74	-0.74	-0.74	-0.74	-0.74	-0.74	-0.74	-0.74
-0.73	-0.73	-0.73	-0.73	-0.73	-0.73	-0.73	-0.73	-0.73
-0.72	-0.72	-0.72	-0.72	-0.72	-0.72	-0.72	-0.72	-0.72
-0.71	-0.71	-0.71	-0.71	-0.71	-0.71	-0.71	-0.71	-0.71
-0.7	-0.7	-0.7	-0.7	-0.7	-0.7	-0.7	-0.7	-0.7
-0.69	-0.69	-0.69	-0.69	-0.69	-0.69	-0.69	-0.69	-0.69
-0.68	-0.68	-0.68	-0.68	-0.68	-0.68	-0.68	-0.68	-0.68
-0.67	-0.67	-0.67	-0.67	-0.67	-0.67	-0.67	-0.67	-0.67
-0.66	-0.66	-0.66	-0.66	-0.66	-0.66	-0.66	-0.66	-0.66
-0.65	-0.65	-0.65	-0.65	-0.65	-0.65	-0.65	-0.65	-0.65
-0.64	-0.64	-0.64	-0.64	-0.64	-0.64	-0.64	-0.64	-0.64
-0.63	-0.63	-0.63	-0.63	-0.63	-0.63	-0.63	-0.63	-0.63
-0.62	-0.62	-0.62	-0.62	-0.62	-0.62	-0.62	-0.62	-0.62
-0.61	-0.61	-0.61	-0.61	-0.61	-0.61	-0.61	-0.61	-0.61
-0.6	-0.6	-0.6	-0.6	-0.6	-0.6	-0.6	-0.6	-0.6
-0.59	-0.59	-0.59	-0.59	-0.59	-0.59	-0.59	-0.59	-0.59
-0.58	-0.58	-0.58	-0.58	-0.58	-0.58	-0.58	-0.58	-0.58
-0.57	-0.57	-0.57	-0.57	-0.57	-0.57	-0.57	-0.57	-0.57
-0.56	-0.56	-0.56	-0.56	-0.56	-0.56	-0.56	-0.56	-0.56
-0.55	-0.55	-0.55	-0.55	-0.55	-0.55	-0.55	-0.55	-0.55
-0.54	-0.54	-0.54	-0.54	-0.54	-0.54	-0.54	-0.54	-0.54
-0.53	-0.53	-0.53	-0.53	-0.53	-0.53	-0.53	-0.53	-0.53
-0.52	-0.52	-0.52	-0.52	-0.52	-0.52	-0.52	-0.52	-0.52
-0.51	-0.51	-0.51	-0.51	-0.51	-0.51	-0.51	-0.51	-0.51

-0.5	-0.5	-0.5	-0.5	-0.5	-0.5	-0.5	-0.5	-0.5
-0.49	-0.49	-0.49	-0.49	-0.49	-0.49	-0.49	-0.49	-0.49
-0.48	-0.48	-0.48	-0.48	-0.48	-0.48	-0.48	-0.48	-0.48
-0.47	-0.47	-0.47	-0.47	-0.47	-0.47	-0.47	-0.47	-0.47
-0.46	-0.46	-0.46	-0.46	-0.46	-0.46	-0.46	-0.46	-0.46
-0.45	-0.45	-0.45	-0.45	-0.45	-0.45	-0.45	-0.45	-0.45
-0.44	-0.44	-0.44	-0.44	-0.44	-0.44	-0.44	-0.44	-0.44
-0.43	-0.43	-0.43	-0.43	-0.43	-0.43	-0.43	-0.43	-0.43
-0.42	-0.42	-0.42	-0.42	-0.42	-0.42	-0.42	-0.42	-0.42
-0.41	-0.41	-0.41	-0.41	-0.41	-0.41	-0.41	-0.41	-0.41
-0.4	-0.4	-0.4	-0.4	-0.4	-0.4	-0.4	-0.4	-0.4
-0.39	-0.39	-0.39	-0.39	-0.39	-0.39	-0.39	-0.39	-0.39
-0.38	-0.38	-0.38	-0.38	-0.38	-0.38	-0.38	-0.38	-0.38
-0.37	-0.37	-0.37	-0.37	-0.37	-0.37	-0.37	-0.37	-0.37
-0.36	-0.36	-0.36	-0.36	-0.36	-0.36	-0.36	-0.36	-0.36
-0.35	-0.35	-0.35	-0.35	-0.35	-0.35	-0.35	-0.35	-0.35
-0.34	-0.34	-0.34	-0.34	-0.34	-0.34	-0.34	-0.34	-0.34
-0.33	-0.33	-0.33	-0.33	-0.33	-0.33	-0.33	-0.33	-0.33
-0.32	-0.32	-0.32	-0.32	-0.32	-0.32	-0.32	-0.32	-0.32
-0.31	-0.31	-0.31	-0.31	-0.31	-0.31	-0.31	-0.31	-0.31
-0.3	-0.3	-0.3	-0.3	-0.3	-0.3	-0.3	-0.3	-0.3
-0.29	-0.29	-0.29	-0.29	-0.29	-0.29	-0.29	-0.29	-0.29
-0.28	-0.28	-0.28	-0.28	-0.28	-0.28	-0.28	-0.28	-0.28
-0.27	-0.27	-0.27	-0.27	-0.27	-0.27	-0.27	-0.27	-0.27
-0.26	-0.26	-0.26	-0.26	-0.26	-0.26	-0.26	-0.26	-0.26
-0.25	-0.25	-0.25	-0.25	-0.25	-0.25	-0.25	-0.25	-0.25
-0.24	-0.24	-0.24	-0.24	-0.24	-0.24	-0.24	-0.24	-0.24
-0.23	-0.23	-0.23	-0.23	-0.23	-0.23	-0.23	-0.23	-0.23
-0.22	-0.22	-0.22	-0.22	-0.22	-0.22	-0.22	-0.22	-0.22
-0.21	-0.21	-0.21	-0.21	-0.21	-0.21	-0.21	-0.21	-0.21
-0.2	-0.2	-0.2	-0.2	-0.2	-0.2	-0.2	-0.2	-0.2
-0.19	-0.19	-0.19	-0.19	-0.19	-0.19	-0.19	-0.19	-0.19
-0.18	-0.18	-0.18	-0.18	-0.18	-0.18	-0.18	-0.18	-0.18
-0.17	-0.17	-0.17	-0.17	-0.17	-0.17	-0.17	-0.17	-0.17
-0.16	-0.16	-0.16	-0.16	-0.16	-0.16	-0.16	-0.16	-0.16
-0.15	-0.15	-0.15	-0.15	-0.15	-0.15	-0.15	-0.15	-0.15
-0.14	-0.14	-0.14	-0.14	-0.14	-0.14	-0.14	-0.14	-0.14
-0.13	-0.13	-0.13	-0.13	-0.13	-0.13	-0.13	-0.13	-0.13
-0.12	-0.12	-0.12	-0.12	-0.12	-0.12	-0.12	-0.12	-0.12
-0.11	-0.11	-0.11	-0.11	-0.11	-0.11	-0.11	-0.11	-0.11
-0.1	-0.1	-0.1	-0.1	-0.1	-0.1	-0.1	-0.1	-0.1
-0.09	-0.09	-0.09	-0.09	-0.09	-0.09	-0.09	-0.09	-0.09
-0.08	-0.08	-0.08	-0.08	-0.08	-0.08	-0.08	-0.08	-0.08
-0.07	-0.07	-0.07	-0.07	-0.07	-0.07	-0.07	-0.07	-0.07
-0.06	-0.06	-0.06	-0.06	-0.06	-0.06	-0.06	-0.06	-0.06
-0.05	-0.05	-0.05	-0.05	-0.05	-0.05	-0.05	-0.05	-0.05
-0.04	-0.04	-0.04	-0.04	-0.04	-0.04	-0.04	-0.04	-0.04
-0.03	-0.03	-0.03	-0.03	-0.03	-0.03	-0.03	-0.03	-0.03
-0.02	-0.02	-0.02	-0.02	-0.02	-0.02	-0.02	-0.02	-0.02
-0.01	-0.01	-0.01	-0.01	-0.01	-0.01	-0.01	-0.01	-0.01

0	0	0	0	0	0	0	0	0
0.01	0.01	0.01	0.01	0.01	0.01	0.01	0.01	0.01
0.02	0.02	0.02	0.02	0.02	0.02	0.02	0.02	0.02
0.03	0.03	0.03	0.03	0.03	0.03	0.03	0.03	0.03
0.04	0.04	0.04	0.04	0.04	0.04	0.04	0.04	0.04
0.05	0.05	0.05	0.05	0.05	0.05	0.05	0.05	0.05
0.06	0.06	0.06	0.06	0.06	0.06	0.06	0.06	0.06
0.07	0.07	0.07	0.07	0.07	0.07	0.07	0.07	0.07
0.08	0.08	0.08	0.08	0.08	0.08	0.08	0.08	0.08
0.09	0.09	0.09	0.09	0.09	0.09	0.09	0.09	0.09
0.1	0.1	0.1	0.1	0.1	0.1	0.1	0.1	0.1
0.11	0.11	0.11	0.11	0.11	0.11	0.11	0.11	0.11
0.12	0.12	0.12	0.12	0.12	0.12	0.12	0.12	0.12
0.13	0.13	0.13	0.13	0.13	0.13	0.13	0.13	0.13
0.14	0.14	0.14	0.14	0.14	0.14	0.14	0.14	0.14
0.15	0.15	0.15	0.15	0.15	0.15	0.15	0.15	0.15
0.16	0.16	0.16	0.16	0.16	0.16	0.16	0.16	0.16
0.17	0.17	0.17	0.17	0.17	0.17	0.17	0.17	0.17
0.18	0.18	0.18	0.18	0.18	0.18	0.18	0.18	0.18
0.19	0.19	0.19	0.19	0.19	0.19	0.19	0.19	0.19
0.2	0.2	0.2	0.2	0.2	0.2	0.2	0.2	0.2
0.21	0.21	0.21	0.21	0.21	0.21	0.21	0.21	0.21
0.22	0.22	0.22	0.22	0.22	0.22	0.22	0.22	0.22
0.23	0.23	0.23	0.23	0.23	0.23	0.23	0.23	0.23
0.24	0.24	0.24	0.24	0.24	0.24	0.24	0.24	0.24
0.25	0.25	0.25	0.25	0.25	0.25	0.25	0.25	0.25
0.26	0.26	0.26	0.26	0.26	0.26	0.26	0.26	0.26
0.27	0.27	0.27	0.27	0.27	0.27	0.27	0.27	0.27
0.28	0.28	0.28	0.28	0.28	0.28	0.28	0.28	0.28
0.29	0.29	0.29	0.29	0.29	0.29	0.29	0.29	0.29
0.3	0.3	0.3	0.3	0.3	0.3	0.3	0.3	0.3
0.31	0.31	0.31	0.31	0.31	0.31	0.31	0.31	0.31
0.32	0.32	0.32	0.32	0.32	0.32	0.32	0.32	0.32
0.33	0.33	0.33	0.33	0.33	0.33	0.33	0.33	0.33
0.34	0.34	0.34	0.34	0.34	0.34	0.34	0.34	0.34
0.35	0.35	0.35	0.35	0.35	0.35	0.35	0.35	0.35
0.36	0.36	0.36	0.36	0.36	0.36	0.36	0.36	0.36
0.37	0.37	0.37	0.37	0.37	0.37	0.37	0.37	0.37
0.38	0.38	0.38	0.38	0.38	0.38	0.38	0.38	0.38
0.39	0.39	0.39	0.39	0.39	0.39	0.39	0.39	0.39
0.4	0.4	0.4	0.4	0.4	0.4	0.4	0.4	0.4
0.41	0.41	0.41	0.41	0.41	0.41	0.41	0.41	0.41
0.42	0.42	0.42	0.42	0.42	0.42	0.42	0.42	0.42
0.43	0.43	0.43	0.43	0.43	0.43	0.43	0.43	0.43
0.44	0.44	0.44	0.44	0.44	0.44	0.44	0.44	0.44
0.45	0.45	0.45	0.45	0.45	0.45	0.45	0.45	0.45
0.46	0.46	0.46	0.46	0.46	0.46	0.46	0.46	0.46
0.47	0.47	0.47	0.47	0.47	0.47	0.47	0.47	0.47
0.48	0.48	0.48	0.48	0.48	0.48	0.48	0.48	0.48
0.49	0.49	0.49	0.49	0.49	0.49	0.49	0.49	0.49

0.5	0.5	0.5	0.5	0.5	0.5	0.5	0.5	0.5
0.51	0.51	0.51	0.51	0.51	0.51	0.51	0.51	0.51
0.52	0.52	0.52	0.52	0.52	0.52	0.52	0.52	0.52
0.53	0.53	0.53	0.53	0.53	0.53	0.53	0.53	0.53
0.54	0.54	0.54	0.54	0.54	0.54	0.54	0.54	0.54
0.55	0.55	0.55	0.55	0.55	0.55	0.55	0.55	0.55
0.56	0.56	0.56	0.56	0.56	0.56	0.56	0.56	0.56
0.57	0.57	0.57	0.57	0.57	0.57	0.57	0.57	0.57
0.58	0.58	0.58	0.58	0.58	0.58	0.58	0.58	0.58
0.59	0.59	0.59	0.59	0.59	0.59	0.59	0.59	0.59
0.6	0.6	0.6	0.6	0.6	0.6	0.6	0.6	0.6
0.61	0.61	0.61	0.61	0.61	0.61	0.61	0.61	0.61
0.62	0.62	0.62	0.62	0.62	0.62	0.62	0.62	0.62
0.63	0.63	0.63	0.63	0.63	0.63	0.63	0.63	0.63
0.64	0.64	0.64	0.64	0.64	0.64	0.64	0.64	0.64
0.65	0.65	0.65	0.65	0.65	0.65	0.65	0.65	0.65
0.66	0.66	0.66	0.66	0.66	0.66	0.66	0.66	0.66
0.67	0.67	0.67	0.67	0.67	0.67	0.67	0.67	0.67
0.68	0.68	0.68	0.68	0.68	0.68	0.68	0.68	0.68
0.69	0.69	0.69	0.69	0.69	0.69	0.69	0.69	0.69
0.7	0.7	0.7	0.7	0.7	0.7	0.7	0.7	0.7
0.71	0.71	0.71	0.71	0.71	0.71	0.71	0.71	0.71
0.72	0.72	0.72	0.72	0.72	0.72	0.72	0.72	0.72
0.73	0.73	0.73	0.73	0.73	0.73	0.73	0.73	0.73
0.74	0.74	0.74	0.74	0.74	0.74	0.74	0.74	0.74
0.75	0.75	0.75	0.75	0.75	0.75	0.75	0.75	0.75
0.76	0.76	0.76	0.76	0.76	0.76	0.76	0.76	0.76
0.77	0.77	0.77	0.77	0.77	0.77	0.77	0.77	0.77
0.78	0.78	0.78	0.78	0.78	0.78	0.78	0.78	0.78
0.79	0.79	0.79	0.79	0.79	0.79	0.79	0.79	0.79
0.8	0.8	0.8	0.8	0.8	0.8	0.8	0.8	0.8
0.81	0.81	0.81	0.81	0.81	0.81	0.81	0.81	0.81
0.82	0.82	0.82	0.82	0.82	0.82	0.82	0.82	0.82
0.83	0.83	0.83	0.83	0.83	0.83	0.83	0.83	0.83
0.84	0.84	0.84	0.84	0.84	0.84	0.84	0.84	0.84
0.85	0.85	0.85	0.85	0.85	0.85	0.85	0.85	0.85
0.86	0.86	0.86	0.86	0.86	0.86	0.86	0.86	0.86
0.87	0.87	0.87	0.87	0.87	0.87	0.87	0.87	0.87
0.88	0.88	0.88	0.88	0.88	0.88	0.88	0.88	0.88
0.89	0.89	0.89	0.89	0.89	0.89	0.89	0.89	0.89
0.9	0.9	0.9	0.9	0.9	0.9	0.9	0.9	0.9
0.91	0.91	0.91	0.91	0.91	0.91	0.91	0.91	0.91
0.92	0.92	0.92	0.92	0.92	0.92	0.92	0.92	0.92
0.93	0.93	0.93	0.93	0.93	0.93	0.93	0.93	0.93
0.94	0.94	0.94	0.94	0.94	0.94	0.94	0.94	0.94
0.95	0.95	0.95	0.95	0.95	0.95	0.95	0.95	0.95
0.96	0.96	0.96	0.96	0.96	0.96	0.96	0.96	0.96
0.97	0.97	0.97	0.97	0.97	0.97	0.97	0.97	0.97
0.98	0.98	0.98	0.98	0.98	0.98	0.98	0.98	0.98
0.99	0.99	0.99	0.99	0.99	0.99	0.99	0.99	0.99

1	1	1	1	1	1	1	1	1
1.01	1.01	1.01	1.01	1.01	1.01	1.01	1.01	1.01
1.02	1.02	1.02	1.02	1.02	1.02	1.02	1.02	1.02
1.03	1.03	1.03	1.03	1.03	1.03	1.03	1.03	1.03
1.04	1.04	1.04	1.04	1.04	1.04	1.04	1.04	1.04
1.05	1.05	1.05	1.05	1.05	1.05	1.05	1.05	1.05
1.06	1.06	1.06	1.06	1.06	1.06	1.06	1.06	1.06
1.07	1.07	1.07	1.07	1.07	1.07	1.07	1.07	1.07
1.08	1.08	1.08	1.08	1.08	1.08	1.08	1.08	1.08
1.09	1.09	1.09	1.09	1.09	1.09	1.09	1.09	1.09
1.1	1.1	1.1	1.1	1.1	1.1	1.1	1.1	1.1
1.11	1.11	1.11	1.11	1.11	1.11	1.11	1.11	1.11
1.12	1.12	1.12	1.12	1.12	1.12	1.12	1.12	1.12
1.13	1.13	1.13	1.13	1.13	1.13	1.13	1.13	1.13
1.14	1.14	1.14	1.14	1.14	1.14	1.14	1.14	1.14
1.15	1.15	1.15	1.15	1.15	1.15	1.15	1.15	1.15
1.16	1.16	1.16	1.16	1.16	1.16	1.16	1.16	1.16
1.17	1.17	1.17	1.17	1.17	1.17	1.17	1.17	1.17
1.18	1.18	1.18	1.18	1.18	1.18	1.18	1.18	1.18
1.19	1.19	1.19	1.19	1.19	1.19	1.19	1.19	1.19
1.2	1.2	1.2	1.2	1.2	1.2	1.2	1.2	1.2
1.21	1.21	1.21	1.21	1.21	1.21	1.21	1.21	1.21
1.22	1.22	1.22	1.22	1.22	1.22	1.22	1.22	1.22
1.23	1.23	1.23	1.23	1.23	1.23	1.23	1.23	1.23
1.24	1.24	1.24	1.24	1.24	1.24	1.24	1.24	1.24
1.25	1.25	1.25	1.25	1.25	1.25	1.25	1.25	1.25
1.26	1.26	1.26	1.26	1.26	1.26	1.26	1.26	1.26
1.27	1.27	1.27	1.27	1.27	1.27	1.27	1.27	1.27
1.28	1.28	1.28	1.28	1.28	1.28	1.28	1.28	1.28
1.29	1.29	1.29	1.29	1.29	1.29	1.29	1.29	1.29
1.3	1.3	1.3	1.3	1.3	1.3	1.3	1.3	1.3
1.31	1.31	1.31	1.31	1.31	1.31	1.31	1.31	1.31
1.32	1.32	1.32	1.32	1.32	1.32	1.32	1.32	1.32
1.33	1.33	1.33	1.33	1.33	1.33	1.33	1.33	1.33
1.34	1.34	1.34	1.34	1.34	1.34	1.34	1.34	1.34
1.35	1.35	1.35	1.35	1.35	1.35	1.35	1.35	1.35
1.36	1.36	1.36	1.36	1.36	1.36	1.36	1.36	1.36
1.37	1.37	1.37	1.37	1.37	1.37	1.37	1.37	1.37
1.38	1.38	1.38	1.38	1.38	1.38	1.38	1.38	1.38
1.39	1.39	1.39	1.39	1.39	1.39	1.39	1.39	1.39
1.4	1.4	1.4	1.4	1.4	1.4	1.4	1.4	1.4
1.41	1.41	1.41	1.41	1.41	1.41	1.41	1.41	1.41
1.42	1.42	1.42	1.42	1.42	1.42	1.42	1.42	1.42
1.43	1.43	1.43	1.43	1.43	1.43	1.43	1.43	1.43
1.44	1.44	1.44	1.44	1.44	1.44	1.44	1.44	1.44
1.45	1.45	1.45	1.45	1.45	1.45	1.45	1.45	1.45
1.46	1.46	1.46	1.46	1.46	1.46	1.46	1.46	1.46
1.47	1.47	1.47	1.47	1.47	1.47	1.47	1.47	1.47
1.48	1.48	1.48	1.48	1.48	1.48	1.48	1.48	1.48
1.49	1.49	1.49	1.49	1.49	1.49	1.49	1.49	1.49

1.5	1.5	1.5	1.5	1.5	1.5	1.5	1.5	1.5
1.51	1.51	1.51	1.51	1.51	1.51	1.51	1.51	1.51
1.52	1.52	1.52	1.52	1.52	1.52	1.52	1.52	1.52
1.53	1.53	1.53	1.53	1.53	1.53	1.53	1.53	1.53
1.54	1.54	1.54	1.54	1.54	1.54	1.54	1.54	1.54
1.55	1.55	1.55	1.55	1.55	1.55	1.55	1.55	1.55
1.56	1.56	1.56	1.56	1.56	1.56	1.56	1.56	1.56
1.57	1.57	1.57	1.57	1.57	1.57	1.57	1.57	1.57
1.58	1.58	1.58	1.58	1.58	1.58	1.58	1.58	1.58
1.59	1.59	1.59	1.59	1.59	1.59	1.59	1.59	1.59
1.6	1.6	1.6	1.6	1.6	1.6	1.6	1.6	1.6
1.61	1.61	1.61	1.61	1.61	1.61	1.61	1.61	1.61
1.62	1.62	1.62	1.62	1.62	1.62	1.62	1.62	1.62
1.63	1.63	1.63	1.63	1.63	1.63	1.63	1.63	1.63
1.64	1.64	1.64	1.64	1.64	1.64	1.64	1.64	1.64
1.65	1.65	1.65	1.65	1.65	1.65	1.65	1.65	1.65
1.66	1.66	1.66	1.66	1.66	1.66	1.66	1.66	1.66
1.67	1.67	1.67	1.67	1.67	1.67	1.67	1.67	1.67
1.68	1.68	1.68	1.68	1.68	1.68	1.68	1.68	1.68
1.69	1.69	1.69	1.69	1.69	1.69	1.69	1.69	1.69
1.7	1.7	1.7	1.7	1.7	1.7	1.7	1.7	1.7
1.71	1.71	1.71	1.71	1.71	1.71	1.71	1.71	1.71
1.72	1.72	1.72	1.72	1.72	1.72	1.72	1.72	1.72
1.73	1.73	1.73	1.73	1.73	1.73	1.73	1.73	1.73
1.74	1.74	1.74	1.74	1.74	1.74	1.74	1.74	1.74
1.75	1.75	1.75	1.75	1.75	1.75	1.75	1.75	1.75
1.76	1.76	1.76	1.76	1.76	1.76	1.76	1.76	1.76
1.77	1.77	1.77	1.77	1.77	1.77	1.77	1.77	1.77
1.78	1.78	1.78	1.78	1.78	1.78	1.78	1.78	1.78
1.79	1.79	1.79	1.79	1.79	1.79	1.79	1.79	1.79
1.8	1.8	1.8	1.8	1.8	1.8	1.8	1.8	1.8
1.81	1.81	1.81	1.81	1.81	1.81	1.81	1.81	1.81
1.82	1.82	1.82	1.82	1.82	1.82	1.82	1.82	1.82
1.83	1.83	1.83	1.83	1.83	1.83	1.83	1.83	1.83
1.84	1.84	1.84	1.84	1.84	1.84	1.84	1.84	1.84
1.85	1.85	1.85	1.85	1.85	1.85	1.85	1.85	1.85
1.86	1.86	1.86	1.86	1.86	1.86	1.86	1.86	1.86
1.87	1.87	1.87	1.87	1.87	1.87	1.87	1.87	1.87
1.88	1.88	1.88	1.88	1.88	1.88	1.88	1.88	1.88
1.89	1.89	1.89	1.89	1.89	1.89	1.89	1.89	1.89
1.9	1.9	1.9	1.9	1.9	1.9	1.9	1.9	1.9
1.91	1.91	1.91	1.91	1.91	1.91	1.91	1.91	1.91
1.92	1.92	1.92	1.92	1.92	1.92	1.92	1.92	1.92
1.93	1.93	1.93	1.93	1.93	1.93	1.93	1.93	1.93
1.94	1.94	1.94	1.94	1.94	1.94	1.94	1.94	1.94
1.95	1.95	1.95	1.95	1.95	1.95	1.95	1.95	1.95
1.96	1.96	1.96	1.96	1.96	1.96	1.96	1.96	1.96
1.97	1.97	1.97	1.97	1.97	1.97	1.97	1.97	1.97
1.98	1.98	1.98	1.98	1.98	1.98	1.98	1.98	1.98
1.99	1.99	1.99	1.99	1.99	1.99	1.99	1.99	1.99

2	2	2	2	2	2	2	2	2
2.01	2.01	2.01	2.01	2.01	2.01	2.01	2.01	2.01
2.02	2.02	2.02	2.02	2.02	2.02	2.02	2.02	2.02
2.03	2.03	2.03	2.03	2.03	2.03	2.03	2.03	2.03
2.04	2.04	2.04	2.04	2.04	2.04	2.04	2.04	2.04
2.05	2.05	2.05	2.05	2.05	2.05	2.05	2.05	2.05
2.06	2.06	2.06	2.06	2.06	2.06	2.06	2.06	2.06
2.07	2.07	2.07	2.07	2.07	2.07	2.07	2.07	2.07
2.08	2.08	2.08	2.08	2.08	2.08	2.08	2.08	2.08
2.09	2.09	2.09	2.09	2.09	2.09	2.09	2.09	2.09
2.1	2.1	2.1	2.1	2.1	2.1	2.1	2.1	2.1
2.11	2.11	2.11	2.11	2.11	2.11	2.11	2.11	2.11
2.12	2.12	2.12	2.12	2.12	2.12	2.12	2.12	2.12
2.13	2.13	2.13	2.13	2.13	2.13	2.13	2.13	2.13
2.14	2.14	2.14	2.14	2.14	2.14	2.14	2.14	2.14
2.15	2.15	2.15	2.15	2.15	2.15	2.15	2.15	2.15
2.16	2.16	2.16	2.16	2.16	2.16	2.16	2.16	2.16
2.17	2.17	2.17	2.17	2.17	2.17	2.17	2.17	2.17
2.18	2.18	2.18	2.18	2.18	2.18	2.18	2.18	2.18
2.19	2.19	2.19	2.19	2.19	2.19	2.19	2.19	2.19
2.2	2.2	2.2	2.2	2.2	2.2	2.2	2.2	2.2
2.21	2.21	2.21	2.21	2.21	2.21	2.21	2.21	2.21
2.22	2.22	2.22	2.22	2.22	2.22	2.22	2.22	2.22
2.23	2.23	2.23	2.23	2.23	2.23	2.23	2.23	2.23
2.24	2.24	2.24	2.24	2.24	2.24	2.24	2.24	2.24
2.25	2.25	2.25	2.25	2.25	2.25	2.25	2.25	2.25
2.26	2.26	2.26	2.26	2.26	2.26	2.26	2.26	2.26
2.27	2.27	2.27	2.27	2.27	2.27	2.27	2.27	2.27
2.28	2.28	2.28	2.28	2.28	2.28	2.28	2.28	2.28
2.29	2.29	2.29	2.29	2.29	2.29	2.29	2.29	2.29
2.3	2.3	2.3	2.3	2.3	2.3	2.3	2.3	2.3
2.31	2.31	2.31	2.31	2.31	2.31	2.31	2.31	2.31
2.32	2.32	2.32	2.32	2.32	2.32	2.32	2.32	2.32
2.33	2.33	2.33	2.33	2.33	2.33	2.33	2.33	2.33
2.34	2.34	2.34	2.34	2.34	2.34	2.34	2.34	2.34
2.35	2.35	2.35	2.35	2.35	2.35	2.35	2.35	2.35
2.36	2.36	2.36	2.36	2.36	2.36	2.36	2.36	2.36
2.37	2.37	2.37	2.37	2.37	2.37	2.37	2.37	2.37
2.38	2.38	2.38	2.38	2.38	2.38	2.38	2.38	2.38
2.39	2.39	2.39	2.39	2.39	2.39	2.39	2.39	2.39
2.4	2.4	2.4	2.4	2.4	2.4	2.4	2.4	2.4
2.41	2.41	2.41	2.41	2.41	2.41	2.41	2.41	2.41
2.42	2.42	2.42	2.42	2.42	2.42	2.42	2.42	2.42
2.43	2.43	2.43	2.43	2.43	2.43	2.43	2.43	2.43
2.44	2.44	2.44	2.44	2.44	2.44	2.44	2.44	2.44
2.45	2.45	2.45	2.45	2.45	2.45	2.45	2.45	2.45
2.46	2.46	2.46	2.46	2.46	2.46	2.46	2.46	2.46
2.47	2.47	2.47	2.47	2.47	2.47	2.47	2.47	2.47
2.48	2.48	2.48	2.48	2.48	2.48	2.48	2.48	2.48
2.49	2.49	2.49	2.49	2.49	2.49	2.49	2.49	2.49

2.5	2.5	2.5	2.5	2.5	2.5	2.5	2.5	2.5
2.51	2.51	2.51	2.51	2.51	2.51	2.51	2.51	2.51
2.52	2.52	2.52	2.52	2.52	2.52	2.52	2.52	2.52
2.53	2.53	2.53	2.53	2.53	2.53	2.53	2.53	2.53
2.54	2.54	2.54	2.54	2.54	2.54	2.54	2.54	2.54
2.55	2.55	2.55	2.55	2.55	2.55	2.55	2.55	2.55
2.56	2.56	2.56	2.56	2.56	2.56	2.56	2.56	2.56
2.57	2.57	2.57	2.57	2.57	2.57	2.57	2.57	2.57
2.58	2.58	2.58	2.58	2.58	2.58	2.58	2.58	2.58
2.59	2.59	2.59	2.59	2.59	2.59	2.59	2.59	2.59
2.6	2.6	2.6	2.6	2.6	2.6	2.6	2.6	2.6
2.61	2.61	2.61	2.61	2.61	2.61	2.61	2.61	2.61
2.62	2.62	2.62	2.62	2.62	2.62	2.62	2.62	2.62
2.63	2.63	2.63	2.63	2.63	2.63	2.63	2.63	2.63
2.64	2.64	2.64	2.64	2.64	2.64	2.64	2.64	2.64
2.65	2.65	2.65	2.65	2.65	2.65	2.65	2.65	2.65
2.66	2.66	2.66	2.66	2.66	2.66	2.66	2.66	2.66
2.67	2.67	2.67	2.67	2.67	2.67	2.67	2.67	2.67
2.68	2.68	2.68	2.68	2.68	2.68	2.68	2.68	2.68
2.69	2.69	2.69	2.69	2.69	2.69	2.69	2.69	2.69
2.7	2.7	2.7	2.7	2.7	2.7	2.7	2.7	2.7
2.71	2.71	2.71	2.71	2.71	2.71	2.71	2.71	2.71
2.72	2.72	2.72	2.72	2.72	2.72	2.72	2.72	2.72
2.73	2.73	2.73	2.73	2.73	2.73	2.73	2.73	2.73
2.74	2.74	2.74	2.74	2.74	2.74	2.74	2.74	2.74
2.75	2.75	2.75	2.75	2.75	2.75	2.75	2.75	2.75
2.76	2.76	2.76	2.76	2.76	2.76	2.76	2.76	2.76
2.77	2.77	2.77	2.77	2.77	2.77	2.77	2.77	2.77
2.78	2.78	2.78	2.78	2.78	2.78	2.78	2.78	2.78
2.79	2.79	2.79	2.79	2.79	2.79	2.79	2.79	2.79
2.8	2.8	2.8	2.8	2.8	2.8	2.8	2.8	2.8
2.81	2.81	2.81	2.81	2.81	2.81	2.81	2.81	2.81
2.82	2.82	2.82	2.82	2.82	2.82	2.82	2.82	2.82
2.83	2.83	2.83	2.83	2.83	2.83	2.83	2.83	2.83
2.84	2.84	2.84	2.84	2.84	2.84	2.84	2.84	2.84
2.85	2.85	2.85	2.85	2.85	2.85	2.85	2.85	2.85
2.86	2.86	2.86	2.86	2.86	2.86	2.86	2.86	2.86
2.87	2.87	2.87	2.87	2.87	2.87	2.87	2.87	2.87
2.88	2.88	2.88	2.88	2.88	2.88	2.88	2.88	2.88
2.89	2.89	2.89	2.89	2.89	2.89	2.89	2.89	2.89
2.9	2.9	2.9	2.9	2.9	2.9	2.9	2.9	2.9
2.91	2.91	2.91	2.91	2.91	2.91	2.91	2.91	2.91
2.92	2.92	2.92	2.92	2.92	2.92	2.92	2.92	2.92
2.93	2.93	2.93	2.93	2.93	2.93	2.93	2.93	2.93
2.94	2.94	2.94	2.94	2.94	2.94	2.94	2.94	2.94
2.95	2.95	2.95	2.95	2.95	2.95	2.95	2.95	2.95
2.96	2.96	2.96	2.96	2.96	2.96	2.96	2.96	2.96
2.97	2.97	2.97	2.97	2.97	2.97	2.97	2.97	2.97
2.98	2.98	2.98	2.98	2.98	2.98	2.98	2.98	2.98
2.99	2.99	2.99	2.99	2.99	2.99	2.99	2.99	2.99

3	3	3	3	3	3	3	3	3
3.01	3.01	3.01	3.01	3.01	3.01	3.01	3.01	3.01
3.02	3.02	3.02	3.02	3.02	3.02	3.02	3.02	3.02
3.03	3.03	3.03	3.03	3.03	3.03	3.03	3.03	3.03
3.04	3.04	3.04	3.04	3.04	3.04	3.04	3.04	3.04
3.05	3.05	3.05	3.05	3.05	3.05	3.05	3.05	3.05
3.06	3.06	3.06	3.06	3.06	3.06	3.06	3.06	3.06
3.07	3.07	3.07	3.07	3.07	3.07	3.07	3.07	3.07
3.08	3.08	3.08	3.08	3.08	3.08	3.08	3.08	3.08
3.09	3.09	3.09	3.09	3.09	3.09	3.09	3.09	3.09
3.1	3.1	3.1	3.1	3.1	3.1	3.1	3.1	3.1
3.11	3.11	3.11	3.11	3.11	3.11	3.11	3.11	3.11
3.12	3.12	3.12	3.12	3.12	3.12	3.12	3.12	3.12
3.13	3.13	3.13	3.13	3.13	3.13	3.13	3.13	3.13
3.14	3.14	3.14	3.14	3.14	3.14	3.14	3.14	3.14
3.15	3.15	3.15	3.15	3.15	3.15	3.15	3.15	3.15
3.16	3.16	3.16	3.16	3.16	3.16	3.16	3.16	3.16
3.17	3.17	3.17	3.17	3.17	3.17	3.17	3.17	3.17
3.18	3.18	3.18	3.18	3.18	3.18	3.18	3.18	3.18
3.19	3.19	3.19	3.19	3.19	3.19	3.19	3.19	3.19
3.2	3.2	3.2	3.2	3.2	3.2	3.2	3.2	3.2
3.21	3.21	3.21	3.21	3.21	3.21	3.21	3.21	3.21
3.22	3.22	3.22	3.22	3.22	3.22	3.22	3.22	3.22
3.23	3.23	3.23	3.23	3.23	3.23	3.23	3.23	3.23
3.24	3.24	3.24	3.24	3.24	3.24	3.24	3.24	3.24
3.25	3.25	3.25	3.25	3.25	3.25	3.25	3.25	3.25
3.26	3.26	3.26	3.26	3.26	3.26	3.26	3.26	3.26
3.27	3.27	3.27	3.27	3.27	3.27	3.27	3.27	3.27
3.28	3.28	3.28	3.28	3.28	3.28	3.28	3.28	3.28
3.29	3.29	3.29	3.29	3.29	3.29	3.29	3.29	3.29
3.3	3.3	3.3	3.3	3.3	3.3	3.3	3.3	3.3
3.31	3.31	3.31	3.31	3.31	3.31	3.31	3.31	3.31
3.32	3.32	3.32	3.32	3.32	3.32	3.32	3.32	3.32
3.33	3.33	3.33	3.33	3.33	3.33	3.33	3.33	3.33
3.34	3.34	3.34	3.34	3.34	3.34	3.34	3.34	3.34
3.35	3.35	3.35	3.35	3.35	3.35	3.35	3.35	3.35
3.36	3.36	3.36	3.36	3.36	3.36	3.36	3.36	3.36
3.37	3.37	3.37	3.37	3.37	3.37	3.37	3.37	3.37
3.38	3.38	3.38	3.38	3.38	3.38	3.38	3.38	3.38
3.39	3.39	3.39	3.39	3.39	3.39	3.39	3.39	3.39
3.4	3.4	3.4	3.4	3.4	3.4	3.4	3.4	3.4
3.41	3.41	3.41	3.41	3.41	3.41	3.41	3.41	3.41
3.42	3.42	3.42	3.42	3.42	3.42	3.42	3.42	3.42
3.43	3.43	3.43	3.43	3.43	3.43	3.43	3.43	3.43
3.44	3.44	3.44	3.44	3.44	3.44	3.44	3.44	3.44
3.45	3.45	3.45	3.45	3.45	3.45	3.45	3.45	3.45
3.46	3.46	3.46	3.46	3.46	3.46	3.46	3.46	3.46
3.47	3.47	3.47	3.47	3.47	3.47	3.47	3.47	3.47
3.48	3.48	3.48	3.48	3.48	3.48	3.48	3.48	3.48
3.49	3.49	3.49	3.49	3.49	3.49	3.49	3.49	3.49

3.5	3.5	3.5	3.5	3.5	3.5	3.5	3.5	3.5
3.51	3.51	3.51	3.51	3.51	3.51	3.51	3.51	3.51
3.52	3.52	3.52	3.52	3.52	3.52	3.52	3.52	3.52
3.53	3.53	3.53	3.53	3.53	3.53	3.53	3.53	3.53
3.54	3.54	3.54	3.54	3.54	3.54	3.54	3.54	3.54
3.55	3.55	3.55	3.55	3.55	3.55	3.55	3.55	3.55
3.56	3.56	3.56	3.56	3.56	3.56	3.56	3.56	3.56
3.57	3.57	3.57	3.57	3.57	3.57	3.57	3.57	3.57
3.58	3.58	3.58	3.58	3.58	3.58	3.58	3.58	3.58
3.59	3.59	3.59	3.59	3.59	3.59	3.59	3.59	3.59
3.6	3.6	3.6	3.6	3.6	3.6	3.6	3.6	3.6
3.61	3.61	3.61	3.61	3.61	3.61	3.61	3.61	3.61
3.62	3.62	3.62	3.62	3.62	3.62	3.62	3.62	3.62
3.63	3.63	3.63	3.63	3.63	3.63	3.63	3.63	3.63
3.64	3.64	3.64	3.64	3.64	3.64	3.64	3.64	3.64
3.65	3.65	3.65	3.65	3.65	3.65	3.65	3.65	3.65
3.66	3.66	3.66	3.66	3.66	3.66	3.66	3.66	3.66
3.67	3.67	3.67	3.67	3.67	3.67	3.67	3.67	3.67
3.68	3.68	3.68	3.68	3.68	3.68	3.68	3.68	3.68
3.69	3.69	3.69	3.69	3.69	3.69	3.69	3.69	3.69
3.7	3.7	3.7	3.7	3.7	3.7	3.7	3.7	3.7
3.71	3.71	3.71	3.71	3.71	3.71	3.71	3.71	3.71
3.72	3.72	3.72	3.72	3.72	3.72	3.72	3.72	3.72
3.73	3.73	3.73	3.73	3.73	3.73	3.73	3.73	3.73
3.74	3.74	3.74	3.74	3.74	3.74	3.74	3.74	3.74
3.75	3.75	3.75	3.75	3.75	3.75	3.75	3.75	3.75
3.76	3.76	3.76	3.76	3.76	3.76	3.76	3.76	3.76
3.77	3.77	3.77	3.77	3.77	3.77	3.77	3.77	3.77
3.78	3.78	3.78	3.78	3.78	3.78	3.78	3.78	3.78
3.79	3.79	3.79	3.79	3.79	3.79	3.79	3.79	3.79
3.8	3.8	3.8	3.8	3.8	3.8	3.8	3.8	3.8
3.81	3.81	3.81	3.81	3.81	3.81	3.81	3.81	3.81
3.82	3.82	3.82	3.82	3.82	3.82	3.82	3.82	3.82
3.83	3.83	3.83	3.83	3.83	3.83	3.83	3.83	3.83
3.84	3.84	3.84	3.84	3.84	3.84	3.84	3.84	3.84
3.85	3.85	3.85	3.85	3.85	3.85	3.85	3.85	3.85
3.86	3.86	3.86	3.86	3.86	3.86	3.86	3.86	3.86
3.87	3.87	3.87	3.87	3.87	3.87	3.87	3.87	3.87
3.88	3.88	3.88	3.88	3.88	3.88	3.88	3.88	3.88
3.89	3.89	3.89	3.89	3.89	3.89	3.89	3.89	3.89
3.9	3.9	3.9	3.9	3.9	3.9	3.9	3.9	3.9
3.91	3.91	3.91	3.91	3.91	3.91	3.91	3.91	3.91
3.92	3.92	3.92	3.92	3.92	3.92	3.92	3.92	3.92
3.93	3.93	3.93	3.93	3.93	3.93	3.93	3.93	3.93
3.94	3.94	3.94	3.94	3.94	3.94	3.94	3.94	3.94
3.95	3.95	3.95	3.95	3.95	3.95	3.95	3.95	3.95
3.96	3.96	3.96	3.96	3.96	3.96	3.96	3.96	3.96
3.97	3.97	3.97	3.97	3.97	3.97	3.97	3.97	3.97
3.98	3.98	3.98	3.98	3.98	3.98	3.98	3.98	3.98
3.99	3.99	3.99	3.99	3.99	3.99	3.99	3.99	3.99

4	4	4	4	4	4	4	4	4
4.01	4.01	4.01	4.01	4.01	4.01	4.01	4.01	4.01
4.02	4.02	4.02	4.02	4.02	4.02	4.02	4.02	4.02
4.03	4.03	4.03	4.03	4.03	4.03	4.03	4.03	4.03
4.04	4.04	4.04	4.04	4.04	4.04	4.04	4.04	4.04
4.05	4.05	4.05	4.05	4.05	4.05	4.05	4.05	4.05
4.06	4.06	4.06	4.06	4.06	4.06	4.06	4.06	4.06
4.07	4.07	4.07	4.07	4.07	4.07	4.07	4.07	4.07
4.08	4.08	4.08	4.08	4.08	4.08	4.08	4.08	4.08
4.09	4.09	4.09	4.09	4.09	4.09	4.09	4.09	4.09
4.1	4.1	4.1	4.1	4.1	4.1	4.1	4.1	4.1
4.11	4.11	4.11	4.11	4.11	4.11	4.11	4.11	4.11
4.12	4.12	4.12	4.12	4.12	4.12	4.12	4.12	4.12
4.13	4.13	4.13	4.13	4.13	4.13	4.13	4.13	4.13
4.14	4.14	4.14	4.14	4.14	4.14	4.14	4.14	4.14
4.15	4.15	4.15	4.15	4.15	4.15	4.15	4.15	4.15
4.16	4.16	4.16	4.16	4.16	4.16	4.16	4.16	4.16
4.17	4.17	4.17	4.17	4.17	4.17	4.17	4.17	4.17
4.18	4.18	4.18	4.18	4.18	4.18	4.18	4.18	4.18
4.19	4.19	4.19	4.19	4.19	4.19	4.19	4.19	4.19
4.2	4.2	4.2	4.2	4.2	4.2	4.2	4.2	4.2
4.21	4.21	4.21	4.21	4.21	4.21	4.21	4.21	4.21
4.22	4.22	4.22	4.22	4.22	4.22	4.22	4.22	4.22
4.23	4.23	4.23	4.23	4.23	4.23	4.23	4.23	4.23
4.24	4.24	4.24	4.24	4.24	4.24	4.24	4.24	4.24
4.25	4.25	4.25	4.25	4.25	4.25	4.25	4.25	4.25
4.26	4.26	4.26	4.26	4.26	4.26	4.26	4.26	4.26
4.27	4.27	4.27	4.27	4.27	4.27	4.27	4.27	4.27
4.28	4.28	4.28	4.28	4.28	4.28	4.28	4.28	4.28
4.29	4.29	4.29	4.29	4.29	4.29	4.29	4.29	4.29
4.3	4.3	4.3	4.3	4.3	4.3	4.3	4.3	4.3
4.31	4.31	4.31	4.31	4.31	4.31	4.31	4.31	4.31
4.32	4.32	4.32	4.32	4.32	4.32	4.32	4.32	4.32
4.33	4.33	4.33	4.33	4.33	4.33	4.33	4.33	4.33
4.34	4.34	4.34	4.34	4.34	4.34	4.34	4.34	4.34
4.35	4.35	4.35	4.35	4.35	4.35	4.35	4.35	4.35
4.36	4.36	4.36	4.36	4.36	4.36	4.36	4.36	4.36
4.37	4.37	4.37	4.37	4.37	4.37	4.37	4.37	4.37
4.38	4.38	4.38	4.38	4.38	4.38	4.38	4.38	4.38
4.39	4.39	4.39	4.39	4.39	4.39	4.39	4.39	4.39
4.4	4.4	4.4	4.4	4.4	4.4	4.4	4.4	4.4
4.41	4.41	4.41	4.41	4.41	4.41	4.41	4.41	4.41
4.42	4.42	4.42	4.42	4.42	4.42	4.42	4.42	4.42
4.43	4.43	4.43	4.43	4.43	4.43	4.43	4.43	4.43
4.44	4.44	4.44	4.44	4.44	4.44	4.44	4.44	4.44
4.45	4.45	4.45	4.45	4.45	4.45	4.45	4.45	4.45
4.46	4.46	4.46	4.46	4.46	4.46	4.46	4.46	4.46
4.47	4.47	4.47	4.47	4.47	4.47	4.47	4.47	4.47
4.48	4.48	4.48	4.48	4.48	4.48	4.48	4.48	4.48
4.49	4.49	4.49	4.49	4.49	4.49	4.49	4.49	4.49

4.5	4.5	4.5	4.5	4.5	4.5	4.5	4.5	4.5
4.51	4.51	4.51	4.51	4.51	4.51	4.51	4.51	4.51
4.52	4.52	4.52	4.52	4.52	4.52	4.52	4.52	4.52
4.53	4.53	4.53	4.53	4.53	4.53	4.53	4.53	4.53
4.54	4.54	4.54	4.54	4.54	4.54	4.54	4.54	4.54
4.55	4.55	4.55	4.55	4.55	4.55	4.55	4.55	4.55
4.56	4.56	4.56	4.56	4.56	4.56	4.56	4.56	4.56
4.57	4.57	4.57	4.57	4.57	4.57	4.57	4.57	4.57
4.58	4.58	4.58	4.58	4.58	4.58	4.58	4.58	4.58
4.59	4.59	4.59	4.59	4.59	4.59	4.59	4.59	4.59
4.6	4.6	4.6	4.6	4.6	4.6	4.6	4.6	4.6
4.61	4.61	4.61	4.61	4.61	4.61	4.61	4.61	4.61
4.62	4.62	4.62	4.62	4.62	4.62	4.62	4.62	4.62
4.63	4.63	4.63	4.63	4.63	4.63	4.63	4.63	4.63
4.64	4.64	4.64	4.64	4.64	4.64	4.64	4.64	4.64
4.65	4.65	4.65	4.65	4.65	4.65	4.65	4.65	4.65
4.66	4.66	4.66	4.66	4.66	4.66	4.66	4.66	4.66
4.67	4.67	4.67	4.67	4.67	4.67	4.67	4.67	4.67
4.68	4.68	4.68	4.68	4.68	4.68	4.68	4.68	4.68
4.69	4.69	4.69	4.69	4.69	4.69	4.69	4.69	4.69
4.7	4.7	4.7	4.7	4.7	4.7	4.7	4.7	4.7
4.71	4.71	4.71	4.71	4.71	4.71	4.71	4.71	4.71
4.72	4.72	4.72	4.72	4.72	4.72	4.72	4.72	4.72
4.73	4.73	4.73	4.73	4.73	4.73	4.73	4.73	4.73
4.74	4.74	4.74	4.74	4.74	4.74	4.74	4.74	4.74
4.75	4.75	4.75	4.75	4.75	4.75	4.75	4.75	4.75
4.76	4.76	4.76	4.76	4.76	4.76	4.76	4.76	4.76
4.77	4.77	4.77	4.77	4.77	4.77	4.77	4.77	4.77
4.78	4.78	4.78	4.78	4.78	4.78	4.78	4.78	4.78
4.79	4.79	4.79	4.79	4.79	4.79	4.79	4.79	4.79
4.8	4.8	4.8	4.8	4.8	4.8	4.8	4.8	4.8
4.81	4.81	4.81	4.81	4.81	4.81	4.81	4.81	4.81
4.82	4.82	4.82	4.82	4.82	4.82	4.82	4.82	4.82
4.83	4.83	4.83	4.83	4.83	4.83	4.83	4.83	4.83
4.84	4.84	4.84	4.84	4.84	4.84	4.84	4.84	4.84
4.85	4.85	4.85	4.85	4.85	4.85	4.85	4.85	4.85
4.86	4.86	4.86	4.86	4.86	4.86	4.86	4.86	4.86
4.87	4.87	4.87	4.87	4.87	4.87	4.87	4.87	4.87
4.88	4.88	4.88	4.88	4.88	4.88	4.88	4.88	4.88
4.89	4.89	4.89	4.89	4.89	4.89	4.89	4.89	4.89
4.9	4.9	4.9	4.9	4.9	4.9	4.9	4.9	4.9
4.91	4.91	4.91	4.91	4.91	4.91	4.91	4.91	4.91
4.92	4.92	4.92	4.92	4.92	4.92	4.92	4.92	4.92
4.93	4.93	4.93	4.93	4.93	4.93	4.93	4.93	4.93
4.94	4.94	4.94	4.94	4.94	4.94	4.94	4.94	4.94
4.95	4.95	4.95	4.95	4.95	4.95	4.95	4.95	4.95
4.96	4.96	4.96	4.96	4.96	4.96	4.96	4.96	4.96
4.97	4.97	4.97	4.97	4.97	4.97	4.97	4.97	4.97
4.98	4.98	4.98	4.98	4.98	4.98	4.98	4.98	4.98
4.99	4.99	4.99	4.99	4.99	4.99	4.99	4.99	4.99

5	5	5	5	5	5	5	5	5
5.01	5.01	5.01	5.01	5.01	5.01	5.01	5.01	5.01
5.02	5.02	5.02	5.02	5.02	5.02	5.02	5.02	5.02
5.03	5.03	5.03	5.03	5.03	5.03	5.03	5.03	5.03
5.04	5.04	5.04	5.04	5.04	5.04	5.04	5.04	5.04
5.05	5.05	5.05	5.05	5.05	5.05	5.05	5.05	5.05
5.06	5.06	5.06	5.06	5.06	5.06	5.06	5.06	5.06
5.07	5.07	5.07	5.07	5.07	5.07	5.07	5.07	5.07
5.08	5.08	5.08	5.08	5.08	5.08	5.08	5.08	5.08
5.09	5.09	5.09	5.09	5.09	5.09	5.09	5.09	5.09
5.1	5.1	5.1	5.1	5.1	5.1	5.1	5.1	5.1
5.11	5.11	5.11	5.11	5.11	5.11	5.11	5.11	5.11
5.12	5.12	5.12	5.12	5.12	5.12	5.12	5.12	5.12
5.13	5.13	5.13	5.13	5.13	5.13	5.13	5.13	5.13
5.14	5.14	5.14	5.14	5.14	5.14	5.14	5.14	5.14
5.15	5.15	5.15	5.15	5.15	5.15	5.15	5.15	5.15
5.16	5.16	5.16	5.16	5.16	5.16	5.16	5.16	5.16
5.17	5.17	5.17	5.17	5.17	5.17	5.17	5.17	5.17
5.18	5.18	5.18	5.18	5.18	5.18	5.18	5.18	5.18
5.19	5.19	5.19	5.19	5.19	5.19	5.19	5.19	5.19
5.2	5.2	5.2	5.2	5.2	5.2	5.2	5.2	5.2
5.21	5.21	5.21	5.21	5.21	5.21	5.21	5.21	5.21
5.22	5.22	5.22	5.22	5.22	5.22	5.22	5.22	5.22
5.23	5.23	5.23	5.23	5.23	5.23	5.23	5.23	5.23
5.24	5.24	5.24	5.24	5.24	5.24	5.24	5.24	5.24
5.25	5.25	5.25	5.25	5.25	5.25	5.25	5.25	5.25
5.26	5.26	5.26	5.26	5.26	5.26	5.26	5.26	5.26
5.27	5.27	5.27	5.27	5.27	5.27	5.27	5.27	5.27
5.28	5.28	5.28	5.28	5.28	5.28	5.28	5.28	5.28
5.29	5.29	5.29	5.29	5.29	5.29	5.29	5.29	5.29
5.3	5.3	5.3	5.3	5.3	5.3	5.3	5.3	5.3
5.31	5.31	5.31	5.31	5.31	5.31	5.31	5.31	5.31
5.32	5.32	5.32	5.32	5.32	5.32	5.32	5.32	5.32
5.33	5.33	5.33	5.33	5.33	5.33	5.33	5.33	5.33
5.34	5.34	5.34	5.34	5.34	5.34	5.34	5.34	5.34
5.35	5.35	5.35	5.35	5.35	5.35	5.35	5.35	5.35
5.36	5.36	5.36	5.36	5.36	5.36	5.36	5.36	5.36
5.37	5.37	5.37	5.37	5.37	5.37	5.37	5.37	5.37
5.38	5.38	5.38	5.38	5.38	5.38	5.38	5.38	5.38
5.39	5.39	5.39	5.39	5.39	5.39	5.39	5.39	5.39
5.4	5.4	5.4	5.4	5.4	5.4	5.4	5.4	5.4
5.41	5.41	5.41	5.41	5.41	5.41	5.41	5.41	5.41
5.42	5.42	5.42	5.42	5.42	5.42	5.42	5.42	5.42
5.43	5.43	5.43	5.43	5.43	5.43	5.43	5.43	5.43
5.44	5.44	5.44	5.44	5.44	5.44	5.44	5.44	5.44
5.45	5.45	5.45	5.45	5.45	5.45	5.45	5.45	5.45
5.46	5.46	5.46	5.46	5.46	5.46	5.46	5.46	5.46
5.47	5.47	5.47	5.47	5.47	5.47	5.47	5.47	5.47
5.48	5.48	5.48	5.48	5.48	5.48	5.48	5.48	5.48
5.49	5.49	5.49	5.49	5.49	5.49	5.49	5.49	5.49

5.5	5.5	5.5	5.5	5.5	5.5	5.5	5.5	5.5
5.51	5.51	5.51	5.51	5.51	5.51	5.51	5.51	5.51
5.52	5.52	5.52	5.52	5.52	5.52	5.52	5.52	5.52
5.53	5.53	5.53	5.53	5.53	5.53	5.53	5.53	5.53
5.54	5.54	5.54	5.54	5.54	5.54	5.54	5.54	5.54
5.55	5.55	5.55	5.55	5.55	5.55	5.55	5.55	5.55
5.56	5.56	5.56	5.56	5.56	5.56	5.56	5.56	5.56
5.57	5.57	5.57	5.57	5.57	5.57	5.57	5.57	5.57
5.58	5.58	5.58	5.58	5.58	5.58	5.58	5.58	5.58
5.59	5.59	5.59	5.59	5.59	5.59	5.59	5.59	5.59
5.6	5.6	5.6	5.6	5.6	5.6	5.6	5.6	5.6
5.61	5.61	5.61	5.61	5.61	5.61	5.61	5.61	5.61
5.62	5.62	5.62	5.62	5.62	5.62	5.62	5.62	5.62
5.63	5.63	5.63	5.63	5.63	5.63	5.63	5.63	5.63
5.64	5.64	5.64	5.64	5.64	5.64	5.64	5.64	5.64
5.65	5.65	5.65	5.65	5.65	5.65	5.65	5.65	5.65
5.66	5.66	5.66	5.66	5.66	5.66	5.66	5.66	5.66
5.67	5.67	5.67	5.67	5.67	5.67	5.67	5.67	5.67
5.68	5.68	5.68	5.68	5.68	5.68	5.68	5.68	5.68
5.69	5.69	5.69	5.69	5.69	5.69	5.69	5.69	5.69
5.7	5.7	5.7	5.7	5.7	5.7	5.7	5.7	5.7
5.71	5.71	5.71	5.71	5.71	5.71	5.71	5.71	5.71
5.72	5.72	5.72	5.72	5.72	5.72	5.72	5.72	5.72
5.73	5.73	5.73	5.73	5.73	5.73	5.73	5.73	5.73
5.74	5.74	5.74	5.74	5.74	5.74	5.74	5.74	5.74
5.75	5.75	5.75	5.75	5.75	5.75	5.75	5.75	5.75
5.76	5.76	5.76	5.76	5.76	5.76	5.76	5.76	5.76
5.77	5.77	5.77	5.77	5.77	5.77	5.77	5.77	5.77
5.78	5.78	5.78	5.78	5.78	5.78	5.78	5.78	5.78
5.79	5.79	5.79	5.79	5.79	5.79	5.79	5.79	5.79
5.8	5.8	5.8	5.8	5.8	5.8	5.8	5.8	5.8
5.81	5.81	5.81	5.81	5.81	5.81	5.81	5.81	5.81
5.82	5.82	5.82	5.82	5.82	5.82	5.82	5.82	5.82
5.83	5.83	5.83	5.83	5.83	5.83	5.83	5.83	5.83
5.84	5.84	5.84	5.84	5.84	5.84	5.84	5.84	5.84
5.85	5.85	5.85	5.85	5.85	5.85	5.85	5.85	5.85
5.86	5.86	5.86	5.86	5.86	5.86	5.86	5.86	5.86
5.87	5.87	5.87	5.87	5.87	5.87	5.87	5.87	5.87
5.88	5.88	5.88	5.88	5.88	5.88	5.88	5.88	5.88
5.89	5.89	5.89	5.89	5.89	5.89	5.89	5.89	5.89
5.9	5.9	5.9	5.9	5.9	5.9	5.9	5.9	5.9
5.91	5.91	5.91	5.91	5.91	5.91	5.91	5.91	5.91
5.92	5.92	5.92	5.92	5.92	5.92	5.92	5.92	5.92
5.93	5.93	5.93	5.93	5.93	5.93	5.93	5.93	5.93
5.94	5.94	5.94	5.94	5.94	5.94	5.94	5.94	5.94
5.95	5.95	5.95	5.95	5.95	5.95	5.95	5.95	5.95
5.96	5.96	5.96	5.96	5.96	5.96	5.96	5.96	5.96
5.97	5.97	5.97	5.97	5.97	5.97	5.97	5.97	5.97
5.98	5.98	5.98	5.98	5.98	5.98	5.98	5.98	5.98
5.99	5.99	5.99	5.99	5.99	5.99	5.99	5.99	5.99

6	6	6	6	6	6	6	6	6
6.01	6.01	6.01	6.01	6.01	6.01	6.01	6.01	6.01
6.02	6.02	6.02	6.02	6.02	6.02	6.02	6.02	6.02
6.03	6.03	6.03	6.03	6.03	6.03	6.03	6.03	6.03
6.04	6.04	6.04	6.04	6.04	6.04	6.04	6.04	6.04
6.05	6.05	6.05	6.05	6.05	6.05	6.05	6.05	6.05
6.06	6.06	6.06	6.06	6.06	6.06	6.06	6.06	6.06
6.07	6.07	6.07	6.07	6.07	6.07	6.07	6.07	6.07
6.08	6.08	6.08	6.08	6.08	6.08	6.08	6.08	6.08
6.09	6.09	6.09	6.09	6.09	6.09	6.09	6.09	6.09
6.1	6.1	6.1	6.1	6.1	6.1	6.1	6.1	6.1
6.11	6.11	6.11	6.11	6.11	6.11	6.11	6.11	6.11
6.12	6.12	6.12	6.12	6.12	6.12	6.12	6.12	6.12
6.13	6.13	6.13	6.13	6.13	6.13	6.13	6.13	6.13
6.14	6.14	6.14	6.14	6.14	6.14	6.14	6.14	6.14
6.15	6.15	6.15	6.15	6.15	6.15	6.15	6.15	6.15
6.16	6.16	6.16	6.16	6.16	6.16	6.16	6.16	6.16
6.17	6.17	6.17	6.17	6.17	6.17	6.17	6.17	6.17
6.18	6.18	6.18	6.18	6.18	6.18	6.18	6.18	6.18
6.19	6.19	6.19	6.19	6.19	6.19	6.19	6.19	6.19
6.2	6.2	6.2	6.2	6.2	6.2	6.2	6.2	6.2
6.21	6.21	6.21	6.21	6.21	6.21	6.21	6.21	6.21
6.22	6.22	6.22	6.22	6.22	6.22	6.22	6.22	6.22
6.23	6.23	6.23	6.23	6.23	6.23	6.23	6.23	6.23
6.24	6.24	6.24	6.24	6.24	6.24	6.24	6.24	6.24
6.25	6.25	6.25	6.25	6.25	6.25	6.25	6.25	6.25
6.26	6.26	6.26	6.26	6.26	6.26	6.26	6.26	6.26
6.27	6.27	6.27	6.27	6.27	6.27	6.27	6.27	6.27
6.28	6.28	6.28	6.28	6.28	6.28	6.28	6.28	6.28
6.29	6.29	6.29	6.29	6.29	6.29	6.29	6.29	6.29
6.3	6.3	6.3	6.3	6.3	6.3	6.3	6.3	6.3
6.31	6.31	6.31	6.31	6.31	6.31	6.31	6.31	6.31
6.32	6.32	6.32	6.32	6.32	6.32	6.32	6.32	6.32
6.33	6.33	6.33	6.33	6.33	6.33	6.33	6.33	6.33
6.34	6.34	6.34	6.34	6.34	6.34	6.34	6.34	6.34
6.35	6.35	6.35	6.35	6.35	6.35	6.35	6.35	6.35
6.36	6.36	6.36	6.36	6.36	6.36	6.36	6.36	6.36
6.37	6.37	6.37	6.37	6.37	6.37	6.37	6.37	6.37
6.38	6.38	6.38	6.38	6.38	6.38	6.38	6.38	6.38
6.39	6.39	6.39	6.39	6.39	6.39	6.39	6.39	6.39
6.4	6.4	6.4	6.4	6.4	6.4	6.4	6.4	6.4
6.41	6.41	6.41	6.41	6.41	6.41	6.41	6.41	6.41
6.42	6.42	6.42	6.42	6.42	6.42	6.42	6.42	6.42
6.43	6.43	6.43	6.43	6.43	6.43	6.43	6.43	6.43
6.44	6.44	6.44	6.44	6.44	6.44	6.44	6.44	6.44
6.45	6.45	6.45	6.45	6.45	6.45	6.45	6.45	6.45
6.46	6.46	6.46	6.46	6.46	6.46	6.46	6.46	6.46
6.47	6.47	6.47	6.47	6.47	6.47	6.47	6.47	6.47
6.48	6.48	6.48	6.48	6.48	6.48	6.48	6.48	6.48
6.49	6.49	6.49	6.49	6.49	6.49	6.49	6.49	6.49

6.5	6.5	6.5	6.5	6.5	6.5	6.5	6.5	6.5
6.51	6.51	6.51	6.51	6.51	6.51	6.51	6.51	6.51
6.52	6.52	6.52	6.52	6.52	6.52	6.52	6.52	6.52
6.53	6.53	6.53	6.53	6.53	6.53	6.53	6.53	6.53
6.54	6.54	6.54	6.54	6.54	6.54	6.54	6.54	6.54
6.55	6.55	6.55	6.55	6.55	6.55	6.55	6.55	6.55
6.56	6.56	6.56	6.56	6.56	6.56	6.56	6.56	6.56
6.57	6.57	6.57	6.57	6.57	6.57	6.57	6.57	6.57
6.58	6.58	6.58	6.58	6.58	6.58	6.58	6.58	6.58
6.59	6.59	6.59	6.59	6.59	6.59	6.59	6.59	6.59
6.6	6.6	6.6	6.6	6.6	6.6	6.6	6.6	6.6
6.61	6.61	6.61	6.61	6.61	6.61	6.61	6.61	6.61
6.62	6.62	6.62	6.62	6.62	6.62	6.62	6.62	6.62
6.63	6.63	6.63	6.63	6.63	6.63	6.63	6.63	6.63
6.64	6.64	6.64	6.64	6.64	6.64	6.64	6.64	6.64
6.65	6.65	6.65	6.65	6.65	6.65	6.65	6.65	6.65
6.66	6.66	6.66	6.66	6.66	6.66	6.66	6.66	6.66
6.67	6.67	6.67	6.67	6.67	6.67	6.67	6.67	6.67
6.68	6.68	6.68	6.68	6.68	6.68	6.68	6.68	6.68
6.69	6.69	6.69	6.69	6.69	6.69	6.69	6.69	6.69
6.7	6.7	6.7	6.7	6.7	6.7	6.7	6.7	6.7
6.71	6.71	6.71	6.71	6.71	6.71	6.71	6.71	6.71
6.72	6.72	6.72	6.72	6.72	6.72	6.72	6.72	6.72
6.73	6.73	6.73	6.73	6.73	6.73	6.73	6.73	6.73
6.74	6.74	6.74	6.74	6.74	6.74	6.74	6.74	6.74
6.75	6.75	6.75	6.75	6.75	6.75	6.75	6.75	6.75
6.76	6.76	6.76	6.76	6.76	6.76	6.76	6.76	6.76
6.77	6.77	6.77	6.77	6.77	6.77	6.77	6.77	6.77
6.78	6.78	6.78	6.78	6.78	6.78	6.78	6.78	6.78
6.79	6.79	6.79	6.79	6.79	6.79	6.79	6.79	6.79
6.8	6.8	6.8	6.8	6.8	6.8	6.8	6.8	6.8
6.81	6.81	6.81	6.81	6.81	6.81	6.81	6.81	6.81
6.82	6.82	6.82	6.82	6.82	6.82	6.82	6.82	6.82
6.83	6.83	6.83	6.83	6.83	6.83	6.83	6.83	6.83
6.84	6.84	6.84	6.84	6.84	6.84	6.84	6.84	6.84
6.85	6.85	6.85	6.85	6.85	6.85	6.85	6.85	6.85
6.86	6.86	6.86	6.86	6.86	6.86	6.86	6.86	6.86
6.87	6.87	6.87	6.87	6.87	6.87	6.87	6.87	6.87
6.88	6.88	6.88	6.88	6.88	6.88	6.88	6.88	6.88
6.89	6.89	6.89	6.89	6.89	6.89	6.89	6.89	6.89
6.9	6.9	6.9	6.9	6.9	6.9	6.9	6.9	6.9
6.91	6.91	6.91	6.91	6.91	6.91	6.91	6.91	6.91
6.92	6.92	6.92	6.92	6.92	6.92	6.92	6.92	6.92
6.93	6.93	6.93	6.93	6.93	6.93	6.93	6.93	6.93
6.94	6.94	6.94	6.94	6.94	6.94	6.94	6.94	6.94
6.95	6.95	6.95	6.95	6.95	6.95	6.95	6.95	6.95
6.96	6.96	6.96	6.96	6.96	6.96	6.96	6.96	6.96
6.97	6.97	6.97	6.97	6.97	6.97	6.97	6.97	6.97
6.98	6.98	6.98	6.98	6.98	6.98	6.98	6.98	6.98
6.99	6.99	6.99	6.99	6.99	6.99	6.99	6.99	6.99

7	7	7	7	7	7	7	7	7
7.01	7.01	7.01	7.01	7.01	7.01	7.01	7.01	7.01
7.02	7.02	7.02	7.02	7.02	7.02	7.02	7.02	7.02
7.03	7.03	7.03	7.03	7.03	7.03	7.03	7.03	7.03
7.04	7.04	7.04	7.04	7.04	7.04	7.04	7.04	7.04
7.05	7.05	7.05	7.05	7.05	7.05	7.05	7.05	7.05
7.06	7.06	7.06	7.06	7.06	7.06	7.06	7.06	7.06
7.07	7.07	7.07	7.07	7.07	7.07	7.07	7.07	7.07
7.08	7.08	7.08	7.08	7.08	7.08	7.08	7.08	7.08
7.09	7.09	7.09	7.09	7.09	7.09	7.09	7.09	7.09
7.1	7.1	7.1	7.1	7.1	7.1	7.1	7.1	7.1
7.11	7.11	7.11	7.11	7.11	7.11	7.11	7.11	7.11
7.12	7.12	7.12	7.12	7.12	7.12	7.12	7.12	7.12
7.13	7.13	7.13	7.13	7.13	7.13	7.13	7.13	7.13
7.14	7.14	7.14	7.14	7.14	7.14	7.14	7.14	7.14
7.15	7.15	7.15	7.15	7.15	7.15	7.15	7.15	7.15
7.16	7.16	7.16	7.16	7.16	7.16	7.16	7.16	7.16
7.17	7.17	7.17	7.17	7.17	7.17	7.17	7.17	7.17
7.18	7.18	7.18	7.18	7.18	7.18	7.18	7.18	7.18
7.19	7.19	7.19	7.19	7.19	7.19	7.19	7.19	7.19
7.2	7.2	7.2	7.2	7.2	7.2	7.2	7.2	7.2
7.21	7.21	7.21	7.21	7.21	7.21	7.21	7.21	7.21
7.22	7.22	7.22	7.22	7.22	7.22	7.22	7.22	7.22
7.23	7.23	7.23	7.23	7.23	7.23	7.23	7.23	7.23
7.24	7.24	7.24	7.24	7.24	7.24	7.24	7.24	7.24
7.25	7.25	7.25	7.25	7.25	7.25	7.25	7.25	7.25
7.26	7.26	7.26	7.26	7.26	7.26	7.26	7.26	7.26
7.27	7.27	7.27	7.27	7.27	7.27	7.27	7.27	7.27
7.28	7.28	7.28	7.28	7.28	7.28	7.28	7.28	7.28
7.29	7.29	7.29	7.29	7.29	7.29	7.29	7.29	7.29
7.3	7.3	7.3	7.3	7.3	7.3	7.3	7.3	7.3
7.31	7.31	7.31	7.31	7.31	7.31	7.31	7.31	7.31
7.32	7.32	7.32	7.32	7.32	7.32	7.32	7.32	7.32
7.33	7.33	7.33	7.33	7.33	7.33	7.33	7.33	7.33
7.34	7.34	7.34	7.34	7.34	7.34	7.34	7.34	7.34
7.35	7.35	7.35	7.35	7.35	7.35	7.35	7.35	7.35
7.36	7.36	7.36	7.36	7.36	7.36	7.36	7.36	7.36
7.37	7.37	7.37	7.37	7.37	7.37	7.37	7.37	7.37
7.38	7.38	7.38	7.38	7.38	7.38	7.38	7.38	7.38
7.39	7.39	7.39	7.39	7.39	7.39	7.39	7.39	7.39
7.4	7.4	7.4	7.4	7.4	7.4	7.4	7.4	7.4
7.41	7.41	7.41	7.41	7.41	7.41	7.41	7.41	7.41
7.42	7.42	7.42	7.42	7.42	7.42	7.42	7.42	7.42
7.43	7.43	7.43	7.43	7.43	7.43	7.43	7.43	7.43
7.44	7.44	7.44	7.44	7.44	7.44	7.44	7.44	7.44
7.45	7.45	7.45	7.45	7.45	7.45	7.45	7.45	7.45
7.46	7.46	7.46	7.46	7.46	7.46	7.46	7.46	7.46
7.47	7.47	7.47	7.47	7.47	7.47	7.47	7.47	7.47
7.48	7.48	7.48	7.48	7.48	7.48	7.48	7.48	7.48
7.49	7.49	7.49	7.49	7.49	7.49	7.49	7.49	7.49

7.5	7.5	7.5	7.5	7.5	7.5	7.5	7.5	7.5
7.51	7.51	7.51	7.51	7.51	7.51	7.51	7.51	7.51
7.52	7.52	7.52	7.52	7.52	7.52	7.52	7.52	7.52
7.53	7.53	7.53	7.53	7.53	7.53	7.53	7.53	7.53
7.54	7.54	7.54	7.54	7.54	7.54	7.54	7.54	7.54
7.55	7.55	7.55	7.55	7.55	7.55	7.55	7.55	7.55
7.56	7.56	7.56	7.56	7.56	7.56	7.56	7.56	7.56
7.57	7.57	7.57	7.57	7.57	7.57	7.57	7.57	7.57
7.58	7.58	7.58	7.58	7.58	7.58	7.58	7.58	7.58
7.59	7.59	7.59	7.59	7.59	7.59	7.59	7.59	7.59
7.6	7.6	7.6	7.6	7.6	7.6	7.6	7.6	7.6
7.61	7.61	7.61	7.61	7.61	7.61	7.61	7.61	7.61
7.62	7.62	7.62	7.62	7.62	7.62	7.62	7.62	7.62
7.63	7.63	7.63	7.63	7.63	7.63	7.63	7.63	7.63
7.64	7.64	7.64	7.64	7.64	7.64	7.64	7.64	7.64
7.65	7.65	7.65	7.65	7.65	7.65	7.65	7.65	7.65
7.66	7.66	7.66	7.66	7.66	7.66	7.66	7.66	7.66
7.67	7.67	7.67	7.67	7.67	7.67	7.67	7.67	7.67
7.68	7.68	7.68	7.68	7.68	7.68	7.68	7.68	7.68
7.69	7.69	7.69	7.69	7.69	7.69	7.69	7.69	7.69
7.7	7.7	7.7	7.7	7.7	7.7	7.7	7.7	7.7
7.71	7.71	7.71	7.71	7.71	7.71	7.71	7.71	7.71
7.72	7.72	7.72	7.72	7.72	7.72	7.72	7.72	7.72
7.73	7.73	7.73	7.73	7.73	7.73	7.73	7.73	7.73
7.74	7.74	7.74	7.74	7.74	7.74	7.74	7.74	7.74
7.75	7.75	7.75	7.75	7.75	7.75	7.75	7.75	7.75
7.76	7.76	7.76	7.76	7.76	7.76	7.76	7.76	7.76
7.77	7.77	7.77	7.77	7.77	7.77	7.77	7.77	7.77
7.78	7.78	7.78	7.78	7.78	7.78	7.78	7.78	7.78
7.79	7.79	7.79	7.79	7.79	7.79	7.79	7.79	7.79
7.8	7.8	7.8	7.8	7.8	7.8	7.8	7.8	7.8
7.81	7.81	7.81	7.81	7.81	7.81	7.81	7.81	7.81
7.82	7.82	7.82	7.82	7.82	7.82	7.82	7.82	7.82
7.83	7.83	7.83	7.83	7.83	7.83	7.83	7.83	7.83
7.84	7.84	7.84	7.84	7.84	7.84	7.84	7.84	7.84
7.85	7.85	7.85	7.85	7.85	7.85	7.85	7.85	7.85
7.86	7.86	7.86	7.86	7.86	7.86	7.86	7.86	7.86
7.87	7.87	7.87	7.87	7.87	7.87	7.87	7.87	7.87
7.88	7.88	7.88	7.88	7.88	7.88	7.88	7.88	7.88
7.89	7.89	7.89	7.89	7.89	7.89	7.89	7.89	7.89
7.9	7.9	7.9	7.9	7.9	7.9	7.9	7.9	7.9
7.91	7.91	7.91	7.91	7.91	7.91	7.91	7.91	7.91
7.92	7.92	7.92	7.92	7.92	7.92	7.92	7.92	7.92
7.93	7.93	7.93	7.93	7.93	7.93	7.93	7.93	7.93
7.94	7.94	7.94	7.94	7.94	7.94	7.94	7.94	7.94
7.95	7.95	7.95	7.95	7.95	7.95	7.95	7.95	7.95
7.96	7.96	7.96	7.96	7.96	7.96	7.96	7.96	7.96
7.97	7.97	7.97	7.97	7.97	7.97	7.97	7.97	7.97
7.98	7.98	7.98	7.98	7.98	7.98	7.98	7.98	7.98
7.99	7.99	7.99	7.99	7.99	7.99	7.99	7.99	7.99

8	8	8	8	8	8	8	8	8
8.01	8.01	8.01	8.01	8.01	8.01	8.01	8.01	8.01
8.02	8.02	8.02	8.02	8.02	8.02	8.02	8.02	8.02
8.03	8.03	8.03	8.03	8.03	8.03	8.03	8.03	8.03
8.04	8.04	8.04	8.04	8.04	8.04	8.04	8.04	8.04
8.05	8.05	8.05	8.05	8.05	8.05	8.05	8.05	8.05
8.06	8.06	8.06	8.06	8.06	8.06	8.06	8.06	8.06
8.07	8.07	8.07	8.07	8.07	8.07	8.07	8.07	8.07
8.08	8.08	8.08	8.08	8.08	8.08	8.08	8.08	8.08
8.09	8.09	8.09	8.09	8.09	8.09	8.09	8.09	8.09
8.1	8.1	8.1	8.1	8.1	8.1	8.1	8.1	8.1
8.11	8.11	8.11	8.11	8.11	8.11	8.11	8.11	8.11
8.12	8.12	8.12	8.12	8.12	8.12	8.12	8.12	8.12
8.13	8.13	8.13	8.13	8.13	8.13	8.13	8.13	8.13
8.14	8.14	8.14	8.14	8.14	8.14	8.14	8.14	8.14
8.15	8.15	8.15	8.15	8.15	8.15	8.15	8.15	8.15
8.16	8.16	8.16	8.16	8.16	8.16	8.16	8.16	8.16
8.17	8.17	8.17	8.17	8.17	8.17	8.17	8.17	8.17
8.18	8.18	8.18	8.18	8.18	8.18	8.18	8.18	8.18
8.19	8.19	8.19	8.19	8.19	8.19	8.19	8.19	8.19
8.2	8.2	8.2	8.2	8.2	8.2	8.2	8.2	8.2
8.21	8.21	8.21	8.21	8.21	8.21	8.21	8.21	8.21
8.22	8.22	8.22	8.22	8.22	8.22	8.22	8.22	8.22
8.23	8.23	8.23	8.23	8.23	8.23	8.23	8.23	8.23
8.24	8.24	8.24	8.24	8.24	8.24	8.24	8.24	8.24
8.25	8.25	8.25	8.25	8.25	8.25	8.25	8.25	8.25
8.26	8.26	8.26	8.26	8.26	8.26	8.26	8.26	8.26
8.27	8.27	8.27	8.27	8.27	8.27	8.27	8.27	8.27
8.28	8.28	8.28	8.28	8.28	8.28	8.28	8.28	8.28
8.29	8.29	8.29	8.29	8.29	8.29	8.29	8.29	8.29
8.3	8.3	8.3	8.3	8.3	8.3	8.3	8.3	8.3
8.31	8.31	8.31	8.31	8.31	8.31	8.31	8.31	8.31
8.32	8.32	8.32	8.32	8.32	8.32	8.32	8.32	8.32
8.33	8.33	8.33	8.33	8.33	8.33	8.33	8.33	8.33
8.34	8.34	8.34	8.34	8.34	8.34	8.34	8.34	8.34
8.35	8.35	8.35	8.35	8.35	8.35	8.35	8.35	8.35
8.36	8.36	8.36	8.36	8.36	8.36	8.36	8.36	8.36
8.37	8.37	8.37	8.37	8.37	8.37	8.37	8.37	8.37
8.38	8.38	8.38	8.38	8.38	8.38	8.38	8.38	8.38
8.39	8.39	8.39	8.39	8.39	8.39	8.39	8.39	8.39
8.4	8.4	8.4	8.4	8.4	8.4	8.4	8.4	8.4
8.41	8.41	8.41	8.41	8.41	8.41	8.41	8.41	8.41
8.42	8.42	8.42	8.42	8.42	8.42	8.42	8.42	8.42
8.43	8.43	8.43	8.43	8.43	8.43	8.43	8.43	8.43
8.44	8.44	8.44	8.44	8.44	8.44	8.44	8.44	8.44
8.45	8.45	8.45	8.45	8.45	8.45	8.45	8.45	8.45
8.46	8.46	8.46	8.46	8.46	8.46	8.46	8.46	8.46
8.47	8.47	8.47	8.47	8.47	8.47	8.47	8.47	8.47
8.48	8.48	8.48	8.48	8.48	8.48	8.48	8.48	8.48
8.49	8.49	8.49	8.49	8.49	8.49	8.49	8.49	8.49

8.5	8.5	8.5	8.5	8.5	8.5	8.5	8.5	8.5
8.51	8.51	8.51	8.51	8.51	8.51	8.51	8.51	8.51
8.52	8.52	8.52	8.52	8.52	8.52	8.52	8.52	8.52
8.53	8.53	8.53	8.53	8.53	8.53	8.53	8.53	8.53
8.54	8.54	8.54	8.54	8.54	8.54	8.54	8.54	8.54
8.55	8.55	8.55	8.55	8.55	8.55	8.55	8.55	8.55
8.56	8.56	8.56	8.56	8.56	8.56	8.56	8.56	8.56
8.57	8.57	8.57	8.57	8.57	8.57	8.57	8.57	8.57
8.58	8.58	8.58	8.58	8.58	8.58	8.58	8.58	8.58
8.59	8.59	8.59	8.59	8.59	8.59	8.59	8.59	8.59
8.6	8.6	8.6	8.6	8.6	8.6	8.6	8.6	8.6
8.61	8.61	8.61	8.61	8.61	8.61	8.61	8.61	8.61
8.62	8.62	8.62	8.62	8.62	8.62	8.62	8.62	8.62
8.63	8.63	8.63	8.63	8.63	8.63	8.63	8.63	8.63
8.64	8.64	8.64	8.64	8.64	8.64	8.64	8.64	8.64
8.65	8.65	8.65	8.65	8.65	8.65	8.65	8.65	8.65
8.66	8.66	8.66	8.66	8.66	8.66	8.66	8.66	8.66
8.67	8.67	8.67	8.67	8.67	8.67	8.67	8.67	8.67
8.68	8.68	8.68	8.68	8.68	8.68	8.68	8.68	8.68
8.69	8.69	8.69	8.69	8.69	8.69	8.69	8.69	8.69
8.7	8.7	8.7	8.7	8.7	8.7	8.7	8.7	8.7
8.71	8.71	8.71	8.71	8.71	8.71	8.71	8.71	8.71
8.72	8.72	8.72	8.72	8.72	8.72	8.72	8.72	8.72
8.73	8.73	8.73	8.73	8.73	8.73	8.73	8.73	8.73
8.74	8.74	8.74	8.74	8.74	8.74	8.74	8.74	8.74
8.75	8.75	8.75	8.75	8.75	8.75	8.75	8.75	8.75
8.76	8.76	8.76	8.76	8.76	8.76	8.76	8.76	8.76
8.77	8.77	8.77	8.77	8.77	8.77	8.77	8.77	8.77
8.78	8.78	8.78	8.78	8.78	8.78	8.78	8.78	8.78
8.79	8.79	8.79	8.79	8.79	8.79	8.79	8.79	8.79
8.8	8.8	8.8	8.8	8.8	8.8	8.8	8.8	8.8
8.81	8.81	8.81	8.81	8.81	8.81	8.81	8.81	8.81
8.82	8.82	8.82	8.82	8.82	8.82	8.82	8.82	8.82
8.83	8.83	8.83	8.83	8.83	8.83	8.83	8.83	8.83
8.84	8.84	8.84	8.84	8.84	8.84	8.84	8.84	8.84
8.85	8.85	8.85	8.85	8.85	8.85	8.85	8.85	8.85
8.86	8.86	8.86	8.86	8.86	8.86	8.86	8.86	8.86
8.87	8.87	8.87	8.87	8.87	8.87	8.87	8.87	8.87
8.88	8.88	8.88	8.88	8.88	8.88	8.88	8.88	8.88
8.89	8.89	8.89	8.89	8.89	8.89	8.89	8.89	8.89
8.9	8.9	8.9	8.9	8.9	8.9	8.9	8.9	8.9
8.91	8.91	8.91	8.91	8.91	8.91	8.91	8.91	8.91
8.92	8.92	8.92	8.92	8.92	8.92	8.92	8.92	8.92
8.93	8.93	8.93	8.93	8.93	8.93	8.93	8.93	8.93
8.94	8.94	8.94	8.94	8.94	8.94	8.94	8.94	8.94
8.95	8.95	8.95	8.95	8.95	8.95	8.95	8.95	8.95
8.96	8.96	8.96	8.96	8.96	8.96	8.96	8.96	8.96
8.97	8.97	8.97	8.97	8.97	8.97	8.97	8.97	8.97
8.98	8.98	8.98	8.98	8.98	8.98	8.98	8.98	8.98
8.99	8.99	8.99	8.99	8.99	8.99	8.99	8.99	8.99

9	9	9	9	9	9	9	9	9
9.01	9.01	9.01	9.01	9.01	9.01	9.01	9.01	9.01
9.02	9.02	9.02	9.02	9.02	9.02	9.02	9.02	9.02
9.03	9.03	9.03	9.03	9.03	9.03	9.03	9.03	9.03
9.04	9.04	9.04	9.04	9.04	9.04	9.04	9.04	9.04
9.05	9.05	9.05	9.05	9.05	9.05	9.05	9.05	9.05
9.06	9.06	9.06	9.06	9.06	9.06	9.06	9.06	9.06
9.07	9.07	9.07	9.07	9.07	9.07	9.07	9.07	9.07
9.08	9.08	9.08	9.08	9.08	9.08	9.08	9.08	9.08
9.09	9.09	9.09	9.09	9.09	9.09	9.09	9.09	9.09
9.1	9.1	9.1	9.1	9.1	9.1	9.1	9.1	9.1
9.11	9.11	9.11	9.11	9.11	9.11	9.11	9.11	9.11
9.12	9.12	9.12	9.12	9.12	9.12	9.12	9.12	9.12
9.13	9.13	9.13	9.13	9.13	9.13	9.13	9.13	9.13
9.14	9.14	9.14	9.14	9.14	9.14	9.14	9.14	9.14
9.15	9.15	9.15	9.15	9.15	9.15	9.15	9.15	9.15
9.16	9.16	9.16	9.16	9.16	9.16	9.16	9.16	9.16
9.17	9.17	9.17	9.17	9.17	9.17	9.17	9.17	9.17
9.18	9.18	9.18	9.18	9.18	9.18	9.18	9.18	9.18
9.19	9.19	9.19	9.19	9.19	9.19	9.19	9.19	9.19
9.2	9.2	9.2	9.2	9.2	9.2	9.2	9.2	9.2
9.21	9.21	9.21	9.21	9.21	9.21	9.21	9.21	9.21
9.22	9.22	9.22	9.22	9.22	9.22	9.22	9.22	9.22
9.23	9.23	9.23	9.23	9.23	9.23	9.23	9.23	9.23
9.24	9.24	9.24	9.24	9.24	9.24	9.24	9.24	9.24
9.25	9.25	9.25	9.25	9.25	9.25	9.25	9.25	9.25
9.26	9.26	9.26	9.26	9.26	9.26	9.26	9.26	9.26
9.27	9.27	9.27	9.27	9.27	9.27	9.27	9.27	9.27
9.28	9.28	9.28	9.28	9.28	9.28	9.28	9.28	9.28
9.29	9.29	9.29	9.29	9.29	9.29	9.29	9.29	9.29
9.3	9.3	9.3	9.3	9.3	9.3	9.3	9.3	9.3
9.31	9.31	9.31	9.31	9.31	9.31	9.31	9.31	9.31
9.32	9.32	9.32	9.32	9.32	9.32	9.32	9.32	9.32
9.33	9.33	9.33	9.33	9.33	9.33	9.33	9.33	9.33
9.34	9.34	9.34	9.34	9.34	9.34	9.34	9.34	9.34
9.35	9.35	9.35	9.35	9.35	9.35	9.35	9.35	9.35
9.36	9.36	9.36	9.36	9.36	9.36	9.36	9.36	9.36
9.37	9.37	9.37	9.37	9.37	9.37	9.37	9.37	9.37
9.38	9.38	9.38	9.38	9.38	9.38	9.38	9.38	9.38
9.39	9.39	9.39	9.39	9.39	9.39	9.39	9.39	9.39
9.4	9.4	9.4	9.4	9.4	9.4	9.4	9.4	9.4
9.41	9.41	9.41	9.41	9.41	9.41	9.41	9.41	9.41
9.42	9.42	9.42	9.42	9.42	9.42	9.42	9.42	9.42
9.43	9.43	9.43	9.43	9.43	9.43	9.43	9.43	9.43
9.44	9.44	9.44	9.44	9.44	9.44	9.44	9.44	9.44
9.45	9.45	9.45	9.45	9.45	9.45	9.45	9.45	9.45
9.46	9.46	9.46	9.46	9.46	9.46	9.46	9.46	9.46
9.47	9.47	9.47	9.47	9.47	9.47	9.47	9.47	9.47
9.48	9.48	9.48	9.48	9.48	9.48	9.48	9.48	9.48
9.49	9.49	9.49	9.49	9.49	9.49	9.49	9.49	9.49

9.5	9.5	9.5	9.5	9.5	9.5	9.5	9.5	9.5
9.51	9.51	9.51	9.51	9.51	9.51	9.51	9.51	9.51
9.52	9.52	9.52	9.52	9.52	9.52	9.52	9.52	9.52
9.53	9.53	9.53	9.53	9.53	9.53	9.53	9.53	9.53
9.54	9.54	9.54	9.54	9.54	9.54	9.54	9.54	9.54
9.55	9.55	9.55	9.55	9.55	9.55	9.55	9.55	9.55
9.56	9.56	9.56	9.56	9.56	9.56	9.56	9.56	9.56
9.57	9.57	9.57	9.57	9.57	9.57	9.57	9.57	9.57
9.58	9.58	9.58	9.58	9.58	9.58	9.58	9.58	9.58
9.59	9.59	9.59	9.59	9.59	9.59	9.59	9.59	9.59
9.6	9.6	9.6	9.6	9.6	9.6	9.6	9.6	9.6
9.61	9.61	9.61	9.61	9.61	9.61	9.61	9.61	9.61
9.62	9.62	9.62	9.62	9.62	9.62	9.62	9.62	9.62
9.63	9.63	9.63	9.63	9.63	9.63	9.63	9.63	9.63
9.64	9.64	9.64	9.64	9.64	9.64	9.64	9.64	9.64
9.65	9.65	9.65	9.65	9.65	9.65	9.65	9.65	9.65
9.66	9.66	9.66	9.66	9.66	9.66	9.66	9.66	9.66
9.67	9.67	9.67	9.67	9.67	9.67	9.67	9.67	9.67
9.68	9.68	9.68	9.68	9.68	9.68	9.68	9.68	9.68
9.69	9.69	9.69	9.69	9.69	9.69	9.69	9.69	9.69
9.7	9.7	9.7	9.7	9.7	9.7	9.7	9.7	9.7
9.71	9.71	9.71	9.71	9.71	9.71	9.71	9.71	9.71
9.72	9.72	9.72	9.72	9.72	9.72	9.72	9.72	9.72
9.73	9.73	9.73	9.73	9.73	9.73	9.73	9.73	9.73
9.74	9.74	9.74	9.74	9.74	9.74	9.74	9.74	9.74
9.75	9.75	9.75	9.75	9.75	9.75	9.75	9.75	9.75
9.76	9.76	9.76	9.76	9.76	9.76	9.76	9.76	9.76
9.77	9.77	9.77	9.77	9.77	9.77	9.77	9.77	9.77
9.78	9.78	9.78	9.78	9.78	9.78	9.78	9.78	9.78
9.79	9.79	9.79	9.79	9.79	9.79	9.79	9.79	9.79
9.8	9.8	9.8	9.8	9.8	9.8	9.8	9.8	9.8
9.81	9.81	9.81	9.81	9.81	9.81	9.81	9.81	9.81
9.82	9.82	9.82	9.82	9.82	9.82	9.82	9.82	9.82
9.83	9.83	9.83	9.83	9.83	9.83	9.83	9.83	9.83
9.84	9.84	9.84	9.84	9.84	9.84	9.84	9.84	9.84
9.85	9.85	9.85	9.85	9.85	9.85	9.85	9.85	9.85
9.86	9.86	9.86	9.86	9.86	9.86	9.86	9.86	9.86
9.87	9.87	9.87	9.87	9.87	9.87	9.87	9.87	9.87
9.88	9.88	9.88	9.88	9.88	9.88	9.88	9.88	9.88
9.89	9.89	9.89	9.89	9.89	9.89	9.89	9.89	9.89
9.9	9.9	9.9	9.9	9.9	9.9	9.9	9.9	9.9
9.91	9.91	9.91	9.91	9.91	9.91	9.91	9.91	9.91
9.92	9.92	9.92	9.92	9.92	9.92	9.92	9.92	9.92
9.93	9.93	9.93	9.93	9.93	9.93	9.93	9.93	9.93
9.94	9.94	9.94	9.94	9.94	9.94	9.94	9.94	9.94
9.95	9.95	9.95	9.95	9.95	9.95	9.95	9.95	9.95
9.96	9.96	9.96	9.96	9.96	9.96	9.96	9.96	9.96
9.97	9.97	9.97	9.97	9.97	9.97	9.97	9.97	9.97
9.98	9.98	9.98	9.98	9.98	9.98	9.98	9.98	9.98
9.99	9.99	9.99	9.99	9.99	9.99	9.99	9.99	9.99

10	10	10	10	10	10	10	10	10
10.01	10.01	10.01	10.01	10.01	10.01	10.01	10.01	10.01
10.02	10.02	10.02	10.02	10.02	10.02	10.02	10.02	10.02
10.03	10.03	10.03	10.03	10.03	10.03	10.03	10.03	10.03
10.04	10.04	10.04	10.04	10.04	10.04	10.04	10.04	10.04
10.05	10.05	10.05	10.05	10.05	10.05	10.05	10.05	10.05
10.06	10.06	10.06	10.06	10.06	10.06	10.06	10.06	10.06
10.07	10.07	10.07	10.07	10.07	10.07	10.07	10.07	10.07
10.08	10.08	10.08	10.08	10.08	10.08	10.08	10.08	10.08
10.09	10.09	10.09	10.09	10.09	10.09	10.09	10.09	10.09
10.1	10.1	10.1	10.1	10.1	10.1	10.1	10.1	10.1
10.11	10.11	10.11	10.11	10.11	10.11	10.11	10.11	10.11
10.12	10.12	10.12	10.12	10.12	10.12	10.12	10.12	10.12
10.13	10.13	10.13	10.13	10.13	10.13	10.13	10.13	10.13
10.14	10.14	10.14	10.14	10.14	10.14	10.14	10.14	10.14
10.15	10.15	10.15	10.15	10.15	10.15	10.15	10.15	10.15
10.16	10.16	10.16	10.16	10.16	10.16	10.16	10.16	10.16
10.17	10.17	10.17	10.17	10.17	10.17	10.17	10.17	10.17
10.18	10.18	10.18	10.18	10.18	10.18	10.18	10.18	10.18
10.19	10.19	10.19	10.19	10.19	10.19	10.19	10.19	10.19
10.2	10.2	10.2	10.2	10.2	10.2	10.2	10.2	10.2
10.21	10.21	10.21	10.21	10.21	10.21	10.21	10.21	10.21
10.22	10.22	10.22	10.22	10.22	10.22	10.22	10.22	10.22
10.23	10.23	10.23	10.23	10.23	10.23	10.23	10.23	10.23
10.24	10.24	10.24	10.24	10.24	10.24	10.24	10.24	10.24
10.25	10.25	10.25	10.25	10.25	10.25	10.25	10.25	10.25
10.26	10.26	10.26	10.26	10.26	10.26	10.26	10.26	10.26
10.27	10.27	10.27	10.27	10.27	10.27	10.27	10.27	10.27
10.28	10.28	10.28	10.28	10.28	10.28	10.28	10.28	10.28
10.29	10.29	10.29	10.29	10.29	10.29	10.29	10.29	10.29
10.3	10.3	10.3	10.3	10.3	10.3	10.3	10.3	10.3
10.31	10.31	10.31	10.31	10.31	10.31	10.31	10.31	10.31
10.32	10.32	10.32	10.32	10.32	10.32	10.32	10.32	10.32
10.33	10.33	10.33	10.33	10.33	10.33	10.33	10.33	10.33
10.34	10.34	10.34	10.34	10.34	10.34	10.34	10.34	10.34
10.35	10.35	10.35	10.35	10.35	10.35	10.35	10.35	10.35
10.36	10.36	10.36	10.36	10.36	10.36	10.36	10.36	10.36
10.37	10.37	10.37	10.37	10.37	10.37	10.37	10.37	10.37
10.38	10.38	10.38	10.38	10.38	10.38	10.38	10.38	10.38
10.39	10.39	10.39	10.39	10.39	10.39	10.39	10.39	10.39
10.4	10.4	10.4	10.4	10.4	10.4	10.4	10.4	10.4
10.41	10.41	10.41	10.41	10.41	10.41	10.41	10.41	10.41
10.42	10.42	10.42	10.42	10.42	10.42	10.42	10.42	10.42
10.43	10.43	10.43	10.43	10.43	10.43	10.43	10.43	10.43
10.44	10.44	10.44	10.44	10.44	10.44	10.44	10.44	10.44
10.45	10.45	10.45	10.45	10.45	10.45	10.45	10.45	10.45
10.46	10.46	10.46	10.46	10.46	10.46	10.46	10.46	10.46
10.47	10.47	10.47	10.47	10.47	10.47	10.47	10.47	10.47
10.48	10.48	10.48	10.48	10.48	10.48	10.48	10.48	10.48
10.49	10.49	10.49	10.49	10.49	10.49	10.49	10.49	10.49

10.5	10.5	10.5	10.5	10.5	10.5	10.5	10.5	10.5
10.51	10.51	10.51	10.51	10.51	10.51	10.51	10.51	10.51
10.52	10.52	10.52	10.52	10.52	10.52	10.52	10.52	10.52
10.53	10.53	10.53	10.53	10.53	10.53	10.53	10.53	10.53
10.54	10.54	10.54	10.54	10.54	10.54	10.54	10.54	10.54
10.55	10.55	10.55	10.55	10.55	10.55	10.55	10.55	10.55
10.56	10.56	10.56	10.56	10.56	10.56	10.56	10.56	10.56
10.57	10.57	10.57	10.57	10.57	10.57	10.57	10.57	10.57
10.58	10.58	10.58	10.58	10.58	10.58	10.58	10.58	10.58
10.59	10.59	10.59	10.59	10.59	10.59	10.59	10.59	10.59
10.6	10.6	10.6	10.6	10.6	10.6	10.6	10.6	10.6
10.61	10.61	10.61	10.61	10.61	10.61	10.61	10.61	10.61
10.62	10.62	10.62	10.62	10.62	10.62	10.62	10.62	10.62
10.63	10.63	10.63	10.63	10.63	10.63	10.63	10.63	10.63
10.64	10.64	10.64	10.64	10.64	10.64	10.64	10.64	10.64
10.65	10.65	10.65	10.65	10.65	10.65	10.65	10.65	10.65
10.66	10.66	10.66	10.66	10.66	10.66	10.66	10.66	10.66
10.67	10.67	10.67	10.67	10.67	10.67	10.67	10.67	10.67
10.68	10.68	10.68	10.68	10.68	10.68	10.68	10.68	10.68
10.69	10.69	10.69	10.69	10.69	10.69	10.69	10.69	10.69
10.7	10.7	10.7	10.7	10.7	10.7	10.7	10.7	10.7
10.71	10.71	10.71	10.71	10.71	10.71	10.71	10.71	10.71
10.72	10.72	10.72	10.72	10.72	10.72	10.72	10.72	10.72
10.73	10.73	10.73	10.73	10.73	10.73	10.73	10.73	10.73
10.74	10.74	10.74	10.74	10.74	10.74	10.74	10.74	10.74
10.75	10.75	10.75	10.75	10.75	10.75	10.75	10.75	10.75
10.76	10.76	10.76	10.76	10.76	10.76	10.76	10.76	10.76
10.77	10.77	10.77	10.77	10.77	10.77	10.77	10.77	10.77
10.78	10.78	10.78	10.78	10.78	10.78	10.78	10.78	10.78
10.79	10.79	10.79	10.79	10.79	10.79	10.79	10.79	10.79
10.8	10.8	10.8	10.8	10.8	10.8	10.8	10.8	10.8
10.81	10.81	10.81	10.81	10.81	10.81	10.81	10.81	10.81
10.82	10.82	10.82	10.82	10.82	10.82	10.82	10.82	10.82
10.83	10.83	10.83	10.83	10.83	10.83	10.83	10.83	10.83
10.84	10.84	10.84	10.84	10.84	10.84	10.84	10.84	10.84
10.85	10.85	10.85	10.85	10.85	10.85	10.85	10.85	10.85
10.86	10.86	10.86	10.86	10.86	10.86	10.86	10.86	10.86
10.87	10.87	10.87	10.87	10.87	10.87	10.87	10.87	10.87
10.88	10.88	10.88	10.88	10.88	10.88	10.88	10.88	10.88
10.89	10.89	10.89	10.89	10.89	10.89	10.89	10.89	10.89
10.9	10.9	10.9	10.9	10.9	10.9	10.9	10.9	10.9
10.91	10.91	10.91	10.91	10.91	10.91	10.91	10.91	10.91
10.92	10.92	10.92	10.92	10.92	10.92	10.92	10.92	10.92
10.93	10.93	10.93	10.93	10.93	10.93	10.93	10.93	10.93
10.94	10.94	10.94	10.94	10.94	10.94	10.94	10.94	10.94
10.95	10.95	10.95	10.95	10.95	10.95	10.95	10.95	10.95
10.96	10.96	10.96	10.96	10.96	10.96	10.96	10.96	10.96
10.97	10.97	10.97	10.97	10.97	10.97	10.97	10.97	10.97
10.98	10.98	10.98	10.98	10.98	10.98	10.98	10.98	10.98
10.99	10.99	10.99	10.99	10.99	10.99	10.99	10.99	10.99

11	11	11	11	11	11	11	11	11
11.01	11.01	11.01	11.01	11.01	11.01	11.01	11.01	11.01
11.02	11.02	11.02	11.02	11.02	11.02	11.02	11.02	11.02
11.03	11.03	11.03	11.03	11.03	11.03	11.03	11.03	11.03
11.04	11.04	11.04	11.04	11.04	11.04	11.04	11.04	11.04
11.05	11.05	11.05	11.05	11.05	11.05	11.05	11.05	11.05
11.06	11.06	11.06	11.06	11.06	11.06	11.06	11.06	11.06
11.07	11.07	11.07	11.07	11.07	11.07	11.07	11.07	11.07
11.08	11.08	11.08	11.08	11.08	11.08	11.08	11.08	11.08
11.09	11.09	11.09	11.09	11.09	11.09	11.09	11.09	11.09
11.1	11.1	11.1	11.1	11.1	11.1	11.1	11.1	11.1
11.11	11.11	11.11	11.11	11.11	11.11	11.11	11.11	11.11
11.12	11.12	11.12	11.12	11.12	11.12	11.12	11.12	11.12
11.13	11.13	11.13	11.13	11.13	11.13	11.13	11.13	11.13
11.14	11.14	11.14	11.14	11.14	11.14	11.14	11.14	11.14
11.15	11.15	11.15	11.15	11.15	11.15	11.15	11.15	11.15
11.16	11.16	11.16	11.16	11.16	11.16	11.16	11.16	11.16
11.17	11.17	11.17	11.17	11.17	11.17	11.17	11.17	11.17
11.18	11.18	11.18	11.18	11.18	11.18	11.18	11.18	11.18
11.19	11.19	11.19	11.19	11.19	11.19	11.19	11.19	11.19
11.2	11.2	11.2	11.2	11.2	11.2	11.2	11.2	11.2
11.21	11.21	11.21	11.21	11.21	11.21	11.21	11.21	11.21
11.22	11.22	11.22	11.22	11.22	11.22	11.22	11.22	11.22
11.23	11.23	11.23	11.23	11.23	11.23	11.23	11.23	11.23
11.24	11.24	11.24	11.24	11.24	11.24	11.24	11.24	11.24
11.25	11.25	11.25	11.25	11.25	11.25	11.25	11.25	11.25
11.26	11.26	11.26	11.26	11.26	11.26	11.26	11.26	11.26
11.27	11.27	11.27	11.27	11.27	11.27	11.27	11.27	11.27
11.28	11.28	11.28	11.28	11.28	11.28	11.28	11.28	11.28
11.29	11.29	11.29	11.29	11.29	11.29	11.29	11.29	11.29
11.3	11.3	11.3	11.3	11.3	11.3	11.3	11.3	11.3
11.31	11.31	11.31	11.31	11.31	11.31	11.31	11.31	11.31
11.32	11.32	11.32	11.32	11.32	11.32	11.32	11.32	11.32
11.33	11.33	11.33	11.33	11.33	11.33	11.33	11.33	11.33
11.34	11.34	11.34	11.34	11.34	11.34	11.34	11.34	11.34
11.35	11.35	11.35	11.35	11.35	11.35	11.35	11.35	11.35
11.36	11.36	11.36	11.36	11.36	11.36	11.36	11.36	11.36
11.37	11.37	11.37	11.37	11.37	11.37	11.37	11.37	11.37
11.38	11.38	11.38	11.38	11.38	11.38	11.38	11.38	11.38
11.39	11.39	11.39	11.39	11.39	11.39	11.39	11.39	11.39
11.4	11.4	11.4	11.4	11.4	11.4	11.4	11.4	11.4
11.41	11.41	11.41	11.41	11.41	11.41	11.41	11.41	11.41
11.42	11.42	11.42	11.42	11.42	11.42	11.42	11.42	11.42
11.43	11.43	11.43	11.43	11.43	11.43	11.43	11.43	11.43
11.44	11.44	11.44	11.44	11.44	11.44	11.44	11.44	11.44
11.45	11.45	11.45	11.45	11.45	11.45	11.45	11.45	11.45
11.46	11.46	11.46	11.46	11.46	11.46	11.46	11.46	11.46
11.47	11.47	11.47	11.47	11.47	11.47	11.47	11.47	11.47
11.48	11.48	11.48	11.48	11.48	11.48	11.48	11.48	11.48
11.49	11.49	11.49	11.49	11.49	11.49	11.49	11.49	11.49

11.5	11.5	11.5	11.5	11.5	11.5	11.5	11.5	11.5
11.51	11.51	11.51	11.51	11.51	11.51	11.51	11.51	11.51
11.52	11.52	11.52	11.52	11.52	11.52	11.52	11.52	11.52
11.53	11.53	11.53	11.53	11.53	11.53	11.53	11.53	11.53
11.54	11.54	11.54	11.54	11.54	11.54	11.54	11.54	11.54
11.55	11.55	11.55	11.55	11.55	11.55	11.55	11.55	11.55
11.56	11.56	11.56	11.56	11.56	11.56	11.56	11.56	11.56
11.57	11.57	11.57	11.57	11.57	11.57	11.57	11.57	11.57
11.58	11.58	11.58	11.58	11.58	11.58	11.58	11.58	11.58
11.59	11.59	11.59	11.59	11.59	11.59	11.59	11.59	11.59
11.6	11.6	11.6	11.6	11.6	11.6	11.6	11.6	11.6
11.61	11.61	11.61	11.61	11.61	11.61	11.61	11.61	11.61
11.62	11.62	11.62	11.62	11.62	11.62	11.62	11.62	11.62
11.63	11.63	11.63	11.63	11.63	11.63	11.63	11.63	11.63
11.64	11.64	11.64	11.64	11.64	11.64	11.64	11.64	11.64
11.65	11.65	11.65	11.65	11.65	11.65	11.65	11.65	11.65
11.66	11.66	11.66	11.66	11.66	11.66	11.66	11.66	11.66
11.67	11.67	11.67	11.67	11.67	11.67	11.67	11.67	11.67
11.68	11.68	11.68	11.68	11.68	11.68	11.68	11.68	11.68
11.69	11.69	11.69	11.69	11.69	11.69	11.69	11.69	11.69
11.7	11.7	11.7	11.7	11.7	11.7	11.7	11.7	11.7
11.71	11.71	11.71	11.71	11.71	11.71	11.71	11.71	11.71
11.72	11.72	11.72	11.72	11.72	11.72	11.72	11.72	11.72
11.73	11.73	11.73	11.73	11.73	11.73	11.73	11.73	11.73
11.74	11.74	11.74	11.74	11.74	11.74	11.74	11.74	11.74
11.75	11.75	11.75	11.75	11.75	11.75	11.75	11.75	11.75
11.76	11.76	11.76	11.76	11.76	11.76	11.76	11.76	11.76
11.77	11.77	11.77	11.77	11.77	11.77	11.77	11.77	11.77
11.78	11.78	11.78	11.78	11.78	11.78	11.78	11.78	11.78
11.79	11.79	11.79	11.79	11.79	11.79	11.79	11.79	11.79
11.8	11.8	11.8	11.8	11.8	11.8	11.8	11.8	11.8
11.81	11.81	11.81	11.81	11.81	11.81	11.81	11.81	11.81
11.82	11.82	11.82	11.82	11.82	11.82	11.82	11.82	11.82
11.83	11.83	11.83	11.83	11.83	11.83	11.83	11.83	11.83
11.84	11.84	11.84	11.84	11.84	11.84	11.84	11.84	11.84
11.85	11.85	11.85	11.85	11.85	11.85	11.85	11.85	11.85
11.86	11.86	11.86	11.86	11.86	11.86	11.86	11.86	11.86
11.87	11.87	11.87	11.87	11.87	11.87	11.87	11.87	11.87
11.88	11.88	11.88	11.88	11.88	11.88	11.88	11.88	11.88
11.89	11.89	11.89	11.89	11.89	11.89	11.89	11.89	11.89
11.9	11.9	11.9	11.9	11.9	11.9	11.9	11.9	11.9
11.91	11.91	11.91	11.91	11.91	11.91	11.91	11.91	11.91
11.92	11.92	11.92	11.92	11.92	11.92	11.92	11.92	11.92
11.93	11.93	11.93	11.93	11.93	11.93	11.93	11.93	11.93
11.94	11.94	11.94	11.94	11.94	11.94	11.94	11.94	11.94
11.95	11.95	11.95	11.95	11.95	11.95	11.95	11.95	11.95
11.96	11.96	11.96	11.96	11.96	11.96	11.96	11.96	11.96
11.97	11.97	11.97	11.97	11.97	11.97	11.97	11.97	11.97
11.98	11.98	11.98	11.98	11.98	11.98	11.98	11.98	11.98
11.99	11.99	11.99	11.99	11.99	11.99	11.99	11.99	11.99

12	12	12	12	12	12	12	12	12

-12	-12	-12	-12	-12	-12	-12	-12	-12
-11.99	-11.99	-11.99	-11.99	-11.99	-11.99	-11.99	-11.99	-11.99
-11.98	-11.98	-11.98	-11.98	-11.98	-11.98	-11.98	-11.98	-11.98
-11.97	-11.97	-11.97	-11.97	-11.97	-11.97	-11.97	-11.97	-11.97
-11.96	-11.96	-11.96	-11.96	-11.96	-11.96	-11.96	-11.96	-11.96
-11.95	-11.95	-11.95	-11.95	-11.95	-11.95	-11.95	-11.95	-11.95
-11.94	-11.94	-11.94	-11.94	-11.94	-11.94	-11.94	-11.94	-11.94
-11.93	-11.93	-11.93	-11.93	-11.93	-11.93	-11.93	-11.93	-11.93
-11.92	-11.92	-11.92	-11.92	-11.92	-11.92	-11.92	-11.92	-11.92
-11.91	-11.91	-11.91	-11.91	-11.91	-11.91	-11.91	-11.91	-11.91
-11.9	-11.9	-11.9	-11.9	-11.9	-11.9	-11.9	-11.9	-11.9
-11.89	-11.89	-11.89	-11.89	-11.89	-11.89	-11.89	-11.89	-11.89
-11.88	-11.88	-11.88	-11.88	-11.88	-11.88	-11.88	-11.88	-11.88
-11.87	-11.87	-11.87	-11.87	-11.87	-11.87	-11.87	-11.87	-11.87
-11.86	-11.86	-11.86	-11.86	-11.86	-11.86	-11.86	-11.86	-11.86
-11.85	-11.85	-11.85	-11.85	-11.85	-11.85	-11.85	-11.85	-11.85
-11.84	-11.84	-11.84	-11.84	-11.84	-11.84	-11.84	-11.84	-11.84
-11.83	-11.83	-11.83	-11.83	-11.83	-11.83	-11.83	-11.83	-11.83
-11.82	-11.82	-11.82	-11.82	-11.82	-11.82	-11.82	-11.82	-11.82
-11.81	-11.81	-11.81	-11.81	-11.81	-11.81	-11.81	-11.81	-11.81
-11.8	-11.8	-11.8	-11.8	-11.8	-11.8	-11.8	-11.8	-11.8
-11.79	-11.79	-11.79	-11.79	-11.79	-11.79	-11.79	-11.79	-11.79
-11.78	-11.78	-11.78	-11.78	-11.78	-11.78	-11.78	-11.78	-11.78
-11.77	-11.77	-11.77	-11.77	-11.77	-11.77	-11.77	-11.77	-11.77
-11.76	-11.76	-11.76	-11.76	-11.76	-11.76	-11.76	-11.76	-11.76
-11.75	-11.75	-11.75	-11.75	-11.75	-11.75	-11.75	-11.75	-11.75
-11.74	-11.74	-11.74	-11.74	-11.74	-11.74	-11.74	-11.74	-11.74
-11.73	-11.73	-11.73	-11.73	-11.73	-11.73	-11.73	-11.73	-11.73
-11.72	-11.72	-11.72	-11.72	-11.72	-11.72	-11.72	-11.72	-11.72
-11.71	-11.71	-11.71	-11.71	-11.71	-11.71	-11.71	-11.71	-11.71
-11.7	-11.7	-11.7	-11.7	-11.7	-11.7	-11.7	-11.7	-11.7
-11.69	-11.69	-11.69	-11.69	-11.69	-11.69	-11.69	-11.69	-11.69
-11.68	-11.68	-11.68	-11.68	-11.68	-11.68	-11.68	-11.68	-11.68
-11.67	-11.67	-11.67	-11.67	-11.67	-11.67	-11.67	-11.67	-11.67
-11.66	-11.66	-11.66	-11.66	-11.66	-11.66	-11.66	-11.66	-11.66
-11.65	-11.65	-11.65	-11.65	-11.65	-11.65	-11.65	-11.65	-11.65
-11.64	-11.64	-11.64	-11.64	-11.64	-11.64	-11.64	-11.64	-11.64
-11.63	-11.63	-11.63	-11.63	-11.63	-11.63	-11.63	-11.63	-11.63
-11.62	-11.62	-11.62	-11.62	-11.62	-11.62	-11.62	-11.62	-11.62
-11.61	-11.61	-11.61	-11.61	-11.61	-11.61	-11.61	-11.61	-11.61
-11.6	-11.6	-11.6	-11.6	-11.6	-11.6	-11.6	-11.6	-11.6
-11.59	-11.59	-11.59	-11.59	-11.59	-11.59	-11.59	-11.59	-11.59
-11.58	-11.58	-11.58	-11.58	-11.58	-11.58	-11.58	-11.58	-11.58
-11.57	-11.57	-11.57	-11.57	-11.57	-11.57	-11.57	-11.57	-11.57
-11.56	-11.56	-11.56	-11.56	-11.56	-11.56	-11.56	-11.56	-11.56
-11.55	-11.55	-11.55	-11.55	-11.55	-11.55	-11.55	-11.55	-11.55
-11.54	-11.54	-11.54	-11.54	-11.54	-11.54	-11.54	-11.54	-11.54
-11.53	-11.53	-11.53	-11.53	-11.53	-11.53	-11.53	-11.53	-11.53
-11.52	-11.52	-11.52	-11.52	-11.52	-11.52	-11.52	-11.52	-11.52
-11.51	-11.51	-11.51	-11.51	-11.51	-11.51	-11.51	-11.51	-11.51

-11.5	-11.5	-11.5	-11.5	-11.5	-11.5	-11.5	-11.5	-11.5
-11.49	-11.49	-11.49	-11.49	-11.49	-11.49	-11.49	-11.49	-11.49
-11.48	-11.48	-11.48	-11.48	-11.48	-11.48	-11.48	-11.48	-11.48
-11.47	-11.47	-11.47	-11.47	-11.47	-11.47	-11.47	-11.47	-11.47
-11.46	-11.46	-11.46	-11.46	-11.46	-11.46	-11.46	-11.46	-11.46
-11.45	-11.45	-11.45	-11.45	-11.45	-11.45	-11.45	-11.45	-11.45
-11.44	-11.44	-11.44	-11.44	-11.44	-11.44	-11.44	-11.44	-11.44
-11.43	-11.43	-11.43	-11.43	-11.43	-11.43	-11.43	-11.43	-11.43
-11.42	-11.42	-11.42	-11.42	-11.42	-11.42	-11.42	-11.42	-11.42
-11.41	-11.41	-11.41	-11.41	-11.41	-11.41	-11.41	-11.41	-11.41
-11.4	-11.4	-11.4	-11.4	-11.4	-11.4	-11.4	-11.4	-11.4
-11.39	-11.39	-11.39	-11.39	-11.39	-11.39	-11.39	-11.39	-11.39
-11.38	-11.38	-11.38	-11.38	-11.38	-11.38	-11.38	-11.38	-11.38
-11.37	-11.37	-11.37	-11.37	-11.37	-11.37	-11.37	-11.37	-11.37
-11.36	-11.36	-11.36	-11.36	-11.36	-11.36	-11.36	-11.36	-11.36
-11.35	-11.35	-11.35	-11.35	-11.35	-11.35	-11.35	-11.35	-11.35
-11.34	-11.34	-11.34	-11.34	-11.34	-11.34	-11.34	-11.34	-11.34
-11.33	-11.33	-11.33	-11.33	-11.33	-11.33	-11.33	-11.33	-11.33
-11.32	-11.32	-11.32	-11.32	-11.32	-11.32	-11.32	-11.32	-11.32
-11.31	-11.31	-11.31	-11.31	-11.31	-11.31	-11.31	-11.31	-11.31
-11.3	-11.3	-11.3	-11.3	-11.3	-11.3	-11.3	-11.3	-11.3
-11.29	-11.29	-11.29	-11.29	-11.29	-11.29	-11.29	-11.29	-11.29
-11.28	-11.28	-11.28	-11.28	-11.28	-11.28	-11.28	-11.28	-11.28
-11.27	-11.27	-11.27	-11.27	-11.27	-11.27	-11.27	-11.27	-11.27
-11.26	-11.26	-11.26	-11.26	-11.26	-11.26	-11.26	-11.26	-11.26
-11.25	-11.25	-11.25	-11.25	-11.25	-11.25	-11.25	-11.25	-11.25
-11.24	-11.24	-11.24	-11.24	-11.24	-11.24	-11.24	-11.24	-11.24
-11.23	-11.23	-11.23	-11.23	-11.23	-11.23	-11.23	-11.23	-11.23
-11.22	-11.22	-11.22	-11.22	-11.22	-11.22	-11.22	-11.22	-11.22
-11.21	-11.21	-11.21	-11.21	-11.21	-11.21	-11.21	-11.21	-11.21
-11.2	-11.2	-11.2	-11.2	-11.2	-11.2	-11.2	-11.2	-11.2
-11.19	-11.19	-11.19	-11.19	-11.19	-11.19	-11.19	-11.19	-11.19
-11.18	-11.18	-11.18	-11.18	-11.18	-11.18	-11.18	-11.18	-11.18
-11.17	-11.17	-11.17	-11.17	-11.17	-11.17	-11.17	-11.17	-11.17
-11.16	-11.16	-11.16	-11.16	-11.16	-11.16	-11.16	-11.16	-11.16
-11.15	-11.15	-11.15	-11.15	-11.15	-11.15	-11.15	-11.15	-11.15
-11.14	-11.14	-11.14	-11.14	-11.14	-11.14	-11.14	-11.14	-11.14
-11.13	-11.13	-11.13	-11.13	-11.13	-11.13	-11.13	-11.13	-11.13
-11.12	-11.12	-11.12	-11.12	-11.12	-11.12	-11.12	-11.12	-11.12
-11.11	-11.11	-11.11	-11.11	-11.11	-11.11	-11.11	-11.11	-11.11
-11.1	-11.1	-11.1	-11.1	-11.1	-11.1	-11.1	-11.1	-11.1
-11.09	-11.09	-11.09	-11.09	-11.09	-11.09	-11.09	-11.09	-11.09
-11.08	-11.08	-11.08	-11.08	-11.08	-11.08	-11.08	-11.08	-11.08
-11.07	-11.07	-11.07	-11.07	-11.07	-11.07	-11.07	-11.07	-11.07
-11.06	-11.06	-11.06	-11.06	-11.06	-11.06	-11.06	-11.06	-11.06
-11.05	-11.05	-11.05	-11.05	-11.05	-11.05	-11.05	-11.05	-11.05
-11.04	-11.04	-11.04	-11.04	-11.04	-11.04	-11.04	-11.04	-11.04
-11.03	-11.03	-11.03	-11.03	-11.03	-11.03	-11.03	-11.03	-11.03
-11.02	-11.02	-11.02	-11.02	-11.02	-11.02	-11.02	-11.02	-11.02
-11.01	-11.01	-11.01	-11.01	-11.01	-11.01	-11.01	-11.01	-11.01

-11	-11	-11	-11	-11	-11	-11	-11	-11
-10.99	-10.99	-10.99	-10.99	-10.99	-10.99	-10.99	-10.99	-10.99
-10.98	-10.98	-10.98	-10.98	-10.98	-10.98	-10.98	-10.98	-10.98
-10.97	-10.97	-10.97	-10.97	-10.97	-10.97	-10.97	-10.97	-10.97
-10.96	-10.96	-10.96	-10.96	-10.96	-10.96	-10.96	-10.96	-10.96
-10.95	-10.95	-10.95	-10.95	-10.95	-10.95	-10.95	-10.95	-10.95
-10.94	-10.94	-10.94	-10.94	-10.94	-10.94	-10.94	-10.94	-10.94
-10.93	-10.93	-10.93	-10.93	-10.93	-10.93	-10.93	-10.93	-10.93
-10.92	-10.92	-10.92	-10.92	-10.92	-10.92	-10.92	-10.92	-10.92
-10.91	-10.91	-10.91	-10.91	-10.91	-10.91	-10.91	-10.91	-10.91
-10.9	-10.9	-10.9	-10.9	-10.9	-10.9	-10.9	-10.9	-10.9
-10.89	-10.89	-10.89	-10.89	-10.89	-10.89	-10.89	-10.89	-10.89
-10.88	-10.88	-10.88	-10.88	-10.88	-10.88	-10.88	-10.88	-10.88
-10.87	-10.87	-10.87	-10.87	-10.87	-10.87	-10.87	-10.87	-10.87
-10.86	-10.86	-10.86	-10.86	-10.86	-10.86	-10.86	-10.86	-10.86
-10.85	-10.85	-10.85	-10.85	-10.85	-10.85	-10.85	-10.85	-10.85
-10.84	-10.84	-10.84	-10.84	-10.84	-10.84	-10.84	-10.84	-10.84
-10.83	-10.83	-10.83	-10.83	-10.83	-10.83	-10.83	-10.83	-10.83
-10.82	-10.82	-10.82	-10.82	-10.82	-10.82	-10.82	-10.82	-10.82
-10.81	-10.81	-10.81	-10.81	-10.81	-10.81	-10.81	-10.81	-10.81
-10.8	-10.8	-10.8	-10.8	-10.8	-10.8	-10.8	-10.8	-10.8
-10.79	-10.79	-10.79	-10.79	-10.79	-10.79	-10.79	-10.79	-10.79
-10.78	-10.78	-10.78	-10.78	-10.78	-10.78	-10.78	-10.78	-10.78
-10.77	-10.77	-10.77	-10.77	-10.77	-10.77	-10.77	-10.77	-10.77
-10.76	-10.76	-10.76	-10.76	-10.76	-10.76	-10.76	-10.76	-10.76
-10.75	-10.75	-10.75	-10.75	-10.75	-10.75	-10.75	-10.75	-10.75
-10.74	-10.74	-10.74	-10.74	-10.74	-10.74	-10.74	-10.74	-10.74
-10.73	-10.73	-10.73	-10.73	-10.73	-10.73	-10.73	-10.73	-10.73
-10.72	-10.72	-10.72	-10.72	-10.72	-10.72	-10.72	-10.72	-10.72
-10.71	-10.71	-10.71	-10.71	-10.71	-10.71	-10.71	-10.71	-10.71
-10.7	-10.7	-10.7	-10.7	-10.7	-10.7	-10.7	-10.7	-10.7
-10.69	-10.69	-10.69	-10.69	-10.69	-10.69	-10.69	-10.69	-10.69
-10.68	-10.68	-10.68	-10.68	-10.68	-10.68	-10.68	-10.68	-10.68
-10.67	-10.67	-10.67	-10.67	-10.67	-10.67	-10.67	-10.67	-10.67
-10.66	-10.66	-10.66	-10.66	-10.66	-10.66	-10.66	-10.66	-10.66
-10.65	-10.65	-10.65	-10.65	-10.65	-10.65	-10.65	-10.65	-10.65
-10.64	-10.64	-10.64	-10.64	-10.64	-10.64	-10.64	-10.64	-10.64
-10.63	-10.63	-10.63	-10.63	-10.63	-10.63	-10.63	-10.63	-10.63
-10.62	-10.62	-10.62	-10.62	-10.62	-10.62	-10.62	-10.62	-10.62
-10.61	-10.61	-10.61	-10.61	-10.61	-10.61	-10.61	-10.61	-10.61
-10.6	-10.6	-10.6	-10.6	-10.6	-10.6	-10.6	-10.6	-10.6
-10.59	-10.59	-10.59	-10.59	-10.59	-10.59	-10.59	-10.59	-10.59
-10.58	-10.58	-10.58	-10.58	-10.58	-10.58	-10.58	-10.58	-10.58
-10.57	-10.57	-10.57	-10.57	-10.57	-10.57	-10.57	-10.57	-10.57
-10.56	-10.56	-10.56	-10.56	-10.56	-10.56	-10.56	-10.56	-10.56
-10.55	-10.55	-10.55	-10.55	-10.55	-10.55	-10.55	-10.55	-10.55
-10.54	-10.54	-10.54	-10.54	-10.54	-10.54	-10.54	-10.54	-10.54
-10.53	-10.53	-10.53	-10.53	-10.53	-10.53	-10.53	-10.53	-10.53
-10.52	-10.52	-10.52	-10.52	-10.52	-10.52	-10.52	-10.52	-10.52
-10.51	-10.51	-10.51	-10.51	-10.51	-10.51	-10.51	-10.51	-10.51

-10.5	-10.5	-10.5	-10.5	-10.5	-10.5	-10.5	-10.5	-10.5
-10.49	-10.49	-10.49	-10.49	-10.49	-10.49	-10.49	-10.49	-10.49
-10.48	-10.48	-10.48	-10.48	-10.48	-10.48	-10.48	-10.48	-10.48
-10.47	-10.47	-10.47	-10.47	-10.47	-10.47	-10.47	-10.47	-10.47
-10.46	-10.46	-10.46	-10.46	-10.46	-10.46	-10.46	-10.46	-10.46
-10.45	-10.45	-10.45	-10.45	-10.45	-10.45	-10.45	-10.45	-10.45
-10.44	-10.44	-10.44	-10.44	-10.44	-10.44	-10.44	-10.44	-10.44
-10.43	-10.43	-10.43	-10.43	-10.43	-10.43	-10.43	-10.43	-10.43
-10.42	-10.42	-10.42	-10.42	-10.42	-10.42	-10.42	-10.42	-10.42
-10.41	-10.41	-10.41	-10.41	-10.41	-10.41	-10.41	-10.41	-10.41
-10.4	-10.4	-10.4	-10.4	-10.4	-10.4	-10.4	-10.4	-10.4
-10.39	-10.39	-10.39	-10.39	-10.39	-10.39	-10.39	-10.39	-10.39
-10.38	-10.38	-10.38	-10.38	-10.38	-10.38	-10.38	-10.38	-10.38
-10.37	-10.37	-10.37	-10.37	-10.37	-10.37	-10.37	-10.37	-10.37
-10.36	-10.36	-10.36	-10.36	-10.36	-10.36	-10.36	-10.36	-10.36
-10.35	-10.35	-10.35	-10.35	-10.35	-10.35	-10.35	-10.35	-10.35
-10.34	-10.34	-10.34	-10.34	-10.34	-10.34	-10.34	-10.34	-10.34
-10.33	-10.33	-10.33	-10.33	-10.33	-10.33	-10.33	-10.33	-10.33
-10.32	-10.32	-10.32	-10.32	-10.32	-10.32	-10.32	-10.32	-10.32
-10.31	-10.31	-10.31	-10.31	-10.31	-10.31	-10.31	-10.31	-10.31
-10.3	-10.3	-10.3	-10.3	-10.3	-10.3	-10.3	-10.3	-10.3
-10.29	-10.29	-10.29	-10.29	-10.29	-10.29	-10.29	-10.29	-10.29
-10.28	-10.28	-10.28	-10.28	-10.28	-10.28	-10.28	-10.28	-10.28
-10.27	-10.27	-10.27	-10.27	-10.27	-10.27	-10.27	-10.27	-10.27
-10.26	-10.26	-10.26	-10.26	-10.26	-10.26	-10.26	-10.26	-10.26
-10.25	-10.25	-10.25	-10.25	-10.25	-10.25	-10.25	-10.25	-10.25
-10.24	-10.24	-10.24	-10.24	-10.24	-10.24	-10.24	-10.24	-10.24
-10.23	-10.23	-10.23	-10.23	-10.23	-10.23	-10.23	-10.23	-10.23
-10.22	-10.22	-10.22	-10.22	-10.22	-10.22	-10.22	-10.22	-10.22
-10.21	-10.21	-10.21	-10.21	-10.21	-10.21	-10.21	-10.21	-10.21
-10.2	-10.2	-10.2	-10.2	-10.2	-10.2	-10.2	-10.2	-10.2
-10.19	-10.19	-10.19	-10.19	-10.19	-10.19	-10.19	-10.19	-10.19
-10.18	-10.18	-10.18	-10.18	-10.18	-10.18	-10.18	-10.18	-10.18
-10.17	-10.17	-10.17	-10.17	-10.17	-10.17	-10.17	-10.17	-10.17
-10.16	-10.16	-10.16	-10.16	-10.16	-10.16	-10.16	-10.16	-10.16
-10.15	-10.15	-10.15	-10.15	-10.15	-10.15	-10.15	-10.15	-10.15
-10.14	-10.14	-10.14	-10.14	-10.14	-10.14	-10.14	-10.14	-10.14
-10.13	-10.13	-10.13	-10.13	-10.13	-10.13	-10.13	-10.13	-10.13
-10.12	-10.12	-10.12	-10.12	-10.12	-10.12	-10.12	-10.12	-10.12
-10.11	-10.11	-10.11	-10.11	-10.11	-10.11	-10.11	-10.11	-10.11
-10.1	-10.1	-10.1	-10.1	-10.1	-10.1	-10.1	-10.1	-10.1
-10.09	-10.09	-10.09	-10.09	-10.09	-10.09	-10.09	-10.09	-10.09
-10.08	-10.08	-10.08	-10.08	-10.08	-10.08	-10.08	-10.08	-10.08
-10.07	-10.07	-10.07	-10.07	-10.07	-10.07	-10.07	-10.07	-10.07
-10.06	-10.06	-10.06	-10.06	-10.06	-10.06	-10.06	-10.06	-10.06
-10.05	-10.05	-10.05	-10.05	-10.05	-10.05	-10.05	-10.05	-10.05
-10.04	-10.04	-10.04	-10.04	-10.04	-10.04	-10.04	-10.04	-10.04
-10.03	-10.03	-10.03	-10.03	-10.03	-10.03	-10.03	-10.03	-10.03
-10.02	-10.02	-10.02	-10.02	-10.02	-10.02	-10.02	-10.02	-10.02
-10.01	-10.01	-10.01	-10.01	-10.01	-10.01	-10.01	-10.01	-10.01

-10	-10	-10	-10	-10	-10	-10	-10	-10
-9.99	-9.99	-9.99	-9.99	-9.99	-9.99	-9.99	-9.99	-9.99
-9.98	-9.98	-9.98	-9.98	-9.98	-9.98	-9.98	-9.98	-9.98
-9.97	-9.97	-9.97	-9.97	-9.97	-9.97	-9.97	-9.97	-9.97
-9.96	-9.96	-9.96	-9.96	-9.96	-9.96	-9.96	-9.96	-9.96
-9.95	-9.95	-9.95	-9.95	-9.95	-9.95	-9.95	-9.95	-9.95
-9.94	-9.94	-9.94	-9.94	-9.94	-9.94	-9.94	-9.94	-9.94
-9.93	-9.93	-9.93	-9.93	-9.93	-9.93	-9.93	-9.93	-9.93
-9.92	-9.92	-9.92	-9.92	-9.92	-9.92	-9.92	-9.92	-9.92
-9.91	-9.91	-9.91	-9.91	-9.91	-9.91	-9.91	-9.91	-9.91
-9.9	-9.9	-9.9	-9.9	-9.9	-9.9	-9.9	-9.9	-9.9
-9.89	-9.89	-9.89	-9.89	-9.89	-9.89	-9.89	-9.89	-9.89
-9.88	-9.88	-9.88	-9.88	-9.88	-9.88	-9.88	-9.88	-9.88
-9.87	-9.87	-9.87	-9.87	-9.87	-9.87	-9.87	-9.87	-9.87
-9.86	-9.86	-9.86	-9.86	-9.86	-9.86	-9.86	-9.86	-9.86
-9.85	-9.85	-9.85	-9.85	-9.85	-9.85	-9.85	-9.85	-9.85
-9.84	-9.84	-9.84	-9.84	-9.84	-9.84	-9.84	-9.84	-9.84
-9.83	-9.83	-9.83	-9.83	-9.83	-9.83	-9.83	-9.83	-9.83
-9.82	-9.82	-9.82	-9.82	-9.82	-9.82	-9.82	-9.82	-9.82
-9.81	-9.81	-9.81	-9.81	-9.81	-9.81	-9.81	-9.81	-9.81
-9.8	-9.8	-9.8	-9.8	-9.8	-9.8	-9.8	-9.8	-9.8
-9.79	-9.79	-9.79	-9.79	-9.79	-9.79	-9.79	-9.79	-9.79
-9.78	-9.78	-9.78	-9.78	-9.78	-9.78	-9.78	-9.78	-9.78
-9.77	-9.77	-9.77	-9.77	-9.77	-9.77	-9.77	-9.77	-9.77
-9.76	-9.76	-9.76	-9.76	-9.76	-9.76	-9.76	-9.76	-9.76
-9.75	-9.75	-9.75	-9.75	-9.75	-9.75	-9.75	-9.75	-9.75
-9.74	-9.74	-9.74	-9.74	-9.74	-9.74	-9.74	-9.74	-9.74
-9.73	-9.73	-9.73	-9.73	-9.73	-9.73	-9.73	-9.73	-9.73
-9.72	-9.72	-9.72	-9.72	-9.72	-9.72	-9.72	-9.72	-9.72
-9.71	-9.71	-9.71	-9.71	-9.71	-9.71	-9.71	-9.71	-9.71
-9.7	-9.7	-9.7	-9.7	-9.7	-9.7	-9.7	-9.7	-9.7
-9.69	-9.69	-9.69	-9.69	-9.69	-9.69	-9.69	-9.69	-9.69
-9.68	-9.68	-9.68	-9.68	-9.68	-9.68	-9.68	-9.68	-9.68
-9.67	-9.67	-9.67	-9.67	-9.67	-9.67	-9.67	-9.67	-9.67
-9.66	-9.66	-9.66	-9.66	-9.66	-9.66	-9.66	-9.66	-9.66
-9.65	-9.65	-9.65	-9.65	-9.65	-9.65	-9.65	-9.65	-9.65
-9.64	-9.64	-9.64	-9.64	-9.64	-9.64	-9.64	-9.64	-9.64
-9.63	-9.63	-9.63	-9.63	-9.63	-9.63	-9.63	-9.63	-9.63
-9.62	-9.62	-9.62	-9.62	-9.62	-9.62	-9.62	-9.62	-9.62
-9.61	-9.61	-9.61	-9.61	-9.61	-9.61	-9.61	-9.61	-9.61
-9.6	-9.6	-9.6	-9.6	-9.6	-9.6	-9.6	-9.6	-9.6
-9.59	-9.59	-9.59	-9.59	-9.59	-9.59	-9.59	-9.59	-9.59
-9.58	-9.58	-9.58	-9.58	-9.58	-9.58	-9.58	-9.58	-9.58
-9.57	-9.57	-9.57	-9.57	-9.57	-9.57	-9.57	-9.57	-9.57
-9.56	-9.56	-9.56	-9.56	-9.56	-9.56	-9.56	-9.56	-9.56
-9.55	-9.55	-9.55	-9.55	-9.55	-9.55	-9.55	-9.55	-9.55
-9.54	-9.54	-9.54	-9.54	-9.54	-9.54	-9.54	-9.54	-9.54
-9.53	-9.53	-9.53	-9.53	-9.53	-9.53	-9.53	-9.53	-9.53
-9.52	-9.52	-9.52	-9.52	-9.52	-9.52	-9.52	-9.52	-9.52
-9.51	-9.51	-9.51	-9.51	-9.51	-9.51	-9.51	-9.51	-9.51

-9.5	-9.5	-9.5	-9.5	-9.5	-9.5	-9.5	-9.5	-9.5
-9.49	-9.49	-9.49	-9.49	-9.49	-9.49	-9.49	-9.49	-9.49
-9.48	-9.48	-9.48	-9.48	-9.48	-9.48	-9.48	-9.48	-9.48
-9.47	-9.47	-9.47	-9.47	-9.47	-9.47	-9.47	-9.47	-9.47
-9.46	-9.46	-9.46	-9.46	-9.46	-9.46	-9.46	-9.46	-9.46
-9.45	-9.45	-9.45	-9.45	-9.45	-9.45	-9.45	-9.45	-9.45
-9.44	-9.44	-9.44	-9.44	-9.44	-9.44	-9.44	-9.44	-9.44
-9.43	-9.43	-9.43	-9.43	-9.43	-9.43	-9.43	-9.43	-9.43
-9.42	-9.42	-9.42	-9.42	-9.42	-9.42	-9.42	-9.42	-9.42
-9.41	-9.41	-9.41	-9.41	-9.41	-9.41	-9.41	-9.41	-9.41
-9.4	-9.4	-9.4	-9.4	-9.4	-9.4	-9.4	-9.4	-9.4
-9.39	-9.39	-9.39	-9.39	-9.39	-9.39	-9.39	-9.39	-9.39
-9.38	-9.38	-9.38	-9.38	-9.38	-9.38	-9.38	-9.38	-9.38
-9.37	-9.37	-9.37	-9.37	-9.37	-9.37	-9.37	-9.37	-9.37
-9.36	-9.36	-9.36	-9.36	-9.36	-9.36	-9.36	-9.36	-9.36
-9.35	-9.35	-9.35	-9.35	-9.35	-9.35	-9.35	-9.35	-9.35
-9.34	-9.34	-9.34	-9.34	-9.34	-9.34	-9.34	-9.34	-9.34
-9.33	-9.33	-9.33	-9.33	-9.33	-9.33	-9.33	-9.33	-9.33
-9.32	-9.32	-9.32	-9.32	-9.32	-9.32	-9.32	-9.32	-9.32
-9.31	-9.31	-9.31	-9.31	-9.31	-9.31	-9.31	-9.31	-9.31
-9.3	-9.3	-9.3	-9.3	-9.3	-9.3	-9.3	-9.3	-9.3
-9.29	-9.29	-9.29	-9.29	-9.29	-9.29	-9.29	-9.29	-9.29
-9.28	-9.28	-9.28	-9.28	-9.28	-9.28	-9.28	-9.28	-9.28
-9.27	-9.27	-9.27	-9.27	-9.27	-9.27	-9.27	-9.27	-9.27
-9.26	-9.26	-9.26	-9.26	-9.26	-9.26	-9.26	-9.26	-9.26
-9.25	-9.25	-9.25	-9.25	-9.25	-9.25	-9.25	-9.25	-9.25
-9.24	-9.24	-9.24	-9.24	-9.24	-9.24	-9.24	-9.24	-9.24
-9.23	-9.23	-9.23	-9.23	-9.23	-9.23	-9.23	-9.23	-9.23
-9.22	-9.22	-9.22	-9.22	-9.22	-9.22	-9.22	-9.22	-9.22
-9.21	-9.21	-9.21	-9.21	-9.21	-9.21	-9.21	-9.21	-9.21
-9.2	-9.2	-9.2	-9.2	-9.2	-9.2	-9.2	-9.2	-9.2
-9.19	-9.19	-9.19	-9.19	-9.19	-9.19	-9.19	-9.19	-9.19
-9.18	-9.18	-9.18	-9.18	-9.18	-9.18	-9.18	-9.18	-9.18
-9.17	-9.17	-9.17	-9.17	-9.17	-9.17	-9.17	-9.17	-9.17
-9.16	-9.16	-9.16	-9.16	-9.16	-9.16	-9.16	-9.16	-9.16
-9.15	-9.15	-9.15	-9.15	-9.15	-9.15	-9.15	-9.15	-9.15
-9.14	-9.14	-9.14	-9.14	-9.14	-9.14	-9.14	-9.14	-9.14
-9.13	-9.13	-9.13	-9.13	-9.13	-9.13	-9.13	-9.13	-9.13
-9.12	-9.12	-9.12	-9.12	-9.12	-9.12	-9.12	-9.12	-9.12
-9.11	-9.11	-9.11	-9.11	-9.11	-9.11	-9.11	-9.11	-9.11
-9.1	-9.1	-9.1	-9.1	-9.1	-9.1	-9.1	-9.1	-9.1
-9.09	-9.09	-9.09	-9.09	-9.09	-9.09	-9.09	-9.09	-9.09
-9.08	-9.08	-9.08	-9.08	-9.08	-9.08	-9.08	-9.08	-9.08
-9.07	-9.07	-9.07	-9.07	-9.07	-9.07	-9.07	-9.07	-9.07
-9.06	-9.06	-9.06	-9.06	-9.06	-9.06	-9.06	-9.06	-9.06
-9.05	-9.05	-9.05	-9.05	-9.05	-9.05	-9.05	-9.05	-9.05
-9.04	-9.04	-9.04	-9.04	-9.04	-9.04	-9.04	-9.04	-9.04
-9.03	-9.03	-9.03	-9.03	-9.03	-9.03	-9.03	-9.03	-9.03
-9.02	-9.02	-9.02	-9.02	-9.02	-9.02	-9.02	-9.02	-9.02
-9.01	-9.01	-9.01	-9.01	-9.01	-9.01	-9.01	-9.01	-9.01

-9	-9	-9	-9	-9	-9	-9	-9	-9
-8.99	-8.99	-8.99	-8.99	-8.99	-8.99	-8.99	-8.99	-8.99
-8.98	-8.98	-8.98	-8.98	-8.98	-8.98	-8.98	-8.98	-8.98
-8.97	-8.97	-8.97	-8.97	-8.97	-8.97	-8.97	-8.97	-8.97
-8.96	-8.96	-8.96	-8.96	-8.96	-8.96	-8.96	-8.96	-8.96
-8.95	-8.95	-8.95	-8.95	-8.95	-8.95	-8.95	-8.95	-8.95
-8.94	-8.94	-8.94	-8.94	-8.94	-8.94	-8.94	-8.94	-8.94
-8.93	-8.93	-8.93	-8.93	-8.93	-8.93	-8.93	-8.93	-8.93
-8.92	-8.92	-8.92	-8.92	-8.92	-8.92	-8.92	-8.92	-8.92
-8.91	-8.91	-8.91	-8.91	-8.91	-8.91	-8.91	-8.91	-8.91
-8.9	-8.9	-8.9	-8.9	-8.9	-8.9	-8.9	-8.9	-8.9
-8.89	-8.89	-8.89	-8.89	-8.89	-8.89	-8.89	-8.89	-8.89
-8.88	-8.88	-8.88	-8.88	-8.88	-8.88	-8.88	-8.88	-8.88
-8.87	-8.87	-8.87	-8.87	-8.87	-8.87	-8.87	-8.87	-8.87
-8.86	-8.86	-8.86	-8.86	-8.86	-8.86	-8.86	-8.86	-8.86
-8.85	-8.85	-8.85	-8.85	-8.85	-8.85	-8.85	-8.85	-8.85
-8.84	-8.84	-8.84	-8.84	-8.84	-8.84	-8.84	-8.84	-8.84
-8.83	-8.83	-8.83	-8.83	-8.83	-8.83	-8.83	-8.83	-8.83
-8.82	-8.82	-8.82	-8.82	-8.82	-8.82	-8.82	-8.82	-8.82
-8.81	-8.81	-8.81	-8.81	-8.81	-8.81	-8.81	-8.81	-8.81
-8.8	-8.8	-8.8	-8.8	-8.8	-8.8	-8.8	-8.8	-8.8
-8.79	-8.79	-8.79	-8.79	-8.79	-8.79	-8.79	-8.79	-8.79
-8.78	-8.78	-8.78	-8.78	-8.78	-8.78	-8.78	-8.78	-8.78
-8.77	-8.77	-8.77	-8.77	-8.77	-8.77	-8.77	-8.77	-8.77
-8.76	-8.76	-8.76	-8.76	-8.76	-8.76	-8.76	-8.76	-8.76
-8.75	-8.75	-8.75	-8.75	-8.75	-8.75	-8.75	-8.75	-8.75
-8.74	-8.74	-8.74	-8.74	-8.74	-8.74	-8.74	-8.74	-8.74
-8.73	-8.73	-8.73	-8.73	-8.73	-8.73	-8.73	-8.73	-8.73
-8.72	-8.72	-8.72	-8.72	-8.72	-8.72	-8.72	-8.72	-8.72
-8.71	-8.71	-8.71	-8.71	-8.71	-8.71	-8.71	-8.71	-8.71
-8.7	-8.7	-8.7	-8.7	-8.7	-8.7	-8.7	-8.7	-8.7
-8.69	-8.69	-8.69	-8.69	-8.69	-8.69	-8.69	-8.69	-8.69
-8.68	-8.68	-8.68	-8.68	-8.68	-8.68	-8.68	-8.68	-8.68
-8.67	-8.67	-8.67	-8.67	-8.67	-8.67	-8.67	-8.67	-8.67
-8.66	-8.66	-8.66	-8.66	-8.66	-8.66	-8.66	-8.66	-8.66
-8.65	-8.65	-8.65	-8.65	-8.65	-8.65	-8.65	-8.65	-8.65
-8.64	-8.64	-8.64	-8.64	-8.64	-8.64	-8.64	-8.64	-8.64
-8.63	-8.63	-8.63	-8.63	-8.63	-8.63	-8.63	-8.63	-8.63
-8.62	-8.62	-8.62	-8.62	-8.62	-8.62	-8.62	-8.62	-8.62
-8.61	-8.61	-8.61	-8.61	-8.61	-8.61	-8.61	-8.61	-8.61
-8.6	-8.6	-8.6	-8.6	-8.6	-8.6	-8.6	-8.6	-8.6
-8.59	-8.59	-8.59	-8.59	-8.59	-8.59	-8.59	-8.59	-8.59
-8.58	-8.58	-8.58	-8.58	-8.58	-8.58	-8.58	-8.58	-8.58
-8.57	-8.57	-8.57	-8.57	-8.57	-8.57	-8.57	-8.57	-8.57
-8.56	-8.56	-8.56	-8.56	-8.56	-8.56	-8.56	-8.56	-8.56
-8.55	-8.55	-8.55	-8.55	-8.55	-8.55	-8.55	-8.55	-8.55
-8.54	-8.54	-8.54	-8.54	-8.54	-8.54	-8.54	-8.54	-8.54
-8.53	-8.53	-8.53	-8.53	-8.53	-8.53	-8.53	-8.53	-8.53
-8.52	-8.52	-8.52	-8.52	-8.52	-8.52	-8.52	-8.52	-8.52
-8.51	-8.51	-8.51	-8.51	-8.51	-8.51	-8.51	-8.51	-8.51

-8.5	-8.5	-8.5	-8.5	-8.5	-8.5	-8.5	-8.5	-8.5
-8.49	-8.49	-8.49	-8.49	-8.49	-8.49	-8.49	-8.49	-8.49
-8.48	-8.48	-8.48	-8.48	-8.48	-8.48	-8.48	-8.48	-8.48
-8.47	-8.47	-8.47	-8.47	-8.47	-8.47	-8.47	-8.47	-8.47
-8.46	-8.46	-8.46	-8.46	-8.46	-8.46	-8.46	-8.46	-8.46
-8.45	-8.45	-8.45	-8.45	-8.45	-8.45	-8.45	-8.45	-8.45
-8.44	-8.44	-8.44	-8.44	-8.44	-8.44	-8.44	-8.44	-8.44
-8.43	-8.43	-8.43	-8.43	-8.43	-8.43	-8.43	-8.43	-8.43
-8.42	-8.42	-8.42	-8.42	-8.42	-8.42	-8.42	-8.42	-8.42
-8.41	-8.41	-8.41	-8.41	-8.41	-8.41	-8.41	-8.41	-8.41
-8.4	-8.4	-8.4	-8.4	-8.4	-8.4	-8.4	-8.4	-8.4
-8.39	-8.39	-8.39	-8.39	-8.39	-8.39	-8.39	-8.39	-8.39
-8.38	-8.38	-8.38	-8.38	-8.38	-8.38	-8.38	-8.38	-8.38
-8.37	-8.37	-8.37	-8.37	-8.37	-8.37	-8.37	-8.37	-8.37
-8.36	-8.36	-8.36	-8.36	-8.36	-8.36	-8.36	-8.36	-8.36
-8.35	-8.35	-8.35	-8.35	-8.35	-8.35	-8.35	-8.35	-8.35
-8.34	-8.34	-8.34	-8.34	-8.34	-8.34	-8.34	-8.34	-8.34
-8.33	-8.33	-8.33	-8.33	-8.33	-8.33	-8.33	-8.33	-8.33
-8.32	-8.32	-8.32	-8.32	-8.32	-8.32	-8.32	-8.32	-8.32
-8.31	-8.31	-8.31	-8.31	-8.31	-8.31	-8.31	-8.31	-8.31
-8.3	-8.3	-8.3	-8.3	-8.3	-8.3	-8.3	-8.3	-8.3
-8.29	-8.29	-8.29	-8.29	-8.29	-8.29	-8.29	-8.29	-8.29
-8.28	-8.28	-8.28	-8.28	-8.28	-8.28	-8.28	-8.28	-8.28
-8.27	-8.27	-8.27	-8.27	-8.27	-8.27	-8.27	-8.27	-8.27
-8.26	-8.26	-8.26	-8.26	-8.26	-8.26	-8.26	-8.26	-8.26
-8.25	-8.25	-8.25	-8.25	-8.25	-8.25	-8.25	-8.25	-8.25
-8.24	-8.24	-8.24	-8.24	-8.24	-8.24	-8.24	-8.24	-8.24
-8.23	-8.23	-8.23	-8.23	-8.23	-8.23	-8.23	-8.23	-8.23
-8.22	-8.22	-8.22	-8.22	-8.22	-8.22	-8.22	-8.22	-8.22
-8.21	-8.21	-8.21	-8.21	-8.21	-8.21	-8.21	-8.21	-8.21
-8.2	-8.2	-8.2	-8.2	-8.2	-8.2	-8.2	-8.2	-8.2
-8.19	-8.19	-8.19	-8.19	-8.19	-8.19	-8.19	-8.19	-8.19
-8.18	-8.18	-8.18	-8.18	-8.18	-8.18	-8.18	-8.18	-8.18
-8.17	-8.17	-8.17	-8.17	-8.17	-8.17	-8.17	-8.17	-8.17
-8.16	-8.16	-8.16	-8.16	-8.16	-8.16	-8.16	-8.16	-8.16
-8.15	-8.15	-8.15	-8.15	-8.15	-8.15	-8.15	-8.15	-8.15
-8.14	-8.14	-8.14	-8.14	-8.14	-8.14	-8.14	-8.14	-8.14
-8.13	-8.13	-8.13	-8.13	-8.13	-8.13	-8.13	-8.13	-8.13
-8.12	-8.12	-8.12	-8.12	-8.12	-8.12	-8.12	-8.12	-8.12
-8.11	-8.11	-8.11	-8.11	-8.11	-8.11	-8.11	-8.11	-8.11
-8.1	-8.1	-8.1	-8.1	-8.1	-8.1	-8.1	-8.1	-8.1
-8.09	-8.09	-8.09	-8.09	-8.09	-8.09	-8.09	-8.09	-8.09
-8.08	-8.08	-8.08	-8.08	-8.08	-8.08	-8.08	-8.08	-8.08
-8.07	-8.07	-8.07	-8.07	-8.07	-8.07	-8.07	-8.07	-8.07
-8.06	-8.06	-8.06	-8.06	-8.06	-8.06	-8.06	-8.06	-8.06
-8.05	-8.05	-8.05	-8.05	-8.05	-8.05	-8.05	-8.05	-8.05
-8.04	-8.04	-8.04	-8.04	-8.04	-8.04	-8.04	-8.04	-8.04
-8.03	-8.03	-8.03	-8.03	-8.03	-8.03	-8.03	-8.03	-8.03
-8.02	-8.02	-8.02	-8.02	-8.02	-8.02	-8.02	-8.02	-8.02
-8.01	-8.01	-8.01	-8.01	-8.01	-8.01	-8.01	-8.01	-8.01

-8	-8	-8	-8	-8	-8	-8	-8	-8
-7.99	-7.99	-7.99	-7.99	-7.99	-7.99	-7.99	-7.99	-7.99
-7.98	-7.98	-7.98	-7.98	-7.98	-7.98	-7.98	-7.98	-7.98
-7.97	-7.97	-7.97	-7.97	-7.97	-7.97	-7.97	-7.97	-7.97
-7.96	-7.96	-7.96	-7.96	-7.96	-7.96	-7.96	-7.96	-7.96
-7.95	-7.95	-7.95	-7.95	-7.95	-7.95	-7.95	-7.95	-7.95
-7.94	-7.94	-7.94	-7.94	-7.94	-7.94	-7.94	-7.94	-7.94
-7.93	-7.93	-7.93	-7.93	-7.93	-7.93	-7.93	-7.93	-7.93
-7.92	-7.92	-7.92	-7.92	-7.92	-7.92	-7.92	-7.92	-7.92
-7.91	-7.91	-7.91	-7.91	-7.91	-7.91	-7.91	-7.91	-7.91
-7.9	-7.9	-7.9	-7.9	-7.9	-7.9	-7.9	-7.9	-7.9
-7.89	-7.89	-7.89	-7.89	-7.89	-7.89	-7.89	-7.89	-7.89
-7.88	-7.88	-7.88	-7.88	-7.88	-7.88	-7.88	-7.88	-7.88
-7.87	-7.87	-7.87	-7.87	-7.87	-7.87	-7.87	-7.87	-7.87
-7.86	-7.86	-7.86	-7.86	-7.86	-7.86	-7.86	-7.86	-7.86
-7.85	-7.85	-7.85	-7.85	-7.85	-7.85	-7.85	-7.85	-7.85
-7.84	-7.84	-7.84	-7.84	-7.84	-7.84	-7.84	-7.84	-7.84
-7.83	-7.83	-7.83	-7.83	-7.83	-7.83	-7.83	-7.83	-7.83
-7.82	-7.82	-7.82	-7.82	-7.82	-7.82	-7.82	-7.82	-7.82
-7.81	-7.81	-7.81	-7.81	-7.81	-7.81	-7.81	-7.81	-7.81
-7.8	-7.8	-7.8	-7.8	-7.8	-7.8	-7.8	-7.8	-7.8
-7.79	-7.79	-7.79	-7.79	-7.79	-7.79	-7.79	-7.79	-7.79
-7.78	-7.78	-7.78	-7.78	-7.78	-7.78	-7.78	-7.78	-7.78
-7.77	-7.77	-7.77	-7.77	-7.77	-7.77	-7.77	-7.77	-7.77
-7.76	-7.76	-7.76	-7.76	-7.76	-7.76	-7.76	-7.76	-7.76
-7.75	-7.75	-7.75	-7.75	-7.75	-7.75	-7.75	-7.75	-7.75
-7.74	-7.74	-7.74	-7.74	-7.74	-7.74	-7.74	-7.74	-7.74
-7.73	-7.73	-7.73	-7.73	-7.73	-7.73	-7.73	-7.73	-7.73
-7.72	-7.72	-7.72	-7.72	-7.72	-7.72	-7.72	-7.72	-7.72
-7.71	-7.71	-7.71	-7.71	-7.71	-7.71	-7.71	-7.71	-7.71
-7.7	-7.7	-7.7	-7.7	-7.7	-7.7	-7.7	-7.7	-7.7
-7.69	-7.69	-7.69	-7.69	-7.69	-7.69	-7.69	-7.69	-7.69
-7.68	-7.68	-7.68	-7.68	-7.68	-7.68	-7.68	-7.68	-7.68
-7.67	-7.67	-7.67	-7.67	-7.67	-7.67	-7.67	-7.67	-7.67
-7.66	-7.66	-7.66	-7.66	-7.66	-7.66	-7.66	-7.66	-7.66
-7.65	-7.65	-7.65	-7.65	-7.65	-7.65	-7.65	-7.65	-7.65
-7.64	-7.64	-7.64	-7.64	-7.64	-7.64	-7.64	-7.64	-7.64
-7.63	-7.63	-7.63	-7.63	-7.63	-7.63	-7.63	-7.63	-7.63
-7.62	-7.62	-7.62	-7.62	-7.62	-7.62	-7.62	-7.62	-7.62
-7.61	-7.61	-7.61	-7.61	-7.61	-7.61	-7.61	-7.61	-7.61
-7.6	-7.6	-7.6	-7.6	-7.6	-7.6	-7.6	-7.6	-7.6
-7.59	-7.59	-7.59	-7.59	-7.59	-7.59	-7.59	-7.59	-7.59
-7.58	-7.58	-7.58	-7.58	-7.58	-7.58	-7.58	-7.58	-7.58
-7.57	-7.57	-7.57	-7.57	-7.57	-7.57	-7.57	-7.57	-7.57
-7.56	-7.56	-7.56	-7.56	-7.56	-7.56	-7.56	-7.56	-7.56
-7.55	-7.55	-7.55	-7.55	-7.55	-7.55	-7.55	-7.55	-7.55
-7.54	-7.54	-7.54	-7.54	-7.54	-7.54	-7.54	-7.54	-7.54
-7.53	-7.53	-7.53	-7.53	-7.53	-7.53	-7.53	-7.53	-7.53
-7.52	-7.52	-7.52	-7.52	-7.52	-7.52	-7.52	-7.52	-7.52
-7.51	-7.51	-7.51	-7.51	-7.51	-7.51	-7.51	-7.51	-7.51

-7.5	-7.5	-7.5	-7.5	-7.5	-7.5	-7.5	-7.5	-7.5
-7.49	-7.49	-7.49	-7.49	-7.49	-7.49	-7.49	-7.49	-7.49
-7.48	-7.48	-7.48	-7.48	-7.48	-7.48	-7.48	-7.48	-7.48
-7.47	-7.47	-7.47	-7.47	-7.47	-7.47	-7.47	-7.47	-7.47
-7.46	-7.46	-7.46	-7.46	-7.46	-7.46	-7.46	-7.46	-7.46
-7.45	-7.45	-7.45	-7.45	-7.45	-7.45	-7.45	-7.45	-7.45
-7.44	-7.44	-7.44	-7.44	-7.44	-7.44	-7.44	-7.44	-7.44
-7.43	-7.43	-7.43	-7.43	-7.43	-7.43	-7.43	-7.43	-7.43
-7.42	-7.42	-7.42	-7.42	-7.42	-7.42	-7.42	-7.42	-7.42
-7.41	-7.41	-7.41	-7.41	-7.41	-7.41	-7.41	-7.41	-7.41
-7.4	-7.4	-7.4	-7.4	-7.4	-7.4	-7.4	-7.4	-7.4
-7.39	-7.39	-7.39	-7.39	-7.39	-7.39	-7.39	-7.39	-7.39
-7.38	-7.38	-7.38	-7.38	-7.38	-7.38	-7.38	-7.38	-7.38
-7.37	-7.37	-7.37	-7.37	-7.37	-7.37	-7.37	-7.37	-7.37
-7.36	-7.36	-7.36	-7.36	-7.36	-7.36	-7.36	-7.36	-7.36
-7.35	-7.35	-7.35	-7.35	-7.35	-7.35	-7.35	-7.35	-7.35
-7.34	-7.34	-7.34	-7.34	-7.34	-7.34	-7.34	-7.34	-7.34
-7.33	-7.33	-7.33	-7.33	-7.33	-7.33	-7.33	-7.33	-7.33
-7.32	-7.32	-7.32	-7.32	-7.32	-7.32	-7.32	-7.32	-7.32
-7.31	-7.31	-7.31	-7.31	-7.31	-7.31	-7.31	-7.31	-7.31
-7.3	-7.3	-7.3	-7.3	-7.3	-7.3	-7.3	-7.3	-7.3
-7.29	-7.29	-7.29	-7.29	-7.29	-7.29	-7.29	-7.29	-7.29
-7.28	-7.28	-7.28	-7.28	-7.28	-7.28	-7.28	-7.28	-7.28
-7.27	-7.27	-7.27	-7.27	-7.27	-7.27	-7.27	-7.27	-7.27
-7.26	-7.26	-7.26	-7.26	-7.26	-7.26	-7.26	-7.26	-7.26
-7.25	-7.25	-7.25	-7.25	-7.25	-7.25	-7.25	-7.25	-7.25
-7.24	-7.24	-7.24	-7.24	-7.24	-7.24	-7.24	-7.24	-7.24
-7.23	-7.23	-7.23	-7.23	-7.23	-7.23	-7.23	-7.23	-7.23
-7.22	-7.22	-7.22	-7.22	-7.22	-7.22	-7.22	-7.22	-7.22
-7.21	-7.21	-7.21	-7.21	-7.21	-7.21	-7.21	-7.21	-7.21
-7.2	-7.2	-7.2	-7.2	-7.2	-7.2	-7.2	-7.2	-7.2
-7.19	-7.19	-7.19	-7.19	-7.19	-7.19	-7.19	-7.19	-7.19
-7.18	-7.18	-7.18	-7.18	-7.18	-7.18	-7.18	-7.18	-7.18
-7.17	-7.17	-7.17	-7.17	-7.17	-7.17	-7.17	-7.17	-7.17
-7.16	-7.16	-7.16	-7.16	-7.16	-7.16	-7.16	-7.16	-7.16
-7.15	-7.15	-7.15	-7.15	-7.15	-7.15	-7.15	-7.15	-7.15
-7.14	-7.14	-7.14	-7.14	-7.14	-7.14	-7.14	-7.14	-7.14
-7.13	-7.13	-7.13	-7.13	-7.13	-7.13	-7.13	-7.13	-7.13
-7.12	-7.12	-7.12	-7.12	-7.12	-7.12	-7.12	-7.12	-7.12
-7.11	-7.11	-7.11	-7.11	-7.11	-7.11	-7.11	-7.11	-7.11
-7.1	-7.1	-7.1	-7.1	-7.1	-7.1	-7.1	-7.1	-7.1
-7.09	-7.09	-7.09	-7.09	-7.09	-7.09	-7.09	-7.09	-7.09
-7.08	-7.08	-7.08	-7.08	-7.08	-7.08	-7.08	-7.08	-7.08
-7.07	-7.07	-7.07	-7.07	-7.07	-7.07	-7.07	-7.07	-7.07
-7.06	-7.06	-7.06	-7.06	-7.06	-7.06	-7.06	-7.06	-7.06
-7.05	-7.05	-7.05	-7.05	-7.05	-7.05	-7.05	-7.05	-7.05
-7.04	-7.04	-7.04	-7.04	-7.04	-7.04	-7.04	-7.04	-7.04
-7.03	-7.03	-7.03	-7.03	-7.03	-7.03	-7.03	-7.03	-7.03
-7.02	-7.02	-7.02	-7.02	-7.02	-7.02	-7.02	-7.02	-7.02
-7.01	-7.01	-7.01	-7.01	-7.01	-7.01	-7.01	-7.01	-7.01

-7	-7	-7	-7	-7	-7	-7	-7	-7
-6.99	-6.99	-6.99	-6.99	-6.99	-6.99	-6.99	-6.99	-6.99
-6.98	-6.98	-6.98	-6.98	-6.98	-6.98	-6.98	-6.98	-6.98
-6.97	-6.97	-6.97	-6.97	-6.97	-6.97	-6.97	-6.97	-6.97
-6.96	-6.96	-6.96	-6.96	-6.96	-6.96	-6.96	-6.96	-6.96
-6.95	-6.95	-6.95	-6.95	-6.95	-6.95	-6.95	-6.95	-6.95
-6.94	-6.94	-6.94	-6.94	-6.94	-6.94	-6.94	-6.94	-6.94
-6.93	-6.93	-6.93	-6.93	-6.93	-6.93	-6.93	-6.93	-6.93
-6.92	-6.92	-6.92	-6.92	-6.92	-6.92	-6.92	-6.92	-6.92
-6.91	-6.91	-6.91	-6.91	-6.91	-6.91	-6.91	-6.91	-6.91
-6.9	-6.9	-6.9	-6.9	-6.9	-6.9	-6.9	-6.9	-6.9
-6.89	-6.89	-6.89	-6.89	-6.89	-6.89	-6.89	-6.89	-6.89
-6.88	-6.88	-6.88	-6.88	-6.88	-6.88	-6.88	-6.88	-6.88
-6.87	-6.87	-6.87	-6.87	-6.87	-6.87	-6.87	-6.87	-6.87
-6.86	-6.86	-6.86	-6.86	-6.86	-6.86	-6.86	-6.86	-6.86
-6.85	-6.85	-6.85	-6.85	-6.85	-6.85	-6.85	-6.85	-6.85
-6.84	-6.84	-6.84	-6.84	-6.84	-6.84	-6.84	-6.84	-6.84
-6.83	-6.83	-6.83	-6.83	-6.83	-6.83	-6.83	-6.83	-6.83
-6.82	-6.82	-6.82	-6.82	-6.82	-6.82	-6.82	-6.82	-6.82
-6.81	-6.81	-6.81	-6.81	-6.81	-6.81	-6.81	-6.81	-6.81
-6.8	-6.8	-6.8	-6.8	-6.8	-6.8	-6.8	-6.8	-6.8
-6.79	-6.79	-6.79	-6.79	-6.79	-6.79	-6.79	-6.79	-6.79
-6.78	-6.78	-6.78	-6.78	-6.78	-6.78	-6.78	-6.78	-6.78
-6.77	-6.77	-6.77	-6.77	-6.77	-6.77	-6.77	-6.77	-6.77
-6.76	-6.76	-6.76	-6.76	-6.76	-6.76	-6.76	-6.76	-6.76
-6.75	-6.75	-6.75	-6.75	-6.75	-6.75	-6.75	-6.75	-6.75
-6.74	-6.74	-6.74	-6.74	-6.74	-6.74	-6.74	-6.74	-6.74
-6.73	-6.73	-6.73	-6.73	-6.73	-6.73	-6.73	-6.73	-6.73
-6.72	-6.72	-6.72	-6.72	-6.72	-6.72	-6.72	-6.72	-6.72
-6.71	-6.71	-6.71	-6.71	-6.71	-6.71	-6.71	-6.71	-6.71
-6.7	-6.7	-6.7	-6.7	-6.7	-6.7	-6.7	-6.7	-6.7
-6.69	-6.69	-6.69	-6.69	-6.69	-6.69	-6.69	-6.69	-6.69
-6.68	-6.68	-6.68	-6.68	-6.68	-6.68	-6.68	-6.68	-6.68
-6.67	-6.67	-6.67	-6.67	-6.67	-6.67	-6.67	-6.67	-6.67
-6.66	-6.66	-6.66	-6.66	-6.66	-6.66	-6.66	-6.66	-6.66
-6.65	-6.65	-6.65	-6.65	-6.65	-6.65	-6.65	-6.65	-6.65
-6.64	-6.64	-6.64	-6.64	-6.64	-6.64	-6.64	-6.64	-6.64
-6.63	-6.63	-6.63	-6.63	-6.63	-6.63	-6.63	-6.63	-6.63
-6.62	-6.62	-6.62	-6.62	-6.62	-6.62	-6.62	-6.62	-6.62
-6.61	-6.61	-6.61	-6.61	-6.61	-6.61	-6.61	-6.61	-6.61
-6.6	-6.6	-6.6	-6.6	-6.6	-6.6	-6.6	-6.6	-6.6
-6.59	-6.59	-6.59	-6.59	-6.59	-6.59	-6.59	-6.59	-6.59
-6.58	-6.58	-6.58	-6.58	-6.58	-6.58	-6.58	-6.58	-6.58
-6.57	-6.57	-6.57	-6.57	-6.57	-6.57	-6.57	-6.57	-6.57
-6.56	-6.56	-6.56	-6.56	-6.56	-6.56	-6.56	-6.56	-6.56
-6.55	-6.55	-6.55	-6.55	-6.55	-6.55	-6.55	-6.55	-6.55
-6.54	-6.54	-6.54	-6.54	-6.54	-6.54	-6.54	-6.54	-6.54
-6.53	-6.53	-6.53	-6.53	-6.53	-6.53	-6.53	-6.53	-6.53
-6.52	-6.52	-6.52	-6.52	-6.52	-6.52	-6.52	-6.52	-6.52
-6.51	-6.51	-6.51	-6.51	-6.51	-6.51	-6.51	-6.51	-6.51

-6.5	-6.5	-6.5	-6.5	-6.5	-6.5	-6.5	-6.5	-6.5
-6.49	-6.49	-6.49	-6.49	-6.49	-6.49	-6.49	-6.49	-6.49
-6.48	-6.48	-6.48	-6.48	-6.48	-6.48	-6.48	-6.48	-6.48
-6.47	-6.47	-6.47	-6.47	-6.47	-6.47	-6.47	-6.47	-6.47
-6.46	-6.46	-6.46	-6.46	-6.46	-6.46	-6.46	-6.46	-6.46
-6.45	-6.45	-6.45	-6.45	-6.45	-6.45	-6.45	-6.45	-6.45
-6.44	-6.44	-6.44	-6.44	-6.44	-6.44	-6.44	-6.44	-6.44
-6.43	-6.43	-6.43	-6.43	-6.43	-6.43	-6.43	-6.43	-6.43
-6.42	-6.42	-6.42	-6.42	-6.42	-6.42	-6.42	-6.42	-6.42
-6.41	-6.41	-6.41	-6.41	-6.41	-6.41	-6.41	-6.41	-6.41
-6.4	-6.4	-6.4	-6.4	-6.4	-6.4	-6.4	-6.4	-6.4
-6.39	-6.39	-6.39	-6.39	-6.39	-6.39	-6.39	-6.39	-6.39
-6.38	-6.38	-6.38	-6.38	-6.38	-6.38	-6.38	-6.38	-6.38
-6.37	-6.37	-6.37	-6.37	-6.37	-6.37	-6.37	-6.37	-6.37
-6.36	-6.36	-6.36	-6.36	-6.36	-6.36	-6.36	-6.36	-6.36
-6.35	-6.35	-6.35	-6.35	-6.35	-6.35	-6.35	-6.35	-6.35
-6.34	-6.34	-6.34	-6.34	-6.34	-6.34	-6.34	-6.34	-6.34
-6.33	-6.33	-6.33	-6.33	-6.33	-6.33	-6.33	-6.33	-6.33
-6.32	-6.32	-6.32	-6.32	-6.32	-6.32	-6.32	-6.32	-6.32
-6.31	-6.31	-6.31	-6.31	-6.31	-6.31	-6.31	-6.31	-6.31
-6.3	-6.3	-6.3	-6.3	-6.3	-6.3	-6.3	-6.3	-6.3
-6.29	-6.29	-6.29	-6.29	-6.29	-6.29	-6.29	-6.29	-6.29
-6.28	-6.28	-6.28	-6.28	-6.28	-6.28	-6.28	-6.28	-6.28
-6.27	-6.27	-6.27	-6.27	-6.27	-6.27	-6.27	-6.27	-6.27
-6.26	-6.26	-6.26	-6.26	-6.26	-6.26	-6.26	-6.26	-6.26
-6.25	-6.25	-6.25	-6.25	-6.25	-6.25	-6.25	-6.25	-6.25
-6.24	-6.24	-6.24	-6.24	-6.24	-6.24	-6.24	-6.24	-6.24
-6.23	-6.23	-6.23	-6.23	-6.23	-6.23	-6.23	-6.23	-6.23
-6.22	-6.22	-6.22	-6.22	-6.22	-6.22	-6.22	-6.22	-6.22
-6.21	-6.21	-6.21	-6.21	-6.21	-6.21	-6.21	-6.21	-6.21
-6.2	-6.2	-6.2	-6.2	-6.2	-6.2	-6.2	-6.2	-6.2
-6.19	-6.19	-6.19	-6.19	-6.19	-6.19	-6.19	-6.19	-6.19
-6.18	-6.18	-6.18	-6.18	-6.18	-6.18	-6.18	-6.18	-6.18
-6.17	-6.17	-6.17	-6.17	-6.17	-6.17	-6.17	-6.17	-6.17
-6.16	-6.16	-6.16	-6.16	-6.16	-6.16	-6.16	-6.16	-6.16
-6.15	-6.15	-6.15	-6.15	-6.15	-6.15	-6.15	-6.15	-6.15
-6.14	-6.14	-6.14	-6.14	-6.14	-6.14	-6.14	-6.14	-6.14
-6.13	-6.13	-6.13	-6.13	-6.13	-6.13	-6.13	-6.13	-6.13
-6.12	-6.12	-6.12	-6.12	-6.12	-6.12	-6.12	-6.12	-6.12
-6.11	-6.11	-6.11	-6.11	-6.11	-6.11	-6.11	-6.11	-6.11
-6.1	-6.1	-6.1	-6.1	-6.1	-6.1	-6.1	-6.1	-6.1
-6.09	-6.09	-6.09	-6.09	-6.09	-6.09	-6.09	-6.09	-6.09
-6.08	-6.08	-6.08	-6.08	-6.08	-6.08	-6.08	-6.08	-6.08
-6.07	-6.07	-6.07	-6.07	-6.07	-6.07	-6.07	-6.07	-6.07
-6.06	-6.06	-6.06	-6.06	-6.06	-6.06	-6.06	-6.06	-6.06
-6.05	-6.05	-6.05	-6.05	-6.05	-6.05	-6.05	-6.05	-6.05
-6.04	-6.04	-6.04	-6.04	-6.04	-6.04	-6.04	-6.04	-6.04
-6.03	-6.03	-6.03	-6.03	-6.03	-6.03	-6.03	-6.03	-6.03
-6.02	-6.02	-6.02	-6.02	-6.02	-6.02	-6.02	-6.02	-6.02
-6.01	-6.01	-6.01	-6.01	-6.01	-6.01	-6.01	-6.01	-6.01

-6	-6	-6	-6	-6	-6	-6	-6	-6
-5.99	-5.99	-5.99	-5.99	-5.99	-5.99	-5.99	-5.99	-5.99
-5.98	-5.98	-5.98	-5.98	-5.98	-5.98	-5.98	-5.98	-5.98
-5.97	-5.97	-5.97	-5.97	-5.97	-5.97	-5.97	-5.97	-5.97
-5.96	-5.96	-5.96	-5.96	-5.96	-5.96	-5.96	-5.96	-5.96
-5.95	-5.95	-5.95	-5.95	-5.95	-5.95	-5.95	-5.95	-5.95
-5.94	-5.94	-5.94	-5.94	-5.94	-5.94	-5.94	-5.94	-5.94
-5.93	-5.93	-5.93	-5.93	-5.93	-5.93	-5.93	-5.93	-5.93
-5.92	-5.92	-5.92	-5.92	-5.92	-5.92	-5.92	-5.92	-5.92
-5.91	-5.91	-5.91	-5.91	-5.91	-5.91	-5.91	-5.91	-5.91
-5.9	-5.9	-5.9	-5.9	-5.9	-5.9	-5.9	-5.9	-5.9
-5.89	-5.89	-5.89	-5.89	-5.89	-5.89	-5.89	-5.89	-5.89
-5.88	-5.88	-5.88	-5.88	-5.88	-5.88	-5.88	-5.88	-5.88
-5.87	-5.87	-5.87	-5.87	-5.87	-5.87	-5.87	-5.87	-5.87
-5.86	-5.86	-5.86	-5.86	-5.86	-5.86	-5.86	-5.86	-5.86
-5.85	-5.85	-5.85	-5.85	-5.85	-5.85	-5.85	-5.85	-5.85
-5.84	-5.84	-5.84	-5.84	-5.84	-5.84	-5.84	-5.84	-5.84
-5.83	-5.83	-5.83	-5.83	-5.83	-5.83	-5.83	-5.83	-5.83
-5.82	-5.82	-5.82	-5.82	-5.82	-5.82	-5.82	-5.82	-5.82
-5.81	-5.81	-5.81	-5.81	-5.81	-5.81	-5.81	-5.81	-5.81
-5.8	-5.8	-5.8	-5.8	-5.8	-5.8	-5.8	-5.8	-5.8
-5.79	-5.79	-5.79	-5.79	-5.79	-5.79	-5.79	-5.79	-5.79
-5.78	-5.78	-5.78	-5.78	-5.78	-5.78	-5.78	-5.78	-5.78
-5.77	-5.77	-5.77	-5.77	-5.77	-5.77	-5.77	-5.77	-5.77
-5.76	-5.76	-5.76	-5.76	-5.76	-5.76	-5.76	-5.76	-5.76
-5.75	-5.75	-5.75	-5.75	-5.75	-5.75	-5.75	-5.75	-5.75
-5.74	-5.74	-5.74	-5.74	-5.74	-5.74	-5.74	-5.74	-5.74
-5.73	-5.73	-5.73	-5.73	-5.73	-5.73	-5.73	-5.73	-5.73
-5.72	-5.72	-5.72	-5.72	-5.72	-5.72	-5.72	-5.72	-5.72
-5.71	-5.71	-5.71	-5.71	-5.71	-5.71	-5.71	-5.71	-5.71
-5.7	-5.7	-5.7	-5.7	-5.7	-5.7	-5.7	-5.7	-5.7
-5.69	-5.69	-5.69	-5.69	-5.69	-5.69	-5.69	-5.69	-5.69
-5.68	-5.68	-5.68	-5.68	-5.68	-5.68	-5.68	-5.68	-5.68
-5.67	-5.67	-5.67	-5.67	-5.67	-5.67	-5.67	-5.67	-5.67
-5.66	-5.66	-5.66	-5.66	-5.66	-5.66	-5.66	-5.66	-5.66
-5.65	-5.65	-5.65	-5.65	-5.65	-5.65	-5.65	-5.65	-5.65
-5.64	-5.64	-5.64	-5.64	-5.64	-5.64	-5.64	-5.64	-5.64
-5.63	-5.63	-5.63	-5.63	-5.63	-5.63	-5.63	-5.63	-5.63
-5.62	-5.62	-5.62	-5.62	-5.62	-5.62	-5.62	-5.62	-5.62
-5.61	-5.61	-5.61	-5.61	-5.61	-5.61	-5.61	-5.61	-5.61
-5.6	-5.6	-5.6	-5.6	-5.6	-5.6	-5.6	-5.6	-5.6
-5.59	-5.59	-5.59	-5.59	-5.59	-5.59	-5.59	-5.59	-5.59
-5.58	-5.58	-5.58	-5.58	-5.58	-5.58	-5.58	-5.58	-5.58
-5.57	-5.57	-5.57	-5.57	-5.57	-5.57	-5.57	-5.57	-5.57
-5.56	-5.56	-5.56	-5.56	-5.56	-5.56	-5.56	-5.56	-5.56
-5.55	-5.55	-5.55	-5.55	-5.55	-5.55	-5.55	-5.55	-5.55
-5.54	-5.54	-5.54	-5.54	-5.54	-5.54	-5.54	-5.54	-5.54
-5.53	-5.53	-5.53	-5.53	-5.53	-5.53	-5.53	-5.53	-5.53
-5.52	-5.52	-5.52	-5.52	-5.52	-5.52	-5.52	-5.52	-5.52
-5.51	-5.51	-5.51	-5.51	-5.51	-5.51	-5.51	-5.51	-5.51

-5.5	-5.5	-5.5	-5.5	-5.5	-5.5	-5.5	-5.5	-5.5
-5.49	-5.49	-5.49	-5.49	-5.49	-5.49	-5.49	-5.49	-5.49
-5.48	-5.48	-5.48	-5.48	-5.48	-5.48	-5.48	-5.48	-5.48
-5.47	-5.47	-5.47	-5.47	-5.47	-5.47	-5.47	-5.47	-5.47
-5.46	-5.46	-5.46	-5.46	-5.46	-5.46	-5.46	-5.46	-5.46
-5.45	-5.45	-5.45	-5.45	-5.45	-5.45	-5.45	-5.45	-5.45
-5.44	-5.44	-5.44	-5.44	-5.44	-5.44	-5.44	-5.44	-5.44
-5.43	-5.43	-5.43	-5.43	-5.43	-5.43	-5.43	-5.43	-5.43
-5.42	-5.42	-5.42	-5.42	-5.42	-5.42	-5.42	-5.42	-5.42
-5.41	-5.41	-5.41	-5.41	-5.41	-5.41	-5.41	-5.41	-5.41
-5.4	-5.4	-5.4	-5.4	-5.4	-5.4	-5.4	-5.4	-5.4
-5.39	-5.39	-5.39	-5.39	-5.39	-5.39	-5.39	-5.39	-5.39
-5.38	-5.38	-5.38	-5.38	-5.38	-5.38	-5.38	-5.38	-5.38
-5.37	-5.37	-5.37	-5.37	-5.37	-5.37	-5.37	-5.37	-5.37
-5.36	-5.36	-5.36	-5.36	-5.36	-5.36	-5.36	-5.36	-5.36
-5.35	-5.35	-5.35	-5.35	-5.35	-5.35	-5.35	-5.35	-5.35
-5.34	-5.34	-5.34	-5.34	-5.34	-5.34	-5.34	-5.34	-5.34
-5.33	-5.33	-5.33	-5.33	-5.33	-5.33	-5.33	-5.33	-5.33
-5.32	-5.32	-5.32	-5.32	-5.32	-5.32	-5.32	-5.32	-5.32
-5.31	-5.31	-5.31	-5.31	-5.31	-5.31	-5.31	-5.31	-5.31
-5.3	-5.3	-5.3	-5.3	-5.3	-5.3	-5.3	-5.3	-5.3
-5.29	-5.29	-5.29	-5.29	-5.29	-5.29	-5.29	-5.29	-5.29
-5.28	-5.28	-5.28	-5.28	-5.28	-5.28	-5.28	-5.28	-5.28
-5.27	-5.27	-5.27	-5.27	-5.27	-5.27	-5.27	-5.27	-5.27
-5.26	-5.26	-5.26	-5.26	-5.26	-5.26	-5.26	-5.26	-5.26
-5.25	-5.25	-5.25	-5.25	-5.25	-5.25	-5.25	-5.25	-5.25
-5.24	-5.24	-5.24	-5.24	-5.24	-5.24	-5.24	-5.24	-5.24
-5.23	-5.23	-5.23	-5.23	-5.23	-5.23	-5.23	-5.23	-5.23
-5.22	-5.22	-5.22	-5.22	-5.22	-5.22	-5.22	-5.22	-5.22
-5.21	-5.21	-5.21	-5.21	-5.21	-5.21	-5.21	-5.21	-5.21
-5.2	-5.2	-5.2	-5.2	-5.2	-5.2	-5.2	-5.2	-5.2
-5.19	-5.19	-5.19	-5.19	-5.19	-5.19	-5.19	-5.19	-5.19
-5.18	-5.18	-5.18	-5.18	-5.18	-5.18	-5.18	-5.18	-5.18
-5.17	-5.17	-5.17	-5.17	-5.17	-5.17	-5.17	-5.17	-5.17
-5.16	-5.16	-5.16	-5.16	-5.16	-5.16	-5.16	-5.16	-5.16
-5.15	-5.15	-5.15	-5.15	-5.15	-5.15	-5.15	-5.15	-5.15
-5.14	-5.14	-5.14	-5.14	-5.14	-5.14	-5.14	-5.14	-5.14
-5.13	-5.13	-5.13	-5.13	-5.13	-5.13	-5.13	-5.13	-5.13
-5.12	-5.12	-5.12	-5.12	-5.12	-5.12	-5.12	-5.12	-5.12
-5.11	-5.11	-5.11	-5.11	-5.11	-5.11	-5.11	-5.11	-5.11
-5.1	-5.1	-5.1	-5.1	-5.1	-5.1	-5.1	-5.1	-5.1
-5.09	-5.09	-5.09	-5.09	-5.09	-5.09	-5.09	-5.09	-5.09
-5.08	-5.08	-5.08	-5.08	-5.08	-5.08	-5.08	-5.08	-5.08
-5.07	-5.07	-5.07	-5.07	-5.07	-5.07	-5.07	-5.07	-5.07
-5.06	-5.06	-5.06	-5.06	-5.06	-5.06	-5.06	-5.06	-5.06
-5.05	-5.05	-5.05	-5.05	-5.05	-5.05	-5.05	-5.05	-5.05
-5.04	-5.04	-5.04	-5.04	-5.04	-5.04	-5.04	-5.04	-5.04
-5.03	-5.03	-5.03	-5.03	-5.03	-5.03	-5.03	-5.03	-5.03
-5.02	-5.02	-5.02	-5.02	-5.02	-5.02	-5.02	-5.02	-5.02
-5.01	-5.01	-5.01	-5.01	-5.01	-5.01	-5.01	-5.01	-5.01

-5	-5	-5	-5	-5	-5	-5	-5	-5
-4.99	-4.99	-4.99	-4.99	-4.99	-4.99	-4.99	-4.99	-4.99
-4.98	-4.98	-4.98	-4.98	-4.98	-4.98	-4.98	-4.98	-4.98
-4.97	-4.97	-4.97	-4.97	-4.97	-4.97	-4.97	-4.97	-4.97
-4.96	-4.96	-4.96	-4.96	-4.96	-4.96	-4.96	-4.96	-4.96
-4.95	-4.95	-4.95	-4.95	-4.95	-4.95	-4.95	-4.95	-4.95
-4.94	-4.94	-4.94	-4.94	-4.94	-4.94	-4.94	-4.94	-4.94
-4.93	-4.93	-4.93	-4.93	-4.93	-4.93	-4.93	-4.93	-4.93
-4.92	-4.92	-4.92	-4.92	-4.92	-4.92	-4.92	-4.92	-4.92
-4.91	-4.91	-4.91	-4.91	-4.91	-4.91	-4.91	-4.91	-4.91
-4.9	-4.9	-4.9	-4.9	-4.9	-4.9	-4.9	-4.9	-4.9
-4.89	-4.89	-4.89	-4.89	-4.89	-4.89	-4.89	-4.89	-4.89
-4.88	-4.88	-4.88	-4.88	-4.88	-4.88	-4.88	-4.88	-4.88
-4.87	-4.87	-4.87	-4.87	-4.87	-4.87	-4.87	-4.87	-4.87
-4.86	-4.86	-4.86	-4.86	-4.86	-4.86	-4.86	-4.86	-4.86
-4.85	-4.85	-4.85	-4.85	-4.85	-4.85	-4.85	-4.85	-4.85
-4.84	-4.84	-4.84	-4.84	-4.84	-4.84	-4.84	-4.84	-4.84
-4.83	-4.83	-4.83	-4.83	-4.83	-4.83	-4.83	-4.83	-4.83
-4.82	-4.82	-4.82	-4.82	-4.82	-4.82	-4.82	-4.82	-4.82
-4.81	-4.81	-4.81	-4.81	-4.81	-4.81	-4.81	-4.81	-4.81
-4.8	-4.8	-4.8	-4.8	-4.8	-4.8	-4.8	-4.8	-4.8
-4.79	-4.79	-4.79	-4.79	-4.79	-4.79	-4.79	-4.79	-4.79
-4.78	-4.78	-4.78	-4.78	-4.78	-4.78	-4.78	-4.78	-4.78
-4.77	-4.77	-4.77	-4.77	-4.77	-4.77	-4.77	-4.77	-4.77
-4.76	-4.76	-4.76	-4.76	-4.76	-4.76	-4.76	-4.76	-4.76
-4.75	-4.75	-4.75	-4.75	-4.75	-4.75	-4.75	-4.75	-4.75
-4.74	-4.74	-4.74	-4.74	-4.74	-4.74	-4.74	-4.74	-4.74
-4.73	-4.73	-4.73	-4.73	-4.73	-4.73	-4.73	-4.73	-4.73
-4.72	-4.72	-4.72	-4.72	-4.72	-4.72	-4.72	-4.72	-4.72
-4.71	-4.71	-4.71	-4.71	-4.71	-4.71	-4.71	-4.71	-4.71
-4.7	-4.7	-4.7	-4.7	-4.7	-4.7	-4.7	-4.7	-4.7
-4.69	-4.69	-4.69	-4.69	-4.69	-4.69	-4.69	-4.69	-4.69
-4.68	-4.68	-4.68	-4.68	-4.68	-4.68	-4.68	-4.68	-4.68
-4.67	-4.67	-4.67	-4.67	-4.67	-4.67	-4.67	-4.67	-4.67
-4.66	-4.66	-4.66	-4.66	-4.66	-4.66	-4.66	-4.66	-4.66
-4.65	-4.65	-4.65	-4.65	-4.65	-4.65	-4.65	-4.65	-4.65
-4.64	-4.64	-4.64	-4.64	-4.64	-4.64	-4.64	-4.64	-4.64
-4.63	-4.63	-4.63	-4.63	-4.63	-4.63	-4.63	-4.63	-4.63
-4.62	-4.62	-4.62	-4.62	-4.62	-4.62	-4.62	-4.62	-4.62
-4.61	-4.61	-4.61	-4.61	-4.61	-4.61	-4.61	-4.61	-4.61
-4.6	-4.6	-4.6	-4.6	-4.6	-4.6	-4.6	-4.6	-4.6
-4.59	-4.59	-4.59	-4.59	-4.59	-4.59	-4.59	-4.59	-4.59
-4.58	-4.58	-4.58	-4.58	-4.58	-4.58	-4.58	-4.58	-4.58
-4.57	-4.57	-4.57	-4.57	-4.57	-4.57	-4.57	-4.57	-4.57
-4.56	-4.56	-4.56	-4.56	-4.56	-4.56	-4.56	-4.56	-4.56
-4.55	-4.55	-4.55	-4.55	-4.55	-4.55	-4.55	-4.55	-4.55
-4.54	-4.54	-4.54	-4.54	-4.54	-4.54	-4.54	-4.54	-4.54
-4.53	-4.53	-4.53	-4.53	-4.53	-4.53	-4.53	-4.53	-4.53
-4.52	-4.52	-4.52	-4.52	-4.52	-4.52	-4.52	-4.52	-4.52
-4.51	-4.51	-4.51	-4.51	-4.51	-4.51	-4.51	-4.51	-4.51

-4.5	-4.5	-4.5	-4.5	-4.5	-4.5	-4.5	-4.5	-4.5
-4.49	-4.49	-4.49	-4.49	-4.49	-4.49	-4.49	-4.49	-4.49
-4.48	-4.48	-4.48	-4.48	-4.48	-4.48	-4.48	-4.48	-4.48
-4.47	-4.47	-4.47	-4.47	-4.47	-4.47	-4.47	-4.47	-4.47
-4.46	-4.46	-4.46	-4.46	-4.46	-4.46	-4.46	-4.46	-4.46
-4.45	-4.45	-4.45	-4.45	-4.45	-4.45	-4.45	-4.45	-4.45
-4.44	-4.44	-4.44	-4.44	-4.44	-4.44	-4.44	-4.44	-4.44
-4.43	-4.43	-4.43	-4.43	-4.43	-4.43	-4.43	-4.43	-4.43
-4.42	-4.42	-4.42	-4.42	-4.42	-4.42	-4.42	-4.42	-4.42
-4.41	-4.41	-4.41	-4.41	-4.41	-4.41	-4.41	-4.41	-4.41
-4.4	-4.4	-4.4	-4.4	-4.4	-4.4	-4.4	-4.4	-4.4
-4.39	-4.39	-4.39	-4.39	-4.39	-4.39	-4.39	-4.39	-4.39
-4.38	-4.38	-4.38	-4.38	-4.38	-4.38	-4.38	-4.38	-4.38
-4.37	-4.37	-4.37	-4.37	-4.37	-4.37	-4.37	-4.37	-4.37
-4.36	-4.36	-4.36	-4.36	-4.36	-4.36	-4.36	-4.36	-4.36
-4.35	-4.35	-4.35	-4.35	-4.35	-4.35	-4.35	-4.35	-4.35
-4.34	-4.34	-4.34	-4.34	-4.34	-4.34	-4.34	-4.34	-4.34
-4.33	-4.33	-4.33	-4.33	-4.33	-4.33	-4.33	-4.33	-4.33
-4.32	-4.32	-4.32	-4.32	-4.32	-4.32	-4.32	-4.32	-4.32
-4.31	-4.31	-4.31	-4.31	-4.31	-4.31	-4.31	-4.31	-4.31
-4.3	-4.3	-4.3	-4.3	-4.3	-4.3	-4.3	-4.3	-4.3
-4.29	-4.29	-4.29	-4.29	-4.29	-4.29	-4.29	-4.29	-4.29
-4.28	-4.28	-4.28	-4.28	-4.28	-4.28	-4.28	-4.28	-4.28
-4.27	-4.27	-4.27	-4.27	-4.27	-4.27	-4.27	-4.27	-4.27
-4.26	-4.26	-4.26	-4.26	-4.26	-4.26	-4.26	-4.26	-4.26
-4.25	-4.25	-4.25	-4.25	-4.25	-4.25	-4.25	-4.25	-4.25
-4.24	-4.24	-4.24	-4.24	-4.24	-4.24	-4.24	-4.24	-4.24
-4.23	-4.23	-4.23	-4.23	-4.23	-4.23	-4.23	-4.23	-4.23
-4.22	-4.22	-4.22	-4.22	-4.22	-4.22	-4.22	-4.22	-4.22
-4.21	-4.21	-4.21	-4.21	-4.21	-4.21	-4.21	-4.21	-4.21
-4.2	-4.2	-4.2	-4.2	-4.2	-4.2	-4.2	-4.2	-4.2
-4.19	-4.19	-4.19	-4.19	-4.19	-4.19	-4.19	-4.19	-4.19
-4.18	-4.18	-4.18	-4.18	-4.18	-4.18	-4.18	-4.18	-4.18
-4.17	-4.17	-4.17	-4.17	-4.17	-4.17	-4.17	-4.17	-4.17
-4.16	-4.16	-4.16	-4.16	-4.16	-4.16	-4.16	-4.16	-4.16
-4.15	-4.15	-4.15	-4.15	-4.15	-4.15	-4.15	-4.15	-4.15
-4.14	-4.14	-4.14	-4.14	-4.14	-4.14	-4.14	-4.14	-4.14
-4.13	-4.13	-4.13	-4.13	-4.13	-4.13	-4.13	-4.13	-4.13
-4.12	-4.12	-4.12	-4.12	-4.12	-4.12	-4.12	-4.12	-4.12
-4.11	-4.11	-4.11	-4.11	-4.11	-4.11	-4.11	-4.11	-4.11
-4.1	-4.1	-4.1	-4.1	-4.1	-4.1	-4.1	-4.1	-4.1
-4.09	-4.09	-4.09	-4.09	-4.09	-4.09	-4.09	-4.09	-4.09
-4.08	-4.08	-4.08	-4.08	-4.08	-4.08	-4.08	-4.08	-4.08
-4.07	-4.07	-4.07	-4.07	-4.07	-4.07	-4.07	-4.07	-4.07
-4.06	-4.06	-4.06	-4.06	-4.06	-4.06	-4.06	-4.06	-4.06
-4.05	-4.05	-4.05	-4.05	-4.05	-4.05	-4.05	-4.05	-4.05
-4.04	-4.04	-4.04	-4.04	-4.04	-4.04	-4.04	-4.04	-4.04
-4.03	-4.03	-4.03	-4.03	-4.03	-4.03	-4.03	-4.03	-4.03
-4.02	-4.02	-4.02	-4.02	-4.02	-4.02	-4.02	-4.02	-4.02
-4.01	-4.01	-4.01	-4.01	-4.01	-4.01	-4.01	-4.01	-4.01

-4	-4	-4	-4	-4	-4	-4	-4	-4
-3.99	-3.99	-3.99	-3.99	-3.99	-3.99	-3.99	-3.99	-3.99
-3.98	-3.98	-3.98	-3.98	-3.98	-3.98	-3.98	-3.98	-3.98
-3.97	-3.97	-3.97	-3.97	-3.97	-3.97	-3.97	-3.97	-3.97
-3.96	-3.96	-3.96	-3.96	-3.96	-3.96	-3.96	-3.96	-3.96
-3.95	-3.95	-3.95	-3.95	-3.95	-3.95	-3.95	-3.95	-3.95
-3.94	-3.94	-3.94	-3.94	-3.94	-3.94	-3.94	-3.94	-3.94
-3.93	-3.93	-3.93	-3.93	-3.93	-3.93	-3.93	-3.93	-3.93
-3.92	-3.92	-3.92	-3.92	-3.92	-3.92	-3.92	-3.92	-3.92
-3.91	-3.91	-3.91	-3.91	-3.91	-3.91	-3.91	-3.91	-3.91
-3.9	-3.9	-3.9	-3.9	-3.9	-3.9	-3.9	-3.9	-3.9
-3.89	-3.89	-3.89	-3.89	-3.89	-3.89	-3.89	-3.89	-3.89
-3.88	-3.88	-3.88	-3.88	-3.88	-3.88	-3.88	-3.88	-3.88
-3.87	-3.87	-3.87	-3.87	-3.87	-3.87	-3.87	-3.87	-3.87
-3.86	-3.86	-3.86	-3.86	-3.86	-3.86	-3.86	-3.86	-3.86
-3.85	-3.85	-3.85	-3.85	-3.85	-3.85	-3.85	-3.85	-3.85
-3.84	-3.84	-3.84	-3.84	-3.84	-3.84	-3.84	-3.84	-3.84
-3.83	-3.83	-3.83	-3.83	-3.83	-3.83	-3.83	-3.83	-3.83
-3.82	-3.82	-3.82	-3.82	-3.82	-3.82	-3.82	-3.82	-3.82
-3.81	-3.81	-3.81	-3.81	-3.81	-3.81	-3.81	-3.81	-3.81
-3.8	-3.8	-3.8	-3.8	-3.8	-3.8	-3.8	-3.8	-3.8
-3.79	-3.79	-3.79	-3.79	-3.79	-3.79	-3.79	-3.79	-3.79
-3.78	-3.78	-3.78	-3.78	-3.78	-3.78	-3.78	-3.78	-3.78
-3.77	-3.77	-3.77	-3.77	-3.77	-3.77	-3.77	-3.77	-3.77
-3.76	-3.76	-3.76	-3.76	-3.76	-3.76	-3.76	-3.76	-3.76
-3.75	-3.75	-3.75	-3.75	-3.75	-3.75	-3.75	-3.75	-3.75
-3.74	-3.74	-3.74	-3.74	-3.74	-3.74	-3.74	-3.74	-3.74
-3.73	-3.73	-3.73	-3.73	-3.73	-3.73	-3.73	-3.73	-3.73
-3.72	-3.72	-3.72	-3.72	-3.72	-3.72	-3.72	-3.72	-3.72
-3.71	-3.71	-3.71	-3.71	-3.71	-3.71	-3.71	-3.71	-3.71
-3.7	-3.7	-3.7	-3.7	-3.7	-3.7	-3.7	-3.7	-3.7
-3.69	-3.69	-3.69	-3.69	-3.69	-3.69	-3.69	-3.69	-3.69
-3.68	-3.68	-3.68	-3.68	-3.68	-3.68	-3.68	-3.68	-3.68
-3.67	-3.67	-3.67	-3.67	-3.67	-3.67	-3.67	-3.67	-3.67
-3.66	-3.66	-3.66	-3.66	-3.66	-3.66	-3.66	-3.66	-3.66
-3.65	-3.65	-3.65	-3.65	-3.65	-3.65	-3.65	-3.65	-3.65
-3.64	-3.64	-3.64	-3.64	-3.64	-3.64	-3.64	-3.64	-3.64
-3.63	-3.63	-3.63	-3.63	-3.63	-3.63	-3.63	-3.63	-3.63
-3.62	-3.62	-3.62	-3.62	-3.62	-3.62	-3.62	-3.62	-3.62
-3.61	-3.61	-3.61	-3.61	-3.61	-3.61	-3.61	-3.61	-3.61
-3.6	-3.6	-3.6	-3.6	-3.6	-3.6	-3.6	-3.6	-3.6
-3.59	-3.59	-3.59	-3.59	-3.59	-3.59	-3.59	-3.59	-3.59
-3.58	-3.58	-3.58	-3.58	-3.58	-3.58	-3.58	-3.58	-3.58
-3.57	-3.57	-3.57	-3.57	-3.57	-3.57	-3.57	-3.57	-3.57
-3.56	-3.56	-3.56	-3.56	-3.56	-3.56	-3.56	-3.56	-3.56
-3.55	-3.55	-3.55	-3.55	-3.55	-3.55	-3.55	-3.55	-3.55
-3.54	-3.54	-3.54	-3.54	-3.54	-3.54	-3.54	-3.54	-3.54
-3.53	-3.53	-3.53	-3.53	-3.53	-3.53	-3.53	-3.53	-3.53
-3.52	-3.52	-3.52	-3.52	-3.52	-3.52	-3.52	-3.52	-3.52
-3.51	-3.51	-3.51	-3.51	-3.51	-3.51	-3.51	-3.51	-3.51

-3.5	-3.5	-3.5	-3.5	-3.5	-3.5	-3.5	-3.5	-3.5
-3.49	-3.49	-3.49	-3.49	-3.49	-3.49	-3.49	-3.49	-3.49
-3.48	-3.48	-3.48	-3.48	-3.48	-3.48	-3.48	-3.48	-3.48
-3.47	-3.47	-3.47	-3.47	-3.47	-3.47	-3.47	-3.47	-3.47
-3.46	-3.46	-3.46	-3.46	-3.46	-3.46	-3.46	-3.46	-3.46
-3.45	-3.45	-3.45	-3.45	-3.45	-3.45	-3.45	-3.45	-3.45
-3.44	-3.44	-3.44	-3.44	-3.44	-3.44	-3.44	-3.44	-3.44
-3.43	-3.43	-3.43	-3.43	-3.43	-3.43	-3.43	-3.43	-3.43
-3.42	-3.42	-3.42	-3.42	-3.42	-3.42	-3.42	-3.42	-3.42
-3.41	-3.41	-3.41	-3.41	-3.41	-3.41	-3.41	-3.41	-3.41
-3.4	-3.4	-3.4	-3.4	-3.4	-3.4	-3.4	-3.4	-3.4
-3.39	-3.39	-3.39	-3.39	-3.39	-3.39	-3.39	-3.39	-3.39
-3.38	-3.38	-3.38	-3.38	-3.38	-3.38	-3.38	-3.38	-3.38
-3.37	-3.37	-3.37	-3.37	-3.37	-3.37	-3.37	-3.37	-3.37
-3.36	-3.36	-3.36	-3.36	-3.36	-3.36	-3.36	-3.36	-3.36
-3.35	-3.35	-3.35	-3.35	-3.35	-3.35	-3.35	-3.35	-3.35
-3.34	-3.34	-3.34	-3.34	-3.34	-3.34	-3.34	-3.34	-3.34
-3.33	-3.33	-3.33	-3.33	-3.33	-3.33	-3.33	-3.33	-3.33
-3.32	-3.32	-3.32	-3.32	-3.32	-3.32	-3.32	-3.32	-3.32
-3.31	-3.31	-3.31	-3.31	-3.31	-3.31	-3.31	-3.31	-3.31
-3.3	-3.3	-3.3	-3.3	-3.3	-3.3	-3.3	-3.3	-3.3
-3.29	-3.29	-3.29	-3.29	-3.29	-3.29	-3.29	-3.29	-3.29
-3.28	-3.28	-3.28	-3.28	-3.28	-3.28	-3.28	-3.28	-3.28
-3.27	-3.27	-3.27	-3.27	-3.27	-3.27	-3.27	-3.27	-3.27
-3.26	-3.26	-3.26	-3.26	-3.26	-3.26	-3.26	-3.26	-3.26
-3.25	-3.25	-3.25	-3.25	-3.25	-3.25	-3.25	-3.25	-3.25
-3.24	-3.24	-3.24	-3.24	-3.24	-3.24	-3.24	-3.24	-3.24
-3.23	-3.23	-3.23	-3.23	-3.23	-3.23	-3.23	-3.23	-3.23
-3.22	-3.22	-3.22	-3.22	-3.22	-3.22	-3.22	-3.22	-3.22
-3.21	-3.21	-3.21	-3.21	-3.21	-3.21	-3.21	-3.21	-3.21
-3.2	-3.2	-3.2	-3.2	-3.2	-3.2	-3.2	-3.2	-3.2
-3.19	-3.19	-3.19	-3.19	-3.19	-3.19	-3.19	-3.19	-3.19
-3.18	-3.18	-3.18	-3.18	-3.18	-3.18	-3.18	-3.18	-3.18
-3.17	-3.17	-3.17	-3.17	-3.17	-3.17	-3.17	-3.17	-3.17
-3.16	-3.16	-3.16	-3.16	-3.16	-3.16	-3.16	-3.16	-3.16
-3.15	-3.15	-3.15	-3.15	-3.15	-3.15	-3.15	-3.15	-3.15
-3.14	-3.14	-3.14	-3.14	-3.14	-3.14	-3.14	-3.14	-3.14
-3.13	-3.13	-3.13	-3.13	-3.13	-3.13	-3.13	-3.13	-3.13
-3.12	-3.12	-3.12	-3.12	-3.12	-3.12	-3.12	-3.12	-3.12
-3.11	-3.11	-3.11	-3.11	-3.11	-3.11	-3.11	-3.11	-3.11
-3.1	-3.1	-3.1	-3.1	-3.1	-3.1	-3.1	-3.1	-3.1
-3.09	-3.09	-3.09	-3.09	-3.09	-3.09	-3.09	-3.09	-3.09
-3.08	-3.08	-3.08	-3.08	-3.08	-3.08	-3.08	-3.08	-3.08
-3.07	-3.07	-3.07	-3.07	-3.07	-3.07	-3.07	-3.07	-3.07
-3.06	-3.06	-3.06	-3.06	-3.06	-3.06	-3.06	-3.06	-3.06
-3.05	-3.05	-3.05	-3.05	-3.05	-3.05	-3.05	-3.05	-3.05
-3.04	-3.04	-3.04	-3.04	-3.04	-3.04	-3.04	-3.04	-3.04
-3.03	-3.03	-3.03	-3.03	-3.03	-3.03	-3.03	-3.03	-3.03
-3.02	-3.02	-3.02	-3.02	-3.02	-3.02	-3.02	-3.02	-3.02
-3.01	-3.01	-3.01	-3.01	-3.01	-3.01	-3.01	-3.01	-3.01

-3	-3	-3	-3	-3	-3	-3	-3	-3
-2.99	-2.99	-2.99	-2.99	-2.99	-2.99	-2.99	-2.99	-2.99
-2.98	-2.98	-2.98	-2.98	-2.98	-2.98	-2.98	-2.98	-2.98
-2.97	-2.97	-2.97	-2.97	-2.97	-2.97	-2.97	-2.97	-2.97
-2.96	-2.96	-2.96	-2.96	-2.96	-2.96	-2.96	-2.96	-2.96
-2.95	-2.95	-2.95	-2.95	-2.95	-2.95	-2.95	-2.95	-2.95
-2.94	-2.94	-2.94	-2.94	-2.94	-2.94	-2.94	-2.94	-2.94
-2.93	-2.93	-2.93	-2.93	-2.93	-2.93	-2.93	-2.93	-2.93
-2.92	-2.92	-2.92	-2.92	-2.92	-2.92	-2.92	-2.92	-2.92
-2.91	-2.91	-2.91	-2.91	-2.91	-2.91	-2.91	-2.91	-2.91
-2.9	-2.9	-2.9	-2.9	-2.9	-2.9	-2.9	-2.9	-2.9
-2.89	-2.89	-2.89	-2.89	-2.89	-2.89	-2.89	-2.89	-2.89
-2.88	-2.88	-2.88	-2.88	-2.88	-2.88	-2.88	-2.88	-2.88
-2.87	-2.87	-2.87	-2.87	-2.87	-2.87	-2.87	-2.87	-2.87
-2.86	-2.86	-2.86	-2.86	-2.86	-2.86	-2.86	-2.86	-2.86
-2.85	-2.85	-2.85	-2.85	-2.85	-2.85	-2.85	-2.85	-2.85
-2.84	-2.84	-2.84	-2.84	-2.84	-2.84	-2.84	-2.84	-2.84
-2.83	-2.83	-2.83	-2.83	-2.83	-2.83	-2.83	-2.83	-2.83
-2.82	-2.82	-2.82	-2.82	-2.82	-2.82	-2.82	-2.82	-2.82
-2.81	-2.81	-2.81	-2.81	-2.81	-2.81	-2.81	-2.81	-2.81
-2.8	-2.8	-2.8	-2.8	-2.8	-2.8	-2.8	-2.8	-2.8
-2.79	-2.79	-2.79	-2.79	-2.79	-2.79	-2.79	-2.79	-2.79
-2.78	-2.78	-2.78	-2.78	-2.78	-2.78	-2.78	-2.78	-2.78
-2.77	-2.77	-2.77	-2.77	-2.77	-2.77	-2.77	-2.77	-2.77
-2.76	-2.76	-2.76	-2.76	-2.76	-2.76	-2.76	-2.76	-2.76
-2.75	-2.75	-2.75	-2.75	-2.75	-2.75	-2.75	-2.75	-2.75
-2.74	-2.74	-2.74	-2.74	-2.74	-2.74	-2.74	-2.74	-2.74
-2.73	-2.73	-2.73	-2.73	-2.73	-2.73	-2.73	-2.73	-2.73
-2.72	-2.72	-2.72	-2.72	-2.72	-2.72	-2.72	-2.72	-2.72
-2.71	-2.71	-2.71	-2.71	-2.71	-2.71	-2.71	-2.71	-2.71
-2.7	-2.7	-2.7	-2.7	-2.7	-2.7	-2.7	-2.7	-2.7
-2.69	-2.69	-2.69	-2.69	-2.69	-2.69	-2.69	-2.69	-2.69
-2.68	-2.68	-2.68	-2.68	-2.68	-2.68	-2.68	-2.68	-2.68
-2.67	-2.67	-2.67	-2.67	-2.67	-2.67	-2.67	-2.67	-2.67
-2.66	-2.66	-2.66	-2.66	-2.66	-2.66	-2.66	-2.66	-2.66
-2.65	-2.65	-2.65	-2.65	-2.65	-2.65	-2.65	-2.65	-2.65
-2.64	-2.64	-2.64	-2.64	-2.64	-2.64	-2.64	-2.64	-2.64
-2.63	-2.63	-2.63	-2.63	-2.63	-2.63	-2.63	-2.63	-2.63
-2.62	-2.62	-2.62	-2.62	-2.62	-2.62	-2.62	-2.62	-2.62
-2.61	-2.61	-2.61	-2.61	-2.61	-2.61	-2.61	-2.61	-2.61
-2.6	-2.6	-2.6	-2.6	-2.6	-2.6	-2.6	-2.6	-2.6
-2.59	-2.59	-2.59	-2.59	-2.59	-2.59	-2.59	-2.59	-2.59
-2.58	-2.58	-2.58	-2.58	-2.58	-2.58	-2.58	-2.58	-2.58
-2.57	-2.57	-2.57	-2.57	-2.57	-2.57	-2.57	-2.57	-2.57
-2.56	-2.56	-2.56	-2.56	-2.56	-2.56	-2.56	-2.56	-2.56
-2.55	-2.55	-2.55	-2.55	-2.55	-2.55	-2.55	-2.55	-2.55
-2.54	-2.54	-2.54	-2.54	-2.54	-2.54	-2.54	-2.54	-2.54
-2.53	-2.53	-2.53	-2.53	-2.53	-2.53	-2.53	-2.53	-2.53
-2.52	-2.52	-2.52	-2.52	-2.52	-2.52	-2.52	-2.52	-2.52
-2.51	-2.51	-2.51	-2.51	-2.51	-2.51	-2.51	-2.51	-2.51

-2.5	-2.5	-2.5	-2.5	-2.5	-2.5	-2.5	-2.5	-2.5
-2.49	-2.49	-2.49	-2.49	-2.49	-2.49	-2.49	-2.49	-2.49
-2.48	-2.48	-2.48	-2.48	-2.48	-2.48	-2.48	-2.48	-2.48
-2.47	-2.47	-2.47	-2.47	-2.47	-2.47	-2.47	-2.47	-2.47
-2.46	-2.46	-2.46	-2.46	-2.46	-2.46	-2.46	-2.46	-2.46
-2.45	-2.45	-2.45	-2.45	-2.45	-2.45	-2.45	-2.45	-2.45
-2.44	-2.44	-2.44	-2.44	-2.44	-2.44	-2.44	-2.44	-2.44
-2.43	-2.43	-2.43	-2.43	-2.43	-2.43	-2.43	-2.43	-2.43
-2.42	-2.42	-2.42	-2.42	-2.42	-2.42	-2.42	-2.42	-2.42
-2.41	-2.41	-2.41	-2.41	-2.41	-2.41	-2.41	-2.41	-2.41
-2.4	-2.4	-2.4	-2.4	-2.4	-2.4	-2.4	-2.4	-2.4
-2.39	-2.39	-2.39	-2.39	-2.39	-2.39	-2.39	-2.39	-2.39
-2.38	-2.38	-2.38	-2.38	-2.38	-2.38	-2.38	-2.38	-2.38
-2.37	-2.37	-2.37	-2.37	-2.37	-2.37	-2.37	-2.37	-2.37
-2.36	-2.36	-2.36	-2.36	-2.36	-2.36	-2.36	-2.36	-2.36
-2.35	-2.35	-2.35	-2.35	-2.35	-2.35	-2.35	-2.35	-2.35
-2.34	-2.34	-2.34	-2.34	-2.34	-2.34	-2.34	-2.34	-2.34
-2.33	-2.33	-2.33	-2.33	-2.33	-2.33	-2.33	-2.33	-2.33
-2.32	-2.32	-2.32	-2.32	-2.32	-2.32	-2.32	-2.32	-2.32
-2.31	-2.31	-2.31	-2.31	-2.31	-2.31	-2.31	-2.31	-2.31
-2.3	-2.3	-2.3	-2.3	-2.3	-2.3	-2.3	-2.3	-2.3
-2.29	-2.29	-2.29	-2.29	-2.29	-2.29	-2.29	-2.29	-2.29
-2.28	-2.28	-2.28	-2.28	-2.28	-2.28	-2.28	-2.28	-2.28
-2.27	-2.27	-2.27	-2.27	-2.27	-2.27	-2.27	-2.27	-2.27
-2.26	-2.26	-2.26	-2.26	-2.26	-2.26	-2.26	-2.26	-2.26
-2.25	-2.25	-2.25	-2.25	-2.25	-2.25	-2.25	-2.25	-2.25
-2.24	-2.24	-2.24	-2.24	-2.24	-2.24	-2.24	-2.24	-2.24
-2.23	-2.23	-2.23	-2.23	-2.23	-2.23	-2.23	-2.23	-2.23
-2.22	-2.22	-2.22	-2.22	-2.22	-2.22	-2.22	-2.22	-2.22
-2.21	-2.21	-2.21	-2.21	-2.21	-2.21	-2.21	-2.21	-2.21
-2.2	-2.2	-2.2	-2.2	-2.2	-2.2	-2.2	-2.2	-2.2
-2.19	-2.19	-2.19	-2.19	-2.19	-2.19	-2.19	-2.19	-2.19
-2.18	-2.18	-2.18	-2.18	-2.18	-2.18	-2.18	-2.18	-2.18
-2.17	-2.17	-2.17	-2.17	-2.17	-2.17	-2.17	-2.17	-2.17
-2.16	-2.16	-2.16	-2.16	-2.16	-2.16	-2.16	-2.16	-2.16
-2.15	-2.15	-2.15	-2.15	-2.15	-2.15	-2.15	-2.15	-2.15
-2.14	-2.14	-2.14	-2.14	-2.14	-2.14	-2.14	-2.14	-2.14
-2.13	-2.13	-2.13	-2.13	-2.13	-2.13	-2.13	-2.13	-2.13
-2.12	-2.12	-2.12	-2.12	-2.12	-2.12	-2.12	-2.12	-2.12
-2.11	-2.11	-2.11	-2.11	-2.11	-2.11	-2.11	-2.11	-2.11
-2.1	-2.1	-2.1	-2.1	-2.1	-2.1	-2.1	-2.1	-2.1
-2.09	-2.09	-2.09	-2.09	-2.09	-2.09	-2.09	-2.09	-2.09
-2.08	-2.08	-2.08	-2.08	-2.08	-2.08	-2.08	-2.08	-2.08
-2.07	-2.07	-2.07	-2.07	-2.07	-2.07	-2.07	-2.07	-2.07
-2.06	-2.06	-2.06	-2.06	-2.06	-2.06	-2.06	-2.06	-2.06
-2.05	-2.05	-2.05	-2.05	-2.05	-2.05	-2.05	-2.05	-2.05
-2.04	-2.04	-2.04	-2.04	-2.04	-2.04	-2.04	-2.04	-2.04
-2.03	-2.03	-2.03	-2.03	-2.03	-2.03	-2.03	-2.03	-2.03
-2.02	-2.02	-2.02	-2.02	-2.02	-2.02	-2.02	-2.02	-2.02
-2.01	-2.01	-2.01	-2.01	-2.01	-2.01	-2.01	-2.01	-2.01

-2	-2	-2	-2	-2	-2	-2	-2	-2
-1.99	-1.99	-1.99	-1.99	-1.99	-1.99	-1.99	-1.99	-1.99
-1.98	-1.98	-1.98	-1.98	-1.98	-1.98	-1.98	-1.98	-1.98
-1.97	-1.97	-1.97	-1.97	-1.97	-1.97	-1.97	-1.97	-1.97
-1.96	-1.96	-1.96	-1.96	-1.96	-1.96	-1.96	-1.96	-1.96
-1.95	-1.95	-1.95	-1.95	-1.95	-1.95	-1.95	-1.95	-1.95
-1.94	-1.94	-1.94	-1.94	-1.94	-1.94	-1.94	-1.94	-1.94
-1.93	-1.93	-1.93	-1.93	-1.93	-1.93	-1.93	-1.93	-1.93
-1.92	-1.92	-1.92	-1.92	-1.92	-1.92	-1.92	-1.92	-1.92
-1.91	-1.91	-1.91	-1.91	-1.91	-1.91	-1.91	-1.91	-1.91
-1.9	-1.9	-1.9	-1.9	-1.9	-1.9	-1.9	-1.9	-1.9
-1.89	-1.89	-1.89	-1.89	-1.89	-1.89	-1.89	-1.89	-1.89
-1.88	-1.88	-1.88	-1.88	-1.88	-1.88	-1.88	-1.88	-1.88
-1.87	-1.87	-1.87	-1.87	-1.87	-1.87	-1.87	-1.87	-1.87
-1.86	-1.86	-1.86	-1.86	-1.86	-1.86	-1.86	-1.86	-1.86
-1.85	-1.85	-1.85	-1.85	-1.85	-1.85	-1.85	-1.85	-1.85
-1.84	-1.84	-1.84	-1.84	-1.84	-1.84	-1.84	-1.84	-1.84
-1.83	-1.83	-1.83	-1.83	-1.83	-1.83	-1.83	-1.83	-1.83
-1.82	-1.82	-1.82	-1.82	-1.82	-1.82	-1.82	-1.82	-1.82
-1.81	-1.81	-1.81	-1.81	-1.81	-1.81	-1.81	-1.81	-1.81
-1.8	-1.8	-1.8	-1.8	-1.8	-1.8	-1.8	-1.8	-1.8
-1.79	-1.79	-1.79	-1.79	-1.79	-1.79	-1.79	-1.79	-1.79
-1.78	-1.78	-1.78	-1.78	-1.78	-1.78	-1.78	-1.78	-1.78
-1.77	-1.77	-1.77	-1.77	-1.77	-1.77	-1.77	-1.77	-1.77
-1.76	-1.76	-1.76	-1.76	-1.76	-1.76	-1.76	-1.76	-1.76
-1.75	-1.75	-1.75	-1.75	-1.75	-1.75	-1.75	-1.75	-1.75
-1.74	-1.74	-1.74	-1.74	-1.74	-1.74	-1.74	-1.74	-1.74
-1.73	-1.73	-1.73	-1.73	-1.73	-1.73	-1.73	-1.73	-1.73
-1.72	-1.72	-1.72	-1.72	-1.72	-1.72	-1.72	-1.72	-1.72
-1.71	-1.71	-1.71	-1.71	-1.71	-1.71	-1.71	-1.71	-1.71
-1.7	-1.7	-1.7	-1.7	-1.7	-1.7	-1.7	-1.7	-1.7
-1.69	-1.69	-1.69	-1.69	-1.69	-1.69	-1.69	-1.69	-1.69
-1.68	-1.68	-1.68	-1.68	-1.68	-1.68	-1.68	-1.68	-1.68
-1.67	-1.67	-1.67	-1.67	-1.67	-1.67	-1.67	-1.67	-1.67
-1.66	-1.66	-1.66	-1.66	-1.66	-1.66	-1.66	-1.66	-1.66
-1.65	-1.65	-1.65	-1.65	-1.65	-1.65	-1.65	-1.65	-1.65
-1.64	-1.64	-1.64	-1.64	-1.64	-1.64	-1.64	-1.64	-1.64
-1.63	-1.63	-1.63	-1.63	-1.63	-1.63	-1.63	-1.63	-1.63
-1.62	-1.62	-1.62	-1.62	-1.62	-1.62	-1.62	-1.62	-1.62
-1.61	-1.61	-1.61	-1.61	-1.61	-1.61	-1.61	-1.61	-1.61
-1.6	-1.6	-1.6	-1.6	-1.6	-1.6	-1.6	-1.6	-1.6
-1.59	-1.59	-1.59	-1.59	-1.59	-1.59	-1.59	-1.59	-1.59
-1.58	-1.58	-1.58	-1.58	-1.58	-1.58	-1.58	-1.58	-1.58
-1.57	-1.57	-1.57	-1.57	-1.57	-1.57	-1.57	-1.57	-1.57
-1.56	-1.56	-1.56	-1.56	-1.56	-1.56	-1.56	-1.56	-1.56
-1.55	-1.55	-1.55	-1.55	-1.55	-1.55	-1.55	-1.55	-1.55
-1.54	-1.54	-1.54	-1.54	-1.54	-1.54	-1.54	-1.54	-1.54
-1.53	-1.53	-1.53	-1.53	-1.53	-1.53	-1.53	-1.53	-1.53
-1.52	-1.52	-1.52	-1.52	-1.52	-1.52	-1.52	-1.52	-1.52
-1.51	-1.51	-1.51	-1.51	-1.51	-1.51	-1.51	-1.51	-1.51

-1.5	-1.5	-1.5	-1.5	-1.5	-1.5	-1.5	-1.5	-1.5
-1.49	-1.49	-1.49	-1.49	-1.49	-1.49	-1.49	-1.49	-1.49
-1.48	-1.48	-1.48	-1.48	-1.48	-1.48	-1.48	-1.48	-1.48
-1.47	-1.47	-1.47	-1.47	-1.47	-1.47	-1.47	-1.47	-1.47
-1.46	-1.46	-1.46	-1.46	-1.46	-1.46	-1.46	-1.46	-1.46
-1.45	-1.45	-1.45	-1.45	-1.45	-1.45	-1.45	-1.45	-1.45
-1.44	-1.44	-1.44	-1.44	-1.44	-1.44	-1.44	-1.44	-1.44
-1.43	-1.43	-1.43	-1.43	-1.43	-1.43	-1.43	-1.43	-1.43
-1.42	-1.42	-1.42	-1.42	-1.42	-1.42	-1.42	-1.42	-1.42
-1.41	-1.41	-1.41	-1.41	-1.41	-1.41	-1.41	-1.41	-1.41
-1.4	-1.4	-1.4	-1.4	-1.4	-1.4	-1.4	-1.4	-1.4
-1.39	-1.39	-1.39	-1.39	-1.39	-1.39	-1.39	-1.39	-1.39
-1.38	-1.38	-1.38	-1.38	-1.38	-1.38	-1.38	-1.38	-1.38
-1.37	-1.37	-1.37	-1.37	-1.37	-1.37	-1.37	-1.37	-1.37
-1.36	-1.36	-1.36	-1.36	-1.36	-1.36	-1.36	-1.36	-1.36
-1.35	-1.35	-1.35	-1.35	-1.35	-1.35	-1.35	-1.35	-1.35
-1.34	-1.34	-1.34	-1.34	-1.34	-1.34	-1.34	-1.34	-1.34
-1.33	-1.33	-1.33	-1.33	-1.33	-1.33	-1.33	-1.33	-1.33
-1.32	-1.32	-1.32	-1.32	-1.32	-1.32	-1.32	-1.32	-1.32
-1.31	-1.31	-1.31	-1.31	-1.31	-1.31	-1.31	-1.31	-1.31
-1.3	-1.3	-1.3	-1.3	-1.3	-1.3	-1.3	-1.3	-1.3
-1.29	-1.29	-1.29	-1.29	-1.29	-1.29	-1.29	-1.29	-1.29
-1.28	-1.28	-1.28	-1.28	-1.28	-1.28	-1.28	-1.28	-1.28
-1.27	-1.27	-1.27	-1.27	-1.27	-1.27	-1.27	-1.27	-1.27
-1.26	-1.26	-1.26	-1.26	-1.26	-1.26	-1.26	-1.26	-1.26
-1.25	-1.25	-1.25	-1.25	-1.25	-1.25	-1.25	-1.25	-1.25
-1.24	-1.24	-1.24	-1.24	-1.24	-1.24	-1.24	-1.24	-1.24
-1.23	-1.23	-1.23	-1.23	-1.23	-1.23	-1.23	-1.23	-1.23
-1.22	-1.22	-1.22	-1.22	-1.22	-1.22	-1.22	-1.22	-1.22
-1.21	-1.21	-1.21	-1.21	-1.21	-1.21	-1.21	-1.21	-1.21
-1.2	-1.2	-1.2	-1.2	-1.2	-1.2	-1.2	-1.2	-1.2
-1.19	-1.19	-1.19	-1.19	-1.19	-1.19	-1.19	-1.19	-1.19
-1.18	-1.18	-1.18	-1.18	-1.18	-1.18	-1.18	-1.18	-1.18
-1.17	-1.17	-1.17	-1.17	-1.17	-1.17	-1.17	-1.17	-1.17
-1.16	-1.16	-1.16	-1.16	-1.16	-1.16	-1.16	-1.16	-1.16
-1.15	-1.15	-1.15	-1.15	-1.15	-1.15	-1.15	-1.15	-1.15
-1.14	-1.14	-1.14	-1.14	-1.14	-1.14	-1.14	-1.14	-1.14
-1.13	-1.13	-1.13	-1.13	-1.13	-1.13	-1.13	-1.13	-1.13
-1.12	-1.12	-1.12	-1.12	-1.12	-1.12	-1.12	-1.12	-1.12
-1.11	-1.11	-1.11	-1.11	-1.11	-1.11	-1.11	-1.11	-1.11
-1.1	-1.1	-1.1	-1.1	-1.1	-1.1	-1.1	-1.1	-1.1
-1.09	-1.09	-1.09	-1.09	-1.09	-1.09	-1.09	-1.09	-1.09
-1.08	-1.08	-1.08	-1.08	-1.08	-1.08	-1.08	-1.08	-1.08
-1.07	-1.07	-1.07	-1.07	-1.07	-1.07	-1.07	-1.07	-1.07
-1.06	-1.06	-1.06	-1.06	-1.06	-1.06	-1.06	-1.06	-1.06
-1.05	-1.05	-1.05	-1.05	-1.05	-1.05	-1.05	-1.05	-1.05
-1.04	-1.04	-1.04	-1.04	-1.04	-1.04	-1.04	-1.04	-1.04
-1.03	-1.03	-1.03	-1.03	-1.03	-1.03	-1.03	-1.03	-1.03
-1.02	-1.02	-1.02	-1.02	-1.02	-1.02	-1.02	-1.02	-1.02
-1.01	-1.01	-1.01	-1.01	-1.01	-1.01	-1.01	-1.01	-1.01

-1	-1	-1	-1	-1	-1	-1	-1	-1
-0.99	-0.99	-0.99	-0.99	-0.99	-0.99	-0.99	-0.99	-0.99
-0.98	-0.98	-0.98	-0.98	-0.98	-0.98	-0.98	-0.98	-0.98
-0.97	-0.97	-0.97	-0.97	-0.97	-0.97	-0.97	-0.97	-0.97
-0.96	-0.96	-0.96	-0.96	-0.96	-0.96	-0.96	-0.96	-0.96
-0.95	-0.95	-0.95	-0.95	-0.95	-0.95	-0.95	-0.95	-0.95
-0.94	-0.94	-0.94	-0.94	-0.94	-0.94	-0.94	-0.94	-0.94
-0.93	-0.93	-0.93	-0.93	-0.93	-0.93	-0.93	-0.93	-0.93
-0.92	-0.92	-0.92	-0.92	-0.92	-0.92	-0.92	-0.92	-0.92
-0.91	-0.91	-0.91	-0.91	-0.91	-0.91	-0.91	-0.91	-0.91
-0.9	-0.9	-0.9	-0.9	-0.9	-0.9	-0.9	-0.9	-0.9
-0.89	-0.89	-0.89	-0.89	-0.89	-0.89	-0.89	-0.89	-0.89
-0.88	-0.88	-0.88	-0.88	-0.88	-0.88	-0.88	-0.88	-0.88
-0.87	-0.87	-0.87	-0.87	-0.87	-0.87	-0.87	-0.87	-0.87
-0.86	-0.86	-0.86	-0.86	-0.86	-0.86	-0.86	-0.86	-0.86
-0.85	-0.85	-0.85	-0.85	-0.85	-0.85	-0.85	-0.85	-0.85
-0.84	-0.84	-0.84	-0.84	-0.84	-0.84	-0.84	-0.84	-0.84
-0.83	-0.83	-0.83	-0.83	-0.83	-0.83	-0.83	-0.83	-0.83
-0.82	-0.82	-0.82	-0.82	-0.82	-0.82	-0.82	-0.82	-0.82
-0.81	-0.81	-0.81	-0.81	-0.81	-0.81	-0.81	-0.81	-0.81
-0.8	-0.8	-0.8	-0.8	-0.8	-0.8	-0.8	-0.8	-0.8
-0.79	-0.79	-0.79	-0.79	-0.79	-0.79	-0.79	-0.79	-0.79
-0.78	-0.78	-0.78	-0.78	-0.78	-0.78	-0.78	-0.78	-0.78
-0.77	-0.77	-0.77	-0.77	-0.77	-0.77	-0.77	-0.77	-0.77
-0.76	-0.76	-0.76	-0.76	-0.76	-0.76	-0.76	-0.76	-0.76
-0.75	-0.75	-0.75	-0.75	-0.75	-0.75	-0.75	-0.75	-0.75
-0.74	-0.74	-0.74	-0.74	-0.74	-0.74	-0.74	-0.74	-0.74
-0.73	-0.73	-0.73	-0.73	-0.73	-0.73	-0.73	-0.73	-0.73
-0.72	-0.72	-0.72	-0.72	-0.72	-0.72	-0.72	-0.72	-0.72
-0.71	-0.71	-0.71	-0.71	-0.71	-0.71	-0.71	-0.71	-0.71
-0.7	-0.7	-0.7	-0.7	-0.7	-0.7	-0.7	-0.7	-0.7
-0.69	-0.69	-0.69	-0.69	-0.69	-0.69	-0.69	-0.69	-0.69
-0.68	-0.68	-0.68	-0.68	-0.68	-0.68	-0.68	-0.68	-0.68
-0.67	-0.67	-0.67	-0.67	-0.67	-0.67	-0.67	-0.67	-0.67
-0.66	-0.66	-0.66	-0.66	-0.66	-0.66	-0.66	-0.66	-0.66
-0.65	-0.65	-0.65	-0.65	-0.65	-0.65	-0.65	-0.65	-0.65
-0.64	-0.64	-0.64	-0.64	-0.64	-0.64	-0.64	-0.64	-0.64
-0.63	-0.63	-0.63	-0.63	-0.63	-0.63	-0.63	-0.63	-0.63
-0.62	-0.62	-0.62	-0.62	-0.62	-0.62	-0.62	-0.62	-0.62
-0.61	-0.61	-0.61	-0.61	-0.61	-0.61	-0.61	-0.61	-0.61
-0.6	-0.6	-0.6	-0.6	-0.6	-0.6	-0.6	-0.6	-0.6
-0.59	-0.59	-0.59	-0.59	-0.59	-0.59	-0.59	-0.59	-0.59
-0.58	-0.58	-0.58	-0.58	-0.58	-0.58	-0.58	-0.58	-0.58
-0.57	-0.57	-0.57	-0.57	-0.57	-0.57	-0.57	-0.57	-0.57
-0.56	-0.56	-0.56	-0.56	-0.56	-0.56	-0.56	-0.56	-0.56
-0.55	-0.55	-0.55	-0.55	-0.55	-0.55	-0.55	-0.55	-0.55
-0.54	-0.54	-0.54	-0.54	-0.54	-0.54	-0.54	-0.54	-0.54
-0.53	-0.53	-0.53	-0.53	-0.53	-0.53	-0.53	-0.53	-0.53
-0.52	-0.52	-0.52	-0.52	-0.52	-0.52	-0.52	-0.52	-0.52
-0.51	-0.51	-0.51	-0.51	-0.51	-0.51	-0.51	-0.51	-0.51

-0.5	-0.5	-0.5	-0.5	-0.5	-0.5	-0.5	-0.5	-0.5
-0.49	-0.49	-0.49	-0.49	-0.49	-0.49	-0.49	-0.49	-0.49
-0.48	-0.48	-0.48	-0.48	-0.48	-0.48	-0.48	-0.48	-0.48
-0.47	-0.47	-0.47	-0.47	-0.47	-0.47	-0.47	-0.47	-0.47
-0.46	-0.46	-0.46	-0.46	-0.46	-0.46	-0.46	-0.46	-0.46
-0.45	-0.45	-0.45	-0.45	-0.45	-0.45	-0.45	-0.45	-0.45
-0.44	-0.44	-0.44	-0.44	-0.44	-0.44	-0.44	-0.44	-0.44
-0.43	-0.43	-0.43	-0.43	-0.43	-0.43	-0.43	-0.43	-0.43
-0.42	-0.42	-0.42	-0.42	-0.42	-0.42	-0.42	-0.42	-0.42
-0.41	-0.41	-0.41	-0.41	-0.41	-0.41	-0.41	-0.41	-0.41
-0.4	-0.4	-0.4	-0.4	-0.4	-0.4	-0.4	-0.4	-0.4
-0.39	-0.39	-0.39	-0.39	-0.39	-0.39	-0.39	-0.39	-0.39
-0.38	-0.38	-0.38	-0.38	-0.38	-0.38	-0.38	-0.38	-0.38
-0.37	-0.37	-0.37	-0.37	-0.37	-0.37	-0.37	-0.37	-0.37
-0.36	-0.36	-0.36	-0.36	-0.36	-0.36	-0.36	-0.36	-0.36
-0.35	-0.35	-0.35	-0.35	-0.35	-0.35	-0.35	-0.35	-0.35
-0.34	-0.34	-0.34	-0.34	-0.34	-0.34	-0.34	-0.34	-0.34
-0.33	-0.33	-0.33	-0.33	-0.33	-0.33	-0.33	-0.33	-0.33
-0.32	-0.32	-0.32	-0.32	-0.32	-0.32	-0.32	-0.32	-0.32
-0.31	-0.31	-0.31	-0.31	-0.31	-0.31	-0.31	-0.31	-0.31
-0.3	-0.3	-0.3	-0.3	-0.3	-0.3	-0.3	-0.3	-0.3
-0.29	-0.29	-0.29	-0.29	-0.29	-0.29	-0.29	-0.29	-0.29
-0.28	-0.28	-0.28	-0.28	-0.28	-0.28	-0.28	-0.28	-0.28
-0.27	-0.27	-0.27	-0.27	-0.27	-0.27	-0.27	-0.27	-0.27
-0.26	-0.26	-0.26	-0.26	-0.26	-0.26	-0.26	-0.26	-0.26
-0.25	-0.25	-0.25	-0.25	-0.25	-0.25	-0.25	-0.25	-0.25
-0.24	-0.24	-0.24	-0.24	-0.24	-0.24	-0.24	-0.24	-0.24
-0.23	-0.23	-0.23	-0.23	-0.23	-0.23	-0.23	-0.23	-0.23
-0.22	-0.22	-0.22	-0.22	-0.22	-0.22	-0.22	-0.22	-0.22
-0.21	-0.21	-0.21	-0.21	-0.21	-0.21	-0.21	-0.21	-0.21
-0.2	-0.2	-0.2	-0.2	-0.2	-0.2	-0.2	-0.2	-0.2
-0.19	-0.19	-0.19	-0.19	-0.19	-0.19	-0.19	-0.19	-0.19
-0.18	-0.18	-0.18	-0.18	-0.18	-0.18	-0.18	-0.18	-0.18
-0.17	-0.17	-0.17	-0.17	-0.17	-0.17	-0.17	-0.17	-0.17
-0.16	-0.16	-0.16	-0.16	-0.16	-0.16	-0.16	-0.16	-0.16
-0.15	-0.15	-0.15	-0.15	-0.15	-0.15	-0.15	-0.15	-0.15
-0.14	-0.14	-0.14	-0.14	-0.14	-0.14	-0.14	-0.14	-0.14
-0.13	-0.13	-0.13	-0.13	-0.13	-0.13	-0.13	-0.13	-0.13
-0.12	-0.12	-0.12	-0.12	-0.12	-0.12	-0.12	-0.12	-0.12
-0.11	-0.11	-0.11	-0.11	-0.11	-0.11	-0.11	-0.11	-0.11
-0.1	-0.1	-0.1	-0.1	-0.1	-0.1	-0.1	-0.1	-0.1
-0.09	-0.09	-0.09	-0.09	-0.09	-0.09	-0.09	-0.09	-0.09
-0.08	-0.08	-0.08	-0.08	-0.08	-0.08	-0.08	-0.08	-0.08
-0.07	-0.07	-0.07	-0.07	-0.07	-0.07	-0.07	-0.07	-0.07
-0.06	-0.06	-0.06	-0.06	-0.06	-0.06	-0.06	-0.06	-0.06
-0.05	-0.05	-0.05	-0.05	-0.05	-0.05	-0.05	-0.05	-0.05
-0.04	-0.04	-0.04	-0.04	-0.04	-0.04	-0.04	-0.04	-0.04
-0.03	-0.03	-0.03	-0.03	-0.03	-0.03	-0.03	-0.03	-0.03
-0.02	-0.02	-0.02	-0.02	-0.02	-0.02	-0.02	-0.02	-0.02
-0.01	-0.01	-0.01	-0.01	-0.01	-0.01	-0.01	-0.01	-0.01

0	0	0	0	0	0	0	0	0
0.01	0.01	0.01	0.01	0.01	0.01	0.01	0.01	0.01
0.02	0.02	0.02	0.02	0.02	0.02	0.02	0.02	0.02
0.03	0.03	0.03	0.03	0.03	0.03	0.03	0.03	0.03
0.04	0.04	0.04	0.04	0.04	0.04	0.04	0.04	0.04
0.05	0.05	0.05	0.05	0.05	0.05	0.05	0.05	0.05
0.06	0.06	0.06	0.06	0.06	0.06	0.06	0.06	0.06
0.07	0.07	0.07	0.07	0.07	0.07	0.07	0.07	0.07
0.08	0.08	0.08	0.08	0.08	0.08	0.08	0.08	0.08
0.09	0.09	0.09	0.09	0.09	0.09	0.09	0.09	0.09
0.1	0.1	0.1	0.1	0.1	0.1	0.1	0.1	0.1
0.11	0.11	0.11	0.11	0.11	0.11	0.11	0.11	0.11
0.12	0.12	0.12	0.12	0.12	0.12	0.12	0.12	0.12
0.13	0.13	0.13	0.13	0.13	0.13	0.13	0.13	0.13
0.14	0.14	0.14	0.14	0.14	0.14	0.14	0.14	0.14
0.15	0.15	0.15	0.15	0.15	0.15	0.15	0.15	0.15
0.16	0.16	0.16	0.16	0.16	0.16	0.16	0.16	0.16
0.17	0.17	0.17	0.17	0.17	0.17	0.17	0.17	0.17
0.18	0.18	0.18	0.18	0.18	0.18	0.18	0.18	0.18
0.19	0.19	0.19	0.19	0.19	0.19	0.19	0.19	0.19
0.2	0.2	0.2	0.2	0.2	0.2	0.2	0.2	0.2
0.21	0.21	0.21	0.21	0.21	0.21	0.21	0.21	0.21
0.22	0.22	0.22	0.22	0.22	0.22	0.22	0.22	0.22
0.23	0.23	0.23	0.23	0.23	0.23	0.23	0.23	0.23
0.24	0.24	0.24	0.24	0.24	0.24	0.24	0.24	0.24
0.25	0.25	0.25	0.25	0.25	0.25	0.25	0.25	0.25
0.26	0.26	0.26	0.26	0.26	0.26	0.26	0.26	0.26
0.27	0.27	0.27	0.27	0.27	0.27	0.27	0.27	0.27
0.28	0.28	0.28	0.28	0.28	0.28	0.28	0.28	0.28
0.29	0.29	0.29	0.29	0.29	0.29	0.29	0.29	0.29
0.3	0.3	0.3	0.3	0.3	0.3	0.3	0.3	0.3
0.31	0.31	0.31	0.31	0.31	0.31	0.31	0.31	0.31
0.32	0.32	0.32	0.32	0.32	0.32	0.32	0.32	0.32
0.33	0.33	0.33	0.33	0.33	0.33	0.33	0.33	0.33
0.34	0.34	0.34	0.34	0.34	0.34	0.34	0.34	0.34
0.35	0.35	0.35	0.35	0.35	0.35	0.35	0.35	0.35
0.36	0.36	0.36	0.36	0.36	0.36	0.36	0.36	0.36
0.37	0.37	0.37	0.37	0.37	0.37	0.37	0.37	0.37
0.38	0.38	0.38	0.38	0.38	0.38	0.38	0.38	0.38
0.39	0.39	0.39	0.39	0.39	0.39	0.39	0.39	0.39
0.4	0.4	0.4	0.4	0.4	0.4	0.4	0.4	0.4
0.41	0.41	0.41	0.41	0.41	0.41	0.41	0.41	0.41
0.42	0.42	0.42	0.42	0.42	0.42	0.42	0.42	0.42
0.43	0.43	0.43	0.43	0.43	0.43	0.43	0.43	0.43
0.44	0.44	0.44	0.44	0.44	0.44	0.44	0.44	0.44
0.45	0.45	0.45	0.45	0.45	0.45	0.45	0.45	0.45
0.46	0.46	0.46	0.46	0.46	0.46	0.46	0.46	0.46
0.47	0.47	0.47	0.47	0.47	0.47	0.47	0.47	0.47
0.48	0.48	0.48	0.48	0.48	0.48	0.48	0.48	0.48
0.49	0.49	0.49	0.49	0.49	0.49	0.49	0.49	0.49

0.5	0.5	0.5	0.5	0.5	0.5	0.5	0.5	0.5
0.51	0.51	0.51	0.51	0.51	0.51	0.51	0.51	0.51
0.52	0.52	0.52	0.52	0.52	0.52	0.52	0.52	0.52
0.53	0.53	0.53	0.53	0.53	0.53	0.53	0.53	0.53
0.54	0.54	0.54	0.54	0.54	0.54	0.54	0.54	0.54
0.55	0.55	0.55	0.55	0.55	0.55	0.55	0.55	0.55
0.56	0.56	0.56	0.56	0.56	0.56	0.56	0.56	0.56
0.57	0.57	0.57	0.57	0.57	0.57	0.57	0.57	0.57
0.58	0.58	0.58	0.58	0.58	0.58	0.58	0.58	0.58
0.59	0.59	0.59	0.59	0.59	0.59	0.59	0.59	0.59
0.6	0.6	0.6	0.6	0.6	0.6	0.6	0.6	0.6
0.61	0.61	0.61	0.61	0.61	0.61	0.61	0.61	0.61
0.62	0.62	0.62	0.62	0.62	0.62	0.62	0.62	0.62
0.63	0.63	0.63	0.63	0.63	0.63	0.63	0.63	0.63
0.64	0.64	0.64	0.64	0.64	0.64	0.64	0.64	0.64
0.65	0.65	0.65	0.65	0.65	0.65	0.65	0.65	0.65
0.66	0.66	0.66	0.66	0.66	0.66	0.66	0.66	0.66
0.67	0.67	0.67	0.67	0.67	0.67	0.67	0.67	0.67
0.68	0.68	0.68	0.68	0.68	0.68	0.68	0.68	0.68
0.69	0.69	0.69	0.69	0.69	0.69	0.69	0.69	0.69
0.7	0.7	0.7	0.7	0.7	0.7	0.7	0.7	0.7
0.71	0.71	0.71	0.71	0.71	0.71	0.71	0.71	0.71
0.72	0.72	0.72	0.72	0.72	0.72	0.72	0.72	0.72
0.73	0.73	0.73	0.73	0.73	0.73	0.73	0.73	0.73
0.74	0.74	0.74	0.74	0.74	0.74	0.74	0.74	0.74
0.75	0.75	0.75	0.75	0.75	0.75	0.75	0.75	0.75
0.76	0.76	0.76	0.76	0.76	0.76	0.76	0.76	0.76
0.77	0.77	0.77	0.77	0.77	0.77	0.77	0.77	0.77
0.78	0.78	0.78	0.78	0.78	0.78	0.78	0.78	0.78
0.79	0.79	0.79	0.79	0.79	0.79	0.79	0.79	0.79
0.8	0.8	0.8	0.8	0.8	0.8	0.8	0.8	0.8
0.81	0.81	0.81	0.81	0.81	0.81	0.81	0.81	0.81
0.82	0.82	0.82	0.82	0.82	0.82	0.82	0.82	0.82
0.83	0.83	0.83	0.83	0.83	0.83	0.83	0.83	0.83
0.84	0.84	0.84	0.84	0.84	0.84	0.84	0.84	0.84
0.85	0.85	0.85	0.85	0.85	0.85	0.85	0.85	0.85
0.86	0.86	0.86	0.86	0.86	0.86	0.86	0.86	0.86
0.87	0.87	0.87	0.87	0.87	0.87	0.87	0.87	0.87
0.88	0.88	0.88	0.88	0.88	0.88	0.88	0.88	0.88
0.89	0.89	0.89	0.89	0.89	0.89	0.89	0.89	0.89
0.9	0.9	0.9	0.9	0.9	0.9	0.9	0.9	0.9
0.91	0.91	0.91	0.91	0.91	0.91	0.91	0.91	0.91
0.92	0.92	0.92	0.92	0.92	0.92	0.92	0.92	0.92
0.93	0.93	0.93	0.93	0.93	0.93	0.93	0.93	0.93
0.94	0.94	0.94	0.94	0.94	0.94	0.94	0.94	0.94
0.95	0.95	0.95	0.95	0.95	0.95	0.95	0.95	0.95
0.96	0.96	0.96	0.96	0.96	0.96	0.96	0.96	0.96
0.97	0.97	0.97	0.97	0.97	0.97	0.97	0.97	0.97
0.98	0.98	0.98	0.98	0.98	0.98	0.98	0.98	0.98
0.99	0.99	0.99	0.99	0.99	0.99	0.99	0.99	0.99

1	1	1	1	1	1	1	1	1
1.01	1.01	1.01	1.01	1.01	1.01	1.01	1.01	1.01
1.02	1.02	1.02	1.02	1.02	1.02	1.02	1.02	1.02
1.03	1.03	1.03	1.03	1.03	1.03	1.03	1.03	1.03
1.04	1.04	1.04	1.04	1.04	1.04	1.04	1.04	1.04
1.05	1.05	1.05	1.05	1.05	1.05	1.05	1.05	1.05
1.06	1.06	1.06	1.06	1.06	1.06	1.06	1.06	1.06
1.07	1.07	1.07	1.07	1.07	1.07	1.07	1.07	1.07
1.08	1.08	1.08	1.08	1.08	1.08	1.08	1.08	1.08
1.09	1.09	1.09	1.09	1.09	1.09	1.09	1.09	1.09
1.1	1.1	1.1	1.1	1.1	1.1	1.1	1.1	1.1
1.11	1.11	1.11	1.11	1.11	1.11	1.11	1.11	1.11
1.12	1.12	1.12	1.12	1.12	1.12	1.12	1.12	1.12
1.13	1.13	1.13	1.13	1.13	1.13	1.13	1.13	1.13
1.14	1.14	1.14	1.14	1.14	1.14	1.14	1.14	1.14
1.15	1.15	1.15	1.15	1.15	1.15	1.15	1.15	1.15
1.16	1.16	1.16	1.16	1.16	1.16	1.16	1.16	1.16
1.17	1.17	1.17	1.17	1.17	1.17	1.17	1.17	1.17
1.18	1.18	1.18	1.18	1.18	1.18	1.18	1.18	1.18
1.19	1.19	1.19	1.19	1.19	1.19	1.19	1.19	1.19
1.2	1.2	1.2	1.2	1.2	1.2	1.2	1.2	1.2
1.21	1.21	1.21	1.21	1.21	1.21	1.21	1.21	1.21
1.22	1.22	1.22	1.22	1.22	1.22	1.22	1.22	1.22
1.23	1.23	1.23	1.23	1.23	1.23	1.23	1.23	1.23
1.24	1.24	1.24	1.24	1.24	1.24	1.24	1.24	1.24
1.25	1.25	1.25	1.25	1.25	1.25	1.25	1.25	1.25
1.26	1.26	1.26	1.26	1.26	1.26	1.26	1.26	1.26
1.27	1.27	1.27	1.27	1.27	1.27	1.27	1.27	1.27
1.28	1.28	1.28	1.28	1.28	1.28	1.28	1.28	1.28
1.29	1.29	1.29	1.29	1.29	1.29	1.29	1.29	1.29
1.3	1.3	1.3	1.3	1.3	1.3	1.3	1.3	1.3
1.31	1.31	1.31	1.31	1.31	1.31	1.31	1.31	1.31
1.32	1.32	1.32	1.32	1.32	1.32	1.32	1.32	1.32
1.33	1.33	1.33	1.33	1.33	1.33	1.33	1.33	1.33
1.34	1.34	1.34	1.34	1.34	1.34	1.34	1.34	1.34
1.35	1.35	1.35	1.35	1.35	1.35	1.35	1.35	1.35
1.36	1.36	1.36	1.36	1.36	1.36	1.36	1.36	1.36
1.37	1.37	1.37	1.37	1.37	1.37	1.37	1.37	1.37
1.38	1.38	1.38	1.38	1.38	1.38	1.38	1.38	1.38
1.39	1.39	1.39	1.39	1.39	1.39	1.39	1.39	1.39
1.4	1.4	1.4	1.4	1.4	1.4	1.4	1.4	1.4
1.41	1.41	1.41	1.41	1.41	1.41	1.41	1.41	1.41
1.42	1.42	1.42	1.42	1.42	1.42	1.42	1.42	1.42
1.43	1.43	1.43	1.43	1.43	1.43	1.43	1.43	1.43
1.44	1.44	1.44	1.44	1.44	1.44	1.44	1.44	1.44
1.45	1.45	1.45	1.45	1.45	1.45	1.45	1.45	1.45
1.46	1.46	1.46	1.46	1.46	1.46	1.46	1.46	1.46
1.47	1.47	1.47	1.47	1.47	1.47	1.47	1.47	1.47
1.48	1.48	1.48	1.48	1.48	1.48	1.48	1.48	1.48
1.49	1.49	1.49	1.49	1.49	1.49	1.49	1.49	1.49

1.5	1.5	1.5	1.5	1.5	1.5	1.5	1.5	1.5
1.51	1.51	1.51	1.51	1.51	1.51	1.51	1.51	1.51
1.52	1.52	1.52	1.52	1.52	1.52	1.52	1.52	1.52
1.53	1.53	1.53	1.53	1.53	1.53	1.53	1.53	1.53
1.54	1.54	1.54	1.54	1.54	1.54	1.54	1.54	1.54
1.55	1.55	1.55	1.55	1.55	1.55	1.55	1.55	1.55
1.56	1.56	1.56	1.56	1.56	1.56	1.56	1.56	1.56
1.57	1.57	1.57	1.57	1.57	1.57	1.57	1.57	1.57
1.58	1.58	1.58	1.58	1.58	1.58	1.58	1.58	1.58
1.59	1.59	1.59	1.59	1.59	1.59	1.59	1.59	1.59
1.6	1.6	1.6	1.6	1.6	1.6	1.6	1.6	1.6
1.61	1.61	1.61	1.61	1.61	1.61	1.61	1.61	1.61
1.62	1.62	1.62	1.62	1.62	1.62	1.62	1.62	1.62
1.63	1.63	1.63	1.63	1.63	1.63	1.63	1.63	1.63
1.64	1.64	1.64	1.64	1.64	1.64	1.64	1.64	1.64
1.65	1.65	1.65	1.65	1.65	1.65	1.65	1.65	1.65
1.66	1.66	1.66	1.66	1.66	1.66	1.66	1.66	1.66
1.67	1.67	1.67	1.67	1.67	1.67	1.67	1.67	1.67
1.68	1.68	1.68	1.68	1.68	1.68	1.68	1.68	1.68
1.69	1.69	1.69	1.69	1.69	1.69	1.69	1.69	1.69
1.7	1.7	1.7	1.7	1.7	1.7	1.7	1.7	1.7
1.71	1.71	1.71	1.71	1.71	1.71	1.71	1.71	1.71
1.72	1.72	1.72	1.72	1.72	1.72	1.72	1.72	1.72
1.73	1.73	1.73	1.73	1.73	1.73	1.73	1.73	1.73
1.74	1.74	1.74	1.74	1.74	1.74	1.74	1.74	1.74
1.75	1.75	1.75	1.75	1.75	1.75	1.75	1.75	1.75
1.76	1.76	1.76	1.76	1.76	1.76	1.76	1.76	1.76
1.77	1.77	1.77	1.77	1.77	1.77	1.77	1.77	1.77
1.78	1.78	1.78	1.78	1.78	1.78	1.78	1.78	1.78
1.79	1.79	1.79	1.79	1.79	1.79	1.79	1.79	1.79
1.8	1.8	1.8	1.8	1.8	1.8	1.8	1.8	1.8
1.81	1.81	1.81	1.81	1.81	1.81	1.81	1.81	1.81
1.82	1.82	1.82	1.82	1.82	1.82	1.82	1.82	1.82
1.83	1.83	1.83	1.83	1.83	1.83	1.83	1.83	1.83
1.84	1.84	1.84	1.84	1.84	1.84	1.84	1.84	1.84
1.85	1.85	1.85	1.85	1.85	1.85	1.85	1.85	1.85
1.86	1.86	1.86	1.86	1.86	1.86	1.86	1.86	1.86
1.87	1.87	1.87	1.87	1.87	1.87	1.87	1.87	1.87
1.88	1.88	1.88	1.88	1.88	1.88	1.88	1.88	1.88
1.89	1.89	1.89	1.89	1.89	1.89	1.89	1.89	1.89
1.9	1.9	1.9	1.9	1.9	1.9	1.9	1.9	1.9
1.91	1.91	1.91	1.91	1.91	1.91	1.91	1.91	1.91
1.92	1.92	1.92	1.92	1.92	1.92	1.92	1.92	1.92
1.93	1.93	1.93	1.93	1.93	1.93	1.93	1.93	1.93
1.94	1.94	1.94	1.94	1.94	1.94	1.94	1.94	1.94
1.95	1.95	1.95	1.95	1.95	1.95	1.95	1.95	1.95
1.96	1.96	1.96	1.96	1.96	1.96	1.96	1.96	1.96
1.97	1.97	1.97	1.97	1.97	1.97	1.97	1.97	1.97
1.98	1.98	1.98	1.98	1.98	1.98	1.98	1.98	1.98
1.99	1.99	1.99	1.99	1.99	1.99	1.99	1.99	1.99

2	2	2	2	2	2	2	2	2
2.01	2.01	2.01	2.01	2.01	2.01	2.01	2.01	2.01
2.02	2.02	2.02	2.02	2.02	2.02	2.02	2.02	2.02
2.03	2.03	2.03	2.03	2.03	2.03	2.03	2.03	2.03
2.04	2.04	2.04	2.04	2.04	2.04	2.04	2.04	2.04
2.05	2.05	2.05	2.05	2.05	2.05	2.05	2.05	2.05
2.06	2.06	2.06	2.06	2.06	2.06	2.06	2.06	2.06
2.07	2.07	2.07	2.07	2.07	2.07	2.07	2.07	2.07
2.08	2.08	2.08	2.08	2.08	2.08	2.08	2.08	2.08
2.09	2.09	2.09	2.09	2.09	2.09	2.09	2.09	2.09
2.1	2.1	2.1	2.1	2.1	2.1	2.1	2.1	2.1
2.11	2.11	2.11	2.11	2.11	2.11	2.11	2.11	2.11
2.12	2.12	2.12	2.12	2.12	2.12	2.12	2.12	2.12
2.13	2.13	2.13	2.13	2.13	2.13	2.13	2.13	2.13
2.14	2.14	2.14	2.14	2.14	2.14	2.14	2.14	2.14
2.15	2.15	2.15	2.15	2.15	2.15	2.15	2.15	2.15
2.16	2.16	2.16	2.16	2.16	2.16	2.16	2.16	2.16
2.17	2.17	2.17	2.17	2.17	2.17	2.17	2.17	2.17
2.18	2.18	2.18	2.18	2.18	2.18	2.18	2.18	2.18
2.19	2.19	2.19	2.19	2.19	2.19	2.19	2.19	2.19
2.2	2.2	2.2	2.2	2.2	2.2	2.2	2.2	2.2
2.21	2.21	2.21	2.21	2.21	2.21	2.21	2.21	2.21
2.22	2.22	2.22	2.22	2.22	2.22	2.22	2.22	2.22
2.23	2.23	2.23	2.23	2.23	2.23	2.23	2.23	2.23
2.24	2.24	2.24	2.24	2.24	2.24	2.24	2.24	2.24
2.25	2.25	2.25	2.25	2.25	2.25	2.25	2.25	2.25
2.26	2.26	2.26	2.26	2.26	2.26	2.26	2.26	2.26
2.27	2.27	2.27	2.27	2.27	2.27	2.27	2.27	2.27
2.28	2.28	2.28	2.28	2.28	2.28	2.28	2.28	2.28
2.29	2.29	2.29	2.29	2.29	2.29	2.29	2.29	2.29
2.3	2.3	2.3	2.3	2.3	2.3	2.3	2.3	2.3
2.31	2.31	2.31	2.31	2.31	2.31	2.31	2.31	2.31
2.32	2.32	2.32	2.32	2.32	2.32	2.32	2.32	2.32
2.33	2.33	2.33	2.33	2.33	2.33	2.33	2.33	2.33
2.34	2.34	2.34	2.34	2.34	2.34	2.34	2.34	2.34
2.35	2.35	2.35	2.35	2.35	2.35	2.35	2.35	2.35
2.36	2.36	2.36	2.36	2.36	2.36	2.36	2.36	2.36
2.37	2.37	2.37	2.37	2.37	2.37	2.37	2.37	2.37
2.38	2.38	2.38	2.38	2.38	2.38	2.38	2.38	2.38
2.39	2.39	2.39	2.39	2.39	2.39	2.39	2.39	2.39
2.4	2.4	2.4	2.4	2.4	2.4	2.4	2.4	2.4
2.41	2.41	2.41	2.41	2.41	2.41	2.41	2.41	2.41
2.42	2.42	2.42	2.42	2.42	2.42	2.42	2.42	2.42
2.43	2.43	2.43	2.43	2.43	2.43	2.43	2.43	2.43
2.44	2.44	2.44	2.44	2.44	2.44	2.44	2.44	2.44
2.45	2.45	2.45	2.45	2.45	2.45	2.45	2.45	2.45
2.46	2.46	2.46	2.46	2.46	2.46	2.46	2.46	2.46
2.47	2.47	2.47	2.47	2.47	2.47	2.47	2.47	2.47
2.48	2.48	2.48	2.48	2.48	2.48	2.48	2.48	2.48
2.49	2.49	2.49	2.49	2.49	2.49	2.49	2.49	2.49

2.5	2.5	2.5	2.5	2.5	2.5	2.5	2.5	2.5
2.51	2.51	2.51	2.51	2.51	2.51	2.51	2.51	2.51
2.52	2.52	2.52	2.52	2.52	2.52	2.52	2.52	2.52
2.53	2.53	2.53	2.53	2.53	2.53	2.53	2.53	2.53
2.54	2.54	2.54	2.54	2.54	2.54	2.54	2.54	2.54
2.55	2.55	2.55	2.55	2.55	2.55	2.55	2.55	2.55
2.56	2.56	2.56	2.56	2.56	2.56	2.56	2.56	2.56
2.57	2.57	2.57	2.57	2.57	2.57	2.57	2.57	2.57
2.58	2.58	2.58	2.58	2.58	2.58	2.58	2.58	2.58
2.59	2.59	2.59	2.59	2.59	2.59	2.59	2.59	2.59
2.6	2.6	2.6	2.6	2.6	2.6	2.6	2.6	2.6
2.61	2.61	2.61	2.61	2.61	2.61	2.61	2.61	2.61
2.62	2.62	2.62	2.62	2.62	2.62	2.62	2.62	2.62
2.63	2.63	2.63	2.63	2.63	2.63	2.63	2.63	2.63
2.64	2.64	2.64	2.64	2.64	2.64	2.64	2.64	2.64
2.65	2.65	2.65	2.65	2.65	2.65	2.65	2.65	2.65
2.66	2.66	2.66	2.66	2.66	2.66	2.66	2.66	2.66
2.67	2.67	2.67	2.67	2.67	2.67	2.67	2.67	2.67
2.68	2.68	2.68	2.68	2.68	2.68	2.68	2.68	2.68
2.69	2.69	2.69	2.69	2.69	2.69	2.69	2.69	2.69
2.7	2.7	2.7	2.7	2.7	2.7	2.7	2.7	2.7
2.71	2.71	2.71	2.71	2.71	2.71	2.71	2.71	2.71
2.72	2.72	2.72	2.72	2.72	2.72	2.72	2.72	2.72
2.73	2.73	2.73	2.73	2.73	2.73	2.73	2.73	2.73
2.74	2.74	2.74	2.74	2.74	2.74	2.74	2.74	2.74
2.75	2.75	2.75	2.75	2.75	2.75	2.75	2.75	2.75
2.76	2.76	2.76	2.76	2.76	2.76	2.76	2.76	2.76
2.77	2.77	2.77	2.77	2.77	2.77	2.77	2.77	2.77
2.78	2.78	2.78	2.78	2.78	2.78	2.78	2.78	2.78
2.79	2.79	2.79	2.79	2.79	2.79	2.79	2.79	2.79
2.8	2.8	2.8	2.8	2.8	2.8	2.8	2.8	2.8
2.81	2.81	2.81	2.81	2.81	2.81	2.81	2.81	2.81
2.82	2.82	2.82	2.82	2.82	2.82	2.82	2.82	2.82
2.83	2.83	2.83	2.83	2.83	2.83	2.83	2.83	2.83
2.84	2.84	2.84	2.84	2.84	2.84	2.84	2.84	2.84
2.85	2.85	2.85	2.85	2.85	2.85	2.85	2.85	2.85
2.86	2.86	2.86	2.86	2.86	2.86	2.86	2.86	2.86
2.87	2.87	2.87	2.87	2.87	2.87	2.87	2.87	2.87
2.88	2.88	2.88	2.88	2.88	2.88	2.88	2.88	2.88
2.89	2.89	2.89	2.89	2.89	2.89	2.89	2.89	2.89
2.9	2.9	2.9	2.9	2.9	2.9	2.9	2.9	2.9
2.91	2.91	2.91	2.91	2.91	2.91	2.91	2.91	2.91
2.92	2.92	2.92	2.92	2.92	2.92	2.92	2.92	2.92
2.93	2.93	2.93	2.93	2.93	2.93	2.93	2.93	2.93
2.94	2.94	2.94	2.94	2.94	2.94	2.94	2.94	2.94
2.95	2.95	2.95	2.95	2.95	2.95	2.95	2.95	2.95
2.96	2.96	2.96	2.96	2.96	2.96	2.96	2.96	2.96
2.97	2.97	2.97	2.97	2.97	2.97	2.97	2.97	2.97
2.98	2.98	2.98	2.98	2.98	2.98	2.98	2.98	2.98
2.99	2.99	2.99	2.99	2.99	2.99	2.99	2.99	2.99

3	3	3	3	3	3	3	3	3
3.01	3.01	3.01	3.01	3.01	3.01	3.01	3.01	3.01
3.02	3.02	3.02	3.02	3.02	3.02	3.02	3.02	3.02
3.03	3.03	3.03	3.03	3.03	3.03	3.03	3.03	3.03
3.04	3.04	3.04	3.04	3.04	3.04	3.04	3.04	3.04
3.05	3.05	3.05	3.05	3.05	3.05	3.05	3.05	3.05
3.06	3.06	3.06	3.06	3.06	3.06	3.06	3.06	3.06
3.07	3.07	3.07	3.07	3.07	3.07	3.07	3.07	3.07
3.08	3.08	3.08	3.08	3.08	3.08	3.08	3.08	3.08
3.09	3.09	3.09	3.09	3.09	3.09	3.09	3.09	3.09
3.1	3.1	3.1	3.1	3.1	3.1	3.1	3.1	3.1
3.11	3.11	3.11	3.11	3.11	3.11	3.11	3.11	3.11
3.12	3.12	3.12	3.12	3.12	3.12	3.12	3.12	3.12
3.13	3.13	3.13	3.13	3.13	3.13	3.13	3.13	3.13
3.14	3.14	3.14	3.14	3.14	3.14	3.14	3.14	3.14
3.15	3.15	3.15	3.15	3.15	3.15	3.15	3.15	3.15
3.16	3.16	3.16	3.16	3.16	3.16	3.16	3.16	3.16
3.17	3.17	3.17	3.17	3.17	3.17	3.17	3.17	3.17
3.18	3.18	3.18	3.18	3.18	3.18	3.18	3.18	3.18
3.19	3.19	3.19	3.19	3.19	3.19	3.19	3.19	3.19
3.2	3.2	3.2	3.2	3.2	3.2	3.2	3.2	3.2
3.21	3.21	3.21	3.21	3.21	3.21	3.21	3.21	3.21
3.22	3.22	3.22	3.22	3.22	3.22	3.22	3.22	3.22
3.23	3.23	3.23	3.23	3.23	3.23	3.23	3.23	3.23
3.24	3.24	3.24	3.24	3.24	3.24	3.24	3.24	3.24
3.25	3.25	3.25	3.25	3.25	3.25	3.25	3.25	3.25
3.26	3.26	3.26	3.26	3.26	3.26	3.26	3.26	3.26
3.27	3.27	3.27	3.27	3.27	3.27	3.27	3.27	3.27
3.28	3.28	3.28	3.28	3.28	3.28	3.28	3.28	3.28
3.29	3.29	3.29	3.29	3.29	3.29	3.29	3.29	3.29
3.3	3.3	3.3	3.3	3.3	3.3	3.3	3.3	3.3
3.31	3.31	3.31	3.31	3.31	3.31	3.31	3.31	3.31
3.32	3.32	3.32	3.32	3.32	3.32	3.32	3.32	3.32
3.33	3.33	3.33	3.33	3.33	3.33	3.33	3.33	3.33
3.34	3.34	3.34	3.34	3.34	3.34	3.34	3.34	3.34
3.35	3.35	3.35	3.35	3.35	3.35	3.35	3.35	3.35
3.36	3.36	3.36	3.36	3.36	3.36	3.36	3.36	3.36
3.37	3.37	3.37	3.37	3.37	3.37	3.37	3.37	3.37
3.38	3.38	3.38	3.38	3.38	3.38	3.38	3.38	3.38
3.39	3.39	3.39	3.39	3.39	3.39	3.39	3.39	3.39
3.4	3.4	3.4	3.4	3.4	3.4	3.4	3.4	3.4
3.41	3.41	3.41	3.41	3.41	3.41	3.41	3.41	3.41
3.42	3.42	3.42	3.42	3.42	3.42	3.42	3.42	3.42
3.43	3.43	3.43	3.43	3.43	3.43	3.43	3.43	3.43
3.44	3.44	3.44	3.44	3.44	3.44	3.44	3.44	3.44
3.45	3.45	3.45	3.45	3.45	3.45	3.45	3.45	3.45
3.46	3.46	3.46	3.46	3.46	3.46	3.46	3.46	3.46
3.47	3.47	3.47	3.47	3.47	3.47	3.47	3.47	3.47
3.48	3.48	3.48	3.48	3.48	3.48	3.48	3.48	3.48
3.49	3.49	3.49	3.49	3.49	3.49	3.49	3.49	3.49

3.5	3.5	3.5	3.5	3.5	3.5	3.5	3.5	3.5
3.51	3.51	3.51	3.51	3.51	3.51	3.51	3.51	3.51
3.52	3.52	3.52	3.52	3.52	3.52	3.52	3.52	3.52
3.53	3.53	3.53	3.53	3.53	3.53	3.53	3.53	3.53
3.54	3.54	3.54	3.54	3.54	3.54	3.54	3.54	3.54
3.55	3.55	3.55	3.55	3.55	3.55	3.55	3.55	3.55
3.56	3.56	3.56	3.56	3.56	3.56	3.56	3.56	3.56
3.57	3.57	3.57	3.57	3.57	3.57	3.57	3.57	3.57
3.58	3.58	3.58	3.58	3.58	3.58	3.58	3.58	3.58
3.59	3.59	3.59	3.59	3.59	3.59	3.59	3.59	3.59
3.6	3.6	3.6	3.6	3.6	3.6	3.6	3.6	3.6
3.61	3.61	3.61	3.61	3.61	3.61	3.61	3.61	3.61
3.62	3.62	3.62	3.62	3.62	3.62	3.62	3.62	3.62
3.63	3.63	3.63	3.63	3.63	3.63	3.63	3.63	3.63
3.64	3.64	3.64	3.64	3.64	3.64	3.64	3.64	3.64
3.65	3.65	3.65	3.65	3.65	3.65	3.65	3.65	3.65
3.66	3.66	3.66	3.66	3.66	3.66	3.66	3.66	3.66
3.67	3.67	3.67	3.67	3.67	3.67	3.67	3.67	3.67
3.68	3.68	3.68	3.68	3.68	3.68	3.68	3.68	3.68
3.69	3.69	3.69	3.69	3.69	3.69	3.69	3.69	3.69
3.7	3.7	3.7	3.7	3.7	3.7	3.7	3.7	3.7
3.71	3.71	3.71	3.71	3.71	3.71	3.71	3.71	3.71
3.72	3.72	3.72	3.72	3.72	3.72	3.72	3.72	3.72
3.73	3.73	3.73	3.73	3.73	3.73	3.73	3.73	3.73
3.74	3.74	3.74	3.74	3.74	3.74	3.74	3.74	3.74
3.75	3.75	3.75	3.75	3.75	3.75	3.75	3.75	3.75
3.76	3.76	3.76	3.76	3.76	3.76	3.76	3.76	3.76
3.77	3.77	3.77	3.77	3.77	3.77	3.77	3.77	3.77
3.78	3.78	3.78	3.78	3.78	3.78	3.78	3.78	3.78
3.79	3.79	3.79	3.79	3.79	3.79	3.79	3.79	3.79
3.8	3.8	3.8	3.8	3.8	3.8	3.8	3.8	3.8
3.81	3.81	3.81	3.81	3.81	3.81	3.81	3.81	3.81
3.82	3.82	3.82	3.82	3.82	3.82	3.82	3.82	3.82
3.83	3.83	3.83	3.83	3.83	3.83	3.83	3.83	3.83
3.84	3.84	3.84	3.84	3.84	3.84	3.84	3.84	3.84
3.85	3.85	3.85	3.85	3.85	3.85	3.85	3.85	3.85
3.86	3.86	3.86	3.86	3.86	3.86	3.86	3.86	3.86
3.87	3.87	3.87	3.87	3.87	3.87	3.87	3.87	3.87
3.88	3.88	3.88	3.88	3.88	3.88	3.88	3.88	3.88
3.89	3.89	3.89	3.89	3.89	3.89	3.89	3.89	3.89
3.9	3.9	3.9	3.9	3.9	3.9	3.9	3.9	3.9
3.91	3.91	3.91	3.91	3.91	3.91	3.91	3.91	3.91
3.92	3.92	3.92	3.92	3.92	3.92	3.92	3.92	3.92
3.93	3.93	3.93	3.93	3.93	3.93	3.93	3.93	3.93
3.94	3.94	3.94	3.94	3.94	3.94	3.94	3.94	3.94
3.95	3.95	3.95	3.95	3.95	3.95	3.95	3.95	3.95
3.96	3.96	3.96	3.96	3.96	3.96	3.96	3.96	3.96
3.97	3.97	3.97	3.97	3.97	3.97	3.97	3.97	3.97
3.98	3.98	3.98	3.98	3.98	3.98	3.98	3.98	3.98
3.99	3.99	3.99	3.99	3.99	3.99	3.99	3.99	3.99

4	4	4	4	4	4	4	4	4
4.01	4.01	4.01	4.01	4.01	4.01	4.01	4.01	4.01
4.02	4.02	4.02	4.02	4.02	4.02	4.02	4.02	4.02
4.03	4.03	4.03	4.03	4.03	4.03	4.03	4.03	4.03
4.04	4.04	4.04	4.04	4.04	4.04	4.04	4.04	4.04
4.05	4.05	4.05	4.05	4.05	4.05	4.05	4.05	4.05
4.06	4.06	4.06	4.06	4.06	4.06	4.06	4.06	4.06
4.07	4.07	4.07	4.07	4.07	4.07	4.07	4.07	4.07
4.08	4.08	4.08	4.08	4.08	4.08	4.08	4.08	4.08
4.09	4.09	4.09	4.09	4.09	4.09	4.09	4.09	4.09
4.1	4.1	4.1	4.1	4.1	4.1	4.1	4.1	4.1
4.11	4.11	4.11	4.11	4.11	4.11	4.11	4.11	4.11
4.12	4.12	4.12	4.12	4.12	4.12	4.12	4.12	4.12
4.13	4.13	4.13	4.13	4.13	4.13	4.13	4.13	4.13
4.14	4.14	4.14	4.14	4.14	4.14	4.14	4.14	4.14
4.15	4.15	4.15	4.15	4.15	4.15	4.15	4.15	4.15
4.16	4.16	4.16	4.16	4.16	4.16	4.16	4.16	4.16
4.17	4.17	4.17	4.17	4.17	4.17	4.17	4.17	4.17
4.18	4.18	4.18	4.18	4.18	4.18	4.18	4.18	4.18
4.19	4.19	4.19	4.19	4.19	4.19	4.19	4.19	4.19
4.2	4.2	4.2	4.2	4.2	4.2	4.2	4.2	4.2
4.21	4.21	4.21	4.21	4.21	4.21	4.21	4.21	4.21
4.22	4.22	4.22	4.22	4.22	4.22	4.22	4.22	4.22
4.23	4.23	4.23	4.23	4.23	4.23	4.23	4.23	4.23
4.24	4.24	4.24	4.24	4.24	4.24	4.24	4.24	4.24
4.25	4.25	4.25	4.25	4.25	4.25	4.25	4.25	4.25
4.26	4.26	4.26	4.26	4.26	4.26	4.26	4.26	4.26
4.27	4.27	4.27	4.27	4.27	4.27	4.27	4.27	4.27
4.28	4.28	4.28	4.28	4.28	4.28	4.28	4.28	4.28
4.29	4.29	4.29	4.29	4.29	4.29	4.29	4.29	4.29
4.3	4.3	4.3	4.3	4.3	4.3	4.3	4.3	4.3
4.31	4.31	4.31	4.31	4.31	4.31	4.31	4.31	4.31
4.32	4.32	4.32	4.32	4.32	4.32	4.32	4.32	4.32
4.33	4.33	4.33	4.33	4.33	4.33	4.33	4.33	4.33
4.34	4.34	4.34	4.34	4.34	4.34	4.34	4.34	4.34
4.35	4.35	4.35	4.35	4.35	4.35	4.35	4.35	4.35
4.36	4.36	4.36	4.36	4.36	4.36	4.36	4.36	4.36
4.37	4.37	4.37	4.37	4.37	4.37	4.37	4.37	4.37
4.38	4.38	4.38	4.38	4.38	4.38	4.38	4.38	4.38
4.39	4.39	4.39	4.39	4.39	4.39	4.39	4.39	4.39
4.4	4.4	4.4	4.4	4.4	4.4	4.4	4.4	4.4
4.41	4.41	4.41	4.41	4.41	4.41	4.41	4.41	4.41
4.42	4.42	4.42	4.42	4.42	4.42	4.42	4.42	4.42
4.43	4.43	4.43	4.43	4.43	4.43	4.43	4.43	4.43
4.44	4.44	4.44	4.44	4.44	4.44	4.44	4.44	4.44
4.45	4.45	4.45	4.45	4.45	4.45	4.45	4.45	4.45
4.46	4.46	4.46	4.46	4.46	4.46	4.46	4.46	4.46
4.47	4.47	4.47	4.47	4.47	4.47	4.47	4.47	4.47
4.48	4.48	4.48	4.48	4.48	4.48	4.48	4.48	4.48
4.49	4.49	4.49	4.49	4.49	4.49	4.49	4.49	4.49

4.5	4.5	4.5	4.5	4.5	4.5	4.5	4.5	4.5
4.51	4.51	4.51	4.51	4.51	4.51	4.51	4.51	4.51
4.52	4.52	4.52	4.52	4.52	4.52	4.52	4.52	4.52
4.53	4.53	4.53	4.53	4.53	4.53	4.53	4.53	4.53
4.54	4.54	4.54	4.54	4.54	4.54	4.54	4.54	4.54
4.55	4.55	4.55	4.55	4.55	4.55	4.55	4.55	4.55
4.56	4.56	4.56	4.56	4.56	4.56	4.56	4.56	4.56
4.57	4.57	4.57	4.57	4.57	4.57	4.57	4.57	4.57
4.58	4.58	4.58	4.58	4.58	4.58	4.58	4.58	4.58
4.59	4.59	4.59	4.59	4.59	4.59	4.59	4.59	4.59
4.6	4.6	4.6	4.6	4.6	4.6	4.6	4.6	4.6
4.61	4.61	4.61	4.61	4.61	4.61	4.61	4.61	4.61
4.62	4.62	4.62	4.62	4.62	4.62	4.62	4.62	4.62
4.63	4.63	4.63	4.63	4.63	4.63	4.63	4.63	4.63
4.64	4.64	4.64	4.64	4.64	4.64	4.64	4.64	4.64
4.65	4.65	4.65	4.65	4.65	4.65	4.65	4.65	4.65
4.66	4.66	4.66	4.66	4.66	4.66	4.66	4.66	4.66
4.67	4.67	4.67	4.67	4.67	4.67	4.67	4.67	4.67
4.68	4.68	4.68	4.68	4.68	4.68	4.68	4.68	4.68
4.69	4.69	4.69	4.69	4.69	4.69	4.69	4.69	4.69
4.7	4.7	4.7	4.7	4.7	4.7	4.7	4.7	4.7
4.71	4.71	4.71	4.71	4.71	4.71	4.71	4.71	4.71
4.72	4.72	4.72	4.72	4.72	4.72	4.72	4.72	4.72
4.73	4.73	4.73	4.73	4.73	4.73	4.73	4.73	4.73
4.74	4.74	4.74	4.74	4.74	4.74	4.74	4.74	4.74
4.75	4.75	4.75	4.75	4.75	4.75	4.75	4.75	4.75
4.76	4.76	4.76	4.76	4.76	4.76	4.76	4.76	4.76
4.77	4.77	4.77	4.77	4.77	4.77	4.77	4.77	4.77
4.78	4.78	4.78	4.78	4.78	4.78	4.78	4.78	4.78
4.79	4.79	4.79	4.79	4.79	4.79	4.79	4.79	4.79
4.8	4.8	4.8	4.8	4.8	4.8	4.8	4.8	4.8
4.81	4.81	4.81	4.81	4.81	4.81	4.81	4.81	4.81
4.82	4.82	4.82	4.82	4.82	4.82	4.82	4.82	4.82
4.83	4.83	4.83	4.83	4.83	4.83	4.83	4.83	4.83
4.84	4.84	4.84	4.84	4.84	4.84	4.84	4.84	4.84
4.85	4.85	4.85	4.85	4.85	4.85	4.85	4.85	4.85
4.86	4.86	4.86	4.86	4.86	4.86	4.86	4.86	4.86
4.87	4.87	4.87	4.87	4.87	4.87	4.87	4.87	4.87
4.88	4.88	4.88	4.88	4.88	4.88	4.88	4.88	4.88
4.89	4.89	4.89	4.89	4.89	4.89	4.89	4.89	4.89
4.9	4.9	4.9	4.9	4.9	4.9	4.9	4.9	4.9
4.91	4.91	4.91	4.91	4.91	4.91	4.91	4.91	4.91
4.92	4.92	4.92	4.92	4.92	4.92	4.92	4.92	4.92
4.93	4.93	4.93	4.93	4.93	4.93	4.93	4.93	4.93
4.94	4.94	4.94	4.94	4.94	4.94	4.94	4.94	4.94
4.95	4.95	4.95	4.95	4.95	4.95	4.95	4.95	4.95
4.96	4.96	4.96	4.96	4.96	4.96	4.96	4.96	4.96
4.97	4.97	4.97	4.97	4.97	4.97	4.97	4.97	4.97
4.98	4.98	4.98	4.98	4.98	4.98	4.98	4.98	4.98
4.99	4.99	4.99	4.99	4.99	4.99	4.99	4.99	4.99

5	5	5	5	5	5	5	5	5
5.01	5.01	5.01	5.01	5.01	5.01	5.01	5.01	5.01
5.02	5.02	5.02	5.02	5.02	5.02	5.02	5.02	5.02
5.03	5.03	5.03	5.03	5.03	5.03	5.03	5.03	5.03
5.04	5.04	5.04	5.04	5.04	5.04	5.04	5.04	5.04
5.05	5.05	5.05	5.05	5.05	5.05	5.05	5.05	5.05
5.06	5.06	5.06	5.06	5.06	5.06	5.06	5.06	5.06
5.07	5.07	5.07	5.07	5.07	5.07	5.07	5.07	5.07
5.08	5.08	5.08	5.08	5.08	5.08	5.08	5.08	5.08
5.09	5.09	5.09	5.09	5.09	5.09	5.09	5.09	5.09
5.1	5.1	5.1	5.1	5.1	5.1	5.1	5.1	5.1
5.11	5.11	5.11	5.11	5.11	5.11	5.11	5.11	5.11
5.12	5.12	5.12	5.12	5.12	5.12	5.12	5.12	5.12
5.13	5.13	5.13	5.13	5.13	5.13	5.13	5.13	5.13
5.14	5.14	5.14	5.14	5.14	5.14	5.14	5.14	5.14
5.15	5.15	5.15	5.15	5.15	5.15	5.15	5.15	5.15
5.16	5.16	5.16	5.16	5.16	5.16	5.16	5.16	5.16
5.17	5.17	5.17	5.17	5.17	5.17	5.17	5.17	5.17
5.18	5.18	5.18	5.18	5.18	5.18	5.18	5.18	5.18
5.19	5.19	5.19	5.19	5.19	5.19	5.19	5.19	5.19
5.2	5.2	5.2	5.2	5.2	5.2	5.2	5.2	5.2
5.21	5.21	5.21	5.21	5.21	5.21	5.21	5.21	5.21
5.22	5.22	5.22	5.22	5.22	5.22	5.22	5.22	5.22
5.23	5.23	5.23	5.23	5.23	5.23	5.23	5.23	5.23
5.24	5.24	5.24	5.24	5.24	5.24	5.24	5.24	5.24
5.25	5.25	5.25	5.25	5.25	5.25	5.25	5.25	5.25
5.26	5.26	5.26	5.26	5.26	5.26	5.26	5.26	5.26
5.27	5.27	5.27	5.27	5.27	5.27	5.27	5.27	5.27
5.28	5.28	5.28	5.28	5.28	5.28	5.28	5.28	5.28
5.29	5.29	5.29	5.29	5.29	5.29	5.29	5.29	5.29
5.3	5.3	5.3	5.3	5.3	5.3	5.3	5.3	5.3
5.31	5.31	5.31	5.31	5.31	5.31	5.31	5.31	5.31
5.32	5.32	5.32	5.32	5.32	5.32	5.32	5.32	5.32
5.33	5.33	5.33	5.33	5.33	5.33	5.33	5.33	5.33
5.34	5.34	5.34	5.34	5.34	5.34	5.34	5.34	5.34
5.35	5.35	5.35	5.35	5.35	5.35	5.35	5.35	5.35
5.36	5.36	5.36	5.36	5.36	5.36	5.36	5.36	5.36
5.37	5.37	5.37	5.37	5.37	5.37	5.37	5.37	5.37
5.38	5.38	5.38	5.38	5.38	5.38	5.38	5.38	5.38
5.39	5.39	5.39	5.39	5.39	5.39	5.39	5.39	5.39
5.4	5.4	5.4	5.4	5.4	5.4	5.4	5.4	5.4
5.41	5.41	5.41	5.41	5.41	5.41	5.41	5.41	5.41
5.42	5.42	5.42	5.42	5.42	5.42	5.42	5.42	5.42
5.43	5.43	5.43	5.43	5.43	5.43	5.43	5.43	5.43
5.44	5.44	5.44	5.44	5.44	5.44	5.44	5.44	5.44
5.45	5.45	5.45	5.45	5.45	5.45	5.45	5.45	5.45
5.46	5.46	5.46	5.46	5.46	5.46	5.46	5.46	5.46
5.47	5.47	5.47	5.47	5.47	5.47	5.47	5.47	5.47
5.48	5.48	5.48	5.48	5.48	5.48	5.48	5.48	5.48
5.49	5.49	5.49	5.49	5.49	5.49	5.49	5.49	5.49

5.5	5.5	5.5	5.5	5.5	5.5	5.5	5.5	5.5
5.51	5.51	5.51	5.51	5.51	5.51	5.51	5.51	5.51
5.52	5.52	5.52	5.52	5.52	5.52	5.52	5.52	5.52
5.53	5.53	5.53	5.53	5.53	5.53	5.53	5.53	5.53
5.54	5.54	5.54	5.54	5.54	5.54	5.54	5.54	5.54
5.55	5.55	5.55	5.55	5.55	5.55	5.55	5.55	5.55
5.56	5.56	5.56	5.56	5.56	5.56	5.56	5.56	5.56
5.57	5.57	5.57	5.57	5.57	5.57	5.57	5.57	5.57
5.58	5.58	5.58	5.58	5.58	5.58	5.58	5.58	5.58
5.59	5.59	5.59	5.59	5.59	5.59	5.59	5.59	5.59
5.6	5.6	5.6	5.6	5.6	5.6	5.6	5.6	5.6
5.61	5.61	5.61	5.61	5.61	5.61	5.61	5.61	5.61
5.62	5.62	5.62	5.62	5.62	5.62	5.62	5.62	5.62
5.63	5.63	5.63	5.63	5.63	5.63	5.63	5.63	5.63
5.64	5.64	5.64	5.64	5.64	5.64	5.64	5.64	5.64
5.65	5.65	5.65	5.65	5.65	5.65	5.65	5.65	5.65
5.66	5.66	5.66	5.66	5.66	5.66	5.66	5.66	5.66
5.67	5.67	5.67	5.67	5.67	5.67	5.67	5.67	5.67
5.68	5.68	5.68	5.68	5.68	5.68	5.68	5.68	5.68
5.69	5.69	5.69	5.69	5.69	5.69	5.69	5.69	5.69
5.7	5.7	5.7	5.7	5.7	5.7	5.7	5.7	5.7
5.71	5.71	5.71	5.71	5.71	5.71	5.71	5.71	5.71
5.72	5.72	5.72	5.72	5.72	5.72	5.72	5.72	5.72
5.73	5.73	5.73	5.73	5.73	5.73	5.73	5.73	5.73
5.74	5.74	5.74	5.74	5.74	5.74	5.74	5.74	5.74
5.75	5.75	5.75	5.75	5.75	5.75	5.75	5.75	5.75
5.76	5.76	5.76	5.76	5.76	5.76	5.76	5.76	5.76
5.77	5.77	5.77	5.77	5.77	5.77	5.77	5.77	5.77
5.78	5.78	5.78	5.78	5.78	5.78	5.78	5.78	5.78
5.79	5.79	5.79	5.79	5.79	5.79	5.79	5.79	5.79
5.8	5.8	5.8	5.8	5.8	5.8	5.8	5.8	5.8
5.81	5.81	5.81	5.81	5.81	5.81	5.81	5.81	5.81
5.82	5.82	5.82	5.82	5.82	5.82	5.82	5.82	5.82
5.83	5.83	5.83	5.83	5.83	5.83	5.83	5.83	5.83
5.84	5.84	5.84	5.84	5.84	5.84	5.84	5.84	5.84
5.85	5.85	5.85	5.85	5.85	5.85	5.85	5.85	5.85
5.86	5.86	5.86	5.86	5.86	5.86	5.86	5.86	5.86
5.87	5.87	5.87	5.87	5.87	5.87	5.87	5.87	5.87
5.88	5.88	5.88	5.88	5.88	5.88	5.88	5.88	5.88
5.89	5.89	5.89	5.89	5.89	5.89	5.89	5.89	5.89
5.9	5.9	5.9	5.9	5.9	5.9	5.9	5.9	5.9
5.91	5.91	5.91	5.91	5.91	5.91	5.91	5.91	5.91
5.92	5.92	5.92	5.92	5.92	5.92	5.92	5.92	5.92
5.93	5.93	5.93	5.93	5.93	5.93	5.93	5.93	5.93
5.94	5.94	5.94	5.94	5.94	5.94	5.94	5.94	5.94
5.95	5.95	5.95	5.95	5.95	5.95	5.95	5.95	5.95
5.96	5.96	5.96	5.96	5.96	5.96	5.96	5.96	5.96
5.97	5.97	5.97	5.97	5.97	5.97	5.97	5.97	5.97
5.98	5.98	5.98	5.98	5.98	5.98	5.98	5.98	5.98
5.99	5.99	5.99	5.99	5.99	5.99	5.99	5.99	5.99

6	6	6	6	6	6	6	6	6
6.01	6.01	6.01	6.01	6.01	6.01	6.01	6.01	6.01
6.02	6.02	6.02	6.02	6.02	6.02	6.02	6.02	6.02
6.03	6.03	6.03	6.03	6.03	6.03	6.03	6.03	6.03
6.04	6.04	6.04	6.04	6.04	6.04	6.04	6.04	6.04
6.05	6.05	6.05	6.05	6.05	6.05	6.05	6.05	6.05
6.06	6.06	6.06	6.06	6.06	6.06	6.06	6.06	6.06
6.07	6.07	6.07	6.07	6.07	6.07	6.07	6.07	6.07
6.08	6.08	6.08	6.08	6.08	6.08	6.08	6.08	6.08
6.09	6.09	6.09	6.09	6.09	6.09	6.09	6.09	6.09
6.1	6.1	6.1	6.1	6.1	6.1	6.1	6.1	6.1
6.11	6.11	6.11	6.11	6.11	6.11	6.11	6.11	6.11
6.12	6.12	6.12	6.12	6.12	6.12	6.12	6.12	6.12
6.13	6.13	6.13	6.13	6.13	6.13	6.13	6.13	6.13
6.14	6.14	6.14	6.14	6.14	6.14	6.14	6.14	6.14
6.15	6.15	6.15	6.15	6.15	6.15	6.15	6.15	6.15
6.16	6.16	6.16	6.16	6.16	6.16	6.16	6.16	6.16
6.17	6.17	6.17	6.17	6.17	6.17	6.17	6.17	6.17
6.18	6.18	6.18	6.18	6.18	6.18	6.18	6.18	6.18
6.19	6.19	6.19	6.19	6.19	6.19	6.19	6.19	6.19
6.2	6.2	6.2	6.2	6.2	6.2	6.2	6.2	6.2
6.21	6.21	6.21	6.21	6.21	6.21	6.21	6.21	6.21
6.22	6.22	6.22	6.22	6.22	6.22	6.22	6.22	6.22
6.23	6.23	6.23	6.23	6.23	6.23	6.23	6.23	6.23
6.24	6.24	6.24	6.24	6.24	6.24	6.24	6.24	6.24
6.25	6.25	6.25	6.25	6.25	6.25	6.25	6.25	6.25
6.26	6.26	6.26	6.26	6.26	6.26	6.26	6.26	6.26
6.27	6.27	6.27	6.27	6.27	6.27	6.27	6.27	6.27
6.28	6.28	6.28	6.28	6.28	6.28	6.28	6.28	6.28
6.29	6.29	6.29	6.29	6.29	6.29	6.29	6.29	6.29
6.3	6.3	6.3	6.3	6.3	6.3	6.3	6.3	6.3
6.31	6.31	6.31	6.31	6.31	6.31	6.31	6.31	6.31
6.32	6.32	6.32	6.32	6.32	6.32	6.32	6.32	6.32
6.33	6.33	6.33	6.33	6.33	6.33	6.33	6.33	6.33
6.34	6.34	6.34	6.34	6.34	6.34	6.34	6.34	6.34
6.35	6.35	6.35	6.35	6.35	6.35	6.35	6.35	6.35
6.36	6.36	6.36	6.36	6.36	6.36	6.36	6.36	6.36
6.37	6.37	6.37	6.37	6.37	6.37	6.37	6.37	6.37
6.38	6.38	6.38	6.38	6.38	6.38	6.38	6.38	6.38
6.39	6.39	6.39	6.39	6.39	6.39	6.39	6.39	6.39
6.4	6.4	6.4	6.4	6.4	6.4	6.4	6.4	6.4
6.41	6.41	6.41	6.41	6.41	6.41	6.41	6.41	6.41
6.42	6.42	6.42	6.42	6.42	6.42	6.42	6.42	6.42
6.43	6.43	6.43	6.43	6.43	6.43	6.43	6.43	6.43
6.44	6.44	6.44	6.44	6.44	6.44	6.44	6.44	6.44
6.45	6.45	6.45	6.45	6.45	6.45	6.45	6.45	6.45
6.46	6.46	6.46	6.46	6.46	6.46	6.46	6.46	6.46
6.47	6.47	6.47	6.47	6.47	6.47	6.47	6.47	6.47
6.48	6.48	6.48	6.48	6.48	6.48	6.48	6.48	6.48
6.49	6.49	6.49	6.49	6.49	6.49	6.49	6.49	6.49

6.5	6.5	6.5	6.5	6.5	6.5	6.5	6.5	6.5
6.51	6.51	6.51	6.51	6.51	6.51	6.51	6.51	6.51
6.52	6.52	6.52	6.52	6.52	6.52	6.52	6.52	6.52
6.53	6.53	6.53	6.53	6.53	6.53	6.53	6.53	6.53
6.54	6.54	6.54	6.54	6.54	6.54	6.54	6.54	6.54
6.55	6.55	6.55	6.55	6.55	6.55	6.55	6.55	6.55
6.56	6.56	6.56	6.56	6.56	6.56	6.56	6.56	6.56
6.57	6.57	6.57	6.57	6.57	6.57	6.57	6.57	6.57
6.58	6.58	6.58	6.58	6.58	6.58	6.58	6.58	6.58
6.59	6.59	6.59	6.59	6.59	6.59	6.59	6.59	6.59
6.6	6.6	6.6	6.6	6.6	6.6	6.6	6.6	6.6
6.61	6.61	6.61	6.61	6.61	6.61	6.61	6.61	6.61
6.62	6.62	6.62	6.62	6.62	6.62	6.62	6.62	6.62
6.63	6.63	6.63	6.63	6.63	6.63	6.63	6.63	6.63
6.64	6.64	6.64	6.64	6.64	6.64	6.64	6.64	6.64
6.65	6.65	6.65	6.65	6.65	6.65	6.65	6.65	6.65
6.66	6.66	6.66	6.66	6.66	6.66	6.66	6.66	6.66
6.67	6.67	6.67	6.67	6.67	6.67	6.67	6.67	6.67
6.68	6.68	6.68	6.68	6.68	6.68	6.68	6.68	6.68
6.69	6.69	6.69	6.69	6.69	6.69	6.69	6.69	6.69
6.7	6.7	6.7	6.7	6.7	6.7	6.7	6.7	6.7
6.71	6.71	6.71	6.71	6.71	6.71	6.71	6.71	6.71
6.72	6.72	6.72	6.72	6.72	6.72	6.72	6.72	6.72
6.73	6.73	6.73	6.73	6.73	6.73	6.73	6.73	6.73
6.74	6.74	6.74	6.74	6.74	6.74	6.74	6.74	6.74
6.75	6.75	6.75	6.75	6.75	6.75	6.75	6.75	6.75
6.76	6.76	6.76	6.76	6.76	6.76	6.76	6.76	6.76
6.77	6.77	6.77	6.77	6.77	6.77	6.77	6.77	6.77
6.78	6.78	6.78	6.78	6.78	6.78	6.78	6.78	6.78
6.79	6.79	6.79	6.79	6.79	6.79	6.79	6.79	6.79
6.8	6.8	6.8	6.8	6.8	6.8	6.8	6.8	6.8
6.81	6.81	6.81	6.81	6.81	6.81	6.81	6.81	6.81
6.82	6.82	6.82	6.82	6.82	6.82	6.82	6.82	6.82
6.83	6.83	6.83	6.83	6.83	6.83	6.83	6.83	6.83
6.84	6.84	6.84	6.84	6.84	6.84	6.84	6.84	6.84
6.85	6.85	6.85	6.85	6.85	6.85	6.85	6.85	6.85
6.86	6.86	6.86	6.86	6.86	6.86	6.86	6.86	6.86
6.87	6.87	6.87	6.87	6.87	6.87	6.87	6.87	6.87
6.88	6.88	6.88	6.88	6.88	6.88	6.88	6.88	6.88
6.89	6.89	6.89	6.89	6.89	6.89	6.89	6.89	6.89
6.9	6.9	6.9	6.9	6.9	6.9	6.9	6.9	6.9
6.91	6.91	6.91	6.91	6.91	6.91	6.91	6.91	6.91
6.92	6.92	6.92	6.92	6.92	6.92	6.92	6.92	6.92
6.93	6.93	6.93	6.93	6.93	6.93	6.93	6.93	6.93
6.94	6.94	6.94	6.94	6.94	6.94	6.94	6.94	6.94
6.95	6.95	6.95	6.95	6.95	6.95	6.95	6.95	6.95
6.96	6.96	6.96	6.96	6.96	6.96	6.96	6.96	6.96
6.97	6.97	6.97	6.97	6.97	6.97	6.97	6.97	6.97
6.98	6.98	6.98	6.98	6.98	6.98	6.98	6.98	6.98
6.99	6.99	6.99	6.99	6.99	6.99	6.99	6.99	6.99

7	7	7	7	7	7	7	7	7
7.01	7.01	7.01	7.01	7.01	7.01	7.01	7.01	7.01
7.02	7.02	7.02	7.02	7.02	7.02	7.02	7.02	7.02
7.03	7.03	7.03	7.03	7.03	7.03	7.03	7.03	7.03
7.04	7.04	7.04	7.04	7.04	7.04	7.04	7.04	7.04
7.05	7.05	7.05	7.05	7.05	7.05	7.05	7.05	7.05
7.06	7.06	7.06	7.06	7.06	7.06	7.06	7.06	7.06
7.07	7.07	7.07	7.07	7.07	7.07	7.07	7.07	7.07
7.08	7.08	7.08	7.08	7.08	7.08	7.08	7.08	7.08
7.09	7.09	7.09	7.09	7.09	7.09	7.09	7.09	7.09
7.1	7.1	7.1	7.1	7.1	7.1	7.1	7.1	7.1
7.11	7.11	7.11	7.11	7.11	7.11	7.11	7.11	7.11
7.12	7.12	7.12	7.12	7.12	7.12	7.12	7.12	7.12
7.13	7.13	7.13	7.13	7.13	7.13	7.13	7.13	7.13
7.14	7.14	7.14	7.14	7.14	7.14	7.14	7.14	7.14
7.15	7.15	7.15	7.15	7.15	7.15	7.15	7.15	7.15
7.16	7.16	7.16	7.16	7.16	7.16	7.16	7.16	7.16
7.17	7.17	7.17	7.17	7.17	7.17	7.17	7.17	7.17
7.18	7.18	7.18	7.18	7.18	7.18	7.18	7.18	7.18
7.19	7.19	7.19	7.19	7.19	7.19	7.19	7.19	7.19
7.2	7.2	7.2	7.2	7.2	7.2	7.2	7.2	7.2
7.21	7.21	7.21	7.21	7.21	7.21	7.21	7.21	7.21
7.22	7.22	7.22	7.22	7.22	7.22	7.22	7.22	7.22
7.23	7.23	7.23	7.23	7.23	7.23	7.23	7.23	7.23
7.24	7.24	7.24	7.24	7.24	7.24	7.24	7.24	7.24
7.25	7.25	7.25	7.25	7.25	7.25	7.25	7.25	7.25
7.26	7.26	7.26	7.26	7.26	7.26	7.26	7.26	7.26
7.27	7.27	7.27	7.27	7.27	7.27	7.27	7.27	7.27
7.28	7.28	7.28	7.28	7.28	7.28	7.28	7.28	7.28
7.29	7.29	7.29	7.29	7.29	7.29	7.29	7.29	7.29
7.3	7.3	7.3	7.3	7.3	7.3	7.3	7.3	7.3
7.31	7.31	7.31	7.31	7.31	7.31	7.31	7.31	7.31
7.32	7.32	7.32	7.32	7.32	7.32	7.32	7.32	7.32
7.33	7.33	7.33	7.33	7.33	7.33	7.33	7.33	7.33
7.34	7.34	7.34	7.34	7.34	7.34	7.34	7.34	7.34
7.35	7.35	7.35	7.35	7.35	7.35	7.35	7.35	7.35
7.36	7.36	7.36	7.36	7.36	7.36	7.36	7.36	7.36
7.37	7.37	7.37	7.37	7.37	7.37	7.37	7.37	7.37
7.38	7.38	7.38	7.38	7.38	7.38	7.38	7.38	7.38
7.39	7.39	7.39	7.39	7.39	7.39	7.39	7.39	7.39
7.4	7.4	7.4	7.4	7.4	7.4	7.4	7.4	7.4
7.41	7.41	7.41	7.41	7.41	7.41	7.41	7.41	7.41
7.42	7.42	7.42	7.42	7.42	7.42	7.42	7.42	7.42
7.43	7.43	7.43	7.43	7.43	7.43	7.43	7.43	7.43
7.44	7.44	7.44	7.44	7.44	7.44	7.44	7.44	7.44
7.45	7.45	7.45	7.45	7.45	7.45	7.45	7.45	7.45
7.46	7.46	7.46	7.46	7.46	7.46	7.46	7.46	7.46
7.47	7.47	7.47	7.47	7.47	7.47	7.47	7.47	7.47
7.48	7.48	7.48	7.48	7.48	7.48	7.48	7.48	7.48
7.49	7.49	7.49	7.49	7.49	7.49	7.49	7.49	7.49

7.5	7.5	7.5	7.5	7.5	7.5	7.5	7.5	7.5
7.51	7.51	7.51	7.51	7.51	7.51	7.51	7.51	7.51
7.52	7.52	7.52	7.52	7.52	7.52	7.52	7.52	7.52
7.53	7.53	7.53	7.53	7.53	7.53	7.53	7.53	7.53
7.54	7.54	7.54	7.54	7.54	7.54	7.54	7.54	7.54
7.55	7.55	7.55	7.55	7.55	7.55	7.55	7.55	7.55
7.56	7.56	7.56	7.56	7.56	7.56	7.56	7.56	7.56
7.57	7.57	7.57	7.57	7.57	7.57	7.57	7.57	7.57
7.58	7.58	7.58	7.58	7.58	7.58	7.58	7.58	7.58
7.59	7.59	7.59	7.59	7.59	7.59	7.59	7.59	7.59
7.6	7.6	7.6	7.6	7.6	7.6	7.6	7.6	7.6
7.61	7.61	7.61	7.61	7.61	7.61	7.61	7.61	7.61
7.62	7.62	7.62	7.62	7.62	7.62	7.62	7.62	7.62
7.63	7.63	7.63	7.63	7.63	7.63	7.63	7.63	7.63
7.64	7.64	7.64	7.64	7.64	7.64	7.64	7.64	7.64
7.65	7.65	7.65	7.65	7.65	7.65	7.65	7.65	7.65
7.66	7.66	7.66	7.66	7.66	7.66	7.66	7.66	7.66
7.67	7.67	7.67	7.67	7.67	7.67	7.67	7.67	7.67
7.68	7.68	7.68	7.68	7.68	7.68	7.68	7.68	7.68
7.69	7.69	7.69	7.69	7.69	7.69	7.69	7.69	7.69
7.7	7.7	7.7	7.7	7.7	7.7	7.7	7.7	7.7
7.71	7.71	7.71	7.71	7.71	7.71	7.71	7.71	7.71
7.72	7.72	7.72	7.72	7.72	7.72	7.72	7.72	7.72
7.73	7.73	7.73	7.73	7.73	7.73	7.73	7.73	7.73
7.74	7.74	7.74	7.74	7.74	7.74	7.74	7.74	7.74
7.75	7.75	7.75	7.75	7.75	7.75	7.75	7.75	7.75
7.76	7.76	7.76	7.76	7.76	7.76	7.76	7.76	7.76
7.77	7.77	7.77	7.77	7.77	7.77	7.77	7.77	7.77
7.78	7.78	7.78	7.78	7.78	7.78	7.78	7.78	7.78
7.79	7.79	7.79	7.79	7.79	7.79	7.79	7.79	7.79
7.8	7.8	7.8	7.8	7.8	7.8	7.8	7.8	7.8
7.81	7.81	7.81	7.81	7.81	7.81	7.81	7.81	7.81
7.82	7.82	7.82	7.82	7.82	7.82	7.82	7.82	7.82
7.83	7.83	7.83	7.83	7.83	7.83	7.83	7.83	7.83
7.84	7.84	7.84	7.84	7.84	7.84	7.84	7.84	7.84
7.85	7.85	7.85	7.85	7.85	7.85	7.85	7.85	7.85
7.86	7.86	7.86	7.86	7.86	7.86	7.86	7.86	7.86
7.87	7.87	7.87	7.87	7.87	7.87	7.87	7.87	7.87
7.88	7.88	7.88	7.88	7.88	7.88	7.88	7.88	7.88
7.89	7.89	7.89	7.89	7.89	7.89	7.89	7.89	7.89
7.9	7.9	7.9	7.9	7.9	7.9	7.9	7.9	7.9
7.91	7.91	7.91	7.91	7.91	7.91	7.91	7.91	7.91
7.92	7.92	7.92	7.92	7.92	7.92	7.92	7.92	7.92
7.93	7.93	7.93	7.93	7.93	7.93	7.93	7.93	7.93
7.94	7.94	7.94	7.94	7.94	7.94	7.94	7.94	7.94
7.95	7.95	7.95	7.95	7.95	7.95	7.95	7.95	7.95
7.96	7.96	7.96	7.96	7.96	7.96	7.96	7.96	7.96
7.97	7.97	7.97	7.97	7.97	7.97	7.97	7.97	7.97
7.98	7.98	7.98	7.98	7.98	7.98	7.98	7.98	7.98
7.99	7.99	7.99	7.99	7.99	7.99	7.99	7.99	7.99

8	8	8	8	8	8	8	8	8
8.01	8.01	8.01	8.01	8.01	8.01	8.01	8.01	8.01
8.02	8.02	8.02	8.02	8.02	8.02	8.02	8.02	8.02
8.03	8.03	8.03	8.03	8.03	8.03	8.03	8.03	8.03
8.04	8.04	8.04	8.04	8.04	8.04	8.04	8.04	8.04
8.05	8.05	8.05	8.05	8.05	8.05	8.05	8.05	8.05
8.06	8.06	8.06	8.06	8.06	8.06	8.06	8.06	8.06
8.07	8.07	8.07	8.07	8.07	8.07	8.07	8.07	8.07
8.08	8.08	8.08	8.08	8.08	8.08	8.08	8.08	8.08
8.09	8.09	8.09	8.09	8.09	8.09	8.09	8.09	8.09
8.1	8.1	8.1	8.1	8.1	8.1	8.1	8.1	8.1
8.11	8.11	8.11	8.11	8.11	8.11	8.11	8.11	8.11
8.12	8.12	8.12	8.12	8.12	8.12	8.12	8.12	8.12
8.13	8.13	8.13	8.13	8.13	8.13	8.13	8.13	8.13
8.14	8.14	8.14	8.14	8.14	8.14	8.14	8.14	8.14
8.15	8.15	8.15	8.15	8.15	8.15	8.15	8.15	8.15
8.16	8.16	8.16	8.16	8.16	8.16	8.16	8.16	8.16
8.17	8.17	8.17	8.17	8.17	8.17	8.17	8.17	8.17
8.18	8.18	8.18	8.18	8.18	8.18	8.18	8.18	8.18
8.19	8.19	8.19	8.19	8.19	8.19	8.19	8.19	8.19
8.2	8.2	8.2	8.2	8.2	8.2	8.2	8.2	8.2
8.21	8.21	8.21	8.21	8.21	8.21	8.21	8.21	8.21
8.22	8.22	8.22	8.22	8.22	8.22	8.22	8.22	8.22
8.23	8.23	8.23	8.23	8.23	8.23	8.23	8.23	8.23
8.24	8.24	8.24	8.24	8.24	8.24	8.24	8.24	8.24
8.25	8.25	8.25	8.25	8.25	8.25	8.25	8.25	8.25
8.26	8.26	8.26	8.26	8.26	8.26	8.26	8.26	8.26
8.27	8.27	8.27	8.27	8.27	8.27	8.27	8.27	8.27
8.28	8.28	8.28	8.28	8.28	8.28	8.28	8.28	8.28
8.29	8.29	8.29	8.29	8.29	8.29	8.29	8.29	8.29
8.3	8.3	8.3	8.3	8.3	8.3	8.3	8.3	8.3
8.31	8.31	8.31	8.31	8.31	8.31	8.31	8.31	8.31
8.32	8.32	8.32	8.32	8.32	8.32	8.32	8.32	8.32
8.33	8.33	8.33	8.33	8.33	8.33	8.33	8.33	8.33
8.34	8.34	8.34	8.34	8.34	8.34	8.34	8.34	8.34
8.35	8.35	8.35	8.35	8.35	8.35	8.35	8.35	8.35
8.36	8.36	8.36	8.36	8.36	8.36	8.36	8.36	8.36
8.37	8.37	8.37	8.37	8.37	8.37	8.37	8.37	8.37
8.38	8.38	8.38	8.38	8.38	8.38	8.38	8.38	8.38
8.39	8.39	8.39	8.39	8.39	8.39	8.39	8.39	8.39
8.4	8.4	8.4	8.4	8.4	8.4	8.4	8.4	8.4
8.41	8.41	8.41	8.41	8.41	8.41	8.41	8.41	8.41
8.42	8.42	8.42	8.42	8.42	8.42	8.42	8.42	8.42
8.43	8.43	8.43	8.43	8.43	8.43	8.43	8.43	8.43
8.44	8.44	8.44	8.44	8.44	8.44	8.44	8.44	8.44
8.45	8.45	8.45	8.45	8.45	8.45	8.45	8.45	8.45
8.46	8.46	8.46	8.46	8.46	8.46	8.46	8.46	8.46
8.47	8.47	8.47	8.47	8.47	8.47	8.47	8.47	8.47
8.48	8.48	8.48	8.48	8.48	8.48	8.48	8.48	8.48
8.49	8.49	8.49	8.49	8.49	8.49	8.49	8.49	8.49

8.5	8.5	8.5	8.5	8.5	8.5	8.5	8.5	8.5
8.51	8.51	8.51	8.51	8.51	8.51	8.51	8.51	8.51
8.52	8.52	8.52	8.52	8.52	8.52	8.52	8.52	8.52
8.53	8.53	8.53	8.53	8.53	8.53	8.53	8.53	8.53
8.54	8.54	8.54	8.54	8.54	8.54	8.54	8.54	8.54
8.55	8.55	8.55	8.55	8.55	8.55	8.55	8.55	8.55
8.56	8.56	8.56	8.56	8.56	8.56	8.56	8.56	8.56
8.57	8.57	8.57	8.57	8.57	8.57	8.57	8.57	8.57
8.58	8.58	8.58	8.58	8.58	8.58	8.58	8.58	8.58
8.59	8.59	8.59	8.59	8.59	8.59	8.59	8.59	8.59
8.6	8.6	8.6	8.6	8.6	8.6	8.6	8.6	8.6
8.61	8.61	8.61	8.61	8.61	8.61	8.61	8.61	8.61
8.62	8.62	8.62	8.62	8.62	8.62	8.62	8.62	8.62
8.63	8.63	8.63	8.63	8.63	8.63	8.63	8.63	8.63
8.64	8.64	8.64	8.64	8.64	8.64	8.64	8.64	8.64
8.65	8.65	8.65	8.65	8.65	8.65	8.65	8.65	8.65
8.66	8.66	8.66	8.66	8.66	8.66	8.66	8.66	8.66
8.67	8.67	8.67	8.67	8.67	8.67	8.67	8.67	8.67
8.68	8.68	8.68	8.68	8.68	8.68	8.68	8.68	8.68
8.69	8.69	8.69	8.69	8.69	8.69	8.69	8.69	8.69
8.7	8.7	8.7	8.7	8.7	8.7	8.7	8.7	8.7
8.71	8.71	8.71	8.71	8.71	8.71	8.71	8.71	8.71
8.72	8.72	8.72	8.72	8.72	8.72	8.72	8.72	8.72
8.73	8.73	8.73	8.73	8.73	8.73	8.73	8.73	8.73
8.74	8.74	8.74	8.74	8.74	8.74	8.74	8.74	8.74
8.75	8.75	8.75	8.75	8.75	8.75	8.75	8.75	8.75
8.76	8.76	8.76	8.76	8.76	8.76	8.76	8.76	8.76
8.77	8.77	8.77	8.77	8.77	8.77	8.77	8.77	8.77
8.78	8.78	8.78	8.78	8.78	8.78	8.78	8.78	8.78
8.79	8.79	8.79	8.79	8.79	8.79	8.79	8.79	8.79
8.8	8.8	8.8	8.8	8.8	8.8	8.8	8.8	8.8
8.81	8.81	8.81	8.81	8.81	8.81	8.81	8.81	8.81
8.82	8.82	8.82	8.82	8.82	8.82	8.82	8.82	8.82
8.83	8.83	8.83	8.83	8.83	8.83	8.83	8.83	8.83
8.84	8.84	8.84	8.84	8.84	8.84	8.84	8.84	8.84
8.85	8.85	8.85	8.85	8.85	8.85	8.85	8.85	8.85
8.86	8.86	8.86	8.86	8.86	8.86	8.86	8.86	8.86
8.87	8.87	8.87	8.87	8.87	8.87	8.87	8.87	8.87
8.88	8.88	8.88	8.88	8.88	8.88	8.88	8.88	8.88
8.89	8.89	8.89	8.89	8.89	8.89	8.89	8.89	8.89
8.9	8.9	8.9	8.9	8.9	8.9	8.9	8.9	8.9
8.91	8.91	8.91	8.91	8.91	8.91	8.91	8.91	8.91
8.92	8.92	8.92	8.92	8.92	8.92	8.92	8.92	8.92
8.93	8.93	8.93	8.93	8.93	8.93	8.93	8.93	8.93
8.94	8.94	8.94	8.94	8.94	8.94	8.94	8.94	8.94
8.95	8.95	8.95	8.95	8.95	8.95	8.95	8.95	8.95
8.96	8.96	8.96	8.96	8.96	8.96	8.96	8.96	8.96
8.97	8.97	8.97	8.97	8.97	8.97	8.97	8.97	8.97
8.98	8.98	8.98	8.98	8.98	8.98	8.98	8.98	8.98
8.99	8.99	8.99	8.99	8.99	8.99	8.99	8.99	8.99

9	9	9	9	9	9	9	9	9
9.01	9.01	9.01	9.01	9.01	9.01	9.01	9.01	9.01
9.02	9.02	9.02	9.02	9.02	9.02	9.02	9.02	9.02
9.03	9.03	9.03	9.03	9.03	9.03	9.03	9.03	9.03
9.04	9.04	9.04	9.04	9.04	9.04	9.04	9.04	9.04
9.05	9.05	9.05	9.05	9.05	9.05	9.05	9.05	9.05
9.06	9.06	9.06	9.06	9.06	9.06	9.06	9.06	9.06
9.07	9.07	9.07	9.07	9.07	9.07	9.07	9.07	9.07
9.08	9.08	9.08	9.08	9.08	9.08	9.08	9.08	9.08
9.09	9.09	9.09	9.09	9.09	9.09	9.09	9.09	9.09
9.1	9.1	9.1	9.1	9.1	9.1	9.1	9.1	9.1
9.11	9.11	9.11	9.11	9.11	9.11	9.11	9.11	9.11
9.12	9.12	9.12	9.12	9.12	9.12	9.12	9.12	9.12
9.13	9.13	9.13	9.13	9.13	9.13	9.13	9.13	9.13
9.14	9.14	9.14	9.14	9.14	9.14	9.14	9.14	9.14
9.15	9.15	9.15	9.15	9.15	9.15	9.15	9.15	9.15
9.16	9.16	9.16	9.16	9.16	9.16	9.16	9.16	9.16
9.17	9.17	9.17	9.17	9.17	9.17	9.17	9.17	9.17
9.18	9.18	9.18	9.18	9.18	9.18	9.18	9.18	9.18
9.19	9.19	9.19	9.19	9.19	9.19	9.19	9.19	9.19
9.2	9.2	9.2	9.2	9.2	9.2	9.2	9.2	9.2
9.21	9.21	9.21	9.21	9.21	9.21	9.21	9.21	9.21
9.22	9.22	9.22	9.22	9.22	9.22	9.22	9.22	9.22
9.23	9.23	9.23	9.23	9.23	9.23	9.23	9.23	9.23
9.24	9.24	9.24	9.24	9.24	9.24	9.24	9.24	9.24
9.25	9.25	9.25	9.25	9.25	9.25	9.25	9.25	9.25
9.26	9.26	9.26	9.26	9.26	9.26	9.26	9.26	9.26
9.27	9.27	9.27	9.27	9.27	9.27	9.27	9.27	9.27
9.28	9.28	9.28	9.28	9.28	9.28	9.28	9.28	9.28
9.29	9.29	9.29	9.29	9.29	9.29	9.29	9.29	9.29
9.3	9.3	9.3	9.3	9.3	9.3	9.3	9.3	9.3
9.31	9.31	9.31	9.31	9.31	9.31	9.31	9.31	9.31
9.32	9.32	9.32	9.32	9.32	9.32	9.32	9.32	9.32
9.33	9.33	9.33	9.33	9.33	9.33	9.33	9.33	9.33
9.34	9.34	9.34	9.34	9.34	9.34	9.34	9.34	9.34
9.35	9.35	9.35	9.35	9.35	9.35	9.35	9.35	9.35
9.36	9.36	9.36	9.36	9.36	9.36	9.36	9.36	9.36
9.37	9.37	9.37	9.37	9.37	9.37	9.37	9.37	9.37
9.38	9.38	9.38	9.38	9.38	9.38	9.38	9.38	9.38
9.39	9.39	9.39	9.39	9.39	9.39	9.39	9.39	9.39
9.4	9.4	9.4	9.4	9.4	9.4	9.4	9.4	9.4
9.41	9.41	9.41	9.41	9.41	9.41	9.41	9.41	9.41
9.42	9.42	9.42	9.42	9.42	9.42	9.42	9.42	9.42
9.43	9.43	9.43	9.43	9.43	9.43	9.43	9.43	9.43
9.44	9.44	9.44	9.44	9.44	9.44	9.44	9.44	9.44
9.45	9.45	9.45	9.45	9.45	9.45	9.45	9.45	9.45
9.46	9.46	9.46	9.46	9.46	9.46	9.46	9.46	9.46
9.47	9.47	9.47	9.47	9.47	9.47	9.47	9.47	9.47
9.48	9.48	9.48	9.48	9.48	9.48	9.48	9.48	9.48
9.49	9.49	9.49	9.49	9.49	9.49	9.49	9.49	9.49

9.5	9.5	9.5	9.5	9.5	9.5	9.5	9.5	9.5
9.51	9.51	9.51	9.51	9.51	9.51	9.51	9.51	9.51
9.52	9.52	9.52	9.52	9.52	9.52	9.52	9.52	9.52
9.53	9.53	9.53	9.53	9.53	9.53	9.53	9.53	9.53
9.54	9.54	9.54	9.54	9.54	9.54	9.54	9.54	9.54
9.55	9.55	9.55	9.55	9.55	9.55	9.55	9.55	9.55
9.56	9.56	9.56	9.56	9.56	9.56	9.56	9.56	9.56
9.57	9.57	9.57	9.57	9.57	9.57	9.57	9.57	9.57
9.58	9.58	9.58	9.58	9.58	9.58	9.58	9.58	9.58
9.59	9.59	9.59	9.59	9.59	9.59	9.59	9.59	9.59
9.6	9.6	9.6	9.6	9.6	9.6	9.6	9.6	9.6
9.61	9.61	9.61	9.61	9.61	9.61	9.61	9.61	9.61
9.62	9.62	9.62	9.62	9.62	9.62	9.62	9.62	9.62
9.63	9.63	9.63	9.63	9.63	9.63	9.63	9.63	9.63
9.64	9.64	9.64	9.64	9.64	9.64	9.64	9.64	9.64
9.65	9.65	9.65	9.65	9.65	9.65	9.65	9.65	9.65
9.66	9.66	9.66	9.66	9.66	9.66	9.66	9.66	9.66
9.67	9.67	9.67	9.67	9.67	9.67	9.67	9.67	9.67
9.68	9.68	9.68	9.68	9.68	9.68	9.68	9.68	9.68
9.69	9.69	9.69	9.69	9.69	9.69	9.69	9.69	9.69
9.7	9.7	9.7	9.7	9.7	9.7	9.7	9.7	9.7
9.71	9.71	9.71	9.71	9.71	9.71	9.71	9.71	9.71
9.72	9.72	9.72	9.72	9.72	9.72	9.72	9.72	9.72
9.73	9.73	9.73	9.73	9.73	9.73	9.73	9.73	9.73
9.74	9.74	9.74	9.74	9.74	9.74	9.74	9.74	9.74
9.75	9.75	9.75	9.75	9.75	9.75	9.75	9.75	9.75
9.76	9.76	9.76	9.76	9.76	9.76	9.76	9.76	9.76
9.77	9.77	9.77	9.77	9.77	9.77	9.77	9.77	9.77
9.78	9.78	9.78	9.78	9.78	9.78	9.78	9.78	9.78
9.79	9.79	9.79	9.79	9.79	9.79	9.79	9.79	9.79
9.8	9.8	9.8	9.8	9.8	9.8	9.8	9.8	9.8
9.81	9.81	9.81	9.81	9.81	9.81	9.81	9.81	9.81
9.82	9.82	9.82	9.82	9.82	9.82	9.82	9.82	9.82
9.83	9.83	9.83	9.83	9.83	9.83	9.83	9.83	9.83
9.84	9.84	9.84	9.84	9.84	9.84	9.84	9.84	9.84
9.85	9.85	9.85	9.85	9.85	9.85	9.85	9.85	9.85
9.86	9.86	9.86	9.86	9.86	9.86	9.86	9.86	9.86
9.87	9.87	9.87	9.87	9.87	9.87	9.87	9.87	9.87
9.88	9.88	9.88	9.88	9.88	9.88	9.88	9.88	9.88
9.89	9.89	9.89	9.89	9.89	9.89	9.89	9.89	9.89
9.9	9.9	9.9	9.9	9.9	9.9	9.9	9.9	9.9
9.91	9.91	9.91	9.91	9.91	9.91	9.91	9.91	9.91
9.92	9.92	9.92	9.92	9.92	9.92	9.92	9.92	9.92
9.93	9.93	9.93	9.93	9.93	9.93	9.93	9.93	9.93
9.94	9.94	9.94	9.94	9.94	9.94	9.94	9.94	9.94
9.95	9.95	9.95	9.95	9.95	9.95	9.95	9.95	9.95
9.96	9.96	9.96	9.96	9.96	9.96	9.96	9.96	9.96
9.97	9.97	9.97	9.97	9.97	9.97	9.97	9.97	9.97
9.98	9.98	9.98	9.98	9.98	9.98	9.98	9.98	9.98
9.99	9.99	9.99	9.99	9.99	9.99	9.99	9.99	9.99

10	10	10	10	10	10	10	10	10
10.01	10.01	10.01	10.01	10.01	10.01	10.01	10.01	10.01
10.02	10.02	10.02	10.02	10.02	10.02	10.02	10.02	10.02
10.03	10.03	10.03	10.03	10.03	10.03	10.03	10.03	10.03
10.04	10.04	10.04	10.04	10.04	10.04	10.04	10.04	10.04
10.05	10.05	10.05	10.05	10.05	10.05	10.05	10.05	10.05
10.06	10.06	10.06	10.06	10.06	10.06	10.06	10.06	10.06
10.07	10.07	10.07	10.07	10.07	10.07	10.07	10.07	10.07
10.08	10.08	10.08	10.08	10.08	10.08	10.08	10.08	10.08
10.09	10.09	10.09	10.09	10.09	10.09	10.09	10.09	10.09
10.1	10.1	10.1	10.1	10.1	10.1	10.1	10.1	10.1
10.11	10.11	10.11	10.11	10.11	10.11	10.11	10.11	10.11
10.12	10.12	10.12	10.12	10.12	10.12	10.12	10.12	10.12
10.13	10.13	10.13	10.13	10.13	10.13	10.13	10.13	10.13
10.14	10.14	10.14	10.14	10.14	10.14	10.14	10.14	10.14
10.15	10.15	10.15	10.15	10.15	10.15	10.15	10.15	10.15
10.16	10.16	10.16	10.16	10.16	10.16	10.16	10.16	10.16
10.17	10.17	10.17	10.17	10.17	10.17	10.17	10.17	10.17
10.18	10.18	10.18	10.18	10.18	10.18	10.18	10.18	10.18
10.19	10.19	10.19	10.19	10.19	10.19	10.19	10.19	10.19
10.2	10.2	10.2	10.2	10.2	10.2	10.2	10.2	10.2
10.21	10.21	10.21	10.21	10.21	10.21	10.21	10.21	10.21
10.22	10.22	10.22	10.22	10.22	10.22	10.22	10.22	10.22
10.23	10.23	10.23	10.23	10.23	10.23	10.23	10.23	10.23
10.24	10.24	10.24	10.24	10.24	10.24	10.24	10.24	10.24
10.25	10.25	10.25	10.25	10.25	10.25	10.25	10.25	10.25
10.26	10.26	10.26	10.26	10.26	10.26	10.26	10.26	10.26
10.27	10.27	10.27	10.27	10.27	10.27	10.27	10.27	10.27
10.28	10.28	10.28	10.28	10.28	10.28	10.28	10.28	10.28
10.29	10.29	10.29	10.29	10.29	10.29	10.29	10.29	10.29
10.3	10.3	10.3	10.3	10.3	10.3	10.3	10.3	10.3
10.31	10.31	10.31	10.31	10.31	10.31	10.31	10.31	10.31
10.32	10.32	10.32	10.32	10.32	10.32	10.32	10.32	10.32
10.33	10.33	10.33	10.33	10.33	10.33	10.33	10.33	10.33
10.34	10.34	10.34	10.34	10.34	10.34	10.34	10.34	10.34
10.35	10.35	10.35	10.35	10.35	10.35	10.35	10.35	10.35
10.36	10.36	10.36	10.36	10.36	10.36	10.36	10.36	10.36
10.37	10.37	10.37	10.37	10.37	10.37	10.37	10.37	10.37
10.38	10.38	10.38	10.38	10.38	10.38	10.38	10.38	10.38
10.39	10.39	10.39	10.39	10.39	10.39	10.39	10.39	10.39
10.4	10.4	10.4	10.4	10.4	10.4	10.4	10.4	10.4
10.41	10.41	10.41	10.41	10.41	10.41	10.41	10.41	10.41
10.42	10.42	10.42	10.42	10.42	10.42	10.42	10.42	10.42
10.43	10.43	10.43	10.43	10.43	10.43	10.43	10.43	10.43
10.44	10.44	10.44	10.44	10.44	10.44	10.44	10.44	10.44
10.45	10.45	10.45	10.45	10.45	10.45	10.45	10.45	10.45
10.46	10.46	10.46	10.46	10.46	10.46	10.46	10.46	10.46
10.47	10.47	10.47	10.47	10.47	10.47	10.47	10.47	10.47
10.48	10.48	10.48	10.48	10.48	10.48	10.48	10.48	10.48
10.49	10.49	10.49	10.49	10.49	10.49	10.49	10.49	10.49

10.5	10.5	10.5	10.5	10.5	10.5	10.5	10.5	10.5
10.51	10.51	10.51	10.51	10.51	10.51	10.51	10.51	10.51
10.52	10.52	10.52	10.52	10.52	10.52	10.52	10.52	10.52
10.53	10.53	10.53	10.53	10.53	10.53	10.53	10.53	10.53
10.54	10.54	10.54	10.54	10.54	10.54	10.54	10.54	10.54
10.55	10.55	10.55	10.55	10.55	10.55	10.55	10.55	10.55
10.56	10.56	10.56	10.56	10.56	10.56	10.56	10.56	10.56
10.57	10.57	10.57	10.57	10.57	10.57	10.57	10.57	10.57
10.58	10.58	10.58	10.58	10.58	10.58	10.58	10.58	10.58
10.59	10.59	10.59	10.59	10.59	10.59	10.59	10.59	10.59
10.6	10.6	10.6	10.6	10.6	10.6	10.6	10.6	10.6
10.61	10.61	10.61	10.61	10.61	10.61	10.61	10.61	10.61
10.62	10.62	10.62	10.62	10.62	10.62	10.62	10.62	10.62
10.63	10.63	10.63	10.63	10.63	10.63	10.63	10.63	10.63
10.64	10.64	10.64	10.64	10.64	10.64	10.64	10.64	10.64
10.65	10.65	10.65	10.65	10.65	10.65	10.65	10.65	10.65
10.66	10.66	10.66	10.66	10.66	10.66	10.66	10.66	10.66
10.67	10.67	10.67	10.67	10.67	10.67	10.67	10.67	10.67
10.68	10.68	10.68	10.68	10.68	10.68	10.68	10.68	10.68
10.69	10.69	10.69	10.69	10.69	10.69	10.69	10.69	10.69
10.7	10.7	10.7	10.7	10.7	10.7	10.7	10.7	10.7
10.71	10.71	10.71	10.71	10.71	10.71	10.71	10.71	10.71
10.72	10.72	10.72	10.72	10.72	10.72	10.72	10.72	10.72
10.73	10.73	10.73	10.73	10.73	10.73	10.73	10.73	10.73
10.74	10.74	10.74	10.74	10.74	10.74	10.74	10.74	10.74
10.75	10.75	10.75	10.75	10.75	10.75	10.75	10.75	10.75
10.76	10.76	10.76	10.76	10.76	10.76	10.76	10.76	10.76
10.77	10.77	10.77	10.77	10.77	10.77	10.77	10.77	10.77
10.78	10.78	10.78	10.78	10.78	10.78	10.78	10.78	10.78
10.79	10.79	10.79	10.79	10.79	10.79	10.79	10.79	10.79
10.8	10.8	10.8	10.8	10.8	10.8	10.8	10.8	10.8
10.81	10.81	10.81	10.81	10.81	10.81	10.81	10.81	10.81
10.82	10.82	10.82	10.82	10.82	10.82	10.82	10.82	10.82
10.83	10.83	10.83	10.83	10.83	10.83	10.83	10.83	10.83
10.84	10.84	10.84	10.84	10.84	10.84	10.84	10.84	10.84
10.85	10.85	10.85	10.85	10.85	10.85	10.85	10.85	10.85
10.86	10.86	10.86	10.86	10.86	10.86	10.86	10.86	10.86
10.87	10.87	10.87	10.87	10.87	10.87	10.87	10.87	10.87
10.88	10.88	10.88	10.88	10.88	10.88	10.88	10.88	10.88
10.89	10.89	10.89	10.89	10.89	10.89	10.89	10.89	10.89
10.9	10.9	10.9	10.9	10.9	10.9	10.9	10.9	10.9
10.91	10.91	10.91	10.91	10.91	10.91	10.91	10.91	10.91
10.92	10.92	10.92	10.92	10.92	10.92	10.92	10.92	10.92
10.93	10.93	10.93	10.93	10.93	10.93	10.93	10.93	10.93
10.94	10.94	10.94	10.94	10.94	10.94	10.94	10.94	10.94
10.95	10.95	10.95	10.95	10.95	10.95	10.95	10.95	10.95
10.96	10.96	10.96	10.96	10.96	10.96	10.96	10.96	10.96
10.97	10.97	10.97	10.97	10.97	10.97	10.97	10.97	10.97
10.98	10.98	10.98	10.98	10.98	10.98	10.98	10.98	10.98
10.99	10.99	10.99	10.99	10.99	10.99	10.99	10.99	10.99

11	11	11	11	11	11	11	11	11
11.01	11.01	11.01	11.01	11.01	11.01	11.01	11.01	11.01
11.02	11.02	11.02	11.02	11.02	11.02	11.02	11.02	11.02
11.03	11.03	11.03	11.03	11.03	11.03	11.03	11.03	11.03
11.04	11.04	11.04	11.04	11.04	11.04	11.04	11.04	11.04
11.05	11.05	11.05	11.05	11.05	11.05	11.05	11.05	11.05
11.06	11.06	11.06	11.06	11.06	11.06	11.06	11.06	11.06
11.07	11.07	11.07	11.07	11.07	11.07	11.07	11.07	11.07
11.08	11.08	11.08	11.08	11.08	11.08	11.08	11.08	11.08
11.09	11.09	11.09	11.09	11.09	11.09	11.09	11.09	11.09
11.1	11.1	11.1	11.1	11.1	11.1	11.1	11.1	11.1
11.11	11.11	11.11	11.11	11.11	11.11	11.11	11.11	11.11
11.12	11.12	11.12	11.12	11.12	11.12	11.12	11.12	11.12
11.13	11.13	11.13	11.13	11.13	11.13	11.13	11.13	11.13
11.14	11.14	11.14	11.14	11.14	11.14	11.14	11.14	11.14
11.15	11.15	11.15	11.15	11.15	11.15	11.15	11.15	11.15
11.16	11.16	11.16	11.16	11.16	11.16	11.16	11.16	11.16
11.17	11.17	11.17	11.17	11.17	11.17	11.17	11.17	11.17
11.18	11.18	11.18	11.18	11.18	11.18	11.18	11.18	11.18
11.19	11.19	11.19	11.19	11.19	11.19	11.19	11.19	11.19
11.2	11.2	11.2	11.2	11.2	11.2	11.2	11.2	11.2
11.21	11.21	11.21	11.21	11.21	11.21	11.21	11.21	11.21
11.22	11.22	11.22	11.22	11.22	11.22	11.22	11.22	11.22
11.23	11.23	11.23	11.23	11.23	11.23	11.23	11.23	11.23
11.24	11.24	11.24	11.24	11.24	11.24	11.24	11.24	11.24
11.25	11.25	11.25	11.25	11.25	11.25	11.25	11.25	11.25
11.26	11.26	11.26	11.26	11.26	11.26	11.26	11.26	11.26
11.27	11.27	11.27	11.27	11.27	11.27	11.27	11.27	11.27
11.28	11.28	11.28	11.28	11.28	11.28	11.28	11.28	11.28
11.29	11.29	11.29	11.29	11.29	11.29	11.29	11.29	11.29
11.3	11.3	11.3	11.3	11.3	11.3	11.3	11.3	11.3
11.31	11.31	11.31	11.31	11.31	11.31	11.31	11.31	11.31
11.32	11.32	11.32	11.32	11.32	11.32	11.32	11.32	11.32
11.33	11.33	11.33	11.33	11.33	11.33	11.33	11.33	11.33
11.34	11.34	11.34	11.34	11.34	11.34	11.34	11.34	11.34
11.35	11.35	11.35	11.35	11.35	11.35	11.35	11.35	11.35
11.36	11.36	11.36	11.36	11.36	11.36	11.36	11.36	11.36
11.37	11.37	11.37	11.37	11.37	11.37	11.37	11.37	11.37
11.38	11.38	11.38	11.38	11.38	11.38	11.38	11.38	11.38
11.39	11.39	11.39	11.39	11.39	11.39	11.39	11.39	11.39
11.4	11.4	11.4	11.4	11.4	11.4	11.4	11.4	11.4
11.41	11.41	11.41	11.41	11.41	11.41	11.41	11.41	11.41
11.42	11.42	11.42	11.42	11.42	11.42	11.42	11.42	11.42
11.43	11.43	11.43	11.43	11.43	11.43	11.43	11.43	11.43
11.44	11.44	11.44	11.44	11.44	11.44	11.44	11.44	11.44
11.45	11.45	11.45	11.45	11.45	11.45	11.45	11.45	11.45
11.46	11.46	11.46	11.46	11.46	11.46	11.46	11.46	11.46
11.47	11.47	11.47	11.47	11.47	11.47	11.47	11.47	11.47
11.48	11.48	11.48	11.48	11.48	11.48	11.48	11.48	11.48
11.49	11.49	11.49	11.49	11.49	11.49	11.49	11.49	11.49

11.5	11.5	11.5	11.5	11.5	11.5	11.5	11.5	11.5
11.51	11.51	11.51	11.51	11.51	11.51	11.51	11.51	11.51
11.52	11.52	11.52	11.52	11.52	11.52	11.52	11.52	11.52
11.53	11.53	11.53	11.53	11.53	11.53	11.53	11.53	11.53
11.54	11.54	11.54	11.54	11.54	11.54	11.54	11.54	11.54
11.55	11.55	11.55	11.55	11.55	11.55	11.55	11.55	11.55
11.56	11.56	11.56	11.56	11.56	11.56	11.56	11.56	11.56
11.57	11.57	11.57	11.57	11.57	11.57	11.57	11.57	11.57
11.58	11.58	11.58	11.58	11.58	11.58	11.58	11.58	11.58
11.59	11.59	11.59	11.59	11.59	11.59	11.59	11.59	11.59
11.6	11.6	11.6	11.6	11.6	11.6	11.6	11.6	11.6
11.61	11.61	11.61	11.61	11.61	11.61	11.61	11.61	11.61
11.62	11.62	11.62	11.62	11.62	11.62	11.62	11.62	11.62
11.63	11.63	11.63	11.63	11.63	11.63	11.63	11.63	11.63
11.64	11.64	11.64	11.64	11.64	11.64	11.64	11.64	11.64
11.65	11.65	11.65	11.65	11.65	11.65	11.65	11.65	11.65
11.66	11.66	11.66	11.66	11.66	11.66	11.66	11.66	11.66
11.67	11.67	11.67	11.67	11.67	11.67	11.67	11.67	11.67
11.68	11.68	11.68	11.68	11.68	11.68	11.68	11.68	11.68
11.69	11.69	11.69	11.69	11.69	11.69	11.69	11.69	11.69
11.7	11.7	11.7	11.7	11.7	11.7	11.7	11.7	11.7
11.71	11.71	11.71	11.71	11.71	11.71	11.71	11.71	11.71
11.72	11.72	11.72	11.72	11.72	11.72	11.72	11.72	11.72
11.73	11.73	11.73	11.73	11.73	11.73	11.73	11.73	11.73
11.74	11.74	11.74	11.74	11.74	11.74	11.74	11.74	11.74
11.75	11.75	11.75	11.75	11.75	11.75	11.75	11.75	11.75
11.76	11.76	11.76	11.76	11.76	11.76	11.76	11.76	11.76
11.77	11.77	11.77	11.77	11.77	11.77	11.77	11.77	11.77
11.78	11.78	11.78	11.78	11.78	11.78	11.78	11.78	11.78
11.79	11.79	11.79	11.79	11.79	11.79	11.79	11.79	11.79
11.8	11.8	11.8	11.8	11.8	11.8	11.8	11.8	11.8
11.81	11.81	11.81	11.81	11.81	11.81	11.81	11.81	11.81
11.82	11.82	11.82	11.82	11.82	11.82	11.82	11.82	11.82
11.83	11.83	11.83	11.83	11.83	11.83	11.83	11.83	11.83
11.84	11.84	11.84	11.84	11.84	11.84	11.84	11.84	11.84
11.85	11.85	11.85	11.85	11.85	11.85	11.85	11.85	11.85
11.86	11.86	11.86	11.86	11.86	11.86	11.86	11.86	11.86
11.87	11.87	11.87	11.87	11.87	11.87	11.87	11.87	11.87
11.88	11.88	11.88	11.88	11.88	11.88	11.88	11.88	11.88
11.89	11.89	11.89	11.89	11.89	11.89	11.89	11.89	11.89
11.9	11.9	11.9	11.9	11.9	11.9	11.9	11.9	11.9
11.91	11.91	11.91	11.91	11.91	11.91	11.91	11.91	11.91
11.92	11.92	11.92	11.92	11.92	11.92	11.92	11.92	11.92
11.93	11.93	11.93	11.93	11.93	11.93	11.93	11.93	11.93
11.94	11.94	11.94	11.94	11.94	11.94	11.94	11.94	11.94
11.95	11.95	11.95	11.95	11.95	11.95	11.95	11.95	11.95
11.96	11.96	11.96	11.96	11.96	11.96	11.96	11.96	11.96
11.97	11.97	11.97	11.97	11.97	11.97	11.97	11.97	11.97
11.98	11.98	11.98	11.98	11.98	11.98	11.98	11.98	11.98
11.99	11.99	11.99	11.99	11.99	11.99	11.99	11.99	11.99

12	12	12	12	12	12	12	12	12

-12	-12	-12	-12	-12	-12	-12	-12	-12
-11.99	-11.99	-11.99	-11.99	-11.99	-11.99	-11.99	-11.99	-11.99
-11.98	-11.98	-11.98	-11.98	-11.98	-11.98	-11.98	-11.98	-11.98
-11.97	-11.97	-11.97	-11.97	-11.97	-11.97	-11.97	-11.97	-11.97
-11.96	-11.96	-11.96	-11.96	-11.96	-11.96	-11.96	-11.96	-11.96
-11.95	-11.95	-11.95	-11.95	-11.95	-11.95	-11.95	-11.95	-11.95
-11.94	-11.94	-11.94	-11.94	-11.94	-11.94	-11.94	-11.94	-11.94
-11.93	-11.93	-11.93	-11.93	-11.93	-11.93	-11.93	-11.93	-11.93
-11.92	-11.92	-11.92	-11.92	-11.92	-11.92	-11.92	-11.92	-11.92
-11.91	-11.91	-11.91	-11.91	-11.91	-11.91	-11.91	-11.91	-11.91
-11.9	-11.9	-11.9	-11.9	-11.9	-11.9	-11.9	-11.9	-11.9
-11.89	-11.89	-11.89	-11.89	-11.89	-11.89	-11.89	-11.89	-11.89
-11.88	-11.88	-11.88	-11.88	-11.88	-11.88	-11.88	-11.88	-11.88
-11.87	-11.87	-11.87	-11.87	-11.87	-11.87	-11.87	-11.87	-11.87
-11.86	-11.86	-11.86	-11.86	-11.86	-11.86	-11.86	-11.86	-11.86
-11.85	-11.85	-11.85	-11.85	-11.85	-11.85	-11.85	-11.85	-11.85
-11.84	-11.84	-11.84	-11.84	-11.84	-11.84	-11.84	-11.84	-11.84
-11.83	-11.83	-11.83	-11.83	-11.83	-11.83	-11.83	-11.83	-11.83
-11.82	-11.82	-11.82	-11.82	-11.82	-11.82	-11.82	-11.82	-11.82
-11.81	-11.81	-11.81	-11.81	-11.81	-11.81	-11.81	-11.81	-11.81
-11.8	-11.8	-11.8	-11.8	-11.8	-11.8	-11.8	-11.8	-11.8
-11.79	-11.79	-11.79	-11.79	-11.79	-11.79	-11.79	-11.79	-11.79
-11.78	-11.78	-11.78	-11.78	-11.78	-11.78	-11.78	-11.78	-11.78
-11.77	-11.77	-11.77	-11.77	-11.77	-11.77	-11.77	-11.77	-11.77
-11.76	-11.76	-11.76	-11.76	-11.76	-11.76	-11.76	-11.76	-11.76
-11.75	-11.75	-11.75	-11.75	-11.75	-11.75	-11.75	-11.75	-11.75
-11.74	-11.74	-11.74	-11.74	-11.74	-11.74	-11.74	-11.74	-11.74
-11.73	-11.73	-11.73	-11.73	-11.73	-11.73	-11.73	-11.73	-11.73
-11.72	-11.72	-11.72	-11.72	-11.72	-11.72	-11.72	-11.72	-11.72
-11.71	-11.71	-11.71	-11.71	-11.71	-11.71	-11.71	-11.71	-11.71
-11.7	-11.7	-11.7	-11.7	-11.7	-11.7	-11.7	-11.7	-11.7
-11.69	-11.69	-11.69	-11.69	-11.69	-11.69	-11.69	-11.69	-11.69
-11.68	-11.68	-11.68	-11.68	-11.68	-11.68	-11.68	-11.68	-11.68
-11.67	-11.67	-11.67	-11.67	-11.67	-11.67	-11.67	-11.67	-11.67
-11.66	-11.66	-11.66	-11.66	-11.66	-11.66	-11.66	-11.66	-11.66
-11.65	-11.65	-11.65	-11.65	-11.65	-11.65	-11.65	-11.65	-11.65
-11.64	-11.64	-11.64	-11.64	-11.64	-11.64	-11.64	-11.64	-11.64
-11.63	-11.63	-11.63	-11.63	-11.63	-11.63	-11.63	-11.63	-11.63
-11.62	-11.62	-11.62	-11.62	-11.62	-11.62	-11.62	-11.62	-11.62
-11.61	-11.61	-11.61	-11.61	-11.61	-11.61	-11.61	-11.61	-11.61
-11.6	-11.6	-11.6	-11.6	-11.6	-11.6	-11.6	-11.6	-11.6
-11.59	-11.59	-11.59	-11.59	-11.59	-11.59	-11.59	-11.59	-11.59
-11.58	-11.58	-11.58	-11.58	-11.58	-11.58	-11.58	-11.58	-11.58
-11.57	-11.57	-11.57	-11.57	-11.57	-11.57	-11.57	-11.57	-11.57
-11.56	-11.56	-11.56	-11.56	-11.56	-11.56	-11.56	-11.56	-11.56
-11.55	-11.55	-11.55	-11.55	-11.55	-11.55	-11.55	-11.55	-11.55
-11.54	-11.54	-11.54	-11.54	-11.54	-11.54	-11.54	-11.54	-11.54
-11.53	-11.53	-11.53	-11.53	-11.53	-11.53	-11.53	-11.53	-11.53
-11.52	-11.52	-11.52	-11.52	-11.52	-11.52	-11.52	-11.52	-11.52
-11.51	-11.51	-11.51	-11.51	-11.51	-11.51	-11.51	-11.51	-11.51

-11.5	-11.5	-11.5	-11.5	-11.5	-11.5	-11.5	-11.5	-11.5
-11.49	-11.49	-11.49	-11.49	-11.49	-11.49	-11.49	-11.49	-11.49
-11.48	-11.48	-11.48	-11.48	-11.48	-11.48	-11.48	-11.48	-11.48
-11.47	-11.47	-11.47	-11.47	-11.47	-11.47	-11.47	-11.47	-11.47
-11.46	-11.46	-11.46	-11.46	-11.46	-11.46	-11.46	-11.46	-11.46
-11.45	-11.45	-11.45	-11.45	-11.45	-11.45	-11.45	-11.45	-11.45
-11.44	-11.44	-11.44	-11.44	-11.44	-11.44	-11.44	-11.44	-11.44
-11.43	-11.43	-11.43	-11.43	-11.43	-11.43	-11.43	-11.43	-11.43
-11.42	-11.42	-11.42	-11.42	-11.42	-11.42	-11.42	-11.42	-11.42
-11.41	-11.41	-11.41	-11.41	-11.41	-11.41	-11.41	-11.41	-11.41
-11.4	-11.4	-11.4	-11.4	-11.4	-11.4	-11.4	-11.4	-11.4
-11.39	-11.39	-11.39	-11.39	-11.39	-11.39	-11.39	-11.39	-11.39
-11.38	-11.38	-11.38	-11.38	-11.38	-11.38	-11.38	-11.38	-11.38
-11.37	-11.37	-11.37	-11.37	-11.37	-11.37	-11.37	-11.37	-11.37
-11.36	-11.36	-11.36	-11.36	-11.36	-11.36	-11.36	-11.36	-11.36
-11.35	-11.35	-11.35	-11.35	-11.35	-11.35	-11.35	-11.35	-11.35
-11.34	-11.34	-11.34	-11.34	-11.34	-11.34	-11.34	-11.34	-11.34
-11.33	-11.33	-11.33	-11.33	-11.33	-11.33	-11.33	-11.33	-11.33
-11.32	-11.32	-11.32	-11.32	-11.32	-11.32	-11.32	-11.32	-11.32
-11.31	-11.31	-11.31	-11.31	-11.31	-11.31	-11.31	-11.31	-11.31
-11.3	-11.3	-11.3	-11.3	-11.3	-11.3	-11.3	-11.3	-11.3
-11.29	-11.29	-11.29	-11.29	-11.29	-11.29	-11.29	-11.29	-11.29
-11.28	-11.28	-11.28	-11.28	-11.28	-11.28	-11.28	-11.28	-11.28
-11.27	-11.27	-11.27	-11.27	-11.27	-11.27	-11.27	-11.27	-11.27
-11.26	-11.26	-11.26	-11.26	-11.26	-11.26	-11.26	-11.26	-11.26
-11.25	-11.25	-11.25	-11.25	-11.25	-11.25	-11.25	-11.25	-11.25
-11.24	-11.24	-11.24	-11.24	-11.24	-11.24	-11.24	-11.24	-11.24
-11.23	-11.23	-11.23	-11.23	-11.23	-11.23	-11.23	-11.23	-11.23
-11.22	-11.22	-11.22	-11.22	-11.22	-11.22	-11.22	-11.22	-11.22
-11.21	-11.21	-11.21	-11.21	-11.21	-11.21	-11.21	-11.21	-11.21
-11.2	-11.2	-11.2	-11.2	-11.2	-11.2	-11.2	-11.2	-11.2
-11.19	-11.19	-11.19	-11.19	-11.19	-11.19	-11.19	-11.19	-11.19
-11.18	-11.18	-11.18	-11.18	-11.18	-11.18	-11.18	-11.18	-11.18
-11.17	-11.17	-11.17	-11.17	-11.17	-11.17	-11.17	-11.17	-11.17
-11.16	-11.16	-11.16	-11.16	-11.16	-11.16	-11.16	-11.16	-11.16
-11.15	-11.15	-11.15	-11.15	-11.15	-11.15	-11.15	-11.15	-11.15
-11.14	-11.14	-11.14	-11.14	-11.14	-11.14	-11.14	-11.14	-11.14
-11.13	-11.13	-11.13	-11.13	-11.13	-11.13	-11.13	-11.13	-11.13
-11.12	-11.12	-11.12	-11.12	-11.12	-11.12	-11.12	-11.12	-11.12
-11.11	-11.11	-11.11	-11.11	-11.11	-11.11	-11.11	-11.11	-11.11
-11.1	-11.1	-11.1	-11.1	-11.1	-11.1	-11.1	-11.1	-11.1
-11.09	-11.09	-11.09	-11.09	-11.09	-11.09	-11.09	-11.09	-11.09
-11.08	-11.08	-11.08	-11.08	-11.08	-11.08	-11.08	-11.08	-11.08
-11.07	-11.07	-11.07	-11.07	-11.07	-11.07	-11.07	-11.07	-11.07
-11.06	-11.06	-11.06	-11.06	-11.06	-11.06	-11.06	-11.06	-11.06
-11.05	-11.05	-11.05	-11.05	-11.05	-11.05	-11.05	-11.05	-11.05
-11.04	-11.04	-11.04	-11.04	-11.04	-11.04	-11.04	-11.04	-11.04
-11.03	-11.03	-11.03	-11.03	-11.03	-11.03	-11.03	-11.03	-11.03
-11.02	-11.02	-11.02	-11.02	-11.02	-11.02	-11.02	-11.02	-11.02
-11.01	-11.01	-11.01	-11.01	-11.01	-11.01	-11.01	-11.01	-11.01

-11	-11	-11	-11	-11	-11	-11	-11	-11
-10.99	-10.99	-10.99	-10.99	-10.99	-10.99	-10.99	-10.99	-10.99
-10.98	-10.98	-10.98	-10.98	-10.98	-10.98	-10.98	-10.98	-10.98
-10.97	-10.97	-10.97	-10.97	-10.97	-10.97	-10.97	-10.97	-10.97
-10.96	-10.96	-10.96	-10.96	-10.96	-10.96	-10.96	-10.96	-10.96
-10.95	-10.95	-10.95	-10.95	-10.95	-10.95	-10.95	-10.95	-10.95
-10.94	-10.94	-10.94	-10.94	-10.94	-10.94	-10.94	-10.94	-10.94
-10.93	-10.93	-10.93	-10.93	-10.93	-10.93	-10.93	-10.93	-10.93
-10.92	-10.92	-10.92	-10.92	-10.92	-10.92	-10.92	-10.92	-10.92
-10.91	-10.91	-10.91	-10.91	-10.91	-10.91	-10.91	-10.91	-10.91
-10.9	-10.9	-10.9	-10.9	-10.9	-10.9	-10.9	-10.9	-10.9
-10.89	-10.89	-10.89	-10.89	-10.89	-10.89	-10.89	-10.89	-10.89
-10.88	-10.88	-10.88	-10.88	-10.88	-10.88	-10.88	-10.88	-10.88
-10.87	-10.87	-10.87	-10.87	-10.87	-10.87	-10.87	-10.87	-10.87
-10.86	-10.86	-10.86	-10.86	-10.86	-10.86	-10.86	-10.86	-10.86
-10.85	-10.85	-10.85	-10.85	-10.85	-10.85	-10.85	-10.85	-10.85
-10.84	-10.84	-10.84	-10.84	-10.84	-10.84	-10.84	-10.84	-10.84
-10.83	-10.83	-10.83	-10.83	-10.83	-10.83	-10.83	-10.83	-10.83
-10.82	-10.82	-10.82	-10.82	-10.82	-10.82	-10.82	-10.82	-10.82
-10.81	-10.81	-10.81	-10.81	-10.81	-10.81	-10.81	-10.81	-10.81
-10.8	-10.8	-10.8	-10.8	-10.8	-10.8	-10.8	-10.8	-10.8
-10.79	-10.79	-10.79	-10.79	-10.79	-10.79	-10.79	-10.79	-10.79
-10.78	-10.78	-10.78	-10.78	-10.78	-10.78	-10.78	-10.78	-10.78
-10.77	-10.77	-10.77	-10.77	-10.77	-10.77	-10.77	-10.77	-10.77
-10.76	-10.76	-10.76	-10.76	-10.76	-10.76	-10.76	-10.76	-10.76
-10.75	-10.75	-10.75	-10.75	-10.75	-10.75	-10.75	-10.75	-10.75
-10.74	-10.74	-10.74	-10.74	-10.74	-10.74	-10.74	-10.74	-10.74
-10.73	-10.73	-10.73	-10.73	-10.73	-10.73	-10.73	-10.73	-10.73
-10.72	-10.72	-10.72	-10.72	-10.72	-10.72	-10.72	-10.72	-10.72
-10.71	-10.71	-10.71	-10.71	-10.71	-10.71	-10.71	-10.71	-10.71
-10.7	-10.7	-10.7	-10.7	-10.7	-10.7	-10.7	-10.7	-10.7
-10.69	-10.69	-10.69	-10.69	-10.69	-10.69	-10.69	-10.69	-10.69
-10.68	-10.68	-10.68	-10.68	-10.68	-10.68	-10.68	-10.68	-10.68
-10.67	-10.67	-10.67	-10.67	-10.67	-10.67	-10.67	-10.67	-10.67
-10.66	-10.66	-10.66	-10.66	-10.66	-10.66	-10.66	-10.66	-10.66
-10.65	-10.65	-10.65	-10.65	-10.65	-10.65	-10.65	-10.65	-10.65
-10.64	-10.64	-10.64	-10.64	-10.64	-10.64	-10.64	-10.64	-10.64
-10.63	-10.63	-10.63	-10.63	-10.63	-10.63	-10.63	-10.63	-10.63
-10.62	-10.62	-10.62	-10.62	-10.62	-10.62	-10.62	-10.62	-10.62
-10.61	-10.61	-10.61	-10.61	-10.61	-10.61	-10.61	-10.61	-10.61
-10.6	-10.6	-10.6	-10.6	-10.6	-10.6	-10.6	-10.6	-10.6
-10.59	-10.59	-10.59	-10.59	-10.59	-10.59	-10.59	-10.59	-10.59
-10.58	-10.58	-10.58	-10.58	-10.58	-10.58	-10.58	-10.58	-10.58
-10.57	-10.57	-10.57	-10.57	-10.57	-10.57	-10.57	-10.57	-10.57
-10.56	-10.56	-10.56	-10.56	-10.56	-10.56	-10.56	-10.56	-10.56
-10.55	-10.55	-10.55	-10.55	-10.55	-10.55	-10.55	-10.55	-10.55
-10.54	-10.54	-10.54	-10.54	-10.54	-10.54	-10.54	-10.54	-10.54
-10.53	-10.53	-10.53	-10.53	-10.53	-10.53	-10.53	-10.53	-10.53
-10.52	-10.52	-10.52	-10.52	-10.52	-10.52	-10.52	-10.52	-10.52
-10.51	-10.51	-10.51	-10.51	-10.51	-10.51	-10.51	-10.51	-10.51

-10.5	-10.5	-10.5	-10.5	-10.5	-10.5	-10.5	-10.5	-10.5
-10.49	-10.49	-10.49	-10.49	-10.49	-10.49	-10.49	-10.49	-10.49
-10.48	-10.48	-10.48	-10.48	-10.48	-10.48	-10.48	-10.48	-10.48
-10.47	-10.47	-10.47	-10.47	-10.47	-10.47	-10.47	-10.47	-10.47
-10.46	-10.46	-10.46	-10.46	-10.46	-10.46	-10.46	-10.46	-10.46
-10.45	-10.45	-10.45	-10.45	-10.45	-10.45	-10.45	-10.45	-10.45
-10.44	-10.44	-10.44	-10.44	-10.44	-10.44	-10.44	-10.44	-10.44
-10.43	-10.43	-10.43	-10.43	-10.43	-10.43	-10.43	-10.43	-10.43
-10.42	-10.42	-10.42	-10.42	-10.42	-10.42	-10.42	-10.42	-10.42
-10.41	-10.41	-10.41	-10.41	-10.41	-10.41	-10.41	-10.41	-10.41
-10.4	-10.4	-10.4	-10.4	-10.4	-10.4	-10.4	-10.4	-10.4
-10.39	-10.39	-10.39	-10.39	-10.39	-10.39	-10.39	-10.39	-10.39
-10.38	-10.38	-10.38	-10.38	-10.38	-10.38	-10.38	-10.38	-10.38
-10.37	-10.37	-10.37	-10.37	-10.37	-10.37	-10.37	-10.37	-10.37
-10.36	-10.36	-10.36	-10.36	-10.36	-10.36	-10.36	-10.36	-10.36
-10.35	-10.35	-10.35	-10.35	-10.35	-10.35	-10.35	-10.35	-10.35
-10.34	-10.34	-10.34	-10.34	-10.34	-10.34	-10.34	-10.34	-10.34
-10.33	-10.33	-10.33	-10.33	-10.33	-10.33	-10.33	-10.33	-10.33
-10.32	-10.32	-10.32	-10.32	-10.32	-10.32	-10.32	-10.32	-10.32
-10.31	-10.31	-10.31	-10.31	-10.31	-10.31	-10.31	-10.31	-10.31
-10.3	-10.3	-10.3	-10.3	-10.3	-10.3	-10.3	-10.3	-10.3
-10.29	-10.29	-10.29	-10.29	-10.29	-10.29	-10.29	-10.29	-10.29
-10.28	-10.28	-10.28	-10.28	-10.28	-10.28	-10.28	-10.28	-10.28
-10.27	-10.27	-10.27	-10.27	-10.27	-10.27	-10.27	-10.27	-10.27
-10.26	-10.26	-10.26	-10.26	-10.26	-10.26	-10.26	-10.26	-10.26
-10.25	-10.25	-10.25	-10.25	-10.25	-10.25	-10.25	-10.25	-10.25
-10.24	-10.24	-10.24	-10.24	-10.24	-10.24	-10.24	-10.24	-10.24
-10.23	-10.23	-10.23	-10.23	-10.23	-10.23	-10.23	-10.23	-10.23
-10.22	-10.22	-10.22	-10.22	-10.22	-10.22	-10.22	-10.22	-10.22
-10.21	-10.21	-10.21	-10.21	-10.21	-10.21	-10.21	-10.21	-10.21
-10.2	-10.2	-10.2	-10.2	-10.2	-10.2	-10.2	-10.2	-10.2
-10.19	-10.19	-10.19	-10.19	-10.19	-10.19	-10.19	-10.19	-10.19
-10.18	-10.18	-10.18	-10.18	-10.18	-10.18	-10.18	-10.18	-10.18
-10.17	-10.17	-10.17	-10.17	-10.17	-10.17	-10.17	-10.17	-10.17
-10.16	-10.16	-10.16	-10.16	-10.16	-10.16	-10.16	-10.16	-10.16
-10.15	-10.15	-10.15	-10.15	-10.15	-10.15	-10.15	-10.15	-10.15
-10.14	-10.14	-10.14	-10.14	-10.14	-10.14	-10.14	-10.14	-10.14
-10.13	-10.13	-10.13	-10.13	-10.13	-10.13	-10.13	-10.13	-10.13
-10.12	-10.12	-10.12	-10.12	-10.12	-10.12	-10.12	-10.12	-10.12
-10.11	-10.11	-10.11	-10.11	-10.11	-10.11	-10.11	-10.11	-10.11
-10.1	-10.1	-10.1	-10.1	-10.1	-10.1	-10.1	-10.1	-10.1
-10.09	-10.09	-10.09	-10.09	-10.09	-10.09	-10.09	-10.09	-10.09
-10.08	-10.08	-10.08	-10.08	-10.08	-10.08	-10.08	-10.08	-10.08
-10.07	-10.07	-10.07	-10.07	-10.07	-10.07	-10.07	-10.07	-10.07
-10.06	-10.06	-10.06	-10.06	-10.06	-10.06	-10.06	-10.06	-10.06
-10.05	-10.05	-10.05	-10.05	-10.05	-10.05	-10.05	-10.05	-10.05
-10.04	-10.04	-10.04	-10.04	-10.04	-10.04	-10.04	-10.04	-10.04
-10.03	-10.03	-10.03	-10.03	-10.03	-10.03	-10.03	-10.03	-10.03
-10.02	-10.02	-10.02	-10.02	-10.02	-10.02	-10.02	-10.02	-10.02
-10.01	-10.01	-10.01	-10.01	-10.01	-10.01	-10.01	-10.01	-10.01

-10	-10	-10	-10	-10	-10	-10	-10	-10
-9.99	-9.99	-9.99	-9.99	-9.99	-9.99	-9.99	-9.99	-9.99
-9.98	-9.98	-9.98	-9.98	-9.98	-9.98	-9.98	-9.98	-9.98
-9.97	-9.97	-9.97	-9.97	-9.97	-9.97	-9.97	-9.97	-9.97
-9.96	-9.96	-9.96	-9.96	-9.96	-9.96	-9.96	-9.96	-9.96
-9.95	-9.95	-9.95	-9.95	-9.95	-9.95	-9.95	-9.95	-9.95
-9.94	-9.94	-9.94	-9.94	-9.94	-9.94	-9.94	-9.94	-9.94
-9.93	-9.93	-9.93	-9.93	-9.93	-9.93	-9.93	-9.93	-9.93
-9.92	-9.92	-9.92	-9.92	-9.92	-9.92	-9.92	-9.92	-9.92
-9.91	-9.91	-9.91	-9.91	-9.91	-9.91	-9.91	-9.91	-9.91
-9.9	-9.9	-9.9	-9.9	-9.9	-9.9	-9.9	-9.9	-9.9
-9.89	-9.89	-9.89	-9.89	-9.89	-9.89	-9.89	-9.89	-9.89
-9.88	-9.88	-9.88	-9.88	-9.88	-9.88	-9.88	-9.88	-9.88
-9.87	-9.87	-9.87	-9.87	-9.87	-9.87	-9.87	-9.87	-9.87
-9.86	-9.86	-9.86	-9.86	-9.86	-9.86	-9.86	-9.86	-9.86
-9.85	-9.85	-9.85	-9.85	-9.85	-9.85	-9.85	-9.85	-9.85
-9.84	-9.84	-9.84	-9.84	-9.84	-9.84	-9.84	-9.84	-9.84
-9.83	-9.83	-9.83	-9.83	-9.83	-9.83	-9.83	-9.83	-9.83
-9.82	-9.82	-9.82	-9.82	-9.82	-9.82	-9.82	-9.82	-9.82
-9.81	-9.81	-9.81	-9.81	-9.81	-9.81	-9.81	-9.81	-9.81
-9.8	-9.8	-9.8	-9.8	-9.8	-9.8	-9.8	-9.8	-9.8
-9.79	-9.79	-9.79	-9.79	-9.79	-9.79	-9.79	-9.79	-9.79
-9.78	-9.78	-9.78	-9.78	-9.78	-9.78	-9.78	-9.78	-9.78
-9.77	-9.77	-9.77	-9.77	-9.77	-9.77	-9.77	-9.77	-9.77
-9.76	-9.76	-9.76	-9.76	-9.76	-9.76	-9.76	-9.76	-9.76
-9.75	-9.75	-9.75	-9.75	-9.75	-9.75	-9.75	-9.75	-9.75
-9.74	-9.74	-9.74	-9.74	-9.74	-9.74	-9.74	-9.74	-9.74
-9.73	-9.73	-9.73	-9.73	-9.73	-9.73	-9.73	-9.73	-9.73
-9.72	-9.72	-9.72	-9.72	-9.72	-9.72	-9.72	-9.72	-9.72
-9.71	-9.71	-9.71	-9.71	-9.71	-9.71	-9.71	-9.71	-9.71
-9.7	-9.7	-9.7	-9.7	-9.7	-9.7	-9.7	-9.7	-9.7
-9.69	-9.69	-9.69	-9.69	-9.69	-9.69	-9.69	-9.69	-9.69
-9.68	-9.68	-9.68	-9.68	-9.68	-9.68	-9.68	-9.68	-9.68
-9.67	-9.67	-9.67	-9.67	-9.67	-9.67	-9.67	-9.67	-9.67
-9.66	-9.66	-9.66	-9.66	-9.66	-9.66	-9.66	-9.66	-9.66
-9.65	-9.65	-9.65	-9.65	-9.65	-9.65	-9.65	-9.65	-9.65
-9.64	-9.64	-9.64	-9.64	-9.64	-9.64	-9.64	-9.64	-9.64
-9.63	-9.63	-9.63	-9.63	-9.63	-9.63	-9.63	-9.63	-9.63
-9.62	-9.62	-9.62	-9.62	-9.62	-9.62	-9.62	-9.62	-9.62
-9.61	-9.61	-9.61	-9.61	-9.61	-9.61	-9.61	-9.61	-9.61
-9.6	-9.6	-9.6	-9.6	-9.6	-9.6	-9.6	-9.6	-9.6
-9.59	-9.59	-9.59	-9.59	-9.59	-9.59	-9.59	-9.59	-9.59
-9.58	-9.58	-9.58	-9.58	-9.58	-9.58	-9.58	-9.58	-9.58
-9.57	-9.57	-9.57	-9.57	-9.57	-9.57	-9.57	-9.57	-9.57
-9.56	-9.56	-9.56	-9.56	-9.56	-9.56	-9.56	-9.56	-9.56
-9.55	-9.55	-9.55	-9.55	-9.55	-9.55	-9.55	-9.55	-9.55
-9.54	-9.54	-9.54	-9.54	-9.54	-9.54	-9.54	-9.54	-9.54
-9.53	-9.53	-9.53	-9.53	-9.53	-9.53	-9.53	-9.53	-9.53
-9.52	-9.52	-9.52	-9.52	-9.52	-9.52	-9.52	-9.52	-9.52
-9.51	-9.51	-9.51	-9.51	-9.51	-9.51	-9.51	-9.51	-9.51

-9.5	-9.5	-9.5	-9.5	-9.5	-9.5	-9.5	-9.5	-9.5
-9.49	-9.49	-9.49	-9.49	-9.49	-9.49	-9.49	-9.49	-9.49
-9.48	-9.48	-9.48	-9.48	-9.48	-9.48	-9.48	-9.48	-9.48
-9.47	-9.47	-9.47	-9.47	-9.47	-9.47	-9.47	-9.47	-9.47
-9.46	-9.46	-9.46	-9.46	-9.46	-9.46	-9.46	-9.46	-9.46
-9.45	-9.45	-9.45	-9.45	-9.45	-9.45	-9.45	-9.45	-9.45
-9.44	-9.44	-9.44	-9.44	-9.44	-9.44	-9.44	-9.44	-9.44
-9.43	-9.43	-9.43	-9.43	-9.43	-9.43	-9.43	-9.43	-9.43
-9.42	-9.42	-9.42	-9.42	-9.42	-9.42	-9.42	-9.42	-9.42
-9.41	-9.41	-9.41	-9.41	-9.41	-9.41	-9.41	-9.41	-9.41
-9.4	-9.4	-9.4	-9.4	-9.4	-9.4	-9.4	-9.4	-9.4
-9.39	-9.39	-9.39	-9.39	-9.39	-9.39	-9.39	-9.39	-9.39
-9.38	-9.38	-9.38	-9.38	-9.38	-9.38	-9.38	-9.38	-9.38
-9.37	-9.37	-9.37	-9.37	-9.37	-9.37	-9.37	-9.37	-9.37
-9.36	-9.36	-9.36	-9.36	-9.36	-9.36	-9.36	-9.36	-9.36
-9.35	-9.35	-9.35	-9.35	-9.35	-9.35	-9.35	-9.35	-9.35
-9.34	-9.34	-9.34	-9.34	-9.34	-9.34	-9.34	-9.34	-9.34
-9.33	-9.33	-9.33	-9.33	-9.33	-9.33	-9.33	-9.33	-9.33
-9.32	-9.32	-9.32	-9.32	-9.32	-9.32	-9.32	-9.32	-9.32
-9.31	-9.31	-9.31	-9.31	-9.31	-9.31	-9.31	-9.31	-9.31
-9.3	-9.3	-9.3	-9.3	-9.3	-9.3	-9.3	-9.3	-9.3
-9.29	-9.29	-9.29	-9.29	-9.29	-9.29	-9.29	-9.29	-9.29
-9.28	-9.28	-9.28	-9.28	-9.28	-9.28	-9.28	-9.28	-9.28
-9.27	-9.27	-9.27	-9.27	-9.27	-9.27	-9.27	-9.27	-9.27
-9.26	-9.26	-9.26	-9.26	-9.26	-9.26	-9.26	-9.26	-9.26
-9.25	-9.25	-9.25	-9.25	-9.25	-9.25	-9.25	-9.25	-9.25
-9.24	-9.24	-9.24	-9.24	-9.24	-9.24	-9.24	-9.24	-9.24
-9.23	-9.23	-9.23	-9.23	-9.23	-9.23	-9.23	-9.23	-9.23
-9.22	-9.22	-9.22	-9.22	-9.22	-9.22	-9.22	-9.22	-9.22
-9.21	-9.21	-9.21	-9.21	-9.21	-9.21	-9.21	-9.21	-9.21
-9.2	-9.2	-9.2	-9.2	-9.2	-9.2	-9.2	-9.2	-9.2
-9.19	-9.19	-9.19	-9.19	-9.19	-9.19	-9.19	-9.19	-9.19
-9.18	-9.18	-9.18	-9.18	-9.18	-9.18	-9.18	-9.18	-9.18
-9.17	-9.17	-9.17	-9.17	-9.17	-9.17	-9.17	-9.17	-9.17
-9.16	-9.16	-9.16	-9.16	-9.16	-9.16	-9.16	-9.16	-9.16
-9.15	-9.15	-9.15	-9.15	-9.15	-9.15	-9.15	-9.15	-9.15
-9.14	-9.14	-9.14	-9.14	-9.14	-9.14	-9.14	-9.14	-9.14
-9.13	-9.13	-9.13	-9.13	-9.13	-9.13	-9.13	-9.13	-9.13
-9.12	-9.12	-9.12	-9.12	-9.12	-9.12	-9.12	-9.12	-9.12
-9.11	-9.11	-9.11	-9.11	-9.11	-9.11	-9.11	-9.11	-9.11
-9.1	-9.1	-9.1	-9.1	-9.1	-9.1	-9.1	-9.1	-9.1
-9.09	-9.09	-9.09	-9.09	-9.09	-9.09	-9.09	-9.09	-9.09
-9.08	-9.08	-9.08	-9.08	-9.08	-9.08	-9.08	-9.08	-9.08
-9.07	-9.07	-9.07	-9.07	-9.07	-9.07	-9.07	-9.07	-9.07
-9.06	-9.06	-9.06	-9.06	-9.06	-9.06	-9.06	-9.06	-9.06
-9.05	-9.05	-9.05	-9.05	-9.05	-9.05	-9.05	-9.05	-9.05
-9.04	-9.04	-9.04	-9.04	-9.04	-9.04	-9.04	-9.04	-9.04
-9.03	-9.03	-9.03	-9.03	-9.03	-9.03	-9.03	-9.03	-9.03
-9.02	-9.02	-9.02	-9.02	-9.02	-9.02	-9.02	-9.02	-9.02
-9.01	-9.01	-9.01	-9.01	-9.01	-9.01	-9.01	-9.01	-9.01

-9	-9	-9	-9	-9	-9	-9	-9	-9
-8.99	-8.99	-8.99	-8.99	-8.99	-8.99	-8.99	-8.99	-8.99
-8.98	-8.98	-8.98	-8.98	-8.98	-8.98	-8.98	-8.98	-8.98
-8.97	-8.97	-8.97	-8.97	-8.97	-8.97	-8.97	-8.97	-8.97
-8.96	-8.96	-8.96	-8.96	-8.96	-8.96	-8.96	-8.96	-8.96
-8.95	-8.95	-8.95	-8.95	-8.95	-8.95	-8.95	-8.95	-8.95
-8.94	-8.94	-8.94	-8.94	-8.94	-8.94	-8.94	-8.94	-8.94
-8.93	-8.93	-8.93	-8.93	-8.93	-8.93	-8.93	-8.93	-8.93
-8.92	-8.92	-8.92	-8.92	-8.92	-8.92	-8.92	-8.92	-8.92
-8.91	-8.91	-8.91	-8.91	-8.91	-8.91	-8.91	-8.91	-8.91
-8.9	-8.9	-8.9	-8.9	-8.9	-8.9	-8.9	-8.9	-8.9
-8.89	-8.89	-8.89	-8.89	-8.89	-8.89	-8.89	-8.89	-8.89
-8.88	-8.88	-8.88	-8.88	-8.88	-8.88	-8.88	-8.88	-8.88
-8.87	-8.87	-8.87	-8.87	-8.87	-8.87	-8.87	-8.87	-8.87
-8.86	-8.86	-8.86	-8.86	-8.86	-8.86	-8.86	-8.86	-8.86
-8.85	-8.85	-8.85	-8.85	-8.85	-8.85	-8.85	-8.85	-8.85
-8.84	-8.84	-8.84	-8.84	-8.84	-8.84	-8.84	-8.84	-8.84
-8.83	-8.83	-8.83	-8.83	-8.83	-8.83	-8.83	-8.83	-8.83
-8.82	-8.82	-8.82	-8.82	-8.82	-8.82	-8.82	-8.82	-8.82
-8.81	-8.81	-8.81	-8.81	-8.81	-8.81	-8.81	-8.81	-8.81
-8.8	-8.8	-8.8	-8.8	-8.8	-8.8	-8.8	-8.8	-8.8
-8.79	-8.79	-8.79	-8.79	-8.79	-8.79	-8.79	-8.79	-8.79
-8.78	-8.78	-8.78	-8.78	-8.78	-8.78	-8.78	-8.78	-8.78
-8.77	-8.77	-8.77	-8.77	-8.77	-8.77	-8.77	-8.77	-8.77
-8.76	-8.76	-8.76	-8.76	-8.76	-8.76	-8.76	-8.76	-8.76
-8.75	-8.75	-8.75	-8.75	-8.75	-8.75	-8.75	-8.75	-8.75
-8.74	-8.74	-8.74	-8.74	-8.74	-8.74	-8.74	-8.74	-8.74
-8.73	-8.73	-8.73	-8.73	-8.73	-8.73	-8.73	-8.73	-8.73
-8.72	-8.72	-8.72	-8.72	-8.72	-8.72	-8.72	-8.72	-8.72
-8.71	-8.71	-8.71	-8.71	-8.71	-8.71	-8.71	-8.71	-8.71
-8.7	-8.7	-8.7	-8.7	-8.7	-8.7	-8.7	-8.7	-8.7
-8.69	-8.69	-8.69	-8.69	-8.69	-8.69	-8.69	-8.69	-8.69
-8.68	-8.68	-8.68	-8.68	-8.68	-8.68	-8.68	-8.68	-8.68
-8.67	-8.67	-8.67	-8.67	-8.67	-8.67	-8.67	-8.67	-8.67
-8.66	-8.66	-8.66	-8.66	-8.66	-8.66	-8.66	-8.66	-8.66
-8.65	-8.65	-8.65	-8.65	-8.65	-8.65	-8.65	-8.65	-8.65
-8.64	-8.64	-8.64	-8.64	-8.64	-8.64	-8.64	-8.64	-8.64
-8.63	-8.63	-8.63	-8.63	-8.63	-8.63	-8.63	-8.63	-8.63
-8.62	-8.62	-8.62	-8.62	-8.62	-8.62	-8.62	-8.62	-8.62
-8.61	-8.61	-8.61	-8.61	-8.61	-8.61	-8.61	-8.61	-8.61
-8.6	-8.6	-8.6	-8.6	-8.6	-8.6	-8.6	-8.6	-8.6
-8.59	-8.59	-8.59	-8.59	-8.59	-8.59	-8.59	-8.59	-8.59
-8.58	-8.58	-8.58	-8.58	-8.58	-8.58	-8.58	-8.58	-8.58
-8.57	-8.57	-8.57	-8.57	-8.57	-8.57	-8.57	-8.57	-8.57
-8.56	-8.56	-8.56	-8.56	-8.56	-8.56	-8.56	-8.56	-8.56
-8.55	-8.55	-8.55	-8.55	-8.55	-8.55	-8.55	-8.55	-8.55
-8.54	-8.54	-8.54	-8.54	-8.54	-8.54	-8.54	-8.54	-8.54
-8.53	-8.53	-8.53	-8.53	-8.53	-8.53	-8.53	-8.53	-8.53
-8.52	-8.52	-8.52	-8.52	-8.52	-8.52	-8.52	-8.52	-8.52
-8.51	-8.51	-8.51	-8.51	-8.51	-8.51	-8.51	-8.51	-8.51

-8.5	-8.5	-8.5	-8.5	-8.5	-8.5	-8.5	-8.5	-8.5
-8.49	-8.49	-8.49	-8.49	-8.49	-8.49	-8.49	-8.49	-8.49
-8.48	-8.48	-8.48	-8.48	-8.48	-8.48	-8.48	-8.48	-8.48
-8.47	-8.47	-8.47	-8.47	-8.47	-8.47	-8.47	-8.47	-8.47
-8.46	-8.46	-8.46	-8.46	-8.46	-8.46	-8.46	-8.46	-8.46
-8.45	-8.45	-8.45	-8.45	-8.45	-8.45	-8.45	-8.45	-8.45
-8.44	-8.44	-8.44	-8.44	-8.44	-8.44	-8.44	-8.44	-8.44
-8.43	-8.43	-8.43	-8.43	-8.43	-8.43	-8.43	-8.43	-8.43
-8.42	-8.42	-8.42	-8.42	-8.42	-8.42	-8.42	-8.42	-8.42
-8.41	-8.41	-8.41	-8.41	-8.41	-8.41	-8.41	-8.41	-8.41
-8.4	-8.4	-8.4	-8.4	-8.4	-8.4	-8.4	-8.4	-8.4
-8.39	-8.39	-8.39	-8.39	-8.39	-8.39	-8.39	-8.39	-8.39
-8.38	-8.38	-8.38	-8.38	-8.38	-8.38	-8.38	-8.38	-8.38
-8.37	-8.37	-8.37	-8.37	-8.37	-8.37	-8.37	-8.37	-8.37
-8.36	-8.36	-8.36	-8.36	-8.36	-8.36	-8.36	-8.36	-8.36
-8.35	-8.35	-8.35	-8.35	-8.35	-8.35	-8.35	-8.35	-8.35
-8.34	-8.34	-8.34	-8.34	-8.34	-8.34	-8.34	-8.34	-8.34
-8.33	-8.33	-8.33	-8.33	-8.33	-8.33	-8.33	-8.33	-8.33
-8.32	-8.32	-8.32	-8.32	-8.32	-8.32	-8.32	-8.32	-8.32
-8.31	-8.31	-8.31	-8.31	-8.31	-8.31	-8.31	-8.31	-8.31
-8.3	-8.3	-8.3	-8.3	-8.3	-8.3	-8.3	-8.3	-8.3
-8.29	-8.29	-8.29	-8.29	-8.29	-8.29	-8.29	-8.29	-8.29
-8.28	-8.28	-8.28	-8.28	-8.28	-8.28	-8.28	-8.28	-8.28
-8.27	-8.27	-8.27	-8.27	-8.27	-8.27	-8.27	-8.27	-8.27
-8.26	-8.26	-8.26	-8.26	-8.26	-8.26	-8.26	-8.26	-8.26
-8.25	-8.25	-8.25	-8.25	-8.25	-8.25	-8.25	-8.25	-8.25
-8.24	-8.24	-8.24	-8.24	-8.24	-8.24	-8.24	-8.24	-8.24
-8.23	-8.23	-8.23	-8.23	-8.23	-8.23	-8.23	-8.23	-8.23
-8.22	-8.22	-8.22	-8.22	-8.22	-8.22	-8.22	-8.22	-8.22
-8.21	-8.21	-8.21	-8.21	-8.21	-8.21	-8.21	-8.21	-8.21
-8.2	-8.2	-8.2	-8.2	-8.2	-8.2	-8.2	-8.2	-8.2
-8.19	-8.19	-8.19	-8.19	-8.19	-8.19	-8.19	-8.19	-8.19
-8.18	-8.18	-8.18	-8.18	-8.18	-8.18	-8.18	-8.18	-8.18
-8.17	-8.17	-8.17	-8.17	-8.17	-8.17	-8.17	-8.17	-8.17
-8.16	-8.16	-8.16	-8.16	-8.16	-8.16	-8.16	-8.16	-8.16
-8.15	-8.15	-8.15	-8.15	-8.15	-8.15	-8.15	-8.15	-8.15
-8.14	-8.14	-8.14	-8.14	-8.14	-8.14	-8.14	-8.14	-8.14
-8.13	-8.13	-8.13	-8.13	-8.13	-8.13	-8.13	-8.13	-8.13
-8.12	-8.12	-8.12	-8.12	-8.12	-8.12	-8.12	-8.12	-8.12
-8.11	-8.11	-8.11	-8.11	-8.11	-8.11	-8.11	-8.11	-8.11
-8.1	-8.1	-8.1	-8.1	-8.1	-8.1	-8.1	-8.1	-8.1
-8.09	-8.09	-8.09	-8.09	-8.09	-8.09	-8.09	-8.09	-8.09
-8.08	-8.08	-8.08	-8.08	-8.08	-8.08	-8.08	-8.08	-8.08
-8.07	-8.07	-8.07	-8.07	-8.07	-8.07	-8.07	-8.07	-8.07
-8.06	-8.06	-8.06	-8.06	-8.06	-8.06	-8.06	-8.06	-8.06
-8.05	-8.05	-8.05	-8.05	-8.05	-8.05	-8.05	-8.05	-8.05
-8.04	-8.04	-8.04	-8.04	-8.04	-8.04	-8.04	-8.04	-8.04
-8.03	-8.03	-8.03	-8.03	-8.03	-8.03	-8.03	-8.03	-8.03
-8.02	-8.02	-8.02	-8.02	-8.02	-8.02	-8.02	-8.02	-8.02
-8.01	-8.01	-8.01	-8.01	-8.01	-8.01	-8.01	-8.01	-8.01

-8	-8	-8	-8	-8	-8	-8	-8	-8
-7.99	-7.99	-7.99	-7.99	-7.99	-7.99	-7.99	-7.99	-7.99
-7.98	-7.98	-7.98	-7.98	-7.98	-7.98	-7.98	-7.98	-7.98
-7.97	-7.97	-7.97	-7.97	-7.97	-7.97	-7.97	-7.97	-7.97
-7.96	-7.96	-7.96	-7.96	-7.96	-7.96	-7.96	-7.96	-7.96
-7.95	-7.95	-7.95	-7.95	-7.95	-7.95	-7.95	-7.95	-7.95
-7.94	-7.94	-7.94	-7.94	-7.94	-7.94	-7.94	-7.94	-7.94
-7.93	-7.93	-7.93	-7.93	-7.93	-7.93	-7.93	-7.93	-7.93
-7.92	-7.92	-7.92	-7.92	-7.92	-7.92	-7.92	-7.92	-7.92
-7.91	-7.91	-7.91	-7.91	-7.91	-7.91	-7.91	-7.91	-7.91
-7.9	-7.9	-7.9	-7.9	-7.9	-7.9	-7.9	-7.9	-7.9
-7.89	-7.89	-7.89	-7.89	-7.89	-7.89	-7.89	-7.89	-7.89
-7.88	-7.88	-7.88	-7.88	-7.88	-7.88	-7.88	-7.88	-7.88
-7.87	-7.87	-7.87	-7.87	-7.87	-7.87	-7.87	-7.87	-7.87
-7.86	-7.86	-7.86	-7.86	-7.86	-7.86	-7.86	-7.86	-7.86
-7.85	-7.85	-7.85	-7.85	-7.85	-7.85	-7.85	-7.85	-7.85
-7.84	-7.84	-7.84	-7.84	-7.84	-7.84	-7.84	-7.84	-7.84
-7.83	-7.83	-7.83	-7.83	-7.83	-7.83	-7.83	-7.83	-7.83
-7.82	-7.82	-7.82	-7.82	-7.82	-7.82	-7.82	-7.82	-7.82
-7.81	-7.81	-7.81	-7.81	-7.81	-7.81	-7.81	-7.81	-7.81
-7.8	-7.8	-7.8	-7.8	-7.8	-7.8	-7.8	-7.8	-7.8
-7.79	-7.79	-7.79	-7.79	-7.79	-7.79	-7.79	-7.79	-7.79
-7.78	-7.78	-7.78	-7.78	-7.78	-7.78	-7.78	-7.78	-7.78
-7.77	-7.77	-7.77	-7.77	-7.77	-7.77	-7.77	-7.77	-7.77
-7.76	-7.76	-7.76	-7.76	-7.76	-7.76	-7.76	-7.76	-7.76
-7.75	-7.75	-7.75	-7.75	-7.75	-7.75	-7.75	-7.75	-7.75
-7.74	-7.74	-7.74	-7.74	-7.74	-7.74	-7.74	-7.74	-7.74
-7.73	-7.73	-7.73	-7.73	-7.73	-7.73	-7.73	-7.73	-7.73
-7.72	-7.72	-7.72	-7.72	-7.72	-7.72	-7.72	-7.72	-7.72
-7.71	-7.71	-7.71	-7.71	-7.71	-7.71	-7.71	-7.71	-7.71
-7.7	-7.7	-7.7	-7.7	-7.7	-7.7	-7.7	-7.7	-7.7
-7.69	-7.69	-7.69	-7.69	-7.69	-7.69	-7.69	-7.69	-7.69
-7.68	-7.68	-7.68	-7.68	-7.68	-7.68	-7.68	-7.68	-7.68
-7.67	-7.67	-7.67	-7.67	-7.67	-7.67	-7.67	-7.67	-7.67
-7.66	-7.66	-7.66	-7.66	-7.66	-7.66	-7.66	-7.66	-7.66
-7.65	-7.65	-7.65	-7.65	-7.65	-7.65	-7.65	-7.65	-7.65
-7.64	-7.64	-7.64	-7.64	-7.64	-7.64	-7.64	-7.64	-7.64
-7.63	-7.63	-7.63	-7.63	-7.63	-7.63	-7.63	-7.63	-7.63
-7.62	-7.62	-7.62	-7.62	-7.62	-7.62	-7.62	-7.62	-7.62
-7.61	-7.61	-7.61	-7.61	-7.61	-7.61	-7.61	-7.61	-7.61
-7.6	-7.6	-7.6	-7.6	-7.6	-7.6	-7.6	-7.6	-7.6
-7.59	-7.59	-7.59	-7.59	-7.59	-7.59	-7.59	-7.59	-7.59
-7.58	-7.58	-7.58	-7.58	-7.58	-7.58	-7.58	-7.58	-7.58
-7.57	-7.57	-7.57	-7.57	-7.57	-7.57	-7.57	-7.57	-7.57
-7.56	-7.56	-7.56	-7.56	-7.56	-7.56	-7.56	-7.56	-7.56
-7.55	-7.55	-7.55	-7.55	-7.55	-7.55	-7.55	-7.55	-7.55
-7.54	-7.54	-7.54	-7.54	-7.54	-7.54	-7.54	-7.54	-7.54
-7.53	-7.53	-7.53	-7.53	-7.53	-7.53	-7.53	-7.53	-7.53
-7.52	-7.52	-7.52	-7.52	-7.52	-7.52	-7.52	-7.52	-7.52
-7.51	-7.51	-7.51	-7.51	-7.51	-7.51	-7.51	-7.51	-7.51

-7.5	-7.5	-7.5	-7.5	-7.5	-7.5	-7.5	-7.5	-7.5
-7.49	-7.49	-7.49	-7.49	-7.49	-7.49	-7.49	-7.49	-7.49
-7.48	-7.48	-7.48	-7.48	-7.48	-7.48	-7.48	-7.48	-7.48
-7.47	-7.47	-7.47	-7.47	-7.47	-7.47	-7.47	-7.47	-7.47
-7.46	-7.46	-7.46	-7.46	-7.46	-7.46	-7.46	-7.46	-7.46
-7.45	-7.45	-7.45	-7.45	-7.45	-7.45	-7.45	-7.45	-7.45
-7.44	-7.44	-7.44	-7.44	-7.44	-7.44	-7.44	-7.44	-7.44
-7.43	-7.43	-7.43	-7.43	-7.43	-7.43	-7.43	-7.43	-7.43
-7.42	-7.42	-7.42	-7.42	-7.42	-7.42	-7.42	-7.42	-7.42
-7.41	-7.41	-7.41	-7.41	-7.41	-7.41	-7.41	-7.41	-7.41
-7.4	-7.4	-7.4	-7.4	-7.4	-7.4	-7.4	-7.4	-7.4
-7.39	-7.39	-7.39	-7.39	-7.39	-7.39	-7.39	-7.39	-7.39
-7.38	-7.38	-7.38	-7.38	-7.38	-7.38	-7.38	-7.38	-7.38
-7.37	-7.37	-7.37	-7.37	-7.37	-7.37	-7.37	-7.37	-7.37
-7.36	-7.36	-7.36	-7.36	-7.36	-7.36	-7.36	-7.36	-7.36
-7.35	-7.35	-7.35	-7.35	-7.35	-7.35	-7.35	-7.35	-7.35
-7.34	-7.34	-7.34	-7.34	-7.34	-7.34	-7.34	-7.34	-7.34
-7.33	-7.33	-7.33	-7.33	-7.33	-7.33	-7.33	-7.33	-7.33
-7.32	-7.32	-7.32	-7.32	-7.32	-7.32	-7.32	-7.32	-7.32
-7.31	-7.31	-7.31	-7.31	-7.31	-7.31	-7.31	-7.31	-7.31
-7.3	-7.3	-7.3	-7.3	-7.3	-7.3	-7.3	-7.3	-7.3
-7.29	-7.29	-7.29	-7.29	-7.29	-7.29	-7.29	-7.29	-7.29
-7.28	-7.28	-7.28	-7.28	-7.28	-7.28	-7.28	-7.28	-7.28
-7.27	-7.27	-7.27	-7.27	-7.27	-7.27	-7.27	-7.27	-7.27
-7.26	-7.26	-7.26	-7.26	-7.26	-7.26	-7.26	-7.26	-7.26
-7.25	-7.25	-7.25	-7.25	-7.25	-7.25	-7.25	-7.25	-7.25
-7.24	-7.24	-7.24	-7.24	-7.24	-7.24	-7.24	-7.24	-7.24
-7.23	-7.23	-7.23	-7.23	-7.23	-7.23	-7.23	-7.23	-7.23
-7.22	-7.22	-7.22	-7.22	-7.22	-7.22	-7.22	-7.22	-7.22
-7.21	-7.21	-7.21	-7.21	-7.21	-7.21	-7.21	-7.21	-7.21
-7.2	-7.2	-7.2	-7.2	-7.2	-7.2	-7.2	-7.2	-7.2
-7.19	-7.19	-7.19	-7.19	-7.19	-7.19	-7.19	-7.19	-7.19
-7.18	-7.18	-7.18	-7.18	-7.18	-7.18	-7.18	-7.18	-7.18
-7.17	-7.17	-7.17	-7.17	-7.17	-7.17	-7.17	-7.17	-7.17
-7.16	-7.16	-7.16	-7.16	-7.16	-7.16	-7.16	-7.16	-7.16
-7.15	-7.15	-7.15	-7.15	-7.15	-7.15	-7.15	-7.15	-7.15
-7.14	-7.14	-7.14	-7.14	-7.14	-7.14	-7.14	-7.14	-7.14
-7.13	-7.13	-7.13	-7.13	-7.13	-7.13	-7.13	-7.13	-7.13
-7.12	-7.12	-7.12	-7.12	-7.12	-7.12	-7.12	-7.12	-7.12
-7.11	-7.11	-7.11	-7.11	-7.11	-7.11	-7.11	-7.11	-7.11
-7.1	-7.1	-7.1	-7.1	-7.1	-7.1	-7.1	-7.1	-7.1
-7.09	-7.09	-7.09	-7.09	-7.09	-7.09	-7.09	-7.09	-7.09
-7.08	-7.08	-7.08	-7.08	-7.08	-7.08	-7.08	-7.08	-7.08
-7.07	-7.07	-7.07	-7.07	-7.07	-7.07	-7.07	-7.07	-7.07
-7.06	-7.06	-7.06	-7.06	-7.06	-7.06	-7.06	-7.06	-7.06
-7.05	-7.05	-7.05	-7.05	-7.05	-7.05	-7.05	-7.05	-7.05
-7.04	-7.04	-7.04	-7.04	-7.04	-7.04	-7.04	-7.04	-7.04
-7.03	-7.03	-7.03	-7.03	-7.03	-7.03	-7.03	-7.03	-7.03
-7.02	-7.02	-7.02	-7.02	-7.02	-7.02	-7.02	-7.02	-7.02
-7.01	-7.01	-7.01	-7.01	-7.01	-7.01	-7.01	-7.01	-7.01

-7	-7	-7	-7	-7	-7	-7	-7	-7
-6.99	-6.99	-6.99	-6.99	-6.99	-6.99	-6.99	-6.99	-6.99
-6.98	-6.98	-6.98	-6.98	-6.98	-6.98	-6.98	-6.98	-6.98
-6.97	-6.97	-6.97	-6.97	-6.97	-6.97	-6.97	-6.97	-6.97
-6.96	-6.96	-6.96	-6.96	-6.96	-6.96	-6.96	-6.96	-6.96
-6.95	-6.95	-6.95	-6.95	-6.95	-6.95	-6.95	-6.95	-6.95
-6.94	-6.94	-6.94	-6.94	-6.94	-6.94	-6.94	-6.94	-6.94
-6.93	-6.93	-6.93	-6.93	-6.93	-6.93	-6.93	-6.93	-6.93
-6.92	-6.92	-6.92	-6.92	-6.92	-6.92	-6.92	-6.92	-6.92
-6.91	-6.91	-6.91	-6.91	-6.91	-6.91	-6.91	-6.91	-6.91
-6.9	-6.9	-6.9	-6.9	-6.9	-6.9	-6.9	-6.9	-6.9
-6.89	-6.89	-6.89	-6.89	-6.89	-6.89	-6.89	-6.89	-6.89
-6.88	-6.88	-6.88	-6.88	-6.88	-6.88	-6.88	-6.88	-6.88
-6.87	-6.87	-6.87	-6.87	-6.87	-6.87	-6.87	-6.87	-6.87
-6.86	-6.86	-6.86	-6.86	-6.86	-6.86	-6.86	-6.86	-6.86
-6.85	-6.85	-6.85	-6.85	-6.85	-6.85	-6.85	-6.85	-6.85
-6.84	-6.84	-6.84	-6.84	-6.84	-6.84	-6.84	-6.84	-6.84
-6.83	-6.83	-6.83	-6.83	-6.83	-6.83	-6.83	-6.83	-6.83
-6.82	-6.82	-6.82	-6.82	-6.82	-6.82	-6.82	-6.82	-6.82
-6.81	-6.81	-6.81	-6.81	-6.81	-6.81	-6.81	-6.81	-6.81
-6.8	-6.8	-6.8	-6.8	-6.8	-6.8	-6.8	-6.8	-6.8
-6.79	-6.79	-6.79	-6.79	-6.79	-6.79	-6.79	-6.79	-6.79
-6.78	-6.78	-6.78	-6.78	-6.78	-6.78	-6.78	-6.78	-6.78
-6.77	-6.77	-6.77	-6.77	-6.77	-6.77	-6.77	-6.77	-6.77
-6.76	-6.76	-6.76	-6.76	-6.76	-6.76	-6.76	-6.76	-6.76
-6.75	-6.75	-6.75	-6.75	-6.75	-6.75	-6.75	-6.75	-6.75
-6.74	-6.74	-6.74	-6.74	-6.74	-6.74	-6.74	-6.74	-6.74
-6.73	-6.73	-6.73	-6.73	-6.73	-6.73	-6.73	-6.73	-6.73
-6.72	-6.72	-6.72	-6.72	-6.72	-6.72	-6.72	-6.72	-6.72
-6.71	-6.71	-6.71	-6.71	-6.71	-6.71	-6.71	-6.71	-6.71
-6.7	-6.7	-6.7	-6.7	-6.7	-6.7	-6.7	-6.7	-6.7
-6.69	-6.69	-6.69	-6.69	-6.69	-6.69	-6.69	-6.69	-6.69
-6.68	-6.68	-6.68	-6.68	-6.68	-6.68	-6.68	-6.68	-6.68
-6.67	-6.67	-6.67	-6.67	-6.67	-6.67	-6.67	-6.67	-6.67
-6.66	-6.66	-6.66	-6.66	-6.66	-6.66	-6.66	-6.66	-6.66
-6.65	-6.65	-6.65	-6.65	-6.65	-6.65	-6.65	-6.65	-6.65
-6.64	-6.64	-6.64	-6.64	-6.64	-6.64	-6.64	-6.64	-6.64
-6.63	-6.63	-6.63	-6.63	-6.63	-6.63	-6.63	-6.63	-6.63
-6.62	-6.62	-6.62	-6.62	-6.62	-6.62	-6.62	-6.62	-6.62
-6.61	-6.61	-6.61	-6.61	-6.61	-6.61	-6.61	-6.61	-6.61
-6.6	-6.6	-6.6	-6.6	-6.6	-6.6	-6.6	-6.6	-6.6
-6.59	-6.59	-6.59	-6.59	-6.59	-6.59	-6.59	-6.59	-6.59
-6.58	-6.58	-6.58	-6.58	-6.58	-6.58	-6.58	-6.58	-6.58
-6.57	-6.57	-6.57	-6.57	-6.57	-6.57	-6.57	-6.57	-6.57
-6.56	-6.56	-6.56	-6.56	-6.56	-6.56	-6.56	-6.56	-6.56
-6.55	-6.55	-6.55	-6.55	-6.55	-6.55	-6.55	-6.55	-6.55
-6.54	-6.54	-6.54	-6.54	-6.54	-6.54	-6.54	-6.54	-6.54
-6.53	-6.53	-6.53	-6.53	-6.53	-6.53	-6.53	-6.53	-6.53
-6.52	-6.52	-6.52	-6.52	-6.52	-6.52	-6.52	-6.52	-6.52
-6.51	-6.51	-6.51	-6.51	-6.51	-6.51	-6.51	-6.51	-6.51

-6.5	-6.5	-6.5	-6.5	-6.5	-6.5	-6.5	-6.5	-6.5
-6.49	-6.49	-6.49	-6.49	-6.49	-6.49	-6.49	-6.49	-6.49
-6.48	-6.48	-6.48	-6.48	-6.48	-6.48	-6.48	-6.48	-6.48
-6.47	-6.47	-6.47	-6.47	-6.47	-6.47	-6.47	-6.47	-6.47
-6.46	-6.46	-6.46	-6.46	-6.46	-6.46	-6.46	-6.46	-6.46
-6.45	-6.45	-6.45	-6.45	-6.45	-6.45	-6.45	-6.45	-6.45
-6.44	-6.44	-6.44	-6.44	-6.44	-6.44	-6.44	-6.44	-6.44
-6.43	-6.43	-6.43	-6.43	-6.43	-6.43	-6.43	-6.43	-6.43
-6.42	-6.42	-6.42	-6.42	-6.42	-6.42	-6.42	-6.42	-6.42
-6.41	-6.41	-6.41	-6.41	-6.41	-6.41	-6.41	-6.41	-6.41
-6.4	-6.4	-6.4	-6.4	-6.4	-6.4	-6.4	-6.4	-6.4
-6.39	-6.39	-6.39	-6.39	-6.39	-6.39	-6.39	-6.39	-6.39
-6.38	-6.38	-6.38	-6.38	-6.38	-6.38	-6.38	-6.38	-6.38
-6.37	-6.37	-6.37	-6.37	-6.37	-6.37	-6.37	-6.37	-6.37
-6.36	-6.36	-6.36	-6.36	-6.36	-6.36	-6.36	-6.36	-6.36
-6.35	-6.35	-6.35	-6.35	-6.35	-6.35	-6.35	-6.35	-6.35
-6.34	-6.34	-6.34	-6.34	-6.34	-6.34	-6.34	-6.34	-6.34
-6.33	-6.33	-6.33	-6.33	-6.33	-6.33	-6.33	-6.33	-6.33
-6.32	-6.32	-6.32	-6.32	-6.32	-6.32	-6.32	-6.32	-6.32
-6.31	-6.31	-6.31	-6.31	-6.31	-6.31	-6.31	-6.31	-6.31
-6.3	-6.3	-6.3	-6.3	-6.3	-6.3	-6.3	-6.3	-6.3
-6.29	-6.29	-6.29	-6.29	-6.29	-6.29	-6.29	-6.29	-6.29
-6.28	-6.28	-6.28	-6.28	-6.28	-6.28	-6.28	-6.28	-6.28
-6.27	-6.27	-6.27	-6.27	-6.27	-6.27	-6.27	-6.27	-6.27
-6.26	-6.26	-6.26	-6.26	-6.26	-6.26	-6.26	-6.26	-6.26
-6.25	-6.25	-6.25	-6.25	-6.25	-6.25	-6.25	-6.25	-6.25
-6.24	-6.24	-6.24	-6.24	-6.24	-6.24	-6.24	-6.24	-6.24
-6.23	-6.23	-6.23	-6.23	-6.23	-6.23	-6.23	-6.23	-6.23
-6.22	-6.22	-6.22	-6.22	-6.22	-6.22	-6.22	-6.22	-6.22
-6.21	-6.21	-6.21	-6.21	-6.21	-6.21	-6.21	-6.21	-6.21
-6.2	-6.2	-6.2	-6.2	-6.2	-6.2	-6.2	-6.2	-6.2
-6.19	-6.19	-6.19	-6.19	-6.19	-6.19	-6.19	-6.19	-6.19
-6.18	-6.18	-6.18	-6.18	-6.18	-6.18	-6.18	-6.18	-6.18
-6.17	-6.17	-6.17	-6.17	-6.17	-6.17	-6.17	-6.17	-6.17
-6.16	-6.16	-6.16	-6.16	-6.16	-6.16	-6.16	-6.16	-6.16
-6.15	-6.15	-6.15	-6.15	-6.15	-6.15	-6.15	-6.15	-6.15
-6.14	-6.14	-6.14	-6.14	-6.14	-6.14	-6.14	-6.14	-6.14
-6.13	-6.13	-6.13	-6.13	-6.13	-6.13	-6.13	-6.13	-6.13
-6.12	-6.12	-6.12	-6.12	-6.12	-6.12	-6.12	-6.12	-6.12
-6.11	-6.11	-6.11	-6.11	-6.11	-6.11	-6.11	-6.11	-6.11
-6.1	-6.1	-6.1	-6.1	-6.1	-6.1	-6.1	-6.1	-6.1
-6.09	-6.09	-6.09	-6.09	-6.09	-6.09	-6.09	-6.09	-6.09
-6.08	-6.08	-6.08	-6.08	-6.08	-6.08	-6.08	-6.08	-6.08
-6.07	-6.07	-6.07	-6.07	-6.07	-6.07	-6.07	-6.07	-6.07
-6.06	-6.06	-6.06	-6.06	-6.06	-6.06	-6.06	-6.06	-6.06
-6.05	-6.05	-6.05	-6.05	-6.05	-6.05	-6.05	-6.05	-6.05
-6.04	-6.04	-6.04	-6.04	-6.04	-6.04	-6.04	-6.04	-6.04
-6.03	-6.03	-6.03	-6.03	-6.03	-6.03	-6.03	-6.03	-6.03
-6.02	-6.02	-6.02	-6.02	-6.02	-6.02	-6.02	-6.02	-6.02
-6.01	-6.01	-6.01	-6.01	-6.01	-6.01	-6.01	-6.01	-6.01

-6	-6	-6	-6	-6	-6	-6	-6	-6
-5.99	-5.99	-5.99	-5.99	-5.99	-5.99	-5.99	-5.99	-5.99
-5.98	-5.98	-5.98	-5.98	-5.98	-5.98	-5.98	-5.98	-5.98
-5.97	-5.97	-5.97	-5.97	-5.97	-5.97	-5.97	-5.97	-5.97
-5.96	-5.96	-5.96	-5.96	-5.96	-5.96	-5.96	-5.96	-5.96
-5.95	-5.95	-5.95	-5.95	-5.95	-5.95	-5.95	-5.95	-5.95
-5.94	-5.94	-5.94	-5.94	-5.94	-5.94	-5.94	-5.94	-5.94
-5.93	-5.93	-5.93	-5.93	-5.93	-5.93	-5.93	-5.93	-5.93
-5.92	-5.92	-5.92	-5.92	-5.92	-5.92	-5.92	-5.92	-5.92
-5.91	-5.91	-5.91	-5.91	-5.91	-5.91	-5.91	-5.91	-5.91
-5.9	-5.9	-5.9	-5.9	-5.9	-5.9	-5.9	-5.9	-5.9
-5.89	-5.89	-5.89	-5.89	-5.89	-5.89	-5.89	-5.89	-5.89
-5.88	-5.88	-5.88	-5.88	-5.88	-5.88	-5.88	-5.88	-5.88
-5.87	-5.87	-5.87	-5.87	-5.87	-5.87	-5.87	-5.87	-5.87
-5.86	-5.86	-5.86	-5.86	-5.86	-5.86	-5.86	-5.86	-5.86
-5.85	-5.85	-5.85	-5.85	-5.85	-5.85	-5.85	-5.85	-5.85
-5.84	-5.84	-5.84	-5.84	-5.84	-5.84	-5.84	-5.84	-5.84
-5.83	-5.83	-5.83	-5.83	-5.83	-5.83	-5.83	-5.83	-5.83
-5.82	-5.82	-5.82	-5.82	-5.82	-5.82	-5.82	-5.82	-5.82
-5.81	-5.81	-5.81	-5.81	-5.81	-5.81	-5.81	-5.81	-5.81
-5.8	-5.8	-5.8	-5.8	-5.8	-5.8	-5.8	-5.8	-5.8
-5.79	-5.79	-5.79	-5.79	-5.79	-5.79	-5.79	-5.79	-5.79
-5.78	-5.78	-5.78	-5.78	-5.78	-5.78	-5.78	-5.78	-5.78
-5.77	-5.77	-5.77	-5.77	-5.77	-5.77	-5.77	-5.77	-5.77
-5.76	-5.76	-5.76	-5.76	-5.76	-5.76	-5.76	-5.76	-5.76
-5.75	-5.75	-5.75	-5.75	-5.75	-5.75	-5.75	-5.75	-5.75
-5.74	-5.74	-5.74	-5.74	-5.74	-5.74	-5.74	-5.74	-5.74
-5.73	-5.73	-5.73	-5.73	-5.73	-5.73	-5.73	-5.73	-5.73
-5.72	-5.72	-5.72	-5.72	-5.72	-5.72	-5.72	-5.72	-5.72
-5.71	-5.71	-5.71	-5.71	-5.71	-5.71	-5.71	-5.71	-5.71
-5.7	-5.7	-5.7	-5.7	-5.7	-5.7	-5.7	-5.7	-5.7
-5.69	-5.69	-5.69	-5.69	-5.69	-5.69	-5.69	-5.69	-5.69
-5.68	-5.68	-5.68	-5.68	-5.68	-5.68	-5.68	-5.68	-5.68
-5.67	-5.67	-5.67	-5.67	-5.67	-5.67	-5.67	-5.67	-5.67
-5.66	-5.66	-5.66	-5.66	-5.66	-5.66	-5.66	-5.66	-5.66
-5.65	-5.65	-5.65	-5.65	-5.65	-5.65	-5.65	-5.65	-5.65
-5.64	-5.64	-5.64	-5.64	-5.64	-5.64	-5.64	-5.64	-5.64
-5.63	-5.63	-5.63	-5.63	-5.63	-5.63	-5.63	-5.63	-5.63
-5.62	-5.62	-5.62	-5.62	-5.62	-5.62	-5.62	-5.62	-5.62
-5.61	-5.61	-5.61	-5.61	-5.61	-5.61	-5.61	-5.61	-5.61
-5.6	-5.6	-5.6	-5.6	-5.6	-5.6	-5.6	-5.6	-5.6
-5.59	-5.59	-5.59	-5.59	-5.59	-5.59	-5.59	-5.59	-5.59
-5.58	-5.58	-5.58	-5.58	-5.58	-5.58	-5.58	-5.58	-5.58
-5.57	-5.57	-5.57	-5.57	-5.57	-5.57	-5.57	-5.57	-5.57
-5.56	-5.56	-5.56	-5.56	-5.56	-5.56	-5.56	-5.56	-5.56
-5.55	-5.55	-5.55	-5.55	-5.55	-5.55	-5.55	-5.55	-5.55
-5.54	-5.54	-5.54	-5.54	-5.54	-5.54	-5.54	-5.54	-5.54
-5.53	-5.53	-5.53	-5.53	-5.53	-5.53	-5.53	-5.53	-5.53
-5.52	-5.52	-5.52	-5.52	-5.52	-5.52	-5.52	-5.52	-5.52
-5.51	-5.51	-5.51	-5.51	-5.51	-5.51	-5.51	-5.51	-5.51

-5.5	-5.5	-5.5	-5.5	-5.5	-5.5	-5.5	-5.5	-5.5
-5.49	-5.49	-5.49	-5.49	-5.49	-5.49	-5.49	-5.49	-5.49
-5.48	-5.48	-5.48	-5.48	-5.48	-5.48	-5.48	-5.48	-5.48
-5.47	-5.47	-5.47	-5.47	-5.47	-5.47	-5.47	-5.47	-5.47
-5.46	-5.46	-5.46	-5.46	-5.46	-5.46	-5.46	-5.46	-5.46
-5.45	-5.45	-5.45	-5.45	-5.45	-5.45	-5.45	-5.45	-5.45
-5.44	-5.44	-5.44	-5.44	-5.44	-5.44	-5.44	-5.44	-5.44
-5.43	-5.43	-5.43	-5.43	-5.43	-5.43	-5.43	-5.43	-5.43
-5.42	-5.42	-5.42	-5.42	-5.42	-5.42	-5.42	-5.42	-5.42
-5.41	-5.41	-5.41	-5.41	-5.41	-5.41	-5.41	-5.41	-5.41
-5.4	-5.4	-5.4	-5.4	-5.4	-5.4	-5.4	-5.4	-5.4
-5.39	-5.39	-5.39	-5.39	-5.39	-5.39	-5.39	-5.39	-5.39
-5.38	-5.38	-5.38	-5.38	-5.38	-5.38	-5.38	-5.38	-5.38
-5.37	-5.37	-5.37	-5.37	-5.37	-5.37	-5.37	-5.37	-5.37
-5.36	-5.36	-5.36	-5.36	-5.36	-5.36	-5.36	-5.36	-5.36
-5.35	-5.35	-5.35	-5.35	-5.35	-5.35	-5.35	-5.35	-5.35
-5.34	-5.34	-5.34	-5.34	-5.34	-5.34	-5.34	-5.34	-5.34
-5.33	-5.33	-5.33	-5.33	-5.33	-5.33	-5.33	-5.33	-5.33
-5.32	-5.32	-5.32	-5.32	-5.32	-5.32	-5.32	-5.32	-5.32
-5.31	-5.31	-5.31	-5.31	-5.31	-5.31	-5.31	-5.31	-5.31
-5.3	-5.3	-5.3	-5.3	-5.3	-5.3	-5.3	-5.3	-5.3
-5.29	-5.29	-5.29	-5.29	-5.29	-5.29	-5.29	-5.29	-5.29
-5.28	-5.28	-5.28	-5.28	-5.28	-5.28	-5.28	-5.28	-5.28
-5.27	-5.27	-5.27	-5.27	-5.27	-5.27	-5.27	-5.27	-5.27
-5.26	-5.26	-5.26	-5.26	-5.26	-5.26	-5.26	-5.26	-5.26
-5.25	-5.25	-5.25	-5.25	-5.25	-5.25	-5.25	-5.25	-5.25
-5.24	-5.24	-5.24	-5.24	-5.24	-5.24	-5.24	-5.24	-5.24
-5.23	-5.23	-5.23	-5.23	-5.23	-5.23	-5.23	-5.23	-5.23
-5.22	-5.22	-5.22	-5.22	-5.22	-5.22	-5.22	-5.22	-5.22
-5.21	-5.21	-5.21	-5.21	-5.21	-5.21	-5.21	-5.21	-5.21
-5.2	-5.2	-5.2	-5.2	-5.2	-5.2	-5.2	-5.2	-5.2
-5.19	-5.19	-5.19	-5.19	-5.19	-5.19	-5.19	-5.19	-5.19
-5.18	-5.18	-5.18	-5.18	-5.18	-5.18	-5.18	-5.18	-5.18
-5.17	-5.17	-5.17	-5.17	-5.17	-5.17	-5.17	-5.17	-5.17
-5.16	-5.16	-5.16	-5.16	-5.16	-5.16	-5.16	-5.16	-5.16
-5.15	-5.15	-5.15	-5.15	-5.15	-5.15	-5.15	-5.15	-5.15
-5.14	-5.14	-5.14	-5.14	-5.14	-5.14	-5.14	-5.14	-5.14
-5.13	-5.13	-5.13	-5.13	-5.13	-5.13	-5.13	-5.13	-5.13
-5.12	-5.12	-5.12	-5.12	-5.12	-5.12	-5.12	-5.12	-5.12
-5.11	-5.11	-5.11	-5.11	-5.11	-5.11	-5.11	-5.11	-5.11
-5.1	-5.1	-5.1	-5.1	-5.1	-5.1	-5.1	-5.1	-5.1
-5.09	-5.09	-5.09	-5.09	-5.09	-5.09	-5.09	-5.09	-5.09
-5.08	-5.08	-5.08	-5.08	-5.08	-5.08	-5.08	-5.08	-5.08
-5.07	-5.07	-5.07	-5.07	-5.07	-5.07	-5.07	-5.07	-5.07
-5.06	-5.06	-5.06	-5.06	-5.06	-5.06	-5.06	-5.06	-5.06
-5.05	-5.05	-5.05	-5.05	-5.05	-5.05	-5.05	-5.05	-5.05
-5.04	-5.04	-5.04	-5.04	-5.04	-5.04	-5.04	-5.04	-5.04
-5.03	-5.03	-5.03	-5.03	-5.03	-5.03	-5.03	-5.03	-5.03
-5.02	-5.02	-5.02	-5.02	-5.02	-5.02	-5.02	-5.02	-5.02
-5.01	-5.01	-5.01	-5.01	-5.01	-5.01	-5.01	-5.01	-5.01

-5	-5	-5	-5	-5	-5	-5	-5	-5
-4.99	-4.99	-4.99	-4.99	-4.99	-4.99	-4.99	-4.99	-4.99
-4.98	-4.98	-4.98	-4.98	-4.98	-4.98	-4.98	-4.98	-4.98
-4.97	-4.97	-4.97	-4.97	-4.97	-4.97	-4.97	-4.97	-4.97
-4.96	-4.96	-4.96	-4.96	-4.96	-4.96	-4.96	-4.96	-4.96
-4.95	-4.95	-4.95	-4.95	-4.95	-4.95	-4.95	-4.95	-4.95
-4.94	-4.94	-4.94	-4.94	-4.94	-4.94	-4.94	-4.94	-4.94
-4.93	-4.93	-4.93	-4.93	-4.93	-4.93	-4.93	-4.93	-4.93
-4.92	-4.92	-4.92	-4.92	-4.92	-4.92	-4.92	-4.92	-4.92
-4.91	-4.91	-4.91	-4.91	-4.91	-4.91	-4.91	-4.91	-4.91
-4.9	-4.9	-4.9	-4.9	-4.9	-4.9	-4.9	-4.9	-4.9
-4.89	-4.89	-4.89	-4.89	-4.89	-4.89	-4.89	-4.89	-4.89
-4.88	-4.88	-4.88	-4.88	-4.88	-4.88	-4.88	-4.88	-4.88
-4.87	-4.87	-4.87	-4.87	-4.87	-4.87	-4.87	-4.87	-4.87
-4.86	-4.86	-4.86	-4.86	-4.86	-4.86	-4.86	-4.86	-4.86
-4.85	-4.85	-4.85	-4.85	-4.85	-4.85	-4.85	-4.85	-4.85
-4.84	-4.84	-4.84	-4.84	-4.84	-4.84	-4.84	-4.84	-4.84
-4.83	-4.83	-4.83	-4.83	-4.83	-4.83	-4.83	-4.83	-4.83
-4.82	-4.82	-4.82	-4.82	-4.82	-4.82	-4.82	-4.82	-4.82
-4.81	-4.81	-4.81	-4.81	-4.81	-4.81	-4.81	-4.81	-4.81
-4.8	-4.8	-4.8	-4.8	-4.8	-4.8	-4.8	-4.8	-4.8
-4.79	-4.79	-4.79	-4.79	-4.79	-4.79	-4.79	-4.79	-4.79
-4.78	-4.78	-4.78	-4.78	-4.78	-4.78	-4.78	-4.78	-4.78
-4.77	-4.77	-4.77	-4.77	-4.77	-4.77	-4.77	-4.77	-4.77
-4.76	-4.76	-4.76	-4.76	-4.76	-4.76	-4.76	-4.76	-4.76
-4.75	-4.75	-4.75	-4.75	-4.75	-4.75	-4.75	-4.75	-4.75
-4.74	-4.74	-4.74	-4.74	-4.74	-4.74	-4.74	-4.74	-4.74
-4.73	-4.73	-4.73	-4.73	-4.73	-4.73	-4.73	-4.73	-4.73
-4.72	-4.72	-4.72	-4.72	-4.72	-4.72	-4.72	-4.72	-4.72
-4.71	-4.71	-4.71	-4.71	-4.71	-4.71	-4.71	-4.71	-4.71
-4.7	-4.7	-4.7	-4.7	-4.7	-4.7	-4.7	-4.7	-4.7
-4.69	-4.69	-4.69	-4.69	-4.69	-4.69	-4.69	-4.69	-4.69
-4.68	-4.68	-4.68	-4.68	-4.68	-4.68	-4.68	-4.68	-4.68
-4.67	-4.67	-4.67	-4.67	-4.67	-4.67	-4.67	-4.67	-4.67
-4.66	-4.66	-4.66	-4.66	-4.66	-4.66	-4.66	-4.66	-4.66
-4.65	-4.65	-4.65	-4.65	-4.65	-4.65	-4.65	-4.65	-4.65
-4.64	-4.64	-4.64	-4.64	-4.64	-4.64	-4.64	-4.64	-4.64
-4.63	-4.63	-4.63	-4.63	-4.63	-4.63	-4.63	-4.63	-4.63
-4.62	-4.62	-4.62	-4.62	-4.62	-4.62	-4.62	-4.62	-4.62
-4.61	-4.61	-4.61	-4.61	-4.61	-4.61	-4.61	-4.61	-4.61
-4.6	-4.6	-4.6	-4.6	-4.6	-4.6	-4.6	-4.6	-4.6
-4.59	-4.59	-4.59	-4.59	-4.59	-4.59	-4.59	-4.59	-4.59
-4.58	-4.58	-4.58	-4.58	-4.58	-4.58	-4.58	-4.58	-4.58
-4.57	-4.57	-4.57	-4.57	-4.57	-4.57	-4.57	-4.57	-4.57
-4.56	-4.56	-4.56	-4.56	-4.56	-4.56	-4.56	-4.56	-4.56
-4.55	-4.55	-4.55	-4.55	-4.55	-4.55	-4.55	-4.55	-4.55
-4.54	-4.54	-4.54	-4.54	-4.54	-4.54	-4.54	-4.54	-4.54
-4.53	-4.53	-4.53	-4.53	-4.53	-4.53	-4.53	-4.53	-4.53
-4.52	-4.52	-4.52	-4.52	-4.52	-4.52	-4.52	-4.52	-4.52
-4.51	-4.51	-4.51	-4.51	-4.51	-4.51	-4.51	-4.51	-4.51

-4.5	-4.5	-4.5	-4.5	-4.5	-4.5	-4.5	-4.5	-4.5
-4.49	-4.49	-4.49	-4.49	-4.49	-4.49	-4.49	-4.49	-4.49
-4.48	-4.48	-4.48	-4.48	-4.48	-4.48	-4.48	-4.48	-4.48
-4.47	-4.47	-4.47	-4.47	-4.47	-4.47	-4.47	-4.47	-4.47
-4.46	-4.46	-4.46	-4.46	-4.46	-4.46	-4.46	-4.46	-4.46
-4.45	-4.45	-4.45	-4.45	-4.45	-4.45	-4.45	-4.45	-4.45
-4.44	-4.44	-4.44	-4.44	-4.44	-4.44	-4.44	-4.44	-4.44
-4.43	-4.43	-4.43	-4.43	-4.43	-4.43	-4.43	-4.43	-4.43
-4.42	-4.42	-4.42	-4.42	-4.42	-4.42	-4.42	-4.42	-4.42
-4.41	-4.41	-4.41	-4.41	-4.41	-4.41	-4.41	-4.41	-4.41
-4.4	-4.4	-4.4	-4.4	-4.4	-4.4	-4.4	-4.4	-4.4
-4.39	-4.39	-4.39	-4.39	-4.39	-4.39	-4.39	-4.39	-4.39
-4.38	-4.38	-4.38	-4.38	-4.38	-4.38	-4.38	-4.38	-4.38
-4.37	-4.37	-4.37	-4.37	-4.37	-4.37	-4.37	-4.37	-4.37
-4.36	-4.36	-4.36	-4.36	-4.36	-4.36	-4.36	-4.36	-4.36
-4.35	-4.35	-4.35	-4.35	-4.35	-4.35	-4.35	-4.35	-4.35
-4.34	-4.34	-4.34	-4.34	-4.34	-4.34	-4.34	-4.34	-4.34
-4.33	-4.33	-4.33	-4.33	-4.33	-4.33	-4.33	-4.33	-4.33
-4.32	-4.32	-4.32	-4.32	-4.32	-4.32	-4.32	-4.32	-4.32
-4.31	-4.31	-4.31	-4.31	-4.31	-4.31	-4.31	-4.31	-4.31
-4.3	-4.3	-4.3	-4.3	-4.3	-4.3	-4.3	-4.3	-4.3
-4.29	-4.29	-4.29	-4.29	-4.29	-4.29	-4.29	-4.29	-4.29
-4.28	-4.28	-4.28	-4.28	-4.28	-4.28	-4.28	-4.28	-4.28
-4.27	-4.27	-4.27	-4.27	-4.27	-4.27	-4.27	-4.27	-4.27
-4.26	-4.26	-4.26	-4.26	-4.26	-4.26	-4.26	-4.26	-4.26
-4.25	-4.25	-4.25	-4.25	-4.25	-4.25	-4.25	-4.25	-4.25
-4.24	-4.24	-4.24	-4.24	-4.24	-4.24	-4.24	-4.24	-4.24
-4.23	-4.23	-4.23	-4.23	-4.23	-4.23	-4.23	-4.23	-4.23
-4.22	-4.22	-4.22	-4.22	-4.22	-4.22	-4.22	-4.22	-4.22
-4.21	-4.21	-4.21	-4.21	-4.21	-4.21	-4.21	-4.21	-4.21
-4.2	-4.2	-4.2	-4.2	-4.2	-4.2	-4.2	-4.2	-4.2
-4.19	-4.19	-4.19	-4.19	-4.19	-4.19	-4.19	-4.19	-4.19
-4.18	-4.18	-4.18	-4.18	-4.18	-4.18	-4.18	-4.18	-4.18
-4.17	-4.17	-4.17	-4.17	-4.17	-4.17	-4.17	-4.17	-4.17
-4.16	-4.16	-4.16	-4.16	-4.16	-4.16	-4.16	-4.16	-4.16
-4.15	-4.15	-4.15	-4.15	-4.15	-4.15	-4.15	-4.15	-4.15
-4.14	-4.14	-4.14	-4.14	-4.14	-4.14	-4.14	-4.14	-4.14
-4.13	-4.13	-4.13	-4.13	-4.13	-4.13	-4.13	-4.13	-4.13
-4.12	-4.12	-4.12	-4.12	-4.12	-4.12	-4.12	-4.12	-4.12
-4.11	-4.11	-4.11	-4.11	-4.11	-4.11	-4.11	-4.11	-4.11
-4.1	-4.1	-4.1	-4.1	-4.1	-4.1	-4.1	-4.1	-4.1
-4.09	-4.09	-4.09	-4.09	-4.09	-4.09	-4.09	-4.09	-4.09
-4.08	-4.08	-4.08	-4.08	-4.08	-4.08	-4.08	-4.08	-4.08
-4.07	-4.07	-4.07	-4.07	-4.07	-4.07	-4.07	-4.07	-4.07
-4.06	-4.06	-4.06	-4.06	-4.06	-4.06	-4.06	-4.06	-4.06
-4.05	-4.05	-4.05	-4.05	-4.05	-4.05	-4.05	-4.05	-4.05
-4.04	-4.04	-4.04	-4.04	-4.04	-4.04	-4.04	-4.04	-4.04
-4.03	-4.03	-4.03	-4.03	-4.03	-4.03	-4.03	-4.03	-4.03
-4.02	-4.02	-4.02	-4.02	-4.02	-4.02	-4.02	-4.02	-4.02
-4.01	-4.01	-4.01	-4.01	-4.01	-4.01	-4.01	-4.01	-4.01

-4	-4	-4	-4	-4	-4	-4	-4	-4
-3.99	-3.99	-3.99	-3.99	-3.99	-3.99	-3.99	-3.99	-3.99
-3.98	-3.98	-3.98	-3.98	-3.98	-3.98	-3.98	-3.98	-3.98
-3.97	-3.97	-3.97	-3.97	-3.97	-3.97	-3.97	-3.97	-3.97
-3.96	-3.96	-3.96	-3.96	-3.96	-3.96	-3.96	-3.96	-3.96
-3.95	-3.95	-3.95	-3.95	-3.95	-3.95	-3.95	-3.95	-3.95
-3.94	-3.94	-3.94	-3.94	-3.94	-3.94	-3.94	-3.94	-3.94
-3.93	-3.93	-3.93	-3.93	-3.93	-3.93	-3.93	-3.93	-3.93
-3.92	-3.92	-3.92	-3.92	-3.92	-3.92	-3.92	-3.92	-3.92
-3.91	-3.91	-3.91	-3.91	-3.91	-3.91	-3.91	-3.91	-3.91
-3.9	-3.9	-3.9	-3.9	-3.9	-3.9	-3.9	-3.9	-3.9
-3.89	-3.89	-3.89	-3.89	-3.89	-3.89	-3.89	-3.89	-3.89
-3.88	-3.88	-3.88	-3.88	-3.88	-3.88	-3.88	-3.88	-3.88
-3.87	-3.87	-3.87	-3.87	-3.87	-3.87	-3.87	-3.87	-3.87
-3.86	-3.86	-3.86	-3.86	-3.86	-3.86	-3.86	-3.86	-3.86
-3.85	-3.85	-3.85	-3.85	-3.85	-3.85	-3.85	-3.85	-3.85
-3.84	-3.84	-3.84	-3.84	-3.84	-3.84	-3.84	-3.84	-3.84
-3.83	-3.83	-3.83	-3.83	-3.83	-3.83	-3.83	-3.83	-3.83
-3.82	-3.82	-3.82	-3.82	-3.82	-3.82	-3.82	-3.82	-3.82
-3.81	-3.81	-3.81	-3.81	-3.81	-3.81	-3.81	-3.81	-3.81
-3.8	-3.8	-3.8	-3.8	-3.8	-3.8	-3.8	-3.8	-3.8
-3.79	-3.79	-3.79	-3.79	-3.79	-3.79	-3.79	-3.79	-3.79
-3.78	-3.78	-3.78	-3.78	-3.78	-3.78	-3.78	-3.78	-3.78
-3.77	-3.77	-3.77	-3.77	-3.77	-3.77	-3.77	-3.77	-3.77
-3.76	-3.76	-3.76	-3.76	-3.76	-3.76	-3.76	-3.76	-3.76
-3.75	-3.75	-3.75	-3.75	-3.75	-3.75	-3.75	-3.75	-3.75
-3.74	-3.74	-3.74	-3.74	-3.74	-3.74	-3.74	-3.74	-3.74
-3.73	-3.73	-3.73	-3.73	-3.73	-3.73	-3.73	-3.73	-3.73
-3.72	-3.72	-3.72	-3.72	-3.72	-3.72	-3.72	-3.72	-3.72
-3.71	-3.71	-3.71	-3.71	-3.71	-3.71	-3.71	-3.71	-3.71
-3.7	-3.7	-3.7	-3.7	-3.7	-3.7	-3.7	-3.7	-3.7
-3.69	-3.69	-3.69	-3.69	-3.69	-3.69	-3.69	-3.69	-3.69
-3.68	-3.68	-3.68	-3.68	-3.68	-3.68	-3.68	-3.68	-3.68
-3.67	-3.67	-3.67	-3.67	-3.67	-3.67	-3.67	-3.67	-3.67
-3.66	-3.66	-3.66	-3.66	-3.66	-3.66	-3.66	-3.66	-3.66
-3.65	-3.65	-3.65	-3.65	-3.65	-3.65	-3.65	-3.65	-3.65
-3.64	-3.64	-3.64	-3.64	-3.64	-3.64	-3.64	-3.64	-3.64
-3.63	-3.63	-3.63	-3.63	-3.63	-3.63	-3.63	-3.63	-3.63
-3.62	-3.62	-3.62	-3.62	-3.62	-3.62	-3.62	-3.62	-3.62
-3.61	-3.61	-3.61	-3.61	-3.61	-3.61	-3.61	-3.61	-3.61
-3.6	-3.6	-3.6	-3.6	-3.6	-3.6	-3.6	-3.6	-3.6
-3.59	-3.59	-3.59	-3.59	-3.59	-3.59	-3.59	-3.59	-3.59
-3.58	-3.58	-3.58	-3.58	-3.58	-3.58	-3.58	-3.58	-3.58
-3.57	-3.57	-3.57	-3.57	-3.57	-3.57	-3.57	-3.57	-3.57
-3.56	-3.56	-3.56	-3.56	-3.56	-3.56	-3.56	-3.56	-3.56
-3.55	-3.55	-3.55	-3.55	-3.55	-3.55	-3.55	-3.55	-3.55
-3.54	-3.54	-3.54	-3.54	-3.54	-3.54	-3.54	-3.54	-3.54
-3.53	-3.53	-3.53	-3.53	-3.53	-3.53	-3.53	-3.53	-3.53
-3.52	-3.52	-3.52	-3.52	-3.52	-3.52	-3.52	-3.52	-3.52
-3.51	-3.51	-3.51	-3.51	-3.51	-3.51	-3.51	-3.51	-3.51

-3.5	-3.5	-3.5	-3.5	-3.5	-3.5	-3.5	-3.5	-3.5
-3.49	-3.49	-3.49	-3.49	-3.49	-3.49	-3.49	-3.49	-3.49
-3.48	-3.48	-3.48	-3.48	-3.48	-3.48	-3.48	-3.48	-3.48
-3.47	-3.47	-3.47	-3.47	-3.47	-3.47	-3.47	-3.47	-3.47
-3.46	-3.46	-3.46	-3.46	-3.46	-3.46	-3.46	-3.46	-3.46
-3.45	-3.45	-3.45	-3.45	-3.45	-3.45	-3.45	-3.45	-3.45
-3.44	-3.44	-3.44	-3.44	-3.44	-3.44	-3.44	-3.44	-3.44
-3.43	-3.43	-3.43	-3.43	-3.43	-3.43	-3.43	-3.43	-3.43
-3.42	-3.42	-3.42	-3.42	-3.42	-3.42	-3.42	-3.42	-3.42
-3.41	-3.41	-3.41	-3.41	-3.41	-3.41	-3.41	-3.41	-3.41
-3.4	-3.4	-3.4	-3.4	-3.4	-3.4	-3.4	-3.4	-3.4
-3.39	-3.39	-3.39	-3.39	-3.39	-3.39	-3.39	-3.39	-3.39
-3.38	-3.38	-3.38	-3.38	-3.38	-3.38	-3.38	-3.38	-3.38
-3.37	-3.37	-3.37	-3.37	-3.37	-3.37	-3.37	-3.37	-3.37
-3.36	-3.36	-3.36	-3.36	-3.36	-3.36	-3.36	-3.36	-3.36
-3.35	-3.35	-3.35	-3.35	-3.35	-3.35	-3.35	-3.35	-3.35
-3.34	-3.34	-3.34	-3.34	-3.34	-3.34	-3.34	-3.34	-3.34
-3.33	-3.33	-3.33	-3.33	-3.33	-3.33	-3.33	-3.33	-3.33
-3.32	-3.32	-3.32	-3.32	-3.32	-3.32	-3.32	-3.32	-3.32
-3.31	-3.31	-3.31	-3.31	-3.31	-3.31	-3.31	-3.31	-3.31
-3.3	-3.3	-3.3	-3.3	-3.3	-3.3	-3.3	-3.3	-3.3
-3.29	-3.29	-3.29	-3.29	-3.29	-3.29	-3.29	-3.29	-3.29
-3.28	-3.28	-3.28	-3.28	-3.28	-3.28	-3.28	-3.28	-3.28
-3.27	-3.27	-3.27	-3.27	-3.27	-3.27	-3.27	-3.27	-3.27
-3.26	-3.26	-3.26	-3.26	-3.26	-3.26	-3.26	-3.26	-3.26
-3.25	-3.25	-3.25	-3.25	-3.25	-3.25	-3.25	-3.25	-3.25
-3.24	-3.24	-3.24	-3.24	-3.24	-3.24	-3.24	-3.24	-3.24
-3.23	-3.23	-3.23	-3.23	-3.23	-3.23	-3.23	-3.23	-3.23
-3.22	-3.22	-3.22	-3.22	-3.22	-3.22	-3.22	-3.22	-3.22
-3.21	-3.21	-3.21	-3.21	-3.21	-3.21	-3.21	-3.21	-3.21
-3.2	-3.2	-3.2	-3.2	-3.2	-3.2	-3.2	-3.2	-3.2
-3.19	-3.19	-3.19	-3.19	-3.19	-3.19	-3.19	-3.19	-3.19
-3.18	-3.18	-3.18	-3.18	-3.18	-3.18	-3.18	-3.18	-3.18
-3.17	-3.17	-3.17	-3.17	-3.17	-3.17	-3.17	-3.17	-3.17
-3.16	-3.16	-3.16	-3.16	-3.16	-3.16	-3.16	-3.16	-3.16
-3.15	-3.15	-3.15	-3.15	-3.15	-3.15	-3.15	-3.15	-3.15
-3.14	-3.14	-3.14	-3.14	-3.14	-3.14	-3.14	-3.14	-3.14
-3.13	-3.13	-3.13	-3.13	-3.13	-3.13	-3.13	-3.13	-3.13
-3.12	-3.12	-3.12	-3.12	-3.12	-3.12	-3.12	-3.12	-3.12
-3.11	-3.11	-3.11	-3.11	-3.11	-3.11	-3.11	-3.11	-3.11
-3.1	-3.1	-3.1	-3.1	-3.1	-3.1	-3.1	-3.1	-3.1
-3.09	-3.09	-3.09	-3.09	-3.09	-3.09	-3.09	-3.09	-3.09
-3.08	-3.08	-3.08	-3.08	-3.08	-3.08	-3.08	-3.08	-3.08
-3.07	-3.07	-3.07	-3.07	-3.07	-3.07	-3.07	-3.07	-3.07
-3.06	-3.06	-3.06	-3.06	-3.06	-3.06	-3.06	-3.06	-3.06
-3.05	-3.05	-3.05	-3.05	-3.05	-3.05	-3.05	-3.05	-3.05
-3.04	-3.04	-3.04	-3.04	-3.04	-3.04	-3.04	-3.04	-3.04
-3.03	-3.03	-3.03	-3.03	-3.03	-3.03	-3.03	-3.03	-3.03
-3.02	-3.02	-3.02	-3.02	-3.02	-3.02	-3.02	-3.02	-3.02
-3.01	-3.01	-3.01	-3.01	-3.01	-3.01	-3.01	-3.01	-3.01

-3	-3	-3	-3	-3	-3	-3	-3	-3
-2.99	-2.99	-2.99	-2.99	-2.99	-2.99	-2.99	-2.99	-2.99
-2.98	-2.98	-2.98	-2.98	-2.98	-2.98	-2.98	-2.98	-2.98
-2.97	-2.97	-2.97	-2.97	-2.97	-2.97	-2.97	-2.97	-2.97
-2.96	-2.96	-2.96	-2.96	-2.96	-2.96	-2.96	-2.96	-2.96
-2.95	-2.95	-2.95	-2.95	-2.95	-2.95	-2.95	-2.95	-2.95
-2.94	-2.94	-2.94	-2.94	-2.94	-2.94	-2.94	-2.94	-2.94
-2.93	-2.93	-2.93	-2.93	-2.93	-2.93	-2.93	-2.93	-2.93
-2.92	-2.92	-2.92	-2.92	-2.92	-2.92	-2.92	-2.92	-2.92
-2.91	-2.91	-2.91	-2.91	-2.91	-2.91	-2.91	-2.91	-2.91
-2.9	-2.9	-2.9	-2.9	-2.9	-2.9	-2.9	-2.9	-2.9
-2.89	-2.89	-2.89	-2.89	-2.89	-2.89	-2.89	-2.89	-2.89
-2.88	-2.88	-2.88	-2.88	-2.88	-2.88	-2.88	-2.88	-2.88
-2.87	-2.87	-2.87	-2.87	-2.87	-2.87	-2.87	-2.87	-2.87
-2.86	-2.86	-2.86	-2.86	-2.86	-2.86	-2.86	-2.86	-2.86
-2.85	-2.85	-2.85	-2.85	-2.85	-2.85	-2.85	-2.85	-2.85
-2.84	-2.84	-2.84	-2.84	-2.84	-2.84	-2.84	-2.84	-2.84
-2.83	-2.83	-2.83	-2.83	-2.83	-2.83	-2.83	-2.83	-2.83
-2.82	-2.82	-2.82	-2.82	-2.82	-2.82	-2.82	-2.82	-2.82
-2.81	-2.81	-2.81	-2.81	-2.81	-2.81	-2.81	-2.81	-2.81
-2.8	-2.8	-2.8	-2.8	-2.8	-2.8	-2.8	-2.8	-2.8
-2.79	-2.79	-2.79	-2.79	-2.79	-2.79	-2.79	-2.79	-2.79
-2.78	-2.78	-2.78	-2.78	-2.78	-2.78	-2.78	-2.78	-2.78
-2.77	-2.77	-2.77	-2.77	-2.77	-2.77	-2.77	-2.77	-2.77
-2.76	-2.76	-2.76	-2.76	-2.76	-2.76	-2.76	-2.76	-2.76
-2.75	-2.75	-2.75	-2.75	-2.75	-2.75	-2.75	-2.75	-2.75
-2.74	-2.74	-2.74	-2.74	-2.74	-2.74	-2.74	-2.74	-2.74
-2.73	-2.73	-2.73	-2.73	-2.73	-2.73	-2.73	-2.73	-2.73
-2.72	-2.72	-2.72	-2.72	-2.72	-2.72	-2.72	-2.72	-2.72
-2.71	-2.71	-2.71	-2.71	-2.71	-2.71	-2.71	-2.71	-2.71
-2.7	-2.7	-2.7	-2.7	-2.7	-2.7	-2.7	-2.7	-2.7
-2.69	-2.69	-2.69	-2.69	-2.69	-2.69	-2.69	-2.69	-2.69
-2.68	-2.68	-2.68	-2.68	-2.68	-2.68	-2.68	-2.68	-2.68
-2.67	-2.67	-2.67	-2.67	-2.67	-2.67	-2.67	-2.67	-2.67
-2.66	-2.66	-2.66	-2.66	-2.66	-2.66	-2.66	-2.66	-2.66
-2.65	-2.65	-2.65	-2.65	-2.65	-2.65	-2.65	-2.65	-2.65
-2.64	-2.64	-2.64	-2.64	-2.64	-2.64	-2.64	-2.64	-2.64
-2.63	-2.63	-2.63	-2.63	-2.63	-2.63	-2.63	-2.63	-2.63
-2.62	-2.62	-2.62	-2.62	-2.62	-2.62	-2.62	-2.62	-2.62
-2.61	-2.61	-2.61	-2.61	-2.61	-2.61	-2.61	-2.61	-2.61
-2.6	-2.6	-2.6	-2.6	-2.6	-2.6	-2.6	-2.6	-2.6
-2.59	-2.59	-2.59	-2.59	-2.59	-2.59	-2.59	-2.59	-2.59
-2.58	-2.58	-2.58	-2.58	-2.58	-2.58	-2.58	-2.58	-2.58
-2.57	-2.57	-2.57	-2.57	-2.57	-2.57	-2.57	-2.57	-2.57
-2.56	-2.56	-2.56	-2.56	-2.56	-2.56	-2.56	-2.56	-2.56
-2.55	-2.55	-2.55	-2.55	-2.55	-2.55	-2.55	-2.55	-2.55
-2.54	-2.54	-2.54	-2.54	-2.54	-2.54	-2.54	-2.54	-2.54
-2.53	-2.53	-2.53	-2.53	-2.53	-2.53	-2.53	-2.53	-2.53
-2.52	-2.52	-2.52	-2.52	-2.52	-2.52	-2.52	-2.52	-2.52
-2.51	-2.51	-2.51	-2.51	-2.51	-2.51	-2.51	-2.51	-2.51

-2.5	-2.5	-2.5	-2.5	-2.5	-2.5	-2.5	-2.5	-2.5
-2.49	-2.49	-2.49	-2.49	-2.49	-2.49	-2.49	-2.49	-2.49
-2.48	-2.48	-2.48	-2.48	-2.48	-2.48	-2.48	-2.48	-2.48
-2.47	-2.47	-2.47	-2.47	-2.47	-2.47	-2.47	-2.47	-2.47
-2.46	-2.46	-2.46	-2.46	-2.46	-2.46	-2.46	-2.46	-2.46
-2.45	-2.45	-2.45	-2.45	-2.45	-2.45	-2.45	-2.45	-2.45
-2.44	-2.44	-2.44	-2.44	-2.44	-2.44	-2.44	-2.44	-2.44
-2.43	-2.43	-2.43	-2.43	-2.43	-2.43	-2.43	-2.43	-2.43
-2.42	-2.42	-2.42	-2.42	-2.42	-2.42	-2.42	-2.42	-2.42
-2.41	-2.41	-2.41	-2.41	-2.41	-2.41	-2.41	-2.41	-2.41
-2.4	-2.4	-2.4	-2.4	-2.4	-2.4	-2.4	-2.4	-2.4
-2.39	-2.39	-2.39	-2.39	-2.39	-2.39	-2.39	-2.39	-2.39
-2.38	-2.38	-2.38	-2.38	-2.38	-2.38	-2.38	-2.38	-2.38
-2.37	-2.37	-2.37	-2.37	-2.37	-2.37	-2.37	-2.37	-2.37
-2.36	-2.36	-2.36	-2.36	-2.36	-2.36	-2.36	-2.36	-2.36
-2.35	-2.35	-2.35	-2.35	-2.35	-2.35	-2.35	-2.35	-2.35
-2.34	-2.34	-2.34	-2.34	-2.34	-2.34	-2.34	-2.34	-2.34
-2.33	-2.33	-2.33	-2.33	-2.33	-2.33	-2.33	-2.33	-2.33
-2.32	-2.32	-2.32	-2.32	-2.32	-2.32	-2.32	-2.32	-2.32
-2.31	-2.31	-2.31	-2.31	-2.31	-2.31	-2.31	-2.31	-2.31
-2.3	-2.3	-2.3	-2.3	-2.3	-2.3	-2.3	-2.3	-2.3
-2.29	-2.29	-2.29	-2.29	-2.29	-2.29	-2.29	-2.29	-2.29
-2.28	-2.28	-2.28	-2.28	-2.28	-2.28	-2.28	-2.28	-2.28
-2.27	-2.27	-2.27	-2.27	-2.27	-2.27	-2.27	-2.27	-2.27
-2.26	-2.26	-2.26	-2.26	-2.26	-2.26	-2.26	-2.26	-2.26
-2.25	-2.25	-2.25	-2.25	-2.25	-2.25	-2.25	-2.25	-2.25
-2.24	-2.24	-2.24	-2.24	-2.24	-2.24	-2.24	-2.24	-2.24
-2.23	-2.23	-2.23	-2.23	-2.23	-2.23	-2.23	-2.23	-2.23
-2.22	-2.22	-2.22	-2.22	-2.22	-2.22	-2.22	-2.22	-2.22
-2.21	-2.21	-2.21	-2.21	-2.21	-2.21	-2.21	-2.21	-2.21
-2.2	-2.2	-2.2	-2.2	-2.2	-2.2	-2.2	-2.2	-2.2
-2.19	-2.19	-2.19	-2.19	-2.19	-2.19	-2.19	-2.19	-2.19
-2.18	-2.18	-2.18	-2.18	-2.18	-2.18	-2.18	-2.18	-2.18
-2.17	-2.17	-2.17	-2.17	-2.17	-2.17	-2.17	-2.17	-2.17
-2.16	-2.16	-2.16	-2.16	-2.16	-2.16	-2.16	-2.16	-2.16
-2.15	-2.15	-2.15	-2.15	-2.15	-2.15	-2.15	-2.15	-2.15
-2.14	-2.14	-2.14	-2.14	-2.14	-2.14	-2.14	-2.14	-2.14
-2.13	-2.13	-2.13	-2.13	-2.13	-2.13	-2.13	-2.13	-2.13
-2.12	-2.12	-2.12	-2.12	-2.12	-2.12	-2.12	-2.12	-2.12
-2.11	-2.11	-2.11	-2.11	-2.11	-2.11	-2.11	-2.11	-2.11
-2.1	-2.1	-2.1	-2.1	-2.1	-2.1	-2.1	-2.1	-2.1
-2.09	-2.09	-2.09	-2.09	-2.09	-2.09	-2.09	-2.09	-2.09
-2.08	-2.08	-2.08	-2.08	-2.08	-2.08	-2.08	-2.08	-2.08
-2.07	-2.07	-2.07	-2.07	-2.07	-2.07	-2.07	-2.07	-2.07
-2.06	-2.06	-2.06	-2.06	-2.06	-2.06	-2.06	-2.06	-2.06
-2.05	-2.05	-2.05	-2.05	-2.05	-2.05	-2.05	-2.05	-2.05
-2.04	-2.04	-2.04	-2.04	-2.04	-2.04	-2.04	-2.04	-2.04
-2.03	-2.03	-2.03	-2.03	-2.03	-2.03	-2.03	-2.03	-2.03
-2.02	-2.02	-2.02	-2.02	-2.02	-2.02	-2.02	-2.02	-2.02
-2.01	-2.01	-2.01	-2.01	-2.01	-2.01	-2.01	-2.01	-2.01

-2	-2	-2	-2	-2	-2	-2	-2	-2
-1.99	-1.99	-1.99	-1.99	-1.99	-1.99	-1.99	-1.99	-1.99
-1.98	-1.98	-1.98	-1.98	-1.98	-1.98	-1.98	-1.98	-1.98
-1.97	-1.97	-1.97	-1.97	-1.97	-1.97	-1.97	-1.97	-1.97
-1.96	-1.96	-1.96	-1.96	-1.96	-1.96	-1.96	-1.96	-1.96
-1.95	-1.95	-1.95	-1.95	-1.95	-1.95	-1.95	-1.95	-1.95
-1.94	-1.94	-1.94	-1.94	-1.94	-1.94	-1.94	-1.94	-1.94
-1.93	-1.93	-1.93	-1.93	-1.93	-1.93	-1.93	-1.93	-1.93
-1.92	-1.92	-1.92	-1.92	-1.92	-1.92	-1.92	-1.92	-1.92
-1.91	-1.91	-1.91	-1.91	-1.91	-1.91	-1.91	-1.91	-1.91
-1.9	-1.9	-1.9	-1.9	-1.9	-1.9	-1.9	-1.9	-1.9
-1.89	-1.89	-1.89	-1.89	-1.89	-1.89	-1.89	-1.89	-1.89
-1.88	-1.88	-1.88	-1.88	-1.88	-1.88	-1.88	-1.88	-1.88
-1.87	-1.87	-1.87	-1.87	-1.87	-1.87	-1.87	-1.87	-1.87
-1.86	-1.86	-1.86	-1.86	-1.86	-1.86	-1.86	-1.86	-1.86
-1.85	-1.85	-1.85	-1.85	-1.85	-1.85	-1.85	-1.85	-1.85
-1.84	-1.84	-1.84	-1.84	-1.84	-1.84	-1.84	-1.84	-1.84
-1.83	-1.83	-1.83	-1.83	-1.83	-1.83	-1.83	-1.83	-1.83
-1.82	-1.82	-1.82	-1.82	-1.82	-1.82	-1.82	-1.82	-1.82
-1.81	-1.81	-1.81	-1.81	-1.81	-1.81	-1.81	-1.81	-1.81
-1.8	-1.8	-1.8	-1.8	-1.8	-1.8	-1.8	-1.8	-1.8
-1.79	-1.79	-1.79	-1.79	-1.79	-1.79	-1.79	-1.79	-1.79
-1.78	-1.78	-1.78	-1.78	-1.78	-1.78	-1.78	-1.78	-1.78
-1.77	-1.77	-1.77	-1.77	-1.77	-1.77	-1.77	-1.77	-1.77
-1.76	-1.76	-1.76	-1.76	-1.76	-1.76	-1.76	-1.76	-1.76
-1.75	-1.75	-1.75	-1.75	-1.75	-1.75	-1.75	-1.75	-1.75
-1.74	-1.74	-1.74	-1.74	-1.74	-1.74	-1.74	-1.74	-1.74
-1.73	-1.73	-1.73	-1.73	-1.73	-1.73	-1.73	-1.73	-1.73
-1.72	-1.72	-1.72	-1.72	-1.72	-1.72	-1.72	-1.72	-1.72
-1.71	-1.71	-1.71	-1.71	-1.71	-1.71	-1.71	-1.71	-1.71
-1.7	-1.7	-1.7	-1.7	-1.7	-1.7	-1.7	-1.7	-1.7
-1.69	-1.69	-1.69	-1.69	-1.69	-1.69	-1.69	-1.69	-1.69
-1.68	-1.68	-1.68	-1.68	-1.68	-1.68	-1.68	-1.68	-1.68
-1.67	-1.67	-1.67	-1.67	-1.67	-1.67	-1.67	-1.67	-1.67
-1.66	-1.66	-1.66	-1.66	-1.66	-1.66	-1.66	-1.66	-1.66
-1.65	-1.65	-1.65	-1.65	-1.65	-1.65	-1.65	-1.65	-1.65
-1.64	-1.64	-1.64	-1.64	-1.64	-1.64	-1.64	-1.64	-1.64
-1.63	-1.63	-1.63	-1.63	-1.63	-1.63	-1.63	-1.63	-1.63
-1.62	-1.62	-1.62	-1.62	-1.62	-1.62	-1.62	-1.62	-1.62
-1.61	-1.61	-1.61	-1.61	-1.61	-1.61	-1.61	-1.61	-1.61
-1.6	-1.6	-1.6	-1.6	-1.6	-1.6	-1.6	-1.6	-1.6
-1.59	-1.59	-1.59	-1.59	-1.59	-1.59	-1.59	-1.59	-1.59
-1.58	-1.58	-1.58	-1.58	-1.58	-1.58	-1.58	-1.58	-1.58
-1.57	-1.57	-1.57	-1.57	-1.57	-1.57	-1.57	-1.57	-1.57
-1.56	-1.56	-1.56	-1.56	-1.56	-1.56	-1.56	-1.56	-1.56
-1.55	-1.55	-1.55	-1.55	-1.55	-1.55	-1.55	-1.55	-1.55
-1.54	-1.54	-1.54	-1.54	-1.54	-1.54	-1.54	-1.54	-1.54
-1.53	-1.53	-1.53	-1.53	-1.53	-1.53	-1.53	-1.53	-1.53
-1.52	-1.52	-1.52	-1.52	-1.52	-1.52	-1.52	-1.52	-1.52
-1.51	-1.51	-1.51	-1.51	-1.51	-1.51	-1.51	-1.51	-1.51

-1.5	-1.5	-1.5	-1.5	-1.5	-1.5	-1.5	-1.5	-1.5
-1.49	-1.49	-1.49	-1.49	-1.49	-1.49	-1.49	-1.49	-1.49
-1.48	-1.48	-1.48	-1.48	-1.48	-1.48	-1.48	-1.48	-1.48
-1.47	-1.47	-1.47	-1.47	-1.47	-1.47	-1.47	-1.47	-1.47
-1.46	-1.46	-1.46	-1.46	-1.46	-1.46	-1.46	-1.46	-1.46
-1.45	-1.45	-1.45	-1.45	-1.45	-1.45	-1.45	-1.45	-1.45
-1.44	-1.44	-1.44	-1.44	-1.44	-1.44	-1.44	-1.44	-1.44
-1.43	-1.43	-1.43	-1.43	-1.43	-1.43	-1.43	-1.43	-1.43
-1.42	-1.42	-1.42	-1.42	-1.42	-1.42	-1.42	-1.42	-1.42
-1.41	-1.41	-1.41	-1.41	-1.41	-1.41	-1.41	-1.41	-1.41
-1.4	-1.4	-1.4	-1.4	-1.4	-1.4	-1.4	-1.4	-1.4
-1.39	-1.39	-1.39	-1.39	-1.39	-1.39	-1.39	-1.39	-1.39
-1.38	-1.38	-1.38	-1.38	-1.38	-1.38	-1.38	-1.38	-1.38
-1.37	-1.37	-1.37	-1.37	-1.37	-1.37	-1.37	-1.37	-1.37
-1.36	-1.36	-1.36	-1.36	-1.36	-1.36	-1.36	-1.36	-1.36
-1.35	-1.35	-1.35	-1.35	-1.35	-1.35	-1.35	-1.35	-1.35
-1.34	-1.34	-1.34	-1.34	-1.34	-1.34	-1.34	-1.34	-1.34
-1.33	-1.33	-1.33	-1.33	-1.33	-1.33	-1.33	-1.33	-1.33
-1.32	-1.32	-1.32	-1.32	-1.32	-1.32	-1.32	-1.32	-1.32
-1.31	-1.31	-1.31	-1.31	-1.31	-1.31	-1.31	-1.31	-1.31
-1.3	-1.3	-1.3	-1.3	-1.3	-1.3	-1.3	-1.3	-1.3
-1.29	-1.29	-1.29	-1.29	-1.29	-1.29	-1.29	-1.29	-1.29
-1.28	-1.28	-1.28	-1.28	-1.28	-1.28	-1.28	-1.28	-1.28
-1.27	-1.27	-1.27	-1.27	-1.27	-1.27	-1.27	-1.27	-1.27
-1.26	-1.26	-1.26	-1.26	-1.26	-1.26	-1.26	-1.26	-1.26
-1.25	-1.25	-1.25	-1.25	-1.25	-1.25	-1.25	-1.25	-1.25
-1.24	-1.24	-1.24	-1.24	-1.24	-1.24	-1.24	-1.24	-1.24
-1.23	-1.23	-1.23	-1.23	-1.23	-1.23	-1.23	-1.23	-1.23
-1.22	-1.22	-1.22	-1.22	-1.22	-1.22	-1.22	-1.22	-1.22
-1.21	-1.21	-1.21	-1.21	-1.21	-1.21	-1.21	-1.21	-1.21
-1.2	-1.2	-1.2	-1.2	-1.2	-1.2	-1.2	-1.2	-1.2
-1.19	-1.19	-1.19	-1.19	-1.19	-1.19	-1.19	-1.19	-1.19
-1.18	-1.18	-1.18	-1.18	-1.18	-1.18	-1.18	-1.18	-1.18
-1.17	-1.17	-1.17	-1.17	-1.17	-1.17	-1.17	-1.17	-1.17
-1.16	-1.16	-1.16	-1.16	-1.16	-1.16	-1.16	-1.16	-1.16
-1.15	-1.15	-1.15	-1.15	-1.15	-1.15	-1.15	-1.15	-1.15
-1.14	-1.14	-1.14	-1.14	-1.14	-1.14	-1.14	-1.14	-1.14
-1.13	-1.13	-1.13	-1.13	-1.13	-1.13	-1.13	-1.13	-1.13
-1.12	-1.12	-1.12	-1.12	-1.12	-1.12	-1.12	-1.12	-1.12
-1.11	-1.11	-1.11	-1.11	-1.11	-1.11	-1.11	-1.11	-1.11
-1.1	-1.1	-1.1	-1.1	-1.1	-1.1	-1.1	-1.1	-1.1
-1.09	-1.09	-1.09	-1.09	-1.09	-1.09	-1.09	-1.09	-1.09
-1.08	-1.08	-1.08	-1.08	-1.08	-1.08	-1.08	-1.08	-1.08
-1.07	-1.07	-1.07	-1.07	-1.07	-1.07	-1.07	-1.07	-1.07
-1.06	-1.06	-1.06	-1.06	-1.06	-1.06	-1.06	-1.06	-1.06
-1.05	-1.05	-1.05	-1.05	-1.05	-1.05	-1.05	-1.05	-1.05
-1.04	-1.04	-1.04	-1.04	-1.04	-1.04	-1.04	-1.04	-1.04
-1.03	-1.03	-1.03	-1.03	-1.03	-1.03	-1.03	-1.03	-1.03
-1.02	-1.02	-1.02	-1.02	-1.02	-1.02	-1.02	-1.02	-1.02
-1.01	-1.01	-1.01	-1.01	-1.01	-1.01	-1.01	-1.01	-1.01

-1	-1	-1	-1	-1	-1	-1	-1	-1
-0.99	-0.99	-0.99	-0.99	-0.99	-0.99	-0.99	-0.99	-0.99
-0.98	-0.98	-0.98	-0.98	-0.98	-0.98	-0.98	-0.98	-0.98
-0.97	-0.97	-0.97	-0.97	-0.97	-0.97	-0.97	-0.97	-0.97
-0.96	-0.96	-0.96	-0.96	-0.96	-0.96	-0.96	-0.96	-0.96
-0.95	-0.95	-0.95	-0.95	-0.95	-0.95	-0.95	-0.95	-0.95
-0.94	-0.94	-0.94	-0.94	-0.94	-0.94	-0.94	-0.94	-0.94
-0.93	-0.93	-0.93	-0.93	-0.93	-0.93	-0.93	-0.93	-0.93
-0.92	-0.92	-0.92	-0.92	-0.92	-0.92	-0.92	-0.92	-0.92
-0.91	-0.91	-0.91	-0.91	-0.91	-0.91	-0.91	-0.91	-0.91
-0.9	-0.9	-0.9	-0.9	-0.9	-0.9	-0.9	-0.9	-0.9
-0.89	-0.89	-0.89	-0.89	-0.89	-0.89	-0.89	-0.89	-0.89
-0.88	-0.88	-0.88	-0.88	-0.88	-0.88	-0.88	-0.88	-0.88
-0.87	-0.87	-0.87	-0.87	-0.87	-0.87	-0.87	-0.87	-0.87
-0.86	-0.86	-0.86	-0.86	-0.86	-0.86	-0.86	-0.86	-0.86
-0.85	-0.85	-0.85	-0.85	-0.85	-0.85	-0.85	-0.85	-0.85
-0.84	-0.84	-0.84	-0.84	-0.84	-0.84	-0.84	-0.84	-0.84
-0.83	-0.83	-0.83	-0.83	-0.83	-0.83	-0.83	-0.83	-0.83
-0.82	-0.82	-0.82	-0.82	-0.82	-0.82	-0.82	-0.82	-0.82
-0.81	-0.81	-0.81	-0.81	-0.81	-0.81	-0.81	-0.81	-0.81
-0.8	-0.8	-0.8	-0.8	-0.8	-0.8	-0.8	-0.8	-0.8
-0.79	-0.79	-0.79	-0.79	-0.79	-0.79	-0.79	-0.79	-0.79
-0.78	-0.78	-0.78	-0.78	-0.78	-0.78	-0.78	-0.78	-0.78
-0.77	-0.77	-0.77	-0.77	-0.77	-0.77	-0.77	-0.77	-0.77
-0.76	-0.76	-0.76	-0.76	-0.76	-0.76	-0.76	-0.76	-0.76
-0.75	-0.75	-0.75	-0.75	-0.75	-0.75	-0.75	-0.75	-0.75
-0.74	-0.74	-0.74	-0.74	-0.74	-0.74	-0.74	-0.74	-0.74
-0.73	-0.73	-0.73	-0.73	-0.73	-0.73	-0.73	-0.73	-0.73
-0.72	-0.72	-0.72	-0.72	-0.72	-0.72	-0.72	-0.72	-0.72
-0.71	-0.71	-0.71	-0.71	-0.71	-0.71	-0.71	-0.71	-0.71
-0.7	-0.7	-0.7	-0.7	-0.7	-0.7	-0.7	-0.7	-0.7
-0.69	-0.69	-0.69	-0.69	-0.69	-0.69	-0.69	-0.69	-0.69
-0.68	-0.68	-0.68	-0.68	-0.68	-0.68	-0.68	-0.68	-0.68
-0.67	-0.67	-0.67	-0.67	-0.67	-0.67	-0.67	-0.67	-0.67
-0.66	-0.66	-0.66	-0.66	-0.66	-0.66	-0.66	-0.66	-0.66
-0.65	-0.65	-0.65	-0.65	-0.65	-0.65	-0.65	-0.65	-0.65
-0.64	-0.64	-0.64	-0.64	-0.64	-0.64	-0.64	-0.64	-0.64
-0.63	-0.63	-0.63	-0.63	-0.63	-0.63	-0.63	-0.63	-0.63
-0.62	-0.62	-0.62	-0.62	-0.62	-0.62	-0.62	-0.62	-0.62
-0.61	-0.61	-0.61	-0.61	-0.61	-0.61	-0.61	-0.61	-0.61
-0.6	-0.6	-0.6	-0.6	-0.6	-0.6	-0.6	-0.6	-0.6
-0.59	-0.59	-0.59	-0.59	-0.59	-0.59	-0.59	-0.59	-0.59
-0.58	-0.58	-0.58	-0.58	-0.58	-0.58	-0.58	-0.58	-0.58
-0.57	-0.57	-0.57	-0.57	-0.57	-0.57	-0.57	-0.57	-0.57
-0.56	-0.56	-0.56	-0.56	-0.56	-0.56	-0.56	-0.56	-0.56
-0.55	-0.55	-0.55	-0.55	-0.55	-0.55	-0.55	-0.55	-0.55
-0.54	-0.54	-0.54	-0.54	-0.54	-0.54	-0.54	-0.54	-0.54
-0.53	-0.53	-0.53	-0.53	-0.53	-0.53	-0.53	-0.53	-0.53
-0.52	-0.52	-0.52	-0.52	-0.52	-0.52	-0.52	-0.52	-0.52
-0.51	-0.51	-0.51	-0.51	-0.51	-0.51	-0.51	-0.51	-0.51

-0.5	-0.5	-0.5	-0.5	-0.5	-0.5	-0.5	-0.5	-0.5
-0.49	-0.49	-0.49	-0.49	-0.49	-0.49	-0.49	-0.49	-0.49
-0.48	-0.48	-0.48	-0.48	-0.48	-0.48	-0.48	-0.48	-0.48
-0.47	-0.47	-0.47	-0.47	-0.47	-0.47	-0.47	-0.47	-0.47
-0.46	-0.46	-0.46	-0.46	-0.46	-0.46	-0.46	-0.46	-0.46
-0.45	-0.45	-0.45	-0.45	-0.45	-0.45	-0.45	-0.45	-0.45
-0.44	-0.44	-0.44	-0.44	-0.44	-0.44	-0.44	-0.44	-0.44
-0.43	-0.43	-0.43	-0.43	-0.43	-0.43	-0.43	-0.43	-0.43
-0.42	-0.42	-0.42	-0.42	-0.42	-0.42	-0.42	-0.42	-0.42
-0.41	-0.41	-0.41	-0.41	-0.41	-0.41	-0.41	-0.41	-0.41
-0.4	-0.4	-0.4	-0.4	-0.4	-0.4	-0.4	-0.4	-0.4
-0.39	-0.39	-0.39	-0.39	-0.39	-0.39	-0.39	-0.39	-0.39
-0.38	-0.38	-0.38	-0.38	-0.38	-0.38	-0.38	-0.38	-0.38
-0.37	-0.37	-0.37	-0.37	-0.37	-0.37	-0.37	-0.37	-0.37
-0.36	-0.36	-0.36	-0.36	-0.36	-0.36	-0.36	-0.36	-0.36
-0.35	-0.35	-0.35	-0.35	-0.35	-0.35	-0.35	-0.35	-0.35
-0.34	-0.34	-0.34	-0.34	-0.34	-0.34	-0.34	-0.34	-0.34
-0.33	-0.33	-0.33	-0.33	-0.33	-0.33	-0.33	-0.33	-0.33
-0.32	-0.32	-0.32	-0.32	-0.32	-0.32	-0.32	-0.32	-0.32
-0.31	-0.31	-0.31	-0.31	-0.31	-0.31	-0.31	-0.31	-0.31
-0.3	-0.3	-0.3	-0.3	-0.3	-0.3	-0.3	-0.3	-0.3
-0.29	-0.29	-0.29	-0.29	-0.29	-0.29	-0.29	-0.29	-0.29
-0.28	-0.28	-0.28	-0.28	-0.28	-0.28	-0.28	-0.28	-0.28
-0.27	-0.27	-0.27	-0.27	-0.27	-0.27	-0.27	-0.27	-0.27
-0.26	-0.26	-0.26	-0.26	-0.26	-0.26	-0.26	-0.26	-0.26
-0.25	-0.25	-0.25	-0.25	-0.25	-0.25	-0.25	-0.25	-0.25
-0.24	-0.24	-0.24	-0.24	-0.24	-0.24	-0.24	-0.24	-0.24
-0.23	-0.23	-0.23	-0.23	-0.23	-0.23	-0.23	-0.23	-0.23
-0.22	-0.22	-0.22	-0.22	-0.22	-0.22	-0.22	-0.22	-0.22
-0.21	-0.21	-0.21	-0.21	-0.21	-0.21	-0.21	-0.21	-0.21
-0.2	-0.2	-0.2	-0.2	-0.2	-0.2	-0.2	-0.2	-0.2
-0.19	-0.19	-0.19	-0.19	-0.19	-0.19	-0.19	-0.19	-0.19
-0.18	-0.18	-0.18	-0.18	-0.18	-0.18	-0.18	-0.18	-0.18
-0.17	-0.17	-0.17	-0.17	-0.17	-0.17	-0.17	-0.17	-0.17
-0.16	-0.16	-0.16	-0.16	-0.16	-0.16	-0.16	-0.16	-0.16
-0.15	-0.15	-0.15	-0.15	-0.15	-0.15	-0.15	-0.15	-0.15
-0.14	-0.14	-0.14	-0.14	-0.14	-0.14	-0.14	-0.14	-0.14
-0.13	-0.13	-0.13	-0.13	-0.13	-0.13	-0.13	-0.13	-0.13
-0.12	-0.12	-0.12	-0.12	-0.12	-0.12	-0.12	-0.12	-0.12
-0.11	-0.11	-0.11	-0.11	-0.11	-0.11	-0.11	-0.11	-0.11
-0.1	-0.1	-0.1	-0.1	-0.1	-0.1	-0.1	-0.1	-0.1
-0.09	-0.09	-0.09	-0.09	-0.09	-0.09	-0.09	-0.09	-0.09
-0.08	-0.08	-0.08	-0.08	-0.08	-0.08	-0.08	-0.08	-0.08
-0.07	-0.07	-0.07	-0.07	-0.07	-0.07	-0.07	-0.07	-0.07
-0.06	-0.06	-0.06	-0.06	-0.06	-0.06	-0.06	-0.06	-0.06
-0.05	-0.05	-0.05	-0.05	-0.05	-0.05	-0.05	-0.05	-0.05
-0.04	-0.04	-0.04	-0.04	-0.04	-0.04	-0.04	-0.04	-0.04
-0.03	-0.03	-0.03	-0.03	-0.03	-0.03	-0.03	-0.03	-0.03
-0.02	-0.02	-0.02	-0.02	-0.02	-0.02	-0.02	-0.02	-0.02
-0.01	-0.01	-0.01	-0.01	-0.01	-0.01	-0.01	-0.01	-0.01

0	0	0	0	0	0	0	0	0
0.01	0.01	0.01	0.01	0.01	0.01	0.01	0.01	0.01
0.02	0.02	0.02	0.02	0.02	0.02	0.02	0.02	0.02
0.03	0.03	0.03	0.03	0.03	0.03	0.03	0.03	0.03
0.04	0.04	0.04	0.04	0.04	0.04	0.04	0.04	0.04
0.05	0.05	0.05	0.05	0.05	0.05	0.05	0.05	0.05
0.06	0.06	0.06	0.06	0.06	0.06	0.06	0.06	0.06
0.07	0.07	0.07	0.07	0.07	0.07	0.07	0.07	0.07
0.08	0.08	0.08	0.08	0.08	0.08	0.08	0.08	0.08
0.09	0.09	0.09	0.09	0.09	0.09	0.09	0.09	0.09
0.1	0.1	0.1	0.1	0.1	0.1	0.1	0.1	0.1
0.11	0.11	0.11	0.11	0.11	0.11	0.11	0.11	0.11
0.12	0.12	0.12	0.12	0.12	0.12	0.12	0.12	0.12
0.13	0.13	0.13	0.13	0.13	0.13	0.13	0.13	0.13
0.14	0.14	0.14	0.14	0.14	0.14	0.14	0.14	0.14
0.15	0.15	0.15	0.15	0.15	0.15	0.15	0.15	0.15
0.16	0.16	0.16	0.16	0.16	0.16	0.16	0.16	0.16
0.17	0.17	0.17	0.17	0.17	0.17	0.17	0.17	0.17
0.18	0.18	0.18	0.18	0.18	0.18	0.18	0.18	0.18
0.19	0.19	0.19	0.19	0.19	0.19	0.19	0.19	0.19
0.2	0.2	0.2	0.2	0.2	0.2	0.2	0.2	0.2
0.21	0.21	0.21	0.21	0.21	0.21	0.21	0.21	0.21
0.22	0.22	0.22	0.22	0.22	0.22	0.22	0.22	0.22
0.23	0.23	0.23	0.23	0.23	0.23	0.23	0.23	0.23
0.24	0.24	0.24	0.24	0.24	0.24	0.24	0.24	0.24
0.25	0.25	0.25	0.25	0.25	0.25	0.25	0.25	0.25
0.26	0.26	0.26	0.26	0.26	0.26	0.26	0.26	0.26
0.27	0.27	0.27	0.27	0.27	0.27	0.27	0.27	0.27
0.28	0.28	0.28	0.28	0.28	0.28	0.28	0.28	0.28
0.29	0.29	0.29	0.29	0.29	0.29	0.29	0.29	0.29
0.3	0.3	0.3	0.3	0.3	0.3	0.3	0.3	0.3
0.31	0.31	0.31	0.31	0.31	0.31	0.31	0.31	0.31
0.32	0.32	0.32	0.32	0.32	0.32	0.32	0.32	0.32
0.33	0.33	0.33	0.33	0.33	0.33	0.33	0.33	0.33
0.34	0.34	0.34	0.34	0.34	0.34	0.34	0.34	0.34
0.35	0.35	0.35	0.35	0.35	0.35	0.35	0.35	0.35
0.36	0.36	0.36	0.36	0.36	0.36	0.36	0.36	0.36
0.37	0.37	0.37	0.37	0.37	0.37	0.37	0.37	0.37
0.38	0.38	0.38	0.38	0.38	0.38	0.38	0.38	0.38
0.39	0.39	0.39	0.39	0.39	0.39	0.39	0.39	0.39
0.4	0.4	0.4	0.4	0.4	0.4	0.4	0.4	0.4
0.41	0.41	0.41	0.41	0.41	0.41	0.41	0.41	0.41
0.42	0.42	0.42	0.42	0.42	0.42	0.42	0.42	0.42
0.43	0.43	0.43	0.43	0.43	0.43	0.43	0.43	0.43
0.44	0.44	0.44	0.44	0.44	0.44	0.44	0.44	0.44
0.45	0.45	0.45	0.45	0.45	0.45	0.45	0.45	0.45
0.46	0.46	0.46	0.46	0.46	0.46	0.46	0.46	0.46
0.47	0.47	0.47	0.47	0.47	0.47	0.47	0.47	0.47
0.48	0.48	0.48	0.48	0.48	0.48	0.48	0.48	0.48
0.49	0.49	0.49	0.49	0.49	0.49	0.49	0.49	0.49

0.5	0.5	0.5	0.5	0.5	0.5	0.5	0.5	0.5
0.51	0.51	0.51	0.51	0.51	0.51	0.51	0.51	0.51
0.52	0.52	0.52	0.52	0.52	0.52	0.52	0.52	0.52
0.53	0.53	0.53	0.53	0.53	0.53	0.53	0.53	0.53
0.54	0.54	0.54	0.54	0.54	0.54	0.54	0.54	0.54
0.55	0.55	0.55	0.55	0.55	0.55	0.55	0.55	0.55
0.56	0.56	0.56	0.56	0.56	0.56	0.56	0.56	0.56
0.57	0.57	0.57	0.57	0.57	0.57	0.57	0.57	0.57
0.58	0.58	0.58	0.58	0.58	0.58	0.58	0.58	0.58
0.59	0.59	0.59	0.59	0.59	0.59	0.59	0.59	0.59
0.6	0.6	0.6	0.6	0.6	0.6	0.6	0.6	0.6
0.61	0.61	0.61	0.61	0.61	0.61	0.61	0.61	0.61
0.62	0.62	0.62	0.62	0.62	0.62	0.62	0.62	0.62
0.63	0.63	0.63	0.63	0.63	0.63	0.63	0.63	0.63
0.64	0.64	0.64	0.64	0.64	0.64	0.64	0.64	0.64
0.65	0.65	0.65	0.65	0.65	0.65	0.65	0.65	0.65
0.66	0.66	0.66	0.66	0.66	0.66	0.66	0.66	0.66
0.67	0.67	0.67	0.67	0.67	0.67	0.67	0.67	0.67
0.68	0.68	0.68	0.68	0.68	0.68	0.68	0.68	0.68
0.69	0.69	0.69	0.69	0.69	0.69	0.69	0.69	0.69
0.7	0.7	0.7	0.7	0.7	0.7	0.7	0.7	0.7
0.71	0.71	0.71	0.71	0.71	0.71	0.71	0.71	0.71
0.72	0.72	0.72	0.72	0.72	0.72	0.72	0.72	0.72
0.73	0.73	0.73	0.73	0.73	0.73	0.73	0.73	0.73
0.74	0.74	0.74	0.74	0.74	0.74	0.74	0.74	0.74
0.75	0.75	0.75	0.75	0.75	0.75	0.75	0.75	0.75
0.76	0.76	0.76	0.76	0.76	0.76	0.76	0.76	0.76
0.77	0.77	0.77	0.77	0.77	0.77	0.77	0.77	0.77
0.78	0.78	0.78	0.78	0.78	0.78	0.78	0.78	0.78
0.79	0.79	0.79	0.79	0.79	0.79	0.79	0.79	0.79
0.8	0.8	0.8	0.8	0.8	0.8	0.8	0.8	0.8
0.81	0.81	0.81	0.81	0.81	0.81	0.81	0.81	0.81
0.82	0.82	0.82	0.82	0.82	0.82	0.82	0.82	0.82
0.83	0.83	0.83	0.83	0.83	0.83	0.83	0.83	0.83
0.84	0.84	0.84	0.84	0.84	0.84	0.84	0.84	0.84
0.85	0.85	0.85	0.85	0.85	0.85	0.85	0.85	0.85
0.86	0.86	0.86	0.86	0.86	0.86	0.86	0.86	0.86
0.87	0.87	0.87	0.87	0.87	0.87	0.87	0.87	0.87
0.88	0.88	0.88	0.88	0.88	0.88	0.88	0.88	0.88
0.89	0.89	0.89	0.89	0.89	0.89	0.89	0.89	0.89
0.9	0.9	0.9	0.9	0.9	0.9	0.9	0.9	0.9
0.91	0.91	0.91	0.91	0.91	0.91	0.91	0.91	0.91
0.92	0.92	0.92	0.92	0.92	0.92	0.92	0.92	0.92
0.93	0.93	0.93	0.93	0.93	0.93	0.93	0.93	0.93
0.94	0.94	0.94	0.94	0.94	0.94	0.94	0.94	0.94
0.95	0.95	0.95	0.95	0.95	0.95	0.95	0.95	0.95
0.96	0.96	0.96	0.96	0.96	0.96	0.96	0.96	0.96
0.97	0.97	0.97	0.97	0.97	0.97	0.97	0.97	0.97
0.98	0.98	0.98	0.98	0.98	0.98	0.98	0.98	0.98
0.99	0.99	0.99	0.99	0.99	0.99	0.99	0.99	0.99

1	1	1	1	1	1	1	1	1
1.01	1.01	1.01	1.01	1.01	1.01	1.01	1.01	1.01
1.02	1.02	1.02	1.02	1.02	1.02	1.02	1.02	1.02
1.03	1.03	1.03	1.03	1.03	1.03	1.03	1.03	1.03
1.04	1.04	1.04	1.04	1.04	1.04	1.04	1.04	1.04
1.05	1.05	1.05	1.05	1.05	1.05	1.05	1.05	1.05
1.06	1.06	1.06	1.06	1.06	1.06	1.06	1.06	1.06
1.07	1.07	1.07	1.07	1.07	1.07	1.07	1.07	1.07
1.08	1.08	1.08	1.08	1.08	1.08	1.08	1.08	1.08
1.09	1.09	1.09	1.09	1.09	1.09	1.09	1.09	1.09
1.1	1.1	1.1	1.1	1.1	1.1	1.1	1.1	1.1
1.11	1.11	1.11	1.11	1.11	1.11	1.11	1.11	1.11
1.12	1.12	1.12	1.12	1.12	1.12	1.12	1.12	1.12
1.13	1.13	1.13	1.13	1.13	1.13	1.13	1.13	1.13
1.14	1.14	1.14	1.14	1.14	1.14	1.14	1.14	1.14
1.15	1.15	1.15	1.15	1.15	1.15	1.15	1.15	1.15
1.16	1.16	1.16	1.16	1.16	1.16	1.16	1.16	1.16
1.17	1.17	1.17	1.17	1.17	1.17	1.17	1.17	1.17
1.18	1.18	1.18	1.18	1.18	1.18	1.18	1.18	1.18
1.19	1.19	1.19	1.19	1.19	1.19	1.19	1.19	1.19
1.2	1.2	1.2	1.2	1.2	1.2	1.2	1.2	1.2
1.21	1.21	1.21	1.21	1.21	1.21	1.21	1.21	1.21
1.22	1.22	1.22	1.22	1.22	1.22	1.22	1.22	1.22
1.23	1.23	1.23	1.23	1.23	1.23	1.23	1.23	1.23
1.24	1.24	1.24	1.24	1.24	1.24	1.24	1.24	1.24
1.25	1.25	1.25	1.25	1.25	1.25	1.25	1.25	1.25
1.26	1.26	1.26	1.26	1.26	1.26	1.26	1.26	1.26
1.27	1.27	1.27	1.27	1.27	1.27	1.27	1.27	1.27
1.28	1.28	1.28	1.28	1.28	1.28	1.28	1.28	1.28
1.29	1.29	1.29	1.29	1.29	1.29	1.29	1.29	1.29
1.3	1.3	1.3	1.3	1.3	1.3	1.3	1.3	1.3
1.31	1.31	1.31	1.31	1.31	1.31	1.31	1.31	1.31
1.32	1.32	1.32	1.32	1.32	1.32	1.32	1.32	1.32
1.33	1.33	1.33	1.33	1.33	1.33	1.33	1.33	1.33
1.34	1.34	1.34	1.34	1.34	1.34	1.34	1.34	1.34
1.35	1.35	1.35	1.35	1.35	1.35	1.35	1.35	1.35
1.36	1.36	1.36	1.36	1.36	1.36	1.36	1.36	1.36
1.37	1.37	1.37	1.37	1.37	1.37	1.37	1.37	1.37
1.38	1.38	1.38	1.38	1.38	1.38	1.38	1.38	1.38
1.39	1.39	1.39	1.39	1.39	1.39	1.39	1.39	1.39
1.4	1.4	1.4	1.4	1.4	1.4	1.4	1.4	1.4
1.41	1.41	1.41	1.41	1.41	1.41	1.41	1.41	1.41
1.42	1.42	1.42	1.42	1.42	1.42	1.42	1.42	1.42
1.43	1.43	1.43	1.43	1.43	1.43	1.43	1.43	1.43
1.44	1.44	1.44	1.44	1.44	1.44	1.44	1.44	1.44
1.45	1.45	1.45	1.45	1.45	1.45	1.45	1.45	1.45
1.46	1.46	1.46	1.46	1.46	1.46	1.46	1.46	1.46
1.47	1.47	1.47	1.47	1.47	1.47	1.47	1.47	1.47
1.48	1.48	1.48	1.48	1.48	1.48	1.48	1.48	1.48
1.49	1.49	1.49	1.49	1.49	1.49	1.49	1.49	1.49

1.5	1.5	1.5	1.5	1.5	1.5	1.5	1.5	1.5
1.51	1.51	1.51	1.51	1.51	1.51	1.51	1.51	1.51
1.52	1.52	1.52	1.52	1.52	1.52	1.52	1.52	1.52
1.53	1.53	1.53	1.53	1.53	1.53	1.53	1.53	1.53
1.54	1.54	1.54	1.54	1.54	1.54	1.54	1.54	1.54
1.55	1.55	1.55	1.55	1.55	1.55	1.55	1.55	1.55
1.56	1.56	1.56	1.56	1.56	1.56	1.56	1.56	1.56
1.57	1.57	1.57	1.57	1.57	1.57	1.57	1.57	1.57
1.58	1.58	1.58	1.58	1.58	1.58	1.58	1.58	1.58
1.59	1.59	1.59	1.59	1.59	1.59	1.59	1.59	1.59
1.6	1.6	1.6	1.6	1.6	1.6	1.6	1.6	1.6
1.61	1.61	1.61	1.61	1.61	1.61	1.61	1.61	1.61
1.62	1.62	1.62	1.62	1.62	1.62	1.62	1.62	1.62
1.63	1.63	1.63	1.63	1.63	1.63	1.63	1.63	1.63
1.64	1.64	1.64	1.64	1.64	1.64	1.64	1.64	1.64
1.65	1.65	1.65	1.65	1.65	1.65	1.65	1.65	1.65
1.66	1.66	1.66	1.66	1.66	1.66	1.66	1.66	1.66
1.67	1.67	1.67	1.67	1.67	1.67	1.67	1.67	1.67
1.68	1.68	1.68	1.68	1.68	1.68	1.68	1.68	1.68
1.69	1.69	1.69	1.69	1.69	1.69	1.69	1.69	1.69
1.7	1.7	1.7	1.7	1.7	1.7	1.7	1.7	1.7
1.71	1.71	1.71	1.71	1.71	1.71	1.71	1.71	1.71
1.72	1.72	1.72	1.72	1.72	1.72	1.72	1.72	1.72
1.73	1.73	1.73	1.73	1.73	1.73	1.73	1.73	1.73
1.74	1.74	1.74	1.74	1.74	1.74	1.74	1.74	1.74
1.75	1.75	1.75	1.75	1.75	1.75	1.75	1.75	1.75
1.76	1.76	1.76	1.76	1.76	1.76	1.76	1.76	1.76
1.77	1.77	1.77	1.77	1.77	1.77	1.77	1.77	1.77
1.78	1.78	1.78	1.78	1.78	1.78	1.78	1.78	1.78
1.79	1.79	1.79	1.79	1.79	1.79	1.79	1.79	1.79
1.8	1.8	1.8	1.8	1.8	1.8	1.8	1.8	1.8
1.81	1.81	1.81	1.81	1.81	1.81	1.81	1.81	1.81
1.82	1.82	1.82	1.82	1.82	1.82	1.82	1.82	1.82
1.83	1.83	1.83	1.83	1.83	1.83	1.83	1.83	1.83
1.84	1.84	1.84	1.84	1.84	1.84	1.84	1.84	1.84
1.85	1.85	1.85	1.85	1.85	1.85	1.85	1.85	1.85
1.86	1.86	1.86	1.86	1.86	1.86	1.86	1.86	1.86
1.87	1.87	1.87	1.87	1.87	1.87	1.87	1.87	1.87
1.88	1.88	1.88	1.88	1.88	1.88	1.88	1.88	1.88
1.89	1.89	1.89	1.89	1.89	1.89	1.89	1.89	1.89
1.9	1.9	1.9	1.9	1.9	1.9	1.9	1.9	1.9
1.91	1.91	1.91	1.91	1.91	1.91	1.91	1.91	1.91
1.92	1.92	1.92	1.92	1.92	1.92	1.92	1.92	1.92
1.93	1.93	1.93	1.93	1.93	1.93	1.93	1.93	1.93
1.94	1.94	1.94	1.94	1.94	1.94	1.94	1.94	1.94
1.95	1.95	1.95	1.95	1.95	1.95	1.95	1.95	1.95
1.96	1.96	1.96	1.96	1.96	1.96	1.96	1.96	1.96
1.97	1.97	1.97	1.97	1.97	1.97	1.97	1.97	1.97
1.98	1.98	1.98	1.98	1.98	1.98	1.98	1.98	1.98
1.99	1.99	1.99	1.99	1.99	1.99	1.99	1.99	1.99

2	2	2	2	2	2	2	2	2
2.01	2.01	2.01	2.01	2.01	2.01	2.01	2.01	2.01
2.02	2.02	2.02	2.02	2.02	2.02	2.02	2.02	2.02
2.03	2.03	2.03	2.03	2.03	2.03	2.03	2.03	2.03
2.04	2.04	2.04	2.04	2.04	2.04	2.04	2.04	2.04
2.05	2.05	2.05	2.05	2.05	2.05	2.05	2.05	2.05
2.06	2.06	2.06	2.06	2.06	2.06	2.06	2.06	2.06
2.07	2.07	2.07	2.07	2.07	2.07	2.07	2.07	2.07
2.08	2.08	2.08	2.08	2.08	2.08	2.08	2.08	2.08
2.09	2.09	2.09	2.09	2.09	2.09	2.09	2.09	2.09
2.1	2.1	2.1	2.1	2.1	2.1	2.1	2.1	2.1
2.11	2.11	2.11	2.11	2.11	2.11	2.11	2.11	2.11
2.12	2.12	2.12	2.12	2.12	2.12	2.12	2.12	2.12
2.13	2.13	2.13	2.13	2.13	2.13	2.13	2.13	2.13
2.14	2.14	2.14	2.14	2.14	2.14	2.14	2.14	2.14
2.15	2.15	2.15	2.15	2.15	2.15	2.15	2.15	2.15
2.16	2.16	2.16	2.16	2.16	2.16	2.16	2.16	2.16
2.17	2.17	2.17	2.17	2.17	2.17	2.17	2.17	2.17
2.18	2.18	2.18	2.18	2.18	2.18	2.18	2.18	2.18
2.19	2.19	2.19	2.19	2.19	2.19	2.19	2.19	2.19
2.2	2.2	2.2	2.2	2.2	2.2	2.2	2.2	2.2
2.21	2.21	2.21	2.21	2.21	2.21	2.21	2.21	2.21
2.22	2.22	2.22	2.22	2.22	2.22	2.22	2.22	2.22
2.23	2.23	2.23	2.23	2.23	2.23	2.23	2.23	2.23
2.24	2.24	2.24	2.24	2.24	2.24	2.24	2.24	2.24
2.25	2.25	2.25	2.25	2.25	2.25	2.25	2.25	2.25
2.26	2.26	2.26	2.26	2.26	2.26	2.26	2.26	2.26
2.27	2.27	2.27	2.27	2.27	2.27	2.27	2.27	2.27
2.28	2.28	2.28	2.28	2.28	2.28	2.28	2.28	2.28
2.29	2.29	2.29	2.29	2.29	2.29	2.29	2.29	2.29
2.3	2.3	2.3	2.3	2.3	2.3	2.3	2.3	2.3
2.31	2.31	2.31	2.31	2.31	2.31	2.31	2.31	2.31
2.32	2.32	2.32	2.32	2.32	2.32	2.32	2.32	2.32
2.33	2.33	2.33	2.33	2.33	2.33	2.33	2.33	2.33
2.34	2.34	2.34	2.34	2.34	2.34	2.34	2.34	2.34
2.35	2.35	2.35	2.35	2.35	2.35	2.35	2.35	2.35
2.36	2.36	2.36	2.36	2.36	2.36	2.36	2.36	2.36
2.37	2.37	2.37	2.37	2.37	2.37	2.37	2.37	2.37
2.38	2.38	2.38	2.38	2.38	2.38	2.38	2.38	2.38
2.39	2.39	2.39	2.39	2.39	2.39	2.39	2.39	2.39
2.4	2.4	2.4	2.4	2.4	2.4	2.4	2.4	2.4
2.41	2.41	2.41	2.41	2.41	2.41	2.41	2.41	2.41
2.42	2.42	2.42	2.42	2.42	2.42	2.42	2.42	2.42
2.43	2.43	2.43	2.43	2.43	2.43	2.43	2.43	2.43
2.44	2.44	2.44	2.44	2.44	2.44	2.44	2.44	2.44
2.45	2.45	2.45	2.45	2.45	2.45	2.45	2.45	2.45
2.46	2.46	2.46	2.46	2.46	2.46	2.46	2.46	2.46
2.47	2.47	2.47	2.47	2.47	2.47	2.47	2.47	2.47
2.48	2.48	2.48	2.48	2.48	2.48	2.48	2.48	2.48
2.49	2.49	2.49	2.49	2.49	2.49	2.49	2.49	2.49

2.5	2.5	2.5	2.5	2.5	2.5	2.5	2.5	2.5
2.51	2.51	2.51	2.51	2.51	2.51	2.51	2.51	2.51
2.52	2.52	2.52	2.52	2.52	2.52	2.52	2.52	2.52
2.53	2.53	2.53	2.53	2.53	2.53	2.53	2.53	2.53
2.54	2.54	2.54	2.54	2.54	2.54	2.54	2.54	2.54
2.55	2.55	2.55	2.55	2.55	2.55	2.55	2.55	2.55
2.56	2.56	2.56	2.56	2.56	2.56	2.56	2.56	2.56
2.57	2.57	2.57	2.57	2.57	2.57	2.57	2.57	2.57
2.58	2.58	2.58	2.58	2.58	2.58	2.58	2.58	2.58
2.59	2.59	2.59	2.59	2.59	2.59	2.59	2.59	2.59
2.6	2.6	2.6	2.6	2.6	2.6	2.6	2.6	2.6
2.61	2.61	2.61	2.61	2.61	2.61	2.61	2.61	2.61
2.62	2.62	2.62	2.62	2.62	2.62	2.62	2.62	2.62
2.63	2.63	2.63	2.63	2.63	2.63	2.63	2.63	2.63
2.64	2.64	2.64	2.64	2.64	2.64	2.64	2.64	2.64
2.65	2.65	2.65	2.65	2.65	2.65	2.65	2.65	2.65
2.66	2.66	2.66	2.66	2.66	2.66	2.66	2.66	2.66
2.67	2.67	2.67	2.67	2.67	2.67	2.67	2.67	2.67
2.68	2.68	2.68	2.68	2.68	2.68	2.68	2.68	2.68
2.69	2.69	2.69	2.69	2.69	2.69	2.69	2.69	2.69
2.7	2.7	2.7	2.7	2.7	2.7	2.7	2.7	2.7
2.71	2.71	2.71	2.71	2.71	2.71	2.71	2.71	2.71
2.72	2.72	2.72	2.72	2.72	2.72	2.72	2.72	2.72
2.73	2.73	2.73	2.73	2.73	2.73	2.73	2.73	2.73
2.74	2.74	2.74	2.74	2.74	2.74	2.74	2.74	2.74
2.75	2.75	2.75	2.75	2.75	2.75	2.75	2.75	2.75
2.76	2.76	2.76	2.76	2.76	2.76	2.76	2.76	2.76
2.77	2.77	2.77	2.77	2.77	2.77	2.77	2.77	2.77
2.78	2.78	2.78	2.78	2.78	2.78	2.78	2.78	2.78
2.79	2.79	2.79	2.79	2.79	2.79	2.79	2.79	2.79
2.8	2.8	2.8	2.8	2.8	2.8	2.8	2.8	2.8
2.81	2.81	2.81	2.81	2.81	2.81	2.81	2.81	2.81
2.82	2.82	2.82	2.82	2.82	2.82	2.82	2.82	2.82
2.83	2.83	2.83	2.83	2.83	2.83	2.83	2.83	2.83
2.84	2.84	2.84	2.84	2.84	2.84	2.84	2.84	2.84
2.85	2.85	2.85	2.85	2.85	2.85	2.85	2.85	2.85
2.86	2.86	2.86	2.86	2.86	2.86	2.86	2.86	2.86
2.87	2.87	2.87	2.87	2.87	2.87	2.87	2.87	2.87
2.88	2.88	2.88	2.88	2.88	2.88	2.88	2.88	2.88
2.89	2.89	2.89	2.89	2.89	2.89	2.89	2.89	2.89
2.9	2.9	2.9	2.9	2.9	2.9	2.9	2.9	2.9
2.91	2.91	2.91	2.91	2.91	2.91	2.91	2.91	2.91
2.92	2.92	2.92	2.92	2.92	2.92	2.92	2.92	2.92
2.93	2.93	2.93	2.93	2.93	2.93	2.93	2.93	2.93
2.94	2.94	2.94	2.94	2.94	2.94	2.94	2.94	2.94
2.95	2.95	2.95	2.95	2.95	2.95	2.95	2.95	2.95
2.96	2.96	2.96	2.96	2.96	2.96	2.96	2.96	2.96
2.97	2.97	2.97	2.97	2.97	2.97	2.97	2.97	2.97
2.98	2.98	2.98	2.98	2.98	2.98	2.98	2.98	2.98
2.99	2.99	2.99	2.99	2.99	2.99	2.99	2.99	2.99

3	3	3	3	3	3	3	3	3
3.01	3.01	3.01	3.01	3.01	3.01	3.01	3.01	3.01
3.02	3.02	3.02	3.02	3.02	3.02	3.02	3.02	3.02
3.03	3.03	3.03	3.03	3.03	3.03	3.03	3.03	3.03
3.04	3.04	3.04	3.04	3.04	3.04	3.04	3.04	3.04
3.05	3.05	3.05	3.05	3.05	3.05	3.05	3.05	3.05
3.06	3.06	3.06	3.06	3.06	3.06	3.06	3.06	3.06
3.07	3.07	3.07	3.07	3.07	3.07	3.07	3.07	3.07
3.08	3.08	3.08	3.08	3.08	3.08	3.08	3.08	3.08
3.09	3.09	3.09	3.09	3.09	3.09	3.09	3.09	3.09
3.1	3.1	3.1	3.1	3.1	3.1	3.1	3.1	3.1
3.11	3.11	3.11	3.11	3.11	3.11	3.11	3.11	3.11
3.12	3.12	3.12	3.12	3.12	3.12	3.12	3.12	3.12
3.13	3.13	3.13	3.13	3.13	3.13	3.13	3.13	3.13
3.14	3.14	3.14	3.14	3.14	3.14	3.14	3.14	3.14
3.15	3.15	3.15	3.15	3.15	3.15	3.15	3.15	3.15
3.16	3.16	3.16	3.16	3.16	3.16	3.16	3.16	3.16
3.17	3.17	3.17	3.17	3.17	3.17	3.17	3.17	3.17
3.18	3.18	3.18	3.18	3.18	3.18	3.18	3.18	3.18
3.19	3.19	3.19	3.19	3.19	3.19	3.19	3.19	3.19
3.2	3.2	3.2	3.2	3.2	3.2	3.2	3.2	3.2
3.21	3.21	3.21	3.21	3.21	3.21	3.21	3.21	3.21
3.22	3.22	3.22	3.22	3.22	3.22	3.22	3.22	3.22
3.23	3.23	3.23	3.23	3.23	3.23	3.23	3.23	3.23
3.24	3.24	3.24	3.24	3.24	3.24	3.24	3.24	3.24
3.25	3.25	3.25	3.25	3.25	3.25	3.25	3.25	3.25
3.26	3.26	3.26	3.26	3.26	3.26	3.26	3.26	3.26
3.27	3.27	3.27	3.27	3.27	3.27	3.27	3.27	3.27
3.28	3.28	3.28	3.28	3.28	3.28	3.28	3.28	3.28
3.29	3.29	3.29	3.29	3.29	3.29	3.29	3.29	3.29
3.3	3.3	3.3	3.3	3.3	3.3	3.3	3.3	3.3
3.31	3.31	3.31	3.31	3.31	3.31	3.31	3.31	3.31
3.32	3.32	3.32	3.32	3.32	3.32	3.32	3.32	3.32
3.33	3.33	3.33	3.33	3.33	3.33	3.33	3.33	3.33
3.34	3.34	3.34	3.34	3.34	3.34	3.34	3.34	3.34
3.35	3.35	3.35	3.35	3.35	3.35	3.35	3.35	3.35
3.36	3.36	3.36	3.36	3.36	3.36	3.36	3.36	3.36
3.37	3.37	3.37	3.37	3.37	3.37	3.37	3.37	3.37
3.38	3.38	3.38	3.38	3.38	3.38	3.38	3.38	3.38
3.39	3.39	3.39	3.39	3.39	3.39	3.39	3.39	3.39
3.4	3.4	3.4	3.4	3.4	3.4	3.4	3.4	3.4
3.41	3.41	3.41	3.41	3.41	3.41	3.41	3.41	3.41
3.42	3.42	3.42	3.42	3.42	3.42	3.42	3.42	3.42
3.43	3.43	3.43	3.43	3.43	3.43	3.43	3.43	3.43
3.44	3.44	3.44	3.44	3.44	3.44	3.44	3.44	3.44
3.45	3.45	3.45	3.45	3.45	3.45	3.45	3.45	3.45
3.46	3.46	3.46	3.46	3.46	3.46	3.46	3.46	3.46
3.47	3.47	3.47	3.47	3.47	3.47	3.47	3.47	3.47
3.48	3.48	3.48	3.48	3.48	3.48	3.48	3.48	3.48
3.49	3.49	3.49	3.49	3.49	3.49	3.49	3.49	3.49

3.5	3.5	3.5	3.5	3.5	3.5	3.5	3.5	3.5
3.51	3.51	3.51	3.51	3.51	3.51	3.51	3.51	3.51
3.52	3.52	3.52	3.52	3.52	3.52	3.52	3.52	3.52
3.53	3.53	3.53	3.53	3.53	3.53	3.53	3.53	3.53
3.54	3.54	3.54	3.54	3.54	3.54	3.54	3.54	3.54
3.55	3.55	3.55	3.55	3.55	3.55	3.55	3.55	3.55
3.56	3.56	3.56	3.56	3.56	3.56	3.56	3.56	3.56
3.57	3.57	3.57	3.57	3.57	3.57	3.57	3.57	3.57
3.58	3.58	3.58	3.58	3.58	3.58	3.58	3.58	3.58
3.59	3.59	3.59	3.59	3.59	3.59	3.59	3.59	3.59
3.6	3.6	3.6	3.6	3.6	3.6	3.6	3.6	3.6
3.61	3.61	3.61	3.61	3.61	3.61	3.61	3.61	3.61
3.62	3.62	3.62	3.62	3.62	3.62	3.62	3.62	3.62
3.63	3.63	3.63	3.63	3.63	3.63	3.63	3.63	3.63
3.64	3.64	3.64	3.64	3.64	3.64	3.64	3.64	3.64
3.65	3.65	3.65	3.65	3.65	3.65	3.65	3.65	3.65
3.66	3.66	3.66	3.66	3.66	3.66	3.66	3.66	3.66
3.67	3.67	3.67	3.67	3.67	3.67	3.67	3.67	3.67
3.68	3.68	3.68	3.68	3.68	3.68	3.68	3.68	3.68
3.69	3.69	3.69	3.69	3.69	3.69	3.69	3.69	3.69
3.7	3.7	3.7	3.7	3.7	3.7	3.7	3.7	3.7
3.71	3.71	3.71	3.71	3.71	3.71	3.71	3.71	3.71
3.72	3.72	3.72	3.72	3.72	3.72	3.72	3.72	3.72
3.73	3.73	3.73	3.73	3.73	3.73	3.73	3.73	3.73
3.74	3.74	3.74	3.74	3.74	3.74	3.74	3.74	3.74
3.75	3.75	3.75	3.75	3.75	3.75	3.75	3.75	3.75
3.76	3.76	3.76	3.76	3.76	3.76	3.76	3.76	3.76
3.77	3.77	3.77	3.77	3.77	3.77	3.77	3.77	3.77
3.78	3.78	3.78	3.78	3.78	3.78	3.78	3.78	3.78
3.79	3.79	3.79	3.79	3.79	3.79	3.79	3.79	3.79
3.8	3.8	3.8	3.8	3.8	3.8	3.8	3.8	3.8
3.81	3.81	3.81	3.81	3.81	3.81	3.81	3.81	3.81
3.82	3.82	3.82	3.82	3.82	3.82	3.82	3.82	3.82
3.83	3.83	3.83	3.83	3.83	3.83	3.83	3.83	3.83
3.84	3.84	3.84	3.84	3.84	3.84	3.84	3.84	3.84
3.85	3.85	3.85	3.85	3.85	3.85	3.85	3.85	3.85
3.86	3.86	3.86	3.86	3.86	3.86	3.86	3.86	3.86
3.87	3.87	3.87	3.87	3.87	3.87	3.87	3.87	3.87
3.88	3.88	3.88	3.88	3.88	3.88	3.88	3.88	3.88
3.89	3.89	3.89	3.89	3.89	3.89	3.89	3.89	3.89
3.9	3.9	3.9	3.9	3.9	3.9	3.9	3.9	3.9
3.91	3.91	3.91	3.91	3.91	3.91	3.91	3.91	3.91
3.92	3.92	3.92	3.92	3.92	3.92	3.92	3.92	3.92
3.93	3.93	3.93	3.93	3.93	3.93	3.93	3.93	3.93
3.94	3.94	3.94	3.94	3.94	3.94	3.94	3.94	3.94
3.95	3.95	3.95	3.95	3.95	3.95	3.95	3.95	3.95
3.96	3.96	3.96	3.96	3.96	3.96	3.96	3.96	3.96
3.97	3.97	3.97	3.97	3.97	3.97	3.97	3.97	3.97
3.98	3.98	3.98	3.98	3.98	3.98	3.98	3.98	3.98
3.99	3.99	3.99	3.99	3.99	3.99	3.99	3.99	3.99

4	4	4	4	4	4	4	4	4
4.01	4.01	4.01	4.01	4.01	4.01	4.01	4.01	4.01
4.02	4.02	4.02	4.02	4.02	4.02	4.02	4.02	4.02
4.03	4.03	4.03	4.03	4.03	4.03	4.03	4.03	4.03
4.04	4.04	4.04	4.04	4.04	4.04	4.04	4.04	4.04
4.05	4.05	4.05	4.05	4.05	4.05	4.05	4.05	4.05
4.06	4.06	4.06	4.06	4.06	4.06	4.06	4.06	4.06
4.07	4.07	4.07	4.07	4.07	4.07	4.07	4.07	4.07
4.08	4.08	4.08	4.08	4.08	4.08	4.08	4.08	4.08
4.09	4.09	4.09	4.09	4.09	4.09	4.09	4.09	4.09
4.1	4.1	4.1	4.1	4.1	4.1	4.1	4.1	4.1
4.11	4.11	4.11	4.11	4.11	4.11	4.11	4.11	4.11
4.12	4.12	4.12	4.12	4.12	4.12	4.12	4.12	4.12
4.13	4.13	4.13	4.13	4.13	4.13	4.13	4.13	4.13
4.14	4.14	4.14	4.14	4.14	4.14	4.14	4.14	4.14
4.15	4.15	4.15	4.15	4.15	4.15	4.15	4.15	4.15
4.16	4.16	4.16	4.16	4.16	4.16	4.16	4.16	4.16
4.17	4.17	4.17	4.17	4.17	4.17	4.17	4.17	4.17
4.18	4.18	4.18	4.18	4.18	4.18	4.18	4.18	4.18
4.19	4.19	4.19	4.19	4.19	4.19	4.19	4.19	4.19
4.2	4.2	4.2	4.2	4.2	4.2	4.2	4.2	4.2
4.21	4.21	4.21	4.21	4.21	4.21	4.21	4.21	4.21
4.22	4.22	4.22	4.22	4.22	4.22	4.22	4.22	4.22
4.23	4.23	4.23	4.23	4.23	4.23	4.23	4.23	4.23
4.24	4.24	4.24	4.24	4.24	4.24	4.24	4.24	4.24
4.25	4.25	4.25	4.25	4.25	4.25	4.25	4.25	4.25
4.26	4.26	4.26	4.26	4.26	4.26	4.26	4.26	4.26
4.27	4.27	4.27	4.27	4.27	4.27	4.27	4.27	4.27
4.28	4.28	4.28	4.28	4.28	4.28	4.28	4.28	4.28
4.29	4.29	4.29	4.29	4.29	4.29	4.29	4.29	4.29
4.3	4.3	4.3	4.3	4.3	4.3	4.3	4.3	4.3
4.31	4.31	4.31	4.31	4.31	4.31	4.31	4.31	4.31
4.32	4.32	4.32	4.32	4.32	4.32	4.32	4.32	4.32
4.33	4.33	4.33	4.33	4.33	4.33	4.33	4.33	4.33
4.34	4.34	4.34	4.34	4.34	4.34	4.34	4.34	4.34
4.35	4.35	4.35	4.35	4.35	4.35	4.35	4.35	4.35
4.36	4.36	4.36	4.36	4.36	4.36	4.36	4.36	4.36
4.37	4.37	4.37	4.37	4.37	4.37	4.37	4.37	4.37
4.38	4.38	4.38	4.38	4.38	4.38	4.38	4.38	4.38
4.39	4.39	4.39	4.39	4.39	4.39	4.39	4.39	4.39
4.4	4.4	4.4	4.4	4.4	4.4	4.4	4.4	4.4
4.41	4.41	4.41	4.41	4.41	4.41	4.41	4.41	4.41
4.42	4.42	4.42	4.42	4.42	4.42	4.42	4.42	4.42
4.43	4.43	4.43	4.43	4.43	4.43	4.43	4.43	4.43
4.44	4.44	4.44	4.44	4.44	4.44	4.44	4.44	4.44
4.45	4.45	4.45	4.45	4.45	4.45	4.45	4.45	4.45
4.46	4.46	4.46	4.46	4.46	4.46	4.46	4.46	4.46
4.47	4.47	4.47	4.47	4.47	4.47	4.47	4.47	4.47
4.48	4.48	4.48	4.48	4.48	4.48	4.48	4.48	4.48
4.49	4.49	4.49	4.49	4.49	4.49	4.49	4.49	4.49

4.5	4.5	4.5	4.5	4.5	4.5	4.5	4.5	4.5
4.51	4.51	4.51	4.51	4.51	4.51	4.51	4.51	4.51
4.52	4.52	4.52	4.52	4.52	4.52	4.52	4.52	4.52
4.53	4.53	4.53	4.53	4.53	4.53	4.53	4.53	4.53
4.54	4.54	4.54	4.54	4.54	4.54	4.54	4.54	4.54
4.55	4.55	4.55	4.55	4.55	4.55	4.55	4.55	4.55
4.56	4.56	4.56	4.56	4.56	4.56	4.56	4.56	4.56
4.57	4.57	4.57	4.57	4.57	4.57	4.57	4.57	4.57
4.58	4.58	4.58	4.58	4.58	4.58	4.58	4.58	4.58
4.59	4.59	4.59	4.59	4.59	4.59	4.59	4.59	4.59
4.6	4.6	4.6	4.6	4.6	4.6	4.6	4.6	4.6
4.61	4.61	4.61	4.61	4.61	4.61	4.61	4.61	4.61
4.62	4.62	4.62	4.62	4.62	4.62	4.62	4.62	4.62
4.63	4.63	4.63	4.63	4.63	4.63	4.63	4.63	4.63
4.64	4.64	4.64	4.64	4.64	4.64	4.64	4.64	4.64
4.65	4.65	4.65	4.65	4.65	4.65	4.65	4.65	4.65
4.66	4.66	4.66	4.66	4.66	4.66	4.66	4.66	4.66
4.67	4.67	4.67	4.67	4.67	4.67	4.67	4.67	4.67
4.68	4.68	4.68	4.68	4.68	4.68	4.68	4.68	4.68
4.69	4.69	4.69	4.69	4.69	4.69	4.69	4.69	4.69
4.7	4.7	4.7	4.7	4.7	4.7	4.7	4.7	4.7
4.71	4.71	4.71	4.71	4.71	4.71	4.71	4.71	4.71
4.72	4.72	4.72	4.72	4.72	4.72	4.72	4.72	4.72
4.73	4.73	4.73	4.73	4.73	4.73	4.73	4.73	4.73
4.74	4.74	4.74	4.74	4.74	4.74	4.74	4.74	4.74
4.75	4.75	4.75	4.75	4.75	4.75	4.75	4.75	4.75
4.76	4.76	4.76	4.76	4.76	4.76	4.76	4.76	4.76
4.77	4.77	4.77	4.77	4.77	4.77	4.77	4.77	4.77
4.78	4.78	4.78	4.78	4.78	4.78	4.78	4.78	4.78
4.79	4.79	4.79	4.79	4.79	4.79	4.79	4.79	4.79
4.8	4.8	4.8	4.8	4.8	4.8	4.8	4.8	4.8
4.81	4.81	4.81	4.81	4.81	4.81	4.81	4.81	4.81
4.82	4.82	4.82	4.82	4.82	4.82	4.82	4.82	4.82
4.83	4.83	4.83	4.83	4.83	4.83	4.83	4.83	4.83
4.84	4.84	4.84	4.84	4.84	4.84	4.84	4.84	4.84
4.85	4.85	4.85	4.85	4.85	4.85	4.85	4.85	4.85
4.86	4.86	4.86	4.86	4.86	4.86	4.86	4.86	4.86
4.87	4.87	4.87	4.87	4.87	4.87	4.87	4.87	4.87
4.88	4.88	4.88	4.88	4.88	4.88	4.88	4.88	4.88
4.89	4.89	4.89	4.89	4.89	4.89	4.89	4.89	4.89
4.9	4.9	4.9	4.9	4.9	4.9	4.9	4.9	4.9
4.91	4.91	4.91	4.91	4.91	4.91	4.91	4.91	4.91
4.92	4.92	4.92	4.92	4.92	4.92	4.92	4.92	4.92
4.93	4.93	4.93	4.93	4.93	4.93	4.93	4.93	4.93
4.94	4.94	4.94	4.94	4.94	4.94	4.94	4.94	4.94
4.95	4.95	4.95	4.95	4.95	4.95	4.95	4.95	4.95
4.96	4.96	4.96	4.96	4.96	4.96	4.96	4.96	4.96
4.97	4.97	4.97	4.97	4.97	4.97	4.97	4.97	4.97
4.98	4.98	4.98	4.98	4.98	4.98	4.98	4.98	4.98
4.99	4.99	4.99	4.99	4.99	4.99	4.99	4.99	4.99

5	5	5	5	5	5	5	5	5
5.01	5.01	5.01	5.01	5.01	5.01	5.01	5.01	5.01
5.02	5.02	5.02	5.02	5.02	5.02	5.02	5.02	5.02
5.03	5.03	5.03	5.03	5.03	5.03	5.03	5.03	5.03
5.04	5.04	5.04	5.04	5.04	5.04	5.04	5.04	5.04
5.05	5.05	5.05	5.05	5.05	5.05	5.05	5.05	5.05
5.06	5.06	5.06	5.06	5.06	5.06	5.06	5.06	5.06
5.07	5.07	5.07	5.07	5.07	5.07	5.07	5.07	5.07
5.08	5.08	5.08	5.08	5.08	5.08	5.08	5.08	5.08
5.09	5.09	5.09	5.09	5.09	5.09	5.09	5.09	5.09
5.1	5.1	5.1	5.1	5.1	5.1	5.1	5.1	5.1
5.11	5.11	5.11	5.11	5.11	5.11	5.11	5.11	5.11
5.12	5.12	5.12	5.12	5.12	5.12	5.12	5.12	5.12
5.13	5.13	5.13	5.13	5.13	5.13	5.13	5.13	5.13
5.14	5.14	5.14	5.14	5.14	5.14	5.14	5.14	5.14
5.15	5.15	5.15	5.15	5.15	5.15	5.15	5.15	5.15
5.16	5.16	5.16	5.16	5.16	5.16	5.16	5.16	5.16
5.17	5.17	5.17	5.17	5.17	5.17	5.17	5.17	5.17
5.18	5.18	5.18	5.18	5.18	5.18	5.18	5.18	5.18
5.19	5.19	5.19	5.19	5.19	5.19	5.19	5.19	5.19
5.2	5.2	5.2	5.2	5.2	5.2	5.2	5.2	5.2
5.21	5.21	5.21	5.21	5.21	5.21	5.21	5.21	5.21
5.22	5.22	5.22	5.22	5.22	5.22	5.22	5.22	5.22
5.23	5.23	5.23	5.23	5.23	5.23	5.23	5.23	5.23
5.24	5.24	5.24	5.24	5.24	5.24	5.24	5.24	5.24
5.25	5.25	5.25	5.25	5.25	5.25	5.25	5.25	5.25
5.26	5.26	5.26	5.26	5.26	5.26	5.26	5.26	5.26
5.27	5.27	5.27	5.27	5.27	5.27	5.27	5.27	5.27
5.28	5.28	5.28	5.28	5.28	5.28	5.28	5.28	5.28
5.29	5.29	5.29	5.29	5.29	5.29	5.29	5.29	5.29
5.3	5.3	5.3	5.3	5.3	5.3	5.3	5.3	5.3
5.31	5.31	5.31	5.31	5.31	5.31	5.31	5.31	5.31
5.32	5.32	5.32	5.32	5.32	5.32	5.32	5.32	5.32
5.33	5.33	5.33	5.33	5.33	5.33	5.33	5.33	5.33
5.34	5.34	5.34	5.34	5.34	5.34	5.34	5.34	5.34
5.35	5.35	5.35	5.35	5.35	5.35	5.35	5.35	5.35
5.36	5.36	5.36	5.36	5.36	5.36	5.36	5.36	5.36
5.37	5.37	5.37	5.37	5.37	5.37	5.37	5.37	5.37
5.38	5.38	5.38	5.38	5.38	5.38	5.38	5.38	5.38
5.39	5.39	5.39	5.39	5.39	5.39	5.39	5.39	5.39
5.4	5.4	5.4	5.4	5.4	5.4	5.4	5.4	5.4
5.41	5.41	5.41	5.41	5.41	5.41	5.41	5.41	5.41
5.42	5.42	5.42	5.42	5.42	5.42	5.42	5.42	5.42
5.43	5.43	5.43	5.43	5.43	5.43	5.43	5.43	5.43
5.44	5.44	5.44	5.44	5.44	5.44	5.44	5.44	5.44
5.45	5.45	5.45	5.45	5.45	5.45	5.45	5.45	5.45
5.46	5.46	5.46	5.46	5.46	5.46	5.46	5.46	5.46
5.47	5.47	5.47	5.47	5.47	5.47	5.47	5.47	5.47
5.48	5.48	5.48	5.48	5.48	5.48	5.48	5.48	5.48
5.49	5.49	5.49	5.49	5.49	5.49	5.49	5.49	5.49

5.5	5.5	5.5	5.5	5.5	5.5	5.5	5.5	5.5
5.51	5.51	5.51	5.51	5.51	5.51	5.51	5.51	5.51
5.52	5.52	5.52	5.52	5.52	5.52	5.52	5.52	5.52
5.53	5.53	5.53	5.53	5.53	5.53	5.53	5.53	5.53
5.54	5.54	5.54	5.54	5.54	5.54	5.54	5.54	5.54
5.55	5.55	5.55	5.55	5.55	5.55	5.55	5.55	5.55
5.56	5.56	5.56	5.56	5.56	5.56	5.56	5.56	5.56
5.57	5.57	5.57	5.57	5.57	5.57	5.57	5.57	5.57
5.58	5.58	5.58	5.58	5.58	5.58	5.58	5.58	5.58
5.59	5.59	5.59	5.59	5.59	5.59	5.59	5.59	5.59
5.6	5.6	5.6	5.6	5.6	5.6	5.6	5.6	5.6
5.61	5.61	5.61	5.61	5.61	5.61	5.61	5.61	5.61
5.62	5.62	5.62	5.62	5.62	5.62	5.62	5.62	5.62
5.63	5.63	5.63	5.63	5.63	5.63	5.63	5.63	5.63
5.64	5.64	5.64	5.64	5.64	5.64	5.64	5.64	5.64
5.65	5.65	5.65	5.65	5.65	5.65	5.65	5.65	5.65
5.66	5.66	5.66	5.66	5.66	5.66	5.66	5.66	5.66
5.67	5.67	5.67	5.67	5.67	5.67	5.67	5.67	5.67
5.68	5.68	5.68	5.68	5.68	5.68	5.68	5.68	5.68
5.69	5.69	5.69	5.69	5.69	5.69	5.69	5.69	5.69
5.7	5.7	5.7	5.7	5.7	5.7	5.7	5.7	5.7
5.71	5.71	5.71	5.71	5.71	5.71	5.71	5.71	5.71
5.72	5.72	5.72	5.72	5.72	5.72	5.72	5.72	5.72
5.73	5.73	5.73	5.73	5.73	5.73	5.73	5.73	5.73
5.74	5.74	5.74	5.74	5.74	5.74	5.74	5.74	5.74
5.75	5.75	5.75	5.75	5.75	5.75	5.75	5.75	5.75
5.76	5.76	5.76	5.76	5.76	5.76	5.76	5.76	5.76
5.77	5.77	5.77	5.77	5.77	5.77	5.77	5.77	5.77
5.78	5.78	5.78	5.78	5.78	5.78	5.78	5.78	5.78
5.79	5.79	5.79	5.79	5.79	5.79	5.79	5.79	5.79
5.8	5.8	5.8	5.8	5.8	5.8	5.8	5.8	5.8
5.81	5.81	5.81	5.81	5.81	5.81	5.81	5.81	5.81
5.82	5.82	5.82	5.82	5.82	5.82	5.82	5.82	5.82
5.83	5.83	5.83	5.83	5.83	5.83	5.83	5.83	5.83
5.84	5.84	5.84	5.84	5.84	5.84	5.84	5.84	5.84
5.85	5.85	5.85	5.85	5.85	5.85	5.85	5.85	5.85
5.86	5.86	5.86	5.86	5.86	5.86	5.86	5.86	5.86
5.87	5.87	5.87	5.87	5.87	5.87	5.87	5.87	5.87
5.88	5.88	5.88	5.88	5.88	5.88	5.88	5.88	5.88
5.89	5.89	5.89	5.89	5.89	5.89	5.89	5.89	5.89
5.9	5.9	5.9	5.9	5.9	5.9	5.9	5.9	5.9
5.91	5.91	5.91	5.91	5.91	5.91	5.91	5.91	5.91
5.92	5.92	5.92	5.92	5.92	5.92	5.92	5.92	5.92
5.93	5.93	5.93	5.93	5.93	5.93	5.93	5.93	5.93
5.94	5.94	5.94	5.94	5.94	5.94	5.94	5.94	5.94
5.95	5.95	5.95	5.95	5.95	5.95	5.95	5.95	5.95
5.96	5.96	5.96	5.96	5.96	5.96	5.96	5.96	5.96
5.97	5.97	5.97	5.97	5.97	5.97	5.97	5.97	5.97
5.98	5.98	5.98	5.98	5.98	5.98	5.98	5.98	5.98
5.99	5.99	5.99	5.99	5.99	5.99	5.99	5.99	5.99

6	6	6	6	6	6	6	6	6
6.01	6.01	6.01	6.01	6.01	6.01	6.01	6.01	6.01
6.02	6.02	6.02	6.02	6.02	6.02	6.02	6.02	6.02
6.03	6.03	6.03	6.03	6.03	6.03	6.03	6.03	6.03
6.04	6.04	6.04	6.04	6.04	6.04	6.04	6.04	6.04
6.05	6.05	6.05	6.05	6.05	6.05	6.05	6.05	6.05
6.06	6.06	6.06	6.06	6.06	6.06	6.06	6.06	6.06
6.07	6.07	6.07	6.07	6.07	6.07	6.07	6.07	6.07
6.08	6.08	6.08	6.08	6.08	6.08	6.08	6.08	6.08
6.09	6.09	6.09	6.09	6.09	6.09	6.09	6.09	6.09
6.1	6.1	6.1	6.1	6.1	6.1	6.1	6.1	6.1
6.11	6.11	6.11	6.11	6.11	6.11	6.11	6.11	6.11
6.12	6.12	6.12	6.12	6.12	6.12	6.12	6.12	6.12
6.13	6.13	6.13	6.13	6.13	6.13	6.13	6.13	6.13
6.14	6.14	6.14	6.14	6.14	6.14	6.14	6.14	6.14
6.15	6.15	6.15	6.15	6.15	6.15	6.15	6.15	6.15
6.16	6.16	6.16	6.16	6.16	6.16	6.16	6.16	6.16
6.17	6.17	6.17	6.17	6.17	6.17	6.17	6.17	6.17
6.18	6.18	6.18	6.18	6.18	6.18	6.18	6.18	6.18
6.19	6.19	6.19	6.19	6.19	6.19	6.19	6.19	6.19
6.2	6.2	6.2	6.2	6.2	6.2	6.2	6.2	6.2
6.21	6.21	6.21	6.21	6.21	6.21	6.21	6.21	6.21
6.22	6.22	6.22	6.22	6.22	6.22	6.22	6.22	6.22
6.23	6.23	6.23	6.23	6.23	6.23	6.23	6.23	6.23
6.24	6.24	6.24	6.24	6.24	6.24	6.24	6.24	6.24
6.25	6.25	6.25	6.25	6.25	6.25	6.25	6.25	6.25
6.26	6.26	6.26	6.26	6.26	6.26	6.26	6.26	6.26
6.27	6.27	6.27	6.27	6.27	6.27	6.27	6.27	6.27
6.28	6.28	6.28	6.28	6.28	6.28	6.28	6.28	6.28
6.29	6.29	6.29	6.29	6.29	6.29	6.29	6.29	6.29
6.3	6.3	6.3	6.3	6.3	6.3	6.3	6.3	6.3
6.31	6.31	6.31	6.31	6.31	6.31	6.31	6.31	6.31
6.32	6.32	6.32	6.32	6.32	6.32	6.32	6.32	6.32
6.33	6.33	6.33	6.33	6.33	6.33	6.33	6.33	6.33
6.34	6.34	6.34	6.34	6.34	6.34	6.34	6.34	6.34
6.35	6.35	6.35	6.35	6.35	6.35	6.35	6.35	6.35
6.36	6.36	6.36	6.36	6.36	6.36	6.36	6.36	6.36
6.37	6.37	6.37	6.37	6.37	6.37	6.37	6.37	6.37
6.38	6.38	6.38	6.38	6.38	6.38	6.38	6.38	6.38
6.39	6.39	6.39	6.39	6.39	6.39	6.39	6.39	6.39
6.4	6.4	6.4	6.4	6.4	6.4	6.4	6.4	6.4
6.41	6.41	6.41	6.41	6.41	6.41	6.41	6.41	6.41
6.42	6.42	6.42	6.42	6.42	6.42	6.42	6.42	6.42
6.43	6.43	6.43	6.43	6.43	6.43	6.43	6.43	6.43
6.44	6.44	6.44	6.44	6.44	6.44	6.44	6.44	6.44
6.45	6.45	6.45	6.45	6.45	6.45	6.45	6.45	6.45
6.46	6.46	6.46	6.46	6.46	6.46	6.46	6.46	6.46
6.47	6.47	6.47	6.47	6.47	6.47	6.47	6.47	6.47
6.48	6.48	6.48	6.48	6.48	6.48	6.48	6.48	6.48
6.49	6.49	6.49	6.49	6.49	6.49	6.49	6.49	6.49

6.5	6.5	6.5	6.5	6.5	6.5	6.5	6.5	6.5
6.51	6.51	6.51	6.51	6.51	6.51	6.51	6.51	6.51
6.52	6.52	6.52	6.52	6.52	6.52	6.52	6.52	6.52
6.53	6.53	6.53	6.53	6.53	6.53	6.53	6.53	6.53
6.54	6.54	6.54	6.54	6.54	6.54	6.54	6.54	6.54
6.55	6.55	6.55	6.55	6.55	6.55	6.55	6.55	6.55
6.56	6.56	6.56	6.56	6.56	6.56	6.56	6.56	6.56
6.57	6.57	6.57	6.57	6.57	6.57	6.57	6.57	6.57
6.58	6.58	6.58	6.58	6.58	6.58	6.58	6.58	6.58
6.59	6.59	6.59	6.59	6.59	6.59	6.59	6.59	6.59
6.6	6.6	6.6	6.6	6.6	6.6	6.6	6.6	6.6
6.61	6.61	6.61	6.61	6.61	6.61	6.61	6.61	6.61
6.62	6.62	6.62	6.62	6.62	6.62	6.62	6.62	6.62
6.63	6.63	6.63	6.63	6.63	6.63	6.63	6.63	6.63
6.64	6.64	6.64	6.64	6.64	6.64	6.64	6.64	6.64
6.65	6.65	6.65	6.65	6.65	6.65	6.65	6.65	6.65
6.66	6.66	6.66	6.66	6.66	6.66	6.66	6.66	6.66
6.67	6.67	6.67	6.67	6.67	6.67	6.67	6.67	6.67
6.68	6.68	6.68	6.68	6.68	6.68	6.68	6.68	6.68
6.69	6.69	6.69	6.69	6.69	6.69	6.69	6.69	6.69
6.7	6.7	6.7	6.7	6.7	6.7	6.7	6.7	6.7
6.71	6.71	6.71	6.71	6.71	6.71	6.71	6.71	6.71
6.72	6.72	6.72	6.72	6.72	6.72	6.72	6.72	6.72
6.73	6.73	6.73	6.73	6.73	6.73	6.73	6.73	6.73
6.74	6.74	6.74	6.74	6.74	6.74	6.74	6.74	6.74
6.75	6.75	6.75	6.75	6.75	6.75	6.75	6.75	6.75
6.76	6.76	6.76	6.76	6.76	6.76	6.76	6.76	6.76
6.77	6.77	6.77	6.77	6.77	6.77	6.77	6.77	6.77
6.78	6.78	6.78	6.78	6.78	6.78	6.78	6.78	6.78
6.79	6.79	6.79	6.79	6.79	6.79	6.79	6.79	6.79
6.8	6.8	6.8	6.8	6.8	6.8	6.8	6.8	6.8
6.81	6.81	6.81	6.81	6.81	6.81	6.81	6.81	6.81
6.82	6.82	6.82	6.82	6.82	6.82	6.82	6.82	6.82
6.83	6.83	6.83	6.83	6.83	6.83	6.83	6.83	6.83
6.84	6.84	6.84	6.84	6.84	6.84	6.84	6.84	6.84
6.85	6.85	6.85	6.85	6.85	6.85	6.85	6.85	6.85
6.86	6.86	6.86	6.86	6.86	6.86	6.86	6.86	6.86
6.87	6.87	6.87	6.87	6.87	6.87	6.87	6.87	6.87
6.88	6.88	6.88	6.88	6.88	6.88	6.88	6.88	6.88
6.89	6.89	6.89	6.89	6.89	6.89	6.89	6.89	6.89
6.9	6.9	6.9	6.9	6.9	6.9	6.9	6.9	6.9
6.91	6.91	6.91	6.91	6.91	6.91	6.91	6.91	6.91
6.92	6.92	6.92	6.92	6.92	6.92	6.92	6.92	6.92
6.93	6.93	6.93	6.93	6.93	6.93	6.93	6.93	6.93
6.94	6.94	6.94	6.94	6.94	6.94	6.94	6.94	6.94
6.95	6.95	6.95	6.95	6.95	6.95	6.95	6.95	6.95
6.96	6.96	6.96	6.96	6.96	6.96	6.96	6.96	6.96
6.97	6.97	6.97	6.97	6.97	6.97	6.97	6.97	6.97
6.98	6.98	6.98	6.98	6.98	6.98	6.98	6.98	6.98
6.99	6.99	6.99	6.99	6.99	6.99	6.99	6.99	6.99

7	7	7	7	7	7	7	7	7
7.01	7.01	7.01	7.01	7.01	7.01	7.01	7.01	7.01
7.02	7.02	7.02	7.02	7.02	7.02	7.02	7.02	7.02
7.03	7.03	7.03	7.03	7.03	7.03	7.03	7.03	7.03
7.04	7.04	7.04	7.04	7.04	7.04	7.04	7.04	7.04
7.05	7.05	7.05	7.05	7.05	7.05	7.05	7.05	7.05
7.06	7.06	7.06	7.06	7.06	7.06	7.06	7.06	7.06
7.07	7.07	7.07	7.07	7.07	7.07	7.07	7.07	7.07
7.08	7.08	7.08	7.08	7.08	7.08	7.08	7.08	7.08
7.09	7.09	7.09	7.09	7.09	7.09	7.09	7.09	7.09
7.1	7.1	7.1	7.1	7.1	7.1	7.1	7.1	7.1
7.11	7.11	7.11	7.11	7.11	7.11	7.11	7.11	7.11
7.12	7.12	7.12	7.12	7.12	7.12	7.12	7.12	7.12
7.13	7.13	7.13	7.13	7.13	7.13	7.13	7.13	7.13
7.14	7.14	7.14	7.14	7.14	7.14	7.14	7.14	7.14
7.15	7.15	7.15	7.15	7.15	7.15	7.15	7.15	7.15
7.16	7.16	7.16	7.16	7.16	7.16	7.16	7.16	7.16
7.17	7.17	7.17	7.17	7.17	7.17	7.17	7.17	7.17
7.18	7.18	7.18	7.18	7.18	7.18	7.18	7.18	7.18
7.19	7.19	7.19	7.19	7.19	7.19	7.19	7.19	7.19
7.2	7.2	7.2	7.2	7.2	7.2	7.2	7.2	7.2
7.21	7.21	7.21	7.21	7.21	7.21	7.21	7.21	7.21
7.22	7.22	7.22	7.22	7.22	7.22	7.22	7.22	7.22
7.23	7.23	7.23	7.23	7.23	7.23	7.23	7.23	7.23
7.24	7.24	7.24	7.24	7.24	7.24	7.24	7.24	7.24
7.25	7.25	7.25	7.25	7.25	7.25	7.25	7.25	7.25
7.26	7.26	7.26	7.26	7.26	7.26	7.26	7.26	7.26
7.27	7.27	7.27	7.27	7.27	7.27	7.27	7.27	7.27
7.28	7.28	7.28	7.28	7.28	7.28	7.28	7.28	7.28
7.29	7.29	7.29	7.29	7.29	7.29	7.29	7.29	7.29
7.3	7.3	7.3	7.3	7.3	7.3	7.3	7.3	7.3
7.31	7.31	7.31	7.31	7.31	7.31	7.31	7.31	7.31
7.32	7.32	7.32	7.32	7.32	7.32	7.32	7.32	7.32
7.33	7.33	7.33	7.33	7.33	7.33	7.33	7.33	7.33
7.34	7.34	7.34	7.34	7.34	7.34	7.34	7.34	7.34
7.35	7.35	7.35	7.35	7.35	7.35	7.35	7.35	7.35
7.36	7.36	7.36	7.36	7.36	7.36	7.36	7.36	7.36
7.37	7.37	7.37	7.37	7.37	7.37	7.37	7.37	7.37
7.38	7.38	7.38	7.38	7.38	7.38	7.38	7.38	7.38
7.39	7.39	7.39	7.39	7.39	7.39	7.39	7.39	7.39
7.4	7.4	7.4	7.4	7.4	7.4	7.4	7.4	7.4
7.41	7.41	7.41	7.41	7.41	7.41	7.41	7.41	7.41
7.42	7.42	7.42	7.42	7.42	7.42	7.42	7.42	7.42
7.43	7.43	7.43	7.43	7.43	7.43	7.43	7.43	7.43
7.44	7.44	7.44	7.44	7.44	7.44	7.44	7.44	7.44
7.45	7.45	7.45	7.45	7.45	7.45	7.45	7.45	7.45
7.46	7.46	7.46	7.46	7.46	7.46	7.46	7.46	7.46
7.47	7.47	7.47	7.47	7.47	7.47	7.47	7.47	7.47
7.48	7.48	7.48	7.48	7.48	7.48	7.48	7.48	7.48
7.49	7.49	7.49	7.49	7.49	7.49	7.49	7.49	7.49

7.5	7.5	7.5	7.5	7.5	7.5	7.5	7.5	7.5
7.51	7.51	7.51	7.51	7.51	7.51	7.51	7.51	7.51
7.52	7.52	7.52	7.52	7.52	7.52	7.52	7.52	7.52
7.53	7.53	7.53	7.53	7.53	7.53	7.53	7.53	7.53
7.54	7.54	7.54	7.54	7.54	7.54	7.54	7.54	7.54
7.55	7.55	7.55	7.55	7.55	7.55	7.55	7.55	7.55
7.56	7.56	7.56	7.56	7.56	7.56	7.56	7.56	7.56
7.57	7.57	7.57	7.57	7.57	7.57	7.57	7.57	7.57
7.58	7.58	7.58	7.58	7.58	7.58	7.58	7.58	7.58
7.59	7.59	7.59	7.59	7.59	7.59	7.59	7.59	7.59
7.6	7.6	7.6	7.6	7.6	7.6	7.6	7.6	7.6
7.61	7.61	7.61	7.61	7.61	7.61	7.61	7.61	7.61
7.62	7.62	7.62	7.62	7.62	7.62	7.62	7.62	7.62
7.63	7.63	7.63	7.63	7.63	7.63	7.63	7.63	7.63
7.64	7.64	7.64	7.64	7.64	7.64	7.64	7.64	7.64
7.65	7.65	7.65	7.65	7.65	7.65	7.65	7.65	7.65
7.66	7.66	7.66	7.66	7.66	7.66	7.66	7.66	7.66
7.67	7.67	7.67	7.67	7.67	7.67	7.67	7.67	7.67
7.68	7.68	7.68	7.68	7.68	7.68	7.68	7.68	7.68
7.69	7.69	7.69	7.69	7.69	7.69	7.69	7.69	7.69
7.7	7.7	7.7	7.7	7.7	7.7	7.7	7.7	7.7
7.71	7.71	7.71	7.71	7.71	7.71	7.71	7.71	7.71
7.72	7.72	7.72	7.72	7.72	7.72	7.72	7.72	7.72
7.73	7.73	7.73	7.73	7.73	7.73	7.73	7.73	7.73
7.74	7.74	7.74	7.74	7.74	7.74	7.74	7.74	7.74
7.75	7.75	7.75	7.75	7.75	7.75	7.75	7.75	7.75
7.76	7.76	7.76	7.76	7.76	7.76	7.76	7.76	7.76
7.77	7.77	7.77	7.77	7.77	7.77	7.77	7.77	7.77
7.78	7.78	7.78	7.78	7.78	7.78	7.78	7.78	7.78
7.79	7.79	7.79	7.79	7.79	7.79	7.79	7.79	7.79
7.8	7.8	7.8	7.8	7.8	7.8	7.8	7.8	7.8
7.81	7.81	7.81	7.81	7.81	7.81	7.81	7.81	7.81
7.82	7.82	7.82	7.82	7.82	7.82	7.82	7.82	7.82
7.83	7.83	7.83	7.83	7.83	7.83	7.83	7.83	7.83
7.84	7.84	7.84	7.84	7.84	7.84	7.84	7.84	7.84
7.85	7.85	7.85	7.85	7.85	7.85	7.85	7.85	7.85
7.86	7.86	7.86	7.86	7.86	7.86	7.86	7.86	7.86
7.87	7.87	7.87	7.87	7.87	7.87	7.87	7.87	7.87
7.88	7.88	7.88	7.88	7.88	7.88	7.88	7.88	7.88
7.89	7.89	7.89	7.89	7.89	7.89	7.89	7.89	7.89
7.9	7.9	7.9	7.9	7.9	7.9	7.9	7.9	7.9
7.91	7.91	7.91	7.91	7.91	7.91	7.91	7.91	7.91
7.92	7.92	7.92	7.92	7.92	7.92	7.92	7.92	7.92
7.93	7.93	7.93	7.93	7.93	7.93	7.93	7.93	7.93
7.94	7.94	7.94	7.94	7.94	7.94	7.94	7.94	7.94
7.95	7.95	7.95	7.95	7.95	7.95	7.95	7.95	7.95
7.96	7.96	7.96	7.96	7.96	7.96	7.96	7.96	7.96
7.97	7.97	7.97	7.97	7.97	7.97	7.97	7.97	7.97
7.98	7.98	7.98	7.98	7.98	7.98	7.98	7.98	7.98
7.99	7.99	7.99	7.99	7.99	7.99	7.99	7.99	7.99

8	8	8	8	8	8	8	8	8
8.01	8.01	8.01	8.01	8.01	8.01	8.01	8.01	8.01
8.02	8.02	8.02	8.02	8.02	8.02	8.02	8.02	8.02
8.03	8.03	8.03	8.03	8.03	8.03	8.03	8.03	8.03
8.04	8.04	8.04	8.04	8.04	8.04	8.04	8.04	8.04
8.05	8.05	8.05	8.05	8.05	8.05	8.05	8.05	8.05
8.06	8.06	8.06	8.06	8.06	8.06	8.06	8.06	8.06
8.07	8.07	8.07	8.07	8.07	8.07	8.07	8.07	8.07
8.08	8.08	8.08	8.08	8.08	8.08	8.08	8.08	8.08
8.09	8.09	8.09	8.09	8.09	8.09	8.09	8.09	8.09
8.1	8.1	8.1	8.1	8.1	8.1	8.1	8.1	8.1
8.11	8.11	8.11	8.11	8.11	8.11	8.11	8.11	8.11
8.12	8.12	8.12	8.12	8.12	8.12	8.12	8.12	8.12
8.13	8.13	8.13	8.13	8.13	8.13	8.13	8.13	8.13
8.14	8.14	8.14	8.14	8.14	8.14	8.14	8.14	8.14
8.15	8.15	8.15	8.15	8.15	8.15	8.15	8.15	8.15
8.16	8.16	8.16	8.16	8.16	8.16	8.16	8.16	8.16
8.17	8.17	8.17	8.17	8.17	8.17	8.17	8.17	8.17
8.18	8.18	8.18	8.18	8.18	8.18	8.18	8.18	8.18
8.19	8.19	8.19	8.19	8.19	8.19	8.19	8.19	8.19
8.2	8.2	8.2	8.2	8.2	8.2	8.2	8.2	8.2
8.21	8.21	8.21	8.21	8.21	8.21	8.21	8.21	8.21
8.22	8.22	8.22	8.22	8.22	8.22	8.22	8.22	8.22
8.23	8.23	8.23	8.23	8.23	8.23	8.23	8.23	8.23
8.24	8.24	8.24	8.24	8.24	8.24	8.24	8.24	8.24
8.25	8.25	8.25	8.25	8.25	8.25	8.25	8.25	8.25
8.26	8.26	8.26	8.26	8.26	8.26	8.26	8.26	8.26
8.27	8.27	8.27	8.27	8.27	8.27	8.27	8.27	8.27
8.28	8.28	8.28	8.28	8.28	8.28	8.28	8.28	8.28
8.29	8.29	8.29	8.29	8.29	8.29	8.29	8.29	8.29
8.3	8.3	8.3	8.3	8.3	8.3	8.3	8.3	8.3
8.31	8.31	8.31	8.31	8.31	8.31	8.31	8.31	8.31
8.32	8.32	8.32	8.32	8.32	8.32	8.32	8.32	8.32
8.33	8.33	8.33	8.33	8.33	8.33	8.33	8.33	8.33
8.34	8.34	8.34	8.34	8.34	8.34	8.34	8.34	8.34
8.35	8.35	8.35	8.35	8.35	8.35	8.35	8.35	8.35
8.36	8.36	8.36	8.36	8.36	8.36	8.36	8.36	8.36
8.37	8.37	8.37	8.37	8.37	8.37	8.37	8.37	8.37
8.38	8.38	8.38	8.38	8.38	8.38	8.38	8.38	8.38
8.39	8.39	8.39	8.39	8.39	8.39	8.39	8.39	8.39
8.4	8.4	8.4	8.4	8.4	8.4	8.4	8.4	8.4
8.41	8.41	8.41	8.41	8.41	8.41	8.41	8.41	8.41
8.42	8.42	8.42	8.42	8.42	8.42	8.42	8.42	8.42
8.43	8.43	8.43	8.43	8.43	8.43	8.43	8.43	8.43
8.44	8.44	8.44	8.44	8.44	8.44	8.44	8.44	8.44
8.45	8.45	8.45	8.45	8.45	8.45	8.45	8.45	8.45
8.46	8.46	8.46	8.46	8.46	8.46	8.46	8.46	8.46
8.47	8.47	8.47	8.47	8.47	8.47	8.47	8.47	8.47
8.48	8.48	8.48	8.48	8.48	8.48	8.48	8.48	8.48
8.49	8.49	8.49	8.49	8.49	8.49	8.49	8.49	8.49

8.5	8.5	8.5	8.5	8.5	8.5	8.5	8.5	8.5
8.51	8.51	8.51	8.51	8.51	8.51	8.51	8.51	8.51
8.52	8.52	8.52	8.52	8.52	8.52	8.52	8.52	8.52
8.53	8.53	8.53	8.53	8.53	8.53	8.53	8.53	8.53
8.54	8.54	8.54	8.54	8.54	8.54	8.54	8.54	8.54
8.55	8.55	8.55	8.55	8.55	8.55	8.55	8.55	8.55
8.56	8.56	8.56	8.56	8.56	8.56	8.56	8.56	8.56
8.57	8.57	8.57	8.57	8.57	8.57	8.57	8.57	8.57
8.58	8.58	8.58	8.58	8.58	8.58	8.58	8.58	8.58
8.59	8.59	8.59	8.59	8.59	8.59	8.59	8.59	8.59
8.6	8.6	8.6	8.6	8.6	8.6	8.6	8.6	8.6
8.61	8.61	8.61	8.61	8.61	8.61	8.61	8.61	8.61
8.62	8.62	8.62	8.62	8.62	8.62	8.62	8.62	8.62
8.63	8.63	8.63	8.63	8.63	8.63	8.63	8.63	8.63
8.64	8.64	8.64	8.64	8.64	8.64	8.64	8.64	8.64
8.65	8.65	8.65	8.65	8.65	8.65	8.65	8.65	8.65
8.66	8.66	8.66	8.66	8.66	8.66	8.66	8.66	8.66
8.67	8.67	8.67	8.67	8.67	8.67	8.67	8.67	8.67
8.68	8.68	8.68	8.68	8.68	8.68	8.68	8.68	8.68
8.69	8.69	8.69	8.69	8.69	8.69	8.69	8.69	8.69
8.7	8.7	8.7	8.7	8.7	8.7	8.7	8.7	8.7
8.71	8.71	8.71	8.71	8.71	8.71	8.71	8.71	8.71
8.72	8.72	8.72	8.72	8.72	8.72	8.72	8.72	8.72
8.73	8.73	8.73	8.73	8.73	8.73	8.73	8.73	8.73
8.74	8.74	8.74	8.74	8.74	8.74	8.74	8.74	8.74
8.75	8.75	8.75	8.75	8.75	8.75	8.75	8.75	8.75
8.76	8.76	8.76	8.76	8.76	8.76	8.76	8.76	8.76
8.77	8.77	8.77	8.77	8.77	8.77	8.77	8.77	8.77
8.78	8.78	8.78	8.78	8.78	8.78	8.78	8.78	8.78
8.79	8.79	8.79	8.79	8.79	8.79	8.79	8.79	8.79
8.8	8.8	8.8	8.8	8.8	8.8	8.8	8.8	8.8
8.81	8.81	8.81	8.81	8.81	8.81	8.81	8.81	8.81
8.82	8.82	8.82	8.82	8.82	8.82	8.82	8.82	8.82
8.83	8.83	8.83	8.83	8.83	8.83	8.83	8.83	8.83
8.84	8.84	8.84	8.84	8.84	8.84	8.84	8.84	8.84
8.85	8.85	8.85	8.85	8.85	8.85	8.85	8.85	8.85
8.86	8.86	8.86	8.86	8.86	8.86	8.86	8.86	8.86
8.87	8.87	8.87	8.87	8.87	8.87	8.87	8.87	8.87
8.88	8.88	8.88	8.88	8.88	8.88	8.88	8.88	8.88
8.89	8.89	8.89	8.89	8.89	8.89	8.89	8.89	8.89
8.9	8.9	8.9	8.9	8.9	8.9	8.9	8.9	8.9
8.91	8.91	8.91	8.91	8.91	8.91	8.91	8.91	8.91
8.92	8.92	8.92	8.92	8.92	8.92	8.92	8.92	8.92
8.93	8.93	8.93	8.93	8.93	8.93	8.93	8.93	8.93
8.94	8.94	8.94	8.94	8.94	8.94	8.94	8.94	8.94
8.95	8.95	8.95	8.95	8.95	8.95	8.95	8.95	8.95
8.96	8.96	8.96	8.96	8.96	8.96	8.96	8.96	8.96
8.97	8.97	8.97	8.97	8.97	8.97	8.97	8.97	8.97
8.98	8.98	8.98	8.98	8.98	8.98	8.98	8.98	8.98
8.99	8.99	8.99	8.99	8.99	8.99	8.99	8.99	8.99

9	9	9	9	9	9	9	9	9
9.01	9.01	9.01	9.01	9.01	9.01	9.01	9.01	9.01
9.02	9.02	9.02	9.02	9.02	9.02	9.02	9.02	9.02
9.03	9.03	9.03	9.03	9.03	9.03	9.03	9.03	9.03
9.04	9.04	9.04	9.04	9.04	9.04	9.04	9.04	9.04
9.05	9.05	9.05	9.05	9.05	9.05	9.05	9.05	9.05
9.06	9.06	9.06	9.06	9.06	9.06	9.06	9.06	9.06
9.07	9.07	9.07	9.07	9.07	9.07	9.07	9.07	9.07
9.08	9.08	9.08	9.08	9.08	9.08	9.08	9.08	9.08
9.09	9.09	9.09	9.09	9.09	9.09	9.09	9.09	9.09
9.1	9.1	9.1	9.1	9.1	9.1	9.1	9.1	9.1
9.11	9.11	9.11	9.11	9.11	9.11	9.11	9.11	9.11
9.12	9.12	9.12	9.12	9.12	9.12	9.12	9.12	9.12
9.13	9.13	9.13	9.13	9.13	9.13	9.13	9.13	9.13
9.14	9.14	9.14	9.14	9.14	9.14	9.14	9.14	9.14
9.15	9.15	9.15	9.15	9.15	9.15	9.15	9.15	9.15
9.16	9.16	9.16	9.16	9.16	9.16	9.16	9.16	9.16
9.17	9.17	9.17	9.17	9.17	9.17	9.17	9.17	9.17
9.18	9.18	9.18	9.18	9.18	9.18	9.18	9.18	9.18
9.19	9.19	9.19	9.19	9.19	9.19	9.19	9.19	9.19
9.2	9.2	9.2	9.2	9.2	9.2	9.2	9.2	9.2
9.21	9.21	9.21	9.21	9.21	9.21	9.21	9.21	9.21
9.22	9.22	9.22	9.22	9.22	9.22	9.22	9.22	9.22
9.23	9.23	9.23	9.23	9.23	9.23	9.23	9.23	9.23
9.24	9.24	9.24	9.24	9.24	9.24	9.24	9.24	9.24
9.25	9.25	9.25	9.25	9.25	9.25	9.25	9.25	9.25
9.26	9.26	9.26	9.26	9.26	9.26	9.26	9.26	9.26
9.27	9.27	9.27	9.27	9.27	9.27	9.27	9.27	9.27
9.28	9.28	9.28	9.28	9.28	9.28	9.28	9.28	9.28
9.29	9.29	9.29	9.29	9.29	9.29	9.29	9.29	9.29
9.3	9.3	9.3	9.3	9.3	9.3	9.3	9.3	9.3
9.31	9.31	9.31	9.31	9.31	9.31	9.31	9.31	9.31
9.32	9.32	9.32	9.32	9.32	9.32	9.32	9.32	9.32
9.33	9.33	9.33	9.33	9.33	9.33	9.33	9.33	9.33
9.34	9.34	9.34	9.34	9.34	9.34	9.34	9.34	9.34
9.35	9.35	9.35	9.35	9.35	9.35	9.35	9.35	9.35
9.36	9.36	9.36	9.36	9.36	9.36	9.36	9.36	9.36
9.37	9.37	9.37	9.37	9.37	9.37	9.37	9.37	9.37
9.38	9.38	9.38	9.38	9.38	9.38	9.38	9.38	9.38
9.39	9.39	9.39	9.39	9.39	9.39	9.39	9.39	9.39
9.4	9.4	9.4	9.4	9.4	9.4	9.4	9.4	9.4
9.41	9.41	9.41	9.41	9.41	9.41	9.41	9.41	9.41
9.42	9.42	9.42	9.42	9.42	9.42	9.42	9.42	9.42
9.43	9.43	9.43	9.43	9.43	9.43	9.43	9.43	9.43
9.44	9.44	9.44	9.44	9.44	9.44	9.44	9.44	9.44
9.45	9.45	9.45	9.45	9.45	9.45	9.45	9.45	9.45
9.46	9.46	9.46	9.46	9.46	9.46	9.46	9.46	9.46
9.47	9.47	9.47	9.47	9.47	9.47	9.47	9.47	9.47
9.48	9.48	9.48	9.48	9.48	9.48	9.48	9.48	9.48
9.49	9.49	9.49	9.49	9.49	9.49	9.49	9.49	9.49

9.5	9.5	9.5	9.5	9.5	9.5	9.5	9.5	9.5
9.51	9.51	9.51	9.51	9.51	9.51	9.51	9.51	9.51
9.52	9.52	9.52	9.52	9.52	9.52	9.52	9.52	9.52
9.53	9.53	9.53	9.53	9.53	9.53	9.53	9.53	9.53
9.54	9.54	9.54	9.54	9.54	9.54	9.54	9.54	9.54
9.55	9.55	9.55	9.55	9.55	9.55	9.55	9.55	9.55
9.56	9.56	9.56	9.56	9.56	9.56	9.56	9.56	9.56
9.57	9.57	9.57	9.57	9.57	9.57	9.57	9.57	9.57
9.58	9.58	9.58	9.58	9.58	9.58	9.58	9.58	9.58
9.59	9.59	9.59	9.59	9.59	9.59	9.59	9.59	9.59
9.6	9.6	9.6	9.6	9.6	9.6	9.6	9.6	9.6
9.61	9.61	9.61	9.61	9.61	9.61	9.61	9.61	9.61
9.62	9.62	9.62	9.62	9.62	9.62	9.62	9.62	9.62
9.63	9.63	9.63	9.63	9.63	9.63	9.63	9.63	9.63
9.64	9.64	9.64	9.64	9.64	9.64	9.64	9.64	9.64
9.65	9.65	9.65	9.65	9.65	9.65	9.65	9.65	9.65
9.66	9.66	9.66	9.66	9.66	9.66	9.66	9.66	9.66
9.67	9.67	9.67	9.67	9.67	9.67	9.67	9.67	9.67
9.68	9.68	9.68	9.68	9.68	9.68	9.68	9.68	9.68
9.69	9.69	9.69	9.69	9.69	9.69	9.69	9.69	9.69
9.7	9.7	9.7	9.7	9.7	9.7	9.7	9.7	9.7
9.71	9.71	9.71	9.71	9.71	9.71	9.71	9.71	9.71
9.72	9.72	9.72	9.72	9.72	9.72	9.72	9.72	9.72
9.73	9.73	9.73	9.73	9.73	9.73	9.73	9.73	9.73
9.74	9.74	9.74	9.74	9.74	9.74	9.74	9.74	9.74
9.75	9.75	9.75	9.75	9.75	9.75	9.75	9.75	9.75
9.76	9.76	9.76	9.76	9.76	9.76	9.76	9.76	9.76
9.77	9.77	9.77	9.77	9.77	9.77	9.77	9.77	9.77
9.78	9.78	9.78	9.78	9.78	9.78	9.78	9.78	9.78
9.79	9.79	9.79	9.79	9.79	9.79	9.79	9.79	9.79
9.8	9.8	9.8	9.8	9.8	9.8	9.8	9.8	9.8
9.81	9.81	9.81	9.81	9.81	9.81	9.81	9.81	9.81
9.82	9.82	9.82	9.82	9.82	9.82	9.82	9.82	9.82
9.83	9.83	9.83	9.83	9.83	9.83	9.83	9.83	9.83
9.84	9.84	9.84	9.84	9.84	9.84	9.84	9.84	9.84
9.85	9.85	9.85	9.85	9.85	9.85	9.85	9.85	9.85
9.86	9.86	9.86	9.86	9.86	9.86	9.86	9.86	9.86
9.87	9.87	9.87	9.87	9.87	9.87	9.87	9.87	9.87
9.88	9.88	9.88	9.88	9.88	9.88	9.88	9.88	9.88
9.89	9.89	9.89	9.89	9.89	9.89	9.89	9.89	9.89
9.9	9.9	9.9	9.9	9.9	9.9	9.9	9.9	9.9
9.91	9.91	9.91	9.91	9.91	9.91	9.91	9.91	9.91
9.92	9.92	9.92	9.92	9.92	9.92	9.92	9.92	9.92
9.93	9.93	9.93	9.93	9.93	9.93	9.93	9.93	9.93
9.94	9.94	9.94	9.94	9.94	9.94	9.94	9.94	9.94
9.95	9.95	9.95	9.95	9.95	9.95	9.95	9.95	9.95
9.96	9.96	9.96	9.96	9.96	9.96	9.96	9.96	9.96
9.97	9.97	9.97	9.97	9.97	9.97	9.97	9.97	9.97
9.98	9.98	9.98	9.98	9.98	9.98	9.98	9.98	9.98
9.99	9.99	9.99	9.99	9.99	9.99	9.99	9.99	9.99

10	10	10	10	10	10	10	10	10
10.01	10.01	10.01	10.01	10.01	10.01	10.01	10.01	10.01
10.02	10.02	10.02	10.02	10.02	10.02	10.02	10.02	10.02
10.03	10.03	10.03	10.03	10.03	10.03	10.03	10.03	10.03
10.04	10.04	10.04	10.04	10.04	10.04	10.04	10.04	10.04
10.05	10.05	10.05	10.05	10.05	10.05	10.05	10.05	10.05
10.06	10.06	10.06	10.06	10.06	10.06	10.06	10.06	10.06
10.07	10.07	10.07	10.07	10.07	10.07	10.07	10.07	10.07
10.08	10.08	10.08	10.08	10.08	10.08	10.08	10.08	10.08
10.09	10.09	10.09	10.09	10.09	10.09	10.09	10.09	10.09
10.1	10.1	10.1	10.1	10.1	10.1	10.1	10.1	10.1
10.11	10.11	10.11	10.11	10.11	10.11	10.11	10.11	10.11
10.12	10.12	10.12	10.12	10.12	10.12	10.12	10.12	10.12
10.13	10.13	10.13	10.13	10.13	10.13	10.13	10.13	10.13
10.14	10.14	10.14	10.14	10.14	10.14	10.14	10.14	10.14
10.15	10.15	10.15	10.15	10.15	10.15	10.15	10.15	10.15
10.16	10.16	10.16	10.16	10.16	10.16	10.16	10.16	10.16
10.17	10.17	10.17	10.17	10.17	10.17	10.17	10.17	10.17
10.18	10.18	10.18	10.18	10.18	10.18	10.18	10.18	10.18
10.19	10.19	10.19	10.19	10.19	10.19	10.19	10.19	10.19
10.2	10.2	10.2	10.2	10.2	10.2	10.2	10.2	10.2
10.21	10.21	10.21	10.21	10.21	10.21	10.21	10.21	10.21
10.22	10.22	10.22	10.22	10.22	10.22	10.22	10.22	10.22
10.23	10.23	10.23	10.23	10.23	10.23	10.23	10.23	10.23
10.24	10.24	10.24	10.24	10.24	10.24	10.24	10.24	10.24
10.25	10.25	10.25	10.25	10.25	10.25	10.25	10.25	10.25
10.26	10.26	10.26	10.26	10.26	10.26	10.26	10.26	10.26
10.27	10.27	10.27	10.27	10.27	10.27	10.27	10.27	10.27
10.28	10.28	10.28	10.28	10.28	10.28	10.28	10.28	10.28
10.29	10.29	10.29	10.29	10.29	10.29	10.29	10.29	10.29
10.3	10.3	10.3	10.3	10.3	10.3	10.3	10.3	10.3
10.31	10.31	10.31	10.31	10.31	10.31	10.31	10.31	10.31
10.32	10.32	10.32	10.32	10.32	10.32	10.32	10.32	10.32
10.33	10.33	10.33	10.33	10.33	10.33	10.33	10.33	10.33
10.34	10.34	10.34	10.34	10.34	10.34	10.34	10.34	10.34
10.35	10.35	10.35	10.35	10.35	10.35	10.35	10.35	10.35
10.36	10.36	10.36	10.36	10.36	10.36	10.36	10.36	10.36
10.37	10.37	10.37	10.37	10.37	10.37	10.37	10.37	10.37
10.38	10.38	10.38	10.38	10.38	10.38	10.38	10.38	10.38
10.39	10.39	10.39	10.39	10.39	10.39	10.39	10.39	10.39
10.4	10.4	10.4	10.4	10.4	10.4	10.4	10.4	10.4
10.41	10.41	10.41	10.41	10.41	10.41	10.41	10.41	10.41
10.42	10.42	10.42	10.42	10.42	10.42	10.42	10.42	10.42
10.43	10.43	10.43	10.43	10.43	10.43	10.43	10.43	10.43
10.44	10.44	10.44	10.44	10.44	10.44	10.44	10.44	10.44
10.45	10.45	10.45	10.45	10.45	10.45	10.45	10.45	10.45
10.46	10.46	10.46	10.46	10.46	10.46	10.46	10.46	10.46
10.47	10.47	10.47	10.47	10.47	10.47	10.47	10.47	10.47
10.48	10.48	10.48	10.48	10.48	10.48	10.48	10.48	10.48
10.49	10.49	10.49	10.49	10.49	10.49	10.49	10.49	10.49

10.5	10.5	10.5	10.5	10.5	10.5	10.5	10.5	10.5
10.51	10.51	10.51	10.51	10.51	10.51	10.51	10.51	10.51
10.52	10.52	10.52	10.52	10.52	10.52	10.52	10.52	10.52
10.53	10.53	10.53	10.53	10.53	10.53	10.53	10.53	10.53
10.54	10.54	10.54	10.54	10.54	10.54	10.54	10.54	10.54
10.55	10.55	10.55	10.55	10.55	10.55	10.55	10.55	10.55
10.56	10.56	10.56	10.56	10.56	10.56	10.56	10.56	10.56
10.57	10.57	10.57	10.57	10.57	10.57	10.57	10.57	10.57
10.58	10.58	10.58	10.58	10.58	10.58	10.58	10.58	10.58
10.59	10.59	10.59	10.59	10.59	10.59	10.59	10.59	10.59
10.6	10.6	10.6	10.6	10.6	10.6	10.6	10.6	10.6
10.61	10.61	10.61	10.61	10.61	10.61	10.61	10.61	10.61
10.62	10.62	10.62	10.62	10.62	10.62	10.62	10.62	10.62
10.63	10.63	10.63	10.63	10.63	10.63	10.63	10.63	10.63
10.64	10.64	10.64	10.64	10.64	10.64	10.64	10.64	10.64
10.65	10.65	10.65	10.65	10.65	10.65	10.65	10.65	10.65
10.66	10.66	10.66	10.66	10.66	10.66	10.66	10.66	10.66
10.67	10.67	10.67	10.67	10.67	10.67	10.67	10.67	10.67
10.68	10.68	10.68	10.68	10.68	10.68	10.68	10.68	10.68
10.69	10.69	10.69	10.69	10.69	10.69	10.69	10.69	10.69
10.7	10.7	10.7	10.7	10.7	10.7	10.7	10.7	10.7
10.71	10.71	10.71	10.71	10.71	10.71	10.71	10.71	10.71
10.72	10.72	10.72	10.72	10.72	10.72	10.72	10.72	10.72
10.73	10.73	10.73	10.73	10.73	10.73	10.73	10.73	10.73
10.74	10.74	10.74	10.74	10.74	10.74	10.74	10.74	10.74
10.75	10.75	10.75	10.75	10.75	10.75	10.75	10.75	10.75
10.76	10.76	10.76	10.76	10.76	10.76	10.76	10.76	10.76
10.77	10.77	10.77	10.77	10.77	10.77	10.77	10.77	10.77
10.78	10.78	10.78	10.78	10.78	10.78	10.78	10.78	10.78
10.79	10.79	10.79	10.79	10.79	10.79	10.79	10.79	10.79
10.8	10.8	10.8	10.8	10.8	10.8	10.8	10.8	10.8
10.81	10.81	10.81	10.81	10.81	10.81	10.81	10.81	10.81
10.82	10.82	10.82	10.82	10.82	10.82	10.82	10.82	10.82
10.83	10.83	10.83	10.83	10.83	10.83	10.83	10.83	10.83
10.84	10.84	10.84	10.84	10.84	10.84	10.84	10.84	10.84
10.85	10.85	10.85	10.85	10.85	10.85	10.85	10.85	10.85
10.86	10.86	10.86	10.86	10.86	10.86	10.86	10.86	10.86
10.87	10.87	10.87	10.87	10.87	10.87	10.87	10.87	10.87
10.88	10.88	10.88	10.88	10.88	10.88	10.88	10.88	10.88
10.89	10.89	10.89	10.89	10.89	10.89	10.89	10.89	10.89
10.9	10.9	10.9	10.9	10.9	10.9	10.9	10.9	10.9
10.91	10.91	10.91	10.91	10.91	10.91	10.91	10.91	10.91
10.92	10.92	10.92	10.92	10.92	10.92	10.92	10.92	10.92
10.93	10.93	10.93	10.93	10.93	10.93	10.93	10.93	10.93
10.94	10.94	10.94	10.94	10.94	10.94	10.94	10.94	10.94
10.95	10.95	10.95	10.95	10.95	10.95	10.95	10.95	10.95
10.96	10.96	10.96	10.96	10.96	10.96	10.96	10.96	10.96
10.97	10.97	10.97	10.97	10.97	10.97	10.97	10.97	10.97
10.98	10.98	10.98	10.98	10.98	10.98	10.98	10.98	10.98
10.99	10.99	10.99	10.99	10.99	10.99	10.99	10.99	10.99

11	11	11	11	11	11	11	11	11
11.01	11.01	11.01	11.01	11.01	11.01	11.01	11.01	11.01
11.02	11.02	11.02	11.02	11.02	11.02	11.02	11.02	11.02
11.03	11.03	11.03	11.03	11.03	11.03	11.03	11.03	11.03
11.04	11.04	11.04	11.04	11.04	11.04	11.04	11.04	11.04
11.05	11.05	11.05	11.05	11.05	11.05	11.05	11.05	11.05
11.06	11.06	11.06	11.06	11.06	11.06	11.06	11.06	11.06
11.07	11.07	11.07	11.07	11.07	11.07	11.07	11.07	11.07
11.08	11.08	11.08	11.08	11.08	11.08	11.08	11.08	11.08
11.09	11.09	11.09	11.09	11.09	11.09	11.09	11.09	11.09
11.1	11.1	11.1	11.1	11.1	11.1	11.1	11.1	11.1
11.11	11.11	11.11	11.11	11.11	11.11	11.11	11.11	11.11
11.12	11.12	11.12	11.12	11.12	11.12	11.12	11.12	11.12
11.13	11.13	11.13	11.13	11.13	11.13	11.13	11.13	11.13
11.14	11.14	11.14	11.14	11.14	11.14	11.14	11.14	11.14
11.15	11.15	11.15	11.15	11.15	11.15	11.15	11.15	11.15
11.16	11.16	11.16	11.16	11.16	11.16	11.16	11.16	11.16
11.17	11.17	11.17	11.17	11.17	11.17	11.17	11.17	11.17
11.18	11.18	11.18	11.18	11.18	11.18	11.18	11.18	11.18
11.19	11.19	11.19	11.19	11.19	11.19	11.19	11.19	11.19
11.2	11.2	11.2	11.2	11.2	11.2	11.2	11.2	11.2
11.21	11.21	11.21	11.21	11.21	11.21	11.21	11.21	11.21
11.22	11.22	11.22	11.22	11.22	11.22	11.22	11.22	11.22
11.23	11.23	11.23	11.23	11.23	11.23	11.23	11.23	11.23
11.24	11.24	11.24	11.24	11.24	11.24	11.24	11.24	11.24
11.25	11.25	11.25	11.25	11.25	11.25	11.25	11.25	11.25
11.26	11.26	11.26	11.26	11.26	11.26	11.26	11.26	11.26
11.27	11.27	11.27	11.27	11.27	11.27	11.27	11.27	11.27
11.28	11.28	11.28	11.28	11.28	11.28	11.28	11.28	11.28
11.29	11.29	11.29	11.29	11.29	11.29	11.29	11.29	11.29
11.3	11.3	11.3	11.3	11.3	11.3	11.3	11.3	11.3
11.31	11.31	11.31	11.31	11.31	11.31	11.31	11.31	11.31
11.32	11.32	11.32	11.32	11.32	11.32	11.32	11.32	11.32
11.33	11.33	11.33	11.33	11.33	11.33	11.33	11.33	11.33
11.34	11.34	11.34	11.34	11.34	11.34	11.34	11.34	11.34
11.35	11.35	11.35	11.35	11.35	11.35	11.35	11.35	11.35
11.36	11.36	11.36	11.36	11.36	11.36	11.36	11.36	11.36
11.37	11.37	11.37	11.37	11.37	11.37	11.37	11.37	11.37
11.38	11.38	11.38	11.38	11.38	11.38	11.38	11.38	11.38
11.39	11.39	11.39	11.39	11.39	11.39	11.39	11.39	11.39
11.4	11.4	11.4	11.4	11.4	11.4	11.4	11.4	11.4
11.41	11.41	11.41	11.41	11.41	11.41	11.41	11.41	11.41
11.42	11.42	11.42	11.42	11.42	11.42	11.42	11.42	11.42
11.43	11.43	11.43	11.43	11.43	11.43	11.43	11.43	11.43
11.44	11.44	11.44	11.44	11.44	11.44	11.44	11.44	11.44
11.45	11.45	11.45	11.45	11.45	11.45	11.45	11.45	11.45
11.46	11.46	11.46	11.46	11.46	11.46	11.46	11.46	11.46
11.47	11.47	11.47	11.47	11.47	11.47	11.47	11.47	11.47
11.48	11.48	11.48	11.48	11.48	11.48	11.48	11.48	11.48
11.49	11.49	11.49	11.49	11.49	11.49	11.49	11.49	11.49

11.5	11.5	11.5	11.5	11.5	11.5	11.5	11.5	11.5
11.51	11.51	11.51	11.51	11.51	11.51	11.51	11.51	11.51
11.52	11.52	11.52	11.52	11.52	11.52	11.52	11.52	11.52
11.53	11.53	11.53	11.53	11.53	11.53	11.53	11.53	11.53
11.54	11.54	11.54	11.54	11.54	11.54	11.54	11.54	11.54
11.55	11.55	11.55	11.55	11.55	11.55	11.55	11.55	11.55
11.56	11.56	11.56	11.56	11.56	11.56	11.56	11.56	11.56
11.57	11.57	11.57	11.57	11.57	11.57	11.57	11.57	11.57
11.58	11.58	11.58	11.58	11.58	11.58	11.58	11.58	11.58
11.59	11.59	11.59	11.59	11.59	11.59	11.59	11.59	11.59
11.6	11.6	11.6	11.6	11.6	11.6	11.6	11.6	11.6
11.61	11.61	11.61	11.61	11.61	11.61	11.61	11.61	11.61
11.62	11.62	11.62	11.62	11.62	11.62	11.62	11.62	11.62
11.63	11.63	11.63	11.63	11.63	11.63	11.63	11.63	11.63
11.64	11.64	11.64	11.64	11.64	11.64	11.64	11.64	11.64
11.65	11.65	11.65	11.65	11.65	11.65	11.65	11.65	11.65
11.66	11.66	11.66	11.66	11.66	11.66	11.66	11.66	11.66
11.67	11.67	11.67	11.67	11.67	11.67	11.67	11.67	11.67
11.68	11.68	11.68	11.68	11.68	11.68	11.68	11.68	11.68
11.69	11.69	11.69	11.69	11.69	11.69	11.69	11.69	11.69
11.7	11.7	11.7	11.7	11.7	11.7	11.7	11.7	11.7
11.71	11.71	11.71	11.71	11.71	11.71	11.71	11.71	11.71
11.72	11.72	11.72	11.72	11.72	11.72	11.72	11.72	11.72
11.73	11.73	11.73	11.73	11.73	11.73	11.73	11.73	11.73
11.74	11.74	11.74	11.74	11.74	11.74	11.74	11.74	11.74
11.75	11.75	11.75	11.75	11.75	11.75	11.75	11.75	11.75
11.76	11.76	11.76	11.76	11.76	11.76	11.76	11.76	11.76
11.77	11.77	11.77	11.77	11.77	11.77	11.77	11.77	11.77
11.78	11.78	11.78	11.78	11.78	11.78	11.78	11.78	11.78
11.79	11.79	11.79	11.79	11.79	11.79	11.79	11.79	11.79
11.8	11.8	11.8	11.8	11.8	11.8	11.8	11.8	11.8
11.81	11.81	11.81	11.81	11.81	11.81	11.81	11.81	11.81
11.82	11.82	11.82	11.82	11.82	11.82	11.82	11.82	11.82
11.83	11.83	11.83	11.83	11.83	11.83	11.83	11.83	11.83
11.84	11.84	11.84	11.84	11.84	11.84	11.84	11.84	11.84
11.85	11.85	11.85	11.85	11.85	11.85	11.85	11.85	11.85
11.86	11.86	11.86	11.86	11.86	11.86	11.86	11.86	11.86
11.87	11.87	11.87	11.87	11.87	11.87	11.87	11.87	11.87
11.88	11.88	11.88	11.88	11.88	11.88	11.88	11.88	11.88
11.89	11.89	11.89	11.89	11.89	11.89	11.89	11.89	11.89
11.9	11.9	11.9	11.9	11.9	11.9	11.9	11.9	11.9
11.91	11.91	11.91	11.91	11.91	11.91	11.91	11.91	11.91
11.92	11.92	11.92	11.92	11.92	11.92	11.92	11.92	11.92
11.93	11.93	11.93	11.93	11.93	11.93	11.93	11.93	11.93
11.94	11.94	11.94	11.94	11.94	11.94	11.94	11.94	11.94
11.95	11.95	11.95	11.95	11.95	11.95	11.95	11.95	11.95
11.96	11.96	11.96	11.96	11.96	11.96	11.96	11.96	11.96
11.97	11.97	11.97	11.97	11.97	11.97	11.97	11.97	11.97
11.98	11.98	11.98	11.98	11.98	11.98	11.98	11.98	11.98
11.99	11.99	11.99	11.99	11.99	11.99	11.99	11.99	11.99

12	12	12	12	12	12	12	12	12

-12	-12	-12	-12	-12	-12	-12	-12	-12
-11.99	-11.99	-11.99	-11.99	-11.99	-11.99	-11.99	-11.99	-11.99
-11.98	-11.98	-11.98	-11.98	-11.98	-11.98	-11.98	-11.98	-11.98
-11.97	-11.97	-11.97	-11.97	-11.97	-11.97	-11.97	-11.97	-11.97
-11.96	-11.96	-11.96	-11.96	-11.96	-11.96	-11.96	-11.96	-11.96
-11.95	-11.95	-11.95	-11.95	-11.95	-11.95	-11.95	-11.95	-11.95
-11.94	-11.94	-11.94	-11.94	-11.94	-11.94	-11.94	-11.94	-11.94
-11.93	-11.93	-11.93	-11.93	-11.93	-11.93	-11.93	-11.93	-11.93
-11.92	-11.92	-11.92	-11.92	-11.92	-11.92	-11.92	-11.92	-11.92
-11.91	-11.91	-11.91	-11.91	-11.91	-11.91	-11.91	-11.91	-11.91
-11.9	-11.9	-11.9	-11.9	-11.9	-11.9	-11.9	-11.9	-11.9
-11.89	-11.89	-11.89	-11.89	-11.89	-11.89	-11.89	-11.89	-11.89
-11.88	-11.88	-11.88	-11.88	-11.88	-11.88	-11.88	-11.88	-11.88
-11.87	-11.87	-11.87	-11.87	-11.87	-11.87	-11.87	-11.87	-11.87
-11.86	-11.86	-11.86	-11.86	-11.86	-11.86	-11.86	-11.86	-11.86
-11.85	-11.85	-11.85	-11.85	-11.85	-11.85	-11.85	-11.85	-11.85
-11.84	-11.84	-11.84	-11.84	-11.84	-11.84	-11.84	-11.84	-11.84
-11.83	-11.83	-11.83	-11.83	-11.83	-11.83	-11.83	-11.83	-11.83
-11.82	-11.82	-11.82	-11.82	-11.82	-11.82	-11.82	-11.82	-11.82
-11.81	-11.81	-11.81	-11.81	-11.81	-11.81	-11.81	-11.81	-11.81
-11.8	-11.8	-11.8	-11.8	-11.8	-11.8	-11.8	-11.8	-11.8
-11.79	-11.79	-11.79	-11.79	-11.79	-11.79	-11.79	-11.79	-11.79
-11.78	-11.78	-11.78	-11.78	-11.78	-11.78	-11.78	-11.78	-11.78
-11.77	-11.77	-11.77	-11.77	-11.77	-11.77	-11.77	-11.77	-11.77
-11.76	-11.76	-11.76	-11.76	-11.76	-11.76	-11.76	-11.76	-11.76
-11.75	-11.75	-11.75	-11.75	-11.75	-11.75	-11.75	-11.75	-11.75
-11.74	-11.74	-11.74	-11.74	-11.74	-11.74	-11.74	-11.74	-11.74
-11.73	-11.73	-11.73	-11.73	-11.73	-11.73	-11.73	-11.73	-11.73
-11.72	-11.72	-11.72	-11.72	-11.72	-11.72	-11.72	-11.72	-11.72
-11.71	-11.71	-11.71	-11.71	-11.71	-11.71	-11.71	-11.71	-11.71
-11.7	-11.7	-11.7	-11.7	-11.7	-11.7	-11.7	-11.7	-11.7
-11.69	-11.69	-11.69	-11.69	-11.69	-11.69	-11.69	-11.69	-11.69
-11.68	-11.68	-11.68	-11.68	-11.68	-11.68	-11.68	-11.68	-11.68
-11.67	-11.67	-11.67	-11.67	-11.67	-11.67	-11.67	-11.67	-11.67
-11.66	-11.66	-11.66	-11.66	-11.66	-11.66	-11.66	-11.66	-11.66
-11.65	-11.65	-11.65	-11.65	-11.65	-11.65	-11.65	-11.65	-11.65
-11.64	-11.64	-11.64	-11.64	-11.64	-11.64	-11.64	-11.64	-11.64
-11.63	-11.63	-11.63	-11.63	-11.63	-11.63	-11.63	-11.63	-11.63
-11.62	-11.62	-11.62	-11.62	-11.62	-11.62	-11.62	-11.62	-11.62
-11.61	-11.61	-11.61	-11.61	-11.61	-11.61	-11.61	-11.61	-11.61
-11.6	-11.6	-11.6	-11.6	-11.6	-11.6	-11.6	-11.6	-11.6
-11.59	-11.59	-11.59	-11.59	-11.59	-11.59	-11.59	-11.59	-11.59
-11.58	-11.58	-11.58	-11.58	-11.58	-11.58	-11.58	-11.58	-11.58
-11.57	-11.57	-11.57	-11.57	-11.57	-11.57	-11.57	-11.57	-11.57
-11.56	-11.56	-11.56	-11.56	-11.56	-11.56	-11.56	-11.56	-11.56
-11.55	-11.55	-11.55	-11.55	-11.55	-11.55	-11.55	-11.55	-11.55
-11.54	-11.54	-11.54	-11.54	-11.54	-11.54	-11.54	-11.54	-11.54
-11.53	-11.53	-11.53	-11.53	-11.53	-11.53	-11.53	-11.53	-11.53
-11.52	-11.52	-11.52	-11.52	-11.52	-11.52	-11.52	-11.52	-11.52
-11.51	-11.51	-11.51	-11.51	-11.51	-11.51	-11.51	-11.51	-11.51

-11.5	-11.5	-11.5	-11.5	-11.5	-11.5	-11.5	-11.5	-11.5
-11.49	-11.49	-11.49	-11.49	-11.49	-11.49	-11.49	-11.49	-11.49
-11.48	-11.48	-11.48	-11.48	-11.48	-11.48	-11.48	-11.48	-11.48
-11.47	-11.47	-11.47	-11.47	-11.47	-11.47	-11.47	-11.47	-11.47
-11.46	-11.46	-11.46	-11.46	-11.46	-11.46	-11.46	-11.46	-11.46
-11.45	-11.45	-11.45	-11.45	-11.45	-11.45	-11.45	-11.45	-11.45
-11.44	-11.44	-11.44	-11.44	-11.44	-11.44	-11.44	-11.44	-11.44
-11.43	-11.43	-11.43	-11.43	-11.43	-11.43	-11.43	-11.43	-11.43
-11.42	-11.42	-11.42	-11.42	-11.42	-11.42	-11.42	-11.42	-11.42
-11.41	-11.41	-11.41	-11.41	-11.41	-11.41	-11.41	-11.41	-11.41
-11.4	-11.4	-11.4	-11.4	-11.4	-11.4	-11.4	-11.4	-11.4
-11.39	-11.39	-11.39	-11.39	-11.39	-11.39	-11.39	-11.39	-11.39
-11.38	-11.38	-11.38	-11.38	-11.38	-11.38	-11.38	-11.38	-11.38
-11.37	-11.37	-11.37	-11.37	-11.37	-11.37	-11.37	-11.37	-11.37
-11.36	-11.36	-11.36	-11.36	-11.36	-11.36	-11.36	-11.36	-11.36
-11.35	-11.35	-11.35	-11.35	-11.35	-11.35	-11.35	-11.35	-11.35
-11.34	-11.34	-11.34	-11.34	-11.34	-11.34	-11.34	-11.34	-11.34
-11.33	-11.33	-11.33	-11.33	-11.33	-11.33	-11.33	-11.33	-11.33
-11.32	-11.32	-11.32	-11.32	-11.32	-11.32	-11.32	-11.32	-11.32
-11.31	-11.31	-11.31	-11.31	-11.31	-11.31	-11.31	-11.31	-11.31
-11.3	-11.3	-11.3	-11.3	-11.3	-11.3	-11.3	-11.3	-11.3
-11.29	-11.29	-11.29	-11.29	-11.29	-11.29	-11.29	-11.29	-11.29
-11.28	-11.28	-11.28	-11.28	-11.28	-11.28	-11.28	-11.28	-11.28
-11.27	-11.27	-11.27	-11.27	-11.27	-11.27	-11.27	-11.27	-11.27
-11.26	-11.26	-11.26	-11.26	-11.26	-11.26	-11.26	-11.26	-11.26
-11.25	-11.25	-11.25	-11.25	-11.25	-11.25	-11.25	-11.25	-11.25
-11.24	-11.24	-11.24	-11.24	-11.24	-11.24	-11.24	-11.24	-11.24
-11.23	-11.23	-11.23	-11.23	-11.23	-11.23	-11.23	-11.23	-11.23
-11.22	-11.22	-11.22	-11.22	-11.22	-11.22	-11.22	-11.22	-11.22
-11.21	-11.21	-11.21	-11.21	-11.21	-11.21	-11.21	-11.21	-11.21
-11.2	-11.2	-11.2	-11.2	-11.2	-11.2	-11.2	-11.2	-11.2
-11.19	-11.19	-11.19	-11.19	-11.19	-11.19	-11.19	-11.19	-11.19
-11.18	-11.18	-11.18	-11.18	-11.18	-11.18	-11.18	-11.18	-11.18
-11.17	-11.17	-11.17	-11.17	-11.17	-11.17	-11.17	-11.17	-11.17
-11.16	-11.16	-11.16	-11.16	-11.16	-11.16	-11.16	-11.16	-11.16
-11.15	-11.15	-11.15	-11.15	-11.15	-11.15	-11.15	-11.15	-11.15
-11.14	-11.14	-11.14	-11.14	-11.14	-11.14	-11.14	-11.14	-11.14
-11.13	-11.13	-11.13	-11.13	-11.13	-11.13	-11.13	-11.13	-11.13
-11.12	-11.12	-11.12	-11.12	-11.12	-11.12	-11.12	-11.12	-11.12
-11.11	-11.11	-11.11	-11.11	-11.11	-11.11	-11.11	-11.11	-11.11
-11.1	-11.1	-11.1	-11.1	-11.1	-11.1	-11.1	-11.1	-11.1
-11.09	-11.09	-11.09	-11.09	-11.09	-11.09	-11.09	-11.09	-11.09
-11.08	-11.08	-11.08	-11.08	-11.08	-11.08	-11.08	-11.08	-11.08
-11.07	-11.07	-11.07	-11.07	-11.07	-11.07	-11.07	-11.07	-11.07
-11.06	-11.06	-11.06	-11.06	-11.06	-11.06	-11.06	-11.06	-11.06
-11.05	-11.05	-11.05	-11.05	-11.05	-11.05	-11.05	-11.05	-11.05
-11.04	-11.04	-11.04	-11.04	-11.04	-11.04	-11.04	-11.04	-11.04
-11.03	-11.03	-11.03	-11.03	-11.03	-11.03	-11.03	-11.03	-11.03
-11.02	-11.02	-11.02	-11.02	-11.02	-11.02	-11.02	-11.02	-11.02
-11.01	-11.01	-11.01	-11.01	-11.01	-11.01	-11.01	-11.01	-11.01

-11	-11	-11	-11	-11	-11	-11	-11	-11
-10.99	-10.99	-10.99	-10.99	-10.99	-10.99	-10.99	-10.99	-10.99
-10.98	-10.98	-10.98	-10.98	-10.98	-10.98	-10.98	-10.98	-10.98
-10.97	-10.97	-10.97	-10.97	-10.97	-10.97	-10.97	-10.97	-10.97
-10.96	-10.96	-10.96	-10.96	-10.96	-10.96	-10.96	-10.96	-10.96
-10.95	-10.95	-10.95	-10.95	-10.95	-10.95	-10.95	-10.95	-10.95
-10.94	-10.94	-10.94	-10.94	-10.94	-10.94	-10.94	-10.94	-10.94
-10.93	-10.93	-10.93	-10.93	-10.93	-10.93	-10.93	-10.93	-10.93
-10.92	-10.92	-10.92	-10.92	-10.92	-10.92	-10.92	-10.92	-10.92
-10.91	-10.91	-10.91	-10.91	-10.91	-10.91	-10.91	-10.91	-10.91
-10.9	-10.9	-10.9	-10.9	-10.9	-10.9	-10.9	-10.9	-10.9
-10.89	-10.89	-10.89	-10.89	-10.89	-10.89	-10.89	-10.89	-10.89
-10.88	-10.88	-10.88	-10.88	-10.88	-10.88	-10.88	-10.88	-10.88
-10.87	-10.87	-10.87	-10.87	-10.87	-10.87	-10.87	-10.87	-10.87
-10.86	-10.86	-10.86	-10.86	-10.86	-10.86	-10.86	-10.86	-10.86
-10.85	-10.85	-10.85	-10.85	-10.85	-10.85	-10.85	-10.85	-10.85
-10.84	-10.84	-10.84	-10.84	-10.84	-10.84	-10.84	-10.84	-10.84
-10.83	-10.83	-10.83	-10.83	-10.83	-10.83	-10.83	-10.83	-10.83
-10.82	-10.82	-10.82	-10.82	-10.82	-10.82	-10.82	-10.82	-10.82
-10.81	-10.81	-10.81	-10.81	-10.81	-10.81	-10.81	-10.81	-10.81
-10.8	-10.8	-10.8	-10.8	-10.8	-10.8	-10.8	-10.8	-10.8
-10.79	-10.79	-10.79	-10.79	-10.79	-10.79	-10.79	-10.79	-10.79
-10.78	-10.78	-10.78	-10.78	-10.78	-10.78	-10.78	-10.78	-10.78
-10.77	-10.77	-10.77	-10.77	-10.77	-10.77	-10.77	-10.77	-10.77
-10.76	-10.76	-10.76	-10.76	-10.76	-10.76	-10.76	-10.76	-10.76
-10.75	-10.75	-10.75	-10.75	-10.75	-10.75	-10.75	-10.75	-10.75
-10.74	-10.74	-10.74	-10.74	-10.74	-10.74	-10.74	-10.74	-10.74
-10.73	-10.73	-10.73	-10.73	-10.73	-10.73	-10.73	-10.73	-10.73
-10.72	-10.72	-10.72	-10.72	-10.72	-10.72	-10.72	-10.72	-10.72
-10.71	-10.71	-10.71	-10.71	-10.71	-10.71	-10.71	-10.71	-10.71
-10.7	-10.7	-10.7	-10.7	-10.7	-10.7	-10.7	-10.7	-10.7
-10.69	-10.69	-10.69	-10.69	-10.69	-10.69	-10.69	-10.69	-10.69
-10.68	-10.68	-10.68	-10.68	-10.68	-10.68	-10.68	-10.68	-10.68
-10.67	-10.67	-10.67	-10.67	-10.67	-10.67	-10.67	-10.67	-10.67
-10.66	-10.66	-10.66	-10.66	-10.66	-10.66	-10.66	-10.66	-10.66
-10.65	-10.65	-10.65	-10.65	-10.65	-10.65	-10.65	-10.65	-10.65
-10.64	-10.64	-10.64	-10.64	-10.64	-10.64	-10.64	-10.64	-10.64
-10.63	-10.63	-10.63	-10.63	-10.63	-10.63	-10.63	-10.63	-10.63
-10.62	-10.62	-10.62	-10.62	-10.62	-10.62	-10.62	-10.62	-10.62
-10.61	-10.61	-10.61	-10.61	-10.61	-10.61	-10.61	-10.61	-10.61
-10.6	-10.6	-10.6	-10.6	-10.6	-10.6	-10.6	-10.6	-10.6
-10.59	-10.59	-10.59	-10.59	-10.59	-10.59	-10.59	-10.59	-10.59
-10.58	-10.58	-10.58	-10.58	-10.58	-10.58	-10.58	-10.58	-10.58
-10.57	-10.57	-10.57	-10.57	-10.57	-10.57	-10.57	-10.57	-10.57
-10.56	-10.56	-10.56	-10.56	-10.56	-10.56	-10.56	-10.56	-10.56
-10.55	-10.55	-10.55	-10.55	-10.55	-10.55	-10.55	-10.55	-10.55
-10.54	-10.54	-10.54	-10.54	-10.54	-10.54	-10.54	-10.54	-10.54
-10.53	-10.53	-10.53	-10.53	-10.53	-10.53	-10.53	-10.53	-10.53
-10.52	-10.52	-10.52	-10.52	-10.52	-10.52	-10.52	-10.52	-10.52
-10.51	-10.51	-10.51	-10.51	-10.51	-10.51	-10.51	-10.51	-10.51

-10.5	-10.5	-10.5	-10.5	-10.5	-10.5	-10.5	-10.5	-10.5
-10.49	-10.49	-10.49	-10.49	-10.49	-10.49	-10.49	-10.49	-10.49
-10.48	-10.48	-10.48	-10.48	-10.48	-10.48	-10.48	-10.48	-10.48
-10.47	-10.47	-10.47	-10.47	-10.47	-10.47	-10.47	-10.47	-10.47
-10.46	-10.46	-10.46	-10.46	-10.46	-10.46	-10.46	-10.46	-10.46
-10.45	-10.45	-10.45	-10.45	-10.45	-10.45	-10.45	-10.45	-10.45
-10.44	-10.44	-10.44	-10.44	-10.44	-10.44	-10.44	-10.44	-10.44
-10.43	-10.43	-10.43	-10.43	-10.43	-10.43	-10.43	-10.43	-10.43
-10.42	-10.42	-10.42	-10.42	-10.42	-10.42	-10.42	-10.42	-10.42
-10.41	-10.41	-10.41	-10.41	-10.41	-10.41	-10.41	-10.41	-10.41
-10.4	-10.4	-10.4	-10.4	-10.4	-10.4	-10.4	-10.4	-10.4
-10.39	-10.39	-10.39	-10.39	-10.39	-10.39	-10.39	-10.39	-10.39
-10.38	-10.38	-10.38	-10.38	-10.38	-10.38	-10.38	-10.38	-10.38
-10.37	-10.37	-10.37	-10.37	-10.37	-10.37	-10.37	-10.37	-10.37
-10.36	-10.36	-10.36	-10.36	-10.36	-10.36	-10.36	-10.36	-10.36
-10.35	-10.35	-10.35	-10.35	-10.35	-10.35	-10.35	-10.35	-10.35
-10.34	-10.34	-10.34	-10.34	-10.34	-10.34	-10.34	-10.34	-10.34
-10.33	-10.33	-10.33	-10.33	-10.33	-10.33	-10.33	-10.33	-10.33
-10.32	-10.32	-10.32	-10.32	-10.32	-10.32	-10.32	-10.32	-10.32
-10.31	-10.31	-10.31	-10.31	-10.31	-10.31	-10.31	-10.31	-10.31
-10.3	-10.3	-10.3	-10.3	-10.3	-10.3	-10.3	-10.3	-10.3
-10.29	-10.29	-10.29	-10.29	-10.29	-10.29	-10.29	-10.29	-10.29
-10.28	-10.28	-10.28	-10.28	-10.28	-10.28	-10.28	-10.28	-10.28
-10.27	-10.27	-10.27	-10.27	-10.27	-10.27	-10.27	-10.27	-10.27
-10.26	-10.26	-10.26	-10.26	-10.26	-10.26	-10.26	-10.26	-10.26
-10.25	-10.25	-10.25	-10.25	-10.25	-10.25	-10.25	-10.25	-10.25
-10.24	-10.24	-10.24	-10.24	-10.24	-10.24	-10.24	-10.24	-10.24
-10.23	-10.23	-10.23	-10.23	-10.23	-10.23	-10.23	-10.23	-10.23
-10.22	-10.22	-10.22	-10.22	-10.22	-10.22	-10.22	-10.22	-10.22
-10.21	-10.21	-10.21	-10.21	-10.21	-10.21	-10.21	-10.21	-10.21
-10.2	-10.2	-10.2	-10.2	-10.2	-10.2	-10.2	-10.2	-10.2
-10.19	-10.19	-10.19	-10.19	-10.19	-10.19	-10.19	-10.19	-10.19
-10.18	-10.18	-10.18	-10.18	-10.18	-10.18	-10.18	-10.18	-10.18
-10.17	-10.17	-10.17	-10.17	-10.17	-10.17	-10.17	-10.17	-10.17
-10.16	-10.16	-10.16	-10.16	-10.16	-10.16	-10.16	-10.16	-10.16
-10.15	-10.15	-10.15	-10.15	-10.15	-10.15	-10.15	-10.15	-10.15
-10.14	-10.14	-10.14	-10.14	-10.14	-10.14	-10.14	-10.14	-10.14
-10.13	-10.13	-10.13	-10.13	-10.13	-10.13	-10.13	-10.13	-10.13
-10.12	-10.12	-10.12	-10.12	-10.12	-10.12	-10.12	-10.12	-10.12
-10.11	-10.11	-10.11	-10.11	-10.11	-10.11	-10.11	-10.11	-10.11
-10.1	-10.1	-10.1	-10.1	-10.1	-10.1	-10.1	-10.1	-10.1
-10.09	-10.09	-10.09	-10.09	-10.09	-10.09	-10.09	-10.09	-10.09
-10.08	-10.08	-10.08	-10.08	-10.08	-10.08	-10.08	-10.08	-10.08
-10.07	-10.07	-10.07	-10.07	-10.07	-10.07	-10.07	-10.07	-10.07
-10.06	-10.06	-10.06	-10.06	-10.06	-10.06	-10.06	-10.06	-10.06
-10.05	-10.05	-10.05	-10.05	-10.05	-10.05	-10.05	-10.05	-10.05
-10.04	-10.04	-10.04	-10.04	-10.04	-10.04	-10.04	-10.04	-10.04
-10.03	-10.03	-10.03	-10.03	-10.03	-10.03	-10.03	-10.03	-10.03
-10.02	-10.02	-10.02	-10.02	-10.02	-10.02	-10.02	-10.02	-10.02
-10.01	-10.01	-10.01	-10.01	-10.01	-10.01	-10.01	-10.01	-10.01

-10	-10	-10	-10	-10	-10	-10	-10	-10
-9.99	-9.99	-9.99	-9.99	-9.99	-9.99	-9.99	-9.99	-9.99
-9.98	-9.98	-9.98	-9.98	-9.98	-9.98	-9.98	-9.98	-9.98
-9.97	-9.97	-9.97	-9.97	-9.97	-9.97	-9.97	-9.97	-9.97
-9.96	-9.96	-9.96	-9.96	-9.96	-9.96	-9.96	-9.96	-9.96
-9.95	-9.95	-9.95	-9.95	-9.95	-9.95	-9.95	-9.95	-9.95
-9.94	-9.94	-9.94	-9.94	-9.94	-9.94	-9.94	-9.94	-9.94
-9.93	-9.93	-9.93	-9.93	-9.93	-9.93	-9.93	-9.93	-9.93
-9.92	-9.92	-9.92	-9.92	-9.92	-9.92	-9.92	-9.92	-9.92
-9.91	-9.91	-9.91	-9.91	-9.91	-9.91	-9.91	-9.91	-9.91
-9.9	-9.9	-9.9	-9.9	-9.9	-9.9	-9.9	-9.9	-9.9
-9.89	-9.89	-9.89	-9.89	-9.89	-9.89	-9.89	-9.89	-9.89
-9.88	-9.88	-9.88	-9.88	-9.88	-9.88	-9.88	-9.88	-9.88
-9.87	-9.87	-9.87	-9.87	-9.87	-9.87	-9.87	-9.87	-9.87
-9.86	-9.86	-9.86	-9.86	-9.86	-9.86	-9.86	-9.86	-9.86
-9.85	-9.85	-9.85	-9.85	-9.85	-9.85	-9.85	-9.85	-9.85
-9.84	-9.84	-9.84	-9.84	-9.84	-9.84	-9.84	-9.84	-9.84
-9.83	-9.83	-9.83	-9.83	-9.83	-9.83	-9.83	-9.83	-9.83
-9.82	-9.82	-9.82	-9.82	-9.82	-9.82	-9.82	-9.82	-9.82
-9.81	-9.81	-9.81	-9.81	-9.81	-9.81	-9.81	-9.81	-9.81
-9.8	-9.8	-9.8	-9.8	-9.8	-9.8	-9.8	-9.8	-9.8
-9.79	-9.79	-9.79	-9.79	-9.79	-9.79	-9.79	-9.79	-9.79
-9.78	-9.78	-9.78	-9.78	-9.78	-9.78	-9.78	-9.78	-9.78
-9.77	-9.77	-9.77	-9.77	-9.77	-9.77	-9.77	-9.77	-9.77
-9.76	-9.76	-9.76	-9.76	-9.76	-9.76	-9.76	-9.76	-9.76
-9.75	-9.75	-9.75	-9.75	-9.75	-9.75	-9.75	-9.75	-9.75
-9.74	-9.74	-9.74	-9.74	-9.74	-9.74	-9.74	-9.74	-9.74
-9.73	-9.73	-9.73	-9.73	-9.73	-9.73	-9.73	-9.73	-9.73
-9.72	-9.72	-9.72	-9.72	-9.72	-9.72	-9.72	-9.72	-9.72
-9.71	-9.71	-9.71	-9.71	-9.71	-9.71	-9.71	-9.71	-9.71
-9.7	-9.7	-9.7	-9.7	-9.7	-9.7	-9.7	-9.7	-9.7
-9.69	-9.69	-9.69	-9.69	-9.69	-9.69	-9.69	-9.69	-9.69
-9.68	-9.68	-9.68	-9.68	-9.68	-9.68	-9.68	-9.68	-9.68
-9.67	-9.67	-9.67	-9.67	-9.67	-9.67	-9.67	-9.67	-9.67
-9.66	-9.66	-9.66	-9.66	-9.66	-9.66	-9.66	-9.66	-9.66
-9.65	-9.65	-9.65	-9.65	-9.65	-9.65	-9.65	-9.65	-9.65
-9.64	-9.64	-9.64	-9.64	-9.64	-9.64	-9.64	-9.64	-9.64
-9.63	-9.63	-9.63	-9.63	-9.63	-9.63	-9.63	-9.63	-9.63
-9.62	-9.62	-9.62	-9.62	-9.62	-9.62	-9.62	-9.62	-9.62
-9.61	-9.61	-9.61	-9.61	-9.61	-9.61	-9.61	-9.61	-9.61
-9.6	-9.6	-9.6	-9.6	-9.6	-9.6	-9.6	-9.6	-9.6
-9.59	-9.59	-9.59	-9.59	-9.59	-9.59	-9.59	-9.59	-9.59
-9.58	-9.58	-9.58	-9.58	-9.58	-9.58	-9.58	-9.58	-9.58
-9.57	-9.57	-9.57	-9.57	-9.57	-9.57	-9.57	-9.57	-9.57
-9.56	-9.56	-9.56	-9.56	-9.56	-9.56	-9.56	-9.56	-9.56
-9.55	-9.55	-9.55	-9.55	-9.55	-9.55	-9.55	-9.55	-9.55
-9.54	-9.54	-9.54	-9.54	-9.54	-9.54	-9.54	-9.54	-9.54
-9.53	-9.53	-9.53	-9.53	-9.53	-9.53	-9.53	-9.53	-9.53
-9.52	-9.52	-9.52	-9.52	-9.52	-9.52	-9.52	-9.52	-9.52
-9.51	-9.51	-9.51	-9.51	-9.51	-9.51	-9.51	-9.51	-9.51

-9.5	-9.5	-9.5	-9.5	-9.5	-9.5	-9.5	-9.5	-9.5
-9.49	-9.49	-9.49	-9.49	-9.49	-9.49	-9.49	-9.49	-9.49
-9.48	-9.48	-9.48	-9.48	-9.48	-9.48	-9.48	-9.48	-9.48
-9.47	-9.47	-9.47	-9.47	-9.47	-9.47	-9.47	-9.47	-9.47
-9.46	-9.46	-9.46	-9.46	-9.46	-9.46	-9.46	-9.46	-9.46
-9.45	-9.45	-9.45	-9.45	-9.45	-9.45	-9.45	-9.45	-9.45
-9.44	-9.44	-9.44	-9.44	-9.44	-9.44	-9.44	-9.44	-9.44
-9.43	-9.43	-9.43	-9.43	-9.43	-9.43	-9.43	-9.43	-9.43
-9.42	-9.42	-9.42	-9.42	-9.42	-9.42	-9.42	-9.42	-9.42
-9.41	-9.41	-9.41	-9.41	-9.41	-9.41	-9.41	-9.41	-9.41
-9.4	-9.4	-9.4	-9.4	-9.4	-9.4	-9.4	-9.4	-9.4
-9.39	-9.39	-9.39	-9.39	-9.39	-9.39	-9.39	-9.39	-9.39
-9.38	-9.38	-9.38	-9.38	-9.38	-9.38	-9.38	-9.38	-9.38
-9.37	-9.37	-9.37	-9.37	-9.37	-9.37	-9.37	-9.37	-9.37
-9.36	-9.36	-9.36	-9.36	-9.36	-9.36	-9.36	-9.36	-9.36
-9.35	-9.35	-9.35	-9.35	-9.35	-9.35	-9.35	-9.35	-9.35
-9.34	-9.34	-9.34	-9.34	-9.34	-9.34	-9.34	-9.34	-9.34
-9.33	-9.33	-9.33	-9.33	-9.33	-9.33	-9.33	-9.33	-9.33
-9.32	-9.32	-9.32	-9.32	-9.32	-9.32	-9.32	-9.32	-9.32
-9.31	-9.31	-9.31	-9.31	-9.31	-9.31	-9.31	-9.31	-9.31
-9.3	-9.3	-9.3	-9.3	-9.3	-9.3	-9.3	-9.3	-9.3
-9.29	-9.29	-9.29	-9.29	-9.29	-9.29	-9.29	-9.29	-9.29
-9.28	-9.28	-9.28	-9.28	-9.28	-9.28	-9.28	-9.28	-9.28
-9.27	-9.27	-9.27	-9.27	-9.27	-9.27	-9.27	-9.27	-9.27
-9.26	-9.26	-9.26	-9.26	-9.26	-9.26	-9.26	-9.26	-9.26
-9.25	-9.25	-9.25	-9.25	-9.25	-9.25	-9.25	-9.25	-9.25
-9.24	-9.24	-9.24	-9.24	-9.24	-9.24	-9.24	-9.24	-9.24
-9.23	-9.23	-9.23	-9.23	-9.23	-9.23	-9.23	-9.23	-9.23
-9.22	-9.22	-9.22	-9.22	-9.22	-9.22	-9.22	-9.22	-9.22
-9.21	-9.21	-9.21	-9.21	-9.21	-9.21	-9.21	-9.21	-9.21
-9.2	-9.2	-9.2	-9.2	-9.2	-9.2	-9.2	-9.2	-9.2
-9.19	-9.19	-9.19	-9.19	-9.19	-9.19	-9.19	-9.19	-9.19
-9.18	-9.18	-9.18	-9.18	-9.18	-9.18	-9.18	-9.18	-9.18
-9.17	-9.17	-9.17	-9.17	-9.17	-9.17	-9.17	-9.17	-9.17
-9.16	-9.16	-9.16	-9.16	-9.16	-9.16	-9.16	-9.16	-9.16
-9.15	-9.15	-9.15	-9.15	-9.15	-9.15	-9.15	-9.15	-9.15
-9.14	-9.14	-9.14	-9.14	-9.14	-9.14	-9.14	-9.14	-9.14
-9.13	-9.13	-9.13	-9.13	-9.13	-9.13	-9.13	-9.13	-9.13
-9.12	-9.12	-9.12	-9.12	-9.12	-9.12	-9.12	-9.12	-9.12
-9.11	-9.11	-9.11	-9.11	-9.11	-9.11	-9.11	-9.11	-9.11
-9.1	-9.1	-9.1	-9.1	-9.1	-9.1	-9.1	-9.1	-9.1
-9.09	-9.09	-9.09	-9.09	-9.09	-9.09	-9.09	-9.09	-9.09
-9.08	-9.08	-9.08	-9.08	-9.08	-9.08	-9.08	-9.08	-9.08
-9.07	-9.07	-9.07	-9.07	-9.07	-9.07	-9.07	-9.07	-9.07
-9.06	-9.06	-9.06	-9.06	-9.06	-9.06	-9.06	-9.06	-9.06
-9.05	-9.05	-9.05	-9.05	-9.05	-9.05	-9.05	-9.05	-9.05
-9.04	-9.04	-9.04	-9.04	-9.04	-9.04	-9.04	-9.04	-9.04
-9.03	-9.03	-9.03	-9.03	-9.03	-9.03	-9.03	-9.03	-9.03
-9.02	-9.02	-9.02	-9.02	-9.02	-9.02	-9.02	-9.02	-9.02
-9.01	-9.01	-9.01	-9.01	-9.01	-9.01	-9.01	-9.01	-9.01

-9	-9	-9	-9	-9	-9	-9	-9	-9
-8.99	-8.99	-8.99	-8.99	-8.99	-8.99	-8.99	-8.99	-8.99
-8.98	-8.98	-8.98	-8.98	-8.98	-8.98	-8.98	-8.98	-8.98
-8.97	-8.97	-8.97	-8.97	-8.97	-8.97	-8.97	-8.97	-8.97
-8.96	-8.96	-8.96	-8.96	-8.96	-8.96	-8.96	-8.96	-8.96
-8.95	-8.95	-8.95	-8.95	-8.95	-8.95	-8.95	-8.95	-8.95
-8.94	-8.94	-8.94	-8.94	-8.94	-8.94	-8.94	-8.94	-8.94
-8.93	-8.93	-8.93	-8.93	-8.93	-8.93	-8.93	-8.93	-8.93
-8.92	-8.92	-8.92	-8.92	-8.92	-8.92	-8.92	-8.92	-8.92
-8.91	-8.91	-8.91	-8.91	-8.91	-8.91	-8.91	-8.91	-8.91
-8.9	-8.9	-8.9	-8.9	-8.9	-8.9	-8.9	-8.9	-8.9
-8.89	-8.89	-8.89	-8.89	-8.89	-8.89	-8.89	-8.89	-8.89
-8.88	-8.88	-8.88	-8.88	-8.88	-8.88	-8.88	-8.88	-8.88
-8.87	-8.87	-8.87	-8.87	-8.87	-8.87	-8.87	-8.87	-8.87
-8.86	-8.86	-8.86	-8.86	-8.86	-8.86	-8.86	-8.86	-8.86
-8.85	-8.85	-8.85	-8.85	-8.85	-8.85	-8.85	-8.85	-8.85
-8.84	-8.84	-8.84	-8.84	-8.84	-8.84	-8.84	-8.84	-8.84
-8.83	-8.83	-8.83	-8.83	-8.83	-8.83	-8.83	-8.83	-8.83
-8.82	-8.82	-8.82	-8.82	-8.82	-8.82	-8.82	-8.82	-8.82
-8.81	-8.81	-8.81	-8.81	-8.81	-8.81	-8.81	-8.81	-8.81
-8.8	-8.8	-8.8	-8.8	-8.8	-8.8	-8.8	-8.8	-8.8
-8.79	-8.79	-8.79	-8.79	-8.79	-8.79	-8.79	-8.79	-8.79
-8.78	-8.78	-8.78	-8.78	-8.78	-8.78	-8.78	-8.78	-8.78
-8.77	-8.77	-8.77	-8.77	-8.77	-8.77	-8.77	-8.77	-8.77
-8.76	-8.76	-8.76	-8.76	-8.76	-8.76	-8.76	-8.76	-8.76
-8.75	-8.75	-8.75	-8.75	-8.75	-8.75	-8.75	-8.75	-8.75
-8.74	-8.74	-8.74	-8.74	-8.74	-8.74	-8.74	-8.74	-8.74
-8.73	-8.73	-8.73	-8.73	-8.73	-8.73	-8.73	-8.73	-8.73
-8.72	-8.72	-8.72	-8.72	-8.72	-8.72	-8.72	-8.72	-8.72
-8.71	-8.71	-8.71	-8.71	-8.71	-8.71	-8.71	-8.71	-8.71
-8.7	-8.7	-8.7	-8.7	-8.7	-8.7	-8.7	-8.7	-8.7
-8.69	-8.69	-8.69	-8.69	-8.69	-8.69	-8.69	-8.69	-8.69
-8.68	-8.68	-8.68	-8.68	-8.68	-8.68	-8.68	-8.68	-8.68
-8.67	-8.67	-8.67	-8.67	-8.67	-8.67	-8.67	-8.67	-8.67
-8.66	-8.66	-8.66	-8.66	-8.66	-8.66	-8.66	-8.66	-8.66
-8.65	-8.65	-8.65	-8.65	-8.65	-8.65	-8.65	-8.65	-8.65
-8.64	-8.64	-8.64	-8.64	-8.64	-8.64	-8.64	-8.64	-8.64
-8.63	-8.63	-8.63	-8.63	-8.63	-8.63	-8.63	-8.63	-8.63
-8.62	-8.62	-8.62	-8.62	-8.62	-8.62	-8.62	-8.62	-8.62
-8.61	-8.61	-8.61	-8.61	-8.61	-8.61	-8.61	-8.61	-8.61
-8.6	-8.6	-8.6	-8.6	-8.6	-8.6	-8.6	-8.6	-8.6
-8.59	-8.59	-8.59	-8.59	-8.59	-8.59	-8.59	-8.59	-8.59
-8.58	-8.58	-8.58	-8.58	-8.58	-8.58	-8.58	-8.58	-8.58
-8.57	-8.57	-8.57	-8.57	-8.57	-8.57	-8.57	-8.57	-8.57
-8.56	-8.56	-8.56	-8.56	-8.56	-8.56	-8.56	-8.56	-8.56
-8.55	-8.55	-8.55	-8.55	-8.55	-8.55	-8.55	-8.55	-8.55
-8.54	-8.54	-8.54	-8.54	-8.54	-8.54	-8.54	-8.54	-8.54
-8.53	-8.53	-8.53	-8.53	-8.53	-8.53	-8.53	-8.53	-8.53
-8.52	-8.52	-8.52	-8.52	-8.52	-8.52	-8.52	-8.52	-8.52
-8.51	-8.51	-8.51	-8.51	-8.51	-8.51	-8.51	-8.51	-8.51

-8.5	-8.5	-8.5	-8.5	-8.5	-8.5	-8.5	-8.5	-8.5
-8.49	-8.49	-8.49	-8.49	-8.49	-8.49	-8.49	-8.49	-8.49
-8.48	-8.48	-8.48	-8.48	-8.48	-8.48	-8.48	-8.48	-8.48
-8.47	-8.47	-8.47	-8.47	-8.47	-8.47	-8.47	-8.47	-8.47
-8.46	-8.46	-8.46	-8.46	-8.46	-8.46	-8.46	-8.46	-8.46
-8.45	-8.45	-8.45	-8.45	-8.45	-8.45	-8.45	-8.45	-8.45
-8.44	-8.44	-8.44	-8.44	-8.44	-8.44	-8.44	-8.44	-8.44
-8.43	-8.43	-8.43	-8.43	-8.43	-8.43	-8.43	-8.43	-8.43
-8.42	-8.42	-8.42	-8.42	-8.42	-8.42	-8.42	-8.42	-8.42
-8.41	-8.41	-8.41	-8.41	-8.41	-8.41	-8.41	-8.41	-8.41
-8.4	-8.4	-8.4	-8.4	-8.4	-8.4	-8.4	-8.4	-8.4
-8.39	-8.39	-8.39	-8.39	-8.39	-8.39	-8.39	-8.39	-8.39
-8.38	-8.38	-8.38	-8.38	-8.38	-8.38	-8.38	-8.38	-8.38
-8.37	-8.37	-8.37	-8.37	-8.37	-8.37	-8.37	-8.37	-8.37
-8.36	-8.36	-8.36	-8.36	-8.36	-8.36	-8.36	-8.36	-8.36
-8.35	-8.35	-8.35	-8.35	-8.35	-8.35	-8.35	-8.35	-8.35
-8.34	-8.34	-8.34	-8.34	-8.34	-8.34	-8.34	-8.34	-8.34
-8.33	-8.33	-8.33	-8.33	-8.33	-8.33	-8.33	-8.33	-8.33
-8.32	-8.32	-8.32	-8.32	-8.32	-8.32	-8.32	-8.32	-8.32
-8.31	-8.31	-8.31	-8.31	-8.31	-8.31	-8.31	-8.31	-8.31
-8.3	-8.3	-8.3	-8.3	-8.3	-8.3	-8.3	-8.3	-8.3
-8.29	-8.29	-8.29	-8.29	-8.29	-8.29	-8.29	-8.29	-8.29
-8.28	-8.28	-8.28	-8.28	-8.28	-8.28	-8.28	-8.28	-8.28
-8.27	-8.27	-8.27	-8.27	-8.27	-8.27	-8.27	-8.27	-8.27
-8.26	-8.26	-8.26	-8.26	-8.26	-8.26	-8.26	-8.26	-8.26
-8.25	-8.25	-8.25	-8.25	-8.25	-8.25	-8.25	-8.25	-8.25
-8.24	-8.24	-8.24	-8.24	-8.24	-8.24	-8.24	-8.24	-8.24
-8.23	-8.23	-8.23	-8.23	-8.23	-8.23	-8.23	-8.23	-8.23
-8.22	-8.22	-8.22	-8.22	-8.22	-8.22	-8.22	-8.22	-8.22
-8.21	-8.21	-8.21	-8.21	-8.21	-8.21	-8.21	-8.21	-8.21
-8.2	-8.2	-8.2	-8.2	-8.2	-8.2	-8.2	-8.2	-8.2
-8.19	-8.19	-8.19	-8.19	-8.19	-8.19	-8.19	-8.19	-8.19
-8.18	-8.18	-8.18	-8.18	-8.18	-8.18	-8.18	-8.18	-8.18
-8.17	-8.17	-8.17	-8.17	-8.17	-8.17	-8.17	-8.17	-8.17
-8.16	-8.16	-8.16	-8.16	-8.16	-8.16	-8.16	-8.16	-8.16
-8.15	-8.15	-8.15	-8.15	-8.15	-8.15	-8.15	-8.15	-8.15
-8.14	-8.14	-8.14	-8.14	-8.14	-8.14	-8.14	-8.14	-8.14
-8.13	-8.13	-8.13	-8.13	-8.13	-8.13	-8.13	-8.13	-8.13
-8.12	-8.12	-8.12	-8.12	-8.12	-8.12	-8.12	-8.12	-8.12
-8.11	-8.11	-8.11	-8.11	-8.11	-8.11	-8.11	-8.11	-8.11
-8.1	-8.1	-8.1	-8.1	-8.1	-8.1	-8.1	-8.1	-8.1
-8.09	-8.09	-8.09	-8.09	-8.09	-8.09	-8.09	-8.09	-8.09
-8.08	-8.08	-8.08	-8.08	-8.08	-8.08	-8.08	-8.08	-8.08
-8.07	-8.07	-8.07	-8.07	-8.07	-8.07	-8.07	-8.07	-8.07
-8.06	-8.06	-8.06	-8.06	-8.06	-8.06	-8.06	-8.06	-8.06
-8.05	-8.05	-8.05	-8.05	-8.05	-8.05	-8.05	-8.05	-8.05
-8.04	-8.04	-8.04	-8.04	-8.04	-8.04	-8.04	-8.04	-8.04
-8.03	-8.03	-8.03	-8.03	-8.03	-8.03	-8.03	-8.03	-8.03
-8.02	-8.02	-8.02	-8.02	-8.02	-8.02	-8.02	-8.02	-8.02
-8.01	-8.01	-8.01	-8.01	-8.01	-8.01	-8.01	-8.01	-8.01

-8	-8	-8	-8	-8	-8	-8	-8	-8
-7.99	-7.99	-7.99	-7.99	-7.99	-7.99	-7.99	-7.99	-7.99
-7.98	-7.98	-7.98	-7.98	-7.98	-7.98	-7.98	-7.98	-7.98
-7.97	-7.97	-7.97	-7.97	-7.97	-7.97	-7.97	-7.97	-7.97
-7.96	-7.96	-7.96	-7.96	-7.96	-7.96	-7.96	-7.96	-7.96
-7.95	-7.95	-7.95	-7.95	-7.95	-7.95	-7.95	-7.95	-7.95
-7.94	-7.94	-7.94	-7.94	-7.94	-7.94	-7.94	-7.94	-7.94
-7.93	-7.93	-7.93	-7.93	-7.93	-7.93	-7.93	-7.93	-7.93
-7.92	-7.92	-7.92	-7.92	-7.92	-7.92	-7.92	-7.92	-7.92
-7.91	-7.91	-7.91	-7.91	-7.91	-7.91	-7.91	-7.91	-7.91
-7.9	-7.9	-7.9	-7.9	-7.9	-7.9	-7.9	-7.9	-7.9
-7.89	-7.89	-7.89	-7.89	-7.89	-7.89	-7.89	-7.89	-7.89
-7.88	-7.88	-7.88	-7.88	-7.88	-7.88	-7.88	-7.88	-7.88
-7.87	-7.87	-7.87	-7.87	-7.87	-7.87	-7.87	-7.87	-7.87
-7.86	-7.86	-7.86	-7.86	-7.86	-7.86	-7.86	-7.86	-7.86
-7.85	-7.85	-7.85	-7.85	-7.85	-7.85	-7.85	-7.85	-7.85
-7.84	-7.84	-7.84	-7.84	-7.84	-7.84	-7.84	-7.84	-7.84
-7.83	-7.83	-7.83	-7.83	-7.83	-7.83	-7.83	-7.83	-7.83
-7.82	-7.82	-7.82	-7.82	-7.82	-7.82	-7.82	-7.82	-7.82
-7.81	-7.81	-7.81	-7.81	-7.81	-7.81	-7.81	-7.81	-7.81
-7.8	-7.8	-7.8	-7.8	-7.8	-7.8	-7.8	-7.8	-7.8
-7.79	-7.79	-7.79	-7.79	-7.79	-7.79	-7.79	-7.79	-7.79
-7.78	-7.78	-7.78	-7.78	-7.78	-7.78	-7.78	-7.78	-7.78
-7.77	-7.77	-7.77	-7.77	-7.77	-7.77	-7.77	-7.77	-7.77
-7.76	-7.76	-7.76	-7.76	-7.76	-7.76	-7.76	-7.76	-7.76
-7.75	-7.75	-7.75	-7.75	-7.75	-7.75	-7.75	-7.75	-7.75
-7.74	-7.74	-7.74	-7.74	-7.74	-7.74	-7.74	-7.74	-7.74
-7.73	-7.73	-7.73	-7.73	-7.73	-7.73	-7.73	-7.73	-7.73
-7.72	-7.72	-7.72	-7.72	-7.72	-7.72	-7.72	-7.72	-7.72
-7.71	-7.71	-7.71	-7.71	-7.71	-7.71	-7.71	-7.71	-7.71
-7.7	-7.7	-7.7	-7.7	-7.7	-7.7	-7.7	-7.7	-7.7
-7.69	-7.69	-7.69	-7.69	-7.69	-7.69	-7.69	-7.69	-7.69
-7.68	-7.68	-7.68	-7.68	-7.68	-7.68	-7.68	-7.68	-7.68
-7.67	-7.67	-7.67	-7.67	-7.67	-7.67	-7.67	-7.67	-7.67
-7.66	-7.66	-7.66	-7.66	-7.66	-7.66	-7.66	-7.66	-7.66
-7.65	-7.65	-7.65	-7.65	-7.65	-7.65	-7.65	-7.65	-7.65
-7.64	-7.64	-7.64	-7.64	-7.64	-7.64	-7.64	-7.64	-7.64
-7.63	-7.63	-7.63	-7.63	-7.63	-7.63	-7.63	-7.63	-7.63
-7.62	-7.62	-7.62	-7.62	-7.62	-7.62	-7.62	-7.62	-7.62
-7.61	-7.61	-7.61	-7.61	-7.61	-7.61	-7.61	-7.61	-7.61
-7.6	-7.6	-7.6	-7.6	-7.6	-7.6	-7.6	-7.6	-7.6
-7.59	-7.59	-7.59	-7.59	-7.59	-7.59	-7.59	-7.59	-7.59
-7.58	-7.58	-7.58	-7.58	-7.58	-7.58	-7.58	-7.58	-7.58
-7.57	-7.57	-7.57	-7.57	-7.57	-7.57	-7.57	-7.57	-7.57
-7.56	-7.56	-7.56	-7.56	-7.56	-7.56	-7.56	-7.56	-7.56
-7.55	-7.55	-7.55	-7.55	-7.55	-7.55	-7.55	-7.55	-7.55
-7.54	-7.54	-7.54	-7.54	-7.54	-7.54	-7.54	-7.54	-7.54
-7.53	-7.53	-7.53	-7.53	-7.53	-7.53	-7.53	-7.53	-7.53
-7.52	-7.52	-7.52	-7.52	-7.52	-7.52	-7.52	-7.52	-7.52
-7.51	-7.51	-7.51	-7.51	-7.51	-7.51	-7.51	-7.51	-7.51

-7.5	-7.5	-7.5	-7.5	-7.5	-7.5	-7.5	-7.5	-7.5
-7.49	-7.49	-7.49	-7.49	-7.49	-7.49	-7.49	-7.49	-7.49
-7.48	-7.48	-7.48	-7.48	-7.48	-7.48	-7.48	-7.48	-7.48
-7.47	-7.47	-7.47	-7.47	-7.47	-7.47	-7.47	-7.47	-7.47
-7.46	-7.46	-7.46	-7.46	-7.46	-7.46	-7.46	-7.46	-7.46
-7.45	-7.45	-7.45	-7.45	-7.45	-7.45	-7.45	-7.45	-7.45
-7.44	-7.44	-7.44	-7.44	-7.44	-7.44	-7.44	-7.44	-7.44
-7.43	-7.43	-7.43	-7.43	-7.43	-7.43	-7.43	-7.43	-7.43
-7.42	-7.42	-7.42	-7.42	-7.42	-7.42	-7.42	-7.42	-7.42
-7.41	-7.41	-7.41	-7.41	-7.41	-7.41	-7.41	-7.41	-7.41
-7.4	-7.4	-7.4	-7.4	-7.4	-7.4	-7.4	-7.4	-7.4
-7.39	-7.39	-7.39	-7.39	-7.39	-7.39	-7.39	-7.39	-7.39
-7.38	-7.38	-7.38	-7.38	-7.38	-7.38	-7.38	-7.38	-7.38
-7.37	-7.37	-7.37	-7.37	-7.37	-7.37	-7.37	-7.37	-7.37
-7.36	-7.36	-7.36	-7.36	-7.36	-7.36	-7.36	-7.36	-7.36
-7.35	-7.35	-7.35	-7.35	-7.35	-7.35	-7.35	-7.35	-7.35
-7.34	-7.34	-7.34	-7.34	-7.34	-7.34	-7.34	-7.34	-7.34
-7.33	-7.33	-7.33	-7.33	-7.33	-7.33	-7.33	-7.33	-7.33
-7.32	-7.32	-7.32	-7.32	-7.32	-7.32	-7.32	-7.32	-7.32
-7.31	-7.31	-7.31	-7.31	-7.31	-7.31	-7.31	-7.31	-7.31
-7.3	-7.3	-7.3	-7.3	-7.3	-7.3	-7.3	-7.3	-7.3
-7.29	-7.29	-7.29	-7.29	-7.29	-7.29	-7.29	-7.29	-7.29
-7.28	-7.28	-7.28	-7.28	-7.28	-7.28	-7.28	-7.28	-7.28
-7.27	-7.27	-7.27	-7.27	-7.27	-7.27	-7.27	-7.27	-7.27
-7.26	-7.26	-7.26	-7.26	-7.26	-7.26	-7.26	-7.26	-7.26
-7.25	-7.25	-7.25	-7.25	-7.25	-7.25	-7.25	-7.25	-7.25
-7.24	-7.24	-7.24	-7.24	-7.24	-7.24	-7.24	-7.24	-7.24
-7.23	-7.23	-7.23	-7.23	-7.23	-7.23	-7.23	-7.23	-7.23
-7.22	-7.22	-7.22	-7.22	-7.22	-7.22	-7.22	-7.22	-7.22
-7.21	-7.21	-7.21	-7.21	-7.21	-7.21	-7.21	-7.21	-7.21
-7.2	-7.2	-7.2	-7.2	-7.2	-7.2	-7.2	-7.2	-7.2
-7.19	-7.19	-7.19	-7.19	-7.19	-7.19	-7.19	-7.19	-7.19
-7.18	-7.18	-7.18	-7.18	-7.18	-7.18	-7.18	-7.18	-7.18
-7.17	-7.17	-7.17	-7.17	-7.17	-7.17	-7.17	-7.17	-7.17
-7.16	-7.16	-7.16	-7.16	-7.16	-7.16	-7.16	-7.16	-7.16
-7.15	-7.15	-7.15	-7.15	-7.15	-7.15	-7.15	-7.15	-7.15
-7.14	-7.14	-7.14	-7.14	-7.14	-7.14	-7.14	-7.14	-7.14
-7.13	-7.13	-7.13	-7.13	-7.13	-7.13	-7.13	-7.13	-7.13
-7.12	-7.12	-7.12	-7.12	-7.12	-7.12	-7.12	-7.12	-7.12
-7.11	-7.11	-7.11	-7.11	-7.11	-7.11	-7.11	-7.11	-7.11
-7.1	-7.1	-7.1	-7.1	-7.1	-7.1	-7.1	-7.1	-7.1
-7.09	-7.09	-7.09	-7.09	-7.09	-7.09	-7.09	-7.09	-7.09
-7.08	-7.08	-7.08	-7.08	-7.08	-7.08	-7.08	-7.08	-7.08
-7.07	-7.07	-7.07	-7.07	-7.07	-7.07	-7.07	-7.07	-7.07
-7.06	-7.06	-7.06	-7.06	-7.06	-7.06	-7.06	-7.06	-7.06
-7.05	-7.05	-7.05	-7.05	-7.05	-7.05	-7.05	-7.05	-7.05
-7.04	-7.04	-7.04	-7.04	-7.04	-7.04	-7.04	-7.04	-7.04
-7.03	-7.03	-7.03	-7.03	-7.03	-7.03	-7.03	-7.03	-7.03
-7.02	-7.02	-7.02	-7.02	-7.02	-7.02	-7.02	-7.02	-7.02
-7.01	-7.01	-7.01	-7.01	-7.01	-7.01	-7.01	-7.01	-7.01

-7	-7	-7	-7	-7	-7	-7	-7	-7
-6.99	-6.99	-6.99	-6.99	-6.99	-6.99	-6.99	-6.99	-6.99
-6.98	-6.98	-6.98	-6.98	-6.98	-6.98	-6.98	-6.98	-6.98
-6.97	-6.97	-6.97	-6.97	-6.97	-6.97	-6.97	-6.97	-6.97
-6.96	-6.96	-6.96	-6.96	-6.96	-6.96	-6.96	-6.96	-6.96
-6.95	-6.95	-6.95	-6.95	-6.95	-6.95	-6.95	-6.95	-6.95
-6.94	-6.94	-6.94	-6.94	-6.94	-6.94	-6.94	-6.94	-6.94
-6.93	-6.93	-6.93	-6.93	-6.93	-6.93	-6.93	-6.93	-6.93
-6.92	-6.92	-6.92	-6.92	-6.92	-6.92	-6.92	-6.92	-6.92
-6.91	-6.91	-6.91	-6.91	-6.91	-6.91	-6.91	-6.91	-6.91
-6.9	-6.9	-6.9	-6.9	-6.9	-6.9	-6.9	-6.9	-6.9
-6.89	-6.89	-6.89	-6.89	-6.89	-6.89	-6.89	-6.89	-6.89
-6.88	-6.88	-6.88	-6.88	-6.88	-6.88	-6.88	-6.88	-6.88
-6.87	-6.87	-6.87	-6.87	-6.87	-6.87	-6.87	-6.87	-6.87
-6.86	-6.86	-6.86	-6.86	-6.86	-6.86	-6.86	-6.86	-6.86
-6.85	-6.85	-6.85	-6.85	-6.85	-6.85	-6.85	-6.85	-6.85
-6.84	-6.84	-6.84	-6.84	-6.84	-6.84	-6.84	-6.84	-6.84
-6.83	-6.83	-6.83	-6.83	-6.83	-6.83	-6.83	-6.83	-6.83
-6.82	-6.82	-6.82	-6.82	-6.82	-6.82	-6.82	-6.82	-6.82
-6.81	-6.81	-6.81	-6.81	-6.81	-6.81	-6.81	-6.81	-6.81
-6.8	-6.8	-6.8	-6.8	-6.8	-6.8	-6.8	-6.8	-6.8
-6.79	-6.79	-6.79	-6.79	-6.79	-6.79	-6.79	-6.79	-6.79
-6.78	-6.78	-6.78	-6.78	-6.78	-6.78	-6.78	-6.78	-6.78
-6.77	-6.77	-6.77	-6.77	-6.77	-6.77	-6.77	-6.77	-6.77
-6.76	-6.76	-6.76	-6.76	-6.76	-6.76	-6.76	-6.76	-6.76
-6.75	-6.75	-6.75	-6.75	-6.75	-6.75	-6.75	-6.75	-6.75
-6.74	-6.74	-6.74	-6.74	-6.74	-6.74	-6.74	-6.74	-6.74
-6.73	-6.73	-6.73	-6.73	-6.73	-6.73	-6.73	-6.73	-6.73
-6.72	-6.72	-6.72	-6.72	-6.72	-6.72	-6.72	-6.72	-6.72
-6.71	-6.71	-6.71	-6.71	-6.71	-6.71	-6.71	-6.71	-6.71
-6.7	-6.7	-6.7	-6.7	-6.7	-6.7	-6.7	-6.7	-6.7
-6.69	-6.69	-6.69	-6.69	-6.69	-6.69	-6.69	-6.69	-6.69
-6.68	-6.68	-6.68	-6.68	-6.68	-6.68	-6.68	-6.68	-6.68
-6.67	-6.67	-6.67	-6.67	-6.67	-6.67	-6.67	-6.67	-6.67
-6.66	-6.66	-6.66	-6.66	-6.66	-6.66	-6.66	-6.66	-6.66
-6.65	-6.65	-6.65	-6.65	-6.65	-6.65	-6.65	-6.65	-6.65
-6.64	-6.64	-6.64	-6.64	-6.64	-6.64	-6.64	-6.64	-6.64
-6.63	-6.63	-6.63	-6.63	-6.63	-6.63	-6.63	-6.63	-6.63
-6.62	-6.62	-6.62	-6.62	-6.62	-6.62	-6.62	-6.62	-6.62
-6.61	-6.61	-6.61	-6.61	-6.61	-6.61	-6.61	-6.61	-6.61
-6.6	-6.6	-6.6	-6.6	-6.6	-6.6	-6.6	-6.6	-6.6
-6.59	-6.59	-6.59	-6.59	-6.59	-6.59	-6.59	-6.59	-6.59
-6.58	-6.58	-6.58	-6.58	-6.58	-6.58	-6.58	-6.58	-6.58
-6.57	-6.57	-6.57	-6.57	-6.57	-6.57	-6.57	-6.57	-6.57
-6.56	-6.56	-6.56	-6.56	-6.56	-6.56	-6.56	-6.56	-6.56
-6.55	-6.55	-6.55	-6.55	-6.55	-6.55	-6.55	-6.55	-6.55
-6.54	-6.54	-6.54	-6.54	-6.54	-6.54	-6.54	-6.54	-6.54
-6.53	-6.53	-6.53	-6.53	-6.53	-6.53	-6.53	-6.53	-6.53
-6.52	-6.52	-6.52	-6.52	-6.52	-6.52	-6.52	-6.52	-6.52
-6.51	-6.51	-6.51	-6.51	-6.51	-6.51	-6.51	-6.51	-6.51

-6.5	-6.5	-6.5	-6.5	-6.5	-6.5	-6.5	-6.5	-6.5
-6.49	-6.49	-6.49	-6.49	-6.49	-6.49	-6.49	-6.49	-6.49
-6.48	-6.48	-6.48	-6.48	-6.48	-6.48	-6.48	-6.48	-6.48
-6.47	-6.47	-6.47	-6.47	-6.47	-6.47	-6.47	-6.47	-6.47
-6.46	-6.46	-6.46	-6.46	-6.46	-6.46	-6.46	-6.46	-6.46
-6.45	-6.45	-6.45	-6.45	-6.45	-6.45	-6.45	-6.45	-6.45
-6.44	-6.44	-6.44	-6.44	-6.44	-6.44	-6.44	-6.44	-6.44
-6.43	-6.43	-6.43	-6.43	-6.43	-6.43	-6.43	-6.43	-6.43
-6.42	-6.42	-6.42	-6.42	-6.42	-6.42	-6.42	-6.42	-6.42
-6.41	-6.41	-6.41	-6.41	-6.41	-6.41	-6.41	-6.41	-6.41
-6.4	-6.4	-6.4	-6.4	-6.4	-6.4	-6.4	-6.4	-6.4
-6.39	-6.39	-6.39	-6.39	-6.39	-6.39	-6.39	-6.39	-6.39
-6.38	-6.38	-6.38	-6.38	-6.38	-6.38	-6.38	-6.38	-6.38
-6.37	-6.37	-6.37	-6.37	-6.37	-6.37	-6.37	-6.37	-6.37
-6.36	-6.36	-6.36	-6.36	-6.36	-6.36	-6.36	-6.36	-6.36
-6.35	-6.35	-6.35	-6.35	-6.35	-6.35	-6.35	-6.35	-6.35
-6.34	-6.34	-6.34	-6.34	-6.34	-6.34	-6.34	-6.34	-6.34
-6.33	-6.33	-6.33	-6.33	-6.33	-6.33	-6.33	-6.33	-6.33
-6.32	-6.32	-6.32	-6.32	-6.32	-6.32	-6.32	-6.32	-6.32
-6.31	-6.31	-6.31	-6.31	-6.31	-6.31	-6.31	-6.31	-6.31
-6.3	-6.3	-6.3	-6.3	-6.3	-6.3	-6.3	-6.3	-6.3
-6.29	-6.29	-6.29	-6.29	-6.29	-6.29	-6.29	-6.29	-6.29
-6.28	-6.28	-6.28	-6.28	-6.28	-6.28	-6.28	-6.28	-6.28
-6.27	-6.27	-6.27	-6.27	-6.27	-6.27	-6.27	-6.27	-6.27
-6.26	-6.26	-6.26	-6.26	-6.26	-6.26	-6.26	-6.26	-6.26
-6.25	-6.25	-6.25	-6.25	-6.25	-6.25	-6.25	-6.25	-6.25
-6.24	-6.24	-6.24	-6.24	-6.24	-6.24	-6.24	-6.24	-6.24
-6.23	-6.23	-6.23	-6.23	-6.23	-6.23	-6.23	-6.23	-6.23
-6.22	-6.22	-6.22	-6.22	-6.22	-6.22	-6.22	-6.22	-6.22
-6.21	-6.21	-6.21	-6.21	-6.21	-6.21	-6.21	-6.21	-6.21
-6.2	-6.2	-6.2	-6.2	-6.2	-6.2	-6.2	-6.2	-6.2
-6.19	-6.19	-6.19	-6.19	-6.19	-6.19	-6.19	-6.19	-6.19
-6.18	-6.18	-6.18	-6.18	-6.18	-6.18	-6.18	-6.18	-6.18
-6.17	-6.17	-6.17	-6.17	-6.17	-6.17	-6.17	-6.17	-6.17
-6.16	-6.16	-6.16	-6.16	-6.16	-6.16	-6.16	-6.16	-6.16
-6.15	-6.15	-6.15	-6.15	-6.15	-6.15	-6.15	-6.15	-6.15
-6.14	-6.14	-6.14	-6.14	-6.14	-6.14	-6.14	-6.14	-6.14
-6.13	-6.13	-6.13	-6.13	-6.13	-6.13	-6.13	-6.13	-6.13
-6.12	-6.12	-6.12	-6.12	-6.12	-6.12	-6.12	-6.12	-6.12
-6.11	-6.11	-6.11	-6.11	-6.11	-6.11	-6.11	-6.11	-6.11
-6.1	-6.1	-6.1	-6.1	-6.1	-6.1	-6.1	-6.1	-6.1
-6.09	-6.09	-6.09	-6.09	-6.09	-6.09	-6.09	-6.09	-6.09
-6.08	-6.08	-6.08	-6.08	-6.08	-6.08	-6.08	-6.08	-6.08
-6.07	-6.07	-6.07	-6.07	-6.07	-6.07	-6.07	-6.07	-6.07
-6.06	-6.06	-6.06	-6.06	-6.06	-6.06	-6.06	-6.06	-6.06
-6.05	-6.05	-6.05	-6.05	-6.05	-6.05	-6.05	-6.05	-6.05
-6.04	-6.04	-6.04	-6.04	-6.04	-6.04	-6.04	-6.04	-6.04
-6.03	-6.03	-6.03	-6.03	-6.03	-6.03	-6.03	-6.03	-6.03
-6.02	-6.02	-6.02	-6.02	-6.02	-6.02	-6.02	-6.02	-6.02
-6.01	-6.01	-6.01	-6.01	-6.01	-6.01	-6.01	-6.01	-6.01

-6	-6	-6	-6	-6	-6	-6	-6	-6
-5.99	-5.99	-5.99	-5.99	-5.99	-5.99	-5.99	-5.99	-5.99
-5.98	-5.98	-5.98	-5.98	-5.98	-5.98	-5.98	-5.98	-5.98
-5.97	-5.97	-5.97	-5.97	-5.97	-5.97	-5.97	-5.97	-5.97
-5.96	-5.96	-5.96	-5.96	-5.96	-5.96	-5.96	-5.96	-5.96
-5.95	-5.95	-5.95	-5.95	-5.95	-5.95	-5.95	-5.95	-5.95
-5.94	-5.94	-5.94	-5.94	-5.94	-5.94	-5.94	-5.94	-5.94
-5.93	-5.93	-5.93	-5.93	-5.93	-5.93	-5.93	-5.93	-5.93
-5.92	-5.92	-5.92	-5.92	-5.92	-5.92	-5.92	-5.92	-5.92
-5.91	-5.91	-5.91	-5.91	-5.91	-5.91	-5.91	-5.91	-5.91
-5.9	-5.9	-5.9	-5.9	-5.9	-5.9	-5.9	-5.9	-5.9
-5.89	-5.89	-5.89	-5.89	-5.89	-5.89	-5.89	-5.89	-5.89
-5.88	-5.88	-5.88	-5.88	-5.88	-5.88	-5.88	-5.88	-5.88
-5.87	-5.87	-5.87	-5.87	-5.87	-5.87	-5.87	-5.87	-5.87
-5.86	-5.86	-5.86	-5.86	-5.86	-5.86	-5.86	-5.86	-5.86
-5.85	-5.85	-5.85	-5.85	-5.85	-5.85	-5.85	-5.85	-5.85
-5.84	-5.84	-5.84	-5.84	-5.84	-5.84	-5.84	-5.84	-5.84
-5.83	-5.83	-5.83	-5.83	-5.83	-5.83	-5.83	-5.83	-5.83
-5.82	-5.82	-5.82	-5.82	-5.82	-5.82	-5.82	-5.82	-5.82
-5.81	-5.81	-5.81	-5.81	-5.81	-5.81	-5.81	-5.81	-5.81
-5.8	-5.8	-5.8	-5.8	-5.8	-5.8	-5.8	-5.8	-5.8
-5.79	-5.79	-5.79	-5.79	-5.79	-5.79	-5.79	-5.79	-5.79
-5.78	-5.78	-5.78	-5.78	-5.78	-5.78	-5.78	-5.78	-5.78
-5.77	-5.77	-5.77	-5.77	-5.77	-5.77	-5.77	-5.77	-5.77
-5.76	-5.76	-5.76	-5.76	-5.76	-5.76	-5.76	-5.76	-5.76
-5.75	-5.75	-5.75	-5.75	-5.75	-5.75	-5.75	-5.75	-5.75
-5.74	-5.74	-5.74	-5.74	-5.74	-5.74	-5.74	-5.74	-5.74
-5.73	-5.73	-5.73	-5.73	-5.73	-5.73	-5.73	-5.73	-5.73
-5.72	-5.72	-5.72	-5.72	-5.72	-5.72	-5.72	-5.72	-5.72
-5.71	-5.71	-5.71	-5.71	-5.71	-5.71	-5.71	-5.71	-5.71
-5.7	-5.7	-5.7	-5.7	-5.7	-5.7	-5.7	-5.7	-5.7
-5.69	-5.69	-5.69	-5.69	-5.69	-5.69	-5.69	-5.69	-5.69
-5.68	-5.68	-5.68	-5.68	-5.68	-5.68	-5.68	-5.68	-5.68
-5.67	-5.67	-5.67	-5.67	-5.67	-5.67	-5.67	-5.67	-5.67
-5.66	-5.66	-5.66	-5.66	-5.66	-5.66	-5.66	-5.66	-5.66
-5.65	-5.65	-5.65	-5.65	-5.65	-5.65	-5.65	-5.65	-5.65
-5.64	-5.64	-5.64	-5.64	-5.64	-5.64	-5.64	-5.64	-5.64
-5.63	-5.63	-5.63	-5.63	-5.63	-5.63	-5.63	-5.63	-5.63
-5.62	-5.62	-5.62	-5.62	-5.62	-5.62	-5.62	-5.62	-5.62
-5.61	-5.61	-5.61	-5.61	-5.61	-5.61	-5.61	-5.61	-5.61
-5.6	-5.6	-5.6	-5.6	-5.6	-5.6	-5.6	-5.6	-5.6
-5.59	-5.59	-5.59	-5.59	-5.59	-5.59	-5.59	-5.59	-5.59
-5.58	-5.58	-5.58	-5.58	-5.58	-5.58	-5.58	-5.58	-5.58
-5.57	-5.57	-5.57	-5.57	-5.57	-5.57	-5.57	-5.57	-5.57
-5.56	-5.56	-5.56	-5.56	-5.56	-5.56	-5.56	-5.56	-5.56
-5.55	-5.55	-5.55	-5.55	-5.55	-5.55	-5.55	-5.55	-5.55
-5.54	-5.54	-5.54	-5.54	-5.54	-5.54	-5.54	-5.54	-5.54
-5.53	-5.53	-5.53	-5.53	-5.53	-5.53	-5.53	-5.53	-5.53
-5.52	-5.52	-5.52	-5.52	-5.52	-5.52	-5.52	-5.52	-5.52
-5.51	-5.51	-5.51	-5.51	-5.51	-5.51	-5.51	-5.51	-5.51

-5.5	-5.5	-5.5	-5.5	-5.5	-5.5	-5.5	-5.5	-5.5
-5.49	-5.49	-5.49	-5.49	-5.49	-5.49	-5.49	-5.49	-5.49
-5.48	-5.48	-5.48	-5.48	-5.48	-5.48	-5.48	-5.48	-5.48
-5.47	-5.47	-5.47	-5.47	-5.47	-5.47	-5.47	-5.47	-5.47
-5.46	-5.46	-5.46	-5.46	-5.46	-5.46	-5.46	-5.46	-5.46
-5.45	-5.45	-5.45	-5.45	-5.45	-5.45	-5.45	-5.45	-5.45
-5.44	-5.44	-5.44	-5.44	-5.44	-5.44	-5.44	-5.44	-5.44
-5.43	-5.43	-5.43	-5.43	-5.43	-5.43	-5.43	-5.43	-5.43
-5.42	-5.42	-5.42	-5.42	-5.42	-5.42	-5.42	-5.42	-5.42
-5.41	-5.41	-5.41	-5.41	-5.41	-5.41	-5.41	-5.41	-5.41
-5.4	-5.4	-5.4	-5.4	-5.4	-5.4	-5.4	-5.4	-5.4
-5.39	-5.39	-5.39	-5.39	-5.39	-5.39	-5.39	-5.39	-5.39
-5.38	-5.38	-5.38	-5.38	-5.38	-5.38	-5.38	-5.38	-5.38
-5.37	-5.37	-5.37	-5.37	-5.37	-5.37	-5.37	-5.37	-5.37
-5.36	-5.36	-5.36	-5.36	-5.36	-5.36	-5.36	-5.36	-5.36
-5.35	-5.35	-5.35	-5.35	-5.35	-5.35	-5.35	-5.35	-5.35
-5.34	-5.34	-5.34	-5.34	-5.34	-5.34	-5.34	-5.34	-5.34
-5.33	-5.33	-5.33	-5.33	-5.33	-5.33	-5.33	-5.33	-5.33
-5.32	-5.32	-5.32	-5.32	-5.32	-5.32	-5.32	-5.32	-5.32
-5.31	-5.31	-5.31	-5.31	-5.31	-5.31	-5.31	-5.31	-5.31
-5.3	-5.3	-5.3	-5.3	-5.3	-5.3	-5.3	-5.3	-5.3
-5.29	-5.29	-5.29	-5.29	-5.29	-5.29	-5.29	-5.29	-5.29
-5.28	-5.28	-5.28	-5.28	-5.28	-5.28	-5.28	-5.28	-5.28
-5.27	-5.27	-5.27	-5.27	-5.27	-5.27	-5.27	-5.27	-5.27
-5.26	-5.26	-5.26	-5.26	-5.26	-5.26	-5.26	-5.26	-5.26
-5.25	-5.25	-5.25	-5.25	-5.25	-5.25	-5.25	-5.25	-5.25
-5.24	-5.24	-5.24	-5.24	-5.24	-5.24	-5.24	-5.24	-5.24
-5.23	-5.23	-5.23	-5.23	-5.23	-5.23	-5.23	-5.23	-5.23
-5.22	-5.22	-5.22	-5.22	-5.22	-5.22	-5.22	-5.22	-5.22
-5.21	-5.21	-5.21	-5.21	-5.21	-5.21	-5.21	-5.21	-5.21
-5.2	-5.2	-5.2	-5.2	-5.2	-5.2	-5.2	-5.2	-5.2
-5.19	-5.19	-5.19	-5.19	-5.19	-5.19	-5.19	-5.19	-5.19
-5.18	-5.18	-5.18	-5.18	-5.18	-5.18	-5.18	-5.18	-5.18
-5.17	-5.17	-5.17	-5.17	-5.17	-5.17	-5.17	-5.17	-5.17
-5.16	-5.16	-5.16	-5.16	-5.16	-5.16	-5.16	-5.16	-5.16
-5.15	-5.15	-5.15	-5.15	-5.15	-5.15	-5.15	-5.15	-5.15
-5.14	-5.14	-5.14	-5.14	-5.14	-5.14	-5.14	-5.14	-5.14
-5.13	-5.13	-5.13	-5.13	-5.13	-5.13	-5.13	-5.13	-5.13
-5.12	-5.12	-5.12	-5.12	-5.12	-5.12	-5.12	-5.12	-5.12
-5.11	-5.11	-5.11	-5.11	-5.11	-5.11	-5.11	-5.11	-5.11
-5.1	-5.1	-5.1	-5.1	-5.1	-5.1	-5.1	-5.1	-5.1
-5.09	-5.09	-5.09	-5.09	-5.09	-5.09	-5.09	-5.09	-5.09
-5.08	-5.08	-5.08	-5.08	-5.08	-5.08	-5.08	-5.08	-5.08
-5.07	-5.07	-5.07	-5.07	-5.07	-5.07	-5.07	-5.07	-5.07
-5.06	-5.06	-5.06	-5.06	-5.06	-5.06	-5.06	-5.06	-5.06
-5.05	-5.05	-5.05	-5.05	-5.05	-5.05	-5.05	-5.05	-5.05
-5.04	-5.04	-5.04	-5.04	-5.04	-5.04	-5.04	-5.04	-5.04
-5.03	-5.03	-5.03	-5.03	-5.03	-5.03	-5.03	-5.03	-5.03
-5.02	-5.02	-5.02	-5.02	-5.02	-5.02	-5.02	-5.02	-5.02
-5.01	-5.01	-5.01	-5.01	-5.01	-5.01	-5.01	-5.01	-5.01

-5	-5	-5	-5	-5	-5	-5	-5	-5
-4.99	-4.99	-4.99	-4.99	-4.99	-4.99	-4.99	-4.99	-4.99
-4.98	-4.98	-4.98	-4.98	-4.98	-4.98	-4.98	-4.98	-4.98
-4.97	-4.97	-4.97	-4.97	-4.97	-4.97	-4.97	-4.97	-4.97
-4.96	-4.96	-4.96	-4.96	-4.96	-4.96	-4.96	-4.96	-4.96
-4.95	-4.95	-4.95	-4.95	-4.95	-4.95	-4.95	-4.95	-4.95
-4.94	-4.94	-4.94	-4.94	-4.94	-4.94	-4.94	-4.94	-4.94
-4.93	-4.93	-4.93	-4.93	-4.93	-4.93	-4.93	-4.93	-4.93
-4.92	-4.92	-4.92	-4.92	-4.92	-4.92	-4.92	-4.92	-4.92
-4.91	-4.91	-4.91	-4.91	-4.91	-4.91	-4.91	-4.91	-4.91
-4.9	-4.9	-4.9	-4.9	-4.9	-4.9	-4.9	-4.9	-4.9
-4.89	-4.89	-4.89	-4.89	-4.89	-4.89	-4.89	-4.89	-4.89
-4.88	-4.88	-4.88	-4.88	-4.88	-4.88	-4.88	-4.88	-4.88
-4.87	-4.87	-4.87	-4.87	-4.87	-4.87	-4.87	-4.87	-4.87
-4.86	-4.86	-4.86	-4.86	-4.86	-4.86	-4.86	-4.86	-4.86
-4.85	-4.85	-4.85	-4.85	-4.85	-4.85	-4.85	-4.85	-4.85
-4.84	-4.84	-4.84	-4.84	-4.84	-4.84	-4.84	-4.84	-4.84
-4.83	-4.83	-4.83	-4.83	-4.83	-4.83	-4.83	-4.83	-4.83
-4.82	-4.82	-4.82	-4.82	-4.82	-4.82	-4.82	-4.82	-4.82
-4.81	-4.81	-4.81	-4.81	-4.81	-4.81	-4.81	-4.81	-4.81
-4.8	-4.8	-4.8	-4.8	-4.8	-4.8	-4.8	-4.8	-4.8
-4.79	-4.79	-4.79	-4.79	-4.79	-4.79	-4.79	-4.79	-4.79
-4.78	-4.78	-4.78	-4.78	-4.78	-4.78	-4.78	-4.78	-4.78
-4.77	-4.77	-4.77	-4.77	-4.77	-4.77	-4.77	-4.77	-4.77
-4.76	-4.76	-4.76	-4.76	-4.76	-4.76	-4.76	-4.76	-4.76
-4.75	-4.75	-4.75	-4.75	-4.75	-4.75	-4.75	-4.75	-4.75
-4.74	-4.74	-4.74	-4.74	-4.74	-4.74	-4.74	-4.74	-4.74
-4.73	-4.73	-4.73	-4.73	-4.73	-4.73	-4.73	-4.73	-4.73
-4.72	-4.72	-4.72	-4.72	-4.72	-4.72	-4.72	-4.72	-4.72
-4.71	-4.71	-4.71	-4.71	-4.71	-4.71	-4.71	-4.71	-4.71
-4.7	-4.7	-4.7	-4.7	-4.7	-4.7	-4.7	-4.7	-4.7
-4.69	-4.69	-4.69	-4.69	-4.69	-4.69	-4.69	-4.69	-4.69
-4.68	-4.68	-4.68	-4.68	-4.68	-4.68	-4.68	-4.68	-4.68
-4.67	-4.67	-4.67	-4.67	-4.67	-4.67	-4.67	-4.67	-4.67
-4.66	-4.66	-4.66	-4.66	-4.66	-4.66	-4.66	-4.66	-4.66
-4.65	-4.65	-4.65	-4.65	-4.65	-4.65	-4.65	-4.65	-4.65
-4.64	-4.64	-4.64	-4.64	-4.64	-4.64	-4.64	-4.64	-4.64
-4.63	-4.63	-4.63	-4.63	-4.63	-4.63	-4.63	-4.63	-4.63
-4.62	-4.62	-4.62	-4.62	-4.62	-4.62	-4.62	-4.62	-4.62
-4.61	-4.61	-4.61	-4.61	-4.61	-4.61	-4.61	-4.61	-4.61
-4.6	-4.6	-4.6	-4.6	-4.6	-4.6	-4.6	-4.6	-4.6
-4.59	-4.59	-4.59	-4.59	-4.59	-4.59	-4.59	-4.59	-4.59
-4.58	-4.58	-4.58	-4.58	-4.58	-4.58	-4.58	-4.58	-4.58
-4.57	-4.57	-4.57	-4.57	-4.57	-4.57	-4.57	-4.57	-4.57
-4.56	-4.56	-4.56	-4.56	-4.56	-4.56	-4.56	-4.56	-4.56
-4.55	-4.55	-4.55	-4.55	-4.55	-4.55	-4.55	-4.55	-4.55
-4.54	-4.54	-4.54	-4.54	-4.54	-4.54	-4.54	-4.54	-4.54
-4.53	-4.53	-4.53	-4.53	-4.53	-4.53	-4.53	-4.53	-4.53
-4.52	-4.52	-4.52	-4.52	-4.52	-4.52	-4.52	-4.52	-4.52
-4.51	-4.51	-4.51	-4.51	-4.51	-4.51	-4.51	-4.51	-4.51

-4.5	-4.5	-4.5	-4.5	-4.5	-4.5	-4.5	-4.5	-4.5
-4.49	-4.49	-4.49	-4.49	-4.49	-4.49	-4.49	-4.49	-4.49
-4.48	-4.48	-4.48	-4.48	-4.48	-4.48	-4.48	-4.48	-4.48
-4.47	-4.47	-4.47	-4.47	-4.47	-4.47	-4.47	-4.47	-4.47
-4.46	-4.46	-4.46	-4.46	-4.46	-4.46	-4.46	-4.46	-4.46
-4.45	-4.45	-4.45	-4.45	-4.45	-4.45	-4.45	-4.45	-4.45
-4.44	-4.44	-4.44	-4.44	-4.44	-4.44	-4.44	-4.44	-4.44
-4.43	-4.43	-4.43	-4.43	-4.43	-4.43	-4.43	-4.43	-4.43
-4.42	-4.42	-4.42	-4.42	-4.42	-4.42	-4.42	-4.42	-4.42
-4.41	-4.41	-4.41	-4.41	-4.41	-4.41	-4.41	-4.41	-4.41
-4.4	-4.4	-4.4	-4.4	-4.4	-4.4	-4.4	-4.4	-4.4
-4.39	-4.39	-4.39	-4.39	-4.39	-4.39	-4.39	-4.39	-4.39
-4.38	-4.38	-4.38	-4.38	-4.38	-4.38	-4.38	-4.38	-4.38
-4.37	-4.37	-4.37	-4.37	-4.37	-4.37	-4.37	-4.37	-4.37
-4.36	-4.36	-4.36	-4.36	-4.36	-4.36	-4.36	-4.36	-4.36
-4.35	-4.35	-4.35	-4.35	-4.35	-4.35	-4.35	-4.35	-4.35
-4.34	-4.34	-4.34	-4.34	-4.34	-4.34	-4.34	-4.34	-4.34
-4.33	-4.33	-4.33	-4.33	-4.33	-4.33	-4.33	-4.33	-4.33
-4.32	-4.32	-4.32	-4.32	-4.32	-4.32	-4.32	-4.32	-4.32
-4.31	-4.31	-4.31	-4.31	-4.31	-4.31	-4.31	-4.31	-4.31
-4.3	-4.3	-4.3	-4.3	-4.3	-4.3	-4.3	-4.3	-4.3
-4.29	-4.29	-4.29	-4.29	-4.29	-4.29	-4.29	-4.29	-4.29
-4.28	-4.28	-4.28	-4.28	-4.28	-4.28	-4.28	-4.28	-4.28
-4.27	-4.27	-4.27	-4.27	-4.27	-4.27	-4.27	-4.27	-4.27
-4.26	-4.26	-4.26	-4.26	-4.26	-4.26	-4.26	-4.26	-4.26
-4.25	-4.25	-4.25	-4.25	-4.25	-4.25	-4.25	-4.25	-4.25
-4.24	-4.24	-4.24	-4.24	-4.24	-4.24	-4.24	-4.24	-4.24
-4.23	-4.23	-4.23	-4.23	-4.23	-4.23	-4.23	-4.23	-4.23
-4.22	-4.22	-4.22	-4.22	-4.22	-4.22	-4.22	-4.22	-4.22
-4.21	-4.21	-4.21	-4.21	-4.21	-4.21	-4.21	-4.21	-4.21
-4.2	-4.2	-4.2	-4.2	-4.2	-4.2	-4.2	-4.2	-4.2
-4.19	-4.19	-4.19	-4.19	-4.19	-4.19	-4.19	-4.19	-4.19
-4.18	-4.18	-4.18	-4.18	-4.18	-4.18	-4.18	-4.18	-4.18
-4.17	-4.17	-4.17	-4.17	-4.17	-4.17	-4.17	-4.17	-4.17
-4.16	-4.16	-4.16	-4.16	-4.16	-4.16	-4.16	-4.16	-4.16
-4.15	-4.15	-4.15	-4.15	-4.15	-4.15	-4.15	-4.15	-4.15
-4.14	-4.14	-4.14	-4.14	-4.14	-4.14	-4.14	-4.14	-4.14
-4.13	-4.13	-4.13	-4.13	-4.13	-4.13	-4.13	-4.13	-4.13
-4.12	-4.12	-4.12	-4.12	-4.12	-4.12	-4.12	-4.12	-4.12
-4.11	-4.11	-4.11	-4.11	-4.11	-4.11	-4.11	-4.11	-4.11
-4.1	-4.1	-4.1	-4.1	-4.1	-4.1	-4.1	-4.1	-4.1
-4.09	-4.09	-4.09	-4.09	-4.09	-4.09	-4.09	-4.09	-4.09
-4.08	-4.08	-4.08	-4.08	-4.08	-4.08	-4.08	-4.08	-4.08
-4.07	-4.07	-4.07	-4.07	-4.07	-4.07	-4.07	-4.07	-4.07
-4.06	-4.06	-4.06	-4.06	-4.06	-4.06	-4.06	-4.06	-4.06
-4.05	-4.05	-4.05	-4.05	-4.05	-4.05	-4.05	-4.05	-4.05
-4.04	-4.04	-4.04	-4.04	-4.04	-4.04	-4.04	-4.04	-4.04
-4.03	-4.03	-4.03	-4.03	-4.03	-4.03	-4.03	-4.03	-4.03
-4.02	-4.02	-4.02	-4.02	-4.02	-4.02	-4.02	-4.02	-4.02
-4.01	-4.01	-4.01	-4.01	-4.01	-4.01	-4.01	-4.01	-4.01

-4	-4	-4	-4	-4	-4	-4	-4	-4
-3.99	-3.99	-3.99	-3.99	-3.99	-3.99	-3.99	-3.99	-3.99
-3.98	-3.98	-3.98	-3.98	-3.98	-3.98	-3.98	-3.98	-3.98
-3.97	-3.97	-3.97	-3.97	-3.97	-3.97	-3.97	-3.97	-3.97
-3.96	-3.96	-3.96	-3.96	-3.96	-3.96	-3.96	-3.96	-3.96
-3.95	-3.95	-3.95	-3.95	-3.95	-3.95	-3.95	-3.95	-3.95
-3.94	-3.94	-3.94	-3.94	-3.94	-3.94	-3.94	-3.94	-3.94
-3.93	-3.93	-3.93	-3.93	-3.93	-3.93	-3.93	-3.93	-3.93
-3.92	-3.92	-3.92	-3.92	-3.92	-3.92	-3.92	-3.92	-3.92
-3.91	-3.91	-3.91	-3.91	-3.91	-3.91	-3.91	-3.91	-3.91
-3.9	-3.9	-3.9	-3.9	-3.9	-3.9	-3.9	-3.9	-3.9
-3.89	-3.89	-3.89	-3.89	-3.89	-3.89	-3.89	-3.89	-3.89
-3.88	-3.88	-3.88	-3.88	-3.88	-3.88	-3.88	-3.88	-3.88
-3.87	-3.87	-3.87	-3.87	-3.87	-3.87	-3.87	-3.87	-3.87
-3.86	-3.86	-3.86	-3.86	-3.86	-3.86	-3.86	-3.86	-3.86
-3.85	-3.85	-3.85	-3.85	-3.85	-3.85	-3.85	-3.85	-3.85
-3.84	-3.84	-3.84	-3.84	-3.84	-3.84	-3.84	-3.84	-3.84
-3.83	-3.83	-3.83	-3.83	-3.83	-3.83	-3.83	-3.83	-3.83
-3.82	-3.82	-3.82	-3.82	-3.82	-3.82	-3.82	-3.82	-3.82
-3.81	-3.81	-3.81	-3.81	-3.81	-3.81	-3.81	-3.81	-3.81
-3.8	-3.8	-3.8	-3.8	-3.8	-3.8	-3.8	-3.8	-3.8
-3.79	-3.79	-3.79	-3.79	-3.79	-3.79	-3.79	-3.79	-3.79
-3.78	-3.78	-3.78	-3.78	-3.78	-3.78	-3.78	-3.78	-3.78
-3.77	-3.77	-3.77	-3.77	-3.77	-3.77	-3.77	-3.77	-3.77
-3.76	-3.76	-3.76	-3.76	-3.76	-3.76	-3.76	-3.76	-3.76
-3.75	-3.75	-3.75	-3.75	-3.75	-3.75	-3.75	-3.75	-3.75
-3.74	-3.74	-3.74	-3.74	-3.74	-3.74	-3.74	-3.74	-3.74
-3.73	-3.73	-3.73	-3.73	-3.73	-3.73	-3.73	-3.73	-3.73
-3.72	-3.72	-3.72	-3.72	-3.72	-3.72	-3.72	-3.72	-3.72
-3.71	-3.71	-3.71	-3.71	-3.71	-3.71	-3.71	-3.71	-3.71
-3.7	-3.7	-3.7	-3.7	-3.7	-3.7	-3.7	-3.7	-3.7
-3.69	-3.69	-3.69	-3.69	-3.69	-3.69	-3.69	-3.69	-3.69
-3.68	-3.68	-3.68	-3.68	-3.68	-3.68	-3.68	-3.68	-3.68
-3.67	-3.67	-3.67	-3.67	-3.67	-3.67	-3.67	-3.67	-3.67
-3.66	-3.66	-3.66	-3.66	-3.66	-3.66	-3.66	-3.66	-3.66
-3.65	-3.65	-3.65	-3.65	-3.65	-3.65	-3.65	-3.65	-3.65
-3.64	-3.64	-3.64	-3.64	-3.64	-3.64	-3.64	-3.64	-3.64
-3.63	-3.63	-3.63	-3.63	-3.63	-3.63	-3.63	-3.63	-3.63
-3.62	-3.62	-3.62	-3.62	-3.62	-3.62	-3.62	-3.62	-3.62
-3.61	-3.61	-3.61	-3.61	-3.61	-3.61	-3.61	-3.61	-3.61
-3.6	-3.6	-3.6	-3.6	-3.6	-3.6	-3.6	-3.6	-3.6
-3.59	-3.59	-3.59	-3.59	-3.59	-3.59	-3.59	-3.59	-3.59
-3.58	-3.58	-3.58	-3.58	-3.58	-3.58	-3.58	-3.58	-3.58
-3.57	-3.57	-3.57	-3.57	-3.57	-3.57	-3.57	-3.57	-3.57
-3.56	-3.56	-3.56	-3.56	-3.56	-3.56	-3.56	-3.56	-3.56
-3.55	-3.55	-3.55	-3.55	-3.55	-3.55	-3.55	-3.55	-3.55
-3.54	-3.54	-3.54	-3.54	-3.54	-3.54	-3.54	-3.54	-3.54
-3.53	-3.53	-3.53	-3.53	-3.53	-3.53	-3.53	-3.53	-3.53
-3.52	-3.52	-3.52	-3.52	-3.52	-3.52	-3.52	-3.52	-3.52
-3.51	-3.51	-3.51	-3.51	-3.51	-3.51	-3.51	-3.51	-3.51

-3.5	-3.5	-3.5	-3.5	-3.5	-3.5	-3.5	-3.5	-3.5
-3.49	-3.49	-3.49	-3.49	-3.49	-3.49	-3.49	-3.49	-3.49
-3.48	-3.48	-3.48	-3.48	-3.48	-3.48	-3.48	-3.48	-3.48
-3.47	-3.47	-3.47	-3.47	-3.47	-3.47	-3.47	-3.47	-3.47
-3.46	-3.46	-3.46	-3.46	-3.46	-3.46	-3.46	-3.46	-3.46
-3.45	-3.45	-3.45	-3.45	-3.45	-3.45	-3.45	-3.45	-3.45
-3.44	-3.44	-3.44	-3.44	-3.44	-3.44	-3.44	-3.44	-3.44
-3.43	-3.43	-3.43	-3.43	-3.43	-3.43	-3.43	-3.43	-3.43
-3.42	-3.42	-3.42	-3.42	-3.42	-3.42	-3.42	-3.42	-3.42
-3.41	-3.41	-3.41	-3.41	-3.41	-3.41	-3.41	-3.41	-3.41
-3.4	-3.4	-3.4	-3.4	-3.4	-3.4	-3.4	-3.4	-3.4
-3.39	-3.39	-3.39	-3.39	-3.39	-3.39	-3.39	-3.39	-3.39
-3.38	-3.38	-3.38	-3.38	-3.38	-3.38	-3.38	-3.38	-3.38
-3.37	-3.37	-3.37	-3.37	-3.37	-3.37	-3.37	-3.37	-3.37
-3.36	-3.36	-3.36	-3.36	-3.36	-3.36	-3.36	-3.36	-3.36
-3.35	-3.35	-3.35	-3.35	-3.35	-3.35	-3.35	-3.35	-3.35
-3.34	-3.34	-3.34	-3.34	-3.34	-3.34	-3.34	-3.34	-3.34
-3.33	-3.33	-3.33	-3.33	-3.33	-3.33	-3.33	-3.33	-3.33
-3.32	-3.32	-3.32	-3.32	-3.32	-3.32	-3.32	-3.32	-3.32
-3.31	-3.31	-3.31	-3.31	-3.31	-3.31	-3.31	-3.31	-3.31
-3.3	-3.3	-3.3	-3.3	-3.3	-3.3	-3.3	-3.3	-3.3
-3.29	-3.29	-3.29	-3.29	-3.29	-3.29	-3.29	-3.29	-3.29
-3.28	-3.28	-3.28	-3.28	-3.28	-3.28	-3.28	-3.28	-3.28
-3.27	-3.27	-3.27	-3.27	-3.27	-3.27	-3.27	-3.27	-3.27
-3.26	-3.26	-3.26	-3.26	-3.26	-3.26	-3.26	-3.26	-3.26
-3.25	-3.25	-3.25	-3.25	-3.25	-3.25	-3.25	-3.25	-3.25
-3.24	-3.24	-3.24	-3.24	-3.24	-3.24	-3.24	-3.24	-3.24
-3.23	-3.23	-3.23	-3.23	-3.23	-3.23	-3.23	-3.23	-3.23
-3.22	-3.22	-3.22	-3.22	-3.22	-3.22	-3.22	-3.22	-3.22
-3.21	-3.21	-3.21	-3.21	-3.21	-3.21	-3.21	-3.21	-3.21
-3.2	-3.2	-3.2	-3.2	-3.2	-3.2	-3.2	-3.2	-3.2
-3.19	-3.19	-3.19	-3.19	-3.19	-3.19	-3.19	-3.19	-3.19
-3.18	-3.18	-3.18	-3.18	-3.18	-3.18	-3.18	-3.18	-3.18
-3.17	-3.17	-3.17	-3.17	-3.17	-3.17	-3.17	-3.17	-3.17
-3.16	-3.16	-3.16	-3.16	-3.16	-3.16	-3.16	-3.16	-3.16
-3.15	-3.15	-3.15	-3.15	-3.15	-3.15	-3.15	-3.15	-3.15
-3.14	-3.14	-3.14	-3.14	-3.14	-3.14	-3.14	-3.14	-3.14
-3.13	-3.13	-3.13	-3.13	-3.13	-3.13	-3.13	-3.13	-3.13
-3.12	-3.12	-3.12	-3.12	-3.12	-3.12	-3.12	-3.12	-3.12
-3.11	-3.11	-3.11	-3.11	-3.11	-3.11	-3.11	-3.11	-3.11
-3.1	-3.1	-3.1	-3.1	-3.1	-3.1	-3.1	-3.1	-3.1
-3.09	-3.09	-3.09	-3.09	-3.09	-3.09	-3.09	-3.09	-3.09
-3.08	-3.08	-3.08	-3.08	-3.08	-3.08	-3.08	-3.08	-3.08
-3.07	-3.07	-3.07	-3.07	-3.07	-3.07	-3.07	-3.07	-3.07
-3.06	-3.06	-3.06	-3.06	-3.06	-3.06	-3.06	-3.06	-3.06
-3.05	-3.05	-3.05	-3.05	-3.05	-3.05	-3.05	-3.05	-3.05
-3.04	-3.04	-3.04	-3.04	-3.04	-3.04	-3.04	-3.04	-3.04
-3.03	-3.03	-3.03	-3.03	-3.03	-3.03	-3.03	-3.03	-3.03
-3.02	-3.02	-3.02	-3.02	-3.02	-3.02	-3.02	-3.02	-3.02
-3.01	-3.01	-3.01	-3.01	-3.01	-3.01	-3.01	-3.01	-3.01

-3	-3	-3	-3	-3	-3	-3	-3	-3
-2.99	-2.99	-2.99	-2.99	-2.99	-2.99	-2.99	-2.99	-2.99
-2.98	-2.98	-2.98	-2.98	-2.98	-2.98	-2.98	-2.98	-2.98
-2.97	-2.97	-2.97	-2.97	-2.97	-2.97	-2.97	-2.97	-2.97
-2.96	-2.96	-2.96	-2.96	-2.96	-2.96	-2.96	-2.96	-2.96
-2.95	-2.95	-2.95	-2.95	-2.95	-2.95	-2.95	-2.95	-2.95
-2.94	-2.94	-2.94	-2.94	-2.94	-2.94	-2.94	-2.94	-2.94
-2.93	-2.93	-2.93	-2.93	-2.93	-2.93	-2.93	-2.93	-2.93
-2.92	-2.92	-2.92	-2.92	-2.92	-2.92	-2.92	-2.92	-2.92
-2.91	-2.91	-2.91	-2.91	-2.91	-2.91	-2.91	-2.91	-2.91
-2.9	-2.9	-2.9	-2.9	-2.9	-2.9	-2.9	-2.9	-2.9
-2.89	-2.89	-2.89	-2.89	-2.89	-2.89	-2.89	-2.89	-2.89
-2.88	-2.88	-2.88	-2.88	-2.88	-2.88	-2.88	-2.88	-2.88
-2.87	-2.87	-2.87	-2.87	-2.87	-2.87	-2.87	-2.87	-2.87
-2.86	-2.86	-2.86	-2.86	-2.86	-2.86	-2.86	-2.86	-2.86
-2.85	-2.85	-2.85	-2.85	-2.85	-2.85	-2.85	-2.85	-2.85
-2.84	-2.84	-2.84	-2.84	-2.84	-2.84	-2.84	-2.84	-2.84
-2.83	-2.83	-2.83	-2.83	-2.83	-2.83	-2.83	-2.83	-2.83
-2.82	-2.82	-2.82	-2.82	-2.82	-2.82	-2.82	-2.82	-2.82
-2.81	-2.81	-2.81	-2.81	-2.81	-2.81	-2.81	-2.81	-2.81
-2.8	-2.8	-2.8	-2.8	-2.8	-2.8	-2.8	-2.8	-2.8
-2.79	-2.79	-2.79	-2.79	-2.79	-2.79	-2.79	-2.79	-2.79
-2.78	-2.78	-2.78	-2.78	-2.78	-2.78	-2.78	-2.78	-2.78
-2.77	-2.77	-2.77	-2.77	-2.77	-2.77	-2.77	-2.77	-2.77
-2.76	-2.76	-2.76	-2.76	-2.76	-2.76	-2.76	-2.76	-2.76
-2.75	-2.75	-2.75	-2.75	-2.75	-2.75	-2.75	-2.75	-2.75
-2.74	-2.74	-2.74	-2.74	-2.74	-2.74	-2.74	-2.74	-2.74
-2.73	-2.73	-2.73	-2.73	-2.73	-2.73	-2.73	-2.73	-2.73
-2.72	-2.72	-2.72	-2.72	-2.72	-2.72	-2.72	-2.72	-2.72
-2.71	-2.71	-2.71	-2.71	-2.71	-2.71	-2.71	-2.71	-2.71
-2.7	-2.7	-2.7	-2.7	-2.7	-2.7	-2.7	-2.7	-2.7
-2.69	-2.69	-2.69	-2.69	-2.69	-2.69	-2.69	-2.69	-2.69
-2.68	-2.68	-2.68	-2.68	-2.68	-2.68	-2.68	-2.68	-2.68
-2.67	-2.67	-2.67	-2.67	-2.67	-2.67	-2.67	-2.67	-2.67
-2.66	-2.66	-2.66	-2.66	-2.66	-2.66	-2.66	-2.66	-2.66
-2.65	-2.65	-2.65	-2.65	-2.65	-2.65	-2.65	-2.65	-2.65
-2.64	-2.64	-2.64	-2.64	-2.64	-2.64	-2.64	-2.64	-2.64
-2.63	-2.63	-2.63	-2.63	-2.63	-2.63	-2.63	-2.63	-2.63
-2.62	-2.62	-2.62	-2.62	-2.62	-2.62	-2.62	-2.62	-2.62
-2.61	-2.61	-2.61	-2.61	-2.61	-2.61	-2.61	-2.61	-2.61
-2.6	-2.6	-2.6	-2.6	-2.6	-2.6	-2.6	-2.6	-2.6
-2.59	-2.59	-2.59	-2.59	-2.59	-2.59	-2.59	-2.59	-2.59
-2.58	-2.58	-2.58	-2.58	-2.58	-2.58	-2.58	-2.58	-2.58
-2.57	-2.57	-2.57	-2.57	-2.57	-2.57	-2.57	-2.57	-2.57
-2.56	-2.56	-2.56	-2.56	-2.56	-2.56	-2.56	-2.56	-2.56
-2.55	-2.55	-2.55	-2.55	-2.55	-2.55	-2.55	-2.55	-2.55
-2.54	-2.54	-2.54	-2.54	-2.54	-2.54	-2.54	-2.54	-2.54
-2.53	-2.53	-2.53	-2.53	-2.53	-2.53	-2.53	-2.53	-2.53
-2.52	-2.52	-2.52	-2.52	-2.52	-2.52	-2.52	-2.52	-2.52
-2.51	-2.51	-2.51	-2.51	-2.51	-2.51	-2.51	-2.51	-2.51

-2.5	-2.5	-2.5	-2.5	-2.5	-2.5	-2.5	-2.5	-2.5
-2.49	-2.49	-2.49	-2.49	-2.49	-2.49	-2.49	-2.49	-2.49
-2.48	-2.48	-2.48	-2.48	-2.48	-2.48	-2.48	-2.48	-2.48
-2.47	-2.47	-2.47	-2.47	-2.47	-2.47	-2.47	-2.47	-2.47
-2.46	-2.46	-2.46	-2.46	-2.46	-2.46	-2.46	-2.46	-2.46
-2.45	-2.45	-2.45	-2.45	-2.45	-2.45	-2.45	-2.45	-2.45
-2.44	-2.44	-2.44	-2.44	-2.44	-2.44	-2.44	-2.44	-2.44
-2.43	-2.43	-2.43	-2.43	-2.43	-2.43	-2.43	-2.43	-2.43
-2.42	-2.42	-2.42	-2.42	-2.42	-2.42	-2.42	-2.42	-2.42
-2.41	-2.41	-2.41	-2.41	-2.41	-2.41	-2.41	-2.41	-2.41
-2.4	-2.4	-2.4	-2.4	-2.4	-2.4	-2.4	-2.4	-2.4
-2.39	-2.39	-2.39	-2.39	-2.39	-2.39	-2.39	-2.39	-2.39
-2.38	-2.38	-2.38	-2.38	-2.38	-2.38	-2.38	-2.38	-2.38
-2.37	-2.37	-2.37	-2.37	-2.37	-2.37	-2.37	-2.37	-2.37
-2.36	-2.36	-2.36	-2.36	-2.36	-2.36	-2.36	-2.36	-2.36
-2.35	-2.35	-2.35	-2.35	-2.35	-2.35	-2.35	-2.35	-2.35
-2.34	-2.34	-2.34	-2.34	-2.34	-2.34	-2.34	-2.34	-2.34
-2.33	-2.33	-2.33	-2.33	-2.33	-2.33	-2.33	-2.33	-2.33
-2.32	-2.32	-2.32	-2.32	-2.32	-2.32	-2.32	-2.32	-2.32
-2.31	-2.31	-2.31	-2.31	-2.31	-2.31	-2.31	-2.31	-2.31
-2.3	-2.3	-2.3	-2.3	-2.3	-2.3	-2.3	-2.3	-2.3
-2.29	-2.29	-2.29	-2.29	-2.29	-2.29	-2.29	-2.29	-2.29
-2.28	-2.28	-2.28	-2.28	-2.28	-2.28	-2.28	-2.28	-2.28
-2.27	-2.27	-2.27	-2.27	-2.27	-2.27	-2.27	-2.27	-2.27
-2.26	-2.26	-2.26	-2.26	-2.26	-2.26	-2.26	-2.26	-2.26
-2.25	-2.25	-2.25	-2.25	-2.25	-2.25	-2.25	-2.25	-2.25
-2.24	-2.24	-2.24	-2.24	-2.24	-2.24	-2.24	-2.24	-2.24
-2.23	-2.23	-2.23	-2.23	-2.23	-2.23	-2.23	-2.23	-2.23
-2.22	-2.22	-2.22	-2.22	-2.22	-2.22	-2.22	-2.22	-2.22
-2.21	-2.21	-2.21	-2.21	-2.21	-2.21	-2.21	-2.21	-2.21
-2.2	-2.2	-2.2	-2.2	-2.2	-2.2	-2.2	-2.2	-2.2
-2.19	-2.19	-2.19	-2.19	-2.19	-2.19	-2.19	-2.19	-2.19
-2.18	-2.18	-2.18	-2.18	-2.18	-2.18	-2.18	-2.18	-2.18
-2.17	-2.17	-2.17	-2.17	-2.17	-2.17	-2.17	-2.17	-2.17
-2.16	-2.16	-2.16	-2.16	-2.16	-2.16	-2.16	-2.16	-2.16
-2.15	-2.15	-2.15	-2.15	-2.15	-2.15	-2.15	-2.15	-2.15
-2.14	-2.14	-2.14	-2.14	-2.14	-2.14	-2.14	-2.14	-2.14
-2.13	-2.13	-2.13	-2.13	-2.13	-2.13	-2.13	-2.13	-2.13
-2.12	-2.12	-2.12	-2.12	-2.12	-2.12	-2.12	-2.12	-2.12
-2.11	-2.11	-2.11	-2.11	-2.11	-2.11	-2.11	-2.11	-2.11
-2.1	-2.1	-2.1	-2.1	-2.1	-2.1	-2.1	-2.1	-2.1
-2.09	-2.09	-2.09	-2.09	-2.09	-2.09	-2.09	-2.09	-2.09
-2.08	-2.08	-2.08	-2.08	-2.08	-2.08	-2.08	-2.08	-2.08
-2.07	-2.07	-2.07	-2.07	-2.07	-2.07	-2.07	-2.07	-2.07
-2.06	-2.06	-2.06	-2.06	-2.06	-2.06	-2.06	-2.06	-2.06
-2.05	-2.05	-2.05	-2.05	-2.05	-2.05	-2.05	-2.05	-2.05
-2.04	-2.04	-2.04	-2.04	-2.04	-2.04	-2.04	-2.04	-2.04
-2.03	-2.03	-2.03	-2.03	-2.03	-2.03	-2.03	-2.03	-2.03
-2.02	-2.02	-2.02	-2.02	-2.02	-2.02	-2.02	-2.02	-2.02
-2.01	-2.01	-2.01	-2.01	-2.01	-2.01	-2.01	-2.01	-2.01

-2	-2	-2	-2	-2	-2	-2	-2	-2
-1.99	-1.99	-1.99	-1.99	-1.99	-1.99	-1.99	-1.99	-1.99
-1.98	-1.98	-1.98	-1.98	-1.98	-1.98	-1.98	-1.98	-1.98
-1.97	-1.97	-1.97	-1.97	-1.97	-1.97	-1.97	-1.97	-1.97
-1.96	-1.96	-1.96	-1.96	-1.96	-1.96	-1.96	-1.96	-1.96
-1.95	-1.95	-1.95	-1.95	-1.95	-1.95	-1.95	-1.95	-1.95
-1.94	-1.94	-1.94	-1.94	-1.94	-1.94	-1.94	-1.94	-1.94
-1.93	-1.93	-1.93	-1.93	-1.93	-1.93	-1.93	-1.93	-1.93
-1.92	-1.92	-1.92	-1.92	-1.92	-1.92	-1.92	-1.92	-1.92
-1.91	-1.91	-1.91	-1.91	-1.91	-1.91	-1.91	-1.91	-1.91
-1.9	-1.9	-1.9	-1.9	-1.9	-1.9	-1.9	-1.9	-1.9
-1.89	-1.89	-1.89	-1.89	-1.89	-1.89	-1.89	-1.89	-1.89
-1.88	-1.88	-1.88	-1.88	-1.88	-1.88	-1.88	-1.88	-1.88
-1.87	-1.87	-1.87	-1.87	-1.87	-1.87	-1.87	-1.87	-1.87
-1.86	-1.86	-1.86	-1.86	-1.86	-1.86	-1.86	-1.86	-1.86
-1.85	-1.85	-1.85	-1.85	-1.85	-1.85	-1.85	-1.85	-1.85
-1.84	-1.84	-1.84	-1.84	-1.84	-1.84	-1.84	-1.84	-1.84
-1.83	-1.83	-1.83	-1.83	-1.83	-1.83	-1.83	-1.83	-1.83
-1.82	-1.82	-1.82	-1.82	-1.82	-1.82	-1.82	-1.82	-1.82
-1.81	-1.81	-1.81	-1.81	-1.81	-1.81	-1.81	-1.81	-1.81
-1.8	-1.8	-1.8	-1.8	-1.8	-1.8	-1.8	-1.8	-1.8
-1.79	-1.79	-1.79	-1.79	-1.79	-1.79	-1.79	-1.79	-1.79
-1.78	-1.78	-1.78	-1.78	-1.78	-1.78	-1.78	-1.78	-1.78
-1.77	-1.77	-1.77	-1.77	-1.77	-1.77	-1.77	-1.77	-1.77
-1.76	-1.76	-1.76	-1.76	-1.76	-1.76	-1.76	-1.76	-1.76
-1.75	-1.75	-1.75	-1.75	-1.75	-1.75	-1.75	-1.75	-1.75
-1.74	-1.74	-1.74	-1.74	-1.74	-1.74	-1.74	-1.74	-1.74
-1.73	-1.73	-1.73	-1.73	-1.73	-1.73	-1.73	-1.73	-1.73
-1.72	-1.72	-1.72	-1.72	-1.72	-1.72	-1.72	-1.72	-1.72
-1.71	-1.71	-1.71	-1.71	-1.71	-1.71	-1.71	-1.71	-1.71
-1.7	-1.7	-1.7	-1.7	-1.7	-1.7	-1.7	-1.7	-1.7
-1.69	-1.69	-1.69	-1.69	-1.69	-1.69	-1.69	-1.69	-1.69
-1.68	-1.68	-1.68	-1.68	-1.68	-1.68	-1.68	-1.68	-1.68
-1.67	-1.67	-1.67	-1.67	-1.67	-1.67	-1.67	-1.67	-1.67
-1.66	-1.66	-1.66	-1.66	-1.66	-1.66	-1.66	-1.66	-1.66
-1.65	-1.65	-1.65	-1.65	-1.65	-1.65	-1.65	-1.65	-1.65
-1.64	-1.64	-1.64	-1.64	-1.64	-1.64	-1.64	-1.64	-1.64
-1.63	-1.63	-1.63	-1.63	-1.63	-1.63	-1.63	-1.63	-1.63
-1.62	-1.62	-1.62	-1.62	-1.62	-1.62	-1.62	-1.62	-1.62
-1.61	-1.61	-1.61	-1.61	-1.61	-1.61	-1.61	-1.61	-1.61
-1.6	-1.6	-1.6	-1.6	-1.6	-1.6	-1.6	-1.6	-1.6
-1.59	-1.59	-1.59	-1.59	-1.59	-1.59	-1.59	-1.59	-1.59
-1.58	-1.58	-1.58	-1.58	-1.58	-1.58	-1.58	-1.58	-1.58
-1.57	-1.57	-1.57	-1.57	-1.57	-1.57	-1.57	-1.57	-1.57
-1.56	-1.56	-1.56	-1.56	-1.56	-1.56	-1.56	-1.56	-1.56
-1.55	-1.55	-1.55	-1.55	-1.55	-1.55	-1.55	-1.55	-1.55
-1.54	-1.54	-1.54	-1.54	-1.54	-1.54	-1.54	-1.54	-1.54
-1.53	-1.53	-1.53	-1.53	-1.53	-1.53	-1.53	-1.53	-1.53
-1.52	-1.52	-1.52	-1.52	-1.52	-1.52	-1.52	-1.52	-1.52
-1.51	-1.51	-1.51	-1.51	-1.51	-1.51	-1.51	-1.51	-1.51

-1.5	-1.5	-1.5	-1.5	-1.5	-1.5	-1.5	-1.5	-1.5
-1.49	-1.49	-1.49	-1.49	-1.49	-1.49	-1.49	-1.49	-1.49
-1.48	-1.48	-1.48	-1.48	-1.48	-1.48	-1.48	-1.48	-1.48
-1.47	-1.47	-1.47	-1.47	-1.47	-1.47	-1.47	-1.47	-1.47
-1.46	-1.46	-1.46	-1.46	-1.46	-1.46	-1.46	-1.46	-1.46
-1.45	-1.45	-1.45	-1.45	-1.45	-1.45	-1.45	-1.45	-1.45
-1.44	-1.44	-1.44	-1.44	-1.44	-1.44	-1.44	-1.44	-1.44
-1.43	-1.43	-1.43	-1.43	-1.43	-1.43	-1.43	-1.43	-1.43
-1.42	-1.42	-1.42	-1.42	-1.42	-1.42	-1.42	-1.42	-1.42
-1.41	-1.41	-1.41	-1.41	-1.41	-1.41	-1.41	-1.41	-1.41
-1.4	-1.4	-1.4	-1.4	-1.4	-1.4	-1.4	-1.4	-1.4
-1.39	-1.39	-1.39	-1.39	-1.39	-1.39	-1.39	-1.39	-1.39
-1.38	-1.38	-1.38	-1.38	-1.38	-1.38	-1.38	-1.38	-1.38
-1.37	-1.37	-1.37	-1.37	-1.37	-1.37	-1.37	-1.37	-1.37
-1.36	-1.36	-1.36	-1.36	-1.36	-1.36	-1.36	-1.36	-1.36
-1.35	-1.35	-1.35	-1.35	-1.35	-1.35	-1.35	-1.35	-1.35
-1.34	-1.34	-1.34	-1.34	-1.34	-1.34	-1.34	-1.34	-1.34
-1.33	-1.33	-1.33	-1.33	-1.33	-1.33	-1.33	-1.33	-1.33
-1.32	-1.32	-1.32	-1.32	-1.32	-1.32	-1.32	-1.32	-1.32
-1.31	-1.31	-1.31	-1.31	-1.31	-1.31	-1.31	-1.31	-1.31
-1.3	-1.3	-1.3	-1.3	-1.3	-1.3	-1.3	-1.3	-1.3
-1.29	-1.29	-1.29	-1.29	-1.29	-1.29	-1.29	-1.29	-1.29
-1.28	-1.28	-1.28	-1.28	-1.28	-1.28	-1.28	-1.28	-1.28
-1.27	-1.27	-1.27	-1.27	-1.27	-1.27	-1.27	-1.27	-1.27
-1.26	-1.26	-1.26	-1.26	-1.26	-1.26	-1.26	-1.26	-1.26
-1.25	-1.25	-1.25	-1.25	-1.25	-1.25	-1.25	-1.25	-1.25
-1.24	-1.24	-1.24	-1.24	-1.24	-1.24	-1.24	-1.24	-1.24
-1.23	-1.23	-1.23	-1.23	-1.23	-1.23	-1.23	-1.23	-1.23
-1.22	-1.22	-1.22	-1.22	-1.22	-1.22	-1.22	-1.22	-1.22
-1.21	-1.21	-1.21	-1.21	-1.21	-1.21	-1.21	-1.21	-1.21
-1.2	-1.2	-1.2	-1.2	-1.2	-1.2	-1.2	-1.2	-1.2
-1.19	-1.19	-1.19	-1.19	-1.19	-1.19	-1.19	-1.19	-1.19
-1.18	-1.18	-1.18	-1.18	-1.18	-1.18	-1.18	-1.18	-1.18
-1.17	-1.17	-1.17	-1.17	-1.17	-1.17	-1.17	-1.17	-1.17
-1.16	-1.16	-1.16	-1.16	-1.16	-1.16	-1.16	-1.16	-1.16
-1.15	-1.15	-1.15	-1.15	-1.15	-1.15	-1.15	-1.15	-1.15
-1.14	-1.14	-1.14	-1.14	-1.14	-1.14	-1.14	-1.14	-1.14
-1.13	-1.13	-1.13	-1.13	-1.13	-1.13	-1.13	-1.13	-1.13
-1.12	-1.12	-1.12	-1.12	-1.12	-1.12	-1.12	-1.12	-1.12
-1.11	-1.11	-1.11	-1.11	-1.11	-1.11	-1.11	-1.11	-1.11
-1.1	-1.1	-1.1	-1.1	-1.1	-1.1	-1.1	-1.1	-1.1
-1.09	-1.09	-1.09	-1.09	-1.09	-1.09	-1.09	-1.09	-1.09
-1.08	-1.08	-1.08	-1.08	-1.08	-1.08	-1.08	-1.08	-1.08
-1.07	-1.07	-1.07	-1.07	-1.07	-1.07	-1.07	-1.07	-1.07
-1.06	-1.06	-1.06	-1.06	-1.06	-1.06	-1.06	-1.06	-1.06
-1.05	-1.05	-1.05	-1.05	-1.05	-1.05	-1.05	-1.05	-1.05
-1.04	-1.04	-1.04	-1.04	-1.04	-1.04	-1.04	-1.04	-1.04
-1.03	-1.03	-1.03	-1.03	-1.03	-1.03	-1.03	-1.03	-1.03
-1.02	-1.02	-1.02	-1.02	-1.02	-1.02	-1.02	-1.02	-1.02
-1.01	-1.01	-1.01	-1.01	-1.01	-1.01	-1.01	-1.01	-1.01

-1	-1	-1	-1	-1	-1	-1	-1	-1
-0.99	-0.99	-0.99	-0.99	-0.99	-0.99	-0.99	-0.99	-0.99
-0.98	-0.98	-0.98	-0.98	-0.98	-0.98	-0.98	-0.98	-0.98
-0.97	-0.97	-0.97	-0.97	-0.97	-0.97	-0.97	-0.97	-0.97
-0.96	-0.96	-0.96	-0.96	-0.96	-0.96	-0.96	-0.96	-0.96
-0.95	-0.95	-0.95	-0.95	-0.95	-0.95	-0.95	-0.95	-0.95
-0.94	-0.94	-0.94	-0.94	-0.94	-0.94	-0.94	-0.94	-0.94
-0.93	-0.93	-0.93	-0.93	-0.93	-0.93	-0.93	-0.93	-0.93
-0.92	-0.92	-0.92	-0.92	-0.92	-0.92	-0.92	-0.92	-0.92
-0.91	-0.91	-0.91	-0.91	-0.91	-0.91	-0.91	-0.91	-0.91
-0.9	-0.9	-0.9	-0.9	-0.9	-0.9	-0.9	-0.9	-0.9
-0.89	-0.89	-0.89	-0.89	-0.89	-0.89	-0.89	-0.89	-0.89
-0.88	-0.88	-0.88	-0.88	-0.88	-0.88	-0.88	-0.88	-0.88
-0.87	-0.87	-0.87	-0.87	-0.87	-0.87	-0.87	-0.87	-0.87
-0.86	-0.86	-0.86	-0.86	-0.86	-0.86	-0.86	-0.86	-0.86
-0.85	-0.85	-0.85	-0.85	-0.85	-0.85	-0.85	-0.85	-0.85
-0.84	-0.84	-0.84	-0.84	-0.84	-0.84	-0.84	-0.84	-0.84
-0.83	-0.83	-0.83	-0.83	-0.83	-0.83	-0.83	-0.83	-0.83
-0.82	-0.82	-0.82	-0.82	-0.82	-0.82	-0.82	-0.82	-0.82
-0.81	-0.81	-0.81	-0.81	-0.81	-0.81	-0.81	-0.81	-0.81
-0.8	-0.8	-0.8	-0.8	-0.8	-0.8	-0.8	-0.8	-0.8
-0.79	-0.79	-0.79	-0.79	-0.79	-0.79	-0.79	-0.79	-0.79
-0.78	-0.78	-0.78	-0.78	-0.78	-0.78	-0.78	-0.78	-0.78
-0.77	-0.77	-0.77	-0.77	-0.77	-0.77	-0.77	-0.77	-0.77
-0.76	-0.76	-0.76	-0.76	-0.76	-0.76	-0.76	-0.76	-0.76
-0.75	-0.75	-0.75	-0.75	-0.75	-0.75	-0.75	-0.75	-0.75
-0.74	-0.74	-0.74	-0.74	-0.74	-0.74	-0.74	-0.74	-0.74
-0.73	-0.73	-0.73	-0.73	-0.73	-0.73	-0.73	-0.73	-0.73
-0.72	-0.72	-0.72	-0.72	-0.72	-0.72	-0.72	-0.72	-0.72
-0.71	-0.71	-0.71	-0.71	-0.71	-0.71	-0.71	-0.71	-0.71
-0.7	-0.7	-0.7	-0.7	-0.7	-0.7	-0.7	-0.7	-0.7
-0.69	-0.69	-0.69	-0.69	-0.69	-0.69	-0.69	-0.69	-0.69
-0.68	-0.68	-0.68	-0.68	-0.68	-0.68	-0.68	-0.68	-0.68
-0.67	-0.67	-0.67	-0.67	-0.67	-0.67	-0.67	-0.67	-0.67
-0.66	-0.66	-0.66	-0.66	-0.66	-0.66	-0.66	-0.66	-0.66
-0.65	-0.65	-0.65	-0.65	-0.65	-0.65	-0.65	-0.65	-0.65
-0.64	-0.64	-0.64	-0.64	-0.64	-0.64	-0.64	-0.64	-0.64
-0.63	-0.63	-0.63	-0.63	-0.63	-0.63	-0.63	-0.63	-0.63
-0.62	-0.62	-0.62	-0.62	-0.62	-0.62	-0.62	-0.62	-0.62
-0.61	-0.61	-0.61	-0.61	-0.61	-0.61	-0.61	-0.61	-0.61
-0.6	-0.6	-0.6	-0.6	-0.6	-0.6	-0.6	-0.6	-0.6
-0.59	-0.59	-0.59	-0.59	-0.59	-0.59	-0.59	-0.59	-0.59
-0.58	-0.58	-0.58	-0.58	-0.58	-0.58	-0.58	-0.58	-0.58
-0.57	-0.57	-0.57	-0.57	-0.57	-0.57	-0.57	-0.57	-0.57
-0.56	-0.56	-0.56	-0.56	-0.56	-0.56	-0.56	-0.56	-0.56
-0.55	-0.55	-0.55	-0.55	-0.55	-0.55	-0.55	-0.55	-0.55
-0.54	-0.54	-0.54	-0.54	-0.54	-0.54	-0.54	-0.54	-0.54
-0.53	-0.53	-0.53	-0.53	-0.53	-0.53	-0.53	-0.53	-0.53
-0.52	-0.52	-0.52	-0.52	-0.52	-0.52	-0.52	-0.52	-0.52
-0.51	-0.51	-0.51	-0.51	-0.51	-0.51	-0.51	-0.51	-0.51

-0.5	-0.5	-0.5	-0.5	-0.5	-0.5	-0.5	-0.5	-0.5
-0.49	-0.49	-0.49	-0.49	-0.49	-0.49	-0.49	-0.49	-0.49
-0.48	-0.48	-0.48	-0.48	-0.48	-0.48	-0.48	-0.48	-0.48
-0.47	-0.47	-0.47	-0.47	-0.47	-0.47	-0.47	-0.47	-0.47
-0.46	-0.46	-0.46	-0.46	-0.46	-0.46	-0.46	-0.46	-0.46
-0.45	-0.45	-0.45	-0.45	-0.45	-0.45	-0.45	-0.45	-0.45
-0.44	-0.44	-0.44	-0.44	-0.44	-0.44	-0.44	-0.44	-0.44
-0.43	-0.43	-0.43	-0.43	-0.43	-0.43	-0.43	-0.43	-0.43
-0.42	-0.42	-0.42	-0.42	-0.42	-0.42	-0.42	-0.42	-0.42
-0.41	-0.41	-0.41	-0.41	-0.41	-0.41	-0.41	-0.41	-0.41
-0.4	-0.4	-0.4	-0.4	-0.4	-0.4	-0.4	-0.4	-0.4
-0.39	-0.39	-0.39	-0.39	-0.39	-0.39	-0.39	-0.39	-0.39
-0.38	-0.38	-0.38	-0.38	-0.38	-0.38	-0.38	-0.38	-0.38
-0.37	-0.37	-0.37	-0.37	-0.37	-0.37	-0.37	-0.37	-0.37
-0.36	-0.36	-0.36	-0.36	-0.36	-0.36	-0.36	-0.36	-0.36
-0.35	-0.35	-0.35	-0.35	-0.35	-0.35	-0.35	-0.35	-0.35
-0.34	-0.34	-0.34	-0.34	-0.34	-0.34	-0.34	-0.34	-0.34
-0.33	-0.33	-0.33	-0.33	-0.33	-0.33	-0.33	-0.33	-0.33
-0.32	-0.32	-0.32	-0.32	-0.32	-0.32	-0.32	-0.32	-0.32
-0.31	-0.31	-0.31	-0.31	-0.31	-0.31	-0.31	-0.31	-0.31
-0.3	-0.3	-0.3	-0.3	-0.3	-0.3	-0.3	-0.3	-0.3
-0.29	-0.29	-0.29	-0.29	-0.29	-0.29	-0.29	-0.29	-0.29
-0.28	-0.28	-0.28	-0.28	-0.28	-0.28	-0.28	-0.28	-0.28
-0.27	-0.27	-0.27	-0.27	-0.27	-0.27	-0.27	-0.27	-0.27
-0.26	-0.26	-0.26	-0.26	-0.26	-0.26	-0.26	-0.26	-0.26
-0.25	-0.25	-0.25	-0.25	-0.25	-0.25	-0.25	-0.25	-0.25
-0.24	-0.24	-0.24	-0.24	-0.24	-0.24	-0.24	-0.24	-0.24
-0.23	-0.23	-0.23	-0.23	-0.23	-0.23	-0.23	-0.23	-0.23
-0.22	-0.22	-0.22	-0.22	-0.22	-0.22	-0.22	-0.22	-0.22
-0.21	-0.21	-0.21	-0.21	-0.21	-0.21	-0.21	-0.21	-0.21
-0.2	-0.2	-0.2	-0.2	-0.2	-0.2	-0.2	-0.2	-0.2
-0.19	-0.19	-0.19	-0.19	-0.19	-0.19	-0.19	-0.19	-0.19
-0.18	-0.18	-0.18	-0.18	-0.18	-0.18	-0.18	-0.18	-0.18
-0.17	-0.17	-0.17	-0.17	-0.17	-0.17	-0.17	-0.17	-0.17
-0.16	-0.16	-0.16	-0.16	-0.16	-0.16	-0.16	-0.16	-0.16
-0.15	-0.15	-0.15	-0.15	-0.15	-0.15	-0.15	-0.15	-0.15
-0.14	-0.14	-0.14	-0.14	-0.14	-0.14	-0.14	-0.14	-0.14
-0.13	-0.13	-0.13	-0.13	-0.13	-0.13	-0.13	-0.13	-0.13
-0.12	-0.12	-0.12	-0.12	-0.12	-0.12	-0.12	-0.12	-0.12
-0.11	-0.11	-0.11	-0.11	-0.11	-0.11	-0.11	-0.11	-0.11
-0.1	-0.1	-0.1	-0.1	-0.1	-0.1	-0.1	-0.1	-0.1
-0.09	-0.09	-0.09	-0.09	-0.09	-0.09	-0.09	-0.09	-0.09
-0.08	-0.08	-0.08	-0.08	-0.08	-0.08	-0.08	-0.08	-0.08
-0.07	-0.07	-0.07	-0.07	-0.07	-0.07	-0.07	-0.07	-0.07
-0.06	-0.06	-0.06	-0.06	-0.06	-0.06	-0.06	-0.06	-0.06
-0.05	-0.05	-0.05	-0.05	-0.05	-0.05	-0.05	-0.05	-0.05
-0.04	-0.04	-0.04	-0.04	-0.04	-0.04	-0.04	-0.04	-0.04
-0.03	-0.03	-0.03	-0.03	-0.03	-0.03	-0.03	-0.03	-0.03
-0.02	-0.02	-0.02	-0.02	-0.02	-0.02	-0.02	-0.02	-0.02
-0.01	-0.01	-0.01	-0.01	-0.01	-0.01	-0.01	-0.01	-0.01

0	0	0	0	0	0	0	0	0
0.01	0.01	0.01	0.01	0.01	0.01	0.01	0.01	0.01
0.02	0.02	0.02	0.02	0.02	0.02	0.02	0.02	0.02
0.03	0.03	0.03	0.03	0.03	0.03	0.03	0.03	0.03
0.04	0.04	0.04	0.04	0.04	0.04	0.04	0.04	0.04
0.05	0.05	0.05	0.05	0.05	0.05	0.05	0.05	0.05
0.06	0.06	0.06	0.06	0.06	0.06	0.06	0.06	0.06
0.07	0.07	0.07	0.07	0.07	0.07	0.07	0.07	0.07
0.08	0.08	0.08	0.08	0.08	0.08	0.08	0.08	0.08
0.09	0.09	0.09	0.09	0.09	0.09	0.09	0.09	0.09
0.1	0.1	0.1	0.1	0.1	0.1	0.1	0.1	0.1
0.11	0.11	0.11	0.11	0.11	0.11	0.11	0.11	0.11
0.12	0.12	0.12	0.12	0.12	0.12	0.12	0.12	0.12
0.13	0.13	0.13	0.13	0.13	0.13	0.13	0.13	0.13
0.14	0.14	0.14	0.14	0.14	0.14	0.14	0.14	0.14
0.15	0.15	0.15	0.15	0.15	0.15	0.15	0.15	0.15
0.16	0.16	0.16	0.16	0.16	0.16	0.16	0.16	0.16
0.17	0.17	0.17	0.17	0.17	0.17	0.17	0.17	0.17
0.18	0.18	0.18	0.18	0.18	0.18	0.18	0.18	0.18
0.19	0.19	0.19	0.19	0.19	0.19	0.19	0.19	0.19
0.2	0.2	0.2	0.2	0.2	0.2	0.2	0.2	0.2
0.21	0.21	0.21	0.21	0.21	0.21	0.21	0.21	0.21
0.22	0.22	0.22	0.22	0.22	0.22	0.22	0.22	0.22
0.23	0.23	0.23	0.23	0.23	0.23	0.23	0.23	0.23
0.24	0.24	0.24	0.24	0.24	0.24	0.24	0.24	0.24
0.25	0.25	0.25	0.25	0.25	0.25	0.25	0.25	0.25
0.26	0.26	0.26	0.26	0.26	0.26	0.26	0.26	0.26
0.27	0.27	0.27	0.27	0.27	0.27	0.27	0.27	0.27
0.28	0.28	0.28	0.28	0.28	0.28	0.28	0.28	0.28
0.29	0.29	0.29	0.29	0.29	0.29	0.29	0.29	0.29
0.3	0.3	0.3	0.3	0.3	0.3	0.3	0.3	0.3
0.31	0.31	0.31	0.31	0.31	0.31	0.31	0.31	0.31
0.32	0.32	0.32	0.32	0.32	0.32	0.32	0.32	0.32
0.33	0.33	0.33	0.33	0.33	0.33	0.33	0.33	0.33
0.34	0.34	0.34	0.34	0.34	0.34	0.34	0.34	0.34
0.35	0.35	0.35	0.35	0.35	0.35	0.35	0.35	0.35
0.36	0.36	0.36	0.36	0.36	0.36	0.36	0.36	0.36
0.37	0.37	0.37	0.37	0.37	0.37	0.37	0.37	0.37
0.38	0.38	0.38	0.38	0.38	0.38	0.38	0.38	0.38
0.39	0.39	0.39	0.39	0.39	0.39	0.39	0.39	0.39
0.4	0.4	0.4	0.4	0.4	0.4	0.4	0.4	0.4
0.41	0.41	0.41	0.41	0.41	0.41	0.41	0.41	0.41
0.42	0.42	0.42	0.42	0.42	0.42	0.42	0.42	0.42
0.43	0.43	0.43	0.43	0.43	0.43	0.43	0.43	0.43
0.44	0.44	0.44	0.44	0.44	0.44	0.44	0.44	0.44
0.45	0.45	0.45	0.45	0.45	0.45	0.45	0.45	0.45
0.46	0.46	0.46	0.46	0.46	0.46	0.46	0.46	0.46
0.47	0.47	0.47	0.47	0.47	0.47	0.47	0.47	0.47
0.48	0.48	0.48	0.48	0.48	0.48	0.48	0.48	0.48
0.49	0.49	0.49	0.49	0.49	0.49	0.49	0.49	0.49

0.5	0.5	0.5	0.5	0.5	0.5	0.5	0.5	0.5
0.51	0.51	0.51	0.51	0.51	0.51	0.51	0.51	0.51
0.52	0.52	0.52	0.52	0.52	0.52	0.52	0.52	0.52
0.53	0.53	0.53	0.53	0.53	0.53	0.53	0.53	0.53
0.54	0.54	0.54	0.54	0.54	0.54	0.54	0.54	0.54
0.55	0.55	0.55	0.55	0.55	0.55	0.55	0.55	0.55
0.56	0.56	0.56	0.56	0.56	0.56	0.56	0.56	0.56
0.57	0.57	0.57	0.57	0.57	0.57	0.57	0.57	0.57
0.58	0.58	0.58	0.58	0.58	0.58	0.58	0.58	0.58
0.59	0.59	0.59	0.59	0.59	0.59	0.59	0.59	0.59
0.6	0.6	0.6	0.6	0.6	0.6	0.6	0.6	0.6
0.61	0.61	0.61	0.61	0.61	0.61	0.61	0.61	0.61
0.62	0.62	0.62	0.62	0.62	0.62	0.62	0.62	0.62
0.63	0.63	0.63	0.63	0.63	0.63	0.63	0.63	0.63
0.64	0.64	0.64	0.64	0.64	0.64	0.64	0.64	0.64
0.65	0.65	0.65	0.65	0.65	0.65	0.65	0.65	0.65
0.66	0.66	0.66	0.66	0.66	0.66	0.66	0.66	0.66
0.67	0.67	0.67	0.67	0.67	0.67	0.67	0.67	0.67
0.68	0.68	0.68	0.68	0.68	0.68	0.68	0.68	0.68
0.69	0.69	0.69	0.69	0.69	0.69	0.69	0.69	0.69
0.7	0.7	0.7	0.7	0.7	0.7	0.7	0.7	0.7
0.71	0.71	0.71	0.71	0.71	0.71	0.71	0.71	0.71
0.72	0.72	0.72	0.72	0.72	0.72	0.72	0.72	0.72
0.73	0.73	0.73	0.73	0.73	0.73	0.73	0.73	0.73
0.74	0.74	0.74	0.74	0.74	0.74	0.74	0.74	0.74
0.75	0.75	0.75	0.75	0.75	0.75	0.75	0.75	0.75
0.76	0.76	0.76	0.76	0.76	0.76	0.76	0.76	0.76
0.77	0.77	0.77	0.77	0.77	0.77	0.77	0.77	0.77
0.78	0.78	0.78	0.78	0.78	0.78	0.78	0.78	0.78
0.79	0.79	0.79	0.79	0.79	0.79	0.79	0.79	0.79
0.8	0.8	0.8	0.8	0.8	0.8	0.8	0.8	0.8
0.81	0.81	0.81	0.81	0.81	0.81	0.81	0.81	0.81
0.82	0.82	0.82	0.82	0.82	0.82	0.82	0.82	0.82
0.83	0.83	0.83	0.83	0.83	0.83	0.83	0.83	0.83
0.84	0.84	0.84	0.84	0.84	0.84	0.84	0.84	0.84
0.85	0.85	0.85	0.85	0.85	0.85	0.85	0.85	0.85
0.86	0.86	0.86	0.86	0.86	0.86	0.86	0.86	0.86
0.87	0.87	0.87	0.87	0.87	0.87	0.87	0.87	0.87
0.88	0.88	0.88	0.88	0.88	0.88	0.88	0.88	0.88
0.89	0.89	0.89	0.89	0.89	0.89	0.89	0.89	0.89
0.9	0.9	0.9	0.9	0.9	0.9	0.9	0.9	0.9
0.91	0.91	0.91	0.91	0.91	0.91	0.91	0.91	0.91
0.92	0.92	0.92	0.92	0.92	0.92	0.92	0.92	0.92
0.93	0.93	0.93	0.93	0.93	0.93	0.93	0.93	0.93
0.94	0.94	0.94	0.94	0.94	0.94	0.94	0.94	0.94
0.95	0.95	0.95	0.95	0.95	0.95	0.95	0.95	0.95
0.96	0.96	0.96	0.96	0.96	0.96	0.96	0.96	0.96
0.97	0.97	0.97	0.97	0.97	0.97	0.97	0.97	0.97
0.98	0.98	0.98	0.98	0.98	0.98	0.98	0.98	0.98
0.99	0.99	0.99	0.99	0.99	0.99	0.99	0.99	0.99

1	1	1	1	1	1	1	1	1
1.01	1.01	1.01	1.01	1.01	1.01	1.01	1.01	1.01
1.02	1.02	1.02	1.02	1.02	1.02	1.02	1.02	1.02
1.03	1.03	1.03	1.03	1.03	1.03	1.03	1.03	1.03
1.04	1.04	1.04	1.04	1.04	1.04	1.04	1.04	1.04
1.05	1.05	1.05	1.05	1.05	1.05	1.05	1.05	1.05
1.06	1.06	1.06	1.06	1.06	1.06	1.06	1.06	1.06
1.07	1.07	1.07	1.07	1.07	1.07	1.07	1.07	1.07
1.08	1.08	1.08	1.08	1.08	1.08	1.08	1.08	1.08
1.09	1.09	1.09	1.09	1.09	1.09	1.09	1.09	1.09
1.1	1.1	1.1	1.1	1.1	1.1	1.1	1.1	1.1
1.11	1.11	1.11	1.11	1.11	1.11	1.11	1.11	1.11
1.12	1.12	1.12	1.12	1.12	1.12	1.12	1.12	1.12
1.13	1.13	1.13	1.13	1.13	1.13	1.13	1.13	1.13
1.14	1.14	1.14	1.14	1.14	1.14	1.14	1.14	1.14
1.15	1.15	1.15	1.15	1.15	1.15	1.15	1.15	1.15
1.16	1.16	1.16	1.16	1.16	1.16	1.16	1.16	1.16
1.17	1.17	1.17	1.17	1.17	1.17	1.17	1.17	1.17
1.18	1.18	1.18	1.18	1.18	1.18	1.18	1.18	1.18
1.19	1.19	1.19	1.19	1.19	1.19	1.19	1.19	1.19
1.2	1.2	1.2	1.2	1.2	1.2	1.2	1.2	1.2
1.21	1.21	1.21	1.21	1.21	1.21	1.21	1.21	1.21
1.22	1.22	1.22	1.22	1.22	1.22	1.22	1.22	1.22
1.23	1.23	1.23	1.23	1.23	1.23	1.23	1.23	1.23
1.24	1.24	1.24	1.24	1.24	1.24	1.24	1.24	1.24
1.25	1.25	1.25	1.25	1.25	1.25	1.25	1.25	1.25
1.26	1.26	1.26	1.26	1.26	1.26	1.26	1.26	1.26
1.27	1.27	1.27	1.27	1.27	1.27	1.27	1.27	1.27
1.28	1.28	1.28	1.28	1.28	1.28	1.28	1.28	1.28
1.29	1.29	1.29	1.29	1.29	1.29	1.29	1.29	1.29
1.3	1.3	1.3	1.3	1.3	1.3	1.3	1.3	1.3
1.31	1.31	1.31	1.31	1.31	1.31	1.31	1.31	1.31
1.32	1.32	1.32	1.32	1.32	1.32	1.32	1.32	1.32
1.33	1.33	1.33	1.33	1.33	1.33	1.33	1.33	1.33
1.34	1.34	1.34	1.34	1.34	1.34	1.34	1.34	1.34
1.35	1.35	1.35	1.35	1.35	1.35	1.35	1.35	1.35
1.36	1.36	1.36	1.36	1.36	1.36	1.36	1.36	1.36
1.37	1.37	1.37	1.37	1.37	1.37	1.37	1.37	1.37
1.38	1.38	1.38	1.38	1.38	1.38	1.38	1.38	1.38
1.39	1.39	1.39	1.39	1.39	1.39	1.39	1.39	1.39
1.4	1.4	1.4	1.4	1.4	1.4	1.4	1.4	1.4
1.41	1.41	1.41	1.41	1.41	1.41	1.41	1.41	1.41
1.42	1.42	1.42	1.42	1.42	1.42	1.42	1.42	1.42
1.43	1.43	1.43	1.43	1.43	1.43	1.43	1.43	1.43
1.44	1.44	1.44	1.44	1.44	1.44	1.44	1.44	1.44
1.45	1.45	1.45	1.45	1.45	1.45	1.45	1.45	1.45
1.46	1.46	1.46	1.46	1.46	1.46	1.46	1.46	1.46
1.47	1.47	1.47	1.47	1.47	1.47	1.47	1.47	1.47
1.48	1.48	1.48	1.48	1.48	1.48	1.48	1.48	1.48
1.49	1.49	1.49	1.49	1.49	1.49	1.49	1.49	1.49

1.5	1.5	1.5	1.5	1.5	1.5	1.5	1.5	1.5
1.51	1.51	1.51	1.51	1.51	1.51	1.51	1.51	1.51
1.52	1.52	1.52	1.52	1.52	1.52	1.52	1.52	1.52
1.53	1.53	1.53	1.53	1.53	1.53	1.53	1.53	1.53
1.54	1.54	1.54	1.54	1.54	1.54	1.54	1.54	1.54
1.55	1.55	1.55	1.55	1.55	1.55	1.55	1.55	1.55
1.56	1.56	1.56	1.56	1.56	1.56	1.56	1.56	1.56
1.57	1.57	1.57	1.57	1.57	1.57	1.57	1.57	1.57
1.58	1.58	1.58	1.58	1.58	1.58	1.58	1.58	1.58
1.59	1.59	1.59	1.59	1.59	1.59	1.59	1.59	1.59
1.6	1.6	1.6	1.6	1.6	1.6	1.6	1.6	1.6
1.61	1.61	1.61	1.61	1.61	1.61	1.61	1.61	1.61
1.62	1.62	1.62	1.62	1.62	1.62	1.62	1.62	1.62
1.63	1.63	1.63	1.63	1.63	1.63	1.63	1.63	1.63
1.64	1.64	1.64	1.64	1.64	1.64	1.64	1.64	1.64
1.65	1.65	1.65	1.65	1.65	1.65	1.65	1.65	1.65
1.66	1.66	1.66	1.66	1.66	1.66	1.66	1.66	1.66
1.67	1.67	1.67	1.67	1.67	1.67	1.67	1.67	1.67
1.68	1.68	1.68	1.68	1.68	1.68	1.68	1.68	1.68
1.69	1.69	1.69	1.69	1.69	1.69	1.69	1.69	1.69
1.7	1.7	1.7	1.7	1.7	1.7	1.7	1.7	1.7
1.71	1.71	1.71	1.71	1.71	1.71	1.71	1.71	1.71
1.72	1.72	1.72	1.72	1.72	1.72	1.72	1.72	1.72
1.73	1.73	1.73	1.73	1.73	1.73	1.73	1.73	1.73
1.74	1.74	1.74	1.74	1.74	1.74	1.74	1.74	1.74
1.75	1.75	1.75	1.75	1.75	1.75	1.75	1.75	1.75
1.76	1.76	1.76	1.76	1.76	1.76	1.76	1.76	1.76
1.77	1.77	1.77	1.77	1.77	1.77	1.77	1.77	1.77
1.78	1.78	1.78	1.78	1.78	1.78	1.78	1.78	1.78
1.79	1.79	1.79	1.79	1.79	1.79	1.79	1.79	1.79
1.8	1.8	1.8	1.8	1.8	1.8	1.8	1.8	1.8
1.81	1.81	1.81	1.81	1.81	1.81	1.81	1.81	1.81
1.82	1.82	1.82	1.82	1.82	1.82	1.82	1.82	1.82
1.83	1.83	1.83	1.83	1.83	1.83	1.83	1.83	1.83
1.84	1.84	1.84	1.84	1.84	1.84	1.84	1.84	1.84
1.85	1.85	1.85	1.85	1.85	1.85	1.85	1.85	1.85
1.86	1.86	1.86	1.86	1.86	1.86	1.86	1.86	1.86
1.87	1.87	1.87	1.87	1.87	1.87	1.87	1.87	1.87
1.88	1.88	1.88	1.88	1.88	1.88	1.88	1.88	1.88
1.89	1.89	1.89	1.89	1.89	1.89	1.89	1.89	1.89
1.9	1.9	1.9	1.9	1.9	1.9	1.9	1.9	1.9
1.91	1.91	1.91	1.91	1.91	1.91	1.91	1.91	1.91
1.92	1.92	1.92	1.92	1.92	1.92	1.92	1.92	1.92
1.93	1.93	1.93	1.93	1.93	1.93	1.93	1.93	1.93
1.94	1.94	1.94	1.94	1.94	1.94	1.94	1.94	1.94
1.95	1.95	1.95	1.95	1.95	1.95	1.95	1.95	1.95
1.96	1.96	1.96	1.96	1.96	1.96	1.96	1.96	1.96
1.97	1.97	1.97	1.97	1.97	1.97	1.97	1.97	1.97
1.98	1.98	1.98	1.98	1.98	1.98	1.98	1.98	1.98
1.99	1.99	1.99	1.99	1.99	1.99	1.99	1.99	1.99

2	2	2	2	2	2	2	2	2
2.01	2.01	2.01	2.01	2.01	2.01	2.01	2.01	2.01
2.02	2.02	2.02	2.02	2.02	2.02	2.02	2.02	2.02
2.03	2.03	2.03	2.03	2.03	2.03	2.03	2.03	2.03
2.04	2.04	2.04	2.04	2.04	2.04	2.04	2.04	2.04
2.05	2.05	2.05	2.05	2.05	2.05	2.05	2.05	2.05
2.06	2.06	2.06	2.06	2.06	2.06	2.06	2.06	2.06
2.07	2.07	2.07	2.07	2.07	2.07	2.07	2.07	2.07
2.08	2.08	2.08	2.08	2.08	2.08	2.08	2.08	2.08
2.09	2.09	2.09	2.09	2.09	2.09	2.09	2.09	2.09
2.1	2.1	2.1	2.1	2.1	2.1	2.1	2.1	2.1
2.11	2.11	2.11	2.11	2.11	2.11	2.11	2.11	2.11
2.12	2.12	2.12	2.12	2.12	2.12	2.12	2.12	2.12
2.13	2.13	2.13	2.13	2.13	2.13	2.13	2.13	2.13
2.14	2.14	2.14	2.14	2.14	2.14	2.14	2.14	2.14
2.15	2.15	2.15	2.15	2.15	2.15	2.15	2.15	2.15
2.16	2.16	2.16	2.16	2.16	2.16	2.16	2.16	2.16
2.17	2.17	2.17	2.17	2.17	2.17	2.17	2.17	2.17
2.18	2.18	2.18	2.18	2.18	2.18	2.18	2.18	2.18
2.19	2.19	2.19	2.19	2.19	2.19	2.19	2.19	2.19
2.2	2.2	2.2	2.2	2.2	2.2	2.2	2.2	2.2
2.21	2.21	2.21	2.21	2.21	2.21	2.21	2.21	2.21
2.22	2.22	2.22	2.22	2.22	2.22	2.22	2.22	2.22
2.23	2.23	2.23	2.23	2.23	2.23	2.23	2.23	2.23
2.24	2.24	2.24	2.24	2.24	2.24	2.24	2.24	2.24
2.25	2.25	2.25	2.25	2.25	2.25	2.25	2.25	2.25
2.26	2.26	2.26	2.26	2.26	2.26	2.26	2.26	2.26
2.27	2.27	2.27	2.27	2.27	2.27	2.27	2.27	2.27
2.28	2.28	2.28	2.28	2.28	2.28	2.28	2.28	2.28
2.29	2.29	2.29	2.29	2.29	2.29	2.29	2.29	2.29
2.3	2.3	2.3	2.3	2.3	2.3	2.3	2.3	2.3
2.31	2.31	2.31	2.31	2.31	2.31	2.31	2.31	2.31
2.32	2.32	2.32	2.32	2.32	2.32	2.32	2.32	2.32
2.33	2.33	2.33	2.33	2.33	2.33	2.33	2.33	2.33
2.34	2.34	2.34	2.34	2.34	2.34	2.34	2.34	2.34
2.35	2.35	2.35	2.35	2.35	2.35	2.35	2.35	2.35
2.36	2.36	2.36	2.36	2.36	2.36	2.36	2.36	2.36
2.37	2.37	2.37	2.37	2.37	2.37	2.37	2.37	2.37
2.38	2.38	2.38	2.38	2.38	2.38	2.38	2.38	2.38
2.39	2.39	2.39	2.39	2.39	2.39	2.39	2.39	2.39
2.4	2.4	2.4	2.4	2.4	2.4	2.4	2.4	2.4
2.41	2.41	2.41	2.41	2.41	2.41	2.41	2.41	2.41
2.42	2.42	2.42	2.42	2.42	2.42	2.42	2.42	2.42
2.43	2.43	2.43	2.43	2.43	2.43	2.43	2.43	2.43
2.44	2.44	2.44	2.44	2.44	2.44	2.44	2.44	2.44
2.45	2.45	2.45	2.45	2.45	2.45	2.45	2.45	2.45
2.46	2.46	2.46	2.46	2.46	2.46	2.46	2.46	2.46
2.47	2.47	2.47	2.47	2.47	2.47	2.47	2.47	2.47
2.48	2.48	2.48	2.48	2.48	2.48	2.48	2.48	2.48
2.49	2.49	2.49	2.49	2.49	2.49	2.49	2.49	2.49

2.5	2.5	2.5	2.5	2.5	2.5	2.5	2.5	2.5
2.51	2.51	2.51	2.51	2.51	2.51	2.51	2.51	2.51
2.52	2.52	2.52	2.52	2.52	2.52	2.52	2.52	2.52
2.53	2.53	2.53	2.53	2.53	2.53	2.53	2.53	2.53
2.54	2.54	2.54	2.54	2.54	2.54	2.54	2.54	2.54
2.55	2.55	2.55	2.55	2.55	2.55	2.55	2.55	2.55
2.56	2.56	2.56	2.56	2.56	2.56	2.56	2.56	2.56
2.57	2.57	2.57	2.57	2.57	2.57	2.57	2.57	2.57
2.58	2.58	2.58	2.58	2.58	2.58	2.58	2.58	2.58
2.59	2.59	2.59	2.59	2.59	2.59	2.59	2.59	2.59
2.6	2.6	2.6	2.6	2.6	2.6	2.6	2.6	2.6
2.61	2.61	2.61	2.61	2.61	2.61	2.61	2.61	2.61
2.62	2.62	2.62	2.62	2.62	2.62	2.62	2.62	2.62
2.63	2.63	2.63	2.63	2.63	2.63	2.63	2.63	2.63
2.64	2.64	2.64	2.64	2.64	2.64	2.64	2.64	2.64
2.65	2.65	2.65	2.65	2.65	2.65	2.65	2.65	2.65
2.66	2.66	2.66	2.66	2.66	2.66	2.66	2.66	2.66
2.67	2.67	2.67	2.67	2.67	2.67	2.67	2.67	2.67
2.68	2.68	2.68	2.68	2.68	2.68	2.68	2.68	2.68
2.69	2.69	2.69	2.69	2.69	2.69	2.69	2.69	2.69
2.7	2.7	2.7	2.7	2.7	2.7	2.7	2.7	2.7
2.71	2.71	2.71	2.71	2.71	2.71	2.71	2.71	2.71
2.72	2.72	2.72	2.72	2.72	2.72	2.72	2.72	2.72
2.73	2.73	2.73	2.73	2.73	2.73	2.73	2.73	2.73
2.74	2.74	2.74	2.74	2.74	2.74	2.74	2.74	2.74
2.75	2.75	2.75	2.75	2.75	2.75	2.75	2.75	2.75
2.76	2.76	2.76	2.76	2.76	2.76	2.76	2.76	2.76
2.77	2.77	2.77	2.77	2.77	2.77	2.77	2.77	2.77
2.78	2.78	2.78	2.78	2.78	2.78	2.78	2.78	2.78
2.79	2.79	2.79	2.79	2.79	2.79	2.79	2.79	2.79
2.8	2.8	2.8	2.8	2.8	2.8	2.8	2.8	2.8
2.81	2.81	2.81	2.81	2.81	2.81	2.81	2.81	2.81
2.82	2.82	2.82	2.82	2.82	2.82	2.82	2.82	2.82
2.83	2.83	2.83	2.83	2.83	2.83	2.83	2.83	2.83
2.84	2.84	2.84	2.84	2.84	2.84	2.84	2.84	2.84
2.85	2.85	2.85	2.85	2.85	2.85	2.85	2.85	2.85
2.86	2.86	2.86	2.86	2.86	2.86	2.86	2.86	2.86
2.87	2.87	2.87	2.87	2.87	2.87	2.87	2.87	2.87
2.88	2.88	2.88	2.88	2.88	2.88	2.88	2.88	2.88
2.89	2.89	2.89	2.89	2.89	2.89	2.89	2.89	2.89
2.9	2.9	2.9	2.9	2.9	2.9	2.9	2.9	2.9
2.91	2.91	2.91	2.91	2.91	2.91	2.91	2.91	2.91
2.92	2.92	2.92	2.92	2.92	2.92	2.92	2.92	2.92
2.93	2.93	2.93	2.93	2.93	2.93	2.93	2.93	2.93
2.94	2.94	2.94	2.94	2.94	2.94	2.94	2.94	2.94
2.95	2.95	2.95	2.95	2.95	2.95	2.95	2.95	2.95
2.96	2.96	2.96	2.96	2.96	2.96	2.96	2.96	2.96
2.97	2.97	2.97	2.97	2.97	2.97	2.97	2.97	2.97
2.98	2.98	2.98	2.98	2.98	2.98	2.98	2.98	2.98
2.99	2.99	2.99	2.99	2.99	2.99	2.99	2.99	2.99

3	3	3	3	3	3	3	3	3
3.01	3.01	3.01	3.01	3.01	3.01	3.01	3.01	3.01
3.02	3.02	3.02	3.02	3.02	3.02	3.02	3.02	3.02
3.03	3.03	3.03	3.03	3.03	3.03	3.03	3.03	3.03
3.04	3.04	3.04	3.04	3.04	3.04	3.04	3.04	3.04
3.05	3.05	3.05	3.05	3.05	3.05	3.05	3.05	3.05
3.06	3.06	3.06	3.06	3.06	3.06	3.06	3.06	3.06
3.07	3.07	3.07	3.07	3.07	3.07	3.07	3.07	3.07
3.08	3.08	3.08	3.08	3.08	3.08	3.08	3.08	3.08
3.09	3.09	3.09	3.09	3.09	3.09	3.09	3.09	3.09
3.1	3.1	3.1	3.1	3.1	3.1	3.1	3.1	3.1
3.11	3.11	3.11	3.11	3.11	3.11	3.11	3.11	3.11
3.12	3.12	3.12	3.12	3.12	3.12	3.12	3.12	3.12
3.13	3.13	3.13	3.13	3.13	3.13	3.13	3.13	3.13
3.14	3.14	3.14	3.14	3.14	3.14	3.14	3.14	3.14
3.15	3.15	3.15	3.15	3.15	3.15	3.15	3.15	3.15
3.16	3.16	3.16	3.16	3.16	3.16	3.16	3.16	3.16
3.17	3.17	3.17	3.17	3.17	3.17	3.17	3.17	3.17
3.18	3.18	3.18	3.18	3.18	3.18	3.18	3.18	3.18
3.19	3.19	3.19	3.19	3.19	3.19	3.19	3.19	3.19
3.2	3.2	3.2	3.2	3.2	3.2	3.2	3.2	3.2
3.21	3.21	3.21	3.21	3.21	3.21	3.21	3.21	3.21
3.22	3.22	3.22	3.22	3.22	3.22	3.22	3.22	3.22
3.23	3.23	3.23	3.23	3.23	3.23	3.23	3.23	3.23
3.24	3.24	3.24	3.24	3.24	3.24	3.24	3.24	3.24
3.25	3.25	3.25	3.25	3.25	3.25	3.25	3.25	3.25
3.26	3.26	3.26	3.26	3.26	3.26	3.26	3.26	3.26
3.27	3.27	3.27	3.27	3.27	3.27	3.27	3.27	3.27
3.28	3.28	3.28	3.28	3.28	3.28	3.28	3.28	3.28
3.29	3.29	3.29	3.29	3.29	3.29	3.29	3.29	3.29
3.3	3.3	3.3	3.3	3.3	3.3	3.3	3.3	3.3
3.31	3.31	3.31	3.31	3.31	3.31	3.31	3.31	3.31
3.32	3.32	3.32	3.32	3.32	3.32	3.32	3.32	3.32
3.33	3.33	3.33	3.33	3.33	3.33	3.33	3.33	3.33
3.34	3.34	3.34	3.34	3.34	3.34	3.34	3.34	3.34
3.35	3.35	3.35	3.35	3.35	3.35	3.35	3.35	3.35
3.36	3.36	3.36	3.36	3.36	3.36	3.36	3.36	3.36
3.37	3.37	3.37	3.37	3.37	3.37	3.37	3.37	3.37
3.38	3.38	3.38	3.38	3.38	3.38	3.38	3.38	3.38
3.39	3.39	3.39	3.39	3.39	3.39	3.39	3.39	3.39
3.4	3.4	3.4	3.4	3.4	3.4	3.4	3.4	3.4
3.41	3.41	3.41	3.41	3.41	3.41	3.41	3.41	3.41
3.42	3.42	3.42	3.42	3.42	3.42	3.42	3.42	3.42
3.43	3.43	3.43	3.43	3.43	3.43	3.43	3.43	3.43
3.44	3.44	3.44	3.44	3.44	3.44	3.44	3.44	3.44
3.45	3.45	3.45	3.45	3.45	3.45	3.45	3.45	3.45
3.46	3.46	3.46	3.46	3.46	3.46	3.46	3.46	3.46
3.47	3.47	3.47	3.47	3.47	3.47	3.47	3.47	3.47
3.48	3.48	3.48	3.48	3.48	3.48	3.48	3.48	3.48
3.49	3.49	3.49	3.49	3.49	3.49	3.49	3.49	3.49

3.5	3.5	3.5	3.5	3.5	3.5	3.5	3.5	3.5
3.51	3.51	3.51	3.51	3.51	3.51	3.51	3.51	3.51
3.52	3.52	3.52	3.52	3.52	3.52	3.52	3.52	3.52
3.53	3.53	3.53	3.53	3.53	3.53	3.53	3.53	3.53
3.54	3.54	3.54	3.54	3.54	3.54	3.54	3.54	3.54
3.55	3.55	3.55	3.55	3.55	3.55	3.55	3.55	3.55
3.56	3.56	3.56	3.56	3.56	3.56	3.56	3.56	3.56
3.57	3.57	3.57	3.57	3.57	3.57	3.57	3.57	3.57
3.58	3.58	3.58	3.58	3.58	3.58	3.58	3.58	3.58
3.59	3.59	3.59	3.59	3.59	3.59	3.59	3.59	3.59
3.6	3.6	3.6	3.6	3.6	3.6	3.6	3.6	3.6
3.61	3.61	3.61	3.61	3.61	3.61	3.61	3.61	3.61
3.62	3.62	3.62	3.62	3.62	3.62	3.62	3.62	3.62
3.63	3.63	3.63	3.63	3.63	3.63	3.63	3.63	3.63
3.64	3.64	3.64	3.64	3.64	3.64	3.64	3.64	3.64
3.65	3.65	3.65	3.65	3.65	3.65	3.65	3.65	3.65
3.66	3.66	3.66	3.66	3.66	3.66	3.66	3.66	3.66
3.67	3.67	3.67	3.67	3.67	3.67	3.67	3.67	3.67
3.68	3.68	3.68	3.68	3.68	3.68	3.68	3.68	3.68
3.69	3.69	3.69	3.69	3.69	3.69	3.69	3.69	3.69
3.7	3.7	3.7	3.7	3.7	3.7	3.7	3.7	3.7
3.71	3.71	3.71	3.71	3.71	3.71	3.71	3.71	3.71
3.72	3.72	3.72	3.72	3.72	3.72	3.72	3.72	3.72
3.73	3.73	3.73	3.73	3.73	3.73	3.73	3.73	3.73
3.74	3.74	3.74	3.74	3.74	3.74	3.74	3.74	3.74
3.75	3.75	3.75	3.75	3.75	3.75	3.75	3.75	3.75
3.76	3.76	3.76	3.76	3.76	3.76	3.76	3.76	3.76
3.77	3.77	3.77	3.77	3.77	3.77	3.77	3.77	3.77
3.78	3.78	3.78	3.78	3.78	3.78	3.78	3.78	3.78
3.79	3.79	3.79	3.79	3.79	3.79	3.79	3.79	3.79
3.8	3.8	3.8	3.8	3.8	3.8	3.8	3.8	3.8
3.81	3.81	3.81	3.81	3.81	3.81	3.81	3.81	3.81
3.82	3.82	3.82	3.82	3.82	3.82	3.82	3.82	3.82
3.83	3.83	3.83	3.83	3.83	3.83	3.83	3.83	3.83
3.84	3.84	3.84	3.84	3.84	3.84	3.84	3.84	3.84
3.85	3.85	3.85	3.85	3.85	3.85	3.85	3.85	3.85
3.86	3.86	3.86	3.86	3.86	3.86	3.86	3.86	3.86
3.87	3.87	3.87	3.87	3.87	3.87	3.87	3.87	3.87
3.88	3.88	3.88	3.88	3.88	3.88	3.88	3.88	3.88
3.89	3.89	3.89	3.89	3.89	3.89	3.89	3.89	3.89
3.9	3.9	3.9	3.9	3.9	3.9	3.9	3.9	3.9
3.91	3.91	3.91	3.91	3.91	3.91	3.91	3.91	3.91
3.92	3.92	3.92	3.92	3.92	3.92	3.92	3.92	3.92
3.93	3.93	3.93	3.93	3.93	3.93	3.93	3.93	3.93
3.94	3.94	3.94	3.94	3.94	3.94	3.94	3.94	3.94
3.95	3.95	3.95	3.95	3.95	3.95	3.95	3.95	3.95
3.96	3.96	3.96	3.96	3.96	3.96	3.96	3.96	3.96
3.97	3.97	3.97	3.97	3.97	3.97	3.97	3.97	3.97
3.98	3.98	3.98	3.98	3.98	3.98	3.98	3.98	3.98
3.99	3.99	3.99	3.99	3.99	3.99	3.99	3.99	3.99

4	4	4	4	4	4	4	4	4
4.01	4.01	4.01	4.01	4.01	4.01	4.01	4.01	4.01
4.02	4.02	4.02	4.02	4.02	4.02	4.02	4.02	4.02
4.03	4.03	4.03	4.03	4.03	4.03	4.03	4.03	4.03
4.04	4.04	4.04	4.04	4.04	4.04	4.04	4.04	4.04
4.05	4.05	4.05	4.05	4.05	4.05	4.05	4.05	4.05
4.06	4.06	4.06	4.06	4.06	4.06	4.06	4.06	4.06
4.07	4.07	4.07	4.07	4.07	4.07	4.07	4.07	4.07
4.08	4.08	4.08	4.08	4.08	4.08	4.08	4.08	4.08
4.09	4.09	4.09	4.09	4.09	4.09	4.09	4.09	4.09
4.1	4.1	4.1	4.1	4.1	4.1	4.1	4.1	4.1
4.11	4.11	4.11	4.11	4.11	4.11	4.11	4.11	4.11
4.12	4.12	4.12	4.12	4.12	4.12	4.12	4.12	4.12
4.13	4.13	4.13	4.13	4.13	4.13	4.13	4.13	4.13
4.14	4.14	4.14	4.14	4.14	4.14	4.14	4.14	4.14
4.15	4.15	4.15	4.15	4.15	4.15	4.15	4.15	4.15
4.16	4.16	4.16	4.16	4.16	4.16	4.16	4.16	4.16
4.17	4.17	4.17	4.17	4.17	4.17	4.17	4.17	4.17
4.18	4.18	4.18	4.18	4.18	4.18	4.18	4.18	4.18
4.19	4.19	4.19	4.19	4.19	4.19	4.19	4.19	4.19
4.2	4.2	4.2	4.2	4.2	4.2	4.2	4.2	4.2
4.21	4.21	4.21	4.21	4.21	4.21	4.21	4.21	4.21
4.22	4.22	4.22	4.22	4.22	4.22	4.22	4.22	4.22
4.23	4.23	4.23	4.23	4.23	4.23	4.23	4.23	4.23
4.24	4.24	4.24	4.24	4.24	4.24	4.24	4.24	4.24
4.25	4.25	4.25	4.25	4.25	4.25	4.25	4.25	4.25
4.26	4.26	4.26	4.26	4.26	4.26	4.26	4.26	4.26
4.27	4.27	4.27	4.27	4.27	4.27	4.27	4.27	4.27
4.28	4.28	4.28	4.28	4.28	4.28	4.28	4.28	4.28
4.29	4.29	4.29	4.29	4.29	4.29	4.29	4.29	4.29
4.3	4.3	4.3	4.3	4.3	4.3	4.3	4.3	4.3
4.31	4.31	4.31	4.31	4.31	4.31	4.31	4.31	4.31
4.32	4.32	4.32	4.32	4.32	4.32	4.32	4.32	4.32
4.33	4.33	4.33	4.33	4.33	4.33	4.33	4.33	4.33
4.34	4.34	4.34	4.34	4.34	4.34	4.34	4.34	4.34
4.35	4.35	4.35	4.35	4.35	4.35	4.35	4.35	4.35
4.36	4.36	4.36	4.36	4.36	4.36	4.36	4.36	4.36
4.37	4.37	4.37	4.37	4.37	4.37	4.37	4.37	4.37
4.38	4.38	4.38	4.38	4.38	4.38	4.38	4.38	4.38
4.39	4.39	4.39	4.39	4.39	4.39	4.39	4.39	4.39
4.4	4.4	4.4	4.4	4.4	4.4	4.4	4.4	4.4
4.41	4.41	4.41	4.41	4.41	4.41	4.41	4.41	4.41
4.42	4.42	4.42	4.42	4.42	4.42	4.42	4.42	4.42
4.43	4.43	4.43	4.43	4.43	4.43	4.43	4.43	4.43
4.44	4.44	4.44	4.44	4.44	4.44	4.44	4.44	4.44
4.45	4.45	4.45	4.45	4.45	4.45	4.45	4.45	4.45
4.46	4.46	4.46	4.46	4.46	4.46	4.46	4.46	4.46
4.47	4.47	4.47	4.47	4.47	4.47	4.47	4.47	4.47
4.48	4.48	4.48	4.48	4.48	4.48	4.48	4.48	4.48
4.49	4.49	4.49	4.49	4.49	4.49	4.49	4.49	4.49

4.5	4.5	4.5	4.5	4.5	4.5	4.5	4.5	4.5
4.51	4.51	4.51	4.51	4.51	4.51	4.51	4.51	4.51
4.52	4.52	4.52	4.52	4.52	4.52	4.52	4.52	4.52
4.53	4.53	4.53	4.53	4.53	4.53	4.53	4.53	4.53
4.54	4.54	4.54	4.54	4.54	4.54	4.54	4.54	4.54
4.55	4.55	4.55	4.55	4.55	4.55	4.55	4.55	4.55
4.56	4.56	4.56	4.56	4.56	4.56	4.56	4.56	4.56
4.57	4.57	4.57	4.57	4.57	4.57	4.57	4.57	4.57
4.58	4.58	4.58	4.58	4.58	4.58	4.58	4.58	4.58
4.59	4.59	4.59	4.59	4.59	4.59	4.59	4.59	4.59
4.6	4.6	4.6	4.6	4.6	4.6	4.6	4.6	4.6
4.61	4.61	4.61	4.61	4.61	4.61	4.61	4.61	4.61
4.62	4.62	4.62	4.62	4.62	4.62	4.62	4.62	4.62
4.63	4.63	4.63	4.63	4.63	4.63	4.63	4.63	4.63
4.64	4.64	4.64	4.64	4.64	4.64	4.64	4.64	4.64
4.65	4.65	4.65	4.65	4.65	4.65	4.65	4.65	4.65
4.66	4.66	4.66	4.66	4.66	4.66	4.66	4.66	4.66
4.67	4.67	4.67	4.67	4.67	4.67	4.67	4.67	4.67
4.68	4.68	4.68	4.68	4.68	4.68	4.68	4.68	4.68
4.69	4.69	4.69	4.69	4.69	4.69	4.69	4.69	4.69
4.7	4.7	4.7	4.7	4.7	4.7	4.7	4.7	4.7
4.71	4.71	4.71	4.71	4.71	4.71	4.71	4.71	4.71
4.72	4.72	4.72	4.72	4.72	4.72	4.72	4.72	4.72
4.73	4.73	4.73	4.73	4.73	4.73	4.73	4.73	4.73
4.74	4.74	4.74	4.74	4.74	4.74	4.74	4.74	4.74
4.75	4.75	4.75	4.75	4.75	4.75	4.75	4.75	4.75
4.76	4.76	4.76	4.76	4.76	4.76	4.76	4.76	4.76
4.77	4.77	4.77	4.77	4.77	4.77	4.77	4.77	4.77
4.78	4.78	4.78	4.78	4.78	4.78	4.78	4.78	4.78
4.79	4.79	4.79	4.79	4.79	4.79	4.79	4.79	4.79
4.8	4.8	4.8	4.8	4.8	4.8	4.8	4.8	4.8
4.81	4.81	4.81	4.81	4.81	4.81	4.81	4.81	4.81
4.82	4.82	4.82	4.82	4.82	4.82	4.82	4.82	4.82
4.83	4.83	4.83	4.83	4.83	4.83	4.83	4.83	4.83
4.84	4.84	4.84	4.84	4.84	4.84	4.84	4.84	4.84
4.85	4.85	4.85	4.85	4.85	4.85	4.85	4.85	4.85
4.86	4.86	4.86	4.86	4.86	4.86	4.86	4.86	4.86
4.87	4.87	4.87	4.87	4.87	4.87	4.87	4.87	4.87
4.88	4.88	4.88	4.88	4.88	4.88	4.88	4.88	4.88
4.89	4.89	4.89	4.89	4.89	4.89	4.89	4.89	4.89
4.9	4.9	4.9	4.9	4.9	4.9	4.9	4.9	4.9
4.91	4.91	4.91	4.91	4.91	4.91	4.91	4.91	4.91
4.92	4.92	4.92	4.92	4.92	4.92	4.92	4.92	4.92
4.93	4.93	4.93	4.93	4.93	4.93	4.93	4.93	4.93
4.94	4.94	4.94	4.94	4.94	4.94	4.94	4.94	4.94
4.95	4.95	4.95	4.95	4.95	4.95	4.95	4.95	4.95
4.96	4.96	4.96	4.96	4.96	4.96	4.96	4.96	4.96
4.97	4.97	4.97	4.97	4.97	4.97	4.97	4.97	4.97
4.98	4.98	4.98	4.98	4.98	4.98	4.98	4.98	4.98
4.99	4.99	4.99	4.99	4.99	4.99	4.99	4.99	4.99

5	5	5	5	5	5	5	5	5
5.01	5.01	5.01	5.01	5.01	5.01	5.01	5.01	5.01
5.02	5.02	5.02	5.02	5.02	5.02	5.02	5.02	5.02
5.03	5.03	5.03	5.03	5.03	5.03	5.03	5.03	5.03
5.04	5.04	5.04	5.04	5.04	5.04	5.04	5.04	5.04
5.05	5.05	5.05	5.05	5.05	5.05	5.05	5.05	5.05
5.06	5.06	5.06	5.06	5.06	5.06	5.06	5.06	5.06
5.07	5.07	5.07	5.07	5.07	5.07	5.07	5.07	5.07
5.08	5.08	5.08	5.08	5.08	5.08	5.08	5.08	5.08
5.09	5.09	5.09	5.09	5.09	5.09	5.09	5.09	5.09
5.1	5.1	5.1	5.1	5.1	5.1	5.1	5.1	5.1
5.11	5.11	5.11	5.11	5.11	5.11	5.11	5.11	5.11
5.12	5.12	5.12	5.12	5.12	5.12	5.12	5.12	5.12
5.13	5.13	5.13	5.13	5.13	5.13	5.13	5.13	5.13
5.14	5.14	5.14	5.14	5.14	5.14	5.14	5.14	5.14
5.15	5.15	5.15	5.15	5.15	5.15	5.15	5.15	5.15
5.16	5.16	5.16	5.16	5.16	5.16	5.16	5.16	5.16
5.17	5.17	5.17	5.17	5.17	5.17	5.17	5.17	5.17
5.18	5.18	5.18	5.18	5.18	5.18	5.18	5.18	5.18
5.19	5.19	5.19	5.19	5.19	5.19	5.19	5.19	5.19
5.2	5.2	5.2	5.2	5.2	5.2	5.2	5.2	5.2
5.21	5.21	5.21	5.21	5.21	5.21	5.21	5.21	5.21
5.22	5.22	5.22	5.22	5.22	5.22	5.22	5.22	5.22
5.23	5.23	5.23	5.23	5.23	5.23	5.23	5.23	5.23
5.24	5.24	5.24	5.24	5.24	5.24	5.24	5.24	5.24
5.25	5.25	5.25	5.25	5.25	5.25	5.25	5.25	5.25
5.26	5.26	5.26	5.26	5.26	5.26	5.26	5.26	5.26
5.27	5.27	5.27	5.27	5.27	5.27	5.27	5.27	5.27
5.28	5.28	5.28	5.28	5.28	5.28	5.28	5.28	5.28
5.29	5.29	5.29	5.29	5.29	5.29	5.29	5.29	5.29
5.3	5.3	5.3	5.3	5.3	5.3	5.3	5.3	5.3
5.31	5.31	5.31	5.31	5.31	5.31	5.31	5.31	5.31
5.32	5.32	5.32	5.32	5.32	5.32	5.32	5.32	5.32
5.33	5.33	5.33	5.33	5.33	5.33	5.33	5.33	5.33
5.34	5.34	5.34	5.34	5.34	5.34	5.34	5.34	5.34
5.35	5.35	5.35	5.35	5.35	5.35	5.35	5.35	5.35
5.36	5.36	5.36	5.36	5.36	5.36	5.36	5.36	5.36
5.37	5.37	5.37	5.37	5.37	5.37	5.37	5.37	5.37
5.38	5.38	5.38	5.38	5.38	5.38	5.38	5.38	5.38
5.39	5.39	5.39	5.39	5.39	5.39	5.39	5.39	5.39
5.4	5.4	5.4	5.4	5.4	5.4	5.4	5.4	5.4
5.41	5.41	5.41	5.41	5.41	5.41	5.41	5.41	5.41
5.42	5.42	5.42	5.42	5.42	5.42	5.42	5.42	5.42
5.43	5.43	5.43	5.43	5.43	5.43	5.43	5.43	5.43
5.44	5.44	5.44	5.44	5.44	5.44	5.44	5.44	5.44
5.45	5.45	5.45	5.45	5.45	5.45	5.45	5.45	5.45
5.46	5.46	5.46	5.46	5.46	5.46	5.46	5.46	5.46
5.47	5.47	5.47	5.47	5.47	5.47	5.47	5.47	5.47
5.48	5.48	5.48	5.48	5.48	5.48	5.48	5.48	5.48
5.49	5.49	5.49	5.49	5.49	5.49	5.49	5.49	5.49

5.5	5.5	5.5	5.5	5.5	5.5	5.5	5.5	5.5
5.51	5.51	5.51	5.51	5.51	5.51	5.51	5.51	5.51
5.52	5.52	5.52	5.52	5.52	5.52	5.52	5.52	5.52
5.53	5.53	5.53	5.53	5.53	5.53	5.53	5.53	5.53
5.54	5.54	5.54	5.54	5.54	5.54	5.54	5.54	5.54
5.55	5.55	5.55	5.55	5.55	5.55	5.55	5.55	5.55
5.56	5.56	5.56	5.56	5.56	5.56	5.56	5.56	5.56
5.57	5.57	5.57	5.57	5.57	5.57	5.57	5.57	5.57
5.58	5.58	5.58	5.58	5.58	5.58	5.58	5.58	5.58
5.59	5.59	5.59	5.59	5.59	5.59	5.59	5.59	5.59
5.6	5.6	5.6	5.6	5.6	5.6	5.6	5.6	5.6
5.61	5.61	5.61	5.61	5.61	5.61	5.61	5.61	5.61
5.62	5.62	5.62	5.62	5.62	5.62	5.62	5.62	5.62
5.63	5.63	5.63	5.63	5.63	5.63	5.63	5.63	5.63
5.64	5.64	5.64	5.64	5.64	5.64	5.64	5.64	5.64
5.65	5.65	5.65	5.65	5.65	5.65	5.65	5.65	5.65
5.66	5.66	5.66	5.66	5.66	5.66	5.66	5.66	5.66
5.67	5.67	5.67	5.67	5.67	5.67	5.67	5.67	5.67
5.68	5.68	5.68	5.68	5.68	5.68	5.68	5.68	5.68
5.69	5.69	5.69	5.69	5.69	5.69	5.69	5.69	5.69
5.7	5.7	5.7	5.7	5.7	5.7	5.7	5.7	5.7
5.71	5.71	5.71	5.71	5.71	5.71	5.71	5.71	5.71
5.72	5.72	5.72	5.72	5.72	5.72	5.72	5.72	5.72
5.73	5.73	5.73	5.73	5.73	5.73	5.73	5.73	5.73
5.74	5.74	5.74	5.74	5.74	5.74	5.74	5.74	5.74
5.75	5.75	5.75	5.75	5.75	5.75	5.75	5.75	5.75
5.76	5.76	5.76	5.76	5.76	5.76	5.76	5.76	5.76
5.77	5.77	5.77	5.77	5.77	5.77	5.77	5.77	5.77
5.78	5.78	5.78	5.78	5.78	5.78	5.78	5.78	5.78
5.79	5.79	5.79	5.79	5.79	5.79	5.79	5.79	5.79
5.8	5.8	5.8	5.8	5.8	5.8	5.8	5.8	5.8
5.81	5.81	5.81	5.81	5.81	5.81	5.81	5.81	5.81
5.82	5.82	5.82	5.82	5.82	5.82	5.82	5.82	5.82
5.83	5.83	5.83	5.83	5.83	5.83	5.83	5.83	5.83
5.84	5.84	5.84	5.84	5.84	5.84	5.84	5.84	5.84
5.85	5.85	5.85	5.85	5.85	5.85	5.85	5.85	5.85
5.86	5.86	5.86	5.86	5.86	5.86	5.86	5.86	5.86
5.87	5.87	5.87	5.87	5.87	5.87	5.87	5.87	5.87
5.88	5.88	5.88	5.88	5.88	5.88	5.88	5.88	5.88
5.89	5.89	5.89	5.89	5.89	5.89	5.89	5.89	5.89
5.9	5.9	5.9	5.9	5.9	5.9	5.9	5.9	5.9
5.91	5.91	5.91	5.91	5.91	5.91	5.91	5.91	5.91
5.92	5.92	5.92	5.92	5.92	5.92	5.92	5.92	5.92
5.93	5.93	5.93	5.93	5.93	5.93	5.93	5.93	5.93
5.94	5.94	5.94	5.94	5.94	5.94	5.94	5.94	5.94
5.95	5.95	5.95	5.95	5.95	5.95	5.95	5.95	5.95
5.96	5.96	5.96	5.96	5.96	5.96	5.96	5.96	5.96
5.97	5.97	5.97	5.97	5.97	5.97	5.97	5.97	5.97
5.98	5.98	5.98	5.98	5.98	5.98	5.98	5.98	5.98
5.99	5.99	5.99	5.99	5.99	5.99	5.99	5.99	5.99

6	6	6	6	6	6	6	6	6
6.01	6.01	6.01	6.01	6.01	6.01	6.01	6.01	6.01
6.02	6.02	6.02	6.02	6.02	6.02	6.02	6.02	6.02
6.03	6.03	6.03	6.03	6.03	6.03	6.03	6.03	6.03
6.04	6.04	6.04	6.04	6.04	6.04	6.04	6.04	6.04
6.05	6.05	6.05	6.05	6.05	6.05	6.05	6.05	6.05
6.06	6.06	6.06	6.06	6.06	6.06	6.06	6.06	6.06
6.07	6.07	6.07	6.07	6.07	6.07	6.07	6.07	6.07
6.08	6.08	6.08	6.08	6.08	6.08	6.08	6.08	6.08
6.09	6.09	6.09	6.09	6.09	6.09	6.09	6.09	6.09
6.1	6.1	6.1	6.1	6.1	6.1	6.1	6.1	6.1
6.11	6.11	6.11	6.11	6.11	6.11	6.11	6.11	6.11
6.12	6.12	6.12	6.12	6.12	6.12	6.12	6.12	6.12
6.13	6.13	6.13	6.13	6.13	6.13	6.13	6.13	6.13
6.14	6.14	6.14	6.14	6.14	6.14	6.14	6.14	6.14
6.15	6.15	6.15	6.15	6.15	6.15	6.15	6.15	6.15
6.16	6.16	6.16	6.16	6.16	6.16	6.16	6.16	6.16
6.17	6.17	6.17	6.17	6.17	6.17	6.17	6.17	6.17
6.18	6.18	6.18	6.18	6.18	6.18	6.18	6.18	6.18
6.19	6.19	6.19	6.19	6.19	6.19	6.19	6.19	6.19
6.2	6.2	6.2	6.2	6.2	6.2	6.2	6.2	6.2
6.21	6.21	6.21	6.21	6.21	6.21	6.21	6.21	6.21
6.22	6.22	6.22	6.22	6.22	6.22	6.22	6.22	6.22
6.23	6.23	6.23	6.23	6.23	6.23	6.23	6.23	6.23
6.24	6.24	6.24	6.24	6.24	6.24	6.24	6.24	6.24
6.25	6.25	6.25	6.25	6.25	6.25	6.25	6.25	6.25
6.26	6.26	6.26	6.26	6.26	6.26	6.26	6.26	6.26
6.27	6.27	6.27	6.27	6.27	6.27	6.27	6.27	6.27
6.28	6.28	6.28	6.28	6.28	6.28	6.28	6.28	6.28
6.29	6.29	6.29	6.29	6.29	6.29	6.29	6.29	6.29
6.3	6.3	6.3	6.3	6.3	6.3	6.3	6.3	6.3
6.31	6.31	6.31	6.31	6.31	6.31	6.31	6.31	6.31
6.32	6.32	6.32	6.32	6.32	6.32	6.32	6.32	6.32
6.33	6.33	6.33	6.33	6.33	6.33	6.33	6.33	6.33
6.34	6.34	6.34	6.34	6.34	6.34	6.34	6.34	6.34
6.35	6.35	6.35	6.35	6.35	6.35	6.35	6.35	6.35
6.36	6.36	6.36	6.36	6.36	6.36	6.36	6.36	6.36
6.37	6.37	6.37	6.37	6.37	6.37	6.37	6.37	6.37
6.38	6.38	6.38	6.38	6.38	6.38	6.38	6.38	6.38
6.39	6.39	6.39	6.39	6.39	6.39	6.39	6.39	6.39
6.4	6.4	6.4	6.4	6.4	6.4	6.4	6.4	6.4
6.41	6.41	6.41	6.41	6.41	6.41	6.41	6.41	6.41
6.42	6.42	6.42	6.42	6.42	6.42	6.42	6.42	6.42
6.43	6.43	6.43	6.43	6.43	6.43	6.43	6.43	6.43
6.44	6.44	6.44	6.44	6.44	6.44	6.44	6.44	6.44
6.45	6.45	6.45	6.45	6.45	6.45	6.45	6.45	6.45
6.46	6.46	6.46	6.46	6.46	6.46	6.46	6.46	6.46
6.47	6.47	6.47	6.47	6.47	6.47	6.47	6.47	6.47
6.48	6.48	6.48	6.48	6.48	6.48	6.48	6.48	6.48
6.49	6.49	6.49	6.49	6.49	6.49	6.49	6.49	6.49

6.5	6.5	6.5	6.5	6.5	6.5	6.5	6.5	6.5
6.51	6.51	6.51	6.51	6.51	6.51	6.51	6.51	6.51
6.52	6.52	6.52	6.52	6.52	6.52	6.52	6.52	6.52
6.53	6.53	6.53	6.53	6.53	6.53	6.53	6.53	6.53
6.54	6.54	6.54	6.54	6.54	6.54	6.54	6.54	6.54
6.55	6.55	6.55	6.55	6.55	6.55	6.55	6.55	6.55
6.56	6.56	6.56	6.56	6.56	6.56	6.56	6.56	6.56
6.57	6.57	6.57	6.57	6.57	6.57	6.57	6.57	6.57
6.58	6.58	6.58	6.58	6.58	6.58	6.58	6.58	6.58
6.59	6.59	6.59	6.59	6.59	6.59	6.59	6.59	6.59
6.6	6.6	6.6	6.6	6.6	6.6	6.6	6.6	6.6
6.61	6.61	6.61	6.61	6.61	6.61	6.61	6.61	6.61
6.62	6.62	6.62	6.62	6.62	6.62	6.62	6.62	6.62
6.63	6.63	6.63	6.63	6.63	6.63	6.63	6.63	6.63
6.64	6.64	6.64	6.64	6.64	6.64	6.64	6.64	6.64
6.65	6.65	6.65	6.65	6.65	6.65	6.65	6.65	6.65
6.66	6.66	6.66	6.66	6.66	6.66	6.66	6.66	6.66
6.67	6.67	6.67	6.67	6.67	6.67	6.67	6.67	6.67
6.68	6.68	6.68	6.68	6.68	6.68	6.68	6.68	6.68
6.69	6.69	6.69	6.69	6.69	6.69	6.69	6.69	6.69
6.7	6.7	6.7	6.7	6.7	6.7	6.7	6.7	6.7
6.71	6.71	6.71	6.71	6.71	6.71	6.71	6.71	6.71
6.72	6.72	6.72	6.72	6.72	6.72	6.72	6.72	6.72
6.73	6.73	6.73	6.73	6.73	6.73	6.73	6.73	6.73
6.74	6.74	6.74	6.74	6.74	6.74	6.74	6.74	6.74
6.75	6.75	6.75	6.75	6.75	6.75	6.75	6.75	6.75
6.76	6.76	6.76	6.76	6.76	6.76	6.76	6.76	6.76
6.77	6.77	6.77	6.77	6.77	6.77	6.77	6.77	6.77
6.78	6.78	6.78	6.78	6.78	6.78	6.78	6.78	6.78
6.79	6.79	6.79	6.79	6.79	6.79	6.79	6.79	6.79
6.8	6.8	6.8	6.8	6.8	6.8	6.8	6.8	6.8
6.81	6.81	6.81	6.81	6.81	6.81	6.81	6.81	6.81
6.82	6.82	6.82	6.82	6.82	6.82	6.82	6.82	6.82
6.83	6.83	6.83	6.83	6.83	6.83	6.83	6.83	6.83
6.84	6.84	6.84	6.84	6.84	6.84	6.84	6.84	6.84
6.85	6.85	6.85	6.85	6.85	6.85	6.85	6.85	6.85
6.86	6.86	6.86	6.86	6.86	6.86	6.86	6.86	6.86
6.87	6.87	6.87	6.87	6.87	6.87	6.87	6.87	6.87
6.88	6.88	6.88	6.88	6.88	6.88	6.88	6.88	6.88
6.89	6.89	6.89	6.89	6.89	6.89	6.89	6.89	6.89
6.9	6.9	6.9	6.9	6.9	6.9	6.9	6.9	6.9
6.91	6.91	6.91	6.91	6.91	6.91	6.91	6.91	6.91
6.92	6.92	6.92	6.92	6.92	6.92	6.92	6.92	6.92
6.93	6.93	6.93	6.93	6.93	6.93	6.93	6.93	6.93
6.94	6.94	6.94	6.94	6.94	6.94	6.94	6.94	6.94
6.95	6.95	6.95	6.95	6.95	6.95	6.95	6.95	6.95
6.96	6.96	6.96	6.96	6.96	6.96	6.96	6.96	6.96
6.97	6.97	6.97	6.97	6.97	6.97	6.97	6.97	6.97
6.98	6.98	6.98	6.98	6.98	6.98	6.98	6.98	6.98
6.99	6.99	6.99	6.99	6.99	6.99	6.99	6.99	6.99

7	7	7	7	7	7	7	7	7
7.01	7.01	7.01	7.01	7.01	7.01	7.01	7.01	7.01
7.02	7.02	7.02	7.02	7.02	7.02	7.02	7.02	7.02
7.03	7.03	7.03	7.03	7.03	7.03	7.03	7.03	7.03
7.04	7.04	7.04	7.04	7.04	7.04	7.04	7.04	7.04
7.05	7.05	7.05	7.05	7.05	7.05	7.05	7.05	7.05
7.06	7.06	7.06	7.06	7.06	7.06	7.06	7.06	7.06
7.07	7.07	7.07	7.07	7.07	7.07	7.07	7.07	7.07
7.08	7.08	7.08	7.08	7.08	7.08	7.08	7.08	7.08
7.09	7.09	7.09	7.09	7.09	7.09	7.09	7.09	7.09
7.1	7.1	7.1	7.1	7.1	7.1	7.1	7.1	7.1
7.11	7.11	7.11	7.11	7.11	7.11	7.11	7.11	7.11
7.12	7.12	7.12	7.12	7.12	7.12	7.12	7.12	7.12
7.13	7.13	7.13	7.13	7.13	7.13	7.13	7.13	7.13
7.14	7.14	7.14	7.14	7.14	7.14	7.14	7.14	7.14
7.15	7.15	7.15	7.15	7.15	7.15	7.15	7.15	7.15
7.16	7.16	7.16	7.16	7.16	7.16	7.16	7.16	7.16
7.17	7.17	7.17	7.17	7.17	7.17	7.17	7.17	7.17
7.18	7.18	7.18	7.18	7.18	7.18	7.18	7.18	7.18
7.19	7.19	7.19	7.19	7.19	7.19	7.19	7.19	7.19
7.2	7.2	7.2	7.2	7.2	7.2	7.2	7.2	7.2
7.21	7.21	7.21	7.21	7.21	7.21	7.21	7.21	7.21
7.22	7.22	7.22	7.22	7.22	7.22	7.22	7.22	7.22
7.23	7.23	7.23	7.23	7.23	7.23	7.23	7.23	7.23
7.24	7.24	7.24	7.24	7.24	7.24	7.24	7.24	7.24
7.25	7.25	7.25	7.25	7.25	7.25	7.25	7.25	7.25
7.26	7.26	7.26	7.26	7.26	7.26	7.26	7.26	7.26
7.27	7.27	7.27	7.27	7.27	7.27	7.27	7.27	7.27
7.28	7.28	7.28	7.28	7.28	7.28	7.28	7.28	7.28
7.29	7.29	7.29	7.29	7.29	7.29	7.29	7.29	7.29
7.3	7.3	7.3	7.3	7.3	7.3	7.3	7.3	7.3
7.31	7.31	7.31	7.31	7.31	7.31	7.31	7.31	7.31
7.32	7.32	7.32	7.32	7.32	7.32	7.32	7.32	7.32
7.33	7.33	7.33	7.33	7.33	7.33	7.33	7.33	7.33
7.34	7.34	7.34	7.34	7.34	7.34	7.34	7.34	7.34
7.35	7.35	7.35	7.35	7.35	7.35	7.35	7.35	7.35
7.36	7.36	7.36	7.36	7.36	7.36	7.36	7.36	7.36
7.37	7.37	7.37	7.37	7.37	7.37	7.37	7.37	7.37
7.38	7.38	7.38	7.38	7.38	7.38	7.38	7.38	7.38
7.39	7.39	7.39	7.39	7.39	7.39	7.39	7.39	7.39
7.4	7.4	7.4	7.4	7.4	7.4	7.4	7.4	7.4
7.41	7.41	7.41	7.41	7.41	7.41	7.41	7.41	7.41
7.42	7.42	7.42	7.42	7.42	7.42	7.42	7.42	7.42
7.43	7.43	7.43	7.43	7.43	7.43	7.43	7.43	7.43
7.44	7.44	7.44	7.44	7.44	7.44	7.44	7.44	7.44
7.45	7.45	7.45	7.45	7.45	7.45	7.45	7.45	7.45
7.46	7.46	7.46	7.46	7.46	7.46	7.46	7.46	7.46
7.47	7.47	7.47	7.47	7.47	7.47	7.47	7.47	7.47
7.48	7.48	7.48	7.48	7.48	7.48	7.48	7.48	7.48
7.49	7.49	7.49	7.49	7.49	7.49	7.49	7.49	7.49

7.5	7.5	7.5	7.5	7.5	7.5	7.5	7.5	7.5
7.51	7.51	7.51	7.51	7.51	7.51	7.51	7.51	7.51
7.52	7.52	7.52	7.52	7.52	7.52	7.52	7.52	7.52
7.53	7.53	7.53	7.53	7.53	7.53	7.53	7.53	7.53
7.54	7.54	7.54	7.54	7.54	7.54	7.54	7.54	7.54
7.55	7.55	7.55	7.55	7.55	7.55	7.55	7.55	7.55
7.56	7.56	7.56	7.56	7.56	7.56	7.56	7.56	7.56
7.57	7.57	7.57	7.57	7.57	7.57	7.57	7.57	7.57
7.58	7.58	7.58	7.58	7.58	7.58	7.58	7.58	7.58
7.59	7.59	7.59	7.59	7.59	7.59	7.59	7.59	7.59
7.6	7.6	7.6	7.6	7.6	7.6	7.6	7.6	7.6
7.61	7.61	7.61	7.61	7.61	7.61	7.61	7.61	7.61
7.62	7.62	7.62	7.62	7.62	7.62	7.62	7.62	7.62
7.63	7.63	7.63	7.63	7.63	7.63	7.63	7.63	7.63
7.64	7.64	7.64	7.64	7.64	7.64	7.64	7.64	7.64
7.65	7.65	7.65	7.65	7.65	7.65	7.65	7.65	7.65
7.66	7.66	7.66	7.66	7.66	7.66	7.66	7.66	7.66
7.67	7.67	7.67	7.67	7.67	7.67	7.67	7.67	7.67
7.68	7.68	7.68	7.68	7.68	7.68	7.68	7.68	7.68
7.69	7.69	7.69	7.69	7.69	7.69	7.69	7.69	7.69
7.7	7.7	7.7	7.7	7.7	7.7	7.7	7.7	7.7
7.71	7.71	7.71	7.71	7.71	7.71	7.71	7.71	7.71
7.72	7.72	7.72	7.72	7.72	7.72	7.72	7.72	7.72
7.73	7.73	7.73	7.73	7.73	7.73	7.73	7.73	7.73
7.74	7.74	7.74	7.74	7.74	7.74	7.74	7.74	7.74
7.75	7.75	7.75	7.75	7.75	7.75	7.75	7.75	7.75
7.76	7.76	7.76	7.76	7.76	7.76	7.76	7.76	7.76
7.77	7.77	7.77	7.77	7.77	7.77	7.77	7.77	7.77
7.78	7.78	7.78	7.78	7.78	7.78	7.78	7.78	7.78
7.79	7.79	7.79	7.79	7.79	7.79	7.79	7.79	7.79
7.8	7.8	7.8	7.8	7.8	7.8	7.8	7.8	7.8
7.81	7.81	7.81	7.81	7.81	7.81	7.81	7.81	7.81
7.82	7.82	7.82	7.82	7.82	7.82	7.82	7.82	7.82
7.83	7.83	7.83	7.83	7.83	7.83	7.83	7.83	7.83
7.84	7.84	7.84	7.84	7.84	7.84	7.84	7.84	7.84
7.85	7.85	7.85	7.85	7.85	7.85	7.85	7.85	7.85
7.86	7.86	7.86	7.86	7.86	7.86	7.86	7.86	7.86
7.87	7.87	7.87	7.87	7.87	7.87	7.87	7.87	7.87
7.88	7.88	7.88	7.88	7.88	7.88	7.88	7.88	7.88
7.89	7.89	7.89	7.89	7.89	7.89	7.89	7.89	7.89
7.9	7.9	7.9	7.9	7.9	7.9	7.9	7.9	7.9
7.91	7.91	7.91	7.91	7.91	7.91	7.91	7.91	7.91
7.92	7.92	7.92	7.92	7.92	7.92	7.92	7.92	7.92
7.93	7.93	7.93	7.93	7.93	7.93	7.93	7.93	7.93
7.94	7.94	7.94	7.94	7.94	7.94	7.94	7.94	7.94
7.95	7.95	7.95	7.95	7.95	7.95	7.95	7.95	7.95
7.96	7.96	7.96	7.96	7.96	7.96	7.96	7.96	7.96
7.97	7.97	7.97	7.97	7.97	7.97	7.97	7.97	7.97
7.98	7.98	7.98	7.98	7.98	7.98	7.98	7.98	7.98
7.99	7.99	7.99	7.99	7.99	7.99	7.99	7.99	7.99

8	8	8	8	8	8	8	8	8
8.01	8.01	8.01	8.01	8.01	8.01	8.01	8.01	8.01
8.02	8.02	8.02	8.02	8.02	8.02	8.02	8.02	8.02
8.03	8.03	8.03	8.03	8.03	8.03	8.03	8.03	8.03
8.04	8.04	8.04	8.04	8.04	8.04	8.04	8.04	8.04
8.05	8.05	8.05	8.05	8.05	8.05	8.05	8.05	8.05
8.06	8.06	8.06	8.06	8.06	8.06	8.06	8.06	8.06
8.07	8.07	8.07	8.07	8.07	8.07	8.07	8.07	8.07
8.08	8.08	8.08	8.08	8.08	8.08	8.08	8.08	8.08
8.09	8.09	8.09	8.09	8.09	8.09	8.09	8.09	8.09
8.1	8.1	8.1	8.1	8.1	8.1	8.1	8.1	8.1
8.11	8.11	8.11	8.11	8.11	8.11	8.11	8.11	8.11
8.12	8.12	8.12	8.12	8.12	8.12	8.12	8.12	8.12
8.13	8.13	8.13	8.13	8.13	8.13	8.13	8.13	8.13
8.14	8.14	8.14	8.14	8.14	8.14	8.14	8.14	8.14
8.15	8.15	8.15	8.15	8.15	8.15	8.15	8.15	8.15
8.16	8.16	8.16	8.16	8.16	8.16	8.16	8.16	8.16
8.17	8.17	8.17	8.17	8.17	8.17	8.17	8.17	8.17
8.18	8.18	8.18	8.18	8.18	8.18	8.18	8.18	8.18
8.19	8.19	8.19	8.19	8.19	8.19	8.19	8.19	8.19
8.2	8.2	8.2	8.2	8.2	8.2	8.2	8.2	8.2
8.21	8.21	8.21	8.21	8.21	8.21	8.21	8.21	8.21
8.22	8.22	8.22	8.22	8.22	8.22	8.22	8.22	8.22
8.23	8.23	8.23	8.23	8.23	8.23	8.23	8.23	8.23
8.24	8.24	8.24	8.24	8.24	8.24	8.24	8.24	8.24
8.25	8.25	8.25	8.25	8.25	8.25	8.25	8.25	8.25
8.26	8.26	8.26	8.26	8.26	8.26	8.26	8.26	8.26
8.27	8.27	8.27	8.27	8.27	8.27	8.27	8.27	8.27
8.28	8.28	8.28	8.28	8.28	8.28	8.28	8.28	8.28
8.29	8.29	8.29	8.29	8.29	8.29	8.29	8.29	8.29
8.3	8.3	8.3	8.3	8.3	8.3	8.3	8.3	8.3
8.31	8.31	8.31	8.31	8.31	8.31	8.31	8.31	8.31
8.32	8.32	8.32	8.32	8.32	8.32	8.32	8.32	8.32
8.33	8.33	8.33	8.33	8.33	8.33	8.33	8.33	8.33
8.34	8.34	8.34	8.34	8.34	8.34	8.34	8.34	8.34
8.35	8.35	8.35	8.35	8.35	8.35	8.35	8.35	8.35
8.36	8.36	8.36	8.36	8.36	8.36	8.36	8.36	8.36
8.37	8.37	8.37	8.37	8.37	8.37	8.37	8.37	8.37
8.38	8.38	8.38	8.38	8.38	8.38	8.38	8.38	8.38
8.39	8.39	8.39	8.39	8.39	8.39	8.39	8.39	8.39
8.4	8.4	8.4	8.4	8.4	8.4	8.4	8.4	8.4
8.41	8.41	8.41	8.41	8.41	8.41	8.41	8.41	8.41
8.42	8.42	8.42	8.42	8.42	8.42	8.42	8.42	8.42
8.43	8.43	8.43	8.43	8.43	8.43	8.43	8.43	8.43
8.44	8.44	8.44	8.44	8.44	8.44	8.44	8.44	8.44
8.45	8.45	8.45	8.45	8.45	8.45	8.45	8.45	8.45
8.46	8.46	8.46	8.46	8.46	8.46	8.46	8.46	8.46
8.47	8.47	8.47	8.47	8.47	8.47	8.47	8.47	8.47
8.48	8.48	8.48	8.48	8.48	8.48	8.48	8.48	8.48
8.49	8.49	8.49	8.49	8.49	8.49	8.49	8.49	8.49

8.5	8.5	8.5	8.5	8.5	8.5	8.5	8.5	8.5
8.51	8.51	8.51	8.51	8.51	8.51	8.51	8.51	8.51
8.52	8.52	8.52	8.52	8.52	8.52	8.52	8.52	8.52
8.53	8.53	8.53	8.53	8.53	8.53	8.53	8.53	8.53
8.54	8.54	8.54	8.54	8.54	8.54	8.54	8.54	8.54
8.55	8.55	8.55	8.55	8.55	8.55	8.55	8.55	8.55
8.56	8.56	8.56	8.56	8.56	8.56	8.56	8.56	8.56
8.57	8.57	8.57	8.57	8.57	8.57	8.57	8.57	8.57
8.58	8.58	8.58	8.58	8.58	8.58	8.58	8.58	8.58
8.59	8.59	8.59	8.59	8.59	8.59	8.59	8.59	8.59
8.6	8.6	8.6	8.6	8.6	8.6	8.6	8.6	8.6
8.61	8.61	8.61	8.61	8.61	8.61	8.61	8.61	8.61
8.62	8.62	8.62	8.62	8.62	8.62	8.62	8.62	8.62
8.63	8.63	8.63	8.63	8.63	8.63	8.63	8.63	8.63
8.64	8.64	8.64	8.64	8.64	8.64	8.64	8.64	8.64
8.65	8.65	8.65	8.65	8.65	8.65	8.65	8.65	8.65
8.66	8.66	8.66	8.66	8.66	8.66	8.66	8.66	8.66
8.67	8.67	8.67	8.67	8.67	8.67	8.67	8.67	8.67
8.68	8.68	8.68	8.68	8.68	8.68	8.68	8.68	8.68
8.69	8.69	8.69	8.69	8.69	8.69	8.69	8.69	8.69
8.7	8.7	8.7	8.7	8.7	8.7	8.7	8.7	8.7
8.71	8.71	8.71	8.71	8.71	8.71	8.71	8.71	8.71
8.72	8.72	8.72	8.72	8.72	8.72	8.72	8.72	8.72
8.73	8.73	8.73	8.73	8.73	8.73	8.73	8.73	8.73
8.74	8.74	8.74	8.74	8.74	8.74	8.74	8.74	8.74
8.75	8.75	8.75	8.75	8.75	8.75	8.75	8.75	8.75
8.76	8.76	8.76	8.76	8.76	8.76	8.76	8.76	8.76
8.77	8.77	8.77	8.77	8.77	8.77	8.77	8.77	8.77
8.78	8.78	8.78	8.78	8.78	8.78	8.78	8.78	8.78
8.79	8.79	8.79	8.79	8.79	8.79	8.79	8.79	8.79
8.8	8.8	8.8	8.8	8.8	8.8	8.8	8.8	8.8
8.81	8.81	8.81	8.81	8.81	8.81	8.81	8.81	8.81
8.82	8.82	8.82	8.82	8.82	8.82	8.82	8.82	8.82
8.83	8.83	8.83	8.83	8.83	8.83	8.83	8.83	8.83
8.84	8.84	8.84	8.84	8.84	8.84	8.84	8.84	8.84
8.85	8.85	8.85	8.85	8.85	8.85	8.85	8.85	8.85
8.86	8.86	8.86	8.86	8.86	8.86	8.86	8.86	8.86
8.87	8.87	8.87	8.87	8.87	8.87	8.87	8.87	8.87
8.88	8.88	8.88	8.88	8.88	8.88	8.88	8.88	8.88
8.89	8.89	8.89	8.89	8.89	8.89	8.89	8.89	8.89
8.9	8.9	8.9	8.9	8.9	8.9	8.9	8.9	8.9
8.91	8.91	8.91	8.91	8.91	8.91	8.91	8.91	8.91
8.92	8.92	8.92	8.92	8.92	8.92	8.92	8.92	8.92
8.93	8.93	8.93	8.93	8.93	8.93	8.93	8.93	8.93
8.94	8.94	8.94	8.94	8.94	8.94	8.94	8.94	8.94
8.95	8.95	8.95	8.95	8.95	8.95	8.95	8.95	8.95
8.96	8.96	8.96	8.96	8.96	8.96	8.96	8.96	8.96
8.97	8.97	8.97	8.97	8.97	8.97	8.97	8.97	8.97
8.98	8.98	8.98	8.98	8.98	8.98	8.98	8.98	8.98
8.99	8.99	8.99	8.99	8.99	8.99	8.99	8.99	8.99

9	9	9	9	9	9	9	9	9
9.01	9.01	9.01	9.01	9.01	9.01	9.01	9.01	9.01
9.02	9.02	9.02	9.02	9.02	9.02	9.02	9.02	9.02
9.03	9.03	9.03	9.03	9.03	9.03	9.03	9.03	9.03
9.04	9.04	9.04	9.04	9.04	9.04	9.04	9.04	9.04
9.05	9.05	9.05	9.05	9.05	9.05	9.05	9.05	9.05
9.06	9.06	9.06	9.06	9.06	9.06	9.06	9.06	9.06
9.07	9.07	9.07	9.07	9.07	9.07	9.07	9.07	9.07
9.08	9.08	9.08	9.08	9.08	9.08	9.08	9.08	9.08
9.09	9.09	9.09	9.09	9.09	9.09	9.09	9.09	9.09
9.1	9.1	9.1	9.1	9.1	9.1	9.1	9.1	9.1
9.11	9.11	9.11	9.11	9.11	9.11	9.11	9.11	9.11
9.12	9.12	9.12	9.12	9.12	9.12	9.12	9.12	9.12
9.13	9.13	9.13	9.13	9.13	9.13	9.13	9.13	9.13
9.14	9.14	9.14	9.14	9.14	9.14	9.14	9.14	9.14
9.15	9.15	9.15	9.15	9.15	9.15	9.15	9.15	9.15
9.16	9.16	9.16	9.16	9.16	9.16	9.16	9.16	9.16
9.17	9.17	9.17	9.17	9.17	9.17	9.17	9.17	9.17
9.18	9.18	9.18	9.18	9.18	9.18	9.18	9.18	9.18
9.19	9.19	9.19	9.19	9.19	9.19	9.19	9.19	9.19
9.2	9.2	9.2	9.2	9.2	9.2	9.2	9.2	9.2
9.21	9.21	9.21	9.21	9.21	9.21	9.21	9.21	9.21
9.22	9.22	9.22	9.22	9.22	9.22	9.22	9.22	9.22
9.23	9.23	9.23	9.23	9.23	9.23	9.23	9.23	9.23
9.24	9.24	9.24	9.24	9.24	9.24	9.24	9.24	9.24
9.25	9.25	9.25	9.25	9.25	9.25	9.25	9.25	9.25
9.26	9.26	9.26	9.26	9.26	9.26	9.26	9.26	9.26
9.27	9.27	9.27	9.27	9.27	9.27	9.27	9.27	9.27
9.28	9.28	9.28	9.28	9.28	9.28	9.28	9.28	9.28
9.29	9.29	9.29	9.29	9.29	9.29	9.29	9.29	9.29
9.3	9.3	9.3	9.3	9.3	9.3	9.3	9.3	9.3
9.31	9.31	9.31	9.31	9.31	9.31	9.31	9.31	9.31
9.32	9.32	9.32	9.32	9.32	9.32	9.32	9.32	9.32
9.33	9.33	9.33	9.33	9.33	9.33	9.33	9.33	9.33
9.34	9.34	9.34	9.34	9.34	9.34	9.34	9.34	9.34
9.35	9.35	9.35	9.35	9.35	9.35	9.35	9.35	9.35
9.36	9.36	9.36	9.36	9.36	9.36	9.36	9.36	9.36
9.37	9.37	9.37	9.37	9.37	9.37	9.37	9.37	9.37
9.38	9.38	9.38	9.38	9.38	9.38	9.38	9.38	9.38
9.39	9.39	9.39	9.39	9.39	9.39	9.39	9.39	9.39
9.4	9.4	9.4	9.4	9.4	9.4	9.4	9.4	9.4
9.41	9.41	9.41	9.41	9.41	9.41	9.41	9.41	9.41
9.42	9.42	9.42	9.42	9.42	9.42	9.42	9.42	9.42
9.43	9.43	9.43	9.43	9.43	9.43	9.43	9.43	9.43
9.44	9.44	9.44	9.44	9.44	9.44	9.44	9.44	9.44
9.45	9.45	9.45	9.45	9.45	9.45	9.45	9.45	9.45
9.46	9.46	9.46	9.46	9.46	9.46	9.46	9.46	9.46
9.47	9.47	9.47	9.47	9.47	9.47	9.47	9.47	9.47
9.48	9.48	9.48	9.48	9.48	9.48	9.48	9.48	9.48
9.49	9.49	9.49	9.49	9.49	9.49	9.49	9.49	9.49

9.5	9.5	9.5	9.5	9.5	9.5	9.5	9.5	9.5
9.51	9.51	9.51	9.51	9.51	9.51	9.51	9.51	9.51
9.52	9.52	9.52	9.52	9.52	9.52	9.52	9.52	9.52
9.53	9.53	9.53	9.53	9.53	9.53	9.53	9.53	9.53
9.54	9.54	9.54	9.54	9.54	9.54	9.54	9.54	9.54
9.55	9.55	9.55	9.55	9.55	9.55	9.55	9.55	9.55
9.56	9.56	9.56	9.56	9.56	9.56	9.56	9.56	9.56
9.57	9.57	9.57	9.57	9.57	9.57	9.57	9.57	9.57
9.58	9.58	9.58	9.58	9.58	9.58	9.58	9.58	9.58
9.59	9.59	9.59	9.59	9.59	9.59	9.59	9.59	9.59
9.6	9.6	9.6	9.6	9.6	9.6	9.6	9.6	9.6
9.61	9.61	9.61	9.61	9.61	9.61	9.61	9.61	9.61
9.62	9.62	9.62	9.62	9.62	9.62	9.62	9.62	9.62
9.63	9.63	9.63	9.63	9.63	9.63	9.63	9.63	9.63
9.64	9.64	9.64	9.64	9.64	9.64	9.64	9.64	9.64
9.65	9.65	9.65	9.65	9.65	9.65	9.65	9.65	9.65
9.66	9.66	9.66	9.66	9.66	9.66	9.66	9.66	9.66
9.67	9.67	9.67	9.67	9.67	9.67	9.67	9.67	9.67
9.68	9.68	9.68	9.68	9.68	9.68	9.68	9.68	9.68
9.69	9.69	9.69	9.69	9.69	9.69	9.69	9.69	9.69
9.7	9.7	9.7	9.7	9.7	9.7	9.7	9.7	9.7
9.71	9.71	9.71	9.71	9.71	9.71	9.71	9.71	9.71
9.72	9.72	9.72	9.72	9.72	9.72	9.72	9.72	9.72
9.73	9.73	9.73	9.73	9.73	9.73	9.73	9.73	9.73
9.74	9.74	9.74	9.74	9.74	9.74	9.74	9.74	9.74
9.75	9.75	9.75	9.75	9.75	9.75	9.75	9.75	9.75
9.76	9.76	9.76	9.76	9.76	9.76	9.76	9.76	9.76
9.77	9.77	9.77	9.77	9.77	9.77	9.77	9.77	9.77
9.78	9.78	9.78	9.78	9.78	9.78	9.78	9.78	9.78
9.79	9.79	9.79	9.79	9.79	9.79	9.79	9.79	9.79
9.8	9.8	9.8	9.8	9.8	9.8	9.8	9.8	9.8
9.81	9.81	9.81	9.81	9.81	9.81	9.81	9.81	9.81
9.82	9.82	9.82	9.82	9.82	9.82	9.82	9.82	9.82
9.83	9.83	9.83	9.83	9.83	9.83	9.83	9.83	9.83
9.84	9.84	9.84	9.84	9.84	9.84	9.84	9.84	9.84
9.85	9.85	9.85	9.85	9.85	9.85	9.85	9.85	9.85
9.86	9.86	9.86	9.86	9.86	9.86	9.86	9.86	9.86
9.87	9.87	9.87	9.87	9.87	9.87	9.87	9.87	9.87
9.88	9.88	9.88	9.88	9.88	9.88	9.88	9.88	9.88
9.89	9.89	9.89	9.89	9.89	9.89	9.89	9.89	9.89
9.9	9.9	9.9	9.9	9.9	9.9	9.9	9.9	9.9
9.91	9.91	9.91	9.91	9.91	9.91	9.91	9.91	9.91
9.92	9.92	9.92	9.92	9.92	9.92	9.92	9.92	9.92
9.93	9.93	9.93	9.93	9.93	9.93	9.93	9.93	9.93
9.94	9.94	9.94	9.94	9.94	9.94	9.94	9.94	9.94
9.95	9.95	9.95	9.95	9.95	9.95	9.95	9.95	9.95
9.96	9.96	9.96	9.96	9.96	9.96	9.96	9.96	9.96
9.97	9.97	9.97	9.97	9.97	9.97	9.97	9.97	9.97
9.98	9.98	9.98	9.98	9.98	9.98	9.98	9.98	9.98
9.99	9.99	9.99	9.99	9.99	9.99	9.99	9.99	9.99

10	10	10	10	10	10	10	10	10
10.01	10.01	10.01	10.01	10.01	10.01	10.01	10.01	10.01
10.02	10.02	10.02	10.02	10.02	10.02	10.02	10.02	10.02
10.03	10.03	10.03	10.03	10.03	10.03	10.03	10.03	10.03
10.04	10.04	10.04	10.04	10.04	10.04	10.04	10.04	10.04
10.05	10.05	10.05	10.05	10.05	10.05	10.05	10.05	10.05
10.06	10.06	10.06	10.06	10.06	10.06	10.06	10.06	10.06
10.07	10.07	10.07	10.07	10.07	10.07	10.07	10.07	10.07
10.08	10.08	10.08	10.08	10.08	10.08	10.08	10.08	10.08
10.09	10.09	10.09	10.09	10.09	10.09	10.09	10.09	10.09
10.1	10.1	10.1	10.1	10.1	10.1	10.1	10.1	10.1
10.11	10.11	10.11	10.11	10.11	10.11	10.11	10.11	10.11
10.12	10.12	10.12	10.12	10.12	10.12	10.12	10.12	10.12
10.13	10.13	10.13	10.13	10.13	10.13	10.13	10.13	10.13
10.14	10.14	10.14	10.14	10.14	10.14	10.14	10.14	10.14
10.15	10.15	10.15	10.15	10.15	10.15	10.15	10.15	10.15
10.16	10.16	10.16	10.16	10.16	10.16	10.16	10.16	10.16
10.17	10.17	10.17	10.17	10.17	10.17	10.17	10.17	10.17
10.18	10.18	10.18	10.18	10.18	10.18	10.18	10.18	10.18
10.19	10.19	10.19	10.19	10.19	10.19	10.19	10.19	10.19
10.2	10.2	10.2	10.2	10.2	10.2	10.2	10.2	10.2
10.21	10.21	10.21	10.21	10.21	10.21	10.21	10.21	10.21
10.22	10.22	10.22	10.22	10.22	10.22	10.22	10.22	10.22
10.23	10.23	10.23	10.23	10.23	10.23	10.23	10.23	10.23
10.24	10.24	10.24	10.24	10.24	10.24	10.24	10.24	10.24
10.25	10.25	10.25	10.25	10.25	10.25	10.25	10.25	10.25
10.26	10.26	10.26	10.26	10.26	10.26	10.26	10.26	10.26
10.27	10.27	10.27	10.27	10.27	10.27	10.27	10.27	10.27
10.28	10.28	10.28	10.28	10.28	10.28	10.28	10.28	10.28
10.29	10.29	10.29	10.29	10.29	10.29	10.29	10.29	10.29
10.3	10.3	10.3	10.3	10.3	10.3	10.3	10.3	10.3
10.31	10.31	10.31	10.31	10.31	10.31	10.31	10.31	10.31
10.32	10.32	10.32	10.32	10.32	10.32	10.32	10.32	10.32
10.33	10.33	10.33	10.33	10.33	10.33	10.33	10.33	10.33
10.34	10.34	10.34	10.34	10.34	10.34	10.34	10.34	10.34
10.35	10.35	10.35	10.35	10.35	10.35	10.35	10.35	10.35
10.36	10.36	10.36	10.36	10.36	10.36	10.36	10.36	10.36
10.37	10.37	10.37	10.37	10.37	10.37	10.37	10.37	10.37
10.38	10.38	10.38	10.38	10.38	10.38	10.38	10.38	10.38
10.39	10.39	10.39	10.39	10.39	10.39	10.39	10.39	10.39
10.4	10.4	10.4	10.4	10.4	10.4	10.4	10.4	10.4
10.41	10.41	10.41	10.41	10.41	10.41	10.41	10.41	10.41
10.42	10.42	10.42	10.42	10.42	10.42	10.42	10.42	10.42
10.43	10.43	10.43	10.43	10.43	10.43	10.43	10.43	10.43
10.44	10.44	10.44	10.44	10.44	10.44	10.44	10.44	10.44
10.45	10.45	10.45	10.45	10.45	10.45	10.45	10.45	10.45
10.46	10.46	10.46	10.46	10.46	10.46	10.46	10.46	10.46
10.47	10.47	10.47	10.47	10.47	10.47	10.47	10.47	10.47
10.48	10.48	10.48	10.48	10.48	10.48	10.48	10.48	10.48
10.49	10.49	10.49	10.49	10.49	10.49	10.49	10.49	10.49

10.5	10.5	10.5	10.5	10.5	10.5	10.5	10.5	10.5
10.51	10.51	10.51	10.51	10.51	10.51	10.51	10.51	10.51
10.52	10.52	10.52	10.52	10.52	10.52	10.52	10.52	10.52
10.53	10.53	10.53	10.53	10.53	10.53	10.53	10.53	10.53
10.54	10.54	10.54	10.54	10.54	10.54	10.54	10.54	10.54
10.55	10.55	10.55	10.55	10.55	10.55	10.55	10.55	10.55
10.56	10.56	10.56	10.56	10.56	10.56	10.56	10.56	10.56
10.57	10.57	10.57	10.57	10.57	10.57	10.57	10.57	10.57
10.58	10.58	10.58	10.58	10.58	10.58	10.58	10.58	10.58
10.59	10.59	10.59	10.59	10.59	10.59	10.59	10.59	10.59
10.6	10.6	10.6	10.6	10.6	10.6	10.6	10.6	10.6
10.61	10.61	10.61	10.61	10.61	10.61	10.61	10.61	10.61
10.62	10.62	10.62	10.62	10.62	10.62	10.62	10.62	10.62
10.63	10.63	10.63	10.63	10.63	10.63	10.63	10.63	10.63
10.64	10.64	10.64	10.64	10.64	10.64	10.64	10.64	10.64
10.65	10.65	10.65	10.65	10.65	10.65	10.65	10.65	10.65
10.66	10.66	10.66	10.66	10.66	10.66	10.66	10.66	10.66
10.67	10.67	10.67	10.67	10.67	10.67	10.67	10.67	10.67
10.68	10.68	10.68	10.68	10.68	10.68	10.68	10.68	10.68
10.69	10.69	10.69	10.69	10.69	10.69	10.69	10.69	10.69
10.7	10.7	10.7	10.7	10.7	10.7	10.7	10.7	10.7
10.71	10.71	10.71	10.71	10.71	10.71	10.71	10.71	10.71
10.72	10.72	10.72	10.72	10.72	10.72	10.72	10.72	10.72
10.73	10.73	10.73	10.73	10.73	10.73	10.73	10.73	10.73
10.74	10.74	10.74	10.74	10.74	10.74	10.74	10.74	10.74
10.75	10.75	10.75	10.75	10.75	10.75	10.75	10.75	10.75
10.76	10.76	10.76	10.76	10.76	10.76	10.76	10.76	10.76
10.77	10.77	10.77	10.77	10.77	10.77	10.77	10.77	10.77
10.78	10.78	10.78	10.78	10.78	10.78	10.78	10.78	10.78
10.79	10.79	10.79	10.79	10.79	10.79	10.79	10.79	10.79
10.8	10.8	10.8	10.8	10.8	10.8	10.8	10.8	10.8
10.81	10.81	10.81	10.81	10.81	10.81	10.81	10.81	10.81
10.82	10.82	10.82	10.82	10.82	10.82	10.82	10.82	10.82
10.83	10.83	10.83	10.83	10.83	10.83	10.83	10.83	10.83
10.84	10.84	10.84	10.84	10.84	10.84	10.84	10.84	10.84
10.85	10.85	10.85	10.85	10.85	10.85	10.85	10.85	10.85
10.86	10.86	10.86	10.86	10.86	10.86	10.86	10.86	10.86
10.87	10.87	10.87	10.87	10.87	10.87	10.87	10.87	10.87
10.88	10.88	10.88	10.88	10.88	10.88	10.88	10.88	10.88
10.89	10.89	10.89	10.89	10.89	10.89	10.89	10.89	10.89
10.9	10.9	10.9	10.9	10.9	10.9	10.9	10.9	10.9
10.91	10.91	10.91	10.91	10.91	10.91	10.91	10.91	10.91
10.92	10.92	10.92	10.92	10.92	10.92	10.92	10.92	10.92
10.93	10.93	10.93	10.93	10.93	10.93	10.93	10.93	10.93
10.94	10.94	10.94	10.94	10.94	10.94	10.94	10.94	10.94
10.95	10.95	10.95	10.95	10.95	10.95	10.95	10.95	10.95
10.96	10.96	10.96	10.96	10.96	10.96	10.96	10.96	10.96
10.97	10.97	10.97	10.97	10.97	10.97	10.97	10.97	10.97
10.98	10.98	10.98	10.98	10.98	10.98	10.98	10.98	10.98
10.99	10.99	10.99	10.99	10.99	10.99	10.99	10.99	10.99

11	11	11	11	11	11	11	11	11
11.01	11.01	11.01	11.01	11.01	11.01	11.01	11.01	11.01
11.02	11.02	11.02	11.02	11.02	11.02	11.02	11.02	11.02
11.03	11.03	11.03	11.03	11.03	11.03	11.03	11.03	11.03
11.04	11.04	11.04	11.04	11.04	11.04	11.04	11.04	11.04
11.05	11.05	11.05	11.05	11.05	11.05	11.05	11.05	11.05
11.06	11.06	11.06	11.06	11.06	11.06	11.06	11.06	11.06
11.07	11.07	11.07	11.07	11.07	11.07	11.07	11.07	11.07
11.08	11.08	11.08	11.08	11.08	11.08	11.08	11.08	11.08
11.09	11.09	11.09	11.09	11.09	11.09	11.09	11.09	11.09
11.1	11.1	11.1	11.1	11.1	11.1	11.1	11.1	11.1
11.11	11.11	11.11	11.11	11.11	11.11	11.11	11.11	11.11
11.12	11.12	11.12	11.12	11.12	11.12	11.12	11.12	11.12
11.13	11.13	11.13	11.13	11.13	11.13	11.13	11.13	11.13
11.14	11.14	11.14	11.14	11.14	11.14	11.14	11.14	11.14
11.15	11.15	11.15	11.15	11.15	11.15	11.15	11.15	11.15
11.16	11.16	11.16	11.16	11.16	11.16	11.16	11.16	11.16
11.17	11.17	11.17	11.17	11.17	11.17	11.17	11.17	11.17
11.18	11.18	11.18	11.18	11.18	11.18	11.18	11.18	11.18
11.19	11.19	11.19	11.19	11.19	11.19	11.19	11.19	11.19
11.2	11.2	11.2	11.2	11.2	11.2	11.2	11.2	11.2
11.21	11.21	11.21	11.21	11.21	11.21	11.21	11.21	11.21
11.22	11.22	11.22	11.22	11.22	11.22	11.22	11.22	11.22
11.23	11.23	11.23	11.23	11.23	11.23	11.23	11.23	11.23
11.24	11.24	11.24	11.24	11.24	11.24	11.24	11.24	11.24
11.25	11.25	11.25	11.25	11.25	11.25	11.25	11.25	11.25
11.26	11.26	11.26	11.26	11.26	11.26	11.26	11.26	11.26
11.27	11.27	11.27	11.27	11.27	11.27	11.27	11.27	11.27
11.28	11.28	11.28	11.28	11.28	11.28	11.28	11.28	11.28
11.29	11.29	11.29	11.29	11.29	11.29	11.29	11.29	11.29
11.3	11.3	11.3	11.3	11.3	11.3	11.3	11.3	11.3
11.31	11.31	11.31	11.31	11.31	11.31	11.31	11.31	11.31
11.32	11.32	11.32	11.32	11.32	11.32	11.32	11.32	11.32
11.33	11.33	11.33	11.33	11.33	11.33	11.33	11.33	11.33
11.34	11.34	11.34	11.34	11.34	11.34	11.34	11.34	11.34
11.35	11.35	11.35	11.35	11.35	11.35	11.35	11.35	11.35
11.36	11.36	11.36	11.36	11.36	11.36	11.36	11.36	11.36
11.37	11.37	11.37	11.37	11.37	11.37	11.37	11.37	11.37
11.38	11.38	11.38	11.38	11.38	11.38	11.38	11.38	11.38
11.39	11.39	11.39	11.39	11.39	11.39	11.39	11.39	11.39
11.4	11.4	11.4	11.4	11.4	11.4	11.4	11.4	11.4
11.41	11.41	11.41	11.41	11.41	11.41	11.41	11.41	11.41
11.42	11.42	11.42	11.42	11.42	11.42	11.42	11.42	11.42
11.43	11.43	11.43	11.43	11.43	11.43	11.43	11.43	11.43
11.44	11.44	11.44	11.44	11.44	11.44	11.44	11.44	11.44
11.45	11.45	11.45	11.45	11.45	11.45	11.45	11.45	11.45
11.46	11.46	11.46	11.46	11.46	11.46	11.46	11.46	11.46
11.47	11.47	11.47	11.47	11.47	11.47	11.47	11.47	11.47
11.48	11.48	11.48	11.48	11.48	11.48	11.48	11.48	11.48
11.49	11.49	11.49	11.49	11.49	11.49	11.49	11.49	11.49

11.5	11.5	11.5	11.5	11.5	11.5	11.5	11.5	11.5
11.51	11.51	11.51	11.51	11.51	11.51	11.51	11.51	11.51
11.52	11.52	11.52	11.52	11.52	11.52	11.52	11.52	11.52
11.53	11.53	11.53	11.53	11.53	11.53	11.53	11.53	11.53
11.54	11.54	11.54	11.54	11.54	11.54	11.54	11.54	11.54
11.55	11.55	11.55	11.55	11.55	11.55	11.55	11.55	11.55
11.56	11.56	11.56	11.56	11.56	11.56	11.56	11.56	11.56
11.57	11.57	11.57	11.57	11.57	11.57	11.57	11.57	11.57
11.58	11.58	11.58	11.58	11.58	11.58	11.58	11.58	11.58
11.59	11.59	11.59	11.59	11.59	11.59	11.59	11.59	11.59
11.6	11.6	11.6	11.6	11.6	11.6	11.6	11.6	11.6
11.61	11.61	11.61	11.61	11.61	11.61	11.61	11.61	11.61
11.62	11.62	11.62	11.62	11.62	11.62	11.62	11.62	11.62
11.63	11.63	11.63	11.63	11.63	11.63	11.63	11.63	11.63
11.64	11.64	11.64	11.64	11.64	11.64	11.64	11.64	11.64
11.65	11.65	11.65	11.65	11.65	11.65	11.65	11.65	11.65
11.66	11.66	11.66	11.66	11.66	11.66	11.66	11.66	11.66
11.67	11.67	11.67	11.67	11.67	11.67	11.67	11.67	11.67
11.68	11.68	11.68	11.68	11.68	11.68	11.68	11.68	11.68
11.69	11.69	11.69	11.69	11.69	11.69	11.69	11.69	11.69
11.7	11.7	11.7	11.7	11.7	11.7	11.7	11.7	11.7
11.71	11.71	11.71	11.71	11.71	11.71	11.71	11.71	11.71
11.72	11.72	11.72	11.72	11.72	11.72	11.72	11.72	11.72
11.73	11.73	11.73	11.73	11.73	11.73	11.73	11.73	11.73
11.74	11.74	11.74	11.74	11.74	11.74	11.74	11.74	11.74
11.75	11.75	11.75	11.75	11.75	11.75	11.75	11.75	11.75
11.76	11.76	11.76	11.76	11.76	11.76	11.76	11.76	11.76
11.77	11.77	11.77	11.77	11.77	11.77	11.77	11.77	11.77
11.78	11.78	11.78	11.78	11.78	11.78	11.78	11.78	11.78
11.79	11.79	11.79	11.79	11.79	11.79	11.79	11.79	11.79
11.8	11.8	11.8	11.8	11.8	11.8	11.8	11.8	11.8
11.81	11.81	11.81	11.81	11.81	11.81	11.81	11.81	11.81
11.82	11.82	11.82	11.82	11.82	11.82	11.82	11.82	11.82
11.83	11.83	11.83	11.83	11.83	11.83	11.83	11.83	11.83
11.84	11.84	11.84	11.84	11.84	11.84	11.84	11.84	11.84
11.85	11.85	11.85	11.85	11.85	11.85	11.85	11.85	11.85
11.86	11.86	11.86	11.86	11.86	11.86	11.86	11.86	11.86
11.87	11.87	11.87	11.87	11.87	11.87	11.87	11.87	11.87
11.88	11.88	11.88	11.88	11.88	11.88	11.88	11.88	11.88
11.89	11.89	11.89	11.89	11.89	11.89	11.89	11.89	11.89
11.9	11.9	11.9	11.9	11.9	11.9	11.9	11.9	11.9
11.91	11.91	11.91	11.91	11.91	11.91	11.91	11.91	11.91
11.92	11.92	11.92	11.92	11.92	11.92	11.92	11.92	11.92
11.93	11.93	11.93	11.93	11.93	11.93	11.93	11.93	11.93
11.94	11.94	11.94	11.94	11.94	11.94	11.94	11.94	11.94
11.95	11.95	11.95	11.95	11.95	11.95	11.95	11.95	11.95
11.96	11.96	11.96	11.96	11.96	11.96	11.96	11.96	11.96
11.97	11.97	11.97	11.97	11.97	11.97	11.97	11.97	11.97
11.98	11.98	11.98	11.98	11.98	11.98	11.98	11.98	11.98
11.99	11.99	11.99	11.99	11.99	11.99	11.99	11.99	11.99

12	12	12	12	12	12	12	12	12

-12	-12	-12	-12	-12	-12	-12	-12	-12
-11.99	-11.99	-11.99	-11.99	-11.99	-11.99	-11.99	-11.99	-11.99
-11.98	-11.98	-11.98	-11.98	-11.98	-11.98	-11.98	-11.98	-11.98
-11.97	-11.97	-11.97	-11.97	-11.97	-11.97	-11.97	-11.97	-11.97
-11.96	-11.96	-11.96	-11.96	-11.96	-11.96	-11.96	-11.96	-11.96
-11.95	-11.95	-11.95	-11.95	-11.95	-11.95	-11.95	-11.95	-11.95
-11.94	-11.94	-11.94	-11.94	-11.94	-11.94	-11.94	-11.94	-11.94
-11.93	-11.93	-11.93	-11.93	-11.93	-11.93	-11.93	-11.93	-11.93
-11.92	-11.92	-11.92	-11.92	-11.92	-11.92	-11.92	-11.92	-11.92
-11.91	-11.91	-11.91	-11.91	-11.91	-11.91	-11.91	-11.91	-11.91
-11.9	-11.9	-11.9	-11.9	-11.9	-11.9	-11.9	-11.9	-11.9
-11.89	-11.89	-11.89	-11.89	-11.89	-11.89	-11.89	-11.89	-11.89
-11.88	-11.88	-11.88	-11.88	-11.88	-11.88	-11.88	-11.88	-11.88
-11.87	-11.87	-11.87	-11.87	-11.87	-11.87	-11.87	-11.87	-11.87
-11.86	-11.86	-11.86	-11.86	-11.86	-11.86	-11.86	-11.86	-11.86
-11.85	-11.85	-11.85	-11.85	-11.85	-11.85	-11.85	-11.85	-11.85
-11.84	-11.84	-11.84	-11.84	-11.84	-11.84	-11.84	-11.84	-11.84
-11.83	-11.83	-11.83	-11.83	-11.83	-11.83	-11.83	-11.83	-11.83
-11.82	-11.82	-11.82	-11.82	-11.82	-11.82	-11.82	-11.82	-11.82
-11.81	-11.81	-11.81	-11.81	-11.81	-11.81	-11.81	-11.81	-11.81
-11.8	-11.8	-11.8	-11.8	-11.8	-11.8	-11.8	-11.8	-11.8
-11.79	-11.79	-11.79	-11.79	-11.79	-11.79	-11.79	-11.79	-11.79
-11.78	-11.78	-11.78	-11.78	-11.78	-11.78	-11.78	-11.78	-11.78
-11.77	-11.77	-11.77	-11.77	-11.77	-11.77	-11.77	-11.77	-11.77
-11.76	-11.76	-11.76	-11.76	-11.76	-11.76	-11.76	-11.76	-11.76
-11.75	-11.75	-11.75	-11.75	-11.75	-11.75	-11.75	-11.75	-11.75
-11.74	-11.74	-11.74	-11.74	-11.74	-11.74	-11.74	-11.74	-11.74
-11.73	-11.73	-11.73	-11.73	-11.73	-11.73	-11.73	-11.73	-11.73
-11.72	-11.72	-11.72	-11.72	-11.72	-11.72	-11.72	-11.72	-11.72
-11.71	-11.71	-11.71	-11.71	-11.71	-11.71	-11.71	-11.71	-11.71
-11.7	-11.7	-11.7	-11.7	-11.7	-11.7	-11.7	-11.7	-11.7
-11.69	-11.69	-11.69	-11.69	-11.69	-11.69	-11.69	-11.69	-11.69
-11.68	-11.68	-11.68	-11.68	-11.68	-11.68	-11.68	-11.68	-11.68
-11.67	-11.67	-11.67	-11.67	-11.67	-11.67	-11.67	-11.67	-11.67
-11.66	-11.66	-11.66	-11.66	-11.66	-11.66	-11.66	-11.66	-11.66
-11.65	-11.65	-11.65	-11.65	-11.65	-11.65	-11.65	-11.65	-11.65
-11.64	-11.64	-11.64	-11.64	-11.64	-11.64	-11.64	-11.64	-11.64
-11.63	-11.63	-11.63	-11.63	-11.63	-11.63	-11.63	-11.63	-11.63
-11.62	-11.62	-11.62	-11.62	-11.62	-11.62	-11.62	-11.62	-11.62
-11.61	-11.61	-11.61	-11.61	-11.61	-11.61	-11.61	-11.61	-11.61
-11.6	-11.6	-11.6	-11.6	-11.6	-11.6	-11.6	-11.6	-11.6
-11.59	-11.59	-11.59	-11.59	-11.59	-11.59	-11.59	-11.59	-11.59
-11.58	-11.58	-11.58	-11.58	-11.58	-11.58	-11.58	-11.58	-11.58
-11.57	-11.57	-11.57	-11.57	-11.57	-11.57	-11.57	-11.57	-11.57
-11.56	-11.56	-11.56	-11.56	-11.56	-11.56	-11.56	-11.56	-11.56
-11.55	-11.55	-11.55	-11.55	-11.55	-11.55	-11.55	-11.55	-11.55
-11.54	-11.54	-11.54	-11.54	-11.54	-11.54	-11.54	-11.54	-11.54
-11.53	-11.53	-11.53	-11.53	-11.53	-11.53	-11.53	-11.53	-11.53
-11.52	-11.52	-11.52	-11.52	-11.52	-11.52	-11.52	-11.52	-11.52
-11.51	-11.51	-11.51	-11.51	-11.51	-11.51	-11.51	-11.51	-11.51

-11.5	-11.5	-11.5	-11.5	-11.5	-11.5	-11.5	-11.5	-11.5
-11.49	-11.49	-11.49	-11.49	-11.49	-11.49	-11.49	-11.49	-11.49
-11.48	-11.48	-11.48	-11.48	-11.48	-11.48	-11.48	-11.48	-11.48
-11.47	-11.47	-11.47	-11.47	-11.47	-11.47	-11.47	-11.47	-11.47
-11.46	-11.46	-11.46	-11.46	-11.46	-11.46	-11.46	-11.46	-11.46
-11.45	-11.45	-11.45	-11.45	-11.45	-11.45	-11.45	-11.45	-11.45
-11.44	-11.44	-11.44	-11.44	-11.44	-11.44	-11.44	-11.44	-11.44
-11.43	-11.43	-11.43	-11.43	-11.43	-11.43	-11.43	-11.43	-11.43
-11.42	-11.42	-11.42	-11.42	-11.42	-11.42	-11.42	-11.42	-11.42
-11.41	-11.41	-11.41	-11.41	-11.41	-11.41	-11.41	-11.41	-11.41
-11.4	-11.4	-11.4	-11.4	-11.4	-11.4	-11.4	-11.4	-11.4
-11.39	-11.39	-11.39	-11.39	-11.39	-11.39	-11.39	-11.39	-11.39
-11.38	-11.38	-11.38	-11.38	-11.38	-11.38	-11.38	-11.38	-11.38
-11.37	-11.37	-11.37	-11.37	-11.37	-11.37	-11.37	-11.37	-11.37
-11.36	-11.36	-11.36	-11.36	-11.36	-11.36	-11.36	-11.36	-11.36
-11.35	-11.35	-11.35	-11.35	-11.35	-11.35	-11.35	-11.35	-11.35
-11.34	-11.34	-11.34	-11.34	-11.34	-11.34	-11.34	-11.34	-11.34
-11.33	-11.33	-11.33	-11.33	-11.33	-11.33	-11.33	-11.33	-11.33
-11.32	-11.32	-11.32	-11.32	-11.32	-11.32	-11.32	-11.32	-11.32
-11.31	-11.31	-11.31	-11.31	-11.31	-11.31	-11.31	-11.31	-11.31
-11.3	-11.3	-11.3	-11.3	-11.3	-11.3	-11.3	-11.3	-11.3
-11.29	-11.29	-11.29	-11.29	-11.29	-11.29	-11.29	-11.29	-11.29
-11.28	-11.28	-11.28	-11.28	-11.28	-11.28	-11.28	-11.28	-11.28
-11.27	-11.27	-11.27	-11.27	-11.27	-11.27	-11.27	-11.27	-11.27
-11.26	-11.26	-11.26	-11.26	-11.26	-11.26	-11.26	-11.26	-11.26
-11.25	-11.25	-11.25	-11.25	-11.25	-11.25	-11.25	-11.25	-11.25
-11.24	-11.24	-11.24	-11.24	-11.24	-11.24	-11.24	-11.24	-11.24
-11.23	-11.23	-11.23	-11.23	-11.23	-11.23	-11.23	-11.23	-11.23
-11.22	-11.22	-11.22	-11.22	-11.22	-11.22	-11.22	-11.22	-11.22
-11.21	-11.21	-11.21	-11.21	-11.21	-11.21	-11.21	-11.21	-11.21
-11.2	-11.2	-11.2	-11.2	-11.2	-11.2	-11.2	-11.2	-11.2
-11.19	-11.19	-11.19	-11.19	-11.19	-11.19	-11.19	-11.19	-11.19
-11.18	-11.18	-11.18	-11.18	-11.18	-11.18	-11.18	-11.18	-11.18
-11.17	-11.17	-11.17	-11.17	-11.17	-11.17	-11.17	-11.17	-11.17
-11.16	-11.16	-11.16	-11.16	-11.16	-11.16	-11.16	-11.16	-11.16
-11.15	-11.15	-11.15	-11.15	-11.15	-11.15	-11.15	-11.15	-11.15
-11.14	-11.14	-11.14	-11.14	-11.14	-11.14	-11.14	-11.14	-11.14
-11.13	-11.13	-11.13	-11.13	-11.13	-11.13	-11.13	-11.13	-11.13
-11.12	-11.12	-11.12	-11.12	-11.12	-11.12	-11.12	-11.12	-11.12
-11.11	-11.11	-11.11	-11.11	-11.11	-11.11	-11.11	-11.11	-11.11
-11.1	-11.1	-11.1	-11.1	-11.1	-11.1	-11.1	-11.1	-11.1
-11.09	-11.09	-11.09	-11.09	-11.09	-11.09	-11.09	-11.09	-11.09
-11.08	-11.08	-11.08	-11.08	-11.08	-11.08	-11.08	-11.08	-11.08
-11.07	-11.07	-11.07	-11.07	-11.07	-11.07	-11.07	-11.07	-11.07
-11.06	-11.06	-11.06	-11.06	-11.06	-11.06	-11.06	-11.06	-11.06
-11.05	-11.05	-11.05	-11.05	-11.05	-11.05	-11.05	-11.05	-11.05
-11.04	-11.04	-11.04	-11.04	-11.04	-11.04	-11.04	-11.04	-11.04
-11.03	-11.03	-11.03	-11.03	-11.03	-11.03	-11.03	-11.03	-11.03
-11.02	-11.02	-11.02	-11.02	-11.02	-11.02	-11.02	-11.02	-11.02
-11.01	-11.01	-11.01	-11.01	-11.01	-11.01	-11.01	-11.01	-11.01

-11	-11	-11	-11	-11	-11	-11	-11	-11
-10.99	-10.99	-10.99	-10.99	-10.99	-10.99	-10.99	-10.99	-10.99
-10.98	-10.98	-10.98	-10.98	-10.98	-10.98	-10.98	-10.98	-10.98
-10.97	-10.97	-10.97	-10.97	-10.97	-10.97	-10.97	-10.97	-10.97
-10.96	-10.96	-10.96	-10.96	-10.96	-10.96	-10.96	-10.96	-10.96
-10.95	-10.95	-10.95	-10.95	-10.95	-10.95	-10.95	-10.95	-10.95
-10.94	-10.94	-10.94	-10.94	-10.94	-10.94	-10.94	-10.94	-10.94
-10.93	-10.93	-10.93	-10.93	-10.93	-10.93	-10.93	-10.93	-10.93
-10.92	-10.92	-10.92	-10.92	-10.92	-10.92	-10.92	-10.92	-10.92
-10.91	-10.91	-10.91	-10.91	-10.91	-10.91	-10.91	-10.91	-10.91
-10.9	-10.9	-10.9	-10.9	-10.9	-10.9	-10.9	-10.9	-10.9
-10.89	-10.89	-10.89	-10.89	-10.89	-10.89	-10.89	-10.89	-10.89
-10.88	-10.88	-10.88	-10.88	-10.88	-10.88	-10.88	-10.88	-10.88
-10.87	-10.87	-10.87	-10.87	-10.87	-10.87	-10.87	-10.87	-10.87
-10.86	-10.86	-10.86	-10.86	-10.86	-10.86	-10.86	-10.86	-10.86
-10.85	-10.85	-10.85	-10.85	-10.85	-10.85	-10.85	-10.85	-10.85
-10.84	-10.84	-10.84	-10.84	-10.84	-10.84	-10.84	-10.84	-10.84
-10.83	-10.83	-10.83	-10.83	-10.83	-10.83	-10.83	-10.83	-10.83
-10.82	-10.82	-10.82	-10.82	-10.82	-10.82	-10.82	-10.82	-10.82
-10.81	-10.81	-10.81	-10.81	-10.81	-10.81	-10.81	-10.81	-10.81
-10.8	-10.8	-10.8	-10.8	-10.8	-10.8	-10.8	-10.8	-10.8
-10.79	-10.79	-10.79	-10.79	-10.79	-10.79	-10.79	-10.79	-10.79
-10.78	-10.78	-10.78	-10.78	-10.78	-10.78	-10.78	-10.78	-10.78
-10.77	-10.77	-10.77	-10.77	-10.77	-10.77	-10.77	-10.77	-10.77
-10.76	-10.76	-10.76	-10.76	-10.76	-10.76	-10.76	-10.76	-10.76
-10.75	-10.75	-10.75	-10.75	-10.75	-10.75	-10.75	-10.75	-10.75
-10.74	-10.74	-10.74	-10.74	-10.74	-10.74	-10.74	-10.74	-10.74
-10.73	-10.73	-10.73	-10.73	-10.73	-10.73	-10.73	-10.73	-10.73
-10.72	-10.72	-10.72	-10.72	-10.72	-10.72	-10.72	-10.72	-10.72
-10.71	-10.71	-10.71	-10.71	-10.71	-10.71	-10.71	-10.71	-10.71
-10.7	-10.7	-10.7	-10.7	-10.7	-10.7	-10.7	-10.7	-10.7
-10.69	-10.69	-10.69	-10.69	-10.69	-10.69	-10.69	-10.69	-10.69
-10.68	-10.68	-10.68	-10.68	-10.68	-10.68	-10.68	-10.68	-10.68
-10.67	-10.67	-10.67	-10.67	-10.67	-10.67	-10.67	-10.67	-10.67
-10.66	-10.66	-10.66	-10.66	-10.66	-10.66	-10.66	-10.66	-10.66
-10.65	-10.65	-10.65	-10.65	-10.65	-10.65	-10.65	-10.65	-10.65
-10.64	-10.64	-10.64	-10.64	-10.64	-10.64	-10.64	-10.64	-10.64
-10.63	-10.63	-10.63	-10.63	-10.63	-10.63	-10.63	-10.63	-10.63
-10.62	-10.62	-10.62	-10.62	-10.62	-10.62	-10.62	-10.62	-10.62
-10.61	-10.61	-10.61	-10.61	-10.61	-10.61	-10.61	-10.61	-10.61
-10.6	-10.6	-10.6	-10.6	-10.6	-10.6	-10.6	-10.6	-10.6
-10.59	-10.59	-10.59	-10.59	-10.59	-10.59	-10.59	-10.59	-10.59
-10.58	-10.58	-10.58	-10.58	-10.58	-10.58	-10.58	-10.58	-10.58
-10.57	-10.57	-10.57	-10.57	-10.57	-10.57	-10.57	-10.57	-10.57
-10.56	-10.56	-10.56	-10.56	-10.56	-10.56	-10.56	-10.56	-10.56
-10.55	-10.55	-10.55	-10.55	-10.55	-10.55	-10.55	-10.55	-10.55
-10.54	-10.54	-10.54	-10.54	-10.54	-10.54	-10.54	-10.54	-10.54
-10.53	-10.53	-10.53	-10.53	-10.53	-10.53	-10.53	-10.53	-10.53
-10.52	-10.52	-10.52	-10.52	-10.52	-10.52	-10.52	-10.52	-10.52
-10.51	-10.51	-10.51	-10.51	-10.51	-10.51	-10.51	-10.51	-10.51

-10.5	-10.5	-10.5	-10.5	-10.5	-10.5	-10.5	-10.5	-10.5
-10.49	-10.49	-10.49	-10.49	-10.49	-10.49	-10.49	-10.49	-10.49
-10.48	-10.48	-10.48	-10.48	-10.48	-10.48	-10.48	-10.48	-10.48
-10.47	-10.47	-10.47	-10.47	-10.47	-10.47	-10.47	-10.47	-10.47
-10.46	-10.46	-10.46	-10.46	-10.46	-10.46	-10.46	-10.46	-10.46
-10.45	-10.45	-10.45	-10.45	-10.45	-10.45	-10.45	-10.45	-10.45
-10.44	-10.44	-10.44	-10.44	-10.44	-10.44	-10.44	-10.44	-10.44
-10.43	-10.43	-10.43	-10.43	-10.43	-10.43	-10.43	-10.43	-10.43
-10.42	-10.42	-10.42	-10.42	-10.42	-10.42	-10.42	-10.42	-10.42
-10.41	-10.41	-10.41	-10.41	-10.41	-10.41	-10.41	-10.41	-10.41
-10.4	-10.4	-10.4	-10.4	-10.4	-10.4	-10.4	-10.4	-10.4
-10.39	-10.39	-10.39	-10.39	-10.39	-10.39	-10.39	-10.39	-10.39
-10.38	-10.38	-10.38	-10.38	-10.38	-10.38	-10.38	-10.38	-10.38
-10.37	-10.37	-10.37	-10.37	-10.37	-10.37	-10.37	-10.37	-10.37
-10.36	-10.36	-10.36	-10.36	-10.36	-10.36	-10.36	-10.36	-10.36
-10.35	-10.35	-10.35	-10.35	-10.35	-10.35	-10.35	-10.35	-10.35
-10.34	-10.34	-10.34	-10.34	-10.34	-10.34	-10.34	-10.34	-10.34
-10.33	-10.33	-10.33	-10.33	-10.33	-10.33	-10.33	-10.33	-10.33
-10.32	-10.32	-10.32	-10.32	-10.32	-10.32	-10.32	-10.32	-10.32
-10.31	-10.31	-10.31	-10.31	-10.31	-10.31	-10.31	-10.31	-10.31
-10.3	-10.3	-10.3	-10.3	-10.3	-10.3	-10.3	-10.3	-10.3
-10.29	-10.29	-10.29	-10.29	-10.29	-10.29	-10.29	-10.29	-10.29
-10.28	-10.28	-10.28	-10.28	-10.28	-10.28	-10.28	-10.28	-10.28
-10.27	-10.27	-10.27	-10.27	-10.27	-10.27	-10.27	-10.27	-10.27
-10.26	-10.26	-10.26	-10.26	-10.26	-10.26	-10.26	-10.26	-10.26
-10.25	-10.25	-10.25	-10.25	-10.25	-10.25	-10.25	-10.25	-10.25
-10.24	-10.24	-10.24	-10.24	-10.24	-10.24	-10.24	-10.24	-10.24
-10.23	-10.23	-10.23	-10.23	-10.23	-10.23	-10.23	-10.23	-10.23
-10.22	-10.22	-10.22	-10.22	-10.22	-10.22	-10.22	-10.22	-10.22
-10.21	-10.21	-10.21	-10.21	-10.21	-10.21	-10.21	-10.21	-10.21
-10.2	-10.2	-10.2	-10.2	-10.2	-10.2	-10.2	-10.2	-10.2
-10.19	-10.19	-10.19	-10.19	-10.19	-10.19	-10.19	-10.19	-10.19
-10.18	-10.18	-10.18	-10.18	-10.18	-10.18	-10.18	-10.18	-10.18
-10.17	-10.17	-10.17	-10.17	-10.17	-10.17	-10.17	-10.17	-10.17
-10.16	-10.16	-10.16	-10.16	-10.16	-10.16	-10.16	-10.16	-10.16
-10.15	-10.15	-10.15	-10.15	-10.15	-10.15	-10.15	-10.15	-10.15
-10.14	-10.14	-10.14	-10.14	-10.14	-10.14	-10.14	-10.14	-10.14
-10.13	-10.13	-10.13	-10.13	-10.13	-10.13	-10.13	-10.13	-10.13
-10.12	-10.12	-10.12	-10.12	-10.12	-10.12	-10.12	-10.12	-10.12
-10.11	-10.11	-10.11	-10.11	-10.11	-10.11	-10.11	-10.11	-10.11
-10.1	-10.1	-10.1	-10.1	-10.1	-10.1	-10.1	-10.1	-10.1
-10.09	-10.09	-10.09	-10.09	-10.09	-10.09	-10.09	-10.09	-10.09
-10.08	-10.08	-10.08	-10.08	-10.08	-10.08	-10.08	-10.08	-10.08
-10.07	-10.07	-10.07	-10.07	-10.07	-10.07	-10.07	-10.07	-10.07
-10.06	-10.06	-10.06	-10.06	-10.06	-10.06	-10.06	-10.06	-10.06
-10.05	-10.05	-10.05	-10.05	-10.05	-10.05	-10.05	-10.05	-10.05
-10.04	-10.04	-10.04	-10.04	-10.04	-10.04	-10.04	-10.04	-10.04
-10.03	-10.03	-10.03	-10.03	-10.03	-10.03	-10.03	-10.03	-10.03
-10.02	-10.02	-10.02	-10.02	-10.02	-10.02	-10.02	-10.02	-10.02
-10.01	-10.01	-10.01	-10.01	-10.01	-10.01	-10.01	-10.01	-10.01

-10	-10	-10	-10	-10	-10	-10	-10	-10
-9.99	-9.99	-9.99	-9.99	-9.99	-9.99	-9.99	-9.99	-9.99
-9.98	-9.98	-9.98	-9.98	-9.98	-9.98	-9.98	-9.98	-9.98
-9.97	-9.97	-9.97	-9.97	-9.97	-9.97	-9.97	-9.97	-9.97
-9.96	-9.96	-9.96	-9.96	-9.96	-9.96	-9.96	-9.96	-9.96
-9.95	-9.95	-9.95	-9.95	-9.95	-9.95	-9.95	-9.95	-9.95
-9.94	-9.94	-9.94	-9.94	-9.94	-9.94	-9.94	-9.94	-9.94
-9.93	-9.93	-9.93	-9.93	-9.93	-9.93	-9.93	-9.93	-9.93
-9.92	-9.92	-9.92	-9.92	-9.92	-9.92	-9.92	-9.92	-9.92
-9.91	-9.91	-9.91	-9.91	-9.91	-9.91	-9.91	-9.91	-9.91
-9.9	-9.9	-9.9	-9.9	-9.9	-9.9	-9.9	-9.9	-9.9
-9.89	-9.89	-9.89	-9.89	-9.89	-9.89	-9.89	-9.89	-9.89
-9.88	-9.88	-9.88	-9.88	-9.88	-9.88	-9.88	-9.88	-9.88
-9.87	-9.87	-9.87	-9.87	-9.87	-9.87	-9.87	-9.87	-9.87
-9.86	-9.86	-9.86	-9.86	-9.86	-9.86	-9.86	-9.86	-9.86
-9.85	-9.85	-9.85	-9.85	-9.85	-9.85	-9.85	-9.85	-9.85
-9.84	-9.84	-9.84	-9.84	-9.84	-9.84	-9.84	-9.84	-9.84
-9.83	-9.83	-9.83	-9.83	-9.83	-9.83	-9.83	-9.83	-9.83
-9.82	-9.82	-9.82	-9.82	-9.82	-9.82	-9.82	-9.82	-9.82
-9.81	-9.81	-9.81	-9.81	-9.81	-9.81	-9.81	-9.81	-9.81
-9.8	-9.8	-9.8	-9.8	-9.8	-9.8	-9.8	-9.8	-9.8
-9.79	-9.79	-9.79	-9.79	-9.79	-9.79	-9.79	-9.79	-9.79
-9.78	-9.78	-9.78	-9.78	-9.78	-9.78	-9.78	-9.78	-9.78
-9.77	-9.77	-9.77	-9.77	-9.77	-9.77	-9.77	-9.77	-9.77
-9.76	-9.76	-9.76	-9.76	-9.76	-9.76	-9.76	-9.76	-9.76
-9.75	-9.75	-9.75	-9.75	-9.75	-9.75	-9.75	-9.75	-9.75
-9.74	-9.74	-9.74	-9.74	-9.74	-9.74	-9.74	-9.74	-9.74
-9.73	-9.73	-9.73	-9.73	-9.73	-9.73	-9.73	-9.73	-9.73
-9.72	-9.72	-9.72	-9.72	-9.72	-9.72	-9.72	-9.72	-9.72
-9.71	-9.71	-9.71	-9.71	-9.71	-9.71	-9.71	-9.71	-9.71
-9.7	-9.7	-9.7	-9.7	-9.7	-9.7	-9.7	-9.7	-9.7
-9.69	-9.69	-9.69	-9.69	-9.69	-9.69	-9.69	-9.69	-9.69
-9.68	-9.68	-9.68	-9.68	-9.68	-9.68	-9.68	-9.68	-9.68
-9.67	-9.67	-9.67	-9.67	-9.67	-9.67	-9.67	-9.67	-9.67
-9.66	-9.66	-9.66	-9.66	-9.66	-9.66	-9.66	-9.66	-9.66
-9.65	-9.65	-9.65	-9.65	-9.65	-9.65	-9.65	-9.65	-9.65
-9.64	-9.64	-9.64	-9.64	-9.64	-9.64	-9.64	-9.64	-9.64
-9.63	-9.63	-9.63	-9.63	-9.63	-9.63	-9.63	-9.63	-9.63
-9.62	-9.62	-9.62	-9.62	-9.62	-9.62	-9.62	-9.62	-9.62
-9.61	-9.61	-9.61	-9.61	-9.61	-9.61	-9.61	-9.61	-9.61
-9.6	-9.6	-9.6	-9.6	-9.6	-9.6	-9.6	-9.6	-9.6
-9.59	-9.59	-9.59	-9.59	-9.59	-9.59	-9.59	-9.59	-9.59
-9.58	-9.58	-9.58	-9.58	-9.58	-9.58	-9.58	-9.58	-9.58
-9.57	-9.57	-9.57	-9.57	-9.57	-9.57	-9.57	-9.57	-9.57
-9.56	-9.56	-9.56	-9.56	-9.56	-9.56	-9.56	-9.56	-9.56
-9.55	-9.55	-9.55	-9.55	-9.55	-9.55	-9.55	-9.55	-9.55
-9.54	-9.54	-9.54	-9.54	-9.54	-9.54	-9.54	-9.54	-9.54
-9.53	-9.53	-9.53	-9.53	-9.53	-9.53	-9.53	-9.53	-9.53
-9.52	-9.52	-9.52	-9.52	-9.52	-9.52	-9.52	-9.52	-9.52
-9.51	-9.51	-9.51	-9.51	-9.51	-9.51	-9.51	-9.51	-9.51

-9.5	-9.5	-9.5	-9.5	-9.5	-9.5	-9.5	-9.5	-9.5
-9.49	-9.49	-9.49	-9.49	-9.49	-9.49	-9.49	-9.49	-9.49
-9.48	-9.48	-9.48	-9.48	-9.48	-9.48	-9.48	-9.48	-9.48
-9.47	-9.47	-9.47	-9.47	-9.47	-9.47	-9.47	-9.47	-9.47
-9.46	-9.46	-9.46	-9.46	-9.46	-9.46	-9.46	-9.46	-9.46
-9.45	-9.45	-9.45	-9.45	-9.45	-9.45	-9.45	-9.45	-9.45
-9.44	-9.44	-9.44	-9.44	-9.44	-9.44	-9.44	-9.44	-9.44
-9.43	-9.43	-9.43	-9.43	-9.43	-9.43	-9.43	-9.43	-9.43
-9.42	-9.42	-9.42	-9.42	-9.42	-9.42	-9.42	-9.42	-9.42
-9.41	-9.41	-9.41	-9.41	-9.41	-9.41	-9.41	-9.41	-9.41
-9.4	-9.4	-9.4	-9.4	-9.4	-9.4	-9.4	-9.4	-9.4
-9.39	-9.39	-9.39	-9.39	-9.39	-9.39	-9.39	-9.39	-9.39
-9.38	-9.38	-9.38	-9.38	-9.38	-9.38	-9.38	-9.38	-9.38
-9.37	-9.37	-9.37	-9.37	-9.37	-9.37	-9.37	-9.37	-9.37
-9.36	-9.36	-9.36	-9.36	-9.36	-9.36	-9.36	-9.36	-9.36
-9.35	-9.35	-9.35	-9.35	-9.35	-9.35	-9.35	-9.35	-9.35
-9.34	-9.34	-9.34	-9.34	-9.34	-9.34	-9.34	-9.34	-9.34
-9.33	-9.33	-9.33	-9.33	-9.33	-9.33	-9.33	-9.33	-9.33
-9.32	-9.32	-9.32	-9.32	-9.32	-9.32	-9.32	-9.32	-9.32
-9.31	-9.31	-9.31	-9.31	-9.31	-9.31	-9.31	-9.31	-9.31
-9.3	-9.3	-9.3	-9.3	-9.3	-9.3	-9.3	-9.3	-9.3
-9.29	-9.29	-9.29	-9.29	-9.29	-9.29	-9.29	-9.29	-9.29
-9.28	-9.28	-9.28	-9.28	-9.28	-9.28	-9.28	-9.28	-9.28
-9.27	-9.27	-9.27	-9.27	-9.27	-9.27	-9.27	-9.27	-9.27
-9.26	-9.26	-9.26	-9.26	-9.26	-9.26	-9.26	-9.26	-9.26
-9.25	-9.25	-9.25	-9.25	-9.25	-9.25	-9.25	-9.25	-9.25
-9.24	-9.24	-9.24	-9.24	-9.24	-9.24	-9.24	-9.24	-9.24
-9.23	-9.23	-9.23	-9.23	-9.23	-9.23	-9.23	-9.23	-9.23
-9.22	-9.22	-9.22	-9.22	-9.22	-9.22	-9.22	-9.22	-9.22
-9.21	-9.21	-9.21	-9.21	-9.21	-9.21	-9.21	-9.21	-9.21
-9.2	-9.2	-9.2	-9.2	-9.2	-9.2	-9.2	-9.2	-9.2
-9.19	-9.19	-9.19	-9.19	-9.19	-9.19	-9.19	-9.19	-9.19
-9.18	-9.18	-9.18	-9.18	-9.18	-9.18	-9.18	-9.18	-9.18
-9.17	-9.17	-9.17	-9.17	-9.17	-9.17	-9.17	-9.17	-9.17
-9.16	-9.16	-9.16	-9.16	-9.16	-9.16	-9.16	-9.16	-9.16
-9.15	-9.15	-9.15	-9.15	-9.15	-9.15	-9.15	-9.15	-9.15
-9.14	-9.14	-9.14	-9.14	-9.14	-9.14	-9.14	-9.14	-9.14
-9.13	-9.13	-9.13	-9.13	-9.13	-9.13	-9.13	-9.13	-9.13
-9.12	-9.12	-9.12	-9.12	-9.12	-9.12	-9.12	-9.12	-9.12
-9.11	-9.11	-9.11	-9.11	-9.11	-9.11	-9.11	-9.11	-9.11
-9.1	-9.1	-9.1	-9.1	-9.1	-9.1	-9.1	-9.1	-9.1
-9.09	-9.09	-9.09	-9.09	-9.09	-9.09	-9.09	-9.09	-9.09
-9.08	-9.08	-9.08	-9.08	-9.08	-9.08	-9.08	-9.08	-9.08
-9.07	-9.07	-9.07	-9.07	-9.07	-9.07	-9.07	-9.07	-9.07
-9.06	-9.06	-9.06	-9.06	-9.06	-9.06	-9.06	-9.06	-9.06
-9.05	-9.05	-9.05	-9.05	-9.05	-9.05	-9.05	-9.05	-9.05
-9.04	-9.04	-9.04	-9.04	-9.04	-9.04	-9.04	-9.04	-9.04
-9.03	-9.03	-9.03	-9.03	-9.03	-9.03	-9.03	-9.03	-9.03
-9.02	-9.02	-9.02	-9.02	-9.02	-9.02	-9.02	-9.02	-9.02
-9.01	-9.01	-9.01	-9.01	-9.01	-9.01	-9.01	-9.01	-9.01

-9	-9	-9	-9	-9	-9	-9	-9	-9
-8.99	-8.99	-8.99	-8.99	-8.99	-8.99	-8.99	-8.99	-8.99
-8.98	-8.98	-8.98	-8.98	-8.98	-8.98	-8.98	-8.98	-8.98
-8.97	-8.97	-8.97	-8.97	-8.97	-8.97	-8.97	-8.97	-8.97
-8.96	-8.96	-8.96	-8.96	-8.96	-8.96	-8.96	-8.96	-8.96
-8.95	-8.95	-8.95	-8.95	-8.95	-8.95	-8.95	-8.95	-8.95
-8.94	-8.94	-8.94	-8.94	-8.94	-8.94	-8.94	-8.94	-8.94
-8.93	-8.93	-8.93	-8.93	-8.93	-8.93	-8.93	-8.93	-8.93
-8.92	-8.92	-8.92	-8.92	-8.92	-8.92	-8.92	-8.92	-8.92
-8.91	-8.91	-8.91	-8.91	-8.91	-8.91	-8.91	-8.91	-8.91
-8.9	-8.9	-8.9	-8.9	-8.9	-8.9	-8.9	-8.9	-8.9
-8.89	-8.89	-8.89	-8.89	-8.89	-8.89	-8.89	-8.89	-8.89
-8.88	-8.88	-8.88	-8.88	-8.88	-8.88	-8.88	-8.88	-8.88
-8.87	-8.87	-8.87	-8.87	-8.87	-8.87	-8.87	-8.87	-8.87
-8.86	-8.86	-8.86	-8.86	-8.86	-8.86	-8.86	-8.86	-8.86
-8.85	-8.85	-8.85	-8.85	-8.85	-8.85	-8.85	-8.85	-8.85
-8.84	-8.84	-8.84	-8.84	-8.84	-8.84	-8.84	-8.84	-8.84
-8.83	-8.83	-8.83	-8.83	-8.83	-8.83	-8.83	-8.83	-8.83
-8.82	-8.82	-8.82	-8.82	-8.82	-8.82	-8.82	-8.82	-8.82
-8.81	-8.81	-8.81	-8.81	-8.81	-8.81	-8.81	-8.81	-8.81
-8.8	-8.8	-8.8	-8.8	-8.8	-8.8	-8.8	-8.8	-8.8
-8.79	-8.79	-8.79	-8.79	-8.79	-8.79	-8.79	-8.79	-8.79
-8.78	-8.78	-8.78	-8.78	-8.78	-8.78	-8.78	-8.78	-8.78
-8.77	-8.77	-8.77	-8.77	-8.77	-8.77	-8.77	-8.77	-8.77
-8.76	-8.76	-8.76	-8.76	-8.76	-8.76	-8.76	-8.76	-8.76
-8.75	-8.75	-8.75	-8.75	-8.75	-8.75	-8.75	-8.75	-8.75
-8.74	-8.74	-8.74	-8.74	-8.74	-8.74	-8.74	-8.74	-8.74
-8.73	-8.73	-8.73	-8.73	-8.73	-8.73	-8.73	-8.73	-8.73
-8.72	-8.72	-8.72	-8.72	-8.72	-8.72	-8.72	-8.72	-8.72
-8.71	-8.71	-8.71	-8.71	-8.71	-8.71	-8.71	-8.71	-8.71
-8.7	-8.7	-8.7	-8.7	-8.7	-8.7	-8.7	-8.7	-8.7
-8.69	-8.69	-8.69	-8.69	-8.69	-8.69	-8.69	-8.69	-8.69
-8.68	-8.68	-8.68	-8.68	-8.68	-8.68	-8.68	-8.68	-8.68
-8.67	-8.67	-8.67	-8.67	-8.67	-8.67	-8.67	-8.67	-8.67
-8.66	-8.66	-8.66	-8.66	-8.66	-8.66	-8.66	-8.66	-8.66
-8.65	-8.65	-8.65	-8.65	-8.65	-8.65	-8.65	-8.65	-8.65
-8.64	-8.64	-8.64	-8.64	-8.64	-8.64	-8.64	-8.64	-8.64
-8.63	-8.63	-8.63	-8.63	-8.63	-8.63	-8.63	-8.63	-8.63
-8.62	-8.62	-8.62	-8.62	-8.62	-8.62	-8.62	-8.62	-8.62
-8.61	-8.61	-8.61	-8.61	-8.61	-8.61	-8.61	-8.61	-8.61
-8.6	-8.6	-8.6	-8.6	-8.6	-8.6	-8.6	-8.6	-8.6
-8.59	-8.59	-8.59	-8.59	-8.59	-8.59	-8.59	-8.59	-8.59
-8.58	-8.58	-8.58	-8.58	-8.58	-8.58	-8.58	-8.58	-8.58
-8.57	-8.57	-8.57	-8.57	-8.57	-8.57	-8.57	-8.57	-8.57
-8.56	-8.56	-8.56	-8.56	-8.56	-8.56	-8.56	-8.56	-8.56
-8.55	-8.55	-8.55	-8.55	-8.55	-8.55	-8.55	-8.55	-8.55
-8.54	-8.54	-8.54	-8.54	-8.54	-8.54	-8.54	-8.54	-8.54
-8.53	-8.53	-8.53	-8.53	-8.53	-8.53	-8.53	-8.53	-8.53
-8.52	-8.52	-8.52	-8.52	-8.52	-8.52	-8.52	-8.52	-8.52
-8.51	-8.51	-8.51	-8.51	-8.51	-8.51	-8.51	-8.51	-8.51

-8.5	-8.5	-8.5	-8.5	-8.5	-8.5	-8.5	-8.5	-8.5
-8.49	-8.49	-8.49	-8.49	-8.49	-8.49	-8.49	-8.49	-8.49
-8.48	-8.48	-8.48	-8.48	-8.48	-8.48	-8.48	-8.48	-8.48
-8.47	-8.47	-8.47	-8.47	-8.47	-8.47	-8.47	-8.47	-8.47
-8.46	-8.46	-8.46	-8.46	-8.46	-8.46	-8.46	-8.46	-8.46
-8.45	-8.45	-8.45	-8.45	-8.45	-8.45	-8.45	-8.45	-8.45
-8.44	-8.44	-8.44	-8.44	-8.44	-8.44	-8.44	-8.44	-8.44
-8.43	-8.43	-8.43	-8.43	-8.43	-8.43	-8.43	-8.43	-8.43
-8.42	-8.42	-8.42	-8.42	-8.42	-8.42	-8.42	-8.42	-8.42
-8.41	-8.41	-8.41	-8.41	-8.41	-8.41	-8.41	-8.41	-8.41
-8.4	-8.4	-8.4	-8.4	-8.4	-8.4	-8.4	-8.4	-8.4
-8.39	-8.39	-8.39	-8.39	-8.39	-8.39	-8.39	-8.39	-8.39
-8.38	-8.38	-8.38	-8.38	-8.38	-8.38	-8.38	-8.38	-8.38
-8.37	-8.37	-8.37	-8.37	-8.37	-8.37	-8.37	-8.37	-8.37
-8.36	-8.36	-8.36	-8.36	-8.36	-8.36	-8.36	-8.36	-8.36
-8.35	-8.35	-8.35	-8.35	-8.35	-8.35	-8.35	-8.35	-8.35
-8.34	-8.34	-8.34	-8.34	-8.34	-8.34	-8.34	-8.34	-8.34
-8.33	-8.33	-8.33	-8.33	-8.33	-8.33	-8.33	-8.33	-8.33
-8.32	-8.32	-8.32	-8.32	-8.32	-8.32	-8.32	-8.32	-8.32
-8.31	-8.31	-8.31	-8.31	-8.31	-8.31	-8.31	-8.31	-8.31
-8.3	-8.3	-8.3	-8.3	-8.3	-8.3	-8.3	-8.3	-8.3
-8.29	-8.29	-8.29	-8.29	-8.29	-8.29	-8.29	-8.29	-8.29
-8.28	-8.28	-8.28	-8.28	-8.28	-8.28	-8.28	-8.28	-8.28
-8.27	-8.27	-8.27	-8.27	-8.27	-8.27	-8.27	-8.27	-8.27
-8.26	-8.26	-8.26	-8.26	-8.26	-8.26	-8.26	-8.26	-8.26
-8.25	-8.25	-8.25	-8.25	-8.25	-8.25	-8.25	-8.25	-8.25
-8.24	-8.24	-8.24	-8.24	-8.24	-8.24	-8.24	-8.24	-8.24
-8.23	-8.23	-8.23	-8.23	-8.23	-8.23	-8.23	-8.23	-8.23
-8.22	-8.22	-8.22	-8.22	-8.22	-8.22	-8.22	-8.22	-8.22
-8.21	-8.21	-8.21	-8.21	-8.21	-8.21	-8.21	-8.21	-8.21
-8.2	-8.2	-8.2	-8.2	-8.2	-8.2	-8.2	-8.2	-8.2
-8.19	-8.19	-8.19	-8.19	-8.19	-8.19	-8.19	-8.19	-8.19
-8.18	-8.18	-8.18	-8.18	-8.18	-8.18	-8.18	-8.18	-8.18
-8.17	-8.17	-8.17	-8.17	-8.17	-8.17	-8.17	-8.17	-8.17
-8.16	-8.16	-8.16	-8.16	-8.16	-8.16	-8.16	-8.16	-8.16
-8.15	-8.15	-8.15	-8.15	-8.15	-8.15	-8.15	-8.15	-8.15
-8.14	-8.14	-8.14	-8.14	-8.14	-8.14	-8.14	-8.14	-8.14
-8.13	-8.13	-8.13	-8.13	-8.13	-8.13	-8.13	-8.13	-8.13
-8.12	-8.12	-8.12	-8.12	-8.12	-8.12	-8.12	-8.12	-8.12
-8.11	-8.11	-8.11	-8.11	-8.11	-8.11	-8.11	-8.11	-8.11
-8.1	-8.1	-8.1	-8.1	-8.1	-8.1	-8.1	-8.1	-8.1
-8.09	-8.09	-8.09	-8.09	-8.09	-8.09	-8.09	-8.09	-8.09
-8.08	-8.08	-8.08	-8.08	-8.08	-8.08	-8.08	-8.08	-8.08
-8.07	-8.07	-8.07	-8.07	-8.07	-8.07	-8.07	-8.07	-8.07
-8.06	-8.06	-8.06	-8.06	-8.06	-8.06	-8.06	-8.06	-8.06
-8.05	-8.05	-8.05	-8.05	-8.05	-8.05	-8.05	-8.05	-8.05
-8.04	-8.04	-8.04	-8.04	-8.04	-8.04	-8.04	-8.04	-8.04
-8.03	-8.03	-8.03	-8.03	-8.03	-8.03	-8.03	-8.03	-8.03
-8.02	-8.02	-8.02	-8.02	-8.02	-8.02	-8.02	-8.02	-8.02
-8.01	-8.01	-8.01	-8.01	-8.01	-8.01	-8.01	-8.01	-8.01

-8	-8	-8	-8	-8	-8	-8	-8	-8
-7.99	-7.99	-7.99	-7.99	-7.99	-7.99	-7.99	-7.99	-7.99
-7.98	-7.98	-7.98	-7.98	-7.98	-7.98	-7.98	-7.98	-7.98
-7.97	-7.97	-7.97	-7.97	-7.97	-7.97	-7.97	-7.97	-7.97
-7.96	-7.96	-7.96	-7.96	-7.96	-7.96	-7.96	-7.96	-7.96
-7.95	-7.95	-7.95	-7.95	-7.95	-7.95	-7.95	-7.95	-7.95
-7.94	-7.94	-7.94	-7.94	-7.94	-7.94	-7.94	-7.94	-7.94
-7.93	-7.93	-7.93	-7.93	-7.93	-7.93	-7.93	-7.93	-7.93
-7.92	-7.92	-7.92	-7.92	-7.92	-7.92	-7.92	-7.92	-7.92
-7.91	-7.91	-7.91	-7.91	-7.91	-7.91	-7.91	-7.91	-7.91
-7.9	-7.9	-7.9	-7.9	-7.9	-7.9	-7.9	-7.9	-7.9
-7.89	-7.89	-7.89	-7.89	-7.89	-7.89	-7.89	-7.89	-7.89
-7.88	-7.88	-7.88	-7.88	-7.88	-7.88	-7.88	-7.88	-7.88
-7.87	-7.87	-7.87	-7.87	-7.87	-7.87	-7.87	-7.87	-7.87
-7.86	-7.86	-7.86	-7.86	-7.86	-7.86	-7.86	-7.86	-7.86
-7.85	-7.85	-7.85	-7.85	-7.85	-7.85	-7.85	-7.85	-7.85
-7.84	-7.84	-7.84	-7.84	-7.84	-7.84	-7.84	-7.84	-7.84
-7.83	-7.83	-7.83	-7.83	-7.83	-7.83	-7.83	-7.83	-7.83
-7.82	-7.82	-7.82	-7.82	-7.82	-7.82	-7.82	-7.82	-7.82
-7.81	-7.81	-7.81	-7.81	-7.81	-7.81	-7.81	-7.81	-7.81
-7.8	-7.8	-7.8	-7.8	-7.8	-7.8	-7.8	-7.8	-7.8
-7.79	-7.79	-7.79	-7.79	-7.79	-7.79	-7.79	-7.79	-7.79
-7.78	-7.78	-7.78	-7.78	-7.78	-7.78	-7.78	-7.78	-7.78
-7.77	-7.77	-7.77	-7.77	-7.77	-7.77	-7.77	-7.77	-7.77
-7.76	-7.76	-7.76	-7.76	-7.76	-7.76	-7.76	-7.76	-7.76
-7.75	-7.75	-7.75	-7.75	-7.75	-7.75	-7.75	-7.75	-7.75
-7.74	-7.74	-7.74	-7.74	-7.74	-7.74	-7.74	-7.74	-7.74
-7.73	-7.73	-7.73	-7.73	-7.73	-7.73	-7.73	-7.73	-7.73
-7.72	-7.72	-7.72	-7.72	-7.72	-7.72	-7.72	-7.72	-7.72
-7.71	-7.71	-7.71	-7.71	-7.71	-7.71	-7.71	-7.71	-7.71
-7.7	-7.7	-7.7	-7.7	-7.7	-7.7	-7.7	-7.7	-7.7
-7.69	-7.69	-7.69	-7.69	-7.69	-7.69	-7.69	-7.69	-7.69
-7.68	-7.68	-7.68	-7.68	-7.68	-7.68	-7.68	-7.68	-7.68
-7.67	-7.67	-7.67	-7.67	-7.67	-7.67	-7.67	-7.67	-7.67
-7.66	-7.66	-7.66	-7.66	-7.66	-7.66	-7.66	-7.66	-7.66
-7.65	-7.65	-7.65	-7.65	-7.65	-7.65	-7.65	-7.65	-7.65
-7.64	-7.64	-7.64	-7.64	-7.64	-7.64	-7.64	-7.64	-7.64
-7.63	-7.63	-7.63	-7.63	-7.63	-7.63	-7.63	-7.63	-7.63
-7.62	-7.62	-7.62	-7.62	-7.62	-7.62	-7.62	-7.62	-7.62
-7.61	-7.61	-7.61	-7.61	-7.61	-7.61	-7.61	-7.61	-7.61
-7.6	-7.6	-7.6	-7.6	-7.6	-7.6	-7.6	-7.6	-7.6
-7.59	-7.59	-7.59	-7.59	-7.59	-7.59	-7.59	-7.59	-7.59
-7.58	-7.58	-7.58	-7.58	-7.58	-7.58	-7.58	-7.58	-7.58
-7.57	-7.57	-7.57	-7.57	-7.57	-7.57	-7.57	-7.57	-7.57
-7.56	-7.56	-7.56	-7.56	-7.56	-7.56	-7.56	-7.56	-7.56
-7.55	-7.55	-7.55	-7.55	-7.55	-7.55	-7.55	-7.55	-7.55
-7.54	-7.54	-7.54	-7.54	-7.54	-7.54	-7.54	-7.54	-7.54
-7.53	-7.53	-7.53	-7.53	-7.53	-7.53	-7.53	-7.53	-7.53
-7.52	-7.52	-7.52	-7.52	-7.52	-7.52	-7.52	-7.52	-7.52
-7.51	-7.51	-7.51	-7.51	-7.51	-7.51	-7.51	-7.51	-7.51

-7.5	-7.5	-7.5	-7.5	-7.5	-7.5	-7.5	-7.5	-7.5
-7.49	-7.49	-7.49	-7.49	-7.49	-7.49	-7.49	-7.49	-7.49
-7.48	-7.48	-7.48	-7.48	-7.48	-7.48	-7.48	-7.48	-7.48
-7.47	-7.47	-7.47	-7.47	-7.47	-7.47	-7.47	-7.47	-7.47
-7.46	-7.46	-7.46	-7.46	-7.46	-7.46	-7.46	-7.46	-7.46
-7.45	-7.45	-7.45	-7.45	-7.45	-7.45	-7.45	-7.45	-7.45
-7.44	-7.44	-7.44	-7.44	-7.44	-7.44	-7.44	-7.44	-7.44
-7.43	-7.43	-7.43	-7.43	-7.43	-7.43	-7.43	-7.43	-7.43
-7.42	-7.42	-7.42	-7.42	-7.42	-7.42	-7.42	-7.42	-7.42
-7.41	-7.41	-7.41	-7.41	-7.41	-7.41	-7.41	-7.41	-7.41
-7.4	-7.4	-7.4	-7.4	-7.4	-7.4	-7.4	-7.4	-7.4
-7.39	-7.39	-7.39	-7.39	-7.39	-7.39	-7.39	-7.39	-7.39
-7.38	-7.38	-7.38	-7.38	-7.38	-7.38	-7.38	-7.38	-7.38
-7.37	-7.37	-7.37	-7.37	-7.37	-7.37	-7.37	-7.37	-7.37
-7.36	-7.36	-7.36	-7.36	-7.36	-7.36	-7.36	-7.36	-7.36
-7.35	-7.35	-7.35	-7.35	-7.35	-7.35	-7.35	-7.35	-7.35
-7.34	-7.34	-7.34	-7.34	-7.34	-7.34	-7.34	-7.34	-7.34
-7.33	-7.33	-7.33	-7.33	-7.33	-7.33	-7.33	-7.33	-7.33
-7.32	-7.32	-7.32	-7.32	-7.32	-7.32	-7.32	-7.32	-7.32
-7.31	-7.31	-7.31	-7.31	-7.31	-7.31	-7.31	-7.31	-7.31
-7.3	-7.3	-7.3	-7.3	-7.3	-7.3	-7.3	-7.3	-7.3
-7.29	-7.29	-7.29	-7.29	-7.29	-7.29	-7.29	-7.29	-7.29
-7.28	-7.28	-7.28	-7.28	-7.28	-7.28	-7.28	-7.28	-7.28
-7.27	-7.27	-7.27	-7.27	-7.27	-7.27	-7.27	-7.27	-7.27
-7.26	-7.26	-7.26	-7.26	-7.26	-7.26	-7.26	-7.26	-7.26
-7.25	-7.25	-7.25	-7.25	-7.25	-7.25	-7.25	-7.25	-7.25
-7.24	-7.24	-7.24	-7.24	-7.24	-7.24	-7.24	-7.24	-7.24
-7.23	-7.23	-7.23	-7.23	-7.23	-7.23	-7.23	-7.23	-7.23
-7.22	-7.22	-7.22	-7.22	-7.22	-7.22	-7.22	-7.22	-7.22
-7.21	-7.21	-7.21	-7.21	-7.21	-7.21	-7.21	-7.21	-7.21
-7.2	-7.2	-7.2	-7.2	-7.2	-7.2	-7.2	-7.2	-7.2
-7.19	-7.19	-7.19	-7.19	-7.19	-7.19	-7.19	-7.19	-7.19
-7.18	-7.18	-7.18	-7.18	-7.18	-7.18	-7.18	-7.18	-7.18
-7.17	-7.17	-7.17	-7.17	-7.17	-7.17	-7.17	-7.17	-7.17
-7.16	-7.16	-7.16	-7.16	-7.16	-7.16	-7.16	-7.16	-7.16
-7.15	-7.15	-7.15	-7.15	-7.15	-7.15	-7.15	-7.15	-7.15
-7.14	-7.14	-7.14	-7.14	-7.14	-7.14	-7.14	-7.14	-7.14
-7.13	-7.13	-7.13	-7.13	-7.13	-7.13	-7.13	-7.13	-7.13
-7.12	-7.12	-7.12	-7.12	-7.12	-7.12	-7.12	-7.12	-7.12
-7.11	-7.11	-7.11	-7.11	-7.11	-7.11	-7.11	-7.11	-7.11
-7.1	-7.1	-7.1	-7.1	-7.1	-7.1	-7.1	-7.1	-7.1
-7.09	-7.09	-7.09	-7.09	-7.09	-7.09	-7.09	-7.09	-7.09
-7.08	-7.08	-7.08	-7.08	-7.08	-7.08	-7.08	-7.08	-7.08
-7.07	-7.07	-7.07	-7.07	-7.07	-7.07	-7.07	-7.07	-7.07
-7.06	-7.06	-7.06	-7.06	-7.06	-7.06	-7.06	-7.06	-7.06
-7.05	-7.05	-7.05	-7.05	-7.05	-7.05	-7.05	-7.05	-7.05
-7.04	-7.04	-7.04	-7.04	-7.04	-7.04	-7.04	-7.04	-7.04
-7.03	-7.03	-7.03	-7.03	-7.03	-7.03	-7.03	-7.03	-7.03
-7.02	-7.02	-7.02	-7.02	-7.02	-7.02	-7.02	-7.02	-7.02
-7.01	-7.01	-7.01	-7.01	-7.01	-7.01	-7.01	-7.01	-7.01

-7	-7	-7	-7	-7	-7	-7	-7	-7
-6.99	-6.99	-6.99	-6.99	-6.99	-6.99	-6.99	-6.99	-6.99
-6.98	-6.98	-6.98	-6.98	-6.98	-6.98	-6.98	-6.98	-6.98
-6.97	-6.97	-6.97	-6.97	-6.97	-6.97	-6.97	-6.97	-6.97
-6.96	-6.96	-6.96	-6.96	-6.96	-6.96	-6.96	-6.96	-6.96
-6.95	-6.95	-6.95	-6.95	-6.95	-6.95	-6.95	-6.95	-6.95
-6.94	-6.94	-6.94	-6.94	-6.94	-6.94	-6.94	-6.94	-6.94
-6.93	-6.93	-6.93	-6.93	-6.93	-6.93	-6.93	-6.93	-6.93
-6.92	-6.92	-6.92	-6.92	-6.92	-6.92	-6.92	-6.92	-6.92
-6.91	-6.91	-6.91	-6.91	-6.91	-6.91	-6.91	-6.91	-6.91
-6.9	-6.9	-6.9	-6.9	-6.9	-6.9	-6.9	-6.9	-6.9
-6.89	-6.89	-6.89	-6.89	-6.89	-6.89	-6.89	-6.89	-6.89
-6.88	-6.88	-6.88	-6.88	-6.88	-6.88	-6.88	-6.88	-6.88
-6.87	-6.87	-6.87	-6.87	-6.87	-6.87	-6.87	-6.87	-6.87
-6.86	-6.86	-6.86	-6.86	-6.86	-6.86	-6.86	-6.86	-6.86
-6.85	-6.85	-6.85	-6.85	-6.85	-6.85	-6.85	-6.85	-6.85
-6.84	-6.84	-6.84	-6.84	-6.84	-6.84	-6.84	-6.84	-6.84
-6.83	-6.83	-6.83	-6.83	-6.83	-6.83	-6.83	-6.83	-6.83
-6.82	-6.82	-6.82	-6.82	-6.82	-6.82	-6.82	-6.82	-6.82
-6.81	-6.81	-6.81	-6.81	-6.81	-6.81	-6.81	-6.81	-6.81
-6.8	-6.8	-6.8	-6.8	-6.8	-6.8	-6.8	-6.8	-6.8
-6.79	-6.79	-6.79	-6.79	-6.79	-6.79	-6.79	-6.79	-6.79
-6.78	-6.78	-6.78	-6.78	-6.78	-6.78	-6.78	-6.78	-6.78
-6.77	-6.77	-6.77	-6.77	-6.77	-6.77	-6.77	-6.77	-6.77
-6.76	-6.76	-6.76	-6.76	-6.76	-6.76	-6.76	-6.76	-6.76
-6.75	-6.75	-6.75	-6.75	-6.75	-6.75	-6.75	-6.75	-6.75
-6.74	-6.74	-6.74	-6.74	-6.74	-6.74	-6.74	-6.74	-6.74
-6.73	-6.73	-6.73	-6.73	-6.73	-6.73	-6.73	-6.73	-6.73
-6.72	-6.72	-6.72	-6.72	-6.72	-6.72	-6.72	-6.72	-6.72
-6.71	-6.71	-6.71	-6.71	-6.71	-6.71	-6.71	-6.71	-6.71
-6.7	-6.7	-6.7	-6.7	-6.7	-6.7	-6.7	-6.7	-6.7
-6.69	-6.69	-6.69	-6.69	-6.69	-6.69	-6.69	-6.69	-6.69
-6.68	-6.68	-6.68	-6.68	-6.68	-6.68	-6.68	-6.68	-6.68
-6.67	-6.67	-6.67	-6.67	-6.67	-6.67	-6.67	-6.67	-6.67
-6.66	-6.66	-6.66	-6.66	-6.66	-6.66	-6.66	-6.66	-6.66
-6.65	-6.65	-6.65	-6.65	-6.65	-6.65	-6.65	-6.65	-6.65
-6.64	-6.64	-6.64	-6.64	-6.64	-6.64	-6.64	-6.64	-6.64
-6.63	-6.63	-6.63	-6.63	-6.63	-6.63	-6.63	-6.63	-6.63
-6.62	-6.62	-6.62	-6.62	-6.62	-6.62	-6.62	-6.62	-6.62
-6.61	-6.61	-6.61	-6.61	-6.61	-6.61	-6.61	-6.61	-6.61
-6.6	-6.6	-6.6	-6.6	-6.6	-6.6	-6.6	-6.6	-6.6
-6.59	-6.59	-6.59	-6.59	-6.59	-6.59	-6.59	-6.59	-6.59
-6.58	-6.58	-6.58	-6.58	-6.58	-6.58	-6.58	-6.58	-6.58
-6.57	-6.57	-6.57	-6.57	-6.57	-6.57	-6.57	-6.57	-6.57
-6.56	-6.56	-6.56	-6.56	-6.56	-6.56	-6.56	-6.56	-6.56
-6.55	-6.55	-6.55	-6.55	-6.55	-6.55	-6.55	-6.55	-6.55
-6.54	-6.54	-6.54	-6.54	-6.54	-6.54	-6.54	-6.54	-6.54
-6.53	-6.53	-6.53	-6.53	-6.53	-6.53	-6.53	-6.53	-6.53
-6.52	-6.52	-6.52	-6.52	-6.52	-6.52	-6.52	-6.52	-6.52
-6.51	-6.51	-6.51	-6.51	-6.51	-6.51	-6.51	-6.51	-6.51

-6.5	-6.5	-6.5	-6.5	-6.5	-6.5	-6.5	-6.5	-6.5
-6.49	-6.49	-6.49	-6.49	-6.49	-6.49	-6.49	-6.49	-6.49
-6.48	-6.48	-6.48	-6.48	-6.48	-6.48	-6.48	-6.48	-6.48
-6.47	-6.47	-6.47	-6.47	-6.47	-6.47	-6.47	-6.47	-6.47
-6.46	-6.46	-6.46	-6.46	-6.46	-6.46	-6.46	-6.46	-6.46
-6.45	-6.45	-6.45	-6.45	-6.45	-6.45	-6.45	-6.45	-6.45
-6.44	-6.44	-6.44	-6.44	-6.44	-6.44	-6.44	-6.44	-6.44
-6.43	-6.43	-6.43	-6.43	-6.43	-6.43	-6.43	-6.43	-6.43
-6.42	-6.42	-6.42	-6.42	-6.42	-6.42	-6.42	-6.42	-6.42
-6.41	-6.41	-6.41	-6.41	-6.41	-6.41	-6.41	-6.41	-6.41
-6.4	-6.4	-6.4	-6.4	-6.4	-6.4	-6.4	-6.4	-6.4
-6.39	-6.39	-6.39	-6.39	-6.39	-6.39	-6.39	-6.39	-6.39
-6.38	-6.38	-6.38	-6.38	-6.38	-6.38	-6.38	-6.38	-6.38
-6.37	-6.37	-6.37	-6.37	-6.37	-6.37	-6.37	-6.37	-6.37
-6.36	-6.36	-6.36	-6.36	-6.36	-6.36	-6.36	-6.36	-6.36
-6.35	-6.35	-6.35	-6.35	-6.35	-6.35	-6.35	-6.35	-6.35
-6.34	-6.34	-6.34	-6.34	-6.34	-6.34	-6.34	-6.34	-6.34
-6.33	-6.33	-6.33	-6.33	-6.33	-6.33	-6.33	-6.33	-6.33
-6.32	-6.32	-6.32	-6.32	-6.32	-6.32	-6.32	-6.32	-6.32
-6.31	-6.31	-6.31	-6.31	-6.31	-6.31	-6.31	-6.31	-6.31
-6.3	-6.3	-6.3	-6.3	-6.3	-6.3	-6.3	-6.3	-6.3
-6.29	-6.29	-6.29	-6.29	-6.29	-6.29	-6.29	-6.29	-6.29
-6.28	-6.28	-6.28	-6.28	-6.28	-6.28	-6.28	-6.28	-6.28
-6.27	-6.27	-6.27	-6.27	-6.27	-6.27	-6.27	-6.27	-6.27
-6.26	-6.26	-6.26	-6.26	-6.26	-6.26	-6.26	-6.26	-6.26
-6.25	-6.25	-6.25	-6.25	-6.25	-6.25	-6.25	-6.25	-6.25
-6.24	-6.24	-6.24	-6.24	-6.24	-6.24	-6.24	-6.24	-6.24
-6.23	-6.23	-6.23	-6.23	-6.23	-6.23	-6.23	-6.23	-6.23
-6.22	-6.22	-6.22	-6.22	-6.22	-6.22	-6.22	-6.22	-6.22
-6.21	-6.21	-6.21	-6.21	-6.21	-6.21	-6.21	-6.21	-6.21
-6.2	-6.2	-6.2	-6.2	-6.2	-6.2	-6.2	-6.2	-6.2
-6.19	-6.19	-6.19	-6.19	-6.19	-6.19	-6.19	-6.19	-6.19
-6.18	-6.18	-6.18	-6.18	-6.18	-6.18	-6.18	-6.18	-6.18
-6.17	-6.17	-6.17	-6.17	-6.17	-6.17	-6.17	-6.17	-6.17
-6.16	-6.16	-6.16	-6.16	-6.16	-6.16	-6.16	-6.16	-6.16
-6.15	-6.15	-6.15	-6.15	-6.15	-6.15	-6.15	-6.15	-6.15
-6.14	-6.14	-6.14	-6.14	-6.14	-6.14	-6.14	-6.14	-6.14
-6.13	-6.13	-6.13	-6.13	-6.13	-6.13	-6.13	-6.13	-6.13
-6.12	-6.12	-6.12	-6.12	-6.12	-6.12	-6.12	-6.12	-6.12
-6.11	-6.11	-6.11	-6.11	-6.11	-6.11	-6.11	-6.11	-6.11
-6.1	-6.1	-6.1	-6.1	-6.1	-6.1	-6.1	-6.1	-6.1
-6.09	-6.09	-6.09	-6.09	-6.09	-6.09	-6.09	-6.09	-6.09
-6.08	-6.08	-6.08	-6.08	-6.08	-6.08	-6.08	-6.08	-6.08
-6.07	-6.07	-6.07	-6.07	-6.07	-6.07	-6.07	-6.07	-6.07
-6.06	-6.06	-6.06	-6.06	-6.06	-6.06	-6.06	-6.06	-6.06
-6.05	-6.05	-6.05	-6.05	-6.05	-6.05	-6.05	-6.05	-6.05
-6.04	-6.04	-6.04	-6.04	-6.04	-6.04	-6.04	-6.04	-6.04
-6.03	-6.03	-6.03	-6.03	-6.03	-6.03	-6.03	-6.03	-6.03
-6.02	-6.02	-6.02	-6.02	-6.02	-6.02	-6.02	-6.02	-6.02
-6.01	-6.01	-6.01	-6.01	-6.01	-6.01	-6.01	-6.01	-6.01

-6	-6	-6	-6	-6	-6	-6	-6	-6
-5.99	-5.99	-5.99	-5.99	-5.99	-5.99	-5.99	-5.99	-5.99
-5.98	-5.98	-5.98	-5.98	-5.98	-5.98	-5.98	-5.98	-5.98
-5.97	-5.97	-5.97	-5.97	-5.97	-5.97	-5.97	-5.97	-5.97
-5.96	-5.96	-5.96	-5.96	-5.96	-5.96	-5.96	-5.96	-5.96
-5.95	-5.95	-5.95	-5.95	-5.95	-5.95	-5.95	-5.95	-5.95
-5.94	-5.94	-5.94	-5.94	-5.94	-5.94	-5.94	-5.94	-5.94
-5.93	-5.93	-5.93	-5.93	-5.93	-5.93	-5.93	-5.93	-5.93
-5.92	-5.92	-5.92	-5.92	-5.92	-5.92	-5.92	-5.92	-5.92
-5.91	-5.91	-5.91	-5.91	-5.91	-5.91	-5.91	-5.91	-5.91
-5.9	-5.9	-5.9	-5.9	-5.9	-5.9	-5.9	-5.9	-5.9
-5.89	-5.89	-5.89	-5.89	-5.89	-5.89	-5.89	-5.89	-5.89
-5.88	-5.88	-5.88	-5.88	-5.88	-5.88	-5.88	-5.88	-5.88
-5.87	-5.87	-5.87	-5.87	-5.87	-5.87	-5.87	-5.87	-5.87
-5.86	-5.86	-5.86	-5.86	-5.86	-5.86	-5.86	-5.86	-5.86
-5.85	-5.85	-5.85	-5.85	-5.85	-5.85	-5.85	-5.85	-5.85
-5.84	-5.84	-5.84	-5.84	-5.84	-5.84	-5.84	-5.84	-5.84
-5.83	-5.83	-5.83	-5.83	-5.83	-5.83	-5.83	-5.83	-5.83
-5.82	-5.82	-5.82	-5.82	-5.82	-5.82	-5.82	-5.82	-5.82
-5.81	-5.81	-5.81	-5.81	-5.81	-5.81	-5.81	-5.81	-5.81
-5.8	-5.8	-5.8	-5.8	-5.8	-5.8	-5.8	-5.8	-5.8
-5.79	-5.79	-5.79	-5.79	-5.79	-5.79	-5.79	-5.79	-5.79
-5.78	-5.78	-5.78	-5.78	-5.78	-5.78	-5.78	-5.78	-5.78
-5.77	-5.77	-5.77	-5.77	-5.77	-5.77	-5.77	-5.77	-5.77
-5.76	-5.76	-5.76	-5.76	-5.76	-5.76	-5.76	-5.76	-5.76
-5.75	-5.75	-5.75	-5.75	-5.75	-5.75	-5.75	-5.75	-5.75
-5.74	-5.74	-5.74	-5.74	-5.74	-5.74	-5.74	-5.74	-5.74
-5.73	-5.73	-5.73	-5.73	-5.73	-5.73	-5.73	-5.73	-5.73
-5.72	-5.72	-5.72	-5.72	-5.72	-5.72	-5.72	-5.72	-5.72
-5.71	-5.71	-5.71	-5.71	-5.71	-5.71	-5.71	-5.71	-5.71
-5.7	-5.7	-5.7	-5.7	-5.7	-5.7	-5.7	-5.7	-5.7
-5.69	-5.69	-5.69	-5.69	-5.69	-5.69	-5.69	-5.69	-5.69
-5.68	-5.68	-5.68	-5.68	-5.68	-5.68	-5.68	-5.68	-5.68
-5.67	-5.67	-5.67	-5.67	-5.67	-5.67	-5.67	-5.67	-5.67
-5.66	-5.66	-5.66	-5.66	-5.66	-5.66	-5.66	-5.66	-5.66
-5.65	-5.65	-5.65	-5.65	-5.65	-5.65	-5.65	-5.65	-5.65
-5.64	-5.64	-5.64	-5.64	-5.64	-5.64	-5.64	-5.64	-5.64
-5.63	-5.63	-5.63	-5.63	-5.63	-5.63	-5.63	-5.63	-5.63
-5.62	-5.62	-5.62	-5.62	-5.62	-5.62	-5.62	-5.62	-5.62
-5.61	-5.61	-5.61	-5.61	-5.61	-5.61	-5.61	-5.61	-5.61
-5.6	-5.6	-5.6	-5.6	-5.6	-5.6	-5.6	-5.6	-5.6
-5.59	-5.59	-5.59	-5.59	-5.59	-5.59	-5.59	-5.59	-5.59
-5.58	-5.58	-5.58	-5.58	-5.58	-5.58	-5.58	-5.58	-5.58
-5.57	-5.57	-5.57	-5.57	-5.57	-5.57	-5.57	-5.57	-5.57
-5.56	-5.56	-5.56	-5.56	-5.56	-5.56	-5.56	-5.56	-5.56
-5.55	-5.55	-5.55	-5.55	-5.55	-5.55	-5.55	-5.55	-5.55
-5.54	-5.54	-5.54	-5.54	-5.54	-5.54	-5.54	-5.54	-5.54
-5.53	-5.53	-5.53	-5.53	-5.53	-5.53	-5.53	-5.53	-5.53
-5.52	-5.52	-5.52	-5.52	-5.52	-5.52	-5.52	-5.52	-5.52
-5.51	-5.51	-5.51	-5.51	-5.51	-5.51	-5.51	-5.51	-5.51

-5.5	-5.5	-5.5	-5.5	-5.5	-5.5	-5.5	-5.5	-5.5
-5.49	-5.49	-5.49	-5.49	-5.49	-5.49	-5.49	-5.49	-5.49
-5.48	-5.48	-5.48	-5.48	-5.48	-5.48	-5.48	-5.48	-5.48
-5.47	-5.47	-5.47	-5.47	-5.47	-5.47	-5.47	-5.47	-5.47
-5.46	-5.46	-5.46	-5.46	-5.46	-5.46	-5.46	-5.46	-5.46
-5.45	-5.45	-5.45	-5.45	-5.45	-5.45	-5.45	-5.45	-5.45
-5.44	-5.44	-5.44	-5.44	-5.44	-5.44	-5.44	-5.44	-5.44
-5.43	-5.43	-5.43	-5.43	-5.43	-5.43	-5.43	-5.43	-5.43
-5.42	-5.42	-5.42	-5.42	-5.42	-5.42	-5.42	-5.42	-5.42
-5.41	-5.41	-5.41	-5.41	-5.41	-5.41	-5.41	-5.41	-5.41
-5.4	-5.4	-5.4	-5.4	-5.4	-5.4	-5.4	-5.4	-5.4
-5.39	-5.39	-5.39	-5.39	-5.39	-5.39	-5.39	-5.39	-5.39
-5.38	-5.38	-5.38	-5.38	-5.38	-5.38	-5.38	-5.38	-5.38
-5.37	-5.37	-5.37	-5.37	-5.37	-5.37	-5.37	-5.37	-5.37
-5.36	-5.36	-5.36	-5.36	-5.36	-5.36	-5.36	-5.36	-5.36
-5.35	-5.35	-5.35	-5.35	-5.35	-5.35	-5.35	-5.35	-5.35
-5.34	-5.34	-5.34	-5.34	-5.34	-5.34	-5.34	-5.34	-5.34
-5.33	-5.33	-5.33	-5.33	-5.33	-5.33	-5.33	-5.33	-5.33
-5.32	-5.32	-5.32	-5.32	-5.32	-5.32	-5.32	-5.32	-5.32
-5.31	-5.31	-5.31	-5.31	-5.31	-5.31	-5.31	-5.31	-5.31
-5.3	-5.3	-5.3	-5.3	-5.3	-5.3	-5.3	-5.3	-5.3
-5.29	-5.29	-5.29	-5.29	-5.29	-5.29	-5.29	-5.29	-5.29
-5.28	-5.28	-5.28	-5.28	-5.28	-5.28	-5.28	-5.28	-5.28
-5.27	-5.27	-5.27	-5.27	-5.27	-5.27	-5.27	-5.27	-5.27
-5.26	-5.26	-5.26	-5.26	-5.26	-5.26	-5.26	-5.26	-5.26
-5.25	-5.25	-5.25	-5.25	-5.25	-5.25	-5.25	-5.25	-5.25
-5.24	-5.24	-5.24	-5.24	-5.24	-5.24	-5.24	-5.24	-5.24
-5.23	-5.23	-5.23	-5.23	-5.23	-5.23	-5.23	-5.23	-5.23
-5.22	-5.22	-5.22	-5.22	-5.22	-5.22	-5.22	-5.22	-5.22
-5.21	-5.21	-5.21	-5.21	-5.21	-5.21	-5.21	-5.21	-5.21
-5.2	-5.2	-5.2	-5.2	-5.2	-5.2	-5.2	-5.2	-5.2
-5.19	-5.19	-5.19	-5.19	-5.19	-5.19	-5.19	-5.19	-5.19
-5.18	-5.18	-5.18	-5.18	-5.18	-5.18	-5.18	-5.18	-5.18
-5.17	-5.17	-5.17	-5.17	-5.17	-5.17	-5.17	-5.17	-5.17
-5.16	-5.16	-5.16	-5.16	-5.16	-5.16	-5.16	-5.16	-5.16
-5.15	-5.15	-5.15	-5.15	-5.15	-5.15	-5.15	-5.15	-5.15
-5.14	-5.14	-5.14	-5.14	-5.14	-5.14	-5.14	-5.14	-5.14
-5.13	-5.13	-5.13	-5.13	-5.13	-5.13	-5.13	-5.13	-5.13
-5.12	-5.12	-5.12	-5.12	-5.12	-5.12	-5.12	-5.12	-5.12
-5.11	-5.11	-5.11	-5.11	-5.11	-5.11	-5.11	-5.11	-5.11
-5.1	-5.1	-5.1	-5.1	-5.1	-5.1	-5.1	-5.1	-5.1
-5.09	-5.09	-5.09	-5.09	-5.09	-5.09	-5.09	-5.09	-5.09
-5.08	-5.08	-5.08	-5.08	-5.08	-5.08	-5.08	-5.08	-5.08
-5.07	-5.07	-5.07	-5.07	-5.07	-5.07	-5.07	-5.07	-5.07
-5.06	-5.06	-5.06	-5.06	-5.06	-5.06	-5.06	-5.06	-5.06
-5.05	-5.05	-5.05	-5.05	-5.05	-5.05	-5.05	-5.05	-5.05
-5.04	-5.04	-5.04	-5.04	-5.04	-5.04	-5.04	-5.04	-5.04
-5.03	-5.03	-5.03	-5.03	-5.03	-5.03	-5.03	-5.03	-5.03
-5.02	-5.02	-5.02	-5.02	-5.02	-5.02	-5.02	-5.02	-5.02
-5.01	-5.01	-5.01	-5.01	-5.01	-5.01	-5.01	-5.01	-5.01

-5	-5	-5	-5	-5	-5	-5	-5	-5
-4.99	-4.99	-4.99	-4.99	-4.99	-4.99	-4.99	-4.99	-4.99
-4.98	-4.98	-4.98	-4.98	-4.98	-4.98	-4.98	-4.98	-4.98
-4.97	-4.97	-4.97	-4.97	-4.97	-4.97	-4.97	-4.97	-4.97
-4.96	-4.96	-4.96	-4.96	-4.96	-4.96	-4.96	-4.96	-4.96
-4.95	-4.95	-4.95	-4.95	-4.95	-4.95	-4.95	-4.95	-4.95
-4.94	-4.94	-4.94	-4.94	-4.94	-4.94	-4.94	-4.94	-4.94
-4.93	-4.93	-4.93	-4.93	-4.93	-4.93	-4.93	-4.93	-4.93
-4.92	-4.92	-4.92	-4.92	-4.92	-4.92	-4.92	-4.92	-4.92
-4.91	-4.91	-4.91	-4.91	-4.91	-4.91	-4.91	-4.91	-4.91
-4.9	-4.9	-4.9	-4.9	-4.9	-4.9	-4.9	-4.9	-4.9
-4.89	-4.89	-4.89	-4.89	-4.89	-4.89	-4.89	-4.89	-4.89
-4.88	-4.88	-4.88	-4.88	-4.88	-4.88	-4.88	-4.88	-4.88
-4.87	-4.87	-4.87	-4.87	-4.87	-4.87	-4.87	-4.87	-4.87
-4.86	-4.86	-4.86	-4.86	-4.86	-4.86	-4.86	-4.86	-4.86
-4.85	-4.85	-4.85	-4.85	-4.85	-4.85	-4.85	-4.85	-4.85
-4.84	-4.84	-4.84	-4.84	-4.84	-4.84	-4.84	-4.84	-4.84
-4.83	-4.83	-4.83	-4.83	-4.83	-4.83	-4.83	-4.83	-4.83
-4.82	-4.82	-4.82	-4.82	-4.82	-4.82	-4.82	-4.82	-4.82
-4.81	-4.81	-4.81	-4.81	-4.81	-4.81	-4.81	-4.81	-4.81
-4.8	-4.8	-4.8	-4.8	-4.8	-4.8	-4.8	-4.8	-4.8
-4.79	-4.79	-4.79	-4.79	-4.79	-4.79	-4.79	-4.79	-4.79
-4.78	-4.78	-4.78	-4.78	-4.78	-4.78	-4.78	-4.78	-4.78
-4.77	-4.77	-4.77	-4.77	-4.77	-4.77	-4.77	-4.77	-4.77
-4.76	-4.76	-4.76	-4.76	-4.76	-4.76	-4.76	-4.76	-4.76
-4.75	-4.75	-4.75	-4.75	-4.75	-4.75	-4.75	-4.75	-4.75
-4.74	-4.74	-4.74	-4.74	-4.74	-4.74	-4.74	-4.74	-4.74
-4.73	-4.73	-4.73	-4.73	-4.73	-4.73	-4.73	-4.73	-4.73
-4.72	-4.72	-4.72	-4.72	-4.72	-4.72	-4.72	-4.72	-4.72
-4.71	-4.71	-4.71	-4.71	-4.71	-4.71	-4.71	-4.71	-4.71
-4.7	-4.7	-4.7	-4.7	-4.7	-4.7	-4.7	-4.7	-4.7
-4.69	-4.69	-4.69	-4.69	-4.69	-4.69	-4.69	-4.69	-4.69
-4.68	-4.68	-4.68	-4.68	-4.68	-4.68	-4.68	-4.68	-4.68
-4.67	-4.67	-4.67	-4.67	-4.67	-4.67	-4.67	-4.67	-4.67
-4.66	-4.66	-4.66	-4.66	-4.66	-4.66	-4.66	-4.66	-4.66
-4.65	-4.65	-4.65	-4.65	-4.65	-4.65	-4.65	-4.65	-4.65
-4.64	-4.64	-4.64	-4.64	-4.64	-4.64	-4.64	-4.64	-4.64
-4.63	-4.63	-4.63	-4.63	-4.63	-4.63	-4.63	-4.63	-4.63
-4.62	-4.62	-4.62	-4.62	-4.62	-4.62	-4.62	-4.62	-4.62
-4.61	-4.61	-4.61	-4.61	-4.61	-4.61	-4.61	-4.61	-4.61
-4.6	-4.6	-4.6	-4.6	-4.6	-4.6	-4.6	-4.6	-4.6
-4.59	-4.59	-4.59	-4.59	-4.59	-4.59	-4.59	-4.59	-4.59
-4.58	-4.58	-4.58	-4.58	-4.58	-4.58	-4.58	-4.58	-4.58
-4.57	-4.57	-4.57	-4.57	-4.57	-4.57	-4.57	-4.57	-4.57
-4.56	-4.56	-4.56	-4.56	-4.56	-4.56	-4.56	-4.56	-4.56
-4.55	-4.55	-4.55	-4.55	-4.55	-4.55	-4.55	-4.55	-4.55
-4.54	-4.54	-4.54	-4.54	-4.54	-4.54	-4.54	-4.54	-4.54
-4.53	-4.53	-4.53	-4.53	-4.53	-4.53	-4.53	-4.53	-4.53
-4.52	-4.52	-4.52	-4.52	-4.52	-4.52	-4.52	-4.52	-4.52
-4.51	-4.51	-4.51	-4.51	-4.51	-4.51	-4.51	-4.51	-4.51

-4.5	-4.5	-4.5	-4.5	-4.5	-4.5	-4.5	-4.5	-4.5
-4.49	-4.49	-4.49	-4.49	-4.49	-4.49	-4.49	-4.49	-4.49
-4.48	-4.48	-4.48	-4.48	-4.48	-4.48	-4.48	-4.48	-4.48
-4.47	-4.47	-4.47	-4.47	-4.47	-4.47	-4.47	-4.47	-4.47
-4.46	-4.46	-4.46	-4.46	-4.46	-4.46	-4.46	-4.46	-4.46
-4.45	-4.45	-4.45	-4.45	-4.45	-4.45	-4.45	-4.45	-4.45
-4.44	-4.44	-4.44	-4.44	-4.44	-4.44	-4.44	-4.44	-4.44
-4.43	-4.43	-4.43	-4.43	-4.43	-4.43	-4.43	-4.43	-4.43
-4.42	-4.42	-4.42	-4.42	-4.42	-4.42	-4.42	-4.42	-4.42
-4.41	-4.41	-4.41	-4.41	-4.41	-4.41	-4.41	-4.41	-4.41
-4.4	-4.4	-4.4	-4.4	-4.4	-4.4	-4.4	-4.4	-4.4
-4.39	-4.39	-4.39	-4.39	-4.39	-4.39	-4.39	-4.39	-4.39
-4.38	-4.38	-4.38	-4.38	-4.38	-4.38	-4.38	-4.38	-4.38
-4.37	-4.37	-4.37	-4.37	-4.37	-4.37	-4.37	-4.37	-4.37
-4.36	-4.36	-4.36	-4.36	-4.36	-4.36	-4.36	-4.36	-4.36
-4.35	-4.35	-4.35	-4.35	-4.35	-4.35	-4.35	-4.35	-4.35
-4.34	-4.34	-4.34	-4.34	-4.34	-4.34	-4.34	-4.34	-4.34
-4.33	-4.33	-4.33	-4.33	-4.33	-4.33	-4.33	-4.33	-4.33
-4.32	-4.32	-4.32	-4.32	-4.32	-4.32	-4.32	-4.32	-4.32
-4.31	-4.31	-4.31	-4.31	-4.31	-4.31	-4.31	-4.31	-4.31
-4.3	-4.3	-4.3	-4.3	-4.3	-4.3	-4.3	-4.3	-4.3
-4.29	-4.29	-4.29	-4.29	-4.29	-4.29	-4.29	-4.29	-4.29
-4.28	-4.28	-4.28	-4.28	-4.28	-4.28	-4.28	-4.28	-4.28
-4.27	-4.27	-4.27	-4.27	-4.27	-4.27	-4.27	-4.27	-4.27
-4.26	-4.26	-4.26	-4.26	-4.26	-4.26	-4.26	-4.26	-4.26
-4.25	-4.25	-4.25	-4.25	-4.25	-4.25	-4.25	-4.25	-4.25
-4.24	-4.24	-4.24	-4.24	-4.24	-4.24	-4.24	-4.24	-4.24
-4.23	-4.23	-4.23	-4.23	-4.23	-4.23	-4.23	-4.23	-4.23
-4.22	-4.22	-4.22	-4.22	-4.22	-4.22	-4.22	-4.22	-4.22
-4.21	-4.21	-4.21	-4.21	-4.21	-4.21	-4.21	-4.21	-4.21
-4.2	-4.2	-4.2	-4.2	-4.2	-4.2	-4.2	-4.2	-4.2
-4.19	-4.19	-4.19	-4.19	-4.19	-4.19	-4.19	-4.19	-4.19
-4.18	-4.18	-4.18	-4.18	-4.18	-4.18	-4.18	-4.18	-4.18
-4.17	-4.17	-4.17	-4.17	-4.17	-4.17	-4.17	-4.17	-4.17
-4.16	-4.16	-4.16	-4.16	-4.16	-4.16	-4.16	-4.16	-4.16
-4.15	-4.15	-4.15	-4.15	-4.15	-4.15	-4.15	-4.15	-4.15
-4.14	-4.14	-4.14	-4.14	-4.14	-4.14	-4.14	-4.14	-4.14
-4.13	-4.13	-4.13	-4.13	-4.13	-4.13	-4.13	-4.13	-4.13
-4.12	-4.12	-4.12	-4.12	-4.12	-4.12	-4.12	-4.12	-4.12
-4.11	-4.11	-4.11	-4.11	-4.11	-4.11	-4.11	-4.11	-4.11
-4.1	-4.1	-4.1	-4.1	-4.1	-4.1	-4.1	-4.1	-4.1
-4.09	-4.09	-4.09	-4.09	-4.09	-4.09	-4.09	-4.09	-4.09
-4.08	-4.08	-4.08	-4.08	-4.08	-4.08	-4.08	-4.08	-4.08
-4.07	-4.07	-4.07	-4.07	-4.07	-4.07	-4.07	-4.07	-4.07
-4.06	-4.06	-4.06	-4.06	-4.06	-4.06	-4.06	-4.06	-4.06
-4.05	-4.05	-4.05	-4.05	-4.05	-4.05	-4.05	-4.05	-4.05
-4.04	-4.04	-4.04	-4.04	-4.04	-4.04	-4.04	-4.04	-4.04
-4.03	-4.03	-4.03	-4.03	-4.03	-4.03	-4.03	-4.03	-4.03
-4.02	-4.02	-4.02	-4.02	-4.02	-4.02	-4.02	-4.02	-4.02
-4.01	-4.01	-4.01	-4.01	-4.01	-4.01	-4.01	-4.01	-4.01

-4	-4	-4	-4	-4	-4	-4	-4	-4
-3.99	-3.99	-3.99	-3.99	-3.99	-3.99	-3.99	-3.99	-3.99
-3.98	-3.98	-3.98	-3.98	-3.98	-3.98	-3.98	-3.98	-3.98
-3.97	-3.97	-3.97	-3.97	-3.97	-3.97	-3.97	-3.97	-3.97
-3.96	-3.96	-3.96	-3.96	-3.96	-3.96	-3.96	-3.96	-3.96
-3.95	-3.95	-3.95	-3.95	-3.95	-3.95	-3.95	-3.95	-3.95
-3.94	-3.94	-3.94	-3.94	-3.94	-3.94	-3.94	-3.94	-3.94
-3.93	-3.93	-3.93	-3.93	-3.93	-3.93	-3.93	-3.93	-3.93
-3.92	-3.92	-3.92	-3.92	-3.92	-3.92	-3.92	-3.92	-3.92
-3.91	-3.91	-3.91	-3.91	-3.91	-3.91	-3.91	-3.91	-3.91
-3.9	-3.9	-3.9	-3.9	-3.9	-3.9	-3.9	-3.9	-3.9
-3.89	-3.89	-3.89	-3.89	-3.89	-3.89	-3.89	-3.89	-3.89
-3.88	-3.88	-3.88	-3.88	-3.88	-3.88	-3.88	-3.88	-3.88
-3.87	-3.87	-3.87	-3.87	-3.87	-3.87	-3.87	-3.87	-3.87
-3.86	-3.86	-3.86	-3.86	-3.86	-3.86	-3.86	-3.86	-3.86
-3.85	-3.85	-3.85	-3.85	-3.85	-3.85	-3.85	-3.85	-3.85
-3.84	-3.84	-3.84	-3.84	-3.84	-3.84	-3.84	-3.84	-3.84
-3.83	-3.83	-3.83	-3.83	-3.83	-3.83	-3.83	-3.83	-3.83
-3.82	-3.82	-3.82	-3.82	-3.82	-3.82	-3.82	-3.82	-3.82
-3.81	-3.81	-3.81	-3.81	-3.81	-3.81	-3.81	-3.81	-3.81
-3.8	-3.8	-3.8	-3.8	-3.8	-3.8	-3.8	-3.8	-3.8
-3.79	-3.79	-3.79	-3.79	-3.79	-3.79	-3.79	-3.79	-3.79
-3.78	-3.78	-3.78	-3.78	-3.78	-3.78	-3.78	-3.78	-3.78
-3.77	-3.77	-3.77	-3.77	-3.77	-3.77	-3.77	-3.77	-3.77
-3.76	-3.76	-3.76	-3.76	-3.76	-3.76	-3.76	-3.76	-3.76
-3.75	-3.75	-3.75	-3.75	-3.75	-3.75	-3.75	-3.75	-3.75
-3.74	-3.74	-3.74	-3.74	-3.74	-3.74	-3.74	-3.74	-3.74
-3.73	-3.73	-3.73	-3.73	-3.73	-3.73	-3.73	-3.73	-3.73
-3.72	-3.72	-3.72	-3.72	-3.72	-3.72	-3.72	-3.72	-3.72
-3.71	-3.71	-3.71	-3.71	-3.71	-3.71	-3.71	-3.71	-3.71
-3.7	-3.7	-3.7	-3.7	-3.7	-3.7	-3.7	-3.7	-3.7
-3.69	-3.69	-3.69	-3.69	-3.69	-3.69	-3.69	-3.69	-3.69
-3.68	-3.68	-3.68	-3.68	-3.68	-3.68	-3.68	-3.68	-3.68
-3.67	-3.67	-3.67	-3.67	-3.67	-3.67	-3.67	-3.67	-3.67
-3.66	-3.66	-3.66	-3.66	-3.66	-3.66	-3.66	-3.66	-3.66
-3.65	-3.65	-3.65	-3.65	-3.65	-3.65	-3.65	-3.65	-3.65
-3.64	-3.64	-3.64	-3.64	-3.64	-3.64	-3.64	-3.64	-3.64
-3.63	-3.63	-3.63	-3.63	-3.63	-3.63	-3.63	-3.63	-3.63
-3.62	-3.62	-3.62	-3.62	-3.62	-3.62	-3.62	-3.62	-3.62
-3.61	-3.61	-3.61	-3.61	-3.61	-3.61	-3.61	-3.61	-3.61
-3.6	-3.6	-3.6	-3.6	-3.6	-3.6	-3.6	-3.6	-3.6
-3.59	-3.59	-3.59	-3.59	-3.59	-3.59	-3.59	-3.59	-3.59
-3.58	-3.58	-3.58	-3.58	-3.58	-3.58	-3.58	-3.58	-3.58
-3.57	-3.57	-3.57	-3.57	-3.57	-3.57	-3.57	-3.57	-3.57
-3.56	-3.56	-3.56	-3.56	-3.56	-3.56	-3.56	-3.56	-3.56
-3.55	-3.55	-3.55	-3.55	-3.55	-3.55	-3.55	-3.55	-3.55
-3.54	-3.54	-3.54	-3.54	-3.54	-3.54	-3.54	-3.54	-3.54
-3.53	-3.53	-3.53	-3.53	-3.53	-3.53	-3.53	-3.53	-3.53
-3.52	-3.52	-3.52	-3.52	-3.52	-3.52	-3.52	-3.52	-3.52
-3.51	-3.51	-3.51	-3.51	-3.51	-3.51	-3.51	-3.51	-3.51

-3.5	-3.5	-3.5	-3.5	-3.5	-3.5	-3.5	-3.5	-3.5
-3.49	-3.49	-3.49	-3.49	-3.49	-3.49	-3.49	-3.49	-3.49
-3.48	-3.48	-3.48	-3.48	-3.48	-3.48	-3.48	-3.48	-3.48
-3.47	-3.47	-3.47	-3.47	-3.47	-3.47	-3.47	-3.47	-3.47
-3.46	-3.46	-3.46	-3.46	-3.46	-3.46	-3.46	-3.46	-3.46
-3.45	-3.45	-3.45	-3.45	-3.45	-3.45	-3.45	-3.45	-3.45
-3.44	-3.44	-3.44	-3.44	-3.44	-3.44	-3.44	-3.44	-3.44
-3.43	-3.43	-3.43	-3.43	-3.43	-3.43	-3.43	-3.43	-3.43
-3.42	-3.42	-3.42	-3.42	-3.42	-3.42	-3.42	-3.42	-3.42
-3.41	-3.41	-3.41	-3.41	-3.41	-3.41	-3.41	-3.41	-3.41
-3.4	-3.4	-3.4	-3.4	-3.4	-3.4	-3.4	-3.4	-3.4
-3.39	-3.39	-3.39	-3.39	-3.39	-3.39	-3.39	-3.39	-3.39
-3.38	-3.38	-3.38	-3.38	-3.38	-3.38	-3.38	-3.38	-3.38
-3.37	-3.37	-3.37	-3.37	-3.37	-3.37	-3.37	-3.37	-3.37
-3.36	-3.36	-3.36	-3.36	-3.36	-3.36	-3.36	-3.36	-3.36
-3.35	-3.35	-3.35	-3.35	-3.35	-3.35	-3.35	-3.35	-3.35
-3.34	-3.34	-3.34	-3.34	-3.34	-3.34	-3.34	-3.34	-3.34
-3.33	-3.33	-3.33	-3.33	-3.33	-3.33	-3.33	-3.33	-3.33
-3.32	-3.32	-3.32	-3.32	-3.32	-3.32	-3.32	-3.32	-3.32
-3.31	-3.31	-3.31	-3.31	-3.31	-3.31	-3.31	-3.31	-3.31
-3.3	-3.3	-3.3	-3.3	-3.3	-3.3	-3.3	-3.3	-3.3
-3.29	-3.29	-3.29	-3.29	-3.29	-3.29	-3.29	-3.29	-3.29
-3.28	-3.28	-3.28	-3.28	-3.28	-3.28	-3.28	-3.28	-3.28
-3.27	-3.27	-3.27	-3.27	-3.27	-3.27	-3.27	-3.27	-3.27
-3.26	-3.26	-3.26	-3.26	-3.26	-3.26	-3.26	-3.26	-3.26
-3.25	-3.25	-3.25	-3.25	-3.25	-3.25	-3.25	-3.25	-3.25
-3.24	-3.24	-3.24	-3.24	-3.24	-3.24	-3.24	-3.24	-3.24
-3.23	-3.23	-3.23	-3.23	-3.23	-3.23	-3.23	-3.23	-3.23
-3.22	-3.22	-3.22	-3.22	-3.22	-3.22	-3.22	-3.22	-3.22
-3.21	-3.21	-3.21	-3.21	-3.21	-3.21	-3.21	-3.21	-3.21
-3.2	-3.2	-3.2	-3.2	-3.2	-3.2	-3.2	-3.2	-3.2
-3.19	-3.19	-3.19	-3.19	-3.19	-3.19	-3.19	-3.19	-3.19
-3.18	-3.18	-3.18	-3.18	-3.18	-3.18	-3.18	-3.18	-3.18
-3.17	-3.17	-3.17	-3.17	-3.17	-3.17	-3.17	-3.17	-3.17
-3.16	-3.16	-3.16	-3.16	-3.16	-3.16	-3.16	-3.16	-3.16
-3.15	-3.15	-3.15	-3.15	-3.15	-3.15	-3.15	-3.15	-3.15
-3.14	-3.14	-3.14	-3.14	-3.14	-3.14	-3.14	-3.14	-3.14
-3.13	-3.13	-3.13	-3.13	-3.13	-3.13	-3.13	-3.13	-3.13
-3.12	-3.12	-3.12	-3.12	-3.12	-3.12	-3.12	-3.12	-3.12
-3.11	-3.11	-3.11	-3.11	-3.11	-3.11	-3.11	-3.11	-3.11
-3.1	-3.1	-3.1	-3.1	-3.1	-3.1	-3.1	-3.1	-3.1
-3.09	-3.09	-3.09	-3.09	-3.09	-3.09	-3.09	-3.09	-3.09
-3.08	-3.08	-3.08	-3.08	-3.08	-3.08	-3.08	-3.08	-3.08
-3.07	-3.07	-3.07	-3.07	-3.07	-3.07	-3.07	-3.07	-3.07
-3.06	-3.06	-3.06	-3.06	-3.06	-3.06	-3.06	-3.06	-3.06
-3.05	-3.05	-3.05	-3.05	-3.05	-3.05	-3.05	-3.05	-3.05
-3.04	-3.04	-3.04	-3.04	-3.04	-3.04	-3.04	-3.04	-3.04
-3.03	-3.03	-3.03	-3.03	-3.03	-3.03	-3.03	-3.03	-3.03
-3.02	-3.02	-3.02	-3.02	-3.02	-3.02	-3.02	-3.02	-3.02
-3.01	-3.01	-3.01	-3.01	-3.01	-3.01	-3.01	-3.01	-3.01

-3	-3	-3	-3	-3	-3	-3	-3	-3
-2.99	-2.99	-2.99	-2.99	-2.99	-2.99	-2.99	-2.99	-2.99
-2.98	-2.98	-2.98	-2.98	-2.98	-2.98	-2.98	-2.98	-2.98
-2.97	-2.97	-2.97	-2.97	-2.97	-2.97	-2.97	-2.97	-2.97
-2.96	-2.96	-2.96	-2.96	-2.96	-2.96	-2.96	-2.96	-2.96
-2.95	-2.95	-2.95	-2.95	-2.95	-2.95	-2.95	-2.95	-2.95
-2.94	-2.94	-2.94	-2.94	-2.94	-2.94	-2.94	-2.94	-2.94
-2.93	-2.93	-2.93	-2.93	-2.93	-2.93	-2.93	-2.93	-2.93
-2.92	-2.92	-2.92	-2.92	-2.92	-2.92	-2.92	-2.92	-2.92
-2.91	-2.91	-2.91	-2.91	-2.91	-2.91	-2.91	-2.91	-2.91
-2.9	-2.9	-2.9	-2.9	-2.9	-2.9	-2.9	-2.9	-2.9
-2.89	-2.89	-2.89	-2.89	-2.89	-2.89	-2.89	-2.89	-2.89
-2.88	-2.88	-2.88	-2.88	-2.88	-2.88	-2.88	-2.88	-2.88
-2.87	-2.87	-2.87	-2.87	-2.87	-2.87	-2.87	-2.87	-2.87
-2.86	-2.86	-2.86	-2.86	-2.86	-2.86	-2.86	-2.86	-2.86
-2.85	-2.85	-2.85	-2.85	-2.85	-2.85	-2.85	-2.85	-2.85
-2.84	-2.84	-2.84	-2.84	-2.84	-2.84	-2.84	-2.84	-2.84
-2.83	-2.83	-2.83	-2.83	-2.83	-2.83	-2.83	-2.83	-2.83
-2.82	-2.82	-2.82	-2.82	-2.82	-2.82	-2.82	-2.82	-2.82
-2.81	-2.81	-2.81	-2.81	-2.81	-2.81	-2.81	-2.81	-2.81
-2.8	-2.8	-2.8	-2.8	-2.8	-2.8	-2.8	-2.8	-2.8
-2.79	-2.79	-2.79	-2.79	-2.79	-2.79	-2.79	-2.79	-2.79
-2.78	-2.78	-2.78	-2.78	-2.78	-2.78	-2.78	-2.78	-2.78
-2.77	-2.77	-2.77	-2.77	-2.77	-2.77	-2.77	-2.77	-2.77
-2.76	-2.76	-2.76	-2.76	-2.76	-2.76	-2.76	-2.76	-2.76
-2.75	-2.75	-2.75	-2.75	-2.75	-2.75	-2.75	-2.75	-2.75
-2.74	-2.74	-2.74	-2.74	-2.74	-2.74	-2.74	-2.74	-2.74
-2.73	-2.73	-2.73	-2.73	-2.73	-2.73	-2.73	-2.73	-2.73
-2.72	-2.72	-2.72	-2.72	-2.72	-2.72	-2.72	-2.72	-2.72
-2.71	-2.71	-2.71	-2.71	-2.71	-2.71	-2.71	-2.71	-2.71
-2.7	-2.7	-2.7	-2.7	-2.7	-2.7	-2.7	-2.7	-2.7
-2.69	-2.69	-2.69	-2.69	-2.69	-2.69	-2.69	-2.69	-2.69
-2.68	-2.68	-2.68	-2.68	-2.68	-2.68	-2.68	-2.68	-2.68
-2.67	-2.67	-2.67	-2.67	-2.67	-2.67	-2.67	-2.67	-2.67
-2.66	-2.66	-2.66	-2.66	-2.66	-2.66	-2.66	-2.66	-2.66
-2.65	-2.65	-2.65	-2.65	-2.65	-2.65	-2.65	-2.65	-2.65
-2.64	-2.64	-2.64	-2.64	-2.64	-2.64	-2.64	-2.64	-2.64
-2.63	-2.63	-2.63	-2.63	-2.63	-2.63	-2.63	-2.63	-2.63
-2.62	-2.62	-2.62	-2.62	-2.62	-2.62	-2.62	-2.62	-2.62
-2.61	-2.61	-2.61	-2.61	-2.61	-2.61	-2.61	-2.61	-2.61
-2.6	-2.6	-2.6	-2.6	-2.6	-2.6	-2.6	-2.6	-2.6
-2.59	-2.59	-2.59	-2.59	-2.59	-2.59	-2.59	-2.59	-2.59
-2.58	-2.58	-2.58	-2.58	-2.58	-2.58	-2.58	-2.58	-2.58
-2.57	-2.57	-2.57	-2.57	-2.57	-2.57	-2.57	-2.57	-2.57
-2.56	-2.56	-2.56	-2.56	-2.56	-2.56	-2.56	-2.56	-2.56
-2.55	-2.55	-2.55	-2.55	-2.55	-2.55	-2.55	-2.55	-2.55
-2.54	-2.54	-2.54	-2.54	-2.54	-2.54	-2.54	-2.54	-2.54
-2.53	-2.53	-2.53	-2.53	-2.53	-2.53	-2.53	-2.53	-2.53
-2.52	-2.52	-2.52	-2.52	-2.52	-2.52	-2.52	-2.52	-2.52
-2.51	-2.51	-2.51	-2.51	-2.51	-2.51	-2.51	-2.51	-2.51

-2.5	-2.5	-2.5	-2.5	-2.5	-2.5	-2.5	-2.5	-2.5
-2.49	-2.49	-2.49	-2.49	-2.49	-2.49	-2.49	-2.49	-2.49
-2.48	-2.48	-2.48	-2.48	-2.48	-2.48	-2.48	-2.48	-2.48
-2.47	-2.47	-2.47	-2.47	-2.47	-2.47	-2.47	-2.47	-2.47
-2.46	-2.46	-2.46	-2.46	-2.46	-2.46	-2.46	-2.46	-2.46
-2.45	-2.45	-2.45	-2.45	-2.45	-2.45	-2.45	-2.45	-2.45
-2.44	-2.44	-2.44	-2.44	-2.44	-2.44	-2.44	-2.44	-2.44
-2.43	-2.43	-2.43	-2.43	-2.43	-2.43	-2.43	-2.43	-2.43
-2.42	-2.42	-2.42	-2.42	-2.42	-2.42	-2.42	-2.42	-2.42
-2.41	-2.41	-2.41	-2.41	-2.41	-2.41	-2.41	-2.41	-2.41
-2.4	-2.4	-2.4	-2.4	-2.4	-2.4	-2.4	-2.4	-2.4
-2.39	-2.39	-2.39	-2.39	-2.39	-2.39	-2.39	-2.39	-2.39
-2.38	-2.38	-2.38	-2.38	-2.38	-2.38	-2.38	-2.38	-2.38
-2.37	-2.37	-2.37	-2.37	-2.37	-2.37	-2.37	-2.37	-2.37
-2.36	-2.36	-2.36	-2.36	-2.36	-2.36	-2.36	-2.36	-2.36
-2.35	-2.35	-2.35	-2.35	-2.35	-2.35	-2.35	-2.35	-2.35
-2.34	-2.34	-2.34	-2.34	-2.34	-2.34	-2.34	-2.34	-2.34
-2.33	-2.33	-2.33	-2.33	-2.33	-2.33	-2.33	-2.33	-2.33
-2.32	-2.32	-2.32	-2.32	-2.32	-2.32	-2.32	-2.32	-2.32
-2.31	-2.31	-2.31	-2.31	-2.31	-2.31	-2.31	-2.31	-2.31
-2.3	-2.3	-2.3	-2.3	-2.3	-2.3	-2.3	-2.3	-2.3
-2.29	-2.29	-2.29	-2.29	-2.29	-2.29	-2.29	-2.29	-2.29
-2.28	-2.28	-2.28	-2.28	-2.28	-2.28	-2.28	-2.28	-2.28
-2.27	-2.27	-2.27	-2.27	-2.27	-2.27	-2.27	-2.27	-2.27
-2.26	-2.26	-2.26	-2.26	-2.26	-2.26	-2.26	-2.26	-2.26
-2.25	-2.25	-2.25	-2.25	-2.25	-2.25	-2.25	-2.25	-2.25
-2.24	-2.24	-2.24	-2.24	-2.24	-2.24	-2.24	-2.24	-2.24
-2.23	-2.23	-2.23	-2.23	-2.23	-2.23	-2.23	-2.23	-2.23
-2.22	-2.22	-2.22	-2.22	-2.22	-2.22	-2.22	-2.22	-2.22
-2.21	-2.21	-2.21	-2.21	-2.21	-2.21	-2.21	-2.21	-2.21
-2.2	-2.2	-2.2	-2.2	-2.2	-2.2	-2.2	-2.2	-2.2
-2.19	-2.19	-2.19	-2.19	-2.19	-2.19	-2.19	-2.19	-2.19
-2.18	-2.18	-2.18	-2.18	-2.18	-2.18	-2.18	-2.18	-2.18
-2.17	-2.17	-2.17	-2.17	-2.17	-2.17	-2.17	-2.17	-2.17
-2.16	-2.16	-2.16	-2.16	-2.16	-2.16	-2.16	-2.16	-2.16
-2.15	-2.15	-2.15	-2.15	-2.15	-2.15	-2.15	-2.15	-2.15
-2.14	-2.14	-2.14	-2.14	-2.14	-2.14	-2.14	-2.14	-2.14
-2.13	-2.13	-2.13	-2.13	-2.13	-2.13	-2.13	-2.13	-2.13
-2.12	-2.12	-2.12	-2.12	-2.12	-2.12	-2.12	-2.12	-2.12
-2.11	-2.11	-2.11	-2.11	-2.11	-2.11	-2.11	-2.11	-2.11
-2.1	-2.1	-2.1	-2.1	-2.1	-2.1	-2.1	-2.1	-2.1
-2.09	-2.09	-2.09	-2.09	-2.09	-2.09	-2.09	-2.09	-2.09
-2.08	-2.08	-2.08	-2.08	-2.08	-2.08	-2.08	-2.08	-2.08
-2.07	-2.07	-2.07	-2.07	-2.07	-2.07	-2.07	-2.07	-2.07
-2.06	-2.06	-2.06	-2.06	-2.06	-2.06	-2.06	-2.06	-2.06
-2.05	-2.05	-2.05	-2.05	-2.05	-2.05	-2.05	-2.05	-2.05
-2.04	-2.04	-2.04	-2.04	-2.04	-2.04	-2.04	-2.04	-2.04
-2.03	-2.03	-2.03	-2.03	-2.03	-2.03	-2.03	-2.03	-2.03
-2.02	-2.02	-2.02	-2.02	-2.02	-2.02	-2.02	-2.02	-2.02
-2.01	-2.01	-2.01	-2.01	-2.01	-2.01	-2.01	-2.01	-2.01

-2	-2	-2	-2	-2	-2	-2	-2	-2
-1.99	-1.99	-1.99	-1.99	-1.99	-1.99	-1.99	-1.99	-1.99
-1.98	-1.98	-1.98	-1.98	-1.98	-1.98	-1.98	-1.98	-1.98
-1.97	-1.97	-1.97	-1.97	-1.97	-1.97	-1.97	-1.97	-1.97
-1.96	-1.96	-1.96	-1.96	-1.96	-1.96	-1.96	-1.96	-1.96
-1.95	-1.95	-1.95	-1.95	-1.95	-1.95	-1.95	-1.95	-1.95
-1.94	-1.94	-1.94	-1.94	-1.94	-1.94	-1.94	-1.94	-1.94
-1.93	-1.93	-1.93	-1.93	-1.93	-1.93	-1.93	-1.93	-1.93
-1.92	-1.92	-1.92	-1.92	-1.92	-1.92	-1.92	-1.92	-1.92
-1.91	-1.91	-1.91	-1.91	-1.91	-1.91	-1.91	-1.91	-1.91
-1.9	-1.9	-1.9	-1.9	-1.9	-1.9	-1.9	-1.9	-1.9
-1.89	-1.89	-1.89	-1.89	-1.89	-1.89	-1.89	-1.89	-1.89
-1.88	-1.88	-1.88	-1.88	-1.88	-1.88	-1.88	-1.88	-1.88
-1.87	-1.87	-1.87	-1.87	-1.87	-1.87	-1.87	-1.87	-1.87
-1.86	-1.86	-1.86	-1.86	-1.86	-1.86	-1.86	-1.86	-1.86
-1.85	-1.85	-1.85	-1.85	-1.85	-1.85	-1.85	-1.85	-1.85
-1.84	-1.84	-1.84	-1.84	-1.84	-1.84	-1.84	-1.84	-1.84
-1.83	-1.83	-1.83	-1.83	-1.83	-1.83	-1.83	-1.83	-1.83
-1.82	-1.82	-1.82	-1.82	-1.82	-1.82	-1.82	-1.82	-1.82
-1.81	-1.81	-1.81	-1.81	-1.81	-1.81	-1.81	-1.81	-1.81
-1.8	-1.8	-1.8	-1.8	-1.8	-1.8	-1.8	-1.8	-1.8
-1.79	-1.79	-1.79	-1.79	-1.79	-1.79	-1.79	-1.79	-1.79
-1.78	-1.78	-1.78	-1.78	-1.78	-1.78	-1.78	-1.78	-1.78
-1.77	-1.77	-1.77	-1.77	-1.77	-1.77	-1.77	-1.77	-1.77
-1.76	-1.76	-1.76	-1.76	-1.76	-1.76	-1.76	-1.76	-1.76
-1.75	-1.75	-1.75	-1.75	-1.75	-1.75	-1.75	-1.75	-1.75
-1.74	-1.74	-1.74	-1.74	-1.74	-1.74	-1.74	-1.74	-1.74
-1.73	-1.73	-1.73	-1.73	-1.73	-1.73	-1.73	-1.73	-1.73
-1.72	-1.72	-1.72	-1.72	-1.72	-1.72	-1.72	-1.72	-1.72
-1.71	-1.71	-1.71	-1.71	-1.71	-1.71	-1.71	-1.71	-1.71
-1.7	-1.7	-1.7	-1.7	-1.7	-1.7	-1.7	-1.7	-1.7
-1.69	-1.69	-1.69	-1.69	-1.69	-1.69	-1.69	-1.69	-1.69
-1.68	-1.68	-1.68	-1.68	-1.68	-1.68	-1.68	-1.68	-1.68
-1.67	-1.67	-1.67	-1.67	-1.67	-1.67	-1.67	-1.67	-1.67
-1.66	-1.66	-1.66	-1.66	-1.66	-1.66	-1.66	-1.66	-1.66
-1.65	-1.65	-1.65	-1.65	-1.65	-1.65	-1.65	-1.65	-1.65
-1.64	-1.64	-1.64	-1.64	-1.64	-1.64	-1.64	-1.64	-1.64
-1.63	-1.63	-1.63	-1.63	-1.63	-1.63	-1.63	-1.63	-1.63
-1.62	-1.62	-1.62	-1.62	-1.62	-1.62	-1.62	-1.62	-1.62
-1.61	-1.61	-1.61	-1.61	-1.61	-1.61	-1.61	-1.61	-1.61
-1.6	-1.6	-1.6	-1.6	-1.6	-1.6	-1.6	-1.6	-1.6
-1.59	-1.59	-1.59	-1.59	-1.59	-1.59	-1.59	-1.59	-1.59
-1.58	-1.58	-1.58	-1.58	-1.58	-1.58	-1.58	-1.58	-1.58
-1.57	-1.57	-1.57	-1.57	-1.57	-1.57	-1.57	-1.57	-1.57
-1.56	-1.56	-1.56	-1.56	-1.56	-1.56	-1.56	-1.56	-1.56
-1.55	-1.55	-1.55	-1.55	-1.55	-1.55	-1.55	-1.55	-1.55
-1.54	-1.54	-1.54	-1.54	-1.54	-1.54	-1.54	-1.54	-1.54
-1.53	-1.53	-1.53	-1.53	-1.53	-1.53	-1.53	-1.53	-1.53
-1.52	-1.52	-1.52	-1.52	-1.52	-1.52	-1.52	-1.52	-1.52
-1.51	-1.51	-1.51	-1.51	-1.51	-1.51	-1.51	-1.51	-1.51

-1.5	-1.5	-1.5	-1.5	-1.5	-1.5	-1.5	-1.5	-1.5
-1.49	-1.49	-1.49	-1.49	-1.49	-1.49	-1.49	-1.49	-1.49
-1.48	-1.48	-1.48	-1.48	-1.48	-1.48	-1.48	-1.48	-1.48
-1.47	-1.47	-1.47	-1.47	-1.47	-1.47	-1.47	-1.47	-1.47
-1.46	-1.46	-1.46	-1.46	-1.46	-1.46	-1.46	-1.46	-1.46
-1.45	-1.45	-1.45	-1.45	-1.45	-1.45	-1.45	-1.45	-1.45
-1.44	-1.44	-1.44	-1.44	-1.44	-1.44	-1.44	-1.44	-1.44
-1.43	-1.43	-1.43	-1.43	-1.43	-1.43	-1.43	-1.43	-1.43
-1.42	-1.42	-1.42	-1.42	-1.42	-1.42	-1.42	-1.42	-1.42
-1.41	-1.41	-1.41	-1.41	-1.41	-1.41	-1.41	-1.41	-1.41
-1.4	-1.4	-1.4	-1.4	-1.4	-1.4	-1.4	-1.4	-1.4
-1.39	-1.39	-1.39	-1.39	-1.39	-1.39	-1.39	-1.39	-1.39
-1.38	-1.38	-1.38	-1.38	-1.38	-1.38	-1.38	-1.38	-1.38
-1.37	-1.37	-1.37	-1.37	-1.37	-1.37	-1.37	-1.37	-1.37
-1.36	-1.36	-1.36	-1.36	-1.36	-1.36	-1.36	-1.36	-1.36
-1.35	-1.35	-1.35	-1.35	-1.35	-1.35	-1.35	-1.35	-1.35
-1.34	-1.34	-1.34	-1.34	-1.34	-1.34	-1.34	-1.34	-1.34
-1.33	-1.33	-1.33	-1.33	-1.33	-1.33	-1.33	-1.33	-1.33
-1.32	-1.32	-1.32	-1.32	-1.32	-1.32	-1.32	-1.32	-1.32
-1.31	-1.31	-1.31	-1.31	-1.31	-1.31	-1.31	-1.31	-1.31
-1.3	-1.3	-1.3	-1.3	-1.3	-1.3	-1.3	-1.3	-1.3
-1.29	-1.29	-1.29	-1.29	-1.29	-1.29	-1.29	-1.29	-1.29
-1.28	-1.28	-1.28	-1.28	-1.28	-1.28	-1.28	-1.28	-1.28
-1.27	-1.27	-1.27	-1.27	-1.27	-1.27	-1.27	-1.27	-1.27
-1.26	-1.26	-1.26	-1.26	-1.26	-1.26	-1.26	-1.26	-1.26
-1.25	-1.25	-1.25	-1.25	-1.25	-1.25	-1.25	-1.25	-1.25
-1.24	-1.24	-1.24	-1.24	-1.24	-1.24	-1.24	-1.24	-1.24
-1.23	-1.23	-1.23	-1.23	-1.23	-1.23	-1.23	-1.23	-1.23
-1.22	-1.22	-1.22	-1.22	-1.22	-1.22	-1.22	-1.22	-1.22
-1.21	-1.21	-1.21	-1.21	-1.21	-1.21	-1.21	-1.21	-1.21
-1.2	-1.2	-1.2	-1.2	-1.2	-1.2	-1.2	-1.2	-1.2
-1.19	-1.19	-1.19	-1.19	-1.19	-1.19	-1.19	-1.19	-1.19
-1.18	-1.18	-1.18	-1.18	-1.18	-1.18	-1.18	-1.18	-1.18
-1.17	-1.17	-1.17	-1.17	-1.17	-1.17	-1.17	-1.17	-1.17
-1.16	-1.16	-1.16	-1.16	-1.16	-1.16	-1.16	-1.16	-1.16
-1.15	-1.15	-1.15	-1.15	-1.15	-1.15	-1.15	-1.15	-1.15
-1.14	-1.14	-1.14	-1.14	-1.14	-1.14	-1.14	-1.14	-1.14
-1.13	-1.13	-1.13	-1.13	-1.13	-1.13	-1.13	-1.13	-1.13
-1.12	-1.12	-1.12	-1.12	-1.12	-1.12	-1.12	-1.12	-1.12
-1.11	-1.11	-1.11	-1.11	-1.11	-1.11	-1.11	-1.11	-1.11
-1.1	-1.1	-1.1	-1.1	-1.1	-1.1	-1.1	-1.1	-1.1
-1.09	-1.09	-1.09	-1.09	-1.09	-1.09	-1.09	-1.09	-1.09
-1.08	-1.08	-1.08	-1.08	-1.08	-1.08	-1.08	-1.08	-1.08
-1.07	-1.07	-1.07	-1.07	-1.07	-1.07	-1.07	-1.07	-1.07
-1.06	-1.06	-1.06	-1.06	-1.06	-1.06	-1.06	-1.06	-1.06
-1.05	-1.05	-1.05	-1.05	-1.05	-1.05	-1.05	-1.05	-1.05
-1.04	-1.04	-1.04	-1.04	-1.04	-1.04	-1.04	-1.04	-1.04
-1.03	-1.03	-1.03	-1.03	-1.03	-1.03	-1.03	-1.03	-1.03
-1.02	-1.02	-1.02	-1.02	-1.02	-1.02	-1.02	-1.02	-1.02
-1.01	-1.01	-1.01	-1.01	-1.01	-1.01	-1.01	-1.01	-1.01

-1	-1	-1	-1	-1	-1	-1	-1	-1
-0.99	-0.99	-0.99	-0.99	-0.99	-0.99	-0.99	-0.99	-0.99
-0.98	-0.98	-0.98	-0.98	-0.98	-0.98	-0.98	-0.98	-0.98
-0.97	-0.97	-0.97	-0.97	-0.97	-0.97	-0.97	-0.97	-0.97
-0.96	-0.96	-0.96	-0.96	-0.96	-0.96	-0.96	-0.96	-0.96
-0.95	-0.95	-0.95	-0.95	-0.95	-0.95	-0.95	-0.95	-0.95
-0.94	-0.94	-0.94	-0.94	-0.94	-0.94	-0.94	-0.94	-0.94
-0.93	-0.93	-0.93	-0.93	-0.93	-0.93	-0.93	-0.93	-0.93
-0.92	-0.92	-0.92	-0.92	-0.92	-0.92	-0.92	-0.92	-0.92
-0.91	-0.91	-0.91	-0.91	-0.91	-0.91	-0.91	-0.91	-0.91
-0.9	-0.9	-0.9	-0.9	-0.9	-0.9	-0.9	-0.9	-0.9
-0.89	-0.89	-0.89	-0.89	-0.89	-0.89	-0.89	-0.89	-0.89
-0.88	-0.88	-0.88	-0.88	-0.88	-0.88	-0.88	-0.88	-0.88
-0.87	-0.87	-0.87	-0.87	-0.87	-0.87	-0.87	-0.87	-0.87
-0.86	-0.86	-0.86	-0.86	-0.86	-0.86	-0.86	-0.86	-0.86
-0.85	-0.85	-0.85	-0.85	-0.85	-0.85	-0.85	-0.85	-0.85
-0.84	-0.84	-0.84	-0.84	-0.84	-0.84	-0.84	-0.84	-0.84
-0.83	-0.83	-0.83	-0.83	-0.83	-0.83	-0.83	-0.83	-0.83
-0.82	-0.82	-0.82	-0.82	-0.82	-0.82	-0.82	-0.82	-0.82
-0.81	-0.81	-0.81	-0.81	-0.81	-0.81	-0.81	-0.81	-0.81
-0.8	-0.8	-0.8	-0.8	-0.8	-0.8	-0.8	-0.8	-0.8
-0.79	-0.79	-0.79	-0.79	-0.79	-0.79	-0.79	-0.79	-0.79
-0.78	-0.78	-0.78	-0.78	-0.78	-0.78	-0.78	-0.78	-0.78
-0.77	-0.77	-0.77	-0.77	-0.77	-0.77	-0.77	-0.77	-0.77
-0.76	-0.76	-0.76	-0.76	-0.76	-0.76	-0.76	-0.76	-0.76
-0.75	-0.75	-0.75	-0.75	-0.75	-0.75	-0.75	-0.75	-0.75
-0.74	-0.74	-0.74	-0.74	-0.74	-0.74	-0.74	-0.74	-0.74
-0.73	-0.73	-0.73	-0.73	-0.73	-0.73	-0.73	-0.73	-0.73
-0.72	-0.72	-0.72	-0.72	-0.72	-0.72	-0.72	-0.72	-0.72
-0.71	-0.71	-0.71	-0.71	-0.71	-0.71	-0.71	-0.71	-0.71
-0.7	-0.7	-0.7	-0.7	-0.7	-0.7	-0.7	-0.7	-0.7
-0.69	-0.69	-0.69	-0.69	-0.69	-0.69	-0.69	-0.69	-0.69
-0.68	-0.68	-0.68	-0.68	-0.68	-0.68	-0.68	-0.68	-0.68
-0.67	-0.67	-0.67	-0.67	-0.67	-0.67	-0.67	-0.67	-0.67
-0.66	-0.66	-0.66	-0.66	-0.66	-0.66	-0.66	-0.66	-0.66
-0.65	-0.65	-0.65	-0.65	-0.65	-0.65	-0.65	-0.65	-0.65
-0.64	-0.64	-0.64	-0.64	-0.64	-0.64	-0.64	-0.64	-0.64
-0.63	-0.63	-0.63	-0.63	-0.63	-0.63	-0.63	-0.63	-0.63
-0.62	-0.62	-0.62	-0.62	-0.62	-0.62	-0.62	-0.62	-0.62
-0.61	-0.61	-0.61	-0.61	-0.61	-0.61	-0.61	-0.61	-0.61
-0.6	-0.6	-0.6	-0.6	-0.6	-0.6	-0.6	-0.6	-0.6
-0.59	-0.59	-0.59	-0.59	-0.59	-0.59	-0.59	-0.59	-0.59
-0.58	-0.58	-0.58	-0.58	-0.58	-0.58	-0.58	-0.58	-0.58
-0.57	-0.57	-0.57	-0.57	-0.57	-0.57	-0.57	-0.57	-0.57
-0.56	-0.56	-0.56	-0.56	-0.56	-0.56	-0.56	-0.56	-0.56
-0.55	-0.55	-0.55	-0.55	-0.55	-0.55	-0.55	-0.55	-0.55
-0.54	-0.54	-0.54	-0.54	-0.54	-0.54	-0.54	-0.54	-0.54
-0.53	-0.53	-0.53	-0.53	-0.53	-0.53	-0.53	-0.53	-0.53
-0.52	-0.52	-0.52	-0.52	-0.52	-0.52	-0.52	-0.52	-0.52
-0.51	-0.51	-0.51	-0.51	-0.51	-0.51	-0.51	-0.51	-0.51

-0.5	-0.5	-0.5	-0.5	-0.5	-0.5	-0.5	-0.5	-0.5
-0.49	-0.49	-0.49	-0.49	-0.49	-0.49	-0.49	-0.49	-0.49
-0.48	-0.48	-0.48	-0.48	-0.48	-0.48	-0.48	-0.48	-0.48
-0.47	-0.47	-0.47	-0.47	-0.47	-0.47	-0.47	-0.47	-0.47
-0.46	-0.46	-0.46	-0.46	-0.46	-0.46	-0.46	-0.46	-0.46
-0.45	-0.45	-0.45	-0.45	-0.45	-0.45	-0.45	-0.45	-0.45
-0.44	-0.44	-0.44	-0.44	-0.44	-0.44	-0.44	-0.44	-0.44
-0.43	-0.43	-0.43	-0.43	-0.43	-0.43	-0.43	-0.43	-0.43
-0.42	-0.42	-0.42	-0.42	-0.42	-0.42	-0.42	-0.42	-0.42
-0.41	-0.41	-0.41	-0.41	-0.41	-0.41	-0.41	-0.41	-0.41
-0.4	-0.4	-0.4	-0.4	-0.4	-0.4	-0.4	-0.4	-0.4
-0.39	-0.39	-0.39	-0.39	-0.39	-0.39	-0.39	-0.39	-0.39
-0.38	-0.38	-0.38	-0.38	-0.38	-0.38	-0.38	-0.38	-0.38
-0.37	-0.37	-0.37	-0.37	-0.37	-0.37	-0.37	-0.37	-0.37
-0.36	-0.36	-0.36	-0.36	-0.36	-0.36	-0.36	-0.36	-0.36
-0.35	-0.35	-0.35	-0.35	-0.35	-0.35	-0.35	-0.35	-0.35
-0.34	-0.34	-0.34	-0.34	-0.34	-0.34	-0.34	-0.34	-0.34
-0.33	-0.33	-0.33	-0.33	-0.33	-0.33	-0.33	-0.33	-0.33
-0.32	-0.32	-0.32	-0.32	-0.32	-0.32	-0.32	-0.32	-0.32
-0.31	-0.31	-0.31	-0.31	-0.31	-0.31	-0.31	-0.31	-0.31
-0.3	-0.3	-0.3	-0.3	-0.3	-0.3	-0.3	-0.3	-0.3
-0.29	-0.29	-0.29	-0.29	-0.29	-0.29	-0.29	-0.29	-0.29
-0.28	-0.28	-0.28	-0.28	-0.28	-0.28	-0.28	-0.28	-0.28
-0.27	-0.27	-0.27	-0.27	-0.27	-0.27	-0.27	-0.27	-0.27
-0.26	-0.26	-0.26	-0.26	-0.26	-0.26	-0.26	-0.26	-0.26
-0.25	-0.25	-0.25	-0.25	-0.25	-0.25	-0.25	-0.25	-0.25
-0.24	-0.24	-0.24	-0.24	-0.24	-0.24	-0.24	-0.24	-0.24
-0.23	-0.23	-0.23	-0.23	-0.23	-0.23	-0.23	-0.23	-0.23
-0.22	-0.22	-0.22	-0.22	-0.22	-0.22	-0.22	-0.22	-0.22
-0.21	-0.21	-0.21	-0.21	-0.21	-0.21	-0.21	-0.21	-0.21
-0.2	-0.2	-0.2	-0.2	-0.2	-0.2	-0.2	-0.2	-0.2
-0.19	-0.19	-0.19	-0.19	-0.19	-0.19	-0.19	-0.19	-0.19
-0.18	-0.18	-0.18	-0.18	-0.18	-0.18	-0.18	-0.18	-0.18
-0.17	-0.17	-0.17	-0.17	-0.17	-0.17	-0.17	-0.17	-0.17
-0.16	-0.16	-0.16	-0.16	-0.16	-0.16	-0.16	-0.16	-0.16
-0.15	-0.15	-0.15	-0.15	-0.15	-0.15	-0.15	-0.15	-0.15
-0.14	-0.14	-0.14	-0.14	-0.14	-0.14	-0.14	-0.14	-0.14
-0.13	-0.13	-0.13	-0.13	-0.13	-0.13	-0.13	-0.13	-0.13
-0.12	-0.12	-0.12	-0.12	-0.12	-0.12	-0.12	-0.12	-0.12
-0.11	-0.11	-0.11	-0.11	-0.11	-0.11	-0.11	-0.11	-0.11
-0.1	-0.1	-0.1	-0.1	-0.1	-0.1	-0.1	-0.1	-0.1
-0.09	-0.09	-0.09	-0.09	-0.09	-0.09	-0.09	-0.09	-0.09
-0.08	-0.08	-0.08	-0.08	-0.08	-0.08	-0.08	-0.08	-0.08
-0.07	-0.07	-0.07	-0.07	-0.07	-0.07	-0.07	-0.07	-0.07
-0.06	-0.06	-0.06	-0.06	-0.06	-0.06	-0.06	-0.06	-0.06
-0.05	-0.05	-0.05	-0.05	-0.05	-0.05	-0.05	-0.05	-0.05
-0.04	-0.04	-0.04	-0.04	-0.04	-0.04	-0.04	-0.04	-0.04
-0.03	-0.03	-0.03	-0.03	-0.03	-0.03	-0.03	-0.03	-0.03
-0.02	-0.02	-0.02	-0.02	-0.02	-0.02	-0.02	-0.02	-0.02
-0.01	-0.01	-0.01	-0.01	-0.01	-0.01	-0.01	-0.01	-0.01

0	0	0	0	0	0	0	0	0
0.01	0.01	0.01	0.01	0.01	0.01	0.01	0.01	0.01
0.02	0.02	0.02	0.02	0.02	0.02	0.02	0.02	0.02
0.03	0.03	0.03	0.03	0.03	0.03	0.03	0.03	0.03
0.04	0.04	0.04	0.04	0.04	0.04	0.04	0.04	0.04
0.05	0.05	0.05	0.05	0.05	0.05	0.05	0.05	0.05
0.06	0.06	0.06	0.06	0.06	0.06	0.06	0.06	0.06
0.07	0.07	0.07	0.07	0.07	0.07	0.07	0.07	0.07
0.08	0.08	0.08	0.08	0.08	0.08	0.08	0.08	0.08
0.09	0.09	0.09	0.09	0.09	0.09	0.09	0.09	0.09
0.1	0.1	0.1	0.1	0.1	0.1	0.1	0.1	0.1
0.11	0.11	0.11	0.11	0.11	0.11	0.11	0.11	0.11
0.12	0.12	0.12	0.12	0.12	0.12	0.12	0.12	0.12
0.13	0.13	0.13	0.13	0.13	0.13	0.13	0.13	0.13
0.14	0.14	0.14	0.14	0.14	0.14	0.14	0.14	0.14
0.15	0.15	0.15	0.15	0.15	0.15	0.15	0.15	0.15
0.16	0.16	0.16	0.16	0.16	0.16	0.16	0.16	0.16
0.17	0.17	0.17	0.17	0.17	0.17	0.17	0.17	0.17
0.18	0.18	0.18	0.18	0.18	0.18	0.18	0.18	0.18
0.19	0.19	0.19	0.19	0.19	0.19	0.19	0.19	0.19
0.2	0.2	0.2	0.2	0.2	0.2	0.2	0.2	0.2
0.21	0.21	0.21	0.21	0.21	0.21	0.21	0.21	0.21
0.22	0.22	0.22	0.22	0.22	0.22	0.22	0.22	0.22
0.23	0.23	0.23	0.23	0.23	0.23	0.23	0.23	0.23
0.24	0.24	0.24	0.24	0.24	0.24	0.24	0.24	0.24
0.25	0.25	0.25	0.25	0.25	0.25	0.25	0.25	0.25
0.26	0.26	0.26	0.26	0.26	0.26	0.26	0.26	0.26
0.27	0.27	0.27	0.27	0.27	0.27	0.27	0.27	0.27
0.28	0.28	0.28	0.28	0.28	0.28	0.28	0.28	0.28
0.29	0.29	0.29	0.29	0.29	0.29	0.29	0.29	0.29
0.3	0.3	0.3	0.3	0.3	0.3	0.3	0.3	0.3
0.31	0.31	0.31	0.31	0.31	0.31	0.31	0.31	0.31
0.32	0.32	0.32	0.32	0.32	0.32	0.32	0.32	0.32
0.33	0.33	0.33	0.33	0.33	0.33	0.33	0.33	0.33
0.34	0.34	0.34	0.34	0.34	0.34	0.34	0.34	0.34
0.35	0.35	0.35	0.35	0.35	0.35	0.35	0.35	0.35
0.36	0.36	0.36	0.36	0.36	0.36	0.36	0.36	0.36
0.37	0.37	0.37	0.37	0.37	0.37	0.37	0.37	0.37
0.38	0.38	0.38	0.38	0.38	0.38	0.38	0.38	0.38
0.39	0.39	0.39	0.39	0.39	0.39	0.39	0.39	0.39
0.4	0.4	0.4	0.4	0.4	0.4	0.4	0.4	0.4
0.41	0.41	0.41	0.41	0.41	0.41	0.41	0.41	0.41
0.42	0.42	0.42	0.42	0.42	0.42	0.42	0.42	0.42
0.43	0.43	0.43	0.43	0.43	0.43	0.43	0.43	0.43
0.44	0.44	0.44	0.44	0.44	0.44	0.44	0.44	0.44
0.45	0.45	0.45	0.45	0.45	0.45	0.45	0.45	0.45
0.46	0.46	0.46	0.46	0.46	0.46	0.46	0.46	0.46
0.47	0.47	0.47	0.47	0.47	0.47	0.47	0.47	0.47
0.48	0.48	0.48	0.48	0.48	0.48	0.48	0.48	0.48
0.49	0.49	0.49	0.49	0.49	0.49	0.49	0.49	0.49

0.5	0.5	0.5	0.5	0.5	0.5	0.5	0.5	0.5
0.51	0.51	0.51	0.51	0.51	0.51	0.51	0.51	0.51
0.52	0.52	0.52	0.52	0.52	0.52	0.52	0.52	0.52
0.53	0.53	0.53	0.53	0.53	0.53	0.53	0.53	0.53
0.54	0.54	0.54	0.54	0.54	0.54	0.54	0.54	0.54
0.55	0.55	0.55	0.55	0.55	0.55	0.55	0.55	0.55
0.56	0.56	0.56	0.56	0.56	0.56	0.56	0.56	0.56
0.57	0.57	0.57	0.57	0.57	0.57	0.57	0.57	0.57
0.58	0.58	0.58	0.58	0.58	0.58	0.58	0.58	0.58
0.59	0.59	0.59	0.59	0.59	0.59	0.59	0.59	0.59
0.6	0.6	0.6	0.6	0.6	0.6	0.6	0.6	0.6
0.61	0.61	0.61	0.61	0.61	0.61	0.61	0.61	0.61
0.62	0.62	0.62	0.62	0.62	0.62	0.62	0.62	0.62
0.63	0.63	0.63	0.63	0.63	0.63	0.63	0.63	0.63
0.64	0.64	0.64	0.64	0.64	0.64	0.64	0.64	0.64
0.65	0.65	0.65	0.65	0.65	0.65	0.65	0.65	0.65
0.66	0.66	0.66	0.66	0.66	0.66	0.66	0.66	0.66
0.67	0.67	0.67	0.67	0.67	0.67	0.67	0.67	0.67
0.68	0.68	0.68	0.68	0.68	0.68	0.68	0.68	0.68
0.69	0.69	0.69	0.69	0.69	0.69	0.69	0.69	0.69
0.7	0.7	0.7	0.7	0.7	0.7	0.7	0.7	0.7
0.71	0.71	0.71	0.71	0.71	0.71	0.71	0.71	0.71
0.72	0.72	0.72	0.72	0.72	0.72	0.72	0.72	0.72
0.73	0.73	0.73	0.73	0.73	0.73	0.73	0.73	0.73
0.74	0.74	0.74	0.74	0.74	0.74	0.74	0.74	0.74
0.75	0.75	0.75	0.75	0.75	0.75	0.75	0.75	0.75
0.76	0.76	0.76	0.76	0.76	0.76	0.76	0.76	0.76
0.77	0.77	0.77	0.77	0.77	0.77	0.77	0.77	0.77
0.78	0.78	0.78	0.78	0.78	0.78	0.78	0.78	0.78
0.79	0.79	0.79	0.79	0.79	0.79	0.79	0.79	0.79
0.8	0.8	0.8	0.8	0.8	0.8	0.8	0.8	0.8
0.81	0.81	0.81	0.81	0.81	0.81	0.81	0.81	0.81
0.82	0.82	0.82	0.82	0.82	0.82	0.82	0.82	0.82
0.83	0.83	0.83	0.83	0.83	0.83	0.83	0.83	0.83
0.84	0.84	0.84	0.84	0.84	0.84	0.84	0.84	0.84
0.85	0.85	0.85	0.85	0.85	0.85	0.85	0.85	0.85
0.86	0.86	0.86	0.86	0.86	0.86	0.86	0.86	0.86
0.87	0.87	0.87	0.87	0.87	0.87	0.87	0.87	0.87
0.88	0.88	0.88	0.88	0.88	0.88	0.88	0.88	0.88
0.89	0.89	0.89	0.89	0.89	0.89	0.89	0.89	0.89
0.9	0.9	0.9	0.9	0.9	0.9	0.9	0.9	0.9
0.91	0.91	0.91	0.91	0.91	0.91	0.91	0.91	0.91
0.92	0.92	0.92	0.92	0.92	0.92	0.92	0.92	0.92
0.93	0.93	0.93	0.93	0.93	0.93	0.93	0.93	0.93
0.94	0.94	0.94	0.94	0.94	0.94	0.94	0.94	0.94
0.95	0.95	0.95	0.95	0.95	0.95	0.95	0.95	0.95
0.96	0.96	0.96	0.96	0.96	0.96	0.96	0.96	0.96
0.97	0.97	0.97	0.97	0.97	0.97	0.97	0.97	0.97
0.98	0.98	0.98	0.98	0.98	0.98	0.98	0.98	0.98
0.99	0.99	0.99	0.99	0.99	0.99	0.99	0.99	0.99

1	1	1	1	1	1	1	1	1
1.01	1.01	1.01	1.01	1.01	1.01	1.01	1.01	1.01
1.02	1.02	1.02	1.02	1.02	1.02	1.02	1.02	1.02
1.03	1.03	1.03	1.03	1.03	1.03	1.03	1.03	1.03
1.04	1.04	1.04	1.04	1.04	1.04	1.04	1.04	1.04
1.05	1.05	1.05	1.05	1.05	1.05	1.05	1.05	1.05
1.06	1.06	1.06	1.06	1.06	1.06	1.06	1.06	1.06
1.07	1.07	1.07	1.07	1.07	1.07	1.07	1.07	1.07
1.08	1.08	1.08	1.08	1.08	1.08	1.08	1.08	1.08
1.09	1.09	1.09	1.09	1.09	1.09	1.09	1.09	1.09
1.1	1.1	1.1	1.1	1.1	1.1	1.1	1.1	1.1
1.11	1.11	1.11	1.11	1.11	1.11	1.11	1.11	1.11
1.12	1.12	1.12	1.12	1.12	1.12	1.12	1.12	1.12
1.13	1.13	1.13	1.13	1.13	1.13	1.13	1.13	1.13
1.14	1.14	1.14	1.14	1.14	1.14	1.14	1.14	1.14
1.15	1.15	1.15	1.15	1.15	1.15	1.15	1.15	1.15
1.16	1.16	1.16	1.16	1.16	1.16	1.16	1.16	1.16
1.17	1.17	1.17	1.17	1.17	1.17	1.17	1.17	1.17
1.18	1.18	1.18	1.18	1.18	1.18	1.18	1.18	1.18
1.19	1.19	1.19	1.19	1.19	1.19	1.19	1.19	1.19
1.2	1.2	1.2	1.2	1.2	1.2	1.2	1.2	1.2
1.21	1.21	1.21	1.21	1.21	1.21	1.21	1.21	1.21
1.22	1.22	1.22	1.22	1.22	1.22	1.22	1.22	1.22
1.23	1.23	1.23	1.23	1.23	1.23	1.23	1.23	1.23
1.24	1.24	1.24	1.24	1.24	1.24	1.24	1.24	1.24
1.25	1.25	1.25	1.25	1.25	1.25	1.25	1.25	1.25
1.26	1.26	1.26	1.26	1.26	1.26	1.26	1.26	1.26
1.27	1.27	1.27	1.27	1.27	1.27	1.27	1.27	1.27
1.28	1.28	1.28	1.28	1.28	1.28	1.28	1.28	1.28
1.29	1.29	1.29	1.29	1.29	1.29	1.29	1.29	1.29
1.3	1.3	1.3	1.3	1.3	1.3	1.3	1.3	1.3
1.31	1.31	1.31	1.31	1.31	1.31	1.31	1.31	1.31
1.32	1.32	1.32	1.32	1.32	1.32	1.32	1.32	1.32
1.33	1.33	1.33	1.33	1.33	1.33	1.33	1.33	1.33
1.34	1.34	1.34	1.34	1.34	1.34	1.34	1.34	1.34
1.35	1.35	1.35	1.35	1.35	1.35	1.35	1.35	1.35
1.36	1.36	1.36	1.36	1.36	1.36	1.36	1.36	1.36
1.37	1.37	1.37	1.37	1.37	1.37	1.37	1.37	1.37
1.38	1.38	1.38	1.38	1.38	1.38	1.38	1.38	1.38
1.39	1.39	1.39	1.39	1.39	1.39	1.39	1.39	1.39
1.4	1.4	1.4	1.4	1.4	1.4	1.4	1.4	1.4
1.41	1.41	1.41	1.41	1.41	1.41	1.41	1.41	1.41
1.42	1.42	1.42	1.42	1.42	1.42	1.42	1.42	1.42
1.43	1.43	1.43	1.43	1.43	1.43	1.43	1.43	1.43
1.44	1.44	1.44	1.44	1.44	1.44	1.44	1.44	1.44
1.45	1.45	1.45	1.45	1.45	1.45	1.45	1.45	1.45
1.46	1.46	1.46	1.46	1.46	1.46	1.46	1.46	1.46
1.47	1.47	1.47	1.47	1.47	1.47	1.47	1.47	1.47
1.48	1.48	1.48	1.48	1.48	1.48	1.48	1.48	1.48
1.49	1.49	1.49	1.49	1.49	1.49	1.49	1.49	1.49

1.5	1.5	1.5	1.5	1.5	1.5	1.5	1.5	1.5
1.51	1.51	1.51	1.51	1.51	1.51	1.51	1.51	1.51
1.52	1.52	1.52	1.52	1.52	1.52	1.52	1.52	1.52
1.53	1.53	1.53	1.53	1.53	1.53	1.53	1.53	1.53
1.54	1.54	1.54	1.54	1.54	1.54	1.54	1.54	1.54
1.55	1.55	1.55	1.55	1.55	1.55	1.55	1.55	1.55
1.56	1.56	1.56	1.56	1.56	1.56	1.56	1.56	1.56
1.57	1.57	1.57	1.57	1.57	1.57	1.57	1.57	1.57
1.58	1.58	1.58	1.58	1.58	1.58	1.58	1.58	1.58
1.59	1.59	1.59	1.59	1.59	1.59	1.59	1.59	1.59
1.6	1.6	1.6	1.6	1.6	1.6	1.6	1.6	1.6
1.61	1.61	1.61	1.61	1.61	1.61	1.61	1.61	1.61
1.62	1.62	1.62	1.62	1.62	1.62	1.62	1.62	1.62
1.63	1.63	1.63	1.63	1.63	1.63	1.63	1.63	1.63
1.64	1.64	1.64	1.64	1.64	1.64	1.64	1.64	1.64
1.65	1.65	1.65	1.65	1.65	1.65	1.65	1.65	1.65
1.66	1.66	1.66	1.66	1.66	1.66	1.66	1.66	1.66
1.67	1.67	1.67	1.67	1.67	1.67	1.67	1.67	1.67
1.68	1.68	1.68	1.68	1.68	1.68	1.68	1.68	1.68
1.69	1.69	1.69	1.69	1.69	1.69	1.69	1.69	1.69
1.7	1.7	1.7	1.7	1.7	1.7	1.7	1.7	1.7
1.71	1.71	1.71	1.71	1.71	1.71	1.71	1.71	1.71
1.72	1.72	1.72	1.72	1.72	1.72	1.72	1.72	1.72
1.73	1.73	1.73	1.73	1.73	1.73	1.73	1.73	1.73
1.74	1.74	1.74	1.74	1.74	1.74	1.74	1.74	1.74
1.75	1.75	1.75	1.75	1.75	1.75	1.75	1.75	1.75
1.76	1.76	1.76	1.76	1.76	1.76	1.76	1.76	1.76
1.77	1.77	1.77	1.77	1.77	1.77	1.77	1.77	1.77
1.78	1.78	1.78	1.78	1.78	1.78	1.78	1.78	1.78
1.79	1.79	1.79	1.79	1.79	1.79	1.79	1.79	1.79
1.8	1.8	1.8	1.8	1.8	1.8	1.8	1.8	1.8
1.81	1.81	1.81	1.81	1.81	1.81	1.81	1.81	1.81
1.82	1.82	1.82	1.82	1.82	1.82	1.82	1.82	1.82
1.83	1.83	1.83	1.83	1.83	1.83	1.83	1.83	1.83
1.84	1.84	1.84	1.84	1.84	1.84	1.84	1.84	1.84
1.85	1.85	1.85	1.85	1.85	1.85	1.85	1.85	1.85
1.86	1.86	1.86	1.86	1.86	1.86	1.86	1.86	1.86
1.87	1.87	1.87	1.87	1.87	1.87	1.87	1.87	1.87
1.88	1.88	1.88	1.88	1.88	1.88	1.88	1.88	1.88
1.89	1.89	1.89	1.89	1.89	1.89	1.89	1.89	1.89
1.9	1.9	1.9	1.9	1.9	1.9	1.9	1.9	1.9
1.91	1.91	1.91	1.91	1.91	1.91	1.91	1.91	1.91
1.92	1.92	1.92	1.92	1.92	1.92	1.92	1.92	1.92
1.93	1.93	1.93	1.93	1.93	1.93	1.93	1.93	1.93
1.94	1.94	1.94	1.94	1.94	1.94	1.94	1.94	1.94
1.95	1.95	1.95	1.95	1.95	1.95	1.95	1.95	1.95
1.96	1.96	1.96	1.96	1.96	1.96	1.96	1.96	1.96
1.97	1.97	1.97	1.97	1.97	1.97	1.97	1.97	1.97
1.98	1.98	1.98	1.98	1.98	1.98	1.98	1.98	1.98
1.99	1.99	1.99	1.99	1.99	1.99	1.99	1.99	1.99

2	2	2	2	2	2	2	2	2
2.01	2.01	2.01	2.01	2.01	2.01	2.01	2.01	2.01
2.02	2.02	2.02	2.02	2.02	2.02	2.02	2.02	2.02
2.03	2.03	2.03	2.03	2.03	2.03	2.03	2.03	2.03
2.04	2.04	2.04	2.04	2.04	2.04	2.04	2.04	2.04
2.05	2.05	2.05	2.05	2.05	2.05	2.05	2.05	2.05
2.06	2.06	2.06	2.06	2.06	2.06	2.06	2.06	2.06
2.07	2.07	2.07	2.07	2.07	2.07	2.07	2.07	2.07
2.08	2.08	2.08	2.08	2.08	2.08	2.08	2.08	2.08
2.09	2.09	2.09	2.09	2.09	2.09	2.09	2.09	2.09
2.1	2.1	2.1	2.1	2.1	2.1	2.1	2.1	2.1
2.11	2.11	2.11	2.11	2.11	2.11	2.11	2.11	2.11
2.12	2.12	2.12	2.12	2.12	2.12	2.12	2.12	2.12
2.13	2.13	2.13	2.13	2.13	2.13	2.13	2.13	2.13
2.14	2.14	2.14	2.14	2.14	2.14	2.14	2.14	2.14
2.15	2.15	2.15	2.15	2.15	2.15	2.15	2.15	2.15
2.16	2.16	2.16	2.16	2.16	2.16	2.16	2.16	2.16
2.17	2.17	2.17	2.17	2.17	2.17	2.17	2.17	2.17
2.18	2.18	2.18	2.18	2.18	2.18	2.18	2.18	2.18
2.19	2.19	2.19	2.19	2.19	2.19	2.19	2.19	2.19
2.2	2.2	2.2	2.2	2.2	2.2	2.2	2.2	2.2
2.21	2.21	2.21	2.21	2.21	2.21	2.21	2.21	2.21
2.22	2.22	2.22	2.22	2.22	2.22	2.22	2.22	2.22
2.23	2.23	2.23	2.23	2.23	2.23	2.23	2.23	2.23
2.24	2.24	2.24	2.24	2.24	2.24	2.24	2.24	2.24
2.25	2.25	2.25	2.25	2.25	2.25	2.25	2.25	2.25
2.26	2.26	2.26	2.26	2.26	2.26	2.26	2.26	2.26
2.27	2.27	2.27	2.27	2.27	2.27	2.27	2.27	2.27
2.28	2.28	2.28	2.28	2.28	2.28	2.28	2.28	2.28
2.29	2.29	2.29	2.29	2.29	2.29	2.29	2.29	2.29
2.3	2.3	2.3	2.3	2.3	2.3	2.3	2.3	2.3
2.31	2.31	2.31	2.31	2.31	2.31	2.31	2.31	2.31
2.32	2.32	2.32	2.32	2.32	2.32	2.32	2.32	2.32
2.33	2.33	2.33	2.33	2.33	2.33	2.33	2.33	2.33
2.34	2.34	2.34	2.34	2.34	2.34	2.34	2.34	2.34
2.35	2.35	2.35	2.35	2.35	2.35	2.35	2.35	2.35
2.36	2.36	2.36	2.36	2.36	2.36	2.36	2.36	2.36
2.37	2.37	2.37	2.37	2.37	2.37	2.37	2.37	2.37
2.38	2.38	2.38	2.38	2.38	2.38	2.38	2.38	2.38
2.39	2.39	2.39	2.39	2.39	2.39	2.39	2.39	2.39
2.4	2.4	2.4	2.4	2.4	2.4	2.4	2.4	2.4
2.41	2.41	2.41	2.41	2.41	2.41	2.41	2.41	2.41
2.42	2.42	2.42	2.42	2.42	2.42	2.42	2.42	2.42
2.43	2.43	2.43	2.43	2.43	2.43	2.43	2.43	2.43
2.44	2.44	2.44	2.44	2.44	2.44	2.44	2.44	2.44
2.45	2.45	2.45	2.45	2.45	2.45	2.45	2.45	2.45
2.46	2.46	2.46	2.46	2.46	2.46	2.46	2.46	2.46
2.47	2.47	2.47	2.47	2.47	2.47	2.47	2.47	2.47
2.48	2.48	2.48	2.48	2.48	2.48	2.48	2.48	2.48
2.49	2.49	2.49	2.49	2.49	2.49	2.49	2.49	2.49

2.5	2.5	2.5	2.5	2.5	2.5	2.5	2.5	2.5
2.51	2.51	2.51	2.51	2.51	2.51	2.51	2.51	2.51
2.52	2.52	2.52	2.52	2.52	2.52	2.52	2.52	2.52
2.53	2.53	2.53	2.53	2.53	2.53	2.53	2.53	2.53
2.54	2.54	2.54	2.54	2.54	2.54	2.54	2.54	2.54
2.55	2.55	2.55	2.55	2.55	2.55	2.55	2.55	2.55
2.56	2.56	2.56	2.56	2.56	2.56	2.56	2.56	2.56
2.57	2.57	2.57	2.57	2.57	2.57	2.57	2.57	2.57
2.58	2.58	2.58	2.58	2.58	2.58	2.58	2.58	2.58
2.59	2.59	2.59	2.59	2.59	2.59	2.59	2.59	2.59
2.6	2.6	2.6	2.6	2.6	2.6	2.6	2.6	2.6
2.61	2.61	2.61	2.61	2.61	2.61	2.61	2.61	2.61
2.62	2.62	2.62	2.62	2.62	2.62	2.62	2.62	2.62
2.63	2.63	2.63	2.63	2.63	2.63	2.63	2.63	2.63
2.64	2.64	2.64	2.64	2.64	2.64	2.64	2.64	2.64
2.65	2.65	2.65	2.65	2.65	2.65	2.65	2.65	2.65
2.66	2.66	2.66	2.66	2.66	2.66	2.66	2.66	2.66
2.67	2.67	2.67	2.67	2.67	2.67	2.67	2.67	2.67
2.68	2.68	2.68	2.68	2.68	2.68	2.68	2.68	2.68
2.69	2.69	2.69	2.69	2.69	2.69	2.69	2.69	2.69
2.7	2.7	2.7	2.7	2.7	2.7	2.7	2.7	2.7
2.71	2.71	2.71	2.71	2.71	2.71	2.71	2.71	2.71
2.72	2.72	2.72	2.72	2.72	2.72	2.72	2.72	2.72
2.73	2.73	2.73	2.73	2.73	2.73	2.73	2.73	2.73
2.74	2.74	2.74	2.74	2.74	2.74	2.74	2.74	2.74
2.75	2.75	2.75	2.75	2.75	2.75	2.75	2.75	2.75
2.76	2.76	2.76	2.76	2.76	2.76	2.76	2.76	2.76
2.77	2.77	2.77	2.77	2.77	2.77	2.77	2.77	2.77
2.78	2.78	2.78	2.78	2.78	2.78	2.78	2.78	2.78
2.79	2.79	2.79	2.79	2.79	2.79	2.79	2.79	2.79
2.8	2.8	2.8	2.8	2.8	2.8	2.8	2.8	2.8
2.81	2.81	2.81	2.81	2.81	2.81	2.81	2.81	2.81
2.82	2.82	2.82	2.82	2.82	2.82	2.82	2.82	2.82
2.83	2.83	2.83	2.83	2.83	2.83	2.83	2.83	2.83
2.84	2.84	2.84	2.84	2.84	2.84	2.84	2.84	2.84
2.85	2.85	2.85	2.85	2.85	2.85	2.85	2.85	2.85
2.86	2.86	2.86	2.86	2.86	2.86	2.86	2.86	2.86
2.87	2.87	2.87	2.87	2.87	2.87	2.87	2.87	2.87
2.88	2.88	2.88	2.88	2.88	2.88	2.88	2.88	2.88
2.89	2.89	2.89	2.89	2.89	2.89	2.89	2.89	2.89
2.9	2.9	2.9	2.9	2.9	2.9	2.9	2.9	2.9
2.91	2.91	2.91	2.91	2.91	2.91	2.91	2.91	2.91
2.92	2.92	2.92	2.92	2.92	2.92	2.92	2.92	2.92
2.93	2.93	2.93	2.93	2.93	2.93	2.93	2.93	2.93
2.94	2.94	2.94	2.94	2.94	2.94	2.94	2.94	2.94
2.95	2.95	2.95	2.95	2.95	2.95	2.95	2.95	2.95
2.96	2.96	2.96	2.96	2.96	2.96	2.96	2.96	2.96
2.97	2.97	2.97	2.97	2.97	2.97	2.97	2.97	2.97
2.98	2.98	2.98	2.98	2.98	2.98	2.98	2.98	2.98
2.99	2.99	2.99	2.99	2.99	2.99	2.99	2.99	2.99

3	3	3	3	3	3	3	3	3
3.01	3.01	3.01	3.01	3.01	3.01	3.01	3.01	3.01
3.02	3.02	3.02	3.02	3.02	3.02	3.02	3.02	3.02
3.03	3.03	3.03	3.03	3.03	3.03	3.03	3.03	3.03
3.04	3.04	3.04	3.04	3.04	3.04	3.04	3.04	3.04
3.05	3.05	3.05	3.05	3.05	3.05	3.05	3.05	3.05
3.06	3.06	3.06	3.06	3.06	3.06	3.06	3.06	3.06
3.07	3.07	3.07	3.07	3.07	3.07	3.07	3.07	3.07
3.08	3.08	3.08	3.08	3.08	3.08	3.08	3.08	3.08
3.09	3.09	3.09	3.09	3.09	3.09	3.09	3.09	3.09
3.1	3.1	3.1	3.1	3.1	3.1	3.1	3.1	3.1
3.11	3.11	3.11	3.11	3.11	3.11	3.11	3.11	3.11
3.12	3.12	3.12	3.12	3.12	3.12	3.12	3.12	3.12
3.13	3.13	3.13	3.13	3.13	3.13	3.13	3.13	3.13
3.14	3.14	3.14	3.14	3.14	3.14	3.14	3.14	3.14
3.15	3.15	3.15	3.15	3.15	3.15	3.15	3.15	3.15
3.16	3.16	3.16	3.16	3.16	3.16	3.16	3.16	3.16
3.17	3.17	3.17	3.17	3.17	3.17	3.17	3.17	3.17
3.18	3.18	3.18	3.18	3.18	3.18	3.18	3.18	3.18
3.19	3.19	3.19	3.19	3.19	3.19	3.19	3.19	3.19
3.2	3.2	3.2	3.2	3.2	3.2	3.2	3.2	3.2
3.21	3.21	3.21	3.21	3.21	3.21	3.21	3.21	3.21
3.22	3.22	3.22	3.22	3.22	3.22	3.22	3.22	3.22
3.23	3.23	3.23	3.23	3.23	3.23	3.23	3.23	3.23
3.24	3.24	3.24	3.24	3.24	3.24	3.24	3.24	3.24
3.25	3.25	3.25	3.25	3.25	3.25	3.25	3.25	3.25
3.26	3.26	3.26	3.26	3.26	3.26	3.26	3.26	3.26
3.27	3.27	3.27	3.27	3.27	3.27	3.27	3.27	3.27
3.28	3.28	3.28	3.28	3.28	3.28	3.28	3.28	3.28
3.29	3.29	3.29	3.29	3.29	3.29	3.29	3.29	3.29
3.3	3.3	3.3	3.3	3.3	3.3	3.3	3.3	3.3
3.31	3.31	3.31	3.31	3.31	3.31	3.31	3.31	3.31
3.32	3.32	3.32	3.32	3.32	3.32	3.32	3.32	3.32
3.33	3.33	3.33	3.33	3.33	3.33	3.33	3.33	3.33
3.34	3.34	3.34	3.34	3.34	3.34	3.34	3.34	3.34
3.35	3.35	3.35	3.35	3.35	3.35	3.35	3.35	3.35
3.36	3.36	3.36	3.36	3.36	3.36	3.36	3.36	3.36
3.37	3.37	3.37	3.37	3.37	3.37	3.37	3.37	3.37
3.38	3.38	3.38	3.38	3.38	3.38	3.38	3.38	3.38
3.39	3.39	3.39	3.39	3.39	3.39	3.39	3.39	3.39
3.4	3.4	3.4	3.4	3.4	3.4	3.4	3.4	3.4
3.41	3.41	3.41	3.41	3.41	3.41	3.41	3.41	3.41
3.42	3.42	3.42	3.42	3.42	3.42	3.42	3.42	3.42
3.43	3.43	3.43	3.43	3.43	3.43	3.43	3.43	3.43
3.44	3.44	3.44	3.44	3.44	3.44	3.44	3.44	3.44
3.45	3.45	3.45	3.45	3.45	3.45	3.45	3.45	3.45
3.46	3.46	3.46	3.46	3.46	3.46	3.46	3.46	3.46
3.47	3.47	3.47	3.47	3.47	3.47	3.47	3.47	3.47
3.48	3.48	3.48	3.48	3.48	3.48	3.48	3.48	3.48
3.49	3.49	3.49	3.49	3.49	3.49	3.49	3.49	3.49

3.5	3.5	3.5	3.5	3.5	3.5	3.5	3.5	3.5
3.51	3.51	3.51	3.51	3.51	3.51	3.51	3.51	3.51
3.52	3.52	3.52	3.52	3.52	3.52	3.52	3.52	3.52
3.53	3.53	3.53	3.53	3.53	3.53	3.53	3.53	3.53
3.54	3.54	3.54	3.54	3.54	3.54	3.54	3.54	3.54
3.55	3.55	3.55	3.55	3.55	3.55	3.55	3.55	3.55
3.56	3.56	3.56	3.56	3.56	3.56	3.56	3.56	3.56
3.57	3.57	3.57	3.57	3.57	3.57	3.57	3.57	3.57
3.58	3.58	3.58	3.58	3.58	3.58	3.58	3.58	3.58
3.59	3.59	3.59	3.59	3.59	3.59	3.59	3.59	3.59
3.6	3.6	3.6	3.6	3.6	3.6	3.6	3.6	3.6
3.61	3.61	3.61	3.61	3.61	3.61	3.61	3.61	3.61
3.62	3.62	3.62	3.62	3.62	3.62	3.62	3.62	3.62
3.63	3.63	3.63	3.63	3.63	3.63	3.63	3.63	3.63
3.64	3.64	3.64	3.64	3.64	3.64	3.64	3.64	3.64
3.65	3.65	3.65	3.65	3.65	3.65	3.65	3.65	3.65
3.66	3.66	3.66	3.66	3.66	3.66	3.66	3.66	3.66
3.67	3.67	3.67	3.67	3.67	3.67	3.67	3.67	3.67
3.68	3.68	3.68	3.68	3.68	3.68	3.68	3.68	3.68
3.69	3.69	3.69	3.69	3.69	3.69	3.69	3.69	3.69
3.7	3.7	3.7	3.7	3.7	3.7	3.7	3.7	3.7
3.71	3.71	3.71	3.71	3.71	3.71	3.71	3.71	3.71
3.72	3.72	3.72	3.72	3.72	3.72	3.72	3.72	3.72
3.73	3.73	3.73	3.73	3.73	3.73	3.73	3.73	3.73
3.74	3.74	3.74	3.74	3.74	3.74	3.74	3.74	3.74
3.75	3.75	3.75	3.75	3.75	3.75	3.75	3.75	3.75
3.76	3.76	3.76	3.76	3.76	3.76	3.76	3.76	3.76
3.77	3.77	3.77	3.77	3.77	3.77	3.77	3.77	3.77
3.78	3.78	3.78	3.78	3.78	3.78	3.78	3.78	3.78
3.79	3.79	3.79	3.79	3.79	3.79	3.79	3.79	3.79
3.8	3.8	3.8	3.8	3.8	3.8	3.8	3.8	3.8
3.81	3.81	3.81	3.81	3.81	3.81	3.81	3.81	3.81
3.82	3.82	3.82	3.82	3.82	3.82	3.82	3.82	3.82
3.83	3.83	3.83	3.83	3.83	3.83	3.83	3.83	3.83
3.84	3.84	3.84	3.84	3.84	3.84	3.84	3.84	3.84
3.85	3.85	3.85	3.85	3.85	3.85	3.85	3.85	3.85
3.86	3.86	3.86	3.86	3.86	3.86	3.86	3.86	3.86
3.87	3.87	3.87	3.87	3.87	3.87	3.87	3.87	3.87
3.88	3.88	3.88	3.88	3.88	3.88	3.88	3.88	3.88
3.89	3.89	3.89	3.89	3.89	3.89	3.89	3.89	3.89
3.9	3.9	3.9	3.9	3.9	3.9	3.9	3.9	3.9
3.91	3.91	3.91	3.91	3.91	3.91	3.91	3.91	3.91
3.92	3.92	3.92	3.92	3.92	3.92	3.92	3.92	3.92
3.93	3.93	3.93	3.93	3.93	3.93	3.93	3.93	3.93
3.94	3.94	3.94	3.94	3.94	3.94	3.94	3.94	3.94
3.95	3.95	3.95	3.95	3.95	3.95	3.95	3.95	3.95
3.96	3.96	3.96	3.96	3.96	3.96	3.96	3.96	3.96
3.97	3.97	3.97	3.97	3.97	3.97	3.97	3.97	3.97
3.98	3.98	3.98	3.98	3.98	3.98	3.98	3.98	3.98
3.99	3.99	3.99	3.99	3.99	3.99	3.99	3.99	3.99

4	4	4	4	4	4	4	4	4
4.01	4.01	4.01	4.01	4.01	4.01	4.01	4.01	4.01
4.02	4.02	4.02	4.02	4.02	4.02	4.02	4.02	4.02
4.03	4.03	4.03	4.03	4.03	4.03	4.03	4.03	4.03
4.04	4.04	4.04	4.04	4.04	4.04	4.04	4.04	4.04
4.05	4.05	4.05	4.05	4.05	4.05	4.05	4.05	4.05
4.06	4.06	4.06	4.06	4.06	4.06	4.06	4.06	4.06
4.07	4.07	4.07	4.07	4.07	4.07	4.07	4.07	4.07
4.08	4.08	4.08	4.08	4.08	4.08	4.08	4.08	4.08
4.09	4.09	4.09	4.09	4.09	4.09	4.09	4.09	4.09
4.1	4.1	4.1	4.1	4.1	4.1	4.1	4.1	4.1
4.11	4.11	4.11	4.11	4.11	4.11	4.11	4.11	4.11
4.12	4.12	4.12	4.12	4.12	4.12	4.12	4.12	4.12
4.13	4.13	4.13	4.13	4.13	4.13	4.13	4.13	4.13
4.14	4.14	4.14	4.14	4.14	4.14	4.14	4.14	4.14
4.15	4.15	4.15	4.15	4.15	4.15	4.15	4.15	4.15
4.16	4.16	4.16	4.16	4.16	4.16	4.16	4.16	4.16
4.17	4.17	4.17	4.17	4.17	4.17	4.17	4.17	4.17
4.18	4.18	4.18	4.18	4.18	4.18	4.18	4.18	4.18
4.19	4.19	4.19	4.19	4.19	4.19	4.19	4.19	4.19
4.2	4.2	4.2	4.2	4.2	4.2	4.2	4.2	4.2
4.21	4.21	4.21	4.21	4.21	4.21	4.21	4.21	4.21
4.22	4.22	4.22	4.22	4.22	4.22	4.22	4.22	4.22
4.23	4.23	4.23	4.23	4.23	4.23	4.23	4.23	4.23
4.24	4.24	4.24	4.24	4.24	4.24	4.24	4.24	4.24
4.25	4.25	4.25	4.25	4.25	4.25	4.25	4.25	4.25
4.26	4.26	4.26	4.26	4.26	4.26	4.26	4.26	4.26
4.27	4.27	4.27	4.27	4.27	4.27	4.27	4.27	4.27
4.28	4.28	4.28	4.28	4.28	4.28	4.28	4.28	4.28
4.29	4.29	4.29	4.29	4.29	4.29	4.29	4.29	4.29
4.3	4.3	4.3	4.3	4.3	4.3	4.3	4.3	4.3
4.31	4.31	4.31	4.31	4.31	4.31	4.31	4.31	4.31
4.32	4.32	4.32	4.32	4.32	4.32	4.32	4.32	4.32
4.33	4.33	4.33	4.33	4.33	4.33	4.33	4.33	4.33
4.34	4.34	4.34	4.34	4.34	4.34	4.34	4.34	4.34
4.35	4.35	4.35	4.35	4.35	4.35	4.35	4.35	4.35
4.36	4.36	4.36	4.36	4.36	4.36	4.36	4.36	4.36
4.37	4.37	4.37	4.37	4.37	4.37	4.37	4.37	4.37
4.38	4.38	4.38	4.38	4.38	4.38	4.38	4.38	4.38
4.39	4.39	4.39	4.39	4.39	4.39	4.39	4.39	4.39
4.4	4.4	4.4	4.4	4.4	4.4	4.4	4.4	4.4
4.41	4.41	4.41	4.41	4.41	4.41	4.41	4.41	4.41
4.42	4.42	4.42	4.42	4.42	4.42	4.42	4.42	4.42
4.43	4.43	4.43	4.43	4.43	4.43	4.43	4.43	4.43
4.44	4.44	4.44	4.44	4.44	4.44	4.44	4.44	4.44
4.45	4.45	4.45	4.45	4.45	4.45	4.45	4.45	4.45
4.46	4.46	4.46	4.46	4.46	4.46	4.46	4.46	4.46
4.47	4.47	4.47	4.47	4.47	4.47	4.47	4.47	4.47
4.48	4.48	4.48	4.48	4.48	4.48	4.48	4.48	4.48
4.49	4.49	4.49	4.49	4.49	4.49	4.49	4.49	4.49

4.5	4.5	4.5	4.5	4.5	4.5	4.5	4.5	4.5
4.51	4.51	4.51	4.51	4.51	4.51	4.51	4.51	4.51
4.52	4.52	4.52	4.52	4.52	4.52	4.52	4.52	4.52
4.53	4.53	4.53	4.53	4.53	4.53	4.53	4.53	4.53
4.54	4.54	4.54	4.54	4.54	4.54	4.54	4.54	4.54
4.55	4.55	4.55	4.55	4.55	4.55	4.55	4.55	4.55
4.56	4.56	4.56	4.56	4.56	4.56	4.56	4.56	4.56
4.57	4.57	4.57	4.57	4.57	4.57	4.57	4.57	4.57
4.58	4.58	4.58	4.58	4.58	4.58	4.58	4.58	4.58
4.59	4.59	4.59	4.59	4.59	4.59	4.59	4.59	4.59
4.6	4.6	4.6	4.6	4.6	4.6	4.6	4.6	4.6
4.61	4.61	4.61	4.61	4.61	4.61	4.61	4.61	4.61
4.62	4.62	4.62	4.62	4.62	4.62	4.62	4.62	4.62
4.63	4.63	4.63	4.63	4.63	4.63	4.63	4.63	4.63
4.64	4.64	4.64	4.64	4.64	4.64	4.64	4.64	4.64
4.65	4.65	4.65	4.65	4.65	4.65	4.65	4.65	4.65
4.66	4.66	4.66	4.66	4.66	4.66	4.66	4.66	4.66
4.67	4.67	4.67	4.67	4.67	4.67	4.67	4.67	4.67
4.68	4.68	4.68	4.68	4.68	4.68	4.68	4.68	4.68
4.69	4.69	4.69	4.69	4.69	4.69	4.69	4.69	4.69
4.7	4.7	4.7	4.7	4.7	4.7	4.7	4.7	4.7
4.71	4.71	4.71	4.71	4.71	4.71	4.71	4.71	4.71
4.72	4.72	4.72	4.72	4.72	4.72	4.72	4.72	4.72
4.73	4.73	4.73	4.73	4.73	4.73	4.73	4.73	4.73
4.74	4.74	4.74	4.74	4.74	4.74	4.74	4.74	4.74
4.75	4.75	4.75	4.75	4.75	4.75	4.75	4.75	4.75
4.76	4.76	4.76	4.76	4.76	4.76	4.76	4.76	4.76
4.77	4.77	4.77	4.77	4.77	4.77	4.77	4.77	4.77
4.78	4.78	4.78	4.78	4.78	4.78	4.78	4.78	4.78
4.79	4.79	4.79	4.79	4.79	4.79	4.79	4.79	4.79
4.8	4.8	4.8	4.8	4.8	4.8	4.8	4.8	4.8
4.81	4.81	4.81	4.81	4.81	4.81	4.81	4.81	4.81
4.82	4.82	4.82	4.82	4.82	4.82	4.82	4.82	4.82
4.83	4.83	4.83	4.83	4.83	4.83	4.83	4.83	4.83
4.84	4.84	4.84	4.84	4.84	4.84	4.84	4.84	4.84
4.85	4.85	4.85	4.85	4.85	4.85	4.85	4.85	4.85
4.86	4.86	4.86	4.86	4.86	4.86	4.86	4.86	4.86
4.87	4.87	4.87	4.87	4.87	4.87	4.87	4.87	4.87
4.88	4.88	4.88	4.88	4.88	4.88	4.88	4.88	4.88
4.89	4.89	4.89	4.89	4.89	4.89	4.89	4.89	4.89
4.9	4.9	4.9	4.9	4.9	4.9	4.9	4.9	4.9
4.91	4.91	4.91	4.91	4.91	4.91	4.91	4.91	4.91
4.92	4.92	4.92	4.92	4.92	4.92	4.92	4.92	4.92
4.93	4.93	4.93	4.93	4.93	4.93	4.93	4.93	4.93
4.94	4.94	4.94	4.94	4.94	4.94	4.94	4.94	4.94
4.95	4.95	4.95	4.95	4.95	4.95	4.95	4.95	4.95
4.96	4.96	4.96	4.96	4.96	4.96	4.96	4.96	4.96
4.97	4.97	4.97	4.97	4.97	4.97	4.97	4.97	4.97
4.98	4.98	4.98	4.98	4.98	4.98	4.98	4.98	4.98
4.99	4.99	4.99	4.99	4.99	4.99	4.99	4.99	4.99

5	5	5	5	5	5	5	5	5
5.01	5.01	5.01	5.01	5.01	5.01	5.01	5.01	5.01
5.02	5.02	5.02	5.02	5.02	5.02	5.02	5.02	5.02
5.03	5.03	5.03	5.03	5.03	5.03	5.03	5.03	5.03
5.04	5.04	5.04	5.04	5.04	5.04	5.04	5.04	5.04
5.05	5.05	5.05	5.05	5.05	5.05	5.05	5.05	5.05
5.06	5.06	5.06	5.06	5.06	5.06	5.06	5.06	5.06
5.07	5.07	5.07	5.07	5.07	5.07	5.07	5.07	5.07
5.08	5.08	5.08	5.08	5.08	5.08	5.08	5.08	5.08
5.09	5.09	5.09	5.09	5.09	5.09	5.09	5.09	5.09
5.1	5.1	5.1	5.1	5.1	5.1	5.1	5.1	5.1
5.11	5.11	5.11	5.11	5.11	5.11	5.11	5.11	5.11
5.12	5.12	5.12	5.12	5.12	5.12	5.12	5.12	5.12
5.13	5.13	5.13	5.13	5.13	5.13	5.13	5.13	5.13
5.14	5.14	5.14	5.14	5.14	5.14	5.14	5.14	5.14
5.15	5.15	5.15	5.15	5.15	5.15	5.15	5.15	5.15
5.16	5.16	5.16	5.16	5.16	5.16	5.16	5.16	5.16
5.17	5.17	5.17	5.17	5.17	5.17	5.17	5.17	5.17
5.18	5.18	5.18	5.18	5.18	5.18	5.18	5.18	5.18
5.19	5.19	5.19	5.19	5.19	5.19	5.19	5.19	5.19
5.2	5.2	5.2	5.2	5.2	5.2	5.2	5.2	5.2
5.21	5.21	5.21	5.21	5.21	5.21	5.21	5.21	5.21
5.22	5.22	5.22	5.22	5.22	5.22	5.22	5.22	5.22
5.23	5.23	5.23	5.23	5.23	5.23	5.23	5.23	5.23
5.24	5.24	5.24	5.24	5.24	5.24	5.24	5.24	5.24
5.25	5.25	5.25	5.25	5.25	5.25	5.25	5.25	5.25
5.26	5.26	5.26	5.26	5.26	5.26	5.26	5.26	5.26
5.27	5.27	5.27	5.27	5.27	5.27	5.27	5.27	5.27
5.28	5.28	5.28	5.28	5.28	5.28	5.28	5.28	5.28
5.29	5.29	5.29	5.29	5.29	5.29	5.29	5.29	5.29
5.3	5.3	5.3	5.3	5.3	5.3	5.3	5.3	5.3
5.31	5.31	5.31	5.31	5.31	5.31	5.31	5.31	5.31
5.32	5.32	5.32	5.32	5.32	5.32	5.32	5.32	5.32
5.33	5.33	5.33	5.33	5.33	5.33	5.33	5.33	5.33
5.34	5.34	5.34	5.34	5.34	5.34	5.34	5.34	5.34
5.35	5.35	5.35	5.35	5.35	5.35	5.35	5.35	5.35
5.36	5.36	5.36	5.36	5.36	5.36	5.36	5.36	5.36
5.37	5.37	5.37	5.37	5.37	5.37	5.37	5.37	5.37
5.38	5.38	5.38	5.38	5.38	5.38	5.38	5.38	5.38
5.39	5.39	5.39	5.39	5.39	5.39	5.39	5.39	5.39
5.4	5.4	5.4	5.4	5.4	5.4	5.4	5.4	5.4
5.41	5.41	5.41	5.41	5.41	5.41	5.41	5.41	5.41
5.42	5.42	5.42	5.42	5.42	5.42	5.42	5.42	5.42
5.43	5.43	5.43	5.43	5.43	5.43	5.43	5.43	5.43
5.44	5.44	5.44	5.44	5.44	5.44	5.44	5.44	5.44
5.45	5.45	5.45	5.45	5.45	5.45	5.45	5.45	5.45
5.46	5.46	5.46	5.46	5.46	5.46	5.46	5.46	5.46
5.47	5.47	5.47	5.47	5.47	5.47	5.47	5.47	5.47
5.48	5.48	5.48	5.48	5.48	5.48	5.48	5.48	5.48
5.49	5.49	5.49	5.49	5.49	5.49	5.49	5.49	5.49

5.5	5.5	5.5	5.5	5.5	5.5	5.5	5.5	5.5
5.51	5.51	5.51	5.51	5.51	5.51	5.51	5.51	5.51
5.52	5.52	5.52	5.52	5.52	5.52	5.52	5.52	5.52
5.53	5.53	5.53	5.53	5.53	5.53	5.53	5.53	5.53
5.54	5.54	5.54	5.54	5.54	5.54	5.54	5.54	5.54
5.55	5.55	5.55	5.55	5.55	5.55	5.55	5.55	5.55
5.56	5.56	5.56	5.56	5.56	5.56	5.56	5.56	5.56
5.57	5.57	5.57	5.57	5.57	5.57	5.57	5.57	5.57
5.58	5.58	5.58	5.58	5.58	5.58	5.58	5.58	5.58
5.59	5.59	5.59	5.59	5.59	5.59	5.59	5.59	5.59
5.6	5.6	5.6	5.6	5.6	5.6	5.6	5.6	5.6
5.61	5.61	5.61	5.61	5.61	5.61	5.61	5.61	5.61
5.62	5.62	5.62	5.62	5.62	5.62	5.62	5.62	5.62
5.63	5.63	5.63	5.63	5.63	5.63	5.63	5.63	5.63
5.64	5.64	5.64	5.64	5.64	5.64	5.64	5.64	5.64
5.65	5.65	5.65	5.65	5.65	5.65	5.65	5.65	5.65
5.66	5.66	5.66	5.66	5.66	5.66	5.66	5.66	5.66
5.67	5.67	5.67	5.67	5.67	5.67	5.67	5.67	5.67
5.68	5.68	5.68	5.68	5.68	5.68	5.68	5.68	5.68
5.69	5.69	5.69	5.69	5.69	5.69	5.69	5.69	5.69
5.7	5.7	5.7	5.7	5.7	5.7	5.7	5.7	5.7
5.71	5.71	5.71	5.71	5.71	5.71	5.71	5.71	5.71
5.72	5.72	5.72	5.72	5.72	5.72	5.72	5.72	5.72
5.73	5.73	5.73	5.73	5.73	5.73	5.73	5.73	5.73
5.74	5.74	5.74	5.74	5.74	5.74	5.74	5.74	5.74
5.75	5.75	5.75	5.75	5.75	5.75	5.75	5.75	5.75
5.76	5.76	5.76	5.76	5.76	5.76	5.76	5.76	5.76
5.77	5.77	5.77	5.77	5.77	5.77	5.77	5.77	5.77
5.78	5.78	5.78	5.78	5.78	5.78	5.78	5.78	5.78
5.79	5.79	5.79	5.79	5.79	5.79	5.79	5.79	5.79
5.8	5.8	5.8	5.8	5.8	5.8	5.8	5.8	5.8
5.81	5.81	5.81	5.81	5.81	5.81	5.81	5.81	5.81
5.82	5.82	5.82	5.82	5.82	5.82	5.82	5.82	5.82
5.83	5.83	5.83	5.83	5.83	5.83	5.83	5.83	5.83
5.84	5.84	5.84	5.84	5.84	5.84	5.84	5.84	5.84
5.85	5.85	5.85	5.85	5.85	5.85	5.85	5.85	5.85
5.86	5.86	5.86	5.86	5.86	5.86	5.86	5.86	5.86
5.87	5.87	5.87	5.87	5.87	5.87	5.87	5.87	5.87
5.88	5.88	5.88	5.88	5.88	5.88	5.88	5.88	5.88
5.89	5.89	5.89	5.89	5.89	5.89	5.89	5.89	5.89
5.9	5.9	5.9	5.9	5.9	5.9	5.9	5.9	5.9
5.91	5.91	5.91	5.91	5.91	5.91	5.91	5.91	5.91
5.92	5.92	5.92	5.92	5.92	5.92	5.92	5.92	5.92
5.93	5.93	5.93	5.93	5.93	5.93	5.93	5.93	5.93
5.94	5.94	5.94	5.94	5.94	5.94	5.94	5.94	5.94
5.95	5.95	5.95	5.95	5.95	5.95	5.95	5.95	5.95
5.96	5.96	5.96	5.96	5.96	5.96	5.96	5.96	5.96
5.97	5.97	5.97	5.97	5.97	5.97	5.97	5.97	5.97
5.98	5.98	5.98	5.98	5.98	5.98	5.98	5.98	5.98
5.99	5.99	5.99	5.99	5.99	5.99	5.99	5.99	5.99

6	6	6	6	6	6	6	6	6
6.01	6.01	6.01	6.01	6.01	6.01	6.01	6.01	6.01
6.02	6.02	6.02	6.02	6.02	6.02	6.02	6.02	6.02
6.03	6.03	6.03	6.03	6.03	6.03	6.03	6.03	6.03
6.04	6.04	6.04	6.04	6.04	6.04	6.04	6.04	6.04
6.05	6.05	6.05	6.05	6.05	6.05	6.05	6.05	6.05
6.06	6.06	6.06	6.06	6.06	6.06	6.06	6.06	6.06
6.07	6.07	6.07	6.07	6.07	6.07	6.07	6.07	6.07
6.08	6.08	6.08	6.08	6.08	6.08	6.08	6.08	6.08
6.09	6.09	6.09	6.09	6.09	6.09	6.09	6.09	6.09
6.1	6.1	6.1	6.1	6.1	6.1	6.1	6.1	6.1
6.11	6.11	6.11	6.11	6.11	6.11	6.11	6.11	6.11
6.12	6.12	6.12	6.12	6.12	6.12	6.12	6.12	6.12
6.13	6.13	6.13	6.13	6.13	6.13	6.13	6.13	6.13
6.14	6.14	6.14	6.14	6.14	6.14	6.14	6.14	6.14
6.15	6.15	6.15	6.15	6.15	6.15	6.15	6.15	6.15
6.16	6.16	6.16	6.16	6.16	6.16	6.16	6.16	6.16
6.17	6.17	6.17	6.17	6.17	6.17	6.17	6.17	6.17
6.18	6.18	6.18	6.18	6.18	6.18	6.18	6.18	6.18
6.19	6.19	6.19	6.19	6.19	6.19	6.19	6.19	6.19
6.2	6.2	6.2	6.2	6.2	6.2	6.2	6.2	6.2
6.21	6.21	6.21	6.21	6.21	6.21	6.21	6.21	6.21
6.22	6.22	6.22	6.22	6.22	6.22	6.22	6.22	6.22
6.23	6.23	6.23	6.23	6.23	6.23	6.23	6.23	6.23
6.24	6.24	6.24	6.24	6.24	6.24	6.24	6.24	6.24
6.25	6.25	6.25	6.25	6.25	6.25	6.25	6.25	6.25
6.26	6.26	6.26	6.26	6.26	6.26	6.26	6.26	6.26
6.27	6.27	6.27	6.27	6.27	6.27	6.27	6.27	6.27
6.28	6.28	6.28	6.28	6.28	6.28	6.28	6.28	6.28
6.29	6.29	6.29	6.29	6.29	6.29	6.29	6.29	6.29
6.3	6.3	6.3	6.3	6.3	6.3	6.3	6.3	6.3
6.31	6.31	6.31	6.31	6.31	6.31	6.31	6.31	6.31
6.32	6.32	6.32	6.32	6.32	6.32	6.32	6.32	6.32
6.33	6.33	6.33	6.33	6.33	6.33	6.33	6.33	6.33
6.34	6.34	6.34	6.34	6.34	6.34	6.34	6.34	6.34
6.35	6.35	6.35	6.35	6.35	6.35	6.35	6.35	6.35
6.36	6.36	6.36	6.36	6.36	6.36	6.36	6.36	6.36
6.37	6.37	6.37	6.37	6.37	6.37	6.37	6.37	6.37
6.38	6.38	6.38	6.38	6.38	6.38	6.38	6.38	6.38
6.39	6.39	6.39	6.39	6.39	6.39	6.39	6.39	6.39
6.4	6.4	6.4	6.4	6.4	6.4	6.4	6.4	6.4
6.41	6.41	6.41	6.41	6.41	6.41	6.41	6.41	6.41
6.42	6.42	6.42	6.42	6.42	6.42	6.42	6.42	6.42
6.43	6.43	6.43	6.43	6.43	6.43	6.43	6.43	6.43
6.44	6.44	6.44	6.44	6.44	6.44	6.44	6.44	6.44
6.45	6.45	6.45	6.45	6.45	6.45	6.45	6.45	6.45
6.46	6.46	6.46	6.46	6.46	6.46	6.46	6.46	6.46
6.47	6.47	6.47	6.47	6.47	6.47	6.47	6.47	6.47
6.48	6.48	6.48	6.48	6.48	6.48	6.48	6.48	6.48
6.49	6.49	6.49	6.49	6.49	6.49	6.49	6.49	6.49

6.5	6.5	6.5	6.5	6.5	6.5	6.5	6.5	6.5
6.51	6.51	6.51	6.51	6.51	6.51	6.51	6.51	6.51
6.52	6.52	6.52	6.52	6.52	6.52	6.52	6.52	6.52
6.53	6.53	6.53	6.53	6.53	6.53	6.53	6.53	6.53
6.54	6.54	6.54	6.54	6.54	6.54	6.54	6.54	6.54
6.55	6.55	6.55	6.55	6.55	6.55	6.55	6.55	6.55
6.56	6.56	6.56	6.56	6.56	6.56	6.56	6.56	6.56
6.57	6.57	6.57	6.57	6.57	6.57	6.57	6.57	6.57
6.58	6.58	6.58	6.58	6.58	6.58	6.58	6.58	6.58
6.59	6.59	6.59	6.59	6.59	6.59	6.59	6.59	6.59
6.6	6.6	6.6	6.6	6.6	6.6	6.6	6.6	6.6
6.61	6.61	6.61	6.61	6.61	6.61	6.61	6.61	6.61
6.62	6.62	6.62	6.62	6.62	6.62	6.62	6.62	6.62
6.63	6.63	6.63	6.63	6.63	6.63	6.63	6.63	6.63
6.64	6.64	6.64	6.64	6.64	6.64	6.64	6.64	6.64
6.65	6.65	6.65	6.65	6.65	6.65	6.65	6.65	6.65
6.66	6.66	6.66	6.66	6.66	6.66	6.66	6.66	6.66
6.67	6.67	6.67	6.67	6.67	6.67	6.67	6.67	6.67
6.68	6.68	6.68	6.68	6.68	6.68	6.68	6.68	6.68
6.69	6.69	6.69	6.69	6.69	6.69	6.69	6.69	6.69
6.7	6.7	6.7	6.7	6.7	6.7	6.7	6.7	6.7
6.71	6.71	6.71	6.71	6.71	6.71	6.71	6.71	6.71
6.72	6.72	6.72	6.72	6.72	6.72	6.72	6.72	6.72
6.73	6.73	6.73	6.73	6.73	6.73	6.73	6.73	6.73
6.74	6.74	6.74	6.74	6.74	6.74	6.74	6.74	6.74
6.75	6.75	6.75	6.75	6.75	6.75	6.75	6.75	6.75
6.76	6.76	6.76	6.76	6.76	6.76	6.76	6.76	6.76
6.77	6.77	6.77	6.77	6.77	6.77	6.77	6.77	6.77
6.78	6.78	6.78	6.78	6.78	6.78	6.78	6.78	6.78
6.79	6.79	6.79	6.79	6.79	6.79	6.79	6.79	6.79
6.8	6.8	6.8	6.8	6.8	6.8	6.8	6.8	6.8
6.81	6.81	6.81	6.81	6.81	6.81	6.81	6.81	6.81
6.82	6.82	6.82	6.82	6.82	6.82	6.82	6.82	6.82
6.83	6.83	6.83	6.83	6.83	6.83	6.83	6.83	6.83
6.84	6.84	6.84	6.84	6.84	6.84	6.84	6.84	6.84
6.85	6.85	6.85	6.85	6.85	6.85	6.85	6.85	6.85
6.86	6.86	6.86	6.86	6.86	6.86	6.86	6.86	6.86
6.87	6.87	6.87	6.87	6.87	6.87	6.87	6.87	6.87
6.88	6.88	6.88	6.88	6.88	6.88	6.88	6.88	6.88
6.89	6.89	6.89	6.89	6.89	6.89	6.89	6.89	6.89
6.9	6.9	6.9	6.9	6.9	6.9	6.9	6.9	6.9
6.91	6.91	6.91	6.91	6.91	6.91	6.91	6.91	6.91
6.92	6.92	6.92	6.92	6.92	6.92	6.92	6.92	6.92
6.93	6.93	6.93	6.93	6.93	6.93	6.93	6.93	6.93
6.94	6.94	6.94	6.94	6.94	6.94	6.94	6.94	6.94
6.95	6.95	6.95	6.95	6.95	6.95	6.95	6.95	6.95
6.96	6.96	6.96	6.96	6.96	6.96	6.96	6.96	6.96
6.97	6.97	6.97	6.97	6.97	6.97	6.97	6.97	6.97
6.98	6.98	6.98	6.98	6.98	6.98	6.98	6.98	6.98
6.99	6.99	6.99	6.99	6.99	6.99	6.99	6.99	6.99

7	7	7	7	7	7	7	7	7
7.01	7.01	7.01	7.01	7.01	7.01	7.01	7.01	7.01
7.02	7.02	7.02	7.02	7.02	7.02	7.02	7.02	7.02
7.03	7.03	7.03	7.03	7.03	7.03	7.03	7.03	7.03
7.04	7.04	7.04	7.04	7.04	7.04	7.04	7.04	7.04
7.05	7.05	7.05	7.05	7.05	7.05	7.05	7.05	7.05
7.06	7.06	7.06	7.06	7.06	7.06	7.06	7.06	7.06
7.07	7.07	7.07	7.07	7.07	7.07	7.07	7.07	7.07
7.08	7.08	7.08	7.08	7.08	7.08	7.08	7.08	7.08
7.09	7.09	7.09	7.09	7.09	7.09	7.09	7.09	7.09
7.1	7.1	7.1	7.1	7.1	7.1	7.1	7.1	7.1
7.11	7.11	7.11	7.11	7.11	7.11	7.11	7.11	7.11
7.12	7.12	7.12	7.12	7.12	7.12	7.12	7.12	7.12
7.13	7.13	7.13	7.13	7.13	7.13	7.13	7.13	7.13
7.14	7.14	7.14	7.14	7.14	7.14	7.14	7.14	7.14
7.15	7.15	7.15	7.15	7.15	7.15	7.15	7.15	7.15
7.16	7.16	7.16	7.16	7.16	7.16	7.16	7.16	7.16
7.17	7.17	7.17	7.17	7.17	7.17	7.17	7.17	7.17
7.18	7.18	7.18	7.18	7.18	7.18	7.18	7.18	7.18
7.19	7.19	7.19	7.19	7.19	7.19	7.19	7.19	7.19
7.2	7.2	7.2	7.2	7.2	7.2	7.2	7.2	7.2
7.21	7.21	7.21	7.21	7.21	7.21	7.21	7.21	7.21
7.22	7.22	7.22	7.22	7.22	7.22	7.22	7.22	7.22
7.23	7.23	7.23	7.23	7.23	7.23	7.23	7.23	7.23
7.24	7.24	7.24	7.24	7.24	7.24	7.24	7.24	7.24
7.25	7.25	7.25	7.25	7.25	7.25	7.25	7.25	7.25
7.26	7.26	7.26	7.26	7.26	7.26	7.26	7.26	7.26
7.27	7.27	7.27	7.27	7.27	7.27	7.27	7.27	7.27
7.28	7.28	7.28	7.28	7.28	7.28	7.28	7.28	7.28
7.29	7.29	7.29	7.29	7.29	7.29	7.29	7.29	7.29
7.3	7.3	7.3	7.3	7.3	7.3	7.3	7.3	7.3
7.31	7.31	7.31	7.31	7.31	7.31	7.31	7.31	7.31
7.32	7.32	7.32	7.32	7.32	7.32	7.32	7.32	7.32
7.33	7.33	7.33	7.33	7.33	7.33	7.33	7.33	7.33
7.34	7.34	7.34	7.34	7.34	7.34	7.34	7.34	7.34
7.35	7.35	7.35	7.35	7.35	7.35	7.35	7.35	7.35
7.36	7.36	7.36	7.36	7.36	7.36	7.36	7.36	7.36
7.37	7.37	7.37	7.37	7.37	7.37	7.37	7.37	7.37
7.38	7.38	7.38	7.38	7.38	7.38	7.38	7.38	7.38
7.39	7.39	7.39	7.39	7.39	7.39	7.39	7.39	7.39
7.4	7.4	7.4	7.4	7.4	7.4	7.4	7.4	7.4
7.41	7.41	7.41	7.41	7.41	7.41	7.41	7.41	7.41
7.42	7.42	7.42	7.42	7.42	7.42	7.42	7.42	7.42
7.43	7.43	7.43	7.43	7.43	7.43	7.43	7.43	7.43
7.44	7.44	7.44	7.44	7.44	7.44	7.44	7.44	7.44
7.45	7.45	7.45	7.45	7.45	7.45	7.45	7.45	7.45
7.46	7.46	7.46	7.46	7.46	7.46	7.46	7.46	7.46
7.47	7.47	7.47	7.47	7.47	7.47	7.47	7.47	7.47
7.48	7.48	7.48	7.48	7.48	7.48	7.48	7.48	7.48
7.49	7.49	7.49	7.49	7.49	7.49	7.49	7.49	7.49

7.5	7.5	7.5	7.5	7.5	7.5	7.5	7.5	7.5
7.51	7.51	7.51	7.51	7.51	7.51	7.51	7.51	7.51
7.52	7.52	7.52	7.52	7.52	7.52	7.52	7.52	7.52
7.53	7.53	7.53	7.53	7.53	7.53	7.53	7.53	7.53
7.54	7.54	7.54	7.54	7.54	7.54	7.54	7.54	7.54
7.55	7.55	7.55	7.55	7.55	7.55	7.55	7.55	7.55
7.56	7.56	7.56	7.56	7.56	7.56	7.56	7.56	7.56
7.57	7.57	7.57	7.57	7.57	7.57	7.57	7.57	7.57
7.58	7.58	7.58	7.58	7.58	7.58	7.58	7.58	7.58
7.59	7.59	7.59	7.59	7.59	7.59	7.59	7.59	7.59
7.6	7.6	7.6	7.6	7.6	7.6	7.6	7.6	7.6
7.61	7.61	7.61	7.61	7.61	7.61	7.61	7.61	7.61
7.62	7.62	7.62	7.62	7.62	7.62	7.62	7.62	7.62
7.63	7.63	7.63	7.63	7.63	7.63	7.63	7.63	7.63
7.64	7.64	7.64	7.64	7.64	7.64	7.64	7.64	7.64
7.65	7.65	7.65	7.65	7.65	7.65	7.65	7.65	7.65
7.66	7.66	7.66	7.66	7.66	7.66	7.66	7.66	7.66
7.67	7.67	7.67	7.67	7.67	7.67	7.67	7.67	7.67
7.68	7.68	7.68	7.68	7.68	7.68	7.68	7.68	7.68
7.69	7.69	7.69	7.69	7.69	7.69	7.69	7.69	7.69
7.7	7.7	7.7	7.7	7.7	7.7	7.7	7.7	7.7
7.71	7.71	7.71	7.71	7.71	7.71	7.71	7.71	7.71
7.72	7.72	7.72	7.72	7.72	7.72	7.72	7.72	7.72
7.73	7.73	7.73	7.73	7.73	7.73	7.73	7.73	7.73
7.74	7.74	7.74	7.74	7.74	7.74	7.74	7.74	7.74
7.75	7.75	7.75	7.75	7.75	7.75	7.75	7.75	7.75
7.76	7.76	7.76	7.76	7.76	7.76	7.76	7.76	7.76
7.77	7.77	7.77	7.77	7.77	7.77	7.77	7.77	7.77
7.78	7.78	7.78	7.78	7.78	7.78	7.78	7.78	7.78
7.79	7.79	7.79	7.79	7.79	7.79	7.79	7.79	7.79
7.8	7.8	7.8	7.8	7.8	7.8	7.8	7.8	7.8
7.81	7.81	7.81	7.81	7.81	7.81	7.81	7.81	7.81
7.82	7.82	7.82	7.82	7.82	7.82	7.82	7.82	7.82
7.83	7.83	7.83	7.83	7.83	7.83	7.83	7.83	7.83
7.84	7.84	7.84	7.84	7.84	7.84	7.84	7.84	7.84
7.85	7.85	7.85	7.85	7.85	7.85	7.85	7.85	7.85
7.86	7.86	7.86	7.86	7.86	7.86	7.86	7.86	7.86
7.87	7.87	7.87	7.87	7.87	7.87	7.87	7.87	7.87
7.88	7.88	7.88	7.88	7.88	7.88	7.88	7.88	7.88
7.89	7.89	7.89	7.89	7.89	7.89	7.89	7.89	7.89
7.9	7.9	7.9	7.9	7.9	7.9	7.9	7.9	7.9
7.91	7.91	7.91	7.91	7.91	7.91	7.91	7.91	7.91
7.92	7.92	7.92	7.92	7.92	7.92	7.92	7.92	7.92
7.93	7.93	7.93	7.93	7.93	7.93	7.93	7.93	7.93
7.94	7.94	7.94	7.94	7.94	7.94	7.94	7.94	7.94
7.95	7.95	7.95	7.95	7.95	7.95	7.95	7.95	7.95
7.96	7.96	7.96	7.96	7.96	7.96	7.96	7.96	7.96
7.97	7.97	7.97	7.97	7.97	7.97	7.97	7.97	7.97
7.98	7.98	7.98	7.98	7.98	7.98	7.98	7.98	7.98
7.99	7.99	7.99	7.99	7.99	7.99	7.99	7.99	7.99

8	8	8	8	8	8	8	8	8
8.01	8.01	8.01	8.01	8.01	8.01	8.01	8.01	8.01
8.02	8.02	8.02	8.02	8.02	8.02	8.02	8.02	8.02
8.03	8.03	8.03	8.03	8.03	8.03	8.03	8.03	8.03
8.04	8.04	8.04	8.04	8.04	8.04	8.04	8.04	8.04
8.05	8.05	8.05	8.05	8.05	8.05	8.05	8.05	8.05
8.06	8.06	8.06	8.06	8.06	8.06	8.06	8.06	8.06
8.07	8.07	8.07	8.07	8.07	8.07	8.07	8.07	8.07
8.08	8.08	8.08	8.08	8.08	8.08	8.08	8.08	8.08
8.09	8.09	8.09	8.09	8.09	8.09	8.09	8.09	8.09
8.1	8.1	8.1	8.1	8.1	8.1	8.1	8.1	8.1
8.11	8.11	8.11	8.11	8.11	8.11	8.11	8.11	8.11
8.12	8.12	8.12	8.12	8.12	8.12	8.12	8.12	8.12
8.13	8.13	8.13	8.13	8.13	8.13	8.13	8.13	8.13
8.14	8.14	8.14	8.14	8.14	8.14	8.14	8.14	8.14
8.15	8.15	8.15	8.15	8.15	8.15	8.15	8.15	8.15
8.16	8.16	8.16	8.16	8.16	8.16	8.16	8.16	8.16
8.17	8.17	8.17	8.17	8.17	8.17	8.17	8.17	8.17
8.18	8.18	8.18	8.18	8.18	8.18	8.18	8.18	8.18
8.19	8.19	8.19	8.19	8.19	8.19	8.19	8.19	8.19
8.2	8.2	8.2	8.2	8.2	8.2	8.2	8.2	8.2
8.21	8.21	8.21	8.21	8.21	8.21	8.21	8.21	8.21
8.22	8.22	8.22	8.22	8.22	8.22	8.22	8.22	8.22
8.23	8.23	8.23	8.23	8.23	8.23	8.23	8.23	8.23
8.24	8.24	8.24	8.24	8.24	8.24	8.24	8.24	8.24
8.25	8.25	8.25	8.25	8.25	8.25	8.25	8.25	8.25
8.26	8.26	8.26	8.26	8.26	8.26	8.26	8.26	8.26
8.27	8.27	8.27	8.27	8.27	8.27	8.27	8.27	8.27
8.28	8.28	8.28	8.28	8.28	8.28	8.28	8.28	8.28
8.29	8.29	8.29	8.29	8.29	8.29	8.29	8.29	8.29
8.3	8.3	8.3	8.3	8.3	8.3	8.3	8.3	8.3
8.31	8.31	8.31	8.31	8.31	8.31	8.31	8.31	8.31
8.32	8.32	8.32	8.32	8.32	8.32	8.32	8.32	8.32
8.33	8.33	8.33	8.33	8.33	8.33	8.33	8.33	8.33
8.34	8.34	8.34	8.34	8.34	8.34	8.34	8.34	8.34
8.35	8.35	8.35	8.35	8.35	8.35	8.35	8.35	8.35
8.36	8.36	8.36	8.36	8.36	8.36	8.36	8.36	8.36
8.37	8.37	8.37	8.37	8.37	8.37	8.37	8.37	8.37
8.38	8.38	8.38	8.38	8.38	8.38	8.38	8.38	8.38
8.39	8.39	8.39	8.39	8.39	8.39	8.39	8.39	8.39
8.4	8.4	8.4	8.4	8.4	8.4	8.4	8.4	8.4
8.41	8.41	8.41	8.41	8.41	8.41	8.41	8.41	8.41
8.42	8.42	8.42	8.42	8.42	8.42	8.42	8.42	8.42
8.43	8.43	8.43	8.43	8.43	8.43	8.43	8.43	8.43
8.44	8.44	8.44	8.44	8.44	8.44	8.44	8.44	8.44
8.45	8.45	8.45	8.45	8.45	8.45	8.45	8.45	8.45
8.46	8.46	8.46	8.46	8.46	8.46	8.46	8.46	8.46
8.47	8.47	8.47	8.47	8.47	8.47	8.47	8.47	8.47
8.48	8.48	8.48	8.48	8.48	8.48	8.48	8.48	8.48
8.49	8.49	8.49	8.49	8.49	8.49	8.49	8.49	8.49

8.5	8.5	8.5	8.5	8.5	8.5	8.5	8.5	8.5
8.51	8.51	8.51	8.51	8.51	8.51	8.51	8.51	8.51
8.52	8.52	8.52	8.52	8.52	8.52	8.52	8.52	8.52
8.53	8.53	8.53	8.53	8.53	8.53	8.53	8.53	8.53
8.54	8.54	8.54	8.54	8.54	8.54	8.54	8.54	8.54
8.55	8.55	8.55	8.55	8.55	8.55	8.55	8.55	8.55
8.56	8.56	8.56	8.56	8.56	8.56	8.56	8.56	8.56
8.57	8.57	8.57	8.57	8.57	8.57	8.57	8.57	8.57
8.58	8.58	8.58	8.58	8.58	8.58	8.58	8.58	8.58
8.59	8.59	8.59	8.59	8.59	8.59	8.59	8.59	8.59
8.6	8.6	8.6	8.6	8.6	8.6	8.6	8.6	8.6
8.61	8.61	8.61	8.61	8.61	8.61	8.61	8.61	8.61
8.62	8.62	8.62	8.62	8.62	8.62	8.62	8.62	8.62
8.63	8.63	8.63	8.63	8.63	8.63	8.63	8.63	8.63
8.64	8.64	8.64	8.64	8.64	8.64	8.64	8.64	8.64
8.65	8.65	8.65	8.65	8.65	8.65	8.65	8.65	8.65
8.66	8.66	8.66	8.66	8.66	8.66	8.66	8.66	8.66
8.67	8.67	8.67	8.67	8.67	8.67	8.67	8.67	8.67
8.68	8.68	8.68	8.68	8.68	8.68	8.68	8.68	8.68
8.69	8.69	8.69	8.69	8.69	8.69	8.69	8.69	8.69
8.7	8.7	8.7	8.7	8.7	8.7	8.7	8.7	8.7
8.71	8.71	8.71	8.71	8.71	8.71	8.71	8.71	8.71
8.72	8.72	8.72	8.72	8.72	8.72	8.72	8.72	8.72
8.73	8.73	8.73	8.73	8.73	8.73	8.73	8.73	8.73
8.74	8.74	8.74	8.74	8.74	8.74	8.74	8.74	8.74
8.75	8.75	8.75	8.75	8.75	8.75	8.75	8.75	8.75
8.76	8.76	8.76	8.76	8.76	8.76	8.76	8.76	8.76
8.77	8.77	8.77	8.77	8.77	8.77	8.77	8.77	8.77
8.78	8.78	8.78	8.78	8.78	8.78	8.78	8.78	8.78
8.79	8.79	8.79	8.79	8.79	8.79	8.79	8.79	8.79
8.8	8.8	8.8	8.8	8.8	8.8	8.8	8.8	8.8
8.81	8.81	8.81	8.81	8.81	8.81	8.81	8.81	8.81
8.82	8.82	8.82	8.82	8.82	8.82	8.82	8.82	8.82
8.83	8.83	8.83	8.83	8.83	8.83	8.83	8.83	8.83
8.84	8.84	8.84	8.84	8.84	8.84	8.84	8.84	8.84
8.85	8.85	8.85	8.85	8.85	8.85	8.85	8.85	8.85
8.86	8.86	8.86	8.86	8.86	8.86	8.86	8.86	8.86
8.87	8.87	8.87	8.87	8.87	8.87	8.87	8.87	8.87
8.88	8.88	8.88	8.88	8.88	8.88	8.88	8.88	8.88
8.89	8.89	8.89	8.89	8.89	8.89	8.89	8.89	8.89
8.9	8.9	8.9	8.9	8.9	8.9	8.9	8.9	8.9
8.91	8.91	8.91	8.91	8.91	8.91	8.91	8.91	8.91
8.92	8.92	8.92	8.92	8.92	8.92	8.92	8.92	8.92
8.93	8.93	8.93	8.93	8.93	8.93	8.93	8.93	8.93
8.94	8.94	8.94	8.94	8.94	8.94	8.94	8.94	8.94
8.95	8.95	8.95	8.95	8.95	8.95	8.95	8.95	8.95
8.96	8.96	8.96	8.96	8.96	8.96	8.96	8.96	8.96
8.97	8.97	8.97	8.97	8.97	8.97	8.97	8.97	8.97
8.98	8.98	8.98	8.98	8.98	8.98	8.98	8.98	8.98
8.99	8.99	8.99	8.99	8.99	8.99	8.99	8.99	8.99

9	9	9	9	9	9	9	9	9
9.01	9.01	9.01	9.01	9.01	9.01	9.01	9.01	9.01
9.02	9.02	9.02	9.02	9.02	9.02	9.02	9.02	9.02
9.03	9.03	9.03	9.03	9.03	9.03	9.03	9.03	9.03
9.04	9.04	9.04	9.04	9.04	9.04	9.04	9.04	9.04
9.05	9.05	9.05	9.05	9.05	9.05	9.05	9.05	9.05
9.06	9.06	9.06	9.06	9.06	9.06	9.06	9.06	9.06
9.07	9.07	9.07	9.07	9.07	9.07	9.07	9.07	9.07
9.08	9.08	9.08	9.08	9.08	9.08	9.08	9.08	9.08
9.09	9.09	9.09	9.09	9.09	9.09	9.09	9.09	9.09
9.1	9.1	9.1	9.1	9.1	9.1	9.1	9.1	9.1
9.11	9.11	9.11	9.11	9.11	9.11	9.11	9.11	9.11
9.12	9.12	9.12	9.12	9.12	9.12	9.12	9.12	9.12
9.13	9.13	9.13	9.13	9.13	9.13	9.13	9.13	9.13
9.14	9.14	9.14	9.14	9.14	9.14	9.14	9.14	9.14
9.15	9.15	9.15	9.15	9.15	9.15	9.15	9.15	9.15
9.16	9.16	9.16	9.16	9.16	9.16	9.16	9.16	9.16
9.17	9.17	9.17	9.17	9.17	9.17	9.17	9.17	9.17
9.18	9.18	9.18	9.18	9.18	9.18	9.18	9.18	9.18
9.19	9.19	9.19	9.19	9.19	9.19	9.19	9.19	9.19
9.2	9.2	9.2	9.2	9.2	9.2	9.2	9.2	9.2
9.21	9.21	9.21	9.21	9.21	9.21	9.21	9.21	9.21
9.22	9.22	9.22	9.22	9.22	9.22	9.22	9.22	9.22
9.23	9.23	9.23	9.23	9.23	9.23	9.23	9.23	9.23
9.24	9.24	9.24	9.24	9.24	9.24	9.24	9.24	9.24
9.25	9.25	9.25	9.25	9.25	9.25	9.25	9.25	9.25
9.26	9.26	9.26	9.26	9.26	9.26	9.26	9.26	9.26
9.27	9.27	9.27	9.27	9.27	9.27	9.27	9.27	9.27
9.28	9.28	9.28	9.28	9.28	9.28	9.28	9.28	9.28
9.29	9.29	9.29	9.29	9.29	9.29	9.29	9.29	9.29
9.3	9.3	9.3	9.3	9.3	9.3	9.3	9.3	9.3
9.31	9.31	9.31	9.31	9.31	9.31	9.31	9.31	9.31
9.32	9.32	9.32	9.32	9.32	9.32	9.32	9.32	9.32
9.33	9.33	9.33	9.33	9.33	9.33	9.33	9.33	9.33
9.34	9.34	9.34	9.34	9.34	9.34	9.34	9.34	9.34
9.35	9.35	9.35	9.35	9.35	9.35	9.35	9.35	9.35
9.36	9.36	9.36	9.36	9.36	9.36	9.36	9.36	9.36
9.37	9.37	9.37	9.37	9.37	9.37	9.37	9.37	9.37
9.38	9.38	9.38	9.38	9.38	9.38	9.38	9.38	9.38
9.39	9.39	9.39	9.39	9.39	9.39	9.39	9.39	9.39
9.4	9.4	9.4	9.4	9.4	9.4	9.4	9.4	9.4
9.41	9.41	9.41	9.41	9.41	9.41	9.41	9.41	9.41
9.42	9.42	9.42	9.42	9.42	9.42	9.42	9.42	9.42
9.43	9.43	9.43	9.43	9.43	9.43	9.43	9.43	9.43
9.44	9.44	9.44	9.44	9.44	9.44	9.44	9.44	9.44
9.45	9.45	9.45	9.45	9.45	9.45	9.45	9.45	9.45
9.46	9.46	9.46	9.46	9.46	9.46	9.46	9.46	9.46
9.47	9.47	9.47	9.47	9.47	9.47	9.47	9.47	9.47
9.48	9.48	9.48	9.48	9.48	9.48	9.48	9.48	9.48
9.49	9.49	9.49	9.49	9.49	9.49	9.49	9.49	9.49

9.5	9.5	9.5	9.5	9.5	9.5	9.5	9.5	9.5
9.51	9.51	9.51	9.51	9.51	9.51	9.51	9.51	9.51
9.52	9.52	9.52	9.52	9.52	9.52	9.52	9.52	9.52
9.53	9.53	9.53	9.53	9.53	9.53	9.53	9.53	9.53
9.54	9.54	9.54	9.54	9.54	9.54	9.54	9.54	9.54
9.55	9.55	9.55	9.55	9.55	9.55	9.55	9.55	9.55
9.56	9.56	9.56	9.56	9.56	9.56	9.56	9.56	9.56
9.57	9.57	9.57	9.57	9.57	9.57	9.57	9.57	9.57
9.58	9.58	9.58	9.58	9.58	9.58	9.58	9.58	9.58
9.59	9.59	9.59	9.59	9.59	9.59	9.59	9.59	9.59
9.6	9.6	9.6	9.6	9.6	9.6	9.6	9.6	9.6
9.61	9.61	9.61	9.61	9.61	9.61	9.61	9.61	9.61
9.62	9.62	9.62	9.62	9.62	9.62	9.62	9.62	9.62
9.63	9.63	9.63	9.63	9.63	9.63	9.63	9.63	9.63
9.64	9.64	9.64	9.64	9.64	9.64	9.64	9.64	9.64
9.65	9.65	9.65	9.65	9.65	9.65	9.65	9.65	9.65
9.66	9.66	9.66	9.66	9.66	9.66	9.66	9.66	9.66
9.67	9.67	9.67	9.67	9.67	9.67	9.67	9.67	9.67
9.68	9.68	9.68	9.68	9.68	9.68	9.68	9.68	9.68
9.69	9.69	9.69	9.69	9.69	9.69	9.69	9.69	9.69
9.7	9.7	9.7	9.7	9.7	9.7	9.7	9.7	9.7
9.71	9.71	9.71	9.71	9.71	9.71	9.71	9.71	9.71
9.72	9.72	9.72	9.72	9.72	9.72	9.72	9.72	9.72
9.73	9.73	9.73	9.73	9.73	9.73	9.73	9.73	9.73
9.74	9.74	9.74	9.74	9.74	9.74	9.74	9.74	9.74
9.75	9.75	9.75	9.75	9.75	9.75	9.75	9.75	9.75
9.76	9.76	9.76	9.76	9.76	9.76	9.76	9.76	9.76
9.77	9.77	9.77	9.77	9.77	9.77	9.77	9.77	9.77
9.78	9.78	9.78	9.78	9.78	9.78	9.78	9.78	9.78
9.79	9.79	9.79	9.79	9.79	9.79	9.79	9.79	9.79
9.8	9.8	9.8	9.8	9.8	9.8	9.8	9.8	9.8
9.81	9.81	9.81	9.81	9.81	9.81	9.81	9.81	9.81
9.82	9.82	9.82	9.82	9.82	9.82	9.82	9.82	9.82
9.83	9.83	9.83	9.83	9.83	9.83	9.83	9.83	9.83
9.84	9.84	9.84	9.84	9.84	9.84	9.84	9.84	9.84
9.85	9.85	9.85	9.85	9.85	9.85	9.85	9.85	9.85
9.86	9.86	9.86	9.86	9.86	9.86	9.86	9.86	9.86
9.87	9.87	9.87	9.87	9.87	9.87	9.87	9.87	9.87
9.88	9.88	9.88	9.88	9.88	9.88	9.88	9.88	9.88
9.89	9.89	9.89	9.89	9.89	9.89	9.89	9.89	9.89
9.9	9.9	9.9	9.9	9.9	9.9	9.9	9.9	9.9
9.91	9.91	9.91	9.91	9.91	9.91	9.91	9.91	9.91
9.92	9.92	9.92	9.92	9.92	9.92	9.92	9.92	9.92
9.93	9.93	9.93	9.93	9.93	9.93	9.93	9.93	9.93
9.94	9.94	9.94	9.94	9.94	9.94	9.94	9.94	9.94
9.95	9.95	9.95	9.95	9.95	9.95	9.95	9.95	9.95
9.96	9.96	9.96	9.96	9.96	9.96	9.96	9.96	9.96
9.97	9.97	9.97	9.97	9.97	9.97	9.97	9.97	9.97
9.98	9.98	9.98	9.98	9.98	9.98	9.98	9.98	9.98
9.99	9.99	9.99	9.99	9.99	9.99	9.99	9.99	9.99

10	10	10	10	10	10	10	10	10
10.01	10.01	10.01	10.01	10.01	10.01	10.01	10.01	10.01
10.02	10.02	10.02	10.02	10.02	10.02	10.02	10.02	10.02
10.03	10.03	10.03	10.03	10.03	10.03	10.03	10.03	10.03
10.04	10.04	10.04	10.04	10.04	10.04	10.04	10.04	10.04
10.05	10.05	10.05	10.05	10.05	10.05	10.05	10.05	10.05
10.06	10.06	10.06	10.06	10.06	10.06	10.06	10.06	10.06
10.07	10.07	10.07	10.07	10.07	10.07	10.07	10.07	10.07
10.08	10.08	10.08	10.08	10.08	10.08	10.08	10.08	10.08
10.09	10.09	10.09	10.09	10.09	10.09	10.09	10.09	10.09
10.1	10.1	10.1	10.1	10.1	10.1	10.1	10.1	10.1
10.11	10.11	10.11	10.11	10.11	10.11	10.11	10.11	10.11
10.12	10.12	10.12	10.12	10.12	10.12	10.12	10.12	10.12
10.13	10.13	10.13	10.13	10.13	10.13	10.13	10.13	10.13
10.14	10.14	10.14	10.14	10.14	10.14	10.14	10.14	10.14
10.15	10.15	10.15	10.15	10.15	10.15	10.15	10.15	10.15
10.16	10.16	10.16	10.16	10.16	10.16	10.16	10.16	10.16
10.17	10.17	10.17	10.17	10.17	10.17	10.17	10.17	10.17
10.18	10.18	10.18	10.18	10.18	10.18	10.18	10.18	10.18
10.19	10.19	10.19	10.19	10.19	10.19	10.19	10.19	10.19
10.2	10.2	10.2	10.2	10.2	10.2	10.2	10.2	10.2
10.21	10.21	10.21	10.21	10.21	10.21	10.21	10.21	10.21
10.22	10.22	10.22	10.22	10.22	10.22	10.22	10.22	10.22
10.23	10.23	10.23	10.23	10.23	10.23	10.23	10.23	10.23
10.24	10.24	10.24	10.24	10.24	10.24	10.24	10.24	10.24
10.25	10.25	10.25	10.25	10.25	10.25	10.25	10.25	10.25
10.26	10.26	10.26	10.26	10.26	10.26	10.26	10.26	10.26
10.27	10.27	10.27	10.27	10.27	10.27	10.27	10.27	10.27
10.28	10.28	10.28	10.28	10.28	10.28	10.28	10.28	10.28
10.29	10.29	10.29	10.29	10.29	10.29	10.29	10.29	10.29
10.3	10.3	10.3	10.3	10.3	10.3	10.3	10.3	10.3
10.31	10.31	10.31	10.31	10.31	10.31	10.31	10.31	10.31
10.32	10.32	10.32	10.32	10.32	10.32	10.32	10.32	10.32
10.33	10.33	10.33	10.33	10.33	10.33	10.33	10.33	10.33
10.34	10.34	10.34	10.34	10.34	10.34	10.34	10.34	10.34
10.35	10.35	10.35	10.35	10.35	10.35	10.35	10.35	10.35
10.36	10.36	10.36	10.36	10.36	10.36	10.36	10.36	10.36
10.37	10.37	10.37	10.37	10.37	10.37	10.37	10.37	10.37
10.38	10.38	10.38	10.38	10.38	10.38	10.38	10.38	10.38
10.39	10.39	10.39	10.39	10.39	10.39	10.39	10.39	10.39
10.4	10.4	10.4	10.4	10.4	10.4	10.4	10.4	10.4
10.41	10.41	10.41	10.41	10.41	10.41	10.41	10.41	10.41
10.42	10.42	10.42	10.42	10.42	10.42	10.42	10.42	10.42
10.43	10.43	10.43	10.43	10.43	10.43	10.43	10.43	10.43
10.44	10.44	10.44	10.44	10.44	10.44	10.44	10.44	10.44
10.45	10.45	10.45	10.45	10.45	10.45	10.45	10.45	10.45
10.46	10.46	10.46	10.46	10.46	10.46	10.46	10.46	10.46
10.47	10.47	10.47	10.47	10.47	10.47	10.47	10.47	10.47
10.48	10.48	10.48	10.48	10.48	10.48	10.48	10.48	10.48
10.49	10.49	10.49	10.49	10.49	10.49	10.49	10.49	10.49

10.5	10.5	10.5	10.5	10.5	10.5	10.5	10.5	10.5
10.51	10.51	10.51	10.51	10.51	10.51	10.51	10.51	10.51
10.52	10.52	10.52	10.52	10.52	10.52	10.52	10.52	10.52
10.53	10.53	10.53	10.53	10.53	10.53	10.53	10.53	10.53
10.54	10.54	10.54	10.54	10.54	10.54	10.54	10.54	10.54
10.55	10.55	10.55	10.55	10.55	10.55	10.55	10.55	10.55
10.56	10.56	10.56	10.56	10.56	10.56	10.56	10.56	10.56
10.57	10.57	10.57	10.57	10.57	10.57	10.57	10.57	10.57
10.58	10.58	10.58	10.58	10.58	10.58	10.58	10.58	10.58
10.59	10.59	10.59	10.59	10.59	10.59	10.59	10.59	10.59
10.6	10.6	10.6	10.6	10.6	10.6	10.6	10.6	10.6
10.61	10.61	10.61	10.61	10.61	10.61	10.61	10.61	10.61
10.62	10.62	10.62	10.62	10.62	10.62	10.62	10.62	10.62
10.63	10.63	10.63	10.63	10.63	10.63	10.63	10.63	10.63
10.64	10.64	10.64	10.64	10.64	10.64	10.64	10.64	10.64
10.65	10.65	10.65	10.65	10.65	10.65	10.65	10.65	10.65
10.66	10.66	10.66	10.66	10.66	10.66	10.66	10.66	10.66
10.67	10.67	10.67	10.67	10.67	10.67	10.67	10.67	10.67
10.68	10.68	10.68	10.68	10.68	10.68	10.68	10.68	10.68
10.69	10.69	10.69	10.69	10.69	10.69	10.69	10.69	10.69
10.7	10.7	10.7	10.7	10.7	10.7	10.7	10.7	10.7
10.71	10.71	10.71	10.71	10.71	10.71	10.71	10.71	10.71
10.72	10.72	10.72	10.72	10.72	10.72	10.72	10.72	10.72
10.73	10.73	10.73	10.73	10.73	10.73	10.73	10.73	10.73
10.74	10.74	10.74	10.74	10.74	10.74	10.74	10.74	10.74
10.75	10.75	10.75	10.75	10.75	10.75	10.75	10.75	10.75
10.76	10.76	10.76	10.76	10.76	10.76	10.76	10.76	10.76
10.77	10.77	10.77	10.77	10.77	10.77	10.77	10.77	10.77
10.78	10.78	10.78	10.78	10.78	10.78	10.78	10.78	10.78
10.79	10.79	10.79	10.79	10.79	10.79	10.79	10.79	10.79
10.8	10.8	10.8	10.8	10.8	10.8	10.8	10.8	10.8
10.81	10.81	10.81	10.81	10.81	10.81	10.81	10.81	10.81
10.82	10.82	10.82	10.82	10.82	10.82	10.82	10.82	10.82
10.83	10.83	10.83	10.83	10.83	10.83	10.83	10.83	10.83
10.84	10.84	10.84	10.84	10.84	10.84	10.84	10.84	10.84
10.85	10.85	10.85	10.85	10.85	10.85	10.85	10.85	10.85
10.86	10.86	10.86	10.86	10.86	10.86	10.86	10.86	10.86
10.87	10.87	10.87	10.87	10.87	10.87	10.87	10.87	10.87
10.88	10.88	10.88	10.88	10.88	10.88	10.88	10.88	10.88
10.89	10.89	10.89	10.89	10.89	10.89	10.89	10.89	10.89
10.9	10.9	10.9	10.9	10.9	10.9	10.9	10.9	10.9
10.91	10.91	10.91	10.91	10.91	10.91	10.91	10.91	10.91
10.92	10.92	10.92	10.92	10.92	10.92	10.92	10.92	10.92
10.93	10.93	10.93	10.93	10.93	10.93	10.93	10.93	10.93
10.94	10.94	10.94	10.94	10.94	10.94	10.94	10.94	10.94
10.95	10.95	10.95	10.95	10.95	10.95	10.95	10.95	10.95
10.96	10.96	10.96	10.96	10.96	10.96	10.96	10.96	10.96
10.97	10.97	10.97	10.97	10.97	10.97	10.97	10.97	10.97
10.98	10.98	10.98	10.98	10.98	10.98	10.98	10.98	10.98
10.99	10.99	10.99	10.99	10.99	10.99	10.99	10.99	10.99

11	11	11	11	11	11	11	11	11
11.01	11.01	11.01	11.01	11.01	11.01	11.01	11.01	11.01
11.02	11.02	11.02	11.02	11.02	11.02	11.02	11.02	11.02
11.03	11.03	11.03	11.03	11.03	11.03	11.03	11.03	11.03
11.04	11.04	11.04	11.04	11.04	11.04	11.04	11.04	11.04
11.05	11.05	11.05	11.05	11.05	11.05	11.05	11.05	11.05
11.06	11.06	11.06	11.06	11.06	11.06	11.06	11.06	11.06
11.07	11.07	11.07	11.07	11.07	11.07	11.07	11.07	11.07
11.08	11.08	11.08	11.08	11.08	11.08	11.08	11.08	11.08
11.09	11.09	11.09	11.09	11.09	11.09	11.09	11.09	11.09
11.1	11.1	11.1	11.1	11.1	11.1	11.1	11.1	11.1
11.11	11.11	11.11	11.11	11.11	11.11	11.11	11.11	11.11
11.12	11.12	11.12	11.12	11.12	11.12	11.12	11.12	11.12
11.13	11.13	11.13	11.13	11.13	11.13	11.13	11.13	11.13
11.14	11.14	11.14	11.14	11.14	11.14	11.14	11.14	11.14
11.15	11.15	11.15	11.15	11.15	11.15	11.15	11.15	11.15
11.16	11.16	11.16	11.16	11.16	11.16	11.16	11.16	11.16
11.17	11.17	11.17	11.17	11.17	11.17	11.17	11.17	11.17
11.18	11.18	11.18	11.18	11.18	11.18	11.18	11.18	11.18
11.19	11.19	11.19	11.19	11.19	11.19	11.19	11.19	11.19
11.2	11.2	11.2	11.2	11.2	11.2	11.2	11.2	11.2
11.21	11.21	11.21	11.21	11.21	11.21	11.21	11.21	11.21
11.22	11.22	11.22	11.22	11.22	11.22	11.22	11.22	11.22
11.23	11.23	11.23	11.23	11.23	11.23	11.23	11.23	11.23
11.24	11.24	11.24	11.24	11.24	11.24	11.24	11.24	11.24
11.25	11.25	11.25	11.25	11.25	11.25	11.25	11.25	11.25
11.26	11.26	11.26	11.26	11.26	11.26	11.26	11.26	11.26
11.27	11.27	11.27	11.27	11.27	11.27	11.27	11.27	11.27
11.28	11.28	11.28	11.28	11.28	11.28	11.28	11.28	11.28
11.29	11.29	11.29	11.29	11.29	11.29	11.29	11.29	11.29
11.3	11.3	11.3	11.3	11.3	11.3	11.3	11.3	11.3
11.31	11.31	11.31	11.31	11.31	11.31	11.31	11.31	11.31
11.32	11.32	11.32	11.32	11.32	11.32	11.32	11.32	11.32
11.33	11.33	11.33	11.33	11.33	11.33	11.33	11.33	11.33
11.34	11.34	11.34	11.34	11.34	11.34	11.34	11.34	11.34
11.35	11.35	11.35	11.35	11.35	11.35	11.35	11.35	11.35
11.36	11.36	11.36	11.36	11.36	11.36	11.36	11.36	11.36
11.37	11.37	11.37	11.37	11.37	11.37	11.37	11.37	11.37
11.38	11.38	11.38	11.38	11.38	11.38	11.38	11.38	11.38
11.39	11.39	11.39	11.39	11.39	11.39	11.39	11.39	11.39
11.4	11.4	11.4	11.4	11.4	11.4	11.4	11.4	11.4
11.41	11.41	11.41	11.41	11.41	11.41	11.41	11.41	11.41
11.42	11.42	11.42	11.42	11.42	11.42	11.42	11.42	11.42
11.43	11.43	11.43	11.43	11.43	11.43	11.43	11.43	11.43
11.44	11.44	11.44	11.44	11.44	11.44	11.44	11.44	11.44
11.45	11.45	11.45	11.45	11.45	11.45	11.45	11.45	11.45
11.46	11.46	11.46	11.46	11.46	11.46	11.46	11.46	11.46
11.47	11.47	11.47	11.47	11.47	11.47	11.47	11.47	11.47
11.48	11.48	11.48	11.48	11.48	11.48	11.48	11.48	11.48
11.49	11.49	11.49	11.49	11.49	11.49	11.49	11.49	11.49

11.5	11.5	11.5	11.5	11.5	11.5	11.5	11.5	11.5
11.51	11.51	11.51	11.51	11.51	11.51	11.51	11.51	11.51
11.52	11.52	11.52	11.52	11.52	11.52	11.52	11.52	11.52
11.53	11.53	11.53	11.53	11.53	11.53	11.53	11.53	11.53
11.54	11.54	11.54	11.54	11.54	11.54	11.54	11.54	11.54
11.55	11.55	11.55	11.55	11.55	11.55	11.55	11.55	11.55
11.56	11.56	11.56	11.56	11.56	11.56	11.56	11.56	11.56
11.57	11.57	11.57	11.57	11.57	11.57	11.57	11.57	11.57
11.58	11.58	11.58	11.58	11.58	11.58	11.58	11.58	11.58
11.59	11.59	11.59	11.59	11.59	11.59	11.59	11.59	11.59
11.6	11.6	11.6	11.6	11.6	11.6	11.6	11.6	11.6
11.61	11.61	11.61	11.61	11.61	11.61	11.61	11.61	11.61
11.62	11.62	11.62	11.62	11.62	11.62	11.62	11.62	11.62
11.63	11.63	11.63	11.63	11.63	11.63	11.63	11.63	11.63
11.64	11.64	11.64	11.64	11.64	11.64	11.64	11.64	11.64
11.65	11.65	11.65	11.65	11.65	11.65	11.65	11.65	11.65
11.66	11.66	11.66	11.66	11.66	11.66	11.66	11.66	11.66
11.67	11.67	11.67	11.67	11.67	11.67	11.67	11.67	11.67
11.68	11.68	11.68	11.68	11.68	11.68	11.68	11.68	11.68
11.69	11.69	11.69	11.69	11.69	11.69	11.69	11.69	11.69
11.7	11.7	11.7	11.7	11.7	11.7	11.7	11.7	11.7
11.71	11.71	11.71	11.71	11.71	11.71	11.71	11.71	11.71
11.72	11.72	11.72	11.72	11.72	11.72	11.72	11.72	11.72
11.73	11.73	11.73	11.73	11.73	11.73	11.73	11.73	11.73
11.74	11.74	11.74	11.74	11.74	11.74	11.74	11.74	11.74
11.75	11.75	11.75	11.75	11.75	11.75	11.75	11.75	11.75
11.76	11.76	11.76	11.76	11.76	11.76	11.76	11.76	11.76
11.77	11.77	11.77	11.77	11.77	11.77	11.77	11.77	11.77
11.78	11.78	11.78	11.78	11.78	11.78	11.78	11.78	11.78
11.79	11.79	11.79	11.79	11.79	11.79	11.79	11.79	11.79
11.8	11.8	11.8	11.8	11.8	11.8	11.8	11.8	11.8
11.81	11.81	11.81	11.81	11.81	11.81	11.81	11.81	11.81
11.82	11.82	11.82	11.82	11.82	11.82	11.82	11.82	11.82
11.83	11.83	11.83	11.83	11.83	11.83	11.83	11.83	11.83
11.84	11.84	11.84	11.84	11.84	11.84	11.84	11.84	11.84
11.85	11.85	11.85	11.85	11.85	11.85	11.85	11.85	11.85
11.86	11.86	11.86	11.86	11.86	11.86	11.86	11.86	11.86
11.87	11.87	11.87	11.87	11.87	11.87	11.87	11.87	11.87
11.88	11.88	11.88	11.88	11.88	11.88	11.88	11.88	11.88
11.89	11.89	11.89	11.89	11.89	11.89	11.89	11.89	11.89
11.9	11.9	11.9	11.9	11.9	11.9	11.9	11.9	11.9
11.91	11.91	11.91	11.91	11.91	11.91	11.91	11.91	11.91
11.92	11.92	11.92	11.92	11.92	11.92	11.92	11.92	11.92
11.93	11.93	11.93	11.93	11.93	11.93	11.93	11.93	11.93
11.94	11.94	11.94	11.94	11.94	11.94	11.94	11.94	11.94
11.95	11.95	11.95	11.95	11.95	11.95	11.95	11.95	11.95
11.96	11.96	11.96	11.96	11.96	11.96	11.96	11.96	11.96
11.97	11.97	11.97	11.97	11.97	11.97	11.97	11.97	11.97
11.98	11.98	11.98	11.98	11.98	11.98	11.98	11.98	11.98
11.99	11.99	11.99	11.99	11.99	11.99	11.99	11.99	11.99

12	12	12	12	12	12	12	12	12

-12	-12	-12	-12	-12	-12	-12	-12	-12
-11.99	-11.99	-11.99	-11.99	-11.99	-11.99	-11.99	-11.99	-11.99
-11.98	-11.98	-11.98	-11.98	-11.98	-11.98	-11.98	-11.98	-11.98
-11.97	-11.97	-11.97	-11.97	-11.97	-11.97	-11.97	-11.97	-11.97
-11.96	-11.96	-11.96	-11.96	-11.96	-11.96	-11.96	-11.96	-11.96
-11.95	-11.95	-11.95	-11.95	-11.95	-11.95	-11.95	-11.95	-11.95
-11.94	-11.94	-11.94	-11.94	-11.94	-11.94	-11.94	-11.94	-11.94
-11.93	-11.93	-11.93	-11.93	-11.93	-11.93	-11.93	-11.93	-11.93
-11.92	-11.92	-11.92	-11.92	-11.92	-11.92	-11.92	-11.92	-11.92
-11.91	-11.91	-11.91	-11.91	-11.91	-11.91	-11.91	-11.91	-11.91
-11.9	-11.9	-11.9	-11.9	-11.9	-11.9	-11.9	-11.9	-11.9
-11.89	-11.89	-11.89	-11.89	-11.89	-11.89	-11.89	-11.89	-11.89
-11.88	-11.88	-11.88	-11.88	-11.88	-11.88	-11.88	-11.88	-11.88
-11.87	-11.87	-11.87	-11.87	-11.87	-11.87	-11.87	-11.87	-11.87
-11.86	-11.86	-11.86	-11.86	-11.86	-11.86	-11.86	-11.86	-11.86
-11.85	-11.85	-11.85	-11.85	-11.85	-11.85	-11.85	-11.85	-11.85
-11.84	-11.84	-11.84	-11.84	-11.84	-11.84	-11.84	-11.84	-11.84
-11.83	-11.83	-11.83	-11.83	-11.83	-11.83	-11.83	-11.83	-11.83
-11.82	-11.82	-11.82	-11.82	-11.82	-11.82	-11.82	-11.82	-11.82
-11.81	-11.81	-11.81	-11.81	-11.81	-11.81	-11.81	-11.81	-11.81
-11.8	-11.8	-11.8	-11.8	-11.8	-11.8	-11.8	-11.8	-11.8
-11.79	-11.79	-11.79	-11.79	-11.79	-11.79	-11.79	-11.79	-11.79
-11.78	-11.78	-11.78	-11.78	-11.78	-11.78	-11.78	-11.78	-11.78
-11.77	-11.77	-11.77	-11.77	-11.77	-11.77	-11.77	-11.77	-11.77
-11.76	-11.76	-11.76	-11.76	-11.76	-11.76	-11.76	-11.76	-11.76
-11.75	-11.75	-11.75	-11.75	-11.75	-11.75	-11.75	-11.75	-11.75
-11.74	-11.74	-11.74	-11.74	-11.74	-11.74	-11.74	-11.74	-11.74
-11.73	-11.73	-11.73	-11.73	-11.73	-11.73	-11.73	-11.73	-11.73
-11.72	-11.72	-11.72	-11.72	-11.72	-11.72	-11.72	-11.72	-11.72
-11.71	-11.71	-11.71	-11.71	-11.71	-11.71	-11.71	-11.71	-11.71
-11.7	-11.7	-11.7	-11.7	-11.7	-11.7	-11.7	-11.7	-11.7
-11.69	-11.69	-11.69	-11.69	-11.69	-11.69	-11.69	-11.69	-11.69
-11.68	-11.68	-11.68	-11.68	-11.68	-11.68	-11.68	-11.68	-11.68
-11.67	-11.67	-11.67	-11.67	-11.67	-11.67	-11.67	-11.67	-11.67
-11.66	-11.66	-11.66	-11.66	-11.66	-11.66	-11.66	-11.66	-11.66
-11.65	-11.65	-11.65	-11.65	-11.65	-11.65	-11.65	-11.65	-11.65
-11.64	-11.64	-11.64	-11.64	-11.64	-11.64	-11.64	-11.64	-11.64
-11.63	-11.63	-11.63	-11.63	-11.63	-11.63	-11.63	-11.63	-11.63
-11.62	-11.62	-11.62	-11.62	-11.62	-11.62	-11.62	-11.62	-11.62
-11.61	-11.61	-11.61	-11.61	-11.61	-11.61	-11.61	-11.61	-11.61
-11.6	-11.6	-11.6	-11.6	-11.6	-11.6	-11.6	-11.6	-11.6
-11.59	-11.59	-11.59	-11.59	-11.59	-11.59	-11.59	-11.59	-11.59
-11.58	-11.58	-11.58	-11.58	-11.58	-11.58	-11.58	-11.58	-11.58
-11.57	-11.57	-11.57	-11.57	-11.57	-11.57	-11.57	-11.57	-11.57
-11.56	-11.56	-11.56	-11.56	-11.56	-11.56	-11.56	-11.56	-11.56
-11.55	-11.55	-11.55	-11.55	-11.55	-11.55	-11.55	-11.55	-11.55
-11.54	-11.54	-11.54	-11.54	-11.54	-11.54	-11.54	-11.54	-11.54
-11.53	-11.53	-11.53	-11.53	-11.53	-11.53	-11.53	-11.53	-11.53
-11.52	-11.52	-11.52	-11.52	-11.52	-11.52	-11.52	-11.52	-11.52
-11.51	-11.51	-11.51	-11.51	-11.51	-11.51	-11.51	-11.51	-11.51

-11.5	-11.5	-11.5	-11.5	-11.5	-11.5	-11.5	-11.5	-11.5
-11.49	-11.49	-11.49	-11.49	-11.49	-11.49	-11.49	-11.49	-11.49
-11.48	-11.48	-11.48	-11.48	-11.48	-11.48	-11.48	-11.48	-11.48
-11.47	-11.47	-11.47	-11.47	-11.47	-11.47	-11.47	-11.47	-11.47
-11.46	-11.46	-11.46	-11.46	-11.46	-11.46	-11.46	-11.46	-11.46
-11.45	-11.45	-11.45	-11.45	-11.45	-11.45	-11.45	-11.45	-11.45
-11.44	-11.44	-11.44	-11.44	-11.44	-11.44	-11.44	-11.44	-11.44
-11.43	-11.43	-11.43	-11.43	-11.43	-11.43	-11.43	-11.43	-11.43
-11.42	-11.42	-11.42	-11.42	-11.42	-11.42	-11.42	-11.42	-11.42
-11.41	-11.41	-11.41	-11.41	-11.41	-11.41	-11.41	-11.41	-11.41
-11.4	-11.4	-11.4	-11.4	-11.4	-11.4	-11.4	-11.4	-11.4
-11.39	-11.39	-11.39	-11.39	-11.39	-11.39	-11.39	-11.39	-11.39
-11.38	-11.38	-11.38	-11.38	-11.38	-11.38	-11.38	-11.38	-11.38
-11.37	-11.37	-11.37	-11.37	-11.37	-11.37	-11.37	-11.37	-11.37
-11.36	-11.36	-11.36	-11.36	-11.36	-11.36	-11.36	-11.36	-11.36
-11.35	-11.35	-11.35	-11.35	-11.35	-11.35	-11.35	-11.35	-11.35
-11.34	-11.34	-11.34	-11.34	-11.34	-11.34	-11.34	-11.34	-11.34
-11.33	-11.33	-11.33	-11.33	-11.33	-11.33	-11.33	-11.33	-11.33
-11.32	-11.32	-11.32	-11.32	-11.32	-11.32	-11.32	-11.32	-11.32
-11.31	-11.31	-11.31	-11.31	-11.31	-11.31	-11.31	-11.31	-11.31
-11.3	-11.3	-11.3	-11.3	-11.3	-11.3	-11.3	-11.3	-11.3
-11.29	-11.29	-11.29	-11.29	-11.29	-11.29	-11.29	-11.29	-11.29
-11.28	-11.28	-11.28	-11.28	-11.28	-11.28	-11.28	-11.28	-11.28
-11.27	-11.27	-11.27	-11.27	-11.27	-11.27	-11.27	-11.27	-11.27
-11.26	-11.26	-11.26	-11.26	-11.26	-11.26	-11.26	-11.26	-11.26
-11.25	-11.25	-11.25	-11.25	-11.25	-11.25	-11.25	-11.25	-11.25
-11.24	-11.24	-11.24	-11.24	-11.24	-11.24	-11.24	-11.24	-11.24
-11.23	-11.23	-11.23	-11.23	-11.23	-11.23	-11.23	-11.23	-11.23
-11.22	-11.22	-11.22	-11.22	-11.22	-11.22	-11.22	-11.22	-11.22
-11.21	-11.21	-11.21	-11.21	-11.21	-11.21	-11.21	-11.21	-11.21
-11.2	-11.2	-11.2	-11.2	-11.2	-11.2	-11.2	-11.2	-11.2
-11.19	-11.19	-11.19	-11.19	-11.19	-11.19	-11.19	-11.19	-11.19
-11.18	-11.18	-11.18	-11.18	-11.18	-11.18	-11.18	-11.18	-11.18
-11.17	-11.17	-11.17	-11.17	-11.17	-11.17	-11.17	-11.17	-11.17
-11.16	-11.16	-11.16	-11.16	-11.16	-11.16	-11.16	-11.16	-11.16
-11.15	-11.15	-11.15	-11.15	-11.15	-11.15	-11.15	-11.15	-11.15
-11.14	-11.14	-11.14	-11.14	-11.14	-11.14	-11.14	-11.14	-11.14
-11.13	-11.13	-11.13	-11.13	-11.13	-11.13	-11.13	-11.13	-11.13
-11.12	-11.12	-11.12	-11.12	-11.12	-11.12	-11.12	-11.12	-11.12
-11.11	-11.11	-11.11	-11.11	-11.11	-11.11	-11.11	-11.11	-11.11
-11.1	-11.1	-11.1	-11.1	-11.1	-11.1	-11.1	-11.1	-11.1
-11.09	-11.09	-11.09	-11.09	-11.09	-11.09	-11.09	-11.09	-11.09
-11.08	-11.08	-11.08	-11.08	-11.08	-11.08	-11.08	-11.08	-11.08
-11.07	-11.07	-11.07	-11.07	-11.07	-11.07	-11.07	-11.07	-11.07
-11.06	-11.06	-11.06	-11.06	-11.06	-11.06	-11.06	-11.06	-11.06
-11.05	-11.05	-11.05	-11.05	-11.05	-11.05	-11.05	-11.05	-11.05
-11.04	-11.04	-11.04	-11.04	-11.04	-11.04	-11.04	-11.04	-11.04
-11.03	-11.03	-11.03	-11.03	-11.03	-11.03	-11.03	-11.03	-11.03
-11.02	-11.02	-11.02	-11.02	-11.02	-11.02	-11.02	-11.02	-11.02
-11.01	-11.01	-11.01	-11.01	-11.01	-11.01	-11.01	-11.01	-11.01

-11	-11	-11	-11	-11	-11	-11	-11	-11
-10.99	-10.99	-10.99	-10.99	-10.99	-10.99	-10.99	-10.99	-10.99
-10.98	-10.98	-10.98	-10.98	-10.98	-10.98	-10.98	-10.98	-10.98
-10.97	-10.97	-10.97	-10.97	-10.97	-10.97	-10.97	-10.97	-10.97
-10.96	-10.96	-10.96	-10.96	-10.96	-10.96	-10.96	-10.96	-10.96
-10.95	-10.95	-10.95	-10.95	-10.95	-10.95	-10.95	-10.95	-10.95
-10.94	-10.94	-10.94	-10.94	-10.94	-10.94	-10.94	-10.94	-10.94
-10.93	-10.93	-10.93	-10.93	-10.93	-10.93	-10.93	-10.93	-10.93
-10.92	-10.92	-10.92	-10.92	-10.92	-10.92	-10.92	-10.92	-10.92
-10.91	-10.91	-10.91	-10.91	-10.91	-10.91	-10.91	-10.91	-10.91
-10.9	-10.9	-10.9	-10.9	-10.9	-10.9	-10.9	-10.9	-10.9
-10.89	-10.89	-10.89	-10.89	-10.89	-10.89	-10.89	-10.89	-10.89
-10.88	-10.88	-10.88	-10.88	-10.88	-10.88	-10.88	-10.88	-10.88
-10.87	-10.87	-10.87	-10.87	-10.87	-10.87	-10.87	-10.87	-10.87
-10.86	-10.86	-10.86	-10.86	-10.86	-10.86	-10.86	-10.86	-10.86
-10.85	-10.85	-10.85	-10.85	-10.85	-10.85	-10.85	-10.85	-10.85
-10.84	-10.84	-10.84	-10.84	-10.84	-10.84	-10.84	-10.84	-10.84
-10.83	-10.83	-10.83	-10.83	-10.83	-10.83	-10.83	-10.83	-10.83
-10.82	-10.82	-10.82	-10.82	-10.82	-10.82	-10.82	-10.82	-10.82
-10.81	-10.81	-10.81	-10.81	-10.81	-10.81	-10.81	-10.81	-10.81
-10.8	-10.8	-10.8	-10.8	-10.8	-10.8	-10.8	-10.8	-10.8
-10.79	-10.79	-10.79	-10.79	-10.79	-10.79	-10.79	-10.79	-10.79
-10.78	-10.78	-10.78	-10.78	-10.78	-10.78	-10.78	-10.78	-10.78
-10.77	-10.77	-10.77	-10.77	-10.77	-10.77	-10.77	-10.77	-10.77
-10.76	-10.76	-10.76	-10.76	-10.76	-10.76	-10.76	-10.76	-10.76
-10.75	-10.75	-10.75	-10.75	-10.75	-10.75	-10.75	-10.75	-10.75
-10.74	-10.74	-10.74	-10.74	-10.74	-10.74	-10.74	-10.74	-10.74
-10.73	-10.73	-10.73	-10.73	-10.73	-10.73	-10.73	-10.73	-10.73
-10.72	-10.72	-10.72	-10.72	-10.72	-10.72	-10.72	-10.72	-10.72
-10.71	-10.71	-10.71	-10.71	-10.71	-10.71	-10.71	-10.71	-10.71
-10.7	-10.7	-10.7	-10.7	-10.7	-10.7	-10.7	-10.7	-10.7
-10.69	-10.69	-10.69	-10.69	-10.69	-10.69	-10.69	-10.69	-10.69
-10.68	-10.68	-10.68	-10.68	-10.68	-10.68	-10.68	-10.68	-10.68
-10.67	-10.67	-10.67	-10.67	-10.67	-10.67	-10.67	-10.67	-10.67
-10.66	-10.66	-10.66	-10.66	-10.66	-10.66	-10.66	-10.66	-10.66
-10.65	-10.65	-10.65	-10.65	-10.65	-10.65	-10.65	-10.65	-10.65
-10.64	-10.64	-10.64	-10.64	-10.64	-10.64	-10.64	-10.64	-10.64
-10.63	-10.63	-10.63	-10.63	-10.63	-10.63	-10.63	-10.63	-10.63
-10.62	-10.62	-10.62	-10.62	-10.62	-10.62	-10.62	-10.62	-10.62
-10.61	-10.61	-10.61	-10.61	-10.61	-10.61	-10.61	-10.61	-10.61
-10.6	-10.6	-10.6	-10.6	-10.6	-10.6	-10.6	-10.6	-10.6
-10.59	-10.59	-10.59	-10.59	-10.59	-10.59	-10.59	-10.59	-10.59
-10.58	-10.58	-10.58	-10.58	-10.58	-10.58	-10.58	-10.58	-10.58
-10.57	-10.57	-10.57	-10.57	-10.57	-10.57	-10.57	-10.57	-10.57
-10.56	-10.56	-10.56	-10.56	-10.56	-10.56	-10.56	-10.56	-10.56
-10.55	-10.55	-10.55	-10.55	-10.55	-10.55	-10.55	-10.55	-10.55
-10.54	-10.54	-10.54	-10.54	-10.54	-10.54	-10.54	-10.54	-10.54
-10.53	-10.53	-10.53	-10.53	-10.53	-10.53	-10.53	-10.53	-10.53
-10.52	-10.52	-10.52	-10.52	-10.52	-10.52	-10.52	-10.52	-10.52
-10.51	-10.51	-10.51	-10.51	-10.51	-10.51	-10.51	-10.51	-10.51

-10.5	-10.5	-10.5	-10.5	-10.5	-10.5	-10.5	-10.5	-10.5
-10.49	-10.49	-10.49	-10.49	-10.49	-10.49	-10.49	-10.49	-10.49
-10.48	-10.48	-10.48	-10.48	-10.48	-10.48	-10.48	-10.48	-10.48
-10.47	-10.47	-10.47	-10.47	-10.47	-10.47	-10.47	-10.47	-10.47
-10.46	-10.46	-10.46	-10.46	-10.46	-10.46	-10.46	-10.46	-10.46
-10.45	-10.45	-10.45	-10.45	-10.45	-10.45	-10.45	-10.45	-10.45
-10.44	-10.44	-10.44	-10.44	-10.44	-10.44	-10.44	-10.44	-10.44
-10.43	-10.43	-10.43	-10.43	-10.43	-10.43	-10.43	-10.43	-10.43
-10.42	-10.42	-10.42	-10.42	-10.42	-10.42	-10.42	-10.42	-10.42
-10.41	-10.41	-10.41	-10.41	-10.41	-10.41	-10.41	-10.41	-10.41
-10.4	-10.4	-10.4	-10.4	-10.4	-10.4	-10.4	-10.4	-10.4
-10.39	-10.39	-10.39	-10.39	-10.39	-10.39	-10.39	-10.39	-10.39
-10.38	-10.38	-10.38	-10.38	-10.38	-10.38	-10.38	-10.38	-10.38
-10.37	-10.37	-10.37	-10.37	-10.37	-10.37	-10.37	-10.37	-10.37
-10.36	-10.36	-10.36	-10.36	-10.36	-10.36	-10.36	-10.36	-10.36
-10.35	-10.35	-10.35	-10.35	-10.35	-10.35	-10.35	-10.35	-10.35
-10.34	-10.34	-10.34	-10.34	-10.34	-10.34	-10.34	-10.34	-10.34
-10.33	-10.33	-10.33	-10.33	-10.33	-10.33	-10.33	-10.33	-10.33
-10.32	-10.32	-10.32	-10.32	-10.32	-10.32	-10.32	-10.32	-10.32
-10.31	-10.31	-10.31	-10.31	-10.31	-10.31	-10.31	-10.31	-10.31
-10.3	-10.3	-10.3	-10.3	-10.3	-10.3	-10.3	-10.3	-10.3
-10.29	-10.29	-10.29	-10.29	-10.29	-10.29	-10.29	-10.29	-10.29
-10.28	-10.28	-10.28	-10.28	-10.28	-10.28	-10.28	-10.28	-10.28
-10.27	-10.27	-10.27	-10.27	-10.27	-10.27	-10.27	-10.27	-10.27
-10.26	-10.26	-10.26	-10.26	-10.26	-10.26	-10.26	-10.26	-10.26
-10.25	-10.25	-10.25	-10.25	-10.25	-10.25	-10.25	-10.25	-10.25
-10.24	-10.24	-10.24	-10.24	-10.24	-10.24	-10.24	-10.24	-10.24
-10.23	-10.23	-10.23	-10.23	-10.23	-10.23	-10.23	-10.23	-10.23
-10.22	-10.22	-10.22	-10.22	-10.22	-10.22	-10.22	-10.22	-10.22
-10.21	-10.21	-10.21	-10.21	-10.21	-10.21	-10.21	-10.21	-10.21
-10.2	-10.2	-10.2	-10.2	-10.2	-10.2	-10.2	-10.2	-10.2
-10.19	-10.19	-10.19	-10.19	-10.19	-10.19	-10.19	-10.19	-10.19
-10.18	-10.18	-10.18	-10.18	-10.18	-10.18	-10.18	-10.18	-10.18
-10.17	-10.17	-10.17	-10.17	-10.17	-10.17	-10.17	-10.17	-10.17
-10.16	-10.16	-10.16	-10.16	-10.16	-10.16	-10.16	-10.16	-10.16
-10.15	-10.15	-10.15	-10.15	-10.15	-10.15	-10.15	-10.15	-10.15
-10.14	-10.14	-10.14	-10.14	-10.14	-10.14	-10.14	-10.14	-10.14
-10.13	-10.13	-10.13	-10.13	-10.13	-10.13	-10.13	-10.13	-10.13
-10.12	-10.12	-10.12	-10.12	-10.12	-10.12	-10.12	-10.12	-10.12
-10.11	-10.11	-10.11	-10.11	-10.11	-10.11	-10.11	-10.11	-10.11
-10.1	-10.1	-10.1	-10.1	-10.1	-10.1	-10.1	-10.1	-10.1
-10.09	-10.09	-10.09	-10.09	-10.09	-10.09	-10.09	-10.09	-10.09
-10.08	-10.08	-10.08	-10.08	-10.08	-10.08	-10.08	-10.08	-10.08
-10.07	-10.07	-10.07	-10.07	-10.07	-10.07	-10.07	-10.07	-10.07
-10.06	-10.06	-10.06	-10.06	-10.06	-10.06	-10.06	-10.06	-10.06
-10.05	-10.05	-10.05	-10.05	-10.05	-10.05	-10.05	-10.05	-10.05
-10.04	-10.04	-10.04	-10.04	-10.04	-10.04	-10.04	-10.04	-10.04
-10.03	-10.03	-10.03	-10.03	-10.03	-10.03	-10.03	-10.03	-10.03
-10.02	-10.02	-10.02	-10.02	-10.02	-10.02	-10.02	-10.02	-10.02
-10.01	-10.01	-10.01	-10.01	-10.01	-10.01	-10.01	-10.01	-10.01

-10	-10	-10	-10	-10	-10	-10	-10	-10
-9.99	-9.99	-9.99	-9.99	-9.99	-9.99	-9.99	-9.99	-9.99
-9.98	-9.98	-9.98	-9.98	-9.98	-9.98	-9.98	-9.98	-9.98
-9.97	-9.97	-9.97	-9.97	-9.97	-9.97	-9.97	-9.97	-9.97
-9.96	-9.96	-9.96	-9.96	-9.96	-9.96	-9.96	-9.96	-9.96
-9.95	-9.95	-9.95	-9.95	-9.95	-9.95	-9.95	-9.95	-9.95
-9.94	-9.94	-9.94	-9.94	-9.94	-9.94	-9.94	-9.94	-9.94
-9.93	-9.93	-9.93	-9.93	-9.93	-9.93	-9.93	-9.93	-9.93
-9.92	-9.92	-9.92	-9.92	-9.92	-9.92	-9.92	-9.92	-9.92
-9.91	-9.91	-9.91	-9.91	-9.91	-9.91	-9.91	-9.91	-9.91
-9.9	-9.9	-9.9	-9.9	-9.9	-9.9	-9.9	-9.9	-9.9
-9.89	-9.89	-9.89	-9.89	-9.89	-9.89	-9.89	-9.89	-9.89
-9.88	-9.88	-9.88	-9.88	-9.88	-9.88	-9.88	-9.88	-9.88
-9.87	-9.87	-9.87	-9.87	-9.87	-9.87	-9.87	-9.87	-9.87
-9.86	-9.86	-9.86	-9.86	-9.86	-9.86	-9.86	-9.86	-9.86
-9.85	-9.85	-9.85	-9.85	-9.85	-9.85	-9.85	-9.85	-9.85
-9.84	-9.84	-9.84	-9.84	-9.84	-9.84	-9.84	-9.84	-9.84
-9.83	-9.83	-9.83	-9.83	-9.83	-9.83	-9.83	-9.83	-9.83
-9.82	-9.82	-9.82	-9.82	-9.82	-9.82	-9.82	-9.82	-9.82
-9.81	-9.81	-9.81	-9.81	-9.81	-9.81	-9.81	-9.81	-9.81
-9.8	-9.8	-9.8	-9.8	-9.8	-9.8	-9.8	-9.8	-9.8
-9.79	-9.79	-9.79	-9.79	-9.79	-9.79	-9.79	-9.79	-9.79
-9.78	-9.78	-9.78	-9.78	-9.78	-9.78	-9.78	-9.78	-9.78
-9.77	-9.77	-9.77	-9.77	-9.77	-9.77	-9.77	-9.77	-9.77
-9.76	-9.76	-9.76	-9.76	-9.76	-9.76	-9.76	-9.76	-9.76
-9.75	-9.75	-9.75	-9.75	-9.75	-9.75	-9.75	-9.75	-9.75
-9.74	-9.74	-9.74	-9.74	-9.74	-9.74	-9.74	-9.74	-9.74
-9.73	-9.73	-9.73	-9.73	-9.73	-9.73	-9.73	-9.73	-9.73
-9.72	-9.72	-9.72	-9.72	-9.72	-9.72	-9.72	-9.72	-9.72
-9.71	-9.71	-9.71	-9.71	-9.71	-9.71	-9.71	-9.71	-9.71
-9.7	-9.7	-9.7	-9.7	-9.7	-9.7	-9.7	-9.7	-9.7
-9.69	-9.69	-9.69	-9.69	-9.69	-9.69	-9.69	-9.69	-9.69
-9.68	-9.68	-9.68	-9.68	-9.68	-9.68	-9.68	-9.68	-9.68
-9.67	-9.67	-9.67	-9.67	-9.67	-9.67	-9.67	-9.67	-9.67
-9.66	-9.66	-9.66	-9.66	-9.66	-9.66	-9.66	-9.66	-9.66
-9.65	-9.65	-9.65	-9.65	-9.65	-9.65	-9.65	-9.65	-9.65
-9.64	-9.64	-9.64	-9.64	-9.64	-9.64	-9.64	-9.64	-9.64
-9.63	-9.63	-9.63	-9.63	-9.63	-9.63	-9.63	-9.63	-9.63
-9.62	-9.62	-9.62	-9.62	-9.62	-9.62	-9.62	-9.62	-9.62
-9.61	-9.61	-9.61	-9.61	-9.61	-9.61	-9.61	-9.61	-9.61
-9.6	-9.6	-9.6	-9.6	-9.6	-9.6	-9.6	-9.6	-9.6
-9.59	-9.59	-9.59	-9.59	-9.59	-9.59	-9.59	-9.59	-9.59
-9.58	-9.58	-9.58	-9.58	-9.58	-9.58	-9.58	-9.58	-9.58
-9.57	-9.57	-9.57	-9.57	-9.57	-9.57	-9.57	-9.57	-9.57
-9.56	-9.56	-9.56	-9.56	-9.56	-9.56	-9.56	-9.56	-9.56
-9.55	-9.55	-9.55	-9.55	-9.55	-9.55	-9.55	-9.55	-9.55
-9.54	-9.54	-9.54	-9.54	-9.54	-9.54	-9.54	-9.54	-9.54
-9.53	-9.53	-9.53	-9.53	-9.53	-9.53	-9.53	-9.53	-9.53
-9.52	-9.52	-9.52	-9.52	-9.52	-9.52	-9.52	-9.52	-9.52
-9.51	-9.51	-9.51	-9.51	-9.51	-9.51	-9.51	-9.51	-9.51

-9.5	-9.5	-9.5	-9.5	-9.5	-9.5	-9.5	-9.5	-9.5
-9.49	-9.49	-9.49	-9.49	-9.49	-9.49	-9.49	-9.49	-9.49
-9.48	-9.48	-9.48	-9.48	-9.48	-9.48	-9.48	-9.48	-9.48
-9.47	-9.47	-9.47	-9.47	-9.47	-9.47	-9.47	-9.47	-9.47
-9.46	-9.46	-9.46	-9.46	-9.46	-9.46	-9.46	-9.46	-9.46
-9.45	-9.45	-9.45	-9.45	-9.45	-9.45	-9.45	-9.45	-9.45
-9.44	-9.44	-9.44	-9.44	-9.44	-9.44	-9.44	-9.44	-9.44
-9.43	-9.43	-9.43	-9.43	-9.43	-9.43	-9.43	-9.43	-9.43
-9.42	-9.42	-9.42	-9.42	-9.42	-9.42	-9.42	-9.42	-9.42
-9.41	-9.41	-9.41	-9.41	-9.41	-9.41	-9.41	-9.41	-9.41
-9.4	-9.4	-9.4	-9.4	-9.4	-9.4	-9.4	-9.4	-9.4
-9.39	-9.39	-9.39	-9.39	-9.39	-9.39	-9.39	-9.39	-9.39
-9.38	-9.38	-9.38	-9.38	-9.38	-9.38	-9.38	-9.38	-9.38
-9.37	-9.37	-9.37	-9.37	-9.37	-9.37	-9.37	-9.37	-9.37
-9.36	-9.36	-9.36	-9.36	-9.36	-9.36	-9.36	-9.36	-9.36
-9.35	-9.35	-9.35	-9.35	-9.35	-9.35	-9.35	-9.35	-9.35
-9.34	-9.34	-9.34	-9.34	-9.34	-9.34	-9.34	-9.34	-9.34
-9.33	-9.33	-9.33	-9.33	-9.33	-9.33	-9.33	-9.33	-9.33
-9.32	-9.32	-9.32	-9.32	-9.32	-9.32	-9.32	-9.32	-9.32
-9.31	-9.31	-9.31	-9.31	-9.31	-9.31	-9.31	-9.31	-9.31
-9.3	-9.3	-9.3	-9.3	-9.3	-9.3	-9.3	-9.3	-9.3
-9.29	-9.29	-9.29	-9.29	-9.29	-9.29	-9.29	-9.29	-9.29
-9.28	-9.28	-9.28	-9.28	-9.28	-9.28	-9.28	-9.28	-9.28
-9.27	-9.27	-9.27	-9.27	-9.27	-9.27	-9.27	-9.27	-9.27
-9.26	-9.26	-9.26	-9.26	-9.26	-9.26	-9.26	-9.26	-9.26
-9.25	-9.25	-9.25	-9.25	-9.25	-9.25	-9.25	-9.25	-9.25
-9.24	-9.24	-9.24	-9.24	-9.24	-9.24	-9.24	-9.24	-9.24
-9.23	-9.23	-9.23	-9.23	-9.23	-9.23	-9.23	-9.23	-9.23
-9.22	-9.22	-9.22	-9.22	-9.22	-9.22	-9.22	-9.22	-9.22
-9.21	-9.21	-9.21	-9.21	-9.21	-9.21	-9.21	-9.21	-9.21
-9.2	-9.2	-9.2	-9.2	-9.2	-9.2	-9.2	-9.2	-9.2
-9.19	-9.19	-9.19	-9.19	-9.19	-9.19	-9.19	-9.19	-9.19
-9.18	-9.18	-9.18	-9.18	-9.18	-9.18	-9.18	-9.18	-9.18
-9.17	-9.17	-9.17	-9.17	-9.17	-9.17	-9.17	-9.17	-9.17
-9.16	-9.16	-9.16	-9.16	-9.16	-9.16	-9.16	-9.16	-9.16
-9.15	-9.15	-9.15	-9.15	-9.15	-9.15	-9.15	-9.15	-9.15
-9.14	-9.14	-9.14	-9.14	-9.14	-9.14	-9.14	-9.14	-9.14
-9.13	-9.13	-9.13	-9.13	-9.13	-9.13	-9.13	-9.13	-9.13
-9.12	-9.12	-9.12	-9.12	-9.12	-9.12	-9.12	-9.12	-9.12
-9.11	-9.11	-9.11	-9.11	-9.11	-9.11	-9.11	-9.11	-9.11
-9.1	-9.1	-9.1	-9.1	-9.1	-9.1	-9.1	-9.1	-9.1
-9.09	-9.09	-9.09	-9.09	-9.09	-9.09	-9.09	-9.09	-9.09
-9.08	-9.08	-9.08	-9.08	-9.08	-9.08	-9.08	-9.08	-9.08
-9.07	-9.07	-9.07	-9.07	-9.07	-9.07	-9.07	-9.07	-9.07
-9.06	-9.06	-9.06	-9.06	-9.06	-9.06	-9.06	-9.06	-9.06
-9.05	-9.05	-9.05	-9.05	-9.05	-9.05	-9.05	-9.05	-9.05
-9.04	-9.04	-9.04	-9.04	-9.04	-9.04	-9.04	-9.04	-9.04
-9.03	-9.03	-9.03	-9.03	-9.03	-9.03	-9.03	-9.03	-9.03
-9.02	-9.02	-9.02	-9.02	-9.02	-9.02	-9.02	-9.02	-9.02
-9.01	-9.01	-9.01	-9.01	-9.01	-9.01	-9.01	-9.01	-9.01

-9	-9	-9	-9	-9	-9	-9	-9	-9
-8.99	-8.99	-8.99	-8.99	-8.99	-8.99	-8.99	-8.99	-8.99
-8.98	-8.98	-8.98	-8.98	-8.98	-8.98	-8.98	-8.98	-8.98
-8.97	-8.97	-8.97	-8.97	-8.97	-8.97	-8.97	-8.97	-8.97
-8.96	-8.96	-8.96	-8.96	-8.96	-8.96	-8.96	-8.96	-8.96
-8.95	-8.95	-8.95	-8.95	-8.95	-8.95	-8.95	-8.95	-8.95
-8.94	-8.94	-8.94	-8.94	-8.94	-8.94	-8.94	-8.94	-8.94
-8.93	-8.93	-8.93	-8.93	-8.93	-8.93	-8.93	-8.93	-8.93
-8.92	-8.92	-8.92	-8.92	-8.92	-8.92	-8.92	-8.92	-8.92
-8.91	-8.91	-8.91	-8.91	-8.91	-8.91	-8.91	-8.91	-8.91
-8.9	-8.9	-8.9	-8.9	-8.9	-8.9	-8.9	-8.9	-8.9
-8.89	-8.89	-8.89	-8.89	-8.89	-8.89	-8.89	-8.89	-8.89
-8.88	-8.88	-8.88	-8.88	-8.88	-8.88	-8.88	-8.88	-8.88
-8.87	-8.87	-8.87	-8.87	-8.87	-8.87	-8.87	-8.87	-8.87
-8.86	-8.86	-8.86	-8.86	-8.86	-8.86	-8.86	-8.86	-8.86
-8.85	-8.85	-8.85	-8.85	-8.85	-8.85	-8.85	-8.85	-8.85
-8.84	-8.84	-8.84	-8.84	-8.84	-8.84	-8.84	-8.84	-8.84
-8.83	-8.83	-8.83	-8.83	-8.83	-8.83	-8.83	-8.83	-8.83
-8.82	-8.82	-8.82	-8.82	-8.82	-8.82	-8.82	-8.82	-8.82
-8.81	-8.81	-8.81	-8.81	-8.81	-8.81	-8.81	-8.81	-8.81
-8.8	-8.8	-8.8	-8.8	-8.8	-8.8	-8.8	-8.8	-8.8
-8.79	-8.79	-8.79	-8.79	-8.79	-8.79	-8.79	-8.79	-8.79
-8.78	-8.78	-8.78	-8.78	-8.78	-8.78	-8.78	-8.78	-8.78
-8.77	-8.77	-8.77	-8.77	-8.77	-8.77	-8.77	-8.77	-8.77
-8.76	-8.76	-8.76	-8.76	-8.76	-8.76	-8.76	-8.76	-8.76
-8.75	-8.75	-8.75	-8.75	-8.75	-8.75	-8.75	-8.75	-8.75
-8.74	-8.74	-8.74	-8.74	-8.74	-8.74	-8.74	-8.74	-8.74
-8.73	-8.73	-8.73	-8.73	-8.73	-8.73	-8.73	-8.73	-8.73
-8.72	-8.72	-8.72	-8.72	-8.72	-8.72	-8.72	-8.72	-8.72
-8.71	-8.71	-8.71	-8.71	-8.71	-8.71	-8.71	-8.71	-8.71
-8.7	-8.7	-8.7	-8.7	-8.7	-8.7	-8.7	-8.7	-8.7
-8.69	-8.69	-8.69	-8.69	-8.69	-8.69	-8.69	-8.69	-8.69
-8.68	-8.68	-8.68	-8.68	-8.68	-8.68	-8.68	-8.68	-8.68
-8.67	-8.67	-8.67	-8.67	-8.67	-8.67	-8.67	-8.67	-8.67
-8.66	-8.66	-8.66	-8.66	-8.66	-8.66	-8.66	-8.66	-8.66
-8.65	-8.65	-8.65	-8.65	-8.65	-8.65	-8.65	-8.65	-8.65
-8.64	-8.64	-8.64	-8.64	-8.64	-8.64	-8.64	-8.64	-8.64
-8.63	-8.63	-8.63	-8.63	-8.63	-8.63	-8.63	-8.63	-8.63
-8.62	-8.62	-8.62	-8.62	-8.62	-8.62	-8.62	-8.62	-8.62
-8.61	-8.61	-8.61	-8.61	-8.61	-8.61	-8.61	-8.61	-8.61
-8.6	-8.6	-8.6	-8.6	-8.6	-8.6	-8.6	-8.6	-8.6
-8.59	-8.59	-8.59	-8.59	-8.59	-8.59	-8.59	-8.59	-8.59
-8.58	-8.58	-8.58	-8.58	-8.58	-8.58	-8.58	-8.58	-8.58
-8.57	-8.57	-8.57	-8.57	-8.57	-8.57	-8.57	-8.57	-8.57
-8.56	-8.56	-8.56	-8.56	-8.56	-8.56	-8.56	-8.56	-8.56
-8.55	-8.55	-8.55	-8.55	-8.55	-8.55	-8.55	-8.55	-8.55
-8.54	-8.54	-8.54	-8.54	-8.54	-8.54	-8.54	-8.54	-8.54
-8.53	-8.53	-8.53	-8.53	-8.53	-8.53	-8.53	-8.53	-8.53
-8.52	-8.52	-8.52	-8.52	-8.52	-8.52	-8.52	-8.52	-8.52
-8.51	-8.51	-8.51	-8.51	-8.51	-8.51	-8.51	-8.51	-8.51

-8.5	-8.5	-8.5	-8.5	-8.5	-8.5	-8.5	-8.5	-8.5
-8.49	-8.49	-8.49	-8.49	-8.49	-8.49	-8.49	-8.49	-8.49
-8.48	-8.48	-8.48	-8.48	-8.48	-8.48	-8.48	-8.48	-8.48
-8.47	-8.47	-8.47	-8.47	-8.47	-8.47	-8.47	-8.47	-8.47
-8.46	-8.46	-8.46	-8.46	-8.46	-8.46	-8.46	-8.46	-8.46
-8.45	-8.45	-8.45	-8.45	-8.45	-8.45	-8.45	-8.45	-8.45
-8.44	-8.44	-8.44	-8.44	-8.44	-8.44	-8.44	-8.44	-8.44
-8.43	-8.43	-8.43	-8.43	-8.43	-8.43	-8.43	-8.43	-8.43
-8.42	-8.42	-8.42	-8.42	-8.42	-8.42	-8.42	-8.42	-8.42
-8.41	-8.41	-8.41	-8.41	-8.41	-8.41	-8.41	-8.41	-8.41
-8.4	-8.4	-8.4	-8.4	-8.4	-8.4	-8.4	-8.4	-8.4
-8.39	-8.39	-8.39	-8.39	-8.39	-8.39	-8.39	-8.39	-8.39
-8.38	-8.38	-8.38	-8.38	-8.38	-8.38	-8.38	-8.38	-8.38
-8.37	-8.37	-8.37	-8.37	-8.37	-8.37	-8.37	-8.37	-8.37
-8.36	-8.36	-8.36	-8.36	-8.36	-8.36	-8.36	-8.36	-8.36
-8.35	-8.35	-8.35	-8.35	-8.35	-8.35	-8.35	-8.35	-8.35
-8.34	-8.34	-8.34	-8.34	-8.34	-8.34	-8.34	-8.34	-8.34
-8.33	-8.33	-8.33	-8.33	-8.33	-8.33	-8.33	-8.33	-8.33
-8.32	-8.32	-8.32	-8.32	-8.32	-8.32	-8.32	-8.32	-8.32
-8.31	-8.31	-8.31	-8.31	-8.31	-8.31	-8.31	-8.31	-8.31
-8.3	-8.3	-8.3	-8.3	-8.3	-8.3	-8.3	-8.3	-8.3
-8.29	-8.29	-8.29	-8.29	-8.29	-8.29	-8.29	-8.29	-8.29
-8.28	-8.28	-8.28	-8.28	-8.28	-8.28	-8.28	-8.28	-8.28
-8.27	-8.27	-8.27	-8.27	-8.27	-8.27	-8.27	-8.27	-8.27
-8.26	-8.26	-8.26	-8.26	-8.26	-8.26	-8.26	-8.26	-8.26
-8.25	-8.25	-8.25	-8.25	-8.25	-8.25	-8.25	-8.25	-8.25
-8.24	-8.24	-8.24	-8.24	-8.24	-8.24	-8.24	-8.24	-8.24
-8.23	-8.23	-8.23	-8.23	-8.23	-8.23	-8.23	-8.23	-8.23
-8.22	-8.22	-8.22	-8.22	-8.22	-8.22	-8.22	-8.22	-8.22
-8.21	-8.21	-8.21	-8.21	-8.21	-8.21	-8.21	-8.21	-8.21
-8.2	-8.2	-8.2	-8.2	-8.2	-8.2	-8.2	-8.2	-8.2
-8.19	-8.19	-8.19	-8.19	-8.19	-8.19	-8.19	-8.19	-8.19
-8.18	-8.18	-8.18	-8.18	-8.18	-8.18	-8.18	-8.18	-8.18
-8.17	-8.17	-8.17	-8.17	-8.17	-8.17	-8.17	-8.17	-8.17
-8.16	-8.16	-8.16	-8.16	-8.16	-8.16	-8.16	-8.16	-8.16
-8.15	-8.15	-8.15	-8.15	-8.15	-8.15	-8.15	-8.15	-8.15
-8.14	-8.14	-8.14	-8.14	-8.14	-8.14	-8.14	-8.14	-8.14
-8.13	-8.13	-8.13	-8.13	-8.13	-8.13	-8.13	-8.13	-8.13
-8.12	-8.12	-8.12	-8.12	-8.12	-8.12	-8.12	-8.12	-8.12
-8.11	-8.11	-8.11	-8.11	-8.11	-8.11	-8.11	-8.11	-8.11
-8.1	-8.1	-8.1	-8.1	-8.1	-8.1	-8.1	-8.1	-8.1
-8.09	-8.09	-8.09	-8.09	-8.09	-8.09	-8.09	-8.09	-8.09
-8.08	-8.08	-8.08	-8.08	-8.08	-8.08	-8.08	-8.08	-8.08
-8.07	-8.07	-8.07	-8.07	-8.07	-8.07	-8.07	-8.07	-8.07
-8.06	-8.06	-8.06	-8.06	-8.06	-8.06	-8.06	-8.06	-8.06
-8.05	-8.05	-8.05	-8.05	-8.05	-8.05	-8.05	-8.05	-8.05
-8.04	-8.04	-8.04	-8.04	-8.04	-8.04	-8.04	-8.04	-8.04
-8.03	-8.03	-8.03	-8.03	-8.03	-8.03	-8.03	-8.03	-8.03
-8.02	-8.02	-8.02	-8.02	-8.02	-8.02	-8.02	-8.02	-8.02
-8.01	-8.01	-8.01	-8.01	-8.01	-8.01	-8.01	-8.01	-8.01

-8	-8	-8	-8	-8	-8	-8	-8	-8
-7.99	-7.99	-7.99	-7.99	-7.99	-7.99	-7.99	-7.99	-7.99
-7.98	-7.98	-7.98	-7.98	-7.98	-7.98	-7.98	-7.98	-7.98
-7.97	-7.97	-7.97	-7.97	-7.97	-7.97	-7.97	-7.97	-7.97
-7.96	-7.96	-7.96	-7.96	-7.96	-7.96	-7.96	-7.96	-7.96
-7.95	-7.95	-7.95	-7.95	-7.95	-7.95	-7.95	-7.95	-7.95
-7.94	-7.94	-7.94	-7.94	-7.94	-7.94	-7.94	-7.94	-7.94
-7.93	-7.93	-7.93	-7.93	-7.93	-7.93	-7.93	-7.93	-7.93
-7.92	-7.92	-7.92	-7.92	-7.92	-7.92	-7.92	-7.92	-7.92
-7.91	-7.91	-7.91	-7.91	-7.91	-7.91	-7.91	-7.91	-7.91
-7.9	-7.9	-7.9	-7.9	-7.9	-7.9	-7.9	-7.9	-7.9
-7.89	-7.89	-7.89	-7.89	-7.89	-7.89	-7.89	-7.89	-7.89
-7.88	-7.88	-7.88	-7.88	-7.88	-7.88	-7.88	-7.88	-7.88
-7.87	-7.87	-7.87	-7.87	-7.87	-7.87	-7.87	-7.87	-7.87
-7.86	-7.86	-7.86	-7.86	-7.86	-7.86	-7.86	-7.86	-7.86
-7.85	-7.85	-7.85	-7.85	-7.85	-7.85	-7.85	-7.85	-7.85
-7.84	-7.84	-7.84	-7.84	-7.84	-7.84	-7.84	-7.84	-7.84
-7.83	-7.83	-7.83	-7.83	-7.83	-7.83	-7.83	-7.83	-7.83
-7.82	-7.82	-7.82	-7.82	-7.82	-7.82	-7.82	-7.82	-7.82
-7.81	-7.81	-7.81	-7.81	-7.81	-7.81	-7.81	-7.81	-7.81
-7.8	-7.8	-7.8	-7.8	-7.8	-7.8	-7.8	-7.8	-7.8
-7.79	-7.79	-7.79	-7.79	-7.79	-7.79	-7.79	-7.79	-7.79
-7.78	-7.78	-7.78	-7.78	-7.78	-7.78	-7.78	-7.78	-7.78
-7.77	-7.77	-7.77	-7.77	-7.77	-7.77	-7.77	-7.77	-7.77
-7.76	-7.76	-7.76	-7.76	-7.76	-7.76	-7.76	-7.76	-7.76
-7.75	-7.75	-7.75	-7.75	-7.75	-7.75	-7.75	-7.75	-7.75
-7.74	-7.74	-7.74	-7.74	-7.74	-7.74	-7.74	-7.74	-7.74
-7.73	-7.73	-7.73	-7.73	-7.73	-7.73	-7.73	-7.73	-7.73
-7.72	-7.72	-7.72	-7.72	-7.72	-7.72	-7.72	-7.72	-7.72
-7.71	-7.71	-7.71	-7.71	-7.71	-7.71	-7.71	-7.71	-7.71
-7.7	-7.7	-7.7	-7.7	-7.7	-7.7	-7.7	-7.7	-7.7
-7.69	-7.69	-7.69	-7.69	-7.69	-7.69	-7.69	-7.69	-7.69
-7.68	-7.68	-7.68	-7.68	-7.68	-7.68	-7.68	-7.68	-7.68
-7.67	-7.67	-7.67	-7.67	-7.67	-7.67	-7.67	-7.67	-7.67
-7.66	-7.66	-7.66	-7.66	-7.66	-7.66	-7.66	-7.66	-7.66
-7.65	-7.65	-7.65	-7.65	-7.65	-7.65	-7.65	-7.65	-7.65
-7.64	-7.64	-7.64	-7.64	-7.64	-7.64	-7.64	-7.64	-7.64
-7.63	-7.63	-7.63	-7.63	-7.63	-7.63	-7.63	-7.63	-7.63
-7.62	-7.62	-7.62	-7.62	-7.62	-7.62	-7.62	-7.62	-7.62
-7.61	-7.61	-7.61	-7.61	-7.61	-7.61	-7.61	-7.61	-7.61
-7.6	-7.6	-7.6	-7.6	-7.6	-7.6	-7.6	-7.6	-7.6
-7.59	-7.59	-7.59	-7.59	-7.59	-7.59	-7.59	-7.59	-7.59
-7.58	-7.58	-7.58	-7.58	-7.58	-7.58	-7.58	-7.58	-7.58
-7.57	-7.57	-7.57	-7.57	-7.57	-7.57	-7.57	-7.57	-7.57
-7.56	-7.56	-7.56	-7.56	-7.56	-7.56	-7.56	-7.56	-7.56
-7.55	-7.55	-7.55	-7.55	-7.55	-7.55	-7.55	-7.55	-7.55
-7.54	-7.54	-7.54	-7.54	-7.54	-7.54	-7.54	-7.54	-7.54
-7.53	-7.53	-7.53	-7.53	-7.53	-7.53	-7.53	-7.53	-7.53
-7.52	-7.52	-7.52	-7.52	-7.52	-7.52	-7.52	-7.52	-7.52
-7.51	-7.51	-7.51	-7.51	-7.51	-7.51	-7.51	-7.51	-7.51

-7.5	-7.5	-7.5	-7.5	-7.5	-7.5	-7.5	-7.5	-7.5
-7.49	-7.49	-7.49	-7.49	-7.49	-7.49	-7.49	-7.49	-7.49
-7.48	-7.48	-7.48	-7.48	-7.48	-7.48	-7.48	-7.48	-7.48
-7.47	-7.47	-7.47	-7.47	-7.47	-7.47	-7.47	-7.47	-7.47
-7.46	-7.46	-7.46	-7.46	-7.46	-7.46	-7.46	-7.46	-7.46
-7.45	-7.45	-7.45	-7.45	-7.45	-7.45	-7.45	-7.45	-7.45
-7.44	-7.44	-7.44	-7.44	-7.44	-7.44	-7.44	-7.44	-7.44
-7.43	-7.43	-7.43	-7.43	-7.43	-7.43	-7.43	-7.43	-7.43
-7.42	-7.42	-7.42	-7.42	-7.42	-7.42	-7.42	-7.42	-7.42
-7.41	-7.41	-7.41	-7.41	-7.41	-7.41	-7.41	-7.41	-7.41
-7.4	-7.4	-7.4	-7.4	-7.4	-7.4	-7.4	-7.4	-7.4
-7.39	-7.39	-7.39	-7.39	-7.39	-7.39	-7.39	-7.39	-7.39
-7.38	-7.38	-7.38	-7.38	-7.38	-7.38	-7.38	-7.38	-7.38
-7.37	-7.37	-7.37	-7.37	-7.37	-7.37	-7.37	-7.37	-7.37
-7.36	-7.36	-7.36	-7.36	-7.36	-7.36	-7.36	-7.36	-7.36
-7.35	-7.35	-7.35	-7.35	-7.35	-7.35	-7.35	-7.35	-7.35
-7.34	-7.34	-7.34	-7.34	-7.34	-7.34	-7.34	-7.34	-7.34
-7.33	-7.33	-7.33	-7.33	-7.33	-7.33	-7.33	-7.33	-7.33
-7.32	-7.32	-7.32	-7.32	-7.32	-7.32	-7.32	-7.32	-7.32
-7.31	-7.31	-7.31	-7.31	-7.31	-7.31	-7.31	-7.31	-7.31
-7.3	-7.3	-7.3	-7.3	-7.3	-7.3	-7.3	-7.3	-7.3
-7.29	-7.29	-7.29	-7.29	-7.29	-7.29	-7.29	-7.29	-7.29
-7.28	-7.28	-7.28	-7.28	-7.28	-7.28	-7.28	-7.28	-7.28
-7.27	-7.27	-7.27	-7.27	-7.27	-7.27	-7.27	-7.27	-7.27
-7.26	-7.26	-7.26	-7.26	-7.26	-7.26	-7.26	-7.26	-7.26
-7.25	-7.25	-7.25	-7.25	-7.25	-7.25	-7.25	-7.25	-7.25
-7.24	-7.24	-7.24	-7.24	-7.24	-7.24	-7.24	-7.24	-7.24
-7.23	-7.23	-7.23	-7.23	-7.23	-7.23	-7.23	-7.23	-7.23
-7.22	-7.22	-7.22	-7.22	-7.22	-7.22	-7.22	-7.22	-7.22
-7.21	-7.21	-7.21	-7.21	-7.21	-7.21	-7.21	-7.21	-7.21
-7.2	-7.2	-7.2	-7.2	-7.2	-7.2	-7.2	-7.2	-7.2
-7.19	-7.19	-7.19	-7.19	-7.19	-7.19	-7.19	-7.19	-7.19
-7.18	-7.18	-7.18	-7.18	-7.18	-7.18	-7.18	-7.18	-7.18
-7.17	-7.17	-7.17	-7.17	-7.17	-7.17	-7.17	-7.17	-7.17
-7.16	-7.16	-7.16	-7.16	-7.16	-7.16	-7.16	-7.16	-7.16
-7.15	-7.15	-7.15	-7.15	-7.15	-7.15	-7.15	-7.15	-7.15
-7.14	-7.14	-7.14	-7.14	-7.14	-7.14	-7.14	-7.14	-7.14
-7.13	-7.13	-7.13	-7.13	-7.13	-7.13	-7.13	-7.13	-7.13
-7.12	-7.12	-7.12	-7.12	-7.12	-7.12	-7.12	-7.12	-7.12
-7.11	-7.11	-7.11	-7.11	-7.11	-7.11	-7.11	-7.11	-7.11
-7.1	-7.1	-7.1	-7.1	-7.1	-7.1	-7.1	-7.1	-7.1
-7.09	-7.09	-7.09	-7.09	-7.09	-7.09	-7.09	-7.09	-7.09
-7.08	-7.08	-7.08	-7.08	-7.08	-7.08	-7.08	-7.08	-7.08
-7.07	-7.07	-7.07	-7.07	-7.07	-7.07	-7.07	-7.07	-7.07
-7.06	-7.06	-7.06	-7.06	-7.06	-7.06	-7.06	-7.06	-7.06
-7.05	-7.05	-7.05	-7.05	-7.05	-7.05	-7.05	-7.05	-7.05
-7.04	-7.04	-7.04	-7.04	-7.04	-7.04	-7.04	-7.04	-7.04
-7.03	-7.03	-7.03	-7.03	-7.03	-7.03	-7.03	-7.03	-7.03
-7.02	-7.02	-7.02	-7.02	-7.02	-7.02	-7.02	-7.02	-7.02
-7.01	-7.01	-7.01	-7.01	-7.01	-7.01	-7.01	-7.01	-7.01

-7	-7	-7	-7	-7	-7	-7	-7	-7
-6.99	-6.99	-6.99	-6.99	-6.99	-6.99	-6.99	-6.99	-6.99
-6.98	-6.98	-6.98	-6.98	-6.98	-6.98	-6.98	-6.98	-6.98
-6.97	-6.97	-6.97	-6.97	-6.97	-6.97	-6.97	-6.97	-6.97
-6.96	-6.96	-6.96	-6.96	-6.96	-6.96	-6.96	-6.96	-6.96
-6.95	-6.95	-6.95	-6.95	-6.95	-6.95	-6.95	-6.95	-6.95
-6.94	-6.94	-6.94	-6.94	-6.94	-6.94	-6.94	-6.94	-6.94
-6.93	-6.93	-6.93	-6.93	-6.93	-6.93	-6.93	-6.93	-6.93
-6.92	-6.92	-6.92	-6.92	-6.92	-6.92	-6.92	-6.92	-6.92
-6.91	-6.91	-6.91	-6.91	-6.91	-6.91	-6.91	-6.91	-6.91
-6.9	-6.9	-6.9	-6.9	-6.9	-6.9	-6.9	-6.9	-6.9
-6.89	-6.89	-6.89	-6.89	-6.89	-6.89	-6.89	-6.89	-6.89
-6.88	-6.88	-6.88	-6.88	-6.88	-6.88	-6.88	-6.88	-6.88
-6.87	-6.87	-6.87	-6.87	-6.87	-6.87	-6.87	-6.87	-6.87
-6.86	-6.86	-6.86	-6.86	-6.86	-6.86	-6.86	-6.86	-6.86
-6.85	-6.85	-6.85	-6.85	-6.85	-6.85	-6.85	-6.85	-6.85
-6.84	-6.84	-6.84	-6.84	-6.84	-6.84	-6.84	-6.84	-6.84
-6.83	-6.83	-6.83	-6.83	-6.83	-6.83	-6.83	-6.83	-6.83
-6.82	-6.82	-6.82	-6.82	-6.82	-6.82	-6.82	-6.82	-6.82
-6.81	-6.81	-6.81	-6.81	-6.81	-6.81	-6.81	-6.81	-6.81
-6.8	-6.8	-6.8	-6.8	-6.8	-6.8	-6.8	-6.8	-6.8
-6.79	-6.79	-6.79	-6.79	-6.79	-6.79	-6.79	-6.79	-6.79
-6.78	-6.78	-6.78	-6.78	-6.78	-6.78	-6.78	-6.78	-6.78
-6.77	-6.77	-6.77	-6.77	-6.77	-6.77	-6.77	-6.77	-6.77
-6.76	-6.76	-6.76	-6.76	-6.76	-6.76	-6.76	-6.76	-6.76
-6.75	-6.75	-6.75	-6.75	-6.75	-6.75	-6.75	-6.75	-6.75
-6.74	-6.74	-6.74	-6.74	-6.74	-6.74	-6.74	-6.74	-6.74
-6.73	-6.73	-6.73	-6.73	-6.73	-6.73	-6.73	-6.73	-6.73
-6.72	-6.72	-6.72	-6.72	-6.72	-6.72	-6.72	-6.72	-6.72
-6.71	-6.71	-6.71	-6.71	-6.71	-6.71	-6.71	-6.71	-6.71
-6.7	-6.7	-6.7	-6.7	-6.7	-6.7	-6.7	-6.7	-6.7
-6.69	-6.69	-6.69	-6.69	-6.69	-6.69	-6.69	-6.69	-6.69
-6.68	-6.68	-6.68	-6.68	-6.68	-6.68	-6.68	-6.68	-6.68
-6.67	-6.67	-6.67	-6.67	-6.67	-6.67	-6.67	-6.67	-6.67
-6.66	-6.66	-6.66	-6.66	-6.66	-6.66	-6.66	-6.66	-6.66
-6.65	-6.65	-6.65	-6.65	-6.65	-6.65	-6.65	-6.65	-6.65
-6.64	-6.64	-6.64	-6.64	-6.64	-6.64	-6.64	-6.64	-6.64
-6.63	-6.63	-6.63	-6.63	-6.63	-6.63	-6.63	-6.63	-6.63
-6.62	-6.62	-6.62	-6.62	-6.62	-6.62	-6.62	-6.62	-6.62
-6.61	-6.61	-6.61	-6.61	-6.61	-6.61	-6.61	-6.61	-6.61
-6.6	-6.6	-6.6	-6.6	-6.6	-6.6	-6.6	-6.6	-6.6
-6.59	-6.59	-6.59	-6.59	-6.59	-6.59	-6.59	-6.59	-6.59
-6.58	-6.58	-6.58	-6.58	-6.58	-6.58	-6.58	-6.58	-6.58
-6.57	-6.57	-6.57	-6.57	-6.57	-6.57	-6.57	-6.57	-6.57
-6.56	-6.56	-6.56	-6.56	-6.56	-6.56	-6.56	-6.56	-6.56
-6.55	-6.55	-6.55	-6.55	-6.55	-6.55	-6.55	-6.55	-6.55
-6.54	-6.54	-6.54	-6.54	-6.54	-6.54	-6.54	-6.54	-6.54
-6.53	-6.53	-6.53	-6.53	-6.53	-6.53	-6.53	-6.53	-6.53
-6.52	-6.52	-6.52	-6.52	-6.52	-6.52	-6.52	-6.52	-6.52
-6.51	-6.51	-6.51	-6.51	-6.51	-6.51	-6.51	-6.51	-6.51

-6.5	-6.5	-6.5	-6.5	-6.5	-6.5	-6.5	-6.5	-6.5
-6.49	-6.49	-6.49	-6.49	-6.49	-6.49	-6.49	-6.49	-6.49
-6.48	-6.48	-6.48	-6.48	-6.48	-6.48	-6.48	-6.48	-6.48
-6.47	-6.47	-6.47	-6.47	-6.47	-6.47	-6.47	-6.47	-6.47
-6.46	-6.46	-6.46	-6.46	-6.46	-6.46	-6.46	-6.46	-6.46
-6.45	-6.45	-6.45	-6.45	-6.45	-6.45	-6.45	-6.45	-6.45
-6.44	-6.44	-6.44	-6.44	-6.44	-6.44	-6.44	-6.44	-6.44
-6.43	-6.43	-6.43	-6.43	-6.43	-6.43	-6.43	-6.43	-6.43
-6.42	-6.42	-6.42	-6.42	-6.42	-6.42	-6.42	-6.42	-6.42
-6.41	-6.41	-6.41	-6.41	-6.41	-6.41	-6.41	-6.41	-6.41
-6.4	-6.4	-6.4	-6.4	-6.4	-6.4	-6.4	-6.4	-6.4
-6.39	-6.39	-6.39	-6.39	-6.39	-6.39	-6.39	-6.39	-6.39
-6.38	-6.38	-6.38	-6.38	-6.38	-6.38	-6.38	-6.38	-6.38
-6.37	-6.37	-6.37	-6.37	-6.37	-6.37	-6.37	-6.37	-6.37
-6.36	-6.36	-6.36	-6.36	-6.36	-6.36	-6.36	-6.36	-6.36
-6.35	-6.35	-6.35	-6.35	-6.35	-6.35	-6.35	-6.35	-6.35
-6.34	-6.34	-6.34	-6.34	-6.34	-6.34	-6.34	-6.34	-6.34
-6.33	-6.33	-6.33	-6.33	-6.33	-6.33	-6.33	-6.33	-6.33
-6.32	-6.32	-6.32	-6.32	-6.32	-6.32	-6.32	-6.32	-6.32
-6.31	-6.31	-6.31	-6.31	-6.31	-6.31	-6.31	-6.31	-6.31
-6.3	-6.3	-6.3	-6.3	-6.3	-6.3	-6.3	-6.3	-6.3
-6.29	-6.29	-6.29	-6.29	-6.29	-6.29	-6.29	-6.29	-6.29
-6.28	-6.28	-6.28	-6.28	-6.28	-6.28	-6.28	-6.28	-6.28
-6.27	-6.27	-6.27	-6.27	-6.27	-6.27	-6.27	-6.27	-6.27
-6.26	-6.26	-6.26	-6.26	-6.26	-6.26	-6.26	-6.26	-6.26
-6.25	-6.25	-6.25	-6.25	-6.25	-6.25	-6.25	-6.25	-6.25
-6.24	-6.24	-6.24	-6.24	-6.24	-6.24	-6.24	-6.24	-6.24
-6.23	-6.23	-6.23	-6.23	-6.23	-6.23	-6.23	-6.23	-6.23
-6.22	-6.22	-6.22	-6.22	-6.22	-6.22	-6.22	-6.22	-6.22
-6.21	-6.21	-6.21	-6.21	-6.21	-6.21	-6.21	-6.21	-6.21
-6.2	-6.2	-6.2	-6.2	-6.2	-6.2	-6.2	-6.2	-6.2
-6.19	-6.19	-6.19	-6.19	-6.19	-6.19	-6.19	-6.19	-6.19
-6.18	-6.18	-6.18	-6.18	-6.18	-6.18	-6.18	-6.18	-6.18
-6.17	-6.17	-6.17	-6.17	-6.17	-6.17	-6.17	-6.17	-6.17
-6.16	-6.16	-6.16	-6.16	-6.16	-6.16	-6.16	-6.16	-6.16
-6.15	-6.15	-6.15	-6.15	-6.15	-6.15	-6.15	-6.15	-6.15
-6.14	-6.14	-6.14	-6.14	-6.14	-6.14	-6.14	-6.14	-6.14
-6.13	-6.13	-6.13	-6.13	-6.13	-6.13	-6.13	-6.13	-6.13
-6.12	-6.12	-6.12	-6.12	-6.12	-6.12	-6.12	-6.12	-6.12
-6.11	-6.11	-6.11	-6.11	-6.11	-6.11	-6.11	-6.11	-6.11
-6.1	-6.1	-6.1	-6.1	-6.1	-6.1	-6.1	-6.1	-6.1
-6.09	-6.09	-6.09	-6.09	-6.09	-6.09	-6.09	-6.09	-6.09
-6.08	-6.08	-6.08	-6.08	-6.08	-6.08	-6.08	-6.08	-6.08
-6.07	-6.07	-6.07	-6.07	-6.07	-6.07	-6.07	-6.07	-6.07
-6.06	-6.06	-6.06	-6.06	-6.06	-6.06	-6.06	-6.06	-6.06
-6.05	-6.05	-6.05	-6.05	-6.05	-6.05	-6.05	-6.05	-6.05
-6.04	-6.04	-6.04	-6.04	-6.04	-6.04	-6.04	-6.04	-6.04
-6.03	-6.03	-6.03	-6.03	-6.03	-6.03	-6.03	-6.03	-6.03
-6.02	-6.02	-6.02	-6.02	-6.02	-6.02	-6.02	-6.02	-6.02
-6.01	-6.01	-6.01	-6.01	-6.01	-6.01	-6.01	-6.01	-6.01

-6	-6	-6	-6	-6	-6	-6	-6	-6
-5.99	-5.99	-5.99	-5.99	-5.99	-5.99	-5.99	-5.99	-5.99
-5.98	-5.98	-5.98	-5.98	-5.98	-5.98	-5.98	-5.98	-5.98
-5.97	-5.97	-5.97	-5.97	-5.97	-5.97	-5.97	-5.97	-5.97
-5.96	-5.96	-5.96	-5.96	-5.96	-5.96	-5.96	-5.96	-5.96
-5.95	-5.95	-5.95	-5.95	-5.95	-5.95	-5.95	-5.95	-5.95
-5.94	-5.94	-5.94	-5.94	-5.94	-5.94	-5.94	-5.94	-5.94
-5.93	-5.93	-5.93	-5.93	-5.93	-5.93	-5.93	-5.93	-5.93
-5.92	-5.92	-5.92	-5.92	-5.92	-5.92	-5.92	-5.92	-5.92
-5.91	-5.91	-5.91	-5.91	-5.91	-5.91	-5.91	-5.91	-5.91
-5.9	-5.9	-5.9	-5.9	-5.9	-5.9	-5.9	-5.9	-5.9
-5.89	-5.89	-5.89	-5.89	-5.89	-5.89	-5.89	-5.89	-5.89
-5.88	-5.88	-5.88	-5.88	-5.88	-5.88	-5.88	-5.88	-5.88
-5.87	-5.87	-5.87	-5.87	-5.87	-5.87	-5.87	-5.87	-5.87
-5.86	-5.86	-5.86	-5.86	-5.86	-5.86	-5.86	-5.86	-5.86
-5.85	-5.85	-5.85	-5.85	-5.85	-5.85	-5.85	-5.85	-5.85
-5.84	-5.84	-5.84	-5.84	-5.84	-5.84	-5.84	-5.84	-5.84
-5.83	-5.83	-5.83	-5.83	-5.83	-5.83	-5.83	-5.83	-5.83
-5.82	-5.82	-5.82	-5.82	-5.82	-5.82	-5.82	-5.82	-5.82
-5.81	-5.81	-5.81	-5.81	-5.81	-5.81	-5.81	-5.81	-5.81
-5.8	-5.8	-5.8	-5.8	-5.8	-5.8	-5.8	-5.8	-5.8
-5.79	-5.79	-5.79	-5.79	-5.79	-5.79	-5.79	-5.79	-5.79
-5.78	-5.78	-5.78	-5.78	-5.78	-5.78	-5.78	-5.78	-5.78
-5.77	-5.77	-5.77	-5.77	-5.77	-5.77	-5.77	-5.77	-5.77
-5.76	-5.76	-5.76	-5.76	-5.76	-5.76	-5.76	-5.76	-5.76
-5.75	-5.75	-5.75	-5.75	-5.75	-5.75	-5.75	-5.75	-5.75
-5.74	-5.74	-5.74	-5.74	-5.74	-5.74	-5.74	-5.74	-5.74
-5.73	-5.73	-5.73	-5.73	-5.73	-5.73	-5.73	-5.73	-5.73
-5.72	-5.72	-5.72	-5.72	-5.72	-5.72	-5.72	-5.72	-5.72
-5.71	-5.71	-5.71	-5.71	-5.71	-5.71	-5.71	-5.71	-5.71
-5.7	-5.7	-5.7	-5.7	-5.7	-5.7	-5.7	-5.7	-5.7
-5.69	-5.69	-5.69	-5.69	-5.69	-5.69	-5.69	-5.69	-5.69
-5.68	-5.68	-5.68	-5.68	-5.68	-5.68	-5.68	-5.68	-5.68
-5.67	-5.67	-5.67	-5.67	-5.67	-5.67	-5.67	-5.67	-5.67
-5.66	-5.66	-5.66	-5.66	-5.66	-5.66	-5.66	-5.66	-5.66
-5.65	-5.65	-5.65	-5.65	-5.65	-5.65	-5.65	-5.65	-5.65
-5.64	-5.64	-5.64	-5.64	-5.64	-5.64	-5.64	-5.64	-5.64
-5.63	-5.63	-5.63	-5.63	-5.63	-5.63	-5.63	-5.63	-5.63
-5.62	-5.62	-5.62	-5.62	-5.62	-5.62	-5.62	-5.62	-5.62
-5.61	-5.61	-5.61	-5.61	-5.61	-5.61	-5.61	-5.61	-5.61
-5.6	-5.6	-5.6	-5.6	-5.6	-5.6	-5.6	-5.6	-5.6
-5.59	-5.59	-5.59	-5.59	-5.59	-5.59	-5.59	-5.59	-5.59
-5.58	-5.58	-5.58	-5.58	-5.58	-5.58	-5.58	-5.58	-5.58
-5.57	-5.57	-5.57	-5.57	-5.57	-5.57	-5.57	-5.57	-5.57
-5.56	-5.56	-5.56	-5.56	-5.56	-5.56	-5.56	-5.56	-5.56
-5.55	-5.55	-5.55	-5.55	-5.55	-5.55	-5.55	-5.55	-5.55
-5.54	-5.54	-5.54	-5.54	-5.54	-5.54	-5.54	-5.54	-5.54
-5.53	-5.53	-5.53	-5.53	-5.53	-5.53	-5.53	-5.53	-5.53
-5.52	-5.52	-5.52	-5.52	-5.52	-5.52	-5.52	-5.52	-5.52
-5.51	-5.51	-5.51	-5.51	-5.51	-5.51	-5.51	-5.51	-5.51

-5.5	-5.5	-5.5	-5.5	-5.5	-5.5	-5.5	-5.5	-5.5
-5.49	-5.49	-5.49	-5.49	-5.49	-5.49	-5.49	-5.49	-5.49
-5.48	-5.48	-5.48	-5.48	-5.48	-5.48	-5.48	-5.48	-5.48
-5.47	-5.47	-5.47	-5.47	-5.47	-5.47	-5.47	-5.47	-5.47
-5.46	-5.46	-5.46	-5.46	-5.46	-5.46	-5.46	-5.46	-5.46
-5.45	-5.45	-5.45	-5.45	-5.45	-5.45	-5.45	-5.45	-5.45
-5.44	-5.44	-5.44	-5.44	-5.44	-5.44	-5.44	-5.44	-5.44
-5.43	-5.43	-5.43	-5.43	-5.43	-5.43	-5.43	-5.43	-5.43
-5.42	-5.42	-5.42	-5.42	-5.42	-5.42	-5.42	-5.42	-5.42
-5.41	-5.41	-5.41	-5.41	-5.41	-5.41	-5.41	-5.41	-5.41
-5.4	-5.4	-5.4	-5.4	-5.4	-5.4	-5.4	-5.4	-5.4
-5.39	-5.39	-5.39	-5.39	-5.39	-5.39	-5.39	-5.39	-5.39
-5.38	-5.38	-5.38	-5.38	-5.38	-5.38	-5.38	-5.38	-5.38
-5.37	-5.37	-5.37	-5.37	-5.37	-5.37	-5.37	-5.37	-5.37
-5.36	-5.36	-5.36	-5.36	-5.36	-5.36	-5.36	-5.36	-5.36
-5.35	-5.35	-5.35	-5.35	-5.35	-5.35	-5.35	-5.35	-5.35
-5.34	-5.34	-5.34	-5.34	-5.34	-5.34	-5.34	-5.34	-5.34
-5.33	-5.33	-5.33	-5.33	-5.33	-5.33	-5.33	-5.33	-5.33
-5.32	-5.32	-5.32	-5.32	-5.32	-5.32	-5.32	-5.32	-5.32
-5.31	-5.31	-5.31	-5.31	-5.31	-5.31	-5.31	-5.31	-5.31
-5.3	-5.3	-5.3	-5.3	-5.3	-5.3	-5.3	-5.3	-5.3
-5.29	-5.29	-5.29	-5.29	-5.29	-5.29	-5.29	-5.29	-5.29
-5.28	-5.28	-5.28	-5.28	-5.28	-5.28	-5.28	-5.28	-5.28
-5.27	-5.27	-5.27	-5.27	-5.27	-5.27	-5.27	-5.27	-5.27
-5.26	-5.26	-5.26	-5.26	-5.26	-5.26	-5.26	-5.26	-5.26
-5.25	-5.25	-5.25	-5.25	-5.25	-5.25	-5.25	-5.25	-5.25
-5.24	-5.24	-5.24	-5.24	-5.24	-5.24	-5.24	-5.24	-5.24
-5.23	-5.23	-5.23	-5.23	-5.23	-5.23	-5.23	-5.23	-5.23
-5.22	-5.22	-5.22	-5.22	-5.22	-5.22	-5.22	-5.22	-5.22
-5.21	-5.21	-5.21	-5.21	-5.21	-5.21	-5.21	-5.21	-5.21
-5.2	-5.2	-5.2	-5.2	-5.2	-5.2	-5.2	-5.2	-5.2
-5.19	-5.19	-5.19	-5.19	-5.19	-5.19	-5.19	-5.19	-5.19
-5.18	-5.18	-5.18	-5.18	-5.18	-5.18	-5.18	-5.18	-5.18
-5.17	-5.17	-5.17	-5.17	-5.17	-5.17	-5.17	-5.17	-5.17
-5.16	-5.16	-5.16	-5.16	-5.16	-5.16	-5.16	-5.16	-5.16
-5.15	-5.15	-5.15	-5.15	-5.15	-5.15	-5.15	-5.15	-5.15
-5.14	-5.14	-5.14	-5.14	-5.14	-5.14	-5.14	-5.14	-5.14
-5.13	-5.13	-5.13	-5.13	-5.13	-5.13	-5.13	-5.13	-5.13
-5.12	-5.12	-5.12	-5.12	-5.12	-5.12	-5.12	-5.12	-5.12
-5.11	-5.11	-5.11	-5.11	-5.11	-5.11	-5.11	-5.11	-5.11
-5.1	-5.1	-5.1	-5.1	-5.1	-5.1	-5.1	-5.1	-5.1
-5.09	-5.09	-5.09	-5.09	-5.09	-5.09	-5.09	-5.09	-5.09
-5.08	-5.08	-5.08	-5.08	-5.08	-5.08	-5.08	-5.08	-5.08
-5.07	-5.07	-5.07	-5.07	-5.07	-5.07	-5.07	-5.07	-5.07
-5.06	-5.06	-5.06	-5.06	-5.06	-5.06	-5.06	-5.06	-5.06
-5.05	-5.05	-5.05	-5.05	-5.05	-5.05	-5.05	-5.05	-5.05
-5.04	-5.04	-5.04	-5.04	-5.04	-5.04	-5.04	-5.04	-5.04
-5.03	-5.03	-5.03	-5.03	-5.03	-5.03	-5.03	-5.03	-5.03
-5.02	-5.02	-5.02	-5.02	-5.02	-5.02	-5.02	-5.02	-5.02
-5.01	-5.01	-5.01	-5.01	-5.01	-5.01	-5.01	-5.01	-5.01

-5	-5	-5	-5	-5	-5	-5	-5	-5
-4.99	-4.99	-4.99	-4.99	-4.99	-4.99	-4.99	-4.99	-4.99
-4.98	-4.98	-4.98	-4.98	-4.98	-4.98	-4.98	-4.98	-4.98
-4.97	-4.97	-4.97	-4.97	-4.97	-4.97	-4.97	-4.97	-4.97
-4.96	-4.96	-4.96	-4.96	-4.96	-4.96	-4.96	-4.96	-4.96
-4.95	-4.95	-4.95	-4.95	-4.95	-4.95	-4.95	-4.95	-4.95
-4.94	-4.94	-4.94	-4.94	-4.94	-4.94	-4.94	-4.94	-4.94
-4.93	-4.93	-4.93	-4.93	-4.93	-4.93	-4.93	-4.93	-4.93
-4.92	-4.92	-4.92	-4.92	-4.92	-4.92	-4.92	-4.92	-4.92
-4.91	-4.91	-4.91	-4.91	-4.91	-4.91	-4.91	-4.91	-4.91
-4.9	-4.9	-4.9	-4.9	-4.9	-4.9	-4.9	-4.9	-4.9
-4.89	-4.89	-4.89	-4.89	-4.89	-4.89	-4.89	-4.89	-4.89
-4.88	-4.88	-4.88	-4.88	-4.88	-4.88	-4.88	-4.88	-4.88
-4.87	-4.87	-4.87	-4.87	-4.87	-4.87	-4.87	-4.87	-4.87
-4.86	-4.86	-4.86	-4.86	-4.86	-4.86	-4.86	-4.86	-4.86
-4.85	-4.85	-4.85	-4.85	-4.85	-4.85	-4.85	-4.85	-4.85
-4.84	-4.84	-4.84	-4.84	-4.84	-4.84	-4.84	-4.84	-4.84
-4.83	-4.83	-4.83	-4.83	-4.83	-4.83	-4.83	-4.83	-4.83
-4.82	-4.82	-4.82	-4.82	-4.82	-4.82	-4.82	-4.82	-4.82
-4.81	-4.81	-4.81	-4.81	-4.81	-4.81	-4.81	-4.81	-4.81
-4.8	-4.8	-4.8	-4.8	-4.8	-4.8	-4.8	-4.8	-4.8
-4.79	-4.79	-4.79	-4.79	-4.79	-4.79	-4.79	-4.79	-4.79
-4.78	-4.78	-4.78	-4.78	-4.78	-4.78	-4.78	-4.78	-4.78
-4.77	-4.77	-4.77	-4.77	-4.77	-4.77	-4.77	-4.77	-4.77
-4.76	-4.76	-4.76	-4.76	-4.76	-4.76	-4.76	-4.76	-4.76
-4.75	-4.75	-4.75	-4.75	-4.75	-4.75	-4.75	-4.75	-4.75
-4.74	-4.74	-4.74	-4.74	-4.74	-4.74	-4.74	-4.74	-4.74
-4.73	-4.73	-4.73	-4.73	-4.73	-4.73	-4.73	-4.73	-4.73
-4.72	-4.72	-4.72	-4.72	-4.72	-4.72	-4.72	-4.72	-4.72
-4.71	-4.71	-4.71	-4.71	-4.71	-4.71	-4.71	-4.71	-4.71
-4.7	-4.7	-4.7	-4.7	-4.7	-4.7	-4.7	-4.7	-4.7
-4.69	-4.69	-4.69	-4.69	-4.69	-4.69	-4.69	-4.69	-4.69
-4.68	-4.68	-4.68	-4.68	-4.68	-4.68	-4.68	-4.68	-4.68
-4.67	-4.67	-4.67	-4.67	-4.67	-4.67	-4.67	-4.67	-4.67
-4.66	-4.66	-4.66	-4.66	-4.66	-4.66	-4.66	-4.66	-4.66
-4.65	-4.65	-4.65	-4.65	-4.65	-4.65	-4.65	-4.65	-4.65
-4.64	-4.64	-4.64	-4.64	-4.64	-4.64	-4.64	-4.64	-4.64
-4.63	-4.63	-4.63	-4.63	-4.63	-4.63	-4.63	-4.63	-4.63
-4.62	-4.62	-4.62	-4.62	-4.62	-4.62	-4.62	-4.62	-4.62
-4.61	-4.61	-4.61	-4.61	-4.61	-4.61	-4.61	-4.61	-4.61
-4.6	-4.6	-4.6	-4.6	-4.6	-4.6	-4.6	-4.6	-4.6
-4.59	-4.59	-4.59	-4.59	-4.59	-4.59	-4.59	-4.59	-4.59
-4.58	-4.58	-4.58	-4.58	-4.58	-4.58	-4.58	-4.58	-4.58
-4.57	-4.57	-4.57	-4.57	-4.57	-4.57	-4.57	-4.57	-4.57
-4.56	-4.56	-4.56	-4.56	-4.56	-4.56	-4.56	-4.56	-4.56
-4.55	-4.55	-4.55	-4.55	-4.55	-4.55	-4.55	-4.55	-4.55
-4.54	-4.54	-4.54	-4.54	-4.54	-4.54	-4.54	-4.54	-4.54
-4.53	-4.53	-4.53	-4.53	-4.53	-4.53	-4.53	-4.53	-4.53
-4.52	-4.52	-4.52	-4.52	-4.52	-4.52	-4.52	-4.52	-4.52
-4.51	-4.51	-4.51	-4.51	-4.51	-4.51	-4.51	-4.51	-4.51

-4.5	-4.5	-4.5	-4.5	-4.5	-4.5	-4.5	-4.5	-4.5
-4.49	-4.49	-4.49	-4.49	-4.49	-4.49	-4.49	-4.49	-4.49
-4.48	-4.48	-4.48	-4.48	-4.48	-4.48	-4.48	-4.48	-4.48
-4.47	-4.47	-4.47	-4.47	-4.47	-4.47	-4.47	-4.47	-4.47
-4.46	-4.46	-4.46	-4.46	-4.46	-4.46	-4.46	-4.46	-4.46
-4.45	-4.45	-4.45	-4.45	-4.45	-4.45	-4.45	-4.45	-4.45
-4.44	-4.44	-4.44	-4.44	-4.44	-4.44	-4.44	-4.44	-4.44
-4.43	-4.43	-4.43	-4.43	-4.43	-4.43	-4.43	-4.43	-4.43
-4.42	-4.42	-4.42	-4.42	-4.42	-4.42	-4.42	-4.42	-4.42
-4.41	-4.41	-4.41	-4.41	-4.41	-4.41	-4.41	-4.41	-4.41
-4.4	-4.4	-4.4	-4.4	-4.4	-4.4	-4.4	-4.4	-4.4
-4.39	-4.39	-4.39	-4.39	-4.39	-4.39	-4.39	-4.39	-4.39
-4.38	-4.38	-4.38	-4.38	-4.38	-4.38	-4.38	-4.38	-4.38
-4.37	-4.37	-4.37	-4.37	-4.37	-4.37	-4.37	-4.37	-4.37
-4.36	-4.36	-4.36	-4.36	-4.36	-4.36	-4.36	-4.36	-4.36
-4.35	-4.35	-4.35	-4.35	-4.35	-4.35	-4.35	-4.35	-4.35
-4.34	-4.34	-4.34	-4.34	-4.34	-4.34	-4.34	-4.34	-4.34
-4.33	-4.33	-4.33	-4.33	-4.33	-4.33	-4.33	-4.33	-4.33
-4.32	-4.32	-4.32	-4.32	-4.32	-4.32	-4.32	-4.32	-4.32
-4.31	-4.31	-4.31	-4.31	-4.31	-4.31	-4.31	-4.31	-4.31
-4.3	-4.3	-4.3	-4.3	-4.3	-4.3	-4.3	-4.3	-4.3
-4.29	-4.29	-4.29	-4.29	-4.29	-4.29	-4.29	-4.29	-4.29
-4.28	-4.28	-4.28	-4.28	-4.28	-4.28	-4.28	-4.28	-4.28
-4.27	-4.27	-4.27	-4.27	-4.27	-4.27	-4.27	-4.27	-4.27
-4.26	-4.26	-4.26	-4.26	-4.26	-4.26	-4.26	-4.26	-4.26
-4.25	-4.25	-4.25	-4.25	-4.25	-4.25	-4.25	-4.25	-4.25
-4.24	-4.24	-4.24	-4.24	-4.24	-4.24	-4.24	-4.24	-4.24
-4.23	-4.23	-4.23	-4.23	-4.23	-4.23	-4.23	-4.23	-4.23
-4.22	-4.22	-4.22	-4.22	-4.22	-4.22	-4.22	-4.22	-4.22
-4.21	-4.21	-4.21	-4.21	-4.21	-4.21	-4.21	-4.21	-4.21
-4.2	-4.2	-4.2	-4.2	-4.2	-4.2	-4.2	-4.2	-4.2
-4.19	-4.19	-4.19	-4.19	-4.19	-4.19	-4.19	-4.19	-4.19
-4.18	-4.18	-4.18	-4.18	-4.18	-4.18	-4.18	-4.18	-4.18
-4.17	-4.17	-4.17	-4.17	-4.17	-4.17	-4.17	-4.17	-4.17
-4.16	-4.16	-4.16	-4.16	-4.16	-4.16	-4.16	-4.16	-4.16
-4.15	-4.15	-4.15	-4.15	-4.15	-4.15	-4.15	-4.15	-4.15
-4.14	-4.14	-4.14	-4.14	-4.14	-4.14	-4.14	-4.14	-4.14
-4.13	-4.13	-4.13	-4.13	-4.13	-4.13	-4.13	-4.13	-4.13
-4.12	-4.12	-4.12	-4.12	-4.12	-4.12	-4.12	-4.12	-4.12
-4.11	-4.11	-4.11	-4.11	-4.11	-4.11	-4.11	-4.11	-4.11
-4.1	-4.1	-4.1	-4.1	-4.1	-4.1	-4.1	-4.1	-4.1
-4.09	-4.09	-4.09	-4.09	-4.09	-4.09	-4.09	-4.09	-4.09
-4.08	-4.08	-4.08	-4.08	-4.08	-4.08	-4.08	-4.08	-4.08
-4.07	-4.07	-4.07	-4.07	-4.07	-4.07	-4.07	-4.07	-4.07
-4.06	-4.06	-4.06	-4.06	-4.06	-4.06	-4.06	-4.06	-4.06
-4.05	-4.05	-4.05	-4.05	-4.05	-4.05	-4.05	-4.05	-4.05
-4.04	-4.04	-4.04	-4.04	-4.04	-4.04	-4.04	-4.04	-4.04
-4.03	-4.03	-4.03	-4.03	-4.03	-4.03	-4.03	-4.03	-4.03
-4.02	-4.02	-4.02	-4.02	-4.02	-4.02	-4.02	-4.02	-4.02
-4.01	-4.01	-4.01	-4.01	-4.01	-4.01	-4.01	-4.01	-4.01

-4	-4	-4	-4	-4	-4	-4	-4	-4
-3.99	-3.99	-3.99	-3.99	-3.99	-3.99	-3.99	-3.99	-3.99
-3.98	-3.98	-3.98	-3.98	-3.98	-3.98	-3.98	-3.98	-3.98
-3.97	-3.97	-3.97	-3.97	-3.97	-3.97	-3.97	-3.97	-3.97
-3.96	-3.96	-3.96	-3.96	-3.96	-3.96	-3.96	-3.96	-3.96
-3.95	-3.95	-3.95	-3.95	-3.95	-3.95	-3.95	-3.95	-3.95
-3.94	-3.94	-3.94	-3.94	-3.94	-3.94	-3.94	-3.94	-3.94
-3.93	-3.93	-3.93	-3.93	-3.93	-3.93	-3.93	-3.93	-3.93
-3.92	-3.92	-3.92	-3.92	-3.92	-3.92	-3.92	-3.92	-3.92
-3.91	-3.91	-3.91	-3.91	-3.91	-3.91	-3.91	-3.91	-3.91
-3.9	-3.9	-3.9	-3.9	-3.9	-3.9	-3.9	-3.9	-3.9
-3.89	-3.89	-3.89	-3.89	-3.89	-3.89	-3.89	-3.89	-3.89
-3.88	-3.88	-3.88	-3.88	-3.88	-3.88	-3.88	-3.88	-3.88
-3.87	-3.87	-3.87	-3.87	-3.87	-3.87	-3.87	-3.87	-3.87
-3.86	-3.86	-3.86	-3.86	-3.86	-3.86	-3.86	-3.86	-3.86
-3.85	-3.85	-3.85	-3.85	-3.85	-3.85	-3.85	-3.85	-3.85
-3.84	-3.84	-3.84	-3.84	-3.84	-3.84	-3.84	-3.84	-3.84
-3.83	-3.83	-3.83	-3.83	-3.83	-3.83	-3.83	-3.83	-3.83
-3.82	-3.82	-3.82	-3.82	-3.82	-3.82	-3.82	-3.82	-3.82
-3.81	-3.81	-3.81	-3.81	-3.81	-3.81	-3.81	-3.81	-3.81
-3.8	-3.8	-3.8	-3.8	-3.8	-3.8	-3.8	-3.8	-3.8
-3.79	-3.79	-3.79	-3.79	-3.79	-3.79	-3.79	-3.79	-3.79
-3.78	-3.78	-3.78	-3.78	-3.78	-3.78	-3.78	-3.78	-3.78
-3.77	-3.77	-3.77	-3.77	-3.77	-3.77	-3.77	-3.77	-3.77
-3.76	-3.76	-3.76	-3.76	-3.76	-3.76	-3.76	-3.76	-3.76
-3.75	-3.75	-3.75	-3.75	-3.75	-3.75	-3.75	-3.75	-3.75
-3.74	-3.74	-3.74	-3.74	-3.74	-3.74	-3.74	-3.74	-3.74
-3.73	-3.73	-3.73	-3.73	-3.73	-3.73	-3.73	-3.73	-3.73
-3.72	-3.72	-3.72	-3.72	-3.72	-3.72	-3.72	-3.72	-3.72
-3.71	-3.71	-3.71	-3.71	-3.71	-3.71	-3.71	-3.71	-3.71
-3.7	-3.7	-3.7	-3.7	-3.7	-3.7	-3.7	-3.7	-3.7
-3.69	-3.69	-3.69	-3.69	-3.69	-3.69	-3.69	-3.69	-3.69
-3.68	-3.68	-3.68	-3.68	-3.68	-3.68	-3.68	-3.68	-3.68
-3.67	-3.67	-3.67	-3.67	-3.67	-3.67	-3.67	-3.67	-3.67
-3.66	-3.66	-3.66	-3.66	-3.66	-3.66	-3.66	-3.66	-3.66
-3.65	-3.65	-3.65	-3.65	-3.65	-3.65	-3.65	-3.65	-3.65
-3.64	-3.64	-3.64	-3.64	-3.64	-3.64	-3.64	-3.64	-3.64
-3.63	-3.63	-3.63	-3.63	-3.63	-3.63	-3.63	-3.63	-3.63
-3.62	-3.62	-3.62	-3.62	-3.62	-3.62	-3.62	-3.62	-3.62
-3.61	-3.61	-3.61	-3.61	-3.61	-3.61	-3.61	-3.61	-3.61
-3.6	-3.6	-3.6	-3.6	-3.6	-3.6	-3.6	-3.6	-3.6
-3.59	-3.59	-3.59	-3.59	-3.59	-3.59	-3.59	-3.59	-3.59
-3.58	-3.58	-3.58	-3.58	-3.58	-3.58	-3.58	-3.58	-3.58
-3.57	-3.57	-3.57	-3.57	-3.57	-3.57	-3.57	-3.57	-3.57
-3.56	-3.56	-3.56	-3.56	-3.56	-3.56	-3.56	-3.56	-3.56
-3.55	-3.55	-3.55	-3.55	-3.55	-3.55	-3.55	-3.55	-3.55
-3.54	-3.54	-3.54	-3.54	-3.54	-3.54	-3.54	-3.54	-3.54
-3.53	-3.53	-3.53	-3.53	-3.53	-3.53	-3.53	-3.53	-3.53
-3.52	-3.52	-3.52	-3.52	-3.52	-3.52	-3.52	-3.52	-3.52
-3.51	-3.51	-3.51	-3.51	-3.51	-3.51	-3.51	-3.51	-3.51

-3.5	-3.5	-3.5	-3.5	-3.5	-3.5	-3.5	-3.5	-3.5
-3.49	-3.49	-3.49	-3.49	-3.49	-3.49	-3.49	-3.49	-3.49
-3.48	-3.48	-3.48	-3.48	-3.48	-3.48	-3.48	-3.48	-3.48
-3.47	-3.47	-3.47	-3.47	-3.47	-3.47	-3.47	-3.47	-3.47
-3.46	-3.46	-3.46	-3.46	-3.46	-3.46	-3.46	-3.46	-3.46
-3.45	-3.45	-3.45	-3.45	-3.45	-3.45	-3.45	-3.45	-3.45
-3.44	-3.44	-3.44	-3.44	-3.44	-3.44	-3.44	-3.44	-3.44
-3.43	-3.43	-3.43	-3.43	-3.43	-3.43	-3.43	-3.43	-3.43
-3.42	-3.42	-3.42	-3.42	-3.42	-3.42	-3.42	-3.42	-3.42
-3.41	-3.41	-3.41	-3.41	-3.41	-3.41	-3.41	-3.41	-3.41
-3.4	-3.4	-3.4	-3.4	-3.4	-3.4	-3.4	-3.4	-3.4
-3.39	-3.39	-3.39	-3.39	-3.39	-3.39	-3.39	-3.39	-3.39
-3.38	-3.38	-3.38	-3.38	-3.38	-3.38	-3.38	-3.38	-3.38
-3.37	-3.37	-3.37	-3.37	-3.37	-3.37	-3.37	-3.37	-3.37
-3.36	-3.36	-3.36	-3.36	-3.36	-3.36	-3.36	-3.36	-3.36
-3.35	-3.35	-3.35	-3.35	-3.35	-3.35	-3.35	-3.35	-3.35
-3.34	-3.34	-3.34	-3.34	-3.34	-3.34	-3.34	-3.34	-3.34
-3.33	-3.33	-3.33	-3.33	-3.33	-3.33	-3.33	-3.33	-3.33
-3.32	-3.32	-3.32	-3.32	-3.32	-3.32	-3.32	-3.32	-3.32
-3.31	-3.31	-3.31	-3.31	-3.31	-3.31	-3.31	-3.31	-3.31
-3.3	-3.3	-3.3	-3.3	-3.3	-3.3	-3.3	-3.3	-3.3
-3.29	-3.29	-3.29	-3.29	-3.29	-3.29	-3.29	-3.29	-3.29
-3.28	-3.28	-3.28	-3.28	-3.28	-3.28	-3.28	-3.28	-3.28
-3.27	-3.27	-3.27	-3.27	-3.27	-3.27	-3.27	-3.27	-3.27
-3.26	-3.26	-3.26	-3.26	-3.26	-3.26	-3.26	-3.26	-3.26
-3.25	-3.25	-3.25	-3.25	-3.25	-3.25	-3.25	-3.25	-3.25
-3.24	-3.24	-3.24	-3.24	-3.24	-3.24	-3.24	-3.24	-3.24
-3.23	-3.23	-3.23	-3.23	-3.23	-3.23	-3.23	-3.23	-3.23
-3.22	-3.22	-3.22	-3.22	-3.22	-3.22	-3.22	-3.22	-3.22
-3.21	-3.21	-3.21	-3.21	-3.21	-3.21	-3.21	-3.21	-3.21
-3.2	-3.2	-3.2	-3.2	-3.2	-3.2	-3.2	-3.2	-3.2
-3.19	-3.19	-3.19	-3.19	-3.19	-3.19	-3.19	-3.19	-3.19
-3.18	-3.18	-3.18	-3.18	-3.18	-3.18	-3.18	-3.18	-3.18
-3.17	-3.17	-3.17	-3.17	-3.17	-3.17	-3.17	-3.17	-3.17
-3.16	-3.16	-3.16	-3.16	-3.16	-3.16	-3.16	-3.16	-3.16
-3.15	-3.15	-3.15	-3.15	-3.15	-3.15	-3.15	-3.15	-3.15
-3.14	-3.14	-3.14	-3.14	-3.14	-3.14	-3.14	-3.14	-3.14
-3.13	-3.13	-3.13	-3.13	-3.13	-3.13	-3.13	-3.13	-3.13
-3.12	-3.12	-3.12	-3.12	-3.12	-3.12	-3.12	-3.12	-3.12
-3.11	-3.11	-3.11	-3.11	-3.11	-3.11	-3.11	-3.11	-3.11
-3.1	-3.1	-3.1	-3.1	-3.1	-3.1	-3.1	-3.1	-3.1
-3.09	-3.09	-3.09	-3.09	-3.09	-3.09	-3.09	-3.09	-3.09
-3.08	-3.08	-3.08	-3.08	-3.08	-3.08	-3.08	-3.08	-3.08
-3.07	-3.07	-3.07	-3.07	-3.07	-3.07	-3.07	-3.07	-3.07
-3.06	-3.06	-3.06	-3.06	-3.06	-3.06	-3.06	-3.06	-3.06
-3.05	-3.05	-3.05	-3.05	-3.05	-3.05	-3.05	-3.05	-3.05
-3.04	-3.04	-3.04	-3.04	-3.04	-3.04	-3.04	-3.04	-3.04
-3.03	-3.03	-3.03	-3.03	-3.03	-3.03	-3.03	-3.03	-3.03
-3.02	-3.02	-3.02	-3.02	-3.02	-3.02	-3.02	-3.02	-3.02
-3.01	-3.01	-3.01	-3.01	-3.01	-3.01	-3.01	-3.01	-3.01

-3	-3	-3	-3	-3	-3	-3	-3	-3
-2.99	-2.99	-2.99	-2.99	-2.99	-2.99	-2.99	-2.99	-2.99
-2.98	-2.98	-2.98	-2.98	-2.98	-2.98	-2.98	-2.98	-2.98
-2.97	-2.97	-2.97	-2.97	-2.97	-2.97	-2.97	-2.97	-2.97
-2.96	-2.96	-2.96	-2.96	-2.96	-2.96	-2.96	-2.96	-2.96
-2.95	-2.95	-2.95	-2.95	-2.95	-2.95	-2.95	-2.95	-2.95
-2.94	-2.94	-2.94	-2.94	-2.94	-2.94	-2.94	-2.94	-2.94
-2.93	-2.93	-2.93	-2.93	-2.93	-2.93	-2.93	-2.93	-2.93
-2.92	-2.92	-2.92	-2.92	-2.92	-2.92	-2.92	-2.92	-2.92
-2.91	-2.91	-2.91	-2.91	-2.91	-2.91	-2.91	-2.91	-2.91
-2.9	-2.9	-2.9	-2.9	-2.9	-2.9	-2.9	-2.9	-2.9
-2.89	-2.89	-2.89	-2.89	-2.89	-2.89	-2.89	-2.89	-2.89
-2.88	-2.88	-2.88	-2.88	-2.88	-2.88	-2.88	-2.88	-2.88
-2.87	-2.87	-2.87	-2.87	-2.87	-2.87	-2.87	-2.87	-2.87
-2.86	-2.86	-2.86	-2.86	-2.86	-2.86	-2.86	-2.86	-2.86
-2.85	-2.85	-2.85	-2.85	-2.85	-2.85	-2.85	-2.85	-2.85
-2.84	-2.84	-2.84	-2.84	-2.84	-2.84	-2.84	-2.84	-2.84
-2.83	-2.83	-2.83	-2.83	-2.83	-2.83	-2.83	-2.83	-2.83
-2.82	-2.82	-2.82	-2.82	-2.82	-2.82	-2.82	-2.82	-2.82
-2.81	-2.81	-2.81	-2.81	-2.81	-2.81	-2.81	-2.81	-2.81
-2.8	-2.8	-2.8	-2.8	-2.8	-2.8	-2.8	-2.8	-2.8
-2.79	-2.79	-2.79	-2.79	-2.79	-2.79	-2.79	-2.79	-2.79
-2.78	-2.78	-2.78	-2.78	-2.78	-2.78	-2.78	-2.78	-2.78
-2.77	-2.77	-2.77	-2.77	-2.77	-2.77	-2.77	-2.77	-2.77
-2.76	-2.76	-2.76	-2.76	-2.76	-2.76	-2.76	-2.76	-2.76
-2.75	-2.75	-2.75	-2.75	-2.75	-2.75	-2.75	-2.75	-2.75
-2.74	-2.74	-2.74	-2.74	-2.74	-2.74	-2.74	-2.74	-2.74
-2.73	-2.73	-2.73	-2.73	-2.73	-2.73	-2.73	-2.73	-2.73
-2.72	-2.72	-2.72	-2.72	-2.72	-2.72	-2.72	-2.72	-2.72
-2.71	-2.71	-2.71	-2.71	-2.71	-2.71	-2.71	-2.71	-2.71
-2.7	-2.7	-2.7	-2.7	-2.7	-2.7	-2.7	-2.7	-2.7
-2.69	-2.69	-2.69	-2.69	-2.69	-2.69	-2.69	-2.69	-2.69
-2.68	-2.68	-2.68	-2.68	-2.68	-2.68	-2.68	-2.68	-2.68
-2.67	-2.67	-2.67	-2.67	-2.67	-2.67	-2.67	-2.67	-2.67
-2.66	-2.66	-2.66	-2.66	-2.66	-2.66	-2.66	-2.66	-2.66
-2.65	-2.65	-2.65	-2.65	-2.65	-2.65	-2.65	-2.65	-2.65
-2.64	-2.64	-2.64	-2.64	-2.64	-2.64	-2.64	-2.64	-2.64
-2.63	-2.63	-2.63	-2.63	-2.63	-2.63	-2.63	-2.63	-2.63
-2.62	-2.62	-2.62	-2.62	-2.62	-2.62	-2.62	-2.62	-2.62
-2.61	-2.61	-2.61	-2.61	-2.61	-2.61	-2.61	-2.61	-2.61
-2.6	-2.6	-2.6	-2.6	-2.6	-2.6	-2.6	-2.6	-2.6
-2.59	-2.59	-2.59	-2.59	-2.59	-2.59	-2.59	-2.59	-2.59
-2.58	-2.58	-2.58	-2.58	-2.58	-2.58	-2.58	-2.58	-2.58
-2.57	-2.57	-2.57	-2.57	-2.57	-2.57	-2.57	-2.57	-2.57
-2.56	-2.56	-2.56	-2.56	-2.56	-2.56	-2.56	-2.56	-2.56
-2.55	-2.55	-2.55	-2.55	-2.55	-2.55	-2.55	-2.55	-2.55
-2.54	-2.54	-2.54	-2.54	-2.54	-2.54	-2.54	-2.54	-2.54
-2.53	-2.53	-2.53	-2.53	-2.53	-2.53	-2.53	-2.53	-2.53
-2.52	-2.52	-2.52	-2.52	-2.52	-2.52	-2.52	-2.52	-2.52
-2.51	-2.51	-2.51	-2.51	-2.51	-2.51	-2.51	-2.51	-2.51

-2.5	-2.5	-2.5	-2.5	-2.5	-2.5	-2.5	-2.5	-2.5
-2.49	-2.49	-2.49	-2.49	-2.49	-2.49	-2.49	-2.49	-2.49
-2.48	-2.48	-2.48	-2.48	-2.48	-2.48	-2.48	-2.48	-2.48
-2.47	-2.47	-2.47	-2.47	-2.47	-2.47	-2.47	-2.47	-2.47
-2.46	-2.46	-2.46	-2.46	-2.46	-2.46	-2.46	-2.46	-2.46
-2.45	-2.45	-2.45	-2.45	-2.45	-2.45	-2.45	-2.45	-2.45
-2.44	-2.44	-2.44	-2.44	-2.44	-2.44	-2.44	-2.44	-2.44
-2.43	-2.43	-2.43	-2.43	-2.43	-2.43	-2.43	-2.43	-2.43
-2.42	-2.42	-2.42	-2.42	-2.42	-2.42	-2.42	-2.42	-2.42
-2.41	-2.41	-2.41	-2.41	-2.41	-2.41	-2.41	-2.41	-2.41
-2.4	-2.4	-2.4	-2.4	-2.4	-2.4	-2.4	-2.4	-2.4
-2.39	-2.39	-2.39	-2.39	-2.39	-2.39	-2.39	-2.39	-2.39
-2.38	-2.38	-2.38	-2.38	-2.38	-2.38	-2.38	-2.38	-2.38
-2.37	-2.37	-2.37	-2.37	-2.37	-2.37	-2.37	-2.37	-2.37
-2.36	-2.36	-2.36	-2.36	-2.36	-2.36	-2.36	-2.36	-2.36
-2.35	-2.35	-2.35	-2.35	-2.35	-2.35	-2.35	-2.35	-2.35
-2.34	-2.34	-2.34	-2.34	-2.34	-2.34	-2.34	-2.34	-2.34
-2.33	-2.33	-2.33	-2.33	-2.33	-2.33	-2.33	-2.33	-2.33
-2.32	-2.32	-2.32	-2.32	-2.32	-2.32	-2.32	-2.32	-2.32
-2.31	-2.31	-2.31	-2.31	-2.31	-2.31	-2.31	-2.31	-2.31
-2.3	-2.3	-2.3	-2.3	-2.3	-2.3	-2.3	-2.3	-2.3
-2.29	-2.29	-2.29	-2.29	-2.29	-2.29	-2.29	-2.29	-2.29
-2.28	-2.28	-2.28	-2.28	-2.28	-2.28	-2.28	-2.28	-2.28
-2.27	-2.27	-2.27	-2.27	-2.27	-2.27	-2.27	-2.27	-2.27
-2.26	-2.26	-2.26	-2.26	-2.26	-2.26	-2.26	-2.26	-2.26
-2.25	-2.25	-2.25	-2.25	-2.25	-2.25	-2.25	-2.25	-2.25
-2.24	-2.24	-2.24	-2.24	-2.24	-2.24	-2.24	-2.24	-2.24
-2.23	-2.23	-2.23	-2.23	-2.23	-2.23	-2.23	-2.23	-2.23
-2.22	-2.22	-2.22	-2.22	-2.22	-2.22	-2.22	-2.22	-2.22
-2.21	-2.21	-2.21	-2.21	-2.21	-2.21	-2.21	-2.21	-2.21
-2.2	-2.2	-2.2	-2.2	-2.2	-2.2	-2.2	-2.2	-2.2
-2.19	-2.19	-2.19	-2.19	-2.19	-2.19	-2.19	-2.19	-2.19
-2.18	-2.18	-2.18	-2.18	-2.18	-2.18	-2.18	-2.18	-2.18
-2.17	-2.17	-2.17	-2.17	-2.17	-2.17	-2.17	-2.17	-2.17
-2.16	-2.16	-2.16	-2.16	-2.16	-2.16	-2.16	-2.16	-2.16
-2.15	-2.15	-2.15	-2.15	-2.15	-2.15	-2.15	-2.15	-2.15
-2.14	-2.14	-2.14	-2.14	-2.14	-2.14	-2.14	-2.14	-2.14
-2.13	-2.13	-2.13	-2.13	-2.13	-2.13	-2.13	-2.13	-2.13
-2.12	-2.12	-2.12	-2.12	-2.12	-2.12	-2.12	-2.12	-2.12
-2.11	-2.11	-2.11	-2.11	-2.11	-2.11	-2.11	-2.11	-2.11
-2.1	-2.1	-2.1	-2.1	-2.1	-2.1	-2.1	-2.1	-2.1
-2.09	-2.09	-2.09	-2.09	-2.09	-2.09	-2.09	-2.09	-2.09
-2.08	-2.08	-2.08	-2.08	-2.08	-2.08	-2.08	-2.08	-2.08
-2.07	-2.07	-2.07	-2.07	-2.07	-2.07	-2.07	-2.07	-2.07
-2.06	-2.06	-2.06	-2.06	-2.06	-2.06	-2.06	-2.06	-2.06
-2.05	-2.05	-2.05	-2.05	-2.05	-2.05	-2.05	-2.05	-2.05
-2.04	-2.04	-2.04	-2.04	-2.04	-2.04	-2.04	-2.04	-2.04
-2.03	-2.03	-2.03	-2.03	-2.03	-2.03	-2.03	-2.03	-2.03
-2.02	-2.02	-2.02	-2.02	-2.02	-2.02	-2.02	-2.02	-2.02
-2.01	-2.01	-2.01	-2.01	-2.01	-2.01	-2.01	-2.01	-2.01

-2	-2	-2	-2	-2	-2	-2	-2	-2
-1.99	-1.99	-1.99	-1.99	-1.99	-1.99	-1.99	-1.99	-1.99
-1.98	-1.98	-1.98	-1.98	-1.98	-1.98	-1.98	-1.98	-1.98
-1.97	-1.97	-1.97	-1.97	-1.97	-1.97	-1.97	-1.97	-1.97
-1.96	-1.96	-1.96	-1.96	-1.96	-1.96	-1.96	-1.96	-1.96
-1.95	-1.95	-1.95	-1.95	-1.95	-1.95	-1.95	-1.95	-1.95
-1.94	-1.94	-1.94	-1.94	-1.94	-1.94	-1.94	-1.94	-1.94
-1.93	-1.93	-1.93	-1.93	-1.93	-1.93	-1.93	-1.93	-1.93
-1.92	-1.92	-1.92	-1.92	-1.92	-1.92	-1.92	-1.92	-1.92
-1.91	-1.91	-1.91	-1.91	-1.91	-1.91	-1.91	-1.91	-1.91
-1.9	-1.9	-1.9	-1.9	-1.9	-1.9	-1.9	-1.9	-1.9
-1.89	-1.89	-1.89	-1.89	-1.89	-1.89	-1.89	-1.89	-1.89
-1.88	-1.88	-1.88	-1.88	-1.88	-1.88	-1.88	-1.88	-1.88
-1.87	-1.87	-1.87	-1.87	-1.87	-1.87	-1.87	-1.87	-1.87
-1.86	-1.86	-1.86	-1.86	-1.86	-1.86	-1.86	-1.86	-1.86
-1.85	-1.85	-1.85	-1.85	-1.85	-1.85	-1.85	-1.85	-1.85
-1.84	-1.84	-1.84	-1.84	-1.84	-1.84	-1.84	-1.84	-1.84
-1.83	-1.83	-1.83	-1.83	-1.83	-1.83	-1.83	-1.83	-1.83
-1.82	-1.82	-1.82	-1.82	-1.82	-1.82	-1.82	-1.82	-1.82
-1.81	-1.81	-1.81	-1.81	-1.81	-1.81	-1.81	-1.81	-1.81
-1.8	-1.8	-1.8	-1.8	-1.8	-1.8	-1.8	-1.8	-1.8
-1.79	-1.79	-1.79	-1.79	-1.79	-1.79	-1.79	-1.79	-1.79
-1.78	-1.78	-1.78	-1.78	-1.78	-1.78	-1.78	-1.78	-1.78
-1.77	-1.77	-1.77	-1.77	-1.77	-1.77	-1.77	-1.77	-1.77
-1.76	-1.76	-1.76	-1.76	-1.76	-1.76	-1.76	-1.76	-1.76
-1.75	-1.75	-1.75	-1.75	-1.75	-1.75	-1.75	-1.75	-1.75
-1.74	-1.74	-1.74	-1.74	-1.74	-1.74	-1.74	-1.74	-1.74
-1.73	-1.73	-1.73	-1.73	-1.73	-1.73	-1.73	-1.73	-1.73
-1.72	-1.72	-1.72	-1.72	-1.72	-1.72	-1.72	-1.72	-1.72
-1.71	-1.71	-1.71	-1.71	-1.71	-1.71	-1.71	-1.71	-1.71
-1.7	-1.7	-1.7	-1.7	-1.7	-1.7	-1.7	-1.7	-1.7
-1.69	-1.69	-1.69	-1.69	-1.69	-1.69	-1.69	-1.69	-1.69
-1.68	-1.68	-1.68	-1.68	-1.68	-1.68	-1.68	-1.68	-1.68
-1.67	-1.67	-1.67	-1.67	-1.67	-1.67	-1.67	-1.67	-1.67
-1.66	-1.66	-1.66	-1.66	-1.66	-1.66	-1.66	-1.66	-1.66
-1.65	-1.65	-1.65	-1.65	-1.65	-1.65	-1.65	-1.65	-1.65
-1.64	-1.64	-1.64	-1.64	-1.64	-1.64	-1.64	-1.64	-1.64
-1.63	-1.63	-1.63	-1.63	-1.63	-1.63	-1.63	-1.63	-1.63
-1.62	-1.62	-1.62	-1.62	-1.62	-1.62	-1.62	-1.62	-1.62
-1.61	-1.61	-1.61	-1.61	-1.61	-1.61	-1.61	-1.61	-1.61
-1.6	-1.6	-1.6	-1.6	-1.6	-1.6	-1.6	-1.6	-1.6
-1.59	-1.59	-1.59	-1.59	-1.59	-1.59	-1.59	-1.59	-1.59
-1.58	-1.58	-1.58	-1.58	-1.58	-1.58	-1.58	-1.58	-1.58
-1.57	-1.57	-1.57	-1.57	-1.57	-1.57	-1.57	-1.57	-1.57
-1.56	-1.56	-1.56	-1.56	-1.56	-1.56	-1.56	-1.56	-1.56
-1.55	-1.55	-1.55	-1.55	-1.55	-1.55	-1.55	-1.55	-1.55
-1.54	-1.54	-1.54	-1.54	-1.54	-1.54	-1.54	-1.54	-1.54
-1.53	-1.53	-1.53	-1.53	-1.53	-1.53	-1.53	-1.53	-1.53
-1.52	-1.52	-1.52	-1.52	-1.52	-1.52	-1.52	-1.52	-1.52
-1.51	-1.51	-1.51	-1.51	-1.51	-1.51	-1.51	-1.51	-1.51

-1.5	-1.5	-1.5	-1.5	-1.5	-1.5	-1.5	-1.5	-1.5
-1.49	-1.49	-1.49	-1.49	-1.49	-1.49	-1.49	-1.49	-1.49
-1.48	-1.48	-1.48	-1.48	-1.48	-1.48	-1.48	-1.48	-1.48
-1.47	-1.47	-1.47	-1.47	-1.47	-1.47	-1.47	-1.47	-1.47
-1.46	-1.46	-1.46	-1.46	-1.46	-1.46	-1.46	-1.46	-1.46
-1.45	-1.45	-1.45	-1.45	-1.45	-1.45	-1.45	-1.45	-1.45
-1.44	-1.44	-1.44	-1.44	-1.44	-1.44	-1.44	-1.44	-1.44
-1.43	-1.43	-1.43	-1.43	-1.43	-1.43	-1.43	-1.43	-1.43
-1.42	-1.42	-1.42	-1.42	-1.42	-1.42	-1.42	-1.42	-1.42
-1.41	-1.41	-1.41	-1.41	-1.41	-1.41	-1.41	-1.41	-1.41
-1.4	-1.4	-1.4	-1.4	-1.4	-1.4	-1.4	-1.4	-1.4
-1.39	-1.39	-1.39	-1.39	-1.39	-1.39	-1.39	-1.39	-1.39
-1.38	-1.38	-1.38	-1.38	-1.38	-1.38	-1.38	-1.38	-1.38
-1.37	-1.37	-1.37	-1.37	-1.37	-1.37	-1.37	-1.37	-1.37
-1.36	-1.36	-1.36	-1.36	-1.36	-1.36	-1.36	-1.36	-1.36
-1.35	-1.35	-1.35	-1.35	-1.35	-1.35	-1.35	-1.35	-1.35
-1.34	-1.34	-1.34	-1.34	-1.34	-1.34	-1.34	-1.34	-1.34
-1.33	-1.33	-1.33	-1.33	-1.33	-1.33	-1.33	-1.33	-1.33
-1.32	-1.32	-1.32	-1.32	-1.32	-1.32	-1.32	-1.32	-1.32
-1.31	-1.31	-1.31	-1.31	-1.31	-1.31	-1.31	-1.31	-1.31
-1.3	-1.3	-1.3	-1.3	-1.3	-1.3	-1.3	-1.3	-1.3
-1.29	-1.29	-1.29	-1.29	-1.29	-1.29	-1.29	-1.29	-1.29
-1.28	-1.28	-1.28	-1.28	-1.28	-1.28	-1.28	-1.28	-1.28
-1.27	-1.27	-1.27	-1.27	-1.27	-1.27	-1.27	-1.27	-1.27
-1.26	-1.26	-1.26	-1.26	-1.26	-1.26	-1.26	-1.26	-1.26
-1.25	-1.25	-1.25	-1.25	-1.25	-1.25	-1.25	-1.25	-1.25
-1.24	-1.24	-1.24	-1.24	-1.24	-1.24	-1.24	-1.24	-1.24
-1.23	-1.23	-1.23	-1.23	-1.23	-1.23	-1.23	-1.23	-1.23
-1.22	-1.22	-1.22	-1.22	-1.22	-1.22	-1.22	-1.22	-1.22
-1.21	-1.21	-1.21	-1.21	-1.21	-1.21	-1.21	-1.21	-1.21
-1.2	-1.2	-1.2	-1.2	-1.2	-1.2	-1.2	-1.2	-1.2
-1.19	-1.19	-1.19	-1.19	-1.19	-1.19	-1.19	-1.19	-1.19
-1.18	-1.18	-1.18	-1.18	-1.18	-1.18	-1.18	-1.18	-1.18
-1.17	-1.17	-1.17	-1.17	-1.17	-1.17	-1.17	-1.17	-1.17
-1.16	-1.16	-1.16	-1.16	-1.16	-1.16	-1.16	-1.16	-1.16
-1.15	-1.15	-1.15	-1.15	-1.15	-1.15	-1.15	-1.15	-1.15
-1.14	-1.14	-1.14	-1.14	-1.14	-1.14	-1.14	-1.14	-1.14
-1.13	-1.13	-1.13	-1.13	-1.13	-1.13	-1.13	-1.13	-1.13
-1.12	-1.12	-1.12	-1.12	-1.12	-1.12	-1.12	-1.12	-1.12
-1.11	-1.11	-1.11	-1.11	-1.11	-1.11	-1.11	-1.11	-1.11
-1.1	-1.1	-1.1	-1.1	-1.1	-1.1	-1.1	-1.1	-1.1
-1.09	-1.09	-1.09	-1.09	-1.09	-1.09	-1.09	-1.09	-1.09
-1.08	-1.08	-1.08	-1.08	-1.08	-1.08	-1.08	-1.08	-1.08
-1.07	-1.07	-1.07	-1.07	-1.07	-1.07	-1.07	-1.07	-1.07
-1.06	-1.06	-1.06	-1.06	-1.06	-1.06	-1.06	-1.06	-1.06
-1.05	-1.05	-1.05	-1.05	-1.05	-1.05	-1.05	-1.05	-1.05
-1.04	-1.04	-1.04	-1.04	-1.04	-1.04	-1.04	-1.04	-1.04
-1.03	-1.03	-1.03	-1.03	-1.03	-1.03	-1.03	-1.03	-1.03
-1.02	-1.02	-1.02	-1.02	-1.02	-1.02	-1.02	-1.02	-1.02
-1.01	-1.01	-1.01	-1.01	-1.01	-1.01	-1.01	-1.01	-1.01

-1	-1	-1	-1	-1	-1	-1	-1	-1
-0.99	-0.99	-0.99	-0.99	-0.99	-0.99	-0.99	-0.99	-0.99
-0.98	-0.98	-0.98	-0.98	-0.98	-0.98	-0.98	-0.98	-0.98
-0.97	-0.97	-0.97	-0.97	-0.97	-0.97	-0.97	-0.97	-0.97
-0.96	-0.96	-0.96	-0.96	-0.96	-0.96	-0.96	-0.96	-0.96
-0.95	-0.95	-0.95	-0.95	-0.95	-0.95	-0.95	-0.95	-0.95
-0.94	-0.94	-0.94	-0.94	-0.94	-0.94	-0.94	-0.94	-0.94
-0.93	-0.93	-0.93	-0.93	-0.93	-0.93	-0.93	-0.93	-0.93
-0.92	-0.92	-0.92	-0.92	-0.92	-0.92	-0.92	-0.92	-0.92
-0.91	-0.91	-0.91	-0.91	-0.91	-0.91	-0.91	-0.91	-0.91
-0.9	-0.9	-0.9	-0.9	-0.9	-0.9	-0.9	-0.9	-0.9
-0.89	-0.89	-0.89	-0.89	-0.89	-0.89	-0.89	-0.89	-0.89
-0.88	-0.88	-0.88	-0.88	-0.88	-0.88	-0.88	-0.88	-0.88
-0.87	-0.87	-0.87	-0.87	-0.87	-0.87	-0.87	-0.87	-0.87
-0.86	-0.86	-0.86	-0.86	-0.86	-0.86	-0.86	-0.86	-0.86
-0.85	-0.85	-0.85	-0.85	-0.85	-0.85	-0.85	-0.85	-0.85
-0.84	-0.84	-0.84	-0.84	-0.84	-0.84	-0.84	-0.84	-0.84
-0.83	-0.83	-0.83	-0.83	-0.83	-0.83	-0.83	-0.83	-0.83
-0.82	-0.82	-0.82	-0.82	-0.82	-0.82	-0.82	-0.82	-0.82
-0.81	-0.81	-0.81	-0.81	-0.81	-0.81	-0.81	-0.81	-0.81
-0.8	-0.8	-0.8	-0.8	-0.8	-0.8	-0.8	-0.8	-0.8
-0.79	-0.79	-0.79	-0.79	-0.79	-0.79	-0.79	-0.79	-0.79
-0.78	-0.78	-0.78	-0.78	-0.78	-0.78	-0.78	-0.78	-0.78
-0.77	-0.77	-0.77	-0.77	-0.77	-0.77	-0.77	-0.77	-0.77
-0.76	-0.76	-0.76	-0.76	-0.76	-0.76	-0.76	-0.76	-0.76
-0.75	-0.75	-0.75	-0.75	-0.75	-0.75	-0.75	-0.75	-0.75
-0.74	-0.74	-0.74	-0.74	-0.74	-0.74	-0.74	-0.74	-0.74
-0.73	-0.73	-0.73	-0.73	-0.73	-0.73	-0.73	-0.73	-0.73
-0.72	-0.72	-0.72	-0.72	-0.72	-0.72	-0.72	-0.72	-0.72
-0.71	-0.71	-0.71	-0.71	-0.71	-0.71	-0.71	-0.71	-0.71
-0.7	-0.7	-0.7	-0.7	-0.7	-0.7	-0.7	-0.7	-0.7
-0.69	-0.69	-0.69	-0.69	-0.69	-0.69	-0.69	-0.69	-0.69
-0.68	-0.68	-0.68	-0.68	-0.68	-0.68	-0.68	-0.68	-0.68
-0.67	-0.67	-0.67	-0.67	-0.67	-0.67	-0.67	-0.67	-0.67
-0.66	-0.66	-0.66	-0.66	-0.66	-0.66	-0.66	-0.66	-0.66
-0.65	-0.65	-0.65	-0.65	-0.65	-0.65	-0.65	-0.65	-0.65
-0.64	-0.64	-0.64	-0.64	-0.64	-0.64	-0.64	-0.64	-0.64
-0.63	-0.63	-0.63	-0.63	-0.63	-0.63	-0.63	-0.63	-0.63
-0.62	-0.62	-0.62	-0.62	-0.62	-0.62	-0.62	-0.62	-0.62
-0.61	-0.61	-0.61	-0.61	-0.61	-0.61	-0.61	-0.61	-0.61
-0.6	-0.6	-0.6	-0.6	-0.6	-0.6	-0.6	-0.6	-0.6
-0.59	-0.59	-0.59	-0.59	-0.59	-0.59	-0.59	-0.59	-0.59
-0.58	-0.58	-0.58	-0.58	-0.58	-0.58	-0.58	-0.58	-0.58
-0.57	-0.57	-0.57	-0.57	-0.57	-0.57	-0.57	-0.57	-0.57
-0.56	-0.56	-0.56	-0.56	-0.56	-0.56	-0.56	-0.56	-0.56
-0.55	-0.55	-0.55	-0.55	-0.55	-0.55	-0.55	-0.55	-0.55
-0.54	-0.54	-0.54	-0.54	-0.54	-0.54	-0.54	-0.54	-0.54
-0.53	-0.53	-0.53	-0.53	-0.53	-0.53	-0.53	-0.53	-0.53
-0.52	-0.52	-0.52	-0.52	-0.52	-0.52	-0.52	-0.52	-0.52
-0.51	-0.51	-0.51	-0.51	-0.51	-0.51	-0.51	-0.51	-0.51

-0.5	-0.5	-0.5	-0.5	-0.5	-0.5	-0.5	-0.5	-0.5
-0.49	-0.49	-0.49	-0.49	-0.49	-0.49	-0.49	-0.49	-0.49
-0.48	-0.48	-0.48	-0.48	-0.48	-0.48	-0.48	-0.48	-0.48
-0.47	-0.47	-0.47	-0.47	-0.47	-0.47	-0.47	-0.47	-0.47
-0.46	-0.46	-0.46	-0.46	-0.46	-0.46	-0.46	-0.46	-0.46
-0.45	-0.45	-0.45	-0.45	-0.45	-0.45	-0.45	-0.45	-0.45
-0.44	-0.44	-0.44	-0.44	-0.44	-0.44	-0.44	-0.44	-0.44
-0.43	-0.43	-0.43	-0.43	-0.43	-0.43	-0.43	-0.43	-0.43
-0.42	-0.42	-0.42	-0.42	-0.42	-0.42	-0.42	-0.42	-0.42
-0.41	-0.41	-0.41	-0.41	-0.41	-0.41	-0.41	-0.41	-0.41
-0.4	-0.4	-0.4	-0.4	-0.4	-0.4	-0.4	-0.4	-0.4
-0.39	-0.39	-0.39	-0.39	-0.39	-0.39	-0.39	-0.39	-0.39
-0.38	-0.38	-0.38	-0.38	-0.38	-0.38	-0.38	-0.38	-0.38
-0.37	-0.37	-0.37	-0.37	-0.37	-0.37	-0.37	-0.37	-0.37
-0.36	-0.36	-0.36	-0.36	-0.36	-0.36	-0.36	-0.36	-0.36
-0.35	-0.35	-0.35	-0.35	-0.35	-0.35	-0.35	-0.35	-0.35
-0.34	-0.34	-0.34	-0.34	-0.34	-0.34	-0.34	-0.34	-0.34
-0.33	-0.33	-0.33	-0.33	-0.33	-0.33	-0.33	-0.33	-0.33
-0.32	-0.32	-0.32	-0.32	-0.32	-0.32	-0.32	-0.32	-0.32
-0.31	-0.31	-0.31	-0.31	-0.31	-0.31	-0.31	-0.31	-0.31
-0.3	-0.3	-0.3	-0.3	-0.3	-0.3	-0.3	-0.3	-0.3
-0.29	-0.29	-0.29	-0.29	-0.29	-0.29	-0.29	-0.29	-0.29
-0.28	-0.28	-0.28	-0.28	-0.28	-0.28	-0.28	-0.28	-0.28
-0.27	-0.27	-0.27	-0.27	-0.27	-0.27	-0.27	-0.27	-0.27
-0.26	-0.26	-0.26	-0.26	-0.26	-0.26	-0.26	-0.26	-0.26
-0.25	-0.25	-0.25	-0.25	-0.25	-0.25	-0.25	-0.25	-0.25
-0.24	-0.24	-0.24	-0.24	-0.24	-0.24	-0.24	-0.24	-0.24
-0.23	-0.23	-0.23	-0.23	-0.23	-0.23	-0.23	-0.23	-0.23
-0.22	-0.22	-0.22	-0.22	-0.22	-0.22	-0.22	-0.22	-0.22
-0.21	-0.21	-0.21	-0.21	-0.21	-0.21	-0.21	-0.21	-0.21
-0.2	-0.2	-0.2	-0.2	-0.2	-0.2	-0.2	-0.2	-0.2
-0.19	-0.19	-0.19	-0.19	-0.19	-0.19	-0.19	-0.19	-0.19
-0.18	-0.18	-0.18	-0.18	-0.18	-0.18	-0.18	-0.18	-0.18
-0.17	-0.17	-0.17	-0.17	-0.17	-0.17	-0.17	-0.17	-0.17
-0.16	-0.16	-0.16	-0.16	-0.16	-0.16	-0.16	-0.16	-0.16
-0.15	-0.15	-0.15	-0.15	-0.15	-0.15	-0.15	-0.15	-0.15
-0.14	-0.14	-0.14	-0.14	-0.14	-0.14	-0.14	-0.14	-0.14
-0.13	-0.13	-0.13	-0.13	-0.13	-0.13	-0.13	-0.13	-0.13
-0.12	-0.12	-0.12	-0.12	-0.12	-0.12	-0.12	-0.12	-0.12
-0.11	-0.11	-0.11	-0.11	-0.11	-0.11	-0.11	-0.11	-0.11
-0.1	-0.1	-0.1	-0.1	-0.1	-0.1	-0.1	-0.1	-0.1
-0.09	-0.09	-0.09	-0.09	-0.09	-0.09	-0.09	-0.09	-0.09
-0.08	-0.08	-0.08	-0.08	-0.08	-0.08	-0.08	-0.08	-0.08
-0.07	-0.07	-0.07	-0.07	-0.07	-0.07	-0.07	-0.07	-0.07
-0.06	-0.06	-0.06	-0.06	-0.06	-0.06	-0.06	-0.06	-0.06
-0.05	-0.05	-0.05	-0.05	-0.05	-0.05	-0.05	-0.05	-0.05
-0.04	-0.04	-0.04	-0.04	-0.04	-0.04	-0.04	-0.04	-0.04
-0.03	-0.03	-0.03	-0.03	-0.03	-0.03	-0.03	-0.03	-0.03
-0.02	-0.02	-0.02	-0.02	-0.02	-0.02	-0.02	-0.02	-0.02
-0.01	-0.01	-0.01	-0.01	-0.01	-0.01	-0.01	-0.01	-0.01

0	0	0	0	0	0	0	0	0
0.01	0.01	0.01	0.01	0.01	0.01	0.01	0.01	0.01
0.02	0.02	0.02	0.02	0.02	0.02	0.02	0.02	0.02
0.03	0.03	0.03	0.03	0.03	0.03	0.03	0.03	0.03
0.04	0.04	0.04	0.04	0.04	0.04	0.04	0.04	0.04
0.05	0.05	0.05	0.05	0.05	0.05	0.05	0.05	0.05
0.06	0.06	0.06	0.06	0.06	0.06	0.06	0.06	0.06
0.07	0.07	0.07	0.07	0.07	0.07	0.07	0.07	0.07
0.08	0.08	0.08	0.08	0.08	0.08	0.08	0.08	0.08
0.09	0.09	0.09	0.09	0.09	0.09	0.09	0.09	0.09
0.1	0.1	0.1	0.1	0.1	0.1	0.1	0.1	0.1
0.11	0.11	0.11	0.11	0.11	0.11	0.11	0.11	0.11
0.12	0.12	0.12	0.12	0.12	0.12	0.12	0.12	0.12
0.13	0.13	0.13	0.13	0.13	0.13	0.13	0.13	0.13
0.14	0.14	0.14	0.14	0.14	0.14	0.14	0.14	0.14
0.15	0.15	0.15	0.15	0.15	0.15	0.15	0.15	0.15
0.16	0.16	0.16	0.16	0.16	0.16	0.16	0.16	0.16
0.17	0.17	0.17	0.17	0.17	0.17	0.17	0.17	0.17
0.18	0.18	0.18	0.18	0.18	0.18	0.18	0.18	0.18
0.19	0.19	0.19	0.19	0.19	0.19	0.19	0.19	0.19
0.2	0.2	0.2	0.2	0.2	0.2	0.2	0.2	0.2
0.21	0.21	0.21	0.21	0.21	0.21	0.21	0.21	0.21
0.22	0.22	0.22	0.22	0.22	0.22	0.22	0.22	0.22
0.23	0.23	0.23	0.23	0.23	0.23	0.23	0.23	0.23
0.24	0.24	0.24	0.24	0.24	0.24	0.24	0.24	0.24
0.25	0.25	0.25	0.25	0.25	0.25	0.25	0.25	0.25
0.26	0.26	0.26	0.26	0.26	0.26	0.26	0.26	0.26
0.27	0.27	0.27	0.27	0.27	0.27	0.27	0.27	0.27
0.28	0.28	0.28	0.28	0.28	0.28	0.28	0.28	0.28
0.29	0.29	0.29	0.29	0.29	0.29	0.29	0.29	0.29
0.3	0.3	0.3	0.3	0.3	0.3	0.3	0.3	0.3
0.31	0.31	0.31	0.31	0.31	0.31	0.31	0.31	0.31
0.32	0.32	0.32	0.32	0.32	0.32	0.32	0.32	0.32
0.33	0.33	0.33	0.33	0.33	0.33	0.33	0.33	0.33
0.34	0.34	0.34	0.34	0.34	0.34	0.34	0.34	0.34
0.35	0.35	0.35	0.35	0.35	0.35	0.35	0.35	0.35
0.36	0.36	0.36	0.36	0.36	0.36	0.36	0.36	0.36
0.37	0.37	0.37	0.37	0.37	0.37	0.37	0.37	0.37
0.38	0.38	0.38	0.38	0.38	0.38	0.38	0.38	0.38
0.39	0.39	0.39	0.39	0.39	0.39	0.39	0.39	0.39
0.4	0.4	0.4	0.4	0.4	0.4	0.4	0.4	0.4
0.41	0.41	0.41	0.41	0.41	0.41	0.41	0.41	0.41
0.42	0.42	0.42	0.42	0.42	0.42	0.42	0.42	0.42
0.43	0.43	0.43	0.43	0.43	0.43	0.43	0.43	0.43
0.44	0.44	0.44	0.44	0.44	0.44	0.44	0.44	0.44
0.45	0.45	0.45	0.45	0.45	0.45	0.45	0.45	0.45
0.46	0.46	0.46	0.46	0.46	0.46	0.46	0.46	0.46
0.47	0.47	0.47	0.47	0.47	0.47	0.47	0.47	0.47
0.48	0.48	0.48	0.48	0.48	0.48	0.48	0.48	0.48
0.49	0.49	0.49	0.49	0.49	0.49	0.49	0.49	0.49

0.5	0.5	0.5	0.5	0.5	0.5	0.5	0.5	0.5
0.51	0.51	0.51	0.51	0.51	0.51	0.51	0.51	0.51
0.52	0.52	0.52	0.52	0.52	0.52	0.52	0.52	0.52
0.53	0.53	0.53	0.53	0.53	0.53	0.53	0.53	0.53
0.54	0.54	0.54	0.54	0.54	0.54	0.54	0.54	0.54
0.55	0.55	0.55	0.55	0.55	0.55	0.55	0.55	0.55
0.56	0.56	0.56	0.56	0.56	0.56	0.56	0.56	0.56
0.57	0.57	0.57	0.57	0.57	0.57	0.57	0.57	0.57
0.58	0.58	0.58	0.58	0.58	0.58	0.58	0.58	0.58
0.59	0.59	0.59	0.59	0.59	0.59	0.59	0.59	0.59
0.6	0.6	0.6	0.6	0.6	0.6	0.6	0.6	0.6
0.61	0.61	0.61	0.61	0.61	0.61	0.61	0.61	0.61
0.62	0.62	0.62	0.62	0.62	0.62	0.62	0.62	0.62
0.63	0.63	0.63	0.63	0.63	0.63	0.63	0.63	0.63
0.64	0.64	0.64	0.64	0.64	0.64	0.64	0.64	0.64
0.65	0.65	0.65	0.65	0.65	0.65	0.65	0.65	0.65
0.66	0.66	0.66	0.66	0.66	0.66	0.66	0.66	0.66
0.67	0.67	0.67	0.67	0.67	0.67	0.67	0.67	0.67
0.68	0.68	0.68	0.68	0.68	0.68	0.68	0.68	0.68
0.69	0.69	0.69	0.69	0.69	0.69	0.69	0.69	0.69
0.7	0.7	0.7	0.7	0.7	0.7	0.7	0.7	0.7
0.71	0.71	0.71	0.71	0.71	0.71	0.71	0.71	0.71
0.72	0.72	0.72	0.72	0.72	0.72	0.72	0.72	0.72
0.73	0.73	0.73	0.73	0.73	0.73	0.73	0.73	0.73
0.74	0.74	0.74	0.74	0.74	0.74	0.74	0.74	0.74
0.75	0.75	0.75	0.75	0.75	0.75	0.75	0.75	0.75
0.76	0.76	0.76	0.76	0.76	0.76	0.76	0.76	0.76
0.77	0.77	0.77	0.77	0.77	0.77	0.77	0.77	0.77
0.78	0.78	0.78	0.78	0.78	0.78	0.78	0.78	0.78
0.79	0.79	0.79	0.79	0.79	0.79	0.79	0.79	0.79
0.8	0.8	0.8	0.8	0.8	0.8	0.8	0.8	0.8
0.81	0.81	0.81	0.81	0.81	0.81	0.81	0.81	0.81
0.82	0.82	0.82	0.82	0.82	0.82	0.82	0.82	0.82
0.83	0.83	0.83	0.83	0.83	0.83	0.83	0.83	0.83
0.84	0.84	0.84	0.84	0.84	0.84	0.84	0.84	0.84
0.85	0.85	0.85	0.85	0.85	0.85	0.85	0.85	0.85
0.86	0.86	0.86	0.86	0.86	0.86	0.86	0.86	0.86
0.87	0.87	0.87	0.87	0.87	0.87	0.87	0.87	0.87
0.88	0.88	0.88	0.88	0.88	0.88	0.88	0.88	0.88
0.89	0.89	0.89	0.89	0.89	0.89	0.89	0.89	0.89
0.9	0.9	0.9	0.9	0.9	0.9	0.9	0.9	0.9
0.91	0.91	0.91	0.91	0.91	0.91	0.91	0.91	0.91
0.92	0.92	0.92	0.92	0.92	0.92	0.92	0.92	0.92
0.93	0.93	0.93	0.93	0.93	0.93	0.93	0.93	0.93
0.94	0.94	0.94	0.94	0.94	0.94	0.94	0.94	0.94
0.95	0.95	0.95	0.95	0.95	0.95	0.95	0.95	0.95
0.96	0.96	0.96	0.96	0.96	0.96	0.96	0.96	0.96
0.97	0.97	0.97	0.97	0.97	0.97	0.97	0.97	0.97
0.98	0.98	0.98	0.98	0.98	0.98	0.98	0.98	0.98
0.99	0.99	0.99	0.99	0.99	0.99	0.99	0.99	0.99

1	1	1	1	1	1	1	1	1
1.01	1.01	1.01	1.01	1.01	1.01	1.01	1.01	1.01
1.02	1.02	1.02	1.02	1.02	1.02	1.02	1.02	1.02
1.03	1.03	1.03	1.03	1.03	1.03	1.03	1.03	1.03
1.04	1.04	1.04	1.04	1.04	1.04	1.04	1.04	1.04
1.05	1.05	1.05	1.05	1.05	1.05	1.05	1.05	1.05
1.06	1.06	1.06	1.06	1.06	1.06	1.06	1.06	1.06
1.07	1.07	1.07	1.07	1.07	1.07	1.07	1.07	1.07
1.08	1.08	1.08	1.08	1.08	1.08	1.08	1.08	1.08
1.09	1.09	1.09	1.09	1.09	1.09	1.09	1.09	1.09
1.1	1.1	1.1	1.1	1.1	1.1	1.1	1.1	1.1
1.11	1.11	1.11	1.11	1.11	1.11	1.11	1.11	1.11
1.12	1.12	1.12	1.12	1.12	1.12	1.12	1.12	1.12
1.13	1.13	1.13	1.13	1.13	1.13	1.13	1.13	1.13
1.14	1.14	1.14	1.14	1.14	1.14	1.14	1.14	1.14
1.15	1.15	1.15	1.15	1.15	1.15	1.15	1.15	1.15
1.16	1.16	1.16	1.16	1.16	1.16	1.16	1.16	1.16
1.17	1.17	1.17	1.17	1.17	1.17	1.17	1.17	1.17
1.18	1.18	1.18	1.18	1.18	1.18	1.18	1.18	1.18
1.19	1.19	1.19	1.19	1.19	1.19	1.19	1.19	1.19
1.2	1.2	1.2	1.2	1.2	1.2	1.2	1.2	1.2
1.21	1.21	1.21	1.21	1.21	1.21	1.21	1.21	1.21
1.22	1.22	1.22	1.22	1.22	1.22	1.22	1.22	1.22
1.23	1.23	1.23	1.23	1.23	1.23	1.23	1.23	1.23
1.24	1.24	1.24	1.24	1.24	1.24	1.24	1.24	1.24
1.25	1.25	1.25	1.25	1.25	1.25	1.25	1.25	1.25
1.26	1.26	1.26	1.26	1.26	1.26	1.26	1.26	1.26
1.27	1.27	1.27	1.27	1.27	1.27	1.27	1.27	1.27
1.28	1.28	1.28	1.28	1.28	1.28	1.28	1.28	1.28
1.29	1.29	1.29	1.29	1.29	1.29	1.29	1.29	1.29
1.3	1.3	1.3	1.3	1.3	1.3	1.3	1.3	1.3
1.31	1.31	1.31	1.31	1.31	1.31	1.31	1.31	1.31
1.32	1.32	1.32	1.32	1.32	1.32	1.32	1.32	1.32
1.33	1.33	1.33	1.33	1.33	1.33	1.33	1.33	1.33
1.34	1.34	1.34	1.34	1.34	1.34	1.34	1.34	1.34
1.35	1.35	1.35	1.35	1.35	1.35	1.35	1.35	1.35
1.36	1.36	1.36	1.36	1.36	1.36	1.36	1.36	1.36
1.37	1.37	1.37	1.37	1.37	1.37	1.37	1.37	1.37
1.38	1.38	1.38	1.38	1.38	1.38	1.38	1.38	1.38
1.39	1.39	1.39	1.39	1.39	1.39	1.39	1.39	1.39
1.4	1.4	1.4	1.4	1.4	1.4	1.4	1.4	1.4
1.41	1.41	1.41	1.41	1.41	1.41	1.41	1.41	1.41
1.42	1.42	1.42	1.42	1.42	1.42	1.42	1.42	1.42
1.43	1.43	1.43	1.43	1.43	1.43	1.43	1.43	1.43
1.44	1.44	1.44	1.44	1.44	1.44	1.44	1.44	1.44
1.45	1.45	1.45	1.45	1.45	1.45	1.45	1.45	1.45
1.46	1.46	1.46	1.46	1.46	1.46	1.46	1.46	1.46
1.47	1.47	1.47	1.47	1.47	1.47	1.47	1.47	1.47
1.48	1.48	1.48	1.48	1.48	1.48	1.48	1.48	1.48
1.49	1.49	1.49	1.49	1.49	1.49	1.49	1.49	1.49

1.5	1.5	1.5	1.5	1.5	1.5	1.5	1.5	1.5
1.51	1.51	1.51	1.51	1.51	1.51	1.51	1.51	1.51
1.52	1.52	1.52	1.52	1.52	1.52	1.52	1.52	1.52
1.53	1.53	1.53	1.53	1.53	1.53	1.53	1.53	1.53
1.54	1.54	1.54	1.54	1.54	1.54	1.54	1.54	1.54
1.55	1.55	1.55	1.55	1.55	1.55	1.55	1.55	1.55
1.56	1.56	1.56	1.56	1.56	1.56	1.56	1.56	1.56
1.57	1.57	1.57	1.57	1.57	1.57	1.57	1.57	1.57
1.58	1.58	1.58	1.58	1.58	1.58	1.58	1.58	1.58
1.59	1.59	1.59	1.59	1.59	1.59	1.59	1.59	1.59
1.6	1.6	1.6	1.6	1.6	1.6	1.6	1.6	1.6
1.61	1.61	1.61	1.61	1.61	1.61	1.61	1.61	1.61
1.62	1.62	1.62	1.62	1.62	1.62	1.62	1.62	1.62
1.63	1.63	1.63	1.63	1.63	1.63	1.63	1.63	1.63
1.64	1.64	1.64	1.64	1.64	1.64	1.64	1.64	1.64
1.65	1.65	1.65	1.65	1.65	1.65	1.65	1.65	1.65
1.66	1.66	1.66	1.66	1.66	1.66	1.66	1.66	1.66
1.67	1.67	1.67	1.67	1.67	1.67	1.67	1.67	1.67
1.68	1.68	1.68	1.68	1.68	1.68	1.68	1.68	1.68
1.69	1.69	1.69	1.69	1.69	1.69	1.69	1.69	1.69
1.7	1.7	1.7	1.7	1.7	1.7	1.7	1.7	1.7
1.71	1.71	1.71	1.71	1.71	1.71	1.71	1.71	1.71
1.72	1.72	1.72	1.72	1.72	1.72	1.72	1.72	1.72
1.73	1.73	1.73	1.73	1.73	1.73	1.73	1.73	1.73
1.74	1.74	1.74	1.74	1.74	1.74	1.74	1.74	1.74
1.75	1.75	1.75	1.75	1.75	1.75	1.75	1.75	1.75
1.76	1.76	1.76	1.76	1.76	1.76	1.76	1.76	1.76
1.77	1.77	1.77	1.77	1.77	1.77	1.77	1.77	1.77
1.78	1.78	1.78	1.78	1.78	1.78	1.78	1.78	1.78
1.79	1.79	1.79	1.79	1.79	1.79	1.79	1.79	1.79
1.8	1.8	1.8	1.8	1.8	1.8	1.8	1.8	1.8
1.81	1.81	1.81	1.81	1.81	1.81	1.81	1.81	1.81
1.82	1.82	1.82	1.82	1.82	1.82	1.82	1.82	1.82
1.83	1.83	1.83	1.83	1.83	1.83	1.83	1.83	1.83
1.84	1.84	1.84	1.84	1.84	1.84	1.84	1.84	1.84
1.85	1.85	1.85	1.85	1.85	1.85	1.85	1.85	1.85
1.86	1.86	1.86	1.86	1.86	1.86	1.86	1.86	1.86
1.87	1.87	1.87	1.87	1.87	1.87	1.87	1.87	1.87
1.88	1.88	1.88	1.88	1.88	1.88	1.88	1.88	1.88
1.89	1.89	1.89	1.89	1.89	1.89	1.89	1.89	1.89
1.9	1.9	1.9	1.9	1.9	1.9	1.9	1.9	1.9
1.91	1.91	1.91	1.91	1.91	1.91	1.91	1.91	1.91
1.92	1.92	1.92	1.92	1.92	1.92	1.92	1.92	1.92
1.93	1.93	1.93	1.93	1.93	1.93	1.93	1.93	1.93
1.94	1.94	1.94	1.94	1.94	1.94	1.94	1.94	1.94
1.95	1.95	1.95	1.95	1.95	1.95	1.95	1.95	1.95
1.96	1.96	1.96	1.96	1.96	1.96	1.96	1.96	1.96
1.97	1.97	1.97	1.97	1.97	1.97	1.97	1.97	1.97
1.98	1.98	1.98	1.98	1.98	1.98	1.98	1.98	1.98
1.99	1.99	1.99	1.99	1.99	1.99	1.99	1.99	1.99

2	2	2	2	2	2	2	2	2
2.01	2.01	2.01	2.01	2.01	2.01	2.01	2.01	2.01
2.02	2.02	2.02	2.02	2.02	2.02	2.02	2.02	2.02
2.03	2.03	2.03	2.03	2.03	2.03	2.03	2.03	2.03
2.04	2.04	2.04	2.04	2.04	2.04	2.04	2.04	2.04
2.05	2.05	2.05	2.05	2.05	2.05	2.05	2.05	2.05
2.06	2.06	2.06	2.06	2.06	2.06	2.06	2.06	2.06
2.07	2.07	2.07	2.07	2.07	2.07	2.07	2.07	2.07
2.08	2.08	2.08	2.08	2.08	2.08	2.08	2.08	2.08
2.09	2.09	2.09	2.09	2.09	2.09	2.09	2.09	2.09
2.1	2.1	2.1	2.1	2.1	2.1	2.1	2.1	2.1
2.11	2.11	2.11	2.11	2.11	2.11	2.11	2.11	2.11
2.12	2.12	2.12	2.12	2.12	2.12	2.12	2.12	2.12
2.13	2.13	2.13	2.13	2.13	2.13	2.13	2.13	2.13
2.14	2.14	2.14	2.14	2.14	2.14	2.14	2.14	2.14
2.15	2.15	2.15	2.15	2.15	2.15	2.15	2.15	2.15
2.16	2.16	2.16	2.16	2.16	2.16	2.16	2.16	2.16
2.17	2.17	2.17	2.17	2.17	2.17	2.17	2.17	2.17
2.18	2.18	2.18	2.18	2.18	2.18	2.18	2.18	2.18
2.19	2.19	2.19	2.19	2.19	2.19	2.19	2.19	2.19
2.2	2.2	2.2	2.2	2.2	2.2	2.2	2.2	2.2
2.21	2.21	2.21	2.21	2.21	2.21	2.21	2.21	2.21
2.22	2.22	2.22	2.22	2.22	2.22	2.22	2.22	2.22
2.23	2.23	2.23	2.23	2.23	2.23	2.23	2.23	2.23
2.24	2.24	2.24	2.24	2.24	2.24	2.24	2.24	2.24
2.25	2.25	2.25	2.25	2.25	2.25	2.25	2.25	2.25
2.26	2.26	2.26	2.26	2.26	2.26	2.26	2.26	2.26
2.27	2.27	2.27	2.27	2.27	2.27	2.27	2.27	2.27
2.28	2.28	2.28	2.28	2.28	2.28	2.28	2.28	2.28
2.29	2.29	2.29	2.29	2.29	2.29	2.29	2.29	2.29
2.3	2.3	2.3	2.3	2.3	2.3	2.3	2.3	2.3
2.31	2.31	2.31	2.31	2.31	2.31	2.31	2.31	2.31
2.32	2.32	2.32	2.32	2.32	2.32	2.32	2.32	2.32
2.33	2.33	2.33	2.33	2.33	2.33	2.33	2.33	2.33
2.34	2.34	2.34	2.34	2.34	2.34	2.34	2.34	2.34
2.35	2.35	2.35	2.35	2.35	2.35	2.35	2.35	2.35
2.36	2.36	2.36	2.36	2.36	2.36	2.36	2.36	2.36
2.37	2.37	2.37	2.37	2.37	2.37	2.37	2.37	2.37
2.38	2.38	2.38	2.38	2.38	2.38	2.38	2.38	2.38
2.39	2.39	2.39	2.39	2.39	2.39	2.39	2.39	2.39
2.4	2.4	2.4	2.4	2.4	2.4	2.4	2.4	2.4
2.41	2.41	2.41	2.41	2.41	2.41	2.41	2.41	2.41
2.42	2.42	2.42	2.42	2.42	2.42	2.42	2.42	2.42
2.43	2.43	2.43	2.43	2.43	2.43	2.43	2.43	2.43
2.44	2.44	2.44	2.44	2.44	2.44	2.44	2.44	2.44
2.45	2.45	2.45	2.45	2.45	2.45	2.45	2.45	2.45
2.46	2.46	2.46	2.46	2.46	2.46	2.46	2.46	2.46
2.47	2.47	2.47	2.47	2.47	2.47	2.47	2.47	2.47
2.48	2.48	2.48	2.48	2.48	2.48	2.48	2.48	2.48
2.49	2.49	2.49	2.49	2.49	2.49	2.49	2.49	2.49

2.5	2.5	2.5	2.5	2.5	2.5	2.5	2.5	2.5
2.51	2.51	2.51	2.51	2.51	2.51	2.51	2.51	2.51
2.52	2.52	2.52	2.52	2.52	2.52	2.52	2.52	2.52
2.53	2.53	2.53	2.53	2.53	2.53	2.53	2.53	2.53
2.54	2.54	2.54	2.54	2.54	2.54	2.54	2.54	2.54
2.55	2.55	2.55	2.55	2.55	2.55	2.55	2.55	2.55
2.56	2.56	2.56	2.56	2.56	2.56	2.56	2.56	2.56
2.57	2.57	2.57	2.57	2.57	2.57	2.57	2.57	2.57
2.58	2.58	2.58	2.58	2.58	2.58	2.58	2.58	2.58
2.59	2.59	2.59	2.59	2.59	2.59	2.59	2.59	2.59
2.6	2.6	2.6	2.6	2.6	2.6	2.6	2.6	2.6
2.61	2.61	2.61	2.61	2.61	2.61	2.61	2.61	2.61
2.62	2.62	2.62	2.62	2.62	2.62	2.62	2.62	2.62
2.63	2.63	2.63	2.63	2.63	2.63	2.63	2.63	2.63
2.64	2.64	2.64	2.64	2.64	2.64	2.64	2.64	2.64
2.65	2.65	2.65	2.65	2.65	2.65	2.65	2.65	2.65
2.66	2.66	2.66	2.66	2.66	2.66	2.66	2.66	2.66
2.67	2.67	2.67	2.67	2.67	2.67	2.67	2.67	2.67
2.68	2.68	2.68	2.68	2.68	2.68	2.68	2.68	2.68
2.69	2.69	2.69	2.69	2.69	2.69	2.69	2.69	2.69
2.7	2.7	2.7	2.7	2.7	2.7	2.7	2.7	2.7
2.71	2.71	2.71	2.71	2.71	2.71	2.71	2.71	2.71
2.72	2.72	2.72	2.72	2.72	2.72	2.72	2.72	2.72
2.73	2.73	2.73	2.73	2.73	2.73	2.73	2.73	2.73
2.74	2.74	2.74	2.74	2.74	2.74	2.74	2.74	2.74
2.75	2.75	2.75	2.75	2.75	2.75	2.75	2.75	2.75
2.76	2.76	2.76	2.76	2.76	2.76	2.76	2.76	2.76
2.77	2.77	2.77	2.77	2.77	2.77	2.77	2.77	2.77
2.78	2.78	2.78	2.78	2.78	2.78	2.78	2.78	2.78
2.79	2.79	2.79	2.79	2.79	2.79	2.79	2.79	2.79
2.8	2.8	2.8	2.8	2.8	2.8	2.8	2.8	2.8
2.81	2.81	2.81	2.81	2.81	2.81	2.81	2.81	2.81
2.82	2.82	2.82	2.82	2.82	2.82	2.82	2.82	2.82
2.83	2.83	2.83	2.83	2.83	2.83	2.83	2.83	2.83
2.84	2.84	2.84	2.84	2.84	2.84	2.84	2.84	2.84
2.85	2.85	2.85	2.85	2.85	2.85	2.85	2.85	2.85
2.86	2.86	2.86	2.86	2.86	2.86	2.86	2.86	2.86
2.87	2.87	2.87	2.87	2.87	2.87	2.87	2.87	2.87
2.88	2.88	2.88	2.88	2.88	2.88	2.88	2.88	2.88
2.89	2.89	2.89	2.89	2.89	2.89	2.89	2.89	2.89
2.9	2.9	2.9	2.9	2.9	2.9	2.9	2.9	2.9
2.91	2.91	2.91	2.91	2.91	2.91	2.91	2.91	2.91
2.92	2.92	2.92	2.92	2.92	2.92	2.92	2.92	2.92
2.93	2.93	2.93	2.93	2.93	2.93	2.93	2.93	2.93
2.94	2.94	2.94	2.94	2.94	2.94	2.94	2.94	2.94
2.95	2.95	2.95	2.95	2.95	2.95	2.95	2.95	2.95
2.96	2.96	2.96	2.96	2.96	2.96	2.96	2.96	2.96
2.97	2.97	2.97	2.97	2.97	2.97	2.97	2.97	2.97
2.98	2.98	2.98	2.98	2.98	2.98	2.98	2.98	2.98
2.99	2.99	2.99	2.99	2.99	2.99	2.99	2.99	2.99

3	3	3	3	3	3	3	3	3
3.01	3.01	3.01	3.01	3.01	3.01	3.01	3.01	3.01
3.02	3.02	3.02	3.02	3.02	3.02	3.02	3.02	3.02
3.03	3.03	3.03	3.03	3.03	3.03	3.03	3.03	3.03
3.04	3.04	3.04	3.04	3.04	3.04	3.04	3.04	3.04
3.05	3.05	3.05	3.05	3.05	3.05	3.05	3.05	3.05
3.06	3.06	3.06	3.06	3.06	3.06	3.06	3.06	3.06
3.07	3.07	3.07	3.07	3.07	3.07	3.07	3.07	3.07
3.08	3.08	3.08	3.08	3.08	3.08	3.08	3.08	3.08
3.09	3.09	3.09	3.09	3.09	3.09	3.09	3.09	3.09
3.1	3.1	3.1	3.1	3.1	3.1	3.1	3.1	3.1
3.11	3.11	3.11	3.11	3.11	3.11	3.11	3.11	3.11
3.12	3.12	3.12	3.12	3.12	3.12	3.12	3.12	3.12
3.13	3.13	3.13	3.13	3.13	3.13	3.13	3.13	3.13
3.14	3.14	3.14	3.14	3.14	3.14	3.14	3.14	3.14
3.15	3.15	3.15	3.15	3.15	3.15	3.15	3.15	3.15
3.16	3.16	3.16	3.16	3.16	3.16	3.16	3.16	3.16
3.17	3.17	3.17	3.17	3.17	3.17	3.17	3.17	3.17
3.18	3.18	3.18	3.18	3.18	3.18	3.18	3.18	3.18
3.19	3.19	3.19	3.19	3.19	3.19	3.19	3.19	3.19
3.2	3.2	3.2	3.2	3.2	3.2	3.2	3.2	3.2
3.21	3.21	3.21	3.21	3.21	3.21	3.21	3.21	3.21
3.22	3.22	3.22	3.22	3.22	3.22	3.22	3.22	3.22
3.23	3.23	3.23	3.23	3.23	3.23	3.23	3.23	3.23
3.24	3.24	3.24	3.24	3.24	3.24	3.24	3.24	3.24
3.25	3.25	3.25	3.25	3.25	3.25	3.25	3.25	3.25
3.26	3.26	3.26	3.26	3.26	3.26	3.26	3.26	3.26
3.27	3.27	3.27	3.27	3.27	3.27	3.27	3.27	3.27
3.28	3.28	3.28	3.28	3.28	3.28	3.28	3.28	3.28
3.29	3.29	3.29	3.29	3.29	3.29	3.29	3.29	3.29
3.3	3.3	3.3	3.3	3.3	3.3	3.3	3.3	3.3
3.31	3.31	3.31	3.31	3.31	3.31	3.31	3.31	3.31
3.32	3.32	3.32	3.32	3.32	3.32	3.32	3.32	3.32
3.33	3.33	3.33	3.33	3.33	3.33	3.33	3.33	3.33
3.34	3.34	3.34	3.34	3.34	3.34	3.34	3.34	3.34
3.35	3.35	3.35	3.35	3.35	3.35	3.35	3.35	3.35
3.36	3.36	3.36	3.36	3.36	3.36	3.36	3.36	3.36
3.37	3.37	3.37	3.37	3.37	3.37	3.37	3.37	3.37
3.38	3.38	3.38	3.38	3.38	3.38	3.38	3.38	3.38
3.39	3.39	3.39	3.39	3.39	3.39	3.39	3.39	3.39
3.4	3.4	3.4	3.4	3.4	3.4	3.4	3.4	3.4
3.41	3.41	3.41	3.41	3.41	3.41	3.41	3.41	3.41
3.42	3.42	3.42	3.42	3.42	3.42	3.42	3.42	3.42
3.43	3.43	3.43	3.43	3.43	3.43	3.43	3.43	3.43
3.44	3.44	3.44	3.44	3.44	3.44	3.44	3.44	3.44
3.45	3.45	3.45	3.45	3.45	3.45	3.45	3.45	3.45
3.46	3.46	3.46	3.46	3.46	3.46	3.46	3.46	3.46
3.47	3.47	3.47	3.47	3.47	3.47	3.47	3.47	3.47
3.48	3.48	3.48	3.48	3.48	3.48	3.48	3.48	3.48
3.49	3.49	3.49	3.49	3.49	3.49	3.49	3.49	3.49

3.5	3.5	3.5	3.5	3.5	3.5	3.5	3.5	3.5
3.51	3.51	3.51	3.51	3.51	3.51	3.51	3.51	3.51
3.52	3.52	3.52	3.52	3.52	3.52	3.52	3.52	3.52
3.53	3.53	3.53	3.53	3.53	3.53	3.53	3.53	3.53
3.54	3.54	3.54	3.54	3.54	3.54	3.54	3.54	3.54
3.55	3.55	3.55	3.55	3.55	3.55	3.55	3.55	3.55
3.56	3.56	3.56	3.56	3.56	3.56	3.56	3.56	3.56
3.57	3.57	3.57	3.57	3.57	3.57	3.57	3.57	3.57
3.58	3.58	3.58	3.58	3.58	3.58	3.58	3.58	3.58
3.59	3.59	3.59	3.59	3.59	3.59	3.59	3.59	3.59
3.6	3.6	3.6	3.6	3.6	3.6	3.6	3.6	3.6
3.61	3.61	3.61	3.61	3.61	3.61	3.61	3.61	3.61
3.62	3.62	3.62	3.62	3.62	3.62	3.62	3.62	3.62
3.63	3.63	3.63	3.63	3.63	3.63	3.63	3.63	3.63
3.64	3.64	3.64	3.64	3.64	3.64	3.64	3.64	3.64
3.65	3.65	3.65	3.65	3.65	3.65	3.65	3.65	3.65
3.66	3.66	3.66	3.66	3.66	3.66	3.66	3.66	3.66
3.67	3.67	3.67	3.67	3.67	3.67	3.67	3.67	3.67
3.68	3.68	3.68	3.68	3.68	3.68	3.68	3.68	3.68
3.69	3.69	3.69	3.69	3.69	3.69	3.69	3.69	3.69
3.7	3.7	3.7	3.7	3.7	3.7	3.7	3.7	3.7
3.71	3.71	3.71	3.71	3.71	3.71	3.71	3.71	3.71
3.72	3.72	3.72	3.72	3.72	3.72	3.72	3.72	3.72
3.73	3.73	3.73	3.73	3.73	3.73	3.73	3.73	3.73
3.74	3.74	3.74	3.74	3.74	3.74	3.74	3.74	3.74
3.75	3.75	3.75	3.75	3.75	3.75	3.75	3.75	3.75
3.76	3.76	3.76	3.76	3.76	3.76	3.76	3.76	3.76
3.77	3.77	3.77	3.77	3.77	3.77	3.77	3.77	3.77
3.78	3.78	3.78	3.78	3.78	3.78	3.78	3.78	3.78
3.79	3.79	3.79	3.79	3.79	3.79	3.79	3.79	3.79
3.8	3.8	3.8	3.8	3.8	3.8	3.8	3.8	3.8
3.81	3.81	3.81	3.81	3.81	3.81	3.81	3.81	3.81
3.82	3.82	3.82	3.82	3.82	3.82	3.82	3.82	3.82
3.83	3.83	3.83	3.83	3.83	3.83	3.83	3.83	3.83
3.84	3.84	3.84	3.84	3.84	3.84	3.84	3.84	3.84
3.85	3.85	3.85	3.85	3.85	3.85	3.85	3.85	3.85
3.86	3.86	3.86	3.86	3.86	3.86	3.86	3.86	3.86
3.87	3.87	3.87	3.87	3.87	3.87	3.87	3.87	3.87
3.88	3.88	3.88	3.88	3.88	3.88	3.88	3.88	3.88
3.89	3.89	3.89	3.89	3.89	3.89	3.89	3.89	3.89
3.9	3.9	3.9	3.9	3.9	3.9	3.9	3.9	3.9
3.91	3.91	3.91	3.91	3.91	3.91	3.91	3.91	3.91
3.92	3.92	3.92	3.92	3.92	3.92	3.92	3.92	3.92
3.93	3.93	3.93	3.93	3.93	3.93	3.93	3.93	3.93
3.94	3.94	3.94	3.94	3.94	3.94	3.94	3.94	3.94
3.95	3.95	3.95	3.95	3.95	3.95	3.95	3.95	3.95
3.96	3.96	3.96	3.96	3.96	3.96	3.96	3.96	3.96
3.97	3.97	3.97	3.97	3.97	3.97	3.97	3.97	3.97
3.98	3.98	3.98	3.98	3.98	3.98	3.98	3.98	3.98
3.99	3.99	3.99	3.99	3.99	3.99	3.99	3.99	3.99

4	4	4	4	4	4	4	4	4
4.01	4.01	4.01	4.01	4.01	4.01	4.01	4.01	4.01
4.02	4.02	4.02	4.02	4.02	4.02	4.02	4.02	4.02
4.03	4.03	4.03	4.03	4.03	4.03	4.03	4.03	4.03
4.04	4.04	4.04	4.04	4.04	4.04	4.04	4.04	4.04
4.05	4.05	4.05	4.05	4.05	4.05	4.05	4.05	4.05
4.06	4.06	4.06	4.06	4.06	4.06	4.06	4.06	4.06
4.07	4.07	4.07	4.07	4.07	4.07	4.07	4.07	4.07
4.08	4.08	4.08	4.08	4.08	4.08	4.08	4.08	4.08
4.09	4.09	4.09	4.09	4.09	4.09	4.09	4.09	4.09
4.1	4.1	4.1	4.1	4.1	4.1	4.1	4.1	4.1
4.11	4.11	4.11	4.11	4.11	4.11	4.11	4.11	4.11
4.12	4.12	4.12	4.12	4.12	4.12	4.12	4.12	4.12
4.13	4.13	4.13	4.13	4.13	4.13	4.13	4.13	4.13
4.14	4.14	4.14	4.14	4.14	4.14	4.14	4.14	4.14
4.15	4.15	4.15	4.15	4.15	4.15	4.15	4.15	4.15
4.16	4.16	4.16	4.16	4.16	4.16	4.16	4.16	4.16
4.17	4.17	4.17	4.17	4.17	4.17	4.17	4.17	4.17
4.18	4.18	4.18	4.18	4.18	4.18	4.18	4.18	4.18
4.19	4.19	4.19	4.19	4.19	4.19	4.19	4.19	4.19
4.2	4.2	4.2	4.2	4.2	4.2	4.2	4.2	4.2
4.21	4.21	4.21	4.21	4.21	4.21	4.21	4.21	4.21
4.22	4.22	4.22	4.22	4.22	4.22	4.22	4.22	4.22
4.23	4.23	4.23	4.23	4.23	4.23	4.23	4.23	4.23
4.24	4.24	4.24	4.24	4.24	4.24	4.24	4.24	4.24
4.25	4.25	4.25	4.25	4.25	4.25	4.25	4.25	4.25
4.26	4.26	4.26	4.26	4.26	4.26	4.26	4.26	4.26
4.27	4.27	4.27	4.27	4.27	4.27	4.27	4.27	4.27
4.28	4.28	4.28	4.28	4.28	4.28	4.28	4.28	4.28
4.29	4.29	4.29	4.29	4.29	4.29	4.29	4.29	4.29
4.3	4.3	4.3	4.3	4.3	4.3	4.3	4.3	4.3
4.31	4.31	4.31	4.31	4.31	4.31	4.31	4.31	4.31
4.32	4.32	4.32	4.32	4.32	4.32	4.32	4.32	4.32
4.33	4.33	4.33	4.33	4.33	4.33	4.33	4.33	4.33
4.34	4.34	4.34	4.34	4.34	4.34	4.34	4.34	4.34
4.35	4.35	4.35	4.35	4.35	4.35	4.35	4.35	4.35
4.36	4.36	4.36	4.36	4.36	4.36	4.36	4.36	4.36
4.37	4.37	4.37	4.37	4.37	4.37	4.37	4.37	4.37
4.38	4.38	4.38	4.38	4.38	4.38	4.38	4.38	4.38
4.39	4.39	4.39	4.39	4.39	4.39	4.39	4.39	4.39
4.4	4.4	4.4	4.4	4.4	4.4	4.4	4.4	4.4
4.41	4.41	4.41	4.41	4.41	4.41	4.41	4.41	4.41
4.42	4.42	4.42	4.42	4.42	4.42	4.42	4.42	4.42
4.43	4.43	4.43	4.43	4.43	4.43	4.43	4.43	4.43
4.44	4.44	4.44	4.44	4.44	4.44	4.44	4.44	4.44
4.45	4.45	4.45	4.45	4.45	4.45	4.45	4.45	4.45
4.46	4.46	4.46	4.46	4.46	4.46	4.46	4.46	4.46
4.47	4.47	4.47	4.47	4.47	4.47	4.47	4.47	4.47
4.48	4.48	4.48	4.48	4.48	4.48	4.48	4.48	4.48
4.49	4.49	4.49	4.49	4.49	4.49	4.49	4.49	4.49

4.5	4.5	4.5	4.5	4.5	4.5	4.5	4.5	4.5
4.51	4.51	4.51	4.51	4.51	4.51	4.51	4.51	4.51
4.52	4.52	4.52	4.52	4.52	4.52	4.52	4.52	4.52
4.53	4.53	4.53	4.53	4.53	4.53	4.53	4.53	4.53
4.54	4.54	4.54	4.54	4.54	4.54	4.54	4.54	4.54
4.55	4.55	4.55	4.55	4.55	4.55	4.55	4.55	4.55
4.56	4.56	4.56	4.56	4.56	4.56	4.56	4.56	4.56
4.57	4.57	4.57	4.57	4.57	4.57	4.57	4.57	4.57
4.58	4.58	4.58	4.58	4.58	4.58	4.58	4.58	4.58
4.59	4.59	4.59	4.59	4.59	4.59	4.59	4.59	4.59
4.6	4.6	4.6	4.6	4.6	4.6	4.6	4.6	4.6
4.61	4.61	4.61	4.61	4.61	4.61	4.61	4.61	4.61
4.62	4.62	4.62	4.62	4.62	4.62	4.62	4.62	4.62
4.63	4.63	4.63	4.63	4.63	4.63	4.63	4.63	4.63
4.64	4.64	4.64	4.64	4.64	4.64	4.64	4.64	4.64
4.65	4.65	4.65	4.65	4.65	4.65	4.65	4.65	4.65
4.66	4.66	4.66	4.66	4.66	4.66	4.66	4.66	4.66
4.67	4.67	4.67	4.67	4.67	4.67	4.67	4.67	4.67
4.68	4.68	4.68	4.68	4.68	4.68	4.68	4.68	4.68
4.69	4.69	4.69	4.69	4.69	4.69	4.69	4.69	4.69
4.7	4.7	4.7	4.7	4.7	4.7	4.7	4.7	4.7
4.71	4.71	4.71	4.71	4.71	4.71	4.71	4.71	4.71
4.72	4.72	4.72	4.72	4.72	4.72	4.72	4.72	4.72
4.73	4.73	4.73	4.73	4.73	4.73	4.73	4.73	4.73
4.74	4.74	4.74	4.74	4.74	4.74	4.74	4.74	4.74
[truncated: 513,872 more chars]
