# Supplementary material for: Artefact-free topography based scleral-asymmetry
Source: PLoS One. 2019 Jul 26;14(7):e0219789. doi: 10.1371/journal.pone.0219789 (PMC6660081; doi:10.1371/journal.pone.0219789)
Supplement: S2 Table — (ZIP) [file Eye mean OD.pdf › Eye mean OD.pdf]

-0.06487	-0.06469
-0.06485	-0.06468
-0.06483	-0.06466
-0.06481	-0.06465
-0.0648	-0.06463
-0.06478	-0.06462
-0.06477	-0.0646
-0.06475	-0.06459
-0.06474	-0.06457
-0.06472	-0.06456
-0.06471	-0.06454
-0.06469	-0.06453
-0.06469	-0.06451
-0.06468	-0.0645
-0.06466	-0.06449
-0.06465	-0.06447
-0.06463	-0.06446
-0.06462	-0.06444
-0.0646	-0.06442
-0.06458	-0.0644
-0.06457	-0.06439
-0.06455	-0.06438
-0.06454	-0.06437
-0.06452	-0.06436

				-0.06451	-0.06434
				-0.0645	-0.06433
				-0.06449	-0.06432
				-0.06448	-0.06431
				-0.06447	-0.06429
				-0.06446	-0.06428
				-0.06444	-0.06427
				-0.06443	-0.06426
				-0.06443	-0.06426
				-0.06442	-0.06425
-0.06509	-0.06492	-0.12807	-0.12774	-0.12742	-0.12709
-0.06508	-0.06491	-0.12806	-0.12773	-0.1274	-0.12708
-0.06507	-0.0649	-0.12804	-0.12771	-0.12738	-0.12706
-0.06506	-0.06489	-0.12802	-0.12769	-0.12737	-0.12704
-0.06505	-0.06488	-0.12801	-0.12768	-0.12735	-0.12702
-0.06504	-0.06487	-0.12799	-0.12766	-0.12733	-0.127
-0.06503	-0.06486	-0.12797	-0.12764	-0.12731	-0.12697
-0.06502	-0.06485	-0.12796	-0.12763	-0.1273	-0.12696
-0.06501	-0.06484	-0.12794	-0.12761	-0.12728	-0.12695
-0.065	-0.06483	-0.12793	-0.1276	-0.12727	-0.12694
-0.065	-0.06482	-0.12791	-0.12758	-0.12725	-0.12693
-0.06499	-0.06482	-0.12789	-0.12756	-0.12724	-0.12691
-0.06498	-0.0648	-0.12788	-0.12755	-0.12722	-0.1269
-0.06497	-0.06479	-0.12786	-0.12753	-0.12721	-0.12688
-0.06496	-0.06479	-0.12785	-0.12752	-0.12719	-0.12686
-0.06496	-0.06478	-0.12784	-0.1275	-0.12717	-0.12685
-0.06495	-0.06478	-0.12783	-0.12749	-0.12716	-0.12683
-0.06494	-0.06477	-0.12782	-0.12748	-0.12715	-0.12681
-0.06493	-0.06476	-0.19188	-0.19138	-0.19087	-0.19036
-0.06492	-0.06475	-0.19187	-0.19137	-0.19087	-0.19036
-0.06492	-0.06474	-0.19185	-0.19136	-0.19086	-0.19036
-0.06491	-0.06474	-0.19185	-0.19134	-0.19084	-0.19034
-0.0649	-0.06473	-0.19184	-0.19133	-0.19082	-0.19033
-0.0649	-0.06473	-0.19184	-0.19133	-0.19082	-0.19033
-0.06489	-0.06472	-0.19184	-0.19133	-0.19083	-0.19034
-0.06489	-0.06472	-0.19184	-0.19133	-0.19083	-0.19033
-0.06488	-0.06472	-0.19185	-0.19134	-0.19083	-0.19032
-0.06487	-0.06471	-0.19184	-0.19134	-0.19083	-0.19032
-0.06487	-0.0647	-0.19182	-0.19133	-0.19082	-0.19032
		-0.13011	-0.12977	-0.19082	-0.19032
		-0.13012	-0.12978	-0.19083	-0.19032
		-0.13012	-0.12978	-0.19083	-0.19033
		-0.13013	-0.12979	-0.19084	-0.19033
		-0.13014	-0.1298	-0.19084	-0.19033
		-0.13014	-0.1298	-0.19083	-0.19033
		-0.13014	-0.12979	-0.19081	-0.19031
		-0.13015	-0.12979	-0.1908	-0.19031
		-0.13015	-0.1298	-0.1908	-0.19031
		-0.13016	-0.12981	-0.19081	-0.19032
		-0.13017	-0.12981	-0.19081	-0.19032

-0.13017	-0.12982	-0.19082	-0.19032
-0.13018	-0.12983	-0.19082	-0.19033
-0.13018	-0.12983	-0.19082	-0.19033
-0.13019	-0.12983	-0.19082	-0.19033
-0.13019	-0.12984	-0.19082	-0.19033
-0.1302	-0.12985	-0.19084	-0.19034
-0.13021	-0.12987	-0.19087	-0.19037
-0.13023	-0.12989	-0.19089	-0.19038
-0.13023	-0.12989	-0.19089	-0.19039
-0.13023	-0.12989	-0.1909	-0.19039
-0.13024	-0.1299	-0.1909	-0.19039
-0.13024	-0.1299	-0.1909	-0.19039
-0.13026	-0.12992	-0.19091	-0.1904
-0.13028	-0.12993	-0.19092	-0.1904
-0.13029	-0.12994	-0.19093	-0.19041
-0.13029	-0.12995	-0.19094	-0.19043
-0.1303	-0.12995	-0.19094	-0.19045
-0.13031	-0.12996	-0.19094	-0.19045
-0.13032	-0.12997	-0.19095	-0.19046
-0.13033	-0.12998	-0.19097	-0.19047
-0.13034	-0.13	-0.191	-0.19049
-0.13036	-0.13002	-0.19102	-0.19051
-0.13038	-0.13004	-0.19103	-0.19051
-0.1304	-0.13005	-0.19104	-0.19052
-0.13041	-0.13005	-0.19105	-0.19054
-0.13042	-0.13006	-0.19106	-0.19056
-0.13043	-0.13008	-0.19108	-0.19058
-0.13044	-0.1301	-0.19111	-0.1906
-0.13046	-0.13011	-0.19113	-0.19063
-0.13048	-0.13013	-0.19115	-0.19065
-0.1305	-0.13015	-0.19117	-0.19067
-0.13052	-0.13017	-0.1912	-0.19069
-0.13053	-0.13019	-0.19122	-0.19071
-0.13055	-0.1302	-0.19124	-0.19071
-0.13057	-0.13022	-0.19125	-0.19072
-0.13059	-0.13024	-0.19128	-0.19075
-0.1306	-0.13026	-0.19131	-0.19079
-0.13062	-0.13027	-0.19134	-0.19082
-0.13064	-0.13029	-0.19135	-0.19085
-0.13066	-0.1303	-0.19138	-0.19087
-0.13067	-0.13032	-0.1914	-0.19089

							-0.06487	-0.06469
							-0.06485	-0.06467
							-0.06482	-0.06464
							-0.06478	-0.06461
							-0.12813	-0.1278
							-0.12807	-0.12774
							-0.12802	-0.12769
							-0.12797	-0.12764
							-0.12792	-0.12759
					-0.06496	-0.06479	-0.12787	-0.12754
					-0.06494	-0.06476	-0.12782	-0.12749
					-0.06491	-0.06473	-0.12776	-0.12744
					-0.06489	-0.06471	-0.12771	-0.12739
					-0.06486	-0.06468	-0.12766	-0.12734
					-0.06484	-0.06466	-0.12761	-0.12729
					-0.06481	-0.06463	-0.12756	-0.12724
					-0.06478	-0.06461	-0.12751	-0.12719
					-0.06476	-0.06458	-0.12746	-0.12714
					-0.06473	-0.06456	-0.12742	-0.12709
					-0.06471	-0.06454	-0.12738	-0.12705
			-0.06503	-0.06486	-0.128	-0.12767	-0.18982	-0.18932
			-0.06501	-0.06483	-0.12795	-0.12763	-0.18976	-0.18926
			-0.06499	-0.06481	-0.1279	-0.12758	-0.1897	-0.18919
			-0.06496	-0.06478	-0.12785	-0.12752	-0.18964	-0.18913
			-0.06493	-0.06476	-0.1278	-0.12747	-0.18957	-0.18907
			-0.06491	-0.06474	-0.12776	-0.12743	-0.18952	-0.18902
			-0.06489	-0.06472	-0.12772	-0.12739	-0.18947	-0.18897
			-0.06487	-0.0647	-0.12768	-0.12734	-0.18941	-0.18892
			-0.06484	-0.06467	-0.12763	-0.12729	-0.18935	-0.18886
			-0.06482	-0.06465	-0.12758	-0.12724	-0.18929	-0.1888
			-0.0648	-0.06463	-0.12754	-0.1272	-0.18925	-0.18876
			-0.06478	-0.06461	-0.12749	-0.12716	-0.18921	-0.18871
			-0.06475	-0.06458	-0.12744	-0.12711	-0.18915	-0.18865
			-0.06473	-0.06455	-0.12739	-0.12706	-0.18909	-0.18859
			-0.06471	-0.06453	-0.12734	-0.12701	-0.18904	-0.18854
			-0.06468	-0.06451	-0.1273	-0.12697	-0.18899	-0.18849
			-0.06466	-0.06449	-0.12726	-0.12693	-0.18894	-0.18845
			-0.06464	-0.06447	-0.12722	-0.12689	-0.1889	-0.18841
-0.06513	-0.06496	-0.0648	-0.12785	-0.12751	-0.18985	-0.18935	-0.18885	-0.18836
-0.06512	-0.06494	-0.06478	-0.12781	-0.12747	-0.1898	-0.1893	-0.18881	-0.1883
-0.0651	-0.06493	-0.06475	-0.12777	-0.12743	-0.18976	-0.18926	-0.18875	-0.18825
-0.06508	-0.06491	-0.06474	-0.12773	-0.12739	-0.18971	-0.1892	-0.1887	-0.18819
-0.06506	-0.06489	-0.06472	-0.12769	-0.12735	-0.18966	-0.18914	-0.18864	-0.18814
-0.06504	-0.06487	-0.0647	-0.12765	-0.1273	-0.1896	-0.18909	-0.18859	-0.1881
-0.06502	-0.06485	-0.06467	-0.1276	-0.12725	-0.18954	-0.18904	-0.18855	-0.18805
-0.065	-0.06483	-0.06465	-0.12756	-0.1272	-0.18949	-0.18899	-0.1885	-0.18801
-0.06498	-0.06481	-0.06464	-0.12752	-0.12717	-0.18945	-0.18895	-0.18846	-0.18797

-0.06496	-0.06479	-0.06462	-0.12749	-0.12714	-0.18943	-0.18891	-0.18841	-0.18792
-0.06494	-0.06477	-0.0646	-0.12745	-0.12711	-0.18939	-0.18888	-0.18838	-0.18788
-0.06492	-0.06475	-0.06458	-0.12741	-0.12708	-0.18936	-0.18885	-0.18835	-0.18785
-0.0649	-0.06473	-0.06456	-0.12737	-0.12704	-0.18931	-0.18882	-0.18832	-0.18781
-0.06488	-0.06471	-0.06455	-0.12733	-0.127	-0.18927	-0.18878	-0.18828	-0.18779
-0.06486	-0.06469	-0.06453	-0.12729	-0.12696	-0.18922	-0.18874	-0.18825	-0.18775
-0.06484	-0.06467	-0.06451	-0.12725	-0.12692	-0.18918	-0.18869	-0.1882	-0.18771
-0.06483	-0.06466	-0.06449	-0.12721	-0.12689	-0.18914	-0.18865	-0.18815	-0.18767
-0.06481	-0.06464	-0.06447	-0.12718	-0.12685	-0.18909	-0.1886	-0.18811	-0.18762
-0.0648	-0.06463	-0.06445	-0.12715	-0.12681	-0.18905	-0.18856	-0.18807	-0.18758
-0.06478	-0.06461	-0.06444	-0.19002	-0.18951	-0.18901	-0.18852	-0.18803	-0.18753
-0.06476	-0.06459	-0.06443	-0.18999	-0.18949	-0.18898	-0.18848	-0.18798	-0.18749
-0.06474	-0.06458	-0.06441	-0.18995	-0.18945	-0.18895	-0.18844	-0.18794	-0.18746
-0.06472	-0.06456	-0.06439	-0.1899	-0.18941	-0.18892	-0.18841	-0.18792	-0.18743
-0.06471	-0.06454	-0.06437	-0.18986	-0.18937	-0.18888	-0.18838	-0.18788	-0.1874
-0.06469	-0.06452	-0.06435	-0.18982	-0.18932	-0.18883	-0.18833	-0.18784	-0.18735
-0.06468	-0.06451	-0.06434	-0.18979	-0.18928	-0.18878	-0.18829	-0.18781	-0.18731
-0.06466	-0.06449	-0.06432	-0.18975	-0.18924	-0.18875	-0.18826	-0.18778	-0.18728
-0.06464	-0.06447	-0.06431	-0.18972	-0.18921	-0.18872	-0.18823	-0.18775	-0.18725
-0.06462	-0.06446	-0.06429	-0.18969	-0.18919	-0.18869	-0.18819	-0.1877	-0.18721
-0.06461	-0.06444	-0.06428	-0.18966	-0.18916	-0.18866	-0.18815	-0.18765	-0.18716
-0.06459	-0.06443	-0.06426	-0.18963	-0.18913	-0.18862	-0.18811	-0.1876	-0.18712
-0.06458	-0.06441	-0.06424	-0.18958	-0.18908	-0.18858	-0.18807	-0.18757	-0.18709
-0.06456	-0.06439	-0.06422	-0.18953	-0.18904	-0.18855	-0.18804	-0.18754	-0.18706
-0.06455	-0.06437	-0.0642	-0.1895	-0.18901	-0.18852	-0.18801	-0.18751	-0.18703
-0.06453	-0.06436	-0.06419	-0.18947	-0.18898	-0.18849	-0.18798	-0.18748	-0.187
-0.19095	-0.19044	-0.18994	-0.18944	-0.18896	-0.18846	-0.18795	-0.18745	-0.18696
-0.19091	-0.19041	-0.18991	-0.18942	-0.18893	-0.18843	-0.18792	-0.18741	-0.18691
-0.19088	-0.19038	-0.18988	-0.18939	-0.1889	-0.1884	-0.18789	-0.18738	-0.18688
-0.19085	-0.19035	-0.18985	-0.18936	-0.18887	-0.18837	-0.18786	-0.18735	-0.18685
-0.19082	-0.19033	-0.18982	-0.18933	-0.18884	-0.18834	-0.18783	-0.18732	-0.18682
-0.1908	-0.1903	-0.18979	-0.1893	-0.18881	-0.18831	-0.1878	-0.18729	-0.18679
-0.19077	-0.19027	-0.18977	-0.18927	-0.18878	-0.18828	-0.18777	-0.18726	-0.18676
-0.19074	-0.19024	-0.18973	-0.18924	-0.18875	-0.18825	-0.18774	-0.18723	-0.18673
-0.19071	-0.19021	-0.1897	-0.18921	-0.18872	-0.18822	-0.18771	-0.18721	-0.1867
-0.19068	-0.19017	-0.18967	-0.18918	-0.18869	-0.18819	-0.18768	-0.18718	-0.18667
-0.19064	-0.19014	-0.18964	-0.18915	-0.18865	-0.18816	-0.18765	-0.18715	-0.18664
-0.19062	-0.19012	-0.18961	-0.18911	-0.18862	-0.18813	-0.18762	-0.18711	-0.18661
-0.1906	-0.19009	-0.18958	-0.18908	-0.18859	-0.18809	-0.18759	-0.18708	-0.18658
-0.19057	-0.19006	-0.18955	-0.18905	-0.18856	-0.18806	-0.18756	-0.18706	-0.18656
-0.19054	-0.19003	-0.18952	-0.18902	-0.18853	-0.18803	-0.18753	-0.18703	-0.18654
-0.19051	-0.19	-0.18949	-0.18899	-0.1885	-0.188	-0.1875	-0.187	-0.18652
-0.19048	-0.18998	-0.18948	-0.18898	-0.18848	-0.18798	-0.18747	-0.18697	-0.18649
-0.19045	-0.18996	-0.18947	-0.18897	-0.18846	-0.18796	-0.18744	-0.18694	-0.18646
-0.19042	-0.18993	-0.18944	-0.18894	-0.18844	-0.18793	-0.18742	-0.18693	-0.18645
-0.19039	-0.1899	-0.18941	-0.18891	-0.1884	-0.1879	-0.18741	-0.18692	-0.18643
-0.19037	-0.18987	-0.18939	-0.18888	-0.18837	-0.18787	-0.18739	-0.1869	-0.18641
-0.19036	-0.18986	-0.18937	-0.18886	-0.18834	-0.18784	-0.18736	-0.18687	-0.18638
-0.19034	-0.18985	-0.18935	-0.18884	-0.18832	-0.18782	-0.18733	-0.18684	-0.18635
-0.19031	-0.18982	-0.18932	-0.18881	-0.18831	-0.18781	-0.18732	-0.18682	-0.18633

-0.19028	-0.18979	-0.18928	-0.18879	-0.18829	-0.1878	-0.1873	-0.1868	-0.1863
-0.19026	-0.18976	-0.18925	-0.18876	-0.18827	-0.18777	-0.18727	-0.18678	-0.18628
-0.19024	-0.18973	-0.18922	-0.18873	-0.18823	-0.18774	-0.18724	-0.18675	-0.18627
-0.19022	-0.18971	-0.18921	-0.18871	-0.18821	-0.18772	-0.18722	-0.18673	-0.18625
-0.19019	-0.18969	-0.1892	-0.1887	-0.18819	-0.1877	-0.18721	-0.18672	-0.18624
-0.19017	-0.18967	-0.18918	-0.18868	-0.18818	-0.18768	-0.18719	-0.18671	-0.18623
-0.19016	-0.18966	-0.18917	-0.18867	-0.18817	-0.18767	-0.18718	-0.18669	-0.1862
-0.19015	-0.18965	-0.18915	-0.18865	-0.18815	-0.18766	-0.18716	-0.18667	-0.18618
-0.19013	-0.18964	-0.18914	-0.18864	-0.18814	-0.18764	-0.18714	-0.18664	-0.18616
-0.19012	-0.18963	-0.18913	-0.18863	-0.18812	-0.18762	-0.18711	-0.18662	-0.18614
-0.19011	-0.18961	-0.18912	-0.18861	-0.18809	-0.18759	-0.18709	-0.1866	-0.18613
-0.1901	-0.1896	-0.1891	-0.18858	-0.18806	-0.18756	-0.18707	-0.1866	-0.18612
-0.19008	-0.18958	-0.18907	-0.18856	-0.18804	-0.18754	-0.18706	-0.18658	-0.1861
-0.19007	-0.18956	-0.18904	-0.18853	-0.18803	-0.18753	-0.18705	-0.18656	-0.18608
-0.19004	-0.18953	-0.18902	-0.18851	-0.18801	-0.18752	-0.18703	-0.18655	-0.18606
-0.19001	-0.18951	-0.18901	-0.1885	-0.18799	-0.1875	-0.18702	-0.18654	-0.18606
-0.18999	-0.18949	-0.18899	-0.18849	-0.18798	-0.18748	-0.18701	-0.18653	-0.18606
-0.18999	-0.18948	-0.18898	-0.18847	-0.18797	-0.18747	-0.18699	-0.18651	-0.18603
-0.18999	-0.18947	-0.18896	-0.18845	-0.18795	-0.18746	-0.18698	-0.1865	-0.18601
-0.18998	-0.18947	-0.18896	-0.18845	-0.18794	-0.18745	-0.18697	-0.18649	-0.186
-0.18997	-0.18947	-0.18896	-0.18844	-0.18793	-0.18743	-0.18696	-0.18648	-0.186
-0.18996	-0.18945	-0.18894	-0.18843	-0.18791	-0.18742	-0.18694	-0.18647	-0.186
-0.18994	-0.18942	-0.18891	-0.1884	-0.1879	-0.18741	-0.18693	-0.18645	-0.18598
-0.18992	-0.1894	-0.18889	-0.18838	-0.18789	-0.1874	-0.18691	-0.18644	-0.18597
-0.18991	-0.1894	-0.18888	-0.18838	-0.18787	-0.18738	-0.1869	-0.18643	-0.18596
-0.1899	-0.1894	-0.18888	-0.18837	-0.18786	-0.18736	-0.18689	-0.18642	-0.18594
-0.18988	-0.18938	-0.18888	-0.18837	-0.18786	-0.18736	-0.18688	-0.1864	-0.18593
-0.18986	-0.18937	-0.18887	-0.18837	-0.18786	-0.18736	-0.18687	-0.18638	-0.18591
-0.18986	-0.18935	-0.18886	-0.18835	-0.18785	-0.18736	-0.18686	-0.18638	-0.18591
-0.18986	-0.18934	-0.18884	-0.18834	-0.18784	-0.18735	-0.18686	-0.18638	-0.18592
-0.18985	-0.18934	-0.18882	-0.18833	-0.18784	-0.18735	-0.18687	-0.18639	-0.18592
-0.18984	-0.18933	-0.18882	-0.18833	-0.18784	-0.18736	-0.18687	-0.18639	-0.18591
-0.18984	-0.18933	-0.18883	-0.18833	-0.18784	-0.18736	-0.18687	-0.18639	-0.1859
-0.18984	-0.18934	-0.18883	-0.18833	-0.18783	-0.18734	-0.18686	-0.18638	-0.1859
-0.18984	-0.18934	-0.18883	-0.18832	-0.18781	-0.18732	-0.18685	-0.18638	-0.18589
-0.18983	-0.18933	-0.18883	-0.18832	-0.18781	-0.18732	-0.18684	-0.18636	-0.18588
-0.18981	-0.18932	-0.18882	-0.18832	-0.18781	-0.18732	-0.18683	-0.18634	-0.18587
-0.18981	-0.1893	-0.18881	-0.18831	-0.18781	-0.18731	-0.18682	-0.18634	-0.18587
-0.18981	-0.18929	-0.18879	-0.18829	-0.1878	-0.18731	-0.18682	-0.18634	-0.18587
-0.18981	-0.18929	-0.18878	-0.18828	-0.18779	-0.18731	-0.18682	-0.18634	-0.18587
-0.18981	-0.18929	-0.18878	-0.18828	-0.18779	-0.18731	-0.18683	-0.18634	-0.18586
-0.18982	-0.1893	-0.18878	-0.18828	-0.1878	-0.18731	-0.18683	-0.18635	-0.18586
-0.18982	-0.1893	-0.18878	-0.18828	-0.1878	-0.18732	-0.18684	-0.18635	-0.18586
-0.18982	-0.1893	-0.18879	-0.18829	-0.1878	-0.18732	-0.18684	-0.18635	-0.18586
-0.18982	-0.18931	-0.18879	-0.18829	-0.18779	-0.18731	-0.18683	-0.18635	-0.18586
-0.18982	-0.18931	-0.18879	-0.18828	-0.18778	-0.18729	-0.18682	-0.18634	-0.18586
-0.18982	-0.18931	-0.1888	-0.18828	-0.18777	-0.18728	-0.18681	-0.18634	-0.18586
-0.18982	-0.18931	-0.1888	-0.18829	-0.18777	-0.18728	-0.18682	-0.18635	-0.18587
-0.18982	-0.18932	-0.1888	-0.18829	-0.18778	-0.18729	-0.18682	-0.18635	-0.18587
-0.18983	-0.18932	-0.18881	-0.18829	-0.18778	-0.18729	-0.18682	-0.18636	-0.18588

-0.18983	-0.18932	-0.18881	-0.1883	-0.18778	-0.18729	-0.18683	-0.18636	-0.18588
-0.18983	-0.18932	-0.18881	-0.1883	-0.18779	-0.1873	-0.18683	-0.18636	-0.18588
-0.18984	-0.18933	-0.18881	-0.1883	-0.18779	-0.1873	-0.18683	-0.18637	-0.18589
-0.18984	-0.18933	-0.18882	-0.1883	-0.18779	-0.1873	-0.18684	-0.18637	-0.18589
-0.18984	-0.18933	-0.18882	-0.18831	-0.18779	-0.1873	-0.18684	-0.18637	-0.1859
-0.18985	-0.18934	-0.18882	-0.18831	-0.18781	-0.18732	-0.18685	-0.18638	-0.1859
-0.18986	-0.18935	-0.18883	-0.18832	-0.18783	-0.18734	-0.18687	-0.1864	-0.18591
-0.18987	-0.18935	-0.18883	-0.18833	-0.18784	-0.18736	-0.18689	-0.18641	-0.18592
-0.18988	-0.18935	-0.18884	-0.18834	-0.18785	-0.18737	-0.18689	-0.18641	-0.18592
-0.18988	-0.18936	-0.18884	-0.18834	-0.18785	-0.18737	-0.18689	-0.18641	-0.18593
-0.18988	-0.18936	-0.18884	-0.18834	-0.18786	-0.18738	-0.1869	-0.18642	-0.18594
-0.18988	-0.18936	-0.18884	-0.18834	-0.18786	-0.18738	-0.1869	-0.18643	-0.18596
-0.18989	-0.18937	-0.18886	-0.18836	-0.18787	-0.18739	-0.1869	-0.18643	-0.18597
-0.18989	-0.18938	-0.18888	-0.18838	-0.18789	-0.1874	-0.18691	-0.18643	-0.18597
-0.1899	-0.1894	-0.1889	-0.1884	-0.1879	-0.18741	-0.18692	-0.18645	-0.18598
-0.18993	-0.18943	-0.18892	-0.18842	-0.18791	-0.18741	-0.18694	-0.18647	-0.18601
-0.18995	-0.18945	-0.18894	-0.18843	-0.18791	-0.18742	-0.18696	-0.18649	-0.18602
-0.18996	-0.18945	-0.18894	-0.18844	-0.18793	-0.18745	-0.18698	-0.18651	-0.18603
-0.18996	-0.18945	-0.18894	-0.18845	-0.18796	-0.18747	-0.187	-0.18652	-0.18604
-0.18997	-0.18946	-0.18895	-0.18845	-0.18797	-0.18748	-0.18701	-0.18653	-0.18606
-0.18999	-0.18947	-0.18895	-0.18846	-0.18797	-0.18749	-0.18701	-0.18654	-0.18607
-0.18999	-0.18948	-0.18897	-0.18847	-0.18799	-0.1875	-0.18702	-0.18654	-0.18608
-0.19	-0.18949	-0.18899	-0.1885	-0.18801	-0.18752	-0.18703	-0.18655	-0.18608
-0.19001	-0.18951	-0.18902	-0.18852	-0.18802	-0.18753	-0.18704	-0.18656	-0.18609
-0.19004	-0.18954	-0.18904	-0.18853	-0.18803	-0.18754	-0.18706	-0.18659	-0.18611
-0.19006	-0.18956	-0.18905	-0.18855	-0.18804	-0.18755	-0.18708	-0.18661	-0.18613
-0.19008	-0.18957	-0.18906	-0.18856	-0.18806	-0.18757	-0.1871	-0.18663	-0.18615
-0.19009	-0.18958	-0.18906	-0.18857	-0.18808	-0.1876	-0.18712	-0.18665	-0.18618
-0.19012	-0.1896	-0.18909	-0.18859	-0.1881	-0.18762	-0.18715	-0.18667	-0.1862
-0.19014	-0.18964	-0.18913	-0.18863	-0.18813	-0.18764	-0.18717	-0.1867	-0.18622
-0.19017	-0.18966	-0.18915	-0.18865	-0.18815	-0.18766	-0.18719	-0.18672	-0.18623
-0.19018	-0.18967	-0.18916	-0.18866	-0.18817	-0.18769	-0.18722	-0.18674	-0.18624
-0.19019	-0.18968	-0.18917	-0.18868	-0.1882	-0.18772	-0.18724	-0.18676	-0.18626
-0.1902	-0.18969	-0.18919	-0.1887	-0.18822	-0.18774	-0.18726	-0.18678	-0.18629
-0.1902	-0.18971	-0.18922	-0.18873	-0.18824	-0.18776	-0.18729	-0.18681	-0.18632
-0.19023	-0.18973	-0.18924	-0.18875	-0.18826	-0.18778	-0.18731	-0.18683	-0.18633
-0.19027	-0.18976	-0.18926	-0.18877	-0.18829	-0.1878	-0.18733	-0.18684	-0.18634
-0.19031	-0.1898	-0.18929	-0.18879	-0.18831	-0.18782	-0.18735	-0.18686	-0.18636
-0.19034	-0.18983	-0.18932	-0.18882	-0.18833	-0.18784	-0.18737	-0.18689	-0.1864
-0.19036	-0.18986	-0.18936	-0.18885	-0.18835	-0.18786	-0.18739	-0.18691	-0.18643
-0.19038	-0.18988	-0.18939	-0.18889	-0.18839	-0.1879	-0.18741	-0.18693	-0.18645
-0.1904	-0.18991	-0.18941	-0.18892	-0.18842	-0.18793	-0.18744	-0.18695	-0.18647
-0.19043	-0.18993	-0.18943	-0.18894	-0.18845	-0.18796	-0.18747	-0.18698	-0.18649
-0.19045	-0.18995	-0.18945	-0.18896	-0.18848	-0.18799	-0.1875	-0.18701	-0.18652
-0.19048	-0.18997	-0.18948	-0.18899	-0.1885	-0.18801	-0.18753	-0.18705	-0.18655
-0.19049	-0.19	-0.1895	-0.18901	-0.18852	-0.18803	-0.18755	-0.18707	-0.18658
-0.19052	-0.19002	-0.18953	-0.18903	-0.18854	-0.18805	-0.18757	-0.18709	-0.18661
-0.19056	-0.19005	-0.18955	-0.18905	-0.18856	-0.18807	-0.18759	-0.18711	-0.18663
-0.1906	-0.19008	-0.18957	-0.18908	-0.18859	-0.1881	-0.18762	-0.18713	-0.18665
-0.19063	-0.19012	-0.18961	-0.18911	-0.18863	-0.18814	-0.18765	-0.18717	-0.18668

-0.19066	-0.19015	-0.18965	-0.18915	-0.18867	-0.18818	-0.18769	-0.1872	-0.1867
-0.1907	-0.19019	-0.18969	-0.18919	-0.18869	-0.1882	-0.18771	-0.18723	-0.18674
-0.19074	-0.19023	-0.18973	-0.18922	-0.18871	-0.18822	-0.18774	-0.18726	-0.18677
-0.19076	-0.19026	-0.18976	-0.18925	-0.18874	-0.18825	-0.18777	-0.18729	-0.1868
-0.19078	-0.19028	-0.18978	-0.18928	-0.18877	-0.18828	-0.1878	-0.18733	-0.18684
-0.1908	-0.19031	-0.18982	-0.18932	-0.18881	-0.18832	-0.18783	-0.18735	-0.18687
-0.19084	-0.19035	-0.18986	-0.18936	-0.18885	-0.18835	-0.18785	-0.18737	-0.18689
-0.12965	-0.12931	-0.12898	-0.18939	-0.18888	-0.18838	-0.18788	-0.18738	-0.18691
-0.12968	-0.12933	-0.12899	-0.18941	-0.18891	-0.18841	-0.18791	-0.18742	-0.18695
-0.1297	-0.12935	-0.129	-0.18943	-0.18893	-0.18844	-0.18794	-0.18745	-0.18698
-0.12973	-0.12938	-0.12903	-0.18946	-0.18897	-0.18847	-0.18798	-0.18749	-0.18701
-0.12976	-0.12941	-0.12906	-0.1895	-0.18901	-0.18851	-0.18801	-0.18752	-0.18703
-0.12978	-0.12943	-0.12908	-0.18954	-0.18905	-0.18855	-0.18805	-0.18755	-0.18706
-0.12979	-0.12945	-0.12911	-0.18958	-0.18909	-0.18859	-0.18809	-0.18759	-0.1871
-0.12981	-0.12948	-0.12914	-0.18962	-0.18913	-0.18863	-0.18812	-0.18762	-0.18713
-0.12984	-0.1295	-0.12917	-0.18966	-0.18916	-0.18866	-0.18816	-0.18766	-0.18717
-0.12987	-0.12953	-0.12919	-0.1897	-0.1892	-0.1887	-0.18819	-0.18769	-0.1872
-0.12989	-0.12956	-0.12922	-0.18974	-0.18924	-0.18874	-0.18823	-0.18773	-0.18724
-0.12992	-0.12959	-0.12925	-0.18978	-0.18928	-0.18878	-0.18827	-0.18776	-0.18727
-0.12994	-0.12961	-0.12927	-0.18982	-0.18932	-0.18881	-0.1883	-0.1878	-0.18731
-0.12997	-0.12963	-0.1293	-0.18986	-0.18936	-0.18885	-0.18834	-0.18783	-0.18734
-0.12999	-0.12966	-0.12932	-0.18989	-0.18939	-0.18889	-0.18837	-0.18786	-0.18737
-0.13001	-0.12968	-0.12935	-0.18993	-0.18943	-0.18892	-0.18841	-0.1879	-0.1874
-0.13004	-0.12971	-0.12937	-0.18997	-0.18947	-0.18896	-0.18844	-0.18793	-0.18744
-0.13007	-0.12974	-0.1294	-0.19	-0.1895	-0.189	-0.18848	-0.18797	-0.18749
			-0.12909	-0.12875	-0.18903	-0.18851	-0.188	-0.18753
			-0.12911	-0.12877	-0.18907	-0.18855	-0.18804	-0.18757
			-0.12913	-0.1288	-0.18911	-0.18858	-0.18808	-0.1876
			-0.12917	-0.12883	-0.18915	-0.18862	-0.18811	-0.18764
			-0.12921	-0.12887	-0.18919	-0.18866	-0.18815	-0.18767
			-0.12924	-0.1289	-0.18924	-0.18871	-0.1882	-0.18772
			-0.12927	-0.12892	-0.18928	-0.18876	-0.18826	-0.18777
			-0.12929	-0.12895	-0.18932	-0.18881	-0.18831	-0.18782
			-0.12932	-0.12897	-0.18935	-0.18885	-0.18835	-0.18786
			-0.12935	-0.129	-0.18939	-0.18889	-0.18839	-0.18789
			-0.12938	-0.12903	-0.18945	-0.18894	-0.18844	-0.18794
			-0.12941	-0.12907	-0.18951	-0.18899	-0.18848	-0.18799
			-0.12944	-0.12911	-0.12876	-0.1284	-0.18852	-0.18803
			-0.12947	-0.12914	-0.12879	-0.12843	-0.18856	-0.18807
			-0.12951	-0.12917	-0.12882	-0.12846	-0.1886	-0.18811
			-0.12953	-0.12919	-0.12884	-0.12849	-0.18866	-0.18816
			-0.06402	-0.06385	-0.12887	-0.12853	-0.18872	-0.18821
			-0.06404	-0.06387	-0.12891	-0.12856	-0.18877	-0.18827
			-0.06405	-0.06388	-0.12895	-0.1286	-0.18882	-0.18832
			-0.06407	-0.0639	-0.12898	-0.12863	-0.18887	-0.18837
			-0.06408	-0.06392	-0.12901	-0.12867	-0.18893	-0.18842
			-0.0641	-0.06394	-0.12905	-0.12871	-0.12836	-0.12801
			-0.06412	-0.06395	-0.12909	-0.12874	-0.12839	-0.12803
			-0.06413	-0.06397	-0.12912	-0.12877	-0.12842	-0.12806
			-0.06415	-0.06399	-0.12915	-0.1288	-0.12844	-0.12809

-0.06417	-0.064	-0.12918	-0.12883	-0.12847	-0.12812
-0.06418	-0.06401	-0.1292	-0.12886	-0.12851	-0.12816
-0.0642	-0.06403	-0.12923	-0.12889	-0.12855	-0.12819
-0.06421	-0.06405	-0.12927	-0.12893	-0.12858	-0.12823
-0.06423	-0.06407	-0.12932	-0.12896	-0.12861	-0.12826
-0.06425	-0.06409	-0.12936	-0.129	-0.12865	-0.1283
-0.06427	-0.0641	-0.12939	-0.12905	-0.1287	-0.12834
-0.06428	-0.06412	-0.12943	-0.12909	-0.12874	-0.12838
-0.0643	-0.06414	-0.12946	-0.12912	-0.12878	-0.12842
-0.06432	-0.06415	-0.12949	-0.12915	-0.12881	-0.12847
		-0.06401	-0.06384	-0.12885	-0.1285
		-0.06402	-0.06386	-0.12888	-0.12853
		-0.06404	-0.06387	-0.12891	-0.12857
		-0.06406	-0.06389	-0.12895	-0.1286
		-0.06407	-0.06391	-0.12899	-0.12864
		-0.0641	-0.06393	-0.12904	-0.12869
		-0.06412	-0.06396	-0.12908	-0.12873
		-0.06414	-0.06397	-0.12911	-0.12877
		-0.06416	-0.06399	-0.12914	-0.1288
		-0.06418	-0.06401	-0.12918	-0.12885
		-0.0642	-0.06403	-0.12923	-0.12889
		-0.06422	-0.06405	-0.12927	-0.12893
		-0.06423	-0.06407	-0.1293	-0.12896
		-0.06425	-0.06409	-0.12934	-0.129
		-0.06428	-0.06411	-0.12938	-0.12904
		-0.0643	-0.06414	-0.06397	-0.0638
		-0.06432	-0.06416	-0.06399	-0.06383
		-0.06434	-0.06418	-0.06402	-0.06385
				-0.06403	-0.06387
				-0.06405	-0.06388
				-0.06407	-0.0639
				-0.06409	-0.06393
				-0.06411	-0.06395
				-0.06414	-0.06397
				-0.06416	-0.064
				-0.06418	-0.06402
				-0.0642	-0.06404
				-0.06423	-0.06406
				-0.06425	-0.06409
				-0.06427	-0.06411
				-0.0643	-0.06413
				-0.06432	-0.06416
				-0.06434	-0.06418
				-0.06437	-0.0642

						-0.065	-0.06483
						-0.06496	-0.0648
						-0.06493	-0.06476
						-0.06489	-0.06472
						-0.06485	-0.06468
						-0.06481	-0.06465
						-0.06478	-0.06461
						-0.06474	-0.06457
						-0.0647	-0.06453
			-0.06502	-0.06485	-0.06467	-0.0645
				-0.06499	-0.06481	-0.06464	-0.06447
				-0.06495	-0.06478	-0.12793	-0.12759
				-0.06491	-0.06474	-0.12786	-0.12752
				-0.06488	-0.06471	-0.12778	-0.12745
				-0.06484	-0.06467	-0.12771	-0.12739
				-0.0648	-0.06463	-0.12764	-0.12732
				-0.06477	-0.0646	-0.12757	-0.12725
				-0.06474	-0.06457	-0.1275	-0.12718
				-0.06471	-0.06453	-0.12744	-0.12712
				-0.06468	-0.0645	-0.12738	-0.12706
				-0.06464	-0.06447	-0.1891	-0.1886
				-0.06462	-0.06445	-0.18902	-0.18852
		-0.06493	-0.06476	-0.12786	-0.12753	-0.29558	-0.29489
		-0.06489	-0.06472	-0.12779	-0.12745	-0.29549	-0.29479
		-0.06485	-0.06469	-0.12772	-0.12738	-0.29539	-0.2947
		-0.06482	-0.06465	-0.12765	-0.12732	-0.29532	-0.29463
		-0.06479	-0.06462	-0.12758	-0.12726	-0.29525	-0.29457
		-0.06476	-0.06459	-0.12752	-0.1272	-0.29517	-0.29449
		-0.06473	-0.06456	-0.12746	-0.12713	-0.29511	-0.29442
		-0.0647	-0.06453	-0.1274	-0.12707	-0.29504	-0.29434
		-0.06467	-0.06449	-0.12733	-0.12701	-0.29498	-0.29428
		-0.06463	-0.06446	-0.12727	-0.12695	-0.29493	-0.29422
		-0.0646	-0.06443	-0.12721	-0.12689	-0.29486	-0.29415
		-0.06457	-0.0644	-0.12715	-0.12682	-0.2948	-0.29408
		-0.12774	-0.12741	-0.18891	-0.18841	-0.29473	-0.29402
		-0.12768	-0.12735	-0.18883	-0.18833	-0.29466	-0.29396
		-0.12762	-0.12729	-0.18875	-0.18826	-0.29461	-0.29391
		-0.12755	-0.12722	-0.18867	-0.18819	-0.29456	-0.29385
-0.06476	-0.06458	-0.12749	-0.12717	-0.1886	-0.18812	-0.29451	-0.2938
-0.06473	-0.06455	-0.12744	-0.12711	-0.18854	-0.18805	-0.29444	-0.29373
-0.0647	-0.06452	-0.12738	-0.12706	-0.18847	-0.18797	-0.29437	-0.29366
-0.06467	-0.0645	-0.12732	-0.127	-0.29575	-0.29502	-0.2943	-0.29358
-0.06464	-0.06447	-0.12726	-0.12694	-0.29569	-0.29495	-0.29422	-0.29351
-0.12787	-0.12753	-0.1272	-0.12687	-0.29563	-0.2949	-0.29417	-0.29345
-0.1278	-0.12747	-0.12714	-0.12681	-0.29556	-0.29485	-0.29413	-0.29341
-0.12774	-0.12741	-0.12708	-0.12676	-0.29551	-0.29479	-0.29407	-0.29335
-0.12769	-0.12735	-0.12703	-0.1267	-0.29545	-0.29472	-0.294	-0.29328
-0.12764	-0.1273	-0.18893	-0.18844	-0.2954	-0.29467	-0.29394	-0.29322
-0.12758	-0.12724	-0.18886	-0.18838	-0.29535	-0.29462	-0.29389	-0.29318

	-0.12752	-0.12719	-0.18879	-0.18831	-0.29531	-0.29458	-0.29385	-0.29315
	-0.12746	-0.12713	-0.18872	-0.18823	-0.29524	-0.29451	-0.2938	-0.2931
	-0.12741	-0.12708	-0.18865	-0.18815	-0.29516	-0.29444	-0.29374	-0.29304
-0.06452	-0.12736	-0.12703	-0.18859	-0.18808	-0.2951	-0.29439	-0.29369	-0.293
-0.06449	-0.12731	-0.12698	-0.18852	-0.18802	-0.29506	-0.29435	-0.29365	-0.29296
-0.06446	-0.12725	-0.12693	-0.18845	-0.18795	-0.29501	-0.29431	-0.29361	-0.29293
-0.06443	-0.12719	-0.12687	-0.18837	-0.18787	-0.29495	-0.29426	-0.29358	-0.2929
-0.12747	-0.29771	-0.297	-0.29629	-0.29558	-0.29489	-0.29422	-0.29354	-0.29287
-0.12741	-0.29766	-0.29694	-0.29624	-0.29553	-0.29484	-0.29416	-0.29349	-0.29281
-0.12736	-0.29761	-0.2969	-0.29619	-0.29549	-0.2948	-0.29411	-0.29343	-0.29275
-0.12731	-0.29756	-0.29685	-0.29614	-0.29545	-0.29476	-0.29407	-0.29338	-0.29271
-0.12726	-0.29751	-0.2968	-0.2961	-0.29541	-0.29473	-0.29403	-0.29335	-0.29267
-0.12721	-0.29747	-0.29675	-0.29606	-0.29537	-0.29469	-0.294	-0.29331	-0.29264
-0.12716	-0.29742	-0.29671	-0.29601	-0.29533	-0.29465	-0.29396	-0.29328	-0.29261
-0.12711	-0.29737	-0.29666	-0.29597	-0.29529	-0.29461	-0.29393	-0.29325	-0.29258
-0.12706	-0.29734	-0.29663	-0.29594	-0.29525	-0.29457	-0.29389	-0.29321	-0.29254
-0.12701	-0.2973	-0.29659	-0.2959	-0.29522	-0.29454	-0.29385	-0.29317	-0.2925
-0.12696	-0.29725	-0.29655	-0.29586	-0.29518	-0.2945	-0.29382	-0.29314	-0.29246
-0.12691	-0.29721	-0.29651	-0.29582	-0.29515	-0.29447	-0.29379	-0.29311	-0.29243
-0.12686	-0.29716	-0.29647	-0.29579	-0.29511	-0.29443	-0.29375	-0.29307	-0.29239
-0.12681	-0.29712	-0.29643	-0.29575	-0.29507	-0.29439	-0.29371	-0.29303	-0.29235
-0.12676	-0.29709	-0.2964	-0.29571	-0.29504	-0.29436	-0.29367	-0.29299	-0.29231
-0.12672	-0.29706	-0.29636	-0.29568	-0.295	-0.29432	-0.29364	-0.29295	-0.29226
-0.18881	-0.29704	-0.29634	-0.29565	-0.29498	-0.2943	-0.29361	-0.29292	-0.29223
-0.18875	-0.29701	-0.29632	-0.29563	-0.29496	-0.29428	-0.29359	-0.2929	-0.29221
-0.18869	-0.29697	-0.2963	-0.29562	-0.29494	-0.29426	-0.29357	-0.29288	-0.2922
-0.18863	-0.29694	-0.29627	-0.29559	-0.29491	-0.29423	-0.29354	-0.29285	-0.29216
-0.18857	-0.29691	-0.29624	-0.29556	-0.29488	-0.29419	-0.29349	-0.2928	-0.29211
-0.18852	-0.29689	-0.29622	-0.29554	-0.29484	-0.29415	-0.29346	-0.29276	-0.29207
-0.18848	-0.29687	-0.2962	-0.29551	-0.29482	-0.29412	-0.29342	-0.29272	-0.29203
-0.18843	-0.29685	-0.29617	-0.29548	-0.29479	-0.29409	-0.29339	-0.29269	-0.292
-0.18837	-0.2968	-0.29613	-0.29544	-0.29476	-0.29406	-0.29336	-0.29267	-0.29198
-0.18831	-0.29676	-0.29608	-0.2954	-0.29472	-0.29403	-0.29334	-0.29264	-0.29195
-0.18826	-0.29673	-0.29604	-0.29536	-0.29468	-0.29399	-0.29329	-0.29259	-0.29191
-0.18821	-0.2967	-0.296	-0.29531	-0.29463	-0.29394	-0.29325	-0.29255	-0.29187
-0.18815	-0.29666	-0.29597	-0.29528	-0.2946	-0.29391	-0.29321	-0.29252	-0.29184
-0.18809	-0.29663	-0.29594	-0.29526	-0.29458	-0.29389	-0.29319	-0.29249	-0.29181
-0.18804	-0.18755	-0.18707	-0.18658	-0.1861	-0.29386	-0.29316	-0.29247	-0.29178
-0.188	-0.18751	-0.18702	-0.18654	-0.18606	-0.29384	-0.29314	-0.29245	-0.29176
-0.18796	-0.18747	-0.18697	-0.18649	-0.18601	-0.2938	-0.29311	-0.29242	-0.29173
-0.18791	-0.18741	-0.18691	-0.18643	-0.18595	-0.29375	-0.29306	-0.29238	-0.29169
-0.18786	-0.18736	-0.18686	-0.18637	-0.18589	-0.2937	-0.29302	-0.29234	-0.29166
-0.1878	-0.18731	-0.18681	-0.18633	-0.18585	-0.29368	-0.29299	-0.29231	-0.29164
-0.18774	-0.18725	-0.18677	-0.18629	-0.1858	-0.29366	-0.29297	-0.29229	-0.29163
-0.18769	-0.18721	-0.18673	-0.18624	-0.18576	-0.29363	-0.29295	-0.29227	-0.29161
-0.18766	-0.18717	-0.18668	-0.1862	-0.18572	-0.29361	-0.29292	-0.29225	-0.29158
-0.18761	-0.18712	-0.18664	-0.18615	-0.18567	-0.29358	-0.2929	-0.29223	-0.29157
-0.18756	-0.18708	-0.1866	-0.18611	-0.18563	-0.29356	-0.29288	-0.29221	-0.29155
-0.18752	-0.18703	-0.18655	-0.18607	-0.18559	-0.29354	-0.29286	-0.29219	-0.29153
-0.18748	-0.18699	-0.18651	-0.18603	-0.18554	-0.29351	-0.29284	-0.29217	-0.29151

-0.18744	-0.18695	-0.18646	-0.18598	-0.1855	-0.29349	-0.29282	-0.29216	-0.2915
-0.18739	-0.18691	-0.18642	-0.18594	-0.18546	-0.29347	-0.2928	-0.29214	-0.29148
-0.18735	-0.18686	-0.18638	-0.1859	-0.18541	-0.29346	-0.29278	-0.29212	-0.29146
-0.18731	-0.18682	-0.18634	-0.18585	-0.18537	-0.29344	-0.29276	-0.2921	-0.29145
-0.18729	-0.18679	-0.18629	-0.18581	-0.18532	-0.29341	-0.29274	-0.29208	-0.29143
-0.18725	-0.18676	-0.18626	-0.18576	-0.18528	-0.29339	-0.29272	-0.29206	-0.29141
-0.18722	-0.18672	-0.18623	-0.18573	-0.18524	-0.29337	-0.29271	-0.29205	-0.2914
-0.18718	-0.18669	-0.18619	-0.1857	-0.1852	-0.29336	-0.29269	-0.29203	-0.29138
-0.18713	-0.18665	-0.18616	-0.18566	-0.18517	-0.29336	-0.29268	-0.29202	-0.29136
-0.18709	-0.1866	-0.18612	-0.18563	-0.18514	-0.29336	-0.29268	-0.292	-0.29135
-0.18705	-0.18656	-0.18607	-0.18558	-0.1851	-0.29334	-0.29267	-0.292	-0.29135
-0.18701	-0.18651	-0.18602	-0.18554	-0.18505	-0.29332	-0.29266	-0.29201	-0.29136
-0.18697	-0.18648	-0.18599	-0.1855	-0.18501	-0.29331	-0.29265	-0.292	-0.29136
-0.18695	-0.18646	-0.18596	-0.18546	-0.18497	-0.29329	-0.29264	-0.29198	-0.29133
-0.18692	-0.18642	-0.18593	-0.18543	-0.18493	-0.29329	-0.29263	-0.29197	-0.29132
-0.18687	-0.18638	-0.18589	-0.1854	-0.1849	-0.29329	-0.29263	-0.29198	-0.29133
-0.18682	-0.18633	-0.18584	-0.18536	-0.18487	-0.29329	-0.29263	-0.29198	-0.29132
-0.18678	-0.18629	-0.1858	-0.18531	-0.18483	-0.29326	-0.29261	-0.29195	-0.2913
-0.18675	-0.18625	-0.18575	-0.18526	-0.18479	-0.29324	-0.29258	-0.29193	-0.29126
-0.18671	-0.18621	-0.18571	-0.18523	-0.18476	-0.29322	-0.29257	-0.29191	-0.29124
-0.18667	-0.18618	-0.18568	-0.1852	-0.18473	-0.29322	-0.29256	-0.29191	-0.29123
-0.18664	-0.18614	-0.18565	-0.18517	-0.1847	-0.18422	-0.18375	-0.29189	-0.29121
-0.18661	-0.18612	-0.18562	-0.18514	-0.18465	-0.18418	-0.18371	-0.29186	-0.29119
-0.18658	-0.18609	-0.1856	-0.18511	-0.18462	-0.18414	-0.18367	-0.29184	-0.29117
-0.18655	-0.18606	-0.18557	-0.18508	-0.18459	-0.18411	-0.18364	-0.29183	-0.29117
-0.18651	-0.18603	-0.18554	-0.18505	-0.18456	-0.18408	-0.18361	-0.29184	-0.29116
-0.18647	-0.18598	-0.1855	-0.18502	-0.18453	-0.18405	-0.18359	-0.29183	-0.29116
-0.18642	-0.18594	-0.18546	-0.18498	-0.1845	-0.18402	-0.18356	-0.29182	-0.29115
-0.18638	-0.1859	-0.18543	-0.18495	-0.18447	-0.184	-0.18353	-0.3485	-0.34764
-0.18635	-0.18587	-0.1854	-0.18493	-0.18444	-0.18397	-0.1835	-0.34847	-0.34761
-0.18632	-0.18584	-0.18538	-0.1849	-0.18442	-0.18394	-0.18347	-0.34844	-0.34758
-0.18629	-0.18581	-0.18535	-0.18488	-0.18439	-0.18391	-0.18344	-0.34841	-0.34755
-0.18626	-0.18579	-0.18532	-0.18485	-0.18437	-0.18389	-0.18341	-0.24194	-0.24126
-0.18623	-0.18576	-0.18529	-0.18482	-0.18434	-0.18386	-0.18339	-0.24189	-0.24121
-0.1862	-0.18573	-0.18526	-0.18479	-0.18431	-0.18383	-0.18336	-0.24185	-0.24117
-0.18618	-0.1857	-0.18523	-0.18476	-0.18428	-0.1838	-0.18333	-0.2418	-0.24112
-0.18615	-0.18567	-0.18521	-0.18473	-0.18425	-0.18377	-0.1833	-0.24176	-0.24108
-0.18612	-0.18564	-0.18518	-0.1847	-0.18422	-0.18374	-0.18327	-0.24171	-0.24103
-0.18608	-0.18561	-0.18515	-0.18467	-0.18419	-0.18372	-0.18325	-0.24166	-0.24099
-0.18606	-0.18559	-0.18512	-0.18465	-0.18417	-0.18369	-0.18322	-0.24162	-0.24095
-0.18605	-0.18558	-0.1851	-0.18462	-0.18414	-0.18366	-0.18319	-0.24158	-0.2409
-0.18604	-0.18556	-0.18509	-0.1846	-0.18411	-0.18363	-0.18316	-0.24153	-0.24086
-0.18602	-0.18554	-0.18506	-0.18457	-0.18408	-0.1836	-0.18313	-0.24149	-0.24081
-0.18599	-0.18551	-0.18503	-0.18454	-0.18405	-0.18357	-0.18311	-0.24145	-0.24077
-0.18597	-0.18548	-0.185	-0.18452	-0.18404	-0.18356	-0.18309	-0.24141	-0.24073
-0.18595	-0.18546	-0.18497	-0.18449	-0.18402	-0.18355	-0.18308	-0.24137	-0.24069
-0.18593	-0.18543	-0.18494	-0.18447	-0.184	-0.18353	-0.18306	-0.24134	-0.24066
-0.18589	-0.1854	-0.18491	-0.18444	-0.18398	-0.18351	-0.18303	-0.24131	-0.24063
-0.18586	-0.18538	-0.18489	-0.18442	-0.18395	-0.18348	-0.183	-0.24127	-0.2406
-0.18583	-0.18535	-0.18488	-0.18441	-0.18394	-0.18346	-0.18298	-0.24122	-0.24055

-0.18581	-0.18533	-0.18487	-0.1844	-0.18392	-0.18344	-0.18295	-0.24118	-0.24051
-0.1858	-0.18532	-0.18485	-0.18438	-0.1839	-0.18342	-0.18293	-0.24115	-0.24048
-0.18579	-0.18531	-0.18484	-0.18436	-0.18387	-0.18339	-0.18292	-0.24114	-0.24046
-0.18578	-0.1853	-0.18482	-0.18433	-0.18385	-0.18337	-0.1829	-0.24111	-0.24043
-0.18576	-0.18528	-0.18479	-0.18431	-0.18384	-0.18336	-0.18289	-0.24107	-0.24039
-0.18574	-0.18526	-0.18477	-0.18429	-0.18382	-0.18335	-0.18287	-0.24103	-0.24035
-0.18571	-0.18523	-0.18476	-0.18429	-0.18381	-0.18333	-0.18285	-0.24099	-0.24032
-0.18569	-0.18522	-0.18475	-0.18428	-0.1838	-0.18331	-0.18282	-0.24096	-0.2403
-0.18568	-0.18521	-0.18474	-0.18426	-0.18377	-0.18328	-0.1828	-0.24094	-0.24027
-0.18567	-0.1852	-0.18472	-0.18423	-0.18374	-0.18326	-0.18278	-0.24092	-0.24024
-0.18566	-0.18518	-0.1847	-0.18421	-0.18372	-0.18324	-0.18277	-0.24088	-0.24021
-0.18564	-0.18516	-0.18467	-0.18418	-0.18371	-0.18323	-0.18276	-0.24085	-0.24017
-0.18562	-0.18514	-0.18465	-0.18417	-0.18369	-0.18322	-0.18274	-0.24083	-0.24015
-0.18559	-0.18512	-0.18464	-0.18416	-0.18368	-0.1832	-0.18273	-0.24082	-0.24015
-0.18558	-0.1851	-0.18463	-0.18415	-0.18367	-0.18319	-0.18272	-0.2408	-0.24013
-0.18558	-0.1851	-0.18462	-0.18414	-0.18366	-0.18318	-0.1827	-0.24076	-0.24009
-0.18558	-0.18509	-0.1846	-0.18412	-0.18364	-0.18317	-0.18269	-0.24073	-0.24006
-0.18555	-0.18507	-0.18459	-0.18411	-0.18363	-0.18315	-0.18268	-0.24072	-0.24005
-0.18553	-0.18505	-0.18458	-0.1841	-0.18361	-0.18313	-0.18266	-0.24071	-0.24004
-0.18552	-0.18504	-0.18456	-0.18408	-0.1836	-0.18312	-0.18265	-0.24067	-0.24001
-0.18552	-0.18503	-0.18454	-0.18406	-0.18358	-0.18311	-0.18264	-0.24064	-0.23997
-0.18552	-0.18503	-0.18454	-0.18405	-0.18357	-0.18309	-0.18262	-0.24061	-0.23994
-0.18551	-0.18503	-0.18454	-0.18405	-0.18356	-0.18308	-0.18261	-0.2406	-0.23993
-0.1855	-0.18502	-0.18454	-0.18405	-0.18355	-0.18307	-0.1826	-0.18214	-0.18168
-0.18548	-0.18501	-0.18453	-0.18404	-0.18355	-0.18306	-0.18259	-0.18212	-0.18166
-0.18547	-0.18499	-0.18452	-0.18403	-0.18355	-0.18306	-0.18258	-0.18211	-0.18165
-0.18545	-0.18498	-0.1845	-0.18402	-0.18354	-0.18306	-0.18258	-0.18211	-0.18165
-0.18544	-0.18496	-0.18448	-0.184	-0.18353	-0.18305	-0.18258	-0.18211	-0.18164
-0.18544	-0.18496	-0.18448	-0.18399	-0.18351	-0.18304	-0.18257	-0.18211	-0.18164
-0.18545	-0.18497	-0.18448	-0.18399	-0.1835	-0.18302	-0.18256	-0.1821	-0.18164
-0.18544	-0.18496	-0.18447	-0.18398	-0.18349	-0.18301	-0.18255	-0.18209	-0.18163
-0.18542	-0.18494	-0.18447	-0.18398	-0.18349	-0.18301	-0.18254	-0.18207	-0.18162
-0.18541	-0.18493	-0.18445	-0.18397	-0.18349	-0.18301	-0.18253	-0.18206	-0.18161
-0.18541	-0.18492	-0.18444	-0.18396	-0.18348	-0.183	-0.18253	-0.18206	-0.1816
-0.18541	-0.18492	-0.18442	-0.18394	-0.18347	-0.183	-0.18253	-0.18206	-0.1816
-0.1854	-0.18491	-0.18442	-0.18393	-0.18345	-0.18298	-0.18252	-0.18206	-0.18159
-0.1854	-0.18491	-0.18442	-0.18393	-0.18344	-0.18296	-0.1825	-0.18205	-0.18159
-0.1854	-0.18491	-0.18442	-0.18393	-0.18344	-0.18296	-0.1825	-0.18205	-0.18159
-0.1854	-0.18492	-0.18443	-0.18394	-0.18344	-0.18296	-0.18251	-0.18205	-0.18159
-0.1854	-0.18492	-0.18443	-0.18394	-0.18344	-0.18296	-0.18251	-0.18205	-0.18159
-0.18538	-0.1849	-0.18442	-0.18393	-0.18344	-0.18296	-0.1825	-0.18203	-0.18157
-0.18537	-0.18489	-0.18441	-0.18393	-0.18344	-0.18296	-0.18249	-0.18202	-0.18156
-0.18537	-0.18489	-0.18441	-0.18393	-0.18345	-0.18297	-0.18249	-0.18202	-0.18156
-0.18537	-0.18489	-0.18442	-0.18394	-0.18345	-0.18297	-0.18249	-0.18202	-0.18156
-0.18537	-0.18489	-0.18442	-0.18394	-0.18345	-0.18297	-0.18249	-0.18202	-0.18157
-0.18538	-0.1849	-0.18442	-0.18394	-0.18346	-0.18298	-0.1825	-0.18203	-0.18157
-0.18538	-0.1849	-0.18443	-0.18395	-0.18346	-0.18298	-0.1825	-0.18203	-0.18157
-0.18539	-0.18491	-0.18443	-0.18395	-0.18347	-0.18299	-0.18251	-0.18203	-0.18157
-0.18539	-0.18491	-0.18443	-0.18395	-0.18347	-0.18299	-0.18251	-0.18204	-0.18158
-0.18539	-0.1849	-0.18441	-0.18393	-0.18345	-0.18298	-0.18251	-0.18204	-0.18158

-0.18539	-0.1849	-0.1844	-0.18392	-0.18344	-0.18297	-0.18251	-0.18204	-0.18158
-0.1854	-0.18491	-0.18442	-0.18393	-0.18346	-0.18298	-0.18251	-0.18205	-0.18159
-0.1854	-0.18492	-0.18444	-0.18396	-0.18347	-0.183	-0.18252	-0.18205	-0.18159
-0.18541	-0.18493	-0.18445	-0.18397	-0.18348	-0.183	-0.18252	-0.18205	-0.1816
-0.18542	-0.18494	-0.18446	-0.18398	-0.18349	-0.18301	-0.18253	-0.18206	-0.1816
-0.18542	-0.18494	-0.18446	-0.18398	-0.18349	-0.18301	-0.18253	-0.18206	-0.18161
-0.18543	-0.18495	-0.18447	-0.18398	-0.18349	-0.18301	-0.18253	-0.18206	-0.18161
-0.18543	-0.18495	-0.18447	-0.18399	-0.1835	-0.18301	-0.18254	-0.18207	-0.18161
-0.18543	-0.18495	-0.18448	-0.18399	-0.1835	-0.18302	-0.18254	-0.18207	-0.18162
-0.18544	-0.18496	-0.18448	-0.184	-0.1835	-0.18302	-0.18254	-0.18208	-0.18162
-0.18546	-0.18498	-0.1845	-0.184	-0.18351	-0.18302	-0.18256	-0.1821	-0.18164
-0.18549	-0.185	-0.18451	-0.18402	-0.18351	-0.18303	-0.18257	-0.18212	-0.18167
-0.1855	-0.18502	-0.18452	-0.18402	-0.18352	-0.18303	-0.18258	-0.18213	-0.18168
-0.18551	-0.18502	-0.18453	-0.18403	-0.18352	-0.18304	-0.18258	-0.18214	-0.18168
-0.18552	-0.18503	-0.18453	-0.18403	-0.18353	-0.18305	-0.18259	-0.18214	-0.18169
-0.18554	-0.18504	-0.18454	-0.18404	-0.18355	-0.18307	-0.18261	-0.18216	-0.1817
-0.18555	-0.18505	-0.18455	-0.18405	-0.18357	-0.18309	-0.18263	-0.18217	-0.18171
-0.18555	-0.18506	-0.18457	-0.18408	-0.18359	-0.18311	-0.18264	-0.18217	-0.18172
-0.18556	-0.18507	-0.18459	-0.1841	-0.18361	-0.18313	-0.18265	-0.18218	-0.18173
-0.18558	-0.18509	-0.18461	-0.18412	-0.18362	-0.18314	-0.18266	-0.1822	-0.18175
-0.1856	-0.18512	-0.18463	-0.18413	-0.18363	-0.18314	-0.18268	-0.18223	-0.18178
-0.18561	-0.18513	-0.18464	-0.18414	-0.18364	-0.18316	-0.1827	-0.18225	-0.1818
-0.18562	-0.18513	-0.18464	-0.18415	-0.18366	-0.18319	-0.18273	-0.18227	-0.18181
-0.18563	-0.18514	-0.18465	-0.18416	-0.18369	-0.18321	-0.18275	-0.18228	-0.18182
-0.18564	-0.18516	-0.18467	-0.18419	-0.18371	-0.18323	-0.18276	-0.18229	-0.18183
-0.18566	-0.18518	-0.18469	-0.18421	-0.18373	-0.18324	-0.18277	-0.1823	-0.18184
-0.18568	-0.1852	-0.18471	-0.18422	-0.18373	-0.18325	-0.18278	-0.18232	-0.18187
-0.1857	-0.18522	-0.18473	-0.18424	-0.18374	-0.18326	-0.1828	-0.18235	-0.18189
-0.18572	-0.18523	-0.18474	-0.18425	-0.18376	-0.18328	-0.18282	-0.24425	-0.24363
-0.18574	-0.18524	-0.18474	-0.18426	-0.18378	-0.18331	-0.18285	-0.24426	-0.24364
-0.18575	-0.18525	-0.18475	-0.18427	-0.1838	-0.18333	-0.18287	-0.24428	-0.24366
-0.18575	-0.18526	-0.18477	-0.1843	-0.18382	-0.18335	-0.18289	-0.24431	-0.24369
-0.18576	-0.18528	-0.1848	-0.18432	-0.18384	-0.18337	-0.18291	-0.24433	-0.24372
-0.18579	-0.1853	-0.18482	-0.18434	-0.18386	-0.1834	-0.18294	-0.24435	-0.24373
-0.18583	-0.18533	-0.18484	-0.18435	-0.18389	-0.18342	-0.18296	-0.24436	-0.24373
-0.18584	-0.18535	-0.18486	-0.18438	-0.1839	-0.18344	-0.18298	-0.24438	-0.24376
-0.18584	-0.18536	-0.18488	-0.1844	-0.18392	-0.18346	-0.183	-0.2444	-0.24379
-0.18587	-0.18538	-0.1849	-0.18442	-0.18394	-0.18348	-0.18302	-0.24442	-0.2438
-0.1859	-0.18541	-0.18492	-0.18444	-0.18397	-0.1835	-0.18304	-0.24443	-0.24381
-0.18594	-0.18544	-0.18495	-0.18446	-0.18399	-0.18352	-0.18305	-0.24444	-0.24382
-0.18597	-0.18547	-0.18498	-0.18449	-0.18401	-0.18353	-0.18307	-0.24446	-0.24385
-0.18599	-0.1855	-0.18501	-0.18452	-0.18403	-0.18355	-0.18309	-0.24448	-0.24388
-0.18601	-0.18552	-0.18504	-0.18455	-0.18406	-0.18358	-0.18311	-0.24449	-0.24389
-0.18603	-0.18554	-0.18506	-0.18458	-0.1841	-0.18362	-0.18314	-0.24451	-0.24391
-0.18606	-0.18557	-0.18508	-0.1846	-0.18412	-0.18365	-0.18317	-0.24454	-0.24393
-0.18609	-0.1856	-0.1851	-0.18462	-0.18415	-0.18368	-0.1832	-0.24458	-0.24396
-0.18612	-0.18563	-0.18513	-0.18465	-0.18417	-0.1837	-0.18323	-0.2446	-0.24399
-0.18615	-0.18566	-0.18517	-0.18468	-0.18419	-0.18371	-0.18325	-0.24462	-0.24402
-0.18618	-0.18569	-0.1852	-0.18471	-0.18422	-0.18373	-0.18327	-0.24464	-0.24404
-0.18619	-0.18571	-0.18523	-0.18474	-0.18425	-0.18376	-0.18329	-0.24465	-0.24405

-0.18621	-0.18573	-0.18525	-0.18477	-0.18428	-0.1838	-0.18332	-0.24467	-0.24407
-0.18624	-0.18576	-0.18527	-0.18479	-0.18431	-0.18383	-0.18334	-0.2447	-0.24411
-0.18628	-0.18578	-0.18529	-0.18481	-0.18433	-0.18385	-0.18338	-0.24474	-0.24414
-0.18632	-0.18582	-0.18532	-0.18484	-0.18436	-0.18389	-0.18341	-0.24478	-0.24417
-0.18635	-0.18586	-0.18536	-0.18488	-0.1844	-0.18393	-0.18345	-0.24481	-0.2442
-0.18639	-0.18589	-0.1854	-0.18491	-0.18443	-0.18396	-0.18348	-0.24484	-0.24422
-0.18641	-0.18593	-0.18544	-0.18495	-0.18446	-0.18397	-0.1835	-0.24486	-0.24425
-0.18644	-0.18596	-0.18548	-0.18498	-0.18448	-0.18399	-0.18352	-0.24489	-0.24428
-0.18648	-0.186	-0.18552	-0.18502	-0.18452	-0.18403	-0.18355	-0.24493	-0.24432
-0.18652	-0.18604	-0.18556	-0.18506	-0.18456	-0.18406	-0.18359	-0.24496	-0.24436
-0.18654	-0.18606	-0.18558	-0.18509	-0.18459	-0.1841	-0.18363	-0.245	-0.24439
-0.18655	-0.18608	-0.18561	-0.18513	-0.18463	-0.18414	-0.18366	-0.24504	-0.24443
-0.18658	-0.18611	-0.18564	-0.18516	-0.18467	-0.18418	-0.1837	-0.24508	-0.24447
-0.18661	-0.18615	-0.18569	-0.18521	-0.18471	-0.18422	-0.18374	-0.24512	-0.2445
-0.18665	-0.18618	-0.18573	-0.18525	-0.18475	-0.18426	-0.18377	-0.24515	-0.24454
-0.18669	-0.18622	-0.18577	-0.18529	-0.18479	-0.1843	-0.18381	-0.24519	-0.24458
-0.18672	-0.18626	-0.18581	-0.18533	-0.18483	-0.18434	-0.18385	-0.24523	-0.24462
-0.18676	-0.1863	-0.18585	-0.18537	-0.18487	-0.18438	-0.18389	-0.24527	-0.24466
-0.1868	-0.18634	-0.18588	-0.18541	-0.18491	-0.18442	-0.18393	-0.24532	-0.2447
-0.18683	-0.18637	-0.18592	-0.18544	-0.18495	-0.18445	-0.18397	-0.24536	-0.24474
-0.18686	-0.1864	-0.18595	-0.18548	-0.18498	-0.18449	-0.184	-0.2454	-0.24478
-0.18689	-0.18643	-0.18598	-0.18551	-0.18502	-0.18452	-0.18404	-0.24544	-0.24482
-0.18692	-0.18646	-0.18601	-0.18554	-0.18505	-0.18456	-0.18408	-0.24548	-0.24486
-0.18696	-0.1865	-0.18605	-0.18558	-0.18508	-0.1846	-0.18411	-0.24552	-0.2449
-0.18702	-0.18656	-0.1861	-0.18562	-0.18512	-0.18463	-0.18415	-0.24556	-0.24494
-0.18707	-0.1866	-0.18614	-0.18566	-0.18516	-0.18467	-0.18419	-0.2456	-0.24498
-0.18711	-0.18664	-0.18617	-0.18569	-0.18519	-0.1847	-0.18422	-0.24564	-0.24501
-0.18714	-0.18667	-0.18621	-0.18572	-0.18523	-0.18474	-0.18426	-0.24568	-0.24506
-0.18717	-0.18671	-0.18624	-0.18576	-0.18527	-0.18479	-0.18431	-0.24573	-0.2451
-0.18721	-0.18674	-0.18627	-0.1858	-0.18533	-0.18485	-0.18436	-0.24579	-0.24515
-0.18725	-0.18677	-0.1863	-0.18584	-0.18537	-0.18489	-0.1844	-0.24584	-0.2452
-0.18729	-0.18681	-0.18634	-0.18587	-0.1854	-0.18493	-0.18444	-0.24588	-0.24525
-0.18733	-0.18685	-0.18638	-0.18591	-0.18544	-0.18497	-0.18448	-0.24593	-0.24531
-0.18737	-0.18689	-0.18643	-0.18596	-0.18549	-0.18501	-0.18453	-0.246	-0.24537
-0.18741	-0.18694	-0.18648	-0.18601	-0.18553	-0.18506	-0.18458	-0.24606	-0.24543
-0.18746	-0.18699	-0.18652	-0.18605	-0.18557	-0.18509	-0.18462	-0.2461	-0.24547
-0.18751	-0.18704	-0.18657	-0.18609	-0.18561	-0.18513	-0.18466	-0.24614	-0.24551
-0.18755	-0.18708	-0.18661	-0.18613	-0.18565	-0.18518	-0.1847	-0.24618	-0.24555
-0.18759	-0.18711	-0.18664	-0.18617	-0.1857	-0.18523	-0.18475	-0.24624	-0.24561
-0.18763	-0.18715	-0.18668	-0.18621	-0.18575	-0.18528	-0.1848	-0.24628	-0.24566
-0.18767	-0.1872	-0.18673	-0.18627	-0.1858	-0.18533	-0.18483	-0.24631	-0.24569
-0.18772	-0.18724	-0.18678	-0.18632	-0.18585	-0.18537	-0.18487	-0.24636	-0.24573
-0.18777	-0.1873	-0.18683	-0.18636	-0.18588	-0.1854	-0.18492	-0.24641	-0.24578
-0.18782	-0.18735	-0.18687	-0.1864	-0.18592	-0.18544	-0.18497	-0.24648	-0.24585
-0.18787	-0.18739	-0.18691	-0.18644	-0.18597	-0.18549	-0.18502	-0.24653	-0.2459
-0.18792	-0.18743	-0.18694	-0.18647	-0.18602	-0.18555	-0.18507	-0.24658	-0.24596
-0.12766	-0.18747	-0.18699	-0.18652	-0.18607	-0.1856	-0.18512	-0.24664	-0.24601
-0.12768	-0.18751	-0.18704	-0.18657	-0.18611	-0.18565	-0.18517	-0.2467	-0.24607
-0.12771	-0.18755	-0.18709	-0.18662	-0.18616	-0.1857	-0.18522	-0.24676	-0.24613
-0.12774	-0.1876	-0.18713	-0.18667	-0.18621	-0.18575	-0.18527	-0.24681	-0.24619

-0.12778	-0.18765	-0.18718	-0.18672	-0.18626	-0.1858	-0.18532	-0.24687	-0.24624
-0.12781	-0.1877	-0.18723	-0.18677	-0.18631	-0.18585	-0.18537	-0.24693	-0.2463
-0.12784	-0.18775	-0.18729	-0.18682	-0.18636	-0.18589	-0.18542	-0.24699	-0.24636
-0.12788	-0.18781	-0.18733	-0.18687	-0.18641	-0.18594	-0.18547	-0.24704	-0.24642
-0.12792	-0.18786	-0.18738	-0.18691	-0.18646	-0.18599	-0.18552	-0.2471	-0.24647
-0.12795	-0.12761	-0.12728	-0.18696	-0.1865	-0.18604	-0.18557	-0.24715	-0.24653
-0.12798	-0.12764	-0.12732	-0.18701	-0.18655	-0.18608	-0.18561	-0.24721	-0.24659
-0.12802	-0.12768	-0.12735	-0.18706	-0.18659	-0.18613	-0.18566	-0.24727	-0.24665
-0.12807	-0.12772	-0.12738	-0.1871	-0.18664	-0.18618	-0.18571	-0.24732	-0.2467
-0.12811	-0.12776	-0.12741	-0.18715	-0.18669	-0.18623	-0.18576	-0.24738	-0.24676
-0.12815	-0.1278	-0.12746	-0.18721	-0.18675	-0.18629	-0.18582	-0.24745	-0.24682
-0.12819	-0.12784	-0.1275	-0.18728	-0.18682	-0.18636	-0.18589	-0.24753	-0.24689
-0.12822	-0.12788	-0.12754	-0.18733	-0.18687	-0.18641	-0.18594	-0.2476	-0.24696
-0.12825	-0.12791	-0.12757	-0.18738	-0.18692	-0.18645	-0.18599	-0.24766	-0.24703
-0.12829	-0.12794	-0.1276	-0.12727	-0.12695	-0.1865	-0.18604	-0.24772	-0.24709
-0.12834	-0.12798	-0.12763	-0.1273	-0.12698	-0.18654	-0.18608	-0.24777	-0.24715
-0.12838	-0.12803	-0.12767	-0.12733	-0.12702	-0.1866	-0.18614	-0.24783	-0.24721
-0.12842	-0.12807	-0.12771	-0.12737	-0.12706	-0.18666	-0.1862	-0.2479	-0.24729
-0.12846	-0.12811	-0.12775	-0.12742	-0.1271	-0.18672	-0.18626	-0.24798	-0.24736
-0.1285	-0.12815	-0.1278	-0.12746	-0.12713	-0.18677	-0.18631	-0.24804	-0.24742
-0.12855	-0.1282	-0.12784	-0.1275	-0.12716	-0.18681	-0.18636	-0.2481	-0.24748
-0.12859	-0.12823	-0.12788	-0.12754	-0.1272	-0.12688	-0.12657	-0.24816	-0.24754
-0.12861	-0.12826	-0.12792	-0.12758	-0.12724	-0.12692	-0.12662	-0.24824	-0.24761
-0.12865	-0.1283	-0.12795	-0.12762	-0.12729	-0.12697	-0.12666	-0.24831	-0.24769
-0.12869	-0.12835	-0.128	-0.12766	-0.12733	-0.12701	-0.1267	-0.24838	-0.24776
-0.06364	-0.12839	-0.12804	-0.12771	-0.12738	-0.12705	-0.12674	-0.24845	-0.24783
-0.06366	-0.12843	-0.12808	-0.12774	-0.12741	-0.12709	-0.12677	-0.2485	-0.24789
-0.06368	-0.12847	-0.12811	-0.12777	-0.12744	-0.12712	-0.1268	-0.24855	-0.24794
-0.0637	-0.12851	-0.12815	-0.12781	-0.12748	-0.12716	-0.12684	-0.24861	-0.24801
-0.06372	-0.12854	-0.1282	-0.12786	-0.12753	-0.1272	-0.12689	-0.24869	-0.24808
-0.06374	-0.12858	-0.12824	-0.1279	-0.12757	-0.12725	-0.12693	-0.19022	-0.18977
-0.06376	-0.12863	-0.12828	-0.12795	-0.12762	-0.12729	-0.12697	-0.19027	-0.18982
-0.06379	-0.12867	-0.12833	-0.12799	-0.12766	-0.12733	-0.12701	-0.19033	-0.18987
-0.06381	-0.12871	-0.12837	-0.12804	-0.1277	-0.12738	-0.12705	-0.19038	-0.18992
-0.06383	-0.12876	-0.12842	-0.12808	-0.12775	-0.12742	-0.1271	-0.19043	-0.18997
-0.06385	-0.1288	-0.12846	-0.12812	-0.12779	-0.12746	-0.12714	-0.19048	-0.19003
-0.06388	-0.12884	-0.1285	-0.12817	-0.12783	-0.1275	-0.12718	-0.19053	-0.19008
-0.0639	-0.12888	-0.12854	-0.12821	-0.12788	-0.12755	-0.12722	-0.19058	-0.19013
-0.06392	-0.12892	-0.12859	-0.12825	-0.12792	-0.12759	-0.12726	-0.19063	-0.19017
-0.06395	-0.12897	-0.12863	-0.12829	-0.12796	-0.12763	-0.12731	-0.19067	-0.19021
-0.06397	-0.12901	-0.12867	-0.12834	-0.12801	-0.12768	-0.12735	-0.19072	-0.19026
-0.06399	-0.12905	-0.12871	-0.12838	-0.12805	-0.12772	-0.1274	-0.12707	-0.12676
-0.06402	-0.12909	-0.12876	-0.12842	-0.12809	-0.12776	-0.12745	-0.12713	-0.12681
-0.06404	-0.12914	-0.1288	-0.12847	-0.12813	-0.12781	-0.1275	-0.12718	-0.12686
	-0.0639	-0.06374	-0.12852	-0.12819	-0.12786	-0.12754	-0.12723	-0.12691
	-0.06393	-0.06377	-0.12858	-0.12824	-0.12791	-0.12759	-0.12727	-0.12695
	-0.06396	-0.06379	-0.12863	-0.12829	-0.12796	-0.12763	-0.12731	-0.12699
	-0.06398	-0.06381	-0.12867	-0.12833	-0.128	-0.12768	-0.12735	-0.12703
	-0.064	-0.06384	-0.12871	-0.12838	-0.12805	-0.12772	-0.1274	-0.12708
	-0.06402	-0.06386	-0.12875	-0.12843	-0.12811	-0.12778	-0.12745	-0.12713

-0.06405	-0.06388	-0.1288	-0.12848	-0.12816	-0.12783	-0.1275	-0.12718
-0.06408	-0.06391	-0.12885	-0.12853	-0.1282	-0.12787	-0.12754	-0.12722
-0.06411	-0.06394	-0.1289	-0.12857	-0.12824	-0.12791	-0.12759	-0.12726
-0.06413	-0.06397	-0.12894	-0.12861	-0.12829	-0.12796	-0.12764	-0.12732
-0.06415	-0.06399	-0.12898	-0.12865	-0.12833	-0.12801	-0.1277	-0.12737
-0.06418	-0.06402	-0.06385	-0.06368	-0.12838	-0.12806	-0.12775	-0.12742
-0.06421	-0.06405	-0.06388	-0.06371	-0.12843	-0.12811	-0.12779	-0.12746
		-0.06391	-0.06375	-0.12848	-0.12816	-0.12783	-0.12751
		-0.06394	-0.06377	-0.12854	-0.12821	-0.12788	-0.12756
		-0.06397	-0.0638	-0.12859	-0.12827	-0.12794	-0.12761
		-0.06399	-0.06383	-0.12864	-0.12832	-0.12799	-0.12767
		-0.06401	-0.06385	-0.12868	-0.12837	-0.12805	-0.12772
		-0.06404	-0.06388	-0.12873	-0.12841	-0.1281	-0.12777
		-0.06407	-0.06391	-0.12878	-0.12846	-0.12814	-0.12782
		-0.0641	-0.06394	-0.06377	-0.0636	-0.12818	-0.12787
		-0.06413	-0.06396	-0.0638	-0.06363	-0.12824	-0.12791
				-0.06383	-0.06366	-0.12829	-0.12796
				-0.06386	-0.06369	-0.12835	-0.12802
				-0.06389	-0.06372	-0.1284	-0.12807
				-0.06392	-0.06375	-0.12846	-0.12813
				-0.06395	-0.06378	-0.12851	-0.12818
				-0.06397	-0.06381	-0.12856	-0.12824
				-0.064	-0.06384	-0.12861	-0.1283
				-0.06403	-0.06387	-0.0637	-0.06354
				-0.06406	-0.0639	-0.06373	-0.06356
				-0.06409	-0.06393	-0.06376	-0.06359
				-0.06412	-0.06396	-0.0638	-0.06363
				-0.06416	-0.064	-0.06383	-0.06366
						-0.06386	-0.0637
						-0.06389	-0.06372
						-0.06392	-0.06375
						-0.06395	-0.06378
						-0.06398	-0.06381
						-0.06401	-0.06385
						-0.06404	-0.06388
						-0.06407	-0.06391
						-0.06411	-0.06394
						-0.06414	-0.06398
						-0.06417	-0.06401

							-0.12793
							-0.12786
							-0.12778
							-0.12769
				-0.06489	-0.06472	-0.06455	-0.18907
				-0.06485	-0.06468	-0.06451	-0.18896
				-0.0648	-0.06464	-0.06447	-0.18885
				-0.06476	-0.0646	-0.06443	-0.18874
				-0.06473	-0.06456	-0.0644	-0.18864
		-0.06502	-0.06485	-0.12818	-0.12786	-0.12755	-0.29302
		-0.06498	-0.06482	-0.12811	-0.12779	-0.12747	-0.2929
		-0.06494	-0.06478	-0.12803	-0.12771	-0.12739	-0.29278
		-0.06491	-0.06474	-0.12796	-0.12763	-0.12731	-0.29265
		-0.06487	-0.06471	-0.12788	-0.12755	-0.12722	-0.29252
		-0.06483	-0.06467	-0.12779	-0.12746	-0.12713	-0.2924
		-0.06479	-0.06462	-0.1277	-0.12738	-0.12705	-0.29229
		-0.06475	-0.06458	-0.29426	-0.29356	-0.29286	-0.29218
		-0.06471	-0.06454	-0.29413	-0.29345	-0.29276	-0.29208
		-0.06467	-0.0645	-0.29402	-0.29334	-0.29266	-0.29197
		-0.06463	-0.06446	-0.29392	-0.29324	-0.29256	-0.29186
-0.06494	-0.06476	-0.12795	-0.12763	-0.29382	-0.29314	-0.29245	-0.29176
-0.0649	-0.06472	-0.12788	-0.12755	-0.29372	-0.29304	-0.29236	-0.29166
-0.06486	-0.06468	-0.1278	-0.12748	-0.29361	-0.29294	-0.29226	-0.29156
-0.06483	-0.06465	-0.12773	-0.12741	-0.29352	-0.29284	-0.29216	-0.29146
-0.06479	-0.06461	-0.12765	-0.12733	-0.29342	-0.29274	-0.29206	-0.29136
-0.06475	-0.06457	-0.29469	-0.29401	-0.29332	-0.29264	-0.29195	-0.29125
-0.0647	-0.06453	-0.29457	-0.2939	-0.29322	-0.29254	-0.29185	-0.29115
-0.06466	-0.0645	-0.29447	-0.2938	-0.29312	-0.29243	-0.29175	-0.29104
-0.06463	-0.06446	-0.29439	-0.2937	-0.29302	-0.29233	-0.29165	-0.29094
-0.0646	-0.06442	-0.29431	-0.29361	-0.29292	-0.29223	-0.29155	-0.29084
-0.06456	-0.06439	-0.29421	-0.29351	-0.29282	-0.29213	-0.29145	-0.29074

	-0.06453	-0.06435	-0.29411	-0.29341	-0.29272	-0.29203	-0.29135	-0.29065
-0.06467	-0.06449	-0.06432	-0.29401	-0.29331	-0.29261	-0.29193	-0.29125	-0.29055
-0.06463	-0.06446	-0.06428	-0.29391	-0.29321	-0.29251	-0.29183	-0.29115	-0.29045
-0.06459	-0.1893	-0.18879	-0.29381	-0.29311	-0.29241	-0.29173	-0.29105	-0.29036
-0.06455	-0.1892	-0.18869	-0.29371	-0.29301	-0.29232	-0.29163	-0.29096	-0.29026
-0.06452	-0.1891	-0.18859	-0.29362	-0.29291	-0.29222	-0.29154	-0.29086	-0.29017
-0.06448	-0.18899	-0.18849	-0.29352	-0.29281	-0.29212	-0.29144	-0.29077	-0.29008
-0.06444	-0.29483	-0.29413	-0.29343	-0.29272	-0.29202	-0.29135	-0.29068	-0.28999
-0.06441	-0.29473	-0.29405	-0.29336	-0.29265	-0.29195	-0.29126	-0.29058	-0.2899
-0.06437	-0.29465	-0.29397	-0.29328	-0.29258	-0.29187	-0.29118	-0.29048	-0.28981
-0.06434	-0.29456	-0.29389	-0.2932	-0.29249	-0.29178	-0.29108	-0.29039	-0.28971
-0.0643	-0.29448	-0.2938	-0.2931	-0.29239	-0.29168	-0.29098	-0.29029	-0.28962
-0.12726	-0.2944	-0.29371	-0.29301	-0.2923	-0.2916	-0.2909	-0.29021	-0.28953
-0.12719	-0.2943	-0.2936	-0.29291	-0.29222	-0.29152	-0.29083	-0.29013	-0.28945
-0.12712	-0.29422	-0.29351	-0.29282	-0.29213	-0.29145	-0.29075	-0.29006	-0.28938
-0.12706	-0.29415	-0.29344	-0.29274	-0.29204	-0.29135	-0.29067	-0.29	-0.28931
-0.127	-0.29407	-0.29336	-0.29266	-0.29195	-0.29126	-0.29059	-0.28992	-0.28925
-0.12694	-0.29398	-0.29328	-0.29258	-0.29188	-0.29119	-0.29051	-0.28984	-0.28916
-0.12687	-0.29389	-0.2932	-0.29251	-0.29181	-0.29112	-0.29043	-0.28975	-0.28908
-0.1268	-0.2938	-0.29311	-0.29242	-0.29173	-0.29105	-0.29036	-0.28968	-0.289
-0.12674	-0.29372	-0.29302	-0.29233	-0.29165	-0.29098	-0.2903	-0.28962	-0.28894
-0.18811	-0.29364	-0.29294	-0.29225	-0.29158	-0.2909	-0.29022	-0.28954	-0.28887
-0.18803	-0.29357	-0.29287	-0.29218	-0.2915	-0.29082	-0.29013	-0.28945	-0.28877
-0.29419	-0.29349	-0.2928	-0.29211	-0.29142	-0.29074	-0.29005	-0.28937	-0.28869
-0.2941	-0.29341	-0.29273	-0.29204	-0.29135	-0.29067	-0.28998	-0.28929	-0.28862
-0.29401	-0.29333	-0.29266	-0.29198	-0.29129	-0.2906	-0.28991	-0.28923	-0.28855
-0.29395	-0.29327	-0.29259	-0.29191	-0.29122	-0.29053	-0.28984	-0.28916	-0.28849
-0.29389	-0.29321	-0.29253	-0.29184	-0.29115	-0.29046	-0.28978	-0.2891	-0.28842
-0.29382	-0.29314	-0.29245	-0.29177	-0.29108	-0.29039	-0.28971	-0.28904	-0.28836
-0.29373	-0.29305	-0.29238	-0.2917	-0.29101	-0.29033	-0.28965	-0.28897	-0.28829
-0.29365	-0.29297	-0.2923	-0.29162	-0.29094	-0.29026	-0.28958	-0.2889	-0.28823
-0.29358	-0.29289	-0.29221	-0.29153	-0.29087	-0.2902	-0.28952	-0.28885	-0.28817
-0.29351	-0.29282	-0.29213	-0.29146	-0.2908	-0.29013	-0.28947	-0.28879	-0.28811
-0.29344	-0.29275	-0.29207	-0.2914	-0.29074	-0.29008	-0.2894	-0.28873	-0.28805
-0.29337	-0.29269	-0.29202	-0.29136	-0.29069	-0.29002	-0.28934	-0.28866	-0.28798
-0.29332	-0.29263	-0.29196	-0.29129	-0.29063	-0.28996	-0.28928	-0.2886	-0.28792
-0.29327	-0.29258	-0.2919	-0.29122	-0.29055	-0.28989	-0.28921	-0.28854	-0.28786
-0.29322	-0.29252	-0.29184	-0.29116	-0.29049	-0.28982	-0.28915	-0.28848	-0.2878
-0.29315	-0.29246	-0.29177	-0.2911	-0.29043	-0.28976	-0.28909	-0.28841	-0.28774
-0.29309	-0.29239	-0.29171	-0.29103	-0.29036	-0.28969	-0.28902	-0.28835	-0.28768
-0.29302	-0.29232	-0.29164	-0.29097	-0.2903	-0.28963	-0.28896	-0.28829	-0.28762
-0.29295	-0.29226	-0.29158	-0.2909	-0.29023	-0.28957	-0.2889	-0.28823	-0.28756
-0.29288	-0.29219	-0.29151	-0.29084	-0.29017	-0.2895	-0.28884	-0.28817	-0.28751
-0.29281	-0.29212	-0.29145	-0.29078	-0.29011	-0.28945	-0.28878	-0.28812	-0.28746
-0.29275	-0.29207	-0.2914	-0.29073	-0.29007	-0.28941	-0.28874	-0.28807	-0.28742
-0.2927	-0.29202	-0.29136	-0.2907	-0.29004	-0.28937	-0.2887	-0.28803	-0.28737
-0.29264	-0.29196	-0.29131	-0.29065	-0.28999	-0.28933	-0.28865	-0.28798	-0.28731
-0.29258	-0.2919	-0.29124	-0.29058	-0.28993	-0.28927	-0.28859	-0.28792	-0.28725
-0.29252	-0.29184	-0.29119	-0.29053	-0.28987	-0.28921	-0.28854	-0.28786	-0.28719
-0.29249	-0.29181	-0.29116	-0.2905	-0.28984	-0.28917	-0.2885	-0.28782	-0.28715

-0.29246	-0.29179	-0.29113	-0.29047	-0.28981	-0.28914	-0.28846	-0.28778	-0.28712
-0.29241	-0.29174	-0.29108	-0.29042	-0.28975	-0.28908	-0.2884	-0.28772	-0.28706
-0.29236	-0.29169	-0.29103	-0.29036	-0.28969	-0.28902	-0.28834	-0.28767	-0.287
-0.29232	-0.29165	-0.29098	-0.29032	-0.28964	-0.28897	-0.28829	-0.28762	-0.28695
-0.29229	-0.29162	-0.29095	-0.29028	-0.2896	-0.28893	-0.28825	-0.28758	-0.28691
-0.29225	-0.29158	-0.29092	-0.29025	-0.28957	-0.28889	-0.28821	-0.28754	-0.28687
-0.29222	-0.29155	-0.29088	-0.29021	-0.28954	-0.28886	-0.28818	-0.2875	-0.28683
-0.29219	-0.29151	-0.29084	-0.29017	-0.2895	-0.28882	-0.28813	-0.28745	-0.28679
-0.29214	-0.29146	-0.2908	-0.29012	-0.28944	-0.28876	-0.28807	-0.28739	-0.28673
-0.29208	-0.29141	-0.29074	-0.29007	-0.28938	-0.28869	-0.28801	-0.28733	-0.28668
-0.29204	-0.29137	-0.2907	-0.29002	-0.28933	-0.28865	-0.28796	-0.28729	-0.35222
-0.29201	-0.29134	-0.29066	-0.28998	-0.28929	-0.28861	-0.28793	-0.28726	-0.35215
-0.29198	-0.2913	-0.29062	-0.28994	-0.28925	-0.28857	-0.28789	-0.28722	-0.35208
-0.29195	-0.29128	-0.29059	-0.2899	-0.28921	-0.28852	-0.28784	-0.28717	-0.352
-0.29192	-0.29124	-0.29055	-0.28986	-0.28917	-0.28848	-0.2878	-0.28713	-0.35193
-0.29188	-0.2912	-0.29051	-0.28982	-0.28913	-0.28844	-0.28776	-0.28709	-0.35185
-0.29183	-0.29116	-0.29047	-0.28978	-0.28909	-0.28841	-0.28772	-0.28705	-0.35178
-0.29179	-0.29111	-0.29043	-0.28974	-0.28905	-0.28837	-0.28768	-0.28701	-0.35171
-0.29175	-0.29107	-0.29038	-0.28969	-0.28901	-0.28833	-0.28765	-0.28698	-0.35165
-0.29171	-0.29103	-0.29034	-0.28965	-0.28897	-0.28829	-0.28761	-0.28695	-0.35158
-0.29167	-0.29099	-0.2903	-0.28962	-0.28893	-0.28825	-0.28758	-0.28692	-0.35152
-0.29163	-0.29094	-0.29026	-0.28958	-0.28889	-0.28822	-0.28755	-0.28689	-0.35145
-0.29158	-0.2909	-0.29022	-0.28954	-0.28885	-0.28817	-0.28751	-0.28686	-0.35139
-0.29154	-0.29086	-0.29018	-0.2895	-0.28882	-0.28814	-0.28748	-0.28682	-0.35132
-0.29153	-0.29085	-0.29017	-0.28949	-0.28881	-0.28813	-0.28746	-0.28679	-0.35125
-0.29151	-0.29083	-0.29015	-0.28947	-0.2888	-0.28812	-0.28744	-0.28676	-0.35119
-0.29147	-0.29079	-0.29011	-0.28943	-0.28876	-0.28809	-0.28742	-0.28675	-0.35116
-0.29143	-0.29075	-0.29007	-0.28939	-0.28872	-0.28806	-0.28739	-0.28674	-0.35112
-0.29139	-0.29071	-0.29003	-0.28936	-0.28869	-0.28802	-0.28737	-0.28672	-0.40588
-0.29135	-0.29067	-0.28999	-0.28932	-0.28865	-0.28799	-0.28734	-0.28669	-0.40578
-0.29132	-0.29064	-0.28996	-0.28929	-0.28863	-0.28797	-0.28732	-0.28667	-0.40569
-0.29129	-0.29062	-0.28995	-0.28928	-0.28861	-0.28795	-0.2873	-0.28665	-0.40561
-0.29126	-0.29059	-0.28992	-0.28926	-0.28859	-0.28793	-0.28728	-0.28664	-0.40553
-0.29123	-0.29055	-0.28989	-0.28922	-0.28856	-0.2879	-0.28725	-0.2866	-0.40545
-0.29119	-0.29052	-0.28985	-0.28919	-0.28853	-0.28787	-0.28722	-0.28657	-0.40537
-0.29116	-0.29049	-0.28982	-0.28916	-0.28851	-0.28785	-0.2872	-0.28655	-0.4053
-0.29114	-0.29047	-0.28981	-0.28914	-0.28849	-0.28783	-0.28718	-0.28653	-0.40523
-0.29111	-0.29044	-0.28978	-0.28912	-0.28846	-0.28781	-0.28716	-0.2865	-0.40515
-0.29107	-0.2904	-0.28974	-0.28908	-0.28843	-0.28777	-0.28712	-0.28647	-0.40505
-0.29104	-0.29037	-0.28971	-0.28906	-0.2884	-0.34403	-0.3432	-0.34237	-0.40497
-0.29101	-0.29035	-0.2897	-0.28904	-0.28838	-0.34398	-0.34315	-0.34232	-0.4049
-0.29099	-0.29033	-0.28968	-0.28903	-0.28836	-0.34394	-0.34311	-0.34228	-0.40484
-0.29098	-0.29032	-0.28967	-0.28901	-0.28835	-0.3439	-0.34307	-0.34224	-0.40478
-0.29097	-0.29031	-0.28965	-0.289	-0.28835	-0.34386	-0.34303	-0.34221	-0.40472
-0.29095	-0.29029	-0.28963	-0.28898	-0.28833	-0.34382	-0.34299	-0.34216	-0.40466
-0.29093	-0.29027	-0.28962	-0.28896	-0.28831	-0.34379	-0.34295	-0.34211	-0.40458
-0.29091	-0.29026	-0.2896	-0.28895	-0.2883	-0.34374	-0.3429	-0.34206	-0.40451
-0.2909	-0.29024	-0.28959	-0.28894	-0.28829	-0.3437	-0.34285	-0.34201	-0.40443
-0.29088	-0.29023	-0.28958	-0.34534	-0.34449	-0.34364	-0.3428	-0.34196	-0.40436
-0.29086	-0.29021	-0.28956	-0.34528	-0.34442	-0.34357	-0.34274	-0.34191	-0.4043

-0.29084	-0.29019	-0.28954	-0.34523	-0.34436	-0.34351	-0.34269	-0.34186	-0.40423
-0.29083	-0.29018	-0.28953	-0.34519	-0.34434	-0.34349	-0.34265	-0.34181	-0.40416
-0.29082	-0.29016	-0.28951	-0.34516	-0.34432	-0.34347	-0.34261	-0.34176	-0.40408
-0.2908	-0.29015	-0.28949	-0.34511	-0.34427	-0.34342	-0.34257	-0.34172	-0.40402
-0.29078	-0.29013	-0.28947	-0.34505	-0.3442	-0.34335	-0.34251	-0.34168	-0.40396
-0.29076	-0.29011	-0.28945	-0.34501	-0.34415	-0.3433	-0.34247	-0.34163	-0.40389
-0.29075	-0.2901	-0.28944	-0.34498	-0.34413	-0.34328	-0.34243	-0.34158	-0.40382
-0.29073	-0.29008	-0.28942	-0.34495	-0.3441	-0.34325	-0.34239	-0.34153	-0.40376
-0.29071	-0.29006	-0.2894	-0.3449	-0.34406	-0.34322	-0.34236	-0.34151	-0.40373
-0.2907	-0.29004	-0.28939	-0.34485	-0.34402	-0.34319	-0.34234	-0.34149	-0.40369
-0.2907	-0.29004	-0.28938	-0.34482	-0.34398	-0.34314	-0.3423	-0.34146	-0.40364
-0.29071	-0.29005	-0.28939	-0.3448	-0.34393	-0.34308	-0.34225	-0.34142	-0.40358
-0.29071	-0.29005	-0.28938	-0.34477	-0.3439	-0.34304	-0.34221	-0.34138	-0.40352
-0.29069	-0.29003	-0.28936	-0.34474	-0.34388	-0.34302	-0.34217	-0.34132	-0.40345
-0.29067	-0.29001	-0.28934	-0.3447	-0.34385	-0.343	-0.34214	-0.34128	-0.40339
-0.29067	-0.29	-0.28932	-0.34465	-0.3438	-0.34296	-0.34211	-0.34127	-0.40336
-0.29066	-0.28999	-0.2893	-0.3446	-0.34375	-0.34291	-0.34208	-0.34125	-0.40333
-0.29063	-0.28996	-0.28929	-0.34457	-0.34371	-0.34286	-0.34203	-0.34121	-0.40328
-0.2906	-0.28994	-0.28927	-0.34454	-0.34367	-0.34282	-0.34199	-0.34117	-0.40322
-0.29057	-0.28991	-0.28925	-0.3445	-0.34363	-0.34278	-0.34196	-0.34114	-0.40318
-0.29055	-0.28988	-0.28921	-0.34445	-0.3436	-0.34276	-0.34194	-0.34112	-0.40314
-0.29053	-0.28986	-0.2892	-0.34442	-0.34357	-0.34274	-0.34191	-0.34109	-0.40309
-0.29052	-0.28986	-0.2892	-0.34439	-0.34355	-0.34271	-0.34187	-0.34104	-0.40303
-0.29051	-0.28985	-0.2892	-0.34437	-0.34353	-0.34269	-0.34184	-0.341	-0.40298
-0.2905	-0.28984	-0.28919	-0.34435	-0.34351	-0.34267	-0.34182	-0.34097	-0.40294
-0.29049	-0.28983	-0.28917	-0.34432	-0.34349	-0.34266	-0.3418	-0.34095	-0.40291
-0.29049	-0.28982	-0.28915	-0.34427	-0.34344	-0.34262	-0.34178	-0.34094	-0.40288
-0.29049	-0.28981	-0.28913	-0.34421	-0.34339	-0.34258	-0.34175	-0.34093	-0.40286
-0.34678	-0.3459	-0.34502	-0.34417	-0.34336	-0.46706	-0.46535	-0.46371	-0.52235
-0.34675	-0.34588	-0.345	-0.34416	-0.34333	-0.46696	-0.46527	-0.46364	-0.52226
-0.34672	-0.34585	-0.34498	-0.34413	-0.34331	-0.46687	-0.46519	-0.46357	-0.52217
-0.34669	-0.34582	-0.34496	-0.34411	-0.34329	-0.46679	-0.46512	-0.4635	-0.52207
-0.24058	-0.3458	-0.34493	-0.34409	-0.34328	-0.46671	-0.46505	-0.46343	-0.52198
-0.24054	-0.34577	-0.34491	-0.34407	-0.34326	-0.46662	-0.46498	-0.46336	-0.5219
-0.24049	-0.34576	-0.34489	-0.34405	-0.34324	-0.46654	-0.46491	-0.4633	-0.52183
-0.24044	-0.34574	-0.34488	-0.34404	-0.34322	-0.46645	-0.46483	-0.46324	-0.52175
-0.2404	-0.34574	-0.34486	-0.34402	-0.34321	-0.46637	-0.46476	-0.46317	-0.52169
-0.24036	-0.34573	-0.34485	-0.34401	-0.34319	-0.46629	-0.46468	-0.4631	-0.52162
-0.24031	-0.34571	-0.34485	-0.344	-0.34318	-0.4662	-0.4646	-0.46303	-0.52156
-0.24027	-0.23959	-0.23891	-0.344	-0.34317	-0.46612	-0.46453	-0.46296	-0.46143
-0.24022	-0.23954	-0.23886	-0.34398	-0.34316	-0.46604	-0.46445	-0.46289	-0.46136
-0.24018	-0.2395	-0.23882	-0.34398	-0.34316	-0.46597	-0.46439	-0.46283	-0.4613
-0.24014	-0.23947	-0.2388	-0.34399	-0.34317	-0.46595	-0.46435	-0.46277	-0.46123
-0.24009	-0.23943	-0.23878	-0.344	-0.34318	-0.46593	-0.46431	-0.46272	-0.46117
-0.24005	-0.23939	-0.23874	-0.23809	-0.23743	-0.46587	-0.46427	-0.46269	-0.57743
-0.24001	-0.23934	-0.23869	-0.23804	-0.23739	-0.46579	-0.46422	-0.46267	-0.57741
-0.23998	-0.23931	-0.23866	-0.238	-0.23735	-0.46572	-0.46417	-0.46263	-0.57735
-0.23996	-0.23929	-0.23863	-0.23797	-0.23731	-0.46566	-0.4641	-0.46255	-0.57724
-0.23994	-0.23927	-0.2386	-0.23793	-0.23727	-0.46562	-0.46405	-0.4625	-0.57715
-0.23989	-0.23923	-0.23856	-0.2379	-0.23725	-0.46561	-0.46405	-0.46249	-0.57709

-0.23985	-0.23919	-0.23852	-0.23787	-0.23723	-0.46559	-0.46404	-0.46248	-0.57703
-0.23982	-0.23915	-0.23848	-0.23782	-0.23719	-0.36457	-0.36316	-0.36177	-0.57696
-0.23978	-0.23911	-0.23844	-0.23778	-0.23714	-0.36447	-0.36307	-0.36168	-0.57689
-0.23975	-0.23908	-0.23841	-0.23775	-0.23711	-0.36441	-0.36301	-0.36163	-0.57685
-0.23971	-0.23904	-0.23839	-0.23774	-0.23709	-0.36436	-0.36297	-0.36159	-0.57683
-0.23968	-0.23902	-0.23837	-0.23771	-0.23706	-0.3643	-0.3629	-0.36153	-0.57677
-0.23966	-0.239	-0.23834	-0.23768	-0.23702	-0.36421	-0.36281	-0.36144	-0.57669
-0.23964	-0.23897	-0.23831	-0.23765	-0.23699	-0.36413	-0.36273	-0.36136	-0.57661
-0.23961	-0.23894	-0.23827	-0.23761	-0.23696	-0.36409	-0.36269	-0.3613	-0.57656
-0.23957	-0.23891	-0.23824	-0.23758	-0.23694	-0.36405	-0.36265	-0.36126	-0.57653
-0.23954	-0.23887	-0.23821	-0.23756	-0.23691	-0.364	-0.3626	-0.36122	-0.48024
-0.2395	-0.23884	-0.23819	-0.23754	-0.23689	-0.36395	-0.36256	-0.36119	-0.48019
-0.23948	-0.23882	-0.23817	-0.23752	-0.23687	-0.36391	-0.36252	-0.36115	-0.48012
-0.23947	-0.23881	-0.23814	-0.23749	-0.23685	-0.36388	-0.36249	-0.3611	-0.48003
-0.23946	-0.23879	-0.23812	-0.23746	-0.23683	-0.36384	-0.36244	-0.36104	-0.47993
-0.23942	-0.23876	-0.2381	-0.23745	-0.23681	-0.36378	-0.36236	-0.36095	-0.47983
-0.23939	-0.23873	-0.23809	-0.23744	-0.23678	-0.36373	-0.36229	-0.36088	-0.47975
-0.23938	-0.23872	-0.23806	-0.23741	-0.23676	-0.3637	-0.36227	-0.36087	-0.47971
-0.23937	-0.2387	-0.23803	-0.23738	-0.23674	-0.36366	-0.36226	-0.36087	-0.47969
-0.23934	-0.23868	-0.23801	-0.23736	-0.23672	-0.36361	-0.36222	-0.36084	-0.47967
-0.2393	-0.23864	-0.238	-0.23735	-0.23669	-0.36355	-0.36216	-0.36079	-0.47965
-0.23928	-0.23862	-0.23797	-0.23732	-0.23666	-0.3635	-0.36211	-0.36074	-0.47958
-0.23927	-0.2386	-0.23794	-0.23729	-0.23664	-0.36346	-0.36207	-0.36069	-0.47947
-0.18121	-0.18075	-0.18028	-0.23726	-0.23662	-0.36342	-0.36203	-0.36064	-0.47939
-0.18121	-0.18075	-0.18028	-0.23725	-0.2366	-0.36335	-0.36197	-0.36059	-0.47937
-0.1812	-0.18074	-0.18028	-0.23725	-0.23658	-0.3633	-0.36191	-0.36055	-0.47935
-0.18119	-0.18073	-0.18027	-0.23724	-0.23657	-0.36329	-0.36188	-0.36049	-0.47928
-0.18118	-0.18072	-0.18026	-0.23723	-0.23657	-0.36329	-0.36185	-0.36044	-0.4792
-0.18117	-0.18071	-0.18025	-0.36519	-0.36399	-0.48492	-0.48296	-0.48102	-0.58404
-0.18117	-0.18071	-0.18024	-0.36513	-0.36397	-0.48489	-0.48296	-0.48103	-0.58401
-0.18117	-0.1807	-0.18023	-0.36509	-0.36392	-0.48483	-0.48292	-0.48101	-0.58397
-0.18117	-0.1807	-0.18023	-0.36507	-0.36387	-0.48473	-0.48284	-0.48098	-0.58394
-0.18116	-0.1807	-0.18023	-0.36505	-0.36382	-0.48466	-0.48278	-0.48092	-0.58389
-0.18114	-0.18068	-0.18022	-0.36505	-0.36382	-0.48465	-0.48273	-0.48084	-0.5838
-0.18113	-0.18067	-0.18021	-0.36504	-0.36382	-0.48465	-0.48269	-0.48078	-0.58372
-0.18112	-0.18067	-0.18021	-0.36504	-0.36382	-0.48466	-0.48269	-0.48076	-0.58369
-0.18112	-0.18067	-0.18022	-0.36503	-0.36382	-0.48467	-0.48269	-0.48076	-0.58369
-0.18113	-0.18067	-0.18021	-0.365	-0.36381	-0.48465	-0.48269	-0.48077	-0.58367
-0.18113	-0.18066	-0.18019	-0.36494	-0.36378	-0.48462	-0.48269	-0.48077	-0.58363
-0.18113	-0.18066	-0.18018	-0.36491	-0.36376	-0.3626	-0.3614	-0.3602	-0.46838
-0.18112	-0.18065	-0.18018	-0.3649	-0.36376	-0.3626	-0.36141	-0.36021	-0.46837
-0.18111	-0.18065	-0.18018	-0.3649	-0.36375	-0.36258	-0.3614	-0.36021	-0.46836
-0.18111	-0.18065	-0.18018	-0.36488	-0.3637	-0.36253	-0.36136	-0.3602	-0.46835
-0.18111	-0.18065	-0.18018	-0.36486	-0.36366	-0.36247	-0.36133	-0.36019	-0.46833
-0.18111	-0.18065	-0.18018	-0.36485	-0.36364	-0.36246	-0.36132	-0.36019	-0.46832
-0.18111	-0.18065	-0.18018	-0.36485	-0.36364	-0.36246	-0.36132	-0.36018	-0.46831
-0.18112	-0.18065	-0.18019	-0.36485	-0.36364	-0.41958	-0.4183	-0.41703	-0.4683
-0.18112	-0.18066	-0.18019	-0.36485	-0.36364	-0.41958	-0.4183	-0.41703	-0.46829
-0.18112	-0.18066	-0.18019	-0.36485	-0.36364	-0.41957	-0.4183	-0.41703	-0.46829
-0.18112	-0.18066	-0.18019	-0.36485	-0.36364	-0.41957	-0.4183	-0.41703	-0.46828

-0.18113	-0.18066	-0.18019	-0.36485	-0.36364	-0.41957	-0.41829	-0.41703	-0.46827
-0.18113	-0.18067	-0.1802	-0.36485	-0.36364	-0.41956	-0.41829	-0.41702	-0.46826
-0.18114	-0.18067	-0.1802	-0.36486	-0.36365	-0.41956	-0.41829	-0.41702	-0.46825
-0.18114	-0.18068	-0.18021	-0.36487	-0.36366	-0.41956	-0.41828	-0.41702	-0.46824
-0.18115	-0.18069	-0.18022	-0.36488	-0.36366	-0.41956	-0.41828	-0.41701	-0.46823
-0.18116	-0.18069	-0.18023	-0.31023	-0.3092	-0.41956	-0.41828	-0.41701	-0.46822
-0.18116	-0.1807	-0.18023	-0.31025	-0.30921	-0.41955	-0.41827	-0.41701	-0.46821
-0.18117	-0.18071	-0.18024	-0.31027	-0.30923	-0.41957	-0.41828	-0.41701	-0.46821
-0.18117	-0.18071	-0.18024	-0.31029	-0.30928	-0.41963	-0.41832	-0.41702	-0.46821
-0.18118	-0.18072	-0.18025	-0.31032	-0.30932	-0.41968	-0.41836	-0.41704	-0.46821
-0.18119	-0.18073	-0.18026	-0.31033	-0.30934	-0.4197	-0.41837	-0.41705	-0.46821
-0.18121	-0.18075	-0.18027	-0.31034	-0.30935	-0.41969	-0.41838	-0.41706	-0.46821
-0.18122	-0.18076	-0.18029	-0.36889	-0.36774	-0.4197	-0.41838	-0.41706	-0.5235
-0.18123	-0.18077	-0.18031	-0.36895	-0.36777	-0.41971	-0.41838	-0.41706	-0.52351
-0.18123	-0.18078	-0.18033	-0.36898	-0.3678	-0.41972	-0.41838	-0.41706	-0.52352
-0.18124	-0.18078	-0.18033	-0.36899	-0.36781	-0.41973	-0.41838	-0.41705	-0.5235
-0.18125	-0.18079	-0.18034	-0.369	-0.36781	-0.41974	-0.41839	-0.41706	-0.5235
-0.18127	-0.18081	-0.18036	-0.36902	-0.36782	-0.41974	-0.41842	-0.41711	-0.52354
-0.18129	-0.18084	-0.18038	-0.36903	-0.36782	-0.41975	-0.41845	-0.41717	-0.52359
-0.18131	-0.18085	-0.18038	-0.36905	-0.36786	-0.41979	-0.41849	-0.4172	-0.52361
-0.18133	-0.18086	-0.18039	-0.36906	-0.3679	-0.41986	-0.41854	-0.41723	-0.52361
-0.18134	-0.18087	-0.1804	-0.36909	-0.36794	-0.41989	-0.41856	-0.41724	-0.52362
-0.18134	-0.18088	-0.18042	-0.36914	-0.36798	-0.41991	-0.41857	-0.41724	-0.52363
-0.18135	-0.18089	-0.18044	-0.30335	-0.30253	-0.35605	-0.35506	-0.35408	-0.52366
-0.18137	-0.18092	-0.18046	-0.30336	-0.30252	-0.35604	-0.35507	-0.35411	-0.52371
-0.18139	-0.18094	-0.18047	-0.30336	-0.30252	-0.35605	-0.35509	-0.35413	-0.52376
-0.18141	-0.18095	-0.18048	-0.30335	-0.30253	-0.35607	-0.35511	-0.35414	-0.52376
-0.18143	-0.18096	-0.18049	-0.30336	-0.30255	-0.3561	-0.35513	-0.35415	-0.52376
-0.24302	-0.2424	-0.24178	-0.30337	-0.30257	-0.41015	-0.40904	-0.40793	-0.52382
-0.24302	-0.2424	-0.2418	-0.30339	-0.30258	-0.41015	-0.40906	-0.40798	-0.52392
-0.24304	-0.24242	-0.24181	-0.30341	-0.3026	-0.41017	-0.40908	-0.408	-0.52396
-0.24308	-0.24246	-0.24184	-0.30343	-0.30262	-0.4102	-0.4091	-0.408	-0.52396
-0.2431	-0.24248	-0.24186	-0.30344	-0.30264	-0.41023	-0.40912	-0.408	-0.52397
-0.2431	-0.24249	-0.24187	-0.30346	-0.30266	-0.41023	-0.40911	-0.40799	-0.52398
-0.24311	-0.24249	-0.24189	-0.30348	-0.30267	-0.41023	-0.40911	-0.40799	-0.52401
-0.24314	-0.24252	-0.24191	-0.3035	-0.30269	-0.41026	-0.40914	-0.40803	-0.52406
-0.24318	-0.24255	-0.24193	-0.30352	-0.30271	-0.4103	-0.40919	-0.40808	-0.52413
-0.24319	-0.24257	-0.24195	-0.30353	-0.30273	-0.41032	-0.40922	-0.40812	-0.52421
-0.24318	-0.24257	-0.24196	-0.30356	-0.30274	-0.41032	-0.40924	-0.40816	-0.52431
-0.2432	-0.24259	-0.24198	-0.30357	-0.30276	-0.35712	-0.35618	-0.35525	-0.52438
-0.24324	-0.24262	-0.242	-0.30359	-0.30278	-0.35715	-0.3562	-0.35526	-0.52441
-0.24327	-0.24265	-0.24203	-0.3036	-0.3028	-0.35717	-0.35622	-0.35527	-0.52444
-0.24329	-0.24268	-0.24206	-0.30364	-0.30282	-0.35718	-0.35623	-0.35529	-0.52449
-0.24331	-0.24271	-0.2421	-0.30368	-0.30284	-0.35719	-0.35625	-0.35532	-0.52455
-0.24333	-0.24272	-0.24212	-0.30371	-0.30287	-0.35723	-0.35629	-0.35536	-0.52464
-0.24334	-0.24274	-0.24214	-0.30374	-0.30291	-0.35728	-0.35634	-0.3554	-0.52474
-0.24337	-0.24276	-0.24216	-0.36349	-0.3625	-0.4155	-0.41439	-0.41328	-0.52481
-0.24341	-0.24279	-0.24218	-0.3635	-0.36253	-0.41554	-0.41441	-0.41328	-0.52484
-0.24344	-0.24283	-0.24221	-0.36353	-0.36256	-0.41558	-0.41444	-0.41331	-0.52488
-0.24346	-0.24285	-0.24224	-0.36358	-0.36262	-0.41564	-0.4145	-0.41336	-0.52494

-0.24348	-0.24288	-0.24228	-0.36363	-0.36267	-0.41569	-0.41456	-0.41342	-0.47449
-0.24352	-0.24292	-0.24232	-0.36367	-0.36269	-0.4157	-0.41458	-0.41345	-0.47456
-0.24355	-0.24296	-0.24236	-0.36371	-0.3627	-0.4157	-0.41459	-0.41348	-0.47465
-0.24357	-0.24298	-0.24238	-0.36375	-0.36274	-0.41574	-0.41464	-0.41354	-0.47473
-0.24359	-0.24299	-0.2424	-0.36379	-0.36278	-0.4158	-0.4147	-0.4136	-0.47483
-0.24361	-0.24302	-0.24243	-0.36384	-0.36284	-0.41586	-0.41474	-0.41363	-0.47488
-0.24364	-0.24305	-0.24247	-0.3639	-0.3629	-0.41591	-0.41477	-0.41363	-0.47491
-0.24368	-0.24309	-0.2425	-0.36394	-0.36294	-0.41596	-0.4148	-0.41365	-0.41254
-0.24372	-0.24312	-0.24252	-0.36395	-0.36297	-0.416	-0.41485	-0.4137	-0.41259
-0.24375	-0.24315	-0.24254	-0.36397	-0.363	-0.41604	-0.4149	-0.41376	-0.41264
-0.24379	-0.24318	-0.24257	-0.36401	-0.36305	-0.41609	-0.41495	-0.41381	-0.4127
-0.24383	-0.24322	-0.24261	-0.36407	-0.36311	-0.41615	-0.41501	-0.41387	-0.41276
-0.24386	-0.30035	-0.29958	-0.41844	-0.41733	-0.41621	-0.41507	-0.41392	-0.4128
-0.2439	-0.30039	-0.29962	-0.4185	-0.41739	-0.41627	-0.41513	-0.41398	-0.41283
-0.24394	-0.30043	-0.29967	-0.41856	-0.41745	-0.41633	-0.41519	-0.41403	-0.41288
-0.24397	-0.30047	-0.29971	-0.41863	-0.41751	-0.41639	-0.41524	-0.41409	-0.41293
-0.24401	-0.30052	-0.29975	-0.41869	-0.41757	-0.41645	-0.4153	-0.41415	-0.41299
-0.24405	-0.30056	-0.29979	-0.41875	-0.41763	-0.4165	-0.41536	-0.41421	-0.41305
-0.24409	-0.3006	-0.29984	-0.4188	-0.41769	-0.41656	-0.41542	-0.41427	-0.41311
-0.24412	-0.30064	-0.29988	-0.41886	-0.41774	-0.41662	-0.41548	-0.41433	-0.41318
-0.24416	-0.30069	-0.29992	-0.41892	-0.4178	-0.41667	-0.41553	-0.41439	-0.41326
-0.2442	-0.30073	-0.29996	-0.41897	-0.41785	-0.41673	-0.41559	-0.41445	-0.41333
-0.24424	-0.30077	-0.3	-0.41903	-0.41791	-0.41679	-0.41564	-0.4145	-0.41339
-0.24428	-0.30082	-0.30005	-0.35929	-0.35836	-0.35742	-0.35647	-0.35552	-0.41344
-0.24432	-0.30087	-0.30011	-0.35939	-0.35844	-0.35749	-0.35653	-0.35557	-0.4135
-0.24436	-0.30092	-0.30018	-0.35948	-0.35852	-0.35756	-0.35659	-0.35563	-0.41356
-0.24439	-0.30096	-0.30022	-0.35954	-0.35858	-0.35761	-0.35664	-0.35568	-0.41361
-0.24443	-0.30101	-0.30026	-0.35959	-0.35863	-0.35767	-0.3567	-0.35573	-0.41367
-0.24447	-0.30105	-0.3003	-0.35965	-0.35868	-0.35772	-0.35676	-0.35581	-0.41376
-0.24451	-0.30109	-0.30034	-0.3597	-0.35873	-0.35778	-0.35684	-0.3559	-0.41386
-0.24457	-0.30115	-0.3004	-0.35976	-0.35879	-0.35783	-0.3569	-0.35598	-0.41394
-0.24463	-0.30122	-0.30046	-0.35984	-0.35885	-0.35788	-0.35695	-0.35603	-0.414
-0.24469	-0.30129	-0.30053	-0.35991	-0.35891	-0.35794	-0.35701	-0.35609	-0.41407
-0.24475	-0.30135	-0.30057	-0.35997	-0.35899	-0.35804	-0.35709	-0.35616	-0.41416
-0.2448	-0.3014	-0.30062	-0.36004	-0.35908	-0.35813	-0.35718	-0.35623	-0.41424
-0.24484	-0.30146	-0.30068	-0.36013	-0.35916	-0.35821	-0.35724	-0.35629	-0.4143
-0.24488	-0.30151	-0.30075	-0.36022	-0.35925	-0.35828	-0.3573	-0.35634	-0.41436
-0.24493	-0.30157	-0.30082	-0.3603	-0.35931	-0.35834	-0.35737	-0.35642	-0.41444
-0.24499	-0.30164	-0.30088	-0.36037	-0.35937	-0.3584	-0.35745	-0.35652	-0.41455
-0.24504	-0.3017	-0.30093	-0.36044	-0.35944	-0.35847	-0.35753	-0.3566	-0.35566
-0.24507	-0.30173	-0.30097	-0.3605	-0.35953	-0.35857	-0.35762	-0.35668	-0.35575
-0.24511	-0.30178	-0.30102	-0.36057	-0.35961	-0.35866	-0.3577	-0.35675	-0.35582
-0.24516	-0.30184	-0.30108	-0.36066	-0.3597	-0.35873	-0.35776	-0.3568	-0.35587
-0.24522	-0.30191	-0.30115	-0.36075	-0.35978	-0.3588	-0.35783	-0.35686	-0.35593
-0.24527	-0.30197	-0.30122	-0.36082	-0.35984	-0.35886	-0.3579	-0.35694	-0.35601
-0.24533	-0.30204	-0.30129	-0.36089	-0.35989	-0.35892	-0.35797	-0.35704	-0.3561
-0.24539	-0.30211	-0.30135	-0.36098	-0.35999	-0.35901	-0.35806	-0.35712	-0.35618
-0.24545	-0.30218	-0.30142	-0.36108	-0.3601	-0.35913	-0.35816	-0.3572	-0.35626
-0.2455	-0.30224	-0.30149	-0.36117	-0.3602	-0.35922	-0.35824	-0.35728	-0.35635
-0.24556	-0.30231	-0.30155	-0.36124	-0.36025	-0.35928	-0.35832	-0.35737	-0.35644

-0.24562	-0.30238	-0.30162	-0.36132	-0.36032	-0.35934	-0.3584	-0.35746	-0.35652
-0.24568	-0.30245	-0.30169	-0.36142	-0.36043	-0.35945	-0.35849	-0.35754	-0.3566
-0.24573	-0.30251	-0.30175	-0.36152	-0.36054	-0.35957	-0.35858	-0.35761	-0.35668
-0.24579	-0.30258	-0.30182	-0.36161	-0.36064	-0.35967	-0.35869	-0.35771	-0.35677
-0.24585	-0.30265	-0.30188	-0.36168	-0.36073	-0.35977	-0.3588	-0.35783	-0.35687
-0.2459	-0.30272	-0.30195	-0.36177	-0.36081	-0.35986	-0.3589	-0.35794	-0.35697
-0.24596	-0.30278	-0.30202	-0.36186	-0.36089	-0.35993	-0.35898	-0.35803	-0.35708
-0.24602	-0.30285	-0.30208	-0.30132	-0.30057	-0.29982	-0.29908	-0.29833	-0.35718
-0.24607	-0.30292	-0.30215	-0.30138	-0.30064	-0.29989	-0.29914	-0.29839	-0.35726
-0.24613	-0.30299	-0.30221	-0.30145	-0.3007	-0.29996	-0.29921	-0.29846	-0.35734
-0.24619	-0.30305	-0.30228	-0.30151	-0.30076	-0.30002	-0.29927	-0.29853	-0.35742
-0.24624	-0.30312	-0.30235	-0.30158	-0.30083	-0.30008	-0.29934	-0.2986	-0.35751
-0.24631	-0.3032	-0.30243	-0.30167	-0.30091	-0.30016	-0.29942	-0.29868	-0.35762
-0.24639	-0.3033	-0.30253	-0.30176	-0.301	-0.30025	-0.29951	-0.29877	-0.35774
-0.24646	-0.30339	-0.30261	-0.30184	-0.30108	-0.30033	-0.29959	-0.29885	-0.35784
-0.24653	-0.30346	-0.30267	-0.3019	-0.30115	-0.3004	-0.29966	-0.29891	-0.35792
-0.24659	-0.30354	-0.30275	-0.30198	-0.30122	-0.30048	-0.29973	-0.29898	-0.358
-0.24667	-0.30364	-0.30284	-0.30207	-0.30132	-0.30057	-0.29982	-0.29907	-0.3581
-0.24675	-0.30373	-0.30294	-0.30216	-0.30141	-0.30065	-0.29991	-0.29916	-0.3582
-0.2468	-0.3038	-0.30301	-0.30224	-0.30147	-0.30072	-0.29998	-0.29923	-0.35831
-0.24685	-0.30386	-0.30308	-0.30231	-0.30154	-0.30078	-0.30004	-0.2993	-0.35841
-0.24692	-0.30394	-0.30317	-0.30239	-0.30161	-0.30085	-0.30011	-0.29938	-0.35852
-0.24699	-0.30403	-0.30326	-0.30248	-0.3017	-0.30094	-0.3002	-0.29946	-0.35863
-0.24706	-0.30413	-0.30335	-0.30257	-0.30179	-0.30103	-0.30028	-0.29954	-0.29879
-0.24714	-0.30422	-0.30344	-0.30267	-0.30189	-0.30112	-0.30035	-0.29959	-0.29885
-0.24721	-0.24659	-0.24597	-0.24535	-0.24473	-0.3012	-0.30042	-0.29965	-0.29891
-0.24727	-0.24665	-0.24603	-0.24541	-0.24479	-0.30127	-0.3005	-0.29974	-0.299
-0.24733	-0.24671	-0.24608	-0.24546	-0.24484	-0.30133	-0.30057	-0.29982	-0.29909
-0.2474	-0.24678	-0.24615	-0.24552	-0.24491	-0.30142	-0.30066	-0.29991	-0.29917
-0.24748	-0.24686	-0.24622	-0.2456	-0.24498	-0.3015	-0.30075	-0.3	-0.29926
-0.18931	-0.24693	-0.2463	-0.24567	-0.24506	-0.30159	-0.30083	-0.30008	-0.29935
-0.18936	-0.24701	-0.24637	-0.24575	-0.24513	-0.30168	-0.30092	-0.30017	-0.29943
-0.18941	-0.24708	-0.24645	-0.24583	-0.24521	-0.30177	-0.30101	-0.30025	-0.29952
-0.18947	-0.24716	-0.24653	-0.2459	-0.24528	-0.30187	-0.3011	-0.30034	-0.2996
-0.18952	-0.24723	-0.2466	-0.24598	-0.24536	-0.30196	-0.30119	-0.30043	-0.29969
-0.18957	-0.24731	-0.24668	-0.24605	-0.24543	-0.30205	-0.30128	-0.30052	-0.29978
-0.18962	-0.24738	-0.24675	-0.24613	-0.24551	-0.30214	-0.30137	-0.3006	-0.29986
-0.18967	-0.24745	-0.24682	-0.2462	-0.24558	-0.30223	-0.30146	-0.30069	-0.29995
-0.18971	-0.24752	-0.24689	-0.24627	-0.24566	-0.30232	-0.30155	-0.30078	-0.30003
-0.18976	-0.24759	-0.24696	-0.24634	-0.24573	-0.30241	-0.30164	-0.30087	-0.30012
-0.1898	-0.24765	-0.24703	-0.24641	-0.2458	-0.3025	-0.30172	-0.30095	-0.3002
-0.12645	-0.12613	-0.12581	-0.24649	-0.24588	-0.24526	-0.24464	-0.24403	-0.3003
-0.1265	-0.12618	-0.12586	-0.24659	-0.24597	-0.24535	-0.24473	-0.24412	-0.30041
-0.12655	-0.12623	-0.12592	-0.24668	-0.24606	-0.24544	-0.24483	-0.24421	-0.30052
-0.12659	-0.12627	-0.12596	-0.24675	-0.24613	-0.24551	-0.2449	-0.24429	-0.30061
-0.12663	-0.12631	-0.126	-0.24682	-0.2462	-0.24558	-0.24497	-0.24437	-0.3007
-0.12667	-0.12635	-0.12603	-0.24689	-0.24627	-0.24565	-0.24505	-0.24444	-0.30079
-0.12671	-0.12639	-0.12607	-0.24696	-0.24634	-0.24573	-0.24512	-0.24451	-0.30088
-0.12675	-0.12644	-0.12612	-0.24704	-0.24642	-0.24581	-0.2452	-0.24459	-0.30097
-0.12681	-0.12649	-0.12617	-0.24712	-0.24652	-0.2459	-0.24529	-0.24468	-0.30109

-0.12686	-0.12654	-0.12622	-0.24721	-0.2466	-0.246	-0.24538	-0.24476	-0.30119
-0.1269	-0.12658	-0.12626	-0.24728	-0.24668	-0.24607	-0.24545	-0.24484	-0.30128
-0.12694	-0.12662	-0.1263	-0.24735	-0.24675	-0.24614	-0.24552	-0.24491	-0.30137
-0.12699	-0.12667	-0.12635	-0.1891	-0.18864	-0.24622	-0.24561	-0.24499	-0.30148
-0.12705	-0.12672	-0.12641	-0.18916	-0.1887	-0.2463	-0.2457	-0.24509	-0.30159
-0.12709	-0.12677	-0.12645	-0.18921	-0.18875	-0.24637	-0.24577	-0.24517	-0.3017
-0.12713	-0.12681	-0.12649	-0.18925	-0.18878	-0.24644	-0.24583	-0.24524	-0.30179
-0.12718	-0.12686	-0.12653	-0.1893	-0.18883	-0.24651	-0.2459	-0.24531	-0.24472
-0.12723	-0.12691	-0.12659	-0.18936	-0.18889	-0.24659	-0.24599	-0.24539	-0.24479
-0.12729	-0.12696	-0.12664	-0.12632	-0.126	-0.18849	-0.18803	-0.18757	-0.24487
-0.12734	-0.12702	-0.12669	-0.12637	-0.12605	-0.18855	-0.18808	-0.18763	-0.24496
-0.12739	-0.12707	-0.12675	-0.12642	-0.1261	-0.1886	-0.18814	-0.18769	-0.24505
-0.12745	-0.12712	-0.1268	-0.12648	-0.12615	-0.18866	-0.1882	-0.18774	-0.24513
-0.1275	-0.12718	-0.12685	-0.12653	-0.1262	-0.18872	-0.18826	-0.1878	-0.24521
-0.12755	-0.12723	-0.1269	-0.12658	-0.12626	-0.18879	-0.18832	-0.18786	-0.1874
-0.12759	-0.12727	-0.12696	-0.12664	-0.12631	-0.18885	-0.18838	-0.18792	-0.18746
-0.12764	-0.12732	-0.12701	-0.12669	-0.12636	-0.18891	-0.18844	-0.18798	-0.18752
-0.12769	-0.12737	-0.12705	-0.12673	-0.12641	-0.18897	-0.1885	-0.18804	-0.18758
-0.12774	-0.12742	-0.12709	-0.12677	-0.12646	-0.18903	-0.18856	-0.1881	-0.18764
-0.1278	-0.12748	-0.12715	-0.12683	-0.12652	-0.18909	-0.18862	-0.18815	-0.18769
-0.12786	-0.12754	-0.12722	-0.1269	-0.12657	-0.18914	-0.18867	-0.1882	-0.18774
-0.12792	-0.1276	-0.12728	-0.12695	-0.12662	-0.1892	-0.18872	-0.18825	-0.18779
-0.12798	-0.12766	-0.12733	-0.127	-0.12667	-0.18925	-0.18877	-0.1883	-0.18784
-0.06337	-0.12771	-0.12738	-0.12705	-0.12673	-0.18931	-0.18883	-0.18835	-0.18789
-0.0634	-0.12775	-0.12742	-0.1271	-0.12677	-0.18936	-0.18888	-0.1884	-0.18794
-0.06342	-0.12779	-0.12747	-0.12715	-0.12682	-0.1265	-0.12618	-0.12586	-0.18799
-0.06346	-0.12785	-0.12752	-0.1272	-0.12687	-0.12655	-0.12623	-0.12591	-0.18803
-0.06349	-0.12791	-0.12757	-0.12725	-0.12692	-0.1266	-0.12628	-0.12596	-0.18809
-0.06353	-0.12796	-0.12762	-0.1273	-0.12698	-0.12666	-0.12634	-0.12602	-0.18815
-0.06356	-0.128	-0.12767	-0.12735	-0.12704	-0.12672	-0.1264	-0.12608	-0.18823
-0.06359	-0.12806	-0.12773	-0.1274	-0.12709	-0.12677	-0.12646	-0.12614	-0.18829
-0.06362	-0.12812	-0.12779	-0.12747	-0.12714	-0.12682	-0.1265	-0.12619	-0.18834
-0.06365	-0.06348	-0.06331	-0.12752	-0.12719	-0.12687	-0.12655	-0.12624	-0.18839
-0.06368	-0.06352	-0.06335	-0.12757	-0.12724	-0.12692	-0.12661	-0.1263	-0.18846
-0.06372	-0.06355	-0.06338	-0.12762	-0.12729	-0.12697	-0.12667	-0.12636	-0.18852
-0.06375	-0.06358	-0.06341	-0.12767	-0.12735	-0.12703	-0.12672	-0.12641	-0.18858
-0.06378	-0.06361	-0.06344	-0.12773	-0.12741	-0.12709	-0.12677	-0.12646	-0.18863
-0.06381	-0.06364	-0.06347	-0.12779	-0.12747	-0.12714	-0.12682	-0.12651	-0.1262
-0.06384	-0.06368	-0.06351	-0.12785	-0.12752	-0.12719	-0.12687	-0.12656	-0.12625
	-0.06371	-0.06354	-0.06337	-0.0632	-0.12725	-0.12693	-0.12661	-0.12631
	-0.06374	-0.06358	-0.06341	-0.06324	-0.12731	-0.12699	-0.12667	-0.12637
	-0.06377	-0.06361	-0.06344	-0.06327	-0.12737	-0.12705	-0.12674	-0.12643
	-0.06381	-0.06364	-0.06347	-0.0633	-0.12742	-0.12711	-0.1268	-0.12649
	-0.06384	-0.06367	-0.0635	-0.06333	-0.12748	-0.12717	-0.12686	-0.12655
	-0.06388	-0.06371	-0.06354	-0.06336	-0.12754	-0.12723	-0.12692	-0.12661
	-0.06391	-0.06374	-0.06357	-0.0634	-0.1276	-0.12729	-0.12698	-0.12667
	-0.06394	-0.06378	-0.06361	-0.06344	-0.12767	-0.12735	-0.12704	-0.12673
	-0.06398	-0.06381	-0.06364	-0.06347	-0.12773	-0.12742	-0.12711	-0.12679
			-0.06368	-0.06351	-0.06334	-0.06318	-0.06302	-0.12685
			-0.06371	-0.06354	-0.06338	-0.06321	-0.06305	-0.12691

-0.06375	-0.06358	-0.06341	-0.06325	-0.06308	-0.12697
-0.06378	-0.06361	-0.06345	-0.06328	-0.06312	-0.12703
-0.06382	-0.06365	-0.06348	-0.06332	-0.06315	-0.12709
-0.06385	-0.06368	-0.06352	-0.06335	-0.06319	-0.12716
-0.06388	-0.06372	-0.06355	-0.06339	-0.06322	-0.12722
-0.06391	-0.06375	-0.06359	-0.06342	-0.06326	-0.0631
-0.06395	-0.06379	-0.06362	-0.06346	-0.0633	-0.06313
-0.06399	-0.06383	-0.06366	-0.0635	-0.06333	-0.06317
-0.06403	-0.06386	-0.0637	-0.06353	-0.06337	-0.0632
-0.06406	-0.0639	-0.06373	-0.06357	-0.06341	-0.06324
-0.0641	-0.06393	-0.06377	-0.06361	-0.06344	-0.06328
-0.06413	-0.06397	-0.0638	-0.06364	-0.06348	-0.06331
		-0.06384	-0.06368	-0.06352	-0.06335
		-0.06387	-0.06371	-0.06355	-0.06339
		-0.06391	-0.06375	-0.06359	-0.06343
		-0.06395	-0.06379	-0.06362	-0.06346
		-0.06399	-0.06383	-0.06366	-0.0635
		-0.06404	-0.06387	-0.0637	-0.06354
		-0.06408	-0.06391	-0.06374	-0.06358
		-0.06411	-0.06395	-0.06378	-0.06362
		-0.06415	-0.06399	-0.06382	-0.06366
		-0.06418	-0.06402	-0.06386	-0.0637
		-0.06422	-0.06406	-0.0639	-0.06374
					-0.06377
					-0.0638
					-0.06384
					-0.06388
					-0.06392
					-0.06396
					-0.06401
					-0.06405
					-0.06408

		-0.12804
		-0.12796
		-0.12787
		-0.12778
		-0.18896
		-0.18882
		-0.18868
		-0.18854
		-0.1884
-0.12784	-0.12752	-0.18825
-0.12775	-0.12743	-0.18811
-0.18896	-0.18846	-0.29105
-0.18885	-0.18836	-0.29093
-0.18873	-0.18825	-0.29081
-0.1886	-0.18812	-0.29065

						-0.18846	-0.18798	-0.29049
			-0.12819	-0.12786	-0.12754	-0.18832	-0.18784	-0.29033
			-0.12811	-0.12778	-0.12745	-0.18819	-0.1877	-0.29017
			-0.18958	-0.18907	-0.18857	-0.29145	-0.29074	-0.29003
			-0.18944	-0.18895	-0.18845	-0.29133	-0.29062	-0.28992
			-0.18931	-0.18882	-0.18833	-0.29119	-0.29049	-0.28978
			-0.18916	-0.18867	-0.18819	-0.29102	-0.29032	-0.28963
			-0.18903	-0.18854	-0.18805	-0.29086	-0.29016	-0.28947
			-0.18891	-0.18842	-0.18792	-0.2907	-0.28999	-0.28931
			-0.18881	-0.1883	-0.18779	-0.29055	-0.28983	-0.28915
	-0.12804	-0.12772	-0.18868	-0.18817	-0.18767	-0.29041	-0.28971	-0.28903
	-0.12795	-0.12763	-0.18854	-0.18804	-0.18755	-0.29028	-0.28959	-0.28892
	-0.18941	-0.18891	-0.29226	-0.29155	-0.29084	-0.29015	-0.28946	-0.28879
	-0.1893	-0.18881	-0.29214	-0.29142	-0.2907	-0.29	-0.28931	-0.28864
	-0.18918	-0.18869	-0.29201	-0.29129	-0.29057	-0.28986	-0.28917	-0.2885
	-0.18905	-0.18855	-0.29186	-0.29114	-0.29043	-0.28974	-0.28906	-0.2884
	-0.18892	-0.18842	-0.2917	-0.29099	-0.2903	-0.28962	-0.28895	-0.2883
	-0.1888	-0.18831	-0.29158	-0.29087	-0.29017	-0.28948	-0.28881	-0.28816
	-0.18869	-0.1882	-0.29146	-0.29075	-0.29004	-0.28935	-0.28867	-0.28802
-0.12762	-0.29274	-0.29204	-0.29133	-0.29062	-0.28991	-0.28922	-0.28855	-0.2879
-0.12754	-0.29262	-0.29191	-0.2912	-0.29049	-0.28979	-0.28911	-0.28845	-0.2878
-0.12746	-0.29249	-0.29178	-0.29107	-0.29037	-0.28967	-0.28901	-0.28835	-0.2877
-0.12737	-0.29235	-0.29165	-0.29095	-0.29026	-0.28958	-0.28891	-0.28824	-0.28758
-0.18858	-0.29221	-0.29152	-0.29083	-0.29016	-0.28948	-0.2888	-0.28813	-0.28746
-0.18847	-0.29209	-0.29141	-0.29072	-0.29004	-0.28936	-0.28868	-0.28801	-0.28734
-0.18836	-0.29198	-0.2913	-0.29061	-0.28992	-0.28923	-0.28856	-0.28789	-0.28722
-0.18826	-0.29187	-0.29118	-0.29049	-0.28979	-0.28911	-0.28844	-0.28778	-0.28711
-0.18816	-0.29175	-0.29105	-0.29036	-0.28967	-0.28899	-0.28833	-0.28767	-0.28701
-0.29233	-0.29163	-0.29092	-0.29023	-0.28955	-0.28887	-0.28822	-0.28756	-0.28689
-0.2922	-0.2915	-0.2908	-0.29011	-0.28944	-0.28877	-0.28811	-0.28744	-0.28676
-0.29207	-0.29137	-0.29068	-0.28999	-0.28933	-0.28866	-0.28799	-0.28731	-0.28663
-0.29194	-0.29125	-0.29057	-0.28989	-0.28921	-0.28854	-0.28786	-0.28718	-0.28651
-0.29182	-0.29113	-0.29046	-0.28979	-0.2891	-0.28842	-0.28774	-0.28706	-0.28639
-0.29171	-0.29103	-0.29036	-0.28969	-0.289	-0.28832	-0.28763	-0.28696	-0.28629
-0.29161	-0.29093	-0.29026	-0.28958	-0.2889	-0.28821	-0.28753	-0.28686	-0.28619
-0.2915	-0.29082	-0.29014	-0.28946	-0.28878	-0.2881	-0.28743	-0.28676	-0.28609
-0.2914	-0.29071	-0.29002	-0.28933	-0.28865	-0.28797	-0.28731	-0.28666	-0.28599
-0.29129	-0.2906	-0.2899	-0.28921	-0.28853	-0.28786	-0.28721	-0.28656	-0.28589
-0.29116	-0.29047	-0.28978	-0.2891	-0.28843	-0.28777	-0.28711	-0.28646	-0.28578
-0.29105	-0.29035	-0.28967	-0.28899	-0.28833	-0.28767	-0.28701	-0.28635	-0.28567
-0.29095	-0.29025	-0.28956	-0.28888	-0.28822	-0.28756	-0.28689	-0.28622	-0.28555
-0.29085	-0.29015	-0.28946	-0.28878	-0.28812	-0.28746	-0.28678	-0.2861	-0.28543
-0.29075	-0.29005	-0.28936	-0.28868	-0.28801	-0.28735	-0.28668	-0.286	-0.28533
-0.29064	-0.28994	-0.28925	-0.28857	-0.28791	-0.28725	-0.28658	-0.2859	-0.28523
-0.29054	-0.28984	-0.28915	-0.28847	-0.28781	-0.28715	-0.28648	-0.2858	-0.28514
-0.29044	-0.28973	-0.28904	-0.28836	-0.2877	-0.28704	-0.28638	-0.28571	-0.28504
-0.29033	-0.28963	-0.28894	-0.28826	-0.2876	-0.28694	-0.28628	-0.28561	-0.28495
-0.29023	-0.28953	-0.28884	-0.28816	-0.2875	-0.28685	-0.28618	-0.28552	-0.28486
-0.29013	-0.28943	-0.28874	-0.28806	-0.2874	-0.28675	-0.28609	-0.28543	-0.28477
-0.29004	-0.28933	-0.28864	-0.28796	-0.28731	-0.28666	-0.28601	-0.28535	-0.2847

-0.28994	-0.28924	-0.28855	-0.28787	-0.28722	-0.28658	-0.28593	-0.28528	-0.28462
-0.28984	-0.28915	-0.28846	-0.28779	-0.28714	-0.2865	-0.28585	-0.28519	-0.28454
-0.28975	-0.28906	-0.28838	-0.28771	-0.28707	-0.28642	-0.28577	-0.28511	-0.28445
-0.28966	-0.28897	-0.28829	-0.28763	-0.28698	-0.28634	-0.28568	-0.28502	-0.28436
-0.28957	-0.28888	-0.2882	-0.28754	-0.28689	-0.28625	-0.28559	-0.28493	-0.28427
-0.28948	-0.28879	-0.28811	-0.28744	-0.2868	-0.28616	-0.28551	-0.28485	-0.28418
-0.28938	-0.2887	-0.28802	-0.28735	-0.28672	-0.28608	-0.28542	-0.28476	-0.28409
-0.28929	-0.28861	-0.28793	-0.28727	-0.28663	-0.28599	-0.28534	-0.28467	-0.28401
-0.28922	-0.28854	-0.28786	-0.28719	-0.28655	-0.28591	-0.28525	-0.28459	-0.28392
-0.28914	-0.28847	-0.28779	-0.28712	-0.28647	-0.28582	-0.28517	-0.28451	-0.28384
-0.28905	-0.28838	-0.28771	-0.28704	-0.28639	-0.28574	-0.28509	-0.28444	-0.28378
-0.28896	-0.28829	-0.28762	-0.28696	-0.2863	-0.28566	-0.28502	-0.28438	-0.28372
-0.28887	-0.2882	-0.28754	-0.28688	-0.28623	-0.28559	-0.28495	-0.28431	-0.28364
-0.28878	-0.28812	-0.28747	-0.28682	-0.28618	-0.28553	-0.28488	-0.28423	-0.28355
-0.2887	-0.28804	-0.2874	-0.28676	-0.28611	-0.28546	-0.2848	-0.28414	-0.28346
-0.28863	-0.28797	-0.28733	-0.28668	-0.28603	-0.28537	-0.28471	-0.28404	-0.28337
-0.28857	-0.2879	-0.28725	-0.2866	-0.28594	-0.28528	-0.28462	-0.28395	-0.28329
-0.28849	-0.28782	-0.28717	-0.28651	-0.28586	-0.28521	-0.28455	-0.28388	-0.28321
-0.28841	-0.28774	-0.28708	-0.28642	-0.28578	-0.28514	-0.28448	-0.28381	-0.28314
-0.28834	-0.28767	-0.287	-0.28635	-0.2857	-0.28506	-0.2844	-0.28374	-0.28308
-0.28827	-0.2876	-0.28694	-0.28628	-0.28562	-0.28497	-0.28432	-0.28367	-0.28302
-0.28819	-0.28753	-0.28687	-0.28621	-0.28555	-0.28489	-0.28424	-0.2836	-0.28294
-0.2881	-0.28744	-0.28679	-0.28614	-0.28548	-0.28482	-0.28417	-0.28352	-0.28286
-0.28801	-0.28736	-0.28672	-0.28607	-0.28542	-0.28476	-0.2841	-0.28344	-0.28278
-0.28795	-0.28729	-0.28663	-0.28598	-0.28534	-0.2847	-0.28404	-0.28337	-0.28271
-0.28788	-0.28722	-0.28655	-0.2859	-0.28527	-0.28463	-0.28397	-0.28331	-0.28265
-0.28782	-0.28715	-0.28648	-0.28583	-0.28519	-0.28455	-0.2839	-0.28324	-0.28259
-0.28775	-0.28708	-0.28642	-0.28577	-0.28511	-0.28446	-0.28381	-0.28318	-0.28253
-0.28769	-0.28702	-0.28636	-0.2857	-0.28504	-0.28438	-0.28374	-0.2831	-0.34665
-0.28763	-0.28696	-0.28629	-0.28562	-0.28497	-0.28432	-0.28367	-0.28302	-0.34652
-0.28757	-0.28689	-0.28622	-0.28555	-0.2849	-0.28425	-0.2836	-0.28295	-0.3464
-0.2875	-0.28682	-0.28616	-0.28549	-0.28484	-0.28418	-0.28354	-0.2829	-0.34631
-0.28743	-0.28676	-0.28609	-0.28543	-0.28478	-0.28412	-0.28348	-0.28284	-0.34621
-0.28737	-0.2867	-0.28603	-0.28537	-0.28472	-0.28408	-0.28343	-0.28278	-0.34611
-0.28731	-0.28664	-0.28597	-0.28531	-0.28467	-0.28403	-0.28338	-0.28273	-0.346
-0.28725	-0.28658	-0.28592	-0.28526	-0.28462	-0.28398	-0.28332	-0.28267	-0.3459
-0.28718	-0.28652	-0.28587	-0.28522	-0.28456	-0.28391	-0.28326	-0.28262	-0.34581
-0.28712	-0.28646	-0.28581	-0.28516	-0.28451	-0.28386	-0.28321	-0.28256	-0.34572
-0.28707	-0.28641	-0.28575	-0.2851	-0.28445	-0.2838	-0.28315	-0.2825	-0.34563
-0.28702	-0.28636	-0.2857	-0.28505	-0.2844	-0.28376	-0.3474	-0.34647	-0.34554
-0.28695	-0.2863	-0.28565	-0.28501	-0.28437	-0.28373	-0.34734	-0.34639	-0.34546
-0.28689	-0.28625	-0.28561	-0.28498	-0.28434	-0.28369	-0.34727	-0.34632	-0.34538
-0.28685	-0.2862	-0.28557	-0.28493	-0.28429	-0.28365	-0.34718	-0.34623	-0.34528
-0.28681	-0.28616	-0.28552	-0.28488	-0.28424	-0.2836	-0.34708	-0.34614	-0.34518
-0.28677	-0.28612	-0.28547	-0.28482	-0.28419	-0.28354	-0.34698	-0.34604	-0.39941
-0.28671	-0.28606	-0.28542	-0.28477	-0.28413	-0.28349	-0.34688	-0.34594	-0.39927
-0.28665	-0.286	-0.28536	-0.28472	-0.28408	-0.28343	-0.34679	-0.34584	-0.39915
-0.28659	-0.28594	-0.2853	-0.28466	-0.28402	-0.28337	-0.3467	-0.34576	-0.39904
-0.28653	-0.28589	-0.28525	-0.34941	-0.34848	-0.34755	-0.34661	-0.34567	-0.51854
-0.2865	-0.28585	-0.28522	-0.34934	-0.34841	-0.34748	-0.34653	-0.34559	-0.51829

-0.28646	-0.28582	-0.28518	-0.34927	-0.34834	-0.34741	-0.34646	-0.3455	-0.51805
-0.28641	-0.28576	-0.28512	-0.34918	-0.34826	-0.34733	-0.34638	-0.34543	-0.51785
-0.28634	-0.2857	-0.28506	-0.34909	-0.34817	-0.34725	-0.34631	-0.34536	-0.51766
-0.28629	-0.28565	-0.28501	-0.349	-0.34808	-0.34716	-0.34622	-0.34527	-0.51744
-0.28625	-0.28561	-0.28497	-0.34893	-0.348	-0.34706	-0.34612	-0.34517	-0.51719
-0.28621	-0.28557	-0.28494	-0.34885	-0.34792	-0.34697	-0.34603	-0.34508	-0.51696
-0.28618	-0.28553	-0.2849	-0.34878	-0.34784	-0.34691	-0.34595	-0.345	-0.51676
-0.28614	-0.28549	-0.28487	-0.3487	-0.34777	-0.34683	-0.4001	-0.39897	-0.51655
-0.28608	-0.28544	-0.28481	-0.3486	-0.34767	-0.34673	-0.39998	-0.39885	-0.51631
-0.28603	-0.28539	-0.28475	-0.34851	-0.34757	-0.34663	-0.39985	-0.39874	-0.51608
-0.35126	-0.35031	-0.34936	-0.34843	-0.34749	-0.34656	-0.39975	-0.39864	-0.51588
-0.35119	-0.35024	-0.34929	-0.34836	-0.34743	-0.3465	-0.39966	-0.39855	-0.51571
-0.35112	-0.35017	-0.34922	-0.40288	-0.40177	-0.40067	-0.51909	-0.5173	-0.57163
-0.35105	-0.3501	-0.34916	-0.40278	-0.40168	-0.40057	-0.5189	-0.51712	-0.57143
-0.35098	-0.35003	-0.34909	-0.40269	-0.40158	-0.40048	-0.51872	-0.51694	-0.57123
-0.3509	-0.34995	-0.34902	-0.40258	-0.40148	-0.40038	-0.51854	-0.51677	-0.57104
-0.35083	-0.40472	-0.40359	-0.40248	-0.40138	-0.40029	-0.51836	-0.51659	-0.57084
-0.35077	-0.40462	-0.40349	-0.40238	-0.40128	-0.40019	-0.51818	-0.51642	-0.57064
-0.3507	-0.40453	-0.4034	-0.40228	-0.40118	-0.4001	-0.51799	-0.51624	-0.57044
-0.35064	-0.40442	-0.4033	-0.40218	-0.40109	-0.40001	-0.51781	-0.51605	-0.57023
-0.35057	-0.40432	-0.40319	-0.40209	-0.401	-0.39992	-0.51763	-0.51587	-0.57003
-0.3505	-0.40421	-0.40309	-0.40199	-0.40091	-0.39983	-0.51745	-0.51569	-0.56982
-0.35044	-0.40412	-0.403	-0.4019	-0.40082	-0.39974	-0.51727	-0.5155	-0.5696
-0.35037	-0.40402	-0.40291	-0.40181	-0.40073	-0.39965	-0.51708	-0.51532	-0.5694
-0.3503	-0.40391	-0.40282	-0.40172	-0.40064	-0.39956	-0.5169	-0.51515	-0.5692
-0.35023	-0.40382	-0.40273	-0.40164	-0.40055	-0.39947	-0.51673	-0.515	-0.56902
-0.35019	-0.40375	-0.40266	-0.40158	-0.40049	-0.39941	-0.5166	-0.51486	-0.56886
-0.35017	-0.40369	-0.4026	-0.40152	-0.40043	-0.39935	-0.51647	-0.51473	-0.56872
-0.40473	-0.40361	-0.40252	-0.52167	-0.51986	-0.51809	-0.57235	-0.57044	-0.73006
-0.40463	-0.40352	-0.40242	-0.52148	-0.51967	-0.5179	-0.57215	-0.57023	-0.72977
-0.40454	-0.40342	-0.40233	-0.5213	-0.51949	-0.51773	-0.57194	-0.57002	-0.7295
-0.40446	-0.40333	-0.40225	-0.52111	-0.51931	-0.51755	-0.57173	-0.56982	-0.72924
-0.40437	-0.40325	-0.40216	-0.52094	-0.51915	-0.51739	-0.57155	-0.56963	-0.77826
-0.40431	-0.40319	-0.40209	-0.5208	-0.51901	-0.51725	-0.57139	-0.56947	-0.77802
-0.40424	-0.40312	-0.40203	-0.52066	-0.51888	-0.51712	-0.57124	-0.56931	-0.77779
-0.40417	-0.40306	-0.40195	-0.52049	-0.5187	-0.51694	-0.57104	-0.56912	-0.77753
-0.4041	-0.40299	-0.40188	-0.5203	-0.5185	-0.51674	-0.57082	-0.56892	-0.77726
-0.40403	-0.40291	-0.4018	-0.52014	-0.51834	-0.51658	-0.57065	-0.56875	-0.77702
-0.40394	-0.40284	-0.40174	-0.52001	-0.51822	-0.51646	-0.5705	-0.5686	-0.7768
-0.40386	-0.40276	-0.40167	-0.51987	-0.5181	-0.51634	-0.57036	-0.56846	-0.77658
-0.40379	-0.40269	-0.4016	-0.51974	-0.51796	-0.5162	-0.57021	-0.5683	-0.77635
-0.40372	-0.40262	-0.40152	-0.51959	-0.51782	-0.51605	-0.62728	-0.6251	-0.77611
-0.40366	-0.40255	-0.40145	-0.51941	-0.51763	-0.51587	-0.62704	-0.62487	-0.77586
-0.40361	-0.40249	-0.40137	-0.51923	-0.51745	-0.51569	-0.6268	-0.62463	-0.77561
-0.40354	-0.52271	-0.52088	-0.57524	-0.57329	-0.57137	-0.6266	-0.62444	-0.77539
-0.40346	-0.52253	-0.52072	-0.57509	-0.57315	-0.57123	-0.62642	-0.62428	-0.7752
-0.40338	-0.52237	-0.52058	-0.57494	-0.573	-0.57108	-0.62625	-0.62411	-0.8226
-0.40331	-0.52223	-0.52046	-0.57479	-0.57285	-0.57092	-0.62607	-0.62392	-0.82238
-0.40324	-0.5221	-0.52033	-0.57464	-0.5727	-0.57077	-0.6259	-0.62375	-0.82216
-0.40318	-0.52198	-0.5202	-0.57449	-0.57256	-0.57064	-0.62573	-0.62358	-0.82196

-0.40313	-0.52185	-0.52006	-0.57434	-0.57241	-0.5705	-0.62556	-0.62342	-0.82175
-0.40306	-0.52172	-0.51993	-0.63194	-0.62974	-0.62756	-0.62539	-0.62326	-0.82155
-0.40298	-0.52158	-0.5198	-0.63175	-0.62956	-0.62739	-0.62523	-0.6231	-0.82136
-0.40292	-0.52145	-0.51967	-0.63157	-0.62938	-0.62721	-0.62506	-0.62294	-0.82116
-0.40286	-0.52132	-0.51954	-0.63141	-0.6292	-0.62703	-0.62489	-0.62278	-0.82096
-0.40279	-0.52119	-0.51941	-0.63123	-0.62903	-0.62686	-0.62472	-0.62262	-0.82076
-0.40273	-0.52106	-0.51928	-0.63104	-0.62886	-0.6267	-0.62456	-0.62246	-0.82056
-0.40268	-0.52094	-0.51917	-0.63087	-0.62869	-0.62654	-0.6244	-0.6223	-0.82036
-0.40264	-0.52086	-0.5191	-0.63075	-0.62853	-0.62635	-0.62422	-0.62214	-0.82016
-0.4026	-0.52078	-0.51902	-0.63064	-0.62838	-0.62618	-0.62405	-0.62199	-0.81996
-0.40255	-0.52067	-0.51891	-0.6305	-0.62826	-0.62605	-0.62391	-0.62183	-0.81975
-0.40249	-0.52055	-0.51878	-0.63034	-0.62814	-0.62596	-0.62379	-0.62167	-0.81954
-0.40244	-0.63457	-0.63236	-0.63017	-0.62799	-0.62583	-0.6743	-0.67204	-0.90776
-0.40238	-0.6344	-0.63217	-0.62997	-0.62781	-0.62568	-0.67418	-0.67195	-0.90763
-0.40233	-0.63425	-0.63201	-0.62981	-0.62764	-0.62551	-0.67403	-0.67183	-0.90749
-0.40229	-0.63414	-0.63191	-0.6297	-0.62749	-0.62533	-0.67385	-0.67168	-0.90733
-0.40226	-0.63401	-0.63179	-0.62958	-0.62734	-0.62516	-0.67368	-0.67154	-0.90717
-0.4022	-0.63382	-0.63162	-0.62943	-0.62721	-0.62504	-0.67355	-0.67141	-0.90704
-0.40214	-0.63362	-0.63143	-0.62926	-0.62707	-0.62492	-0.67343	-0.67129	-0.90689
-0.4021	-0.63346	-0.63126	-0.62907	-0.6269	-0.62477	-0.67327	-0.67111	-0.90669
-0.40207	-0.63334	-0.63108	-0.62887	-0.62672	-0.6246	-0.67309	-0.67092	-0.90645
-0.40203	-0.6332	-0.63093	-0.62872	-0.62657	-0.62446	-0.67294	-0.67076	-0.90625
-0.40198	-0.63306	-0.63081	-0.62861	-0.62645	-0.62434	-0.67282	-0.67066	-0.9061
-0.40194	-0.63292	-0.6307	-0.6285	-0.62634	-0.62422	-0.72263	-0.72037	-0.94867
-0.4019	-0.63281	-0.63059	-0.62839	-0.62622	-0.62409	-0.7225	-0.72025	-0.94853
-0.40186	-0.6327	-0.63048	-0.62828	-0.6261	-0.62396	-0.72236	-0.72012	-0.9484
-0.40183	-0.63258	-0.63037	-0.62816	-0.62595	-0.6238	-0.72217	-0.71995	-0.94822
-0.4018	-0.63247	-0.63025	-0.62804	-0.6258	-0.62363	-0.72198	-0.71978	-0.94805
-0.5206	-0.63236	-0.63014	-0.62793	-0.62568	-0.6235	-0.81432	-0.8119	-0.99278
-0.52051	-0.63225	-0.63003	-0.62781	-0.62557	-0.62339	-0.81421	-0.81179	-0.99263
-0.52041	-0.6321	-0.62989	-0.62768	-0.62545	-0.62329	-0.8141	-0.81169	-0.99248
-0.52029	-0.6319	-0.62971	-0.62753	-0.62532	-0.62318	-0.81398	-0.81158	-0.99234
-0.52017	-0.63173	-0.62955	-0.62738	-0.6252	-0.62307	-0.85838	-0.85587	-1.03963
-0.52008	-0.6316	-0.62942	-0.62726	-0.62509	-0.62298	-0.85826	-0.85574	-1.03946
-0.52	-0.63149	-0.62931	-0.62715	-0.62499	-0.62288	-0.85813	-0.85561	-1.03929
-0.51993	-0.63138	-0.62919	-0.62703	-0.62488	-0.62279	-0.85801	-0.85549	-1.03913
-0.51987	-0.63128	-0.62908	-0.62692	-0.62478	-0.6227	-0.85789	-0.85537	-1.03897
-0.51983	-0.63123	-0.62902	-0.62684	-0.62469	-0.6226	-0.85777	-0.85525	-1.03882
-0.51979	-0.6312	-0.62898	-0.62679	-0.62461	-0.6225	-0.85764	-0.85513	-1.03866
-0.45992	-0.57392	-0.57195	-0.62673	-0.62453	-0.62242	-0.85751	-0.85501	-1.0385
-0.45985	-0.57383	-0.57187	-0.62663	-0.62445	-0.62234	-0.85739	-0.85488	-1.03833
-0.45979	-0.57373	-0.57177	-0.62653	-0.62437	-0.62227	-0.85729	-0.85477	-1.03817
-0.45972	-0.57363	-0.57168	-0.62644	-0.62431	-0.62224	-0.85724	-0.85471	-1.03805
-0.45966	-0.57354	-0.57158	-0.62634	-0.62425	-0.62222	-0.8572	-0.85466	-1.03794
-0.57545	-0.57347	-0.5715	-0.62624	-0.62416	-0.62214	-0.85711	-0.85458	-1.03782
-0.57541	-0.57341	-0.57142	-0.62614	-0.62406	-0.62204	-0.85699	-0.85448	-1.03768
-0.57534	-0.67292	-0.67066	-0.76949	-0.76703	-0.76464	-0.85687	-0.85437	-1.03755
-0.57525	-0.67284	-0.67057	-0.76938	-0.76692	-0.76453	-0.85674	-0.85424	-1.03742
-0.57515	-0.67276	-0.6705	-0.7693	-0.76684	-0.76445	-0.85663	-0.85413	-1.03728
-0.57506	-0.67268	-0.67045	-0.76925	-0.76681	-0.76442	-0.85657	-0.85403	-1.03713

-0.57499	-0.67261	-0.67041	-0.76922	-0.76678	-0.76438	-0.8565	-0.85394	-1.037
-0.57494	-0.67259	-0.6704	-0.76918	-0.76672	-0.7643	-0.85642	-0.85388	-1.03691
-0.57491	-0.67258	-0.67038	-0.76914	-0.76664	-0.76421	-0.85633	-0.85383	-1.03685
-0.57488	-0.67255	-0.67033	-0.76907	-0.76658	-0.76415	-0.85628	-0.85379	-1.03677
-0.57484	-0.6725	-0.67025	-0.76897	-0.76651	-0.76411	-0.85625	-0.85375	-1.03668
-0.57478	-0.67244	-0.67018	-0.71535	-0.71313	-0.71096	-0.8562	-0.85369	-1.03658
-0.57469	-0.67237	-0.67013	-0.71531	-0.7131	-0.71093	-0.85613	-0.85359	-1.03647
-0.57462	-0.67231	-0.6701	-0.71528	-0.71306	-0.71088	-0.85605	-0.8535	-1.03636
-0.57459	-0.67229	-0.67009	-0.71524	-0.71299	-0.71078	-0.85596	-0.85344	-1.03629
-0.57457	-0.67228	-0.67006	-0.7152	-0.71291	-0.71069	-0.85588	-0.85339	-1.03622
-0.47841	-0.67224	-0.67	-0.76417	-0.76179	-0.75948	-0.85583	-0.85335	-1.03613
-0.47834	-0.67219	-0.66991	-0.76406	-0.76172	-0.75944	-0.8558	-0.85331	-1.03604
-0.47824	-0.76882	-0.76629	-0.81196	-0.8095	-0.80711	-0.90226	-0.89961	-1.03594
-0.47813	-0.76873	-0.76626	-0.81194	-0.80947	-0.80706	-0.90217	-0.89948	-1.03581
-0.47804	-0.76868	-0.76622	-0.81191	-0.80944	-0.80702	-0.90212	-0.89942	-1.03574
-0.47799	-0.76866	-0.76618	-0.81186	-0.80941	-0.80701	-0.90213	-0.89946	-1.03574
-0.47794	-0.68017	-0.67779	-0.81182	-0.80938	-0.80699	-0.90212	-0.89946	-1.03573
-0.47786	-0.68008	-0.67774	-0.8118	-0.80935	-0.80695	-0.90205	-0.89937	-1.03563
-0.47779	-0.67999	-0.67768	-0.81179	-0.80933	-0.80692	-0.90198	-0.89927	-1.03554
-0.47777	-0.67996	-0.67762	-0.81174	-0.80931	-0.80691	-0.90197	-0.89927	-1.03552
-0.47778	-0.67995	-0.67756	-0.81169	-0.80928	-0.80692	-0.902	-0.89931	-1.03555
-0.47771	-0.72998	-0.72747	-0.72506	-0.72277	-0.72053	-0.90199	-0.89932	-1.03555
-0.47759	-0.72984	-0.7274	-0.72502	-0.72271	-0.72044	-0.90194	-0.89928	-1.03553
-0.4775	-0.67973	-0.67745	-0.67522	-0.67303	-0.67089	-0.85187	-0.84944	-0.9453
-0.47749	-0.67969	-0.67737	-0.67512	-0.67297	-0.67086	-0.85185	-0.84939	-0.94521
-0.47748	-0.67967	-0.67731	-0.67504	-0.67291	-0.67082	-0.85182	-0.84935	-0.94514
-0.47745	-0.67966	-0.6773	-0.67503	-0.67286	-0.67074	-0.7671	-0.76476	-0.94512
-0.4774	-0.67963	-0.67731	-0.67504	-0.67282	-0.67067	-0.76702	-0.76472	-0.94512
-0.58197	-0.67956	-0.67727	-0.77866	-0.77611	-0.77362	-0.81863	-0.81616	-0.94509
-0.58187	-0.67945	-0.6772	-0.77862	-0.77611	-0.77363	-0.8186	-0.81607	-0.94504
-0.58181	-0.67939	-0.67715	-0.77856	-0.77607	-0.77361	-0.81857	-0.81601	-0.86391
-0.58181	-0.67938	-0.67709	-0.77846	-0.776	-0.77358	-0.81855	-0.816	-0.8639
-0.58179	-0.67937	-0.67705	-0.77839	-0.77595	-0.77355	-0.81853	-0.81599	-0.86389
-0.58173	-0.67934	-0.67702	-0.77836	-0.77595	-0.77355	-0.81854	-0.816	-0.86387
-0.58167	-0.67931	-0.67702	-0.77836	-0.77594	-0.77354	-0.81854	-0.816	-0.86384
-0.58165	-0.67932	-0.67703	-0.77835	-0.77588	-0.77345	-0.81847	-0.81596	-0.81342
-0.58165	-0.67933	-0.67704	-0.77834	-0.77581	-0.77334	-0.81837	-0.81591	-0.8134
-0.5816	-0.67929	-0.67702	-0.77832	-0.77578	-0.77329	-0.8183	-0.81583	-0.81334
-0.58152	-0.67922	-0.67697	-0.77829	-0.77577	-0.77329	-0.81826	-0.81573	-0.81324
-0.46691	-0.57941	-0.57744	-0.68305	-0.68081	-0.67859	-0.81825	-0.81568	-0.81318
-0.4669	-0.57941	-0.57744	-0.68305	-0.68081	-0.67859	-0.81826	-0.81569	-0.81319
-0.4669	-0.57941	-0.57743	-0.68302	-0.68079	-0.67858	-0.81827	-0.8157	-0.8132
-0.46689	-0.57939	-0.57738	-0.68293	-0.68072	-0.67853	-0.81824	-0.81568	-0.81319
-0.46688	-0.57937	-0.57732	-0.68284	-0.68064	-0.67848	-0.81822	-0.81566	-0.81318
-0.46687	-0.57937	-0.57731	-0.68281	-0.68062	-0.67845	-0.81821	-0.81566	-0.81315
-0.46687	-0.57937	-0.57731	-0.68281	-0.68061	-0.67845	-0.81822	-0.81566	-0.81313
-0.46686	-0.63213	-0.62992	-0.68281	-0.68061	-0.67844	-0.81822	-0.81567	-0.8635
-0.46685	-0.63213	-0.62992	-0.68281	-0.6806	-0.67843	-0.81823	-0.81567	-0.8635
-0.46685	-0.63213	-0.62991	-0.6828	-0.6806	-0.67842	-0.81824	-0.81568	-0.86351
-0.46684	-0.63212	-0.62991	-0.6828	-0.68059	-0.67842	-0.81824	-0.81568	-0.86351

-0.46683	-0.63212	-0.62991	-0.68279	-0.68059	-0.67841	-0.81824	-0.81568	-0.86351
-0.46683	-0.63211	-0.6299	-0.68278	-0.68057	-0.6784	-0.81825	-0.81569	-0.86351
-0.46682	-0.6321	-0.62988	-0.68276	-0.68056	-0.67838	-0.81825	-0.81569	-0.86351
-0.46681	-0.63208	-0.62987	-0.68274	-0.68054	-0.67836	-0.81825	-0.8157	-0.86352
-0.4668	-0.63207	-0.62986	-0.68273	-0.68052	-0.67834	-0.81826	-0.81573	-0.86354
-0.46679	-0.63206	-0.62984	-0.68271	-0.6805	-0.67832	-0.81826	-0.81574	-0.86357
-0.46679	-0.63205	-0.62983	-0.68269	-0.68048	-0.6783	-0.81826	-0.81574	-0.86361
-0.46678	-0.63205	-0.62983	-0.6827	-0.68048	-0.6783	-0.81826	-0.81575	-0.86363
-0.46678	-0.63206	-0.62989	-0.68278	-0.68054	-0.67832	-0.81828	-0.81575	-0.86363
-0.46677	-0.63207	-0.62994	-0.68285	-0.68059	-0.67835	-0.8183	-0.81576	-0.86362
-0.46676	-0.63205	-0.62993	-0.68285	-0.68059	-0.67835	-0.81832	-0.81575	-0.86361
-0.46676	-0.63203	-0.62991	-0.68282	-0.68057	-0.67834	-0.81833	-0.81576	-0.86361
-0.5219	-0.63208	-0.62994	-0.68283	-0.68057	-0.67833	-0.86902	-0.8663	-0.86366
-0.52196	-0.63217	-0.63	-0.68287	-0.68057	-0.67832	-0.86905	-0.86639	-0.86377
-0.52199	-0.63221	-0.63004	-0.68289	-0.68059	-0.67834	-0.86911	-0.86647	-0.86385
-0.52198	-0.6322	-0.63003	-0.6829	-0.68064	-0.67842	-0.86921	-0.86655	-0.8639
-0.52197	-0.63219	-0.63001	-0.68289	-0.68067	-0.67848	-0.86928	-0.86661	-0.86395
-0.52199	-0.63221	-0.63	-0.68286	-0.68065	-0.67846	-0.86929	-0.86663	-0.864
-0.52201	-0.63222	-0.63	-0.68284	-0.68062	-0.67844	-0.86928	-0.86664	-0.86403
-0.522	-0.63223	-0.63002	-0.68288	-0.68065	-0.67845	-0.86931	-0.86664	-0.86403
-0.522	-0.63222	-0.63007	-0.68295	-0.6807	-0.67848	-0.86935	-0.86665	-0.86401
-0.52202	-0.63226	-0.63011	-0.68299	-0.68072	-0.67848	-0.86939	-0.86669	-0.86406
-0.52207	-0.63233	-0.63016	-0.68301	-0.68071	-0.67846	-0.86942	-0.86677	-0.86415
-0.52211	-0.52054	-0.51895	-0.68301	-0.68072	-0.67848	-0.82289	-0.82037	-0.86423
-0.52214	-0.52054	-0.51893	-0.683	-0.68075	-0.67855	-0.82299	-0.82045	-0.86428
-0.52215	-0.52054	-0.51893	-0.683	-0.68079	-0.67862	-0.82309	-0.82053	-0.86435
-0.52214	-0.52055	-0.51897	-0.68306	-0.68083	-0.67865	-0.82316	-0.82062	-0.86447
-0.52214	-0.52056	-0.51901	-0.68313	-0.68088	-0.67868	-0.82322	-0.82071	-0.8646
-0.5222	-0.5206	-0.51904	-0.68316	-0.68092	-0.67873	-0.8233	-0.8208	-0.86468
-0.52229	-0.52068	-0.51906	-0.68318	-0.68097	-0.6788	-0.82338	-0.82087	-0.86473
-0.52234	-0.52072	-0.51909	-0.68323	-0.68102	-0.67885	-0.82344	-0.82091	-0.86477
-0.52233	-0.52072	-0.51914	-0.6833	-0.68107	-0.67887	-0.82346	-0.8209	-0.8648
-0.52234	-0.52074	-0.51918	-0.68337	-0.68112	-0.67891	-0.82353	-0.82096	-0.86486
-0.52239	-0.5208	-0.5192	-0.6834	-0.68117	-0.67899	-0.82366	-0.82111	-0.86501
-0.52245	-0.52086	-0.51923	-0.68343	-0.68122	-0.67906	-0.82379	-0.82127	-0.86515
-0.52247	-0.52088	-0.51927	-0.68351	-0.68128	-0.6791	-0.82384	-0.8213	-0.86521
-0.52249	-0.52089	-0.51933	-0.68359	-0.68135	-0.67914	-0.82389	-0.82132	-0.86525
-0.52256	-0.52095	-0.51937	-0.68365	-0.68141	-0.67921	-0.824	-0.82143	-0.86536
-0.52267	-0.52103	-0.5194	-0.68368	-0.68147	-0.6793	-0.82417	-0.82161	-0.86551
-0.52276	-0.52112	-0.51946	-0.57512	-0.57347	-0.57184	-0.7308	-0.72853	-0.86566
-0.52284	-0.52122	-0.51956	-0.57522	-0.57351	-0.57185	-0.73085	-0.72862	-0.86583
-0.52289	-0.52129	-0.51966	-0.57532	-0.57358	-0.57188	-0.73091	-0.72869	-0.86597
-0.52291	-0.52132	-0.51972	-0.57542	-0.57368	-0.57197	-0.73101	-0.72873	-0.86604
-0.52293	-0.52134	-0.51978	-0.5755	-0.57378	-0.57208	-0.73112	-0.72879	-0.86612
-0.52301	-0.5214	-0.51981	-0.57553	-0.57385	-0.57217	-0.73124	-0.72891	-0.86625
-0.52312	-0.52148	-0.51984	-0.57554	-0.57389	-0.57225	-0.73137	-0.72905	-0.86641
-0.52321	-0.57941	-0.57755	-0.63189	-0.63003	-0.62821	-0.73145	-0.72917	-0.86657
-0.52328	-0.57951	-0.57766	-0.63201	-0.63009	-0.62822	-0.73149	-0.72925	-0.86674
-0.52333	-0.57958	-0.57776	-0.63211	-0.63017	-0.62827	-0.73156	-0.72932	-0.86687
-0.52334	-0.5796	-0.57782	-0.63221	-0.63028	-0.62838	-0.73167	-0.72936	-0.86696

-0.47301	-0.57963	-0.57787	-0.63229	-0.63038	-0.62849	-0.73178	-0.72943	-0.86706
-0.47309	-0.57971	-0.57791	-0.63232	-0.63044	-0.62857	-0.73188	-0.72954	-0.86721
-0.47318	-0.5798	-0.57796	-0.63234	-0.63048	-0.62865	-0.73199	-0.72967	-0.86738
-0.47327	-0.5799	-0.57804	-0.63243	-0.63057	-0.62874	-0.73212	-0.7298	-0.86754
-0.47336	-0.58001	-0.57816	-0.63255	-0.63069	-0.62885	-0.73226	-0.72994	-0.86769
-0.47343	-0.58011	-0.57826	-0.57647	-0.57476	-0.57307	-0.57141	-0.56975	-0.72775
-0.47348	-0.58019	-0.57836	-0.57657	-0.57486	-0.57318	-0.57151	-0.56986	-0.72789
-0.41144	-0.58028	-0.57846	-0.57667	-0.57496	-0.57327	-0.5716	-0.56994	-0.72801
-0.41149	-0.58038	-0.57856	-0.57677	-0.57502	-0.5733	-0.57164	-0.57	-0.72811
-0.41155	-0.58049	-0.57867	-0.57687	-0.57508	-0.57333	-0.57168	-0.57007	-0.72821
-0.41161	-0.53142	-0.52978	-0.57698	-0.57517	-0.57342	-0.57176	-0.57015	-0.72833
-0.41166	-0.53152	-0.52987	-0.57709	-0.57528	-0.57352	-0.57186	-0.57025	-0.72847
-0.41169	-0.53157	-0.52994	-0.57718	-0.57538	-0.57363	-0.57196	-0.57034	-0.7286
-0.41169	-0.53158	-0.52998	-0.57726	-0.57547	-0.57373	-0.57207	-0.57044	-0.72873
-0.41172	-0.53164	-0.53005	-0.57734	-0.57557	-0.57383	-0.57217	-0.57054	-0.72887
-0.41177	-0.53173	-0.53014	-0.57744	-0.57567	-0.57393	-0.57226	-0.57063	-0.729
-0.41183	-0.53183	-0.53024	-0.57754	-0.57577	-0.57403	-0.57236	-0.57073	-0.72914
-0.41188	-0.53192	-0.53033	-0.57765	-0.57588	-0.57413	-0.57247	-0.57083	-0.72927
-0.41195	-0.53202	-0.53043	-0.57776	-0.57598	-0.57424	-0.57257	-0.57093	-0.72941
-0.41204	-0.53217	-0.53057	-0.52896	-0.52735	-0.52576	-0.57267	-0.57103	-0.72955
-0.41215	-0.53235	-0.53072	-0.52909	-0.52745	-0.52585	-0.57277	-0.57113	-0.72968
-0.41222	-0.53248	-0.53084	-0.5292	-0.52756	-0.52595	-0.57286	-0.57123	-0.72982
-0.41228	-0.53257	-0.53094	-0.52931	-0.52765	-0.52604	-0.57296	-0.57133	-0.72995
-0.41234	-0.47164	-0.47032	-0.52941	-0.52777	-0.52615	-0.57308	-0.57144	-0.62534
-0.4124	-0.4717	-0.47038	-0.52952	-0.52791	-0.52632	-0.57325	-0.57158	-0.62547
-0.41245	-0.47177	-0.47045	-0.52962	-0.52804	-0.52647	-0.5734	-0.57171	-0.62559
-0.41251	-0.47183	-0.47051	-0.52972	-0.52814	-0.52658	-0.57352	-0.57183	-0.62572
-0.41256	-0.47189	-0.47057	-0.52982	-0.52824	-0.52667	-0.57363	-0.57194	-0.62584
-0.41264	-0.47197	-0.47064	-0.52991	-0.52833	-0.52677	-0.52522	-0.52367	-0.62597
-0.41273	-0.47206	-0.47072	-0.53	-0.52843	-0.52688	-0.52532	-0.52377	-0.62611
-0.4128	-0.47215	-0.47081	-0.53014	-0.52856	-0.527	-0.52544	-0.5239	-0.62627
-0.41286	-0.47223	-0.47091	-0.53031	-0.52871	-0.52713	-0.52559	-0.52406	-0.62646
-0.41293	-0.47232	-0.47102	-0.53046	-0.52885	-0.52725	-0.52571	-0.5242	-0.62662
-0.41303	-0.47244	-0.47113	-0.5306	-0.52896	-0.52735	-0.52581	-0.5243	-0.62673
-0.41314	-0.47255	-0.47123	-0.53073	-0.52907	-0.52745	-0.52591	-0.52439	-0.62685
-0.4132	-0.47263	-0.47131	-0.53083	-0.52921	-0.52761	-0.52606	-0.52453	-0.57972
-0.41326	-0.4727	-0.47138	-0.53094	-0.52936	-0.52778	-0.52622	-0.52467	-0.57985
-0.41334	-0.47279	-0.47148	-0.53109	-0.52951	-0.52793	-0.52635	-0.52478	-0.57998
-0.41343	-0.4729	-0.47159	-0.53126	-0.52967	-0.52807	-0.52647	-0.52487	-0.58011
-0.35472	-0.47301	-0.47171	-0.47041	-0.4691	-0.46779	-0.52659	-0.525	-0.52347
-0.35482	-0.47314	-0.47182	-0.47051	-0.46918	-0.46786	-0.52671	-0.52517	-0.52363
-0.3549	-0.47325	-0.47192	-0.4706	-0.46926	-0.46794	-0.52685	-0.52533	-0.52379
-0.35496	-0.47332	-0.47199	-0.47068	-0.46936	-0.46806	-0.52701	-0.52546	-0.52392
-0.35501	-0.47339	-0.47207	-0.47076	-0.46947	-0.46818	-0.52717	-0.52559	-0.52406
-0.35508	-0.47349	-0.47217	-0.47087	-0.46957	-0.46829	-0.52732	-0.52576	-0.52422
-0.35516	-0.47359	-0.47229	-0.47099	-0.46968	-0.46839	-0.52746	-0.52592	-0.52438
-0.35525	-0.47371	-0.4724	-0.4711	-0.4698	-0.46851	-0.52762	-0.52607	-0.52453
-0.35534	-0.47383	-0.47251	-0.4712	-0.46991	-0.46863	-0.52777	-0.5262	-0.52466
-0.35543	-0.47395	-0.47262	-0.47131	-0.47003	-0.46875	-0.52793	-0.52635	-0.52481
-0.35551	-0.47406	-0.47274	-0.47144	-0.47014	-0.46885	-0.52807	-0.52652	-0.52497

-0.35559	-0.47416	-0.47286	-0.47155	-0.47025	-0.46895	-0.52822	-0.52667	-0.52513
-0.35568	-0.47428	-0.47296	-0.47165	-0.47035	-0.46906	-0.52837	-0.5268	-0.52526
-0.35577	-0.4744	-0.47307	-0.47175	-0.47046	-0.46918	-0.52851	-0.52693	-0.52539
-0.35584	-0.4745	-0.47317	-0.47186	-0.47056	-0.46928	-0.52866	-0.52708	-0.52554
-0.35592	-0.4746	-0.47328	-0.47198	-0.47067	-0.46938	-0.5288	-0.52725	-0.5257
-0.35602	-0.47472	-0.47339	-0.47208	-0.47077	-0.46948	-0.46821	-0.46695	-0.52584
-0.35613	-0.47486	-0.4735	-0.47217	-0.47088	-0.46959	-0.46831	-0.46704	-0.52596
-0.35624	-0.41745	-0.41626	-0.47229	-0.471	-0.46972	-0.46843	-0.46715	-0.52611
-0.35633	-0.41756	-0.41639	-0.47244	-0.47115	-0.46987	-0.46858	-0.4673	-0.52628
-0.35642	-0.41768	-0.41652	-0.47259	-0.4713	-0.47002	-0.46873	-0.46745	-0.52646
-0.35649	-0.41777	-0.41663	-0.47272	-0.47141	-0.47012	-0.46885	-0.46757	-0.52664
-0.35657	-0.41786	-0.41673	-0.47284	-0.47152	-0.47022	-0.46895	-0.46769	-0.52681
-0.35667	-0.41798	-0.41683	-0.47294	-0.47162	-0.47033	-0.46905	-0.46779	-0.52696
-0.35679	-0.41811	-0.41693	-0.47303	-0.47173	-0.47044	-0.46915	-0.46788	-0.52709
-0.3569	-0.41823	-0.41705	-0.47316	-0.47185	-0.47057	-0.46928	-0.468	-0.52725
-0.35699	-0.41835	-0.41718	-0.47332	-0.47201	-0.47072	-0.46943	-0.46815	-0.52744
-0.35708	-0.41846	-0.41731	-0.47347	-0.47216	-0.47086	-0.46957	-0.46829	-0.52762
-0.35715	-0.41855	-0.41741	-0.47359	-0.47226	-0.47096	-0.46967	-0.4684	-0.52777
-0.35724	-0.41865	-0.41751	-0.47371	-0.47237	-0.47106	-0.46978	-0.46852	-0.52793
-0.35734	-0.41878	-0.41764	-0.47385	-0.47251	-0.4712	-0.46992	-0.46866	-0.46739
-0.35745	-0.41892	-0.41778	-0.47401	-0.47267	-0.47135	-0.47007	-0.46881	-0.46753
-0.35756	-0.41905	-0.41792	-0.47416	-0.47282	-0.4715	-0.47022	-0.46896	-0.46768
-0.35768	-0.41919	-0.41806	-0.47432	-0.47298	-0.47165	-0.47037	-0.46911	-0.46783
-0.29805	-0.35685	-0.35593	-0.355	-0.35406	-0.35312	-0.47051	-0.46924	-0.46796
-0.29811	-0.35693	-0.356	-0.35507	-0.35414	-0.35321	-0.47061	-0.46933	-0.46806
-0.29818	-0.35703	-0.35609	-0.35515	-0.35423	-0.35331	-0.47073	-0.46944	-0.46816
-0.29827	-0.35714	-0.3562	-0.35526	-0.35434	-0.35342	-0.47088	-0.46958	-0.4683
-0.29836	-0.35725	-0.35631	-0.35538	-0.35446	-0.35353	-0.47103	-0.46973	-0.46846
-0.29845	-0.35737	-0.35643	-0.3555	-0.35457	-0.35365	-0.47119	-0.46989	-0.46861
-0.29853	-0.35748	-0.35654	-0.35561	-0.35469	-0.35377	-0.47134	-0.47004	-0.46876
-0.29862	-0.35759	-0.35665	-0.35572	-0.3548	-0.35388	-0.4715	-0.47019	-0.46891
-0.29871	-0.35771	-0.35677	-0.35583	-0.35492	-0.354	-0.47165	-0.47034	-0.46906
-0.29879	-0.35782	-0.35688	-0.35595	-0.35503	-0.35411	-0.4718	-0.47049	-0.46921
-0.29888	-0.35794	-0.35699	-0.35606	-0.35514	-0.35423	-0.47195	-0.47065	-0.46936
-0.29896	-0.35805	-0.35711	-0.35617	-0.35526	-0.35434	-0.4721	-0.4708	-0.46951
-0.29905	-0.35816	-0.35722	-0.35628	-0.35537	-0.35445	-0.47225	-0.47095	-0.46966
-0.29913	-0.35827	-0.35732	-0.35639	-0.35548	-0.35456	-0.4724	-0.47109	-0.46981
-0.29922	-0.35838	-0.35743	-0.3565	-0.35558	-0.35467	-0.47254	-0.47124	-0.46995
-0.2993	-0.35848	-0.35754	-0.3566	-0.35569	-0.35478	-0.47269	-0.47138	-0.4701
-0.29938	-0.35859	-0.35764	-0.35671	-0.3558	-0.35489	-0.35396	-0.35302	-0.47025
-0.29946	-0.3587	-0.35775	-0.35681	-0.3559	-0.355	-0.35406	-0.35313	-0.47039
-0.29955	-0.29881	-0.29807	-0.35693	-0.35602	-0.35511	-0.35418	-0.35325	-0.47055
-0.29966	-0.29892	-0.29818	-0.35707	-0.35615	-0.35524	-0.35432	-0.3534	-0.47075
-0.29976	-0.29902	-0.29828	-0.35721	-0.35629	-0.35536	-0.35445	-0.35354	-0.47094
-0.29985	-0.29911	-0.29837	-0.35733	-0.3564	-0.35547	-0.35457	-0.35366	-0.4711
-0.29994	-0.29919	-0.29845	-0.35744	-0.35651	-0.35558	-0.35468	-0.35377	-0.47125
-0.30002	-0.29927	-0.29854	-0.35756	-0.35663	-0.35572	-0.3548	-0.3539	-0.47142
-0.30011	-0.29936	-0.29862	-0.35767	-0.35677	-0.35587	-0.35495	-0.35403	-0.4716
-0.30021	-0.29946	-0.29871	-0.35779	-0.35689	-0.356	-0.35508	-0.35415	-0.47177
-0.30033	-0.29958	-0.29882	-0.35792	-0.35701	-0.35611	-0.35518	-0.35426	-0.47191

-0.30044	-0.29969	-0.29893	-0.35804	-0.35712	-0.35621	-0.35529	-0.35437	-0.47206
-0.30053	-0.29978	-0.29902	-0.35815	-0.35722	-0.35631	-0.35541	-0.35451	-0.47225
-0.30062	-0.29986	-0.2991	-0.35827	-0.35733	-0.35642	-0.35553	-0.35465	-0.47244
-0.30072	-0.29997	-0.29921	-0.35841	-0.35746	-0.35654	-0.35564	-0.35477	-0.4156
-0.30083	-0.30008	-0.29932	-0.35857	-0.35761	-0.35666	-0.35576	-0.35487	-0.41572
-0.30094	-0.30019	-0.29943	-0.35871	-0.35775	-0.3568	-0.35589	-0.35499	-0.41586
-0.30105	-0.3003	-0.29954	-0.35884	-0.35789	-0.35695	-0.35602	-0.35511	-0.41602
-0.24413	-0.30041	-0.29965	-0.2989	-0.29814	-0.2974	-0.35616	-0.35525	-0.35434
-0.2442	-0.30051	-0.29976	-0.29901	-0.29826	-0.2975	-0.3563	-0.35539	-0.35448
-0.24428	-0.3006	-0.29987	-0.29912	-0.29836	-0.2976	-0.35643	-0.35553	-0.35462
-0.24436	-0.3007	-0.29996	-0.29921	-0.29845	-0.29769	-0.35654	-0.35564	-0.35474
-0.24445	-0.3008	-0.30005	-0.2993	-0.29853	-0.29777	-0.35664	-0.35574	-0.35486
-0.24453	-0.30091	-0.30016	-0.2994	-0.29863	-0.29788	-0.35677	-0.35586	-0.35497
-0.24462	-0.30102	-0.30027	-0.29951	-0.29875	-0.29799	-0.35692	-0.35599	-0.35508
-0.18695	-0.30113	-0.30038	-0.29962	-0.29886	-0.2981	-0.35706	-0.35613	-0.35522
-0.18701	-0.30124	-0.30049	-0.29973	-0.29897	-0.29821	-0.3572	-0.35627	-0.35537
-0.18707	-0.30134	-0.3006	-0.29984	-0.29908	-0.29832	-0.35734	-0.35641	-0.35552
-0.18713	-0.30145	-0.30071	-0.29995	-0.29919	-0.29844	-0.35748	-0.35655	-0.35564
-0.18718	-0.30156	-0.30081	-0.30006	-0.2993	-0.29855	-0.35763	-0.35668	-0.35576
-0.18724	-0.30167	-0.30092	-0.30017	-0.29941	-0.29865	-0.29789	-0.29714	-0.3559
-0.18729	-0.30177	-0.30103	-0.30028	-0.29951	-0.29875	-0.29799	-0.29724	-0.35604
-0.18734	-0.2453	-0.24471	-0.30038	-0.29962	-0.29886	-0.2981	-0.29736	-0.3562
-0.18739	-0.24537	-0.24478	-0.30049	-0.29973	-0.29897	-0.29822	-0.29748	-0.35635
-0.18744	-0.24544	-0.24485	-0.30059	-0.29984	-0.29909	-0.29834	-0.2976	-0.3565
-0.18748	-0.24551	-0.24492	-0.3007	-0.29996	-0.29922	-0.29847	-0.29771	-0.35662
-0.18753	-0.18709	-0.18665	-0.30081	-0.30008	-0.29935	-0.29858	-0.29782	-0.35676
-0.18758	-0.18714	-0.18671	-0.30092	-0.30019	-0.29945	-0.29869	-0.29793	-0.3569
-0.18763	-0.18719	-0.18677	-0.30104	-0.3003	-0.29955	-0.29879	-0.29804	-0.35705
-0.1877	-0.18725	-0.18683	-0.30114	-0.3004	-0.29965	-0.2989	-0.29816	-0.29741
-0.18777	-0.18732	-0.18688	-0.30123	-0.3005	-0.29976	-0.29902	-0.29829	-0.29754
-0.18782	-0.18737	-0.18693	-0.24481	-0.24422	-0.24364	-0.29914	-0.2984	-0.29765
-0.18787	-0.18742	-0.18698	-0.24488	-0.24431	-0.24373	-0.29926	-0.2985	-0.29775
-0.18793	-0.18747	-0.18704	-0.24496	-0.24439	-0.24382	-0.29937	-0.29861	-0.29786
-0.18799	-0.18754	-0.1871	-0.24505	-0.24447	-0.24389	-0.29947	-0.29872	-0.29797
-0.18805	-0.1876	-0.18716	-0.24514	-0.24455	-0.24397	-0.29958	-0.29883	-0.29809
-0.1881	-0.18765	-0.18722	-0.24522	-0.24464	-0.24406	-0.2997	-0.29896	-0.29821
-0.18815	-0.1877	-0.18728	-0.24531	-0.24473	-0.24415	-0.29983	-0.29909	-0.29833
-0.12589	-0.18777	-0.18733	-0.24539	-0.24481	-0.24423	-0.29995	-0.29921	-0.29846
-0.12596	-0.18784	-0.18739	-0.24546	-0.24487	-0.2443	-0.30006	-0.29934	-0.29859
-0.12602	-0.18792	-0.18746	-0.24554	-0.24496	-0.24437	-0.30017	-0.29945	-0.2987
-0.12608	-0.18799	-0.18754	-0.24565	-0.24505	-0.24446	-0.30027	-0.29954	-0.2988
-0.12614	-0.12583	-0.12552	-0.18716	-0.18672	-0.18628	-0.24396	-0.24336	-0.2989
-0.12619	-0.12588	-0.12557	-0.18722	-0.18679	-0.18635	-0.24405	-0.24345	-0.29901
-0.12624	-0.12593	-0.12562	-0.18729	-0.18685	-0.18642	-0.24415	-0.24354	-0.29914
-0.1263	-0.12599	-0.12568	-0.18736	-0.18693	-0.18649	-0.24424	-0.24363	-0.29926
-0.12636	-0.12605	-0.12574	-0.18743	-0.187	-0.18656	-0.24434	-0.24373	-0.29939
-0.12642	-0.12611	-0.12581	-0.1255	-0.1252	-0.12489	-0.24444	-0.24382	-0.29951
-0.12648	-0.12617	-0.12587	-0.12556	-0.12526	-0.12495	-0.24454	-0.24391	-0.29963
-0.12654	-0.12623	-0.12593	-0.12563	-0.12532	-0.12502	-0.24464	-0.24401	-0.29976
-0.1266	-0.12629	-0.12599	-0.12569	-0.12539	-0.12508	-0.24474	-0.24411	-0.29989

-0.12666	-0.12635	-0.12605	-0.12575	-0.12545	-0.12514	-0.24485	-0.24421	-0.30002
-0.12672	-0.12641	-0.12611	-0.12581	-0.12551	-0.12521	-0.24495	-0.24431	-0.30014
-0.12678	-0.12647	-0.12617	-0.12587	-0.12557	-0.12527	-0.24505	-0.2444	-0.30027
-0.12684	-0.12653	-0.12623	-0.12593	-0.12563	-0.12533	-0.18668	-0.1862	-0.24387
-0.1269	-0.12659	-0.12629	-0.12599	-0.12569	-0.12539	-0.18674	-0.18626	-0.24396
-0.06293	-0.12665	-0.12635	-0.12605	-0.12575	-0.12545	-0.12513	-0.12481	-0.24405
-0.06297	-0.12671	-0.1264	-0.1261	-0.12581	-0.12551	-0.12519	-0.12487	-0.24415
-0.063	-0.12677	-0.12646	-0.12616	-0.12587	-0.12557	-0.12525	-0.12493	-0.24424
-0.06304	-0.12683	-0.12652	-0.12622	-0.12592	-0.12562	-0.12531	-0.12499	-0.24433
-0.06307	-0.12689	-0.12658	-0.12628	-0.12598	-0.12568	-0.12537	-0.12505	-0.24443
-0.06311	-0.12695	-0.12664	-0.12633	-0.12604	-0.12574	-0.12544	-0.12512	-0.24455
-0.06315	-0.12701	-0.1267	-0.12639	-0.1261	-0.1258	-0.1255	-0.12519	-0.24467
-0.06319	-0.12708	-0.12677	-0.12646	-0.12616	-0.12586	-0.12556	-0.12526	-0.18635
-0.06323	-0.12716	-0.12684	-0.12652	-0.12622	-0.12591	-0.12561	-0.12531	-0.18641
-0.06327	-0.06311	-0.06294	-0.1266	-0.12628	-0.12598	-0.12567	-0.12537	-0.12506
-0.0633	-0.06314	-0.06298	-0.12667	-0.12636	-0.12604	-0.12574	-0.12543	-0.12512
-0.06334	-0.06318	-0.06302	-0.12674	-0.12643	-0.12611	-0.1258	-0.12549	-0.12518
-0.06337	-0.06321	-0.06306	-0.1268	-0.12649	-0.12617	-0.12585	-0.12554	-0.12524
-0.06341	-0.06325	-0.06309	-0.12686	-0.12655	-0.12623	-0.12591	-0.1256	-0.1253
-0.06345	-0.06329	-0.06313	-0.12693	-0.12661	-0.1263	-0.12599	-0.12567	-0.12536
-0.06349	-0.06333	-0.06317	-0.12699	-0.12668	-0.12637	-0.12606	-0.12574	-0.12543
-0.06353	-0.06337	-0.06321	-0.06304	-0.06289	-0.06273	-0.12613	-0.12581	-0.1255
-0.06357	-0.06341	-0.06325	-0.06308	-0.06292	-0.06277	-0.12619	-0.12589	-0.12557
-0.06361	-0.06345	-0.06329	-0.06313	-0.06296	-0.0628	-0.12625	-0.12595	-0.12564
-0.06365	-0.06349	-0.06333	-0.06317	-0.06301	-0.06284	-0.12632	-0.12601	-0.1257
-0.06368	-0.06352	-0.06337	-0.06321	-0.06305	-0.06288	-0.06272	-0.06256	-0.12577
-0.06372	-0.06356	-0.0634	-0.06324	-0.06308	-0.06293	-0.06277	-0.0626	-0.12583
-0.06375	-0.06359	-0.06343	-0.06328	-0.06312	-0.06297	-0.06281	-0.06265	-0.1259
-0.0638	-0.06364	-0.06348	-0.06332	-0.06316	-0.063	-0.06284	-0.06269	-0.12598
-0.06385	-0.06368	-0.06352	-0.06336	-0.0632	-0.06304	-0.06288	-0.06273	-0.12605
-0.06389	-0.06372	-0.06356	-0.0634	-0.06324	-0.06307	-0.06292	-0.06276	-0.06261
-0.06392	-0.06376	-0.0636	-0.06344	-0.06328	-0.06312	-0.06296	-0.0628	-0.06264
	-0.0638	-0.06364	-0.06348	-0.06332	-0.06316	-0.063	-0.06284	-0.06268
	-0.06383	-0.06367	-0.06351	-0.06335	-0.0632	-0.06304	-0.06288	-0.06273
	-0.06387	-0.06371	-0.06355	-0.06339	-0.06324	-0.06308	-0.06293	-0.06277
	-0.06392	-0.06375	-0.06359	-0.06343	-0.06328	-0.06312	-0.06296	-0.06281
	-0.06396	-0.0638	-0.06364	-0.06348	-0.06332	-0.06316	-0.063	-0.06284
	-0.064	-0.06384	-0.06368	-0.06352	-0.06336	-0.0632	-0.06304	-0.06288
	-0.06404	-0.06388	-0.06371	-0.06355	-0.0634	-0.06324	-0.06309	-0.06293
	-0.06409	-0.06392	-0.06375	-0.06359	-0.06344	-0.06328	-0.06313	-0.06297
	-0.06413	-0.06397	-0.0638	-0.06364	-0.06348	-0.06332	-0.06316	-0.06301
			-0.06385	-0.06368	-0.06352	-0.06336	-0.0632	-0.06304
			-0.06388	-0.06372	-0.06356	-0.0634	-0.06325	-0.06309
			-0.06392	-0.06376	-0.0636	-0.06345	-0.06329	-0.06314
			-0.06396	-0.0638	-0.06365	-0.06349	-0.06334	-0.06318
			-0.06401	-0.06385	-0.06369	-0.06354	-0.06339	-0.06323
			-0.06406	-0.0639	-0.06374	-0.06358	-0.06342	-0.06327
			-0.06411	-0.06394	-0.06378	-0.06362	-0.06346	-0.0633
			-0.06416	-0.06399	-0.06382	-0.06366	-0.0635	-0.06334
			-0.06419	-0.06402	-0.06386	-0.0637	-0.06354	-0.06339

-0.06375	-0.06359	-0.06344
-0.06379	-0.06364	-0.06348
-0.06384	-0.06368	-0.06352
-0.06388	-0.06372	-0.06356
-0.06392	-0.06375	-0.0636
-0.06396	-0.06379	-0.06364
-0.064	-0.06384	-0.06368
		-0.06373
		-0.06378
		-0.06382
		-0.06387
		-0.06391

								-0.06519
								-0.06515
								-0.0651
								-0.06505
								-0.065
								-0.12796
								-0.12784
								-0.12773
								-0.12763
						-0.12817	-0.12785	-0.18853
						-0.12807	-0.12775	-0.18838
						-0.12797	-0.12765	-0.18823
						-0.12787	-0.12755	-0.18808
						-0.12777	-0.12744	-0.18793
						-0.12766	-0.12734	-0.18778
						-0.12756	-0.12723	-0.18762
			-0.1284	-0.12808	-0.12777	-0.18845	-0.18796	-0.35511
			-0.12828	-0.12796	-0.12764	-0.18827	-0.18777	-0.35484
			-0.12817	-0.12785	-0.12752	-0.18809	-0.1876	-0.35459
			-0.12806	-0.12774	-0.12742	-0.18794	-0.18745	-0.35437
			-0.12795	-0.12763	-0.12731	-0.28982	-0.28914	-0.35416
			-0.12785	-0.12753	-0.12721	-0.28965	-0.28897	-0.35395
			-0.12775	-0.12743	-0.12711	-0.28949	-0.28881	-0.35374
			-0.12765	-0.12733	-0.12701	-0.28932	-0.28865	-0.35354
			-0.12755	-0.12723	-0.12691	-0.28916	-0.2885	-0.35333
	-0.12808	-0.12776	-0.18852	-0.18804	-0.18756	-0.35487	-0.354	-0.35313
	-0.12798	-0.12766	-0.18837	-0.18789	-0.18741	-0.35466	-0.3538	-0.35292
	-0.12788	-0.12756	-0.29069	-0.29002	-0.28936	-0.35446	-0.35359	-0.35272
	-0.12778	-0.12746	-0.29054	-0.28987	-0.2892	-0.35425	-0.35338	-0.35251
	-0.12768	-0.12737	-0.2904	-0.28972	-0.28905	-0.35404	-0.35318	-0.3523
	-0.12759	-0.12727	-0.29024	-0.28956	-0.28889	-0.35383	-0.35297	-0.35209
	-0.12749	-0.12717	-0.29009	-0.28941	-0.28873	-0.35362	-0.35275	-0.35188
-0.12772	-0.18849	-0.188	-0.28993	-0.28925	-0.28858	-0.35342	-0.35254	-0.35167
-0.12763	-0.18836	-0.18787	-0.28978	-0.2891	-0.28842	-0.35321	-0.35233	-0.35145
-0.12754	-0.29104	-0.29033	-0.28963	-0.28895	-0.28827	-0.353	-0.35212	-0.35124
-0.12746	-0.2909	-0.2902	-0.28949	-0.2888	-0.28811	-0.35279	-0.3519	-0.35103
-0.18847	-0.29077	-0.29006	-0.28936	-0.28866	-0.28797	-0.35259	-0.3517	-0.35082
-0.18834	-0.29062	-0.28992	-0.28922	-0.28854	-0.28785	-0.35244	-0.35153	-0.35063
-0.1882	-0.29046	-0.28977	-0.28908	-0.28841	-0.28773	-0.35228	-0.35136	-0.35045
-0.18805	-0.35652	-0.35563	-0.35475	-0.35387	-0.35299	-0.35209	-0.35118	-0.35028
-0.18791	-0.35631	-0.35542	-0.35453	-0.35365	-0.35277	-0.35189	-0.351	-0.35011
-0.18777	-0.3561	-0.35521	-0.35432	-0.35344	-0.35256	-0.35168	-0.3508	-0.34992
-0.18763	-0.35588	-0.355	-0.35411	-0.35323	-0.35235	-0.35147	-0.35059	-0.34972
-0.29036	-0.35568	-0.35479	-0.3539	-0.35302	-0.35214	-0.35126	-0.35039	-0.34952
-0.29022	-0.35549	-0.35458	-0.35368	-0.3528	-0.35192	-0.35105	-0.35018	-0.34932
-0.29008	-0.3553	-0.35439	-0.35349	-0.3526	-0.35172	-0.35085	-0.34998	-0.34912
-0.28994	-0.35513	-0.35423	-0.35333	-0.35244	-0.35154	-0.35066	-0.34978	-0.34892

-0.28979	-0.35495	-0.35407	-0.35318	-0.35228	-0.35137	-0.35048	-0.34959	-0.34872
-0.28963	-0.35475	-0.35387	-0.35299	-0.3521	-0.3512	-0.35031	-0.34941	-0.34854
-0.28947	-0.35453	-0.35366	-0.35279	-0.35191	-0.35104	-0.35014	-0.34925	-0.34836
-0.28933	-0.35434	-0.35346	-0.35259	-0.35172	-0.35085	-0.34997	-0.34908	-0.3482
-0.28921	-0.35416	-0.35326	-0.35238	-0.35151	-0.35065	-0.34978	-0.34891	-0.34804
-0.28908	-0.35399	-0.35309	-0.3522	-0.35133	-0.35047	-0.3496	-0.34873	-0.34787
-0.28894	-0.35382	-0.35295	-0.35207	-0.35119	-0.35031	-0.34942	-0.34854	-0.34767
-0.28879	-0.35365	-0.3528	-0.35194	-0.35105	-0.35015	-0.34925	-0.34835	-0.34747
-0.28864	-0.35345	-0.35261	-0.35175	-0.35087	-0.34999	-0.3491	-0.34821	-0.34733
-0.28849	-0.35325	-0.35241	-0.35155	-0.35069	-0.34982	-0.34895	-0.34807	-0.34719
-0.28837	-0.35309	-0.35223	-0.35138	-0.35051	-0.34964	-0.34876	-0.34788	-0.34701
-0.28826	-0.35295	-0.35208	-0.35121	-0.35032	-0.34944	-0.34855	-0.34767	-0.3468
-0.28814	-0.35279	-0.35192	-0.35104	-0.35015	-0.34926	-0.34837	-0.34748	-0.34661
-0.28799	-0.3526	-0.35174	-0.35088	-0.35	-0.34912	-0.34822	-0.34731	-0.34642
-0.28786	-0.35242	-0.35157	-0.35071	-0.34984	-0.34896	-0.34805	-0.34714	-0.34625
-0.28776	-0.35228	-0.35141	-0.35054	-0.34965	-0.34876	-0.34786	-0.34696	-0.34609
-0.28766	-0.35213	-0.35126	-0.35037	-0.34946	-0.34856	-0.34767	-0.34679	-0.34593
-0.28752	-0.35195	-0.35108	-0.3502	-0.3493	-0.3484	-0.34751	-0.34663	-0.34577
-0.28738	-0.35177	-0.3509	-0.35003	-0.34915	-0.34826	-0.34736	-0.34647	-0.34561
-0.28726	-0.35161	-0.35074	-0.34986	-0.34897	-0.34808	-0.34719	-0.34631	-0.34545
-0.28717	-0.35146	-0.35058	-0.34969	-0.34878	-0.34787	-0.34699	-0.34614	-0.3453
-0.28705	-0.3513	-0.35042	-0.34952	-0.34861	-0.3477	-0.34683	-0.34598	-0.34515
-0.28692	-0.35112	-0.35024	-0.34936	-0.34847	-0.34757	-0.3467	-0.34584	-0.345
-0.28679	-0.35094	-0.35008	-0.34921	-0.34832	-0.34744	-0.34657	-0.3457	-0.34486
-0.28667	-0.3508	-0.34994	-0.34906	-0.34818	-0.34729	-0.34641	-0.34555	-0.34471
-0.28656	-0.35066	-0.34979	-0.34891	-0.34802	-0.34713	-0.34626	-0.3454	-0.34458
-0.28645	-0.35049	-0.34961	-0.34873	-0.34784	-0.34696	-0.34611	-0.34527	-0.34445
-0.28633	-0.35031	-0.34941	-0.34853	-0.34766	-0.3468	-0.34597	-0.34515	-0.34432
-0.2862	-0.35014	-0.34925	-0.34836	-0.3475	-0.34666	-0.34583	-0.34501	-0.40171
-0.28607	-0.34998	-0.3491	-0.34822	-0.34737	-0.34653	-0.34569	-0.34487	-0.40152
-0.28595	-0.34983	-0.34895	-0.34809	-0.34723	-0.34639	-0.34555	-0.34472	-0.40134
-0.28584	-0.34969	-0.34882	-0.34795	-0.34709	-0.34623	-0.34539	-0.34457	-0.40116
-0.28573	-0.34955	-0.34868	-0.34781	-0.34694	-0.34608	-0.34524	-0.34443	-0.46222
-0.28563	-0.34941	-0.34853	-0.34766	-0.34679	-0.34593	-0.34511	-0.34431	-0.46203
-0.28552	-0.34926	-0.34838	-0.3475	-0.34664	-0.34579	-0.34498	-0.34419	-0.46185
-0.28541	-0.34911	-0.34823	-0.34736	-0.34651	-0.34567	-0.34486	-0.34407	-0.46164
-0.2853	-0.34895	-0.34808	-0.34722	-0.34638	-0.34556	-0.34475	-0.34395	-0.46142
-0.2852	-0.34881	-0.34794	-0.34708	-0.34626	-0.34544	-0.34463	-0.34381	-0.46119
-0.2851	-0.34868	-0.34781	-0.34696	-0.34613	-0.34532	-0.34449	-0.34366	-0.46095
-0.28499	-0.34854	-0.34768	-0.34683	-0.34601	-0.34519	-0.40705	-0.40592	-0.46072
-0.28487	-0.34839	-0.34754	-0.34671	-0.34589	-0.34507	-0.40687	-0.40575	-0.46052
-0.28476	-0.34824	-0.34741	-0.34658	-0.34576	-0.34494	-0.40669	-0.40557	-0.46032
-0.28466	-0.34811	-0.34728	-0.34646	-0.34562	-0.34479	-0.40649	-0.40537	-0.46009
-0.28457	-0.34799	-0.34716	-0.34633	-0.34548	-0.34464	-0.40628	-0.40517	-0.45986
-0.28448	-0.34786	-0.34704	-0.34621	-0.34535	-0.3445	-0.4061	-0.40499	-0.51743
-0.28439	-0.34774	-0.34692	-0.34608	-0.34523	-0.34437	-0.40594	-0.40483	-0.51721
-0.2843	-0.34762	-0.3468	-0.34596	-0.34511	-0.34425	-0.40577	-0.40467	-0.46245
-0.28421	-0.3475	-0.34668	-0.34585	-0.34499	-0.34414	-0.4056	-0.4045	-0.46225
-0.28412	-0.34738	-0.34656	-0.40884	-0.4077	-0.40656	-0.40544	-0.40433	-0.46204
-0.28404	-0.34727	-0.34644	-0.40867	-0.40753	-0.40639	-0.40527	-0.40416	-0.46184

-0.28396	-0.34715	-0.34632	-0.4085	-0.40736	-0.40622	-0.4051	-0.40399	-0.46164
-0.28388	-0.34703	-0.3462	-0.40832	-0.40719	-0.40605	-0.40493	-0.40382	-0.57795
-0.28379	-0.34691	-0.34607	-0.40815	-0.40702	-0.40588	-0.40476	-0.40366	-0.57763
-0.2837	-0.34678	-0.34595	-0.40798	-0.40685	-0.40571	-0.4046	-0.4035	-0.5773
-0.28361	-0.34666	-0.34583	-0.40781	-0.40668	-0.40555	-0.40443	-0.40333	-0.57698
-0.28352	-0.28286	-0.28221	-0.34566	-0.34471	-0.34376	-0.40427	-0.40317	-0.57666
-0.28343	-0.28277	-0.28212	-0.34552	-0.34458	-0.34364	-0.40412	-0.40302	-0.57634
-0.28334	-0.28268	-0.28203	-0.34539	-0.34445	-0.34351	-0.40397	-0.40287	-0.62567
-0.28326	-0.2826	-0.28195	-0.34526	-0.34433	-0.34339	-0.40381	-0.40271	-0.6253
-0.28318	-0.28252	-0.28187	-0.34513	-0.3442	-0.34327	-0.40366	-0.40256	-0.62494
-0.28311	-0.34685	-0.34592	-0.345	-0.34408	-0.34316	-0.40353	-0.40243	-0.62461
-0.28304	-0.34674	-0.34579	-0.34486	-0.34395	-0.34304	-0.4034	-0.40231	-0.6243
-0.28296	-0.34661	-0.34566	-0.34472	-0.34382	-0.34292	-0.57837	-0.57643	-0.83134
-0.28287	-0.34648	-0.34552	-0.34458	-0.34368	-0.34279	-0.57804	-0.57612	-0.8309
-0.28278	-0.34636	-0.3454	-0.34446	-0.34355	-0.34266	-0.57773	-0.57582	-0.83048
-0.2827	-0.34625	-0.3453	-0.34437	-0.34344	-0.34254	-0.57743	-0.57555	-0.8301
-0.28263	-0.34614	-0.3452	-0.34427	-0.34334	-0.34242	-0.57713	-0.57529	-0.82971
-0.28255	-0.34601	-0.34509	-0.34417	-0.34324	-0.34232	-0.62646	-0.62444	-0.82926
-0.28247	-0.34587	-0.34497	-0.34407	-0.34314	-0.34222	-0.62613	-0.62408	-0.82879
-0.28241	-0.34576	-0.34485	-0.34395	-0.34302	-0.3421	-0.62578	-0.62371	-0.82834
-0.28235	-0.34566	-0.34473	-0.34381	-0.3429	-0.34199	-0.62542	-0.62335	-0.82789
-0.28228	-0.34555	-0.34461	-0.34368	-0.34277	-0.34187	-0.6251	-0.62303	-0.82747
-0.2822	-0.34542	-0.34448	-0.34355	-0.34265	-0.34177	-0.62482	-0.62275	-0.82709
-0.28212	-0.3453	-0.34435	-0.39785	-0.39678	-0.39573	-0.6202	-0.61816	-0.82672
-0.28205	-0.34519	-0.34425	-0.3977	-0.39663	-0.39558	-0.61988	-0.61788	-0.82637
-0.28199	-0.34508	-0.34416	-0.39757	-0.39649	-0.39542	-0.61956	-0.61759	-0.82601
-0.28193	-0.34499	-0.34406	-0.39743	-0.39635	-0.39528	-0.61923	-0.61725	-0.82559
-0.28188	-0.3449	-0.34396	-0.3973	-0.39622	-0.39515	-0.61892	-0.6169	-0.82514
-0.34573	-0.3448	-0.34386	-0.57257	-0.57064	-0.56873	-0.72315	-0.72084	-0.87215
-0.34562	-0.3447	-0.34377	-0.5723	-0.57037	-0.56846	-0.72282	-0.72053	-0.87176
-0.3455	-0.34459	-0.34367	-0.57202	-0.5701	-0.56819	-0.72249	-0.72021	-0.87137
-0.34539	-0.34447	-0.34355	-0.57175	-0.56983	-0.56793	-0.72216	-0.71988	-0.87099
-0.34528	-0.39878	-0.39769	-0.57148	-0.56957	-0.56767	-0.72184	-0.71955	-0.87062
-0.34519	-0.39866	-0.39756	-0.57121	-0.5693	-0.5674	-0.72151	-0.71923	-0.87024
-0.3451	-0.39854	-0.39744	-0.57094	-0.56903	-0.56714	-0.72118	-0.71891	-0.86987
-0.34499	-0.39839	-0.3973	-0.57067	-0.56877	-0.56688	-0.72086	-0.71859	-0.91983
-0.34488	-0.39823	-0.39716	-0.5704	-0.5685	-0.56661	-0.72053	-0.71826	-0.91942
-0.34478	-0.3981	-0.39703	-0.57013	-0.56823	-0.56635	-0.72021	-0.71795	-0.86945
-0.3447	-0.39798	-0.3969	-0.56986	-0.56796	-0.56608	-0.71989	-0.71765	-0.86907
-0.34462	-0.51706	-0.51529	-0.56958	-0.56768	-0.56581	-0.71956	-0.71735	-0.8687
-0.34453	-0.51681	-0.51503	-0.5693	-0.56741	-0.56555	-0.71925	-0.71703	-0.86831
-0.34443	-0.51656	-0.51478	-0.56902	-0.56715	-0.5653	-0.71894	-0.71671	-0.86793
-0.34432	-0.5163	-0.51453	-0.56875	-0.56688	-0.56505	-0.71863	-0.71641	-0.86754
-0.34421	-0.51603	-0.51428	-0.56848	-0.56663	-0.56481	-0.71833	-0.71611	-0.86717
-0.39826	-0.57202	-0.5701	-0.56822	-0.56639	-0.56459	-0.71806	-0.71586	-0.86685
-0.39813	-0.57174	-0.56983	-0.56797	-0.56616	-0.56438	-0.71781	-0.71563	-0.86656
-0.39801	-0.5715	-0.56959	-0.73029	-0.72804	-0.72581	-0.87153	-0.86886	-0.86623
-0.39792	-0.5713	-0.56939	-0.73001	-0.72774	-0.72551	-0.87113	-0.86847	-0.86585
-0.51673	-0.57109	-0.56917	-0.72972	-0.72745	-0.72521	-0.87074	-0.86808	-0.86547
-0.51649	-0.57082	-0.56891	-0.72941	-0.72714	-0.72492	-0.87037	-0.86771	-0.8651

-0.51625	-0.57056	-0.56865	-0.7291	-0.72684	-0.72462	-0.87001	-0.86735	-0.86474
-0.51604	-0.57033	-0.56845	-0.72885	-0.72658	-0.72435	-0.86968	-0.86704	-0.86443
-0.51585	-0.57012	-0.56825	-0.72861	-0.72633	-0.72409	-0.86937	-0.86674	-0.86415
-0.51563	-0.56988	-0.56802	-0.72833	-0.72605	-0.7238	-0.86901	-0.86641	-0.86382
-0.5154	-0.56963	-0.56777	-0.72801	-0.72574	-0.7235	-0.86864	-0.86605	-0.86347
-0.51519	-0.56939	-0.56753	-0.72771	-0.72545	-0.72323	-0.8683	-0.86572	-0.86314
-0.515	-0.56919	-0.56733	-0.72746	-0.72521	-0.72299	-0.868	-0.86543	-0.86285
-0.51481	-0.56898	-0.56713	-0.78081	-0.77831	-0.77585	-0.8677	-0.86513	-0.86256
-0.51457	-0.56873	-0.5669	-0.78051	-0.77802	-0.77558	-0.8674	-0.86483	-0.86227
-0.51433	-0.56847	-0.56667	-0.78019	-0.77772	-0.7753	-0.86709	-0.86451	-0.86196
-0.51414	-0.56825	-0.56643	-0.77984	-0.77738	-0.77497	-0.86673	-0.86416	-0.86161
-0.51397	-0.56804	-0.5662	-0.77947	-0.77703	-0.77462	-0.86636	-0.86379	-0.86125
-0.56973	-0.56785	-0.56599	-0.87391	-0.87124	-0.86862	-0.86602	-0.86346	-0.86093
-0.56954	-0.56766	-0.56579	-0.8736	-0.87093	-0.86831	-0.86573	-0.86319	-0.86066
-0.56934	-0.56747	-0.5656	-0.8733	-0.87063	-0.86802	-0.86545	-0.86291	-0.86038
-0.56915	-0.56727	-0.56541	-0.87299	-0.87034	-0.86774	-0.86517	-0.86263	-0.8601
-0.56895	-0.6241	-0.62197	-0.8727	-0.87005	-0.86746	-0.86489	-0.86235	-0.85982
-0.56875	-0.62387	-0.62174	-0.8724	-0.86977	-0.86718	-0.86461	-0.86206	-0.85954
-0.56855	-0.62363	-0.62152	-0.87211	-0.86948	-0.86689	-0.86432	-0.86178	-0.85925
-0.56835	-0.6234	-0.62129	-0.87181	-0.86918	-0.8666	-0.86404	-0.8615	-0.98886
-0.56815	-0.62317	-0.62106	-0.87151	-0.86889	-0.86631	-0.86375	-0.86122	-0.98854
-0.56794	-0.62293	-0.62083	-0.8712	-0.86859	-0.86602	-0.86347	-0.86094	-0.98823
-0.56773	-0.62269	-0.62059	-0.8709	-0.8683	-0.86574	-0.86319	-0.86065	-0.9879
-0.56753	-0.67365	-0.67144	-0.87059	-0.86801	-0.86546	-0.8629	-0.86036	-0.98758
-0.56732	-0.67338	-0.67119	-0.87029	-0.86771	-0.86517	-0.86261	-0.86007	-0.98725
-0.56712	-0.67312	-0.67094	-0.87	-0.86742	-0.86488	-0.86232	-0.85978	-0.98693
-0.56696	-0.67291	-0.67071	-0.86971	-0.86712	-0.86458	-0.86204	-0.85951	-0.98663
-0.56682	-0.67271	-0.67049	-0.86942	-0.86683	-0.86429	-0.86177	-0.85926	-0.98634
-0.7276	-0.82189	-0.8193	-0.86918	-0.86659	-0.86405	-0.86153	-0.85904	-0.98608
-0.72732	-0.82161	-0.81905	-0.86895	-0.86638	-0.86385	-0.86133	-0.85883	-0.98585
-0.72706	-0.82133	-0.8188	-0.8687	-0.86614	-0.8636	-0.94662	-0.94392	-1.11481
-0.72681	-0.82106	-0.81852	-0.86842	-0.86585	-0.86331	-0.94632	-0.94363	-1.11444
-0.77573	-0.8208	-0.81827	-0.86814	-0.86556	-0.86303	-0.94602	-0.94334	-1.11406
-0.77551	-0.82058	-0.81805	-0.86791	-0.86531	-0.86275	-0.94573	-0.94305	-1.11368
-0.77529	-0.82036	-0.81784	-0.86768	-0.86507	-0.86249	-0.94544	-0.94277	-1.11331
-0.77504	-0.8201	-0.81759	-0.86743	-0.86483	-0.86227	-0.9452	-0.94252	-1.11298
-0.77476	-0.81981	-0.81732	-0.86718	-0.86461	-0.86207	-0.94498	-0.94228	-1.11267
-0.77452	-0.81957	-0.81709	-0.86695	-0.86439	-0.86185	-0.94474	-0.94203	-1.11232
-0.77431	-0.81935	-0.81689	-0.86675	-0.86419	-0.86164	-0.94449	-0.94175	-1.11195
-0.77408	-0.81911	-0.81666	-0.86652	-0.86396	-0.8614	-0.94425	-0.9415	-1.11161
-0.77383	-0.81884	-0.81637	-0.86623	-0.86368	-0.86115	-0.94399	-0.94126	-1.11133
-0.77359	-0.81857	-0.81611	-0.95191	-0.94917	-0.94646	-1.02906	-1.02611	-1.11105
-0.77337	-0.81836	-0.8159	-0.95168	-0.94894	-0.94623	-1.02879	-1.02583	-1.11075
-0.77315	-0.81816	-0.81571	-0.95146	-0.94873	-0.94601	-1.02852	-1.02555	-1.11043
-0.77296	-0.81796	-0.81551	-0.95124	-0.94851	-0.94579	-1.02824	-1.02525	-1.11008
-0.77277	-0.81776	-0.81532	-0.95102	-0.94828	-0.94556	-1.02795	-1.02493	-1.10972
-0.82005	-0.81755	-0.81511	-0.95079	-0.94805	-0.94531	-1.07046	-1.06732	-1.10939
-0.81982	-0.81733	-0.81489	-0.95056	-0.94779	-0.94504	-1.07014	-1.06704	-1.10911
-0.81961	-0.81711	-0.81467	-0.95031	-0.94754	-0.94478	-1.06984	-1.06676	-1.10883
-0.81942	-0.81691	-0.81443	-0.95003	-0.94729	-0.94457	-1.06958	-1.06648	-1.10854

-0.81923	-0.81672	-0.81421	-0.94977	-0.94705	-0.94436	-1.06932	-1.06621	-1.10825
-0.81903	-0.90454	-0.90185	-1.03525	-1.03227	-1.02931	-1.06905	-1.06595	-1.10798
-0.81883	-0.90435	-0.9017	-1.03508	-1.03206	-1.02905	-1.06877	-1.0657	-1.10771
-0.81864	-0.90414	-0.90148	-1.03481	-1.0318	-1.02881	-1.06851	-1.06543	-1.10743
-0.81844	-0.90393	-0.90123	-1.03449	-1.03151	-1.02856	-1.06825	-1.06515	-1.10715
-0.81825	-0.90374	-0.90103	-1.03423	-1.03126	-1.02831	-1.06798	-1.06488	-1.10686
-0.81806	-0.90357	-0.90089	-1.03407	-1.03105	-1.02805	-1.06769	-1.06462	-1.10659
-0.81786	-0.90339	-0.90074	-0.98527	-0.98235	-0.97944	-1.06609	-1.06302	-1.10632
-0.81767	-0.90319	-0.90055	-0.98507	-0.98217	-0.97928	-1.06589	-1.06277	-1.10606
-0.81748	-0.90298	-0.90035	-0.98485	-0.98199	-0.97913	-1.0657	-1.06254	-1.10581
-0.81727	-0.90277	-0.90014	-0.9846	-0.98176	-0.97893	-1.06548	-1.06234	-1.10563
-0.81706	-0.90256	-0.89993	-0.98435	-0.98152	-0.9787	-1.06525	-1.06216	-1.10546
-0.90504	-0.98987	-0.98698	-0.98412	-0.98127	-0.97845	-1.06499	-1.06194	-1.10525
-0.90487	-0.98963	-0.98676	-0.9839	-0.98103	-0.97819	-1.06471	-1.06169	-1.10501
-0.90471	-0.98943	-0.98656	-0.9837	-0.98083	-0.97797	-1.06445	-1.06143	-1.10476
-0.90457	-0.98927	-0.9864	-0.98354	-0.98067	-0.97782	-1.06425	-1.06119	-1.10451
-0.90444	-0.98911	-0.98623	-1.03051	-1.02752	-1.02454	-1.06405	-1.06097	-1.10428
-0.90433	-0.98897	-0.98604	-1.03027	-1.02729	-1.02433	-1.06384	-1.06077	-1.10409
-0.9042	-0.98882	-0.98584	-1.03002	-1.02705	-1.02411	-1.06364	-1.0606	-1.10392
-0.90402	-0.98862	-0.98567	-1.02984	-1.02685	-1.02391	-1.06345	-1.06043	-1.10375
-0.9038	-0.98841	-0.98549	-1.02968	-1.02668	-1.02372	-1.06326	-1.06028	-1.10358
-0.90359	-0.98817	-0.98529	-1.02948	-1.02648	-1.0235	-1.06304	-1.06008	-1.10339
-0.9034	-0.98792	-0.98506	-1.02925	-1.02624	-1.02326	-1.06279	-1.05984	-1.10317
-0.94583	-1.035	-1.03201	-1.02903	-1.02602	-1.02303	-1.06256	-1.05962	-1.10297
-0.9457	-1.03484	-1.03185	-1.02886	-1.02583	-1.02285	-1.06236	-1.05943	-1.1028
-0.94557	-1.0347	-1.03169	-1.0287	-1.02567	-1.02267	-1.06218	-1.05925	-1.10261
-0.94543	-1.03455	-1.03155	-1.02854	-1.02551	-1.0225	-1.06198	-1.05902	-1.10238
-0.94529	-1.0344	-1.0314	-1.02839	-1.02535	-1.02233	-1.06177	-1.05878	-1.10214
-0.98989	-1.03423	-1.03123	-1.02823	-1.02519	-1.02218	-1.0616	-1.0586	-1.10195
-0.98973	-1.03405	-1.03104	-1.02804	-1.02502	-1.02203	-1.06145	-1.05844	-1.10179
-0.98958	-1.03387	-1.03084	-1.02782	-1.02481	-1.02184	-1.06127	-1.05828	-1.10163
-0.98944	-1.0337	-1.03061	-1.02755	-1.02455	-1.0216	-1.06105	-1.05812	-1.10149
-1.0366	-1.03353	-1.0304	-1.02732	-1.02431	-1.02138	-1.06085	-1.05796	-1.10134
-1.03644	-1.03337	-1.03025	-1.02716	-1.02415	-1.0212	-1.06067	-1.05779	-1.10119
-1.03627	-1.03321	-1.0301	-1.02702	-1.024	-1.02105	-1.0605	-1.05763	-1.10103
-1.03611	-1.03305	-1.02994	-1.02687	-1.02385	-1.0209	-1.06034	-1.05747	-1.14458
-1.03594	-1.03288	-1.02977	-1.02671	-1.02369	-1.02075	-1.06019	-1.05732	-1.14442
-1.03579	-1.03273	-1.02963	-1.02656	-1.02355	-1.0206	-1.06004	-1.05718	-1.14426
-1.03565	-1.0326	-1.0295	-1.02643	-1.0234	-1.02045	-1.05988	-1.05703	-1.1441
-1.03551	-1.03247	-1.02939	-1.02633	-1.0233	-1.02034	-1.05975	-1.05688	-1.09856
-1.03534	-1.03233	-1.0293	-1.02628	-1.02325	-1.02027	-1.05964	-1.05674	-1.09839
-1.03518	-1.03218	-1.0292	-1.02621	-1.02319	-1.0202	-1.05954	-1.05661	-1.09823
-1.03503	-1.03202	-1.02904	-1.02607	-1.02306	-1.02009	-1.05943	-1.05649	-1.09809
-1.03489	-1.03186	-1.02887	-1.0259	-1.02292	-1.01997	-1.05931	-1.05638	-1.09796
-1.03475	-1.03171	-1.02872	-1.02574	-1.02277	-1.01984	-1.05919	-1.05629	-1.09788
-1.03461	-1.03158	-1.02857	-1.02559	-1.02262	-1.0197	-1.05907	-1.05622	-1.09781
-1.0345	-1.07424	-1.07111	-1.06801	-1.06491	-1.06187	-1.1036	-1.10063	-1.13945
-1.03442	-1.07417	-1.07105	-1.06793	-1.06481	-1.06175	-1.10345	-1.10047	-1.13929
-1.03431	-1.03133	-1.02832	-1.02532	-1.02232	-1.01937	-1.10333	-1.10034	-1.13917
-1.03415	-1.03117	-1.02817	-1.02518	-1.02222	-1.0193	-1.10326	-1.10027	-1.13909

-1.03401	-1.03101	-1.02801	-1.02504	-1.0221	-1.01922	-1.10318	-1.10019	-1.13901
-1.03392	-1.03092	-1.02789	-1.02491	-1.02197	-1.01909	-1.10303	-1.10006	-1.1389
-1.03385	-1.03083	-1.02779	-1.02479	-1.02184	-1.01896	-1.10288	-1.09991	-1.13878
-1.03375	-1.03073	-1.02769	-1.0247	-1.02176	-1.01887	-1.10277	-1.09979	-1.13865
-1.03363	-1.0306	-1.0276	-1.02463	-1.0217	-1.01882	-1.10268	-1.09967	-1.13853
-1.03353	-1.03051	-1.02752	-1.02457	-1.02163	-1.01875	-1.10259	-1.09958	-1.13844
-1.03345	-1.03045	-1.02747	-1.02451	-1.02155	-1.01864	-1.10248	-1.09951	-1.13839
-1.03338	-1.03039	-1.0274	-1.02443	-1.02146	-1.01855	-1.10239	-1.09945	-1.09654
-1.0333	-1.03031	-1.02729	-1.02431	-1.02138	-1.01851	-1.10234	-1.0994	-1.09649
-1.03322	-1.03021	-1.02718	-1.0242	-1.0213	-1.01847	-1.1023	-1.09935	-1.09644
-1.03311	-1.0301	-1.0271	-1.02414	-1.02125	-1.01843	-1.10224	-1.09929	-1.09638
-1.033	-1.02999	-1.02703	-1.0241	-1.02121	-1.01838	-1.10217	-1.09922	-1.09631
-1.03291	-1.02992	-1.02697	-1.06975	-1.06673	-1.06376	-1.10211	-1.09916	-1.09623
-1.03284	-1.02988	-1.02692	-1.06969	-1.06667	-1.0637	-1.10205	-1.09909	-1.09615
-1.03278	-1.02983	-1.02687	-1.06964	-1.06662	-1.06365	-1.10198	-1.09901	-1.09606
-1.03273	-1.02976	-1.02682	-1.06959	-1.06658	-1.06361	-1.10191	-1.0989	-1.09595
-1.03268	-1.0297	-1.02677	-1.06954	-1.06654	-1.06357	-1.10185	-1.09882	-1.09587
-1.03263	-1.02966	-1.02672	-1.06948	-1.06647	-1.06351	-1.10181	-1.09881	-1.09586
-1.03259	-1.02964	-1.02668	-1.06942	-1.06641	-1.06345	-1.10178	-1.09882	-1.09587
-1.03254	-1.02957	-1.02663	-1.06937	-1.06637	-1.06342	-1.10172	-1.09875	-1.09579
-1.03249	-1.0295	-1.02658	-1.06934	-1.06635	-1.0634	-1.10166	-1.09864	-1.09569
-1.03248	-1.02948	-1.02655	-1.06928	-1.0663	-1.06336	-1.10163	-1.09861	-1.09565
-1.03251	-1.02951	-1.02652	-1.06922	-1.06624	-1.06331	-1.10161	-1.09865	-1.09568
-0.94254	-0.93977	-0.93699	-1.06916	-1.06618	-1.06327	-1.10159	-1.09866	-1.0957
-0.94242	-0.93966	-0.93693	-1.06912	-1.06615	-1.06323	-1.10155	-1.09862	-1.09569
-0.94231	-0.93956	-0.93688	-1.06909	-1.06611	-1.06319	-1.1015	-1.09857	-1.09566
-0.94231	-0.93955	-0.93685	-1.06903	-1.06607	-1.06316	-1.10146	-1.09852	-1.0956
-0.94234	-0.93958	-0.93683	-1.06898	-1.06603	-1.06314	-1.10143	-1.09847	-1.09555
-0.94234	-0.98713	-0.98422	-1.06899	-1.06604	-1.06315	-1.10144	-1.09848	-1.09555
-0.94232	-0.98712	-0.98424	-1.06903	-1.06608	-1.06319	-1.10148	-1.09855	-1.09558
-0.8613	-0.98713	-0.98427	-1.06908	-1.06611	-1.06318	-1.10148	-1.09856	-1.09559
-0.86129	-0.98716	-0.98432	-1.06914	-1.06612	-1.06314	-1.10142	-1.09852	-1.09558
-0.86127	-0.98716	-0.98433	-1.06917	-1.06612	-1.06312	-1.10138	-1.09847	-1.09555
-0.86118	-0.98709	-0.98428	-1.06914	-1.06613	-1.06316	-1.10139	-1.09841	-1.09547
-0.8611	-0.98702	-0.98422	-1.06908	-1.06613	-1.0632	-1.1014	-1.09836	-1.09541
-0.81087	-0.95245	-0.94958	-1.11052	-1.10746	-1.10444	-1.1014	-1.09838	-1.09541
-0.81087	-0.95244	-0.94951	-1.11046	-1.1074	-1.1044	-1.1014	-1.09842	-1.09545
-0.81084	-0.9524	-0.94947	-1.11045	-1.10738	-1.10438	-1.10141	-1.09846	-1.09549
-0.81078	-0.95235	-0.94944	-1.11047	-1.1074	-1.10439	-1.10142	-1.0985	-1.09553
-0.81074	-0.95232	-0.94942	-1.03567	-1.03266	-1.0297	-1.10141	-1.0985	-1.09555
-0.81074	-0.95232	-0.94943	-1.03567	-1.03262	-1.02962	-1.10136	-1.09847	-1.09554
-0.81074	-0.95232	-0.94943	-1.03568	-1.03259	-1.02956	-1.10133	-1.09844	-1.09554
-0.81075	-0.95232	-0.94944	-1.03569	-1.0326	-1.02956	-1.10135	-1.09847	-1.09557
-0.81074	-0.95231	-0.94943	-1.0357	-1.03261	-1.02957	-1.1014	-1.09851	-1.09561
-0.81069	-0.90401	-0.90128	-1.03566	-1.03259	-1.02956	-1.02664	-1.02379	-1.09564
-0.81061	-0.90391	-0.90119	-1.0356	-1.03255	-1.02955	-1.02663	-1.02377	-1.09567
-0.8608	-0.90386	-0.90115	-1.03557	-1.03253	-1.02954	-1.02661	-1.02373	-1.09565
-0.8608	-0.90386	-0.90115	-1.03557	-1.03254	-1.02954	-1.02658	-1.02365	-1.0956
-0.86081	-0.90387	-0.90115	-1.03557	-1.03254	-1.02954	-1.02656	-1.0236	-1.02073
-0.86081	-0.90387	-0.90115	-1.03558	-1.03255	-1.02954	-1.02655	-1.02359	-1.02072

-0.86081	-0.90387	-0.90115	-0.98935	-0.98647	-0.98363	-1.02657	-1.02361	-1.02073
-0.86082	-0.90388	-0.90116	-0.98937	-0.9865	-0.98367	-1.0266	-1.02364	-1.02075
-0.86082	-0.90388	-0.90117	-0.9894	-0.98654	-0.98371	-1.02665	-1.02368	-1.02079
-0.86083	-0.90388	-0.90117	-0.98942	-0.98656	-0.98374	-1.0267	-1.02376	-1.02087
-0.86084	-0.90388	-0.90117	-0.98944	-0.98658	-0.98377	-1.02676	-1.02386	-1.02098
-0.86088	-0.90393	-0.90121	-0.89854	-0.8959	-0.89332	-1.02679	-1.02391	-1.02104
-0.86098	-0.90405	-0.90131	-0.8986	-0.89594	-0.89333	-1.0268	-1.02392	-1.02105
-0.86104	-0.90413	-0.90138	-0.89866	-0.89598	-0.89334	-1.02682	-1.02393	-1.02106
-0.86105	-0.90415	-0.90142	-0.8987	-0.896	-0.89334	-1.02684	-1.02394	-1.02107
-0.86104	-0.90414	-0.90144	-0.89874	-0.89603	-0.89337	-1.02688	-1.02396	-1.02109
-0.86103	-0.90414	-0.90144	-0.89876	-0.89609	-0.89345	-0.9812	-0.97844	-1.02114
-0.86103	-0.90414	-0.90145	-0.89878	-0.89615	-0.89355	-0.98131	-0.97853	-1.02121
-0.86109	-0.90419	-0.90147	-0.89879	-0.89617	-0.89358	-0.98136	-0.97858	-1.02126
-0.86119	-0.90427	-0.9015	-0.89879	-0.89616	-0.89358	-0.98138	-0.97861	-1.02129
-0.86125	-0.90432	-0.90155	-0.89884	-0.8962	-0.89361	-0.98142	-0.97865	-1.02134
-0.86127	-0.90434	-0.90162	-0.89894	-0.89631	-0.89371	-0.9815	-0.9787	-1.02139
-0.86131	-0.90439	-0.9017	-0.89904	-0.8964	-0.89378	-0.98158	-0.97876	-1.02146
-0.86138	-0.90448	-0.90179	-0.89912	-0.89645	-0.89381	-0.98164	-0.97884	-1.02156
-0.86146	-0.90457	-0.90187	-0.89918	-0.89648	-0.89383	-0.9817	-0.97893	-1.02167
-0.86147	-0.90458	-0.90188	-0.8992	-0.89653	-0.89389	-0.98179	-0.97902	-1.02176
-0.86145	-0.90456	-0.90187	-0.8992	-0.89656	-0.89396	-0.98188	-0.97911	-1.02185
-0.86149	-0.90458	-0.90187	-0.89919	-0.89658	-0.894	-0.89145	-0.88893	-0.97642
-0.86157	-0.90464	-0.90188	-0.89917	-0.89656	-0.894	-0.89146	-0.88894	-0.97648
-0.86163	-0.90468	-0.90191	-0.8992	-0.89659	-0.89403	-0.89148	-0.88897	-0.97655
-0.86164	-0.9047	-0.90198	-0.8993	-0.89667	-0.89408	-0.89154	-0.88904	-0.97664
-0.86168	-0.90474	-0.90206	-0.8994	-0.89676	-0.89415	-0.89161	-0.88912	-0.97673
-0.86183	-0.90487	-0.90214	-0.89945	-0.89682	-0.89423	-0.89169	-0.8892	-0.97682
-0.86199	-0.90503	-0.90223	-0.8995	-0.89688	-0.89432	-0.89178	-0.88928	-0.9769
-0.86205	-0.90509	-0.90231	-0.89959	-0.89697	-0.89439	-0.89184	-0.88931	-1.02073
-0.86206	-0.9051	-0.90239	-0.89972	-0.89707	-0.89446	-0.89187	-0.88932	-1.02077
-0.86211	-0.90516	-0.90247	-0.8998	-0.89716	-0.89454	-0.89195	-0.8894	-1.02088
-0.8622	-0.90527	-0.90254	-0.89984	-0.89721	-0.89463	-0.89208	-0.88956	-1.02105
-0.86229	-0.90537	-0.90261	-0.89991	-0.89729	-0.89471	-0.89218	-0.88968	-1.02118
-0.86237	-0.90543	-0.90271	-0.90003	-0.89739	-0.89479	-0.89222	-0.8897	-1.02122
-0.86245	-0.90549	-0.90281	-0.90015	-0.8975	-0.89487	-0.89228	-0.88972	-1.02128
-0.86256	-0.90561	-0.9029	-0.90022	-0.89758	-0.89498	-0.8924	-0.88985	-1.02142
-0.86266	-0.90574	-0.90298	-0.90028	-0.89767	-0.8951	-0.89255	-0.89002	-1.0216
-0.86275	-0.90582	-0.90307	-0.90038	-0.89776	-0.89519	-0.89266	-0.89016	-1.02176
-0.86283	-0.90587	-0.90317	-0.9005	-0.89785	-0.89525	-0.89273	-0.89027	-1.02191
-0.86295	-0.90598	-0.90327	-0.9006	-0.89794	-0.89533	-0.89282	-0.89036	-0.93334
-0.86314	-0.90615	-0.90337	-0.90065	-0.89802	-0.89545	-0.89292	-0.89042	-0.9334
-0.86331	-0.90633	-0.90349	-0.90073	-0.89811	-0.89555	-0.89301	-0.8905	-0.93347
-0.86344	-0.90649	-0.90367	-0.9009	-0.89822	-0.89561	-0.89307	-0.8906	-0.93358
-0.86354	-0.90662	-0.90384	-0.90109	-0.89835	-0.89568	-0.89315	-0.89071	-0.93368
-0.86362	-0.9067	-0.90396	-0.90124	-0.89851	-0.89583	-0.89328	-0.8908	-0.93377
-0.8637	-0.90675	-0.90405	-0.90136	-0.89867	-0.89602	-0.89343	-0.8909	-0.93385
-0.86384	-0.90688	-0.90415	-0.90146	-0.89879	-0.89616	-0.93927	-0.93658	-0.93397
-0.86404	-0.90706	-0.90427	-0.90153	-0.89888	-0.89627	-0.9394	-0.93674	-0.93414
-0.8642	-0.90723	-0.9044	-0.90164	-0.89899	-0.89641	-0.93955	-0.9369	-0.9343
-0.86432	-0.90738	-0.90456	-0.90181	-0.89916	-0.89658	-0.93973	-0.93708	-0.93445

-0.86445	-0.90753	-0.90473	-0.90199	-0.89933	-0.89674	-0.93988	-0.93724	-0.93459
-0.86462	-0.90771	-0.90493	-0.90218	-0.89948	-0.89684	-0.93998	-0.93736	-0.93472
-0.86479	-0.90789	-0.90512	-0.90236	-0.89962	-0.89694	-0.94007	-0.93747	-0.93486
-0.86491	-0.90801	-0.90525	-0.90251	-0.89977	-0.89708	-0.94021	-0.93761	-0.93501
-0.86501	-0.9081	-0.90536	-0.90264	-0.89992	-0.89724	-0.94037	-0.93777	-0.93517
-0.72541	-0.8625	-0.8599	-0.85731	-0.85472	-0.85217	-0.94052	-0.9379	-0.93529
-0.72555	-0.86268	-0.86008	-0.85749	-0.85489	-0.85234	-0.94066	-0.93798	-0.93536
-0.72568	-0.86286	-0.86026	-0.85767	-0.85507	-0.85251	-0.94081	-0.9381	-0.93547
-0.7258	-0.86303	-0.86044	-0.85786	-0.85526	-0.85269	-0.94099	-0.93827	-0.93563
-0.72592	-0.8632	-0.86061	-0.85803	-0.85543	-0.85286	-0.94116	-0.93845	-0.93581
-0.72605	-0.86336	-0.86073	-0.85813	-0.85554	-0.85299	-0.94131	-0.93861	-0.93598
-0.72619	-0.86352	-0.86085	-0.85821	-0.85563	-0.85311	-0.94145	-0.93878	-0.93616
-0.72632	-0.91087	-0.90804	-0.90527	-0.90256	-0.89991	-0.94161	-0.93894	-0.93633
-0.72646	-0.91106	-0.90824	-0.90546	-0.90274	-0.90008	-0.94178	-0.93912	-0.9365
-0.72659	-0.91124	-0.90843	-0.90565	-0.90292	-0.90025	-0.94195	-0.93929	-0.93667
-0.72672	-0.91142	-0.9086	-0.90583	-0.90311	-0.90043	-0.94214	-0.93947	-0.93685
-0.72686	-0.9116	-0.90879	-0.90601	-0.90329	-0.90062	-0.94232	-0.93964	-0.93702
-0.727	-0.9118	-0.90897	-0.90619	-0.90346	-0.90079	-0.94249	-0.93981	-0.93718
-0.72714	-0.912	-0.90917	-0.90639	-0.90365	-0.90097	-0.94267	-0.93998	-0.93735
-0.72728	-0.9122	-0.90941	-0.90665	-0.9039	-0.90119	-0.94287	-0.94016	-0.93752
-0.72741	-0.9124	-0.90966	-0.90693	-0.90416	-0.90142	-0.94309	-0.94034	-0.93768
-0.72754	-0.86692	-0.86432	-0.90715	-0.90439	-0.90164	-0.9433	-0.94053	-0.93785
-0.72768	-0.86709	-0.86448	-0.90732	-0.90458	-0.90185	-0.9435	-0.94071	-0.93802
-0.62353	-0.77546	-0.7731	-0.9075	-0.90476	-0.90204	-0.94371	-0.94092	-0.93823
-0.62364	-0.77559	-0.77324	-0.90767	-0.90494	-0.90223	-0.94393	-0.94118	-0.9385
-0.62376	-0.77573	-0.77337	-0.90786	-0.90512	-0.90241	-0.94414	-0.94142	-0.93875
-0.62386	-0.77586	-0.7735	-0.90805	-0.9053	-0.90258	-0.94432	-0.9416	-0.93892
-0.62398	-0.77601	-0.77364	-0.90825	-0.9055	-0.90278	-0.94451	-0.94176	-0.93908
-0.62414	-0.77621	-0.77383	-0.90847	-0.90574	-0.90303	-0.94476	-0.942	-0.93929
-0.62432	-0.77645	-0.77404	-0.90872	-0.906	-0.90331	-0.94504	-0.94226	-0.93953
-0.62449	-0.77666	-0.77422	-0.90893	-0.90622	-0.90353	-0.94527	-0.94248	-0.93975
-0.62465	-0.77685	-0.77438	-0.90911	-0.90638	-0.90369	-0.94544	-0.94267	-0.93995
-0.62479	-0.77702	-0.77456	-0.90933	-0.90659	-0.90389	-0.94563	-0.94285	-0.94015
-0.62491	-0.77719	-0.77476	-0.90961	-0.90687	-0.90414	-0.94586	-0.94304	-0.94032
-0.62502	-0.77734	-0.77496	-0.90988	-0.90714	-0.9044	-0.9461	-0.94325	-0.94052
-0.578	-0.67525	-0.67334	-0.86449	-0.86186	-0.85924	-0.94634	-0.94352	-0.94079
-0.57811	-0.67537	-0.67345	-0.86469	-0.86205	-0.85943	-0.94659	-0.94381	-0.94108
-0.57826	-0.67554	-0.6736	-0.86491	-0.86228	-0.85967	-0.94685	-0.94407	-0.94134
-0.57844	-0.67574	-0.67378	-0.86515	-0.86253	-0.85994	-0.94713	-0.94431	-0.94158
-0.52197	-0.67592	-0.67395	-0.77348	-0.77111	-0.76875	-0.94738	-0.94454	-0.9418
-0.52211	-0.67608	-0.67414	-0.77372	-0.77133	-0.76895	-0.94762	-0.94474	-0.94199
-0.52225	-0.67625	-0.67432	-0.77395	-0.77154	-0.76913	-0.94785	-0.94496	-0.9422
-0.5224	-0.67644	-0.67449	-0.77415	-0.77171	-0.76928	-0.94809	-0.94524	-0.94249
-0.52256	-0.67663	-0.67466	-0.77434	-0.77188	-0.76945	-0.94833	-0.94554	-0.94279
-0.5227	-0.63066	-0.62879	-0.67281	-0.67085	-0.66889	-0.90409	-0.90139	-0.94306
-0.52284	-0.63081	-0.62891	-0.67294	-0.67101	-0.66909	-0.90438	-0.90164	-0.94332
-0.52299	-0.63098	-0.62908	-0.67313	-0.67119	-0.66927	-0.90465	-0.90191	-0.94361
-0.52315	-0.63117	-0.62931	-0.67337	-0.67139	-0.66942	-0.9049	-0.90222	-0.94392
-0.5233	-0.63134	-0.62948	-0.67356	-0.67156	-0.66958	-0.90517	-0.9025	-0.94422
-0.52344	-0.63148	-0.62959	-0.67368	-0.67172	-0.66978	-0.90545	-0.90274	-0.94447

-0.52359	-0.63163	-0.62972	-0.62785	-0.62605	-0.62427	-0.8158	-0.81329	-0.90034
-0.52374	-0.63181	-0.62992	-0.62805	-0.62621	-0.6244	-0.81601	-0.81353	-0.90062
-0.52389	-0.632	-0.63013	-0.62827	-0.62639	-0.62454	-0.81623	-0.81377	-0.9009
-0.52402	-0.63216	-0.63031	-0.62846	-0.62659	-0.62474	-0.81649	-0.81399	-0.90116
-0.52415	-0.63229	-0.63047	-0.62864	-0.6268	-0.62496	-0.81677	-0.81421	-0.90141
-0.52429	-0.63245	-0.63062	-0.6288	-0.62697	-0.62515	-0.66894	-0.66694	-0.9017
-0.52444	-0.63263	-0.63077	-0.62893	-0.62712	-0.62532	-0.66915	-0.66717	-0.90205
-0.52459	-0.63279	-0.63092	-0.62907	-0.62727	-0.62547	-0.66934	-0.66739	-0.90237
-0.52473	-0.63293	-0.63109	-0.62925	-0.62742	-0.6256	-0.6695	-0.66758	-0.90266
-0.52487	-0.63309	-0.63127	-0.62944	-0.62759	-0.62574	-0.66965	-0.66776	-0.90293
-0.52505	-0.6333	-0.63148	-0.62965	-0.6278	-0.62594	-0.62413	-0.62235	-0.81326
-0.52524	-0.63352	-0.6317	-0.62987	-0.62801	-0.62616	-0.62432	-0.62249	-0.81347
-0.5254	-0.63372	-0.63187	-0.63003	-0.62818	-0.62634	-0.6245	-0.62268	-0.81374
-0.52555	-0.6339	-0.63202	-0.63015	-0.62832	-0.62651	-0.62469	-0.62288	-0.81405
-0.52572	-0.6341	-0.6322	-0.63032	-0.62848	-0.62666	-0.62485	-0.62307	-0.81434
-0.52592	-0.63432	-0.63242	-0.63053	-0.62865	-0.62679	-0.62499	-0.62323	-0.81458
-0.5261	-0.63451	-0.63263	-0.63074	-0.62883	-0.62695	-0.62515	-0.62341	-0.67161
-0.52623	-0.63465	-0.6328	-0.63094	-0.62903	-0.62715	-0.62536	-0.62362	-0.67183
-0.52636	-0.63479	-0.63296	-0.63112	-0.62924	-0.62737	-0.62559	-0.62384	-0.67206
-0.46611	-0.63497	-0.63315	-0.63133	-0.62945	-0.62759	-0.6258	-0.62405	-0.67229
-0.46625	-0.63517	-0.63335	-0.63152	-0.62966	-0.6278	-0.62601	-0.62425	-0.67251
-0.4664	-0.63537	-0.63352	-0.63168	-0.62983	-0.62799	-0.62619	-0.62442	-0.67269
-0.46655	-0.63557	-0.63367	-0.6318	-0.62998	-0.62817	-0.62636	-0.62455	-0.67283
-0.46669	-0.52571	-0.52413	-0.57926	-0.5776	-0.57595	-0.62654	-0.62472	-0.67302
-0.46679	-0.52587	-0.52431	-0.57947	-0.57781	-0.57616	-0.62675	-0.62493	-0.67324
-0.46691	-0.52604	-0.52449	-0.57968	-0.57802	-0.57637	-0.62697	-0.62515	-0.67347
-0.46704	-0.52623	-0.52468	-0.57989	-0.57823	-0.57659	-0.62718	-0.62536	-0.6737
-0.46719	-0.52642	-0.52487	-0.58009	-0.57844	-0.5768	-0.6274	-0.62557	-0.67393
-0.46734	-0.46607	-0.4648	-0.5803	-0.57865	-0.577	-0.62761	-0.62578	-0.67416
-0.46749	-0.46622	-0.46494	-0.5805	-0.57885	-0.57721	-0.62782	-0.62599	-0.67439
-0.46764	-0.46637	-0.46509	-0.58071	-0.57906	-0.57742	-0.62802	-0.62621	-0.67461
-0.46779	-0.46651	-0.46523	-0.58092	-0.57926	-0.57762	-0.62823	-0.62642	-0.67484
-0.46794	-0.46666	-0.46538	-0.58112	-0.57947	-0.57783	-0.62844	-0.62662	-0.67507
-0.46808	-0.46681	-0.46552	-0.58132	-0.57967	-0.57803	-0.62865	-0.62683	-0.67529
-0.46823	-0.46695	-0.46566	-0.58152	-0.57987	-0.57823	-0.62885	-0.62703	-0.67551
-0.46838	-0.4671	-0.4658	-0.52263	-0.52123	-0.51983	-0.62905	-0.62723	-0.67573
-0.46853	-0.46725	-0.46595	-0.52279	-0.52138	-0.51999	-0.62925	-0.62743	-0.67595
-0.46868	-0.46739	-0.46609	-0.52295	-0.52154	-0.52014	-0.62945	-0.62764	-0.67617
-0.46883	-0.46754	-0.46624	-0.52311	-0.5217	-0.52029	-0.62966	-0.62785	-0.6764
-0.46897	-0.46769	-0.4664	-0.46512	-0.46387	-0.46263	-0.5776	-0.57594	-0.62631
-0.46912	-0.46785	-0.46659	-0.46533	-0.46406	-0.46281	-0.57785	-0.57622	-0.62659
-0.46928	-0.46801	-0.46676	-0.46551	-0.46424	-0.46298	-0.57809	-0.57647	-0.62685
-0.46946	-0.46819	-0.46693	-0.46567	-0.46439	-0.46313	-0.57829	-0.57668	-0.62705
-0.46965	-0.46836	-0.46708	-0.46581	-0.46453	-0.46327	-0.57849	-0.57687	-0.62725
-0.4698	-0.46851	-0.46723	-0.46596	-0.46467	-0.4634	-0.51994	-0.51857	-0.62745
-0.46995	-0.46866	-0.46738	-0.4661	-0.46482	-0.46355	-0.5201	-0.51873	-0.62766
-0.47013	-0.46884	-0.46755	-0.46627	-0.46499	-0.46372	-0.52029	-0.51891	-0.62789
-0.47033	-0.46905	-0.46775	-0.46646	-0.46518	-0.46391	-0.5205	-0.5191	-0.62813
-0.4705	-0.46922	-0.46792	-0.46663	-0.46535	-0.46408	-0.52068	-0.51927	-0.62836
-0.47065	-0.46937	-0.46807	-0.46677	-0.46549	-0.46422	-0.52084	-0.51943	-0.62856

-0.4708	-0.46952	-0.46822	-0.46693	-0.46565	-0.46437	-0.52101	-0.51959	-0.62878
-0.47098	-0.4697	-0.46841	-0.46712	-0.46583	-0.46454	-0.5212	-0.5198	-0.62905
-0.47116	-0.46988	-0.4686	-0.46732	-0.46602	-0.46472	-0.52141	-0.52001	-0.62933
-0.4145	-0.47002	-0.46876	-0.46748	-0.46619	-0.46491	-0.52163	-0.52022	-0.62958
-0.41462	-0.47016	-0.46889	-0.46763	-0.46637	-0.46511	-0.52185	-0.52042	-0.62982
-0.41477	-0.47033	-0.46906	-0.46779	-0.46653	-0.46528	-0.46401	-0.46274	-0.5192
-0.41494	-0.47052	-0.46923	-0.46796	-0.46669	-0.46542	-0.46415	-0.46288	-0.51936
-0.35344	-0.4707	-0.46942	-0.46813	-0.46686	-0.46558	-0.46431	-0.46303	-0.51953
-0.35358	-0.47088	-0.4696	-0.46833	-0.46704	-0.46576	-0.46448	-0.46322	-0.51975
-0.35371	-0.47106	-0.46979	-0.46852	-0.46723	-0.46594	-0.46467	-0.46342	-0.51996
-0.35385	-0.47125	-0.46997	-0.46869	-0.46741	-0.46613	-0.46486	-0.4636	-0.52017
-0.35398	-0.47145	-0.47016	-0.46887	-0.4676	-0.46633	-0.46505	-0.46377	-0.52037
-0.3541	-0.41482	-0.41372	-0.46906	-0.46779	-0.46651	-0.46523	-0.46396	-0.52057
-0.3542	-0.41494	-0.41388	-0.46926	-0.46798	-0.46669	-0.46541	-0.46415	-0.52079
-0.35433	-0.4151	-0.41404	-0.46944	-0.46816	-0.46688	-0.4656	-0.46433	-0.52099
-0.3545	-0.4153	-0.41421	-0.46961	-0.46834	-0.46708	-0.4658	-0.46451	-0.52119
-0.35464	-0.41547	-0.41438	-0.46979	-0.46853	-0.46727	-0.46598	-0.46469	-0.5214
-0.35475	-0.41561	-0.41453	-0.46997	-0.46871	-0.46744	-0.46616	-0.46489	-0.52162
-0.35486	-0.41574	-0.41468	-0.47016	-0.46888	-0.46761	-0.46634	-0.46507	-0.52184
-0.35501	-0.35412	-0.35324	-0.47033	-0.46906	-0.46779	-0.46652	-0.46525	-0.52204
-0.35517	-0.35428	-0.35338	-0.4705	-0.46924	-0.46797	-0.4667	-0.46542	-0.52224
-0.35531	-0.35442	-0.35351	-0.47069	-0.46942	-0.46815	-0.46687	-0.4656	-0.46434
-0.35544	-0.35453	-0.35364	-0.47088	-0.4696	-0.46832	-0.46705	-0.46578	-0.46454
-0.35557	-0.35466	-0.35377	-0.41441	-0.4133	-0.41219	-0.46724	-0.46598	-0.46475
-0.35571	-0.3548	-0.3539	-0.41456	-0.41345	-0.41235	-0.46744	-0.46621	-0.46497
-0.35584	-0.35493	-0.35403	-0.41471	-0.41361	-0.41252	-0.46764	-0.46642	-0.46517
-0.35597	-0.35506	-0.35415	-0.41487	-0.41379	-0.41271	-0.46784	-0.4666	-0.46534
-0.35611	-0.35519	-0.35429	-0.41504	-0.41397	-0.4129	-0.46804	-0.46677	-0.46551
-0.29666	-0.35534	-0.35444	-0.35356	-0.35268	-0.35179	-0.46821	-0.46694	-0.46569
-0.29679	-0.35551	-0.35461	-0.35372	-0.35282	-0.35192	-0.46838	-0.46712	-0.46589
-0.2969	-0.35566	-0.35477	-0.35388	-0.35297	-0.35207	-0.46858	-0.46732	-0.46609
-0.297	-0.3558	-0.35493	-0.35404	-0.35314	-0.35223	-0.46881	-0.46755	-0.46629
-0.29711	-0.35595	-0.35507	-0.35419	-0.35329	-0.35239	-0.46903	-0.46777	-0.4665
-0.29723	-0.35611	-0.35521	-0.35431	-0.35342	-0.35253	-0.46924	-0.46799	-0.46673
-0.29735	-0.35626	-0.35534	-0.35443	-0.35354	-0.35266	-0.35179	-0.35093	-0.46695
-0.29746	-0.35639	-0.35547	-0.35457	-0.35368	-0.35281	-0.35193	-0.35105	-0.46712
-0.29757	-0.35652	-0.35561	-0.35471	-0.35383	-0.35296	-0.35207	-0.35117	-0.46728
-0.2977	-0.35667	-0.35576	-0.35486	-0.35399	-0.35312	-0.35222	-0.35131	-0.46748
-0.29783	-0.35684	-0.35593	-0.35503	-0.35415	-0.35328	-0.35238	-0.35147	-0.46769
-0.29795	-0.357	-0.35609	-0.35519	-0.3543	-0.35342	-0.35252	-0.35161	-0.46789
-0.29805	-0.35715	-0.35625	-0.35535	-0.35444	-0.35354	-0.35264	-0.35175	-0.46808
-0.29816	-0.29744	-0.29673	-0.35551	-0.35459	-0.35367	-0.35277	-0.35188	-0.46827
-0.29828	-0.29756	-0.29685	-0.35567	-0.35475	-0.35383	-0.35293	-0.35204	-0.46849
-0.2984	-0.29768	-0.29698	-0.35583	-0.35491	-0.35399	-0.35309	-0.3522	-0.4687
-0.29852	-0.29781	-0.2971	-0.356	-0.35507	-0.35415	-0.35325	-0.35235	-0.35148
-0.29865	-0.29793	-0.29723	-0.35616	-0.35523	-0.35431	-0.3534	-0.35251	-0.35164
-0.29877	-0.29805	-0.29736	-0.35632	-0.3554	-0.35447	-0.35356	-0.35267	-0.35179
-0.29889	-0.29818	-0.29748	-0.35649	-0.35556	-0.35464	-0.35373	-0.35283	-0.35195
-0.29902	-0.2983	-0.29761	-0.35665	-0.35573	-0.3548	-0.35389	-0.35299	-0.3521
-0.29914	-0.29843	-0.29773	-0.35681	-0.35589	-0.35497	-0.35406	-0.35315	-0.35227

-0.29927	-0.29855	-0.29786	-0.35698	-0.35606	-0.35513	-0.35422	-0.35332	-0.35243
-0.29939	-0.29867	-0.29798	-0.35714	-0.35622	-0.3553	-0.35439	-0.35348	-0.35259
-0.29951	-0.29879	-0.29809	-0.35729	-0.35638	-0.35546	-0.35455	-0.35365	-0.35275
-0.24324	-0.2989	-0.29821	-0.29751	-0.2968	-0.29608	-0.35471	-0.35381	-0.35291
-0.24333	-0.29901	-0.29832	-0.29762	-0.29691	-0.2962	-0.35487	-0.35397	-0.35308
-0.24342	-0.29913	-0.29843	-0.29773	-0.29703	-0.29632	-0.35503	-0.35413	-0.35324
-0.24351	-0.29925	-0.29854	-0.29785	-0.29714	-0.29644	-0.35519	-0.35429	-0.35339
-0.2436	-0.24299	-0.24242	-0.29796	-0.29726	-0.29656	-0.29585	-0.29513	-0.35356
-0.24369	-0.24308	-0.2425	-0.29808	-0.29739	-0.29671	-0.296	-0.29528	-0.35373
-0.24378	-0.24317	-0.24259	-0.2982	-0.29752	-0.29685	-0.29614	-0.29542	-0.35391
-0.2439	-0.24328	-0.24268	-0.29831	-0.29764	-0.29697	-0.29626	-0.29554	-0.35407
-0.24402	-0.24339	-0.24278	-0.29843	-0.29775	-0.29708	-0.29638	-0.29566	-0.35424
-0.18587	-0.24349	-0.24287	-0.24228	-0.24173	-0.24119	-0.2965	-0.2958	-0.29509
-0.18593	-0.24358	-0.24296	-0.24236	-0.24181	-0.24127	-0.29663	-0.29595	-0.29524
-0.12475	-0.24369	-0.24306	-0.24246	-0.24189	-0.24135	-0.29675	-0.29609	-0.29539
-0.12482	-0.2438	-0.24316	-0.24255	-0.24198	-0.24142	-0.29686	-0.29621	-0.29552
-0.12488	-0.2439	-0.24327	-0.24266	-0.24207	-0.2415	-0.29697	-0.29632	-0.29564
-0.12494	-0.244	-0.24337	-0.24276	-0.24217	-0.2416	-0.29708	-0.29642	-0.29575
-0.125	-0.24409	-0.24348	-0.24287	-0.24228	-0.24169	-0.2972	-0.29653	-0.29585
-0.12506	-0.24418	-0.24357	-0.24297	-0.24238	-0.2418	-0.29734	-0.29666	-0.29597
-0.12512	-0.24427	-0.24366	-0.24306	-0.24248	-0.24191	-0.29748	-0.29679	-0.2961
-0.12519	-0.12488	-0.12458	-0.24315	-0.24257	-0.242	-0.29761	-0.29692	-0.29623
-0.12526	-0.12495	-0.12464	-0.24324	-0.24265	-0.24208	-0.29774	-0.29706	-0.29637
-0.12533	-0.12502	-0.12471	-0.24335	-0.24275	-0.24217	-0.24161	-0.24107	-0.2965
-0.1254	-0.1251	-0.12479	-0.24348	-0.24288	-0.24228	-0.2417	-0.24115	-0.29661
-0.12547	-0.12517	-0.12487	-0.18405	-0.18359	-0.18312	-0.2418	-0.24123	-0.29673
-0.12553	-0.12522	-0.12493	-0.18414	-0.18368	-0.18323	-0.24191	-0.24133	-0.29685
-0.12559	-0.12528	-0.12498	-0.18422	-0.18378	-0.18333	-0.24201	-0.24143	-0.29698
-0.12567	-0.12536	-0.12505	-0.18431	-0.18386	-0.18342	-0.24209	-0.24152	-0.29712
-0.12575	-0.12544	-0.12513	-0.18441	-0.18395	-0.1835	-0.24217	-0.24161	-0.29726
-0.06245	-0.12551	-0.1252	-0.12489	-0.12459	-0.12429	-0.18315	-0.18271	-0.2974
-0.06249	-0.12557	-0.12526	-0.12496	-0.12467	-0.12437	-0.18327	-0.18282	-0.29753
-0.06253	-0.12563	-0.12533	-0.12503	-0.12474	-0.12445	-0.18337	-0.18292	-0.29767
-0.06257	-0.12572	-0.12541	-0.1251	-0.1248	-0.1245	-0.18346	-0.18302	-0.29783
-0.06262	-0.1258	-0.12548	-0.12517	-0.12487	-0.12456	-0.18355	-0.18312	-0.29798
-0.06265	-0.12586	-0.12555	-0.12524	-0.12494	-0.12464	-0.18366	-0.18322	-0.29811
-0.06269	-0.12592	-0.12562	-0.12531	-0.12502	-0.12472	-0.18377	-0.18332	-0.29824
-0.06273	-0.06257	-0.06242	-0.12538	-0.12508	-0.12478	-0.12449	-0.12419	-0.18296
-0.06278	-0.06262	-0.06246	-0.12545	-0.12514	-0.12484	-0.12454	-0.12425	-0.18307
-0.06282	-0.06266	-0.0625	-0.12552	-0.12521	-0.12491	-0.12461	-0.12433	-0.18318
-0.06285	-0.0627	-0.06254	-0.12559	-0.12529	-0.12499	-0.12469	-0.12441	-0.18329
-0.06289	-0.06273	-0.06258	-0.06242	-0.06226	-0.06211	-0.12477	-0.12448	-0.18339
-0.06294	-0.06278	-0.06262	-0.06247	-0.06231	-0.06215	-0.12483	-0.12453	-0.18347
-0.06298	-0.06283	-0.06267	-0.06251	-0.06235	-0.06219	-0.12489	-0.12459	-0.18356
-0.06303	-0.06287	-0.06271	-0.06255	-0.06239	-0.06223	-0.12496	-0.12466	-0.18367
-0.06307	-0.06291	-0.06274	-0.06258	-0.06243	-0.06227	-0.12504	-0.12474	-0.18378
-0.06311	-0.06295	-0.06279	-0.06263	-0.06247	-0.06231	-0.06216	-0.062	-0.12452
-0.06315	-0.06299	-0.06283	-0.06267	-0.06252	-0.06236	-0.0622	-0.06204	-0.12458
-0.06319	-0.06304	-0.06288	-0.06272	-0.06256	-0.0624	-0.06224	-0.06208	-0.12465
-0.06324	-0.06308	-0.06293	-0.06277	-0.06261	-0.06244	-0.06228	-0.06212	-0.12472

-0.06328	-0.06313	-0.06297	-0.06281	-0.06265	-0.06248	-0.06232	-0.06216	-0.06201
-0.06332	-0.06317	-0.06301	-0.06285	-0.06269	-0.06252	-0.06236	-0.06221	-0.06205
-0.06336	-0.06321	-0.06304	-0.06288	-0.06272	-0.06257	-0.06241	-0.06225	-0.0621
-0.0634	-0.06325	-0.06309	-0.06293	-0.06277	-0.06261	-0.06245	-0.0623	-0.06214
-0.06344	-0.06329	-0.06313	-0.06298	-0.06282	-0.06266	-0.0625	-0.06234	-0.06219
-0.06348	-0.06333	-0.06318	-0.06302	-0.06286	-0.06271	-0.06255	-0.06239	-0.06223
-0.06353	-0.06338	-0.06322	-0.06307	-0.06291	-0.06275	-0.06259	-0.06243	-0.06227
-0.06357	-0.06342	-0.06327	-0.06311	-0.06296	-0.0628	-0.06264	-0.06248	-0.06232
-0.06362	-0.06347	-0.06331	-0.06316	-0.063	-0.06285	-0.06268	-0.06252	-0.06236
-0.06366	-0.06351	-0.06336	-0.0632	-0.06305	-0.06289	-0.06273	-0.06256	-0.0624
-0.06371	-0.06355	-0.0634	-0.06324	-0.06308	-0.06292	-0.06276	-0.0626	-0.06244
-0.06375	-0.06359	-0.06344	-0.06328	-0.06312	-0.06296	-0.0628	-0.06264	-0.06248
	-0.06364	-0.06348	-0.06332	-0.06316	-0.063	-0.06284	-0.06268	-0.06253
	-0.06367	-0.06352	-0.06336	-0.0632	-0.06305	-0.06289	-0.06273	-0.06257
	-0.06371	-0.06356	-0.0634	-0.06325	-0.06309	-0.06294	-0.06278	-0.06262
	-0.06376	-0.0636	-0.06345	-0.06329	-0.06314	-0.06298	-0.06283	-0.06267
			-0.06349	-0.06334	-0.06318	-0.06303	-0.06287	-0.06271
			-0.06353	-0.06338	-0.06323	-0.06308	-0.06292	-0.06276
			-0.06358	-0.06343	-0.06327	-0.06312	-0.06296	-0.06281
			-0.06362	-0.06347	-0.06332	-0.06317	-0.06301	-0.06285
			-0.06367	-0.06351	-0.06336	-0.06321	-0.06305	-0.0629
			-0.06371	-0.06356	-0.06341	-0.06325	-0.0631	-0.06294
			-0.06376	-0.0636	-0.06345	-0.0633	-0.06315	-0.06299
						-0.06334	-0.06319	-0.06304
						-0.06339	-0.06324	-0.06308
						-0.06343	-0.06328	-0.06313
						-0.06348	-0.06333	-0.06317
						-0.06352	-0.06337	-0.06322
						-0.06357	-0.06342	-0.06326
						-0.06361	-0.06346	-0.06331
						-0.06366	-0.06351	-0.06336
						-0.06372	-0.06357	-0.06341
								-0.06347
								-0.06351
								-0.06355
								-0.0636
								-0.06364
								-0.06369
								-0.06373

							-0.12841
							-0.12829
							-0.25812
							-0.25791
							-0.25771
							-0.25746
							-0.25721
					-0.12825	-0.12792	-0.25695
					-0.12813	-0.1278	-0.2567
					-0.2579	-0.25718	-0.25647
					-0.2577	-0.25698	-0.25627
			-0.0652	-0.06505	-0.25749	-0.25677	-0.25606
			-0.06514	-0.06498	-0.25725	-0.25653	-0.25582
			-0.06508	-0.06492	-0.25699	-0.25628	-0.25557
			-0.06502	-0.06486	-0.25674	-0.25604	-0.35349
			-0.06495	-0.06479	-0.2565	-0.25581	-0.35324
			-0.12786	-0.12753	-0.25627	-0.25559	-0.35299
			-0.12775	-0.12742	-0.25606	-0.25536	-0.35273
-0.06528	-0.06511	-0.06495	-0.25728	-0.25655	-0.35425	-0.35336	-0.35248
-0.06521	-0.06505	-0.06489	-0.25702	-0.2563	-0.35398	-0.3531	-0.35224
-0.06515	-0.06499	-0.06483	-0.25678	-0.25606	-0.35372	-0.35285	-0.352
-0.0651	-0.06494	-0.06478	-0.25657	-0.25585	-0.35349	-0.35261	-0.35176

	-0.06505	-0.06489	-0.06473	-0.25637	-0.25565	-0.35326	-0.35238	-0.35151
	-0.065	-0.06484	-0.06468	-0.25617	-0.25546	-0.35304	-0.35216	-0.35129
	-0.06495	-0.06479	-0.06463	-0.25598	-0.25526	-0.35282	-0.35195	-0.35108
-0.06504	-0.18898	-0.18849	-0.18799	-0.35438	-0.35346	-0.35257	-0.3517	-0.35084
-0.06499	-0.18881	-0.18831	-0.18781	-0.3541	-0.35319	-0.3523	-0.35143	-0.35057
-0.06494	-0.18864	-0.18814	-0.18764	-0.35383	-0.35293	-0.35205	-0.35119	-0.35033
-0.06489	-0.1885	-0.18799	-0.18749	-0.35361	-0.35272	-0.35184	-0.35098	-0.35012
-0.06484	-0.18834	-0.18784	-0.18734	-0.35338	-0.3525	-0.35162	-0.35077	-0.34991
-0.12764	-0.25677	-0.25605	-0.25534	-0.35311	-0.35223	-0.35137	-0.35054	-0.3497
-0.12751	-0.25652	-0.2558	-0.25509	-0.35282	-0.35195	-0.35111	-0.3503	-0.34948
-0.1274	-0.2563	-0.25558	-0.25488	-0.35259	-0.35172	-0.35088	-0.35007	-0.40354
-0.1273	-0.25611	-0.25539	-0.25469	-0.35238	-0.35152	-0.35067	-0.34984	-0.40325
-0.18803	-0.35487	-0.35395	-0.35305	-0.35217	-0.35132	-0.35047	-0.34963	-0.40298
-0.18788	-0.35464	-0.35373	-0.35284	-0.35197	-0.35111	-0.35026	-0.34942	-0.40273
-0.18773	-0.35442	-0.35352	-0.35263	-0.35176	-0.35091	-0.35006	-0.34922	-0.46341
-0.18758	-0.35421	-0.35331	-0.35242	-0.35156	-0.35071	-0.34987	-0.34902	-0.46312
-0.18743	-0.35399	-0.35309	-0.35221	-0.35135	-0.35051	-0.34966	-0.34881	-0.46281
-0.18727	-0.35375	-0.35284	-0.35196	-0.3511	-0.35026	-0.34941	-0.34855	-0.46245
-0.18711	-0.3535	-0.35258	-0.35169	-0.35083	-0.34999	-0.34914	-0.34829	-0.46206
-0.35418	-0.35326	-0.35235	-0.35147	-0.35061	-0.34976	-0.34891	-0.34805	-0.46173
-0.35393	-0.35303	-0.35214	-0.35127	-0.35041	-0.34956	-0.3487	-0.34784	-0.46143
-0.35369	-0.35281	-0.35193	-0.35107	-0.35021	-0.34935	-0.34849	-0.34763	-0.46114
-0.35348	-0.3526	-0.35173	-0.35086	-0.35	-0.34914	-0.34828	-0.34742	-0.46085
-0.35328	-0.3524	-0.35153	-0.35066	-0.3498	-0.34894	-0.34807	-0.34721	-0.46056
-0.35308	-0.3522	-0.35133	-0.35046	-0.34959	-0.34873	-0.34786	-0.347	-0.46027
-0.35287	-0.352	-0.35113	-0.35026	-0.34939	-0.34852	-0.34766	-0.34679	-0.45997
-0.35267	-0.3518	-0.35093	-0.35005	-0.34918	-0.34831	-0.46213	-0.4609	-0.45969
-0.35246	-0.35159	-0.35072	-0.34984	-0.34897	-0.3481	-0.46184	-0.46061	-0.4594
-0.35226	-0.35139	-0.35051	-0.34964	-0.34877	-0.3479	-0.46155	-0.46032	-0.57026
-0.35205	-0.35118	-0.3503	-0.34943	-0.34856	-0.34769	-0.46126	-0.46004	-0.56991
-0.35184	-0.35097	-0.35009	-0.34922	-0.34836	-0.34749	-0.46097	-0.45975	-0.56955
-0.35163	-0.35076	-0.34988	-0.34901	-0.34815	-0.34729	-0.46069	-0.45947	-0.5692
-0.35142	-0.35054	-0.34967	-0.34881	-0.34794	-0.34708	-0.46041	-0.45919	-0.56885
-0.35121	-0.35033	-0.34946	-0.3486	-0.34774	-0.34688	-0.46013	-0.45892	-0.5685
-0.351	-0.35012	-0.34925	-0.34839	-0.34754	-0.34668	-0.45985	-0.45865	-0.56816
-0.35079	-0.34991	-0.34905	-0.34819	-0.34733	-0.34648	-0.45957	-0.45838	-0.56781
-0.35058	-0.3497	-0.34884	-0.34798	-0.34713	-0.34628	-0.45929	-0.4581	-0.56746
-0.35037	-0.3495	-0.34864	-0.34778	-0.34693	-0.34608	-0.45902	-0.45783	-0.56711
-0.35016	-0.34929	-0.34843	-0.34758	-0.34673	-0.34588	-0.45874	-0.45755	-0.56676
-0.34995	-0.34908	-0.34823	-0.34738	-0.34653	-0.34569	-0.51407	-0.51273	-0.56641
-0.34974	-0.34887	-0.34802	-0.34718	-0.34633	-0.34549	-0.51375	-0.51241	-0.56606
-0.34955	-0.34867	-0.34782	-0.34698	-0.34614	-0.34529	-0.51343	-0.51209	-0.5657
-0.34937	-0.34849	-0.34763	-0.34678	-0.34593	-0.3451	-0.51311	-0.51176	-0.56535
-0.34921	-0.34832	-0.34744	-0.34658	-0.34574	-0.3449	-0.51279	-0.51144	-0.56499
-0.34903	-0.40237	-0.40133	-0.4003	-0.45945	-0.45826	-0.51246	-0.51112	-0.67356
-0.34886	-0.40218	-0.40114	-0.40011	-0.45919	-0.45799	-0.51213	-0.51079	-0.67312
-0.34867	-0.40198	-0.40095	-0.39992	-0.45896	-0.45775	-0.51184	-0.51048	-0.6727
-0.34847	-0.40176	-0.40075	-0.39974	-0.45876	-0.45755	-0.51159	-0.5102	-0.67231
-0.34827	-0.40154	-0.40054	-0.39954	-0.51409	-0.51273	-0.51134	-0.50993	-0.67193
-0.34807	-0.40131	-0.40032	-0.39932	-0.51379	-0.51243	-0.51106	-0.50966	-0.67157

-0.34788	-0.40108	-0.40009	-0.39909	-0.51346	-0.51212	-0.51076	-0.50939	-0.6712
-0.34768	-0.40085	-0.39985	-0.39885	-0.51314	-0.5118	-0.51045	-0.5091	-0.72562
-0.34749	-0.40061	-0.39962	-0.39862	-0.51281	-0.51147	-0.51013	-0.5088	-0.72517
-0.34733	-0.40041	-0.39941	-0.3984	-0.5125	-0.51115	-0.50982	-0.50849	-0.72471
-0.34718	-0.40024	-0.39922	-0.3982	-0.51219	-0.51083	-0.50949	-0.50817	-0.72422
-0.34702	-0.40005	-0.39903	-0.398	-0.51191	-0.51054	-0.5092	-0.50788	-0.72377
-0.34681	-0.39983	-0.39882	-0.39782	-0.51167	-0.51031	-0.50896	-0.50761	-0.72336
-0.34661	-0.39961	-0.39862	-0.39762	-0.51142	-0.51007	-0.62057	-0.61885	-0.72297
-0.34645	-0.39941	-0.3984	-0.3974	-0.51111	-0.50978	-0.62021	-0.61852	-0.72258
-0.3463	-0.39921	-0.39819	-0.39718	-0.5108	-0.50948	-0.61985	-0.61819	-0.72218
-0.34613	-0.45575	-0.45457	-0.45341	-0.51054	-0.50921	-0.61949	-0.61783	-0.72175
-0.34594	-0.45553	-0.45438	-0.45323	-0.51031	-0.50895	-0.61915	-0.61746	-0.72131
-0.34575	-0.45529	-0.45415	-0.45302	-0.51006	-0.50871	-0.72487	-0.72287	-0.82584
-0.34556	-0.45501	-0.45389	-0.45277	-0.5098	-0.50847	-0.72448	-0.72251	-0.82542
-0.34538	-0.45476	-0.45364	-0.45252	-0.50952	-0.50821	-0.72408	-0.72212	-0.82497
-0.34524	-0.45457	-0.45343	-0.45229	-0.50923	-0.50791	-0.72362	-0.72166	-0.82445
-0.3451	-0.45437	-0.45322	-0.45207	-0.50894	-0.50761	-0.72317	-0.7212	-0.82393
-0.34494	-0.45414	-0.453	-0.45186	-0.6205	-0.6188	-0.72278	-0.7208	-0.96769
-0.34477	-0.4539	-0.45278	-0.45165	-0.62019	-0.61851	-0.72241	-0.72043	-0.96704
-0.34462	-0.4537	-0.45257	-0.45144	-0.61985	-0.61817	-0.72201	-0.72004	-0.96642
-0.34449	-0.45352	-0.45237	-0.45121	-0.61949	-0.6178	-0.72158	-0.71965	-0.96583
-0.34435	-0.45332	-0.45216	-0.45101	-0.61917	-0.61747	-0.72119	-0.71928	-0.96525
-0.34419	-0.45309	-0.45196	-0.45082	-0.61888	-0.61719	-0.72084	-0.71894	-0.96469
-0.34404	-0.51285	-0.51147	-0.51009	-0.61859	-0.61691	-0.87865	-0.876	-0.96409
-0.34391	-0.51262	-0.51124	-0.50986	-0.61829	-0.61659	-0.87809	-0.87541	-0.96343
-0.34378	-0.51238	-0.511	-0.50962	-0.61798	-0.61627	-0.87751	-0.87482	-0.96277
-0.34364	-0.51213	-0.51073	-0.50934	-0.61764	-0.61595	-0.87697	-0.87429	-0.96217
-0.3435	-0.51186	-0.51045	-0.50905	-0.61729	-0.61563	-0.87644	-0.87379	-0.96159
-0.40071	-0.62203	-0.62033	-0.61865	-0.72248	-0.72054	-0.91993	-0.91713	-0.961
-0.40052	-0.62172	-0.62004	-0.61837	-0.72214	-0.72019	-0.91938	-0.91657	-0.9604
-0.40035	-0.62142	-0.61975	-0.61809	-0.72179	-0.71983	-0.91882	-0.91601	-0.95981
-0.40019	-0.62115	-0.61947	-0.6178	-0.72142	-0.71946	-0.91825	-0.91544	-0.95922
-0.461	-0.62088	-0.61919	-0.6175	-0.87922	-0.87651	-0.91768	-0.91488	-0.95864
-0.46081	-0.6206	-0.61889	-0.61719	-0.87868	-0.87598	-0.91713	-0.91435	-0.95809
-0.46061	-0.62031	-0.61859	-0.61688	-0.87815	-0.87545	-0.91658	-0.91382	-0.95754
-0.46039	-0.61999	-0.61827	-0.61656	-0.87761	-0.87494	-0.91604	-0.91327	-0.95694
-0.46016	-0.61965	-0.61794	-0.61624	-0.87709	-0.87445	-0.9155	-0.9127	-0.95633
-0.45992	-0.61932	-0.61763	-0.61595	-0.87659	-0.87396	-0.91498	-0.91216	-0.95576
-0.45968	-0.619	-0.61733	-0.61568	-0.87612	-0.87348	-0.91447	-0.91166	-0.95524
-0.45945	-0.61869	-0.61704	-0.6154	-0.87563	-0.87298	-0.91394	-0.91114	-0.95471
-0.45924	-0.6184	-0.61675	-0.61511	-0.87512	-0.87246	-0.91337	-0.91059	-0.95414
-0.45904	-0.61812	-0.61647	-0.61482	-0.87459	-0.87192	-0.91281	-0.91004	-0.95359
-0.45883	-0.72775	-0.72546	-0.72319	-0.91784	-0.91503	-0.91227	-0.90954	-0.95309
-0.45863	-0.72734	-0.72503	-0.72273	-0.91726	-0.91448	-0.91175	-0.90907	-0.9526
-0.51603	-0.72694	-0.72462	-0.72231	-0.91673	-0.91395	-0.91124	-0.90858	-0.95209
-0.51581	-0.72656	-0.72424	-0.72193	-0.91623	-0.91345	-0.91074	-0.90809	-0.95157
-0.4612	-0.88023	-0.87749	-0.87477	-0.91573	-0.91295	-0.9566	-0.95383	-0.99932
-0.46101	-0.87976	-0.87702	-0.8743	-0.91523	-0.91245	-0.95608	-0.95332	-0.99878
-0.46081	-0.87928	-0.87655	-0.87383	-0.91473	-0.91195	-0.95557	-0.95281	-1.03942
-0.46061	-0.87881	-0.87608	-0.87337	-0.91423	-0.91146	-0.95505	-0.95231	-1.03885

-0.46041	-0.87834	-0.87562	-0.87291	-0.91374	-0.91097	-0.95454	-0.95181	-1.0383
-0.57609	-0.92177	-0.91891	-0.91607	-0.91326	-0.9105	-0.95406	-0.95134	-1.03776
-0.57576	-0.92127	-0.91841	-0.91558	-0.91279	-0.91005	-0.95358	-0.95087	-1.03723
-0.57545	-0.92077	-0.91792	-0.91509	-0.91231	-0.90958	-0.95309	-0.9504	-1.03669
-0.57513	-0.92029	-0.91744	-0.9146	-0.91183	-0.9091	-0.9526	-0.94991	-1.03614
-0.57482	-0.91979	-0.91694	-0.91411	-0.91134	-0.90862	-0.9521	-0.94943	-1.03559
-0.57449	-0.91928	-0.91643	-0.91361	-0.91085	-0.90814	-0.95161	-0.94895	-1.03504
-0.62367	-0.91877	-0.91593	-0.91311	-0.91036	-0.90766	-0.95111	-0.94847	-1.0345
-0.6233	-0.91826	-0.91542	-0.91261	-0.90987	-0.90719	-0.95062	-0.94799	-1.03396
-0.62294	-0.91776	-0.91492	-0.91211	-0.90938	-0.90672	-0.95014	-0.94752	-1.03342
-0.6226	-0.91726	-0.91444	-0.91165	-0.90892	-0.90626	-0.99773	-0.99491	-1.03288
-0.62226	-0.91677	-0.91397	-0.9112	-0.90848	-0.90582	-0.99722	-0.99441	-1.03235
-0.82872	-0.91629	-0.91352	-0.91078	-0.95424	-0.95144	-0.99673	-0.99391	-1.03182
-0.82827	-0.91582	-0.91309	-0.91039	-0.95384	-0.95104	-0.99626	-0.99341	-1.0313
-0.82785	-0.91537	-0.91266	-0.90998	-0.95342	-0.95062	-0.99579	-0.99291	-1.07392
-0.82746	-0.91493	-0.9122	-0.90951	-0.95294	-0.95017	-0.99529	-0.99239	-1.07335
-0.82707	-0.9145	-0.91174	-0.90904	-0.95247	-0.94973	-0.99481	-0.9919	-1.0728
-0.82664	-0.91404	-0.91128	-0.90857	-0.95201	-0.94932	-0.99439	-0.99146	-1.0723
-0.8262	-0.91358	-0.91082	-0.90812	-0.95156	-0.94892	-0.99397	-0.99104	-1.07181
-0.82578	-0.91316	-0.91041	-0.90772	-0.99926	-0.99639	-0.99349	-0.99056	-1.07129
-0.82537	-0.91276	-0.91005	-0.90737	-0.9988	-0.99588	-0.99297	-0.99006	-1.07075
-0.82496	-0.91233	-0.90965	-0.907	-0.99835	-0.9954	-0.99248	-0.98959	-1.07025
-0.82454	-0.91187	-0.90922	-0.90661	-0.99791	-0.99496	-0.99204	-0.98916	-1.0698
-0.82414	-0.91142	-0.9088	-0.9062	-0.99745	-0.9945	-0.99159	-0.98871	-1.06933
-0.82379	-0.91103	-0.90837	-0.90575	-0.99694	-0.99401	-0.9911	-0.98822	-1.06881
-0.82345	-0.91065	-0.90796	-0.90531	-0.99644	-0.99353	-0.99062	-0.98772	-1.06829
-0.82307	-0.95965	-0.95674	-0.95387	-0.99599	-0.99308	-0.99017	-0.98728	-1.02804
-0.82268	-0.95924	-0.95635	-0.95348	-0.99558	-0.99266	-0.98975	-0.98686	-1.02758
-0.86959	-0.95878	-0.95592	-0.95309	-0.99516	-0.99224	-0.98933	-0.98644	-1.02714
-0.86914	-0.95827	-0.95547	-0.95269	-0.99476	-0.99183	-0.98892	-0.98602	-1.02671
-0.86874	-0.9578	-0.95501	-0.95226	-0.99432	-0.99141	-1.03227	-1.02927	-1.02629
-0.86838	-0.95737	-0.95455	-0.95176	-0.99384	-0.99098	-1.03185	-1.02884	-1.02587
-0.86802	-0.95695	-0.95409	-0.95128	-0.99335	-0.99054	-1.03141	-1.02841	-1.02545
-0.86764	-0.95652	-0.95367	-0.95086	-0.99288	-0.99005	-1.03093	-1.02797	-1.02504
-0.86726	-0.9561	-0.95326	-0.95045	-0.99243	-0.98956	-1.03043	-1.02752	-1.02463
-0.917	-0.9557	-0.95286	-0.95005	-0.99199	-0.98912	-1.02994	-1.02704	-1.14079
-0.91661	-0.95531	-0.95246	-0.94965	-0.99158	-0.9887	-1.02948	-1.02654	-1.14025
-0.8668	-0.95492	-0.95207	-0.94926	-0.99117	-0.98829	-1.02905	-1.0261	-1.13977
-0.86642	-0.95452	-0.95168	-0.94888	-0.99077	-0.9879	-1.02865	-1.02573	-1.13937
-0.86605	-0.95413	-0.9513	-0.94851	-0.99038	-0.98753	-1.02826	-1.02534	-1.13894
-0.86568	-0.95374	-0.95092	-0.94814	-0.99002	-0.98718	-1.02789	-1.02495	-1.13849
-0.86531	-0.95335	-0.95055	-0.94779	-0.98965	-0.98683	-1.02751	-1.02455	-1.13804
-0.86494	-0.95298	-0.9502	-0.94745	-0.98929	-0.98645	-1.02709	-1.02413	-1.1376
-0.86458	-0.95262	-0.94986	-0.94712	-0.98892	-0.98605	-1.02666	-1.02371	-1.13715
-0.86426	-0.95227	-0.94951	-0.94676	-0.98852	-0.98564	-1.10427	-1.10116	-1.18042
-0.86396	-0.95193	-0.94914	-0.94638	-0.9881	-0.98522	-1.10382	-1.10072	-1.17995
-0.86363	-0.949	-0.94621	-0.94343	-1.03132	-1.02831	-1.10339	-1.1003	-1.17949
-0.86327	-0.94861	-0.94581	-0.94303	-1.03087	-1.02787	-1.10295	-1.09989	-1.17905
-0.8629	-0.94821	-0.94541	-0.94263	-1.03044	-1.02744	-1.14121	-1.13808	-1.17863
-0.86254	-0.94782	-0.94502	-0.94224	-1.03001	-1.02703	-1.14078	-1.13769	-1.17825

-0.86219	-0.94745	-0.94464	-0.94186	-1.02959	-1.02662	-1.14035	-1.1373	-1.17786
-0.86188	-0.9471	-0.94431	-0.94154	-1.02922	-1.02624	-1.13993	-1.13688	-1.17743
-0.86158	-0.94678	-0.944	-0.94124	-1.02887	-1.02587	-1.1395	-1.13645	-1.17697
-0.86125	-0.94641	-0.94364	-0.94088	-1.10674	-1.10356	-1.18279	-1.17962	-1.17652
-0.86089	-0.94602	-0.94325	-0.9405	-1.10631	-1.10313	-1.18232	-1.17916	-1.17608
-0.86056	-0.94567	-0.9429	-0.94015	-1.10591	-1.10275	-1.18189	-1.17874	-1.17567
-0.86027	-0.94536	-0.94259	-0.93985	-1.10557	-1.10242	-1.18152	-1.17836	-1.17528
-0.85999	-0.94505	-0.94228	-0.93954	-1.06074	-1.05772	-1.18115	-1.17799	-1.1749
-0.85972	-0.94476	-0.94198	-0.93922	-1.06039	-1.05739	-1.18077	-1.17763	-1.17454
-0.85943	-0.94445	-0.94165	-0.93889	-1.06003	-1.05704	-1.18039	-1.17725	-1.17417
-0.8591	-1.06888	-1.06575	-1.06266	-1.14355	-1.14036	-1.17995	-1.17682	-1.17374
-0.85874	-1.06845	-1.06531	-1.06222	-1.14307	-1.1399	-1.1795	-1.17637	-1.1733
-0.85843	-1.06806	-1.06492	-1.06183	-1.14264	-1.13949	-1.17908	-1.17597	-1.1729
-0.85815	-1.06771	-1.06458	-1.06149	-1.14226	-1.13911	-1.17871	-1.17561	-1.17255
-0.85787	-1.06736	-1.06423	-1.06115	-1.14188	-1.13874	-1.17834	-1.17525	-1.21119
-0.8576	-1.06702	-1.06389	-1.0608	-1.14149	-1.13836	-1.17797	-1.1749	-1.21085
-0.85732	-1.06668	-1.06354	-1.06045	-1.14111	-1.13799	-1.1776	-1.17455	-1.21052
-0.85704	-1.06634	-1.0632	-1.06011	-1.14073	-1.13762	-1.17723	-1.1742	-1.21017
-0.85676	-1.066	-1.06286	-1.05977	-1.14035	-1.13725	-1.17687	-1.17384	-1.20983
-0.986	-1.14984	-1.14647	-1.14316	-1.13998	-1.13689	-1.1765	-1.17348	-1.20949
-0.98568	-1.14944	-1.14609	-1.14279	-1.13961	-1.13653	-1.17614	-1.17313	-1.20914
-0.98536	-1.14905	-1.14571	-1.14242	-1.13925	-1.13617	-1.13319	-1.13028	-1.2088
-0.98504	-1.14866	-1.14533	-1.14205	-1.13889	-1.13582	-1.13284	-1.12994	-1.20845
-0.98472	-1.14828	-1.14495	-1.14169	-1.13854	-1.13548	-1.1325	-1.1296	-1.2081
-0.9844	-1.14789	-1.14457	-1.14131	-1.13818	-1.13514	-1.13216	-1.12926	-1.20775
-0.98407	-1.14749	-1.14418	-1.14094	-1.13783	-1.1348	-1.13183	-1.12891	-1.2074
-0.98374	-1.14708	-1.1438	-1.14058	-1.13747	-1.13445	-1.13148	-1.12857	-1.20705
-0.98343	-1.14668	-1.14341	-1.14021	-1.13712	-1.1341	-1.13114	-1.12823	-1.20671
-0.98316	-1.14634	-1.14306	-1.13985	-1.13676	-1.13375	-1.1308	-1.1279	-1.20637
-0.98294	-1.14604	-1.14273	-1.13949	-1.13641	-1.13341	-1.13047	-1.12757	-1.20604
-1.11154	-1.14571	-1.1424	-1.13917	-1.13609	-1.13311	-1.17014	-1.16715	-1.24272
-1.11116	-1.14535	-1.14207	-1.13888	-1.13582	-1.13284	-1.16989	-1.1669	-1.24246
-1.11078	-1.14498	-1.14174	-1.13858	-1.13553	-1.13257	-1.16962	-1.16664	-1.24216
-1.1104	-1.1446	-1.14137	-1.13824	-1.1352	-1.13225	-1.1693	-1.16631	-1.2418
-1.11003	-1.14423	-1.14102	-1.13789	-1.13487	-1.13193	-1.16899	-1.16598	-1.24143
-1.10972	-1.14392	-1.1407	-1.13758	-1.13458	-1.13165	-1.16872	-1.1657	-1.2411
-1.10942	-1.14364	-1.14041	-1.13728	-1.1343	-1.13139	-1.16847	-1.16544	-1.2408
-1.10909	-1.1433	-1.14009	-1.13696	-1.13398	-1.13108	-1.16817	-1.16515	-1.24047
-1.10871	-1.14292	-1.13973	-1.13662	-1.13364	-1.13074	-1.16783	-1.16483	-1.24014
-1.10837	-1.14259	-1.1394	-1.1363	-1.13332	-1.13041	-1.16752	-1.16453	-1.23982
-1.10809	-1.14231	-1.13913	-1.13603	-1.13304	-1.13013	-1.16725	-1.16427	-1.23955
-1.10782	-1.14204	-1.13886	-1.13577	-1.13277	-1.12984	-1.16697	-1.16401	-1.23925
-1.10754	-1.14177	-1.13859	-1.13549	-1.13248	-1.12953	-1.16665	-1.16368	-1.2389
-1.10725	-1.1415	-1.13832	-1.13521	-1.13219	-1.12922	-1.16633	-1.16335	-1.23853
-1.10691	-1.14116	-1.13801	-1.13492	-1.1319	-1.12895	-1.16606	-1.16307	-1.23823
-1.10654	-1.14079	-1.13767	-1.13461	-1.13163	-1.12869	-1.16582	-1.16283	-1.23796
-1.10622	-1.14048	-1.13737	-1.13433	-1.17161	-1.16859	-1.16558	-1.16259	-1.23769
-1.10594	-1.14021	-1.13711	-1.13408	-1.17137	-1.16834	-1.16534	-1.16236	-1.23743
-1.10567	-1.13995	-1.13685	-1.13382	-1.17112	-1.1681	-1.24353	-1.24035	-1.23717
-1.1054	-1.13968	-1.13659	-1.13356	-1.17087	-1.16786	-1.24326	-1.24008	-1.2369

-1.10512	-1.13941	-1.13633	-1.1333	-1.17063	-1.16762	-1.24299	-1.2398	-1.23662
-1.10485	-1.13914	-1.13608	-1.13307	-1.17039	-1.16738	-1.24271	-1.23952	-1.23634
-1.10458	-1.13888	-1.13583	-1.13284	-1.17016	-1.16713	-1.24243	-1.23924	-1.23606
-1.10432	-1.13862	-1.13558	-1.1326	-1.16993	-1.1669	-1.24217	-1.23897	-1.23578
-1.10406	-1.13837	-1.13533	-1.13235	-1.16969	-1.16668	-1.24192	-1.23871	-1.23549
-1.10379	-1.13812	-1.13509	-1.13211	-1.16946	-1.16645	-1.24166	-1.23845	-1.23524
-1.10352	-1.13785	-1.13484	-1.13188	-1.16923	-1.1662	-1.2414	-1.23821	-1.23502
-1.10325	-1.1376	-1.13461	-1.13165	-1.169	-1.16595	-1.24113	-1.23796	-1.23479
-1.10301	-1.13736	-1.13436	-1.1314	-1.16875	-1.16573	-1.24087	-1.23768	-1.23453
-1.10279	-1.13714	-1.13412	-1.13115	-1.16851	-1.1655	-1.24062	-1.23741	-1.23425
-1.10261	-1.13696	-1.13395	-1.13097	-1.1683	-1.16525	-1.24034	-1.23715	-1.234
-1.10245	-1.13681	-1.13381	-1.13083	-1.16811	-1.16501	-1.24005	-1.23689	-1.23375
-1.10224	-1.1366	-1.13362	-1.13065	-1.16793	-1.16481	-1.23982	-1.23663	-1.23348
-1.102	-1.13636	-1.13339	-1.13044	-1.16776	-1.16466	-1.23962	-1.23637	-1.23319
-1.10176	-1.13613	-1.13316	-1.13021	-1.20949	-1.20631	-1.23942	-1.23614	-1.27112
-1.10154	-1.13592	-1.13292	-1.12995	-1.20924	-1.20608	-1.23921	-1.23595	-1.27093
-1.10134	-1.13573	-1.13271	-1.12972	-1.20899	-1.20584	-1.23898	-1.23577	-1.27074
-1.10116	-1.13555	-1.13254	-1.12955	-1.20878	-1.2056	-1.23873	-1.23555	-1.27054
-1.10098	-1.13538	-1.13239	-1.12941	-1.2086	-1.20536	-1.23847	-1.23532	-1.27033
-1.10078	-1.13516	-1.13218	-1.12922	-1.2084	-1.20517	-1.23827	-1.23512	-1.27013
-1.10057	-1.13492	-1.13195	-1.12901	-1.20822	-1.20501	-1.2381	-1.23494	-1.26995
-1.10037	-1.13471	-1.13176	-1.12882	-1.20804	-1.20485	-1.23792	-1.23474	-1.26973
-1.10019	-1.13455	-1.13159	-1.12864	-1.20787	-1.20469	-1.23773	-1.2345	-1.26947
-1.10002	-1.13439	-1.13141	-1.12845	-1.20767	-1.20451	-1.23753	-1.23428	-1.26924
-1.09986	-1.13423	-1.13121	-1.12822	-1.20744	-1.20429	-1.23732	-1.23409	-1.26906
-1.09969	-1.13406	-1.13102	-1.128	-1.2072	-1.20407	-1.23711	-1.23391	-1.26889
-1.09948	-1.17673	-1.17357	-1.17044	-1.20702	-1.2039	-1.23693	-1.23375	-1.26871
-1.09927	-1.17652	-1.17338	-1.17027	-1.20685	-1.20372	-1.23675	-1.23358	-1.26853
-1.09908	-1.17633	-1.1732	-1.17009	-1.20666	-1.2035	-1.23652	-1.23337	-1.26832
-1.09893	-1.17616	-1.17304	-1.16992	-1.20645	-1.20325	-1.23625	-1.23312	-1.2681
-1.09877	-1.21321	-1.21	-1.20678	-1.24255	-1.23923	-1.23603	-1.23291	-1.26791
-1.09862	-1.21304	-1.20982	-1.20661	-1.24238	-1.23907	-1.23586	-1.23274	-1.26773
-1.09848	-1.21287	-1.20965	-1.20644	-1.24222	-1.23891	-1.23571	-1.23259	-1.26757
-1.09833	-1.21271	-1.20949	-1.20627	-1.24206	-1.23875	-1.23555	-1.23243	-1.26742
-1.09819	-1.21254	-1.20932	-1.2061	-1.2419	-1.23859	-1.23539	-1.23228	-1.26727
-1.14162	-1.21237	-1.20916	-1.20594	-1.24175	-1.23844	-1.23524	-1.23213	-1.26711
-1.14145	-1.2122	-1.20899	-1.20579	-1.2416	-1.23829	-1.23509	-1.23198	-1.26695
-1.14129	-1.21203	-1.20883	-1.20562	-1.24145	-1.23814	-1.23494	-1.23183	-1.2668
-1.14114	-1.21187	-1.20866	-1.20545	-1.24128	-1.23798	-1.23479	-1.23169	-1.26665
-1.09572	-1.2117	-1.2085	-1.20529	-1.24113	-1.23783	-1.23464	-1.23154	-1.2665
-1.09555	-1.21153	-1.20835	-1.20516	-1.241	-1.23769	-1.2345	-1.23139	-1.26634
-1.09539	-1.21136	-1.20819	-1.20502	-1.24088	-1.23758	-1.23438	-1.23125	-1.26619
-1.09524	-1.21119	-1.20803	-1.20487	-1.24078	-1.23752	-1.23432	-1.23118	-1.26609
-1.09509	-1.21102	-1.20786	-1.20473	-1.24068	-1.23747	-1.23428	-1.23112	-1.266
-1.095	-1.2109	-1.20772	-1.20457	-1.24054	-1.23734	-1.23416	-1.231	-1.26588
-1.09493	-1.2108	-1.2076	-1.20442	-1.24037	-1.23718	-1.23401	-1.23087	-1.26575
-1.13646	-1.25008	-1.24676	-1.24346	-1.24022	-1.23702	-1.23386	-1.23073	-1.26562
-1.13632	-1.24994	-1.24662	-1.24332	-1.24008	-1.23687	-1.23371	-1.23059	-1.26549
-1.1362	-1.24981	-1.2465	-1.24321	-1.23996	-1.23674	-1.23358	-1.23045	-1.26536
-1.13609	-1.24968	-1.2464	-1.24314	-1.23989	-1.23666	-1.23348	-1.23033	-1.26521

-1.13599	-1.24957	-1.24632	-1.24308	-1.23983	-1.23659	-1.23339	-1.23022	-1.26509
-1.13591	-1.24951	-1.24625	-1.24301	-1.23975	-1.23649	-1.23329	-1.23014	-1.26501
-1.13582	-1.24944	-1.24619	-1.24293	-1.23966	-1.23639	-1.2332	-1.23007	-1.26496
-1.13571	-1.24933	-1.24608	-1.24283	-1.23956	-1.23631	-1.23313	-1.23001	-1.26489
-1.13557	-1.24918	-1.24594	-1.2427	-1.23946	-1.23623	-1.23307	-1.22995	-1.26481
-1.13548	-1.24908	-1.24584	-1.24261	-1.23938	-1.23616	-1.233	-1.22987	-1.26472
-1.13542	-1.24902	-1.24578	-1.24255	-1.23932	-1.2361	-1.23292	-1.22977	-1.26461
-1.09368	-1.12916	-1.12624	-1.12333	-1.20426	-1.20113	-1.23283	-1.22967	-1.26451
-1.09362	-1.12908	-1.12616	-1.12324	-1.20416	-1.20102	-1.23274	-1.2296	-1.26445
-1.09356	-1.12901	-1.12608	-1.12315	-1.20405	-1.2009	-1.23264	-1.22953	-1.2644
-1.0935	-1.12893	-1.12598	-1.12304	-1.20395	-1.20081	-1.23256	-1.22946	-1.26432
-1.09344	-1.12885	-1.12587	-1.12292	-1.20385	-1.20074	-1.2325	-1.22939	-1.26423
-1.09335	-1.12874	-1.12576	-1.12282	-1.20377	-1.20067	-1.23242	-1.2293	-1.26413
-1.09323	-1.1286	-1.12565	-1.12273	-1.2037	-1.2006	-1.23235	-1.22921	-1.26403
-1.09313	-1.1285	-1.12556	-1.12265	-1.20363	-1.20052	-1.23226	-1.22912	-1.26395
-1.09306	-1.12844	-1.12551	-1.12259	-1.20353	-1.20038	-1.23215	-1.22906	-1.26391
-1.093	-1.12839	-1.12546	-1.12253	-1.20346	-1.20029	-1.23207	-1.229	-1.26386
-1.09296	-1.12832	-1.1254	-1.12249	-1.20346	-1.20031	-1.23207	-1.22896	-1.2638
-1.09292	-1.12825	-1.12534	-1.12245	-1.20347	-1.20035	-1.2321	-1.22894	-1.26374
-1.09287	-1.12819	-1.12528	-1.12238	-1.16012	-1.15715	-1.23209	-1.22894	-1.26374
-1.0928	-1.12815	-1.12522	-1.12231	-1.16004	-1.15707	-1.23206	-1.22896	-1.26376
-1.09275	-1.12809	-1.12515	-1.12224	-1.15998	-1.15701	-1.23201	-1.22891	-1.26371
-1.09272	-1.12801	-1.12509	-1.1222	-1.15995	-1.15697	-1.23194	-1.2288	-1.26359
-1.09271	-1.12797	-1.12504	-1.12214	-1.1599	-1.15693	-1.23189	-1.22873	-1.26353
-1.09275	-1.128	-1.12501	-1.12206	-1.15982	-1.15688	-1.23189	-1.22876	-1.26355
-1.09277	-1.12803	-1.12499	-1.122	-1.15976	-1.15683	-1.23188	-1.22879	-1.26359
-1.09273	-1.12801	-1.12501	-1.12202	-1.15975	-1.15677	-1.23183	-1.22877	-1.30092
-1.09267	-1.12797	-1.12502	-1.12207	-1.15977	-1.15672	-1.23177	-1.22873	-1.3009
-1.09263	-1.1279	-1.12497	-1.12205	-1.15978	-1.15673	-1.18937	-1.18641	-1.30085
-1.09259	-1.12782	-1.12489	-1.12199	-1.15978	-1.15677	-1.1894	-1.18637	-1.30078
-1.09259	-1.12779	-1.12484	-1.12193	-1.15974	-1.15677	-1.18941	-1.18636	-1.30076
-1.09264	-1.12784	-1.12482	-1.12185	-1.15966	-1.15672	-1.1894	-1.1864	-1.30079
-1.09265	-1.12786	-1.12481	-1.1218	-1.15961	-1.15667	-1.18939	-1.18642	-1.30083
-1.09259	-1.12783	-1.12482	-1.12184	-1.15962	-1.15663	-1.18935	-1.18641	-1.30084
-1.09253	-1.12778	-1.12483	-1.12188	-1.15965	-1.15661	-1.18932	-1.1864	-1.30085
-1.0925	-1.12773	-1.1248	-1.12188	-1.15966	-1.15663	-1.22819	-1.22517	-1.30088
-1.09249	-1.12768	-1.12476	-1.12186	-1.15967	-1.15667	-1.22823	-1.2252	-1.30091
-1.0925	-1.12767	-1.12475	-1.12186	-1.15971	-1.15672	-1.22827	-1.2252	-1.30089
-1.09254	-1.1277	-1.12478	-1.12189	-1.15976	-1.15678	-1.22831	-1.22518	-1.30085
-1.09257	-1.12773	-1.12479	-1.12189	-1.15977	-1.1568	-1.22833	-1.22519	-1.26
-1.0926	-1.12776	-1.12478	-1.12184	-1.15973	-1.15678	-1.22833	-1.22521	-1.26004
-1.09263	-1.12779	-1.12479	-1.12182	-1.19938	-1.19636	-1.22835	-1.22525	-1.26008
-1.09267	-1.12784	-1.12483	-1.12186	-1.19942	-1.1964	-1.2284	-1.2253	-1.26011
-1.09272	-1.12788	-1.12488	-1.12192	-1.19948	-1.19645	-1.22847	-1.22536	-1.26015
-1.09276	-1.12793	-1.12493	-1.12197	-1.19954	-1.19651	-1.22852	-1.22541	-1.26019
-1.0928	-1.12798	-1.12498	-1.12202	-1.19959	-1.19656	-1.22858	-1.22547	-1.26025
-1.0928	-1.128	-1.12502	-1.12207	-1.23858	-1.23544	-1.26673	-1.26351	-1.2603
-1.09277	-1.12798	-1.12504	-1.12212	-1.23865	-1.2355	-1.2668	-1.26357	-1.26036
-1.01794	-1.12797	-1.12506	-1.12216	-1.23871	-1.23556	-1.26686	-1.26363	-1.26042
-1.01793	-1.12799	-1.12509	-1.12221	-1.23876	-1.23561	-1.26692	-1.26369	-1.26047

-1.01793	-1.16872	-1.1657	-1.16271	-1.23881	-1.23566	-1.26697	-1.26374	-1.26052
-1.01794	-1.16874	-1.16574	-1.16275	-1.23885	-1.23571	-1.26703	-1.2638	-1.26058
-1.01797	-1.16878	-1.16577	-1.16279	-1.2389	-1.23576	-1.26709	-1.26386	-1.26064
-1.01803	-1.09669	-1.09372	-1.09077	-1.23895	-1.23581	-1.26714	-1.26391	-1.2607
-1.01813	-1.09676	-1.09375	-1.09078	-1.23899	-1.23586	-1.26721	-1.26398	-1.26076
-1.01818	-1.05816	-1.05522	-1.05232	-1.0495	-1.04673	-1.19405	-1.19102	-1.26083
-1.0182	-1.05817	-1.05523	-1.05233	-1.04951	-1.04674	-1.1941	-1.19109	-1.2609
-1.01822	-1.05819	-1.05525	-1.05235	-1.04953	-1.04675	-1.12348	-1.12052	-1.26098
-1.01824	-1.05821	-1.05528	-1.05237	-1.04954	-1.04676	-1.12348	-1.12053	-1.26107
-1.01826	-1.05824	-1.05531	-1.05242	-1.04958	-1.04678	-1.1235	-1.12055	-1.26115
-1.01829	-1.05826	-1.05536	-1.0525	-1.04967	-1.04686	-1.12357	-1.1206	-1.26123
-1.01832	-1.05827	-1.05542	-1.0526	-1.04978	-1.04697	-1.12366	-1.12066	-1.2613
-1.01835	-1.0583	-1.05545	-1.05264	-1.04982	-1.04702	-1.12372	-1.12075	-1.19321
-1.01838	-1.05832	-1.05546	-1.05263	-1.04982	-1.04703	-1.12377	-1.12085	-1.19334
-1.01843	-1.05838	-1.0555	-1.05267	-1.04985	-1.04706	-1.12382	-1.12091	-1.19343
-1.01851	-1.05847	-1.0556	-1.05276	-1.04992	-1.0471	-1.12384	-1.12092	-1.19345
-1.0186	-1.05857	-1.05569	-1.05284	-1.04999	-1.04715	-1.12388	-1.12095	-1.19348
-1.0187	-1.05866	-1.05576	-1.05289	-1.05004	-1.04722	-1.12397	-1.12103	-1.19356
-1.01881	-1.05875	-1.05583	-1.05294	-1.0501	-1.0473	-1.12407	-1.12112	-1.19365
-1.01888	-1.05881	-1.0559	-1.05302	-1.05019	-1.04738	-1.12415	-1.12118	-1.19373
-1.01894	-1.05886	-1.05597	-1.05311	-1.05028	-1.04747	-1.12422	-1.12124	-1.1938
-0.97368	-1.05894	-1.05606	-1.0532	-1.05036	-1.04754	-1.08287	-1.08	-1.19389
-0.97376	-1.05904	-1.05616	-1.05329	-1.05043	-1.04758	-1.08291	-1.08007	-1.19401
-0.97385	-1.05914	-1.05624	-1.05337	-1.05049	-1.04764	-1.08297	-1.08014	-1.19411
-0.97395	-1.05923	-1.05631	-1.05342	-1.05055	-1.04771	-1.08305	-1.08023	-1.1942
-0.97404	-1.05932	-1.05638	-1.05348	-1.05062	-1.04779	-1.08314	-1.0803	-1.19429
-0.9741	-1.05938	-1.05646	-1.05357	-1.05072	-1.0479	-1.08322	-1.08035	-1.19435
-0.97416	-1.05945	-1.05655	-1.05369	-1.05084	-1.04801	-1.08331	-1.0804	-1.19442
-1.0179	-1.05954	-1.05665	-1.05379	-1.0927	-1.08976	-1.12424	-1.12123	-1.19457
-1.01799	-1.05966	-1.05676	-1.05389	-1.09281	-1.08986	-1.12436	-1.12139	-1.19477
-1.01809	-1.05977	-1.05687	-1.05399	-1.0929	-1.08995	-1.12446	-1.1215	-1.1949
-1.01821	-1.05987	-1.05697	-1.05409	-1.093	-1.09005	-1.12453	-1.12154	-1.19497
-1.01832	-1.05998	-1.05707	-1.05419	-1.09309	-1.09012	-1.12459	-1.1216	-1.15515
-1.01841	-1.0601	-1.05719	-1.0543	-1.09317	-1.09016	-1.12463	-1.12168	-1.15526
-1.0185	-1.06022	-1.05731	-1.05441	-1.09326	-1.09023	-1.1247	-1.12178	-1.15538
-1.01861	-1.01582	-1.01308	-1.01034	-1.09341	-1.09039	-1.12485	-1.12188	-1.15547
-1.01873	-1.01591	-1.01317	-1.01046	-1.09357	-1.09059	-1.12503	-1.122	-1.15557
-1.01889	-1.01606	-1.01329	-1.01055	-1.09369	-1.09073	-1.12519	-1.12216	-1.1192
-1.01908	-1.01625	-1.01342	-1.01062	-1.09377	-1.09083	-1.12533	-1.12236	-1.1194
-0.93075	-1.01642	-1.01357	-1.01075	-1.05208	-1.04924	-1.08457	-1.08175	-1.11957
-0.93085	-1.01655	-1.01374	-1.01094	-1.05225	-1.04934	-1.08466	-1.08185	-1.1197
-0.93093	-1.01666	-1.0139	-1.01112	-1.05242	-1.04946	-1.08476	-1.08195	-1.11981
-0.93101	-1.01675	-1.014	-1.01125	-1.05258	-1.04965	-1.08492	-1.08205	-1.1199
-0.93109	-1.01684	-1.01409	-1.01135	-1.05272	-1.04984	-1.0851	-1.08217	-1.11999
-0.93118	-1.01696	-1.01419	-1.01144	-1.00871	-1.006	-1.08525	-1.08233	-1.12015
-0.93129	-1.0171	-1.0143	-1.01152	-1.00879	-1.0061	-1.08538	-1.08252	-1.12035
-0.93143	-1.01725	-1.01445	-1.01166	-1.00891	-1.00619	-1.08549	-1.08265	-1.1205
-0.9316	-1.01743	-1.01465	-1.01186	-1.00906	-1.00627	-1.08556	-1.08275	-1.12063
-0.93173	-1.01758	-1.01481	-1.01203	-1.00921	-1.0064	-1.08568	-1.08285	-1.12075
-0.93182	-1.01767	-1.01491	-1.01216	-1.00937	-1.00659	-1.08586	-1.08297	-1.12085

-0.93192	-1.01777	-1.01501	-1.01227	-1.00952	-1.00677	-1.04292	-1.04012	-1.12097
-0.93207	-1.01794	-1.01514	-1.01238	-1.00963	-1.00692	-1.04308	-1.04028	-1.12115
-0.93224	-1.01814	-1.0153	-1.01249	-1.00975	-1.00705	-1.04324	-1.04046	-1.12135
-0.93241	-1.01834	-1.01548	-1.01266	-1.00992	-1.00723	-1.04343	-1.04065	-1.12155
-0.93257	-1.01854	-1.01569	-1.01287	-1.01013	-1.00743	-1.04363	-1.04085	-1.12174
-0.93271	-0.93013	-0.92753	-0.92495	-1.01032	-1.00762	-1.00492	-1.00224	-1.12195
-0.9328	-0.93024	-0.92766	-0.9251	-1.0105	-1.0078	-1.00511	-1.00244	-1.12217
-0.93292	-0.93038	-0.92781	-0.92526	-1.01068	-1.00796	-1.00527	-1.00261	-1.12237
-0.93308	-0.93053	-0.92797	-0.92542	-1.01084	-1.00808	-1.00539	-1.00274	-1.12253
-0.93325	-0.9307	-0.92814	-0.92559	-1.011	-1.00822	-1.00551	-1.00287	-1.1227
-0.93342	-0.93087	-0.92831	-0.92576	-1.01119	-1.0084	-1.00569	-1.00305	-1.08069
-0.93359	-0.93104	-0.92849	-0.92594	-1.0114	-1.00861	-1.00589	-1.00324	-1.08089
-0.93377	-0.93121	-0.92866	-0.92611	-1.0116	-1.00881	-1.00609	-1.00343	-1.04309
-0.93394	-0.93138	-0.92883	-0.92628	-1.01179	-1.009	-1.00629	-1.00363	-1.0433
-0.93411	-0.93155	-0.929	-0.92645	-1.01199	-1.0092	-1.00648	-1.00383	-1.0435
-0.93428	-0.93172	-0.92917	-0.92661	-1.01218	-1.00939	-1.00668	-1.00402	-1.04369
-0.93445	-0.93189	-0.92933	-0.92678	-1.01237	-1.00958	-1.00686	-1.0042	-1.04389
-0.93462	-0.93206	-0.9295	-0.92694	-1.01254	-1.00976	-1.00704	-1.00438	-1.04408
-0.93478	-0.93222	-0.92966	-0.9271	-1.01272	-1.00994	-1.00722	-1.00456	-1.04426
-0.93495	-0.93239	-0.92983	-0.92728	-1.01292	-1.01013	-1.00741	-1.00474	-1.04444
-0.93511	-0.93255	-0.93	-0.92746	-1.01312	-1.01034	-1.00761	-1.00492	-1.04463
-0.93527	-0.93271	-0.93017	-0.92763	-0.92508	-0.92253	-1.00785	-1.00515	-1.04485
-0.93543	-0.93287	-0.93033	-0.9278	-0.92528	-0.92278	-1.00812	-1.00542	-1.04512
-0.93563	-0.93306	-0.9305	-0.92797	-0.92547	-0.92299	-1.00836	-1.00566	-1.04536
-0.93588	-0.93329	-0.93069	-0.92813	-0.92562	-0.92314	-1.00853	-1.00584	-1.04557
-0.93612	-0.93351	-0.93089	-0.92831	-0.92579	-0.9233	-1.00872	-1.00602	-1.04578
-0.9363	-0.9337	-0.93111	-0.92855	-0.92603	-0.92353	-1.00895	-1.00623	-1.04597
-0.93646	-0.93388	-0.93133	-0.9288	-0.92628	-0.92377	-1.00919	-1.00644	-1.04617
-0.93664	-0.93404	-0.9315	-0.92898	-0.92647	-0.92396	-1.00941	-1.00667	-1.04643
-0.93684	-0.93421	-0.93166	-0.92913	-0.92662	-0.92413	-1.00962	-1.00692	-1.04671
-0.93706	-0.93442	-0.93186	-0.92933	-0.9268	-0.92429	-1.00982	-1.00713	-1.04693
-0.9373	-0.93468	-0.93211	-0.92957	-0.92701	-0.92446	-1.01	-1.00729	-1.0471
-0.93752	-0.93492	-0.93235	-0.92979	-0.92722	-0.92465	-0.92213	-0.91965	-1.04731
-0.9377	-0.93511	-0.93254	-0.92998	-0.92743	-0.92489	-0.92238	-0.91989	-1.04758
-0.9379	-0.93531	-0.93273	-0.93018	-0.92765	-0.92515	-0.92264	-0.92015	-1.04785
-0.93814	-0.93553	-0.93296	-0.93042	-0.9279	-0.92539	-0.92287	-0.92035	-1.0481
-0.9384	-0.93578	-0.93321	-0.93068	-0.92815	-0.92563	-0.92309	-0.92055	-1.04834
-0.93867	-0.93604	-0.93347	-0.93093	-0.92838	-0.92584	-0.92331	-0.92078	-1.04863
-0.93893	-0.93632	-0.93374	-0.93118	-0.9286	-0.92603	-0.92352	-0.92105	-1.04894
-0.93917	-0.93656	-0.93397	-0.9314	-0.92882	-0.92626	-0.92376	-0.9213	-1.04924
-0.93936	-0.93675	-0.93416	-0.93159	-0.92905	-0.92653	-0.92403	-0.92154	-1.04952
-0.93956	-0.93696	-0.93437	-0.93181	-0.92929	-0.9268	-0.92429	-0.92179	-0.96351
-0.93981	-0.93719	-0.93462	-0.93208	-0.92955	-0.92703	-0.92453	-0.92204	-0.96379
-0.94008	-0.93745	-0.9349	-0.93237	-0.92982	-0.92727	-0.92477	-0.9223	-0.96406
-0.94037	-0.93773	-0.93516	-0.93261	-0.93007	-0.92755	-0.92504	-0.92256	-0.96431
-0.94066	-0.93804	-0.93542	-0.93285	-0.93033	-0.92783	-0.92533	-0.92282	-0.96455
-0.94094	-0.93831	-0.93569	-0.93312	-0.93059	-0.92809	-0.92558	-0.92308	-0.96483
-0.94121	-0.93855	-0.93596	-0.93341	-0.93086	-0.92832	-0.92582	-0.92336	-0.96515
-0.94148	-0.93881	-0.93623	-0.93368	-0.93112	-0.92857	-0.92607	-0.92362	-0.96543
-0.94176	-0.9391	-0.93649	-0.93392	-0.93137	-0.92885	-0.92634	-0.92385	-0.96567

-0.89776	-0.93938	-0.93675	-0.93416	-0.93163	-0.92912	-0.92661	-0.92409	-0.96592
-0.898	-0.93963	-0.93702	-0.93444	-0.93189	-0.92936	-0.92685	-0.92436	-0.96619
-0.89825	-0.93987	-0.93728	-0.93472	-0.93215	-0.9296	-0.92709	-0.92462	-0.96647
-0.89851	-0.94014	-0.93753	-0.93496	-0.9324	-0.92986	-0.92735	-0.92487	-0.92239
-0.89878	-0.94043	-0.93779	-0.93519	-0.93265	-0.93014	-0.92762	-0.9251	-0.92261
-0.89908	-0.94075	-0.93809	-0.93547	-0.93292	-0.9304	-0.92787	-0.92535	-0.92286
-0.89942	-0.9411	-0.93844	-0.93582	-0.93322	-0.93065	-0.92812	-0.92563	-0.92313
-0.89973	-0.94141	-0.93876	-0.93615	-0.93353	-0.93093	-0.9284	-0.92592	-0.92343
-0.89998	-0.94166	-0.93903	-0.93644	-0.93383	-0.93125	-0.92873	-0.92625	-0.92376
-0.90022	-0.9419	-0.93929	-0.93672	-0.93413	-0.93157	-0.92905	-0.92657	-0.92408
-0.81079	-0.89786	-0.89534	-0.89286	-0.9344	-0.93186	-0.92933	-0.92682	-0.92433
-0.81101	-0.89814	-0.89559	-0.89309	-0.93466	-0.93214	-0.92961	-0.92707	-0.92457
-0.81128	-0.89845	-0.89588	-0.89337	-0.93496	-0.93246	-0.92993	-0.92738	-0.92486
-0.81158	-0.89879	-0.89621	-0.89369	-0.93529	-0.9328	-0.93027	-0.92773	-0.92519
-0.81184	-0.89908	-0.8965	-0.89398	-0.93559	-0.93309	-0.93057	-0.92804	-0.9255
-0.81206	-0.89934	-0.89676	-0.89425	-0.93586	-0.93333	-0.93081	-0.92831	-0.92579
-0.66968	-0.80984	-0.8075	-0.80522	-0.93615	-0.9336	-0.93108	-0.92859	-0.92609
-0.66989	-0.81014	-0.80779	-0.80549	-0.93648	-0.93392	-0.9314	-0.92891	-0.92641
-0.67012	-0.81045	-0.80808	-0.80576	-0.9368	-0.93425	-0.93172	-0.92922	-0.92672
-0.67035	-0.81076	-0.80833	-0.80597	-0.93708	-0.93454	-0.932	-0.92948	-0.92698
-0.67058	-0.81106	-0.80858	-0.80618	-0.93735	-0.93482	-0.93227	-0.92974	-0.92722
-0.67078	-0.81134	-0.80885	-0.80643	-0.80416	-0.80197	-0.93258	-0.93004	-0.92752
-0.67094	-0.8116	-0.80913	-0.80673	-0.80445	-0.80224	-0.93291	-0.93038	-0.92786
-0.67114	-0.81187	-0.80942	-0.80703	-0.80474	-0.80251	-0.93325	-0.93071	-0.92819
-0.67136	-0.81217	-0.80971	-0.80731	-0.80502	-0.80279	-0.93359	-0.93104	-0.92852
-0.67159	-0.66971	-0.66781	-0.66595	-0.8053	-0.80306	-0.84654	-0.8442	-0.92885
-0.67182	-0.66993	-0.66803	-0.66616	-0.80558	-0.80334	-0.84682	-0.84449	-0.92918
-0.67205	-0.67016	-0.66824	-0.66637	-0.80587	-0.80361	-0.84711	-0.84477	-0.92952
-0.67227	-0.67038	-0.66846	-0.66658	-0.80615	-0.80389	-0.8474	-0.84506	-0.92986
-0.6725	-0.67061	-0.66868	-0.66679	-0.80644	-0.80417	-0.84769	-0.84535	-0.9302
-0.67273	-0.67083	-0.6689	-0.66701	-0.80673	-0.80445	-0.84799	-0.84565	-0.93054
-0.67295	-0.67106	-0.66912	-0.66722	-0.80702	-0.80474	-0.84829	-0.84594	-0.93088
-0.67318	-0.67128	-0.66934	-0.66744	-0.66563	-0.66387	-0.80275	-0.80051	-0.84393
-0.6734	-0.6715	-0.66956	-0.66765	-0.66584	-0.66407	-0.80303	-0.80079	-0.84422
-0.67362	-0.67172	-0.66977	-0.66786	-0.66604	-0.66427	-0.8033	-0.80107	-0.84452
-0.67384	-0.67194	-0.66999	-0.66808	-0.66625	-0.66447	-0.80358	-0.80134	-0.8448
-0.67406	-0.67215	-0.67021	-0.66829	-0.66646	-0.66467	-0.80385	-0.80161	-0.84509
-0.67428	-0.67237	-0.67043	-0.66851	-0.66667	-0.66488	-0.80412	-0.80188	-0.79967
-0.6745	-0.67259	-0.67065	-0.66873	-0.66688	-0.66508	-0.8044	-0.80216	-0.79995
-0.62455	-0.67283	-0.6709	-0.66898	-0.66713	-0.66531	-0.80471	-0.80247	-0.80026
-0.6248	-0.67309	-0.67118	-0.66928	-0.6674	-0.66555	-0.80504	-0.80282	-0.80062
-0.62504	-0.67334	-0.67144	-0.66956	-0.66765	-0.66578	-0.664	-0.6623	-0.80095
-0.62524	-0.67357	-0.67167	-0.66978	-0.66787	-0.66598	-0.6642	-0.6625	-0.80122
-0.62544	-0.67378	-0.67189	-0.67	-0.66807	-0.66617	-0.66439	-0.66269	-0.80148
-0.62564	-0.674	-0.67211	-0.67021	-0.66828	-0.66637	-0.66458	-0.66287	-0.80175
-0.62586	-0.67423	-0.67233	-0.67043	-0.6685	-0.66658	-0.66478	-0.66306	-0.80202
-0.6261	-0.6745	-0.67259	-0.67068	-0.66874	-0.66683	-0.66502	-0.66328	-0.80231
-0.62637	-0.67481	-0.67287	-0.67094	-0.66902	-0.66712	-0.6653	-0.66353	-0.80263
-0.62662	-0.67508	-0.67313	-0.67118	-0.66927	-0.66739	-0.66556	-0.66379	-0.76072
-0.62682	-0.6753	-0.67335	-0.67141	-0.66949	-0.66761	-0.6658	-0.66405	-0.76106

-0.62703	-0.67553	-0.67359	-0.67166	-0.66973	-0.66784	-0.66604	-0.6643	-0.76137
-0.62727	-0.67578	-0.67386	-0.67195	-0.67002	-0.6681	-0.66628	-0.66452	-0.76164
-0.62753	-0.67605	-0.67415	-0.67225	-0.67031	-0.66838	-0.66653	-0.66474	-0.76189
-0.6278	-0.67635	-0.67443	-0.67252	-0.67059	-0.66867	-0.66681	-0.66499	-0.66322
-0.62807	-0.67666	-0.67472	-0.67279	-0.67087	-0.66898	-0.6671	-0.66525	-0.66347
-0.51785	-0.62655	-0.62477	-0.623	-0.67116	-0.66926	-0.66738	-0.66551	-0.66372
-0.518	-0.62675	-0.62502	-0.62329	-0.67147	-0.66954	-0.66765	-0.66579	-0.66399
-0.51816	-0.62696	-0.62525	-0.62354	-0.67175	-0.66982	-0.66793	-0.66606	-0.66425
-0.51836	-0.62718	-0.62546	-0.62374	-0.67201	-0.67012	-0.66823	-0.66633	-0.6645
-0.51856	-0.62742	-0.62567	-0.62394	-0.67225	-0.67041	-0.66852	-0.66659	-0.66476
-0.51877	-0.6277	-0.62592	-0.62416	-0.67248	-0.67063	-0.66876	-0.66687	-0.66505
-0.51898	-0.62798	-0.62618	-0.6244	-0.67271	-0.67085	-0.66899	-0.66715	-0.66534
-0.51918	-0.62823	-0.62644	-0.62467	-0.67299	-0.6711	-0.66923	-0.66739	-0.66559
-0.51938	-0.62847	-0.6267	-0.62495	-0.67328	-0.67137	-0.66949	-0.66762	-0.66581
-0.51958	-0.62872	-0.62696	-0.62521	-0.67357	-0.67166	-0.66977	-0.6679	-0.66608
-0.51979	-0.629	-0.62721	-0.62544	-0.67384	-0.67195	-0.67008	-0.66823	-0.66641
-0.51999	-0.5186	-0.51722	-0.51585	-0.67412	-0.67225	-0.67039	-0.66855	-0.66671
-0.5202	-0.5188	-0.51742	-0.51606	-0.67442	-0.67257	-0.67069	-0.66882	-0.66696
-0.52041	-0.519	-0.51763	-0.51627	-0.67473	-0.67287	-0.67099	-0.66908	-0.66721
-0.52061	-0.51922	-0.51786	-0.51651	-0.67503	-0.67314	-0.67125	-0.66935	-0.66749
-0.52082	-0.51944	-0.51809	-0.51675	-0.67533	-0.6734	-0.67151	-0.66963	-0.66777
-0.46311	-0.51967	-0.51832	-0.51696	-0.62531	-0.62352	-0.67178	-0.6699	-0.66803
-0.46332	-0.51992	-0.51853	-0.51715	-0.62554	-0.62378	-0.67207	-0.67017	-0.66829
-0.46353	-0.52014	-0.51874	-0.51734	-0.62579	-0.62403	-0.67234	-0.67044	-0.66856
-0.46372	-0.52033	-0.51894	-0.51755	-0.62604	-0.62426	-0.67259	-0.67071	-0.66886
-0.4639	-0.52054	-0.51915	-0.51777	-0.51637	-0.51499	-0.67286	-0.671	-0.66916
-0.46408	-0.52075	-0.51938	-0.51801	-0.51662	-0.51522	-0.67318	-0.67132	-0.66947
-0.46426	-0.52098	-0.51962	-0.51826	-0.51687	-0.51547	-0.6735	-0.67164	-0.66977
-0.46447	-0.52122	-0.51985	-0.51847	-0.51708	-0.51569	-0.67381	-0.67191	-0.67003
-0.46468	-0.52147	-0.52006	-0.51866	-0.51728	-0.5159	-0.6741	-0.67218	-0.67029
-0.46487	-0.52168	-0.52027	-0.51887	-0.51749	-0.51613	-0.67442	-0.67248	-0.67059
-0.46505	-0.52187	-0.52046	-0.51908	-0.51772	-0.51637	-0.67475	-0.67282	-0.67093
-0.46524	-0.52208	-0.52068	-0.5193	-0.51794	-0.5166	-0.61888	-0.61721	-0.67125
-0.46546	-0.52233	-0.52093	-0.51954	-0.51816	-0.51678	-0.61908	-0.61743	-0.67154
-0.46568	-0.52258	-0.52118	-0.51979	-0.51838	-0.51698	-0.61931	-0.61766	-0.67183
-0.46586	-0.52281	-0.52142	-0.52004	-0.51863	-0.51722	-0.61958	-0.61793	-0.67215
-0.46604	-0.52303	-0.52165	-0.52028	-0.51887	-0.51747	-0.61988	-0.61822	-0.67247
-0.46623	-0.52325	-0.52186	-0.52048	-0.51908	-0.51769	-0.51631	-0.51495	-0.67279
-0.46644	-0.52348	-0.52206	-0.52066	-0.51927	-0.5179	-0.51654	-0.51519	-0.67312
-0.46665	-0.46541	-0.46417	-0.46294	-0.51948	-0.51812	-0.51677	-0.51543	-0.67345
-0.46685	-0.46563	-0.46438	-0.46315	-0.51972	-0.51836	-0.51701	-0.51567	-0.67378
-0.46706	-0.46584	-0.4646	-0.46336	-0.51997	-0.5186	-0.51725	-0.51592	-0.61798
-0.46728	-0.46606	-0.46482	-0.46358	-0.52021	-0.51884	-0.51749	-0.51616	-0.61826
-0.46749	-0.46627	-0.46503	-0.4638	-0.52046	-0.51909	-0.51774	-0.5164	-0.61854
-0.35063	-0.46649	-0.46525	-0.46401	-0.52071	-0.51934	-0.51798	-0.51665	-0.61882
-0.35078	-0.4667	-0.46546	-0.46423	-0.52096	-0.51958	-0.51823	-0.51689	-0.61911
-0.35094	-0.46691	-0.46567	-0.46444	-0.5212	-0.51983	-0.51848	-0.51714	-0.6194
-0.35109	-0.46711	-0.46587	-0.46465	-0.52145	-0.52007	-0.51872	-0.51739	-0.61969
-0.35124	-0.46732	-0.46608	-0.46485	-0.52169	-0.52031	-0.51896	-0.51763	-0.56884
-0.3514	-0.46753	-0.46629	-0.46506	-0.52192	-0.52055	-0.51919	-0.51786	-0.5691

-0.35155	-0.35068	-0.3498	-0.34892	-0.52216	-0.52078	-0.51943	-0.51809	-0.56936
-0.35171	-0.35083	-0.34995	-0.34907	-0.5224	-0.52102	-0.51967	-0.51833	-0.56963
-0.35186	-0.35098	-0.3501	-0.34922	-0.52264	-0.52126	-0.51991	-0.51857	-0.5699
-0.35202	-0.35114	-0.35025	-0.34937	-0.52288	-0.52151	-0.52015	-0.51881	-0.57017
-0.35218	-0.35129	-0.3504	-0.34952	-0.52312	-0.52175	-0.52039	-0.51905	-0.57045
-0.35234	-0.35145	-0.35055	-0.34967	-0.46508	-0.46388	-0.52063	-0.51929	-0.51793
-0.3525	-0.35161	-0.3507	-0.34982	-0.46528	-0.46409	-0.52087	-0.51953	-0.51818
-0.35266	-0.35177	-0.35087	-0.34998	-0.4655	-0.4643	-0.52111	-0.51977	-0.51842
-0.35283	-0.35193	-0.35105	-0.35017	-0.46575	-0.46452	-0.52136	-0.51999	-0.51865
-0.353	-0.3521	-0.35122	-0.35035	-0.34948	-0.34861	-0.52162	-0.52024	-0.51888
-0.35316	-0.35226	-0.35138	-0.35051	-0.34965	-0.34879	-0.52191	-0.52051	-0.51913
-0.35333	-0.35243	-0.35154	-0.35066	-0.34981	-0.34897	-0.52221	-0.52079	-0.5194
-0.29435	-0.3526	-0.3517	-0.35081	-0.34996	-0.34913	-0.52247	-0.52105	-0.51965
-0.29451	-0.35278	-0.35186	-0.35097	-0.35011	-0.34928	-0.52271	-0.52129	-0.51989
-0.29466	-0.35298	-0.35205	-0.35113	-0.35027	-0.34943	-0.52296	-0.52156	-0.52016
-0.29481	-0.35319	-0.35225	-0.35132	-0.35043	-0.34958	-0.52322	-0.52185	-0.52047
-0.29494	-0.35338	-0.35244	-0.35151	-0.35061	-0.34974	-0.40966	-0.40866	-0.52075
-0.29506	-0.35355	-0.35263	-0.3517	-0.3508	-0.3499	-0.40985	-0.40884	-0.52098
-0.29516	-0.3537	-0.3528	-0.3519	-0.35098	-0.35008	-0.41004	-0.40901	-0.52121
-0.29528	-0.29458	-0.29386	-0.29314	-0.35116	-0.35026	-0.41027	-0.40922	-0.52146
-0.2954	-0.29469	-0.29398	-0.29327	-0.35134	-0.35045	-0.4105	-0.40943	-0.52173
-0.29553	-0.29482	-0.2941	-0.29339	-0.3515	-0.35061	-0.34974	-0.34888	-0.40861
-0.29566	-0.29495	-0.29423	-0.29351	-0.35165	-0.35076	-0.3499	-0.34905	-0.40883
-0.2958	-0.29509	-0.29437	-0.29365	-0.35182	-0.35093	-0.35006	-0.34922	-0.40904
-0.29594	-0.29525	-0.29454	-0.29382	-0.35203	-0.35111	-0.35022	-0.34937	-0.40923
-0.29606	-0.29539	-0.29469	-0.29398	-0.35223	-0.3513	-0.3504	-0.34953	-0.40943
-0.29618	-0.2955	-0.29481	-0.29411	-0.35241	-0.35149	-0.3506	-0.34973	-0.40966
-0.2963	-0.29561	-0.29492	-0.29422	-0.35258	-0.35168	-0.3508	-0.34994	-0.4099
-0.29644	-0.29575	-0.29505	-0.29435	-0.29363	-0.29291	-0.35097	-0.3501	-0.4101
-0.29659	-0.2959	-0.29519	-0.29447	-0.29375	-0.29303	-0.35111	-0.35025	-0.41028
-0.29672	-0.29603	-0.29532	-0.29461	-0.29389	-0.29318	-0.3513	-0.35041	-0.41048
-0.29684	-0.29614	-0.29545	-0.29476	-0.29406	-0.29335	-0.35151	-0.3506	-0.41068
-0.29697	-0.29627	-0.29559	-0.2949	-0.2942	-0.2935	-0.35171	-0.35078	-0.41089
-0.29713	-0.29643	-0.29573	-0.29503	-0.29433	-0.29362	-0.35187	-0.35097	-0.41113
-0.29728	-0.29658	-0.29588	-0.29517	-0.29446	-0.29374	-0.29303	-0.29233	-0.35029
-0.2974	-0.2967	-0.296	-0.2953	-0.2946	-0.2939	-0.2932	-0.29249	-0.3505
-0.29752	-0.29682	-0.29613	-0.29544	-0.29475	-0.29406	-0.29336	-0.29266	-0.35071
-0.18251	-0.29697	-0.29628	-0.2956	-0.2949	-0.2942	-0.29349	-0.29279	-0.35088
-0.18263	-0.29713	-0.29644	-0.29576	-0.29505	-0.29433	-0.29361	-0.2929	-0.35103
-0.18273	-0.29727	-0.29658	-0.29589	-0.29518	-0.29446	-0.29375	-0.29304	-0.29233
-0.18284	-0.2974	-0.29669	-0.29599	-0.29529	-0.29459	-0.29389	-0.29319	-0.2925
-0.18294	-0.24011	-0.23951	-0.23891	-0.29541	-0.29473	-0.29404	-0.29335	-0.29266
-0.18303	-0.24025	-0.23966	-0.23907	-0.29557	-0.29489	-0.29419	-0.29349	-0.29279
-0.18312	-0.2404	-0.23981	-0.23923	-0.29573	-0.29504	-0.29434	-0.29363	-0.29292
-0.18323	-0.24056	-0.23996	-0.23936	-0.29586	-0.29516	-0.29446	-0.29376	-0.29306
-0.18335	-0.24071	-0.2401	-0.2395	-0.29598	-0.29527	-0.29458	-0.29389	-0.29321
-0.12423	-0.183	-0.18255	-0.1821	-0.23904	-0.23845	-0.29472	-0.29404	-0.29336
-0.12429	-0.18308	-0.18264	-0.1822	-0.23919	-0.2386	-0.29488	-0.2942	-0.29352
-0.12435	-0.18317	-0.18274	-0.1823	-0.23934	-0.23876	-0.29504	-0.29436	-0.29367
-0.12443	-0.18329	-0.18285	-0.18242	-0.2395	-0.23892	-0.2952	-0.29451	-0.29382

-0.06186	-0.1834	-0.18296	-0.18253	-0.23966	-0.23908	-0.2385	-0.23792	-0.29396
-0.06191	-0.18351	-0.18308	-0.18264	-0.23982	-0.23924	-0.23865	-0.23806	-0.29409
-0.06195	-0.18363	-0.18319	-0.18275	-0.23998	-0.23939	-0.2388	-0.23819	-0.29421
-0.06199	-0.12445	-0.12415	-0.12386	-0.18241	-0.18197	-0.23892	-0.23832	-0.29435
-0.06204	-0.12452	-0.12422	-0.12393	-0.18249	-0.18204	-0.23904	-0.23846	-0.29449
-0.06208	-0.06193	-0.06177	-0.06162	-0.18258	-0.18214	-0.23918	-0.2386	-0.23803
-0.06212	-0.06196	-0.06181	-0.06166	-0.18269	-0.18225	-0.23934	-0.23876	-0.23818
-0.06216	-0.062	-0.06185	-0.0617	-0.1828	-0.18236	-0.18192	-0.18149	-0.23834
-0.0622	-0.06204	-0.06189	-0.06174	-0.18291	-0.18247	-0.18204	-0.1816	-0.23849
-0.06224	-0.06208	-0.06193	-0.06178	-0.122	-0.1217	-0.18215	-0.18171	-0.23865
-0.06228	-0.06212	-0.06197	-0.06183	-0.12208	-0.12178	-0.18226	-0.18182	-0.2388
-0.06232	-0.06217	-0.06202	-0.06187	-0.12216	-0.12186	-0.18237	-0.18193	-0.23896
-0.06237	-0.06221	-0.06206	-0.06191	-0.06177	-0.06162	-0.18248	-0.18205	-0.18161
-0.06241	-0.06226	-0.06211	-0.06196	-0.06181	-0.06166	-0.18259	-0.18216	-0.18172
-0.06246	-0.0623	-0.06215	-0.062	-0.06185	-0.06171	-0.1218	-0.1215	-0.18183
-0.0625	-0.06235	-0.06219	-0.06204	-0.06189	-0.06175	-0.12188	-0.12158	-0.18194
-0.06255	-0.06239	-0.06224	-0.06209	-0.06194	-0.06179	-0.12196	-0.12165	-0.12135
-0.0626	-0.06244	-0.06228	-0.06213	-0.06198	-0.06183	-0.12203	-0.12173	-0.12143
-0.06264	-0.06249	-0.06233	-0.06217	-0.06202	-0.06188	-0.12211	-0.12181	-0.12151
-0.06269	-0.06253	-0.06237	-0.06222	-0.06207	-0.06192	-0.06177	-0.06163	-0.12159
-0.06274	-0.06258	-0.06242	-0.06226	-0.06211	-0.06196	-0.06182	-0.06167	-0.12167
-0.06279	-0.06263	-0.06247	-0.06231	-0.06216	-0.06201	-0.06186	-0.06171	-0.12175
-0.06283	-0.06267	-0.06251	-0.06235	-0.0622	-0.06205	-0.0619	-0.06176	-0.12183
-0.06288	-0.06272	-0.06256	-0.0624	-0.06225	-0.06209	-0.06195	-0.0618	-0.06165
-0.06293	-0.06277	-0.06261	-0.06245	-0.06229	-0.06214	-0.06199	-0.06184	-0.0617
-0.06297	-0.06281	-0.06265	-0.06249	-0.06234	-0.06218	-0.06203	-0.06189	-0.06174
-0.06302	-0.06286	-0.0627	-0.06254	-0.06238	-0.06223	-0.06208	-0.06193	-0.06178
-0.06306	-0.06291	-0.06275	-0.06259	-0.06243	-0.06227	-0.06212	-0.06197	-0.06182
-0.06311	-0.06295	-0.06279	-0.06264	-0.06248	-0.06232	-0.06217	-0.06202	-0.06187
-0.06316	-0.063	-0.06284	-0.06268	-0.06252	-0.06236	-0.06221	-0.06206	-0.06191
-0.06321	-0.06305	-0.06289	-0.06273	-0.06257	-0.06241	-0.06225	-0.0621	-0.06195
-0.06326	-0.0631	-0.06294	-0.06278	-0.06262	-0.06245	-0.0623	-0.06215	-0.062
-0.06331	-0.06315	-0.06299	-0.06283	-0.06267	-0.06251	-0.06235	-0.0622	-0.06204
-0.06336	-0.0632	-0.06305	-0.06289	-0.06273	-0.06257	-0.06241	-0.06225	-0.0621
-0.06341	-0.06326	-0.0631	-0.06294	-0.06279	-0.06262	-0.06247	-0.06231	-0.06215
-0.06345	-0.0633	-0.06315	-0.06299	-0.06283	-0.06267	-0.06251	-0.06235	-0.0622
-0.06349	-0.06334	-0.06319	-0.06304	-0.06288	-0.06272	-0.06256	-0.0624	-0.06224
-0.06354	-0.06339	-0.06324	-0.06308	-0.06292	-0.06276	-0.0626	-0.06244	-0.06229
-0.06358	-0.06343	-0.06328	-0.06313	-0.06297	-0.06281	-0.06265	-0.06249	-0.06233
	-0.06347	-0.06332	-0.06317	-0.06302	-0.06286	-0.0627	-0.06254	-0.06238
	-0.06352	-0.06337	-0.06322	-0.06306	-0.0629	-0.06275	-0.06259	-0.06244
	-0.06357	-0.06342	-0.06327	-0.06311	-0.06295	-0.0628	-0.06264	-0.06249
	-0.06362	-0.06347	-0.06332	-0.06317	-0.06301	-0.06285	-0.0627	-0.06254
	-0.06367	-0.06352	-0.06337	-0.06322	-0.06307	-0.06291	-0.06275	-0.06259
	-0.06372	-0.06357	-0.06342	-0.06327	-0.06311	-0.06296	-0.0628	-0.06264
	-0.06377	-0.06361	-0.06346	-0.06331	-0.06316	-0.063	-0.06284	-0.06268
	-0.06382	-0.06366	-0.06351	-0.06336	-0.06321	-0.06306	-0.0629	-0.06274
	-0.06387	-0.06372	-0.06357	-0.06342	-0.06327	-0.06311	-0.06296	-0.0628
				-0.06347	-0.06332	-0.06316	-0.06301	-0.06285
				-0.06351	-0.06336	-0.06321	-0.06305	-0.0629

-0.06355	-0.0634	-0.06325	-0.0631	-0.06294
-0.0636	-0.06345	-0.0633	-0.06315	-0.06299
-0.06364	-0.06349	-0.06334	-0.0632	-0.06305
-0.0637	-0.06355	-0.0634	-0.06325	-0.0631
-0.06376	-0.0636	-0.06345	-0.0633	-0.06315
		-0.0635	-0.06335	-0.0632
		-0.06354	-0.0634	-0.06325
		-0.06359	-0.06345	-0.0633
		-0.06365	-0.0635	-0.06335
				-0.0634
				-0.06346
				-0.06351
				-0.06356
				-0.0636

								-0.25713
								-0.25687
								-0.25661
								-0.25634
								-0.25608
						-0.06538	-0.06522	-0.31469
						-0.06532	-0.06516	-0.31438
						-0.06525	-0.06509	-0.31407
						-0.06519	-0.06503	-0.31376
				-0.06543	-0.06528	-0.31526	-0.31435	-0.31345
				-0.06537	-0.06522	-0.31495	-0.31404	-0.31314
				-0.06531	-0.06515	-0.31464	-0.31373	-0.31284
				-0.06525	-0.06509	-0.31433	-0.31343	-0.31254
				-0.06519	-0.06503	-0.31402	-0.31312	-0.31224
				-0.06512	-0.06496	-0.31371	-0.31281	-0.31193
				-0.06506	-0.0649	-0.3134	-0.31251	-0.31164
	-0.06546	-0.06531	-0.06515	-0.25731	-0.25659	-0.3131	-0.31222	-0.31135
	-0.0654	-0.06525	-0.06509	-0.25705	-0.25634	-0.3128	-0.31193	-0.31107
	-0.06534	-0.06518	-0.06502	-0.2568	-0.25609	-0.31251	-0.31164	-0.46728
	-0.06528	-0.06512	-0.06496	-0.25655	-0.25585	-0.31221	-0.31134	-0.46688
	-0.25843	-0.25772	-0.257	-0.2563	-0.2556	-0.31191	-0.31105	-0.4665
	-0.25818	-0.25747	-0.25675	-0.25605	-0.25535	-0.31162	-0.31078	-0.46615
	-0.25793	-0.25722	-0.25651	-0.2558	-0.2551	-0.31134	-0.31052	-0.4658
	-0.25768	-0.25696	-0.25626	-0.31279	-0.31192	-0.40613	-0.40511	-0.52254
	-0.25742	-0.25671	-0.25601	-0.31251	-0.31166	-0.40583	-0.40481	-0.52215
-0.12809	-0.25719	-0.25648	-0.25579	-0.31224	-0.31139	-0.40553	-0.40451	-0.52174
-0.12797	-0.25697	-0.25628	-0.25559	-0.31199	-0.31113	-0.40522	-0.40419	-0.52133
-0.25743	-0.25675	-0.25606	-0.25538	-0.40703	-0.40596	-0.46641	-0.46517	-0.52091
-0.25722	-0.25652	-0.25583	-0.25514	-0.40669	-0.40563	-0.46602	-0.46478	-0.52049
-0.25699	-0.25629	-0.25559	-0.2549	-0.40635	-0.4053	-0.46563	-0.4644	-0.52009
-0.25675	-0.25604	-0.25534	-0.25465	-0.40602	-0.40497	-0.46526	-0.46405	-0.57512
-0.2565	-0.2558	-0.2551	-0.25441	-0.4057	-0.40467	-0.4649	-0.46371	-0.57472
-0.25624	-0.25555	-0.25486	-0.25419	-0.40541	-0.40439	-0.46457	-0.46337	-0.57429
-0.25599	-0.2553	-0.25463	-0.25398	-0.40515	-0.40413	-0.46425	-0.46303	-0.57384
-0.25576	-0.35295	-0.35209	-0.35124	-0.40486	-0.40385	-0.4639	-0.46267	-0.57338
-0.25556	-0.35273	-0.35186	-0.35099	-0.40456	-0.40353	-0.46352	-0.46228	-0.5729
-0.25535	-0.3525	-0.35162	-0.35075	-0.40425	-0.40322	-0.46314	-0.4619	-0.57244
-0.25511	-0.35223	-0.35135	-0.35049	-0.40393	-0.4029	-0.46276	-0.46154	-0.57203
-0.25488	-0.35196	-0.35109	-0.35023	-0.40363	-0.4026	-0.4624	-0.4612	-0.57162
-0.35261	-0.40649	-0.40544	-0.4044	-0.40336	-0.40232	-0.46207	-0.46085	-0.57118
-0.35238	-0.40624	-0.4052	-0.40415	-0.40311	-0.40207	-0.46174	-0.4605	-0.57073
-0.35213	-0.40595	-0.4049	-0.40386	-0.46387	-0.46263	-0.5733	-0.57178	-0.73446
-0.35187	-0.40562	-0.40458	-0.40354	-0.46348	-0.46223	-0.57282	-0.57131	-0.73385
-0.35162	-0.40533	-0.40428	-0.40324	-0.46313	-0.46187	-0.57238	-0.57088	-0.73328
-0.3514	-0.40508	-0.40404	-0.40299	-0.46282	-0.46157	-0.572	-0.57051	-0.73277
-0.35117	-0.40482	-0.40378	-0.40273	-0.46251	-0.46125	-0.57161	-0.57012	-0.73224
-0.35092	-0.4045	-0.40346	-0.40242	-0.46213	-0.46087	-0.57115	-0.56966	-0.73164

-0.35066	-0.40418	-0.40314	-0.4021	-0.46174	-0.46049	-0.57068	-0.5692	-0.73104
-0.35044	-0.40392	-0.40288	-0.40183	-0.46142	-0.46017	-0.57029	-0.56881	-0.73051
-0.35023	-0.40367	-0.40263	-0.40158	-0.46113	-0.45988	-0.56993	-0.56846	-0.73002
-0.34999	-0.40339	-0.40235	-0.4013	-0.46079	-0.45954	-0.56952	-0.56805	-0.72947
-0.34972	-0.40307	-0.40203	-0.40098	-0.4604	-0.45916	-0.56905	-0.56759	-0.72887
-0.34948	-0.46379	-0.46254	-0.46129	-0.57161	-0.57011	-0.56862	-0.56716	-0.72831
-0.34927	-0.46349	-0.46224	-0.46099	-0.57123	-0.56973	-0.56825	-0.56679	-0.72781
-0.34906	-0.46319	-0.46194	-0.4607	-0.57086	-0.56937	-0.56789	-0.56644	-0.72733
-0.34886	-0.4629	-0.46165	-0.46041	-0.57051	-0.56901	-0.67733	-0.67557	-0.72684
-0.34864	-0.4626	-0.46135	-0.4601	-0.57014	-0.56865	-0.67687	-0.67511	-0.72634
-0.40252	-0.46224	-0.461	-0.45975	-0.56971	-0.56823	-0.67636	-0.6746	-0.72578
-0.40221	-0.46186	-0.46061	-0.45936	-0.56924	-0.56777	-0.67581	-0.67406	-0.72519
-0.40193	-0.46151	-0.46026	-0.45902	-0.56882	-0.56735	-0.72845	-0.72654	-0.72465
-0.40168	-0.46121	-0.45996	-0.45872	-0.56845	-0.56699	-0.72795	-0.72605	-0.72417
-0.46216	-0.57256	-0.57106	-0.56956	-0.56809	-0.56663	-0.72746	-0.72557	-0.72369
-0.46187	-0.57221	-0.5707	-0.56921	-0.56773	-0.56628	-0.72698	-0.72509	-0.72321
-0.46157	-0.57183	-0.57033	-0.56884	-0.56737	-0.56591	-0.72647	-0.72459	-0.72271
-0.4612	-0.57138	-0.56988	-0.5684	-0.6765	-0.67475	-0.72591	-0.72403	-0.77563
-0.46081	-0.57091	-0.56942	-0.56794	-0.67596	-0.67422	-0.72533	-0.72345	-0.775
-0.46048	-0.5705	-0.56901	-0.56754	-0.67549	-0.67375	-0.72481	-0.72294	-0.77444
-0.46018	-0.57014	-0.56866	-0.56718	-0.67506	-0.67332	-0.72435	-0.72248	-0.77392
-0.4599	-0.56979	-0.5683	-0.56684	-0.67463	-0.6729	-0.72388	-0.72202	-0.77341
-0.45961	-0.56944	-0.56796	-0.5665	-0.67421	-0.67248	-0.72342	-0.72156	-0.7729
-0.45933	-0.56909	-0.56762	-0.56616	-0.6738	-0.67206	-0.77657	-0.77448	-0.7724
-0.45904	-0.56874	-0.56727	-0.56581	-0.67338	-0.67164	-0.77605	-0.77396	-0.77188
-0.45876	-0.56838	-0.56692	-0.56547	-0.67296	-0.67122	-0.77554	-0.77345	-0.77137
-0.45848	-0.56803	-0.56658	-0.56513	-0.67255	-0.67081	-0.77503	-0.77295	-0.82475
-0.45819	-0.56769	-0.56623	-0.56479	-0.67213	-0.67041	-0.77454	-0.77244	-0.8242
-0.56879	-0.67694	-0.67519	-0.67345	-0.72443	-0.72256	-0.77403	-0.77192	-0.82364
-0.56844	-0.67651	-0.67476	-0.67303	-0.72397	-0.7221	-0.77352	-0.7714	-0.82305
-0.56809	-0.67608	-0.67434	-0.67261	-0.72351	-0.72163	-0.77299	-0.77086	-0.82246
-0.56774	-0.67566	-0.67392	-0.67218	-0.72304	-0.72115	-0.77244	-0.77031	-0.82186
-0.56739	-0.67523	-0.67349	-0.67176	-0.77615	-0.77401	-0.77188	-0.76975	-0.82126
-0.56704	-0.6748	-0.67306	-0.67133	-0.77561	-0.77347	-0.77133	-0.76919	-0.82067
-0.5667	-0.67437	-0.67264	-0.6709	-0.77508	-0.77294	-0.77079	-0.76864	-0.82008
-0.56635	-0.67395	-0.67221	-0.67048	-0.77458	-0.77245	-0.82413	-0.82182	-0.96317
-0.566	-0.67353	-0.67179	-0.67006	-0.77408	-0.77198	-0.82364	-0.82134	-0.96252
-0.56566	-0.6731	-0.67137	-0.66964	-0.77357	-0.77149	-0.82313	-0.82083	-0.96183
-0.5653	-0.67267	-0.67094	-0.66922	-0.77306	-0.77098	-0.82258	-0.82029	-0.96111
-0.56495	-0.67224	-0.67052	-0.6688	-0.77254	-0.77046	-0.82204	-0.81975	-0.96039
-0.5646	-0.67182	-0.67009	-0.66838	-0.77204	-0.76996	-0.82151	-0.81923	-0.95968
-0.56425	-0.67139	-0.66967	-0.66796	-0.77153	-0.76947	-0.82099	-0.81872	-0.95898
-0.5639	-0.72574	-0.7238	-0.72187	-0.8249	-0.8227	-0.96427	-0.9613	-0.95835
-0.56354	-0.72526	-0.72332	-0.72141	-0.82436	-0.82217	-0.96358	-0.96066	-0.95775
-0.67182	-0.72478	-0.72285	-0.72094	-0.82382	-0.82164	-0.96287	-0.95998	-0.9571
-0.67139	-0.72429	-0.72237	-0.72047	-0.82328	-0.8211	-0.96215	-0.95928	-0.95642
-0.67096	-0.77515	-0.77308	-0.77104	-0.82274	-0.82056	-0.96143	-0.95856	-0.95572
-0.67054	-0.77461	-0.77255	-0.77053	-0.8222	-0.82002	-0.9607	-0.95784	-0.95501
-0.67014	-0.7741	-0.77205	-0.77003	-0.82166	-0.81949	-0.95998	-0.95712	-0.95429
-0.66977	-0.77365	-0.77158	-0.76954	-0.82112	-0.81894	-0.95924	-0.95639	-0.95357

-0.66942	-0.77323	-0.77114	-0.76908	-0.82061	-0.81842	-0.95852	-0.95566	-0.95285
-0.72364	-0.82682	-0.82459	-0.82237	-0.96368	-0.96075	-0.95785	-0.95495	-0.95212
-0.72323	-0.82638	-0.82417	-0.82196	-0.96311	-0.96015	-0.9572	-0.95426	-0.9514
-0.72279	-0.82589	-0.82369	-0.82149	-0.96249	-0.95953	-0.95658	-0.95362	-0.99212
-0.72231	-0.82535	-0.82316	-0.82098	-0.96181	-0.9589	-0.95598	-0.95303	-0.99148
-0.72186	-0.82481	-0.82263	-0.82045	-0.96111	-0.95824	-0.95534	-0.95244	-0.99086
-0.72143	-0.82428	-0.82208	-0.81991	-0.96038	-0.95752	-0.95468	-0.95183	-0.99027
-0.72102	-0.96834	-0.96542	-0.96252	-0.95965	-0.95681	-0.95399	-0.9512	-0.98965
-0.72064	-0.96774	-0.96479	-0.96187	-0.95898	-0.95611	-0.95328	-0.95051	-0.98892
-0.72027	-0.96716	-0.9642	-0.96125	-0.95833	-0.95543	-0.95259	-0.94981	-0.98818
-0.71984	-0.96649	-0.96355	-0.96063	-0.95772	-0.95483	-0.95199	-0.9492	-1.03482
-0.71938	-0.96577	-0.96287	-0.96	-0.95714	-0.95428	-0.95145	-0.94864	-1.03413
-0.82361	-0.9651	-0.96221	-0.95935	-0.9565	-0.95367	-0.95086	-0.94806	-1.03347
-0.82318	-0.96447	-0.96155	-0.95866	-0.95581	-0.95299	-0.95021	-0.94746	-1.03285
-0.82273	-0.96385	-0.96091	-0.95802	-0.95516	-0.95234	-0.99098	-0.988	-1.03219
-0.82224	-0.96323	-0.96033	-0.95746	-0.9546	-0.95177	-0.99034	-0.98734	-1.03147
-0.82175	-0.96259	-0.95974	-0.9569	-0.95405	-0.95121	-0.98971	-0.9867	-1.03076
-0.96477	-0.96189	-0.95906	-0.95625	-0.95342	-0.9506	-0.9891	-0.98612	-1.03015
-0.96409	-0.96119	-0.95837	-0.95558	-0.95278	-0.94999	-0.98849	-0.98556	-1.02957
-0.96348	-0.96058	-0.95777	-0.95498	-0.95218	-0.94939	-1.03512	-1.03201	-1.0289
-0.96292	-0.96005	-0.95723	-0.95443	-0.95162	-0.94881	-1.03442	-1.03128	-1.02818
-0.96235	-0.95948	-0.95665	-0.95384	-0.95102	-0.94822	-1.03374	-1.03062	-1.02753
-0.96177	-0.95887	-0.95601	-0.95318	-0.95039	-0.94762	-1.03311	-1.03003	-1.02694
-0.96117	-0.95827	-0.95539	-0.95255	-0.94977	-0.94704	-1.03248	-1.02942	-1.02634
-0.96054	-0.95767	-0.95483	-0.95201	-0.94923	-0.94648	-1.03182	-1.02874	-1.02567
-0.95991	-0.95707	-0.95426	-0.95147	-0.94869	-0.94593	-1.03117	-1.02807	-1.025
-0.9593	-0.95645	-0.95363	-0.95084	-0.99623	-0.99331	-1.03055	-1.02746	-1.02441
-0.95869	-0.95582	-0.95298	-0.95017	-0.99555	-0.99269	-1.02995	-1.02688	-1.02385
-0.95809	-0.95522	-0.95238	-0.9496	-0.99495	-0.9921	-1.02935	-1.02628	-1.0663
-0.95749	-0.95462	-0.95183	-0.94908	-0.9944	-0.99154	-1.02875	-1.02565	-1.06561
-0.9569	-0.95405	-0.95129	-0.94857	-1.03432	-1.03124	-1.02814	-1.02503	-1.06495
-0.95634	-0.95352	-0.95077	-0.94807	-1.03373	-1.03062	-1.02751	-1.02441	-1.06431
-0.95579	-0.95298	-0.95024	-0.94753	-1.03313	-1.03	-1.02689	-1.02381	-1.10666
-0.95523	-0.95241	-0.94965	-0.94694	-1.03247	-1.02939	-1.02631	-1.02324	-1.10605
-0.95467	-0.95184	-0.94906	-0.94633	-1.03181	-1.02878	-1.02574	-1.02269	-1.10545
-0.95408	-0.99959	-0.99663	-0.99373	-1.03122	-1.0282	-1.02516	-1.0221	-1.1048
-0.95347	-0.99897	-0.99606	-0.9932	-1.03066	-1.02763	-1.02457	-1.02148	-1.10412
-0.95291	-0.99838	-0.99549	-0.99265	-1.03009	-1.02703	-1.06699	-1.06377	-1.10346
-0.95239	-0.99782	-0.99494	-0.99209	-1.02949	-1.0264	-1.06632	-1.06312	-1.10282
-0.95188	-0.99727	-0.99438	-0.99152	-1.02888	-1.02578	-1.06567	-1.06249	-1.1022
-0.95136	-0.99673	-0.99382	-0.99093	-1.02824	-1.02515	-1.06504	-1.06189	-1.1016
-0.95085	-0.9962	-0.99327	-0.99035	-1.02763	-1.02455	-1.06443	-1.06131	-1.10102
-0.95036	-0.99568	-0.99275	-0.98984	-1.02709	-1.02401	-1.06387	-1.06076	-1.10046
-0.94987	-0.99515	-0.99224	-0.98934	-1.02658	-1.02349	-1.06332	-1.06023	-1.0999
-0.94935	-0.99457	-0.99167	-0.98878	-1.06909	-1.06591	-1.06278	-1.05969	-1.09934
-0.94881	-0.99395	-0.99105	-0.98819	-1.06848	-1.06534	-1.06223	-1.05915	-1.09879
-0.99633	-0.99338	-0.99048	-0.98763	-1.06789	-1.06477	-1.06167	-1.05859	-1.09821
-0.9958	-0.99286	-0.98996	-0.9871	-1.06731	-1.0642	-1.0611	-1.05799	-1.09759
-1.0362	-1.03302	-1.02989	-1.02681	-1.06675	-1.06365	-1.06054	-1.05743	-1.13588
-1.03565	-1.03248	-1.02937	-1.02629	-1.06621	-1.06311	-1.06002	-1.05692	-1.13534

-1.0351	-1.03195	-1.02884	-1.02578	-1.06567	-1.06258	-1.0595	-1.05643	-1.13482
-1.03457	-1.03142	-1.02831	-1.02524	-1.06512	-1.06203	-1.05896	-1.05592	-1.13427
-1.03404	-1.03088	-1.02777	-1.0247	-1.06455	-1.06147	-1.05842	-1.05539	-1.13371
-1.0335	-1.07353	-1.0703	-1.06712	-1.06399	-1.06092	-1.05788	-1.05487	-1.13316
-1.03296	-1.07296	-1.06974	-1.06656	-1.06344	-1.06037	-1.05734	-1.05435	-1.1326
-1.03241	-1.0724	-1.06917	-1.066	-1.06289	-1.05984	-1.05683	-1.05385	-1.20468
-1.03187	-1.07183	-1.06861	-1.06544	-1.06235	-1.05932	-1.05634	-1.05339	-1.20416
-1.03133	-1.07127	-1.06806	-1.0649	-1.06182	-1.05882	-1.09541	-1.09238	-1.20363
-1.0308	-1.07073	-1.06751	-1.06436	-1.0613	-1.05831	-1.09489	-1.09188	-1.20309
-1.03027	-1.07018	-1.06697	-1.06382	-1.06077	-1.0578	-1.09437	-1.09136	-1.20254
-1.02975	-1.06963	-1.06642	-1.06327	-1.06023	-1.05728	-1.16806	-1.1649	-1.202
-1.02922	-1.06908	-1.06587	-1.06271	-1.05969	-1.05676	-1.16751	-1.16437	-1.20145
-1.02872	-1.06858	-1.06536	-1.06219	-1.05917	-1.05625	-1.16697	-1.16385	-1.24304
-1.02825	-1.06813	-1.0649	-1.06172	-1.05868	-1.05575	-1.16644	-1.16334	-1.2425
-1.07079	-1.06764	-1.06442	-1.06123	-1.05819	-1.05525	-1.16592	-1.16285	-1.24199
-1.07023	-1.0671	-1.0639	-1.06073	-1.0577	-1.05476	-1.1654	-1.16238	-1.24151
-1.06967	-1.06655	-1.06337	-1.06022	-1.0572	-1.05428	-1.16489	-1.16191	-1.24102
-1.06914	-1.06601	-1.06284	-1.05971	-1.0567	-1.05378	-1.16436	-1.16141	-1.27685
-1.06863	-1.06547	-1.06232	-1.05922	-1.05622	-1.05331	-1.16384	-1.16089	-1.27626
-1.06809	-1.06493	-1.06182	-1.05877	-1.16963	-1.16644	-1.16337	-1.1604	-1.2757
-1.06753	-1.06438	-1.06134	-1.05835	-1.1692	-1.16601	-1.16292	-1.15993	-1.27515
-1.06703	-1.06388	-1.06086	-1.0579	-1.16874	-1.16558	-1.20531	-1.20217	-1.27461
-1.06657	-1.06342	-1.06037	-1.05741	-1.16825	-1.16516	-1.20488	-1.20172	-1.27409
-1.06612	-1.06298	-1.05991	-1.05693	-1.16775	-1.16471	-1.20442	-1.20125	-1.27357
-1.06566	-1.06256	-1.05949	-1.0565	-1.16726	-1.16421	-1.20389	-1.20073	-1.27301
-1.06519	-1.06212	-1.05907	-1.05608	-1.16678	-1.16371	-1.20335	-1.20021	-1.27247
-1.0251	-1.13867	-1.13556	-1.1325	-1.16634	-1.16326	-1.20288	-1.19976	-1.27199
-1.0246	-1.1381	-1.13505	-1.13206	-1.16592	-1.16284	-1.20245	-1.19934	-1.27154
-1.02415	-1.1376	-1.13456	-1.1316	-1.20832	-1.20516	-1.20202	-1.19891	-1.31093
-1.02374	-1.13715	-1.13409	-1.1311	-1.20782	-1.20472	-1.20161	-1.19847	-1.31044
-1.02334	-1.13671	-1.13363	-1.13064	-1.20732	-1.20425	-1.20115	-1.19803	-1.30994
-1.02293	-1.13628	-1.1332	-1.1302	-1.20682	-1.20372	-1.20063	-1.19757	-1.30947
-1.02252	-1.13585	-1.13278	-1.12977	-1.20633	-1.2032	-1.27563	-1.27241	-1.30896
-1.02213	-1.13542	-1.13236	-1.12937	-1.20589	-1.20274	-1.27511	-1.27187	-1.30839
-1.02172	-1.13498	-1.13195	-1.12897	-1.20546	-1.2023	-1.27461	-1.27134	-1.30782
-1.13764	-1.13455	-1.13153	-1.12856	-1.20503	-1.20188	-1.27414	-1.27085	-1.3073
-1.13716	-1.13412	-1.13111	-1.12815	-1.2046	-1.20146	-1.2737	-1.27038	-1.30681
-1.13671	-1.17734	-1.17423	-1.17115	-1.20416	-1.20103	-1.31312	-1.30971	-1.30633
-1.13628	-1.17687	-1.17378	-1.17072	-1.20372	-1.20058	-1.31262	-1.30922	-1.30585
-1.13584	-1.17641	-1.17332	-1.17028	-1.20328	-1.20013	-1.31212	-1.30873	-1.30536
-1.1354	-1.17595	-1.17286	-1.16982	-1.20283	-1.1997	-1.31163	-1.30823	-1.30486
-1.13496	-1.17549	-1.17241	-1.16937	-1.20238	-1.19927	-1.31115	-1.30773	-1.30436
-1.13452	-1.17503	-1.17196	-1.16894	-1.16593	-1.16294	-1.31063	-1.30723	-1.30387
-1.13408	-1.17457	-1.17152	-1.16851	-1.1655	-1.1625	-1.31012	-1.30673	-1.30338
-1.17725	-1.17414	-1.17109	-1.16808	-1.16507	-1.16207	-1.30963	-1.30623	-1.30288
-1.1768	-1.17371	-1.17067	-1.16766	-1.16465	-1.16166	-1.30916	-1.30574	-1.30239
-1.17636	-1.17328	-1.17024	-1.16723	-1.16425	-1.16128	-1.30872	-1.30531	-1.33744
-1.17592	-1.17284	-1.1698	-1.16681	-1.16385	-1.16092	-1.30833	-1.30493	-1.33707
-1.1755	-1.17242	-1.16939	-1.16641	-1.24209	-1.23897	-1.34359	-1.34011	-1.33667
-1.17513	-1.17206	-1.16903	-1.16604	-1.24166	-1.23851	-1.34309	-1.33963	-1.33622

-1.17477	-1.1717	-1.16867	-1.16566	-1.24122	-1.23804	-1.34259	-1.33914	-1.33574
-1.17435	-1.1713	-1.16826	-1.16525	-1.24076	-1.23758	-1.34211	-1.33866	-1.33527
-1.17392	-1.17089	-1.16785	-1.16482	-1.2403	-1.23713	-1.34165	-1.33819	-1.3348
-1.17351	-1.1705	-1.16746	-1.16443	-1.27699	-1.27375	-1.34123	-1.33777	-1.33438
-1.1731	-1.17012	-1.16709	-1.16407	-1.27661	-1.27337	-1.34083	-1.33738	-1.334
-1.17269	-1.20847	-1.20536	-1.20224	-1.2762	-1.27294	-1.30725	-1.30396	-1.33359
-1.17227	-1.20803	-1.20493	-1.20183	-1.27574	-1.27246	-1.30676	-1.30348	-1.33313
-1.17187	-1.20762	-1.20452	-1.20143	-1.27529	-1.27201	-1.30629	-1.30303	-1.33269
-1.1715	-1.20725	-1.20415	-1.20106	-1.2749	-1.27163	-1.3059	-1.30264	-1.3323
-1.17114	-1.20689	-1.20378	-1.20069	-1.27451	-1.27127	-1.30553	-1.30227	-1.33194
-1.17072	-1.20648	-1.20337	-1.20027	-1.27406	-1.27083	-1.3051	-1.30187	-1.33157
-1.17028	-1.20606	-1.20294	-1.19984	-1.27359	-1.27036	-1.30464	-1.30146	-1.33119
-1.16989	-1.20568	-1.20256	-1.19945	-1.27317	-1.26994	-1.30421	-1.30103	-1.33077
-1.16955	-1.20534	-1.20222	-1.19911	-1.27279	-1.26956	-1.3038	-1.30058	-1.33031
-1.20809	-1.2823	-1.27899	-1.27569	-1.31003	-1.30669	-1.3034	-1.30017	-1.29698
-1.20775	-1.28192	-1.27861	-1.27531	-1.30965	-1.30632	-1.30304	-1.2998	-1.29662
-1.20741	-1.28155	-1.27824	-1.27494	-1.30927	-1.30595	-1.30268	-1.29945	-1.29627
-1.20707	-1.28118	-1.27787	-1.27457	-1.30889	-1.30557	-1.30231	-1.2991	-1.29593
-1.20673	-1.2808	-1.2775	-1.2742	-1.3085	-1.3052	-1.30194	-1.29875	-1.29559
-1.20639	-1.28043	-1.27712	-1.27383	-1.30812	-1.30482	-1.30158	-1.29839	-1.29523
-1.20605	-1.28006	-1.27675	-1.27345	-1.30773	-1.30444	-1.30121	-1.29802	-1.29487
-1.2057	-1.27969	-1.27637	-1.27307	-1.30734	-1.30406	-1.30083	-1.29765	-1.29451
-1.20535	-1.2793	-1.27599	-1.27269	-1.30695	-1.30367	-1.30045	-1.29728	-1.29414
-1.20501	-1.27892	-1.2756	-1.27231	-1.30655	-1.30329	-1.30007	-1.29691	-1.29378
-1.20466	-1.27854	-1.27522	-1.27193	-1.30616	-1.3029	-1.29969	-1.29655	-1.29342
-1.20431	-1.27817	-1.27484	-1.27154	-1.30577	-1.30251	-1.29931	-1.29618	-1.29306
-1.20396	-1.27778	-1.27446	-1.27117	-1.30538	-1.30213	-1.29894	-1.29581	-1.29269
-1.20361	-1.2774	-1.27408	-1.27079	-1.305	-1.30176	-1.29857	-1.29544	-1.29232
-1.20327	-1.27703	-1.27372	-1.27044	-1.30463	-1.30139	-1.2982	-1.29507	-1.29196
-1.20294	-1.27666	-1.27336	-1.27009	-1.30427	-1.30101	-1.29782	-1.2947	-1.29159
-1.23952	-1.27633	-1.27304	-1.26978	-1.30394	-1.30068	-1.29748	-1.29435	-1.29123
-1.23924	-1.27604	-1.27276	-1.26951	-1.30367	-1.3004	-1.29719	-1.29401	-1.29088
-1.23894	-1.31341	-1.31002	-1.30667	-1.30337	-1.30011	-1.29689	-1.2937	-1.32407
-1.23858	-1.31302	-1.30964	-1.3063	-1.30301	-1.29976	-1.29656	-1.29341	-1.32379
-1.23821	-1.31262	-1.30925	-1.30591	-1.30263	-1.2994	-1.29623	-1.29312	-1.32351
-1.23786	-1.31223	-1.30886	-1.30553	-1.30226	-1.29905	-1.29589	-1.29278	-1.32318
-1.23752	-1.31186	-1.30848	-1.30516	-1.3019	-1.2987	-1.29554	-1.29244	-1.32282
-1.2372	-1.27224	-1.26896	-1.26574	-1.30158	-1.2984	-1.29524	-1.29213	-1.3225
-1.2369	-1.27194	-1.26867	-1.26544	-1.30131	-1.29814	-1.29499	-1.29185	-1.32221
-1.2366	-1.27164	-1.26836	-1.26514	-1.301	-1.29785	-1.2947	-1.29156	-1.3219
-1.23631	-1.27133	-1.26803	-1.26479	-1.30064	-1.29749	-1.29435	-1.29122	-1.32155
-1.23602	-1.27101	-1.26771	-1.26446	-1.3003	-1.29715	-1.29401	-1.29089	-1.32121
-1.23567	-1.27067	-1.2674	-1.26418	-1.30002	-1.29687	-1.29373	-1.29061	-1.32092
-1.23533	-1.27034	-1.2671	-1.26392	-1.29977	-1.29662	-1.29348	-1.29034	-1.32063
-1.23503	-1.27004	-1.26682	-1.26365	-1.29951	-1.29636	-1.29321	-1.29007	-1.32034
-1.23476	-1.26977	-1.26655	-1.26339	-1.29924	-1.2961	-1.29295	-1.2898	-1.32004
-1.2345	-1.2695	-1.26629	-1.26313	-1.26002	-1.25697	-1.29267	-1.2895	-1.31972
-1.23425	-1.26924	-1.26603	-1.26286	-1.25976	-1.2567	-1.29238	-1.28918	-1.31938
-1.23399	-1.26897	-1.26576	-1.2626	-1.25949	-1.25641	-1.32562	-1.32232	-1.31906
-1.23372	-1.2687	-1.26549	-1.26233	-1.2592	-1.2561	-1.32529	-1.32202	-1.31879

-1.23345	-1.26842	-1.26521	-1.26205	-1.2589	-1.25579	-1.32497	-1.32174	-1.31853
-1.23317	-1.26813	-1.26491	-1.26174	-1.25861	-1.25552	-1.3247	-1.32146	-1.31825
-1.23289	-1.26783	-1.2646	-1.26142	-1.25832	-1.25527	-1.32444	-1.32119	-1.31798
-1.23259	-1.26751	-1.2643	-1.26114	-1.25805	-1.255	-1.32417	-1.32092	-1.31771
-1.23227	-1.26717	-1.264	-1.26088	-1.2578	-1.25474	-1.32389	-1.32065	-1.31744
-1.23202	-1.26691	-1.26374	-1.26063	-1.25754	-1.25448	-1.28552	-1.28237	-1.31718
-1.23183	-1.26673	-1.26352	-1.26037	-1.25727	-1.25423	-1.28526	-1.28211	-1.31691
-1.23164	-1.26655	-1.26332	-1.26014	-1.25703	-1.25398	-1.285	-1.28185	-1.31665
-1.2314	-1.26633	-1.26314	-1.25997	-1.25683	-1.25373	-1.28472	-1.28159	-1.31639
-1.23115	-1.26609	-1.26294	-1.25981	-1.25665	-1.25349	-1.28446	-1.28132	-1.31613
-1.23089	-1.26584	-1.26271	-1.2596	-1.25646	-1.2533	-1.28425	-1.28108	-1.31586
-1.23063	-1.26556	-1.26245	-1.25937	-1.25626	-1.25314	-1.28407	-1.28085	-1.31559
-1.23037	-1.2653	-1.2622	-1.25912	-1.25603	-1.25294	-1.28387	-1.28065	-1.31538
-1.23011	-1.26505	-1.26194	-1.25885	-1.25577	-1.2527	-1.28365	-1.28047	-1.31521
-1.26794	-1.2648	-1.26169	-1.2586	-1.25552	-1.25245	-1.28341	-1.28027	-1.27715
-1.26771	-1.26454	-1.26144	-1.25837	-1.25527	-1.25218	-1.28313	-1.28002	-1.27693
-1.26749	-1.2643	-1.26121	-1.25816	-1.25505	-1.25193	-1.28287	-1.27976	-1.2767
-1.26729	-1.26411	-1.26103	-1.25798	-1.25488	-1.25176	-1.28268	-1.27953	-1.27645
-1.26711	-1.26395	-1.26086	-1.2578	-1.25471	-1.25161	-1.2825	-1.27931	-1.2762
-1.26694	-1.26378	-1.26067	-1.25758	-1.25449	-1.2514	-1.2823	-1.27912	-1.27601
-1.26677	-1.26362	-1.26046	-1.25733	-1.25424	-1.25118	-1.28209	-1.27894	-1.27586
-1.26657	-1.26343	-1.26027	-1.25712	-1.25403	-1.25098	-1.2819	-1.27877	-1.2757
-1.26633	-1.26321	-1.26007	-1.25695	-1.25386	-1.25081	-1.28173	-1.27861	-1.27553
-1.26611	-1.263	-1.25988	-1.25678	-1.25369	-1.25062	-1.28153	-1.27842	-1.27535
-1.26593	-1.26283	-1.25971	-1.25661	-1.25349	-1.25038	-1.28127	-1.27819	-1.27514
-1.26575	-1.26264	-1.25954	-1.25644	-1.2533	-1.25015	-1.28103	-1.27796	-1.27494
-1.26553	-1.26239	-1.25931	-1.25623	-1.25311	-1.24997	-1.28084	-1.27778	-1.27475
-1.26531	-1.26214	-1.25907	-1.25602	-1.25292	-1.24981	-1.28068	-1.2776	-1.27457
-1.26511	-1.26195	-1.25888	-1.25584	-1.25275	-1.24965	-1.2805	-1.2774	-1.27435
-1.26493	-1.2618	-1.25873	-1.25568	-1.25259	-1.24949	-1.28032	-1.27718	-1.27411
-1.26476	-1.29601	-1.29284	-1.28969	-1.28651	-1.28332	-1.28015	-1.27699	-1.27392
-1.2646	-1.29584	-1.29268	-1.28954	-1.28636	-1.28317	-1.28	-1.27684	-1.27377
-1.26444	-1.29568	-1.29253	-1.28938	-1.2862	-1.28302	-1.27985	-1.27669	-1.27363
-1.26429	-1.29553	-1.29237	-1.28922	-1.28605	-1.28287	-1.2797	-1.27655	-1.27348
-1.26414	-1.29537	-1.29221	-1.28906	-1.28589	-1.28272	-1.27956	-1.27641	-1.27334
-1.26399	-1.29521	-1.29205	-1.28891	-1.28574	-1.28257	-1.27941	-1.27626	-1.2732
-1.26383	-1.29505	-1.2919	-1.28876	-1.28559	-1.28241	-1.27926	-1.27611	-1.27306
-1.26368	-1.29489	-1.29174	-1.2886	-1.28544	-1.28226	-1.27911	-1.27597	-1.27291
-1.26353	-1.29473	-1.29158	-1.28845	-1.28528	-1.28212	-1.27897	-1.27583	-1.27278
-1.26338	-1.29458	-1.29143	-1.28829	-1.28513	-1.28197	-1.27882	-1.27569	-1.27264
-1.26322	-1.29443	-1.29128	-1.28814	-1.28498	-1.28181	-1.27867	-1.27554	-1.27249
-1.26308	-1.29427	-1.29113	-1.28799	-1.28483	-1.28166	-1.27852	-1.27539	-1.27235
-1.26294	-1.29411	-1.29096	-1.28783	-1.28468	-1.28153	-1.27839	-1.27527	-1.27222
-1.26281	-1.29395	-1.29079	-1.28767	-1.28453	-1.28139	-1.27827	-1.27515	-1.27211
-1.26269	-1.2938	-1.29064	-1.28752	-1.28438	-1.28125	-1.27814	-1.27506	-1.30799
-1.26256	-1.29368	-1.2905	-1.28736	-1.28422	-1.2811	-1.27802	-1.27498	-1.30793
-1.26246	-1.29358	-1.2904	-1.28725	-1.2841	-1.28097	-1.27789	-1.27487	-1.30783
-1.26238	-1.29352	-1.29035	-1.28719	-1.28401	-1.28085	-1.27776	-1.27473	-1.30768
-1.26227	-1.29343	-1.29026	-1.2871	-1.28392	-1.28075	-1.27765	-1.27461	-1.30756
-1.26212	-1.29328	-1.29012	-1.28696	-1.28382	-1.28068	-1.2776	-1.27455	-1.30748

-1.26199	-1.29313	-1.28997	-1.28682	-1.2837	-1.2806	-1.27753	-1.27449	-1.30741
-1.26191	-1.29304	-1.28985	-1.28669	-1.28356	-1.28046	-1.27739	-1.27437	-1.3073
-1.26184	-1.29296	-1.28975	-1.28656	-1.28342	-1.2803	-1.27724	-1.27422	-1.30717
-1.26176	-1.29286	-1.28966	-1.28647	-1.28332	-1.2802	-1.31323	-1.3101	-1.30704
-1.26167	-1.29275	-1.28956	-1.2864	-1.28325	-1.28013	-1.31314	-1.30999	-1.30691
-1.26157	-1.29266	-1.28948	-1.28633	-1.28318	-1.28004	-1.31304	-1.3099	-1.30681
-1.26149	-1.29259	-1.28942	-1.28626	-1.28309	-1.27994	-1.31294	-1.30981	-1.30675
-1.26141	-1.29251	-1.28934	-1.28617	-1.283	-1.27983	-1.31284	-1.30974	-1.30668
-1.26135	-1.29244	-1.28925	-1.28606	-1.28289	-1.27975	-1.31277	-1.30968	-1.30662
-1.26128	-1.29236	-1.28914	-1.28594	-1.28279	-1.27968	-1.31271	-1.30961	-1.30654
-1.26119	-1.29225	-1.28905	-1.28586	-1.319	-1.31579	-1.31263	-1.30951	-1.30644
-1.26108	-1.29213	-1.28895	-1.28579	-1.31894	-1.31572	-1.31254	-1.30941	-1.30634
-1.26099	-1.29205	-1.28888	-1.28573	-1.31886	-1.31562	-1.31244	-1.30932	-1.30627
-1.26093	-1.292	-1.28884	-1.28568	-1.31878	-1.3155	-1.31232	-1.30925	-1.30622
-1.26087	-1.29195	-1.28879	-1.28563	-1.31872	-1.31543	-1.31226	-1.30919	-1.30618
-1.26082	-1.2919	-1.28874	-1.28558	-1.3187	-1.31545	-1.31227	-1.30917	-1.30613
-1.26077	-1.29185	-1.28869	-1.28554	-1.31867	-1.31544	-1.31227	-1.30914	-1.30608
-1.26072	-1.2918	-1.28864	-1.28548	-1.31859	-1.31533	-1.31216	-1.30907	-1.30604
-1.26067	-1.29175	-1.28859	-1.28542	-1.3185	-1.31521	-1.31205	-1.309	-1.30599
-1.26064	-1.32826	-1.325	-1.32175	-1.31848	-1.31521	-1.31204	-1.30895	-1.30593
-1.26061	-1.3282	-1.32495	-1.32172	-1.31848	-1.31525	-1.31207	-1.30893	-1.30587
-1.26056	-1.32815	-1.3249	-1.32167	-1.31845	-1.31524	-1.31207	-1.30892	-1.30584
-1.26049	-1.3281	-1.32484	-1.32161	-1.31838	-1.31518	-1.31203	-1.30892	-1.30586
-1.26043	-1.29452	-1.29135	-1.2882	-1.28506	-1.28195	-1.31197	-1.30889	-1.30583
-1.26041	-1.29445	-1.29129	-1.28815	-1.28502	-1.28191	-1.31191	-1.30878	-1.30571
-1.2604	-1.2944	-1.29124	-1.28811	-1.28498	-1.28186	-1.31185	-1.30869	-1.30561
-1.29766	-1.29443	-1.29122	-1.28804	-1.28491	-1.28182	-1.31182	-1.30869	-1.30562
-1.29769	-1.29447	-1.29122	-1.28799	-1.28485	-1.28177	-1.3118	-1.30871	-1.30565
-1.29767	-1.29448	-1.29123	-1.288	-1.28484	-1.28173	-1.31175	-1.30868	-1.30564
-1.29761	-1.29445	-1.29125	-1.28805	-1.28486	-1.28169	-1.31168	-1.30863	-1.3056
-1.29757	-1.29441	-1.29123	-1.28806	-1.28487	-1.28168	-1.31166	-1.30859	-1.30556
-1.29754	-1.29433	-1.29117	-1.28803	-1.28487	-1.28171	-1.31169	-1.30856	-1.30551
-1.29754	-1.29429	-1.29112	-1.28798	-1.28485	-1.28173	-1.31172	-1.30856	-1.30549
-1.29756	-1.29432	-1.29111	-1.28794	-1.28481	-1.28172	-1.31172	-1.30857	-1.30551
-1.29761	-1.29437	-1.29112	-1.2879	-1.28478	-1.28171	-1.31172	-1.3086	-1.30555
-1.29765	-1.29442	-1.29116	-1.28792	-1.28479	-1.28172	-1.31174	-1.30864	-1.30558
-1.2977	-1.29448	-1.29121	-1.28797	-1.28482	-1.28174	-1.31176	-1.30868	-1.30561
-1.2977	-1.2945	-1.29125	-1.28801	-1.28484	-1.28174	-1.31175	-1.30868	-1.30562
-1.29767	-1.2945	-1.29127	-1.28805	-1.28486	-1.28171	-1.31171	-1.30865	-1.30561
-1.25694	-1.2945	-1.2913	-1.2881	-1.2849	-1.28171	-1.27864	-1.27566	-1.30561
-1.25696	-1.29453	-1.29134	-1.28815	-1.28495	-1.28175	-1.27866	-1.27568	-1.30564
-1.25698	-1.29453	-1.29136	-1.28819	-1.28499	-1.28179	-1.2787	-1.2757	-1.30565
-1.25697	-1.29449	-1.29133	-1.28819	-1.28501	-1.28183	-1.27872	-1.27568	-1.3056
-1.25696	-1.29446	-1.29131	-1.28818	-1.28503	-1.28187	-1.27875	-1.27566	-1.30556
-1.25699	-1.29448	-1.29133	-1.28821	-1.28506	-1.28191	-1.27879	-1.27569	-1.30559
-1.25705	-1.29454	-1.29138	-1.28826	-1.28511	-1.28196	-1.27884	-1.27575	-1.30564
-1.2571	-1.29459	-1.29143	-1.28831	-1.28516	-1.28202	-1.2789	-1.2758	-1.30569
-1.25716	-1.29465	-1.29149	-1.28837	-1.28522	-1.28207	-1.27895	-1.27585	-1.30574
-1.25721	-1.29471	-1.29155	-1.28843	-1.28528	-1.28213	-1.279	-1.2759	-1.30579
-1.25726	-1.29476	-1.2916	-1.28848	-1.28533	-1.28218	-1.27906	-1.27595	-1.30584

-1.25731	-1.25415	-1.25109	-1.24807	-1.28538	-1.28223	-1.27911	-1.27601	-1.3059
-1.25736	-1.2542	-1.25114	-1.24812	-1.28544	-1.28229	-1.27917	-1.27606	-1.30596
-1.25742	-1.25426	-1.2512	-1.24818	-1.28549	-1.28235	-1.27923	-1.27612	-1.30603
-1.25748	-1.25432	-1.25126	-1.24823	-1.28555	-1.2824	-1.27928	-1.27618	-1.30608
-1.25754	-1.25438	-1.25132	-1.24829	-1.28562	-1.28247	-1.27934	-1.27623	-1.30614
-1.25761	-1.25445	-1.25138	-1.24835	-1.28568	-1.28253	-1.27941	-1.2763	-1.27329
-1.25769	-1.25453	-1.25144	-1.2484	-1.28573	-1.2826	-1.27948	-1.27638	-1.27336
-1.25779	-1.25463	-1.25153	-1.24847	-1.2858	-1.28267	-1.27956	-1.27648	-1.27347
-1.25792	-1.25479	-1.25168	-1.24859	-1.2859	-1.28274	-1.27964	-1.2766	-1.27361
-1.25804	-1.25493	-1.25182	-1.24872	-1.28601	-1.28282	-1.27973	-1.27672	-1.27375
-1.25811	-1.25501	-1.2519	-1.2488	-1.2457	-1.24262	-1.2798	-1.2768	-1.27382
-1.25817	-1.25505	-1.25196	-1.24887	-1.24578	-1.24269	-1.27988	-1.27686	-1.27388
-1.19011	-1.25515	-1.25203	-1.24894	-1.24586	-1.24281	-1.28	-1.27697	-1.31549
-1.19022	-1.25527	-1.25213	-1.24901	-1.24596	-1.24294	-1.28016	-1.27711	-1.31563
-1.1903	-1.18719	-1.18409	-1.18103	-1.24604	-1.24305	-1.28028	-1.27722	-1.31575
-1.19034	-1.18725	-1.18415	-1.18108	-1.24611	-1.2431	-1.28034	-1.2773	-1.31585
-1.19038	-1.1873	-1.18421	-1.18115	-1.2462	-1.24317	-1.24019	-1.23726	-1.31595
-1.19045	-1.18736	-1.18429	-1.18125	-1.24634	-1.24331	-1.24031	-1.23736	-1.31604
-1.19052	-1.18743	-1.18438	-1.18137	-1.24649	-1.24346	-1.24045	-1.23747	-1.31613
-1.19061	-1.18753	-1.18449	-1.18147	-1.17846	-1.17547	-1.24056	-1.23759	-1.31629
-1.19071	-1.18764	-1.1846	-1.18157	-1.17855	-1.17554	-1.24067	-1.23773	-1.31647
-1.19081	-1.18774	-1.18469	-1.18165	-1.17863	-1.17563	-1.24079	-1.23787	-1.31663
-1.19091	-1.18784	-1.18477	-1.18172	-1.17871	-1.17574	-1.24094	-1.23801	-1.31677
-1.19101	-1.18792	-1.18485	-1.18181	-1.17881	-1.17585	-1.17293	-1.17004	-1.31691
-1.19108	-1.18799	-1.18494	-1.18193	-1.17894	-1.17598	-1.17304	-1.17013	-1.31702
-1.19116	-1.18807	-1.18505	-1.18206	-1.17907	-1.17611	-1.17317	-1.17023	-1.27782
-1.19125	-1.18819	-1.18517	-1.18218	-1.1792	-1.17624	-1.17331	-1.1704	-1.27804
-1.19136	-1.18832	-1.1853	-1.1823	-1.17932	-1.17637	-1.17346	-1.17058	-1.27828
-1.19149	-1.18844	-1.18541	-1.18241	-1.17944	-1.17648	-1.17357	-1.17069	-1.24924
-1.19164	-1.18856	-1.18553	-1.18253	-1.17955	-1.17658	-1.17366	-1.17076	-1.24933
-1.19177	-1.18868	-1.18565	-1.18265	-1.17965	-1.17667	-1.17374	-1.17085	-1.24945
-1.19188	-1.18882	-1.18579	-1.18278	-1.17975	-1.17674	-1.1738	-1.17095	-1.2496
-1.15219	-1.18895	-1.18592	-1.1829	-1.17986	-1.17684	-1.17391	-1.17107	-1.24974
-1.1523	-1.18907	-1.18604	-1.18302	-1.18001	-1.17701	-1.17408	-1.1712	-1.24985
-1.15242	-1.18918	-1.18615	-1.18314	-1.18015	-1.17718	-1.17424	-1.17133	-1.24996
-1.15252	-1.18931	-1.18627	-1.18326	-1.18025	-1.17725	-1.17432	-1.17143	-1.2501
-1.15262	-1.18943	-1.1864	-1.18337	-1.18033	-1.17731	-1.17438	-1.17153	-1.25024
-1.11631	-1.18954	-1.18651	-1.1835	-1.18047	-1.17746	-1.17452	-1.17165	-1.25035
-1.11645	-1.18964	-1.18662	-1.18364	-1.18064	-1.17766	-1.17471	-1.17178	-1.25045
-1.1166	-1.18979	-1.18675	-1.18376	-1.18079	-1.17784	-1.17489	-1.17194	-1.2506
-1.11677	-1.19001	-1.18691	-1.18386	-1.18088	-1.17796	-1.17505	-1.17214	-1.25082
-1.11693	-1.15043	-1.14742	-1.14444	-1.181	-1.17808	-1.17519	-1.17231	-1.25103
-1.11703	-1.15058	-1.1476	-1.14464	-1.18118	-1.1782	-1.17528	-1.17243	-1.2512
-1.11713	-1.15071	-1.14777	-1.14484	-1.18136	-1.17833	-1.17539	-1.17254	-1.25135
-1.11724	-1.15081	-1.14788	-1.14498	-1.2264	-1.22313	-1.25782	-1.25462	-1.25148
-1.11737	-1.1509	-1.14798	-1.14509	-1.22659	-1.22336	-1.25804	-1.25478	-1.2516
-1.11753	-1.15106	-1.14811	-1.14521	-1.22676	-1.22356	-1.25825	-1.25497	-1.25177
-1.11771	-1.15127	-1.14828	-1.14534	-1.22689	-1.22374	-1.25845	-1.25519	-1.252
-1.11787	-1.15146	-1.14846	-1.14549	-1.22707	-1.22392	-1.25866	-1.25541	-1.25222
-1.11799	-1.15162	-1.14864	-1.14567	-1.22729	-1.22414	-1.25888	-1.25564	-1.25244

-1.11812	-1.15178	-1.14882	-1.14587	-1.22751	-1.22434	-1.25908	-1.25585	-1.25265
-1.11829	-1.15197	-1.14902	-1.14608	-1.22772	-1.22449	-1.25922	-1.25603	-1.25284
-1.11847	-1.15215	-1.14921	-1.14629	-1.22792	-1.22465	-1.25936	-1.25619	-1.25303
-1.11863	-1.1523	-1.14937	-1.14645	-1.22812	-1.22485	-1.25956	-1.25639	-1.25323
-1.11878	-1.15242	-1.1495	-1.1466	-1.22833	-1.22508	-1.2598	-1.2566	-1.25343
-1.11896	-1.11602	-1.11319	-1.11039	-1.1897	-1.18657	-1.26002	-1.25679	-1.2536
-1.11917	-1.11623	-1.11339	-1.11059	-1.18993	-1.18679	-1.26023	-1.25694	-1.25373
-1.11938	-1.11644	-1.1136	-1.11079	-1.19016	-1.18702	-1.26045	-1.25713	-1.2539
-1.11957	-1.11665	-1.1138	-1.11099	-1.19039	-1.18726	-1.26069	-1.25736	-1.25412
-1.11975	-1.11685	-1.11401	-1.11119	-1.19063	-1.18749	-1.26094	-1.25761	-1.25437
-1.07787	-1.11706	-1.11421	-1.11139	-1.19086	-1.18772	-1.26119	-1.25785	-1.2546
-1.07806	-1.11727	-1.11442	-1.1116	-1.1911	-1.18796	-1.26143	-1.2581	-1.25485
-1.04036	-1.11747	-1.11462	-1.1118	-1.19133	-1.18819	-1.26168	-1.25834	-1.25508
-1.04056	-1.11767	-1.11482	-1.11199	-1.19156	-1.18842	-1.26192	-1.25858	-1.25532
-1.04075	-1.11788	-1.11502	-1.11219	-1.19179	-1.18865	-1.26216	-1.25881	-1.25555
-1.04095	-1.11808	-1.11522	-1.11239	-1.19202	-1.18888	-1.2624	-1.25905	-1.25579
-1.04114	-1.07626	-1.0735	-1.07078	-1.19225	-1.1891	-1.26264	-1.25929	-1.25602
-1.04133	-1.07645	-1.07369	-1.07097	-1.19247	-1.18933	-1.26288	-1.25952	-1.25625
-1.04151	-1.07664	-1.07388	-1.07115	-1.1927	-1.18955	-1.26312	-1.25976	-1.25649
-1.04169	-1.07682	-1.07407	-1.07134	-1.19293	-1.18979	-1.26337	-1.26001	-1.25674
-1.04188	-1.07701	-1.07425	-1.07153	-1.19316	-1.19003	-1.26363	-1.26028	-1.257
-1.04209	-1.07721	-1.07444	-1.07171	-1.11666	-1.11375	-1.22575	-1.22254	-1.25731
-1.04232	-1.07742	-1.07463	-1.07189	-1.11685	-1.11395	-1.22601	-1.22285	-1.25767
-1.04256	-1.07765	-1.07486	-1.07211	-1.11707	-1.11416	-1.22627	-1.22313	-1.25797
-1.04281	-1.07794	-1.07514	-1.07237	-1.11733	-1.11439	-1.22651	-1.22336	-1.25821
-1.04304	-1.0782	-1.0754	-1.07263	-1.11758	-1.11463	-1.18741	-1.18438	-1.25846
-1.04323	-1.07839	-1.0756	-1.07283	-1.11784	-1.11491	-1.18772	-1.18468	-1.25876
-1.04342	-1.07858	-1.07579	-1.07303	-1.11809	-1.1152	-1.18804	-1.18498	-1.25907
-1.04367	-1.08304	-1.08019	-1.07738	-1.15855	-1.15554	-1.18828	-1.18523	-1.22134
-1.04394	-1.0833	-1.08043	-1.0776	-1.15876	-1.15574	-1.1885	-1.18546	-1.2216
-1.04417	-1.08354	-1.08068	-1.07785	-1.15903	-1.15599	-1.18875	-1.1857	-1.22186
-1.04435	-1.08375	-1.08092	-1.07813	-1.15936	-1.15629	-1.18903	-1.18596	-1.22211
-1.04455	-1.08396	-1.08116	-1.07839	-1.15966	-1.15658	-1.18932	-1.18623	-1.18319
-1.04479	-1.08417	-1.08137	-1.0786	-1.1599	-1.15684	-1.18961	-1.18654	-1.18351
-1.04504	-1.08441	-1.0816	-1.07883	-1.16016	-1.1571	-1.18991	-1.18686	-1.18384
-1.04531	-1.0847	-1.08188	-1.0791	-1.16046	-1.15741	-1.19023	-1.18717	-1.18413
-1.04559	-1.08502	-1.08219	-1.0794	-1.16079	-1.15774	-1.19055	-1.18747	-1.18441
-1.04587	-1.08532	-1.08249	-1.07969	-1.11389	-1.11107	-1.10827	-1.10548	-1.18469
-1.04617	-1.08561	-1.08279	-1.07999	-1.11419	-1.11135	-1.10853	-1.10572	-1.18496
-1.04646	-1.08591	-1.08308	-1.08027	-1.11447	-1.11161	-1.10878	-1.10597	-1.18526
-1.04675	-1.0862	-1.08337	-1.08055	-1.11473	-1.11185	-1.10903	-1.10625	-1.1856
-0.96097	-1.0865	-1.08364	-1.08081	-1.11498	-1.1121	-1.10929	-1.10654	-1.18594
-0.96123	-1.08678	-1.0839	-1.08105	-1.11524	-1.11239	-1.10958	-1.10682	-1.18625
-0.96149	-1.08705	-1.08415	-1.0813	-1.11551	-1.11269	-1.10988	-1.10711	-1.18656
-0.9617	-1.08728	-1.08441	-1.08158	-1.1158	-1.11297	-1.11017	-1.1074	-1.18691
-0.96191	-1.08752	-1.08468	-1.08188	-1.11611	-1.11326	-1.11046	-1.1077	-1.18726
-0.96219	-1.08785	-1.08498	-1.08217	-1.1164	-1.11356	-1.11075	-1.10799	-1.18758
-0.96254	-1.08824	-1.08532	-1.08245	-1.11668	-1.11386	-1.11106	-1.10827	-1.18788
-0.96285	-1.0041	-1.00138	-0.9987	-1.11699	-1.11416	-1.11134	-1.10855	-1.1882
-0.96311	-1.0044	-1.00171	-0.99904	-1.11733	-1.11443	-1.1116	-1.10883	-1.18854

-0.96336	-1.00468	-1.00202	-0.99937	-1.11768	-1.11474	-1.11188	-1.10912	-1.18887
-0.96361	-1.00493	-1.00229	-0.99967	-1.11803	-1.1151	-1.11223	-1.10941	-1.18916
-0.96386	-1.00516	-1.00254	-0.99994	-1.11836	-1.11548	-1.1126	-1.10972	-1.18947
-0.9199	-1.00543	-1.0028	-1.00019	-1.11866	-1.1158	-1.11294	-1.11006	-1.15393
-0.92015	-1.00572	-1.00305	-1.00042	-1.11894	-1.1161	-1.11326	-1.11043	-1.15435
-0.9204	-1.00598	-1.00331	-1.00067	-1.11923	-1.11639	-1.11357	-1.11076	-1.15472
-0.92063	-1.00622	-1.00357	-1.00095	-1.11952	-1.11666	-1.11384	-1.11105	-1.15507
-0.92091	-1.0065	-1.00384	-1.00123	-1.03716	-1.03449	-1.11412	-1.11134	-1.1554
-0.92124	-1.00685	-1.00414	-1.00148	-1.03741	-1.03477	-1.11443	-1.11163	-1.1557
-0.92157	-1.0072	-1.00446	-1.00176	-1.03769	-1.03507	-1.11476	-1.11194	-1.15602
-0.92184	-1.00753	-1.00478	-1.00208	-1.03802	-1.0354	-1.11511	-1.11229	-1.1564
-0.9221	-1.00783	-1.00511	-1.00242	-1.03837	-1.03574	-1.11547	-1.11265	-1.1568
-0.92237	-1.00811	-1.0054	-1.00272	-1.03867	-1.03602	-1.11578	-1.11298	-1.15716
-0.92264	-1.00836	-1.00566	-1.00301	-1.03895	-1.03627	-1.11607	-1.11328	-1.1575
-0.92293	-1.00867	-1.00597	-1.00332	-1.03926	-1.03656	-1.03392	-1.03134	-1.15786
-0.92325	-1.00903	-1.00633	-1.00367	-1.03961	-1.0369	-1.03425	-1.03167	-1.15823
-0.92357	-1.0094	-1.0067	-1.00402	-1.03996	-1.03725	-1.03459	-1.03198	-1.15859
-0.92389	-1.00975	-1.00705	-1.00437	-1.04032	-1.0376	-1.03491	-1.03226	-1.15889
-0.9242	-1.0101	-1.00739	-1.00471	-1.04067	-1.03794	-1.03524	-1.03254	-1.15921
-0.92448	-0.92199	-0.91953	-0.91709	-0.95864	-0.95615	-1.03555	-1.03286	-1.03022
-0.92474	-0.92226	-0.91978	-0.91733	-0.95889	-0.95642	-1.03586	-1.03319	-1.03057
-0.92504	-0.92256	-0.92008	-0.91762	-0.95919	-0.95672	-1.0362	-1.03354	-1.03092
-0.92537	-0.92289	-0.92041	-0.91795	-0.95953	-0.95705	-1.03655	-1.03388	-1.03127
-0.9257	-0.92322	-0.92073	-0.91828	-0.95987	-0.95738	-1.0369	-1.03424	-1.03162
-0.92603	-0.92355	-0.92107	-0.91861	-0.96021	-0.95771	-1.03725	-1.03459	-1.03197
-0.92636	-0.92388	-0.9214	-0.91894	-0.96055	-0.95805	-1.03761	-1.03494	-1.03232
-0.92669	-0.92421	-0.92173	-0.91927	-0.9609	-0.95839	-1.03797	-1.0353	-1.03267
-0.92702	-0.92454	-0.92206	-0.9196	-0.96125	-0.95873	-1.03833	-1.03565	-1.03302
-0.92736	-0.92487	-0.92239	-0.91993	-0.96159	-0.95908	-1.03869	-1.03601	-1.03337
-0.92769	-0.92521	-0.92272	-0.92026	-0.96193	-0.95942	-1.03906	-1.03637	-1.03373
-0.92803	-0.92554	-0.92306	-0.92059	-0.96228	-0.95976	-1.03942	-1.03673	-1.03408
-0.92837	-0.92587	-0.92339	-0.92092	-0.96262	-0.96009	-1.03978	-1.03709	-1.03443
-0.84166	-0.92621	-0.92372	-0.92125	-0.91881	-0.9164	-1.04013	-1.03744	-1.03479
-0.84195	-0.92654	-0.92405	-0.92158	-0.91913	-0.91671	-1.04049	-1.03779	-1.03514
-0.84224	-0.92688	-0.92439	-0.92191	-0.91946	-0.91703	-1.04084	-1.03815	-1.03549
-0.84253	-0.92722	-0.92472	-0.92224	-0.91979	-0.91736	-1.04121	-1.0385	-1.03584
-0.84281	-0.92756	-0.92505	-0.92257	-0.92012	-0.9177	-1.04158	-1.03887	-1.03621
-0.79751	-0.9279	-0.92539	-0.9229	-0.92045	-0.91802	-1.00334	-1.00074	-1.03662
-0.79778	-0.92825	-0.92574	-0.92324	-0.92078	-0.91834	-1.00371	-1.00116	-1.03707
-0.79808	-0.92862	-0.92612	-0.92362	-0.92114	-0.91869	-1.00409	-1.00154	-1.03747
-0.79842	-0.92902	-0.92653	-0.92405	-0.92155	-0.91907	-1.00448	-1.0019	-1.03782
-0.79874	-0.79656	-0.79442	-0.79229	-0.92195	-0.91945	-0.96162	-0.95913	-1.03818
-0.79902	-0.79685	-0.7947	-0.79256	-0.9223	-0.91979	-0.96198	-0.95947	-1.03854
-0.7993	-0.79714	-0.79498	-0.79283	-0.92265	-0.92014	-0.96235	-0.95984	-1.03891
-0.79961	-0.79748	-0.79531	-0.79314	-0.92304	-0.92055	-0.96277	-0.96023	-1.03933
-0.79993	-0.79783	-0.79565	-0.79347	-0.92344	-0.92097	-0.9632	-0.96064	-1.03975
-0.80023	-0.79813	-0.79595	-0.79376	-0.9238	-0.92134	-0.96359	-0.96102	-1.04014
-0.80051	-0.79839	-0.79622	-0.79404	-0.92415	-0.92168	-0.96395	-0.96137	-1.04051
-0.75867	-0.79867	-0.79651	-0.79435	-0.79219	-0.79004	-0.96433	-0.96175	-1.04092
-0.75899	-0.79898	-0.79684	-0.79471	-0.79254	-0.79037	-0.96475	-0.96219	-1.04139

-0.75928	-0.79928	-0.79716	-0.79504	-0.79289	-0.79071	-0.96519	-0.96263	-1.04185
-0.75953	-0.79956	-0.79742	-0.79531	-0.79319	-0.79107	-0.96564	-0.96305	-1.04229
-0.75979	-0.79983	-0.79769	-0.79557	-0.79348	-0.79141	-0.96608	-0.96346	-1.0427
-0.66151	-0.75803	-0.75598	-0.75396	-0.79377	-0.7917	-0.83484	-0.83257	-1.00061
-0.66177	-0.75836	-0.7563	-0.75427	-0.79407	-0.79197	-0.83512	-0.83286	-1.00097
-0.66202	-0.75867	-0.75662	-0.75459	-0.79439	-0.79227	-0.83543	-0.83319	-1.00139
-0.66226	-0.75897	-0.75693	-0.75492	-0.79474	-0.7926	-0.83578	-0.83356	-1.00186
-0.66251	-0.75927	-0.75724	-0.75523	-0.75321	-0.75118	-0.79081	-0.7887	-0.91896
-0.66276	-0.75959	-0.75755	-0.75553	-0.75351	-0.7515	-0.79116	-0.78904	-0.91934
-0.66303	-0.75992	-0.75786	-0.75583	-0.75382	-0.75182	-0.79151	-0.78937	-0.91972
-0.66329	-0.76024	-0.75818	-0.75616	-0.75414	-0.75213	-0.79183	-0.78972	-0.92013
-0.66355	-0.76056	-0.7585	-0.75648	-0.75445	-0.75243	-0.79215	-0.79006	-0.92054
-0.66381	-0.76089	-0.75882	-0.75679	-0.75476	-0.75274	-0.79249	-0.79039	-0.92092
-0.66407	-0.76123	-0.75914	-0.75709	-0.75507	-0.75307	-0.79284	-0.79071	-0.92128
-0.66433	-0.66261	-0.66089	-0.65919	-0.75537	-0.75338	-0.75137	-0.74936	-0.83625
-0.6646	-0.66284	-0.66112	-0.65944	-0.75568	-0.75367	-0.75167	-0.74967	-0.83661
-0.66487	-0.66308	-0.66137	-0.65969	-0.75599	-0.75397	-0.75197	-0.75	-0.837
-0.66513	-0.66334	-0.66161	-0.65992	-0.75629	-0.75428	-0.7523	-0.75034	-0.83741
-0.66539	-0.66361	-0.66186	-0.66015	-0.7566	-0.7546	-0.75263	-0.75069	-0.83781
-0.66565	-0.66385	-0.66211	-0.66041	-0.75694	-0.75496	-0.75298	-0.75101	-0.79071
-0.6659	-0.66408	-0.66235	-0.66067	-0.75729	-0.75533	-0.75334	-0.75133	-0.79102
-0.66616	-0.66434	-0.66262	-0.66095	-0.75763	-0.75566	-0.75366	-0.75164	-0.79135
-0.66642	-0.66461	-0.6629	-0.66123	-0.75797	-0.75596	-0.75395	-0.75194	-0.79169
-0.66671	-0.6649	-0.66318	-0.6615	-0.6598	-0.65812	-0.75425	-0.75225	-0.75025
-0.66703	-0.66523	-0.66347	-0.66175	-0.66005	-0.65837	-0.75456	-0.75254	-0.75053
-0.66734	-0.66553	-0.66375	-0.662	-0.6603	-0.65864	-0.7549	-0.75286	-0.75084
-0.66762	-0.66579	-0.66401	-0.66226	-0.66058	-0.65893	-0.75528	-0.75324	-0.75122
-0.66789	-0.66605	-0.66427	-0.66253	-0.66085	-0.65921	-0.75565	-0.75361	-0.7516
-0.66816	-0.66632	-0.66455	-0.66282	-0.66112	-0.65946	-0.75597	-0.75394	-0.75193
-0.66843	-0.66661	-0.66485	-0.66311	-0.66139	-0.65969	-0.75625	-0.75424	-0.75225
-0.66874	-0.66692	-0.66513	-0.66337	-0.66164	-0.65993	-0.65826	-0.65661	-0.75258
-0.66908	-0.66724	-0.66541	-0.66361	-0.66187	-0.66018	-0.65852	-0.65688	-0.75293
-0.6694	-0.66757	-0.6657	-0.66387	-0.66213	-0.66045	-0.6588	-0.65716	-0.75329
-0.66972	-0.66789	-0.66602	-0.66417	-0.66242	-0.66074	-0.65908	-0.65746	-0.75366
-0.67003	-0.66821	-0.66633	-0.66448	-0.66273	-0.66104	-0.65937	-0.65774	-0.75402
-0.67034	-0.66853	-0.66665	-0.66479	-0.66303	-0.66134	-0.65965	-0.65799	-0.75431
-0.67065	-0.66883	-0.66696	-0.6651	-0.66334	-0.66164	-0.65993	-0.65823	-0.7546
-0.67095	-0.6691	-0.66724	-0.6654	-0.66364	-0.66194	-0.66022	-0.6585	-0.75493
-0.67122	-0.66935	-0.6675	-0.66569	-0.66394	-0.66223	-0.66052	-0.6588	-0.7553
-0.67153	-0.66964	-0.6678	-0.666	-0.66425	-0.66254	-0.66082	-0.65909	-0.6574
-0.67186	-0.66997	-0.66812	-0.66632	-0.66456	-0.66284	-0.66112	-0.65939	-0.6577
-0.61631	-0.6703	-0.66845	-0.66664	-0.66488	-0.66315	-0.66142	-0.65969	-0.65799
-0.61658	-0.67061	-0.66876	-0.66695	-0.66518	-0.66345	-0.66172	-0.65999	-0.65829
-0.61685	-0.67093	-0.66908	-0.66727	-0.66549	-0.66375	-0.66201	-0.66028	-0.65858
-0.61713	-0.67126	-0.66941	-0.66759	-0.66581	-0.66405	-0.6623	-0.66057	-0.65887
-0.61742	-0.67159	-0.66974	-0.66792	-0.66613	-0.66435	-0.6626	-0.66086	-0.65916
-0.6177	-0.67192	-0.67007	-0.66825	-0.66645	-0.66467	-0.66291	-0.66116	-0.65945
-0.61799	-0.67225	-0.6704	-0.66858	-0.66678	-0.665	-0.66323	-0.66147	-0.65975
-0.5673	-0.6166	-0.61496	-0.61336	-0.66711	-0.66533	-0.66355	-0.66178	-0.66005
-0.56757	-0.61688	-0.61524	-0.61364	-0.66743	-0.66565	-0.66387	-0.66209	-0.66035

-0.56783	-0.61716	-0.61552	-0.61391	-0.66776	-0.66598	-0.66419	-0.66239	-0.66064
-0.5681	-0.61743	-0.6158	-0.61419	-0.66808	-0.6663	-0.6645	-0.66269	-0.66093
-0.56836	-0.61772	-0.61607	-0.61446	-0.66841	-0.66662	-0.66482	-0.663	-0.66123
-0.56863	-0.618	-0.61636	-0.61474	-0.66873	-0.66695	-0.66515	-0.66335	-0.66158
-0.5689	-0.6183	-0.61665	-0.61502	-0.66906	-0.66728	-0.66549	-0.66371	-0.66195
-0.51655	-0.61863	-0.61697	-0.61532	-0.6694	-0.66761	-0.66582	-0.66405	-0.66229
-0.51683	-0.61898	-0.61731	-0.61564	-0.66975	-0.66794	-0.66614	-0.66436	-0.6626
-0.51709	-0.56832	-0.56679	-0.56527	-0.61431	-0.61271	-0.66646	-0.66468	-0.66291
-0.51732	-0.56858	-0.56705	-0.56552	-0.61459	-0.61298	-0.66677	-0.66499	-0.66323
-0.51754	-0.56884	-0.56732	-0.56579	-0.61488	-0.61326	-0.6671	-0.66531	-0.66355
-0.51779	-0.56911	-0.56761	-0.56612	-0.61522	-0.61358	-0.66745	-0.66564	-0.66387
-0.51804	-0.56938	-0.56791	-0.56645	-0.61557	-0.61392	-0.66783	-0.66599	-0.6642
-0.51827	-0.56965	-0.56819	-0.56673	-0.61589	-0.61426	-0.66823	-0.66636	-0.66454
-0.51851	-0.56992	-0.56845	-0.567	-0.61619	-0.6146	-0.66865	-0.66676	-0.6649
-0.51878	-0.5702	-0.56872	-0.56726	-0.56581	-0.56437	-0.61329	-0.61166	-0.66529
-0.51907	-0.57051	-0.56901	-0.56753	-0.56607	-0.56462	-0.61357	-0.61198	-0.6657
-0.51935	-0.51795	-0.51658	-0.51523	-0.56634	-0.56489	-0.61384	-0.61228	-0.66607
-0.51961	-0.51824	-0.51689	-0.51554	-0.56667	-0.56518	-0.61412	-0.61254	-0.66638
-0.51986	-0.51852	-0.51719	-0.51585	-0.56701	-0.56548	-0.61442	-0.6128	-0.66669
-0.52011	-0.51876	-0.51743	-0.5161	-0.56731	-0.5658	-0.56431	-0.56282	-0.66704
-0.52035	-0.51899	-0.51766	-0.51633	-0.5676	-0.56613	-0.56464	-0.56314	-0.66741
-0.40758	-0.51925	-0.5179	-0.51656	-0.56787	-0.56641	-0.56493	-0.56345	-0.6678
-0.4078	-0.51951	-0.51815	-0.5168	-0.56813	-0.56667	-0.56521	-0.56375	-0.66821
-0.40801	-0.5198	-0.51843	-0.51707	-0.56843	-0.56696	-0.56549	-0.56404	-0.5626
-0.40822	-0.5201	-0.51875	-0.5174	-0.56879	-0.56727	-0.56578	-0.5643	-0.56285
-0.40842	-0.40743	-0.40644	-0.40543	-0.51633	-0.51495	-0.56608	-0.56459	-0.56312
-0.40864	-0.40762	-0.40662	-0.40561	-0.51658	-0.51522	-0.56641	-0.56493	-0.56345
-0.40885	-0.40782	-0.4068	-0.40579	-0.51682	-0.51549	-0.56674	-0.56527	-0.56379
-0.40906	-0.40804	-0.40703	-0.40603	-0.51712	-0.51576	-0.56705	-0.56556	-0.56407
-0.40927	-0.40826	-0.40728	-0.40628	-0.51743	-0.51604	-0.56734	-0.56583	-0.56435
-0.40946	-0.40846	-0.40748	-0.40649	-0.5177	-0.51633	-0.56767	-0.56616	-0.56467
-0.40965	-0.40864	-0.40765	-0.40667	-0.51795	-0.51661	-0.56802	-0.56653	-0.56504
-0.40986	-0.40884	-0.40785	-0.40687	-0.40586	-0.40485	-0.56835	-0.56687	-0.56538
-0.4101	-0.40908	-0.4081	-0.40711	-0.40609	-0.40506	-0.56864	-0.56714	-0.56565
-0.34944	-0.40932	-0.40833	-0.40734	-0.40631	-0.40527	-0.56894	-0.56743	-0.56594
-0.34963	-0.40954	-0.40852	-0.40752	-0.4065	-0.40548	-0.56927	-0.56777	-0.56629
-0.34982	-0.40974	-0.40871	-0.4077	-0.40669	-0.40569	-0.5696	-0.56812	-0.56664
-0.34999	-0.40996	-0.40893	-0.40792	-0.40692	-0.40592	-0.40492	-0.40389	-0.56696
-0.35016	-0.41018	-0.40916	-0.40815	-0.40716	-0.40617	-0.40516	-0.40411	-0.56726
-0.29165	-0.41041	-0.40939	-0.40837	-0.40737	-0.40638	-0.40537	-0.40433	-0.56758
-0.29181	-0.41067	-0.40962	-0.40858	-0.40756	-0.40656	-0.40555	-0.40454	-0.56792
-0.29197	-0.4109	-0.40984	-0.40879	-0.40776	-0.40676	-0.40575	-0.40476	-0.45888
-0.29209	-0.41109	-0.41004	-0.40901	-0.408	-0.40699	-0.40599	-0.405	-0.45918
-0.29221	-0.41129	-0.41026	-0.40925	-0.40824	-0.40724	-0.40624	-0.40524	-0.45946
-0.29236	-0.29167	-0.29099	-0.29033	-0.40848	-0.40748	-0.40647	-0.40545	-0.4597
-0.29252	-0.29184	-0.29116	-0.29049	-0.40873	-0.40772	-0.4067	-0.40566	-0.45993
-0.29268	-0.292	-0.29132	-0.29065	-0.40897	-0.40794	-0.40691	-0.40587	-0.46019
-0.29284	-0.29216	-0.29149	-0.29081	-0.4092	-0.40815	-0.40711	-0.40608	-0.46046
-0.29299	-0.29231	-0.29163	-0.29095	-0.40941	-0.40836	-0.40732	-0.4063	-0.4053
-0.29312	-0.29243	-0.29174	-0.29105	-0.40959	-0.40857	-0.40755	-0.40654	-0.40554

-0.29325	-0.29255	-0.29186	-0.29117	-0.35257	-0.35173	-0.40779	-0.40678	-0.40578
-0.29338	-0.29268	-0.292	-0.29132	-0.35277	-0.35192	-0.40803	-0.40702	-0.40602
-0.29351	-0.29282	-0.29215	-0.29148	-0.35298	-0.35213	-0.40828	-0.40727	-0.40627
-0.29366	-0.29297	-0.2923	-0.29164	-0.35319	-0.35234	-0.40853	-0.40752	-0.40651
-0.29381	-0.29313	-0.29246	-0.29179	-0.3534	-0.35254	-0.40877	-0.40776	-0.40675
-0.23746	-0.29329	-0.29262	-0.29195	-0.29128	-0.29061	-0.40902	-0.40801	-0.40699
-0.23761	-0.29344	-0.29277	-0.2921	-0.29144	-0.29077	-0.40926	-0.40825	-0.40722
-0.23777	-0.29359	-0.29292	-0.29226	-0.29159	-0.29093	-0.40951	-0.40848	-0.40744
-0.23792	-0.29375	-0.29308	-0.29241	-0.29175	-0.29108	-0.40974	-0.40869	-0.40764
-0.23807	-0.2375	-0.23694	-0.23638	-0.2919	-0.29123	-0.35268	-0.35178	-0.40785
-0.23823	-0.23766	-0.23709	-0.23653	-0.29205	-0.29138	-0.35288	-0.35198	-0.40809
-0.23838	-0.23781	-0.23724	-0.23668	-0.2922	-0.29153	-0.35308	-0.35218	-0.40834
-0.18118	-0.23796	-0.23739	-0.23683	-0.29234	-0.29168	-0.35329	-0.35239	-0.40859
-0.18129	-0.23811	-0.23755	-0.23698	-0.29249	-0.29184	-0.35349	-0.35259	-0.40884
-0.1814	-0.23827	-0.2377	-0.23713	-0.29264	-0.29197	-0.35367	-0.35278	-0.40908
-0.18151	-0.23842	-0.23785	-0.23728	-0.29278	-0.2921	-0.35384	-0.35295	-0.40931
-0.12105	-0.23858	-0.23801	-0.23744	-0.23686	-0.23628	-0.29156	-0.29088	-0.35227
-0.12113	-0.23873	-0.23816	-0.23759	-0.23702	-0.23645	-0.29173	-0.29105	-0.35249
-0.12121	-0.23889	-0.23831	-0.23774	-0.23718	-0.23661	-0.2919	-0.29122	-0.35271
-0.12129	-0.12099	-0.12068	-0.12038	-0.23733	-0.23677	-0.29205	-0.29139	-0.35295
-0.12137	-0.12107	-0.12076	-0.12046	-0.23748	-0.23692	-0.2922	-0.29155	-0.35318
-0.12145	-0.12115	-0.12084	-0.12054	-0.23764	-0.23707	-0.23651	-0.23596	-0.35339
-0.12153	-0.12123	-0.12093	-0.12062	-0.23779	-0.23722	-0.23666	-0.23611	-0.35358
-0.0615	-0.12131	-0.12101	-0.1207	-0.1204	-0.1201	-0.23681	-0.23626	-0.35377
-0.06155	-0.12139	-0.12109	-0.12078	-0.12048	-0.12018	-0.23696	-0.2364	-0.35396
-0.06159	-0.12147	-0.12117	-0.12086	-0.12056	-0.12026	-0.17832	-0.17789	-0.236
-0.06164	-0.12155	-0.12125	-0.12094	-0.12064	-0.12033	-0.17845	-0.17801	-0.23615
-0.06168	-0.12163	-0.12133	-0.12103	-0.12072	-0.12041	-0.17857	-0.17813	-0.2363
-0.06172	-0.06158	-0.06143	-0.06127	-0.1208	-0.1205	-0.12019	-0.1199	-0.17782
-0.06177	-0.06162	-0.06147	-0.06132	-0.12088	-0.12058	-0.12028	-0.11998	-0.17794
-0.06181	-0.06166	-0.06151	-0.06136	-0.12096	-0.12066	-0.12036	-0.12006	-0.17807
-0.06185	-0.06171	-0.06156	-0.06141	-0.12104	-0.12074	-0.12043	-0.12013	-0.17819
-0.0619	-0.06175	-0.06161	-0.06146	-0.12113	-0.12083	-0.12052	-0.12022	-0.11992
-0.06195	-0.06181	-0.06166	-0.06151	-0.12123	-0.12093	-0.12063	-0.12032	-0.12002
-0.06201	-0.06186	-0.06171	-0.06157	-0.06142	-0.06126	-0.12073	-0.12042	-0.12012
-0.06205	-0.0619	-0.06176	-0.06161	-0.06146	-0.06131	-0.12081	-0.1205	-0.1202
-0.06209	-0.06194	-0.0618	-0.06165	-0.06151	-0.06135	-0.12089	-0.12058	-0.12028
-0.06214	-0.06199	-0.06184	-0.0617	-0.06155	-0.0614	-0.12097	-0.12066	-0.12036
-0.06218	-0.06203	-0.06188	-0.06174	-0.06159	-0.06144	-0.12105	-0.12074	-0.12044
-0.06223	-0.06208	-0.06193	-0.06179	-0.06164	-0.06149	-0.06134	-0.06119	-0.12053
-0.06228	-0.06214	-0.06199	-0.06184	-0.0617	-0.06155	-0.0614	-0.06125	-0.12064
-0.06234	-0.06219	-0.06204	-0.06189	-0.06175	-0.0616	-0.06145	-0.0613	-0.12073
-0.06238	-0.06223	-0.06208	-0.06194	-0.06179	-0.06165	-0.0615	-0.06135	-0.12081
-0.06243	-0.06228	-0.06212	-0.06198	-0.06183	-0.06169	-0.06154	-0.06139	-0.06124
-0.06248	-0.06232	-0.06217	-0.06202	-0.06187	-0.06173	-0.06158	-0.06143	-0.06128
-0.06252	-0.06236	-0.06221	-0.06206	-0.06192	-0.06177	-0.06163	-0.06148	-0.06133
-0.06258	-0.06242	-0.06226	-0.06211	-0.06197	-0.06182	-0.06168	-0.06153	-0.06138
-0.06264	-0.06248	-0.06232	-0.06217	-0.06202	-0.06188	-0.06173	-0.06159	-0.06144
-0.06269	-0.06253	-0.06237	-0.06222	-0.06207	-0.06193	-0.06178	-0.06164	-0.06149
-0.06273	-0.06257	-0.06242	-0.06226	-0.06211	-0.06197	-0.06182	-0.06168	-0.06153

-0.06278	-0.06262	-0.06247	-0.06231	-0.06216	-0.06201	-0.06187	-0.06172	-0.06158
-0.06284	-0.06268	-0.06252	-0.06237	-0.06221	-0.06207	-0.06192	-0.06178	-0.06163
-0.0629	-0.06274	-0.06258	-0.06242	-0.06227	-0.06212	-0.06197	-0.06183	-0.06169
-0.06294	-0.06279	-0.06263	-0.06247	-0.06231	-0.06216	-0.06202	-0.06187	-0.06173
-0.06299	-0.06283	-0.06267	-0.06251	-0.06236	-0.06221	-0.06206	-0.06192	-0.06177
-0.06305	-0.06289	-0.06273	-0.06257	-0.06241	-0.06226	-0.06211	-0.06196	-0.06182
-0.0631	-0.06295	-0.06279	-0.06263	-0.06247	-0.06232	-0.06216	-0.06202	-0.06187
-0.06315	-0.063	-0.06284	-0.06268	-0.06252	-0.06237	-0.06222	-0.06207	-0.06193
-0.0632	-0.06305	-0.06289	-0.06273	-0.06257	-0.06241	-0.06226	-0.06211	-0.06197
-0.06325	-0.06309	-0.06294	-0.06278	-0.06262	-0.06246	-0.06231	-0.06216	-0.06202
-0.0633	-0.06315	-0.06299	-0.06284	-0.06268	-0.06252	-0.06236	-0.06221	-0.06207
-0.06336	-0.06321	-0.06305	-0.0629	-0.06274	-0.06258	-0.06242	-0.06227	-0.06212
-0.06341	-0.06326	-0.0631	-0.06295	-0.06279	-0.06263	-0.06248	-0.06232	-0.06217
-0.06345	-0.0633	-0.06315	-0.063	-0.06285	-0.06269	-0.06254	-0.06238	-0.06223
	-0.06335	-0.0632	-0.06305	-0.0629	-0.06274	-0.06259	-0.06243	-0.06228
	-0.06341	-0.06325	-0.0631	-0.06295	-0.06279	-0.06263	-0.06248	-0.06233
	-0.06346	-0.06331	-0.06316	-0.063	-0.06284	-0.06268	-0.06253	-0.06238
	-0.06352	-0.06337	-0.06321	-0.06306	-0.0629	-0.06274	-0.06258	-0.06243
	-0.06357	-0.06342	-0.06327	-0.06312	-0.06296	-0.0628	-0.06264	-0.06248
	-0.06362	-0.06347	-0.06333	-0.06317	-0.06302	-0.06286	-0.0627	-0.06254
	-0.06367	-0.06353	-0.06338	-0.06323	-0.06307	-0.06292	-0.06276	-0.0626
				-0.06328	-0.06313	-0.06297	-0.06282	-0.06266
				-0.06332	-0.06318	-0.06303	-0.06287	-0.06272
				-0.06337	-0.06323	-0.06308	-0.06293	-0.06277
				-0.06343	-0.06328	-0.06313	-0.06298	-0.06283
						-0.06318	-0.06303	-0.06288
						-0.06324	-0.06308	-0.06293
						-0.06329	-0.06314	-0.06298
						-0.06335	-0.06319	-0.06304
						-0.0634	-0.06325	-0.0631
								-0.06315
								-0.06321
								-0.06327
								-0.06332
								-0.06338
								-0.06343
								-0.06349

						-0.25678
						-0.25649
						-0.25621
						-0.31493
						-0.3146
						-0.31428
						-0.31395
				-0.31536	-0.31449	-0.37817
				-0.31502	-0.31414	-0.37774
				-0.31468	-0.3138	-0.52662
				-0.31433	-0.31346	-0.52612
				-0.31398	-0.31312	-0.52563
		-0.25639	-0.25565	-0.31366	-0.3128	-0.52517
		-0.25612	-0.2554	-0.31335	-0.3125	-0.52472
		-0.25586	-0.25514	-0.40754	-0.40647	-0.52424
		-0.25561	-0.25488	-0.40718	-0.4061	-0.52375
-0.25679	-0.25607	-0.31416	-0.31327	-0.46877	-0.46748	-0.52328
-0.25652	-0.25578	-0.31381	-0.31294	-0.46833	-0.46705	-0.52283
-0.25625	-0.2555	-0.40822	-0.40714	-0.5251	-0.52373	-0.52239
-0.25599	-0.25524	-0.40784	-0.40675	-0.5246	-0.52326	-0.52193

		-0.25573	-0.25498	-0.40747	-0.40637	-0.5241	-0.52279	-0.52149
		-0.25547	-0.25473	-0.40711	-0.40602	-0.52366	-0.52236	-0.52107
		-0.25521	-0.25448	-0.40677	-0.40569	-0.52324	-0.52195	-0.52066
-0.2564	-0.25568	-0.31366	-0.31278	-0.40643	-0.40536	-0.52281	-0.52151	-0.52023
-0.25614	-0.25542	-0.31335	-0.31247	-0.4061	-0.40502	-0.52237	-0.52106	-0.51976
-0.25588	-0.25516	-0.31304	-0.31216	-0.52467	-0.52329	-0.52194	-0.52061	-0.5193
-0.25561	-0.25488	-0.3127	-0.31184	-0.52422	-0.52287	-0.52152	-0.52019	-0.51888
-0.25534	-0.25461	-0.31237	-0.31152	-0.52377	-0.52244	-0.52111	-0.51978	-0.51845
-0.3138	-0.31291	-0.31206	-0.31122	-0.52335	-0.52202	-0.52069	-0.51935	-0.68472
-0.31349	-0.31261	-0.31176	-0.31093	-0.52293	-0.5216	-0.52027	-0.51893	-0.68413
-0.31318	-0.3123	-0.40612	-0.40511	-0.52251	-0.52118	-0.51984	-0.51849	-0.68353
-0.31287	-0.312	-0.40579	-0.40478	-0.52209	-0.52075	-0.51941	-0.51805	-0.68293
-0.31256	-0.3117	-0.40546	-0.40446	-0.52167	-0.52033	-0.51898	-0.51762	-0.73635
-0.31226	-0.3114	-0.40514	-0.40414	-0.52124	-0.5199	-0.51854	-0.51718	-0.7357
-0.31196	-0.31111	-0.52344	-0.52214	-0.52081	-0.51947	-0.51811	-0.51675	-0.73505
-0.31167	-0.31081	-0.52301	-0.52171	-0.52038	-0.51903	-0.51768	-0.51632	-0.73442
-0.31137	-0.31052	-0.52259	-0.52128	-0.51995	-0.5186	-0.51725	-0.51589	-0.73379
-0.31108	-0.31023	-0.52216	-0.52085	-0.51951	-0.51817	-0.68302	-0.68122	-0.73317
-0.31078	-0.30994	-0.52174	-0.52042	-0.51909	-0.51775	-0.68245	-0.68066	-0.73256
-0.3105	-0.30967	-0.52134	-0.52001	-0.51868	-0.51733	-0.68188	-0.68009	-0.73195
-0.31024	-0.30942	-0.52097	-0.51963	-0.51828	-0.51692	-0.6813	-0.67953	-0.73133
-0.46607	-0.46488	-0.52057	-0.51923	-0.51787	-0.5165	-0.68073	-0.67896	-0.73072
-0.46568	-0.46449	-0.52015	-0.51882	-0.51746	-0.51609	-0.68017	-0.6784	-0.7301
-0.4653	-0.4641	-0.51973	-0.5184	-0.68322	-0.6814	-0.73333	-0.7314	-0.72948
-0.46494	-0.46373	-0.51933	-0.51798	-0.68265	-0.68083	-0.73271	-0.73079	-0.72889
-0.46459	-0.46337	-0.51893	-0.51756	-0.68207	-0.68026	-0.7321	-0.73019	-0.72831
-0.52121	-0.51987	-0.51851	-0.51714	-0.6815	-0.67971	-0.7315	-0.72961	-0.72774
-0.5208	-0.51945	-0.51809	-0.51672	-0.68093	-0.67916	-0.73091	-0.72904	-0.72719
-0.52039	-0.51903	-0.57286	-0.57135	-0.73416	-0.73223	-0.73034	-0.72848	-0.72663
-0.51997	-0.51861	-0.57239	-0.57089	-0.73357	-0.73166	-0.72977	-0.72792	-0.72607
-0.51955	-0.5182	-0.57193	-0.57044	-0.73298	-0.73109	-0.72921	-0.72735	-0.72549
-0.51914	-0.51779	-0.57149	-0.57002	-0.73242	-0.73052	-0.72864	-0.72677	-0.7249
-0.51874	-0.5174	-0.57105	-0.5696	-0.73184	-0.72995	-0.72806	-0.72618	-0.72429
-0.57362	-0.57213	-0.73512	-0.73318	-0.73125	-0.72934	-0.72745	-0.72557	-0.72369
-0.57323	-0.57174	-0.73457	-0.7326	-0.73065	-0.72873	-0.72683	-0.72496	-0.72309
-0.5728	-0.5713	-0.73397	-0.73199	-0.73005	-0.72813	-0.72624	-0.72436	-0.77568
-0.57233	-0.57082	-0.73333	-0.73137	-0.72944	-0.72754	-0.72566	-0.72378	-0.77505
-0.57186	-0.57035	-0.7327	-0.73075	-0.72885	-0.72697	-0.7251	-0.72323	-0.77445
-0.57138	-0.56987	-0.73208	-0.73015	-0.72827	-0.72642	-0.72457	-0.72273	-0.7739
-0.57092	-0.56942	-0.7315	-0.72958	-0.72771	-0.72587	-0.72404	-0.7222	-0.77331
-0.57053	-0.56905	-0.73098	-0.72907	-0.72718	-0.72533	-0.72347	-0.72162	-0.77266
-0.57014	-0.56867	-0.73047	-0.72855	-0.72666	-0.72478	-0.7229	-0.72103	-0.772
-0.5697	-0.56823	-0.72988	-0.72798	-0.72608	-0.72419	-0.77555	-0.77344	-0.77136
-0.56924	-0.56776	-0.72927	-0.72739	-0.7255	-0.72361	-0.77491	-0.77281	-0.77072
-0.7325	-0.73057	-0.72868	-0.72682	-0.72495	-0.72307	-0.77433	-0.77222	-0.82397
-0.7319	-0.72999	-0.72812	-0.72628	-0.72443	-0.72258	-0.77379	-0.77168	-0.82337
-0.73134	-0.72943	-0.72757	-0.72573	-0.72389	-0.72205	-0.77322	-0.7711	-0.82274
-0.73084	-0.72893	-0.72704	-0.72517	-0.72332	-0.72147	-0.77258	-0.77047	-0.82207
-0.73031	-0.7284	-0.7265	-0.72461	-0.72275	-0.7209	-0.77196	-0.76985	-0.82141
-0.72972	-0.72782	-0.72592	-0.72403	-0.72217	-0.72033	-0.77135	-0.76927	-0.82083

-0.72913	-0.72723	-0.72534	-0.72345	-0.7216	-0.71978	-0.77075	-0.76871	-0.82024
-0.72861	-0.72671	-0.72482	-0.72294	-0.77428	-0.77221	-0.77015	-0.7681	-0.8196
-0.72811	-0.72622	-0.72433	-0.72246	-0.77374	-0.77164	-0.76955	-0.76748	-0.81892
-0.72757	-0.72568	-0.72381	-0.72195	-0.77318	-0.77108	-0.82275	-0.82052	-0.91858
-0.72697	-0.72509	-0.72324	-0.72141	-0.7726	-0.77054	-0.82219	-0.81997	-0.91788
-0.72641	-0.72454	-0.7227	-0.72087	-0.77202	-0.76996	-0.82159	-0.81938	-0.91713
-0.72592	-0.72404	-0.72218	-0.72033	-0.77141	-0.76934	-0.82091	-0.81871	-0.91628
-0.72543	-0.72355	-0.72167	-0.7198	-0.77081	-0.76872	-0.82025	-0.81806	-0.91545
-0.72495	-0.72307	-0.77447	-0.77236	-0.77027	-0.76817	-0.81966	-0.81748	-0.91473
-0.72445	-0.72257	-0.77392	-0.77182	-0.76973	-0.76764	-0.81909	-0.81692	-0.91403
-0.7239	-0.72202	-0.77331	-0.77122	-0.8229	-0.82067	-0.91872	-0.91595	-0.95636
-0.7233	-0.72143	-0.77267	-0.77058	-0.82223	-0.82001	-0.91785	-0.9151	-0.95548
-0.72277	-0.7209	-0.77209	-0.77001	-0.82162	-0.8194	-0.91708	-0.91434	-0.95469
-0.72229	-0.72043	-0.77157	-0.76949	-0.82107	-0.81887	-0.91641	-0.91368	-0.954
-0.72182	-0.71996	-0.77105	-0.76897	-0.82052	-0.81833	-0.95896	-0.95612	-0.95332
-0.72134	-0.71948	-0.77052	-0.76845	-0.81997	-0.81779	-0.95825	-0.95543	-0.95265
-0.72085	-0.71899	-0.76997	-0.7679	-0.8194	-0.81722	-0.9575	-0.95471	-0.95195
-0.77353	-0.77144	-0.76936	-0.7673	-0.81875	-0.81658	-0.95665	-0.95388	-0.95114
-0.77291	-0.77082	-0.76874	-0.76667	-0.81809	-0.81592	-0.95577	-0.95302	-0.9503
-0.77234	-0.77026	-0.8219	-0.81969	-0.96066	-0.9578	-0.955	-0.95227	-0.94955
-0.77183	-0.76975	-0.82135	-0.81915	-0.95995	-0.95711	-0.95432	-0.95159	-0.94888
-0.77133	-0.76925	-0.82082	-0.81862	-0.95924	-0.95642	-0.95364	-0.95092	-0.98872
-0.77082	-0.76875	-0.82028	-0.81809	-0.95854	-0.95573	-0.95296	-0.95024	-0.98799
-0.77031	-0.76824	-0.81975	-0.81756	-0.95783	-0.95504	-0.95228	-0.94956	-0.98726
-0.76981	-0.76774	-0.81921	-0.81703	-0.95712	-0.95435	-0.95159	-0.94886	-0.98652
-0.7693	-0.76724	-0.81867	-0.8165	-0.95641	-0.95365	-0.9509	-0.94816	-0.98575
-0.82253	-0.82031	-0.96139	-0.95852	-0.95571	-0.95295	-0.9502	-0.94743	-0.98494
-0.82197	-0.81975	-0.96065	-0.9578	-0.955	-0.95225	-0.94948	-0.94668	-0.9841
-0.82139	-0.81916	-0.95986	-0.95701	-0.95421	-0.95147	-0.9894	-0.98633	-1.02774
-0.82079	-0.81855	-0.95901	-0.95614	-0.95334	-0.95061	-0.98852	-0.9855	-1.02693
-0.82019	-0.81795	-0.9582	-0.95533	-0.95254	-0.94982	-0.98769	-0.98471	-1.02614
-0.81959	-0.81734	-0.95742	-0.9546	-0.95184	-0.94912	-0.98693	-0.98398	-1.0254
-0.819	-0.81676	-0.95666	-0.9539	-0.95115	-0.94844	-0.9862	-0.98326	-1.02466
-0.81843	-0.81622	-0.95595	-0.9532	-0.95046	-0.94775	-0.98547	-0.98254	-1.02392
-0.81787	-0.81569	-0.95526	-0.95251	-0.94977	-0.94707	-0.98475	-0.98183	-1.02318
-0.96024	-0.95737	-0.95457	-0.95182	-0.94909	-0.94639	-0.98402	-0.98111	-1.02244
-0.95957	-0.95668	-0.95389	-0.95114	-0.94842	-0.94571	-0.9833	-0.9804	-1.0217
-0.95888	-0.95599	-0.9532	-0.95046	-0.98849	-0.98552	-1.02697	-1.02395	-1.11009
-0.95817	-0.95529	-0.9525	-0.94978	-0.98777	-0.98481	-1.02623	-1.02321	-1.10927
-0.95746	-0.95459	-0.95181	-0.94909	-0.98704	-0.9841	-1.02549	-1.02248	-1.10845
-0.95674	-0.95387	-0.9511	-0.94839	-0.98632	-0.98338	-1.02476	-1.02175	-1.10764
-0.95604	-0.95317	-0.9504	-0.9477	-0.98558	-0.98267	-1.02402	-1.02102	-1.10683
-0.95542	-0.95253	-0.94973	-0.947	-0.98483	-0.98194	-1.02328	-1.02029	-1.10602
-0.95482	-0.95193	-0.94909	-0.94631	-0.98408	-0.98121	-1.02253	-1.01956	-1.10521
-0.9542	-0.95133	-0.98944	-0.98637	-0.98339	-0.9805	-1.11093	-1.10764	-1.10439
-0.95357	-0.95074	-0.98884	-0.98575	-0.98274	-0.9798	-1.11008	-1.1068	-1.10357
-0.95291	-0.95011	-0.9882	-0.98514	-0.98212	-0.97914	-1.10929	-1.10599	-1.10276
-0.95221	-0.94944	-0.98754	-0.98453	-0.98153	-0.97855	-1.10855	-1.10522	-1.10195
-0.9515	-0.94875	-0.98685	-0.9839	-1.07132	-1.06801	-1.10784	-1.10449	-1.10121
-0.95079	-0.94806	-0.98613	-0.98321	-1.0706	-1.06734	-1.10719	-1.10386	-1.10059

-0.95008	-0.94736	-0.9854	-0.9825	-1.06985	-1.06665	-1.10652	-1.10323	-1.09999
-0.94936	-0.94666	-0.98466	-0.98179	-1.06907	-1.06589	-1.10576	-1.1025	-1.09929
-0.94865	-0.94596	-0.98393	-0.98107	-1.06827	-1.0651	-1.10494	-1.10171	-1.09854
-0.98908	-0.98611	-1.0303	-1.02725	-1.11065	-1.10737	-1.10412	-1.10091	-1.09777
-0.98841	-0.98542	-1.02954	-1.02648	-1.10981	-1.10653	-1.1033	-1.10009	-1.09697
-0.98779	-0.98479	-1.02884	-1.02576	-1.10901	-1.10573	-1.10249	-1.09929	-1.09619
-0.98724	-0.98425	-1.02824	-1.02511	-1.10825	-1.10494	-1.10169	-1.0985	-1.09541
-0.98666	-0.9837	-1.02764	-1.02449	-1.10754	-1.1042	-1.10094	-1.09775	-1.09468
-0.98598	-0.98306	-1.02698	-1.02387	-1.1069	-1.10359	-1.10033	-1.09713	-1.09404
-0.98526	-0.98238	-1.0263	-1.02325	-1.10627	-1.10299	-1.09976	-1.09655	-1.09345
-1.0317	-1.02863	-1.02557	-1.02253	-1.10552	-1.10227	-1.09908	-1.09593	-1.09287
-1.03098	-1.02788	-1.02481	-1.02177	-1.1047	-1.10149	-1.09835	-1.09529	-1.09231
-1.03032	-1.02721	-1.02412	-1.02107	-1.10392	-1.10071	-1.0976	-1.0946	-1.13077
-1.02973	-1.02664	-1.02353	-1.02043	-1.10318	-1.09994	-1.09682	-1.09384	-1.13001
-1.0291	-1.02602	-1.06606	-1.06281	-1.10249	-1.09923	-1.09611	-1.09314	-1.1293
-1.02838	-1.02532	-1.06536	-1.06216	-1.10186	-1.09863	-1.09552	-1.09253	-1.12864
-1.02767	-1.02461	-1.06465	-1.0615	-1.10122	-1.09804	-1.09495	-1.09196	-1.12802
-1.02704	-1.02396	-1.10696	-1.1037	-1.10051	-1.09738	-1.09433	-1.09137	-1.17037
-1.02644	-1.02333	-1.10623	-1.10296	-1.09978	-1.0967	-1.0937	-1.0908	-1.16978
-1.02579	-1.0227	-1.10556	-1.10231	-1.09916	-1.0961	-1.09312	-1.09023	-1.16917
-1.02511	-1.02207	-1.10493	-1.10171	-1.09859	-1.09556	-1.09259	-1.08968	-1.16855
-1.02446	-1.02143	-1.10424	-1.10104	-1.09794	-1.09494	-1.13101	-1.12802	-1.20592
-1.02385	-1.02078	-1.10349	-1.10028	-1.09719	-1.09422	-1.13031	-1.12739	-1.20529
-1.02323	-1.02014	-1.10278	-1.09956	-1.09648	-1.09355	-1.12964	-1.12676	-1.20465
-1.0226	-1.01955	-1.10216	-1.09895	-1.09589	-1.09298	-1.12905	-1.12616	-1.20398
-1.02197	-1.01897	-1.10156	-1.09837	-1.09533	-1.09243	-1.12847	-1.12556	-1.20331
-1.0214	-1.01842	-1.10097	-1.09778	-1.09474	-1.09183	-1.12784	-1.12496	-1.20268
-1.02084	-1.01786	-1.10037	-1.09718	-1.09414	-1.09121	-1.1272	-1.12436	-1.20207
-1.06316	-1.06005	-1.09972	-1.09658	-1.13255	-1.12954	-1.12661	-1.12377	-1.20143
-1.06244	-1.05932	-1.09901	-1.09596	-1.13195	-1.12898	-1.12606	-1.12319	-1.20076
-1.06176	-1.05864	-1.09836	-1.09536	-1.13136	-1.12841	-1.1255	-1.12261	-1.24216
-1.06114	-1.05805	-1.09777	-1.09479	-1.13078	-1.12782	-1.12491	-1.12203	-1.24152
-1.1034	-1.10023	-1.09717	-1.09421	-1.13018	-1.12723	-1.12432	-1.12147	-1.24089
-1.10278	-1.09958	-1.09652	-1.09356	-1.12953	-1.12664	-1.12377	-1.12094	-1.2403
-1.10216	-1.09894	-1.09587	-1.09291	-1.12888	-1.12605	-1.12323	-1.12042	-1.2397
-1.10152	-1.09832	-1.09528	-1.09235	-1.12831	-1.1255	-1.20321	-1.20019	-1.23905
-1.10088	-1.09773	-1.09473	-1.09184	-1.12779	-1.12497	-1.20262	-1.19957	-1.23838
-1.10026	-1.09716	-1.13311	-1.13015	-1.12725	-1.12441	-1.202	-1.19895	-1.23773
-1.09967	-1.09661	-1.13256	-1.12962	-1.1267	-1.12383	-1.20136	-1.19833	-1.23709
-1.09909	-1.09606	-1.132	-1.12906	-1.12614	-1.12326	-1.24272	-1.23959	-1.30393
-1.09852	-1.09551	-1.13142	-1.12846	-1.12555	-1.12269	-1.24209	-1.23899	-1.30333
-1.09795	-1.09495	-1.13083	-1.12787	-1.12497	-1.12213	-1.24147	-1.23839	-1.30274
-1.09737	-1.09435	-1.13021	-1.12728	-1.1244	-1.12159	-1.24087	-1.2378	-1.30214
-1.09678	-1.09376	-1.1296	-1.12671	-1.12385	-1.12106	-1.24028	-1.23721	-1.30153
-1.09624	-1.09322	-1.12905	-1.12615	-1.12331	-1.12052	-1.30736	-1.30411	-1.30088
-1.09572	-1.09272	-1.12852	-1.12562	-1.12278	-1.11999	-1.30672	-1.30345	-1.3002
-1.09517	-1.0922	-1.12799	-1.1251	-1.20337	-1.20035	-1.30606	-1.30278	-1.29954
-1.09458	-1.09165	-1.12744	-1.12457	-1.20277	-1.1997	-1.30537	-1.30211	-1.2989
-1.1328	-1.12981	-1.1269	-1.12406	-1.27144	-1.26818	-1.30471	-1.30148	-1.29829
-1.13227	-1.12929	-1.1264	-1.12356	-1.27087	-1.26761	-1.30412	-1.30088	-1.2977

-1.13175	-1.12878	-1.12589	-1.12307	-1.27031	-1.26706	-1.30355	-1.30031	-1.29713
-1.13122	-1.12826	-1.19757	-1.19457	-1.30962	-1.30627	-1.30298	-1.29974	-1.29656
-1.13069	-1.12774	-1.19704	-1.19404	-1.30903	-1.30569	-1.3024	-1.29918	-1.29601
-1.13015	-1.12721	-1.1965	-1.19351	-1.30843	-1.3051	-1.30183	-1.29862	-1.29546
-1.12961	-1.12668	-1.19596	-1.19298	-1.30783	-1.30452	-1.30126	-1.29806	-1.29491
-1.20153	-1.19846	-1.27383	-1.27065	-1.30724	-1.30393	-1.30068	-1.2975	-1.29436
-1.20101	-1.19793	-1.27325	-1.27007	-1.30665	-1.30335	-1.3001	-1.29692	-1.29378
-1.20049	-1.19741	-1.27267	-1.2695	-1.30606	-1.30276	-1.29952	-1.29634	-1.29321
-1.19995	-1.19688	-1.2721	-1.26893	-1.30547	-1.30217	-1.29893	-1.29575	-1.29263
-1.19942	-1.19635	-1.27152	-1.26836	-1.30488	-1.30157	-1.29834	-1.29517	-1.29205
-1.19888	-1.19582	-1.27094	-1.26779	-1.30429	-1.301	-1.29777	-1.2946	-1.29148
-1.19834	-1.19529	-1.27037	-1.26722	-1.30371	-1.30043	-1.2972	-1.29404	-1.29093
-1.23982	-1.23666	-1.26979	-1.26665	-1.30313	-1.29985	-1.29663	-1.29348	-1.29036
-1.23927	-1.23611	-1.26921	-1.26608	-1.30254	-1.29926	-1.29605	-1.2929	-1.2898
-1.23875	-1.23557	-1.26865	-1.26552	-1.30196	-1.29869	-1.29547	-1.29233	-1.28923
-1.23827	-1.23507	-1.26811	-1.26496	-1.30138	-1.29811	-1.2949	-1.29175	-1.28865
-1.23778	-1.23457	-1.26757	-1.26441	-1.3008	-1.29756	-1.29436	-1.2912	-1.28809
-1.27352	-1.27022	-1.30676	-1.30348	-1.30024	-1.29705	-1.29388	-1.29071	-1.28759
-1.27295	-1.26966	-1.30618	-1.30289	-1.29969	-1.29655	-1.29341	-1.29023	-1.2871
-1.27242	-1.26916	-1.30565	-1.30235	-1.29914	-1.29602	-1.29288	-1.28971	-1.31921
-1.27192	-1.26871	-1.30516	-1.30183	-1.2986	-1.29545	-1.2923	-1.28916	-1.31866
-1.2714	-1.2682	-1.30464	-1.3013	-1.29805	-1.29489	-1.29175	-1.28864	-1.35218
-1.27086	-1.26766	-1.30407	-1.30074	-1.2975	-1.29432	-1.2912	-1.28816	-1.35174
-1.27032	-1.2671	-1.30352	-1.30022	-1.29698	-1.29379	-1.29068	-1.28767	-1.35128
-1.26975	-1.26654	-1.30299	-1.29975	-1.29653	-1.29332	-1.29019	-1.28715	-1.35073
-1.26921	-1.266	-1.30246	-1.29928	-1.29608	-1.29287	-1.28972	-1.28663	-1.35018
-1.26872	-1.26549	-1.30192	-1.29874	-1.29556	-1.29241	-1.32406	-1.32087	-1.34967
-1.26826	-1.26501	-1.30139	-1.29817	-1.29502	-1.29194	-1.32363	-1.32042	-1.34919
-1.30754	-1.30419	-1.30089	-1.29765	-1.2945	-1.29143	-1.32314	-1.31995	-1.31678
-1.30706	-1.30373	-1.30042	-1.29716	-1.29398	-1.29087	-1.3226	-1.31948	-1.31636
-1.30657	-1.30324	-1.29994	-1.29669	-1.29349	-1.29036	-1.32208	-1.31898	-1.31588
-1.30608	-1.30273	-1.29946	-1.29623	-1.29304	-1.28989	-1.32159	-1.31845	-1.31533
-1.30557	-1.30221	-1.29896	-1.29576	-1.29258	-1.28943	-1.32112	-1.31795	-1.3148
-1.30502	-1.30168	-1.29842	-1.29522	-1.29207	-1.28896	-1.32067	-1.31749	-1.31434
-1.30446	-1.30115	-1.29789	-1.29468	-1.29155	-1.28849	-1.32024	-1.31705	-1.31389
-1.30396	-1.30066	-1.29743	-1.29425	-1.32612	-1.32295	-1.31979	-1.31661	-1.34627
-1.30347	-1.30019	-1.297	-1.29386	-1.32573	-1.32252	-1.31933	-1.31617	-1.34583
-1.30299	-1.2997	-1.29652	-1.29339	-1.32528	-1.32208	-1.31889	-1.31573	-1.34538
-1.30251	-1.29921	-1.296	-1.29285	-1.32476	-1.32162	-1.31847	-1.31529	-1.34491
-1.30202	-1.29873	-1.2955	-1.29233	-1.32425	-1.32114	-1.31801	-1.31485	-1.34446
-1.30153	-1.29825	-1.29502	-1.29184	-1.32374	-1.32061	-1.31749	-1.31439	-1.34404
-1.30104	-1.29777	-1.29455	-1.29138	-1.32326	-1.3201	-1.31699	-1.31394	-1.3436
-1.30056	-1.29729	-1.29409	-1.29093	-1.32284	-1.3197	-1.31658	-1.31351	-1.34314
-1.30007	-1.29682	-1.29363	-1.2905	-1.32245	-1.31932	-1.3162	-1.31308	-1.34267
-1.29959	-1.29635	-1.3284	-1.32521	-1.32204	-1.3189	-1.31577	-1.31265	-1.34222
-1.29911	-1.2959	-1.32798	-1.32481	-1.32163	-1.31846	-1.31532	-1.31222	-1.34179
-1.33408	-1.33078	-1.32755	-1.32438	-1.3212	-1.31802	-1.34768	-1.34449	-1.34133
-1.33369	-1.33038	-1.32712	-1.32391	-1.32073	-1.31757	-1.34722	-1.34403	-1.34086
-1.33329	-1.32996	-1.32669	-1.32346	-1.32027	-1.31712	-1.34676	-1.34357	-1.3404
-1.33285	-1.32953	-1.32626	-1.32303	-1.31983	-1.31667	-1.34629	-1.34311	-1.33996

-1.33239	-1.32909	-1.32583	-1.32262	-1.31942	-1.31623	-1.34585	-1.34268	-1.33954
-1.33193	-1.32865	-1.32542	-1.32223	-1.31905	-1.31587	-1.34547	-1.3423	-1.33916
-1.33147	-1.32821	-1.32501	-1.32186	-1.3187	-1.31554	-1.34513	-1.34195	-1.33879
-1.33106	-1.3278	-1.3246	-1.32144	-1.31829	-1.31514	-1.34472	-1.34154	-1.33839
-1.33068	-1.3274	-1.32418	-1.32099	-1.31783	-1.3147	-1.34427	-1.3411	-1.33795
-1.33026	-1.32699	-1.32375	-1.32056	-1.35024	-1.34701	-1.34381	-1.34065	-1.33753
-1.32981	-1.32653	-1.32331	-1.32012	-1.34978	-1.34654	-1.34335	-1.34021	-1.3371
-1.32937	-1.32611	-1.32289	-1.31971	-1.34936	-1.34611	-1.34292	-1.33979	-1.33669
-1.329	-1.32574	-1.32253	-1.31936	-1.34899	-1.34575	-1.34256	-1.33942	-1.33631
-1.32865	-1.3254	-1.3222	-1.31902	-1.34865	-1.34542	-1.34222	-1.33907	-1.33595
-1.32829	-1.32505	-1.32184	-1.31867	-1.34828	-1.34506	-1.34187	-1.33872	-1.3356
-1.32793	-1.32469	-1.32148	-1.3183	-1.3479	-1.34469	-1.34151	-1.33836	-1.33525
-1.32752	-1.32429	-1.32107	-1.31788	-1.34748	-1.34428	-1.3411	-1.33796	-1.33486
-1.32706	-1.32384	-1.32063	-1.31743	-1.34702	-1.34383	-1.34066	-1.33752	-1.33441
-1.29386	-1.29075	-1.32022	-1.31702	-1.3466	-1.34341	-1.34025	-1.3371	-1.334
-1.29349	-1.29039	-1.31986	-1.31667	-1.34624	-1.34304	-1.33988	-1.33675	-1.33366
-1.29315	-1.29005	-1.3524	-1.34913	-1.34589	-1.34269	-1.33953	-1.3364	-1.33333
-1.29281	-1.2897	-1.35205	-1.34877	-1.34554	-1.34235	-1.33919	-1.33607	-1.33299
-1.29247	-1.28937	-1.3517	-1.34842	-1.34519	-1.34201	-1.33886	-1.33574	-1.33266
-1.29212	-1.28901	-1.35134	-1.34807	-1.34484	-1.34166	-1.33852	-1.3354	-1.33234
-1.29176	-1.28866	-1.35098	-1.34771	-1.34449	-1.34132	-1.33818	-1.33507	-1.33201
-1.2914	-1.2883	-1.31856	-1.31537	-1.34414	-1.34097	-1.33783	-1.33474	-1.33169
-1.29104	-1.28794	-1.31819	-1.315	-1.34378	-1.34062	-1.33749	-1.33441	-1.33137
-1.29067	-1.28758	-1.31782	-1.31463	-1.34342	-1.34027	-1.33716	-1.33408	-1.33105
-1.29031	-1.28722	-1.31745	-1.31427	-1.34308	-1.33993	-1.33682	-1.33375	-1.33072
-1.28995	-1.28685	-1.31708	-1.31391	-1.34273	-1.33959	-1.33648	-1.33341	-1.33039
-1.28959	-1.28649	-1.31671	-1.31354	-1.31042	-1.30735	-1.33614	-1.33308	-1.33006
-1.28922	-1.28613	-1.31634	-1.31317	-1.31006	-1.30699	-1.3358	-1.33275	-1.32974
-1.28886	-1.28577	-1.31596	-1.3128	-1.30969	-1.30664	-1.33546	-1.33241	-1.32941
-1.28849	-1.2854	-1.31559	-1.31243	-1.30933	-1.30629	-1.33512	-1.33207	-1.32907
-1.28813	-1.28504	-1.31522	-1.31207	-1.30898	-1.30595	-1.30295	-1.29997	-1.32875
-1.28777	-1.28469	-1.31486	-1.31172	-1.30864	-1.3056	-1.3026	-1.29965	-1.32844
-1.32087	-1.31769	-1.31453	-1.3114	-1.30832	-1.30528	-1.30229	-1.29934	-1.32815
-1.32059	-1.31741	-1.31425	-1.31113	-1.30805	-1.30502	-1.30203	-1.29909	-1.32791
-1.32031	-1.31713	-1.31398	-1.31086	-1.30779	-1.30475	-1.30177	-1.29883	-1.32766
-1.31998	-1.3168	-1.31365	-1.31053	-1.30746	-1.30443	-1.30145	-1.29851	-1.35811
-1.31963	-1.31645	-1.3133	-1.31018	-1.30711	-1.30408	-1.3011	-1.29817	-1.35777
-1.31931	-1.31615	-1.313	-1.30987	-1.30679	-1.30375	-1.30077	-1.29783	-1.32619
-1.31904	-1.31588	-1.31273	-1.30959	-1.3065	-1.30344	-1.30044	-1.29749	-1.32584
-1.31873	-1.31558	-1.31243	-1.3093	-1.30621	-1.30314	-1.30014	-1.2972	-1.32554
-1.31837	-1.31521	-1.31208	-1.30898	-1.30591	-1.30288	-1.29989	-1.29696	-1.32529
-1.31802	-1.31485	-1.31174	-1.30866	-1.30562	-1.30261	-1.29965	-1.29673	-1.32506
-1.31772	-1.31455	-1.31144	-1.30838	-1.30535	-1.30235	-1.2994	-1.29649	-1.32481
-1.31743	-1.31427	-1.31116	-1.3081	-1.30507	-1.30208	-1.29913	-1.29623	-1.32455
-1.31711	-1.31393	-1.31082	-1.30777	-1.30475	-1.30177	-1.29883	-1.29593	-1.32424
-1.31678	-1.31357	-1.31046	-1.30741	-1.3044	-1.30143	-1.2985	-1.2956	-1.3239
-1.31647	-1.31326	-1.31015	-1.30711	-1.3041	-1.30114	-1.29822	-1.29532	-1.32361
-1.31616	-1.31299	-1.30989	-1.30685	-1.30385	-1.3009	-1.29798	-1.29508	-1.32337
-1.31587	-1.31272	-1.30963	-1.30659	-1.3036	-1.30065	-1.29774	-1.29485	-1.32312
-1.31559	-1.31244	-1.30936	-1.30634	-1.30335	-1.3004	-1.29749	-1.2946	-1.32287

-1.31532	-1.31217	-1.3091	-1.30608	-1.3031	-1.30016	-1.29724	-1.29436	-1.32261
-1.31505	-1.31191	-1.30884	-1.30583	-1.30285	-1.29991	-1.29701	-1.29413	-1.32236
-1.31479	-1.31165	-1.30858	-1.30557	-1.3026	-1.29968	-1.29677	-1.2939	-1.32211
-1.31452	-1.31139	-1.30833	-1.30532	-1.30236	-1.29943	-1.29654	-1.29366	-1.32188
-1.31426	-1.31113	-1.30807	-1.30508	-1.30212	-1.29919	-1.2963	-1.29343	-1.32165
-1.314	-1.31087	-1.30783	-1.30484	-1.30188	-1.29895	-1.29606	-1.29319	-1.32141
-1.31374	-1.31062	-1.30758	-1.30459	-1.30164	-1.29872	-1.29583	-1.29296	-1.32116
-1.31349	-1.31037	-1.30734	-1.30435	-1.3014	-1.29849	-1.2956	-1.29274	-1.32092
-1.31324	-1.31013	-1.3071	-1.30412	-1.30117	-1.29825	-1.29537	-1.29251	-1.3207
-1.31299	-1.30989	-1.30686	-1.30389	-1.30094	-1.29802	-1.29513	-1.29229	-1.32049
-1.31272	-1.30964	-1.30662	-1.30365	-1.3007	-1.29778	-1.2949	-1.29206	-1.32025
-1.31245	-1.30939	-1.30637	-1.3034	-1.30046	-1.29756	-1.29468	-1.29183	-1.32001
-1.31223	-1.30914	-1.30613	-1.30317	-1.30023	-1.29732	-1.29445	-1.2916	-1.31978
-1.31203	-1.30891	-1.30589	-1.30295	-1.30001	-1.29709	-1.29421	-1.29138	-1.31955
-1.27406	-1.27103	-1.3057	-1.30275	-1.29981	-1.29689	-1.29401	-1.29116	-1.31932
-1.27388	-1.27087	-1.30555	-1.3026	-1.29967	-1.29676	-1.29386	-1.29096	-1.31907
-1.27368	-1.27071	-1.30539	-1.30244	-1.29951	-1.29661	-1.2937	-1.29077	-1.31885
-1.27345	-1.27051	-1.30517	-1.3022	-1.29928	-1.29639	-1.29351	-1.29061	-1.3187
-1.27322	-1.27029	-1.30494	-1.30196	-1.29904	-1.29616	-1.2933	-1.29045	-1.31856
-1.27304	-1.27011	-1.30476	-1.30179	-1.29885	-1.29594	-1.29308	-1.29025	-1.31838
-1.27288	-1.26994	-1.30461	-1.30165	-1.29869	-1.29574	-1.29285	-1.29004	-1.31817
-1.2727	-1.26975	-1.26685	-1.264	-1.29852	-1.29556	-1.29267	-1.28986	-1.35206
-1.2725	-1.26952	-1.26662	-1.26378	-1.29832	-1.29539	-1.29252	-1.28971	-1.35191
-1.2723	-1.26931	-1.26642	-1.26359	-1.29814	-1.29524	-1.29237	-1.28956	-1.35173
-1.27211	-1.26914	-1.26625	-1.26343	-1.29798	-1.2951	-1.29223	-1.28937	-1.3515
-1.27193	-1.26898	-1.26609	-1.26326	-1.29781	-1.29494	-1.29207	-1.28919	-1.35128
-1.27177	-1.26883	-1.26592	-1.26304	-1.29759	-1.29474	-1.29188	-1.28902	-1.35113
-1.27161	-1.26867	-1.26573	-1.26282	-1.29735	-1.29452	-1.29169	-1.28887	-1.35099
-1.2714	-1.26848	-1.26555	-1.26264	-1.29718	-1.29435	-1.3263	-1.3234	-1.35085
-1.27116	-1.26826	-1.26537	-1.2625	-1.29705	-1.29422	-1.32616	-1.32326	-1.3507
-1.27097	-1.26808	-1.2652	-1.26236	-1.25957	-1.25682	-1.32602	-1.32313	-1.35056
-1.27082	-1.26793	-1.26505	-1.2622	-1.25941	-1.25667	-1.32586	-1.323	-1.35043
-1.27068	-1.26779	-1.2649	-1.26205	-1.29478	-1.29194	-1.32569	-1.32284	-1.35029
-1.27054	-1.26765	-1.26476	-1.2619	-1.29461	-1.29174	-1.32547	-1.32264	-1.3501
-1.2704	-1.26751	-1.26462	-1.26176	-1.29444	-1.29155	-1.32526	-1.32243	-1.3499
-1.27026	-1.26737	-1.26448	-1.26162	-1.2943	-1.2914	-1.3251	-1.32228	-1.34975
-1.27012	-1.26723	-1.26435	-1.26148	-1.29416	-1.29127	-1.32497	-1.32215	-1.34962
-1.26998	-1.26709	-1.29991	-1.29696	-1.29404	-1.29115	-1.28833	-1.28558	-1.34947
-1.26984	-1.26695	-1.29978	-1.29684	-1.29393	-1.29105	-1.28822	-1.28545	-1.34932
-1.2697	-1.26682	-1.29964	-1.29672	-1.29382	-1.29096	-1.28813	-1.28535	-1.34919
-1.26956	-1.26668	-1.29951	-1.29659	-1.29372	-1.29089	-1.28808	-1.2853	-1.34912
-1.26942	-1.26655	-1.29938	-1.29646	-1.29361	-1.29081	-1.28802	-1.28523	-1.28245
-1.26928	-1.2664	-1.29923	-1.29633	-1.29349	-1.2907	-1.28791	-1.28511	-1.28233
-1.26916	-1.26626	-1.29909	-1.2962	-1.29336	-1.29056	-1.28777	-1.28498	-1.28219
-1.30494	-1.30195	-1.29899	-1.29608	-1.29323	-1.29043	-1.28763	-1.28485	-1.28207
-1.30489	-1.30189	-1.29891	-1.29597	-1.2931	-1.29029	-1.2875	-1.28472	-1.28195
-1.3048	-1.3018	-1.29883	-1.29589	-1.29302	-1.29019	-1.28739	-1.2846	-1.28183
-1.30466	-1.30168	-1.29874	-1.29584	-1.29298	-1.29014	-1.28731	-1.28449	-1.28171
-1.30453	-1.30155	-1.29864	-1.29577	-1.29291	-1.29006	-1.28723	-1.28438	-1.32026
-1.30443	-1.30144	-1.29851	-1.29564	-1.29278	-1.28994	-1.28711	-1.28427	-1.32016

-1.30435	-1.30134	-1.2984	-1.29551	-1.29265	-1.28982	-1.28699	-1.28417	-1.32007
-1.30426	-1.30127	-1.29834	-1.29544	-1.29257	-1.28972	-1.2869	-1.28409	-1.32002
-1.30417	-1.30122	-1.29829	-1.29538	-1.2925	-1.28964	-1.28681	-1.28402	-1.31999
-1.30405	-1.3011	-1.29818	-1.29528	-1.29241	-1.28956	-1.28674	-1.28396	-1.31994
-1.30391	-1.30096	-1.29804	-1.29516	-1.29231	-1.28949	-1.28669	-1.2839	-1.31988
-1.3038	-1.30085	-1.29792	-1.29504	-1.2922	-1.28939	-1.2866	-1.28382	-1.31981
-1.30374	-1.30077	-1.29783	-1.29492	-1.29207	-1.28926	-1.28647	-1.28371	-1.31972
-1.30367	-1.3007	-1.29774	-1.29483	-1.29196	-1.28915	-1.28637	-1.28362	-1.31965
-1.30359	-1.3006	-1.29766	-1.29477	-1.29191	-1.28911	-1.28632	-1.28356	-1.3196
-1.3035	-1.30051	-1.29759	-1.29472	-1.29188	-1.28907	-1.28628	-1.28351	-1.31956
-1.30342	-1.30045	-1.29754	-1.29468	-1.29184	-1.28903	-1.32535	-1.32242	-1.31951
-1.30334	-1.3004	-1.2975	-1.29464	-1.2918	-1.28898	-1.32532	-1.32238	-1.31945
-1.30329	-1.30036	-1.29746	-1.2946	-1.29175	-1.28892	-1.32526	-1.32231	-1.31938
-1.30326	-1.30032	-1.29742	-1.29455	-1.29169	-1.28886	-1.32519	-1.32222	-1.31928
-1.30321	-1.30029	-1.29738	-1.2945	-1.29163	-1.28879	-1.32511	-1.32214	-1.35378
-1.30317	-1.30024	-1.29734	-1.29446	-1.29158	-1.28869	-1.32502	-1.32208	-1.35375
-1.30312	-1.3002	-1.2973	-1.29442	-1.29153	-1.28863	-1.32496	-1.32204	-1.35372
-1.30308	-1.30015	-1.29725	-1.29438	-1.2915	-1.28862	-1.32497	-1.32202	-1.35367
-1.30303	-1.30011	-1.29721	-1.29433	-1.29147	-1.28862	-1.32498	-1.32201	-1.35363
-1.30297	-1.30006	-1.29716	-1.29429	-1.33096	-1.32792	-1.32492	-1.32197	-1.3536
-1.30291	-1.3	-1.29711	-1.29424	-1.3309	-1.32783	-1.32484	-1.32192	-1.35357
-1.30287	-1.29995	-1.29706	-1.2942	-1.33088	-1.32782	-1.32482	-1.3219	-1.35354
-1.30285	-1.29989	-1.297	-1.29416	-1.33089	-1.32787	-1.32487	-1.32189	-1.35348
-1.30281	-1.29984	-1.29695	-1.29412	-1.33089	-1.3279	-1.32491	-1.32191	-1.35347
-1.30272	-1.29978	-1.2969	-1.29407	-1.33085	-1.32788	-1.32492	-1.32196	-1.35353
-1.30264	-1.29972	-1.29685	-1.29402	-1.33081	-1.32785	-1.32491	-1.32199	-1.35358
-1.30261	-1.29966	-1.29679	-1.29397	-1.33078	-1.32782	-1.32488	-1.32198	-1.35358
-1.3026	-1.29961	-1.29673	-1.29392	-1.33075	-1.32778	-1.32485	-1.32195	-1.35357
-1.3026	-1.29961	-1.29671	-1.29388	-1.33072	-1.32776	-1.32483	-1.32192	-1.35353
-1.30261	-1.29965	-1.29673	-1.29385	-1.33068	-1.32775	-1.32483	-1.3219	-1.35349
-1.30259	-1.29966	-1.33675	-1.33368	-1.33069	-1.32777	-1.35953	-1.3565	-1.35349
-1.30256	-1.29965	-1.33679	-1.33374	-1.33075	-1.32782	-1.35958	-1.35656	-1.35355
-1.30254	-1.29964	-1.33682	-1.3338	-1.33081	-1.32786	-1.3596	-1.3566	-1.35359
-1.30256	-1.29966	-1.33687	-1.33386	-1.33085	-1.32784	-1.35956	-1.35657	-1.35358
-1.30258	-1.29967	-1.3369	-1.33391	-1.33088	-1.32783	-1.35951	-1.35653	-1.35355
-1.30258	-1.29963	-1.33687	-1.3339	-1.33088	-1.32785	-1.35952	-1.3565	-1.3535
-1.30256	-1.29957	-1.33682	-1.33386	-1.33087	-1.32788	-1.35955	-1.35648	-1.35345
-1.30257	-1.29957	-1.3368	-1.33382	-1.33084	-1.32787	-1.35955	-1.35648	-1.35344
-1.30258	-1.2996	-1.33681	-1.33377	-1.33078	-1.32784	-1.35954	-1.35649	-1.35347
-1.30261	-1.29964	-1.33684	-1.33377	-1.33077	-1.32784	-1.35955	-1.35651	-1.3535
-1.30265	-1.2997	-1.33691	-1.33383	-1.33083	-1.32789	-1.35959	-1.35655	-1.35353
-1.30267	-1.29974	-1.33698	-1.3339	-1.33089	-1.32793	-1.35962	-1.35659	-1.35356
-1.30264	-1.29973	-1.337	-1.33394	-1.33093	-1.32797	-1.35965	-1.35663	-1.3536
-1.30261	-1.29971	-1.33701	-1.33398	-1.33097	-1.32798	-1.35967	-1.35665	-1.35364
-1.30263	-1.29973	-1.33706	-1.33402	-1.33099	-1.32797	-1.35963	-1.35663	-1.35364
-1.30268	-1.29978	-1.33712	-1.33407	-1.33101	-1.32794	-1.35957	-1.35659	-1.35362
-1.30273	-1.29983	-1.33717	-1.33413	-1.36586	-1.36267	-1.35957	-1.35659	-1.35363
-1.30278	-1.29987	-1.33723	-1.33418	-1.36591	-1.36271	-1.35961	-1.35662	-1.35366
-1.30282	-1.29992	-1.33729	-1.33424	-1.36596	-1.36276	-1.35965	-1.35667	-1.3537
-1.30288	-1.29997	-1.33736	-1.3343	-1.36602	-1.36281	-1.3597	-1.35671	-1.35375

-1.30293	-1.30003	-1.33742	-1.33437	-1.36608	-1.36286	-1.35975	-1.35677	-1.3538
-1.30299	-1.30008	-1.33749	-1.33442	-1.36613	-1.36291	-1.3598	-1.35682	-1.35385
-1.30305	-1.30014	-1.33755	-1.33448	-1.36618	-1.36297	-1.35986	-1.35687	-1.3539
-1.30311	-1.30019	-1.3376	-1.33454	-1.36624	-1.36303	-1.35991	-1.35691	-1.35394
-1.30316	-1.30024	-1.33766	-1.33461	-1.36631	-1.3631	-1.35998	-1.35697	-1.35398
-1.27039	-1.26755	-1.33772	-1.33467	-1.36639	-1.3632	-1.36009	-1.35706	-1.35406
-1.27045	-1.2676	-1.33778	-1.33474	-1.36648	-1.36335	-1.36025	-1.3572	-1.35417
-1.27054	-1.26767	-1.33785	-1.3348	-1.36656	-1.36346	-1.36037	-1.35731	-1.35427
-1.27067	-1.26778	-1.33793	-1.33486	-1.36661	-1.36351	-1.36043	-1.35738	-1.35434
-1.2708	-1.26788	-1.33802	-1.33492	-1.36665	-1.36355	-1.36048	-1.35743	-1.3544
-1.27088	-1.26796	-1.33811	-1.33499	-1.36671	-1.3636	-1.36052	-1.35748	-1.35447
-1.27094	-1.26804	-1.3382	-1.33508	-1.36678	-1.36366	-1.36057	-1.35754	-1.35453
-1.31237	-1.30928	-1.37327	-1.37007	-1.3669	-1.36376	-1.36066	-1.35761	-1.3546
-1.31247	-1.30937	-1.37338	-1.37021	-1.36704	-1.36389	-1.36077	-1.35769	-1.35466
-1.31259	-1.30949	-1.37351	-1.37035	-1.36718	-1.36402	-1.36089	-1.3578	-1.35475
-1.31274	-1.30966	-1.37368	-1.3705	-1.36731	-1.36413	-1.361	-1.35793	-1.3549
-1.31286	-1.30981	-1.37382	-1.37063	-1.36743	-1.36424	-1.36112	-1.35807	-1.35504
-1.31293	-1.30988	-1.3739	-1.37071	-1.36754	-1.36439	-1.36127	-1.35821	-1.35518
-1.31301	-1.30995	-1.37396	-1.37079	-1.36764	-1.36452	-1.36143	-1.35835	-1.3553
-1.31315	-1.31007	-1.37407	-1.37088	-1.36773	-1.36461	-1.36153	-1.35846	-1.35542
-1.31333	-1.31022	-1.3742	-1.37097	-1.36781	-1.36469	-1.3616	-1.35855	-1.35553
-1.31347	-1.31035	-1.34279	-1.33966	-1.36794	-1.36481	-1.36172	-1.35868	-1.35566
-1.31359	-1.31046	-1.34292	-1.33982	-1.36811	-1.36498	-1.36188	-1.35883	-1.35581
-1.31372	-1.3106	-1.34307	-1.33999	-1.36829	-1.36515	-1.36205	-1.35899	-1.35595
-1.31387	-1.31078	-1.34325	-1.34016	-1.36847	-1.36533	-1.36222	-1.35914	-1.35608
-1.27479	-1.27182	-1.34343	-1.34033	-1.36864	-1.3655	-1.36238	-1.35929	-1.35621
-1.27498	-1.27198	-1.34359	-1.34049	-1.3688	-1.36565	-1.36252	-1.35941	-1.35632
-1.27518	-1.27214	-1.34375	-1.34065	-1.36895	-1.36579	-1.36264	-1.35952	-1.35644
-1.24612	-1.24305	-1.34393	-1.34082	-1.3691	-1.36591	-1.36276	-1.35965	-1.35658
-1.24625	-1.24321	-1.34413	-1.34099	-1.36926	-1.36603	-1.36287	-1.35979	-1.35674
-1.24638	-1.24336	-1.28002	-1.27694	-1.36943	-1.3662	-1.36304	-1.35995	-1.3569
-1.24652	-1.24348	-1.28017	-1.2771	-1.36964	-1.36644	-1.36327	-1.36015	-1.35706
-1.24665	-1.24361	-1.24062	-1.23767	-1.33839	-1.33527	-1.36347	-1.36032	-1.35722
-1.24678	-1.24375	-1.24075	-1.23778	-1.33851	-1.33537	-1.36356	-1.36045	-1.35738
-1.2469	-1.24389	-1.24088	-1.23789	-1.33864	-1.33548	-1.36367	-1.36058	-1.35753
-1.24703	-1.244	-1.241	-1.23802	-1.27454	-1.27146	-1.36387	-1.36075	-1.35767
-1.24715	-1.2441	-1.24111	-1.23816	-1.27471	-1.27167	-1.36411	-1.36093	-1.35781
-1.24727	-1.24422	-1.24123	-1.23828	-1.27485	-1.27183	-1.30119	-1.29809	-1.35801
-1.24738	-1.24436	-1.24135	-1.23838	-1.27497	-1.27197	-1.30136	-1.2983	-1.35826
-1.24751	-1.24448	-1.24147	-1.23849	-1.27509	-1.2721	-1.30151	-1.29847	-1.29544
-1.24767	-1.24458	-1.24157	-1.23862	-1.27521	-1.27221	-1.30162	-1.2986	-1.2956
-1.24784	-1.2447	-1.24169	-1.23875	-1.23581	-1.2329	-1.26935	-1.26642	-1.29574
-1.24803	-1.24491	-1.24186	-1.23886	-1.23592	-1.23303	-1.2695	-1.26654	-1.29585
-1.24822	-1.24513	-1.24204	-1.239	-1.23605	-1.23319	-1.26966	-1.26667	-1.29597
-1.24838	-1.24531	-1.24223	-1.23918	-1.23623	-1.23337	-1.26985	-1.26686	-1.29616
-1.24852	-1.24548	-1.24242	-1.23939	-1.23644	-1.23356	-1.27005	-1.26707	-1.29638
-1.2487	-1.24566	-1.24262	-1.2396	-1.23663	-1.23371	-1.2702	-1.26724	-1.29656
-1.24891	-1.24586	-1.24282	-1.23981	-1.23681	-1.23383	-1.2703	-1.26736	-1.29671
-1.2491	-1.24603	-1.243	-1.24	-1.23699	-1.23399	-1.23106	-1.22821	-1.26457
-1.24927	-1.24616	-1.24313	-1.24016	-1.23717	-1.23418	-1.23124	-1.22835	-1.26468

-1.24945	-1.2463	-1.24328	-1.24033	-1.23736	-1.23439	-1.23145	-1.22851	-1.26482
-1.24965	-1.2465	-1.24348	-1.24052	-1.23755	-1.23461	-1.23166	-1.2287	-1.26501
-1.24986	-1.24673	-1.24369	-1.2407	-1.23774	-1.23481	-1.23188	-1.22892	-1.26522
-1.25007	-1.24695	-1.24388	-1.24085	-1.23789	-1.23498	-1.23206	-1.22911	-1.26542
-1.25029	-1.24716	-1.24405	-1.24098	-1.23802	-1.23513	-1.23223	-1.2293	-1.26562
-1.25047	-1.24736	-1.24424	-1.24116	-1.23819	-1.23531	-1.23242	-1.2295	-1.26583
-1.25061	-1.24753	-1.24444	-1.24138	-1.23841	-1.23552	-1.23263	-1.22971	-1.26606
-1.25078	-1.24772	-1.24464	-1.2416	-1.23863	-1.23573	-1.23283	-1.22992	-1.22703
-1.251	-1.24793	-1.24486	-1.24181	-1.23884	-1.23594	-1.23303	-1.23013	-1.22724
-1.25124	-1.24816	-1.24507	-1.24202	-1.23904	-1.23612	-1.23322	-1.23033	-1.22745
-1.25147	-1.24839	-1.24529	-1.24223	-1.23922	-1.23627	-1.23335	-1.23048	-1.22762
-1.25171	-1.24862	-1.24552	-1.24244	-1.2394	-1.23641	-1.23347	-1.23062	-1.22778
-1.25194	-1.24885	-1.24574	-1.24266	-1.23961	-1.23659	-1.23365	-1.2308	-1.22797
-1.25217	-1.24908	-1.24597	-1.24288	-1.23983	-1.23682	-1.23388	-1.23102	-1.22818
-1.25241	-1.24931	-1.2462	-1.24311	-1.24006	-1.23705	-1.23411	-1.23124	-1.2284
-1.25264	-1.24954	-1.24643	-1.24334	-1.24029	-1.23729	-1.23434	-1.23146	-1.22861
-1.25287	-1.24977	-1.24666	-1.24357	-1.24054	-1.23755	-1.2346	-1.2317	-1.22884
-1.2531	-1.25	-1.24689	-1.24382	-1.2408	-1.23785	-1.23491	-1.23199	-1.2291
-1.25334	-1.25023	-1.24713	-1.24405	-1.24106	-1.23814	-1.23522	-1.23228	-1.22936
-1.25357	-1.25046	-1.24735	-1.24428	-1.2413	-1.23838	-1.23546	-1.23253	-1.22961
-1.25382	-1.25069	-1.24758	-1.24451	-1.24151	-1.23859	-1.23567	-1.23276	-1.22985
-1.25412	-1.25096	-1.24783	-1.24475	-1.24174	-1.23881	-1.23589	-1.23298	-1.23008
-1.25445	-1.25127	-1.24812	-1.24501	-1.24198	-1.23903	-1.2361	-1.23319	-1.23031
-1.25476	-1.25156	-1.24841	-1.24529	-1.24225	-1.23928	-1.23634	-1.23343	-1.23055
-1.255	-1.25183	-1.2487	-1.24562	-1.24258	-1.23959	-1.23662	-1.23368	-1.23078
-1.25525	-1.25209	-1.24899	-1.24593	-1.24289	-1.23988	-1.2369	-1.23394	-1.23103
-1.25553	-1.25234	-1.24923	-1.24617	-1.24313	-1.24012	-1.23716	-1.23423	-1.23133
-1.25581	-1.2526	-1.24947	-1.24639	-1.24335	-1.24035	-1.2374	-1.23452	-1.23164
-1.21821	-1.21511	-1.24977	-1.24667	-1.24361	-1.2406	-1.23764	-1.23475	-1.23188
-1.21851	-1.21544	-1.25011	-1.24698	-1.2439	-1.24085	-1.23787	-1.23496	-1.23208
-1.21878	-1.21572	-1.25041	-1.24727	-1.24418	-1.24113	-1.23814	-1.23522	-1.23232
-1.21901	-1.21596	-1.25066	-1.24755	-1.24448	-1.24144	-1.23846	-1.23552	-1.23261
-1.18022	-1.17727	-1.25094	-1.24785	-1.24478	-1.24176	-1.23876	-1.23581	-1.23288
-1.18052	-1.17756	-1.25127	-1.24818	-1.24511	-1.24206	-1.23905	-1.23608	-1.23314
-1.18083	-1.17785	-1.25159	-1.24851	-1.24543	-1.24236	-1.23934	-1.23635	-1.23341
-1.1811	-1.1781	-1.25185	-1.24877	-1.24569	-1.24263	-1.23961	-1.23665	-1.23373
-1.18136	-1.17835	-1.25211	-1.24902	-1.24594	-1.24289	-1.23989	-1.23696	-1.23406
-1.18165	-1.17865	-1.25243	-1.24932	-1.24624	-1.2432	-1.24021	-1.23728	-1.23437
-1.18195	-1.17899	-1.25279	-1.24965	-1.24657	-1.24353	-1.24054	-1.23759	-1.23467
-1.18227	-1.17931	-1.25314	-1.25	-1.24691	-1.24386	-1.24086	-1.23791	-1.23499
-1.18259	-1.17961	-1.25348	-1.25037	-1.24727	-1.24419	-1.24118	-1.23823	-1.23531
-1.18291	-1.17993	-1.177	-1.1741	-1.24762	-1.24453	-1.2415	-1.23855	-1.23562
-1.18324	-1.18027	-1.17732	-1.17441	-1.24795	-1.24488	-1.24185	-1.23888	-1.23593
-1.18357	-1.1806	-1.17765	-1.17471	-1.24829	-1.24523	-1.2422	-1.23921	-1.23624
-1.18389	-1.18092	-1.17796	-1.17504	-1.24864	-1.24556	-1.24253	-1.23954	-1.23657
-1.18422	-1.18122	-1.17828	-1.17538	-1.249	-1.2459	-1.24285	-1.23987	-1.23692
-1.18454	-1.18154	-1.1786	-1.1757	-1.24934	-1.24624	-1.24319	-1.2402	-1.23723
-1.18487	-1.18188	-1.17892	-1.17599	-1.24967	-1.24659	-1.24354	-1.24053	-1.23753
-1.18519	-1.18221	-1.17924	-1.1763	-1.17341	-1.17055	-1.24388	-1.24086	-1.23785
-1.1855	-1.1825	-1.17954	-1.17663	-1.17372	-1.17084	-1.24419	-1.24119	-1.2382

-1.18582	-1.1828	-1.17985	-1.17694	-1.17403	-1.17114	-1.24451	-1.24152	-1.23854
-1.18613	-1.18313	-1.18017	-1.17723	-1.17433	-1.17145	-1.24485	-1.24183	-1.23885
-1.18644	-1.18347	-1.18048	-1.17753	-1.17462	-1.17176	-1.24519	-1.24215	-1.23915
-1.15097	-1.14805	-1.1808	-1.17785	-1.17494	-1.17207	-1.20751	-1.20458	-1.23949
-1.15131	-1.14834	-1.18111	-1.17819	-1.17527	-1.17237	-1.20781	-1.20491	-1.23985
-1.15168	-1.14868	-1.18147	-1.17856	-1.17563	-1.17272	-1.20817	-1.20528	-1.24022
-1.15205	-1.14908	-1.18188	-1.17894	-1.17602	-1.17311	-1.20858	-1.20567	-1.24062
-1.15241	-1.14946	-1.18226	-1.1793	-1.17637	-1.17348	-1.17062	-1.1678	-1.20314
-1.15273	-1.1498	-1.18259	-1.1796	-1.17667	-1.17379	-1.17094	-1.1681	-1.20344
-1.15307	-1.15014	-1.18293	-1.17991	-1.17697	-1.1741	-1.17124	-1.16839	-1.20374
-1.15344	-1.15051	-1.18332	-1.18029	-1.17733	-1.17441	-1.17153	-1.16869	-1.20406
-1.15383	-1.15089	-1.18372	-1.18069	-1.1777	-1.17473	-1.17183	-1.16901	-1.20441
-1.15417	-1.15121	-1.18406	-1.18106	-1.17807	-1.17509	-1.17219	-1.16937	-1.20478
-1.15448	-1.1515	-1.18438	-1.1814	-1.17843	-1.17549	-1.1726	-1.16976	-1.20518
-1.15483	-1.15184	-1.18474	-1.18176	-1.17881	-1.17588	-1.17299	-1.17015	-1.20557
-1.15521	-1.15223	-1.18514	-1.18215	-1.17919	-1.17627	-1.17338	-1.17053	-1.20597
-1.15558	-1.1526	-1.18552	-1.18251	-1.17955	-1.17664	-1.17375	-1.17089	-1.20633
-1.1559	-1.15294	-1.18585	-1.18283	-1.17987	-1.17697	-1.17408	-1.1712	-1.20664
-1.15622	-1.15328	-1.1862	-1.18316	-1.18021	-1.17731	-1.17442	-1.17152	-1.20695
-1.02768	-1.02516	-1.15068	-1.14774	-1.1806	-1.1777	-1.1748	-1.17189	-1.16903
-1.02802	-1.0255	-1.15108	-1.14815	-1.18102	-1.1781	-1.1752	-1.17229	-1.16943
-1.02836	-1.02583	-1.15148	-1.14855	-1.18143	-1.17851	-1.1756	-1.17269	-1.16982
-1.0287	-1.02617	-1.15189	-1.14895	-1.18185	-1.17892	-1.17601	-1.1731	-1.17022
-1.02905	-1.02651	-1.07213	-1.06939	-1.18226	-1.17933	-1.17641	-1.1735	-1.20777
-1.02939	-1.02685	-1.0725	-1.06976	-1.18267	-1.17974	-1.17682	-1.1739	-1.20817
-1.02974	-1.02719	-1.07286	-1.07012	-1.18309	-1.18015	-1.17722	-1.1743	-1.20858
-1.03008	-1.02753	-1.07323	-1.07048	-1.1835	-1.18056	-1.17763	-1.1747	-1.20899
-1.03042	-1.02786	-1.0736	-1.07085	-1.18391	-1.18097	-1.17803	-1.17511	-1.2094
-1.03077	-1.0282	-1.07397	-1.07121	-1.18432	-1.18137	-1.17844	-1.17551	-1.20981
-1.03112	-1.02855	-1.07434	-1.07158	-1.18474	-1.18178	-1.17884	-1.17591	-1.21021
-1.03147	-1.02889	-1.07471	-1.07195	-1.06923	-1.06656	-1.17924	-1.17631	-1.21062
-1.03182	-1.02924	-1.07508	-1.07232	-1.06959	-1.06691	-1.17965	-1.17671	-1.21103
-1.03217	-1.02959	-1.07545	-1.07268	-1.06996	-1.06727	-1.18005	-1.17711	-1.21144
-1.03252	-1.02993	-1.07582	-1.07305	-1.07032	-1.06763	-1.18045	-1.17751	-1.21184
-1.03287	-1.03028	-1.07619	-1.07341	-1.07068	-1.06799	-1.18087	-1.17792	-1.21226
-1.03322	-1.03064	-1.02807	-1.02552	-1.07105	-1.06836	-1.18129	-1.17833	-1.21268
-1.03359	-1.031	-1.02843	-1.02588	-1.07142	-1.06874	-1.18172	-1.17876	-1.21311
-1.034	-1.0314	-1.02883	-1.02628	-1.07186	-1.06916	-1.18219	-1.17924	-1.2136
-1.03444	-1.03182	-1.02926	-1.02672	-1.07233	-1.0696	-1.18267	-1.17973	-1.21412
-1.03483	-1.03222	-1.02965	-1.02712	-1.07275	-1.07	-1.0673	-1.06466	-1.21459
-1.03519	-1.03258	-1.03001	-1.02746	-1.07312	-1.07037	-1.06767	-1.06503	-1.21502
-1.03554	-1.03293	-1.03036	-1.02781	-1.07348	-1.07074	-1.06804	-1.0654	-1.21545
-1.03589	-1.03328	-1.03071	-1.02817	-1.07386	-1.0711	-1.0684	-1.06575	-1.21587
-1.03627	-1.03365	-1.03108	-1.02854	-1.07425	-1.07149	-1.06877	-1.06612	-1.21631
-1.03669	-1.03409	-1.0315	-1.02894	-1.07468	-1.07194	-1.06922	-1.06653	-1.21678
-1.03713	-1.03455	-1.03194	-1.02935	-1.07512	-1.07241	-1.0697	-1.06697	-1.21727
-1.03752	-1.03494	-1.03235	-1.02978	-1.07558	-1.07286	-1.07014	-1.06742	-1.1407
-1.03787	-1.03529	-1.03273	-1.0302	-1.07605	-1.07328	-1.07055	-1.06786	-1.1412
-1.03826	-1.03566	-1.03311	-1.0306	-1.07648	-1.07368	-1.07094	-1.06827	-1.14166
-1.0387	-1.03605	-1.03348	-1.03095	-1.07685	-1.07405	-1.07131	-1.06864	-1.14205

-1.03914	-1.03647	-1.03387	-1.03131	-1.02875	-1.02619	-1.0717	-1.06902	-1.10584
-1.03959	-1.03694	-1.03431	-1.03171	-1.02914	-1.02661	-1.07216	-1.06945	-1.10627
-1.04004	-1.03741	-1.03475	-1.03212	-1.02956	-1.02705	-1.07263	-1.06989	-1.10671
-0.99806	-0.99553	-1.03517	-1.03257	-1.03	-1.02747	-1.07307	-1.07035	-1.10719
-0.99839	-0.99587	-1.03557	-1.03303	-1.03046	-1.02788	-1.07349	-1.07081	-1.10768
-0.99879	-0.99625	-1.03596	-1.03344	-1.03088	-1.02831	-1.07396	-1.07126	-1.10814
-0.99923	-0.99665	-1.03634	-1.03378	-1.03125	-1.02874	-1.07445	-1.07171	-1.10857
-0.91654	-0.91416	-1.03675	-1.03416	-1.03162	-1.02914	-1.07491	-1.07215	-1.10902
-0.91693	-0.91456	-1.03721	-1.03458	-1.03201	-1.02949	-1.07529	-1.07259	-1.10951
-0.91733	-0.91496	-1.03768	-1.03502	-1.03242	-1.02987	-1.07569	-1.07303	-1.11
-0.91772	-0.91533	-1.03813	-1.03548	-1.03289	-1.03034	-1.07618	-1.07348	-1.11043
-0.91811	-0.9157	-1.03858	-1.03595	-1.03337	-1.03083	-1.0767	-1.07394	-1.11086
-0.91849	-0.91609	-1.03906	-1.03645	-1.03385	-1.03128	-1.07716	-1.07439	-1.11133
-0.91886	-0.91648	-1.03954	-1.03695	-1.03433	-1.0317	-1.07758	-1.07485	-1.11181
-0.83402	-0.83182	-0.95844	-0.95605	-1.03476	-1.03212	-1.07805	-1.0753	-1.11226
-0.83436	-0.83214	-0.95876	-0.95636	-1.03513	-1.03255	-1.07854	-1.07574	-1.11268
-0.83472	-0.83248	-0.95913	-0.95671	-1.03553	-1.03299	-1.03043	-1.02785	-1.11312
-0.83514	-0.83289	-0.95959	-0.95715	-1.036	-1.03342	-1.03085	-1.02828	-1.1136
-0.83555	-0.8333	-0.96005	-0.95759	-1.03648	-1.03386	-1.03128	-1.02872	-1.11411
-0.7886	-0.7865	-0.91647	-0.9141	-0.95554	-0.95314	-1.03173	-1.02919	-1.11464
-0.78892	-0.78683	-0.91685	-0.91446	-0.95592	-0.95354	-1.03219	-1.02967	-1.11519
-0.78925	-0.78716	-0.91722	-0.91484	-0.95633	-0.95396	-1.03267	-1.03013	-1.11568
-0.78957	-0.78747	-0.91759	-0.91523	-0.95676	-0.9544	-1.03315	-1.03057	-1.11613
-0.74824	-0.74625	-0.83285	-0.83069	-0.95718	-0.95481	-0.95244	-0.95005	-1.11661
-0.74854	-0.74656	-0.83323	-0.83108	-0.95761	-0.95519	-0.9528	-0.95043	-1.1171
-0.74885	-0.74689	-0.83361	-0.83146	-0.95802	-0.95558	-0.95317	-0.95082	-1.03984
-0.74922	-0.74725	-0.83398	-0.83179	-0.95839	-0.95596	-0.95357	-0.95123	-1.04032
-0.7496	-0.74762	-0.83435	-0.83212	-0.95876	-0.95636	-0.95399	-0.95165	-1.04079
-0.74996	-0.74799	-0.83476	-0.83252	-0.95921	-0.9568	-0.95441	-0.95204	-1.04122
-0.7503	-0.74835	-0.83519	-0.83294	-0.95968	-0.95726	-0.95484	-0.95243	-1.04163
-0.75062	-0.74866	-0.74668	-0.74469	-0.91752	-0.91519	-0.95529	-0.95286	-1.04212
-0.75093	-0.74894	-0.74696	-0.745	-0.91794	-0.91562	-0.95576	-0.95333	-1.04266
-0.75127	-0.74925	-0.74727	-0.74532	-0.91835	-0.91603	-0.95618	-0.95376	-1.04318
-0.75164	-0.74963	-0.74764	-0.74568	-0.91876	-0.91639	-0.95655	-0.95414	-1.04365
-0.752	-0.74999	-0.74801	-0.74605	-0.83266	-0.83047	-0.95692	-0.95454	-0.99444
-0.75231	-0.75032	-0.74836	-0.74642	-0.83308	-0.83087	-0.95737	-0.95498	-0.9949
-0.7526	-0.75063	-0.7487	-0.74678	-0.8335	-0.83128	-0.95784	-0.95544	-0.99537
-0.75294	-0.75097	-0.74905	-0.74714	-0.83392	-0.83169	-0.95831	-0.9559	-0.99584
-0.7533	-0.75133	-0.74941	-0.7475	-0.83433	-0.8321	-0.95877	-0.95636	-0.99632
-0.65575	-0.65413	-0.74977	-0.74787	-0.74591	-0.74393	-0.9166	-0.91428	-0.99679
-0.65604	-0.65442	-0.75013	-0.74823	-0.74627	-0.74428	-0.91703	-0.9147	-0.99725
-0.65633	-0.65471	-0.7505	-0.74859	-0.74663	-0.74464	-0.91747	-0.91514	-0.99772
-0.65662	-0.65499	-0.75086	-0.74895	-0.74699	-0.745	-0.91793	-0.91559	-0.9982
-0.65691	-0.65527	-0.75122	-0.74931	-0.74736	-0.74537	-0.91839	-0.91604	-0.99869
-0.6572	-0.65556	-0.75158	-0.74967	-0.74771	-0.74573	-0.91884	-0.91649	-0.99917
-0.65749	-0.65585	-0.75194	-0.75003	-0.74807	-0.74609	-0.91929	-0.91693	-0.99965
-0.65778	-0.65614	-0.65454	-0.65297	-0.74843	-0.74645	-0.83492	-0.83279	-0.95831
-0.65808	-0.65644	-0.65484	-0.65325	-0.7488	-0.74681	-0.83532	-0.83318	-0.95877
-0.65837	-0.65673	-0.65512	-0.65354	-0.74916	-0.74717	-0.83572	-0.83358	-0.95922
-0.65867	-0.65702	-0.65541	-0.65382	-0.74952	-0.74754	-0.83612	-0.83398	-0.95969

-0.65896	-0.65731	-0.65569	-0.6541	-0.65249	-0.65087	-0.74594	-0.74401	-0.96015
-0.65924	-0.65759	-0.65597	-0.65437	-0.65276	-0.65114	-0.7463	-0.74436	-0.9606
-0.65953	-0.65787	-0.65625	-0.65465	-0.65304	-0.65143	-0.74667	-0.74473	-0.96107
-0.65986	-0.65819	-0.65655	-0.65494	-0.65333	-0.65174	-0.74709	-0.74514	-0.96158
-0.66022	-0.65851	-0.65685	-0.65523	-0.65364	-0.65208	-0.74753	-0.74557	-0.96211
-0.66054	-0.65882	-0.65715	-0.65552	-0.65393	-0.65238	-0.65082	-0.64924	-0.87947
-0.66084	-0.65912	-0.65744	-0.65581	-0.65421	-0.65266	-0.65111	-0.64956	-0.87999
-0.66116	-0.65944	-0.65776	-0.65611	-0.65451	-0.65294	-0.65139	-0.64987	-0.88047
-0.66151	-0.65981	-0.65811	-0.65644	-0.65481	-0.65322	-0.65166	-0.65014	-0.8809
-0.66185	-0.66016	-0.65846	-0.65678	-0.65513	-0.65352	-0.65194	-0.65041	-0.88131
-0.66216	-0.66047	-0.65879	-0.65713	-0.65548	-0.65383	-0.65223	-0.65068	-0.88173
-0.66246	-0.66077	-0.65911	-0.65748	-0.65583	-0.65416	-0.65254	-0.65096	-0.88215
-0.66278	-0.66106	-0.65941	-0.65779	-0.65615	-0.6545	-0.65287	-0.65128	-0.69854
-0.6631	-0.66136	-0.65969	-0.65807	-0.65645	-0.65484	-0.65323	-0.65162	-0.69889
-0.66346	-0.66168	-0.65999	-0.65836	-0.65675	-0.65515	-0.65355	-0.65195	-0.69925
-0.66384	-0.66202	-0.6603	-0.65865	-0.65702	-0.65543	-0.65385	-0.65228	-0.69963
-0.66421	-0.66238	-0.66065	-0.65898	-0.65734	-0.65573	-0.65415	-0.65259	-0.65104
-0.66456	-0.66276	-0.66105	-0.65937	-0.65771	-0.65606	-0.65445	-0.65287	-0.65131
-0.6649	-0.66314	-0.66143	-0.65976	-0.65808	-0.65641	-0.65476	-0.65316	-0.65159
-0.66524	-0.66347	-0.66176	-0.66008	-0.65841	-0.65676	-0.65512	-0.65349	-0.65189
-0.66558	-0.6638	-0.66206	-0.66037	-0.65872	-0.65711	-0.65548	-0.65384	-0.65222
-0.66598	-0.66419	-0.66243	-0.6607	-0.65904	-0.65742	-0.6558	-0.65416	-0.65256
-0.66642	-0.66463	-0.66283	-0.66106	-0.65935	-0.65771	-0.65609	-0.65448	-0.6529
-0.56117	-0.55975	-0.6632	-0.66141	-0.6597	-0.65804	-0.65642	-0.65481	-0.65324
-0.56142	-0.56	-0.66353	-0.66176	-0.66007	-0.65843	-0.65679	-0.65516	-0.65355
-0.56168	-0.56026	-0.66386	-0.66211	-0.66042	-0.65879	-0.65715	-0.6555	-0.65387
-0.56199	-0.56054	-0.66419	-0.66243	-0.66073	-0.65909	-0.65746	-0.65583	-0.65423
-0.5623	-0.56083	-0.66454	-0.66276	-0.66105	-0.6594	-0.65777	-0.65617	-0.65459
-0.5626	-0.56114	-0.61613	-0.6145	-0.66146	-0.65979	-0.65815	-0.65652	-0.65491
-0.56289	-0.56146	-0.61653	-0.61491	-0.66189	-0.66021	-0.65854	-0.65687	-0.65523
-0.56321	-0.56177	-0.56035	-0.55894	-0.66225	-0.66056	-0.65888	-0.6572	-0.65556
-0.56355	-0.56207	-0.56062	-0.5592	-0.66255	-0.66085	-0.65917	-0.65753	-0.6559
-0.56387	-0.56238	-0.56092	-0.55949	-0.6143	-0.61271	-0.6595	-0.65786	-0.65624
-0.56416	-0.56269	-0.56125	-0.55983	-0.61469	-0.6131	-0.65988	-0.6582	-0.65654
-0.56447	-0.56302	-0.56158	-0.56016	-0.61508	-0.61348	-0.61188	-0.61028	-0.65687
-0.56482	-0.56337	-0.56191	-0.56047	-0.61542	-0.61379	-0.61219	-0.61061	-0.65723
-0.56518	-0.56371	-0.56224	-0.56077	-0.61575	-0.61411	-0.6125	-0.61095	-0.65761
-0.56548	-0.56401	-0.56254	-0.56109	-0.61613	-0.61448	-0.61288	-0.61133	-0.6098
-0.56576	-0.56427	-0.56283	-0.5614	-0.61653	-0.6149	-0.6133	-0.61173	-0.61019
-0.56607	-0.56459	-0.56315	-0.56174	-0.56033	-0.55893	-0.6137	-0.6121	-0.61053
-0.56643	-0.56496	-0.56352	-0.5621	-0.56069	-0.55929	-0.61411	-0.61246	-0.61085
-0.45772	-0.45656	-0.56387	-0.56244	-0.56101	-0.55961	-0.61448	-0.61281	-0.61118
-0.45802	-0.45686	-0.56421	-0.56273	-0.56129	-0.55987	-0.61479	-0.61314	-0.61153
-0.4583	-0.45714	-0.56454	-0.56303	-0.56157	-0.56014	-0.61511	-0.61349	-0.61191
-0.45854	-0.45737	-0.56482	-0.56334	-0.56189	-0.56047	-0.61551	-0.61389	-0.6123
-0.45875	-0.45759	-0.56511	-0.56366	-0.56223	-0.56082	-0.61593	-0.61431	-0.61271
-0.45901	-0.45785	-0.56544	-0.564	-0.56258	-0.56117	-0.55977	-0.55837	-0.61311
-0.4593	-0.45815	-0.56581	-0.56436	-0.56294	-0.56153	-0.56012	-0.55872	-0.61351
-0.4043	-0.40331	-0.45729	-0.45615	-0.56329	-0.56188	-0.56047	-0.55906	-0.61391
-0.40454	-0.40356	-0.45758	-0.45644	-0.56365	-0.56223	-0.56081	-0.55941	-0.6143

-0.40479	-0.40379	-0.45787	-0.45673	-0.564	-0.56256	-0.56113	-0.55972	-0.55831
-0.40503	-0.40403	-0.45816	-0.45702	-0.56434	-0.56287	-0.56142	-0.55999	-0.55856
-0.40527	-0.40427	-0.45844	-0.45729	-0.56467	-0.56317	-0.5617	-0.56026	-0.55883
-0.40551	-0.4045	-0.4587	-0.45754	-0.50918	-0.50785	-0.56201	-0.56058	-0.55916
-0.40574	-0.40473	-0.45896	-0.45777	-0.50944	-0.50813	-0.56234	-0.56093	-0.55951
-0.40596	-0.40494	-0.4592	-0.45802	-0.50972	-0.50843	-0.56269	-0.56128	-0.55987
-0.40617	-0.40512	-0.45944	-0.45827	-0.51002	-0.50874	-0.56305	-0.56163	-0.56022
-0.40638	-0.40533	-0.40431	-0.40331	-0.4574	-0.45628	-0.50778	-0.50652	-0.56057
-0.40659	-0.40555	-0.40454	-0.40355	-0.45768	-0.45656	-0.50809	-0.50683	-0.56092
-0.4068	-0.40578	-0.40477	-0.40379	-0.45796	-0.45684	-0.5084	-0.50714	-0.56127
-0.40704	-0.40602	-0.40501	-0.40403	-0.45825	-0.45712	-0.50871	-0.50745	-0.56162
-0.4073	-0.40627	-0.40526	-0.40426	-0.45853	-0.4574	-0.50901	-0.50775	-0.56197
-0.40755	-0.40652	-0.4055	-0.4045	-0.4588	-0.45767	-0.50931	-0.50806	-0.56232
-0.4078	-0.40676	-0.40574	-0.40473	-0.45908	-0.45795	-0.50961	-0.50836	-0.56266
-0.40804	-0.40701	-0.40598	-0.40497	-0.40397	-0.40299	-0.4571	-0.456	-0.50741
-0.40828	-0.40726	-0.40623	-0.4052	-0.4042	-0.40322	-0.45738	-0.45627	-0.50771
-0.35142	-0.35056	-0.40647	-0.40545	-0.40445	-0.40346	-0.45766	-0.45655	-0.508
-0.35162	-0.35076	-0.40672	-0.4057	-0.40469	-0.4037	-0.45794	-0.45682	-0.5083
-0.35184	-0.35096	-0.40697	-0.40595	-0.40494	-0.40394	-0.45822	-0.4571	-0.5086
-0.35207	-0.35118	-0.40723	-0.4062	-0.40518	-0.40418	-0.45849	-0.45737	-0.45625
-0.3523	-0.35141	-0.4075	-0.40646	-0.40544	-0.40442	-0.45878	-0.45764	-0.45652
-0.35252	-0.35165	-0.35078	-0.34991	-0.40574	-0.40471	-0.45911	-0.45795	-0.45681
-0.35274	-0.35189	-0.35104	-0.35018	-0.40606	-0.40503	-0.45946	-0.45827	-0.45711
-0.35294	-0.35211	-0.35127	-0.35042	-0.40635	-0.40531	-0.40428	-0.40324	-0.45742
-0.35314	-0.35231	-0.35147	-0.35062	-0.4066	-0.40556	-0.40453	-0.40352	-0.45776
-0.23545	-0.2349	-0.35167	-0.35082	-0.34996	-0.34909	-0.40479	-0.40378	-0.45809
-0.2356	-0.23505	-0.35186	-0.35102	-0.35016	-0.34929	-0.40504	-0.40402	-0.45837
-0.23575	-0.2352	-0.35206	-0.35122	-0.35037	-0.3495	-0.40529	-0.40427	-0.45865
-0.1774	-0.17697	-0.35225	-0.35141	-0.35057	-0.34971	-0.34884	-0.34798	-0.45894
-0.17752	-0.17709	-0.35243	-0.35161	-0.35077	-0.34991	-0.34905	-0.34818	-0.45922
-0.17763	-0.17721	-0.24156	-0.24094	-0.35097	-0.35012	-0.34926	-0.34838	-0.40398
-0.17775	-0.17733	-0.24173	-0.24111	-0.35116	-0.35033	-0.34947	-0.34859	-0.40423
-0.11963	-0.11934	-0.24192	-0.2413	-0.35138	-0.35055	-0.3497	-0.34882	-0.34795
-0.11973	-0.11944	-0.24213	-0.24151	-0.35163	-0.3508	-0.34995	-0.34908	-0.34822
-0.11982	-0.11953	-0.24234	-0.24171	-0.24109	-0.24048	-0.35019	-0.34933	-0.34847
-0.1199	-0.11961	-0.24252	-0.24189	-0.24127	-0.24065	-0.35039	-0.34954	-0.34868
-0.11998	-0.11969	-0.24269	-0.24206	-0.24144	-0.24082	-0.35058	-0.34973	-0.34887
-0.12006	-0.11976	-0.18598	-0.18549	-0.24161	-0.24099	-0.35077	-0.34992	-0.34907
-0.12014	-0.11984	-0.18611	-0.18562	-0.24179	-0.24117	-0.35096	-0.35012	-0.34928
-0.12023	-0.11993	-0.11964	-0.11935	-0.24198	-0.24136	-0.24074	-0.24013	-0.34951
-0.12033	-0.12003	-0.11974	-0.11945	-0.24219	-0.24157	-0.24095	-0.24034	-0.34976
-0.12043	-0.12012	-0.11983	-0.11954	-0.18559	-0.18511	-0.24115	-0.24054	-0.35
-0.12051	-0.1202	-0.11991	-0.11962	-0.18571	-0.18524	-0.24133	-0.24071	-0.35019
-0.06108	-0.06092	-0.11999	-0.11969	-0.18585	-0.18537	-0.2415	-0.24089	-0.24028
-0.06113	-0.06097	-0.12007	-0.11978	-0.18599	-0.18552	-0.24171	-0.24109	-0.24048
-0.06117	-0.06102	-0.12015	-0.11986	-0.18614	-0.18567	-0.24192	-0.2413	-0.24069
-0.06123	-0.06107	-0.12024	-0.11995	-0.18628	-0.18581	-0.2421	-0.24149	-0.24087
-0.06128	-0.06113	-0.12034	-0.12004	-0.18641	-0.18593	-0.24227	-0.24165	-0.24104
-0.06134	-0.06118	-0.12043	-0.12012	-0.18654	-0.18606	-0.18559	-0.18512	-0.24123
-0.06138	-0.06123	-0.12051	-0.1202	-0.18668	-0.1862	-0.18573	-0.18527	-0.24144

-0.06143	-0.06128	-0.06112	-0.06096	-0.12	-0.11971	-0.18588	-0.18541	-0.24163
-0.06148	-0.06133	-0.06118	-0.06102	-0.12009	-0.1198	-0.18601	-0.18554	-0.2418
-0.06154	-0.06139	-0.06123	-0.06108	-0.12019	-0.1199	-0.18615	-0.18567	-0.24198
-0.06158	-0.06143	-0.06128	-0.06113	-0.12029	-0.11999	-0.18631	-0.18582	-0.18534
-0.06162	-0.06148	-0.06133	-0.06118	-0.12038	-0.12009	-0.18647	-0.18598	-0.18549
-0.06167	-0.06153	-0.06138	-0.06123	-0.06108	-0.06093	-0.18661	-0.18612	-0.18563
-0.06173	-0.06158	-0.06144	-0.06128	-0.06113	-0.06097	-0.18674	-0.18624	-0.18576
-0.06178	-0.06164	-0.06149	-0.06134	-0.06118	-0.06103	-0.18688	-0.18639	-0.1859
-0.06183	-0.06169	-0.06155	-0.0614	-0.06124	-0.06108	-0.18705	-0.18655	-0.18606
-0.06188	-0.06174	-0.0616	-0.06145	-0.0613	-0.06114	-0.06099	-0.06084	-0.18622
-0.06193	-0.06179	-0.06164	-0.06149	-0.06134	-0.06119	-0.06104	-0.06089	-0.18638
-0.06197	-0.06183	-0.06168	-0.06153	-0.06139	-0.06124	-0.06109	-0.06094	-0.18654
-0.06202	-0.06188	-0.06173	-0.06159	-0.06144	-0.06129	-0.06114	-0.06099	-0.18668
-0.06208	-0.06193	-0.06179	-0.06164	-0.0615	-0.06135	-0.06119	-0.06104	-0.18681
-0.06213	-0.06198	-0.06184	-0.0617	-0.06155	-0.0614	-0.06125	-0.06109	-0.12917
-0.06218	-0.06204	-0.06189	-0.06175	-0.06161	-0.06146	-0.0613	-0.06115	-0.1293
-0.06223	-0.06209	-0.06194	-0.0618	-0.06166	-0.06151	-0.06136	-0.06121	-0.06105
-0.06228	-0.06213	-0.06199	-0.06185	-0.06171	-0.06156	-0.06142	-0.06126	-0.06111
-0.06233	-0.06217	-0.06203	-0.06189	-0.06176	-0.06162	-0.06147	-0.06132	-0.06117
-0.06238	-0.06223	-0.06208	-0.06194	-0.0618	-0.06166	-0.06152	-0.06137	-0.06122
-0.06244	-0.06229	-0.06214	-0.06199	-0.06185	-0.06171	-0.06157	-0.06143	-0.06128
-0.0625	-0.06234	-0.06219	-0.06204	-0.0619	-0.06176	-0.06162	-0.06148	-0.06133
-0.06256	-0.0624	-0.06225	-0.0621	-0.06195	-0.06181	-0.06167	-0.06153	-0.06138
-0.06261	-0.06246	-0.0623	-0.06215	-0.062	-0.06186	-0.06172	-0.06157	-0.06143
-0.06267	-0.06251	-0.06236	-0.06221	-0.06206	-0.06191	-0.06177	-0.06162	-0.06148
-0.06273	-0.06257	-0.06242	-0.06226	-0.06211	-0.06197	-0.06182	-0.06168	-0.06153
-0.06278	-0.06263	-0.06247	-0.06232	-0.06217	-0.06202	-0.06188	-0.06173	-0.06159
-0.06283	-0.06268	-0.06253	-0.06237	-0.06222	-0.06208	-0.06193	-0.06179	-0.06164
-0.06289	-0.06274	-0.06258	-0.06243	-0.06228	-0.06213	-0.06198	-0.06184	-0.06169
-0.06295	-0.0628	-0.06264	-0.06249	-0.06234	-0.06218	-0.06204	-0.06189	-0.06175
-0.063	-0.06285	-0.0627	-0.06254	-0.06239	-0.06224	-0.06209	-0.06195	-0.0618
-0.06306	-0.0629	-0.06275	-0.0626	-0.06244	-0.06229	-0.06215	-0.062	-0.06185
-0.06311	-0.06296	-0.0628	-0.06265	-0.0625	-0.06235	-0.0622	-0.06205	-0.06191
-0.06317	-0.06301	-0.06286	-0.06271	-0.06256	-0.06241	-0.06226	-0.06211	-0.06196
-0.06322	-0.06307	-0.06292	-0.06277	-0.06262	-0.06247	-0.06231	-0.06216	-0.06202
-0.06328	-0.06313	-0.06298	-0.06283	-0.06268	-0.06252	-0.06237	-0.06222	-0.06207
-0.06334	-0.06319	-0.06304	-0.06289	-0.06274	-0.06258	-0.06243	-0.06228	-0.06213
		-0.06309	-0.06295	-0.0628	-0.06264	-0.06249	-0.06234	-0.06219
		-0.06315	-0.063	-0.06285	-0.0627	-0.06255	-0.0624	-0.06224
		-0.06321	-0.06306	-0.06291	-0.06276	-0.06261	-0.06246	-0.0623
		-0.06326	-0.06312	-0.06297	-0.06282	-0.06267	-0.06251	-0.06236
				-0.06303	-0.06288	-0.06273	-0.06257	-0.06242
				-0.06308	-0.06294	-0.06278	-0.06263	-0.06248
				-0.06314	-0.06299	-0.06284	-0.06269	-0.06254
				-0.0632	-0.06305	-0.0629	-0.06275	-0.0626
				-0.06326	-0.06311	-0.06296	-0.0628	-0.06265
						-0.06301	-0.06286	-0.06272
						-0.06307	-0.06292	-0.06278
						-0.06313	-0.06298	-0.06284
						-0.06318	-0.06304	-0.06289

-0.06324	-0.0631	-0.06295
-0.06331	-0.06316	-0.06301
-0.06338	-0.06322	-0.06307
		-0.06313
		-0.06319
		-0.06325
		-0.06331
		-0.06337
		-0.06343
		-0.06349
		-0.06356
		-0.06362

						-0.06307	-0.06287	-0.06267
						-0.06301	-0.0628	-0.0626
						-0.06294	-0.06273	-0.06253
						-0.06286	-0.06266	-0.06246
				-0.06318	-0.06298	-0.3833	-0.38225	-0.3812
				-0.06311	-0.06291	-0.38291	-0.38186	-0.3808
				-0.06304	-0.06284	-0.38252	-0.38146	-0.3804
				-0.06297	-0.06277	-0.38209	-0.38103	-0.37997
				-0.06289	-0.06269	-0.38165	-0.38059	-0.37952
				-0.06282	-0.06262	-0.43748	-0.43626	-0.43506
				-0.06275	-0.06256	-0.437	-0.4358	-0.43461
		-0.06308	-0.06288	-0.38255	-0.38148	-0.43654	-0.43534	-0.43416
		-0.06301	-0.06281	-0.38214	-0.38108	-0.43609	-0.43489	-0.4337
		-0.06294	-0.06274	-0.43803	-0.43683	-0.52485	-0.52347	-0.52211
		-0.06287	-0.06267	-0.43758	-0.43637	-0.52435	-0.52299	-0.52164
		-0.0628	-0.0626	-0.43712	-0.43591	-0.52386	-0.52251	-0.52118
		-0.25573	-0.25501	-0.43666	-0.43545	-0.52337	-0.52204	-0.52071
		-0.25545	-0.25474	-0.43619	-0.435	-0.52289	-0.52156	-0.52025
		-0.31401	-0.31314	-0.52511	-0.52376	-0.52242	-0.5211	-0.51979
		-0.31367	-0.3128	-0.52463	-0.52329	-0.52195	-0.52063	-0.51932
-0.25605	-0.25533	-0.43722	-0.43603	-0.52415	-0.52281	-0.52149	-0.52017	-0.51886
-0.25577	-0.25506	-0.43676	-0.43556	-0.52366	-0.52234	-0.52102	-0.5197	-0.51838
-0.25549	-0.25478	-0.4363	-0.4351	-0.52317	-0.52186	-0.52055	-0.51923	-0.5179
-0.31405	-0.31317	-0.52537	-0.52402	-0.52269	-0.52139	-0.69012	-0.68834	-0.68656
-0.31372	-0.31283	-0.52489	-0.52355	-0.52222	-0.52091	-0.68947	-0.68769	-0.68591
-0.31339	-0.31251	-0.52441	-0.52307	-0.52174	-0.52044	-0.68881	-0.68704	-0.68527
-0.31307	-0.31219	-0.52395	-0.52259	-0.52126	-0.51996	-0.68815	-0.68639	-0.68463
-0.37707	-0.37598	-0.52349	-0.52213	-0.5208	-0.5195	-0.68749	-0.68573	-0.68398
-0.37666	-0.37558	-0.52302	-0.52169	-0.52036	-0.51903	-0.68684	-0.68507	-0.68332
-0.52524	-0.52389	-0.52256	-0.52124	-0.63417	-0.63252	-0.68621	-0.68443	-0.68267
-0.52475	-0.5234	-0.52208	-0.52078	-0.6336	-0.63197	-0.68561	-0.68381	-0.68202
-0.52427	-0.52293	-0.52161	-0.52032	-0.63302	-0.63142	-0.68502	-0.6832	-0.68138
-0.52381	-0.52247	-0.52116	-0.51986	-0.68792	-0.68616	-0.7363	-0.73435	-0.7324
-0.52337	-0.52203	-0.52071	-0.5194	-0.68727	-0.68551	-0.7356	-0.73368	-0.73176
-0.52291	-0.52159	-0.52027	-0.51896	-0.68665	-0.68487	-0.7349	-0.733	-0.73109
-0.52243	-0.52113	-0.51983	-0.51854	-0.68606	-0.68425	-0.73419	-0.73229	-0.7304
-0.52196	-0.52068	-0.51939	-0.5181	-0.73742	-0.73545	-0.7335	-0.73159	-0.7297
-0.52151	-0.52021	-0.51893	-0.51766	-0.73673	-0.73479	-0.73285	-0.73091	-0.729
-0.52107	-0.51976	-0.63227	-0.63068	-0.73604	-0.73412	-0.7322	-0.73025	-0.72831
-0.52063	-0.51933	-0.63172	-0.63011	-0.73534	-0.73344	-0.73152	-0.72959	-0.72767

-0.5202	-0.51891	-0.63117	-0.62954	-0.73464	-0.73274	-0.73085	-0.72894	-0.72704
-0.51978	-0.51849	-0.73787	-0.7359	-0.73397	-0.73208	-0.73019	-0.7283	-0.72642
-0.51937	-0.51808	-0.73722	-0.73525	-0.73332	-0.73142	-0.72953	-0.72767	-0.7258
-0.51894	-0.51764	-0.73656	-0.7346	-0.73266	-0.73075	-0.72886	-0.72701	-0.72516
-0.51847	-0.51718	-0.73589	-0.73394	-0.732	-0.73007	-0.72817	-0.72633	-0.72451
-0.51802	-0.51673	-0.73522	-0.73328	-0.73135	-0.72941	-0.72751	-0.72568	-0.72386
-0.51758	-0.51629	-0.73457	-0.73265	-0.73072	-0.72878	-0.72689	-0.72505	-0.72323
-0.51716	-0.51586	-0.73392	-0.73201	-0.7301	-0.72817	-0.72627	-0.72443	-0.7226
-0.68297	-0.68124	-0.73327	-0.73139	-0.72948	-0.72755	-0.72565	-0.72378	-0.72194
-0.68237	-0.68063	-0.73262	-0.73075	-0.72886	-0.72693	-0.72502	-0.72313	-0.72127
-0.68176	-0.68001	-0.73195	-0.73009	-0.72821	-0.72631	-0.72441	-0.72251	-0.72064
-0.68114	-0.67938	-0.73126	-0.72942	-0.72756	-0.72568	-0.7238	-0.72191	-0.72003
-0.73439	-0.73247	-0.7306	-0.72877	-0.72692	-0.72506	-0.72319	-0.7213	-0.71942
-0.73375	-0.73184	-0.72998	-0.72815	-0.72631	-0.72445	-0.72258	-0.72068	-0.7188
-0.73312	-0.73122	-0.72937	-0.72754	-0.72569	-0.72383	-0.72196	-0.72007	-0.71819
-0.73249	-0.7306	-0.72875	-0.72693	-0.72508	-0.72321	-0.72134	-0.71946	-0.71758
-0.73187	-0.72998	-0.72814	-0.72632	-0.72447	-0.7226	-0.72072	-0.71884	-0.71698
-0.73125	-0.72937	-0.72752	-0.7257	-0.72386	-0.72198	-0.7201	-0.71823	-0.71637
-0.73064	-0.72876	-0.72691	-0.72509	-0.72324	-0.72136	-0.71949	-0.71762	-0.71576
-0.73003	-0.72814	-0.72629	-0.72447	-0.72262	-0.72075	-0.8253	-0.82304	-0.82081
-0.72942	-0.72753	-0.72567	-0.72385	-0.722	-0.72015	-0.8246	-0.82235	-0.82013
-0.72881	-0.72692	-0.72506	-0.72323	-0.72139	-0.71954	-0.8239	-0.82166	-0.81945
-0.7282	-0.72632	-0.72446	-0.72261	-0.72077	-0.71894	-0.8232	-0.82098	-0.81878
-0.7276	-0.72573	-0.72387	-0.72201	-0.72017	-0.71834	-0.8225	-0.8203	-0.81811
-0.72702	-0.72516	-0.72331	-0.72145	-0.7196	-0.71775	-0.8218	-0.81961	-0.81743
-0.72645	-0.72461	-0.72276	-0.7209	-0.71904	-0.71717	-0.82111	-0.81892	-0.81676
-0.72589	-0.72405	-0.72219	-0.72033	-0.82491	-0.82265	-0.82042	-0.81824	-0.81608
-0.72534	-0.72349	-0.72162	-0.71975	-0.82422	-0.82197	-0.81975	-0.81756	-0.81541
-0.72477	-0.72291	-0.72104	-0.71916	-0.82353	-0.82128	-0.81907	-0.81689	-0.81475
-0.7242	-0.72232	-0.72044	-0.71856	-0.82282	-0.82058	-0.81838	-0.81622	-0.8141
-0.72361	-0.72173	-0.77262	-0.77052	-0.82212	-0.8199	-0.81771	-0.81556	-0.81345
-0.72301	-0.72113	-0.77196	-0.76986	-0.82143	-0.81922	-0.81705	-0.81493	-0.81283
-0.72241	-0.72053	-0.77131	-0.76921	-0.82074	-0.81856	-0.81641	-0.81429	-0.8122
-0.72182	-0.71994	-0.77068	-0.76858	-0.82007	-0.81791	-0.91552	-0.91284	-0.91022
-0.72123	-0.71937	-0.77006	-0.76796	-0.81942	-0.81727	-0.91471	-0.91206	-0.90946
-0.77361	-0.77155	-0.82324	-0.82101	-0.81881	-0.81665	-0.91391	-0.91126	-0.90866
-0.77299	-0.77095	-0.82263	-0.82042	-0.81823	-0.81605	-0.91313	-0.91046	-0.90785
-0.77239	-0.77034	-0.82198	-0.81978	-0.91787	-0.91505	-0.95551	-0.95272	-0.94999
-0.77181	-0.76973	-0.82131	-0.81909	-0.91695	-0.91416	-0.9546	-0.95185	-0.94916
-0.77122	-0.76912	-0.82063	-0.8184	-0.91604	-0.91328	-0.95371	-0.95101	-0.94835
-0.77058	-0.76849	-0.81996	-0.81773	-0.91517	-0.91243	-0.95285	-0.95019	-0.94757
-0.76993	-0.76786	-0.8193	-0.81708	-0.91434	-0.91163	-0.95202	-0.94939	-0.9468
-0.7693	-0.76725	-0.81868	-0.81649	-0.91362	-0.91093	-0.95128	-0.94864	-0.94603
-0.76868	-0.76665	-0.81808	-0.81593	-0.91294	-0.91027	-0.95057	-0.94789	-0.94524
-0.82177	-0.81959	-0.91758	-0.91484	-0.95531	-0.95252	-0.94978	-0.9471	-0.94444
-0.82115	-0.81895	-0.91671	-0.91396	-0.95439	-0.95164	-0.94893	-0.94626	-0.94361
-0.82051	-0.8183	-0.91585	-0.9131	-0.9535	-0.95078	-0.99366	-0.99088	-0.98811
-0.81984	-0.81763	-0.91498	-0.91226	-0.95263	-0.94992	-0.99278	-0.99003	-0.9873
-0.81919	-0.81698	-0.91417	-0.91147	-0.95181	-0.94911	-0.99193	-0.9892	-0.98648
-0.81861	-0.81642	-0.91347	-0.91079	-0.9511	-0.9484	-0.99117	-0.9884	-0.98566

-0.81804	-0.81586	-0.91278	-0.91013	-0.9504	-0.94771	-0.99042	-0.98761	-0.98482
-0.8174	-0.81522	-0.91199	-0.90936	-0.94961	-0.94691	-1.02872	-1.02562	-1.02257
-0.81673	-0.81456	-0.91114	-0.90854	-0.94877	-0.94607	-1.02777	-1.02468	-1.02165
-0.91578	-0.91305	-0.95343	-0.95071	-0.948	-0.94531	-1.0269	-1.02383	-1.0208
-0.9151	-0.91238	-0.95272	-0.95001	-0.94731	-0.94462	-1.02612	-1.02305	-1.02002
-0.91437	-0.91167	-0.95197	-0.94927	-1.03145	-1.02836	-1.06963	-1.06644	-1.06328
-0.91355	-0.91087	-0.95114	-0.94843	-1.0305	-1.02741	-1.06865	-1.06546	-1.06231
-0.91275	-0.91009	-0.95033	-0.94762	-1.02957	-1.02649	-1.06769	-1.06451	-1.06138
-0.91205	-0.90942	-0.94962	-0.94691	-1.02876	-1.02568	-1.06685	-1.0637	-1.06058
-0.91137	-0.90876	-0.94893	-0.94622	-1.02798	-1.0249	-1.06605	-1.06292	-1.05984
-0.95359	-0.95087	-0.94814	-0.94543	-1.02709	-1.02402	-1.06514	-1.06206	-1.05902
-0.95273	-0.95002	-0.9473	-0.94459	-1.02614	-1.02308	-1.06419	-1.06116	-1.05818
-0.95195	-0.94925	-0.98681	-0.98382	-1.02527	-1.02223	-1.10838	-1.10519	-1.10205
-0.95127	-0.94856	-0.98605	-0.98308	-1.0245	-1.02146	-1.10751	-1.10429	-1.10112
-0.95059	-0.94789	-0.98531	-0.98235	-1.02375	-1.02071	-1.10667	-1.10342	-1.10023
-0.94992	-0.94721	-0.98459	-0.98163	-1.02301	-1.01999	-1.10586	-1.10261	-1.09941
-0.94921	-0.9465	-0.98383	-0.98088	-1.02224	-1.01923	-1.10503	-1.10178	-1.09859
-0.94841	-0.94569	-1.02744	-1.02439	-1.06549	-1.06237	-1.10408	-1.10085	-1.09767
-0.94756	-0.94484	-1.02649	-1.02345	-1.06453	-1.06142	-1.1031	-1.09988	-1.09671
-0.94682	-0.9441	-1.02565	-1.02262	-1.06367	-1.06058	-1.14917	-1.14567	-1.14224
-0.94615	-0.94344	-1.0249	-1.02187	-1.06289	-1.05981	-1.14827	-1.1448	-1.14139
-0.98572	-0.98275	-1.02416	-1.02114	-1.10709	-1.10383	-1.14739	-1.14393	-1.14055
-0.98501	-0.98205	-1.02344	-1.02042	-1.10629	-1.10303	-1.14653	-1.14308	-1.13972
-0.98429	-0.98134	-1.0227	-1.0197	-1.10549	-1.10223	-1.14567	-1.14224	-1.13889
-0.98356	-0.98063	-1.02197	-1.01897	-1.10469	-1.10143	-1.14481	-1.1414	-1.13807
-0.9828	-0.97988	-1.0212	-1.01821	-1.10385	-1.1006	-1.14393	-1.14053	-1.13723
-0.98197	-0.97904	-1.02032	-1.01735	-1.10291	-1.09968	-1.14295	-1.13959	-1.13632
-0.9811	-0.97816	-1.01939	-1.01643	-1.10191	-1.0987	-1.14193	-1.13861	-1.13538
-1.02463	-1.02157	-1.1075	-1.10424	-1.10102	-1.09782	-1.141	-1.13772	-1.13453
-1.02385	-1.02081	-1.10667	-1.10342	-1.1002	-1.09703	-1.14016	-1.13691	-1.13376
-1.02309	-1.02007	-1.10586	-1.10261	-1.09941	-1.09625	-1.13933	-1.13612	-1.13301
-1.02236	-1.01936	-1.10507	-1.10182	-1.09862	-1.09547	-1.13851	-1.13534	-1.13227
-1.02164	-1.01865	-1.10429	-1.10104	-1.09785	-1.09471	-1.13771	-1.13457	-1.13154
-1.02091	-1.01794	-1.1035	-1.10026	-1.09709	-1.09399	-1.13695	-1.13384	-1.13082
-1.02019	-1.01722	-1.10271	-1.09949	-1.09635	-1.09328	-1.1362	-1.13312	-1.13012
-1.01945	-1.01649	-1.10191	-1.09871	-1.09559	-1.09256	-1.21614	-1.21288	-1.20971
-1.01872	-1.01576	-1.10111	-1.09793	-1.09484	-1.09184	-1.21534	-1.21211	-1.20897
-1.1068	-1.10353	-1.10032	-1.09715	-1.09409	-1.09112	-1.21454	-1.21135	-1.20822
-1.10598	-1.10273	-1.09952	-1.09638	-1.09334	-1.09042	-1.21376	-1.21059	-1.20748
-1.10517	-1.10193	-1.09874	-1.09561	-1.09261	-1.08972	-1.2506	-1.24736	-1.24418
-1.10436	-1.10113	-1.09796	-1.09486	-1.09188	-1.08903	-1.24979	-1.24658	-1.24341
-1.10356	-1.10034	-1.09719	-1.09412	-1.09117	-1.08834	-1.24899	-1.2458	-1.24264
-1.10277	-1.09956	-1.09643	-1.09339	-1.09046	-1.08766	-1.24818	-1.245	-1.24186
-1.10197	-1.09878	-1.09568	-1.09267	-1.08977	-1.08698	-1.24738	-1.2442	-1.24106
-1.10116	-1.098	-1.09493	-1.09195	-1.08908	-1.08631	-1.24658	-1.2434	-1.24026
-1.10035	-1.09721	-1.09418	-1.09124	-1.08839	-1.08564	-1.24577	-1.2426	-1.23946
-1.09955	-1.09642	-1.09342	-1.09052	-1.16969	-1.16676	-1.24498	-1.2418	-1.23867
-1.09874	-1.09562	-1.09265	-1.0898	-1.16894	-1.16603	-1.24419	-1.24102	-1.23788
-1.098	-1.09488	-1.09192	-1.08909	-1.1682	-1.16531	-1.24339	-1.24023	-1.23709
-1.09737	-1.09424	-1.09126	-1.08841	-1.16745	-1.16458	-1.24259	-1.23942	-1.23629

-1.09677	-1.09363	-1.09064	-1.08776	-1.16673	-1.16385	-1.24178	-1.23862	-1.2355
-1.09612	-1.09303	-1.09004	-1.08715	-1.20417	-1.20118	-1.24097	-1.23782	-1.23471
-1.09545	-1.09243	-1.08946	-1.08657	-1.20349	-1.20045	-1.24016	-1.23702	-1.23393
-1.09473	-1.09177	-1.08885	-1.08599	-1.20284	-1.19976	-1.27368	-1.27045	-1.26729
-1.09397	-1.09106	-1.08821	-1.08542	-1.20222	-1.19913	-1.27294	-1.26969	-1.26651
-1.09322	-1.09035	-1.16946	-1.16652	-1.20157	-1.19849	-1.27225	-1.26899	-1.26581
-1.09248	-1.08964	-1.16872	-1.16581	-1.20087	-1.19785	-1.2716	-1.26839	-1.26522
-1.09176	-1.08894	-1.16798	-1.16509	-1.20015	-1.19718	-1.27093	-1.26775	-1.26461
-1.09111	-1.08828	-1.16725	-1.16436	-1.19939	-1.19644	-1.27015	-1.26701	-1.26391
-1.0905	-1.08765	-1.16654	-1.16364	-1.19865	-1.19569	-1.26934	-1.26624	-1.26317
-1.08995	-1.08711	-1.16594	-1.16301	-1.19795	-1.19498	-1.26856	-1.26546	-1.26241
-1.08944	-1.08663	-1.1654	-1.16242	-1.19729	-1.19429	-1.26779	-1.26468	-1.26163
-1.12784	-1.12497	-1.20279	-1.19971	-1.19668	-1.19368	-1.26711	-1.26398	-1.26091
-1.12713	-1.1243	-1.20211	-1.1991	-1.19611	-1.19315	-1.26654	-1.26337	-1.26024
-1.12643	-1.12363	-1.20141	-1.19844	-1.19549	-1.19256	-1.30637	-1.30309	-1.29984
-1.12575	-1.12294	-1.20065	-1.19769	-1.19476	-1.19186	-1.30563	-1.3024	-1.2992
-1.12512	-1.12228	-1.19992	-1.19695	-1.19402	-1.19114	-1.30486	-1.30169	-1.29854
-1.16737	-1.16442	-1.1993	-1.19629	-1.19333	-1.19044	-1.30405	-1.3009	-1.29778
-1.16681	-1.16388	-1.19872	-1.19567	-1.19268	-1.18975	-1.30325	-1.3001	-1.29699
-1.1662	-1.16326	-1.19809	-1.19506	-1.26811	-1.26499	-1.30257	-1.29942	-1.2963
-1.16555	-1.1626	-1.19743	-1.19445	-1.2675	-1.26442	-1.30197	-1.29882	-1.29569
-1.20284	-1.1998	-1.1968	-1.19384	-1.26685	-1.26378	-1.3013	-1.29815	-1.29502
-1.20225	-1.19923	-1.19622	-1.19322	-1.26615	-1.26306	-1.30054	-1.29739	-1.29428
-1.20163	-1.19863	-1.23755	-1.23443	-1.26547	-1.26236	-1.29981	-1.29666	-1.29355
-1.20094	-1.19792	-1.23684	-1.23377	-1.26482	-1.26175	-1.29917	-1.29599	-1.29285
-1.20025	-1.19722	-1.23613	-1.2331	-1.26418	-1.26114	-1.29853	-1.29533	-1.29217
-1.19964	-1.19663	-1.2355	-1.23245	-1.26349	-1.26045	-1.36214	-1.35884	-1.35556
-1.19907	-1.19608	-1.2349	-1.2318	-1.26279	-1.25972	-1.36136	-1.3581	-1.35487
-1.19843	-1.19545	-1.23424	-1.23116	-1.26214	-1.25907	-1.36066	-1.3574	-1.35417
-1.19774	-1.19476	-1.23354	-1.23052	-1.26152	-1.25849	-1.36002	-1.35673	-1.35346
-1.23902	-1.23593	-1.23289	-1.22991	-1.32666	-1.3235	-1.35936	-1.35606	-1.35277
-1.2384	-1.23533	-1.23231	-1.22933	-1.32604	-1.32286	-1.35868	-1.35538	-1.3521
-1.2378	-1.23474	-1.23172	-1.22873	-1.3254	-1.32221	-1.35799	-1.3547	-1.35145
-1.23718	-1.2341	-1.23105	-1.22805	-1.3247	-1.32154	-1.35732	-1.35405	-1.35081
-1.23656	-1.23344	-1.23038	-1.22736	-1.32398	-1.32087	-1.35666	-1.35341	-1.35017
-1.23592	-1.2328	-1.29683	-1.29368	-1.32334	-1.32025	-1.35603	-1.35277	-1.34952
-1.23526	-1.23217	-1.29619	-1.29307	-1.32274	-1.31966	-1.3554	-1.35212	-1.34886
-1.23462	-1.23155	-1.29555	-1.29243	-1.32208	-1.31899	-1.35471	-1.35144	-1.34819
-1.234	-1.23093	-1.29491	-1.29176	-1.32137	-1.31826	-1.35395	-1.35073	-1.34753
-1.30068	-1.29747	-1.29428	-1.29112	-1.32069	-1.31757	-1.35324	-1.35004	-1.34687
-1.30006	-1.29682	-1.29364	-1.2905	-1.32008	-1.31697	-1.35262	-1.34942	-1.34625
-1.29946	-1.2962	-1.29302	-1.2899	-1.31948	-1.3164	-1.35202	-1.34881	-1.34562
-1.29887	-1.29562	-1.29245	-1.28932	-1.31889	-1.31582	-1.35141	-1.34818	-1.34497
-1.29829	-1.29506	-1.29189	-1.28875	-1.31831	-1.31523	-1.3508	-1.34754	-1.34431
-1.29766	-1.29446	-1.2913	-1.28819	-1.31772	-1.31462	-1.35014	-1.34689	-1.34367
-1.29699	-1.29382	-1.2907	-1.28762	-1.31714	-1.314	-1.34946	-1.34623	-1.34304
-1.29635	-1.29321	-1.29012	-1.28706	-1.31657	-1.31341	-1.31028	-1.30722	-1.30421
-1.29575	-1.29263	-1.28956	-1.28651	-1.31602	-1.31286	-1.30975	-1.30668	-1.30368
-1.29516	-1.29206	-1.28899	-1.28593	-1.31543	-1.31229	-1.30919	-1.30614	-1.30314
-1.29458	-1.29149	-1.28839	-1.2853	-1.31477	-1.31165	-1.30857	-1.30555	-1.30258

-1.29401	-1.29092	-1.2878	-1.28466	-1.31411	-1.311	-1.30795	-1.30496	-1.30203
-1.29345	-1.29037	-1.28724	-1.2841	-1.31353	-1.31042	-1.30737	-1.3044	-1.30147
-1.2929	-1.28982	-1.2867	-1.28356	-1.31297	-1.30986	-1.30681	-1.30384	-1.30092
-1.29236	-1.28928	-1.28616	-1.28302	-1.31242	-1.30931	-1.30626	-1.30331	-1.30038
-1.29182	-1.28875	-1.28563	-1.28249	-1.31188	-1.30878	-1.30574	-1.30278	-1.29987
-1.29127	-1.2882	-1.28508	-1.28195	-1.31134	-1.30824	-1.3052	-1.30226	-1.29935
-1.2907	-1.28763	-1.28452	-1.2814	-1.31078	-1.30769	-1.30467	-1.30173	-1.29883
-1.29013	-1.28706	-1.28396	-1.28084	-1.31021	-1.30714	-1.33688	-1.33387	-1.3309
-1.28955	-1.28649	-1.28339	-1.28028	-1.30965	-1.30658	-1.33632	-1.33333	-1.33036
-1.28898	-1.28592	-1.28283	-1.27972	-1.30908	-1.30603	-1.33576	-1.33278	-1.32983
-1.28842	-1.28537	-1.28227	-1.27917	-1.30852	-1.30548	-1.3352	-1.33223	-1.32929
-1.28786	-1.28482	-1.28172	-1.27862	-1.30797	-1.30494	-1.33464	-1.33168	-1.32875
-1.2873	-1.28426	-1.28117	-1.27807	-1.30741	-1.30439	-1.36693	-1.36391	-1.36091
-1.28674	-1.2837	-1.28062	-1.27752	-1.30685	-1.30384	-1.36637	-1.36336	-1.36037
-1.28618	-1.28315	-1.28007	-1.27698	-1.33909	-1.33599	-1.36582	-1.36281	-1.35983
-1.28562	-1.28261	-1.27955	-1.27647	-1.33854	-1.33544	-1.36528	-1.36228	-1.35931
-1.28507	-1.28207	-1.27903	-1.27597	-1.33802	-1.33491	-1.36476	-1.36176	-1.35879
-1.28454	-1.28154	-1.27852	-1.27551	-1.37059	-1.3674	-1.36428	-1.36126	-1.35827
-1.28403	-1.281	-1.27801	-1.27505	-1.37013	-1.36694	-1.36381	-1.36077	-1.35775
-1.31604	-1.31292	-1.34365	-1.34058	-1.36962	-1.36644	-1.36332	-1.36026	-1.35724
-1.31547	-1.31234	-1.34308	-1.34003	-1.36908	-1.36592	-1.36281	-1.35975	-1.35673
-1.34891	-1.34568	-1.34256	-1.3395	-1.36854	-1.36542	-1.36233	-1.35927	-1.35625
-1.34847	-1.34522	-1.34207	-1.33898	-1.36801	-1.36495	-1.3619	-1.35882	-1.35579
-1.34802	-1.34477	-1.37381	-1.37061	-1.36749	-1.36446	-1.36143	-1.35836	-1.35531
-1.34752	-1.34432	-1.37335	-1.37012	-1.36699	-1.36394	-1.3609	-1.35784	-1.3548
-1.34701	-1.34386	-1.37288	-1.36964	-1.36649	-1.36341	-1.36036	-1.35731	-1.3543
-1.34649	-1.34334	-1.37235	-1.36913	-1.36597	-1.36288	-1.35984	-1.35683	-1.35385
-1.34598	-1.3428	-1.3718	-1.36861	-1.36546	-1.36236	-1.35932	-1.35636	-1.35342
-1.31364	-1.31052	-1.34012	-1.33702	-1.365	-1.36188	-1.35883	-1.35586	-1.35293
-1.3132	-1.31007	-1.33967	-1.33659	-1.36456	-1.36144	-1.35837	-1.35536	-1.35239
-1.31273	-1.3096	-1.33919	-1.33612	-1.36409	-1.361	-1.35793	-1.35489	-1.35191
-1.31219	-1.30908	-1.33866	-1.33559	-1.36358	-1.36055	-1.35753	-1.35449	-1.35148
-1.31168	-1.30859	-1.33815	-1.33507	-1.36307	-1.36008	-1.3571	-1.35407	-1.35107
-1.31123	-1.30816	-1.3377	-1.33462	-1.36259	-1.35957	-1.35658	-1.3536	-1.35065
-1.3108	-1.30775	-1.33728	-1.33419	-1.36212	-1.35905	-1.35604	-1.35312	-1.35023
-1.34309	-1.33995	-1.33684	-1.33375	-1.36167	-1.35858	-1.35556	-1.35263	-1.34975
-1.34264	-1.3395	-1.33639	-1.33332	-1.36122	-1.35813	-1.3551	-1.35214	-1.34924
-1.3422	-1.33905	-1.33595	-1.33289	-1.36078	-1.35769	-1.35466	-1.35168	-1.34877
-1.34175	-1.33862	-1.33552	-1.33246	-1.36034	-1.35726	-1.35423	-1.35125	-1.34833
-1.34131	-1.33818	-1.33509	-1.33202	-1.3599	-1.35682	-1.35379	-1.35082	-1.3479
-1.34088	-1.33774	-1.33465	-1.33159	-1.35945	-1.35637	-1.35335	-1.35039	-1.34748
-1.34045	-1.3373	-1.33421	-1.33115	-1.35901	-1.35593	-1.35291	-1.34996	-1.34707
-1.33999	-1.33686	-1.33377	-1.33072	-1.35856	-1.35549	-1.35248	-1.34954	-1.34666
-1.33953	-1.33642	-1.33334	-1.33029	-1.35812	-1.35506	-1.35206	-1.34913	-1.34625
-1.33908	-1.33597	-1.3329	-1.32985	-1.35768	-1.35463	-1.35164	-1.34872	-1.34585
-1.33864	-1.33552	-1.33245	-1.32941	-1.35723	-1.3542	-1.35122	-1.34832	-1.34547
-1.33819	-1.33507	-1.332	-1.32897	-1.35679	-1.35377	-1.35082	-1.34793	-1.3451
-1.33772	-1.33462	-1.33156	-1.32853	-1.35635	-1.35336	-1.35042	-1.34757	-1.34476
-1.33727	-1.33417	-1.33112	-1.3281	-1.35593	-1.35295	-1.35004	-1.34721	-1.34441
-1.33683	-1.33373	-1.33068	-1.32768	-1.35551	-1.35257	-1.34967	-1.34683	-1.34401

-1.33641	-1.3333	-1.33026	-1.32727	-1.35511	-1.35219	-1.3493	-1.34644	-1.34361
-1.33602	-1.33293	-1.32988	-1.32689	-1.35471	-1.35179	-1.3489	-1.34604	-1.34321
-1.33566	-1.33257	-1.32952	-1.32652	-1.35432	-1.35138	-1.34849	-1.34563	-1.34281
-1.33526	-1.33218	-1.32913	-1.32612	-1.35391	-1.35097	-1.4227	-1.41963	-1.41661
-1.33484	-1.33177	-1.32871	-1.3257	-1.35347	-1.35054	-1.42227	-1.41921	-1.41619
-1.33443	-1.33137	-1.32833	-1.32531	-1.35308	-1.35016	-1.42188	-1.41883	-1.41582
-1.33404	-1.33101	-1.32799	-1.32499	-1.35276	-1.34984	-1.42156	-1.41851	-1.41551
-1.33365	-1.33064	-1.32764	-1.32466	-1.35243	-1.3495	-1.42122	-1.41818	-1.41518
-1.33325	-1.33023	-1.32724	-1.32428	-1.35204	-1.34911	-1.42081	-1.41778	-1.41479
-1.33286	-1.32982	-1.32683	-1.3239	-1.35165	-1.34872	-1.42041	-1.41738	-1.41439
-1.33251	-1.32947	-1.32648	-1.32355	-1.3513	-1.34839	-1.42007	-1.41704	-1.41405
-1.33218	-1.32914	-1.32616	-1.32322	-1.35097	-1.34808	-1.41977	-1.41673	-1.41372
-1.33179	-1.32877	-1.3258	-1.32286	-1.3506	-1.34772	-1.38155	-1.37861	-1.3757
-1.33136	-1.32836	-1.32539	-1.32247	-1.3502	-1.34732	-1.38114	-1.3782	-1.37529
-1.33097	-1.32798	-1.32502	-1.32211	-1.34983	-1.34695	-1.38076	-1.37782	-1.37492
-1.33063	-1.32765	-1.3247	-1.32179	-1.34952	-1.34664	-1.38044	-1.3775	-1.3746
-1.3303	-1.32733	-1.32439	-1.32149	-1.34921	-1.34633	-1.38012	-1.37719	-1.3743
-1.32997	-1.327	-1.32407	-1.32118	-1.34889	-1.34601	-1.3798	-1.37687	-1.37398
-1.32965	-1.32668	-1.32376	-1.32088	-1.34858	-1.3457	-1.37947	-1.37655	-1.37366
-1.32932	-1.32636	-1.32344	-1.32056	-1.34826	-1.34538	-1.37914	-1.37623	-1.37335
-1.329	-1.32604	-1.32313	-1.32025	-1.34793	-1.34506	-1.37882	-1.37591	-1.37303
-1.32869	-1.32573	-1.32282	-1.31994	-1.34762	-1.34474	-1.37849	-1.37558	-1.37271
-1.32837	-1.32542	-1.32251	-1.31964	-1.34731	-1.34443	-1.37817	-1.37526	-1.37239
-1.32806	-1.32511	-1.3222	-1.31934	-1.34699	-1.34411	-1.37785	-1.37494	-1.37207
-1.32774	-1.3248	-1.32189	-1.31903	-1.34667	-1.34379	-1.37752	-1.37462	-1.37176
-1.32741	-1.32448	-1.32158	-1.31872	-1.34635	-1.34347	-1.3772	-1.37431	-1.37145
-1.32709	-1.32416	-1.32127	-1.31841	-1.34604	-1.34317	-1.37689	-1.374	-1.37114
-1.32677	-1.32385	-1.32096	-1.31811	-1.34573	-1.34286	-1.37658	-1.37369	-1.37083
-1.32645	-1.32353	-1.32065	-1.3178	-1.34541	-1.34255	-1.37626	-1.37338	-1.37052
-1.32612	-1.32321	-1.32033	-1.31748	-1.34509	-1.34223	-1.37595	-1.37307	-1.37022
-1.3258	-1.3229	-1.32002	-1.31717	-1.34477	-1.34192	-1.37563	-1.37276	-1.36992
-1.32548	-1.32257	-1.31969	-1.31686	-1.34446	-1.34161	-1.37532	-1.37245	-1.36961
-1.32519	-1.32227	-1.31939	-1.31656	-1.34415	-1.34131	-1.37501	-1.37214	-1.36931
-1.32495	-1.32203	-1.31913	-1.31627	-1.34383	-1.34099	-1.37469	-1.37184	-1.36901
-1.32472	-1.32179	-1.31888	-1.31599	-1.34353	-1.3407	-1.3744	-1.37155	-1.36874
-1.3551	-1.35211	-1.34913	-1.34618	-1.34329	-1.34045	-1.41206	-1.40912	-1.40622
-1.35476	-1.35179	-1.34885	-1.34594	-1.34307	-1.34024	-1.41184	-1.40891	-1.406
-1.32326	-1.32036	-1.34855	-1.34566	-1.3428	-1.33998	-1.41158	-1.40865	-1.40574
-1.32292	-1.32004	-1.34823	-1.34534	-1.34249	-1.33969	-1.4113	-1.40836	-1.40544
-1.32262	-1.31975	-1.34794	-1.34504	-1.3422	-1.33941	-1.41103	-1.40808	-1.40517
-1.32237	-1.31949	-1.34769	-1.3448	-1.34196	-1.33917	-1.41078	-1.40784	-1.40493
-1.32213	-1.31924	-1.34745	-1.34455	-1.34171	-1.33892	-1.41052	-1.40759	-1.40469
-1.32189	-1.31899	-1.34719	-1.34427	-1.34142	-1.33863	-1.41022	-1.4073	-1.4044
-1.32163	-1.31874	-1.34693	-1.34399	-1.34113	-1.33834	-1.40992	-1.407	-1.40411
-1.32133	-1.31845	-1.31557	-1.31272	-1.34088	-1.3381	-1.33535	-1.33263	-1.32994
-1.321	-1.31814	-1.3153	-1.31248	-1.34067	-1.33789	-1.33514	-1.33242	-1.32972
-1.32072	-1.31786	-1.31503	-1.31223	-1.34045	-1.33768	-1.33493	-1.33221	-1.3295
-1.32047	-1.31761	-1.31478	-1.31199	-1.34022	-1.33746	-1.33472	-1.33199	-1.32927
-1.32022	-1.31736	-1.31453	-1.31172	-1.30897	-1.30625	-1.33448	-1.33175	-1.32904
-1.31997	-1.3171	-1.31426	-1.31143	-1.30866	-1.30594	-1.33418	-1.33147	-1.32877

-1.31971	-1.31684	-1.31398	-1.31115	-1.30836	-1.30563	-1.33389	-1.33119	-1.32851
-1.31944	-1.31654	-1.31369	-1.31088	-1.30811	-1.30539	-1.36753	-1.36473	-1.36195
-1.31917	-1.31625	-1.31341	-1.31063	-1.30788	-1.30517	-1.36732	-1.36451	-1.36172
-1.31894	-1.31603	-1.31319	-1.31041	-1.30766	-1.30494	-1.3671	-1.36429	-1.3615
-1.31875	-1.31587	-1.31302	-1.3102	-1.30743	-1.30471	-1.36688	-1.36407	-1.36129
-1.31852	-1.31565	-1.3128	-1.30998	-1.30721	-1.30449	-1.36916	-1.3663	-1.36347
-1.31824	-1.31535	-1.31251	-1.30973	-1.30698	-1.30426	-1.36892	-1.36606	-1.36322
-1.31799	-1.31508	-1.31226	-1.30949	-1.30675	-1.30404	-1.36868	-1.36582	-1.36297
-1.3178	-1.31491	-1.31207	-1.30927	-1.30651	-1.3038	-1.36843	-1.36557	-1.36274
-1.31762	-1.31476	-1.31191	-1.30906	-1.30628	-1.30356	-1.36818	-1.36533	-1.3625
-1.3174	-1.31456	-1.31172	-1.3089	-1.30611	-1.30335	-1.36793	-1.36508	-1.36226
-1.31716	-1.31433	-1.31153	-1.30875	-1.30596	-1.30315	-1.36768	-1.36482	-1.362
-1.31693	-1.31411	-1.31132	-1.30856	-1.33947	-1.33659	-1.40299	-1.39998	-1.39701
-1.3167	-1.31387	-1.31109	-1.30833	-1.33927	-1.33642	-1.40285	-1.39979	-1.39677
-1.31646	-1.31364	-1.31086	-1.3081	-1.33905	-1.33623	-1.40268	-1.39961	-1.39657
-1.31622	-1.31341	-1.31063	-1.30787	-1.33881	-1.336	-1.40247	-1.39944	-1.39643
-1.31599	-1.31319	-1.34424	-1.3414	-1.33858	-1.33577	-1.40225	-1.39926	-1.39628
-1.31581	-1.31296	-1.344	-1.34118	-1.33835	-1.33553	-1.40201	-1.39904	-1.39608
-1.31564	-1.31275	-1.34379	-1.34097	-1.33815	-1.33531	-1.40177	-1.3988	-1.39586
-1.31547	-1.31258	-1.34361	-1.3408	-1.37185	-1.36891	-1.4016	-1.3986	-1.39564
-1.3153	-1.31245	-1.34347	-1.34064	-1.37168	-1.36876	-1.40147	-1.39842	-1.39541
-1.34913	-1.34621	-1.34332	-1.34046	-1.4074	-1.40435	-1.40131	-1.39825	-1.39523
-1.34898	-1.34607	-1.34316	-1.34026	-1.40718	-1.40414	-1.40112	-1.39809	-1.39509
-1.34881	-1.34592	-1.343	-1.34008	-1.40698	-1.40395	-1.40094	-1.39794	-1.39496
-1.3486	-1.34572	-1.34282	-1.33992	-1.40684	-1.4038	-1.40079	-1.3978	-1.39482
-1.34839	-1.34553	-1.34265	-1.33977	-1.4067	-1.40365	-1.40063	-1.39765	-1.39468
-1.34823	-1.34537	-1.3425	-1.33964	-1.40655	-1.40346	-1.40042	-1.39745	-1.39449
-1.34809	-1.34521	-1.34235	-1.3395	-1.4064	-1.40327	-1.4002	-1.39724	-1.3943
-1.34792	-1.34502	-1.34216	-1.33932	-1.40623	-1.40311	-1.40004	-1.39708	-1.39414
-1.34774	-1.34482	-1.34195	-1.33912	-1.40605	-1.40296	-1.39992	-1.39696	-1.39401
-1.34759	-1.34464	-1.34177	-1.33894	-1.40589	-1.40282	-1.39979	-1.39682	-1.39387
-1.34746	-1.34451	-1.34163	-1.3388	-1.40575	-1.40269	-1.39966	-1.39668	-1.39373
-1.34733	-1.34438	-1.37837	-1.37539	-1.40562	-1.40255	-1.39952	-1.39653	-1.39356
-1.34717	-1.34425	-1.37826	-1.37528	-1.40549	-1.40242	-1.39937	-1.39634	-1.39335
-1.34701	-1.34412	-1.37815	-1.37516	-1.40536	-1.40228	-1.39922	-1.39616	-1.39314
-1.34687	-1.34399	-1.37803	-1.37504	-1.40523	-1.40216	-1.39909	-1.39602	-1.39299
-1.34674	-1.34387	-1.37791	-1.37491	-1.4051	-1.40204	-1.39897	-1.3959	-1.39287
-1.34659	-1.34373	-1.37778	-1.37479	-1.40497	-1.4019	-1.39884	-1.39577	-1.39273
-1.34644	-1.34358	-1.37765	-1.37465	-1.40483	-1.40176	-1.3987	-1.39563	-1.3926
-1.3463	-1.34343	-1.37751	-1.37452	-1.40469	-1.40163	-1.39857	-1.3955	-1.39247
-1.3462	-1.34329	-1.37737	-1.37439	-1.40456	-1.4015	-1.39845	-1.39539	-1.39235
-1.27967	-1.27691	-1.34779	-1.34489	-1.40442	-1.40138	-1.39834	-1.39528	-1.39225
-1.27954	-1.27677	-1.34766	-1.34477	-1.40429	-1.40125	-1.39822	-1.39521	-1.3922
-1.2794	-1.27663	-1.34753	-1.34465	-1.40416	-1.40113	-1.39811	-1.39513	-1.39216
-1.27928	-1.27651	-1.34741	-1.34452	-1.40402	-1.40099	-1.39798	-1.39501	-1.39206
-1.27917	-1.27641	-1.3473	-1.34439	-1.40387	-1.40084	-1.39784	-1.39488	-1.39192
-1.27907	-1.27633	-1.3118	-1.30897	-1.40376	-1.40073	-1.39772	-1.39474	-1.39178
-1.27897	-1.27627	-1.31178	-1.30894	-1.4037	-1.40063	-1.39759	-1.3946	-1.39163
-1.3174	-1.31457	-1.3461	-1.34315	-1.43559	-1.4324	-1.4632	-1.45997	-1.45678
-1.31728	-1.31445	-1.34598	-1.34305	-1.43551	-1.43235	-1.46316	-1.45992	-1.45671

-1.31719	-1.31434	-1.34586	-1.34294	-1.43541	-1.43229	-1.46311	-1.45987	-1.45664
-1.31713	-1.31428	-1.34578	-1.34284	-1.4353	-1.43218	-1.463	-1.45976	-1.45654
-1.3171	-1.31423	-1.34571	-1.34275	-1.43518	-1.43205	-1.46286	-1.45963	-1.45642
-1.31704	-1.31416	-1.34563	-1.34269	-1.40152	-1.39846	-1.46275	-1.45951	-1.4563
-1.31697	-1.31408	-1.34556	-1.34264	-1.40147	-1.39841	-1.46267	-1.4594	-1.45616
-1.3169	-1.31401	-1.3455	-1.34259	-1.40142	-1.39834	-1.46258	-1.45931	-1.45607
-1.31683	-1.31397	-1.34545	-1.34253	-1.40135	-1.39825	-1.46248	-1.45924	-1.45602
-1.31678	-1.31392	-1.3454	-1.34246	-1.36875	-1.36575	-1.43117	-1.42804	-1.42493
-1.31673	-1.31387	-1.34533	-1.34238	-1.36866	-1.36568	-1.4311	-1.42797	-1.42486
-1.31668	-1.31382	-1.34525	-1.3423	-1.36858	-1.36562	-1.43104	-1.4279	-1.42478
-1.31661	-1.31374	-1.34518	-1.34224	-1.36851	-1.36556	-1.43096	-1.42781	-1.42468
-1.31654	-1.31366	-1.3451	-1.34218	-1.36846	-1.36549	-1.43088	-1.42772	-1.42459
-1.31647	-1.3136	-1.34504	-1.34213	-1.36839	-1.36542	-1.39742	-1.39436	-1.39132
-1.3164	-1.31355	-1.34499	-1.34207	-1.36833	-1.36533	-1.39732	-1.39428	-1.39127
-1.35082	-1.34788	-1.34494	-1.342	-1.36824	-1.36524	-1.39724	-1.39422	-1.39122
-1.35079	-1.34784	-1.34487	-1.3419	-1.36814	-1.36517	-1.39719	-1.39417	-1.39118
-1.35076	-1.34781	-1.34482	-1.34184	-1.36807	-1.36512	-1.39714	-1.39413	-1.39114
-1.35071	-1.34778	-1.34481	-1.34185	-1.36807	-1.36509	-1.3971	-1.39409	-1.3911
-1.35067	-1.34775	-1.3448	-1.34186	-1.36808	-1.36507	-1.39706	-1.39404	-1.39105
-1.35064	-1.34771	-1.34476	-1.3418	-1.36802	-1.36502	-1.39701	-1.394	-1.39101
-1.35062	-1.34767	-1.34469	-1.3417	-1.36792	-1.36496	-1.39697	-1.39396	-1.39097
-1.35058	-1.34764	-1.34466	-1.34168	-1.36788	-1.36492	-1.39692	-1.39392	-1.39093
-1.35053	-1.34762	-1.34467	-1.34172	-1.36792	-1.3649	-1.39688	-1.39387	-1.39089
-1.35051	-1.34759	-1.34466	-1.34174	-1.36794	-1.3649	-1.39686	-1.39384	-1.39085
-1.35052	-1.34755	-1.34462	-1.34172	-1.36794	-1.36494	-1.3969	-1.39384	-1.3908
-1.35054	-1.34753	-1.34459	-1.3417	-1.36793	-1.36497	-1.39694	-1.39384	-1.39078
-1.35057	-1.34756	-1.34459	-1.34166	-1.36788	-1.36494	-1.39694	-1.39387	-1.39081
-1.35059	-1.34761	-1.34462	-1.34163	-1.36783	-1.3649	-1.39692	-1.39389	-1.39086
-1.35057	-1.34762	-1.34464	-1.34165	-1.36784	-1.36488	-1.39689	-1.39387	-1.39087
-1.35053	-1.3476	-1.34465	-1.34171	-1.36788	-1.36486	-1.39684	-1.39383	-1.39085
-1.35052	-1.34757	-1.34464	-1.34172	-1.3679	-1.36487	-1.39683	-1.39381	-1.39082
-1.35053	-1.34753	-1.34459	-1.34169	-1.36789	-1.3649	-1.39687	-1.39381	-1.39077
-1.35054	-1.34751	-1.34455	-1.34165	-1.36787	-1.36492	-1.3969	-1.39381	-1.39075
-1.35056	-1.34755	-1.34457	-1.34162	-1.36781	-1.36488	-1.39689	-1.39383	-1.39079
-1.35057	-1.34759	-1.34459	-1.3416	-1.36777	-1.36484	-1.39687	-1.39385	-1.39082
-1.35054	-1.34759	-1.3446	-1.34161	-1.36777	-1.3648	-1.39682	-1.39382	-1.39082
-1.3505	-1.34757	-1.34461	-1.34164	-1.36779	-1.36478	-1.39676	-1.39377	-1.3908
-1.35049	-1.34758	-1.34463	-1.34168	-1.36783	-1.36479	-1.39676	-1.39377	-1.3908
-1.35052	-1.34759	-1.34466	-1.34173	-1.36788	-1.36484	-1.3968	-1.3938	-1.39083
-1.35053	-1.34758	-1.34466	-1.34174	-1.36791	-1.36487	-1.39683	-1.39381	-1.39083
-1.35052	-1.34754	-1.34461	-1.34172	-1.36791	-1.3649	-1.39685	-1.3938	-1.39079
-1.35052	-1.34751	-1.34459	-1.34171	-1.3679	-1.36492	-1.39687	-1.3938	-1.39076
-1.35056	-1.34754	-1.3446	-1.34172	-1.36792	-1.36496	-1.39692	-1.39384	-1.39079
-1.3506	-1.34757	-1.34463	-1.34173	-1.36793	-1.36498	-1.39696	-1.39389	-1.39084
-1.35062	-1.3476	-1.34464	-1.34171	-1.3679	-1.36497	-1.39696	-1.39391	-1.39088
-1.35063	-1.34763	-1.34465	-1.34168	-1.36786	-1.36493	-1.39695	-1.39392	-1.39092
-1.35065	-1.34767	-1.34468	-1.34168	-1.36786	-1.36494	-1.39696	-1.39395	-1.39094
-1.35069	-1.34771	-1.34472	-1.34173	-1.3679	-1.36497	-1.39699	-1.39397	-1.39096
-1.35073	-1.34776	-1.34477	-1.34177	-1.36794	-1.36502	-1.39702	-1.39401	-1.39099
-1.35078	-1.34781	-1.34482	-1.34183	-1.36799	-1.36507	-1.39707	-1.39405	-1.39103

-1.35083	-1.34786	-1.34487	-1.34188	-1.36805	-1.36512	-1.39712	-1.3941	-1.39109
-1.35088	-1.34791	-1.34492	-1.34192	-1.36809	-1.36516	-1.39717	-1.39415	-1.39114
-1.35093	-1.34795	-1.34496	-1.34197	-1.36814	-1.36521	-1.39722	-1.39421	-1.3912
-1.35097	-1.348	-1.34501	-1.34202	-1.36819	-1.36525	-1.39726	-1.39426	-1.39127
-1.35101	-1.34806	-1.34507	-1.34208	-1.36824	-1.3653	-1.3973	-1.39431	-1.39134
-1.35108	-1.34811	-1.34514	-1.34217	-1.33925	-1.33636	-1.39737	-1.39438	-1.39139
-1.35116	-1.34817	-1.34522	-1.3423	-1.33939	-1.33649	-1.39748	-1.39445	-1.39144
-1.35124	-1.34824	-1.3453	-1.3424	-1.3395	-1.33659	-1.39758	-1.39452	-1.3915
-1.3513	-1.34829	-1.34535	-1.34245	-1.33955	-1.33665	-1.39764	-1.39459	-1.39155
-1.35137	-1.34837	-1.34541	-1.3425	-1.33959	-1.3367	-1.3977	-1.39466	-1.39163
-1.35147	-1.34849	-1.34553	-1.3426	-1.33968	-1.33677	-1.39778	-1.39476	-1.39176
-1.35157	-1.34863	-1.34567	-1.34272	-1.33978	-1.33684	-1.39785	-1.39487	-1.3919
-1.35164	-1.34871	-1.34575	-1.3428	-1.33985	-1.33691	-1.39792	-1.39494	-1.39198
-1.35168	-1.34874	-1.34579	-1.34285	-1.33991	-1.33697	-1.39799	-1.395	-1.39202
-1.35177	-1.34881	-1.34586	-1.34291	-1.33998	-1.33706	-1.39808	-1.39508	-1.3921
-1.3519	-1.34893	-1.34596	-1.343	-1.34007	-1.33718	-1.39821	-1.39521	-1.39222
-1.35204	-1.34906	-1.34607	-1.3431	-1.34018	-1.3373	-1.39835	-1.39534	-1.39233
-1.35215	-1.34916	-1.34618	-1.34323	-1.34031	-1.33743	-1.39847	-1.39545	-1.39243
-1.35227	-1.34926	-1.34629	-1.34336	-1.34045	-1.33755	-1.39858	-1.39555	-1.39253
-1.35239	-1.34939	-1.34642	-1.34349	-1.34056	-1.33765	-1.39868	-1.39566	-1.39264
-1.35252	-1.34953	-1.34656	-1.34361	-1.34067	-1.33774	-1.39878	-1.39576	-1.39276
-1.35266	-1.34967	-1.34669	-1.34372	-1.34078	-1.33785	-1.39889	-1.39588	-1.39288
-1.3528	-1.3498	-1.34681	-1.34382	-1.34088	-1.33798	-1.39903	-1.39601	-1.393
-1.35293	-1.34992	-1.34692	-1.34393	-1.34099	-1.3381	-1.39916	-1.39613	-1.39311
-1.35304	-1.35002	-1.34702	-1.34406	-1.34112	-1.33822	-1.39928	-1.39623	-1.3932
-1.35315	-1.35012	-1.34714	-1.34418	-1.34125	-1.33834	-1.39939	-1.39633	-1.3933
-1.35328	-1.35026	-1.34727	-1.34432	-1.34137	-1.33844	-1.39948	-1.39644	-1.39342
-1.35341	-1.35041	-1.34742	-1.34445	-1.34149	-1.33853	-1.39958	-1.39656	-1.39356
-1.35355	-1.35055	-1.34755	-1.34455	-1.34159	-1.33865	-1.39971	-1.3967	-1.3937
-1.35371	-1.3507	-1.34767	-1.34464	-1.34168	-1.33877	-1.39986	-1.39685	-1.39385
-1.35387	-1.35086	-1.34783	-1.3448	-1.34183	-1.33892	-1.40001	-1.397	-1.394
-1.35404	-1.35103	-1.34802	-1.34501	-1.34203	-1.33908	-1.40016	-1.39714	-1.39414
-1.35419	-1.3512	-1.34819	-1.34519	-1.34221	-1.33924	-1.40031	-1.39729	-1.39429
-1.35435	-1.35133	-1.34831	-1.34528	-1.34231	-1.33937	-1.40047	-1.39744	-1.39444
-1.35449	-1.35147	-1.34843	-1.34539	-1.34241	-1.3395	-1.40062	-1.3976	-1.39459
-1.35464	-1.35164	-1.34861	-1.34558	-1.3426	-1.33966	-1.40076	-1.39774	-1.39474
-1.35479	-1.3518	-1.3488	-1.34581	-1.34282	-1.33982	-1.40091	-1.39788	-1.39488
-1.35496	-1.35195	-1.34896	-1.34599	-1.34301	-1.34002	-1.33707	-1.33418	-1.33132
-1.35515	-1.35209	-1.34909	-1.34615	-1.3432	-1.34025	-1.33731	-1.33436	-1.33145
-1.2924	-1.28938	-1.34926	-1.3463	-1.34336	-1.34044	-1.33752	-1.33456	-1.33163
-1.29259	-1.2896	-1.34947	-1.34645	-1.34349	-1.34059	-1.33769	-1.33477	-1.33186
-1.29276	-1.2898	-1.34968	-1.34662	-1.34364	-1.34074	-1.33785	-1.33496	-1.33208
-1.29289	-1.28995	-1.34988	-1.34685	-1.34385	-1.3409	-1.33798	-1.3351	-1.33224
-1.29302	-1.29009	-1.35007	-1.34708	-1.34408	-1.34107	-1.33812	-1.33524	-1.33239
-1.29317	-1.29021	-1.28728	-1.28438	-1.34427	-1.34128	-1.33833	-1.33542	-1.33254
-1.29334	-1.29033	-1.28739	-1.28451	-1.34445	-1.3415	-1.33856	-1.33561	-1.3327
-1.29351	-1.29049	-1.28754	-1.28464	-1.28177	-1.27892	-1.33877	-1.33582	-1.33289
-1.2937	-1.29071	-1.28773	-1.28478	-1.28189	-1.27907	-1.33896	-1.33603	-1.33312
-1.26167	-1.25879	-1.28792	-1.28494	-1.28204	-1.27923	-1.33915	-1.33624	-1.33335
-1.2618	-1.25895	-1.2881	-1.28513	-1.28224	-1.27942	-1.33936	-1.33647	-1.33358

-1.26194	-1.25911	-1.28828	-1.28533	-1.28244	-1.2796	-1.27677	-1.27396	-1.27116
-1.26213	-1.2593	-1.28848	-1.28553	-1.28262	-1.27974	-1.2769	-1.2741	-1.27132
-1.26233	-1.25948	-1.28867	-1.28573	-1.2828	-1.27988	-1.27702	-1.27424	-1.27148
-1.2625	-1.25962	-1.28882	-1.2859	-1.28298	-1.28006	-1.2772	-1.27442	-1.27166
-1.26266	-1.25974	-1.28895	-1.28605	-1.28315	-1.28027	-1.27742	-1.27464	-1.27187
-1.26285	-1.25991	-1.28912	-1.28623	-1.28334	-1.28047	-1.27763	-1.27484	-1.27207
-1.26307	-1.26013	-1.28934	-1.28644	-1.28355	-1.28067	-1.27783	-1.27504	-1.27226
-1.22415	-1.2213	-1.25748	-1.25466	-1.28376	-1.28088	-1.27802	-1.27522	-1.27243
-1.22436	-1.22151	-1.25769	-1.25487	-1.28398	-1.28108	-1.27821	-1.27537	-1.27255
-1.22457	-1.22172	-1.25791	-1.25509	-1.2842	-1.28129	-1.2784	-1.27553	-1.27269
-1.22476	-1.22192	-1.25813	-1.2553	-1.28441	-1.2815	-1.27861	-1.27573	-1.27288
-1.22494	-1.22213	-1.25834	-1.25551	-1.28463	-1.28172	-1.27882	-1.27594	-1.27309
-1.22514	-1.22233	-1.25856	-1.25573	-1.28485	-1.28193	-1.27903	-1.27615	-1.27329
-1.22535	-1.22255	-1.25878	-1.25594	-1.28507	-1.28215	-1.27924	-1.27635	-1.2735
-1.22556	-1.22275	-1.21998	-1.21724	-1.25331	-1.25047	-1.27946	-1.27657	-1.27371
-1.22577	-1.22296	-1.22019	-1.21744	-1.25352	-1.25068	-1.27968	-1.27679	-1.27393
-1.22599	-1.22317	-1.22039	-1.21764	-1.25374	-1.2509	-1.27991	-1.27703	-1.27417
-1.22622	-1.22338	-1.22059	-1.21784	-1.25395	-1.25111	-1.28015	-1.2773	-1.27447
-1.22646	-1.2236	-1.2208	-1.21805	-1.25416	-1.25132	-1.28038	-1.27756	-1.27475
-1.2267	-1.22382	-1.22101	-1.21825	-1.25436	-1.25152	-1.2806	-1.27778	-1.27498
-1.22694	-1.22405	-1.22123	-1.21846	-1.25457	-1.25173	-1.2808	-1.27798	-1.27518
-1.22719	-1.22432	-1.2215	-1.2187	-1.25481	-1.25195	-1.28102	-1.27819	-1.27538
-1.22746	-1.22463	-1.2218	-1.21899	-1.25508	-1.25219	-1.28125	-1.2784	-1.27558
-1.22771	-1.2249	-1.22207	-1.21924	-1.21644	-1.21365	-1.2496	-1.24682	-1.24407
-1.22792	-1.2251	-1.22228	-1.21946	-1.21668	-1.21391	-1.24989	-1.2471	-1.24433
-1.22816	-1.22532	-1.22249	-1.21968	-1.21691	-1.21417	-1.25016	-1.24737	-1.24458
-1.22845	-1.22559	-1.22274	-1.21991	-1.21712	-1.21437	-1.25038	-1.24758	-1.24481
-1.22875	-1.22588	-1.22301	-1.22015	-1.21734	-1.21457	-1.25058	-1.24779	-1.24503
-1.22899	-1.22612	-1.22326	-1.22042	-1.21761	-1.21482	-1.25082	-1.24801	-1.24525
-1.2292	-1.22635	-1.22352	-1.22071	-1.2179	-1.2151	-1.25108	-1.24825	-1.24547
-1.22944	-1.22657	-1.22375	-1.22096	-1.21816	-1.21535	-1.21258	-1.20985	-1.20716
-1.22969	-1.2268	-1.22396	-1.22117	-1.21837	-1.21558	-1.21282	-1.21011	-1.20744
-1.22996	-1.22706	-1.22421	-1.2214	-1.21861	-1.21582	-1.21308	-1.21038	-1.20771
-1.23024	-1.22736	-1.22451	-1.22169	-1.21889	-1.21611	-1.21336	-1.21065	-1.20797
-1.23053	-1.22768	-1.22483	-1.22199	-1.21918	-1.2164	-1.21365	-1.21092	-1.20823
-1.23084	-1.22797	-1.22513	-1.2223	-1.21948	-1.21668	-1.21391	-1.21118	-1.20847
-1.23115	-1.22827	-1.22542	-1.2226	-1.21978	-1.21696	-1.21417	-1.21142	-1.20871
-1.23147	-1.22858	-1.22572	-1.22289	-1.22005	-1.21722	-1.21443	-1.21168	-1.20898
-1.23178	-1.2289	-1.22602	-1.22316	-1.22031	-1.21746	-1.21467	-1.21196	-1.20928
-1.23208	-1.2292	-1.22631	-1.22343	-1.22057	-1.21773	-1.21495	-1.21224	-1.20957
-1.23239	-1.22948	-1.22657	-1.22368	-1.22083	-1.21802	-1.21525	-1.21253	-1.20985
-1.23268	-1.22975	-1.22684	-1.22394	-1.2211	-1.21831	-1.25044	-1.24762	-1.24483
-1.23296	-1.23001	-1.22711	-1.22424	-1.2214	-1.21861	-1.25074	-1.24792	-1.24514
-1.23325	-1.23029	-1.22739	-1.22454	-1.22171	-1.2189	-1.25104	-1.24823	-1.24544
-1.23359	-1.23064	-1.22772	-1.22485	-1.22201	-1.21921	-1.25135	-1.24853	-1.24574
-1.23396	-1.23101	-1.22808	-1.22516	-1.22231	-1.21952	-1.25167	-1.24883	-1.24603
-1.23427	-1.23132	-1.22838	-1.22547	-1.22261	-1.21982	-1.25198	-1.24914	-1.24633
-1.23453	-1.23156	-1.22864	-1.22577	-1.22293	-1.22011	-1.25227	-1.24944	-1.24664
-1.23484	-1.23186	-1.22895	-1.22608	-1.22324	-1.22042	-1.25258	-1.24974	-1.24694
-1.23522	-1.23225	-1.22931	-1.22639	-1.22354	-1.22074	-1.25291	-1.25005	-1.24722

-1.23559	-1.23264	-1.22968	-1.22673	-1.22386	-1.22108	-1.25326	-1.25038	-1.24753
-1.23591	-1.23298	-1.23004	-1.22711	-1.22424	-1.22145	-1.25364	-1.25076	-1.24791
-1.23622	-1.23331	-1.2304	-1.22749	-1.22463	-1.22183	-1.25402	-1.25115	-1.2483
-1.23654	-1.23362	-1.23071	-1.22782	-1.22497	-1.22215	-1.25434	-1.25148	-1.24864
-1.23687	-1.23391	-1.231	-1.22812	-1.22527	-1.22244	-1.25464	-1.25179	-1.24896
-1.23723	-1.23426	-1.23133	-1.22844	-1.22557	-1.22275	-1.25496	-1.2521	-1.24927
-1.23764	-1.23467	-1.23171	-1.22875	-1.22587	-1.22307	-1.2553	-1.25242	-1.24957
-1.20027	-1.1974	-1.23208	-1.22909	-1.26142	-1.25853	-1.29421	-1.29122	-1.28826
-1.20058	-1.19774	-1.23244	-1.22947	-1.26182	-1.25891	-1.2946	-1.29162	-1.28867
-1.20089	-1.19806	-1.23279	-1.22986	-1.26222	-1.25929	-1.29499	-1.29202	-1.28908
-1.2012	-1.19836	-1.19552	-1.1927	-1.26258	-1.25963	-1.29533	-1.29237	-1.28944
-1.20152	-1.19866	-1.19583	-1.19303	-1.26293	-1.25997	-1.29567	-1.29272	-1.28979
-1.20188	-1.19901	-1.19618	-1.19339	-1.2633	-1.26034	-1.29606	-1.2931	-1.29016
-1.20228	-1.1994	-1.19656	-1.19376	-1.26369	-1.26074	-1.29648	-1.2935	-1.29055
-1.20267	-1.19978	-1.19693	-1.1941	-1.22722	-1.22437	-1.29686	-1.29387	-1.29091
-1.20306	-1.20016	-1.19728	-1.19441	-1.22752	-1.22468	-1.29721	-1.2942	-1.29123
-1.20342	-1.20053	-1.19763	-1.19473	-1.22785	-1.22502	-1.29757	-1.29456	-1.29157
-1.20374	-1.20087	-1.19798	-1.19511	-1.22824	-1.2254	-1.29798	-1.29497	-1.29198
-1.20406	-1.20121	-1.19835	-1.1955	-1.22865	-1.2258	-1.2984	-1.29539	-1.29241
-1.16623	-1.16347	-1.19872	-1.19588	-1.22905	-1.2262	-1.26276	-1.25984	-1.25694
-1.16661	-1.16384	-1.1991	-1.19626	-1.22945	-1.22659	-1.26317	-1.26025	-1.25735
-1.167	-1.16421	-1.19949	-1.19665	-1.22984	-1.22699	-1.26358	-1.26066	-1.25776
-1.16739	-1.16459	-1.19988	-1.19703	-1.23024	-1.22738	-1.264	-1.26107	-1.25816
-1.20483	-1.20192	-1.23647	-1.23353	-1.23063	-1.22778	-1.26441	-1.26148	-1.25857
-1.20522	-1.20231	-1.23687	-1.23393	-1.23103	-1.22817	-1.26482	-1.26189	-1.25898
-1.20562	-1.20269	-1.23727	-1.23432	-1.23142	-1.22856	-1.26523	-1.2623	-1.25939
-1.20602	-1.20308	-1.23767	-1.23472	-1.23182	-1.22896	-1.26564	-1.26271	-1.25979
-1.20642	-1.20347	-1.23807	-1.23512	-1.23222	-1.22936	-1.26605	-1.26312	-1.2602
-1.20682	-1.20386	-1.20097	-1.19812	-1.23262	-1.22976	-1.26647	-1.26353	-1.26061
-1.20722	-1.20426	-1.20135	-1.1985	-1.23302	-1.23016	-1.26689	-1.26395	-1.26103
-1.20762	-1.20465	-1.20175	-1.19889	-1.23342	-1.23056	-1.26731	-1.26436	-1.26144
-1.20803	-1.20506	-1.20215	-1.19928	-1.23382	-1.23096	-1.26772	-1.26478	-1.26186
-1.20843	-1.20546	-1.20255	-1.19968	-1.23422	-1.23136	-1.26814	-1.2652	-1.26227
-1.20884	-1.20587	-1.20294	-1.20007	-1.23462	-1.23176	-1.26855	-1.2656	-1.26268
-1.20925	-1.20627	-1.20335	-1.20046	-1.23503	-1.23216	-1.26897	-1.26602	-1.26309
-1.20967	-1.20669	-1.20375	-1.20086	-1.23544	-1.23257	-1.26939	-1.26644	-1.26351
-1.2101	-1.20711	-1.20417	-1.20127	-1.23586	-1.23298	-1.26982	-1.26687	-1.26395
-1.21057	-1.20757	-1.20462	-1.20172	-1.19888	-1.19608	-1.27029	-1.26735	-1.26443
-1.21107	-1.20805	-1.2051	-1.20221	-1.19935	-1.19654	-1.27078	-1.26785	-1.26494
-1.21154	-1.20851	-1.20555	-1.20265	-1.19979	-1.19697	-1.27124	-1.26832	-1.26541
-1.21197	-1.20894	-1.20597	-1.20304	-1.20018	-1.19736	-1.27167	-1.26875	-1.26583
-1.21241	-1.20939	-1.20641	-1.20346	-1.20058	-1.19778	-1.27212	-1.26919	-1.26627
-1.21287	-1.20989	-1.2069	-1.20392	-1.20103	-1.19823	-1.27262	-1.26967	-1.26674
-1.21334	-1.21038	-1.20738	-1.20438	-1.20148	-1.19867	-1.27311	-1.27014	-1.26721
-1.21379	-1.21082	-1.20781	-1.2048	-1.20188	-1.19907	-1.23681	-1.23395	-1.23112
-1.21425	-1.21125	-1.20823	-1.20521	-1.20229	-1.19945	-1.23721	-1.23435	-1.23153
-1.13787	-1.13505	-1.20869	-1.20569	-1.20276	-1.1999	-1.23765	-1.2348	-1.23198
-1.13834	-1.1355	-1.20919	-1.20622	-1.20328	-1.20039	-1.23815	-1.23529	-1.23247
-1.13879	-1.13593	-1.20967	-1.2067	-1.20377	-1.20088	-1.19804	-1.19529	-1.19256
-1.13918	-1.13632	-1.21009	-1.20711	-1.2042	-1.20136	-1.19855	-1.19577	-1.19301

-1.10306	-1.1003	-1.13392	-1.13113	-1.20464	-1.20182	-1.19903	-1.19623	-1.19347
-1.1035	-1.10076	-1.13439	-1.13156	-1.20509	-1.20224	-1.19943	-1.19668	-1.19394
-1.10396	-1.10124	-1.13487	-1.13202	-1.20556	-1.20266	-1.19983	-1.19711	-1.19441
-1.10442	-1.10168	-1.13534	-1.13251	-1.20606	-1.20312	-1.20026	-1.19752	-1.19482
-1.10489	-1.10211	-1.13581	-1.13301	-1.20659	-1.20361	-1.20072	-1.19792	-1.19519
-1.10535	-1.10257	-1.13629	-1.13348	-1.13068	-1.12788	-1.20119	-1.19836	-1.1956
-1.1058	-1.10306	-1.13678	-1.13393	-1.13113	-1.12835	-1.20169	-1.19884	-1.19604
-1.10626	-1.10352	-1.13726	-1.1344	-1.13159	-1.12881	-1.20218	-1.19931	-1.1965
-1.10673	-1.10395	-1.13772	-1.1349	-1.13208	-1.12927	-1.20266	-1.1998	-1.19699
-1.10719	-1.10439	-1.13819	-1.13539	-1.13257	-1.12973	-1.12695	-1.12429	-1.12168
-1.10764	-1.10486	-1.13867	-1.13585	-1.13303	-1.1302	-1.12744	-1.12475	-1.12212
-1.10809	-1.10534	-1.13916	-1.1363	-1.13348	-1.13068	-1.12793	-1.12522	-1.12256
-1.10854	-1.10578	-1.13962	-1.13677	-1.13395	-1.13115	-1.1284	-1.12568	-1.12303
-1.10899	-1.10619	-1.14006	-1.13725	-1.13444	-1.13162	-1.12885	-1.12614	-1.1235
-1.10944	-1.10663	-1.14052	-1.13772	-1.13491	-1.13209	-1.12932	-1.12662	-1.12398
-1.10988	-1.10711	-1.14101	-1.13818	-1.13536	-1.13256	-1.12981	-1.12713	-1.12449
-1.11033	-1.10757	-1.1048	-1.10204	-1.13583	-1.13305	-1.13031	-1.12763	-1.12497
-1.11078	-1.10799	-1.10523	-1.10249	-1.13633	-1.13357	-1.13084	-1.12811	-1.12542
-1.11125	-1.10844	-1.10567	-1.10296	-1.13683	-1.13409	-1.13135	-1.12859	-1.12587
-1.11178	-1.10895	-1.10618	-1.10346	-1.13734	-1.13457	-1.13182	-1.12905	-1.12632
-1.11232	-1.10949	-1.10671	-1.10397	-1.13784	-1.13504	-1.13227	-1.1295	-1.12679
-1.11282	-1.10999	-1.1072	-1.10444	-1.13831	-1.13551	-1.13274	-1.12999	-1.12729
-1.11328	-1.11047	-1.10766	-1.10487	-1.13877	-1.13599	-1.13323	-1.1305	-1.12781
-1.11376	-1.11096	-1.10815	-1.10535	-1.13927	-1.13649	-1.13374	-1.131	-1.1283
-1.11427	-1.11148	-1.10868	-1.10589	-1.13982	-1.13703	-1.13425	-1.13148	-1.12875
-1.03721	-1.03462	-1.10919	-1.10641	-1.14037	-1.13757	-1.13478	-1.13198	-1.12923
-1.03765	-1.03502	-1.10964	-1.10687	-1.14088	-1.13811	-1.13534	-1.13254	-1.12978
-1.03808	-1.03541	-1.11009	-1.10734	-1.14138	-1.13863	-1.13588	-1.1331	-1.13033
-1.0385	-1.03583	-1.11058	-1.10784	-1.10512	-1.10243	-1.13636	-1.13359	-1.13084
-1.03892	-1.03628	-1.1111	-1.10836	-1.10562	-1.10288	-1.1368	-1.13405	-1.13132
-1.0394	-1.03675	-1.03417	-1.03166	-1.10614	-1.10338	-1.13731	-1.13455	-1.13183
-1.03992	-1.03725	-1.03467	-1.03215	-1.10669	-1.10392	-1.13786	-1.13509	-1.13235
-1.04043	-1.03775	-1.03515	-1.0326	-1.10719	-1.10442	-1.1384	-1.13563	-1.13288
-1.04093	-1.03825	-1.03561	-1.033	-1.10763	-1.10488	-1.13891	-1.13616	-1.13343
-0.99203	-0.98962	-1.03608	-1.03342	-1.03082	-1.02828	-1.13942	-1.13669	-1.13398
-0.99248	-0.99008	-1.03657	-1.03389	-1.03128	-1.02873	-1.13995	-1.13723	-1.13452
-0.99295	-0.99055	-1.03707	-1.03439	-1.03176	-1.0292	-1.14049	-1.13777	-1.13507
-0.99342	-0.99101	-1.03758	-1.03488	-1.03225	-1.02968	-1.10421	-1.10158	-1.09896
-0.99389	-0.99148	-1.03809	-1.03538	-1.03275	-1.03017	-1.10476	-1.10211	-1.09949
-0.99436	-0.99194	-1.0386	-1.03588	-1.03324	-1.03067	-1.02815	-1.02571	-1.02329
-0.99482	-0.99241	-1.03912	-1.03639	-1.03374	-1.03116	-1.02863	-1.02618	-1.02376
-0.99529	-0.99288	-1.03964	-1.0369	-1.03424	-1.03165	-1.02912	-1.02666	-1.02422
-0.99577	-0.99335	-1.04016	-1.03741	-1.03474	-1.03214	-1.0296	-1.02712	-1.02468
-0.99625	-0.99383	-1.04068	-1.03793	-1.03525	-1.03263	-1.03008	-1.02759	-1.02513
-0.99672	-0.9943	-1.0412	-1.03844	-1.03575	-1.03313	-1.03057	-1.02807	-1.0256
-0.9972	-0.99477	-1.04171	-1.03894	-1.03625	-1.03363	-1.03106	-1.02855	-1.02606
-0.95595	-0.95361	-1.04223	-1.03945	-1.03675	-1.03412	-1.03154	-1.02903	-1.02653
-0.9564	-0.95405	-1.04273	-1.03994	-1.03724	-1.03461	-1.03203	-1.02951	-1.02701
-0.95685	-0.9545	-1.04325	-1.04045	-1.03774	-1.0351	-1.03251	-1.02999	-1.0275
-0.95731	-0.95496	-1.04377	-1.04097	-1.03825	-1.0356	-1.033	-1.03048	-1.028

-0.95777	-0.95541	-0.95302	-0.95061	-1.03879	-1.03612	-1.03351	-1.03098	-1.0285
-0.95822	-0.95587	-0.95351	-0.95114	-1.03941	-1.0367	-1.03406	-1.0315	-1.02899
-0.95868	-0.95633	-0.95399	-0.95167	-1.04004	-1.03729	-1.03461	-1.03202	-1.02948
-0.95916	-0.95678	-0.95444	-0.95213	-1.04058	-1.03782	-1.03513	-1.03251	-1.02996
-0.95965	-0.95724	-0.95488	-0.95257	-1.04109	-1.03834	-1.03564	-1.03301	-1.03044
-0.87722	-0.875	-0.95535	-0.95303	-1.00089	-0.99825	-1.03621	-1.03356	-1.03096
-0.87772	-0.87547	-0.95585	-0.95349	-1.00142	-0.99882	-1.03682	-1.03415	-1.03151
-0.8782	-0.87593	-0.95633	-0.95395	-1.00193	-0.99936	-1.0374	-1.03473	-1.03208
-0.87862	-0.87634	-0.95679	-0.9544	-1.00241	-0.99984	-1.03792	-1.0353	-1.03269
-0.87904	-0.87677	-0.95727	-0.95486	-1.00291	-1.00032	-0.99778	-0.99528	-0.9928
-0.87948	-0.87725	-0.95779	-0.95536	-1.00343	-1.00082	-0.99826	-0.99576	-0.99328
-0.87993	-0.87773	-0.95833	-0.95588	-1.00398	-1.00134	-0.99876	-0.99624	-0.99376
-0.69689	-0.69525	-0.92068	-0.91836	-1.00459	-1.00196	-0.99936	-0.9968	-0.99427
-0.6972	-0.69554	-0.92114	-0.91887	-1.00523	-1.00263	-1.00002	-0.9974	-0.99481
-0.69755	-0.69587	-0.92161	-0.91934	-1.00579	-1.0032	-1.0006	-0.99797	-0.99536
-0.69792	-0.69621	-0.92208	-0.91977	-1.00627	-1.00367	-1.00109	-0.99851	-0.99594
-0.64946	-0.64788	-0.83282	-0.83074	-0.95602	-0.95368	-1.0016	-0.99904	-0.99649
-0.64976	-0.64822	-0.83333	-0.83126	-0.9566	-0.95421	-1.00213	-0.99953	-0.99697
-0.65005	-0.64854	-0.83382	-0.83176	-0.95718	-0.95475	-1.00268	-1.00004	-0.99745
-0.65035	-0.64883	-0.83423	-0.83217	-0.95767	-0.95528	-1.00328	-1.00061	-0.99799
-0.65065	-0.64911	-0.83461	-0.83255	-0.95814	-0.95579	-1.00389	-1.0012	-0.99854
-0.65099	-0.64945	-0.64791	-0.64637	-0.92044	-0.91818	-1.00442	-1.00176	-0.99912
-0.65136	-0.64983	-0.64827	-0.6467	-0.92091	-0.91861	-1.0049	-1.0023	-0.99973
-0.6517	-0.65016	-0.6486	-0.64702	-0.92142	-0.91911	-1.00546	-1.00287	-1.0003
-0.65198	-0.65044	-0.64889	-0.64735	-0.92197	-0.91969	-1.00612	-1.00347	-1.00085
-0.65229	-0.65074	-0.6492	-0.64768	-0.92251	-0.92023	-1.00674	-1.00406	-1.00141
-0.65266	-0.65111	-0.64956	-0.64801	-0.92299	-0.92069	-1.00727	-1.00462	-1.00201
-0.65303	-0.65148	-0.64991	-0.64833	-0.92348	-0.92114	-1.00779	-1.00519	-1.00262
-0.65334	-0.65178	-0.65022	-0.64865	-0.7398	-0.73804	-0.91937	-0.91713	-0.91491
-0.65362	-0.65205	-0.6505	-0.64897	-0.74019	-0.73845	-0.91993	-0.91769	-0.91545
-0.65395	-0.65237	-0.65082	-0.64929	-0.74055	-0.7388	-0.92041	-0.91816	-0.91593
-0.65431	-0.65273	-0.65116	-0.64959	-0.74088	-0.73911	-0.92082	-0.91857	-0.91635
-0.65464	-0.65307	-0.65149	-0.64991	-0.69707	-0.69539	-0.78555	-0.78372	-0.78191
-0.65493	-0.65335	-0.65179	-0.65024	-0.69745	-0.69578	-0.78601	-0.78417	-0.78235
-0.65525	-0.65366	-0.65211	-0.65059	-0.69783	-0.69618	-0.78647	-0.78461	-0.78277
-0.65561	-0.65402	-0.65247	-0.65095	-0.69823	-0.69658	-0.78691	-0.78502	-0.78314
-0.656	-0.65441	-0.65285	-0.65131	-0.69861	-0.69697	-0.78734	-0.78541	-0.7835
-0.6083	-0.60681	-0.65321	-0.65163	-0.69894	-0.69729	-0.73973	-0.73795	-0.73619
-0.60868	-0.60718	-0.65355	-0.65194	-0.69925	-0.69759	-0.74004	-0.7383	-0.73657
-0.60901	-0.60751	-0.65388	-0.65226	-0.6996	-0.69794	-0.74042	-0.73869	-0.73697
-0.60929	-0.60778	-0.65418	-0.65259	-0.69998	-0.69833	-0.74086	-0.73912	-0.7374
-0.60961	-0.60808	-0.60659	-0.60514	-0.70037	-0.69873	-0.74129	-0.73955	-0.73781
-0.60997	-0.60845	-0.60696	-0.6055	-0.70078	-0.69913	-0.74171	-0.73997	-0.73823
-0.61035	-0.60883	-0.60734	-0.60587	-0.70118	-0.69952	-0.74211	-0.74037	-0.73863
-0.61074	-0.60921	-0.60771	-0.60624	-0.70156	-0.69987	-0.69819	-0.69653	-0.69489
-0.61114	-0.60959	-0.60808	-0.6066	-0.70194	-0.7002	-0.6985	-0.69682	-0.69517
-0.61153	-0.60997	-0.60844	-0.60693	-0.65551	-0.65387	-0.69884	-0.69717	-0.69552
-0.61192	-0.61035	-0.60878	-0.60724	-0.65583	-0.65421	-0.6992	-0.69755	-0.69592
-0.61229	-0.61069	-0.60911	-0.60755	-0.65618	-0.65457	-0.69958	-0.69794	-0.69631
-0.61265	-0.61101	-0.60942	-0.60788	-0.65654	-0.65494	-0.69998	-0.69833	-0.6967

-0.55686	-0.55543	-0.60975	-0.60822	-0.60672	-0.60527	-0.70038	-0.69873	-0.69709
-0.55713	-0.55571	-0.61009	-0.60857	-0.60708	-0.60563	-0.70078	-0.69912	-0.69748
-0.5574	-0.556	-0.61045	-0.60893	-0.60744	-0.60599	-0.70119	-0.69952	-0.69787
-0.55773	-0.55633	-0.61083	-0.6093	-0.60781	-0.60635	-0.65494	-0.65337	-0.65181
-0.55809	-0.55668	-0.61123	-0.60968	-0.60817	-0.60671	-0.65532	-0.65375	-0.65219
-0.55844	-0.55702	-0.55564	-0.55429	-0.60854	-0.60707	-0.65571	-0.65414	-0.65258
-0.55879	-0.55737	-0.55598	-0.55461	-0.60891	-0.60743	-0.6561	-0.65453	-0.65297
-0.55914	-0.55772	-0.55631	-0.55493	-0.60928	-0.60779	-0.6565	-0.65492	-0.65336
-0.55949	-0.55807	-0.55665	-0.55525	-0.60965	-0.60816	-0.65689	-0.65531	-0.65375
-0.55984	-0.55842	-0.557	-0.55558	-0.61002	-0.60852	-0.65727	-0.6557	-0.65414
-0.5602	-0.55877	-0.55735	-0.55592	-0.6104	-0.60888	-0.65766	-0.65608	-0.65451
-0.56055	-0.55913	-0.5577	-0.55627	-0.61079	-0.60925	-0.65805	-0.65645	-0.65488
-0.5609	-0.55948	-0.55805	-0.55661	-0.55522	-0.55387	-0.65843	-0.65684	-0.65526
-0.56125	-0.55983	-0.55839	-0.55695	-0.55555	-0.55419	-0.65883	-0.65723	-0.65565
-0.50615	-0.50489	-0.55874	-0.5573	-0.55589	-0.55451	-0.65922	-0.65761	-0.65603
-0.50645	-0.50519	-0.55909	-0.55765	-0.55624	-0.55484	-0.65962	-0.658	-0.65641
-0.50675	-0.50549	-0.55943	-0.558	-0.55658	-0.55517	-0.60956	-0.60807	-0.60661
-0.50704	-0.50578	-0.55977	-0.55834	-0.55691	-0.55549	-0.60992	-0.60842	-0.60695
-0.50733	-0.50607	-0.5601	-0.55868	-0.55725	-0.55582	-0.61029	-0.60877	-0.6073
-0.45515	-0.45404	-0.56044	-0.55902	-0.5576	-0.55617	-0.61069	-0.60915	-0.60766
-0.45541	-0.45431	-0.56078	-0.55937	-0.55795	-0.55652	-0.61109	-0.60953	-0.60802
-0.45569	-0.45458	-0.5057	-0.50445	-0.55829	-0.55686	-0.55546	-0.55408	-0.55275
-0.45597	-0.45486	-0.506	-0.50475	-0.55863	-0.5572	-0.55579	-0.55441	-0.55306
-0.45629	-0.45517	-0.45406	-0.45296	-0.55901	-0.55759	-0.55618	-0.55478	-0.55341
-0.45663	-0.45552	-0.45441	-0.4533	-0.55945	-0.55803	-0.55662	-0.5552	-0.55382
-0.45696	-0.45585	-0.45474	-0.45363	-0.45252	-0.45141	-0.55703	-0.55561	-0.55422
-0.45725	-0.45613	-0.45502	-0.45392	-0.45281	-0.4517	-0.55739	-0.55596	-0.55456
-0.45752	-0.4564	-0.45529	-0.45419	-0.45308	-0.45197	-0.55772	-0.5563	-0.5549
-0.45779	-0.45667	-0.45556	-0.45445	-0.45335	-0.45224	-0.55807	-0.55664	-0.55524
-0.45807	-0.45694	-0.45582	-0.45472	-0.45362	-0.45251	-0.5584	-0.55698	-0.55557
-0.40298	-0.40199	-0.45609	-0.45499	-0.45389	-0.45279	-0.45168	-0.45057	-0.44946
-0.40322	-0.40223	-0.45637	-0.45527	-0.45416	-0.45306	-0.45196	-0.45085	-0.44974
-0.3471	-0.34626	-0.45669	-0.45558	-0.45447	-0.45337	-0.45226	-0.45116	-0.45005
-0.34736	-0.34651	-0.45705	-0.45593	-0.45482	-0.45371	-0.45261	-0.4515	-0.4504
-0.34761	-0.34676	-0.40212	-0.40116	-0.45516	-0.45405	-0.45294	-0.45184	-0.45073
-0.34782	-0.34697	-0.40237	-0.40139	-0.45544	-0.45433	-0.45322	-0.45212	-0.45102
-0.34802	-0.34716	-0.4026	-0.40162	-0.45571	-0.4546	-0.4535	-0.4524	-0.4513
-0.34822	-0.34736	-0.34652	-0.34569	-0.45599	-0.45488	-0.45377	-0.45269	-0.45161
-0.34842	-0.34757	-0.34672	-0.34589	-0.45628	-0.45516	-0.45406	-0.45299	-0.45193
-0.34865	-0.3478	-0.34696	-0.34612	-0.4014	-0.40043	-0.45438	-0.4533	-0.45224
-0.34891	-0.34806	-0.34722	-0.34638	-0.40171	-0.40073	-0.45473	-0.45363	-0.45253
-0.34915	-0.3483	-0.34746	-0.34661	-0.34578	-0.34496	-0.45506	-0.45394	-0.45282
-0.34935	-0.3485	-0.34766	-0.34681	-0.34598	-0.34515	-0.45534	-0.45422	-0.4531
-0.23966	-0.23905	-0.34786	-0.34701	-0.34618	-0.34535	-0.45564	-0.45451	-0.4534
-0.23987	-0.23925	-0.34811	-0.34726	-0.34643	-0.3456	-0.45599	-0.45486	-0.45374
-0.24008	-0.23946	-0.34836	-0.34752	-0.34668	-0.34585	-0.45635	-0.45522	-0.4541
-0.24026	-0.23965	-0.29333	-0.29261	-0.34689	-0.34606	-0.34523	-0.3444	-0.34359
-0.24043	-0.23982	-0.29349	-0.29278	-0.34709	-0.34625	-0.34542	-0.34459	-0.34377
-0.24062	-0.24	-0.23939	-0.23877	-0.29225	-0.29153	-0.34564	-0.34481	-0.34399
-0.24082	-0.24021	-0.23959	-0.23898	-0.29246	-0.29174	-0.34589	-0.34506	-0.34424

-0.24102	-0.2404	-0.23979	-0.23917	-0.29265	-0.29194	-0.34612	-0.3453	-0.34448
-0.24119	-0.24057	-0.23996	-0.23934	-0.29281	-0.2921	-0.34631	-0.34551	-0.34471
-0.24136	-0.24075	-0.24013	-0.23952	-0.29297	-0.29226	-0.3465	-0.34572	-0.34493
-0.18486	-0.18439	-0.24033	-0.23972	-0.2391	-0.23848	-0.29176	-0.29108	-0.2904
-0.18502	-0.18454	-0.24054	-0.23993	-0.23931	-0.23869	-0.29197	-0.29127	-0.29058
-0.18516	-0.18469	-0.24075	-0.24014	-0.23952	-0.2389	-0.29217	-0.29147	-0.29078
-0.18529	-0.18483	-0.24095	-0.24034	-0.23973	-0.23911	-0.29238	-0.29168	-0.29099
-0.18543	-0.18497	-0.1845	-0.18403	-0.23992	-0.2393	-0.23869	-0.23809	-0.23748
-0.18558	-0.18511	-0.18463	-0.18415	-0.24009	-0.23947	-0.23886	-0.23827	-0.23768
-0.18573	-0.18525	-0.18477	-0.18428	-0.24026	-0.23965	-0.23904	-0.23845	-0.23786
-0.1859	-0.18541	-0.18492	-0.18444	-0.24046	-0.23985	-0.23924	-0.23864	-0.23805
-0.18605	-0.18557	-0.18508	-0.18459	-0.24068	-0.24006	-0.23945	-0.23884	-0.23823
-0.1862	-0.18571	-0.18523	-0.18475	-0.24089	-0.24027	-0.23966	-0.23904	-0.23843
-0.18633	-0.18586	-0.18538	-0.18491	-0.2411	-0.24048	-0.23987	-0.23925	-0.23864
-0.12883	-0.12849	-0.18552	-0.18505	-0.18457	-0.18409	-0.24007	-0.23946	-0.23885
-0.12894	-0.12859	-0.18566	-0.18517	-0.1847	-0.18423	-0.24027	-0.23967	-0.23906
-0.0609	-0.06075	-0.1858	-0.18531	-0.18484	-0.18437	-0.24047	-0.23987	-0.23927
-0.06095	-0.0608	-0.18596	-0.18547	-0.18499	-0.18451	-0.24065	-0.24006	-0.23947
-0.06101	-0.06086	-0.1286	-0.12825	-0.18515	-0.18466	-0.18419	-0.18373	-0.18327
-0.06107	-0.06091	-0.12872	-0.12837	-0.18531	-0.18482	-0.18434	-0.18387	-0.18341
-0.06113	-0.06097	-0.12884	-0.12849	-0.18547	-0.18499	-0.18451	-0.18402	-0.18355
-0.06118	-0.06103	-0.12896	-0.12861	-0.12826	-0.12792	-0.18467	-0.18418	-0.1837
-0.06123	-0.06108	-0.12909	-0.12873	-0.12838	-0.12803	-0.18482	-0.18434	-0.18387
-0.06129	-0.06114	-0.12921	-0.12885	-0.1285	-0.12815	-0.18498	-0.1845	-0.18403
-0.06133	-0.06119	-0.12932	-0.12897	-0.12862	-0.12827	-0.18514	-0.18466	-0.18419
-0.06138	-0.06124	-0.06109	-0.06094	-0.12874	-0.12839	-0.12804	-0.1277	-0.12736
-0.06144	-0.06129	-0.06114	-0.06099	-0.12885	-0.12851	-0.12816	-0.12782	-0.12747
-0.06149	-0.06134	-0.06119	-0.06105	-0.12897	-0.12862	-0.12828	-0.12793	-0.12759
-0.06154	-0.0614	-0.06125	-0.0611	-0.12909	-0.12874	-0.1284	-0.12805	-0.12771
-0.0616	-0.06145	-0.0613	-0.06116	-0.12921	-0.12887	-0.12852	-0.12817	-0.12782
-0.06165	-0.06151	-0.06136	-0.06121	-0.12934	-0.12899	-0.12863	-0.12829	-0.12794
-0.06171	-0.06156	-0.06142	-0.06127	-0.12946	-0.1291	-0.12875	-0.1284	-0.12805
-0.06176	-0.06161	-0.06147	-0.06133	-0.06118	-0.06104	-0.12887	-0.12852	-0.12817
-0.06181	-0.06167	-0.06153	-0.06138	-0.06124	-0.06109	-0.12899	-0.12864	-0.12829
-0.06187	-0.06172	-0.06158	-0.06144	-0.0613	-0.06115	-0.12911	-0.12876	-0.12841
-0.06192	-0.06178	-0.06164	-0.06149	-0.06135	-0.06121	-0.12924	-0.12888	-0.12852
-0.06198	-0.06183	-0.06169	-0.06155	-0.06141	-0.06126	-0.12936	-0.129	-0.12864
-0.06204	-0.06189	-0.06175	-0.0616	-0.06146	-0.06132	-0.06117	-0.06103	-0.06088
-0.06209	-0.06195	-0.0618	-0.06166	-0.06152	-0.06137	-0.06123	-0.06108	-0.06094
-0.06215	-0.06201	-0.06186	-0.06172	-0.06157	-0.06143	-0.06128	-0.06114	-0.06099
-0.06221	-0.06207	-0.06192	-0.06178	-0.06163	-0.06149	-0.06134	-0.06119	-0.06105
-0.06227	-0.06213	-0.06198	-0.06184	-0.06169	-0.06154	-0.06139	-0.06125	-0.06111
-0.06233	-0.06219	-0.06204	-0.0619	-0.06175	-0.0616	-0.06145	-0.06131	-0.06117
-0.06239	-0.06225	-0.0621	-0.06195	-0.0618	-0.06165	-0.0615	-0.06136	-0.06123
-0.06245	-0.0623	-0.06216	-0.06201	-0.06186	-0.06171	-0.06156	-0.06142	-0.06128
-0.06251	-0.06236	-0.06222	-0.06207	-0.06192	-0.06176	-0.06161	-0.06147	-0.06133
-0.06257	-0.06243	-0.06228	-0.06213	-0.06198	-0.06182	-0.06167	-0.06153	-0.06139
-0.06264	-0.06249	-0.06234	-0.06219	-0.06204	-0.06189	-0.06174	-0.06159	-0.06145
-0.0627	-0.06255	-0.0624	-0.06225	-0.0621	-0.06195	-0.0618	-0.06165	-0.06151
-0.06275	-0.06261	-0.06246	-0.06231	-0.06215	-0.062	-0.06185	-0.06171	-0.06156

-0.06281	-0.06266	-0.06252	-0.06237	-0.06221	-0.06206	-0.06191	-0.06176	-0.06161
-0.06286	-0.06272	-0.06257	-0.06243	-0.06228	-0.06213	-0.06198	-0.06182	-0.06167
-0.06292	-0.06277	-0.06263	-0.06249	-0.06234	-0.06219	-0.06204	-0.06188	-0.06172
-0.06298	-0.06283	-0.06269	-0.06255	-0.0624	-0.06225	-0.0621	-0.06194	-0.06179
-0.06304	-0.06289	-0.06275	-0.06261	-0.06246	-0.06231	-0.06216	-0.062	-0.06185
-0.0631	-0.06295	-0.06281	-0.06267	-0.06252	-0.06237	-0.06222	-0.06207	-0.06192
-0.06316	-0.06301	-0.06287	-0.06272	-0.06258	-0.06243	-0.06228	-0.06213	-0.06198
-0.06322	-0.06307	-0.06292	-0.06278	-0.06263	-0.06249	-0.06235	-0.0622	-0.06204
-0.06328	-0.06313	-0.06298	-0.06284	-0.06269	-0.06255	-0.06241	-0.06226	-0.0621
-0.06334	-0.06319	-0.06304	-0.0629	-0.06275	-0.06261	-0.06247	-0.06232	-0.06216
-0.0634	-0.06325	-0.0631	-0.06295	-0.06281	-0.06267	-0.06253	-0.06238	-0.06223
-0.06347	-0.06331	-0.06316	-0.06301	-0.06287	-0.06272	-0.06258	-0.06244	-0.06229
		-0.06322	-0.06307	-0.06292	-0.06278	-0.06264	-0.0625	-0.06235
		-0.06328	-0.06313	-0.06298	-0.06284	-0.0627	-0.06256	-0.06242
		-0.06334	-0.06319	-0.06304	-0.0629	-0.06276	-0.06262	-0.06248
		-0.0634	-0.06325	-0.0631	-0.06296	-0.06282	-0.06268	-0.06254
		-0.06347	-0.06331	-0.06316	-0.06302	-0.06287	-0.06274	-0.0626
		-0.06353	-0.06337	-0.06322	-0.06307	-0.06293	-0.0628	-0.06266
				-0.06329	-0.06314	-0.06299	-0.06285	-0.06271
				-0.06335	-0.0632	-0.06305	-0.06291	-0.06277
				-0.06342	-0.06326	-0.06311	-0.06297	-0.06283
				-0.06348	-0.06333	-0.06318	-0.06303	-0.06289
				-0.06355	-0.06339	-0.06324	-0.06309	-0.06295
						-0.0633	-0.06315	-0.06301
						-0.06337	-0.06321	-0.06307
						-0.06343	-0.06328	-0.06313
						-0.0635	-0.06334	-0.06319
						-0.06356	-0.06341	-0.06325

				-0.06286	-0.06266	-0.06247
				-0.06278	-0.06258	-0.06239
				-0.0627	-0.06251	-0.06231
				-0.06263	-0.06243	-0.06224
				-0.06255	-0.06235	-0.06216
		-0.06287	-0.06267	-0.38224	-0.3812	-0.38019
		-0.06279	-0.06259	-0.38181	-0.38078	-0.37976
		-0.06272	-0.06252	-0.38138	-0.38035	-0.37934
		-0.06264	-0.06244	-0.38095	-0.37993	-0.37892
-0.06297	-0.06276	-0.38262	-0.38157	-0.38053	-0.37951	-0.3785
-0.0629	-0.06268	-0.38218	-0.38114	-0.3801	-0.37909	-0.37809

		-0.06282	-0.06261	-0.38176	-0.38071	-0.37968	-0.37867	-0.37767
		-0.06274	-0.06253	-0.38133	-0.38029	-0.52389	-0.52259	-0.5213
		-0.06266	-0.06245	-0.38091	-0.37987	-0.52338	-0.52207	-0.52078
		-0.06259	-0.06238	-0.3805	-0.37947	-0.52288	-0.52157	-0.52027
		-0.06251	-0.06232	-0.38009	-0.37908	-0.5224	-0.52109	-0.51978
-0.06283	-0.06264	-0.38173	-0.3807	-0.43577	-0.43462	-0.52191	-0.52059	-0.51928
-0.06276	-0.06256	-0.38134	-0.38031	-0.4353	-0.43414	-0.52139	-0.52007	-0.51875
-0.06269	-0.06249	-0.38095	-0.3799	-0.43482	-0.43366	-0.52086	-0.51954	-0.51824
-0.06262	-0.06242	-0.38053	-0.37948	-0.52297	-0.52165	-0.69092	-0.68916	-0.68741
-0.06254	-0.06235	-0.3801	-0.37905	-0.52244	-0.52112	-0.6902	-0.68847	-0.68675
-0.38179	-0.38074	-0.43576	-0.43456	-0.52193	-0.52062	-0.68951	-0.68778	-0.68607
-0.3814	-0.38035	-0.43531	-0.43412	-0.52145	-0.52013	-0.68882	-0.68708	-0.68536
-0.381	-0.37994	-0.52364	-0.52228	-0.57923	-0.57774	-0.68814	-0.68639	-0.68466
-0.38057	-0.3795	-0.52308	-0.52174	-0.57862	-0.57715	-0.68744	-0.6857	-0.68397
-0.43625	-0.43503	-0.52255	-0.52121	-0.57803	-0.57657	-0.68676	-0.68502	-0.68329
-0.43579	-0.43458	-0.52204	-0.52071	-0.57747	-0.57603	-0.68612	-0.68438	-0.68264
-0.43533	-0.43413	-0.52155	-0.52022	-0.57692	-0.5755	-0.68547	-0.68373	-0.68199
-0.52376	-0.52238	-0.5793	-0.57781	-0.68822	-0.6865	-0.68479	-0.68306	-0.68132
-0.52322	-0.52186	-0.57872	-0.57724	-0.68754	-0.68582	-0.6841	-0.68237	-0.68064
-0.52271	-0.52136	-0.57816	-0.57669	-0.68688	-0.68516	-0.73539	-0.73352	-0.73166
-0.52222	-0.52089	-0.57763	-0.57618	-0.68624	-0.68452	-0.7347	-0.73282	-0.73094
-0.52174	-0.52042	-0.5771	-0.57566	-0.6856	-0.68388	-0.734	-0.73212	-0.73024
-0.52126	-0.51995	-0.57657	-0.57514	-0.68497	-0.68324	-0.73329	-0.73141	-0.72954
-0.57897	-0.5775	-0.68778	-0.68606	-0.68434	-0.68259	-0.73257	-0.7307	-0.72883
-0.57844	-0.57697	-0.68714	-0.68542	-0.68368	-0.68193	-0.73185	-0.72998	-0.72812
-0.57791	-0.57645	-0.68651	-0.68477	-0.68302	-0.68126	-0.73112	-0.72926	-0.7274
-0.57738	-0.57592	-0.68586	-0.68411	-0.73419	-0.73227	-0.73038	-0.72851	-0.72666
-0.57685	-0.5754	-0.6852	-0.68344	-0.73345	-0.73153	-0.72962	-0.72775	-0.72589
-0.68807	-0.6863	-0.68453	-0.68276	-0.73271	-0.73079	-0.72889	-0.72702	-0.72517
-0.6874	-0.68564	-0.68386	-0.68208	-0.73196	-0.73006	-0.72818	-0.72633	-0.7245
-0.68674	-0.68496	-0.73506	-0.73313	-0.73123	-0.72934	-0.72748	-0.72564	-0.72382
-0.68607	-0.68427	-0.73431	-0.7324	-0.73051	-0.72864	-0.72679	-0.72496	-0.72314
-0.6854	-0.68359	-0.73358	-0.73168	-0.72981	-0.72795	-0.72611	-0.72428	-0.72246
-0.68476	-0.68297	-0.73289	-0.73098	-0.72911	-0.72726	-0.72542	-0.7236	-0.72179
-0.68413	-0.68236	-0.73223	-0.7303	-0.72841	-0.72656	-0.72474	-0.72293	-0.72113
-0.73538	-0.73347	-0.73155	-0.72964	-0.72775	-0.72589	-0.72406	-0.72225	-0.72045
-0.73468	-0.73277	-0.73088	-0.729	-0.72712	-0.72524	-0.72338	-0.72157	-0.71977
-0.73398	-0.73208	-0.7302	-0.72834	-0.72647	-0.7246	-0.72274	-0.72091	-0.71911
-0.73327	-0.73139	-0.72952	-0.72766	-0.72581	-0.72396	-0.72212	-0.72028	-0.71844
-0.73257	-0.7307	-0.72883	-0.72698	-0.72514	-0.72332	-0.72149	-0.71964	-0.7178
-0.73186	-0.72999	-0.72814	-0.7263	-0.72447	-0.72265	-0.72083	-0.71901	-0.71718
-0.73116	-0.72929	-0.72744	-0.72561	-0.72379	-0.72197	-0.72016	-0.71836	-0.71657
-0.73049	-0.72859	-0.72674	-0.72492	-0.72311	-0.7213	-0.7195	-0.71771	-0.71592
-0.72983	-0.72791	-0.72604	-0.72422	-0.72241	-0.72062	-0.71883	-0.71704	-0.71525
-0.72917	-0.72725	-0.72538	-0.72355	-0.72173	-0.71994	-0.82395	-0.82179	-0.81965
-0.72851	-0.72663	-0.72475	-0.72289	-0.72105	-0.71926	-0.82315	-0.82101	-0.81888
-0.72783	-0.72598	-0.72411	-0.72224	-0.7204	-0.7186	-0.86827	-0.86599	-0.86373
-0.72713	-0.72529	-0.72345	-0.72161	-0.71978	-0.71795	-0.86743	-0.86516	-0.86291
-0.72644	-0.72461	-0.72278	-0.72098	-0.77139	-0.76933	-0.86661	-0.86434	-0.86209
-0.72579	-0.72393	-0.72212	-0.72033	-0.7707	-0.76865	-0.86584	-0.86356	-0.8613

-0.72515	-0.72328	-0.72146	-0.71968	-0.77	-0.76798	-0.86509	-0.8628	-0.86052
-0.72453	-0.72265	-0.72083	-0.71905	-0.76932	-0.76731	-0.86433	-0.86202	-0.85972
-0.72392	-0.72204	-0.72022	-0.71844	-0.76863	-0.76664	-0.86358	-0.86124	-0.85891
-0.7233	-0.72143	-0.77197	-0.76994	-0.82154	-0.8194	-0.86278	-0.86045	-0.85813
-0.72266	-0.72081	-0.77128	-0.76923	-0.82077	-0.81863	-0.86195	-0.85967	-0.85738
-0.72203	-0.7202	-0.77061	-0.76854	-0.8657	-0.8634	-0.95958	-0.95673	-0.95393
-0.72142	-0.7196	-0.76995	-0.76787	-0.86491	-0.86259	-0.95851	-0.95568	-0.95291
-0.72079	-0.71899	-0.76929	-0.76721	-0.86414	-0.86179	-0.95746	-0.95465	-0.95191
-0.72014	-0.71835	-0.7686	-0.76654	-0.86339	-0.86104	-0.9565	-0.9537	-0.95098
-0.71947	-0.71769	-0.7679	-0.76588	-0.86265	-0.86032	-0.95557	-0.95278	-0.95007
-0.7712	-0.76921	-0.82079	-0.81865	-0.86191	-0.85958	-0.95464	-0.95185	-0.94914
-0.77052	-0.76852	-0.82007	-0.81796	-0.86117	-0.85884	-0.9537	-0.95091	-0.9482
-0.76984	-0.76782	-0.81933	-0.81724	-0.95856	-0.95564	-0.9987	-0.99578	-0.99293
-0.76915	-0.76711	-0.81857	-0.81649	-0.95751	-0.95466	-0.99773	-0.99484	-0.99202
-0.82214	-0.81995	-0.81783	-0.81576	-0.9565	-0.9537	-0.99678	-0.99392	-0.99111
-0.82144	-0.81925	-0.81713	-0.81507	-0.95555	-0.95279	-0.99585	-0.993	-0.99019
-0.82074	-0.81856	-0.81645	-0.81439	-0.95463	-0.9519	-0.99493	-0.99209	-0.98928
-0.82003	-0.81788	-0.86107	-0.85886	-0.95372	-0.95101	-0.99401	-0.99119	-0.98838
-0.81933	-0.81719	-0.86034	-0.85814	-0.95281	-0.95013	-0.9931	-0.99029	-0.98748
-0.81864	-0.81651	-0.8596	-0.85742	-0.9519	-0.94925	-1.03917	-1.03623	-1.0333
-0.81796	-0.81583	-0.85887	-0.85669	-0.951	-0.94838	-1.03823	-1.03529	-1.03236
-0.81728	-0.81515	-0.95549	-0.95278	-0.99589	-0.99314	-1.03728	-1.03436	-1.03142
-0.81661	-0.81448	-0.95459	-0.95191	-0.99498	-0.99223	-1.03635	-1.03342	-1.03048
-0.81595	-0.81381	-0.9537	-0.95104	-0.99408	-0.99133	-1.11708	-1.11376	-1.11044
-0.81528	-0.81316	-0.95282	-0.95017	-0.99317	-0.99044	-1.11604	-1.11272	-1.1094
-0.81463	-0.81253	-0.95196	-0.94931	-0.99227	-0.98955	-1.115	-1.11168	-1.10837
-0.81398	-0.8119	-0.95114	-0.94851	-0.99142	-0.98867	-1.11395	-1.11064	-1.10733
-0.81333	-0.81128	-0.95034	-0.94773	-0.99058	-0.98779	-1.1129	-1.10958	-1.10629
-0.95485	-0.95216	-0.99523	-0.99248	-0.98971	-0.98691	-1.11184	-1.10853	-1.10525
-0.95397	-0.95129	-0.99432	-0.99159	-0.98882	-0.98601	-1.11079	-1.10748	-1.1042
-0.95311	-0.95044	-0.99343	-0.99069	-1.07115	-1.06795	-1.1915	-1.18791	-1.18436
-0.95229	-0.94965	-0.99258	-0.98981	-1.07013	-1.06693	-1.19036	-1.18678	-1.18327
-0.95148	-0.94885	-0.99172	-0.98892	-1.06911	-1.06592	-1.18923	-1.18569	-1.18221
-0.95066	-0.94803	-0.99084	-0.98802	-1.0681	-1.06492	-1.18813	-1.18464	-1.18122
-0.94984	-0.94719	-0.98994	-0.98713	-1.06709	-1.06394	-1.18704	-1.18361	-1.18025
-0.949	-0.94634	-0.98905	-0.98625	-1.11117	-1.10792	-1.23149	-1.2278	-1.22419
-0.94814	-0.94549	-0.98817	-0.98538	-1.11018	-1.10695	-1.23038	-1.22671	-1.22311
-0.99286	-0.99008	-0.9873	-0.98453	-1.10921	-1.106	-1.22927	-1.22561	-1.22202
-0.99199	-0.9892	-0.98643	-0.9837	-1.10827	-1.10507	-1.22816	-1.2245	-1.2209
-0.99115	-0.98836	-1.06858	-1.06548	-1.18878	-1.1853	-1.22704	-1.22339	-1.2198
-0.99037	-0.98761	-1.06772	-1.06458	-1.18772	-1.18423	-1.22591	-1.22226	-1.21869
-0.98959	-0.98685	-1.06685	-1.06366	-1.18665	-1.18315	-1.22477	-1.22114	-1.2176
-0.98875	-0.98599	-1.06587	-1.06268	-1.18555	-1.18208	-1.22363	-1.22003	-1.21652
-0.98789	-0.9851	-1.06486	-1.06168	-1.18444	-1.18101	-1.22251	-1.21894	-1.21546
-0.98702	-0.98421	-1.06386	-1.0607	-1.18337	-1.17999	-1.22146	-1.21793	-1.2145
-0.98614	-0.98333	-1.06288	-1.05973	-1.18232	-1.179	-1.22047	-1.21701	-1.21362
-1.06831	-1.06513	-1.10703	-1.10371	-1.18134	-1.17805	-1.21946	-1.21604	-1.2127
-1.06744	-1.0643	-1.10617	-1.10287	-1.18046	-1.17714	-1.21845	-1.21504	-1.2117
-1.06655	-1.06344	-1.10528	-1.102	-1.22458	-1.22096	-1.25504	-1.25154	-1.24813
-1.0656	-1.06249	-1.10427	-1.10101	-1.22349	-1.2199	-1.25397	-1.25048	-1.24709

-1.06464	-1.06152	-1.10325	-1.10002	-1.2224	-1.21885	-1.25291	-1.24944	-1.24607
-1.0637	-1.06062	-1.18333	-1.17995	-1.22144	-1.21791	-1.25196	-1.24851	-1.24516
-1.06277	-1.05975	-1.18242	-1.17909	-1.22054	-1.21703	-1.25106	-1.24763	-1.24429
-1.06189	-1.05888	-1.18146	-1.17817	-1.21957	-1.21608	-1.25009	-1.24669	-1.24337
-1.06105	-1.058	-1.18047	-1.17717	-1.21853	-1.21505	-1.24905	-1.24568	-1.24239
-1.10508	-1.10184	-1.17948	-1.1762	-1.21752	-1.21408	-1.24808	-1.24474	-1.24147
-1.10405	-1.10085	-1.17846	-1.17524	-1.21657	-1.2132	-1.24723	-1.2439	-1.24065
-1.10307	-1.09989	-1.17748	-1.17431	-1.21562	-1.21231	-1.24635	-1.24305	-1.23981
-1.10224	-1.09906	-1.22143	-1.21799	-1.25204	-1.24867	-1.28256	-1.2792	-1.2759
-1.10146	-1.09827	-1.2205	-1.21701	-1.25102	-1.24763	-1.2815	-1.27816	-1.27489
-1.10065	-1.09749	-1.21962	-1.21613	-1.2501	-1.24672	-1.28057	-1.27725	-1.274
-1.09982	-1.09672	-1.21877	-1.21529	-1.24925	-1.24588	-1.27971	-1.27641	-1.27318
-1.09894	-1.09586	-1.21783	-1.21437	-1.24831	-1.24497	-1.27878	-1.2755	-1.27229
-1.09799	-1.09491	-1.21677	-1.21335	-1.24729	-1.24398	-1.27778	-1.27452	-1.27132
-1.09709	-1.09401	-1.25317	-1.24969	-1.28348	-1.28011	-1.27682	-1.27359	-1.27041
-1.09628	-1.0932	-1.25222	-1.24878	-1.28257	-1.27923	-1.27597	-1.27277	-1.26962
-1.09546	-1.0924	-1.25128	-1.24787	-1.28166	-1.27836	-1.27512	-1.27195	-1.26883
-1.09455	-1.0915	-1.25025	-1.24687	-1.28066	-1.27738	-1.27418	-1.27105	-1.26798
-1.09362	-1.09059	-1.2492	-1.24586	-1.27964	-1.27638	-1.27321	-1.27012	-1.2671
-1.21782	-1.21436	-1.24827	-1.24496	-1.27873	-1.27549	-1.27233	-1.26924	-1.26622
-1.21694	-1.21351	-1.24742	-1.24414	-1.27791	-1.27469	-1.27153	-1.2684	-1.26532
-1.21608	-1.21268	-1.24659	-1.24334	-1.2771	-1.2739	-1.27074	-1.2676	-1.2645
-1.21523	-1.21187	-1.24578	-1.24256	-1.27631	-1.27313	-1.26998	-1.26686	-1.26377
-1.25168	-1.24827	-1.28203	-1.27875	-1.27552	-1.27235	-1.26922	-1.26611	-1.26304
-1.25081	-1.24744	-1.28119	-1.27795	-1.27474	-1.27157	-1.26845	-1.26535	-1.2623
-1.24993	-1.24658	-1.28034	-1.27712	-1.27393	-1.27076	-1.26764	-1.26456	-1.26152
-1.24896	-1.24565	-1.27939	-1.27619	-1.27301	-1.26984	-1.26672	-1.26365	-1.26062
-1.24796	-1.24468	-1.27841	-1.27521	-1.27204	-1.26888	-1.26576	-1.26269	-1.25967
-1.24706	-1.24381	-1.27752	-1.27434	-1.27117	-1.26801	-1.2649	-1.26184	-1.25882
-1.24625	-1.24303	-1.27672	-1.27354	-1.27038	-1.26724	-1.26413	-1.26107	-1.25805
-1.28245	-1.27917	-1.27594	-1.27276	-1.26961	-1.26647	-1.26338	-1.26032	-1.2573
-1.28164	-1.27839	-1.27517	-1.27198	-1.26883	-1.26571	-1.26263	-1.25959	-1.25658
-1.28083	-1.27761	-1.2744	-1.27121	-1.26806	-1.26495	-1.26189	-1.25886	-1.25586
-1.28004	-1.27683	-1.27362	-1.27043	-1.26728	-1.26418	-1.26113	-1.25811	-1.25513
-1.27925	-1.27605	-1.27285	-1.26965	-1.26651	-1.26341	-1.26036	-1.25736	-1.2544
-1.27847	-1.27527	-1.27207	-1.26889	-1.26575	-1.26266	-1.25961	-1.25662	-1.25366
-1.27768	-1.27449	-1.2713	-1.26813	-1.265	-1.26191	-1.25886	-1.25587	-1.25292
-1.2769	-1.2737	-1.27052	-1.26736	-1.26424	-1.26116	-1.25812	-1.25513	-1.25217
-1.27611	-1.27291	-1.26973	-1.26659	-1.26348	-1.26042	-1.25739	-1.2544	-1.25143
-1.27532	-1.27212	-1.26895	-1.26581	-1.26272	-1.25968	-1.25666	-1.25366	-1.25067
-1.27454	-1.27133	-1.26817	-1.26504	-1.26195	-1.25892	-1.25591	-1.25288	-1.24987
-1.27376	-1.27055	-1.26739	-1.26427	-1.26119	-1.25817	-1.25515	-1.25211	-1.24907
-1.27296	-1.26976	-1.26661	-1.2635	-1.26043	-1.25741	-1.29467	-1.29151	-1.28835
-1.27215	-1.26897	-1.26583	-1.26273	-1.25967	-1.25667	-1.29389	-1.29074	-1.28759
-1.27135	-1.26817	-1.26505	-1.26197	-1.25892	-1.25592	-1.29312	-1.28997	-1.28682
-1.27054	-1.26738	-1.26427	-1.2612	-1.25817	-1.25519	-1.29235	-1.28921	-1.28606
-1.26973	-1.26659	-1.26349	-1.26044	-1.25743	-1.25445	-1.29159	-1.28847	-1.28534
-1.26893	-1.2658	-1.26271	-1.25968	-1.25668	-1.25372	-1.29084	-1.28776	-1.28467
-1.26814	-1.26501	-1.26194	-1.25893	-1.2962	-1.29314	-1.29009	-1.28704	-1.28399
-1.26734	-1.26423	-1.26118	-1.25817	-1.29541	-1.29236	-1.28931	-1.28628	-1.28324

-1.26654	-1.26346	-1.26042	-1.25741	-1.29463	-1.29157	-1.28853	-1.28551	-1.28248
-1.26575	-1.26269	-1.25966	-1.25667	-1.29385	-1.29079	-1.28776	-1.28473	-1.28172
-1.26497	-1.26192	-1.2589	-1.25592	-1.29307	-1.29002	-1.28698	-1.28396	-1.28095
-1.26419	-1.26115	-1.29844	-1.29537	-1.2923	-1.28924	-1.2862	-1.28318	-1.28019
-1.26342	-1.26039	-1.29766	-1.2946	-1.29154	-1.28848	-1.28543	-1.28241	-1.27942
-1.26271	-1.25967	-1.29689	-1.29383	-1.29076	-1.2877	-1.28466	-1.28165	-1.27866
-1.26209	-1.25901	-1.29616	-1.29305	-1.28997	-1.28692	-1.2839	-1.28089	-1.27791
-1.30185	-1.29863	-1.29545	-1.2923	-1.2892	-1.28615	-1.28313	-1.28013	-1.27716
-1.30116	-1.29798	-1.2948	-1.29161	-1.28848	-1.2854	-1.28236	-1.27936	-1.2764
-1.30044	-1.29732	-1.29415	-1.29095	-1.2878	-1.28468	-1.28162	-1.27861	-1.27566
-1.29967	-1.29658	-1.29345	-1.29029	-1.28716	-1.28405	-1.34426	-1.34111	-1.33801
-1.29886	-1.29578	-1.2927	-1.28962	-1.28654	-1.28346	-1.34368	-1.3405	-1.33737
-1.29809	-1.29501	-1.29194	-1.28889	-1.28584	-1.28279	-1.34301	-1.33986	-1.33674
-1.29735	-1.29423	-1.29115	-1.28809	-1.28505	-1.28203	-1.34227	-1.33917	-1.33611
-1.29666	-1.29352	-1.29041	-1.28734	-1.3478	-1.34463	-1.34151	-1.33845	-1.33544
-1.29605	-1.29292	-1.2898	-1.28668	-1.34709	-1.34388	-1.34073	-1.33767	-1.33467
-1.29542	-1.29231	-1.28918	-1.28604	-1.34642	-1.34317	-1.34	-1.33692	-1.33392
-1.29467	-1.29158	-1.28849	-1.28539	-1.34578	-1.34256	-1.33939	-1.3363	-1.33326
-1.29389	-1.29081	-1.28776	-1.28473	-1.34514	-1.34197	-1.33883	-1.33572	-1.33265
-1.29319	-1.2901	-1.35077	-1.34758	-1.34441	-1.34127	-1.33816	-1.33507	-1.33203
-1.29255	-1.28942	-1.35003	-1.34679	-1.34361	-1.34049	-1.33741	-1.33439	-1.33141
-1.3559	-1.35261	-1.34934	-1.34609	-1.3429	-1.33977	-1.37225	-1.36915	-1.3661
-1.35519	-1.35196	-1.34873	-1.3455	-1.3423	-1.33913	-1.37154	-1.3684	-1.36533
-1.35447	-1.35128	-1.34807	-1.34486	-1.34167	-1.33848	-1.37085	-1.3677	-1.36462
-1.35372	-1.3505	-1.3473	-1.34411	-1.34095	-1.33782	-1.37021	-1.36709	-1.36402
-1.35297	-1.34973	-1.34652	-1.34335	-1.34022	-1.33714	-1.36957	-1.36648	-1.36344
-1.3523	-1.34906	-1.34584	-1.34265	-1.33951	-1.33642	-1.36884	-1.36578	-1.36276
-1.35165	-1.34843	-1.34521	-1.34198	-1.33881	-1.33571	-1.36809	-1.36504	-1.36206
-1.35095	-1.34774	-1.34453	-1.34132	-1.33816	-1.33506	-1.36744	-1.3644	-1.36143
-1.35022	-1.34701	-1.34382	-1.34067	-1.33756	-1.33448	-1.36686	-1.36384	-1.36087
-1.34952	-1.34631	-1.34314	-1.34003	-1.33695	-1.33389	-1.36626	-1.36324	-1.36026
-1.34886	-1.34566	-1.34252	-1.33942	-1.33635	-1.33329	-1.36562	-1.36258	-1.35959
-1.34822	-1.34502	-1.34189	-1.3388	-1.33573	-1.33267	-1.36498	-1.36192	-1.35892
-1.34757	-1.34436	-1.3412	-1.3381	-1.33503	-1.33202	-1.36435	-1.36132	-1.35833
-1.34692	-1.34369	-1.34052	-1.33739	-1.33434	-1.33138	-1.36374	-1.36073	-1.35776
-1.34627	-1.34305	-1.33988	-1.33677	-1.33374	-1.3308	-1.36316	-1.36015	-1.35716
-1.34562	-1.34242	-1.33928	-1.3362	-1.33319	-1.33026	-1.36259	-1.35957	-1.35656
-1.34498	-1.3418	-1.33867	-1.3356	-1.3326	-1.32966	-1.36197	-1.35895	-1.35595
-1.34434	-1.34118	-1.33806	-1.33498	-1.33196	-1.32899	-1.36128	-1.3583	-1.35535
-1.34371	-1.34056	-1.33744	-1.33436	-1.33134	-1.32837	-1.39742	-1.39436	-1.39135
-1.34307	-1.33991	-1.3368	-1.33374	-1.33074	-1.32779	-1.39683	-1.39378	-1.39076
-1.34243	-1.33926	-1.33617	-1.33314	-1.33016	-1.32724	-1.39625	-1.3932	-1.39016
-1.34178	-1.33863	-1.33556	-1.33256	-1.36151	-1.35852	-1.42631	-1.42316	-1.42002
-1.34114	-1.33802	-1.33498	-1.332	-1.36094	-1.35796	-1.42572	-1.42254	-1.41938
-1.34052	-1.33743	-1.36642	-1.36338	-1.36037	-1.35738	-1.46125	-1.45798	-1.45473
-1.33992	-1.33686	-1.36585	-1.36281	-1.35979	-1.35676	-1.46057	-1.45731	-1.45408
-1.30127	-1.2984	-1.36527	-1.36222	-1.35919	-1.35615	-1.45992	-1.45667	-1.45347
-1.30075	-1.29788	-1.3647	-1.36161	-1.35856	-1.35553	-1.45929	-1.45607	-1.4529
-1.33291	-1.32997	-1.36412	-1.36101	-1.35795	-1.35494	-1.45868	-1.45548	-1.45233
-1.33235	-1.32941	-1.36356	-1.36046	-1.3574	-1.35438	-1.45809	-1.45491	-1.45177

-1.33178	-1.32884	-1.36298	-1.3599	-1.35685	-1.35382	-1.4575	-1.45433	-1.4512
-1.33119	-1.32823	-1.36234	-1.35928	-1.35625	-1.35325	-1.48685	-1.48362	-1.48044
-1.33059	-1.32761	-1.36168	-1.35864	-1.35563	-1.35267	-1.48628	-1.48306	-1.47987
-1.33003	-1.32704	-1.36109	-1.35804	-1.38675	-1.38374	-1.48571	-1.48247	-1.47927
-1.3295	-1.32651	-1.36054	-1.35749	-1.3862	-1.38321	-1.48514	-1.48187	-1.47863
-1.32897	-1.32598	-1.35999	-1.35693	-1.45827	-1.45508	-1.48458	-1.48129	-1.47803
-1.32843	-1.32545	-1.35944	-1.35638	-1.45771	-1.45453	-1.48402	-1.48073	-1.47748
-1.3279	-1.32492	-1.39077	-1.38764	-1.45716	-1.45399	-1.48346	-1.48019	-1.47695
-1.32738	-1.3244	-1.39024	-1.38712	-1.45663	-1.45347	-1.48293	-1.47966	-1.47642
-1.32685	-1.32388	-1.38971	-1.3866	-1.45611	-1.45295	-1.4824	-1.47914	-1.47591
-1.35894	-1.3559	-1.38917	-1.38607	-1.45557	-1.45242	-1.48187	-1.47862	-1.47539
-1.35841	-1.35537	-1.38863	-1.38553	-1.45502	-1.45189	-1.48133	-1.47809	-1.47488
-1.35787	-1.35484	-1.38809	-1.385	-1.45449	-1.45136	-1.4808	-1.47758	-1.47438
-1.35734	-1.35431	-1.38755	-1.38448	-1.45396	-1.45085	-1.48028	-1.47707	-1.47388
-1.3568	-1.35378	-1.35082	-1.34791	-1.45342	-1.45033	-1.47975	-1.47655	-1.47338
-1.35628	-1.35325	-1.3503	-1.3474	-1.45288	-1.44979	-1.47922	-1.47603	-1.47286
-1.35576	-1.35273	-1.34978	-1.34688	-1.45233	-1.44926	-1.47868	-1.4755	-1.47235
-1.35524	-1.35222	-1.34927	-1.34637	-1.45179	-1.44873	-1.47816	-1.475	-1.47185
-1.35473	-1.35173	-1.34878	-1.34587	-1.45126	-1.44822	-1.47764	-1.4745	-1.47138
-1.35424	-1.35128	-1.34834	-1.34544	-1.45078	-1.44773	-1.47713	-1.47402	-1.47093
-1.35378	-1.35086	-1.34795	-1.34504	-1.45034	-1.44725	-1.47663	-1.47355	-1.47051
-1.35331	-1.35041	-1.42145	-1.41834	-1.48246	-1.47926	-1.47612	-1.47307	-1.47005
-1.35283	-1.34991	-1.42094	-1.41785	-1.48195	-1.47874	-1.47561	-1.47257	-1.46957
-1.35234	-1.34941	-1.42043	-1.41738	-1.48147	-1.47826	-1.47512	-1.47208	-1.46908
-1.35183	-1.34891	-1.41994	-1.41696	-1.48106	-1.47784	-1.47468	-1.47161	-1.46859
-1.35133	-1.34842	-1.41947	-1.41654	-1.48064	-1.47743	-1.47426	-1.47116	-1.4681
-1.35089	-1.34797	-1.419	-1.41605	-1.48014	-1.47698	-1.47384	-1.47073	-1.46764
-1.35047	-1.34754	-1.41854	-1.41555	-1.4796	-1.4765	-1.47341	-1.4703	-1.4672
-1.35001	-1.34711	-1.41811	-1.41509	-1.4791	-1.47601	-1.47294	-1.46982	-1.46673
-1.34951	-1.34668	-1.41769	-1.41467	-1.47862	-1.47551	-1.47242	-1.46931	-1.46623
-1.34903	-1.34622	-1.41724	-1.41422	-1.44422	-1.44121	-1.47189	-1.46881	-1.46576
-1.34858	-1.34574	-1.41673	-1.41372	-1.4437	-1.44068	-1.47136	-1.46833	-1.46533
-1.34814	-1.34527	-1.41624	-1.41324	-1.44321	-1.44018	-1.47085	-1.46785	-1.46489
-1.34774	-1.34485	-1.41582	-1.41282	-1.44278	-1.43975	-1.4704	-1.46737	-1.46438
-1.34733	-1.34445	-1.41542	-1.41243	-1.44237	-1.43935	-1.46997	-1.4669	-1.46387
-1.34688	-1.34405	-1.41502	-1.41203	-1.44195	-1.43893	-1.46955	-1.46646	-1.46342
-1.34642	-1.34364	-1.41463	-1.41162	-1.44153	-1.43851	-1.46913	-1.46605	-1.463
-1.34595	-1.34319	-1.41419	-1.41121	-1.44112	-1.4381	-1.46871	-1.46563	-1.46258
-1.34548	-1.34269	-1.41369	-1.41079	-1.44072	-1.43771	-1.4683	-1.46522	-1.46218
-1.34503	-1.34222	-1.41322	-1.41034	-1.44029	-1.43729	-1.46788	-1.46481	-1.46177
-1.34462	-1.3418	-1.41277	-1.40986	-1.43979	-1.43683	-1.46746	-1.4644	-1.46135
-1.34422	-1.3414	-1.41234	-1.4094	-1.43931	-1.43638	-1.46703	-1.46398	-1.46094
-1.34381	-1.341	-1.41194	-1.40902	-1.43893	-1.43599	-1.46662	-1.46357	-1.46053
-1.34341	-1.34061	-1.41157	-1.40868	-1.43858	-1.43561	-1.46621	-1.46316	-1.46013
-1.34303	-1.34026	-1.41121	-1.40832	-1.43819	-1.43521	-1.46579	-1.46275	-1.45972
-1.34268	-1.33993	-1.41087	-1.40795	-1.43779	-1.4348	-1.46538	-1.46233	-1.45931
-1.34232	-1.33958	-1.41051	-1.40757	-1.43738	-1.4344	-1.43144	-1.42852	-1.42562
-1.34197	-1.3392	-1.4101	-1.40717	-1.43698	-1.434	-1.43105	-1.42813	-1.42523
-1.3416	-1.33881	-1.4097	-1.40677	-1.43657	-1.43361	-1.43066	-1.42774	-1.42484
-1.34121	-1.33843	-1.40931	-1.40638	-1.43617	-1.4332	-1.43026	-1.42735	-1.42445

-1.34081	-1.33804	-1.40892	-1.406	-1.43577	-1.4328	-1.42986	-1.42695	-1.42407
-1.34041	-1.33765	-1.40853	-1.40562	-1.43538	-1.43241	-1.42947	-1.42657	-1.42369
-1.34002	-1.33726	-1.40814	-1.40524	-1.435	-1.43204	-1.4291	-1.4262	-1.42333
-1.41363	-1.41068	-1.44059	-1.43762	-1.43466	-1.4317	-1.42877	-1.42586	-1.42298
-1.41323	-1.41031	-1.44023	-1.43729	-1.43435	-1.4314	-1.42847	-1.42555	-1.42266
-1.41286	-1.40994	-1.43986	-1.43693	-1.434	-1.43106	-1.42813	-1.42521	-1.42231
-1.41254	-1.40961	-1.43949	-1.43654	-1.4336	-1.43067	-1.42774	-1.42483	-1.42193
-1.41221	-1.40926	-1.43911	-1.43615	-1.4332	-1.43027	-1.42735	-1.42444	-1.42155
-1.41182	-1.40887	-1.43871	-1.43574	-1.43279	-1.42986	-1.42694	-1.42404	-1.42116
-1.41143	-1.40849	-1.43832	-1.43535	-1.4324	-1.42946	-1.42655	-1.42366	-1.42079
-1.41109	-1.40816	-1.43797	-1.435	-1.43205	-1.42912	-1.42621	-1.42331	-1.42044
-1.41077	-1.40785	-1.43766	-1.43468	-1.43173	-1.42882	-1.42591	-1.423	-1.4201
-1.37284	-1.37003	-1.43731	-1.43435	-1.43141	-1.4285	-1.42559	-1.42268	-1.41978
-1.37244	-1.36963	-1.43693	-1.43401	-1.4311	-1.42819	-1.42528	-1.42237	-1.41946
-1.37207	-1.36927	-1.43656	-1.43365	-1.43074	-1.42784	-1.42493	-1.42202	-1.41912
-1.37175	-1.36893	-1.43619	-1.43326	-1.43034	-1.42744	-1.42454	-1.42162	-1.41871
-1.37144	-1.36861	-1.43584	-1.43288	-1.42995	-1.42705	-1.42415	-1.42124	-1.41833
-1.37112	-1.3683	-1.43552	-1.43255	-1.42962	-1.42672	-1.42382	-1.42091	-1.41801
-1.37082	-1.368	-1.43522	-1.43225	-1.42931	-1.42641	-1.42351	-1.42061	-1.41772
-1.3705	-1.36769	-1.4349	-1.43194	-1.42901	-1.4261	-1.45694	-1.45393	-1.45094
-1.37019	-1.36738	-1.43458	-1.43163	-1.4287	-1.42579	-1.45661	-1.45361	-1.45061
-1.36987	-1.36707	-1.43426	-1.43132	-1.42839	-1.42548	-1.45628	-1.45328	-1.45029
-1.36955	-1.36675	-1.43395	-1.431	-1.42807	-1.42516	-1.45595	-1.45295	-1.44996
-1.36924	-1.36644	-1.43363	-1.43068	-1.42776	-1.42485	-1.45562	-1.45263	-1.44964
-1.36893	-1.36614	-1.43332	-1.43038	-1.42745	-1.42454	-1.4553	-1.4523	-1.44931
-1.36862	-1.36583	-1.433	-1.43007	-1.42715	-1.42424	-1.45497	-1.45197	-1.44899
-1.36832	-1.36553	-1.43269	-1.42976	-1.42684	-1.42392	-1.45465	-1.45165	-1.44866
-1.36801	-1.36522	-1.43238	-1.42944	-1.42652	-1.42361	-1.45431	-1.45132	-1.44834
-1.36771	-1.36492	-1.43207	-1.42914	-1.42621	-1.4233	-1.45399	-1.45099	-1.44801
-1.36741	-1.36463	-1.43177	-1.42884	-1.42592	-1.42299	-1.45366	-1.45066	-1.44768
-1.40035	-1.39747	-1.43146	-1.42853	-1.42561	-1.42268	-1.48571	-1.48259	-1.4795
-1.40003	-1.39716	-1.43115	-1.42822	-1.4253	-1.42238	-1.48541	-1.48228	-1.47918
-1.39972	-1.39686	-1.43085	-1.42791	-1.42499	-1.42207	-1.51458	-1.51137	-1.50817
-1.39941	-1.39654	-1.43054	-1.42761	-1.42469	-1.42176	-1.51426	-1.51105	-1.50786
-1.39912	-1.39625	-1.43025	-1.42733	-1.4244	-1.42146	-1.51395	-1.51074	-1.50755
-1.43592	-1.43295	-1.43001	-1.42709	-1.45788	-1.45482	-1.54299	-1.5397	-1.53643
-1.4357	-1.43273	-1.42979	-1.42686	-1.45764	-1.45458	-1.54275	-1.53943	-1.53614
-1.43544	-1.43247	-1.42952	-1.42659	-1.45735	-1.4543	-1.54247	-1.53916	-1.53588
-1.43513	-1.43218	-1.42922	-1.42627	-1.45702	-1.45398	-1.54218	-1.5389	-1.53565
-1.43485	-1.4319	-1.42895	-1.42598	-1.54847	-1.54516	-1.54187	-1.53862	-1.53539
-1.43461	-1.43167	-1.42871	-1.42574	-1.5482	-1.54486	-1.54155	-1.53829	-1.53507
-1.43436	-1.43142	-1.42847	-1.4255	-1.54794	-1.54458	-1.54125	-1.53798	-1.53475
-1.43405	-1.43111	-1.42816	-1.42521	-1.54766	-1.54431	-1.541	-1.53773	-1.5345
-1.43374	-1.43079	-1.42784	-1.42491	-1.54738	-1.54406	-1.54077	-1.5375	-1.53425
-1.36091	-1.35815	-1.42758	-1.42465	-1.54713	-1.54382	-1.54051	-1.53722	-1.53396
-1.36068	-1.35792	-1.42734	-1.42441	-1.5469	-1.54357	-1.54025	-1.53694	-1.53365
-1.36043	-1.35766	-1.49114	-1.48803	-1.54662	-1.5433	-1.53998	-1.53666	-1.53337
-1.36018	-1.35738	-1.49084	-1.48772	-1.5463	-1.543	-1.5397	-1.53641	-1.53314
-1.35992	-1.35711	-1.49057	-1.48744	-1.54601	-1.54272	-1.53944	-1.53616	-1.53291
-1.35966	-1.35687	-1.49035	-1.48722	-1.54577	-1.54248	-1.53921	-1.53594	-1.53269

-1.35941	-1.35664	-1.49014	-1.48701	-1.54556	-1.54226	-1.53898	-1.53571	-1.53247
-1.39143	-1.38859	-1.51973	-1.51652	-1.54534	-1.54204	-1.53875	-1.53548	-1.53223
-1.3912	-1.38837	-1.51951	-1.51632	-1.54512	-1.54182	-1.53853	-1.53525	-1.53199
-1.39097	-1.38814	-1.5193	-1.5161	-1.54489	-1.54159	-1.5383	-1.53501	-1.53174
-1.39075	-1.38792	-1.51909	-1.51589	-1.54467	-1.54136	-1.53806	-1.53476	-1.53148
-1.42505	-1.42211	-1.51888	-1.51567	-1.54444	-1.54113	-1.53782	-1.5345	-1.5312
-1.42484	-1.42189	-1.51865	-1.51545	-1.54421	-1.54089	-1.53757	-1.53422	-1.53091
-1.42462	-1.42167	-1.51842	-1.51523	-1.54397	-1.54065	-1.53732	-1.53398	-1.53065
-1.4244	-1.42145	-1.51819	-1.51499	-1.54373	-1.54042	-1.53711	-1.53379	-1.53049
-1.42418	-1.42123	-1.51797	-1.51476	-1.54349	-1.54019	-1.53691	-1.53362	-1.53035
-1.39454	-1.39163	-1.48251	-1.47939	-1.54325	-1.53996	-1.53668	-1.53341	-1.53016
-1.39431	-1.39139	-1.48227	-1.47916	-1.54302	-1.53972	-1.53645	-1.53319	-1.52996
-1.39407	-1.39116	-1.48204	-1.47893	-1.54278	-1.53948	-1.53621	-1.53296	-1.52974
-1.39383	-1.39093	-1.48182	-1.4787	-1.54254	-1.53925	-1.53597	-1.53273	-1.5295
-1.39362	-1.3907	-1.45319	-1.45011	-1.54231	-1.53901	-1.53573	-1.53249	-1.52927
-1.39344	-1.39048	-1.45294	-1.44988	-1.54208	-1.53877	-1.53548	-1.53224	-1.52903
-1.42474	-1.42166	-1.48582	-1.48265	-1.54188	-1.53855	-1.53526	-1.53201	-1.5288
-1.42457	-1.4215	-1.48566	-1.48249	-1.54171	-1.5384	-1.5351	-1.53182	-1.52857
-1.42438	-1.42136	-1.48552	-1.48233	-1.54156	-1.53825	-1.53495	-1.53164	-1.52835
-1.42416	-1.42116	-1.4853	-1.4821	-1.54134	-1.53805	-1.53476	-1.53147	-1.5282
-1.42392	-1.42094	-1.48506	-1.48185	-1.5411	-1.53782	-1.53455	-1.5313	-1.52808
-1.42373	-1.42075	-1.48486	-1.48165	-1.51348	-1.51021	-1.53433	-1.5311	-1.5279
-1.4236	-1.4206	-1.4847	-1.4815	-1.51332	-1.50999	-1.53409	-1.53087	-1.52768
-1.42345	-1.42043	-1.48452	-1.48133	-1.51315	-1.5098	-1.53389	-1.53066	-1.52747
-1.42328	-1.42022	-1.48428	-1.48112	-1.51296	-1.50963	-1.53374	-1.53051	-1.52731
-1.42311	-1.42001	-1.48406	-1.48091	-1.51277	-1.50947	-1.5336	-1.53036	-1.52715
-1.42294	-1.41986	-1.45041	-1.44734	-1.47759	-1.47441	-1.50604	-1.5028	-1.49958
-1.42278	-1.41973	-1.45027	-1.44719	-1.47742	-1.47424	-1.50588	-1.5026	-1.49935
-1.42263	-1.41958	-1.4501	-1.44699	-1.4772	-1.47404	-1.50569	-1.50242	-1.49917
-1.42249	-1.41944	-1.44991	-1.44677	-1.47696	-1.47381	-1.50548	-1.50224	-1.49903
-1.42236	-1.4193	-1.44975	-1.44659	-1.47676	-1.47362	-1.5053	-1.50209	-1.49889
-1.42223	-1.41918	-1.44963	-1.44645	-1.47662	-1.47347	-1.50516	-1.50194	-1.49875
-1.42207	-1.41904	-1.44949	-1.44632	-1.47649	-1.47334	-1.50502	-1.5018	-1.4986
-1.42185	-1.41885	-1.44931	-1.44617	-1.47635	-1.4732	-1.50488	-1.50165	-1.49844
-1.42164	-1.41865	-1.44913	-1.44602	-1.47622	-1.47307	-1.50475	-1.50151	-1.49828
-1.42148	-1.41849	-1.44897	-1.44588	-1.47608	-1.47293	-1.50461	-1.50137	-1.49813
-1.42135	-1.41836	-1.44884	-1.44574	-1.47595	-1.47279	-1.50447	-1.50123	-1.49799
-1.42122	-1.41822	-1.44869	-1.4456	-1.4758	-1.47265	-1.50433	-1.50109	-1.49785
-1.42108	-1.41809	-1.44855	-1.44545	-1.47565	-1.4725	-1.50419	-1.50095	-1.49771
-1.42095	-1.41795	-1.4484	-1.44531	-1.4755	-1.47235	-1.50404	-1.5008	-1.49756
-1.42082	-1.41781	-1.44826	-1.44517	-1.47536	-1.47221	-1.5039	-1.50065	-1.49741
-1.42072	-1.4177	-1.44813	-1.44504	-1.44196	-1.4389	-1.46892	-1.46575	-1.46258
-1.42067	-1.41763	-1.44803	-1.44491	-1.44181	-1.43876	-1.46877	-1.46561	-1.46245
-1.42063	-1.41757	-1.44794	-1.44478	-1.44167	-1.43861	-1.46863	-1.46547	-1.46232
-1.42054	-1.41747	-1.44783	-1.44466	-1.44154	-1.43847	-1.46849	-1.46534	-1.46219
-1.4204	-1.41735	-1.44771	-1.44454	-1.44142	-1.43834	-1.46834	-1.4652	-1.46206
-1.42027	-1.41723	-1.4476	-1.44445	-1.44134	-1.43825	-1.46823	-1.46507	-1.46193
-1.42012	-1.41709	-1.4475	-1.4444	-1.4413	-1.4382	-1.46816	-1.46496	-1.46179
-1.45362	-1.45049	-1.44739	-1.44432	-1.44123	-1.43812	-1.46807	-1.46485	-1.46165
-1.45353	-1.45037	-1.44725	-1.44417	-1.44108	-1.43798	-1.46794	-1.46472	-1.46152

-1.45344	-1.45027	-1.44713	-1.44404	-1.44094	-1.43785	-1.46781	-1.4646	-1.46141
-1.45336	-1.45021	-1.44708	-1.44397	-1.44086	-1.43776	-1.46771	-1.46452	-1.46134
-1.45327	-1.45016	-1.44704	-1.44392	-1.4408	-1.43767	-1.46761	-1.46444	-1.4613
-1.45315	-1.45004	-1.44693	-1.44382	-1.44071	-1.43759	-1.46754	-1.46438	-1.46123
-1.45301	-1.44989	-1.44678	-1.44368	-1.44059	-1.43751	-1.46749	-1.46432	-1.46116
-1.45291	-1.44978	-1.44666	-1.44356	-1.44047	-1.4374	-1.46739	-1.46422	-1.46107
-1.45285	-1.44971	-1.44657	-1.44343	-1.44032	-1.43725	-1.46724	-1.46409	-1.46095
-1.4528	-1.44964	-1.44649	-1.44333	-1.44021	-1.43713	-1.43407	-1.43103	-1.428
-1.45271	-1.44955	-1.44639	-1.44325	-1.44014	-1.43706	-1.434	-1.43096	-1.42794
-1.45263	-1.44945	-1.44631	-1.44319	-1.44009	-1.43699	-1.43392	-1.43089	-1.42787
-1.45255	-1.4494	-1.44626	-1.44314	-1.44002	-1.43689	-1.4338	-1.43075	-1.42774
-1.45248	-1.44935	-1.44622	-1.44309	-1.43995	-1.43679	-1.43367	-1.43061	-1.42759
-1.45242	-1.44931	-1.44618	-1.44305	-1.43991	-1.43675	-1.43362	-1.43054	-1.42751
-1.45238	-1.44926	-1.44614	-1.44301	-1.43989	-1.43676	-1.43364	-1.43054	-1.42747
-1.45234	-1.44922	-1.44609	-1.44297	-1.43984	-1.43672	-1.43361	-1.43051	-1.42743
-1.4523	-1.44918	-1.44604	-1.4429	-1.43976	-1.43662	-1.43351	-1.43043	-1.42737
-1.45226	-1.44913	-1.44598	-1.44282	-1.43967	-1.43652	-1.43341	-1.43035	-1.42732
-1.45223	-1.44908	-1.44591	-1.44272	-1.43956	-1.43644	-1.43335	-1.4303	-1.42727
-1.45219	-1.44903	-1.44584	-1.44262	-1.43946	-1.43637	-1.4333	-1.43025	-1.42723
-1.41958	-1.41651	-1.44581	-1.44261	-1.43945	-1.43633	-1.43325	-1.43021	-1.42719
-1.41954	-1.41649	-1.44581	-1.44263	-1.43947	-1.43632	-1.43321	-1.43016	-1.42714
-1.41951	-1.41645	-1.44576	-1.44258	-1.43942	-1.43627	-1.43316	-1.43011	-1.42709
-1.41947	-1.4164	-1.44569	-1.44248	-1.43931	-1.4362	-1.43311	-1.43006	-1.42703
-1.41943	-1.41636	-1.44564	-1.44242	-1.43925	-1.43614	-1.43306	-1.43001	-1.42698
-1.41939	-1.41634	-1.44564	-1.44245	-1.43927	-1.43612	-1.43301	-1.42995	-1.42693
-1.41935	-1.41631	-1.44564	-1.44247	-1.43931	-1.43612	-1.43298	-1.42991	-1.42689
-1.41936	-1.41628	-1.4456	-1.44246	-1.43931	-1.43615	-1.43301	-1.4299	-1.42683
-1.41939	-1.41625	-1.44555	-1.44242	-1.4393	-1.43617	-1.43305	-1.42991	-1.42679
-1.41941	-1.41627	-1.41317	-1.4101	-1.43926	-1.43615	-1.43305	-1.42991	-1.4268
-1.41943	-1.41632	-1.4132	-1.41007	-1.4392	-1.4361	-1.43302	-1.42993	-1.42684
-1.41943	-1.41634	-1.41321	-1.41007	-1.43918	-1.43607	-1.43298	-1.42991	-1.42684
-1.41938	-1.41632	-1.41323	-1.41011	-1.43922	-1.43606	-1.43293	-1.42986	-1.42682
-1.41935	-1.41629	-1.41322	-1.41013	-1.43925	-1.43606	-1.43291	-1.42983	-1.42678
-1.41937	-1.41626	-1.41317	-1.41011	-1.43926	-1.43609	-1.43294	-1.42983	-1.42674
-1.41939	-1.41623	-1.41313	-1.41008	-1.43924	-1.43611	-1.43298	-1.42984	-1.42671
-1.41939	-1.41624	-1.41313	-1.41004	-1.40699	-1.40397	-1.43298	-1.42985	-1.42674
-1.4194	-1.41627	-1.41314	-1.41001	-1.40694	-1.40394	-1.43297	-1.42987	-1.42678
-1.41943	-1.41631	-1.41318	-1.41003	-1.40695	-1.40395	-1.43298	-1.4299	-1.42682
-1.41947	-1.41638	-1.41325	-1.41009	-1.407	-1.40399	-1.43302	-1.42993	-1.42686
-1.38786	-1.38489	-1.41329	-1.41014	-1.40704	-1.404	-1.43302	-1.42994	-1.42687
-1.38782	-1.38487	-1.4133	-1.41016	-1.40706	-1.40398	-1.43296	-1.42989	-1.42685
-1.3878	-1.38487	-1.41331	-1.41019	-1.40709	-1.40398	-1.43294	-1.42987	-1.42684
-1.38783	-1.3849	-1.41335	-1.41024	-1.40714	-1.40403	-1.43298	-1.42991	-1.42688
-1.38788	-1.38494	-1.4134	-1.4103	-1.4072	-1.40409	-1.43305	-1.42998	-1.42694
-1.38792	-1.38498	-1.41345	-1.41036	-1.40726	-1.40415	-1.40109	-1.3981	-1.39516
-1.38795	-1.38501	-1.41349	-1.41041	-1.40732	-1.40421	-1.40114	-1.39816	-1.39521
-1.38797	-1.385	-1.41349	-1.41042	-1.40735	-1.40425	-1.4012	-1.39821	-1.39526
-1.38795	-1.38496	-1.41344	-1.41039	-1.40734	-1.40428	-1.40124	-1.39826	-1.39531
-1.38797	-1.38495	-1.41343	-1.41039	-1.40736	-1.40432	-1.4013	-1.39832	-1.39536
-1.38801	-1.38499	-1.41347	-1.41044	-1.40741	-1.40437	-1.40135	-1.39838	-1.39542

-1.38807	-1.38505	-1.38207	-1.37912	-1.40746	-1.40442	-1.40141	-1.39843	-1.39547
-1.38812	-1.38511	-1.38213	-1.37916	-1.4075	-1.40447	-1.40145	-1.39848	-1.39552
-1.38819	-1.38518	-1.38219	-1.37921	-1.40755	-1.40451	-1.4015	-1.39852	-1.39556
-1.38828	-1.38529	-1.3823	-1.3793	-1.40763	-1.40457	-1.40154	-1.39856	-1.3956
-1.38837	-1.38541	-1.38243	-1.37942	-1.40772	-1.40463	-1.40157	-1.39859	-1.39564
-1.38844	-1.3855	-1.38252	-1.3795	-1.4078	-1.40468	-1.40161	-1.39863	-1.39568
-1.38849	-1.38555	-1.38257	-1.37955	-1.40785	-1.40473	-1.40165	-1.39867	-1.39573
-1.38853	-1.38559	-1.38261	-1.37959	-1.4079	-1.40478	-1.4017	-1.39872	-1.39577
-1.38858	-1.38563	-1.38264	-1.37964	-1.40795	-1.40483	-1.40176	-1.39876	-1.39581
-1.38865	-1.38568	-1.38269	-1.37968	-1.40801	-1.4049	-1.40183	-1.39883	-1.39586
-1.38876	-1.38578	-1.38277	-1.37975	-1.37674	-1.37374	-1.40195	-1.39894	-1.39596
-1.3889	-1.3859	-1.38287	-1.37982	-1.37681	-1.37384	-1.40208	-1.39907	-1.39608
-1.38899	-1.38598	-1.38294	-1.37988	-1.37687	-1.3739	-1.40216	-1.39915	-1.39617
-1.38903	-1.38604	-1.383	-1.37994	-1.37692	-1.37394	-1.4022	-1.3992	-1.39624
-1.38911	-1.38611	-1.38308	-1.38003	-1.377	-1.37401	-1.40226	-1.39927	-1.3963
-1.38921	-1.3862	-1.38317	-1.38014	-1.37712	-1.37413	-1.40237	-1.39935	-1.39636
-1.38932	-1.3863	-1.38328	-1.38025	-1.37724	-1.37424	-1.37126	-1.36832	-1.3654
-1.38942	-1.38642	-1.3834	-1.38037	-1.37735	-1.37434	-1.37135	-1.36841	-1.36551
-1.38953	-1.38654	-1.38353	-1.38049	-1.37746	-1.37443	-1.37144	-1.36852	-1.36563
-1.38965	-1.38666	-1.38364	-1.38059	-1.37756	-1.37454	-1.37155	-1.36863	-1.36575
-1.38977	-1.38677	-1.38373	-1.38068	-1.37765	-1.37464	-1.37168	-1.36875	-1.36586
-1.38988	-1.38687	-1.38383	-1.38078	-1.37775	-1.37475	-1.37179	-1.36886	-1.36596
-1.38998	-1.38696	-1.38393	-1.38089	-1.37787	-1.37487	-1.37189	-1.36895	-1.36604
-1.39008	-1.38706	-1.38403	-1.381	-1.37798	-1.37497	-1.37199	-1.36905	-1.36614
-1.39018	-1.38718	-1.38415	-1.38112	-1.37809	-1.37507	-1.37208	-1.36915	-1.36627
-1.39029	-1.3873	-1.38428	-1.38123	-1.3782	-1.37517	-1.37218	-1.36927	-1.3664
-1.39042	-1.38743	-1.38439	-1.38132	-1.37828	-1.37527	-1.3723	-1.3694	-1.36654
-1.39056	-1.38756	-1.38451	-1.38142	-1.37837	-1.37538	-1.37243	-1.36954	-1.36669
-1.39071	-1.38771	-1.38466	-1.38158	-1.37853	-1.37553	-1.37257	-1.36968	-1.36682
-1.39086	-1.38787	-1.38484	-1.38177	-1.37873	-1.37569	-1.37271	-1.36981	-1.36696
-1.39101	-1.38801	-1.38498	-1.38192	-1.37887	-1.37584	-1.37285	-1.36995	-1.3671
-1.39115	-1.38815	-1.3851	-1.38201	-1.37896	-1.37595	-1.37299	-1.3701	-1.36724
-1.39129	-1.38829	-1.38524	-1.38214	-1.37909	-1.37609	-1.40362	-1.40065	-1.39772
-1.39145	-1.38846	-1.38542	-1.38235	-1.3793	-1.37626	-1.40377	-1.4008	-1.39786
-1.39161	-1.38862	-1.38561	-1.38256	-1.37951	-1.37645	-1.40394	-1.40095	-1.39801
-1.39175	-1.38878	-1.38577	-1.38273	-1.3797	-1.37665	-1.40414	-1.40113	-1.39816
-1.3919	-1.38893	-1.38592	-1.38289	-1.37987	-1.37684	-1.40436	-1.40132	-1.39831
-1.35728	-1.35439	-1.38606	-1.38304	-1.38001	-1.37699	-1.40451	-1.40147	-1.39847
-1.35741	-1.35453	-1.3862	-1.38317	-1.38014	-1.3771	-1.40461	-1.4016	-1.39863
-1.35757	-1.35468	-1.38634	-1.38332	-1.3803	-1.37726	-1.40478	-1.40178	-1.39882
-1.35777	-1.35482	-1.38649	-1.38348	-1.38048	-1.37747	-1.40502	-1.40202	-1.39906
-1.35798	-1.35499	-1.38665	-1.38365	-1.38066	-1.37768	-1.40525	-1.40225	-1.39927
-1.35817	-1.35521	-1.38686	-1.38382	-1.38082	-1.37784	-1.40542	-1.40241	-1.39943
-1.35836	-1.35544	-1.38708	-1.38401	-1.38099	-1.37801	-1.40559	-1.40257	-1.39958
-1.35852	-1.35562	-1.38729	-1.38423	-1.4119	-1.40885	-1.43944	-1.43633	-1.43324
-1.35867	-1.3558	-1.38749	-1.38446	-1.41216	-1.40909	-1.43968	-1.43658	-1.43349
-1.35887	-1.356	-1.38771	-1.38469	-1.41239	-1.40931	-1.43988	-1.43678	-1.43369
-1.35911	-1.35622	-1.38793	-1.38492	-1.41262	-1.40949	-1.44004	-1.43693	-1.43386
-1.35933	-1.35641	-1.38813	-1.38513	-1.41283	-1.40968	-1.44022	-1.4371	-1.43402
-1.35952	-1.35657	-1.38829	-1.38531	-1.41302	-1.4099	-1.44045	-1.43729	-1.43417

-1.35972	-1.35673	-1.38845	-1.38548	-1.41322	-1.41012	-1.44069	-1.4375	-1.43435
-1.35991	-1.35694	-1.38867	-1.3857	-1.41344	-1.41035	-1.44094	-1.43776	-1.43459
-1.36012	-1.35717	-1.38891	-1.38591	-1.41365	-1.41058	-1.4412	-1.43801	-1.43485
-1.29873	-1.29586	-1.38912	-1.3861	-1.41383	-1.41077	-1.4414	-1.43824	-1.43509
-1.29894	-1.29606	-1.38932	-1.38626	-1.41399	-1.41094	-1.44159	-1.43845	-1.43533
-1.29914	-1.29627	-1.32913	-1.32612	-1.41419	-1.41114	-1.4418	-1.43867	-1.43556
-1.29935	-1.29648	-1.32935	-1.32634	-1.41443	-1.41139	-1.44205	-1.43892	-1.43579
-1.29953	-1.29668	-1.32956	-1.32656	-1.41469	-1.41163	-1.44231	-1.43917	-1.43603
-1.29966	-1.29683	-1.32974	-1.32677	-1.41494	-1.41188	-1.44256	-1.43942	-1.43629
-1.29979	-1.29698	-1.32993	-1.32698	-1.41519	-1.41213	-1.44281	-1.43967	-1.43654
-1.29999	-1.29717	-1.36208	-1.35906	-1.39102	-1.38793	-1.44306	-1.43993	-1.43679
-1.3002	-1.29738	-1.3623	-1.35928	-1.39125	-1.38817	-1.44332	-1.44018	-1.43704
-1.30041	-1.29759	-1.36253	-1.35951	-1.39148	-1.3884	-1.38532	-1.38224	-1.37917
-1.30062	-1.2978	-1.36275	-1.35973	-1.39171	-1.38862	-1.38554	-1.38246	-1.37939
-1.30083	-1.298	-1.36297	-1.35995	-1.39193	-1.38885	-1.38576	-1.38269	-1.37961
-1.30104	-1.29821	-1.36318	-1.36016	-1.39216	-1.38906	-1.38598	-1.38291	-1.37984
-1.30128	-1.29843	-1.36341	-1.36037	-1.39237	-1.38928	-1.3862	-1.38313	-1.38007
-1.30157	-1.29869	-1.36366	-1.3606	-1.39259	-1.38949	-1.38641	-1.38335	-1.38031
-1.30186	-1.29896	-1.36393	-1.36083	-1.39281	-1.38971	-1.38663	-1.38358	-1.38055
-1.30209	-1.2992	-1.32887	-1.32586	-1.39304	-1.38992	-1.38684	-1.38379	-1.38077
-1.3023	-1.29942	-1.3291	-1.32609	-1.39328	-1.39015	-1.38706	-1.384	-1.38098
-1.30251	-1.29964	-1.32934	-1.32635	-1.39356	-1.39042	-1.38731	-1.38424	-1.3812
-1.30272	-1.29985	-1.32958	-1.32663	-1.39388	-1.39074	-1.38761	-1.38451	-1.38144
-1.30295	-1.30007	-1.32981	-1.32689	-1.39417	-1.39102	-1.38789	-1.38479	-1.38171
-1.3032	-1.30029	-1.33003	-1.32709	-1.39439	-1.39126	-1.38815	-1.38507	-1.38201
-1.33626	-1.33324	-1.36614	-1.3631	-1.39462	-1.3915	-1.3884	-1.38534	-1.3823
-1.33653	-1.33354	-1.36645	-1.36339	-1.3949	-1.39175	-1.38863	-1.38557	-1.38254
-1.33679	-1.33383	-1.36677	-1.3637	-1.3952	-1.39202	-1.38888	-1.3858	-1.38276
-1.33703	-1.33408	-1.36703	-1.36397	-1.39549	-1.39231	-1.38917	-1.38609	-1.38305
-1.33726	-1.33431	-1.36727	-1.36422	-1.39578	-1.39262	-1.38949	-1.38641	-1.38337
-1.30686	-1.30398	-1.36755	-1.36451	-1.39609	-1.39294	-1.3898	-1.38671	-1.38366
-1.30715	-1.30427	-1.36787	-1.36484	-1.39642	-1.39326	-1.39012	-1.387	-1.38391
-1.30744	-1.30455	-1.36817	-1.36513	-1.39673	-1.39356	-1.39042	-1.38729	-1.38418
-1.30771	-1.30479	-1.36841	-1.36537	-1.39697	-1.39383	-1.3907	-1.38759	-1.3845
-1.30798	-1.30504	-1.36867	-1.36562	-1.39724	-1.3941	-1.39099	-1.3879	-1.38483
-1.24035	-1.23761	-1.33898	-1.336	-1.36289	-1.35986	-1.39132	-1.38823	-1.38516
-1.24062	-1.23791	-1.33931	-1.33633	-1.36323	-1.3602	-1.39165	-1.38856	-1.3855
-1.2409	-1.23819	-1.33964	-1.33666	-1.36357	-1.36053	-1.392	-1.3889	-1.38583
-1.2412	-1.23848	-1.33996	-1.33699	-1.36391	-1.36087	-1.39234	-1.38923	-1.38615
-1.2415	-1.23878	-1.30372	-1.30084	-1.33434	-1.33137	-1.39268	-1.38956	-1.38647
-1.24179	-1.23908	-1.30404	-1.30115	-1.33466	-1.33169	-1.39301	-1.38988	-1.38678
-1.24209	-1.23938	-1.30436	-1.30146	-1.33498	-1.332	-1.39333	-1.3902	-1.38708
-1.24239	-1.23967	-1.30467	-1.30178	-1.33531	-1.33232	-1.39366	-1.39049	-1.38735
-1.24269	-1.23995	-1.30497	-1.30209	-1.33563	-1.33265	-1.39399	-1.3908	-1.38764
-1.24298	-1.24025	-1.30529	-1.3024	-1.33595	-1.33297	-1.39435	-1.39117	-1.38802
-1.24328	-1.24055	-1.3056	-1.3027	-1.33627	-1.3333	-1.39472	-1.39157	-1.38843
-1.24357	-1.24084	-1.30591	-1.30301	-1.30012	-1.29725	-1.36577	-1.3627	-1.35965
-1.24387	-1.24113	-1.30622	-1.30333	-1.30044	-1.29755	-1.3661	-1.36304	-1.36
-1.24416	-1.24142	-1.30653	-1.30364	-1.30075	-1.29786	-1.36643	-1.36337	-1.36034
-1.24445	-1.24171	-1.30684	-1.30394	-1.30105	-1.29817	-1.36676	-1.3637	-1.36066

-1.24475	-1.24202	-1.27722	-1.27439	-1.30136	-1.29848	-1.3671	-1.36403	-1.36099
-1.2451	-1.24232	-1.27752	-1.27471	-1.30168	-1.29879	-1.36742	-1.36435	-1.36132
-1.24545	-1.24263	-1.27784	-1.27504	-1.30203	-1.29911	-1.36776	-1.36469	-1.36165
-1.2458	-1.24298	-1.27821	-1.27541	-1.3024	-1.29949	-1.33239	-1.32938	-1.32641
-1.24615	-1.24336	-1.27861	-1.2758	-1.30279	-1.2999	-1.3328	-1.32976	-1.32674
-1.24647	-1.24371	-1.27897	-1.27614	-1.30314	-1.30025	-1.33318	-1.33013	-1.32711
-1.24678	-1.24403	-1.2793	-1.27644	-1.30344	-1.30056	-1.33353	-1.33052	-1.32752
-1.28537	-1.28251	-1.27964	-1.27678	-1.30377	-1.30089	-1.33387	-1.33088	-1.32791
-1.28577	-1.2829	-1.28003	-1.27717	-1.30416	-1.30123	-1.3342	-1.33121	-1.32824
-1.28617	-1.28328	-1.28041	-1.27756	-1.30455	-1.30158	-1.33454	-1.33154	-1.32858
-1.28652	-1.28361	-1.28074	-1.27791	-1.30491	-1.30195	-1.33494	-1.33193	-1.32896
-1.28685	-1.28393	-1.28106	-1.27824	-1.30526	-1.30233	-1.33536	-1.33234	-1.32935
-1.28722	-1.2843	-1.28143	-1.2786	-1.30563	-1.30271	-1.33575	-1.33271	-1.32969
-1.28761	-1.2847	-1.28182	-1.27897	-1.30601	-1.3031	-1.33615	-1.33307	-1.33001
-1.28797	-1.28506	-1.28218	-1.27931	-1.30635	-1.30345	-1.33652	-1.33344	-1.33037
-1.28829	-1.28538	-1.28249	-1.27962	-1.30666	-1.30377	-1.33687	-1.33382	-1.33078
-1.28864	-1.28573	-1.28283	-1.27995	-1.2771	-1.27429	-1.33724	-1.33421	-1.33119
-1.28904	-1.28612	-1.28322	-1.28033	-1.27748	-1.27467	-1.33764	-1.33462	-1.33161
-1.28945	-1.28653	-1.28362	-1.28074	-1.27788	-1.27506	-1.33806	-1.33503	-1.33202
-1.28986	-1.28693	-1.28402	-1.28113	-1.27828	-1.27545	-1.33847	-1.33544	-1.33244
-1.29028	-1.28734	-1.28442	-1.28153	-1.27867	-1.27584	-1.33889	-1.33586	-1.33285
-1.29069	-1.28775	-1.28483	-1.28193	-1.27906	-1.27623	-1.33931	-1.33628	-1.33327
-1.29111	-1.28816	-1.28523	-1.28233	-1.27946	-1.27662	-1.33973	-1.3367	-1.33368
-1.25568	-1.25282	-1.28564	-1.28273	-1.27985	-1.27702	-1.34015	-1.33712	-1.3341
-1.25609	-1.25323	-1.28605	-1.28313	-1.28025	-1.27742	-1.34057	-1.33754	-1.33452
-1.2565	-1.25363	-1.25078	-1.24794	-1.28065	-1.27781	-1.34099	-1.33796	-1.33494
-1.25691	-1.25404	-1.25118	-1.24834	-1.28106	-1.27821	-1.34141	-1.33837	-1.33535
-1.25731	-1.25444	-1.25158	-1.24873	-1.28146	-1.27861	-1.34183	-1.33879	-1.33577
-1.25772	-1.25485	-1.25198	-1.24913	-1.28186	-1.27901	-1.34225	-1.33922	-1.33619
-1.25813	-1.25526	-1.25239	-1.24953	-1.28227	-1.27941	-1.34268	-1.33964	-1.33662
-1.25854	-1.25566	-1.25279	-1.24992	-1.2471	-1.24432	-1.3431	-1.34006	-1.33703
-1.25895	-1.25607	-1.25319	-1.25032	-1.24749	-1.2447	-1.34353	-1.34048	-1.33745
-1.25936	-1.25648	-1.2536	-1.25072	-1.24789	-1.2451	-1.27982	-1.27693	-1.27405
-1.25978	-1.2569	-1.25401	-1.25113	-1.24829	-1.24549	-1.28023	-1.27734	-1.27446
-1.26019	-1.25732	-1.25444	-1.25156	-1.24871	-1.24591	-1.28065	-1.27776	-1.27489
-1.26061	-1.25774	-1.25487	-1.25202	-1.24919	-1.24637	-1.28113	-1.27824	-1.27537
-1.26104	-1.25816	-1.25531	-1.25249	-1.24967	-1.24684	-1.28161	-1.27873	-1.27586
-1.26151	-1.25861	-1.25575	-1.25293	-1.2501	-1.24726	-1.28205	-1.27917	-1.2763
-1.26201	-1.25909	-1.2562	-1.25333	-1.25048	-1.24764	-1.28245	-1.27957	-1.27672
-1.26248	-1.25955	-1.25664	-1.25375	-1.25088	-1.24804	-1.28286	-1.27999	-1.27713
-1.2629	-1.25997	-1.25706	-1.25417	-1.25129	-1.24844	-1.28328	-1.2804	-1.27755
-1.26334	-1.26043	-1.25752	-1.25461	-1.25173	-1.24888	-1.28373	-1.28085	-1.27799
-1.26383	-1.26094	-1.25803	-1.25511	-1.25222	-1.24937	-1.28423	-1.28133	-1.27846
-1.26432	-1.26144	-1.25854	-1.25561	-1.25272	-1.24988	-1.28475	-1.28184	-1.27895
-1.26474	-1.26187	-1.25899	-1.2561	-1.25323	-1.25037	-1.28524	-1.28234	-1.27946
-1.26515	-1.26226	-1.25941	-1.25658	-1.25373	-1.25084	-1.28573	-1.28284	-1.27997
-1.26561	-1.26271	-1.25985	-1.25702	-1.25417	-1.25128	-1.28618	-1.28329	-1.28043
-1.26611	-1.26318	-1.2603	-1.25744	-1.25457	-1.25169	-1.2866	-1.28371	-1.28085
-1.22698	-1.22416	-1.26077	-1.25789	-1.25501	-1.25213	-1.24929	-1.24648	-1.24372
-1.22745	-1.22465	-1.26128	-1.25837	-1.25549	-1.25262	-1.24978	-1.24696	-1.24417

-1.22793	-1.22513	-1.26178	-1.25887	-1.25598	-1.25312	-1.25027	-1.24745	-1.24465
-1.22842	-1.2256	-1.26227	-1.25938	-1.25649	-1.25361	-1.25075	-1.24793	-1.24514
-1.2289	-1.22606	-1.26275	-1.25988	-1.257	-1.2541	-1.25123	-1.24842	-1.24564
-1.1921	-1.18939	-1.26326	-1.26037	-1.25749	-1.2546	-1.25173	-1.24892	-1.24613
-1.1925	-1.18985	-1.26377	-1.26086	-1.25797	-1.2551	-1.25225	-1.24941	-1.2466
-1.19291	-1.19026	-1.26424	-1.26134	-1.25846	-1.25559	-1.25274	-1.24991	-1.24709
-1.19331	-1.19063	-1.26466	-1.26183	-1.25898	-1.25609	-1.25322	-1.2504	-1.2476
-1.19374	-1.19104	-1.2651	-1.2623	-1.25947	-1.25658	-1.25371	-1.25089	-1.24809
-1.19422	-1.1915	-1.26556	-1.26272	-1.25989	-1.25705	-1.25422	-1.25139	-1.24856
-1.19471	-1.19198	-1.26605	-1.26318	-1.26034	-1.25753	-1.25472	-1.25188	-1.24904
-1.1952	-1.19247	-1.26659	-1.26372	-1.26087	-1.25803	-1.2552	-1.25235	-1.24952
-1.19569	-1.19297	-1.26715	-1.2643	-1.26143	-1.25854	-1.25567	-1.25282	-1.25
-1.1962	-1.19349	-1.2677	-1.26482	-1.26194	-1.25904	-1.25616	-1.25331	-1.25048
-1.19672	-1.19401	-1.26825	-1.26533	-1.26242	-1.25953	-1.25666	-1.2538	-1.25096
-1.12141	-1.11888	-1.23219	-1.22935	-1.26293	-1.26003	-1.25716	-1.25429	-1.25144
-1.12188	-1.1193	-1.23267	-1.22984	-1.26344	-1.26053	-1.25764	-1.25477	-1.25194
-1.12234	-1.11973	-1.23316	-1.23033	-1.26395	-1.26103	-1.25814	-1.25528	-1.25246
-1.12278	-1.12019	-1.23366	-1.23082	-1.26444	-1.26154	-1.25866	-1.25582	-1.253
-1.12323	-1.12065	-1.23418	-1.23132	-1.26496	-1.26206	-1.2592	-1.25636	-1.25355
-1.12371	-1.12114	-1.16047	-1.1578	-1.26555	-1.26264	-1.25976	-1.25689	-1.25405
-1.12419	-1.12165	-1.16101	-1.15835	-1.26617	-1.26325	-1.26034	-1.25743	-1.25455
-1.12467	-1.12211	-1.1615	-1.15885	-1.26674	-1.26383	-1.26092	-1.25801	-1.25511
-1.12516	-1.12256	-1.16195	-1.15931	-1.26728	-1.2644	-1.26152	-1.25861	-1.25571
-1.12563	-1.12301	-1.16242	-1.15979	-1.26783	-1.26495	-1.26207	-1.25917	-1.25627
-1.12608	-1.12346	-1.1629	-1.1603	-1.26838	-1.26548	-1.26258	-1.25967	-1.25679
-1.12655	-1.12393	-1.1634	-1.16079	-1.15817	-1.15552	-1.26308	-1.26018	-1.25731
-1.12708	-1.12443	-1.16389	-1.16124	-1.1586	-1.15597	-1.26361	-1.26073	-1.25787
-1.12762	-1.12494	-1.16439	-1.16169	-1.15904	-1.15643	-1.26415	-1.26129	-1.25844
-1.12813	-1.12546	-1.16493	-1.16222	-1.15955	-1.15692	-1.2647	-1.26185	-1.25901
-1.12864	-1.12599	-1.16548	-1.16277	-1.16008	-1.15743	-1.26526	-1.26241	-1.25957
-1.12913	-1.12647	-1.12383	-1.12123	-1.16059	-1.15794	-1.19312	-1.19043	-1.18776
-1.12961	-1.12691	-1.12426	-1.12168	-1.16108	-1.15845	-1.19365	-1.19092	-1.18821
-1.13012	-1.12738	-1.12473	-1.12216	-1.16158	-1.15896	-1.19418	-1.19143	-1.18869
-1.13067	-1.12792	-1.12526	-1.12267	-1.16211	-1.15949	-1.19473	-1.19197	-1.18922
-1.13122	-1.12848	-1.12581	-1.1232	-1.16265	-1.16002	-1.1574	-1.15475	-1.1521
-1.13177	-1.12903	-1.12635	-1.12373	-1.1632	-1.16057	-1.15794	-1.15527	-1.15262
-1.13232	-1.12958	-1.1269	-1.12427	-1.16375	-1.16111	-1.15847	-1.1558	-1.15314
-1.13287	-1.13013	-1.12745	-1.12481	-1.16431	-1.16165	-1.159	-1.15632	-1.15366
-1.13342	-1.13068	-1.12799	-1.12534	-1.16486	-1.16219	-1.15953	-1.15685	-1.15417
-1.13397	-1.13122	-1.12853	-1.12588	-1.16542	-1.16274	-1.16007	-1.15738	-1.15469
-1.13451	-1.13177	-1.12908	-1.12642	-1.16598	-1.1633	-1.16062	-1.15791	-1.15522
-1.13506	-1.13231	-1.12962	-1.12695	-1.16653	-1.16385	-1.16116	-1.15845	-1.15575
-1.1356	-1.13285	-1.13015	-1.12748	-1.16708	-1.16439	-1.1617	-1.15898	-1.15627
-1.13613	-1.13338	-1.13068	-1.12801	-1.16763	-1.16493	-1.16224	-1.15952	-1.15681
-1.06121	-1.05866	-1.13121	-1.12854	-1.16819	-1.1655	-1.16281	-1.16007	-1.15735
-1.0617	-1.05914	-1.13175	-1.12907	-1.16875	-1.16607	-1.16338	-1.16064	-1.15791
-1.06219	-1.05962	-1.13228	-1.1296	-1.12697	-1.1244	-1.16395	-1.16122	-1.15851
-1.06268	-1.0601	-1.13281	-1.13013	-1.1275	-1.12493	-1.16451	-1.16183	-1.15915
-1.025	-1.02251	-1.05809	-1.05559	-1.12804	-1.12546	-1.16507	-1.16241	-1.15975
-1.02553	-1.02307	-1.05868	-1.05614	-1.12862	-1.12601	-1.16562	-1.16296	-1.16031

-1.02604	-1.02361	-1.05924	-1.05668	-1.12919	-1.12655	-1.16617	-1.16351	-1.16087
-1.02653	-1.02409	-1.05974	-1.05718	-1.12973	-1.12708	-1.16671	-1.16406	-1.16143
-1.027	-1.02455	-1.06023	-1.05768	-1.13028	-1.12761	-1.16726	-1.16461	-1.16198
-1.02746	-1.02502	-1.06074	-1.05822	-1.05571	-1.05321	-1.16785	-1.16516	-1.16252
-1.02793	-1.02548	-1.06126	-1.05878	-1.05629	-1.05376	-1.16845	-1.16573	-1.16306
-1.02842	-1.02595	-1.06175	-1.0593	-1.05682	-1.05431	-1.16911	-1.16635	-1.16364
-1.02893	-1.02641	-1.06223	-1.05977	-1.05732	-1.05487	-1.1698	-1.16702	-1.16426
-1.02947	-1.02691	-1.06272	-1.06025	-1.05781	-1.05539	-1.05297	-1.0505	-1.04804
-1.03007	-1.02746	-1.06325	-1.06074	-1.05827	-1.05586	-1.05346	-1.05103	-1.0486
-1.03065	-1.02802	-1.06381	-1.06125	-1.05875	-1.05633	-1.05393	-1.05154	-1.04915
-1.0312	-1.02862	-1.06442	-1.06181	-1.05927	-1.05682	-1.0544	-1.05201	-1.04963
-1.03174	-1.02921	-1.06504	-1.0624	-1.05983	-1.05734	-1.05489	-1.05248	-1.0501
-1.03226	-1.02973	-1.06561	-1.06299	-1.06043	-1.05793	-1.05548	-1.05303	-1.05061
-1.03277	-1.03021	-1.06613	-1.06357	-1.06105	-1.05857	-1.05611	-1.05362	-1.05114
-0.99281	-0.9903	-1.02819	-1.02572	-1.06159	-1.05913	-1.05667	-1.05418	-1.05169
-0.99337	-0.99082	-1.0287	-1.02618	-1.06206	-1.0596	-1.05715	-1.05471	-1.05227
-0.99392	-0.99136	-1.02924	-1.02671	-1.06258	-1.0601	-1.05765	-1.05523	-1.05281
-0.99444	-0.99194	-1.02987	-1.02734	-1.06323	-1.06068	-1.05818	-1.05573	-1.0533
-0.99495	-0.99249	-1.03048	-1.02797	-1.06387	-1.06128	-1.05873	-1.05625	-1.05381
-0.99545	-0.99298	-0.99053	-0.98811	-1.06443	-1.06186	-1.05933	-1.05685	-1.05441
-0.99597	-0.99346	-0.99099	-0.98857	-1.06495	-1.06243	-1.05993	-1.05747	-1.05503
-0.99656	-0.99403	-0.99154	-0.98908	-1.06549	-1.06295	-1.06046	-1.058	-1.05557
-0.99718	-0.99467	-0.99214	-0.98962	-1.06603	-1.06345	-1.06093	-1.05848	-1.05606
-0.99776	-0.99524	-0.9927	-0.99017	-1.06661	-1.06402	-1.06148	-1.05903	-1.05661
-0.99827	-0.99573	-0.99321	-0.99072	-1.06725	-1.06466	-1.06212	-1.05966	-1.05723
-0.99881	-0.99625	-0.99375	-0.9913	-0.98886	-0.98643	-1.06273	-1.06026	-1.05782
-0.99942	-0.99686	-0.99436	-0.99191	-0.98945	-0.98697	-1.06328	-1.06077	-1.0583
-1.00004	-0.99749	-0.99497	-0.9925	-0.99002	-0.9875	-1.06382	-1.06127	-1.05878
-1.00064	-0.99805	-0.99551	-0.99302	-0.99053	-0.98803	-1.06443	-1.06187	-1.05937
-1.00122	-0.99859	-0.99602	-0.9935	-0.99102	-0.98857	-1.06506	-1.06251	-1.06
-1.00178	-0.99916	-0.99659	-0.99406	-0.99158	-0.98916	-1.0657	-1.06312	-1.06058
-1.00232	-0.99975	-0.99721	-0.99469	-0.99221	-0.98978	-1.06635	-1.06372	-1.06111
-1.0029	-1.00036	-0.99782	-0.99529	-0.99279	-0.99034	-1.06695	-1.06429	-1.06167
-1.00354	-1.00101	-0.99844	-0.99585	-0.9933	-0.99081	-1.06746	-1.06483	-1.06225
-1.00416	-1.00162	-0.99902	-0.9964	-0.99382	-0.99131	-0.98884	-0.98642	-0.98403
-1.00468	-1.00211	-0.99953	-0.99695	-0.9944	-0.9919	-0.98944	-0.98701	-0.98462
-1.00519	-1.0026	-1.00004	-0.99751	-0.99501	-0.99253	-0.99006	-0.98762	-0.98521
-0.78204	-0.78022	-1.00064	-0.99813	-0.99563	-0.99316	-0.99069	-0.98821	-0.98575
-0.78248	-0.78067	-1.00129	-0.99876	-0.99626	-0.99379	-0.99131	-0.98878	-0.98627
-0.78292	-0.78111	-1.00191	-0.99936	-0.99683	-0.99435	-0.99186	-0.98932	-0.98681
-0.78337	-0.78156	-1.00253	-0.99992	-0.99735	-0.99482	-0.99232	-0.98983	-0.98737
-0.78381	-0.78197	-1.00312	-1.00048	-0.99788	-0.99534	-0.99283	-0.99037	-0.98795
-0.78422	-0.78234	-1.00366	-1.00104	-0.99847	-0.99594	-0.99344	-0.99098	-0.98855
-0.78461	-0.78271	-1.00421	-1.00162	-0.99907	-0.99656	-0.99407	-0.9916	-0.98916
-0.73719	-0.73544	-0.82744	-0.82553	-0.99969	-0.99718	-0.99469	-0.99222	-0.98977
-0.73751	-0.7358	-0.82789	-0.82598	-1.00031	-0.9978	-0.99532	-0.99284	-0.99038
-0.7379	-0.73619	-0.82834	-0.82644	-1.00093	-0.99843	-0.99594	-0.99346	-0.991
-0.73831	-0.7366	-0.8288	-0.82689	-1.00155	-0.99904	-0.99656	-0.99409	-0.99163
-0.73873	-0.737	-0.73531	-0.73364	-1.00217	-0.99967	-0.99719	-0.99471	-0.99225
-0.73914	-0.73742	-0.73571	-0.73404	-1.0028	-1.0003	-0.99782	-0.99534	-0.99287

-0.73956	-0.73783	-0.73612	-0.73444	-0.96616	-0.96374	-0.99845	-0.99597	-0.99348
-0.73997	-0.73824	-0.73653	-0.73485	-0.96677	-0.96434	-0.99907	-0.99658	-0.9941
-0.74038	-0.73865	-0.73694	-0.73525	-0.96738	-0.96494	-0.99968	-0.99719	-0.99469
-0.69661	-0.69498	-0.73736	-0.73566	-0.78111	-0.77934	-1.00029	-0.99776	-0.99523
-0.697	-0.69538	-0.73777	-0.73606	-0.78153	-0.77976	-1.00089	-0.99831	-0.99575
-0.6974	-0.69577	-0.73818	-0.73646	-0.78196	-0.78018	-1.0015	-0.99891	-0.99633
-0.69779	-0.69616	-0.73859	-0.73687	-0.78238	-0.7806	-1.00212	-0.99953	-0.99696
-0.6518	-0.65024	-0.739	-0.73727	-0.78281	-0.78103	-0.96544	-0.96295	-0.96046
-0.65218	-0.65062	-0.73941	-0.73768	-0.78325	-0.78146	-0.96605	-0.96354	-0.96104
-0.65257	-0.651	-0.64944	-0.64789	-0.73639	-0.73471	-0.78009	-0.77827	-0.77646
-0.65294	-0.65138	-0.64982	-0.64827	-0.7368	-0.73511	-0.78052	-0.77872	-0.77693
-0.65332	-0.65176	-0.6502	-0.64865	-0.73721	-0.73551	-0.78094	-0.77917	-0.7774
-0.6537	-0.65214	-0.65059	-0.64904	-0.69241	-0.69078	-0.78135	-0.77958	-0.77783
-0.65408	-0.65253	-0.65097	-0.64942	-0.69281	-0.69117	-0.78176	-0.77998	-0.77823
-0.65446	-0.65291	-0.65135	-0.6498	-0.69321	-0.69156	-0.68994	-0.68836	-0.68681
-0.65484	-0.65329	-0.65173	-0.65017	-0.69361	-0.69196	-0.69034	-0.68875	-0.68719
-0.65522	-0.65367	-0.65211	-0.65055	-0.69402	-0.69237	-0.69074	-0.68915	-0.68758
-0.6556	-0.65404	-0.65248	-0.65092	-0.69442	-0.69277	-0.69115	-0.68954	-0.68796
-0.65597	-0.65442	-0.65286	-0.6513	-0.69482	-0.69318	-0.69155	-0.68993	-0.68833
-0.65635	-0.65479	-0.65323	-0.65168	-0.65013	-0.64858	-0.69195	-0.69032	-0.68871
-0.65673	-0.65517	-0.65361	-0.65206	-0.65051	-0.64895	-0.69234	-0.69071	-0.68909
-0.65711	-0.65554	-0.65398	-0.65243	-0.65088	-0.64933	-0.69274	-0.6911	-0.68948
-0.6575	-0.65593	-0.65436	-0.65281	-0.65126	-0.64971	-0.69315	-0.69151	-0.68988
-0.65794	-0.65636	-0.65479	-0.65324	-0.65169	-0.65014	-0.69361	-0.69197	-0.69034
-0.65844	-0.65684	-0.65527	-0.65372	-0.65218	-0.65063	-0.69414	-0.6925	-0.69087
-0.65891	-0.65731	-0.65573	-0.65418	-0.65264	-0.65109	-0.64954	-0.648	-0.64646
-0.65933	-0.65771	-0.65612	-0.65457	-0.65302	-0.65148	-0.64994	-0.64839	-0.64685
-0.65974	-0.65809	-0.65649	-0.65494	-0.65339	-0.65185	-0.65031	-0.64876	-0.64722
-0.6054	-0.60392	-0.65688	-0.65532	-0.65376	-0.65222	-0.65068	-0.64914	-0.64759
-0.60576	-0.60427	-0.65727	-0.6557	-0.65414	-0.6526	-0.65106	-0.64951	-0.64798
-0.60613	-0.60462	-0.65765	-0.65608	-0.65452	-0.65297	-0.65143	-0.64991	-0.64839
-0.60649	-0.60498	-0.65805	-0.65646	-0.65489	-0.65334	-0.65181	-0.65032	-0.64883
-0.55524	-0.55385	-0.65849	-0.65689	-0.65532	-0.65376	-0.65223	-0.65074	-0.64927
-0.55567	-0.55428	-0.65901	-0.65739	-0.6558	-0.65424	-0.65271	-0.65119	-0.64969
-0.44963	-0.44853	-0.65951	-0.65787	-0.65627	-0.65471	-0.65317	-0.65163	-0.65009
-0.44992	-0.44882	-0.65994	-0.65829	-0.65667	-0.65511	-0.65356	-0.65201	-0.65047
-0.4502	-0.4491	-0.66038	-0.6587	-0.65708	-0.6555	-0.65395	-0.65239	-0.65085
-0.45052	-0.44943	-0.60601	-0.60451	-0.65755	-0.65596	-0.6544	-0.65285	-0.65131
-0.45086	-0.44978	-0.60648	-0.60496	-0.65804	-0.65645	-0.65489	-0.65333	-0.65179
-0.45116	-0.45009	-0.50281	-0.50157	-0.65849	-0.65689	-0.65531	-0.65376	-0.65222
-0.45145	-0.45036	-0.50311	-0.50187	-0.65889	-0.65727	-0.65569	-0.65413	-0.65259
-0.45172	-0.45063	-0.50342	-0.50219	-0.60688	-0.60539	-0.6561	-0.65454	-0.653
-0.452	-0.4509	-0.50373	-0.50253	-0.60734	-0.60585	-0.6566	-0.65503	-0.65348
-0.45229	-0.45119	-0.50407	-0.50288	-0.5017	-0.50053	-0.65709	-0.65551	-0.65395
-0.45263	-0.45153	-0.50444	-0.50323	-0.50203	-0.50084	-0.65749	-0.6559	-0.65434
-0.45299	-0.45188	-0.50482	-0.50358	-0.50236	-0.50114	-0.65788	-0.65628	-0.65471
-0.4533	-0.45221	-0.50519	-0.50396	-0.50273	-0.50149	-0.60596	-0.60449	-0.60305
-0.45359	-0.45252	-0.50556	-0.50434	-0.50312	-0.50188	-0.60642	-0.60493	-0.60349
-0.4539	-0.45283	-0.50591	-0.50469	-0.50346	-0.50222	-0.501	-0.49981	-0.49865
-0.45424	-0.45313	-0.50623	-0.50499	-0.50376	-0.50252	-0.5013	-0.5001	-0.49893

-0.39983	-0.39885	-0.45234	-0.45123	-0.50408	-0.50284	-0.50163	-0.50042	-0.49924
-0.40012	-0.39915	-0.45269	-0.45158	-0.50446	-0.50323	-0.502	-0.50079	-0.4996
-0.40041	-0.39945	-0.45304	-0.45192	-0.50485	-0.50361	-0.50239	-0.50116	-0.49996
-0.40067	-0.3997	-0.45333	-0.45221	-0.50518	-0.50396	-0.50274	-0.50153	-0.50033
-0.40092	-0.39994	-0.45361	-0.45248	-0.50549	-0.50429	-0.50309	-0.50189	-0.5007
-0.29008	-0.28938	-0.39924	-0.39825	-0.50585	-0.50464	-0.50343	-0.50222	-0.50103
-0.29029	-0.28959	-0.39954	-0.39855	-0.50624	-0.505	-0.50376	-0.50254	-0.50133
-0.29049	-0.28979	-0.34649	-0.34563	-0.50664	-0.50537	-0.50412	-0.50288	-0.50166
-0.29069	-0.29	-0.34677	-0.3459	-0.50705	-0.50577	-0.50451	-0.50327	-0.50204
-0.23728	-0.23669	-0.34703	-0.34616	-0.40189	-0.40088	-0.5049	-0.50366	-0.50243
-0.23745	-0.23686	-0.34725	-0.34641	-0.40219	-0.40119	-0.50532	-0.50407	-0.50282
-0.23763	-0.23703	-0.34747	-0.34664	-0.40248	-0.4015	-0.50572	-0.50447	-0.50322
-0.23782	-0.23722	-0.29004	-0.28937	-0.40277	-0.40179	-0.45528	-0.45415	-0.45301
-0.23803	-0.23742	-0.29024	-0.28956	-0.40305	-0.40207	-0.45562	-0.4545	-0.45338
-0.23824	-0.23763	-0.23703	-0.23643	-0.40335	-0.40236	-0.40139	-0.40042	-0.39946
-0.23845	-0.23784	-0.23723	-0.23663	-0.40366	-0.40265	-0.40167	-0.4007	-0.39975
-0.23866	-0.23805	-0.23744	-0.23684	-0.28947	-0.28878	-0.40196	-0.40099	-0.40003
-0.23886	-0.23826	-0.23765	-0.23705	-0.28968	-0.28899	-0.40227	-0.40128	-0.4003
-0.23907	-0.23847	-0.23786	-0.23725	-0.23665	-0.23606	-0.40259	-0.40159	-0.40059
-0.23926	-0.23866	-0.23806	-0.23746	-0.23686	-0.23626	-0.40291	-0.4019	-0.4009
-0.23945	-0.23886	-0.23827	-0.23766	-0.23706	-0.23647	-0.40322	-0.40222	-0.40122
-0.23965	-0.23907	-0.23847	-0.23787	-0.23727	-0.23667	-0.34719	-0.34636	-0.34552
-0.23986	-0.23928	-0.23868	-0.23808	-0.23748	-0.23688	-0.34745	-0.34661	-0.34578
-0.18356	-0.1831	-0.23888	-0.23828	-0.23768	-0.23708	-0.29584	-0.29509	-0.29434
-0.18371	-0.18324	-0.23907	-0.23848	-0.23788	-0.23729	-0.29609	-0.29534	-0.2946
-0.18386	-0.18338	-0.23926	-0.23868	-0.23809	-0.23749	-0.29635	-0.2956	-0.29485
-0.18402	-0.18354	-0.23947	-0.23889	-0.2383	-0.2377	-0.29661	-0.29585	-0.2951
-0.18418	-0.1837	-0.23969	-0.2391	-0.23851	-0.2379	-0.29686	-0.2961	-0.29535
-0.18433	-0.18386	-0.2399	-0.23932	-0.23872	-0.23811	-0.29712	-0.29636	-0.2956
-0.18449	-0.18402	-0.24011	-0.23953	-0.23894	-0.23833	-0.29738	-0.29662	-0.29585
-0.12759	-0.12725	-0.18371	-0.18325	-0.23915	-0.23854	-0.29764	-0.29687	-0.29611
-0.12771	-0.12737	-0.18386	-0.18341	-0.23936	-0.23875	-0.29791	-0.29713	-0.29636
-0.12782	-0.12748	-0.18402	-0.18357	-0.23957	-0.23896	-0.29817	-0.29739	-0.29662
-0.12794	-0.1276	-0.18417	-0.18372	-0.23978	-0.23918	-0.29843	-0.29765	-0.29687
-0.12806	-0.12772	-0.12738	-0.12706	-0.18342	-0.18295	-0.23878	-0.23815	-0.23753
-0.12818	-0.12783	-0.1275	-0.12717	-0.18357	-0.18311	-0.23899	-0.23837	-0.23774
-0.12829	-0.12795	-0.12762	-0.12729	-0.18373	-0.18327	-0.2392	-0.23858	-0.23796
-0.12842	-0.12808	-0.12775	-0.12742	-0.18389	-0.18342	-0.23941	-0.23879	-0.23817
-0.12854	-0.12821	-0.12788	-0.12754	-0.18406	-0.18358	-0.23962	-0.239	-0.23839
-0.12866	-0.12834	-0.128	-0.12766	-0.12732	-0.12698	-0.18326	-0.18279	-0.18231
-0.12878	-0.12845	-0.12812	-0.12778	-0.12744	-0.12709	-0.18342	-0.18295	-0.18249
-0.06097	-0.06083	-0.12823	-0.12789	-0.12755	-0.12721	-0.18358	-0.18312	-0.18266
-0.06103	-0.06089	-0.12835	-0.12801	-0.12767	-0.12732	-0.18373	-0.18327	-0.18281
-0.06109	-0.06095	-0.12848	-0.12813	-0.12779	-0.12744	-0.12711	-0.12677	-0.12644
-0.06114	-0.06101	-0.1286	-0.12825	-0.12791	-0.12757	-0.12723	-0.1269	-0.12657
-0.0612	-0.06106	-0.12871	-0.12837	-0.12803	-0.1277	-0.12737	-0.12703	-0.12669
-0.06125	-0.06111	-0.12884	-0.12849	-0.12816	-0.12783	-0.12749	-0.12715	-0.12681
-0.06131	-0.06117	-0.12896	-0.12862	-0.12829	-0.12795	-0.12762	-0.12727	-0.12693
-0.06136	-0.06122	-0.06108	-0.06095	-0.12841	-0.12808	-0.12774	-0.12739	-0.12705
-0.06141	-0.06127	-0.06113	-0.061	-0.12853	-0.12819	-0.12785	-0.12752	-0.12719

-0.06147	-0.06132	-0.06119	-0.06106	-0.12865	-0.12831	-0.12797	-0.12764	-0.12731
-0.06152	-0.06138	-0.06124	-0.06111	-0.12878	-0.12843	-0.12809	-0.12776	-0.12743
-0.06158	-0.06144	-0.0613	-0.06117	-0.1289	-0.12856	-0.12821	-0.12788	-0.12755
-0.06164	-0.0615	-0.06136	-0.06122	-0.12903	-0.12868	-0.12834	-0.12801	-0.12767
-0.0617	-0.06156	-0.06141	-0.06127	-0.12915	-0.12881	-0.12848	-0.12814	-0.12781
-0.06176	-0.06161	-0.06147	-0.06133	-0.12928	-0.12894	-0.12861	-0.12827	-0.12794
-0.06183	-0.06167	-0.06152	-0.06138	-0.12942	-0.12908	-0.12874	-0.1284	-0.12807
-0.06189	-0.06173	-0.06158	-0.06144	-0.0613	-0.06117	-0.12887	-0.12853	-0.1282
-0.06195	-0.06179	-0.06164	-0.0615	-0.06136	-0.06123	-0.129	-0.12866	-0.12833
-0.06201	-0.06185	-0.0617	-0.06156	-0.06142	-0.06129	-0.12914	-0.12879	-0.12845
-0.06207	-0.06192	-0.06177	-0.06162	-0.06148	-0.06134	-0.12926	-0.12892	-0.12857
-0.06214	-0.06198	-0.06183	-0.06168	-0.06153	-0.0614	-0.12939	-0.12904	-0.12869
-0.0622	-0.06205	-0.0619	-0.06174	-0.06159	-0.06145	-0.12951	-0.12916	-0.12882
-0.06227	-0.06212	-0.06196	-0.0618	-0.06165	-0.0615	-0.12963	-0.12929	-0.12895
-0.06233	-0.06218	-0.06202	-0.06187	-0.06171	-0.06156	-0.06142	-0.06128	-0.06116
-0.06239	-0.06225	-0.06209	-0.06193	-0.06177	-0.06162	-0.06148	-0.06134	-0.06121
-0.06246	-0.06231	-0.06215	-0.06199	-0.06183	-0.06168	-0.06154	-0.0614	-0.06127
-0.06251	-0.06236	-0.06221	-0.06205	-0.0619	-0.06174	-0.0616	-0.06146	-0.06132
-0.06257	-0.06242	-0.06227	-0.06212	-0.06196	-0.06181	-0.06166	-0.06151	-0.06138
-0.06263	-0.06248	-0.06234	-0.06218	-0.06203	-0.06187	-0.06172	-0.06157	-0.06143
-0.06269	-0.06255	-0.0624	-0.06225	-0.06209	-0.06193	-0.06178	-0.06163	-0.06149
-0.06275	-0.06261	-0.06247	-0.06231	-0.06216	-0.062	-0.06184	-0.06169	-0.06155
-0.06281	-0.06267	-0.06253	-0.06238	-0.06223	-0.06207	-0.06191	-0.06176	-0.06161
-0.06287	-0.06273	-0.06259	-0.06245	-0.0623	-0.06214	-0.06198	-0.06182	-0.06167
-0.06292	-0.06279	-0.06265	-0.06251	-0.06236	-0.06221	-0.06205	-0.06189	-0.06174
-0.06298	-0.06284	-0.0627	-0.06256	-0.06242	-0.06227	-0.06212	-0.06196	-0.06181
-0.06304	-0.0629	-0.06276	-0.06262	-0.06248	-0.06234	-0.06218	-0.06203	-0.06187
-0.06311	-0.06296	-0.06282	-0.06268	-0.06254	-0.0624	-0.06225	-0.06209	-0.06194
-0.06317	-0.06302	-0.06288	-0.06274	-0.0626	-0.06246	-0.06231	-0.06216	-0.062
-0.06323	-0.06308	-0.06294	-0.0628	-0.06266	-0.06252	-0.06237	-0.06222	-0.06207
-0.06329	-0.06314	-0.063	-0.06286	-0.06272	-0.06258	-0.06243	-0.06228	-0.06213
-0.06336	-0.0632	-0.06306	-0.06292	-0.06278	-0.06264	-0.0625	-0.06235	-0.0622
-0.06342	-0.06327	-0.06312	-0.06298	-0.06284	-0.0627	-0.06256	-0.06241	-0.06226
-0.0635	-0.06334	-0.06319	-0.06304	-0.0629	-0.06276	-0.06262	-0.06248	-0.06233
		-0.06325	-0.06311	-0.06296	-0.06282	-0.06268	-0.06254	-0.0624
		-0.06332	-0.06317	-0.06302	-0.06288	-0.06274	-0.0626	-0.06246
		-0.06338	-0.06323	-0.06308	-0.06294	-0.0628	-0.06266	-0.06252
		-0.06345	-0.0633	-0.06315	-0.063	-0.06286	-0.06272	-0.06258
		-0.06353	-0.06337	-0.06321	-0.06307	-0.06292	-0.06279	-0.06265
				-0.06328	-0.06313	-0.06299	-0.06285	-0.06271
				-0.06334	-0.06319	-0.06304	-0.06291	-0.06277
				-0.06341	-0.06325	-0.06311	-0.06297	-0.06283
				-0.06348	-0.06332	-0.06317	-0.06303	-0.06289
						-0.06324	-0.06309	-0.06295
						-0.06331	-0.06316	-0.06301
						-0.06338	-0.06323	-0.06308
						-0.06344	-0.06329	-0.06314
						-0.06351	-0.06335	-0.0632

							-0.06247	-0.06228
							-0.06239	-0.06221
							-0.06231	-0.06213
							-0.06223	-0.06204
							-0.06214	-0.06195
				-0.06266	-0.06245	-0.06226	-0.25776	-0.25706
				-0.06257	-0.06237	-0.06218	-0.25745	-0.25677
				-0.06249	-0.0623	-0.06211	-0.25716	-0.25648
				-0.06241	-0.06222	-0.06203	-0.25688	-0.2562
		-0.06272	-0.06253	-0.25864	-0.25797	-0.25729	-0.37893	-0.37791
		-0.06265	-0.06245	-0.25836	-0.25768	-0.257	-0.3785	-0.37747
		-0.06257	-0.06238	-0.38112	-0.38011	-0.37909	-0.49486	-0.49353
		-0.06249	-0.0623	-0.3807	-0.37968	-0.37865	-0.49427	-0.49292
		-0.06242	-0.06223	-0.38027	-0.37924	-0.3782	-0.49367	-0.49232
-0.06273	-0.06254	-0.25859	-0.25791	-0.37983	-0.37878	-0.37773	-0.57873	-0.57725
-0.06266	-0.06246	-0.25831	-0.25761	-0.37938	-0.37831	-0.37725	-0.57808	-0.57662
-0.06258	-0.06239	-0.38103	-0.38	-0.49595	-0.49456	-0.49319	-0.68973	-0.68798
-0.0625	-0.06231	-0.38059	-0.37956	-0.49539	-0.49401	-0.49264	-0.68902	-0.68727
-0.06243	-0.06223	-0.38015	-0.37912	-0.58079	-0.57927	-0.57776	-0.68831	-0.68657
-0.06235	-0.06215	-0.3797	-0.37865	-0.58012	-0.57862	-0.57714	-0.68757	-0.68586
-0.38131	-0.38029	-0.37925	-0.37819	-0.57947	-0.57799	-0.57653	-0.68684	-0.68514
-0.38088	-0.37986	-0.37882	-0.37776	-0.57886	-0.57739	-0.57594	-0.68613	-0.68443
-0.38045	-0.37943	-0.37839	-0.37734	-0.57827	-0.57681	-0.57536	-0.68542	-0.68372
-0.38002	-0.379	-0.58069	-0.57917	-0.68993	-0.68817	-0.68643	-0.68472	-0.68301
-0.37959	-0.37858	-0.58009	-0.57858	-0.68922	-0.68746	-0.68572	-0.68401	-0.68231
-0.37917	-0.37815	-0.57949	-0.57799	-0.6885	-0.68674	-0.68501	-0.6833	-0.6816
-0.37874	-0.37773	-0.57889	-0.5774	-0.68778	-0.68602	-0.68429	-0.68259	-0.6809
-0.5226	-0.52131	-0.69065	-0.68885	-0.68706	-0.68531	-0.68358	-0.68188	-0.68019
-0.52209	-0.5208	-0.68993	-0.68813	-0.68635	-0.6846	-0.68286	-0.68117	-0.67949
-0.52158	-0.52029	-0.68922	-0.68742	-0.68564	-0.68388	-0.68215	-0.7323	-0.73049
-0.52106	-0.51979	-0.68851	-0.6867	-0.68492	-0.68317	-0.68144	-0.73153	-0.72973

-0.52055	-0.51928	-0.6878	-0.68599	-0.6842	-0.68245	-0.68073	-0.73077	-0.72897
-0.69056	-0.68884	-0.68708	-0.68527	-0.68348	-0.68174	-0.68002	-0.73	-0.72822
-0.68984	-0.68812	-0.68636	-0.68455	-0.68277	-0.68103	-0.67932	-0.72925	-0.72747
-0.68912	-0.6874	-0.68564	-0.68384	-0.73402	-0.73213	-0.73029	-0.72849	-0.72673
-0.68842	-0.68667	-0.68492	-0.68315	-0.73327	-0.73139	-0.72953	-0.72774	-0.72599
-0.68771	-0.68596	-0.68421	-0.68246	-0.73255	-0.73066	-0.7288	-0.72701	-0.72525
-0.68701	-0.68525	-0.68351	-0.6818	-0.73185	-0.72997	-0.72811	-0.7263	-0.72452
-0.68631	-0.68455	-0.68283	-0.68114	-0.73114	-0.72927	-0.72741	-0.72559	-0.7238
-0.68565	-0.68389	-0.68216	-0.68046	-0.73041	-0.72854	-0.72668	-0.72486	-0.72306
-0.685	-0.68324	-0.6815	-0.67978	-0.72965	-0.72779	-0.72594	-0.72412	-0.72232
-0.68432	-0.68256	-0.73262	-0.73076	-0.7289	-0.72705	-0.72521	-0.72339	-0.72158
-0.68362	-0.68187	-0.73187	-0.73	-0.72814	-0.72631	-0.72448	-0.72266	-0.72085
-0.68293	-0.6812	-0.73115	-0.72927	-0.72741	-0.72559	-0.72377	-0.77028	-0.76831
-0.68226	-0.68056	-0.73046	-0.72858	-0.72672	-0.7249	-0.72309	-0.76953	-0.76755
-0.73343	-0.73161	-0.72976	-0.72788	-0.72602	-0.7242	-0.72239	-0.76878	-0.76679
-0.7327	-0.73087	-0.72901	-0.72714	-0.72529	-0.72346	-0.72165	-0.76798	-0.76602
-0.73197	-0.73011	-0.72826	-0.72641	-0.72456	-0.72273	-0.72091	-0.76719	-0.76525
-0.73124	-0.72936	-0.72751	-0.72569	-0.72387	-0.72203	-0.72021	-0.76644	-0.76452
-0.7305	-0.72861	-0.72677	-0.72498	-0.72318	-0.72135	-0.71953	-0.76571	-0.7638
-0.72977	-0.72788	-0.72604	-0.72424	-0.72245	-0.72064	-0.71883	-0.76495	-0.76304
-0.72906	-0.72718	-0.72533	-0.7235	-0.72169	-0.71989	-0.71811	-0.76418	-0.76225
-0.72836	-0.72648	-0.72463	-0.72278	-0.7692	-0.76727	-0.76535	-0.86817	-0.86595
-0.72766	-0.72579	-0.72394	-0.72211	-0.76846	-0.76654	-0.76463	-0.86735	-0.86514
-0.72696	-0.72509	-0.72325	-0.72144	-0.76774	-0.76582	-0.7639	-0.95788	-0.95538
-0.72626	-0.7244	-0.72256	-0.72076	-0.76703	-0.7651	-0.76318	-0.95696	-0.95447
-0.72555	-0.7237	-0.72187	-0.72008	-0.7663	-0.76437	-0.76244	-0.95603	-0.95355
-0.72481	-0.72298	-0.72116	-0.71937	-0.76554	-0.76361	-0.76169	-0.95506	-0.95258
-0.72406	-0.72225	-0.72044	-0.71864	-0.76474	-0.76283	-0.76093	-0.95407	-0.95157
-0.72335	-0.72155	-0.76785	-0.76591	-0.86715	-0.86489	-0.86264	-0.95311	-0.95063
-0.72268	-0.72087	-0.76712	-0.76519	-0.86632	-0.86405	-0.86179	-0.9522	-0.94974
-0.72201	-0.7202	-0.7664	-0.76447	-0.8655	-0.86322	-0.86095	-0.99754	-0.99495
-0.72133	-0.71953	-0.76568	-0.76375	-0.86465	-0.86238	-0.86012	-0.99659	-0.994
-0.72066	-0.71887	-0.76496	-0.76304	-0.86381	-0.86155	-0.8593	-0.99564	-0.99306
-0.71999	-0.7182	-0.86755	-0.86526	-0.86298	-0.86071	-0.85846	-0.99469	-0.99212
-0.71933	-0.71753	-0.86671	-0.86443	-0.86216	-0.85988	-0.85762	-0.99373	-0.99119
-0.71866	-0.71687	-0.86588	-0.8636	-0.95419	-0.9517	-0.94924	-1.12907	-1.12591
-0.71799	-0.71621	-0.86505	-0.86276	-0.95327	-0.9508	-0.94836	-1.12789	-1.12474
-0.86877	-0.8665	-0.86421	-0.86192	-0.95236	-0.94991	-0.94747	-1.12672	-1.12358
-0.86793	-0.86565	-0.86337	-0.86108	-0.95146	-0.94901	-0.94657	-1.12555	-1.12244
-0.8671	-0.86481	-0.86252	-0.86025	-1.08943	-1.08631	-1.08323	-1.12439	-1.12131
-0.86631	-0.86399	-0.86169	-0.85941	-1.08824	-1.08515	-1.0821	-1.12323	-1.12016
-0.86553	-0.86318	-0.86086	-0.85858	-1.08706	-1.08399	-1.08098	-1.12208	-1.11901
-0.86473	-0.86239	-0.86008	-0.85778	-1.08591	-1.08286	-1.07986	-1.16395	-1.16079
-0.86392	-0.86162	-0.85932	-0.857	-1.08479	-1.08173	-1.07874	-1.16278	-1.15963
-0.8631	-0.86082	-0.85853	-0.85623	-1.08371	-1.08065	-1.07764	-1.1616	-1.15846
-0.86227	-0.85999	-0.85772	-0.85548	-1.08268	-1.07959	-1.07655	-1.16041	-1.15728
-0.86145	-0.85916	-1.04765	-1.04464	-1.08163	-1.07855	-1.07551	-1.15927	-1.15611
-0.86063	-0.85835	-1.04653	-1.04355	-1.08053	-1.07752	-1.07453	-1.15818	-1.15496
-0.85983	-0.85756	-1.04545	-1.04247	-1.12333	-1.12026	-1.11719	-1.20392	-1.2003
-0.85905	-0.8568	-1.04443	-1.04143	-1.12219	-1.11913	-1.11609	-1.20271	-1.19912

-0.85828	-0.85606	-1.04342	-1.0404	-1.12106	-1.11799	-1.11496	-1.20149	-1.19796
-0.95504	-0.95229	-1.08233	-1.07926	-1.11999	-1.1169	-1.11384	-1.20023	-1.19674
-0.95399	-0.95125	-1.08121	-1.07823	-1.11896	-1.11583	-1.11272	-1.19894	-1.1955
-0.953	-0.95025	-1.0801	-1.07716	-1.16108	-1.15784	-1.15462	-1.1977	-1.19426
-0.95207	-0.9493	-1.079	-1.07607	-1.15993	-1.15675	-1.15358	-1.19652	-1.19302
-1.0441	-1.04106	-1.07793	-1.07499	-1.15877	-1.15564	-1.15251	-1.2294	-1.22566
-1.04305	-1.04008	-1.07692	-1.07393	-1.15761	-1.1545	-1.1514	-1.22815	-1.22446
-1.04201	-1.03911	-1.07593	-1.07291	-1.15648	-1.15338	-1.1503	-1.22692	-1.22329
-1.04103	-1.03816	-1.11865	-1.1155	-1.20212	-1.19867	-1.19527	-1.26756	-1.2638
-1.04006	-1.03722	-1.11764	-1.1145	-1.20097	-1.19751	-1.1941	-1.2663	-1.2626
-1.03905	-1.03623	-1.11659	-1.11348	-1.23417	-1.23045	-1.22681	-1.26498	-1.26132
-1.03802	-1.03521	-1.11551	-1.11244	-1.23292	-1.22917	-1.2255	-1.2636	-1.26
-1.03702	-1.03422	-1.11446	-1.11143	-1.2317	-1.22795	-1.22426	-1.26228	-1.25868
-1.03608	-1.03327	-1.11344	-1.11043	-1.23053	-1.22678	-1.22308	-1.26101	-1.25736
-1.07956	-1.07662	-1.19514	-1.19157	-1.27132	-1.26742	-1.26356	-1.25979	-1.25609
-1.07854	-1.07556	-1.19383	-1.1903	-1.27001	-1.26617	-1.26237	-1.25862	-1.25494
-1.07753	-1.07451	-1.19253	-1.18903	-1.26869	-1.26492	-1.26119	-1.25748	-1.25382
-1.07655	-1.07352	-1.1913	-1.1878	-1.26739	-1.26367	-1.25998	-1.25631	-1.25269
-1.07558	-1.07255	-1.19011	-1.18661	-1.2661	-1.26242	-1.25878	-1.25514	-1.25155
-1.14941	-1.14603	-1.2317	-1.22803	-1.26487	-1.26122	-1.2576	-1.25399	-1.25043
-1.14832	-1.14496	-1.23049	-1.22685	-1.26368	-1.26004	-1.25645	-1.25286	-1.24932
-1.14724	-1.14388	-1.22928	-1.22567	-1.2625	-1.25888	-1.2553	-1.32747	-1.32367
-1.14615	-1.14279	-1.22806	-1.22448	-1.2613	-1.25771	-1.25416	-1.32627	-1.32253
-1.23441	-1.23056	-1.22686	-1.2233	-1.26012	-1.25655	-1.25304	-1.32509	-1.3214
-1.23317	-1.22935	-1.22567	-1.22213	-1.25895	-1.25541	-1.25193	-1.32391	-1.32026
-1.23195	-1.22815	-1.22449	-1.22097	-1.25779	-1.25428	-1.25083	-1.32274	-1.31913
-1.23074	-1.22697	-1.22333	-1.21983	-1.29375	-1.29019	-1.28669	-1.35775	-1.35409
-1.22954	-1.2258	-1.22218	-1.21871	-1.29258	-1.28906	-1.28559	-1.35658	-1.35295
-1.22834	-1.22463	-1.22104	-1.21758	-1.29142	-1.28793	-1.2845	-1.35543	-1.35182
-1.22714	-1.22346	-1.2199	-1.21646	-1.29025	-1.28681	-1.28342	-1.3543	-1.35073
-1.22597	-1.22234	-1.21881	-1.21538	-1.28913	-1.28571	-1.28235	-1.35318	-1.34963
-1.22485	-1.22128	-1.21778	-1.21437	-1.28806	-1.28464	-1.28127	-1.35204	-1.34853
-1.22375	-1.22023	-1.21678	-1.21338	-1.28701	-1.28357	-1.28021	-1.35091	-1.34744
-1.22271	-1.21921	-1.25347	-1.25	-1.32277	-1.31926	-1.31582	-1.3498	-1.34636
-1.22169	-1.21819	-1.25242	-1.24896	-1.32168	-1.31818	-1.31476	-1.3487	-1.34529
-1.22064	-1.21714	-1.25135	-1.24791	-1.32059	-1.31711	-1.31371	-1.34763	-1.34424
-1.21956	-1.21607	-1.25026	-1.24684	-1.31948	-1.31604	-1.31267	-1.34656	-1.3432
-1.21847	-1.21499	-1.28861	-1.28512	-1.31838	-1.31497	-1.31164	-1.34552	-1.3422
-1.21737	-1.2139	-1.28747	-1.28401	-1.31728	-1.3139	-1.31062	-1.34451	-1.34125
-1.21628	-1.21284	-1.28637	-1.28294	-1.3162	-1.31287	-1.30963	-1.34352	-1.3403
-1.21521	-1.21182	-1.28531	-1.2819	-1.31516	-1.31189	-1.3087	-1.34255	-1.33933
-1.21417	-1.21082	-1.2843	-1.28092	-1.31417	-1.31094	-1.30778	-1.34159	-1.33835
-1.25069	-1.24733	-1.32002	-1.31661	-1.31328	-1.31003	-1.30685	-1.3406	-1.33736
-1.24965	-1.24637	-1.31907	-1.31572	-1.31241	-1.30914	-1.30592	-1.33961	-1.33636
-1.24867	-1.2454	-1.31807	-1.31475	-1.31146	-1.30819	-1.30496	-1.33862	-1.33537
-1.24776	-1.24444	-1.31702	-1.31371	-1.31043	-1.30719	-1.30399	-1.33762	-1.33438
-1.24681	-1.24348	-1.31599	-1.31267	-1.30941	-1.30618	-1.303	-1.33663	-1.33343
-1.24581	-1.24252	-1.31497	-1.31164	-1.30836	-1.30515	-1.302	-1.33565	-1.33253
-1.2821	-1.27877	-1.31398	-1.31065	-1.30738	-1.30418	-1.30105	-1.3347	-1.33163
-1.28107	-1.27781	-1.31307	-1.30979	-1.30655	-1.30335	-1.30021	-1.3338	-1.3307

-1.28007	-1.27687	-1.31217	-1.30894	-1.30574	-1.30255	-1.29939	-1.33292	-1.32977
-1.27912	-1.27592	-1.3112	-1.308	-1.30482	-1.30164	-1.2985	-1.33197	-1.3288
-1.2782	-1.27497	-1.3102	-1.307	-1.30384	-1.30069	-1.29759	-1.33101	-1.32783
-1.31583	-1.31248	-1.3092	-1.306	-1.30285	-1.29976	-1.29671	-1.33011	-1.32693
-1.3148	-1.31146	-1.30818	-1.30498	-1.30186	-1.29884	-1.29586	-1.32926	-1.3261
-1.31384	-1.31051	-1.30724	-1.30404	-1.30093	-1.29795	-1.295	-1.32837	-1.32522
-1.31299	-1.30968	-1.30642	-1.30322	-1.3001	-1.29709	-1.29412	-1.32743	-1.32427
-1.31212	-1.30883	-1.30559	-1.3024	-1.29927	-1.29623	-1.29322	-1.32647	-1.3233
-1.31113	-1.30787	-1.30465	-1.30148	-1.29837	-1.29532	-1.29231	-1.32551	-1.32238
-1.31013	-1.30689	-1.30369	-1.30055	-1.29746	-1.29441	-1.29141	-1.32457	-1.32146
-1.30923	-1.306	-1.30283	-1.29972	-1.29664	-1.29359	-1.29057	-1.32366	-1.32054
-1.30839	-1.30517	-1.30201	-1.29893	-1.29587	-1.29282	-1.28978	-1.32278	-1.31962
-1.30749	-1.30428	-1.30114	-1.29807	-1.29502	-1.29197	-1.28894	-1.36089	-1.35762
-1.30651	-1.30331	-1.30018	-1.29712	-1.29408	-1.29104	-1.28802	-1.3599	-1.35667
-1.30559	-1.3024	-1.29928	-1.29623	-1.29319	-1.29016	-1.28715	-1.35894	-1.35573
-1.30479	-1.30162	-1.2985	-1.29544	-1.2924	-1.28936	-1.28635	-1.35801	-1.35476
-1.30401	-1.30086	-1.29775	-1.29468	-1.29164	-1.2886	-1.28558	-1.35712	-1.35383
-1.30319	-1.30009	-1.297	-1.29393	-1.29088	-1.28784	-1.28482	-1.35628	-1.353
-1.30235	-1.2993	-1.29624	-1.29317	-1.29012	-1.28708	-1.28406	-1.35545	-1.35221
-1.30145	-1.29842	-1.29537	-1.29231	-1.32896	-1.3258	-1.32268	-1.35451	-1.3513
-1.3005	-1.29745	-1.2944	-1.29135	-1.32797	-1.32482	-1.32169	-1.35349	-1.35032
-1.29963	-1.29657	-1.29352	-1.29047	-1.32705	-1.32391	-1.32079	-1.35256	-1.3494
-1.29887	-1.29579	-1.29273	-1.28969	-1.32624	-1.3231	-1.32	-1.35173	-1.34858
-1.29812	-1.29503	-1.29196	-1.28893	-1.32545	-1.32232	-1.31922	-1.35092	-1.34778
-1.29737	-1.29427	-1.29121	-1.28817	-1.32466	-1.32155	-1.31847	-1.35013	-1.347
-1.29658	-1.2935	-1.29044	-1.2874	-1.32385	-1.32075	-1.31769	-1.34932	-1.3462
-1.29567	-1.2926	-1.28955	-1.28652	-1.32293	-1.31985	-1.31679	-1.3484	-1.34529
-1.29471	-1.29166	-1.28862	-1.28559	-1.32197	-1.31889	-1.31584	-1.34742	-1.34433
-1.29384	-1.2908	-1.32732	-1.3242	-1.3211	-1.31803	-1.31498	-1.34653	-1.34345
-1.29305	-1.29001	-1.32651	-1.3234	-1.32031	-1.31724	-1.31421	-1.34573	-1.34265
-1.29228	-1.28925	-1.32572	-1.32261	-1.31953	-1.31647	-1.31344	-1.34493	-1.34186
-1.29153	-1.28849	-1.32493	-1.32183	-1.31876	-1.31571	-1.31269	-1.34416	-1.34109
-1.29079	-1.28775	-1.32415	-1.32106	-1.31799	-1.31495	-1.31194	-1.34338	-1.34032
-1.29005	-1.28701	-1.32338	-1.32029	-1.31722	-1.31418	-1.31118	-1.34259	-1.33954
-1.28931	-1.28628	-1.32261	-1.31951	-1.31645	-1.3134	-1.3104	-1.34179	-1.33875
-1.32808	-1.32495	-1.32183	-1.31874	-1.31566	-1.3126	-1.30956	-1.30659	-1.30367
-1.32728	-1.32415	-1.32104	-1.31795	-1.31487	-1.31176	-1.30869	-1.3057	-1.30276
-1.32646	-1.32331	-1.32019	-1.3171	-1.31401	-1.31091	-1.30785	-1.30486	-1.30193
-1.32562	-1.32243	-1.31927	-1.31616	-1.31308	-1.31004	-1.30704	-1.30408	-1.30116
-1.32477	-1.32155	-1.31838	-1.31526	-1.31219	-1.30919	-1.30624	-1.30331	-1.30042
-1.3239	-1.3207	-1.31755	-1.31444	-1.31139	-1.30841	-1.30547	-1.30256	-1.29969
-1.32304	-1.31987	-1.31675	-1.31366	-1.31063	-1.30765	-1.30472	-1.30183	-1.29897
-1.32224	-1.31907	-1.31595	-1.31289	-1.30987	-1.3069	-1.30398	-1.36235	-1.3594
-1.32145	-1.31828	-1.31517	-1.31213	-1.30913	-1.30617	-1.30325	-1.36163	-1.35868
-1.32066	-1.31749	-1.31439	-1.31136	-1.30838	-1.30543	-1.30252	-1.3609	-1.35795
-1.31987	-1.31669	-1.3136	-1.3106	-1.30763	-1.3047	-1.3018	-1.36017	-1.35722
-1.31913	-1.31594	-1.31284	-1.30984	-1.36841	-1.36539	-1.3624	-1.39616	-1.39311
-1.31845	-1.31523	-1.31211	-1.30908	-1.36764	-1.36463	-1.36166	-1.3954	-1.39236
-1.31776	-1.31453	-1.31139	-1.30834	-1.36687	-1.36387	-1.36091	-1.39464	-1.39161
-1.31701	-1.3138	-1.31067	-1.3076	-1.3661	-1.36311	-1.36016	-1.39387	-1.39084

-1.31625	-1.31308	-1.30996	-1.30689	-1.36536	-1.36236	-1.35941	-1.39309	-1.39008
-1.31551	-1.31239	-1.37097	-1.36778	-1.36467	-1.36164	-1.35867	-1.39233	-1.38933
-1.31477	-1.31171	-1.37031	-1.36713	-1.364	-1.36095	-1.35795	-1.39157	-1.38858
-1.31401	-1.31098	-1.36961	-1.36647	-1.36336	-1.36028	-1.35726	-1.39085	-1.38785
-1.31323	-1.31021	-1.36887	-1.3658	-1.36274	-1.35966	-1.35661	-1.39015	-1.38712
-1.3743	-1.37118	-1.36812	-1.3651	-1.36208	-1.35901	-1.35597	-1.38948	-1.38643
-1.37352	-1.37041	-1.36736	-1.36436	-1.36136	-1.35835	-1.35535	-1.38885	-1.38579
-1.37273	-1.36965	-1.3666	-1.36361	-1.39741	-1.39434	-1.39127	-1.38821	-1.38516
-1.37195	-1.36888	-1.36585	-1.36286	-1.39665	-1.39359	-1.39055	-1.38753	-1.38453
-1.37118	-1.36812	-1.3651	-1.36212	-1.39589	-1.39283	-1.3898	-1.38683	-1.3839
-1.37045	-1.36738	-1.36435	-1.36137	-1.39512	-1.39207	-1.38906	-1.38611	-1.38322
-1.36975	-1.36665	-1.3636	-1.36062	-1.39435	-1.39132	-1.38833	-1.38539	-1.3825
-1.3691	-1.36599	-1.36294	-1.35994	-1.39363	-1.39059	-1.3876	-1.42034	-1.41737
-1.36851	-1.36543	-1.36237	-1.35933	-1.39297	-1.38989	-1.38687	-1.41961	-1.41665
-1.36785	-1.3648	-1.36176	-1.35872	-1.39232	-1.38924	-1.38621	-1.41894	-1.41597
-1.36709	-1.36408	-1.36108	-1.35809	-1.39172	-1.38867	-1.38565	-1.41837	-1.41536
-1.36632	-1.36333	-1.36037	-1.35743	-1.39108	-1.38808	-1.38509	-1.41781	-1.41477
-1.36562	-1.3626	-1.35963	-1.3567	-1.39035	-1.38738	-1.38443	-1.41718	-1.41418
-1.36494	-1.36189	-1.3589	-1.35596	-1.38959	-1.38664	-1.38372	-1.41652	-1.41357
-1.36432	-1.36127	-1.39498	-1.39192	-1.42487	-1.42181	-1.4188	-1.41584	-1.41291
-1.36374	-1.36072	-1.39442	-1.39133	-1.42425	-1.42116	-1.41811	-1.41513	-1.4122
-1.36308	-1.3601	-1.39379	-1.39072	-1.42364	-1.42054	-1.41749	-1.41449	-1.41155
-1.36232	-1.35937	-1.39309	-1.39007	-1.42305	-1.41999	-1.41696	-1.41395	-1.41098
-1.36161	-1.35867	-1.39239	-1.38941	-1.42242	-1.41939	-1.41638	-1.41338	-1.41041
-1.36101	-1.35804	-1.39171	-1.3887	-1.4217	-1.41869	-1.41571	-1.41275	-1.40982
-1.36042	-1.35743	-1.39105	-1.38801	-1.42099	-1.41798	-1.41501	-1.41209	-1.40922
-1.35977	-1.35681	-1.39044	-1.38741	-1.4204	-1.41736	-1.41437	-1.41144	-1.40857
-1.35911	-1.35619	-1.38984	-1.38685	-1.41986	-1.41679	-1.41376	-1.4108	-1.4079
-1.39521	-1.39219	-1.42535	-1.42228	-1.41923	-1.41617	-1.41315	-1.41019	-1.4073
-1.3946	-1.39155	-1.42468	-1.42159	-1.41853	-1.41551	-1.41254	-1.40962	-1.40676
-1.39397	-1.3909	-1.42404	-1.42094	-1.41789	-1.41489	-1.41193	-1.40903	-1.40618
-1.39329	-1.39027	-1.42345	-1.42038	-1.41734	-1.41431	-1.41133	-1.40841	-1.40554
-1.39262	-1.38963	-1.42284	-1.4198	-1.41677	-1.41373	-1.41074	-1.4078	-1.40493
-1.39199	-1.38898	-1.42218	-1.41913	-1.41611	-1.41311	-1.41016	-1.40725	-1.4044
-1.39138	-1.38833	-1.42151	-1.41844	-1.41543	-1.41249	-1.40959	-1.40672	-1.40388
-1.42715	-1.42399	-1.42089	-1.41783	-1.41483	-1.41191	-1.40904	-1.43584	-1.43294
-1.42653	-1.42339	-1.4203	-1.41725	-1.41427	-1.41136	-1.40849	-1.43529	-1.43237
-1.42593	-1.4228	-1.41972	-1.41667	-1.44334	-1.44035	-1.43739	-1.46366	-1.46068
-1.42534	-1.42223	-1.41915	-1.41608	-1.4427	-1.43969	-1.43672	-1.46303	-1.46011
-1.45466	-1.45149	-1.44833	-1.44518	-1.47149	-1.46841	-1.46539	-1.46244	-1.45954
-1.45404	-1.45086	-1.4477	-1.44457	-1.47091	-1.46785	-1.46484	-1.4619	-1.459
-1.45342	-1.45022	-1.44707	-1.44397	-1.47035	-1.46731	-1.46433	-1.46137	-1.45846
-1.45279	-1.44961	-1.44648	-1.4434	-1.46981	-1.46679	-1.46381	-1.46085	-1.45791
-1.45216	-1.449	-1.4459	-1.44285	-1.4693	-1.46628	-1.46329	-1.46031	-1.45735
-1.45154	-1.44841	-1.4749	-1.47181	-1.46876	-1.46573	-1.46273	-1.45975	-1.45679
-1.45093	-1.44782	-1.47434	-1.47127	-1.46821	-1.46516	-1.46214	-1.45916	-1.45621
-1.45033	-1.44725	-1.47378	-1.4707	-1.46764	-1.46459	-1.46157	-1.4586	-1.45567
-1.44977	-1.44669	-1.47322	-1.47012	-1.46705	-1.46402	-1.46102	-1.45808	-1.45517
-1.47893	-1.47577	-1.47264	-1.46953	-1.46647	-1.46345	-1.46048	-1.45756	-1.45468
-1.47835	-1.47517	-1.47203	-1.46893	-1.46589	-1.4629	-1.45995	-1.45705	-1.45418

-1.47778	-1.47457	-1.47142	-1.46834	-1.46532	-1.46236	-1.45944	-1.45654	-1.45367
-1.47723	-1.47403	-1.47089	-1.46782	-1.4648	-1.46185	-1.45894	-1.45603	-1.45314
-1.47668	-1.47351	-1.47039	-1.46732	-1.4643	-1.46135	-1.45844	-1.45551	-1.45259
-1.47609	-1.47295	-1.46985	-1.4668	-1.46378	-1.46083	-1.45789	-1.45495	-1.45203
-1.47546	-1.47234	-1.46928	-1.46626	-1.46327	-1.46028	-1.45731	-1.45437	-1.45147
-1.47487	-1.47177	-1.46872	-1.46574	-1.46276	-1.45975	-1.45677	-1.45383	-1.45093
-1.47432	-1.47122	-1.46819	-1.46523	-1.46226	-1.45926	-1.45627	-1.45333	-1.45042
-1.47379	-1.4707	-1.46767	-1.46472	-1.46177	-1.45877	-1.45579	-1.45285	-1.44993
-1.47327	-1.47019	-1.46718	-1.46424	-1.46129	-1.4583	-1.45531	-1.45237	-1.44946
-1.47277	-1.46969	-1.46669	-1.46375	-1.46081	-1.45782	-1.45484	-1.4519	-1.44898
-1.47226	-1.46919	-1.46618	-1.46325	-1.46031	-1.45733	-1.45435	-1.45141	-1.44849
-1.47175	-1.46868	-1.46568	-1.46275	-1.45981	-1.45683	-1.45386	-1.45091	-1.448
-1.47125	-1.46818	-1.46518	-1.46226	-1.45933	-1.45635	-1.45337	-1.45043	-1.44751
-1.47076	-1.4677	-1.46471	-1.46178	-1.45885	-1.45587	-1.4529	-1.44996	-1.44704
-1.47027	-1.46721	-1.46422	-1.4613	-1.45837	-1.45539	-1.45242	-1.44948	-1.44656
-1.46976	-1.46671	-1.46372	-1.4608	-1.45787	-1.4549	-1.45193	-1.44899	-1.44607
-1.46925	-1.46621	-1.46323	-1.46031	-1.45738	-1.4544	-1.45143	-1.4485	-1.44559
-1.46876	-1.46572	-1.46273	-1.45982	-1.45689	-1.45392	-1.45095	-1.44802	-1.44511
-1.46829	-1.46523	-1.46225	-1.45934	-1.45641	-1.45344	-1.45047	-1.44754	-1.44464
-1.46785	-1.46478	-1.46178	-1.45886	-1.45592	-1.45295	-1.44999	-1.44705	-1.44415
-1.46743	-1.46436	-1.46134	-1.45838	-1.45543	-1.45246	-1.44951	-1.44657	-1.44365
-1.46699	-1.46391	-1.46089	-1.45791	-1.45495	-1.452	-1.44907	-1.44613	-1.4432
-1.46652	-1.46345	-1.46043	-1.45744	-1.45448	-1.45158	-1.44868	-1.44574	-1.4428
-1.46605	-1.463	-1.45998	-1.45698	-1.45402	-1.45114	-1.44826	-1.44533	-1.44238
-1.46557	-1.46257	-1.45957	-1.45656	-1.45359	-1.45068	-1.44778	-1.44485	-1.44192
-1.4651	-1.46214	-1.45916	-1.45614	-1.45315	-1.45021	-1.44729	-1.44437	-1.44146
-1.46464	-1.46168	-1.45869	-1.45568	-1.4527	-1.44975	-1.44682	-1.44392	-1.44104
-1.46417	-1.46119	-1.45821	-1.45522	-1.45224	-1.44928	-1.44635	-1.44348	-1.44063
-1.46369	-1.46069	-1.45771	-1.45476	-1.45181	-1.44885	-1.44591	-1.47594	-1.47297
-1.46319	-1.46017	-1.45722	-1.45431	-1.4514	-1.44844	-1.44548	-1.47546	-1.47245
-1.46272	-1.4597	-1.45674	-1.45386	-1.45095	-1.44801	-1.44507	-1.475	-1.47197
-1.46231	-1.45928	-1.4563	-1.45338	-1.45047	-1.44757	-1.44467	-1.47458	-1.47153
-1.46188	-1.45886	-1.45588	-1.45291	-1.44998	-1.44711	-1.44424	-1.47414	-1.47109
-1.4614	-1.45844	-1.45547	-1.45249	-1.44954	-1.44664	-1.44375	-1.47362	-1.4706
-1.46091	-1.45801	-1.45506	-1.45207	-1.4491	-1.44617	-1.44325	-1.47311	-1.47011
-1.46045	-1.45753	-1.4546	-1.45165	-1.4487	-1.44575	-1.44281	-1.47265	-1.46965
-1.46	-1.45704	-1.45411	-1.45122	-1.44831	-1.44536	-1.44241	-1.47222	-1.46921
-1.45956	-1.45658	-1.45364	-1.45075	-1.44786	-1.44493	-1.442	-1.4718	-1.46878
-1.45915	-1.45615	-1.45319	-1.45025	-1.44735	-1.44447	-1.4416	-1.47139	-1.46837
-1.45875	-1.45574	-1.45276	-1.44979	-1.47988	-1.47691	-1.47394	-1.47095	-1.46796
-1.45833	-1.45534	-1.45235	-1.44938	-1.47943	-1.47642	-1.47344	-1.47047	-1.46753
-1.45792	-1.45494	-1.45195	-1.44897	-1.47899	-1.47594	-1.47294	-1.46999	-1.46708
-1.45752	-1.45452	-1.45154	-1.44856	-1.47854	-1.47549	-1.47248	-1.46952	-1.4666
-1.45711	-1.45411	-1.45112	-1.44814	-1.4781	-1.47506	-1.47204	-1.46906	-1.46612
-1.45671	-1.45371	-1.45072	-1.44775	-1.47769	-1.47464	-1.47161	-1.46862	-1.46566
-1.45631	-1.45332	-1.45033	-1.44736	-1.47728	-1.47423	-1.4712	-1.4682	-1.46524
-1.4559	-1.45292	-1.44993	-1.44696	-1.47686	-1.47381	-1.47079	-1.4678	-1.46484
-1.4555	-1.4525	-1.44952	-1.44655	-1.47644	-1.4734	-1.47039	-1.46741	-1.46446
-1.4551	-1.4521	-1.44911	-1.44615	-1.47602	-1.473	-1.47	-1.46703	-1.46409
-1.45471	-1.45171	-1.44873	-1.44575	-1.4756	-1.47259	-1.4696	-1.46665	-1.46372

-1.45433	-1.45134	-1.44836	-1.44537	-1.4752	-1.47219	-1.46921	-1.46627	-1.46335
-1.45396	-1.45099	-1.48101	-1.47792	-1.47487	-1.47184	-1.46885	-1.52473	-1.52163
-1.4536	-1.45065	-1.48067	-1.4776	-1.47455	-1.47151	-1.46849	-1.52436	-1.52123
-1.45324	-1.45029	-1.48029	-1.47723	-1.47417	-1.47113	-1.4681	-1.52396	-1.52083
-1.45289	-1.44992	-1.47989	-1.47681	-1.47375	-1.47071	-1.46769	-1.52355	-1.52042
-1.45252	-1.44954	-1.47948	-1.4764	-1.47333	-1.4703	-1.46729	-1.52315	-1.52001
-1.45213	-1.44916	-1.47907	-1.47599	-1.47293	-1.4699	-1.46689	-1.52275	-1.51959
-1.48486	-1.48176	-1.47868	-1.4756	-1.47255	-1.46952	-1.46651	-1.52238	-1.51921
-1.48447	-1.4814	-1.47833	-1.47526	-1.47221	-1.46918	-1.46616	-1.52206	-1.5189
-1.48409	-1.48104	-1.47798	-1.47492	-1.47188	-1.46883	-1.46581	-1.52174	-1.51859
-1.4837	-1.48064	-1.47758	-1.47453	-1.5309	-1.52769	-1.5245	-1.52135	-1.51821
-1.4833	-1.48022	-1.47716	-1.47411	-1.5305	-1.52729	-1.52409	-1.52093	-1.51779
-1.48294	-1.47985	-1.47678	-1.47373	-1.53013	-1.52692	-1.52373	-1.52056	-1.51742
-1.4826	-1.47952	-1.47645	-1.47338	-1.52978	-1.52659	-1.52341	-1.52025	-1.5171
-1.44974	-1.44677	-1.47608	-1.47301	-1.5294	-1.52623	-1.52307	-1.5199	-1.51674
-1.44932	-1.44635	-1.47566	-1.47259	-1.52899	-1.52582	-1.52265	-1.51948	-1.51633
-1.44892	-1.44594	-1.53506	-1.53182	-1.5286	-1.52542	-1.52226	-1.51909	-1.51593
-1.44858	-1.4456	-1.53471	-1.53148	-1.52827	-1.52509	-1.52192	-1.51875	-1.51559
-1.44827	-1.44529	-1.53439	-1.53117	-1.52797	-1.52479	-1.52161	-1.51844	-1.51528
-1.44795	-1.44497	-1.53407	-1.53085	-1.52765	-1.52447	-1.5213	-1.54642	-1.5432
-1.44763	-1.44466	-1.53377	-1.53054	-1.52733	-1.52415	-1.52099	-1.5461	-1.54289
-1.44731	-1.44434	-1.53346	-1.53023	-1.52702	-1.52384	-1.52067	-1.54578	-1.54257
-1.44698	-1.44401	-1.53313	-1.52991	-1.5267	-1.52352	-1.52035	-1.54546	-1.54224
-1.47861	-1.47554	-1.53281	-1.52959	-1.55483	-1.55159	-1.54836	-1.54513	-1.54192
-1.47831	-1.47524	-1.5325	-1.52927	-1.55451	-1.55127	-1.54804	-1.54482	-1.54161
-1.478	-1.47494	-1.53218	-1.52895	-1.55418	-1.55094	-1.54772	-1.5445	-1.5413
-1.50694	-1.5038	-1.53187	-1.52864	-1.55387	-1.55062	-1.5474	-1.54418	-1.54098
-1.50662	-1.50348	-1.53155	-1.52833	-1.55355	-1.5503	-1.54707	-1.54385	-1.54066
-1.5063	-1.50317	-1.55978	-1.5565	-1.55323	-1.54999	-1.54675	-1.54354	-1.54033
-1.50598	-1.50284	-1.55944	-1.55617	-1.55291	-1.54967	-1.54644	-1.54322	-1.54001
-1.5348	-1.5316	-1.52842	-1.52525	-1.55258	-1.54935	-1.54612	-1.5429	-1.53969
-1.53447	-1.53128	-1.52809	-1.52492	-1.55225	-1.54902	-1.5458	-1.54259	-1.53938
-1.53414	-1.53095	-1.52777	-1.5246	-1.55192	-1.54869	-1.54547	-1.54227	-1.53907
-1.53382	-1.53062	-1.52744	-1.52427	-1.55159	-1.54836	-1.54515	-1.54194	-1.53874
-1.53351	-1.5303	-1.52711	-1.52395	-1.55126	-1.54803	-1.54482	-1.54161	-1.53842
-1.5332	-1.52999	-1.5268	-1.52364	-1.55094	-1.54771	-1.54449	-1.54129	-1.5381
-1.53289	-1.52969	-1.52651	-1.52334	-1.55063	-1.5474	-1.54418	-1.54098	-1.5378
-1.53264	-1.52943	-1.52625	-1.52309	-1.55037	-1.54713	-1.5439	-1.54071	-1.53754
-1.53242	-1.52921	-1.52603	-1.52289	-1.55016	-1.54691	-1.54367	-1.54048	-1.53731
-1.53217	-1.52896	-1.52578	-1.52264	-1.54992	-1.54666	-1.54342	-1.54022	-1.53705
-1.53185	-1.52866	-1.52548	-1.52233	-1.5496	-1.54636	-1.54314	-1.53993	-1.53673
-1.53154	-1.52836	-1.52519	-1.52203	-1.54929	-1.54607	-1.54286	-1.53964	-1.53641
-1.53129	-1.52812	-1.52495	-1.52178	-1.54903	-1.54583	-1.54263	-1.53939	-1.53616
-1.53105	-1.52788	-1.52471	-1.52154	-1.54878	-1.54558	-1.54238	-1.53915	-1.53592
-1.53075	-1.52757	-1.52441	-1.52125	-1.5485	-1.54528	-1.54208	-1.53886	-1.53564
-1.53042	-1.52724	-1.52408	-1.52094	-1.54819	-1.54496	-1.54174	-1.53854	-1.53534
-1.53014	-1.52696	-1.5238	-1.52067	-1.51754	-1.5144	-1.51128	-1.53825	-1.53507
-1.52992	-1.52674	-1.52358	-1.52044	-1.5173	-1.51417	-1.51105	-1.53801	-1.53483
-1.5297	-1.52653	-1.52336	-1.52021	-1.51706	-1.51392	-1.51079	-1.53774	-1.53456
-1.52948	-1.52631	-1.52314	-1.51998	-1.51682	-1.51365	-1.5105	-1.53743	-1.53424

-1.52926	-1.52608	-1.52291	-1.51974	-1.51656	-1.51338	-1.5102	-1.53712	-1.53392
-1.52902	-1.52584	-1.52265	-1.51947	-1.51629	-1.51311	-1.50995	-1.53687	-1.53367
-1.52877	-1.52558	-1.52238	-1.51919	-1.51601	-1.51286	-1.50973	-1.53664	-1.53344
-1.52851	-1.52531	-1.52211	-1.51892	-1.51576	-1.51262	-1.5095	-1.53641	-1.53321
-1.52824	-1.52502	-1.52183	-1.51868	-1.51553	-1.51239	-1.50927	-1.53618	-1.53299
-1.52796	-1.52475	-1.52158	-1.51844	-1.51531	-1.51217	-1.50905	-1.53595	-1.53276
-1.52768	-1.52451	-1.52135	-1.51822	-1.51509	-1.51195	-1.50882	-1.53572	-1.53252
-1.52743	-1.52427	-1.52113	-1.518	-1.51487	-1.51172	-1.50859	-1.53548	-1.53229
-1.52725	-1.52405	-1.52088	-1.51776	-1.51464	-1.5115	-1.50837	-1.53525	-1.53206
-1.52709	-1.52384	-1.52065	-1.51753	-1.5144	-1.51127	-1.50814	-1.53502	-1.53183
-1.52692	-1.52368	-1.52048	-1.51731	-1.51416	-1.51102	-1.5079	-1.53478	-1.53158
-1.52674	-1.52355	-1.52034	-1.51711	-1.51392	-1.51078	-1.50766	-1.53454	-1.53133
-1.52654	-1.52336	-1.52016	-1.51693	-1.51373	-1.51056	-1.50742	-1.50431	-1.50121
-1.52631	-1.52314	-1.51996	-1.51678	-1.51358	-1.51037	-1.50718	-1.50406	-1.50098
-1.52608	-1.52292	-1.51975	-1.51659	-1.51341	-1.51019	-1.50698	-1.50384	-1.50075
-1.52584	-1.52267	-1.51952	-1.51637	-1.51321	-1.51002	-1.50684	-1.50367	-1.50052
-1.52561	-1.52244	-1.51928	-1.51614	-1.51299	-1.50984	-1.50668	-1.5035	-1.50032
-1.52537	-1.52221	-1.51905	-1.5159	-1.51275	-1.50962	-1.50648	-1.50332	-1.50016
-1.52515	-1.522	-1.51884	-1.51567	-1.51251	-1.50938	-1.50626	-1.50314	-1.50001
-1.525	-1.52184	-1.51868	-1.51551	-1.51234	-1.50918	-1.50603	-1.50291	-1.49981
-1.52488	-1.52171	-1.51854	-1.51537	-1.5122	-1.50898	-1.50579	-1.50267	-1.49958
-1.52471	-1.52152	-1.51835	-1.51519	-1.51201	-1.5088	-1.50559	-1.50246	-1.49936
-1.52448	-1.52128	-1.5181	-1.51496	-1.5118	-1.50862	-1.50545	-1.50228	-1.49914
-1.52426	-1.52106	-1.51788	-1.51472	-1.51157	-1.50843	-1.50528	-1.50211	-1.49895
-1.52411	-1.52091	-1.51771	-1.5145	-1.51133	-1.5082	-1.50509	-1.50194	-1.4988
-1.52394	-1.52075	-1.51754	-1.5143	-1.51111	-1.50799	-1.50489	-1.50178	-1.49866
-1.52372	-1.52054	-1.51735	-1.51413	-1.51095	-1.50783	-1.50473	-1.50162	-1.49852
-1.52349	-1.52033	-1.51716	-1.51397	-1.51081	-1.50768	-1.50457	-1.50147	-1.49837
-1.52332	-1.52016	-1.517	-1.51382	-1.51066	-1.5075	-1.50437	-1.50127	-1.49819
-1.52319	-1.52003	-1.51685	-1.51367	-1.51049	-1.5073	-1.50414	-1.50105	-1.49799
-1.49573	-1.49258	-1.51671	-1.51352	-1.51034	-1.50713	-1.50396	-1.50087	-1.49782
-1.49557	-1.49242	-1.51656	-1.51339	-1.51021	-1.507	-1.50383	-1.50074	-1.49769
-1.49541	-1.49224	-1.51638	-1.51322	-1.51006	-1.50687	-1.5037	-1.50061	-1.49756
-1.49522	-1.49201	-1.51615	-1.51301	-1.50987	-1.50671	-1.50357	-1.50049	-1.49743
-1.49503	-1.49178	-1.51592	-1.51279	-1.50968	-1.50655	-1.50344	-1.50037	-1.49731
-1.49487	-1.49162	-1.51576	-1.51263	-1.50952	-1.50641	-1.50331	-1.50024	-1.49719
-1.49474	-1.49149	-1.51563	-1.51249	-1.50938	-1.50627	-1.50318	-1.50012	-1.49707
-1.49459	-1.49134	-1.48816	-1.48506	-1.50924	-1.50614	-1.50305	-1.49999	-1.49694
-1.49445	-1.4912	-1.48802	-1.48492	-1.50911	-1.50601	-1.50293	-1.49986	-1.49681
-1.49431	-1.49105	-1.48787	-1.48477	-1.50898	-1.50588	-1.5028	-1.49973	-1.49668
-1.49415	-1.4909	-1.48773	-1.48464	-1.50885	-1.50575	-1.50267	-1.4996	-1.49654
-1.49401	-1.49076	-1.48759	-1.4845	-1.50872	-1.50562	-1.50253	-1.49947	-1.49641
-1.49388	-1.49063	-1.48746	-1.48436	-1.50858	-1.50548	-1.5024	-1.49934	-1.49627
-1.49376	-1.49052	-1.48735	-1.48423	-1.50845	-1.50535	-1.50227	-1.4992	-1.49614
-1.49365	-1.49044	-1.48728	-1.48416	-1.48105	-1.47797	-1.4749	-1.49907	-1.496
-1.49355	-1.49038	-1.48723	-1.4841	-1.48098	-1.47787	-1.47477	-1.49893	-1.49586
-1.49343	-1.49028	-1.48714	-1.48401	-1.48089	-1.47776	-1.47464	-1.49879	-1.49572
-1.49327	-1.49013	-1.487	-1.48389	-1.48077	-1.47764	-1.47452	-1.49866	-1.49557
-1.49313	-1.48999	-1.48686	-1.48376	-1.48065	-1.47753	-1.47443	-1.47135	-1.46829
-1.49299	-1.48983	-1.48671	-1.48362	-1.48053	-1.47746	-1.47438	-1.47131	-1.46823

-1.49287	-1.48971	-1.48657	-1.48348	-1.48041	-1.47737	-1.47433	-1.47125	-1.46816
-1.49281	-1.48963	-1.48649	-1.48338	-1.48029	-1.47725	-1.4742	-1.47113	-1.46805
-1.49277	-1.48958	-1.48642	-1.48329	-1.48018	-1.47712	-1.47406	-1.47099	-1.46792
-1.4581	-1.45499	-1.48634	-1.48321	-1.48011	-1.47703	-1.47396	-1.47088	-1.46779
-1.45801	-1.45489	-1.48624	-1.48314	-1.48005	-1.47697	-1.47389	-1.47078	-1.46766
-1.45793	-1.4548	-1.48616	-1.48307	-1.47999	-1.47689	-1.4738	-1.47068	-1.46756
-1.45784	-1.45473	-1.4861	-1.483	-1.4799	-1.47679	-1.47368	-1.47058	-1.46749
-1.45775	-1.45466	-1.48603	-1.48291	-1.4798	-1.47669	-1.47358	-1.47049	-1.46742
-1.45769	-1.45459	-1.48595	-1.48281	-1.4797	-1.47659	-1.4735	-1.47042	-1.46734
-1.45761	-1.45451	-1.48586	-1.48272	-1.4796	-1.47651	-1.47343	-1.47035	-1.46726
-1.45749	-1.45441	-1.48576	-1.48263	-1.47952	-1.47643	-1.47335	-1.47025	-1.46715
-1.45735	-1.45429	-1.48567	-1.48256	-1.47946	-1.47636	-1.47327	-1.47015	-1.46704
-1.45726	-1.45421	-1.45116	-1.44812	-1.47938	-1.47627	-1.47316	-1.47006	-1.46696
-1.45721	-1.45414	-1.45109	-1.44804	-1.47929	-1.47616	-1.47305	-1.46996	-1.4669
-1.45716	-1.45409	-1.45102	-1.44795	-1.47919	-1.47606	-1.47295	-1.46989	-1.46684
-1.45711	-1.45403	-1.45094	-1.44784	-1.47907	-1.47597	-1.47289	-1.46983	-1.4668
-1.45706	-1.45398	-1.45088	-1.44777	-1.47898	-1.47589	-1.47283	-1.46978	-1.46675
-1.45702	-1.45394	-1.45086	-1.44776	-1.47898	-1.47587	-1.47278	-1.46972	-1.46669
-1.45698	-1.4539	-1.45083	-1.44776	-1.47899	-1.47584	-1.47272	-1.46966	-1.46662
-1.45693	-1.45385	-1.45076	-1.44767	-1.4446	-1.44155	-1.43852	-1.4696	-1.46656
-1.45688	-1.45379	-1.45068	-1.44756	-1.44447	-1.44145	-1.43846	-1.46955	-1.4665
-1.45683	-1.45374	-1.45063	-1.4475	-1.44442	-1.4414	-1.43841	-1.46949	-1.46645
-1.45677	-1.45369	-1.4506	-1.44751	-1.44444	-1.44138	-1.43836	-1.46944	-1.46641
-1.42397	-1.42099	-1.45056	-1.44748	-1.44441	-1.44134	-1.4383	-1.46938	-1.46636
-1.42392	-1.42093	-1.45048	-1.44737	-1.44429	-1.44125	-1.43824	-1.46932	-1.4663
-1.42387	-1.42087	-1.45041	-1.44728	-1.44419	-1.44117	-1.43819	-1.46926	-1.46624
-1.42381	-1.42082	-1.45038	-1.44727	-1.44418	-1.44114	-1.43813	-1.46919	-1.46618
-1.42376	-1.42078	-1.45036	-1.44728	-1.44421	-1.44113	-1.43808	-1.46913	-1.46612
-1.42374	-1.42074	-1.45031	-1.44725	-1.4442	-1.44113	-1.43808	-1.43509	-1.43214
-1.42376	-1.4207	-1.45025	-1.44721	-1.44417	-1.44114	-1.43811	-1.4351	-1.4321
-1.42377	-1.42069	-1.45023	-1.44717	-1.44413	-1.44112	-1.43812	-1.46669	-1.4636
-1.42377	-1.42072	-1.45026	-1.44714	-1.44408	-1.44108	-1.4381	-1.46669	-1.46363
-1.42375	-1.42074	-1.45028	-1.44714	-1.44405	-1.44106	-1.43809	-1.4667	-1.46366
-1.42371	-1.42072	-1.45027	-1.44715	-1.44408	-1.44109	-1.43812	-1.46674	-1.4637
-1.42368	-1.4207	-1.45027	-1.44718	-1.44413	-1.44112	-1.43814	-1.46676	-1.46373
-1.4237	-1.42072	-1.4503	-1.44722	-1.44416	-1.44112	-1.43811	-1.49439	-1.4913
-1.42375	-1.42076	-1.45034	-1.44727	-1.44419	-1.44111	-1.43806	-1.49433	-1.49125
-1.42378	-1.42076	-1.45033	-1.44727	-1.4442	-1.44112	-1.43806	-1.49431	-1.4912
-1.42379	-1.42073	-1.45029	-1.44724	-1.4442	-1.44114	-1.43809	-1.49431	-1.49116
-1.4238	-1.42073	-1.41772	-1.41477	-1.4442	-1.44116	-1.43813	-1.49433	-1.49115
-1.4238	-1.42076	-1.41776	-1.4148	-1.44423	-1.4412	-1.43818	-1.49438	-1.49118
-1.42381	-1.42079	-1.4178	-1.41482	-1.44425	-1.44123	-1.43821	-1.49442	-1.49122
-1.42385	-1.42083	-1.41782	-1.41481	-1.44421	-1.4412	-1.4382	-1.49443	-1.49125
-1.42391	-1.42088	-1.41784	-1.4148	-1.44418	-1.44117	-1.43819	-1.49443	-1.49128
-1.42396	-1.42093	-1.41789	-1.41483	-1.44421	-1.4412	-1.43822	-1.49447	-1.49133
-1.42402	-1.42099	-1.41795	-1.41489	-1.44427	-1.44126	-1.43827	-1.49452	-1.49138
-1.42407	-1.42104	-1.418	-1.41495	-1.50405	-1.50087	-1.49772	-1.49457	-1.49142
-1.42412	-1.42109	-1.41805	-1.41499	-1.5041	-1.50092	-1.49776	-1.49461	-1.49146
-1.42417	-1.42114	-1.4181	-1.41504	-1.50415	-1.50097	-1.49781	-1.49466	-1.49151
-1.42422	-1.4212	-1.41816	-1.4151	-1.50421	-1.50103	-1.49787	-1.49471	-1.49156

-1.39253	-1.38959	-1.41822	-1.41516	-1.50427	-1.50108	-1.49792	-1.49476	-1.49161
-1.39257	-1.38963	-1.41826	-1.4152	-1.50432	-1.50113	-1.49797	-1.49481	-1.49166
-1.39262	-1.38967	-1.4183	-1.41524	-1.50436	-1.50118	-1.49802	-1.49486	-1.49171
-1.39265	-1.38971	-1.41834	-1.41528	-1.5044	-1.50122	-1.49806	-1.49491	-1.49175
-1.39269	-1.38975	-1.41837	-1.41531	-1.50444	-1.50126	-1.4981	-1.49495	-1.49179
-1.39274	-1.38979	-1.41842	-1.41536	-1.47307	-1.46997	-1.46691	-1.49499	-1.49184
-1.39278	-1.38985	-1.41848	-1.41542	-1.47313	-1.47002	-1.46695	-1.49504	-1.4919
-1.39283	-1.3899	-1.41854	-1.41549	-1.47322	-1.4701	-1.46701	-1.4951	-1.49197
-1.39287	-1.38996	-1.41862	-1.41561	-1.47336	-1.47023	-1.46713	-1.4952	-1.49204
-1.39293	-1.39002	-1.41869	-1.41571	-1.47349	-1.47036	-1.46724	-1.4953	-1.49211
-1.39301	-1.39008	-1.38716	-1.38427	-1.47354	-1.47042	-1.46731	-1.49536	-1.49217
-1.3931	-1.39015	-1.38721	-1.3843	-1.47357	-1.47046	-1.46736	-1.49542	-1.49223
-1.3932	-1.39025	-1.44939	-1.4463	-1.47366	-1.47054	-1.46743	-1.49551	-1.49234
-1.3933	-1.39038	-1.44954	-1.44644	-1.47379	-1.47064	-1.46752	-1.49561	-1.49247
-1.39337	-1.39047	-1.44964	-1.44655	-1.44347	-1.44041	-1.43737	-1.49574	-1.49261
-1.39342	-1.39051	-1.44969	-1.44661	-1.44355	-1.44052	-1.43751	-1.49589	-1.49276
-1.39349	-1.39057	-1.44975	-1.44667	-1.44362	-1.44061	-1.43762	-1.49602	-1.49288
-1.3936	-1.39068	-1.44985	-1.44675	-1.44368	-1.44066	-1.43767	-1.49607	-1.49295
-1.39373	-1.3908	-1.44996	-1.44683	-1.44374	-1.44071	-1.43771	-1.49612	-1.49301
-1.39385	-1.3909	-1.45006	-1.44694	-1.44386	-1.44082	-1.43781	-1.4962	-1.49307
-1.39395	-1.39099	-1.45016	-1.44707	-1.444	-1.44096	-1.43794	-1.49631	-1.49313
-1.39405	-1.3911	-1.45028	-1.4472	-1.44414	-1.44108	-1.43805	-1.46521	-1.46213
-1.39415	-1.39122	-1.45042	-1.44734	-1.44426	-1.44119	-1.43814	-1.46532	-1.46227
-1.42398	-1.42099	-1.45056	-1.44746	-1.44437	-1.44129	-1.43825	-1.46545	-1.46241
-1.42413	-1.42113	-1.45068	-1.44755	-1.44445	-1.4414	-1.43838	-1.46559	-1.46257
-1.39378	-1.39087	-1.45082	-1.44767	-1.44456	-1.44152	-1.43852	-1.43555	-1.43261
-1.39393	-1.39102	-1.45099	-1.44786	-1.44476	-1.4417	-1.43867	-1.43569	-1.43274
-1.39408	-1.39117	-1.45117	-1.44807	-1.44498	-1.44189	-1.43883	-1.43583	-1.43288
-1.42749	-1.42449	-1.45131	-1.44821	-1.44511	-1.44203	-1.43898	-1.47209	-1.46903
-1.42763	-1.42463	-1.45144	-1.4483	-1.4452	-1.44215	-1.43913	-1.47225	-1.46917
-1.42777	-1.42477	-1.45159	-1.44845	-1.44535	-1.4423	-1.43928	-1.4724	-1.46933
-1.42792	-1.42492	-1.45176	-1.44866	-1.44556	-1.44248	-1.43943	-1.47255	-1.46949
-1.42807	-1.42507	-1.42207	-1.41906	-1.44574	-1.44265	-1.43958	-1.4727	-1.46965
-1.42822	-1.42522	-1.4222	-1.41916	-1.44584	-1.44276	-1.43972	-1.47285	-1.4698
-1.42837	-1.42537	-1.42234	-1.41928	-1.44594	-1.44289	-1.43987	-1.473	-1.46994
-1.42852	-1.42552	-1.4225	-1.41947	-1.44615	-1.44307	-1.44002	-1.47314	-1.47008
-1.42867	-1.42567	-1.42268	-1.41968	-1.44638	-1.44327	-1.44018	-1.47329	-1.47023
-1.42882	-1.42582	-1.42283	-1.41985	-1.44658	-1.44347	-1.44038	-1.47347	-1.47039
-1.42899	-1.42597	-1.42298	-1.42	-1.44675	-1.44368	-1.44062	-1.47367	-1.47054
-1.42919	-1.42617	-1.42316	-1.42016	-1.44691	-1.44387	-1.44083	-1.47388	-1.47072
-1.42943	-1.42641	-1.42338	-1.42034	-1.44706	-1.44402	-1.441	-1.47408	-1.47096
-1.42965	-1.42664	-1.42359	-1.42052	-1.41749	-1.41452	-1.41158	-1.47427	-1.47118
-1.42981	-1.4268	-1.42378	-1.42074	-1.41771	-1.41471	-1.41173	-1.47442	-1.47135
-1.42997	-1.42695	-1.42394	-1.42095	-1.41794	-1.4149	-1.41189	-1.47456	-1.4715
-1.43017	-1.42711	-1.42409	-1.42111	-1.45505	-1.45193	-1.44882	-1.47475	-1.47166
-1.43038	-1.42728	-1.42424	-1.42126	-1.45521	-1.45213	-1.44905	-1.47496	-1.47183
-1.43059	-1.42748	-1.42443	-1.42142	-1.45537	-1.45231	-1.44925	-1.47517	-1.47203
-1.43078	-1.42771	-1.42466	-1.42161	-1.45552	-1.45246	-1.44943	-1.47538	-1.47226
-1.43096	-1.42792	-1.42487	-1.4218	-1.4557	-1.45265	-1.44962	-1.44659	-1.44357
-1.43111	-1.42808	-1.42506	-1.42202	-1.45593	-1.45287	-1.44984	-1.44681	-1.44379

-1.43128	-1.42826	-1.42525	-1.42224	-1.45617	-1.4531	-1.45004	-1.44702	-1.44401
-1.43152	-1.4285	-1.42548	-1.42247	-1.45639	-1.45328	-1.4502	-1.44717	-1.44418
-1.43177	-1.42875	-1.42572	-1.4227	-1.4566	-1.45346	-1.45036	-1.44733	-1.44434
-1.43202	-1.42899	-1.42596	-1.42294	-1.45684	-1.45369	-1.45057	-1.44754	-1.44455
-1.43225	-1.42922	-1.42619	-1.42318	-1.45709	-1.45394	-1.45082	-1.44778	-1.44478
-1.43247	-1.42941	-1.42638	-1.42338	-1.45731	-1.45417	-1.45106	-1.44802	-1.44502
-1.43268	-1.42958	-1.42653	-1.42354	-1.45748	-1.45438	-1.4513	-1.44827	-1.44526
-1.4329	-1.42977	-1.42671	-1.42372	-1.45767	-1.4546	-1.45154	-1.44851	-1.44551
-1.43315	-1.43002	-1.42695	-1.42395	-1.4579	-1.45483	-1.45178	-1.44875	-1.44575
-1.4334	-1.43027	-1.4272	-1.4242	-1.45814	-1.45507	-1.45202	-1.44899	-1.44598
-1.43365	-1.43052	-1.42745	-1.42444	-1.45839	-1.45531	-1.45226	-1.44923	-1.44622
-1.4339	-1.43076	-1.42769	-1.42469	-1.45863	-1.45556	-1.4525	-1.44947	-1.44646
-1.43414	-1.431	-1.42793	-1.42493	-1.45887	-1.4558	-1.45275	-1.44972	-1.4467
-1.43439	-1.43125	-1.42817	-1.42517	-1.45911	-1.45604	-1.45299	-1.44996	-1.44694
-1.43464	-1.4315	-1.42842	-1.42541	-1.45935	-1.45628	-1.45323	-1.4502	-1.44718
-1.43489	-1.43175	-1.42868	-1.42565	-1.45959	-1.45652	-1.45347	-1.45044	-1.44742
-1.37701	-1.37395	-1.42895	-1.42592	-1.45984	-1.45677	-1.45371	-1.45068	-1.44766
-1.37727	-1.37425	-1.42928	-1.42624	-1.46014	-1.45704	-1.45395	-1.45091	-1.4479
-1.37753	-1.37454	-1.4296	-1.42656	-1.46045	-1.45731	-1.4542	-1.45115	-1.44813
-1.37776	-1.37478	-1.3718	-1.36883	-1.46072	-1.45757	-1.45445	-1.45139	-1.44837
-1.37797	-1.37499	-1.37202	-1.36906	-1.46098	-1.45784	-1.45472	-1.45165	-1.44861
-1.37819	-1.37521	-1.37224	-1.36929	-1.46125	-1.45812	-1.45502	-1.45194	-1.4489
-1.37841	-1.37542	-1.37246	-1.36951	-1.46152	-1.45843	-1.45535	-1.45228	-1.44923
-1.41707	-1.41398	-1.41091	-1.40786	-1.40484	-1.40185	-1.39886	-1.45258	-1.44953
-1.41738	-1.41428	-1.41118	-1.40811	-1.40507	-1.40206	-1.39908	-1.45283	-1.44979
-1.41769	-1.41457	-1.41147	-1.40838	-1.40532	-1.4023	-1.39931	-1.39633	-1.39338
-1.41793	-1.41484	-1.41175	-1.40867	-1.40561	-1.40259	-1.39959	-1.39659	-1.39362
-1.41817	-1.4151	-1.41203	-1.40897	-1.40592	-1.4029	-1.39988	-1.39687	-1.39386
-1.41847	-1.4154	-1.41234	-1.40927	-1.40622	-1.40318	-1.40015	-1.39714	-1.39414
-1.41879	-1.41572	-1.41265	-1.40957	-1.40651	-1.40345	-1.40042	-1.39741	-1.39444
-1.41906	-1.41599	-1.41292	-1.40985	-1.40679	-1.40373	-1.4007	-1.3977	-1.39473
-1.4193	-1.41622	-1.41315	-1.4101	-1.40706	-1.40402	-1.401	-1.398	-1.39502
-1.41957	-1.41648	-1.41341	-1.41037	-1.40734	-1.40432	-1.4013	-1.3983	-1.3953
-1.41989	-1.4168	-1.41373	-1.41068	-1.40764	-1.40461	-1.40159	-1.39857	-1.39557
-1.42023	-1.41714	-1.41406	-1.411	-1.40795	-1.40491	-1.40187	-1.39885	-1.39584
-1.42057	-1.41747	-1.41438	-1.41131	-1.40824	-1.40518	-1.40214	-1.39912	-1.39613
-1.4209	-1.4178	-1.4147	-1.41161	-1.40853	-1.40546	-1.40241	-1.3994	-1.39644
-1.42123	-1.41811	-1.415	-1.41189	-1.40881	-1.40574	-1.4027	-1.39971	-1.39675
-1.42155	-1.41842	-1.41529	-1.41217	-1.40908	-1.40603	-1.40301	-1.40002	-1.39706
-1.42185	-1.41871	-1.41558	-1.41245	-1.40937	-1.40633	-1.40333	-1.40034	-1.39738
-1.42215	-1.41898	-1.41585	-1.41276	-1.40969	-1.40665	-1.40365	-1.40067	-1.39771
-1.42244	-1.41927	-1.41615	-1.41307	-1.41001	-1.40698	-1.40397	-1.40099	-1.39803
-1.42272	-1.41958	-1.41647	-1.41339	-1.41034	-1.4073	-1.40429	-1.40131	-1.39835
-1.42302	-1.41991	-1.4168	-1.41372	-1.41066	-1.40763	-1.40462	-1.40163	-1.39866
-1.42339	-1.42024	-1.41712	-1.41405	-1.41099	-1.40796	-1.40494	-1.40195	-1.39898
-1.42378	-1.42059	-1.41745	-1.41438	-1.41133	-1.40829	-1.40527	-1.40227	-1.3993
-1.42418	-1.42097	-1.41782	-1.41472	-1.41165	-1.40861	-1.40559	-1.40259	-1.39962
-1.42457	-1.42139	-1.41823	-1.41507	-1.41197	-1.40892	-1.40591	-1.40291	-1.39993
-1.38645	-1.38339	-1.38033	-1.37726	-1.41232	-1.40926	-1.40624	-1.40323	-1.40026
-1.38679	-1.38375	-1.3807	-1.37766	-1.41273	-1.40963	-1.40657	-1.40356	-1.40059

-1.38713	-1.38409	-1.38106	-1.37805	-1.41314	-1.41001	-1.40692	-1.40391	-1.40094
-1.38746	-1.38442	-1.38139	-1.37839	-1.41351	-1.41041	-1.40733	-1.40431	-1.40133
-1.3878	-1.38475	-1.38172	-1.37873	-1.41387	-1.4108	-1.40775	-1.40473	-1.40173
-1.35896	-1.35598	-1.38206	-1.37906	-1.4142	-1.41115	-1.40811	-1.40508	-1.40207
-1.35929	-1.35632	-1.38241	-1.3794	-1.41453	-1.41148	-1.40845	-1.40541	-1.40239
-1.35967	-1.35669	-1.38279	-1.37978	-1.41492	-1.41186	-1.40882	-1.40578	-1.40275
-1.3601	-1.3571	-1.3832	-1.3802	-1.41535	-1.41228	-1.40923	-1.40619	-1.40316
-1.36049	-1.35749	-1.3836	-1.3806	-1.41575	-1.41267	-1.40961	-1.40657	-1.40355
-1.36084	-1.35782	-1.38394	-1.38094	-1.4161	-1.41302	-1.40995	-1.40692	-1.4039
-1.36119	-1.35817	-1.3843	-1.38129	-1.41645	-1.41337	-1.4103	-1.40727	-1.40426
-1.32601	-1.32308	-1.35559	-1.35265	-1.41685	-1.41377	-1.41071	-1.40768	-1.40466
-1.3264	-1.32348	-1.35599	-1.35304	-1.41725	-1.41418	-1.41113	-1.40809	-1.40507
-1.32673	-1.32381	-1.35634	-1.35338	-1.41761	-1.41454	-1.4115	-1.40846	-1.40543
-1.32704	-1.32412	-1.35666	-1.3537	-1.41794	-1.41488	-1.41183	-1.40879	-1.40576
-1.32739	-1.32447	-1.32157	-1.3187	-1.38984	-1.38685	-1.38388	-1.40917	-1.40615
-1.3278	-1.32486	-1.32196	-1.31909	-1.39025	-1.38726	-1.38429	-1.4096	-1.40658
-1.32821	-1.32527	-1.32236	-1.31949	-1.39067	-1.38768	-1.38471	-1.41003	-1.40702
-1.32863	-1.32567	-1.32276	-1.31989	-1.39109	-1.3881	-1.38512	-1.41046	-1.40745
-1.32904	-1.32609	-1.32317	-1.3203	-1.39151	-1.38852	-1.38555	-1.4109	-1.40788
-1.32945	-1.32649	-1.32358	-1.3207	-1.35722	-1.35431	-1.35142	-1.38299	-1.38004
-1.32986	-1.3269	-1.32398	-1.3211	-1.35762	-1.35471	-1.35182	-1.38341	-1.38046
-1.33028	-1.32731	-1.32439	-1.3215	-1.35803	-1.35511	-1.35222	-1.38382	-1.38087
-1.33069	-1.32772	-1.3248	-1.3219	-1.35843	-1.35551	-1.35262	-1.38424	-1.38129
-1.33111	-1.32813	-1.3252	-1.3223	-1.35883	-1.35591	-1.35302	-1.38465	-1.3817
-1.33152	-1.32854	-1.32561	-1.3227	-1.35924	-1.35632	-1.35342	-1.38506	-1.38211
-1.33193	-1.32895	-1.32601	-1.3231	-1.35964	-1.35672	-1.35382	-1.35093	-1.34807
-1.33235	-1.32937	-1.32642	-1.32351	-1.36005	-1.35712	-1.35421	-1.35133	-1.34846
-1.33277	-1.32978	-1.32683	-1.32391	-1.36045	-1.35752	-1.35461	-1.35172	-1.34885
-1.33319	-1.3302	-1.32724	-1.32432	-1.36086	-1.35792	-1.35501	-1.35212	-1.34924
-1.33361	-1.33061	-1.32765	-1.32472	-1.36126	-1.35832	-1.35541	-1.35251	-1.34963
-1.33402	-1.33103	-1.32807	-1.32513	-1.32223	-1.31938	-1.31656	-1.35291	-1.35003
-1.33444	-1.33144	-1.32848	-1.32553	-1.32263	-1.31978	-1.31695	-1.3533	-1.35042
-1.33486	-1.33186	-1.32889	-1.32595	-1.32304	-1.32018	-1.31735	-1.3537	-1.35082
-1.33529	-1.33229	-1.32932	-1.32637	-1.32346	-1.32059	-1.31775	-1.35411	-1.35123
-1.33574	-1.33274	-1.32976	-1.32682	-1.3239	-1.32102	-1.31817	-1.35454	-1.35166
-1.33624	-1.33323	-1.33025	-1.32731	-1.32439	-1.3215	-1.31865	-1.35501	-1.35214
-1.33675	-1.33374	-1.33076	-1.32782	-1.3249	-1.322	-1.31913	-1.3555	-1.35262
-1.30312	-1.30019	-1.33122	-1.32827	-1.32535	-1.32245	-1.31958	-1.38376	-1.38081
-1.30355	-1.30062	-1.33166	-1.3287	-1.32576	-1.32287	-1.32	-1.38421	-1.38123
-1.30399	-1.30108	-1.29818	-1.29529	-1.32622	-1.32332	-1.32046	-1.38468	-1.3817
-1.30444	-1.30157	-1.29868	-1.29578	-1.32671	-1.32381	-1.32094	-1.3852	-1.3822
-1.3049	-1.30204	-1.29916	-1.29625	-1.32719	-1.32429	-1.32141	-1.38569	-1.3827
-1.30535	-1.30247	-1.29957	-1.29667	-1.32763	-1.32471	-1.32182	-1.38613	-1.38314
-1.30582	-1.3029	-1.29999	-1.2971	-1.32807	-1.32514	-1.32224	-1.38658	-1.38359
-1.30633	-1.30339	-1.30048	-1.29758	-1.29471	-1.29186	-1.28903	-1.38708	-1.38409
-1.30686	-1.30391	-1.30098	-1.29809	-1.29521	-1.29235	-1.28952	-1.38761	-1.38462
-1.30734	-1.30441	-1.30149	-1.29858	-1.2957	-1.29285	-1.29002	-1.34944	-1.34653
-1.30779	-1.3049	-1.302	-1.29907	-1.29618	-1.29334	-1.29052	-1.34997	-1.34704
-1.30825	-1.30537	-1.30246	-1.29953	-1.29664	-1.2938	-1.29099	-1.31734	-1.3145
-1.30871	-1.30579	-1.30287	-1.29994	-1.29705	-1.29421	-1.2914	-1.31782	-1.31502

-1.30918	-1.30624	-1.3033	-1.30038	-1.29749	-1.29464	-1.29183	-1.31828	-1.31552
-1.3097	-1.30674	-1.3038	-1.30088	-1.29799	-1.29512	-1.29229	-1.31875	-1.31595
-1.31022	-1.30726	-1.30432	-1.3014	-1.2985	-1.29562	-1.29277	-1.31921	-1.31639
-1.31074	-1.30778	-1.30484	-1.30191	-1.299	-1.29612	-1.29327	-1.31972	-1.31687
-1.31125	-1.3083	-1.30536	-1.30242	-1.2995	-1.29662	-1.29377	-1.32024	-1.31738
-1.31177	-1.30882	-1.30587	-1.30293	-1.30001	-1.29713	-1.29428	-1.32076	-1.3179
-1.31229	-1.30933	-1.30638	-1.30344	-1.30052	-1.29763	-1.29477	-1.32128	-1.31843
-1.31281	-1.30985	-1.30689	-1.30395	-1.30104	-1.29814	-1.29528	-1.32181	-1.31898
-1.31332	-1.31036	-1.30741	-1.30446	-1.30154	-1.29865	-1.2958	-1.32236	-1.31953
-1.31383	-1.31087	-1.30792	-1.30497	-1.30205	-1.29917	-1.29632	-1.32291	-1.32008
-1.31434	-1.31138	-1.30843	-1.30549	-1.30258	-1.29971	-1.29687	-1.32346	-1.3206
-1.31484	-1.31189	-1.30894	-1.30601	-1.30311	-1.30025	-1.29741	-1.32401	-1.32112
-1.31536	-1.31241	-1.30948	-1.30656	-1.30366	-1.30078	-1.29792	-1.32453	-1.32164
-1.31588	-1.31294	-1.31002	-1.30711	-1.30421	-1.30131	-1.29842	-1.32504	-1.32216
-1.24866	-1.24592	-1.31057	-1.30766	-1.30475	-1.30183	-1.29892	-1.32556	-1.32267
-1.24917	-1.24644	-1.31111	-1.30819	-1.30526	-1.30234	-1.29944	-1.32609	-1.32318
-1.24969	-1.24696	-1.31165	-1.30871	-1.30579	-1.30287	-1.29998	-1.32663	-1.32372
-1.25022	-1.24745	-1.31217	-1.30924	-1.30632	-1.30343	-1.30055	-1.32723	-1.32431
-1.25074	-1.24795	-1.3127	-1.30977	-1.30687	-1.30399	-1.30112	-1.32783	-1.32491
-1.25123	-1.24844	-1.31323	-1.31032	-1.30742	-1.30453	-1.30164	-1.32837	-1.32545
-1.25172	-1.24893	-1.31377	-1.31087	-1.30797	-1.30506	-1.30215	-1.32889	-1.32597
-1.25227	-1.24947	-1.31432	-1.31141	-1.3085	-1.30557	-1.30266	-1.32943	-1.32652
-1.25286	-1.25003	-1.31488	-1.31193	-1.309	-1.30608	-1.30318	-1.32999	-1.3271
-1.25342	-1.25059	-1.31546	-1.31249	-1.30955	-1.30662	-1.30372	-1.33055	-1.32766
-1.25394	-1.25114	-1.31606	-1.31309	-1.31014	-1.30719	-1.30427	-1.33109	-1.32817
-1.25447	-1.25167	-1.31663	-1.31368	-1.31072	-1.30777	-1.30483	-1.33165	-1.32871
-1.25501	-1.25216	-1.31715	-1.3142	-1.31126	-1.30833	-1.30541	-1.33226	-1.32931
-1.25556	-1.25267	-1.31767	-1.31471	-1.31178	-1.30888	-1.30599	-1.33286	-1.32992
-1.25613	-1.25323	-1.31826	-1.31528	-1.31233	-1.30941	-1.30651	-1.3334	-1.33047
-1.2567	-1.25382	-1.31888	-1.31588	-1.31289	-1.30993	-1.307	-1.33391	-1.33099
-1.25723	-1.25436	-1.28945	-1.28652	-1.31348	-1.3105	-1.30755	-1.33448	-1.33156
-1.25772	-1.25488	-1.29001	-1.28711	-1.3141	-1.31111	-1.30816	-1.33509	-1.33215
-1.25824	-1.25541	-1.29059	-1.2877	-1.31471	-1.31172	-1.30876	-1.3357	-1.33275
-1.25881	-1.25598	-1.29119	-1.2883	-1.31532	-1.31232	-1.30935	-1.3363	-1.33335
-1.18707	-1.18441	-1.29179	-1.28889	-1.31593	-1.31292	-1.30994	-1.3369	-1.33395
-1.18761	-1.18495	-1.2924	-1.2895	-1.31654	-1.31353	-1.31054	-1.33751	-1.33455
-1.18816	-1.1855	-1.29301	-1.2901	-1.31715	-1.31414	-1.31114	-1.33812	-1.33515
-1.18869	-1.18603	-1.29362	-1.29071	-1.31777	-1.31475	-1.31174	-1.33873	-1.33576
-1.18922	-1.18657	-1.29422	-1.29131	-1.31838	-1.31535	-1.31234	-1.33935	-1.33637
-1.18976	-1.1871	-1.22393	-1.22119	-1.319	-1.31596	-1.31295	-1.33996	-1.33698
-1.1903	-1.18764	-1.22449	-1.22175	-1.31961	-1.31658	-1.31355	-1.34059	-1.3376
-1.15312	-1.15054	-1.22506	-1.22232	-1.32023	-1.31719	-1.31416	-1.34121	-1.33821
-1.15364	-1.15106	-1.22562	-1.22287	-1.32084	-1.31779	-1.31476	-1.34182	-1.33882
-1.15417	-1.15158	-1.22618	-1.22343	-1.32145	-1.3184	-1.31537	-1.34244	-1.33943
-1.1547	-1.1521	-1.22675	-1.22401	-1.25244	-1.24955	-1.24668	-1.31299	-1.31004
-1.15524	-1.15263	-1.22732	-1.22459	-1.25303	-1.25014	-1.24726	-1.31361	-1.31064
-1.15584	-1.1532	-1.18826	-1.18564	-1.22242	-1.21963	-1.21684	-1.24506	-1.24224
-1.15647	-1.15379	-1.18884	-1.1862	-1.22302	-1.22027	-1.21751	-1.24574	-1.24288
-1.15706	-1.15437	-1.18941	-1.18675	-1.2236	-1.22087	-1.21814	-1.24639	-1.24353
-1.15762	-1.15491	-1.18996	-1.18728	-1.22415	-1.22144	-1.21873	-1.24702	-1.24419

-1.15818	-1.15547	-1.15282	-1.15021	-1.22472	-1.222	-1.2193	-1.24763	-1.24484
-1.15877	-1.1561	-1.15345	-1.15081	-1.22533	-1.22258	-1.21986	-1.24821	-1.24543
-1.15935	-1.15673	-1.15408	-1.15142	-1.22596	-1.22318	-1.22042	-1.24878	-1.246
-1.1599	-1.15729	-1.15467	-1.15203	-1.22663	-1.22381	-1.22102	-1.24938	-1.24659
-1.16043	-1.15783	-1.15523	-1.15265	-1.22732	-1.22448	-1.22165	-1.24999	-1.24718
-1.16098	-1.15837	-1.15578	-1.15322	-1.22796	-1.22513	-1.2223	-1.25064	-1.2478
-1.16156	-1.15891	-1.15631	-1.15374	-1.22856	-1.22578	-1.22299	-1.25132	-1.24843
-1.16217	-1.15948	-1.15685	-1.15427	-1.15171	-1.14919	-1.14666	-1.25199	-1.24908
-1.16282	-1.16009	-1.15741	-1.1548	-1.15222	-1.1497	-1.14718	-1.25263	-1.24976
-1.16346	-1.16071	-1.15801	-1.15535	-1.15275	-1.15021	-1.1477	-1.25324	-1.25042
-1.16407	-1.16136	-1.15866	-1.15596	-1.15332	-1.15074	-1.1482	-1.25382	-1.25101
-1.16465	-1.162	-1.15932	-1.1566	-1.15392	-1.15131	-1.14874	-1.2544	-1.2516
-1.09187	-1.0894	-1.1599	-1.15722	-1.15456	-1.15195	-1.14937	-1.25507	-1.25224
-1.09238	-1.08988	-1.16046	-1.15783	-1.15522	-1.15263	-1.15005	-1.25579	-1.2529
-1.09294	-1.09041	-1.16103	-1.15841	-1.15582	-1.15324	-1.15066	-1.22017	-1.21737
-1.09355	-1.09098	-1.16163	-1.15897	-1.15634	-1.15376	-1.1512	-1.2208	-1.21805
-1.05037	-1.04791	-1.08902	-1.08654	-1.15693	-1.15433	-1.15176	-1.18174	-1.17914
-1.05088	-1.04846	-1.08964	-1.08717	-1.15762	-1.15497	-1.15235	-1.1823	-1.17966
-1.0514	-1.04902	-1.09025	-1.0878	-1.1583	-1.15562	-1.15295	-1.18289	-1.18022
-1.05197	-1.04956	-1.09079	-1.08832	-1.15888	-1.15622	-1.15358	-1.18355	-1.18089
-1.05256	-1.05009	-1.09132	-1.08882	-1.15943	-1.15681	-1.15422	-1.18424	-1.1816
-1.05311	-1.05064	-1.0919	-1.08941	-1.08694	-1.08449	-1.08206	-1.18486	-1.18221
-1.05363	-1.05121	-1.09252	-1.09005	-1.08757	-1.08507	-1.08259	-1.18544	-1.18278
-1.05418	-1.05175	-1.0931	-1.09062	-1.08814	-1.08563	-1.08315	-1.15342	-1.15082
-1.05476	-1.05228	-1.09362	-1.09112	-1.08863	-1.08617	-1.08373	-1.15407	-1.15148
-1.05532	-1.05281	-1.09417	-1.09165	-1.08917	-1.08673	-1.08432	-1.0819	-1.0795
-1.05583	-1.05337	-1.09478	-1.09227	-1.08979	-1.08736	-1.08494	-1.08249	-1.08004
-1.05634	-1.05393	-1.09541	-1.09291	-1.09043	-1.08799	-1.08555	-1.08307	-1.08058
-1.05693	-1.05454	-1.05213	-1.04971	-1.09099	-1.08852	-1.08607	-1.0836	-1.08113
-1.05755	-1.05515	-1.05271	-1.05025	-1.09152	-1.08902	-1.08655	-1.08411	-1.0817
-1.0581	-1.05568	-1.05324	-1.05078	-1.09209	-1.08959	-1.08712	-1.08469	-1.08229
-1.0586	-1.05614	-1.05371	-1.0513	-1.09269	-1.09021	-1.08776	-1.08533	-1.08291
-1.05914	-1.05667	-1.05424	-1.05186	-1.0933	-1.09084	-1.08839	-1.08595	-1.08351
-1.05974	-1.05728	-1.05485	-1.05247	-1.09395	-1.09149	-1.08904	-1.08656	-1.08407
-1.06035	-1.0579	-1.05547	-1.05307	-1.09457	-1.0921	-1.08964	-1.08714	-1.08462
-1.061	-1.05854	-1.05608	-1.05363	-1.09512	-1.09262	-1.09014	-1.08766	-1.08519
-1.06163	-1.05915	-1.05667	-1.05417	-1.09565	-1.09313	-1.09064	-1.08819	-1.08577
-1.06218	-1.05968	-1.05719	-1.05471	-1.09624	-1.09372	-1.09123	-1.08879	-1.08638
-1.06269	-1.06015	-1.05767	-1.05525	-1.09685	-1.09435	-1.09187	-1.08943	-1.08701
-1.06327	-1.06071	-1.05823	-1.05583	-1.05344	-1.05106	-1.0487	-1.09006	-1.08764
-1.06391	-1.06135	-1.05886	-1.05645	-1.05405	-1.05167	-1.0493	-1.09069	-1.08826
-0.98553	-0.98313	-1.0595	-1.05707	-1.05466	-1.05227	-1.0499	-1.09131	-1.08885
-0.98612	-0.98371	-1.06013	-1.05768	-1.05527	-1.05288	-1.05049	-1.0919	-1.0894
-0.98672	-0.9843	-1.06076	-1.05831	-1.05587	-1.05347	-1.05107	-1.09248	-1.08994
-0.98733	-0.98491	-1.06141	-1.05894	-1.05648	-1.05404	-1.05161	-1.09304	-1.09051
-0.98794	-0.98551	-1.06207	-1.05957	-1.05708	-1.0546	-1.05213	-1.0936	-1.09109
-0.98856	-0.98613	-1.02305	-1.02059	-1.05764	-1.05515	-1.05268	-1.09419	-1.09169
-0.98918	-0.98674	-1.02366	-1.02114	-1.05818	-1.05569	-1.05324	-1.09479	-1.09231
-0.98978	-0.98732	-1.02424	-1.02168	-1.05874	-1.05626	-1.05383	-1.05143	-1.04906
-0.99036	-0.98784	-1.02477	-1.02222	-1.05931	-1.05685	-1.05443	-1.05204	-1.04967

-0.99095	-0.98839	-0.98588	-0.98342	-1.02032	-1.01794	-1.01561	-1.05265	-1.05029
-0.99156	-0.98899	-0.98648	-0.984	-1.02091	-1.01853	-1.01621	-1.05327	-1.0509
-0.99216	-0.9896	-0.98708	-0.98459	-1.02153	-1.01914	-1.0168	-1.05389	-1.05152
-0.9927	-0.99017	-0.98767	-0.98519	-1.02216	-1.01975	-1.0174	-1.01507	-1.01278
-0.99322	-0.99072	-0.98825	-0.9858	-1.02281	-1.02038	-1.01799	-1.01566	-1.01336
-0.9938	-0.99131	-0.98885	-0.98641	-1.02345	-1.021	-1.01859	-1.01625	-1.01394
-0.99442	-0.99192	-0.98945	-0.98701	-1.02408	-1.02161	-1.01919	-1.01684	-1.01452
-0.99504	-0.99253	-0.99006	-0.98761	-1.02471	-1.02223	-1.01979	-1.01743	-1.01511
-0.99564	-0.99312	-0.99065	-0.98821	-1.02534	-1.02285	-1.02039	-1.01801	-1.01568
-0.99626	-0.99374	-0.99125	-0.98882	-1.02598	-1.02347	-1.021	-1.01861	-1.01626
-0.99695	-0.9944	-0.99189	-0.98943	-1.0266	-1.02408	-1.02161	-1.0192	-1.01685
-0.99765	-0.99508	-0.99255	-0.99006	-1.02725	-1.02472	-1.02224	-1.01982	-1.01745
-0.96115	-0.9587	-0.99327	-0.99078	-0.98831	-0.98584	-0.98341	-1.02045	-1.01805
-0.96176	-0.95937	-0.99401	-0.99154	-0.98907	-0.98656	-0.98407	-1.02111	-1.01867
-0.87565	-0.87355	-0.99467	-0.99222	-0.98975	-0.98725	-0.98477	-1.02182	-1.01936
-0.87614	-0.87403	-0.99526	-0.99282	-0.99038	-0.98793	-0.98549	-1.0226	-1.02013
-0.7882	-0.78629	-0.95876	-0.95641	-0.99099	-0.98857	-0.98617	-1.02332	-1.02085
-0.78867	-0.78675	-0.95933	-0.95698	-0.99159	-0.98917	-0.98677	-1.02395	-1.02147
-0.78914	-0.78722	-0.9599	-0.95755	-0.99218	-0.98976	-0.98735	-1.02457	-1.02209
-0.68715	-0.68562	-0.83192	-0.8299	-0.99278	-0.99035	-0.98794	-1.02519	-1.02271
-0.68752	-0.68599	-0.83241	-0.83039	-0.99337	-0.99095	-0.98853	-1.02582	-1.02334
-0.68791	-0.68637	-0.8329	-0.83088	-0.95444	-0.95208	-0.94974	-1.02644	-1.02396
-0.6883	-0.68675	-0.8334	-0.83137	-0.95503	-0.95267	-0.95032	-1.02707	-1.02459
-0.68875	-0.68719	-0.74094	-0.73914	-0.8299	-0.8279	-0.82591	-0.94864	-0.94632
-0.68927	-0.68769	-0.74151	-0.7397	-0.83053	-0.82852	-0.82653	-0.94938	-0.94705
-0.68975	-0.68815	-0.68659	-0.68506	-0.83112	-0.8291	-0.8271	-0.95007	-0.94773
-0.69015	-0.68855	-0.68698	-0.68545	-0.83163	-0.8296	-0.82759	-0.95066	-0.94832
-0.69054	-0.68893	-0.68735	-0.68582	-0.83211	-0.83007	-0.82805	-0.95122	-0.94889
-0.69094	-0.68932	-0.68774	-0.68619	-0.78642	-0.7845	-0.78261	-0.86576	-0.86369
-0.69135	-0.68972	-0.68813	-0.68658	-0.78688	-0.78497	-0.78308	-0.86628	-0.8642
-0.69181	-0.69019	-0.6886	-0.68703	-0.78743	-0.78552	-0.78362	-0.86687	-0.86478
-0.69231	-0.69071	-0.68912	-0.68754	-0.78805	-0.78612	-0.78422	-0.86753	-0.86542
-0.69278	-0.69118	-0.68959	-0.688	-0.68644	-0.68491	-0.68339	-0.78286	-0.78097
-0.6932	-0.69159	-0.68999	-0.68839	-0.68682	-0.68529	-0.68377	-0.78333	-0.78143
-0.64856	-0.64703	-0.69037	-0.68877	-0.6872	-0.68566	-0.68415	-0.7838	-0.7819
-0.64893	-0.6474	-0.69076	-0.68915	-0.68757	-0.68603	-0.68451	-0.78428	-0.78243
-0.64931	-0.64778	-0.69117	-0.68956	-0.68797	-0.68642	-0.68489	-0.78478	-0.78297
-0.64977	-0.64824	-0.69166	-0.69004	-0.68845	-0.68688	-0.68535	-0.78533	-0.78349
-0.65025	-0.64872	-0.69218	-0.69055	-0.68896	-0.68739	-0.68584	-0.78589	-0.78401
-0.65068	-0.64915	-0.69263	-0.69101	-0.6894	-0.68782	-0.68627	-0.68475	-0.68326
-0.65105	-0.64952	-0.69303	-0.6914	-0.68979	-0.68821	-0.68665	-0.68515	-0.68368
-0.65146	-0.64993	-0.6484	-0.64687	-0.69023	-0.68863	-0.68706	-0.68556	-0.6841
-0.65193	-0.65039	-0.64886	-0.64733	-0.69073	-0.68912	-0.68754	-0.68601	-0.68451
-0.65239	-0.65084	-0.64931	-0.64779	-0.69121	-0.68961	-0.68802	-0.68647	-0.68494
-0.65278	-0.65123	-0.64969	-0.64817	-0.69163	-0.69005	-0.68849	-0.68694	-0.68541
-0.65315	-0.65161	-0.65007	-0.64853	-0.69203	-0.69047	-0.68894	-0.6874	-0.68588
-0.6536	-0.65205	-0.65051	-0.64898	-0.64747	-0.64599	-0.64453	-0.68783	-0.68629
-0.65408	-0.65253	-0.65099	-0.64946	-0.64794	-0.64643	-0.64493	-0.68823	-0.68667
-0.60246	-0.60106	-0.65146	-0.64993	-0.6484	-0.64688	-0.64537	-0.68868	-0.6871
-0.60282	-0.60146	-0.65191	-0.65039	-0.64887	-0.64735	-0.64584	-0.68917	-0.68759

-0.6032	-0.60184	-0.65234	-0.65082	-0.64931	-0.64779	-0.64628	-0.64478	-0.64329
-0.60361	-0.60222	-0.65274	-0.65121	-0.64968	-0.64816	-0.64665	-0.64517	-0.64371
-0.60404	-0.60261	-0.65314	-0.6516	-0.65006	-0.64853	-0.64702	-0.64556	-0.64412
-0.49916	-0.49802	-0.60161	-0.60019	-0.65051	-0.64898	-0.64747	-0.64599	-0.64453
-0.49953	-0.49837	-0.60205	-0.60063	-0.65099	-0.64946	-0.64794	-0.64643	-0.64494
-0.49986	-0.49871	-0.60247	-0.60106	-0.59966	-0.59826	-0.59687	-0.64689	-0.64538
-0.50016	-0.49903	-0.60289	-0.6015	-0.6001	-0.59869	-0.59729	-0.64736	-0.64584
-0.50049	-0.49935	-0.55472	-0.55343	-0.6005	-0.59909	-0.5977	-0.64782	-0.64631
-0.50085	-0.49968	-0.55506	-0.55375	-0.60083	-0.59946	-0.5981	-0.64827	-0.64678
-0.50122	-0.50002	-0.49885	-0.49771	-0.60118	-0.59983	-0.59849	-0.59714	-0.59578
-0.5016	-0.50038	-0.4992	-0.49805	-0.60158	-0.6002	-0.59885	-0.59751	-0.59618
-0.50198	-0.50076	-0.49957	-0.49841	-0.60201	-0.60059	-0.59921	-0.59787	-0.59656
-0.50238	-0.50115	-0.49995	-0.49877	-0.55401	-0.5527	-0.55141	-0.59826	-0.59693
-0.50279	-0.50155	-0.50034	-0.49915	-0.55444	-0.55312	-0.55182	-0.59866	-0.59729
-0.50318	-0.50195	-0.50073	-0.49953	-0.49836	-0.49723	-0.49612	-0.55094	-0.54967
-0.50357	-0.50235	-0.50113	-0.49991	-0.49872	-0.49758	-0.49646	-0.55134	-0.55007
-0.39907	-0.39812	-0.50151	-0.50028	-0.49908	-0.49793	-0.49681	-0.55174	-0.55047
-0.39934	-0.3984	-0.50188	-0.50066	-0.49946	-0.49829	-0.49715	-0.55215	-0.55087
-0.39962	-0.39867	-0.45177	-0.45066	-0.49983	-0.49865	-0.4975	-0.49639	-0.49532
-0.39992	-0.39895	-0.45209	-0.45099	-0.5002	-0.49902	-0.49786	-0.49675	-0.49566
-0.40023	-0.39924	-0.45241	-0.45133	-0.50057	-0.49939	-0.49823	-0.4971	-0.496
-0.40054	-0.39955	-0.39857	-0.39763	-0.45061	-0.44952	-0.44844	-0.49745	-0.49634
-0.40086	-0.39987	-0.39889	-0.39795	-0.45097	-0.44987	-0.44878	-0.49781	-0.49668
-0.40118	-0.40019	-0.39921	-0.39826	-0.39731	-0.39635	-0.3954	-0.44806	-0.44701
-0.40149	-0.4005	-0.39953	-0.39858	-0.39763	-0.39667	-0.39571	-0.44841	-0.44734
-0.35133	-0.35042	-0.39985	-0.3989	-0.39794	-0.39698	-0.39601	-0.44876	-0.44768
-0.35164	-0.35073	-0.40015	-0.3992	-0.39824	-0.39728	-0.39632	-0.4491	-0.44801
-0.35195	-0.35104	-0.40045	-0.3995	-0.39855	-0.39759	-0.39662	-0.44945	-0.44835
-0.29485	-0.29411	-0.40076	-0.39981	-0.39885	-0.39789	-0.39693	-0.39595	-0.39497
-0.2951	-0.29436	-0.40106	-0.40011	-0.39916	-0.3982	-0.39723	-0.39625	-0.39526
-0.29535	-0.29461	-0.35107	-0.35021	-0.39947	-0.39851	-0.39754	-0.39655	-0.39556
-0.2956	-0.29485	-0.35138	-0.35051	-0.39977	-0.39881	-0.39785	-0.39686	-0.39587
-0.29585	-0.29511	-0.29438	-0.29367	-0.34995	-0.34907	-0.34819	-0.39716	-0.39617
-0.29611	-0.29536	-0.29463	-0.29392	-0.35026	-0.34938	-0.3485	-0.39747	-0.39648
-0.29636	-0.29562	-0.29489	-0.29417	-0.35057	-0.34969	-0.3488	-0.39777	-0.39679
-0.29663	-0.29589	-0.29516	-0.29445	-0.3509	-0.35001	-0.34911	-0.39807	-0.39709
-0.2969	-0.29617	-0.29544	-0.29472	-0.35123	-0.35033	-0.34942	-0.39838	-0.39739
-0.29717	-0.29643	-0.2957	-0.29497	-0.29425	-0.2935	-0.29276	-0.34883	-0.34794
-0.29743	-0.29668	-0.29595	-0.29522	-0.29449	-0.29375	-0.29302	-0.34915	-0.34828
-0.29771	-0.29695	-0.29621	-0.29547	-0.29474	-0.29401	-0.29328	-0.34949	-0.34861
-0.298	-0.29724	-0.29648	-0.29573	-0.29499	-0.29427	-0.29356	-0.34983	-0.34895
-0.29829	-0.29752	-0.29675	-0.29599	-0.29524	-0.29453	-0.29382	-0.35016	-0.34927
-0.29855	-0.29778	-0.29702	-0.29625	-0.2955	-0.29478	-0.29407	-0.35047	-0.34958
-0.29881	-0.29805	-0.29728	-0.29651	-0.29576	-0.29503	-0.29432	-0.2936	-0.29287
-0.29909	-0.29832	-0.29755	-0.29678	-0.29603	-0.29531	-0.29458	-0.29385	-0.29312
-0.29937	-0.29859	-0.29782	-0.29706	-0.29632	-0.29558	-0.29485	-0.29411	-0.29337
-0.18909	-0.18858	-0.2981	-0.29735	-0.2966	-0.29585	-0.29511	-0.29437	-0.29363
-0.18927	-0.18877	-0.29839	-0.29763	-0.29688	-0.29612	-0.29537	-0.29463	-0.2939
-0.18945	-0.18895	-0.29867	-0.29791	-0.29715	-0.29639	-0.29563	-0.2949	-0.29417
-0.18964	-0.18914	-0.29895	-0.29818	-0.29742	-0.29666	-0.29592	-0.29518	-0.29445

-0.12698	-0.12664	-0.18882	-0.18831	-0.29769	-0.29694	-0.2962	-0.29545	-0.29472
-0.12709	-0.12675	-0.18899	-0.18849	-0.29797	-0.29722	-0.29648	-0.29572	-0.29497
-0.12721	-0.12687	-0.18917	-0.18867	-0.29826	-0.29751	-0.29675	-0.29599	-0.29523
-0.12734	-0.127	-0.18936	-0.18886	-0.24471	-0.24408	-0.24346	-0.29626	-0.29551
-0.12747	-0.12713	-0.18954	-0.18904	-0.24495	-0.24431	-0.24368	-0.29653	-0.29579
-0.1276	-0.12726	-0.18973	-0.18922	-0.18872	-0.18822	-0.18772	-0.24329	-0.24268
-0.12773	-0.12739	-0.18991	-0.1894	-0.18889	-0.18839	-0.1879	-0.24354	-0.24293
-0.12786	-0.12751	-0.1901	-0.18958	-0.18907	-0.18857	-0.18809	-0.1876	-0.18712
-0.12798	-0.12763	-0.19027	-0.18976	-0.18925	-0.18876	-0.18827	-0.18779	-0.1873
-0.1281	-0.12776	-0.19045	-0.18994	-0.18944	-0.18894	-0.18846	-0.18797	-0.18748
-0.12823	-0.12789	-0.19064	-0.19013	-0.18963	-0.18913	-0.18864	-0.18815	-0.18767
-0.12835	-0.12802	-0.19084	-0.19033	-0.18982	-0.18931	-0.18881	-0.18833	-0.18785
-0.12848	-0.12815	-0.19103	-0.19051	-0.19001	-0.1895	-0.189	-0.18851	-0.18802
-0.12862	-0.12828	-0.19122	-0.1907	-0.19019	-0.18968	-0.18918	-0.18869	-0.1882
-0.12874	-0.1284	-0.19141	-0.19089	-0.19038	-0.18987	-0.18937	-0.18887	-0.18838
-0.12887	-0.12853	-0.1916	-0.19109	-0.19058	-0.19006	-0.18955	-0.18905	-0.18856
-0.129	-0.12866	-0.19179	-0.19128	-0.19077	-0.19025	-0.18973	-0.18923	-0.18874
-0.12912	-0.12879	-0.19198	-0.19147	-0.19096	-0.19044	-0.18993	-0.18942	-0.18891
-0.12925	-0.12892	-0.12858	-0.12824	-0.19115	-0.19063	-0.19012	-0.18961	-0.18909
-0.12937	-0.12904	-0.12871	-0.12837	-0.19134	-0.19083	-0.19031	-0.18979	-0.18928
-0.1295	-0.12917	-0.12883	-0.1285	-0.19153	-0.19102	-0.1905	-0.18998	-0.18947
-0.12963	-0.12929	-0.12896	-0.12863	-0.19172	-0.19121	-0.1907	-0.19018	-0.18966
-0.12976	-0.12941	-0.12908	-0.12876	-0.19192	-0.19141	-0.19089	-0.19037	-0.18985
-0.06153	-0.06139	-0.12921	-0.12889	-0.19211	-0.19161	-0.1911	-0.19058	-0.19006
-0.06159	-0.06145	-0.12935	-0.12902	-0.19231	-0.19181	-0.19131	-0.19079	-0.19027
-0.06166	-0.06152	-0.12948	-0.12915	-0.19251	-0.19201	-0.19151	-0.191	-0.19048
-0.06172	-0.06158	-0.12961	-0.12928	-0.19269	-0.1922	-0.1917	-0.19119	-0.19067
-0.06179	-0.06164	-0.12973	-0.1294	-0.19288	-0.19238	-0.19189	-0.19138	-0.19087
-0.06185	-0.0617	-0.12986	-0.12953	-0.19306	-0.19257	-0.19207	-0.19157	-0.19106
-0.06191	-0.06176	-0.12999	-0.12965	-0.19325	-0.19275	-0.19226	-0.19176	-0.19126
-0.06198	-0.06182	-0.06167	-0.06153	-0.12946	-0.12913	-0.1288	-0.19196	-0.19146
-0.06204	-0.06189	-0.06174	-0.06159	-0.1296	-0.12926	-0.12893	-0.19216	-0.19166
-0.06211	-0.06195	-0.0618	-0.06166	-0.12973	-0.1294	-0.12907	-0.19236	-0.19186
-0.06218	-0.06202	-0.06187	-0.06172	-0.12986	-0.12952	-0.1292	-0.19254	-0.19204
-0.06225	-0.0621	-0.06194	-0.06179	-0.06164	-0.0615	-0.06136	-0.19273	-0.19223
-0.06231	-0.06217	-0.06201	-0.06186	-0.0617	-0.06156	-0.06142	-0.19293	-0.19243
-0.06238	-0.06223	-0.06208	-0.06192	-0.06177	-0.06162	-0.06148	-0.19313	-0.19264
-0.06244	-0.0623	-0.06215	-0.06199	-0.06184	-0.06169	-0.06154	-0.19332	-0.19283
-0.06251	-0.06236	-0.06221	-0.06205	-0.0619	-0.06175	-0.0616	-0.19351	-0.19301
-0.06257	-0.06243	-0.06228	-0.06212	-0.06197	-0.06181	-0.06166	-0.06152	-0.06138
-0.06264	-0.06249	-0.06235	-0.06219	-0.06204	-0.06188	-0.06173	-0.06158	-0.06144
-0.0627	-0.06256	-0.06241	-0.06226	-0.06211	-0.06195	-0.0618	-0.06165	-0.06151
-0.06275	-0.06262	-0.06247	-0.06233	-0.06218	-0.06203	-0.06187	-0.06172	-0.06157
-0.06281	-0.06268	-0.06254	-0.0624	-0.06225	-0.0621	-0.06194	-0.06179	-0.06164
-0.06287	-0.06274	-0.0626	-0.06246	-0.06231	-0.06216	-0.062	-0.06185	-0.0617
-0.06294	-0.0628	-0.06266	-0.06252	-0.06237	-0.06222	-0.06207	-0.06192	-0.06177
-0.063	-0.06286	-0.06272	-0.06258	-0.06244	-0.06229	-0.06214	-0.06199	-0.06184
-0.06306	-0.06292	-0.06279	-0.06265	-0.06251	-0.06236	-0.06221	-0.06206	-0.06191
-0.06312	-0.06299	-0.06285	-0.06271	-0.06257	-0.06243	-0.06228	-0.06213	-0.06198
-0.06319	-0.06305	-0.06291	-0.06277	-0.06263	-0.06249	-0.06235	-0.0622	-0.06205

-0.06325	-0.06311	-0.06297	-0.06283	-0.0627	-0.06256	-0.06241	-0.06227	-0.06212
-0.06332	-0.06318	-0.06304	-0.0629	-0.06276	-0.06262	-0.06248	-0.06234	-0.06219
-0.0634	-0.06325	-0.0631	-0.06296	-0.06282	-0.06268	-0.06254	-0.0624	-0.06226
		-0.06317	-0.06302	-0.06288	-0.06274	-0.06261	-0.06247	-0.06233
		-0.06323	-0.06309	-0.06294	-0.06281	-0.06267	-0.06253	-0.0624
		-0.0633	-0.06315	-0.06301	-0.06287	-0.06273	-0.0626	-0.06246
		-0.06338	-0.06322	-0.06307	-0.06293	-0.06279	-0.06266	-0.06253
				-0.06314	-0.063	-0.06286	-0.06272	-0.06259
				-0.0632	-0.06306	-0.06292	-0.06278	-0.06265
				-0.06327	-0.06312	-0.06298	-0.06285	-0.06272
				-0.06334	-0.06319	-0.06304	-0.06291	-0.06278
				-0.06341	-0.06326	-0.06311	-0.06297	-0.06284
							-0.06304	-0.0629
							-0.06311	-0.06297
							-0.06317	-0.06303
							-0.06324	-0.06309

							-0.13226	-0.13183
							-0.13207	-0.13164
							-0.19507	-0.1945
							-0.19478	-0.19422
				-0.06247	-0.06226	-0.06207	-0.25725	-0.25658
				-0.06238	-0.06218	-0.06199	-0.25697	-0.25631
				-0.0623	-0.06211	-0.06192	-0.2567	-0.25603
				-0.25844	-0.25775	-0.25707	-0.25639	-0.25572
				-0.25815	-0.25745	-0.25676	-0.25608	-0.2554
		-0.06245	-0.06224	-0.25784	-0.25715	-0.25646	-0.50629	-0.5049
		-0.06236	-0.06216	-0.25753	-0.25686	-0.25618	-0.50568	-0.50431
		-0.25858	-0.2579	-0.25723	-0.25656	-0.25588	-0.55853	-0.55706
		-0.2583	-0.25762	-0.25694	-0.25625	-0.25556	-0.55782	-0.55635
-0.0625	-0.0623	-0.25801	-0.25733	-0.50799	-0.50657	-0.50515	-0.60878	-0.60719
-0.06241	-0.06222	-0.25769	-0.25701	-0.50734	-0.50594	-0.50454	-0.60806	-0.60648
-0.06232	-0.06213	-0.25737	-0.2567	-0.50669	-0.50531	-0.50394	-0.60736	-0.60578
-0.25844	-0.25776	-0.25709	-0.25641	-0.61145	-0.60985	-0.60825	-0.68762	-0.68589
-0.25817	-0.25749	-0.25682	-0.25614	-0.61074	-0.60914	-0.60755	-0.68688	-0.68514
-0.25789	-0.25721	-0.50767	-0.50625	-0.61001	-0.6084	-0.60682	-0.68612	-0.6844
-0.25761	-0.25693	-0.50705	-0.50563	-0.60925	-0.60764	-0.60605	-0.68533	-0.68366
-0.25731	-0.25663	-0.61183	-0.61016	-0.68976	-0.68799	-0.68625	-0.68457	-0.68294
-0.25698	-0.2563	-0.61104	-0.60941	-0.689	-0.68725	-0.68554	-0.68387	-0.68223
-0.25666	-0.25597	-0.61026	-0.60867	-0.68824	-0.68653	-0.68483	-0.68316	-0.68151
-0.37862	-0.37759	-0.69096	-0.68922	-0.68749	-0.68578	-0.68408	-0.6824	-0.68074
-0.37818	-0.37716	-0.69023	-0.68848	-0.68674	-0.68503	-0.68333	-0.68164	-0.67995
-0.6114	-0.60977	-0.68949	-0.68773	-0.686	-0.68428	-0.68258	-0.6809	-0.67922
-0.61069	-0.60906	-0.68875	-0.68699	-0.68525	-0.68354	-0.68185	-0.68017	-0.67851
-0.69156	-0.68977	-0.688	-0.68625	-0.68451	-0.68281	-0.68113	-0.73132	-0.72952
-0.69079	-0.689	-0.68724	-0.6855	-0.68378	-0.6821	-0.68043	-0.73055	-0.72873
-0.69002	-0.68824	-0.68649	-0.68476	-0.68306	-0.68139	-0.67972	-0.77878	-0.77679
-0.68925	-0.68748	-0.68575	-0.68405	-0.68237	-0.68069	-0.67901	-0.77792	-0.77591
-0.68848	-0.68674	-0.68502	-0.68335	-0.68167	-0.67998	-0.67828	-0.77705	-0.77502
-0.68772	-0.68601	-0.68431	-0.68263	-0.73291	-0.73107	-0.72921	-0.77616	-0.77414
-0.68698	-0.68529	-0.68361	-0.68192	-0.73212	-0.73026	-0.72839	-0.77527	-0.77327
-0.68626	-0.68458	-0.6829	-0.68119	-0.73131	-0.72944	-0.72757	-0.8781	-0.87582
-0.68556	-0.68388	-0.68218	-0.68045	-0.73048	-0.72862	-0.72676	-0.87715	-0.87491
-0.68485	-0.68317	-0.73344	-0.73156	-0.72968	-0.72782	-0.72597	-0.87621	-0.874
-0.68415	-0.68246	-0.73266	-0.73078	-0.72891	-0.72705	-0.7252	-0.87529	-0.87309
-0.68344	-0.68173	-0.73187	-0.73	-0.82994	-0.82779	-0.82566	-0.87437	-0.87219
-0.68271	-0.68098	-0.73104	-0.72918	-0.82902	-0.82689	-0.8248	-0.87347	-0.87129
-0.68199	-0.68023	-0.73021	-0.72836	-0.82809	-0.82601	-0.82395	-0.87257	-0.87039
-0.68128	-0.67952	-0.72943	-0.72758	-0.82721	-0.82514	-0.82309	-0.87165	-0.86945
-0.68058	-0.67882	-0.72867	-0.72682	-0.82634	-0.82428	-0.82225	-0.87072	-0.8685
-0.73169	-0.72979	-0.72792	-0.72607	-0.82548	-0.82343	-0.8214	-0.86981	-0.86757
-0.73092	-0.72903	-0.72717	-0.72533	-0.82464	-0.82259	-0.82057	-0.86891	-0.86668
-0.73016	-0.72828	-0.77516	-0.77318	-0.87459	-0.87241	-0.87024	-0.86802	-0.86578
-0.7294	-0.72752	-0.77435	-0.77239	-0.8737	-0.87151	-0.86934	-0.86713	-0.86489
-0.72864	-0.72678	-0.77356	-0.7716	-0.8728	-0.87062	-0.86845	-0.96093	-0.95845
-0.72789	-0.72603	-0.77276	-0.77081	-0.87191	-0.86973	-0.86756	-0.95994	-0.95745

-0.72714	-0.7253	-0.77197	-0.77002	-0.87102	-0.86884	-0.86666	-0.95894	-0.95645
-0.7264	-0.72457	-0.82381	-0.8217	-0.87012	-0.86794	-0.86577	-0.95795	-0.95546
-0.72567	-0.72384	-0.82297	-0.82086	-0.86923	-0.86705	-0.86487	-0.95696	-0.95446
-0.72493	-0.72311	-0.82213	-0.82002	-0.92002	-0.91771	-0.91539	-1.04775	-1.04493
-0.72419	-0.72237	-0.82128	-0.81917	-0.91907	-0.91676	-0.91444	-1.04664	-1.04383
-0.77194	-0.76997	-0.87098	-0.86875	-0.91812	-0.9158	-0.91349	-1.04554	-1.04273
-0.77114	-0.76917	-0.87008	-0.86785	-0.91717	-0.91485	-0.91254	-1.04443	-1.04164
-0.77034	-0.76837	-0.86918	-0.86695	-0.91622	-0.9139	-0.9116	-1.04333	-1.04056
-0.76954	-0.76757	-0.91998	-0.91761	-1.05043	-1.0477	-1.04499	-1.13363	-1.13037
-0.76874	-0.76678	-0.91903	-0.91666	-1.04932	-1.04659	-1.04389	-1.13234	-1.12908
-0.76795	-0.76601	-0.91812	-0.91575	-1.04823	-1.04551	-1.0428	-1.13104	-1.1278
-0.76716	-0.76526	-0.91724	-0.91486	-1.04718	-1.04443	-1.04172	-1.12975	-1.12652
-0.81875	-0.81672	-0.91632	-0.91395	-1.04611	-1.04336	-1.04064	-1.12847	-1.12528
-0.81794	-0.81589	-0.91537	-0.91301	-1.04503	-1.04228	-1.03957	-1.12721	-1.12408
-0.86975	-0.86754	-1.04949	-1.04672	-1.13563	-1.13234	-1.12911	-1.12597	-1.12288
-0.86887	-0.86665	-1.04839	-1.04567	-1.13441	-1.13114	-1.12791	-1.12475	-1.12164
-0.868	-0.86577	-1.0473	-1.04462	-1.13319	-1.12995	-1.12671	-1.12354	-1.1204
-0.91636	-0.914	-1.04624	-1.04357	-1.13191	-1.12868	-1.12547	-1.1654	-1.16216
-0.91549	-0.91312	-1.0452	-1.0425	-1.13059	-1.12739	-1.12421	-1.1641	-1.16087
-1.00397	-1.00131	-1.04415	-1.04144	-1.1293	-1.12614	-1.123	-1.16287	-1.15963
-1.00292	-1.00029	-1.04309	-1.04038	-1.12802	-1.12491	-1.12184	-1.1617	-1.15845
-1.00191	-0.9993	-1.04206	-1.03935	-1.12678	-1.1237	-1.12065	-1.16048	-1.15724
-1.00097	-0.99835	-1.04106	-1.03836	-1.1256	-1.12249	-1.1194	-1.15921	-1.156
-1.04554	-1.04279	-1.1308	-1.1276	-1.12444	-1.12129	-1.11817	-1.15794	-1.15476
-1.04455	-1.0418	-1.12961	-1.12643	-1.12328	-1.12014	-1.11702	-1.15675	-1.15358
-1.04354	-1.0408	-1.12842	-1.12526	-1.12212	-1.11899	-1.11588	-1.15557	-1.1524
-1.04248	-1.03976	-1.12718	-1.12402	-1.16392	-1.16072	-1.15753	-1.20069	-1.19717
-1.04138	-1.03869	-1.1259	-1.12275	-1.16262	-1.15943	-1.15627	-1.19932	-1.19578
-1.04033	-1.03766	-1.12467	-1.12153	-1.16138	-1.15821	-1.15505	-1.19799	-1.19445
-1.03933	-1.03665	-1.12347	-1.12037	-1.16021	-1.15703	-1.15386	-1.19669	-1.19319
-1.12863	-1.12544	-1.1223	-1.11922	-1.15904	-1.15585	-1.15268	-1.1954	-1.19194
-1.12745	-1.12428	-1.12116	-1.11807	-1.15785	-1.15468	-1.15152	-1.19412	-1.1907
-1.12627	-1.12314	-1.12003	-1.11693	-1.15666	-1.15351	-1.15037	-1.19286	-1.18945
-1.1251	-1.12198	-1.16184	-1.15865	-1.20204	-1.19853	-1.19505	-1.19161	-1.1882
-1.12394	-1.12082	-1.16065	-1.15746	-1.20071	-1.19724	-1.1938	-1.19036	-1.18695
-1.12278	-1.11967	-1.15946	-1.15625	-1.19936	-1.19589	-1.19247	-1.27224	-1.26844
-1.12162	-1.11853	-1.15827	-1.15502	-1.19797	-1.1945	-1.19108	-1.27074	-1.26703
-1.16348	-1.1603	-1.20385	-1.20024	-1.19667	-1.19318	-1.18976	-1.26928	-1.26565
-1.16231	-1.15913	-1.20259	-1.19901	-1.19546	-1.19196	-1.18852	-1.26788	-1.26429
-1.16115	-1.15796	-1.20132	-1.19776	-1.23691	-1.23322	-1.22959	-1.26652	-1.26295
-1.15997	-1.15679	-1.20001	-1.19645	-1.23553	-1.2319	-1.22834	-1.26522	-1.26164
-1.15879	-1.15562	-1.1987	-1.19514	-1.23416	-1.23059	-1.2271	-1.26394	-1.26035
-1.20451	-1.20097	-1.19745	-1.19392	-1.2329	-1.22933	-1.22583	-1.29447	-1.29073
-1.20322	-1.19971	-1.19622	-1.19275	-1.23169	-1.2281	-1.22457	-1.29309	-1.28942
-1.20193	-1.19843	-1.23767	-1.23405	-1.27119	-1.26739	-1.26367	-1.36499	-1.36115
-1.20065	-1.19716	-1.23633	-1.23276	-1.26986	-1.26614	-1.26247	-1.36364	-1.35974
-1.19936	-1.19589	-1.23502	-1.23147	-1.3007	-1.29684	-1.29302	-1.36231	-1.35841
-1.19806	-1.19462	-1.23372	-1.23019	-1.2993	-1.29547	-1.29171	-1.361	-1.35718
-1.19679	-1.19337	-1.26549	-1.26177	-1.29791	-1.2941	-1.29037	-1.35968	-1.35596
-1.19559	-1.19213	-1.26407	-1.26041	-1.29652	-1.29274	-1.28903	-1.35831	-1.35468

-1.19443	-1.19092	-1.26268	-1.25906	-1.29514	-1.29138	-1.28769	-1.35695	-1.35338
-1.23584	-1.23216	-1.26142	-1.25776	-1.36694	-1.36305	-1.35926	-1.35561	-1.35208
-1.2346	-1.23101	-1.26024	-1.25651	-1.36548	-1.36164	-1.35792	-1.3543	-1.35078
-1.26645	-1.26272	-1.25901	-1.25529	-1.36412	-1.36029	-1.3566	-1.35302	-1.34955
-1.26506	-1.2614	-1.25775	-1.25411	-1.36285	-1.35902	-1.3553	-1.35177	-1.34839
-1.26372	-1.26008	-1.29617	-1.29238	-1.36157	-1.35776	-1.35406	-1.35057	-1.34726
-1.26245	-1.25876	-1.29477	-1.29104	-1.36023	-1.3565	-1.35288	-1.34945	-1.34616
-1.26122	-1.25748	-1.29341	-1.28972	-1.35891	-1.35527	-1.35173	-1.34834	-1.34507
-1.26004	-1.2563	-1.29215	-1.28849	-1.35766	-1.35407	-1.35059	-1.34721	-1.34392
-1.25888	-1.25515	-1.29095	-1.28731	-1.35643	-1.35289	-1.34946	-1.34608	-1.34277
-1.25766	-1.25399	-1.36261	-1.35883	-1.35518	-1.3517	-1.34833	-1.34498	-1.34168
-1.2564	-1.25281	-1.36135	-1.35757	-1.35393	-1.3505	-1.34719	-1.34389	-1.34063
-1.25512	-1.25161	-1.36006	-1.35632	-1.35272	-1.34932	-1.34603	-1.34277	-1.33955
-1.25381	-1.25037	-1.35874	-1.35508	-1.35154	-1.34814	-1.34484	-1.3416	-1.33843
-1.25256	-1.24918	-1.35747	-1.35389	-1.35039	-1.34699	-1.34368	-1.34046	-1.33731
-1.25143	-1.24807	-1.35627	-1.35274	-1.34929	-1.3459	-1.34259	-1.33936	-1.33621
-1.25034	-1.247	-1.3551	-1.35162	-1.34821	-1.34483	-1.34152	-1.33828	-1.3351
-1.32322	-1.31976	-1.35396	-1.35053	-1.34715	-1.34378	-1.34045	-1.33718	-1.33397
-1.32205	-1.31863	-1.35281	-1.34944	-1.34609	-1.34273	-1.33939	-1.33609	-1.33284
-1.32087	-1.31745	-1.3516	-1.34828	-1.34499	-1.34165	-1.33833	-1.33503	-1.33177
-1.31968	-1.31624	-1.35034	-1.34708	-1.34385	-1.34056	-1.33727	-1.33401	-1.33076
-1.35619	-1.35259	-1.34915	-1.34592	-1.34272	-1.33947	-1.33622	-1.33297	-1.32975
-1.35505	-1.35148	-1.34805	-1.34481	-1.34161	-1.33838	-1.33516	-1.33193	-1.32872
-1.35393	-1.35039	-1.34698	-1.34372	-1.34052	-1.3373	-1.3341	-1.3309	-1.32771
-1.3528	-1.3493	-1.34591	-1.34266	-1.33945	-1.33625	-1.33307	-1.32988	-1.32671
-1.35167	-1.34821	-1.34485	-1.3416	-1.33841	-1.33522	-1.33205	-1.32888	-1.32572
-1.35055	-1.34711	-1.34377	-1.34054	-1.33735	-1.33419	-1.33104	-1.32789	-1.32474
-1.34943	-1.34601	-1.34269	-1.33947	-1.3363	-1.33316	-1.33004	-1.32691	-1.32377
-1.34833	-1.34493	-1.34162	-1.33841	-1.33526	-1.33214	-1.32904	-1.32592	-1.32279
-1.34725	-1.34386	-1.34056	-1.33737	-1.33423	-1.33112	-1.32804	-1.32493	-1.32181
-1.34617	-1.3428	-1.33951	-1.33633	-1.33321	-1.33011	-1.32704	-1.32394	-1.32082
-1.3451	-1.34175	-1.33848	-1.33531	-1.33219	-1.32911	-1.32605	-1.32295	-1.31984
-1.34405	-1.34073	-1.33748	-1.33432	-1.3312	-1.32813	-1.32507	-1.32197	-1.31886
-1.34301	-1.33975	-1.33654	-1.33339	-1.33027	-1.32718	-1.3241	-1.32099	-1.31788
-1.34199	-1.33879	-1.33562	-1.33248	-1.32936	-1.32624	-1.32312	-1.32001	-1.31691
-1.34096	-1.33778	-1.33463	-1.33152	-1.3284	-1.32527	-1.32214	-1.31902	-1.31593
-1.33993	-1.33674	-1.33361	-1.33052	-1.32743	-1.32429	-1.32114	-1.31803	-1.31494
-1.33895	-1.33577	-1.33263	-1.32954	-1.32645	-1.3233	-1.32015	-1.31704	-1.31396
-1.33803	-1.33485	-1.3317	-1.32858	-1.32546	-1.32231	-1.31916	-1.31606	-1.313
-1.3371	-1.33393	-1.33076	-1.32761	-1.32446	-1.32131	-1.31818	-1.3151	-1.31206
-1.33613	-1.33296	-1.32979	-1.32662	-1.32346	-1.32032	-1.31721	-1.31416	-1.31117
-1.33515	-1.33198	-1.32881	-1.32563	-1.32247	-1.31934	-1.31625	-1.31324	-1.3103
-1.33415	-1.33098	-1.32781	-1.32463	-1.32148	-1.31838	-1.31532	-1.35123	-1.34821
-1.33314	-1.32997	-1.3268	-1.32364	-1.32051	-1.31744	-1.31442	-1.3503	-1.34729
-1.33215	-1.32898	-1.32582	-1.32268	-1.31958	-1.31652	-1.3135	-1.34934	-1.34633
-1.33117	-1.328	-1.32486	-1.32176	-1.31868	-1.31561	-1.31257	-1.34837	-1.34536
-1.33025	-1.32707	-1.32394	-1.32084	-1.35681	-1.35362	-1.35047	-1.3474	-1.34439
-1.32939	-1.32624	-1.32309	-1.31995	-1.35583	-1.35264	-1.3495	-1.34643	-1.34343
-1.32851	-1.32537	-1.32222	-1.31904	-1.35484	-1.35166	-1.34853	-1.34548	-1.34249
-1.32757	-1.32442	-1.32126	-1.31807	-1.35384	-1.35066	-1.34755	-1.34452	-1.34156

-1.3266	-1.32343	-1.32027	-1.31709	-1.35284	-1.34968	-1.3466	-1.34359	-1.34066
-1.32562	-1.32244	-1.31929	-1.31614	-1.35189	-1.34878	-1.34574	-1.34277	-1.33985
-1.32464	-1.32145	-1.31831	-1.31521	-1.35097	-1.34792	-1.34493	-1.34199	-1.33908
-1.32374	-1.32055	-1.35637	-1.35317	-1.35005	-1.34702	-1.34406	-1.34113	-1.33824
-1.32292	-1.31973	-1.35549	-1.35228	-1.34914	-1.3461	-1.34314	-1.34021	-1.33733
-1.32205	-1.31885	-1.35457	-1.35136	-1.34822	-1.34518	-1.34221	-1.33929	-1.33641
-1.32108	-1.31786	-1.35355	-1.35037	-1.34726	-1.34424	-1.34127	-1.33836	-1.3355
-1.32011	-1.31689	-1.35255	-1.34941	-1.34633	-1.34333	-1.34038	-1.33748	-1.33462
-1.35818	-1.35488	-1.35166	-1.34855	-1.3455	-1.34252	-1.33959	-1.33669	-1.33382
-1.35726	-1.354	-1.35081	-1.34772	-1.34469	-1.34173	-1.33883	-1.33593	-1.33305
-1.3563	-1.35306	-1.3499	-1.34682	-1.3438	-1.34086	-1.33797	-1.33508	-1.33219
-1.35533	-1.3521	-1.34895	-1.34588	-1.34288	-1.33995	-1.33706	-1.33417	-1.33128
-1.35439	-1.3512	-1.34808	-1.34503	-1.34203	-1.33911	-1.33622	-1.33333	-1.33044
-1.35349	-1.35037	-1.34729	-1.34426	-1.34127	-1.33835	-1.33546	-1.33257	-1.32969
-1.35258	-1.3495	-1.34645	-1.34344	-1.34047	-1.33755	-1.33466	-1.36893	-1.36595
-1.3516	-1.34852	-1.3455	-1.34251	-1.33956	-1.33665	-1.33375	-1.368	-1.36502
-1.35065	-1.34757	-1.34455	-1.34159	-1.33865	-1.33574	-1.33285	-1.36706	-1.36409
-1.34982	-1.34674	-1.34372	-1.34076	-1.33784	-1.33493	-1.33204	-1.36623	-1.36325
-1.34904	-1.34595	-1.34293	-1.33998	-1.33706	-1.33415	-1.33126	-1.36543	-1.36245
-1.34816	-1.34508	-1.34206	-1.3391	-1.33618	-1.33328	-1.33039	-1.36453	-1.36156
-1.3472	-1.34414	-1.34113	-1.33817	-1.33525	-1.33234	-1.32945	-1.36357	-1.3606
-1.34631	-1.34327	-1.34027	-1.33731	-1.37167	-1.36865	-1.36566	-1.36268	-1.35972
-1.3455	-1.34248	-1.33949	-1.33654	-1.37087	-1.36786	-1.36486	-1.3619	-1.35896
-1.34471	-1.34169	-1.33872	-1.33577	-1.37009	-1.36707	-1.36408	-1.36112	-1.3582
-1.34393	-1.34091	-1.33794	-1.335	-1.36931	-1.36629	-1.3633	-1.36035	-1.35744
-1.34313	-1.34011	-1.33713	-1.3342	-1.36849	-1.36548	-1.3625	-1.35956	-1.35666
-1.34223	-1.33921	-1.33624	-1.3333	-1.36757	-1.36456	-1.36158	-1.41689	-1.41391
-1.34128	-1.33827	-1.3353	-1.33236	-1.36658	-1.36358	-1.36061	-1.41593	-1.41297
-1.34041	-1.33741	-1.33445	-1.33151	-1.36571	-1.36271	-1.35975	-1.41509	-1.41214
-1.33961	-1.33663	-1.33367	-1.33074	-1.36492	-1.36194	-1.35899	-1.41433	-1.4114
-1.33883	-1.33586	-1.37018	-1.36715	-1.42266	-1.41959	-1.41656	-1.44779	-1.4448
-1.33807	-1.3351	-1.3694	-1.36638	-1.4219	-1.41884	-1.41582	-1.44707	-1.44409
-1.3373	-1.33434	-1.36862	-1.3656	-1.42113	-1.41809	-1.41508	-1.44635	-1.44338
-1.33654	-1.33358	-1.36785	-1.36483	-1.42035	-1.41732	-1.41434	-1.44563	-1.44267
-1.33575	-1.3328	-1.36705	-1.36403	-1.41955	-1.41653	-1.41357	-1.44489	-1.44194
-1.30078	-1.29794	-1.42483	-1.42172	-1.41866	-1.41566	-1.41271	-1.44406	-1.44112
-1.29987	-1.29704	-1.42388	-1.42078	-1.41773	-1.41474	-1.4118	-1.44317	-1.44025
-1.33693	-1.33399	-1.42301	-1.41993	-1.4514	-1.44834	-1.44533	-1.44237	-1.43946
-1.33615	-1.33322	-1.42223	-1.41917	-1.45067	-1.44762	-1.44462	-1.44167	-1.43875
-1.39531	-1.39228	-1.42146	-1.41842	-1.44994	-1.44692	-1.44393	-1.44098	-1.43807
-1.39456	-1.39153	-1.42068	-1.41766	-1.44921	-1.4462	-1.44324	-1.4403	-1.4374
-1.39382	-1.39078	-1.41992	-1.4169	-1.44848	-1.44549	-1.44255	-1.43962	-1.43673
-1.39308	-1.39005	-1.41917	-1.41616	-1.44776	-1.44478	-1.44184	-1.43893	-1.43605
-1.39234	-1.38932	-1.41842	-1.41543	-1.44705	-1.44407	-1.44114	-1.43824	-1.43538
-1.39159	-1.38859	-1.41768	-1.4147	-1.44635	-1.44337	-1.44044	-1.43755	-1.4347
-1.39085	-1.38786	-1.41695	-1.41399	-1.44565	-1.44269	-1.43976	-1.43687	-1.43401
-1.3901	-1.38713	-1.45097	-1.44794	-1.44495	-1.442	-1.43908	-1.43619	-1.43333
-1.38936	-1.38641	-1.45023	-1.44721	-1.44423	-1.4413	-1.4384	-1.43552	-1.43267
-1.38862	-1.38567	-1.44949	-1.44648	-1.44351	-1.44059	-1.43771	-1.43485	-1.43201
-1.38787	-1.38494	-1.44875	-1.44575	-1.4428	-1.43988	-1.43701	-1.43415	-1.43133

-1.38712	-1.3842	-1.44801	-1.44502	-1.44208	-1.43917	-1.4363	-1.43346	-1.43065
-1.38637	-1.38347	-1.44726	-1.4443	-1.44136	-1.43847	-1.4356	-1.43277	-1.42997
-1.38564	-1.38275	-1.44652	-1.44357	-1.44065	-1.43776	-1.43491	-1.43209	-1.4293
-1.4204	-1.41743	-1.41451	-1.41164	-1.43993	-1.43705	-1.43421	-1.4314	-1.42862
-1.41967	-1.41673	-1.41382	-1.41095	-1.43922	-1.43634	-1.43351	-1.43071	-1.42794
-1.41898	-1.41603	-1.41313	-1.41027	-1.43851	-1.43564	-1.43281	-1.45843	-1.45563
-1.41832	-1.41535	-1.41243	-1.40958	-1.43781	-1.43495	-1.43213	-1.45777	-1.45496
-1.41769	-1.4147	-1.41177	-1.40892	-1.43713	-1.43428	-1.43146	-1.4571	-1.45429
-1.4171	-1.41413	-1.4112	-1.40832	-1.43649	-1.43361	-1.43078	-1.45643	-1.45364
-1.41651	-1.41357	-1.41065	-1.40775	-1.43587	-1.43297	-1.43011	-1.45578	-1.45299
-1.41585	-1.41293	-1.41004	-1.40716	-1.43528	-1.43236	-1.42949	-1.45515	-1.45235
-1.41515	-1.41225	-1.40938	-1.40656	-1.43469	-1.43179	-1.42891	-1.45456	-1.45172
-1.41444	-1.41156	-1.40872	-1.40593	-1.43407	-1.43119	-1.42833	-1.454	-1.45117
-1.41373	-1.41087	-1.40805	-1.40526	-1.4334	-1.43058	-1.42777	-1.4535	-1.45068
-1.41305	-1.41019	-1.40737	-1.40459	-1.4614	-1.45856	-1.45575	-1.45294	-1.45015
-1.41241	-1.40951	-1.40668	-1.40391	-1.46072	-1.45789	-1.45509	-1.4523	-1.44952
-1.41179	-1.40886	-1.40601	-1.40325	-1.46004	-1.45721	-1.45441	-1.45162	-1.44886
-1.4112	-1.40826	-1.40539	-1.40261	-1.45938	-1.45654	-1.45374	-1.45095	-1.44818
-1.41063	-1.40769	-1.40482	-1.40201	-1.45875	-1.4559	-1.45308	-1.45028	-1.4475
-1.40999	-1.4071	-1.40425	-1.40145	-1.4279	-1.42512	-1.42237	-1.44965	-1.44685
-1.40932	-1.40649	-1.40369	-1.40093	-1.4274	-1.4246	-1.42182	-1.44905	-1.44621
-1.40868	-1.40587	-1.43242	-1.42963	-1.42685	-1.42405	-1.42127	-1.44848	-1.44563
-1.40807	-1.40523	-1.43177	-1.42898	-1.42622	-1.42346	-1.42071	-1.44793	-1.44511
-1.40749	-1.40462	-1.43116	-1.42837	-1.4256	-1.42286	-1.42014	-1.44736	-1.44456
-1.40694	-1.40409	-1.43065	-1.42783	-1.42503	-1.42226	-1.41951	-1.44671	-1.44391
-1.40637	-1.40357	-1.43014	-1.4273	-1.42447	-1.42167	-1.4189	-1.44606	-1.44326
-1.40573	-1.40295	-1.42954	-1.42673	-1.42392	-1.42113	-1.41836	-1.44549	-1.44267
-1.40506	-1.40229	-1.42892	-1.42614	-1.42337	-1.42061	-1.41786	-1.44496	-1.4421
-1.43396	-1.43112	-1.42831	-1.42554	-1.42278	-1.42003	-1.41729	-1.44438	-1.44153
-1.43343	-1.43056	-1.42772	-1.42492	-1.42215	-1.4194	-1.41668	-1.44376	-1.44096
-1.43287	-1.43	-1.42716	-1.42434	-1.42156	-1.41882	-1.4161	-1.44318	-1.44038
-1.43225	-1.42942	-1.42661	-1.42382	-1.42105	-1.41831	-1.4156	-1.44264	-1.43982
-1.43166	-1.42887	-1.42608	-1.4233	-1.44915	-1.44634	-1.44355	-1.47002	-1.46712
-1.43115	-1.42837	-1.42558	-1.42278	-1.44859	-1.44576	-1.44294	-1.4694	-1.46653
-1.43066	-1.42787	-1.42507	-1.42225	-1.44802	-1.44516	-1.44232	-1.46878	-1.46594
-1.45894	-1.45606	-1.45319	-1.45031	-1.44745	-1.4446	-1.44177	-1.46821	-1.46536
-1.45832	-1.45541	-1.45253	-1.44969	-1.44687	-1.44406	-1.44126	-1.46767	-1.46479
-1.45774	-1.45483	-1.45195	-1.44911	-1.4463	-1.44349	-1.44069	-1.43789	-1.4351
-1.45719	-1.4543	-1.45143	-1.44857	-1.44572	-1.44289	-1.44007	-1.4373	-1.43454
-1.45665	-1.45377	-1.45089	-1.44801	-1.44515	-1.44231	-1.43949	-1.46966	-1.46682
-1.4561	-1.45321	-1.45032	-1.44744	-1.44459	-1.44177	-1.43897	-1.46913	-1.4663
-1.45554	-1.45264	-1.44976	-1.44689	-1.44404	-1.44124	-1.43846	-1.46859	-1.46575
-1.45499	-1.45208	-1.44921	-1.44636	-1.44353	-1.44071	-1.43791	-1.46799	-1.46513
-1.45443	-1.45153	-1.44867	-1.44584	-1.44302	-1.44018	-1.43736	-1.46737	-1.46449
-1.45387	-1.45099	-1.44814	-1.4453	-1.44248	-1.43964	-1.43681	-1.46679	-1.46393
-1.45331	-1.45046	-1.4476	-1.44475	-1.44191	-1.43908	-1.43627	-1.46625	-1.46342
-1.45278	-1.44992	-1.44707	-1.44421	-1.47442	-1.47149	-1.46858	-1.46572	-1.4629
-1.45227	-1.44938	-1.44651	-1.44365	-1.47385	-1.47094	-1.46806	-1.4652	-1.46237
-1.45178	-1.44886	-1.44598	-1.44312	-1.4733	-1.4704	-1.46754	-1.46468	-1.46183
-1.45129	-1.44838	-1.44549	-1.44262	-1.47277	-1.46988	-1.46701	-1.46414	-1.46127

-1.45078	-1.44789	-1.44501	-1.44214	-1.47225	-1.46935	-1.46648	-1.46359	-1.4607
-1.45025	-1.44736	-1.44449	-1.44164	-1.47173	-1.46881	-1.46591	-1.46301	-1.46013
-1.44969	-1.44681	-1.44395	-1.44113	-1.4712	-1.46826	-1.46532	-1.46242	-1.45955
-1.44914	-1.44627	-1.44344	-1.44064	-1.47068	-1.46773	-1.46479	-1.46189	-1.45901
-1.44859	-1.44575	-1.44293	-1.44014	-1.47017	-1.46723	-1.46431	-1.4614	-1.45851
-1.44807	-1.44524	-1.44243	-1.43963	-1.46962	-1.4667	-1.46378	-1.46088	-1.458
-1.44756	-1.44475	-1.44192	-1.43909	-1.46902	-1.4661	-1.4632	-1.46033	-1.45748
-1.44707	-1.44426	-1.4744	-1.47142	-1.46845	-1.46553	-1.46264	-1.45979	-1.45696
-1.4466	-1.44379	-1.4739	-1.47091	-1.46795	-1.46502	-1.46213	-1.45927	-1.45645
-1.44612	-1.44332	-1.47341	-1.47043	-1.46747	-1.46454	-1.46163	-1.45877	-1.45594
-1.44564	-1.44283	-1.4729	-1.46992	-1.46697	-1.46404	-1.46114	-1.45828	-1.45545
-1.44514	-1.44234	-1.47238	-1.46941	-1.46646	-1.46354	-1.46064	-1.45778	-1.45495
-1.44466	-1.44186	-1.47187	-1.46891	-1.46595	-1.46303	-1.46014	-1.45728	-1.45445
-1.44419	-1.44139	-1.47137	-1.4684	-1.46545	-1.46252	-1.45962	-1.45677	-1.45394
-1.44371	-1.44091	-1.47087	-1.4679	-1.46494	-1.46201	-1.45911	-1.45626	-1.45344
-1.44322	-1.44043	-1.47036	-1.4674	-1.46444	-1.46151	-1.45861	-1.45576	-1.45294
-1.44274	-1.43995	-1.46986	-1.46689	-1.46394	-1.46101	-1.45811	-1.45526	-1.45244
-1.44226	-1.43947	-1.46935	-1.46638	-1.46343	-1.4605	-1.4576	-1.45475	-1.45193
-1.44179	-1.43899	-1.46884	-1.46587	-1.46292	-1.45999	-1.45709	-1.45424	-1.45143
-1.4742	-1.47128	-1.46834	-1.46538	-1.46242	-1.45949	-1.45659	-1.45374	-1.45093
-1.47368	-1.47077	-1.46785	-1.46489	-1.46194	-1.45901	-1.4561	-1.45324	-1.45043
-1.47319	-1.47028	-1.46735	-1.46442	-1.46148	-1.45855	-1.45563	-1.45276	-1.44994
-1.47275	-1.4698	-1.46687	-1.46396	-1.46105	-1.45812	-1.45519	-1.45231	-1.44945
-1.47229	-1.46932	-1.46638	-1.46349	-1.4606	-1.45767	-1.45474	-1.48115	-1.4782
-1.4718	-1.46881	-1.46587	-1.46299	-1.46011	-1.45718	-1.45426	-1.48068	-1.47773
-1.47131	-1.46831	-1.46537	-1.46248	-1.4596	-1.45668	-1.45377	-1.48021	-1.47726
-1.47087	-1.46786	-1.46489	-1.46199	-1.45909	-1.45618	-1.45327	-1.50619	-1.50316
-1.47044	-1.46742	-1.46443	-1.4615	-1.45859	-1.45567	-1.45277	-1.50568	-1.50265
-1.46996	-1.46695	-1.46396	-1.46102	-1.48771	-1.48473	-1.48176	-1.50524	-1.50219
-1.46945	-1.46646	-1.46349	-1.46054	-1.48725	-1.4843	-1.48137	-1.50485	-1.50179
-1.46896	-1.46599	-1.46303	-1.46008	-1.4868	-1.48386	-1.48095	-1.53186	-1.52875
-1.46853	-1.46556	-1.4626	-1.45965	-1.48637	-1.48341	-1.48047	-1.5314	-1.52835
-1.46809	-1.46512	-1.46217	-1.45922	-1.48596	-1.48297	-1.48002	-1.53094	-1.52793
-1.4676	-1.46463	-1.46169	-1.4588	-1.48558	-1.48258	-1.4796	-1.53048	-1.52743
-1.46712	-1.46414	-1.46122	-1.45836	-1.4852	-1.4822	-1.4792	-1.53003	-1.52694
-1.46666	-1.46369	-1.49064	-1.48769	-1.51279	-1.50974	-1.5067	-1.52963	-1.52652
-1.46622	-1.46326	-1.49021	-1.48722	-1.5123	-1.5093	-1.50632	-1.52926	-1.52614
-1.4658	-1.46284	-1.4898	-1.48679	-1.51184	-1.50885	-1.50588	-1.52883	-1.52573
-1.46539	-1.46243	-1.48941	-1.48639	-1.5114	-1.50837	-1.50538	-1.52835	-1.52531
-1.46498	-1.46202	-1.51717	-1.51407	-1.51098	-1.50793	-1.50491	-1.52788	-1.52486
-1.46457	-1.46161	-1.51676	-1.51366	-1.51059	-1.50753	-1.5045	-1.52743	-1.52438
-1.46415	-1.4612	-1.51634	-1.51326	-1.5102	-1.50714	-1.5041	-1.527	-1.52391
-1.46369	-1.46078	-1.51596	-1.51287	-1.50979	-1.50673	-1.50369	-1.52658	-1.52349
-1.46321	-1.46035	-1.51557	-1.51247	-1.50938	-1.50632	-1.50329	-1.52618	-1.52309
-1.52119	-1.51817	-1.51513	-1.51206	-1.50899	-1.50593	-1.50289	-1.52577	-1.52268
-1.52074	-1.5177	-1.51466	-1.51163	-1.5086	-1.50555	-1.5025	-1.52537	-1.52228
-1.52034	-1.51728	-1.51424	-1.51122	-1.5082	-1.50516	-1.50211	-1.52497	-1.52188
-1.51999	-1.51693	-1.51387	-1.51083	-1.50778	-1.50474	-1.50171	-1.52456	-1.52147
-1.51964	-1.51657	-1.51351	-1.51043	-1.50737	-1.50433	-1.5013	-1.52416	-1.52106
-1.51928	-1.5162	-1.51312	-1.51004	-1.50697	-1.50392	-1.50089	-1.52373	-1.52065

-1.51892	-1.51582	-1.51272	-1.50964	-1.50657	-1.50352	-1.50048	-1.52331	-1.52023
-1.51852	-1.51541	-1.51231	-1.50923	-1.50616	-1.50312	-1.50009	-1.55035	-1.5472
-1.5181	-1.51499	-1.51189	-1.50881	-1.50575	-1.50272	-1.4997	-1.54994	-1.5468
-1.5177	-1.51458	-1.51148	-1.5084	-1.50535	-1.50231	-1.49929	-1.54953	-1.5464
-1.51729	-1.51417	-1.51108	-1.508	-1.50495	-1.5019	-1.49887	-1.54911	-1.546
-1.51689	-1.51377	-1.51069	-1.50763	-1.5326	-1.52949	-1.5264	-1.54873	-1.54563
-1.51647	-1.51338	-1.51032	-1.50729	-1.53227	-1.52919	-1.5261	-1.54841	-1.54527
-1.51608	-1.513	-1.50995	-1.50694	-1.53193	-1.52886	-1.52578	-1.54807	-1.54491
-1.51576	-1.51266	-1.50958	-1.50655	-1.53152	-1.52845	-1.52538	-1.54768	-1.54452
-1.51545	-1.51231	-1.50921	-1.50615	-1.5311	-1.52802	-1.52496	-1.54726	-1.54413
-1.51507	-1.51193	-1.53692	-1.5338	-1.5307	-1.52761	-1.52454	-1.54684	-1.54372
-1.51466	-1.51152	-1.53652	-1.53341	-1.53032	-1.52721	-1.52412	-1.54641	-1.54332
-1.51429	-1.51117	-1.53616	-1.53306	-1.52996	-1.52685	-1.52376	-1.54604	-1.54294
-1.51397	-1.51086	-1.53585	-1.53274	-1.52964	-1.52654	-1.52345	-1.54571	-1.54258
-1.5418	-1.53865	-1.53552	-1.53241	-1.52931	-1.52622	-1.52314	-1.54538	-1.54224
-1.5414	-1.53826	-1.53514	-1.53205	-1.52897	-1.52589	-1.52282	-1.54506	-1.5419
-1.541	-1.53787	-1.53477	-1.53169	-1.52862	-1.52555	-1.52247	-1.5447	-1.54155
-1.54065	-1.53752	-1.5344	-1.53131	-1.52823	-1.52515	-1.52207	-1.54429	-1.54113
-1.54033	-1.53718	-1.53404	-1.53093	-1.52782	-1.52474	-1.52166	-1.54387	-1.5407
-1.54001	-1.53685	-1.53371	-1.53058	-1.52747	-1.52438	-1.5213	-1.5435	-1.54034
-1.5397	-1.53653	-1.53338	-1.53026	-1.52715	-1.52406	-1.52098	-1.54317	-1.54002
-1.53938	-1.53621	-1.55872	-1.55552	-1.55233	-1.54916	-1.54599	-1.54284	-1.53969
-1.53905	-1.53589	-1.5584	-1.55519	-1.552	-1.54882	-1.54565	-1.5425	-1.53936
-1.53873	-1.53557	-1.55807	-1.55486	-1.55166	-1.54848	-1.54532	-1.5738	-1.57049
-1.53842	-1.53525	-1.55774	-1.55453	-1.55133	-1.54815	-1.54499	-1.57346	-1.57014
-1.5381	-1.53493	-1.55741	-1.5542	-1.551	-1.54783	-1.54467	-1.57311	-1.56979
-1.53779	-1.53462	-1.55709	-1.55387	-1.55067	-1.5475	-1.54434	-1.57277	-1.56945
-1.53748	-1.53431	-1.55678	-1.55355	-1.55034	-1.54716	-1.54401	-1.57243	-1.56911
-1.53715	-1.53399	-1.55646	-1.55323	-1.55002	-1.54684	-1.54367	-1.57208	-1.56877
-1.53683	-1.53367	-1.55613	-1.55291	-1.5497	-1.54651	-1.54334	-1.57173	-1.56842
-1.53651	-1.53335	-1.55581	-1.55259	-1.54938	-1.54619	-1.54302	-1.57139	-1.56808
-1.5362	-1.53303	-1.55548	-1.55226	-1.54906	-1.54587	-1.5427	-1.57105	-1.56773
-1.53588	-1.53271	-1.55515	-1.55193	-1.54873	-1.54555	-1.54238	-1.57071	-1.56739
-1.53556	-1.53239	-1.55483	-1.5516	-1.5484	-1.54522	-1.54205	-1.57037	-1.56705
-1.53524	-1.53208	-1.55451	-1.55128	-1.54807	-1.54488	-1.54172	-1.57003	-1.56671
-1.53493	-1.53176	-1.55418	-1.55095	-1.54774	-1.54455	-1.54138	-1.56967	-1.56636
-1.53462	-1.53144	-1.55385	-1.55063	-1.54742	-1.54423	-1.54104	-1.56932	-1.56601
-1.53435	-1.53116	-1.55355	-1.55032	-1.54711	-1.5439	-1.54071	-1.56898	-1.56567
-1.53412	-1.53092	-1.55329	-1.55003	-1.54679	-1.54358	-1.54039	-1.56864	-1.56533
-1.53386	-1.53065	-1.55301	-1.54975	-1.54651	-1.54329	-1.5401	-1.56834	-1.56503
-1.53354	-1.53035	-1.55273	-1.54949	-1.54627	-1.54305	-1.53985	-1.56808	-1.56478
-1.53322	-1.53004	-1.55244	-1.54922	-1.54601	-1.54279	-1.53959	-1.56781	-1.56452
-1.53294	-1.52974	-1.55211	-1.5489	-1.5457	-1.54249	-1.53928	-1.56749	-1.56419
-1.53268	-1.52944	-1.55179	-1.54858	-1.54538	-1.54217	-1.53898	-1.56716	-1.56385
-1.53241	-1.52918	-1.55153	-1.54831	-1.5451	-1.5419	-1.53872	-1.56688	-1.56356
-1.53214	-1.52894	-1.55129	-1.54807	-1.54486	-1.54166	-1.53848	-1.56663	-1.5633
-1.53189	-1.5287	-1.55104	-1.5478	-1.54458	-1.54138	-1.5382	-1.56634	-1.56301
-1.53164	-1.52844	-1.55077	-1.54751	-1.54427	-1.54106	-1.53787	-1.56601	-1.5627
-1.53137	-1.52817	-1.55049	-1.54723	-1.54398	-1.54076	-1.53756	-1.56571	-1.56241
-1.53105	-1.52787	-1.55021	-1.54696	-1.54373	-1.54052	-1.53732	-1.56546	-1.56217

-1.53074	-1.52758	-1.54994	-1.54671	-1.5435	-1.54029	-1.53709	-1.56522	-1.56193
-1.53049	-1.52734	-1.54969	-1.54647	-1.54326	-1.54006	-1.53687	-1.56498	-1.56168
-1.53027	-1.52712	-1.54946	-1.54625	-1.54304	-1.53984	-1.53665	-1.56474	-1.56142
-1.53004	-1.52689	-1.54923	-1.54602	-1.54281	-1.53961	-1.53641	-1.56448	-1.56113
-1.52981	-1.52666	-1.549	-1.54579	-1.54258	-1.53938	-1.53617	-1.5642	-1.56083
-1.52958	-1.52643	-1.54877	-1.54556	-1.54236	-1.53915	-1.53594	-1.56396	-1.56057
-1.52935	-1.5262	-1.54854	-1.54533	-1.54212	-1.53892	-1.53572	-1.56376	-1.5604
-1.52912	-1.52597	-1.54831	-1.5451	-1.54189	-1.53869	-1.5355	-1.56354	-1.5602
-1.52889	-1.52574	-1.54807	-1.54486	-1.54166	-1.53846	-1.53526	-1.56328	-1.55991
-1.52866	-1.52551	-1.54784	-1.54463	-1.54143	-1.53822	-1.53502	-1.56301	-1.55962
-1.52841	-1.52527	-1.54759	-1.54438	-1.54118	-1.53798	-1.53478	-1.56278	-1.5594
-1.52817	-1.52503	-1.54735	-1.54413	-1.54093	-1.53773	-1.53455	-1.56256	-1.55921
-1.52792	-1.52478	-1.5471	-1.54388	-1.54068	-1.53749	-1.5343	-1.5623	-1.55896
-1.52769	-1.52454	-1.54684	-1.54364	-1.54043	-1.53723	-1.53403	-1.56201	-1.55865
-1.52745	-1.52429	-1.5466	-1.54339	-1.54019	-1.53699	-1.53379	-1.56176	-1.5584
-1.5272	-1.52406	-1.54636	-1.54314	-1.53993	-1.53675	-1.53357	-1.56156	-1.55822
-1.52697	-1.52383	-1.54612	-1.5429	-1.53969	-1.53651	-1.53335	-1.56135	-1.55804
-1.52679	-1.5236	-1.54587	-1.54266	-1.53946	-1.53627	-1.5331	-1.5611	-1.55781
-1.52663	-1.52339	-1.54563	-1.54243	-1.53925	-1.53604	-1.53285	-1.56084	-1.55756
-1.52644	-1.52321	-1.54545	-1.54225	-1.53906	-1.53586	-1.53267	-1.58945	-1.58605
-1.52624	-1.52306	-1.54531	-1.5421	-1.5389	-1.53571	-1.53253	-1.58927	-1.58582
-1.52602	-1.52287	-1.54513	-1.5419	-1.53869	-1.53552	-1.53235	-1.58907	-1.58562
-1.52578	-1.52264	-1.54491	-1.54167	-1.53845	-1.53528	-1.53213	-1.58887	-1.58546
-1.49585	-1.49281	-1.54471	-1.54147	-1.53824	-1.53506	-1.5319	-1.58865	-1.58528
-1.49571	-1.49266	-1.54454	-1.54131	-1.53808	-1.53486	-1.53166	-1.58839	-1.58505
-1.49557	-1.49251	-1.54437	-1.54115	-1.53793	-1.53468	-1.53143	-1.58815	-1.58482
-1.49542	-1.49231	-1.54415	-1.54095	-1.53774	-1.5345	-1.53127	-1.58797	-1.58463
-1.49525	-1.49211	-1.54392	-1.54073	-1.53755	-1.53433	-1.53113	-1.58782	-1.58446
-1.49508	-1.49195	-1.54375	-1.54055	-1.53737	-1.53417	-1.53098	-1.58765	-1.58425
-1.49491	-1.49181	-1.54362	-1.54041	-1.5372	-1.53401	-1.53083	-1.58746	-1.58402
-1.49475	-1.49167	-1.54348	-1.54023	-1.53701	-1.53383	-1.53065	-1.58727	-1.58382
-1.49461	-1.49153	-1.54332	-1.54003	-1.53678	-1.5336	-1.53044	-1.58708	-1.58366
-1.49448	-1.49139	-1.51411	-1.5109	-1.53657	-1.53339	-1.53025	-1.5869	-1.58351
-1.49436	-1.49127	-1.51398	-1.51077	-1.53643	-1.53324	-1.53009	-1.58674	-1.58337
-1.49424	-1.49115	-1.51386	-1.51065	-1.53631	-1.53311	-1.52994	-1.58659	-1.58322
-1.49411	-1.49102	-1.51373	-1.51052	-1.56558	-1.5623	-1.55905	-1.58644	-1.58307
-1.49399	-1.4909	-1.5136	-1.51039	-1.56544	-1.56216	-1.55891	-1.5863	-1.58292
-1.49386	-1.49077	-1.51346	-1.51025	-1.5653	-1.56201	-1.55877	-1.58615	-1.58277
-1.49373	-1.49064	-1.51333	-1.51012	-1.56515	-1.56187	-1.55862	-1.586	-1.58261
-1.4936	-1.4905	-1.51319	-1.50998	-1.565	-1.56172	-1.55848	-1.58585	-1.58246
-1.49346	-1.49036	-1.51305	-1.50983	-1.56485	-1.56157	-1.55832	-1.5857	-1.58232
-1.49332	-1.49022	-1.5129	-1.50969	-1.56469	-1.56141	-1.55817	-1.58556	-1.58218
-1.49318	-1.49008	-1.51275	-1.50954	-1.56454	-1.56126	-1.55802	-1.58541	-1.58204
-1.49305	-1.48993	-1.51261	-1.50939	-1.56438	-1.5611	-1.55786	-1.58525	-1.58189
-1.49291	-1.48979	-1.51246	-1.50925	-1.53588	-1.5327	-1.52957	-1.58509	-1.58174
-1.49276	-1.48965	-1.51232	-1.50912	-1.53575	-1.53256	-1.52942	-1.58493	-1.58158
-1.49262	-1.48951	-1.5122	-1.50902	-1.53566	-1.53247	-1.52931	-1.5848	-1.58143
-1.49248	-1.48938	-1.51208	-1.50894	-1.53561	-1.53241	-1.52923	-1.58469	-1.58128
-1.49235	-1.48925	-1.51197	-1.50885	-1.53552	-1.53233	-1.52914	-1.58458	-1.58115
-1.49227	-1.48914	-1.51183	-1.5087	-1.53537	-1.53219	-1.52902	-1.58446	-1.58101

-1.4922	-1.48903	-1.51171	-1.50857	-1.53523	-1.53205	-1.52889	-1.58434	-1.58089
-1.4921	-1.48896	-1.54158	-1.53835	-1.53514	-1.53195	-1.52877	-1.60914	-1.60565
-1.492	-1.48889	-1.54152	-1.5383	-1.53508	-1.53187	-1.52867	-1.60904	-1.60558
-1.46472	-1.46167	-1.54141	-1.53819	-1.53498	-1.53178	-1.52859	-1.60897	-1.60552
-1.46458	-1.46152	-1.54125	-1.53805	-1.53486	-1.53169	-1.52853	-1.60892	-1.60545
-1.46447	-1.4614	-1.54113	-1.53792	-1.53473	-1.53157	-1.52843	-1.60882	-1.60535
-1.46441	-1.46134	-1.54105	-1.53781	-1.53459	-1.53141	-1.52827	-1.60866	-1.60522
-1.46434	-1.46127	-1.51476	-1.51154	-1.53447	-1.53129	-1.52813	-1.60852	-1.60508
-1.46426	-1.46117	-1.51466	-1.51146	-1.53441	-1.53122	-1.52805	-1.60842	-1.60495
-1.46416	-1.46107	-1.51456	-1.51139	-1.53436	-1.53117	-1.52799	-1.60833	-1.60483
-1.46406	-1.46099	-1.51449	-1.51132	-1.50816	-1.505	-1.50184	-1.58108	-1.5777
-1.46397	-1.46092	-1.51444	-1.51126	-1.50809	-1.5049	-1.50173	-1.58099	-1.57764
-1.4639	-1.46086	-1.51438	-1.51118	-1.53434	-1.53109	-1.52785	-1.58092	-1.57759
-1.46385	-1.46082	-1.51432	-1.51109	-1.53423	-1.53099	-1.52779	-1.58088	-1.57755
-1.49475	-1.49163	-1.51428	-1.51104	-1.53417	-1.53093	-1.52773	-1.55566	-1.55237
-1.4947	-1.49159	-1.51425	-1.51104	-1.53419	-1.53093	-1.5277	-1.55561	-1.55232
-1.49465	-1.49154	-1.51421	-1.51102	-1.53419	-1.53092	-1.52766	-1.55557	-1.55227
-1.49459	-1.49148	-1.51414	-1.51094	-1.5341	-1.53085	-1.52761	-1.55553	-1.55223
-1.49451	-1.4914	-1.51406	-1.51084	-1.53399	-1.53076	-1.52756	-1.55549	-1.55218
-1.49444	-1.4913	-1.54043	-1.53716	-1.53391	-1.5307	-1.52751	-1.55544	-1.55213
-1.49436	-1.49119	-1.54031	-1.53707	-1.53385	-1.53065	-1.52746	-1.55539	-1.55208
-1.49431	-1.49114	-1.54027	-1.53701	-1.53378	-1.53058	-1.5274	-1.55534	-1.55203
-1.49429	-1.49116	-1.54029	-1.53699	-1.53372	-1.53051	-1.52734	-1.55529	-1.55198
-1.49426	-1.49116	-1.5403	-1.53698	-1.53369	-1.53047	-1.52728	-1.55523	-1.55193
-1.4942	-1.49111	-1.54027	-1.53699	-1.53372	-1.53046	-1.52723	-1.55517	-1.55189
-1.49414	-1.49105	-1.54024	-1.537	-1.53375	-1.53047	-1.5272	-1.55513	-1.55186
-1.52111	-1.51796	-1.54019	-1.53696	-1.53374	-1.53048	-1.52722	-1.55514	-1.55181
-1.52105	-1.5179	-1.54013	-1.53691	-1.53369	-1.53048	-1.52726	-1.55516	-1.55178
-1.52099	-1.51785	-1.54008	-1.53686	-1.53364	-1.53044	-1.52725	-1.55517	-1.55179
-1.52094	-1.5178	-1.54004	-1.5368	-1.53357	-1.53038	-1.5272	-1.55517	-1.55184
-1.52093	-1.51779	-1.54003	-1.53679	-1.53356	-1.53034	-1.52715	-1.58801	-1.58463
-1.52096	-1.51782	-1.54005	-1.53683	-1.53359	-1.53034	-1.5271	-1.58795	-1.58459
-1.521	-1.51783	-1.54006	-1.53684	-1.53361	-1.53034	-1.52707	-1.58791	-1.58455
-1.52102	-1.5178	-1.54001	-1.5368	-1.53359	-1.53035	-1.52711	-1.58792	-1.5845
-1.52103	-1.51778	-1.53996	-1.53675	-1.53354	-1.53034	-1.52714	-1.58794	-1.58447
-1.48817	-1.48501	-1.53997	-1.53671	-1.53348	-1.53029	-1.52712	-1.58794	-1.58449
-1.48814	-1.48503	-1.54	-1.53669	-1.53342	-1.53023	-1.52706	-1.58793	-1.58452
-1.4881	-1.48501	-1.54	-1.53669	-1.53341	-1.53019	-1.52701	-1.5879	-1.58452
-1.48804	-1.48497	-1.53997	-1.5367	-1.53343	-1.53018	-1.52696	-1.58784	-1.58449
-1.48802	-1.48495	-1.53996	-1.53672	-1.53347	-1.5302	-1.52695	-1.5571	-1.55391
-1.48805	-1.48498	-1.53999	-1.53676	-1.53353	-1.53026	-1.527	-1.55715	-1.55395
-1.48809	-1.485	-1.54	-1.53679	-1.56716	-1.5638	-1.56046	-1.5572	-1.55399
-1.4881	-1.48497	-1.53996	-1.53676	-1.56714	-1.56381	-1.5605	-1.55724	-1.55403
-1.48812	-1.48495	-1.53992	-1.53672	-1.56712	-1.56382	-1.56053	-1.55728	-1.55405
-1.48816	-1.48498	-1.50773	-1.50459	-1.56713	-1.56385	-1.56056	-1.55729	-1.55403
-1.48821	-1.48503	-1.50778	-1.50465	-1.56717	-1.56389	-1.5606	-1.5573	-1.55399
-1.48826	-1.48508	-1.50783	-1.50469	-1.56721	-1.56392	-1.56063	-1.55732	-1.554
-1.4883	-1.48512	-1.50788	-1.50474	-1.56725	-1.56397	-1.56068	-1.55737	-1.55404
-1.48834	-1.48516	-1.50793	-1.50479	-1.5673	-1.56401	-1.56072	-1.55741	-1.55409
-1.48839	-1.48521	-1.50798	-1.50484	-1.56735	-1.56406	-1.56077	-1.55746	-1.55414

-1.48844	-1.48526	-1.54228	-1.53905	-1.53584	-1.53262	-1.52939	-1.55751	-1.55419
-1.48849	-1.48531	-1.54233	-1.5391	-1.53589	-1.53266	-1.52943	-1.55756	-1.55423
-1.48854	-1.48536	-1.54238	-1.53915	-1.53594	-1.53271	-1.52948	-1.5576	-1.55428
-1.48859	-1.4854	-1.54242	-1.53919	-1.53598	-1.53276	-1.52953	-1.55765	-1.55433
-1.48863	-1.48544	-1.54246	-1.53923	-1.53602	-1.5328	-1.52958	-1.5577	-1.55439
-1.48868	-1.4855	-1.54251	-1.53928	-1.53606	-1.53285	-1.52964	-1.55777	-1.55448
-1.48874	-1.48556	-1.54257	-1.53933	-1.53611	-1.5329	-1.5297	-1.55786	-1.55461
-1.48881	-1.48565	-1.54267	-1.53941	-1.53617	-1.53296	-1.52976	-1.55794	-1.55471
-1.48889	-1.48577	-1.54282	-1.53955	-1.53629	-1.53304	-1.52981	-1.55798	-1.55476
-1.48898	-1.48589	-1.54296	-1.53968	-1.53641	-1.53313	-1.52986	-1.55802	-1.5548
-1.48903	-1.48595	-1.54303	-1.53976	-1.53649	-1.5332	-1.52992	-1.52671	-1.52355
-1.48909	-1.486	-1.54307	-1.53982	-1.53655	-1.53327	-1.52999	-1.52677	-1.5236
-1.52408	-1.52088	-1.54317	-1.53989	-1.53663	-1.53335	-1.53009	-1.52688	-1.5237
-1.52422	-1.52101	-1.54329	-1.53999	-1.5367	-1.53345	-1.53023	-1.52702	-1.52384
-1.52436	-1.52114	-1.5434	-1.54009	-1.53681	-1.53357	-1.53036	-1.52715	-1.52395
-1.52449	-1.52124	-1.54351	-1.54022	-1.53695	-1.53372	-1.5305	-1.52727	-1.52405
-1.5246	-1.52134	-1.54362	-1.54035	-1.5371	-1.53386	-1.53062	-1.52739	-1.52415
-1.5247	-1.52147	-1.54377	-1.5405	-1.53723	-1.53397	-1.53072	-1.52749	-1.52428
-1.52478	-1.52159	-1.54391	-1.54063	-1.53736	-1.53408	-1.53082	-1.5276	-1.52441
-1.52483	-1.52165	-1.54399	-1.54072	-1.53746	-1.5342	-1.53094	-1.52773	-1.52454
-1.52487	-1.52168	-1.54404	-1.54079	-1.53755	-1.53431	-1.53108	-1.52787	-1.52467
-1.52498	-1.52179	-1.54415	-1.54091	-1.53767	-1.53444	-1.53121	-1.52799	-1.52478
-1.52514	-1.52195	-1.54431	-1.54106	-1.53781	-1.53457	-1.53133	-1.5281	-1.52486
-1.5253	-1.52211	-1.54447	-1.54121	-1.53795	-1.5347	-1.53145	-1.5282	-1.52496
-1.52546	-1.52226	-1.54463	-1.54136	-1.53808	-1.53481	-1.53154	-1.5283	-1.52508
-1.52561	-1.52241	-1.54478	-1.54149	-1.53821	-1.53492	-1.53164	-1.52841	-1.52521
-1.52575	-1.52254	-1.54491	-1.5416	-1.53831	-1.53503	-1.53178	-1.52855	-1.52536
-1.52588	-1.52267	-1.54502	-1.5417	-1.53841	-1.53515	-1.53192	-1.52871	-1.52551
-1.49697	-1.49381	-1.54512	-1.54182	-1.53854	-1.53529	-1.53207	-1.52886	-1.52567
-1.4971	-1.4939	-1.54522	-1.54194	-1.53868	-1.53545	-1.53222	-1.52902	-1.52583
-1.49726	-1.49406	-1.54538	-1.5421	-1.53884	-1.5356	-1.53238	-1.52917	-1.52598
-1.49746	-1.4943	-1.54563	-1.54229	-1.53899	-1.53575	-1.53254	-1.52933	-1.52613
-1.49764	-1.49451	-1.54585	-1.54249	-1.53916	-1.53591	-1.53269	-1.52948	-1.52628
-1.49779	-1.49466	-1.54604	-1.54272	-1.5394	-1.53612	-1.53285	-1.52963	-1.52644
-1.49793	-1.49481	-1.54621	-1.54292	-1.53962	-1.53631	-1.53301	-1.52979	-1.52661
-1.49808	-1.49495	-1.54635	-1.54304	-1.53973	-1.53644	-1.53317	-1.52995	-1.52676
-1.49823	-1.4951	-1.54648	-1.54314	-1.53981	-1.53655	-1.53332	-1.53011	-1.52692
-1.46732	-1.46428	-1.51819	-1.51493	-1.53999	-1.53672	-1.53347	-1.53025	-1.52707
-1.46746	-1.46443	-1.51838	-1.51516	-1.54024	-1.53692	-1.53362	-1.53039	-1.52722
-1.46763	-1.46459	-1.51855	-1.51535	-1.54046	-1.53713	-1.53381	-1.53057	-1.52738
-1.46785	-1.46475	-1.5187	-1.51551	-1.54064	-1.53735	-1.53406	-1.53079	-1.52755
-1.46806	-1.46493	-1.51887	-1.51568	-1.5125	-1.50932	-1.50613	-1.53102	-1.52774
-1.46825	-1.46515	-1.5191	-1.51586	-1.51265	-1.50948	-1.50633	-1.53124	-1.52798
-1.46844	-1.46537	-1.51934	-1.51606	-1.51282	-1.50965	-1.50652	-1.53146	-1.52823
-1.46859	-1.46555	-1.51955	-1.51628	-1.51304	-1.50988	-1.50674	-1.5317	-1.52849
-1.46875	-1.46572	-1.51976	-1.51652	-1.5133	-1.51013	-1.50698	-1.53194	-1.52874
-1.46895	-1.46593	-1.51998	-1.51676	-1.51355	-1.51035	-1.50717	-1.50403	-1.50091
-1.46919	-1.46616	-1.52022	-1.51701	-1.5138	-1.51056	-1.50733	-1.50418	-1.50107
-1.46942	-1.46636	-1.52044	-1.51724	-1.51403	-1.51077	-1.50752	-1.50435	-1.50124
-1.46963	-1.46653	-1.5206	-1.51741	-1.51422	-1.51099	-1.50776	-1.50456	-1.5014

-1.46983	-1.4667	-1.49028	-1.48718	-1.51442	-1.51122	-1.50801	-1.50479	-1.50159
-1.47003	-1.46692	-1.4905	-1.4874	-1.51465	-1.51146	-1.50827	-1.50505	-1.50183
-1.47022	-1.46714	-1.49075	-1.48764	-1.51488	-1.5117	-1.50853	-1.50532	-1.5021
-1.47044	-1.46737	-1.49098	-1.48783	-1.51506	-1.51189	-1.50874	-1.52764	-1.5244
-1.47067	-1.4676	-1.4912	-1.48802	-1.51522	-1.51205	-1.50892	-1.52787	-1.52467
-1.44202	-1.43903	-1.49144	-1.48824	-1.51543	-1.51226	-1.50913	-1.52813	-1.52494
-1.44226	-1.43927	-1.4917	-1.48849	-1.51569	-1.51252	-1.50938	-1.5284	-1.52521
-1.4425	-1.43951	-1.49195	-1.48875	-1.51595	-1.51278	-1.50964	-1.52869	-1.52549
-1.44274	-1.43975	-1.4922	-1.489	-1.51621	-1.51304	-1.5099	-1.52897	-1.52576
-1.44298	-1.43999	-1.49246	-1.48926	-1.51647	-1.5133	-1.51016	-1.52926	-1.52604
-1.44322	-1.44022	-1.49271	-1.48951	-1.51673	-1.51356	-1.51042	-1.52955	-1.52633
-1.44346	-1.44046	-1.49297	-1.48977	-1.51699	-1.51381	-1.51067	-1.52983	-1.52663
-1.44369	-1.4407	-1.4649	-1.46178	-1.53975	-1.53651	-1.53331	-1.53011	-1.52691
-1.44393	-1.44093	-1.46514	-1.46202	-1.54003	-1.53679	-1.53359	-1.53038	-1.52718
-1.44417	-1.44117	-1.46539	-1.46226	-1.54031	-1.53707	-1.53386	-1.53065	-1.52745
-1.44441	-1.44141	-1.46563	-1.4625	-1.54058	-1.53734	-1.53414	-1.53093	-1.52772
-1.44465	-1.44164	-1.43863	-1.43558	-1.4564	-1.45339	-1.45042	-1.5312	-1.52799
-1.44489	-1.44189	-1.43887	-1.43583	-1.45667	-1.45365	-1.45067	-1.53146	-1.52826
-1.44512	-1.44213	-1.43912	-1.43609	-1.45696	-1.45393	-1.45093	-1.53174	-1.52853
-1.44536	-1.44237	-1.43938	-1.43637	-1.45729	-1.45426	-1.45124	-1.53205	-1.52882
-1.4456	-1.44262	-1.43964	-1.43667	-1.45764	-1.45461	-1.45158	-1.53238	-1.52912
-1.44588	-1.44288	-1.43989	-1.43692	-1.45793	-1.45491	-1.45188	-1.50559	-1.5024
-1.44618	-1.44315	-1.44013	-1.43715	-1.45819	-1.45517	-1.45216	-1.5059	-1.50275
-1.44648	-1.44343	-1.46456	-1.46151	-1.45847	-1.45545	-1.45244	-1.50621	-1.50306
-1.44677	-1.44375	-1.46491	-1.46186	-1.45881	-1.45575	-1.45271	-1.50648	-1.50334
-1.44704	-1.44404	-1.46525	-1.4622	-1.45914	-1.45607	-1.45301	-1.50678	-1.50363
-1.44729	-1.44429	-1.46553	-1.46249	-1.45945	-1.45639	-1.45333	-1.50713	-1.50399
-1.44754	-1.44453	-1.4658	-1.46279	-1.45977	-1.45672	-1.45367	-1.5075	-1.50436
-1.44784	-1.44483	-1.46613	-1.46312	-1.4601	-1.45706	-1.45402	-1.50785	-1.50468
-1.44817	-1.44516	-1.46649	-1.46347	-1.46045	-1.45741	-1.45436	-1.50819	-1.50499
-1.39177	-1.38882	-1.4668	-1.46378	-1.46076	-1.45772	-1.45469	-1.50854	-1.50534
-1.39204	-1.38907	-1.46708	-1.46405	-1.46102	-1.45801	-1.455	-1.50889	-1.50571
-1.39232	-1.38933	-1.41162	-1.40866	-1.46132	-1.45831	-1.45532	-1.47893	-1.47585
-1.39259	-1.38962	-1.41196	-1.409	-1.46169	-1.45866	-1.45565	-1.47928	-1.47621
-1.39287	-1.38992	-1.4123	-1.40935	-1.46206	-1.45903	-1.45599	-1.47963	-1.47656
-1.39317	-1.39024	-1.41264	-1.40969	-1.40674	-1.40378	-1.40082	-1.47998	-1.47688
-1.39349	-1.39055	-1.41297	-1.41003	-1.40708	-1.40412	-1.40116	-1.48034	-1.47721
-1.3938	-1.39087	-1.41332	-1.41037	-1.40742	-1.40447	-1.40151	-1.48073	-1.4776
-1.39412	-1.39119	-1.41366	-1.41071	-1.40776	-1.40481	-1.40186	-1.48114	-1.47803
-1.39443	-1.3915	-1.414	-1.41105	-1.4081	-1.40515	-1.40221	-1.42684	-1.42381
-1.39476	-1.39182	-1.41434	-1.4114	-1.40845	-1.40549	-1.40254	-1.42717	-1.42411
-1.39508	-1.39214	-1.41469	-1.41174	-1.40879	-1.40584	-1.40287	-1.42751	-1.42444
-1.3954	-1.39246	-1.41503	-1.41208	-1.40913	-1.40618	-1.40322	-1.42789	-1.42484
-1.39571	-1.39278	-1.41537	-1.41242	-1.40947	-1.40652	-1.40358	-1.42827	-1.42524
-1.39603	-1.39309	-1.41571	-1.41276	-1.40981	-1.40686	-1.40391	-1.42861	-1.42556
-1.39635	-1.39341	-1.41605	-1.4131	-1.41015	-1.40719	-1.40424	-1.42893	-1.42586
-1.39666	-1.39372	-1.41639	-1.41344	-1.41049	-1.40754	-1.40458	-1.4293	-1.42624
-1.39698	-1.39404	-1.41673	-1.41378	-1.41083	-1.40789	-1.40495	-1.4297	-1.42667
-1.3973	-1.39435	-1.41707	-1.41411	-1.41117	-1.40823	-1.4053	-1.43008	-1.42707
-1.39762	-1.39467	-1.4174	-1.41444	-1.4115	-1.40856	-1.40563	-1.43043	-1.42743

-1.39797	-1.395	-1.41775	-1.41479	-1.41184	-1.4089	-1.40597	-1.43078	-1.42778
-1.39836	-1.39539	-1.41815	-1.41515	-1.41218	-1.40924	-1.40632	-1.43115	-1.42814
-1.39875	-1.39579	-1.41857	-1.41553	-1.41252	-1.40959	-1.40669	-1.43153	-1.42851
-1.39909	-1.39614	-1.41895	-1.41593	-1.41293	-1.41	-1.40708	-1.43195	-1.42893
-1.39941	-1.39646	-1.41932	-1.41634	-1.41337	-1.41043	-1.4075	-1.43238	-1.42938
-1.39976	-1.39679	-1.41968	-1.41671	-1.41376	-1.41082	-1.40788	-1.43278	-1.42979
-1.40014	-1.39713	-1.42002	-1.41706	-1.41411	-1.41116	-1.40823	-1.43315	-1.43017
-1.40052	-1.39749	-1.42039	-1.41743	-1.41448	-1.41154	-1.4086	-1.43354	-1.43056
-1.40089	-1.39788	-1.42082	-1.41785	-1.4149	-1.41196	-1.40903	-1.43398	-1.43099
-1.40127	-1.39828	-1.42127	-1.41827	-1.4153	-1.41237	-1.40946	-1.43442	-1.43141
-1.40167	-1.39869	-1.4217	-1.41867	-1.41567	-1.41273	-1.40982	-1.4348	-1.43179
-1.40207	-1.3991	-1.42212	-1.41906	-1.41604	-1.41309	-1.41017	-1.43516	-1.43216
-1.40244	-1.39947	-1.42253	-1.41948	-1.41645	-1.4135	-1.41057	-1.43558	-1.43258
-1.40278	-1.39983	-1.42294	-1.41991	-1.41691	-1.41395	-1.41101	-1.43603	-1.43304
-1.40316	-1.40021	-1.42336	-1.42035	-1.41736	-1.4144	-1.41145	-1.40853	-1.40563
-1.40359	-1.40063	-1.42379	-1.42079	-1.4178	-1.41484	-1.4119	-1.40897	-1.40606
-1.40403	-1.40105	-1.42424	-1.42124	-1.41825	-1.41529	-1.41235	-1.40942	-1.4065
-1.40445	-1.40148	-1.42469	-1.42169	-1.4187	-1.41574	-1.41279	-1.40986	-1.40694
-1.40488	-1.40191	-1.42514	-1.42214	-1.41916	-1.41619	-1.41323	-1.4103	-1.40738
-1.40531	-1.40234	-1.4256	-1.42259	-1.41961	-1.41663	-1.41368	-1.41074	-1.40782
-1.40574	-1.40276	-1.42605	-1.42305	-1.42006	-1.41708	-1.41412	-1.41118	-1.40826
-1.40617	-1.40319	-1.4265	-1.4235	-1.4205	-1.41753	-1.41457	-1.41163	-1.4087
-1.4066	-1.40361	-1.42695	-1.42394	-1.42095	-1.41797	-1.41501	-1.41207	-1.40914
-1.40702	-1.40404	-1.42739	-1.42439	-1.4214	-1.41842	-1.41545	-1.41251	-1.40958
-1.40744	-1.40445	-1.42783	-1.42483	-1.42184	-1.41886	-1.41589	-1.41294	-1.41001
-1.37957	-1.37665	-1.42827	-1.42527	-1.42228	-1.4193	-1.41633	-1.41338	-1.41045
-1.37998	-1.37705	-1.42871	-1.42571	-1.42271	-1.41973	-1.41677	-1.41382	-1.41088
-1.38038	-1.37745	-1.42915	-1.42614	-1.42315	-1.42017	-1.4172	-1.41425	-1.41132
-1.38078	-1.37786	-1.42959	-1.42658	-1.42358	-1.4206	-1.41763	-1.41468	-1.41175
-1.38119	-1.37826	-1.43002	-1.42701	-1.42402	-1.42103	-1.41807	-1.41512	-1.41218
-1.3816	-1.37867	-1.4027	-1.39975	-1.42446	-1.42147	-1.41851	-1.41555	-1.41262
-1.38201	-1.37908	-1.40314	-1.40019	-1.42491	-1.42192	-1.41895	-1.41599	-1.41306
-1.38242	-1.3795	-1.40358	-1.40064	-1.42538	-1.42239	-1.41941	-1.41646	-1.41353
-1.38285	-1.37992	-1.40404	-1.40113	-1.4259	-1.42291	-1.41993	-1.41697	-1.41404
-1.38329	-1.38036	-1.4045	-1.40161	-1.42641	-1.42342	-1.42043	-1.41748	-1.41455
-1.34927	-1.3464	-1.40497	-1.40206	-1.39915	-1.39623	-1.3933	-1.41793	-1.415
-1.34974	-1.34687	-1.40544	-1.4025	-1.39957	-1.39664	-1.39372	-1.41836	-1.41543
-1.37787	-1.37494	-1.4059	-1.40294	-1.4	-1.39706	-1.39415	-1.39126	-1.3884
-1.37829	-1.37538	-1.40635	-1.40338	-1.40043	-1.39749	-1.39458	-1.39169	-1.38882
-1.37875	-1.37585	-1.40684	-1.40386	-1.4009	-1.39796	-1.39505	-1.39215	-1.38928
-1.37926	-1.37636	-1.40737	-1.40438	-1.40142	-1.39848	-1.39556	-1.39266	-1.38977
-1.37975	-1.37685	-1.37395	-1.37107	-1.40193	-1.399	-1.39607	-1.39317	-1.39027
-1.38019	-1.37727	-1.37439	-1.37154	-1.40246	-1.39951	-1.39658	-1.39367	-1.39079
-1.38063	-1.37771	-1.37483	-1.37201	-1.40297	-1.40002	-1.39707	-1.39416	-1.39129
-1.38113	-1.37819	-1.37529	-1.37245	-1.40342	-1.40046	-1.39752	-1.39461	-1.39174
-1.38165	-1.37869	-1.37578	-1.37289	-1.40384	-1.40089	-1.39796	-1.39505	-1.39216
-1.38216	-1.37921	-1.37628	-1.37338	-1.3705	-1.36764	-1.3648	-1.39553	-1.39263
-1.38267	-1.37973	-1.3768	-1.37389	-1.37101	-1.36815	-1.3653	-1.39604	-1.39313
-1.3832	-1.38025	-1.37732	-1.37441	-1.37152	-1.36865	-1.3658	-1.39655	-1.39363
-1.38375	-1.38077	-1.37783	-1.37492	-1.37203	-1.36915	-1.36629	-1.39706	-1.39415

-1.38427	-1.38129	-1.37834	-1.37543	-1.37254	-1.36965	-1.36679	-1.36394	-1.36112
-1.38474	-1.38181	-1.37888	-1.37595	-1.37304	-1.37016	-1.36729	-1.36445	-1.36161
-1.38519	-1.38231	-1.3794	-1.37646	-1.37354	-1.37066	-1.3678	-1.36495	-1.36211
-1.35324	-1.35041	-1.37988	-1.37696	-1.37405	-1.37117	-1.3683	-1.36544	-1.3626
-1.35373	-1.35086	-1.38033	-1.37745	-1.37458	-1.37168	-1.36879	-1.36594	-1.3631
-1.35425	-1.35137	-1.34853	-1.34572	-1.37509	-1.37219	-1.36929	-1.36643	-1.3636
-1.35481	-1.35194	-1.34909	-1.34624	-1.37559	-1.37269	-1.3698	-1.36694	-1.36409
-1.31617	-1.3134	-1.34963	-1.34675	-1.3761	-1.37319	-1.37031	-1.36745	-1.3646
-1.31671	-1.31391	-1.35014	-1.34726	-1.37662	-1.37371	-1.37083	-1.36796	-1.36512
-1.31725	-1.31442	-1.35064	-1.34777	-1.34492	-1.34208	-1.33927	-1.36849	-1.36565
-1.31776	-1.31493	-1.35116	-1.34828	-1.34542	-1.34259	-1.33979	-1.36903	-1.36621
-1.31827	-1.31544	-1.35168	-1.34879	-1.34593	-1.34311	-1.34031	-1.36958	-1.36677
-1.31878	-1.31595	-1.35219	-1.34931	-1.34646	-1.34364	-1.34086	-1.33808	-1.33533
-1.31929	-1.31646	-1.3527	-1.34984	-1.347	-1.34419	-1.34141	-1.33862	-1.33583
-1.31981	-1.31697	-1.35324	-1.35038	-1.34755	-1.34474	-1.34194	-1.33914	-1.33633
-1.32032	-1.3175	-1.35379	-1.35094	-1.3481	-1.34528	-1.34246	-1.33964	-1.33684
-1.32085	-1.31804	-1.31525	-1.31249	-1.34865	-1.34581	-1.34297	-1.34016	-1.33736
-1.32144	-1.3186	-1.31579	-1.31301	-1.34917	-1.34633	-1.3435	-1.3407	-1.33791
-1.32202	-1.31916	-1.31633	-1.31354	-1.34969	-1.34685	-1.34403	-1.34123	-1.33846
-1.32255	-1.31968	-1.31685	-1.31406	-1.35023	-1.34739	-1.34457	-1.34177	-1.33898
-1.32307	-1.3202	-1.31737	-1.31458	-1.35077	-1.34794	-1.34512	-1.3423	-1.33948
-1.32363	-1.32076	-1.31793	-1.31513	-1.35132	-1.34847	-1.34564	-1.34281	-1.33999
-1.32423	-1.32136	-1.31853	-1.31571	-1.35189	-1.349	-1.34614	-1.34331	-1.34051
-1.32478	-1.32193	-1.3191	-1.31628	-1.35246	-1.34956	-1.34668	-1.34385	-1.34105
-1.32529	-1.32246	-1.31964	-1.31685	-1.35306	-1.35016	-1.34728	-1.34443	-1.34161
-1.32582	-1.32299	-1.32019	-1.31742	-1.35365	-1.35076	-1.34787	-1.34501	-1.34218
-1.32641	-1.32356	-1.32075	-1.31798	-1.35424	-1.35135	-1.34846	-1.34559	-1.34274
-1.32701	-1.32415	-1.32132	-1.31854	-1.35481	-1.35192	-1.34903	-1.34616	-1.34331
-1.32757	-1.32471	-1.32188	-1.31908	-1.31629	-1.3135	-1.31073	-1.34672	-1.34388
-1.32811	-1.32528	-1.32244	-1.3196	-1.31678	-1.314	-1.31124	-1.34726	-1.34445
-1.32868	-1.32585	-1.32302	-1.32016	-1.31733	-1.31454	-1.31178	-1.34782	-1.34502
-1.32927	-1.32644	-1.3236	-1.32075	-1.31791	-1.31511	-1.31235	-1.3484	-1.3456
-1.32986	-1.32703	-1.32419	-1.32133	-1.31849	-1.31569	-1.31292	-1.34898	-1.34618
-1.33046	-1.32762	-1.32478	-1.32191	-1.31907	-1.31627	-1.3135	-1.34957	-1.34676
-1.33105	-1.32821	-1.32536	-1.32249	-1.31965	-1.31685	-1.31408	-1.35016	-1.34735
-1.33164	-1.3288	-1.32595	-1.32308	-1.32023	-1.31743	-1.31466	-1.35075	-1.34793
-1.33224	-1.32939	-1.32653	-1.32366	-1.32082	-1.31801	-1.31523	-1.35133	-1.34851
-1.33284	-1.32998	-1.32712	-1.32425	-1.3214	-1.31859	-1.31581	-1.31306	-1.31033
-1.33345	-1.33058	-1.32771	-1.32484	-1.32199	-1.31918	-1.31639	-1.31363	-1.3109
-1.33405	-1.33118	-1.32831	-1.32543	-1.32257	-1.31976	-1.31697	-1.31421	-1.31147
-1.33466	-1.33178	-1.3289	-1.32602	-1.32315	-1.32033	-1.31754	-1.31478	-1.31204
-1.33528	-1.33239	-1.3295	-1.32661	-1.32374	-1.32091	-1.31812	-1.31535	-1.31262
-1.33588	-1.33299	-1.33011	-1.32721	-1.32433	-1.32149	-1.31868	-1.31592	-1.3132
-1.33649	-1.3336	-1.33071	-1.32782	-1.32494	-1.32209	-1.31927	-1.3165	-1.31378
-1.30716	-1.30434	-1.33134	-1.32846	-1.3256	-1.32273	-1.31989	-1.31711	-1.31436
-1.30775	-1.30494	-1.33196	-1.32912	-1.32627	-1.3234	-1.32054	-1.31772	-1.31494
-1.30838	-1.30554	-1.33256	-1.32973	-1.32689	-1.32402	-1.32115	-1.31832	-1.31552
-1.30903	-1.30614	-1.33315	-1.33031	-1.32749	-1.32462	-1.32175	-1.31891	-1.31609
-1.3097	-1.30677	-1.33377	-1.33092	-1.32809	-1.32524	-1.32239	-1.31954	-1.3167
-1.3104	-1.30744	-1.33442	-1.33155	-1.32871	-1.32589	-1.32308	-1.32023	-1.31736

-1.24203	-1.23922	-1.33507	-1.33217	-1.32932	-1.32652	-1.32374	-1.3209	-1.31803
-1.24262	-1.23979	-1.33569	-1.33277	-1.3299	-1.32711	-1.32435	-1.32154	-1.3187
-1.2432	-1.24037	-1.33632	-1.33338	-1.33049	-1.3277	-1.32494	-1.32215	-1.31936
-1.2438	-1.241	-1.33701	-1.33404	-1.33113	-1.3283	-1.32552	-1.32274	-1.31997
-1.2444	-1.24165	-1.33772	-1.33473	-1.33179	-1.32893	-1.3261	-1.32331	-1.32054
-1.245	-1.24226	-1.27054	-1.26772	-1.33247	-1.32958	-1.32673	-1.32391	-1.32112
-1.2456	-1.24283	-1.27113	-1.26835	-1.33317	-1.33027	-1.32738	-1.32454	-1.32172
-1.24623	-1.24342	-1.27172	-1.26896	-1.33383	-1.33093	-1.32805	-1.3252	-1.32236
-1.2469	-1.24405	-1.27233	-1.26954	-1.33443	-1.33157	-1.32874	-1.32591	-1.32309
-1.24756	-1.24469	-1.27297	-1.27014	-1.26738	-1.26469	-1.26204	-1.3266	-1.32379
-1.24819	-1.24535	-1.27365	-1.2708	-1.26799	-1.26527	-1.26258	-1.32718	-1.32439
-1.2488	-1.24601	-1.27434	-1.27147	-1.26863	-1.26586	-1.26313	-1.32776	-1.32496
-1.24942	-1.24662	-1.27496	-1.27211	-1.26929	-1.26652	-1.26379	-1.32843	-1.32561
-1.25004	-1.2472	-1.27556	-1.27275	-1.26997	-1.26723	-1.2645	-1.32917	-1.32629
-1.25072	-1.24787	-1.27622	-1.27341	-1.27062	-1.26787	-1.26514	-1.26241	-1.25969
-1.25145	-1.24861	-1.27696	-1.27409	-1.27125	-1.26846	-1.26571	-1.26299	-1.26032
-1.25213	-1.2493	-1.27766	-1.27476	-1.27189	-1.26908	-1.26632	-1.26361	-1.26095
-1.2527	-1.24988	-1.27828	-1.27542	-1.27259	-1.2698	-1.26703	-1.26429	-1.26158
-1.25328	-1.25046	-1.27888	-1.27606	-1.27326	-1.27049	-1.26774	-1.26497	-1.26221
-1.25399	-1.25112	-1.27952	-1.27667	-1.27386	-1.27109	-1.26834	-1.26559	-1.26285
-1.25472	-1.25181	-1.28017	-1.27728	-1.27444	-1.27166	-1.26892	-1.26619	-1.26349
-1.21911	-1.21629	-1.28086	-1.27797	-1.27512	-1.27233	-1.26958	-1.26684	-1.26412
-1.21973	-1.21695	-1.28158	-1.2787	-1.27586	-1.27307	-1.2703	-1.26752	-1.26475
-1.22039	-1.21763	-1.2823	-1.27942	-1.27656	-1.27375	-1.27096	-1.26817	-1.26538
-1.2211	-1.21835	-1.28304	-1.2801	-1.2772	-1.27435	-1.27155	-1.26877	-1.26603
-1.2218	-1.21904	-1.25247	-1.24957	-1.27785	-1.27498	-1.27217	-1.2694	-1.26669
-1.22243	-1.21966	-1.2531	-1.25022	-1.27853	-1.27568	-1.27287	-1.27011	-1.26739
-1.22305	-1.22027	-1.25374	-1.25088	-1.27922	-1.27638	-1.27358	-1.27081	-1.26808
-1.11252	-1.11004	-1.25445	-1.25161	-1.27995	-1.27708	-1.27424	-1.27144	-1.2687
-1.11312	-1.11066	-1.2552	-1.25235	-1.28068	-1.27777	-1.27488	-1.27206	-1.26928
-1.11373	-1.11126	-1.21956	-1.21679	-1.28135	-1.27844	-1.27555	-1.27272	-1.26995
-1.11435	-1.11183	-1.22018	-1.21739	-1.28197	-1.27909	-1.27624	-1.27344	-1.27067
-1.08105	-1.07859	-1.15088	-1.14827	-1.28265	-1.27978	-1.27696	-1.27416	-1.27139
-1.0816	-1.07915	-1.15151	-1.14892	-1.28341	-1.28054	-1.2777	-1.27489	-1.27211
-1.08215	-1.07972	-1.15215	-1.14958	-1.28419	-1.28131	-1.27845	-1.27563	-1.27284
-1.08275	-1.08033	-1.15282	-1.15026	-1.28496	-1.28207	-1.2792	-1.27637	-1.27356
-1.08336	-1.08095	-1.1535	-1.15095	-1.28573	-1.28282	-1.27994	-1.27709	-1.27427
-1.08397	-1.08157	-1.15418	-1.15162	-1.18203	-1.17936	-1.1767	-1.27775	-1.27491
-1.0846	-1.08219	-1.15485	-1.15228	-1.18268	-1.17998	-1.17727	-1.27837	-1.27551
-1.08522	-1.08281	-1.15551	-1.15291	-1.18331	-1.18059	-1.17788	-1.27905	-1.27618
-1.08584	-1.08343	-1.15615	-1.15352	-1.1839	-1.18119	-1.17851	-1.27978	-1.27692
-1.08642	-1.084	-1.11547	-1.11294	-1.15148	-1.14887	-1.14629	-1.21328	-1.2106
-1.08692	-1.08448	-1.11597	-1.11348	-1.15209	-1.14951	-1.14694	-1.21397	-1.21129
-1.08744	-1.08498	-1.11649	-1.11403	-1.15272	-1.15016	-1.14761	-1.21466	-1.21198
-1.08802	-1.08556	-1.11709	-1.11463	-1.15336	-1.15081	-1.14828	-1.21537	-1.21268
-1.08862	-1.08618	-1.11773	-1.11526	-1.15401	-1.15147	-1.14895	-1.21608	-1.21338
-1.08923	-1.08679	-1.11837	-1.11589	-1.15467	-1.15214	-1.14962	-1.17989	-1.17728
-1.08985	-1.08742	-1.11902	-1.11654	-1.15535	-1.15281	-1.15029	-1.18056	-1.17795
-1.09048	-1.08805	-1.11967	-1.11718	-1.15602	-1.15348	-1.15095	-1.18123	-1.17863
-1.09112	-1.08868	-1.12032	-1.11783	-1.1567	-1.15414	-1.15161	-1.18189	-1.1793

-1.09177	-1.08932	-1.12097	-1.11847	-1.15738	-1.15481	-1.15227	-1.18256	-1.17997
-1.09241	-1.08996	-1.12162	-1.11912	-1.15806	-1.15548	-1.15293	-1.18322	-1.18063
-1.09306	-1.09059	-1.12227	-1.11977	-1.15874	-1.15616	-1.1536	-1.18388	-1.18129
-1.0937	-1.09123	-1.12293	-1.12042	-1.11793	-1.11546	-1.11301	-1.15171	-1.14919
-1.09433	-1.09186	-1.12358	-1.12107	-1.11857	-1.11609	-1.11362	-1.15236	-1.14984
-1.05097	-1.0486	-1.12423	-1.12171	-1.1192	-1.11671	-1.11425	-1.15302	-1.15049
-1.05157	-1.0492	-1.12487	-1.12234	-1.11983	-1.11735	-1.11488	-1.15368	-1.15115
-1.0128	-1.0105	-1.12552	-1.12298	-1.12047	-1.11798	-1.11552	-1.15435	-1.1518
-1.01337	-1.01107	-1.12616	-1.12362	-1.12111	-1.11862	-1.11615	-1.15501	-1.15246
-1.01394	-1.01164	-1.09253	-1.09008	-1.12176	-1.11926	-1.11679	-1.15568	-1.15311
-1.01452	-1.01222	-1.09317	-1.09071	-1.1224	-1.1199	-1.11742	-1.15634	-1.15377
-1.01511	-1.0128	-1.09381	-1.09134	-1.12304	-1.12054	-1.11805	-1.157	-1.15443
-1.01569	-1.01339	-1.05585	-1.05345	-1.12368	-1.12117	-1.11867	-1.1162	-1.11375
-1.01629	-1.01398	-1.05647	-1.05406	-1.12432	-1.12181	-1.11931	-1.11684	-1.11439
-1.01697	-1.01465	-1.01234	-1.01003	-1.12506	-1.12254	-1.12004	-1.11756	-1.11511
-1.01773	-1.0154	-1.01308	-1.01076	-1.12589	-1.12336	-1.12086	-1.11838	-1.11591
-1.01844	-1.01609	-1.01376	-1.01145	-1.08877	-1.08631	-1.08387	-1.11913	-1.11666
-1.01906	-1.0167	-1.01436	-1.01205	-1.08942	-1.08695	-1.0845	-1.11978	-1.11731
-1.01967	-1.01729	-1.01495	-1.01263	-1.09005	-1.08757	-1.08511	-1.1204	-1.11793
-1.02028	-1.0179	-1.01554	-1.01321	-1.09068	-1.08819	-1.08572	-1.12104	-1.11856
-1.0209	-1.0185	-1.01613	-1.01379	-1.09131	-1.08881	-1.08634	-1.12167	-1.11918
-1.02152	-1.0191	-1.01671	-1.01437	-1.01205	-1.00973	-1.00744	-1.0845	-1.08209
-1.02213	-1.01969	-1.01729	-1.01495	-1.01263	-1.01031	-1.00801	-1.08512	-1.08269
-1.02282	-1.02036	-1.01796	-1.01561	-1.01328	-1.01096	-1.00865	-1.08581	-1.08338
-1.02358	-1.02112	-1.01871	-1.01635	-1.01402	-1.01169	-1.00939	-1.08661	-1.08417
-0.98582	-0.98342	-1.01942	-1.01706	-1.01471	-1.01239	-1.01008	-1.08736	-1.08491
-0.98644	-0.98405	-1.02005	-1.01767	-1.01531	-1.01298	-1.01067	-1.08801	-1.08555
-0.98704	-0.98465	-1.02065	-1.01825	-1.01588	-1.01355	-1.01124	-1.08863	-1.08616
-0.98764	-0.98525	-0.98287	-0.98053	-1.01646	-1.01412	-1.01182	-1.08925	-1.08677
-0.98826	-0.98585	-0.98347	-0.98112	-1.01705	-1.01471	-1.0124	-1.08988	-1.08739
-0.98895	-0.98654	-0.98416	-0.98179	-0.97947	-0.97719	-0.97494	-1.01075	-1.00845
-0.98972	-0.98731	-0.98492	-0.98255	-0.98021	-0.97792	-0.97566	-1.01148	-1.00918
-0.86392	-0.86186	-0.98562	-0.98323	-0.98089	-0.97859	-0.97633	-0.97408	-0.97186
-0.86443	-0.86237	-0.98622	-0.98383	-0.98148	-0.97917	-0.9769	-0.97466	-0.97244
-0.86497	-0.8629	-0.98686	-0.98447	-0.98211	-0.97979	-0.97751	-0.97526	-0.97304
-0.86559	-0.86353	-0.98762	-0.98522	-0.98286	-0.98053	-0.97823	-0.97596	-0.97373
-0.86621	-0.86415	-0.98838	-0.98598	-0.98361	-0.98126	-0.97895	-0.97667	-0.97443
-0.82174	-0.81979	-0.98902	-0.98662	-0.98424	-0.98188	-0.97955	-0.97726	-0.97502
-0.82225	-0.82027	-0.98961	-0.9872	-0.98482	-0.98245	-0.98011	-0.97781	-0.97556
-0.78264	-0.78075	-0.90596	-0.90381	-0.98549	-0.98312	-0.98077	-0.97847	-0.97621
-0.78319	-0.7813	-0.90663	-0.90448	-0.98625	-0.98387	-0.98152	-0.97921	-0.97694
-0.78372	-0.78183	-0.86317	-0.86112	-0.98694	-0.98456	-0.98221	-0.97989	-0.97761
-0.78419	-0.7823	-0.8637	-0.86164	-0.98754	-0.98515	-0.9828	-0.98048	-0.97819
-0.73819	-0.7364	-0.821	-0.81901	-0.94584	-0.94356	-0.94131	-0.98108	-0.97878
-0.73874	-0.73695	-0.8216	-0.81961	-0.94655	-0.94426	-0.942	-0.98178	-0.97947
-0.7393	-0.73751	-0.82222	-0.82022	-0.9473	-0.945	-0.94273	-0.98253	-0.98018
-0.73978	-0.73798	-0.82276	-0.82077	-0.86199	-0.85996	-0.85794	-0.98327	-0.98092
-0.74021	-0.73839	-0.82324	-0.8213	-0.86259	-0.86057	-0.85856	-0.98401	-0.98167
-0.68556	-0.68404	-0.7371	-0.73537	-0.86317	-0.86114	-0.85913	-0.94254	-0.94031
-0.68604	-0.68451	-0.73764	-0.73587	-0.86372	-0.86167	-0.85964	-0.94311	-0.94087

-0.68652	-0.68499	-0.73819	-0.73639	-0.86431	-0.86223	-0.86018	-0.94372	-0.94147
-0.68698	-0.68547	-0.73876	-0.73695	-0.86497	-0.86287	-0.8608	-0.94442	-0.94216
-0.68744	-0.68593	-0.73931	-0.7375	-0.86563	-0.86353	-0.86144	-0.94515	-0.94287
-0.68785	-0.68632	-0.73978	-0.738	-0.86627	-0.86417	-0.86209	-0.86003	-0.858
-0.68826	-0.6867	-0.74022	-0.73848	-0.8669	-0.86481	-0.86273	-0.86067	-0.85864
-0.68871	-0.68713	-0.6856	-0.68414	-0.73722	-0.73547	-0.73372	-0.8613	-0.85927
-0.68919	-0.6876	-0.68605	-0.68454	-0.73766	-0.73593	-0.73422	-0.86192	-0.8599
-0.6448	-0.64329	-0.68651	-0.68497	-0.73814	-0.73641	-0.7347	-0.86252	-0.8605
-0.64527	-0.64376	-0.687	-0.68545	-0.73869	-0.73691	-0.73516	-0.86307	-0.86109
-0.64574	-0.64423	-0.68749	-0.68594	-0.73924	-0.73743	-0.73564	-0.77898	-0.7772
-0.64619	-0.64469	-0.68799	-0.68642	-0.73979	-0.73797	-0.73618	-0.77951	-0.77769
-0.64662	-0.64514	-0.68848	-0.6869	-0.74035	-0.73852	-0.73673	-0.78007	-0.77819
-0.64703	-0.64557	-0.68894	-0.68736	-0.74089	-0.73907	-0.73726	-0.78064	-0.77875
-0.64742	-0.64598	-0.6894	-0.68782	-0.74144	-0.73961	-0.7378	-0.78122	-0.77933
-0.59634	-0.59503	-0.64497	-0.6435	-0.68674	-0.6852	-0.68367	-0.73654	-0.73476
-0.59676	-0.59546	-0.64544	-0.64397	-0.68724	-0.68568	-0.68414	-0.73709	-0.7353
-0.59718	-0.59586	-0.64588	-0.64441	-0.68772	-0.68616	-0.68461	-0.73763	-0.73585
-0.59758	-0.59623	-0.64627	-0.64482	-0.68817	-0.68661	-0.68506	-0.73816	-0.73638
-0.55	-0.54874	-0.59525	-0.59394	-0.68863	-0.68707	-0.68552	-0.7387	-0.73691
-0.5504	-0.54913	-0.59567	-0.59436	-0.68912	-0.68756	-0.686	-0.73925	-0.73745
-0.5508	-0.54954	-0.59609	-0.59478	-0.68961	-0.68805	-0.68648	-0.73981	-0.73799
-0.5512	-0.54993	-0.59651	-0.5952	-0.59389	-0.59254	-0.59118	-0.68541	-0.68386
-0.55159	-0.55033	-0.59692	-0.59561	-0.5943	-0.59295	-0.5916	-0.68588	-0.68433
-0.49593	-0.49486	-0.54947	-0.54827	-0.5947	-0.59336	-0.592	-0.68636	-0.68479
-0.49627	-0.4952	-0.54986	-0.54864	-0.59509	-0.59375	-0.59241	-0.68682	-0.68524
-0.44664	-0.44563	-0.55024	-0.54902	-0.59549	-0.59415	-0.59281	-0.59145	-0.59008
-0.44696	-0.44594	-0.55062	-0.54939	-0.59589	-0.59455	-0.59321	-0.59185	-0.59048
-0.44728	-0.44626	-0.551	-0.54977	-0.59629	-0.59496	-0.59362	-0.59226	-0.59089
-0.44761	-0.44657	-0.44557	-0.44462	-0.50028	-0.49913	-0.49799	-0.59267	-0.5913
-0.44795	-0.4469	-0.44589	-0.44493	-0.50064	-0.49949	-0.49835	-0.59307	-0.59171
-0.39461	-0.39369	-0.44624	-0.44528	-0.50103	-0.49986	-0.4987	-0.54596	-0.5447
-0.39492	-0.39401	-0.44661	-0.44563	-0.50142	-0.50023	-0.49906	-0.54633	-0.54507
-0.39522	-0.39431	-0.44696	-0.44597	-0.44499	-0.44399	-0.44299	-0.49825	-0.49712
-0.39552	-0.3946	-0.44729	-0.44629	-0.4453	-0.44429	-0.4433	-0.49862	-0.49751
-0.39583	-0.39489	-0.39398	-0.3931	-0.44561	-0.44461	-0.44361	-0.49899	-0.4979
-0.39612	-0.39518	-0.39426	-0.39337	-0.44593	-0.44492	-0.44393	-0.49935	-0.49825
-0.39643	-0.39548	-0.39455	-0.39365	-0.44625	-0.44524	-0.44425	-0.49972	-0.49861
-0.39676	-0.39581	-0.39487	-0.39395	-0.39305	-0.39218	-0.39132	-0.50012	-0.49899
-0.3971	-0.39615	-0.3952	-0.39426	-0.39334	-0.39247	-0.39162	-0.50055	-0.49938
-0.34774	-0.34686	-0.39552	-0.39456	-0.39364	-0.39276	-0.39191	-0.44431	-0.44333
-0.34806	-0.34717	-0.39582	-0.39487	-0.39395	-0.39306	-0.3922	-0.44464	-0.44365
-0.34838	-0.34748	-0.3466	-0.34574	-0.39426	-0.39336	-0.39248	-0.39162	-0.39076
-0.34869	-0.34778	-0.3469	-0.34604	-0.39455	-0.39365	-0.39277	-0.3919	-0.39106
-0.34899	-0.34809	-0.34721	-0.34635	-0.39486	-0.39394	-0.39305	-0.39219	-0.39135
-0.3493	-0.34841	-0.34754	-0.34667	-0.39519	-0.39425	-0.39334	-0.39246	-0.39162
-0.34962	-0.34874	-0.34787	-0.347	-0.39552	-0.39457	-0.39363	-0.39274	-0.39189
-0.34995	-0.34907	-0.3482	-0.34732	-0.34645	-0.34558	-0.34472	-0.39304	-0.39218
-0.35028	-0.3494	-0.34852	-0.34763	-0.34676	-0.34589	-0.34504	-0.39335	-0.39248
-0.29344	-0.29272	-0.34883	-0.34795	-0.34707	-0.34621	-0.34536	-0.34453	-0.3437
-0.29371	-0.29297	-0.34914	-0.34826	-0.3474	-0.34654	-0.34569	-0.34485	-0.34403

-0.29397	-0.29322	-0.34946	-0.34859	-0.34773	-0.34687	-0.34602	-0.34518	-0.34435
-0.29423	-0.29349	-0.3498	-0.34892	-0.34806	-0.3472	-0.34635	-0.34551	-0.34467
-0.29449	-0.29376	-0.35014	-0.34926	-0.34839	-0.34753	-0.34668	-0.34583	-0.34498
-0.29477	-0.29404	-0.29331	-0.29259	-0.34872	-0.34786	-0.347	-0.34615	-0.34529
-0.29505	-0.29432	-0.29359	-0.29286	-0.34906	-0.34819	-0.34732	-0.34646	-0.34559
-0.29533	-0.2946	-0.29387	-0.29314	-0.29241	-0.29168	-0.29096	-0.34677	-0.3459
-0.29562	-0.29488	-0.29414	-0.29341	-0.29267	-0.29194	-0.2912	-0.34707	-0.34622
-0.24255	-0.24193	-0.29442	-0.29368	-0.29293	-0.29219	-0.29146	-0.34738	-0.34654
-0.24279	-0.24217	-0.29469	-0.29393	-0.29318	-0.29244	-0.29172	-0.34771	-0.34686
-0.24303	-0.24241	-0.29497	-0.2942	-0.29344	-0.29271	-0.29198	-0.29127	-0.29056
-0.24327	-0.24265	-0.29525	-0.29449	-0.29374	-0.29299	-0.29225	-0.29153	-0.29082
-0.2435	-0.24289	-0.29553	-0.29478	-0.29403	-0.29327	-0.29252	-0.29179	-0.29108
-0.18754	-0.18706	-0.2425	-0.24187	-0.2943	-0.29354	-0.2928	-0.29206	-0.29135
-0.18772	-0.18724	-0.24274	-0.2421	-0.29455	-0.29381	-0.29308	-0.29234	-0.29162
-0.18789	-0.18742	-0.24298	-0.24234	-0.24171	-0.24109	-0.24047	-0.29263	-0.2919
-0.18807	-0.18759	-0.24321	-0.24259	-0.24197	-0.24135	-0.24074	-0.29293	-0.29219
-0.18825	-0.18777	-0.18729	-0.18682	-0.24223	-0.24161	-0.24099	-0.29322	-0.29248
-0.18842	-0.18794	-0.18747	-0.187	-0.24247	-0.24185	-0.24123	-0.2935	-0.29276
-0.1886	-0.18812	-0.18764	-0.18717	-0.24271	-0.24209	-0.24147	-0.24085	-0.24022
-0.18878	-0.18829	-0.18782	-0.18735	-0.24294	-0.24233	-0.24171	-0.24109	-0.24046
-0.18896	-0.18847	-0.18799	-0.18752	-0.24317	-0.24256	-0.24195	-0.24133	-0.2407
-0.18915	-0.18865	-0.18816	-0.1877	-0.18723	-0.18676	-0.18628	-0.24157	-0.24095
-0.18934	-0.18883	-0.18834	-0.18787	-0.18741	-0.18694	-0.18646	-0.24181	-0.24119
-0.18954	-0.18902	-0.18853	-0.18806	-0.18759	-0.18712	-0.18665	-0.24206	-0.24144
-0.18975	-0.18923	-0.18873	-0.18825	-0.18777	-0.18731	-0.18684	-0.24231	-0.2417
-0.18995	-0.18943	-0.18892	-0.18843	-0.18796	-0.18749	-0.18703	-0.18656	-0.18607
-0.19015	-0.18962	-0.18911	-0.18862	-0.18814	-0.18767	-0.18721	-0.18673	-0.18625
-0.19035	-0.18982	-0.1893	-0.1888	-0.18832	-0.18785	-0.18738	-0.18691	-0.18643
-0.19054	-0.19001	-0.1895	-0.18899	-0.1885	-0.18803	-0.18757	-0.1871	-0.18662
-0.19074	-0.19021	-0.18969	-0.18919	-0.18869	-0.18822	-0.18776	-0.18729	-0.18682
-0.19095	-0.19042	-0.1899	-0.18939	-0.18889	-0.18841	-0.18794	-0.18747	-0.187
-0.19115	-0.19064	-0.19012	-0.18961	-0.1891	-0.1886	-0.18812	-0.18764	-0.18717
-0.19135	-0.19084	-0.19033	-0.18982	-0.18931	-0.18881	-0.18831	-0.18783	-0.18736
-0.19154	-0.19103	-0.19053	-0.19002	-0.18952	-0.18902	-0.18852	-0.18803	-0.18756
-0.19173	-0.19123	-0.19072	-0.19022	-0.18972	-0.18922	-0.18872	-0.18823	-0.18775
-0.19194	-0.19143	-0.19093	-0.19042	-0.18992	-0.18941	-0.18891	-0.18842	-0.18795
-0.19214	-0.19164	-0.19114	-0.19062	-0.19011	-0.1896	-0.1891	-0.18861	-0.18814
-0.19233	-0.19184	-0.19135	-0.19083	-0.19032	-0.18981	-0.1893	-0.18881	-0.18833
-0.19252	-0.19204	-0.19155	-0.19104	-0.19054	-0.19003	-0.18952	-0.18902	-0.18852
-0.19272	-0.19223	-0.19174	-0.19124	-0.19074	-0.19023	-0.18973	-0.18922	-0.18872
-0.19292	-0.19242	-0.19193	-0.19142	-0.19092	-0.19043	-0.18994	-0.18943	-0.18892
-0.19312	-0.19262	-0.19212	-0.19162	-0.19112	-0.19063	-0.19015	-0.18964	-0.18913
-0.19333	-0.19282	-0.19232	-0.19182	-0.19133	-0.19084	-0.19035	-0.18985	-0.18933
-0.19354	-0.19303	-0.19253	-0.19203	-0.19153	-0.19105	-0.19056	-0.19005	-0.18954
-0.19375	-0.19324	-0.19273	-0.19223	-0.19174	-0.19125	-0.19076	-0.19026	-0.18974
-0.19397	-0.19345	-0.19294	-0.19244	-0.19194	-0.19146	-0.19097	-0.19046	-0.18995
-0.06169	-0.06154	-0.19315	-0.19265	-0.19215	-0.19166	-0.19117	-0.19067	-0.19016
-0.06176	-0.06161	-0.19336	-0.19285	-0.19235	-0.19187	-0.19138	-0.19088	-0.19036
-0.06183	-0.06168	-0.19358	-0.19306	-0.19256	-0.19207	-0.19159	-0.19108	-0.19057
-0.0619	-0.06174	-0.19379	-0.19327	-0.19277	-0.19228	-0.19179	-0.19129	-0.19077

-0.06197	-0.06181	-0.19401	-0.19349	-0.19298	-0.19249	-0.192	-0.19149	-0.19098
-0.06204	-0.06188	-0.19423	-0.1937	-0.19319	-0.1927	-0.19221	-0.1917	-0.19119
-0.06211	-0.06195	-0.19445	-0.19392	-0.1934	-0.19291	-0.19242	-0.19191	-0.19139
-0.06218	-0.06202	-0.19467	-0.19414	-0.19362	-0.19312	-0.19262	-0.19212	-0.1916
-0.06225	-0.06209	-0.19489	-0.19436	-0.19384	-0.19333	-0.19284	-0.19232	-0.19181
-0.06232	-0.06216	-0.06201	-0.06185	-0.19406	-0.19355	-0.19305	-0.19254	-0.19202
-0.06238	-0.06223	-0.06208	-0.06192	-0.19428	-0.19377	-0.19326	-0.19275	-0.19224
-0.06245	-0.0623	-0.06215	-0.06199	-0.1945	-0.19399	-0.19348	-0.19297	-0.19246
-0.06251	-0.06237	-0.06222	-0.06206	-0.19472	-0.19421	-0.1937	-0.19319	-0.19269
-0.06258	-0.06243	-0.06229	-0.06213	-0.19495	-0.19443	-0.19392	-0.19342	-0.19291
-0.06264	-0.0625	-0.06236	-0.06221	-0.19519	-0.19466	-0.19414	-0.19363	-0.19313
-0.06271	-0.06257	-0.06243	-0.06228	-0.19543	-0.19489	-0.19437	-0.19385	-0.19334
-0.06277	-0.06264	-0.0625	-0.06235	-0.12831	-0.12797	-0.12762	-0.19406	-0.19355
-0.06283	-0.0627	-0.06256	-0.06242	-0.12846	-0.12811	-0.12777	-0.19428	-0.19377
-0.06289	-0.06276	-0.06263	-0.06249	-0.06234	-0.06219	-0.06203	-0.1945	-0.19399
-0.06295	-0.06282	-0.06269	-0.06255	-0.06241	-0.06226	-0.06211	-0.19474	-0.19421
-0.06302	-0.06288	-0.06275	-0.06262	-0.06248	-0.06234	-0.06219	-0.1279	-0.12755
-0.06309	-0.06295	-0.06281	-0.06268	-0.06255	-0.06241	-0.06226	-0.12806	-0.12771
-0.06316	-0.06301	-0.06287	-0.06274	-0.06261	-0.06248	-0.06233	-0.12821	-0.12787
-0.06323	-0.06308	-0.06294	-0.06281	-0.06267	-0.06254	-0.0624	-0.12835	-0.12802
-0.06331	-0.06315	-0.06301	-0.06287	-0.06274	-0.0626	-0.06246	-0.12849	-0.12817
		-0.06308	-0.06294	-0.0628	-0.06266	-0.06253	-0.12863	-0.12831
		-0.06315	-0.06301	-0.06287	-0.06273	-0.0626	-0.12877	-0.12845
		-0.06322	-0.06308	-0.06294	-0.0628	-0.06266	-0.06252	-0.06238
		-0.06329	-0.06314	-0.063	-0.06287	-0.06273	-0.06259	-0.06245
				-0.06307	-0.06293	-0.0628	-0.06266	-0.06252
				-0.06314	-0.063	-0.06286	-0.06273	-0.06259
				-0.0632	-0.06306	-0.06293	-0.0628	-0.06267
				-0.06328	-0.06313	-0.06299	-0.06286	-0.06273
				-0.06335	-0.0632	-0.06305	-0.06292	-0.06279
				-0.06343	-0.06327	-0.06313	-0.06299	-0.06285
				-0.06351	-0.06336	-0.06321	-0.06306	-0.06292
							-0.06313	-0.06299
							-0.0632	-0.06306
							-0.06327	-0.06312
							-0.06334	-0.06319
							-0.06342	-0.06326
							-0.0635	-0.06334
							-0.06358	-0.06342
							-0.06365	-0.06349
							-0.06373	-0.06357

							-0.06209	-0.06189
							-0.06199	-0.0618
							-0.13137	-0.13094
							-0.13117	-0.13074
					-0.06211	-0.06191	-0.25674	-0.25607
					-0.06201	-0.06181	-0.25642	-0.25574
					-0.13146	-0.13102	-0.32154	-0.32068
					-0.13128	-0.13086	-0.32119	-0.32034
					-0.1311	-0.1307	-0.32084	-0.32001
		-0.13219	-0.13176	-0.13134	-0.25657	-0.25592	-0.50554	-0.50421
		-0.13202	-0.13159	-0.13117	-0.25627	-0.25561	-0.50489	-0.50355
		-0.13184	-0.13141	-0.13098	-0.32138	-0.32052	-0.50423	-0.50289
		-0.13163	-0.13121	-0.13078	-0.32096	-0.32012	-0.50355	-0.50223
-0.1323	-0.13186	-0.25733	-0.25665	-0.25598	-0.50559	-0.50423	-0.5029	-0.50157
-0.1321	-0.13167	-0.257	-0.25633	-0.25567	-0.50494	-0.50358	-0.50225	-0.50094
-0.13191	-0.13148	-0.25669	-0.25603	-0.25536	-0.5043	-0.50296	-0.50163	-0.50032
-0.19498	-0.19442	-0.32198	-0.32113	-0.32028	-0.50372	-0.50238	-0.55425	-0.55282

-0.19475	-0.19419	-0.32164	-0.32079	-0.31995	-0.50315	-0.50182	-0.55364	-0.55221
-0.25719	-0.25652	-0.50658	-0.50521	-0.50386	-0.55582	-0.5544	-0.60436	-0.60281
-0.25687	-0.25621	-0.5059	-0.50454	-0.5032	-0.55512	-0.55369	-0.60358	-0.60204
-0.32216	-0.32131	-0.50523	-0.50388	-0.50255	-0.60592	-0.60436	-0.68312	-0.68142
-0.32174	-0.3209	-0.50457	-0.50323	-0.5019	-0.60515	-0.6036	-0.6823	-0.6806
-0.50665	-0.50528	-0.55733	-0.55589	-0.55446	-0.6044	-0.60286	-0.88739	-0.88515
-0.50605	-0.50469	-0.5567	-0.55526	-0.55383	-0.60372	-0.60218	-0.88644	-0.8842
-0.50546	-0.5041	-0.55607	-0.55463	-0.55321	-0.60303	-0.6015	-0.88549	-0.88324
-0.55827	-0.55682	-0.60694	-0.60538	-0.60383	-0.68265	-0.68096	-0.88446	-0.8822
-0.55756	-0.55612	-0.60616	-0.60461	-0.60307	-0.68184	-0.68015	-0.8834	-0.88113
-0.60856	-0.607	-0.68615	-0.68446	-0.68278	-0.68109	-0.6794	-0.8824	-0.88011
-0.60787	-0.60631	-0.68543	-0.68375	-0.68207	-0.68039	-0.6787	-0.88144	-0.87912
-0.60715	-0.60559	-0.68467	-0.683	-0.68133	-0.88502	-0.88275	-0.88045	-0.87813
-0.60638	-0.60483	-0.68386	-0.68219	-0.68053	-0.88397	-0.88171	-0.87943	-0.87713
-0.68642	-0.68475	-0.68308	-0.68141	-0.67974	-0.88294	-0.88068	-0.87841	-0.87613
-0.68567	-0.68401	-0.68235	-0.68069	-0.67903	-0.88198	-0.87972	-0.87744	-0.87514
-0.68494	-0.68329	-0.68164	-0.67998	-0.67831	-0.88102	-0.87876	-0.87647	-0.87415
-0.68419	-0.68252	-0.83479	-0.8327	-0.83061	-0.87998	-0.87771	-0.87544	-0.87316
-0.68343	-0.68174	-0.83378	-0.83169	-0.82959	-0.8789	-0.87663	-0.87439	-0.87216
-0.68269	-0.681	-0.83284	-0.83073	-0.82863	-0.87789	-0.87562	-0.9178	-0.91547
-0.68199	-0.6803	-0.83194	-0.82984	-0.82773	-0.87695	-0.87467	-0.91679	-0.91444
-0.73331	-0.7315	-0.88277	-0.8805	-0.87823	-0.87596	-0.8737	-0.91577	-0.91343
-0.73255	-0.73071	-0.88177	-0.87946	-0.87716	-0.8749	-0.87268	-0.91473	-0.91244
-0.73175	-0.72991	-0.88077	-0.87843	-0.87611	-0.87387	-0.87167	-0.91371	-0.91147
-0.83246	-0.83032	-0.87977	-0.87745	-0.87515	-0.9173	-0.915	-0.96086	-0.95849
-0.83148	-0.82936	-0.87879	-0.8765	-0.87423	-0.91634	-0.91404	-0.95984	-0.95748
-0.83055	-0.82843	-0.87782	-0.87556	-0.87331	-0.91537	-0.91307	-1.0085	-1.006
-0.82964	-0.82752	-0.87687	-0.87462	-0.87239	-0.91441	-0.91209	-1.00735	-1.00482
-0.82872	-0.82658	-0.87588	-0.87364	-0.87143	-0.91341	-0.91112	-1.00625	-1.00372
-0.82778	-0.82561	-0.87485	-0.87261	-0.87041	-0.91238	-0.91015	-1.00519	-1.00268
-0.87844	-0.87613	-0.91827	-0.91592	-0.91362	-0.91138	-0.90919	-1.00414	-1.00165
-0.87744	-0.87514	-0.91725	-0.91494	-0.91267	-0.91045	-0.90826	-1.00309	-1.00061
-0.87645	-0.87417	-0.91626	-0.91398	-0.91174	-0.90952	-0.90732	-1.00203	-0.99955
-0.87547	-0.87323	-0.9153	-0.91304	-0.9108	-1.00592	-1.0034	-1.04564	-1.04296
-0.87451	-0.87231	-0.91435	-0.9121	-0.90985	-1.00483	-1.00227	-1.04442	-1.04171
-0.87359	-0.8714	-0.9134	-0.91114	-0.90889	-1.00372	-1.00116	-1.04324	-1.04053
-0.87269	-0.87051	-0.91246	-0.91018	-0.9079	-1.0026	-1.00006	-1.04209	-1.0394
-0.87179	-0.8696	-0.96219	-0.95976	-0.95733	-1.04637	-1.04365	-1.04096	-1.03829
-0.87088	-0.86866	-0.96114	-0.95871	-0.95629	-1.04523	-1.04253	-1.03985	-1.0372
-0.91418	-0.91185	-0.96011	-0.95768	-0.95527	-1.0441	-1.04142	-1.03876	-1.03612
-0.91326	-0.91093	-0.95913	-0.95669	-0.95427	-1.043	-1.04033	-1.03768	-1.03503
-0.91232	-0.91001	-0.95816	-0.95571	-0.95329	-1.0419	-1.03925	-1.03659	-1.03394
-0.96204	-0.95958	-1.04897	-1.04622	-1.04349	-1.0408	-1.03814	-1.12587	-1.12272
-0.96097	-0.95853	-1.04781	-1.04509	-1.04238	-1.03969	-1.03702	-1.12452	-1.12136
-0.95995	-0.95751	-1.04667	-1.04396	-1.04128	-1.03858	-1.03589	-1.16636	-1.1631
-0.95895	-0.95651	-1.04555	-1.04285	-1.04016	-1.03746	-1.03475	-1.16494	-1.16171
-0.95796	-0.95551	-1.04444	-1.04175	-1.03907	-1.12686	-1.12364	-1.16357	-1.16036
-0.95697	-0.95453	-1.04333	-1.04065	-1.03798	-1.12556	-1.12235	-1.16225	-1.15905
-1.04771	-1.04495	-1.1338	-1.13061	-1.12747	-1.12428	-1.12109	-1.16095	-1.15776
-1.04659	-1.04384	-1.13247	-1.12931	-1.12619	-1.12301	-1.11983	-1.15966	-1.15647

-1.04548	-1.04273	-1.13115	-1.12802	-1.12491	-1.12175	-1.11859	-1.15838	-1.15519
-1.04437	-1.04163	-1.12984	-1.12673	-1.12364	-1.16357	-1.16031	-1.2004	-1.19694
-1.04326	-1.04054	-1.12854	-1.12544	-1.12236	-1.16227	-1.15902	-1.19901	-1.19558
-1.13364	-1.13041	-1.12725	-1.12416	-1.1211	-1.16097	-1.15773	-1.19762	-1.19421
-1.13233	-1.12912	-1.12597	-1.1229	-1.11984	-1.15968	-1.15644	-1.19623	-1.19284
-1.13104	-1.12783	-1.1679	-1.16475	-1.16161	-1.15839	-1.15515	-1.19485	-1.19149
-1.12974	-1.12654	-1.16658	-1.16344	-1.16032	-1.15711	-1.15389	-1.1935	-1.19016
-1.12844	-1.12525	-1.16526	-1.16214	-1.15903	-1.15584	-1.15262	-1.19216	-1.18884
-1.12715	-1.12397	-1.16394	-1.16083	-1.15773	-1.15455	-1.15135	-1.23562	-1.23196
-1.12586	-1.12269	-1.16263	-1.15952	-1.15642	-1.15326	-1.15009	-1.23415	-1.23053
-1.16778	-1.16452	-1.16132	-1.15822	-1.15513	-1.19488	-1.19147	-1.23269	-1.22909
-1.16648	-1.16322	-1.16003	-1.15692	-1.15385	-1.19351	-1.19012	-1.23123	-1.22767
-1.16523	-1.16197	-1.15877	-1.15566	-1.15258	-1.23711	-1.2334	-1.26818	-1.26457
-1.16402	-1.16077	-1.15756	-1.15442	-1.15132	-1.23563	-1.23195	-1.26672	-1.26314
-1.1628	-1.15955	-1.15634	-1.15318	-1.15006	-1.23416	-1.23051	-1.30613	-1.30242
-1.16152	-1.15828	-1.15507	-1.15192	-1.14881	-1.2327	-1.22909	-1.30462	-1.30093
-1.16023	-1.15699	-1.1538	-1.15066	-1.14757	-1.23126	-1.2277	-1.30315	-1.29947
-1.15894	-1.15573	-1.19868	-1.19518	-1.19174	-1.22986	-1.22637	-1.30176	-1.29808
-1.15766	-1.15447	-1.19731	-1.19382	-1.1904	-1.22848	-1.22507	-1.3004	-1.29672
-1.15643	-1.15326	-1.23798	-1.23424	-1.23059	-1.30627	-1.30265	-1.299	-1.29534
-1.15525	-1.15209	-1.2366	-1.23288	-1.22923	-1.30481	-1.30119	-1.29756	-1.29393
-1.20033	-1.19683	-1.23518	-1.23149	-1.22787	-1.30335	-1.29973	-1.29613	-1.29256
-1.19898	-1.19546	-1.23371	-1.23006	-1.22649	-1.30187	-1.29826	-1.29471	-1.29123
-1.19764	-1.19411	-1.27407	-1.27029	-1.26659	-1.30043	-1.29683	-1.29332	-1.2899
-1.19634	-1.19282	-1.27263	-1.26891	-1.26527	-1.29909	-1.29548	-1.29196	-1.28853
-1.19505	-1.19156	-1.27122	-1.26755	-1.26396	-1.29777	-1.29417	-1.29064	-1.28717
-1.19369	-1.19025	-1.26977	-1.26613	-1.26258	-1.29641	-1.29287	-1.35663	-1.35292
-1.19231	-1.18892	-1.26831	-1.26468	-1.26115	-1.29502	-1.29159	-1.35531	-1.35161
-1.27445	-1.27064	-1.30442	-1.30073	-1.29714	-1.29366	-1.29026	-1.3539	-1.35024
-1.27305	-1.26929	-1.30308	-1.2994	-1.29581	-1.2923	-1.28887	-1.35242	-1.34882
-1.27166	-1.26795	-1.30174	-1.29808	-1.2945	-1.3583	-1.3546	-1.35099	-1.34746
-1.27029	-1.2666	-1.30038	-1.29677	-1.29323	-1.35695	-1.35325	-1.34967	-1.3462
-1.26891	-1.26524	-1.29901	-1.29544	-1.29194	-1.35559	-1.35191	-1.34837	-1.34496
-1.26751	-1.26385	-1.3287	-1.32497	-1.32131	-1.35414	-1.35052	-1.34703	-1.34365
-1.26611	-1.26246	-1.32722	-1.32348	-1.31983	-1.35267	-1.34912	-1.34568	-1.34234
-1.30214	-1.29844	-1.36239	-1.3586	-1.3549	-1.3513	-1.3478	-1.3444	-1.3411
-1.30075	-1.29709	-1.36098	-1.35722	-1.35356	-1.35	-1.34654	-1.34318	-1.33991
-1.33059	-1.32678	-1.35957	-1.35586	-1.35224	-1.34872	-1.3453	-1.34198	-1.33875
-1.32916	-1.32538	-1.35817	-1.35452	-1.35096	-1.34748	-1.34409	-1.3408	-1.33762
-1.36436	-1.36053	-1.35679	-1.35319	-1.34968	-1.34624	-1.34289	-1.33964	-1.33649
-1.36292	-1.35913	-1.35543	-1.35185	-1.34836	-1.34499	-1.34172	-1.33851	-1.33536
-1.36151	-1.35776	-1.3541	-1.35053	-1.34707	-1.34377	-1.34057	-1.33738	-1.33424
-1.36014	-1.35642	-1.3528	-1.34929	-1.34587	-1.34259	-1.3394	-1.33624	-1.33312
-1.35879	-1.35511	-1.35154	-1.34808	-1.34471	-1.34144	-1.33824	-1.3351	-1.33201
-1.35742	-1.35379	-1.35027	-1.34686	-1.34354	-1.34029	-1.3371	-1.33398	-1.3309
-1.35601	-1.35246	-1.349	-1.34563	-1.34235	-1.33914	-1.33599	-1.33288	-1.3298
-1.35469	-1.35117	-1.34775	-1.34442	-1.34118	-1.33799	-1.33487	-1.33177	-1.32869
-1.35349	-1.34993	-1.34649	-1.34322	-1.34003	-1.33685	-1.33372	-1.33064	-1.3276
-1.35231	-1.34873	-1.34529	-1.34204	-1.33888	-1.33571	-1.33258	-1.32951	-1.3265
-1.35111	-1.34761	-1.34421	-1.34093	-1.33773	-1.33458	-1.33147	-1.32841	-1.32539

-1.3499	-1.34651	-1.34317	-1.33985	-1.3366	-1.33345	-1.33038	-1.32732	-1.32428
-1.34866	-1.34535	-1.34207	-1.33878	-1.33554	-1.33237	-1.32927	-1.32621	-1.32319
-1.3474	-1.34417	-1.34095	-1.33772	-1.33452	-1.33132	-1.32816	-1.32509	-1.32212
-1.34621	-1.34299	-1.3398	-1.33662	-1.33345	-1.33026	-1.3271	-1.32403	-1.32105
-1.34507	-1.34182	-1.33862	-1.33546	-1.33234	-1.3292	-1.32609	-1.32302	-1.31999
-1.34396	-1.3407	-1.33749	-1.33433	-1.33122	-1.32812	-1.32505	-1.322	-1.31895
-1.3429	-1.33967	-1.33646	-1.33325	-1.33008	-1.327	-1.32397	-1.32096	-1.31797
-1.34183	-1.33863	-1.33543	-1.33218	-1.32897	-1.32589	-1.32289	-1.31992	-1.31698
-1.34068	-1.33751	-1.33434	-1.33112	-1.32793	-1.32484	-1.3218	-1.31884	-1.31595
-1.33952	-1.33636	-1.33322	-1.33006	-1.32692	-1.32381	-1.32073	-1.31777	-1.31491
-1.33843	-1.33523	-1.33207	-1.32895	-1.32586	-1.32277	-1.31972	-1.31675	-1.31387
-1.33737	-1.3341	-1.3309	-1.32781	-1.32476	-1.32174	-1.31874	-1.31578	-1.31285
-1.3363	-1.33301	-1.3298	-1.3267	-1.32366	-1.32068	-1.31774	-1.31479	-1.31185
-1.33522	-1.33197	-1.32876	-1.32562	-1.32255	-1.31959	-1.3167	-1.3138	-1.31089
-1.33414	-1.33093	-1.32775	-1.32458	-1.32148	-1.31854	-1.31569	-1.31281	-1.30995
-1.33305	-1.32989	-1.32675	-1.32359	-1.3205	-1.31756	-1.3147	-1.31185	-1.30901
-1.33195	-1.32884	-1.32573	-1.32261	-1.31954	-1.31659	-1.31373	-1.31088	-1.30807
-1.33082	-1.32774	-1.32468	-1.3216	-1.31856	-1.31561	-1.31271	-1.30987	-1.30708
-1.32969	-1.32664	-1.32361	-1.32059	-1.31759	-1.31462	-1.31169	-1.30884	-1.30606
-1.32862	-1.32559	-1.32259	-1.3196	-1.31663	-1.31366	-1.31071	-1.34679	-1.34389
-1.32762	-1.32459	-1.32159	-1.31863	-1.31568	-1.31272	-1.30976	-1.34577	-1.34282
-1.32661	-1.32357	-1.32057	-1.31764	-1.31472	-1.31177	-1.30883	-1.34478	-1.3418
-1.32558	-1.32251	-1.31951	-1.3166	-1.31372	-1.3108	-1.30789	-1.34382	-1.34084
-1.32457	-1.32148	-1.31847	-1.31557	-1.31272	-1.34882	-1.34584	-1.38035	-1.37728
-1.32358	-1.32049	-1.31748	-1.31459	-1.31174	-1.34782	-1.34487	-1.37936	-1.3763
-1.3226	-1.31952	-1.31652	-1.31362	-1.31077	-1.34683	-1.3439	-1.37838	-1.37533
-1.32163	-1.31856	-1.31556	-1.31267	-1.30982	-1.34584	-1.34293	-1.37739	-1.37435
-1.32067	-1.31761	-1.31461	-1.31172	-1.30888	-1.34487	-1.34196	-1.3764	-1.37337
-1.31971	-1.31665	-1.31366	-1.31078	-1.30794	-1.3439	-1.341	-1.37542	-1.37241
-1.31873	-1.31568	-1.3127	-1.30984	-1.30702	-1.34295	-1.34006	-1.37446	-1.37145
-1.31775	-1.31471	-1.35072	-1.34778	-1.34488	-1.34199	-1.33912	-1.3735	-1.3705
-1.31677	-1.31373	-1.34971	-1.34678	-1.3439	-1.34103	-1.33817	-1.37254	-1.36956
-1.31579	-1.31276	-1.34871	-1.34579	-1.34293	-1.34007	-1.33723	-1.37158	-1.36861
-1.31482	-1.3118	-1.34772	-1.34482	-1.34196	-1.37649	-1.37356	-1.37061	-1.36765
-1.31386	-1.31085	-1.34674	-1.34385	-1.34101	-1.37551	-1.37259	-1.36965	-1.3667
-1.31289	-1.3099	-1.34577	-1.34289	-1.34005	-1.37454	-1.37163	-1.3687	-1.36574
-1.31192	-1.30896	-1.34481	-1.34193	-1.33911	-1.37357	-1.37067	-1.36775	-1.3648
-1.34985	-1.34683	-1.34388	-1.34102	-1.3382	-1.37262	-1.36972	-1.36679	-1.36385
-1.34887	-1.34591	-1.343	-1.34015	-1.33732	-1.3717	-1.36877	-1.36584	-1.3629
-1.34793	-1.345	-1.34211	-1.33927	-1.33644	-1.37077	-1.36782	-1.36489	-1.36195
-1.34704	-1.34411	-1.34122	-1.33836	-1.33553	-1.36982	-1.36687	-1.36393	-1.361
-1.34615	-1.34322	-1.34032	-1.33745	-1.3346	-1.36886	-1.36591	-1.36298	-1.36005
-1.34524	-1.34231	-1.33941	-1.33653	-1.33368	-1.36789	-1.36495	-1.36202	-1.3591
-1.34431	-1.34139	-1.33849	-1.33561	-1.33274	-1.36693	-1.36399	-1.36107	-1.35817
-1.34337	-1.34045	-1.37488	-1.37189	-1.36893	-1.36598	-1.36305	-1.36015	-1.35728
-1.3424	-1.3395	-1.37391	-1.37094	-1.36798	-1.36503	-1.36211	-1.35925	-1.35645
-1.34144	-1.33856	-1.37295	-1.36998	-1.36704	-1.36411	-1.36121	-1.39782	-1.39491
-1.34049	-1.33763	-1.37199	-1.36903	-1.3661	-1.3632	-1.36034	-1.39693	-1.39403
-1.33957	-1.33672	-1.37107	-1.36812	-1.3652	-1.36232	-1.35947	-1.39604	-1.39314
-1.33867	-1.33586	-1.37022	-1.36729	-1.36438	-1.36148	-1.3586	-1.39511	-1.3922

-1.3378	-1.33503	-1.36938	-1.36648	-1.36358	-1.36065	-1.35772	-1.39417	-1.39126
-1.33699	-1.3342	-1.3685	-1.36559	-1.36268	-1.35975	-1.35683	-1.39324	-1.39033
-1.33621	-1.33337	-1.36761	-1.36466	-1.36174	-1.35882	-1.35593	-1.39232	-1.38942
-1.33536	-1.3325	-1.36669	-1.36373	-1.36079	-1.35789	-1.35503	-1.39141	-1.38853
-1.33445	-1.3316	-1.36576	-1.36279	-1.35984	-1.35695	-1.35411	-1.3905	-1.38769
-1.37075	-1.3678	-1.36486	-1.36189	-1.35896	-1.35608	-1.35326	-1.38963	-1.38684
-1.36982	-1.36692	-1.36401	-1.36109	-1.35818	-1.35532	-1.3525	-1.38882	-1.386
-1.36893	-1.36604	-1.36317	-1.36028	-1.3574	-1.35455	-1.35173	-1.388	-1.38514
-1.36809	-1.36518	-1.36228	-1.35939	-1.35653	-1.35369	-1.35088	-1.44348	-1.44052
-1.36727	-1.36431	-1.36138	-1.35849	-1.35564	-1.35281	-1.35001	-1.44259	-1.43964
-1.36638	-1.3634	-1.36047	-1.35761	-1.35479	-1.35199	-1.34922	-1.44179	-1.43884
-1.36544	-1.36247	-1.35956	-1.35675	-1.35397	-1.35122	-1.34849	-1.44104	-1.43809
-1.36457	-1.36161	-1.3587	-1.3559	-1.35314	-1.4461	-1.44317	-1.50079	-1.49771
-1.36379	-1.36082	-1.35791	-1.35508	-1.35229	-1.44521	-1.44227	-1.49991	-1.49684
-1.36297	-1.36001	-1.35709	-1.35424	-1.35143	-1.44432	-1.44139	-1.49904	-1.49599
-1.36205	-1.35911	-1.3562	-1.35335	-1.35055	-1.44343	-1.44053	-1.49822	-1.49521
-1.36114	-1.35821	-1.35532	-1.35248	-1.34969	-1.44256	-1.43969	-1.4974	-1.49443
-1.36031	-1.35741	-1.41245	-1.40953	-1.40666	-1.47479	-1.47183	-1.49656	-1.49359
-1.35952	-1.35663	-1.41168	-1.40879	-1.40593	-1.47402	-1.47102	-1.49573	-1.49275
-1.35863	-1.35576	-1.41083	-1.40798	-1.40515	-1.47324	-1.47025	-1.49496	-1.49198
-1.35768	-1.35482	-1.40992	-1.40711	-1.40433	-1.47245	-1.4695	-1.49424	-1.49128
-1.41489	-1.41195	-1.44292	-1.44006	-1.43723	-1.47162	-1.4687	-1.49345	-1.4905
-1.41414	-1.4112	-1.44217	-1.43927	-1.4364	-1.47075	-1.46783	-1.49258	-1.48964
-1.41339	-1.41046	-1.44144	-1.43851	-1.43561	-1.46991	-1.46698	-1.49173	-1.4888
-1.41264	-1.40972	-1.44072	-1.4378	-1.43491	-1.46917	-1.46625	-1.49099	-1.48807
-1.41186	-1.40896	-1.43998	-1.43707	-1.4342	-1.46845	-1.46552	-1.49026	-1.48735
-1.44501	-1.44206	-1.43914	-1.43624	-1.43338	-1.46762	-1.4647	-1.48945	-1.48655
-1.44409	-1.44115	-1.43824	-1.43537	-1.43253	-1.46675	-1.46385	-1.48861	-1.48572
-1.44328	-1.44034	-1.43745	-1.43458	-1.43175	-1.46597	-1.46309	-1.48785	-1.48497
-1.44256	-1.43963	-1.43673	-1.43387	-1.43105	-1.46525	-1.4624	-1.48716	-1.48428
-1.44185	-1.43892	-1.43603	-1.43317	-1.43035	-1.46454	-1.4617	-1.48646	-1.48359
-1.44114	-1.43823	-1.43535	-1.43249	-1.42967	-1.46384	-1.46101	-1.48578	-1.48292
-1.44044	-1.43754	-1.43466	-1.4318	-1.42898	-1.46312	-1.46029	-1.48506	-1.48221
-1.43974	-1.43684	-1.43396	-1.43109	-1.42825	-1.46235	-1.4595	-1.48424	-1.48139
-1.43902	-1.43612	-1.43324	-1.43036	-1.42751	-1.46156	-1.45869	-1.48341	-1.48056
-1.43821	-1.43533	-1.43246	-1.42961	-1.42678	-1.46083	-1.45797	-1.50946	-1.50656
-1.43735	-1.43448	-1.43164	-1.42884	-1.42606	-1.46012	-1.4573	-1.50881	-1.5059
-1.43657	-1.43372	-1.4309	-1.42812	-1.42537	-1.45943	-1.45662	-1.50815	-1.50525
-1.43588	-1.43303	-1.43022	-1.42745	-1.42471	-1.45874	-1.45595	-1.50749	-1.5046
-1.4352	-1.43236	-1.42956	-1.42679	-1.42406	-1.48543	-1.48259	-1.50683	-1.50395
-1.43453	-1.4317	-1.4289	-1.42615	-1.42342	-1.48479	-1.48195	-1.50617	-1.50329
-1.43387	-1.43104	-1.42825	-1.4255	-1.42279	-1.48415	-1.48131	-1.50552	-1.50263
-1.43321	-1.43039	-1.4276	-1.42486	-1.42214	-1.4835	-1.48067	-1.50487	-1.50199
-1.43254	-1.42974	-1.42696	-1.42422	-1.4215	-1.48285	-1.48002	-1.50423	-1.50136
-1.43187	-1.42908	-1.4544	-1.45161	-1.44885	-1.4822	-1.47938	-1.50358	-1.50071
-1.43119	-1.42841	-1.45376	-1.45099	-1.44823	-1.48156	-1.47873	-1.50292	-1.50005
-1.43052	-1.42775	-1.45312	-1.45035	-1.44761	-1.50807	-1.50516	-1.50227	-1.49938
-1.42986	-1.42709	-1.45247	-1.4497	-1.44696	-1.50741	-1.50451	-1.50161	-1.49872
-1.45743	-1.45461	-1.45182	-1.44906	-1.44632	-1.50674	-1.50385	-1.50094	-1.49804
-1.45678	-1.45397	-1.45119	-1.44843	-1.44569	-1.50608	-1.50317	-1.50025	-1.49732

-1.45613	-1.45334	-1.45056	-1.44781	-1.44506	-1.50541	-1.5025	-1.49956	-1.4966
-1.45547	-1.45268	-1.44991	-1.44716	-1.44443	-1.50475	-1.50183	-1.49889	-1.49593
-1.45481	-1.45202	-1.44925	-1.44651	-1.44379	-1.50408	-1.50118	-1.49824	-1.49528
-1.45415	-1.45136	-1.4486	-1.44586	-1.44314	-1.50341	-1.50052	-1.4976	-1.49466
-1.45349	-1.45071	-1.44795	-1.44521	-1.44248	-1.50273	-1.49985	-1.49696	-1.49407
-1.45283	-1.45006	-1.44731	-1.44456	-1.44183	-1.50205	-1.49918	-1.49631	-1.49345
-1.45217	-1.44941	-1.44666	-1.44392	-1.4412	-1.50138	-1.49851	-1.49564	-1.49279
-1.45151	-1.44876	-1.44602	-1.44328	-1.44056	-1.5007	-1.49783	-1.52692	-1.52394
-1.45086	-1.4481	-1.44536	-1.44263	-1.43991	-1.50003	-1.49716	-1.52622	-1.52325
-1.45021	-1.44745	-1.4447	-1.44197	-1.43925	-1.49934	-1.49648	-1.52552	-1.52256
-1.44956	-1.4468	-1.44405	-1.44132	-1.43861	-1.49867	-1.4958	-1.52481	-1.52185
-1.44892	-1.44615	-1.44341	-1.44068	-1.43797	-1.49799	-1.49512	-1.52408	-1.52113
-1.44835	-1.44556	-1.47052	-1.4677	-1.46489	-1.49731	-1.49444	-1.52337	-1.52041
-1.44785	-1.44502	-1.46992	-1.46706	-1.46423	-1.49662	-1.49377	-1.52267	-1.51971
-1.44732	-1.44447	-1.46934	-1.46645	-1.4636	-1.49596	-1.49311	-1.52197	-1.519
-1.44672	-1.44391	-1.4688	-1.4659	-1.46302	-1.49532	-1.49243	-1.52124	-1.51828
-1.44609	-1.44332	-1.46824	-1.46534	-1.46245	-1.49471	-1.4918	-1.52055	-1.51758
-1.44542	-1.44267	-1.46761	-1.46475	-1.4619	-1.526	-1.52297	-1.51996	-1.51694
-1.44474	-1.442	-1.46694	-1.46414	-1.46134	-1.52543	-1.52242	-1.51939	-1.51634
-1.44407	-1.44133	-1.46626	-1.46348	-1.46071	-1.52477	-1.52178	-1.51876	-1.51572
-1.44342	-1.44066	-1.46558	-1.46279	-1.46002	-1.52405	-1.52108	-1.51809	-1.51509
-1.44282	-1.44005	-1.46493	-1.46213	-1.45935	-1.52332	-1.52037	-1.5174	-1.51443
-1.44229	-1.43949	-1.46433	-1.4615	-1.45869	-1.52259	-1.51964	-1.51668	-1.51372
-1.44175	-1.43894	-1.49624	-1.49328	-1.49035	-1.52191	-1.51893	-1.51597	-1.51301
-1.44113	-1.43836	-1.49565	-1.49271	-1.48978	-1.5213	-1.51828	-1.51529	-1.51231
-1.4405	-1.43776	-1.49506	-1.49214	-1.48921	-1.5207	-1.51766	-1.51464	-1.51163
-1.43989	-1.43714	-1.49438	-1.49147	-1.48857	-1.52007	-1.51705	-1.51404	-1.51104
-1.43929	-1.43651	-1.49368	-1.49078	-1.4879	-1.51942	-1.51645	-1.51347	-1.51048
-1.47463	-1.47174	-1.52773	-1.52472	-1.52173	-1.54491	-1.54188	-1.53885	-1.53583
-1.47407	-1.47121	-1.52715	-1.52411	-1.52108	-1.54421	-1.54115	-1.53812	-1.53512
-1.50082	-1.4979	-1.55285	-1.54974	-1.54664	-1.54355	-1.54049	-1.53746	-1.53448
-1.5002	-1.49726	-1.55217	-1.54909	-1.54603	-1.54297	-1.53993	-1.53691	-1.53392
-1.53147	-1.52842	-1.55151	-1.54845	-1.54541	-1.54237	-1.53934	-1.53633	-1.53334
-1.53087	-1.52783	-1.55091	-1.54783	-1.54476	-1.5417	-1.53866	-1.53564	-1.53265
-1.53027	-1.52725	-1.55032	-1.54721	-1.54411	-1.54103	-1.53796	-1.53494	-1.53195
-1.52963	-1.52661	-1.54967	-1.54658	-1.54349	-1.54041	-1.53735	-1.53432	-1.53131
-1.52898	-1.52594	-1.54899	-1.54593	-1.54288	-1.53983	-1.53679	-1.53374	-1.5307
-1.52836	-1.52532	-1.54836	-1.5453	-1.54225	-1.5392	-1.53616	-1.53312	-1.53008
-1.52777	-1.52475	-1.54777	-1.54468	-1.5416	-1.53853	-1.53548	-1.53246	-1.52947
-1.5272	-1.52417	-1.54718	-1.54407	-1.54097	-1.53789	-1.53484	-1.53184	-1.52888
-1.52663	-1.52359	-1.54658	-1.54347	-1.54037	-1.53731	-1.53428	-1.53128	-1.52832
-1.52604	-1.52299	-1.54598	-1.54287	-1.53979	-1.53674	-1.53372	-1.53072	-1.52775
-1.52539	-1.52237	-1.54538	-1.54228	-1.53921	-1.53615	-1.53311	-1.5301	-1.52711
-1.52472	-1.52175	-1.54478	-1.5417	-1.53862	-1.53555	-1.53249	-1.52946	-1.52644
-1.55029	-1.54725	-1.54419	-1.54111	-1.53802	-1.53495	-1.53189	-1.56144	-1.55826
-1.54975	-1.54668	-1.54361	-1.54051	-1.53742	-1.53436	-1.53131	-1.56085	-1.55768
-1.54918	-1.5461	-1.54301	-1.53991	-1.53682	-1.53377	-1.53074	-1.56026	-1.5571
-1.5486	-1.54549	-1.54239	-1.53929	-1.53622	-1.53319	-1.53019	-1.55968	-1.55651
-1.52058	-1.51752	-1.54178	-1.53869	-1.53563	-1.53262	-1.52963	-1.55909	-1.55591
-1.51999	-1.51693	-1.54118	-1.53812	-1.53508	-1.53207	-1.52908	-1.55849	-1.55528

-1.51939	-1.51635	-1.5406	-1.53756	-1.53453	-1.53152	-1.52852	-1.55787	-1.55465
-1.51879	-1.51577	-1.54003	-1.53699	-1.53397	-1.53094	-1.52792	-1.55723	-1.554
-1.51821	-1.51521	-1.53946	-1.53643	-1.53339	-1.53035	-1.5273	-1.55656	-1.55335
-1.51765	-1.51466	-1.5389	-1.53585	-1.5328	-1.52974	-1.52669	-1.55592	-1.55272
-1.51711	-1.51413	-1.53835	-1.53526	-1.53218	-1.52912	-1.52608	-1.5553	-1.55212
-1.51657	-1.51359	-1.53779	-1.53469	-1.53159	-1.52853	-1.52549	-1.60705	-1.60377
-1.51604	-1.51305	-1.53723	-1.53414	-1.53105	-1.52798	-1.52492	-1.60644	-1.60316
-1.5155	-1.5125	-1.5095	-1.50649	-1.50348	-1.52743	-1.52436	-1.60584	-1.60255
-1.51495	-1.51192	-1.5089	-1.5059	-1.5029	-1.52685	-1.5238	-1.60524	-1.60191
-1.5144	-1.51134	-1.50831	-1.5053	-1.50232	-1.52628	-1.52325	-1.60466	-1.60129
-1.51388	-1.51081	-1.50776	-1.50475	-1.50177	-1.61077	-1.60745	-1.60409	-1.60071
-1.51337	-1.5103	-1.50725	-1.50423	-1.50123	-1.6102	-1.60689	-1.60354	-1.60017
-1.51284	-1.50978	-1.50673	-1.5037	-1.5007	-1.60963	-1.60633	-1.60298	-1.59961
-1.51231	-1.50925	-1.5062	-1.50317	-1.50017	-1.60906	-1.60575	-1.60241	-1.59904
-1.51178	-1.50872	-1.56047	-1.5573	-1.55417	-1.60849	-1.60519	-1.60185	-1.59848
-1.51126	-1.5082	-1.55995	-1.55679	-1.55366	-1.60793	-1.60463	-1.6013	-1.59794
-1.51074	-1.50767	-1.55943	-1.55627	-1.55314	-1.60737	-1.60408	-1.60075	-1.59739
-1.51021	-1.50714	-1.55891	-1.55574	-1.55261	-1.58126	-1.57803	-1.60018	-1.59683
-1.50967	-1.50661	-1.55837	-1.55521	-1.55209	-1.5807	-1.57748	-1.59962	-1.59627
-1.50358	-1.50056	-1.52478	-1.52173	-1.5187	-1.58015	-1.57694	-1.59907	-1.59571
-1.50309	-1.50006	-1.52428	-1.52123	-1.51821	-1.57961	-1.5764	-1.59852	-1.59517
-1.52996	-1.52686	-1.52378	-1.52073	-1.5177	-1.57906	-1.57587	-1.59798	-1.59463
-1.52945	-1.52635	-1.52327	-1.52021	-1.51719	-1.57851	-1.57533	-1.59744	-1.59409
-1.52895	-1.52587	-1.5228	-1.51973	-1.5167	-1.57798	-1.5748	-1.59691	-1.59358
-1.52848	-1.52544	-1.52239	-1.51932	-1.51627	-1.57747	-1.57427	-1.59638	-1.59309
-1.52801	-1.52501	-1.52199	-1.51891	-1.51585	-1.57698	-1.57375	-1.59585	-1.5926
-1.5275	-1.52452	-1.52152	-1.51846	-1.51539	-1.57647	-1.57322	-1.57001	-1.56683
-1.52698	-1.52401	-1.52102	-1.51798	-1.51493	-1.57596	-1.57269	-1.56946	-1.56629
-1.52651	-1.52352	-1.52053	-1.51751	-1.51449	-1.57548	-1.57218	-1.56894	-1.56575
-1.52607	-1.52305	-1.52004	-1.51705	-1.51406	-1.57501	-1.57169	-1.56843	-1.56521
-1.52566	-1.5226	-1.51957	-1.51659	-1.51361	-1.57452	-1.57119	-1.56792	-1.56468
-1.52528	-1.52219	-1.51913	-1.51613	-1.51313	-1.574	-1.57068	-1.5674	-1.56415
-1.52487	-1.52177	-1.51869	-1.51566	-1.51263	-1.57348	-1.57018	-1.5669	-1.56364
-1.52437	-1.52128	-1.51821	-1.51516	-1.51213	-1.57297	-1.56973	-1.56646	-1.56318
-1.52386	-1.52079	-1.51773	-1.51467	-1.51164	-1.57246	-1.56927	-1.56602	-1.56273
-1.52343	-1.52036	-1.5173	-1.51424	-1.5112	-1.57196	-1.56876	-1.56553	-1.56228
-1.52304	-1.51997	-1.51691	-1.51384	-1.51078	-1.57146	-1.56822	-1.565	-1.56182
-1.52265	-1.51957	-1.5165	-1.51344	-1.51038	-1.57101	-1.56772	-1.59062	-1.5874
-1.52224	-1.51916	-1.51609	-1.51304	-1.51001	-1.57059	-1.56728	-1.59013	-1.58688
-1.52181	-1.51872	-1.51565	-1.51261	-1.50958	-1.57014	-1.56684	-1.58967	-1.58638
-1.52131	-1.51823	-1.51516	-1.51211	-1.50908	-1.56964	-1.56639	-1.58924	-1.58593
-1.52083	-1.51775	-1.51469	-1.51163	-1.50859	-1.56914	-1.56594	-1.5888	-1.58548
-1.5204	-1.51733	-1.54629	-1.54306	-1.53985	-1.59484	-1.59158	-1.5883	-1.58503
-1.52	-1.51693	-1.54587	-1.54264	-1.53943	-1.59436	-1.59107	-1.58781	-1.58458
-1.5196	-1.51652	-1.57222	-1.56894	-1.56569	-1.59394	-1.59065	-1.58738	-1.58415
-1.51919	-1.5161	-1.57177	-1.56851	-1.56527	-1.59354	-1.59028	-1.587	-1.58372
-1.51879	-1.5157	-1.57133	-1.56807	-1.56483	-1.5931	-1.58984	-1.58657	-1.58329
-1.51839	-1.5153	-1.57091	-1.56763	-1.56438	-1.59259	-1.58932	-1.58607	-1.58284
-1.54532	-1.54219	-1.57049	-1.56721	-1.56394	-1.59213	-1.58884	-1.5856	-1.58239
-1.5449	-1.54177	-1.57006	-1.56679	-1.56354	-1.59174	-1.58845	-1.58519	-1.58195

-1.54448	-1.54135	-1.56963	-1.56639	-1.56316	-1.59136	-1.58808	-1.5848	-1.58151
-1.54407	-1.54095	-1.56922	-1.56599	-1.56277	-1.59095	-1.58766	-1.58437	-1.58109
-1.54367	-1.54056	-1.56882	-1.56559	-1.56238	-1.59053	-1.58723	-1.58394	-1.58068
-1.54328	-1.54019	-1.56844	-1.5652	-1.56197	-1.59011	-1.58679	-1.58351	-1.58025
-1.54291	-1.53983	-1.56807	-1.56481	-1.56156	-1.58967	-1.58636	-1.58307	-1.5798
-1.54254	-1.53946	-1.56767	-1.5644	-1.56114	-1.58923	-1.58592	-1.58263	-1.57936
-1.54215	-1.53906	-1.56724	-1.56397	-1.56072	-1.5888	-1.58547	-1.58219	-1.57893
-1.54177	-1.53866	-1.5668	-1.56354	-1.56029	-1.58836	-1.58504	-1.61005	-1.60672
-1.54139	-1.53827	-1.56638	-1.5631	-1.55984	-1.58792	-1.58462	-1.60962	-1.60628
-1.541	-1.53787	-1.56596	-1.56268	-1.55941	-1.58748	-1.58421	-1.6092	-1.60586
-1.54061	-1.53751	-1.56559	-1.56231	-1.55904	-1.5871	-1.58383	-1.60881	-1.60548
-1.54023	-1.53715	-1.56524	-1.56198	-1.55872	-1.58676	-1.58346	-1.60844	-1.60513
-1.53985	-1.53677	-1.56484	-1.56159	-1.55834	-1.58637	-1.58306	-1.60803	-1.60473
-1.53946	-1.53635	-1.56439	-1.56115	-1.55792	-1.58594	-1.58263	-1.60759	-1.6043
-1.5391	-1.53597	-1.56398	-1.56074	-1.55751	-1.58553	-1.58222	-1.60717	-1.60388
-1.53877	-1.53564	-1.56363	-1.56037	-1.55713	-1.58515	-1.58186	-1.6068	-1.6035
-1.53842	-1.5353	-1.56327	-1.56	-1.55675	-1.58476	-1.58149	-1.60642	-1.60311
-1.538	-1.53489	-1.56286	-1.55959	-1.55633	-1.58433	-1.58107	-1.60599	-1.60266
-1.53757	-1.53447	-1.56243	-1.55916	-1.55591	-1.58391	-1.58064	-1.60554	-1.60221
-1.53721	-1.5341	-1.56205	-1.55879	-1.55554	-1.58353	-1.58026	-1.60515	-1.60182
-1.53688	-1.53376	-1.56169	-1.55844	-1.55521	-1.58318	-1.57991	-1.60479	-1.60146
-1.53656	-1.53343	-1.56135	-1.5581	-1.55487	-1.58283	-1.57956	-1.60443	-1.6011
-1.53623	-1.53311	-1.56101	-1.55776	-1.55453	-1.58248	-1.57922	-1.60407	-1.60075
-1.5988	-1.59544	-1.59209	-1.58875	-1.58543	-1.61039	-1.60705	-1.60372	-1.6004
-1.59844	-1.59509	-1.59174	-1.58841	-1.58509	-1.61004	-1.60669	-1.60336	-1.60004
-1.59808	-1.59473	-1.59139	-1.58806	-1.58475	-1.60969	-1.60633	-1.60299	-1.59967
-1.59773	-1.59437	-1.59103	-1.5877	-1.58439	-1.60932	-1.60596	-1.60262	-1.5993
-1.59738	-1.59402	-1.59067	-1.58733	-1.58402	-1.60894	-1.60558	-1.60224	-1.59892
-1.59703	-1.59366	-1.59031	-1.58698	-1.58365	-1.60854	-1.60517	-1.60182	-1.5985
-1.59667	-1.59331	-1.58996	-1.58662	-1.58329	-1.60814	-1.60472	-1.60137	-1.59806
-1.59632	-1.59296	-1.58961	-1.58628	-1.58294	-1.60778	-1.60436	-1.601	-1.59769
-1.59596	-1.59261	-1.58927	-1.58594	-1.58262	-1.60748	-1.60409	-1.60074	-1.59741
-1.59561	-1.59225	-1.58892	-1.5856	-1.58229	-1.60717	-1.60381	-1.60047	-1.59712
-1.59526	-1.5919	-1.58856	-1.58525	-1.58195	-1.60681	-1.60347	-1.60012	-1.59679
-1.59492	-1.59155	-1.58821	-1.58489	-1.5816	-1.60645	-1.6031	-1.59976	-1.59644
-1.59456	-1.5912	-1.58786	-1.58455	-1.58125	-1.6061	-1.60275	-1.59941	-1.59608
-1.59421	-1.59086	-1.58752	-1.5842	-1.5809	-1.60574	-1.6024	-1.59905	-1.59572
-1.59386	-1.59051	-1.58718	-1.58386	-1.58056	-1.60539	-1.60204	-1.5987	-1.59536
-1.59352	-1.59017	-1.58684	-1.58353	-1.58023	-1.60503	-1.60167	-1.59833	-1.595
-1.59321	-1.58986	-1.61484	-1.61146	-1.60809	-1.60472	-1.60134	-1.59799	-1.59466
-1.59296	-1.5896	-1.61457	-1.6112	-1.60784	-1.60445	-1.60106	-1.59769	-1.59433
-1.5927	-1.58933	-1.61429	-1.61093	-1.60757	-1.60418	-1.60078	-1.59739	-1.59401
-1.59237	-1.58901	-1.61397	-1.61059	-1.60722	-1.60384	-1.60046	-1.59709	-1.59373
-1.59202	-1.58869	-1.61364	-1.61024	-1.60686	-1.60349	-1.60013	-1.59678	-1.59345
-1.59172	-1.5884	-1.61336	-1.60995	-1.60654	-1.60316	-1.59979	-1.59644	-1.59312
-1.59145	-1.58814	-1.61309	-1.60967	-1.60626	-1.60284	-1.59944	-1.59608	-1.59276
-1.59116	-1.58784	-1.61278	-1.60937	-1.60596	-1.60254	-1.59914	-1.59578	-1.59245
-1.59084	-1.5875	-1.61242	-1.60903	-1.60565	-1.60226	-1.59887	-1.59552	-1.59219
-1.59054	-1.58719	-1.61209	-1.60872	-1.60536	-1.60198	-1.59861	-1.59526	-1.59194
-1.59029	-1.58693	-1.61182	-1.60844	-1.60508	-1.6017	-1.59833	-1.595	-1.5917

-1.59005	-1.58669	-1.61156	-1.60818	-1.60481	-1.60142	-1.59805	-1.59472	-1.59143
-1.58979	-1.58645	-1.61132	-1.60792	-1.60452	-1.60112	-1.59773	-1.59439	-1.59109
-1.58952	-1.5862	-1.61107	-1.60765	-1.60423	-1.60081	-1.59741	-1.59405	-1.59074
-1.61756	-1.61416	-1.61076	-1.60734	-1.60393	-1.60052	-1.59712	-1.59377	-1.59044
-1.61721	-1.61379	-1.61039	-1.607	-1.60362	-1.60024	-1.59687	-1.59352	-1.5902
-1.61692	-1.61348	-1.61007	-1.6067	-1.60334	-1.59997	-1.59661	-1.59327	-1.58995
-1.61673	-1.61324	-1.6098	-1.60642	-1.60308	-1.59971	-1.59634	-1.59301	-1.58971
-1.61651	-1.613	-1.60954	-1.60617	-1.60282	-1.59945	-1.59609	-1.59276	-1.58947
-1.61622	-1.61274	-1.6093	-1.60592	-1.60255	-1.5992	-1.59585	-1.59253	-1.58922
-1.61592	-1.61248	-1.60907	-1.60567	-1.60229	-1.59894	-1.59561	-1.59229	-1.58896
-1.61568	-1.61223	-1.6088	-1.60541	-1.60204	-1.59869	-1.59535	-1.59203	-1.58871
-1.61548	-1.61198	-1.60853	-1.60515	-1.60179	-1.59843	-1.59508	-1.59176	-1.58846
-1.61521	-1.61172	-1.60828	-1.60489	-1.60153	-1.59817	-1.59483	-1.5915	-1.5882
-1.61491	-1.61147	-1.60805	-1.60464	-1.60126	-1.5979	-1.59458	-1.59125	-1.58794
-1.61465	-1.61125	-1.60785	-1.60443	-1.60103	-1.59766	-1.59432	-1.59099	-1.58768
-1.61448	-1.61107	-1.60767	-1.60427	-1.60087	-1.59746	-1.59405	-1.59071	-1.58741
-1.6143	-1.61088	-1.60747	-1.60409	-1.6007	-1.59726	-1.59381	-1.61444	-1.61108
-1.61406	-1.61063	-1.60723	-1.60386	-1.60049	-1.59707	-1.59364	-1.61425	-1.61083
-1.6138	-1.61037	-1.60697	-1.6036	-1.60025	-1.59687	-1.59349	-1.61408	-1.6106
-1.61354	-1.61012	-1.60673	-1.60335	-1.59998	-1.59662	-1.59327	-1.61387	-1.6104
-1.61328	-1.60989	-1.6065	-1.60309	-1.59969	-1.59635	-1.59302	-1.61365	-1.61023
-1.61307	-1.60968	-1.60629	-1.60288	-1.59948	-1.59611	-1.59277	-1.6134	-1.61002
-1.61292	-1.60951	-1.60611	-1.60271	-1.59932	-1.59591	-1.5925	-1.61312	-1.60976
-1.61275	-1.60932	-1.60591	-1.60253	-1.59914	-1.59571	-1.59227	-1.61287	-1.60951
-1.61252	-1.60907	-1.60565	-1.60228	-1.59892	-1.59551	-1.5921	-1.61269	-1.60927
-1.61229	-1.60884	-1.60541	-1.60204	-1.59867	-1.5953	-1.59193	-1.61251	-1.60904
-1.6121	-1.60866	-1.60523	-1.60181	-1.59841	-1.61914	-1.61575	-1.61232	-1.60886
-1.61192	-1.60849	-1.60506	-1.6016	-1.59816	-1.61889	-1.61552	-1.61212	-1.6087
-1.58089	-1.57758	-1.57425	-1.57089	-1.56755	-1.61869	-1.61532	-1.61194	-1.60853
-1.58064	-1.57735	-1.57405	-1.57071	-1.56739	-1.61853	-1.61514	-1.61175	-1.60837
-1.58045	-1.57715	-1.59854	-1.59516	-1.59179	-1.61835	-1.61493	-1.61154	-1.60818
-1.5803	-1.577	-1.59838	-1.595	-1.59163	-1.61815	-1.61469	-1.6113	-1.60795
-1.58016	-1.57685	-1.59822	-1.59485	-1.59147	-1.61797	-1.61448	-1.61108	-1.60774
-1.58002	-1.57671	-1.59808	-1.59471	-1.59133	-1.61781	-1.61432	-1.61091	-1.60757
-1.57988	-1.57657	-1.59793	-1.59456	-1.59119	-1.61767	-1.61419	-1.61077	-1.60741
-1.57973	-1.57641	-1.59778	-1.59442	-1.59106	-1.61753	-1.61404	-1.61063	-1.60727
-1.57957	-1.57625	-1.59761	-1.59427	-1.59092	-1.61739	-1.61391	-1.61049	-1.60713
-1.5794	-1.57605	-1.5974	-1.59406	-1.59073	-1.61722	-1.61376	-1.61035	-1.60699
-1.57922	-1.57583	-1.59715	-1.59382	-1.5905	-1.61703	-1.6136	-1.61021	-1.60685
-1.60386	-1.60038	-1.59695	-1.59361	-1.5903	-1.61684	-1.61344	-1.61006	-1.6067
-1.60372	-1.60024	-1.5968	-1.59344	-1.59012	-1.61666	-1.61328	-1.6099	-1.60654
-1.60359	-1.60012	-1.59669	-1.59331	-1.58997	-1.6165	-1.61312	-1.60974	-1.60638
-1.60347	-1.60003	-1.59662	-1.59324	-1.58988	-1.61638	-1.61296	-1.60957	-1.60621
-1.60334	-1.59995	-1.59656	-1.59318	-1.5898	-1.61626	-1.61281	-1.6094	-1.60604
-1.60319	-1.59981	-1.59644	-1.59306	-1.58968	-1.61612	-1.61265	-1.60924	-1.60587
-1.60303	-1.59965	-1.59628	-1.59291	-1.58953	-1.61598	-1.61249	-1.60907	-1.60571
-1.60287	-1.59949	-1.59612	-1.59276	-1.58939	-1.61583	-1.61234	-1.63988	-1.63642
-1.60271	-1.59933	-1.59596	-1.5926	-1.58924	-1.61568	-1.61219	-1.63971	-1.63624
-1.60255	-1.59917	-1.5958	-1.59245	-1.58909	-1.61554	-1.61207	-1.63958	-1.6361
-1.6024	-1.59901	-1.59564	-1.59229	-1.58894	-1.61543	-1.61199	-1.63951	-1.63602

-1.60228	-1.59887	-1.59549	-1.59213	-1.5888	-1.61531	-1.61191	-1.63944	-1.63594
-1.6022	-1.59879	-1.59539	-1.59201	-1.58866	-1.61515	-1.61175	-1.63928	-1.63581
-1.60214	-1.59872	-1.59532	-1.59191	-1.58853	-1.615	-1.61159	-1.63911	-1.63565
-1.60207	-1.59864	-1.59522	-1.59182	-1.58843	-1.6149	-1.61148	-1.63898	-1.63551
-1.60199	-1.59854	-1.59512	-1.59173	-1.58837	-1.61483	-1.6114	-1.63887	-1.63536
-1.6019	-1.59845	-1.59503	-1.59166	-1.5883	-1.64582	-1.64229	-1.63877	-1.63525
-1.60179	-1.59838	-1.59498	-1.5916	-1.58823	-1.64572	-1.64218	-1.63867	-1.63517
-1.60167	-1.59828	-1.5949	-1.59152	-1.58815	-1.64563	-1.64209	-1.63858	-1.6351
-1.60152	-1.59813	-1.59475	-1.5914	-1.58805	-1.64554	-1.64201	-1.63851	-1.63503
-1.60139	-1.59799	-1.59461	-1.59127	-1.58795	-1.64546	-1.64195	-1.63844	-1.63495
-1.60131	-1.59791	-1.59454	-1.5912	-1.58788	-1.64538	-1.64187	-1.63836	-1.63485
-1.60127	-1.59787	-1.5945	-1.59115	-1.58782	-1.64531	-1.64179	-1.63827	-1.63475
-1.60122	-1.59783	-1.62619	-1.62275	-1.61932	-1.64523	-1.64169	-1.63817	-1.63465
-1.60118	-1.59779	-1.62615	-1.62269	-1.61925	-1.64513	-1.64158	-1.63807	-1.63457
-1.60114	-1.59774	-1.62609	-1.62262	-1.61916	-1.64503	-1.64149	-1.63798	-1.6345
-1.60109	-1.59769	-1.62601	-1.62252	-1.61905	-1.64494	-1.64143	-1.63793	-1.63445
-1.57632	-1.57294	-1.62592	-1.62243	-1.61895	-1.64486	-1.64137	-1.63788	-1.6344
-1.57626	-1.57285	-1.62581	-1.62234	-1.61889	-1.6448	-1.64131	-1.63783	-1.63436
-1.5762	-1.57276	-1.62571	-1.62226	-1.61884	-1.64475	-1.64126	-1.63778	-1.63432
-1.57616	-1.57273	-1.62569	-1.62222	-1.61878	-1.6447	-1.64121	-1.63774	-1.63427
-1.57613	-1.57274	-1.62572	-1.62221	-1.61873	-1.64464	-1.64118	-1.6377	-1.63421
-1.58148	-1.5781	-1.60143	-1.59796	-1.59452	-1.64459	-1.64112	-1.63764	-1.63415
-1.5814	-1.57798	-1.6013	-1.59787	-1.59447	-1.64454	-1.64104	-1.63756	-1.63409
-1.58134	-1.57791	-1.60123	-1.59781	-1.59442	-1.64448	-1.64097	-1.63749	-1.63403
-1.58133	-1.57793	-1.60125	-1.59778	-1.59434	-1.64439	-1.64092	-1.63743	-1.63395
-1.5813	-1.57795	-1.60127	-1.59777	-1.59429	-1.64433	-1.64086	-1.63737	-1.63388
-1.58124	-1.5779	-1.60126	-1.59779	-1.59432	-1.62059	-1.61709	-1.63729	-1.63382
-1.58117	-1.57784	-1.60123	-1.5978	-1.59437	-1.6206	-1.61704	-1.63722	-1.63377
-1.58117	-1.57781	-1.57447	-1.57116	-1.56785	-1.62061	-1.61704	-1.63721	-1.63372
-1.58121	-1.57778	-1.5744	-1.5711	-1.56782	-1.62061	-1.61709	-1.63724	-1.63367
-1.58123	-1.57779	-1.57439	-1.57107	-1.56777	-1.62058	-1.6171	-1.61357	-1.61003
-1.58122	-1.57783	-1.57444	-1.57106	-1.5677	-1.6205	-1.61705	-1.61357	-1.61006
-1.58119	-1.57784	-1.57447	-1.57106	-1.56767	-1.62045	-1.617	-1.61353	-1.61007
-1.58112	-1.57779	-1.57445	-1.57108	-1.5677	-1.62044	-1.61692	-1.61345	-1.61001
-1.58107	-1.57774	-1.57442	-1.57109	-1.56774	-1.62045	-1.61687	-1.61337	-1.60995
-1.58107	-1.57771	-1.57436	-1.57105	-1.56773	-1.62045	-1.61687	-1.61336	-1.60989
-1.58111	-1.57768	-1.5743	-1.571	-1.56771	-1.62046	-1.61692	-1.61338	-1.60986
-1.58112	-1.57769	-1.5743	-1.571	-1.56771	-1.62049	-1.61696	-1.61343	-1.60987
-1.58111	-1.57772	-1.57435	-1.57104	-1.56774	-1.62052	-1.61702	-1.61349	-1.60992
-1.5507	-1.54747	-1.5744	-1.57106	-1.56774	-1.59452	-1.59114	-1.61352	-1.60997
-1.55073	-1.54751	-1.57443	-1.57105	-1.56769	-1.59448	-1.59111	-1.61353	-1.61
-1.55077	-1.54755	-1.57447	-1.57106	-1.56767	-1.59445	-1.59109	-1.61353	-1.61004
-1.55081	-1.54759	-1.57454	-1.57111	-1.56771	-1.59448	-1.59112	-1.61356	-1.61008
-1.55083	-1.54763	-1.57459	-1.57117	-1.56777	-1.59453	-1.59116	-1.6136	-1.61013
-1.5508	-1.5476	-1.57459	-1.57119	-1.56781	-1.59457	-1.5912	-1.61364	-1.61018
-1.55074	-1.54755	-1.57457	-1.5712	-1.56784	-1.59461	-1.59122	-1.61367	-1.61023
-1.55074	-1.54755	-1.57458	-1.57123	-1.56787	-1.59463	-1.59122	-1.61366	-1.61023
-1.55078	-1.54759	-1.57463	-1.57127	-1.56791	-1.59463	-1.59117	-1.61361	-1.6102
-1.55083	-1.54763	-1.57468	-1.57132	-1.56796	-1.59465	-1.59117	-1.6136	-1.61019
-1.55088	-1.54768	-1.57474	-1.57138	-1.56801	-1.5947	-1.59121	-1.61364	-1.61022

-1.55093	-1.54773	-1.57479	-1.57143	-1.56806	-1.59476	-1.59127	-1.58787	-1.58453
-1.55097	-1.54777	-1.57485	-1.57149	-1.56813	-1.59482	-1.59133	-1.58792	-1.58457
-1.55102	-1.54782	-1.5749	-1.57155	-1.56819	-1.59489	-1.59141	-1.58799	-1.58463
-1.55107	-1.54786	-1.57494	-1.5716	-1.56825	-1.59498	-1.59153	-1.58812	-1.58474
-1.55112	-1.5479	-1.57499	-1.57165	-1.56832	-1.59508	-1.59167	-1.58827	-1.58488
-1.55122	-1.54799	-1.54477	-1.54155	-1.53835	-1.56506	-1.56176	-1.58837	-1.58498
-1.55136	-1.54812	-1.54487	-1.54162	-1.53839	-1.56509	-1.5618	-1.58842	-1.58504
-1.55148	-1.54823	-1.54497	-1.54169	-1.53843	-1.56513	-1.56184	-1.58846	-1.58509
-1.55153	-1.54829	-1.54504	-1.54175	-1.53848	-1.56518	-1.56189	-1.5885	-1.58514
-1.55158	-1.54835	-1.5451	-1.54182	-1.53855	-1.56526	-1.56195	-1.58856	-1.5852
-1.55162	-1.54839	-1.54516	-1.54192	-1.53867	-1.56539	-1.56206	-1.58865	-1.58527
-1.55166	-1.54844	-1.54523	-1.54202	-1.53881	-1.56554	-1.56219	-1.58876	-1.58534
-1.55174	-1.54851	-1.54529	-1.54208	-1.53889	-1.56563	-1.56229	-1.6136	-1.61008
-1.55186	-1.54859	-1.54534	-1.54212	-1.53892	-1.56568	-1.56236	-1.61367	-1.61015
-1.55197	-1.54869	-1.54543	-1.5422	-1.53899	-1.56576	-1.56243	-1.61376	-1.61025
-1.55206	-1.5488	-1.54555	-1.54233	-1.53911	-1.56586	-1.56251	-1.61384	-1.61037
-1.52095	-1.51777	-1.54569	-1.54246	-1.53923	-1.56597	-1.56261	-1.61395	-1.6105
-1.52108	-1.51791	-1.54581	-1.54256	-1.53931	-1.56608	-1.56274	-1.61408	-1.61063
-1.52122	-1.51804	-1.54592	-1.54265	-1.5394	-1.56619	-1.56288	-1.61422	-1.61076
-1.52134	-1.51814	-1.54601	-1.54276	-1.53952	-1.56633	-1.56302	-1.61435	-1.61088
-1.52146	-1.51824	-1.5461	-1.54286	-1.53965	-1.56648	-1.56316	-1.61447	-1.61098
-1.52157	-1.51835	-1.51515	-1.51199	-1.50883	-1.53657	-1.53337	-1.61459	-1.6111
-1.52165	-1.51846	-1.51529	-1.51212	-1.50896	-1.53668	-1.53346	-1.61469	-1.61123
-1.52176	-1.51858	-1.51542	-1.51225	-1.50908	-1.53678	-1.53356	-1.6339	-1.63042
-1.52189	-1.51871	-1.51554	-1.51234	-1.50916	-1.53687	-1.53369	-1.63408	-1.6306
-1.52202	-1.51884	-1.51565	-1.51244	-1.50926	-1.50612	-1.50301	-1.68348	-1.67987
-1.52215	-1.51894	-1.51573	-1.51255	-1.50938	-1.50626	-1.50316	-1.68367	-1.68006
-1.52229	-1.51904	-1.51583	-1.51267	-1.50953	-1.50641	-1.50331	-1.68385	-1.68026
-1.52245	-1.51922	-1.51601	-1.51283	-1.50968	-1.50656	-1.50346	-1.68405	-1.68045
-1.52263	-1.51943	-1.51623	-1.51302	-1.50983	-1.50671	-1.50362	-1.68424	-1.68064
-1.52278	-1.51958	-1.51638	-1.51317	-1.50999	-1.52768	-1.52456	-1.65619	-1.65263
-1.52291	-1.51967	-1.51646	-1.51329	-1.51014	-1.52786	-1.52473	-1.65638	-1.65283
-1.52305	-1.5198	-1.51658	-1.51342	-1.51029	-1.52803	-1.52491	-1.65657	-1.65302
-1.52323	-1.52001	-1.5168	-1.5136	-1.51043	-1.52819	-1.52508	-1.65675	-1.6532
-1.52342	-1.52023	-1.51703	-1.5138	-1.51059	-1.52837	-1.52525	-1.65694	-1.65338
-1.52358	-1.52041	-1.51722	-1.51401	-1.51081	-1.52859	-1.52543	-1.62876	-1.62534
-1.52373	-1.52057	-1.5174	-1.51423	-1.51106	-1.52883	-1.52561	-1.62893	-1.62552
-1.52389	-1.52072	-1.53866	-1.53547	-1.53228	-1.52906	-1.52584	-1.62914	-1.6257
-1.52405	-1.52087	-1.53883	-1.53565	-1.53248	-1.52929	-1.52611	-1.62939	-1.62589
-1.5242	-1.52103	-1.53902	-1.53583	-1.53266	-1.5295	-1.52634	-1.62963	-1.62611
-1.52434	-1.52118	-1.53921	-1.53601	-1.53283	-1.52967	-1.52653	-1.62985	-1.62636
-1.52452	-1.52137	-1.53942	-1.53622	-1.53303	-1.52986	-1.52671	-1.63006	-1.6266
-1.52477	-1.52161	-1.53968	-1.53648	-1.53329	-1.53009	-1.52689	-1.63023	-1.62679
-1.52503	-1.52185	-1.53994	-1.53675	-1.53356	-1.53032	-1.52708	-1.63041	-1.62698
-1.52527	-1.52205	-1.54015	-1.53696	-1.53377	-1.53055	-1.52732	-1.63064	-1.62717
-1.52551	-1.52224	-1.54033	-1.53714	-1.53397	-1.53077	-1.52758	-1.63088	-1.62737
-1.54715	-1.54384	-1.54057	-1.53735	-1.53416	-1.53098	-1.52781	-1.63112	-1.6276
-1.54736	-1.5441	-1.54084	-1.53758	-1.53434	-1.53116	-1.52801	-1.63135	-1.62786
-1.54756	-1.54433	-1.54109	-1.53781	-1.53455	-1.53137	-1.52823	-1.6316	-1.62812
-1.54774	-1.54452	-1.54131	-1.53806	-1.53482	-1.53164	-1.52849	-1.63186	-1.62839

-1.54794	-1.54473	-1.54153	-1.53831	-1.5351	-1.53191	-1.52874	-1.60286	-1.59948
-1.54821	-1.545	-1.54179	-1.53858	-1.53536	-1.53215	-1.52894	-1.60306	-1.59969
-1.5485	-1.54529	-1.54207	-1.53885	-1.53562	-1.53238	-1.52914	-1.60326	-1.5999
-1.54879	-1.54558	-1.54236	-1.53913	-1.5359	-1.53265	-1.5294	-1.60351	-1.60015
-1.54909	-1.54587	-1.54265	-1.53943	-1.5362	-1.53294	-1.52969	-1.60379	-1.60043
-1.52177	-1.51862	-1.54293	-1.53971	-1.53648	-1.53323	-1.52998	-1.60407	-1.60072
-1.52204	-1.51889	-1.54321	-1.54	-1.53678	-1.53352	-1.53026	-1.60436	-1.60101
-1.52231	-1.51914	-1.54345	-1.54025	-1.53704	-1.53379	-1.53054	-1.60464	-1.60129
-1.52256	-1.51935	-1.54363	-1.54044	-1.53725	-1.53403	-1.53082	-1.60494	-1.60158
-1.52282	-1.51957	-1.54383	-1.54063	-1.53746	-1.53428	-1.5311	-1.60523	-1.60186
-1.5231	-1.51985	-1.5441	-1.54089	-1.53771	-1.53454	-1.53138	-1.60552	-1.60215
-1.5234	-1.52015	-1.54441	-1.54118	-1.53799	-1.53481	-1.53166	-1.6058	-1.60244
-1.5237	-1.52048	-1.51728	-1.51412	-1.51098	-1.53511	-1.53194	-1.60608	-1.60272
-1.52399	-1.52082	-1.51765	-1.51448	-1.51132	-1.53543	-1.53222	-1.60634	-1.60299
-1.52428	-1.52112	-1.51797	-1.5148	-1.51164	-1.53574	-1.53249	-1.60661	-1.60326
-1.52454	-1.52139	-1.51824	-1.51508	-1.51192	-1.53602	-1.53276	-1.60688	-1.60353
-1.52481	-1.52165	-1.5185	-1.51535	-1.5122	-1.53629	-1.53304	-1.60715	-1.6038
-1.52508	-1.52192	-1.51876	-1.51562	-1.51247	-1.53658	-1.53332	-1.60743	-1.60406
-1.52535	-1.52218	-1.51903	-1.51589	-1.51274	-1.53686	-1.53362	-1.60773	-1.60435
-1.52562	-1.52245	-1.51929	-1.51615	-1.51302	-1.53717	-1.53396	-1.60808	-1.60469
-1.5259	-1.52272	-1.51956	-1.51642	-1.5133	-1.53749	-1.53432	-1.60846	-1.60506
-1.52623	-1.52304	-1.51987	-1.51671	-1.51358	-1.51048	-1.5074	-1.60878	-1.60538
-1.52659	-1.5234	-1.52021	-1.51703	-1.51386	-1.51074	-1.50765	-1.60906	-1.60568
-1.52692	-1.52373	-1.52055	-1.51735	-1.51418	-1.51105	-1.50795	-1.60936	-1.60598
-1.5272	-1.52403	-1.52086	-1.51769	-1.51453	-1.51141	-1.5083	-1.60971	-1.60629
-1.5275	-1.52434	-1.52118	-1.51803	-1.51489	-1.51177	-1.50865	-1.61005	-1.60661
-1.52786	-1.5247	-1.52154	-1.51839	-1.51525	-1.51211	-1.50898	-1.61039	-1.60695
-1.52822	-1.52506	-1.5219	-1.51875	-1.5156	-1.51244	-1.5093	-1.61073	-1.60731
-1.50154	-1.49843	-1.52222	-1.51907	-1.51593	-1.51278	-1.50965	-1.58511	-1.58176
-1.50184	-1.49872	-1.52251	-1.51937	-1.51625	-1.51313	-1.51001	-1.58548	-1.58213
-1.50218	-1.49906	-1.52285	-1.51972	-1.5166	-1.51348	-1.51036	-1.56057	-1.5573
-1.50257	-1.49945	-1.52324	-1.5201	-1.51697	-1.51384	-1.51072	-1.56092	-1.55762
-1.50295	-1.49984	-1.49674	-1.49365	-1.49056	-1.5142	-1.51106	-1.56126	-1.55796
-1.50332	-1.50022	-1.49712	-1.49402	-1.49092	-1.51454	-1.51138	-1.56159	-1.55831
-1.50368	-1.50058	-1.49748	-1.49438	-1.49128	-1.51489	-1.51172	-1.56193	-1.55868
-1.50399	-1.50088	-1.49779	-1.49469	-1.4916	-1.51523	-1.51207	-1.5623	-1.55905
-1.5043	-1.50118	-1.49808	-1.495	-1.49193	-1.51558	-1.51244	-1.56268	-1.55944
-1.50469	-1.50154	-1.49843	-1.49535	-1.49228	-1.48921	-1.48615	-1.56307	-1.55982
-1.50513	-1.50196	-1.49882	-1.49573	-1.49265	-1.48959	-1.48653	-1.56346	-1.56021
-1.50552	-1.50235	-1.49921	-1.49611	-1.49303	-1.48997	-1.48691	-1.56385	-1.5606
-1.50585	-1.5027	-1.49958	-1.49649	-1.49342	-1.49034	-1.48728	-1.56423	-1.56098
-1.45255	-1.4495	-1.49996	-1.49688	-1.4938	-1.49072	-1.48765	-1.53881	-1.53564
-1.45295	-1.44987	-1.50034	-1.49725	-1.49418	-1.4911	-1.48803	-1.53919	-1.53601
-1.45336	-1.45025	-1.50072	-1.49763	-1.49456	-1.49148	-1.48841	-1.53957	-1.53639
-1.45369	-1.4506	-1.44754	-1.44452	-1.44152	-1.49184	-1.48878	-1.53994	-1.53677
-1.454	-1.45095	-1.44792	-1.44488	-1.44186	-1.49221	-1.48915	-1.54032	-1.53714
-1.45439	-1.45136	-1.44833	-1.44529	-1.44226	-1.49262	-1.48953	-1.5407	-1.53753
-1.45485	-1.4518	-1.44876	-1.44574	-1.44271	-1.49306	-1.48993	-1.54109	-1.53792
-1.42408	-1.4211	-1.44916	-1.44615	-1.44313	-1.44008	-1.43702	-1.54152	-1.53835
-1.42444	-1.42146	-1.44953	-1.44651	-1.44351	-1.44048	-1.43746	-1.542	-1.53882

-1.42479	-1.42182	-1.44989	-1.44687	-1.44387	-1.44087	-1.43788	-1.49085	-1.48774
-1.42515	-1.42218	-1.45027	-1.44723	-1.44422	-1.44122	-1.43825	-1.49123	-1.48813
-1.42552	-1.42256	-1.45065	-1.4476	-1.44457	-1.44157	-1.4386	-1.4916	-1.4885
-1.42594	-1.42297	-1.50785	-1.50464	-1.50144	-1.49825	-1.49509	-1.49196	-1.48887
-1.42639	-1.42341	-1.5083	-1.50509	-1.50189	-1.49868	-1.49548	-1.49233	-1.48924
-1.4268	-1.42381	-1.50872	-1.50551	-1.50232	-1.4991	-1.49589	-1.49275	-1.48965
-1.42718	-1.42418	-1.50911	-1.50591	-1.50272	-1.49953	-1.49635	-1.4932	-1.49009
-1.42757	-1.42457	-1.50953	-1.50633	-1.50315	-1.49997	-1.4968	-1.49365	-1.49052
-1.428	-1.42501	-1.51	-1.50679	-1.5036	-1.50042	-1.49725	-1.49408	-1.49092
-1.42843	-1.42545	-1.51046	-1.50724	-1.50404	-1.50085	-1.49768	-1.4945	-1.49132
-1.4288	-1.42583	-1.51086	-1.50764	-1.50444	-1.50126	-1.49809	-1.49492	-1.49175
-1.42917	-1.4262	-1.51125	-1.50803	-1.50483	-1.50165	-1.4985	-1.49534	-1.4922
-1.4296	-1.42662	-1.48148	-1.47835	-1.47524	-1.50209	-1.49893	-1.49578	-1.49265
-1.43005	-1.42707	-1.48193	-1.47881	-1.4757	-1.50256	-1.49939	-1.49624	-1.49311
-1.43051	-1.42752	-1.48239	-1.47927	-1.47616	-1.50303	-1.49985	-1.4967	-1.49357
-1.43096	-1.42797	-1.48285	-1.47973	-1.47662	-1.5035	-1.50032	-1.49717	-1.49404
-1.43142	-1.42843	-1.48332	-1.48019	-1.47708	-1.50396	-1.50079	-1.49764	-1.4945
-1.43187	-1.42888	-1.48379	-1.48065	-1.47753	-1.50442	-1.50125	-1.4981	-1.49496
-1.43232	-1.42934	-1.48425	-1.48111	-1.47797	-1.50487	-1.5017	-1.49855	-1.49541
-1.43278	-1.4298	-1.48471	-1.48157	-1.47842	-1.50531	-1.50212	-1.49895	-1.49581
-1.43324	-1.43025	-1.48518	-1.48203	-1.47888	-1.50576	-1.50253	-1.49934	-1.49619
-1.4337	-1.43071	-1.48564	-1.48249	-1.47935	-1.50624	-1.50302	-1.49983	-1.49667
-1.43415	-1.43115	-1.48611	-1.48296	-1.47983	-1.50675	-1.50356	-1.50039	-1.49723
-1.4346	-1.4316	-1.48657	-1.48343	-1.4803	-1.50725	-1.50408	-1.50091	-1.49776
-1.43505	-1.43205	-1.48703	-1.48389	-1.48076	-1.50773	-1.50456	-1.5014	-1.49825
-1.4355	-1.4325	-1.4875	-1.48435	-1.48122	-1.5082	-1.50503	-1.50187	-1.49873
-1.43595	-1.43296	-1.48796	-1.48481	-1.48168	-1.50867	-1.5055	-1.50235	-1.4992
-1.43641	-1.43341	-1.48843	-1.48528	-1.48215	-1.50914	-1.50598	-1.50282	-1.49967
-1.43686	-1.43386	-1.4889	-1.48574	-1.48261	-1.4795	-1.47642	-1.50329	-1.50013
-1.4373	-1.4343	-1.48936	-1.48621	-1.48308	-1.47997	-1.47688	-1.50375	-1.5006
-1.43776	-1.43476	-1.48984	-1.48668	-1.48355	-1.48043	-1.47734	-1.50422	-1.50107
-1.43822	-1.43522	-1.49031	-1.48716	-1.48403	-1.48091	-1.4778	-1.5047	-1.50156
-1.43871	-1.4357	-1.49082	-1.48767	-1.48453	-1.4814	-1.4783	-1.5052	-1.50207
-1.43924	-1.43623	-1.49136	-1.48821	-1.48507	-1.48195	-1.47884	-1.50575	-1.50262
-1.43977	-1.43676	-1.49191	-1.48875	-1.48561	-1.48249	-1.47938	-1.5063	-1.50316
-1.44024	-1.43724	-1.49241	-1.48925	-1.4861	-1.48298	-1.47987	-1.50681	-1.50366
-1.4407	-1.43771	-1.49291	-1.48974	-1.48659	-1.48347	-1.48037	-1.5073	-1.50414
-1.44117	-1.43821	-1.49344	-1.49028	-1.48712	-1.484	-1.48089	-1.4778	-1.47472
-1.44165	-1.43872	-1.494	-1.49084	-1.48767	-1.48455	-1.48144	-1.47834	-1.47526
-1.44214	-1.43921	-1.49451	-1.49135	-1.48819	-1.48506	-1.48195	-1.47885	-1.47577
-1.44264	-1.43967	-1.49498	-1.49183	-1.48868	-1.48555	-1.48242	-1.47932	-1.47625
-1.41613	-1.41321	-1.49547	-1.49232	-1.48919	-1.48605	-1.48292	-1.47982	-1.47675
-1.41667	-1.41373	-1.49601	-1.49286	-1.48973	-1.48659	-1.48347	-1.48037	-1.47728
-1.4172	-1.41426	-1.49657	-1.49342	-1.49028	-1.48715	-1.48403	-1.48092	-1.47783
-1.38889	-1.38606	-1.4388	-1.43581	-1.43283	-1.42986	-1.4269	-1.48148	-1.47838
-1.38933	-1.38654	-1.43934	-1.43635	-1.43336	-1.43039	-1.42744	-1.48204	-1.47894
-1.38978	-1.38699	-1.41287	-1.40997	-1.40707	-1.43094	-1.42797	-1.4826	-1.4795
-1.39025	-1.38742	-1.41331	-1.41045	-1.4076	-1.43149	-1.42851	-1.48316	-1.48006
-1.39074	-1.38788	-1.41376	-1.41092	-1.40809	-1.43201	-1.42905	-1.48372	-1.48062
-1.39125	-1.38837	-1.41425	-1.41138	-1.40853	-1.43249	-1.42958	-1.48429	-1.48119

-1.39176	-1.38887	-1.41475	-1.41185	-1.40898	-1.40615	-1.40334	-1.48485	-1.48174
-1.39227	-1.38937	-1.41528	-1.41236	-1.40948	-1.40663	-1.4038	-1.48536	-1.48229
-1.39277	-1.38988	-1.41581	-1.41289	-1.40999	-1.40712	-1.40427	-1.48587	-1.48283
-1.39329	-1.39039	-1.41633	-1.41341	-1.41051	-1.40765	-1.40481	-1.48645	-1.4834
-1.3938	-1.39089	-1.41685	-1.41393	-1.41105	-1.40821	-1.40539	-1.48705	-1.48397
-1.36077	-1.35795	-1.41738	-1.41446	-1.41157	-1.40872	-1.4059	-1.46171	-1.45869
-1.36126	-1.35844	-1.41792	-1.41498	-1.41207	-1.4092	-1.40636	-1.4622	-1.45921
-1.36176	-1.35895	-1.41846	-1.41551	-1.41259	-1.40971	-1.40686	-1.46272	-1.45974
-1.36228	-1.35945	-1.41899	-1.41604	-1.41313	-1.41026	-1.40741	-1.46328	-1.46028
-1.36281	-1.35997	-1.41954	-1.4166	-1.4137	-1.41082	-1.40798	-1.46385	-1.46083
-1.36338	-1.36054	-1.42015	-1.41719	-1.41427	-1.41138	-1.40851	-1.4644	-1.46138
-1.36395	-1.36112	-1.42077	-1.41779	-1.41484	-1.41193	-1.40905	-1.46496	-1.46194
-1.36447	-1.36164	-1.42134	-1.41836	-1.41539	-1.41248	-1.40959	-1.46552	-1.46251
-1.36496	-1.36213	-1.42188	-1.41889	-1.41593	-1.41302	-1.41013	-1.46609	-1.46309
-1.36547	-1.36263	-1.38939	-1.38649	-1.38363	-1.41357	-1.41069	-1.46667	-1.46366
-1.36598	-1.36313	-1.3899	-1.38702	-1.38416	-1.41413	-1.41125	-1.46725	-1.46423
-1.33457	-1.33179	-1.39045	-1.38757	-1.38472	-1.38189	-1.37909	-1.46783	-1.46479
-1.33514	-1.33236	-1.39106	-1.38817	-1.38529	-1.38244	-1.37962	-1.46838	-1.46535
-1.3357	-1.33294	-1.39168	-1.38877	-1.38588	-1.38301	-1.38016	-1.46896	-1.46592
-1.33621	-1.33345	-1.36092	-1.3581	-1.35529	-1.3836	-1.38074	-1.46957	-1.46652
-1.3367	-1.33395	-1.36145	-1.35866	-1.35587	-1.3842	-1.38135	-1.47021	-1.46713
-1.33721	-1.33447	-1.362	-1.3592	-1.35641	-1.35363	-1.35085	-1.47082	-1.46776
-1.33774	-1.33501	-1.36256	-1.35973	-1.35692	-1.35413	-1.35137	-1.47143	-1.46839
-1.33828	-1.33556	-1.36313	-1.36029	-1.35746	-1.35467	-1.35191	-1.47204	-1.46902
-1.33884	-1.33612	-1.36372	-1.36088	-1.35805	-1.35525	-1.35247	-1.47266	-1.46964
-1.33939	-1.33666	-1.33393	-1.33119	-1.32846	-1.35582	-1.35303	-1.41141	-1.40854
-1.33993	-1.33716	-1.33441	-1.33168	-1.32896	-1.35636	-1.35359	-1.41199	-1.40909
-1.34048	-1.33767	-1.3349	-1.33216	-1.32946	-1.35691	-1.35415	-1.41257	-1.40963
-1.34105	-1.33823	-1.33544	-1.33271	-1.33	-1.35748	-1.35473	-1.41317	-1.41022
-1.34163	-1.33881	-1.33602	-1.33328	-1.33056	-1.35806	-1.35532	-1.41378	-1.41082
-1.34221	-1.33938	-1.33659	-1.33384	-1.33113	-1.35865	-1.3559	-1.41438	-1.41143
-1.34278	-1.33995	-1.33716	-1.33441	-1.3317	-1.35924	-1.35648	-1.41499	-1.41203
-1.34336	-1.34053	-1.33773	-1.33498	-1.33227	-1.35983	-1.35707	-1.4156	-1.41264
-1.34394	-1.34111	-1.33831	-1.33556	-1.33284	-1.36042	-1.35766	-1.41621	-1.41324
-1.34453	-1.34169	-1.33888	-1.33613	-1.33341	-1.36101	-1.35825	-1.41682	-1.41385
-1.3451	-1.34226	-1.33945	-1.3367	-1.33397	-1.3616	-1.35883	-1.41743	-1.41445
-1.34568	-1.34284	-1.34002	-1.33726	-1.33453	-1.36218	-1.35941	-1.41803	-1.41505
-1.34626	-1.34341	-1.34059	-1.33783	-1.33509	-1.36277	-1.36	-1.41863	-1.41565
-1.34684	-1.34398	-1.34116	-1.33839	-1.33566	-1.36335	-1.36058	-1.41924	-1.41625
-1.34742	-1.34456	-1.34173	-1.33896	-1.33622	-1.36394	-1.36117	-1.41985	-1.41686
-1.348	-1.34513	-1.3423	-1.33952	-1.33677	-1.36452	-1.36176	-1.42047	-1.41747
-1.30987	-1.30711	-1.3429	-1.3401	-1.33734	-1.33464	-1.33197	-1.4211	-1.41811
-1.31047	-1.30773	-1.34354	-1.34073	-1.33795	-1.33523	-1.33254	-1.42175	-1.41879
-1.31106	-1.30834	-1.34419	-1.34137	-1.33857	-1.33582	-1.33312	-1.42239	-1.41947
-1.31163	-1.30892	-1.34478	-1.34196	-1.33915	-1.3364	-1.33368	-1.42302	-1.42011
-1.3122	-1.30948	-1.34536	-1.34254	-1.33974	-1.33698	-1.33424	-1.42364	-1.42073
-1.31276	-1.31005	-1.30736	-1.30465	-1.30196	-1.3376	-1.33484	-1.42428	-1.42136
-1.31333	-1.31062	-1.30794	-1.30527	-1.30261	-1.33826	-1.33548	-1.42494	-1.42198
-1.31392	-1.3112	-1.30851	-1.30586	-1.30321	-1.33889	-1.33612	-1.42563	-1.42264
-1.31455	-1.31178	-1.30906	-1.30641	-1.30378	-1.33951	-1.33678	-1.42635	-1.42333

-1.31519	-1.31238	-1.30963	-1.30697	-1.30434	-1.3401	-1.33741	-1.42706	-1.42402
-1.31587	-1.31303	-1.31024	-1.30754	-1.30488	-1.34065	-1.33798	-1.42774	-1.42473
-1.31654	-1.31369	-1.31088	-1.30812	-1.30543	-1.3412	-1.33853	-1.42839	-1.42543
-1.31717	-1.31434	-1.31153	-1.30876	-1.30603	-1.30337	-1.30077	-1.42902	-1.42608
-1.31776	-1.31498	-1.3122	-1.30941	-1.30665	-1.30396	-1.30132	-1.42964	-1.42671
-1.31835	-1.31558	-1.31282	-1.31004	-1.30729	-1.30458	-1.30191	-1.43028	-1.42733
-1.31892	-1.31615	-1.31339	-1.31066	-1.30793	-1.30522	-1.30253	-1.43094	-1.42795
-1.31955	-1.31675	-1.31399	-1.31126	-1.30855	-1.30584	-1.30315	-1.40189	-1.39897
-1.32026	-1.31742	-1.31461	-1.31184	-1.30911	-1.30643	-1.30378	-1.4026	-1.39969
-1.32096	-1.31809	-1.31525	-1.31245	-1.30969	-1.30701	-1.30439	-1.36612	-1.36332
-1.32157	-1.31874	-1.31591	-1.31309	-1.3103	-1.3076	-1.30495	-1.36671	-1.36392
-1.32216	-1.31937	-1.31657	-1.31374	-1.31094	-1.3082	-1.3055	-1.36729	-1.3645
-1.32279	-1.31998	-1.31718	-1.31437	-1.31159	-1.30886	-1.30615	-1.36796	-1.36514
-1.32343	-1.32058	-1.31777	-1.315	-1.31226	-1.30954	-1.30686	-1.36867	-1.36581
-1.3241	-1.32124	-1.31842	-1.31565	-1.3129	-1.31019	-1.3075	-1.36935	-1.36648
-1.32479	-1.32196	-1.31914	-1.31633	-1.31354	-1.31079	-1.30808	-1.36998	-1.36714
-1.25828	-1.25561	-1.31983	-1.31699	-1.31418	-1.31142	-1.3087	-1.37065	-1.36783
-1.25887	-1.25619	-1.32044	-1.31763	-1.31485	-1.31211	-1.3094	-1.37139	-1.36855
-1.25948	-1.25677	-1.32107	-1.31828	-1.31552	-1.31279	-1.31009	-1.37211	-1.36926
-1.26013	-1.25743	-1.32176	-1.31894	-1.31615	-1.3134	-1.31068	-1.37273	-1.36988
-1.2608	-1.25811	-1.32247	-1.31962	-1.31678	-1.31399	-1.31124	-1.37333	-1.37048
-1.26142	-1.25873	-1.25604	-1.25334	-1.25066	-1.31465	-1.3119	-1.37402	-1.37116
-1.26201	-1.2593	-1.25662	-1.25394	-1.2513	-1.31535	-1.3126	-1.37477	-1.37189
-1.26264	-1.25993	-1.25724	-1.25458	-1.25195	-1.31603	-1.31328	-1.37547	-1.37257
-1.26331	-1.26061	-1.25793	-1.25527	-1.25262	-1.31671	-1.31391	-1.37612	-1.3732
-1.26398	-1.26129	-1.2586	-1.25593	-1.25326	-1.25059	-1.24793	-1.3768	-1.37386
-1.26466	-1.26193	-1.25921	-1.25651	-1.25384	-1.25118	-1.24856	-1.37752	-1.37459
-1.26533	-1.26256	-1.25982	-1.2571	-1.25442	-1.25179	-1.2492	-1.37827	-1.37534
-1.26595	-1.2632	-1.26047	-1.25777	-1.25509	-1.25246	-1.24986	-1.37902	-1.37608
-1.26655	-1.26384	-1.26115	-1.25847	-1.25581	-1.25316	-1.25053	-1.37978	-1.37682
-1.26721	-1.26452	-1.26184	-1.25917	-1.25651	-1.25386	-1.25121	-1.31603	-1.31321
-1.26793	-1.26523	-1.26254	-1.25987	-1.25722	-1.25455	-1.25189	-1.31672	-1.31387
-1.26865	-1.26594	-1.26324	-1.26055	-1.25788	-1.2552	-1.25252	-1.31739	-1.31453
-1.26937	-1.26665	-1.26394	-1.2612	-1.25848	-1.25577	-1.25308	-1.31801	-1.31518
-1.27007	-1.26735	-1.26461	-1.26185	-1.25909	-1.25636	-1.25367	-1.31866	-1.31586
-1.27078	-1.26801	-1.26525	-1.26248	-1.25974	-1.25702	-1.25435	-1.31939	-1.31659
-1.27146	-1.26866	-1.26587	-1.26312	-1.2604	-1.25772	-1.25506	-1.32014	-1.31733
-1.27209	-1.2693	-1.26653	-1.26379	-1.26108	-1.2584	-1.25576	-1.32089	-1.31807
-1.2727	-1.26995	-1.26721	-1.26448	-1.26178	-1.2591	-1.25646	-1.32164	-1.31882
-1.27337	-1.27063	-1.2679	-1.26518	-1.26248	-1.25981	-1.25716	-1.32239	-1.31956
-1.2741	-1.27134	-1.26859	-1.26588	-1.26318	-1.26052	-1.25787	-1.32314	-1.3203
-1.27485	-1.27206	-1.2693	-1.26659	-1.26389	-1.26122	-1.25857	-1.32389	-1.32104
-1.27559	-1.27279	-1.27003	-1.2673	-1.2646	-1.26192	-1.25927	-1.32463	-1.32177
-1.27634	-1.27354	-1.27076	-1.26802	-1.26531	-1.26263	-1.25996	-1.32538	-1.3225
-1.27709	-1.27428	-1.2715	-1.26874	-1.26602	-1.26333	-1.26066	-1.32612	-1.32325
-1.27785	-1.27502	-1.27223	-1.26947	-1.26674	-1.26403	-1.26135	-1.32685	-1.32399
-1.21141	-1.20875	-1.27298	-1.27021	-1.26746	-1.26474	-1.26203	-1.32757	-1.32468
-1.21211	-1.20945	-1.27373	-1.27095	-1.26819	-1.26544	-1.26271	-1.32826	-1.32533
-1.21281	-1.21015	-1.27448	-1.27169	-1.26891	-1.26615	-1.2634	-1.32898	-1.32603
-1.21351	-1.21084	-1.27523	-1.27242	-1.26963	-1.26687	-1.26411	-1.32973	-1.32676

-1.2142	-1.21153	-1.20888	-1.20624	-1.20362	-1.26758	-1.26483	-1.3305	-1.32753
-1.21489	-1.21221	-1.20955	-1.20691	-1.20428	-1.26831	-1.26555	-1.33129	-1.32834
-1.21557	-1.21289	-1.21022	-1.20758	-1.20495	-1.26903	-1.26628	-1.33209	-1.32916
-1.17936	-1.17676	-1.2109	-1.20825	-1.20562	-1.26975	-1.26699	-1.33286	-1.32995
-1.18001	-1.17742	-1.21158	-1.20892	-1.20628	-1.27046	-1.26769	-1.33362	-1.33072
-1.18066	-1.17807	-1.21225	-1.20959	-1.20695	-1.20432	-1.20172	-1.26567	-1.26298
-1.18131	-1.17871	-1.21292	-1.21025	-1.2076	-1.20498	-1.20237	-1.26638	-1.26367
-1.18195	-1.17936	-1.21359	-1.21092	-1.20827	-1.20563	-1.20303	-1.20045	-1.1979
-1.1826	-1.18001	-1.21427	-1.21159	-1.20893	-1.20629	-1.20368	-1.20109	-1.19853
-1.18325	-1.18065	-1.21494	-1.21226	-1.2096	-1.20695	-1.20433	-1.20173	-1.19917
-1.1839	-1.1813	-1.21561	-1.21293	-1.21026	-1.20761	-1.20498	-1.20239	-1.19982
-1.18455	-1.18195	-1.21629	-1.2136	-1.21092	-1.20827	-1.20564	-1.20305	-1.20047
-1.15253	-1.15	-1.18	-1.1774	-1.17481	-1.20893	-1.2063	-1.2037	-1.20112
-1.15319	-1.15065	-1.18065	-1.17805	-1.17546	-1.2096	-1.20697	-1.20435	-1.20177
-1.15395	-1.1514	-1.18137	-1.17875	-1.17614	-1.21029	-1.20763	-1.205	-1.20241
-1.1548	-1.15223	-1.18218	-1.17951	-1.17685	-1.21098	-1.20829	-1.20565	-1.20306
-1.15558	-1.15301	-1.18294	-1.18026	-1.17758	-1.21173	-1.20901	-1.20636	-1.20377
-1.15627	-1.1537	-1.18364	-1.18099	-1.17835	-1.21255	-1.20985	-1.2072	-1.2046
-1.15693	-1.15436	-1.18431	-1.1817	-1.1791	-1.21335	-1.21066	-1.20801	-1.2054
-1.11609	-1.11365	-1.18496	-1.18235	-1.17976	-1.17716	-1.17456	-1.20871	-1.2061
-1.11671	-1.11425	-1.1856	-1.18299	-1.18039	-1.17779	-1.1752	-1.20938	-1.20675
-1.11733	-1.11487	-1.18624	-1.18363	-1.18103	-1.17844	-1.17585	-1.21005	-1.20742
-1.11795	-1.11549	-1.1869	-1.18428	-1.18168	-1.17908	-1.1765	-1.21072	-1.20809
-1.11866	-1.1162	-1.15515	-1.15259	-1.15005	-1.1798	-1.17722	-1.21147	-1.20883
-1.11947	-1.117	-1.15599	-1.15342	-1.15087	-1.18061	-1.17803	-1.21231	-1.20966
-1.08248	-1.08008	-1.15679	-1.15421	-1.15164	-1.18138	-1.17879	-1.17621	-1.17365
-1.08311	-1.08071	-1.15747	-1.15488	-1.15231	-1.18203	-1.17944	-1.17686	-1.1743
-1.08372	-1.08131	-1.15813	-1.15554	-1.15297	-1.18269	-1.18009	-1.17751	-1.17496
-1.08432	-1.08191	-1.11719	-1.11477	-1.11236	-1.18346	-1.18087	-1.1783	-1.17574
-1.08494	-1.08252	-1.11784	-1.11545	-1.11308	-1.18425	-1.18169	-1.17912	-1.17655
-1.08566	-1.08323	-1.08085	-1.07851	-1.0762	-1.18498	-1.18241	-1.17983	-1.17725
-1.08645	-1.08402	-1.08161	-1.07923	-1.07687	-1.18568	-1.18307	-1.18047	-1.17788
-1.08718	-1.08474	-1.08231	-1.0799	-1.07751	-1.18638	-1.18376	-1.18116	-1.17857
-1.08782	-1.08536	-1.08293	-1.08051	-1.07813	-1.18709	-1.1845	-1.18192	-1.17936
-1.05194	-1.04954	-1.08357	-1.08116	-1.07878	-1.07645	-1.07415	-1.18267	-1.18012
-1.05269	-1.05029	-1.08432	-1.0819	-1.07951	-1.07715	-1.07481	-1.18336	-1.18078
-1.05345	-1.05104	-1.08508	-1.08266	-1.08026	-1.07786	-1.07548	-1.18406	-1.18144
-1.00918	-1.00688	-1.04931	-1.04697	-1.04465	-1.07858	-1.0762	-1.18485	-1.18221
-1.00974	-1.00745	-1.04992	-1.04762	-1.04533	-1.0793	-1.07695	-1.18568	-1.18302
-1.01041	-1.0081	-1.0506	-1.04829	-1.04599	-1.07996	-1.07761	-1.1501	-1.14751
-1.01115	-1.00883	-1.05134	-1.04897	-1.04662	-1.08057	-1.07819	-1.15069	-1.14811
-0.97535	-0.97312	-1.00723	-1.00494	-1.00267	-1.04495	-1.04261	-1.15136	-1.14877
-0.97595	-0.97376	-1.00793	-1.00565	-1.00339	-1.0457	-1.04336	-1.15214	-1.14956
-0.97655	-0.97439	-1.0086	-1.00634	-1.00408	-1.04644	-1.04408	-1.04176	-1.03945
-0.97721	-0.97501	-1.00921	-1.00693	-1.00466	-1.04704	-1.04469	-1.04239	-1.04012
-0.97789	-0.97564	-1.00983	-1.00751	-1.00522	-1.04763	-1.04528	-1.043	-1.04076
-0.97861	-0.97634	-1.01053	-1.0082	-1.0059	-1.00362	-1.00137	-1.04367	-1.04141
-0.97936	-0.97708	-1.01129	-1.00896	-1.00664	-1.00435	-1.00208	-1.04438	-1.04207
-0.98003	-0.97777	-1.01202	-1.00969	-1.00738	-1.00507	-1.00279	-1.0451	-1.04277
-0.98064	-0.9784	-1.01271	-1.01041	-1.00812	-1.0058	-1.0035	-1.04584	-1.0435

-0.98127	-0.97904	-1.01337	-1.01109	-1.0088	-1.0065	-1.00419	-1.00193	-0.99971
-0.98195	-0.97967	-1.01399	-1.01168	-1.0094	-1.00713	-1.00487	-1.00264	-1.00043
-0.98265	-0.98032	-1.01462	-1.01229	-1.01	-1.00775	-1.00554	-1.00333	-1.00113
-0.94132	-0.93908	-1.01536	-1.013	-1.01068	-1.00841	-1.00618	-1.00397	-1.00179
-0.94205	-0.93981	-1.01613	-1.01375	-1.0114	-1.00909	-1.00681	-1.00459	-1.00242
-0.94277	-0.94052	-1.01689	-1.0145	-1.01214	-1.0098	-1.0075	-1.00525	-1.00306
-0.9435	-0.94124	-1.01764	-1.01525	-1.01288	-1.01053	-1.00822	-1.00594	-1.00369
-0.85849	-0.85648	-0.93971	-0.9375	-0.93531	-1.01127	-1.00895	-1.00665	-1.00437
-0.8591	-0.85711	-0.94043	-0.9382	-0.93599	-1.01201	-1.00968	-1.00738	-1.0051
-0.85969	-0.85771	-0.94114	-0.9389	-0.93669	-1.01275	-1.01042	-1.00811	-1.00583
-0.86023	-0.85828	-0.94182	-0.93961	-0.9374	-1.01352	-1.01116	-1.00884	-1.00655
-0.86077	-0.85884	-0.94248	-0.94029	-0.93811	-1.01428	-1.01191	-1.00958	-1.00727
-0.86136	-0.8594	-0.85746	-0.85552	-0.8536	-0.97393	-0.97167	-1.01031	-1.008
-0.86198	-0.85996	-0.85798	-0.85606	-0.85417	-0.97461	-0.97237	-1.01105	-1.00873
-0.77804	-0.7762	-0.85856	-0.85665	-0.85476	-0.97532	-0.97308	-1.01178	-1.00946
-0.77862	-0.77677	-0.8592	-0.85727	-0.85537	-0.97604	-0.97378	-1.0125	-1.01018
-0.7792	-0.77735	-0.85983	-0.8579	-0.85599	-0.89297	-0.89093	-0.97225	-0.97001
-0.77978	-0.77791	-0.86045	-0.85851	-0.8566	-0.89361	-0.89157	-0.97297	-0.97072
-0.78035	-0.77847	-0.86107	-0.85913	-0.85721	-0.85527	-0.85332	-0.9737	-0.97142
-0.78093	-0.77905	-0.8617	-0.85975	-0.85783	-0.85588	-0.85393	-0.97441	-0.97212
-0.78151	-0.77962	-0.86233	-0.86037	-0.85844	-0.85649	-0.85454	-0.97513	-0.97281
-0.78209	-0.7802	-0.77836	-0.77658	-0.77482	-0.85708	-0.85514	-0.89206	-0.88999
-0.78267	-0.78077	-0.77892	-0.77713	-0.77536	-0.85767	-0.85573	-0.89268	-0.89061
-0.78324	-0.78134	-0.77949	-0.77768	-0.7759	-0.85828	-0.85633	-0.89331	-0.89124
-0.78381	-0.78191	-0.78005	-0.77824	-0.77646	-0.85889	-0.85694	-0.89394	-0.89188
-0.68414	-0.68262	-0.78062	-0.77881	-0.77703	-0.8153	-0.81343	-0.89457	-0.89251
-0.68461	-0.68308	-0.78119	-0.77938	-0.77759	-0.81588	-0.814	-0.89518	-0.89313
-0.68509	-0.68355	-0.78178	-0.77996	-0.77816	-0.81647	-0.81457	-0.8958	-0.89374
-0.63556	-0.63416	-0.78241	-0.78057	-0.77875	-0.81707	-0.81515	-0.85734	-0.85538
-0.63601	-0.63462	-0.78307	-0.7812	-0.77935	-0.81767	-0.81574	-0.85794	-0.85598
-0.59078	-0.58949	-0.73009	-0.72848	-0.72689	-0.77807	-0.77621	-0.81444	-0.81259
-0.59118	-0.58988	-0.73059	-0.72899	-0.72739	-0.77864	-0.77678	-0.81505	-0.81322
-0.54423	-0.54301	-0.68213	-0.68064	-0.67915	-0.77923	-0.77738	-0.81569	-0.81385
-0.54466	-0.54343	-0.68264	-0.68112	-0.67962	-0.77983	-0.77802	-0.81635	-0.81448
-0.49679	-0.49568	-0.59217	-0.5908	-0.58945	-0.73271	-0.73104	-0.817	-0.8151
-0.49715	-0.49604	-0.5926	-0.59122	-0.58986	-0.73324	-0.73157	-0.8176	-0.81569
-0.4975	-0.49639	-0.59303	-0.59166	-0.59029	-0.73379	-0.7321	-0.81818	-0.81628
-0.49786	-0.49675	-0.54374	-0.5425	-0.54126	-0.63483	-0.63344	-0.77854	-0.77669
-0.49824	-0.4971	-0.54412	-0.5429	-0.54169	-0.63533	-0.63392	-0.77914	-0.77728
-0.49861	-0.49748	-0.49637	-0.49527	-0.49417	-0.59035	-0.58901	-0.73204	-0.73031
-0.49899	-0.49788	-0.49677	-0.49567	-0.49455	-0.59079	-0.58943	-0.7326	-0.7309
-0.49939	-0.49828	-0.49717	-0.49605	-0.49493	-0.59123	-0.58987	-0.64462	-0.64307
-0.49981	-0.49868	-0.49756	-0.49643	-0.4953	-0.59169	-0.59033	-0.64518	-0.6436
-0.44372	-0.44276	-0.49794	-0.4968	-0.49567	-0.54523	-0.544	-0.64574	-0.64413
-0.44404	-0.44307	-0.4983	-0.49718	-0.49606	-0.54568	-0.54444	-0.64627	-0.64463
-0.44436	-0.44339	-0.49867	-0.49756	-0.49646	-0.54613	-0.54488	-0.64681	-0.64514
-0.39133	-0.39049	-0.49907	-0.49796	-0.49685	-0.49573	-0.49459	-0.59081	-0.58945
-0.39162	-0.39079	-0.4995	-0.49837	-0.49725	-0.4961	-0.49496	-0.59126	-0.58992
-0.39192	-0.39108	-0.44347	-0.4425	-0.44154	-0.49648	-0.49535	-0.54493	-0.5437
-0.39223	-0.39138	-0.44381	-0.44283	-0.44185	-0.49686	-0.49575	-0.5454	-0.54415

-0.34352	-0.3427	-0.39082	-0.38997	-0.38912	-0.44123	-0.4403	-0.54586	-0.5446
-0.34383	-0.343	-0.3911	-0.39024	-0.3894	-0.44157	-0.44064	-0.5463	-0.54506
-0.34414	-0.3433	-0.39137	-0.39053	-0.3897	-0.44192	-0.44097	-0.54675	-0.54552
-0.34444	-0.3436	-0.34277	-0.34196	-0.34117	-0.38916	-0.38834	-0.5472	-0.54597
-0.34474	-0.34391	-0.34309	-0.34228	-0.34148	-0.38946	-0.38863	-0.54765	-0.54641
-0.34506	-0.34422	-0.3434	-0.34259	-0.34179	-0.34099	-0.34019	-0.49324	-0.49218
-0.34538	-0.34454	-0.34372	-0.3429	-0.34209	-0.34129	-0.3405	-0.49363	-0.49258
-0.3457	-0.34486	-0.34403	-0.34321	-0.3424	-0.3416	-0.3408	-0.34001	-0.33923
-0.34602	-0.34518	-0.34435	-0.34352	-0.34271	-0.3419	-0.3411	-0.34031	-0.33953
-0.34633	-0.34549	-0.34466	-0.34383	-0.34301	-0.34221	-0.34141	-0.34062	-0.33983
-0.34665	-0.34581	-0.34498	-0.34415	-0.34333	-0.34252	-0.34172	-0.34092	-0.34013
-0.34697	-0.34613	-0.3453	-0.34447	-0.34364	-0.34283	-0.34202	-0.34123	-0.34043
-0.34729	-0.34646	-0.34562	-0.34479	-0.34396	-0.34314	-0.34233	-0.34153	-0.34074
-0.34762	-0.34678	-0.34594	-0.3451	-0.34427	-0.34345	-0.34264	-0.34183	-0.34104
-0.29118	-0.29047	-0.34626	-0.34542	-0.34459	-0.34377	-0.34295	-0.34214	-0.34134
-0.29146	-0.29074	-0.34657	-0.34574	-0.34491	-0.34408	-0.34325	-0.34245	-0.34165
-0.29174	-0.29101	-0.3469	-0.34607	-0.34524	-0.34441	-0.34358	-0.34277	-0.34197
-0.29202	-0.29129	-0.34726	-0.34642	-0.34559	-0.34475	-0.34392	-0.34311	-0.3423
-0.2923	-0.29158	-0.29087	-0.29017	-0.28948	-0.34509	-0.34426	-0.34344	-0.34263
-0.29257	-0.29185	-0.29113	-0.29043	-0.28974	-0.34542	-0.34459	-0.34376	-0.34294
-0.29285	-0.29211	-0.29139	-0.29069	-0.29	-0.34574	-0.34491	-0.34408	-0.34325
-0.24032	-0.23969	-0.29166	-0.29095	-0.29026	-0.28957	-0.28889	-0.34441	-0.34358
-0.24057	-0.23993	-0.29192	-0.29121	-0.29052	-0.28984	-0.28917	-0.34476	-0.34393
-0.24082	-0.24018	-0.23956	-0.23896	-0.23838	-0.29011	-0.28944	-0.34509	-0.34426
-0.24108	-0.24045	-0.23983	-0.23922	-0.23863	-0.29039	-0.2897	-0.34541	-0.34458
-0.24133	-0.24071	-0.24009	-0.23947	-0.23887	-0.23828	-0.2377	-0.28928	-0.28859
-0.24157	-0.24095	-0.24033	-0.23971	-0.2391	-0.23851	-0.23792	-0.28954	-0.28886
-0.2418	-0.24119	-0.24058	-0.23995	-0.23934	-0.23874	-0.23816	-0.28981	-0.28914
-0.24206	-0.24144	-0.24083	-0.24021	-0.23959	-0.23899	-0.23841	-0.23783	-0.23725
-0.24231	-0.2417	-0.24109	-0.24047	-0.23985	-0.23925	-0.23866	-0.23807	-0.23748
-0.18652	-0.18604	-0.24133	-0.24071	-0.2401	-0.2395	-0.23891	-0.23832	-0.23772
-0.1867	-0.18621	-0.24155	-0.24094	-0.24034	-0.23975	-0.23916	-0.23857	-0.23797
-0.18688	-0.1864	-0.24179	-0.24119	-0.24059	-0.23999	-0.2394	-0.23881	-0.23821
-0.18708	-0.18659	-0.24205	-0.24144	-0.24084	-0.24024	-0.23964	-0.23904	-0.23845
-0.18727	-0.18679	-0.18631	-0.18583	-0.18536	-0.24049	-0.23988	-0.23928	-0.23868
-0.18747	-0.18699	-0.1865	-0.18603	-0.18555	-0.24074	-0.24013	-0.23953	-0.23894
-0.18766	-0.18718	-0.1867	-0.18622	-0.18575	-0.241	-0.24038	-0.23978	-0.23919
-0.18784	-0.18736	-0.18688	-0.18641	-0.18594	-0.18546	-0.18498	-0.24003	-0.23944
-0.18803	-0.18754	-0.18707	-0.1866	-0.18614	-0.18566	-0.18518	-0.24029	-0.23969
-0.18822	-0.18773	-0.18726	-0.18679	-0.18633	-0.18585	-0.18537	-0.24054	-0.23994
-0.18843	-0.18794	-0.18746	-0.18699	-0.18653	-0.18605	-0.18557	-0.24079	-0.2402
-0.18863	-0.18814	-0.18766	-0.18719	-0.18673	-0.18625	-0.18576	-0.18528	-0.18481
-0.18883	-0.18834	-0.18786	-0.18739	-0.18693	-0.18645	-0.18596	-0.18548	-0.185
-0.18903	-0.18854	-0.18806	-0.18759	-0.18713	-0.18664	-0.18616	-0.18567	-0.1852
-0.18924	-0.18874	-0.18826	-0.18779	-0.18732	-0.18684	-0.18635	-0.18587	-0.18539
-0.18944	-0.18895	-0.18846	-0.18799	-0.18752	-0.18704	-0.18655	-0.18607	-0.18559
-0.18965	-0.18915	-0.18866	-0.18819	-0.18772	-0.18723	-0.18675	-0.18626	-0.18579
-0.18986	-0.18935	-0.18886	-0.18839	-0.18791	-0.18743	-0.18694	-0.18646	-0.18598
-0.19006	-0.18956	-0.18906	-0.18859	-0.18811	-0.18763	-0.18714	-0.18665	-0.18618
-0.19027	-0.18976	-0.18927	-0.18879	-0.18831	-0.18782	-0.18733	-0.18685	-0.18637

-0.19047	-0.18997	-0.18948	-0.18899	-0.18851	-0.18802	-0.18753	-0.18704	-0.18657
-0.19068	-0.19018	-0.18968	-0.1892	-0.18871	-0.18822	-0.18772	-0.18724	-0.18676
-0.19089	-0.19039	-0.18989	-0.1894	-0.18892	-0.18842	-0.18792	-0.18743	-0.18696
-0.19109	-0.19059	-0.1901	-0.18961	-0.18912	-0.18862	-0.18812	-0.18763	-0.18715
-0.1913	-0.1908	-0.1903	-0.18982	-0.18933	-0.18883	-0.18833	-0.18784	-0.18735
-0.19151	-0.191	-0.19051	-0.19002	-0.18953	-0.18904	-0.18854	-0.18805	-0.18756
-0.19172	-0.19121	-0.19071	-0.19022	-0.18974	-0.18925	-0.18876	-0.18827	-0.18778
-0.19194	-0.19143	-0.19092	-0.19043	-0.18995	-0.18946	-0.18897	-0.18848	-0.18799
-0.19218	-0.19165	-0.19114	-0.19064	-0.19015	-0.18967	-0.18918	-0.18869	-0.18821
-0.1924	-0.19188	-0.19136	-0.19086	-0.19036	-0.18987	-0.18939	-0.1889	-0.18842
-0.19261	-0.19209	-0.19158	-0.19106	-0.19056	-0.19007	-0.18959	-0.18911	-0.18863
-0.19282	-0.19231	-0.19179	-0.19128	-0.19077	-0.19028	-0.1898	-0.18931	-0.18883
-0.19304	-0.19253	-0.19202	-0.1915	-0.19099	-0.19049	-0.19	-0.18952	-0.18903
-0.19326	-0.19276	-0.19225	-0.19173	-0.19122	-0.19071	-0.19021	-0.18972	-0.18924
-0.19348	-0.19297	-0.19247	-0.19196	-0.19145	-0.19094	-0.19043	-0.18994	-0.18945
-0.19369	-0.19318	-0.19268	-0.19218	-0.19168	-0.19117	-0.19066	-0.19016	-0.18967
-0.19391	-0.1934	-0.19289	-0.19239	-0.1919	-0.1914	-0.19089	-0.19039	-0.18989
-0.19415	-0.19362	-0.1931	-0.19261	-0.19211	-0.19161	-0.19111	-0.19062	-0.19013
-0.19438	-0.19385	-0.19333	-0.19282	-0.19232	-0.19183	-0.19133	-0.19084	-0.19036
-0.19462	-0.19409	-0.19356	-0.19305	-0.19254	-0.19204	-0.19154	-0.19105	-0.19057
-0.19485	-0.19433	-0.1938	-0.19328	-0.19276	-0.19225	-0.19175	-0.19126	-0.19077
-0.12799	-0.12766	-0.19404	-0.19352	-0.19301	-0.19249	-0.19198	-0.19148	-0.19098
-0.12813	-0.12781	-0.19428	-0.19377	-0.19326	-0.19274	-0.19222	-0.19171	-0.1912
-0.12828	-0.12795	-0.1945	-0.194	-0.19349	-0.19298	-0.19245	-0.19194	-0.19143
-0.12841	-0.12809	-0.1947	-0.1942	-0.1937	-0.19319	-0.19268	-0.19217	-0.19168
-0.12856	-0.12824	-0.19492	-0.19442	-0.19391	-0.19341	-0.1929	-0.1924	-0.19191
-0.12872	-0.1284	-0.19515	-0.19464	-0.19413	-0.19362	-0.19312	-0.19262	-0.19212
-0.12888	-0.12856	-0.12823	-0.12789	-0.12755	-0.19384	-0.19334	-0.19284	-0.19233
-0.12902	-0.1287	-0.12838	-0.12805	-0.12772	-0.19409	-0.19359	-0.19308	-0.19257
-0.12915	-0.12884	-0.12853	-0.12821	-0.12789	-0.19434	-0.19384	-0.19333	-0.19281
-0.06272	-0.06258	-0.12867	-0.12835	-0.12804	-0.19457	-0.19407	-0.19355	-0.19304
-0.06279	-0.06265	-0.12881	-0.12849	-0.12818	-0.19479	-0.19428	-0.19377	-0.19326
-0.06285	-0.06272	-0.12896	-0.12864	-0.12833	-0.12801	-0.12769	-0.19401	-0.19349
-0.06292	-0.06279	-0.12912	-0.12881	-0.1285	-0.12818	-0.12786	-0.19427	-0.19374
-0.06299	-0.06286	-0.06272	-0.06259	-0.06245	-0.12834	-0.12802	-0.19451	-0.19398
-0.06305	-0.06292	-0.06279	-0.06265	-0.06252	-0.12848	-0.12817	-0.19474	-0.19421
-0.06312	-0.06298	-0.06285	-0.06272	-0.06258	-0.12862	-0.12831	-0.19497	-0.19444
-0.06319	-0.06305	-0.06292	-0.06279	-0.06266	-0.12878	-0.12847	-0.12815	-0.12782
-0.06327	-0.06313	-0.06299	-0.06286	-0.06273	-0.12894	-0.12863	-0.12831	-0.12798
-0.06334	-0.0632	-0.06306	-0.06292	-0.06279	-0.06266	-0.06253	-0.12846	-0.12814
-0.06341	-0.06327	-0.06312	-0.06299	-0.06285	-0.06272	-0.06259	-0.1286	-0.12828
-0.06349	-0.06334	-0.06319	-0.06305	-0.06292	-0.06279	-0.06266	-0.12875	-0.12844
-0.06358	-0.06342	-0.06327	-0.06313	-0.06299	-0.06286	-0.06273	-0.12891	-0.1286
-0.06366	-0.0635	-0.06335	-0.0632	-0.06306	-0.06293	-0.0628	-0.06267	-0.06253
-0.06373	-0.06357	-0.06342	-0.06327	-0.06313	-0.06299	-0.06286	-0.06273	-0.0626
-0.0638	-0.06365	-0.06349	-0.06334	-0.0632	-0.06306	-0.06292	-0.06279	-0.06266
		-0.06357	-0.06342	-0.06327	-0.06313	-0.06299	-0.06286	-0.06273
		-0.06366	-0.06351	-0.06335	-0.06321	-0.06307	-0.06293	-0.0628
		-0.06375	-0.06359	-0.06343	-0.06329	-0.06314	-0.06301	-0.06287
		-0.06383	-0.06368	-0.06352	-0.06337	-0.06322	-0.06308	-0.06295

-0.06345	-0.0633	-0.06315	-0.06302
-0.06352	-0.06337	-0.06322	-0.06308
-0.06359	-0.06344	-0.06329	-0.06314
-0.06367	-0.06352	-0.06337	-0.06322
-0.06376	-0.06361	-0.06345	-0.0633
		-0.06354	-0.06339
		-0.06362	-0.06347
		-0.0637	-0.06355
		-0.06378	-0.06363

							-0.06182	-0.06162
							-0.06173	-0.06153
							-0.25648	-0.25584
							-0.25616	-0.25552
					-0.06183	-0.06165	-0.38315	-0.3822
					-0.06174	-0.06155	-0.38268	-0.38173
					-0.06165	-0.06146	-0.3822	-0.38125
					-0.25621	-0.25555	-0.50513	-0.50382
					-0.25589	-0.25523	-0.50446	-0.50314
		-0.13138	-0.13095	-0.13053	-0.3208	-0.31995	-0.50379	-0.50248
		-0.13119	-0.13076	-0.13033	-0.32039	-0.31955	-0.50313	-0.50182
		-0.25695	-0.25627	-0.2556	-0.50515	-0.50381	-0.50248	-0.50117
		-0.25662	-0.25595	-0.25528	-0.50449	-0.50316	-0.50183	-0.50052
-0.13146	-0.13104	-0.32175	-0.32089	-0.32004	-0.50384	-0.50251	-0.66909	-0.66731
-0.13127	-0.13085	-0.32134	-0.32049	-0.31965	-0.50319	-0.50186	-0.66824	-0.66647
-0.13108	-0.13066	-0.32094	-0.32009	-0.31925	-0.50254	-0.50122	-0.6674	-0.66565
-0.25669	-0.25602	-0.50592	-0.50456	-0.50322	-0.50189	-0.50058	-0.71781	-0.71592
-0.25637	-0.2557	-0.50525	-0.5039	-0.50256	-0.50124	-0.49994	-0.71691	-0.71506
-0.32145	-0.32059	-0.50458	-0.50324	-0.50192	-0.6684	-0.66664	-0.716	-0.71416
-0.32103	-0.32018	-0.50391	-0.50258	-0.50127	-0.66755	-0.66578	-0.71507	-0.71324
-0.50596	-0.5046	-0.50326	-0.50195	-0.50065	-0.71798	-0.71607	-0.83659	-0.8345
-0.5053	-0.50395	-0.50263	-0.50134	-0.50006	-0.71716	-0.71527	-0.8357	-0.83359
-0.50467	-0.50333	-0.6704	-0.66866	-0.66693	-0.71633	-0.71445	-0.88389	-0.88165
-0.50408	-0.50275	-0.66964	-0.66788	-0.66614	-0.71546	-0.71358	-0.88286	-0.88062
-0.50351	-0.50218	-0.66888	-0.66711	-0.66535	-0.71457	-0.7127	-0.88181	-0.87958
-0.50288	-0.50156	-0.71942	-0.7175	-0.71558	-0.83608	-0.83395	-0.93084	-0.92847
-0.50222	-0.50091	-0.7185	-0.71659	-0.71469	-0.83508	-0.83295	-0.9297	-0.92733
-0.55482	-0.55339	-0.84049	-0.83833	-0.83619	-0.88308	-0.88084	-0.92854	-0.92618
-0.5541	-0.55267	-0.83944	-0.83728	-0.83514	-0.88198	-0.87975	-0.92737	-0.92501
-0.71955	-0.71763	-0.88762	-0.88537	-0.88314	-0.8809	-0.87867	-0.92622	-0.92386
-0.71863	-0.71671	-0.88655	-0.88431	-0.88207	-0.87983	-0.8776	-0.92508	-0.92272
-0.71773	-0.71583	-0.88553	-0.88328	-0.88104	-0.8788	-0.87657	-0.92399	-0.92162
-0.83981	-0.83766	-0.88458	-0.88234	-0.88009	-0.9277	-0.92533	-0.96655	-0.9641

-0.83893	-0.83678	-0.88366	-0.88141	-0.87917	-0.92671	-0.92435	-0.96553	-0.96309
-0.88714	-0.88489	-0.88265	-0.8804	-0.87816	-0.92563	-0.92327	-0.96441	-0.96198
-0.88608	-0.88382	-0.88158	-0.87934	-0.8771	-0.9245	-0.92214	-0.96323	-0.96081
-0.88501	-0.88276	-0.88051	-0.87827	-0.87604	-0.96696	-0.9645	-0.96207	-0.95965
-0.88395	-0.88169	-0.87944	-0.8772	-0.87497	-0.96577	-0.96333	-0.9609	-0.95849
-0.88291	-0.88066	-0.87841	-0.87617	-0.87394	-0.96463	-0.9622	-1.00957	-1.00702
-0.88195	-0.8797	-0.87746	-0.87522	-0.87299	-0.96358	-0.96116	-1.00847	-1.00594
-0.88099	-0.87874	-0.8765	-0.87427	-0.87205	-0.96253	-0.96011	-1.00738	-1.00486
-0.87994	-0.8777	-0.96874	-0.96628	-0.96383	-0.9614	-0.95898	-1.0062	-1.00368
-0.87887	-0.87663	-0.96755	-0.9651	-0.96266	-0.96024	-0.95783	-1.00498	-1.00247
-0.87783	-0.87558	-0.96637	-0.96392	-0.96149	-1.00884	-1.0063	-1.00378	-1.00127
-0.87682	-0.87454	-0.96518	-0.96274	-0.96032	-1.00761	-1.00508	-1.00257	-1.00007
-0.87582	-0.87354	-0.96406	-0.96162	-0.95922	-1.00645	-1.00394	-1.00144	-0.99895
-0.87485	-0.87261	-0.96304	-0.96061	-0.95821	-1.00539	-1.00289	-1.0004	-0.99792
-0.96691	-0.96444	-0.96199	-0.95958	-0.95718	-1.00432	-1.00182	-1.04364	-1.04099
-0.96578	-0.9633	-0.96084	-0.95844	-0.95606	-1.00314	-1.00065	-1.04242	-1.03978
-0.96465	-0.96215	-0.95969	-0.9573	-0.95494	-1.00197	-0.99949	-1.0412	-1.03855
-0.96356	-0.96109	-1.00838	-1.00586	-1.00337	-1.04538	-1.04272	-1.04007	-1.03742
-0.96248	-0.96006	-1.00733	-1.00482	-1.00233	-1.04427	-1.04163	-1.03898	-1.03632
-0.96137	-0.95897	-1.00619	-1.00369	-1.00121	-1.04309	-1.04045	-1.0378	-1.03515
-0.96023	-0.95781	-1.00497	-1.00248	-1.00001	-1.04182	-1.03918	-1.03655	-1.03392
-1.00889	-1.00633	-1.04858	-1.04591	-1.04327	-1.04062	-1.03797	-1.03534	-1.03273
-1.0078	-1.00526	-1.04745	-1.0448	-1.04216	-1.03951	-1.03685	-1.03422	-1.03161
-1.00673	-1.00421	-1.04634	-1.04368	-1.04104	-1.03839	-1.03572	-1.03309	-1.03048
-1.00567	-1.00313	-1.04518	-1.04249	-1.03982	-1.03716	-1.03451	-1.20916	-1.20567
-1.00461	-1.00205	-1.04402	-1.04129	-1.03858	-1.03591	-1.03328	-1.20748	-1.20403
-1.04831	-1.0456	-1.04288	-1.04015	-1.03743	-1.03476	-1.03214	-1.20595	-1.20252
-1.04708	-1.04442	-1.04176	-1.03905	-1.03634	-1.03368	-1.03106	-1.2045	-1.2011
-1.04592	-1.04328	-1.04063	-1.03794	-1.03525	-1.21001	-1.20646	-1.20299	-1.19961
-1.04483	-1.04216	-1.0395	-1.03683	-1.03416	-1.20851	-1.20492	-1.20143	-1.19802
-1.04373	-1.04104	-1.03835	-1.03569	-1.03303	-1.20697	-1.2034	-1.19991	-1.1965
-1.04262	-1.03989	-1.03717	-1.03448	-1.03183	-1.20537	-1.20189	-1.19847	-1.19509
-1.04148	-1.03872	-1.03598	-1.03329	-1.03064	-1.2038	-1.20041	-1.19705	-1.1937
-1.04027	-1.03754	-1.12521	-1.12204	-1.11891	-1.20234	-1.19898	-1.19562	-1.19228
-1.03903	-1.03635	-1.12385	-1.12073	-1.11766	-1.20094	-1.19756	-1.1942	-1.19085
-1.03786	-1.03523	-1.20988	-1.20639	-1.20294	-1.19951	-1.19612	-1.19276	-1.18942
-1.03675	-1.03415	-1.20845	-1.20497	-1.20152	-1.19808	-1.19466	-1.1913	-1.188
-1.12614	-1.12305	-1.20699	-1.20352	-1.20007	-1.19663	-1.19322	-1.27316	-1.26947
-1.12483	-1.12175	-1.20551	-1.20203	-1.19857	-1.19515	-1.19178	-1.27159	-1.26795
-1.16677	-1.16356	-1.20401	-1.20053	-1.19708	-1.19369	-1.19036	-1.27004	-1.26645
-1.16542	-1.1622	-1.20251	-1.19903	-1.19561	-1.19227	-1.189	-1.26853	-1.26497
-1.16406	-1.16084	-1.201	-1.19755	-1.19416	-1.19087	-1.18767	-1.26703	-1.2635
-1.16267	-1.15945	-1.1995	-1.1961	-1.19277	-1.27273	-1.26912	-1.26554	-1.262
-1.16126	-1.15805	-1.198	-1.19467	-1.19141	-1.27122	-1.26762	-1.26405	-1.26049
-1.20346	-1.19997	-1.19657	-1.19328	-1.19006	-1.2697	-1.26611	-1.26254	-1.25898
-1.20198	-1.19854	-1.19518	-1.19192	-1.18872	-1.26818	-1.26458	-1.261	-1.25746
-1.20053	-1.19714	-1.27757	-1.2739	-1.27029	-1.26668	-1.26308	-1.25952	-1.25598
-1.19911	-1.19577	-1.27605	-1.27241	-1.26881	-1.26522	-1.26166	-1.2581	-1.25455
-1.19771	-1.1944	-1.27455	-1.2709	-1.26731	-1.26375	-1.26022	-1.29719	-1.2935
-1.1963	-1.19302	-1.273	-1.26936	-1.26575	-1.2622	-1.2587	-1.29565	-1.292

-1.19492	-1.19165	-1.27147	-1.26781	-1.2642	-1.26065	-1.25716	-1.2941	-1.2905
-1.19357	-1.19032	-1.26997	-1.26633	-1.26272	-1.25917	-1.25568	-1.29258	-1.28903
-1.19224	-1.18901	-1.2685	-1.26488	-1.26129	-1.25774	-1.25424	-1.29109	-1.28756
-1.23567	-1.23212	-1.26704	-1.26344	-1.25986	-1.29682	-1.29319	-1.28963	-1.28613
-1.23419	-1.23068	-1.2656	-1.26202	-1.25844	-1.29535	-1.29172	-1.28819	-1.28473
-1.27123	-1.26768	-1.26416	-1.26059	-1.25703	-1.29389	-1.29028	-1.32402	-1.32054
-1.26974	-1.26622	-1.26272	-1.25916	-1.25561	-1.29244	-1.28884	-1.32258	-1.31918
-1.26826	-1.26476	-1.26129	-1.25774	-1.25419	-1.29099	-1.28741	-1.32117	-1.31783
-1.2668	-1.26333	-1.3005	-1.29682	-1.29315	-1.28955	-1.286	-1.31975	-1.31646
-1.26536	-1.2619	-1.29903	-1.29536	-1.2917	-1.28812	-1.28461	-1.31835	-1.31509
-1.30473	-1.30114	-1.29757	-1.29392	-1.29027	-1.32394	-1.32039	-1.317	-1.31377
-1.30322	-1.29966	-1.29611	-1.29248	-1.28886	-1.32253	-1.31905	-1.3157	-1.31247
-1.30173	-1.29819	-1.29466	-1.29105	-1.28746	-1.32114	-1.31771	-1.31441	-1.31124
-1.30026	-1.29673	-1.29322	-1.28963	-1.28607	-1.31974	-1.31637	-1.31314	-1.31009
-1.2988	-1.29528	-1.32908	-1.32544	-1.32183	-1.31838	-1.31505	-1.34142	-1.33834
-1.29733	-1.29383	-1.32762	-1.32402	-1.32047	-1.31706	-1.31378	-1.34013	-1.33708
-1.29588	-1.2924	-1.32618	-1.32263	-1.31913	-1.31577	-1.31253	-1.33886	-1.33582
-1.29448	-1.291	-1.32474	-1.32123	-1.31779	-1.34411	-1.34078	-1.3376	-1.3346
-1.29312	-1.28961	-1.32331	-1.31985	-1.31647	-1.34278	-1.3395	-1.33637	-1.3334
-1.29175	-1.28824	-1.35187	-1.34832	-1.34487	-1.34152	-1.33827	-1.33517	-1.33221
-1.29038	-1.28688	-1.35042	-1.34696	-1.34361	-1.34031	-1.33709	-1.33399	-1.33103
-1.32621	-1.32265	-1.34903	-1.34563	-1.34235	-1.33909	-1.3359	-1.33281	-1.32985
-1.3249	-1.32137	-1.3477	-1.34432	-1.34106	-1.33785	-1.33469	-1.33164	-1.32868
-1.3536	-1.34994	-1.34638	-1.34302	-1.33978	-1.3366	-1.3335	-1.33047	-1.32752
-1.35214	-1.34853	-1.34503	-1.3417	-1.33849	-1.33535	-1.33229	-1.32929	-1.32636
-1.35067	-1.34713	-1.34369	-1.3404	-1.33722	-1.33412	-1.3311	-1.32813	-1.32521
-1.34932	-1.34584	-1.34246	-1.33919	-1.33602	-1.33295	-1.32997	-1.32702	-1.3241
-1.34804	-1.34462	-1.34129	-1.33803	-1.33486	-1.33182	-1.32888	-1.32594	-1.32301
-1.34672	-1.34335	-1.34007	-1.33684	-1.3337	-1.33068	-1.32774	-1.36758	-1.36448
-1.34535	-1.34203	-1.33879	-1.33563	-1.33254	-1.32952	-1.32657	-1.36634	-1.36327
-1.34404	-1.34075	-1.33754	-1.33443	-1.3314	-1.32839	-1.32543	-1.36515	-1.36209
-1.34283	-1.33953	-1.33634	-1.33327	-1.33028	-1.3273	-1.32435	-1.364	-1.36096
-1.34162	-1.33834	-1.33516	-1.33212	-1.32916	-1.3262	-1.32327	-1.36288	-1.35983
-1.34035	-1.33713	-1.33398	-1.33095	-1.32799	-1.32507	-1.32219	-1.36177	-1.35869
-1.33909	-1.33592	-1.33282	-1.32979	-1.32681	-1.32392	-1.3211	-1.36065	-1.35755
-1.33788	-1.33476	-1.33169	-1.32867	-1.32569	-1.3228	-1.31997	-1.35947	-1.35638
-1.33673	-1.33363	-1.33059	-1.32758	-1.3246	-1.32168	-1.3188	-1.35824	-1.35519
-1.3356	-1.33252	-1.32949	-1.32649	-1.32352	-1.32059	-1.31768	-1.35705	-1.35403
-1.33449	-1.33141	-1.32838	-1.3254	-1.32246	-1.31953	-1.31661	-1.35592	-1.35292
-1.33338	-1.33031	-1.32729	-1.32432	-1.32139	-1.31847	-1.31555	-1.35481	-1.35182
-1.33227	-1.32922	-1.32622	-1.32325	-1.32032	-1.3174	-1.31451	-1.35373	-1.35072
-1.33115	-1.32814	-1.32516	-1.32218	-1.31924	-1.31634	-1.31348	-1.35264	-1.34962
-1.33005	-1.32704	-1.32407	-1.32111	-1.31818	-1.35757	-1.35454	-1.3892	-1.38608
-1.32897	-1.32595	-1.32297	-1.32004	-1.31713	-1.35646	-1.35341	-1.38803	-1.38492
-1.32787	-1.32486	-1.32189	-1.31896	-1.31606	-1.35533	-1.35227	-1.38686	-1.38378
-1.32676	-1.32377	-1.32082	-1.31788	-1.31497	-1.35418	-1.35114	-1.3857	-1.38266
-1.32567	-1.32269	-1.31974	-1.3168	-1.31388	-1.35304	-1.35002	-1.38456	-1.38154
-1.32459	-1.32161	-1.31865	-1.3157	-1.31278	-1.3519	-1.34892	-1.38345	-1.38043
-1.32351	-1.32053	-1.31758	-1.31463	-1.31171	-1.3508	-1.34784	-1.38235	-1.37933
-1.3224	-1.31946	-1.31654	-1.31362	-1.31073	-1.34975	-1.34676	-1.38123	-1.37825

-1.3213	-1.31839	-1.31551	-1.31263	-1.30977	-1.34873	-1.3457	-1.38013	-1.37718
-1.32023	-1.31733	-1.35672	-1.35371	-1.35072	-1.38525	-1.38214	-1.41664	-1.41356
-1.31918	-1.31628	-1.35561	-1.35262	-1.34966	-1.3842	-1.38113	-1.4156	-1.41245
-1.31812	-1.31523	-1.35451	-1.35153	-1.34858	-1.38311	-1.38008	-1.41453	-1.41139
-1.31703	-1.31417	-1.35344	-1.35046	-1.3475	-1.382	-1.37901	-1.41344	-1.41035
-1.31599	-1.31314	-1.35236	-1.34938	-1.34642	-1.41847	-1.4154	-1.41234	-1.40931
-1.31502	-1.31212	-1.35124	-1.34828	-1.34536	-1.41734	-1.41426	-1.41122	-1.40825
-1.31406	-1.31113	-1.35015	-1.3472	-1.3443	-1.4162	-1.41312	-1.41011	-1.40717
-1.31306	-1.31016	-1.38673	-1.38364	-1.3806	-1.41504	-1.412	-1.40901	-1.40608
-1.31205	-1.3092	-1.38571	-1.38258	-1.37949	-1.41389	-1.41089	-1.40792	-1.40499
-1.31102	-1.3082	-1.42231	-1.41909	-1.4159	-1.4128	-1.40978	-1.40682	-1.40392
-1.30997	-1.30718	-1.42119	-1.41803	-1.41488	-1.41177	-1.40869	-1.40572	-1.40286
-1.30897	-1.30616	-1.42004	-1.41692	-1.41383	-1.41073	-1.40765	-1.40468	-1.40184
-1.30801	-1.30516	-1.41885	-1.41577	-1.41273	-1.40969	-1.40667	-1.40372	-1.40086
-1.34605	-1.34305	-1.41772	-1.41465	-1.41165	-1.40866	-1.40568	-1.43125	-1.42829
-1.34509	-1.34209	-1.41667	-1.41361	-1.41061	-1.40764	-1.4047	-1.43026	-1.42726
-1.34413	-1.34114	-1.41566	-1.41258	-1.40957	-1.40661	-1.4037	-1.42926	-1.42622
-1.38059	-1.37755	-1.41463	-1.41154	-1.4085	-1.40555	-1.40267	-1.42825	-1.42522
-1.37952	-1.37652	-1.4136	-1.41049	-1.40743	-1.40448	-1.40162	-1.42722	-1.42424
-1.37842	-1.37547	-1.41255	-1.40946	-1.40641	-1.40345	-1.40056	-1.42616	-1.42323
-1.37731	-1.37439	-1.41148	-1.40844	-1.40542	-1.40243	-1.39948	-1.42507	-1.42219
-1.37625	-1.37336	-1.41043	-1.40743	-1.40444	-1.40144	-1.39846	-1.42403	-1.42118
-1.37526	-1.37238	-1.40941	-1.40643	-1.40347	-1.40048	-1.39749	-1.42306	-1.4202
-1.37429	-1.37141	-1.40841	-1.40544	-1.4025	-1.39952	-1.39654	-1.4221	-1.41922
-1.37332	-1.37045	-1.40742	-1.40447	-1.40153	-1.39856	-1.39557	-1.42112	-1.4182
-1.37236	-1.36949	-1.40642	-1.40348	-1.40055	-1.39759	-1.39461	-1.42014	-1.4172
-1.37138	-1.36849	-1.40536	-1.40244	-1.39954	-1.42521	-1.42218	-1.41919	-1.41624
-1.3704	-1.36748	-1.40428	-1.40137	-1.3985	-1.42421	-1.42122	-1.41826	-1.41531
-1.40934	-1.40626	-1.40325	-1.40034	-1.39748	-1.42321	-1.42027	-1.41733	-1.41439
-1.40835	-1.40527	-1.40226	-1.39935	-1.39649	-1.42223	-1.41932	-1.41639	-1.41346
-1.40736	-1.40429	-1.40128	-1.39837	-1.39551	-1.42126	-1.41837	-1.41546	-1.41254
-1.40638	-1.40332	-1.40031	-1.3974	-1.39455	-1.42031	-1.41743	-1.41453	-1.41161
-1.4054	-1.40234	-1.39934	-1.39644	-1.39359	-1.41936	-1.41649	-1.4136	-1.4107
-1.40441	-1.40136	-1.39836	-1.39547	-1.39264	-1.41842	-1.41556	-1.41269	-1.40979
-1.40342	-1.40037	-1.39739	-1.39451	-1.39169	-1.41748	-1.41464	-1.41177	-1.40889
-1.40243	-1.39938	-1.42521	-1.42227	-1.4194	-1.41655	-1.41371	-1.41086	-1.40799
-1.40144	-1.39839	-1.42422	-1.4213	-1.41845	-1.41561	-1.41279	-1.40996	-1.40709
-1.40046	-1.39742	-1.42326	-1.42035	-1.41751	-1.41468	-1.41187	-1.40904	-1.40619
-1.39948	-1.39646	-1.42232	-1.41942	-1.41657	-1.41374	-1.41094	-1.40812	-1.40529
-1.39851	-1.39552	-1.42141	-1.41851	-1.41567	-1.41283	-1.41002	-1.43982	-1.43695
-1.39754	-1.39462	-1.42056	-1.41766	-1.41481	-1.41196	-1.40913	-1.43894	-1.43607
-1.39658	-1.39372	-1.4197	-1.41682	-1.41395	-1.41109	-1.40824	-1.43806	-1.43519
-1.3956	-1.39276	-1.41878	-1.41591	-1.41306	-1.4102	-1.40735	-1.43717	-1.43431
-1.39463	-1.3918	-1.41783	-1.41498	-1.41215	-1.4093	-1.40645	-1.43629	-1.43343
-1.39374	-1.3909	-1.41693	-1.41407	-1.41124	-1.44117	-1.43827	-1.4354	-1.43257
-1.39289	-1.39005	-1.41606	-1.41318	-1.41032	-1.44026	-1.43737	-1.43452	-1.43171
-1.421	-1.41808	-1.41518	-1.41228	-1.4094	-1.43936	-1.43648	-1.48738	-1.48455
-1.42012	-1.41719	-1.41428	-1.41138	-1.4085	-1.43847	-1.4356	-1.48654	-1.48375
-1.41923	-1.41629	-1.41338	-1.41048	-1.40761	-1.49148	-1.48856	-1.48572	-1.48296
-1.4183	-1.41537	-1.41246	-1.40957	-1.40672	-1.49064	-1.48777	-1.48495	-1.48219

-1.41736	-1.41443	-1.41153	-1.40867	-1.40584	-1.48981	-1.48699	-1.48419	-1.48142
-1.41643	-1.41351	-1.49778	-1.4948	-1.49187	-1.48901	-1.48619	-1.50946	-1.50663
-1.41552	-1.4126	-1.49689	-1.49395	-1.49106	-1.48821	-1.48539	-1.50866	-1.50582
-1.46993	-1.46694	-1.49607	-1.49313	-1.49024	-1.48739	-1.48457	-1.50784	-1.50499
-1.46912	-1.46616	-1.49532	-1.49235	-1.48942	-1.48655	-1.48372	-1.50699	-1.50414
-1.46829	-1.46535	-1.49452	-1.49155	-1.4886	-1.5119	-1.50902	-1.50616	-1.50331
-1.46742	-1.46447	-1.49366	-1.4907	-1.48777	-1.51108	-1.50819	-1.50533	-1.50248
-1.46653	-1.46358	-1.49278	-1.48985	-1.48696	-1.51029	-1.5074	-1.50453	-1.50169
-1.49787	-1.49486	-1.4919	-1.48901	-1.48617	-1.50955	-1.50669	-1.50384	-1.50099
-1.49699	-1.49398	-1.49104	-1.48819	-1.48539	-1.50882	-1.506	-1.50316	-1.50031
-1.4962	-1.49319	-1.49025	-1.4874	-1.48459	-1.50803	-1.50522	-1.50239	-1.49954
-1.49546	-1.49246	-1.48952	-1.48663	-1.48379	-1.50721	-1.50438	-1.50155	-1.49872
-1.49467	-1.49168	-1.48873	-1.48584	-1.48298	-1.50639	-1.50356	-1.50073	-1.49793
-1.49382	-1.49084	-1.4879	-1.48501	-1.48216	-1.50558	-1.50273	-1.49992	-1.49715
-1.49299	-1.49003	-1.4871	-1.48422	-1.48138	-1.5048	-1.50195	-1.49914	-1.49639
-1.49224	-1.48929	-1.48639	-1.48351	-1.48067	-1.50411	-1.50126	-1.49844	-1.49565
-1.49148	-1.48856	-1.48566	-1.4828	-1.47996	-1.50341	-1.50057	-1.49773	-1.4949
-1.49065	-1.48774	-1.48485	-1.48199	-1.47915	-1.50262	-1.49977	-1.49694	-1.49412
-1.4898	-1.48689	-1.48401	-1.48116	-1.47833	-1.5018	-1.49895	-1.49612	-1.49332
-1.48904	-1.48614	-1.48326	-1.48041	-1.47758	-1.50107	-1.49821	-1.49539	-1.49259
-1.48835	-1.48545	-1.48258	-1.47972	-1.47689	-1.50039	-1.49753	-1.4947	-1.4919
-1.48759	-1.4847	-1.48183	-1.47899	-1.47615	-1.49966	-1.49681	-1.49398	-1.49117
-1.48674	-1.48387	-1.48102	-1.47817	-1.47534	-1.49886	-1.49602	-1.49319	-1.49038
-1.48591	-1.48306	-1.48022	-1.47738	-1.47455	-1.49809	-1.49525	-1.49243	-1.48962
-1.48518	-1.48233	-1.4795	-1.47667	-1.47386	-1.49741	-1.49458	-1.49177	-1.48896
-1.48446	-1.48161	-1.47878	-1.47596	-1.47316	-1.49674	-1.49393	-1.49112	-1.48832
-1.48367	-1.48081	-1.50449	-1.50163	-1.49879	-1.49599	-1.49321	-1.49044	-1.48766
-1.48285	-1.47999	-1.50367	-1.5008	-1.49797	-1.4952	-1.49247	-1.48973	-1.48699
-1.4821	-1.47925	-1.50294	-1.50007	-1.49723	-1.49446	-1.49173	-1.52101	-1.51817
-1.48142	-1.47857	-1.50229	-1.49942	-1.49657	-1.49376	-1.49098	-1.52018	-1.51734
-1.50738	-1.5045	-1.50163	-1.49876	-1.49591	-1.49307	-1.49026	-1.51941	-1.51655
-1.50673	-1.50384	-1.50097	-1.49811	-1.49526	-1.49243	-1.48961	-1.51872	-1.51584
-1.50604	-1.50315	-1.50027	-1.49741	-1.49457	-1.49175	-1.48894	-1.518	-1.51512
-1.50523	-1.50235	-1.49948	-1.49663	-1.49379	-1.49097	-1.48816	-1.51719	-1.51431
-1.5044	-1.50154	-1.49868	-1.49583	-1.49299	-1.49017	-1.48737	-1.51636	-1.51348
-1.50368	-1.50081	-1.49796	-1.49512	-1.49228	-1.52142	-1.51851	-1.51562	-1.51274
-1.50301	-1.50014	-1.49729	-1.49446	-1.49164	-1.52074	-1.51783	-1.51493	-1.51205
-1.50236	-1.49949	-1.49664	-1.49381	-1.491	-1.52007	-1.51716	-1.51426	-1.51137
-1.50172	-1.49885	-1.49599	-1.49316	-1.49035	-1.51939	-1.51649	-1.5136	-1.5107
-1.50108	-1.49821	-1.49535	-1.49252	-1.48971	-1.51872	-1.51582	-1.51293	-1.51004
-1.50042	-1.49756	-1.49472	-1.49189	-1.48907	-1.51804	-1.51514	-1.51225	-1.50937
-1.49976	-1.49691	-1.49408	-1.49125	-1.48843	-1.51737	-1.51446	-1.51157	-1.50869
-1.49912	-1.49628	-1.52547	-1.52254	-1.51961	-1.51669	-1.51378	-1.51088	-1.508
-1.49849	-1.49564	-1.5248	-1.52186	-1.51893	-1.516	-1.51308	-1.51018	-1.5073
-1.49784	-1.49499	-1.52412	-1.52117	-1.51823	-1.51528	-1.51234	-1.50941	-1.50652
-1.49718	-1.49433	-1.52343	-1.52047	-1.51752	-1.51454	-1.51155	-1.5086	-1.50568
-1.49651	-1.49364	-1.52269	-1.51972	-1.51675	-1.51377	-1.51078	-1.50782	-1.50489
-1.49582	-1.49292	-1.52188	-1.5189	-1.51593	-1.51296	-1.51001	-1.50708	-1.50417
-1.49512	-1.49219	-1.52109	-1.5181	-1.51512	-1.51218	-1.50927	-1.50636	-1.50347
-1.49439	-1.49147	-1.52034	-1.51735	-1.51439	-1.51146	-1.50855	-1.50566	-1.50278

-1.49367	-1.49075	-1.5196	-1.51663	-1.51368	-1.51075	-1.50785	-1.50497	-1.5021
-1.52492	-1.52188	-1.51888	-1.51591	-1.51298	-1.51006	-1.50716	-1.50428	-1.50141
-1.52423	-1.52118	-1.51817	-1.51522	-1.5123	-1.50938	-1.50648	-1.50359	-1.50072
-1.52358	-1.52052	-1.51749	-1.51453	-1.5116	-1.50868	-1.50578	-1.53612	-1.53305
-1.52298	-1.5199	-1.51685	-1.51385	-1.5109	-1.50797	-1.50508	-1.53538	-1.53232
-1.52234	-1.51926	-1.5162	-1.51318	-1.51019	-1.50727	-1.50439	-1.53463	-1.53158
-1.52164	-1.51858	-1.51553	-1.51249	-1.50949	-1.50656	-1.50369	-1.53388	-1.53083
-1.52094	-1.51791	-1.51487	-1.51183	-1.50881	-1.50588	-1.503	-1.53314	-1.53009
-1.52027	-1.51726	-1.51425	-1.5112	-1.50817	-1.50521	-1.50231	-1.53238	-1.52934
-1.5196	-1.51662	-1.51363	-1.51059	-1.50755	-1.50457	-1.50163	-1.53163	-1.52861
-1.5189	-1.51594	-1.51297	-1.50996	-1.50695	-1.50396	-1.50099	-1.53092	-1.52787
-1.51818	-1.51523	-1.51228	-1.50932	-1.50636	-1.50338	-1.50039	-1.53024	-1.52715
-1.51746	-1.51452	-1.51158	-1.50865	-1.50572	-1.50276	-1.49979	-1.52958	-1.52647
-1.51675	-1.5138	-1.51086	-1.50794	-1.50503	-1.50212	-1.4992	-1.52897	-1.52584
-1.51604	-1.51309	-1.51015	-1.50723	-1.50434	-1.53449	-1.53143	-1.52833	-1.5252
-1.51532	-1.51238	-1.50944	-1.50654	-1.50364	-1.53376	-1.53071	-1.52764	-1.52456
-1.51462	-1.51167	-1.50874	-1.50583	-1.50295	-1.53301	-1.52997	-1.52693	-1.5239
-1.51395	-1.51097	-1.50802	-1.50511	-1.50224	-1.53226	-1.52923	-1.5262	-1.52319
-1.5133	-1.51028	-1.5073	-1.5044	-1.50153	-1.5315	-1.52848	-1.52546	-1.52246
-1.51268	-1.50964	-1.50664	-1.50372	-1.50084	-1.53076	-1.52773	-1.52472	-1.52172
-1.51208	-1.50904	-1.50602	-1.50307	-1.50017	-1.53002	-1.52698	-1.52397	-1.52097
-1.51144	-1.50842	-1.50542	-1.50247	-1.49954	-1.52934	-1.52629	-1.52325	-1.52024
-1.51076	-1.5078	-1.50484	-1.50191	-1.49899	-1.52874	-1.52565	-1.52258	-1.51954
-1.51007	-1.50715	-1.50423	-1.50132	-1.49842	-1.55338	-1.55022	-1.54708	-1.54396
-1.50935	-1.50644	-1.50354	-1.50065	-1.49778	-1.55271	-1.54958	-1.54646	-1.54335
-1.50866	-1.50574	-1.50284	-1.49996	-1.49711	-1.552	-1.54892	-1.54583	-1.54273
-1.53401	-1.531	-1.5606	-1.55746	-1.55434	-1.55125	-1.54817	-1.5451	-1.54203
-1.53343	-1.5304	-1.55992	-1.55674	-1.55359	-1.55048	-1.5474	-1.54433	-1.54127
-1.5328	-1.52978	-1.55926	-1.55609	-1.55293	-1.5498	-1.5467	-1.54361	-1.54054
-1.53213	-1.52915	-1.55862	-1.55548	-1.55234	-1.5492	-1.54605	-1.54292	-1.53982
-1.5315	-1.52853	-1.55797	-1.55484	-1.55171	-1.54856	-1.5454	-1.54226	-1.53914
-1.53092	-1.52792	-1.55729	-1.55413	-1.55099	-1.54786	-1.54474	-1.54163	-1.53853
-1.53033	-1.5273	-1.55662	-1.55344	-1.55029	-1.54717	-1.54408	-1.541	-1.53792
-1.52967	-1.52668	-1.55598	-1.55283	-1.54968	-1.54655	-1.54343	-1.54032	-1.53722
-1.52898	-1.52604	-1.55534	-1.55221	-1.54908	-1.54593	-1.54278	-1.53964	-1.53651
-1.56089	-1.55775	-1.55463	-1.55151	-1.54839	-1.54526	-1.54212	-1.56493	-1.56177
-1.5602	-1.55702	-1.55387	-1.55076	-1.54766	-1.54455	-1.54145	-1.56431	-1.56119
-1.55955	-1.55636	-1.5532	-1.55009	-1.547	-1.54389	-1.54079	-1.58907	-1.5859
-1.55894	-1.55576	-1.55261	-1.5495	-1.5464	-1.54327	-1.54013	-1.58839	-1.5852
-1.55833	-1.55516	-1.552	-1.54888	-1.54576	-1.54262	-1.53946	-1.58772	-1.58452
-1.55773	-1.55453	-1.55135	-1.54818	-1.54504	-1.5419	-1.53879	-1.58708	-1.58391
-1.55711	-1.5539	-1.55069	-1.5475	-1.54432	-1.5412	-1.53811	-1.58644	-1.58328
-1.55642	-1.55324	-1.55006	-1.54687	-1.54369	-1.59214	-1.58894	-1.58576	-1.5826
-1.55572	-1.55258	-1.54943	-1.54626	-1.54308	-1.59152	-1.58828	-1.58508	-1.58191
-1.5551	-1.55196	-1.54881	-1.54564	-1.54245	-1.59089	-1.58764	-1.58444	-1.58128
-1.55452	-1.55137	-1.5482	-1.54501	-1.54181	-1.59025	-1.58703	-1.58384	-1.5807
-1.55393	-1.55075	-1.59947	-1.59616	-1.59287	-1.58962	-1.58642	-1.61174	-1.60853
-1.55333	-1.55012	-1.59881	-1.59551	-1.59224	-1.58902	-1.58583	-1.61115	-1.60796
-1.55271	-1.54948	-1.59816	-1.59487	-1.59162	-1.58841	-1.58523	-1.61054	-1.60735
-1.55207	-1.54884	-1.59752	-1.59425	-1.59101	-1.5878	-1.5846	-1.60988	-1.60666

-1.55142	-1.54819	-1.5969	-1.59364	-1.59041	-1.58718	-1.58397	-1.60921	-1.60596
-1.60288	-1.59957	-1.59628	-1.59301	-1.58976	-1.58654	-1.58334	-1.60857	-1.60534
-1.60226	-1.59897	-1.59569	-1.59239	-1.58911	-1.58589	-1.58272	-1.60796	-1.60476
-1.60166	-1.59838	-1.59511	-1.59181	-1.58853	-1.58531	-1.58214	-1.60737	-1.60417
-1.60108	-1.59782	-1.59455	-1.59128	-1.58801	-1.58478	-1.58158	-1.60679	-1.6036
-1.60049	-1.59722	-1.59396	-1.5907	-1.58745	-1.58422	-1.58103	-1.60621	-1.603
-1.59988	-1.59658	-1.5933	-1.59006	-1.58683	-1.58363	-1.58046	-1.60562	-1.60238
-1.59925	-1.59594	-1.59265	-1.58942	-1.58622	-1.58305	-1.5799	-1.60504	-1.60177
-1.5986	-1.5953	-1.59204	-1.58883	-1.58565	-1.58249	-1.57935	-1.60449	-1.60122
-1.59796	-1.59468	-1.59144	-1.58825	-1.58509	-1.58194	-1.5788	-1.60393	-1.60068
-1.59738	-1.5941	-1.59087	-1.58769	-1.58454	-1.58137	-1.5782	-1.60331	-1.60008
-1.59683	-1.59355	-1.59031	-1.58714	-1.58399	-1.5808	-1.57759	-1.60267	-1.59945
-1.59628	-1.59299	-1.58976	-1.58659	-1.58344	-1.60863	-1.60533	-1.60206	-1.59885
-1.59572	-1.59244	-1.5892	-1.58604	-1.58289	-1.60807	-1.60476	-1.6015	-1.59827
-1.59516	-1.59189	-1.58866	-1.58549	-1.58235	-1.60751	-1.60421	-1.60094	-1.59771
-1.59462	-1.59135	-1.58812	-1.58496	-1.58181	-1.60696	-1.60366	-1.6004	-1.59716
-1.59407	-1.59081	-1.58758	-1.58442	-1.58127	-1.60641	-1.60311	-1.59985	-1.59662
-1.59352	-1.59025	-1.58703	-1.58386	-1.58072	-1.60584	-1.60255	-1.59929	-1.59606
-1.59296	-1.58969	-1.58647	-1.58331	-1.58016	-1.60527	-1.60198	-1.59872	-1.59549
-1.5924	-1.58914	-1.58592	-1.58276	-1.57961	-1.60471	-1.60142	-1.62313	-1.61986
-1.59186	-1.5886	-1.58538	-1.58221	-1.57906	-1.60415	-1.60087	-1.62258	-1.6193
-1.59132	-1.58805	-1.58483	-1.58166	-1.57852	-1.60359	-1.60031	-1.62202	-1.61875
-1.59078	-1.58751	-1.58427	-1.58111	-1.57797	-1.60303	-1.59976	-1.62146	-1.61819
-1.59027	-1.58698	-1.58373	-1.58056	-1.57742	-1.62752	-1.6242	-1.6209	-1.61764
-1.5898	-1.58649	-1.58322	-1.58003	-1.57687	-1.62695	-1.62364	-1.62034	-1.61707
-1.58932	-1.586	-1.58271	-1.5795	-1.57632	-1.62639	-1.62309	-1.6198	-1.61651
-1.5888	-1.58548	-1.58219	-1.57897	-1.57579	-1.62585	-1.6226	-1.61933	-1.61602
-1.58824	-1.58493	-1.58165	-1.57843	-1.57526	-1.62534	-1.62213	-1.61887	-1.61556
-1.58768	-1.58439	-1.63467	-1.63133	-1.62803	-1.6248	-1.62161	-1.61837	-1.61506
-1.58712	-1.58383	-1.63411	-1.63077	-1.62746	-1.62424	-1.62107	-1.61783	-1.61453
-1.58657	-1.58331	-1.63359	-1.63025	-1.62693	-1.6237	-1.62052	-1.61729	-1.61402
-1.58603	-1.58282	-1.63312	-1.62978	-1.62645	-1.62318	-1.61996	-1.61674	-1.61351
-1.58648	-1.5833	-1.63264	-1.6293	-1.62597	-1.62269	-1.61943	-1.6162	-1.613
-1.58599	-1.5828	-1.6321	-1.6288	-1.62552	-1.62223	-1.61894	-1.61568	-1.61246
-1.58553	-1.5823	-1.63155	-1.62829	-1.62507	-1.62178	-1.61846	-1.61517	-1.61192
-1.58508	-1.58183	-1.63103	-1.62777	-1.62454	-1.62125	-1.61794	-1.61465	-1.61137
-1.58465	-1.58138	-1.63053	-1.62724	-1.62398	-1.62069	-1.6174	-1.61411	-1.61082
-1.58417	-1.58092	-1.60595	-1.60271	-1.59948	-1.62017	-1.6169	-1.61361	-1.61031
-1.58365	-1.58046	-1.60552	-1.60226	-1.59901	-1.61968	-1.61642	-1.61314	-1.60982
-1.58315	-1.57998	-1.60505	-1.60178	-1.59852	-1.61918	-1.61592	-1.61264	-1.60934
-1.58267	-1.57948	-1.60452	-1.60126	-1.598	-1.61864	-1.61537	-1.61211	-1.60887
-1.58221	-1.57898	-1.60399	-1.60073	-1.5975	-1.61812	-1.61482	-1.61157	-1.60838
-1.61011	-1.6068	-1.60351	-1.60026	-1.59703	-1.59379	-1.59054	-1.61107	-1.60785
-1.60967	-1.60635	-1.60305	-1.5998	-1.59657	-1.59333	-1.59009	-1.61058	-1.60732
-1.60922	-1.6059	-1.60261	-1.59935	-1.59611	-1.59287	-1.58963	-1.61011	-1.60683
-1.60877	-1.60547	-1.60218	-1.59891	-1.59565	-1.59241	-1.58918	-1.60965	-1.60636
-1.60832	-1.60503	-1.60174	-1.59846	-1.5952	-1.59196	-1.58873	-1.60918	-1.6059
-1.60789	-1.60458	-1.60129	-1.59802	-1.59477	-1.59152	-1.58827	-1.60872	-1.60544
-1.60744	-1.60414	-1.60084	-1.59758	-1.59434	-1.59108	-1.58783	-1.58459	-1.58138
-1.60697	-1.60368	-1.6004	-1.59713	-1.59388	-1.59063	-1.58739	-1.58416	-1.58094

-1.6065	-1.60322	-1.59995	-1.59668	-1.59342	-1.59018	-1.58695	-1.58373	-1.58049
-1.60607	-1.60278	-1.5995	-1.59623	-1.59297	-1.58973	-1.58649	-1.58326	-1.58004
-1.60564	-1.60234	-1.59905	-1.59578	-1.59253	-1.58927	-1.58602	-1.58279	-1.57959
-1.6052	-1.6019	-1.59861	-1.59534	-1.59208	-1.58882	-1.58557	-1.58234	-1.57913
-1.60474	-1.60146	-1.59819	-1.5949	-1.59163	-1.58837	-1.58513	-1.5819	-1.57868
-1.60429	-1.60102	-1.59775	-1.59447	-1.59119	-1.58793	-1.58469	-1.58146	-1.57824
-1.60385	-1.60056	-1.59729	-1.59402	-1.59076	-1.5875	-1.58424	-1.58101	-1.57781
-1.60341	-1.60012	-1.59684	-1.59359	-1.59035	-1.58709	-1.58382	-1.58059	-1.5774
-1.60297	-1.59969	-1.59643	-1.59319	-1.58997	-1.58672	-1.58346	-1.58024	-1.57703
-1.60255	-1.59928	-1.59603	-1.5928	-1.58958	-1.58635	-1.58311	-1.57988	-1.57666
-1.60218	-1.5989	-1.59564	-1.59239	-1.58916	-1.58592	-1.58268	-1.57946	-1.57625
-1.60183	-1.59854	-1.59525	-1.59198	-1.58872	-1.58547	-1.58223	-1.57901	-1.57581
-1.60144	-1.59814	-1.59484	-1.59156	-1.58829	-1.58503	-1.58178	-1.57856	-1.57537
-1.601	-1.59769	-1.5944	-1.59113	-1.58787	-1.5846	-1.58133	-1.57811	-1.57493
-1.60058	-1.59727	-1.59398	-1.59071	-1.58745	-1.58419	-1.58092	-1.5777	-1.57452
-1.6002	-1.5969	-1.5936	-1.59032	-1.58705	-1.5838	-1.58056	-1.57734	-1.57416
-1.5998	-1.59651	-1.59322	-1.58993	-1.58664	-1.5834	-1.58018	-1.57698	-1.5738
-1.59936	-1.59607	-1.59279	-1.58949	-1.5862	-1.58296	-1.57975	-1.57657	-1.57341
-1.5989	-1.59563	-1.59235	-1.58905	-1.58576	-1.58251	-1.5793	-1.57613	-1.57301
-1.59851	-1.59523	-1.59195	-1.58866	-1.58537	-1.58212	-1.57891	-1.57572	-1.57259
-1.59815	-1.59486	-1.59158	-1.5883	-1.58503	-1.58177	-1.57853	-1.57533	-1.57215
-1.59779	-1.59449	-1.59121	-1.58794	-1.58468	-1.58142	-1.57817	-1.57495	-1.57176
-1.59744	-1.59414	-1.59085	-1.58758	-1.58432	-1.58107	-1.57783	-1.57461	-1.57141
-1.59709	-1.59378	-1.59049	-1.58722	-1.58396	-1.58071	-1.57748	-1.57426	-1.57106
-1.59673	-1.59343	-1.59014	-1.58687	-1.58361	-1.58036	-1.57713	-1.57391	-1.57071
-1.59637	-1.59307	-1.58979	-1.58651	-1.58325	-1.58	-1.57677	-1.57355	-1.57035
-1.59599	-1.5927	-1.58942	-1.58615	-1.58289	-1.57964	-1.5764	-1.59681	-1.59356
-1.59561	-1.59231	-1.58903	-1.58577	-1.58252	-1.57927	-1.57602	-1.59642	-1.59317
-1.5952	-1.5919	-1.58861	-1.58535	-1.5821	-1.57885	-1.5756	-1.59599	-1.59273
-1.59476	-1.59145	-1.58816	-1.5849	-1.58165	-1.57839	-1.57514	-1.59553	-1.59227
-1.59439	-1.59109	-1.5878	-1.58453	-1.58127	-1.578	-1.57474	-1.59511	-1.59185
-1.5941	-1.59082	-1.58754	-1.58426	-1.58098	-1.57769	-1.57441	-1.59476	-1.59147
-1.59381	-1.59054	-1.58727	-1.58398	-1.58068	-1.57739	-1.5741	-1.59443	-1.59113
-1.59348	-1.5902	-1.58693	-1.58365	-1.58036	-1.57709	-1.57382	-1.59416	-1.59085
-1.59313	-1.58985	-1.58657	-1.58329	-1.58002	-1.57677	-1.57353	-1.59388	-1.59058
-1.59277	-1.58949	-1.58621	-1.58294	-1.57967	-1.57643	-1.57319	-1.59354	-1.59026
-1.59241	-1.58912	-1.58585	-1.58258	-1.57931	-1.57606	-1.57282	-1.59317	-1.58991
-1.59205	-1.58876	-1.58548	-1.58221	-1.57895	-1.59939	-1.59609	-1.59281	-1.58955
-1.59169	-1.5884	-1.58512	-1.58184	-1.57858	-1.59902	-1.59572	-1.59244	-1.58918
-1.59134	-1.58805	-1.58476	-1.58149	-1.57823	-1.59866	-1.59537	-1.62002	-1.61667
-1.59099	-1.58769	-1.58441	-1.58114	-1.57788	-1.59831	-1.59501	-1.61966	-1.6163
-1.59066	-1.58736	-1.58408	-1.58082	-1.57756	-1.59798	-1.59468	-1.61931	-1.61596
-1.59039	-1.58708	-1.5838	-1.58053	-1.57728	-1.5977	-1.59439	-1.61902	-1.61568
-1.59012	-1.58681	-1.58352	-1.58026	-1.57701	-1.59742	-1.59411	-1.61873	-1.6154
-1.5898	-1.58649	-1.60705	-1.60372	-1.60041	-1.5971	-1.5938	-1.61841	-1.61507
-1.58945	-1.58616	-1.60672	-1.60338	-1.60006	-1.59675	-1.59347	-1.61807	-1.61471
-1.58915	-1.58586	-1.60641	-1.60307	-1.59974	-1.59644	-1.59317	-1.61776	-1.61439
-1.58889	-1.5856	-1.60614	-1.6028	-1.59947	-1.59618	-1.5929	-1.61749	-1.61412
-1.58863	-1.58533	-1.60586	-1.60252	-1.59918	-1.59589	-1.59262	-1.61719	-1.61382
-1.58839	-1.58508	-1.60559	-1.60222	-1.59887	-1.59555	-1.59227	-1.61685	-1.61349

-1.58813	-1.58481	-1.60531	-1.60192	-1.59855	-1.59523	-1.59194	-1.61651	-1.61316
-1.5878	-1.58449	-1.605	-1.60163	-1.59828	-1.59496	-1.59166	-1.61622	-1.61288
-1.58744	-1.58416	-1.60469	-1.60135	-1.59802	-1.59471	-1.59142	-1.61596	-1.6126
-1.58715	-1.58388	-1.60441	-1.60108	-1.59776	-1.59446	-1.59116	-1.61569	-1.61233
-1.5869	-1.58363	-1.60415	-1.60083	-1.59751	-1.59421	-1.59092	-1.61543	-1.61206
-1.61056	-1.60722	-1.6039	-1.60058	-1.59726	-1.59396	-1.59065	-1.61515	-1.61177
-1.61032	-1.60697	-1.60363	-1.60032	-1.59701	-1.5937	-1.59038	-1.61485	-1.61146
-1.61007	-1.60672	-1.60338	-1.60006	-1.59675	-1.59343	-1.5901	-1.61455	-1.61114
-1.60981	-1.60647	-1.60314	-1.59982	-1.5965	-1.59315	-1.58979	-1.61423	-1.61084
-1.60955	-1.60623	-1.60291	-1.59958	-1.59624	-1.59288	-1.5895	-1.61392	-1.61056
-1.6093	-1.60597	-1.60264	-1.59932	-1.59599	-1.59263	-1.58926	-1.64377	-1.6403
-1.60904	-1.6057	-1.60237	-1.59904	-1.59573	-1.5924	-1.58906	-1.64355	-1.64004
-1.60878	-1.60544	-1.6021	-1.59878	-1.59546	-1.59213	-1.58879	-1.64326	-1.63976
-1.60852	-1.60518	-1.60185	-1.59852	-1.59519	-1.59183	-1.58846	-1.64292	-1.63947
-1.60825	-1.60492	-1.60159	-1.59826	-1.59493	-1.59157	-1.58819	-1.64263	-1.63919
-1.60799	-1.60465	-1.60131	-1.598	-1.59468	-1.59134	-1.588	-1.64241	-1.63892
-1.60773	-1.60438	-1.60104	-1.59773	-1.59443	-1.59112	-1.5878	-1.6422	-1.63867
-1.60745	-1.60411	-1.60079	-1.59747	-1.59416	-1.59086	-1.58756	-1.64197	-1.63847
-1.60718	-1.60385	-1.60053	-1.59721	-1.59388	-1.59059	-1.58731	-1.64174	-1.63828
-1.60697	-1.60361	-1.60027	-1.59694	-1.59363	-1.59033	-1.58705	-1.64146	-1.63803
-1.60681	-1.60339	-1.6	-1.59669	-1.5934	-1.59009	-1.58678	-1.64117	-1.63776
-1.60662	-1.60319	-1.59979	-1.59647	-1.59319	-1.62047	-1.61707	-1.64093	-1.6375
-1.60639	-1.603	-1.59963	-1.59631	-1.593	-1.62028	-1.6169	-1.64073	-1.63724
-1.60616	-1.6028	-1.59945	-1.59612	-1.5928	-1.62007	-1.6167	-1.64052	-1.63701
-1.60588	-1.60255	-1.59922	-1.59588	-1.59254	-1.6198	-1.61645	-1.64029	-1.63681
-1.60563	-1.60231	-1.59899	-1.59564	-1.59229	-1.61954	-1.61618	-1.64004	-1.63661
-1.60545	-1.60212	-1.5988	-1.59545	-1.59211	-1.61932	-1.61591	-1.63976	-1.63634
-1.6053	-1.60195	-1.59861	-1.59527	-1.59194	-1.61911	-1.61565	-1.63946	-1.63607
-1.60514	-1.60175	-1.59838	-1.59505	-1.59173	-1.61889	-1.61543	-1.63924	-1.63584
-1.60497	-1.60153	-1.59813	-1.5948	-1.59149	-1.61868	-1.61525	-1.63905	-1.63564
-1.60479	-1.60134	-1.59792	-1.59459	-1.59129	-1.61847	-1.61507	-1.63886	-1.63542
-1.60458	-1.60117	-1.59777	-1.59443	-1.59111	-1.61829	-1.61488	-1.63866	-1.63517
-1.60439	-1.601	-1.62837	-1.62493	-1.62151	-1.6181	-1.6147	-1.63846	-1.63495
-1.60421	-1.60084	-1.6282	-1.62476	-1.62133	-1.61793	-1.61454	-1.63829	-1.63476
-1.60405	-1.60067	-1.62803	-1.62458	-1.62114	-1.61774	-1.61436	-1.63812	-1.63459
-1.6039	-1.60052	-1.65545	-1.65187	-1.64831	-1.64482	-1.64137	-1.6379	-1.6344
-1.60376	-1.60036	-1.65526	-1.65164	-1.64803	-1.64453	-1.64111	-1.63766	-1.6342
-1.60361	-1.60022	-1.65509	-1.65144	-1.64782	-1.64431	-1.64088	-1.63745	-1.63401
-1.60347	-1.60007	-1.65493	-1.65128	-1.64764	-1.64413	-1.64069	-1.63726	-1.63383
-1.63417	-1.63069	-1.65476	-1.65111	-1.64748	-1.64395	-1.64051	-1.63708	-1.63365
-1.63401	-1.63053	-1.65459	-1.65094	-1.64731	-1.64379	-1.64034	-1.6369	-1.63348
-1.63384	-1.63036	-1.65442	-1.65078	-1.64715	-1.64362	-1.64017	-1.63673	-1.6333
-1.63366	-1.63019	-1.65426	-1.65062	-1.64699	-1.64346	-1.64001	-1.63656	-1.63313
-1.63349	-1.63003	-1.6541	-1.65047	-1.64685	-1.64332	-1.63985	-1.6364	-1.63296
-1.63332	-1.62986	-1.65394	-1.65034	-1.64675	-1.64323	-1.63974	-1.63627	-1.63281
-1.63314	-1.62969	-1.65378	-1.65022	-1.64668	-1.64316	-1.63966	-1.63616	-1.63266
-1.63297	-1.62952	-1.65361	-1.65008	-1.64656	-1.64305	-1.63954	-1.63603	-1.63252
-1.63279	-1.62934	-1.65343	-1.6499	-1.64639	-1.64289	-1.6394	-1.6359	-1.63237
-1.63264	-1.62918	-1.65325	-1.64973	-1.64622	-1.64273	-1.63925	-1.63576	-1.63224
-1.63253	-1.62904	-1.65309	-1.64955	-1.64604	-1.64255	-1.63909	-1.63562	-1.63214

-1.63244	-1.62892	-1.65294	-1.6494	-1.64588	-1.64239	-1.63893	-1.63548	-1.63203
-1.63232	-1.62883	-1.65286	-1.64931	-1.64579	-1.64228	-1.63878	-1.70566	-1.70201
-1.63219	-1.62874	-1.65279	-1.64925	-1.64571	-1.64218	-1.63865	-1.70548	-1.70182
-1.63204	-1.62859	-1.65266	-1.64912	-1.6456	-1.64207	-1.63855	-1.70536	-1.70168
-1.63188	-1.62842	-1.65248	-1.64896	-1.64546	-1.64196	-1.63848	-1.70527	-1.70159
-1.63176	-1.62829	-1.65235	-1.64882	-1.64532	-1.64183	-1.63836	-1.70514	-1.70146
-1.63169	-1.62822	-1.65226	-1.6487	-1.64516	-1.64166	-1.63819	-1.70495	-1.70129
-1.63162	-1.62814	-1.65216	-1.64859	-1.64503	-1.64152	-1.63804	-1.70478	-1.70113
-1.63154	-1.62804	-1.65205	-1.64849	-1.64495	-1.64144	-1.63795	-1.70466	-1.70098
-1.63145	-1.62794	-1.65194	-1.64839	-1.64487	-1.64137	-1.63788	-1.70454	-1.70083
-1.63135	-1.62785	-1.62437	-1.62092	-1.61749	-1.61406	-1.61063	-1.70442	-1.70071
-1.63124	-1.62776	-1.6243	-1.62085	-1.61741	-1.61397	-1.61053	-1.70429	-1.7006
-1.63116	-1.62768	-1.62422	-1.62077	-1.61732	-1.61388	-1.61044	-1.70419	-1.70051
-1.63109	-1.62761	-1.62415	-1.62067	-1.61721	-1.61378	-1.61038	-1.70411	-1.70044
-1.63102	-1.62753	-1.62405	-1.62058	-1.61712	-1.6137	-1.61031	-1.70404	-1.70037
-1.63095	-1.62743	-1.62394	-1.62048	-1.61705	-1.61364	-1.61025	-1.70398	-1.70031
-1.6309	-1.62736	-1.62385	-1.62041	-1.61699	-1.68516	-1.68156	-1.70391	-1.70024
-1.63087	-1.62736	-1.62386	-1.62038	-1.61694	-1.68508	-1.68148	-1.70383	-1.70016
-1.63085	-1.62736	-1.62387	-1.62037	-1.61688	-1.68501	-1.68141	-1.70374	-1.70006
-1.63079	-1.62728	-1.62378	-1.6203	-1.61683	-1.68494	-1.68133	-1.70365	-1.69995
-1.6307	-1.62717	-1.62366	-1.62021	-1.61678	-1.68487	-1.68126	-1.70356	-1.69982
-1.63064	-1.62711	-1.6236	-1.62015	-1.61672	-1.6848	-1.68119	-1.70349	-1.69976
-1.6306	-1.6271	-1.6236	-1.62012	-1.61665	-1.68472	-1.68113	-1.70345	-1.69974
-1.63055	-1.62705	-1.62356	-1.62006	-1.61659	-1.68464	-1.68106	-1.70339	-1.69971
-1.63046	-1.62693	-1.62343	-1.61997	-1.61653	-1.68456	-1.68098	-1.70331	-1.69964
-1.63037	-1.62683	-1.62332	-1.61988	-1.61647	-1.68449	-1.6809	-1.70323	-1.69956
-1.63034	-1.62682	-1.62332	-1.61984	-1.6164	-1.68439	-1.68082	-1.70315	-1.69948
-1.63032	-1.62683	-1.62334	-1.61983	-1.61634	-1.68431	-1.68074	-1.70307	-1.69939
-1.63025	-1.62679	-1.62333	-1.61983	-1.61634	-1.68427	-1.68066	-1.70298	-1.6993
-1.63016	-1.62672	-1.62329	-1.61984	-1.61639	-1.68426	-1.68059	-1.70288	-1.69923
-1.63013	-1.62667	-1.69521	-1.69156	-1.68791	-1.68424	-1.68056	-1.70283	-1.69916
-1.63017	-1.62664	-1.69511	-1.69147	-1.68785	-1.68422	-1.68057	-1.70282	-1.6991
-1.60658	-1.60304	-1.69504	-1.6914	-1.68779	-1.68418	-1.68057	-1.70282	-1.69906
-1.60655	-1.60305	-1.69504	-1.69136	-1.68772	-1.68411	-1.68053	-1.70281	-1.69906
-1.60651	-1.60306	-1.69506	-1.69135	-1.68766	-1.68405	-1.68049	-1.70279	-1.69907
-1.60645	-1.60303	-1.69503	-1.69134	-1.68766	-1.68405	-1.68049	-1.7028	-1.6991
-1.60638	-1.60298	-1.695	-1.69134	-1.68768	-1.68407	-1.6805	-1.70281	-1.69913
-1.60638	-1.60297	-1.67249	-1.66887	-1.66526	-1.68408	-1.68047	-1.70278	-1.69911
-1.60642	-1.603	-1.67249	-1.66888	-1.66528	-1.68407	-1.68043	-1.70271	-1.69907
-1.60646	-1.60301	-1.67247	-1.66887	-1.66528	-1.68407	-1.6804	-1.70268	-1.69904
-1.60649	-1.603	-1.67241	-1.66881	-1.66524	-1.68405	-1.68041	-1.70269	-1.69905
-1.60653	-1.603	-1.67238	-1.66877	-1.66521	-1.66163	-1.65806	-1.70271	-1.69904
-1.60659	-1.60305	-1.6724	-1.66879	-1.66522	-1.66164	-1.65807	-1.70271	-1.69901
-1.60664	-1.60311	-1.67244	-1.66882	-1.66525	-1.66167	-1.65809	-1.70271	-1.69897
-1.6067	-1.60317	-1.67248	-1.66886	-1.66528	-1.6617	-1.65812	-1.70274	-1.69899
-1.60675	-1.60322	-1.67252	-1.6689	-1.66532	-1.66174	-1.65816	-1.70277	-1.69902
-1.65646	-1.6528	-1.67255	-1.66893	-1.66535	-1.66177	-1.6582	-1.69768	-1.69396
-1.65644	-1.65282	-1.67258	-1.66896	-1.66537	-1.6618	-1.65824	-1.69774	-1.69403
-1.65644	-1.65284	-1.67261	-1.66898	-1.66537	-1.6618	-1.65825	-1.69779	-1.69409
-1.65647	-1.65288	-1.67264	-1.66898	-1.66533	-1.66175	-1.6582	-1.6978	-1.69414

-1.65651	-1.65292	-1.67268	-1.66899	-1.66533	-1.67903	-1.67547	-1.69783	-1.69419
-1.65655	-1.65297	-1.67274	-1.66908	-1.66543	-1.67916	-1.67557	-1.69792	-1.69427
-1.6566	-1.65302	-1.6728	-1.66918	-1.66556	-1.67932	-1.67571	-1.69803	-1.69435
-1.65669	-1.65309	-1.67286	-1.66924	-1.66564	-1.67943	-1.67581	-1.69812	-1.69443
-1.6568	-1.65316	-1.6729	-1.66927	-1.66568	-1.6795	-1.6759	-1.69821	-1.6945
-1.65688	-1.65322	-1.67293	-1.66931	-1.66571	-1.67957	-1.67598	-1.67237	-1.66875
-1.65694	-1.65327	-1.67297	-1.66934	-1.66574	-1.67964	-1.67605	-1.67245	-1.66883
-1.65699	-1.65332	-1.69061	-1.68694	-1.68332	-1.67971	-1.67613	-1.67253	-1.66893
-1.65705	-1.65338	-1.69069	-1.68701	-1.68338	-1.67978	-1.67621	-1.67264	-1.66907
-1.65711	-1.65345	-1.69079	-1.68711	-1.68346	-1.67986	-1.6763	-1.67274	-1.6692
-1.63226	-1.62873	-1.69094	-1.68726	-1.6836	-1.67998	-1.6764	-1.67283	-1.66929
-1.63233	-1.62884	-1.6911	-1.68743	-1.68377	-1.68013	-1.67651	-1.67292	-1.66936
-1.65624	-1.65267	-1.69121	-1.68754	-1.68389	-1.68026	-1.67664	-1.67305	-1.66948
-1.6563	-1.65272	-1.69128	-1.68763	-1.684	-1.68039	-1.6768	-1.67322	-1.66964
-1.65639	-1.6528	-1.69138	-1.68773	-1.6841	-1.6805	-1.67693	-1.67334	-1.66976
-1.65653	-1.65294	-1.69153	-1.68786	-1.6842	-1.68058	-1.677	-1.67342	-1.66985
-1.67485	-1.67123	-1.69169	-1.68799	-1.68431	-1.68068	-1.67709	-1.67351	-1.66995
-1.67499	-1.67136	-1.69181	-1.68812	-1.68446	-1.68083	-1.67723	-1.67364	-1.67005
-1.67514	-1.67148	-1.69194	-1.68827	-1.68462	-1.68099	-1.67739	-1.67378	-1.67017
-1.67527	-1.67162	-1.69209	-1.68842	-1.68478	-1.68114	-1.67752	-1.67391	-1.67031
-1.6754	-1.67177	-1.69226	-1.68859	-1.68493	-1.68128	-1.67765	-1.67403	-1.67045
-1.67555	-1.67193	-1.66833	-1.66474	-1.66116	-1.68142	-1.67779	-1.67418	-1.67061
-1.67571	-1.6721	-1.66849	-1.66488	-1.66129	-1.68156	-1.67795	-1.67435	-1.67078
-1.67588	-1.67226	-1.66864	-1.66503	-1.66143	-1.68171	-1.67812	-1.67453	-1.67096
-1.67606	-1.6724	-1.66876	-1.66516	-1.66159	-1.68189	-1.6783	-1.67472	-1.67115
-1.67624	-1.67256	-1.66891	-1.66532	-1.66176	-1.68207	-1.67848	-1.6749	-1.67133
-1.67644	-1.67279	-1.66914	-1.66553	-1.66195	-1.68225	-1.67866	-1.67507	-1.6715
-1.67665	-1.67303	-1.6694	-1.66577	-1.66215	-1.68243	-1.67884	-1.67524	-1.67165
-1.67684	-1.6732	-1.66957	-1.66594	-1.66234	-1.68262	-1.67901	-1.6754	-1.67179
-1.67701	-1.67334	-1.66969	-1.66609	-1.66252	-1.68282	-1.67919	-1.67555	-1.67192
-1.6772	-1.67353	-1.66988	-1.66628	-1.66271	-1.65915	-1.65561	-1.67574	-1.6721
-1.6774	-1.67376	-1.67013	-1.6665	-1.66289	-1.65933	-1.65581	-1.67597	-1.67235
-1.6776	-1.67397	-1.67034	-1.6667	-1.66307	-1.65951	-1.656	-1.67617	-1.67258
-1.67776	-1.6741	-1.67046	-1.66684	-1.66325	-1.65969	-1.65617	-1.67635	-1.67277
-1.67792	-1.67425	-1.67059	-1.66699	-1.66342	-1.65987	-1.65635	-1.67653	-1.67295
-1.67812	-1.67446	-1.67081	-1.66719	-1.66359	-1.66004	-1.65652	-1.67671	-1.67312
-1.67832	-1.6747	-1.67107	-1.66742	-1.66378	-1.66021	-1.6567	-1.67689	-1.67329
-1.6785	-1.6749	-1.67129	-1.66765	-1.66401	-1.66042	-1.65688	-1.67706	-1.67347
-1.67866	-1.67508	-1.67149	-1.66789	-1.66427	-1.66067	-1.65707	-1.67723	-1.67365
-1.65077	-1.64722	-1.67168	-1.66809	-1.66451	-1.6609	-1.65729	-1.65373	-1.65022
-1.65103	-1.64743	-1.67184	-1.66826	-1.66471	-1.66113	-1.65755	-1.65398	-1.65043
-1.65129	-1.64765	-1.67202	-1.66844	-1.66489	-1.66134	-1.65779	-1.65424	-1.65066
-1.65151	-1.6479	-1.67228	-1.66866	-1.66508	-1.66153	-1.65801	-1.65447	-1.65091
-1.65173	-1.64815	-1.67254	-1.6689	-1.66527	-1.66172	-1.65822	-1.6547	-1.65117
-1.65192	-1.64836	-1.67278	-1.66914	-1.66552	-1.66197	-1.65847	-1.65496	-1.65144
-1.65211	-1.64857	-1.673	-1.6694	-1.6658	-1.66225	-1.65874	-1.65522	-1.65172
-1.65235	-1.64881	-1.64528	-1.64175	-1.63822	-1.66251	-1.65897	-1.65545	-1.65195
-1.65264	-1.64909	-1.64555	-1.64202	-1.63849	-1.66276	-1.65918	-1.65564	-1.65215
-1.65292	-1.64935	-1.64579	-1.64227	-1.63875	-1.663	-1.65941	-1.65586	-1.65238
-1.65319	-1.64958	-1.646	-1.64247	-1.63896	-1.66323	-1.65966	-1.65613	-1.65264

-1.62517	-1.62166	-1.64622	-1.64269	-1.63919	-1.63569	-1.63219	-1.6564	-1.6529
-1.62539	-1.62191	-1.64649	-1.64297	-1.63947	-1.63597	-1.63247	-1.65666	-1.65312
-1.62562	-1.62217	-1.64679	-1.64327	-1.63976	-1.63625	-1.63275	-1.65692	-1.65334
-1.62588	-1.62244	-1.64709	-1.64356	-1.64004	-1.63653	-1.63304	-1.6572	-1.65361
-1.62615	-1.62272	-1.64738	-1.64384	-1.64032	-1.63681	-1.63333	-1.65749	-1.6539
-1.62644	-1.623	-1.64766	-1.6441	-1.64056	-1.63706	-1.63358	-1.63008	-1.62656
-1.62672	-1.62328	-1.64794	-1.64434	-1.64077	-1.63725	-1.63378	-1.63031	-1.62683
-1.62701	-1.62356	-1.64822	-1.6446	-1.641	-1.63748	-1.63402	-1.63056	-1.6271
-1.62729	-1.62384	-1.64852	-1.6449	-1.64129	-1.63776	-1.63429	-1.63083	-1.62739
-1.62757	-1.62412	-1.64882	-1.6452	-1.64158	-1.63805	-1.63458	-1.63112	-1.62769
-1.62786	-1.62441	-1.64912	-1.64549	-1.64187	-1.63834	-1.63487	-1.63141	-1.62797
-1.62815	-1.62469	-1.64941	-1.64578	-1.64216	-1.63863	-1.63516	-1.6317	-1.62826
-1.62843	-1.62498	-1.64972	-1.64608	-1.64247	-1.63893	-1.63545	-1.63199	-1.62854
-1.62871	-1.62526	-1.65003	-1.64639	-1.64277	-1.63923	-1.63574	-1.63227	-1.62882
-1.62899	-1.62555	-1.65034	-1.64672	-1.64312	-1.63956	-1.63606	-1.63257	-1.62911
-1.62927	-1.62584	-1.65066	-1.64708	-1.6435	-1.63995	-1.63642	-1.6329	-1.62941
-1.62954	-1.62612	-1.6227	-1.61927	-1.61583	-1.64031	-1.63677	-1.63323	-1.62971
-1.62981	-1.62639	-1.62298	-1.61955	-1.61612	-1.64062	-1.63708	-1.63355	-1.63001
-1.63008	-1.62667	-1.62325	-1.61982	-1.61639	-1.64091	-1.63738	-1.63385	-1.63031
-1.63041	-1.62697	-1.62353	-1.62009	-1.61666	-1.6412	-1.63768	-1.63416	-1.63065
-1.63076	-1.62729	-1.62382	-1.62037	-1.61694	-1.64148	-1.63797	-1.63447	-1.63099
-1.6311	-1.62762	-1.62415	-1.6207	-1.61726	-1.64181	-1.63827	-1.63477	-1.63131
-1.63142	-1.62797	-1.62452	-1.62107	-1.61762	-1.64216	-1.63859	-1.63506	-1.63159
-1.63173	-1.6283	-1.62486	-1.62142	-1.61796	-1.64251	-1.63892	-1.63539	-1.63191
-1.63202	-1.62858	-1.62515	-1.62172	-1.61828	-1.64286	-1.63929	-1.63576	-1.63228
-1.63232	-1.62888	-1.62546	-1.62203	-1.61862	-1.64323	-1.63967	-1.63615	-1.63265
-1.63268	-1.62924	-1.62582	-1.6224	-1.61898	-1.64362	-1.64006	-1.63652	-1.633
-1.63305	-1.62961	-1.62619	-1.62277	-1.61935	-1.644	-1.64044	-1.63688	-1.63334
-1.63341	-1.62995	-1.62651	-1.62308	-1.61966	-1.64433	-1.64078	-1.63724	-1.63369
-1.63377	-1.63028	-1.62681	-1.62336	-1.61994	-1.64464	-1.64111	-1.63759	-1.63407
-1.60982	-1.6064	-1.62714	-1.6237	-1.62028	-1.61686	-1.61347	-1.63796	-1.63445
-1.61015	-1.60676	-1.62752	-1.62409	-1.62067	-1.61725	-1.61384	-1.63835	-1.63484
-1.6105	-1.60712	-1.6279	-1.62448	-1.62106	-1.61764	-1.61422	-1.63874	-1.63522
-1.61088	-1.60751	-1.62829	-1.62486	-1.62144	-1.61801	-1.61459	-1.63912	-1.63558
-1.61128	-1.6079	-1.62869	-1.62525	-1.62182	-1.61839	-1.61496	-1.63949	-1.63594
-1.58581	-1.58251	-1.62908	-1.62564	-1.6222	-1.61875	-1.6153	-1.63984	-1.63631
-1.5862	-1.5829	-1.62948	-1.62603	-1.62258	-1.61911	-1.61564	-1.6402	-1.6367
-1.58659	-1.58329	-1.62988	-1.62643	-1.62297	-1.64969	-1.64613	-1.67018	-1.66658
-1.58699	-1.58368	-1.63027	-1.62682	-1.62338	-1.65012	-1.64659	-1.67067	-1.66702
-1.58738	-1.58407	-1.63067	-1.62722	-1.62377	-1.65052	-1.64699	-1.67109	-1.66745
-1.58776	-1.58445	-1.63105	-1.6276	-1.62415	-1.65088	-1.64732	-1.67144	-1.66784
-1.58815	-1.58483	-1.60607	-1.60268	-1.59929	-1.65126	-1.64769	-1.67183	-1.66824
-1.58854	-1.58522	-1.60646	-1.60308	-1.5997	-1.6517	-1.64815	-1.67231	-1.66868
-1.58893	-1.58561	-1.60686	-1.60348	-1.60012	-1.65215	-1.64863	-1.6728	-1.66914
-1.58932	-1.586	-1.60725	-1.60387	-1.60051	-1.65257	-1.64907	-1.67328	-1.66963
-1.5897	-1.58639	-1.60765	-1.60427	-1.6009	-1.65298	-1.64949	-1.67374	-1.67013
-1.53436	-1.53119	-1.58348	-1.58019	-1.57692	-1.65338	-1.6499	-1.64642	-1.64295
-1.53475	-1.53158	-1.58386	-1.58058	-1.57731	-1.65379	-1.6503	-1.64683	-1.64336
-1.53518	-1.532	-1.58429	-1.58099	-1.57771	-1.6542	-1.65071	-1.64724	-1.64377
-1.53564	-1.53247	-1.58476	-1.58143	-1.57811	-1.6546	-1.65112	-1.64764	-1.64417

-1.53609	-1.53293	-1.56009	-1.55679	-1.5535	-1.63021	-1.62679	-1.64807	-1.6446
-1.53651	-1.53334	-1.56052	-1.55724	-1.55398	-1.6307	-1.62727	-1.64855	-1.64508
-1.53691	-1.53373	-1.56093	-1.55769	-1.55446	-1.6312	-1.62776	-1.64904	-1.64558
-1.48578	-1.48268	-1.56133	-1.5581	-1.55488	-1.63164	-1.62819	-1.64948	-1.64603
-1.48615	-1.48306	-1.56172	-1.55849	-1.55528	-1.63205	-1.62861	-1.64991	-1.64646
-1.48656	-1.48347	-1.51162	-1.50847	-1.50532	-1.6325	-1.62907	-1.65038	-1.64692
-1.487	-1.48392	-1.51209	-1.50892	-1.50576	-1.63299	-1.62956	-1.65088	-1.6474
-1.48742	-1.48434	-1.51252	-1.50935	-1.50619	-1.5605	-1.55718	-1.6266	-1.62318
-1.48779	-1.48471	-1.5129	-1.50973	-1.50657	-1.56089	-1.55757	-1.62701	-1.62359
-1.48818	-1.48509	-1.51329	-1.51012	-1.50696	-1.56129	-1.55797	-1.62742	-1.62401
-1.48862	-1.48552	-1.51373	-1.51056	-1.5074	-1.56174	-1.55841	-1.62789	-1.62448
-1.48908	-1.48598	-1.51418	-1.51102	-1.50786	-1.56222	-1.55888	-1.62838	-1.62498
-1.48953	-1.48643	-1.51464	-1.51148	-1.50832	-1.53762	-1.53439	-1.55604	-1.55276
-1.48999	-1.48689	-1.51511	-1.51194	-1.50878	-1.53809	-1.53486	-1.55652	-1.55323
-1.49045	-1.48735	-1.51558	-1.5124	-1.50924	-1.53855	-1.53533	-1.55699	-1.5537
-1.49091	-1.48781	-1.51604	-1.51286	-1.50969	-1.53901	-1.53578	-1.55745	-1.55415
-1.49138	-1.48826	-1.51651	-1.51332	-1.51015	-1.53946	-1.53622	-1.55789	-1.55459
-1.49183	-1.48871	-1.51696	-1.51377	-1.5106	-1.5399	-1.53662	-1.55828	-1.55498
-1.49228	-1.48915	-1.5174	-1.51422	-1.51104	-1.54033	-1.53702	-1.55867	-1.55538
-1.49267	-1.48954	-1.5178	-1.51462	-1.51145	-1.54075	-1.53746	-1.55913	-1.55583
-1.49306	-1.48994	-1.51821	-1.51502	-1.51186	-1.54119	-1.53793	-1.55962	-1.55631
-1.49354	-1.49042	-1.5187	-1.51551	-1.51234	-1.54169	-1.53845	-1.56016	-1.55685
-1.49409	-1.49096	-1.51926	-1.51606	-1.51288	-1.54224	-1.53901	-1.56073	-1.55742
-1.49461	-1.49149	-1.51979	-1.51659	-1.5134	-1.54277	-1.53954	-1.56127	-1.55796
-1.49511	-1.49197	-1.52028	-1.51708	-1.51389	-1.54327	-1.54003	-1.56176	-1.55846
-1.49559	-1.49245	-1.52076	-1.51756	-1.51438	-1.54376	-1.54051	-1.53728	-1.53408
-1.49606	-1.49292	-1.52124	-1.51804	-1.51485	-1.54424	-1.54099	-1.53776	-1.53455
-1.49653	-1.49339	-1.52171	-1.51851	-1.51533	-1.54472	-1.54146	-1.53823	-1.53501
-1.49699	-1.49386	-1.49073	-1.48762	-1.48452	-1.5452	-1.54194	-1.53871	-1.53549
-1.49746	-1.49433	-1.4912	-1.48808	-1.48498	-1.54568	-1.54242	-1.53919	-1.53598
-1.49793	-1.4948	-1.49168	-1.48856	-1.48545	-1.54616	-1.54291	-1.53969	-1.5365
-1.49842	-1.49528	-1.49215	-1.48903	-1.48593	-1.54665	-1.5434	-1.54019	-1.53704
-1.49893	-1.49579	-1.49265	-1.48953	-1.48643	-1.54718	-1.54392	-1.54071	-1.53757
-1.49948	-1.49634	-1.4932	-1.49009	-1.48698	-1.54775	-1.54448	-1.54126	-1.53809
-1.50002	-1.49688	-1.49375	-1.49063	-1.48753	-1.54831	-1.54503	-1.54179	-1.53859
-1.50051	-1.49738	-1.49425	-1.49113	-1.48802	-1.54882	-1.54555	-1.5423	-1.53908
-1.501	-1.49787	-1.49475	-1.49163	-1.48851	-1.54933	-1.54606	-1.54281	-1.53958
-1.50153	-1.49841	-1.49529	-1.49217	-1.48905	-1.54988	-1.54661	-1.54335	-1.54011
-1.50208	-1.49897	-1.49585	-1.49273	-1.48962	-1.55046	-1.54718	-1.54392	-1.54068
-1.50261	-1.49949	-1.49638	-1.49327	-1.49017	-1.55105	-1.54776	-1.54449	-1.54126
-1.50309	-1.49996	-1.49685	-1.49379	-1.49073	-1.55163	-1.54834	-1.54507	-1.54184
-1.5036	-1.50046	-1.49735	-1.49429	-1.49126	-1.55219	-1.54889	-1.54562	-1.5424
-1.50414	-1.501	-1.49788	-1.4948	-1.49173	-1.55268	-1.54939	-1.54613	-1.54289
-1.50469	-1.50155	-1.49842	-1.49531	-1.49221	-1.55317	-1.5499	-1.54664	-1.54338
-1.50525	-1.50211	-1.49897	-1.49585	-1.49274	-1.55372	-1.55044	-1.54718	-1.54393
-1.50582	-1.50267	-1.49953	-1.49641	-1.4933	-1.55429	-1.55101	-1.54775	-1.5445
-1.50639	-1.50323	-1.50009	-1.49696	-1.49385	-1.55486	-1.55158	-1.54832	-1.54507
-1.50696	-1.5038	-1.50065	-1.49752	-1.49441	-1.55544	-1.55217	-1.5489	-1.54566
-1.47753	-1.47444	-1.50121	-1.49807	-1.49496	-1.55602	-1.55275	-1.54949	-1.54624
-1.47809	-1.475	-1.50177	-1.49863	-1.49552	-1.5566	-1.55333	-1.55007	-1.54683

-1.47865	-1.47555	-1.50233	-1.49919	-1.49607	-1.55718	-1.55391	-1.55066	-1.54741
-1.47921	-1.47611	-1.5029	-1.49975	-1.49663	-1.55777	-1.55449	-1.55123	-1.54798
-1.47977	-1.47668	-1.50347	-1.50032	-1.49719	-1.55835	-1.55507	-1.5518	-1.54856
-1.48033	-1.47723	-1.50403	-1.50088	-1.49774	-1.55892	-1.55564	-1.55238	-1.54913
-1.48088	-1.47778	-1.50459	-1.50143	-1.4983	-1.55949	-1.55621	-1.55295	-1.54971
-1.48143	-1.47833	-1.50515	-1.50199	-1.49885	-1.56007	-1.55679	-1.55353	-1.5503
-1.48198	-1.47888	-1.50571	-1.50255	-1.49941	-1.56066	-1.55737	-1.55412	-1.5509
-1.48253	-1.47943	-1.50628	-1.50311	-1.49997	-1.49686	-1.49379	-1.52207	-1.51897
-1.48309	-1.47999	-1.50685	-1.50368	-1.50052	-1.49742	-1.49436	-1.52267	-1.51958
-1.45781	-1.45479	-1.50744	-1.50426	-1.5011	-1.498	-1.49494	-1.52327	-1.52019
-1.45837	-1.45537	-1.50806	-1.5049	-1.50174	-1.49862	-1.49554	-1.52388	-1.52079
-1.45894	-1.45595	-1.50869	-1.50554	-1.50238	-1.49926	-1.49616	-1.52449	-1.52138
-1.45951	-1.45652	-1.47928	-1.47621	-1.47313	-1.49985	-1.49674	-1.52508	-1.52197
-1.46009	-1.45708	-1.47985	-1.47677	-1.4737	-1.50043	-1.49732	-1.52566	-1.52255
-1.46066	-1.45764	-1.45463	-1.45163	-1.44863	-1.50104	-1.49793	-1.52629	-1.52317
-1.46121	-1.4582	-1.45519	-1.45221	-1.44923	-1.50169	-1.49859	-1.52696	-1.52382
-1.46177	-1.45876	-1.45576	-1.45279	-1.44982	-1.47712	-1.47408	-1.52761	-1.52447
-1.46233	-1.45933	-1.45634	-1.45336	-1.45038	-1.47768	-1.47464	-1.52822	-1.5251
-1.4629	-1.4599	-1.45692	-1.45392	-1.45093	-1.47824	-1.47521	-1.52882	-1.52573
-1.46348	-1.46047	-1.45747	-1.45447	-1.45149	-1.47883	-1.47581	-1.52945	-1.52633
-1.46408	-1.46103	-1.45801	-1.45502	-1.45206	-1.47942	-1.47642	-1.53008	-1.52692
-1.4647	-1.46164	-1.45861	-1.45561	-1.45264	-1.48003	-1.47703	-1.50601	-1.5029
-1.46534	-1.46228	-1.45923	-1.45622	-1.45325	-1.48064	-1.47765	-1.50665	-1.50354
-1.46598	-1.46291	-1.45986	-1.45684	-1.45385	-1.48125	-1.47824	-1.50725	-1.50415
-1.46661	-1.46354	-1.46049	-1.45746	-1.45446	-1.48185	-1.4788	-1.50781	-1.50472
-1.46721	-1.46415	-1.46111	-1.45808	-1.45507	-1.48245	-1.47938	-1.50839	-1.5053
-1.46777	-1.46472	-1.46169	-1.45869	-1.45569	-1.48309	-1.48001	-1.50903	-1.50593
-1.46833	-1.46529	-1.46228	-1.45929	-1.45632	-1.48374	-1.48065	-1.50969	-1.50658
-1.4395	-1.43653	-1.46289	-1.45991	-1.45695	-1.45395	-1.45094	-1.56898	-1.56572
-1.44012	-1.43715	-1.46352	-1.46054	-1.45758	-1.45458	-1.45157	-1.56967	-1.56641
-1.4085	-1.40561	-1.43482	-1.4319	-1.429	-1.45521	-1.45219	-1.57035	-1.56709
-1.4091	-1.40621	-1.43545	-1.43252	-1.42961	-1.45583	-1.45282	-1.57104	-1.56777
-1.40971	-1.40682	-1.43607	-1.43314	-1.43023	-1.42729	-1.42433	-1.57172	-1.56845
-1.41031	-1.40742	-1.43669	-1.43377	-1.43085	-1.4279	-1.42495	-1.5724	-1.56913
-1.41092	-1.40803	-1.40516	-1.40231	-1.39946	-1.42852	-1.42556	-1.57309	-1.56982
-1.41152	-1.40862	-1.40575	-1.4029	-1.40006	-1.42913	-1.42617	-1.57377	-1.5705
-1.41211	-1.40922	-1.40635	-1.4035	-1.40066	-1.42974	-1.42678	-1.57445	-1.57117
-1.41271	-1.40982	-1.40695	-1.4041	-1.40126	-1.43036	-1.4274	-1.54789	-1.54467
-1.41331	-1.41042	-1.40755	-1.4047	-1.40187	-1.43099	-1.42803	-1.54857	-1.54534
-1.41391	-1.41101	-1.40814	-1.4053	-1.40248	-1.43164	-1.42868	-1.48754	-1.48448
-1.41452	-1.4116	-1.40872	-1.4059	-1.4031	-1.4323	-1.42937	-1.48827	-1.48519
-1.41515	-1.41222	-1.40933	-1.4065	-1.4037	-1.43294	-1.43004	-1.48898	-1.48588
-1.41584	-1.41289	-1.40997	-1.40712	-1.4043	-1.43355	-1.43066	-1.48964	-1.48654
-1.41653	-1.41357	-1.41064	-1.40775	-1.4049	-1.43416	-1.43128	-1.49029	-1.4872
-1.41719	-1.41425	-1.41132	-1.40842	-1.40554	-1.4027	-1.39988	-1.49096	-1.48791
-1.41783	-1.41492	-1.41201	-1.4091	-1.40619	-1.40333	-1.4005	-1.49164	-1.48862
-1.41845	-1.41555	-1.41265	-1.40973	-1.40682	-1.40394	-1.4011	-1.4613	-1.45838
-1.41905	-1.41616	-1.41326	-1.41034	-1.40742	-1.40454	-1.40169	-1.46192	-1.45901
-1.41968	-1.41677	-1.41388	-1.41097	-1.40806	-1.40518	-1.40232	-1.46257	-1.45964
-1.42034	-1.4174	-1.4145	-1.41161	-1.40874	-1.40587	-1.40299	-1.46325	-1.46029

-1.42102	-1.41806	-1.41513	-1.41225	-1.4094	-1.40654	-1.40367	-1.46396	-1.46097
-1.42173	-1.41874	-1.41578	-1.41288	-1.41001	-1.40717	-1.40435	-1.46471	-1.46173
-1.42245	-1.41943	-1.41645	-1.41351	-1.41062	-1.40779	-1.40501	-1.46545	-1.46249
-1.42312	-1.42013	-1.41714	-1.41419	-1.41126	-1.40841	-1.40562	-1.46609	-1.46315
-1.42377	-1.42081	-1.41785	-1.41488	-1.41192	-1.40904	-1.40621	-1.46669	-1.46377
-1.42439	-1.42145	-1.41851	-1.41554	-1.41259	-1.40969	-1.40684	-1.46733	-1.4644
-1.42499	-1.42205	-1.41912	-1.41619	-1.41327	-1.41038	-1.4075	-1.468	-1.46503
-1.42564	-1.42269	-1.41975	-1.41683	-1.41393	-1.41104	-1.40817	-1.4687	-1.46572
-1.42638	-1.42339	-1.42042	-1.41747	-1.41454	-1.41167	-1.40883	-1.46943	-1.46647
-1.42711	-1.4241	-1.42111	-1.41813	-1.41519	-1.41231	-1.4095	-1.47015	-1.46721
-1.42777	-1.42479	-1.42182	-1.41886	-1.41592	-1.41302	-1.41017	-1.47084	-1.46788
-1.42841	-1.42547	-1.42253	-1.41959	-1.41666	-1.41374	-1.41085	-1.47152	-1.46854
-1.42907	-1.42612	-1.42319	-1.42025	-1.41733	-1.41442	-1.41153	-1.47224	-1.46927
-1.42974	-1.42676	-1.42381	-1.42087	-1.41796	-1.41507	-1.4122	-1.47298	-1.47002
-1.43044	-1.42746	-1.4245	-1.42157	-1.41866	-1.41576	-1.41288	-1.47368	-1.47072
-1.43118	-1.42821	-1.42526	-1.42233	-1.41941	-1.41648	-1.41356	-1.47436	-1.47138
-1.39538	-1.39251	-1.426	-1.42306	-1.42013	-1.41718	-1.41424	-1.47506	-1.47206
-1.39612	-1.39321	-1.42669	-1.42372	-1.42077	-1.41783	-1.41491	-1.4758	-1.47282
-1.39683	-1.3939	-1.42737	-1.42438	-1.42142	-1.41849	-1.41559	-1.47654	-1.47356
-1.39749	-1.39457	-1.42808	-1.4251	-1.42214	-1.41919	-1.41627	-1.41339	-1.41057
-1.39813	-1.39524	-1.42878	-1.42583	-1.42288	-1.41992	-1.41695	-1.41405	-1.41119
-1.39883	-1.39592	-1.39303	-1.39015	-1.38729	-1.4206	-1.41765	-1.41475	-1.41189
-1.39956	-1.39661	-1.39368	-1.39078	-1.38791	-1.42126	-1.41836	-1.41548	-1.41263
-1.40026	-1.3973	-1.39436	-1.39146	-1.38859	-1.42196	-1.41908	-1.41621	-1.41336
-1.40092	-1.39799	-1.39508	-1.39219	-1.38933	-1.42272	-1.41983	-1.41696	-1.41411
-1.40161	-1.3987	-1.39581	-1.39293	-1.39007	-1.38723	-1.3844	-1.41767	-1.41482
-1.40236	-1.39944	-1.39655	-1.39368	-1.39081	-1.38795	-1.38508	-1.41834	-1.41546
-1.40313	-1.40019	-1.39728	-1.3944	-1.39154	-1.38865	-1.38576	-1.41901	-1.41611
-1.40389	-1.40092	-1.39798	-1.39508	-1.39219	-1.38931	-1.38644	-1.41972	-1.41683
-1.40464	-1.40164	-1.39866	-1.39573	-1.39283	-1.38996	-1.38712	-1.42044	-1.41757
-1.40537	-1.40235	-1.39937	-1.39643	-1.39353	-1.39067	-1.38785	-1.38504	-1.38226
-1.40609	-1.40307	-1.40009	-1.39716	-1.39428	-1.39143	-1.3886	-1.3858	-1.38302
-1.34359	-1.34073	-1.40084	-1.39792	-1.39505	-1.39219	-1.38936	-1.38655	-1.38376
-1.3443	-1.34146	-1.40162	-1.39871	-1.39583	-1.39297	-1.39013	-1.3873	-1.3845
-1.34504	-1.34221	-1.40242	-1.3995	-1.39661	-1.39374	-1.39089	-1.38804	-1.38522
-1.34579	-1.34296	-1.40321	-1.40029	-1.39739	-1.39451	-1.39163	-1.38877	-1.38592
-1.34656	-1.34372	-1.40401	-1.40108	-1.39816	-1.39526	-1.39237	-1.38949	-1.38661
-1.31529	-1.31254	-1.34167	-1.33887	-1.3361	-1.396	-1.39308	-1.39018	-1.3873
-1.31603	-1.31327	-1.34241	-1.33961	-1.33682	-1.39673	-1.39378	-1.39087	-1.38799
-1.31676	-1.31398	-1.34315	-1.34032	-1.33751	-1.33469	-1.33187	-1.39157	-1.3887
-1.31749	-1.3147	-1.34388	-1.34102	-1.33817	-1.33535	-1.33254	-1.39229	-1.38944
-1.3182	-1.3154	-1.34459	-1.34171	-1.33885	-1.33602	-1.33323	-1.39303	-1.39019
-1.31892	-1.31607	-1.34526	-1.34238	-1.33953	-1.33672	-1.33395	-1.39379	-1.39096
-1.31963	-1.31675	-1.34595	-1.34307	-1.34023	-1.33743	-1.33467	-1.39456	-1.39172
-1.32037	-1.31748	-1.34669	-1.3438	-1.34095	-1.33815	-1.3354	-1.33268	-1.32997
-1.32111	-1.31821	-1.34745	-1.34455	-1.34169	-1.33889	-1.33614	-1.33341	-1.3307
-1.32179	-1.3189	-1.34818	-1.34528	-1.34243	-1.33963	-1.33688	-1.33415	-1.33143
-1.32243	-1.31955	-1.34889	-1.34602	-1.34319	-1.34039	-1.33762	-1.33488	-1.33215
-1.32312	-1.32024	-1.31741	-1.31464	-1.31192	-1.34114	-1.33836	-1.33561	-1.33288
-1.32384	-1.32095	-1.31811	-1.31535	-1.31263	-1.34188	-1.3391	-1.33634	-1.33361

-1.3246	-1.3217	-1.31885	-1.31608	-1.31334	-1.34262	-1.33983	-1.33708	-1.33434
-1.32542	-1.32251	-1.31964	-1.31683	-1.31406	-1.34335	-1.34056	-1.33779	-1.33506
-1.32625	-1.32334	-1.32046	-1.31761	-1.3148	-1.34411	-1.3413	-1.33853	-1.33579
-1.32705	-1.32417	-1.3213	-1.31846	-1.31563	-1.34495	-1.3421	-1.3393	-1.33653
-1.32783	-1.32498	-1.32215	-1.31932	-1.3165	-1.34582	-1.34294	-1.34009	-1.33729
-1.3286	-1.32576	-1.32293	-1.32011	-1.3173	-1.34667	-1.34378	-1.34093	-1.33812
-1.32934	-1.32649	-1.32366	-1.32085	-1.31806	-1.34749	-1.34464	-1.34182	-1.33902
-1.33007	-1.32722	-1.32439	-1.32158	-1.3188	-1.31604	-1.3133	-1.34265	-1.33985
-1.33081	-1.32795	-1.32512	-1.32231	-1.31953	-1.31676	-1.31402	-1.34341	-1.34062
-1.33155	-1.32868	-1.32585	-1.32304	-1.32025	-1.31748	-1.31473	-1.34415	-1.34136
-1.3323	-1.32942	-1.32658	-1.32376	-1.32097	-1.3182	-1.31545	-1.3449	-1.3421
-1.33306	-1.33016	-1.3273	-1.32448	-1.32169	-1.31892	-1.31617	-1.34565	-1.34284
-1.26952	-1.26678	-1.32804	-1.32521	-1.32241	-1.31963	-1.31688	-1.34638	-1.34358
-1.27021	-1.26747	-1.32877	-1.32594	-1.32313	-1.32034	-1.31758	-1.34712	-1.34432
-1.2709	-1.26815	-1.32951	-1.32667	-1.32385	-1.32106	-1.31828	-1.34786	-1.34506
-1.2716	-1.26884	-1.33025	-1.3274	-1.32458	-1.32178	-1.319	-1.3486	-1.3458
-1.27236	-1.26959	-1.26685	-1.26416	-1.2615	-1.32255	-1.31977	-1.3494	-1.34659
-1.27324	-1.27046	-1.26771	-1.265	-1.26233	-1.32343	-1.32064	-1.35029	-1.34747
-1.27409	-1.27131	-1.26855	-1.26584	-1.26315	-1.3243	-1.32149	-1.35117	-1.34834
-1.27485	-1.27206	-1.2693	-1.26657	-1.26386	-1.32507	-1.32226	-1.31948	-1.31672
-1.27557	-1.27277	-1.27	-1.26726	-1.26455	-1.32582	-1.32301	-1.32022	-1.31745
-1.27629	-1.27348	-1.2707	-1.26795	-1.26524	-1.26258	-1.25996	-1.32102	-1.31826
-1.277	-1.27418	-1.2714	-1.26865	-1.26594	-1.2633	-1.26072	-1.32186	-1.31912
-1.27779	-1.27497	-1.27218	-1.26942	-1.26671	-1.26406	-1.26147	-1.32266	-1.31992
-1.27868	-1.27586	-1.27306	-1.27029	-1.26755	-1.26486	-1.26222	-1.32343	-1.32066
-1.27953	-1.2767	-1.2739	-1.27112	-1.26836	-1.26564	-1.26295	-1.2603	-1.25767
-1.28028	-1.27745	-1.27464	-1.27185	-1.26908	-1.26635	-1.26365	-1.26097	-1.25832
-1.28103	-1.2782	-1.27539	-1.27259	-1.26982	-1.26707	-1.26436	-1.26167	-1.259
-1.2819	-1.27906	-1.27624	-1.27344	-1.27066	-1.2679	-1.26516	-1.26246	-1.25979
-1.28279	-1.27995	-1.27713	-1.27432	-1.27153	-1.26876	-1.266	-1.26329	-1.26061
-1.28359	-1.28074	-1.27792	-1.2751	-1.27231	-1.26952	-1.26675	-1.26403	-1.26134
-1.28431	-1.28147	-1.27864	-1.27582	-1.27301	-1.27022	-1.26744	-1.26471	-1.26201
-1.17602	-1.17349	-1.27942	-1.27659	-1.27378	-1.27099	-1.26821	-1.26546	-1.26275
-1.17681	-1.17427	-1.28029	-1.27747	-1.27466	-1.27186	-1.26907	-1.26632	-1.26359
-1.17758	-1.17503	-1.28113	-1.2783	-1.27549	-1.2727	-1.26991	-1.26715	-1.26442
-1.17822	-1.17566	-1.28185	-1.27902	-1.27621	-1.27341	-1.27063	-1.26789	-1.26518
-1.17885	-1.17628	-1.28257	-1.27973	-1.27691	-1.27412	-1.27134	-1.26862	-1.26593
-1.1796	-1.17703	-1.24776	-1.245	-1.24226	-1.27492	-1.27214	-1.2694	-1.26669
-1.1804	-1.17783	-1.24861	-1.24584	-1.24309	-1.27578	-1.27299	-1.27022	-1.26745
-1.18111	-1.17853	-1.24938	-1.24663	-1.2439	-1.27663	-1.27384	-1.27106	-1.26827
-1.18174	-1.17916	-1.2501	-1.24738	-1.24469	-1.27748	-1.27471	-1.27194	-1.26915
-1.18243	-1.17984	-1.1773	-1.17482	-1.17238	-1.24278	-1.2401	-1.27275	-1.26996
-1.18323	-1.18062	-1.17805	-1.17553	-1.17304	-1.24348	-1.24078	-1.27345	-1.27067
-1.14778	-1.14524	-1.17883	-1.17627	-1.17373	-1.2442	-1.24148	-1.27416	-1.27138
-1.14852	-1.146	-1.17963	-1.17706	-1.1745	-1.24502	-1.24228	-1.27498	-1.27219
-1.14924	-1.14674	-1.18043	-1.17786	-1.1753	-1.24586	-1.24312	-1.27585	-1.27304
-1.11513	-1.11269	-1.14493	-1.14246	-1.14	-1.24669	-1.24396	-1.27671	-1.2739
-1.11578	-1.11331	-1.14555	-1.14311	-1.1407	-1.24751	-1.2448	-1.27759	-1.27478
-1.11649	-1.114	-1.11155	-1.10915	-1.10679	-1.17499	-1.17252	-1.24289	-1.24018
-1.11726	-1.11474	-1.11226	-1.10983	-1.10744	-1.17566	-1.17322	-1.24369	-1.241

-1.07566	-1.07327	-1.11301	-1.11054	-1.10811	-1.10575	-1.10342	-1.24446	-1.24179
-1.07641	-1.07401	-1.11378	-1.11129	-1.10883	-1.10643	-1.10407	-1.24521	-1.24255
-1.07716	-1.07475	-1.11456	-1.11205	-1.10957	-1.10714	-1.10474	-1.24595	-1.24329
-1.07788	-1.07548	-1.11534	-1.11282	-1.11033	-1.10787	-1.10544	-1.17878	-1.17622
-1.07858	-1.0762	-1.11613	-1.1136	-1.11109	-1.10862	-1.10617	-1.17954	-1.17695
-1.04551	-1.04326	-1.11688	-1.11437	-1.11186	-1.10937	-1.10691	-1.18033	-1.17771
-1.04616	-1.04391	-1.11762	-1.11513	-1.11263	-1.11014	-1.10766	-1.18114	-1.1785
-1.04686	-1.04462	-1.11838	-1.1159	-1.11341	-1.11092	-1.10842	-1.10596	-1.10354
-1.04762	-1.04537	-1.11919	-1.1167	-1.11421	-1.11171	-1.1092	-1.10672	-1.10428
-1.0036	-1.00144	-1.07742	-1.07507	-1.07272	-1.11249	-1.10997	-1.10748	-1.10502
-1.0043	-1.0021	-1.07808	-1.07574	-1.0734	-1.11324	-1.11072	-1.10823	-1.10576
-1.005	-1.00275	-1.07875	-1.07641	-1.07409	-1.11399	-1.11148	-1.10899	-1.1065
-1.00572	-1.00346	-1.07948	-1.07714	-1.07483	-1.11478	-1.11227	-1.10977	-1.10726
-1.00644	-1.00418	-1.08024	-1.0779	-1.07559	-1.11558	-1.11308	-1.11057	-1.10804
-1.00716	-1.0049	-1.08099	-1.07865	-1.07633	-1.11637	-1.11386	-1.11135	-1.10881
-1.00788	-1.00561	-1.08172	-1.07938	-1.07706	-1.11714	-1.11464	-1.11212	-1.10957
-1.0086	-1.00632	-1.03831	-1.03607	-1.03386	-1.07544	-1.07307	-1.11289	-1.11033
-1.00931	-1.00703	-1.03901	-1.03676	-1.03454	-1.07617	-1.07381	-1.11367	-1.11109
-0.96919	-0.967	-1.0397	-1.03744	-1.03522	-1.07689	-1.07454	-1.11444	-1.11185
-0.96987	-0.96767	-1.04038	-1.03812	-1.03589	-1.0776	-1.07525	-1.1152	-1.11262
-0.97055	-0.96835	-1.04106	-1.03879	-1.03655	-1.0783	-1.07595	-1.11596	-1.11339
-0.97125	-0.96903	-1.00758	-1.00538	-1.00322	-1.07901	-1.07665	-1.07427	-1.07186
-0.97196	-0.96973	-1.00829	-1.00608	-1.00392	-1.07973	-1.07736	-1.07497	-1.07256
-0.88921	-0.88722	-0.96827	-0.96616	-0.96407	-1.08047	-1.07807	-1.07568	-1.07328
-0.88986	-0.88789	-0.96904	-0.96691	-0.9648	-1.08122	-1.0788	-1.07639	-1.07399
-0.89049	-0.88854	-0.96979	-0.96764	-0.96551	-0.99792	-0.9957	-1.07712	-1.07473
-0.89111	-0.88915	-0.97049	-0.96833	-0.9662	-0.99859	-0.99636	-1.07785	-1.0755
-0.89173	-0.88976	-0.97118	-0.96902	-0.96688	-0.99926	-0.99702	-1.07859	-1.07627
-0.89235	-0.89038	-0.88844	-0.88649	-0.88456	-0.99992	-0.99767	-1.04034	-1.03811
-0.89297	-0.89101	-0.88906	-0.8871	-0.88515	-1.00059	-0.99833	-1.04104	-1.03879
-0.89364	-0.89167	-0.8897	-0.88773	-0.88576	-1.00128	-0.99904	-1.04179	-1.03953
-0.89434	-0.89235	-0.89036	-0.88836	-0.88637	-1.00199	-0.99978	-1.04258	-1.04029
-0.89501	-0.89301	-0.89101	-0.889	-0.88702	-0.88509	-0.8832	-0.9983	-0.9961
-0.89567	-0.89365	-0.89165	-0.88966	-0.8877	-0.88576	-0.88383	-0.99899	-0.99677
-0.81322	-0.81137	-0.89227	-0.8903	-0.88836	-0.8864	-0.88445	-0.99968	-0.99745
-0.8138	-0.81193	-0.89287	-0.8909	-0.88897	-0.88701	-0.88506	-1.00038	-0.99817
-0.81438	-0.8125	-0.89348	-0.8915	-0.88957	-0.88761	-0.88566	-1.00108	-0.99889
-0.81498	-0.81311	-0.89415	-0.89217	-0.89021	-0.88824	-0.88626	-0.92004	-0.91806
-0.81559	-0.81375	-0.89486	-0.89286	-0.89087	-0.88887	-0.88687	-0.92062	-0.91862
-0.81623	-0.81438	-0.85237	-0.85044	-0.84853	-0.88951	-0.88751	-0.92125	-0.91924
-0.8169	-0.81501	-0.853	-0.85105	-0.84912	-0.89014	-0.88818	-0.92192	-0.91989
-0.77085	-0.76904	-0.85364	-0.85168	-0.84975	-0.89079	-0.88885	-0.88691	-0.88496
-0.77144	-0.76961	-0.85427	-0.85233	-0.85042	-0.89148	-0.88952	-0.88757	-0.88564
-0.68677	-0.6851	-0.81495	-0.81312	-0.81132	-0.8492	-0.84731	-0.88824	-0.88631
-0.6873	-0.68565	-0.81561	-0.81376	-0.81193	-0.84984	-0.84797	-0.88891	-0.88696
-0.68783	-0.68621	-0.81629	-0.81441	-0.81255	-0.85049	-0.84863	-0.88959	-0.8876
-0.64408	-0.64253	-0.72705	-0.72535	-0.72366	-0.85115	-0.84927	-0.89024	-0.88824
-0.64465	-0.64307	-0.72762	-0.72593	-0.72427	-0.85183	-0.84991	-0.89089	-0.88887
-0.6452	-0.6436	-0.72822	-0.72652	-0.72485	-0.76464	-0.76287	-0.84862	-0.84671
-0.64574	-0.64415	-0.72884	-0.72711	-0.72541	-0.76522	-0.76343	-0.84924	-0.84735

-0.60071	-0.5992	-0.64312	-0.64155	-0.64	-0.72425	-0.72257	-0.76226	-0.76055
-0.60129	-0.59975	-0.64364	-0.64205	-0.64049	-0.72481	-0.72315	-0.76287	-0.76114
-0.60187	-0.6003	-0.64418	-0.64257	-0.64099	-0.72539	-0.72373	-0.76349	-0.76173
-0.54473	-0.54348	-0.5993	-0.59778	-0.5963	-0.72599	-0.7243	-0.76408	-0.76234
-0.54517	-0.54394	-0.59987	-0.59835	-0.59686	-0.72662	-0.72487	-0.76467	-0.76296
-0.54563	-0.54439	-0.60041	-0.5989	-0.59741	-0.6839	-0.68223	-0.76531	-0.76357
-0.54608	-0.54484	-0.60094	-0.59943	-0.59796	-0.68452	-0.68285	-0.76599	-0.76418
-0.50106	-0.49986	-0.54405	-0.54283	-0.54162	-0.64074	-0.63919	-0.76666	-0.76482
-0.50151	-0.50032	-0.54451	-0.54328	-0.54206	-0.64128	-0.63976	-0.76734	-0.7655
-0.39334	-0.39244	-0.49959	-0.4984	-0.49721	-0.59804	-0.59662	-0.63879	-0.63724
-0.39369	-0.39279	-0.50004	-0.49885	-0.49767	-0.59858	-0.59712	-0.63931	-0.6378
-0.39404	-0.39315	-0.50048	-0.4993	-0.49813	-0.59912	-0.59762	-0.63984	-0.63834
-0.3944	-0.3935	-0.50094	-0.49976	-0.49858	-0.4974	-0.49623	-0.6404	-0.63889
-0.39476	-0.39385	-0.50141	-0.50022	-0.49903	-0.49785	-0.49669	-0.64098	-0.63943
-0.39512	-0.39421	-0.3933	-0.39241	-0.39153	-0.49832	-0.49716	-0.54171	-0.54044
-0.39548	-0.39456	-0.39365	-0.39276	-0.39188	-0.49879	-0.49764	-0.54224	-0.54096
-0.39585	-0.39493	-0.39402	-0.39313	-0.39225	-0.39137	-0.39051	-0.49695	-0.49579
-0.39624	-0.39532	-0.39441	-0.39351	-0.39262	-0.39174	-0.39086	-0.49739	-0.49623
-0.39662	-0.3957	-0.39479	-0.39388	-0.39299	-0.3921	-0.39122	-0.44285	-0.44186
-0.39699	-0.39607	-0.39516	-0.39425	-0.39334	-0.39245	-0.39157	-0.44325	-0.44225
-0.39736	-0.39644	-0.39552	-0.39461	-0.3937	-0.39281	-0.39192	-0.44366	-0.44265
-0.34277	-0.34198	-0.3959	-0.39499	-0.39408	-0.39318	-0.39229	-0.39141	-0.39052
-0.34311	-0.34231	-0.39629	-0.39537	-0.39447	-0.39356	-0.39267	-0.39178	-0.3909
-0.34344	-0.34263	-0.39666	-0.39575	-0.39484	-0.39393	-0.39304	-0.39215	-0.39127
-0.34375	-0.34293	-0.39703	-0.39611	-0.3952	-0.39429	-0.39339	-0.3925	-0.39162
-0.34408	-0.34325	-0.3974	-0.39648	-0.39557	-0.39466	-0.39375	-0.39286	-0.39198
-0.34442	-0.34359	-0.3978	-0.39687	-0.39596	-0.39504	-0.39414	-0.39324	-0.39236
-0.34476	-0.34393	-0.3982	-0.39727	-0.39635	-0.39544	-0.39453	-0.39363	-0.39273
-0.28872	-0.28803	-0.39859	-0.39766	-0.39674	-0.39583	-0.39492	-0.39401	-0.39312
-0.28898	-0.28829	-0.39896	-0.39804	-0.39713	-0.39622	-0.39531	-0.3944	-0.3935
-0.23714	-0.23655	-0.34408	-0.34329	-0.3425	-0.39659	-0.39568	-0.39478	-0.39388
-0.23738	-0.2368	-0.34443	-0.34361	-0.34281	-0.39695	-0.39605	-0.39515	-0.39426
-0.23762	-0.23704	-0.23647	-0.23589	-0.23533	-0.39733	-0.39642	-0.39553	-0.39465
-0.23786	-0.23729	-0.23672	-0.23613	-0.23555	-0.39772	-0.39681	-0.39591	-0.39503
-0.2381	-0.23753	-0.23697	-0.23638	-0.23579	-0.29268	-0.29202	-0.3963	-0.39541
-0.23835	-0.23779	-0.23721	-0.23662	-0.23602	-0.29297	-0.2923	-0.39669	-0.3958
-0.23861	-0.23804	-0.23746	-0.23686	-0.23627	-0.29327	-0.2926	-0.39708	-0.39618
-0.23886	-0.23828	-0.23771	-0.23711	-0.23651	-0.23594	-0.23539	-0.39746	-0.39656
-0.2391	-0.23853	-0.23795	-0.23736	-0.23676	-0.23618	-0.23562	-0.39784	-0.39693
-0.23935	-0.23878	-0.2382	-0.2376	-0.23701	-0.23643	-0.23586	-0.2928	-0.29212
-0.23961	-0.23903	-0.23845	-0.23785	-0.23725	-0.23667	-0.2361	-0.2931	-0.29243
-0.23986	-0.23928	-0.2387	-0.2381	-0.2375	-0.23691	-0.23634	-0.29341	-0.29274
-0.24011	-0.23953	-0.23894	-0.23834	-0.23774	-0.23716	-0.23658	-0.29372	-0.29304
-0.24036	-0.23978	-0.23919	-0.23859	-0.23799	-0.2374	-0.23683	-0.29403	-0.29334
-0.24062	-0.24004	-0.23945	-0.23884	-0.23823	-0.23765	-0.23707	-0.29434	-0.29365
-0.24087	-0.24029	-0.2397	-0.23909	-0.23848	-0.23789	-0.23732	-0.29465	-0.29395
-0.18532	-0.18486	-0.23996	-0.23934	-0.23873	-0.23814	-0.23756	-0.23699	-0.23644
-0.18551	-0.18505	-0.24021	-0.23959	-0.23898	-0.23838	-0.2378	-0.23724	-0.23668
-0.18571	-0.18525	-0.24047	-0.23985	-0.23923	-0.23863	-0.23805	-0.23748	-0.23692
-0.18591	-0.18545	-0.24072	-0.24011	-0.23949	-0.23889	-0.23829	-0.23772	-0.23716

-0.1861	-0.18564	-0.18518	-0.18471	-0.18423	-0.23915	-0.23855	-0.23797	-0.23741
-0.1863	-0.18584	-0.18538	-0.18491	-0.18444	-0.23943	-0.23882	-0.23823	-0.23766
-0.18649	-0.18603	-0.18557	-0.18512	-0.18466	-0.23971	-0.23909	-0.23849	-0.23791
-0.18668	-0.18622	-0.18577	-0.18532	-0.18486	-0.18438	-0.18389	-0.23875	-0.23816
-0.18687	-0.18642	-0.18597	-0.18551	-0.18505	-0.18458	-0.1841	-0.23901	-0.23841
-0.18708	-0.18662	-0.18616	-0.18571	-0.18525	-0.18478	-0.18431	-0.23928	-0.23868
-0.18729	-0.18682	-0.18635	-0.1859	-0.18544	-0.18498	-0.18452	-0.23957	-0.23896
-0.18751	-0.18703	-0.18656	-0.1861	-0.18564	-0.18518	-0.18472	-0.18426	-0.18378
-0.18773	-0.18725	-0.18678	-0.1863	-0.18584	-0.18538	-0.18492	-0.18446	-0.18399
-0.18794	-0.18747	-0.18699	-0.18651	-0.18604	-0.18558	-0.18512	-0.18466	-0.18419
-0.18815	-0.18768	-0.1872	-0.18673	-0.18626	-0.18578	-0.18532	-0.18485	-0.18439
-0.18835	-0.18788	-0.18741	-0.18694	-0.18647	-0.186	-0.18552	-0.18505	-0.18459
-0.18856	-0.18808	-0.18761	-0.18715	-0.18668	-0.18621	-0.18573	-0.18526	-0.18479
-0.18876	-0.18829	-0.18782	-0.18735	-0.18689	-0.18642	-0.18595	-0.18547	-0.18499
-0.18897	-0.18849	-0.18802	-0.18755	-0.18709	-0.18663	-0.18616	-0.18569	-0.18521
-0.18918	-0.18869	-0.18821	-0.18775	-0.18728	-0.18682	-0.18637	-0.1859	-0.18542
-0.1894	-0.18891	-0.18842	-0.18795	-0.18748	-0.18702	-0.18657	-0.18611	-0.18564
-0.18964	-0.18915	-0.18865	-0.18816	-0.18768	-0.18722	-0.18676	-0.1863	-0.18584
-0.18988	-0.18938	-0.18888	-0.18838	-0.18789	-0.18742	-0.18696	-0.1865	-0.18604
-0.19008	-0.18959	-0.1891	-0.18861	-0.18813	-0.18765	-0.18717	-0.18671	-0.18624
-0.19028	-0.1898	-0.18932	-0.18884	-0.18836	-0.18788	-0.18739	-0.18692	-0.18645
-0.19049	-0.19001	-0.18953	-0.18905	-0.18857	-0.18809	-0.18761	-0.18713	-0.18667
-0.1907	-0.19021	-0.18973	-0.18925	-0.18877	-0.18829	-0.18781	-0.18735	-0.18689
-0.19093	-0.19043	-0.18995	-0.18946	-0.18897	-0.18849	-0.18802	-0.18755	-0.1871
-0.19118	-0.19068	-0.19018	-0.18968	-0.18919	-0.1887	-0.18822	-0.18775	-0.18729
-0.19141	-0.19091	-0.1904	-0.1899	-0.18941	-0.18892	-0.18844	-0.18796	-0.1875
-0.19162	-0.19113	-0.19063	-0.19014	-0.18965	-0.18916	-0.18868	-0.1882	-0.18773
-0.19184	-0.19135	-0.19086	-0.19037	-0.18989	-0.1894	-0.18892	-0.18844	-0.18796
-0.19206	-0.19157	-0.19107	-0.19059	-0.19011	-0.18962	-0.18913	-0.18865	-0.18817
-0.19229	-0.19179	-0.19129	-0.1908	-0.19032	-0.18983	-0.18935	-0.18886	-0.18838
-0.19253	-0.19202	-0.19153	-0.19104	-0.19056	-0.19007	-0.18959	-0.1891	-0.18861
-0.19276	-0.19227	-0.19178	-0.19129	-0.19081	-0.19032	-0.18984	-0.18935	-0.18886
-0.19299	-0.1925	-0.19201	-0.19152	-0.19104	-0.19056	-0.19007	-0.18958	-0.18909
-0.19322	-0.19271	-0.19221	-0.19173	-0.19125	-0.19077	-0.19028	-0.18979	-0.1893
-0.19345	-0.19294	-0.19243	-0.19195	-0.19147	-0.19099	-0.1905	-0.19002	-0.18952
-0.19369	-0.19318	-0.19267	-0.19219	-0.19171	-0.19123	-0.19075	-0.19026	-0.18977
-0.19393	-0.19342	-0.19292	-0.19243	-0.19195	-0.19147	-0.19099	-0.19051	-0.19002
-0.19416	-0.19364	-0.19313	-0.19264	-0.19216	-0.19168	-0.19121	-0.19073	-0.19024
-0.1944	-0.19387	-0.19334	-0.19285	-0.19236	-0.19189	-0.19141	-0.19094	-0.19045
-0.19465	-0.19411	-0.19359	-0.19308	-0.19259	-0.19211	-0.19164	-0.19116	-0.19068
-0.1949	-0.19437	-0.19385	-0.19334	-0.19284	-0.19236	-0.19188	-0.19141	-0.19093
-0.19516	-0.19463	-0.19411	-0.1936	-0.19309	-0.1926	-0.19212	-0.19165	-0.19118
-0.19542	-0.1949	-0.19438	-0.19386	-0.19334	-0.19285	-0.19236	-0.19189	-0.19142
-0.12845	-0.12813	-0.19464	-0.19411	-0.19359	-0.19308	-0.1926	-0.19212	-0.19164
-0.12859	-0.12828	-0.19487	-0.19434	-0.19382	-0.19331	-0.19281	-0.19233	-0.19185
-0.12873	-0.12842	-0.1951	-0.19458	-0.19405	-0.19354	-0.19303	-0.19254	-0.19206
-0.12889	-0.12858	-0.19537	-0.19484	-0.19431	-0.19379	-0.19328	-0.19278	-0.1923
-0.12906	-0.12874	-0.19564	-0.19511	-0.19459	-0.19406	-0.19354	-0.19303	-0.19255
-0.12922	-0.12891	-0.1286	-0.12828	-0.12797	-0.19433	-0.1938	-0.19329	-0.19279
-0.12938	-0.12907	-0.12876	-0.12845	-0.12813	-0.1946	-0.19407	-0.19355	-0.19304

-0.06288	-0.06275	-0.12892	-0.1286	-0.12829	-0.19485	-0.19432	-0.1938	-0.19328
-0.06294	-0.06281	-0.12905	-0.12874	-0.12843	-0.19508	-0.19455	-0.19403	-0.1935
-0.06301	-0.06287	-0.1292	-0.12888	-0.12857	-0.12826	-0.12794	-0.19426	-0.19373
-0.06308	-0.06295	-0.12936	-0.12904	-0.12873	-0.12842	-0.1281	-0.19453	-0.194
-0.06316	-0.06302	-0.12952	-0.12921	-0.1289	-0.12859	-0.12827	-0.19481	-0.19427
-0.06324	-0.0631	-0.06296	-0.06283	-0.0627	-0.12875	-0.12844	-0.19508	-0.19455
-0.06332	-0.06317	-0.06303	-0.0629	-0.06277	-0.12892	-0.1286	-0.19535	-0.19482
-0.0634	-0.06325	-0.06311	-0.06297	-0.06284	-0.12908	-0.12877	-0.19562	-0.1951
-0.06348	-0.06333	-0.06319	-0.06305	-0.06292	-0.12925	-0.12893	-0.19589	-0.19537
-0.06356	-0.06341	-0.06326	-0.06312	-0.06299	-0.12941	-0.1291	-0.12878	-0.12847
-0.06364	-0.06348	-0.06333	-0.06319	-0.06306	-0.12956	-0.12926	-0.12895	-0.12864
-0.06371	-0.06355	-0.0634	-0.06326	-0.06312	-0.12971	-0.12941	-0.12911	-0.1288
-0.06379	-0.06363	-0.06348	-0.06334	-0.0632	-0.06306	-0.06293	-0.12925	-0.12894
-0.06387	-0.06372	-0.06357	-0.06342	-0.06327	-0.06313	-0.06299	-0.12939	-0.12909
		-0.06365	-0.0635	-0.06335	-0.06321	-0.06306	-0.12954	-0.12924
		-0.06374	-0.06358	-0.06343	-0.06328	-0.06314	-0.12971	-0.12941
		-0.06382	-0.06367	-0.06351	-0.06337	-0.06322	-0.12988	-0.12957
		-0.0639	-0.06375	-0.0636	-0.06345	-0.0633	-0.13005	-0.12973
					-0.06353	-0.06338	-0.13021	-0.12989
					-0.06361	-0.06346	-0.13038	-0.13006
					-0.0637	-0.06355	-0.13055	-0.13022
					-0.06378	-0.06363	-0.06348	-0.06333
					-0.06386	-0.06371	-0.06356	-0.06341
							-0.06364	-0.06349
							-0.06373	-0.06358

							-0.06156	-0.06137
							-0.06146	-0.06128
					-0.06175	-0.06156	-0.19349	-0.19295
					-0.06166	-0.06147	-0.19323	-0.19268
					-0.06157	-0.06138	-0.38225	-0.38129
					-0.06147	-0.06129	-0.38175	-0.3808
					-0.06138	-0.0612	-0.38125	-0.3803
			-0.06167	-0.06147	-0.19322	-0.19268	-0.44283	-0.44172
			-0.06158	-0.06139	-0.19296	-0.19242	-0.44228	-0.44116
			-0.19379	-0.19324	-0.38176	-0.38078	-0.56202	-0.56054
			-0.1935	-0.19297	-0.38124	-0.38029	-0.56128	-0.55979
-0.06187	-0.06168	-0.0615	-0.38265	-0.38169	-0.50389	-0.5026	-0.61268	-0.61108
-0.06178	-0.06159	-0.0614	-0.38216	-0.38119	-0.50319	-0.50193	-0.61188	-0.61029
-0.19432	-0.19376	-0.1932	-0.38167	-0.3807	-0.56193	-0.56049	-0.71836	-0.71646
-0.19406	-0.1935	-0.19294	-0.3812	-0.38023	-0.56119	-0.55975	-0.71743	-0.71555
-0.19379	-0.19324	-0.19268	-0.38073	-0.37977	-0.56046	-0.55901	-0.71649	-0.71462
-0.38321	-0.38221	-0.38122	-0.50318	-0.50187	-0.61179	-0.61022	-0.71552	-0.71367
-0.38271	-0.38173	-0.38075	-0.50253	-0.50121	-0.61097	-0.60939	-0.71453	-0.71269
-0.50585	-0.50453	-0.50321	-0.56121	-0.55969	-0.71738	-0.71546	-0.71358	-0.71172
-0.50518	-0.50388	-0.50258	-0.56049	-0.55898	-0.71648	-0.71455	-0.71264	-0.71077
-0.50452	-0.50323	-0.50194	-0.66811	-0.66632	-0.71557	-0.71364	-0.86325	-0.86095
-0.50387	-0.50259	-0.5013	-0.66728	-0.66548	-0.71467	-0.71273	-0.86214	-0.85985
-0.50321	-0.50194	-0.50066	-0.66643	-0.66464	-0.71376	-0.71183	-0.86103	-0.85876
-0.56198	-0.56053	-0.55907	-0.71672	-0.71477	-0.71284	-0.71092	-0.97384	-0.97136
-0.5612	-0.55976	-0.55832	-0.71578	-0.71384	-0.71193	-0.71001	-0.97262	-0.97015
-0.669	-0.66729	-0.66558	-0.71484	-0.71293	-0.86227	-0.85996	-0.97143	-0.96897
-0.66813	-0.66641	-0.66471	-0.7139	-0.71201	-0.86116	-0.85888	-0.97028	-0.96783
-0.71856	-0.7167	-0.71485	-0.71298	-0.71111	-0.97407	-0.97159	-0.96912	-0.96667
-0.71763	-0.71578	-0.71394	-0.71208	-0.71022	-0.97288	-0.97041	-0.96796	-0.96552
-0.71671	-0.71486	-0.71303	-0.88219	-0.88001	-0.9717	-0.96924	-0.96679	-0.96436
-0.7158	-0.71395	-0.71212	-0.88113	-0.87896	-0.97053	-0.96808	-0.96563	-0.96319
-0.71491	-0.71306	-0.71122	-0.88007	-0.87791	-0.96936	-0.96691	-0.96446	-0.96202
-0.71406	-0.7122	-0.71035	-0.97302	-0.9706	-0.96816	-0.96571	-0.96327	-0.96083
-0.71321	-0.71135	-0.70949	-0.97185	-0.9694	-0.96696	-0.96451	-0.96207	-0.95963
-0.88357	-0.88138	-0.87918	-0.97068	-0.96822	-0.96577	-0.96333	-1.01073	-1.00816
-0.88248	-0.88031	-0.87813	-0.96951	-0.96705	-0.9646	-0.96216	-1.0095	-1.00693
-0.93162	-0.92931	-0.927	-0.96834	-0.96588	-0.96343	-0.96099	-1.00827	-1.00572
-0.93053	-0.9282	-0.92588	-0.96716	-0.9647	-0.96226	-0.95982	-1.00704	-1.00451
-0.92945	-0.9271	-0.92476	-0.96598	-0.96353	-1.01093	-1.00837	-1.00583	-1.0033
-0.92834	-0.92598	-0.92363	-0.96481	-0.96236	-1.0097	-1.00715	-1.00462	-1.0021
-0.92723	-0.92487	-0.92251	-0.96364	-0.9612	-1.00848	-1.00594	-1.00341	-1.00091
-0.96983	-0.96736	-0.96491	-0.96246	-0.96003	-1.00726	-1.00472	-1.0022	-0.9997
-0.96865	-0.96618	-0.96372	-0.96128	-0.95886	-1.00603	-1.0035	-1.00099	-0.9985
-0.96744	-0.96498	-0.96254	-1.00989	-1.00734	-1.00481	-1.0023	-0.9998	-0.99731
-0.96622	-0.96378	-0.96134	-1.00864	-1.0061	-1.00359	-1.0011	-0.99862	-0.99614
-0.96502	-0.96258	-0.96016	-1.00741	-1.00489	-1.0024	-0.99994	-1.04138	-1.03874
-0.96384	-0.9614	-0.95899	-1.00619	-1.0037	-1.00124	-0.99882	-1.04023	-1.03763
-0.9627	-0.96027	-0.95786	-1.00502	-1.00254	-1.00011	-0.99772	-1.03908	-1.0365
-0.96166	-0.95924	-0.95684	-1.00395	-1.00147	-0.99903	-0.99661	-1.03789	-1.0353

-0.96066	-0.95825	-0.95585	-1.00291	-1.00042	-0.99795	-0.9955	-1.03668	-1.03406
-1.00932	-1.00679	-1.00427	-1.00177	-0.99929	-1.04073	-1.03808	-1.03545	-1.03283
-1.0081	-1.00558	-1.00307	-1.00058	-0.9981	-1.03947	-1.03683	-1.0342	-1.03159
-1.00689	-1.00437	-1.00187	-0.99939	-0.99691	-1.03822	-1.03558	-1.16862	-1.16518
-1.00567	-1.00316	-1.00067	-0.99819	-0.99572	-1.03696	-1.03433	-1.16696	-1.16356
-1.0045	-1.002	-0.99951	-1.04104	-1.03839	-1.03576	-1.03313	-1.16536	-1.16201
-1.00343	-1.00093	-0.99845	-1.03991	-1.03727	-1.03464	-1.03202	-1.16391	-1.16058
-1.00235	-0.99986	-0.99738	-1.03878	-1.03614	-1.03352	-1.03092	-1.16246	-1.15916
-1.00118	-0.9987	-0.99622	-1.03756	-1.03493	-1.16766	-1.16426	-1.20324	-1.19984
-0.99998	-0.9975	-0.99504	-1.0363	-1.03369	-1.166	-1.16264	-1.2016	-1.19822
-1.04299	-1.04033	-1.03769	-1.03507	-1.03246	-1.16438	-1.16105	-1.23976	-1.23631
-1.04173	-1.03909	-1.03646	-1.03384	-1.03124	-1.16277	-1.15946	-1.23809	-1.23464
-1.04055	-1.03791	-1.03529	-1.168	-1.16461	-1.2036	-1.20019	-1.23651	-1.23305
-1.03946	-1.03683	-1.03421	-1.16659	-1.16322	-1.20218	-1.19878	-1.23506	-1.23157
-1.03835	-1.03572	-1.03311	-1.16514	-1.1618	-1.24052	-1.23704	-1.23357	-1.23009
-1.03714	-1.03451	-1.0319	-1.16353	-1.16022	-1.23886	-1.23541	-1.23197	-1.22856
-1.03591	-1.03328	-1.03067	-1.1619	-1.15861	-1.23716	-1.23373	-1.23035	-1.22701
-1.03477	-1.03212	-1.02949	-1.20262	-1.1992	-1.23549	-1.23209	-1.22874	-1.22544
-1.03366	-1.031	-1.02835	-1.20105	-1.19759	-1.23385	-1.23047	-1.22715	-1.22388
-1.21014	-1.20656	-1.20301	-1.19952	-1.19609	-1.23232	-1.22898	-1.22568	-1.22243
-1.20847	-1.20495	-1.20147	-1.19805	-1.19468	-1.23092	-1.2276	-1.22432	-1.22109
-1.20686	-1.20338	-1.19994	-1.23622	-1.23281	-1.22946	-1.22616	-1.2661	-1.26257
-1.20535	-1.20186	-1.1984	-1.23464	-1.23122	-1.22787	-1.22461	-1.26443	-1.26092
-1.20383	-1.20034	-1.19688	-1.23307	-1.22964	-1.22632	-1.22308	-1.26278	-1.2593
-1.20222	-1.19877	-1.19537	-1.23156	-1.22819	-1.2249	-1.22168	-1.26126	-1.2578
-1.20061	-1.19722	-1.19387	-1.23008	-1.22677	-1.22351	-1.22031	-1.25976	-1.25633
-1.19912	-1.19574	-1.19239	-1.22857	-1.22527	-1.26513	-1.26163	-1.25816	-1.25473
-1.19772	-1.19431	-1.19094	-1.22704	-1.22372	-1.26344	-1.25995	-1.2565	-1.25307
-1.19624	-1.19286	-1.18951	-1.26907	-1.26542	-1.26186	-1.25838	-1.25493	-1.25151
-1.19467	-1.19137	-1.18811	-1.26755	-1.26393	-1.26039	-1.25692	-1.25348	-1.25006
-1.23269	-1.22937	-1.22609	-1.26604	-1.26246	-1.25894	-1.25545	-1.2923	-1.28874
-1.23127	-1.22797	-1.22473	-1.26455	-1.26101	-1.25747	-1.25395	-1.2907	-1.28711
-1.22986	-1.22658	-1.22334	-1.26303	-1.25952	-1.25599	-1.25244	-1.2891	-1.2855
-1.27214	-1.2685	-1.26492	-1.2614	-1.25793	-1.25443	-1.25092	-1.28756	-1.28399
-1.27053	-1.26685	-1.26325	-1.25974	-1.2563	-1.25285	-1.24941	-1.28605	-1.28254
-1.26894	-1.26529	-1.26171	-1.25821	-1.25477	-1.29162	-1.28807	-1.28457	-1.28111
-1.26737	-1.26379	-1.26026	-1.25677	-1.25332	-1.29012	-1.28659	-1.28312	-1.2797
-1.26585	-1.2623	-1.25881	-1.25531	-1.25185	-1.28858	-1.28506	-1.31891	-1.31549
-1.26437	-1.26085	-1.25736	-1.25385	-1.25034	-1.28698	-1.28344	-1.3173	-1.314
-1.2629	-1.25939	-1.2559	-1.29269	-1.28901	-1.32277	-1.31917	-1.31576	-1.31258
-1.26144	-1.25791	-1.2544	-1.29112	-1.28747	-1.32124	-1.31773	-1.3144	-1.31129
-1.25997	-1.25642	-1.25288	-1.28957	-1.28596	-1.31977	-1.31636	-1.31311	-1.31004
-1.25847	-1.25491	-1.25138	-1.28804	-1.28448	-1.31832	-1.315	-1.35595	-1.35271
-1.25694	-1.2534	-1.24989	-1.28655	-1.28304	-1.31691	-1.31366	-1.3546	-1.35139
-1.29589	-1.29223	-1.28862	-1.3224	-1.31887	-1.31552	-1.31232	-1.35323	-1.35007
-1.29435	-1.29072	-1.28715	-1.32094	-1.31748	-1.31417	-1.31099	-1.35185	-1.34876
-1.29283	-1.28923	-1.2857	-1.31949	-1.31609	-1.31282	-1.30969	-1.35052	-1.34748
-1.29134	-1.28776	-1.28426	-1.31804	-1.31468	-1.31148	-1.30843	-1.34926	-1.34624
-1.32725	-1.32361	-1.32007	-1.31663	-1.3133	-1.31017	-1.30721	-1.34802	-1.34503
-1.32575	-1.32216	-1.31865	-1.31527	-1.31201	-1.30893	-1.30602	-1.34679	-1.34383

-1.32427	-1.32072	-1.31726	-1.31395	-1.31076	-1.30773	-1.30486	-1.34559	-1.34265
-1.32279	-1.31928	-1.31587	-1.31262	-1.30951	-1.35041	-1.34737	-1.34439	-1.34146
-1.32132	-1.31784	-1.31448	-1.3113	-1.30828	-1.34916	-1.34617	-1.34321	-1.34027
-1.31989	-1.31645	-1.31314	-1.31003	-1.30707	-1.34793	-1.34497	-1.34202	-1.33907
-1.31849	-1.31511	-1.31185	-1.3088	-1.30589	-1.34671	-1.34377	-1.34082	-1.33783
-1.31714	-1.31381	-1.31061	-1.3076	-1.30473	-1.3455	-1.34257	-1.36791	-1.3648
-1.31585	-1.31258	-1.30942	-1.30644	-1.30358	-1.34429	-1.34138	-1.36669	-1.36358
-1.31457	-1.31137	-1.30826	-1.30529	-1.30244	-1.3431	-1.3402	-1.36547	-1.36238
-1.31326	-1.31013	-1.30709	-1.34791	-1.34488	-1.37028	-1.36728	-1.36425	-1.36118
-1.31194	-1.30889	-1.30592	-1.34671	-1.34369	-1.36905	-1.36605	-1.36304	-1.35999
-1.31064	-1.30764	-1.30472	-1.37398	-1.37087	-1.3678	-1.36479	-1.36177	-1.35874
-1.30936	-1.30637	-1.30348	-1.37267	-1.36961	-1.36655	-1.3635	-1.36047	-1.35747
-1.33755	-1.33445	-1.33144	-1.3714	-1.36837	-1.36532	-1.36225	-1.35922	-1.35623
-1.33643	-1.33334	-1.33031	-1.3702	-1.36717	-1.36412	-1.36106	-1.35803	-1.35502
-1.33529	-1.33222	-1.32919	-1.36901	-1.36598	-1.36293	-1.35988	-1.35685	-1.35384
-1.33407	-1.33103	-1.32804	-1.36782	-1.3648	-1.36175	-1.3587	-1.35567	-1.35266
-1.33283	-1.32984	-1.32689	-1.36664	-1.36362	-1.36058	-1.35754	-1.35452	-1.35151
-1.37475	-1.3716	-1.36849	-1.36545	-1.36244	-1.35942	-1.35642	-1.35342	-1.3504
-1.37351	-1.37038	-1.36728	-1.36425	-1.36125	-1.35827	-1.35531	-1.35234	-1.34932
-1.37227	-1.36916	-1.36608	-1.36305	-1.36006	-1.3571	-1.35416	-1.35121	-1.3482
-1.37102	-1.36794	-1.36488	-1.36185	-1.35885	-1.35589	-1.35297	-1.35003	-1.34705
-1.36979	-1.36672	-1.36368	-1.36065	-1.35764	-1.35468	-1.35177	-1.38638	-1.38332
-1.36856	-1.3655	-1.36247	-1.35943	-1.35641	-1.35345	-1.35057	-1.38516	-1.38214
-1.36733	-1.3643	-1.36128	-1.35823	-1.3552	-1.35224	-1.34937	-1.38395	-1.38096
-1.36613	-1.36313	-1.36015	-1.3571	-1.35403	-1.35107	-1.34818	-1.38272	-1.37977
-1.36493	-1.36197	-1.35902	-1.35597	-1.35289	-1.34991	-1.347	-1.38151	-1.3786
-1.36376	-1.36079	-1.35783	-1.35479	-1.35173	-1.3863	-1.38328	-1.38034	-1.37748
-1.36259	-1.35958	-1.3566	-1.35358	-1.35057	-1.38512	-1.38212	-1.3792	-1.37639
-1.36141	-1.35838	-1.35538	-1.35239	-1.34943	-1.38398	-1.38098	-1.37807	-1.37528
-1.36022	-1.35717	-1.35416	-1.35122	-1.34833	-1.38287	-1.37988	-1.37697	-1.37415
-1.35905	-1.35599	-1.35298	-1.38766	-1.38471	-1.38174	-1.37877	-1.37586	-1.37302
-1.35792	-1.35486	-1.35185	-1.38648	-1.38351	-1.38056	-1.37762	-1.37473	-1.37188
-1.35679	-1.35375	-1.35073	-1.38533	-1.38233	-1.37939	-1.37648	-1.37361	-1.37076
-1.35564	-1.35263	-1.34965	-1.38421	-1.38122	-1.37828	-1.3754	-1.43796	-1.43492
-1.35449	-1.35151	-1.34858	-1.38312	-1.38012	-1.3772	-1.37434	-1.43685	-1.43381
-1.39107	-1.388	-1.38497	-1.38196	-1.37899	-1.37607	-1.37322	-1.43569	-1.43267
-1.38987	-1.38679	-1.38375	-1.38077	-1.37783	-1.37493	-1.37206	-1.43448	-1.4315
-1.3887	-1.38562	-1.38259	-1.37963	-1.37671	-1.43937	-1.43632	-1.43332	-1.43038
-1.38757	-1.38452	-1.3815	-1.37855	-1.37565	-1.43826	-1.43521	-1.43222	-1.4293
-1.38645	-1.38342	-1.38042	-1.37748	-1.37458	-1.43715	-1.43412	-1.43114	-1.42823
-1.38531	-1.38232	-1.37936	-1.37642	-1.3735	-1.43604	-1.43306	-1.43011	-1.42717
-1.38417	-1.38121	-1.37829	-1.37534	-1.37241	-1.43492	-1.43199	-1.42906	-1.42611
-1.38302	-1.38005	-1.37713	-1.41168	-1.40867	-1.43376	-1.43084	-1.42792	-1.42499
-1.38187	-1.37888	-1.37594	-1.41046	-1.4075	-1.4326	-1.42967	-1.42675	-1.42385
-1.38075	-1.37776	-1.37482	-1.43748	-1.43447	-1.43149	-1.42855	-1.42564	-1.42275
-1.37965	-1.37668	-1.37374	-1.43637	-1.4334	-1.43043	-1.42749	-1.42457	-1.42168
-1.41614	-1.41309	-1.41007	-1.43529	-1.43232	-1.42937	-1.42643	-1.42352	-1.42063
-1.415	-1.41197	-1.40899	-1.43421	-1.43125	-1.42831	-1.42539	-1.42249	-1.4196
-1.41386	-1.41086	-1.40791	-1.43314	-1.43018	-1.42725	-1.42435	-1.42146	-1.41857
-1.41276	-1.40976	-1.40681	-1.43206	-1.42913	-1.4262	-1.42329	-1.42039	-1.41753

-1.41166	-1.40866	-1.40571	-1.43099	-1.42808	-1.42516	-1.42222	-1.41932	-1.41649
-1.41053	-1.40756	-1.40464	-1.42993	-1.42702	-1.4241	-1.42117	-1.41829	-1.41546
-1.4094	-1.40647	-1.4036	-1.42888	-1.42594	-1.42303	-1.42014	-1.41727	-1.41443
-1.4367	-1.4337	-1.43076	-1.42781	-1.42488	-1.42198	-1.4191	-1.41625	-1.41342
-1.43569	-1.43266	-1.42967	-1.42673	-1.42384	-1.42094	-1.41806	-1.41522	-1.41242
-1.43469	-1.43163	-1.42862	-1.42568	-1.42279	-1.4199	-1.41703	-1.4142	-1.41142
-1.43367	-1.43064	-1.42764	-1.42467	-1.42172	-1.41883	-1.416	-1.41319	-1.41041
-1.43263	-1.42965	-1.42667	-1.42367	-1.42067	-1.41778	-1.41497	-1.41219	-1.40941
-1.43155	-1.4286	-1.42566	-1.42268	-1.41971	-1.4168	-1.41396	-1.41116	-1.4084
-1.43046	-1.42753	-1.42462	-1.42169	-1.41877	-1.41586	-1.41296	-1.41013	-1.40739
-1.4294	-1.42647	-1.42355	-1.42066	-1.41778	-1.41489	-1.412	-1.40917	-1.40642
-1.42836	-1.4254	-1.42246	-1.41959	-1.41676	-1.41392	-1.41108	-1.40827	-1.40547
-1.42735	-1.42438	-1.42144	-1.41857	-1.41574	-1.41292	-1.41012	-1.40733	-1.40453
-1.42636	-1.42342	-1.4205	-1.41759	-1.4147	-1.41187	-1.40911	-1.40636	-1.40361
-1.42535	-1.42244	-1.41954	-1.41661	-1.41369	-1.41086	-1.40811	-1.40538	-1.40267
-1.4243	-1.42141	-1.41855	-1.41565	-1.41276	-1.40992	-1.40712	-1.40438	-1.4017
-1.42324	-1.42037	-1.41753	-1.41468	-1.41183	-1.40899	-1.40615	-1.40339	-1.40072
-1.42225	-1.41935	-1.41649	-1.41366	-1.41085	-1.40804	-1.40523	-1.40247	-1.39977
-1.42128	-1.41833	-1.41543	-1.41261	-1.40985	-1.40708	-1.40432	-1.40158	-1.39884
-1.4203	-1.41736	-1.41445	-1.41162	-1.40884	-1.40609	-1.40338	-1.40066	-1.39793
-1.41931	-1.41641	-1.41351	-1.41065	-1.40782	-1.40507	-1.4024	-1.39973	-1.39704
-1.41833	-1.41546	-1.41259	-1.40971	-1.40685	-1.4041	-1.40145	-1.39881	-1.39615
-1.41736	-1.41451	-1.41167	-1.40881	-1.40596	-1.40321	-1.40054	-1.39789	-1.39527
-1.41637	-1.41355	-1.41075	-1.40791	-1.40508	-1.40232	-1.39963	-1.42897	-1.42633
-1.41534	-1.41255	-1.40977	-1.40697	-1.40417	-1.40141	-1.39869	-1.42804	-1.42542
-1.41432	-1.41154	-1.40879	-1.40603	-1.40326	-1.40051	-1.39777	-1.42712	-1.4245
-1.41336	-1.41058	-1.40784	-1.4051	-1.40237	-1.39963	-1.39688	-1.42623	-1.42358
-1.41243	-1.40965	-1.40691	-1.40418	-1.40148	-1.39876	-1.39602	-1.42537	-1.42268
-1.4115	-1.4087	-1.40595	-1.40324	-1.40056	-1.43002	-1.42726	-1.42453	-1.42182
-1.41057	-1.40774	-1.40497	-1.40227	-1.39962	-1.42912	-1.42641	-1.4237	-1.42099
-1.40965	-1.40681	-1.40402	-1.40133	-1.3987	-1.42824	-1.42556	-1.42287	-1.42016
-1.40873	-1.4059	-1.40312	-1.40043	-1.39781	-1.42737	-1.42471	-1.42204	-1.41933
-1.40783	-1.405	-1.40222	-1.39955	-1.39694	-1.42652	-1.42387	-1.4212	-1.4185
-1.40693	-1.4041	-1.40134	-1.43096	-1.42832	-1.42567	-1.42302	-1.42036	-1.41767
-1.40604	-1.40322	-1.40045	-1.4301	-1.42746	-1.42482	-1.42218	-1.41952	-1.41683
-1.43762	-1.43476	-1.43195	-1.42925	-1.42661	-1.42397	-1.42133	-1.56368	-1.5605
-1.43675	-1.4339	-1.4311	-1.42841	-1.42577	-1.42313	-1.4205	-1.56275	-1.55957
-1.43587	-1.43303	-1.43025	-1.42756	-1.42493	-1.47555	-1.47286	-1.56183	-1.55864
-1.43499	-1.43216	-1.42939	-1.42671	-1.42409	-1.47473	-1.47203	-1.5609	-1.55772
-1.43411	-1.4313	-1.42854	-1.42586	-1.42325	-1.49951	-1.49677	-1.55996	-1.5568
-1.43323	-1.43043	-1.42768	-1.42501	-1.4224	-1.49869	-1.49595	-1.55902	-1.55588
-1.43236	-1.42956	-1.42682	-1.42416	-1.42156	-1.49786	-1.49512	-1.55807	-1.55496
-1.43149	-1.42871	-1.42599	-1.47674	-1.47406	-1.49702	-1.49429	-1.55712	-1.55404
-1.43062	-1.42787	-1.42516	-1.47591	-1.47322	-1.49619	-1.49346	-1.55616	-1.55312
-1.48339	-1.4806	-1.47785	-1.50088	-1.49814	-1.49539	-1.49265	-1.55524	-1.5522
-1.48256	-1.47981	-1.47709	-1.50014	-1.49738	-1.49461	-1.49185	-1.55433	-1.5513
-1.48176	-1.47903	-1.47632	-1.49938	-1.4966	-1.49382	-1.49104	-1.55343	-1.55041
-1.48099	-1.47826	-1.47554	-1.49859	-1.4958	-1.49301	-1.49022	-1.55253	-1.54952
-1.50615	-1.50337	-1.50059	-1.49779	-1.49499	-1.49219	-1.4894	-1.55163	-1.54864
-1.50539	-1.50258	-1.49978	-1.49697	-1.49416	-1.49137	-1.48859	-1.55074	-1.54777

-1.50461	-1.50179	-1.49897	-1.49615	-1.49334	-1.49055	-1.48778	-1.54985	-1.5469
-1.50381	-1.50098	-1.49815	-1.49534	-1.49253	-1.48975	-1.48698	-1.58009	-1.57708
-1.50298	-1.50015	-1.49733	-1.49452	-1.49173	-1.48896	-1.48619	-1.5792	-1.57623
-1.50215	-1.49932	-1.49651	-1.49373	-1.49096	-1.48821	-1.48546	-1.57837	-1.57537
-1.50131	-1.49849	-1.49569	-1.49294	-1.49022	-1.4875	-1.48478	-1.57759	-1.57453
-1.50048	-1.49768	-1.4949	-1.49217	-1.48947	-1.51894	-1.51612	-1.57676	-1.57365
-1.49967	-1.4969	-1.49415	-1.49142	-1.48872	-1.51813	-1.5153	-1.57585	-1.57272
-1.49889	-1.49613	-1.4934	-1.49067	-1.48796	-1.5173	-1.51446	-1.57493	-1.57179
-1.49817	-1.4954	-1.49265	-1.4899	-1.48717	-1.51647	-1.51362	-1.574	-1.57084
-1.49748	-1.49467	-1.49189	-1.48912	-1.48637	-1.51562	-1.51279	-1.57308	-1.56992
-1.49671	-1.49389	-1.4911	-1.48832	-1.48557	-1.5148	-1.51199	-1.57222	-1.56907
-1.4959	-1.49309	-1.49029	-1.48752	-1.48477	-1.51399	-1.51121	-1.57139	-1.56829
-1.49513	-1.49233	-1.48955	-1.51887	-1.51602	-1.51321	-1.51043	-1.57053	-1.56743
-1.4944	-1.49163	-1.48887	-1.51816	-1.5153	-1.51246	-1.50964	-1.56962	-1.5665
-1.49365	-1.49091	-1.48817	-1.51742	-1.51455	-1.51169	-1.50884	-1.60133	-1.59799
-1.49288	-1.49013	-1.48738	-1.5166	-1.51373	-1.51087	-1.50802	-1.60036	-1.597
-1.4921	-1.48933	-1.48658	-1.51576	-1.51291	-1.51006	-1.50721	-1.59943	-1.59606
-1.49131	-1.48853	-1.48577	-1.51494	-1.51212	-1.5093	-1.50647	-1.59858	-1.59524
-1.49053	-1.48774	-1.48497	-1.51414	-1.51136	-1.50856	-1.50575	-1.59777	-1.59445
-1.4898	-1.48701	-1.48425	-1.51337	-1.51058	-1.50778	-1.50498	-1.6264	-1.62302
-1.48911	-1.48634	-1.48358	-1.51263	-1.5098	-1.50698	-1.50416	-1.62545	-1.62206
-1.52044	-1.51757	-1.51472	-1.51187	-1.50903	-1.5062	-1.50338	-1.62455	-1.62116
-1.5196	-1.51673	-1.51389	-1.51107	-1.50826	-1.50546	-1.50265	-1.62374	-1.62036
-1.5188	-1.51593	-1.51309	-1.51028	-1.5075	-1.53822	-1.53521	-1.62289	-1.61951
-1.51811	-1.51523	-1.51237	-1.50954	-1.50672	-1.53736	-1.53435	-1.62193	-1.61855
-1.51744	-1.51455	-1.51168	-1.50882	-1.50597	-1.53651	-1.53348	-1.62096	-1.61757
-1.51676	-1.51388	-1.511	-1.50813	-1.50526	-1.53574	-1.5327	-1.62008	-1.61669
-1.51608	-1.5132	-1.51032	-1.50745	-1.50459	-1.53499	-1.53196	-1.61924	-1.61585
-1.51532	-1.51244	-1.50956	-1.50669	-1.50383	-1.53417	-1.53113	-1.61832	-1.61493
-1.51448	-1.5116	-1.50872	-1.50585	-1.503	-1.53328	-1.53024	-1.61731	-1.61393
-1.51369	-1.51081	-1.50794	-1.50508	-1.50223	-1.53245	-1.52942	-1.61638	-1.61299
-1.51298	-1.51012	-1.50726	-1.5044	-1.50155	-1.53172	-1.52869	-1.61556	-1.61216
-1.51225	-1.50941	-1.50658	-1.53709	-1.53404	-1.531	-1.52797	-1.61476	-1.61135
-1.51145	-1.50863	-1.50584	-1.53632	-1.5333	-1.53028	-1.52725	-1.61395	-1.61055
-1.51064	-1.50784	-1.50507	-1.53552	-1.53255	-1.52954	-1.52651	-1.61311	-1.60974
-1.50989	-1.50708	-1.5043	-1.53468	-1.53171	-1.61896	-1.61556	-1.61218	-1.60882
-1.50918	-1.50634	-1.50352	-1.53381	-1.53081	-1.61794	-1.61455	-1.61119	-1.60785
-1.50849	-1.50564	-1.50279	-1.53301	-1.53	-1.61702	-1.61363	-1.61028	-1.60697
-1.50783	-1.50497	-1.50213	-1.5323	-1.52928	-1.6162	-1.6128	-1.60945	-1.60616
-1.50716	-1.50431	-1.50147	-1.53158	-1.52857	-1.61539	-1.61199	-1.60864	-1.60537
-1.5065	-1.50364	-1.5008	-1.53086	-1.52783	-1.61458	-1.61118	-1.60783	-1.60457
-1.50582	-1.50297	-1.50012	-1.53012	-1.52709	-1.61375	-1.61036	-1.60702	-1.60375
-1.50514	-1.50229	-1.49944	-1.52938	-1.52634	-1.61291	-1.6095	-1.60614	-1.60285
-1.50443	-1.50158	-1.49874	-1.52862	-1.52557	-1.61206	-1.60862	-1.60524	-1.60193
-1.53692	-1.53386	-1.53082	-1.52778	-1.52474	-1.61116	-1.60776	-1.6044	-1.60109
-1.53601	-1.53295	-1.5299	-1.52687	-1.52385	-1.61023	-1.60691	-1.6036	-1.6003
-1.53516	-1.5321	-1.52907	-1.52604	-1.52302	-1.60936	-1.60608	-1.6028	-1.59952
-1.5344	-1.53135	-1.52832	-1.52529	-1.52228	-1.60855	-1.60527	-1.602	-1.59873
-1.53366	-1.53062	-1.52759	-1.52457	-1.52155	-1.60776	-1.60448	-1.60121	-1.59795
-1.53294	-1.52989	-1.52686	-1.52384	-1.52084	-1.607	-1.60372	-1.60044	-1.59717

-1.53222	-1.52917	-1.52614	-1.52313	-1.52013	-1.60625	-1.60296	-1.59967	-1.59639
-1.53148	-1.52844	-1.52542	-1.55257	-1.5495	-1.60548	-1.60218	-1.59889	-1.59562
-1.53074	-1.52771	-1.5247	-1.55183	-1.54877	-1.6047	-1.60139	-1.59811	-1.59485
-1.53001	-1.52698	-1.52398	-1.5511	-1.54803	-1.60392	-1.60061	-1.59732	-1.59407
-1.52928	-1.52626	-1.52325	-1.55036	-1.5473	-1.60314	-1.59982	-1.59654	-1.5933
-1.52854	-1.52553	-1.52252	-1.54962	-1.54656	-1.60236	-1.59903	-1.59575	-1.59252
-1.5278	-1.52479	-1.52179	-1.54887	-1.54582	-1.60156	-1.59823	-1.59495	-1.59173
-1.52706	-1.52405	-1.52106	-1.54812	-1.54507	-1.62455	-1.62117	-1.61784	-1.61458
-1.52633	-1.52332	-1.52033	-1.54737	-1.54432	-1.62374	-1.62038	-1.61706	-1.61381
-1.5256	-1.52259	-1.5196	-1.54662	-1.54357	-1.62294	-1.6196	-1.61629	-1.61303
-1.52485	-1.52185	-1.51887	-1.54587	-1.54282	-1.62214	-1.61881	-1.61551	-1.61226
-1.5241	-1.52111	-1.51814	-1.54512	-1.54207	-1.62134	-1.61802	-1.61473	-1.61148
-1.5234	-1.52039	-1.51741	-1.56899	-1.56589	-1.62054	-1.61724	-1.61396	-1.61071
-1.52274	-1.51969	-1.51667	-1.56821	-1.56512	-1.61976	-1.61646	-1.61318	-1.60993
-1.52209	-1.51902	-1.51597	-1.56746	-1.56436	-1.61898	-1.61568	-1.61241	-1.60914
-1.52148	-1.51839	-1.51531	-1.56675	-1.56362	-1.6182	-1.61489	-1.61161	-1.60835
-1.52085	-1.51776	-1.51468	-1.56607	-1.56291	-1.61745	-1.61413	-1.61083	-1.60756
-1.52017	-1.51712	-1.51407	-1.56544	-1.56228	-1.6168	-1.61343	-1.61009	-1.60678
-1.51946	-1.51646	-1.51346	-1.56483	-1.56169	-1.61617	-1.61276	-1.60938	-1.60602
-1.54384	-1.5408	-1.53776	-1.56413	-1.56101	-1.61549	-1.61209	-1.63282	-1.6294
-1.54307	-1.54003	-1.537	-1.56336	-1.56027	-1.61477	-1.61141	-1.63218	-1.62875
-1.54231	-1.53927	-1.53624	-1.56259	-1.55952	-1.614	-1.61067	-1.65535	-1.65189
-1.54156	-1.5385	-1.53546	-1.5618	-1.55874	-1.61318	-1.60987	-1.65459	-1.65121
-1.54086	-1.53778	-1.53473	-1.56103	-1.55796	-1.63661	-1.63328	-1.65381	-1.65049
-1.54024	-1.53714	-1.53405	-1.56031	-1.55721	-1.63581	-1.63248	-1.653	-1.6497
-1.53963	-1.53652	-1.53341	-1.55962	-1.55648	-1.63503	-1.63169	-1.6522	-1.64891
-1.53895	-1.53586	-1.53277	-1.58416	-1.581	-1.65826	-1.65482	-1.65144	-1.64814
-1.53822	-1.53518	-1.53215	-1.58357	-1.58042	-1.65762	-1.65414	-1.65072	-1.6474
-1.53749	-1.53447	-1.53145	-1.58289	-1.57976	-1.65693	-1.65346	-1.65006	-1.64674
-1.53674	-1.53371	-1.5307	-1.58214	-1.57903	-1.65619	-1.65281	-1.64946	-1.64615
-1.59084	-1.58769	-1.58457	-1.60621	-1.60303	-1.65544	-1.65211	-1.64879	-1.6455
-1.59025	-1.58711	-1.58399	-1.60559	-1.60236	-1.65469	-1.65133	-1.64801	-1.64474
-1.61459	-1.6114	-1.6082	-1.60496	-1.60171	-1.65397	-1.65059	-1.64727	-1.644
-1.61386	-1.61066	-1.60747	-1.60426	-1.60106	-1.65333	-1.64997	-1.64664	-1.64334
-1.61312	-1.6099	-1.60671	-1.60354	-1.6004	-1.6527	-1.64937	-1.64604	-1.64271
-1.61247	-1.60923	-1.60601	-1.60283	-1.59968	-1.65197	-1.64865	-1.64534	-1.64203
-1.61187	-1.6086	-1.60535	-1.60212	-1.59893	-1.65118	-1.64788	-1.64459	-1.64133
-1.61121	-1.60795	-1.60469	-1.60146	-1.59826	-1.65048	-1.64716	-1.64387	-1.64061
-1.61049	-1.60726	-1.60404	-1.60084	-1.59766	-1.64985	-1.64649	-1.64316	-1.63987
-1.6098	-1.60658	-1.60339	-1.60021	-1.59704	-1.6492	-1.64583	-1.64249	-1.63918
-1.60918	-1.60594	-1.60271	-1.59952	-1.59635	-1.6485	-1.64518	-1.64187	-1.63858
-1.60855	-1.60529	-1.60204	-1.59884	-1.59567	-1.64781	-1.64453	-1.64127	-1.638
-1.60785	-1.60462	-1.60141	-1.59822	-1.59506	-1.64718	-1.64392	-1.64065	-1.63736
-1.60715	-1.60396	-1.60079	-1.59762	-1.59447	-1.64656	-1.6433	-1.64002	-1.63671
-1.60651	-1.60333	-1.60017	-1.597	-1.59385	-1.6459	-1.64262	-1.63934	-1.63606
-1.60592	-1.60274	-1.59956	-1.59638	-1.5932	-1.6452	-1.6419	-1.63863	-1.6354
-1.60534	-1.60214	-1.59895	-1.59576	-1.59258	-1.64454	-1.64123	-1.63797	-1.63477
-1.60477	-1.60154	-1.59832	-1.59513	-1.59196	-1.64393	-1.64064	-1.63738	-1.63418
-1.66014	-1.65674	-1.65334	-1.64997	-1.64663	-1.64332	-1.64003	-1.63677	-1.63356
-1.6594	-1.65604	-1.65268	-1.64934	-1.64601	-1.64269	-1.63938	-1.6361	-1.63287

-1.65867	-1.65535	-1.65203	-1.64871	-1.64538	-1.64206	-1.63873	-1.63543	-1.63217
-1.65804	-1.65473	-1.65142	-1.64808	-1.64474	-1.64142	-1.6381	-1.63481	-1.63156
-1.65745	-1.65414	-1.65081	-1.64746	-1.6441	-1.64077	-1.63748	-1.63422	-1.63098
-1.65685	-1.65351	-1.65017	-1.64682	-1.64346	-1.64015	-1.63688	-1.63362	-1.63039
-1.65625	-1.65287	-1.6495	-1.64616	-1.64283	-1.63954	-1.63628	-1.63303	-1.6298
-1.6556	-1.65221	-1.64884	-1.64551	-1.64221	-1.63893	-1.63568	-1.65631	-1.65302
-1.65491	-1.65153	-1.64819	-1.64487	-1.64159	-1.63833	-1.63509	-1.6557	-1.65237
-1.65424	-1.65088	-1.64755	-1.64425	-1.64098	-1.66166	-1.65838	-1.65507	-1.65172
-1.65366	-1.65029	-1.64695	-1.64366	-1.64039	-1.66105	-1.65772	-1.65439	-1.65105
-1.65309	-1.64972	-1.64637	-1.64308	-1.63982	-1.66044	-1.65706	-1.65371	-1.65039
-1.65248	-1.64912	-1.64579	-1.64251	-1.63924	-1.65985	-1.65645	-1.65309	-1.64976
-1.65185	-1.64852	-1.64522	-1.64193	-1.63865	-1.65925	-1.65587	-1.6525	-1.64916
-1.65123	-1.64792	-1.64463	-1.66532	-1.66197	-1.65861	-1.65524	-1.65189	-1.64856
-1.65061	-1.64732	-1.64402	-1.66468	-1.66129	-1.65792	-1.65458	-1.65126	-1.64796
-1.67413	-1.6708	-1.66746	-1.66406	-1.66064	-1.65727	-1.65394	-1.65063	-1.64736
-1.67355	-1.67022	-1.66687	-1.66348	-1.66006	-1.65668	-1.65334	-1.65003	-1.64677
-1.67297	-1.66964	-1.6663	-1.6629	-1.65948	-1.6561	-1.65275	-1.64944	-1.64618
-1.67238	-1.66905	-1.66571	-1.66231	-1.65889	-1.65551	-1.65215	-1.64884	-1.64559
-1.67178	-1.66846	-1.66512	-1.66172	-1.65829	-1.65491	-1.65156	-1.64825	-1.645
-1.6712	-1.66788	-1.66454	-1.66114	-1.65771	-1.65432	-1.65097	-1.64766	-1.64442
-1.67062	-1.6673	-1.66396	-1.66056	-1.65713	-1.65374	-1.65038	-1.64707	-1.64384
-1.67004	-1.66671	-1.66338	-1.65997	-1.65654	-1.65315	-1.64979	-1.64649	-1.64326
-1.66946	-1.66613	-1.66279	-1.65939	-1.65595	-1.65257	-1.64921	-1.64591	-1.64268
-1.66887	-1.66554	-1.6622	-1.65879	-1.65536	-1.65197	-1.64862	-1.64533	-1.6421
-1.66827	-1.66493	-1.66159	-1.65818	-1.65474	-1.65136	-1.64802	-1.64474	-1.64152
-1.66767	-1.66433	-1.66099	-1.65757	-1.65413	-1.65076	-1.64743	-1.64415	-1.64093
-1.66713	-1.66377	-1.6604	-1.65698	-1.65354	-1.65016	-1.64683	-1.64355	-1.64035
-1.66662	-1.66322	-1.65982	-1.65639	-1.65296	-1.64958	-1.64624	-1.64296	-1.63976
-1.66608	-1.66265	-1.65924	-1.65583	-1.65243	-1.64906	-1.6457	-1.64241	-1.6392
-1.66553	-1.66208	-1.65865	-1.65528	-1.65193	-1.64857	-1.6452	-1.64189	-1.63865
-1.665	-1.66153	-1.65808	-1.65472	-1.65139	-1.64804	-1.64468	-1.69049	-1.68708
-1.6645	-1.66101	-1.65753	-1.65414	-1.65079	-1.64746	-1.64413	-1.68992	-1.68649
-1.66397	-1.66047	-1.65698	-1.65357	-1.6502	-1.64687	-1.64358	-1.68936	-1.68591
-1.6634	-1.6599	-1.65642	-1.65299	-1.64961	-1.64629	-1.64305	-1.68883	-1.68537
-1.6628	-1.65933	-1.65587	-1.65243	-1.64904	-1.64574	-1.64253	-1.6883	-1.68483
-1.66222	-1.65879	-1.65537	-1.65194	-1.64853	-1.64521	-1.64199	-1.68772	-1.68426
-1.66164	-1.65826	-1.65489	-1.65148	-1.64805	-1.64471	-1.64144	-1.68713	-1.68367
-1.66108	-1.65771	-1.65436	-1.65097	-1.64758	-1.64424	-1.64093	-1.68657	-1.68313
-1.66054	-1.65713	-1.65376	-1.65043	-1.64712	-1.64379	-1.64045	-1.68605	-1.68262
-1.66003	-1.65659	-1.65319	-1.64988	-1.64661	-1.64329	-1.63994	-1.68551	-1.68208
-1.65957	-1.6561	-1.65266	-1.64932	-1.64603	-1.64271	-1.63937	-1.68492	-1.68148
-1.65909	-1.65561	-1.65216	-1.64879	-1.64547	-1.64214	-1.63882	-1.68435	-1.68089
-1.65853	-1.65511	-1.6517	-1.64832	-1.64497	-1.64164	-1.63833	-1.68383	-1.68035
-1.65795	-1.65459	-1.65124	-1.64787	-1.64451	-1.64117	-1.63786	-1.68333	-1.67984
-1.65741	-1.65405	-1.65071	-1.64737	-1.64404	-1.68977	-1.6863	-1.68282	-1.67934
-1.65689	-1.65349	-1.65013	-1.64684	-1.64358	-1.68931	-1.68581	-1.68232	-1.67885
-1.65639	-1.65297	-1.6496	-1.64631	-1.64308	-1.68879	-1.68531	-1.68183	-1.67836
-1.65592	-1.65248	-1.6491	-1.64578	-1.64251	-1.68822	-1.6848	-1.68135	-1.67787
-1.65545	-1.65201	-1.64862	-1.64528	-1.64199	-1.68769	-1.68429	-1.68086	-1.67737
-1.65496	-1.65154	-1.64817	-1.64485	-1.64157	-1.68722	-1.68379	-1.68034	-1.67687

-1.65448	-1.65108	-1.64772	-1.64442	-1.64115	-1.68676	-1.68329	-1.67982	-1.67637
-1.65401	-1.65061	-1.64724	-1.64393	-1.64064	-1.68624	-1.68279	-1.67933	-1.67587
-1.65355	-1.65014	-1.64676	-1.64342	-1.64012	-1.6857	-1.68229	-1.67885	-1.67537
-1.57595	-1.5728	-1.56968	-1.5899	-1.58675	-1.68522	-1.68179	-1.67835	-1.67488
-1.5755	-1.57237	-1.56926	-1.58949	-1.58637	-1.68478	-1.68131	-1.67784	-1.67438
-1.57506	-1.57194	-1.56885	-1.63969	-1.63642	-1.68431	-1.68082	-1.67734	-1.67389
-1.57465	-1.57154	-1.56846	-1.63925	-1.63595	-1.68381	-1.68033	-1.67685	-1.67339
-1.57424	-1.57114	-1.56806	-1.61625	-1.61298	-1.6833	-1.67982	-1.67635	-1.67288
-1.57386	-1.57074	-1.56763	-1.61579	-1.61252	-1.6828	-1.67931	-1.67583	-1.67238
-1.57347	-1.57033	-1.5672	-1.61533	-1.61205	-1.68231	-1.6788	-1.67531	-1.67187
-1.57306	-1.56991	-1.56678	-1.61488	-1.61159	-1.68181	-1.67831	-1.67483	-1.67137
-1.57263	-1.5695	-1.56637	-1.61443	-1.61113	-1.68132	-1.67784	-1.67436	-1.67088
-1.57221	-1.56909	-1.56597	-1.61401	-1.61071	-1.68088	-1.6774	-1.67393	-1.67044
-1.57179	-1.56868	-1.56559	-1.61363	-1.61034	-1.68048	-1.677	-1.67352	-1.67004
-1.62299	-1.61974	-1.61649	-1.61322	-1.60993	-1.65838	-1.65495	-1.67308	-1.66962
-1.62259	-1.61931	-1.61604	-1.61276	-1.60948	-1.65791	-1.65447	-1.6726	-1.66915
-1.6222	-1.61889	-1.6156	-1.61231	-1.60903	-1.65745	-1.65401	-1.67213	-1.66869
-1.62181	-1.61851	-1.6152	-1.6119	-1.60861	-1.65702	-1.65359	-1.67171	-1.66827
-1.62142	-1.61812	-1.61481	-1.61151	-1.60821	-1.6566	-1.65319	-1.67131	-1.66785
-1.62097	-1.61767	-1.61437	-1.61106	-1.60776	-1.65614	-1.65274	-1.67085	-1.66739
-1.62049	-1.61719	-1.6139	-1.61059	-1.60729	-1.65565	-1.65225	-1.67036	-1.66689
-1.62006	-1.61676	-1.61346	-1.61016	-1.60687	-1.65521	-1.65181	-1.66992	-1.66645
-1.61968	-1.61637	-1.61306	-1.60977	-1.60649	-1.65482	-1.65141	-1.66951	-1.66604
-1.61931	-1.61598	-1.61268	-1.60939	-1.60611	-1.65443	-1.65103	-1.64762	-1.64422
-1.61894	-1.61561	-1.61229	-1.609	-1.60574	-1.65404	-1.65064	-1.64725	-1.64385
-1.61857	-1.61523	-1.61191	-1.60862	-1.60536	-1.65365	-1.65026	-1.64687	-1.64347
-1.61818	-1.61485	-1.61153	-1.60824	-1.60497	-1.65325	-1.64987	-1.69802	-1.69447
-1.61779	-1.61445	-1.61113	-1.60784	-1.60458	-1.65284	-1.64946	-1.69759	-1.69404
-1.61734	-1.61401	-1.61069	-1.60741	-1.60415	-1.6524	-1.649	-1.6971	-1.69357
-1.61687	-1.61353	-1.61021	-1.60694	-1.60369	-1.65192	-1.64851	-1.69658	-1.69306
-1.61643	-1.61309	-1.60978	-1.60651	-1.60326	-1.65148	-1.64807	-1.69611	-1.69258
-1.61603	-1.6127	-1.6094	-1.60613	-1.60288	-1.65108	-1.64767	-1.69569	-1.69216
-1.61567	-1.61234	-1.60904	-1.60576	-1.60251	-1.6507	-1.6473	-1.69529	-1.69175
-1.61537	-1.61201	-1.60868	-1.60539	-1.60214	-1.65033	-1.64693	-1.69489	-1.69134
-1.61509	-1.6117	-1.60834	-1.60505	-1.60179	-1.64998	-1.64658	-1.69452	-1.69095
-1.61477	-1.6114	-1.60805	-1.60475	-1.60149	-1.59824	-1.59501	-1.69419	-1.69062
-1.61443	-1.6111	-1.60778	-1.60448	-1.60121	-1.59796	-1.59473	-1.69389	-1.69032
-1.61408	-1.61076	-1.60746	-1.60417	-1.60089	-1.62739	-1.62408	-1.69354	-1.68998
-1.6137	-1.61039	-1.6071	-1.60382	-1.60054	-1.62703	-1.6237	-1.69314	-1.6896
-1.61333	-1.61002	-1.60673	-1.60345	-1.60018	-1.62665	-1.62332	-1.69273	-1.68921
-1.61297	-1.60965	-1.60635	-1.60307	-1.59981	-1.62627	-1.62293	-1.69232	-1.68879
-1.61263	-1.6093	-1.60599	-1.60271	-1.59944	-1.64917	-1.64574	-1.69191	-1.68839
-1.61235	-1.60901	-1.60569	-1.60238	-1.59908	-1.64877	-1.64535	-1.69151	-1.68799
-1.61207	-1.60874	-1.60541	-1.60207	-1.59874	-1.64839	-1.64497	-1.69113	-1.68761
-1.61174	-1.60841	-1.60509	-1.60176	-1.59843	-1.64806	-1.64464	-1.69079	-1.68728
-1.61137	-1.60806	-1.60476	-1.60145	-1.59815	-1.64776	-1.64433	-1.69048	-1.68698
-1.61104	-1.60772	-1.60441	-1.63093	-1.62756	-1.64742	-1.644	-1.6406	-1.63722
-1.61075	-1.6074	-1.60406	-1.63056	-1.62718	-1.64704	-1.64363	-1.64023	-1.63683
-1.61046	-1.60709	-1.60373	-1.63021	-1.62684	-1.64668	-1.64328	-1.63987	-1.63647
-1.61014	-1.60679	-1.60345	-1.62993	-1.62655	-1.64638	-1.64296	-1.63956	-1.63615

-1.60983	-1.6065	-1.60318	-1.62966	-1.62628	-1.64609	-1.64266	-1.63924	-1.63584
-1.60954	-1.60622	-1.60291	-1.62938	-1.62601	-1.64581	-1.64236	-1.63892	-1.63549
-1.60926	-1.60594	-1.60262	-1.62909	-1.62573	-1.64551	-1.64205	-1.6386	-1.63514
-1.63898	-1.63555	-1.63213	-1.62875	-1.6254	-1.64516	-1.64171	-1.63826	-1.63481
-1.63869	-1.63522	-1.63178	-1.62838	-1.62501	-1.64477	-1.64135	-1.63792	-1.63449
-1.63838	-1.63491	-1.63145	-1.65132	-1.64786	-1.64442	-1.64101	-1.63759	-1.63417
-1.63804	-1.63459	-1.63117	-1.65103	-1.64757	-1.64413	-1.64071	-1.63729	-1.63388
-1.63772	-1.6343	-1.63089	-1.65075	-1.64728	-1.64384	-1.64041	-1.63699	-1.63358
-1.63743	-1.63401	-1.63062	-1.65047	-1.647	-1.64356	-1.64012	-1.6367	-1.63329
-1.63715	-1.63374	-1.63034	-1.65018	-1.64673	-1.64328	-1.63984	-1.63641	-1.633
-1.63686	-1.63346	-1.63007	-1.6499	-1.64644	-1.64299	-1.63955	-1.63612	-1.63271
-1.63658	-1.63318	-1.6298	-1.64961	-1.64614	-1.64269	-1.63926	-1.63583	-1.63242
-1.6363	-1.6329	-1.62952	-1.64932	-1.64585	-1.6424	-1.63897	-1.68218	-1.67864
-1.63604	-1.63263	-1.62923	-1.64903	-1.64557	-1.64212	-1.63868	-1.68187	-1.67833
-1.63576	-1.63235	-1.62895	-1.64874	-1.64529	-1.64183	-1.63839	-1.68156	-1.67802
-1.63546	-1.63206	-1.62868	-1.64846	-1.64499	-1.64154	-1.6381	-1.68125	-1.67771
-1.63519	-1.63178	-1.62841	-1.64817	-1.6447	-1.64125	-1.63781	-1.68094	-1.6774
-1.63499	-1.63155	-1.62813	-1.64788	-1.64442	-1.64097	-1.63751	-1.68061	-1.67708
-1.63481	-1.63133	-1.62786	-1.6476	-1.64416	-1.6407	-1.63723	-1.6803	-1.67676
-1.63459	-1.63112	-1.62766	-1.64739	-1.64394	-1.64048	-1.63701	-1.68005	-1.67647
-1.63434	-1.63091	-1.62749	-1.64721	-1.64374	-1.64028	-1.63682	-1.67982	-1.6762
-1.65743	-1.65394	-1.65046	-1.64698	-1.6435	-1.68675	-1.68317	-1.67958	-1.67594
-1.65714	-1.65366	-1.65019	-1.64671	-1.64323	-1.68646	-1.68289	-1.67931	-1.67571
-1.65688	-1.6534	-1.64995	-1.64646	-1.64297	-1.68618	-1.6826	-1.67903	-1.67545
-1.65669	-1.65321	-1.64974	-1.64626	-1.64278	-1.68593	-1.68231	-1.67871	-1.67516
-1.6565	-1.65301	-1.64953	-1.64605	-1.64258	-1.68569	-1.68202	-1.6784	-1.67486
-1.65624	-1.65275	-1.64925	-1.64579	-1.64232	-1.68543	-1.68177	-1.67815	-1.67458
-1.65596	-1.65246	-1.64896	-1.6455	-1.64204	-1.68515	-1.68154	-1.67792	-1.67431
-1.65572	-1.65222	-1.64872	-1.64524	-1.64176	-1.68486	-1.68127	-1.67767	-1.67406
-1.65551	-1.65202	-1.64852	-1.64499	-1.64146	-1.68454	-1.68098	-1.67741	-1.67383
-1.65526	-1.65178	-1.64829	-1.64476	-1.64121	-1.68428	-1.68073	-1.67718	-1.67361
-1.65498	-1.6515	-1.64803	-1.64452	-1.64101	-1.68407	-1.68051	-1.67696	-1.6734
-1.65472	-1.65124	-1.64778	-1.69105	-1.68744	-1.68386	-1.68031	-1.67675	-1.67319
-1.65452	-1.65103	-1.64757	-1.69083	-1.68723	-1.68365	-1.6801	-1.67654	-1.67299
-1.65434	-1.65085	-1.64738	-1.69062	-1.68702	-1.68344	-1.67988	-1.67632	-1.67278
-1.65415	-1.65066	-1.64718	-1.69041	-1.68681	-1.68321	-1.67962	-1.67605	-1.67252
-1.65397	-1.65048	-1.647	-1.6902	-1.68659	-1.68297	-1.67935	-1.67576	-1.67224
-1.65379	-1.6503	-1.64681	-1.69	-1.68638	-1.68275	-1.67911	-1.67552	-1.67199
-1.65361	-1.65012	-1.64663	-1.6898	-1.68618	-1.68255	-1.67891	-1.67532	-1.67179
-1.65342	-1.64993	-1.64644	-1.6896	-1.68598	-1.68236	-1.67872	-1.67512	-1.67158
-1.65324	-1.64975	-1.64626	-1.6894	-1.68578	-1.68216	-1.67852	-1.67492	-1.67139
-1.70007	-1.69644	-1.69282	-1.68921	-1.68559	-1.68196	-1.67832	-1.67473	-1.6712
-1.69987	-1.69625	-1.69263	-1.68901	-1.68539	-1.68176	-1.67812	-1.67453	-1.671
-1.69967	-1.69605	-1.69243	-1.68881	-1.6852	-1.68157	-1.67793	-1.67434	-1.6708
-1.69947	-1.69585	-1.69223	-1.68862	-1.68501	-1.68139	-1.67775	-1.67415	-1.6706
-1.69927	-1.69564	-1.69203	-1.68843	-1.68482	-1.68121	-1.67757	-1.67397	-1.67041
-1.69909	-1.69545	-1.69184	-1.68823	-1.68464	-1.68104	-1.67743	-1.67383	-1.67026
-1.6989	-1.69526	-1.69164	-1.68804	-1.68447	-1.68089	-1.67731	-1.67374	-1.67016
-1.69875	-1.69509	-1.69147	-1.68787	-1.68429	-1.68073	-1.67717	-1.67361	-1.67003
-1.69864	-1.69498	-1.69134	-1.68771	-1.68411	-1.68053	-1.67699	-1.67343	-1.66986

-1.69853	-1.69487	-1.69121	-1.68756	-1.68393	-1.68034	-1.67679	-1.67324	-1.66968
-1.69835	-1.6947	-1.69105	-1.6874	-1.68375	-1.7112	-1.70738	-1.70356	-1.69974
-1.69816	-1.69452	-1.69089	-1.68724	-1.68358	-1.711	-1.70716	-1.70334	-1.69952
-1.69802	-1.69437	-1.69074	-1.68709	-1.68345	-1.71085	-1.707	-1.70317	-1.69933
-1.69791	-1.69424	-1.69058	-1.68696	-1.68335	-1.71074	-1.70688	-1.70303	-1.69915
-1.69779	-1.69411	-1.69046	-1.68684	-1.68324	-1.71062	-1.70675	-1.70287	-1.69899
-1.69765	-1.694	-1.69036	-1.68675	-1.68315	-1.71048	-1.70659	-1.70271	-1.69884
-1.69749	-1.69386	-1.69024	-1.68664	-1.68303	-1.71034	-1.70643	-1.70255	-1.6987
-1.69732	-1.69368	-1.69006	-1.68646	-1.68288	-1.71017	-1.70629	-1.70243	-1.69858
-1.69715	-1.6935	-1.68988	-1.68629	-1.6827	-1.71	-1.70615	-1.7023	-1.69844
-1.69703	-1.69338	-1.68976	-1.68614	-1.68253	-1.70979	-1.70595	-1.70211	-1.69827
-1.69694	-1.69329	-1.68965	-1.686	-1.68236	-1.70958	-1.70574	-1.70191	-1.69809
-1.69685	-1.69318	-1.68952	-1.68587	-1.68223	-1.70943	-1.70559	-1.70176	-1.69794
-1.69676	-1.69307	-1.68938	-1.68574	-1.68213	-1.70933	-1.70548	-1.70164	-1.69782
-1.69669	-1.69299	-1.68929	-1.68566	-1.68205	-1.70923	-1.70537	-1.70153	-1.6977
-1.69664	-1.69296	-1.68928	-1.68562	-1.68198	-1.70912	-1.70526	-1.70142	-1.69758
-1.69658	-1.69291	-1.68925	-1.68558	-1.68192	-1.70902	-1.70516	-1.70131	-1.69745
-1.69649	-1.69281	-1.68914	-1.68548	-1.68184	-1.70893	-1.70506	-1.70119	-1.6973
-1.69638	-1.69269	-1.68902	-1.68538	-1.68176	-1.70885	-1.70496	-1.70108	-1.69717
-1.69625	-1.69257	-1.68891	-1.68529	-1.68168	-1.70875	-1.70487	-1.70098	-1.69708
-1.69612	-1.69246	-1.68882	-1.6852	-1.6816	-1.70865	-1.70477	-1.7009	-1.69703
-1.69604	-1.69237	-1.68873	-1.68511	-1.68151	-1.70855	-1.70468	-1.7161	-1.71221
-1.69604	-1.69233	-1.68864	-1.68502	-1.68143	-1.70846	-1.70457	-1.71601	-1.7121
-1.69601	-1.69229	-1.68858	-1.68494	-1.68134	-1.70836	-1.70447	-1.71595	-1.71203
-1.69596	-1.69226	-1.68857	-1.6849	-1.68126	-1.70825	-1.70438	-1.71592	-1.71203
-1.69589	-1.69221	-1.68854	-1.68485	-1.68118	-1.70814	-1.70429	-1.71589	-1.71203
-1.6958	-1.6921	-1.68841	-1.68474	-1.68108	-1.70804	-1.70419	-1.71583	-1.712
-1.69569	-1.69197	-1.68826	-1.68461	-1.68099	-1.70795	-1.70409	-1.71576	-1.71194
-1.69562	-1.69191	-1.6882	-1.68454	-1.68091	-1.7234	-1.71954	-1.71571	-1.71189
-1.69557	-1.69188	-1.68819	-1.68451	-1.68084	-1.72334	-1.71951	-1.71568	-1.71183
-1.69549	-1.69183	-1.68816	-1.68448	-1.6808	-1.72333	-1.7195	-1.71567	-1.71182
-1.6954	-1.69174	-1.68811	-1.68446	-1.68081	-1.72338	-1.71953	-1.7157	-1.71186
-1.69534	-1.69168	-1.68805	-1.68442	-1.68081	-1.7234	-1.71955	-1.71571	-1.71188
-1.69533	-1.69163	-1.68797	-1.68435	-1.68075	-1.72337	-1.7195	-1.71566	-1.71185
-1.69534	-1.69161	-1.68791	-1.68428	-1.6807	-1.72335	-1.71946	-1.71561	-1.7118
-1.69538	-1.69163	-1.6879	-1.70041	-1.69682	-1.72337	-1.71949	-1.71562	-1.71177
-1.69542	-1.69167	-1.68793	-1.70046	-1.69686	-1.7234	-1.71953	-1.71565	-1.71175
-1.69543	-1.69168	-1.68795	-1.7005	-1.69687	-1.72339	-1.71954	-1.71567	-1.71175
-1.6954	-1.69169	-1.68797	-1.70053	-1.69686	-1.72336	-1.71952	-1.71567	-1.71178
-1.69539	-1.6917	-1.688	-1.70058	-1.69688	-1.72335	-1.71952	-1.71568	-1.71182
-1.69539	-1.69172	-1.68805	-1.70065	-1.69694	-1.72339	-1.71955	-1.71571	-1.71186
-1.71176	-1.7081	-1.70443	-1.70072	-1.697	-1.72342	-1.71956	-1.71572	-1.71189
-1.71174	-1.70809	-1.70444	-1.70075	-1.69705	-1.72345	-1.71955	-1.71569	-1.71187
-1.71172	-1.70807	-1.70444	-1.70078	-1.6971	-1.72348	-1.71954	-1.71566	-1.71185
-1.71176	-1.70811	-1.70448	-1.70083	-1.69716	-1.72354	-1.71958	-1.71569	-1.71188
-1.71183	-1.70818	-1.70454	-1.70089	-1.69723	-1.7236	-1.71964	-1.71575	-1.71194
-1.7119	-1.70824	-1.70461	-1.70096	-1.69731	-1.72367	-1.71971	-1.71581	-1.712
-1.71197	-1.70832	-1.70469	-1.70104	-1.69739	-1.72374	-1.71978	-1.71588	-1.71207
-1.71204	-1.7084	-1.70477	-1.70113	-1.69747	-1.72381	-1.71985	-1.71595	-1.71214
-1.71211	-1.70848	-1.70485	-1.7012	-1.69755	-1.72388	-1.71992	-1.71602	-1.71221

-1.69057	-1.68697	-1.68337	-1.70128	-1.69762	-1.72395	-1.71999	-1.71609	-1.71228
-1.69064	-1.68704	-1.68344	-1.70135	-1.6977	-1.72402	-1.72005	-1.71615	-1.71234
-1.69071	-1.6871	-1.68351	-1.70142	-1.69776	-1.72408	-1.72011	-1.71621	-1.7124
-1.69077	-1.68717	-1.68358	-1.70149	-1.69783	-1.72414	-1.72017	-1.71627	-1.71246
-1.69084	-1.68724	-1.68365	-1.70156	-1.6979	-1.7242	-1.72023	-1.71633	-1.71252
-1.69091	-1.68731	-1.68372	-1.6801	-1.67647	-1.72427	-1.7203	-1.7164	-1.71258
-1.69099	-1.68738	-1.68378	-1.68016	-1.67655	-1.72435	-1.72038	-1.71647	-1.71264
-1.6911	-1.68747	-1.68386	-1.68024	-1.67663	-1.72443	-1.72048	-1.71658	-1.71274
-1.69126	-1.68762	-1.68399	-1.68035	-1.67671	-1.72453	-1.72062	-1.71674	-1.71288
-1.69141	-1.68777	-1.68412	-1.68046	-1.6768	-1.72462	-1.72075	-1.71689	-1.71303
-1.6915	-1.68787	-1.68422	-1.68055	-1.67688	-1.7036	-1.69977	-1.71698	-1.71312
-1.69158	-1.68795	-1.68432	-1.68065	-1.67697	-1.70368	-1.69984	-1.71704	-1.7132
-1.69169	-1.68805	-1.68441	-1.68075	-1.67709	-1.70381	-1.69996	-1.74405	-1.74012
-1.69182	-1.68815	-1.6845	-1.68086	-1.67725	-1.70398	-1.70011	-1.74419	-1.74024
-1.69194	-1.68825	-1.68458	-1.68096	-1.67737	-1.70411	-1.70025	-1.72364	-1.71972
-1.69203	-1.68834	-1.68466	-1.68103	-1.67744	-1.7042	-1.70036	-1.72376	-1.71985
-1.69213	-1.68844	-1.68476	-1.68113	-1.67754	-1.7043	-1.70047	-1.72388	-1.71999
-1.69223	-1.68856	-1.6849	-1.68127	-1.67768	-1.70444	-1.70062	-1.72403	-1.72013
-1.69234	-1.68869	-1.68505	-1.68143	-1.67783	-1.70458	-1.70076	-1.72416	-1.72027
-1.69248	-1.68883	-1.6852	-1.68157	-1.67795	-1.70468	-1.70085	-1.72425	-1.72037
-1.69263	-1.68899	-1.68535	-1.6817	-1.67805	-1.70476	-1.70093	-1.72432	-1.72045
-1.69279	-1.68914	-1.68549	-1.68183	-1.67818	-1.7049	-1.70105	-1.72444	-1.72057
-1.69296	-1.68928	-1.6856	-1.68196	-1.67834	-1.70507	-1.70122	-1.7246	-1.72072
-1.69314	-1.68945	-1.68576	-1.68212	-1.67851	-1.70524	-1.7014	-1.72476	-1.72087
-1.69333	-1.68966	-1.686	-1.68234	-1.6787	-1.70542	-1.70157	-1.72492	-1.72102
-1.69352	-1.68987	-1.68622	-1.68255	-1.67888	-1.73295	-1.72901	-1.72508	-1.72117
-1.69368	-1.69002	-1.68636	-1.68271	-1.67906	-1.73313	-1.72918	-1.72524	-1.7213
-1.69383	-1.69015	-1.68649	-1.68285	-1.67924	-1.73332	-1.72936	-1.72541	-1.72143
-1.69395	-1.69029	-1.68664	-1.68302	-1.67941	-1.7335	-1.72954	-1.72559	-1.72162
-1.69407	-1.69043	-1.68681	-1.68319	-1.67959	-1.73368	-1.72972	-1.72578	-1.72184
-1.69425	-1.6906	-1.68697	-1.68337	-1.67977	-1.73386	-1.7299	-1.72595	-1.72201
-1.69449	-1.69081	-1.68715	-1.68353	-1.67995	-1.73404	-1.73008	-1.72611	-1.72214
-1.69473	-1.69103	-1.68734	-1.68372	-1.68012	-1.73422	-1.73025	-1.72629	-1.7223
-1.69494	-1.69126	-1.68758	-1.68393	-1.6803	-1.73438	-1.73044	-1.72649	-1.72254
-1.69514	-1.69147	-1.68781	-1.68414	-1.68047	-1.73455	-1.73062	-1.7267	-1.72278
-1.66953	-1.66594	-1.66235	-1.68428	-1.68064	-1.70475	-1.70108	-1.69742	-1.69376
-1.66969	-1.66607	-1.66246	-1.68441	-1.6808	-1.70492	-1.70125	-1.69759	-1.69394
-1.66987	-1.66626	-1.66265	-1.6846	-1.68097	-1.70509	-1.70142	-1.69777	-1.69412
-1.67008	-1.66649	-1.66289	-1.68482	-1.68115	-1.70526	-1.70161	-1.69796	-1.6943
-1.67028	-1.6667	-1.66313	-1.68505	-1.68137	-1.70547	-1.70182	-1.69817	-1.69452
-1.67045	-1.66689	-1.66334	-1.68529	-1.68164	-1.70574	-1.70208	-1.69843	-1.69479
-1.67066	-1.66711	-1.66357	-1.71343	-1.70971	-1.70601	-1.70233	-1.69867	-1.69504
-1.67093	-1.66737	-1.66383	-1.7137	-1.70998	-1.70627	-1.70255	-1.69886	-1.69523
-1.67122	-1.66764	-1.66408	-1.71395	-1.71025	-1.70651	-1.70276	-1.69905	-1.69542
-1.67149	-1.66788	-1.66428	-1.71416	-1.71046	-1.70674	-1.703	-1.69929	-1.69563
-1.67177	-1.66811	-1.66447	-1.71433	-1.71065	-1.70695	-1.70325	-1.69955	-1.69584
-1.64843	-1.64486	-1.6413	-1.71454	-1.71083	-1.70714	-1.70347	-1.69978	-1.69608
-1.64865	-1.6451	-1.64155	-1.71477	-1.71101	-1.70731	-1.70366	-1.7	-1.69633
-1.64888	-1.64536	-1.64182	-1.71502	-1.71123	-1.70753	-1.70389	-1.70024	-1.69659
-1.64914	-1.64563	-1.64211	-1.71531	-1.71151	-1.7078	-1.70416	-1.70051	-1.69685

-1.64939	-1.64589	-1.64238	-1.69045	-1.68673	-1.70808	-1.70444	-1.70079	-1.69713
-1.64959	-1.6461	-1.6426	-1.6907	-1.687	-1.70837	-1.70471	-1.70106	-1.69741
-1.6498	-1.6463	-1.64282	-1.69093	-1.68727	-1.70864	-1.70498	-1.70133	-1.69768
-1.65005	-1.64656	-1.64307	-1.66821	-1.66463	-1.7089	-1.70521	-1.70154	-1.6979
-1.65034	-1.64684	-1.64335	-1.66849	-1.66489	-1.70915	-1.70541	-1.70172	-1.69808
-1.67954	-1.67595	-1.67236	-1.66877	-1.66516	-1.70941	-1.70565	-1.70195	-1.69831
-1.67983	-1.67624	-1.67266	-1.66906	-1.66545	-1.7097	-1.70593	-1.70222	-1.69858
-1.68013	-1.67654	-1.67295	-1.66934	-1.66573	-1.70999	-1.70622	-1.7025	-1.69886
-1.68042	-1.67683	-1.67324	-1.66963	-1.66602	-1.71028	-1.7065	-1.70279	-1.69914
-1.68071	-1.67712	-1.67353	-1.66992	-1.6663	-1.71057	-1.70679	-1.70307	-1.69943
-1.68099	-1.6774	-1.67381	-1.6702	-1.66658	-1.68583	-1.68212	-1.70335	-1.69971
-1.68126	-1.67768	-1.67408	-1.67048	-1.66686	-1.68611	-1.6824	-1.70363	-1.69998
-1.65442	-1.65088	-1.64733	-1.67076	-1.66715	-1.6864	-1.68269	-1.70391	-1.70026
-1.6547	-1.65116	-1.64762	-1.67104	-1.66743	-1.6867	-1.68298	-1.70421	-1.70054
-1.65498	-1.65144	-1.6479	-1.67131	-1.66772	-1.66412	-1.66051	-1.70454	-1.70086
-1.65525	-1.65171	-1.64817	-1.67159	-1.66802	-1.66444	-1.66086	-1.70493	-1.70124
-1.65553	-1.65198	-1.64845	-1.67187	-1.6683	-1.66475	-1.6612	-1.70529	-1.7016
-1.65582	-1.65226	-1.64872	-1.67214	-1.66858	-1.66503	-1.6615	-1.7056	-1.70191
-1.65612	-1.65256	-1.64902	-1.67242	-1.66887	-1.66532	-1.66178	-1.70589	-1.70221
-1.65648	-1.65291	-1.64936	-1.64582	-1.64228	-1.6656	-1.66205	-1.68127	-1.67768
-1.65685	-1.65329	-1.64974	-1.64617	-1.6426	-1.66589	-1.66234	-1.68156	-1.67797
-1.65718	-1.65363	-1.65009	-1.64652	-1.64295	-1.66623	-1.66267	-1.72981	-1.72607
-1.65747	-1.65395	-1.65043	-1.64688	-1.64332	-1.6666	-1.66304	-1.73018	-1.72641
-1.65779	-1.65428	-1.65077	-1.64724	-1.6437	-1.64016	-1.63663	-1.73055	-1.72677
-1.65817	-1.65465	-1.65115	-1.64762	-1.64408	-1.64053	-1.63698	-1.73091	-1.72715
-1.65853	-1.65501	-1.65151	-1.64798	-1.64445	-1.6409	-1.63734	-1.70916	-1.70549
-1.65885	-1.65532	-1.65181	-1.6483	-1.64479	-1.64126	-1.63771	-1.70955	-1.70588
-1.65917	-1.65563	-1.65212	-1.64862	-1.64513	-1.64162	-1.63809	-1.70994	-1.70628
-1.65953	-1.65599	-1.65248	-1.64898	-1.64551	-1.642	-1.63847	-1.68437	-1.68073
-1.65992	-1.65638	-1.65287	-1.64937	-1.64589	-1.64238	-1.63885	-1.68476	-1.68109
-1.66031	-1.65675	-1.65322	-1.64972	-1.64623	-1.64273	-1.63921	-1.68513	-1.68147
-1.6607	-1.65711	-1.65354	-1.65002	-1.64653	-1.64303	-1.63953	-1.68548	-1.68185
-1.66107	-1.65747	-1.65389	-1.65036	-1.64686	-1.64337	-1.63988	-1.68585	-1.68224
-1.66142	-1.65784	-1.65428	-1.65075	-1.64725	-1.64376	-1.64027	-1.68626	-1.68265
-1.66178	-1.65822	-1.65468	-1.65116	-1.64766	-1.64417	-1.64068	-1.68667	-1.68307
-1.66218	-1.65862	-1.65509	-1.65158	-1.64807	-1.64458	-1.64109	-1.68709	-1.68349
-1.66259	-1.65904	-1.65551	-1.652	-1.64849	-1.64499	-1.6415	-1.68751	-1.68391
-1.663	-1.65946	-1.65593	-1.65241	-1.6489	-1.69515	-1.69153	-1.68793	-1.68432
-1.66342	-1.65987	-1.65634	-1.65282	-1.64931	-1.69556	-1.69195	-1.68835	-1.68474
-1.66384	-1.66029	-1.65676	-1.65323	-1.64972	-1.69598	-1.69237	-1.68876	-1.68515
-1.66426	-1.66071	-1.65717	-1.65365	-1.65013	-1.69641	-1.69279	-1.68917	-1.68557
-1.66468	-1.66113	-1.65759	-1.65406	-1.65055	-1.69683	-1.6932	-1.68959	-1.68599
-1.66509	-1.66154	-1.65801	-1.65448	-1.65096	-1.69725	-1.69362	-1.69001	-1.6864
-1.66552	-1.66196	-1.65843	-1.6549	-1.65137	-1.69767	-1.69405	-1.69043	-1.68682
-1.66601	-1.66242	-1.65885	-1.65531	-1.65179	-1.69809	-1.69446	-1.69084	-1.68723
-1.66652	-1.66289	-1.65928	-1.65573	-1.65222	-1.69853	-1.69487	-1.69124	-1.68764
-1.667	-1.66338	-1.65977	-1.65621	-1.65269	-1.69901	-1.69535	-1.69171	-1.68808
-1.66745	-1.66387	-1.66029	-1.65674	-1.65319	-1.69952	-1.69588	-1.69222	-1.68854
-1.66788	-1.66433	-1.66077	-1.65722	-1.65366	-1.70001	-1.69637	-1.69271	-1.68902
-1.66829	-1.66474	-1.6612	-1.65764	-1.65409	-1.70044	-1.69681	-1.69317	-1.68951

-1.66872	-1.66516	-1.66161	-1.65806	-1.65451	-1.70087	-1.69724	-1.69361	-1.69
-1.66922	-1.66562	-1.66203	-1.65847	-1.65493	-1.7013	-1.69766	-1.69403	-1.69043
-1.66973	-1.66609	-1.66246	-1.65889	-1.65536	-1.70175	-1.69809	-1.69446	-1.69087
-1.67021	-1.66658	-1.66295	-1.65938	-1.65584	-1.70224	-1.69858	-1.69495	-1.69136
-1.67068	-1.66707	-1.66348	-1.6599	-1.65634	-1.70276	-1.69911	-1.69548	-1.69187
-1.67116	-1.66758	-1.66399	-1.6604	-1.65682	-1.70323	-1.69959	-1.69596	-1.69235
-1.67166	-1.66808	-1.6645	-1.66088	-1.65726	-1.70366	-1.70002	-1.6964	-1.69278
-1.67213	-1.66857	-1.66499	-1.66136	-1.65772	-1.70413	-1.70049	-1.69686	-1.69325
-1.67257	-1.669	-1.66544	-1.66184	-1.65823	-1.70466	-1.70101	-1.69738	-1.69377
-1.67301	-1.66945	-1.6659	-1.66232	-1.65875	-1.70521	-1.70155	-1.69791	-1.6943
-1.64573	-1.64228	-1.63884	-1.66283	-1.65927	-1.70575	-1.70209	-1.69845	-1.69483
-1.64624	-1.64278	-1.63934	-1.66336	-1.6598	-1.7063	-1.70264	-1.69899	-1.69536
-1.64674	-1.64328	-1.63983	-1.66387	-1.66032	-1.70684	-1.70317	-1.69952	-1.69589
-1.64723	-1.64377	-1.64031	-1.66438	-1.66083	-1.70737	-1.7037	-1.70005	-1.69641
-1.57518	-1.57184	-1.56852	-1.66485	-1.66131	-1.70785	-1.70418	-1.70053	-1.69689
-1.57563	-1.57226	-1.5689	-1.66527	-1.66173	-1.70829	-1.70461	-1.70095	-1.69733
-1.55128	-1.54795	-1.54463	-1.67131	-1.66772	-1.70875	-1.70507	-1.7507	-1.74688
-1.55169	-1.54838	-1.54509	-1.67182	-1.66822	-1.70926	-1.70558	-1.75122	-1.7474
-1.5521	-1.54883	-1.54556	-1.67234	-1.66873	-1.7098	-1.70612	-1.75177	-1.74795
-1.55255	-1.54929	-1.54604	-1.67287	-1.66925	-1.66564	-1.66203	-1.75233	-1.7485
-1.55303	-1.54977	-1.54653	-1.67341	-1.66979	-1.66617	-1.66257	-1.7529	-1.74907
-1.55356	-1.5503	-1.54705	-1.62641	-1.62289	-1.66675	-1.66315	-1.71028	-1.7065
-1.55413	-1.55086	-1.54761	-1.62701	-1.6235	-1.66737	-1.66378	-1.71094	-1.70714
-1.55467	-1.5514	-1.54813	-1.62758	-1.62407	-1.66796	-1.66436	-1.71155	-1.70774
-1.55517	-1.55189	-1.54862	-1.6281	-1.62459	-1.6685	-1.6649	-1.7121	-1.70829
-1.55566	-1.55237	-1.5491	-1.60013	-1.59675	-1.64598	-1.64242	-1.71263	-1.70883
-1.55614	-1.55285	-1.54957	-1.60062	-1.59723	-1.64649	-1.64293	-1.71316	-1.70936
-1.55661	-1.55333	-1.55005	-1.60111	-1.59772	-1.647	-1.64344	-1.71369	-1.70989
-1.5571	-1.55381	-1.55053	-1.6016	-1.59821	-1.64752	-1.64396	-1.71423	-1.71043
-1.5576	-1.5543	-1.55101	-1.6021	-1.59871	-1.64805	-1.64449	-1.71478	-1.71099
-1.55813	-1.55483	-1.55154	-1.60265	-1.59925	-1.62422	-1.62072	-1.69312	-1.68938
-1.55871	-1.55541	-1.55211	-1.60323	-1.59984	-1.62482	-1.62132	-1.69375	-1.69001
-1.55925	-1.55596	-1.55267	-1.65795	-1.65432	-1.67827	-1.67452	-1.69436	-1.69062
-1.55975	-1.55647	-1.55318	-1.65849	-1.65484	-1.67882	-1.67507	-1.69492	-1.69117
-1.56025	-1.55697	-1.55369	-1.65904	-1.65538	-1.67937	-1.67563	-1.69549	-1.69172
-1.56075	-1.5575	-1.55425	-1.65964	-1.65597	-1.67998	-1.67625	-1.69612	-1.69234
-1.56125	-1.55803	-1.55481	-1.66025	-1.65657	-1.6806	-1.67687	-1.69675	-1.69296
-1.56179	-1.55855	-1.55532	-1.66079	-1.65712	-1.68117	-1.67743	-1.67371	-1.66999
-1.56235	-1.55908	-1.55581	-1.66131	-1.65765	-1.68171	-1.67796	-1.67424	-1.67053
-1.56293	-1.55963	-1.55635	-1.66187	-1.65821	-1.6823	-1.67854	-1.67481	-1.67112
-1.56353	-1.56022	-1.55693	-1.66247	-1.65881	-1.68292	-1.67917	-1.67544	-1.67176
-1.53918	-1.53597	-1.53276	-1.6088	-1.60537	-1.60195	-1.59855	-1.67609	-1.6724
-1.53967	-1.5365	-1.53334	-1.60941	-1.60597	-1.60255	-1.59915	-1.67674	-1.67304
-1.54017	-1.53703	-1.5339	-1.61002	-1.60658	-1.60316	-1.59976	-1.67739	-1.67369
-1.54071	-1.53755	-1.53441	-1.61058	-1.60719	-1.60378	-1.60037	-1.67803	-1.67433
-1.54127	-1.53807	-1.5349	-1.61113	-1.60778	-1.6044	-1.60098	-1.67866	-1.67497
-1.54184	-1.53863	-1.53544	-1.61168	-1.60834	-1.60497	-1.60157	-1.6793	-1.6756
-1.54243	-1.53921	-1.53601	-1.61225	-1.60887	-1.60551	-1.60215	-1.67995	-1.67624
-1.54301	-1.53979	-1.53658	-1.61284	-1.60946	-1.60609	-1.60274	-1.68059	-1.67688
-1.54359	-1.54037	-1.53717	-1.61348	-1.61012	-1.60674	-1.60336	-1.68121	-1.67751

-1.54417	-1.54095	-1.53775	-1.61411	-1.61076	-1.60738	-1.60397	-1.65407	-1.65049
-1.54475	-1.54152	-1.53832	-1.61468	-1.61131	-1.60794	-1.60456	-1.6547	-1.6511
-1.54532	-1.5421	-1.53889	-1.61526	-1.61187	-1.6085	-1.60514	-1.65532	-1.65172
-1.5459	-1.54269	-1.53948	-1.61589	-1.6125	-1.60912	-1.60575	-1.65594	-1.65233
-1.54649	-1.54328	-1.54009	-1.61654	-1.61316	-1.60977	-1.60636	-1.65656	-1.65294
-1.54709	-1.54389	-1.54071	-1.61719	-1.6138	-1.61039	-1.60696	-1.65718	-1.65355
-1.54769	-1.54452	-1.54134	-1.61784	-1.61442	-1.61099	-1.60756	-1.65781	-1.65417
-1.54831	-1.54514	-1.54196	-1.59444	-1.59108	-1.61159	-1.60816	-1.65844	-1.6548
-1.54894	-1.54575	-1.54256	-1.59505	-1.59169	-1.6122	-1.60875	-1.65907	-1.65544
-1.54956	-1.54636	-1.54315	-1.59566	-1.59231	-1.61283	-1.60938	-1.65972	-1.6561
-1.55016	-1.54695	-1.54375	-1.5963	-1.59297	-1.61352	-1.61007	-1.66045	-1.65681
-1.55075	-1.54755	-1.54436	-1.59695	-1.59364	-1.61422	-1.61078	-1.66119	-1.65752
-1.55136	-1.54816	-1.54498	-1.65327	-1.6497	-1.66927	-1.66557	-1.66187	-1.65818
-1.55196	-1.54878	-1.54561	-1.65394	-1.65035	-1.66992	-1.66622	-1.66253	-1.65882
-1.55259	-1.5494	-1.54622	-1.65459	-1.65099	-1.67059	-1.6669	-1.66321	-1.65951
-1.55323	-1.55002	-1.54681	-1.65522	-1.65163	-1.67126	-1.6676	-1.66393	-1.66024
-1.55388	-1.55065	-1.54743	-1.65588	-1.6523	-1.64873	-1.64517	-1.66463	-1.66096
-1.55455	-1.55132	-1.5481	-1.6566	-1.653	-1.64941	-1.64581	-1.66527	-1.66162
-1.5552	-1.55199	-1.54877	-1.65732	-1.65371	-1.6501	-1.64647	-1.66593	-1.66229
-1.5558	-1.55259	-1.54938	-1.65801	-1.65442	-1.65081	-1.64717	-1.66665	-1.66301
-1.55639	-1.55317	-1.54998	-1.65868	-1.65511	-1.65152	-1.6479	-1.66739	-1.66374
-1.55703	-1.5538	-1.5506	-1.60341	-1.6001	-1.59677	-1.59343	-1.66809	-1.66445
-1.5577	-1.55446	-1.55123	-1.60404	-1.6007	-1.59736	-1.59403	-1.66876	-1.66515
-1.53414	-1.53097	-1.5278	-1.60469	-1.60133	-1.59799	-1.59467	-1.66945	-1.66586
-1.53474	-1.53159	-1.52844	-1.60538	-1.60201	-1.59867	-1.59535	-1.67017	-1.66659
-1.53535	-1.53222	-1.5291	-1.60608	-1.60271	-1.59936	-1.59604	-1.64773	-1.64425
-1.53599	-1.53287	-1.52975	-1.60678	-1.6034	-1.60006	-1.59673	-1.64845	-1.64496
-1.53665	-1.53353	-1.53041	-1.60748	-1.6041	-1.60075	-1.59743	-1.64917	-1.64568
-1.5947	-1.59143	-1.58817	-1.58486	-1.58154	-1.60145	-1.59812	-1.64988	-1.64639
-1.5954	-1.59212	-1.58885	-1.58554	-1.58222	-1.60214	-1.59882	-1.6506	-1.64711
-1.59609	-1.5928	-1.58953	-1.58622	-1.58289	-1.5796	-1.57633	-1.65132	-1.64783
-1.59678	-1.59349	-1.59021	-1.58689	-1.58356	-1.58027	-1.57701	-1.65205	-1.64855
-1.56521	-1.56203	-1.55885	-1.58757	-1.58424	-1.58095	-1.57769	-1.65278	-1.64928
-1.5659	-1.56271	-1.55953	-1.58827	-1.58493	-1.58163	-1.57837	-1.65352	-1.65002
-1.56658	-1.56339	-1.56021	-1.58896	-1.58562	-1.58232	-1.57905	-1.63185	-1.62839
-1.56726	-1.56408	-1.5609	-1.58966	-1.58631	-1.583	-1.57972	-1.63256	-1.62911
-1.56794	-1.56476	-1.56159	-1.59037	-1.58702	-1.5837	-1.58041	-1.63328	-1.62983
-1.56862	-1.56545	-1.56228	-1.5911	-1.58777	-1.58446	-1.58114	-1.57787	-1.57466
-1.5693	-1.56613	-1.56298	-1.59184	-1.58855	-1.58524	-1.5819	-1.5786	-1.57536
-1.54286	-1.53973	-1.53664	-1.59256	-1.58929	-1.58598	-1.58263	-1.57932	-1.57605
-1.54358	-1.54043	-1.5373	-1.59325	-1.58999	-1.58669	-1.58335	-1.58003	-1.57674
-1.54429	-1.54111	-1.53797	-1.56186	-1.55872	-1.5874	-1.58407	-1.58076	-1.57746
-1.54498	-1.54178	-1.53863	-1.56253	-1.55941	-1.58813	-1.58484	-1.58155	-1.57823
-1.54568	-1.54247	-1.5393	-1.56321	-1.56009	-1.58885	-1.5856	-1.58233	-1.57901
-1.54645	-1.54322	-1.54002	-1.53689	-1.5338	-1.58954	-1.58632	-1.58307	-1.57978
-1.54722	-1.54399	-1.54076	-1.5376	-1.53448	-1.59023	-1.58701	-1.58378	-1.58054
-1.54796	-1.54474	-1.54153	-1.53835	-1.53519	-1.56447	-1.56128	-1.58447	-1.58126
-1.54866	-1.54549	-1.54232	-1.53913	-1.53593	-1.56519	-1.56197	-1.58514	-1.58193
-1.51989	-1.51684	-1.51377	-1.53988	-1.53667	-1.56595	-1.56269	-1.55947	-1.5563
-1.52055	-1.51748	-1.51443	-1.54059	-1.53744	-1.56674	-1.56345	-1.5602	-1.55699

-1.52124	-1.51816	-1.5151	-1.5413	-1.53818	-1.5675	-1.56422	-1.56096	-1.55773
-1.52204	-1.51891	-1.51581	-1.54199	-1.53886	-1.56822	-1.56499	-1.56176	-1.55855
-1.52284	-1.51968	-1.51653	-1.54269	-1.53953	-1.56892	-1.56574	-1.56256	-1.55936
-1.49118	-1.48814	-1.4851	-1.54344	-1.54024	-1.53711	-1.53403	-1.56325	-1.56008
-1.49184	-1.48884	-1.48583	-1.5442	-1.54098	-1.53782	-1.53469	-1.56392	-1.56075
-1.46148	-1.45857	-1.45566	-1.54494	-1.54173	-1.53857	-1.53544	-1.56467	-1.56148
-1.46209	-1.45918	-1.45628	-1.54565	-1.5425	-1.53936	-1.53624	-1.56548	-1.56226
-1.46276	-1.45984	-1.45693	-1.51701	-1.51396	-1.54011	-1.537	-1.56626	-1.56303
-1.46352	-1.46056	-1.45761	-1.51769	-1.51461	-1.54077	-1.53767	-1.56697	-1.56379
-1.46426	-1.46128	-1.45831	-1.5184	-1.5153	-1.54146	-1.53836	-1.56768	-1.56452
-1.46493	-1.46197	-1.45903	-1.51917	-1.51607	-1.54223	-1.53909	-1.5684	-1.56522
-1.46559	-1.46266	-1.45974	-1.51996	-1.51687	-1.54303	-1.53984	-1.56913	-1.56592
-1.46631	-1.46337	-1.46044	-1.52069	-1.51759	-1.54377	-1.54059	-1.56991	-1.56669
-1.46706	-1.46409	-1.46112	-1.52139	-1.51829	-1.5445	-1.54136	-1.57072	-1.56752
-1.46776	-1.46479	-1.46183	-1.49168	-1.48866	-1.54528	-1.54215	-1.53904	-1.53593
-1.46842	-1.46547	-1.46255	-1.49246	-1.48944	-1.54611	-1.54298	-1.53985	-1.53671
-1.4691	-1.46619	-1.46329	-1.49322	-1.4902	-1.54691	-1.54377	-1.54063	-1.53748
-1.46987	-1.46695	-1.46405	-1.49398	-1.49093	-1.54765	-1.54449	-1.54135	-1.53822
-1.47062	-1.4677	-1.46479	-1.49473	-1.49166	-1.54839	-1.54522	-1.54208	-1.53898
-1.40778	-1.40503	-1.40229	-1.46253	-1.45958	-1.48936	-1.48636	-1.54288	-1.53979
-1.40839	-1.40564	-1.40291	-1.46321	-1.4603	-1.49013	-1.48714	-1.54371	-1.54061
-1.40907	-1.40632	-1.4036	-1.46394	-1.46104	-1.49089	-1.48788	-1.54447	-1.54136
-1.40981	-1.40705	-1.40432	-1.4647	-1.4618	-1.49165	-1.48861	-1.54521	-1.54206
-1.41054	-1.40777	-1.40501	-1.46541	-1.4625	-1.49237	-1.48934	-1.51612	-1.51304
-1.41127	-1.40846	-1.40567	-1.46607	-1.46315	-1.49306	-1.49007	-1.5169	-1.51384
-1.41198	-1.40914	-1.40633	-1.46676	-1.46383	-1.49377	-1.49082	-1.5177	-1.51466
-1.41262	-1.4098	-1.40701	-1.4675	-1.46459	-1.49455	-1.4916	-1.5185	-1.51547
-1.41326	-1.41047	-1.40772	-1.46827	-1.46537	-1.49536	-1.49239	-1.51931	-1.51628
-1.41398	-1.4112	-1.40845	-1.46905	-1.46616	-1.49616	-1.49318	-1.52011	-1.51705
-1.41474	-1.41195	-1.40919	-1.46985	-1.46695	-1.49697	-1.49397	-1.52089	-1.51779
-1.4155	-1.4127	-1.40993	-1.47063	-1.46773	-1.49775	-1.49473	-1.52165	-1.51853
-1.41626	-1.41345	-1.41067	-1.4714	-1.46849	-1.49851	-1.49545	-1.52237	-1.51927
-1.41701	-1.41418	-1.41137	-1.47213	-1.46921	-1.49924	-1.49617	-1.52311	-1.52002
-1.41774	-1.41487	-1.41202	-1.4728	-1.46985	-1.49991	-1.49688	-1.52387	-1.52079
-1.38241	-1.3796	-1.37681	-1.47347	-1.47051	-1.5006	-1.49761	-1.49463	-1.49165
-1.38309	-1.38027	-1.37748	-1.47421	-1.47125	-1.50137	-1.49838	-1.4954	-1.49243
-1.38376	-1.38095	-1.37816	-1.47496	-1.47202	-1.50216	-1.49917	-1.49619	-1.49322
-1.38445	-1.38165	-1.37888	-1.44099	-1.43813	-1.50296	-1.49996	-1.49698	-1.49402
-1.38515	-1.38237	-1.37962	-1.44178	-1.43891	-1.50378	-1.50077	-1.49778	-1.49481
-1.38588	-1.38311	-1.38037	-1.44257	-1.4397	-1.5046	-1.50158	-1.49859	-1.49561
-1.38663	-1.38386	-1.38112	-1.44337	-1.44049	-1.50542	-1.5024	-1.49939	-1.49641
-1.38739	-1.38462	-1.38188	-1.44416	-1.44127	-1.47212	-1.46917	-1.5002	-1.4972
-1.38815	-1.38538	-1.38263	-1.44496	-1.44206	-1.47293	-1.46997	-1.50101	-1.49801
-1.38892	-1.38614	-1.38338	-1.44575	-1.44285	-1.47374	-1.47077	-1.50182	-1.49881
-1.38968	-1.38689	-1.38413	-1.44654	-1.44364	-1.47455	-1.47158	-1.50263	-1.49961
-1.39044	-1.38765	-1.38488	-1.44734	-1.44443	-1.47536	-1.47238	-1.50344	-1.50042
-1.32873	-1.32606	-1.32341	-1.44813	-1.44521	-1.47616	-1.47318	-1.47022	-1.46726
-1.32945	-1.32678	-1.32412	-1.44892	-1.446	-1.47696	-1.47397	-1.471	-1.46805
-1.33018	-1.32749	-1.32483	-1.44971	-1.44678	-1.47776	-1.47477	-1.47179	-1.46884
-1.33091	-1.32822	-1.32555	-1.4505	-1.44756	-1.47856	-1.47556	-1.47259	-1.46963

-1.33163	-1.32893	-1.32626	-1.39114	-1.38832	-1.44542	-1.44252	-1.47338	-1.47042
-1.33235	-1.32965	-1.32696	-1.3919	-1.38907	-1.44619	-1.44329	-1.47417	-1.47121
-1.33306	-1.33036	-1.32768	-1.39266	-1.38982	-1.44697	-1.44406	-1.47496	-1.47199
-1.33378	-1.33108	-1.32839	-1.39342	-1.39057	-1.38775	-1.38496	-1.47575	-1.47278
-1.33452	-1.33181	-1.32913	-1.3942	-1.39133	-1.38851	-1.38571	-1.47655	-1.47357
-1.33535	-1.33263	-1.32994	-1.39507	-1.39219	-1.38934	-1.38651	-1.47738	-1.47438
-1.33625	-1.33353	-1.33083	-1.39601	-1.39313	-1.39024	-1.38736	-1.47825	-1.47521
-1.33709	-1.33436	-1.33165	-1.3969	-1.39401	-1.39112	-1.38821	-1.42028	-1.41734
-1.33785	-1.33512	-1.3324	-1.3977	-1.39483	-1.39195	-1.38907	-1.42119	-1.41827
-1.33859	-1.33585	-1.33313	-1.39849	-1.39562	-1.39276	-1.38991	-1.42208	-1.41916
-1.33932	-1.33657	-1.33385	-1.39926	-1.39639	-1.39353	-1.39069	-1.42288	-1.41997
-1.34005	-1.33729	-1.33456	-1.40002	-1.39715	-1.39429	-1.39144	-1.42366	-1.42075
-1.34079	-1.33802	-1.33528	-1.40078	-1.39791	-1.39505	-1.39221	-1.42445	-1.42154
-1.34153	-1.33876	-1.33601	-1.40155	-1.39867	-1.39581	-1.39298	-1.42524	-1.42233
-1.34228	-1.33952	-1.33679	-1.40239	-1.39952	-1.39666	-1.39382	-1.42611	-1.4232
-1.34303	-1.34031	-1.33761	-1.4033	-1.40044	-1.39759	-1.39474	-1.42705	-1.42414
-1.34382	-1.3411	-1.33842	-1.33575	-1.33309	-1.39846	-1.39561	-1.39277	-1.38995
-1.34468	-1.34193	-1.33921	-1.3365	-1.33382	-1.39923	-1.39638	-1.39354	-1.39072
-1.34552	-1.34274	-1.33998	-1.33724	-1.33453	-1.39998	-1.39712	-1.39429	-1.39147
-1.3463	-1.3435	-1.34072	-1.33797	-1.33524	-1.40072	-1.39786	-1.39503	-1.39222
-1.34706	-1.34426	-1.34148	-1.33871	-1.33597	-1.40149	-1.39862	-1.39578	-1.39297
-1.34791	-1.3451	-1.34231	-1.33954	-1.33678	-1.40236	-1.39948	-1.39663	-1.39382
-1.34881	-1.346	-1.3432	-1.34042	-1.33766	-1.40329	-1.4004	-1.39754	-1.39472
-1.34965	-1.34682	-1.34402	-1.34123	-1.33847	-1.40415	-1.40125	-1.39838	-1.39556
-1.3504	-1.34758	-1.34477	-1.34199	-1.33922	-1.40495	-1.40202	-1.39915	-1.39631
-1.35115	-1.34832	-1.34552	-1.34274	-1.33999	-1.40577	-1.40284	-1.39995	-1.39711
-1.35189	-1.34906	-1.34626	-1.34351	-1.34079	-1.40665	-1.40374	-1.40086	-1.39801
-1.35266	-1.34982	-1.34703	-1.34429	-1.34159	-1.40755	-1.40465	-1.40177	-1.3989
-1.29087	-1.28818	-1.28552	-1.34512	-1.3424	-1.33968	-1.33697	-1.40258	-1.3997
-1.29172	-1.28901	-1.28634	-1.34597	-1.34321	-1.34047	-1.33773	-1.40337	-1.40048
-1.29249	-1.28977	-1.28709	-1.34678	-1.34403	-1.34128	-1.33855	-1.40424	-1.40134
-1.29319	-1.29047	-1.2878	-1.34757	-1.34484	-1.34212	-1.33941	-1.40518	-1.40226
-1.29396	-1.29123	-1.28856	-1.28595	-1.28338	-1.34293	-1.34022	-1.40605	-1.40312
-1.29481	-1.29207	-1.28937	-1.28672	-1.2841	-1.34369	-1.34096	-1.40684	-1.40388
-1.26172	-1.25906	-1.25644	-1.28752	-1.28486	-1.28224	-1.27965	-1.40765	-1.40469
-1.26251	-1.25986	-1.25725	-1.28837	-1.2857	-1.28306	-1.28045	-1.40858	-1.40561
-1.26328	-1.26065	-1.25805	-1.28921	-1.28653	-1.28389	-1.28127	-1.40953	-1.40657
-1.26401	-1.26137	-1.25875	-1.28995	-1.28728	-1.28465	-1.28205	-1.34159	-1.33887
-1.26472	-1.26205	-1.25941	-1.29065	-1.28798	-1.28537	-1.28282	-1.34244	-1.33973
-1.26552	-1.26282	-1.26016	-1.29142	-1.28875	-1.28613	-1.28357	-1.28102	-1.27847
-1.26639	-1.26367	-1.26098	-1.29226	-1.28957	-1.28692	-1.2843	-1.28171	-1.27915
-1.2672	-1.26448	-1.26179	-1.2931	-1.2904	-1.28773	-1.28508	-1.28246	-1.27987
-1.26792	-1.26522	-1.26256	-1.29395	-1.29126	-1.28858	-1.28591	-1.28328	-1.28068
-1.26865	-1.26597	-1.26333	-1.29477	-1.29211	-1.28943	-1.28676	-1.28411	-1.2815
-1.26944	-1.26674	-1.26408	-1.29557	-1.29292	-1.29026	-1.2876	-1.28496	-1.28233
-1.27027	-1.26753	-1.26484	-1.29636	-1.29371	-1.29106	-1.28842	-1.28579	-1.28316
-1.27112	-1.26836	-1.26565	-1.263	-1.2604	-1.29181	-1.28917	-1.28654	-1.28393
-1.27198	-1.26921	-1.26648	-1.26378	-1.26113	-1.29253	-1.28987	-1.28724	-1.28466
-1.27285	-1.27007	-1.26731	-1.26459	-1.2619	-1.29331	-1.29062	-1.28798	-1.2854
-1.27371	-1.27093	-1.26816	-1.26542	-1.26271	-1.29414	-1.29143	-1.28876	-1.28613

-1.27456	-1.27178	-1.26901	-1.26626	-1.26354	-1.29499	-1.29225	-1.28956	-1.2869
-1.27537	-1.27262	-1.26988	-1.26713	-1.26438	-1.29586	-1.29311	-1.29039	-1.28772
-1.27616	-1.27345	-1.27073	-1.26798	-1.26523	-1.29674	-1.29397	-1.29125	-1.28856
-1.245	-1.24235	-1.2397	-1.26882	-1.26608	-1.29764	-1.29484	-1.29209	-1.28939
-1.24577	-1.24312	-1.2405	-1.26964	-1.26694	-1.29854	-1.29572	-1.29294	-1.29022
-1.24656	-1.2439	-1.24127	-1.23863	-1.236	-1.26506	-1.26233	-1.29381	-1.29107
-1.24738	-1.24468	-1.24201	-1.23939	-1.23678	-1.26586	-1.26316	-1.29469	-1.29193
-1.17668	-1.17411	-1.17159	-1.24018	-1.23759	-1.23497	-1.23232	-1.29557	-1.2928
-1.17747	-1.1749	-1.17237	-1.24102	-1.23843	-1.23581	-1.23317	-1.29646	-1.29367
-1.17827	-1.17569	-1.17316	-1.20732	-1.2048	-1.23665	-1.23402	-1.26627	-1.2635
-1.17907	-1.17648	-1.17395	-1.20814	-1.20562	-1.2375	-1.23487	-1.26715	-1.26438
-1.17988	-1.17727	-1.17473	-1.20896	-1.20644	-1.23835	-1.23571	-1.26804	-1.26525
-1.18071	-1.17808	-1.17551	-1.20976	-1.20725	-1.23919	-1.23656	-1.26893	-1.26613
-1.18155	-1.17889	-1.1763	-1.21057	-1.20804	-1.24001	-1.23739	-1.26981	-1.26701
-1.10632	-1.10388	-1.1015	-1.17457	-1.17212	-1.20629	-1.20374	-1.27069	-1.26788
-1.10707	-1.10462	-1.10223	-1.17535	-1.17288	-1.20708	-1.20453	-1.27155	-1.26876
-1.10781	-1.10535	-1.10295	-1.17614	-1.17365	-1.20787	-1.20532	-1.23721	-1.23453
-1.10855	-1.10608	-1.10367	-1.17693	-1.17444	-1.20868	-1.20612	-1.23804	-1.23536
-1.1093	-1.10682	-1.1044	-1.17773	-1.17523	-1.20949	-1.20692	-1.20434	-1.20173
-1.11007	-1.10758	-1.10514	-1.17854	-1.17601	-1.2103	-1.20771	-1.20512	-1.20253
-1.11085	-1.10835	-1.1059	-1.17936	-1.17681	-1.2111	-1.2085	-1.20591	-1.20332
-1.11163	-1.10915	-1.1067	-1.18022	-1.17764	-1.17508	-1.17254	-1.2067	-1.20411
-1.11242	-1.10995	-1.10752	-1.18112	-1.1785	-1.1759	-1.17333	-1.2075	-1.20489
-1.11319	-1.11074	-1.10831	-1.1059	-1.10351	-1.17672	-1.17412	-1.20831	-1.20572
-1.11395	-1.1115	-1.10907	-1.10665	-1.10424	-1.17752	-1.17492	-1.20915	-1.20658
-1.07237	-1.07004	-1.06774	-1.1074	-1.10498	-1.17835	-1.17574	-1.21001	-1.20745
-1.07316	-1.07082	-1.0685	-1.10819	-1.10575	-1.1792	-1.1766	-1.2109	-1.20831
-1.07394	-1.0716	-1.06926	-1.10898	-1.10652	-1.18005	-1.17746	-1.21179	-1.20917
-1.07467	-1.07233	-1.06999	-1.10974	-1.10726	-1.18087	-1.17827	-1.1757	-1.17316
-1.07537	-1.07304	-1.07071	-1.11049	-1.10799	-1.18168	-1.17908	-1.1765	-1.17395
-1.03727	-1.03501	-1.03275	-1.06905	-1.06669	-1.10635	-1.10398	-1.17733	-1.17476
-1.038	-1.0357	-1.03342	-1.06977	-1.06746	-1.10717	-1.10477	-1.17818	-1.17558
-1.03873	-1.03642	-1.03413	-1.03189	-1.02968	-1.10797	-1.10555	-1.17902	-1.17641
-1.03943	-1.03715	-1.03489	-1.03264	-1.0304	-1.10873	-1.1063	-1.17986	-1.17726
-1.04015	-1.0379	-1.03565	-1.03339	-1.03112	-1.06737	-1.06504	-1.1807	-1.17812
-1.04093	-1.03866	-1.03639	-1.03411	-1.03182	-1.06809	-1.06577	-1.18156	-1.17897
-1.0417	-1.03941	-1.03712	-1.03482	-1.03253	-1.06882	-1.06652	-1.18243	-1.17982
-0.99737	-0.99518	-0.993	-1.03554	-1.03326	-1.03102	-1.02879	-1.10701	-1.10462
-0.99801	-0.99583	-0.99366	-1.03626	-1.03401	-1.03177	-1.02953	-1.10781	-1.10538
-0.9987	-0.99652	-0.99435	-1.037	-1.03475	-1.0325	-1.03025	-1.02801	-1.02579
-0.99943	-0.99724	-0.99507	-1.03775	-1.03549	-1.03322	-1.03094	-1.02869	-1.02649
-0.91853	-0.91654	-0.91456	-0.99361	-0.99143	-1.03393	-1.03165	-1.02941	-1.0272
-0.9192	-0.91719	-0.91518	-0.99429	-0.9921	-1.03464	-1.03239	-1.03016	-1.02794
-0.91985	-0.91782	-0.9158	-0.99498	-0.99277	-1.03536	-1.03313	-1.03091	-1.02868
-0.92048	-0.91843	-0.9164	-0.99567	-0.99347	-1.03611	-1.03387	-1.03164	-1.02943
-0.9211	-0.91904	-0.917	-0.99636	-0.99419	-1.03687	-1.03461	-1.03238	-1.03018
-0.92172	-0.91967	-0.91764	-0.99708	-0.9949	-0.99273	-0.99058	-1.03312	-1.03092
-0.92236	-0.92032	-0.9183	-0.99782	-0.99562	-0.99344	-0.99129	-1.03388	-1.03165
-0.88754	-0.8856	-0.88368	-0.91692	-0.91492	-0.99416	-0.992	-1.03462	-1.03238
-0.88822	-0.88625	-0.88431	-0.91755	-0.91557	-0.9949	-0.99271	-1.03536	-1.03313

-0.88889	-0.88691	-0.88495	-0.91819	-0.91622	-0.99563	-0.99343	-1.03611	-1.03387
-0.88954	-0.88758	-0.88564	-0.91886	-0.91684	-0.99634	-0.99416	-1.03689	-1.03463
-0.8902	-0.88826	-0.88632	-0.91952	-0.91747	-0.99706	-0.99488	-1.03766	-1.03538
-0.80684	-0.80502	-0.80321	-0.88059	-0.87861	-0.91612	-0.91413	-1.03841	-1.03613
-0.8075	-0.80565	-0.80381	-0.88122	-0.87926	-0.91677	-0.91475	-1.03916	-1.03689
-0.76184	-0.7601	-0.75837	-0.88187	-0.87988	-0.87791	-0.87596	-0.99485	-0.99268
-0.76241	-0.7607	-0.759	-0.88253	-0.88048	-0.8785	-0.87658	-0.9956	-0.9934
-0.76303	-0.76133	-0.75964	-0.7579	-0.75615	-0.87916	-0.87724	-0.87532	-0.87338
-0.76371	-0.76199	-0.76029	-0.75855	-0.75681	-0.87989	-0.87794	-0.876	-0.87406
-0.76438	-0.76264	-0.76093	-0.7592	-0.75747	-0.83629	-0.83442	-0.87666	-0.87473
-0.76504	-0.76327	-0.76152	-0.7598	-0.7581	-0.83692	-0.83501	-0.87728	-0.87535
-0.76568	-0.76389	-0.76211	-0.7604	-0.75871	-0.83754	-0.8356	-0.87789	-0.87596
-0.7239	-0.7222	-0.72052	-0.761	-0.75929	-0.83814	-0.83622	-0.83433	-0.83248
-0.72449	-0.72281	-0.72114	-0.76161	-0.75986	-0.83874	-0.83685	-0.83496	-0.83306
-0.72512	-0.72344	-0.72177	-0.72009	-0.71842	-0.75876	-0.75708	-0.8356	-0.83368
-0.72579	-0.72409	-0.72242	-0.72074	-0.71906	-0.75941	-0.75772	-0.83626	-0.83434
-0.63963	-0.63811	-0.63661	-0.72136	-0.71969	-0.71804	-0.71643	-0.83688	-0.83498
-0.6402	-0.63865	-0.63713	-0.72195	-0.72029	-0.71863	-0.71699	-0.83746	-0.83557
-0.5412	-0.53994	-0.5387	-0.72255	-0.72089	-0.71923	-0.71757	-0.79465	-0.79287
-0.54167	-0.54042	-0.53918	-0.72317	-0.72152	-0.71986	-0.7182	-0.79528	-0.79349
-0.54214	-0.5409	-0.53967	-0.7238	-0.72215	-0.72049	-0.71883	-0.79593	-0.79413
-0.48889	-0.48781	-0.48674	-0.58408	-0.58277	-0.72109	-0.71944	-0.71781	-0.71621
-0.48938	-0.48828	-0.48719	-0.58461	-0.58328	-0.72167	-0.72003	-0.71841	-0.71682
-0.43833	-0.43734	-0.43636	-0.53278	-0.5316	-0.72228	-0.72064	-0.71901	-0.71742
-0.43873	-0.43775	-0.43678	-0.5333	-0.53211	-0.72293	-0.72126	-0.71961	-0.71799
-0.39111	-0.39026	-0.3894	-0.48751	-0.48643	-0.53143	-0.53026	-0.72023	-0.71858
-0.39148	-0.39061	-0.38975	-0.48795	-0.48688	-0.53192	-0.53076	-0.72089	-0.71922
-0.39185	-0.39097	-0.3901	-0.48839	-0.48733	-0.53241	-0.53127	-0.72155	-0.71987
-0.39223	-0.39134	-0.39047	-0.43741	-0.43646	-0.53291	-0.53177	-0.58613	-0.58471
-0.39261	-0.39172	-0.39085	-0.43783	-0.43687	-0.53342	-0.53227	-0.58672	-0.58528
-0.39299	-0.3921	-0.39122	-0.43825	-0.43728	-0.48359	-0.48255	-0.53163	-0.53045
-0.39338	-0.39249	-0.3916	-0.43867	-0.43769	-0.48404	-0.483	-0.53213	-0.53096
-0.39376	-0.39287	-0.39198	-0.3911	-0.39024	-0.43714	-0.4362	-0.48239	-0.48134
-0.39414	-0.39325	-0.39235	-0.39147	-0.3906	-0.43754	-0.43658	-0.4828	-0.48175
-0.39453	-0.39363	-0.39273	-0.39184	-0.39097	-0.43794	-0.43697	-0.48322	-0.48217
-0.39491	-0.39401	-0.39311	-0.39222	-0.39134	-0.43835	-0.43737	-0.48367	-0.4826
-0.39529	-0.39439	-0.39349	-0.3926	-0.39172	-0.43876	-0.43778	-0.48412	-0.48305
-0.39565	-0.39475	-0.39384	-0.39296	-0.39208	-0.39121	-0.39034	-0.43721	-0.43625
-0.39601	-0.3951	-0.3942	-0.39332	-0.39245	-0.39158	-0.39072	-0.43763	-0.43666
-0.39641	-0.39549	-0.39459	-0.3937	-0.39283	-0.39196	-0.3911	-0.43805	-0.43708
-0.39683	-0.39591	-0.39499	-0.3941	-0.39322	-0.39235	-0.3915	-0.4385	-0.43752
-0.29207	-0.2914	-0.29074	-0.39449	-0.3936	-0.39273	-0.39188	-0.39103	-0.39016
-0.29236	-0.29169	-0.29102	-0.39488	-0.39398	-0.39311	-0.39225	-0.3914	-0.39053
-0.29266	-0.29198	-0.29131	-0.39527	-0.39437	-0.39348	-0.39262	-0.39176	-0.3909
-0.29296	-0.29227	-0.29159	-0.39565	-0.39475	-0.39387	-0.393	-0.39213	-0.39127
-0.29326	-0.29256	-0.29188	-0.39604	-0.39514	-0.39425	-0.39338	-0.39251	-0.39164
-0.29356	-0.29286	-0.29217	-0.29151	-0.29086	-0.39463	-0.39376	-0.39289	-0.39203
-0.29387	-0.29316	-0.29247	-0.2918	-0.29114	-0.39502	-0.39415	-0.39328	-0.39242
-0.29418	-0.29348	-0.29278	-0.2921	-0.29144	-0.29079	-0.29016	-0.39367	-0.39281
-0.2945	-0.2938	-0.2931	-0.29242	-0.29174	-0.29108	-0.29044	-0.39405	-0.39319

-0.29482	-0.29412	-0.29342	-0.29273	-0.29204	-0.29138	-0.29073	-0.39444	-0.39357
-0.29513	-0.29443	-0.29373	-0.29304	-0.29234	-0.29167	-0.29102	-0.39482	-0.39395
-0.29544	-0.29474	-0.29404	-0.29334	-0.29265	-0.29197	-0.29131	-0.39521	-0.39434
-0.29575	-0.29505	-0.29436	-0.29366	-0.29297	-0.29229	-0.29162	-0.29096	-0.29031
-0.29607	-0.29537	-0.29468	-0.29399	-0.29329	-0.29261	-0.29193	-0.29126	-0.29061
-0.29639	-0.29569	-0.29499	-0.2943	-0.29361	-0.29293	-0.29225	-0.29157	-0.29091
-0.29673	-0.29601	-0.2953	-0.29461	-0.29392	-0.29324	-0.29256	-0.29189	-0.29122
-0.23863	-0.23804	-0.23746	-0.29492	-0.29424	-0.29355	-0.29288	-0.2922	-0.29154
-0.23891	-0.23831	-0.23772	-0.29524	-0.29454	-0.29386	-0.29318	-0.29252	-0.29185
-0.18373	-0.18326	-0.1828	-0.29557	-0.29486	-0.29417	-0.29349	-0.29282	-0.29216
-0.18393	-0.18346	-0.183	-0.29591	-0.29519	-0.29449	-0.2938	-0.29312	-0.29246
-0.18412	-0.18366	-0.1832	-0.29625	-0.29553	-0.29481	-0.29412	-0.29343	-0.29276
-0.18432	-0.18386	-0.1834	-0.29659	-0.29587	-0.29516	-0.29446	-0.29377	-0.29308
-0.18452	-0.18405	-0.18359	-0.29693	-0.29621	-0.29551	-0.29481	-0.29412	-0.29342
-0.18473	-0.18426	-0.18379	-0.2431	-0.24251	-0.29584	-0.29514	-0.29444	-0.29375
-0.18495	-0.18447	-0.18399	-0.24337	-0.24277	-0.29616	-0.29545	-0.29476	-0.29407
-0.18516	-0.18469	-0.18421	-0.24365	-0.24305	-0.29649	-0.29578	-0.29508	-0.29439
-0.18538	-0.18491	-0.18444	-0.24397	-0.24335	-0.29685	-0.29612	-0.29541	-0.29471
-0.18559	-0.18513	-0.18467	-0.1842	-0.18374	-0.24304	-0.24243	-0.29574	-0.29504
-0.18578	-0.18533	-0.18487	-0.1844	-0.18394	-0.24332	-0.24271	-0.29609	-0.29539
-0.18598	-0.18552	-0.18506	-0.18459	-0.18413	-0.24359	-0.24299	-0.29644	-0.29574
-0.1862	-0.18574	-0.18527	-0.1848	-0.18433	-0.24387	-0.24326	-0.29677	-0.29607
-0.18643	-0.18596	-0.18549	-0.18501	-0.18454	-0.24414	-0.24353	-0.2971	-0.29639
-0.18664	-0.18617	-0.1857	-0.18523	-0.18476	-0.24443	-0.24383	-0.24322	-0.24263
-0.18683	-0.18637	-0.18591	-0.18545	-0.18499	-0.24475	-0.24414	-0.24354	-0.24294
-0.18704	-0.18658	-0.18612	-0.18566	-0.18521	-0.24505	-0.24445	-0.24384	-0.24324
-0.18726	-0.1868	-0.18634	-0.18587	-0.18541	-0.24533	-0.24472	-0.24411	-0.24351
-0.18749	-0.18702	-0.18655	-0.18608	-0.18561	-0.18515	-0.18469	-0.24439	-0.24378
-0.1877	-0.18722	-0.18676	-0.18629	-0.18584	-0.18538	-0.18492	-0.2447	-0.24409
-0.1879	-0.18743	-0.18696	-0.18651	-0.18606	-0.1856	-0.18515	-0.24501	-0.2444
-0.18813	-0.18765	-0.18718	-0.18672	-0.18626	-0.18581	-0.18535	-0.2453	-0.24469
-0.18837	-0.18788	-0.18739	-0.18692	-0.18646	-0.186	-0.18555	-0.24557	-0.24496
-0.1886	-0.1881	-0.18761	-0.18713	-0.18667	-0.18621	-0.18576	-0.24586	-0.24525
-0.18881	-0.18831	-0.18783	-0.18735	-0.18689	-0.18644	-0.18599	-0.24617	-0.24557
-0.18903	-0.18854	-0.18805	-0.18758	-0.18712	-0.18667	-0.18622	-0.24649	-0.24588
-0.18928	-0.18878	-0.18829	-0.18782	-0.18735	-0.18689	-0.18645	-0.24679	-0.24618
-0.18952	-0.18903	-0.18853	-0.18805	-0.18758	-0.18711	-0.18667	-0.24709	-0.24647
-0.18975	-0.18925	-0.18875	-0.18826	-0.18778	-0.18732	-0.18687	-0.18641	-0.18595
-0.18996	-0.18946	-0.18897	-0.18848	-0.18799	-0.18752	-0.18706	-0.18661	-0.18616
-0.1902	-0.1897	-0.18921	-0.18871	-0.18822	-0.18774	-0.18728	-0.18682	-0.18638
-0.19045	-0.18996	-0.18946	-0.18897	-0.18847	-0.18799	-0.18752	-0.18706	-0.18661
-0.1907	-0.19021	-0.18972	-0.18922	-0.18873	-0.18824	-0.18776	-0.18729	-0.18684
-0.19094	-0.19045	-0.18997	-0.18947	-0.18898	-0.18849	-0.188	-0.18752	-0.18706
-0.19117	-0.19069	-0.1902	-0.18971	-0.18922	-0.18872	-0.18823	-0.18775	-0.18729
-0.19138	-0.1909	-0.19042	-0.18993	-0.18944	-0.18894	-0.18845	-0.18796	-0.18749
-0.19159	-0.19112	-0.19064	-0.19015	-0.18966	-0.18916	-0.18867	-0.18818	-0.1877
-0.19182	-0.19135	-0.19087	-0.19039	-0.1899	-0.18941	-0.18891	-0.18842	-0.18794
-0.19207	-0.19159	-0.19112	-0.19064	-0.19015	-0.18966	-0.18917	-0.18868	-0.18819
-0.19231	-0.19183	-0.19136	-0.19088	-0.1904	-0.18991	-0.18942	-0.18893	-0.18844
-0.19255	-0.19207	-0.1916	-0.19112	-0.19064	-0.19016	-0.18967	-0.18918	-0.18869

-0.19278	-0.1923	-0.19182	-0.19135	-0.19087	-0.19039	-0.18991	-0.18943	-0.18893
-0.19299	-0.1925	-0.19203	-0.19155	-0.19108	-0.1906	-0.19013	-0.18966	-0.18918
-0.19322	-0.19272	-0.19224	-0.19176	-0.19129	-0.19082	-0.19035	-0.18989	-0.18941
-0.19348	-0.19297	-0.19248	-0.192	-0.19152	-0.19105	-0.19059	-0.19011	-0.18963
-0.19375	-0.19324	-0.19273	-0.19225	-0.19177	-0.1913	-0.19082	-0.19034	-0.18985
-0.19402	-0.1935	-0.19299	-0.19249	-0.19202	-0.19154	-0.19106	-0.19058	-0.19009
-0.1943	-0.19376	-0.19324	-0.19274	-0.19226	-0.19178	-0.19131	-0.19083	-0.19034
-0.19457	-0.19403	-0.19351	-0.193	-0.19251	-0.19203	-0.19155	-0.19107	-0.19059
-0.19484	-0.19431	-0.19378	-0.19327	-0.19276	-0.19227	-0.19179	-0.19132	-0.19084
-0.19511	-0.19458	-0.19405	-0.19353	-0.19302	-0.19252	-0.19204	-0.19156	-0.19109
-0.19538	-0.19485	-0.19432	-0.1938	-0.19327	-0.19277	-0.19228	-0.1918	-0.19133
-0.19564	-0.19512	-0.19459	-0.19406	-0.19353	-0.19303	-0.19253	-0.19205	-0.19157
-0.1959	-0.19538	-0.19486	-0.19433	-0.1938	-0.19329	-0.19278	-0.19229	-0.19181
-0.19614	-0.19564	-0.19513	-0.1946	-0.19408	-0.19356	-0.19304	-0.19254	-0.19206
-0.12894	-0.12865	-0.12835	-0.19488	-0.19435	-0.19382	-0.1933	-0.19279	-0.1923
-0.12911	-0.12881	-0.12851	-0.19515	-0.19462	-0.19409	-0.19356	-0.19305	-0.19255
-0.12927	-0.12897	-0.12867	-0.19541	-0.19488	-0.19436	-0.19383	-0.19331	-0.19281
-0.12942	-0.12912	-0.12882	-0.19566	-0.19515	-0.19463	-0.19411	-0.19358	-0.19307
-0.12957	-0.12927	-0.12897	-0.12867	-0.12838	-0.1949	-0.19438	-0.19386	-0.19334
-0.12974	-0.12943	-0.12913	-0.12884	-0.12854	-0.19517	-0.19465	-0.19412	-0.1936
-0.1299	-0.1296	-0.1293	-0.129	-0.1287	-0.19543	-0.19491	-0.19439	-0.19386
-0.13007	-0.12976	-0.12946	-0.12916	-0.12887	-0.19569	-0.19518	-0.19466	-0.19413
-0.13024	-0.12993	-0.12962	-0.12932	-0.12903	-0.19595	-0.19544	-0.19492	-0.1944
-0.13041	-0.1301	-0.12979	-0.12949	-0.12919	-0.19621	-0.1957	-0.19519	-0.19467
-0.13059	-0.13027	-0.12996	-0.12965	-0.12936	-0.19647	-0.19596	-0.19545	-0.19493
-0.13077	-0.13045	-0.13013	-0.12982	-0.12952	-0.12921	-0.1289	-0.19571	-0.19519
-0.13096	-0.13064	-0.13032	-0.13	-0.12969	-0.12937	-0.12906	-0.19597	-0.19546
-0.06369	-0.06355	-0.0634	-0.13018	-0.12986	-0.12954	-0.12923	-0.19623	-0.19572
-0.06377	-0.06363	-0.06348	-0.13036	-0.13003	-0.12971	-0.12939	-0.19649	-0.19598
-0.06385	-0.06371	-0.06356	-0.13053	-0.1302	-0.12988	-0.12956	-0.19675	-0.19624
			-0.13071	-0.13037	-0.13005	-0.12973	-0.12941	-0.1291
			-0.13088	-0.13055	-0.13022	-0.1299	-0.12958	-0.12927
			-0.06366	-0.06351	-0.13039	-0.13007	-0.12976	-0.12945
			-0.06374	-0.06359	-0.13057	-0.13025	-0.12994	-0.12963
					-0.13075	-0.13043	-0.13012	-0.12981
					-0.13093	-0.13061	-0.13029	-0.12998
					-0.1311	-0.13078	-0.13046	-0.13015
					-0.06378	-0.06364	-0.13065	-0.13033
					-0.06387	-0.06372	-0.13084	-0.13051
							-0.13102	-0.13069
							-0.1312	-0.13087
							-0.06382	-0.06368
							-0.06391	-0.06376

							-0.06139	-0.06121
							-0.06129	-0.0611
							-0.06119	-0.061
							-0.0611	-0.06091
					-0.06138	-0.06119	-0.44375	-0.44259
					-0.06129	-0.0611	-0.44315	-0.442
					-0.06119	-0.061	-0.44255	-0.4414
					-0.0611	-0.0609	-0.56267	-0.56117
					-0.061	-0.06081	-0.56188	-0.56038
			-0.06128	-0.06109	-0.44303	-0.4419	-0.56108	-0.55959
			-0.06118	-0.061	-0.44244	-0.4413	-0.56028	-0.55881
-0.06166	-0.06147	-0.06127	-0.38197	-0.38097	-0.56246	-0.56096	-0.76767	-0.76567
-0.06157	-0.06138	-0.06119	-0.38146	-0.38048	-0.56169	-0.56018	-0.76661	-0.76462
-0.06148	-0.06129	-0.0611	-0.38095	-0.37999	-0.56093	-0.55942	-0.76556	-0.76358
-0.06138	-0.06119	-0.06101	-0.44242	-0.44128	-0.61228	-0.61067	-0.76456	-0.76257

-0.06128	-0.0611	-0.06091	-0.44183	-0.44069	-0.61144	-0.60987	-0.76357	-0.76157
-0.3823	-0.38134	-0.38039	-0.56155	-0.56006	-0.76655	-0.76456	-0.81613	-0.81397
-0.38179	-0.38082	-0.37986	-0.56074	-0.55928	-0.7655	-0.76353	-0.81504	-0.81291
-0.44343	-0.44228	-0.44115	-0.61206	-0.61051	-0.76446	-0.76251	-0.81392	-0.81182
-0.44287	-0.44172	-0.44058	-0.61123	-0.60969	-0.76344	-0.76148	-0.81278	-0.81069
-0.56288	-0.56137	-0.55987	-0.71732	-0.71549	-0.81593	-0.81378	-0.86154	-0.85935
-0.56212	-0.56062	-0.55913	-0.71639	-0.71456	-0.81484	-0.81272	-0.86042	-0.85819
-0.56136	-0.55988	-0.5584	-0.71548	-0.71364	-0.81376	-0.81165	-0.85931	-0.85706
-0.61277	-0.61119	-0.60961	-0.71457	-0.71271	-0.86257	-0.86037	-0.85817	-0.85593
-0.61196	-0.61039	-0.60884	-0.71366	-0.71177	-0.86138	-0.8592	-0.85702	-0.85482
-0.71831	-0.71646	-0.71461	-0.81475	-0.81257	-0.86022	-0.85803	-0.90087	-0.8986
-0.71734	-0.71549	-0.71366	-0.81367	-0.81152	-0.8591	-0.85687	-0.89962	-0.89737
-0.7164	-0.71454	-0.71271	-0.86245	-0.86021	-0.85797	-0.85572	-0.96659	-0.96423
-0.71548	-0.7136	-0.71174	-0.86124	-0.85904	-0.85682	-0.8546	-0.96539	-0.96299
-0.71457	-0.71267	-0.71078	-0.86004	-0.85785	-0.969	-0.96659	-0.96419	-0.96178
-0.71366	-0.71175	-0.70984	-0.85886	-0.85666	-0.96771	-0.96535	-0.96299	-0.96061
-0.71275	-0.71084	-0.70893	-0.85772	-0.85549	-0.96644	-0.96411	-0.96178	-0.95944
-0.86346	-0.86117	-0.85888	-0.97011	-0.96764	-0.96523	-0.96288	-0.96055	-0.95824
-0.86228	-0.86003	-0.85779	-0.96895	-0.96648	-0.96405	-0.96166	-0.9593	-0.95701
-0.86108	-0.85888	-0.85668	-0.96777	-0.96533	-0.9629	-0.96048	-1.0078	-1.00538
-0.85987	-0.8577	-0.85555	-0.96658	-0.96417	-0.96176	-0.95933	-1.00658	-1.00415
-0.97247	-0.97008	-0.96775	-0.96538	-0.96301	-0.96061	-0.95819	-1.00536	-1.00291
-0.97128	-0.9689	-0.96656	-0.9642	-0.96184	-0.95945	-0.95702	-1.00412	-1.00164
-0.97011	-0.96772	-0.96536	-0.963	-0.96065	-0.95827	-0.95586	-1.00289	-1.0004
-0.96891	-0.9665	-0.96412	-0.96177	-0.95944	-1.00671	-1.00419	-1.00168	-0.99919
-0.96771	-0.96528	-0.96288	-0.96053	-0.95822	-1.00546	-1.00298	-1.00049	-0.99801
-0.96654	-0.96411	-0.9617	-1.00911	-1.00668	-1.00423	-1.00177	-0.9993	-0.99682
-0.96539	-0.96296	-0.96055	-1.00789	-1.00546	-1.00301	-1.00056	-0.99811	-0.99565
-0.96424	-0.96181	-0.9594	-1.00667	-1.00423	-1.0018	-0.99936	-1.04048	-1.03785
-0.96309	-0.96065	-0.95823	-1.00545	-1.00301	-1.00059	-0.99817	-1.03923	-1.03659
-1.01182	-1.00925	-1.0067	-1.00421	-1.00177	-0.99935	-0.99695	-1.03796	-1.03534
-1.01059	-1.00801	-1.00545	-1.00294	-1.00047	-0.99805	-0.99569	-1.03666	-1.03408
-1.00935	-1.00677	-1.0042	-1.00168	-0.99918	-0.99677	-0.99444	-1.03536	-1.03282
-1.00809	-1.00553	-1.00297	-1.00046	-0.99797	-1.0391	-1.0366	-1.03411	-1.03158
-1.00684	-1.00429	-1.00176	-0.99926	-0.9968	-1.03787	-1.03537	-1.03287	-1.03035
-1.00561	-1.00307	-1.00056	-0.99808	-0.99563	-1.03664	-1.03414	-1.03164	-1.02913
-1.00439	-1.00187	-0.99936	-0.9969	-0.99447	-1.03541	-1.03291	-1.03042	-1.02791
-1.00319	-1.00067	-0.99818	-1.03934	-1.03673	-1.03418	-1.03168	-1.16418	-1.16089
-1.00199	-0.99949	-0.99701	-1.03811	-1.03549	-1.03294	-1.03044	-1.16251	-1.15924
-1.00079	-0.99831	-0.99584	-1.03687	-1.03426	-1.0317	-1.02921	-1.24319	-1.23977
-0.99961	-0.99713	-0.99467	-1.03563	-1.03302	-1.03047	-1.02797	-1.24145	-1.23807
-0.99842	-0.99595	-0.99349	-1.03439	-1.0318	-1.02925	-1.02674	-1.23973	-1.23639
-1.04103	-1.03838	-1.03576	-1.03317	-1.0306	-1.16251	-1.15926	-1.2381	-1.23477
-1.03975	-1.03713	-1.03454	-1.03197	-1.02943	-1.16097	-1.15775	-1.23652	-1.23318
-1.03851	-1.03591	-1.03334	-1.0308	-1.02828	-1.24168	-1.23829	-1.23493	-1.23158
-1.0373	-1.03472	-1.03218	-1.02965	-1.02714	-1.2401	-1.23669	-1.23332	-1.22998
-1.03613	-1.03356	-1.03102	-1.16297	-1.15971	-1.2385	-1.23509	-1.23171	-1.22838
-1.03503	-1.03244	-1.02986	-1.1614	-1.15814	-1.23684	-1.23346	-1.23011	-1.22678
-1.03391	-1.0313	-1.0287	-1.15983	-1.15657	-1.23519	-1.23183	-1.2285	-1.2252
-1.0327	-1.03011	-1.02752	-1.24038	-1.23695	-1.23356	-1.23022	-1.22691	-1.22361

-1.03146	-1.02888	-1.02632	-1.23873	-1.23532	-1.23195	-1.22862	-1.22531	-1.22202
-1.16507	-1.16171	-1.15841	-1.23707	-1.23368	-1.23033	-1.22701	-1.26686	-1.26326
-1.16342	-1.16009	-1.15683	-1.23541	-1.23204	-1.22871	-1.2254	-1.26511	-1.26153
-1.20417	-1.20076	-1.1974	-1.23377	-1.23042	-1.22709	-1.22379	-1.26336	-1.2598
-1.20253	-1.19914	-1.19581	-1.23214	-1.22879	-1.22547	-1.22218	-1.2616	-1.25807
-1.2408	-1.23736	-1.23395	-1.23058	-1.22724	-1.22393	-1.22065	-1.25994	-1.25643
-1.23931	-1.23588	-1.2325	-1.22914	-1.22582	-1.22254	-1.21928	-1.25845	-1.25497
-1.23782	-1.23441	-1.23104	-1.22771	-1.22441	-1.22114	-1.21791	-1.25698	-1.25352
-1.23621	-1.23282	-1.22947	-1.22615	-1.22287	-1.26239	-1.25886	-1.25538	-1.25195
-1.23456	-1.23118	-1.22784	-1.22454	-1.22128	-1.26069	-1.25719	-1.25374	-1.25033
-1.2329	-1.22954	-1.22622	-1.22295	-1.21971	-1.259	-1.25554	-1.2521	-1.24871
-1.23124	-1.2279	-1.22461	-1.22136	-1.21815	-1.25733	-1.25388	-1.25046	-1.24707
-1.22964	-1.22631	-1.22303	-1.26264	-1.25914	-1.25567	-1.25224	-1.24884	-1.24546
-1.22814	-1.22477	-1.22147	-1.26094	-1.25747	-1.25403	-1.25061	-1.24723	-1.24385
-1.22665	-1.22328	-1.21998	-1.25932	-1.25587	-1.25245	-1.24904	-1.33093	-1.32719
-1.22518	-1.22188	-1.21862	-1.25785	-1.25442	-1.251	-1.2476	-1.32934	-1.32563
-1.22372	-1.22048	-1.21728	-1.25641	-1.25299	-1.24958	-1.24618	-1.32778	-1.3241
-1.26534	-1.2618	-1.25831	-1.25486	-1.25144	-1.24804	-1.24463	-1.3261	-1.32249
-1.26364	-1.26014	-1.25668	-1.25325	-1.24984	-1.24644	-1.24303	-1.32437	-1.32085
-1.26207	-1.25859	-1.25516	-1.25173	-1.24833	-1.33014	-1.32636	-1.32271	-1.31924
-1.2606	-1.25714	-1.25372	-1.2503	-1.24689	-1.32855	-1.32474	-1.32109	-1.31766
-1.25908	-1.25564	-1.25222	-1.2488	-1.24539	-1.32693	-1.32317	-1.31957	-1.3162
-1.25745	-1.25402	-1.25061	-1.2472	-1.2438	-1.32525	-1.32165	-1.31819	-1.31491
-1.25585	-1.25242	-1.24901	-1.24561	-1.24222	-1.32359	-1.32014	-1.31682	-1.31364
-1.25437	-1.25094	-1.24753	-1.28408	-1.28054	-1.32197	-1.31857	-1.35178	-1.34863
-1.25291	-1.24949	-1.24607	-1.28257	-1.27904	-1.3204	-1.31704	-1.35026	-1.34722
-1.25133	-1.24791	-1.24453	-1.32607	-1.32242	-1.35545	-1.35211	-1.34892	-1.34594
-1.24967	-1.24628	-1.24293	-1.32443	-1.32094	-1.35406	-1.35082	-1.3477	-1.34474
-1.28825	-1.28474	-1.28129	-1.32286	-1.31946	-1.35264	-1.34947	-1.34641	-1.34347
-1.28676	-1.28327	-1.27985	-1.32137	-1.318	-1.35117	-1.34806	-1.34505	-1.34211
-1.28523	-1.28178	-1.27842	-1.3564	-1.353	-1.34977	-1.34672	-1.34373	-1.3408
-1.28361	-1.28023	-1.27696	-1.35493	-1.35164	-1.34849	-1.34547	-1.34251	-1.33959
-1.28201	-1.27871	-1.27554	-1.3535	-1.35034	-1.34725	-1.34426	-1.34131	-1.33839
-1.31784	-1.31453	-1.31137	-1.35216	-1.34908	-1.34603	-1.34302	-1.34005	-1.3371
-1.31641	-1.31317	-1.31009	-1.35087	-1.34785	-1.34482	-1.34178	-1.33878	-1.3358
-1.35924	-1.35585	-1.35261	-1.34951	-1.34654	-1.34355	-1.34053	-1.33754	-1.33456
-1.35776	-1.3544	-1.35119	-1.34812	-1.34519	-1.34224	-1.33927	-1.33632	-1.33337
-1.35631	-1.35301	-1.34985	-1.34681	-1.34389	-1.34096	-1.33803	-1.33511	-1.33216
-1.35489	-1.35169	-1.34861	-1.3456	-1.34267	-1.33974	-1.33681	-1.33389	-1.33093
-1.35351	-1.3504	-1.34739	-1.34439	-1.34144	-1.3385	-1.33557	-1.37113	-1.36809
-1.3522	-1.34914	-1.34617	-1.34317	-1.34017	-1.3372	-1.33426	-1.36978	-1.3668
-1.3509	-1.34789	-1.34494	-1.34193	-1.3389	-1.3359	-1.33295	-1.36844	-1.36554
-1.34959	-1.34657	-1.34362	-1.34063	-1.33763	-1.33466	-1.33173	-1.3672	-1.36432
-1.34827	-1.34524	-1.34227	-1.33931	-1.33638	-1.33346	-1.33056	-1.36602	-1.36315
-1.34699	-1.34397	-1.34099	-1.33806	-1.33516	-1.33227	-1.3294	-1.39218	-1.38921
-1.34574	-1.34274	-1.33978	-1.33686	-1.33397	-1.33111	-1.32827	-1.39097	-1.38798
-1.3445	-1.34153	-1.33859	-1.33568	-1.3328	-1.39577	-1.39274	-1.38974	-1.38675
-1.34329	-1.34034	-1.33742	-1.33452	-1.33164	-1.39451	-1.39147	-1.38846	-1.38551
-1.34209	-1.33916	-1.33625	-1.36146	-1.35847	-1.39325	-1.39019	-1.38719	-1.38428
-1.34091	-1.33798	-1.33508	-1.36023	-1.35723	-1.39196	-1.38893	-1.38596	-1.38308

-1.33973	-1.33681	-1.33389	-1.35898	-1.35597	-1.39067	-1.38768	-1.38475	-1.38189
-1.33854	-1.3356	-1.33267	-1.35772	-1.35473	-1.38941	-1.38646	-1.38357	-1.38073
-1.33734	-1.33438	-1.33143	-1.35644	-1.35349	-1.38817	-1.38526	-1.3824	-1.37958
-1.36429	-1.36122	-1.35818	-1.3552	-1.35228	-1.38695	-1.38407	-1.38123	-1.37841
-1.36298	-1.35994	-1.35694	-1.35399	-1.3511	-1.38575	-1.38289	-1.38005	-1.37723
-1.36171	-1.35869	-1.35572	-1.39044	-1.38748	-1.38457	-1.3817	-1.37886	-1.37603
-1.3605	-1.35748	-1.35452	-1.38922	-1.38629	-1.38338	-1.38049	-1.37763	-1.37482
-1.35931	-1.3563	-1.35333	-1.38801	-1.38512	-1.38221	-1.37929	-1.37642	-1.37362
-1.35813	-1.35512	-1.35216	-1.38682	-1.38394	-1.38105	-1.37814	-1.37527	-1.37247
-1.35696	-1.35396	-1.35101	-1.38564	-1.38277	-1.3799	-1.37702	-1.37417	-1.37135
-1.35574	-1.35278	-1.34985	-1.38446	-1.3816	-1.37875	-1.37588	-1.37305	-1.37024
-1.3545	-1.35158	-1.3487	-1.38329	-1.38043	-1.37759	-1.37475	-1.37193	-1.36913
-1.39095	-1.38795	-1.38501	-1.38211	-1.37924	-1.3764	-1.37358	-1.37079	-1.36801
-1.3897	-1.38673	-1.38381	-1.38091	-1.37802	-1.37518	-1.37238	-1.36961	-1.36687
-1.38847	-1.38552	-1.38262	-1.37972	-1.37682	-1.37398	-1.37119	-1.43333	-1.43045
-1.38726	-1.38432	-1.38144	-1.37855	-1.37567	-1.37284	-1.37005	-1.43213	-1.42927
-1.38607	-1.38314	-1.38027	-1.3774	-1.37454	-1.37171	-1.36892	-1.43094	-1.4281
-1.38492	-1.38199	-1.37913	-1.37626	-1.37341	-1.37059	-1.3678	-1.42977	-1.42694
-1.38379	-1.38086	-1.378	-1.37513	-1.37228	-1.36946	-1.36669	-1.42862	-1.42579
-1.38264	-1.37972	-1.37686	-1.374	-1.37116	-1.43331	-1.43036	-1.42746	-1.42463
-1.38147	-1.37856	-1.37572	-1.37287	-1.37005	-1.43215	-1.4292	-1.4263	-1.42348
-1.38031	-1.37741	-1.37458	-1.37176	-1.36896	-1.43103	-1.42807	-1.42516	-1.42234
-1.37916	-1.37628	-1.37345	-1.37067	-1.36793	-1.42996	-1.42699	-1.42406	-1.42121
-1.37802	-1.37515	-1.37233	-1.43467	-1.4318	-1.42889	-1.4259	-1.42296	-1.42008
-1.37686	-1.374	-1.37119	-1.43349	-1.43064	-1.42774	-1.42477	-1.42184	-1.41896
-1.37572	-1.37287	-1.37005	-1.4323	-1.42946	-1.42657	-1.42362	-1.4207	-1.41783
-1.37464	-1.37179	-1.36896	-1.43114	-1.42829	-1.4254	-1.42246	-1.41956	-1.4167
-1.37359	-1.37074	-1.36789	-1.43	-1.42712	-1.42422	-1.4213	-1.41841	-1.41558
-1.43784	-1.43482	-1.4318	-1.42885	-1.42596	-1.42306	-1.42014	-1.41727	-1.41446
-1.43665	-1.43365	-1.43066	-1.42771	-1.4248	-1.42189	-1.419	-1.41614	-1.41334
-1.43545	-1.43249	-1.42954	-1.4266	-1.42367	-1.42077	-1.41789	-1.41506	-1.41226
-1.43426	-1.43134	-1.42846	-1.42553	-1.42259	-1.4197	-1.41686	-1.41405	-1.41125
-1.43308	-1.43021	-1.42737	-1.42446	-1.42151	-1.41863	-1.41583	-1.41304	-1.41024
-1.43194	-1.42907	-1.42623	-1.42333	-1.4204	-1.41753	-1.41473	-1.41195	-1.40917
-1.43082	-1.42793	-1.42507	-1.42218	-1.41929	-1.41643	-1.41362	-1.41084	-1.4081
-1.42969	-1.42679	-1.42392	-1.42106	-1.41821	-1.41538	-1.41256	-1.40978	-1.40707
-1.42856	-1.42565	-1.42277	-1.41995	-1.41717	-1.41437	-1.41154	-1.40875	-1.40606
-1.42746	-1.42454	-1.42165	-1.41884	-1.4161	-1.41332	-1.41049	-1.40772	-1.40503
-1.42639	-1.42347	-1.42058	-1.41775	-1.41498	-1.41221	-1.40942	-1.40667	-1.40398
-1.42533	-1.42242	-1.41952	-1.41668	-1.41388	-1.41111	-1.40836	-1.40563	-1.40294
-1.42426	-1.42137	-1.41851	-1.41566	-1.41283	-1.41006	-1.40734	-1.40464	-1.40195
-1.42318	-1.42032	-1.41748	-1.41464	-1.41179	-1.40902	-1.40632	-1.40365	-1.40097
-1.42208	-1.41922	-1.41638	-1.41355	-1.41073	-1.40797	-1.40527	-1.50789	-1.5047
-1.42097	-1.4181	-1.41525	-1.41244	-1.40966	-1.40691	-1.40421	-1.50662	-1.50346
-1.41988	-1.41702	-1.41417	-1.41138	-1.40862	-1.4059	-1.40319	-1.50543	-1.50229
-1.41882	-1.41596	-1.41313	-1.41036	-1.40763	-1.40492	-1.40223	-1.50428	-1.50117
-1.41777	-1.41492	-1.41211	-1.40935	-1.40663	-1.40394	-1.40126	-1.50316	-1.50005
-1.41673	-1.41391	-1.41112	-1.40836	-1.40564	-1.40295	-1.4003	-1.50206	-1.49893
-1.4157	-1.4129	-1.41013	-1.40738	-1.40466	-1.40198	-1.39935	-1.50095	-1.49782
-1.41469	-1.41189	-1.40913	-1.4064	-1.4037	-1.40103	-1.39839	-1.4998	-1.49669

-1.41368	-1.41089	-1.40812	-1.40541	-1.40275	-1.40009	-1.39743	-1.49865	-1.49556
-1.41266	-1.40988	-1.40714	-1.40444	-1.40178	-1.39913	-1.39649	-1.49753	-1.49446
-1.41163	-1.40888	-1.40616	-1.40347	-1.4008	-1.39816	-1.39554	-1.49644	-1.49336
-1.41063	-1.40789	-1.40518	-1.4025	-1.39983	-1.50154	-1.49841	-1.52509	-1.52198
-1.40965	-1.40691	-1.4042	-1.40152	-1.39888	-1.50042	-1.49724	-1.52392	-1.52083
-1.40867	-1.40594	-1.40323	-1.40056	-1.39792	-1.49928	-1.49607	-1.55464	-1.55149
-1.40767	-1.40495	-1.40227	-1.3996	-1.39696	-1.4981	-1.4949	-1.55347	-1.55037
-1.40667	-1.40397	-1.40131	-1.39865	-1.396	-1.49694	-1.49377	-1.55236	-1.54927
-1.40568	-1.40299	-1.40033	-1.39768	-1.39506	-1.52578	-1.52262	-1.55132	-1.54823
-1.4047	-1.40201	-1.39935	-1.39672	-1.39412	-1.52471	-1.52161	-1.55033	-1.54721
-1.40371	-1.40103	-1.39838	-1.39576	-1.39317	-1.52359	-1.52056	-1.5493	-1.54622
-1.40272	-1.40005	-1.39742	-1.39481	-1.39222	-1.52245	-1.51947	-1.54825	-1.54525
-1.40178	-1.3991	-1.39646	-1.39386	-1.39128	-1.52135	-1.51841	-1.54719	-1.54424
-1.40088	-1.39818	-1.3955	-1.3929	-1.39034	-1.52031	-1.51735	-1.54611	-1.54318
-1.39998	-1.39726	-1.39457	-1.49338	-1.49045	-1.51926	-1.5163	-1.54503	-1.5421
-1.39903	-1.39635	-1.39369	-1.49234	-1.48939	-1.51818	-1.51527	-1.544	-1.54103
-1.39808	-1.39544	-1.39282	-1.49133	-1.48836	-1.51712	-1.51424	-1.54296	-1.53996
-1.39711	-1.3945	-1.39191	-1.49034	-1.48739	-1.51612	-1.51322	-1.54189	-1.53889
-1.39614	-1.39354	-1.39099	-1.48937	-1.48646	-1.51516	-1.5122	-1.54081	-1.53782
-1.42709	-1.42444	-1.42183	-1.48835	-1.48549	-1.51418	-1.51121	-1.61176	-1.60865
-1.42624	-1.42355	-1.4209	-1.48732	-1.48451	-1.5132	-1.51024	-1.61079	-1.60767
-1.42539	-1.42269	-1.42001	-1.48632	-1.48355	-1.54387	-1.54084	-1.60981	-1.60667
-1.42455	-1.42186	-1.41918	-1.48538	-1.48261	-1.54287	-1.53985	-1.60881	-1.60562
-1.49329	-1.49028	-1.48732	-1.48445	-1.48166	-1.61409	-1.61096	-1.6078	-1.60457
-1.49231	-1.48933	-1.48638	-1.4835	-1.48066	-1.61303	-1.60991	-1.60677	-1.60355
-1.49131	-1.48837	-1.48545	-1.48254	-1.47966	-1.61196	-1.60884	-1.60571	-1.60253
-1.49029	-1.48738	-1.4845	-1.4816	-1.4787	-1.61094	-1.60778	-1.60462	-1.60147
-1.48925	-1.48637	-1.48354	-1.48067	-1.47777	-1.60995	-1.60672	-1.60352	-1.6004
-1.48828	-1.48541	-1.48259	-1.55486	-1.55185	-1.60898	-1.60572	-1.6025	-1.59939
-1.48737	-1.48448	-1.48164	-1.55393	-1.55094	-1.60803	-1.60477	-1.60157	-1.59847
-1.56192	-1.55891	-1.55595	-1.55298	-1.55001	-1.60707	-1.60383	-1.60063	-1.59753
-1.56101	-1.55795	-1.55494	-1.55197	-1.54904	-1.60608	-1.60287	-1.59969	-1.59656
-1.5601	-1.557	-1.55395	-1.55098	-1.54808	-1.6051	-1.60193	-1.59876	-1.59561
-1.55919	-1.55609	-1.55302	-1.55005	-1.54715	-1.60415	-1.601	-1.6279	-1.62465
-1.55828	-1.55518	-1.55213	-1.54915	-1.54625	-1.60321	-1.60009	-1.62696	-1.62372
-1.55735	-1.55426	-1.55122	-1.54826	-1.54536	-1.60228	-1.59918	-1.62603	-1.62278
-1.55642	-1.55334	-1.55032	-1.54737	-1.54449	-1.60137	-1.59828	-1.62509	-1.62184
-1.58572	-1.58256	-1.57945	-1.60659	-1.60354	-1.63054	-1.62736	-1.62415	-1.6209
-1.58479	-1.58163	-1.57853	-1.60567	-1.60264	-1.6296	-1.62641	-1.6232	-1.61994
-1.58386	-1.58072	-1.57763	-1.60476	-1.60174	-1.62866	-1.62547	-1.62224	-1.61898
-1.58294	-1.57981	-1.57674	-1.60386	-1.60083	-1.62772	-1.62451	-1.62129	-1.61802
-1.58202	-1.57891	-1.57585	-1.60296	-1.59992	-1.62676	-1.62355	-1.62033	-1.61706
-1.5811	-1.57801	-1.57496	-1.63211	-1.62897	-1.62579	-1.62259	-1.61936	-1.61611
-1.58017	-1.57709	-1.57405	-1.63115	-1.628	-1.62482	-1.62161	-1.61839	-1.61515
-1.57924	-1.57618	-1.57315	-1.63018	-1.62701	-1.62383	-1.62062	-1.6486	-1.64519
-1.57831	-1.57526	-1.57223	-1.6292	-1.62602	-1.62282	-1.61961	-1.64752	-1.64415
-1.60804	-1.60491	-1.60181	-1.62825	-1.62505	-1.62184	-1.61861	-1.64646	-1.64311
-1.60711	-1.60401	-1.60093	-1.62735	-1.62412	-1.62088	-1.61763	-1.6454	-1.64206
-1.60618	-1.60309	-1.60003	-1.62642	-1.62318	-1.65124	-1.64776	-1.64435	-1.64103
-1.60524	-1.60214	-1.59905	-1.62543	-1.62218	-1.65018	-1.64672	-1.64332	-1.64

-1.60431	-1.60119	-1.59807	-1.62441	-1.62117	-1.64911	-1.64567	-1.64229	-1.63899
-1.60342	-1.60028	-1.59713	-1.62343	-1.62016	-1.64802	-1.64462	-1.64127	-1.63799
-1.60255	-1.5994	-1.5962	-1.62245	-1.61914	-1.64693	-1.64357	-1.64026	-1.637
-1.63132	-1.62806	-1.62477	-1.65283	-1.6493	-1.64587	-1.64254	-1.63927	-1.63604
-1.63037	-1.62708	-1.62378	-1.65176	-1.64824	-1.64483	-1.64153	-1.63828	-1.63511
-1.6294	-1.62609	-1.62278	-1.6507	-1.64721	-1.64382	-1.64055	-1.63733	-1.63418
-1.62841	-1.62508	-1.62175	-1.64962	-1.64619	-1.64285	-1.63961	-1.63642	-1.63326
-1.62742	-1.62407	-1.62072	-1.64857	-1.64519	-1.64189	-1.63869	-1.63551	-1.63234
-1.65806	-1.65449	-1.65095	-1.64753	-1.64422	-1.64096	-1.63776	-1.63458	-1.63139
-1.65703	-1.65345	-1.64991	-1.64653	-1.64327	-1.64003	-1.63683	-1.63364	-1.63042
-1.65611	-1.65254	-1.64901	-1.64562	-1.64233	-1.63908	-1.63588	-1.63267	-1.62944
-1.65526	-1.65172	-1.64819	-1.64476	-1.64141	-1.63812	-1.6349	-1.63169	-1.62846
-1.65432	-1.6508	-1.64729	-1.64386	-1.64048	-1.63717	-1.63393	-1.63071	-1.62751
-1.65327	-1.64978	-1.64631	-1.6429	-1.63954	-1.63623	-1.63297	-1.62975	-1.62659
-1.65223	-1.64876	-1.64532	-1.64194	-1.63861	-1.63532	-1.63205	-1.62883	-1.62569
-1.65119	-1.64773	-1.64433	-1.64099	-1.63771	-1.63445	-1.63122	-1.62801	-1.62482
-1.6502	-1.64675	-1.64336	-1.64005	-1.6368	-1.63358	-1.63038	-1.62718	-1.62395
-1.64935	-1.6459	-1.64249	-1.63916	-1.63588	-1.63264	-1.62945	-1.62625	-1.62303
-1.64855	-1.64509	-1.64166	-1.63828	-1.63495	-1.63168	-1.62848	-1.62529	-1.62208
-1.64763	-1.64418	-1.64074	-1.63736	-1.63401	-1.63073	-1.62752	-1.62434	-1.62116
-1.64665	-1.6432	-1.63978	-1.6364	-1.63305	-1.62977	-1.62656	-1.62339	-1.62025
-1.64572	-1.64228	-1.63886	-1.63549	-1.63216	-1.62889	-1.62567	-1.6225	-1.61937
-1.6449	-1.64145	-1.63802	-1.63466	-1.63136	-1.62809	-1.62487	-1.62167	-1.61851
-1.64403	-1.64058	-1.63715	-1.6338	-1.63052	-1.62727	-1.62404	-1.62084	-1.61764
-1.64304	-1.63958	-1.63616	-1.63284	-1.62958	-1.62635	-1.62313	-1.61992	-1.61672
-1.64203	-1.63858	-1.63517	-1.63186	-1.62863	-1.62542	-1.62221	-1.61901	-1.6158
-1.64114	-1.63769	-1.63429	-1.63099	-1.62778	-1.62457	-1.62138	-1.61818	-1.61497
-1.64029	-1.63685	-1.63346	-1.63017	-1.62696	-1.62376	-1.62058	-1.61739	-1.61419
-1.63935	-1.63593	-1.63256	-1.62928	-1.62607	-1.62287	-1.61969	-1.61651	-1.61333
-1.63834	-1.63495	-1.6316	-1.62832	-1.6251	-1.62189	-1.61871	-1.61555	-1.61241
-1.60964	-1.60637	-1.60314	-1.59995	-1.59681	-1.62099	-1.6178	-1.61464	-1.61151
-1.60881	-1.60555	-1.60235	-1.59918	-1.59604	-1.6202	-1.61699	-1.61381	-1.61064
-1.608	-1.60476	-1.60158	-1.59842	-1.59528	-1.61941	-1.6162	-1.61299	-1.60979
-1.60721	-1.60398	-1.6008	-1.59764	-1.5945	-1.61862	-1.6154	-1.61219	-1.60899
-1.60641	-1.60318	-1.6	-1.59683	-1.59369	-1.61779	-1.61458	-1.61138	-1.60819
-1.60551	-1.60228	-1.5991	-1.59594	-1.5928	-1.61687	-1.61366	-1.61046	-1.60728
-1.60456	-1.60134	-1.59816	-1.59499	-1.59185	-1.61589	-1.61268	-1.60948	-1.60631
-1.6037	-1.60048	-1.59729	-1.59413	-1.59098	-1.615	-1.61179	-1.6086	-1.60544
-1.60291	-1.5997	-1.59651	-1.59334	-1.5902	-1.61421	-1.61101	-1.60783	-1.60467
-1.60213	-1.59892	-1.59573	-1.59256	-1.58942	-1.61341	-1.61022	-1.60705	-1.60389
-1.60134	-1.59813	-1.59495	-1.59179	-1.58864	-1.61262	-1.60943	-1.60627	-1.60312
-1.60052	-1.59732	-1.59414	-1.59097	-1.58783	-1.61179	-1.60861	-1.60545	-1.60231
-1.59961	-1.5964	-1.59322	-1.59006	-1.58692	-1.61087	-1.6077	-1.60455	-1.60141
-1.59867	-1.59545	-1.59227	-1.58912	-1.58598	-1.60992	-1.60676	-1.60361	-1.60047
-1.59782	-1.5946	-1.59143	-1.58828	-1.58515	-1.60907	-1.60592	-1.60277	-1.59963
-1.59704	-1.59383	-1.59065	-1.58751	-1.58439	-1.60829	-1.60514	-1.60199	-1.59885
-1.59626	-1.59305	-1.58988	-1.58674	-1.58363	-1.60752	-1.60437	-1.60122	-1.59808
-1.59549	-1.59229	-1.58912	-1.58598	-1.58287	-1.60675	-1.60361	-1.60047	-1.59732
-1.59472	-1.59152	-1.58836	-1.58522	-1.58211	-1.60598	-1.60284	-1.59971	-1.59656
-1.59393	-1.59074	-1.58759	-1.58446	-1.58136	-1.60522	-1.60208	-1.59894	-1.5958

-1.59315	-1.58996	-1.58682	-1.58371	-1.58061	-1.60446	-1.60132	-1.59818	-1.59505
-1.59238	-1.5892	-1.58606	-1.58294	-1.57985	-1.60369	-1.60056	-1.59742	-1.59429
-1.59162	-1.58844	-1.5853	-1.58218	-1.57909	-1.60292	-1.59979	-1.59667	-1.59354
-1.59086	-1.58768	-1.58454	-1.58143	-1.57834	-1.60216	-1.59903	-1.61884	-1.61567
-1.59009	-1.58692	-1.58379	-1.58069	-1.5776	-1.6014	-1.59827	-1.61807	-1.6149
-1.61294	-1.60973	-1.60656	-1.60341	-1.60029	-1.62365	-1.62047	-1.66382	-1.66059
-1.61215	-1.60896	-1.60579	-1.60265	-1.59953	-1.62287	-1.6197	-1.66306	-1.65983
-1.61137	-1.60818	-1.60501	-1.60188	-1.59876	-1.6221	-1.61894	-1.6623	-1.65907
-1.61059	-1.60739	-1.60423	-1.6011	-1.59799	-1.62132	-1.61816	-1.66153	-1.65829
-1.60981	-1.60661	-1.60344	-1.60031	-1.59722	-1.62054	-1.61738	-1.66075	-1.65751
-1.60903	-1.60583	-1.60267	-1.59954	-1.59645	-1.66642	-1.66321	-1.65998	-1.65673
-1.60826	-1.60506	-1.6019	-1.59877	-1.59567	-1.66564	-1.66243	-1.6592	-1.65595
-1.60748	-1.60429	-1.60112	-1.64556	-1.6424	-1.63925	-1.63612	-1.65842	-1.65517
-1.6067	-1.6035	-1.60034	-1.64479	-1.64163	-1.63849	-1.63536	-1.65764	-1.65439
-1.60591	-1.60272	-1.59956	-1.64402	-1.64087	-1.63772	-1.63459	-1.65686	-1.65361
-1.60512	-1.60194	-1.59879	-1.64326	-1.6401	-1.63695	-1.63381	-1.65606	-1.65282
-1.60433	-1.60115	-1.59801	-1.6425	-1.63933	-1.63618	-1.63303	-1.65526	-1.65204
-1.65135	-1.6481	-1.64491	-1.64173	-1.63857	-1.63541	-1.63226	-1.65448	-1.65126
-1.65056	-1.64731	-1.64413	-1.64096	-1.6378	-1.63465	-1.6315	-1.6537	-1.65049
-1.64982	-1.64656	-1.64337	-1.64019	-1.63703	-1.63388	-1.63073	-1.65291	-1.6497
-1.64914	-1.64584	-1.64261	-1.63941	-1.63626	-1.63311	-1.62996	-1.65213	-1.64892
-1.64848	-1.64517	-1.64193	-1.63871	-1.63553	-1.63236	-1.6292	-1.65135	-1.64814
-1.64786	-1.64458	-1.64134	-1.63809	-1.63486	-1.63164	-1.62845	-1.65056	-1.64736
-1.6472	-1.64395	-1.64071	-1.63746	-1.6342	-1.63096	-1.62773	-1.64982	-1.64661
-1.64644	-1.64321	-1.64	-1.63678	-1.63355	-1.63033	-1.62708	-1.64913	-1.64588
-1.64566	-1.64244	-1.63925	-1.63607	-1.6329	-1.62969	-1.62645	-1.64846	-1.64518
-1.64489	-1.64167	-1.63849	-1.63532	-1.63216	-1.62899	-1.62578	-1.64781	-1.64454
-1.64413	-1.64091	-1.63772	-1.63454	-1.63139	-1.62824	-1.6251	-1.64716	-1.64392
-1.64346	-1.64021	-1.63699	-1.6338	-1.63064	-1.62749	-1.62436	-1.64644	-1.64322
-1.64287	-1.63958	-1.63631	-1.63309	-1.62989	-1.62672	-1.62359	-1.64565	-1.64244
-1.64222	-1.63893	-1.63565	-1.63242	-1.62921	-1.62602	-1.62287	-1.64489	-1.64167
-1.64149	-1.63825	-1.63502	-1.63182	-1.62862	-1.62542	-1.62222	-1.64418	-1.64091
-1.64077	-1.63756	-1.63437	-1.63119	-1.62801	-1.62481	-1.62158	-1.6435	-1.64018
-1.64008	-1.63684	-1.63364	-1.63045	-1.62729	-1.6241	-1.62091	-1.64285	-1.63955
-1.6394	-1.63613	-1.6329	-1.6297	-1.62654	-1.62338	-1.62023	-1.64219	-1.63893
-1.63874	-1.63548	-1.63225	-1.62904	-1.62585	-1.62267	-1.61951	-1.66396	-1.66066
-1.63808	-1.63486	-1.63165	-1.62843	-1.6252	-1.62198	-1.61876	-1.66315	-1.65986
-1.63738	-1.63418	-1.63099	-1.62777	-1.62454	-1.62132	-1.61809	-1.66243	-1.65912
-1.63662	-1.63342	-1.63025	-1.62707	-1.62389	-1.6207	-1.61749	-1.6618	-1.65845
-1.63591	-1.63272	-1.62956	-1.6264	-1.62323	-1.62005	-1.61685	-1.68772	-1.68427
-1.63533	-1.63213	-1.62896	-1.62576	-1.62255	-1.61934	-1.61612	-1.68695	-1.68354
-1.63475	-1.63154	-1.62835	-1.62513	-1.62188	-1.61864	-1.6154	-1.68619	-1.6828
-1.63409	-1.63087	-1.62766	-1.62444	-1.62122	-1.61799	-1.61477	-1.70804	-1.7046
-1.63342	-1.63017	-1.62695	-1.62374	-1.62056	-1.61737	-1.61417	-1.70739	-1.70392
-1.63279	-1.62954	-1.62631	-1.62309	-1.61989	-1.64033	-1.63708	-1.68274	-1.67935
-1.63218	-1.62895	-1.62572	-1.62248	-1.61923	-1.63962	-1.63635	-1.68197	-1.67863
-1.63157	-1.62834	-1.6251	-1.62184	-1.61857	-1.66595	-1.6626	-1.68127	-1.67796
-1.63098	-1.62772	-1.62445	-1.62118	-1.61791	-1.66529	-1.66195	-1.68063	-1.67733
-1.63035	-1.62708	-1.6238	-1.64422	-1.64092	-1.66463	-1.66131	-1.67998	-1.67668
-1.62964	-1.6264	-1.62316	-1.6436	-1.6403	-1.66399	-1.66064	-1.67927	-1.67596

-1.62894	-1.62573	-1.62253	-1.64298	-1.63969	-1.66334	-1.65997	-1.67857	-1.67524
-1.65214	-1.64889	-1.64564	-1.64235	-1.63905	-1.68471	-1.68127	-1.67788	-1.67456
-1.65156	-1.6483	-1.64503	-1.64172	-1.63839	-1.68402	-1.68059	-1.67721	-1.6739
-1.65096	-1.64768	-1.64438	-1.64107	-1.63774	-1.68334	-1.67993	-1.67656	-1.67325
-1.65035	-1.64703	-1.6437	-1.64039	-1.63708	-1.68266	-1.67929	-1.67594	-1.67259
-1.64972	-1.64638	-1.64304	-1.66677	-1.66339	-1.68201	-1.67864	-1.67529	-1.67193
-1.64904	-1.64571	-1.6424	-1.66612	-1.66277	-1.68138	-1.67799	-1.67461	-1.67126
-1.64837	-1.64506	-1.64176	-1.6655	-1.66217	-1.68077	-1.67735	-1.67395	-1.67061
-1.64772	-1.64442	-1.64115	-1.66488	-1.66157	-1.68017	-1.67675	-1.67336	-1.66999
-1.64709	-1.6438	-1.64054	-1.66427	-1.66097	-1.67956	-1.67616	-1.67277	-1.66939
-1.64646	-1.64319	-1.63994	-1.66364	-1.66031	-1.67888	-1.6755	-1.67213	-1.66877
-1.64585	-1.64259	-1.63935	-1.66301	-1.65963	-1.67817	-1.6748	-1.67146	-1.66814
-1.64526	-1.642	-1.63876	-1.68439	-1.68093	-1.67752	-1.67415	-1.67082	-1.66751
-1.64468	-1.64143	-1.63819	-1.68379	-1.68035	-1.67693	-1.67355	-1.6702	-1.66689
-1.67123	-1.66789	-1.66456	-1.68316	-1.67974	-1.67633	-1.67294	-1.66958	-1.66627
-1.67063	-1.66726	-1.66388	-1.68248	-1.67907	-1.67569	-1.67233	-1.66897	-1.66563
-1.67003	-1.66663	-1.66322	-1.68179	-1.67841	-1.67505	-1.67171	-1.66837	-1.66499
-1.66943	-1.66602	-1.66261	-1.68116	-1.67778	-1.67442	-1.6711	-1.69148	-1.68803
-1.66883	-1.66543	-1.66202	-1.68055	-1.67716	-1.6738	-1.67048	-1.69087	-1.68742
-1.69031	-1.68685	-1.68338	-1.67996	-1.67655	-1.67319	-1.66988	-1.69027	-1.68682
-1.68971	-1.68626	-1.68279	-1.67937	-1.67596	-1.6726	-1.66929	-1.68967	-1.68623
-1.68911	-1.68566	-1.6822	-1.67877	-1.67537	-1.672	-1.66869	-1.71152	-1.70802
-1.6885	-1.68506	-1.6816	-1.67817	-1.67476	-1.6714	-1.66809	-1.7109	-1.7074
-1.6879	-1.68446	-1.681	-1.67757	-1.67416	-1.67079	-1.66749	-1.71027	-1.70678
-1.68729	-1.68385	-1.68039	-1.67696	-1.67355	-1.67019	-1.66688	-1.70964	-1.70616
-1.68668	-1.68324	-1.67978	-1.67635	-1.67294	-1.66958	-1.66627	-1.70901	-1.70554
-1.68607	-1.68264	-1.67917	-1.67574	-1.67232	-1.66896	-1.66566	-1.68507	-1.68166
-1.68547	-1.68203	-1.67857	-1.67513	-1.6717	-1.66834	-1.66504	-1.68444	-1.68104
-1.68487	-1.68146	-1.67802	-1.67458	-1.67114	-1.66776	-1.66446	-1.68383	-1.68043
-1.68429	-1.6809	-1.67751	-1.67408	-1.67062	-1.66722	-1.66389	-1.68324	-1.67983
-1.68369	-1.68033	-1.67697	-1.67354	-1.67007	-1.66667	-1.66332	-1.68265	-1.67926
-1.68308	-1.67972	-1.67637	-1.67296	-1.66951	-1.6661	-1.66274	-1.68206	-1.67871
-1.68248	-1.67912	-1.67577	-1.67237	-1.66894	-1.68832	-1.68488	-1.68148	-1.67815
-1.68192	-1.67853	-1.67516	-1.67178	-1.66841	-1.6878	-1.68434	-1.68093	-1.67758
-1.68136	-1.67794	-1.67454	-1.67119	-1.66787	-1.68727	-1.68381	-1.68038	-1.677
-1.68079	-1.67734	-1.67392	-1.67058	-1.66729	-1.68668	-1.68323	-1.6798	-1.67641
-1.68021	-1.67675	-1.67331	-1.66996	-1.66668	-1.68607	-1.68263	-1.6792	-1.6758
-1.67968	-1.67621	-1.67275	-1.66939	-1.66609	-1.68547	-1.68206	-1.7067	-1.7032
-1.67918	-1.67571	-1.67224	-1.66885	-1.6655	-1.68487	-1.68151	-1.70616	-1.70266
-1.67865	-1.6752	-1.67174	-1.66833	-1.66496	-1.6843	-1.68095	-1.7056	-1.70208
-1.67805	-1.67465	-1.67127	-1.66786	-1.66445	-1.68375	-1.68036	-1.70497	-1.70147
-1.67746	-1.6741	-1.67076	-1.66737	-1.66396	-1.68322	-1.67979	-1.70436	-1.70086
-1.6769	-1.67352	-1.67018	-1.66682	-1.66346	-1.68272	-1.67927	-1.7038	-1.70031
-1.67637	-1.67295	-1.66957	-1.66624	-1.66295	-1.68222	-1.67877	-1.70327	-1.69978
-1.67587	-1.67243	-1.66902	-1.66568	-1.66239	-1.68166	-1.67824	-1.70275	-1.69925
-1.67539	-1.67194	-1.66851	-1.66513	-1.66179	-1.68105	-1.67769	-1.70223	-1.69873
-1.6749	-1.67145	-1.66801	-1.68733	-1.68388	-1.70853	-1.7051	-1.70166	-1.69818
-1.67439	-1.67096	-1.66754	-1.68682	-1.68335	-1.70795	-1.70448	-1.70103	-1.69761
-1.6739	-1.67047	-1.66706	-1.68632	-1.68283	-1.70739	-1.70388	-1.70041	-1.69702
-1.67341	-1.66998	-1.66656	-1.68582	-1.68233	-1.70687	-1.70332	-1.69983	-1.69641

-1.67292	-1.66949	-1.66606	-1.68531	-1.68183	-1.70635	-1.70279	-1.69927	-1.69582
-1.67242	-1.66899	-1.66557	-1.6848	-1.68131	-1.70581	-1.70225	-1.69873	-1.69527
-1.6719	-1.66848	-1.66508	-1.68429	-1.68079	-1.70527	-1.70173	-1.69821	-1.69474
-1.67141	-1.66798	-1.66457	-1.68377	-1.68027	-1.70474	-1.70119	-1.69768	-1.69422
-1.67093	-1.66749	-1.66406	-1.68324	-1.67975	-1.70421	-1.70066	-1.69715	-1.6937
-1.67044	-1.66699	-1.66355	-1.68272	-1.67923	-1.70369	-1.70014	-1.69663	-1.69318
-1.66993	-1.66649	-1.66306	-1.68221	-1.67872	-1.70316	-1.69963	-1.69613	-1.69265
-1.66943	-1.666	-1.66257	-1.70976	-1.70619	-1.70264	-1.69914	-1.69564	-1.69216
-1.66893	-1.6655	-1.66208	-1.70924	-1.70568	-1.70216	-1.69868	-1.6952	-1.69173
-1.66844	-1.665	-1.66158	-1.70872	-1.70519	-1.70169	-1.69822	-1.69477	-1.6913
-1.66793	-1.6645	-1.66109	-1.70823	-1.70471	-1.70122	-1.69774	-1.69428	-1.69082
-1.66742	-1.664	-1.66062	-1.70774	-1.70424	-1.70075	-1.69725	-1.69376	-1.6903
-1.68969	-1.68619	-1.68272	-1.70725	-1.70375	-1.70024	-1.69674	-1.72419	-1.72047
-1.68929	-1.68577	-1.68226	-1.70676	-1.70323	-1.69971	-1.69622	-1.72364	-1.71991
-1.68886	-1.68532	-1.6818	-1.70626	-1.70271	-1.69919	-1.6957	-1.72307	-1.71935
-1.68838	-1.68484	-1.6813	-1.70574	-1.7022	-1.69867	-1.69516	-1.72249	-1.71878
-1.71607	-1.71245	-1.70883	-1.70525	-1.7017	-1.69817	-1.69466	-1.72193	-1.71823
-1.71562	-1.71201	-1.7084	-1.70482	-1.70126	-1.69773	-1.69423	-1.72147	-1.71776
-1.71517	-1.71157	-1.70798	-1.7044	-1.70083	-1.6973	-1.69382	-1.72102	-1.7173
-1.71467	-1.71108	-1.7075	-1.70392	-1.70035	-1.69682	-1.69335	-1.72052	-1.71681
-1.71415	-1.71057	-1.707	-1.70343	-1.69985	-1.69632	-1.69285	-1.71999	-1.71631
-1.71367	-1.71009	-1.70653	-1.70296	-1.69939	-1.69587	-1.69239	-1.71947	-1.7158
-1.71324	-1.70966	-1.7061	-1.70254	-1.69898	-1.69546	-1.69196	-1.71898	-1.71527
-1.71283	-1.70924	-1.70567	-1.70212	-1.69858	-1.69506	-1.69155	-1.76205	-1.7582
-1.71242	-1.70883	-1.70526	-1.70171	-1.69817	-1.69465	-1.69115	-1.76159	-1.75773
-1.71201	-1.70842	-1.70485	-1.7013	-1.69777	-1.69425	-1.69075	-1.76113	-1.75727
-1.7116	-1.708	-1.70443	-1.70088	-1.69736	-1.69384	-1.69034	-1.76065	-1.7568
-1.71117	-1.70758	-1.704	-1.70045	-1.69693	-1.69342	-1.68992	-1.76016	-1.75632
-1.7107	-1.7071	-1.70352	-1.69998	-1.69647	-1.72368	-1.71991	-1.75962	-1.75578
-1.71019	-1.70659	-1.703	-1.69946	-1.69596	-1.72314	-1.71935	-1.75903	-1.75521
-1.68907	-1.68553	-1.68201	-1.69899	-1.69549	-1.72262	-1.71883	-1.75849	-1.75468
-1.68864	-1.68511	-1.6816	-1.69858	-1.69506	-1.72215	-1.71837	-1.75801	-1.75419
-1.68822	-1.68471	-1.6812	-1.69817	-1.69465	-1.76529	-1.7614	-1.75754	-1.75372
-1.68781	-1.68429	-1.6808	-1.69776	-1.69423	-1.76481	-1.76092	-1.75706	-1.75324
-1.68741	-1.6839	-1.6804	-1.69736	-1.69383	-1.76435	-1.76045	-1.75658	-1.75277
-1.68707	-1.68353	-1.68001	-1.67652	-1.67304	-1.76389	-1.75999	-1.75613	-1.75231
-1.68675	-1.68319	-1.67963	-1.67613	-1.67266	-1.76344	-1.75955	-1.75569	-1.75187
-1.68641	-1.68285	-1.6793	-1.67579	-1.67231	-1.76303	-1.75915	-1.7553	-1.75147
-1.68606	-1.68252	-1.67899	-1.67549	-1.672	-1.76266	-1.75879	-1.75494	-1.75111
-1.68569	-1.68217	-1.67865	-1.67515	-1.67166	-1.74292	-1.73911	-1.75455	-1.75072
-1.68528	-1.68176	-1.67826	-1.67476	-1.67128	-1.74248	-1.73867	-1.75411	-1.75028
-1.68487	-1.68136	-1.67786	-1.74974	-1.74587	-1.74203	-1.73822	-1.75365	-1.74982
-1.68448	-1.68097	-1.67746	-1.74929	-1.74542	-1.74157	-1.73775	-1.75317	-1.74935
-1.6841	-1.68059	-1.67708	-1.74885	-1.74497	-1.74112	-1.7373	-1.75271	-1.74888
-1.68377	-1.68025	-1.67674	-1.74845	-1.74458	-1.74074	-1.7369	-1.75229	-1.74845
-1.68348	-1.67995	-1.67643	-1.74808	-1.74423	-1.74038	-1.73654	-1.75191	-1.74804
-1.68313	-1.6796	-1.67608	-1.74768	-1.74382	-1.73997	-1.73614	-1.73232	-1.72851
-1.68272	-1.67921	-1.6757	-1.74723	-1.74336	-1.73951	-1.7357	-1.73191	-1.72812
-1.68234	-1.67883	-1.67533	-1.7468	-1.74291	-1.73906	-1.73526	-1.73148	-1.72772
-1.68202	-1.67851	-1.67501	-1.74642	-1.74252	-1.73865	-1.73482	-1.73102	-1.72726

-1.68169	-1.67818	-1.67468	-1.74603	-1.74212	-1.73825	-1.73439	-1.73058	-1.72681
-1.68132	-1.6778	-1.67429	-1.74559	-1.74169	-1.73783	-1.734	-1.7302	-1.72643
-1.68094	-1.6774	-1.67389	-1.74513	-1.74126	-1.73743	-1.73364	-1.72986	-1.72609
-1.72578	-1.72211	-1.71847	-1.74473	-1.74088	-1.73706	-1.73328	-1.72951	-1.72574
-1.72545	-1.72179	-1.71814	-1.74437	-1.74053	-1.73671	-1.73293	-1.72916	-1.72539
-1.67727	-1.67376	-1.67027	-1.74402	-1.74018	-1.73636	-1.73258	-1.72881	-1.72504
-1.67696	-1.67345	-1.66996	-1.74369	-1.73983	-1.73601	-1.73224	-1.72846	-1.72468
-1.67666	-1.67315	-1.66965	-1.74335	-1.73948	-1.76048	-1.75663	-1.78988	-1.7859
-1.67635	-1.67284	-1.66935	-1.74299	-1.73914	-1.76013	-1.75626	-1.78949	-1.78548
-1.67604	-1.67253	-1.66903	-1.74263	-1.73878	-1.75976	-1.75588	-1.78907	-1.78506
-1.67573	-1.67221	-1.66871	-1.74227	-1.73842	-1.75938	-1.75548	-1.78865	-1.78465
-1.67541	-1.67189	-1.66838	-1.74191	-1.73804	-1.75899	-1.75506	-1.78822	-1.78425
-1.6751	-1.67158	-1.66806	-1.74154	-1.73765	-1.75858	-1.75467	-1.78782	-1.78387
-1.6748	-1.67127	-1.66774	-1.74115	-1.73724	-1.75816	-1.75428	-1.78746	-1.7835
-1.67449	-1.67096	-1.66744	-1.74081	-1.73688	-1.7578	-1.75392	-1.78709	-1.78314
-1.67418	-1.67066	-1.66714	-1.74052	-1.73662	-1.75752	-1.75358	-1.78671	-1.78278
-1.67387	-1.67035	-1.66685	-1.69359	-1.68986	-1.75725	-1.75327	-1.78637	-1.78243
-1.67355	-1.67004	-1.66654	-1.69326	-1.68955	-1.75696	-1.75301	-1.78609	-1.78211
-1.67324	-1.66972	-1.66623	-1.69291	-1.68922	-1.75666	-1.75275	-1.78584	-1.78181
-1.67292	-1.6694	-1.66591	-1.69257	-1.68888	-1.75632	-1.75243	-1.78555	-1.78152
-1.6726	-1.66908	-1.6656	-1.69223	-1.68853	-1.75596	-1.75209	-1.78523	-1.78124
-1.70317	-1.69937	-1.69562	-1.69189	-1.6882	-1.75561	-1.75175	-1.78488	-1.78093
-1.70293	-1.69909	-1.69529	-1.69155	-1.68788	-1.75529	-1.75139	-1.78451	-1.78058
-1.70267	-1.69882	-1.69499	-1.69125	-1.68758	-1.75498	-1.75107	-1.78417	-1.78024
-1.70237	-1.69855	-1.69474	-1.69101	-1.68732	-1.75472	-1.75082	-1.78391	-1.77993
-1.70206	-1.69827	-1.69449	-1.69076	-1.68706	-1.75445	-1.75056	-1.78364	-1.77963
-1.70172	-1.69794	-1.69419	-1.69046	-1.68674	-1.70846	-1.70472	-1.78334	-1.77935
-1.70138	-1.69761	-1.69388	-1.69014	-1.68641	-1.70812	-1.70438	-1.78302	-1.77907
-1.7011	-1.69733	-1.69359	-1.68986	-1.68612	-1.70782	-1.70405	-1.78267	-1.77875
-1.70086	-1.69708	-1.69333	-1.6896	-1.68587	-1.70755	-1.70373	-1.7823	-1.7784
-1.70061	-1.69682	-1.69305	-1.68931	-1.6856	-1.70726	-1.70344	-1.78199	-1.7781
-1.70037	-1.69654	-1.69273	-1.68899	-1.68529	-1.70697	-1.70318	-1.78176	-1.77787
-1.70013	-1.69628	-1.69244	-1.68869	-1.685	-1.70669	-1.70293	-1.78153	-1.77764
-1.6999	-1.69604	-1.6922	-1.68844	-1.68475	-1.70644	-1.70269	-1.7813	-1.77741
-1.69967	-1.69582	-1.69198	-1.68822	-1.68452	-1.7062	-1.70245	-1.78106	-1.77717
-1.69939	-1.69556	-1.69175	-1.68799	-1.68429	-1.70597	-1.70221	-1.73656	-1.73274
-1.6991	-1.6953	-1.69152	-1.68778	-1.68407	-1.70575	-1.70198	-1.7363	-1.73245
-1.69883	-1.69506	-1.69129	-1.68756	-1.68385	-1.70552	-1.70175	-1.73606	-1.73219
-1.69859	-1.69482	-1.69107	-1.68733	-1.68362	-1.70529	-1.70152	-1.73583	-1.73196
-1.69836	-1.69459	-1.69084	-1.68711	-1.68339	-1.70506	-1.7013	-1.73561	-1.73173
-1.69814	-1.69437	-1.69062	-1.68689	-1.68317	-1.70484	-1.70108	-1.73539	-1.73151
-1.69792	-1.69416	-1.6904	-1.68667	-1.68295	-1.70461	-1.70086	-1.73516	-1.73128
-1.6977	-1.69393	-1.69018	-1.68645	-1.68273	-1.70439	-1.70063	-1.73494	-1.73106
-1.69748	-1.69371	-1.68996	-1.68622	-1.68251	-1.70416	-1.70041	-1.73471	-1.73083
-1.69725	-1.69348	-1.68973	-1.686	-1.68229	-1.70394	-1.70019	-1.73448	-1.73059
-1.69702	-1.69325	-1.6895	-1.68577	-1.68207	-1.70372	-1.69996	-1.73425	-1.73036
-1.69683	-1.69304	-1.68928	-1.68555	-1.68184	-1.7035	-1.69975	-1.73403	-1.73014
-1.69667	-1.69285	-1.68906	-1.68531	-1.68162	-1.70328	-1.69953	-1.73381	-1.72992
-1.69651	-1.69267	-1.68884	-1.68509	-1.6814	-1.70306	-1.69932	-1.73361	-1.72973
-1.69633	-1.69247	-1.68864	-1.68488	-1.68117	-1.70283	-1.6991	-1.73341	-1.72957

-1.69613	-1.69229	-1.68845	-1.68469	-1.68097	-1.70262	-1.6989	-1.73321	-1.7294
-1.69593	-1.69211	-1.68831	-1.68455	-1.68082	-1.70246	-1.69871	-1.733	-1.72919
-1.69572	-1.69194	-1.68818	-1.68443	-1.6807	-1.70232	-1.69854	-1.73279	-1.72897
-1.69551	-1.69174	-1.68799	-1.68425	-1.68053	-1.70215	-1.69838	-1.74645	-1.74261
-1.69531	-1.69152	-1.68777	-1.68404	-1.68033	-1.70198	-1.69823	-1.74636	-1.7425
-1.69514	-1.69135	-1.68759	-1.68385	-1.68014	-1.71617	-1.71242	-1.74623	-1.74237
-1.69501	-1.69121	-1.68745	-1.6837	-1.67995	-1.716	-1.71224	-1.74604	-1.74219
-1.69488	-1.69108	-1.68731	-1.68354	-1.67978	-1.71585	-1.71208	-1.74587	-1.74203
-1.69474	-1.69094	-1.68715	-1.68339	-1.67964	-1.71575	-1.71198	-1.74576	-1.74189
-1.69461	-1.69079	-1.687	-1.68324	-1.67951	-1.71566	-1.71189	-1.74565	-1.74177
-1.69445	-1.69064	-1.68686	-1.68311	-1.67938	-1.71556	-1.71178	-1.74553	-1.74166
-1.69429	-1.6905	-1.68673	-1.68298	-1.67926	-1.71546	-1.71167	-1.74541	-1.74155
-1.69414	-1.69036	-1.6866	-1.68285	-1.67911	-1.71534	-1.71156	-1.7453	-1.74146
-1.69401	-1.69024	-1.68648	-1.68271	-1.67896	-1.71522	-1.71145	-1.74521	-1.74137
-1.69388	-1.6901	-1.68634	-1.68257	-1.67881	-1.71511	-1.71136	-1.74513	-1.74128
-1.69375	-1.68995	-1.68617	-1.68242	-1.67869	-1.71503	-1.71129	-1.74505	-1.74118
-1.7087	-1.70488	-1.70109	-1.69734	-1.69364	-1.71497	-1.71123	-1.74499	-1.7411
-1.70857	-1.70476	-1.701	-1.69726	-1.69357	-1.7149	-1.71117	-1.74494	-1.74107
-1.70846	-1.70466	-1.70092	-1.6972	-1.6935	-1.71485	-1.71112	-1.74489	-1.74105
-1.70842	-1.70461	-1.70086	-1.69713	-1.69345	-1.71479	-1.71106	-1.74483	-1.74098
-1.70842	-1.70459	-1.70081	-1.69707	-1.69339	-1.71473	-1.71101	-1.74476	-1.74088
-1.70835	-1.70453	-1.70075	-1.69702	-1.69333	-1.71467	-1.71095	-1.77015	-1.76619
-1.70823	-1.70443	-1.70068	-1.69696	-1.69327	-1.71461	-1.71088	-1.77008	-1.76615
-1.70815	-1.70436	-1.70062	-1.6969	-1.69321	-1.71455	-1.71082	-1.77001	-1.7661
-1.70816	-1.70434	-1.70055	-1.69682	-1.69314	-1.71448	-1.71075	-1.76993	-1.76598
-1.70818	-1.70433	-1.7005	-1.69676	-1.69308	-1.71442	-1.71069	-1.76985	-1.76589
-1.70817	-1.70434	-1.70053	-1.69676	-1.69302	-1.71434	-1.71063	-1.7698	-1.76586
-1.70814	-1.70435	-1.70057	-1.69677	-1.69299	-1.71428	-1.71057	-1.76975	-1.76584
-1.70809	-1.70432	-1.70056	-1.69678	-1.69299	-1.71427	-1.71052	-1.76968	-1.76579
-1.70802	-1.70426	-1.70052	-1.69678	-1.69303	-1.7143	-1.71049	-1.76959	-1.76572
-1.708	-1.70422	-1.70048	-1.69674	-1.69303	-1.74046	-1.73656	-1.76956	-1.76567
-1.70804	-1.70422	-1.70041	-1.69668	-1.69299	-1.74044	-1.73658	-1.76959	-1.76564
-1.70806	-1.70421	-1.70038	-1.69663	-1.69294	-1.75903	-1.75515	-1.78761	-1.78358
-1.70805	-1.70423	-1.70041	-1.69664	-1.69289	-1.75895	-1.75509	-1.78758	-1.78358
-1.70801	-1.70422	-1.70044	-1.69665	-1.69286	-1.75889	-1.75502	-1.78753	-1.78357
-1.70795	-1.70418	-1.70043	-1.69665	-1.69287	-1.75888	-1.75499	-1.78747	-1.78354
-1.70788	-1.70411	-1.70039	-1.69664	-1.69289	-1.75891	-1.75496	-1.78742	-1.78349
-1.70787	-1.70408	-1.70034	-1.69661	-1.69289	-1.75891	-1.75497	-1.78742	-1.7835
-1.70791	-1.70408	-1.70029	-1.69655	-1.69287	-1.7589	-1.75499	-1.78746	-1.78353
-1.70796	-1.70411	-1.70028	-1.69655	-1.69287	-1.75891	-1.75502	-1.7875	-1.78356
-1.70802	-1.70417	-1.70034	-1.6966	-1.69292	-1.75895	-1.75507	-1.78754	-1.78355
-1.70807	-1.70423	-1.7004	-1.72327	-1.7195	-1.759	-1.75512	-1.75123	-1.74732
-1.70807	-1.70425	-1.70045	-1.72332	-1.71955	-1.75905	-1.75518	-1.7513	-1.74737
-1.70807	-1.70428	-1.7005	-1.72336	-1.71958	-1.75908	-1.75523	-1.75135	-1.74742
-1.7081	-1.70432	-1.70055	-1.72339	-1.71958	-1.75906	-1.75522	-1.75135	-1.74744
-1.70816	-1.70437	-1.70059	-1.7234	-1.71955	-1.75902	-1.75518	-1.75133	-1.74745
-1.70822	-1.70443	-1.70065	-1.72344	-1.71957	-1.75903	-1.75519	-1.75135	-1.74749
-1.70829	-1.7045	-1.70072	-1.7235	-1.71963	-1.75908	-1.75523	-1.75139	-1.74754
-1.70836	-1.70457	-1.70079	-1.72356	-1.71969	-1.75914	-1.75529	-1.75144	-1.74759
-1.70843	-1.70464	-1.70086	-1.72363	-1.71975	-1.7592	-1.75535	-1.7515	-1.74765

-1.7353	-1.73143	-1.72756	-1.72369	-1.71981	-1.75926	-1.75541	-1.75156	-1.7477
-1.73535	-1.73148	-1.72762	-1.72374	-1.71986	-1.75931	-1.75545	-1.75161	-1.74775
-1.73541	-1.73154	-1.72767	-1.72379	-1.71991	-1.75936	-1.7555	-1.75165	-1.74779
-1.73546	-1.7316	-1.72774	-1.72386	-1.71998	-1.75942	-1.75555	-1.7517	-1.74784
-1.73552	-1.73166	-1.72781	-1.72394	-1.72006	-1.7595	-1.75561	-1.75174	-1.74789
-1.73557	-1.73172	-1.72788	-1.72403	-1.72017	-1.73527	-1.73145	-1.72763	-1.72383
-1.73562	-1.73178	-1.72795	-1.72413	-1.72031	-1.73543	-1.73159	-1.72776	-1.72392
-1.73569	-1.73184	-1.72802	-1.72422	-1.72043	-1.73556	-1.73172	-1.72787	-1.72401
-1.73582	-1.73193	-1.72808	-1.72427	-1.72049	-1.73563	-1.7318	-1.72796	-1.72409
-1.73594	-1.73203	-1.72814	-1.72432	-1.72054	-1.73569	-1.73187	-1.72803	-1.72417
-1.73604	-1.73212	-1.72822	-1.72438	-1.72059	-1.73574	-1.73192	-1.72811	-1.72427
-1.73612	-1.7322	-1.7283	-1.72445	-1.72065	-1.73579	-1.73198	-1.72818	-1.72437
-1.73621	-1.73231	-1.72842	-1.72457	-1.72076	-1.73589	-1.73206	-1.72825	-1.72445
-1.73631	-1.73243	-1.72857	-1.72473	-1.72091	-1.73602	-1.73216	-1.72832	-1.7245
-1.73643	-1.73256	-1.72871	-1.72487	-1.72104	-1.73615	-1.73227	-1.72842	-1.72459
-1.73658	-1.73271	-1.72886	-1.72501	-1.72116	-1.73626	-1.7324	-1.72855	-1.72473
-1.73673	-1.73286	-1.729	-1.72514	-1.72128	-1.73638	-1.73253	-1.7287	-1.72488
-1.73687	-1.73299	-1.72912	-1.72526	-1.72141	-1.73652	-1.73267	-1.72884	-1.725
-1.73701	-1.73311	-1.72923	-1.72537	-1.72154	-1.73666	-1.73281	-1.72897	-1.72512
-1.7165	-1.71265	-1.70881	-1.7255	-1.72168	-1.7368	-1.73294	-1.72909	-1.72524
-1.7166	-1.71277	-1.70894	-1.72564	-1.72182	-1.73693	-1.73305	-1.72919	-1.72536
-1.71673	-1.7129	-1.70908	-1.70527	-1.70148	-1.73705	-1.73317	-1.72931	-1.72549
-1.71687	-1.71303	-1.70922	-1.7054	-1.70158	-1.73715	-1.73328	-1.72944	-1.72563
-1.717	-1.71316	-1.70934	-1.70552	-1.7017	-1.73727	-1.73342	-1.72958	-1.72576
-1.71714	-1.71329	-1.70945	-1.70563	-1.70183	-1.73742	-1.73358	-1.72974	-1.72589
-1.71728	-1.71341	-1.70957	-1.70576	-1.70198	-1.71743	-1.71364	-1.77375	-1.76976
-1.71738	-1.71352	-1.70971	-1.70591	-1.70214	-1.7176	-1.7138	-1.77392	-1.76997
-1.71749	-1.71365	-1.70986	-1.70608	-1.70232	-1.71778	-1.71397	-1.77411	-1.77018
-1.71769	-1.71383	-1.71003	-1.70624	-1.70248	-1.71795	-1.71414	-1.7548	-1.75089
-1.71792	-1.71405	-1.71021	-1.70641	-1.70265	-1.71812	-1.71432	-1.75496	-1.75103
-1.7181	-1.71422	-1.71038	-1.70659	-1.70283	-1.7183	-1.71449	-1.75513	-1.7512
-1.71821	-1.71436	-1.71055	-1.70677	-1.703	-1.71847	-1.71465	-1.75531	-1.75141
-1.71836	-1.71452	-1.71073	-1.70694	-1.70318	-1.71864	-1.71482	-1.75549	-1.7516
-1.71861	-1.71473	-1.7109	-1.7071	-1.70334	-1.71882	-1.71499	-1.75565	-1.75173
-1.71887	-1.71497	-1.71109	-1.70728	-1.70352	-1.71899	-1.71517	-1.75583	-1.75188
-1.71909	-1.71521	-1.71134	-1.70751	-1.70369	-1.71915	-1.71533	-1.75602	-1.7521
-1.7193	-1.71545	-1.71162	-1.70776	-1.70389	-1.71931	-1.71551	-1.75622	-1.75233
-1.71948	-1.71566	-1.71185	-1.70799	-1.70412	-1.76426	-1.76031	-1.7564	-1.75253
-1.71965	-1.71584	-1.71204	-1.70822	-1.70439	-1.76454	-1.76052	-1.75657	-1.75271
-1.71987	-1.71604	-1.71223	-1.70842	-1.70462	-1.76479	-1.76076	-1.77949	-1.77555
-1.72015	-1.71627	-1.71241	-1.70859	-1.70481	-1.76501	-1.76102	-1.77977	-1.77578
-1.72041	-1.7165	-1.71261	-1.70878	-1.70499	-1.76522	-1.76128	-1.78004	-1.77602
-1.72063	-1.71675	-1.71287	-1.70902	-1.70519	-1.76541	-1.76149	-1.78028	-1.77627
-1.72084	-1.71699	-1.71315	-1.70928	-1.7054	-1.76561	-1.7617	-1.78051	-1.77654
-1.72103	-1.7172	-1.71338	-1.70952	-1.70565	-1.76587	-1.76196	-1.78077	-1.77682
-1.72121	-1.71739	-1.7136	-1.70977	-1.70592	-1.76616	-1.76224	-1.78104	-1.77711
-1.72145	-1.71762	-1.71384	-1.75565	-1.7517	-1.76645	-1.76249	-1.78127	-1.77735
-1.72173	-1.7179	-1.7141	-1.75592	-1.75199	-1.76673	-1.76272	-1.78147	-1.77755
-1.72201	-1.71816	-1.71433	-1.75617	-1.75225	-1.767	-1.76297	-1.78171	-1.77778
-1.72228	-1.7184	-1.71454	-1.75637	-1.75247	-1.76724	-1.76324	-1.78199	-1.77805

-1.72256	-1.71865	-1.71475	-1.75658	-1.7527	-1.76749	-1.76351	-1.78228	-1.77832
-1.72286	-1.71893	-1.71502	-1.75685	-1.75297	-1.76777	-1.7638	-1.78256	-1.77857
-1.72315	-1.71922	-1.71531	-1.75715	-1.75326	-1.76806	-1.7641	-1.78285	-1.77882
-1.72339	-1.71949	-1.7156	-1.75745	-1.75356	-1.76836	-1.7644	-1.78315	-1.7791
-1.72361	-1.71974	-1.71589	-1.75776	-1.75387	-1.76867	-1.76471	-1.78346	-1.77941
-1.76993	-1.76595	-1.76199	-1.75807	-1.75418	-1.76898	-1.76502	-1.78377	-1.77972
-1.77022	-1.76624	-1.76229	-1.75837	-1.75449	-1.76929	-1.76534	-1.78408	-1.78003
-1.77051	-1.76654	-1.76259	-1.75868	-1.75479	-1.7696	-1.76565	-1.7844	-1.78034
-1.77082	-1.76684	-1.76289	-1.75898	-1.75509	-1.76991	-1.76596	-1.78471	-1.78065
-1.77112	-1.76715	-1.76319	-1.75928	-1.75539	-1.77021	-1.76626	-1.78501	-1.78096
-1.77142	-1.76744	-1.76349	-1.75957	-1.75569	-1.77051	-1.76656	-1.78531	-1.78126
-1.77172	-1.76774	-1.76379	-1.75987	-1.75598	-1.77081	-1.76685	-1.78561	-1.78156
-1.77202	-1.76804	-1.76409	-1.76017	-1.75628	-1.77111	-1.76716	-1.78592	-1.78187
-1.77231	-1.76833	-1.76438	-1.76046	-1.75657	-1.77141	-1.76747	-1.78624	-1.78219
-1.7441	-1.74034	-1.7366	-1.76076	-1.75687	-1.77171	-1.76777	-1.78656	-1.78253
-1.74447	-1.74067	-1.7369	-1.76105	-1.75716	-1.77201	-1.76808	-1.78688	-1.7829
-1.74483	-1.741	-1.7372	-1.76134	-1.75745	-1.77231	-1.76838	-1.7872	-1.78324
-1.74515	-1.74132	-1.7375	-1.76164	-1.75774	-1.77259	-1.76867	-1.7875	-1.78355
-1.74547	-1.74164	-1.73781	-1.76197	-1.75806	-1.7729	-1.76897	-1.7878	-1.78384
-1.74579	-1.74198	-1.73818	-1.76235	-1.75843	-1.77326	-1.7693	-1.7881	-1.78414
-1.7461	-1.74233	-1.73856	-1.76276	-1.75883	-1.77364	-1.76964	-1.78842	-1.78445
-1.74641	-1.74265	-1.73891	-1.76313	-1.7592	-1.77402	-1.77002	-1.78879	-1.78481
-1.74672	-1.74295	-1.73921	-1.76346	-1.75956	-1.7744	-1.77041	-1.78919	-1.78522
-1.74707	-1.74329	-1.73955	-1.76382	-1.75993	-1.77478	-1.77081	-1.7896	-1.78561
-1.74746	-1.74368	-1.73994	-1.76421	-1.76033	-1.7752	-1.77122	-1.79	-1.78598
-1.74787	-1.74408	-1.74032	-1.7646	-1.76072	-1.77559	-1.77161	-1.83403	-1.82985
-1.74826	-1.74445	-1.74066	-1.76494	-1.76105	-1.77591	-1.77196	-1.8344	-1.83023
-1.74865	-1.74482	-1.741	-1.76527	-1.76137	-1.77624	-1.7723	-1.83477	-1.83061
-1.74902	-1.74519	-1.74138	-1.76567	-1.76176	-1.75785	-1.75397	-1.81733	-1.81323
-1.74937	-1.74556	-1.74179	-1.76611	-1.76219	-1.75828	-1.75438	-1.81774	-1.81364
-1.70017	-1.6965	-1.69286	-1.76654	-1.76261	-1.75869	-1.75479	-1.81814	-1.81402
-1.70057	-1.69691	-1.69327	-1.76697	-1.76304	-1.75911	-1.75519	-1.81854	-1.8144
-1.67864	-1.67506	-1.6715	-1.7674	-1.76346	-1.80464	-1.80055	-1.81892	-1.81477
-1.67905	-1.67548	-1.67191	-1.76782	-1.76386	-1.80502	-1.80092	-1.81928	-1.81515
-1.67947	-1.67589	-1.67231	-1.76824	-1.76425	-1.80539	-1.80128	-1.81965	-1.81555
-1.67989	-1.6763	-1.67272	-1.76771	-1.76367	-1.82832	-1.82417	-1.82005	-1.81596
-1.68031	-1.67672	-1.67313	-1.76812	-1.76405	-1.82868	-1.82457	-1.82048	-1.81639
-1.68073	-1.67714	-1.67355	-1.76859	-1.76452	-1.82914	-1.825	-1.82089	-1.81681
-1.68114	-1.67755	-1.67398	-1.76909	-1.76506	-1.82967	-1.82547	-1.82131	-1.81723
-1.68155	-1.67797	-1.6744	-1.81504	-1.81086	-1.80668	-1.8025	-1.82179	-1.81768
-1.68197	-1.67838	-1.67481	-1.81549	-1.81133	-1.80718	-1.80304	-1.82233	-1.81816
-1.68239	-1.6788	-1.67521	-1.81593	-1.81179	-1.80766	-1.80356	-1.82286	-1.81866
-1.68281	-1.67921	-1.67563	-1.81639	-1.81223	-1.80811	-1.80403	-1.82334	-1.81917
-1.68322	-1.67962	-1.67604	-1.81684	-1.81268	-1.80855	-1.80448	-1.8238	-1.81968
-1.68363	-1.68004	-1.67645	-1.74515	-1.74137	-1.80901	-1.80493	-1.80085	-1.79678
-1.68405	-1.68045	-1.67687	-1.74557	-1.7418	-1.80946	-1.80537	-1.80129	-1.79723
-1.68447	-1.68087	-1.67729	-1.746	-1.74222	-1.8099	-1.80581	-1.80173	-1.79767
-1.68489	-1.6813	-1.67773	-1.74644	-1.74263	-1.81033	-1.80625	-1.80218	-1.7981
-1.68535	-1.68176	-1.6782	-1.74691	-1.7431	-1.81082	-1.80672	-1.80263	-1.79855
-1.68588	-1.68229	-1.67871	-1.74744	-1.74363	-1.81137	-1.8072	-1.80307	-1.79899

-1.73511	-1.73135	-1.7276	-1.74795	-1.74416	-1.81193	-1.80772	-1.80355	-1.79948
-1.73557	-1.7318	-1.72805	-1.74841	-1.74463	-1.81247	-1.80826	-1.8041	-1.80002
-1.73602	-1.73225	-1.72849	-1.74887	-1.7451	-1.813	-1.80883	-1.80468	-1.80057
-1.73652	-1.73275	-1.729	-1.74938	-1.7456	-1.81355	-1.8094	-1.80525	-1.8011
-1.73703	-1.73327	-1.72953	-1.74991	-1.74613	-1.81411	-1.80997	-1.80582	-1.80162
-1.73751	-1.73375	-1.73002	-1.7504	-1.74661	-1.77098	-1.76699	-1.80635	-1.80215
-1.73795	-1.73419	-1.73046	-1.75084	-1.74705	-1.77144	-1.76746	-1.80685	-1.80269
-1.73842	-1.73466	-1.73093	-1.75132	-1.74753	-1.77194	-1.76796	-1.80738	-1.80324
-1.73896	-1.7352	-1.73146	-1.75186	-1.74807	-1.77252	-1.76852	-1.80794	-1.80382
-1.73951	-1.73574	-1.73199	-1.75241	-1.74862	-1.7731	-1.76909	-1.80851	-1.80439
-1.74005	-1.73627	-1.73252	-1.75294	-1.74917	-1.74539	-1.7416	-1.80907	-1.80496
-1.74058	-1.73679	-1.73304	-1.75347	-1.74969	-1.74591	-1.74212	-1.80962	-1.80551
-1.74111	-1.73729	-1.7335	-1.75393	-1.75016	-1.74638	-1.74259	-1.76666	-1.76269
-1.74163	-1.73777	-1.73394	-1.75436	-1.7506	-1.74682	-1.74302	-1.76711	-1.76315
-1.74213	-1.73826	-1.73442	-1.75484	-1.75108	-1.7473	-1.7435	-1.76762	-1.76365
-1.74259	-1.73876	-1.73495	-1.75538	-1.75161	-1.74783	-1.74403	-1.76818	-1.76421
-1.74308	-1.73927	-1.73549	-1.75594	-1.75215	-1.74837	-1.74458	-1.76876	-1.76478
-1.7436	-1.7398	-1.73602	-1.75648	-1.7527	-1.74891	-1.74512	-1.76934	-1.76535
-1.74414	-1.74034	-1.73656	-1.75703	-1.75325	-1.74946	-1.74568	-1.76992	-1.76592
-1.74469	-1.74089	-1.73711	-1.75758	-1.7538	-1.75001	-1.74623	-1.77051	-1.76651
-1.74525	-1.74146	-1.73768	-1.75816	-1.75436	-1.75058	-1.7468	-1.77112	-1.7671
-1.74586	-1.74207	-1.7383	-1.75878	-1.75498	-1.75119	-1.74742	-1.77178	-1.76775
-1.74651	-1.74273	-1.73896	-1.75946	-1.75565	-1.75186	-1.74808	-1.77248	-1.76844
-1.74713	-1.74334	-1.73958	-1.76008	-1.75627	-1.75247	-1.74869	-1.74491	-1.74111
-1.7477	-1.74391	-1.74014	-1.76065	-1.75684	-1.75304	-1.74925	-1.74546	-1.74166
-1.70506	-1.70132	-1.69761	-1.7612	-1.7574	-1.75359	-1.7498	-1.74601	-1.74221
-1.70559	-1.70185	-1.69814	-1.76176	-1.75794	-1.75414	-1.75035	-1.74656	-1.74276
-1.70612	-1.70238	-1.69868	-1.76231	-1.75849	-1.75468	-1.7509	-1.74712	-1.74333
-1.70666	-1.70293	-1.69921	-1.72045	-1.71669	-1.75524	-1.75146	-1.74769	-1.74392
-1.70722	-1.70348	-1.69976	-1.721	-1.71724	-1.75582	-1.75203	-1.74826	-1.74454
-1.70782	-1.70407	-1.70034	-1.72159	-1.71784	-1.71409	-1.71036	-1.74886	-1.74514
-1.70847	-1.70471	-1.70096	-1.72223	-1.71848	-1.71474	-1.71099	-1.7495	-1.74574
-1.6869	-1.68318	-1.67948	-1.72284	-1.71909	-1.71534	-1.71159	-1.70785	-1.70413
-1.68743	-1.68373	-1.68004	-1.7234	-1.71964	-1.71589	-1.71214	-1.7084	-1.70467
-1.68798	-1.68428	-1.68061	-1.72397	-1.7202	-1.71644	-1.7127	-1.70897	-1.70523
-1.68858	-1.6849	-1.68124	-1.72461	-1.72083	-1.71706	-1.71332	-1.70958	-1.70583
-1.6892	-1.68552	-1.68187	-1.72527	-1.72148	-1.71771	-1.71396	-1.71021	-1.70646
-1.68978	-1.6861	-1.68245	-1.70416	-1.70046	-1.71837	-1.71461	-1.71084	-1.70709
-1.69035	-1.68665	-1.68299	-1.70474	-1.7011	-1.71904	-1.71526	-1.71148	-1.70773
-1.69095	-1.68724	-1.68357	-1.70533	-1.7017	-1.69802	-1.6943	-1.71212	-1.70836
-1.6916	-1.68788	-1.68418	-1.70592	-1.70224	-1.69856	-1.69489	-1.71276	-1.70899
-1.69225	-1.68852	-1.68481	-1.70653	-1.70281	-1.69912	-1.69547	-1.71336	-1.70959
-1.69289	-1.68917	-1.68546	-1.70718	-1.70342	-1.6997	-1.69602	-1.71388	-1.71013
-1.69354	-1.68982	-1.68611	-1.70783	-1.70406	-1.70031	-1.69658	-1.71441	-1.71067
-1.67064	-1.66698	-1.66333	-1.70848	-1.70471	-1.70095	-1.6972	-1.69347	-1.68978
-1.67129	-1.66762	-1.66396	-1.70914	-1.70537	-1.70161	-1.69785	-1.69411	-1.69041
-1.67192	-1.66826	-1.6646	-1.7098	-1.70603	-1.70226	-1.69849	-1.69475	-1.69104
-1.67255	-1.6689	-1.66526	-1.71047	-1.70668	-1.70291	-1.69914	-1.69539	-1.69168
-1.67319	-1.66954	-1.6659	-1.71113	-1.70734	-1.70356	-1.69978	-1.69604	-1.69232
-1.67384	-1.67017	-1.66652	-1.71178	-1.70799	-1.70421	-1.70043	-1.69669	-1.69298

-1.67447	-1.67081	-1.66715	-1.68935	-1.68563	-1.70486	-1.70109	-1.69735	-1.69365
-1.67509	-1.67142	-1.66777	-1.68998	-1.68626	-1.70551	-1.70175	-1.69803	-1.69435
-1.67571	-1.67204	-1.66839	-1.69061	-1.6869	-1.70616	-1.70243	-1.69872	-1.69506
-1.67634	-1.67266	-1.66901	-1.69125	-1.68755	-1.70684	-1.70312	-1.69942	-1.69575
-1.67698	-1.67329	-1.66963	-1.6919	-1.68821	-1.70753	-1.70383	-1.70014	-1.69643
-1.67762	-1.67393	-1.67027	-1.69256	-1.68889	-1.70823	-1.70453	-1.70082	-1.69709
-1.67825	-1.67457	-1.67093	-1.69324	-1.68959	-1.70894	-1.7052	-1.70147	-1.69773
-1.67891	-1.67524	-1.67161	-1.69393	-1.69028	-1.68661	-1.68291	-1.70212	-1.69837
-1.67959	-1.67593	-1.6723	-1.69463	-1.69095	-1.68726	-1.68356	-1.70277	-1.69901
-1.65252	-1.64899	-1.64547	-1.69531	-1.6916	-1.6879	-1.68422	-1.70345	-1.69968
-1.6532	-1.64964	-1.6461	-1.69597	-1.69225	-1.68856	-1.6849	-1.70417	-1.70041
-1.65388	-1.65029	-1.64672	-1.69661	-1.69291	-1.68923	-1.6856	-1.7049	-1.70116
-1.65452	-1.65092	-1.64736	-1.6973	-1.6936	-1.68993	-1.68627	-1.70558	-1.70185
-1.65515	-1.65156	-1.64801	-1.698	-1.69432	-1.69064	-1.68695	-1.70623	-1.70251
-1.65584	-1.65224	-1.6487	-1.69873	-1.69506	-1.69138	-1.68766	-1.68397	-1.68031
-1.65658	-1.65297	-1.64941	-1.69947	-1.69581	-1.69214	-1.68843	-1.68474	-1.68107
-1.65731	-1.6537	-1.65013	-1.70022	-1.69655	-1.69286	-1.68917	-1.68549	-1.68181
-1.658	-1.65441	-1.65085	-1.70096	-1.69723	-1.69353	-1.68984	-1.68617	-1.68252
-1.65869	-1.65512	-1.65158	-1.7017	-1.69793	-1.69419	-1.69051	-1.68685	-1.68322
-1.6594	-1.65584	-1.6523	-1.67638	-1.67266	-1.66898	-1.66535	-1.68755	-1.6839
-1.66012	-1.65655	-1.65301	-1.67712	-1.67341	-1.66973	-1.66608	-1.68827	-1.68459
-1.66083	-1.65724	-1.65367	-1.6501	-1.64653	-1.67045	-1.66681	-1.68901	-1.68532
-1.66154	-1.65792	-1.6543	-1.65073	-1.64718	-1.67115	-1.66754	-1.68977	-1.68609
-1.66227	-1.65862	-1.65498	-1.6514	-1.64786	-1.67187	-1.66828	-1.69054	-1.68685
-1.663	-1.65936	-1.65571	-1.65212	-1.64858	-1.67262	-1.66903	-1.69131	-1.68762
-1.66374	-1.6601	-1.65645	-1.65286	-1.64931	-1.67337	-1.66979	-1.69208	-1.68839
-1.66447	-1.66083	-1.65718	-1.65359	-1.65004	-1.67413	-1.67054	-1.69285	-1.68916
-1.6652	-1.66156	-1.65791	-1.65432	-1.65077	-1.67489	-1.6713	-1.69362	-1.68992
-1.66593	-1.66229	-1.65864	-1.65505	-1.6515	-1.67564	-1.67206	-1.69439	-1.69068
-1.66666	-1.66302	-1.65937	-1.65578	-1.65223	-1.6764	-1.67281	-1.69515	-1.69144
-1.66739	-1.66375	-1.6601	-1.65651	-1.65296	-1.67715	-1.67356	-1.69591	-1.69219
-1.66812	-1.66448	-1.66083	-1.65724	-1.65369	-1.6779	-1.67431	-1.69667	-1.69294
-1.66886	-1.66522	-1.66157	-1.65797	-1.65442	-1.67866	-1.67507	-1.69743	-1.69371
-1.66961	-1.66597	-1.66232	-1.65872	-1.65515	-1.67941	-1.67583	-1.69821	-1.69448
-1.67037	-1.66674	-1.6631	-1.65949	-1.6559	-1.65236	-1.6489	-1.69899	-1.69527
-1.67113	-1.66753	-1.66393	-1.66031	-1.65669	-1.65314	-1.64965	-1.69978	-1.69609
-1.67189	-1.66833	-1.66476	-1.66115	-1.6575	-1.65392	-1.65041	-1.70056	-1.69691
-1.62711	-1.62368	-1.62026	-1.66193	-1.65828	-1.65468	-1.65114	-1.70133	-1.69769
-1.62782	-1.62439	-1.62097	-1.66268	-1.65904	-1.65544	-1.65188	-1.70208	-1.69845
-1.62853	-1.6251	-1.6217	-1.66345	-1.65983	-1.65624	-1.65266	-1.70288	-1.69923
-1.62924	-1.62582	-1.62242	-1.66424	-1.66067	-1.65709	-1.65348	-1.70372	-1.70004
-1.62999	-1.62655	-1.62315	-1.64309	-1.6396	-1.6579	-1.65429	-1.70455	-1.70084
-1.63078	-1.6273	-1.62386	-1.64381	-1.64034	-1.65866	-1.65504	-1.70534	-1.70161
-1.63159	-1.62807	-1.62459	-1.64454	-1.64106	-1.65941	-1.6558	-1.70615	-1.7024
-1.63243	-1.62889	-1.62536	-1.6453	-1.6418	-1.6383	-1.63478	-1.70701	-1.70326
-1.63328	-1.62973	-1.62617	-1.64608	-1.64255	-1.63905	-1.63557	-1.70789	-1.70414
-1.63406	-1.63053	-1.62699	-1.62348	-1.61998	-1.63982	-1.63633	-1.70871	-1.70499
-1.63478	-1.63131	-1.62782	-1.62431	-1.62079	-1.64061	-1.63707	-1.70949	-1.70582
-1.63551	-1.63205	-1.62859	-1.6251	-1.6216	-1.64142	-1.63786	-1.6892	-1.68558
-1.63621	-1.63275	-1.6293	-1.62585	-1.6224	-1.64226	-1.63868	-1.69005	-1.68639

-1.58081	-1.57759	-1.57438	-1.62659	-1.62318	-1.64307	-1.63951	-1.63597	-1.63247
-1.58164	-1.57838	-1.57513	-1.62733	-1.62389	-1.64382	-1.64032	-1.63682	-1.63334
-1.58247	-1.57918	-1.57589	-1.62809	-1.62461	-1.64456	-1.64111	-1.63766	-1.63421
-1.5569	-1.55369	-1.55048	-1.62891	-1.62539	-1.64534	-1.64187	-1.63842	-1.63497
-1.55759	-1.55443	-1.55126	-1.62974	-1.62621	-1.64613	-1.64262	-1.63914	-1.63568
-1.55831	-1.55515	-1.55199	-1.6052	-1.60174	-1.62353	-1.62009	-1.63995	-1.63648
-1.55905	-1.55586	-1.55267	-1.60594	-1.60253	-1.62437	-1.62096	-1.64084	-1.63733
-1.55981	-1.5566	-1.55341	-1.60672	-1.60333	-1.59996	-1.5966	-1.64168	-1.63816
-1.5606	-1.55742	-1.55424	-1.60757	-1.60413	-1.60072	-1.59734	-1.64244	-1.63895
-1.56137	-1.55821	-1.55505	-1.6084	-1.60493	-1.6015	-1.59809	-1.61844	-1.61502
-1.56205	-1.55891	-1.55576	-1.60918	-1.60574	-1.60233	-1.59894	-1.61929	-1.61584
-1.56273	-1.55959	-1.55647	-1.60995	-1.60655	-1.60317	-1.59979	-1.62016	-1.61668
-1.56351	-1.56037	-1.55725	-1.61077	-1.60735	-1.60395	-1.60057	-1.59719	-1.59381
-1.56434	-1.56121	-1.55808	-1.61162	-1.60815	-1.60472	-1.60132	-1.59794	-1.5946
-1.56514	-1.56199	-1.55885	-1.61243	-1.60896	-1.60553	-1.60212	-1.59875	-1.59542
-1.5659	-1.56272	-1.55956	-1.6132	-1.60979	-1.60639	-1.60298	-1.59961	-1.59629
-1.56667	-1.56348	-1.56031	-1.61401	-1.61064	-1.60725	-1.60384	-1.60045	-1.59711
-1.56746	-1.56428	-1.56112	-1.61489	-1.61152	-1.60812	-1.60466	-1.60123	-1.59786
-1.56826	-1.56509	-1.56194	-1.61575	-1.61238	-1.60896	-1.60548	-1.60202	-1.59862
-1.56909	-1.56589	-1.56271	-1.61654	-1.61315	-1.60973	-1.60629	-1.60287	-1.59947
-1.56991	-1.56669	-1.56346	-1.61732	-1.6139	-1.61049	-1.6071	-1.60373	-1.60036
-1.53825	-1.53513	-1.53203	-1.61816	-1.61474	-1.61134	-1.60797	-1.6046	-1.60124
-1.53894	-1.53586	-1.5328	-1.61905	-1.61564	-1.61224	-1.60886	-1.60548	-1.60212
-1.5397	-1.53664	-1.5336	-1.61995	-1.61654	-1.61313	-1.60972	-1.60633	-1.60295
-1.54054	-1.53747	-1.53442	-1.62086	-1.61745	-1.61402	-1.61056	-1.60712	-1.6037
-1.54138	-1.5383	-1.53525	-1.62176	-1.61833	-1.61487	-1.61139	-1.60792	-1.60448
-1.54223	-1.53915	-1.53608	-1.62264	-1.61915	-1.61568	-1.61221	-1.60876	-1.60533
-1.54305	-1.53997	-1.53689	-1.62349	-1.61997	-1.61648	-1.61303	-1.60961	-1.6062
-1.54381	-1.5407	-1.5376	-1.59296	-1.58957	-1.61732	-1.6139	-1.61049	-1.60709
-1.54453	-1.54139	-1.53827	-1.5937	-1.59037	-1.61819	-1.61478	-1.61139	-1.608
-1.51544	-1.51237	-1.50933	-1.59452	-1.59121	-1.61906	-1.61567	-1.61229	-1.60891
-1.51619	-1.51314	-1.51012	-1.59539	-1.59207	-1.61994	-1.61657	-1.6132	-1.60983
-1.51696	-1.51393	-1.51092	-1.5376	-1.53456	-1.62084	-1.61747	-1.6141	-1.61073
-1.51775	-1.51473	-1.51173	-1.53843	-1.53539	-1.62175	-1.61837	-1.61499	-1.61162
-1.51854	-1.51553	-1.51254	-1.53926	-1.53622	-1.62265	-1.61927	-1.61589	-1.61252
-1.51935	-1.51634	-1.51335	-1.54009	-1.53705	-1.62356	-1.62018	-1.6168	-1.61342
-1.52016	-1.51715	-1.51415	-1.54092	-1.53787	-1.62447	-1.62109	-1.6177	-1.61432
-1.52097	-1.51796	-1.51496	-1.51198	-1.509	-1.59408	-1.59079	-1.6186	-1.61522
-1.52179	-1.51877	-1.51577	-1.51279	-1.50981	-1.59497	-1.59167	-1.61949	-1.61611
-1.5226	-1.51958	-1.51658	-1.51359	-1.51061	-1.59585	-1.59255	-1.62038	-1.617
-1.52341	-1.52038	-1.51738	-1.51439	-1.51142	-1.59674	-1.59343	-1.62128	-1.6179
-1.52422	-1.52119	-1.51819	-1.5152	-1.51222	-1.59762	-1.59431	-1.62219	-1.6188
-1.52504	-1.52201	-1.519	-1.516	-1.51302	-1.59851	-1.5952	-1.62309	-1.6197
-1.52586	-1.52282	-1.5198	-1.5168	-1.51382	-1.59939	-1.59608	-1.62399	-1.62061
-1.49662	-1.49365	-1.49071	-1.5176	-1.51462	-1.57141	-1.56817	-1.59365	-1.59037
-1.49742	-1.49444	-1.49149	-1.51839	-1.51541	-1.57228	-1.56903	-1.59453	-1.59125
-1.49822	-1.49523	-1.49227	-1.51919	-1.5162	-1.57314	-1.56989	-1.59541	-1.59212
-1.49902	-1.49603	-1.49306	-1.51999	-1.51699	-1.574	-1.57075	-1.5963	-1.593
-1.49982	-1.49682	-1.49384	-1.52079	-1.51779	-1.5148	-1.51184	-1.59718	-1.59388
-1.50061	-1.49761	-1.49463	-1.5216	-1.51859	-1.51561	-1.51264	-1.59806	-1.59476

-1.46747	-1.46455	-1.46164	-1.52241	-1.5194	-1.51641	-1.51343	-1.5701	-1.56686
-1.46826	-1.46533	-1.46242	-1.52322	-1.5202	-1.5172	-1.51423	-1.57096	-1.56772
-1.46905	-1.46612	-1.4632	-1.52403	-1.52101	-1.518	-1.51502	-1.57182	-1.56858
-1.46983	-1.4669	-1.46398	-1.49484	-1.49189	-1.5188	-1.51581	-1.57268	-1.56943
-1.47061	-1.46768	-1.46477	-1.49564	-1.49269	-1.51961	-1.51661	-1.57354	-1.57029
-1.47141	-1.46847	-1.46556	-1.46266	-1.45978	-1.52042	-1.51742	-1.57442	-1.57116
-1.47221	-1.46926	-1.46636	-1.46345	-1.46056	-1.52124	-1.51823	-1.57531	-1.57205
-1.47307	-1.47012	-1.46721	-1.4643	-1.46141	-1.52212	-1.51911	-1.57628	-1.57301
-1.47403	-1.47108	-1.46816	-1.46525	-1.46235	-1.52311	-1.52008	-1.57736	-1.57407
-1.41627	-1.41344	-1.41063	-1.46616	-1.46326	-1.49087	-1.48792	-1.5784	-1.57509
-1.41708	-1.41424	-1.41142	-1.46698	-1.46408	-1.49171	-1.48876	-1.57933	-1.57601
-1.41787	-1.41501	-1.41219	-1.46777	-1.46487	-1.49252	-1.48956	-1.58022	-1.5769
-1.41865	-1.41579	-1.41295	-1.41014	-1.40736	-1.49333	-1.49037	-1.58112	-1.57779
-1.41943	-1.41656	-1.41372	-1.4109	-1.40811	-1.49414	-1.49117	-1.58201	-1.57868
-1.4203	-1.41742	-1.41456	-1.41174	-1.40895	-1.46443	-1.46155	-1.55086	-1.54759
-1.42124	-1.41835	-1.41549	-1.41266	-1.40987	-1.46538	-1.4625	-1.55192	-1.54865
-1.42213	-1.41924	-1.41637	-1.41354	-1.41073	-1.40795	-1.40519	-1.55292	-1.54965
-1.42292	-1.42004	-1.41717	-1.41433	-1.41152	-1.40873	-1.40597	-1.55382	-1.55056
-1.4237	-1.42082	-1.41795	-1.4151	-1.41228	-1.40949	-1.40673	-1.55471	-1.55145
-1.42447	-1.42158	-1.41871	-1.41587	-1.41304	-1.41024	-1.40748	-1.50025	-1.49714
-1.42525	-1.42237	-1.4195	-1.41664	-1.41381	-1.41101	-1.40824	-1.50111	-1.49804
-1.42612	-1.42324	-1.42037	-1.41752	-1.41467	-1.41187	-1.40909	-1.47152	-1.46851
-1.42704	-1.42417	-1.42131	-1.41845	-1.4156	-1.41278	-1.40999	-1.47249	-1.46943
-1.4279	-1.42503	-1.42217	-1.41932	-1.41647	-1.41364	-1.41083	-1.47342	-1.47032
-1.42868	-1.42581	-1.42296	-1.4201	-1.41725	-1.41442	-1.4116	-1.47429	-1.47116
-1.4295	-1.42663	-1.42377	-1.42092	-1.41807	-1.41523	-1.41241	-1.47521	-1.47205
-1.43041	-1.42753	-1.42467	-1.42182	-1.41896	-1.41613	-1.4133	-1.47623	-1.47305
-1.43132	-1.42843	-1.42556	-1.4227	-1.41985	-1.41702	-1.41419	-1.47725	-1.47406
-1.39686	-1.39405	-1.39127	-1.42349	-1.42064	-1.41781	-1.41498	-1.47817	-1.47499
-1.39762	-1.3948	-1.39201	-1.42426	-1.4214	-1.41857	-1.41575	-1.47906	-1.47591
-1.39846	-1.39563	-1.39284	-1.4251	-1.42225	-1.41941	-1.41659	-1.41379	-1.41105
-1.39936	-1.39653	-1.39372	-1.42601	-1.42316	-1.42032	-1.41748	-1.41467	-1.41188
-1.40021	-1.39736	-1.39455	-1.42688	-1.42404	-1.42121	-1.41839	-1.41557	-1.41275
-1.40097	-1.39811	-1.39531	-1.42768	-1.42489	-1.42209	-1.4193	-1.4165	-1.41368
-1.40177	-1.3989	-1.39609	-1.39335	-1.39066	-1.42294	-1.42017	-1.41739	-1.41458
-1.40268	-1.3998	-1.39696	-1.39418	-1.39145	-1.42373	-1.42095	-1.41817	-1.41541
-1.40364	-1.40073	-1.39786	-1.39504	-1.39226	-1.42452	-1.42172	-1.41894	-1.41621
-1.40459	-1.40168	-1.39879	-1.39594	-1.39313	-1.4254	-1.42258	-1.41978	-1.41703
-1.40554	-1.40263	-1.39973	-1.39687	-1.39404	-1.42632	-1.42348	-1.42066	-1.41786
-1.40641	-1.40349	-1.40058	-1.39773	-1.39491	-1.42723	-1.42439	-1.42156	-1.41873
-1.40719	-1.40426	-1.40135	-1.39852	-1.39575	-1.42813	-1.4253	-1.42248	-1.41965
-1.40803	-1.40508	-1.40217	-1.39934	-1.39658	-1.39384	-1.39112	-1.42338	-1.42056
-1.40896	-1.406	-1.40307	-1.4002	-1.39739	-1.39464	-1.39194	-1.42425	-1.42146
-1.34992	-1.3471	-1.34431	-1.4011	-1.39823	-1.39544	-1.39275	-1.4251	-1.42233
-1.35081	-1.34799	-1.34519	-1.40202	-1.39912	-1.3963	-1.39355	-1.4259	-1.42315
-1.35169	-1.34888	-1.34609	-1.40296	-1.40003	-1.39717	-1.39436	-1.4267	-1.42396
-1.35255	-1.34976	-1.34697	-1.4039	-1.40096	-1.39807	-1.39522	-1.42755	-1.42478
-1.3534	-1.35063	-1.34786	-1.40484	-1.40189	-1.39899	-1.39612	-1.42844	-1.42561
-1.28284	-1.2803	-1.27778	-1.34594	-1.34314	-1.39991	-1.39703	-1.42935	-1.42648
-1.28355	-1.28103	-1.27854	-1.34679	-1.34402	-1.40086	-1.39796	-1.43029	-1.42738

-1.2843	-1.28178	-1.2793	-1.34765	-1.3449	-1.4018	-1.39889	-1.39601	-1.39318
-1.2851	-1.28258	-1.28009	-1.34852	-1.3458	-1.40276	-1.39982	-1.39693	-1.39408
-1.28593	-1.28338	-1.28087	-1.34938	-1.34667	-1.40371	-1.40076	-1.39785	-1.39498
-1.28674	-1.28415	-1.28161	-1.35018	-1.3475	-1.34478	-1.342	-1.39878	-1.3959
-1.28755	-1.28492	-1.28234	-1.35096	-1.3483	-1.3456	-1.34286	-1.39972	-1.39683
-1.28837	-1.28572	-1.28312	-1.3518	-1.34914	-1.34645	-1.34373	-1.40066	-1.39776
-1.28922	-1.28655	-1.28394	-1.35268	-1.35001	-1.34732	-1.34461	-1.4016	-1.39869
-1.29006	-1.28738	-1.28476	-1.35357	-1.35088	-1.34818	-1.34548	-1.34276	-1.34
-1.29092	-1.28822	-1.28558	-1.35445	-1.35175	-1.34905	-1.34635	-1.34363	-1.34086
-1.29177	-1.28906	-1.2864	-1.35533	-1.35263	-1.34992	-1.34721	-1.34448	-1.34172
-1.29264	-1.28991	-1.28723	-1.3562	-1.35348	-1.35077	-1.34806	-1.34535	-1.34258
-1.2935	-1.29076	-1.28806	-1.35709	-1.35434	-1.35162	-1.34892	-1.3462	-1.34344
-1.26336	-1.26064	-1.25797	-1.35799	-1.35523	-1.35248	-1.34977	-1.34706	-1.3443
-1.26423	-1.2615	-1.25883	-1.35891	-1.35612	-1.35336	-1.35063	-1.3479	-1.34516
-1.2651	-1.26237	-1.25968	-1.25706	-1.2545	-1.35425	-1.35149	-1.34875	-1.34601
-1.26598	-1.26324	-1.26054	-1.25791	-1.25535	-1.35515	-1.35236	-1.34959	-1.34686
-1.26685	-1.2641	-1.26139	-1.25876	-1.25619	-1.32634	-1.32358	-1.35045	-1.34771
-1.26773	-1.26497	-1.26224	-1.2596	-1.25702	-1.32724	-1.32446	-1.35131	-1.34856
-1.2686	-1.26585	-1.26312	-1.26047	-1.25787	-1.32815	-1.32536	-1.32259	-1.31986
-1.26949	-1.26676	-1.26406	-1.2614	-1.25877	-1.3291	-1.32628	-1.32351	-1.32079
-1.27037	-1.26768	-1.26502	-1.26235	-1.25968	-1.33006	-1.32722	-1.32443	-1.32173
-1.27123	-1.26856	-1.26591	-1.26324	-1.26057	-1.33101	-1.32814	-1.32533	-1.32262
-1.27208	-1.26941	-1.26677	-1.26411	-1.26144	-1.33196	-1.32906	-1.32623	-1.32349
-1.23906	-1.23645	-1.23387	-1.26499	-1.26231	-1.33291	-1.33002	-1.32719	-1.32442
-1.23999	-1.23736	-1.23474	-1.26588	-1.26318	-1.33387	-1.33102	-1.3282	-1.32541
-1.2409	-1.23825	-1.23562	-1.23298	-1.23035	-1.26141	-1.2588	-1.32921	-1.32639
-1.24178	-1.23912	-1.23648	-1.23385	-1.23124	-1.26234	-1.25971	-1.33019	-1.32735
-1.24266	-1.23998	-1.23733	-1.23472	-1.23213	-1.26326	-1.26061	-1.33116	-1.32831
-1.20739	-1.2048	-1.20223	-1.23554	-1.23297	-1.23039	-1.22782	-1.33213	-1.3293
-1.20821	-1.2056	-1.20301	-1.23637	-1.23379	-1.23122	-1.22866	-1.33309	-1.33029
-1.20903	-1.20642	-1.20385	-1.23724	-1.23465	-1.23206	-1.22948	-1.26059	-1.25801
-1.20986	-1.20727	-1.20472	-1.23815	-1.23554	-1.23292	-1.2303	-1.26141	-1.25882
-1.17384	-1.17133	-1.16886	-1.23905	-1.23641	-1.23378	-1.23115	-1.22856	-1.22604
-1.1747	-1.17217	-1.16968	-1.23992	-1.23727	-1.23464	-1.23204	-1.22947	-1.22693
-1.17556	-1.17301	-1.17049	-1.2408	-1.23814	-1.23552	-1.23294	-1.23038	-1.22782
-1.1764	-1.17384	-1.1713	-1.2417	-1.23904	-1.23642	-1.23384	-1.23127	-1.22872
-1.17724	-1.17467	-1.17212	-1.2426	-1.23996	-1.23734	-1.23474	-1.23217	-1.22962
-1.17807	-1.1755	-1.17296	-1.17046	-1.168	-1.23825	-1.23565	-1.23307	-1.2305
-1.1789	-1.17634	-1.17381	-1.17132	-1.16885	-1.23918	-1.23657	-1.23398	-1.23139
-1.17975	-1.17719	-1.17467	-1.17217	-1.16971	-1.24011	-1.23749	-1.23488	-1.23226
-1.1806	-1.17805	-1.17553	-1.17303	-1.17056	-1.24105	-1.23841	-1.23577	-1.23312
-1.1452	-1.14271	-1.14026	-1.17389	-1.17141	-1.20584	-1.20326	-1.23665	-1.23399
-1.14607	-1.14357	-1.1411	-1.17473	-1.17223	-1.20669	-1.20409	-1.23751	-1.23487
-1.06929	-1.06701	-1.06476	-1.13948	-1.13705	-1.17056	-1.16805	-1.23839	-1.23577
-1.07009	-1.06779	-1.06552	-1.14033	-1.13789	-1.17142	-1.16891	-1.23933	-1.23668
-1.0709	-1.06858	-1.06629	-1.14117	-1.13873	-1.17229	-1.16978	-1.24029	-1.23761
-1.02873	-1.02654	-1.02438	-1.14202	-1.13957	-1.17315	-1.17065	-1.2051	-1.20251
-1.02944	-1.02727	-1.02511	-1.14288	-1.14042	-1.17401	-1.17151	-1.20603	-1.20344
-1.03017	-1.02799	-1.02584	-1.06635	-1.0641	-1.13881	-1.13638	-1.20693	-1.20435
-1.03092	-1.02872	-1.02655	-1.06711	-1.06487	-1.13964	-1.13721	-1.20782	-1.20524

-1.03166	-1.02945	-1.02727	-1.06789	-1.06563	-1.14048	-1.13804	-1.1734	-1.17089
-1.03239	-1.03019	-1.02802	-1.06867	-1.06639	-1.14133	-1.13888	-1.17429	-1.17177
-1.03313	-1.03093	-1.02877	-1.06945	-1.06715	-1.14217	-1.13972	-1.17517	-1.17264
-1.03388	-1.03166	-1.02948	-1.0273	-1.02515	-1.06567	-1.06344	-1.13812	-1.13569
-1.03463	-1.03239	-1.03018	-1.02801	-1.02588	-1.06645	-1.0642	-1.13895	-1.13652
-1.03538	-1.03314	-1.03092	-1.02874	-1.02659	-1.06721	-1.06495	-1.13979	-1.13735
-1.03613	-1.03389	-1.03168	-1.02948	-1.02729	-1.06795	-1.06571	-1.14063	-1.13818
-1.03693	-1.03468	-1.03247	-1.03025	-1.02804	-1.06874	-1.0665	-1.1415	-1.13905
-1.03778	-1.03552	-1.03327	-1.03105	-1.02885	-1.0696	-1.06733	-1.14241	-1.13995
-0.92024	-0.91822	-0.91623	-1.03184	-1.02964	-1.02747	-1.02531	-1.06588	-1.06366
-0.92092	-0.91889	-0.91687	-1.03258	-1.03038	-1.0282	-1.02603	-1.06664	-1.06441
-0.92158	-0.91954	-0.91751	-1.03333	-1.03112	-1.02893	-1.02675	-1.06741	-1.06516
-0.87465	-0.87273	-0.87084	-0.99712	-0.99497	-1.02971	-1.02752	-1.06822	-1.06597
-0.87523	-0.87333	-0.87147	-0.99789	-0.99575	-1.03051	-1.02832	-1.06906	-1.0668
-0.87588	-0.87397	-0.87211	-0.91756	-0.9156	-0.99434	-0.9922	-0.99009	-0.988
-0.87658	-0.87465	-0.87276	-0.91823	-0.91623	-0.99503	-0.9929	-0.99079	-0.98869
-0.8331	-0.83127	-0.82946	-0.87149	-0.86958	-0.9149	-0.91297	-0.99151	-0.9894
-0.83371	-0.83188	-0.83008	-0.87215	-0.87024	-0.91561	-0.91366	-0.99228	-0.99017
-0.83432	-0.83249	-0.83068	-0.82886	-0.82704	-0.9163	-0.91433	-0.99303	-0.99092
-0.83496	-0.8331	-0.83126	-0.82943	-0.82761	-0.91694	-0.91495	-0.99372	-0.99161
-0.83561	-0.83372	-0.83183	-0.82999	-0.82818	-0.91757	-0.91556	-0.9944	-0.9923
-0.83627	-0.83436	-0.83246	-0.83061	-0.82879	-0.87082	-0.8689	-0.91424	-0.91234
-0.83693	-0.83502	-0.83311	-0.83125	-0.82942	-0.87148	-0.86956	-0.91493	-0.91301
-0.75318	-0.75146	-0.74975	-0.83188	-0.83006	-0.82822	-0.82638	-0.91563	-0.91369
-0.75372	-0.75202	-0.75033	-0.83252	-0.83069	-0.82885	-0.82701	-0.91633	-0.91437
-0.71696	-0.71537	-0.7138	-0.74925	-0.74759	-0.82949	-0.82764	-0.87408	-0.87219
-0.71758	-0.71598	-0.71441	-0.74985	-0.74819	-0.83013	-0.82828	-0.87476	-0.87287
-0.71822	-0.71661	-0.71502	-0.75045	-0.74879	-0.83077	-0.82891	-0.87544	-0.87354
-0.71885	-0.71723	-0.71565	-0.75105	-0.74936	-0.79149	-0.78972	-0.82769	-0.82591
-0.71948	-0.71786	-0.71627	-0.75165	-0.74993	-0.79206	-0.7903	-0.8283	-0.82652
-0.67912	-0.67751	-0.67593	-0.75227	-0.75054	-0.74884	-0.74717	-0.82893	-0.82715
-0.67979	-0.67818	-0.67658	-0.7529	-0.75117	-0.74947	-0.74779	-0.82957	-0.82779
-0.58561	-0.58422	-0.58284	-0.75352	-0.7518	-0.75009	-0.7484	-0.79044	-0.78874
-0.58617	-0.58478	-0.5834	-0.75414	-0.75242	-0.75071	-0.74902	-0.79109	-0.78938
-0.53122	-0.53008	-0.52895	-0.67159	-0.66996	-0.75133	-0.74965	-0.74799	-0.74636
-0.53171	-0.53057	-0.52944	-0.6722	-0.67057	-0.75195	-0.75027	-0.74861	-0.74698
-0.5322	-0.53106	-0.52994	-0.67283	-0.67118	-0.75258	-0.7509	-0.74923	-0.7476
-0.48243	-0.48141	-0.4804	-0.6237	-0.62221	-0.7132	-0.71152	-0.74987	-0.74822
-0.48288	-0.48185	-0.48084	-0.62429	-0.62282	-0.7139	-0.7122	-0.75052	-0.74885
-0.48334	-0.48231	-0.48129	-0.48027	-0.47926	-0.67151	-0.66989	-0.71118	-0.70953
-0.48382	-0.48277	-0.48173	-0.48071	-0.4797	-0.67212	-0.6705	-0.71182	-0.71016
-0.43696	-0.43601	-0.43508	-0.48115	-0.48014	-0.57506	-0.57368	-0.71245	-0.71079
-0.43738	-0.43642	-0.43548	-0.48159	-0.48058	-0.57558	-0.57419	-0.71307	-0.71141
-0.43779	-0.43683	-0.43588	-0.48203	-0.48102	-0.5761	-0.57471	-0.57333	-0.57197
-0.4382	-0.43723	-0.43628	-0.48248	-0.48146	-0.57663	-0.57525	-0.57387	-0.5725
-0.43861	-0.43764	-0.43668	-0.48292	-0.48191	-0.57716	-0.5758	-0.57443	-0.57305
-0.43905	-0.43807	-0.43711	-0.48339	-0.48236	-0.48135	-0.48034	-0.57496	-0.5736
-0.4395	-0.43852	-0.43754	-0.48386	-0.48282	-0.48179	-0.48078	-0.57548	-0.57414
-0.39196	-0.39111	-0.39026	-0.43703	-0.43608	-0.48225	-0.48123	-0.57601	-0.57467
-0.39234	-0.3915	-0.39066	-0.43748	-0.43652	-0.48272	-0.48169	-0.57653	-0.57518

-0.39272	-0.39188	-0.39105	-0.43793	-0.43696	-0.48321	-0.48216	-0.52178	-0.52066
-0.3931	-0.39226	-0.39143	-0.43836	-0.43741	-0.48371	-0.48264	-0.52224	-0.5211
-0.39348	-0.39263	-0.3918	-0.43879	-0.43785	-0.4842	-0.48314	-0.52272	-0.52156
-0.39385	-0.393	-0.39217	-0.4392	-0.43827	-0.48468	-0.48363	-0.5232	-0.52204
-0.39424	-0.39338	-0.39254	-0.43961	-0.43869	-0.48514	-0.48411	-0.5237	-0.52253
-0.29026	-0.28963	-0.28901	-0.44002	-0.4391	-0.43817	-0.43725	-0.48354	-0.4825
-0.29057	-0.28992	-0.28929	-0.44044	-0.4395	-0.43858	-0.43766	-0.484	-0.48298
-0.29088	-0.29023	-0.28959	-0.39284	-0.392	-0.43899	-0.43807	-0.48446	-0.48344
-0.2912	-0.29055	-0.28991	-0.39326	-0.3924	-0.43942	-0.43848	-0.48491	-0.48389
-0.29151	-0.29086	-0.29022	-0.28958	-0.28894	-0.43986	-0.43891	-0.48538	-0.48435
-0.2918	-0.29116	-0.29052	-0.28988	-0.28924	-0.44032	-0.43938	-0.48589	-0.48484
-0.2921	-0.29145	-0.29081	-0.29017	-0.28954	-0.44079	-0.43984	-0.48639	-0.48534
-0.29241	-0.29175	-0.2911	-0.29046	-0.28983	-0.33937	-0.33864	-0.43932	-0.43838
-0.29273	-0.29206	-0.2914	-0.29075	-0.29012	-0.3397	-0.33897	-0.43974	-0.43881
-0.29306	-0.29238	-0.29172	-0.29107	-0.29043	-0.2898	-0.28917	-0.39265	-0.39182
-0.29339	-0.29272	-0.29206	-0.29141	-0.29076	-0.29012	-0.28948	-0.39305	-0.39219
-0.29371	-0.29304	-0.29238	-0.29173	-0.29108	-0.29043	-0.28978	-0.33926	-0.33852
-0.29402	-0.29335	-0.29268	-0.29202	-0.29137	-0.29073	-0.29008	-0.33962	-0.33889
-0.29434	-0.29366	-0.29299	-0.29233	-0.29167	-0.29102	-0.29038	-0.28976	-0.28915
-0.29469	-0.29401	-0.29333	-0.29265	-0.29198	-0.29132	-0.29068	-0.29005	-0.28943
-0.29505	-0.29436	-0.29367	-0.29298	-0.2923	-0.29163	-0.29098	-0.29034	-0.28972
-0.29537	-0.29468	-0.29399	-0.2933	-0.29262	-0.29195	-0.2913	-0.29066	-0.29003
-0.29569	-0.295	-0.29431	-0.29363	-0.29295	-0.29228	-0.29163	-0.291	-0.29036
-0.29603	-0.29534	-0.29465	-0.29396	-0.29327	-0.2926	-0.29195	-0.29131	-0.29067
-0.29641	-0.2957	-0.295	-0.2943	-0.2936	-0.29291	-0.29225	-0.29159	-0.29096
-0.29676	-0.29605	-0.29535	-0.29464	-0.29393	-0.29324	-0.29256	-0.2919	-0.29126
-0.29708	-0.29637	-0.29567	-0.29497	-0.29427	-0.29358	-0.2929	-0.29224	-0.29159
-0.24318	-0.24258	-0.242	-0.2953	-0.29461	-0.29393	-0.29324	-0.29257	-0.29192
-0.24348	-0.24288	-0.24229	-0.29566	-0.29497	-0.29427	-0.29358	-0.29289	-0.29223
-0.2438	-0.24319	-0.2426	-0.29602	-0.29532	-0.29462	-0.29391	-0.29321	-0.29253
-0.24408	-0.24347	-0.24287	-0.24228	-0.2417	-0.29495	-0.29425	-0.29355	-0.29287
-0.24435	-0.24374	-0.24314	-0.24254	-0.24196	-0.29528	-0.29459	-0.2939	-0.29322
-0.24464	-0.24403	-0.24343	-0.24283	-0.24225	-0.29563	-0.29494	-0.29426	-0.29358
-0.24496	-0.24435	-0.24375	-0.24315	-0.24256	-0.296	-0.29531	-0.29462	-0.29394
-0.24527	-0.24466	-0.24405	-0.24345	-0.24286	-0.24227	-0.24169	-0.29497	-0.29429
-0.24556	-0.24494	-0.24433	-0.24373	-0.24313	-0.24254	-0.24195	-0.2953	-0.29461
-0.24585	-0.24523	-0.24461	-0.244	-0.2434	-0.24281	-0.24222	-0.29562	-0.29493
-0.24615	-0.24554	-0.24492	-0.24432	-0.24371	-0.24312	-0.24253	-0.29599	-0.29529
-0.24645	-0.24585	-0.24524	-0.24464	-0.24404	-0.24344	-0.24285	-0.29637	-0.29567
-0.24676	-0.24616	-0.24556	-0.24496	-0.24436	-0.24376	-0.24316	-0.24257	-0.24199
-0.24708	-0.24648	-0.24588	-0.24528	-0.24468	-0.24408	-0.24348	-0.24289	-0.2423
-0.24739	-0.24679	-0.2462	-0.2456	-0.24499	-0.24439	-0.24379	-0.2432	-0.24261
-0.24771	-0.24711	-0.24651	-0.24591	-0.24531	-0.24471	-0.24411	-0.24351	-0.24292
-0.24801	-0.24741	-0.24681	-0.24621	-0.24561	-0.24501	-0.24441	-0.24381	-0.24322
-0.24829	-0.24768	-0.24708	-0.24648	-0.24588	-0.24528	-0.24468	-0.24409	-0.24349
-0.24857	-0.24796	-0.24736	-0.24676	-0.24616	-0.24556	-0.24496	-0.24436	-0.24376
-0.18746	-0.187	-0.18655	-0.24706	-0.24646	-0.24587	-0.24527	-0.24467	-0.24407
-0.18771	-0.18724	-0.18678	-0.24738	-0.24678	-0.24619	-0.24559	-0.24499	-0.24439
-0.18795	-0.18748	-0.18702	-0.2477	-0.2471	-0.2465	-0.2459	-0.24531	-0.24471
-0.1882	-0.18772	-0.18725	-0.24802	-0.24742	-0.24682	-0.24622	-0.24562	-0.24502

-0.18845	-0.18796	-0.18749	-0.24834	-0.24774	-0.24714	-0.24654	-0.24594	-0.24534
-0.18869	-0.18821	-0.18773	-0.24867	-0.24806	-0.24745	-0.24685	-0.24626	-0.24566
-0.18893	-0.18845	-0.18797	-0.1875	-0.18704	-0.24777	-0.24717	-0.24657	-0.24597
-0.18915	-0.18867	-0.1882	-0.18774	-0.18728	-0.2481	-0.2475	-0.24689	-0.24629
-0.18937	-0.18889	-0.18843	-0.18797	-0.18752	-0.24843	-0.24782	-0.24721	-0.24661
-0.18961	-0.18913	-0.18866	-0.1882	-0.18774	-0.24874	-0.24814	-0.24753	-0.24693
-0.18986	-0.18938	-0.18889	-0.18842	-0.18796	-0.24905	-0.24846	-0.24786	-0.24725
-0.19011	-0.18962	-0.18914	-0.18866	-0.18819	-0.24936	-0.24877	-0.24818	-0.24757
-0.19036	-0.18988	-0.18939	-0.18891	-0.18843	-0.24968	-0.24908	-0.24849	-0.24789
-0.19061	-0.19012	-0.18964	-0.18915	-0.18867	-0.25001	-0.2494	-0.24881	-0.24821
-0.19085	-0.19037	-0.18989	-0.1894	-0.18892	-0.25034	-0.24974	-0.24914	-0.24854
-0.19109	-0.19061	-0.19013	-0.18965	-0.18917	-0.25068	-0.25008	-0.24947	-0.24887
-0.19133	-0.19086	-0.19038	-0.18989	-0.18941	-0.18894	-0.18849	-0.24981	-0.2492
-0.19158	-0.1911	-0.19062	-0.19014	-0.18966	-0.18919	-0.18873	-0.25014	-0.24954
-0.19182	-0.19134	-0.19086	-0.19038	-0.1899	-0.18943	-0.18898	-0.25048	-0.24987
-0.19206	-0.19158	-0.19111	-0.19062	-0.19014	-0.18967	-0.18922	-0.25081	-0.2502
-0.19231	-0.19183	-0.19135	-0.19087	-0.19039	-0.18992	-0.18947	-0.25115	-0.25054
-0.19257	-0.19208	-0.1916	-0.19111	-0.19064	-0.19017	-0.18971	-0.25148	-0.25087
-0.19282	-0.19233	-0.19184	-0.19136	-0.19089	-0.19042	-0.18996	-0.1895	-0.18904
-0.19308	-0.19258	-0.19209	-0.19161	-0.19115	-0.19069	-0.19022	-0.18976	-0.18929
-0.19335	-0.19284	-0.19234	-0.19187	-0.19141	-0.19095	-0.19049	-0.19002	-0.18954
-0.19361	-0.1931	-0.19259	-0.19212	-0.19166	-0.1912	-0.19074	-0.19027	-0.18979
-0.19388	-0.19336	-0.19285	-0.19237	-0.19191	-0.19145	-0.19098	-0.19051	-0.19004
-0.19415	-0.19363	-0.19311	-0.19263	-0.19216	-0.19169	-0.19123	-0.19076	-0.19028
-0.19441	-0.19389	-0.19338	-0.19289	-0.19241	-0.19194	-0.19148	-0.19101	-0.19053
-0.19468	-0.19416	-0.19366	-0.19316	-0.19267	-0.1922	-0.19173	-0.19125	-0.19077
-0.19494	-0.19444	-0.19394	-0.19345	-0.19295	-0.19247	-0.19198	-0.1915	-0.19102
-0.19521	-0.19471	-0.19422	-0.19373	-0.19323	-0.19273	-0.19224	-0.19175	-0.19127
-0.19547	-0.19498	-0.19449	-0.19399	-0.1935	-0.193	-0.1925	-0.192	-0.19153
-0.19574	-0.19525	-0.19475	-0.19426	-0.19376	-0.19326	-0.19275	-0.19226	-0.19179
-0.19602	-0.19552	-0.19503	-0.19453	-0.19403	-0.19352	-0.19301	-0.19252	-0.19205
-0.1963	-0.1958	-0.19531	-0.1948	-0.19429	-0.19378	-0.19327	-0.19278	-0.1923
-0.19658	-0.19608	-0.19558	-0.19507	-0.19455	-0.19404	-0.19354	-0.19305	-0.19257
-0.19686	-0.19635	-0.19584	-0.19533	-0.19481	-0.19431	-0.19382	-0.19333	-0.19285
-0.12951	-0.12919	-0.12887	-0.19559	-0.19508	-0.19458	-0.19409	-0.19361	-0.19312
-0.12967	-0.12936	-0.12904	-0.19586	-0.19536	-0.19486	-0.19437	-0.19388	-0.19339
-0.12984	-0.12953	-0.12921	-0.19614	-0.19564	-0.19514	-0.19465	-0.19415	-0.19366
-0.13002	-0.1297	-0.12938	-0.19642	-0.19592	-0.19542	-0.19492	-0.19442	-0.19392
-0.13019	-0.12987	-0.12956	-0.19671	-0.19621	-0.1957	-0.19519	-0.19469	-0.1942
-0.13037	-0.13006	-0.12974	-0.12944	-0.12913	-0.19598	-0.19546	-0.19496	-0.19447
-0.13056	-0.13024	-0.12993	-0.12962	-0.1293	-0.19625	-0.19574	-0.19524	-0.19475
-0.13074	-0.13042	-0.13011	-0.1298	-0.12948	-0.19653	-0.19603	-0.19553	-0.19503
-0.13091	-0.13059	-0.13028	-0.12997	-0.12965	-0.19682	-0.19632	-0.19581	-0.1953
-0.13109	-0.13077	-0.13046	-0.13014	-0.12983	-0.12952	-0.12922	-0.19609	-0.19557
-0.13127	-0.13095	-0.13063	-0.13031	-0.13	-0.12969	-0.12939	-0.19637	-0.19585
-0.13146	-0.13114	-0.13081	-0.13049	-0.13017	-0.12986	-0.12956	-0.19665	-0.19614
-0.06396	-0.06381	-0.06367	-0.13067	-0.13036	-0.13005	-0.12974	-0.12943	-0.12912
-0.06404	-0.06389	-0.06375	-0.13086	-0.13055	-0.13024	-0.12992	-0.12961	-0.12931
			-0.0637	-0.06355	-0.13042	-0.13011	-0.12979	-0.12949
			-0.06378	-0.06364	-0.1306	-0.13029	-0.12998	-0.12967

-0.06387	-0.06373	-0.13079	-0.13048	-0.13016	-0.12984
-0.06396	-0.06381	-0.13098	-0.13066	-0.13034	-0.13002
		-0.06376	-0.06361	-0.13051	-0.13019
		-0.06384	-0.06369	-0.13069	-0.13038
		-0.06392	-0.06378	-0.13088	-0.13057
		-0.064	-0.06386	-0.06372	-0.06358
		-0.06409	-0.06395	-0.06381	-0.06366
				-0.06389	-0.06375
				-0.06398	-0.06384
				-0.06407	-0.06392
				-0.06415	-0.06401
				-0.06424	-0.0641

								-0.06087
								-0.06077
								-0.06067
					-0.06113	-0.06095	-0.06076	-0.25744
					-0.06103	-0.06084	-0.06066	-0.25704
					-0.06094	-0.06075	-0.06056	-0.44428
					-0.06085	-0.06066	-0.06046	-0.44365
			-0.06113	-0.06095	-0.25808	-0.25736	-0.25664	-0.55911
			-0.06103	-0.06084	-0.25768	-0.25697	-0.25626	-0.55832
	-0.06131	-0.06112	-0.19239	-0.19185	-0.56189	-0.56041	-0.55894	-0.71475
	-0.06121	-0.06102	-0.1921	-0.19157	-0.56108	-0.5596	-0.55815	-0.71374
	-0.06112	-0.06093	-0.19183	-0.1913	-0.56028	-0.55881	-0.55737	-0.71274
	-0.06102	-0.06083	-0.56247	-0.56096	-0.7173	-0.71544	-0.71361	-0.71179
	-0.06093	-0.06073	-0.56167	-0.56017	-0.71631	-0.71447	-0.71265	-0.71085
-0.06102	-0.19206	-0.19153	-0.56085	-0.55936	-0.7153	-0.71347	-0.71166	-0.81302
-0.06092	-0.19176	-0.19124	-0.56001	-0.55854	-0.71427	-0.71245	-0.71065	-0.81187
-0.06082	-0.56216	-0.56066	-0.71696	-0.71511	-0.76317	-0.76121	-0.75928	-0.81072
-0.06072	-0.56137	-0.55988	-0.71598	-0.71413	-0.76213	-0.76017	-0.75825	-0.80958
-0.44144	-0.56058	-0.5591	-0.76504	-0.76306	-0.81469	-0.81258	-0.8105	-0.85828
-0.44085	-0.55978	-0.55831	-0.76398	-0.76202	-0.81357	-0.81149	-0.80942	-0.85712
-0.44027	-0.55899	-0.55754	-0.76294	-0.76099	-0.81245	-0.8104	-0.80834	-0.85597
-0.55968	-0.71577	-0.71392	-0.81553	-0.81344	-0.81136	-0.8093	-0.80725	-0.85481
-0.5589	-0.7148	-0.71298	-0.81443	-0.81235	-0.81028	-0.80821	-0.80614	-0.85364
-0.55813	-0.76379	-0.76184	-0.81332	-0.81125	-0.85902	-0.85683	-0.85465	-0.89748
-0.55737	-0.76276	-0.76082	-0.81221	-0.81014	-0.85784	-0.85567	-0.8535	-0.89628
-0.7637	-0.81528	-0.81319	-0.8111	-0.80903	-0.85667	-0.85451	-0.85235	-0.89507
-0.76266	-0.81416	-0.81208	-0.81	-0.80794	-0.85552	-0.85335	-0.85119	-0.89387
-0.76163	-0.81305	-0.81097	-0.8089	-0.80685	-0.85437	-0.8522	-0.85003	-0.89266
-0.76061	-0.81193	-0.80986	-0.85753	-0.85536	-0.89819	-0.89593	-0.89369	-0.89146

-0.75959	-0.81081	-0.80876	-0.85637	-0.85419	-0.89696	-0.89472	-0.89249	-0.89027
-0.81183	-0.85954	-0.85736	-0.90024	-0.89798	-0.89574	-0.8935	-0.89128	-1.00612
-0.81079	-0.85841	-0.85617	-0.89898	-0.89674	-0.89451	-0.89228	-0.89007	-1.00481
-0.80972	-0.85727	-0.85502	-0.89777	-0.89552	-0.96101	-0.95866	-0.95633	-1.00352
-0.80861	-0.85614	-0.85392	-0.8966	-0.89432	-0.95974	-0.95741	-0.95511	-1.00223
-0.85717	-0.90001	-0.89773	-0.96334	-0.9609	-1.00825	-1.0058	-1.00337	-1.00095
-0.85599	-0.89878	-0.89654	-0.96213	-0.95972	-1.00701	-1.00452	-1.00207	-0.99966
-0.85482	-0.89756	-0.89534	-0.96091	-0.95855	-1.00578	-1.00327	-1.00079	-0.99839
-0.85371	-0.96438	-0.96201	-0.95966	-0.95734	-1.00453	-1.00204	-0.99957	-0.99715
-0.85262	-0.96318	-0.96077	-0.95842	-0.95611	-1.00327	-1.00082	-0.99838	-0.99594
-0.89633	-0.96197	-0.95956	-1.00686	-1.00442	-1.00199	-0.99958	-0.99716	-0.99473
-0.89513	-0.96076	-0.95839	-1.00561	-1.00314	-1.0007	-0.99831	-0.99593	-0.99353
-0.96189	-0.95955	-0.95721	-1.00437	-1.00189	-0.99944	-0.99706	-0.99469	-1.0787
-0.96063	-0.9583	-0.95601	-1.00314	-1.00069	-0.99825	-0.99583	-0.99342	-1.07731
-0.9594	-1.00672	-1.00432	-1.0019	-0.99948	-0.99705	-0.9946	-0.99217	-1.07593
-0.95824	-1.00547	-1.00304	-1.00062	-0.99822	-0.99581	-0.99338	-0.99095	-1.07457
-0.9571	-1.00424	-1.00176	-0.99933	-0.99695	-0.99456	-0.99216	-0.98975	-1.07323
-0.95593	-1.00301	-1.00053	-0.99809	-0.99568	-1.07985	-1.07721	-1.07457	-1.1161
-0.95473	-1.00178	-0.99931	-0.99686	-0.99442	-1.07845	-1.07582	-1.07322	-1.11473
-1.00298	-1.00055	-0.99811	-0.99566	-0.99319	-1.07709	-1.07446	-1.07186	-1.11333
-1.00174	-0.99933	-0.99692	-0.99448	-0.99201	-1.07578	-1.07311	-1.07049	-1.11191
-1.00049	-0.99809	-0.9957	-1.03646	-1.03383	-1.07448	-1.0718	-1.06916	-1.23973
-0.99921	-0.99682	-0.99446	-1.03517	-1.03259	-1.07322	-1.07053	-1.06787	-1.23793
-0.99794	-0.99556	-0.99321	-1.03386	-1.03133	-1.07195	-1.06926	-1.0666	-1.23618
-0.99673	-0.99432	-0.99195	-1.07584	-1.07324	-1.24505	-1.24148	-1.23795	-1.2345
-0.99553	-0.9931	-0.9907	-1.07445	-1.07187	-1.24319	-1.23971	-1.23627	-1.23287
-0.99436	-1.03507	-1.03247	-1.07313	-1.07055	-1.24139	-1.23798	-1.2346	-1.23123
-0.99319	-1.03384	-1.03124	-1.07184	-1.06925	-1.23962	-1.23627	-1.23295	-1.22961
-1.03521	-1.0326	-1.03	-1.24475	-1.24125	-1.23784	-1.23455	-1.23129	-1.22799
-1.03396	-1.03135	-1.02875	-1.24295	-1.23944	-1.23605	-1.2328	-1.22959	-1.22635
-1.03271	-1.03011	-1.02751	-1.24118	-1.23768	-1.23431	-1.23109	-1.22792	-1.22472
-1.03148	-1.02889	-1.0263	-1.23948	-1.236	-1.23265	-1.22944	-1.22628	-1.22312
-1.03026	-1.02768	-1.0251	-1.23779	-1.23436	-1.23102	-1.2278	-1.22464	-1.2215
-1.02904	-1.24316	-1.23959	-1.23611	-1.23271	-1.22938	-1.22613	-1.22295	-1.21982
-1.02782	-1.24143	-1.23789	-1.23444	-1.23107	-1.22774	-1.22446	-1.22125	-1.21812
-1.02661	-1.23972	-1.23622	-1.2328	-1.22944	-1.22612	-1.22284	-1.21962	-1.25888
-1.02539	-1.23803	-1.23457	-1.23117	-1.22782	-1.22452	-1.22125	-1.21804	-1.25716
-1.15762	-1.23635	-1.23292	-1.22954	-1.22622	-1.22293	-1.21968	-1.21647	-1.25543
-1.156	-1.23466	-1.23126	-1.22792	-1.22462	-1.22135	-1.21811	-1.21491	-1.25369
-1.23638	-1.23299	-1.22963	-1.22631	-1.22303	-1.26252	-1.25896	-1.25545	-1.25198
-1.23472	-1.23136	-1.22801	-1.22471	-1.22145	-1.2608	-1.25727	-1.25377	-1.25032
-1.23308	-1.22975	-1.22642	-1.22313	-1.21988	-1.25909	-1.25558	-1.25211	-1.2487
-1.23146	-1.22814	-1.22484	-1.26447	-1.26091	-1.25738	-1.2539	-1.25046	-1.24706
-1.22986	-1.22655	-1.22326	-1.26276	-1.25921	-1.25569	-1.25223	-1.24881	-1.24543
-1.22826	-1.22496	-1.22169	-1.26105	-1.25751	-1.25402	-1.25057	-1.24718	-1.28943
-1.22666	-1.22337	-1.22011	-1.25934	-1.25583	-1.25236	-1.24894	-1.24556	-1.28773
-1.22507	-1.26473	-1.26116	-1.25763	-1.25415	-1.25071	-1.24731	-1.24395	-1.28605
-1.22349	-1.26301	-1.25945	-1.25594	-1.25248	-1.24905	-1.24566	-1.24233	-1.28436
-1.22192	-1.2613	-1.25776	-1.25426	-1.25082	-1.24741	-1.24404	-1.24073	-1.2827
-1.22034	-1.25958	-1.25606	-1.25259	-1.24917	-1.2916	-1.28801	-1.28451	-1.28112

-1.21876	-1.25787	-1.25438	-1.25093	-1.24753	-1.28989	-1.28634	-1.28289	-1.27958
-1.25969	-1.25618	-1.25271	-1.24929	-1.24591	-1.28821	-1.28471	-1.28133	-1.31686
-1.25798	-1.25449	-1.25104	-1.24765	-1.2443	-1.28655	-1.28313	-1.27983	-1.31538
-1.25629	-1.25282	-1.2494	-1.29192	-1.28838	-1.32397	-1.3205	-1.31713	-1.31391
-1.25459	-1.25116	-1.24778	-1.29024	-1.28677	-1.32237	-1.31894	-1.31563	-1.31245
-1.25298	-1.24958	-1.24623	-1.28862	-1.28519	-1.32078	-1.3174	-1.31414	-1.34753
-1.25153	-1.24814	-1.2448	-1.28709	-1.28365	-1.31918	-1.31586	-1.31267	-1.34607
-1.2501	-1.24671	-1.24337	-1.28557	-1.28212	-1.3176	-1.31434	-1.31121	-1.34464
-1.24855	-1.24518	-1.24184	-1.32298	-1.31944	-1.31606	-1.31285	-1.30978	-1.34321
-1.24695	-1.24359	-1.24027	-1.32131	-1.31785	-1.31454	-1.31139	-1.30837	-1.34181
-1.24534	-1.32681	-1.32315	-1.31964	-1.31627	-1.34955	-1.34641	-1.34337	-1.34044
-1.24372	-1.32504	-1.32143	-1.31798	-1.31469	-1.34802	-1.34496	-1.342	-1.33909
-1.24212	-1.32332	-1.31977	-1.31639	-1.31319	-1.34654	-1.34355	-1.34064	-1.33776
-1.24053	-1.32163	-1.31817	-1.31488	-1.31174	-1.34512	-1.34217	-1.33929	-1.33642
-1.32354	-1.32003	-1.31666	-1.34993	-1.3468	-1.34377	-1.34085	-1.33799	-1.33514
-1.32201	-1.31857	-1.31528	-1.34859	-1.34554	-1.34256	-1.33967	-1.33681	-1.33396
-1.32054	-1.31717	-1.31395	-1.3473	-1.34432	-1.34139	-1.3385	-1.33565	-1.3328
-1.31901	-1.31571	-1.31257	-1.34593	-1.343	-1.3401	-1.33722	-1.33437	-1.33154
-1.31747	-1.31425	-1.31118	-1.34455	-1.34164	-1.33877	-1.33589	-1.33305	-1.33022
-1.31594	-1.34923	-1.34617	-1.34318	-1.3403	-1.33743	-1.33457	-1.33172	-1.36729
-1.31441	-1.34774	-1.34478	-1.34184	-1.33896	-1.33609	-1.33323	-1.33039	-1.36591
-1.31301	-1.34637	-1.34347	-1.34056	-1.33768	-1.33481	-1.33196	-1.32912	-1.36461
-1.31179	-1.34515	-1.34227	-1.33938	-1.3365	-1.33363	-1.33078	-1.32795	-1.36342
-1.31059	-1.34395	-1.34108	-1.33819	-1.33531	-1.33244	-1.32958	-1.32676	-1.36222
-1.34561	-1.34266	-1.33978	-1.3369	-1.33402	-1.36961	-1.36666	-1.36375	-1.36091
-1.34429	-1.34136	-1.33846	-1.33558	-1.33271	-1.36826	-1.36531	-1.3624	-1.35957
-1.34305	-1.34013	-1.33723	-1.33434	-1.33147	-1.36699	-1.36407	-1.36118	-1.35835
-1.34185	-1.33893	-1.33603	-1.33314	-1.33027	-1.36577	-1.36289	-1.36004	-1.3572
-1.34058	-1.33767	-1.33477	-1.33188	-1.32901	-1.36449	-1.36164	-1.35883	-1.35602
-1.33922	-1.33632	-1.33343	-1.33055	-1.3277	-1.36314	-1.36032	-1.35754	-1.35481
-1.33791	-1.33501	-1.33213	-1.36765	-1.36472	-1.36185	-1.35904	-1.35629	-1.3536
-1.3367	-1.33381	-1.33094	-1.36644	-1.36355	-1.36069	-1.35786	-1.35508	-1.35238
-1.33549	-1.33261	-1.32975	-1.36524	-1.36238	-1.35954	-1.3567	-1.35391	-1.35118
-1.33419	-1.33131	-1.32846	-1.36393	-1.3611	-1.3583	-1.35552	-1.35277	-1.35006
-1.33287	-1.32999	-1.32716	-1.3626	-1.3598	-1.35705	-1.35434	-1.35165	-1.34897
-1.33163	-1.36712	-1.36423	-1.3614	-1.35862	-1.35588	-1.35317	-1.35049	-1.3747
-1.33045	-1.36594	-1.36309	-1.36028	-1.35751	-1.35476	-1.35202	-1.34929	-1.37344
-1.32925	-1.36471	-1.36188	-1.35909	-1.35635	-1.38065	-1.37781	-1.375	-1.37224
-1.32801	-1.36342	-1.36058	-1.35781	-1.35511	-1.37941	-1.37663	-1.37386	-1.3711
-1.36508	-1.36216	-1.35932	-1.38367	-1.38092	-1.37818	-1.37545	-1.37272	-1.36997
-1.36387	-1.361	-1.35819	-1.3825	-1.37975	-1.37702	-1.37431	-1.37159	-1.36882
-1.36269	-1.35987	-1.35709	-1.38137	-1.3786	-1.37586	-1.37316	-1.37045	-1.36767
-1.36151	-1.38588	-1.38305	-1.38023	-1.37743	-1.37466	-1.37194	-1.36923	-1.36649
-1.36033	-1.38469	-1.3819	-1.37908	-1.37625	-1.37345	-1.37069	-1.36797	-1.36529
-1.38629	-1.38345	-1.38066	-1.37786	-1.37505	-1.37227	-1.3695	-1.36679	-1.36415
-1.38504	-1.38217	-1.37936	-1.37659	-1.37385	-1.37112	-1.36837	-1.36567	-1.36305
-1.38381	-1.38093	-1.37812	-1.37536	-1.37267	-1.36997	-1.36723	-1.36453	-1.38922
-1.38261	-1.37977	-1.37698	-1.37422	-1.37152	-1.36881	-1.36606	-1.36336	-1.38802
-1.38143	-1.37862	-1.37585	-1.3731	-1.37037	-1.39514	-1.39233	-1.38955	-1.42369
-1.38025	-1.37747	-1.37472	-1.37196	-1.3692	-1.39395	-1.39117	-1.38842	-1.42252

-1.37909	-1.37632	-1.37359	-1.37082	-1.36803	-1.39277	-1.39001	-1.3873	-1.42137
-1.37794	-1.37516	-1.37241	-1.36964	-1.36686	-1.39161	-1.38888	-1.38619	-1.52915
-1.37679	-1.37399	-1.37121	-1.36844	-1.36569	-1.39047	-1.38776	-1.38509	-1.52775
-1.37562	-1.37281	-1.37002	-1.39488	-1.39208	-1.42621	-1.42344	-1.42068	-1.52632
-1.37441	-1.37162	-1.36884	-1.39372	-1.39096	-1.42506	-1.4223	-1.41954	-1.52489
-1.37322	-1.37043	-1.36768	-1.42953	-1.42671	-1.42391	-1.42114	-1.41838	-1.55651
-1.37203	-1.36928	-1.36655	-1.42836	-1.42556	-1.42277	-1.41996	-1.41716	-1.55502
-1.37086	-1.36813	-1.36543	-1.4272	-1.42442	-1.42162	-1.41877	-1.41596	-1.55354
-1.36972	-1.39472	-1.39197	-1.42605	-1.42328	-1.42048	-1.41763	-1.41481	-1.55212
-1.36859	-1.39359	-1.39087	-1.42492	-1.42213	-1.41933	-1.41649	-1.41369	-1.55073
-1.36747	-1.42937	-1.42657	-1.42376	-1.42094	-1.52653	-1.5231	-1.51972	-1.54934
-1.36637	-1.42822	-1.42542	-1.42258	-1.41972	-1.52502	-1.52165	-1.51834	-1.54797
-1.36526	-1.42707	-1.42427	-1.42142	-1.41854	-1.52358	-1.52025	-1.51698	-1.54661
-1.36416	-1.42592	-1.42312	-1.42028	-1.41741	-1.52222	-1.51889	-1.51564	-1.54527
-1.42759	-1.42476	-1.42195	-1.41911	-1.41626	-1.52086	-1.51755	-1.51431	-1.54393
-1.42643	-1.42358	-1.42072	-1.41789	-1.41508	-1.5195	-1.51622	-1.51301	-1.54262
-1.42527	-1.42239	-1.4195	-1.41667	-1.4139	-1.51813	-1.5149	-1.51173	-1.5413
-1.42412	-1.42123	-1.41833	-1.41551	-1.41275	-1.54969	-1.54642	-1.54319	-1.53998
-1.42297	-1.42009	-1.4172	-1.41438	-1.41162	-1.54832	-1.54508	-1.54188	-1.53865
-1.42182	-1.41895	-1.41607	-1.41326	-1.41051	-1.54697	-1.54376	-1.54058	-1.53735
-1.42068	-1.41782	-1.41495	-1.41216	-1.40942	-1.54564	-1.54245	-1.53928	-1.53608
-1.41953	-1.41669	-1.41384	-1.51805	-1.51476	-1.54433	-1.54115	-1.53799	-1.53481
-1.41839	-1.41556	-1.41274	-1.51675	-1.51347	-1.54302	-1.53986	-1.53673	-1.53356
-1.41725	-1.41444	-1.41167	-1.51548	-1.51221	-1.54174	-1.53859	-1.53547	-1.53231
-1.41612	-1.41336	-1.41064	-1.51428	-1.51099	-1.54048	-1.53733	-1.53422	-1.53107
-1.41501	-1.41229	-1.40963	-1.5131	-1.50979	-1.53924	-1.53609	-1.53298	-1.52983
-1.4139	-1.41121	-1.40857	-1.54465	-1.54128	-1.538	-1.53484	-1.53172	-1.5286
-1.4128	-1.41012	-1.4075	-1.54335	-1.54001	-1.53675	-1.53359	-1.53047	-1.52737
-1.4117	-1.40904	-1.40643	-1.54204	-1.53874	-1.5355	-1.53234	-1.52923	-1.52617
-1.4106	-1.40795	-1.40536	-1.54073	-1.53745	-1.53424	-1.53109	-1.52801	-1.52501
-1.40952	-1.40688	-1.40429	-1.53942	-1.53617	-1.53298	-1.52985	-1.52679	-1.52384
-1.40849	-1.40583	-1.40323	-1.5381	-1.53489	-1.53172	-1.52861	-1.52556	-1.52263
-1.40747	-1.40479	-1.40217	-1.5368	-1.53362	-1.53049	-1.52739	-1.52435	-1.52142
-1.40643	-1.50924	-1.50607	-1.53554	-1.53243	-1.52933	-1.52624	-1.52319	-1.52024
-1.40539	-1.508	-1.50484	-1.53431	-1.53126	-1.5282	-1.5251	-1.52204	-1.51907
-1.40438	-1.50679	-1.50363	-1.53309	-1.53006	-1.52701	-1.52394	-1.52088	-1.5179
-1.40339	-1.50561	-1.50246	-1.53188	-1.52884	-1.5258	-1.52275	-1.51971	-1.51672
-1.40238	-1.53703	-1.53381	-1.53067	-1.52761	-1.52458	-1.52157	-1.51858	-1.51557
-1.40132	-1.53577	-1.53258	-1.52945	-1.52638	-1.52337	-1.52042	-1.51748	-1.51446
-1.40028	-1.53453	-1.53137	-1.52826	-1.52518	-1.52218	-1.51926	-1.51635	-1.56533
-1.3993	-1.53335	-1.53024	-1.52713	-1.52405	-1.52102	-1.51808	-1.51514	-1.56415
-1.39832	-1.53219	-1.5291	-1.52601	-1.52293	-1.51989	-1.51691	-1.51393	-1.56297
-1.50156	-1.53097	-1.52789	-1.52484	-1.52182	-1.57109	-1.56799	-1.56491	-1.56187
-1.50035	-1.52973	-1.52665	-1.52363	-1.52069	-1.57002	-1.5669	-1.5638	-1.56078
-1.4992	-1.52855	-1.52546	-1.52245	-1.51952	-1.56888	-1.56576	-1.56267	-1.55966
-1.4981	-1.52742	-1.52431	-1.52127	-1.5183	-1.56767	-1.56458	-1.56151	-1.5585
-1.49698	-1.52628	-1.52318	-1.57265	-1.56958	-1.56651	-1.56345	-1.5604	-1.55739
-1.49585	-1.52515	-1.52209	-1.5716	-1.5685	-1.56543	-1.56239	-1.55936	-1.55633
-1.49472	-1.55313	-1.55007	-1.57055	-1.56745	-1.56438	-1.56134	-1.55831	-1.55528
-1.49362	-1.55203	-1.54897	-1.56947	-1.56641	-1.56336	-1.5603	-1.55726	-1.55425

-1.49253	-1.55093	-1.54785	-1.56839	-1.56537	-1.56233	-1.55925	-1.55619	-1.5532
-1.49143	-1.54981	-1.54673	-1.56727	-1.56425	-1.56122	-1.55815	-1.5551	-1.59945
-1.49031	-1.54868	-1.5456	-1.56613	-1.56309	-1.56006	-1.55701	-1.55398	-1.59833
-1.51891	-1.54757	-1.5445	-1.56504	-1.56199	-1.55895	-1.55593	-1.55291	-1.62768
-1.5178	-1.54647	-1.54344	-1.564	-1.56094	-1.5579	-1.55488	-1.55187	-1.62661
-1.54842	-1.54538	-1.54239	-1.56296	-1.5599	-1.60438	-1.60132	-1.59828	-1.62555
-1.54733	-1.54431	-1.54133	-1.56192	-1.55888	-1.60338	-1.60029	-1.59723	-1.62448
-1.54625	-1.54325	-1.54027	-1.56089	-1.55786	-1.60237	-1.59926	-1.59618	-1.62342
-1.54518	-1.54219	-1.53924	-1.55987	-1.55681	-1.60132	-1.59822	-1.59515	-1.62235
-1.54414	-1.54115	-1.53821	-1.55884	-1.55576	-1.60027	-1.59718	-1.59413	-1.62127
-1.54316	-1.54015	-1.53716	-1.60548	-1.60235	-1.59924	-1.59616	-1.5931	-1.62021
-1.54224	-1.53918	-1.5361	-1.60441	-1.60132	-1.59823	-1.59513	-1.59208	-1.61916
-1.54127	-1.53819	-1.53507	-1.60338	-1.60027	-1.62747	-1.62431	-1.62118	-1.61813
-1.54023	-1.53719	-1.53409	-1.60239	-1.5992	-1.62633	-1.62322	-1.62016	-1.61711
-1.53917	-1.60775	-1.60459	-1.6014	-1.59816	-1.62523	-1.62215	-1.61912	-1.61608
-1.53806	-1.60667	-1.60355	-1.60039	-1.59719	-1.62422	-1.6211	-1.61804	-1.61502
-1.53695	-1.60557	-1.60248	-1.59936	-1.59622	-1.62323	-1.62007	-1.61697	-1.61397
-1.53587	-1.60449	-1.60138	-1.59828	-1.59521	-1.62221	-1.61908	-1.61598	-1.61295
-1.53481	-1.60342	-1.60026	-1.59718	-1.59417	-1.62119	-1.6181	-1.61502	-1.61195
-1.60555	-1.60239	-1.59922	-1.62632	-1.62321	-1.62013	-1.61708	-1.61403	-1.61096
-1.60456	-1.6014	-1.59824	-1.62527	-1.62212	-1.61903	-1.61602	-1.61302	-1.60998
-1.60352	-1.60038	-1.59725	-1.62423	-1.62108	-1.61799	-1.61498	-1.61199	-1.63994
-1.60243	-1.59931	-1.59623	-1.62321	-1.62009	-1.617	-1.61396	-1.61094	-1.63882
-1.60136	-1.59825	-1.59521	-1.62218	-1.6191	-1.61603	-1.61296	-1.6099	-1.6377
-1.60035	-1.59723	-1.59416	-1.62111	-1.61807	-1.61503	-1.61198	-1.60892	-1.63662
-1.59936	-1.59622	-1.59313	-1.62004	-1.61704	-1.61402	-1.611	-1.60795	-1.63556
-1.59834	-1.59523	-1.59216	-1.61903	-1.616	-1.61298	-1.60998	-1.60695	-1.63451
-1.59731	-1.59426	-1.59124	-1.61804	-1.61497	-1.61193	-1.60894	-1.60594	-1.63347
-1.59634	-1.62327	-1.62018	-1.61708	-1.61399	-1.64192	-1.63878	-1.63564	-1.63243
-1.59542	-1.62232	-1.61924	-1.61614	-1.61304	-1.64091	-1.63776	-1.63461	-1.63141
-1.59448	-1.62135	-1.61828	-1.61519	-1.6121	-1.6399	-1.63672	-1.63355	-1.63035
-1.59349	-1.62033	-1.61729	-1.61423	-1.61115	-1.63888	-1.63564	-1.63243	-1.62924
-1.59251	-1.61932	-1.6163	-1.61325	-1.61019	-1.63786	-1.63458	-1.63132	-1.62814
-1.62145	-1.61836	-1.61533	-1.61229	-1.60925	-1.63687	-1.63357	-1.63028	-1.62707
-1.62051	-1.61741	-1.61436	-1.61132	-1.60829	-1.63587	-1.63257	-1.62928	-1.62603
-1.61956	-1.61643	-1.61335	-1.6412	-1.63804	-1.63484	-1.63156	-1.62828	-1.62501
-1.61861	-1.61544	-1.61231	-1.64009	-1.63696	-1.63379	-1.63054	-1.62729	-1.62403
-1.61765	-1.61447	-1.61132	-1.63903	-1.63591	-1.63275	-1.62954	-1.62631	-1.62305
-1.6167	-1.61352	-1.61038	-1.63804	-1.6349	-1.63174	-1.62855	-1.62534	-1.62208
-1.61574	-1.64354	-1.64026	-1.63705	-1.6339	-1.63074	-1.62756	-1.62436	-1.6211
-1.61478	-1.64252	-1.63926	-1.63606	-1.63292	-1.62976	-1.62657	-1.62337	-1.62012
-1.61381	-1.64151	-1.63827	-1.63507	-1.63194	-1.62878	-1.62559	-1.62237	-1.61912
-1.61287	-1.64051	-1.63727	-1.63409	-1.63096	-1.6278	-1.6246	-1.62138	-1.61812
-1.61193	-1.63952	-1.63628	-1.6331	-1.62997	-1.62681	-1.6236	-1.62038	-1.61712
-1.64182	-1.63853	-1.63529	-1.63211	-1.62898	-1.62582	-1.62261	-1.61939	-1.61614
-1.64082	-1.63754	-1.63431	-1.63112	-1.62798	-1.62481	-1.62161	-1.6184	-1.61516
-1.6398	-1.63654	-1.63331	-1.63012	-1.62697	-1.62381	-1.62061	-1.61741	-1.61419
-1.63878	-1.63552	-1.6323	-1.62911	-1.62596	-1.6228	-1.61961	-1.61642	-1.61322
-1.63776	-1.63451	-1.6313	-1.62811	-1.62495	-1.62179	-1.6186	-1.61542	-1.61225
-1.63674	-1.6335	-1.63029	-1.62711	-1.62394	-1.62078	-1.6176	-1.61443	-1.61129

-1.63574	-1.63251	-1.62931	-1.62612	-1.62295	-1.61978	-1.6166	-1.61345	-1.61032
-1.63475	-1.63155	-1.62837	-1.62518	-1.622	-1.61882	-1.61564	-1.61247	-1.60935
-1.63379	-1.63061	-1.62745	-1.62428	-1.62108	-1.61789	-1.61469	-1.61151	-1.60839
-1.63286	-1.62969	-1.62653	-1.62334	-1.62014	-1.61694	-1.61373	-1.61055	-1.60744
-1.63195	-1.62878	-1.6256	-1.62239	-1.61917	-1.61596	-1.61276	-1.6096	-1.60649
-1.63104	-1.62786	-1.62465	-1.62143	-1.61819	-1.61497	-1.61179	-1.60864	-1.60554
-1.63011	-1.62691	-1.62368	-1.62044	-1.61719	-1.61397	-1.61081	-1.60769	-1.60459
-1.62916	-1.62594	-1.6227	-1.61946	-1.6162	-1.61299	-1.60985	-1.60674	-1.60365
-1.62819	-1.62495	-1.62171	-1.61847	-1.61523	-1.61204	-1.6089	-1.60579	-1.60272
-1.6272	-1.62396	-1.62071	-1.61749	-1.61429	-1.61112	-1.60797	-1.60486	-1.60182
-1.62621	-1.62297	-1.61973	-1.61654	-1.61338	-1.61024	-1.60711	-1.60401	-1.60096
-1.62524	-1.622	-1.61878	-1.61561	-1.6125	-1.6094	-1.6063	-1.60321	-1.60012
-1.62431	-1.62109	-1.61788	-1.61473	-1.61162	-1.60852	-1.60543	-1.60234	-1.59922
-1.62344	-1.62026	-1.61708	-1.61391	-1.61076	-1.60762	-1.60449	-1.60138	-1.59827
-1.62256	-1.6194	-1.61623	-1.61305	-1.60987	-1.6067	-1.60355	-1.60042	-1.59732
-1.62165	-1.61847	-1.6153	-1.61212	-1.60893	-1.60576	-1.60261	-1.59948	-1.59638
-1.62072	-1.61753	-1.61435	-1.61117	-1.60799	-1.60482	-1.60168	-1.59856	-1.59546
-1.6198	-1.61659	-1.6134	-1.61021	-1.60704	-1.60389	-1.60078	-1.5977	-1.59463
-1.61887	-1.61566	-1.61245	-1.60927	-1.6061	-1.60298	-1.59991	-1.59686	-1.59383
-1.61799	-1.61479	-1.6116	-1.60842	-1.60524	-1.60212	-1.59904	-1.596	-1.59297
-1.61712	-1.61397	-1.61081	-1.60764	-1.60445	-1.6013	-1.59819	-1.59511	-1.59206
-1.61625	-1.61311	-1.60997	-1.6068	-1.60361	-1.60044	-1.59732	-1.59422	-1.59115
-1.61536	-1.6122	-1.60904	-1.60587	-1.60268	-1.59952	-1.59639	-1.5933	-1.59023
-1.61446	-1.61129	-1.60812	-1.60495	-1.60178	-1.59863	-1.59551	-1.59242	-1.58934
-1.61353	-1.61037	-1.60724	-1.60411	-1.60097	-1.59784	-1.59473	-1.59163	-1.58855
-1.61261	-1.60948	-1.60638	-1.60327	-1.60017	-1.59706	-1.59395	-1.59085	-1.58776
-1.61178	-1.60862	-1.6055	-1.60238	-1.59928	-1.59618	-1.59307	-1.58997	-1.58689
-1.61099	-1.6078	-1.60462	-1.60148	-1.59837	-1.59526	-1.59215	-1.58906	-1.58598
-1.61015	-1.60697	-1.60379	-1.60064	-1.59753	-1.59442	-1.59131	-1.58821	-1.58514
-1.60928	-1.60614	-1.60301	-1.59987	-1.59674	-1.59363	-1.59052	-1.58743	-1.58436
-1.6084	-1.60528	-1.60217	-1.59904	-1.59591	-1.59279	-1.58968	-1.58659	-1.58353
-1.60749	-1.60436	-1.60124	-1.59811	-1.59498	-1.59186	-1.58875	-1.58566	-1.5826
-1.60661	-1.60346	-1.60032	-1.59719	-1.59407	-1.59096	-1.58784	-1.58475	-1.58169
-1.6058	-1.60265	-1.59951	-1.59638	-1.59326	-1.59014	-1.58703	-1.58394	-1.58088
-1.60501	-1.60185	-1.59872	-1.59558	-1.59246	-1.58934	-1.58624	-1.58316	-1.58009
-1.60411	-1.60096	-1.59782	-1.59469	-1.59156	-1.58845	-1.58535	-1.58227	-1.6031
-1.60316	-1.60001	-1.59688	-1.59375	-1.59062	-1.58751	-1.58442	-1.58134	-1.60215
-1.6023	-1.59915	-1.59602	-1.59289	-1.58976	-1.58665	-1.58356	-1.58049	-1.60128
-1.60152	-1.59838	-1.59525	-1.59211	-1.58898	-1.58587	-1.58278	-1.57972	-1.6005
-1.60075	-1.59761	-1.59448	-1.59134	-1.5882	-1.58509	-1.58201	-1.57895	-1.6455
-1.59998	-1.59684	-1.5937	-1.59057	-1.58743	-1.58432	-1.58125	-1.57819	-1.64472
-1.59918	-1.59604	-1.59289	-1.58976	-1.58663	-1.58353	-1.58045	-1.57739	-1.64391
-1.59827	-1.59513	-1.59198	-1.58885	-1.58572	-1.60655	-1.60341	-1.6003	-1.643
-1.59733	-1.59418	-1.59103	-1.5879	-1.58478	-1.60559	-1.60246	-1.59936	-1.64207
-1.59649	-1.59334	-1.59019	-1.58706	-1.58394	-1.65073	-1.64754	-1.64437	-1.64123
-1.59571	-1.59256	-1.58941	-1.58629	-1.58318	-1.64995	-1.64677	-1.64361	-1.64046
-1.59494	-1.5918	-1.58865	-1.58552	-1.58242	-1.64919	-1.646	-1.64284	-1.63969
-1.59418	-1.59104	-1.5879	-1.58478	-1.58168	-1.64843	-1.64525	-1.64208	-1.63893
-1.59342	-1.59028	-1.58715	-1.63088	-1.62772	-1.64768	-1.64449	-1.64132	-1.63817
-1.59267	-1.58953	-1.5864	-1.63014	-1.62697	-1.64691	-1.64373	-1.64056	-1.63741

-1.59191	-1.58878	-1.58564	-1.62938	-1.62621	-1.64614	-1.64296	-1.6398	-1.63665
-1.59116	-1.58802	-1.58489	-1.62863	-1.62546	-1.66701	-1.6638	-1.6606	-1.65741
-1.5904	-1.58727	-1.58413	-1.62787	-1.62469	-1.66621	-1.66299	-1.65979	-1.65661
-1.6125	-1.65563	-1.65238	-1.64913	-1.64588	-1.66535	-1.66211	-1.6589	-1.68214
-1.61173	-1.65486	-1.6516	-1.64832	-1.64502	-1.66443	-1.66116	-1.65794	-1.68116
-1.65735	-1.65408	-1.65078	-1.64748	-1.64416	-1.66353	-1.66026	-1.65704	-1.68024
-1.65658	-1.65327	-1.64992	-1.64661	-1.6433	-1.66267	-1.65944	-1.65624	-1.67942
-1.6558	-1.65246	-1.64908	-1.64575	-1.64246	-1.66184	-1.65864	-1.65547	-1.67863
-1.65502	-1.65167	-1.64829	-1.64496	-1.64167	-1.66103	-1.65784	-1.65468	-1.67783
-1.65423	-1.65089	-1.64752	-1.64419	-1.6409	-1.66023	-1.65705	-1.6539	-1.67705
-1.65345	-1.65011	-1.64674	-1.64341	-1.64013	-1.68597	-1.68271	-1.67949	-1.67626
-1.65267	-1.64932	-1.64595	-1.64264	-1.63937	-1.68518	-1.68193	-1.67871	-1.67548
-1.65189	-1.64855	-1.64518	-1.64187	-1.63861	-1.68439	-1.68114	-1.67792	-1.6747
-1.65111	-1.64778	-1.64441	-1.64111	-1.63785	-1.6836	-1.68034	-1.67712	-1.6739
-1.65034	-1.64703	-1.64369	-1.64039	-1.63712	-1.68282	-1.67955	-1.67632	-1.6731
-1.64958	-1.64631	-1.64303	-1.63974	-1.63645	-1.68209	-1.67877	-1.6755	-1.6723
-1.64882	-1.64559	-1.64236	-1.6391	-1.63579	-1.68137	-1.67802	-1.67472	-1.6715
-1.64805	-1.64484	-1.64163	-1.66093	-1.65761	-1.6807	-1.67732	-1.67399	-1.67074
-1.64728	-1.64407	-1.64086	-1.66018	-1.65693	-1.68003	-1.67665	-1.67329	-1.67
-1.6465	-1.64329	-1.64009	-1.65941	-1.65618	-1.6793	-1.67595	-1.67261	-1.6693
-1.64571	-1.64251	-1.63932	-1.65862	-1.6554	-1.67853	-1.67524	-1.67195	-1.66864
-1.64494	-1.64174	-1.63854	-1.68432	-1.68102	-1.67774	-1.67449	-1.67124	-1.69084
-1.64418	-1.64097	-1.63776	-1.68349	-1.68019	-1.67691	-1.67369	-1.67047	-1.69011
-1.64342	-1.6402	-1.63698	-1.68267	-1.67936	-1.67609	-1.67288	-1.66969	-1.68936
-1.64265	-1.63942	-1.63619	-1.68184	-1.67853	-1.67527	-1.67208	-1.66891	-1.68857
-1.64191	-1.63866	-1.63543	-1.68104	-1.67772	-1.67446	-1.67128	-1.66812	-1.68776
-1.64127	-1.66048	-1.65716	-1.68028	-1.67694	-1.67367	-1.67048	-1.66731	-1.68693
-1.64067	-1.65985	-1.65649	-1.67956	-1.6762	-1.6729	-1.66969	-1.66651	-1.68611
-1.63998	-1.65916	-1.6558	-1.67886	-1.67551	-1.67221	-1.66898	-1.66577	-1.70721
-1.63922	-1.65842	-1.65512	-1.67821	-1.67489	-1.6716	-1.66833	-1.66508	-1.70644
-1.63844	-1.65765	-1.65439	-1.67749	-1.67421	-1.69325	-1.68994	-1.68664	-1.70572
-1.63764	-1.65682	-1.65357	-1.67668	-1.67344	-1.69252	-1.68925	-1.68597	-1.70506
-1.63689	-1.6825	-1.67917	-1.67589	-1.67266	-1.69176	-1.68852	-1.68528	-1.70437
-1.63627	-1.68182	-1.67845	-1.67514	-1.67189	-1.69096	-1.68772	-1.68448	-1.70357
-1.63567	-1.68117	-1.67776	-1.67441	-1.67113	-1.69018	-1.68692	-1.68367	-1.70274
-1.65736	-1.68046	-1.67707	-1.69611	-1.69279	-1.68949	-1.68621	-1.68293	-1.70195
-1.65658	-1.67972	-1.67639	-1.69546	-1.69216	-1.68886	-1.68554	-1.68222	-1.70119
-1.65584	-1.7014	-1.69805	-1.69473	-1.69145	-1.71064	-1.70725	-1.70386	-1.70047
-1.65512	-1.70063	-1.69726	-1.69394	-1.69067	-1.70986	-1.70652	-1.70317	-1.6998
-1.68084	-1.69989	-1.69652	-1.69319	-1.68991	-1.7091	-1.70577	-1.70244	-1.6991
-1.68015	-1.69923	-1.69588	-1.69255	-1.68923	-1.70835	-1.70499	-1.70164	-1.6983
-1.67946	-1.69856	-1.69523	-1.6919	-1.68855	-1.70762	-1.70423	-1.70085	-1.69752
-1.70119	-1.69782	-1.69449	-1.69116	-1.68783	-1.70691	-1.70354	-1.70018	-1.69686
-1.70047	-1.69708	-1.69372	-1.6904	-1.6871	-1.7062	-1.70286	-1.69953	-1.69622
-1.67599	-1.69639	-1.69303	-1.6897	-1.68639	-1.70547	-1.70213	-1.6988	-1.69548
-1.67532	-1.69573	-1.69239	-1.68905	-1.6857	-1.70472	-1.70136	-1.69801	-1.6947
-1.67467	-1.69508	-1.69174	-1.68838	-1.68502	-1.70401	-1.70064	-1.69729	-1.69397
-1.67406	-1.69444	-1.69107	-1.6877	-1.68434	-1.70333	-1.69998	-1.69664	-1.69333
-1.6734	-1.69377	-1.69039	-1.68703	-1.68368	-1.70266	-1.69931	-1.69598	-1.69266
-1.67268	-1.69305	-1.6897	-1.68636	-1.68303	-1.70199	-1.69862	-1.69527	-1.69192

-1.67195	-1.69232	-1.68902	-1.6857	-1.68238	-1.70132	-1.69793	-1.69455	-1.69118
-1.67128	-1.69165	-1.68836	-1.68505	-1.68172	-1.70064	-1.69724	-1.69386	-1.71806
-1.67063	-1.69099	-1.6877	-1.68438	-1.68104	-1.69995	-1.69656	-1.69319	-1.71739
-1.66996	-1.69029	-1.68698	-1.70603	-1.70264	-1.69926	-1.69589	-1.69253	-1.75609
-1.66927	-1.68956	-1.68622	-1.70526	-1.70192	-1.69859	-1.69524	-1.69188	-1.75537
-1.66859	-1.68886	-1.68551	-1.70455	-1.70124	-1.69792	-1.69458	-1.69122	-1.75465
-1.66794	-1.68821	-1.68488	-1.70391	-1.7006	-1.69727	-1.6939	-1.69053	-1.7539
-1.66729	-1.68757	-1.68427	-1.70328	-1.69995	-1.72428	-1.72083	-1.71737	-1.75317
-1.66666	-1.68694	-1.68364	-1.70263	-1.69927	-1.72357	-1.72011	-1.71666	-1.75246
-1.66604	-1.68631	-1.68301	-1.70196	-1.69858	-1.72284	-1.7194	-1.71597	-1.75176
-1.66543	-1.68568	-1.68235	-1.70128	-1.69789	-1.72214	-1.71872	-1.7153	-1.75108
-1.66483	-1.68505	-1.68168	-1.70058	-1.69721	-1.72146	-1.71805	-1.71465	-1.7504
-1.66422	-1.68444	-1.68105	-1.69994	-1.69656	-1.72079	-1.71739	-1.71399	-1.7497
-1.66361	-1.68384	-1.68047	-1.69934	-1.69596	-1.72016	-1.71676	-1.71335	-1.74899
-1.66298	-1.70555	-1.70212	-1.69872	-1.69534	-1.71952	-1.71612	-1.7127	-1.77804
-1.6623	-1.70485	-1.70145	-1.69807	-1.69472	-1.7189	-1.71548	-1.71205	-1.77731
-1.66163	-1.70416	-1.70077	-1.69741	-1.69409	-1.71826	-1.71483	-1.71138	-1.77657
-1.68458	-1.70351	-1.70013	-1.72442	-1.72102	-1.7176	-1.71413	-1.71066	-1.77577
-1.68398	-1.70289	-1.6995	-1.72376	-1.72037	-1.71693	-1.71343	-1.70992	-1.77495
-1.68339	-1.70229	-1.69889	-1.72313	-1.71973	-1.75554	-1.75191	-1.74829	-1.7742
-1.6828	-1.70169	-1.69829	-1.72251	-1.71911	-1.75489	-1.75126	-1.74763	-1.77348
-1.70452	-1.70109	-1.69769	-1.72189	-1.71848	-1.75423	-1.7506	-1.74697	-1.77277
-1.70392	-1.70048	-1.69708	-1.72126	-1.71785	-1.75358	-1.74995	-1.74632	-1.77207
-1.7033	-1.69987	-1.69647	-1.72063	-1.71722	-1.78292	-1.77904	-1.77518	-1.81326
-1.70269	-1.69926	-1.69585	-1.72	-1.71658	-1.78221	-1.77833	-1.77447	-1.81252
-1.70207	-1.69864	-1.69523	-1.71936	-1.71594	-1.78149	-1.77761	-1.77376	-1.81178
-1.67827	-1.69801	-1.6946	-1.7187	-1.71528	-1.78076	-1.77689	-1.77303	-1.81103
-1.67764	-1.69737	-1.69396	-1.71805	-1.71463	-1.78002	-1.77616	-1.77231	-1.81028
-1.67704	-1.72435	-1.72086	-1.7174	-1.71398	-1.77931	-1.77545	-1.7716	-1.80954
-1.67644	-1.72372	-1.72021	-1.71676	-1.71335	-1.77862	-1.77476	-1.77091	-1.80881
-1.67588	-1.72313	-1.7196	-1.71614	-1.71273	-1.77793	-1.7741	-1.77026	-1.80812
-1.67536	-1.72259	-1.71904	-1.71556	-1.71212	-1.77725	-1.77347	-1.76967	-1.80749
-1.67483	-1.72204	-1.71848	-1.74569	-1.742	-1.81854	-1.81467	-1.81079	-1.80684
-1.67425	-1.72143	-1.71788	-1.74503	-1.74132	-1.8178	-1.81395	-1.81008	-1.80613
-1.67365	-1.7208	-1.71728	-1.74438	-1.74065	-1.81707	-1.81321	-1.80935	-1.8054
-1.67304	-1.72019	-1.7167	-1.74377	-1.74002	-1.81637	-1.8125	-1.80862	-1.82801
-1.67243	-1.71958	-1.71614	-1.74318	-1.73941	-1.81568	-1.81179	-1.8079	-1.82728
-1.69973	-1.71898	-1.71554	-1.74255	-1.73878	-1.81498	-1.81107	-1.80717	-1.82656
-1.69916	-1.71838	-1.71492	-1.74187	-1.73812	-1.81427	-1.81034	-1.80643	-1.82585
-1.69858	-1.71777	-1.71429	-1.74121	-1.73748	-1.81359	-1.80964	-1.80572	-1.82516
-1.69797	-1.71715	-1.71366	-1.74054	-1.73688	-1.81297	-1.80901	-1.80506	-1.82446
-1.69738	-1.71654	-1.71304	-1.73989	-1.73627	-1.81235	-1.80838	-1.80442	-1.82378
-1.69683	-1.69337	-1.68993	-1.73926	-1.73561	-1.81166	-1.80774	-1.80382	-1.82315
-1.69631	-1.69284	-1.68939	-1.73864	-1.73495	-1.81095	-1.80709	-1.80322	-1.82254
-1.69577	-1.69231	-1.68887	-1.73806	-1.73433	-1.81027	-1.80641	-1.80254	-1.82188
-1.69524	-1.69179	-1.68836	-1.7375	-1.73375	-1.80961	-1.80571	-1.80183	-1.8212
-1.69471	-1.69126	-1.68783	-1.73693	-1.73317	-1.80897	-1.80504	-1.80114	-1.82053
-1.69419	-1.69075	-1.6873	-1.73634	-1.7326	-1.80836	-1.80441	-1.80049	-1.81984
-1.69364	-1.72105	-1.71735	-1.81563	-1.81169	-1.83116	-1.82714	-1.82314	-1.81917
-1.69303	-1.72043	-1.71679	-1.81504	-1.81107	-1.83052	-1.82651	-1.82251	-1.81854

-1.69241	-1.71979	-1.71621	-1.81443	-1.81046	-1.82989	-1.82589	-1.8219	-1.81793
-1.69185	-1.71917	-1.71558	-1.81377	-1.80983	-1.82928	-1.82527	-1.82127	-1.8173
-1.69131	-1.71855	-1.71493	-1.81309	-1.8092	-1.82867	-1.82465	-1.82064	-1.81668
-1.69078	-1.71798	-1.71436	-1.81248	-1.80859	-1.82805	-1.82402	-1.82002	-1.81607
-1.69028	-1.71746	-1.71386	-1.81192	-1.80798	-1.82741	-1.8234	-1.81941	-1.81545
-1.68976	-1.7169	-1.7133	-1.81131	-1.80737	-1.82678	-1.82278	-1.81879	-1.81484
-1.68921	-1.71628	-1.71267	-1.81065	-1.80675	-1.82617	-1.82216	-1.81816	-1.81422
-1.6887	-1.71569	-1.71206	-1.78911	-1.78529	-1.82556	-1.82154	-1.81754	-1.8136
-1.68826	-1.7152	-1.71154	-1.78853	-1.78468	-1.82493	-1.82092	-1.81693	-1.81299
-1.68784	-1.71472	-1.71103	-1.78795	-1.78408	-1.8243	-1.8203	-1.81632	-1.81239
-1.68736	-1.71418	-1.71048	-1.78735	-1.78347	-1.82368	-1.81969	-1.81572	-1.81179
-1.68684	-1.71362	-1.70991	-1.78674	-1.78286	-1.82305	-1.81908	-1.81512	-1.81121
-1.71677	-1.75648	-1.75267	-1.78614	-1.78225	-1.82243	-1.81847	-1.81454	-1.81065
-1.7162	-1.7559	-1.7521	-1.78555	-1.78164	-1.82181	-1.81786	-1.81396	-1.8101
-1.71564	-1.75534	-1.75156	-1.8087	-1.80472	-1.82123	-1.81729	-1.8134	-1.80955
-1.71509	-1.7548	-1.75104	-1.80819	-1.80421	-1.82072	-1.81677	-1.81286	-1.80899
-1.71456	-1.75426	-1.75052	-1.80766	-1.80369	-1.8202	-1.81624	-1.81231	-1.80842
-1.71407	-1.75373	-1.74997	-1.80707	-1.80312	-1.81963	-1.81566	-1.81173	-1.80783
-1.71359	-1.7532	-1.74941	-1.80647	-1.80253	-1.81904	-1.81508	-1.81114	-1.80725
-1.71312	-1.7527	-1.7489	-1.80593	-1.80199	-1.8185	-1.81454	-1.81061	-1.80671
-1.71265	-1.75222	-1.74842	-1.80543	-1.80147	-1.81798	-1.81403	-1.81011	-1.80621
-1.71213	-1.7517	-1.7479	-1.80489	-1.80092	-1.81742	-1.81349	-1.80957	-1.80566
-1.71157	-1.75112	-1.74734	-1.8043	-1.80034	-1.81683	-1.8129	-1.80899	-1.80507
-1.75437	-1.75058	-1.74681	-1.80375	-1.79978	-1.79585	-1.79198	-1.78813	-1.80452
-1.7539	-1.7501	-1.74632	-1.80324	-1.79929	-1.79536	-1.79149	-1.78763	-1.80401
-1.75344	-1.74963	-1.74585	-1.80275	-1.79881	-1.79489	-1.791	-1.78714	-1.80352
-1.75297	-1.74915	-1.74536	-1.80225	-1.79832	-1.7944	-1.79052	-1.78665	-1.80303
-1.75249	-1.74867	-1.74488	-1.80175	-1.79782	-1.79391	-1.79002	-1.78615	-1.80253
-1.75196	-1.74815	-1.74434	-1.80119	-1.79727	-1.79336	-1.78947	-1.7856	-1.84152
-1.7514	-1.74759	-1.74377	-1.80059	-1.79669	-1.79279	-1.78889	-1.78501	-1.84092
-1.75088	-1.74706	-1.74325	-1.80004	-1.79614	-1.79224	-1.78834	-1.78446	-1.84036
-1.75039	-1.74658	-1.74277	-1.79955	-1.79563	-1.79173	-1.78784	-1.78396	-1.83985
-1.74992	-1.74611	-1.74231	-1.79906	-1.79514	-1.79124	-1.78735	-1.78348	-1.83935
-1.74944	-1.74564	-1.74184	-1.79858	-1.79466	-1.79075	-1.78686	-1.78299	-1.83886
-1.74898	-1.74518	-1.74138	-1.7981	-1.79418	-1.79028	-1.78639	-1.78251	-1.83837
-1.74852	-1.74472	-1.74093	-1.79763	-1.7937	-1.78979	-1.7859	-1.78203	-1.83787
-1.74807	-1.74428	-1.74049	-1.79716	-1.79322	-1.7893	-1.78541	-1.78154	-1.83737
-1.74766	-1.74387	-1.7401	-1.79675	-1.79279	-1.78886	-1.78495	-1.78107	-1.81722
-1.74729	-1.74351	-1.73974	-1.79638	-1.79241	-1.78846	-1.78451	-1.7806	-1.81673
-1.7469	-1.74311	-1.73935	-1.79597	-1.792	-1.78805	-1.78409	-1.78016	-1.81629
-1.74646	-1.74267	-1.7389	-1.79551	-1.79156	-1.78762	-1.78369	-1.77979	-1.8159
-1.74601	-1.76646	-1.76261	-1.79504	-1.7911	-1.78718	-1.78328	-1.7794	-1.81551
-1.74555	-1.766	-1.76214	-1.79456	-1.79062	-1.7867	-1.78281	-1.77894	-1.81504
-1.74508	-1.76552	-1.76167	-1.79407	-1.79013	-1.78622	-1.78233	-1.77846	-1.81456
-1.74463	-1.76506	-1.7612	-1.79359	-1.78966	-1.78574	-1.78185	-1.77799	-1.81408
-1.74419	-1.7646	-1.76074	-1.79312	-1.78919	-1.78528	-1.78139	-1.77752	-1.81361
-1.72471	-1.76418	-1.76032	-1.79268	-1.78876	-1.78485	-1.78096	-1.77709	-1.81318
-1.72435	-1.76382	-1.75994	-1.7923	-1.78838	-1.78448	-1.78058	-1.77671	-1.81279
-1.72397	-1.76343	-1.75955	-1.79189	-1.78798	-1.78408	-1.78018	-1.77631	-1.81238
-1.72351	-1.76297	-1.75911	-1.79144	-1.78752	-1.78362	-1.77973	-1.77587	-1.81193

-1.72307	-1.76252	-1.75867	-1.79099	-1.78706	-1.78315	-1.77929	-1.77544	-1.81148
-1.72269	-1.74343	-1.73963	-1.79058	-1.78664	-1.78274	-1.77889	-1.77505	-1.81108
-1.72233	-1.74306	-1.73925	-1.79019	-1.78625	-1.78234	-1.7785	-1.77467	-1.81069
-1.72198	-1.7427	-1.73887	-1.78978	-1.78582	-1.78191	-1.77806	-1.77424	-1.81025
-1.72163	-1.74233	-1.7385	-1.78937	-1.78538	-1.78145	-1.7776	-1.77377	-1.80979
-1.72127	-1.74195	-1.7381	-1.77087	-1.76693	-1.78103	-1.77717	-1.77334	-1.80936
-1.72089	-1.74155	-1.73768	-1.77045	-1.76654	-1.78065	-1.77679	-1.77297	-1.80899
-1.78192	-1.77794	-1.77397	-1.79005	-1.78611	-1.82044	-1.81648	-1.81254	-1.82543
-1.78149	-1.77752	-1.77358	-1.78967	-1.78574	-1.82006	-1.81612	-1.81219	-1.82507
-1.78106	-1.77711	-1.7732	-1.78931	-1.78538	-1.8197	-1.81576	-1.81184	-1.82472
-1.78067	-1.77673	-1.77283	-1.78895	-1.78502	-1.8019	-1.798	-1.79412	-1.82436
-1.7803	-1.77637	-1.77246	-1.78859	-1.78468	-1.80155	-1.79764	-1.79376	-1.824
-1.77993	-1.77601	-1.77211	-1.78824	-1.78432	-1.80119	-1.79729	-1.7934	-1.82364
-1.77957	-1.77566	-1.77177	-1.78789	-1.78397	-1.80083	-1.79693	-1.79305	-1.82327
-1.77921	-1.77531	-1.77142	-1.78754	-1.78362	-1.80048	-1.79658	-1.7927	-1.80584
-1.77887	-1.77496	-1.77106	-1.78718	-1.78328	-1.80013	-1.79622	-1.79233	-1.80547
-1.77852	-1.77461	-1.77071	-1.78683	-1.78293	-1.79978	-1.79587	-1.79197	-1.80511
-1.77815	-1.77424	-1.77036	-1.78648	-1.78258	-1.79942	-1.79551	-1.79161	-1.80474
-1.7778	-1.77388	-1.77001	-1.78614	-1.78223	-1.79906	-1.79515	-1.79126	-1.80437
-1.77751	-1.77357	-1.76967	-1.78579	-1.78189	-1.79872	-1.79479	-1.79089	-1.804
-1.77726	-1.77329	-1.76934	-1.78544	-1.78156	-1.79839	-1.79444	-1.79051	-1.80362
-1.77697	-1.77301	-1.76905	-1.78514	-1.78127	-1.79809	-1.79414	-1.7902	-1.80328
-1.77666	-1.77273	-1.7688	-1.78491	-1.78102	-1.79783	-1.79389	-1.78994	-1.80298
-1.77633	-1.77243	-1.76853	-1.78464	-1.78074	-1.81469	-1.81071	-1.80672	-1.80267
-1.77598	-1.77208	-1.76822	-1.78433	-1.78041	-1.81434	-1.81037	-1.80639	-1.80237
-1.77565	-1.77175	-1.76791	-1.78402	-1.78008	-1.814	-1.81002	-1.80605	-1.80206
-1.77539	-1.77149	-1.76764	-1.78375	-1.77982	-1.81372	-1.8097	-1.80569	-1.80172
-1.77514	-1.77125	-1.76738	-1.78349	-1.77957	-1.81346	-1.80938	-1.80533	-1.80137
-1.77485	-1.77095	-1.76707	-1.78318	-1.77927	-1.81316	-1.80909	-1.80504	-1.8128
-1.77453	-1.77063	-1.76673	-1.78284	-1.77894	-1.81284	-1.80881	-1.8048	-1.81259
-1.77424	-1.77034	-1.76643	-1.78252	-1.77861	-1.8125	-1.8085	-1.80452	-1.81234
-1.774	-1.77009	-1.76618	-1.78224	-1.77827	-1.81213	-1.80815	-1.80419	-1.81205
-1.77376	-1.76986	-1.76594	-1.78198	-1.77797	-1.81181	-1.80783	-1.80388	-1.81178
-1.77353	-1.76963	-1.76572	-1.78176	-1.77774	-1.81155	-1.80757	-1.80362	-1.81156
-1.77328	-1.76939	-1.76549	-1.78154	-1.77752	-1.81132	-1.80734	-1.80338	-1.81135
-1.72894	-1.76908	-1.76521	-1.78127	-1.77728	-1.8111	-1.80711	-1.80315	-1.81116
-1.72861	-1.76875	-1.7649	-1.78099	-1.77704	-1.81088	-1.80689	-1.80292	-1.81097
-1.72834	-1.78964	-1.78573	-1.8308	-1.82674	-1.82271	-1.81871	-1.81473	-1.81077
-1.7281	-1.7894	-1.78549	-1.8306	-1.82654	-1.8225	-1.8185	-1.81452	-1.81057
-1.72788	-1.78918	-1.78526	-1.8304	-1.82633	-1.82229	-1.81827	-1.81427	-1.81033
-1.72765	-1.78896	-1.78504	-1.83021	-1.82613	-1.82207	-1.818	-1.81397	-1.81004
-1.72743	-1.8014	-1.79747	-1.83002	-1.82594	-1.82185	-1.81775	-1.81369	-1.8332
-1.7272	-1.8012	-1.79728	-1.82982	-1.82574	-1.82165	-1.81754	-1.81348	-1.83297
-1.72697	-1.80101	-1.79708	-1.82963	-1.82555	-1.82146	-1.81735	-1.81329	-1.83277
-1.72673	-1.80081	-1.79689	-1.82943	-1.82536	-1.82128	-1.81718	-1.81312	-1.83258
-1.72649	-1.80061	-1.79669	-1.82923	-1.82517	-1.82111	-1.81701	-1.81296	-1.8324
-1.72627	-1.75752	-1.7537	-1.82904	-1.82499	-1.82094	-1.81688	-1.81284	-1.83227
-1.72605	-1.75732	-1.75349	-1.82884	-1.8248	-1.82078	-1.81676	-1.81277	-1.83218
-1.72587	-1.75717	-1.75332	-1.82866	-1.82462	-1.8442	-1.84015	-1.83611	-1.83207
-1.72574	-1.75706	-1.75319	-1.82851	-1.82444	-1.84399	-1.83995	-1.83593	-1.8319

-1.72559	-1.75695	-1.75307	-1.82837	-1.82427	-1.84379	-1.83975	-1.83574	-1.83172
-1.72538	-1.75678	-1.75291	-1.82821	-1.8241	-1.84361	-1.83956	-1.83554	-1.83152
-1.72516	-1.75659	-1.75273	-1.82805	-1.82394	-1.84344	-1.83938	-1.83535	-1.83133
-1.73878	-1.77432	-1.7704	-1.78626	-1.78231	-1.84332	-1.83925	-1.83521	-1.83118
-1.73866	-1.77417	-1.77022	-1.78609	-1.78219	-1.84324	-1.83917	-1.83511	-1.83105
-1.73852	-1.77401	-1.77005	-1.78592	-1.78204	-1.8023	-1.79835	-1.79441	-1.83093
-1.73835	-1.77384	-1.76987	-1.78575	-1.78186	-1.80212	-1.7982	-1.79428	-1.83083
-1.73819	-1.77369	-1.76972	-1.80985	-1.80589	-1.80196	-1.79805	-1.79415	-1.83073
-1.73804	-1.77355	-1.76961	-1.80974	-1.80579	-1.80185	-1.79795	-1.79405	-1.83064
-1.73791	-1.77342	-1.76951	-1.80965	-1.80569	-1.80175	-1.79785	-1.79394	-1.83055
-1.7378	-1.77332	-1.76942	-1.80955	-1.80556	-1.8016	-1.79768	-1.79378	-1.8304
-1.73772	-1.77324	-1.76934	-1.80944	-1.80542	-1.80144	-1.79751	-1.79359	-1.83024
-1.73763	-1.77314	-1.76923	-1.78984	-1.78587	-1.78195	-1.77806	-1.7742	-1.83013
-1.73754	-1.77304	-1.7691	-1.78971	-1.78576	-1.78185	-1.77799	-1.77413	-1.83005
-1.73744	-1.79758	-1.79356	-1.78959	-1.78567	-1.78177	-1.77791	-1.77405	-1.82997
-1.73732	-1.79745	-1.79346	-1.78951	-1.7856	-1.78171	-1.77783	-1.77396	-1.82989
-1.73722	-1.79735	-1.79338	-1.78944	-1.78554	-1.78164	-1.77775	-1.77387	-1.78921
-1.73721	-1.79732	-1.79332	-1.78937	-1.78546	-1.78155	-1.77766	-1.77376	-1.78909
-1.73721	-1.79729	-1.79327	-1.7893	-1.78537	-1.78146	-1.77756	-1.77365	-1.78896
-1.73713	-1.79721	-1.7932	-1.78923	-1.7853	-1.78138	-1.77744	-1.77352	-1.78883
-1.73701	-1.79709	-1.79312	-1.78916	-1.78524	-1.7813	-1.77732	-1.77337	-1.7887
-1.76224	-1.79703	-1.79305	-1.78909	-1.78516	-1.78122	-1.77725	-1.77329	-1.82847
-1.76223	-1.797	-1.79299	-1.78902	-1.78508	-1.78115	-1.77721	-1.77328	-1.82844
-1.76218	-1.79694	-1.79292	-1.78894	-1.78501	-1.78108	-1.77715	-1.77323	-1.82838
-1.76205	-1.79682	-1.79282	-1.78886	-1.78493	-1.78099	-1.77703	-1.77308	-1.82825
-1.76194	-1.79671	-1.79273	-1.78878	-1.78485	-1.7809	-1.77692	-1.77296	-1.82814
-1.76192	-1.79667	-1.79266	-1.78869	-1.78477	-1.78084	-1.77689	-1.77294	-1.80939
-1.76194	-1.79666	-1.7926	-1.78862	-1.7847	-1.78079	-1.77687	-1.77295	-1.80937
-1.76191	-1.79665	-1.7926	-1.7886	-1.78465	-1.78072	-1.77682	-1.77292	-1.80936
-1.76186	-1.79664	-1.79263	-1.78862	-1.78461	-1.78064	-1.77675	-1.77287	-1.80936
-1.7618	-1.79659	-1.79262	-1.78862	-1.78459	-1.78061	-1.7767	-1.77281	-1.80931
-1.76172	-1.7965	-1.79256	-1.78859	-1.78461	-1.78064	-1.77667	-1.77273	-1.80923
-1.77956	-1.79644	-1.7925	-1.78855	-1.78461	-1.82134	-1.81723	-1.81316	-1.82995
-1.77959	-1.79643	-1.79244	-1.78848	-1.78455	-1.82131	-1.81724	-1.81318	-1.82993
-1.77962	-1.79644	-1.79239	-1.78841	-1.78449	-1.82126	-1.81724	-1.81321	-1.82993
-1.77961	-1.79645	-1.7924	-1.7884	-1.78444	-1.8212	-1.8172	-1.8132	-1.82993
-1.77959	-1.79646	-1.79244	-1.78843	-1.78442	-1.82114	-1.81715	-1.81318	-1.82993
-1.7796	-1.79648	-1.79249	-1.78847	-1.78443	-1.82113	-1.81715	-1.81318	-1.82995
-1.77962	-1.79652	-1.79255	-1.78854	-1.78448	-1.82117	-1.81717	-1.81319	-1.82998
-1.77962	-1.79653	-1.79258	-1.78858	-1.78452	-1.82121	-1.81718	-1.81318	-1.82997
-1.77959	-1.79649	-1.79256	-1.78857	-1.78455	-1.82124	-1.81717	-1.81313	-1.82992
-1.74343	-1.76097	-1.75713	-1.78857	-1.78458	-1.82127	-1.81717	-1.8131	-1.82988
-1.74345	-1.76098	-1.75714	-1.78859	-1.78462	-1.82133	-1.81722	-1.81314	-1.82991
-1.7435	-1.76101	-1.75715	-1.78861	-1.78466	-1.82139	-1.81727	-1.81319	-1.82996
-1.74353	-1.76102	-1.75713	-1.78858	-1.78464	-1.82139	-1.8173	-1.81323	-1.83001
-1.74357	-1.76104	-1.75711	-1.78853	-1.7846	-1.82136	-1.81731	-1.81328	-1.83005
-1.74362	-1.76108	-1.75712	-1.82942	-1.8254	-1.84228	-1.83819	-1.83412	-1.83006
-1.74368	-1.76114	-1.75717	-1.82947	-1.82544	-1.84232	-1.83823	-1.83415	-1.83006
-1.74373	-1.76119	-1.75723	-1.82953	-1.82549	-1.84236	-1.83827	-1.83419	-1.83007
-1.74379	-1.76125	-1.75728	-1.82959	-1.82555	-1.84242	-1.83833	-1.83424	-1.83012

-1.74384	-1.7613	-1.75733	-1.82964	-1.8256	-1.84247	-1.83838	-1.83429	-1.83018
-1.74388	-1.76134	-1.75737	-1.82969	-1.82565	-1.84252	-1.83844	-1.83435	-1.83024
-1.74393	-1.76138	-1.75741	-1.82973	-1.8257	-1.84257	-1.8385	-1.83442	-1.83032
-1.74398	-1.76144	-1.75747	-1.82979	-1.82574	-1.84262	-1.83856	-1.8345	-1.83043
-1.74404	-1.76151	-1.75755	-1.82986	-1.8258	-1.84267	-1.83862	-1.83458	-1.83055
-1.72003	-1.76158	-1.75765	-1.82998	-1.8259	-1.84276	-1.83869	-1.83465	-1.83064
-1.7201	-1.76167	-1.75778	-1.83014	-1.82605	-1.84289	-1.83878	-1.8347	-1.8307
-1.72017	-1.76176	-1.75789	-1.83028	-1.82618	-1.843	-1.83887	-1.83477	-1.83075
-1.72023	-1.76182	-1.75795	-1.83035	-1.82627	-1.84309	-1.83895	-1.83484	-1.83081
-1.72032	-1.76189	-1.75801	-1.83041	-1.82634	-1.84317	-1.83904	-1.83493	-1.83089
-1.72044	-1.80423	-1.80023	-1.85152	-1.84737	-1.84325	-1.83915	-1.83508	-1.83104
-1.72058	-1.80439	-1.80037	-1.85163	-1.84745	-1.84332	-1.83927	-1.83524	-1.83121
-1.72066	-1.80449	-1.80047	-1.85173	-1.84753	-1.8434	-1.83935	-1.83534	-1.83132
-1.72071	-1.80454	-1.80054	-1.85181	-1.84761	-1.84347	-1.83942	-1.83539	-1.83139
-1.72079	-1.80463	-1.80063	-1.8519	-1.84771	-1.84358	-1.83952	-1.83549	-1.83147
-1.72093	-1.80476	-1.80073	-1.852	-1.84785	-1.84374	-1.83968	-1.83563	-1.83159
-1.72107	-1.80489	-1.80084	-1.85212	-1.848	-1.8439	-1.83983	-1.83578	-1.83171
-1.72118	-1.80502	-1.80099	-1.85228	-1.84816	-1.84405	-1.83997	-1.83589	-1.83184
-1.72129	-1.80515	-1.80114	-1.85244	-1.84832	-1.8442	-1.8401	-1.83601	-1.83197
-1.72142	-1.7845	-1.78057	-1.83273	-1.82865	-1.84433	-1.84023	-1.83615	-1.83212
-1.72155	-1.78466	-1.78073	-1.83288	-1.82878	-1.84445	-1.84036	-1.83631	-1.83228
-1.72169	-1.7848	-1.78086	-1.83302	-1.82891	-1.84459	-1.84051	-1.83647	-1.83243
-1.72183	-1.78494	-1.78098	-1.83313	-1.82905	-1.84474	-1.84068	-1.83662	-1.83256
-1.72196	-1.78507	-1.78111	-1.83327	-1.82921	-1.84491	-1.84083	-1.83677	-1.8327
-1.72206	-1.78518	-1.78125	-1.83345	-1.82939	-1.84509	-1.84097	-1.83687	-1.83282
-1.7658	-1.80724	-1.80328	-1.83363	-1.82958	-1.84528	-1.84113	-1.83701	-1.83297
-1.76603	-1.80746	-1.80347	-1.83381	-1.82977	-1.84548	-1.84135	-1.83725	-1.83319
-1.76626	-1.8077	-1.80367	-1.834	-1.82996	-1.84568	-1.84159	-1.83751	-1.83343
-1.74701	-1.80786	-1.80385	-1.83419	-1.83015	-1.84586	-1.84176	-1.83767	-1.83359
-1.74712	-1.80799	-1.80401	-1.83439	-1.83034	-1.84604	-1.8419	-1.83778	-1.83373
-1.74729	-1.80816	-1.80419	-1.83457	-1.83054	-1.84624	-1.84209	-1.83798	-1.83392
-1.74752	-1.80839	-1.80439	-1.83476	-1.83073	-1.84646	-1.84236	-1.83826	-1.83417
-1.74772	-1.78979	-1.78581	-1.83494	-1.83093	-1.84667	-1.8426	-1.83853	-1.83442
-1.74783	-1.78992	-1.78597	-1.83513	-1.83112	-1.84687	-1.84281	-1.83876	-1.83468
-1.74796	-1.79006	-1.78614	-1.83532	-1.83131	-1.84707	-1.84301	-1.83896	-1.83493
-1.74819	-1.76686	-1.76297	-1.78241	-1.77854	-1.84726	-1.8432	-1.83916	-1.83514
-1.74846	-1.7671	-1.76317	-1.78258	-1.77871	-1.84744	-1.84339	-1.83935	-1.83533
-1.74868	-1.76734	-1.76341	-1.78282	-1.77892	-1.84764	-1.84359	-1.83955	-1.83553
-1.74888	-1.76758	-1.76369	-1.7831	-1.77914	-1.84783	-1.84379	-1.83977	-1.83572
-1.77165	-1.76779	-1.76394	-1.78336	-1.77939	-1.83016	-1.82615	-1.82216	-1.83596
-1.77183	-1.76795	-1.76413	-1.78359	-1.77967	-1.83047	-1.82645	-1.82244	-1.83626
-1.77203	-1.76814	-1.76432	-1.7838	-1.77992	-1.83077	-1.82673	-1.82271	-1.83654
-1.77229	-1.76838	-1.76451	-1.78399	-1.78013	-1.831	-1.82696	-1.82292	-1.83675
-1.77258	-1.76864	-1.76472	-1.78419	-1.78034	-1.83122	-1.82717	-1.82313	-1.83695
-1.77288	-1.76892	-1.76498	-1.78444	-1.78059	-1.83149	-1.82745	-1.8234	-1.85943
-1.77318	-1.76923	-1.76527	-1.78472	-1.78086	-1.83176	-1.82773	-1.82369	-1.85968
-1.77344	-1.7695	-1.76555	-1.785	-1.7811	-1.832	-1.82798	-1.82395	-1.85992
-1.77366	-1.76974	-1.76582	-1.78527	-1.78133	-1.8322	-1.82818	-1.82417	-1.86018
-1.77389	-1.76999	-1.7661	-1.78555	-1.78158	-1.81237	-1.80842	-1.80447	-1.86044
-1.77415	-1.77027	-1.7664	-1.78585	-1.78187	-1.81266	-1.80871	-1.80476	-1.86073

-1.7744	-1.77052	-1.76667	-1.78614	-1.78216	-1.85394	-1.84985	-1.84577	-1.86103
-1.77461	-1.77073	-1.76689	-1.78638	-1.78243	-1.85423	-1.85014	-1.84606	-1.86132
-1.77482	-1.77093	-1.76709	-1.78661	-1.7827	-1.85453	-1.85042	-1.84633	-1.8616
-1.77509	-1.77119	-1.76735	-1.78687	-1.78297	-1.85481	-1.85067	-1.84654	-1.86182
-1.77539	-1.77148	-1.76763	-1.78715	-1.78326	-1.85509	-1.85089	-1.84673	-1.86201
-1.7757	-1.77178	-1.76792	-1.78745	-1.78355	-1.85538	-1.85116	-1.84696	-1.86224
-1.776	-1.77208	-1.76822	-1.78775	-1.78385	-1.85568	-1.85145	-1.84725	-1.86253
-1.77631	-1.77239	-1.76852	-1.78805	-1.78415	-1.85599	-1.85176	-1.84756	-1.86284
-1.77662	-1.77269	-1.76882	-1.78835	-1.78446	-1.85631	-1.85208	-1.84788	-1.86316
-1.77692	-1.77299	-1.76912	-1.78866	-1.78476	-1.85663	-1.85242	-1.84823	-1.8635
-1.77722	-1.77329	-1.76941	-1.83126	-1.82723	-1.85697	-1.8528	-1.84863	-1.8639
-1.77752	-1.77359	-1.76971	-1.83157	-1.82754	-1.8573	-1.85318	-1.84905	-1.86432
-1.77784	-1.77389	-1.77001	-1.83186	-1.82783	-1.85762	-1.85351	-1.8494	-1.86469
-1.77816	-1.77421	-1.7703	-1.83215	-1.82813	-1.85792	-1.85382	-1.84971	-1.86501
-1.77852	-1.77456	-1.77064	-1.80966	-1.80568	-1.8244	-1.82038	-1.81638	-1.86532
-1.77892	-1.77497	-1.77103	-1.81002	-1.80599	-1.82469	-1.82067	-1.81667	-1.86563
-1.7793	-1.81854	-1.81444	-1.81036	-1.8063	-1.82498	-1.82096	-1.81697	-1.86595
-1.77961	-1.81886	-1.81476	-1.81069	-1.80662	-1.82529	-1.82126	-1.81726	-1.86626
-1.7799	-1.81916	-1.81508	-1.81101	-1.80694	-1.82562	-1.82159	-1.81758	-1.8666
-1.7802	-1.81947	-1.8154	-1.81135	-1.80731	-1.826	-1.82197	-1.81796	-1.867
-1.78051	-1.81978	-1.81574	-1.81171	-1.80769	-1.82641	-1.82238	-1.81837	-1.86742
-1.78086	-1.82014	-1.81611	-1.81209	-1.80808	-1.82681	-1.82276	-1.81873	-1.86779
-1.78127	-1.82055	-1.81652	-1.8125	-1.80848	-1.8272	-1.82312	-1.81906	-1.86811
-1.78165	-1.82092	-1.81689	-1.81286	-1.80885	-1.82758	-1.82349	-1.81942	-1.86847
-1.78199	-1.82124	-1.81719	-1.81317	-1.80918	-1.82793	-1.82387	-1.81981	-1.86888
-1.82568	-1.82157	-1.81751	-1.81349	-1.80952	-1.8283	-1.82425	-1.82021	-1.86929
-1.82607	-1.82196	-1.81789	-1.81387	-1.8099	-1.82869	-1.82465	-1.82061	-1.86969
-1.82647	-1.82236	-1.81828	-1.81426	-1.81029	-1.82908	-1.82504	-1.821	-1.87008
-1.80914	-1.82274	-1.81864	-1.8146	-1.81061	-1.80664	-1.80268	-1.79872	-1.85097
-1.80954	-1.82312	-1.81899	-1.81492	-1.81091	-1.80694	-1.803	-1.79906	-1.85135
-1.80991	-1.8235	-1.81937	-1.81529	-1.81127	-1.80729	-1.80336	-1.79943	-1.85175
-1.81027	-1.82387	-1.81977	-1.81571	-1.81168	-1.8077	-1.80375	-1.79982	-1.85218
-1.81065	-1.82426	-1.82019	-1.81613	-1.8121	-1.80811	-1.80416	-1.80023	-1.85261
-1.81105	-1.82468	-1.82061	-1.81656	-1.81253	-1.80853	-1.80458	-1.80064	-1.85304
-1.81147	-1.82511	-1.82104	-1.81698	-1.81295	-1.80895	-1.805	-1.80106	-1.85348
-1.81189	-1.82554	-1.82147	-1.81741	-1.81337	-1.80937	-1.80542	-1.80148	-1.82016
-1.81232	-1.82598	-1.82191	-1.81785	-1.8138	-1.80979	-1.80583	-1.8019	-1.82058
-1.81275	-1.82641	-1.82234	-1.81827	-1.81422	-1.81021	-1.80625	-1.80231	-1.82101
-1.81319	-1.82685	-1.82277	-1.8187	-1.81465	-1.81063	-1.80667	-1.80273	-1.82143
-1.81361	-1.82727	-1.82319	-1.81913	-1.81507	-1.81105	-1.80709	-1.80314	-1.82184
-1.81402	-1.82767	-1.82361	-1.81955	-1.8155	-1.81148	-1.80751	-1.80356	-1.82226
-1.81447	-1.82811	-1.82404	-1.81998	-1.81592	-1.81191	-1.80793	-1.80397	-1.82268
-1.81501	-1.82861	-1.82447	-1.82038	-1.81634	-1.81233	-1.80835	-1.80438	-1.8231
-1.81555	-1.82912	-1.82492	-1.8208	-1.81677	-1.81276	-1.80877	-1.8048	-1.82352
-1.79271	-1.81189	-1.80775	-1.8213	-1.81722	-1.81319	-1.8092	-1.80523	-1.80126
-1.79317	-1.81237	-1.80828	-1.82183	-1.81769	-1.81362	-1.80964	-1.80568	-1.8017
-1.79361	-1.81282	-1.80876	-1.82232	-1.81819	-1.81412	-1.81013	-1.80616	-1.80218
-1.79403	-1.81324	-1.80919	-1.82279	-1.81871	-1.81467	-1.81066	-1.80668	-1.8027
-1.79448	-1.79044	-1.78643	-1.82324	-1.81921	-1.81518	-1.81116	-1.80716	-1.8032
-1.79494	-1.7909	-1.78687	-1.82367	-1.81965	-1.81564	-1.81161	-1.80761	-1.80364

-1.79543	-1.79137	-1.78733	-1.80127	-1.79728	-1.8161	-1.81208	-1.80807	-1.80411
-1.79596	-1.7919	-1.78786	-1.80181	-1.79781	-1.8166	-1.81259	-1.8086	-1.80462
-1.79649	-1.79244	-1.78841	-1.80235	-1.79833	-1.81711	-1.81312	-1.80913	-1.80514
-1.79698	-1.79292	-1.7889	-1.80284	-1.79881	-1.81757	-1.81358	-1.8096	-1.80561
-1.79745	-1.79337	-1.78936	-1.8033	-1.79927	-1.81801	-1.81402	-1.81004	-1.80604
-1.79797	-1.79389	-1.78988	-1.80382	-1.79978	-1.79577	-1.7918	-1.78783	-1.80653
-1.79854	-1.79447	-1.79044	-1.80438	-1.80034	-1.79633	-1.79234	-1.78837	-1.80705
-1.79912	-1.79505	-1.79101	-1.80494	-1.8009	-1.79689	-1.79289	-1.78891	-1.78495
-1.79971	-1.79563	-1.79159	-1.80552	-1.80147	-1.79744	-1.79343	-1.78945	-1.78549
-1.80029	-1.79621	-1.79216	-1.80609	-1.80203	-1.79799	-1.79396	-1.78997	-1.78601
-1.80086	-1.79678	-1.79272	-1.80664	-1.80259	-1.79853	-1.79446	-1.79043	-1.78647
-1.80142	-1.79733	-1.79325	-1.80719	-1.80313	-1.79907	-1.79496	-1.79088	-1.78691
-1.75874	-1.79783	-1.79375	-1.80768	-1.80364	-1.79958	-1.79547	-1.79141	-1.78743
-1.75921	-1.7983	-1.79421	-1.80815	-1.80411	-1.80007	-1.79601	-1.79197	-1.78799
-1.75971	-1.79882	-1.79472	-1.80866	-1.80462	-1.80059	-1.79655	-1.79254	-1.78855
-1.76026	-1.79938	-1.79529	-1.80923	-1.80518	-1.80114	-1.79711	-1.7931	-1.78911
-1.76082	-1.79995	-1.79587	-1.80981	-1.80575	-1.8017	-1.79768	-1.79367	-1.78968
-1.76138	-1.80052	-1.79644	-1.81038	-1.80632	-1.80227	-1.79824	-1.79422	-1.79023
-1.76195	-1.8011	-1.79702	-1.81096	-1.80689	-1.80284	-1.7988	-1.79479	-1.79079
-1.76252	-1.77913	-1.77511	-1.81155	-1.80747	-1.80341	-1.79938	-1.79536	-1.79135
-1.7631	-1.77973	-1.77571	-1.81215	-1.80805	-1.80399	-1.79996	-1.79595	-1.79194
-1.76374	-1.78037	-1.77635	-1.81279	-1.80869	-1.80462	-1.8006	-1.79659	-1.79257
-1.76441	-1.78105	-1.77704	-1.81348	-1.80936	-1.80529	-1.80127	-1.79726	-1.79324
-1.73731	-1.76107	-1.75712	-1.77366	-1.76965	-1.80591	-1.80189	-1.79789	-1.79387
-1.73787	-1.76164	-1.75768	-1.77423	-1.77021	-1.80648	-1.80246	-1.79845	-1.79443
-1.73842	-1.76221	-1.75824	-1.77479	-1.77077	-1.80705	-1.80302	-1.79901	-1.79499
-1.73897	-1.76279	-1.75881	-1.77536	-1.77134	-1.80762	-1.80358	-1.79956	-1.79556
-1.73954	-1.76338	-1.75939	-1.77594	-1.77192	-1.8082	-1.80416	-1.80014	-1.79614
-1.74016	-1.76404	-1.76003	-1.77658	-1.77256	-1.7868	-1.78281	-1.77884	-1.79677
-1.74082	-1.76473	-1.76069	-1.77725	-1.77324	-1.78748	-1.78347	-1.7795	-1.79744
-1.74142	-1.76536	-1.76132	-1.77789	-1.77386	-1.7881	-1.78409	-1.78012	-1.79806
-1.74198	-1.76595	-1.76193	-1.7785	-1.77444	-1.78867	-1.78467	-1.78071	-1.79864
-1.70042	-1.76655	-1.76255	-1.77913	-1.77506	-1.78928	-1.78529	-1.78133	-1.77738
-1.70095	-1.76716	-1.76321	-1.77982	-1.77572	-1.78995	-1.78596	-1.78201	-1.77805
-1.7015	-1.76778	-1.76386	-1.78048	-1.77639	-1.79061	-1.78663	-1.78267	-1.77873
-1.70209	-1.72699	-1.72309	-1.78108	-1.77699	-1.79123	-1.78722	-1.78326	-1.77935
-1.70271	-1.7276	-1.72366	-1.78165	-1.7776	-1.79184	-1.78781	-1.78383	-1.77996
-1.70334	-1.72825	-1.72429	-1.7823	-1.77826	-1.77424	-1.77025	-1.76631	-1.78058
-1.70398	-1.72894	-1.72496	-1.783	-1.77896	-1.77494	-1.77093	-1.76697	-1.78119
-1.70461	-1.70087	-1.69714	-1.72169	-1.71778	-1.77563	-1.77162	-1.76764	-1.78184
-1.70522	-1.7015	-1.69779	-1.72238	-1.71845	-1.77633	-1.77231	-1.76834	-1.78253
-1.70582	-1.7021	-1.6984	-1.72304	-1.71912	-1.73633	-1.73238	-1.72847	-1.78323
-1.70638	-1.70266	-1.69895	-1.72364	-1.71977	-1.73702	-1.73307	-1.72914	-1.78393
-1.70695	-1.70322	-1.69951	-1.72425	-1.72042	-1.73772	-1.73375	-1.72981	-1.78462
-1.68611	-1.70386	-1.70018	-1.72496	-1.7211	-1.73839	-1.73443	-1.7305	-1.74531
-1.68674	-1.70453	-1.70088	-1.72569	-1.72179	-1.73905	-1.73511	-1.73119	-1.74599
-1.68737	-1.68373	-1.68012	-1.72635	-1.72245	-1.73974	-1.7358	-1.73187	-1.74667
-1.68799	-1.68433	-1.68069	-1.72696	-1.72311	-1.74044	-1.73648	-1.73254	-1.74736
-1.68863	-1.68496	-1.68131	-1.72762	-1.72378	-1.71993	-1.71605	-1.71219	-1.74804
-1.6893	-1.68565	-1.68202	-1.72836	-1.72447	-1.72059	-1.71672	-1.71287	-1.74873

-1.68999	-1.68635	-1.68273	-1.70812	-1.70423	-1.72126	-1.71739	-1.71355	-1.74943
-1.6907	-1.68704	-1.6834	-1.7088	-1.70491	-1.72194	-1.71807	-1.71422	-1.75012
-1.69141	-1.68773	-1.68405	-1.70948	-1.70559	-1.72262	-1.71874	-1.7149	-1.75081
-1.69208	-1.68839	-1.6847	-1.68103	-1.67737	-1.7024	-1.69856	-1.69476	-1.7328
-1.69272	-1.68903	-1.68534	-1.68167	-1.67801	-1.70307	-1.69925	-1.69546	-1.73353
-1.69337	-1.68967	-1.68599	-1.68232	-1.67866	-1.70377	-1.69996	-1.69618	-1.71321
-1.694	-1.6903	-1.68662	-1.68296	-1.67932	-1.70447	-1.70068	-1.69691	-1.71392
-1.69464	-1.69094	-1.68727	-1.68362	-1.67999	-1.70519	-1.7014	-1.69763	-1.69383
-1.69526	-1.69157	-1.68792	-1.68429	-1.68068	-1.70593	-1.70212	-1.69832	-1.69452
-1.69592	-1.69224	-1.68859	-1.68497	-1.68138	-1.70667	-1.70283	-1.69901	-1.69521
-1.69667	-1.69297	-1.68931	-1.68567	-1.68205	-1.70736	-1.70352	-1.6997	-1.69592
-1.69743	-1.69373	-1.69005	-1.68638	-1.68273	-1.70806	-1.70421	-1.7004	-1.69663
-1.69814	-1.69445	-1.69078	-1.68712	-1.68346	-1.70883	-1.70497	-1.70114	-1.69735
-1.69881	-1.69515	-1.69151	-1.68787	-1.68422	-1.70964	-1.70576	-1.70191	-1.69807
-1.67667	-1.69586	-1.69222	-1.68857	-1.68493	-1.71041	-1.70654	-1.70268	-1.69883
-1.6774	-1.69657	-1.69288	-1.68923	-1.68559	-1.71115	-1.70731	-1.70348	-1.69962
-1.67814	-1.69729	-1.69358	-1.68991	-1.68628	-1.71189	-1.70807	-1.70426	-1.70041
-1.67888	-1.69805	-1.69434	-1.69066	-1.687	-1.71266	-1.70884	-1.70502	-1.70119
-1.6796	-1.6988	-1.69509	-1.6914	-1.68774	-1.71343	-1.70959	-1.70577	-1.70194
-1.68027	-1.69948	-1.69578	-1.69211	-1.68846	-1.68483	-1.68122	-1.67761	-1.70264
-1.68093	-1.70014	-1.69646	-1.69281	-1.68919	-1.68556	-1.6819	-1.67826	-1.70332
-1.68164	-1.70087	-1.69719	-1.69354	-1.68993	-1.6863	-1.68262	-1.67896	-1.70407
-1.68241	-1.70164	-1.69795	-1.6943	-1.69069	-1.68706	-1.68338	-1.67971	-1.70486
-1.68317	-1.67952	-1.67589	-1.69507	-1.69146	-1.68782	-1.68413	-1.68046	-1.70565
-1.68394	-1.68029	-1.67666	-1.69585	-1.69223	-1.68859	-1.6849	-1.68122	-1.70646
-1.68471	-1.68106	-1.67743	-1.69663	-1.69301	-1.68936	-1.68566	-1.68198	-1.70726
-1.68547	-1.68182	-1.67819	-1.6974	-1.69377	-1.69012	-1.68642	-1.68273	-1.70805
-1.68623	-1.68257	-1.67895	-1.69817	-1.69454	-1.69088	-1.68717	-1.68347	-1.70884
-1.68698	-1.68333	-1.6797	-1.69893	-1.6953	-1.69163	-1.68791	-1.68421	-1.70961
-1.68773	-1.68408	-1.68045	-1.69969	-1.69605	-1.69238	-1.68865	-1.68494	-1.71039
-1.68848	-1.68482	-1.6812	-1.70045	-1.69681	-1.69314	-1.68941	-1.68569	-1.71118
-1.68922	-1.68557	-1.68194	-1.7012	-1.69757	-1.6939	-1.69017	-1.68645	-1.71198
-1.68998	-1.68632	-1.68269	-1.70196	-1.69834	-1.69468	-1.69096	-1.68725	-1.68356
-1.69075	-1.68708	-1.68343	-1.70272	-1.69911	-1.69547	-1.69178	-1.6881	-1.6844
-1.69155	-1.68786	-1.6842	-1.68059	-1.67704	-1.69625	-1.6926	-1.68894	-1.68524
-1.6924	-1.6887	-1.68502	-1.68138	-1.6778	-1.697	-1.69337	-1.68972	-1.68602
-1.69326	-1.68956	-1.68584	-1.68218	-1.67855	-1.69775	-1.69412	-1.69048	-1.68679
-1.69406	-1.69036	-1.68664	-1.68295	-1.6793	-1.69849	-1.69487	-1.69125	-1.68759
-1.69483	-1.69113	-1.68741	-1.68372	-1.68004	-1.69924	-1.69562	-1.69201	-1.68839
-1.6956	-1.69193	-1.68823	-1.68454	-1.68084	-1.70003	-1.69638	-1.69276	-1.68916
-1.69639	-1.69274	-1.68909	-1.68541	-1.6817	-1.70086	-1.69716	-1.6935	-1.68989
-1.69717	-1.69353	-1.68993	-1.68626	-1.68256	-1.70172	-1.69797	-1.69427	-1.69065
-1.69792	-1.69429	-1.6907	-1.68708	-1.68342	-1.70262	-1.69885	-1.6951	-1.69143
-1.69869	-1.69506	-1.69147	-1.68787	-1.68426	-1.70351	-1.69973	-1.69596	-1.69224
-1.69953	-1.69586	-1.69224	-1.68863	-1.68503	-1.68141	-1.67774	-1.67407	-1.69312
-1.7004	-1.69669	-1.69302	-1.68939	-1.68578	-1.68218	-1.67856	-1.67494	-1.69402
-1.70128	-1.69758	-1.69391	-1.69025	-1.68661	-1.68298	-1.67936	-1.67575	-1.69486
-1.70217	-1.69852	-1.69486	-1.69118	-1.68748	-1.6838	-1.68014	-1.6765	-1.69566
-1.682	-1.69938	-1.69574	-1.69206	-1.68835	-1.68465	-1.68095	-1.6773	-1.69647
-1.68276	-1.70014	-1.69652	-1.69287	-1.68921	-1.68553	-1.68182	-1.67813	-1.69728

-1.62901	-1.67995	-1.67636	-1.69367	-1.69004	-1.68638	-1.68268	-1.67899	-1.67533
-1.62988	-1.68083	-1.67718	-1.69448	-1.69081	-1.68716	-1.68351	-1.67987	-1.67623
-1.63075	-1.68172	-1.67803	-1.69529	-1.69159	-1.68793	-1.68433	-1.68074	-1.67713
-1.63152	-1.68255	-1.67888	-1.69616	-1.69243	-1.68875	-1.68513	-1.68154	-1.67793
-1.63225	-1.68335	-1.67973	-1.69704	-1.6933	-1.6896	-1.68594	-1.6823	-1.6787
-1.63302	-1.68416	-1.68054	-1.67689	-1.67323	-1.73083	-1.72706	-1.72332	-1.71961
-1.63382	-1.68497	-1.68132	-1.67768	-1.67407	-1.73174	-1.728	-1.72428	-1.72058
-1.63464	-1.68582	-1.68216	-1.67852	-1.67492	-1.71233	-1.70864	-1.70496	-1.72149
-1.63548	-1.68673	-1.68308	-1.67944	-1.67579	-1.71317	-1.70946	-1.70576	-1.72229
-1.61162	-1.68762	-1.68399	-1.68034	-1.67666	-1.71403	-1.71029	-1.70658	-1.72312
-1.61241	-1.68844	-1.6848	-1.68116	-1.67751	-1.71494	-1.71121	-1.70751	-1.72406
-1.6132	-1.68926	-1.6856	-1.68198	-1.67837	-1.71586	-1.71215	-1.70845	-1.72503
-1.59043	-1.69014	-1.68649	-1.68287	-1.67928	-1.71678	-1.71305	-1.70933	-1.70565
-1.59127	-1.69106	-1.68741	-1.68379	-1.6802	-1.7177	-1.71394	-1.71018	-1.70647
-1.59211	-1.61233	-1.60889	-1.68467	-1.68106	-1.71858	-1.71482	-1.71107	-1.70735
-1.59297	-1.61317	-1.6097	-1.68549	-1.68185	-1.7194	-1.71568	-1.71198	-1.70829
-1.59378	-1.61399	-1.61051	-1.6625	-1.65889	-1.72027	-1.71658	-1.71291	-1.70924
-1.59451	-1.61477	-1.61134	-1.6634	-1.6598	-1.72121	-1.71753	-1.71386	-1.7102
-1.59526	-1.61557	-1.61218	-1.66431	-1.66073	-1.72218	-1.71848	-1.71479	-1.71113
-1.59611	-1.59278	-1.58949	-1.66524	-1.66167	-1.72312	-1.71938	-1.71566	-1.71197
-1.597	-1.59367	-1.59035	-1.66616	-1.66259	-1.72405	-1.72027	-1.7165	-1.71278
-1.59788	-1.59453	-1.59118	-1.66702	-1.66342	-1.70178	-1.69805	-1.69434	-1.71367
-1.59876	-1.59537	-1.59197	-1.66783	-1.66421	-1.70258	-1.6989	-1.69524	-1.71461
-1.59957	-1.59617	-1.59277	-1.66869	-1.66506	-1.70346	-1.6998	-1.69617	-1.69255
-1.6003	-1.59693	-1.59357	-1.66959	-1.66598	-1.70442	-1.70077	-1.69715	-1.69352
-1.60107	-1.59772	-1.5944	-1.59109	-1.58778	-1.7054	-1.70176	-1.69812	-1.69449
-1.60193	-1.59858	-1.59527	-1.59196	-1.58866	-1.7064	-1.70274	-1.69909	-1.69545
-1.60283	-1.59948	-1.59616	-1.59285	-1.58956	-1.70739	-1.70372	-1.70006	-1.6964
-1.60372	-1.60038	-1.59706	-1.59375	-1.59046	-1.70838	-1.7047	-1.70101	-1.69732
-1.60464	-1.60129	-1.59796	-1.59465	-1.59136	-1.70936	-1.70566	-1.70195	-1.69821
-1.60555	-1.6022	-1.59886	-1.59554	-1.59223	-1.71031	-1.70659	-1.70285	-1.6991
-1.60646	-1.6031	-1.59975	-1.59642	-1.5931	-1.71124	-1.70748	-1.70372	-1.69998
-1.60737	-1.604	-1.60063	-1.59728	-1.59393	-1.71215	-1.70837	-1.70461	-1.70089
-1.60826	-1.60488	-1.6015	-1.59812	-1.59473	-1.71302	-1.70925	-1.70551	-1.70182
-1.60914	-1.60575	-1.60235	-1.59894	-1.59553	-1.63657	-1.63309	-1.62964	-1.70277
-1.61003	-1.6066	-1.60314	-1.59972	-1.59633	-1.63741	-1.63396	-1.63053	-1.70374
-1.61092	-1.60744	-1.60394	-1.60051	-1.59713	-1.63828	-1.63485	-1.63144	-1.70472
-1.61181	-1.60833	-1.60481	-1.60136	-1.59799	-1.63916	-1.63575	-1.63236	-1.70571
-1.61271	-1.60923	-1.60571	-1.60226	-1.59886	-1.64007	-1.63666	-1.63328	-1.7067
-1.6136	-1.61013	-1.60662	-1.60316	-1.59975	-1.64097	-1.63757	-1.63419	-1.70768
-1.6145	-1.61102	-1.60752	-1.60406	-1.60064	-1.64189	-1.63848	-1.6351	-1.70865
-1.61539	-1.61192	-1.60842	-1.60496	-1.60153	-1.6428	-1.63939	-1.63601	-1.68753
-1.6163	-1.61283	-1.60933	-1.60586	-1.6024	-1.6437	-1.64029	-1.63691	-1.68848
-1.61722	-1.61375	-1.61026	-1.60678	-1.60331	-1.64462	-1.64121	-1.63783	-1.68944
-1.58708	-1.61469	-1.61125	-1.60778	-1.60429	-1.6456	-1.64215	-1.63874	-1.69039
-1.58796	-1.61564	-1.61225	-1.6088	-1.60529	-1.64661	-1.64312	-1.63967	-1.69137
-1.58885	-1.61655	-1.61318	-1.60976	-1.6063	-1.64767	-1.64417	-1.64071	-1.63731
-1.58972	-1.61745	-1.61408	-1.61069	-1.6073	-1.64876	-1.64528	-1.64182	-1.63841
-1.5906	-1.61834	-1.61497	-1.61161	-1.60825	-1.64978	-1.6463	-1.64285	-1.63944
-1.59147	-1.61923	-1.61587	-1.6125	-1.60915	-1.6507	-1.64723	-1.64378	-1.64037

-1.56365	-1.58907	-1.5858	-1.6134	-1.61003	-1.65162	-1.64814	-1.64469	-1.64129
-1.56451	-1.58994	-1.58667	-1.61429	-1.61093	-1.65254	-1.64906	-1.6456	-1.64219
-1.56536	-1.59082	-1.58755	-1.61518	-1.61182	-1.65346	-1.64997	-1.6465	-1.64309
-1.56621	-1.59169	-1.58842	-1.61607	-1.61271	-1.65438	-1.65088	-1.64742	-1.64399
-1.56707	-1.59256	-1.58929	-1.61696	-1.61359	-1.65529	-1.6518	-1.64833	-1.6449
-1.56794	-1.59345	-1.59017	-1.61786	-1.61448	-1.65621	-1.65272	-1.64925	-1.64581
-1.56881	-1.59433	-1.59106	-1.61876	-1.61538	-1.65714	-1.65365	-1.65018	-1.64673
-1.56976	-1.56653	-1.56332	-1.58873	-1.58545	-1.65814	-1.65465	-1.65118	-1.64773
-1.57081	-1.56756	-1.56434	-1.58977	-1.58649	-1.65926	-1.65577	-1.65229	-1.64883
-1.57182	-1.56857	-1.56534	-1.59079	-1.5875	-1.66033	-1.65684	-1.65337	-1.6499
-1.57272	-1.56946	-1.56623	-1.59169	-1.58839	-1.66127	-1.65779	-1.65431	-1.65085
-1.5736	-1.57033	-1.56708	-1.59256	-1.58925	-1.66218	-1.65869	-1.65522	-1.65176
-1.57448	-1.5712	-1.56795	-1.59343	-1.59012	-1.63299	-1.62959	-1.62621	-1.65267
-1.57537	-1.57209	-1.56882	-1.59432	-1.591	-1.6339	-1.63048	-1.62709	-1.65358
-1.54435	-1.57306	-1.56979	-1.59529	-1.59197	-1.6349	-1.63147	-1.62807	-1.65458
-1.5454	-1.57412	-1.57084	-1.59637	-1.59303	-1.63599	-1.63255	-1.62914	-1.65567
-1.5464	-1.57514	-1.57185	-1.56857	-1.5653	-1.63703	-1.63358	-1.63016	-1.62679
-1.54731	-1.57607	-1.57277	-1.56948	-1.5662	-1.63798	-1.63451	-1.63108	-1.62771
-1.54822	-1.577	-1.5737	-1.57041	-1.56712	-1.63895	-1.63547	-1.63202	-1.62864
-1.49406	-1.54598	-1.54278	-1.57145	-1.56815	-1.64003	-1.63654	-1.63309	-1.62969
-1.495	-1.54698	-1.54381	-1.57251	-1.5692	-1.64114	-1.63764	-1.63418	-1.63077
-1.46554	-1.49285	-1.48979	-1.54154	-1.5383	-1.64215	-1.63865	-1.63518	-1.63175
-1.46641	-1.49372	-1.49065	-1.54243	-1.53919	-1.64311	-1.63961	-1.63613	-1.63268
-1.46727	-1.49459	-1.49153	-1.54336	-1.54014	-1.61612	-1.61268	-1.60926	-1.63369
-1.46809	-1.49546	-1.49244	-1.54434	-1.54117	-1.61722	-1.61377	-1.61034	-1.63479
-1.46897	-1.49636	-1.49335	-1.49032	-1.48728	-1.53896	-1.53572	-1.53249	-1.58959
-1.46993	-1.49731	-1.49425	-1.49119	-1.48814	-1.53985	-1.53661	-1.53338	-1.59051
-1.47092	-1.49827	-1.49516	-1.49206	-1.489	-1.54074	-1.53751	-1.53428	-1.59145
-1.47186	-1.49925	-1.49613	-1.49303	-1.48996	-1.48687	-1.48376	-1.48066	-1.56127
-1.4728	-1.50023	-1.49713	-1.49403	-1.49095	-1.48786	-1.48477	-1.48168	-1.56235
-1.40833	-1.50116	-1.49803	-1.49492	-1.49183	-1.48876	-1.48571	-1.48266	-1.53419
-1.40911	-1.50204	-1.49887	-1.49574	-1.49265	-1.4896	-1.48661	-1.48362	-1.53521
-1.40995	-1.47244	-1.46931	-1.49664	-1.49353	-1.49049	-1.48751	-1.48454	-1.48151
-1.41087	-1.47347	-1.4703	-1.49762	-1.49451	-1.49143	-1.48842	-1.48541	-1.48236
-1.41178	-1.4745	-1.47131	-1.49864	-1.49551	-1.49241	-1.48935	-1.48631	-1.48324
-1.41264	-1.47552	-1.47233	-1.49969	-1.49654	-1.49342	-1.49035	-1.48729	-1.48421
-1.41348	-1.47651	-1.47333	-1.50072	-1.49756	-1.49444	-1.49136	-1.48829	-1.48521
-1.41428	-1.47743	-1.47425	-1.50166	-1.49852	-1.49541	-1.49234	-1.48928	-1.48622
-1.41507	-1.47831	-1.47512	-1.50257	-1.49944	-1.49636	-1.49331	-1.49027	-1.48722
-1.41593	-1.41314	-1.41037	-1.47294	-1.46988	-1.49726	-1.4942	-1.49116	-1.48814
-1.41684	-1.41403	-1.41124	-1.47391	-1.47077	-1.49812	-1.49502	-1.49198	-1.48899
-1.41775	-1.41494	-1.41213	-1.47491	-1.47171	-1.49904	-1.49591	-1.49284	-1.48987
-1.41866	-1.41586	-1.41305	-1.47595	-1.47272	-1.50005	-1.49689	-1.4938	-1.49077
-1.41956	-1.41676	-1.41396	-1.477	-1.47376	-1.5011	-1.49791	-1.49478	-1.49171
-1.42042	-1.41765	-1.41487	-1.47805	-1.47481	-1.50217	-1.49895	-1.49579	-1.49269
-1.42126	-1.41851	-1.41576	-1.4791	-1.47587	-1.50325	-1.5	-1.49681	-1.49369
-1.42206	-1.41933	-1.41662	-1.48011	-1.47691	-1.50433	-1.50106	-1.49784	-1.49469
-1.42284	-1.42012	-1.41745	-1.48109	-1.47794	-1.5054	-1.50212	-1.49888	-1.49571
-1.42366	-1.42094	-1.41827	-1.41556	-1.41285	-1.47579	-1.4726	-1.46944	-1.49673
-1.42453	-1.42177	-1.41906	-1.41636	-1.41367	-1.47677	-1.47361	-1.47045	-1.49777

-1.3904	-1.42262	-1.41988	-1.41718	-1.41452	-1.47776	-1.47462	-1.47148	-1.49881
-1.39128	-1.42352	-1.42076	-1.41805	-1.41539	-1.47877	-1.47564	-1.47251	-1.49986
-1.39217	-1.42443	-1.42166	-1.41894	-1.41628	-1.47979	-1.47667	-1.47354	-1.50091
-1.39307	-1.42534	-1.42256	-1.41983	-1.41717	-1.4808	-1.4777	-1.47458	-1.50195
-1.39397	-1.42626	-1.42346	-1.42073	-1.41806	-1.48182	-1.47872	-1.4756	-1.503
-1.39488	-1.42718	-1.42437	-1.42163	-1.41896	-1.41628	-1.41357	-1.41084	-1.50405
-1.3958	-1.4281	-1.42527	-1.42252	-1.41986	-1.41718	-1.41447	-1.41173	-1.50509
-1.33725	-1.42903	-1.42618	-1.42343	-1.42075	-1.41807	-1.41536	-1.41263	-1.50614
-1.33811	-1.42996	-1.4271	-1.42433	-1.42164	-1.41895	-1.41625	-1.41352	-1.5072
-1.33896	-1.4309	-1.42803	-1.42524	-1.42253	-1.41983	-1.41714	-1.41442	-1.47754
-1.33983	-1.43185	-1.42896	-1.42616	-1.42343	-1.42072	-1.41802	-1.41531	-1.47856
-1.34069	-1.4328	-1.4299	-1.42708	-1.42433	-1.42161	-1.4189	-1.41619	-1.47958
-1.34155	-1.33884	-1.33617	-1.428	-1.42524	-1.4225	-1.41979	-1.41707	-1.48059
-1.34242	-1.3397	-1.33702	-1.42892	-1.42615	-1.4234	-1.42068	-1.41795	-1.48159
-1.34328	-1.34058	-1.33792	-1.4299	-1.4271	-1.42433	-1.42158	-1.41884	-1.41609
-1.34415	-1.34148	-1.33885	-1.43094	-1.42809	-1.42528	-1.4225	-1.41974	-1.41698
-1.34501	-1.34236	-1.33976	-1.37343	-1.37072	-1.42624	-1.42343	-1.42064	-1.41789
-1.34584	-1.34321	-1.34062	-1.37433	-1.37162	-1.42719	-1.42435	-1.42155	-1.41882
-1.31718	-1.34406	-1.34147	-1.37522	-1.37251	-1.42815	-1.4253	-1.42249	-1.41977
-1.31812	-1.34498	-1.34237	-1.37612	-1.3734	-1.42911	-1.42628	-1.42348	-1.42075
-1.31907	-1.34591	-1.34326	-1.37703	-1.37429	-1.43006	-1.42726	-1.42449	-1.42173
-1.31996	-1.31731	-1.31469	-1.37793	-1.37517	-1.37246	-1.36983	-1.36722	-1.42266
-1.32081	-1.31816	-1.31555	-1.37882	-1.37604	-1.37332	-1.37068	-1.36806	-1.42358
-1.32171	-1.31904	-1.31641	-1.31378	-1.31116	-1.37424	-1.37158	-1.36894	-1.4245
-1.32265	-1.31994	-1.31728	-1.31466	-1.31208	-1.37521	-1.37252	-1.36984	-1.42544
-1.32359	-1.32086	-1.31818	-1.31556	-1.313	-1.34736	-1.34466	-1.34198	-1.33933
-1.32454	-1.3218	-1.31912	-1.31649	-1.31391	-1.34828	-1.34557	-1.34289	-1.34025
-1.3255	-1.32276	-1.32007	-1.31742	-1.31481	-1.34919	-1.34648	-1.3438	-1.34118
-1.3265	-1.32374	-1.32101	-1.31833	-1.31569	-1.3501	-1.34741	-1.34475	-1.34212
-1.3275	-1.32472	-1.32195	-1.31924	-1.31657	-1.35101	-1.34835	-1.34571	-1.34307
-1.25544	-1.32566	-1.32289	-1.32017	-1.31749	-1.35195	-1.34929	-1.34664	-1.34398
-1.25627	-1.32658	-1.32382	-1.32111	-1.31844	-1.35293	-1.35023	-1.34755	-1.34488
-1.22355	-1.32753	-1.32478	-1.32207	-1.31939	-1.31674	-1.31411	-1.31151	-1.34578
-1.22442	-1.32851	-1.32578	-1.32305	-1.32034	-1.31765	-1.315	-1.31239	-1.3467
-1.22529	-1.22281	-1.22035	-1.32402	-1.32128	-1.31857	-1.3159	-1.31327	-1.34763
-1.22619	-1.22368	-1.2212	-1.32497	-1.3222	-1.31948	-1.31682	-1.3142	-1.34858
-1.22708	-1.22456	-1.22205	-1.32591	-1.32312	-1.3204	-1.31774	-1.31513	-1.34953
-1.22795	-1.22541	-1.22289	-1.29452	-1.29181	-1.32135	-1.31868	-1.31605	-1.35048
-1.22881	-1.22625	-1.22372	-1.29544	-1.29276	-1.32234	-1.31963	-1.31698	-1.35143
-1.22966	-1.2271	-1.22457	-1.29638	-1.2937	-1.32331	-1.3206	-1.31792	-1.31531
-1.2305	-1.22794	-1.22542	-1.29734	-1.29466	-1.32429	-1.32157	-1.31888	-1.31624
-1.23137	-1.22881	-1.22629	-1.22379	-1.22134	-1.29294	-1.29028	-1.28765	-1.31719
-1.23227	-1.2297	-1.22715	-1.22465	-1.2222	-1.29391	-1.29124	-1.28859	-1.31815
-1.23318	-1.23059	-1.22803	-1.22552	-1.22307	-1.29487	-1.29219	-1.28954	-1.28693
-1.23406	-1.23148	-1.22893	-1.22642	-1.22393	-1.2958	-1.29314	-1.29049	-1.28787
-1.23496	-1.23238	-1.22984	-1.22731	-1.2248	-1.29674	-1.29409	-1.29145	-1.28881
-1.19994	-1.2333	-1.23073	-1.22819	-1.22568	-1.2977	-1.29503	-1.29239	-1.28974
-1.20087	-1.23424	-1.23163	-1.22907	-1.22656	-1.29868	-1.29599	-1.29332	-1.29067
-1.20178	-1.19922	-1.19669	-1.22999	-1.22748	-1.22499	-1.22251	-1.22005	-1.29166
-1.20268	-1.20013	-1.19761	-1.23096	-1.22844	-1.22594	-1.22346	-1.221	-1.2927

-1.1684	-1.20103	-1.19852	-1.2319	-1.22938	-1.22688	-1.2244	-1.22193	-1.29372
-1.16927	-1.20191	-1.19939	-1.23281	-1.23027	-1.22776	-1.22528	-1.22282	-1.2947
-1.17013	-1.20279	-1.20026	-1.2337	-1.23116	-1.22864	-1.22615	-1.22369	-1.29566
-1.13328	-1.16851	-1.16605	-1.19863	-1.19615	-1.22951	-1.22701	-1.22453	-1.29659
-1.1341	-1.16938	-1.1669	-1.19949	-1.197	-1.23038	-1.22787	-1.22538	-1.29753
-1.13492	-1.17025	-1.16776	-1.16531	-1.1629	-1.2313	-1.22877	-1.22627	-1.22381
-1.13575	-1.17112	-1.16863	-1.16619	-1.16379	-1.23225	-1.22971	-1.2272	-1.22473
-1.13661	-1.17202	-1.16953	-1.16708	-1.16466	-1.16226	-1.15987	-1.15751	-1.22561
-1.13752	-1.17297	-1.17045	-1.16798	-1.16553	-1.1631	-1.1607	-1.15832	-1.22646
-1.06146	-1.13596	-1.13354	-1.16887	-1.16639	-1.16395	-1.16153	-1.15915	-1.19315
-1.06219	-1.13679	-1.13436	-1.16974	-1.16725	-1.16481	-1.1624	-1.16002	-1.19406
-1.06293	-1.13761	-1.13519	-1.17061	-1.16813	-1.16568	-1.16328	-1.1609	-1.19497
-1.06373	-1.1385	-1.13607	-1.17153	-1.16903	-1.16657	-1.16415	-1.16175	-1.19584
-1.06455	-1.1394	-1.13696	-1.17247	-1.16996	-1.16748	-1.16502	-1.1626	-1.1967
-0.98594	-1.0631	-1.0609	-1.1734	-1.17089	-1.1684	-1.16593	-1.16348	-1.16106
-0.98661	-1.06385	-1.06166	-1.17432	-1.17183	-1.16935	-1.16686	-1.16439	-1.16196
-0.98732	-1.02868	-1.02654	-1.13714	-1.13476	-1.17026	-1.16777	-1.1653	-1.16286
-0.98808	-1.02947	-1.02732	-1.13801	-1.13559	-1.17114	-1.16864	-1.16617	-1.16375
-0.98883	-0.98677	-0.98473	-1.02594	-1.0238	-1.17203	-1.16953	-1.16706	-1.16465
-0.98954	-0.9875	-0.98548	-1.02675	-1.02459	-1.17296	-1.17046	-1.16799	-1.16557
-0.99024	-0.98822	-0.98623	-1.02756	-1.0254	-1.17392	-1.17141	-1.16894	-1.1665
-0.91047	-0.98896	-0.98698	-0.98496	-0.98291	-1.14102	-1.13855	-1.1361	-1.13368
-0.91113	-0.98971	-0.98773	-0.98571	-0.98366	-1.14197	-1.13949	-1.13702	-1.13456
-0.91179	-0.90995	-0.90814	-0.98645	-0.9844	-1.02564	-1.02349	-1.02136	-1.13547
-0.91245	-0.9106	-0.9088	-0.9872	-0.98515	-1.02644	-1.02429	-1.02214	-1.1364
-0.87034	-0.91125	-0.90943	-0.98791	-0.98587	-0.98384	-0.98183	-0.97981	-1.13733
-0.871	-0.9119	-0.91002	-0.98857	-0.98655	-0.98454	-0.98254	-0.98054	-1.13827
-0.87167	-0.91255	-0.91062	-0.98924	-0.98723	-0.98523	-0.98325	-0.98127	-1.1392
-0.82415	-0.87046	-0.8686	-0.90939	-0.90755	-0.98594	-0.98397	-0.98199	-1.02313
-0.82477	-0.87112	-0.86925	-0.91007	-0.90819	-0.98667	-0.98469	-0.98272	-1.02391
-0.82539	-0.87179	-0.86991	-0.91074	-0.90885	-0.907	-0.9052	-0.90339	-0.98142
-0.82602	-0.87246	-0.87056	-0.91142	-0.90953	-0.90767	-0.90585	-0.90403	-0.98215
-0.78705	-0.87314	-0.87122	-0.86937	-0.86757	-0.90834	-0.90651	-0.90469	-0.98289
-0.78768	-0.87382	-0.8719	-0.87003	-0.86821	-0.909	-0.90718	-0.90536	-0.98367
-0.74475	-0.78659	-0.78486	-0.87072	-0.86887	-0.86707	-0.86532	-0.86359	-0.9042
-0.74536	-0.78724	-0.78554	-0.87146	-0.86958	-0.86774	-0.86597	-0.86422	-0.90486
-0.74598	-0.78789	-0.78621	-0.87221	-0.87029	-0.86843	-0.86663	-0.86485	-0.90552
-0.74659	-0.78853	-0.78685	-0.83429	-0.83245	-0.8691	-0.86728	-0.86549	-0.86373
-0.7472	-0.78915	-0.78747	-0.83496	-0.83312	-0.86979	-0.86794	-0.86613	-0.86436
-0.7079	-0.7462	-0.7446	-0.83564	-0.83382	-0.83202	-0.83023	-0.82847	-0.86501
-0.70854	-0.74682	-0.74521	-0.83634	-0.83454	-0.83274	-0.83092	-0.82913	-0.86566
-0.70916	-0.70756	-0.70598	-0.78767	-0.786	-0.83344	-0.8316	-0.82979	-0.86632
-0.70978	-0.70817	-0.70658	-0.78828	-0.78661	-0.8341	-0.83227	-0.83045	-0.86697
-0.57063	-0.70879	-0.70719	-0.70561	-0.70405	-0.83477	-0.83294	-0.83113	-0.82934
-0.57115	-0.70941	-0.70781	-0.70622	-0.70466	-0.83545	-0.83365	-0.83185	-0.83004
-0.57168	-0.71005	-0.70843	-0.70684	-0.70527	-0.83613	-0.83436	-0.83258	-0.83076
-0.57223	-0.61559	-0.61418	-0.70746	-0.70588	-0.74848	-0.74685	-0.74523	-0.83146
-0.57279	-0.61618	-0.61475	-0.70808	-0.70648	-0.74909	-0.74746	-0.74584	-0.83216
-0.57333	-0.57198	-0.57063	-0.70874	-0.70711	-0.70551	-0.70396	-0.70243	-0.79484
-0.57384	-0.57251	-0.57118	-0.70943	-0.70777	-0.70614	-0.70457	-0.70302	-0.7955

-0.51954	-0.57303	-0.57172	-0.61508	-0.61366	-0.7068	-0.7052	-0.70363	-0.74607
-0.51998	-0.57353	-0.57222	-0.61563	-0.61424	-0.70748	-0.70585	-0.70424	-0.74668
-0.52042	-0.57404	-0.57272	-0.61618	-0.61481	-0.70816	-0.70651	-0.70488	-0.74733
-0.52089	-0.57457	-0.57322	-0.5719	-0.57061	-0.61397	-0.61258	-0.61121	-0.70399
-0.52136	-0.57511	-0.57373	-0.57239	-0.57109	-0.61449	-0.61314	-0.6118	-0.70469
-0.48145	-0.52069	-0.51955	-0.57295	-0.57162	-0.61503	-0.61368	-0.61234	-0.65966
-0.48195	-0.52119	-0.52006	-0.57354	-0.57217	-0.61558	-0.6142	-0.61285	-0.6602
-0.48242	-0.52166	-0.52053	-0.5194	-0.51827	-0.57137	-0.57006	-0.56877	-0.61205
-0.48287	-0.5221	-0.52097	-0.51985	-0.51873	-0.57194	-0.57063	-0.56932	-0.61262
-0.48333	-0.52255	-0.52142	-0.5203	-0.51919	-0.5725	-0.57118	-0.56986	-0.61319
-0.48381	-0.52302	-0.52187	-0.52074	-0.51962	-0.57301	-0.57168	-0.57037	-0.61375
-0.48429	-0.5235	-0.52233	-0.52119	-0.52006	-0.57352	-0.57219	-0.57088	-0.6143
-0.43744	-0.48374	-0.48271	-0.52169	-0.52056	-0.51944	-0.51832	-0.51723	-0.57009
-0.43789	-0.48425	-0.48323	-0.52221	-0.52107	-0.51994	-0.5188	-0.51767	-0.57058
-0.39099	-0.48472	-0.4837	-0.52269	-0.52155	-0.52041	-0.51927	-0.51814	-0.51703
-0.39136	-0.48516	-0.48415	-0.52314	-0.522	-0.52087	-0.51975	-0.51863	-0.51753
-0.3378	-0.39092	-0.39011	-0.52362	-0.52248	-0.52135	-0.52023	-0.51911	-0.518
-0.33816	-0.39135	-0.39053	-0.52415	-0.523	-0.52186	-0.52071	-0.51957	-0.51844
-0.28854	-0.33781	-0.3371	-0.47933	-0.47827	-0.52235	-0.52119	-0.52004	-0.5189
-0.28882	-0.33813	-0.33743	-0.47976	-0.4787	-0.52282	-0.52167	-0.52053	-0.5194
-0.2891	-0.33846	-0.33775	-0.48018	-0.47913	-0.52329	-0.52216	-0.52103	-0.51991
-0.28942	-0.33882	-0.3381	-0.39129	-0.39048	-0.47855	-0.4775	-0.47646	-0.5204
-0.28974	-0.33919	-0.33847	-0.39172	-0.3909	-0.47903	-0.47797	-0.47692	-0.52088
-0.29005	-0.28943	-0.28882	-0.3381	-0.33739	-0.47948	-0.47842	-0.47737	-0.47631
-0.29033	-0.28971	-0.2891	-0.33842	-0.33772	-0.47991	-0.47885	-0.47781	-0.47676
-0.29063	-0.29001	-0.28939	-0.28879	-0.28819	-0.42848	-0.42754	-0.42661	-0.47722
-0.29096	-0.29033	-0.28971	-0.2891	-0.28851	-0.42891	-0.42797	-0.42703	-0.47769
-0.29129	-0.29065	-0.29003	-0.28942	-0.28882	-0.33806	-0.33736	-0.33667	-0.4265
-0.29158	-0.29094	-0.29032	-0.28971	-0.2891	-0.33839	-0.33769	-0.33699	-0.42688
-0.29187	-0.29123	-0.29061	-0.29	-0.28939	-0.33872	-0.33801	-0.33731	-0.42725
-0.2922	-0.29156	-0.29093	-0.29031	-0.2897	-0.2891	-0.2885	-0.28792	-0.42766
-0.29256	-0.29191	-0.29127	-0.29065	-0.29003	-0.28942	-0.28883	-0.28824	-0.4281
-0.29291	-0.29225	-0.29161	-0.29098	-0.29036	-0.28975	-0.28915	-0.28856	-0.42853
-0.29326	-0.2926	-0.29195	-0.29131	-0.29069	-0.29008	-0.28948	-0.28888	-0.42897
-0.2936	-0.29293	-0.29228	-0.29164	-0.29101	-0.2904	-0.28979	-0.28918	-0.38157
-0.29392	-0.29325	-0.29258	-0.29193	-0.2913	-0.29068	-0.29007	-0.28947	-0.38192
-0.29424	-0.29356	-0.29289	-0.29224	-0.2916	-0.29097	-0.29035	-0.28975	-0.38228
-0.2946	-0.29392	-0.29324	-0.29257	-0.29193	-0.29129	-0.29067	-0.29007	-0.28947
-0.29497	-0.29428	-0.2936	-0.29293	-0.29227	-0.29163	-0.29101	-0.2904	-0.2898
-0.24141	-0.29465	-0.29396	-0.29329	-0.29262	-0.29197	-0.29134	-0.29073	-0.29012
-0.24172	-0.29503	-0.29434	-0.29365	-0.29298	-0.29232	-0.29168	-0.29106	-0.29044
-0.24202	-0.2954	-0.29471	-0.29402	-0.29334	-0.29267	-0.29202	-0.29139	-0.29077
-0.24233	-0.29577	-0.29508	-0.29439	-0.2937	-0.29303	-0.29237	-0.29172	-0.2911
-0.24263	-0.24204	-0.24146	-0.29475	-0.29406	-0.29338	-0.29272	-0.29206	-0.29144
-0.2429	-0.24231	-0.24173	-0.29508	-0.29441	-0.29373	-0.29307	-0.29241	-0.29177
-0.24317	-0.24259	-0.24201	-0.29542	-0.29475	-0.29408	-0.29342	-0.29276	-0.29211
-0.24348	-0.24289	-0.2423	-0.24173	-0.24116	-0.29443	-0.29376	-0.2931	-0.29245
-0.2438	-0.24321	-0.24261	-0.24203	-0.24146	-0.29477	-0.2941	-0.29344	-0.29279
-0.24411	-0.24352	-0.24293	-0.24234	-0.24176	-0.29512	-0.29445	-0.29378	-0.29313
-0.24443	-0.24383	-0.24324	-0.24265	-0.24206	-0.29549	-0.2948	-0.29413	-0.29346

-0.24474	-0.24415	-0.24355	-0.24296	-0.24237	-0.24179	-0.24121	-0.24063	-0.2938
-0.24506	-0.24446	-0.24387	-0.24327	-0.24268	-0.2421	-0.24151	-0.24093	-0.29415
-0.24537	-0.24478	-0.24418	-0.24359	-0.243	-0.24241	-0.24182	-0.24123	-0.24066
-0.24569	-0.24509	-0.24449	-0.2439	-0.24331	-0.24272	-0.24213	-0.24154	-0.24096
-0.24601	-0.24541	-0.24481	-0.24421	-0.24362	-0.24303	-0.24244	-0.24185	-0.24128
-0.24633	-0.24572	-0.24512	-0.24453	-0.24393	-0.24334	-0.24275	-0.24216	-0.24158
-0.24665	-0.24604	-0.24544	-0.24484	-0.24425	-0.24365	-0.24306	-0.24248	-0.24189
-0.24697	-0.24636	-0.24576	-0.24516	-0.24456	-0.24397	-0.24337	-0.24279	-0.2422
-0.24729	-0.24668	-0.24608	-0.24547	-0.24488	-0.24428	-0.24369	-0.2431	-0.24251
-0.24761	-0.24701	-0.2464	-0.24579	-0.24519	-0.24459	-0.244	-0.24341	-0.24283
-0.24794	-0.24733	-0.24672	-0.24611	-0.24551	-0.24491	-0.24431	-0.24372	-0.24314
-0.24827	-0.24766	-0.24704	-0.24644	-0.24583	-0.24523	-0.24463	-0.24404	-0.24346
-0.2486	-0.24798	-0.24737	-0.24676	-0.24615	-0.24554	-0.24495	-0.24436	-0.24378
-0.24893	-0.24831	-0.2477	-0.24708	-0.24647	-0.24586	-0.24527	-0.24468	-0.24411
-0.24926	-0.24864	-0.24802	-0.24741	-0.24679	-0.24619	-0.24559	-0.245	-0.24443
-0.24959	-0.24897	-0.24835	-0.24773	-0.24712	-0.24651	-0.24591	-0.24532	-0.24474
-0.24992	-0.2493	-0.24868	-0.24806	-0.24745	-0.24684	-0.24624	-0.24565	-0.24506
-0.25025	-0.24963	-0.249	-0.24839	-0.24778	-0.24717	-0.24658	-0.24599	-0.2454
-0.18858	-0.24996	-0.24933	-0.24872	-0.24811	-0.2475	-0.24691	-0.24632	-0.24573
-0.18882	-0.25029	-0.24967	-0.24905	-0.24844	-0.24783	-0.24724	-0.24665	-0.24605
-0.18907	-0.25062	-0.25	-0.24938	-0.24877	-0.24817	-0.24757	-0.24697	-0.24637
-0.18931	-0.25096	-0.25034	-0.24972	-0.24912	-0.24851	-0.24791	-0.24731	-0.2467
-0.18956	-0.2513	-0.25068	-0.25007	-0.24947	-0.24886	-0.24826	-0.24765	-0.24704
-0.1898	-0.25164	-0.25102	-0.25041	-0.24981	-0.2492	-0.2486	-0.24799	-0.24737
-0.19004	-0.25198	-0.25136	-0.25074	-0.25014	-0.24953	-0.24892	-0.24831	-0.2477
-0.19029	-0.25232	-0.2517	-0.25109	-0.25048	-0.24987	-0.24926	-0.24864	-0.24803
-0.19054	-0.25268	-0.25206	-0.25144	-0.25083	-0.25021	-0.24959	-0.24898	-0.24836
-0.19079	-0.19033	-0.18986	-0.2518	-0.25118	-0.25056	-0.24993	-0.24931	-0.2487
-0.19106	-0.19059	-0.19013	-0.25215	-0.25152	-0.25089	-0.25027	-0.24965	-0.24904
-0.19133	-0.19086	-0.19039	-0.2525	-0.25186	-0.25123	-0.25061	-0.25	-0.24939
-0.19158	-0.19111	-0.19064	-0.25284	-0.25221	-0.25158	-0.25096	-0.25035	-0.24974
-0.19183	-0.19136	-0.19089	-0.25319	-0.25256	-0.25193	-0.25131	-0.2507	-0.25009
-0.19209	-0.19162	-0.19115	-0.25354	-0.25291	-0.25228	-0.25166	-0.25105	-0.25044
-0.19237	-0.19188	-0.1914	-0.2539	-0.25327	-0.25264	-0.25202	-0.2514	-0.25078
-0.19264	-0.19215	-0.19166	-0.19119	-0.19072	-0.253	-0.25238	-0.25175	-0.25113
-0.1929	-0.19241	-0.19193	-0.19146	-0.19099	-0.25336	-0.25273	-0.2521	-0.25148
-0.19316	-0.19268	-0.1922	-0.19173	-0.19126	-0.25372	-0.25309	-0.25246	-0.25183
-0.19343	-0.19295	-0.19247	-0.192	-0.19152	-0.25408	-0.25345	-0.25282	-0.25219
-0.19371	-0.19323	-0.19275	-0.19226	-0.19178	-0.25443	-0.2538	-0.25318	-0.25255
-0.19399	-0.1935	-0.19302	-0.19253	-0.19204	-0.19156	-0.19109	-0.19062	-0.25291
-0.19426	-0.19377	-0.19328	-0.19279	-0.19231	-0.19184	-0.19136	-0.19089	-0.25326
-0.19453	-0.19404	-0.19355	-0.19306	-0.19259	-0.19211	-0.19163	-0.19115	-0.25362
-0.1948	-0.1943	-0.19382	-0.19334	-0.19286	-0.19238	-0.19189	-0.19141	-0.25398
-0.19506	-0.19457	-0.19409	-0.19361	-0.19313	-0.19265	-0.19216	-0.19167	-0.19119
-0.19534	-0.19485	-0.19436	-0.19388	-0.19339	-0.19291	-0.19243	-0.19194	-0.19147
-0.19563	-0.19513	-0.19464	-0.19415	-0.19365	-0.19317	-0.19269	-0.19222	-0.19175
-0.12882	-0.19541	-0.19491	-0.19441	-0.19392	-0.19344	-0.19297	-0.1925	-0.19202
-0.129	-0.19569	-0.19518	-0.19468	-0.19419	-0.19371	-0.19324	-0.19277	-0.1923
-0.12918	-0.19597	-0.19545	-0.19495	-0.19447	-0.19399	-0.19352	-0.19304	-0.19257
-0.12935	-0.19625	-0.19574	-0.19524	-0.19475	-0.19427	-0.19379	-0.19332	-0.19284

-0.12952	-0.12921	-0.1289	-0.19553	-0.19503	-0.19454	-0.19406	-0.19359	-0.19312
-0.1297	-0.12939	-0.12909	-0.19582	-0.19531	-0.19482	-0.19434	-0.19386	-0.19339
-0.12988	-0.12958	-0.12928	-0.19611	-0.1956	-0.1951	-0.19462	-0.19414	-0.19366
-0.13007	-0.12976	-0.12945	-0.19641	-0.19589	-0.19539	-0.19489	-0.19441	-0.19393
-0.13026	-0.12995	-0.12963	-0.1967	-0.19619	-0.19568	-0.19518	-0.19468	-0.1942
-0.06344	-0.13013	-0.12982	-0.12951	-0.1292	-0.19597	-0.19546	-0.19496	-0.19448
-0.06352	-0.13031	-0.13	-0.12969	-0.12938	-0.19627	-0.19575	-0.19525	-0.19475
-0.06361	-0.13049	-0.13018	-0.12987	-0.12956	-0.19657	-0.19605	-0.19554	-0.19504
-0.0637	-0.13068	-0.13036	-0.13005	-0.12974	-0.19686	-0.19635	-0.19583	-0.19532
-0.06378	-0.06364	-0.0635	-0.13023	-0.12993	-0.12962	-0.12931	-0.129	-0.19562
-0.06387	-0.06373	-0.06358	-0.13041	-0.13011	-0.1298	-0.12949	-0.12918	-0.19591
-0.06395	-0.06381	-0.06367	-0.1306	-0.13029	-0.12997	-0.12967	-0.12937	-0.19621
	-0.0639	-0.06376	-0.13078	-0.13047	-0.13016	-0.12985	-0.12954	-0.19652
	-0.06398	-0.06384	-0.13096	-0.13065	-0.13034	-0.13003	-0.12972	-0.19683
			-0.06379	-0.06365	-0.13053	-0.13022	-0.12991	-0.12961
			-0.06389	-0.06374	-0.13072	-0.13041	-0.1301	-0.1298
			-0.06397	-0.06383	-0.13091	-0.1306	-0.13029	-0.12998
			-0.06406	-0.06392	-0.13108	-0.13077	-0.13047	-0.13016
					-0.06386	-0.06372	-0.06358	-0.13034
					-0.06395	-0.06381	-0.06367	-0.13052
					-0.06403	-0.06389	-0.06375	-0.13071
								-0.13089
								-0.13108
								-0.06388
								-0.06397

								-0.06076
								-0.06067
								-0.06057
								-0.06047
								-0.06038
					-0.0608	-0.06063	-0.06046	-0.38287
					-0.06071	-0.06053	-0.06036	-0.38228
					-0.06061	-0.06044	-0.06027	-0.3817
					-0.06052	-0.06034	-0.06017	-0.38112
			-0.0608	-0.06061	-0.38364	-0.38259	-0.38154	-0.44149
			-0.0607	-0.06051	-0.38306	-0.382	-0.38097	-0.44083
			-0.06061	-0.06042	-0.38246	-0.38142	-0.38039	-0.44017
	-0.06087	-0.06069	-0.38393	-0.3829	-0.44308	-0.44188	-0.44069	-0.71714
	-0.06077	-0.0606	-0.38333	-0.38232	-0.44241	-0.44123	-0.44004	-0.7161
	-0.06068	-0.0605	-0.38274	-0.38173	-0.66444	-0.66273	-0.66102	-0.81262
	-0.06058	-0.0604	-0.38216	-0.38116	-0.6635	-0.66181	-0.66013	-0.81149
-0.06067	-0.38368	-0.38262	-0.5589	-0.55746	-0.76777	-0.76582	-0.76388	-0.86048
-0.06057	-0.38309	-0.38204	-0.55808	-0.55664	-0.76661	-0.76468	-0.76277	-0.85924
-0.06047	-0.38251	-0.38147	-0.55728	-0.55584	-0.76548	-0.76356	-0.76167	-0.85801
-0.25673	-0.55939	-0.55794	-0.71338	-0.71154	-0.81301	-0.81096	-0.80894	-0.8568
-0.25634	-0.55858	-0.55717	-0.71242	-0.7106	-0.81189	-0.80983	-0.8078	-0.8556
-0.44308	-0.66475	-0.66307	-0.76625	-0.76428	-0.86088	-0.85868	-0.85652	-0.8544
-0.44245	-0.66384	-0.66215	-0.76513	-0.76321	-0.85971	-0.85752	-0.85534	-0.85321
-0.55765	-0.71306	-0.71123	-0.81262	-0.8106	-0.85852	-0.85633	-0.85414	-0.85201
-0.55689	-0.7121	-0.71027	-0.81147	-0.80945	-0.85729	-0.85511	-0.85293	-0.8508
-0.71295	-0.76585	-0.76385	-0.86035	-0.8582	-0.85606	-0.8539	-0.85174	-0.89457
-0.71194	-0.76474	-0.76277	-0.85913	-0.85697	-0.85483	-0.85271	-0.8506	-0.89337
-0.71094	-0.76365	-0.7617	-0.85793	-0.85576	-0.85362	-0.85153	-0.84946	-0.89218
-0.70998	-0.81108	-0.80901	-0.85675	-0.85458	-0.85243	-0.85034	-0.84826	-0.89094
-0.70906	-0.80998	-0.80793	-0.8556	-0.85343	-0.85127	-0.84916	-0.84705	-0.88968
-0.81095	-0.85877	-0.8566	-0.85444	-0.85228	-0.89509	-0.89287	-0.89066	-0.93969
-0.80985	-0.85762	-0.85543	-0.85327	-0.85114	-0.89391	-0.89169	-0.88947	-0.93844
-0.80873	-0.85643	-0.85424	-0.89711	-0.8949	-0.8927	-0.89049	-0.8883	-0.9372
-0.80757	-0.8552	-0.85303	-0.89585	-0.89363	-0.89144	-0.88928	-0.88714	-0.93598
-0.85613	-0.85397	-0.85183	-0.89459	-0.89237	-0.94151	-0.93925	-0.93702	-1.00001
-0.85495	-0.85279	-0.85065	-0.89337	-0.89115	-0.94021	-0.93795	-0.93571	-0.99866
-0.85379	-0.85163	-0.84949	-0.89216	-0.88995	-0.93893	-0.93665	-0.93439	-0.99729
-0.85263	-0.8954	-0.89317	-0.89096	-0.88877	-1.00317	-1.00077	-0.99837	-0.99599
-0.85149	-0.8942	-0.89198	-0.88978	-0.88761	-1.0019	-0.9995	-0.9971	-0.99471
-0.89524	-0.89301	-0.89079	-1.00546	-1.00303	-1.00062	-0.99822	-0.99583	-0.99344
-0.89403	-0.89182	-0.88962	-1.00417	-1.00174	-0.99933	-0.99694	-0.99457	-0.99219
-0.89283	-0.94195	-0.93963	-1.00287	-1.00045	-0.99805	-0.99568	-0.99331	-1.07697
-0.89164	-0.94068	-0.93836	-1.00157	-0.99918	-0.99679	-0.99442	-0.99205	-1.07559
-0.89045	-0.93942	-0.9371	-1.00028	-0.9979	-0.99554	-0.99317	-0.9908	-1.07422
-0.88926	-1.00382	-1.0014	-0.99901	-0.99664	-1.03862	-1.03616	-1.03369	-1.11715

-0.88807	-1.00254	-1.00013	-0.99775	-0.99539	-1.03732	-1.03486	-1.0324	-1.1157
-1.00367	-1.00126	-0.99887	-0.9965	-0.99414	-1.07797	-1.07534	-1.07271	-1.11424
-1.00238	-0.99998	-0.99762	-0.99525	-0.99289	-1.07658	-1.07395	-1.07133	-1.11277
-1.0011	-0.99871	-0.99635	-1.03831	-1.03586	-1.1196	-1.11686	-1.11411	-1.11135
-0.99982	-0.99744	-0.99507	-1.03698	-1.03454	-1.11816	-1.11542	-1.11269	-1.10996
-0.99855	-0.99617	-0.9938	-1.07764	-1.07505	-1.11672	-1.11399	-1.11127	-1.19507
-0.99728	-0.99491	-0.99255	-1.07628	-1.07368	-1.11527	-1.11255	-1.10984	-1.19334
-0.99602	-0.99366	-0.99131	-1.07491	-1.0723	-1.11382	-1.11112	-1.10842	-1.19163
-0.99476	-1.07874	-1.07614	-1.11783	-1.1151	-1.19949	-1.1963	-1.19314	-1.23229
-0.99351	-1.07736	-1.07475	-1.11638	-1.11365	-1.19777	-1.19461	-1.19149	-1.23058
-0.9923	-1.07601	-1.07338	-1.11493	-1.11221	-1.23885	-1.23546	-1.23212	-1.22887
-0.99113	-1.0747	-1.07202	-1.11348	-1.11077	-1.237	-1.23365	-1.23037	-1.22715
-1.07606	-1.11766	-1.11483	-1.199	-1.1958	-1.23518	-1.23188	-1.22864	-1.22545
-1.07469	-1.11625	-1.11347	-1.19739	-1.19417	-1.2334	-1.23013	-1.22693	-1.22375
-1.07331	-1.11484	-1.11211	-1.23855	-1.23507	-1.23167	-1.22842	-1.22523	-1.22206
-1.07193	-1.1134	-1.11072	-1.23678	-1.23338	-1.23003	-1.22676	-1.22353	-1.22036
-1.07056	-1.11197	-1.10932	-1.23501	-1.23171	-1.22842	-1.22512	-1.22185	-1.21866
-1.11332	-1.24	-1.23658	-1.23324	-1.22999	-1.22675	-1.22349	-1.22024	-1.21703
-1.112	-1.23826	-1.23481	-1.23147	-1.22825	-1.22506	-1.22186	-1.21866	-1.21544
-1.11064	-1.23653	-1.23311	-1.22978	-1.22655	-1.22336	-1.22019	-1.21703	-1.25582
-1.10926	-1.2348	-1.23147	-1.22817	-1.22488	-1.22165	-1.21849	-1.21536	-1.25407
-1.23636	-1.23305	-1.22981	-1.22654	-1.22324	-1.21998	-1.21681	-1.21369	-1.25229
-1.23455	-1.23127	-1.22809	-1.22488	-1.22162	-1.21839	-1.21519	-1.21203	-1.25047
-1.23279	-1.22953	-1.22638	-1.22322	-1.22003	-1.21683	-1.2136	-1.21039	-1.24867
-1.23113	-1.22788	-1.22474	-1.2216	-1.21845	-1.25746	-1.25389	-1.25035	-1.29261
-1.22952	-1.22628	-1.22312	-1.21998	-1.21686	-1.25575	-1.25222	-1.2487	-1.29082
-1.22791	-1.22466	-1.22147	-1.26081	-1.25741	-1.25399	-1.25051	-1.24705	-1.28908
-1.2263	-1.22303	-1.21981	-1.25897	-1.25557	-1.25218	-1.24878	-1.24541	-1.28737
-1.2247	-1.22143	-1.21818	-1.25718	-1.25375	-1.25037	-1.24704	-1.24374	-1.28565
-1.22311	-1.21986	-1.2166	-1.25543	-1.25195	-1.24855	-1.24526	-1.24202	-1.28392
-1.22153	-1.26082	-1.25724	-1.25371	-1.25021	-1.2925	-1.28899	-1.28557	-1.28217
-1.21996	-1.25912	-1.25554	-1.25202	-1.24853	-1.29072	-1.28718	-1.28374	-1.2804
-1.21837	-1.25741	-1.25385	-1.25035	-1.2469	-1.28899	-1.28542	-1.28196	-1.27866
-1.21672	-1.25565	-1.25215	-1.24869	-1.24527	-1.28729	-1.28374	-1.28029	-1.27704
-1.21504	-1.25388	-1.25045	-1.24705	-1.24366	-1.28561	-1.2821	-1.27871	-1.27549
-1.25552	-1.25215	-1.2488	-1.29108	-1.28748	-1.28394	-1.28047	-1.27712	-1.27394
-1.25379	-1.25046	-1.24717	-1.28939	-1.28581	-1.28229	-1.27886	-1.27555	-1.2724
-1.25206	-1.24875	-1.24551	-1.28768	-1.28414	-1.28067	-1.2773	-1.27405	-1.30939
-1.25029	-1.247	-1.24379	-1.28592	-1.28247	-1.27909	-1.2758	-1.27262	-1.30797
-1.24857	-1.29096	-1.28757	-1.28419	-1.28082	-1.27752	-1.27432	-1.27122	-1.34315
-1.24694	-1.28923	-1.28585	-1.2825	-1.27919	-1.27596	-1.27282	-1.26979	-1.3417
-1.24534	-1.28755	-1.28417	-1.28085	-1.27759	-1.27443	-1.27136	-1.2684	-1.34027
-1.24373	-1.28586	-1.28251	-1.27925	-1.27607	-1.31151	-1.30838	-1.30536	-1.37767
-1.24212	-1.28419	-1.28088	-1.27768	-1.27459	-1.31006	-1.30699	-1.30402	-1.37627
-1.28591	-1.28253	-1.27927	-1.3148	-1.31166	-1.34517	-1.3421	-1.33911	-1.37488
-1.28423	-1.28088	-1.27767	-1.31319	-1.31012	-1.34366	-1.34067	-1.33774	-1.37351
-1.28257	-1.31807	-1.31479	-1.34827	-1.34519	-1.3422	-1.33927	-1.33639	-1.37214
-1.28093	-1.31643	-1.31323	-1.34675	-1.34374	-1.3408	-1.3379	-1.33505	-1.37077
-1.27934	-1.31484	-1.31173	-1.34527	-1.34233	-1.33943	-1.33656	-1.33372	-1.3694
-1.27784	-1.31336	-1.3103	-1.34384	-1.34094	-1.37682	-1.37386	-1.37094	-1.36805

-1.2764	-1.31194	-1.30892	-1.34246	-1.33958	-1.37543	-1.37249	-1.36958	-1.36671
-1.31364	-1.3471	-1.34405	-1.34109	-1.33823	-1.37404	-1.37112	-1.36823	-1.36537
-1.31223	-1.34569	-1.34269	-1.33976	-1.33689	-1.37266	-1.36975	-1.36688	-1.36404
-1.31081	-1.34428	-1.34132	-1.33841	-1.33556	-1.37129	-1.3684	-1.36554	-1.36271
-1.3094	-1.34286	-1.33995	-1.33707	-1.33423	-1.36992	-1.36705	-1.36421	-1.3614
-1.34445	-1.34147	-1.33858	-1.33573	-1.3329	-1.36857	-1.36571	-1.36289	-1.3601
-1.34304	-1.3401	-1.33724	-1.3344	-1.33159	-1.36722	-1.36437	-1.36156	-1.35879
-1.34166	-1.33875	-1.3359	-1.33308	-1.33028	-1.36588	-1.36304	-1.36025	-1.35749
-1.34029	-1.3374	-1.33456	-1.37026	-1.36739	-1.36454	-1.36173	-1.35895	-1.35621
-1.33893	-1.33606	-1.33323	-1.3689	-1.36604	-1.36322	-1.36042	-1.35767	-1.35496
-1.33758	-1.33474	-1.33192	-1.36755	-1.3647	-1.36189	-1.35912	-1.35639	-1.35372
-1.33625	-1.33342	-1.33062	-1.36621	-1.36336	-1.36056	-1.35781	-1.35511	-1.3525
-1.33492	-1.33212	-1.32936	-1.36493	-1.3621	-1.35932	-1.35658	-1.35391	-1.35131
-1.3336	-1.33084	-1.32813	-1.36372	-1.36094	-1.3582	-1.35548	-1.3528	-1.35017
-1.33232	-1.368	-1.36524	-1.36249	-1.35976	-1.35705	-1.35434	-1.35166	-1.34902
-1.33115	-1.36677	-1.36396	-1.3612	-1.35848	-1.35579	-1.35311	-1.35045	-1.3478
-1.32998	-1.36553	-1.36268	-1.3599	-1.35718	-1.3545	-1.35184	-1.3492	-1.34657
-1.32872	-1.36423	-1.36139	-1.3586	-1.35589	-1.35321	-1.35057	-1.34794	-1.34532
-1.32742	-1.3629	-1.36008	-1.35731	-1.35461	-1.35193	-1.34929	-1.34668	-1.34407
-1.3644	-1.36156	-1.35877	-1.35603	-1.35333	-1.35067	-1.34804	-1.34543	-1.37978
-1.36303	-1.36021	-1.35745	-1.35473	-1.35207	-1.34942	-1.3468	-1.34419	-1.3785
-1.36174	-1.35894	-1.35621	-1.35351	-1.35087	-1.34824	-1.34562	-1.34302	-1.37728
-1.36058	-1.3578	-1.35508	-1.3524	-1.34977	-1.34715	-1.34454	-1.34194	-1.37617
-1.35941	-1.35666	-1.35395	-1.35128	-1.34866	-1.34604	-1.34344	-1.34084	-1.37504
-1.35812	-1.3554	-1.35273	-1.35008	-1.34746	-1.34485	-1.34223	-1.33963	-1.51262
-1.35681	-1.35411	-1.35147	-1.34884	-1.34622	-1.34361	-1.34099	-1.33839	-1.51097
-1.35557	-1.35287	-1.35023	-1.3476	-1.34499	-1.34238	-1.33976	-1.33715	-1.50934
-1.35439	-1.35167	-1.349	-1.34636	-1.34375	-1.34114	-1.33852	-1.33591	-1.50773
-1.35324	-1.35052	-1.34785	-1.3452	-1.34259	-1.51648	-1.51299	-1.50955	-1.50623
-1.35212	-1.34944	-1.34677	-1.34413	-1.34151	-1.51502	-1.51155	-1.50815	-1.50486
-1.35097	-1.34831	-1.34566	-1.36985	-1.36717	-1.51352	-1.51009	-1.50672	-1.52827
-1.34973	-1.34708	-1.34445	-1.36861	-1.36594	-1.51191	-1.50853	-1.50521	-1.52678
-1.3485	-1.34586	-1.34325	-1.36738	-1.36471	-1.51032	-1.50699	-1.50372	-1.5253
-1.34739	-1.37157	-1.3689	-1.36624	-1.36359	-1.53386	-1.5305	-1.52719	-1.52388
-1.34631	-1.37046	-1.36778	-1.36513	-1.36249	-1.53244	-1.52911	-1.52582	-1.5225
-1.37197	-1.36926	-1.36658	-1.5127	-1.50932	-1.53096	-1.52765	-1.52438	-1.55543
-1.37071	-1.36801	-1.36533	-1.5111	-1.50776	-1.52943	-1.52614	-1.52288	-1.55394
-1.36951	-1.36681	-1.36414	-1.53465	-1.53129	-1.52797	-1.52469	-1.52145	-1.55249
-1.36837	-1.36568	-1.36305	-1.53324	-1.5299	-1.5266	-1.52334	-1.52011	-1.55108
-1.36723	-1.36456	-1.36194	-1.53181	-1.52849	-1.55965	-1.55631	-1.55299	-1.54966
-1.36606	-1.36337	-1.36075	-1.53028	-1.52698	-1.5581	-1.55477	-1.55146	-1.5482
-1.36489	-1.3622	-1.35958	-1.52877	-1.52548	-1.55656	-1.55323	-1.54995	-1.54674
-1.36376	-1.38831	-1.38563	-1.56184	-1.55847	-1.55514	-1.55184	-1.54858	-1.54536
-1.36265	-1.38723	-1.38457	-1.56045	-1.55709	-1.55377	-1.5505	-1.54726	-1.54401
-1.36156	-1.53137	-1.52797	-1.559	-1.55563	-1.55231	-1.54908	-1.54587	-1.54265
-1.36047	-1.52997	-1.52654	-1.55749	-1.55409	-1.55077	-1.54757	-1.54442	-1.54127
-1.38658	-1.56297	-1.55946	-1.55601	-1.55261	-1.5493	-1.54612	-1.54301	-1.53992
-1.38537	-1.56139	-1.55796	-1.55457	-1.55122	-1.54795	-1.54478	-1.54168	-1.53862
-1.42093	-1.55984	-1.55647	-1.55314	-1.54985	-1.54662	-1.54346	-1.54035	-1.5373
-1.41977	-1.55838	-1.55502	-1.55172	-1.54849	-1.54529	-1.54211	-1.53897	-1.5359

-1.41865	-1.55695	-1.55358	-1.5503	-1.54711	-1.54395	-1.54075	-1.53759	-1.53448
-1.52581	-1.55553	-1.55215	-1.54885	-1.54566	-1.54251	-1.53935	-1.53622	-1.53313
-1.52443	-1.55411	-1.55071	-1.5474	-1.54417	-1.54101	-1.53792	-1.53488	-1.53182
-1.52299	-1.55265	-1.54928	-1.54598	-1.54276	-1.53961	-1.53656	-1.53355	-1.53052
-1.5215	-1.55114	-1.54784	-1.5446	-1.54142	-1.5383	-1.53526	-1.53225	-1.52923
-1.55303	-1.54968	-1.54643	-1.54323	-1.54009	-1.537	-1.53397	-1.53096	-1.52794
-1.5516	-1.54829	-1.54505	-1.54188	-1.53878	-1.53571	-1.53269	-1.52967	-1.52663
-1.5502	-1.54692	-1.5437	-1.54055	-1.53747	-1.53443	-1.5314	-1.52837	-1.5253
-1.5488	-1.54555	-1.54238	-1.53925	-1.53619	-1.53314	-1.53007	-1.52702	-1.52396
-1.54742	-1.5442	-1.54107	-1.53797	-1.5349	-1.53184	-1.52874	-1.52565	-1.52261
-1.54605	-1.54286	-1.53975	-1.53665	-1.53357	-1.53049	-1.52739	-1.52432	-1.55059
-1.54472	-1.54153	-1.53842	-1.53531	-1.53219	-1.5291	-1.52604	-1.52301	-1.54929
-1.54339	-1.54021	-1.53707	-1.53395	-1.53083	-1.52775	-1.52472	-1.52173	-1.57067
-1.54208	-1.53888	-1.5357	-1.53256	-1.52946	-1.52642	-1.52342	-1.52047	-1.56942
-1.54077	-1.53757	-1.53435	-1.53121	-1.52814	-1.52512	-1.52216	-1.51924	-1.56818
-1.53946	-1.53627	-1.53308	-1.52995	-1.52688	-1.52387	-1.52095	-1.51806	-1.56695
-1.53814	-1.53498	-1.53183	-1.52872	-1.52566	-1.52266	-1.51976	-1.51688	-1.56573
-1.53679	-1.53364	-1.53052	-1.52745	-1.52442	-1.5447	-1.54177	-1.53886	-1.56453
-1.53544	-1.5323	-1.52921	-1.52618	-1.52319	-1.54351	-1.54059	-1.53768	-1.56332
-1.53414	-1.53101	-1.52794	-1.52493	-1.52198	-1.54233	-1.53938	-1.53645	-1.56206
-1.5329	-1.52978	-1.52671	-1.52371	-1.52079	-1.54115	-1.53815	-1.53517	-1.56075
-1.53165	-1.52854	-1.52548	-1.5225	-1.5196	-1.53997	-1.53695	-1.53393	-1.55949
-1.53041	-1.52731	-1.52427	-1.52129	-1.5184	-1.53878	-1.53577	-1.53276	-1.55828
-1.52917	-1.52608	-1.52305	-1.52009	-1.51721	-1.5376	-1.5346	-1.5316	-1.55709
-1.52793	-1.52487	-1.52185	-1.5189	-1.51603	-1.53643	-1.53342	-1.53042	-1.55589
-1.52671	-1.52365	-1.52065	-1.51771	-1.51484	-1.53525	-1.53225	-1.52925	-1.55469
-1.52549	-1.52245	-1.51945	-1.53991	-1.53701	-1.56261	-1.55955	-1.55649	-1.58096
-1.52429	-1.52125	-1.51825	-1.53872	-1.53582	-1.56141	-1.55836	-1.55532	-1.57981
-1.52314	-1.52011	-1.5171	-1.53756	-1.53465	-1.58777	-1.58471	-1.58166	-1.57866
-1.52205	-1.51903	-1.51599	-1.53643	-1.53349	-1.58658	-1.58354	-1.58052	-1.57753
-1.52092	-1.51792	-1.51488	-1.5353	-1.53234	-1.5854	-1.58238	-1.57938	-1.57639
-1.51973	-1.51676	-1.51373	-1.53418	-1.5312	-1.58423	-1.58121	-1.57823	-1.57525
-1.51853	-1.51557	-1.51257	-1.53303	-1.53005	-1.58306	-1.58007	-1.57709	-1.57412
-1.51733	-1.51437	-1.51138	-1.56	-1.55701	-1.58191	-1.57896	-1.57602	-1.60392
-1.51614	-1.51316	-1.51017	-1.55882	-1.55585	-1.58076	-1.57785	-1.57496	-1.60282
-1.51494	-1.56373	-1.56066	-1.55765	-1.55469	-1.57958	-1.5767	-1.57383	-1.60165
-1.51374	-1.56255	-1.5595	-1.55649	-1.55354	-1.57839	-1.57552	-1.57266	-1.60044
-1.51257	-1.56141	-1.55838	-1.55539	-1.55242	-1.6082	-1.60523	-1.60227	-1.59923
-1.51144	-1.56031	-1.55732	-1.55434	-1.55136	-1.60704	-1.60404	-1.60105	-1.59801
-1.56222	-1.5592	-1.55625	-1.55328	-1.5503	-1.60588	-1.60285	-1.59984	-1.5968
-1.56106	-1.55805	-1.5551	-1.55215	-1.54919	-1.60471	-1.60166	-1.59863	-1.59561
-1.55992	-1.55692	-1.55396	-1.55102	-1.54809	-1.60355	-1.60048	-1.59743	-1.59443
-1.55885	-1.55585	-1.55287	-1.5813	-1.57831	-1.60242	-1.59933	-1.59625	-1.63857
-1.5578	-1.5548	-1.55181	-1.58019	-1.57721	-1.60129	-1.59819	-1.59509	-1.6374
-1.55669	-1.5537	-1.55071	-1.57905	-1.57606	-1.64554	-1.64242	-1.63931	-1.66714
-1.55552	-1.55255	-1.54958	-1.57788	-1.57486	-1.64432	-1.64122	-1.63813	-1.66587
-1.55439	-1.5828	-1.57977	-1.62302	-1.61997	-1.64314	-1.64005	-1.63697	-1.66464
-1.55332	-1.58169	-1.57866	-1.62194	-1.61888	-1.64202	-1.63893	-1.63586	-1.66347
-1.55227	-1.62697	-1.62393	-1.62087	-1.61781	-1.6721	-1.66881	-1.66555	-1.6623
-1.55126	-1.62593	-1.62286	-1.6198	-1.61677	-1.67095	-1.66764	-1.66435	-1.66112

-1.55024	-1.62488	-1.62178	-1.61873	-1.61572	-1.6698	-1.66646	-1.66314	-1.65993
-1.59645	-1.62376	-1.62067	-1.61762	-1.6146	-1.66857	-1.66524	-1.66194	-1.65874
-1.59532	-1.6226	-1.61954	-1.61649	-1.61344	-1.66728	-1.66401	-1.66076	-1.65754
-1.62456	-1.62148	-1.61845	-1.6154	-1.61234	-1.66607	-1.66282	-1.6596	-1.65639
-1.62349	-1.62042	-1.61739	-1.61435	-1.61129	-1.66491	-1.66167	-1.65847	-1.65527
-1.62243	-1.61936	-1.61633	-1.64473	-1.64148	-1.66377	-1.66055	-1.65735	-1.65416
-1.62139	-1.61832	-1.61528	-1.64359	-1.64038	-1.66267	-1.65944	-1.65624	-1.65305
-1.62035	-1.61729	-1.61423	-1.64247	-1.63929	-1.66157	-1.65834	-1.65513	-1.65194
-1.61929	-1.61624	-1.6132	-1.64137	-1.63819	-1.66045	-1.65723	-1.65402	-1.65084
-1.61822	-1.61519	-1.61218	-1.64028	-1.63709	-1.65931	-1.6561	-1.65291	-1.64973
-1.61717	-1.61415	-1.61115	-1.63918	-1.636	-1.65819	-1.65498	-1.65178	-1.64859
-1.61614	-1.61312	-1.61012	-1.63807	-1.63491	-1.65708	-1.65385	-1.65063	-1.64743
-1.61511	-1.6121	-1.60909	-1.63698	-1.63381	-1.63065	-1.62747	-1.62431	-1.64632
-1.61408	-1.61107	-1.60807	-1.6359	-1.63271	-1.62953	-1.62637	-1.62323	-1.64525
-1.61306	-1.64115	-1.63798	-1.63481	-1.63161	-1.62843	-1.62528	-1.62216	-1.64417
-1.61203	-1.64005	-1.63687	-1.63369	-1.63052	-1.62735	-1.62421	-1.62108	-1.64305
-1.611	-1.63894	-1.63575	-1.63257	-1.62942	-1.62627	-1.62313	-1.62001	-1.64194
-1.60994	-1.63781	-1.63464	-1.63147	-1.62829	-1.62515	-1.62204	-1.61896	-1.64089
-1.60888	-1.63668	-1.63353	-1.63036	-1.62716	-1.62401	-1.62094	-1.61791	-1.63985
-1.60788	-1.63559	-1.63242	-1.62924	-1.62604	-1.6229	-1.61985	-1.61684	-1.63878
-1.60692	-1.63455	-1.6313	-1.62809	-1.62494	-1.62183	-1.61877	-1.61575	-1.63768
-1.63675	-1.6335	-1.63022	-1.627	-1.62385	-1.64581	-1.6427	-1.63962	-1.63658
-1.63566	-1.63244	-1.62919	-1.62597	-1.62279	-1.64469	-1.64159	-1.63853	-1.6355
-1.63455	-1.63136	-1.62814	-1.62494	-1.62174	-1.6436	-1.6405	-1.63744	-1.63441
-1.63342	-1.63023	-1.62705	-1.62388	-1.62072	-1.64258	-1.63943	-1.63634	-1.63331
-1.63229	-1.6291	-1.62594	-1.62281	-1.61971	-1.64156	-1.63839	-1.63525	-1.63223
-1.63124	-1.62802	-1.62485	-1.64677	-1.64364	-1.64051	-1.63736	-1.63423	-1.63122
-1.63022	-1.62699	-1.62377	-1.64564	-1.64254	-1.63945	-1.63634	-1.63326	-1.63025
-1.6292	-1.62597	-1.62273	-1.64458	-1.64148	-1.6384	-1.63533	-1.63228	-1.62927
-1.62819	-1.62497	-1.62174	-1.64356	-1.64045	-1.63738	-1.63433	-1.6313	-1.62826
-1.62715	-1.62395	-1.62074	-1.64254	-1.63941	-1.63633	-1.6333	-1.6303	-1.62725
-1.62608	-1.62291	-1.61973	-1.6415	-1.63833	-1.63521	-1.63221	-1.62924	-1.62624
-1.62499	-1.62185	-1.61871	-1.64046	-1.63726	-1.63412	-1.63113	-1.62818	-1.62522
-1.6239	-1.62076	-1.61767	-1.63942	-1.63624	-1.63311	-1.63009	-1.62711	-1.62416
-1.62281	-1.61968	-1.61661	-1.63837	-1.63524	-1.63214	-1.62908	-1.62606	-1.62311
-1.62178	-1.61865	-1.61559	-1.63735	-1.63426	-1.63118	-1.62811	-1.62506	-1.62211
-1.62079	-1.61765	-1.61458	-1.63633	-1.63328	-1.63024	-1.62716	-1.62411	-1.62114
-1.6198	-1.61665	-1.61356	-1.63528	-1.63227	-1.62926	-1.6262	-1.62316	-1.62016
-1.61882	-1.61563	-1.6125	-1.63419	-1.63122	-1.62824	-1.62522	-1.6222	-1.61918
-1.61784	-1.61464	-1.61148	-1.63314	-1.63018	-1.62723	-1.62424	-1.62124	-1.6182
-1.61686	-1.61367	-1.61053	-1.63217	-1.62921	-1.62625	-1.62327	-1.62028	-1.61723
-1.61587	-1.6127	-1.6096	-1.63123	-1.62825	-1.62528	-1.6223	-1.61931	-1.61626
-1.61488	-1.61173	-1.60865	-1.63027	-1.62729	-1.62432	-1.62134	-1.61835	-1.6153
-1.61389	-1.61076	-1.60771	-1.62932	-1.62634	-1.62336	-1.62038	-1.61738	-1.61433
-1.61291	-1.60979	-1.60676	-1.62836	-1.62538	-1.6224	-1.61941	-1.61641	-1.61336
-1.61194	-1.60884	-1.60581	-1.62739	-1.62441	-1.62144	-1.61845	-1.61544	-1.61239
-1.61099	-1.60789	-1.60487	-1.62642	-1.62345	-1.62047	-1.61748	-1.61446	-1.61142
-1.61005	-1.60696	-1.60394	-1.62546	-1.62248	-1.6195	-1.6165	-1.61349	-1.61045
-1.60911	-1.60603	-1.603	-1.6245	-1.62151	-1.61853	-1.61553	-1.61252	-1.60948
-1.60816	-1.60509	-1.60205	-1.62352	-1.62054	-1.61755	-1.61456	-1.61155	-1.60851

-1.60722	-1.60414	-1.6011	-1.62254	-1.61956	-1.61657	-1.61358	-1.61058	-1.60755
-1.60627	-1.6032	-1.60016	-1.62157	-1.61857	-1.61559	-1.6126	-1.60961	-1.60658
-1.60531	-1.60225	-1.59922	-1.6206	-1.61759	-1.6146	-1.61162	-1.60864	-1.60562
-1.60437	-1.60132	-1.59831	-1.61968	-1.61666	-1.61365	-1.61066	-1.60767	-1.60465
-1.60342	-1.6004	-1.59743	-1.61879	-1.61577	-1.61274	-1.60972	-1.6067	-1.60368
-1.60248	-1.59947	-1.59652	-1.61787	-1.61485	-1.61182	-1.60878	-1.60574	-1.60271
-1.60152	-1.59852	-1.59556	-1.6169	-1.61389	-1.61086	-1.60782	-1.60478	-1.60175
-1.60059	-1.59758	-1.59461	-1.61592	-1.61291	-1.6099	-1.60686	-1.60381	-1.6008
-1.59969	-1.59668	-1.59371	-1.61498	-1.61195	-1.60893	-1.60589	-1.60286	-1.59985
-1.59881	-1.59581	-1.59281	-1.61405	-1.611	-1.60795	-1.60492	-1.60191	-1.59891
-1.59792	-1.61922	-1.61616	-1.61309	-1.61003	-1.60698	-1.60396	-1.60096	-1.59799
-1.59703	-1.61827	-1.61519	-1.61212	-1.60906	-1.60602	-1.60301	-1.60003	-1.59709
-1.59611	-1.61732	-1.61423	-1.61116	-1.6081	-1.60507	-1.60208	-1.59912	-1.5962
-1.59518	-1.61637	-1.61328	-1.6102	-1.60715	-1.60414	-1.60118	-1.59825	-1.59533
-1.59425	-1.61543	-1.61235	-1.60929	-1.60625	-1.60325	-1.6003	-1.59738	-1.59445
-1.59331	-1.61449	-1.61146	-1.60844	-1.60543	-1.60244	-1.59947	-1.5965	-1.59355
-1.5924	-1.61359	-1.61058	-1.60759	-1.60461	-1.60163	-1.59862	-1.59562	-1.59263
-1.59159	-1.61274	-1.60971	-1.6067	-1.60371	-1.60072	-1.59771	-1.5947	-1.5917
-1.59081	-1.61192	-1.60884	-1.60578	-1.60277	-1.59977	-1.59677	-1.59377	-1.59077
-1.58995	-1.61104	-1.60794	-1.60487	-1.60184	-1.59883	-1.59583	-1.59284	-1.58985
-1.58903	-1.6101	-1.60702	-1.60396	-1.60091	-1.59789	-1.59488	-1.5919	-1.58895
-1.58811	-1.60917	-1.60612	-1.60308	-1.60004	-1.59701	-1.594	-1.59102	-1.61127
-1.58719	-1.60825	-1.60524	-1.60223	-1.59923	-1.59623	-1.59322	-1.59021	-1.61042
-1.5863	-1.60735	-1.60436	-1.60138	-1.5984	-1.59542	-1.5924	-1.58938	-1.63656
-1.58549	-1.6065	-1.60348	-1.60048	-1.59749	-1.5945	-1.59149	-1.58847	-1.63561
-1.5847	-1.60565	-1.60259	-1.59956	-1.59655	-1.59355	-1.59054	-1.58754	-1.63466
-1.58383	-1.60475	-1.60168	-1.59863	-1.59561	-1.61585	-1.61284	-1.60984	-1.65545
-1.58293	-1.60383	-1.60075	-1.5977	-1.59467	-1.61489	-1.61191	-1.60896	-1.6546
-1.58209	-1.60297	-1.59989	-1.59684	-1.5938	-1.63615	-1.63314	-1.63016	-1.69712
-1.58131	-1.60217	-1.5991	-1.59604	-1.593	-1.63534	-1.63229	-1.62927	-1.69615
-1.58048	-1.60133	-1.59826	-1.61849	-1.61539	-1.63449	-1.63142	-1.62837	-1.69518
-1.57956	-1.60039	-1.59731	-1.61753	-1.61444	-1.63355	-1.63048	-1.62743	-1.69421
-1.57866	-1.59946	-1.59639	-1.63882	-1.63573	-1.65934	-1.65619	-1.65306	-1.71601
-1.57785	-1.59864	-1.59558	-1.63802	-1.63493	-1.65852	-1.65537	-1.65223	-1.71509
-1.57705	-1.59783	-1.59478	-1.63724	-1.63416	-1.65773	-1.65458	-1.65144	-1.7142
-1.60001	-1.64257	-1.63945	-1.63637	-1.63332	-1.6569	-1.65378	-1.65065	-1.71334
-1.59906	-1.64163	-1.63851	-1.63545	-1.63244	-1.65605	-1.65296	-1.64985	-1.7125
-1.5982	-1.64077	-1.63765	-1.63459	-1.63158	-1.65518	-1.65209	-1.64899	-1.71158
-1.59742	-1.63999	-1.63688	-1.63379	-1.63074	-1.65427	-1.65116	-1.64804	-1.71056
-1.64235	-1.63922	-1.63611	-1.65972	-1.65655	-1.6534	-1.65027	-1.64714	-1.72964
-1.64158	-1.63845	-1.63535	-1.65894	-1.65576	-1.6526	-1.64945	-1.64632	-1.72875
-1.64078	-1.63766	-1.63455	-1.65813	-1.65495	-1.65178	-1.64863	-1.64549	-1.72786
-1.63987	-1.63675	-1.63364	-1.6572	-1.65402	-1.71683	-1.71348	-1.71016	-1.72685
-1.63894	-1.63581	-1.6327	-1.65623	-1.65305	-1.71579	-1.71246	-1.70913	-1.72582
-1.6381	-1.63497	-1.63186	-1.65536	-1.65218	-1.71487	-1.71154	-1.70822	-1.7249
-1.63733	-1.6342	-1.63109	-1.65457	-1.65139	-1.71402	-1.7107	-1.70739	-1.72406
-1.63656	-1.66017	-1.65697	-1.65378	-1.65061	-1.73328	-1.72992	-1.72657	-1.72323
-1.63581	-1.6594	-1.65619	-1.65301	-1.64984	-1.73245	-1.7291	-1.72576	-1.72242
-1.63505	-1.65862	-1.65541	-1.67329	-1.67008	-1.73162	-1.72827	-1.72494	-1.72161
-1.63428	-1.65783	-1.65462	-1.67249	-1.6693	-1.73079	-1.72744	-1.72411	-1.72078

-1.63351	-1.65703	-1.65382	-1.67167	-1.66849	-1.72992	-1.72658	-1.72324	-1.7199
-1.65423	-1.67732	-1.67404	-1.6708	-1.66758	-1.72894	-1.72559	-1.72226	-1.71893
-1.65343	-1.67649	-1.67318	-1.6699	-1.66664	-1.72791	-1.72456	-1.72124	-1.7179
-1.67889	-1.67561	-1.6723	-1.69199	-1.68867	-1.72698	-1.72363	-1.72031	-1.71697
-1.67792	-1.67468	-1.67142	-1.69114	-1.68785	-1.72614	-1.72279	-1.71945	-1.71611
-1.67702	-1.67379	-1.67058	-1.6903	-1.68703	-1.72531	-1.72196	-1.71861	-1.71528
-1.67619	-1.67298	-1.66978	-1.68949	-1.68623	-1.72448	-1.72113	-1.71779	-1.71445
-1.6754	-1.67218	-1.66899	-1.73038	-1.72701	-1.72366	-1.72031	-1.71696	-1.71362
-1.67461	-1.6714	-1.66821	-1.72954	-1.72619	-1.72283	-1.71948	-1.71613	-1.71279
-1.67383	-1.67063	-1.66743	-1.72872	-1.72536	-1.72201	-1.71865	-1.7153	-1.71195
-1.67305	-1.66985	-1.66666	-1.72789	-1.72454	-1.74465	-1.74123	-1.7378	-1.75952
-1.67227	-1.66907	-1.66589	-1.72707	-1.72371	-1.74381	-1.74037	-1.73694	-1.75864
-1.67149	-1.69123	-1.68797	-1.72624	-1.72288	-1.74296	-1.73952	-1.73609	-1.75776
-1.6707	-1.69042	-1.68718	-1.72541	-1.72205	-1.7421	-1.73866	-1.73523	-1.75689
-1.66991	-1.68962	-1.68637	-1.72457	-1.7212	-1.74123	-1.7378	-1.73437	-1.75602
-1.66912	-1.68881	-1.68557	-1.72373	-1.72036	-1.74037	-1.73694	-1.73351	-1.75515
-1.66834	-1.68801	-1.68475	-1.72288	-1.71951	-1.7395	-1.73607	-1.73265	-1.75428
-1.66754	-1.68718	-1.68394	-1.72204	-1.71866	-1.73863	-1.7352	-1.73178	-1.7738
-1.66676	-1.68636	-1.68313	-1.72119	-1.71781	-1.73776	-1.73433	-1.73091	-1.77291
-1.66603	-1.68558	-1.68232	-1.72034	-1.71696	-1.7577	-1.75423	-1.75078	-1.77202
-1.66534	-1.68483	-1.68152	-1.71948	-1.7161	-1.75682	-1.75335	-1.7499	-1.77113
-1.68747	-1.68411	-1.68076	-1.73983	-1.7364	-1.75594	-1.75247	-1.74903	-1.79637
-1.68679	-1.68343	-1.68005	-1.73905	-1.73557	-1.75507	-1.7516	-1.74816	-1.79546
-1.68608	-1.72412	-1.72061	-1.73829	-1.73479	-1.77905	-1.77549	-1.77197	-1.79457
-1.6853	-1.72334	-1.71988	-1.73759	-1.73411	-1.77833	-1.77474	-1.77117	-1.79371
-1.6845	-1.72254	-1.71913	-1.73687	-1.73342	-1.77762	-1.774	-1.77039	-1.79287
-1.68369	-1.74293	-1.73949	-1.73606	-1.73262	-1.77682	-1.77322	-1.76963	-1.79208
-1.68287	-1.74207	-1.73863	-1.7352	-1.73177	-1.77596	-1.77242	-1.76887	-1.79132
-1.7039	-1.7412	-1.73776	-1.73433	-1.73091	-1.80129	-1.7977	-1.79412	-1.79051
-1.70309	-1.74033	-1.73688	-1.73346	-1.73004	-1.80036	-1.79679	-1.79324	-1.78968
-1.70234	-1.73953	-1.73606	-1.73262	-1.72919	-1.79945	-1.79589	-1.79235	-1.8177
-1.70169	-1.73882	-1.73532	-1.73183	-1.72837	-1.79855	-1.79498	-1.79144	-1.81673
-1.70101	-1.73811	-1.73458	-1.73108	-1.7276	-1.7977	-1.79411	-1.79056	-1.81577
-1.70024	-1.73734	-1.73385	-1.73037	-1.72691	-1.79696	-1.79334	-1.78974	-1.81485
-1.69943	-1.73653	-1.7331	-1.72967	-1.72624	-1.79625	-1.79259	-1.78895	-1.81397
-1.69862	-1.73569	-1.73227	-1.75577	-1.75229	-1.79543	-1.7918	-1.78817	-1.81315
-1.69781	-1.73483	-1.73141	-1.75489	-1.75142	-1.79456	-1.79098	-1.78741	-1.81236
-1.69708	-1.73404	-1.7306	-1.75405	-1.75057	-1.82282	-1.81902	-1.81525	-1.85224
-1.69643	-1.73335	-1.72985	-1.75323	-1.74971	-1.82183	-1.81803	-1.81426	-1.85126
-1.69574	-1.73263	-1.72911	-1.7771	-1.77348	-1.82092	-1.8171	-1.81332	-1.85029
-1.69497	-1.73185	-1.72838	-1.77636	-1.77276	-1.82014	-1.81628	-1.81245	-1.84935
-1.69421	-1.73109	-1.72764	-1.77561	-1.77205	-1.81935	-1.81547	-1.8116	-1.84843
-1.69354	-1.73038	-1.72691	-1.77482	-1.77124	-1.81847	-1.8146	-1.81076	-1.84758
-1.6929	-1.72968	-1.72618	-1.77402	-1.77041	-1.81756	-1.81373	-1.80993	-1.84676
-1.69217	-1.75581	-1.75224	-1.8028	-1.79896	-1.85762	-1.85367	-1.84976	-1.8664
-1.69139	-1.755	-1.75147	-1.80199	-1.79818	-1.8568	-1.85283	-1.84889	-1.86548
-1.69067	-1.75423	-1.7507	-1.80115	-1.79735	-1.85593	-1.85197	-1.84802	-1.8646
-1.69002	-1.7535	-1.74993	-1.80028	-1.79645	-1.855	-1.85107	-1.84716	-1.86376
-1.68934	-1.75276	-1.74917	-1.79944	-1.79559	-1.8541	-1.85018	-1.8463	-1.8629
-1.68859	-1.75197	-1.74843	-1.79865	-1.79482	-1.85327	-1.84933	-1.84542	-1.86199

-1.68783	-1.75119	-1.7477	-1.79788	-1.79406	-1.85246	-1.8485	-1.84455	-1.86108
-1.71465	-1.75048	-1.74701	-1.79712	-1.79329	-1.85163	-1.84765	-1.8437	-1.86022
-1.71398	-1.7498	-1.74632	-1.79636	-1.79251	-1.8508	-1.84681	-1.84286	-1.85939
-1.75257	-1.77894	-1.77521	-1.79553	-1.79169	-1.84996	-1.84599	-1.84205	-1.85856
-1.75181	-1.77809	-1.77433	-1.79464	-1.79085	-1.84911	-1.84517	-1.84125	-1.85774
-1.75107	-1.77728	-1.77351	-1.83516	-1.83128	-1.84828	-1.84435	-1.84043	-1.8569
-1.75033	-1.77652	-1.77276	-1.83438	-1.8305	-1.84748	-1.84353	-1.83959	-1.85605
-1.74962	-1.77577	-1.77203	-1.83361	-1.82972	-1.84667	-1.8427	-1.83874	-1.85521
-1.74893	-1.77504	-1.77131	-1.83283	-1.8289	-1.84582	-1.84185	-1.8379	-1.85439
-1.74825	-1.77431	-1.77057	-1.83203	-1.82808	-1.84497	-1.841	-1.83708	-1.85358
-1.74757	-1.77356	-1.7698	-1.8312	-1.82725	-1.84413	-1.84019	-1.83629	-1.85279
-1.74689	-1.7728	-1.769	-1.83035	-1.82641	-1.84331	-1.83939	-1.83552	-1.85202
-1.74617	-1.772	-1.7682	-1.82952	-1.8256	-1.8425	-1.8386	-1.83474	-1.85122
-1.74541	-1.77118	-1.76741	-1.8287	-1.8248	-1.84172	-1.83782	-1.83395	-1.8504
-1.77419	-1.77039	-1.76663	-1.82789	-1.82402	-1.84094	-1.83704	-1.83315	-1.84957
-1.77346	-1.76966	-1.7659	-1.82712	-1.82326	-1.84016	-1.83623	-1.83231	-1.84872
-1.77273	-1.76894	-1.76517	-1.82635	-1.8225	-1.83939	-1.83543	-1.83149	-1.84789
-1.77196	-1.81001	-1.80616	-1.82559	-1.82174	-1.83862	-1.83467	-1.83074	-1.84712
-1.77117	-1.80923	-1.80541	-1.82484	-1.82097	-1.83784	-1.83391	-1.83	-1.84637
-1.77042	-1.83184	-1.82795	-1.82407	-1.82017	-1.83702	-1.8331	-1.82921	-1.8456
-1.7697	-1.83108	-1.82719	-1.82329	-1.81935	-1.83615	-1.83226	-1.8284	-1.84481
-1.76899	-1.83033	-1.82644	-1.82253	-1.81856	-1.8557	-1.85177	-1.84789	-1.84404
-1.76829	-1.82959	-1.82571	-1.82179	-1.81782	-1.85494	-1.85101	-1.84713	-1.84328
-1.80937	-1.82885	-1.82497	-1.82105	-1.81709	-1.85418	-1.85025	-1.84637	-1.84252
-1.80864	-1.8281	-1.82422	-1.82031	-1.81635	-1.85344	-1.84951	-1.84562	-1.84177
-1.8079	-1.82735	-1.82348	-1.81957	-1.81562	-1.85269	-1.84877	-1.84488	-1.84102
-1.80715	-1.82659	-1.82272	-1.81882	-1.81488	-1.85194	-1.84801	-1.84413	-1.85894
-1.8064	-1.82583	-1.82196	-1.81806	-1.81413	-1.85118	-1.84726	-1.84338	-1.85819
-1.80565	-1.82507	-1.82121	-1.81731	-1.81339	-1.85043	-1.84651	-1.84263	-1.85743
-1.80491	-1.82432	-1.82045	-1.81657	-1.81266	-1.84968	-1.84576	-1.84188	-1.85667
-1.8042	-1.82359	-1.81972	-1.81583	-1.81193	-1.82861	-1.82473	-1.82089	-1.83599
-1.80354	-1.8229	-1.81899	-1.8151	-1.81121	-1.82788	-1.824	-1.82015	-1.83524
-1.80287	-1.8222	-1.81827	-1.81437	-1.8105	-1.82719	-1.82329	-1.81943	-1.83451
-1.80217	-1.82148	-1.81755	-1.81367	-1.80984	-1.82655	-1.82264	-1.81875	-1.8338
-1.80144	-1.82075	-1.81683	-1.81297	-1.80919	-1.82591	-1.822	-1.81809	-1.83311
-1.82399	-1.82002	-1.8161	-1.81225	-1.80849	-1.84424	-1.84028	-1.83631	-1.83239
-1.82326	-1.81929	-1.81537	-1.81152	-1.80777	-1.84351	-1.83957	-1.83561	-1.83167
-1.82258	-1.81862	-1.81469	-1.81083	-1.80707	-1.84279	-1.83887	-1.83493	-1.83099
-1.82194	-1.818	-1.81405	-1.81018	-1.80638	-1.84206	-1.83818	-1.83429	-1.83035
-1.82127	-1.81735	-1.81342	-1.80954	-1.8057	-1.84134	-1.83748	-1.83361	-1.82968
-1.82055	-1.81666	-1.81279	-1.80893	-1.80506	-1.84065	-1.83676	-1.83288	-1.82895
-1.81984	-1.81596	-1.81214	-1.8083	-1.80441	-1.83996	-1.83605	-1.83214	-1.82822
-1.81917	-1.81528	-1.81145	-1.8076	-1.80372	-1.83925	-1.83532	-1.8314	-1.82751
-1.81853	-1.81461	-1.81074	-1.80688	-1.80302	-1.83855	-1.8346	-1.83067	-1.82681
-1.81791	-1.81398	-1.8101	-1.80623	-1.80237	-1.83789	-1.83393	-1.82999	-1.82612
-1.8173	-1.81339	-1.80949	-1.80561	-1.80176	-1.83727	-1.8333	-1.82935	-1.82544
-1.81664	-1.81276	-1.80888	-1.82552	-1.82161	-1.83663	-1.83267	-1.82872	-1.82479
-1.81594	-1.81208	-1.80825	-1.82491	-1.82098	-1.83599	-1.83204	-1.82811	-1.82417
-1.81525	-1.8114	-1.80761	-1.84328	-1.8393	-1.83533	-1.83139	-1.82747	-1.82353
-1.81461	-1.81073	-1.80691	-1.84256	-1.83859	-1.83463	-1.83069	-1.82677	-1.82286

-1.81398	-1.81008	-1.80622	-1.84184	-1.83789	-1.83395	-1.83001	-1.82608	-1.8222
-1.81337	-1.80946	-1.80558	-1.84118	-1.83724	-1.83331	-1.8294	-1.8255	-1.82162
-1.81276	-1.80886	-1.80498	-1.84056	-1.83661	-1.83269	-1.82881	-1.82495	-1.82105
-1.81215	-1.8288	-1.82487	-1.83994	-1.83598	-1.83205	-1.82817	-1.8243	-1.82042
-1.81153	-1.82818	-1.82426	-1.83932	-1.83535	-1.8314	-1.82748	-1.82359	-1.81974
-1.81092	-1.82756	-1.82364	-1.83869	-1.83472	-1.83076	-1.82682	-1.8229	-1.83537
-1.81031	-1.82695	-1.82302	-1.83806	-1.83409	-1.83013	-1.82618	-1.82226	-1.83469
-1.80971	-1.82634	-1.82241	-1.83743	-1.83346	-1.82951	-1.82556	-1.82163	-1.83405
-1.80909	-1.82572	-1.82179	-1.83682	-1.83284	-1.82888	-1.82494	-1.82103	-1.83346
-1.80848	-1.8251	-1.82118	-1.8362	-1.83223	-1.82827	-1.82435	-1.82045	-1.83289
-1.80789	-1.8245	-1.82057	-1.83558	-1.83162	-1.82768	-1.82377	-1.81989	-1.83232
-1.80732	-1.82392	-1.81997	-1.83498	-1.83103	-1.8271	-1.82321	-1.81933	-1.83175
-1.80678	-1.82338	-1.81943	-1.83442	-1.83047	-1.82654	-1.82264	-1.81876	-1.83116
-1.80626	-1.82287	-1.81893	-1.83392	-1.82994	-1.82599	-1.82206	-1.81815	-1.83054
-1.80572	-1.84135	-1.83736	-1.83338	-1.82939	-1.84183	-1.83783	-1.83386	-1.82992
-1.80515	-1.84075	-1.83676	-1.83278	-1.82879	-1.84122	-1.83722	-1.83324	-1.8293
-1.80456	-1.84014	-1.83615	-1.83216	-1.82817	-1.84061	-1.83661	-1.83263	-1.82869
-1.80397	-1.83952	-1.83553	-1.83154	-1.82757	-1.84001	-1.836	-1.83201	-1.82808
-1.80338	-1.83892	-1.83492	-1.83093	-1.82697	-1.83942	-1.8354	-1.83141	-1.82749
-1.80284	-1.83837	-1.83437	-1.83039	-1.82642	-1.83886	-1.83486	-1.83088	-1.82696
-1.80232	-1.83784	-1.83386	-1.82988	-1.8259	-1.83833	-1.83435	-1.8304	-1.82647
-1.80176	-1.83728	-1.83331	-1.82933	-1.82534	-1.83776	-1.8338	-1.82987	-1.82595
-1.80117	-1.83667	-1.83271	-1.82873	-1.82474	-1.83716	-1.83321	-1.82928	-1.82539
-1.80061	-1.8361	-1.83213	-1.82816	-1.82417	-1.83659	-1.83263	-1.82872	-1.82484
-1.80011	-1.83559	-1.83162	-1.82765	-1.82366	-1.83608	-1.83211	-1.82818	-1.82428
-1.79962	-1.83509	-1.83112	-1.82714	-1.82317	-1.83558	-1.83161	-1.82766	-1.82373
-1.79914	-1.83459	-1.83061	-1.82664	-1.82267	-1.83509	-1.83112	-1.82717	-1.82323
-1.79864	-1.83409	-1.8301	-1.82613	-1.82218	-1.8346	-1.83064	-1.82668	-1.82274
-1.83753	-1.83353	-1.82953	-1.82556	-1.82162	-1.83406	-1.83011	-1.82618	-1.82225
-1.83693	-1.83293	-1.82892	-1.82495	-1.82102	-1.83346	-1.82956	-1.82566	-1.82175
-1.83638	-1.83237	-1.82837	-1.84084	-1.83685	-1.8329	-1.829	-1.82511	-1.8212
-1.83586	-1.83186	-1.82786	-1.84033	-1.83633	-1.83236	-1.82843	-1.82452	-1.8206
-1.83536	-1.83137	-1.82737	-1.83984	-1.83583	-1.83184	-1.82788	-1.82394	-1.82003
-1.83487	-1.83087	-1.82688	-1.83935	-1.83534	-1.83135	-1.82738	-1.82344	-1.81951
-1.83438	-1.83039	-1.82639	-1.83886	-1.83485	-1.83086	-1.8269	-1.82295	-1.81902
-1.83389	-1.82989	-1.8259	-1.83836	-1.83436	-1.83037	-1.8264	-1.82245	-1.81852
-1.83339	-1.8294	-1.82541	-1.83787	-1.83386	-1.82988	-1.82591	-1.82196	-1.81803
-1.8133	-1.82891	-1.82493	-1.83738	-1.83337	-1.82938	-1.82541	-1.82147	-1.81754
-1.81281	-1.82843	-1.82446	-1.8369	-1.83288	-1.82887	-1.82491	-1.82096	-1.81703
-1.81236	-1.82798	-1.82402	-1.83646	-1.83243	-1.82841	-1.82443	-1.82047	-1.81654
-1.81197	-1.82759	-1.82364	-1.83608	-1.83203	-1.828	-1.82398	-1.81999	-1.81605
-1.81156	-1.82719	-1.82324	-1.83568	-1.83163	-1.82759	-1.82355	-1.81954	-1.81558
-1.8111	-1.82673	-1.82278	-1.83522	-1.83118	-1.82715	-1.82313	-1.81914	-1.81518
-1.81063	-1.82625	-1.82229	-1.83473	-1.8307	-1.82669	-1.82271	-1.81874	-1.81479
-1.81016	-1.84228	-1.83825	-1.83423	-1.83021	-1.82621	-1.82223	-1.81828	-1.81434
-1.80969	-1.8418	-1.83776	-1.83372	-1.8297	-1.8257	-1.82173	-1.81778	-1.81385
-1.80927	-1.84137	-1.83731	-1.83326	-1.82922	-1.82521	-1.82123	-1.81728	-1.81336
-1.80889	-1.84098	-1.83692	-1.83285	-1.82877	-1.82473	-1.82074	-1.81679	-1.81286
-1.80848	-1.84057	-1.83651	-1.83244	-1.82834	-1.82428	-1.82028	-1.81632	-1.81241
-1.80801	-1.8401	-1.83606	-1.83201	-1.82793	-1.82388	-1.81988	-1.81592	-1.81201

-1.80755	-1.83964	-1.83562	-1.83159	-1.82753	-1.8235	-1.8195	-1.81553	-1.81161
-1.80714	-1.83923	-1.83522	-1.8312	-1.82714	-1.8231	-1.81908	-1.81509	-1.81116
-1.80674	-1.83884	-1.83483	-1.83081	-1.82675	-1.82271	-1.81867	-1.81465	-1.8107
-1.80632	-1.81912	-1.81516	-1.83037	-1.82633	-1.82229	-1.81826	-1.81425	-1.8103
-1.80587	-1.81866	-1.81468	-1.82989	-1.82587	-1.82187	-1.81786	-1.81388	-1.80994
-1.80545	-1.81823	-1.81425	-1.82946	-1.82545	-1.82146	-1.81747	-1.8135	-1.80957
-1.80508	-1.81786	-1.81388	-1.82908	-1.82507	-1.82107	-1.81708	-1.81312	-1.8092
-1.82147	-1.81749	-1.81352	-1.82872	-1.82469	-1.82068	-1.81669	-1.81272	-1.81928
-1.82111	-1.81713	-1.81315	-1.82834	-1.82431	-1.82028	-1.81627	-1.81228	-1.81886
-1.82075	-1.81677	-1.81279	-1.82797	-1.82392	-1.81988	-1.81585	-1.81184	-1.81845
-1.8204	-1.81642	-1.81242	-1.82759	-1.82351	-1.81945	-1.81543	-1.81144	-1.81809
-1.82004	-1.81606	-1.81206	-1.82721	-1.82309	-1.81902	-1.81502	-1.81107	-1.81777
-1.81968	-1.8157	-1.8117	-1.82684	-1.82273	-1.81866	-1.81466	-1.8107	-1.81744
-1.81931	-1.81533	-1.81133	-1.8265	-1.82242	-1.81837	-1.81434	-1.81035	-1.81712
-1.80192	-1.81496	-1.81097	-1.82613	-1.82207	-1.8288	-1.82475	-1.82074	-1.83936
-1.80155	-1.81459	-1.81059	-1.82574	-1.82165	-1.8284	-1.82439	-1.82041	-1.83903
-1.80118	-1.81422	-1.81022	-1.82536	-1.82125	-1.82804	-1.82404	-1.82009	-1.83869
-1.80081	-1.81385	-1.80986	-1.82502	-1.82094	-1.82778	-1.82375	-1.81976	-1.83834
-1.80043	-1.81348	-1.8095	-1.82469	-1.82066	-1.82755	-1.82348	-1.81944	-1.83801
-1.80006	-1.79613	-1.7922	-1.80517	-1.80121	-1.82728	-1.82323	-1.8192	-1.83772
-1.7997	-1.79576	-1.79183	-1.8048	-1.80086	-1.82699	-1.82299	-1.81899	-1.83747
-1.79933	-1.79539	-1.79146	-1.80444	-1.8005	-1.84937	-1.84533	-1.84129	-1.83721
-1.79896	-1.795	-1.79109	-1.80407	-1.80014	-1.84904	-1.84501	-1.84098	-1.83694
-1.79862	-1.80618	-1.80226	-1.79835	-1.79445	-1.84872	-1.84469	-1.84067	-1.83665
-1.79835	-1.80592	-1.80195	-1.79803	-1.79415	-1.84841	-1.84436	-1.84033	-1.83632
-1.79807	-1.80568	-1.80167	-1.79773	-1.79386	-1.84811	-1.84406	-1.84001	-1.83599
-1.79776	-1.80542	-1.80144	-1.7975	-1.79363	-1.84786	-1.84382	-1.83977	-1.83572
-1.79745	-1.80518	-1.80122	-1.7973	-1.79341	-1.84763	-1.84359	-1.83956	-1.83547
-1.80888	-1.80495	-1.80101	-1.82038	-1.81639	-1.83123	-1.82724	-1.82325	-1.85501
-1.80865	-1.80473	-1.80081	-1.82016	-1.81612	-1.83093	-1.82694	-1.82297	-1.85475
-1.8084	-1.80448	-1.80058	-1.81993	-1.81587	-1.83067	-1.82668	-1.82271	-1.8545
-1.8081	-1.80419	-1.80031	-1.81966	-1.81564	-1.83046	-1.82646	-1.82247	-1.85427
-1.80783	-1.80393	-1.80005	-1.81941	-1.81542	-1.81143	-1.80746	-1.8035	-1.83824
-1.80761	-1.80371	-1.79983	-1.81918	-1.8152	-1.81122	-1.8072	-1.80321	-1.83794
-1.80741	-1.8035	-1.7996	-1.81894	-1.81498	-1.81099	-1.80695	-1.80292	-1.83764
-1.80721	-1.82663	-1.82263	-1.81865	-1.8147	-1.81073	-1.8067	-1.80267	-1.87444
-1.80701	-1.82639	-1.82234	-1.81834	-1.8144	-1.81045	-1.80645	-1.80246	-1.87422
-1.80681	-1.82618	-1.82211	-1.81809	-1.81415	-1.8102	-1.80622	-1.80224	-1.87397
-1.80663	-1.82599	-1.82191	-1.81789	-1.81394	-1.80999	-1.806	-1.80202	-1.87371
-1.80641	-1.82577	-1.82171	-1.81769	-1.81374	-1.83016	-1.82612	-1.82208	-1.87348
-1.80614	-1.82552	-1.8215	-1.8175	-1.81355	-1.82995	-1.82592	-1.82188	-1.87326
-1.82925	-1.82527	-1.82129	-1.81731	-1.81335	-1.82975	-1.82572	-1.82167	-1.87306
-1.82902	-1.82505	-1.82108	-1.81711	-1.81315	-1.82955	-1.82551	-1.82147	-1.87286
-1.82881	-1.82484	-1.82087	-1.81691	-1.81295	-1.82934	-1.82531	-1.82127	-1.87266
-1.82861	-1.82463	-1.82067	-1.81671	-1.81275	-1.8669	-1.86278	-1.85866	-1.85451
-1.82841	-1.82443	-1.82046	-1.8165	-1.81255	-1.86669	-1.86257	-1.85845	-1.8543
-1.82826	-1.82426	-1.82027	-1.81629	-1.81234	-1.86648	-1.86237	-1.85825	-1.85411
-1.82815	-1.82411	-1.82008	-1.81608	-1.81213	-1.86627	-1.86217	-1.85806	-1.85393
-1.82803	-1.82397	-1.8199	-1.81588	-1.81193	-1.86606	-1.86197	-1.85787	-1.85376
-1.82787	-1.8238	-1.81972	-1.81569	-1.81173	-1.86584	-1.86176	-1.85769	-1.85361

-1.82769	-1.82364	-1.81956	-1.81553	-1.81155	-1.86565	-1.86157	-1.85751	-1.85346
-1.82751	-1.82348	-1.81944	-1.81543	-1.81144	-1.86552	-1.86142	-1.85733	-1.85328
-1.82733	-1.82333	-1.81933	-1.81534	-1.81136	-1.86542	-1.86129	-1.85717	-1.8531
-1.82716	-1.82316	-1.81917	-1.87351	-1.86939	-1.86528	-1.86116	-1.85705	-1.85297
-1.827	-1.82297	-1.81897	-1.8733	-1.8692	-1.86511	-1.86104	-1.85697	-1.85289
-1.82687	-1.82284	-1.81883	-1.87314	-1.86903	-1.86494	-1.86088	-1.85683	-1.85276
-1.82678	-1.82275	-1.81873	-1.87302	-1.86887	-1.86476	-1.86069	-1.85663	-1.85257
-1.82669	-1.82266	-1.81864	-1.8729	-1.86873	-1.8646	-1.86052	-1.85646	-1.85239
-1.8266	-1.82256	-1.81851	-1.87276	-1.86862	-1.86449	-1.86041	-1.85634	-1.85225
-1.82651	-1.82245	-1.81839	-1.87263	-1.86851	-1.8644	-1.86032	-1.85623	-1.85212
-1.82638	-1.82233	-1.81828	-1.87253	-1.86841	-1.8643	-1.8602	-1.8561	-1.85199
-1.82624	-1.82221	-1.81818	-1.87244	-1.86832	-1.8642	-1.86007	-1.85595	-1.85186
-1.82613	-1.82211	-1.81808	-1.87233	-1.8682	-1.86407	-1.85995	-1.85584	-1.85176
-1.82603	-1.82201	-1.81799	-1.87223	-1.86807	-1.86393	-1.85983	-1.85574	-1.85166
-1.82594	-1.8605	-1.85637	-1.8721	-1.86794	-1.8638	-1.85972	-1.85565	-1.85156
-1.82585	-1.86039	-1.85623	-1.87196	-1.86782	-1.8637	-1.85962	-1.85555	-1.85145
-1.78527	-1.88021	-1.876	-1.87183	-1.86771	-1.86361	-1.85953	-1.85544	-1.85133
-1.78513	-1.88007	-1.87588	-1.87174	-1.86763	-1.86353	-1.85941	-1.85529	-1.85119
-1.78499	-1.87994	-1.87579	-1.87166	-1.86757	-1.86346	-1.85931	-1.85516	-1.85107
-1.78488	-1.87985	-1.87571	-1.87159	-1.8675	-1.8634	-1.85926	-1.85513	-1.85103
-1.78478	-1.87977	-1.87564	-1.87152	-1.86743	-1.86334	-1.85924	-1.85514	-1.85102
-1.82445	-1.8409	-1.83689	-1.87145	-1.86736	-1.86327	-1.85917	-1.85507	-1.85095
-1.82438	-1.84082	-1.8368	-1.87138	-1.86729	-1.86319	-1.85905	-1.85492	-1.85083
-1.8243	-1.84073	-1.83672	-1.87131	-1.86722	-1.86312	-1.85898	-1.85483	-1.85074
-1.82421	-1.84065	-1.83664	-1.87124	-1.86716	-1.86307	-1.85896	-1.85485	-1.85073
-1.82413	-1.84057	-1.83656	-1.87117	-1.86709	-1.86302	-1.85895	-1.85487	-1.85073
-1.80539	-1.84048	-1.83648	-1.87111	-1.86703	-1.86295	-1.85889	-1.85482	-1.85071
-1.80532	-1.8404	-1.8364	-1.87104	-1.86695	-1.86288	-1.85882	-1.85476	-1.85068
-1.80531	-1.84037	-1.83633	-1.87096	-1.86688	-1.86281	-1.85874	-1.85468	-1.8506
-1.80535	-1.84038	-1.83627	-1.87087	-1.8668	-1.86274	-1.85867	-1.85459	-1.85049
-1.80534	-1.84037	-1.83625	-1.83219	-1.8282	-1.86266	-1.85859	-1.85452	-1.85042
-1.80528	-1.84035	-1.83627	-1.83221	-1.82815	-1.86256	-1.8585	-1.85446	-1.85039
-1.82594	-1.84031	-1.83628	-1.83222	-1.82813	-1.86252	-1.85844	-1.8544	-1.85036
-1.82588	-1.84024	-1.83624	-1.83221	-1.82814	-1.86254	-1.85842	-1.85433	-1.8503
-1.82583	-1.84018	-1.8362	-1.83219	-1.82817	-1.86259	-1.85842	-1.85429	-1.85025
-1.82584	-1.8402	-1.8362	-1.83221	-1.82821	-1.8242	-1.82013	-1.81608	-1.85026
-1.82588	-1.84024	-1.83623	-1.83223	-1.82825	-1.82425	-1.82019	-1.81613	-1.85031
-1.82591	-1.84026	-1.83621	-1.83219	-1.82823	-1.82425	-1.82021	-1.81617	-1.85034
-1.82595	-1.84028	-1.83617	-1.83212	-1.82815	-1.82419	-1.8202	-1.81622	-1.85036
-1.82596	-1.84028	-1.83615	-1.83209	-1.8281	-1.82413	-1.82018	-1.81622	-1.85038
-1.82592	-1.84026	-1.83617	-1.8321	-1.82806	-1.82405	-1.82011	-1.81618	-1.85039
-1.8259	-1.82189	-1.81787	-1.83213	-1.82805	-1.82401	-1.82007	-1.81616	-1.85041
-1.82593	-1.82192	-1.81792	-1.83218	-1.82809	-1.82405	-1.8201	-1.81619	-1.85045
-1.82597	-1.82197	-1.81797	-1.83225	-1.82815	-1.82411	-1.82016	-1.81624	-1.85051
-1.82601	-1.82202	-1.81803	-1.83231	-1.82821	-1.82416	-1.82021	-1.8163	-1.85058
-1.82604	-1.82206	-1.81808	-1.83238	-1.82828	-1.82423	-1.82028	-1.81636	-1.85065
-1.82603	-1.82205	-1.81809	-1.8324	-1.82832	-1.82429	-1.82034	-1.81642	-1.85072
-1.82599	-1.82199	-1.81804	-1.83238	-1.82835	-1.82435	-1.82041	-1.81648	-1.85079
-1.82597	-1.82197	-1.81803	-1.83239	-1.82839	-1.82441	-1.82047	-1.81654	-1.85086
-1.82601	-1.822	-1.81806	-1.83243	-1.82844	-1.82447	-1.82053	-1.8166	-1.85093

-1.82607	-1.82206	-1.81811	-1.81417	-1.81024	-1.82453	-1.82059	-1.81666	-1.81272
-1.82614	-1.82212	-1.81816	-1.81422	-1.81029	-1.82459	-1.82065	-1.81671	-1.81278
-1.82624	-1.82221	-1.81823	-1.81428	-1.81035	-1.82465	-1.82071	-1.81677	-1.81284
-1.82637	-1.82235	-1.81836	-1.81439	-1.81042	-1.82471	-1.82076	-1.81683	-1.81289
-1.82654	-1.82253	-1.81853	-1.81452	-1.81051	-1.82476	-1.82081	-1.81688	-1.81295
-1.82665	-1.82265	-1.81865	-1.81463	-1.81059	-1.82482	-1.82087	-1.81694	-1.813
-1.82672	-1.82272	-1.81872	-1.8147	-1.81065	-1.82487	-1.82092	-1.817	-1.81306
-1.82677	-1.82278	-1.81878	-1.81477	-1.81071	-1.8067	-1.80279	-1.79891	-1.81312
-1.82682	-1.82283	-1.81884	-1.81483	-1.81078	-1.80677	-1.80285	-1.79895	-1.81317
-1.82689	-1.82289	-1.81891	-1.8149	-1.81087	-1.80687	-1.80293	-1.79902	-1.81323
-1.82703	-1.82301	-1.81901	-1.815	-1.81099	-1.80701	-1.80307	-1.79915	-1.81335
-1.82719	-1.82316	-1.81912	-1.81511	-1.81113	-1.80717	-1.80323	-1.79931	-1.81348
-1.82731	-1.82329	-1.81926	-1.81526	-1.81128	-1.80732	-1.80337	-1.79943	-1.8136
-1.82739	-1.8234	-1.81941	-1.81543	-1.81145	-1.80747	-1.80348	-1.79952	-1.81371
-1.82748	-1.8235	-1.81953	-1.81555	-1.81157	-1.80759	-1.80358	-1.7996	-1.81381
-1.82756	-1.82356	-1.81958	-1.81561	-1.81164	-1.80767	-1.80366	-1.79967	-1.81388
-1.82766	-1.82365	-1.81966	-1.81568	-1.81172	-1.80775	-1.80375	-1.79977	-1.81398
-1.8278	-1.82379	-1.8198	-1.81581	-1.81182	-1.80784	-1.80386	-1.7999	-1.81412
-1.82795	-1.82395	-1.81996	-1.81596	-1.81194	-1.80795	-1.80399	-1.80006	-1.81429
-1.82811	-1.82409	-1.82008	-1.81608	-1.81207	-1.80809	-1.80415	-1.80023	-1.79631
-1.82827	-1.82424	-1.8202	-1.81619	-1.81222	-1.80826	-1.80434	-1.80042	-1.79646
-1.8284	-1.82437	-1.82034	-1.81634	-1.81238	-1.80845	-1.80452	-1.8006	-1.83536
-1.82851	-1.82449	-1.82049	-1.81651	-1.81257	-1.80864	-1.80471	-1.80079	-1.83557
-1.82865	-1.82463	-1.82065	-1.81669	-1.81276	-1.80883	-1.80489	-1.80097	-1.83575
-1.8288	-1.82481	-1.82084	-1.81688	-1.81293	-1.80899	-1.80506	-1.80113	-1.83589
-1.82897	-1.82499	-1.82103	-1.81707	-1.81311	-1.84817	-1.84411	-1.84006	-1.83601
-1.82916	-1.82517	-1.82122	-1.81726	-1.8133	-1.84834	-1.84425	-1.84017	-1.83612
-1.82936	-1.82536	-1.8214	-1.81744	-1.81349	-1.84851	-1.84439	-1.84028	-1.83625
-1.82955	-1.82555	-1.82159	-1.81763	-1.81367	-1.84869	-1.84458	-1.84048	-1.83644
-1.82972	-1.82574	-1.82178	-1.81782	-1.81386	-1.84887	-1.8448	-1.84074	-1.83667
-1.82991	-1.82592	-1.82196	-1.85718	-1.85311	-1.84904	-1.84498	-1.84091	-1.83685
-1.8301	-1.82609	-1.82214	-1.85735	-1.85329	-1.84921	-1.8451	-1.84101	-1.83696
-1.83032	-1.8263	-1.82232	-1.85753	-1.85346	-1.84939	-1.84526	-1.84115	-1.83711
-1.83061	-1.82656	-1.82253	-1.85769	-1.85364	-1.84958	-1.84549	-1.84141	-1.83734
-1.8309	-1.82684	-1.82275	-1.85788	-1.85382	-1.84977	-1.84573	-1.84167	-1.83759
-1.83112	-1.82708	-1.82302	-1.85813	-1.85402	-1.84994	-1.84591	-1.84188	-1.83782
-1.83132	-1.82731	-1.8233	-1.85842	-1.85425	-1.85012	-1.84608	-1.84207	-1.83805
-1.83151	-1.86688	-1.86278	-1.85866	-1.85449	-1.85036	-1.84629	-1.84224	-1.83824
-1.83169	-1.86705	-1.86297	-1.85887	-1.85476	-1.85065	-1.84652	-1.84242	-1.8384
-1.83192	-1.86727	-1.86315	-1.85906	-1.85499	-1.8509	-1.84676	-1.84263	-1.8386
-1.83223	-1.86753	-1.86335	-1.85922	-1.85516	-1.8511	-1.84701	-1.84292	-1.83884
-1.83251	-1.86779	-1.86356	-1.85941	-1.85534	-1.85129	-1.84724	-1.84319	-1.83909
-1.83273	-1.86803	-1.86383	-1.85968	-1.85556	-1.85147	-1.84743	-1.84341	-1.83934
-1.83292	-1.86825	-1.86411	-1.85996	-1.85579	-1.85166	-1.84763	-1.84363	-1.83959
-1.85529	-1.86844	-1.86433	-1.8602	-1.85604	-1.85191	-1.84788	-1.84387	-1.83985
-1.85547	-1.86862	-1.86453	-1.86043	-1.8563	-1.8522	-1.84815	-1.84412	-1.84012
-1.85571	-1.86886	-1.86477	-1.86067	-1.85657	-1.85248	-1.8484	-1.84434	-1.84034
-1.856	-1.86914	-1.86503	-1.86094	-1.85685	-1.85276	-1.84863	-1.84453	-1.84052
-1.85628	-1.85215	-1.84805	-1.86117	-1.8571	-1.85302	-1.84887	-1.84475	-1.84073
-1.85657	-1.85241	-1.84825	-1.86135	-1.85729	-1.85323	-1.84912	-1.84502	-1.841

-1.85686	-1.85267	-1.84847	-1.86156	-1.85751	-1.85346	-1.84938	-1.8453	-1.84128
-1.85716	-1.85297	-1.84875	-1.86183	-1.85778	-1.85373	-1.84965	-1.84558	-1.84152
-1.85745	-1.85327	-1.84905	-1.86213	-1.85807	-1.85402	-1.84994	-1.84587	-1.84177
-1.85769	-1.85353	-1.84934	-1.84523	-1.8412	-1.85432	-1.85024	-1.84616	-1.84205
-1.8579	-1.85377	-1.84963	-1.84554	-1.84151	-1.85462	-1.85054	-1.84646	-1.84235
-1.85815	-1.85403	-1.84992	-1.84584	-1.84181	-1.85493	-1.85085	-1.84676	-1.84265
-1.85843	-1.85432	-1.85022	-1.84615	-1.84211	-1.85523	-1.85115	-1.84706	-1.84294
-1.85873	-1.85462	-1.85052	-1.84645	-1.84242	-1.83838	-1.83433	-1.83026	-1.84324
-1.85903	-1.85491	-1.85082	-1.84675	-1.84272	-1.83868	-1.83462	-1.83056	-1.84353
-1.85935	-1.85523	-1.85113	-1.84705	-1.84302	-1.83897	-1.83492	-1.83085	-1.84383
-1.85973	-1.85558	-1.85144	-1.84735	-1.84331	-1.83927	-1.83522	-1.83116	-1.84414
-1.86013	-1.85594	-1.85176	-1.84764	-1.8436	-1.83957	-1.83552	-1.83147	-1.84446
-1.86049	-1.85629	-1.85208	-1.84795	-1.8439	-1.83986	-1.83582	-1.83178	-1.82772
-1.86082	-1.85662	-1.85242	-1.84827	-1.8442	-1.84015	-1.83611	-1.83208	-1.82806
-1.86115	-1.85697	-1.85278	-1.84864	-1.84455	-1.84048	-1.83643	-1.8324	-1.82839
-1.86147	-1.85732	-1.85318	-1.84906	-1.84496	-1.84087	-1.83678	-1.83272	-1.82869
-1.86179	-1.85767	-1.85356	-1.84945	-1.84535	-1.84125	-1.83715	-1.83306	-1.82902
-1.86212	-1.85799	-1.85388	-1.84977	-1.84567	-1.84159	-1.83751	-1.83346	-1.82941
-1.86247	-1.85833	-1.85419	-1.85008	-1.84599	-1.84192	-1.83787	-1.83385	-1.82981
-1.86287	-1.85873	-1.85458	-1.85045	-1.84633	-1.84224	-1.83819	-1.83417	-1.83013
-1.8633	-1.85915	-1.855	-1.85084	-1.84668	-1.84256	-1.83849	-1.83446	-1.83043
-1.86366	-1.85951	-1.85536	-1.85121	-1.84705	-1.84293	-1.83885	-1.8348	-1.83076
-1.86396	-1.85981	-1.85568	-1.85155	-1.84743	-1.84333	-1.83926	-1.83519	-1.83112
-1.8643	-1.86014	-1.856	-1.85188	-1.84778	-1.8437	-1.83963	-1.83556	-1.83148
-1.8647	-1.86051	-1.85633	-1.85219	-1.84808	-1.844	-1.83995	-1.83591	-1.83185
-1.8651	-1.86089	-1.85668	-1.85252	-1.8484	-1.84432	-1.84028	-1.83626	-1.83222
-1.86547	-1.86126	-1.85707	-1.85292	-1.8488	-1.84471	-1.84067	-1.83665	-1.83261
-1.86584	-1.86165	-1.85748	-1.85334	-1.84923	-1.84514	-1.84108	-1.83705	-1.83301
-1.8468	-1.86206	-1.85791	-1.85377	-1.84965	-1.84555	-1.84148	-1.83744	-1.83339
-1.84721	-1.86249	-1.85834	-1.8542	-1.85006	-1.84595	-1.84188	-1.83783	-1.83376
-1.84764	-1.86293	-1.85877	-1.85462	-1.85047	-1.84635	-1.84226	-1.8382	-1.83413
-1.84807	-1.86336	-1.8592	-1.85503	-1.85087	-1.84673	-1.84262	-1.83854	-1.83449
-1.84851	-1.8638	-1.85962	-1.85544	-1.85126	-1.84711	-1.84299	-1.8389	-1.83487
-1.84894	-1.86424	-1.86006	-1.85586	-1.85163	-1.84745	-1.84335	-1.8393	-1.83528
-1.84938	-1.86468	-1.86049	-1.85628	-1.85202	-1.84782	-1.84374	-1.83971	-1.8357
-1.81615	-1.86512	-1.86093	-1.85672	-1.85248	-1.84829	-1.84419	-1.84013	-1.83612
-1.81658	-1.86556	-1.86137	-1.85717	-1.85297	-1.8488	-1.84466	-1.84057	-1.83654
-1.817	-1.866	-1.86181	-1.8576	-1.85339	-1.84921	-1.84507	-1.84099	-1.83697
-1.81741	-1.86644	-1.86224	-1.85802	-1.85376	-1.84956	-1.84546	-1.84141	-1.83739
-1.81783	-1.86688	-1.86268	-1.85847	-1.85421	-1.85	-1.84589	-1.84184	-1.83783
-1.81825	-1.86732	-1.86313	-1.85894	-1.85473	-1.85054	-1.84639	-1.84228	-1.83826
-1.81866	-1.86775	-1.86358	-1.8594	-1.85523	-1.85107	-1.84689	-1.84275	-1.83873
-1.81908	-1.86819	-1.86401	-1.85985	-1.85569	-1.85155	-1.84741	-1.8433	-1.83927
-1.8195	-1.86863	-1.86446	-1.86029	-1.85614	-1.85201	-1.84791	-1.84384	-1.83981
-1.79729	-1.84957	-1.84547	-1.86075	-1.85658	-1.85245	-1.84836	-1.8443	-1.84028
-1.79772	-1.85002	-1.84593	-1.86122	-1.85703	-1.85288	-1.84879	-1.84473	-1.84071
-1.7982	-1.85053	-1.84644	-1.86173	-1.85755	-1.85339	-1.84926	-1.84517	-1.84114
-1.79874	-1.85109	-1.84698	-1.86227	-1.85811	-1.85394	-1.84976	-1.84561	-1.84158
-1.79924	-1.85161	-1.84748	-1.86278	-1.85862	-1.85446	-1.85027	-1.8461	-1.84206
-1.79969	-1.85207	-1.84792	-1.86323	-1.85908	-1.85493	-1.85078	-1.84665	-1.8426

-1.80015	-1.85254	-1.84839	-1.86369	-1.85953	-1.85539	-1.85127	-1.84719	-1.84314
-1.80066	-1.85307	-1.84893	-1.86422	-1.86	-1.85583	-1.85173	-1.84766	-1.84361
-1.80116	-1.85361	-1.84947	-1.86476	-1.86049	-1.85629	-1.85219	-1.84813	-1.84408
-1.80162	-1.85409	-1.84996	-1.86527	-1.86102	-1.85682	-1.85272	-1.84866	-1.84461
-1.80205	-1.85454	-1.85043	-1.86577	-1.86157	-1.8574	-1.8533	-1.84923	-1.84517
-1.80254	-1.85505	-1.85094	-1.86631	-1.86213	-1.85797	-1.85386	-1.84978	-1.84573
-1.80307	-1.85562	-1.8515	-1.86688	-1.8627	-1.85854	-1.85442	-1.85033	-1.84628
-1.78101	-1.83402	-1.82997	-1.84795	-1.84384	-1.8591	-1.85496	-1.85085	-1.8468
-1.78156	-1.83457	-1.83052	-1.8485	-1.8444	-1.85966	-1.85548	-1.85132	-1.84725
-1.78208	-1.8351	-1.83104	-1.84903	-1.84494	-1.8602	-1.85599	-1.85179	-1.84771
-1.78254	-1.83557	-1.83149	-1.8495	-1.84541	-1.86071	-1.85651	-1.85234	-1.84825
-1.78299	-1.83601	-1.83193	-1.84995	-1.84588	-1.8612	-1.85705	-1.85291	-1.84883
-1.7835	-1.81455	-1.8105	-1.85047	-1.8464	-1.86174	-1.8576	-1.85348	-1.8494
-1.78404	-1.8151	-1.81106	-1.85103	-1.84696	-1.86231	-1.85817	-1.85406	-1.84997
-1.78459	-1.81566	-1.81162	-1.83	-1.82595	-1.86288	-1.85875	-1.85463	-1.85054
-1.78514	-1.81623	-1.81219	-1.83058	-1.82652	-1.86345	-1.85931	-1.8552	-1.85111
-1.7857	-1.8168	-1.81275	-1.83115	-1.8271	-1.86402	-1.85989	-1.85577	-1.85168
-1.78625	-1.81736	-1.81331	-1.83171	-1.82766	-1.86459	-1.86046	-1.85635	-1.85226
-1.78681	-1.81793	-1.81387	-1.83228	-1.82823	-1.86516	-1.86105	-1.85696	-1.85286
-1.78736	-1.8185	-1.81444	-1.83286	-1.8288	-1.84452	-1.84049	-1.83646	-1.85352
-1.78794	-1.81909	-1.81503	-1.83346	-1.82938	-1.84512	-1.84112	-1.83714	-1.8542
-1.78856	-1.78459	-1.78065	-1.81164	-1.80762	-1.82598	-1.82204	-1.81812	-1.83372
-1.78922	-1.78525	-1.78132	-1.81232	-1.80828	-1.82663	-1.82264	-1.81868	-1.83428
-1.78985	-1.78588	-1.78194	-1.81296	-1.8089	-1.82725	-1.82323	-1.81925	-1.83485
-1.79042	-1.78645	-1.7825	-1.81353	-1.80947	-1.82782	-1.8238	-1.81981	-1.83543
-1.79099	-1.78701	-1.78305	-1.8141	-1.81004	-1.8284	-1.82437	-1.82038	-1.83601
-1.79156	-1.78758	-1.78361	-1.81466	-1.81061	-1.82898	-1.82494	-1.82094	-1.83662
-1.79215	-1.78816	-1.78418	-1.81525	-1.8112	-1.82958	-1.82553	-1.82152	-1.83724
-1.7928	-1.78881	-1.78481	-1.81589	-1.81184	-1.83024	-1.82617	-1.82215	-1.86969
-1.79347	-1.78948	-1.78548	-1.81657	-1.81252	-1.83093	-1.82684	-1.82279	-1.87032
-1.79409	-1.7901	-1.7861	-1.8172	-1.81315	-1.83156	-1.82747	-1.82341	-1.87093
-1.79466	-1.79067	-1.78669	-1.81781	-1.81374	-1.83214	-1.82805	-1.824	-1.87152
-1.77344	-1.79128	-1.78731	-1.81844	-1.81436	-1.83276	-1.82867	-1.82462	-1.85295
-1.77411	-1.79195	-1.78798	-1.81913	-1.81504	-1.83345	-1.82935	-1.82528	-1.85362
-1.77479	-1.79264	-1.78866	-1.81983	-1.81574	-1.83416	-1.83005	-1.82597	-1.85431
-1.77547	-1.79332	-1.78933	-1.82052	-1.81645	-1.83489	-1.83076	-1.82667	-1.85501
-1.77613	-1.794	-1.78999	-1.82119	-1.81714	-1.8356	-1.83148	-1.82738	-1.85573
-1.77673	-1.79461	-1.79061	-1.78663	-1.78267	-1.81373	-1.8097	-1.8057	-1.85645
-1.77729	-1.79517	-1.79119	-1.78722	-1.78324	-1.81432	-1.81035	-1.80641	-1.85718
-1.77791	-1.77399	-1.77008	-1.78785	-1.78386	-1.81494	-1.81099	-1.80706	-1.85787
-1.77859	-1.77466	-1.77075	-1.78852	-1.78453	-1.81561	-1.81161	-1.80765	-1.8585
-1.77928	-1.77535	-1.77143	-1.7892	-1.78521	-1.84949	-1.84537	-1.84129	-1.85914
-1.77998	-1.77604	-1.77211	-1.78989	-1.7859	-1.85021	-1.84607	-1.84196	-1.8598
-1.78067	-1.77673	-1.7728	-1.79058	-1.7866	-1.85093	-1.84679	-1.84267	-1.86048
-1.74141	-1.77742	-1.77349	-1.79128	-1.7873	-1.85167	-1.84752	-1.8434	-1.86122
-1.74208	-1.77811	-1.77418	-1.79198	-1.78801	-1.8524	-1.84826	-1.84416	-1.862
-1.74276	-1.73887	-1.73502	-1.77095	-1.76703	-1.85313	-1.84902	-1.84493	-1.86278
-1.74345	-1.73955	-1.73568	-1.77162	-1.7677	-1.85386	-1.84978	-1.84572	-1.86357
-1.74413	-1.74023	-1.73635	-1.73248	-1.72863	-1.85457	-1.8505	-1.84645	-1.8643
-1.74481	-1.74091	-1.73703	-1.73316	-1.72929	-1.85524	-1.85115	-1.84708	-1.86496

-1.74549	-1.7416	-1.73774	-1.73387	-1.72997	-1.7619	-1.75799	-1.75413	-1.84369
-1.74619	-1.74231	-1.73847	-1.73462	-1.73073	-1.76266	-1.75873	-1.75484	-1.84441
-1.7469	-1.74304	-1.73921	-1.73537	-1.73149	-1.76343	-1.75949	-1.75556	-1.84516
-1.72895	-1.74376	-1.73991	-1.73605	-1.73218	-1.76413	-1.76018	-1.75626	-1.80888
-1.72969	-1.74448	-1.7406	-1.73671	-1.73283	-1.7648	-1.76086	-1.75694	-1.8096
-1.70943	-1.74519	-1.74129	-1.73739	-1.7335	-1.76547	-1.76154	-1.75763	-1.78936
-1.71012	-1.74589	-1.74198	-1.73808	-1.73416	-1.76613	-1.76221	-1.75832	-1.79009
-1.69005	-1.74659	-1.7427	-1.77484	-1.77083	-1.76685	-1.76293	-1.75904	-1.79083
-1.69074	-1.74732	-1.74345	-1.77562	-1.77162	-1.76765	-1.76371	-1.75979	-1.79156
-1.69144	-1.7279	-1.72407	-1.77639	-1.77241	-1.76845	-1.76449	-1.76055	-1.79232
-1.69216	-1.72863	-1.72479	-1.77711	-1.77314	-1.76919	-1.76525	-1.76133	-1.79312
-1.69289	-1.72936	-1.72549	-1.77782	-1.77385	-1.76991	-1.766	-1.7621	-1.79393
-1.69358	-1.73007	-1.72619	-1.77854	-1.77459	-1.77066	-1.76673	-1.76282	-1.79466
-1.69426	-1.73076	-1.72689	-1.77927	-1.77535	-1.77142	-1.76745	-1.7635	-1.79535
-1.69499	-1.73149	-1.72762	-1.76047	-1.7566	-1.77219	-1.76821	-1.76424	-1.7961
-1.69577	-1.73227	-1.72838	-1.76123	-1.75736	-1.77297	-1.769	-1.76503	-1.79691
-1.69656	-1.71202	-1.70818	-1.762	-1.75812	-1.75424	-1.75032	-1.74641	-1.7619
-1.69735	-1.7128	-1.70895	-1.76276	-1.75883	-1.75493	-1.75102	-1.74714	-1.76265
-1.69812	-1.71358	-1.70972	-1.76353	-1.75956	-1.75563	-1.75173	-1.74786	-1.7634
-1.69883	-1.71433	-1.71049	-1.76433	-1.76035	-1.7564	-1.75249	-1.74863	-1.76417
-1.69954	-1.71506	-1.71126	-1.76515	-1.76116	-1.7572	-1.75329	-1.74942	-1.76496
-1.70028	-1.71582	-1.71204	-1.76596	-1.76196	-1.75799	-1.75408	-1.75021	-1.74638
-1.70106	-1.7166	-1.71283	-1.76677	-1.76276	-1.75879	-1.75488	-1.75101	-1.74718
-1.70185	-1.75489	-1.75103	-1.76758	-1.76357	-1.75959	-1.75568	-1.75181	-1.74797
-1.70264	-1.7557	-1.75184	-1.76839	-1.76438	-1.76039	-1.75648	-1.7526	-1.74877
-1.70344	-1.75651	-1.75265	-1.76921	-1.76518	-1.7612	-1.75728	-1.7534	-1.74957
-1.70422	-1.75731	-1.75344	-1.77001	-1.76598	-1.76199	-1.75807	-1.7542	-1.75037
-1.705	-1.7581	-1.75424	-1.77082	-1.76678	-1.76279	-1.75887	-1.755	-1.75118
-1.70577	-1.75889	-1.75504	-1.77163	-1.7676	-1.76361	-1.75968	-1.75579	-1.75197
-1.70654	-1.75968	-1.75584	-1.77245	-1.76843	-1.76444	-1.76049	-1.75659	-1.75277
-1.70732	-1.76048	-1.75664	-1.77329	-1.7693	-1.76532	-1.76135	-1.75742	-1.75358
-1.7081	-1.76127	-1.75745	-1.77413	-1.77019	-1.76624	-1.76225	-1.75829	-1.75442
-1.67991	-1.74328	-1.73949	-1.75442	-1.75057	-1.76712	-1.76313	-1.75915	-1.75524
-1.68073	-1.74412	-1.74029	-1.7552	-1.75137	-1.76794	-1.76396	-1.75998	-1.75605
-1.68154	-1.74496	-1.74109	-1.75599	-1.75217	-1.76876	-1.76479	-1.76082	-1.75688
-1.68232	-1.74579	-1.74188	-1.75677	-1.75296	-1.76959	-1.76566	-1.76173	-1.75778
-1.6831	-1.74662	-1.74268	-1.75757	-1.75376	-1.77042	-1.76654	-1.76264	-1.7587
-1.68392	-1.74752	-1.74354	-1.7584	-1.75457	-1.77123	-1.76736	-1.7635	-1.75959
-1.68477	-1.74845	-1.74444	-1.75928	-1.75539	-1.77202	-1.76816	-1.76432	-1.76046
-1.68557	-1.74935	-1.74535	-1.76018	-1.75625	-1.77286	-1.76898	-1.76513	-1.76129
-1.68632	-1.75021	-1.74626	-1.76111	-1.75716	-1.77375	-1.76981	-1.76592	-1.76208
-1.68707	-1.75105	-1.74714	-1.76202	-1.75807	-1.75415	-1.75024	-1.74637	-1.76288
-1.68781	-1.75185	-1.74797	-1.76288	-1.75898	-1.75507	-1.75115	-1.74724	-1.76372
-1.68857	-1.75267	-1.74879	-1.76372	-1.75986	-1.75599	-1.75206	-1.74814	-1.76459
-1.68942	-1.72483	-1.72116	-1.76455	-1.76068	-1.75682	-1.75294	-1.74905	-1.7655
-1.6903	-1.72568	-1.72196	-1.76538	-1.7615	-1.75764	-1.7538	-1.74996	-1.76644
-1.69117	-1.72659	-1.72286	-1.74752	-1.74364	-1.7585	-1.75465	-1.75081	-1.76732
-1.69204	-1.72752	-1.72382	-1.74854	-1.74459	-1.7594	-1.75549	-1.75162	-1.76817
-1.69287	-1.72839	-1.72471	-1.74948	-1.74552	-1.76032	-1.75638	-1.75248	-1.74864
-1.69363	-1.72915	-1.72549	-1.75034	-1.74643	-1.76126	-1.75731	-1.75339	-1.74951

-1.6717	-1.72995	-1.72629	-1.72264	-1.719	-1.76218	-1.75824	-1.75431	-1.75041
-1.67261	-1.73087	-1.72716	-1.72347	-1.7198	-1.76304	-1.75913	-1.75524	-1.75137
-1.67352	-1.73179	-1.72805	-1.72433	-1.72063	-1.76391	-1.76003	-1.75617	-1.75233
-1.67434	-1.73266	-1.72893	-1.72522	-1.72154	-1.76489	-1.76098	-1.7571	-1.75323
-1.67513	-1.73351	-1.72981	-1.72613	-1.72248	-1.76592	-1.76197	-1.75803	-1.75413
-1.71594	-1.7344	-1.7307	-1.72701	-1.72336	-1.76686	-1.7629	-1.75896	-1.75506
-1.7169	-1.73534	-1.73159	-1.72787	-1.72418	-1.76773	-1.76381	-1.75991	-1.75604
-1.71779	-1.71409	-1.71038	-1.72874	-1.72505	-1.7405	-1.73682	-1.73316	-1.75699
-1.7186	-1.71492	-1.71124	-1.72964	-1.72598	-1.74145	-1.73774	-1.73403	-1.75788
-1.71944	-1.71576	-1.7121	-1.73053	-1.7269	-1.74238	-1.73865	-1.73492	-1.7588
-1.72037	-1.71667	-1.71297	-1.73138	-1.72773	-1.74321	-1.73951	-1.73583	-1.75979
-1.72132	-1.71759	-1.71384	-1.73223	-1.72855	-1.74404	-1.74039	-1.73675	-1.76082
-1.70197	-1.71846	-1.71473	-1.73315	-1.72948	-1.72583	-1.72223	-1.71865	-1.76183
-1.70278	-1.71931	-1.71564	-1.73412	-1.73045	-1.72681	-1.72321	-1.71961	-1.76285
-1.70367	-1.70003	-1.69644	-1.73508	-1.73143	-1.72778	-1.72415	-1.72053	-1.73593
-1.70462	-1.70099	-1.69739	-1.73606	-1.7324	-1.72874	-1.72507	-1.7214	-1.73677
-1.70558	-1.70194	-1.69832	-1.71485	-1.71124	-1.72965	-1.72597	-1.72228	-1.73765
-1.70654	-1.70287	-1.69919	-1.7157	-1.71205	-1.73047	-1.72682	-1.72319	-1.73859
-1.70747	-1.70377	-1.70006	-1.71656	-1.71288	-1.7313	-1.72769	-1.72411	-1.73954
-1.70829	-1.70462	-1.70094	-1.6973	-1.69367	-1.73224	-1.72864	-1.72507	-1.74051
-1.70909	-1.70545	-1.70183	-1.69823	-1.69463	-1.73323	-1.72963	-1.72604	-1.74149
-1.70999	-1.70636	-1.70277	-1.69918	-1.69559	-1.71442	-1.71084	-1.70727	-1.74243
-1.71094	-1.70733	-1.70374	-1.70016	-1.69657	-1.7154	-1.7118	-1.70819	-1.74334
-1.68895	-1.70829	-1.7047	-1.70111	-1.69753	-1.69395	-1.69037	-1.68679	-1.72481
-1.68991	-1.70925	-1.70564	-1.70205	-1.69847	-1.69488	-1.69126	-1.68764	-1.72568
-1.69086	-1.68726	-1.68368	-1.70295	-1.69936	-1.69576	-1.69213	-1.6885	-1.72656
-1.69181	-1.68818	-1.68454	-1.70378	-1.70016	-1.69656	-1.69295	-1.68937	-1.72747
-1.69274	-1.68907	-1.68539	-1.7046	-1.70096	-1.69735	-1.69379	-1.69025	-1.72841
-1.69362	-1.68993	-1.68625	-1.70547	-1.70185	-1.69826	-1.6947	-1.69117	-1.70997
-1.69448	-1.69079	-1.68713	-1.70638	-1.70278	-1.69922	-1.69566	-1.69212	-1.71093
-1.69536	-1.69168	-1.68804	-1.68444	-1.68091	-1.70017	-1.69662	-1.69308	-1.68957
-1.69626	-1.6926	-1.68897	-1.68539	-1.68187	-1.70114	-1.69759	-1.69405	-1.69053
-1.69719	-1.69354	-1.68993	-1.68635	-1.68283	-1.67931	-1.6758	-1.67231	-1.6915
-1.69816	-1.69452	-1.6909	-1.68733	-1.68378	-1.68026	-1.67676	-1.67327	-1.69246
-1.69913	-1.6955	-1.69189	-1.6883	-1.68475	-1.68121	-1.67771	-1.67422	-1.67074
-1.70011	-1.69648	-1.69287	-1.68928	-1.68571	-1.68217	-1.67866	-1.67517	-1.67169
-1.70109	-1.69747	-1.69385	-1.69025	-1.68667	-1.68312	-1.67961	-1.67612	-1.67264
-1.70208	-1.69845	-1.69483	-1.69123	-1.68764	-1.68408	-1.68057	-1.67708	-1.67359
-1.70306	-1.69943	-1.69581	-1.6922	-1.6886	-1.68504	-1.68153	-1.67803	-1.67454
-1.70404	-1.70041	-1.69678	-1.69317	-1.68957	-1.686	-1.68248	-1.67899	-1.67549
-1.70502	-1.70139	-1.69776	-1.69415	-1.69054	-1.68697	-1.68344	-1.67994	-1.67645
-1.68398	-1.70236	-1.69874	-1.69512	-1.69151	-1.68793	-1.6844	-1.68089	-1.6774
-1.68493	-1.70333	-1.69971	-1.6961	-1.69249	-1.6889	-1.68535	-1.68183	-1.67834
-1.68588	-1.7043	-1.70068	-1.69707	-1.69346	-1.68987	-1.68631	-1.68277	-1.67928
-1.68683	-1.70527	-1.70165	-1.69803	-1.69442	-1.69083	-1.68726	-1.68372	-1.68021
-1.68781	-1.70626	-1.70262	-1.699	-1.69539	-1.6918	-1.68822	-1.68466	-1.68115
-1.63393	-1.70734	-1.70367	-1.70002	-1.69639	-1.69278	-1.68919	-1.68563	-1.68211
-1.63502	-1.70847	-1.70477	-1.70108	-1.69741	-1.69377	-1.69017	-1.68661	-1.68309
-1.63604	-1.6875	-1.68386	-1.70217	-1.6985	-1.69486	-1.69125	-1.68768	-1.68416
-1.63699	-1.68853	-1.68494	-1.70331	-1.69967	-1.69605	-1.69245	-1.68887	-1.68533

-1.63791	-1.68952	-1.68597	-1.7044	-1.70078	-1.69718	-1.69358	-1.69	-1.68645
-1.63881	-1.69047	-1.68693	-1.70538	-1.70178	-1.69818	-1.69459	-1.69101	-1.68745
-1.6397	-1.69141	-1.68787	-1.70634	-1.70273	-1.69914	-1.69555	-1.69198	-1.68841
-1.6406	-1.69234	-1.6888	-1.70729	-1.70369	-1.70009	-1.6965	-1.69292	-1.68935
-1.64149	-1.69327	-1.68973	-1.70823	-1.70463	-1.70103	-1.69744	-1.69385	-1.69027
-1.6424	-1.66767	-1.6642	-1.68713	-1.68359	-1.70199	-1.69839	-1.69481	-1.69123
-1.64331	-1.66862	-1.66514	-1.68808	-1.68454	-1.70296	-1.69936	-1.69578	-1.6922
-1.6443	-1.66964	-1.66615	-1.68911	-1.68556	-1.704	-1.7004	-1.69682	-1.69324
-1.6454	-1.67076	-1.66727	-1.69024	-1.68669	-1.70514	-1.70155	-1.69796	-1.69439
-1.64646	-1.64305	-1.63968	-1.69135	-1.68779	-1.70626	-1.70266	-1.69908	-1.6955
-1.64741	-1.644	-1.64062	-1.69233	-1.68878	-1.70728	-1.70367	-1.70007	-1.6965
-1.64832	-1.64491	-1.64153	-1.69328	-1.68975	-1.70827	-1.70466	-1.70105	-1.69748
-1.64923	-1.64582	-1.64244	-1.69425	-1.69075	-1.68725	-1.68372	-1.68019	-1.69857
-1.65014	-1.64674	-1.64336	-1.69523	-1.69177	-1.6883	-1.68481	-1.6813	-1.69971
-1.65115	-1.64774	-1.64436	-1.66974	-1.66633	-1.68933	-1.68583	-1.68233	-1.70076
-1.65224	-1.64883	-1.64544	-1.67082	-1.66736	-1.69033	-1.6868	-1.68329	-1.70173
-1.62346	-1.64986	-1.64647	-1.67186	-1.66837	-1.69132	-1.68777	-1.68424	-1.70271
-1.62437	-1.6508	-1.6474	-1.67283	-1.66932	-1.69227	-1.68872	-1.68518	-1.7037
-1.6253	-1.65175	-1.64835	-1.67381	-1.67031	-1.69327	-1.68971	-1.68617	-1.70473
-1.62634	-1.6528	-1.6494	-1.67491	-1.67141	-1.6944	-1.69083	-1.68729	-1.68379
-1.62739	-1.65387	-1.65048	-1.67602	-1.67254	-1.69555	-1.692	-1.68845	-1.6849
-1.62836	-1.62501	-1.62171	-1.67704	-1.67356	-1.67009	-1.66664	-1.66319	-1.68602
-1.62927	-1.62592	-1.62261	-1.678	-1.67452	-1.67107	-1.66766	-1.66426	-1.68714
-1.63026	-1.6269	-1.6236	-1.67904	-1.67556	-1.67211	-1.66871	-1.66531	-1.68821
-1.63135	-1.62798	-1.62468	-1.68019	-1.6767	-1.67322	-1.66978	-1.66634	-1.68921
-1.58628	-1.62904	-1.62575	-1.6522	-1.64885	-1.67435	-1.67087	-1.6674	-1.69027
-1.58721	-1.63003	-1.62677	-1.65327	-1.64994	-1.6755	-1.67203	-1.66855	-1.69144
-1.58815	-1.63102	-1.62777	-1.65431	-1.651	-1.67661	-1.67316	-1.66969	-1.69261
-1.55802	-1.63205	-1.62876	-1.62549	-1.62225	-1.67761	-1.67415	-1.6707	-1.66726
-1.55908	-1.63312	-1.62974	-1.62642	-1.62316	-1.67856	-1.67511	-1.67168	-1.66827
-1.53099	-1.63422	-1.6308	-1.62745	-1.62417	-1.67962	-1.67617	-1.67273	-1.66932
-1.53204	-1.63536	-1.63192	-1.62856	-1.62526	-1.68076	-1.6773	-1.67385	-1.6704
-1.47847	-1.60609	-1.60273	-1.62965	-1.62635	-1.6819	-1.67844	-1.67498	-1.67151
-1.47931	-1.60707	-1.60376	-1.63072	-1.62744	-1.68307	-1.6796	-1.67614	-1.67267
-1.48019	-1.55597	-1.5528	-1.60152	-1.59829	-1.62522	-1.62198	-1.61875	-1.67382
-1.48115	-1.55696	-1.55374	-1.60247	-1.59921	-1.62614	-1.62294	-1.61976	-1.67492
-1.48215	-1.55798	-1.5547	-1.60341	-1.60011	-1.62704	-1.62387	-1.62074	-1.676
-1.48315	-1.52976	-1.52653	-1.60446	-1.60112	-1.62803	-1.62483	-1.62168	-1.67702
-1.48416	-1.53082	-1.52759	-1.60555	-1.60218	-1.62906	-1.62581	-1.62262	-1.67801
-1.48512	-1.53185	-1.52863	-1.55461	-1.55133	-1.59994	-1.59671	-1.59353	-1.64988
-1.48604	-1.53286	-1.52968	-1.55569	-1.55241	-1.60104	-1.59777	-1.59455	-1.65089
-1.48694	-1.48396	-1.48096	-1.55675	-1.55348	-1.55025	-1.54706	-1.54391	-1.62235
-1.4878	-1.48483	-1.48188	-1.55779	-1.55454	-1.55131	-1.5481	-1.54493	-1.62342
-1.48869	-1.48573	-1.48281	-1.55882	-1.55559	-1.55237	-1.54915	-1.54596	-1.59447
-1.48965	-1.48668	-1.48378	-1.55987	-1.55666	-1.55344	-1.55021	-1.54699	-1.59552
-1.49063	-1.48766	-1.48475	-1.56092	-1.55772	-1.55451	-1.55126	-1.54803	-1.59658
-1.49161	-1.48861	-1.48567	-1.53256	-1.52946	-1.55554	-1.5523	-1.54908	-1.54591
-1.4926	-1.48955	-1.48656	-1.53349	-1.53041	-1.55655	-1.55334	-1.55014	-1.54696
-1.4936	-1.49052	-1.4875	-1.53446	-1.53141	-1.55759	-1.55439	-1.5512	-1.54801
-1.49462	-1.49153	-1.48849	-1.53548	-1.53243	-1.55864	-1.55545	-1.55225	-1.54905

-1.49564	-1.49253	-1.48948	-1.53651	-1.53346	-1.5597	-1.55651	-1.55331	-1.5501
-1.49666	-1.49354	-1.49048	-1.53755	-1.5345	-1.56076	-1.55758	-1.55438	-1.55116
-1.49769	-1.49455	-1.49147	-1.53859	-1.53554	-1.56182	-1.55864	-1.55544	-1.55222
-1.49871	-1.49555	-1.49247	-1.53963	-1.53657	-1.53351	-1.53041	-1.52729	-1.55328
-1.49974	-1.49657	-1.49346	-1.54066	-1.5376	-1.53454	-1.53144	-1.52832	-1.55434
-1.50078	-1.49759	-1.49447	-1.5417	-1.53863	-1.53557	-1.53247	-1.52935	-1.5554
-1.50183	-1.49863	-1.4955	-1.54276	-1.53967	-1.53659	-1.53349	-1.53038	-1.55646
-1.50288	-1.49967	-1.49652	-1.49346	-1.49049	-1.53761	-1.53451	-1.5314	-1.55751
-1.50392	-1.50071	-1.49754	-1.49446	-1.49148	-1.53865	-1.53554	-1.53242	-1.55856
-1.47435	-1.50175	-1.49856	-1.49547	-1.49248	-1.53969	-1.53656	-1.53344	-1.53032
-1.47539	-1.5028	-1.49959	-1.49648	-1.49347	-1.54072	-1.53759	-1.53448	-1.53135
-1.47642	-1.50387	-1.50064	-1.49751	-1.49449	-1.54176	-1.53863	-1.53551	-1.53238
-1.47744	-1.50494	-1.50176	-1.49863	-1.49556	-1.54283	-1.53968	-1.53654	-1.53344
-1.47846	-1.50602	-1.50291	-1.49979	-1.49667	-1.54394	-1.54074	-1.53758	-1.5345
-1.41334	-1.50709	-1.50399	-1.50088	-1.49774	-1.54503	-1.5418	-1.53862	-1.53555
-1.41424	-1.50814	-1.50503	-1.50191	-1.49878	-1.54611	-1.54287	-1.53967	-1.53658
-1.41517	-1.50923	-1.5061	-1.50297	-1.49983	-1.5472	-1.54396	-1.54076	-1.53765
-1.41613	-1.51037	-1.50722	-1.50406	-1.50088	-1.54829	-1.54508	-1.54191	-1.53878
-1.41709	-1.5115	-1.50833	-1.50516	-1.50196	-1.54941	-1.54621	-1.54306	-1.53991
-1.41804	-1.51258	-1.5094	-1.50624	-1.50308	-1.55061	-1.54739	-1.54419	-1.54101
-1.41898	-1.51365	-1.51045	-1.5073	-1.50421	-1.55182	-1.54856	-1.54532	-1.54211
-1.41991	-1.41716	-1.41442	-1.50834	-1.50525	-1.55294	-1.54968	-1.54643	-1.54323
-1.42083	-1.41807	-1.41531	-1.50936	-1.50626	-1.55401	-1.55076	-1.54753	-1.54435
-1.42174	-1.41899	-1.41623	-1.51043	-1.50732	-1.50423	-1.50112	-1.49804	-1.54544
-1.42266	-1.41991	-1.41719	-1.51157	-1.50842	-1.50529	-1.50215	-1.49904	-1.54649
-1.33671	-1.42085	-1.41816	-1.5127	-1.50952	-1.50636	-1.50321	-1.5001	-1.54758
-1.33764	-1.42183	-1.41912	-1.51381	-1.5106	-1.50743	-1.50431	-1.50122	-1.54874
-1.33858	-1.42281	-1.42007	-1.51491	-1.51168	-1.50851	-1.50541	-1.50236	-1.54993
-1.33951	-1.39606	-1.39332	-1.41826	-1.41553	-1.50961	-1.50651	-1.50345	-1.55109
-1.34043	-1.39703	-1.39426	-1.41919	-1.41649	-1.51073	-1.50762	-1.50454	-1.55226
-1.34134	-1.33871	-1.3361	-1.39252	-1.38984	-1.51186	-1.50873	-1.50565	-1.55342
-1.34222	-1.33961	-1.33704	-1.39353	-1.39083	-1.513	-1.50986	-1.50678	-1.55457
-1.34313	-1.34053	-1.33797	-1.39454	-1.39183	-1.48802	-1.48489	-1.48179	-1.55572
-1.34406	-1.34146	-1.33889	-1.39553	-1.39284	-1.48924	-1.48606	-1.48291	-1.55684
-1.345	-1.3424	-1.33982	-1.39652	-1.39385	-1.39117	-1.38847	-1.38577	-1.48089
-1.34594	-1.34334	-1.34076	-1.39752	-1.39482	-1.39213	-1.38945	-1.38677	-1.48206
-1.34689	-1.34428	-1.3417	-1.39852	-1.3958	-1.3931	-1.39042	-1.38776	-1.48325
-1.34784	-1.34522	-1.34263	-1.34005	-1.33749	-1.39409	-1.3914	-1.38872	-1.48437
-1.34879	-1.34616	-1.34355	-1.34097	-1.33843	-1.3951	-1.39238	-1.38966	-1.48548
-1.31275	-1.3471	-1.34448	-1.3419	-1.33935	-1.39609	-1.39337	-1.39065	-1.48665
-1.31365	-1.34802	-1.34542	-1.34284	-1.34026	-1.39705	-1.39436	-1.39168	-1.48789
-1.31457	-1.34896	-1.34636	-1.34377	-1.34118	-1.33862	-1.33608	-1.33355	-1.4891
-1.31553	-1.34993	-1.34729	-1.34468	-1.34211	-1.33954	-1.33699	-1.33445	-1.49027
-1.28436	-1.3509	-1.34823	-1.34561	-1.34303	-1.34047	-1.3379	-1.33535	-1.39193
-1.28527	-1.35186	-1.34919	-1.34656	-1.34395	-1.34137	-1.33881	-1.33626	-1.3929
-1.28619	-1.35283	-1.35016	-1.34752	-1.34488	-1.34228	-1.33971	-1.33717	-1.39387
-1.28711	-1.31668	-1.31409	-1.34846	-1.34582	-1.34321	-1.34064	-1.33808	-1.33553
-1.28805	-1.31764	-1.31502	-1.3494	-1.34678	-1.34417	-1.34157	-1.33899	-1.33644
-1.28904	-1.28643	-1.28386	-1.3504	-1.34777	-1.34516	-1.34254	-1.33995	-1.33739
-1.29007	-1.28745	-1.28486	-1.35146	-1.34881	-1.34618	-1.34356	-1.34096	-1.33839

-1.29107	-1.28844	-1.28584	-1.32093	-1.31832	-1.31573	-1.31317	-1.31064	-1.33938
-1.29204	-1.2894	-1.28678	-1.32189	-1.31925	-1.31664	-1.31409	-1.31156	-1.34032
-1.29299	-1.29034	-1.2877	-1.32283	-1.32017	-1.31755	-1.315	-1.31247	-1.34124
-1.29394	-1.29128	-1.28862	-1.32378	-1.32111	-1.31848	-1.31589	-1.31334	-1.31081
-1.29489	-1.29222	-1.28955	-1.32473	-1.32206	-1.31941	-1.3168	-1.31422	-1.31168
-1.22137	-1.2932	-1.29053	-1.32574	-1.32305	-1.32039	-1.31776	-1.31517	-1.31262
-1.22228	-1.2942	-1.29154	-1.32679	-1.32407	-1.32139	-1.31876	-1.31617	-1.3136
-1.22315	-1.29516	-1.29252	-1.28988	-1.28724	-1.32236	-1.31973	-1.31714	-1.31457
-1.22397	-1.29607	-1.29344	-1.29081	-1.28816	-1.32331	-1.32068	-1.31809	-1.31553
-1.19071	-1.22237	-1.21995	-1.29175	-1.2891	-1.32428	-1.32163	-1.31904	-1.31649
-1.19162	-1.22329	-1.22085	-1.29273	-1.29008	-1.32529	-1.32263	-1.32	-1.31742
-1.19253	-1.22421	-1.22174	-1.29371	-1.29106	-1.32631	-1.32363	-1.32098	-1.31837
-1.19339	-1.19096	-1.18855	-1.29465	-1.29201	-1.32731	-1.32463	-1.32198	-1.31935
-1.19424	-1.1918	-1.18937	-1.29558	-1.29296	-1.32831	-1.32564	-1.32299	-1.32035
-1.15867	-1.19267	-1.19024	-1.18784	-1.1855	-1.32932	-1.32666	-1.32402	-1.32137
-1.15956	-1.19359	-1.19113	-1.18873	-1.18638	-1.33035	-1.3277	-1.32507	-1.32241
-1.16045	-1.19451	-1.19205	-1.18963	-1.18727	-1.29923	-1.29664	-1.29406	-1.3234
-1.16136	-1.19546	-1.19299	-1.19055	-1.18816	-1.30024	-1.29759	-1.29498	-1.32434
-1.16227	-1.1964	-1.19392	-1.19148	-1.18906	-1.18667	-1.18431	-1.18198	-1.29331
-1.16318	-1.19734	-1.19485	-1.19239	-1.18996	-1.18756	-1.18519	-1.18285	-1.2943
-1.16408	-1.19827	-1.19577	-1.19331	-1.19087	-1.18847	-1.18609	-1.18374	-1.29532
-1.13127	-1.16253	-1.16013	-1.19421	-1.19178	-1.18937	-1.18698	-1.18461	-1.29632
-1.13212	-1.16339	-1.16098	-1.19511	-1.19269	-1.19029	-1.18787	-1.18549	-1.29733
-1.13301	-1.1306	-1.12824	-1.16306	-1.16069	-1.1912	-1.18878	-1.18637	-1.22395
-1.13393	-1.13151	-1.12914	-1.16398	-1.16161	-1.19213	-1.18968	-1.18726	-1.22486
-1.13486	-1.13243	-1.13004	-1.1277	-1.1254	-1.16015	-1.15774	-1.15535	-1.19366
-1.13579	-1.13335	-1.13095	-1.1286	-1.12629	-1.16107	-1.15866	-1.15626	-1.19459
-1.13672	-1.13428	-1.13187	-1.12951	-1.12719	-1.162	-1.15958	-1.15717	-1.19554
-1.02099	-1.13521	-1.1328	-1.13043	-1.1281	-1.16293	-1.1605	-1.15808	-1.15571
-1.02177	-1.13614	-1.13373	-1.13135	-1.12902	-1.16388	-1.16143	-1.159	-1.15661
-0.9794	-1.13705	-1.13464	-1.13227	-1.12994	-1.12761	-1.12526	-1.12292	-1.15757
-0.98011	-1.13797	-1.13556	-1.13319	-1.13086	-1.12854	-1.12622	-1.12389	-1.15858
-0.98086	-1.02199	-1.01989	-1.13411	-1.13178	-1.12946	-1.12715	-1.12484	-1.1225
-0.98165	-1.02282	-1.02069	-1.13504	-1.1327	-1.13037	-1.12806	-1.12575	-1.12341
-0.90237	-0.98041	-0.97838	-1.13597	-1.13361	-1.13127	-1.12896	-1.12666	-1.12431
-0.90304	-0.98116	-0.97911	-1.13688	-1.1345	-1.13215	-1.12985	-1.12755	-1.12523
-0.9037	-0.98191	-0.97985	-1.13781	-1.13541	-1.13304	-1.13074	-1.12844	-1.12613
-0.86199	-0.90252	-0.90067	-1.13878	-1.13635	-1.13397	-1.13164	-1.12934	-1.12704
-0.86262	-0.90319	-0.90136	-1.13977	-1.13732	-1.13492	-1.13256	-1.13024	-1.12794
-0.86325	-0.8615	-0.85976	-0.90022	-0.89841	-1.13589	-1.13351	-1.13116	-1.12884
-0.86388	-0.86213	-0.86039	-0.90089	-0.8991	-1.13689	-1.13448	-1.1321	-1.12975
-0.86452	-0.86276	-0.86102	-0.90155	-0.89977	-1.0638	-1.06154	-1.0593	-1.13069
-0.86516	-0.86339	-0.86165	-0.90221	-0.90042	-1.06468	-1.06244	-1.06022	-1.13167
-0.82758	-0.86403	-0.86229	-0.86056	-0.85884	-0.94199	-0.94014	-0.93829	-1.05889
-0.82825	-0.86469	-0.86293	-0.86119	-0.85946	-0.9427	-0.94084	-0.93899	-1.05978
-0.82894	-0.86537	-0.86358	-0.86183	-0.8601	-0.94342	-0.94154	-0.93968	-1.06066
-0.82965	-0.82788	-0.82613	-0.86248	-0.86073	-0.85901	-0.85731	-0.85564	-1.06151
-0.83039	-0.82861	-0.82682	-0.86315	-0.86138	-0.85964	-0.85793	-0.85624	-1.06235
-0.79312	-0.82931	-0.82752	-0.86385	-0.86208	-0.86033	-0.85859	-0.85687	-0.89866
-0.79378	-0.82999	-0.82823	-0.8646	-0.86283	-0.86107	-0.85929	-0.85754	-0.89934

-0.74446	-0.79274	-0.79104	-0.8272	-0.8255	-0.86178	-0.85999	-0.85822	-0.90007
-0.74507	-0.79339	-0.79169	-0.82784	-0.82614	-0.86244	-0.86069	-0.85894	-0.90086
-0.7457	-0.79406	-0.79234	-0.82848	-0.82677	-0.86309	-0.86136	-0.85964	-0.90163
-0.70244	-0.79479	-0.79305	-0.79133	-0.78963	-0.82571	-0.82404	-0.82239	-0.85855
-0.70313	-0.79555	-0.79377	-0.79202	-0.79029	-0.82635	-0.82466	-0.82299	-0.85917
-0.65819	-0.75325	-0.75157	-0.79275	-0.79102	-0.78931	-0.78764	-0.78599	-0.822
-0.65875	-0.75393	-0.75228	-0.7935	-0.79178	-0.79007	-0.78839	-0.78672	-0.82269
-0.61072	-0.65787	-0.65644	-0.79419	-0.79248	-0.79077	-0.78908	-0.7874	-0.82336
-0.61126	-0.65841	-0.65696	-0.79481	-0.7931	-0.7914	-0.78971	-0.78803	-0.82401
-0.61181	-0.65899	-0.65752	-0.79547	-0.79376	-0.79206	-0.79037	-0.78868	-0.78703
-0.61239	-0.65962	-0.65815	-0.79622	-0.79451	-0.79281	-0.7911	-0.78941	-0.78772
-0.61296	-0.66024	-0.65877	-0.79698	-0.79526	-0.79355	-0.79184	-0.79013	-0.78841
-0.5688	-0.61213	-0.61079	-0.71004	-0.70848	-0.79421	-0.7925	-0.79079	-0.78908
-0.56927	-0.61262	-0.61129	-0.71063	-0.70907	-0.79486	-0.79314	-0.79144	-0.78975
-0.51594	-0.56852	-0.56727	-0.7113	-0.70973	-0.75182	-0.75019	-0.74857	-0.79045
-0.51643	-0.5691	-0.56784	-0.71201	-0.71044	-0.75254	-0.7509	-0.74927	-0.79117
-0.5169	-0.56964	-0.56837	-0.61171	-0.61038	-0.70953	-0.70798	-0.70642	-0.74828
-0.51734	-0.57013	-0.56886	-0.61222	-0.6109	-0.71014	-0.70858	-0.70702	-0.74891
-0.51779	-0.5167	-0.51564	-0.5681	-0.56684	-0.71077	-0.7092	-0.70764	-0.7061
-0.51829	-0.51719	-0.51612	-0.56866	-0.5674	-0.71147	-0.70989	-0.70833	-0.70678
-0.51879	-0.51769	-0.51661	-0.56922	-0.56795	-0.71217	-0.71059	-0.70902	-0.70746
-0.51927	-0.51816	-0.51707	-0.51599	-0.51493	-0.5672	-0.56594	-0.56469	-0.70808
-0.51974	-0.51862	-0.5175	-0.51641	-0.51534	-0.56768	-0.56642	-0.56517	-0.70868
-0.47526	-0.51909	-0.51797	-0.51687	-0.5158	-0.56821	-0.56694	-0.56569	-0.6621
-0.47572	-0.51959	-0.51847	-0.51737	-0.51629	-0.56878	-0.5675	-0.56625	-0.66276
-0.47618	-0.47515	-0.47413	-0.51788	-0.51679	-0.51571	-0.51465	-0.5136	-0.56555
-0.47665	-0.47562	-0.47459	-0.51838	-0.51728	-0.5162	-0.51513	-0.51408	-0.56612
-0.42556	-0.47607	-0.47505	-0.51886	-0.51776	-0.51668	-0.5156	-0.51454	-0.5135
-0.42594	-0.47651	-0.47548	-0.51932	-0.51821	-0.51712	-0.51604	-0.51498	-0.51393
-0.42632	-0.47694	-0.4759	-0.51977	-0.51866	-0.51756	-0.51648	-0.5154	-0.51434
-0.42673	-0.4258	-0.42487	-0.47533	-0.47431	-0.51804	-0.51695	-0.51587	-0.5148
-0.42716	-0.42622	-0.42529	-0.47581	-0.47478	-0.51854	-0.51745	-0.51636	-0.51529
-0.42759	-0.42665	-0.42571	-0.42479	-0.42386	-0.47423	-0.47321	-0.47221	-0.51578
-0.42802	-0.42707	-0.42614	-0.42521	-0.42428	-0.4747	-0.47368	-0.47267	-0.51627
-0.38073	-0.42749	-0.42656	-0.42563	-0.4247	-0.42378	-0.42286	-0.42196	-0.47214
-0.38108	-0.42789	-0.42696	-0.42604	-0.42512	-0.4242	-0.42328	-0.42237	-0.47261
-0.38143	-0.42828	-0.42737	-0.42645	-0.42553	-0.42462	-0.4237	-0.42278	-0.47307
-0.28888	-0.4287	-0.42777	-0.42684	-0.42592	-0.425	-0.42408	-0.42316	-0.42226
-0.2892	-0.42913	-0.42818	-0.42724	-0.4263	-0.42537	-0.42445	-0.42353	-0.42264
-0.28952	-0.38176	-0.38092	-0.42766	-0.42672	-0.42578	-0.42485	-0.42393	-0.42303
-0.28984	-0.38216	-0.38132	-0.4281	-0.42716	-0.42621	-0.42527	-0.42434	-0.42342
-0.29017	-0.33272	-0.33198	-0.42854	-0.42759	-0.42664	-0.4257	-0.42476	-0.42383
-0.29049	-0.33306	-0.33232	-0.42897	-0.42801	-0.42706	-0.42612	-0.42518	-0.42426
-0.29082	-0.29022	-0.28962	-0.38164	-0.38079	-0.42749	-0.42655	-0.42561	-0.42468
-0.29115	-0.29054	-0.28994	-0.38203	-0.38118	-0.42793	-0.42698	-0.42604	-0.42511
-0.29148	-0.29086	-0.29026	-0.38244	-0.38158	-0.42837	-0.42742	-0.42648	-0.42553
-0.29182	-0.29119	-0.29059	-0.33295	-0.3322	-0.42881	-0.42786	-0.42691	-0.42596
-0.29215	-0.29153	-0.29091	-0.3333	-0.33254	-0.42926	-0.42829	-0.42734	-0.42639
-0.29249	-0.29186	-0.29124	-0.33365	-0.33289	-0.38094	-0.38007	-0.37922	-0.42683
-0.29281	-0.29218	-0.29156	-0.33401	-0.33324	-0.38134	-0.38047	-0.37961	-0.42727

-0.29315	-0.29251	-0.29189	-0.33436	-0.3336	-0.33284	-0.33209	-0.33134	-0.42771
-0.29348	-0.29284	-0.29221	-0.33472	-0.33395	-0.33319	-0.33243	-0.33168	-0.42815
-0.24009	-0.29318	-0.29254	-0.29192	-0.29132	-0.33355	-0.33278	-0.33203	-0.37993
-0.2404	-0.29354	-0.29289	-0.29226	-0.29165	-0.3339	-0.33314	-0.33238	-0.38033
-0.24071	-0.2939	-0.29325	-0.29261	-0.29198	-0.33426	-0.33349	-0.33273	-0.38073
-0.24101	-0.29427	-0.29361	-0.29296	-0.29232	-0.33463	-0.33385	-0.33308	-0.33232
-0.24132	-0.29464	-0.29398	-0.29333	-0.29267	-0.335	-0.33422	-0.33344	-0.33267
-0.24163	-0.24106	-0.24051	-0.29369	-0.29303	-0.33538	-0.33458	-0.3338	-0.33303
-0.24194	-0.24137	-0.24081	-0.29406	-0.29338	-0.33576	-0.33495	-0.33416	-0.33338
-0.24225	-0.24168	-0.24112	-0.29442	-0.29374	-0.29307	-0.29242	-0.29179	-0.33375
-0.24256	-0.24199	-0.24143	-0.2948	-0.29411	-0.29344	-0.29278	-0.29213	-0.33412
-0.24288	-0.24231	-0.24174	-0.29517	-0.29449	-0.29381	-0.29314	-0.29247	-0.33449
-0.24321	-0.24264	-0.24207	-0.24149	-0.24092	-0.29418	-0.29349	-0.29282	-0.33487
-0.24354	-0.24297	-0.2424	-0.24182	-0.24123	-0.29455	-0.29385	-0.29318	-0.33525
-0.24386	-0.24329	-0.24271	-0.24213	-0.24154	-0.29492	-0.29423	-0.29354	-0.33565
-0.24417	-0.24359	-0.24302	-0.24244	-0.24185	-0.2953	-0.2946	-0.29392	-0.33607
-0.24449	-0.24391	-0.24333	-0.24275	-0.24216	-0.24158	-0.241	-0.24044	-0.33649
-0.24482	-0.24423	-0.24365	-0.24306	-0.24247	-0.24189	-0.24131	-0.24075	-0.33689
-0.24515	-0.24456	-0.24396	-0.24337	-0.24279	-0.24221	-0.24163	-0.24106	-0.29438
-0.24546	-0.24487	-0.24427	-0.24369	-0.24311	-0.24253	-0.24196	-0.24139	-0.29478
-0.24577	-0.24518	-0.24458	-0.244	-0.24343	-0.24286	-0.24229	-0.24172	-0.29518
-0.2461	-0.2455	-0.2449	-0.24432	-0.24375	-0.24318	-0.24261	-0.24205	-0.24147
-0.24642	-0.24582	-0.24523	-0.24464	-0.24408	-0.24351	-0.24294	-0.24237	-0.2418
-0.24675	-0.24615	-0.24556	-0.24497	-0.2444	-0.24383	-0.24326	-0.24269	-0.24211
-0.24708	-0.24648	-0.24589	-0.24531	-0.24473	-0.24415	-0.24357	-0.243	-0.24242
-0.24742	-0.24682	-0.24622	-0.24563	-0.24505	-0.24447	-0.24389	-0.24331	-0.24274
-0.24775	-0.24715	-0.24655	-0.24595	-0.24536	-0.24478	-0.2442	-0.24362	-0.24306
-0.24809	-0.24748	-0.24688	-0.24628	-0.24568	-0.2451	-0.24452	-0.24395	-0.24338
-0.24844	-0.24783	-0.24722	-0.24662	-0.24602	-0.24543	-0.24484	-0.24427	-0.24371
-0.24879	-0.24818	-0.24758	-0.24697	-0.24636	-0.24576	-0.24518	-0.2446	-0.24403
-0.24914	-0.24853	-0.24792	-0.24731	-0.24671	-0.24611	-0.24552	-0.24494	-0.24436
-0.24948	-0.24887	-0.24826	-0.24766	-0.24706	-0.24646	-0.24587	-0.24528	-0.24469
-0.24983	-0.24922	-0.24861	-0.24801	-0.24741	-0.24681	-0.24621	-0.24562	-0.24503
-0.25017	-0.24956	-0.24896	-0.24835	-0.24775	-0.24715	-0.24655	-0.24595	-0.24536
-0.25051	-0.2499	-0.2493	-0.2487	-0.2481	-0.2475	-0.2469	-0.2463	-0.24571
-0.25086	-0.25025	-0.24965	-0.24904	-0.24844	-0.24784	-0.24724	-0.24664	-0.24606
-0.25122	-0.2506	-0.24999	-0.24938	-0.24878	-0.24818	-0.24758	-0.24699	-0.24641
-0.25157	-0.25095	-0.25034	-0.24973	-0.24912	-0.24852	-0.24793	-0.24735	-0.24676
-0.25192	-0.2513	-0.25068	-0.25007	-0.24946	-0.24887	-0.24828	-0.2477	-0.24711
-0.25227	-0.25165	-0.25103	-0.25042	-0.24982	-0.24922	-0.24863	-0.24805	-0.24746
-0.25263	-0.252	-0.25138	-0.25077	-0.25017	-0.24958	-0.24898	-0.24839	-0.2478
-0.25298	-0.25236	-0.25174	-0.25113	-0.25053	-0.24993	-0.24933	-0.24873	-0.24814
-0.25335	-0.25272	-0.2521	-0.25149	-0.25088	-0.25027	-0.24968	-0.24908	-0.24848
-0.19072	-0.25309	-0.25247	-0.25185	-0.25124	-0.25062	-0.25003	-0.24943	-0.24883
-0.191	-0.25346	-0.25284	-0.25221	-0.2516	-0.25098	-0.25038	-0.24978	-0.24918
-0.19128	-0.25384	-0.2532	-0.25258	-0.25196	-0.25135	-0.25074	-0.25013	-0.24953
-0.19155	-0.25421	-0.25358	-0.25295	-0.25233	-0.25171	-0.25109	-0.25048	-0.24988
-0.19182	-0.25458	-0.25396	-0.25333	-0.2527	-0.25207	-0.25145	-0.25084	-0.25022
-0.1921	-0.25495	-0.25433	-0.2537	-0.25307	-0.25244	-0.25181	-0.2512	-0.25058
-0.19237	-0.25532	-0.25469	-0.25407	-0.25344	-0.25281	-0.25218	-0.25156	-0.25095

-0.19265	-0.19217	-0.1917	-0.25444	-0.25381	-0.25318	-0.25255	-0.25193	-0.25132
-0.19292	-0.19245	-0.19197	-0.25481	-0.25418	-0.25355	-0.25292	-0.2523	-0.25169
-0.19319	-0.19272	-0.19225	-0.25519	-0.25455	-0.25392	-0.25329	-0.25267	-0.25205
-0.19346	-0.19299	-0.19252	-0.25556	-0.25493	-0.2543	-0.25367	-0.25304	-0.25241
-0.19373	-0.19326	-0.19279	-0.25593	-0.2553	-0.25468	-0.25404	-0.2534	-0.25278
-0.194	-0.19353	-0.19306	-0.19259	-0.19212	-0.25505	-0.25441	-0.25378	-0.25315
-0.19427	-0.1938	-0.19333	-0.19286	-0.19239	-0.25542	-0.25479	-0.25416	-0.25352
-0.19454	-0.19407	-0.1936	-0.19313	-0.19266	-0.25579	-0.25516	-0.25453	-0.2539
-0.19483	-0.19434	-0.19387	-0.1934	-0.19293	-0.25617	-0.25554	-0.25491	-0.25427
-0.19511	-0.19462	-0.19414	-0.19367	-0.1932	-0.19274	-0.19227	-0.19181	-0.25466
-0.1954	-0.1949	-0.19441	-0.19393	-0.19347	-0.19301	-0.19255	-0.19209	-0.25506
-0.1957	-0.19519	-0.19469	-0.19421	-0.19374	-0.19328	-0.19283	-0.19238	-0.25546
-0.19601	-0.1955	-0.19499	-0.1945	-0.19403	-0.19356	-0.19311	-0.19265	-0.25583
-0.19632	-0.19581	-0.1953	-0.1948	-0.19432	-0.19385	-0.19338	-0.19292	-0.25621
-0.12931	-0.19612	-0.1956	-0.19509	-0.1946	-0.19412	-0.19365	-0.19319	-0.25658
-0.1295	-0.19641	-0.19589	-0.19538	-0.19488	-0.19439	-0.19392	-0.19346	-0.25695
-0.12968	-0.19671	-0.19619	-0.19567	-0.19516	-0.19467	-0.1942	-0.19373	-0.19327
-0.12986	-0.19701	-0.19649	-0.19597	-0.19545	-0.19495	-0.19448	-0.19401	-0.19355
-0.13004	-0.12973	-0.12943	-0.19627	-0.19575	-0.19525	-0.19477	-0.1943	-0.19383
-0.13023	-0.12992	-0.12962	-0.1966	-0.19608	-0.19557	-0.19507	-0.19458	-0.1941
-0.13041	-0.13012	-0.12981	-0.19692	-0.1964	-0.19589	-0.19537	-0.19486	-0.19438
-0.1306	-0.1303	-0.13	-0.19722	-0.19672	-0.19621	-0.19569	-0.19517	-0.19468
-0.13078	-0.13048	-0.13017	-0.19752	-0.19702	-0.19652	-0.196	-0.19549	-0.19499
-0.06374	-0.13066	-0.13036	-0.13006	-0.12976	-0.19682	-0.19631	-0.19579	-0.19529
-0.06383	-0.13084	-0.13054	-0.13025	-0.12995	-0.19712	-0.19661	-0.19609	-0.19559
	-0.13103	-0.13073	-0.13044	-0.13014	-0.19742	-0.19691	-0.1964	-0.1959
	-0.13122	-0.13092	-0.13062	-0.13031	-0.19771	-0.19722	-0.19672	-0.19621
	-0.06396	-0.06381	-0.1308	-0.13049	-0.13019	-0.1299	-0.12961	-0.19652
	-0.06405	-0.06391	-0.13099	-0.13068	-0.13038	-0.13009	-0.1298	-0.19684
	-0.06414	-0.064	-0.13119	-0.13088	-0.13058	-0.13028	-0.12999	-0.19715
			-0.13138	-0.13107	-0.13077	-0.13047	-0.13018	-0.19745
			-0.13157	-0.13127	-0.13097	-0.13066	-0.13036	-0.19774
			-0.06412	-0.06399	-0.13116	-0.13085	-0.13055	-0.13024
			-0.06421	-0.06408	-0.13136	-0.13105	-0.13074	-0.13043
					-0.13156	-0.13125	-0.13094	-0.13063
					-0.13176	-0.13145	-0.13114	-0.13082
					-0.13197	-0.13165	-0.13133	-0.13102
								-0.13121
								-0.1314
								-0.1316
								-0.1318

					-0.06039
					-0.0603
					-0.06021
					-0.06012
			-0.06039	-0.06021	-0.38206
			-0.0603	-0.06012	-0.38146
			-0.06021	-0.06003	-0.38085
-0.06064	-0.06047	-0.06029	-0.06011	-0.05993	-0.44273

			-0.06055	-0.06038	-0.0602	-0.06002	-0.05983	-0.44197
	-0.0608	-0.06063	-0.06046	-0.06028	-0.06011	-0.38112	-0.38006	-0.61557
	-0.0607	-0.06053	-0.06036	-0.06019	-0.06001	-0.38051	-0.37946	-0.61457
	-0.06061	-0.06044	-0.383	-0.38198	-0.38095	-0.61702	-0.61529	-0.66399
	-0.06051	-0.06034	-0.3824	-0.38137	-0.38034	-0.61598	-0.61422	-0.6629
-0.06059	-0.06042	-0.06025	-0.3818	-0.38077	-0.37973	-0.66547	-0.66361	-0.86083
-0.06049	-0.06032	-0.06015	-0.3812	-0.38017	-0.37914	-0.66444	-0.66262	-0.85958
-0.0604	-0.06022	-0.06005	-0.38061	-0.37958	-0.37855	-0.66343	-0.66164	-0.85836
-0.0603	-0.38203	-0.38103	-0.61712	-0.6154	-0.61369	-0.71368	-0.71185	-0.85712
-0.06021	-0.38145	-0.38043	-0.61612	-0.61438	-0.61267	-0.71258	-0.7108	-0.85587
-0.38187	-0.44182	-0.44065	-0.71715	-0.71524	-0.71335	-0.85884	-0.85671	-0.85463
-0.38129	-0.44116	-0.43998	-0.71609	-0.7142	-0.71234	-0.8576	-0.85547	-0.85339
-0.38071	-0.66722	-0.66544	-0.81268	-0.81057	-0.80849	-0.8564	-0.85426	-0.85217
-0.38013	-0.66619	-0.66444	-0.81151	-0.80945	-0.80741	-0.85525	-0.85309	-0.85099
-0.44034	-0.71663	-0.71483	-0.86053	-0.8584	-0.85626	-0.85409	-0.85193	-0.84981
-0.43968	-0.71558	-0.71379	-0.85928	-0.85717	-0.85505	-0.85289	-0.85073	-0.84861
-0.43902	-0.71453	-0.71276	-0.85803	-0.85593	-0.85382	-0.85168	-0.84953	-0.84741
-0.71529	-0.81082	-0.80883	-0.85679	-0.8547	-0.8526	-0.85047	-0.84832	-0.94255
-0.71425	-0.80964	-0.80766	-0.85555	-0.85347	-0.85138	-0.84925	-0.84711	-0.94119
-0.81052	-0.8585	-0.8564	-0.85431	-0.85224	-0.85016	-0.84804	-0.8459	-0.93985
-0.80939	-0.85728	-0.85517	-0.85308	-0.85101	-0.84894	-0.84682	-0.8447	-0.93851
-0.85825	-0.85607	-0.85395	-0.85185	-0.84979	-0.84773	-0.94185	-0.9395	-0.93718
-0.85703	-0.85486	-0.85273	-0.85063	-0.84858	-0.84653	-0.94051	-0.93816	-0.93585
-0.85582	-0.85366	-0.85153	-0.84943	-0.84738	-0.84534	-0.93919	-0.93685	-0.93455
-0.85466	-0.85251	-0.85036	-0.89319	-0.89105	-0.88891	-0.9379	-0.9356	-0.93331
-0.8535	-0.85137	-0.84921	-0.89198	-0.88981	-0.88767	-0.93662	-0.93438	-0.9321
-0.85232	-0.8502	-0.84804	-0.94213	-0.93983	-0.93755	-0.93532	-0.93311	-0.93085
-0.85111	-0.84899	-0.84685	-0.94081	-0.9385	-0.93622	-0.93401	-0.93181	-0.92957
-0.8499	-0.89269	-0.89049	-0.93952	-0.93721	-0.93493	-0.93271	-0.93052	-0.97407
-0.84869	-0.89144	-0.88929	-0.93829	-0.93597	-0.93367	-0.93145	-0.92925	-0.97276
-0.89236	-0.94155	-0.9393	-0.93703	-0.93472	-0.93242	-0.93018	-0.92798	-1.07992
-0.89115	-0.94025	-0.938	-0.93574	-0.93344	-0.93115	-0.9289	-0.92668	-1.07845
-0.88995	-0.93896	-0.9367	-0.93443	-0.93215	-0.92988	-0.92763	-0.9254	-1.07699
-0.88872	-0.93766	-0.93539	-0.93314	-0.9309	-0.92866	-1.03531	-1.03283	-1.11614
-0.88748	-0.93635	-0.93408	-0.93185	-0.92965	-0.92746	-1.03401	-1.03153	-1.11469
-0.93738	-0.93509	-0.93282	-0.99553	-0.9932	-0.99088	-1.07792	-1.07536	-1.11324
-0.93615	-0.93387	-0.9316	-0.99425	-0.99188	-0.98954	-1.07648	-1.07398	-1.1118
-0.93492	-0.99777	-0.99537	-1.03729	-1.0348	-1.03234	-1.11563	-1.113	-1.11034
-0.93369	-0.99649	-0.99411	-1.03598	-1.03348	-1.03101	-1.11414	-1.11149	-1.10885
-0.99759	-0.9952	-0.99282	-1.12088	-1.11812	-1.11538	-1.11268	-1.11001	-1.10737
-0.99625	-0.99387	-0.99149	-1.11935	-1.11665	-1.11396	-1.11127	-1.10859	-1.10596
-0.99491	-0.99253	-0.99014	-1.11781	-1.11517	-1.11253	-1.10986	-1.10719	-1.10457
-0.9936	-1.07725	-1.0746	-1.11633	-1.11368	-1.11105	-1.10839	-1.10574	-1.23122
-0.99233	-1.07584	-1.07321	-1.11487	-1.11219	-1.10954	-1.1069	-1.10429	-1.22934
-0.99106	-1.11888	-1.11614	-1.11343	-1.11074	-1.10808	-1.23413	-1.2308	-1.22753
-0.98982	-1.11745	-1.11472	-1.11201	-1.10933	-1.10668	-1.23231	-1.22901	-1.22577
-1.07435	-1.11602	-1.11329	-1.1106	-1.10793	-1.1053	-1.23052	-1.22725	-1.22404
-1.07298	-1.11459	-1.11187	-1.10919	-1.10654	-1.10393	-1.22878	-1.22555	-1.22234
-1.0716	-1.11315	-1.11045	-1.10779	-1.10516	-1.10257	-1.22706	-1.22385	-1.22066
-1.11441	-1.11171	-1.10904	-1.23519	-1.23184	-1.22854	-1.22532	-1.22215	-1.21897

-1.11295	-1.11027	-1.10763	-1.23338	-1.23004	-1.22677	-1.22358	-1.22044	-1.21728
-1.11148	-1.19545	-1.19234	-1.2315	-1.22822	-1.22498	-1.22181	-1.21867	-1.21553
-1.11	-1.19365	-1.19055	-1.2296	-1.22637	-1.22319	-1.22001	-1.21686	-1.21374
-1.10858	-1.2344	-1.23105	-1.22777	-1.22459	-1.22144	-1.21827	-1.21512	-1.25372
-1.10724	-1.2327	-1.22933	-1.22605	-1.22287	-1.21973	-1.21659	-1.21347	-1.2519
-1.19191	-1.23097	-1.22762	-1.22435	-1.22118	-1.21804	-1.21493	-1.21183	-1.29582
-1.19018	-1.22913	-1.22587	-1.22266	-1.21951	-1.21639	-1.21328	-1.21019	-1.2939
-1.18847	-1.22732	-1.22413	-1.22098	-1.21785	-1.21475	-1.21164	-1.20854	-1.292
-1.22893	-1.22565	-1.22245	-1.21929	-1.21618	-1.21308	-1.25148	-1.2481	-1.2901
-1.22733	-1.22407	-1.22081	-1.21761	-1.2145	-1.21141	-1.24968	-1.24631	-1.28823
-1.22567	-1.22244	-1.2192	-1.216	-1.21286	-1.20975	-1.29346	-1.28987	-1.28637
-1.22398	-1.2208	-1.21763	-1.21445	-1.21125	-1.20809	-1.29152	-1.288	-1.28453
-1.22229	-1.21914	-1.21601	-1.25467	-1.25114	-1.24766	-1.28965	-1.28616	-1.28272
-1.22061	-1.21747	-1.21434	-1.25289	-1.24943	-1.246	-1.28787	-1.28434	-1.28091
-1.21892	-1.21579	-1.21266	-1.29698	-1.29333	-1.28971	-1.28612	-1.28258	-1.27916
-1.21722	-1.2141	-1.211	-1.29504	-1.29146	-1.28791	-1.28438	-1.28089	-1.27748
-1.21553	-1.21242	-1.20934	-1.29311	-1.28958	-1.2861	-1.28265	-1.27923	-1.27585
-1.21387	-1.25243	-1.24905	-1.29123	-1.28773	-1.28429	-1.28088	-1.27753	-1.27422
-1.21223	-1.2506	-1.24727	-1.28937	-1.28589	-1.28247	-1.27911	-1.27582	-1.27261
-1.25228	-1.2946	-1.29103	-1.28752	-1.28407	-1.28068	-1.27736	-1.27412	-1.31139
-1.25061	-1.29282	-1.2892	-1.28568	-1.28226	-1.27892	-1.27563	-1.27242	-1.3097
-1.2489	-1.29104	-1.28741	-1.28389	-1.28049	-1.27719	-1.27394	-1.2708	-1.30808
-1.24713	-1.28922	-1.28566	-1.28218	-1.27876	-1.27545	-1.27229	-1.26926	-1.30656
-1.24536	-1.2874	-1.28393	-1.2805	-1.27708	-1.27378	-1.2707	-1.26777	-1.30509
-1.28905	-1.28557	-1.28217	-1.27882	-1.27546	-1.27221	-1.26917	-1.26627	-1.3036
-1.28723	-1.28377	-1.28043	-1.27715	-1.27389	-1.27073	-1.26771	-1.26482	-1.30215
-1.28549	-1.28205	-1.27875	-1.27553	-1.27238	-1.26931	-1.26635	-1.2635	-1.37401
-1.2838	-1.28039	-1.27711	-1.27395	-1.27092	-1.26797	-1.26508	-1.26227	-1.37262
-1.28213	-1.27875	-1.2755	-1.27239	-1.26944	-1.26659	-1.30388	-1.30104	-1.37126
-1.28048	-1.27715	-1.2739	-1.27082	-1.26794	-1.26517	-1.30251	-1.29978	-1.36995
-1.27883	-1.27556	-1.27235	-1.26931	-1.26648	-1.26378	-1.37432	-1.37147	-1.36861
-1.27716	-1.27398	-1.27087	-1.2679	-1.26509	-1.2624	-1.37283	-1.37005	-1.36724
-1.27551	-1.27243	-1.26944	-1.26655	-1.26375	-1.26106	-1.37136	-1.36861	-1.36584
-1.27394	-1.27094	-1.26805	-1.3788	-1.37574	-1.37277	-1.36992	-1.36716	-1.36442
-1.27243	-1.26949	-1.26668	-1.37736	-1.37435	-1.37141	-1.36852	-1.36571	-1.36298
-1.27091	-1.30637	-1.30352	-1.37592	-1.37297	-1.37005	-1.36717	-1.36434	-1.3616
-1.2694	-1.30485	-1.3021	-1.37448	-1.37159	-1.36872	-1.36586	-1.36303	-1.36026
-1.30631	-1.37876	-1.3759	-1.37306	-1.37022	-1.36739	-1.36455	-1.36173	-1.39644
-1.30494	-1.37733	-1.37448	-1.37166	-1.36886	-1.36606	-1.36325	-1.36044	-1.39512
-1.34011	-1.37593	-1.37307	-1.37025	-1.36748	-1.36473	-1.36194	-1.35915	-1.39381
-1.33872	-1.37452	-1.37164	-1.36882	-1.36608	-1.36337	-1.36061	-1.35786	-1.39249
-1.33736	-1.37313	-1.37022	-1.36739	-1.36469	-1.362	-1.35929	-1.35657	-1.39117
-1.37469	-1.37175	-1.36884	-1.36603	-1.36333	-1.36067	-1.35799	-1.35531	-1.38989
-1.37331	-1.37039	-1.3675	-1.36469	-1.362	-1.35934	-1.35669	-1.35405	-1.38862
-1.37194	-1.36904	-1.36617	-1.36336	-1.36065	-1.35799	-1.35536	-1.35277	-1.38732
-1.37059	-1.3677	-1.36485	-1.36204	-1.35929	-1.3566	-1.35399	-1.35144	-1.38598
-1.36924	-1.36637	-1.36353	-1.36073	-1.35796	-1.35525	-1.38993	-1.38731	-1.38467
-1.36788	-1.36503	-1.36221	-1.35943	-1.35667	-1.35398	-1.38862	-1.38601	-1.38339
-1.36653	-1.36369	-1.3609	-1.35814	-1.3554	-1.35273	-1.38734	-1.38473	-1.38212
-1.3652	-1.36238	-1.35959	-1.35685	-1.35413	-1.35148	-1.38606	-1.38346	-1.38087

-1.36387	-1.36107	-1.35829	-1.35556	-1.35287	-1.35024	-1.38479	-1.3822	-1.37963
-1.36255	-1.35976	-1.357	-1.35429	-1.35161	-1.349	-1.38352	-1.38095	-1.37838
-1.36123	-1.35846	-1.35572	-1.35302	-1.35036	-1.34776	-1.38225	-1.37969	-1.37713
-1.35992	-1.35717	-1.35445	-1.38901	-1.38627	-1.38358	-1.38099	-1.37844	-1.37588
-1.35863	-1.35589	-1.35319	-1.38773	-1.38499	-1.38231	-1.37972	-1.37719	-1.37463
-1.35734	-1.35462	-1.35194	-1.38645	-1.38372	-1.38105	-1.37847	-1.37593	-1.48776
-1.35605	-1.35334	-1.35068	-1.38517	-1.38246	-1.3798	-1.37721	-1.37467	-1.4861
-1.35477	-1.35208	-1.34944	-1.38389	-1.38121	-1.37857	-1.37597	-1.37342	-1.48446
-1.35351	-1.35084	-1.3482	-1.38263	-1.37999	-1.37738	-1.37478	-1.37221	-1.48287
-1.35228	-1.34963	-1.34699	-1.3814	-1.37879	-1.37621	-1.37361	-1.37102	-1.4813
-1.35109	-1.34847	-1.34586	-1.38023	-1.37762	-1.37502	-1.48637	-1.483	-1.47972
-1.34993	-1.34736	-1.34478	-1.37912	-1.37646	-1.37381	-1.48475	-1.48138	-1.47815
-1.34877	-1.38327	-1.38062	-1.37796	-1.37526	-1.37259	-1.48315	-1.47981	-1.47661
-1.34759	-1.38203	-1.37938	-1.37671	-1.37402	-1.37135	-1.48157	-1.47828	-1.47511
-1.3464	-1.38078	-1.37811	-1.4902	-1.48673	-1.48331	-1.48	-1.47677	-1.49835
-1.34518	-1.37951	-1.37683	-1.48853	-1.48505	-1.48164	-1.4784	-1.47527	-1.49687
-1.34396	-1.37824	-1.37555	-1.48686	-1.48338	-1.48	-1.47682	-1.47377	-1.49541
-1.34272	-1.37696	-1.37426	-1.4852	-1.48178	-1.47846	-1.50002	-1.49696	-1.52518
-1.34147	-1.37568	-1.37298	-1.48356	-1.48024	-1.477	-1.49855	-1.49548	-1.52366
-1.37709	-1.48877	-1.4853	-1.50664	-1.50338	-1.5002	-1.49708	-1.49402	-1.54607
-1.3758	-1.4871	-1.48365	-1.50501	-1.50181	-1.49869	-1.49561	-1.49258	-1.54458
-1.37458	-1.51024	-1.50682	-1.50351	-1.50035	-1.49727	-1.51866	-1.51559	-1.57687
-1.37347	-1.50881	-1.50546	-1.5022	-1.49905	-1.49597	-1.51737	-1.51431	-1.57552
-1.37234	-1.50738	-1.5041	-1.50088	-1.49775	-1.49467	-1.51607	-1.51301	-1.57414
-1.50917	-1.50584	-1.50261	-1.52406	-1.52088	-1.51775	-1.54532	-1.54219	-1.57266
-1.50755	-1.50426	-1.50108	-1.52256	-1.51941	-1.5163	-1.54383	-1.5407	-1.57113
-1.50598	-1.50272	-1.49957	-1.52106	-1.51793	-1.51482	-1.57598	-1.57278	-1.56959
-1.50444	-1.50122	-1.49807	-1.51955	-1.51643	-1.51334	-1.57442	-1.57122	-1.56805
-1.50299	-1.52451	-1.5213	-1.55248	-1.54928	-1.54611	-1.57296	-1.56978	-1.56661
-1.50165	-1.52318	-1.52	-1.55115	-1.54796	-1.5448	-1.57162	-1.56845	-1.5653
-1.52502	-1.52181	-1.51865	-1.54976	-1.54659	-1.54345	-1.57024	-1.56708	-1.56395
-1.52354	-1.52034	-1.51719	-1.54827	-1.54511	-1.54199	-1.56875	-1.56561	-1.56251
-1.52206	-1.51886	-1.51571	-1.54675	-1.54361	-1.5405	-1.56724	-1.56412	-1.56105
-1.52061	-1.55166	-1.54842	-1.57523	-1.57203	-1.56887	-1.56574	-1.56264	-1.55959
-1.51918	-1.55016	-1.54692	-1.5737	-1.57051	-1.56736	-1.56425	-1.56118	-1.55814
-1.55204	-1.54874	-1.54552	-1.57227	-1.5691	-1.56597	-1.56288	-1.55983	-1.55681
-1.55063	-1.54738	-1.54419	-1.57094	-1.56779	-1.56468	-1.56161	-1.55857	-1.55556
-1.54921	-1.54599	-1.54281	-1.56954	-1.56641	-1.56332	-1.56027	-1.55724	-1.55424
-1.54778	-1.54454	-1.54136	-1.56805	-1.56494	-1.56187	-1.55882	-1.55581	-1.55282
-1.54635	-1.54312	-1.53995	-1.5666	-1.56351	-1.56045	-1.55742	-1.55441	-1.55142
-1.54496	-1.54178	-1.53864	-1.56528	-1.5622	-1.55916	-1.55613	-1.55312	-1.55014
-1.54358	-1.54045	-1.53735	-1.56398	-1.56091	-1.55787	-1.55484	-1.55184	-1.54887
-1.54218	-1.53905	-1.53596	-1.56256	-1.5595	-1.55646	-1.55344	-1.55045	-1.54749
-1.54078	-1.53762	-1.53454	-1.5611	-1.55804	-1.55501	-1.55199	-1.54901	-1.54607
-1.53943	-1.56601	-1.56285	-1.55975	-1.55668	-1.55366	-1.55065	-1.54767	-1.54474
-1.53814	-1.56472	-1.56158	-1.55848	-1.55542	-1.55239	-1.54939	-1.54641	-1.54349
-1.53685	-1.56342	-1.56028	-1.55717	-1.5541	-1.55107	-1.54807	-1.54511	-1.56436
-1.53559	-1.56211	-1.55893	-1.55578	-1.55268	-1.54963	-1.54665	-1.54372	-1.56299
-1.53428	-1.56077	-1.55757	-1.5544	-1.55129	-1.54823	-1.54526	-1.54235	-1.56165
-1.53287	-1.55933	-1.55618	-1.55306	-1.54997	-1.54693	-1.54398	-1.54109	-1.56041

-1.53143	-1.55787	-1.55479	-1.55173	-1.54868	-1.54567	-1.54274	-1.53985	-1.55919
-1.53008	-1.5565	-1.55345	-1.55042	-1.54741	-1.54443	-1.56377	-1.56082	-1.55789
-1.52878	-1.55518	-1.55213	-1.54911	-1.54614	-1.54319	-1.56252	-1.55952	-1.55656
-1.52749	-1.55386	-1.55079	-1.54777	-1.54482	-1.54188	-1.56123	-1.55824	-1.55528
-1.52622	-1.55254	-1.54944	-1.54639	-1.54342	-1.54048	-1.55989	-1.55697	-1.55405
-1.52492	-1.55121	-1.5481	-1.56748	-1.56447	-1.5615	-1.55859	-1.55572	-1.55283
-1.52358	-1.54984	-1.54677	-1.5662	-1.56321	-1.56027	-1.55737	-1.55451	-1.55163
-1.52223	-1.54847	-1.54546	-1.56494	-1.56199	-1.55907	-1.55617	-1.55328	-1.5504
-1.52091	-1.54715	-1.54418	-1.56371	-1.56078	-1.55786	-1.55494	-1.55201	-1.5491
-1.51961	-1.54586	-1.54292	-1.56249	-1.55957	-1.55666	-1.5537	-1.55073	-1.54779
-1.54757	-1.56716	-1.5642	-1.56127	-1.55834	-1.55541	-1.55244	-1.54947	-1.59241
-1.54631	-1.56594	-1.563	-1.56005	-1.55708	-1.55412	-1.55116	-1.54822	-1.59121
-1.56768	-1.56471	-1.56176	-1.5588	-1.55582	-1.55286	-1.54992	-1.547	-1.61627
-1.56645	-1.56347	-1.56049	-1.55752	-1.55456	-1.55162	-1.54871	-1.54582	-1.61515
-1.56521	-1.56222	-1.55922	-1.55625	-1.55331	-1.5504	-1.54752	-1.54465	-1.6442
-1.56394	-1.56094	-1.55796	-1.55501	-1.5521	-1.54921	-1.54634	-1.54347	-1.643
-1.56267	-1.55967	-1.55672	-1.55379	-1.5509	-1.54803	-1.54515	-1.54228	-1.64178
-1.56147	-1.55847	-1.55551	-1.55259	-1.54972	-1.54685	-1.57106	-1.56813	-1.64052
-1.56029	-1.55729	-1.55432	-1.5514	-1.54854	-1.54567	-1.56986	-1.56688	-1.63925
-1.55905	-1.55607	-1.55313	-1.57744	-1.57456	-1.57166	-1.64417	-1.64108	-1.63804
-1.55777	-1.55483	-1.55193	-1.57628	-1.5734	-1.5705	-1.643	-1.63991	-1.63687
-1.55652	-1.58091	-1.57801	-1.57512	-1.57224	-1.56934	-1.64183	-1.63876	-1.63571
-1.55531	-1.57972	-1.57684	-1.57396	-1.57108	-1.56818	-1.64068	-1.63761	-1.63457
-1.55412	-1.57855	-1.57567	-1.60379	-1.6008	-1.59781	-1.63949	-1.63645	-1.63342
-1.55293	-1.57739	-1.57452	-1.60256	-1.59954	-1.59652	-1.63825	-1.63524	-1.63223
-1.55176	-1.57624	-1.57337	-1.60135	-1.59829	-1.59524	-1.637	-1.63401	-1.63103
-1.57801	-1.57509	-1.57223	-1.60016	-1.59709	-1.59403	-1.63581	-1.63281	-1.66058
-1.57686	-1.57395	-1.5711	-1.599	-1.59593	-1.59286	-1.63466	-1.63162	-1.65931
-1.57571	-1.60374	-1.6008	-1.59782	-1.59476	-1.59168	-1.63349	-1.63044	-1.65806
-1.57457	-1.60255	-1.59961	-1.59663	-1.59357	-1.59049	-1.63232	-1.62927	-1.6568
-1.57343	-1.60137	-1.59842	-1.6404	-1.63731	-1.63422	-1.66192	-1.65869	-1.65556
-1.57228	-1.60017	-1.59723	-1.63923	-1.63613	-1.63303	-1.66066	-1.65746	-1.65434
-1.57115	-1.59899	-1.59603	-1.63805	-1.63494	-1.63184	-1.6594	-1.65623	-1.65312
-1.60086	-1.59784	-1.59485	-1.63688	-1.63376	-1.63065	-1.65814	-1.65499	-1.6519
-1.59974	-1.59669	-1.59366	-1.63571	-1.63258	-1.62948	-1.6569	-1.65375	-1.65068
-1.59856	-1.59551	-1.59247	-1.66548	-1.6622	-1.65894	-1.65574	-1.65258	-1.6495
-1.59735	-1.5943	-1.59126	-1.66423	-1.66102	-1.65783	-1.65464	-1.65145	-1.64834
-1.59615	-1.59309	-1.59004	-1.66295	-1.6598	-1.65666	-1.65348	-1.65029	-1.64717
-1.59494	-1.59187	-1.5888	-1.66164	-1.65852	-1.65542	-1.65226	-1.64909	-1.64598
-1.59376	-1.66689	-1.66358	-1.66036	-1.65725	-1.65416	-1.65103	-1.64789	-1.64479
-1.59261	-1.66569	-1.66238	-1.65914	-1.65602	-1.65292	-1.6498	-1.64667	-1.64359
-1.59146	-1.6645	-1.66118	-1.65794	-1.6548	-1.65169	-1.64858	-1.64548	-1.64241
-1.63556	-1.66323	-1.65995	-1.65673	-1.65358	-1.65047	-1.64739	-1.64435	-1.64129
-1.63434	-1.66194	-1.65872	-1.65553	-1.65237	-1.64926	-1.64623	-1.64324	-1.64021
-1.66389	-1.66068	-1.65751	-1.65436	-1.6512	-1.64809	-1.64507	-1.64209	-1.63907
-1.6626	-1.65943	-1.65634	-1.65323	-1.65007	-1.64694	-1.6439	-1.6409	-1.63788
-1.66138	-1.65822	-1.65515	-1.65206	-1.64892	-1.64579	-1.64273	-1.63971	-1.6367
-1.66022	-1.65704	-1.65394	-1.65083	-1.64771	-1.64461	-1.64156	-1.63853	-1.63553
-1.65908	-1.65589	-1.65274	-1.64962	-1.64652	-1.64345	-1.6404	-1.63738	-1.63438
-1.65794	-1.65477	-1.6516	-1.64847	-1.6454	-1.64235	-1.6393	-1.63627	-1.63329

-1.65679	-1.65364	-1.65046	-1.64734	-1.64428	-1.64125	-1.63821	-1.63519	-1.63223
-1.65559	-1.65244	-1.64928	-1.64617	-1.64312	-1.6401	-1.63708	-1.63408	-1.63112
-1.65436	-1.6512	-1.64807	-1.64498	-1.64193	-1.63892	-1.63593	-1.63296	-1.63001
-1.65319	-1.65004	-1.64693	-1.64385	-1.64081	-1.6378	-1.63482	-1.63188	-1.62893
-1.65209	-1.64895	-1.64585	-1.64278	-1.63974	-1.63673	-1.63377	-1.63083	-1.62789
-1.65098	-1.64786	-1.64478	-1.64172	-1.63868	-1.63568	-1.63271	-1.62978	-1.62684
-1.64988	-1.64677	-1.64369	-1.64064	-1.63762	-1.63463	-1.63166	-1.62871	-1.62578
-1.64878	-1.64567	-1.64261	-1.63957	-1.63656	-1.63357	-1.63059	-1.62763	-1.6247
-1.64767	-1.64457	-1.64153	-1.6385	-1.63548	-1.63248	-1.62949	-1.62652	-1.62359
-1.64656	-1.64346	-1.64044	-1.63742	-1.6344	-1.63139	-1.62839	-1.62541	-1.62245
-1.64542	-1.64232	-1.6393	-1.63629	-1.63328	-1.63029	-1.6273	-1.62432	-1.62137
-1.64426	-1.64116	-1.63812	-1.63512	-1.63215	-1.62918	-1.62622	-1.62326	-1.62031
-1.64316	-1.64005	-1.63701	-1.634	-1.63104	-1.62809	-1.62514	-1.62219	-1.61925
-1.64213	-1.63903	-1.63595	-1.63292	-1.62996	-1.62703	-1.62407	-1.62113	-1.61818
-1.64107	-1.63798	-1.63489	-1.63185	-1.6289	-1.62596	-1.62301	-1.62006	-1.61712
-1.63994	-1.63686	-1.6338	-1.63079	-1.62783	-1.62489	-1.62195	-1.61901	-1.61605
-1.63881	-1.63574	-1.63272	-1.62973	-1.62677	-1.62382	-1.62088	-1.61795	-1.61498
-1.63777	-1.6347	-1.63167	-1.62867	-1.62571	-1.62277	-1.61982	-1.61687	-1.66087
-1.63677	-1.6337	-1.63063	-1.62761	-1.62465	-1.62171	-1.61875	-1.61578	-1.65973
-1.63573	-1.63267	-1.62962	-1.62659	-1.62361	-1.62064	-1.61767	-1.6147	-1.6586
-1.63466	-1.63164	-1.62864	-1.62562	-1.62258	-1.61955	-1.61657	-1.61362	-1.65747
-1.63358	-1.63059	-1.62762	-1.62462	-1.62156	-1.61851	-1.61552	-1.61254	-1.65634
-1.63249	-1.62951	-1.62656	-1.62358	-1.62057	-1.61755	-1.6145	-1.61146	-1.65521
-1.63141	-1.62843	-1.62549	-1.62253	-1.61957	-1.61657	-1.6135	-1.61041	-1.6541
-1.63034	-1.62737	-1.62441	-1.62146	-1.61851	-1.61554	-1.61249	-1.60943	-1.65305
-1.62929	-1.62633	-1.62335	-1.62039	-1.61744	-1.61448	-1.61148	-1.60846	-1.65203
-1.62828	-1.62532	-1.62235	-1.61938	-1.61639	-1.6134	-1.61043	-1.60746	-1.65101
-1.6273	-1.62434	-1.62139	-1.6184	-1.61535	-1.61232	-1.60936	-1.60643	-1.64999
-1.62628	-1.62332	-1.62038	-1.6174	-1.61434	-1.61129	-1.60832	-1.60539	-1.64892
-1.62523	-1.62225	-1.61933	-1.61638	-1.61336	-1.61034	-1.60732	-1.60432	-1.64781
-1.62421	-1.62122	-1.61829	-1.61534	-1.61235	-1.60936	-1.65294	-1.6498	-1.64674
-1.62323	-1.62023	-1.61723	-1.61425	-1.61129	-1.60833	-1.65192	-1.6488	-1.64572
-1.62226	-1.61925	-1.6162	-1.61319	-1.61024	-1.6073	-1.65088	-1.6478	-1.6447
-1.62124	-1.61825	-1.61521	-1.61219	-1.60921	-1.60625	-1.6498	-1.64676	-1.66889
-1.62021	-1.61724	-1.61424	-1.61122	-1.6082	-1.6052	-1.64872	-1.6457	-1.66785
-1.6192	-1.61626	-1.61328	-1.61027	-1.60723	-1.60421	-1.64765	-1.64462	-1.66677
-1.61822	-1.61527	-1.61232	-1.60933	-1.60629	-1.60325	-1.64661	-1.64353	-1.66567
-1.6172	-1.61426	-1.61132	-1.60836	-1.60533	-1.6023	-1.64559	-1.64249	-1.71363
-1.61616	-1.6132	-1.61028	-1.60733	-1.60435	-1.60135	-1.6446	-1.64149	-1.71256
-1.61514	-1.61216	-1.60924	-1.60631	-1.60337	-1.6004	-1.64362	-1.64049	-1.71149
-1.61416	-1.61117	-1.60825	-1.60533	-1.6024	-1.59945	-1.64262	-1.63949	-1.7104
-1.61319	-1.6102	-1.60726	-1.60434	-1.60143	-1.59849	-1.64162	-1.63849	-1.70932
-1.61222	-1.6092	-1.60624	-1.6033	-1.6004	-1.59749	-1.6406	-1.63749	-1.70827
-1.61125	-1.60821	-1.6052	-1.60224	-1.59935	-1.59646	-1.63957	-1.63649	-1.70722
-1.61028	-1.60724	-1.60422	-1.60125	-1.59836	-1.59547	-1.63855	-1.63549	-1.70617
-1.60932	-1.60628	-1.60327	-1.60031	-1.5974	-1.5945	-1.63755	-1.6345	-1.70512
-1.60835	-1.60532	-1.60232	-1.64562	-1.6426	-1.63958	-1.63655	-1.6335	-1.70407
-1.60739	-1.60436	-1.60136	-1.64462	-1.6416	-1.6386	-1.63556	-1.63252	-1.70302
-1.60642	-1.6034	-1.6004	-1.64362	-1.64061	-1.63761	-1.68406	-1.68086	-1.72739
-1.60546	-1.60244	-1.59945	-1.64263	-1.63962	-1.63662	-1.68302	-1.67981	-1.72631

-1.60449	-1.60148	-1.59849	-1.64164	-1.63863	-1.63563	-1.68197	-1.67875	-1.72523
-1.60354	-1.60053	-1.59754	-1.64064	-1.63764	-1.63463	-1.68093	-1.6777	-1.72415
-1.60259	-1.59958	-1.59659	-1.63964	-1.63664	-1.63364	-1.67988	-1.67666	-1.72307
-1.60163	-1.59862	-1.59563	-1.63865	-1.63564	-1.63264	-1.70464	-1.70135	-1.76908
-1.60066	-1.59766	-1.59468	-1.63765	-1.63464	-1.63163	-1.70356	-1.70027	-1.76796
-1.59969	-1.5967	-1.59372	-1.63666	-1.63364	-1.63062	-1.70248	-1.6992	-1.76686
-1.59874	-1.59575	-1.59278	-1.63566	-1.63264	-1.62961	-1.70141	-1.69814	-1.76576
-1.5978	-1.59482	-1.59185	-1.63469	-1.63165	-1.62862	-1.70035	-1.69708	-1.76466
-1.59686	-1.59389	-1.59095	-1.63377	-1.63073	-1.62768	-1.72078	-1.71747	-1.76355
-1.59593	-1.59298	-1.59007	-1.63287	-1.62981	-1.62674	-1.71974	-1.7164	-1.76244
-1.59504	-1.5921	-1.58919	-1.65856	-1.65541	-1.65225	-1.71868	-1.71533	-1.76134
-1.59417	-1.59125	-1.58831	-1.6576	-1.65442	-1.65125	-1.71762	-1.71427	-1.76024
-1.5933	-1.63621	-1.63313	-1.72662	-1.72326	-1.7199	-1.71655	-1.71321	-1.77863
-1.59242	-1.63526	-1.63216	-1.72555	-1.72218	-1.71881	-1.71548	-1.71216	-1.77756
-1.59152	-1.6343	-1.63118	-1.72448	-1.7211	-1.71774	-1.74061	-1.73723	-1.7765
-1.59059	-1.63332	-1.63019	-1.7234	-1.72003	-1.71668	-1.73953	-1.73615	-1.77544
-1.58965	-1.63233	-1.6292	-1.72234	-1.71898	-1.71565	-1.73848	-1.73511	-1.77441
-1.58871	-1.6801	-1.67687	-1.72129	-1.71797	-1.71467	-1.75736	-1.754	-1.77345
-1.58777	-1.67909	-1.67586	-1.72026	-1.71698	-1.71373	-1.75643	-1.7531	-1.77251
-1.58688	-1.67814	-1.67494	-1.73952	-1.73623	-1.73296	-1.75544	-1.7521	-1.77149
-1.58601	-1.67726	-1.67407	-1.73861	-1.73529	-1.73197	-1.7544	-1.75104	-1.77041
-1.60832	-1.69727	-1.69406	-1.73765	-1.7343	-1.73096	-1.77272	-1.76931	-1.78835
-1.60743	-1.6963	-1.69308	-1.73661	-1.73326	-1.72991	-1.77164	-1.76822	-1.78725
-1.63345	-1.71814	-1.71483	-1.73557	-1.73221	-1.72887	-1.77056	-1.76716	-1.7862
-1.63249	-1.71711	-1.7138	-1.73451	-1.73116	-1.72783	-1.76952	-1.76615	-1.78521
-1.63155	-1.7161	-1.71279	-1.73348	-1.73013	-1.72682	-1.7685	-1.76516	-1.78425
-1.65233	-1.7152	-1.71188	-1.7523	-1.74891	-1.74555	-1.76752	-1.76417	-1.78324
-1.65152	-1.71435	-1.71103	-1.75141	-1.74801	-1.74464	-1.76656	-1.76317	-1.78221
-1.69393	-1.73747	-1.73408	-1.75043	-1.74703	-1.74365	-1.76554	-1.76214	-1.78116
-1.69294	-1.73643	-1.73305	-1.74938	-1.74598	-1.7426	-1.76447	-1.76108	-1.78009
-1.69197	-1.73543	-1.73206	-1.7738	-1.77032	-1.76687	-1.78593	-1.78249	-1.77907
-1.69104	-1.73448	-1.73114	-1.77288	-1.7694	-1.76595	-1.785	-1.78157	-1.77815
-1.7128	-1.75328	-1.74993	-1.77194	-1.76847	-1.76501	-1.78406	-1.78063	-1.77722
-1.71184	-1.75227	-1.74891	-1.77089	-1.76742	-1.76397	-1.78302	-1.7796	-1.77619
-1.71089	-1.75127	-1.74788	-1.76983	-1.76637	-1.76293	-1.78197	-1.77856	-1.77515
-1.71001	-1.75035	-1.74694	-1.76887	-1.76542	-1.76199	-1.78103	-1.77761	-1.80045
-1.70917	-1.74947	-1.74606	-1.76798	-1.76453	-1.76111	-1.78013	-1.77672	-1.79954
-1.70825	-1.74852	-1.74511	-1.767	-1.76357	-1.76015	-1.77916	-1.77575	-1.79854
-1.70724	-1.74748	-1.74406	-1.76594	-1.76251	-1.75911	-1.77811	-1.77469	-1.79746
-1.72628	-1.74648	-1.74307	-1.76493	-1.76151	-1.75811	-1.7771	-1.77368	-1.79643
-1.7254	-1.74558	-1.74219	-1.76404	-1.76062	-1.75722	-1.7762	-1.77278	-1.79551
-1.7245	-1.74468	-1.7413	-1.76315	-1.75974	-1.75635	-1.77532	-1.7719	-1.79461
-1.72351	-1.74367	-1.7403	-1.76214	-1.75877	-1.75542	-1.77441	-1.77101	-1.79371
-1.72248	-1.74263	-1.73927	-1.76111	-1.75778	-1.75447	-1.77348	-1.77011	-1.79281
-1.72156	-1.76698	-1.76354	-1.76014	-1.75681	-1.7535	-1.77249	-1.76912	-1.7918
-1.72072	-1.76609	-1.76265	-1.75923	-1.75585	-1.7525	-1.77145	-1.76807	-1.79071
-1.71989	-1.76522	-1.76178	-1.75835	-1.75494	-1.75156	-1.79662	-1.79316	-1.7897
-1.71909	-1.76439	-1.76094	-1.7575	-1.75408	-1.75068	-1.79571	-1.79224	-1.78878
-1.71828	-1.76354	-1.76009	-1.75665	-1.75321	-1.74981	-1.7948	-1.79133	-1.78787
-1.71745	-1.76267	-1.75922	-1.75578	-1.75235	-1.74894	-1.7939	-1.79043	-1.78696

-1.71658	-1.76177	-1.75831	-1.75487	-1.75145	-1.74805	-1.79297	-1.78949	-1.78603
-1.7156	-1.76074	-1.75729	-1.75385	-1.75044	-1.74705	-1.79193	-1.78846	-1.81372
-1.71456	-1.75966	-1.75621	-1.75278	-1.74938	-1.746	-1.79085	-1.78738	-1.81256
-1.71363	-1.75868	-1.75523	-1.75181	-1.74842	-1.74505	-1.78986	-1.78639	-1.83077
-1.71278	-1.75779	-1.75434	-1.75092	-1.74753	-1.74417	-1.78895	-1.78548	-1.82978
-1.71194	-1.75692	-1.75347	-1.75005	-1.74667	-1.7433	-1.8077	-1.80419	-1.82882
-1.71111	-1.75606	-1.75261	-1.74919	-1.74581	-1.74245	-1.8068	-1.8033	-1.82785
-1.71029	-1.7552	-1.75175	-1.7748	-1.77135	-1.76792	-1.83436	-1.83062	-1.86705
-1.70945	-1.75433	-1.75089	-1.77392	-1.77048	-1.76705	-1.8334	-1.82967	-1.86607
-1.70861	-1.75346	-1.75004	-1.77305	-1.76961	-1.76618	-1.83245	-1.82872	-1.86509
-1.75604	-1.75259	-1.74918	-1.81382	-1.81029	-1.80677	-1.8315	-1.82777	-1.86411
-1.75516	-1.75172	-1.74832	-1.81293	-1.80939	-1.80587	-1.83054	-1.82681	-1.86312
-1.75429	-1.77123	-1.7678	-1.81203	-1.8085	-1.80498	-1.82957	-1.82585	-1.86214
-1.75342	-1.77036	-1.76694	-1.81114	-1.8076	-1.80408	-1.82861	-1.8249	-1.86115
-1.75255	-1.79562	-1.79213	-1.8389	-1.83513	-1.83138	-1.82765	-1.82394	-1.86016
-1.75169	-1.79473	-1.79124	-1.83793	-1.83416	-1.83042	-1.82669	-1.82298	-1.85917
-1.75082	-1.79384	-1.79035	-1.83695	-1.8332	-1.82945	-1.82573	-1.82201	-1.85817
-1.77032	-1.79294	-1.78946	-1.83598	-1.83223	-1.82848	-1.86481	-1.86099	-1.85718
-1.76944	-1.79205	-1.78856	-1.83501	-1.83125	-1.82751	-1.86382	-1.86	-1.85619
-1.76857	-1.79116	-1.78767	-1.83403	-1.83028	-1.82654	-1.86282	-1.85901	-1.85521
-1.76769	-1.79026	-1.78677	-1.83305	-1.8293	-1.82557	-1.86182	-1.85802	-1.85423
-1.79286	-1.81835	-1.81463	-1.83207	-1.82833	-1.8246	-1.86082	-1.85703	-1.85324
-1.79196	-1.81738	-1.81366	-1.83109	-1.82735	-1.82364	-1.85982	-1.85603	-1.85226
-1.79106	-1.81641	-1.81269	-1.8703	-1.86646	-1.86264	-1.85883	-1.85503	-1.85127
-1.79016	-1.81543	-1.81172	-1.8693	-1.86546	-1.86164	-1.85783	-1.85403	-1.85027
-1.78928	-1.81447	-1.81076	-1.8683	-1.86446	-1.86064	-1.85683	-1.85303	-1.84927
-1.78846	-1.81355	-1.80981	-1.86729	-1.86345	-1.85964	-1.85583	-1.85204	-1.84828
-1.78767	-1.81266	-1.80888	-1.86628	-1.86244	-1.85863	-1.85483	-1.85104	-1.84729
-1.78689	-1.81182	-1.80801	-1.86533	-1.86147	-1.85764	-1.85383	-1.85005	-1.8463
-1.78611	-1.81101	-1.80718	-1.86443	-1.86052	-1.85665	-1.85283	-1.84905	-1.84531
-1.81394	-1.85089	-1.84697	-1.86355	-1.85962	-1.85573	-1.8519	-1.84811	-1.84435
-1.81299	-1.84995	-1.84609	-1.8627	-1.8588	-1.85492	-1.85107	-1.84724	-1.84344
-1.81203	-1.84899	-1.84518	-1.86183	-1.85796	-1.85409	-1.85023	-1.84638	-1.84255
-1.81108	-1.84801	-1.84421	-1.86085	-1.85701	-1.85317	-1.84934	-1.84552	-1.84172
-1.81016	-1.84704	-1.84323	-1.85986	-1.85603	-1.85221	-1.84842	-1.84465	-1.84089
-1.80934	-1.84617	-1.84233	-1.85893	-1.85507	-1.85125	-1.84746	-1.84371	-1.83996
-1.80859	-1.84537	-1.84148	-1.85803	-1.85413	-1.85027	-1.84648	-1.84272	-1.83899
-1.84838	-1.86501	-1.86107	-1.85715	-1.85325	-1.84938	-1.84557	-1.8418	-1.83805
-1.84743	-1.86408	-1.86018	-1.8563	-1.85243	-1.84859	-1.84475	-1.84094	-1.83714
-1.84647	-1.86312	-1.85926	-1.85541	-1.85157	-1.84775	-1.84391	-1.84008	-1.85488
-1.84549	-1.86213	-1.85827	-1.85443	-1.85062	-1.84682	-1.84302	-1.83923	-1.85404
-1.84456	-1.86117	-1.85731	-1.85348	-1.84967	-1.84588	-1.84212	-1.83836	-1.85319
-1.84372	-1.86033	-1.85648	-1.85264	-1.84881	-1.84499	-1.8412	-1.83742	-1.85224
-1.84292	-1.85953	-1.85568	-1.85183	-1.84796	-1.84411	-1.84028	-1.83647	-1.85126
-1.8625	-1.85863	-1.85478	-1.85094	-1.84709	-1.84325	-1.83943	-1.83561	-1.85037
-1.86155	-1.85767	-1.85383	-1.85	-1.8462	-1.8424	-1.83861	-1.83481	-1.84954
-1.86066	-1.85677	-1.85291	-1.84908	-1.84529	-1.84151	-1.85636	-1.85253	-1.84867
-1.85984	-1.85593	-1.85204	-1.84818	-1.84435	-1.84056	-1.8554	-1.85159	-1.84776
-1.85899	-1.85509	-1.85118	-1.8473	-1.84345	-1.83964	-1.85448	-1.85068	-1.84686
-1.85807	-1.85418	-1.8503	-1.84645	-1.84263	-1.83882	-1.85365	-1.84985	-1.84602

-1.85715	-1.85327	-1.84945	-1.84563	-1.84182	-1.83802	-1.85284	-1.84903	-1.84517
-1.8563	-1.85243	-1.84863	-1.84482	-1.84099	-1.83717	-1.85195	-1.84811	-1.84426
-1.85548	-1.85163	-1.84783	-1.84402	-1.84017	-1.83631	-1.85104	-1.84717	-1.84333
-1.85464	-1.85078	-1.84697	-1.84316	-1.83931	-1.83546	-1.85017	-1.84629	-1.84245
-1.85379	-1.8499	-1.84606	-1.84224	-1.83843	-1.83462	-1.84934	-1.84546	-1.8416
-1.85295	-1.84905	-1.8452	-1.86005	-1.85621	-1.85237	-1.8485	-1.84463	-1.84075
-1.85211	-1.84823	-1.8444	-1.85926	-1.85541	-1.85156	-1.84767	-1.84378	-1.8399
-1.8513	-1.84743	-1.84362	-1.85847	-1.85461	-1.85074	-1.84683	-1.84293	-1.83904
-1.85051	-1.84666	-1.84285	-1.85768	-1.85378	-1.84988	-1.84597	-1.84207	-1.83821
-1.84974	-1.8459	-1.84207	-1.85687	-1.85295	-1.84902	-1.84511	-1.84122	-1.83737
-1.84896	-1.84511	-1.84125	-1.85602	-1.85208	-1.84816	-1.84426	-1.84039	-1.83655
-1.84817	-1.8443	-1.84041	-1.85514	-1.85121	-1.8473	-1.84343	-1.83958	-1.83574
-1.84735	-1.84347	-1.83957	-1.85429	-1.85037	-1.84647	-1.8426	-1.83875	-1.83491
-1.84651	-1.84262	-1.83873	-1.85346	-1.84957	-1.84569	-1.84179	-1.8379	-1.83405
-1.84567	-1.84178	-1.83791	-1.85265	-1.84878	-1.84491	-1.84099	-1.83708	-1.83324
-1.84483	-1.84096	-1.83712	-1.85187	-1.84801	-1.84414	-1.84023	-1.83632	-1.83248
-1.84401	-1.84016	-1.83634	-1.8511	-1.84723	-1.84335	-1.83946	-1.83556	-1.83172
-1.84324	-1.85805	-1.85419	-1.85032	-1.84642	-1.84252	-1.83863	-1.83476	-1.83094
-1.84249	-1.85729	-1.85343	-1.84955	-1.8456	-1.84167	-1.83779	-1.83394	-1.83015
-1.84174	-1.85653	-1.85267	-1.84877	-1.84482	-1.84088	-1.837	-1.83316	-1.84521
-1.84098	-1.85577	-1.8519	-1.848	-1.84405	-1.84012	-1.83624	-1.8324	-1.84443
-1.84022	-1.855	-1.85109	-1.84718	-1.84325	-1.83933	-1.83547	-1.83163	-1.84365
-1.83946	-1.8542	-1.85025	-1.84632	-1.8424	-1.83851	-1.83467	-1.83086	-1.84286
-1.8387	-1.85342	-1.84943	-1.84548	-1.84156	-1.83769	-1.83388	-1.8301	-1.84208
-1.83794	-1.85264	-1.84864	-1.84468	-1.84077	-1.83691	-1.83312	-1.82935	-1.84132
-1.83719	-1.85187	-1.84788	-1.84391	-1.84001	-1.83614	-1.83236	-1.82861	-1.84058
-1.85506	-1.85112	-1.84712	-1.84316	-1.83925	-1.83539	-1.84749	-1.8437	-1.83983
-1.85431	-1.85037	-1.84637	-1.84241	-1.83851	-1.83466	-1.84675	-1.84295	-1.83909
-1.85355	-1.84961	-1.84562	-1.84166	-1.83777	-1.83391	-1.846	-1.84221	-1.83834
-1.85279	-1.84886	-1.84486	-1.84091	-1.83702	-1.83318	-1.84526	-1.84146	-1.83759
-1.83216	-1.84811	-1.84412	-1.84017	-1.83628	-1.83244	-1.84451	-1.84071	-1.83684
-1.83142	-1.84737	-1.84339	-1.83944	-1.83554	-1.83169	-1.84376	-1.83996	-1.83609
-1.83069	-1.84664	-1.84267	-1.83874	-1.83483	-1.83097	-1.84302	-1.83922	-1.83534
-1.82996	-1.84592	-1.842	-1.83809	-1.83417	-1.83029	-1.84232	-1.83849	-1.83459
-1.82924	-1.8452	-1.84132	-1.83744	-1.83352	-1.82963	-1.84162	-1.83776	-1.83386
-1.8285	-1.84445	-1.8406	-1.83673	-1.83282	-1.82892	-1.8409	-1.83703	-1.83314
-1.82776	-1.84369	-1.83985	-1.83598	-1.83208	-1.82819	-1.84016	-1.83629	-1.83243
-1.82706	-1.84297	-1.83911	-1.8512	-1.84726	-1.84333	-1.83943	-1.83555	-1.8317
-1.82639	-1.84228	-1.83839	-1.85046	-1.84652	-1.84259	-1.83868	-1.83479	-1.83094
-1.82572	-1.84158	-1.83767	-1.84972	-1.8458	-1.84189	-1.83798	-1.83407	-1.83022
-1.825	-1.84086	-1.83694	-1.84899	-1.84513	-1.84126	-1.83734	-1.83342	-1.82953
-1.8243	-1.84015	-1.83622	-1.84828	-1.84445	-1.84062	-1.83671	-1.83277	-1.82885
-1.82364	-1.8395	-1.83556	-1.84761	-1.84375	-1.8399	-1.83602	-1.83213	-1.82821
-1.82298	-1.83886	-1.83492	-1.84695	-1.84305	-1.83918	-1.83533	-1.83148	-1.82756
-1.82229	-1.81845	-1.8146	-1.84632	-1.8424	-1.83849	-1.83463	-1.83077	-1.82685
-1.82157	-1.81774	-1.81396	-1.8457	-1.84177	-1.83784	-1.83393	-1.83002	-1.82609
-1.82088	-1.81705	-1.81329	-1.84504	-1.84112	-1.83719	-1.83326	-1.82932	-1.82538
-1.82024	-1.81638	-1.81258	-1.84432	-1.84045	-1.83656	-1.83262	-1.82865	-1.82472
-1.8196	-1.83197	-1.82809	-1.84361	-1.83976	-1.8359	-1.83195	-1.82798	-1.82405
-1.81897	-1.83134	-1.82745	-1.84294	-1.83906	-1.83517	-1.83122	-1.82726	-1.82332

-1.81835	-1.83073	-1.82683	-1.84229	-1.83837	-1.83444	-1.83049	-1.82654	-1.8226
-1.81774	-1.83011	-1.82621	-1.84165	-1.83772	-1.83378	-1.82983	-1.82587	-1.82193
-1.81714	-1.82949	-1.82559	-1.84103	-1.8371	-1.83315	-1.82919	-1.82523	-1.82128
-1.81653	-1.82888	-1.82498	-1.84041	-1.83647	-1.83252	-1.82856	-1.82459	-1.82064
-1.81591	-1.82828	-1.82437	-1.83979	-1.83585	-1.8319	-1.82793	-1.82396	-1.82002
-1.83152	-1.82764	-1.82375	-1.81985	-1.81595	-1.81205	-1.8273	-1.82334	-1.81939
-1.83082	-1.82695	-1.8231	-1.81924	-1.81534	-1.81143	-1.82668	-1.82271	-1.81876
-1.83015	-1.82629	-1.82247	-1.81862	-1.81472	-1.8108	-1.82605	-1.82208	-1.81814
-1.82958	-1.82572	-1.82187	-1.818	-1.8141	-1.81018	-1.82542	-1.82146	-1.81752
-1.82903	-1.82516	-1.82128	-1.81738	-1.81348	-1.80956	-1.8248	-1.82084	-1.81691
-1.82845	-1.82457	-1.82067	-1.81677	-1.81285	-1.80894	-1.82418	-1.82022	-1.81629
-1.82786	-1.82396	-1.82006	-1.81615	-1.81224	-1.80832	-1.82355	-1.8196	-1.81567
-1.82725	-1.82335	-1.81944	-1.81553	-1.81161	-1.8077	-1.82293	-1.81898	-1.81506
-1.82663	-1.82272	-1.81882	-1.81491	-1.81099	-1.80708	-1.82231	-1.81837	-1.81446
-1.82601	-1.8221	-1.81821	-1.8143	-1.81038	-1.80646	-1.80256	-1.79868	-1.81387
-1.82539	-1.82149	-1.81761	-1.81371	-1.80977	-1.80585	-1.80196	-1.79809	-1.8133
-1.82479	-1.8209	-1.81702	-1.81313	-1.80919	-1.80526	-1.80137	-1.79751	-1.81273
-1.82419	-1.82031	-1.81646	-1.81259	-1.80867	-1.80474	-1.80084	-1.79697	-1.81217
-1.8236	-1.81974	-1.81591	-1.81206	-1.80816	-1.80424	-1.80033	-1.79643	-1.8116
-1.82306	-1.81919	-1.81535	-1.81148	-1.80759	-1.80369	-1.79977	-1.79586	-1.81102
-1.82255	-1.81865	-1.81477	-1.81089	-1.807	-1.8031	-1.79918	-1.79527	-1.81043
-1.82204	-1.81813	-1.81423	-1.81033	-1.80644	-1.80254	-1.79863	-1.79473	-1.80988
-1.82152	-1.81763	-1.81373	-1.80983	-1.80592	-1.80202	-1.79812	-1.79423	-1.80937
-1.82098	-1.8171	-1.8132	-1.8093	-1.80538	-1.80147	-1.79759	-1.79372	-1.80885
-1.8204	-1.81651	-1.81261	-1.80871	-1.80479	-1.80088	-1.797	-1.79314	-1.80827
-1.81982	-1.81592	-1.81203	-1.80813	-1.80421	-1.8003	-1.79643	-1.79256	-1.80769
-1.81931	-1.8154	-1.81151	-1.80761	-1.8037	-1.79979	-1.79591	-1.79205	-1.78819
-1.81882	-1.81491	-1.81101	-1.80711	-1.8032	-1.79931	-1.79543	-1.79156	-1.7877
-1.81832	-1.81441	-1.81051	-1.8066	-1.80271	-1.79882	-1.79493	-1.79106	-1.7872
-1.81783	-1.81391	-1.81	-1.8061	-1.80221	-1.79833	-1.79444	-1.79056	-1.7867
-1.81729	-1.81338	-1.80946	-1.80555	-1.80167	-1.79779	-1.7939	-1.79002	-1.78616
-1.8167	-1.81278	-1.80886	-1.80495	-1.80107	-1.7972	-1.79331	-1.78942	-1.78557
-1.81612	-1.81221	-1.80829	-1.80438	-1.8005	-1.79663	-1.79274	-1.78886	-1.785
-1.8156	-1.81169	-1.80777	-1.80387	-1.79999	-1.79611	-1.79223	-1.78835	-1.78449
-1.81511	-1.81119	-1.80728	-1.80338	-1.7995	-1.79562	-1.79174	-1.78786	-1.78401
-1.81461	-1.8107	-1.80679	-1.80289	-1.799	-1.79512	-1.79124	-1.78737	-1.79317
-1.81412	-1.81021	-1.8063	-1.8024	-1.79851	-1.79464	-1.79076	-1.78689	-1.79271
-1.81362	-1.80971	-1.8058	-1.8019	-1.79802	-1.79414	-1.79027	-1.78639	-1.79225
-1.81312	-1.80921	-1.8053	-1.8014	-1.79752	-1.79365	-1.78977	-1.7859	-1.79179
-1.81263	-1.80872	-1.80481	-1.80092	-1.79703	-1.79315	-1.78927	-1.7854	-1.79133
-1.81213	-1.80823	-1.80433	-1.80044	-1.79654	-1.79264	-1.78877	-1.7849	-1.79086
-1.81167	-1.80776	-1.80387	-1.79998	-1.79607	-1.79216	-1.79815	-1.79427	-1.79041
-1.81126	-1.80736	-1.80347	-1.79958	-1.79566	-1.79175	-1.79774	-1.79383	-1.78996
-1.81087	-1.80696	-1.80308	-1.79919	-1.79527	-1.79134	-1.79735	-1.79341	-1.78953
-1.81042	-1.80651	-1.80263	-1.79873	-1.79482	-1.7909	-1.79696	-1.79303	-1.867
-1.80993	-1.80603	-1.80213	-1.79824	-1.79434	-1.79044	-1.79657	-1.79268	-1.86664
-1.80945	-1.80554	-1.80165	-1.80782	-1.80393	-1.80004	-1.79615	-1.79228	-1.86623
-1.80897	-1.80506	-1.80116	-1.80736	-1.80348	-1.7996	-1.79571	-1.79183	-1.86576
-1.80852	-1.80461	-1.8007	-1.80694	-1.80306	-1.79919	-1.79529	-1.79141	-1.8653
-1.80812	-1.80422	-1.8003	-1.80658	-1.8027	-1.79883	-1.79493	-1.79104	-1.86489

-1.80773	-1.80382	-1.79991	-1.80622	-1.80234	-1.79847	-1.79457	-1.79067	-1.86449
-1.80727	-1.81362	-1.80971	-1.80582	-1.80193	-1.79804	-1.87224	-1.86816	-1.86409
-1.8068	-1.81318	-1.8093	-1.8054	-1.8015	-1.79761	-1.87178	-1.86774	-1.86368
-1.80639	-1.81281	-1.80892	-1.80503	-1.80112	-1.79722	-1.87138	-1.86734	-1.8633
-1.80603	-1.81248	-1.80858	-1.80468	-1.80076	-1.79687	-1.871	-1.86697	-1.86293
-1.80567	-1.81215	-1.80824	-1.82672	-1.82273	-1.81876	-1.87059	-1.86655	-1.86251
-1.80531	-1.81183	-1.8079	-1.82635	-1.82233	-1.81833	-1.87012	-1.86607	-1.86204
-1.81539	-1.81147	-1.80754	-1.82597	-1.82194	-1.81793	-1.86969	-1.86563	-1.8616
-1.81496	-1.81105	-1.80713	-1.82558	-1.82156	-1.81756	-1.86931	-1.86525	-1.86123
-1.81453	-1.81062	-1.80673	-1.82518	-1.82119	-1.81722	-1.86897	-1.86491	-1.86088
-1.81417	-1.83274	-1.82878	-1.8809	-1.87679	-1.87269	-1.86862	-1.86456	-1.86052
-1.81385	-1.83241	-1.82844	-1.88054	-1.87644	-1.87235	-1.86827	-1.86421	-1.86018
-1.81353	-1.83207	-1.82809	-1.88019	-1.87609	-1.872	-1.86792	-1.86386	-1.85983
-1.81321	-1.83174	-1.82776	-1.87984	-1.87573	-1.87164	-1.86757	-1.86351	-1.85947
-1.83539	-1.83141	-1.82743	-1.87949	-1.87539	-1.87129	-1.86722	-1.86317	-1.85913
-1.83505	-1.83107	-1.82709	-1.87914	-1.87504	-1.87095	-1.86688	-1.86282	-1.85879
-1.83472	-1.83074	-1.82676	-1.8788	-1.8747	-1.87061	-1.86654	-1.86248	-1.85845
-1.83437	-1.8304	-1.82642	-1.87845	-1.87435	-1.87026	-1.8662	-1.86215	-1.85811
-1.83403	-1.83006	-1.82608	-1.8781	-1.874	-1.86992	-1.86585	-1.86181	-1.85778
-1.8337	-1.82971	-1.82574	-1.87775	-1.87366	-1.86958	-1.86551	-1.86147	-1.85744
-1.83339	-1.82936	-1.8254	-1.8774	-1.87331	-1.86924	-1.86517	-1.86113	-1.8571
-1.83311	-1.8488	-1.84475	-1.87706	-1.87298	-1.86891	-1.86484	-1.86079	-1.85676
-1.83289	-1.84856	-1.84446	-1.87672	-1.87265	-1.86859	-1.86452	-1.86045	-1.85642
-1.83263	-1.88479	-1.88058	-1.87642	-1.87235	-1.8683	-1.86423	-1.86015	-1.85611
-1.83232	-1.8845	-1.88033	-1.8762	-1.87212	-1.86806	-1.864	-1.85993	-1.85586
-1.832	-1.8842	-1.88008	-1.87598	-1.87189	-1.86781	-1.86376	-1.85971	-1.85561
-1.83168	-1.88386	-1.87977	-1.87568	-1.87159	-1.8675	-1.86345	-1.85941	-1.85533
-1.83138	-1.88353	-1.87945	-1.87537	-1.87127	-1.86718	-1.86313	-1.85908	-1.85503
-1.85087	-1.88326	-1.87918	-1.8751	-1.87101	-1.86692	-1.86284	-1.85876	-1.85472
-1.85065	-1.88304	-1.87894	-1.87486	-1.87078	-1.8667	-1.86257	-1.85844	-1.85439
-1.85042	-1.88282	-1.87868	-1.87458	-1.87052	-1.86645	-1.86231	-1.85816	-1.8541
-1.85021	-1.88258	-1.87839	-1.87426	-1.8702	-1.86616	-1.86205	-1.85793	-1.85388
-1.83423	-1.88233	-1.87812	-1.87397	-1.86991	-1.86587	-1.8618	-1.85772	-1.85366
-1.83394	-1.88206	-1.87787	-1.87374	-1.86967	-1.86563	-1.86157	-1.85751	-1.85343
-1.83365	-1.88179	-1.87764	-1.87352	-1.86944	-1.86538	-1.86133	-1.85728	-1.85318
-1.87036	-1.88154	-1.87742	-1.87331	-1.86919	-1.86509	-1.86104	-1.85701	-1.85292
-1.87012	-1.8813	-1.8772	-1.87308	-1.86892	-1.86478	-1.86073	-1.85672	-1.85266
-1.86984	-1.86575	-1.8617	-1.87282	-1.86866	-1.86453	-1.86048	-1.85647	-1.85242
-1.86954	-1.86543	-1.86139	-1.87253	-1.86842	-1.86432	-1.86028	-1.85625	-1.85221
-1.86927	-1.86515	-1.86112	-1.87228	-1.8682	-1.86412	-1.86008	-1.85604	-1.852
-1.86906	-1.86494	-1.8609	-1.87206	-1.86799	-1.86393	-1.85988	-1.85584	-1.8518
-1.86885	-1.86473	-1.86069	-1.87186	-1.86779	-1.86373	-1.85969	-1.85565	-1.8516
-1.86865	-1.86453	-1.86049	-1.87166	-1.86759	-1.86354	-1.85949	-1.85545	-1.8514
-1.86845	-1.86434	-1.8603	-1.87147	-1.8674	-1.86334	-1.85929	-1.85525	-1.8512
-1.85036	-1.86414	-1.8601	-1.85607	-1.85203	-1.848	-1.8591	-1.85505	-1.85101
-1.85016	-1.86394	-1.85991	-1.85588	-1.85184	-1.8478	-1.85889	-1.85485	-1.85081
-1.84997	-1.86375	-1.85971	-1.85568	-1.85163	-1.8476	-1.85869	-1.85465	-1.85061
-1.84979	-1.86357	-1.85951	-1.85547	-1.85143	-1.84739	-1.85849	-1.85445	-1.85041
-1.84963	-1.86342	-1.85935	-1.85529	-1.85123	-1.84719	-1.84317	-1.83915	-1.85021
-1.84953	-1.86334	-1.85925	-1.85517	-1.85107	-1.84699	-1.84296	-1.83895	-1.85002

-1.84942	-1.86324	-1.85915	-1.85504	-1.8509	-1.84679	-1.84275	-1.83875	-1.84982
-1.84924	-1.8452	-1.84116	-1.85488	-1.85073	-1.8466	-1.84255	-1.83854	-1.84962
-1.84905	-1.84501	-1.84098	-1.8547	-1.85055	-1.84643	-1.84237	-1.83835	-1.84943
-1.84891	-1.84485	-1.8408	-1.85452	-1.8504	-1.8463	-1.84225	-1.83822	-1.86818
-1.84881	-1.84472	-1.84062	-1.85433	-1.85027	-1.84622	-1.84217	-1.83813	-1.86806
-1.84867	-1.84457	-1.84044	-1.85415	-1.8501	-1.84608	-1.84204	-1.838	-1.86792
-1.84849	-1.84439	-1.84026	-1.85396	-1.84991	-1.84588	-1.84185	-1.83783	-1.86778
-1.84832	-1.84422	-1.8401	-1.85381	-1.84974	-1.8457	-1.84167	-1.83766	-1.86762
-1.84815	-1.84406	-1.83997	-1.8537	-1.84964	-1.84559	-1.84153	-1.83749	-1.86743
-1.848	-1.84391	-1.83986	-1.85361	-1.84955	-1.84549	-1.84141	-1.83733	-1.86726
-1.84788	-1.84381	-1.83976	-1.85352	-1.84943	-1.84536	-1.84128	-1.83722	-1.86714
-1.84779	-1.84372	-1.83966	-1.85341	-1.84931	-1.84522	-1.84116	-1.83713	-1.86705
-1.84769	-1.84362	-1.83954	-1.83548	-1.83142	-1.82739	-1.84105	-1.83703	-1.86694
-1.84759	-1.84351	-1.83941	-1.83534	-1.8313	-1.82729	-1.84097	-1.83694	-1.86682
-1.84748	-1.84339	-1.83928	-1.83522	-1.8312	-1.82721	-1.84087	-1.83683	-1.8667
-1.84734	-1.84325	-1.83918	-1.83513	-1.83113	-1.82713	-1.84077	-1.83669	-1.86655
-1.8472	-1.84313	-1.83908	-1.83506	-1.83107	-1.82706	-1.84069	-1.83658	-1.86644
-1.8471	-1.84304	-1.83901	-1.83499	-1.831	-1.82701	-1.84066	-1.83658	-1.86642
-1.84701	-1.84297	-1.83894	-1.83493	-1.83094	-1.82695	-1.84063	-1.83658	-1.86641
-1.84695	-1.8429	-1.83887	-1.83486	-1.83086	-1.82687	-1.84055	-1.83649	-1.86632
-1.8469	-1.84283	-1.8388	-1.83479	-1.83078	-1.82677	-1.84043	-1.83635	-1.8662
-1.84683	-1.84276	-1.83874	-1.83471	-1.83068	-1.82665	-1.84031	-1.83624	-1.8661
-1.84675	-1.8427	-1.83867	-1.83463	-1.83056	-1.8265	-1.84018	-1.83615	-1.86602
-1.84668	-1.84264	-1.83861	-1.83457	-1.83048	-1.82642	-1.8401	-1.83607	-1.86594
-1.84662	-1.84256	-1.83855	-1.83453	-1.83049	-1.82644	-1.84009	-1.83601	-1.86585
-1.84658	-1.8425	-1.83849	-1.83449	-1.83049	-1.82647	-1.8401	-1.83597	-1.86579
-1.84658	-1.8425	-1.83845	-1.83442	-1.83044	-1.82645	-1.8224	-1.81834	-1.86579
-1.84659	-1.8425	-1.83841	-1.83436	-1.83037	-1.8264	-1.8224	-1.81839	-1.86581
-1.84651	-1.84243	-1.83835	-1.8343	-1.83031	-1.82634	-1.82236	-1.81838	-1.86581
-1.84637	-1.8423	-1.83828	-1.83427	-1.83026	-1.82626	-1.82229	-1.81832	-1.86579
-1.84631	-1.84226	-1.83826	-1.83426	-1.83025	-1.82625	-1.82225	-1.81826	-1.86574
-1.84633	-1.8423	-1.83829	-1.83429	-1.8303	-1.8263	-1.82224	-1.81819	-1.86566
-1.84634	-1.84232	-1.83831	-1.8343	-1.83032	-1.82632	-1.82224	-1.81815	-1.86561
-1.8463	-1.84229	-1.83827	-1.83426	-1.83028	-1.82629	-1.82225	-1.81819	-1.86561
-1.84627	-1.84226	-1.83823	-1.83422	-1.83023	-1.82624	-1.82224	-1.81822	-1.86563
-1.84628	-1.84227	-1.83826	-1.83424	-1.8302	-1.82617	-1.82218	-1.81819	-1.86561
-1.8463	-1.8423	-1.83831	-1.83427	-1.83018	-1.8261	-1.8221	-1.81813	-1.86559
-1.8463	-1.84228	-1.8383	-1.83428	-1.83018	-1.8261	-1.82207	-1.81807	-1.84832
-1.84627	-1.84223	-1.83826	-1.83426	-1.83021	-1.82614	-1.82207	-1.81802	-1.84826
-1.84627	-1.84223	-1.83826	-1.83427	-1.83024	-1.82619	-1.8221	-1.81801	-1.84823
-1.84631	-1.84228	-1.83829	-1.8343	-1.83029	-1.82626	-1.82216	-1.81805	-1.84826
-1.84636	-1.84233	-1.83832	-1.83431	-1.83032	-1.82631	-1.82222	-1.81811	-1.8483
-1.84641	-1.84236	-1.8383	-1.83427	-1.83029	-1.8263	-1.82223	-1.81815	-1.84832
-1.84647	-1.84239	-1.83829	-1.83424	-1.83026	-1.82628	-1.82225	-1.8182	-1.84834
-1.84653	-1.84245	-1.83833	-1.83426	-1.83028	-1.82631	-1.82229	-1.81826	-1.84839
-1.8466	-1.84251	-1.83839	-1.83433	-1.83034	-1.82637	-1.82235	-1.81832	-1.84844
-1.84667	-1.84258	-1.83846	-1.83439	-1.8304	-1.82643	-1.82242	-1.81839	-1.88424
-1.84673	-1.84265	-1.83852	-1.83446	-1.83047	-1.82649	-1.82248	-1.81846	-1.88429
-1.8468	-1.84272	-1.83859	-1.83452	-1.83054	-1.82656	-1.82255	-1.81853	-1.88435
-1.84687	-1.84278	-1.83866	-1.83459	-1.8306	-1.82663	-1.82262	-1.81859	-1.88439

-1.80879	-1.84285	-1.83873	-1.83465	-1.83067	-1.82669	-1.85946	-1.85534	-1.88444
-1.80884	-1.84291	-1.83879	-1.83471	-1.83073	-1.82675	-1.85951	-1.85538	-1.88448
-1.8089	-1.84297	-1.83884	-1.83477	-1.83078	-1.82681	-1.85955	-1.85542	-1.88452
-1.80895	-1.84303	-1.8389	-1.83483	-1.83084	-1.82686	-1.85959	-1.85547	-1.88455
-1.80901	-1.84309	-1.83896	-1.83488	-1.8309	-1.82692	-1.85963	-1.85551	-1.88459
-1.80907	-1.84316	-1.83902	-1.83495	-1.83095	-1.82697	-1.85967	-1.85555	-1.85139
-1.80913	-1.84323	-1.8391	-1.83502	-1.83101	-1.82702	-1.85971	-1.8556	-1.85145
-1.80919	-1.80524	-1.80125	-1.87208	-1.86797	-1.86387	-1.85976	-1.85565	-1.85151
-1.80925	-1.80533	-1.80139	-1.87224	-1.86812	-1.86399	-1.85985	-1.85571	-1.85159
-1.80932	-1.80542	-1.80151	-1.8724	-1.86826	-1.86411	-1.85994	-1.85577	-1.85167
-1.80941	-1.80549	-1.80158	-1.87247	-1.86834	-1.86419	-1.86001	-1.85582	-1.85172
-1.8095	-1.80556	-1.80164	-1.87251	-1.86839	-1.86426	-1.86008	-1.85589	-1.85178
-1.80963	-1.80569	-1.80176	-1.87261	-1.86847	-1.86433	-1.86017	-1.85601	-1.8519
-1.80978	-1.80585	-1.80193	-1.87275	-1.86857	-1.86441	-1.86028	-1.85617	-1.85207
-1.8099	-1.84406	-1.84003	-1.83599	-1.83192	-1.82787	-1.86041	-1.85632	-1.85223
-1.80997	-1.84412	-1.84012	-1.8361	-1.83205	-1.82802	-1.86058	-1.85648	-1.85237
-1.81006	-1.8442	-1.84021	-1.83621	-1.83219	-1.82817	-1.86074	-1.85662	-1.8525
-1.81021	-1.84434	-1.84036	-1.83637	-1.83235	-1.82832	-1.86087	-1.85674	-1.85262
-1.81037	-1.84449	-1.84051	-1.83652	-1.8325	-1.82847	-1.861	-1.85685	-1.85274
-1.7924	-1.84458	-1.84059	-1.8366	-1.83259	-1.82858	-1.86113	-1.85699	-1.85289
-1.7925	-1.84465	-1.84065	-1.83665	-1.83267	-1.82868	-1.86127	-1.85715	-1.85306
-1.83129	-1.84482	-1.84078	-1.83677	-1.83279	-1.82881	-1.86142	-1.85731	-1.85321
-1.83154	-1.84507	-1.84099	-1.83694	-1.83295	-1.82896	-1.86157	-1.85746	-1.85334
-1.83174	-1.84527	-1.84118	-1.83712	-1.8331	-1.8291	-1.8251	-1.82109	-1.85347
-1.83186	-1.8454	-1.84133	-1.83728	-1.83326	-1.82925	-1.82522	-1.82119	-1.85359
-1.83197	-1.84552	-1.84148	-1.83744	-1.83342	-1.82939	-1.82534	-1.82131	-1.85372
-1.83211	-1.84568	-1.84165	-1.83761	-1.83354	-1.82948	-1.82545	-1.82145	-1.85389
-1.83226	-1.84585	-1.84183	-1.83778	-1.83368	-1.82959	-1.82558	-1.82161	-1.85407
-1.83243	-1.84602	-1.842	-1.83796	-1.83388	-1.82981	-1.82578	-1.82178	-1.85425
-1.83262	-1.8462	-1.84218	-1.83816	-1.83414	-1.8301	-1.82603	-1.82197	-1.85442
-1.8328	-1.82879	-1.82482	-1.83835	-1.83435	-1.83033	-1.82626	-1.82218	-1.85463
-1.83295	-1.82896	-1.825	-1.83854	-1.83453	-1.83052	-1.82648	-1.82244	-1.85488
-1.83311	-1.82913	-1.82517	-1.83871	-1.8347	-1.83069	-1.82667	-1.82265	-1.81862
-1.83329	-1.82928	-1.82533	-1.83888	-1.83487	-1.83086	-1.82681	-1.82275	-1.81874
-1.83349	-1.82946	-1.8255	-1.83905	-1.83504	-1.83102	-1.82695	-1.82287	-1.81886
-1.83375	-1.82971	-1.82569	-1.83921	-1.83521	-1.8312	-1.82716	-1.8231	-1.81908
-1.83403	-1.82998	-1.8259	-1.83939	-1.83538	-1.83139	-1.82738	-1.82337	-1.81934
-1.83425	-1.83021	-1.82614	-1.8221	-1.81811	-1.81413	-1.82757	-1.82359	-1.81957
-1.83442	-1.83043	-1.82641	-1.82239	-1.81833	-1.8143	-1.82774	-1.82377	-1.81979
-1.83461	-1.83062	-1.82664	-1.82264	-1.81857	-1.81452	-1.82794	-1.82396	-1.81999
-1.83479	-1.83078	-1.82682	-1.82285	-1.81883	-1.8148	-1.82818	-1.82414	-1.82016
-1.83499	-1.83097	-1.82701	-1.82304	-1.81907	-1.81507	-1.82844	-1.82436	-1.82036
-1.83526	-1.83122	-1.82721	-1.82322	-1.81926	-1.8153	-1.82869	-1.82463	-1.82064
-1.83554	-1.83149	-1.82742	-1.82341	-1.81946	-1.81552	-1.82895	-1.82492	-1.82092
-1.83583	-1.83178	-1.82769	-1.82365	-1.8197	-1.81576	-1.8118	-1.80783	-1.82119
-1.83612	-1.83207	-1.82797	-1.82392	-1.81996	-1.81601	-1.81206	-1.80811	-1.82145
-1.83636	-1.83233	-1.82824	-1.82419	-1.8202	-1.81623	-1.81228	-1.80834	-1.82169
-1.83656	-1.83255	-1.8285	-1.82446	-1.82042	-1.81641	-1.81246	-1.80854	-1.82191
-1.83678	-1.83279	-1.82877	-1.82474	-1.82067	-1.81664	-1.81268	-1.80877	-1.82216
-1.83703	-1.83305	-1.82906	-1.82504	-1.82097	-1.81691	-1.81296	-1.80904	-1.82244

-1.83728	-1.83329	-1.82933	-1.82532	-1.82126	-1.81721	-1.81325	-1.80932	-1.80537
-1.83748	-1.83348	-1.82953	-1.82555	-1.82152	-1.8175	-1.81354	-1.80962	-1.80567
-1.83768	-1.83367	-1.82972	-1.82576	-1.82177	-1.81779	-1.81385	-1.80991	-1.80596
-1.83794	-1.83392	-1.82997	-1.82602	-1.82205	-1.81808	-1.81414	-1.81021	-1.80626
-1.83824	-1.83421	-1.83025	-1.8263	-1.82234	-1.81838	-1.81444	-1.8105	-1.80655
-1.83853	-1.83449	-1.83054	-1.82658	-1.82262	-1.81867	-1.81473	-1.81079	-1.80684
-1.83882	-1.83478	-1.83083	-1.82688	-1.82292	-1.81896	-1.81502	-1.81108	-1.80712
-1.83911	-1.83507	-1.83112	-1.82717	-1.82321	-1.81925	-1.81531	-1.81137	-1.80742
-1.8394	-1.83536	-1.83141	-1.82746	-1.8235	-1.81955	-1.8156	-1.81166	-1.80771
-1.8397	-1.83566	-1.83171	-1.82775	-1.82379	-1.81984	-1.8159	-1.81196	-1.808
-1.84001	-1.83597	-1.832	-1.82804	-1.82408	-1.82013	-1.81619	-1.81225	-1.8083
-1.84034	-1.83629	-1.8323	-1.82833	-1.82437	-1.82042	-1.81648	-1.81255	-1.8086
-1.82366	-1.83667	-1.83267	-1.82868	-1.8247	-1.82073	-1.81678	-1.81284	-1.80889
-1.82405	-1.83708	-1.83307	-1.82907	-1.82505	-1.82105	-1.81708	-1.81313	-1.80918
-1.82439	-1.82041	-1.81644	-1.82943	-1.82541	-1.8214	-1.81742	-1.81346	-1.80951
-1.82469	-1.82071	-1.81675	-1.82976	-1.82578	-1.8218	-1.81782	-1.81385	-1.80988
-1.825	-1.82102	-1.81706	-1.83009	-1.82613	-1.82217	-1.8182	-1.81423	-1.83998
-1.82539	-1.82138	-1.81739	-1.8304	-1.82643	-1.82247	-1.81851	-1.81456	-1.84033
-1.82577	-1.82175	-1.81774	-1.83072	-1.82674	-1.82277	-1.81882	-1.81488	-1.84068
-1.82611	-1.8221	-1.8181	-1.81413	-1.81017	-1.80623	-1.81916	-1.8152	-1.84099
-1.82642	-1.82243	-1.81848	-1.81453	-1.81058	-1.80662	-1.81953	-1.81553	-1.8413
-1.82673	-1.82275	-1.81882	-1.81488	-1.81094	-1.80698	-1.80301	-1.79904	-1.84165
-1.82706	-1.82306	-1.81912	-1.81519	-1.81126	-1.80733	-1.80338	-1.79942	-1.84205
-1.82741	-1.8234	-1.81945	-1.81552	-1.81161	-1.8077	-1.80376	-1.79981	-1.82604
-1.82779	-1.82379	-1.81984	-1.81591	-1.812	-1.80809	-1.80415	-1.8002	-1.82642
-1.82818	-1.82419	-1.82023	-1.81629	-1.81238	-1.80847	-1.80453	-1.80058	-1.8268
-1.82858	-1.82457	-1.82058	-1.81662	-1.81269	-1.80877	-1.80485	-1.80092	-1.82716
-1.82897	-1.82495	-1.82093	-1.81695	-1.813	-1.80908	-1.80517	-1.80126	-1.82753
-1.82934	-1.82531	-1.82131	-1.81733	-1.81338	-1.80946	-1.80554	-1.80164	-1.82792
-1.82969	-1.82567	-1.82169	-1.81774	-1.8138	-1.80988	-1.80596	-1.80204	-1.82834
-1.83007	-1.82606	-1.8221	-1.81815	-1.81422	-1.81029	-1.80637	-1.80245	-1.82875
-1.83046	-1.82647	-1.82252	-1.81858	-1.81464	-1.81071	-1.80679	-1.80288	-1.82918
-1.83087	-1.8269	-1.82295	-1.81901	-1.81507	-1.81113	-1.83759	-1.8336	-1.84944
-1.8313	-1.82733	-1.82337	-1.81943	-1.81549	-1.81155	-1.83802	-1.83402	-1.84986
-1.83172	-1.82776	-1.8238	-1.81985	-1.81591	-1.81198	-1.83844	-1.83443	-1.85028
-1.83215	-1.82818	-1.82422	-1.82028	-1.81633	-1.8124	-1.83886	-1.83486	-1.85071
-1.83257	-1.8286	-1.82465	-1.8207	-1.81675	-1.81281	-1.83928	-1.83528	-1.85114
-1.83299	-1.82902	-1.82507	-1.82112	-1.81717	-1.81323	-1.83971	-1.8357	-1.85156
-1.83341	-1.82944	-1.82548	-1.82153	-1.81759	-1.81365	-1.84013	-1.83611	-1.85199
-1.83385	-1.82988	-1.82591	-1.82195	-1.818	-1.81407	-1.84055	-1.83654	-1.85241
-1.8343	-1.83032	-1.82633	-1.82236	-1.81842	-1.81448	-1.84098	-1.83696	-1.85284
-1.83477	-1.83079	-1.82679	-1.8228	-1.81885	-1.8149	-1.84141	-1.8374	-1.85327
-1.83529	-1.8313	-1.82731	-1.82331	-1.81931	-1.81532	-1.84183	-1.83783	-1.85371
-1.83581	-1.83182	-1.82783	-1.82383	-1.81979	-1.81577	-1.84228	-1.83829	-1.85416
-1.83627	-1.83228	-1.82829	-1.8243	-1.82028	-1.81628	-1.8428	-1.8388	-1.85467
-1.83671	-1.83271	-1.82872	-1.82474	-1.82078	-1.81682	-1.84334	-1.83932	-1.8552
-1.83716	-1.83316	-1.82916	-1.85591	-1.85188	-1.84787	-1.84383	-1.8398	-1.85568
-1.83761	-1.83361	-1.8296	-1.85635	-1.85233	-1.84832	-1.84428	-1.84024	-1.85612
-1.83808	-1.83409	-1.83008	-1.85683	-1.85282	-1.8488	-1.84476	-1.84072	-1.85659
-1.83861	-1.83461	-1.83061	-1.85738	-1.85335	-1.84932	-1.84529	-1.84125	-1.8571

-1.83912	-1.83512	-1.83114	-1.85792	-1.85387	-1.84983	-1.8458	-1.84177	-1.85762
-1.83958	-1.83558	-1.8316	-1.85839	-1.85433	-1.85029	-1.84627	-1.84225	-1.85812
-1.84004	-1.83604	-1.83206	-1.85885	-1.8548	-1.85075	-1.84673	-1.84273	-1.85862
-1.84057	-1.83656	-1.83258	-1.85938	-1.85532	-1.85127	-1.84724	-1.84323	-1.85915
-1.84113	-1.83712	-1.83314	-1.85994	-1.85587	-1.85181	-1.84778	-1.84376	-1.85968
-1.84169	-1.83768	-1.83368	-1.86049	-1.85642	-1.85236	-1.84829	-1.84424	-1.84023
-1.84225	-1.83823	-1.83423	-1.86103	-1.85697	-1.8529	-1.8488	-1.8447	-1.84066
-1.84277	-1.83875	-1.83473	-1.86155	-1.85749	-1.85342	-1.8493	-1.84518	-1.84114
-1.84323	-1.83921	-1.83518	-1.862	-1.85795	-1.85389	-1.84981	-1.84572	-1.84168
-1.8437	-1.83967	-1.83565	-1.86247	-1.85842	-1.85438	-1.85032	-1.84627	-1.84224
-1.84423	-1.8402	-1.83617	-1.86301	-1.85896	-1.85491	-1.85087	-1.84682	-1.8428
-1.84478	-1.84075	-1.83673	-1.86358	-1.85952	-1.85547	-1.85142	-1.84738	-1.84336
-1.84534	-1.87233	-1.86822	-1.86414	-1.86008	-1.85603	-1.85198	-1.84794	-1.84392
-1.84591	-1.8729	-1.8688	-1.86471	-1.86064	-1.85659	-1.85254	-1.8485	-1.84448
-1.84648	-1.87347	-1.86936	-1.86528	-1.86121	-1.85716	-1.8531	-1.84906	-1.84504
-1.84704	-1.87404	-1.86993	-1.86584	-1.86177	-1.85771	-1.85366	-1.84961	-1.84559
-1.84761	-1.87462	-1.8705	-1.86641	-1.86234	-1.85828	-1.85422	-1.85017	-1.84615
-1.84818	-1.87519	-1.87109	-1.86699	-1.86291	-1.85884	-1.85479	-1.85075	-1.84672
-1.84877	-1.87579	-1.87169	-1.8676	-1.8635	-1.85942	-1.85537	-1.85134	-1.84731
-1.84942	-1.87645	-1.87236	-1.86826	-1.86416	-1.86007	-1.85602	-1.85199	-1.84795
-1.8501	-1.87713	-1.87304	-1.86895	-1.86484	-1.86074	-1.85669	-1.85265	-1.84861
-1.82965	-1.87775	-1.87366	-1.86956	-1.86545	-1.86135	-1.85729	-1.85325	-1.84921
-1.83023	-1.87833	-1.87423	-1.87013	-1.86601	-1.86192	-1.85785	-1.8538	-1.84976
-1.83081	-1.87891	-1.8748	-1.87069	-1.86658	-1.86249	-1.85841	-1.85436	-1.85032
-1.83139	-1.87948	-1.87537	-1.87126	-1.86715	-1.86306	-1.85898	-1.85491	-1.85087
-1.83199	-1.85959	-1.85549	-1.87185	-1.86775	-1.86367	-1.85957	-1.85549	-1.86642
-1.83265	-1.86027	-1.85616	-1.8725	-1.86841	-1.86433	-1.86022	-1.85613	-1.86707
-1.83331	-1.86096	-1.85685	-1.87317	-1.86908	-1.86499	-1.86089	-1.85679	-1.86773
-1.86566	-1.86158	-1.85748	-1.87379	-1.86969	-1.86559	-1.86149	-1.85739	-1.86834
-1.86625	-1.86217	-1.85808	-1.8744	-1.87028	-1.86617	-1.86207	-1.85799	-1.86893
-1.86684	-1.86277	-1.85871	-1.85464	-1.85054	-1.84645	-1.86269	-1.85862	-1.86957
-1.86742	-1.86336	-1.85936	-1.85532	-1.85122	-1.84712	-1.86336	-1.8593	-1.87026
-1.8489	-1.86398	-1.85999	-1.85597	-1.85187	-1.84777	-1.864	-1.85994	-1.87093
-1.84956	-1.86463	-1.8606	-1.85654	-1.85246	-1.84837	-1.86459	-1.86052	-1.87155
-1.85025	-1.86531	-1.86122	-1.85714	-1.85306	-1.849	-1.86519	-1.86112	-1.87216
-1.85094	-1.866	-1.8619	-1.85781	-1.85373	-1.84967	-1.8456	-1.84155	-1.85775
-1.85165	-1.86671	-1.86261	-1.85852	-1.85444	-1.85036	-1.8463	-1.84224	-1.8584
-1.85235	-1.86742	-1.86332	-1.85922	-1.85514	-1.85106	-1.847	-1.84294	-1.85907
-1.85306	-1.86813	-1.86402	-1.85993	-1.85585	-1.85178	-1.84771	-1.84365	-1.85976
-1.85377	-1.84969	-1.84564	-1.86064	-1.85656	-1.85249	-1.84842	-1.84436	-1.84031
-1.85446	-1.8504	-1.84634	-1.86135	-1.85726	-1.85319	-1.84912	-1.84507	-1.84102
-1.85513	-1.85109	-1.84704	-1.86206	-1.85797	-1.85389	-1.84983	-1.84577	-1.84172
-1.85575	-1.85172	-1.84771	-1.86277	-1.85868	-1.8546	-1.85054	-1.84648	-1.84243
-1.85639	-1.85236	-1.84839	-1.86348	-1.85939	-1.85531	-1.85124	-1.84718	-1.84313
-1.85714	-1.85309	-1.8491	-1.86418	-1.86009	-1.85601	-1.85194	-1.84789	-1.84383
-1.85792	-1.85386	-1.84982	-1.86489	-1.86079	-1.85671	-1.85264	-1.84859	-1.84453
-1.85868	-1.8546	-1.85053	-1.86559	-1.8615	-1.85741	-1.85334	-1.84928	-1.84523
-1.85942	-1.85531	-1.85124	-1.8663	-1.8622	-1.85811	-1.85404	-1.84998	-1.84592
-1.86015	-1.85603	-1.85195	-1.86701	-1.86291	-1.85883	-1.85475	-1.85068	-1.84663
-1.86084	-1.85674	-1.85266	-1.86774	-1.86364	-1.85955	-1.85546	-1.85138	-1.84733

-1.83965	-1.85746	-1.85338	-1.84932	-1.84528	-1.84126	-1.8562	-1.85211	-1.84806
-1.84037	-1.85818	-1.8541	-1.85005	-1.84604	-1.84205	-1.85701	-1.85292	-1.84886
-1.84109	-1.85891	-1.85483	-1.8508	-1.84681	-1.84284	-1.85782	-1.85373	-1.84966
-1.80487	-1.85965	-1.85559	-1.85156	-1.84757	-1.84358	-1.85857	-1.85449	-1.85041
-1.8056	-1.86041	-1.85637	-1.85233	-1.84831	-1.8443	-1.85929	-1.85522	-1.85114
-1.78543	-1.82481	-1.82082	-1.85308	-1.84905	-1.84502	-1.86004	-1.85598	-1.85191
-1.78618	-1.82557	-1.82155	-1.85381	-1.84977	-1.84576	-1.86081	-1.85678	-1.85273
-1.78691	-1.82631	-1.82227	-1.81826	-1.8143	-1.81037	-1.86158	-1.85755	-1.85351
-1.78761	-1.82702	-1.82298	-1.81899	-1.81504	-1.81112	-1.86234	-1.85829	-1.85424
-1.78834	-1.7844	-1.78049	-1.81972	-1.81579	-1.81186	-1.82748	-1.82347	-1.85497
-1.78913	-1.78518	-1.78125	-1.82048	-1.81651	-1.81256	-1.82819	-1.82419	-1.85572
-1.78994	-1.78598	-1.78202	-1.82125	-1.81723	-1.81325	-1.82889	-1.82492	-1.85649
-1.79069	-1.78674	-1.7828	-1.82205	-1.81802	-1.81401	-1.82966	-1.82568	-1.85728
-1.7914	-1.78748	-1.78359	-1.82288	-1.81883	-1.81481	-1.83046	-1.82647	-1.85809
-1.79215	-1.78824	-1.78437	-1.8032	-1.79922	-1.79525	-1.81164	-1.8077	-1.8233
-1.79294	-1.78902	-1.78515	-1.804	-1.80002	-1.79606	-1.81244	-1.80848	-1.82406
-1.75802	-1.78979	-1.7859	-1.80476	-1.80079	-1.79684	-1.81323	-1.80927	-1.82484
-1.75878	-1.79056	-1.78661	-1.80546	-1.8015	-1.79756	-1.81399	-1.81006	-1.82565
-1.75956	-1.79133	-1.78734	-1.80616	-1.8022	-1.79828	-1.81475	-1.81085	-1.82646
-1.76034	-1.79213	-1.78812	-1.78415	-1.78025	-1.7764	-1.7952	-1.79137	-1.82728
-1.76113	-1.79294	-1.78892	-1.78495	-1.78103	-1.77717	-1.79598	-1.79217	-1.82809
-1.74258	-1.79375	-1.78973	-1.78575	-1.78182	-1.77795	-1.79678	-1.79296	-1.8289
-1.74338	-1.79456	-1.79054	-1.78655	-1.78262	-1.77874	-1.79758	-1.79376	-1.82971
-1.74417	-1.77658	-1.77259	-1.78736	-1.78342	-1.77954	-1.79839	-1.79456	-1.81055
-1.74496	-1.77739	-1.77339	-1.78816	-1.78423	-1.78034	-1.7992	-1.79536	-1.81136
-1.74576	-1.77821	-1.77421	-1.78897	-1.78504	-1.78115	-1.80001	-1.79616	-1.81217
-1.74657	-1.77904	-1.77504	-1.7898	-1.78585	-1.78194	-1.80082	-1.79697	-1.81299
-1.74739	-1.77988	-1.77589	-1.79065	-1.78668	-1.78276	-1.80163	-1.79779	-1.81382
-1.7482	-1.78074	-1.77678	-1.77282	-1.76887	-1.76495	-1.80248	-1.79862	-1.79473
-1.749	-1.78159	-1.77767	-1.77374	-1.76977	-1.76584	-1.80335	-1.79945	-1.79558
-1.7498	-1.74601	-1.74222	-1.77461	-1.77064	-1.76669	-1.76281	-1.75898	-1.79639
-1.75059	-1.74679	-1.74301	-1.77542	-1.77147	-1.76752	-1.76362	-1.75976	-1.79718
-1.75138	-1.74757	-1.74379	-1.77624	-1.77231	-1.76838	-1.76446	-1.76058	-1.798
-1.75216	-1.74834	-1.74458	-1.77707	-1.77318	-1.76928	-1.76536	-1.76144	-1.79885
-1.75297	-1.74913	-1.74537	-1.7779	-1.77404	-1.77017	-1.76624	-1.76231	-1.78143
-1.75385	-1.74997	-1.74616	-1.77869	-1.77485	-1.771	-1.76708	-1.76314	-1.78226
-1.75475	-1.75085	-1.74698	-1.77949	-1.77565	-1.77181	-1.7679	-1.76398	-1.7831
-1.75567	-1.75176	-1.74785	-1.78034	-1.77647	-1.77262	-1.76874	-1.76486	-1.784
-1.75659	-1.75269	-1.74877	-1.78124	-1.77732	-1.77344	-1.76959	-1.76577	-1.78493
-1.75746	-1.75358	-1.74968	-1.78217	-1.77821	-1.77429	-1.77045	-1.76664	-1.76279
-1.75827	-1.75444	-1.75059	-1.78314	-1.77915	-1.77519	-1.7713	-1.76746	-1.76362
-1.75907	-1.75526	-1.75146	-1.78406	-1.78007	-1.7761	-1.77218	-1.7683	-1.76447
-1.75986	-1.75603	-1.75225	-1.78491	-1.78098	-1.77704	-1.77311	-1.76919	-1.76533
-1.76067	-1.75682	-1.75304	-1.78574	-1.78186	-1.77796	-1.77404	-1.7701	-1.7662
-1.76155	-1.75767	-1.75385	-1.75006	-1.74628	-1.74252	-1.77491	-1.77102	-1.76713
-1.76247	-1.75855	-1.75469	-1.75086	-1.74707	-1.74331	-1.77578	-1.77194	-1.76806
-1.76338	-1.75948	-1.75561	-1.75177	-1.74795	-1.74417	-1.77664	-1.77281	-1.76895
-1.7643	-1.76044	-1.7566	-1.75275	-1.7489	-1.74507	-1.77751	-1.77365	-1.7698
-1.74485	-1.76133	-1.75751	-1.75367	-1.74982	-1.74598	-1.77843	-1.77455	-1.77071
-1.74567	-1.76214	-1.75832	-1.7545	-1.7507	-1.74691	-1.77941	-1.77554	-1.77168

-1.74654	-1.763	-1.75916	-1.75535	-1.75157	-1.74781	-1.78036	-1.7765	-1.81434
-1.74751	-1.76396	-1.76007	-1.75622	-1.75241	-1.74863	-1.7812	-1.77736	-1.81525
-1.74849	-1.76495	-1.76102	-1.75713	-1.75328	-1.74946	-1.78205	-1.77822	-1.81615
-1.74939	-1.76586	-1.76195	-1.75807	-1.75422	-1.75041	-1.78302	-1.77918	-1.81711
-1.75025	-1.76676	-1.76289	-1.75905	-1.75522	-1.75141	-1.78404	-1.78017	-1.81808
-1.7512	-1.76772	-1.76387	-1.76003	-1.75619	-1.75236	-1.74856	-1.74478	-1.81902
-1.7522	-1.76874	-1.76488	-1.76102	-1.75714	-1.75328	-1.74945	-1.74564	-1.81995
-1.75315	-1.7697	-1.76584	-1.76197	-1.75809	-1.75422	-1.75037	-1.74655	-1.82091
-1.75401	-1.77057	-1.76671	-1.76286	-1.759	-1.75516	-1.75134	-1.74754	-1.82194
-1.75491	-1.75106	-1.74727	-1.76377	-1.75994	-1.75612	-1.75231	-1.74851	-1.82295
-1.75591	-1.75207	-1.74826	-1.76476	-1.76093	-1.75711	-1.75327	-1.74944	-1.8239
-1.75695	-1.7531	-1.74928	-1.76576	-1.76192	-1.75808	-1.75422	-1.75036	-1.82484
-1.75798	-1.75411	-1.75025	-1.76671	-1.76284	-1.75898	-1.75514	-1.7513	-1.79054
-1.75899	-1.75511	-1.7512	-1.76763	-1.76372	-1.75985	-1.75603	-1.75225	-1.79154
-1.73228	-1.75605	-1.75214	-1.76859	-1.76467	-1.7608	-1.75699	-1.75323	-1.81335
-1.73309	-1.75693	-1.75308	-1.76959	-1.76569	-1.76182	-1.75801	-1.75423	-1.81438
-1.73395	-1.75786	-1.75404	-1.77059	-1.76671	-1.76285	-1.75902	-1.75521	-1.81538
-1.73491	-1.75888	-1.75505	-1.77161	-1.76774	-1.76387	-1.76	-1.75614	-1.81632
-1.73589	-1.75991	-1.75606	-1.7726	-1.76873	-1.76486	-1.76096	-1.75707	-1.81725
-1.73686	-1.76092	-1.75702	-1.77354	-1.76963	-1.76574	-1.76186	-1.75799	-1.81823
-1.73782	-1.76192	-1.75798	-1.77446	-1.77051	-1.7666	-1.76274	-1.75893	-1.81923
-1.73874	-1.76288	-1.75893	-1.77543	-1.77147	-1.76756	-1.76371	-1.7599	-1.82025
-1.73963	-1.76381	-1.75988	-1.77642	-1.77249	-1.76859	-1.76473	-1.76091	-1.82129
-1.72113	-1.76477	-1.76086	-1.77744	-1.77352	-1.76964	-1.76577	-1.76193	-1.82234
-1.722	-1.76575	-1.76188	-1.77849	-1.77459	-1.7707	-1.76683	-1.76297	-1.82341
-1.7229	-1.73866	-1.73504	-1.75907	-1.75525	-1.75143	-1.76789	-1.76402	-1.82449
-1.72384	-1.73963	-1.73602	-1.76012	-1.75629	-1.75247	-1.76894	-1.76507	-1.82557
-1.7248	-1.74062	-1.73701	-1.76117	-1.75734	-1.75351	-1.77	-1.76613	-1.82665
-1.70643	-1.72219	-1.71863	-1.76223	-1.75839	-1.75456	-1.77106	-1.76718	-1.82772
-1.7074	-1.72317	-1.7196	-1.76328	-1.75944	-1.75561	-1.77212	-1.76823	-1.8288
-1.68609	-1.72413	-1.72058	-1.76783	-1.76396	-1.7601	-1.83808	-1.83398	-1.82988
-1.68704	-1.7251	-1.72154	-1.76888	-1.76502	-1.76116	-1.83919	-1.83507	-1.83096
-1.68799	-1.72607	-1.72251	-1.76992	-1.76607	-1.76222	-1.82248	-1.8184	-1.83205
-1.68895	-1.72704	-1.72347	-1.77096	-1.76712	-1.76327	-1.82357	-1.81949	-1.83315
-1.66727	-1.72801	-1.72443	-1.77199	-1.76815	-1.76431	-1.80485	-1.80083	-1.83425
-1.66821	-1.72898	-1.72539	-1.77303	-1.76918	-1.76533	-1.8059	-1.80189	-1.83535
-1.66916	-1.72995	-1.72635	-1.77407	-1.7702	-1.76634	-1.80694	-1.80294	-1.83643
-1.67011	-1.68661	-1.68311	-1.74671	-1.74307	-1.73943	-1.80798	-1.80398	-1.8197
-1.67106	-1.68756	-1.68406	-1.74769	-1.74404	-1.74039	-1.80902	-1.80501	-1.82075
-1.67201	-1.68852	-1.68501	-1.74866	-1.745	-1.74135	-1.81006	-1.80604	-1.8218
-1.67297	-1.68947	-1.68596	-1.74962	-1.74596	-1.74231	-1.8111	-1.80708	-1.82285
-1.67392	-1.69043	-1.68691	-1.72901	-1.72538	-1.72176	-1.81214	-1.80811	-1.82389
-1.67486	-1.69138	-1.68786	-1.72999	-1.72634	-1.72271	-1.81318	-1.80915	-1.82494
-1.6758	-1.69234	-1.68882	-1.73096	-1.72731	-1.72366	-1.81422	-1.81018	-1.82598
-1.67674	-1.69328	-1.68976	-1.70996	-1.70636	-1.70276	-1.76698	-1.7632	-1.80721
-1.67768	-1.69423	-1.69072	-1.71093	-1.70732	-1.70372	-1.76797	-1.76418	-1.80825
-1.67863	-1.69519	-1.69168	-1.7119	-1.7083	-1.7047	-1.76898	-1.76518	-1.78885
-1.67961	-1.69616	-1.69265	-1.71289	-1.7093	-1.7057	-1.77001	-1.7662	-1.78993
-1.68066	-1.69722	-1.69371	-1.71396	-1.71037	-1.70678	-1.77112	-1.76731	-1.7911
-1.68182	-1.69839	-1.69487	-1.71515	-1.71156	-1.70796	-1.77235	-1.76852	-1.79238

-1.68293	-1.6995	-1.69598	-1.71627	-1.71268	-1.70909	-1.75225	-1.74848	-1.76588
-1.68391	-1.70048	-1.69696	-1.71727	-1.71368	-1.71008	-1.75329	-1.74952	-1.76693
-1.68485	-1.70142	-1.6979	-1.71822	-1.71464	-1.71104	-1.75429	-1.75052	-1.76795
-1.68579	-1.68227	-1.6788	-1.71918	-1.7156	-1.71201	-1.75529	-1.75152	-1.76897
-1.68671	-1.68319	-1.67972	-1.72013	-1.71656	-1.71299	-1.7563	-1.75253	-1.76999
-1.68766	-1.68414	-1.68067	-1.7211	-1.71756	-1.71402	-1.75738	-1.75364	-1.77112
-1.68863	-1.68511	-1.68163	-1.7221	-1.71859	-1.71509	-1.75853	-1.75483	-1.77233
-1.68967	-1.68615	-1.68266	-1.72314	-1.71964	-1.71614	-1.75963	-1.75594	-1.77346
-1.69082	-1.68729	-1.68378	-1.72427	-1.72071	-1.71717	-1.76066	-1.75696	-1.77449
-1.69194	-1.6884	-1.68487	-1.70565	-1.7021	-1.69856	-1.76168	-1.75796	-1.75423
-1.69294	-1.6894	-1.68586	-1.70665	-1.70308	-1.69954	-1.76268	-1.75895	-1.75521
-1.69393	-1.69038	-1.68684	-1.70764	-1.70408	-1.70053	-1.7637	-1.75996	-1.75622
-1.69502	-1.74079	-1.73708	-1.75702	-1.75329	-1.74957	-1.76484	-1.76109	-1.75735
-1.69615	-1.74195	-1.73824	-1.75821	-1.75447	-1.75074	-1.76602	-1.76228	-1.75854
-1.6972	-1.74303	-1.73933	-1.75932	-1.75557	-1.75184	-1.76712	-1.76338	-1.75964
-1.69817	-1.74405	-1.74034	-1.76034	-1.75659	-1.75286	-1.76814	-1.7644	-1.76065
-1.69917	-1.7451	-1.74139	-1.76141	-1.75766	-1.75393	-1.76922	-1.76547	-1.76172
-1.7002	-1.7462	-1.74253	-1.76258	-1.75883	-1.75509	-1.77039	-1.76664	-1.7629
-1.70125	-1.7473	-1.74365	-1.76374	-1.75999	-1.75624	-1.77154	-1.7678	-1.76406
-1.68032	-1.74835	-1.7447	-1.76479	-1.76104	-1.75729	-1.7726	-1.76885	-1.76511
-1.68136	-1.74941	-1.74573	-1.76583	-1.76207	-1.75832	-1.77364	-1.76988	-1.76615
-1.68247	-1.75056	-1.74686	-1.76696	-1.7632	-1.75946	-1.75574	-1.75203	-1.7673
-1.68362	-1.75177	-1.74804	-1.76814	-1.76439	-1.76066	-1.75695	-1.75324	-1.76851
-1.68469	-1.7529	-1.74914	-1.76925	-1.76551	-1.76179	-1.75809	-1.75441	-1.7697
-1.68568	-1.75394	-1.75016	-1.77028	-1.76654	-1.76283	-1.75917	-1.75554	-1.77087
-1.68672	-1.75503	-1.75124	-1.77137	-1.76761	-1.76391	-1.76026	-1.75664	-1.77201
-1.68788	-1.75623	-1.75242	-1.77255	-1.76878	-1.76506	-1.76137	-1.75772	-1.77306
-1.68905	-1.75746	-1.75363	-1.77377	-1.76999	-1.76624	-1.76252	-1.75882	-1.77414
-1.66382	-1.73724	-1.7335	-1.77502	-1.77123	-1.76747	-1.76373	-1.76001	-1.77532
-1.66488	-1.7384	-1.7347	-1.77628	-1.77249	-1.76872	-1.76497	-1.76125	-1.77655
-1.66592	-1.7395	-1.7358	-1.77743	-1.77365	-1.7699	-1.76617	-1.76247	-1.77778
-1.66694	-1.74055	-1.73683	-1.7785	-1.77475	-1.77103	-1.76735	-1.76369	-1.77903
-1.66803	-1.74166	-1.73793	-1.77963	-1.77586	-1.77214	-1.76847	-1.76482	-1.76116
-1.66919	-1.74287	-1.73912	-1.78081	-1.77698	-1.7732	-1.7695	-1.76585	-1.76221
-1.67035	-1.71849	-1.71478	-1.76093	-1.75711	-1.75334	-1.77058	-1.7669	-1.76327
-1.6715	-1.7197	-1.716	-1.76217	-1.75834	-1.75455	-1.77176	-1.76804	-1.76438
-1.67261	-1.7209	-1.71721	-1.76342	-1.75959	-1.75579	-1.77299	-1.76923	-1.76551
-1.67367	-1.72205	-1.71839	-1.74024	-1.73649	-1.73275	-1.75036	-1.74664	-1.76669
-1.6747	-1.72316	-1.71955	-1.74146	-1.73772	-1.73398	-1.75159	-1.74785	-1.7679
-1.64664	-1.72423	-1.72067	-1.74264	-1.73892	-1.7352	-1.75282	-1.74906	-1.76911
-1.64759	-1.72525	-1.72175	-1.74378	-1.74011	-1.73641	-1.75406	-1.75027	-1.77033
-1.61906	-1.72631	-1.72282	-1.7193	-1.71573	-1.71213	-1.73389	-1.73016	-1.77157
-1.62011	-1.72741	-1.72386	-1.72033	-1.71681	-1.71326	-1.73507	-1.73137	-1.77281
-1.59127	-1.70028	-1.69676	-1.72138	-1.71789	-1.71439	-1.73624	-1.73257	-1.77406
-1.59231	-1.70142	-1.69788	-1.72251	-1.71903	-1.71554	-1.73742	-1.73377	-1.77529
-1.59336	-1.70257	-1.69903	-1.72368	-1.72018	-1.71669	-1.73861	-1.73497	-1.77653
-1.54278	-1.67499	-1.6715	-1.69668	-1.69327	-1.68986	-1.7398	-1.73617	-1.77777
-1.54381	-1.67614	-1.67264	-1.69783	-1.69442	-1.69101	-1.741	-1.73737	-1.77901
-1.54484	-1.62811	-1.6247	-1.67033	-1.66698	-1.66362	-1.71667	-1.7131	-1.75914
-1.54586	-1.62923	-1.62582	-1.67148	-1.66811	-1.66475	-1.71783	-1.71427	-1.76035

-1.54689	-1.63035	-1.62694	-1.62358	-1.62029	-1.61701	-1.66248	-1.65906	-1.76155
-1.54794	-1.63149	-1.62806	-1.62469	-1.6214	-1.61813	-1.66362	-1.66019	-1.76276
-1.54899	-1.63263	-1.62918	-1.62581	-1.62252	-1.61924	-1.66476	-1.66133	-1.76397
-1.55005	-1.63378	-1.63032	-1.62692	-1.62363	-1.62035	-1.61702	-1.61368	-1.68745
-1.55111	-1.63494	-1.63145	-1.62805	-1.62475	-1.62146	-1.61813	-1.61478	-1.68861
-1.55217	-1.54899	-1.54586	-1.57366	-1.57057	-1.56749	-1.61924	-1.61589	-1.61252
-1.55323	-1.55004	-1.54689	-1.57472	-1.57162	-1.56855	-1.62035	-1.617	-1.61364
-1.55429	-1.55111	-1.54797	-1.57584	-1.57272	-1.56963	-1.62147	-1.61812	-1.61476
-1.55536	-1.55221	-1.54911	-1.57704	-1.57388	-1.57074	-1.62261	-1.61925	-1.61588
-1.5272	-1.5533	-1.55023	-1.57822	-1.57503	-1.57184	-1.62375	-1.62038	-1.61701
-1.52823	-1.55434	-1.55128	-1.57932	-1.57611	-1.57292	-1.62488	-1.62151	-1.61816
-1.52926	-1.5554	-1.55233	-1.5804	-1.57719	-1.57399	-1.62601	-1.62263	-1.61931
-1.53036	-1.55652	-1.55343	-1.58152	-1.57827	-1.57505	-1.62712	-1.62374	-1.62042
-1.53147	-1.55768	-1.55455	-1.58265	-1.57937	-1.57611	-1.62823	-1.62486	-1.62153
-1.53253	-1.55876	-1.55563	-1.58377	-1.58046	-1.5772	-1.62938	-1.62601	-1.62268
-1.53354	-1.55979	-1.55667	-1.58487	-1.58157	-1.5783	-1.63055	-1.6272	-1.62386
-1.53459	-1.53153	-1.52847	-1.58597	-1.58268	-1.57943	-1.63174	-1.62838	-1.62503
-1.53568	-1.53259	-1.52951	-1.58707	-1.58382	-1.5806	-1.63296	-1.62957	-1.62619
-1.53678	-1.53367	-1.53057	-1.58819	-1.58497	-1.58176	-1.63417	-1.63075	-1.62735
-1.53786	-1.53474	-1.53166	-1.58936	-1.58613	-1.58291	-1.63535	-1.63191	-1.62851
-1.53893	-1.53582	-1.53276	-1.59055	-1.5873	-1.58405	-1.63653	-1.63307	-1.62968
-1.54005	-1.53693	-1.53385	-1.56009	-1.55696	-1.55384	-1.63773	-1.63428	-1.63088
-1.5412	-1.53806	-1.53494	-1.56117	-1.55802	-1.55491	-1.63894	-1.63551	-1.6321
-1.5423	-1.53916	-1.53602	-1.56226	-1.55911	-1.55599	-1.64015	-1.63673	-1.6333
-1.54335	-1.54022	-1.53708	-1.56335	-1.56022	-1.55711	-1.64136	-1.63792	-1.63447
-1.54444	-1.5413	-1.53817	-1.53507	-1.53201	-1.52896	-1.64256	-1.6391	-1.63565
-1.54557	-1.54242	-1.5393	-1.5362	-1.53311	-1.53004	-1.64375	-1.64028	-1.63683
-1.54673	-1.54356	-1.54044	-1.53733	-1.53421	-1.5311	-1.64494	-1.64146	-1.63803
-1.5479	-1.54473	-1.54158	-1.53843	-1.53528	-1.53215	-1.64615	-1.64269	-1.63926
-1.54909	-1.54591	-1.54272	-1.53953	-1.53635	-1.53321	-1.64736	-1.64393	-1.6405
-1.55024	-1.54704	-1.54383	-1.54063	-1.53745	-1.53432	-1.6486	-1.64515	-1.64171
-1.55137	-1.54816	-1.54493	-1.54174	-1.53858	-1.53547	-1.64986	-1.64635	-1.64292
-1.55248	-1.54926	-1.54604	-1.54286	-1.53972	-1.53661	-1.59184	-1.58853	-1.64415
-1.55357	-1.55034	-1.54716	-1.544	-1.54085	-1.53773	-1.59304	-1.58974	-1.64542
-1.47776	-1.52623	-1.52308	-1.54515	-1.54199	-1.53886	-1.59425	-1.59095	-1.64669
-1.47894	-1.52745	-1.52425	-1.54628	-1.54314	-1.54002	-1.59546	-1.59213	-1.64793
-1.48013	-1.52867	-1.52543	-1.54743	-1.5443	-1.54118	-1.59667	-1.59331	-1.64918
-1.48123	-1.52983	-1.5266	-1.52342	-1.52028	-1.51717	-1.59788	-1.59452	-1.62243
-1.48231	-1.53098	-1.52779	-1.52462	-1.52145	-1.51831	-1.59909	-1.59576	-1.62369
-1.48346	-1.53219	-1.52902	-1.52585	-1.52266	-1.5195	-1.57606	-1.57272	-1.59361
-1.48469	-1.53346	-1.53028	-1.5271	-1.52391	-1.52074	-1.57734	-1.57395	-1.59482
-1.4859	-1.48277	-1.4797	-1.52832	-1.52514	-1.52197	-1.57863	-1.5752	-1.57185
-1.48709	-1.48395	-1.48085	-1.52951	-1.52634	-1.52318	-1.57994	-1.57651	-1.57316
-1.38928	-1.48512	-1.48199	-1.53069	-1.52752	-1.52437	-1.58123	-1.57782	-1.57446
-1.39024	-1.48627	-1.48314	-1.53189	-1.5287	-1.52553	-1.58248	-1.5791	-1.57574
-1.39119	-1.48741	-1.4843	-1.5331	-1.52987	-1.52668	-1.58371	-1.58036	-1.577
-1.333	-1.48857	-1.48543	-1.53427	-1.53105	-1.52786	-1.58496	-1.58159	-1.57823
-1.33392	-1.48974	-1.48656	-1.53544	-1.53223	-1.52905	-1.58621	-1.5828	-1.57945
-1.33487	-1.43485	-1.43177	-1.48461	-1.48154	-1.4785	-1.5875	-1.58407	-1.58072
-1.33585	-1.43603	-1.43298	-1.48586	-1.48277	-1.4797	-1.58882	-1.58539	-1.58202

-1.33682	-1.33427	-1.33175	-1.48709	-1.48397	-1.48088	-1.59014	-1.58671	-1.58332
-1.33774	-1.33519	-1.33266	-1.48828	-1.48513	-1.48202	-1.59142	-1.588	-1.58459
-1.33865	-1.3361	-1.33358	-1.48946	-1.4863	-1.48317	-1.59273	-1.5893	-1.58587
-1.30829	-1.30581	-1.30337	-1.43454	-1.4315	-1.4285	-1.53325	-1.53007	-1.5872
-1.30918	-1.30672	-1.30429	-1.43569	-1.43268	-1.42968	-1.53455	-1.53135	-1.58855
-1.31011	-1.30763	-1.30518	-1.33674	-1.33421	-1.33171	-1.50823	-1.5051	-1.58992
-1.31106	-1.30855	-1.30606	-1.33762	-1.3351	-1.33261	-1.50945	-1.50635	-1.5913
-1.31203	-1.3095	-1.30698	-1.3045	-1.30207	-1.29967	-1.40071	-1.39788	-1.56599
-1.31301	-1.31048	-1.30795	-1.30545	-1.303	-1.30057	-1.40177	-1.3989	-1.56723
-1.31397	-1.31144	-1.30891	-1.3064	-1.30393	-1.30149	-1.3329	-1.33041	-1.56849
-1.31488	-1.31235	-1.30982	-1.30732	-1.30486	-1.30242	-1.33386	-1.33136	-1.56982
-1.31579	-1.31325	-1.31073	-1.30825	-1.3058	-1.30337	-1.33484	-1.33233	-1.57118
-1.31675	-1.3142	-1.31168	-1.3092	-1.30676	-1.30433	-1.33582	-1.3333	-1.51918
-1.31773	-1.31517	-1.31265	-1.31016	-1.30772	-1.30528	-1.3368	-1.33427	-1.52049
-1.31873	-1.31615	-1.31361	-1.31111	-1.30863	-1.30617	-1.30372	-1.30128	-1.4687
-1.31975	-1.31714	-1.31458	-1.31204	-1.30951	-1.30701	-1.30456	-1.30214	-1.46988
-1.32074	-1.31812	-1.31555	-1.31299	-1.31043	-1.3079	-1.30544	-1.30302	-1.47107
-1.32169	-1.31909	-1.31653	-1.31398	-1.31141	-1.30887	-1.30639	-1.30395	-1.47228
-1.29073	-1.32006	-1.31752	-1.31497	-1.3124	-1.30985	-1.30735	-1.3049	-1.33643
-1.29172	-1.32106	-1.31851	-1.31595	-1.31337	-1.31081	-1.30831	-1.30584	-1.3374
-1.29273	-1.32208	-1.31951	-1.31694	-1.31435	-1.31179	-1.30927	-1.3068	-1.33838
-1.29373	-1.3231	-1.32052	-1.31794	-1.31535	-1.31278	-1.31025	-1.30777	-1.33936
-1.29474	-1.32413	-1.32153	-1.31895	-1.31635	-1.31377	-1.31124	-1.30874	-1.34036
-1.22157	-1.29318	-1.29064	-1.31995	-1.31734	-1.31475	-1.31222	-1.30971	-1.30726
-1.22246	-1.29416	-1.29163	-1.32095	-1.31832	-1.31572	-1.31318	-1.31068	-1.30823
-1.19129	-1.29516	-1.29263	-1.29009	-1.28753	-1.28498	-1.31417	-1.31167	-1.3092
-1.1922	-1.29618	-1.29365	-1.29112	-1.28857	-1.28604	-1.31523	-1.31269	-1.3102
-1.19312	-1.2972	-1.29467	-1.29215	-1.28962	-1.2871	-1.3163	-1.31372	-1.3112
-1.15339	-1.19167	-1.18938	-1.26232	-1.25983	-1.25734	-1.28557	-1.28305	-1.31219
-1.15427	-1.19257	-1.19027	-1.2633	-1.26081	-1.25833	-1.28657	-1.28403	-1.31319
-1.1552	-1.19352	-1.19119	-1.26429	-1.2618	-1.25931	-1.2568	-1.25429	-1.2825
-1.15617	-1.1945	-1.19212	-1.26529	-1.26279	-1.26029	-1.25776	-1.25524	-1.28345
-1.12016	-1.19547	-1.19305	-1.26628	-1.26378	-1.26129	-1.25876	-1.25623	-1.25376
-1.12106	-1.19643	-1.19397	-1.26728	-1.26477	-1.26229	-1.25979	-1.25729	-1.25481
-1.12196	-1.15659	-1.15421	-1.19249	-1.19017	-1.18789	-1.2608	-1.25834	-1.25585
-1.12289	-1.15755	-1.15514	-1.19343	-1.19108	-1.18877	-1.26179	-1.25935	-1.25688
-1.12382	-1.15852	-1.15609	-1.19439	-1.192	-1.18966	-1.26278	-1.26034	-1.2579
-1.12474	-1.12243	-1.12009	-1.19542	-1.19297	-1.19058	-1.26376	-1.26131	-1.25886
-1.12566	-1.12336	-1.12105	-1.19647	-1.19397	-1.19151	-1.26475	-1.26227	-1.25981
-1.12656	-1.12426	-1.12197	-1.15666	-1.15426	-1.1519	-1.19011	-1.18779	-1.26079
-1.12744	-1.12514	-1.12287	-1.1576	-1.15523	-1.15288	-1.19111	-1.18873	-1.26179
-1.12835	-1.12604	-1.12376	-1.12149	-1.11921	-1.11694	-1.1921	-1.18969	-1.26284
-1.1293	-1.12696	-1.12466	-1.12237	-1.12009	-1.11784	-1.19309	-1.19069	-1.26396
-1.05665	-1.12791	-1.12557	-1.12326	-1.12098	-1.11873	-1.15338	-1.1511	-1.18935
-1.05756	-1.12889	-1.12652	-1.12418	-1.12188	-1.1196	-1.15427	-1.15198	-1.19028
-1.05847	-1.12987	-1.12749	-1.12513	-1.1228	-1.12049	-1.15518	-1.15287	-1.19119
-1.05933	-1.05714	-1.05493	-1.12613	-1.12379	-1.12147	-1.11917	-1.1169	-1.19214
-1.06018	-1.05801	-1.05584	-1.12715	-1.12482	-1.12249	-1.12015	-1.11784	-1.1931
-0.8969	-1.05886	-1.05671	-1.05455	-1.05238	-1.0502	-1.12112	-1.11879	-1.19412
-0.89756	-1.05968	-1.05754	-1.05539	-1.05323	-1.05107	-1.12209	-1.11978	-1.1952

-0.89826	-0.97865	-0.97669	-1.05623	-1.05407	-1.05192	-1.04976	-1.04761	-1.11845
-0.89903	-0.97949	-0.97748	-1.05706	-1.05489	-1.05273	-1.05057	-1.04843	-1.11933
-0.8998	-0.98032	-0.97828	-1.05792	-1.05573	-1.05356	-1.0514	-1.04926	-1.12023
-0.85684	-0.89868	-0.89687	-1.01997	-1.01785	-1.01574	-1.0523	-1.05016	-1.04801
-0.85746	-0.89938	-0.89759	-1.02086	-1.01874	-1.01664	-1.05323	-1.05107	-1.04892
-0.82033	-0.90006	-0.89826	-0.97869	-0.9767	-0.97473	-1.01538	-1.0133	-1.04977
-0.82098	-0.90073	-0.8989	-0.97942	-0.97744	-0.97548	-1.01619	-1.0141	-1.0506
-0.82164	-0.81995	-0.81829	-0.86099	-0.85929	-0.85762	-0.97431	-0.97235	-1.01288
-0.82233	-0.82066	-0.819	-0.86176	-0.86004	-0.85835	-0.97516	-0.9732	-1.01378
-0.78539	-0.82135	-0.81969	-0.86251	-0.86078	-0.85907	-0.94079	-0.9389	-1.01466
-0.78604	-0.82197	-0.82031	-0.86319	-0.86145	-0.85973	-0.94155	-0.93966	-1.01548
-0.7867	-0.82261	-0.82094	-0.86388	-0.86213	-0.8604	-0.94231	-0.94042	-1.01631
-0.78738	-0.78572	-0.78408	-0.81999	-0.81836	-0.81674	-0.85944	-0.85775	-0.93935
-0.78808	-0.78643	-0.7848	-0.82071	-0.81908	-0.81746	-0.86021	-0.8585	-0.94019
-0.78876	-0.78709	-0.78546	-0.78384	-0.78224	-0.78064	-0.86092	-0.85919	-0.8575
-0.78942	-0.78773	-0.78608	-0.78445	-0.78285	-0.78126	-0.86158	-0.85985	-0.85814
-0.74661	-0.78841	-0.78674	-0.78511	-0.7835	-0.78191	-0.82564	-0.82393	-0.85884
-0.74728	-0.78913	-0.78746	-0.78582	-0.78422	-0.78262	-0.82642	-0.8247	-0.85959
-0.70456	-0.74635	-0.74476	-0.78655	-0.78494	-0.78334	-0.78174	-0.78014	-0.82378
-0.70525	-0.74705	-0.74546	-0.78729	-0.78567	-0.78406	-0.78246	-0.78086	-0.82456
-0.70592	-0.74773	-0.74614	-0.78801	-0.78638	-0.78477	-0.78316	-0.78156	-0.82533
-0.70653	-0.74835	-0.74675	-0.74518	-0.74362	-0.74208	-0.7838	-0.7822	-0.82603
-0.70712	-0.74894	-0.74734	-0.74576	-0.74419	-0.74264	-0.7844	-0.7828	-0.8267
-0.66066	-0.70622	-0.70469	-0.7464	-0.74483	-0.74327	-0.74174	-0.74022	-0.78186
-0.66131	-0.70691	-0.70537	-0.7471	-0.74552	-0.74395	-0.74241	-0.74088	-0.78257
-0.5643	-0.66054	-0.65913	-0.70454	-0.70303	-0.70154	-0.74308	-0.74154	-0.74002
-0.56487	-0.66121	-0.65979	-0.70523	-0.70371	-0.70221	-0.74377	-0.74222	-0.74069
-0.51247	-0.56418	-0.56295	-0.65901	-0.65762	-0.65624	-0.74443	-0.74287	-0.74133
-0.51289	-0.56467	-0.56343	-0.65957	-0.65818	-0.6568	-0.74503	-0.74346	-0.74192
-0.5133	-0.56515	-0.5639	-0.66013	-0.65873	-0.65734	-0.74562	-0.74405	-0.7425
-0.51376	-0.56568	-0.56443	-0.66075	-0.65934	-0.65795	-0.65657	-0.6552	-0.74314
-0.51424	-0.56625	-0.56499	-0.66142	-0.65999	-0.65858	-0.6572	-0.65583	-0.74382
-0.51472	-0.51367	-0.51264	-0.56431	-0.56308	-0.56186	-0.65783	-0.65646	-0.65509
-0.5152	-0.51414	-0.51311	-0.56487	-0.56362	-0.56239	-0.65846	-0.65709	-0.65572
-0.47114	-0.51462	-0.51358	-0.51255	-0.51153	-0.51051	-0.61571	-0.61441	-0.65635
-0.4716	-0.51512	-0.51407	-0.51303	-0.512	-0.51099	-0.61633	-0.61501	-0.65699
-0.47207	-0.51561	-0.51455	-0.51351	-0.51248	-0.51146	-0.61695	-0.61562	-0.65763
-0.42138	-0.47153	-0.47055	-0.51399	-0.51296	-0.51194	-0.5634	-0.56218	-0.65827
-0.42176	-0.47198	-0.471	-0.51447	-0.51344	-0.51241	-0.56397	-0.56273	-0.65892
-0.42214	-0.42126	-0.42039	-0.47046	-0.4695	-0.46856	-0.51186	-0.51085	-0.61614
-0.42252	-0.42163	-0.42075	-0.47087	-0.46992	-0.46898	-0.51232	-0.51133	-0.61677
-0.42291	-0.42201	-0.42113	-0.4713	-0.47035	-0.46941	-0.46848	-0.46756	-0.51079
-0.42334	-0.42243	-0.42154	-0.47175	-0.47077	-0.46982	-0.46889	-0.46798	-0.51124
-0.42376	-0.42285	-0.42195	-0.42106	-0.42018	-0.41932	-0.46932	-0.46841	-0.46749
-0.42418	-0.42327	-0.42236	-0.42146	-0.42058	-0.41972	-0.46977	-0.46885	-0.46793
-0.4246	-0.42368	-0.42277	-0.42187	-0.42099	-0.42013	-0.47023	-0.4693	-0.46837
-0.42503	-0.4241	-0.42319	-0.42229	-0.4214	-0.42053	-0.41969	-0.41885	-0.46882
-0.42545	-0.42453	-0.42361	-0.4227	-0.42181	-0.42094	-0.42009	-0.41925	-0.46927
-0.42589	-0.42495	-0.42403	-0.42312	-0.42223	-0.42135	-0.42049	-0.41965	-0.41882
-0.42632	-0.42539	-0.42445	-0.42354	-0.42264	-0.42176	-0.4209	-0.42006	-0.41922

-0.42676	-0.42582	-0.42488	-0.42396	-0.42306	-0.42217	-0.42131	-0.42047	-0.41962
-0.42719	-0.42624	-0.42531	-0.42438	-0.42348	-0.42259	-0.42173	-0.42088	-0.42003
-0.37907	-0.42668	-0.42573	-0.42481	-0.4239	-0.42301	-0.42214	-0.42129	-0.42044
-0.37946	-0.42711	-0.42617	-0.42524	-0.42432	-0.42343	-0.42256	-0.4217	-0.42085
-0.37986	-0.42756	-0.42661	-0.42567	-0.42475	-0.42385	-0.42298	-0.42212	-0.42126
-0.33157	-0.428	-0.42704	-0.4261	-0.42519	-0.42429	-0.42341	-0.42254	-0.42168
-0.33191	-0.42844	-0.42748	-0.42654	-0.42564	-0.42474	-0.42386	-0.42298	-0.42211
-0.33227	-0.38018	-0.37932	-0.42698	-0.42607	-0.42518	-0.42429	-0.42341	-0.42253
-0.33262	-0.38057	-0.37971	-0.42742	-0.42651	-0.42561	-0.42471	-0.42383	-0.42294
-0.33298	-0.33223	-0.33148	-0.42788	-0.42695	-0.42604	-0.42514	-0.42425	-0.42336
-0.33335	-0.3326	-0.33186	-0.42836	-0.42743	-0.4265	-0.42559	-0.42468	-0.42378
-0.33372	-0.33297	-0.33224	-0.42884	-0.4279	-0.42697	-0.42604	-0.42511	-0.4242
-0.33409	-0.33333	-0.3326	-0.38053	-0.37968	-0.37884	-0.42647	-0.42554	-0.42462
-0.33446	-0.3337	-0.33296	-0.38093	-0.38008	-0.37923	-0.42691	-0.42596	-0.42504
-0.33485	-0.33408	-0.33334	-0.3326	-0.33185	-0.33111	-0.42735	-0.4264	-0.42548
-0.33527	-0.33449	-0.33373	-0.33297	-0.33222	-0.33147	-0.4278	-0.42685	-0.42593
-0.33567	-0.33488	-0.33411	-0.33335	-0.33259	-0.33183	-0.37958	-0.37874	-0.42639
-0.33607	-0.33526	-0.33448	-0.33371	-0.33295	-0.3322	-0.37999	-0.37915	-0.42684
-0.29372	-0.33566	-0.33486	-0.33408	-0.33332	-0.33257	-0.33183	-0.3311	-0.4273
-0.29411	-0.33608	-0.33526	-0.33446	-0.3337	-0.33295	-0.33221	-0.33148	-0.42777
-0.2945	-0.33652	-0.33567	-0.33486	-0.33408	-0.33332	-0.33258	-0.33185	-0.42825
-0.2409	-0.33695	-0.3361	-0.33527	-0.33448	-0.33371	-0.33295	-0.33222	-0.37997
-0.24122	-0.33738	-0.33653	-0.3357	-0.33489	-0.33409	-0.33333	-0.33258	-0.38038
-0.24153	-0.28484	-0.2841	-0.33612	-0.3353	-0.33448	-0.3337	-0.33294	-0.33219
-0.24185	-0.2852	-0.28446	-0.33654	-0.3357	-0.33488	-0.33408	-0.33331	-0.33256
-0.24217	-0.2416	-0.24103	-0.33697	-0.33612	-0.33529	-0.33448	-0.33369	-0.33293
-0.24249	-0.24193	-0.24136	-0.33742	-0.33656	-0.33571	-0.33489	-0.33409	-0.33332
-0.24282	-0.24226	-0.24169	-0.28485	-0.2841	-0.28336	-0.33531	-0.3345	-0.33372
-0.24314	-0.24258	-0.24201	-0.28523	-0.28447	-0.28373	-0.33574	-0.33492	-0.33413
-0.24347	-0.2429	-0.24233	-0.2856	-0.28485	-0.2841	-0.33618	-0.33536	-0.33455
-0.24379	-0.24322	-0.24265	-0.24209	-0.24153	-0.24098	-0.33663	-0.3358	-0.33498
-0.24412	-0.24355	-0.24298	-0.24242	-0.24186	-0.24131	-0.33709	-0.33625	-0.33542
-0.24445	-0.24388	-0.24331	-0.24275	-0.24219	-0.24164	-0.28453	-0.28379	-0.33586
-0.24478	-0.24421	-0.24365	-0.24309	-0.24253	-0.24196	-0.28491	-0.28417	-0.33629
-0.24512	-0.24455	-0.24399	-0.24342	-0.24285	-0.24229	-0.24172	-0.24116	-0.33673
-0.24548	-0.2449	-0.24433	-0.24376	-0.24318	-0.24261	-0.24204	-0.24148	-0.33717
-0.24583	-0.24525	-0.24467	-0.24409	-0.24351	-0.24294	-0.24237	-0.2418	-0.33762
-0.24618	-0.24559	-0.24501	-0.24442	-0.24384	-0.24326	-0.24269	-0.24212	-0.28494
-0.24652	-0.24593	-0.24535	-0.24476	-0.24417	-0.24359	-0.24301	-0.24245	-0.28533
-0.24687	-0.24628	-0.24568	-0.24509	-0.24451	-0.24392	-0.24335	-0.24278	-0.28573
-0.24721	-0.24662	-0.24602	-0.24543	-0.24484	-0.24426	-0.24369	-0.24313	-0.28614
-0.24755	-0.24695	-0.24636	-0.24577	-0.24519	-0.24461	-0.24403	-0.24347	-0.2429
-0.24788	-0.24729	-0.2467	-0.24611	-0.24554	-0.24496	-0.24438	-0.2438	-0.24323
-0.24822	-0.24763	-0.24704	-0.24646	-0.24588	-0.24531	-0.24472	-0.24414	-0.24357
-0.24858	-0.24799	-0.2474	-0.24681	-0.24623	-0.24564	-0.24506	-0.24449	-0.24391
-0.24894	-0.24835	-0.24775	-0.24716	-0.24657	-0.24598	-0.24541	-0.24483	-0.24426
-0.24928	-0.24869	-0.2481	-0.24751	-0.24692	-0.24633	-0.24575	-0.24518	-0.2446
-0.24962	-0.24902	-0.24844	-0.24786	-0.24727	-0.24669	-0.2461	-0.24552	-0.24494
-0.24998	-0.24938	-0.2488	-0.24822	-0.24763	-0.24705	-0.24646	-0.24588	-0.2453
-0.25036	-0.24976	-0.24917	-0.24858	-0.248	-0.24743	-0.24684	-0.24625	-0.24566

-0.25072	-0.25013	-0.24953	-0.24895	-0.24837	-0.24779	-0.2472	-0.24661	-0.24602
-0.25108	-0.25048	-0.24989	-0.2493	-0.24871	-0.24813	-0.24754	-0.24696	-0.24637
-0.25143	-0.25083	-0.25024	-0.24965	-0.24906	-0.24847	-0.24789	-0.24731	-0.24672
-0.2518	-0.25119	-0.25059	-0.25	-0.24941	-0.24882	-0.24824	-0.24765	-0.24707
-0.25216	-0.25155	-0.25095	-0.25035	-0.24976	-0.24917	-0.24858	-0.24799	-0.24741
-0.25253	-0.25191	-0.2513	-0.2507	-0.25011	-0.24953	-0.24893	-0.24834	-0.24776
-0.25289	-0.25227	-0.25166	-0.25106	-0.25047	-0.24988	-0.24929	-0.2487	-0.24812
-0.25326	-0.25263	-0.25203	-0.25143	-0.25083	-0.25024	-0.24965	-0.24906	-0.24848
-0.25363	-0.25301	-0.2524	-0.25179	-0.2512	-0.25061	-0.25003	-0.24944	-0.24885
-0.25402	-0.25339	-0.25277	-0.25216	-0.25157	-0.25098	-0.2504	-0.24981	-0.24922
-0.25443	-0.25379	-0.25316	-0.25254	-0.25194	-0.25134	-0.25075	-0.25016	-0.24956
-0.25482	-0.25419	-0.25355	-0.25292	-0.2523	-0.2517	-0.2511	-0.25051	-0.24991
-0.2552	-0.25456	-0.25392	-0.25329	-0.25267	-0.25206	-0.25146	-0.25087	-0.25028
-0.25557	-0.25493	-0.2543	-0.25367	-0.25304	-0.25243	-0.25183	-0.25124	-0.25065
-0.25594	-0.25531	-0.25467	-0.25405	-0.25342	-0.2528	-0.2522	-0.2516	-0.25101
-0.25631	-0.25568	-0.25505	-0.25443	-0.2538	-0.25318	-0.25257	-0.25196	-0.25137
-0.1928	-0.25607	-0.25544	-0.25481	-0.25418	-0.25356	-0.25294	-0.25233	-0.25173
-0.19308	-0.25647	-0.25584	-0.2552	-0.25456	-0.25393	-0.25331	-0.25269	-0.25209
-0.19337	-0.25686	-0.25623	-0.25559	-0.25495	-0.25431	-0.25368	-0.25307	-0.25246
-0.19364	-0.25724	-0.25662	-0.25599	-0.25535	-0.25471	-0.25408	-0.25346	-0.25285
-0.19391	-0.25762	-0.257	-0.25638	-0.25574	-0.25511	-0.25448	-0.25385	-0.25324
-0.1942	-0.19373	-0.19327	-0.25675	-0.25613	-0.2555	-0.25487	-0.25424	-0.25362
-0.1945	-0.19402	-0.19355	-0.25713	-0.2565	-0.25588	-0.25525	-0.25463	-0.254
-0.1948	-0.19431	-0.19383	-0.25752	-0.25689	-0.25627	-0.25564	-0.25502	-0.25439
-0.19509	-0.19461	-0.19413	-0.25792	-0.25729	-0.25667	-0.25604	-0.25541	-0.25478
-0.19539	-0.1949	-0.19441	-0.19394	-0.19347	-0.193	-0.25643	-0.2558	-0.25517
-0.19569	-0.19519	-0.19469	-0.19421	-0.19375	-0.19329	-0.25682	-0.25618	-0.25554
-0.196	-0.19549	-0.19498	-0.1945	-0.19404	-0.19358	-0.25721	-0.25657	-0.25593
-0.19632	-0.1958	-0.19529	-0.1948	-0.19434	-0.19388	-0.25761	-0.25697	-0.25633
-0.19663	-0.19612	-0.19561	-0.19511	-0.19464	-0.19417	-0.25802	-0.25737	-0.25673
-0.19694	-0.19643	-0.19592	-0.19543	-0.19495	-0.19447	-0.25842	-0.25778	-0.25714
-0.19723	-0.19673	-0.19623	-0.19574	-0.19526	-0.19478	-0.25884	-0.25818	-0.25754
-0.12993	-0.19704	-0.19655	-0.19606	-0.19557	-0.19509	-0.19459	-0.1941	-0.25795
-0.13013	-0.19736	-0.19687	-0.19638	-0.19589	-0.1954	-0.1949	-0.1944	-0.25836
-0.13032	-0.19767	-0.19718	-0.19669	-0.19621	-0.19571	-0.19521	-0.19471	-0.25877
-0.13051	-0.19798	-0.1975	-0.19701	-0.19652	-0.19603	-0.19552	-0.19501	-0.25919
-0.13071	-0.1983	-0.19781	-0.19732	-0.19683	-0.19633	-0.19583	-0.19532	-0.2596
-0.1309	-0.1306	-0.13031	-0.19764	-0.19713	-0.19663	-0.19612	-0.19562	-0.19512
-0.1311	-0.1308	-0.1305	-0.19795	-0.19743	-0.19692	-0.19641	-0.19591	-0.19542
-0.13129	-0.131	-0.1307	-0.19826	-0.19775	-0.19723	-0.19672	-0.19622	-0.19573
-0.13149	-0.13119	-0.1309	-0.19858	-0.19807	-0.19755	-0.19704	-0.19653	-0.19604
	-0.13139	-0.13109	-0.13079	-0.13049	-0.13018	-0.19737	-0.19685	-0.19636
	-0.13159	-0.13129	-0.13099	-0.13069	-0.13039	-0.19771	-0.19719	-0.19668
			-0.13119	-0.13089	-0.13059	-0.19804	-0.19752	-0.19701
			-0.13139	-0.13109	-0.13079	-0.19836	-0.19784	-0.19733
			-0.13159	-0.13129	-0.13099	-0.19868	-0.19816	-0.19765
			-0.13179	-0.13149	-0.13119	-0.13088	-0.13057	-0.19798
			-0.13199	-0.13169	-0.13139	-0.13108	-0.13078	-0.19832
						-0.13129	-0.13098	-0.13067
						-0.13149	-0.13118	-0.13087

-0.13107
-0.13128
-0.06402
-0.06411
-0.0642

								-0.05994
								-0.05984
								-0.05974
								-0.44376
								-0.44304
						-0.19256	-0.19199	-0.4423
						-0.19227	-0.19169	-0.44156
			-0.1937	-0.19312	-0.19253	-0.44321	-0.442	-0.61454
			-0.19336	-0.19277	-0.19219	-0.4424	-0.44118	-0.61344
			-0.19301	-0.19243	-0.19186	-0.4416	-0.44039	-0.61236
	-0.06023	-0.06005	-0.44452	-0.44329	-0.44207	-0.61471	-0.61302	-0.66155
	-0.06013	-0.05996	-0.44378	-0.44256	-0.44134	-0.61373	-0.61206	-0.66057
-0.06021	-0.19325	-0.19268	-0.44304	-0.44182	-0.44062	-0.61275	-0.61111	-0.7618
-0.06013	-0.19295	-0.19237	-0.4423	-0.44109	-0.43991	-0.61179	-0.61017	-0.76069
-0.06004	-0.444	-0.44278	-0.61582	-0.61412	-0.61245	-0.66102	-0.65935	-0.85582
-0.05995	-0.44327	-0.44205	-0.61483	-0.61314	-0.61149	-0.66004	-0.65839	-0.85461
-0.38104	-0.61724	-0.61552	-0.6642	-0.66244	-0.66071	-0.85747	-0.85541	-0.85336
-0.38042	-0.61622	-0.61451	-0.66315	-0.66139	-0.65967	-0.85614	-0.85409	-0.85204
-0.37981	-0.61519	-0.61349	-0.6621	-0.66035	-0.65864	-0.85483	-0.85278	-0.85073
-0.44147	-0.66455	-0.66277	-0.76328	-0.76133	-0.75942	-0.85359	-0.85154	-0.8495

-0.44069	-0.66344	-0.66168	-0.76204	-0.76015	-0.7583	-0.85239	-0.85035	-0.8483
-0.61379	-0.6624	-0.66067	-0.8573	-0.85525	-0.85323	-0.85119	-0.84914	-0.89208
-0.61283	-0.66143	-0.65972	-0.85609	-0.85405	-0.85203	-0.84998	-0.84794	-0.89081
-0.66219	-0.85909	-0.85697	-0.85489	-0.85285	-0.85083	-0.84878	-0.84673	-0.94096
-0.66115	-0.85783	-0.85574	-0.85369	-0.85166	-0.84963	-0.84758	-0.84551	-0.9396
-0.85868	-0.85658	-0.85453	-0.85249	-0.85046	-0.84843	-0.89131	-0.88914	-0.93823
-0.85745	-0.85536	-0.85332	-0.8513	-0.84926	-0.84721	-0.89003	-0.88785	-0.93687
-0.85623	-0.85415	-0.85212	-0.8501	-0.84804	-0.84598	-0.88874	-0.88656	-0.9355
-0.85502	-0.85295	-0.85092	-0.89393	-0.89177	-0.88961	-0.93872	-0.93642	-0.98021
-0.85381	-0.85175	-0.84971	-0.89264	-0.89049	-0.88832	-0.93734	-0.93503	-0.97878
-0.85258	-0.85054	-0.84849	-0.94288	-0.94058	-0.93828	-0.93597	-0.93366	-0.97736
-0.85136	-0.84932	-0.84727	-0.94151	-0.93921	-0.93691	-0.9346	-0.93229	-0.97595
-0.85013	-0.89309	-0.89095	-0.94014	-0.93785	-0.93555	-0.97928	-0.97689	-1.02158
-0.84893	-0.89181	-0.88967	-0.93879	-0.93651	-0.93423	-0.97792	-0.97554	-1.02017
-0.84772	-0.94198	-0.9397	-0.93744	-0.93518	-0.93292	-0.97657	-0.97421	-1.01878
-0.84652	-0.94062	-0.93835	-0.93609	-0.93385	-0.93162	-0.97523	-0.97289	-1.01739
-0.84531	-0.93927	-0.937	-0.93476	-0.93255	-0.93034	-0.9739	-0.97157	-1.016
-0.94022	-0.93793	-0.93568	-0.97947	-0.97718	-0.9749	-1.01948	-1.01703	-1.01462
-0.93887	-0.9366	-0.93437	-0.97812	-0.97584	-0.97357	-1.01809	-1.01565	-1.01323
-0.93754	-0.93528	-0.93307	-0.97678	-0.97451	-0.97224	-1.0167	-1.01426	-1.07296
-0.93621	-0.93396	-0.93177	-0.97544	-0.97318	-0.97092	-1.01531	-1.01286	-1.07153
-0.93489	-0.97863	-0.97635	-1.02107	-1.0187	-1.01633	-1.07519	-1.07263	-1.11018
-0.93358	-0.97727	-0.97501	-1.01966	-1.0173	-1.01493	-1.07376	-1.0712	-1.10867
-0.93229	-0.97592	-0.97367	-1.01825	-1.0159	-1.01354	-1.07233	-1.06978	-1.10717
-0.93104	-0.97461	-0.97234	-1.01684	-1.01449	-1.01214	-1.07091	-1.06837	-1.1057
-0.92981	-0.97331	-0.97102	-1.01543	-1.01309	-1.01075	-1.0695	-1.06697	-1.10424
-0.92856	-1.01884	-1.01641	-1.07544	-1.073	-1.07057	-1.10811	-1.10547	-1.10282
-0.92729	-1.01745	-1.01502	-1.07399	-1.07156	-1.06916	-1.10665	-1.10406	-1.10144
-0.97172	-1.07764	-1.07509	-1.11289	-1.1103	-1.10774	-1.10518	-1.10262	-1.18573
-0.97046	-1.07629	-1.07373	-1.11143	-1.10882	-1.10624	-1.10369	-1.10114	-1.18398
-1.07743	-1.1154	-1.11267	-1.10998	-1.10735	-1.10475	-1.18822	-1.18524	-1.22393
-1.07599	-1.11389	-1.11119	-1.10851	-1.10586	-1.10325	-1.18643	-1.18349	-1.22208
-1.07454	-1.11238	-1.10971	-1.10705	-1.10439	-1.10177	-1.18467	-1.18177	-1.22026
-1.11351	-1.11088	-1.10825	-1.10562	-1.10297	-1.10035	-1.22471	-1.22159	-1.21847
-1.11202	-1.10939	-1.1068	-1.10422	-1.10158	-1.09896	-1.22293	-1.21979	-1.21669
-1.11055	-1.10792	-1.10535	-1.23099	-1.22765	-1.22435	-1.22114	-1.21798	-1.2149
-1.10911	-1.10647	-1.10389	-1.22908	-1.22579	-1.22254	-1.21933	-1.21617	-1.21309
-1.10766	-1.19141	-1.18833	-1.2272	-1.22397	-1.22077	-1.21758	-1.21442	-1.21134
-1.1062	-1.18965	-1.18657	-1.22533	-1.22218	-1.21907	-1.21591	-1.21275	-1.20966
-1.10476	-1.23004	-1.22673	-1.22351	-1.22041	-1.21736	-1.21424	-1.2111	-1.29471
-1.10337	-1.22826	-1.22499	-1.22178	-1.21867	-1.21559	-1.21252	-1.20945	-1.29283
-1.102	-1.22651	-1.22329	-1.2201	-1.21694	-1.21382	-1.21078	-1.20779	-1.29096
-1.22794	-1.22472	-1.22156	-1.21842	-1.21525	-1.21211	-1.25043	-1.24717	-1.28901
-1.22609	-1.22291	-1.21982	-1.21673	-1.21357	-1.21043	-1.24856	-1.24528	-1.28701
-1.2243	-1.22114	-1.21807	-1.215	-1.21186	-1.20873	-1.29207	-1.28853	-1.28505
-1.22258	-1.21942	-1.2163	-1.2132	-1.21009	-1.207	-1.29009	-1.28655	-1.28311
-1.22087	-1.21771	-1.21457	-1.25307	-1.2497	-1.24635	-1.28815	-1.28462	-1.32206
-1.21917	-1.21602	-1.21289	-1.25124	-1.24788	-1.24457	-1.28628	-1.28278	-1.32014
-1.21749	-1.21434	-1.21123	-1.24944	-1.24609	-1.24279	-1.28442	-1.28096	-1.31825
-1.21581	-1.25443	-1.25103	-1.29314	-1.28956	-1.28603	-1.28257	-1.27916	-1.31642

-1.21412	-1.25259	-1.24923	-1.29126	-1.28767	-1.28414	-1.28071	-1.27737	-1.31461
-1.2124	-1.25072	-1.24737	-1.28929	-1.28575	-1.28226	-1.27885	-1.27553	-1.31275
-1.21065	-1.24884	-1.24548	-1.28728	-1.2838	-1.28038	-1.27699	-1.27365	-1.31088
-1.25036	-1.29247	-1.28888	-1.28535	-1.28191	-1.27854	-1.31577	-1.31234	-1.30909
-1.24854	-1.29055	-1.28699	-1.2835	-1.28009	-1.27674	-1.31395	-1.31058	-1.30738
-1.29221	-1.28865	-1.28513	-1.28168	-1.2783	-1.27498	-1.31218	-1.30888	-1.30574
-1.29029	-1.28676	-1.2833	-1.2799	-1.27653	-1.27324	-1.31045	-1.30724	-1.30417
-1.28841	-1.2849	-1.28149	-1.27813	-1.2748	-1.27155	-1.30877	-1.30565	-1.30266
-1.28657	-1.2831	-1.2797	-1.31699	-1.31358	-1.31028	-1.30711	-1.30407	-1.30117
-1.28476	-1.28133	-1.27794	-1.31518	-1.31183	-1.3086	-1.3055	-1.30253	-1.29971
-1.28294	-1.27955	-1.2762	-1.31342	-1.31014	-1.30698	-1.30396	-1.30107	-1.29831
-1.28112	-1.27777	-1.27448	-1.31171	-1.30851	-1.30543	-1.30249	-1.29969	-1.29697
-1.27933	-1.31661	-1.31327	-1.31003	-1.30691	-1.30391	-1.30105	-1.29832	-1.36868
-1.27757	-1.31483	-1.31153	-1.30836	-1.30533	-1.30242	-1.29964	-1.29696	-1.36726
-1.27584	-1.31309	-1.30985	-1.30674	-1.30379	-1.30097	-1.29825	-1.29562	-1.40351
-1.27415	-1.31137	-1.30822	-1.3052	-1.30233	-1.29957	-1.29689	-1.29429	-1.40206
-1.27251	-1.3097	-1.30664	-1.30371	-1.3009	-1.29819	-1.29555	-1.29298	-1.40062
-1.27096	-1.30815	-1.30513	-1.30224	-1.29949	-1.29684	-1.3671	-1.36436	-1.39919
-1.26948	-1.30669	-1.30368	-1.3008	-1.29811	-1.29552	-1.36569	-1.36295	-1.39776
-1.30826	-1.30523	-1.30229	-1.37268	-1.36981	-1.36704	-1.36428	-1.36157	-1.39635
-1.30668	-1.30376	-1.30096	-1.37132	-1.36844	-1.36562	-1.36288	-1.36021	-1.39497
-1.30515	-1.30232	-1.29962	-1.36996	-1.36708	-1.36424	-1.39905	-1.39631	-1.39358
-1.30369	-1.30092	-1.29827	-1.36855	-1.36573	-1.36293	-1.39769	-1.3949	-1.39217
-1.30227	-1.29954	-1.29693	-1.36713	-1.36437	-1.36163	-1.39634	-1.39352	-1.39079
-1.30087	-1.37126	-1.36846	-1.36571	-1.36299	-1.3603	-1.395	-1.3922	-1.38945
-1.29951	-1.36983	-1.36704	-1.36429	-1.36161	-1.35895	-1.39365	-1.3909	-1.38815
-1.37119	-1.3684	-1.36563	-1.40051	-1.39774	-1.395	-1.39227	-1.38956	-1.38683
-1.36976	-1.36696	-1.36424	-1.39911	-1.39633	-1.39357	-1.39086	-1.38819	-1.3855
-1.36838	-1.36558	-1.36289	-1.39774	-1.39496	-1.39221	-1.38949	-1.38682	-1.38416
-1.36707	-1.36428	-1.36159	-1.3964	-1.39365	-1.39091	-1.38817	-1.38544	-1.38278
-1.36576	-1.40059	-1.3978	-1.39504	-1.39231	-1.3896	-1.38685	-1.3841	-1.38144
-1.36443	-1.39923	-1.39641	-1.39363	-1.39092	-1.38825	-1.38554	-1.38283	-1.38016
-1.36309	-1.39787	-1.39502	-1.39222	-1.38953	-1.38688	-1.38422	-1.38156	-1.3789
-1.36172	-1.39649	-1.39367	-1.39089	-1.38817	-1.3855	-1.38286	-1.38025	-1.37763
-1.36033	-1.39511	-1.39234	-1.38959	-1.38683	-1.38412	-1.38149	-1.37892	-1.37635
-1.35894	-1.39368	-1.39097	-1.38825	-1.38551	-1.3828	-1.38017	-1.3776	-1.44474
-1.35754	-1.39223	-1.38956	-1.38689	-1.38421	-1.38153	-1.37888	-1.37627	-1.44328
-1.3936	-1.39084	-1.3882	-1.38557	-1.38292	-1.38028	-1.37763	-1.37498	-1.44186
-1.39229	-1.38955	-1.3869	-1.38428	-1.38166	-1.37904	-1.3764	-1.37376	-1.4405
-1.391	-1.38826	-1.3856	-1.38298	-1.38038	-1.37779	-1.41128	-1.4085	-1.51596
-1.38968	-1.38694	-1.38426	-1.38163	-1.37906	-1.37651	-1.40996	-1.40721	-1.51435
-1.38837	-1.38562	-1.38292	-1.38028	-1.37773	-1.37521	-1.40862	-1.40592	-1.51275
-1.3871	-1.38435	-1.38165	-1.37899	-1.37642	-1.37388	-1.44067	-1.43793	-1.51112
-1.38585	-1.38311	-1.38041	-1.37774	-1.37513	-1.37255	-1.43922	-1.43652	-1.50949
-1.38458	-1.38187	-1.37917	-1.41271	-1.40991	-1.40716	-1.51425	-1.51105	-1.50786
-1.3833	-1.38062	-1.37795	-1.41142	-1.40863	-1.40588	-1.51262	-1.50941	-1.50625
-1.38203	-1.37938	-1.37673	-1.48887	-1.48548	-1.48214	-1.51099	-1.50777	-1.50465
-1.38077	-1.37815	-1.3755	-1.48728	-1.48389	-1.48055	-1.50935	-1.50613	-1.50303
-1.37953	-1.37691	-1.37428	-1.48569	-1.4823	-1.47897	-1.50773	-1.50452	-1.50144
-1.37829	-1.41184	-1.40905	-1.48409	-1.48072	-1.47742	-1.50616	-1.50298	-1.49991

-1.37706	-1.41054	-1.40775	-1.48249	-1.47915	-1.47589	-1.50462	-1.50146	-1.49841
-1.37582	-1.48765	-1.48424	-1.51296	-1.5096	-1.50631	-1.5031	-1.49997	-1.49691
-1.37457	-1.48602	-1.48261	-1.51131	-1.50801	-1.50479	-1.50162	-1.4985	-1.49543
-1.37332	-1.48439	-1.481	-1.50969	-1.50644	-1.50327	-1.50013	-1.49703	-1.49396
-1.37206	-1.48276	-1.47942	-1.5081	-1.50489	-1.50176	-1.49865	-1.49558	-1.49253
-1.48444	-1.48114	-1.47785	-1.50654	-1.50336	-1.50025	-1.49717	-1.49413	-1.54915
-1.48279	-1.47952	-1.47631	-1.50501	-1.50186	-1.49876	-1.4957	-1.49267	-1.54764
-1.48116	-1.47793	-1.47478	-1.5035	-1.50037	-1.49728	-1.49423	-1.49121	-1.54613
-1.47959	-1.50832	-1.50513	-1.50199	-1.49889	-1.49583	-1.55095	-1.54777	-1.56798
-1.47805	-1.50677	-1.50361	-1.5005	-1.49743	-1.49438	-1.54944	-1.54626	-1.56645
-1.47653	-1.50524	-1.50211	-1.49902	-1.49597	-1.49293	-1.5713	-1.56809	-1.56492
-1.47502	-1.50373	-1.50063	-1.49756	-1.49452	-1.49148	-1.56977	-1.56657	-1.5634
-1.47352	-1.52649	-1.52339	-1.57787	-1.57465	-1.57145	-1.56825	-1.56505	-1.56189
-1.47205	-1.52497	-1.52191	-1.57634	-1.57312	-1.56991	-1.56671	-1.56353	-1.5604
-1.49528	-1.54732	-1.54421	-1.57482	-1.5716	-1.56838	-1.56518	-1.56201	-1.55893
-1.49384	-1.54583	-1.54272	-1.57328	-1.57006	-1.56685	-1.56366	-1.5605	-1.55747
-1.4924	-1.54435	-1.54124	-1.57175	-1.56853	-1.56533	-1.56215	-1.55902	-1.55604
-1.52212	-1.5766	-1.5734	-1.57021	-1.567	-1.56381	-1.56069	-1.55763	-1.5547
-1.52064	-1.57507	-1.57187	-1.56868	-1.56547	-1.5623	-1.55926	-1.5563	-1.55341
-1.54298	-1.57354	-1.57037	-1.56722	-1.56403	-1.56089	-1.55788	-1.55497	-1.55209
-1.54147	-1.57201	-1.56891	-1.56582	-1.56267	-1.55955	-1.55655	-1.55363	-1.55076
-1.57368	-1.57054	-1.56746	-1.5644	-1.56129	-1.55821	-1.55521	-1.55227	-1.54941
-1.57232	-1.56916	-1.56604	-1.56294	-1.55987	-1.55683	-1.55384	-1.5509	-1.54804
-1.57094	-1.56776	-1.5646	-1.56148	-1.55844	-1.55544	-1.55247	-1.54953	-1.54668
-1.56946	-1.56629	-1.56312	-1.56002	-1.557	-1.55403	-1.55109	-1.54818	-1.54533
-1.56794	-1.56478	-1.56164	-1.55856	-1.55556	-1.55262	-1.54971	-1.54683	-1.54398
-1.56642	-1.56328	-1.56018	-1.55713	-1.55414	-1.55121	-1.54832	-1.54547	-1.54262
-1.56489	-1.56178	-1.55873	-1.55571	-1.55275	-1.54982	-1.54694	-1.5441	-1.54126
-1.56347	-1.56039	-1.55737	-1.55439	-1.55144	-1.54852	-1.54565	-1.54282	-1.53997
-1.56218	-1.55912	-1.55612	-1.55315	-1.55023	-1.54734	-1.54447	-1.54162	-1.53877
-1.56085	-1.55781	-1.55482	-1.55188	-1.54898	-1.5461	-1.54323	-1.54038	-1.53751
-1.55945	-1.55643	-1.55345	-1.55051	-1.54761	-1.54475	-1.54188	-1.53903	-1.53616
-1.55802	-1.55502	-1.55204	-1.54911	-1.54622	-1.54336	-1.5405	-1.53765	-1.53478
-1.55657	-1.55359	-1.55063	-1.54771	-1.54482	-1.54196	-1.53911	-1.53625	-1.55509
-1.55514	-1.55217	-1.54923	-1.54632	-1.54344	-1.54057	-1.53772	-1.53487	-1.55372
-1.55382	-1.55086	-1.54793	-1.54503	-1.54215	-1.53929	-1.55822	-1.55533	-1.59757
-1.55258	-1.54962	-1.54671	-1.54381	-1.54094	-1.53808	-1.55702	-1.55413	-1.59641
-1.55126	-1.54832	-1.54541	-1.54252	-1.53965	-1.5368	-1.55574	-1.55286	-1.59517
-1.54985	-1.54691	-1.54401	-1.54113	-1.53827	-1.53543	-1.55438	-1.55151	-1.59385
-1.54847	-1.54553	-1.54262	-1.56168	-1.55877	-1.55588	-1.553	-1.55014	-1.59252
-1.54719	-1.54425	-1.5413	-1.56033	-1.55739	-1.55448	-1.5516	-1.54877	-1.59117
-1.54593	-1.54298	-1.54	-1.559	-1.55603	-1.5531	-1.55024	-1.54742	-1.58985
-1.54457	-1.56369	-1.56069	-1.55771	-1.55476	-1.55185	-1.54899	-1.54617	-1.58862
-1.54316	-1.56232	-1.55938	-1.55645	-1.55354	-1.55065	-1.5478	-1.54498	-1.58744
-1.54184	-1.56101	-1.55808	-1.55516	-1.55225	-1.54937	-1.59188	-1.58903	-1.61702
-1.5406	-1.55976	-1.55678	-1.55383	-1.5509	-1.54801	-1.59054	-1.58769	-1.61563
-1.56143	-1.55848	-1.55549	-1.55253	-1.5496	-1.54671	-1.58925	-1.58641	-1.64157
-1.56007	-1.55714	-1.5542	-1.55127	-1.54837	-1.5455	-1.58806	-1.58522	-1.64032
-1.55873	-1.55582	-1.55291	-1.59555	-1.59262	-1.58972	-1.61767	-1.61473	-1.63905
-1.55751	-1.55459	-1.55165	-1.59427	-1.59133	-1.58842	-1.61631	-1.61338	-1.6377

-1.5563	-1.55337	-1.55039	-1.59299	-1.59003	-1.5871	-1.61494	-1.61203	-1.63634
-1.55499	-1.55207	-1.54911	-1.59175	-1.5888	-1.58589	-1.61368	-1.61078	-1.66011
-1.55363	-1.55071	-1.54782	-1.59054	-1.58762	-1.58473	-1.61249	-1.6096	-1.65894
-1.55234	-1.59514	-1.59223	-1.62016	-1.61718	-1.61422	-1.61129	-1.60838	-1.65771
-1.55113	-1.59394	-1.59103	-1.61893	-1.61597	-1.61302	-1.61007	-1.60712	-1.65642
-1.54993	-1.59275	-1.58983	-1.61767	-1.61471	-1.61177	-1.6612	-1.65816	-1.68559
-1.54874	-1.59157	-1.58861	-1.61635	-1.61339	-1.61045	-1.6599	-1.65692	-1.68429
-1.54753	-1.59037	-1.58738	-1.61504	-1.61207	-1.60914	-1.65862	-1.65568	-1.68299
-1.54621	-1.58908	-1.58614	-1.63943	-1.63645	-1.6335	-1.65741	-1.65448	-1.6817
-1.54486	-1.58778	-1.5849	-1.63821	-1.63525	-1.63232	-1.65621	-1.65327	-1.68039
-1.58946	-1.64292	-1.63996	-1.63701	-1.63404	-1.63109	-1.65495	-1.652	-1.67903
-1.58829	-1.64173	-1.63878	-1.63582	-1.63283	-1.62984	-1.65365	-1.65067	-1.67763
-1.61335	-1.64055	-1.63759	-1.63461	-1.63161	-1.62862	-1.6826	-1.67943	-1.6763
-1.61224	-1.63938	-1.63638	-1.63339	-1.6304	-1.62743	-1.68134	-1.67818	-1.67506
-1.6412	-1.63819	-1.63517	-1.63218	-1.6292	-1.62625	-1.68009	-1.67695	-1.67384
-1.63997	-1.63696	-1.63395	-1.63096	-1.62801	-1.62508	-1.67884	-1.67572	-1.67262
-1.63873	-1.63571	-1.63272	-1.62975	-1.62683	-1.62393	-1.67759	-1.67448	-1.67139
-1.63749	-1.63449	-1.63151	-1.68574	-1.68259	-1.67946	-1.67631	-1.67317	-1.67007
-1.63625	-1.63327	-1.63032	-1.68447	-1.68134	-1.67821	-1.67504	-1.67185	-1.66873
-1.63504	-1.63207	-1.62915	-1.68321	-1.68009	-1.67696	-1.67378	-1.67059	-1.66746
-1.63386	-1.63089	-1.62798	-1.68196	-1.67884	-1.67572	-1.67255	-1.66938	-1.66627
-1.63269	-1.66051	-1.65743	-1.68071	-1.67758	-1.67445	-1.6713	-1.66815	-1.66506
-1.63154	-1.65928	-1.6562	-1.67947	-1.6763	-1.67314	-1.67	-1.6669	-1.66384
-1.63039	-1.65805	-1.65497	-1.67823	-1.67503	-1.67185	-1.66873	-1.66565	-1.66263
-1.62923	-1.65682	-1.65375	-1.677	-1.67381	-1.67064	-1.66753	-1.66446	-1.66145
-1.62807	-1.6556	-1.65253	-1.67576	-1.67259	-1.66944	-1.66634	-1.66328	-1.66028
-1.65746	-1.65436	-1.65126	-1.67446	-1.67132	-1.66819	-1.66512	-1.66209	-1.6591
-1.65622	-1.65311	-1.64997	-1.67313	-1.67	-1.66692	-1.66389	-1.66089	-1.65792
-1.65498	-1.65187	-1.64871	-1.67185	-1.66874	-1.66568	-1.66267	-1.6597	-1.65672
-1.65374	-1.65064	-1.64749	-1.67063	-1.66752	-1.66447	-1.66146	-1.6585	-1.65551
-1.65251	-1.64942	-1.64628	-1.66942	-1.66632	-1.66327	-1.66027	-1.65731	-1.65432
-1.65129	-1.64821	-1.64508	-1.66821	-1.66513	-1.66208	-1.6591	-1.65614	-1.65315
-1.65008	-1.647	-1.64389	-1.66702	-1.66395	-1.66091	-1.65793	-1.65498	-1.652
-1.64887	-1.64581	-1.64271	-1.66585	-1.66278	-1.65975	-1.65677	-1.65383	-1.65085
-1.64767	-1.64463	-1.64154	-1.66468	-1.66162	-1.65859	-1.65562	-1.65268	-1.64971
-1.64647	-1.64343	-1.64036	-1.6635	-1.66044	-1.65742	-1.65446	-1.65153	-1.64857
-1.64528	-1.64222	-1.63917	-1.66231	-1.65926	-1.65624	-1.65329	-1.65037	-1.64742
-1.6441	-1.64104	-1.63799	-1.66113	-1.65809	-1.65507	-1.65212	-1.64922	-1.64627
-1.64292	-1.63986	-1.63682	-1.65997	-1.65692	-1.65391	-1.65096	-1.64806	-1.64511
-1.64173	-1.6387	-1.63568	-1.65883	-1.65578	-1.65277	-1.64982	-1.64692	-1.64397
-1.64054	-1.63753	-1.63456	-1.65774	-1.6547	-1.65168	-1.64872	-1.6458	-1.64285
-1.63937	-1.63638	-1.63345	-1.65665	-1.65363	-1.6506	-1.64763	-1.64468	-1.64173
-1.63825	-1.63526	-1.63232	-1.65551	-1.65249	-1.64948	-1.6465	-1.64356	-1.71296
-1.63716	-1.63415	-1.63119	-1.65435	-1.65134	-1.64833	-1.64537	-1.64242	-1.71177
-1.63603	-1.63302	-1.63005	-1.6532	-1.65019	-1.6472	-1.64423	-1.64128	-1.71057
-1.63486	-1.63187	-1.6289	-1.65205	-1.64905	-1.64607	-1.6431	-1.64014	-1.70934
-1.6337	-1.63072	-1.62776	-1.6509	-1.64793	-1.64497	-1.68844	-1.68536	-1.70814
-1.63254	-1.62957	-1.62662	-1.64977	-1.64684	-1.64392	-1.68734	-1.68425	-1.70698
-1.63141	-1.62845	-1.6255	-1.64865	-1.64575	-1.64286	-1.68624	-1.68313	-1.72988
-1.63035	-1.62741	-1.62446	-1.64759	-1.64467	-1.64175	-1.68508	-1.68197	-1.72864

-1.62931	-1.62638	-1.62343	-1.64655	-1.64359	-1.64064	-1.68391	-1.6808	-1.72741
-1.6282	-1.65142	-1.64848	-1.69207	-1.68898	-1.68588	-1.68279	-1.67969	-1.72623
-1.62708	-1.6503	-1.64741	-1.691	-1.6879	-1.68478	-1.68168	-1.6786	-1.72506
-1.62599	-1.64921	-1.64633	-1.68987	-1.68677	-1.68364	-1.70633	-1.70316	-1.72386
-1.62495	-1.64814	-1.64522	-1.68868	-1.68559	-1.68247	-1.70513	-1.70195	-1.72262
-1.62391	-1.69367	-1.69059	-1.68751	-1.68442	-1.68132	-1.70394	-1.70074	-1.7214
-1.62287	-1.69258	-1.68949	-1.6864	-1.6833	-1.6802	-1.70278	-1.69958	-1.72021
-1.62181	-1.69147	-1.68837	-1.68526	-1.68216	-1.67906	-1.70161	-1.6984	-1.71902
-1.62068	-1.69027	-1.68717	-1.68406	-1.68096	-1.67785	-1.72426	-1.72099	-1.71778
-1.61952	-1.68905	-1.68595	-1.68284	-1.67973	-1.67661	-1.72298	-1.71973	-1.71653
-1.61843	-1.68788	-1.68477	-1.68166	-1.67854	-1.67543	-1.72176	-1.71853	-1.71535
-1.61737	-1.68675	-1.68363	-1.68051	-1.67739	-1.67428	-1.72058	-1.71737	-1.7142
-1.61631	-1.68563	-1.6825	-1.67937	-1.67625	-1.67315	-1.7194	-1.71622	-1.71305
-1.61525	-1.68451	-1.68136	-1.67823	-1.67512	-1.67203	-1.71823	-1.71506	-1.71191
-1.61418	-1.68338	-1.68023	-1.6771	-1.674	-1.67092	-1.71707	-1.71391	-1.71077
-1.61309	-1.68222	-1.6791	-1.67598	-1.67288	-1.66981	-1.71592	-1.71277	-1.70963
-1.612	-1.68106	-1.67796	-1.67486	-1.67177	-1.66871	-1.71477	-1.71163	-1.70849
-1.65776	-1.67992	-1.67682	-1.72322	-1.72	-1.7168	-1.71363	-1.71049	-1.70735
-1.65663	-1.67879	-1.67567	-1.72203	-1.71883	-1.71566	-1.7125	-1.70935	-1.70622
-1.65551	-1.67766	-1.67455	-1.72086	-1.71767	-1.71451	-1.71136	-1.70821	-1.73185
-1.65438	-1.67653	-1.67344	-1.7197	-1.71652	-1.71336	-1.71021	-1.70707	-1.73068
-1.65326	-1.67541	-1.67234	-1.71855	-1.71537	-1.71221	-1.70907	-1.70593	-1.72952
-1.65215	-1.67431	-1.67123	-1.71739	-1.71422	-1.71107	-1.70793	-1.70479	-1.72836
-1.65105	-1.67321	-1.67014	-1.71624	-1.71308	-1.70993	-1.70679	-1.70365	-1.7272
-1.64996	-1.67209	-1.66905	-1.71512	-1.71195	-1.7088	-1.70565	-1.70252	-1.72603
-1.64889	-1.671	-1.66797	-1.714	-1.71083	-1.70766	-1.70452	-1.70139	-1.72487
-1.64788	-1.66997	-1.66691	-1.71285	-1.70968	-1.70653	-1.70339	-1.70026	-1.72372
-1.6469	-1.66899	-1.66585	-1.7117	-1.70853	-1.7054	-1.70226	-1.69913	-1.72257
-1.64588	-1.71714	-1.71385	-1.7106	-1.70742	-1.70426	-1.70112	-1.69799	-1.72141
-1.64481	-1.71606	-1.7128	-1.70956	-1.70633	-1.70312	-1.69997	-1.69685	-1.72025
-1.64374	-1.71494	-1.71172	-1.7085	-1.70525	-1.70201	-1.69884	-1.69572	-1.74392
-1.64266	-1.71378	-1.71059	-1.7074	-1.70417	-1.70094	-1.69775	-1.69459	-1.74273
-1.6416	-1.71263	-1.70945	-1.70628	-1.70308	-1.69989	-1.69668	-1.69348	-1.74157
-1.66574	-1.71154	-1.70833	-1.70514	-1.70197	-1.69881	-1.69562	-1.69242	-1.74047
-1.66473	-1.71047	-1.70722	-1.704	-1.70085	-1.69772	-1.69456	-1.69139	-1.73939
-1.66369	-1.7094	-1.70614	-1.70292	-1.69977	-1.69665	-1.74488	-1.74158	-1.7591
-1.66263	-1.70832	-1.70509	-1.70188	-1.69873	-1.69559	-1.74379	-1.74051	-1.75799
-1.71044	-1.70725	-1.70403	-1.70083	-1.69766	-1.6945	-1.74266	-1.7394	-1.75686
-1.70937	-1.70617	-1.70298	-1.69978	-1.69657	-1.69337	-1.74149	-1.73825	-1.75572
-1.70828	-1.70509	-1.7019	-1.72406	-1.72076	-1.71747	-1.76113	-1.75785	-1.75456
-1.70717	-1.70396	-1.70079	-1.72294	-1.71965	-1.71637	-1.75997	-1.75665	-1.75336
-1.70606	-1.70284	-1.69968	-1.72183	-1.71856	-1.71529	-1.75883	-1.75547	-1.75217
-1.70498	-1.70175	-1.6986	-1.72073	-1.71748	-1.71424	-1.75773	-1.75435	-1.75104
-1.70392	-1.70068	-1.69752	-1.71963	-1.71642	-1.7132	-1.75664	-1.75325	-1.74994
-1.70286	-1.72497	-1.72172	-1.76548	-1.76218	-1.75888	-1.75552	-1.75215	-1.74883
-1.7018	-1.72386	-1.72057	-1.76428	-1.76099	-1.75771	-1.75438	-1.75103	-1.74772
-1.70074	-1.72277	-1.71946	-1.76312	-1.75983	-1.75656	-1.75325	-1.74993	-1.74662
-1.69969	-1.7217	-1.71838	-1.762	-1.75872	-1.75546	-1.75216	-1.74885	-1.74555
-1.72399	-1.76768	-1.76426	-1.7609	-1.75762	-1.75436	-1.75107	-1.74778	-1.74448
-1.72292	-1.76658	-1.76317	-1.75981	-1.75653	-1.75327	-1.74999	-1.74671	-1.74341

-1.72184	-1.76548	-1.76208	-1.75872	-1.75544	-1.75219	-1.74892	-1.74564	-1.74236
-1.72076	-1.76437	-1.76098	-1.75763	-1.75436	-1.75111	-1.74785	-1.74458	-1.76051
-1.71968	-1.76327	-1.75988	-1.75654	-1.75328	-1.75004	-1.74679	-1.74352	-1.75947
-1.7656	-1.76217	-1.75878	-1.75545	-1.75219	-1.74896	-1.76499	-1.76172	-1.75843
-1.76449	-1.76107	-1.75769	-1.75437	-1.75111	-1.74788	-1.76394	-1.76068	-1.75738
-1.76339	-1.75997	-1.7566	-1.75328	-1.75003	-1.7468	-1.76287	-1.75962	-1.75633
-1.7623	-1.75888	-1.75551	-1.75219	-1.74895	-1.74573	-1.76181	-1.75855	-1.75525
-1.7612	-1.75779	-1.75442	-1.7511	-1.74786	-1.74465	-1.76074	-1.75747	-1.75418
-1.76011	-1.7567	-1.75332	-1.76939	-1.76614	-1.76292	-1.75967	-1.75641	-1.75311
-1.75901	-1.75561	-1.75223	-1.7683	-1.76506	-1.76184	-1.7586	-1.75534	-1.75205
-1.75791	-1.77395	-1.77056	-1.76722	-1.76398	-1.76076	-1.75752	-1.75427	-1.75098
-1.75682	-1.77287	-1.76948	-1.76615	-1.7629	-1.75968	-1.75644	-1.75319	-1.74991
-1.7752	-1.7718	-1.76844	-1.76512	-1.76187	-1.75863	-1.75538	-1.75212	-1.74883
-1.77414	-1.77077	-1.76744	-1.76414	-1.76088	-1.75763	-1.75435	-1.75105	-1.74775
-1.7731	-1.76975	-1.76645	-1.76316	-1.75989	-1.75661	-1.75331	-1.74999	-1.77308
-1.77209	-1.76876	-1.76547	-1.76217	-1.75887	-1.75556	-1.75224	-1.74892	-1.772
-1.77109	-1.76778	-1.76448	-1.76117	-1.75783	-1.75449	-1.75117	-1.74786	-1.77094
-1.77009	-1.76676	-1.76344	-1.76012	-1.75677	-1.75343	-1.75011	-1.74681	-1.76989
-1.7691	-1.76572	-1.76238	-1.75904	-1.7557	-1.75237	-1.74906	-1.74578	-1.76885
-1.76807	-1.76468	-1.76132	-1.77688	-1.7735	-1.77015	-1.76683	-1.76354	-1.78626
-1.76701	-1.76362	-1.76027	-1.77581	-1.77244	-1.7691	-1.76581	-1.76256	-1.78527
-1.78492	-1.78152	-1.77814	-1.77478	-1.77143	-1.7681	-1.79089	-1.78758	-1.8132
-1.78383	-1.78045	-1.77713	-1.77382	-1.7705	-1.76719	-1.78994	-1.78658	-1.81209
-1.78279	-1.77943	-1.77613	-1.77284	-1.76955	-1.76625	-1.78896	-1.78557	-1.85182
-1.78184	-1.77848	-1.77515	-1.77183	-1.76852	-1.76521	-1.78789	-1.78449	-1.85062
-1.7809	-1.77754	-1.77417	-1.77081	-1.76747	-1.76414	-1.7868	-1.78341	-1.84943
-1.77989	-1.77652	-1.77314	-1.79593	-1.79251	-1.7891	-1.78571	-1.78232	-1.84824
-1.77883	-1.77547	-1.7721	-1.79487	-1.79144	-1.78802	-1.78462	-1.78124	-1.84707
-1.77778	-1.77441	-1.77107	-1.79385	-1.79044	-1.78703	-1.81246	-1.80884	-1.84595
-1.7767	-1.77336	-1.77007	-1.79287	-1.7895	-1.78613	-1.81149	-1.80784	-1.84488
-1.77569	-1.79856	-1.79522	-1.79188	-1.78853	-1.78517	-1.85128	-1.84752	-1.84378
-1.77477	-1.7976	-1.79422	-1.79086	-1.78749	-1.78412	-1.85012	-1.84636	-1.84262
-1.77383	-1.79663	-1.79322	-1.78982	-1.78645	-1.78307	-1.84897	-1.84522	-1.84148
-1.7728	-1.79558	-1.79216	-1.78877	-1.78541	-1.78206	-1.84787	-1.84416	-1.84044
-1.77176	-1.79451	-1.7911	-1.78772	-1.78438	-1.78105	-1.84679	-1.84311	-1.8394
-1.79699	-1.79355	-1.79013	-1.78674	-1.78339	-1.78005	-1.84567	-1.84198	-1.83828
-1.79608	-1.79264	-1.78921	-1.7858	-1.78242	-1.77905	-1.84454	-1.84082	-1.8371
-1.79509	-1.79165	-1.78822	-1.85456	-1.85085	-1.84715	-1.84344	-1.83972	-1.836
-1.79401	-1.79057	-1.78715	-1.8534	-1.84971	-1.84605	-1.84237	-1.83868	-1.83499
-1.79299	-1.78955	-1.78612	-1.85227	-1.8486	-1.84495	-1.84128	-1.83761	-1.85249
-1.79206	-1.78862	-1.78519	-1.85121	-1.84751	-1.84382	-1.84014	-1.83645	-1.85132
-1.79116	-1.78772	-1.78428	-1.85019	-1.84644	-1.84272	-1.839	-1.8353	-1.85017
-1.79026	-1.78682	-1.78338	-1.84917	-1.84541	-1.84167	-1.85655	-1.85281	-1.8491
-1.78935	-1.78591	-1.78247	-1.84814	-1.84439	-1.84064	-1.85551	-1.85176	-1.84806
-1.78834	-1.81367	-1.81	-1.84702	-1.84327	-1.83953	-1.85439	-1.85065	-1.84694
-1.78725	-1.81251	-1.80884	-1.84584	-1.84209	-1.83835	-1.85322	-1.84948	-1.84579
-1.78624	-1.85226	-1.84849	-1.86338	-1.85961	-1.85586	-1.85212	-1.8484	-1.8447
-1.78532	-1.85124	-1.84747	-1.86236	-1.85859	-1.85484	-1.85111	-1.8474	-1.84371
-1.78441	-1.86892	-1.86513	-1.86135	-1.85759	-1.85384	-1.85012	-1.84642	-1.84273
-1.78351	-1.86791	-1.86412	-1.86035	-1.85659	-1.85285	-1.84913	-1.84544	-1.84176

-1.78258	-1.86687	-1.86308	-1.85931	-1.85556	-1.85183	-1.84812	-1.84443	-1.84075
-1.81004	-1.86572	-1.86194	-1.85818	-1.85443	-1.8507	-1.847	-1.84331	-1.83964
-1.80888	-1.86454	-1.86075	-1.85699	-1.85325	-1.84952	-1.84582	-1.84215	-1.83847
-1.82706	-1.86344	-1.85966	-1.8559	-1.85216	-1.84844	-1.84475	-1.84107	-1.83739
-1.82608	-1.86242	-1.85865	-1.85489	-1.85116	-1.84744	-1.84374	-1.84007	-1.83639
-1.82511	-1.86142	-1.85765	-1.8539	-1.85017	-1.84646	-1.84276	-1.83909	-1.83541
-1.82416	-1.86044	-1.85667	-1.85293	-1.8492	-1.8455	-1.84181	-1.83813	-1.83445
-1.86324	-1.85946	-1.8557	-1.85196	-1.84824	-1.84454	-1.84085	-1.83717	-1.83349
-1.86226	-1.85848	-1.85472	-1.85098	-1.84728	-1.84358	-1.83989	-1.8362	-1.83252
-1.86129	-1.8575	-1.85374	-1.85001	-1.8463	-1.84262	-1.83892	-1.83523	-1.83153
-1.86031	-1.85652	-1.85276	-1.84903	-1.84532	-1.84163	-1.83793	-1.83423	-1.84908
-1.85932	-1.85554	-1.85178	-1.84804	-1.84433	-1.84062	-1.83692	-1.83321	-1.84803
-1.85834	-1.85456	-1.8508	-1.84705	-1.84331	-1.83958	-1.83585	-1.83213	-1.84693
-1.85735	-1.85357	-1.8498	-1.84603	-1.84226	-1.83848	-1.83472	-1.83098	-1.84576
-1.85636	-1.85257	-1.84879	-1.845	-1.8412	-1.8374	-1.83362	-1.82987	-1.84465
-1.85536	-1.85155	-1.84774	-1.84393	-1.84012	-1.83633	-1.83257	-1.82884	-1.84362
-1.85436	-1.85054	-1.84669	-1.84287	-1.83906	-1.83529	-1.83155	-1.82784	-1.84262
-1.85337	-1.84954	-1.84569	-1.84185	-1.83805	-1.83428	-1.83055	-1.82685	-1.84163
-1.85239	-1.84856	-1.84471	-1.84087	-1.83706	-1.83329	-1.82957	-1.82588	-1.84065
-1.85141	-1.84759	-1.84373	-1.83989	-1.83608	-1.8323	-1.84711	-1.84338	-1.83966
-1.85044	-1.84663	-1.84278	-1.83893	-1.83511	-1.83132	-1.84611	-1.84238	-1.83867
-1.84947	-1.84567	-1.84185	-1.83802	-1.83419	-1.83038	-1.84514	-1.8414	-1.83767
-1.8485	-1.84473	-1.84096	-1.83716	-1.83332	-1.82949	-1.8442	-1.84041	-1.83667
-1.84753	-1.84378	-1.84005	-1.83628	-1.83245	-1.82862	-1.84329	-1.83945	-1.8357
-1.84654	-1.84281	-1.83909	-1.83536	-1.83157	-1.82777	-1.84242	-1.83856	-1.83477
-1.84554	-1.84183	-1.83812	-1.83441	-1.83067	-1.82691	-1.84157	-1.83769	-1.83388
-1.84456	-1.84084	-1.83714	-1.85202	-1.84826	-1.84448	-1.84066	-1.83683	-1.83301
-1.84357	-1.83986	-1.83615	-1.85102	-1.84727	-1.84351	-1.83974	-1.83596	-1.83217
-1.84258	-1.83887	-1.83516	-1.85002	-1.84627	-1.84253	-1.83878	-1.83504	-1.83127
-1.84159	-1.83788	-1.83417	-1.84902	-1.84527	-1.84153	-1.8378	-1.83407	-1.83035
-1.84062	-1.8369	-1.83319	-1.84802	-1.84428	-1.84054	-1.83682	-1.8331	-1.8452
-1.83966	-1.83592	-1.8322	-1.84701	-1.84327	-1.83954	-1.83582	-1.83212	-1.84421
-1.83876	-1.85359	-1.8498	-1.84603	-1.84229	-1.83855	-1.83483	-1.83113	-1.84322
-1.83794	-1.85275	-1.84893	-1.84512	-1.84133	-1.83757	-1.83383	-1.83013	-1.84222
-1.83713	-1.85194	-1.84809	-1.84424	-1.84041	-1.83661	-1.83286	-1.82915	-1.84122
-1.83623	-1.85104	-1.84721	-1.84338	-1.83956	-1.83575	-1.83198	-1.82824	-1.84027
-1.83527	-1.8501	-1.84632	-1.84253	-1.83874	-1.83494	-1.83116	-1.82738	-1.83936
-1.83432	-1.84914	-1.84537	-1.84161	-1.83784	-1.83407	-1.83029	-1.82651	-1.83846
-1.83337	-1.84814	-1.84437	-1.84061	-1.83686	-1.83312	-1.82938	-1.82564	-1.8376
-1.85103	-1.8472	-1.84341	-1.83964	-1.83589	-1.83216	-1.82844	-1.82474	-1.83671
-1.85021	-1.84637	-1.84254	-1.83873	-1.83494	-1.83118	-1.82747	-1.82378	-1.83576
-1.84937	-1.84554	-1.84168	-1.83784	-1.83402	-1.83024	-1.82651	-1.82282	-1.83481
-1.84843	-1.84462	-1.84079	-1.83697	-1.83318	-1.82941	-1.84142	-1.83765	-1.83388
-1.84745	-1.84366	-1.83988	-1.83611	-1.83236	-1.82861	-1.8406	-1.83678	-1.83296
-1.84654	-1.84274	-1.83895	-1.83518	-1.83145	-1.82772	-1.83971	-1.8359	-1.83209
-1.84567	-1.84183	-1.838	-1.8342	-1.83047	-1.82676	-1.83878	-1.83502	-1.83124
-1.8448	-1.84094	-1.8371	-1.8333	-1.82956	-1.82584	-1.83785	-1.8341	-1.83033
-1.84391	-1.84009	-1.83628	-1.83249	-1.82871	-1.82495	-1.83692	-1.83312	-1.82934
-1.84303	-1.83923	-1.83544	-1.84749	-1.84366	-1.83983	-1.83601	-1.83219	-1.82839
-1.84217	-1.83834	-1.83453	-1.84656	-1.84275	-1.83895	-1.83515	-1.83135	-1.82754

-1.8413	-1.83744	-1.83361	-1.84563	-1.84184	-1.83807	-1.83429	-1.83051	-1.8267
-1.84041	-1.83658	-1.83277	-1.84479	-1.84098	-1.83719	-1.8334	-1.82961	-1.8258
-1.83951	-1.83572	-1.83195	-1.84398	-1.84015	-1.83632	-1.8325	-1.82869	-1.82489
-1.83863	-1.83483	-1.83107	-1.8431	-1.83928	-1.83546	-1.83164	-1.82782	-1.82403
-1.83774	-1.83393	-1.83015	-1.84217	-1.83839	-1.83461	-1.83081	-1.82701	-1.82323
-1.83688	-1.83305	-1.82927	-1.84129	-1.83753	-1.83378	-1.82999	-1.82618	-1.82239
-1.83602	-1.8322	-1.82845	-1.84048	-1.83674	-1.83299	-1.82917	-1.82533	-1.82151
-1.83518	-1.84722	-1.84343	-1.83967	-1.83592	-1.83216	-1.82834	-1.82447	-1.82062
-1.83437	-1.8464	-1.8426	-1.83881	-1.83503	-1.83125	-1.82744	-1.8236	-1.81977
-1.83357	-1.84559	-1.84176	-1.83794	-1.83413	-1.83033	-1.82653	-1.82274	-1.81893
-1.83274	-1.84476	-1.84092	-1.8371	-1.8333	-1.8295	-1.82571	-1.82191	-1.81811
-1.83191	-1.84392	-1.84009	-1.83627	-1.83249	-1.82872	-1.82492	-1.82111	-1.81729
-1.83108	-1.84309	-1.83927	-1.83548	-1.8317	-1.82793	-1.82412	-1.82029	-1.81645
-1.83025	-1.84226	-1.83849	-1.83471	-1.83093	-1.82714	-1.8233	-1.81944	-1.8156
-1.82944	-1.84146	-1.8377	-1.83394	-1.83014	-1.82632	-1.82247	-1.81859	-1.81475
-1.82868	-1.84068	-1.83693	-1.83315	-1.82932	-1.82547	-1.8216	-1.81773	-1.81391
-1.82792	-1.83991	-1.83614	-1.83234	-1.82848	-1.8246	-1.82073	-1.81688	-1.81308
-1.82716	-1.83914	-1.83532	-1.83149	-1.82761	-1.82373	-1.81989	-1.81607	-1.81229
-1.8264	-1.83837	-1.8345	-1.83063	-1.82675	-1.82288	-1.81906	-1.81528	-1.81151
-1.84142	-1.8376	-1.83371	-1.82983	-1.82594	-1.82207	-1.81826	-1.81449	-1.81073
-1.84065	-1.83682	-1.83294	-1.82906	-1.82517	-1.8213	-1.81749	-1.81373	-1.80994
-1.83985	-1.83601	-1.83213	-1.82826	-1.82438	-1.82053	-1.81672	-1.81295	-1.80916
-1.83901	-1.83515	-1.83128	-1.82742	-1.82358	-1.81975	-1.81596	-1.81218	-1.80839
-1.83819	-1.83431	-1.83044	-1.8266	-1.82278	-1.81898	-1.81518	-1.81139	-1.8076
-1.83742	-1.83353	-1.82966	-1.82581	-1.822	-1.8182	-1.81438	-1.81056	-1.80675
-1.83667	-1.83278	-1.82889	-1.82504	-1.82123	-1.81743	-1.81358	-1.80972	-1.80591
-1.83592	-1.83202	-1.82813	-1.82427	-1.82046	-1.81666	-1.81281	-1.80894	-1.80513
-1.83517	-1.83127	-1.82738	-1.82351	-1.8197	-1.8159	-1.81205	-1.80819	-1.80437
-1.83442	-1.83052	-1.82662	-1.82275	-1.81894	-1.81514	-1.8113	-1.80744	-1.80362
-1.83367	-1.82976	-1.82586	-1.82199	-1.81819	-1.81439	-1.81055	-1.80669	-1.80288
-1.83292	-1.82901	-1.8251	-1.82124	-1.81743	-1.81364	-1.8098	-1.80595	-1.80213
-1.83216	-1.82824	-1.82434	-1.82048	-1.81668	-1.81289	-1.80905	-1.8052	-1.80139
-1.8314	-1.82749	-1.82359	-1.81972	-1.81593	-1.81214	-1.80831	-1.80446	-1.80065
-1.83066	-1.82674	-1.82283	-1.81896	-1.81518	-1.81141	-1.80758	-1.80374	-1.79992
-1.82993	-1.82601	-1.82209	-1.81822	-1.81443	-1.81068	-1.80686	-1.80302	-1.7992
-1.82925	-1.82534	-1.8214	-1.81751	-1.81372	-1.80995	-1.80616	-1.80235	-1.79854
-1.82858	-1.82469	-1.82075	-1.81684	-1.81302	-1.80923	-1.80546	-1.8017	-1.7979
-1.82787	-1.824	-1.82006	-1.81615	-1.8123	-1.8085	-1.80474	-1.80101	-1.79721
-1.82713	-1.82327	-1.81934	-1.81543	-1.81158	-1.80775	-1.804	-1.80027	-1.79648
-1.82639	-1.82254	-1.81863	-1.81474	-1.81088	-1.80704	-1.80327	-1.79954	-1.79574
-1.82566	-1.8218	-1.81795	-1.8141	-1.81023	-1.80637	-1.80257	-1.79881	-1.79499
-1.82494	-1.82107	-1.81726	-1.81343	-1.80957	-1.8057	-1.80188	-1.79808	-1.79426
-1.82427	-1.82038	-1.81655	-1.81272	-1.80886	-1.805	-1.80116	-1.79735	-1.79353
-1.8236	-1.81969	-1.81583	-1.81199	-1.80814	-1.80429	-1.80045	-1.79662	-1.79282
-1.82289	-1.81897	-1.8151	-1.81125	-1.80743	-1.80362	-1.79979	-1.79595	-1.79213
-1.82215	-1.81824	-1.81436	-1.81052	-1.80674	-1.80298	-1.79916	-1.79531	-1.79147
-1.82146	-1.81755	-1.81366	-1.80982	-1.80605	-1.8023	-1.7985	-1.79467	-1.79082
-1.82081	-1.81691	-1.81302	-1.80916	-1.80536	-1.80158	-1.7978	-1.79402	-1.79019
-1.82014	-1.81625	-1.81237	-1.80851	-1.80468	-1.80088	-1.7971	-1.79335	-1.78952
-1.81941	-1.81553	-1.81171	-1.8079	-1.80406	-1.80022	-1.79642	-1.79262	-1.78879

-1.81868	-1.81482	-1.81105	-1.80726	-1.80343	-1.79958	-1.79574	-1.7919	-1.78805
-1.818	-1.81414	-1.81035	-1.80656	-1.80276	-1.79895	-1.79511	-1.79125	-1.82405
-1.81736	-1.81347	-1.80964	-1.80583	-1.80208	-1.79832	-1.79449	-1.79062	-1.8234
-1.81672	-1.81283	-1.80897	-1.80515	-1.80139	-1.79763	-1.79382	-1.78998	-1.84194
-1.81609	-1.81219	-1.80833	-1.8045	-1.80069	-1.7969	-1.79312	-1.78934	-1.8413
-1.81546	-1.81157	-1.8077	-1.80385	-1.80002	-1.79621	-1.79243	-1.78868	-1.84064
-1.81484	-1.81094	-1.80707	-1.80323	-1.79939	-1.79557	-1.79176	-1.78797	-1.83991
-1.81422	-1.81032	-1.80645	-1.80261	-1.79877	-1.79495	-1.79112	-1.7873	-1.83922
-1.8136	-1.80971	-1.80584	-1.80199	-1.79815	-1.79433	-1.79052	-1.78672	-1.83863
-1.81299	-1.80909	-1.80523	-1.80138	-1.79754	-1.79372	-1.78993	-1.78615	-1.83804
-1.81237	-1.80848	-1.80461	-1.80076	-1.79692	-1.79309	-1.78929	-1.78551	-1.83738
-1.81176	-1.80787	-1.804	-1.80015	-1.7963	-1.79247	-1.78865	-1.78483	-1.83668
-1.81116	-1.80727	-1.8034	-1.79954	-1.7957	-1.79187	-1.82475	-1.82085	-1.83605
-1.81056	-1.80668	-1.8028	-1.79893	-1.7951	-1.79128	-1.82417	-1.82029	-1.83547
-1.80999	-1.80611	-1.80223	-1.79837	-1.79453	-1.79071	-1.84275	-1.83883	-1.83488
-1.80945	-1.8056	-1.80173	-1.79787	-1.79401	-1.79017	-1.84217	-1.83822	-1.83426
-1.80891	-1.80507	-1.80122	-1.83429	-1.83033	-1.82638	-1.84159	-1.83761	-1.83363
-1.80833	-1.80449	-1.80063	-1.8337	-1.82974	-1.82579	-1.84098	-1.837	-1.83301
-1.80775	-1.80389	-1.80002	-1.83308	-1.82913	-1.82519	-1.84037	-1.83639	-1.8324
-1.80715	-1.80329	-1.79942	-1.83246	-1.82852	-1.82459	-1.83978	-1.83582	-1.8402
-1.80656	-1.80269	-1.79882	-1.83184	-1.82791	-1.82399	-1.8392	-1.83527	-1.83971
-1.806	-1.80213	-1.79826	-1.85052	-1.84653	-1.84256	-1.83861	-1.83469	-1.83917
-1.80548	-1.80161	-1.79775	-1.85	-1.846	-1.842	-1.83803	-1.83408	-1.83858
-1.80495	-1.80107	-1.79722	-1.84946	-1.84544	-1.84143	-1.84593	-1.84196	-1.85887
-1.80436	-1.80048	-1.79664	-1.84885	-1.84483	-1.84081	-1.84534	-1.84137	-1.85826
-1.80379	-1.79991	-1.79606	-1.84825	-1.84422	-1.8402	-1.84477	-1.8408	-1.85768
-1.78433	-1.79939	-1.79553	-1.85629	-1.85227	-1.84826	-1.84427	-1.84029	-1.85716
-1.78385	-1.7989	-1.79503	-1.8558	-1.85179	-1.84779	-1.8438	-1.83982	-1.85667
-1.78336	-1.7984	-1.79453	-1.85531	-1.85131	-1.84731	-1.86426	-1.86021	-1.85618
-1.78287	-1.7979	-1.79403	-1.85481	-1.85082	-1.84684	-1.86377	-1.85972	-1.85569
-1.78233	-1.80664	-1.80274	-1.85428	-1.8503	-1.84633	-1.86324	-1.85918	-1.85515
-1.78174	-1.80608	-1.80219	-1.8537	-1.84972	-1.84575	-1.86265	-1.8586	-1.85457
-1.78117	-1.86118	-1.85715	-1.87419	-1.87016	-1.86614	-1.86209	-1.85804	-1.854
-1.78066	-1.86067	-1.85665	-1.87369	-1.86965	-1.86563	-1.86158	-1.85753	-1.8535
-1.78016	-1.86019	-1.85617	-1.87321	-1.86917	-1.86514	-1.8611	-1.85706	-1.85302
-1.78931	-1.85971	-1.8557	-1.87273	-1.86869	-1.86466	-1.86062	-1.85658	-1.85255
-1.78886	-1.85923	-1.85523	-1.87225	-1.86822	-1.86419	-1.86015	-1.85611	-1.85208
-1.7884	-1.86221	-1.8582	-1.87177	-1.86774	-1.86372	-1.85968	-1.85563	-1.8516
-1.78794	-1.86172	-1.85772	-1.87129	-1.86726	-1.86324	-1.8592	-1.85516	-1.85113
-1.78748	-1.86124	-1.85724	-1.87081	-1.86679	-1.86277	-1.85873	-1.85468	-1.85066
-1.78702	-1.86076	-1.85676	-1.87032	-1.86631	-1.86229	-1.85825	-1.8542	-1.85018
-1.78657	-1.86028	-1.85628	-1.86985	-1.86583	-1.86182	-1.85778	-1.85373	-1.84971
-1.78612	-1.8598	-1.85582	-1.86939	-1.86536	-1.86134	-1.85731	-1.85327	-1.84924
-1.78569	-1.85935	-1.85537	-1.86894	-1.86492	-1.86089	-1.85686	-1.85284	-1.8488
-1.86296	-1.85895	-1.85498	-1.85101	-1.84702	-1.84303	-1.85647	-1.85245	-1.84842
-1.8626	-1.85859	-1.85462	-1.85066	-1.84667	-1.84268	-1.85611	-1.8521	-1.84806
-1.86219	-1.85819	-1.85422	-1.85025	-1.84626	-1.84227	-1.8557	-1.85169	-1.84766
-1.86174	-1.85774	-1.85376	-1.84979	-1.84581	-1.84182	-1.85524	-1.85122	-1.8472
-1.86128	-1.85728	-1.8533	-1.84933	-1.84535	-1.84137	-1.85478	-1.85076	-1.84674
-1.86084	-1.85682	-1.85284	-1.84886	-1.84489	-1.84092	-1.85432	-1.85029	-1.84627

-1.86041	-1.85637	-1.85239	-1.84841	-1.84445	-1.84048	-1.85388	-1.84984	-1.84582
-1.86001	-1.85598	-1.85199	-1.84802	-1.84406	-1.8401	-1.85349	-1.84944	-1.84543
-1.85963	-1.85561	-1.85162	-1.84764	-1.84368	-1.83972	-1.85311	-1.84907	-1.84506
-1.85925	-1.85522	-1.85122	-1.84722	-1.84325	-1.83928	-1.85268	-1.84865	-1.84464
-1.85888	-1.85484	-1.8508	-1.84677	-1.84278	-1.8388	-1.85221	-1.84819	-1.84419
-1.85848	-1.85444	-1.85039	-1.84636	-1.84236	-1.83838	-1.83441	-1.83045	-1.84377
-1.85803	-1.85402	-1.85	-1.84598	-1.842	-1.83802	-1.83404	-1.83007	-1.84339
-1.85761	-1.85361	-1.84962	-1.84562	-1.84164	-1.83767	-1.83369	-1.8297	-1.84301
-1.85723	-1.85324	-1.84926	-1.84528	-1.84129	-1.83731	-1.83332	-1.82933	-1.84263
-1.85687	-1.85288	-1.8489	-1.84493	-1.84094	-1.83695	-1.83295	-1.82896	-1.84223
-1.85652	-1.85253	-1.84855	-1.84457	-1.84058	-1.83658	-1.83256	-1.82855	-1.84182
-1.85617	-1.85218	-1.8482	-1.84421	-1.84021	-1.83619	-1.83216	-1.82813	-1.8414
-1.85582	-1.85182	-1.84783	-1.84384	-1.83982	-1.83579	-1.83176	-1.82774	-1.84102
-1.85546	-1.85145	-1.84746	-1.84345	-1.83941	-1.83537	-1.83135	-1.82737	-1.84067
-1.85511	-1.85109	-1.84707	-1.84305	-1.839	-1.83496	-1.83097	-1.82701	-1.84033
-1.85476	-1.85072	-1.84666	-1.84261	-1.83859	-1.83459	-1.83062	-1.82667	-1.83999
-1.85443	-1.85038	-1.84629	-1.84222	-1.83821	-1.83423	-1.83027	-1.82634	-1.82241
-1.8541	-1.85006	-1.846	-1.84195	-1.83791	-1.83389	-1.82993	-1.826	-1.82208
-1.85377	-1.84975	-1.84574	-1.84172	-1.83764	-1.83357	-1.82959	-1.82567	-1.82175
-1.85343	-1.84943	-1.84543	-1.84143	-1.83737	-1.83332	-1.82931	-1.82534	-1.8214
-1.85309	-1.84909	-1.8451	-1.84111	-1.83711	-1.8331	-1.82907	-1.82503	-1.82106
-1.85276	-1.84876	-1.84477	-1.84079	-1.83682	-1.83283	-1.8288	-1.82475	-1.82077
-1.85242	-1.84842	-1.84443	-1.84045	-1.83649	-1.83252	-1.82853	-1.82453	-1.82053
-1.85209	-1.84809	-1.8441	-1.84012	-1.83616	-1.8322	-1.82824	-1.82428	-1.85319
-1.85177	-1.84773	-1.84375	-1.83978	-1.83582	-1.83187	-1.82792	-1.82397	-1.8529
-1.85147	-1.8474	-1.84341	-1.83945	-1.83548	-1.83153	-1.82759	-1.82364	-1.8526
-1.85122	-1.84715	-1.84311	-1.83911	-1.83515	-1.83121	-1.82726	-1.82331	-1.85226
-1.85098	-1.84692	-1.84283	-1.83879	-1.83483	-1.83091	-1.82695	-1.82298	-1.85192
-1.8507	-1.84666	-1.84257	-1.83853	-1.83457	-1.83064	-1.82668	-1.82272	-1.85163
-1.85039	-1.84638	-1.84233	-1.83832	-1.83435	-1.8304	-1.82645	-1.82251	-1.85139
-1.85012	-1.84612	-1.8421	-1.8381	-1.83411	-1.83013	-1.85924	-1.8552	-1.85112
-1.84988	-1.84588	-1.84189	-1.83789	-1.83385	-1.82983	-1.85892	-1.8549	-1.85085
-1.84963	-1.84562	-1.84165	-1.83766	-1.8336	-1.82954	-1.85862	-1.85461	-1.85058
-1.84934	-1.84531	-1.84135	-1.83738	-1.83334	-1.82931	-1.85838	-1.85436	-1.85033
-1.84905	-1.84501	-1.84105	-1.8371	-1.83309	-1.8291	-1.85815	-1.85411	-1.85008
-1.84881	-1.84476	-1.8408	-1.83684	-1.83287	-1.82888	-1.8579	-1.85382	-1.84978
-1.84858	-1.84455	-1.84057	-1.8366	-1.83264	-1.82867	-1.85765	-1.85352	-1.84946
-1.84836	-1.84433	-1.84031	-1.83632	-1.83236	-1.82841	-1.85738	-1.85325	-1.8492
-1.84815	-1.8441	-1.84003	-1.836	-1.83205	-1.82811	-1.85711	-1.85302	-1.84898
-1.84795	-1.84388	-1.83978	-1.86901	-1.86495	-1.86091	-1.85686	-1.8528	-1.84878
-1.84774	-1.84367	-1.83958	-1.86878	-1.86472	-1.86069	-1.85664	-1.85259	-1.84857
-1.84754	-1.84347	-1.83938	-1.86857	-1.86451	-1.86048	-1.85644	-1.85239	-1.84837
-1.84734	-1.84327	-1.83918	-1.86837	-1.86431	-1.86028	-1.85623	-1.85219	-1.84817
-1.84715	-1.84308	-1.83898	-1.86817	-1.86411	-1.86008	-1.85603	-1.85199	-1.84796
-1.84696	-1.84289	-1.83879	-1.86796	-1.8639	-1.85988	-1.85583	-1.85179	-1.84776
-1.84677	-1.8427	-1.8386	-1.86776	-1.8637	-1.85967	-1.85563	-1.85159	-1.84756
-1.84657	-1.84251	-1.8384	-1.86756	-1.8635	-1.85947	-1.85543	-1.85139	-1.84737
-1.84637	-1.84231	-1.83821	-1.86735	-1.86329	-1.85927	-1.85523	-1.8512	-1.84718
-1.84618	-1.84212	-1.83802	-1.86716	-1.8631	-1.85907	-1.85503	-1.851	-1.84698
-1.84599	-1.84193	-1.83784	-1.86697	-1.86289	-1.85885	-1.85482	-1.8508	-1.84678

-1.8458	-1.84175	-1.83767	-1.86681	-1.86272	-1.85867	-1.85462	-1.8506	-1.84659
-1.8456	-1.84158	-1.83755	-1.8667	-1.86262	-1.85855	-1.85448	-1.85042	-1.8464
-1.84542	-1.84142	-1.83743	-1.86662	-1.86253	-1.85845	-1.85436	-1.85027	-1.84622
-1.86408	-1.87461	-1.87053	-1.86646	-1.86238	-1.85831	-1.85422	-1.85015	-1.8461
-1.86391	-1.87443	-1.87034	-1.86626	-1.86219	-1.85813	-1.85409	-1.85005	-1.84602
-1.86377	-1.87429	-1.87019	-1.8661	-1.86203	-1.85796	-1.85393	-1.84991	-1.84589
-1.86367	-1.8742	-1.87009	-1.86599	-1.86188	-1.85778	-1.85374	-1.84972	-1.8457
-1.86354	-1.85946	-1.85538	-1.86587	-1.86174	-1.85763	-1.85357	-1.84955	-1.84553
-1.86336	-1.85928	-1.85522	-1.86573	-1.86162	-1.85752	-1.85347	-1.84944	-1.84541
-1.86317	-1.85911	-1.85506	-1.86559	-1.86151	-1.85743	-1.85338	-1.84934	-1.84529
-1.86306	-1.85899	-1.85494	-1.86548	-1.86141	-1.85733	-1.85327	-1.84921	-1.84516
-1.86296	-1.85889	-1.85484	-1.86538	-1.86131	-1.85724	-1.85316	-1.84908	-1.84502
-1.86283	-1.85875	-1.85469	-1.85065	-1.84661	-1.84258	-1.85304	-1.84896	-1.84492
-1.86268	-1.85857	-1.8545	-1.85046	-1.84643	-1.84242	-1.85291	-1.84886	-1.84483
-1.86255	-1.85843	-1.85436	-1.85031	-1.84629	-1.84229	-1.8528	-1.84876	-1.84474
-1.86243	-1.85834	-1.85427	-1.85023	-1.84621	-1.8422	-1.8527	-1.84867	-1.84463
-1.86234	-1.85826	-1.8542	-1.85016	-1.84613	-1.8421	-1.83809	-1.83409	-1.84452
-1.86229	-1.85819	-1.85413	-1.85008	-1.84604	-1.84201	-1.83798	-1.83395	-1.84439
-1.86224	-1.85812	-1.85405	-1.85	-1.84596	-1.84192	-1.83787	-1.83381	-1.84425
-1.86217	-1.85805	-1.85399	-1.84993	-1.84585	-1.84179	-1.83774	-1.8337	-1.84416
-1.86208	-1.85799	-1.85392	-1.84985	-1.84574	-1.84165	-1.83761	-1.83361	-1.84409
-1.86201	-1.85793	-1.85386	-1.84978	-1.84568	-1.84158	-1.83754	-1.83353	-1.82955
-1.86194	-1.85786	-1.85379	-1.84972	-1.84566	-1.84159	-1.83753	-1.83349	-1.82948
-1.86187	-1.8578	-1.85372	-1.84966	-1.8456	-1.84155	-1.83749	-1.83342	-1.82941
-1.86179	-1.85772	-1.85365	-1.84958	-1.84549	-1.84141	-1.83736	-1.83334	-1.82935
-1.86171	-1.85764	-1.85358	-1.8495	-1.84539	-1.84128	-1.83725	-1.83326	-1.82928
-1.86166	-1.85756	-1.8535	-1.84944	-1.84535	-1.84127	-1.83722	-1.83319	-1.82919
-1.86162	-1.85748	-1.85343	-1.84938	-1.84534	-1.84129	-1.83722	-1.83313	-1.82911
-1.86162	-1.85747	-1.85338	-1.84931	-1.84528	-1.84127	-1.83721	-1.83313	-1.86376
-1.86165	-1.85751	-1.85334	-1.84923	-1.84521	-1.84121	-1.83719	-1.83316	-1.86377
-1.86164	-1.85751	-1.85333	-1.8492	-1.84516	-1.84115	-1.83715	-1.83315	-1.86376
-1.86158	-1.85748	-1.85335	-1.84924	-1.84514	-1.84107	-1.83706	-1.83309	-1.86372
-1.86152	-1.85743	-1.85335	-1.84925	-1.84513	-1.84103	-1.8718	-1.86773	-1.86366
-1.86146	-1.85735	-1.85328	-1.84922	-1.84513	-1.84105	-1.87178	-1.86765	-1.86355
-1.86142	-1.85728	-1.85322	-1.84917	-1.84513	-1.84108	-1.87177	-1.86758	-1.86347
-1.86143	-1.85728	-1.85318	-1.84911	-1.84508	-1.84106	-1.87176	-1.86758	-1.86344
-1.86145	-1.85731	-1.85315	-1.84904	-1.84503	-1.84104	-1.87175	-1.86761	-1.86346
-1.84427	-1.85732	-1.85315	-1.88404	-1.87994	-1.87587	-1.87177	-1.86764	-1.86349
-1.84423	-1.8573	-1.85318	-1.88407	-1.87997	-1.87588	-1.87179	-1.86769	-1.86354
-1.84421	-1.85731	-1.85321	-1.88411	-1.87997	-1.87586	-1.87177	-1.86769	-1.86356
-1.84423	-1.85734	-1.85325	-1.88414	-1.87996	-1.8758	-1.8717	-1.86764	-1.86353
-1.84425	-1.8925	-1.88833	-1.88416	-1.87994	-1.87575	-1.87165	-1.8676	-1.86351
-1.84422	-1.89244	-1.88828	-1.88413	-1.87994	-1.87576	-1.87166	-1.8676	-1.86352
-1.84419	-1.89238	-1.88823	-1.88409	-1.87994	-1.87579	-1.87169	-1.8676	-1.86353
-1.84422	-1.87567	-1.87157	-1.88411	-1.87996	-1.87582	-1.87168	-1.86756	-1.86347
-1.84427	-1.87571	-1.87161	-1.88414	-1.88	-1.87585	-1.87168	-1.8675	-1.86339
-1.87996	-1.87576	-1.87165	-1.88419	-1.88004	-1.87589	-1.8717	-1.86749	-1.86337
-1.88001	-1.87581	-1.8717	-1.88425	-1.8801	-1.87594	-1.87174	-1.86752	-1.8634
-1.88007	-1.87586	-1.87176	-1.88431	-1.88015	-1.87599	-1.87178	-1.86756	-1.86343
-1.88012	-1.87591	-1.87181	-1.88436	-1.88021	-1.87604	-1.87184	-1.86761	-1.86348

-1.88016	-1.87596	-1.87185	-1.88441	-1.88026	-1.8761	-1.87189	-1.86766	-1.86353
-1.8802	-1.876	-1.87189	-1.88445	-1.8803	-1.87614	-1.87193	-1.86771	-1.86358
-1.88024	-1.87604	-1.87193	-1.8845	-1.88035	-1.87618	-1.87198	-1.86776	-1.86363
-1.88027	-1.87607	-1.87197	-1.88454	-1.88039	-1.87623	-1.87203	-1.86781	-1.86368
-1.88031	-1.87611	-1.872	-1.88458	-1.88044	-1.87629	-1.87209	-1.86788	-1.86375
-1.84721	-1.8431	-1.83907	-1.86794	-1.86384	-1.85974	-1.87217	-1.86799	-1.86386
-1.84727	-1.84316	-1.83911	-1.86797	-1.86388	-1.85979	-1.87227	-1.86814	-1.86403
-1.84736	-1.84325	-1.83919	-1.86803	-1.86393	-1.85985	-1.87235	-1.86826	-1.86417
-1.84749	-1.84341	-1.83934	-1.86816	-1.86402	-1.85991	-1.87241	-1.86832	-1.86423
-1.84762	-1.84356	-1.83949	-1.86828	-1.86412	-1.85999	-1.87248	-1.86838	-1.86429
-1.84768	-1.84363	-1.83957	-1.86837	-1.86424	-1.86012	-1.87262	-1.86851	-1.89113
-1.84773	-1.84367	-1.83962	-1.86844	-1.86435	-1.86028	-1.87278	-1.86866	-1.89125
-1.84784	-1.84377	-1.8397	-1.86851	-1.86443	-1.86036	-1.87288	-1.86876	-1.89134
-1.848	-1.84391	-1.83981	-1.86858	-1.86448	-1.86041	-1.87294	-1.86883	-1.89141
-1.84814	-1.84404	-1.83993	-1.86868	-1.86458	-1.86049	-1.87302	-1.8689	-1.89149
-1.84825	-1.84415	-1.84006	-1.86884	-1.86473	-1.86063	-1.87314	-1.86899	-1.89158
-1.84837	-1.84428	-1.84021	-1.869	-1.86488	-1.86077	-1.87326	-1.8691	-1.89169
-1.84852	-1.84444	-1.84037	-1.86915	-1.86501	-1.86087	-1.87338	-1.86924	-1.89184
-1.84867	-1.8446	-1.84053	-1.86929	-1.86513	-1.86098	-1.87351	-1.8694	-1.892
-1.84883	-1.84475	-1.84066	-1.83657	-1.83251	-1.82846	-1.85703	-1.85296	-1.89215
-1.84898	-1.84489	-1.84077	-1.83668	-1.83264	-1.82862	-1.85719	-1.85311	-1.89228
-1.84911	-1.84501	-1.8409	-1.83682	-1.83278	-1.82877	-1.85733	-1.85324	-1.8924
-1.84922	-1.84512	-1.84104	-1.83697	-1.83294	-1.82892	-1.85746	-1.85334	-1.89251
-1.84935	-1.84526	-1.84119	-1.83714	-1.83309	-1.82905	-1.85758	-1.85346	-1.89264
-1.8495	-1.84543	-1.84137	-1.83731	-1.83322	-1.82915	-1.85769	-1.85361	-1.89282
-1.84967	-1.84561	-1.84155	-1.83748	-1.83338	-1.82928	-1.85784	-1.85378	-1.893
-1.84984	-1.84579	-1.84174	-1.83768	-1.8336	-1.82952	-1.85806	-1.85397	-1.89318
-1.85003	-1.84598	-1.84193	-1.83788	-1.83383	-1.82978	-1.85829	-1.85416	-1.89336
-1.85021	-1.84616	-1.84211	-1.83805	-1.83399	-1.82992	-1.82587	-1.82184	-1.87732
-1.85039	-1.84634	-1.84229	-1.83822	-1.83412	-1.83002	-1.82599	-1.822	-1.8775
-1.85057	-1.84652	-1.84247	-1.83841	-1.8343	-1.8302	-1.82617	-1.82217	-1.87767
-1.85077	-1.84669	-1.84265	-1.83861	-1.83454	-1.83047	-1.8264	-1.82235	-1.87782
-1.8146	-1.84686	-1.84283	-1.8388	-1.83476	-1.83072	-1.82665	-1.82256	-1.87801
-1.81475	-1.84704	-1.843	-1.83897	-1.83495	-1.83093	-1.82688	-1.82283	-1.87826
-1.81491	-1.84723	-1.84318	-1.83914	-1.83512	-1.83111	-1.82711	-1.8231	-1.87853
-1.81509	-1.8474	-1.84336	-1.83933	-1.8353	-1.83128	-1.85525	-1.85119	-1.87876
-1.81529	-1.84758	-1.84356	-1.83954	-1.83549	-1.83145	-1.85541	-1.85137	-1.87898
-1.81554	-1.84783	-1.84381	-1.83978	-1.83573	-1.83169	-1.85563	-1.85155	-1.8475
-1.81581	-1.84814	-1.84409	-1.84005	-1.83602	-1.83198	-1.85587	-1.85173	-1.84765
-1.81604	-1.84841	-1.84434	-1.86846	-1.86436	-1.86025	-1.8561	-1.85195	-1.84786
-1.81623	-1.84861	-1.84453	-1.86864	-1.86454	-1.86045	-1.85634	-1.85222	-1.84812
-1.81644	-1.84883	-1.84475	-1.86884	-1.86473	-1.86064	-1.85656	-1.85249	-1.84838
-1.8167	-1.8491	-1.84502	-1.86911	-1.86496	-1.86082	-1.85675	-1.85271	-1.84862
-1.81696	-1.84937	-1.84531	-1.86939	-1.86519	-1.86102	-1.85694	-1.85293	-1.84887
-1.81718	-1.81321	-1.80928	-1.86963	-1.86545	-1.86128	-1.8572	-1.85318	-1.84913
-1.81739	-1.81339	-1.80947	-1.86986	-1.86571	-1.86157	-1.85749	-1.85345	-1.84941
-1.81762	-1.81362	-1.8097	-1.87011	-1.86598	-1.86186	-1.85776	-1.85369	-1.84963
-1.81788	-1.8139	-1.80997	-1.87038	-1.86627	-1.86215	-1.85802	-1.85389	-1.84981
-1.81816	-1.81417	-1.81022	-1.87062	-1.86652	-1.86241	-1.85827	-1.85412	-1.85004
-1.81843	-1.81443	-1.81043	-1.87081	-1.86671	-1.86263	-1.85852	-1.8544	-1.85033

-1.80142	-1.8147	-1.81067	-1.83567	-1.83168	-1.82772	-1.85877	-1.85469	-1.85064
-1.80171	-1.81499	-1.81094	-1.83594	-1.83194	-1.82799	-1.85906	-1.85499	-1.85095
-1.802	-1.81529	-1.81124	-1.83623	-1.83223	-1.82827	-1.85936	-1.8553	-1.85126
-1.80229	-1.84473	-1.8406	-1.83652	-1.83252	-1.82856	-1.85966	-1.8556	-1.85156
-1.80258	-1.84503	-1.8409	-1.83682	-1.83282	-1.82886	-1.85997	-1.85592	-1.85187
-1.80287	-1.84532	-1.8412	-1.83711	-1.83311	-1.82915	-1.86028	-1.85622	-1.85218
-1.80316	-1.84562	-1.84149	-1.83741	-1.83341	-1.82945	-1.86059	-1.85653	-1.85249
-1.80345	-1.82905	-1.82497	-1.83771	-1.83371	-1.82974	-1.8609	-1.85684	-1.87122
-1.80375	-1.82934	-1.82526	-1.83801	-1.83401	-1.83004	-1.8612	-1.85715	-1.87153
-1.80404	-1.82963	-1.82556	-1.83831	-1.83431	-1.83035	-1.86151	-1.85745	-1.87183
-1.80434	-1.82994	-1.82587	-1.83862	-1.83461	-1.83064	-1.86182	-1.85775	-1.87214
-1.80465	-1.83025	-1.82618	-1.83895	-1.83493	-1.83095	-1.86213	-1.85806	-1.87244
-1.80494	-1.83057	-1.82654	-1.83933	-1.83531	-1.83131	-1.82733	-1.82336	-1.87276
-1.80524	-1.83089	-1.82691	-1.83974	-1.83573	-1.83172	-1.8277	-1.82369	-1.87308
-1.80556	-1.83122	-1.82725	-1.82329	-1.81933	-1.81536	-1.84706	-1.843	-1.87344
-1.80589	-1.83153	-1.82756	-1.8236	-1.81964	-1.8157	-1.84742	-1.84339	-1.87387
-1.8359	-1.83187	-1.82788	-1.82392	-1.81996	-1.81602	-1.84777	-1.84376	-1.88752
-1.83629	-1.83227	-1.82828	-1.8243	-1.82031	-1.81634	-1.84808	-1.84407	-1.88785
-1.83667	-1.83267	-1.82868	-1.82468	-1.82067	-1.81667	-1.8484	-1.84438	-1.88818
-1.83699	-1.83299	-1.82901	-1.82503	-1.82104	-1.81706	-1.84879	-1.84476	-1.88855
-1.83728	-1.83328	-1.82932	-1.82536	-1.8214	-1.81746	-1.84921	-1.84517	-1.88895
-1.83763	-1.83362	-1.82964	-1.84505	-1.84105	-1.83707	-1.83309	-1.8291	-1.85516
-1.83802	-1.83399	-1.82997	-1.84535	-1.84134	-1.83736	-1.83339	-1.82944	-1.85553
-1.82203	-1.83436	-1.83032	-1.8457	-1.84169	-1.8377	-1.83374	-1.8298	-1.85592
-1.82238	-1.83471	-1.8307	-1.84609	-1.84209	-1.83812	-1.83415	-1.83019	-1.85632
-1.82275	-1.83508	-1.83109	-1.84651	-1.84252	-1.83855	-1.83457	-1.83059	-1.85672
-1.82313	-1.83548	-1.83151	-1.84694	-1.84294	-1.83896	-1.83498	-1.83099	-1.85711
-1.82352	-1.8359	-1.83193	-1.84737	-1.84337	-1.83937	-1.83538	-1.83139	-1.8575
-1.82394	-1.81997	-1.81604	-1.84779	-1.84378	-1.83977	-1.83576	-1.83175	-1.84164
-1.82436	-1.8204	-1.81645	-1.84819	-1.84418	-1.84016	-1.83614	-1.8321	-1.842
-1.82478	-1.82081	-1.81685	-1.83253	-1.82853	-1.82454	-1.8365	-1.83247	-1.84238
-1.82519	-1.82121	-1.81724	-1.83291	-1.8289	-1.82489	-1.83686	-1.83286	-1.8428
-1.84539	-1.84135	-1.83731	-1.83328	-1.82926	-1.82525	-1.83546	-1.83148	-1.84322
-1.84581	-1.84175	-1.83767	-1.83361	-1.82961	-1.82563	-1.83587	-1.8319	-1.84365
-1.84624	-1.84217	-1.83805	-1.83397	-1.82998	-1.82603	-1.83629	-1.83232	-1.84408
-1.84667	-1.84261	-1.83852	-1.83445	-1.83043	-1.82645	-1.83669	-1.83274	-1.82879
-1.8471	-1.84306	-1.83901	-1.83497	-1.83092	-1.82688	-1.83711	-1.83315	-1.8292
-1.84753	-1.8435	-1.83948	-1.83545	-1.8314	-1.82737	-1.83759	-1.83358	-1.82961
-1.84796	-1.84394	-1.83991	-1.8359	-1.8319	-1.82791	-1.83809	-1.83403	-1.83003
-1.84839	-1.84437	-1.84035	-1.83634	-1.83237	-1.82839	-1.83859	-1.83451	-1.83049
-1.84881	-1.84479	-1.84078	-1.83678	-1.8328	-1.82884	-1.83907	-1.83503	-1.831
-1.84923	-1.84521	-1.8412	-1.83721	-1.83323	-1.82927	-1.83954	-1.83553	-1.83151
-1.84966	-1.84563	-1.84162	-1.83762	-1.83365	-1.82969	-1.83998	-1.83598	-1.83198
-1.8501	-1.84606	-1.84204	-1.83804	-1.83407	-1.83012	-1.84041	-1.83641	-1.83244
-1.85062	-1.84656	-1.8425	-1.83847	-1.83449	-1.83054	-1.84084	-1.83685	-1.83287
-1.85116	-1.84709	-1.84298	-1.83891	-1.83493	-1.83098	-1.84128	-1.83728	-1.83331
-1.85165	-1.84758	-1.84348	-1.8538	-1.84977	-1.84577	-1.84177	-1.83778	-1.83381
-1.85208	-1.84804	-1.84399	-1.85436	-1.85032	-1.8463	-1.84231	-1.83833	-1.83436
-1.85253	-1.84849	-1.84448	-1.85488	-1.85083	-1.8468	-1.84281	-1.83884	-1.83486
-1.85299	-1.84892	-1.84492	-1.85534	-1.85129	-1.84726	-1.84326	-1.83929	-1.83531

-1.85347	-1.84939	-1.84539	-1.85581	-1.85177	-1.84773	-1.84374	-1.83976	-1.83578
-1.85399	-1.84992	-1.84592	-1.85635	-1.85231	-1.84827	-1.84427	-1.84029	-1.83631
-1.85454	-1.85049	-1.84648	-1.85691	-1.85286	-1.84883	-1.84483	-1.84085	-1.83685
-1.85508	-1.85104	-1.84702	-1.85745	-1.85341	-1.84938	-1.84538	-1.84139	-1.83739
-1.85563	-1.85159	-1.84756	-1.85799	-1.85395	-1.84993	-1.84592	-1.84192	-1.83792
-1.83624	-1.83225	-1.82827	-1.85848	-1.85445	-1.85043	-1.84641	-1.8424	-1.8384
-1.83668	-1.83269	-1.82871	-1.85892	-1.85489	-1.85087	-1.84685	-1.84284	-1.83884
-1.83715	-1.83317	-1.82919	-1.85941	-1.85537	-1.85135	-1.84732	-1.84331	-1.83932
-1.83769	-1.8337	-1.82972	-1.85995	-1.85591	-1.85188	-1.84786	-1.84385	-1.83986
-1.83824	-1.83425	-1.83027	-1.86052	-1.85647	-1.85244	-1.84841	-1.8444	-1.84041
-1.83879	-1.8348	-1.83082	-1.86108	-1.85703	-1.853	-1.84897	-1.84496	-1.84097
-1.83935	-1.83536	-1.83138	-1.86165	-1.8576	-1.85357	-1.84954	-1.84552	-1.84153
-1.83991	-1.83592	-1.83193	-1.86221	-1.85817	-1.85413	-1.8501	-1.84608	-1.84208
-1.84047	-1.83648	-1.83249	-1.86278	-1.85873	-1.85469	-1.85066	-1.84664	-1.84264
-1.84103	-1.83703	-1.83305	-1.86334	-1.85929	-1.85526	-1.85122	-1.8472	-1.84319
-1.84158	-1.83759	-1.8336	-1.8639	-1.85986	-1.85582	-1.85178	-1.84775	-1.84374
-1.84214	-1.83814	-1.83416	-1.86448	-1.86043	-1.85639	-1.85235	-1.84832	-1.84431
-1.8427	-1.8387	-1.83472	-1.86506	-1.861	-1.85695	-1.85292	-1.84889	-1.84488
-1.84328	-1.83928	-1.8353	-1.86566	-1.86159	-1.85754	-1.85351	-1.84949	-1.84548
-1.84392	-1.83991	-1.83594	-1.84674	-1.84273	-1.83872	-1.85415	-1.85014	-1.84613
-1.84458	-1.84057	-1.8366	-1.84741	-1.84339	-1.83938	-1.85482	-1.85081	-1.84679
-1.84518	-1.84117	-1.83719	-1.84801	-1.84399	-1.83999	-1.85543	-1.85141	-1.8474
-1.84573	-1.84173	-1.83774	-1.84856	-1.84455	-1.84055	-1.85599	-1.85197	-1.84795
-1.84629	-1.84229	-1.8383	-1.84912	-1.84511	-1.84111	-1.85656	-1.85253	-1.84852
-1.84686	-1.84285	-1.83884	-1.84966	-1.84566	-1.84168	-1.85712	-1.85309	-1.84907
-1.86238	-1.85833	-1.85427	-1.85024	-1.84625	-1.84227	-1.85772	-1.85368	-1.84967
-1.86303	-1.85898	-1.85492	-1.85089	-1.8469	-1.84293	-1.85839	-1.85435	-1.85034
-1.86369	-1.85964	-1.85558	-1.85154	-1.84756	-1.8436	-1.85908	-1.85505	-1.85104
-1.86429	-1.86024	-1.85619	-1.85216	-1.84817	-1.8442	-1.85971	-1.85572	-1.85172
-1.86486	-1.86081	-1.85677	-1.85275	-1.84875	-1.84477	-1.86032	-1.85638	-1.8524
-1.86549	-1.86144	-1.8574	-1.85339	-1.84938	-1.84539	-1.86094	-1.857	-1.85301
-1.86618	-1.86212	-1.85808	-1.85406	-1.85004	-1.84605	-1.86156	-1.85756	-1.85356
-1.86687	-1.86281	-1.85876	-1.85473	-1.85072	-1.84672	-1.86221	-1.85818	-1.85416
-1.86754	-1.8635	-1.85944	-1.8554	-1.8514	-1.84741	-1.8629	-1.85886	-1.85483
-1.86819	-1.86417	-1.8601	-1.85605	-1.85206	-1.84808	-1.86359	-1.85955	-1.85551
-1.85377	-1.84978	-1.84576	-1.85666	-1.85265	-1.84867	-1.84471	-1.84077	-1.85621
-1.85436	-1.85034	-1.84634	-1.85725	-1.85324	-1.84925	-1.84532	-1.84143	-1.8569
-1.85501	-1.85098	-1.84697	-1.8579	-1.85388	-1.84989	-1.84596	-1.84206	-1.85754
-1.8557	-1.85166	-1.84765	-1.85857	-1.85456	-1.85057	-1.8466	-1.84267	-1.85815
-1.83627	-1.85235	-1.84832	-1.85925	-1.85524	-1.85125	-1.84729	-1.84334	-1.91067
-1.83697	-1.85303	-1.84901	-1.85995	-1.85594	-1.85195	-1.84801	-1.8441	-1.91144
-1.83768	-1.83365	-1.82966	-1.86065	-1.85663	-1.85265	-1.84871	-1.84481	-1.91219
-1.83838	-1.83436	-1.83036	-1.86133	-1.85732	-1.85333	-1.84937	-1.84544	-1.91285
-1.83909	-1.83506	-1.83106	-1.86202	-1.858	-1.85401	-1.85004	-1.84608	-1.91352
-1.83979	-1.83576	-1.83175	-1.84775	-1.84378	-1.83983	-1.85075	-1.8468	-1.91426
-1.84048	-1.83645	-1.83246	-1.84844	-1.84448	-1.84054	-1.85149	-1.84755	-1.91502
-1.84118	-1.83716	-1.83317	-1.8292	-1.82528	-1.82137	-1.85223	-1.84827	-1.91577
-1.84188	-1.83787	-1.83389	-1.82993	-1.82603	-1.82214	-1.85297	-1.84898	-1.91649
-1.8426	-1.83859	-1.83462	-1.83068	-1.82677	-1.82288	-1.83407	-1.83009	-1.89843
-1.84331	-1.83933	-1.83538	-1.83144	-1.82752	-1.8236	-1.83479	-1.8308	-1.89915

-1.84405	-1.84007	-1.83613	-1.83219	-1.82825	-1.82431	-1.82038	-1.81644	-1.89989
-1.84482	-1.84082	-1.83686	-1.83291	-1.82897	-1.82503	-1.82111	-1.8172	-1.90068
-1.8456	-1.84157	-1.83759	-1.83362	-1.82968	-1.82575	-1.82185	-1.81797	-1.90148
-1.84634	-1.8423	-1.8383	-1.83434	-1.8304	-1.82649	-1.87719	-1.87311	-1.88346
-1.84706	-1.84302	-1.83902	-1.83505	-1.83114	-1.82725	-1.87795	-1.87386	-1.8842
-1.84784	-1.8438	-1.83979	-1.83581	-1.83188	-1.82798	-1.8787	-1.8746	-1.88496
-1.84867	-1.84463	-1.8406	-1.83659	-1.83263	-1.82869	-1.87943	-1.87533	-1.88571
-1.84947	-1.84543	-1.8414	-1.83739	-1.8334	-1.82943	-1.88019	-1.87609	-1.88648
-1.8502	-1.84618	-1.84219	-1.8382	-1.83422	-1.83025	-1.88102	-1.87689	-1.88726
-1.85094	-1.84693	-1.84296	-1.83899	-1.83502	-1.83105	-1.88184	-1.87771	-1.88806
-1.85172	-1.84771	-1.84371	-1.83972	-1.83575	-1.8318	-1.88264	-1.87853	-1.8889
-1.85251	-1.8485	-1.84446	-1.84045	-1.83647	-1.83253	-1.88343	-1.87936	-1.88976
-1.85331	-1.84931	-1.84526	-1.84124	-1.83725	-1.8333	-1.88423	-1.88018	-1.89059
-1.85412	-1.85012	-1.84608	-1.84206	-1.83807	-1.8341	-1.88504	-1.88099	-1.89142
-1.81937	-1.85088	-1.84686	-1.84285	-1.83886	-1.8349	-1.88584	-1.88177	-1.8922
-1.82008	-1.85159	-1.84758	-1.84359	-1.83964	-1.83569	-1.88664	-1.88251	-1.89292
-1.82082	-1.81684	-1.81291	-1.84436	-1.84043	-1.8365	-1.88745	-1.88328	-1.89368
-1.82161	-1.81763	-1.81369	-1.84516	-1.84124	-1.83732	-1.8883	-1.88411	-1.89451
-1.82242	-1.81843	-1.81449	-1.84598	-1.84206	-1.83815	-1.88915	-1.88495	-1.89535
-1.82323	-1.81924	-1.81529	-1.86835	-1.86432	-1.8603	-1.89	-1.8858	-1.8962
-1.82404	-1.82005	-1.8161	-1.86919	-1.86516	-1.86113	-1.89086	-1.88665	-1.89706
-1.82486	-1.82086	-1.81691	-1.87002	-1.86599	-1.86197	-1.85786	-1.85371	-1.89791
-1.82567	-1.82167	-1.81771	-1.87086	-1.86683	-1.8628	-1.85868	-1.85453	-1.89876
-1.80656	-1.82248	-1.81852	-1.87169	-1.86766	-1.86363	-1.85951	-1.85536	-1.85125
-1.80737	-1.82328	-1.81932	-1.87253	-1.86849	-1.86446	-1.86035	-1.8562	-1.85208
-1.80818	-1.8241	-1.82013	-1.87336	-1.86933	-1.8653	-1.8612	-1.85706	-1.85294
-1.809	-1.82492	-1.82094	-1.87418	-1.87015	-1.86614	-1.86207	-1.85797	-1.85386
-1.80984	-1.82576	-1.82176	-1.87502	-1.87099	-1.86698	-1.86294	-1.85887	-1.85477
-1.79085	-1.80677	-1.8028	-1.8759	-1.87184	-1.86781	-1.86377	-1.85973	-1.85564
-1.79174	-1.80769	-1.8037	-1.8768	-1.8727	-1.86863	-1.86459	-1.86056	-1.85648
-1.79258	-1.80856	-1.80457	-1.87769	-1.87355	-1.86945	-1.86541	-1.86139	-1.85734
-1.79338	-1.80939	-1.80541	-1.87857	-1.8744	-1.87027	-1.86622	-1.86223	-1.8582
-1.79419	-1.81021	-1.80627	-1.86045	-1.85632	-1.85221	-1.86707	-1.86306	-1.85904
-1.79501	-1.81105	-1.80716	-1.86141	-1.85727	-1.85313	-1.86795	-1.86388	-1.85985
-1.77758	-1.87043	-1.8664	-1.86235	-1.85822	-1.85408	-1.86886	-1.86474	-1.86068
-1.77838	-1.87125	-1.86725	-1.86324	-1.85916	-1.85506	-1.86983	-1.86566	-1.86156
-1.77919	-1.87208	-1.86808	-1.86409	-1.86006	-1.85602	-1.87081	-1.86662	-1.86247
-1.78006	-1.85576	-1.85174	-1.86491	-1.86091	-1.8569	-1.85283	-1.84873	-1.86342
-1.78098	-1.85668	-1.8526	-1.86571	-1.86171	-1.85773	-1.85372	-1.84969	-1.8644
-1.75891	-1.85763	-1.8535	-1.84943	-1.84543	-1.84147	-1.85457	-1.85058	-1.86532
-1.75981	-1.85861	-1.85446	-1.85035	-1.84631	-1.8423	-1.85538	-1.85139	-1.86619
-1.76068	-1.85956	-1.85542	-1.8513	-1.84723	-1.84319	-1.85625	-1.85225	-1.84824
-1.7615	-1.86043	-1.85634	-1.85228	-1.84822	-1.84419	-1.85722	-1.85317	-1.84914
-1.76233	-1.8613	-1.85726	-1.85323	-1.84921	-1.84518	-1.8582	-1.85412	-1.85005
-1.76323	-1.84277	-1.83875	-1.8541	-1.85008	-1.84607	-1.84204	-1.83802	-1.85102
-1.76415	-1.84371	-1.83963	-1.85496	-1.85092	-1.84692	-1.84293	-1.83895	-1.85199
-1.76507	-1.82254	-1.81852	-1.85592	-1.85185	-1.84782	-1.84381	-1.83983	-1.83585
-1.76599	-1.82353	-1.81953	-1.85695	-1.85284	-1.84876	-1.8447	-1.84066	-1.83667
-1.76691	-1.82449	-1.82049	-1.85793	-1.85381	-1.8497	-1.84562	-1.84157	-1.83756
-1.76783	-1.82541	-1.82138	-1.85884	-1.85474	-1.85066	-1.84661	-1.84257	-1.83855

-1.81034	-1.82634	-1.8223	-1.85978	-1.85569	-1.85163	-1.84758	-1.84355	-1.83952
-1.81127	-1.82732	-1.82329	-1.86081	-1.85668	-1.85258	-1.8485	-1.84446	-1.84043
-1.81221	-1.82829	-1.82429	-1.86184	-1.85768	-1.85354	-1.84944	-1.84537	-1.84136
-1.81314	-1.82921	-1.8252	-1.8212	-1.81718	-1.81318	-1.85043	-1.84636	-1.84235
-1.81406	-1.83012	-1.82609	-1.82209	-1.81811	-1.81415	-1.85145	-1.84739	-1.84338
-1.81501	-1.83107	-1.82704	-1.82305	-1.81909	-1.81515	-1.85247	-1.84839	-1.84436
-1.81597	-1.83206	-1.82805	-1.82405	-1.8201	-1.81615	-1.85348	-1.84937	-1.84531
-1.81695	-1.83306	-1.82904	-1.82504	-1.82107	-1.81711	-1.85446	-1.85035	-1.84627
-1.81797	-1.83408	-1.83002	-1.82598	-1.82197	-1.81799	-1.85539	-1.85133	-1.84728
-1.81897	-1.83508	-1.83099	-1.82693	-1.82289	-1.81889	-1.81495	-1.81104	-1.84829
-1.81989	-1.836	-1.83195	-1.82792	-1.82389	-1.81989	-1.81594	-1.81203	-1.8493
-1.8208	-1.83693	-1.83292	-1.82893	-1.82492	-1.82092	-1.81696	-1.81303	-1.8503
-1.78659	-1.83794	-1.83394	-1.82995	-1.82595	-1.82196	-1.81798	-1.81401	-1.85129
-1.7876	-1.83899	-1.83498	-1.83098	-1.82699	-1.823	-1.81899	-1.81498	-1.85227
-1.80936	-1.84004	-1.836	-1.83197	-1.82796	-1.82396	-1.81995	-1.81594	-1.85327
-1.81041	-1.84109	-1.837	-1.83292	-1.82887	-1.82485	-1.82085	-1.81689	-1.85429
-1.8114	-1.8421	-1.83798	-1.83388	-1.82981	-1.82578	-1.82181	-1.81787	-1.83356
-1.8123	-1.84302	-1.83893	-1.83487	-1.83083	-1.82681	-1.82284	-1.81891	-1.83461
-1.81321	-1.84395	-1.8399	-1.83588	-1.83187	-1.82787	-1.82391	-1.81997	-1.83568
-1.8142	-1.84497	-1.84093	-1.83692	-1.83292	-1.82895	-1.82498	-1.82103	-1.83675
-1.81522	-1.84603	-1.84199	-1.83798	-1.83399	-1.83003	-1.82606	-1.82211	-1.83781
-1.81626	-1.84711	-1.84307	-1.83905	-1.83506	-1.83109	-1.82712	-1.82316	-1.81919
-1.81732	-1.8482	-1.84416	-1.84013	-1.83614	-1.83215	-1.82817	-1.82419	-1.8202
-1.81838	-1.81443	-1.81048	-1.84121	-1.8372	-1.8332	-1.82919	-1.82519	-1.82118
-1.81944	-1.81548	-1.81154	-1.84229	-1.83826	-1.83423	-1.83019	-1.82615	-1.82213
-1.8205	-1.81653	-1.81258	-1.84335	-1.8393	-1.83524	-1.83118	-1.82712	-1.82309
-1.82156	-1.81757	-1.8136	-1.84438	-1.84029	-1.8362	-1.83213	-1.82808	-1.82407
-1.82261	-1.8186	-1.81461	-1.84538	-1.84127	-1.83716	-1.83309	-1.82906	-1.82507
-1.82366	-1.81962	-1.81559	-1.84635	-1.84223	-1.83813	-1.83408	-1.83007	-1.8261
-1.82471	-1.82063	-1.81655	-1.8473	-1.84318	-1.83911	-1.83509	-1.83111	-1.82716
-1.82577	-1.82167	-1.81756	-1.84831	-1.84419	-1.84012	-1.83612	-1.83217	-1.82823
-1.82685	-1.82273	-1.81861	-1.84937	-1.84524	-1.84117	-1.83717	-1.83324	-1.82931
-1.82793	-1.82381	-1.81968	-1.85045	-1.84631	-1.84222	-1.83823	-1.8343	-1.83037
-1.82903	-1.8249	-1.82077	-1.85156	-1.84739	-1.84329	-1.83929	-1.83536	-1.83143
-1.83012	-1.826	-1.82188	-1.85269	-1.84852	-1.84439	-1.84037	-1.83642	-1.83248
-1.83123	-1.82712	-1.82302	-1.85389	-1.8497	-1.84556	-1.84149	-1.83749	-1.83352
-1.83232	-1.82824	-1.82417	-1.85509	-1.85091	-1.84675	-1.84265	-1.83859	-1.83459
-1.81564	-1.82932	-1.82527	-1.85623	-1.85209	-1.84797	-1.84388	-1.8398	-1.83578
-1.81669	-1.8304	-1.82633	-1.85733	-1.85326	-1.8492	-1.84514	-1.84108	-1.83703
-1.81775	-1.81371	-1.80968	-1.82338	-1.81942	-1.81549	-1.84631	-1.84227	-1.83823
-1.8188	-1.81477	-1.81074	-1.82444	-1.82047	-1.81654	-1.8474	-1.84339	-1.83939
-1.81985	-1.81582	-1.81179	-1.8255	-1.82152	-1.81758	-1.84847	-1.84448	-1.8405
-1.82089	-1.81686	-1.81284	-1.82657	-1.82258	-1.81863	-1.84953	-1.84554	-1.84156
-1.82193	-1.8179	-1.8139	-1.82764	-1.82364	-1.81967	-1.85059	-1.84659	-1.84261
-1.80321	-1.81894	-1.81494	-1.81095	-1.80698	-1.80304	-1.85165	-1.84764	-1.84365
-1.80425	-1.81999	-1.81598	-1.81199	-1.80802	-1.80407	-1.85271	-1.8487	-1.8447
-1.78487	-1.82105	-1.81704	-1.81305	-1.80907	-1.80512	-1.83662	-1.83264	-1.84578
-1.78594	-1.82213	-1.81812	-1.81413	-1.81015	-1.8062	-1.83771	-1.83373	-1.84688
-1.78709	-1.80317	-1.79919	-1.81531	-1.81132	-1.80736	-1.80344	-1.79956	-1.84807
-1.78835	-1.80443	-1.80046	-1.81658	-1.8126	-1.80863	-1.80471	-1.80081	-1.84936

-1.76209	-1.80566	-1.80169	-1.8178	-1.81382	-1.80985	-1.80592	-1.80202	-1.83345
-1.76314	-1.80678	-1.8028	-1.8189	-1.81492	-1.81096	-1.80702	-1.8031	-1.83456
-1.76416	-1.80788	-1.80388	-1.81997	-1.81599	-1.81203	-1.80809	-1.80416	-1.83563
-1.76518	-1.80898	-1.80496	-1.80098	-1.79703	-1.79311	-1.80916	-1.80522	-1.83672
-1.7662	-1.81009	-1.80605	-1.80206	-1.79811	-1.79419	-1.81024	-1.8063	-1.83781
-1.76733	-1.79116	-1.78717	-1.80324	-1.79929	-1.79537	-1.81142	-1.80747	-1.80356
-1.76854	-1.79243	-1.78844	-1.8045	-1.80055	-1.79662	-1.81267	-1.80873	-1.80482
-1.76966	-1.76589	-1.76214	-1.80571	-1.80175	-1.79782	-1.7939	-1.78999	-1.80601
-1.77069	-1.76692	-1.76317	-1.80683	-1.80287	-1.79894	-1.79502	-1.79111	-1.80713
-1.75049	-1.76793	-1.76418	-1.80794	-1.80398	-1.80006	-1.79615	-1.79225	-1.78836
-1.75147	-1.76893	-1.76519	-1.80903	-1.80511	-1.80122	-1.79734	-1.79347	-1.78959
-1.75248	-1.76995	-1.76621	-1.81015	-1.80624	-1.80238	-1.79853	-1.79469	-1.79083
-1.75361	-1.77109	-1.76734	-1.81137	-1.80743	-1.80354	-1.79967	-1.79583	-1.79196
-1.7548	-1.77229	-1.76854	-1.81264	-1.80865	-1.8047	-1.80079	-1.79693	-1.79305
-1.75589	-1.77339	-1.76964	-1.7864	-1.78265	-1.77892	-1.80197	-1.79809	-1.79421
-1.75691	-1.77442	-1.77067	-1.78747	-1.78374	-1.78005	-1.80319	-1.79932	-1.79544
-1.75799	-1.75428	-1.75058	-1.78858	-1.78486	-1.78118	-1.80438	-1.8005	-1.79661
-1.75917	-1.75546	-1.75176	-1.78977	-1.78601	-1.78227	-1.80551	-1.80159	-1.79769
-1.76034	-1.75663	-1.75293	-1.79097	-1.78717	-1.78339	-1.80666	-1.80271	-1.7988
-1.7614	-1.75771	-1.75405	-1.77157	-1.76786	-1.76416	-1.80792	-1.80394	-1.80001
-1.76244	-1.75877	-1.75515	-1.77272	-1.76904	-1.76535	-1.80919	-1.80518	-1.80125
-1.76358	-1.75989	-1.75625	-1.77382	-1.77012	-1.76643	-1.78312	-1.77935	-1.80241
-1.76478	-1.76106	-1.75736	-1.77489	-1.77116	-1.76745	-1.78415	-1.78038	-1.80353
-1.76598	-1.76225	-1.75853	-1.75481	-1.75111	-1.74743	-1.78527	-1.78149	-1.8047
-1.76719	-1.76348	-1.75976	-1.75604	-1.75232	-1.74862	-1.78647	-1.78268	-1.80593
-1.76834	-1.76466	-1.76096	-1.75725	-1.75353	-1.74983	-1.7877	-1.7839	-1.8072
-1.76939	-1.76572	-1.76206	-1.7584	-1.75473	-1.75105	-1.78897	-1.78516	-1.80853
-1.77045	-1.76678	-1.76315	-1.75953	-1.75589	-1.75224	-1.79021	-1.78641	-1.80986
-1.7716	-1.76791	-1.76426	-1.76061	-1.75696	-1.75331	-1.79134	-1.78758	-1.78383
-1.77281	-1.76908	-1.76537	-1.76168	-1.75799	-1.75433	-1.79241	-1.78871	-1.785
-1.77404	-1.77029	-1.76655	-1.76283	-1.75912	-1.75544	-1.77266	-1.769	-1.78613
-1.77528	-1.77154	-1.76779	-1.76405	-1.76031	-1.7566	-1.77379	-1.77008	-1.78721
-1.75748	-1.77275	-1.76901	-1.76527	-1.76153	-1.75779	-1.77496	-1.77122	-1.78834
-1.7586	-1.77392	-1.77022	-1.76651	-1.76276	-1.75901	-1.77618	-1.77241	-1.7895
-1.75969	-1.77505	-1.7714	-1.76772	-1.76398	-1.76023	-1.7565	-1.75278	-1.76988
-1.76076	-1.77612	-1.77251	-1.76887	-1.76517	-1.76145	-1.75772	-1.75398	-1.77109
-1.76184	-1.7772	-1.7736	-1.76999	-1.76634	-1.76266	-1.75894	-1.7552	-1.77231
-1.76299	-1.77832	-1.7747	-1.77108	-1.76746	-1.76382	-1.76013	-1.75641	-1.77354
-1.76418	-1.77948	-1.77581	-1.77216	-1.76856	-1.76496	-1.7613	-1.75761	-1.77479
-1.76538	-1.76168	-1.75802	-1.77331	-1.76972	-1.76613	-1.76249	-1.75883	-1.77602
-1.76659	-1.76289	-1.75923	-1.77451	-1.77092	-1.76733	-1.7637	-1.76004	-1.77726
-1.76781	-1.7641	-1.76043	-1.77573	-1.77212	-1.76854	-1.76491	-1.76126	-1.77849
-1.76903	-1.76531	-1.76164	-1.77694	-1.77334	-1.76976	-1.76613	-1.76247	-1.77971
-1.77026	-1.76652	-1.76284	-1.77815	-1.77456	-1.77098	-1.76735	-1.76368	-1.75997
-1.77148	-1.76773	-1.76404	-1.77936	-1.77577	-1.77219	-1.76856	-1.76489	-1.76119
-1.7727	-1.76894	-1.76525	-1.78056	-1.77697	-1.7734	-1.76977	-1.76611	-1.7624
-1.77393	-1.77015	-1.76645	-1.76282	-1.75926	-1.75573	-1.77097	-1.76731	-1.76361
-1.77515	-1.77137	-1.76767	-1.76402	-1.76045	-1.75691	-1.77216	-1.76851	-1.76481
-1.75535	-1.7726	-1.76888	-1.76522	-1.76165	-1.7581	-1.77335	-1.76971	-1.76601
-1.75656	-1.77383	-1.77009	-1.76642	-1.76284	-1.75929	-1.77455	-1.77091	-1.76722

-1.75777	-1.77506	-1.77131	-1.76762	-1.76403	-1.76048	-1.75689	-1.75329	-1.76842
-1.75898	-1.77628	-1.77252	-1.76882	-1.76523	-1.76168	-1.75808	-1.75448	-1.76962
-1.76019	-1.77751	-1.77373	-1.77003	-1.76643	-1.76287	-1.75928	-1.75567	-1.77082
-1.6839	-1.77874	-1.77496	-1.77124	-1.76763	-1.76406	-1.76047	-1.75687	-1.77203
-1.68507	-1.77999	-1.7762	-1.77248	-1.76885	-1.76526	-1.76166	-1.75807	-1.77324
-1.60914	-1.7601	-1.7564	-1.77378	-1.77012	-1.7665	-1.76289	-1.75928	-1.75567
-1.61026	-1.76134	-1.7577	-1.77512	-1.77144	-1.76778	-1.76413	-1.76051	-1.7569
-1.61138	-1.63423	-1.63089	-1.77642	-1.77272	-1.76903	-1.76537	-1.76172	-1.75812
-1.61249	-1.63537	-1.63203	-1.77765	-1.77395	-1.77026	-1.76659	-1.76294	-1.75932
-1.61364	-1.63653	-1.63318	-1.70644	-1.70288	-1.69932	-1.76782	-1.76417	-1.76055
-1.61483	-1.63777	-1.6344	-1.70769	-1.7041	-1.70052	-1.76907	-1.76543	-1.76181
-1.61603	-1.63901	-1.63561	-1.70894	-1.70533	-1.70174	-1.77034	-1.76672	-1.7631
-1.61716	-1.64017	-1.63678	-1.63338	-1.62998	-1.62659	-1.77164	-1.768	-1.76436
-1.61826	-1.64129	-1.63792	-1.63454	-1.63118	-1.62782	-1.77295	-1.76928	-1.76563
-1.61939	-1.64244	-1.63905	-1.63568	-1.63233	-1.62898	-1.70195	-1.69841	-1.76688
-1.62054	-1.64359	-1.64018	-1.63679	-1.63344	-1.6301	-1.70312	-1.69959	-1.76812
-1.62169	-1.64476	-1.64133	-1.63793	-1.63457	-1.63123	-1.6507	-1.6473	-1.69727
-1.62283	-1.64593	-1.64252	-1.63913	-1.63575	-1.63239	-1.65186	-1.64843	-1.69843
-1.62399	-1.62065	-1.61734	-1.64033	-1.63694	-1.63355	-1.65304	-1.6496	-1.69961
-1.62516	-1.62183	-1.61852	-1.64152	-1.63811	-1.63471	-1.65423	-1.65081	-1.70086
-1.62635	-1.62302	-1.61969	-1.64271	-1.63928	-1.63587	-1.65544	-1.65205	-1.70214
-1.62753	-1.62418	-1.62083	-1.64387	-1.64046	-1.63707	-1.63372	-1.6304	-1.6499
-1.62871	-1.62533	-1.62196	-1.64502	-1.64164	-1.63828	-1.63493	-1.6316	-1.65114
-1.62988	-1.62648	-1.62312	-1.64621	-1.64284	-1.63949	-1.63614	-1.63281	-1.65237
-1.63103	-1.62764	-1.6243	-1.64744	-1.64405	-1.64068	-1.63734	-1.63403	-1.65361
-1.63221	-1.62882	-1.62549	-1.64867	-1.64527	-1.64189	-1.63855	-1.63525	-1.65484
-1.63342	-1.63004	-1.62668	-1.64988	-1.6465	-1.64314	-1.63978	-1.63644	-1.65606
-1.63464	-1.63126	-1.62788	-1.65109	-1.64773	-1.64439	-1.64101	-1.63764	-1.65728
-1.63586	-1.63248	-1.62911	-1.65235	-1.64896	-1.64559	-1.64222	-1.63885	-1.63551
-1.63708	-1.63369	-1.63035	-1.65362	-1.65019	-1.64678	-1.64341	-1.64007	-1.63672
-1.6383	-1.63492	-1.63156	-1.65485	-1.65142	-1.64801	-1.64463	-1.64127	-1.63792
-1.63954	-1.63615	-1.63276	-1.65606	-1.65266	-1.64927	-1.64587	-1.64247	-1.63911
-1.64076	-1.63737	-1.63397	-1.65729	-1.65388	-1.65049	-1.64709	-1.64369	-1.64032
-1.64197	-1.63856	-1.63519	-1.65854	-1.6551	-1.65168	-1.64829	-1.64492	-1.64156
-1.6432	-1.63977	-1.6364	-1.63304	-1.62966	-1.6263	-1.64949	-1.64615	-1.64278
-1.64446	-1.64102	-1.63761	-1.63423	-1.63087	-1.62752	-1.65072	-1.64734	-1.64397
-1.64571	-1.64226	-1.63883	-1.63543	-1.63207	-1.62874	-1.65196	-1.64854	-1.64515
-1.61898	-1.64347	-1.64004	-1.63664	-1.63328	-1.62993	-1.65318	-1.64977	-1.64638
-1.62018	-1.64467	-1.64126	-1.63787	-1.63448	-1.63111	-1.65439	-1.65102	-1.64763
-1.59026	-1.64593	-1.64249	-1.63908	-1.63569	-1.63232	-1.65562	-1.65223	-1.64885
-1.59149	-1.64725	-1.64374	-1.64029	-1.6369	-1.63355	-1.65685	-1.65342	-1.65004
-1.56855	-1.59683	-1.59338	-1.64155	-1.63816	-1.63481	-1.65813	-1.65467	-1.65128
-1.56986	-1.59819	-1.59472	-1.6429	-1.63949	-1.63612	-1.65947	-1.656	-1.6526
-1.57115	-1.59954	-1.59607	-1.62095	-1.61753	-1.61415	-1.63404	-1.63067	-1.65392
-1.5724	-1.60083	-1.59737	-1.62227	-1.61882	-1.61541	-1.63529	-1.63194	-1.65521
-1.57364	-1.60212	-1.59867	-1.6236	-1.62012	-1.61667	-1.63654	-1.6332	-1.6565
-1.57489	-1.60344	-1.6	-1.59658	-1.59314	-1.58974	-1.61461	-1.61127	-1.63498
-1.57615	-1.60478	-1.60136	-1.59794	-1.59449	-1.59107	-1.61595	-1.61259	-1.63631
-1.57742	-1.60611	-1.60268	-1.59926	-1.59581	-1.59238	-1.61726	-1.61388	-1.63762
-1.5787	-1.60743	-1.60398	-1.60054	-1.59711	-1.59369	-1.61856	-1.61513	-1.6389

-1.57997	-1.57664	-1.57333	-1.60183	-1.5984	-1.59499	-1.61988	-1.61643	-1.64022
-1.58119	-1.57784	-1.57455	-1.60312	-1.59971	-1.59631	-1.62124	-1.61778	-1.6416
-1.58244	-1.57907	-1.57579	-1.60442	-1.60101	-1.59762	-1.62259	-1.61914	-1.64299
-1.58376	-1.58038	-1.57707	-1.60573	-1.6023	-1.5989	-1.59551	-1.59216	-1.61708
-1.58512	-1.58173	-1.57837	-1.60707	-1.6036	-1.60016	-1.59678	-1.59345	-1.61841
-1.58649	-1.58308	-1.57971	-1.60843	-1.60495	-1.6015	-1.59811	-1.59476	-1.61976
-1.58786	-1.58445	-1.58106	-1.60983	-1.60633	-1.60287	-1.59946	-1.59609	-1.62111
-1.56261	-1.55925	-1.55591	-1.61123	-1.60772	-1.60425	-1.60082	-1.59744	-1.62246
-1.56386	-1.56052	-1.55721	-1.61263	-1.60912	-1.60563	-1.60219	-1.59879	-1.6238
-1.56513	-1.5618	-1.55849	-1.58796	-1.58449	-1.58105	-1.60355	-1.60014	-1.59674
-1.56644	-1.56307	-1.55973	-1.58924	-1.58577	-1.58234	-1.60487	-1.60147	-1.59806
-1.56776	-1.56436	-1.56098	-1.59052	-1.58706	-1.58364	-1.6062	-1.60281	-1.59939
-1.51589	-1.56569	-1.56228	-1.55892	-1.5556	-1.55233	-1.58159	-1.57824	-1.60074
-1.51718	-1.56703	-1.56362	-1.56024	-1.55691	-1.55362	-1.58293	-1.57957	-1.60208
-1.46555	-1.56838	-1.56496	-1.56157	-1.55823	-1.55493	-1.58429	-1.58091	-1.57752
-1.46676	-1.56974	-1.56631	-1.56291	-1.55956	-1.55625	-1.58566	-1.58226	-1.57886
-1.46798	-1.57109	-1.56765	-1.56424	-1.56088	-1.55757	-1.58703	-1.58362	-1.58019
-1.46919	-1.57243	-1.56898	-1.56555	-1.56219	-1.55887	-1.58839	-1.58496	-1.58152
-1.33401	-1.52032	-1.51697	-1.56687	-1.56351	-1.56018	-1.58976	-1.58633	-1.58287
-1.33496	-1.52161	-1.51826	-1.56821	-1.56484	-1.56151	-1.59116	-1.58772	-1.58425
-1.33593	-1.52291	-1.51955	-1.56956	-1.56618	-1.56285	-1.59256	-1.58913	-1.58565
-1.3369	-1.52421	-1.52084	-1.57092	-1.56752	-1.56418	-1.56088	-1.55761	-1.58707
-1.33787	-1.52552	-1.52215	-1.57228	-1.56887	-1.56551	-1.56221	-1.55896	-1.5885
-1.30486	-1.4735	-1.47029	-1.52018	-1.51687	-1.5136	-1.56356	-1.5603	-1.58992
-1.30582	-1.47481	-1.47163	-1.5216	-1.51825	-1.51494	-1.56494	-1.56165	-1.59132
-1.30679	-1.47612	-1.47294	-1.52298	-1.51961	-1.51627	-1.5663	-1.56298	-1.59271
-1.30775	-1.47741	-1.4742	-1.52429	-1.52092	-1.51757	-1.56763	-1.56429	-1.59406
-1.30871	-1.47871	-1.47546	-1.52559	-1.52223	-1.51889	-1.56899	-1.5656	-1.59543
-1.30969	-1.48003	-1.47675	-1.52694	-1.52359	-1.52027	-1.51694	-1.51364	-1.56362
-1.31067	-1.48137	-1.47806	-1.5283	-1.52497	-1.52167	-1.51832	-1.51497	-1.56497
-1.28003	-1.30917	-1.30674	-1.4761	-1.47291	-1.46975	-1.51965	-1.51628	-1.56633
-1.28098	-1.31013	-1.3077	-1.4774	-1.47418	-1.47099	-1.52095	-1.51759	-1.56769
-1.25132	-1.27953	-1.27714	-1.47872	-1.47546	-1.47224	-1.52229	-1.51895	-1.51564
-1.25233	-1.28052	-1.2781	-1.48004	-1.47675	-1.47352	-1.52366	-1.52036	-1.51708
-1.25336	-1.2509	-1.2485	-1.37863	-1.37587	-1.37315	-1.52502	-1.52175	-1.51848
-1.25441	-1.25195	-1.2495	-1.37976	-1.37696	-1.37421	-1.52635	-1.52306	-1.51979
-1.25544	-1.25299	-1.25052	-1.38091	-1.37807	-1.37528	-1.52768	-1.52436	-1.52108
-1.25643	-1.25399	-1.25154	-1.32219	-1.3194	-1.31666	-1.52905	-1.52568	-1.52237
-1.25738	-1.25496	-1.25255	-1.32338	-1.32055	-1.31776	-1.53044	-1.52702	-1.52368
-1.25836	-1.25594	-1.25353	-1.25112	-1.24869	-1.24627	-1.31618	-1.3135	-1.52508
-1.25933	-1.2569	-1.25449	-1.2521	-1.24971	-1.24733	-1.31739	-1.31467	-1.52657
-1.26037	-1.25792	-1.25548	-1.25308	-1.2507	-1.24834	-1.31855	-1.31583	-1.47336
-1.26148	-1.259	-1.25652	-1.25407	-1.25166	-1.24928	-1.31965	-1.31695	-1.47482
-1.18707	-1.26008	-1.25757	-1.25509	-1.25266	-1.25026	-1.32076	-1.31807	-1.37402
-1.18799	-1.26112	-1.25864	-1.25619	-1.25374	-1.25132	-1.32193	-1.3192	-1.37519
-1.1889	-1.26214	-1.25971	-1.25728	-1.25484	-1.2524	-1.32312	-1.32034	-1.37637
-1.18981	-1.26312	-1.2607	-1.25829	-1.25585	-1.25342	-1.25099	-1.24858	-1.31877
-1.19071	-1.26409	-1.26166	-1.25924	-1.25682	-1.25441	-1.25201	-1.24962	-1.32
-1.1917	-1.18934	-1.18708	-1.26022	-1.25779	-1.25537	-1.25298	-1.2506	-1.32114
-1.19275	-1.19036	-1.18803	-1.26122	-1.25875	-1.25631	-1.2539	-1.25151	-1.3222

-1.11618	-1.19136	-1.18898	-1.26225	-1.25976	-1.25731	-1.25488	-1.25249	-1.32333
-1.11706	-1.19232	-1.18994	-1.26332	-1.26084	-1.2584	-1.25598	-1.25358	-1.32456
-1.11796	-1.19329	-1.19091	-1.2644	-1.26193	-1.25949	-1.25707	-1.25467	-1.32579
-1.04587	-1.12406	-1.12177	-1.18951	-1.18722	-1.18498	-1.25806	-1.25566	-1.32696
-1.04677	-1.12503	-1.12268	-1.19046	-1.18813	-1.18586	-1.25904	-1.25664	-1.32812
-1.04762	-1.08838	-1.08611	-1.12133	-1.11912	-1.11697	-1.2601	-1.25771	-1.25531
-1.04845	-1.08927	-1.08703	-1.1223	-1.12008	-1.11791	-1.26123	-1.25883	-1.25642
-1.01079	-1.04719	-1.04506	-1.12324	-1.121	-1.1188	-1.11667	-1.11458	-1.25747
-1.01168	-1.04808	-1.04592	-1.12414	-1.12187	-1.11965	-1.1175	-1.1154	-1.25844
-1.01256	-1.01045	-1.00834	-1.08749	-1.08528	-1.08312	-1.11837	-1.11626	-1.11416
-1.01339	-1.01129	-1.0092	-1.08843	-1.08622	-1.08404	-1.11931	-1.11718	-1.11507
-1.01422	-1.01214	-1.01007	-1.08939	-1.08718	-1.085	-1.12029	-1.11813	-1.11601
-0.93746	-1.01305	-1.01097	-1.05265	-1.05051	-1.04839	-1.08383	-1.08173	-1.11696
-0.9383	-1.01396	-1.01187	-1.0536	-1.05146	-1.04934	-1.08479	-1.08268	-1.11793
-0.85582	-0.93719	-0.93529	-1.01066	-1.00862	-1.0066	-1.04813	-1.04606	-1.11882
-0.85645	-0.93793	-0.93603	-1.01146	-1.00941	-1.00738	-1.04894	-1.04686	-1.11967
-0.85714	-0.93871	-0.93682	-0.97815	-0.97614	-0.97415	-1.04981	-1.04772	-1.04565
-0.85789	-0.93954	-0.93765	-0.97904	-0.97703	-0.97503	-1.05073	-1.04863	-1.04655
-0.82208	-0.85694	-0.85527	-0.93661	-0.93471	-0.93282	-0.97395	-0.97199	-1.04746
-0.82285	-0.8577	-0.85602	-0.93746	-0.93557	-0.93368	-0.97485	-0.97288	-1.0484
-0.82361	-0.85845	-0.85676	-0.9383	-0.93641	-0.93453	-0.97575	-0.97375	-1.04932
-0.82431	-0.82261	-0.82094	-0.85575	-0.85408	-0.85243	-0.93341	-0.93153	-1.01694
-0.82498	-0.82328	-0.82159	-0.85638	-0.85471	-0.85306	-0.93415	-0.93225	-1.01776
-0.78026	-0.824	-0.8223	-0.82063	-0.81898	-0.81734	-0.93493	-0.93303	-0.97419
-0.78097	-0.82477	-0.82306	-0.82137	-0.81971	-0.81807	-0.93575	-0.93385	-0.97507
-0.73851	-0.82554	-0.82383	-0.82213	-0.82046	-0.81881	-0.81716	-0.81552	-0.93281
-0.73917	-0.82633	-0.82461	-0.82291	-0.82122	-0.81955	-0.81789	-0.81626	-0.93364
-0.73981	-0.73831	-0.73681	-0.82368	-0.82198	-0.82029	-0.81863	-0.817	-0.81538
-0.7404	-0.7389	-0.73742	-0.82444	-0.82274	-0.82105	-0.81939	-0.81775	-0.81613
-0.74097	-0.73947	-0.73802	-0.82518	-0.82349	-0.8218	-0.82014	-0.8185	-0.81687
-0.7416	-0.74009	-0.73862	-0.73715	-0.73567	-0.73419	-0.82084	-0.81922	-0.8176
-0.74227	-0.74073	-0.73922	-0.73772	-0.73622	-0.73474	-0.82152	-0.81992	-0.81833
-0.65374	-0.7414	-0.73986	-0.73834	-0.73682	-0.73533	-0.78007	-0.77854	-0.81902
-0.65436	-0.74208	-0.74054	-0.739	-0.73748	-0.73597	-0.78075	-0.77918	-0.81969
-0.65499	-0.74276	-0.74121	-0.73968	-0.73814	-0.73662	-0.73512	-0.73364	-0.82038
-0.65562	-0.74345	-0.74189	-0.74034	-0.73881	-0.73728	-0.73578	-0.73429	-0.82112
-0.65626	-0.74413	-0.74257	-0.74102	-0.73948	-0.73795	-0.73645	-0.73496	-0.82187
-0.6569	-0.65553	-0.65417	-0.7417	-0.74015	-0.73862	-0.73711	-0.73562	-0.73413
-0.65754	-0.65617	-0.65481	-0.7424	-0.74084	-0.73929	-0.73777	-0.73628	-0.73479
-0.61482	-0.65681	-0.65544	-0.65408	-0.65272	-0.65136	-0.73844	-0.73694	-0.73545
-0.61543	-0.65745	-0.65608	-0.65472	-0.65335	-0.65199	-0.73911	-0.7376	-0.73611
-0.50978	-0.61472	-0.61343	-0.65535	-0.65398	-0.65262	-0.73978	-0.73827	-0.73677
-0.51025	-0.61533	-0.61403	-0.65599	-0.65462	-0.65326	-0.74046	-0.73894	-0.73744
-0.46658	-0.61595	-0.61463	-0.65663	-0.65526	-0.65389	-0.65253	-0.65119	-0.7381
-0.46702	-0.61658	-0.61525	-0.65728	-0.6559	-0.65453	-0.65317	-0.65182	-0.73877
-0.46746	-0.61721	-0.61586	-0.65793	-0.65654	-0.65517	-0.65381	-0.65245	-0.73945
-0.4679	-0.46698	-0.46604	-0.61514	-0.61383	-0.61254	-0.65445	-0.65309	-0.69507
-0.46835	-0.46742	-0.46648	-0.61576	-0.61444	-0.61313	-0.65508	-0.65372	-0.69572
-0.41799	-0.46786	-0.46692	-0.56526	-0.56413	-0.56301	-0.61244	-0.61116	-0.65302
-0.41838	-0.46831	-0.46737	-0.5658	-0.56467	-0.56354	-0.61304	-0.61175	-0.65366

-0.41878	-0.46876	-0.46782	-0.52321	-0.52215	-0.52109	-0.61365	-0.61235	-0.65431
-0.41918	-0.46922	-0.46826	-0.52372	-0.52266	-0.5216	-0.61425	-0.61295	-0.65495
-0.41958	-0.41873	-0.41789	-0.46777	-0.46682	-0.46589	-0.56402	-0.56291	-0.6123
-0.41999	-0.41914	-0.41828	-0.46822	-0.46727	-0.46634	-0.56456	-0.56346	-0.61293
-0.42041	-0.41955	-0.41869	-0.46867	-0.46772	-0.46679	-0.56512	-0.56402	-0.61358
-0.42082	-0.41996	-0.41909	-0.46913	-0.46818	-0.46724	-0.52261	-0.52159	-0.56347
-0.42123	-0.42036	-0.41949	-0.46959	-0.46864	-0.4677	-0.52313	-0.5221	-0.56401
-0.42164	-0.42077	-0.4199	-0.41905	-0.4182	-0.41737	-0.46725	-0.46633	-0.56456
-0.42206	-0.42118	-0.42032	-0.41946	-0.41862	-0.4178	-0.46773	-0.46681	-0.56512
-0.42247	-0.42159	-0.42073	-0.41988	-0.41905	-0.41822	-0.46822	-0.46728	-0.52266
-0.42289	-0.42201	-0.42116	-0.42031	-0.41948	-0.41865	-0.46869	-0.46775	-0.52319
-0.4233	-0.42243	-0.42158	-0.42074	-0.4199	-0.41907	-0.46917	-0.46822	-0.52372
-0.42372	-0.42284	-0.42199	-0.42115	-0.42031	-0.41948	-0.41864	-0.41779	-0.46774
-0.42414	-0.42326	-0.4224	-0.42156	-0.42072	-0.41989	-0.41905	-0.4182	-0.46822
-0.42458	-0.4237	-0.42283	-0.42198	-0.42113	-0.42029	-0.41946	-0.41862	-0.41779
-0.42504	-0.42415	-0.42327	-0.4224	-0.42155	-0.4207	-0.41987	-0.41904	-0.41821
-0.42549	-0.4246	-0.42371	-0.42283	-0.42197	-0.42112	-0.42028	-0.41946	-0.41863
-0.42594	-0.42504	-0.42414	-0.42326	-0.4224	-0.42154	-0.4207	-0.41987	-0.41904
-0.42638	-0.42548	-0.42458	-0.4237	-0.42283	-0.42197	-0.42112	-0.42028	-0.41944
-0.42684	-0.42593	-0.42503	-0.42414	-0.42327	-0.4224	-0.42154	-0.42069	-0.41985
-0.4273	-0.42638	-0.42548	-0.42459	-0.4237	-0.42282	-0.42196	-0.4211	-0.42026
-0.37911	-0.42682	-0.42591	-0.42502	-0.42413	-0.42325	-0.42238	-0.42152	-0.42067
-0.37951	-0.42725	-0.42634	-0.42545	-0.42456	-0.42369	-0.42281	-0.42194	-0.42109
-0.33146	-0.42772	-0.4268	-0.42589	-0.425	-0.42413	-0.42325	-0.42237	-0.42151
-0.33184	-0.4282	-0.42727	-0.42635	-0.42546	-0.42457	-0.42369	-0.4228	-0.42193
-0.33221	-0.37993	-0.37909	-0.42682	-0.42591	-0.42502	-0.42413	-0.42324	-0.42236
-0.33259	-0.38035	-0.37951	-0.42729	-0.42637	-0.42547	-0.42456	-0.42367	-0.42279
-0.33298	-0.33224	-0.33152	-0.42776	-0.42683	-0.42591	-0.42501	-0.42411	-0.47634
-0.33336	-0.33262	-0.33189	-0.42824	-0.4273	-0.42636	-0.42545	-0.42455	-0.47687
-0.33376	-0.333	-0.33227	-0.42871	-0.42776	-0.42682	-0.4259	-0.425	-0.47741
-0.33417	-0.33339	-0.33264	-0.38034	-0.37949	-0.37865	-0.42636	-0.42546	-0.47796
-0.33459	-0.3338	-0.33302	-0.38075	-0.3799	-0.37907	-0.42683	-0.42592	-0.47851
-0.33502	-0.33421	-0.33342	-0.33265	-0.33191	-0.3312	-0.4273	-0.42639	-0.47906
-0.33545	-0.33463	-0.33382	-0.33304	-0.3323	-0.33158	-0.42777	-0.42685	-0.47961
-0.33588	-0.33505	-0.33423	-0.33344	-0.33269	-0.33196	-0.37949	-0.37867	-0.48016
-0.33632	-0.33549	-0.33466	-0.33386	-0.33308	-0.33233	-0.37991	-0.37909	-0.48072
-0.33676	-0.33592	-0.33509	-0.33428	-0.33349	-0.33272	-0.38033	-0.37951	-0.48128
-0.2842	-0.33637	-0.33554	-0.33472	-0.33391	-0.33312	-0.33237	-0.33165	-0.48184
-0.28459	-0.33683	-0.33599	-0.33516	-0.33434	-0.33354	-0.33277	-0.33203	-0.48239
-0.28498	-0.28423	-0.2835	-0.3356	-0.33478	-0.33396	-0.33318	-0.33242	-0.4352
-0.28537	-0.28462	-0.28389	-0.33605	-0.33522	-0.3344	-0.3336	-0.33283	-0.43573
-0.24233	-0.28502	-0.28428	-0.33651	-0.33567	-0.33484	-0.33403	-0.33324	-0.38901
-0.24267	-0.28543	-0.28467	-0.33696	-0.33612	-0.33529	-0.33447	-0.33366	-0.38951
-0.243	-0.28584	-0.28507	-0.28432	-0.28359	-0.28287	-0.33492	-0.3341	-0.39003
-0.24334	-0.28625	-0.28548	-0.28473	-0.28398	-0.28325	-0.33537	-0.33454	-0.39056
-0.24368	-0.28666	-0.2859	-0.28514	-0.28438	-0.28364	-0.33582	-0.335	-0.39111
-0.24402	-0.28707	-0.28631	-0.28555	-0.28478	-0.28403	-0.28331	-0.28259	-0.39167
-0.24436	-0.28749	-0.28672	-0.28596	-0.2852	-0.28444	-0.28371	-0.28298	-0.39224
-0.24472	-0.28791	-0.28714	-0.28638	-0.28562	-0.28486	-0.28412	-0.28338	-0.39282
-0.24509	-0.28835	-0.28757	-0.2868	-0.28604	-0.28529	-0.28454	-0.28379	-0.3934

-0.24544	-0.24487	-0.24429	-0.28722	-0.28646	-0.28571	-0.28495	-0.2842	-0.34211
-0.24579	-0.2452	-0.24463	-0.28763	-0.28687	-0.28611	-0.28535	-0.28461	-0.34262
-0.24613	-0.24554	-0.24497	-0.28804	-0.28727	-0.28651	-0.28576	-0.28501	-0.34314
-0.24648	-0.24589	-0.24531	-0.28847	-0.28769	-0.28692	-0.28616	-0.28542	-0.34367
-0.24683	-0.24625	-0.24566	-0.2889	-0.28811	-0.28733	-0.28658	-0.28583	-0.34421
-0.24719	-0.24661	-0.24602	-0.28934	-0.28855	-0.28777	-0.287	-0.28625	-0.34474
-0.24754	-0.24696	-0.24638	-0.28978	-0.289	-0.28821	-0.28744	-0.28666	-0.34526
-0.2479	-0.24732	-0.24674	-0.24616	-0.24559	-0.24501	-0.28786	-0.28709	-0.34581
-0.24826	-0.24767	-0.24709	-0.24651	-0.24593	-0.24535	-0.28829	-0.28752	-0.34638
-0.24862	-0.24803	-0.24744	-0.24686	-0.24628	-0.2457	-0.28872	-0.28796	-0.34695
-0.24897	-0.24838	-0.24779	-0.24721	-0.24663	-0.24605	-0.28916	-0.28839	-0.34749
-0.24932	-0.24873	-0.24814	-0.24756	-0.24698	-0.24641	-0.2896	-0.28882	-0.34804
-0.24969	-0.2491	-0.24851	-0.24792	-0.24734	-0.24675	-0.24617	-0.2456	-0.34861
-0.25006	-0.24947	-0.24888	-0.24829	-0.24769	-0.2471	-0.24652	-0.24594	-0.3492
-0.25043	-0.24984	-0.24925	-0.24865	-0.24806	-0.24746	-0.24688	-0.2463	-0.34979
-0.25078	-0.25019	-0.24961	-0.24902	-0.24843	-0.24784	-0.24725	-0.24667	-0.35039
-0.25114	-0.25055	-0.24997	-0.24938	-0.24879	-0.2482	-0.24762	-0.24703	-0.30828
-0.25151	-0.25092	-0.25032	-0.24973	-0.24915	-0.24856	-0.24797	-0.24739	-0.30878
-0.25188	-0.25128	-0.25069	-0.25009	-0.2495	-0.24892	-0.24834	-0.24776	-0.30929
-0.25225	-0.25165	-0.25106	-0.25047	-0.24988	-0.24929	-0.24871	-0.24813	-0.30982
-0.25263	-0.25202	-0.25143	-0.25084	-0.25025	-0.24967	-0.24909	-0.2485	-0.31035
-0.253	-0.25239	-0.25179	-0.2512	-0.25062	-0.25004	-0.24946	-0.24888	-0.31088
-0.25337	-0.25275	-0.25215	-0.25156	-0.25098	-0.25041	-0.24983	-0.24925	-0.31141
-0.25375	-0.25313	-0.25252	-0.25193	-0.25135	-0.25078	-0.2502	-0.24961	-0.31193
-0.25415	-0.25352	-0.25291	-0.25231	-0.25172	-0.25115	-0.25056	-0.24996	-0.31244
-0.25453	-0.25391	-0.25329	-0.25269	-0.2521	-0.25152	-0.25092	-0.25033	-0.24973
-0.25491	-0.25429	-0.25368	-0.25308	-0.25249	-0.25189	-0.2513	-0.2507	-0.25011
-0.25529	-0.25467	-0.25407	-0.25347	-0.25287	-0.25228	-0.25168	-0.25108	-0.25049
-0.25569	-0.25507	-0.25447	-0.25386	-0.25326	-0.25266	-0.25206	-0.25146	-0.25087
-0.2561	-0.25548	-0.25486	-0.25426	-0.25365	-0.25305	-0.25244	-0.25184	-0.25124
-0.2565	-0.25588	-0.25527	-0.25465	-0.25404	-0.25344	-0.25282	-0.25222	-0.25162
-0.25691	-0.25628	-0.25567	-0.25505	-0.25444	-0.25382	-0.25321	-0.2526	-0.252
-0.25732	-0.25669	-0.25607	-0.25546	-0.25484	-0.25422	-0.2536	-0.25298	-0.25238
-0.25772	-0.25709	-0.25648	-0.25586	-0.25524	-0.25461	-0.25399	-0.25337	-0.25276
-0.25813	-0.2575	-0.25688	-0.25626	-0.25564	-0.25501	-0.25438	-0.25376	-0.25315
-0.25854	-0.25791	-0.25729	-0.25667	-0.25604	-0.25541	-0.25478	-0.25415	-0.25353
-0.25896	-0.25832	-0.2577	-0.25708	-0.25645	-0.25582	-0.25518	-0.25454	-0.25392
-0.19464	-0.25874	-0.25811	-0.25749	-0.25686	-0.25622	-0.25558	-0.25494	-0.25432
-0.19494	-0.25915	-0.25853	-0.2579	-0.25727	-0.25663	-0.25599	-0.25535	-0.25472
-0.19525	-0.25957	-0.25894	-0.25831	-0.25768	-0.25705	-0.25641	-0.25577	-0.25513
-0.19556	-0.25999	-0.25936	-0.25873	-0.2581	-0.25747	-0.25683	-0.2562	-0.25556
-0.19588	-0.1954	-0.19492	-0.25914	-0.25851	-0.25789	-0.25725	-0.25662	-0.25598
-0.19619	-0.19571	-0.19523	-0.25956	-0.25893	-0.2583	-0.25767	-0.25704	-0.2564
-0.19651	-0.19601	-0.19553	-0.25997	-0.25934	-0.25872	-0.25809	-0.25746	-0.25682
-0.19681	-0.19632	-0.19584	-0.26039	-0.25976	-0.25913	-0.2585	-0.25787	-0.25724
-0.19713	-0.19663	-0.19615	-0.26081	-0.26018	-0.25954	-0.25891	-0.25828	-0.25765
-0.19746	-0.19696	-0.19647	-0.19599	-0.19551	-0.19504	-0.25933	-0.2587	-0.25807
-0.1978	-0.19729	-0.19678	-0.19629	-0.19581	-0.19534	-0.25974	-0.25911	-0.25848
-0.13036	-0.19761	-0.1971	-0.1966	-0.19612	-0.19565	-0.26016	-0.25952	-0.25889
-0.13056	-0.19793	-0.19741	-0.19691	-0.19642	-0.19595	-0.26057	-0.25994	-0.25931

-0.13076	-0.19826	-0.19774	-0.19724	-0.19674	-0.19626	-0.19578	-0.19531	-0.25972
-0.13097	-0.1986	-0.1981	-0.19759	-0.19708	-0.19658	-0.19609	-0.19561	-0.26013
-0.06389	-0.13087	-0.13056	-0.19794	-0.19742	-0.19691	-0.19641	-0.19593	-0.26056
-0.06398	-0.13107	-0.13076	-0.19826	-0.19775	-0.19724	-0.19675	-0.19626	-0.26101
-0.06407	-0.13127	-0.13096	-0.19858	-0.19808	-0.19757	-0.19708	-0.19659	-0.26146
	-0.13147	-0.13116	-0.13086	-0.13055	-0.13024	-0.1974	-0.19691	-0.19643
	-0.13167	-0.13137	-0.13106	-0.13075	-0.13044	-0.19771	-0.19721	-0.19673
			-0.13126	-0.13095	-0.13064	-0.13033	-0.13002	-0.19704
			-0.13146	-0.13115	-0.13084	-0.13053	-0.13022	-0.19735
			-0.06412	-0.06398	-0.06384	-0.13074	-0.13042	-0.19766
			-0.06421	-0.06407	-0.06394	-0.13094	-0.13063	-0.19799
						-0.06389	-0.06375	-0.13052
						-0.06399	-0.06386	-0.13076
						-0.0641	-0.06396	-0.13099
								-0.06392
								-0.06401
								-0.0641
								-0.06419

								-0.12971
								-0.12945
						-0.12997	-0.12959	-0.44216
						-0.12972	-0.12934	-0.44142
						-0.12946	-0.12909	-0.44067
				-0.12996	-0.12959	-0.44223	-0.44106	-0.61291
				-0.12972	-0.12934	-0.44146	-0.44028	-0.61182
				-0.25846	-0.25768	-0.6141	-0.61244	-0.71424
				-0.25796	-0.2572	-0.61303	-0.6114	-0.71312
	-0.25981	-0.25903	-0.25825	-0.44152	-0.44033	-0.61198	-0.61037	-0.76114
	-0.25937	-0.25859	-0.25781	-0.4408	-0.4396	-0.61098	-0.60935	-0.7599
-0.05977	-0.44367	-0.44248	-0.44128	-0.61333	-0.61164	-0.71335	-0.71152	-0.7587
-0.05967	-0.44289	-0.44171	-0.44053	-0.61232	-0.61066	-0.71227	-0.71047	-0.75758
-0.05958	-0.44211	-0.44094	-0.43977	-0.61131	-0.60968	-0.71121	-0.70943	-0.75648
-0.44255	-0.61519	-0.61354	-0.6119	-0.66032	-0.65863	-0.75905	-0.75717	-0.75532
-0.44182	-0.61419	-0.61251	-0.61085	-0.65924	-0.65757	-0.75783	-0.75598	-0.75413
-0.44108	-0.61319	-0.61152	-0.60987	-0.76043	-0.75855	-0.75668	-0.75484	-0.84852
-0.44035	-0.61221	-0.61056	-0.60895	-0.75933	-0.75746	-0.7556	-0.75376	-0.84733
-0.61286	-0.66134	-0.65964	-0.65796	-0.75819	-0.75633	-0.85016	-0.84813	-0.89861
-0.61177	-0.66024	-0.65856	-0.65691	-0.75698	-0.75514	-0.84886	-0.84682	-0.89719
-0.61072	-0.65916	-0.65751	-0.65588	-0.75579	-0.75396	-0.84757	-0.84552	-0.89579
-0.65985	-0.76024	-0.75838	-0.75653	-0.85041	-0.84839	-0.84635	-0.8443	-0.93839
-0.65889	-0.75914	-0.7573	-0.75547	-0.84923	-0.84719	-0.84515	-0.8431	-0.93705
-0.75992	-0.85412	-0.85209	-0.85007	-0.84803	-0.84599	-0.94026	-0.93798	-0.9357
-0.75883	-0.85293	-0.8509	-0.84886	-0.84682	-0.84478	-0.93891	-0.93661	-0.93434
-0.85378	-0.85173	-0.84967	-0.84761	-0.94204	-0.93976	-0.93747	-0.93518	-0.979
-0.85258	-0.85052	-0.84842	-0.84631	-0.94055	-0.93823	-0.93594	-0.93367	-0.97744
-0.85133	-0.84927	-0.84715	-0.84503	-0.93909	-0.93676	-0.98059	-0.97825	-1.02315
-0.85001	-0.84796	-0.84586	-0.84376	-0.93769	-0.93537	-0.97917	-0.97686	-1.02171
-0.84869	-0.84665	-0.84458	-0.84252	-0.93631	-0.93402	-0.9778	-0.97552	-1.02032
-0.84745	-0.94182	-0.93953	-0.93724	-0.93495	-0.93269	-0.97643	-0.97419	-1.01893

-0.84624	-0.94046	-0.93816	-0.93587	-0.93361	-0.93138	-0.9751	-0.97288	-1.01756
-0.88992	-0.93909	-0.93679	-0.93451	-0.97829	-0.976	-1.02081	-1.01849	-1.01619
-0.88864	-0.93772	-0.93543	-0.93316	-0.97692	-0.97466	-1.01941	-1.01712	-1.01483
-0.93866	-0.98254	-0.98017	-0.97783	-0.97555	-0.9733	-1.01797	-1.01569	-1.01339
-0.93729	-0.98113	-0.97879	-0.97647	-0.97418	-0.97192	-1.01648	-1.01417	-1.01185
-0.93594	-0.97973	-0.9774	-0.9751	-1.01977	-1.01738	-1.01501	-1.01268	-1.01035
-0.93458	-0.97833	-0.97601	-0.97371	-1.01832	-1.01596	-1.0136	-1.01127	-1.00893
-0.93322	-0.97692	-0.97461	-0.97233	-1.01689	-1.01455	-1.01221	-1.00988	-1.00755
-0.97785	-0.97552	-0.97323	-0.97098	-1.01547	-1.01315	-1.01082	-1.00849	-1.10632
-0.97643	-0.97413	-0.97187	-0.96964	-1.01407	-1.01176	-1.00942	-1.00709	-1.1048
-0.97504	-1.01969	-1.01734	-1.01501	-1.01268	-1.01036	-1.06865	-1.06619	-1.1033
-0.97366	-1.01827	-1.01594	-1.01362	-1.01129	-1.00897	-1.06721	-1.06476	-1.1018
-1.01921	-1.01686	-1.01454	-1.01223	-1.07068	-1.06823	-1.10548	-1.10288	-1.10031
-1.0178	-1.01545	-1.01314	-1.01083	-1.06924	-1.06679	-1.10397	-1.10138	-1.09882
-1.0164	-1.0751	-1.07267	-1.07025	-1.10765	-1.10506	-1.10247	-1.09989	-1.18298
-1.01501	-1.07368	-1.07124	-1.06882	-1.10614	-1.10355	-1.10098	-1.09843	-1.18124
-1.01363	-1.07226	-1.06982	-1.06738	-1.10464	-1.10205	-1.0995	-1.09698	-1.17953
-1.01224	-1.07083	-1.06838	-1.06595	-1.10314	-1.10057	-1.18371	-1.18077	-1.17783
-1.01085	-1.0694	-1.06695	-1.06452	-1.10164	-1.09911	-1.18198	-1.17905	-1.17614
-1.07045	-1.10788	-1.10528	-1.1027	-1.10016	-1.09765	-1.18024	-1.17732	-1.21547
-1.06901	-1.10637	-1.10378	-1.10122	-1.09869	-1.09618	-1.17849	-1.17557	-1.21359
-1.1075	-1.10487	-1.1023	-1.09976	-1.18265	-1.17972	-1.21803	-1.21487	-1.21174
-1.10601	-1.10339	-1.10085	-1.09833	-1.18096	-1.17806	-1.21626	-1.21309	-1.20995
-1.10453	-1.10193	-1.0994	-1.09691	-1.17929	-1.17642	-1.2145	-1.21133	-1.2082
-1.10305	-1.18635	-1.18339	-1.18048	-1.17761	-1.17476	-1.21273	-1.20956	-1.24735
-1.10159	-1.1846	-1.18166	-1.17878	-1.17593	-1.1731	-1.21096	-1.20781	-1.24546
-1.10017	-1.1829	-1.17997	-1.1771	-1.21533	-1.21229	-1.2092	-1.20606	-1.32973
-1.09879	-1.18125	-1.17832	-1.17545	-1.21355	-1.21052	-1.20744	-1.20433	-1.32765
-1.18263	-1.22107	-1.21792	-1.21482	-1.21178	-1.20876	-1.29182	-1.28815	-1.3256
-1.18093	-1.21928	-1.21612	-1.21302	-1.21	-1.20702	-1.2898	-1.28617	-1.32357
-1.22069	-1.21749	-1.21434	-1.21124	-1.20824	-1.20528	-1.32893	-1.32524	-1.32159
-1.21888	-1.2157	-1.21256	-1.20947	-1.20648	-1.20355	-1.32689	-1.32329	-1.3197
-1.21709	-1.21394	-1.2108	-1.20772	-1.20474	-1.20183	-1.32487	-1.32136	-1.31783
-1.21535	-1.21224	-1.20911	-1.20603	-1.28876	-1.28531	-1.32285	-1.3194	-1.31592
-1.21365	-1.21058	-1.20746	-1.20437	-1.28679	-1.28334	-1.32083	-1.31742	-1.31398
-1.2119	-1.2957	-1.29201	-1.28837	-1.28483	-1.28138	-1.31882	-1.31545	-1.31205
-1.21011	-1.29364	-1.29001	-1.28642	-1.28289	-1.27944	-1.31683	-1.31348	-1.31012
-1.20835	-1.2916	-1.28804	-1.28451	-1.32189	-1.31832	-1.31488	-1.31154	-1.30823
-1.20663	-1.28957	-1.2861	-1.28265	-1.31999	-1.31643	-1.31298	-1.30964	-1.30639
-1.2911	-1.28758	-1.28417	-1.2808	-1.3181	-1.31455	-1.31112	-1.30777	-1.30457
-1.2892	-1.28566	-1.28226	-1.2789	-1.31616	-1.31265	-1.30924	-1.30591	-1.30277
-1.28733	-1.28378	-1.28037	-1.277	-1.31423	-1.31078	-1.30741	-1.30411	-1.30102
-1.28542	-1.3228	-1.31925	-1.31577	-1.31235	-1.30899	-1.30569	-1.30246	-1.29944
-1.28351	-1.32086	-1.31731	-1.31385	-1.3105	-1.30725	-1.30405	-1.30091	-1.29797
-1.28163	-1.31896	-1.31542	-1.31197	-1.30869	-1.30552	-1.30241	-1.29938	-1.33551
-1.27978	-1.31711	-1.31359	-1.31015	-1.3069	-1.30378	-1.30076	-1.29785	-1.33402
-1.31865	-1.31524	-1.31175	-1.30835	-1.30515	-1.30209	-1.29917	-1.29638	-1.33258
-1.3167	-1.3133	-1.30988	-1.30657	-1.30343	-1.30044	-1.29763	-1.29499	-1.33123
-1.31478	-1.31139	-1.30806	-1.30486	-1.30179	-1.29887	-1.29616	-1.29363	-1.32989
-1.31296	-1.3096	-1.30637	-1.30326	-1.30028	-1.29743	-1.33371	-1.3311	-1.36439

-1.31118	-1.30789	-1.30475	-1.30174	-1.29885	-1.29608	-1.33232	-1.32968	-1.36294
-1.30942	-1.30622	-1.30315	-1.30022	-1.29742	-1.29474	-1.33098	-1.32832	-1.39703
-1.30769	-1.3046	-1.30159	-1.29871	-1.29602	-1.29344	-1.32967	-1.32701	-1.39559
-1.306	-1.303	-1.30007	-1.29726	-1.29463	-1.2921	-1.39977	-1.39695	-1.39418
-1.30434	-1.30141	-1.29859	-1.29586	-1.29324	-1.29069	-1.39825	-1.39553	-1.39281
-1.30274	-1.29987	-1.29715	-1.2945	-1.40233	-1.39949	-1.39675	-1.3941	-1.39142
-1.30124	-1.29843	-1.29577	-1.29317	-1.40087	-1.39804	-1.3953	-1.39263	-1.38996
-1.2998	-1.29705	-1.29442	-1.29186	-1.39945	-1.39663	-1.39388	-1.39118	-1.38851
-1.29839	-1.29571	-1.2931	-1.29055	-1.39802	-1.39524	-1.39249	-1.38979	-1.38712
-1.29703	-1.2944	-1.29181	-1.28926	-1.39659	-1.39386	-1.39113	-1.38843	-1.38577
-1.29567	-1.40362	-1.40076	-1.39795	-1.39519	-1.39247	-1.38977	-1.38709	-1.38444
-1.29433	-1.40212	-1.39931	-1.39654	-1.3938	-1.39109	-1.38841	-1.38576	-1.38313
-1.36586	-1.40067	-1.39788	-1.39514	-1.39242	-1.38973	-1.38706	-1.38442	-1.38181
-1.36449	-1.39928	-1.3965	-1.39375	-1.39105	-1.38839	-1.38573	-1.38308	-1.38047
-1.40069	-1.3979	-1.39512	-1.39238	-1.38969	-1.38705	-1.38439	-1.38173	-1.44946
-1.39926	-1.39648	-1.39375	-1.39104	-1.38835	-1.38568	-1.38301	-1.38035	-1.44794
-1.39782	-1.39507	-1.39238	-1.3897	-1.387	-1.3843	-1.38162	-1.37897	-1.44642
-1.39642	-1.39369	-1.39101	-1.38834	-1.38563	-1.38291	-1.38025	-1.37763	-1.44495
-1.39504	-1.39234	-1.38965	-1.38696	-1.38424	-1.38153	-1.3789	-1.37631	-1.44352
-1.39365	-1.39097	-1.38828	-1.3856	-1.3829	-1.38021	-1.44771	-1.44487	-1.44209
-1.39226	-1.38958	-1.38691	-1.38426	-1.3816	-1.37895	-1.44631	-1.44345	-1.44065
-1.39087	-1.3882	-1.38555	-1.38291	-1.38029	-1.37767	-1.44492	-1.44206	-1.43926
-1.3895	-1.38684	-1.38419	-1.38156	-1.37896	-1.37638	-1.44353	-1.44073	-1.43794
-1.38813	-1.38549	-1.38284	-1.38021	-1.37763	-1.37508	-1.44214	-1.43939	-1.43662
-1.38676	-1.3841	-1.38147	-1.37888	-1.37632	-1.37377	-1.44072	-1.43799	-1.5117
-1.38539	-1.38271	-1.38012	-1.37757	-1.37501	-1.37247	-1.43929	-1.43658	-1.51004
-1.38408	-1.3814	-1.37879	-1.37623	-1.44336	-1.44062	-1.51477	-1.51155	-1.50835
-1.38282	-1.38015	-1.3775	-1.37489	-1.44191	-1.43921	-1.5131	-1.50985	-1.50663
-1.38152	-1.37888	-1.37623	-1.37362	-1.4405	-1.43779	-1.51141	-1.50818	-1.50498
-1.38019	-1.3776	-1.375	-1.37242	-1.43915	-1.43637	-1.50968	-1.50653	-1.50341
-1.37886	-1.44617	-1.44339	-1.44062	-1.51455	-1.5112	-1.50799	-1.5049	-1.50182
-1.37754	-1.4447	-1.44196	-1.43923	-1.51293	-1.50961	-1.50639	-1.50326	-1.50018
-1.37625	-1.44326	-1.44053	-1.43782	-1.51131	-1.50805	-1.50483	-1.50164	-1.49856
-1.375	-1.4419	-1.43913	-1.43639	-1.50962	-1.50644	-1.50327	-1.50011	-1.49703
-1.37378	-1.44058	-1.43775	-1.43497	-1.50792	-1.50482	-1.50172	-1.49862	-1.49554
-1.44199	-1.51613	-1.51275	-1.50944	-1.50627	-1.50318	-1.50012	-1.49709	-1.49404
-1.44056	-1.51447	-1.51115	-1.50787	-1.50466	-1.50153	-1.49848	-1.49552	-1.49254
-1.43913	-1.51276	-1.50952	-1.50629	-1.50309	-1.49994	-1.49689	-1.49394	-1.491
-1.43771	-1.51101	-1.50784	-1.50471	-1.50157	-1.49845	-1.49538	-1.49235	-1.4894
-1.51257	-1.50929	-1.50617	-1.5031	-1.50003	-1.49696	-1.49389	-1.49081	-1.48785
-1.51095	-1.50766	-1.50452	-1.50145	-1.49842	-1.49542	-1.4924	-1.48937	-1.48641
-1.50937	-1.50608	-1.5029	-1.49981	-1.49681	-1.49388	-1.49092	-1.48796	-1.48501
-1.5078	-1.50455	-1.50137	-1.49827	-1.49528	-1.49237	-1.48945	-1.48655	-1.51819
-1.50625	-1.50305	-1.49988	-1.49679	-1.4938	-1.49088	-1.48799	-1.48513	-1.51676
-1.5047	-1.50155	-1.49841	-1.49531	-1.49231	-1.48938	-1.52113	-1.51823	-1.51531
-1.50316	-1.50007	-1.49694	-1.49384	-1.49082	-1.48787	-1.51957	-1.5167	-1.51382
-1.50161	-1.49857	-1.49547	-1.49239	-1.48937	-1.48639	-1.51804	-1.51516	-1.53528
-1.50004	-1.49704	-1.49399	-1.49095	-1.48795	-1.48497	-1.51654	-1.51361	-1.53371
-1.49848	-1.49551	-1.49252	-1.48953	-1.48654	-1.48357	-1.51507	-1.51209	-1.53216
-1.49696	-1.49402	-1.49107	-1.48812	-1.51969	-1.51663	-1.53667	-1.53363	-1.55412

-1.49546	-1.49253	-1.48962	-1.4867	-1.51824	-1.51519	-1.53523	-1.53221	-1.55269
-1.49394	-1.52569	-1.52273	-1.51978	-1.53991	-1.53683	-1.5572	-1.55419	-1.55127
-1.49241	-1.52409	-1.52116	-1.51824	-1.5384	-1.53536	-1.55575	-1.55278	-1.54986
-1.49092	-1.54585	-1.54287	-1.53991	-1.5603	-1.55729	-1.55432	-1.55138	-1.54848
-1.48949	-1.54435	-1.54136	-1.53841	-1.55881	-1.55584	-1.5529	-1.54998	-1.54711
-1.54597	-1.56625	-1.56324	-1.56028	-1.55733	-1.55441	-1.5515	-1.54861	-1.54576
-1.54447	-1.56474	-1.56172	-1.55877	-1.55586	-1.553	-1.55013	-1.54726	-1.54443
-1.54296	-1.56323	-1.56022	-1.55728	-1.55442	-1.55161	-1.54877	-1.54593	-1.54311
-1.56481	-1.56173	-1.55875	-1.55583	-1.553	-1.55021	-1.54741	-1.54461	-1.57109
-1.56329	-1.56023	-1.55729	-1.55442	-1.5516	-1.54883	-1.54606	-1.5433	-1.56975
-1.56177	-1.55873	-1.55584	-1.55301	-1.55022	-1.54747	-1.54472	-1.54197	-1.56838
-1.56024	-1.55722	-1.55438	-1.55162	-1.54886	-1.54614	-1.54338	-1.54061	-1.56697
-1.55876	-1.55576	-1.55296	-1.55022	-1.54751	-1.5448	-1.54205	-1.53926	-1.56557
-1.55733	-1.55437	-1.55156	-1.54883	-1.54614	-1.54348	-1.54073	-1.53791	-1.56415
-1.55593	-1.55302	-1.55021	-1.54747	-1.5448	-1.54215	-1.56862	-1.56568	-1.60695
-1.55456	-1.55172	-1.54894	-1.54619	-1.54349	-1.5408	-1.56721	-1.56427	-1.60556
-1.55321	-1.55043	-1.54768	-1.54494	-1.54219	-1.53944	-1.56579	-1.56286	-1.60416
-1.55189	-1.54912	-1.54638	-1.54364	-1.54087	-1.53807	-1.56437	-1.56144	-1.60277
-1.55058	-1.5478	-1.54507	-1.54233	-1.53953	-1.53671	-1.56295	-1.56001	-1.60138
-1.54927	-1.54648	-1.54373	-1.54098	-1.53817	-1.53533	-1.60593	-1.60293	-1.62022
-1.54796	-1.54517	-1.54239	-1.5396	-1.53678	-1.53395	-1.60452	-1.60153	-1.61884
-1.54663	-1.54384	-1.54103	-1.53822	-1.58071	-1.57783	-1.62344	-1.62043	-1.61748
-1.54527	-1.54249	-1.53967	-1.53684	-1.57933	-1.57644	-1.62204	-1.61907	-1.61614
-1.54391	-1.54112	-1.5383	-1.53546	-1.57795	-1.57505	-1.62065	-1.61772	-1.61482
-1.54254	-1.53973	-1.5369	-1.53406	-1.60482	-1.60185	-1.61927	-1.61637	-1.6135
-1.54116	-1.53833	-1.5355	-1.53266	-1.60341	-1.60045	-1.6179	-1.61503	-1.61219
-1.53978	-1.58247	-1.57958	-1.5767	-1.62243	-1.61946	-1.61656	-1.6137	-1.61088
-1.5384	-1.58111	-1.5782	-1.57532	-1.62106	-1.61813	-1.61524	-1.61239	-1.60959
-1.53711	-1.60073	-1.59779	-1.59487	-1.61978	-1.61687	-1.614	-1.61116	-1.60836
-1.5359	-1.59955	-1.59661	-1.5937	-1.6186	-1.61571	-1.61285	-1.61002	-1.60721
-1.53465	-1.59832	-1.59539	-1.5925	-1.61738	-1.61451	-1.61166	-1.60883	-1.63634
-1.53329	-1.59698	-1.59408	-1.59121	-1.61608	-1.61321	-1.61036	-1.60754	-1.63498
-1.53191	-1.59563	-1.59275	-1.5899	-1.61475	-1.61188	-1.60903	-1.6062	-1.63358
-1.55218	-1.59428	-1.59141	-1.58857	-1.61341	-1.61055	-1.6077	-1.60486	-1.66336
-1.55083	-1.59296	-1.59008	-1.58723	-1.61206	-1.60921	-1.60635	-1.60351	-1.66187
-1.59465	-1.61957	-1.61659	-1.61364	-1.61074	-1.60787	-1.63523	-1.63229	-1.66039
-1.59352	-1.61842	-1.6154	-1.6124	-1.60945	-1.60654	-1.63383	-1.63089	-1.65892
-1.59231	-1.6172	-1.61418	-1.61117	-1.63851	-1.63549	-1.66369	-1.66061	-1.65754
-1.591	-1.61589	-1.61293	-1.60998	-1.63729	-1.63427	-1.6624	-1.65932	-1.65626
-1.58967	-1.61456	-1.61165	-1.60875	-1.63603	-1.63302	-1.6611	-1.65802	-1.65495
-1.58833	-1.6132	-1.61031	-1.60742	-1.63466	-1.63168	-1.65969	-1.65661	-1.65353
-1.587	-1.61185	-1.60897	-1.60609	-1.6333	-1.63035	-1.65828	-1.6552	-1.65212
-1.58578	-1.64092	-1.63794	-1.63498	-1.66314	-1.66007	-1.65699	-1.65391	-1.6751
-1.5846	-1.63968	-1.63671	-1.63375	-1.66186	-1.6588	-1.65573	-1.65267	-1.67387
-1.61409	-1.63836	-1.63539	-1.63245	-1.66048	-1.65742	-1.67872	-1.67564	-1.67255
-1.61271	-1.63697	-1.63401	-1.63107	-1.65902	-1.65596	-1.67727	-1.67421	-1.67115
-1.6386	-1.63564	-1.63269	-1.62976	-1.68203	-1.67893	-1.67585	-1.67278	-1.66974
-1.63736	-1.63441	-1.63147	-1.62854	-1.68072	-1.67757	-1.67446	-1.67137	-1.66832
-1.6361	-1.68878	-1.68568	-1.68257	-1.6794	-1.67623	-1.6731	-1.66999	-1.66694
-1.63475	-1.68737	-1.68428	-1.68118	-1.67803	-1.67488	-1.67178	-1.6687	-1.66567

-1.6334	-1.68596	-1.68287	-1.67977	-1.67666	-1.67354	-1.67047	-1.66742	-1.66443
-1.65716	-1.68466	-1.68157	-1.67846	-1.67533	-1.6722	-1.66912	-1.66606	-1.66309
-1.65601	-1.68343	-1.68033	-1.6772	-1.67404	-1.67086	-1.66774	-1.66467	-1.66169
-1.65477	-1.68211	-1.679	-1.67588	-1.67272	-1.66955	-1.66644	-1.66337	-1.66041
-1.65346	-1.6807	-1.67759	-1.67448	-1.67137	-1.66827	-1.66522	-1.6622	-1.65925
-1.68245	-1.67932	-1.67621	-1.67312	-1.67005	-1.667	-1.66399	-1.66101	-1.65807
-1.68114	-1.67802	-1.67492	-1.67184	-1.66878	-1.66574	-1.66274	-1.65978	-1.65683
-1.67984	-1.67672	-1.67362	-1.67055	-1.6675	-1.66447	-1.66148	-1.65853	-1.65558
-1.67851	-1.67536	-1.67224	-1.66916	-1.66613	-1.66314	-1.66019	-1.65727	-1.65434
-1.67717	-1.67398	-1.67084	-1.66775	-1.66475	-1.66182	-1.6589	-1.656	-1.6531
-1.67583	-1.67266	-1.66953	-1.66645	-1.66348	-1.66057	-1.65766	-1.65477	-1.699
-1.6745	-1.67139	-1.66828	-1.66522	-1.66228	-1.65938	-1.65647	-1.65356	-1.69772
-1.67321	-1.67014	-1.66706	-1.66402	-1.66107	-1.65817	-1.65524	-1.65231	-1.72251
-1.67198	-1.66892	-1.66587	-1.66285	-1.65987	-1.65691	-1.65395	-1.65099	-1.7211
-1.67076	-1.6677	-1.66467	-1.66166	-1.65866	-1.65567	-1.65269	-1.64971	-1.71974
-1.66955	-1.66649	-1.66346	-1.66045	-1.65744	-1.65445	-1.65148	-1.64853	-1.71849
-1.66832	-1.66527	-1.66224	-1.65922	-1.65623	-1.65325	-1.6503	-1.64738	-1.71726
-1.667	-1.66397	-1.66097	-1.65799	-1.65502	-1.65206	-1.69609	-1.69307	-1.71601
-1.66566	-1.66264	-1.65969	-1.65676	-1.65382	-1.65087	-1.69486	-1.69183	-1.71474
-1.6644	-1.66139	-1.65846	-1.65555	-1.65262	-1.64968	-1.71967	-1.71656	-1.76531
-1.66321	-1.66021	-1.65728	-1.65436	-1.65143	-1.6485	-1.71839	-1.71525	-1.76393
-1.66203	-1.65903	-1.65609	-1.65317	-1.65023	-1.64729	-1.71709	-1.71395	-1.76258
-1.66084	-1.65786	-1.65492	-1.65198	-1.64903	-1.64607	-1.71578	-1.71266	-1.76125
-1.65966	-1.6567	-1.65374	-1.65079	-1.64782	-1.64485	-1.71448	-1.71139	-1.75994
-1.65849	-1.65553	-1.65254	-1.64957	-1.6466	-1.64365	-1.71321	-1.71015	-1.75866
-1.65732	-1.65435	-1.65134	-1.64834	-1.64539	-1.64246	-1.71196	-1.70893	-1.75739
-1.65615	-1.65318	-1.65017	-1.64717	-1.7169	-1.71379	-1.73503	-1.73193	-1.75611
-1.65496	-1.652	-1.64901	-1.64603	-1.71567	-1.71256	-1.73377	-1.73068	-1.75483
-1.65375	-1.65078	-1.6478	-1.64485	-1.71442	-1.71132	-1.75988	-1.75672	-1.75355
-1.6525	-1.64951	-1.64656	-1.64362	-1.71315	-1.71009	-1.75859	-1.75544	-1.75228
-1.65129	-1.64829	-1.64534	-1.64243	-1.71189	-1.70886	-1.7573	-1.75414	-1.75098
-1.65013	-1.64714	-1.64418	-1.64127	-1.71066	-1.70764	-1.75599	-1.7528	-1.74962
-1.64899	-1.646	-1.64305	-1.64013	-1.70945	-1.70643	-1.75469	-1.75147	-1.74828
-1.64785	-1.71753	-1.71439	-1.71129	-1.73245	-1.72934	-1.75343	-1.7502	-1.74701
-1.64671	-1.71631	-1.71318	-1.71009	-1.73122	-1.72812	-1.75218	-1.74895	-1.74576
-1.64557	-1.71509	-1.71196	-1.70888	-1.72997	-1.72688	-1.75092	-1.74769	-1.7445
-1.64442	-1.71387	-1.71075	-1.70766	-1.72872	-1.72564	-1.74965	-1.74642	-1.74323
-1.64327	-1.71265	-1.70953	-1.70645	-1.72747	-1.72439	-1.74838	-1.74516	-1.74198
-1.64213	-1.71143	-1.70832	-1.70524	-1.72623	-1.72315	-1.74711	-1.7439	-1.74072
-1.64099	-1.71022	-1.70711	-1.70404	-1.75218	-1.74903	-1.74585	-1.74264	-1.73947
-1.63987	-1.70901	-1.7059	-1.70283	-1.75091	-1.74777	-1.74459	-1.7414	-1.73824
-1.63877	-1.70783	-1.70471	-1.70162	-1.74964	-1.7465	-1.74334	-1.74015	-1.737
-1.70982	-1.7308	-1.72758	-1.72439	-1.74839	-1.74524	-1.74207	-1.7389	-1.73576
-1.70868	-1.72964	-1.72641	-1.7232	-1.74715	-1.74397	-1.7408	-1.73765	-1.73451
-1.7075	-1.72843	-1.7252	-1.72198	-1.7459	-1.74271	-1.73954	-1.7364	-1.73326
-1.70628	-1.72719	-1.72396	-1.72074	-1.74464	-1.74147	-1.7383	-1.73515	-1.73202
-1.70508	-1.72594	-1.72272	-1.71952	-1.7434	-1.74023	-1.73707	-1.73394	-1.73081
-1.70389	-1.72471	-1.72148	-1.71829	-1.74215	-1.739	-1.73587	-1.73277	-1.72966
-1.72667	-1.72347	-1.72027	-1.71709	-1.74093	-1.73778	-1.73467	-1.7316	-1.72849
-1.72543	-1.72224	-1.71908	-1.71595	-1.73976	-1.73659	-1.73347	-1.73037	-1.72726

-1.72419	-1.72101	-1.71791	-1.71482	-1.7386	-1.73541	-1.73226	-1.72913	-1.726
-1.72298	-1.7198	-1.7167	-1.71362	-1.73738	-1.73419	-1.73103	-1.72789	-1.72475
-1.72179	-1.71859	-1.71548	-1.71239	-1.73613	-1.73296	-1.7298	-1.72665	-1.72351
-1.72059	-1.74443	-1.74123	-1.73806	-1.7349	-1.73176	-1.7286	-1.72545	-1.72231
-1.71937	-1.7432	-1.73998	-1.73681	-1.73369	-1.73058	-1.72744	-1.72428	-1.72115
-1.71818	-1.74199	-1.73877	-1.73559	-1.73247	-1.72939	-1.72626	-1.7231	-1.74479
-1.71702	-1.74082	-1.7376	-1.7344	-1.73126	-1.72815	-1.72501	-1.72187	-1.74353
-1.71586	-1.73965	-1.73643	-1.73323	-1.73006	-1.72691	-1.72377	-1.72064	-1.74229
-1.71463	-1.73842	-1.73524	-1.73208	-1.72891	-1.72574	-1.7226	-1.71947	-1.7411
-1.71339	-1.73717	-1.73405	-1.73093	-1.72776	-1.72458	-1.72144	-1.71831	-1.73992
-1.7122	-1.73595	-1.73283	-1.72971	-1.72655	-1.72337	-1.74506	-1.74186	-1.73868
-1.71104	-1.73474	-1.73159	-1.72844	-1.72529	-1.72214	-1.7438	-1.74059	-1.73741
-1.70989	-1.73356	-1.73039	-1.72722	-1.72407	-1.72093	-1.74257	-1.73937	-1.73619
-1.70876	-1.73241	-1.72922	-1.72605	-1.7229	-1.71977	-1.74139	-1.73819	-1.73502
-1.70764	-1.73126	-1.72807	-1.72489	-1.72174	-1.7186	-1.7402	-1.73702	-1.73386
-1.7065	-1.7301	-1.72691	-1.72374	-1.72058	-1.71744	-1.73902	-1.73585	-1.73269
-1.70537	-1.72895	-1.72576	-1.72258	-1.71941	-1.71626	-1.73782	-1.73466	-1.73151
-1.70423	-1.72778	-1.72459	-1.7214	-1.74301	-1.73976	-1.73657	-1.73341	-1.751
-1.7031	-1.72661	-1.72341	-1.72021	-1.74177	-1.7385	-1.73529	-1.73214	-1.74971
-1.72865	-1.72544	-1.72222	-1.719	-1.74054	-1.73728	-1.73408	-1.73093	-1.74848
-1.72748	-1.72426	-1.72101	-1.71778	-1.73932	-1.7361	-1.73292	-1.72977	-1.7473
-1.72631	-1.72309	-1.71984	-1.71661	-1.73813	-1.73492	-1.75249	-1.7493	-1.74613
-1.72516	-1.72195	-1.71872	-1.7155	-1.73698	-1.73375	-1.75129	-1.74812	-1.74497
-1.724	-1.7208	-1.71759	-1.71438	-1.73583	-1.73259	-1.7501	-1.74694	-1.7438
-1.72283	-1.71961	-1.71639	-1.71318	-1.73463	-1.73142	-1.74893	-1.74577	-1.74264
-1.72165	-1.71843	-1.71519	-1.71197	-1.73343	-1.73026	-1.74777	-1.7446	-1.74147
-1.7205	-1.74208	-1.73878	-1.73551	-1.75301	-1.74978	-1.74659	-1.74344	-1.74032
-1.71937	-1.74094	-1.73767	-1.73442	-1.75188	-1.74861	-1.74542	-1.74229	-1.73918
-1.71822	-1.73978	-1.73654	-1.73332	-1.75076	-1.74749	-1.74429	-1.74115	-1.73804
-1.71705	-1.73859	-1.73539	-1.73219	-1.74965	-1.74641	-1.7432	-1.74002	-1.73688
-1.74066	-1.75822	-1.75498	-1.75175	-1.74852	-1.7453	-1.7421	-1.73891	-1.7547
-1.73949	-1.75705	-1.7538	-1.75056	-1.74735	-1.74417	-1.74099	-1.73782	-1.75361
-1.73835	-1.7559	-1.75263	-1.74939	-1.74619	-1.74302	-1.73987	-1.73674	-1.75253
-1.73725	-1.75478	-1.75152	-1.74828	-1.74506	-1.74187	-1.73872	-1.73562	-1.75144
-1.73617	-1.75368	-1.75043	-1.74718	-1.74394	-1.74072	-1.73757	-1.73448	-1.75033
-1.7558	-1.75252	-1.74928	-1.74605	-1.74282	-1.73961	-1.75544	-1.75231	-1.77555
-1.75464	-1.75134	-1.7481	-1.7449	-1.7417	-1.73853	-1.75435	-1.75117	-1.77436
-1.7535	-1.75019	-1.74694	-1.74373	-1.74057	-1.73742	-1.75325	-1.75006	-1.77322
-1.75239	-1.74907	-1.74579	-1.74255	-1.7394	-1.73629	-1.75215	-1.74899	-1.77213
-1.75125	-1.74796	-1.74465	-1.7414	-1.73825	-1.73515	-1.75104	-1.7479	-1.77103
-1.75008	-1.74682	-1.74354	-1.7403	-1.73712	-1.73398	-1.74987	-1.74677	-1.7699
-1.74892	-1.74568	-1.74245	-1.73923	-1.73602	-1.73283	-1.74872	-1.74562	-1.76876
-1.74779	-1.74457	-1.74137	-1.73817	-1.75399	-1.75077	-1.74761	-1.74448	-1.76759
-1.74668	-1.74347	-1.74029	-1.73712	-1.75296	-1.74972	-1.74652	-1.74334	-1.76642
-1.74556	-1.74234	-1.73919	-1.73604	-1.7519	-1.74867	-1.77179	-1.76852	-1.79472
-1.74443	-1.74121	-1.73806	-1.73492	-1.75082	-1.74762	-1.77072	-1.76744	-1.79355
-1.74333	-1.75921	-1.75606	-1.75292	-1.74975	-1.74657	-1.81197	-1.80856	-1.83378
-1.74225	-1.75814	-1.75499	-1.75186	-1.7487	-1.74554	-1.81087	-1.80744	-1.83255
-1.74118	-1.75708	-1.75391	-1.75077	-1.74763	-1.74447	-1.80975	-1.80632	-1.83133
-1.74011	-1.75601	-1.75281	-1.74964	-1.74649	-1.74336	-1.80861	-1.8052	-1.83014

-1.73905	-1.75495	-1.7517	-1.74849	-1.74535	-1.74223	-1.80745	-1.80408	-1.82895
-1.75719	-1.7539	-1.75063	-1.74741	-1.77059	-1.76741	-1.83493	-1.83135	-1.82775
-1.75615	-1.75286	-1.74958	-1.74635	-1.7695	-1.76632	-1.83372	-1.83014	-1.82654
-1.7551	-1.7518	-1.74853	-1.74529	-1.81069	-1.80739	-1.83252	-1.82895	-1.82535
-1.75405	-1.75074	-1.74746	-1.74423	-1.80957	-1.80627	-1.83133	-1.82776	-1.82416
-1.75299	-1.74968	-1.7464	-1.74316	-1.80845	-1.80515	-1.83013	-1.82657	-1.82298
-1.75192	-1.74861	-1.74534	-1.7421	-1.80733	-1.80403	-1.82893	-1.82537	-1.82179
-1.75085	-1.74754	-1.74427	-1.74105	-1.80622	-1.80291	-1.82773	-1.82418	-1.8206
-1.74978	-1.77287	-1.76954	-1.76625	-1.8336	-1.83008	-1.82654	-1.82298	-1.8194
-1.74871	-1.77178	-1.76845	-1.76517	-1.8324	-1.82888	-1.82534	-1.82179	-1.81821
-1.74764	-1.7707	-1.76737	-1.76408	-1.8312	-1.82769	-1.82415	-1.8206	-1.81702
-1.74659	-1.76962	-1.76629	-1.763	-1.82999	-1.82649	-1.82296	-1.81942	-1.81584
-1.74552	-1.76854	-1.76521	-1.76192	-1.8288	-1.8253	-1.82177	-1.81822	-1.81465
-1.74445	-1.76746	-1.76414	-1.76085	-1.8276	-1.82409	-1.82055	-1.81701	-1.81344
-1.76972	-1.83724	-1.8336	-1.82999	-1.82643	-1.82291	-1.81936	-1.8158	-1.81223
-1.76866	-1.83608	-1.83246	-1.82887	-1.82532	-1.82178	-1.81821	-1.81461	-1.81103
-1.76762	-1.83494	-1.83135	-1.82777	-1.82421	-1.82065	-1.81705	-1.81343	-1.80983
-1.7666	-1.83383	-1.83025	-1.82667	-1.8405	-1.83687	-1.83322	-1.82956	-1.82592
-1.7656	-1.83274	-1.82915	-1.82555	-1.83934	-1.83567	-1.83201	-1.82834	-1.82472
-1.78298	-1.84911	-1.84548	-1.84183	-1.83815	-1.83446	-1.83079	-1.82714	-1.82352
-1.78197	-1.84797	-1.8443	-1.84062	-1.83693	-1.83323	-1.82957	-1.82594	-1.82234
-1.80965	-1.8468	-1.84311	-1.83942	-1.83572	-1.83202	-1.82837	-1.82475	-1.82117
-1.8085	-1.84559	-1.84189	-1.8382	-1.8345	-1.83082	-1.82718	-1.82357	-1.82001
-1.8481	-1.84439	-1.84069	-1.837	-1.83331	-1.82965	-1.82603	-1.82244	-1.8189
-1.8469	-1.8432	-1.83949	-1.83581	-1.83216	-1.82853	-1.82495	-1.82139	-1.81785
-1.84572	-1.84202	-1.83832	-1.83465	-1.83102	-1.82744	-1.82388	-1.82036	-1.81681
-1.84457	-1.8409	-1.83722	-1.83356	-1.82994	-1.82635	-1.82278	-1.81924	-1.81568
-1.84344	-1.83981	-1.83616	-1.83252	-1.82888	-1.82525	-1.82165	-1.81808	-1.8145
-1.84232	-1.83869	-1.83504	-1.8314	-1.82776	-1.82413	-1.82052	-1.81692	-1.8317
-1.84121	-1.83754	-1.83387	-1.83021	-1.82658	-1.82297	-1.81936	-1.81576	-1.83054
-1.84008	-1.83638	-1.8327	-1.82904	-1.82542	-1.82184	-1.83666	-1.83305	-1.82943
-1.8389	-1.8352	-1.83152	-1.82787	-1.82429	-1.82075	-1.8356	-1.83202	-1.82842
-1.83776	-1.83406	-1.83037	-1.82673	-1.84163	-1.83809	-1.83454	-1.83098	-1.84596
-1.83671	-1.833	-1.82932	-1.82568	-1.84056	-1.83699	-1.83342	-1.82985	-1.84481
-1.83567	-1.83197	-1.8283	-1.82466	-1.8395	-1.83589	-1.83228	-1.82869	-1.84362
-1.83455	-1.83085	-1.82719	-1.82355	-1.83838	-1.83475	-1.83113	-1.82752	-1.84244
-1.83338	-1.82969	-1.82603	-1.8224	-1.83723	-1.83359	-1.82996	-1.82634	-1.84126
-1.83229	-1.84712	-1.84345	-1.83979	-1.83614	-1.8325	-1.82886	-1.82523	-1.84013
-1.83127	-1.8461	-1.84244	-1.83879	-1.83514	-1.83149	-1.82784	-1.8242	-1.83906
-1.84876	-1.84505	-1.84139	-1.83774	-1.83409	-1.83044	-1.84533	-1.84164	-1.83797
-1.84761	-1.84391	-1.84025	-1.83661	-1.83295	-1.8293	-1.84418	-1.84048	-1.83681
-1.84646	-1.84278	-1.83912	-1.83547	-1.83182	-1.82817	-1.84303	-1.83934	-1.83567
-1.84541	-1.84174	-1.83808	-1.83442	-1.83077	-1.82712	-1.84197	-1.83829	-1.83463
-1.84438	-1.84071	-1.83705	-1.8334	-1.82974	-1.82609	-1.84094	-1.83726	-1.83362
-1.84328	-1.83961	-1.83595	-1.83229	-1.82864	-1.82499	-1.83983	-1.83616	-1.83253
-1.84212	-1.83846	-1.8348	-1.83114	-1.82748	-1.82383	-1.83865	-1.835	-1.8314
-1.84104	-1.83737	-1.83371	-1.83006	-1.84493	-1.84123	-1.83756	-1.83391	-1.83031
-1.84004	-1.83638	-1.83272	-1.82906	-1.84392	-1.84023	-1.83655	-1.8329	-1.82929
-1.83906	-1.83539	-1.83173	-1.82808	-1.84293	-1.83923	-1.83556	-1.83191	-1.84407
-1.83808	-1.83442	-1.83075	-1.8271	-1.84194	-1.83825	-1.83458	-1.83093	-1.84308

-1.83708	-1.83341	-1.82975	-1.82609	-1.84092	-1.83724	-1.83358	-1.82993	-1.84206
-1.83596	-1.83229	-1.82863	-1.82498	-1.83979	-1.83612	-1.83246	-1.82882	-1.84094
-1.8348	-1.83113	-1.82747	-1.82381	-1.83862	-1.83495	-1.83129	-1.82766	-1.83976
-1.83372	-1.84863	-1.84492	-1.84122	-1.83754	-1.83387	-1.83023	-1.8266	-1.83868
-1.83273	-1.84762	-1.84392	-1.84022	-1.83654	-1.83288	-1.82924	-1.82561	-1.83769
-1.83174	-1.84663	-1.84293	-1.83924	-1.83556	-1.8319	-1.84403	-1.84037	-1.8367
-1.83078	-1.84565	-1.84195	-1.83827	-1.8346	-1.83094	-1.84306	-1.8394	-1.83573
-1.82981	-1.84467	-1.84097	-1.83729	-1.83362	-1.82997	-1.84208	-1.83842	-1.83475
-1.82883	-1.84366	-1.83997	-1.83629	-1.83263	-1.82898	-1.84108	-1.83742	-1.83375
-1.82783	-1.84263	-1.83894	-1.83526	-1.8316	-1.82797	-1.84006	-1.83639	-1.83272
-1.8453	-1.84155	-1.83782	-1.83413	-1.83047	-1.82684	-1.83892	-1.83526	-1.83159
-1.84421	-1.84042	-1.83665	-1.83293	-1.82928	-1.82566	-1.83773	-1.83407	-1.8304
-1.84311	-1.83932	-1.83555	-1.83183	-1.84394	-1.84029	-1.83662	-1.83296	-1.82929
-1.842	-1.83825	-1.83452	-1.83082	-1.84293	-1.83927	-1.8356	-1.83194	-1.82826
-1.84092	-1.83721	-1.83351	-1.82983	-1.84193	-1.83827	-1.8346	-1.83094	-1.82726
-1.8399	-1.8362	-1.83252	-1.82886	-1.84095	-1.83729	-1.83362	-1.82995	-1.82627
-1.8389	-1.83521	-1.83154	-1.82789	-1.83997	-1.83631	-1.83265	-1.82897	-1.82529
-1.83791	-1.83422	-1.83056	-1.82692	-1.83899	-1.83533	-1.83166	-1.82798	-1.82429
-1.83693	-1.83324	-1.82959	-1.82595	-1.83801	-1.83434	-1.83067	-1.82698	-1.82329
-1.83594	-1.84807	-1.84438	-1.8407	-1.83703	-1.83336	-1.82968	-1.82599	-1.82229
-1.83495	-1.84707	-1.84339	-1.83972	-1.83605	-1.83238	-1.8287	-1.825	-1.82129
-1.83397	-1.84608	-1.8424	-1.83873	-1.83507	-1.8314	-1.82771	-1.824	-1.82029
-1.83298	-1.8451	-1.84142	-1.83775	-1.83408	-1.8304	-1.82671	-1.823	-1.81929
-1.83201	-1.84412	-1.84043	-1.83676	-1.83309	-1.82941	-1.82571	-1.822	-1.81829
-1.83105	-1.84312	-1.83944	-1.83577	-1.8321	-1.82841	-1.82471	-1.82099	-1.81728
-1.83011	-1.84214	-1.83845	-1.83479	-1.8311	-1.82741	-1.8237	-1.81998	-1.81627
-1.82922	-1.84122	-1.83749	-1.83379	-1.8301	-1.82641	-1.8227	-1.81897	-1.81526
-1.82836	-1.84032	-1.83654	-1.8328	-1.82909	-1.8254	-1.82169	-1.81797	-1.81426
-1.8275	-1.83945	-1.83564	-1.83186	-1.82811	-1.8244	-1.82068	-1.81696	-1.81326
-1.82663	-1.8386	-1.83478	-1.83097	-1.82717	-1.82339	-1.81965	-1.81594	-1.81225
-1.84147	-1.83771	-1.83391	-1.83009	-1.82626	-1.82244	-1.81867	-1.81495	-1.81125
-1.8405	-1.83677	-1.83301	-1.82923	-1.82539	-1.82154	-1.81773	-1.81397	-1.81025
-1.83952	-1.83581	-1.83209	-1.82834	-1.82451	-1.82066	-1.81683	-1.81303	-1.80929
-1.83852	-1.83482	-1.83112	-1.82739	-1.8236	-1.81978	-1.81599	-1.8122	-1.80845
-1.83753	-1.83383	-1.83012	-1.8264	-1.82266	-1.81889	-1.81514	-1.81139	-1.80764
-1.83654	-1.83282	-1.82911	-1.8254	-1.82167	-1.81793	-1.8142	-1.81048	-1.80675
-1.83557	-1.83181	-1.82809	-1.82438	-1.82065	-1.81692	-1.81321	-1.80951	-1.80582
-1.83464	-1.83085	-1.8271	-1.82337	-1.81963	-1.81591	-1.81221	-1.80853	-1.80486
-1.83377	-1.82995	-1.82615	-1.82237	-1.81861	-1.81488	-1.81118	-1.80752	-1.80387
-1.83289	-1.82907	-1.82524	-1.82142	-1.81765	-1.8139	-1.8102	-1.80653	-1.80288
-1.83199	-1.8282	-1.82439	-1.82059	-1.81679	-1.813	-1.80926	-1.80556	-1.80189
-1.83107	-1.82732	-1.82354	-1.81974	-1.81593	-1.81212	-1.80836	-1.80463	-1.80094
-1.83011	-1.82634	-1.82257	-1.8188	-1.81502	-1.81124	-1.8075	-1.80379	-1.80009
-1.82915	-1.82536	-1.82158	-1.81783	-1.81409	-1.81037	-1.80666	-1.80298	-1.79928
-1.82827	-1.82447	-1.82068	-1.81691	-1.81316	-1.80944	-1.80574	-1.80207	-1.79839
-1.82745	-1.82365	-1.81982	-1.81601	-1.81223	-1.80848	-1.80477	-1.80111	-1.79746
-1.82655	-1.82276	-1.81894	-1.81512	-1.81134	-1.80758	-1.80387	-1.8002	-1.83308
-1.82557	-1.82179	-1.81802	-1.81425	-1.8105	-1.80677	-1.80305	-1.79934	-1.83217
-1.82461	-1.82084	-1.81709	-1.81335	-1.80964	-1.80593	-1.80221	-1.79848	-1.83127
-1.82371	-1.81991	-1.81614	-1.81239	-1.80869	-1.80501	-1.80131	-1.7976	-1.83041

-1.82284	-1.81901	-1.81521	-1.81144	-1.80775	-1.80409	-1.80041	-1.79673	-1.82955
-1.82197	-1.81815	-1.81436	-1.8106	-1.80691	-1.80326	-1.79957	-1.79588	-1.82866
-1.8211	-1.81732	-1.81355	-1.8098	-1.80612	-1.80246	-1.79876	-1.79503	-1.82776
-1.82027	-1.8165	-1.81273	-1.80897	-1.80527	-1.80158	-1.79788	-1.79416	-1.82688
-1.81947	-1.8157	-1.8119	-1.80812	-1.80437	-1.80065	-1.79695	-1.79327	-1.82601
-1.81862	-1.81484	-1.81105	-1.80726	-1.8035	-1.79977	-1.79606	-1.79238	-1.82512
-1.8177	-1.81391	-1.81014	-1.8064	-1.80267	-1.79896	-1.79524	-1.79152	-1.82421
-1.81679	-1.81299	-1.80925	-1.80553	-1.80183	-1.79813	-1.7944	-1.79066	-1.82331
-1.81594	-1.81214	-1.80838	-1.80465	-1.80093	-1.79722	-1.7935	-1.78978	-1.82244
-1.81512	-1.81132	-1.80754	-1.80377	-1.80003	-1.79631	-1.79261	-1.78892	-1.82159
-1.81428	-1.81047	-1.80669	-1.80292	-1.79919	-1.79548	-1.79178	-1.78809	-1.82075
-1.81345	-1.80963	-1.80585	-1.8021	-1.79839	-1.79471	-1.791	-1.78729	-1.81992
-1.81261	-1.8088	-1.80503	-1.8013	-1.7976	-1.79392	-1.82672	-1.8229	-1.81907
-1.81176	-1.80797	-1.80424	-1.80053	-1.79684	-1.79314	-1.8259	-1.82204	-1.81819
-1.81093	-1.80716	-1.80345	-1.79976	-1.79605	-1.79234	-1.82506	-1.82118	-1.81732
-1.81013	-1.80639	-1.80269	-1.79898	-1.79525	-1.7915	-1.82419	-1.82031	-1.81645
-1.80935	-1.80562	-1.80191	-1.79819	-1.79443	-1.79065	-1.82332	-1.81945	-1.81561
-1.80857	-1.80484	-1.8011	-1.79735	-1.79358	-1.7898	-1.82247	-1.81863	-1.83389
-1.80778	-1.80404	-1.80027	-1.7965	-1.79273	-1.78895	-1.82164	-1.81783	-1.83308
-1.80697	-1.80321	-1.79943	-1.79566	-1.7919	-1.78814	-1.82083	-1.81704	-1.83228
-1.80614	-1.80235	-1.79858	-1.79482	-1.79108	-1.78736	-1.82005	-1.81625	-1.83149
-1.80534	-1.80154	-1.79777	-1.79402	-1.79029	-1.78658	-1.81925	-1.81543	-1.83066
-1.80458	-1.80078	-1.79701	-1.79325	-1.78953	-1.78581	-1.81845	-1.81457	-1.82977
-1.8038	-1.80001	-1.79624	-1.79249	-1.82531	-1.8215	-1.81764	-1.81374	-1.82891
-1.80296	-1.7992	-1.79546	-1.79172	-1.82453	-1.82072	-1.81686	-1.81296	-1.82812
-1.80213	-1.79838	-1.79467	-1.79096	-1.82375	-1.81992	-1.81606	-1.81218	-1.82734
-1.80135	-1.7976	-1.7939	-1.79019	-1.82294	-1.81907	-1.8343	-1.83039	-1.82651
-1.80059	-1.79684	-1.79313	-1.78941	-1.82211	-1.8182	-1.83342	-1.82952	-1.82566
-1.79984	-1.79608	-1.79237	-1.78864	-1.82132	-1.81739	-1.83259	-1.8287	-1.82486
-1.7991	-1.79534	-1.79162	-1.78789	-1.82054	-1.81661	-1.8318	-1.82792	-1.82407
-1.79835	-1.79459	-1.79087	-1.78713	-1.81977	-1.81584	-1.83102	-1.82714	-1.8233
-1.79761	-1.79385	-1.79012	-1.78637	-1.81901	-1.81508	-1.83025	-1.82637	-1.82253
-1.79686	-1.7931	-1.78937	-1.78562	-1.81824	-1.81431	-1.82948	-1.8256	-1.82176
-1.79612	-1.79234	-1.78861	-1.78486	-1.81747	-1.81354	-1.8287	-1.82483	-1.821
-1.79538	-1.7916	-1.78786	-1.7841	-1.8167	-1.81277	-1.82792	-1.82406	-1.82023
-1.7947	-1.7909	-1.78713	-1.78336	-1.81594	-1.81202	-1.82716	-1.82329	-1.81946
-1.79405	-1.79022	-1.78642	-1.78261	-1.81519	-1.81128	-1.82641	-1.82252	-1.81869
-1.79335	-1.78951	-1.78569	-1.78188	-1.83353	-1.82961	-1.8257	-1.82181	-1.81796
-1.79262	-1.78877	-1.78495	-1.78114	-1.83282	-1.82894	-1.82504	-1.82114	-1.81727
-1.79188	-1.82464	-1.82073	-1.81685	-1.83209	-1.82825	-1.82436	-1.82045	-1.81657
-1.79114	-1.82389	-1.81996	-1.81608	-1.83132	-1.8275	-1.82363	-1.81972	-1.81584
-1.79041	-1.82316	-1.81922	-1.81532	-1.83056	-1.82675	-1.82289	-1.819	-1.81513
-1.78972	-1.82248	-1.81854	-1.81462	-1.82983	-1.826	-1.82216	-1.81832	-1.81446
-1.78905	-1.82182	-1.81787	-1.81393	-1.82911	-1.82526	-1.82143	-1.81764	-1.81381
-1.78834	-1.84025	-1.83626	-1.83228	-1.82836	-1.82449	-1.82068	-1.81691	-1.8131
-1.78763	-1.83949	-1.8355	-1.83154	-1.82761	-1.82373	-1.81991	-1.81616	-1.81236
-1.78695	-1.83876	-1.83479	-1.83084	-1.82691	-1.82301	-1.81918	-1.81542	-1.81928
-1.7863	-1.83809	-1.83412	-1.83018	-1.82626	-1.82235	-1.8185	-1.81468	-1.81856
-1.78563	-1.83739	-1.83344	-1.8295	-1.82559	-1.82171	-1.81783	-1.81397	-1.81786
-1.78491	-1.83666	-1.83269	-1.82876	-1.8249	-1.82107	-1.8172	-1.81332	-1.81721

-1.78419	-1.83593	-1.83196	-1.82803	-1.8242	-1.82042	-1.81657	-1.81268	-1.81658
-1.8201	-1.83525	-1.83128	-1.82735	-1.82351	-1.8197	-1.81587	-1.81204	-1.81598
-1.81945	-1.8346	-1.83063	-1.8267	-1.82281	-1.81897	-1.81516	-1.81138	-1.81539
-1.83793	-1.83395	-1.82999	-1.82606	-1.82215	-1.81828	-1.82232	-1.81854	-1.81473
-1.8373	-1.83331	-1.82936	-1.82542	-1.82152	-1.81763	-1.82167	-1.81784	-1.81403
-1.83665	-1.83268	-1.82873	-1.82479	-1.82088	-1.81699	-1.82103	-1.81718	-1.81336
-1.83598	-1.83204	-1.8281	-1.82416	-1.82025	-1.81636	-1.82043	-1.81656	-1.81272
-1.83532	-1.8314	-1.82747	-1.82353	-1.81963	-1.81573	-1.81982	-1.81595	-1.83268
-1.83469	-1.83076	-1.82683	-1.8229	-1.819	-1.8151	-1.81923	-1.81535	-1.83206
-1.83408	-1.83012	-1.82619	-1.82227	-1.81836	-1.81447	-1.81863	-1.81475	-1.83144
-1.83343	-1.82949	-1.82556	-1.82164	-1.8258	-1.82191	-1.81802	-1.81415	-1.83083
-1.83277	-1.82886	-1.82493	-1.82101	-1.8252	-1.8213	-1.81742	-1.81355	-1.83022
-1.83214	-1.82822	-1.8243	-1.82037	-1.82459	-1.82069	-1.83745	-1.83352	-1.8296
-1.83153	-1.82759	-1.82366	-1.81973	-1.82399	-1.82009	-1.83683	-1.8329	-1.82898
-1.83091	-1.83515	-1.83121	-1.82729	-1.8441	-1.84015	-1.83621	-1.83228	-1.82837
-1.83028	-1.83455	-1.83061	-1.82669	-1.84348	-1.83953	-1.83559	-1.83167	-1.82777
-1.82965	-1.83395	-1.83001	-1.82609	-1.84287	-1.83891	-1.83498	-1.83107	-1.82717
-1.82902	-1.83335	-1.82942	-1.8255	-1.84226	-1.8383	-1.83437	-1.83047	-1.8266
-1.82841	-1.83277	-1.82884	-1.82492	-1.84167	-1.83771	-1.83378	-1.82989	-1.82603
-1.83622	-1.85308	-1.84908	-1.8451	-1.84113	-1.83717	-1.83325	-1.82935	-1.82548
-1.83574	-1.85259	-1.84858	-1.84459	-1.84062	-1.83668	-1.83275	-1.82883	-1.82495
-1.8352	-1.85204	-1.84803	-1.84403	-1.84008	-1.83614	-1.83221	-1.82829	-1.82439
-1.8346	-1.85143	-1.84743	-1.84345	-1.83949	-1.83556	-1.83163	-1.82771	-1.82382
-1.85484	-1.85082	-1.84683	-1.84286	-1.83891	-1.83499	-1.83107	-1.82716	-1.82327
-1.85422	-1.85021	-1.84622	-1.84226	-1.83835	-1.83447	-1.83058	-1.82668	-1.82278
-1.85363	-1.84962	-1.84563	-1.84169	-1.8378	-1.83395	-1.83007	-1.82619	-1.8223
-1.85312	-1.8491	-1.84511	-1.84116	-1.83726	-1.83339	-1.82951	-1.82563	-1.82174
-1.85264	-1.84862	-1.84462	-1.84065	-1.83673	-1.83283	-1.82894	-1.82506	-1.82117
-1.85215	-1.84814	-1.84413	-1.84015	-1.83622	-1.83232	-1.82842	-1.82454	-1.82066
-1.85167	-1.84766	-1.84365	-1.83966	-1.83574	-1.83184	-1.82795	-1.82407	-1.82018
-1.85114	-1.84714	-1.84312	-1.83914	-1.83522	-1.83133	-1.82745	-1.82358	-1.81969
-1.85056	-1.84655	-1.84255	-1.83857	-1.83465	-1.83077	-1.82691	-1.82308	-1.81922
-1.84999	-1.84599	-1.84199	-1.83802	-1.8341	-1.83023	-1.82638	-1.82256	-1.81871
-1.84949	-1.84548	-1.84149	-1.83753	-1.83361	-1.82972	-1.82585	-1.822	-1.81814
-1.849	-1.845	-1.84102	-1.83706	-1.83313	-1.82923	-1.82534	-1.82145	-1.81756
-1.84853	-1.84452	-1.84054	-1.83659	-1.83266	-1.82876	-1.82485	-1.82095	-1.81706
-1.84806	-1.84406	-1.84008	-1.83612	-1.8322	-1.8283	-1.82439	-1.82048	-1.81658
-1.84759	-1.84359	-1.83962	-1.83566	-1.83174	-1.82783	-1.82392	-1.82001	-1.81611
-1.84712	-1.84312	-1.83915	-1.83521	-1.83128	-1.82737	-1.82346	-1.81955	-1.81564
-1.84664	-1.84265	-1.83869	-1.83475	-1.83082	-1.82691	-1.82299	-1.81908	-1.81518
-1.84617	-1.84218	-1.83822	-1.83428	-1.83035	-1.82644	-1.82253	-1.81862	-1.81472
-1.84569	-1.84171	-1.83775	-1.83381	-1.82989	-1.82597	-1.82206	-1.81816	-1.81426
-1.84522	-1.84122	-1.83727	-1.83334	-1.82942	-1.8255	-1.82159	-1.81769	-1.81379
-1.84476	-1.84075	-1.8368	-1.83287	-1.82895	-1.82504	-1.82113	-1.81722	-1.81332
-1.84437	-1.84034	-1.83637	-1.83242	-1.82849	-1.82458	-1.82067	-1.81676	-1.81286
-1.84402	-1.83998	-1.83596	-1.83197	-1.82803	-1.82412	-1.82022	-1.81632	-1.81242
-1.84362	-1.83959	-1.83557	-1.83157	-1.82763	-1.82372	-1.81982	-1.81592	-1.84393
-1.84318	-1.83917	-1.83518	-1.83122	-1.82728	-1.82337	-1.81946	-1.81557	-1.84356
-1.84273	-1.83874	-1.83478	-1.83083	-1.8269	-1.82299	-1.81908	-1.81519	-1.84316
-1.84227	-1.83829	-1.83433	-1.83038	-1.82646	-1.82254	-1.81864	-1.81474	-1.84271

-1.84183	-1.83786	-1.83389	-1.82994	-1.82601	-1.82209	-1.81818	-1.81428	-1.84223
-1.84145	-1.83748	-1.83351	-1.82955	-1.82559	-1.82165	-1.81772	-1.81382	-1.84175
-1.84108	-1.83711	-1.83314	-1.82918	-1.8252	-1.82122	-1.81727	-1.81336	-1.84128
-1.84066	-1.83669	-1.83273	-1.82878	-1.8248	-1.82081	-1.84887	-1.84486	-1.84086
-1.8402	-1.83623	-1.83229	-1.82835	-1.8244	-1.82044	-1.84849	-1.84448	-1.84049
-1.83978	-1.83581	-1.83188	-1.82796	-1.82401	-1.82006	-1.84811	-1.84408	-1.84008
-1.8394	-1.83544	-1.83151	-1.82759	-1.82365	-1.8197	-1.84772	-1.84366	-1.83963
-1.83902	-1.83506	-1.83112	-1.8272	-1.82326	-1.81932	-1.84732	-1.84324	-1.8392
-1.83861	-1.83462	-1.83068	-1.82675	-1.82282	-1.8189	-1.8469	-1.84284	-1.83883
-1.83819	-1.83419	-1.83024	-1.82631	-1.82239	-1.81848	-1.84649	-1.84247	-1.83847
-1.8378	-1.83381	-1.82986	-1.82593	-1.85413	-1.85012	-1.84611	-1.84211	-1.83813
-1.83742	-1.83345	-1.82951	-1.82558	-1.85377	-1.84975	-1.84575	-1.84177	-1.83779
-1.83706	-1.8331	-1.82916	-1.82524	-1.85341	-1.84939	-1.8454	-1.84142	-1.87186
-1.83672	-1.83277	-1.82882	-1.82489	-1.85306	-1.84905	-1.84505	-1.84107	-1.87149
-1.83638	-1.83243	-1.82848	-1.82455	-1.8527	-1.8487	-1.84471	-1.84072	-1.87112
-1.83604	-1.83209	-1.82814	-1.82421	-1.85235	-1.84834	-1.84435	-1.84037	-1.87074
-1.8185	-1.8641	-1.86006	-1.85602	-1.852	-1.84799	-1.844	-1.84003	-1.87037
-1.81817	-1.86376	-1.85971	-1.85567	-1.85165	-1.84764	-1.84366	-1.83969	-1.87004
-1.81784	-1.86342	-1.85936	-1.85532	-1.8513	-1.8473	-1.84332	-1.83936	-1.86972
-1.8175	-1.86307	-1.85901	-1.85497	-1.85096	-1.84696	-1.84298	-1.83902	-1.86938
-1.81716	-1.86271	-1.85867	-1.85463	-1.85062	-1.84662	-1.84265	-1.83869	-1.86904
-1.81683	-1.86236	-1.85832	-1.85429	-1.85028	-1.84628	-1.84231	-1.83835	-1.86869
-1.81653	-1.86201	-1.85796	-1.85394	-1.84993	-1.84595	-1.84197	-1.83801	-1.86833
-1.84906	-1.84498	-1.84096	-1.83698	-1.8496	-1.84562	-1.84164	-1.83767	-1.86798
-1.84881	-1.84474	-1.84067	-1.83664	-1.84926	-1.84529	-1.84131	-1.83733	-1.86762
-1.84855	-1.8445	-1.8404	-1.83634	-1.84895	-1.84499	-1.84101	-1.83702	-1.86729
-1.84824	-1.8442	-1.84014	-1.8361	-1.84871	-1.84475	-1.84076	-1.83678	-1.89294
-1.8479	-1.84389	-1.83988	-1.83589	-1.8485	-1.84451	-1.84054	-1.83656	-1.89268
-1.84758	-1.84356	-1.83958	-1.83562	-1.83165	-1.8277	-1.84025	-1.83628	-1.89239
-1.84728	-1.84323	-1.83925	-1.8353	-1.83134	-1.82739	-1.83993	-1.83596	-1.89207
-1.847	-1.84294	-1.83897	-1.83503	-1.83107	-1.82711	-1.83964	-1.83565	-1.89174
-1.84675	-1.84271	-1.83874	-1.83479	-1.83083	-1.82688	-1.83938	-1.83534	-1.8914
-1.84652	-1.84249	-1.8385	-1.83453	-1.83058	-1.82664	-1.83912	-1.83506	-1.89108
-1.84629	-1.84226	-1.83823	-1.83422	-1.83027	-1.82635	-1.83885	-1.83481	-1.89083
-1.84606	-1.84202	-1.83796	-1.83392	-1.82997	-1.82606	-1.83859	-1.83457	-1.89059
-1.84577	-1.84175	-1.83769	-1.83366	-1.82972	-1.82581	-1.83835	-1.83435	-1.89033
-1.84546	-1.84146	-1.83744	-1.83344	-1.82949	-1.82557	-1.83811	-1.83412	-1.89007
-1.8452	-1.84121	-1.8372	-1.83321	-1.82924	-1.82529	-1.82135	-1.81742	-1.8898
-1.84498	-1.84099	-1.83698	-1.83298	-1.82897	-1.82498	-1.82102	-1.81711	-1.8895
-1.84478	-1.84078	-1.83677	-1.83276	-1.82873	-1.8247	-1.82074	-1.81683	-1.88923
-1.84457	-1.84057	-1.83657	-1.83256	-1.82852	-1.82448	-1.82051	-1.81661	-1.88899
-1.84436	-1.84036	-1.83636	-1.83235	-1.82831	-1.82427	-1.8203	-1.81639	-1.87315
-1.84416	-1.84016	-1.83616	-1.83215	-1.8281	-1.82406	-1.82009	-1.81619	-1.87293
-1.84396	-1.83995	-1.83596	-1.83195	-1.8279	-1.82386	-1.81989	-1.81599	-1.87271
-1.84376	-1.83975	-1.83576	-1.83175	-1.8277	-1.82365	-1.81969	-1.81579	-1.8725
-1.84356	-1.83956	-1.83556	-1.83156	-1.82751	-1.82346	-1.81949	-1.81559	-1.87228
-1.84336	-1.83936	-1.83537	-1.83136	-1.82731	-1.82326	-1.81929	-1.81538	-1.87206
-1.84317	-1.83917	-1.83517	-1.83117	-1.82712	-1.82307	-1.81909	-1.81518	-1.87185
-1.84298	-1.83897	-1.83498	-1.83098	-1.82693	-1.82288	-1.81891	-1.81499	-1.87163
-1.84278	-1.83878	-1.83479	-1.83079	-1.82675	-1.8227	-1.81872	-1.81478	-1.87141

-1.84259	-1.83859	-1.83461	-1.83061	-1.82658	-1.82255	-1.81856	-1.8146	-1.8712
-1.84239	-1.8384	-1.83442	-1.83044	-1.82644	-1.82244	-1.81846	-1.81449	-1.87105
-1.84221	-1.83822	-1.83424	-1.83026	-1.8263	-1.82234	-1.81837	-1.8144	-1.87092
-1.84208	-1.83808	-1.83407	-1.83008	-1.82612	-1.82218	-1.81822	-1.81426	-1.87074
-1.842	-1.83797	-1.83391	-1.82988	-1.82592	-1.82199	-1.81803	-1.81407	-1.87055
-1.84187	-1.83783	-1.83375	-1.8297	-1.82573	-1.82179	-1.84479	-1.84076	-1.87036
-1.84169	-1.83766	-1.83359	-1.82953	-1.82554	-1.82158	-1.84457	-1.84056	-1.87018
-1.84152	-1.83749	-1.83343	-1.82938	-1.82538	-1.82141	-1.84438	-1.84037	-1.87
-1.84137	-1.83733	-1.8333	-1.82928	-1.82528	-1.82131	-1.84424	-1.84019	-1.86979
-1.84123	-1.83718	-1.83318	-1.82919	-1.8252	-1.82121	-1.84412	-1.84003	-1.8696
-1.8411	-1.83707	-1.83308	-1.82909	-1.82509	-1.82108	-1.84398	-1.8399	-1.90153
-1.841	-1.83698	-1.83299	-1.82899	-1.82497	-1.82094	-1.84384	-1.83979	-1.90142
-1.8409	-1.83689	-1.83288	-1.82887	-1.82484	-1.82082	-1.84372	-1.83969	-1.9013
-1.84081	-1.83679	-1.83275	-1.82872	-1.82471	-1.82071	-1.84362	-1.83959	-1.90117
-1.84071	-1.83668	-1.83263	-1.82859	-1.82459	-1.82061	-1.87654	-1.8724	-1.86825
-1.84058	-1.83654	-1.8325	-1.82848	-1.82449	-1.82051	-1.87641	-1.87224	-1.86809
-1.84046	-1.83641	-1.8324	-1.82839	-1.8244	-1.82041	-1.87627	-1.87208	-1.86793
-1.84034	-1.83632	-1.83232	-1.82832	-1.82429	-1.82027	-1.8761	-1.87195	-1.86782
-1.84024	-1.83624	-1.83225	-1.82826	-1.8242	-1.82013	-1.87596	-1.87183	-1.86772
-1.84016	-1.83617	-1.83219	-1.82819	-1.85807	-1.85391	-1.87589	-1.87175	-1.86763
-1.8401	-1.83611	-1.83212	-1.82813	-1.85803	-1.85391	-1.87587	-1.87168	-1.86754
-1.82559	-1.83605	-1.83206	-1.82806	-1.85795	-1.85383	-1.87579	-1.8716	-1.86745
-1.82552	-1.83597	-1.83199	-1.82799	-1.85783	-1.85368	-1.87564	-1.87149	-1.86736
-1.82545	-1.87005	-1.86598	-1.86188	-1.85773	-1.85356	-1.87553	-1.8714	-1.86728
-1.82538	-1.86997	-1.86589	-1.8618	-1.85768	-1.85354	-1.87549	-1.87132	-1.86719
-1.82531	-1.86988	-1.8658	-1.86172	-1.85761	-1.8535	-1.87544	-1.87124	-1.8671
-1.82523	-1.85578	-1.85173	-1.84766	-1.8575	-1.85336	-1.8753	-1.87114	-1.86701
-1.82515	-1.8557	-1.85164	-1.84756	-1.85738	-1.85321	-1.87515	-1.87102	-1.86691
-1.85967	-1.8556	-1.85155	-1.84749	-1.84338	-1.83925	-1.87509	-1.87094	-1.86681
-1.85961	-1.8555	-1.85146	-1.84742	-1.84335	-1.83926	-1.87508	-1.87087	-1.86671
-1.85958	-1.85545	-1.85139	-1.84734	-1.84329	-1.83924	-1.87506	-1.87084	-1.86667
-1.85959	-1.85547	-1.85135	-1.84725	-1.8432	-1.83917	-1.87503	-1.87085	-1.86668
-1.85958	-1.85547	-1.85132	-1.84719	-1.86963	-1.86554	-1.86143	-1.85733	-1.87895
-1.8595	-1.85543	-1.85131	-1.8472	-1.8696	-1.86546	-1.86135	-1.85728	-1.87893
-1.85942	-1.85536	-1.8513	-1.84722	-1.86958	-1.8654	-1.86129	-1.85723	-1.87892
-1.85935	-1.85528	-1.85124	-1.84719	-1.86957	-1.8654	-1.86129	-1.85723	-1.87893
-1.85931	-1.8552	-1.85117	-1.84715	-1.86956	-1.86542	-1.86132	-1.85725	-1.87894
-1.85931	-1.85518	-1.85116	-1.84714	-1.86957	-1.86545	-1.86133	-1.85721	-1.86555
-1.85935	-1.85521	-1.85117	-1.84716	-1.86959	-1.86547	-1.86132	-1.85715	-1.86545
-1.85938	-1.85524	-1.85117	-1.84714	-1.86957	-1.86547	-1.86131	-1.85712	-1.8654
-1.85939	-1.85525	-1.85115	-1.84706	-1.8695	-1.86542	-1.86129	-1.85712	-1.86539
-1.8594	-1.85528	-1.85113	-1.84701	-1.86944	-1.86538	-1.86127	-1.85713	-1.86541
-1.85943	-1.85532	-1.85116	-1.84702	-1.86944	-1.86537	-1.86128	-1.85716	-1.86545
-1.85946	-1.85535	-1.85119	-1.84704	-1.86944	-1.86536	-1.86128	-1.85719	-1.86549
-1.85942	-1.85533	-1.85119	-1.84706	-1.86943	-1.86531	-1.86122	-1.85715	-1.86548
-1.85935	-1.85529	-1.85119	-1.84708	-1.86941	-1.86524	-1.86114	-1.85708	-1.86544
-1.85933	-1.88193	-1.87778	-1.87361	-1.86941	-1.86522	-1.86111	-1.85706	-1.87804
-1.85936	-1.88195	-1.87781	-1.87365	-1.86945	-1.86525	-1.86113	-1.85709	-1.87806
-1.85939	-1.88198	-1.87784	-1.87368	-1.86948	-1.86528	-1.86117	-1.85712	-1.87809
-1.85944	-1.88203	-1.87789	-1.87373	-1.86953	-1.86533	-1.86121	-1.85717	-1.87814

-1.85949	-1.88207	-1.87793	-1.87377	-1.86957	-1.86537	-1.86126	-1.85722	-1.87819
-1.85954	-1.88212	-1.87798	-1.87382	-1.86962	-1.86542	-1.8613	-1.85726	-1.87824
-1.85959	-1.88217	-1.87802	-1.87386	-1.86966	-1.86546	-1.86135	-1.8573	-1.87828
-1.85963	-1.8822	-1.87807	-1.87391	-1.86971	-1.86551	-1.8614	-1.85735	-1.87833
-1.85968	-1.88224	-1.87811	-1.87397	-1.86977	-1.86558	-1.86146	-1.85739	-1.87837
-1.85978	-1.88232	-1.87818	-1.87402	-1.86985	-1.86568	-1.86156	-1.85748	-1.87844
-1.85994	-1.88245	-1.87826	-1.87409	-1.86995	-1.86584	-1.86173	-1.85763	-1.87856
-1.86007	-1.88256	-1.87835	-1.87416	-1.87004	-1.86596	-1.86186	-1.85775	-1.87866
-1.86014	-1.88263	-1.87841	-1.87421	-1.8701	-1.86601	-1.86192	-1.85781	-1.87873
-1.86021	-1.8827	-1.87849	-1.87429	-1.87017	-1.86607	-1.86197	-1.85787	-1.8788
-1.88695	-1.88277	-1.8786	-1.87444	-1.87031	-1.8662	-1.8746	-1.87044	-1.87889
-1.88703	-1.88285	-1.87872	-1.8746	-1.87048	-1.86636	-1.87473	-1.87054	-1.87899
-1.8871	-1.88291	-1.8788	-1.8747	-1.87058	-1.86646	-1.87484	-1.87067	-1.87914
-1.88717	-1.88298	-1.87885	-1.87475	-1.87064	-1.86654	-1.87495	-1.87083	-1.87931
-1.88727	-1.88308	-1.87895	-1.87484	-1.87072	-1.86661	-1.87504	-1.87093	-1.87943
-1.8874	-1.88324	-1.8791	-1.87497	-1.87082	-1.86668	-1.87509	-1.87098	-1.87948
-1.88754	-1.88339	-1.87925	-1.8751	-1.87093	-1.86676	-1.87516	-1.87105	-1.87954
-1.8877	-1.88354	-1.87937	-1.8752	-1.87105	-1.8669	-1.8753	-1.87118	-1.87966
-1.88785	-1.88368	-1.87949	-1.87531	-1.87117	-1.86705	-1.87546	-1.87133	-1.87979
-1.88797	-1.88379	-1.87961	-1.87544	-1.87131	-1.8672	-1.87561	-1.87144	-1.8799
-1.88807	-1.88389	-1.87973	-1.8756	-1.87147	-1.86735	-1.87574	-1.87156	-1.88002
-1.8882	-1.88402	-1.87989	-1.87576	-1.87162	-1.86748	-1.87586	-1.87168	-1.88016
-1.88834	-1.88419	-1.88007	-1.87593	-1.87176	-1.86757	-1.87596	-1.87183	-1.88033
-1.8885	-1.88437	-1.88025	-1.87611	-1.88452	-1.88029	-1.88883	-1.88466	-1.8805
-1.88868	-1.88455	-1.88043	-1.8763	-1.88474	-1.88054	-1.88906	-1.88485	-1.88068
-1.88887	-1.88474	-1.88062	-1.87649	-1.88495	-1.88077	-1.88929	-1.88505	-1.88085
-1.88905	-1.88492	-1.8808	-1.87666	-1.8851	-1.8809	-1.88943	-1.88522	-1.88104
-1.88923	-1.8851	-1.88097	-1.87683	-1.88524	-1.88101	-1.88955	-1.88538	-1.88122
-1.87323	-1.88528	-1.88115	-1.87701	-1.88543	-1.88121	-1.88975	-1.88557	-1.8814
-1.87341	-1.88545	-1.88132	-1.87719	-1.88565	-1.88146	-1.88999	-1.88577	-1.88158
-1.87357	-1.88562	-1.88149	-1.87736	-1.88583	-1.88164	-1.89017	-1.88594	-1.88176
-1.87373	-1.88579	-1.88166	-1.87752	-1.88596	-1.88174	-1.89029	-1.8861	-1.88194
-1.8739	-1.88596	-1.88183	-1.8777	-1.88612	-1.88189	-1.89044	-1.88627	-1.88212
-1.8741	-1.88613	-1.88201	-1.87789	-1.88635	-1.88214	-1.89067	-1.88645	-1.88227
-1.87431	-1.88632	-1.88219	-1.87808	-1.88658	-1.8824	-1.89092	-1.88666	-1.88245
-1.87456	-1.88658	-1.88241	-1.87826	-1.88676	-1.88262	-1.89116	-1.88692	-1.89972
-1.87483	-1.88687	-1.88264	-1.87844	-1.88693	-1.88281	-1.89139	-1.88719	-1.9
-1.84347	-1.87091	-1.86673	-1.86257	-1.88715	-1.88299	-1.89158	-1.88741	-1.90024
-1.84364	-1.87112	-1.86699	-1.86284	-1.88739	-1.88316	-1.89174	-1.8876	-1.90046
-1.84383	-1.88422	-1.88008	-1.87593	-1.88763	-1.88339	-1.90905	-1.90486	-1.90067
-1.84402	-1.88438	-1.88025	-1.87613	-1.88788	-1.88367	-1.90931	-1.90506	-1.90084
-1.84424	-1.88457	-1.88044	-1.87632	-1.88811	-1.88394	-1.90958	-1.90529	-1.90106
-1.8445	-1.88483	-1.88065	-1.8765	-1.88831	-1.88417	-1.90984	-1.90557	-1.90134
-1.84477	-1.88511	-1.88088	-1.87669	-1.88851	-1.8844	-1.9101	-1.90586	-1.90165
-1.84506	-1.85426	-1.85011	-1.84598	-1.90616	-1.90201	-1.91039	-1.90617	-1.90194
-1.84536	-1.85458	-1.85042	-1.8463	-1.90648	-1.90233	-1.9107	-1.90647	-1.90223
-1.8456	-1.85484	-1.85071	-1.8466	-1.9068	-1.90263	-1.91101	-1.90679	-1.90254
-1.84579	-1.85507	-1.85098	-1.8469	-1.90711	-1.90294	-1.91132	-1.90711	-1.90287
-1.84603	-1.85532	-1.85125	-1.84719	-1.86115	-1.85707	-1.91159	-1.9074	-1.90317
-1.84632	-1.85561	-1.85154	-1.84747	-1.86139	-1.85727	-1.91179	-1.90762	-1.90342

-1.84662	-1.87404	-1.86993	-1.86581	-1.86165	-1.85748	-1.91201	-1.90785	-1.90367
-1.84692	-1.87435	-1.87024	-1.86611	-1.86194	-1.85777	-1.9123	-1.90814	-1.90397
-1.84723	-1.87466	-1.87055	-1.86642	-1.86225	-1.85808	-1.91262	-1.90846	-1.90428
-1.84754	-1.88829	-1.88413	-1.87996	-1.87574	-1.87153	-1.86741	-1.86335	-1.9046
-1.84785	-1.8886	-1.88444	-1.88027	-1.87606	-1.87185	-1.86772	-1.86366	-1.90492
-1.84815	-1.88892	-1.88475	-1.88058	-1.87637	-1.87216	-1.86804	-1.86397	-1.90524
-1.84846	-1.88923	-1.88506	-1.88089	-1.87668	-1.87247	-1.86834	-1.86428	-1.90556
-1.86715	-1.88954	-1.88538	-1.8812	-1.87699	-1.87278	-1.86865	-1.86459	-1.87636
-1.86745	-1.88985	-1.88569	-1.88151	-1.8773	-1.87308	-1.86896	-1.86489	-1.87667
-1.86776	-1.89016	-1.886	-1.88183	-1.87761	-1.8734	-1.86927	-1.8652	-1.87699
-1.86806	-1.89047	-1.88631	-1.88214	-1.87793	-1.87372	-1.86958	-1.86551	-1.8773
-1.86837	-1.89078	-1.88663	-1.88247	-1.87826	-1.87405	-1.86992	-1.86583	-1.87762
-1.86867	-1.87776	-1.87365	-1.86955	-1.86542	-1.86129	-1.87031	-1.86621	-1.87798
-1.86898	-1.87806	-1.87396	-1.86987	-1.86579	-1.86171	-1.87075	-1.86663	-1.87839
-1.86934	-1.87841	-1.8743	-1.87021	-1.86614	-1.86208	-1.87113	-1.86702	-1.86289
-1.86976	-1.87882	-1.87467	-1.87054	-1.86645	-1.8624	-1.87145	-1.86736	-1.86326
-1.88337	-1.87921	-1.87504	-1.87089	-1.8668	-1.86273	-1.87179	-1.86769	-1.86361
-1.88371	-1.87957	-1.87543	-1.87131	-1.86721	-1.86313	-1.87216	-1.86803	-1.86393
-1.88404	-1.87992	-1.87582	-1.87172	-1.86763	-1.86354	-1.87255	-1.86839	-1.86426
-1.88439	-1.88025	-1.87615	-1.87206	-1.86799	-1.86391	-1.87293	-1.86878	-1.86465
-1.88475	-1.88058	-1.87647	-1.87238	-1.86832	-1.86427	-1.87332	-1.86919	-1.86507
-1.85108	-1.88097	-1.87685	-1.87276	-1.8687	-1.86466	-1.87372	-1.8696	-1.86548
-1.85147	-1.88139	-1.87727	-1.87317	-1.86912	-1.86508	-1.87414	-1.87001	-1.86588
-1.85187	-1.88181	-1.87767	-1.87356	-1.8695	-1.86546	-1.87452	-1.8704	-1.86627
-1.85227	-1.88221	-1.87804	-1.8739	-1.86982	-1.86577	-1.87484	-1.87074	-1.86664
-1.85267	-1.8826	-1.87841	-1.87426	-1.87016	-1.8661	-1.87518	-1.87111	-1.86702
-1.85303	-1.88297	-1.8788	-1.87466	-1.87057	-1.86651	-1.87559	-1.87152	-1.86744
-1.8534	-1.88334	-1.8792	-1.87509	-1.87101	-1.86695	-1.87603	-1.87195	-1.86787
-1.83759	-1.84973	-1.84572	-1.84173	-1.87145	-1.86739	-1.87647	-1.87238	-1.8683
-1.83799	-1.85015	-1.84615	-1.84217	-1.8719	-1.86783	-1.87692	-1.87283	-1.86874
-1.8384	-1.85059	-1.84659	-1.8426	-1.87234	-1.86828	-1.87736	-1.87326	-1.90076
-1.83883	-1.85103	-1.84702	-1.84302	-1.87278	-1.86872	-1.8778	-1.8737	-1.90119
-1.83925	-1.85146	-1.84745	-1.84345	-1.87322	-1.86916	-1.87824	-1.87413	-1.90161
-1.83968	-1.85189	-1.84788	-1.84389	-1.87367	-1.8696	-1.87868	-1.87457	-1.90203
-1.8401	-1.85232	-1.84831	-1.84432	-1.87411	-1.87005	-1.87913	-1.87501	-1.90246
-1.82485	-1.83656	-1.8326	-1.82866	-1.87456	-1.87049	-1.87958	-1.87547	-1.90293
-1.82527	-1.83699	-1.83303	-1.82908	-1.87501	-1.87094	-1.88003	-1.87594	-1.90342
-1.82568	-1.83741	-1.83345	-1.82951	-1.87545	-1.87138	-1.88048	-1.87639	-1.90389
-1.82609	-1.83783	-1.83388	-1.82994	-1.87589	-1.87182	-1.88093	-1.87684	-1.90434
-1.82652	-1.82259	-1.81867	-1.81477	-1.82643	-1.8225	-1.8682	-1.86416	-1.90479
-1.82698	-1.82301	-1.8191	-1.8152	-1.82686	-1.82293	-1.86863	-1.8646	-1.90524
-1.82746	-1.82346	-1.81955	-1.81566	-1.81175	-1.80783	-1.86909	-1.86505	-1.90569
-1.82797	-1.82399	-1.82007	-1.81617	-1.81226	-1.80836	-1.86961	-1.86551	-1.90614
-1.82847	-1.82452	-1.8206	-1.81668	-1.81278	-1.80888	-1.87015	-1.866	-1.9066
-1.82892	-1.82498	-1.82105	-1.81714	-1.81324	-1.80934	-1.8729	-1.86874	-1.92221
-1.82936	-1.82542	-1.82149	-1.81757	-1.81367	-1.80977	-1.87339	-1.86928	-1.9228
-1.82985	-1.82591	-1.82198	-1.81805	-1.81413	-1.81021	-1.87384	-1.86977	-1.9092
-1.83038	-1.82643	-1.8225	-1.81858	-1.81461	-1.81065	-1.87427	-1.87022	-1.90968
-1.83088	-1.82692	-1.823	-1.81907	-1.8151	-1.81112	-1.87476	-1.87072	-1.89523
-1.83132	-1.82736	-1.82344	-1.81953	-1.81559	-1.81165	-1.87531	-1.87126	-1.89579

-1.83179	-1.82783	-1.8239	-1.82	-1.81609	-1.81219	-1.87588	-1.87181	-1.89635
-1.83231	-1.82834	-1.82441	-1.8205	-1.81661	-1.81273	-1.87644	-1.87236	-1.89691
-1.83285	-1.82888	-1.82493	-1.82101	-1.81712	-1.81325	-1.87699	-1.8729	-1.89745
-1.83339	-1.8294	-1.82542	-1.82147	-1.81758	-1.81372	-1.84371	-1.83973	-1.885
-1.83393	-1.82993	-1.82591	-1.82191	-1.81801	-1.81414	-1.84415	-1.84017	-1.88545
-1.83442	-1.83043	-1.8264	-1.82241	-1.81849	-1.81463	-1.84463	-1.84066	-1.88596
-1.83487	-1.83089	-1.82691	-1.82294	-1.81903	-1.81515	-1.84516	-1.84119	-1.88651
-1.83535	-1.83139	-1.82743	-1.82349	-1.81958	-1.81569	-1.8457	-1.84173	-1.88708
-1.83588	-1.83192	-1.82797	-1.82404	-1.82012	-1.81623	-1.84625	-1.84228	-1.88764
-1.83643	-1.83247	-1.82852	-1.82459	-1.82067	-1.81678	-1.8468	-1.84283	-1.88821
-1.83699	-1.83302	-1.82907	-1.82513	-1.85535	-1.85135	-1.86361	-1.85958	-1.88878
-1.83754	-1.83358	-1.82962	-1.82568	-1.85591	-1.8519	-1.86418	-1.86014	-1.88935
-1.8381	-1.83413	-1.83017	-1.82623	-1.85647	-1.85246	-1.86473	-1.86069	-1.85667
-1.83865	-1.83467	-1.83071	-1.82677	-1.85702	-1.85301	-1.86529	-1.86125	-1.85722
-1.8392	-1.83522	-1.83126	-1.82732	-1.85757	-1.85357	-1.86586	-1.86182	-1.85779
-1.83975	-1.83578	-1.83181	-1.82786	-1.85813	-1.85413	-1.86643	-1.86239	-1.85836
-1.84031	-1.83633	-1.83236	-1.82842	-1.85869	-1.85469	-1.86701	-1.86299	-1.85896
-1.84088	-1.83689	-1.83292	-1.82897	-1.85925	-1.85526	-1.86761	-1.86363	-1.85963
-1.84147	-1.83747	-1.8335	-1.82956	-1.85983	-1.85583	-1.86821	-1.86427	-1.86028
-1.84211	-1.83812	-1.83414	-1.83019	-1.86047	-1.85646	-1.86883	-1.86486	-1.86087
-1.84278	-1.83878	-1.8348	-1.83084	-1.86112	-1.8571	-1.86945	-1.86544	-1.86143
-1.84339	-1.83939	-1.83541	-1.83145	-1.87818	-1.8741	-1.87005	-1.86602	-1.86199
-1.84395	-1.83996	-1.83598	-1.83202	-1.87878	-1.8747	-1.87064	-1.86659	-1.86255
-1.84452	-1.87511	-1.87102	-1.86695	-1.87938	-1.87533	-1.87126	-1.8672	-1.86315
-1.84508	-1.87569	-1.8716	-1.86753	-1.88	-1.87599	-1.87193	-1.86786	-1.86382
-1.84568	-1.87631	-1.8722	-1.86814	-1.88063	-1.87663	-1.87258	-1.86851	-1.86447
-1.84635	-1.87697	-1.87287	-1.8688	-1.88126	-1.87722	-1.87316	-1.86911	-1.86507
-1.84703	-1.87766	-1.87356	-1.86948	-1.8819	-1.8778	-1.87374	-1.8697	-1.86566
-1.84771	-1.89499	-1.89084	-1.88671	-1.88257	-1.87845	-1.87437	-1.87034	-1.8663
-1.84837	-1.89567	-1.89153	-1.8874	-1.88325	-1.87911	-1.87503	-1.871	-1.86697
-1.84899	-1.89631	-1.89216	-1.88802	-1.88388	-1.87974	-1.87566	-1.87162	-1.86759
-1.84956	-1.89691	-1.89274	-1.88859	-1.88445	-1.88033	-1.87625	-1.87219	-1.86817
-1.85016	-1.89753	-1.89336	-1.8892	-1.88507	-1.88095	-1.87686	-1.87279	-1.86878
-1.85081	-1.89819	-1.89403	-1.88988	-1.88574	-1.88161	-1.87751	-1.87344	-1.86941
-1.85148	-1.89887	-1.89472	-1.89058	-1.88643	-1.88229	-1.87819	-1.87411	-1.87007
-1.85217	-1.89957	-1.89541	-1.89127	-1.88712	-1.88298	-1.87888	-1.8748	-1.87074
-1.85286	-1.90027	-1.89611	-1.89196	-1.88782	-1.88369	-1.87958	-1.87549	-1.87142
-1.85353	-1.90098	-1.89681	-1.89266	-1.88852	-1.88439	-1.88028	-1.87619	-1.87211
-1.85418	-1.90168	-1.89752	-1.89336	-1.88921	-1.88508	-1.88097	-1.87688	-1.87281
-1.90653	-1.90238	-1.89822	-1.89406	-1.88991	-1.88578	-1.88167	-1.87758	-1.8735
-1.90727	-1.90309	-1.89892	-1.89475	-1.89061	-1.88648	-1.88237	-1.87826	-1.87418
-1.90799	-1.9038	-1.89962	-1.89545	-1.8913	-1.88718	-1.88306	-1.87896	-1.87488
-1.90868	-1.90451	-1.90033	-1.89615	-1.892	-1.88787	-1.88376	-1.87965	-1.87558
-1.90937	-1.90522	-1.90103	-1.89685	-1.8927	-1.88857	-1.88445	-1.88036	-1.87629
-1.9101	-1.90593	-1.90173	-1.89755	-1.8934	-1.88927	-1.88516	-1.88107	-1.87702
-1.91083	-1.90664	-1.90243	-1.89824	-1.8941	-1.88998	-1.88588	-1.88179	-1.87775
-1.91155	-1.90734	-1.90313	-1.89895	-1.89481	-1.8907	-1.88661	-1.88253	-1.87849
-1.91226	-1.90804	-1.90384	-1.89966	-1.89553	-1.89143	-1.88735	-1.8833	-1.87926
-1.89424	-1.90875	-1.90455	-1.90038	-1.89626	-1.89217	-1.8881	-1.88405	-1.88
-1.89494	-1.90946	-1.90527	-1.90111	-1.89701	-1.89293	-1.88886	-1.8848	-1.88073

-1.89568	-1.9102	-1.90601	-1.90186	-1.89776	-1.89369	-1.88962	-1.88554	-1.88146
-1.89649	-1.91102	-1.90681	-1.90263	-1.89852	-1.89443	-1.89035	-1.88626	-1.8822
-1.89731	-1.91185	-1.90761	-1.90341	-1.89927	-1.89517	-1.89107	-1.88699	-1.88294
-1.87931	-1.91261	-1.90837	-1.90415	-1.90001	-1.8959	-1.89182	-1.88776	-1.88371
-1.88005	-1.91335	-1.9091	-1.90489	-1.90074	-1.89664	-1.89257	-1.88855	-1.8845
-1.88082	-1.91412	-1.90989	-1.90568	-1.90153	-1.89741	-1.89335	-1.88934	-1.88529
-1.88161	-1.91493	-1.91072	-1.90652	-1.90235	-1.89822	-1.89415	-1.89014	-1.8861
-1.88238	-1.89727	-1.8931	-1.88893	-1.90316	-1.89903	-1.89495	-1.89092	-1.88687
-1.88313	-1.89801	-1.89385	-1.8897	-1.90394	-1.89983	-1.89574	-1.89166	-1.88758
-1.8839	-1.89877	-1.89461	-1.89047	-1.90473	-1.90064	-1.89652	-1.8924	-1.88831
-1.88472	-1.89958	-1.89541	-1.89128	-1.90552	-1.90144	-1.89733	-1.8932	-1.8891
-1.88556	-1.90042	-1.89624	-1.8921	-1.90632	-1.90223	-1.89812	-1.89401	-1.8899
-1.8864	-1.88223	-1.8781	-1.87399	-1.8888	-1.8847	-1.89886	-1.89476	-1.89066
-1.88724	-1.88307	-1.87893	-1.8748	-1.88958	-1.88544	-1.89957	-1.89548	-1.89141
-1.88803	-1.88388	-1.87974	-1.87562	-1.89039	-1.88623	-1.90034	-1.89624	-1.89218
-1.88877	-1.88464	-1.88054	-1.87645	-1.89123	-1.88706	-1.90115	-1.89704	-1.89298
-1.88953	-1.88542	-1.88135	-1.87728	-1.89208	-1.8879	-1.88377	-1.87968	-1.89379
-1.89035	-1.88623	-1.88217	-1.87812	-1.89292	-1.88874	-1.88461	-1.88052	-1.89461
-1.89118	-1.88706	-1.88301	-1.87895	-1.89377	-1.88959	-1.88546	-1.88136	-1.89544
-1.89203	-1.88791	-1.88385	-1.87979	-1.89461	-1.89043	-1.8863	-1.8822	-1.87815
-1.89289	-1.88876	-1.88469	-1.88063	-1.89546	-1.89128	-1.88714	-1.88304	-1.87899
-1.89374	-1.88961	-1.88554	-1.88148	-1.87736	-1.87323	-1.888	-1.88388	-1.87983
-1.89459	-1.89046	-1.8864	-1.88233	-1.87822	-1.87409	-1.88886	-1.88473	-1.88067
-1.84717	-1.89131	-1.88725	-1.88319	-1.8791	-1.87499	-1.88976	-1.88562	-1.88154
-1.84799	-1.89215	-1.8881	-1.88406	-1.88	-1.87593	-1.89071	-1.88655	-1.88246
-1.84883	-1.85975	-1.85575	-1.85178	-1.88089	-1.87685	-1.89165	-1.88749	-1.88337
-1.84973	-1.86063	-1.8566	-1.8526	-1.88174	-1.87772	-1.89254	-1.88838	-1.88426
-1.85063	-1.86152	-1.85746	-1.85343	-1.88258	-1.87857	-1.89341	-1.88927	-1.88515
-1.8515	-1.86239	-1.8583	-1.85425	-1.85028	-1.84634	-1.87539	-1.87133	-1.88609
-1.85236	-1.86326	-1.85914	-1.85507	-1.8511	-1.84717	-1.87628	-1.87226	-1.88704
-1.85325	-1.84916	-1.84506	-1.84101	-1.85195	-1.848	-1.84406	-1.84013	-1.88794
-1.85416	-1.85009	-1.846	-1.84192	-1.85283	-1.84883	-1.84487	-1.84095	-1.88881
-1.85503	-1.851	-1.84693	-1.84285	-1.85374	-1.84969	-1.84571	-1.84179	-1.85714
-1.85587	-1.85187	-1.84784	-1.84381	-1.85469	-1.85061	-1.84659	-1.84262	-1.85798
-1.85669	-1.85271	-1.84873	-1.84474	-1.85564	-1.85155	-1.8475	-1.84349	-1.83952
-1.85752	-1.85351	-1.84956	-1.84561	-1.85656	-1.8525	-1.84845	-1.8444	-1.8404
-1.85836	-1.85432	-1.85037	-1.84644	-1.85744	-1.85344	-1.8494	-1.84533	-1.8413
-1.85927	-1.85518	-1.8512	-1.84725	-1.85828	-1.85431	-1.8503	-1.84626	-1.84224
-1.86022	-1.85609	-1.85204	-1.84804	-1.85909	-1.85515	-1.85118	-1.84719	-1.84319
-1.86116	-1.85702	-1.85294	-1.8489	-1.85993	-1.85598	-1.85203	-1.84807	-1.84409
-1.86209	-1.85799	-1.85389	-1.84981	-1.8608	-1.85681	-1.85284	-1.84889	-1.84494
-1.84425	-1.85894	-1.85483	-1.85074	-1.84672	-1.84273	-1.85371	-1.84975	-1.8458
-1.84512	-1.85982	-1.85575	-1.8517	-1.84769	-1.84371	-1.85469	-1.85068	-1.8467
-1.84601	-1.86071	-1.85667	-1.85266	-1.84867	-1.8447	-1.85567	-1.85163	-1.84761
-1.84699	-1.8617	-1.85764	-1.8536	-1.84959	-1.8456	-1.85659	-1.85257	-1.84857
-1.84798	-1.86271	-1.85861	-1.85453	-1.85049	-1.84649	-1.85749	-1.85351	-1.84953
-1.8319	-1.84488	-1.84086	-1.83685	-1.85145	-1.84744	-1.85845	-1.85445	-1.85047
-1.83272	-1.84574	-1.84177	-1.8378	-1.85244	-1.84844	-1.85944	-1.8554	-1.85139
-1.8336	-1.82966	-1.82573	-1.82181	-1.83478	-1.83085	-1.8604	-1.85635	-1.85233
-1.83454	-1.83056	-1.8266	-1.82266	-1.83563	-1.83172	-1.86131	-1.8573	-1.8533

-1.83549	-1.83148	-1.8275	-1.82355	-1.83652	-1.83262	-1.84721	-1.84324	-1.85426
-1.83643	-1.83245	-1.82848	-1.82454	-1.83751	-1.83361	-1.84818	-1.84419	-1.85519
-1.8374	-1.83344	-1.82949	-1.82555	-1.83852	-1.8346	-1.84916	-1.84513	-1.85612
-1.8384	-1.83444	-1.83047	-1.82651	-1.82259	-1.81868	-1.83157	-1.82763	-1.8571
-1.83942	-1.83545	-1.83145	-1.82746	-1.8235	-1.81957	-1.83248	-1.82858	-1.8581
-1.84037	-1.83639	-1.8324	-1.82843	-1.82446	-1.82052	-1.81663	-1.81278	-1.82566
-1.84128	-1.83728	-1.83333	-1.8294	-1.82545	-1.82152	-1.81763	-1.81378	-1.82665
-1.84222	-1.83822	-1.83429	-1.83038	-1.82644	-1.82251	-1.81861	-1.81474	-1.82761
-1.84323	-1.83923	-1.8353	-1.83139	-1.82743	-1.82346	-1.81954	-1.81564	-1.8285
-1.84424	-1.84024	-1.8363	-1.83237	-1.8284	-1.82442	-1.82048	-1.81656	-1.81267
-1.84524	-1.84121	-1.83723	-1.83327	-1.82932	-1.82539	-1.82147	-1.81756	-1.81368
-1.84622	-1.84217	-1.83815	-1.83417	-1.83025	-1.82637	-1.82248	-1.8186	-1.81473
-1.84721	-1.84316	-1.83914	-1.83517	-1.83126	-1.82739	-1.82352	-1.81965	-1.81578
-1.8482	-1.84417	-1.84017	-1.83622	-1.83232	-1.82845	-1.82457	-1.82071	-1.81684
-1.84922	-1.8452	-1.84123	-1.83729	-1.83338	-1.82949	-1.82562	-1.82175	-1.81787
-1.85026	-1.84627	-1.8423	-1.83836	-1.83444	-1.83054	-1.82665	-1.82278	-1.81888
-1.82962	-1.84734	-1.84337	-1.83942	-1.83548	-1.83155	-1.82764	-1.82375	-1.81985
-1.83068	-1.84841	-1.84443	-1.84045	-1.83647	-1.8325	-1.82856	-1.82464	-1.82073
-1.83174	-1.84948	-1.84547	-1.84147	-1.83746	-1.83345	-1.82948	-1.82554	-1.82164
-1.83278	-1.85052	-1.84648	-1.84245	-1.83842	-1.83442	-1.83045	-1.82652	-1.82263
-1.83382	-1.85154	-1.84747	-1.84341	-1.83939	-1.83539	-1.83145	-1.82755	-1.82368
-1.81522	-1.85253	-1.84845	-1.84439	-1.84038	-1.8364	-1.83248	-1.82859	-1.82473
-1.81619	-1.85351	-1.84942	-1.84537	-1.84138	-1.83744	-1.83353	-1.82966	-1.82579
-1.81716	-1.8545	-1.85042	-1.84639	-1.84241	-1.83848	-1.83458	-1.83072	-1.82686
-1.81811	-1.8555	-1.85145	-1.84744	-1.84348	-1.83955	-1.83565	-1.83178	-1.82791
-1.81909	-1.83473	-1.83076	-1.82684	-1.84455	-1.84061	-1.83671	-1.83284	-1.82897
-1.8201	-1.83577	-1.83182	-1.8279	-1.84563	-1.84168	-1.83778	-1.8339	-1.83003
-1.82113	-1.83683	-1.83289	-1.82897	-1.84671	-1.84276	-1.83884	-1.83496	-1.83109
-1.82217	-1.8379	-1.83396	-1.83004	-1.84779	-1.84384	-1.83991	-1.83602	-1.83215
-1.82324	-1.83898	-1.83504	-1.83111	-1.84888	-1.84492	-1.84099	-1.83709	-1.83321
-1.82431	-1.84006	-1.83612	-1.83218	-1.84996	-1.846	-1.84206	-1.83815	-1.83426
-1.82538	-1.84115	-1.83719	-1.83325	-1.85105	-1.84708	-1.84313	-1.83921	-1.83532
-1.82645	-1.84222	-1.83826	-1.83432	-1.8304	-1.82649	-1.84421	-1.84027	-1.83638
-1.8275	-1.8433	-1.83933	-1.83538	-1.83146	-1.82755	-1.84529	-1.84135	-1.83744
-1.82856	-1.84437	-1.8404	-1.83645	-1.83252	-1.82862	-1.84637	-1.84242	-1.83851
-1.8296	-1.84542	-1.84145	-1.8375	-1.83358	-1.82967	-1.84744	-1.84349	-1.83958
-1.83066	-1.84649	-1.84252	-1.83857	-1.83464	-1.83073	-1.84852	-1.84457	-1.84065
-1.83181	-1.82785	-1.82391	-1.82	-1.83571	-1.8318	-1.84961	-1.84567	-1.84175
-1.833	-1.829	-1.82503	-1.82109	-1.83681	-1.8329	-1.85073	-1.84679	-1.84286
-1.83419	-1.83018	-1.82621	-1.82227	-1.838	-1.83409	-1.83019	-1.82631	-1.84406
-1.83538	-1.83141	-1.82746	-1.82354	-1.83929	-1.83537	-1.83147	-1.82758	-1.84535
-1.83652	-1.83257	-1.82865	-1.82474	-1.84051	-1.83658	-1.83268	-1.82879	-1.84658
-1.83759	-1.83364	-1.82973	-1.82583	-1.84162	-1.83769	-1.83379	-1.82989	-1.8477
-1.83863	-1.83469	-1.83078	-1.82689	-1.84269	-1.83876	-1.83486	-1.83096	-1.84879
-1.83968	-1.83573	-1.83182	-1.82792	-1.84373	-1.83982	-1.83591	-1.83201	-1.82813
-1.84072	-1.83677	-1.83285	-1.82895	-1.84477	-1.84086	-1.83695	-1.83305	-1.82917
-1.84179	-1.83784	-1.8339	-1.83	-1.82612	-1.82226	-1.83801	-1.83412	-1.83023
-1.84289	-1.83892	-1.83498	-1.83107	-1.82718	-1.82332	-1.83909	-1.8352	-1.83131
-1.84407	-1.8401	-1.83615	-1.83223	-1.82834	-1.82447	-1.84026	-1.83636	-1.83247
-1.84536	-1.84138	-1.83743	-1.8335	-1.8296	-1.82573	-1.84152	-1.83762	-1.83373

-1.82948	-1.84262	-1.83865	-1.83472	-1.83081	-1.82694	-1.84274	-1.83884	-1.83494
-1.83059	-1.84374	-1.83976	-1.83582	-1.83191	-1.82803	-1.84384	-1.83994	-1.83606
-1.83167	-1.84483	-1.84084	-1.83689	-1.83297	-1.82909	-1.84491	-1.84102	-1.83714
-1.83275	-1.8288	-1.82484	-1.82092	-1.83404	-1.83015	-1.82629	-1.82245	-1.83824
-1.83384	-1.82989	-1.82593	-1.822	-1.83513	-1.83123	-1.82736	-1.82353	-1.83935
-1.79969	-1.83107	-1.82712	-1.82318	-1.83632	-1.83241	-1.82853	-1.82469	-1.84052
-1.80093	-1.83234	-1.82839	-1.82445	-1.83759	-1.83367	-1.82978	-1.82593	-1.84174
-1.80212	-1.83355	-1.8296	-1.82566	-1.82174	-1.81784	-1.83097	-1.82711	-1.84292
-1.80323	-1.83468	-1.83072	-1.82678	-1.82285	-1.81894	-1.83208	-1.82821	-1.84403
-1.78448	-1.83584	-1.83188	-1.82793	-1.82399	-1.82007	-1.8162	-1.81236	-1.84517
-1.78571	-1.83709	-1.83312	-1.82917	-1.82522	-1.8213	-1.81742	-1.81357	-1.84642
-1.78694	-1.83835	-1.83437	-1.83041	-1.82646	-1.82253	-1.81865	-1.8148	-1.84767
-1.78807	-1.80404	-1.80017	-1.79631	-1.82761	-1.82367	-1.81978	-1.81593	-1.8321
-1.78915	-1.80513	-1.80125	-1.79739	-1.82871	-1.82478	-1.82088	-1.81702	-1.83322
-1.79031	-1.78644	-1.78259	-1.77877	-1.82991	-1.82598	-1.82207	-1.8182	-1.81439
-1.79155	-1.78767	-1.78383	-1.78	-1.83118	-1.82726	-1.82335	-1.81946	-1.81561
-1.79273	-1.78886	-1.78501	-1.78119	-1.81317	-1.8093	-1.82461	-1.82072	-1.81685
-1.79381	-1.78994	-1.78609	-1.78227	-1.81433	-1.8105	-1.82585	-1.82198	-1.81812
-1.79491	-1.79105	-1.7872	-1.78338	-1.81548	-1.81169	-1.82705	-1.8232	-1.81936
-1.79613	-1.79227	-1.7884	-1.78457	-1.81667	-1.81284	-1.82819	-1.82433	-1.82048
-1.79738	-1.79352	-1.78965	-1.78581	-1.81789	-1.814	-1.82931	-1.82543	-1.82157
-1.79856	-1.79473	-1.79089	-1.78706	-1.78324	-1.77943	-1.81137	-1.8075	-1.82277
-1.79971	-1.79591	-1.79212	-1.78834	-1.78453	-1.78071	-1.81267	-1.80878	-1.82403
-1.80087	-1.79706	-1.79329	-1.78952	-1.78572	-1.78193	-1.81393	-1.81005	-1.80619
-1.80203	-1.79817	-1.79436	-1.79059	-1.78683	-1.78308	-1.81516	-1.81133	-1.80749
-1.80323	-1.79933	-1.79549	-1.7917	-1.78794	-1.78422	-1.81635	-1.81254	-1.80873
-1.80452	-1.80058	-1.79672	-1.7929	-1.7891	-1.78533	-1.81746	-1.81364	-1.80985
-1.80584	-1.80187	-1.79798	-1.79413	-1.79029	-1.78647	-1.81859	-1.81475	-1.81096
-1.78007	-1.80318	-1.79927	-1.79539	-1.79153	-1.78771	-1.78391	-1.78014	-1.81214
-1.78129	-1.8045	-1.80057	-1.79667	-1.79281	-1.78899	-1.78517	-1.78137	-1.81336
-1.78246	-1.80577	-1.80185	-1.79795	-1.79409	-1.79026	-1.78644	-1.78262	-1.81462
-1.78358	-1.807	-1.80312	-1.79924	-1.79538	-1.79154	-1.78771	-1.7839	-1.81592
-1.78469	-1.8082	-1.80434	-1.80048	-1.79664	-1.79281	-1.78899	-1.78517	-1.81721
-1.7858	-1.80935	-1.8055	-1.80168	-1.79788	-1.79408	-1.79027	-1.78644	-1.81851
-1.76617	-1.81053	-1.80667	-1.80286	-1.79909	-1.79533	-1.79153	-1.78771	-1.78391
-1.76736	-1.81178	-1.80787	-1.80402	-1.80026	-1.79653	-1.79276	-1.78897	-1.78518
-1.76857	-1.81306	-1.80909	-1.8052	-1.80143	-1.79773	-1.79398	-1.79022	-1.78645
-1.76979	-1.78681	-1.78308	-1.77941	-1.80268	-1.79896	-1.79522	-1.79149	-1.78772
-1.77102	-1.78805	-1.78432	-1.78064	-1.80396	-1.80021	-1.79647	-1.79275	-1.789
-1.77224	-1.76851	-1.76483	-1.7612	-1.80525	-1.80148	-1.79774	-1.79402	-1.79026
-1.77346	-1.76972	-1.76604	-1.76242	-1.80656	-1.80277	-1.79901	-1.79528	-1.79152
-1.77468	-1.77093	-1.76725	-1.76363	-1.80786	-1.80406	-1.80029	-1.79655	-1.79277
-1.77589	-1.77213	-1.76845	-1.76483	-1.80915	-1.80536	-1.80157	-1.79781	-1.79403
-1.75622	-1.77334	-1.76965	-1.76602	-1.76247	-1.75896	-1.80285	-1.79907	-1.79528
-1.75743	-1.77456	-1.77086	-1.76722	-1.76366	-1.76015	-1.80413	-1.80033	-1.79653
-1.75864	-1.77579	-1.77207	-1.76841	-1.76485	-1.76134	-1.80541	-1.80159	-1.79777
-1.75985	-1.77702	-1.77328	-1.76961	-1.76604	-1.76253	-1.80668	-1.80285	-1.79902
-1.76106	-1.77824	-1.7745	-1.77081	-1.76724	-1.76372	-1.80796	-1.80412	-1.80028
-1.76227	-1.77947	-1.77572	-1.77202	-1.76844	-1.76491	-1.78199	-1.7784	-1.80155
-1.76348	-1.7807	-1.77693	-1.77323	-1.76963	-1.76609	-1.78318	-1.7796	-1.80283

-1.76468	-1.78193	-1.77818	-1.77448	-1.77086	-1.76731	-1.76375	-1.7602	-1.80411
-1.76589	-1.78318	-1.77947	-1.7758	-1.77217	-1.76856	-1.76498	-1.76141	-1.80539
-1.7671	-1.78443	-1.78076	-1.77712	-1.77347	-1.76983	-1.76621	-1.76261	-1.80668
-1.76831	-1.76465	-1.76106	-1.75749	-1.7747	-1.77105	-1.76742	-1.76381	-1.78081
-1.76954	-1.76587	-1.76227	-1.75869	-1.77592	-1.77226	-1.76863	-1.76501	-1.78203
-1.75203	-1.76714	-1.76353	-1.75992	-1.77715	-1.77348	-1.76983	-1.76621	-1.78324
-1.75331	-1.76845	-1.76482	-1.76118	-1.7784	-1.7747	-1.77104	-1.7674	-1.78444
-1.75456	-1.76972	-1.76607	-1.76242	-1.77965	-1.77594	-1.77227	-1.76864	-1.76506
-1.75576	-1.77092	-1.76729	-1.76365	-1.7809	-1.77717	-1.77352	-1.76991	-1.76634
-1.75697	-1.75338	-1.74979	-1.7462	-1.78215	-1.77843	-1.77479	-1.77119	-1.76763
-1.75822	-1.75462	-1.75101	-1.74741	-1.78341	-1.77973	-1.77609	-1.77248	-1.7689
-1.75948	-1.75587	-1.75225	-1.74864	-1.78469	-1.78104	-1.7774	-1.77377	-1.77016
-1.76074	-1.75712	-1.7535	-1.7499	-1.78597	-1.78233	-1.77868	-1.77504	-1.77143
-1.76199	-1.75837	-1.75477	-1.75118	-1.78727	-1.78361	-1.77995	-1.77631	-1.7727
-1.76325	-1.75963	-1.75604	-1.75245	-1.76753	-1.76392	-1.78122	-1.77759	-1.77398
-1.76451	-1.76091	-1.75731	-1.75372	-1.76878	-1.76516	-1.78249	-1.77887	-1.77527
-1.69382	-1.76217	-1.75857	-1.75497	-1.75137	-1.74778	-1.78376	-1.78015	-1.77655
-1.69497	-1.76337	-1.75978	-1.7562	-1.75261	-1.74903	-1.78504	-1.78143	-1.77782
-1.69614	-1.76458	-1.76101	-1.75744	-1.75387	-1.75029	-1.78632	-1.78269	-1.77908
-1.69737	-1.76585	-1.76228	-1.75872	-1.75514	-1.75156	-1.78759	-1.78394	-1.78032
-1.69862	-1.76713	-1.76356	-1.76	-1.75641	-1.75282	-1.78886	-1.78519	-1.78157
-1.64652	-1.69641	-1.69295	-1.68951	-1.75766	-1.75405	-1.79012	-1.78646	-1.78284
-1.64777	-1.69768	-1.6942	-1.69072	-1.75889	-1.75527	-1.79138	-1.78773	-1.78413
-1.649	-1.67146	-1.66802	-1.66459	-1.68843	-1.68496	-1.77433	-1.77073	-1.78542
-1.65021	-1.67267	-1.6692	-1.66575	-1.68963	-1.6862	-1.77564	-1.77203	-1.78672
-1.65144	-1.64803	-1.64461	-1.64122	-1.69085	-1.68744	-1.73321	-1.72968	-1.78803
-1.65268	-1.64929	-1.64588	-1.64248	-1.69212	-1.68866	-1.73447	-1.73097	-1.78935
-1.65392	-1.65055	-1.64714	-1.64374	-1.69339	-1.6899	-1.73574	-1.73226	-1.79067
-1.6322	-1.65174	-1.64832	-1.64492	-1.66727	-1.66387	-1.71018	-1.70674	-1.73004
-1.63338	-1.65292	-1.64949	-1.64609	-1.66847	-1.6651	-1.71146	-1.70798	-1.73129
-1.63458	-1.65415	-1.65072	-1.64731	-1.64395	-1.64063	-1.66295	-1.65963	-1.70579
-1.6358	-1.65541	-1.65199	-1.64858	-1.64518	-1.64181	-1.66415	-1.66086	-1.70707
-1.637	-1.65664	-1.65324	-1.64985	-1.64644	-1.64305	-1.66541	-1.66212	-1.70838
-1.63819	-1.65785	-1.65448	-1.65113	-1.64774	-1.64436	-1.66673	-1.66342	-1.70972
-1.63939	-1.63604	-1.63276	-1.62948	-1.64901	-1.64565	-1.64234	-1.63907	-1.68422
-1.64059	-1.63725	-1.63393	-1.63064	-1.65022	-1.6469	-1.64359	-1.64029	-1.68548
-1.6418	-1.63846	-1.63511	-1.63179	-1.65141	-1.64812	-1.64481	-1.64149	-1.68672
-1.64302	-1.63966	-1.63631	-1.63299	-1.65262	-1.64931	-1.646	-1.64269	-1.68797
-1.64424	-1.64087	-1.63754	-1.63422	-1.65384	-1.6505	-1.64719	-1.64391	-1.68922
-1.64547	-1.64211	-1.63879	-1.63548	-1.65512	-1.65176	-1.64845	-1.64517	-1.64189
-1.6467	-1.64338	-1.64007	-1.63677	-1.65644	-1.65308	-1.64976	-1.64648	-1.6432
-1.64795	-1.64464	-1.64134	-1.63804	-1.63473	-1.63143	-1.65104	-1.64775	-1.64446
-1.64925	-1.64591	-1.64258	-1.63925	-1.63594	-1.63264	-1.65227	-1.64895	-1.64567
-1.65054	-1.64717	-1.64381	-1.64047	-1.63716	-1.63387	-1.6535	-1.65018	-1.64689
-1.65179	-1.6484	-1.64505	-1.64172	-1.63842	-1.63514	-1.6548	-1.65147	-1.64817
-1.65304	-1.64963	-1.64629	-1.64298	-1.63968	-1.63641	-1.6561	-1.65277	-1.64946
-1.63152	-1.65092	-1.64757	-1.64424	-1.64093	-1.63764	-1.65735	-1.65402	-1.65072
-1.63286	-1.65224	-1.64887	-1.64552	-1.64217	-1.63885	-1.65858	-1.65525	-1.65195
-1.63418	-1.63077	-1.62741	-1.62406	-1.64345	-1.64011	-1.63681	-1.63354	-1.65321
-1.63548	-1.6321	-1.62874	-1.62541	-1.64478	-1.64143	-1.63811	-1.63481	-1.65449

-1.63681	-1.63342	-1.63006	-1.62672	-1.62337	-1.62003	-1.61672	-1.61343	-1.65579
-1.63816	-1.63475	-1.63134	-1.62796	-1.62462	-1.62131	-1.61802	-1.61475	-1.65712
-1.63953	-1.63609	-1.63264	-1.62922	-1.62588	-1.62258	-1.6193	-1.61605	-1.65844
-1.61372	-1.63746	-1.63399	-1.63055	-1.62718	-1.62386	-1.62055	-1.61727	-1.63741
-1.61507	-1.63885	-1.63537	-1.63191	-1.62851	-1.62514	-1.6218	-1.61848	-1.63863
-1.61643	-1.64025	-1.63676	-1.63329	-1.62987	-1.62647	-1.6231	-1.61976	-1.61648
-1.61779	-1.64165	-1.63817	-1.63469	-1.63125	-1.62783	-1.62444	-1.62108	-1.61778
-1.61911	-1.64299	-1.63952	-1.63606	-1.63262	-1.62919	-1.62579	-1.62242	-1.6191
-1.62039	-1.64427	-1.64082	-1.63739	-1.63397	-1.63055	-1.62715	-1.62376	-1.62043
-1.59334	-1.61827	-1.61492	-1.6116	-1.63531	-1.63192	-1.62851	-1.6251	-1.62176
-1.59464	-1.61958	-1.61622	-1.6129	-1.63665	-1.63327	-1.62986	-1.62643	-1.62307
-1.59596	-1.62091	-1.61754	-1.6142	-1.638	-1.63463	-1.63121	-1.62776	-1.62438
-1.59729	-1.62226	-1.61888	-1.61554	-1.63937	-1.636	-1.63258	-1.62911	-1.62572
-1.59864	-1.62362	-1.62023	-1.61689	-1.64075	-1.63738	-1.63394	-1.63047	-1.62707
-1.57411	-1.62499	-1.62159	-1.61823	-1.64212	-1.63875	-1.63531	-1.63183	-1.62842
-1.57544	-1.62635	-1.62294	-1.61958	-1.64349	-1.64012	-1.63668	-1.6332	-1.62978
-1.57676	-1.60226	-1.59888	-1.59556	-1.64486	-1.64149	-1.63805	-1.63457	-1.63114
-1.57807	-1.60358	-1.6002	-1.59687	-1.64623	-1.64285	-1.63941	-1.63593	-1.63249
-1.5794	-1.57598	-1.57265	-1.56938	-1.59491	-1.59166	-1.64078	-1.63731	-1.63387
-1.58075	-1.57731	-1.57396	-1.57067	-1.59623	-1.59299	-1.64219	-1.63877	-1.63533
-1.58212	-1.57866	-1.57529	-1.57199	-1.59756	-1.59432	-1.64361	-1.64023	-1.63681
-1.58356	-1.5801	-1.57668	-1.57334	-1.59891	-1.59565	-1.62005	-1.61672	-1.63822
-1.58503	-1.58157	-1.57811	-1.57471	-1.60027	-1.59698	-1.6214	-1.61808	-1.6396
-1.58647	-1.58301	-1.57952	-1.57608	-1.60163	-1.59831	-1.62275	-1.61943	-1.61608
-1.58789	-1.58443	-1.58092	-1.57745	-1.603	-1.59964	-1.6241	-1.62078	-1.61745
-1.58928	-1.58583	-1.58233	-1.57885	-1.57545	-1.57212	-1.59767	-1.59442	-1.61879
-1.59065	-1.58722	-1.58376	-1.5803	-1.57686	-1.57346	-1.599	-1.59571	-1.6201
-1.59202	-1.58861	-1.58518	-1.58175	-1.5783	-1.57485	-1.60037	-1.59703	-1.62142
-1.56035	-1.58998	-1.58658	-1.58317	-1.57974	-1.57631	-1.60183	-1.59841	-1.62279
-1.56167	-1.59136	-1.58795	-1.58456	-1.58118	-1.57779	-1.60332	-1.59984	-1.62419
-1.56299	-1.59272	-1.58932	-1.58594	-1.58257	-1.57921	-1.60478	-1.6013	-1.62567
-1.56431	-1.59408	-1.59067	-1.58729	-1.58393	-1.58058	-1.60621	-1.60279	-1.6272
-1.51238	-1.59554	-1.59209	-1.58868	-1.5853	-1.58194	-1.60762	-1.60424	-1.60083
-1.51381	-1.5971	-1.59358	-1.5901	-1.58668	-1.58329	-1.609	-1.60563	-1.60225
-1.51522	-1.59864	-1.59508	-1.59155	-1.58808	-1.58467	-1.5813	-1.57799	-1.60364
-1.51654	-1.60011	-1.59656	-1.59304	-1.58954	-1.58607	-1.58267	-1.57932	-1.60498
-1.51784	-1.60155	-1.59803	-1.59452	-1.591	-1.5875	-1.58406	-1.58068	-1.60634
-1.51913	-1.5159	-1.51268	-1.50947	-1.59246	-1.58899	-1.58556	-1.58217	-1.60783
-1.52041	-1.51717	-1.51394	-1.51075	-1.59391	-1.5905	-1.58709	-1.5837	-1.60937
-1.52176	-1.51848	-1.51523	-1.51203	-1.59533	-1.59192	-1.58853	-1.58515	-1.61085
-1.52317	-1.51982	-1.51654	-1.5133	-1.59672	-1.59329	-1.58989	-1.58653	-1.61231
-1.46999	-1.52123	-1.51792	-1.51467	-1.54572	-1.54237	-1.59131	-1.58794	-1.58459
-1.47146	-1.52273	-1.51942	-1.51615	-1.54726	-1.54387	-1.59282	-1.58938	-1.58598
-1.37121	-1.46962	-1.46638	-1.46318	-1.51436	-1.51112	-1.59433	-1.59084	-1.5874
-1.37235	-1.47098	-1.46773	-1.46453	-1.51572	-1.51248	-1.59582	-1.59232	-1.58887
-1.3735	-1.47233	-1.46908	-1.46587	-1.51708	-1.51383	-1.5973	-1.59379	-1.59035
-1.31605	-1.47382	-1.47054	-1.46729	-1.49213	-1.48887	-1.5204	-1.51701	-1.59176
-1.31727	-1.47537	-1.47204	-1.46874	-1.49353	-1.49019	-1.52177	-1.51834	-1.59315
-1.31842	-1.40723	-1.40432	-1.40142	-1.46683	-1.46352	-1.52323	-1.51979	-1.56971
-1.3195	-1.40842	-1.4055	-1.40261	-1.46824	-1.46496	-1.52478	-1.52134	-1.5713

-1.32062	-1.40966	-1.40673	-1.40383	-1.46967	-1.46638	-1.46313	-1.45992	-1.51947
-1.32184	-1.41098	-1.40804	-1.40511	-1.47111	-1.46777	-1.46449	-1.46126	-1.52088
-1.32305	-1.4123	-1.40934	-1.40639	-1.47257	-1.46918	-1.46587	-1.46263	-1.52233
-1.3242	-1.32147	-1.31876	-1.31607	-1.40468	-1.40175	-1.46734	-1.46409	-1.49638
-1.32534	-1.32259	-1.31989	-1.31721	-1.40595	-1.40303	-1.46886	-1.46559	-1.49795
-1.25292	-1.3238	-1.32108	-1.31839	-1.40726	-1.40435	-1.40147	-1.39861	-1.49953
-1.25402	-1.32504	-1.32232	-1.31962	-1.4086	-1.40569	-1.4028	-1.39993	-1.50111
-1.25506	-1.32625	-1.3235	-1.32079	-1.40988	-1.40697	-1.40406	-1.40118	-1.46676
-1.25604	-1.32741	-1.32464	-1.3219	-1.4111	-1.40817	-1.40525	-1.40235	-1.46812
-1.11208	-1.3286	-1.32581	-1.32305	-1.32034	-1.31766	-1.40647	-1.40355	-1.40067
-1.11299	-1.32984	-1.32705	-1.32428	-1.32155	-1.31885	-1.40777	-1.40484	-1.40195
-1.11392	-1.33111	-1.3283	-1.32551	-1.32276	-1.32003	-1.40907	-1.40613	-1.40323
-1.11486	-1.19073	-1.18838	-1.18606	-1.32391	-1.32116	-1.41028	-1.40734	-1.40443
-1.11581	-1.19176	-1.18938	-1.18701	-1.32506	-1.32226	-1.41147	-1.40854	-1.40562
-1.11668	-1.11453	-1.11237	-1.11022	-1.26158	-1.25887	-1.41274	-1.4098	-1.40688
-1.1175	-1.11535	-1.11322	-1.11111	-1.26275	-1.26001	-1.41406	-1.41112	-1.40819
-1.04361	-1.11622	-1.11411	-1.11201	-1.18881	-1.18627	-1.35397	-1.3511	-1.40953
-1.04449	-1.11715	-1.11503	-1.11293	-1.18991	-1.1874	-1.35533	-1.35243	-1.4109
-1.04539	-1.04335	-1.04137	-1.03941	-1.19103	-1.18854	-1.26101	-1.25835	-1.38292
-1.04631	-1.04427	-1.04227	-1.0403	-1.19217	-1.18968	-1.26226	-1.25958	-1.38428
-1.04723	-1.04518	-1.04316	-1.04117	-1.1933	-1.1908	-1.26348	-1.26079	-1.38562
-1.0149	-1.04601	-1.04398	-1.04197	-1.0779	-1.07589	-1.18933	-1.18684	-1.3869
-1.01571	-1.04682	-1.04477	-1.04275	-1.0787	-1.07668	-1.19032	-1.18784	-1.38813
-0.97224	-1.01454	-1.01254	-1.01057	-1.0416	-1.03964	-1.15615	-1.15374	-1.31893
-0.9731	-1.01544	-1.01343	-1.01145	-1.04248	-1.0405	-1.1572	-1.15477	-1.32009
-0.93095	-1.01636	-1.01435	-1.01236	-1.01039	-1.00844	-1.12178	-1.11941	-1.18899
-0.93178	-1.0173	-1.01528	-1.01328	-1.0113	-1.00934	-1.12289	-1.12048	-1.19009
-0.81377	-0.93079	-0.92898	-0.9272	-1.01221	-1.01025	-1.00831	-1.00639	-1.15542
-0.81451	-0.93163	-0.92981	-0.92802	-1.01314	-1.01116	-1.0092	-1.00726	-1.15651
-0.81525	-0.93248	-0.93065	-0.92884	-1.01407	-1.01208	-1.0101	-1.00814	-1.15762
-0.816	-0.89854	-0.89672	-0.89492	-0.97294	-0.97106	-1.01102	-1.00904	-1.12786
-0.81674	-0.8994	-0.89757	-0.89575	-0.97382	-0.97193	-1.01193	-1.00995	-1.12902
-0.81745	-0.81587	-0.81427	-0.81268	-0.89481	-0.89304	-1.01285	-1.01086	-1.04729
-0.81814	-0.81659	-0.815	-0.81342	-0.89565	-0.89387	-1.01378	-1.01177	-1.04821
-0.81883	-0.81729	-0.81572	-0.81415	-0.81257	-0.811	-0.93872	-0.93684	-1.04914
-0.81952	-0.81794	-0.8164	-0.81485	-0.81329	-0.81171	-0.9396	-0.93772	-1.05009
-0.82022	-0.81862	-0.81708	-0.81556	-0.814	-0.81243	-0.94048	-0.93859	-1.05104
-0.73266	-0.81934	-0.8178	-0.81628	-0.81472	-0.81315	-0.89539	-0.8936	-0.97751
-0.73331	-0.8201	-0.81854	-0.817	-0.81545	-0.81388	-0.89622	-0.89442	-0.97841
-0.73397	-0.77872	-0.77722	-0.77574	-0.81618	-0.81461	-0.85549	-0.85382	-0.97931
-0.73463	-0.77945	-0.77793	-0.77643	-0.81691	-0.81535	-0.85625	-0.85456	-0.98021
-0.73529	-0.73384	-0.73243	-0.73103	-0.77564	-0.77414	-0.85701	-0.85531	-0.85365
-0.73596	-0.7345	-0.73308	-0.73167	-0.77634	-0.77483	-0.85777	-0.85607	-0.85441
-0.73662	-0.73516	-0.73374	-0.73232	-0.77705	-0.77553	-0.81738	-0.81576	-0.85516
-0.73728	-0.73582	-0.7344	-0.73298	-0.77777	-0.77623	-0.8181	-0.81647	-0.85591
-0.73795	-0.73648	-0.73506	-0.73364	-0.77849	-0.77693	-0.81882	-0.81719	-0.85666
-0.69367	-0.73714	-0.73571	-0.73429	-0.73285	-0.7314	-0.81956	-0.81791	-0.81629
-0.6943	-0.73781	-0.73637	-0.73495	-0.7335	-0.73205	-0.8203	-0.81864	-0.81701
-0.65168	-0.69359	-0.69224	-0.6909	-0.73417	-0.73271	-0.82105	-0.81937	-0.81774
-0.65236	-0.69428	-0.69292	-0.69156	-0.73484	-0.73337	-0.8218	-0.82012	-0.81849

-0.65301	-0.69496	-0.69358	-0.69221	-0.73551	-0.73403	-0.777	-0.77543	-0.81924
-0.65365	-0.6956	-0.69422	-0.69285	-0.73618	-0.73469	-0.7777	-0.77613	-0.82
-0.61106	-0.65298	-0.65167	-0.65037	-0.69209	-0.6907	-0.7784	-0.77684	-0.82077
-0.61168	-0.65363	-0.65231	-0.65099	-0.69272	-0.69132	-0.7791	-0.77757	-0.82158
-0.61232	-0.65429	-0.65295	-0.65162	-0.69335	-0.69195	-0.77983	-0.77832	-0.8224
-0.56239	-0.61166	-0.61039	-0.60913	-0.694	-0.69262	-0.73677	-0.73536	-0.77753
-0.56292	-0.61226	-0.61098	-0.60972	-0.69466	-0.6933	-0.73749	-0.73606	-0.77824
-0.56346	-0.61287	-0.61158	-0.61031	-0.69881	-0.69746	-0.73819	-0.73674	-0.73529
-0.564	-0.61347	-0.61217	-0.61089	-0.69946	-0.69812	-0.73885	-0.73741	-0.73595
-0.52159	-0.56341	-0.5623	-0.5612	-0.6578	-0.65652	-0.73952	-0.73808	-0.73662
-0.52211	-0.56396	-0.56284	-0.56175	-0.65847	-0.65716	-0.7402	-0.73874	-0.73729
-0.52264	-0.56452	-0.5634	-0.56231	-0.65914	-0.65781	-0.74089	-0.73941	-0.73797
-0.4668	-0.56509	-0.56398	-0.56287	-0.6104	-0.60924	-0.70008	-0.69869	-0.73867
-0.46728	-0.56568	-0.56455	-0.56344	-0.61097	-0.6098	-0.70072	-0.69936	-0.73938
-0.41696	-0.52324	-0.52217	-0.52112	-0.61156	-0.61037	-0.6584	-0.6571	-0.69864
-0.41738	-0.52378	-0.52271	-0.52165	-0.61215	-0.61096	-0.65907	-0.65775	-0.69928
-0.41779	-0.41696	-0.41612	-0.41529	-0.5708	-0.56968	-0.61039	-0.60924	-0.69992
-0.4182	-0.41736	-0.41653	-0.4157	-0.57138	-0.57025	-0.61098	-0.6098	-0.70057
-0.41861	-0.41777	-0.41694	-0.41611	-0.57196	-0.57082	-0.61157	-0.61038	-0.60921
-0.41902	-0.41819	-0.41735	-0.41652	-0.57253	-0.57138	-0.61216	-0.61097	-0.6098
-0.41943	-0.4186	-0.41776	-0.41693	-0.57309	-0.57195	-0.61276	-0.61157	-0.61039
-0.41984	-0.41901	-0.41817	-0.41734	-0.46847	-0.46756	-0.57139	-0.57026	-0.61098
-0.42025	-0.41941	-0.41857	-0.41774	-0.46891	-0.46801	-0.57196	-0.57084	-0.61158
-0.42066	-0.41981	-0.41898	-0.41815	-0.46938	-0.46847	-0.52394	-0.52292	-0.57029
-0.42107	-0.42022	-0.41939	-0.41856	-0.46986	-0.46893	-0.52447	-0.52345	-0.57088
-0.42149	-0.42063	-0.4198	-0.41897	-0.47034	-0.46939	-0.46847	-0.46757	-0.52295
-0.42192	-0.42106	-0.42022	-0.41939	-0.47082	-0.46987	-0.46893	-0.46802	-0.52346
-0.47531	-0.47429	-0.47328	-0.47229	-0.47131	-0.47035	-0.4694	-0.46847	-0.52396
-0.47584	-0.47481	-0.4738	-0.4728	-0.47181	-0.47083	-0.46986	-0.46891	-0.52447
-0.47636	-0.47533	-0.47432	-0.47332	-0.47231	-0.47131	-0.47033	-0.46936	-0.52498
-0.47691	-0.47587	-0.47484	-0.47382	-0.47281	-0.47179	-0.4708	-0.46983	-0.46888
-0.47746	-0.47642	-0.47537	-0.47433	-0.4733	-0.47227	-0.47128	-0.4703	-0.46935
-0.47801	-0.47696	-0.4759	-0.47485	-0.47381	-0.47278	-0.47177	-0.47079	-0.46983
-0.47854	-0.47749	-0.47644	-0.4754	-0.47435	-0.4733	-0.47228	-0.47128	-0.4703
-0.47909	-0.47803	-0.47698	-0.47593	-0.47488	-0.47383	-0.4728	-0.47178	-0.4708
-0.47964	-0.47857	-0.47751	-0.47646	-0.47541	-0.47437	-0.47334	-0.47232	-0.47133
-0.4802	-0.47912	-0.47805	-0.47698	-0.47593	-0.4749	-0.47387	-0.47286	-0.47186
-0.48075	-0.47966	-0.47859	-0.47753	-0.47647	-0.47542	-0.47439	-0.47338	-0.47238
-0.48129	-0.48021	-0.47914	-0.47808	-0.47701	-0.47595	-0.47491	-0.47389	-0.47288
-0.43419	-0.48077	-0.47969	-0.47862	-0.47755	-0.47649	-0.47544	-0.47441	-0.4734
-0.43472	-0.48135	-0.48025	-0.47915	-0.47809	-0.47703	-0.47598	-0.47494	-0.47391
-0.3881	-0.43424	-0.43322	-0.43222	-0.47864	-0.47758	-0.47652	-0.47547	-0.47444
-0.38858	-0.43475	-0.43374	-0.43274	-0.4792	-0.47811	-0.47705	-0.476	-0.47497
-0.38908	-0.43527	-0.43426	-0.43327	-0.47976	-0.47866	-0.47759	-0.47655	-0.47551
-0.38959	-0.43581	-0.43479	-0.43378	-0.48032	-0.47923	-0.47816	-0.47711	-0.47607
-0.39012	-0.43637	-0.43532	-0.4343	-0.4809	-0.47981	-0.47874	-0.47768	-0.47663
-0.39068	-0.3897	-0.38875	-0.38782	-0.43383	-0.43284	-0.47932	-0.47824	-0.47718
-0.39124	-0.39026	-0.38929	-0.38834	-0.43439	-0.43339	-0.47991	-0.4788	-0.47772
-0.39181	-0.39081	-0.38982	-0.38886	-0.43494	-0.43393	-0.48049	-0.47937	-0.47828
-0.39237	-0.39135	-0.39035	-0.38938	-0.43549	-0.43445	-0.48107	-0.47996	-0.47887

-0.34117	-0.39191	-0.39089	-0.3899	-0.38894	-0.38801	-0.43397	-0.43296	-0.47947
-0.34168	-0.39247	-0.39144	-0.39043	-0.38946	-0.38852	-0.43452	-0.43351	-0.48006
-0.3422	-0.34126	-0.34032	-0.33941	-0.39	-0.38904	-0.43508	-0.43406	-0.48065
-0.34273	-0.34179	-0.34084	-0.33992	-0.39056	-0.38959	-0.43564	-0.4346	-0.48124
-0.34326	-0.34232	-0.34137	-0.34044	-0.39114	-0.39015	-0.43622	-0.43515	-0.48183
-0.34378	-0.34283	-0.34188	-0.34095	-0.39171	-0.39071	-0.38972	-0.38875	-0.43466
-0.34429	-0.34333	-0.34239	-0.34146	-0.39229	-0.39129	-0.39029	-0.38929	-0.43524
-0.34483	-0.34386	-0.34291	-0.34198	-0.34106	-0.34015	-0.39084	-0.38983	-0.38886
-0.3454	-0.34442	-0.34346	-0.34251	-0.34157	-0.34063	-0.39138	-0.39037	-0.3894
-0.34596	-0.34498	-0.34401	-0.34305	-0.34209	-0.34114	-0.34021	-0.33931	-0.38995
-0.3465	-0.34552	-0.34456	-0.3436	-0.34264	-0.34168	-0.34074	-0.33983	-0.39053
-0.34705	-0.34607	-0.34511	-0.34415	-0.34318	-0.34222	-0.34128	-0.34036	-0.39112
-0.34762	-0.34663	-0.34565	-0.34467	-0.34371	-0.34275	-0.34181	-0.34088	-0.3917
-0.3482	-0.3472	-0.34619	-0.3452	-0.34423	-0.34328	-0.34234	-0.34141	-0.39228
-0.34879	-0.34778	-0.34676	-0.34576	-0.34478	-0.34383	-0.34288	-0.34195	-0.34102
-0.34938	-0.34837	-0.34735	-0.34635	-0.34536	-0.34439	-0.34344	-0.34251	-0.34157
-0.30746	-0.34894	-0.34793	-0.34692	-0.34593	-0.34496	-0.344	-0.34306	-0.34211
-0.30794	-0.34949	-0.34848	-0.34748	-0.3465	-0.34554	-0.34457	-0.34361	-0.34265
-0.30843	-0.35007	-0.34905	-0.34806	-0.34708	-0.34611	-0.34514	-0.34416	-0.34318
-0.30895	-0.35068	-0.34965	-0.34864	-0.34766	-0.34669	-0.3457	-0.3447	-0.34371
-0.30948	-0.3513	-0.35026	-0.34924	-0.34825	-0.34727	-0.34627	-0.34525	-0.34426
-0.31001	-0.35192	-0.35087	-0.34985	-0.34884	-0.34785	-0.34684	-0.34582	-0.34482
-0.31054	-0.35254	-0.35149	-0.35046	-0.34945	-0.34844	-0.34743	-0.34641	-0.3454
-0.31105	-0.3102	-0.30937	-0.30854	-0.35006	-0.34904	-0.34801	-0.34699	-0.34598
-0.31156	-0.31071	-0.30988	-0.30906	-0.35067	-0.34964	-0.3486	-0.34757	-0.34655
-0.24914	-0.24856	-0.24799	-0.24742	-0.35129	-0.35025	-0.3492	-0.34815	-0.34713
-0.24952	-0.24894	-0.24836	-0.24779	-0.35191	-0.35086	-0.34981	-0.34875	-0.34772
-0.2499	-0.24932	-0.24874	-0.24817	-0.30981	-0.30898	-0.35042	-0.34935	-0.34831
-0.25028	-0.2497	-0.24912	-0.24854	-0.31033	-0.3095	-0.35103	-0.34996	-0.34892
-0.25066	-0.25007	-0.2495	-0.24892	-0.31085	-0.31002	-0.35165	-0.35058	-0.34953
-0.25103	-0.25045	-0.24988	-0.2493	-0.3114	-0.31056	-0.35229	-0.35122	-0.35017
-0.25141	-0.25083	-0.25026	-0.24968	-0.31196	-0.3111	-0.35294	-0.35188	-0.35082
-0.25179	-0.25121	-0.25063	-0.25006	-0.31252	-0.31165	-0.3108	-0.30997	-0.35146
-0.25217	-0.25159	-0.25101	-0.25044	-0.31308	-0.3122	-0.31134	-0.3105	-0.35208
-0.25255	-0.25196	-0.25139	-0.25082	-0.25025	-0.24967	-0.31189	-0.31103	-0.35272
-0.25293	-0.25234	-0.25177	-0.2512	-0.25062	-0.25005	-0.31245	-0.31158	-0.35338
-0.25332	-0.25273	-0.25215	-0.25158	-0.251	-0.25043	-0.31301	-0.31213	-0.35406
-0.2537	-0.25311	-0.25253	-0.25195	-0.25138	-0.25081	-0.31359	-0.3127	-0.31182
-0.2541	-0.25349	-0.25291	-0.25233	-0.25176	-0.25119	-0.31416	-0.31327	-0.31239
-0.2545	-0.25389	-0.2533	-0.25271	-0.25214	-0.25157	-0.31473	-0.31384	-0.31296
-0.25492	-0.2543	-0.25369	-0.2531	-0.25252	-0.25195	-0.3153	-0.31441	-0.31353
-0.25534	-0.25471	-0.25409	-0.25349	-0.2529	-0.25233	-0.25175	-0.25118	-0.3141
-0.25577	-0.25514	-0.25451	-0.25389	-0.25329	-0.25271	-0.25213	-0.25156	-0.31467
-0.25619	-0.25556	-0.25493	-0.2543	-0.25369	-0.2531	-0.25252	-0.25195	-0.31524
-0.25661	-0.25598	-0.25534	-0.25472	-0.2541	-0.2535	-0.25291	-0.25233	-0.3158
-0.25702	-0.25639	-0.25576	-0.25514	-0.25452	-0.25391	-0.25331	-0.25273	-0.31639
-0.25743	-0.2568	-0.25617	-0.25555	-0.25493	-0.25432	-0.25372	-0.25314	-0.25256
-0.25785	-0.25722	-0.25658	-0.25596	-0.25534	-0.25473	-0.25414	-0.25357	-0.25299
-0.25826	-0.25763	-0.257	-0.25637	-0.25575	-0.25514	-0.25454	-0.25396	-0.25339
-0.25868	-0.25805	-0.25741	-0.25678	-0.25615	-0.25553	-0.25493	-0.25434	-0.25376

-0.2591	-0.25847	-0.25783	-0.25719	-0.25656	-0.25594	-0.25532	-0.25472	-0.25414
-0.25951	-0.25888	-0.25824	-0.25761	-0.25698	-0.25634	-0.25572	-0.25511	-0.25452
-0.25993	-0.2593	-0.25867	-0.25803	-0.25739	-0.25676	-0.25613	-0.25551	-0.25491
-0.26038	-0.25975	-0.25911	-0.25847	-0.25782	-0.25718	-0.25654	-0.25592	-0.2553
-0.26083	-0.2602	-0.25955	-0.25891	-0.25826	-0.25761	-0.25697	-0.25633	-0.25571
-0.19597	-0.26062	-0.25999	-0.25935	-0.25871	-0.25806	-0.25742	-0.25679	-0.25616
-0.19626	-0.26103	-0.26041	-0.25979	-0.25916	-0.25853	-0.2579	-0.25726	-0.25663
-0.19655	-0.26144	-0.26082	-0.26021	-0.25959	-0.25897	-0.25834	-0.2577	-0.25707
-0.19685	-0.26183	-0.26122	-0.2606	-0.25999	-0.25937	-0.25875	-0.25812	-0.25748
-0.19716	-0.19668	-0.19621	-0.19575	-0.26039	-0.25977	-0.25915	-0.25853	-0.2579
-0.19748	-0.19698	-0.19651	-0.19604	-0.26079	-0.26018	-0.25956	-0.25894	-0.25832
-0.13021	-0.19731	-0.19682	-0.19634	-0.19588	-0.19543	-0.25998	-0.25936	-0.25874
-0.13044	-0.19767	-0.19717	-0.19668	-0.19621	-0.19576	-0.26042	-0.25981	-0.2592
-0.13067	-0.19804	-0.19753	-0.19703	-0.19655	-0.19609	-0.26087	-0.26026	-0.25965
-0.06378	-0.13057	-0.13026	-0.12995	-0.19687	-0.19639	-0.26129	-0.26068	-0.26007
-0.06387	-0.13078	-0.13046	-0.13015	-0.19719	-0.19669	-0.26169	-0.26108	-0.26047
-0.06396	-0.06383	-0.06369	-0.06355	-0.19752	-0.19702	-0.19654	-0.19608	-0.26089
-0.06405	-0.06392	-0.06378	-0.06365	-0.19788	-0.19738	-0.19689	-0.19641	-0.26133
	-0.06401	-0.06388	-0.06375	-0.1305	-0.1302	-0.19724	-0.19674	-0.19627
	-0.06412	-0.06398	-0.06385	-0.13073	-0.13041	-0.19757	-0.19707	-0.19658
	-0.06422	-0.06408	-0.06395	-0.13094	-0.13062	-0.1979	-0.19739	-0.1969
				-0.0639	-0.06376	-0.13052	-0.13021	-0.19724
				-0.06399	-0.06385	-0.13074	-0.13043	-0.19759
						-0.06382	-0.06369	-0.19794
						-0.06392	-0.06379	-0.1983
						-0.06402	-0.06389	-0.06375
						-0.06411	-0.06398	-0.06385
								-0.06395
								-0.06404
								-0.06413

								-0.44159
								-0.44079
						-0.44238	-0.44118	-0.44001
						-0.44159	-0.44041	-0.43925
				-0.12955	-0.12916	-0.44082	-0.43965	-0.71401
				-0.1293	-0.12892	-0.44009	-0.43893	-0.7129
				-0.12906	-0.12868	-0.43935	-0.4382	-0.71176
	-0.12993	-0.12956	-0.12918	-0.44088	-0.43973	-0.61085	-0.60921	-0.71056
	-0.12968	-0.12931	-0.12894	-0.44012	-0.43896	-0.60977	-0.60812	-0.70934
	-0.44284	-0.44167	-0.44051	-0.61198	-0.61034	-0.71184	-0.70999	-0.75726
	-0.44209	-0.4409	-0.43973	-0.6109	-0.60925	-0.71064	-0.70882	-0.75604
-0.12932	-0.44132	-0.44013	-0.43895	-0.71314	-0.7113	-0.75862	-0.75672	-0.75486
-0.12906	-0.44053	-0.43934	-0.43818	-0.71196	-0.71018	-0.75744	-0.75558	-0.75374
-0.44095	-0.61266	-0.611	-0.60935	-0.75998	-0.75812	-0.75627	-0.75443	-0.7526
-0.44022	-0.61167	-0.61	-0.60835	-0.75878	-0.75691	-0.75506	-0.75323	-0.75141
-0.43948	-0.61067	-0.60901	-0.60737	-0.75759	-0.75571	-0.75386	-0.75204	-0.75023
-0.61125	-0.7129	-0.71105	-0.70923	-0.75637	-0.75451	-0.75268	-0.75089	-0.79757
-0.61015	-0.71168	-0.70986	-0.70807	-0.75515	-0.75332	-0.75152	-0.74976	-0.79643
-0.71239	-0.75965	-0.75774	-0.75586	-0.75401	-0.75219	-0.7504	-0.74863	-0.89399
-0.71131	-0.75853	-0.75665	-0.75478	-0.75294	-0.75112	-0.74931	-0.74751	-0.89262
-0.75925	-0.75737	-0.75551	-0.75366	-0.75182	-0.75	-0.89554	-0.89341	-0.98127
-0.75802	-0.75616	-0.75431	-0.75247	-0.75064	-0.74882	-0.89417	-0.89206	-0.97983
-0.75683	-0.75498	-0.75314	-0.7513	-0.89707	-0.89493	-0.98292	-0.98065	-0.97838
-0.75573	-0.75389	-0.75205	-0.75021	-0.89577	-0.89362	-0.98148	-0.97918	-0.97689
-0.75464	-0.7528	-0.75096	-0.74912	-0.89448	-0.89231	-0.98004	-0.97771	-0.9754
-0.75348	-0.84702	-0.84499	-0.84295	-0.93695	-0.93469	-0.97855	-0.97625	-1.02114
-0.75229	-0.84571	-0.84367	-0.84163	-0.93548	-0.93322	-0.97704	-0.9748	-1.01968
-0.84649	-0.94088	-0.9386	-0.93633	-0.98022	-0.97787	-0.97559	-0.97338	-1.01819
-0.8453	-0.93954	-0.93726	-0.93498	-0.97881	-0.97647	-0.9742	-0.97197	-1.01669
-0.89643	-0.93815	-0.93586	-0.93359	-0.97738	-0.97508	-1.0199	-1.01756	-1.01525
-0.895	-0.93665	-0.93438	-0.93213	-0.9759	-0.97368	-1.01848	-1.01619	-1.01391
-0.89359	-0.93518	-0.93292	-0.93069	-0.97445	-0.97229	-1.01706	-1.01481	-1.01256
-0.93609	-0.97993	-0.97759	-0.97529	-1.02014	-1.01786	-1.01559	-1.01335	-1.01109
-0.93475	-0.97855	-0.97623	-0.97395	-1.01872	-1.01642	-1.01413	-1.01188	-1.0096
-0.93342	-0.97717	-0.97488	-0.97263	-1.01734	-1.01504	-1.01275	-1.01048	-1.00819
-0.93209	-0.97582	-0.97355	-0.97133	-1.01598	-1.0137	-1.01141	-1.00912	-1.00681
-0.97668	-1.02152	-1.01917	-1.01685	-1.01456	-1.01228	-1.00999	-1.00767	-1.00535
-0.97516	-1.01996	-1.01765	-1.01536	-1.01307	-1.01078	-1.00847	-1.00614	-1.0038
-1.02078	-1.01846	-1.01617	-1.0139	-1.01161	-1.0093	-1.00698	-1.00463	-1.04284
-1.01937	-1.01707	-1.01479	-1.01252	-1.01022	-1.00791	-1.00557	-1.00321	-1.04133
-1.018	-1.01571	-1.01343	-1.01115	-1.00885	-1.00652	-1.00417	-1.0018	-1.03986
-1.01664	-1.01435	-1.01206	-1.00976	-1.00744	-1.00511	-1.04338	-1.04086	-1.09688

-1.01528	-1.01299	-1.01068	-1.00836	-1.00602	-1.00367	-1.04187	-1.03937	-1.09538
-1.01391	-1.01161	-1.00926	-1.0069	-1.0453	-1.04278	-1.09895	-1.09637	-1.17921
-1.01253	-1.0102	-1.00781	-1.00539	-1.04369	-1.04115	-1.09729	-1.09476	-1.17733
-1.01108	-1.00874	-1.00633	-1.00391	-1.10093	-1.09829	-1.18135	-1.17842	-1.17555
-1.00954	-1.0072	-1.00483	-1.00244	-1.09939	-1.0968	-1.17962	-1.17672	-1.17388
-1.00802	-1.10566	-1.10305	-1.10044	-1.09788	-1.09534	-1.17793	-1.17506	-1.21621
-1.0066	-1.10411	-1.10151	-1.09893	-1.0964	-1.0939	-1.17624	-1.17343	-1.21451
-1.0052	-1.10261	-1.10002	-1.09745	-1.09494	-1.09247	-1.17457	-1.1718	-1.21282
-1.10371	-1.10111	-1.09854	-1.09601	-1.17863	-1.17576	-1.17294	-1.17017	-1.25081
-1.1022	-1.09963	-1.09709	-1.09458	-1.17696	-1.17412	-1.17131	-1.16853	-1.24893
-1.10072	-1.18406	-1.18109	-1.17816	-1.17527	-1.17243	-1.25329	-1.25011	-1.28673
-1.09924	-1.18232	-1.17937	-1.17647	-1.17357	-1.17069	-1.25128	-1.24808	-1.28451
-1.09777	-1.18057	-1.17766	-1.17476	-1.17186	-1.16896	-1.24932	-1.24612	-1.32628
-1.0963	-1.17884	-1.17593	-1.17303	-1.17013	-1.16724	-1.24741	-1.24427	-1.32414
-1.18003	-1.1771	-1.1742	-1.17131	-1.25194	-1.24871	-1.32933	-1.32566	-1.32206
-1.17829	-1.17536	-1.17246	-1.16958	-1.25003	-1.24686	-1.32721	-1.3236	-1.32004
-1.17656	-1.17362	-1.17073	-1.16787	-1.24816	-1.24505	-1.32514	-1.32157	-1.31806
-1.17488	-1.2128	-1.20968	-1.20661	-1.28609	-1.28271	-1.32307	-1.31955	-1.31608
-1.17322	-1.21104	-1.20793	-1.20487	-1.28411	-1.28076	-1.32103	-1.31755	-1.31411
-1.21234	-1.25036	-1.24694	-1.24358	-1.32608	-1.32252	-1.31902	-1.31557	-1.31216
-1.21048	-1.24836	-1.24499	-1.24168	-1.32401	-1.32049	-1.31703	-1.31362	-1.31024
-1.20864	-1.33285	-1.32915	-1.32553	-1.32197	-1.31849	-1.31506	-1.31168	-1.30833
-1.20686	-1.33067	-1.32703	-1.32346	-1.31995	-1.31651	-1.31311	-1.30975	-1.30643
-1.2051	-1.32853	-1.32494	-1.32142	-1.31796	-1.31455	-1.31118	-1.30784	-1.30456
-1.24394	-1.32643	-1.32287	-1.31941	-1.31598	-1.3126	-1.30926	-1.30595	-1.3027
-1.24206	-1.32436	-1.32085	-1.31742	-1.31402	-1.31067	-1.30735	-1.30408	-1.30087
-1.32599	-1.32235	-1.31886	-1.31546	-1.31208	-1.30875	-1.30547	-1.30224	-1.33813
-1.32396	-1.32037	-1.31691	-1.31352	-1.31016	-1.30685	-1.30361	-1.30044	-1.33635
-1.32195	-1.31839	-1.31496	-1.3116	-1.30827	-1.30499	-1.3018	-1.29871	-1.33466
-1.31994	-1.31641	-1.313	-1.30967	-1.30638	-1.30315	-1.30004	-1.29706	-1.33306
-1.31799	-1.31447	-1.31108	-1.30778	-1.30453	-1.30137	-1.33737	-1.3344	-1.33152
-1.31611	-1.31259	-1.3092	-1.30591	-1.30273	-1.29965	-1.33569	-1.33282	-1.33002
-1.31425	-1.31075	-1.30736	-1.30408	-1.30096	-1.29799	-1.33407	-1.33129	-1.32856
-1.31237	-1.3089	-1.30553	-1.30228	-1.29924	-1.29636	-1.33249	-1.32979	-1.36319
-1.31048	-1.30706	-1.30372	-1.30053	-1.29756	-1.29478	-1.33095	-1.32834	-1.36175
-1.30861	-1.30524	-1.30197	-1.29885	-1.33493	-1.33215	-1.32948	-1.32692	-1.36031
-1.30673	-1.30343	-1.30026	-1.29724	-1.33337	-1.33069	-1.32807	-1.32553	-1.35889
-1.30493	-1.30173	-1.29865	-1.29572	-1.33187	-1.32925	-1.36267	-1.36008	-1.35748
-1.30322	-1.30015	-1.29716	-1.2943	-1.33045	-1.32784	-1.36122	-1.35866	-1.35609
-1.30154	-1.33766	-1.33467	-1.33178	-1.36508	-1.3624	-1.35979	-1.35724	-1.38997
-1.29983	-1.33601	-1.33315	-1.33036	-1.36364	-1.36096	-1.35835	-1.35583	-1.38852
-1.29817	-1.3344	-1.33165	-1.32897	-1.36223	-1.35953	-1.35693	-1.35443	-1.38708
-1.29665	-1.33289	-1.33022	-1.32761	-1.36085	-1.35816	-1.39086	-1.38826	-1.38566
-1.29522	-1.33146	-1.32885	-1.32629	-1.35951	-1.35682	-1.38946	-1.38685	-1.38426
-1.33271	-1.36603	-1.36338	-1.36079	-1.39353	-1.39076	-1.38806	-1.38543	-1.41768
-1.33126	-1.36454	-1.36193	-1.35936	-1.39207	-1.38933	-1.38665	-1.38402	-1.41622
-1.32985	-1.3987	-1.39598	-1.39332	-1.39062	-1.38793	-1.38527	-1.38264	-1.41479
-1.32852	-1.39724	-1.39451	-1.39184	-1.38919	-1.38657	-1.38395	-1.38131	-1.41341
-1.3272	-1.39582	-1.39307	-1.39039	-1.38777	-1.38521	-1.38262	-1.37999	-1.41204
-1.3617	-1.39445	-1.39169	-1.38899	-1.38637	-1.38381	-1.41596	-1.41329	-1.4463

-1.36034	-1.39309	-1.39034	-1.38761	-1.38498	-1.3824	-1.41451	-1.41188	-1.44483
-1.39435	-1.39167	-1.38893	-1.38622	-1.38359	-1.38102	-1.4131	-1.41052	-1.44342
-1.39288	-1.39019	-1.38749	-1.38482	-1.38221	-1.37965	-1.41171	-1.40919	-1.44204
-1.39145	-1.38876	-1.3861	-1.38347	-1.41555	-1.41291	-1.44595	-1.4433	-1.44063
-1.39008	-1.38739	-1.38476	-1.38217	-1.41421	-1.41157	-1.44451	-1.44183	-1.43915
-1.3887	-1.38602	-1.38342	-1.38085	-1.41287	-1.41024	-1.4431	-1.44039	-1.43769
-1.38728	-1.38463	-1.38204	-1.37948	-1.4115	-1.40894	-1.44174	-1.439	-1.43628
-1.38587	-1.38325	-1.38066	-1.37811	-1.41011	-1.40761	-1.44036	-1.43761	-1.43488
-1.3845	-1.38191	-1.37933	-1.37678	-1.44424	-1.44159	-1.43889	-1.43616	-1.43346
-1.38317	-1.38059	-1.37802	-1.37547	-1.44278	-1.44008	-1.43739	-1.4347	-1.43202
-1.38184	-1.44964	-1.44685	-1.44409	-1.44135	-1.43863	-1.43594	-1.43327	-1.5066
-1.38052	-1.44819	-1.44543	-1.44269	-1.43995	-1.43723	-1.43453	-1.43186	-1.50492
-1.37921	-1.44676	-1.44401	-1.44128	-1.43855	-1.43584	-1.50954	-1.50638	-1.50325
-1.37792	-1.44536	-1.4426	-1.43986	-1.43715	-1.43447	-1.5079	-1.50471	-1.50159
-1.44669	-1.44393	-1.44117	-1.43843	-1.43573	-1.43307	-1.50625	-1.50307	-1.49997
-1.44516	-1.44241	-1.43967	-1.43695	-1.43426	-1.43159	-1.50453	-1.50146	-1.49843
-1.44363	-1.44089	-1.43817	-1.43547	-1.43278	-1.43011	-1.50279	-1.49986	-1.4969
-1.44218	-1.43944	-1.43674	-1.43405	-1.5073	-1.50414	-1.5011	-1.4982	-1.49526
-1.44077	-1.43805	-1.43534	-1.43267	-1.50564	-1.50249	-1.49945	-1.49651	-1.49357
-1.43936	-1.51349	-1.51029	-1.50713	-1.50399	-1.50088	-1.49785	-1.49488	-1.49193
-1.43794	-1.51182	-1.50861	-1.50544	-1.50235	-1.49933	-1.49631	-1.49331	-1.49034
-1.43654	-1.51014	-1.50695	-1.50379	-1.50075	-1.49776	-1.49476	-1.49175	-1.48878
-1.43517	-1.50843	-1.50529	-1.50221	-1.49917	-1.49617	-1.49318	-1.4902	-1.48727
-1.43381	-1.50675	-1.50366	-1.50064	-1.4976	-1.49458	-1.4916	-1.48866	-1.48576
-1.5084	-1.50518	-1.50207	-1.49902	-1.496	-1.49301	-1.49005	-1.48712	-1.48424
-1.50682	-1.50363	-1.50049	-1.4974	-1.4944	-1.49146	-1.48852	-1.48558	-1.48271
-1.50516	-1.502	-1.49889	-1.49583	-1.49283	-1.48988	-1.48696	-1.48407	-1.51257
-1.50345	-1.50032	-1.49729	-1.49429	-1.49128	-1.48828	-1.48538	-1.48257	-1.51107
-1.50182	-1.49872	-1.4957	-1.49272	-1.48973	-1.48674	-1.48387	-1.48109	-1.54358
-1.50029	-1.4972	-1.49414	-1.49111	-1.48816	-1.48528	-1.48243	-1.47962	-1.54199
-1.49876	-1.49569	-1.4926	-1.48955	-1.48664	-1.48381	-1.51559	-1.51269	-1.54044
-1.49717	-1.49417	-1.49112	-1.48809	-1.48515	-1.48229	-1.51404	-1.51121	-1.53894
-1.49559	-1.49264	-1.48964	-1.48665	-1.4837	-1.4808	-1.51251	-1.50975	-1.53745
-1.49402	-1.49105	-1.48811	-1.4852	-1.48229	-1.47941	-1.54169	-1.53879	-1.53592
-1.49246	-1.48945	-1.48657	-1.48374	-1.48089	-1.47806	-1.54024	-1.53729	-1.53439
-1.49097	-1.48797	-1.48508	-1.48226	-1.51396	-1.51111	-1.53878	-1.53584	-1.53294
-1.48954	-1.48656	-1.48363	-1.48075	-1.51243	-1.50964	-1.53732	-1.53444	-1.53156
-1.48806	-1.51979	-1.51677	-1.5138	-1.51095	-1.50818	-1.53583	-1.533	-1.53014
-1.48654	-1.51829	-1.51534	-1.5124	-1.50953	-1.50671	-1.5343	-1.5315	-1.52869
-1.48502	-1.51677	-1.51388	-1.51101	-1.50813	-1.50527	-1.55499	-1.55214	-1.5493
-1.48353	-1.5152	-1.51236	-1.50955	-1.50672	-1.5039	-1.55355	-1.55065	-1.54778
-1.48206	-1.51364	-1.51083	-1.50807	-1.50531	-1.50255	-1.55213	-1.54917	-1.54627
-1.51516	-1.51219	-1.50936	-1.50659	-1.55648	-1.5536	-1.57373	-1.57077	-1.56786
-1.51376	-1.5108	-1.50793	-1.50511	-1.55493	-1.55208	-1.57223	-1.56933	-1.56645
-1.51234	-1.53228	-1.52932	-1.52641	-1.57646	-1.57359	-1.57072	-1.56788	-1.56503
-1.51091	-1.53087	-1.52793	-1.52503	-1.57498	-1.57206	-1.5692	-1.56641	-1.56361
-1.53236	-1.55291	-1.54999	-1.54708	-1.57353	-1.57059	-1.56774	-1.56497	-1.60567
-1.53086	-1.55146	-1.54857	-1.54569	-1.57212	-1.5692	-1.56635	-1.56358	-1.60432
-1.52937	-1.55	-1.54716	-1.54432	-1.57073	-1.56783	-1.56499	-1.5622	-1.60295
-1.55133	-1.57806	-1.57517	-1.57227	-1.56937	-1.56647	-1.56361	-1.56078	-1.60153

-1.54989	-1.57658	-1.57373	-1.5709	-1.56802	-1.56511	-1.56223	-1.55935	-1.60011
-1.54844	-1.57507	-1.57226	-1.56948	-1.56663	-1.56375	-1.60452	-1.60159	-1.59873
-1.547	-1.57355	-1.57077	-1.56803	-1.56522	-1.56239	-1.6032	-1.60027	-1.59739
-1.54561	-1.5721	-1.56931	-1.56656	-1.56378	-1.56099	-1.60185	-1.59894	-1.59607
-1.54428	-1.57074	-1.56789	-1.56507	-1.56229	-1.55954	-1.60047	-1.5976	-1.59477
-1.54296	-1.56938	-1.56649	-1.56362	-1.60467	-1.60189	-1.59909	-1.59627	-1.61327
-1.54164	-1.56803	-1.56512	-1.56224	-1.60329	-1.60049	-1.59771	-1.59495	-1.61199
-1.54033	-1.56667	-1.56376	-1.56088	-1.60192	-1.5991	-1.59633	-1.59362	-1.6107
-1.5682	-1.5653	-1.56239	-1.55949	-1.60054	-1.59769	-1.59493	-1.59225	-1.60937
-1.56684	-1.56392	-1.561	-1.55809	-1.59915	-1.59629	-1.59353	-1.59087	-1.60803
-1.56546	-1.60658	-1.60361	-1.60067	-1.59777	-1.59493	-1.61208	-1.6094	-1.60671
-1.56406	-1.6052	-1.60222	-1.59927	-1.59641	-1.59361	-1.61079	-1.60811	-1.60542
-1.56264	-1.60381	-1.60084	-1.5979	-1.61505	-1.61225	-1.60951	-1.60684	-1.63552
-1.56122	-1.60241	-1.59947	-1.59658	-1.61374	-1.61095	-1.60822	-1.60558	-1.63424
-1.60396	-1.62112	-1.61818	-1.61529	-1.61245	-1.60965	-1.60694	-1.60431	-1.66427
-1.60256	-1.61972	-1.61682	-1.61397	-1.61115	-1.60837	-1.60565	-1.60301	-1.66284
-1.60116	-1.61834	-1.61546	-1.61265	-1.60985	-1.60709	-1.60437	-1.60169	-1.66139
-1.5998	-1.617	-1.61414	-1.61133	-1.60855	-1.6058	-1.60307	-1.60038	-1.68926
-1.59845	-1.6157	-1.61284	-1.61002	-1.60724	-1.6045	-1.60176	-1.59905	-1.68775
-1.61728	-1.61438	-1.61153	-1.60872	-1.60594	-1.60318	-1.66197	-1.65901	-1.68625
-1.61593	-1.61306	-1.61023	-1.60743	-1.60463	-1.60185	-1.66049	-1.65754	-1.68475
-1.61459	-1.61175	-1.60893	-1.60614	-1.60333	-1.60053	-1.65902	-1.65606	-1.68324
-1.61327	-1.61042	-1.60762	-1.60483	-1.60202	-1.5992	-1.65755	-1.65457	-1.68172
-1.61195	-1.60912	-1.60631	-1.60352	-1.60071	-1.5979	-1.65611	-1.6531	-1.68022
-1.61066	-1.60783	-1.60501	-1.6022	-1.66075	-1.65774	-1.68493	-1.68184	-1.67878
-1.60937	-1.60656	-1.60373	-1.6009	-1.65935	-1.65636	-1.68353	-1.68044	-1.67736
-1.60812	-1.60534	-1.60252	-1.59969	-1.65799	-1.65497	-1.6821	-1.679	-1.67593
-1.60688	-1.60414	-1.60135	-1.59854	-1.65666	-1.65357	-1.68065	-1.67755	-1.67449
-1.60564	-1.66446	-1.66143	-1.65837	-1.65527	-1.65215	-1.67919	-1.6761	-1.67306
-1.60443	-1.66306	-1.65999	-1.65692	-1.65381	-1.6507	-1.67772	-1.67465	-1.67162
-1.63341	-1.66163	-1.65854	-1.65545	-1.65235	-1.64925	-1.67626	-1.6732	-1.76696
-1.63204	-1.66016	-1.65707	-1.65397	-1.65088	-1.6478	-1.6748	-1.67176	-1.76545
-1.63064	-1.65868	-1.65559	-1.6525	-1.64943	-1.64637	-1.67336	-1.67033	-1.76396
-1.66028	-1.6572	-1.65411	-1.65104	-1.648	-1.64497	-1.69553	-1.69247	-1.76247
-1.65879	-1.65571	-1.65265	-1.6496	-1.64659	-1.6436	-1.69416	-1.69111	-1.76102
-1.65731	-1.65424	-1.65119	-1.64817	-1.66926	-1.66631	-1.69285	-1.68984	-1.75969
-1.65585	-1.65279	-1.64975	-1.64675	-1.6679	-1.66501	-1.6916	-1.68866	-1.75845
-1.65446	-1.67557	-1.67252	-1.66951	-1.66658	-1.66371	-1.73726	-1.73426	-1.75715
-1.6532	-1.67433	-1.67129	-1.66828	-1.66532	-1.6624	-1.73585	-1.73287	-1.75575
-1.65189	-1.67304	-1.67001	-1.66701	-1.66403	-1.66109	-1.76041	-1.75737	-1.75432
-1.65047	-1.67164	-1.66862	-1.66564	-1.66268	-1.65977	-1.75896	-1.75591	-1.75285
-1.64905	-1.67022	-1.6672	-1.66424	-1.66132	-1.65844	-1.75751	-1.75444	-1.75138
-1.67197	-1.66888	-1.66583	-1.66285	-1.68938	-1.68647	-1.75607	-1.75299	-1.77423
-1.67071	-1.66758	-1.66449	-1.66147	-1.68799	-1.68512	-1.75464	-1.75156	-1.77276
-1.66941	-1.6663	-1.66323	-1.66022	-1.68672	-1.68386	-1.75328	-1.7502	-1.77136
-1.66809	-1.66506	-1.66205	-1.65909	-1.68556	-1.68266	-1.75199	-1.74892	-1.77004
-1.66673	-1.66375	-1.66081	-1.6579	-1.75683	-1.75374	-1.77498	-1.77182	-1.76866
-1.66531	-1.66235	-1.65946	-1.65662	-1.75544	-1.75233	-1.77352	-1.77033	-1.76715
-1.66393	-1.66098	-1.65814	-1.65534	-1.75405	-1.75094	-1.77207	-1.76887	-1.76567
-1.6627	-1.65978	-1.65694	-1.65413	-1.75273	-1.74963	-1.77072	-1.76751	-1.7643

-1.6615	-1.65861	-1.65575	-1.65292	-1.75141	-1.74833	-1.76938	-1.76617	-1.76296
-1.6602	-1.65734	-1.65448	-1.65163	-1.75	-1.74691	-1.76792	-1.76473	-1.76153
-1.65885	-1.65602	-1.65316	-1.65031	-1.74854	-1.74543	-1.7664	-1.76322	-1.76007
-1.65758	-1.72834	-1.72529	-1.72225	-1.77135	-1.76814	-1.76494	-1.76177	-1.75862
-1.65639	-1.72704	-1.724	-1.72095	-1.76999	-1.76677	-1.76356	-1.76036	-1.7572
-1.65518	-1.72571	-1.72267	-1.71962	-1.76859	-1.76538	-1.76217	-1.75896	-1.7558
-1.65391	-1.72431	-1.72125	-1.71821	-1.7671	-1.76391	-1.76073	-1.75757	-1.75445
-1.65263	-1.72292	-1.71985	-1.7168	-1.76563	-1.76246	-1.75931	-1.75619	-1.7531
-1.65139	-1.74962	-1.74648	-1.74337	-1.76427	-1.76111	-1.75797	-1.75485	-1.75175
-1.65017	-1.74829	-1.74516	-1.74206	-1.76294	-1.75981	-1.75666	-1.75352	-1.75039
-1.69595	-1.74692	-1.74377	-1.74067	-1.76152	-1.75842	-1.7553	-1.75218	-1.74907
-1.69463	-1.7455	-1.74234	-1.73922	-1.76005	-1.75697	-1.7539	-1.75084	-1.74778
-1.71934	-1.76821	-1.76497	-1.76177	-1.75866	-1.7556	-1.75255	-1.74953	-1.74648
-1.71798	-1.76683	-1.76362	-1.76046	-1.75736	-1.75432	-1.75127	-1.74824	-1.74519
-1.71666	-1.76548	-1.76231	-1.75917	-1.75608	-1.75303	-1.74997	-1.74693	-1.74387
-1.71542	-1.76418	-1.76103	-1.7579	-1.75479	-1.7517	-1.74861	-1.74554	-1.74247
-1.7142	-1.7629	-1.75975	-1.75662	-1.75349	-1.75036	-1.74725	-1.74416	-1.74108
-1.71293	-1.76155	-1.7584	-1.75527	-1.75215	-1.74905	-1.74595	-1.74286	-1.73978
-1.71165	-1.76018	-1.75702	-1.75389	-1.7508	-1.74774	-1.74468	-1.74161	-1.73853
-1.76205	-1.75883	-1.75568	-1.75257	-1.7495	-1.74646	-1.74341	-1.74035	-1.73727
-1.76069	-1.7575	-1.75438	-1.7513	-1.74824	-1.7452	-1.74214	-1.73908	-1.73599
-1.75936	-1.7562	-1.7531	-1.75003	-1.74696	-1.7439	-1.74083	-1.73776	-1.73467
-1.75807	-1.75493	-1.75183	-1.74875	-1.74565	-1.74255	-1.73945	-1.73634	-1.73326
-1.7568	-1.75367	-1.75056	-1.74746	-1.74433	-1.7412	-1.73808	-1.73495	-1.75688
-1.75553	-1.75241	-1.74928	-1.74615	-1.74301	-1.73987	-1.73675	-1.73365	-1.75557
-1.75427	-1.75114	-1.74799	-1.74483	-1.74169	-1.73855	-1.73546	-1.73239	-1.7543
-1.75297	-1.74982	-1.74667	-1.74352	-1.74039	-1.73728	-1.73419	-1.73115	-1.75303
-1.75164	-1.74847	-1.74533	-1.7422	-1.7391	-1.73602	-1.73296	-1.72992	-1.75177
-1.75035	-1.74717	-1.74403	-1.74091	-1.73783	-1.73477	-1.73172	-1.72868	-1.7505
-1.74909	-1.74592	-1.74277	-1.73965	-1.73657	-1.73353	-1.73048	-1.72743	-1.74924
-1.74781	-1.74464	-1.7415	-1.73839	-1.73531	-1.73227	-1.72922	-1.72616	-1.74795
-1.74647	-1.74333	-1.74022	-1.73713	-1.73406	-1.73101	-1.72795	-1.72487	-1.74662
-1.74514	-1.74202	-1.73893	-1.73587	-1.7328	-1.72973	-1.72666	-1.72356	-1.74528
-1.74386	-1.74075	-1.73767	-1.73461	-1.73152	-1.72843	-1.75024	-1.74708	-1.74396
-1.74261	-1.73949	-1.73642	-1.73335	-1.73024	-1.72711	-1.74889	-1.74575	-1.74265
-1.74135	-1.73824	-1.73517	-1.7321	-1.72898	-1.72584	-1.74759	-1.74446	-1.74137
-1.74009	-1.73698	-1.73392	-1.73086	-1.72774	-1.7246	-1.74632	-1.74319	-1.74011
-1.73884	-1.73573	-1.73267	-1.72961	-1.7265	-1.72336	-1.74506	-1.74193	-1.73885
-1.73759	-1.73449	-1.73143	-1.72837	-1.72526	-1.72213	-1.7438	-1.74069	-1.73761
-1.73634	-1.73324	-1.73018	-1.72713	-1.72402	-1.72089	-1.74256	-1.73944	-1.73637
-1.73511	-1.73201	-1.72895	-1.7259	-1.7228	-1.71967	-1.74131	-1.7382	-1.73513
-1.73387	-1.73077	-1.72772	-1.72467	-1.72157	-1.71845	-1.74007	-1.73697	-1.7339
-1.73263	-1.72954	-1.72648	-1.72343	-1.74515	-1.74197	-1.73883	-1.73573	-1.73267
-1.73139	-1.7283	-1.72524	-1.7222	-1.7439	-1.74072	-1.73759	-1.7345	-1.73144
-1.73014	-1.72705	-1.724	-1.72097	-1.74265	-1.73948	-1.73636	-1.73326	-1.73021
-1.72889	-1.7258	-1.72276	-1.71974	-1.7414	-1.73824	-1.73512	-1.73203	-1.72898
-1.72768	-1.72458	-1.72153	-1.71851	-1.74015	-1.737	-1.73388	-1.73079	-1.72774
-1.72651	-1.72339	-1.72032	-1.71727	-1.73889	-1.73574	-1.73263	-1.72955	-1.7265
-1.72534	-1.7222	-1.71911	-1.71604	-1.73763	-1.73449	-1.73138	-1.7283	-1.74597
-1.7241	-1.72097	-1.71787	-1.7148	-1.73637	-1.73324	-1.73013	-1.72705	-1.74469

-1.72285	-1.71973	-1.71664	-1.71357	-1.73513	-1.732	-1.7289	-1.72581	-1.74343
-1.72162	-1.74329	-1.74014	-1.73701	-1.7339	-1.73081	-1.72772	-1.72462	-1.7422
-1.72039	-1.74204	-1.73889	-1.73577	-1.73269	-1.72964	-1.72656	-1.72345	-1.74099
-1.7192	-1.74083	-1.73768	-1.73455	-1.73148	-1.72844	-1.74606	-1.7429	-1.73977
-1.71804	-1.73966	-1.7365	-1.73336	-1.73027	-1.72721	-1.7448	-1.74165	-1.73854
-1.74162	-1.73846	-1.7353	-1.73216	-1.72905	-1.72597	-1.74355	-1.74042	-1.73734
-1.74036	-1.7372	-1.73405	-1.73092	-1.72782	-1.72473	-1.7423	-1.73924	-1.73619
-1.73911	-1.73595	-1.73282	-1.7297	-1.72659	-1.7235	-1.74107	-1.73805	-1.73503
-1.73792	-1.73476	-1.73163	-1.72852	-1.74608	-1.74293	-1.73984	-1.73684	-1.73382
-1.73674	-1.73358	-1.73045	-1.72734	-1.74488	-1.74172	-1.73863	-1.73561	-1.73259
-1.73551	-1.73236	-1.72923	-1.72612	-1.74364	-1.74049	-1.73741	-1.73438	-1.73136
-1.73425	-1.73111	-1.72798	-1.72486	-1.74237	-1.73926	-1.73618	-1.73315	-1.73015
-1.73304	-1.75063	-1.74744	-1.74428	-1.74115	-1.73806	-1.73499	-1.73196	-1.72895
-1.73187	-1.74945	-1.74626	-1.74309	-1.73997	-1.7369	-1.73384	-1.7308	-1.72778
-1.73071	-1.74826	-1.74508	-1.74192	-1.73881	-1.73575	-1.73269	-1.72965	-1.72661
-1.72954	-1.74707	-1.74391	-1.74077	-1.73767	-1.7346	-1.73155	-1.72851	-1.72547
-1.72836	-1.74588	-1.74273	-1.73961	-1.73651	-1.73344	-1.73039	-1.72736	-1.72431
-1.7478	-1.74463	-1.7415	-1.7384	-1.73531	-1.73225	-1.72919	-1.72615	-1.7231
-1.74653	-1.74338	-1.74026	-1.73717	-1.73409	-1.73103	-1.72798	-1.72492	-1.72186
-1.74531	-1.74217	-1.73907	-1.73598	-1.73291	-1.72986	-1.72679	-1.72373	-1.74702
-1.74414	-1.741	-1.73791	-1.73483	-1.73177	-1.7287	-1.72564	-1.72257	-1.74585
-1.74298	-1.73985	-1.73677	-1.7337	-1.73062	-1.72756	-1.75093	-1.7478	-1.7631
-1.74183	-1.73872	-1.73564	-1.73257	-1.72949	-1.72642	-1.74978	-1.74665	-1.76195
-1.74069	-1.73759	-1.73451	-1.73143	-1.72836	-1.72528	-1.76708	-1.76394	-1.79003
-1.73954	-1.73646	-1.73337	-1.73029	-1.72721	-1.72413	-1.76593	-1.76278	-1.78878
-1.73839	-1.73532	-1.73223	-1.72915	-1.72606	-1.72297	-1.76477	-1.76162	-1.78752
-1.73725	-1.73417	-1.73109	-1.728	-1.76993	-1.76676	-1.76361	-1.76046	-1.78628
-1.7361	-1.73303	-1.72995	-1.72686	-1.76878	-1.76561	-1.76245	-1.75931	-1.78505
-1.73496	-1.75078	-1.74768	-1.74457	-1.76762	-1.76445	-1.76129	-1.75815	-1.7838
-1.73381	-1.74964	-1.74652	-1.7434	-1.76645	-1.76328	-1.76012	-1.75698	-1.78254
-1.75159	-1.7748	-1.77163	-1.76845	-1.76528	-1.76211	-1.78802	-1.78464	-1.78129
-1.75046	-1.77362	-1.77045	-1.76729	-1.76412	-1.76095	-1.78676	-1.7834	-1.78005
-1.74934	-1.77246	-1.76928	-1.76612	-1.76295	-1.75978	-1.78551	-1.78216	-1.77882
-1.74826	-1.77135	-1.76814	-1.76494	-1.76177	-1.75861	-1.78426	-1.78091	-1.77757
-1.74718	-1.77027	-1.76701	-1.76376	-1.76058	-1.75744	-1.78302	-1.77966	-1.77632
-1.77236	-1.76915	-1.7659	-1.76265	-1.78851	-1.78512	-1.78175	-1.7784	-1.77507
-1.7712	-1.76802	-1.7648	-1.76157	-1.78732	-1.78387	-1.78048	-1.77714	-1.77383
-1.77004	-1.76686	-1.76367	-1.76047	-1.78613	-1.78266	-1.77925	-1.77591	-1.77258
-1.76889	-1.76567	-1.76249	-1.75932	-1.78493	-1.78149	-1.77808	-1.77468	-1.77133
-1.76775	-1.7939	-1.7905	-1.78711	-1.78372	-1.78031	-1.7769	-1.77348	-1.77011
-1.76666	-1.79273	-1.78928	-1.78586	-1.78247	-1.7791	-1.77571	-1.7723	-1.76894
-1.76555	-1.79157	-1.78809	-1.78462	-1.78123	-1.77789	-1.77452	-1.77113	-1.76779
-1.76441	-1.79037	-1.7869	-1.78343	-1.78005	-1.77672	-1.77336	-1.76999	-1.76664
-1.76325	-1.78915	-1.78571	-1.78228	-1.7789	-1.77555	-1.7722	-1.76885	-1.7655
-1.79134	-1.82922	-1.82569	-1.82215	-1.81865	-1.81516	-1.8117	-1.80826	-1.84427
-1.79017	-1.82801	-1.82449	-1.82095	-1.81741	-1.81387	-1.8104	-1.807	-1.84302
-1.83027	-1.82676	-1.82325	-1.81973	-1.81618	-1.81261	-1.80913	-1.80573	-1.84174
-1.829	-1.82547	-1.82198	-1.81849	-1.81495	-1.8114	-1.8079	-1.80446	-1.84042
-1.82774	-1.82419	-1.82071	-1.81724	-1.81373	-1.8102	-1.8067	-1.80322	-1.83912
-1.82652	-1.82296	-1.81947	-1.81601	-1.81253	-1.80903	-1.80553	-1.80203	-1.83789

-1.82532	-1.82174	-1.81824	-1.81478	-1.81132	-1.80786	-1.80437	-1.80086	-1.83668
-1.82411	-1.82052	-1.817	-1.81352	-1.81006	-1.80663	-1.80317	-1.79967	-1.83547
-1.82291	-1.8193	-1.81575	-1.81223	-1.80879	-1.80539	-1.80195	-1.79849	-1.83428
-1.82171	-1.8181	-1.81453	-1.811	-1.80756	-1.80417	-1.80076	-1.79731	-1.83308
-1.82053	-1.81691	-1.81334	-1.80981	-1.80637	-1.80299	-1.79958	-1.79614	-1.83189
-1.81934	-1.81573	-1.81216	-1.80863	-1.80519	-1.80182	-1.79841	-1.79497	-1.84929
-1.81816	-1.81455	-1.81098	-1.80746	-1.80402	-1.80064	-1.79722	-1.79379	-1.84807
-1.81698	-1.81338	-1.80981	-1.80629	-1.80285	-1.79946	-1.79604	-1.7926	-1.84684
-1.81578	-1.81219	-1.80864	-1.80513	-1.80169	-1.79829	-1.79486	-1.79142	-1.84561
-1.81459	-1.811	-1.80746	-1.80396	-1.80052	-1.79712	-1.79369	-1.79023	-1.84438
-1.8134	-1.80981	-1.80627	-1.80278	-1.79934	-1.79594	-1.7925	-1.78904	-1.84315
-1.81222	-1.80863	-1.80509	-1.80159	-1.79815	-1.79475	-1.79132	-1.78785	-1.84193
-1.81103	-1.80744	-1.8039	-1.8004	-1.79696	-1.79356	-1.80921	-1.8057	-1.84071
-1.80984	-1.80625	-1.80272	-1.79922	-1.79577	-1.79237	-1.80799	-1.80448	-1.83948
-1.80864	-1.80507	-1.80153	-1.79803	-1.81176	-1.80831	-1.82364	-1.82011	-1.88757
-1.80744	-1.80387	-1.80034	-1.79684	-1.81056	-1.80711	-1.82243	-1.81889	-1.88632
-1.80624	-1.80268	-1.79916	-1.79566	-1.80936	-1.80592	-1.82121	-1.81767	-1.88507
-1.82232	-1.81874	-1.81518	-1.81165	-1.80816	-1.80471	-1.83799	-1.83444	-1.88383
-1.82113	-1.81756	-1.814	-1.81047	-1.80698	-1.80351	-1.83676	-1.83323	-1.88259
-1.81995	-1.81639	-1.81286	-1.80934	-1.82464	-1.82111	-1.83557	-1.83202	-1.88135
-1.81877	-1.81524	-1.81173	-1.80824	-1.82353	-1.81997	-1.83441	-1.83082	-1.8801
-1.81763	-1.81411	-1.81062	-1.80713	-1.84042	-1.83683	-1.83323	-1.82962	-1.87887
-1.8165	-1.81301	-1.80952	-1.80602	-1.83927	-1.83565	-1.83203	-1.82842	-1.87764
-1.8154	-1.83024	-1.82672	-1.82318	-1.83809	-1.83445	-1.88382	-1.88011	-1.87643
-1.81433	-1.82913	-1.82556	-1.82199	-1.83687	-1.83323	-1.88259	-1.87889	-1.87524
-1.81325	-1.82801	-1.8244	-1.82079	-1.83565	-1.83202	-1.88136	-1.8777	-1.87407
-1.81211	-1.84543	-1.84176	-1.83809	-1.83445	-1.83082	-1.88015	-1.87653	-1.87294
-1.81092	-1.84423	-1.84057	-1.83691	-1.83327	-1.82965	-1.87897	-1.87539	-1.87182
-1.82809	-1.84304	-1.8394	-1.83577	-1.83216	-1.82856	-1.87785	-1.87427	-1.87069
-1.82692	-1.84185	-1.83825	-1.83468	-1.8311	-1.82754	-1.8768	-1.87317	-1.86954
-1.8258	-1.84071	-1.83713	-1.83357	-1.83001	-1.82647	-1.87569	-1.87203	-1.86837
-1.82478	-1.83965	-1.83604	-1.83245	-1.82888	-1.82532	-1.87451	-1.87083	-1.86716
-1.84227	-1.83859	-1.83494	-1.83131	-1.82772	-1.82416	-1.8733	-1.86963	-1.86596
-1.84113	-1.83745	-1.83379	-1.83015	-1.82655	-1.82299	-1.8721	-1.86843	-1.86478
-1.83996	-1.8363	-1.83264	-1.82899	-1.8254	-1.82184	-1.87092	-1.86725	-1.86361
-1.83881	-1.83519	-1.83155	-1.82794	-1.84012	-1.83651	-1.86983	-1.86616	-1.86251
-1.83767	-1.83409	-1.83051	-1.82694	-1.83912	-1.83551	-1.8688	-1.86513	-1.86146
-1.83654	-1.83298	-1.82943	-1.82588	-1.83805	-1.83443	-1.86771	-1.86402	-1.86034
-1.83544	-1.83185	-1.8283	-1.82475	-1.83691	-1.83329	-1.86654	-1.86285	-1.85915
-1.83433	-1.83072	-1.82717	-1.82364	-1.83578	-1.83217	-1.86541	-1.86171	-1.858
-1.83316	-1.82956	-1.82604	-1.82255	-1.83472	-1.83114	-1.86438	-1.86066	-1.85694
-1.83203	-1.82844	-1.82494	-1.82147	-1.83365	-1.8301	-1.86333	-1.8596	-1.85588
-1.831	-1.84319	-1.83963	-1.83609	-1.83253	-1.82898	-1.86218	-1.85844	-1.85471
-1.83001	-1.84219	-1.83859	-1.835	-1.83141	-1.82782	-1.86099	-1.85725	-1.85352
-1.82896	-1.84115	-1.83756	-1.83396	-1.83036	-1.82674	-1.85988	-1.85615	-1.85242
-1.82787	-1.84009	-1.83653	-1.83296	-1.82935	-1.82571	-1.85883	-1.8551	-1.85137
-1.8268	-1.83901	-1.83545	-1.83189	-1.82827	-1.82462	-1.85771	-1.85398	-1.85026
-1.82574	-1.8379	-1.83431	-1.83072	-1.82709	-1.82343	-1.85651	-1.85278	-1.84906
-1.84043	-1.83681	-1.8332	-1.82958	-1.82594	-1.82228	-1.85533	-1.8516	-1.84789
-1.83943	-1.8358	-1.83217	-1.82853	-1.82488	-1.82122	-1.85426	-1.85053	-1.84682

-1.83842	-1.83477	-1.83113	-1.82748	-1.82383	-1.82016	-1.85319	-1.84946	-1.84575
-1.83729	-1.83364	-1.82999	-1.82633	-1.82267	-1.81901	-1.85202	-1.84829	-1.84459
-1.83611	-1.83246	-1.8288	-1.82514	-1.82148	-1.81782	-1.85081	-1.84709	-1.84339
-1.83503	-1.83138	-1.82771	-1.82405	-1.82039	-1.81673	-1.84971	-1.84598	-1.84229
-1.83403	-1.83037	-1.8267	-1.82304	-1.81937	-1.81572	-1.84868	-1.84497	-1.84127
-1.83304	-1.82937	-1.8257	-1.82204	-1.81837	-1.81472	-1.84768	-1.84397	-1.84028
-1.83207	-1.8284	-1.82472	-1.82105	-1.81739	-1.81374	-1.84669	-1.84299	-1.8393
-1.83108	-1.82741	-1.82373	-1.82006	-1.8164	-1.81275	-1.8457	-1.842	-1.83831
-1.83008	-1.82641	-1.82273	-1.81906	-1.8154	-1.81175	-1.84468	-1.84099	-1.83731
-1.82905	-1.82537	-1.82169	-1.81802	-1.81436	-1.81071	-1.84363	-1.83994	-1.83627
-1.82791	-1.82423	-1.82055	-1.81688	-1.81322	-1.80957	-1.84247	-1.83879	-1.83512
-1.82671	-1.82303	-1.81935	-1.81568	-1.81202	-1.80838	-1.84126	-1.83758	-1.83391
-1.8256	-1.82192	-1.81824	-1.81457	-1.81091	-1.80727	-1.84014	-1.83646	-1.83279
-1.82457	-1.82089	-1.8172	-1.81353	-1.80988	-1.80625	-1.8391	-1.83542	-1.83176
-1.82357	-1.81988	-1.81619	-1.81252	-1.80887	-1.80525	-1.83808	-1.83441	-1.83075
-1.82257	-1.81888	-1.81519	-1.81152	-1.80788	-1.80426	-1.83709	-1.83342	-1.82975
-1.82159	-1.81789	-1.8142	-1.81053	-1.80689	-1.80328	-1.83609	-1.83243	-1.82876
-1.82059	-1.81689	-1.81321	-1.80954	-1.8059	-1.8023	-1.8351	-1.83144	-1.82777
-1.81959	-1.8159	-1.81221	-1.80854	-1.80492	-1.80132	-1.8341	-1.83045	-1.82678
-1.81859	-1.8149	-1.81122	-1.80756	-1.84045	-1.83678	-1.83311	-1.82945	-1.82579
-1.81759	-1.81389	-1.81022	-1.80657	-1.83945	-1.83578	-1.83212	-1.82846	-1.8248
-1.81658	-1.81289	-1.80923	-1.80559	-1.83845	-1.83479	-1.83112	-1.82747	-1.8238
-1.81558	-1.81189	-1.80824	-1.8046	-1.83746	-1.83379	-1.83013	-1.82646	-1.8228
-1.81458	-1.81089	-1.80724	-1.80362	-1.83646	-1.8328	-1.82913	-1.82546	-1.82179
-1.81357	-1.80989	-1.80625	-1.80264	-1.83547	-1.83181	-1.82814	-1.82447	-1.82079
-1.81256	-1.80889	-1.80526	-1.80165	-1.83448	-1.83082	-1.82715	-1.82347	-1.81978
-1.81157	-1.80789	-1.80427	-1.80067	-1.83348	-1.82982	-1.82615	-1.82246	-1.81877
-1.81057	-1.80691	-1.80328	-1.79968	-1.83249	-1.82883	-1.82515	-1.82146	-1.81776
-1.80958	-1.80592	-1.8023	-1.7987	-1.83148	-1.82782	-1.82414	-1.82045	-1.81675
-1.80858	-1.80493	-1.80131	-1.79771	-1.83048	-1.82681	-1.82313	-1.81944	-1.81574
-1.80759	-1.80395	-1.80033	-1.79672	-1.82948	-1.8258	-1.82212	-1.81842	-1.81473
-1.80658	-1.80295	-1.79934	-1.79574	-1.82847	-1.82479	-1.8211	-1.81741	-1.81372
-1.80562	-1.83854	-1.83485	-1.83116	-1.82747	-1.82378	-1.82009	-1.8164	-1.81271
-1.80475	-1.83762	-1.83388	-1.83016	-1.82645	-1.82277	-1.81908	-1.81538	-1.8117
-1.8039	-1.83672	-1.83293	-1.82917	-1.82545	-1.82176	-1.81806	-1.81437	-1.81068
-1.80303	-1.83584	-1.83203	-1.82824	-1.82449	-1.82076	-1.81705	-1.81335	-1.80966
-1.80214	-1.83496	-1.83116	-1.82735	-1.82356	-1.81979	-1.81605	-1.81233	-1.80864
-1.80121	-1.83404	-1.83026	-1.82647	-1.82268	-1.81889	-1.81513	-1.81139	-1.80767
-1.80023	-1.83308	-1.82934	-1.8256	-1.82184	-1.81808	-1.81431	-1.81052	-1.80675
-1.79925	-1.8321	-1.82839	-1.82468	-1.82095	-1.81721	-1.81344	-1.80964	-1.82485
-1.79825	-1.83109	-1.82739	-1.82369	-1.81997	-1.81625	-1.81251	-1.80875	-1.82396
-1.79729	-1.8301	-1.82639	-1.82268	-1.81897	-1.81526	-1.81155	-1.80783	-1.82306
-1.79641	-1.82918	-1.82542	-1.82168	-1.81795	-1.81424	-1.81054	-1.80685	-1.8221
-1.79558	-1.82828	-1.82448	-1.8207	-1.81695	-1.81322	-1.80952	-1.80584	-1.82111
-1.7947	-1.8274	-1.82361	-1.81981	-1.81604	-1.81228	-1.80855	-1.80485	-1.8201
-1.79381	-1.82654	-1.82278	-1.819	-1.8152	-1.81139	-1.80762	-1.80388	-1.81908
-1.82936	-1.82562	-1.82187	-1.81811	-1.81432	-1.8105	-1.82573	-1.82192	-1.81814
-1.82839	-1.82463	-1.82088	-1.81713	-1.81338	-1.80962	-1.82486	-1.82108	-1.81731
-1.82747	-1.82368	-1.81992	-1.81617	-1.81243	-1.8087	-1.82396	-1.82021	-1.81645
-1.82663	-1.82285	-1.81905	-1.81526	-1.81149	-1.80773	-1.82297	-1.81923	-1.81548

-1.82579	-1.82201	-1.81819	-1.81436	-1.81056	-1.80677	-1.82198	-1.81824	-1.81451
-1.82488	-1.82109	-1.81728	-1.81347	-1.80968	-1.80591	-1.8211	-1.81733	-1.81357
-1.82394	-1.82014	-1.81635	-1.81258	-1.80883	-1.80509	-1.82027	-1.81645	-1.81266
-1.82306	-1.81925	-1.81546	-1.81169	-1.82695	-1.82316	-1.81936	-1.81555	-1.81176
-1.82222	-1.81842	-1.8146	-1.81079	-1.82601	-1.8222	-1.8184	-1.81463	-1.81088
-1.82134	-1.81754	-1.81372	-1.8099	-1.8251	-1.82128	-1.81749	-1.81373	-1.80999
-1.82039	-1.81659	-1.81279	-1.80901	-1.82423	-1.82044	-1.81665	-1.81285	-1.80908
-1.81947	-1.81565	-1.81187	-1.80812	-1.82335	-1.81958	-1.81579	-1.81198	-1.80818
-1.81861	-1.8148	-1.811	-1.80722	-1.82242	-1.81863	-1.81485	-1.81107	-1.80731
-1.81778	-1.81396	-1.81014	-1.80633	-1.8215	-1.81769	-1.81391	-1.81018	-1.80646
-1.81693	-1.83218	-1.82831	-1.82445	-1.82063	-1.81683	-1.81307	-1.80935	-1.80562
-1.81607	-1.83131	-1.82744	-1.82359	-1.81979	-1.81603	-1.81228	-1.80854	-1.8048
-1.81521	-1.83043	-1.82657	-1.82275	-1.81897	-1.81522	-1.81147	-1.80772	-1.80396
-1.81433	-1.82955	-1.82572	-1.82193	-1.81817	-1.81443	-1.81067	-1.80688	-1.80309
-1.81347	-1.82868	-1.82487	-1.82109	-1.81733	-1.81358	-1.80981	-1.80602	-1.80223
-1.81263	-1.82785	-1.82402	-1.82021	-1.81642	-1.81264	-1.80887	-1.80511	-1.80136
-1.8118	-1.82701	-1.82317	-1.81933	-1.8155	-1.8117	-1.80794	-1.80423	-1.80051
-1.83001	-1.82614	-1.82229	-1.81845	-1.81464	-1.81086	-1.80711	-1.8034	-1.79967
-1.82917	-1.82528	-1.82142	-1.81759	-1.81381	-1.81005	-1.80631	-1.80259	-1.79885
-1.82837	-1.82447	-1.8206	-1.81677	-1.81299	-1.80925	-1.8055	-1.80175	-1.798
-1.82759	-1.8237	-1.81983	-1.81598	-1.8122	-1.80845	-1.80469	-1.80091	-1.79713
-1.82677	-1.82289	-1.81903	-1.8152	-1.8114	-1.80763	-1.80385	-1.80005	-1.79627
-1.8259	-1.82205	-1.81822	-1.81442	-1.8106	-1.80678	-1.80297	-1.79918	-1.79541
-1.82505	-1.82121	-1.81742	-1.81363	-1.8098	-1.80594	-1.80213	-1.79834	-1.79459
-1.82425	-1.82041	-1.81662	-1.81284	-1.80902	-1.80517	-1.80135	-1.79756	-1.7938
-1.82346	-1.81962	-1.81582	-1.81204	-1.80822	-1.80439	-1.80058	-1.79679	-1.79303
-1.82266	-1.81882	-1.815	-1.81119	-1.80737	-1.80355	-1.79976	-1.796	-1.79226
-1.82185	-1.81804	-1.81418	-1.81032	-1.80649	-1.80269	-1.79893	-1.79521	-1.7915
-1.82106	-1.81726	-1.81338	-1.8095	-1.80567	-1.80188	-1.79813	-1.79443	-1.7979
-1.82029	-1.81649	-1.81261	-1.80873	-1.8049	-1.8011	-1.79735	-1.79365	-1.79714
-1.81952	-1.81572	-1.81185	-1.80796	-1.80413	-1.80033	-1.79658	-1.79288	-1.79638
-1.81875	-1.81496	-1.81108	-1.8072	-1.80337	-1.79958	-1.79582	-1.79212	-1.79564
-1.81799	-1.81419	-1.81032	-1.80645	-1.80262	-1.79882	-1.80235	-1.79864	-1.7949
-1.81722	-1.81343	-1.80956	-1.80569	-1.80186	-1.79807	-1.80161	-1.79791	-1.79417
-1.81646	-1.81267	-1.8088	-1.80493	-1.80111	-1.79731	-1.80088	-1.79717	-1.79343
-1.81569	-1.8119	-1.80804	-1.80417	-1.80035	-1.79655	-1.80015	-1.79644	-1.7927
-1.81492	-1.81114	-1.80728	-1.80341	-1.79959	-1.79579	-1.79941	-1.7957	-1.79197
-1.81418	-1.81038	-1.80653	-1.80267	-1.80624	-1.80243	-1.79867	-1.79497	-1.79124
-1.81345	-1.80963	-1.80578	-1.80192	-1.80552	-1.8017	-1.79793	-1.79424	-1.79052
-1.81272	-1.80888	-1.80505	-1.80121	-1.80483	-1.80099	-1.79722	-1.79352	-1.7898
-1.81198	-1.80814	-1.80434	-1.80055	-1.8042	-1.80035	-1.79655	-1.79282	-1.7891
-1.81125	-1.80741	-1.80364	-1.79988	-1.80357	-1.7997	-1.79589	-1.79212	-1.78839
-1.81058	-1.81428	-1.81049	-1.80671	-1.80287	-1.79901	-1.79519	-1.79141	-1.78766
-1.80992	-1.81363	-1.80981	-1.806	-1.80216	-1.79832	-1.7945	-1.79069	-1.78694
-1.80922	-1.81295	-1.80911	-1.80528	-1.80147	-1.79766	-1.79385	-1.79004	-1.78626
-1.80849	-1.81226	-1.8084	-1.80456	-1.80078	-1.79702	-1.79324	-1.78942	-1.78562
-1.81544	-1.81159	-1.80773	-1.80388	-1.8001	-1.79636	-1.79258	-1.78876	-1.80522
-1.81477	-1.81096	-1.8071	-1.80323	-1.79943	-1.79566	-1.79187	-1.78807	-1.80452
-1.8141	-1.81031	-1.80645	-1.80257	-1.79875	-1.79496	-1.8115	-1.80767	-1.80384
-1.81341	-1.80959	-1.80574	-1.80188	-1.79805	-1.79424	-1.81079	-1.80701	-1.80321

-1.81273	-1.80889	-1.80503	-1.80119	-1.79736	-1.79354	-1.81009	-1.80635	-1.80258
-1.81211	-1.80825	-1.80439	-1.80055	-1.81712	-1.81324	-1.80942	-1.80566	-1.80189
-1.81152	-1.80764	-1.80378	-1.79993	-1.81649	-1.81262	-1.80878	-1.80497	-1.80118
-1.81089	-1.80705	-1.80318	-1.79933	-1.81587	-1.81201	-1.80815	-1.80432	-1.80051
-1.81026	-1.80646	-1.8026	-1.79873	-1.81527	-1.81139	-1.80754	-1.8037	-1.79988
-1.8096	-1.82633	-1.82244	-1.81855	-1.81466	-1.81079	-1.80693	-1.80309	-1.79925
-1.80891	-1.82561	-1.82178	-1.81794	-1.81407	-1.81019	-1.80633	-1.80248	-1.79864
-1.82878	-1.82491	-1.8211	-1.8173	-1.81345	-1.80959	-1.80573	-1.80187	-1.79803
-1.82814	-1.82426	-1.82041	-1.81659	-1.81278	-1.80897	-1.80513	-1.80128	-1.79742
-1.82752	-1.82362	-1.81974	-1.81588	-1.81209	-1.80833	-1.80452	-1.80067	-1.79682
-1.8269	-1.823	-1.8191	-1.81523	-1.81143	-1.80766	-1.80386	-1.80004	-1.79622
-1.82629	-1.82238	-1.81849	-1.81462	-1.81078	-1.80697	-1.80318	-1.7994	-1.79561
-1.82568	-1.82177	-1.81789	-1.81402	-1.81018	-1.80637	-1.80257	-1.7988	-1.79501
-1.82507	-1.82118	-1.8173	-1.81344	-1.80963	-1.80584	-1.80204	-1.79823	-1.79442
-1.82447	-1.82059	-1.81673	-1.81289	-1.80908	-1.80529	-1.80149	-1.79765	-1.79382
-1.82387	-1.82	-1.81617	-1.81236	-1.80854	-1.80473	-1.8009	-1.79706	-1.79321
-1.82329	-1.81944	-1.81562	-1.81182	-1.808	-1.80416	-1.80031	-1.79645	-1.79261
-1.82274	-1.81891	-1.81508	-1.81126	-1.80742	-1.80356	-1.7997	-1.79585	-1.79201
-1.82221	-1.81838	-1.81454	-1.81069	-1.80682	-1.80295	-1.79909	-1.79525	-1.79141
-1.82166	-1.81783	-1.81397	-1.8101	-1.80623	-1.80235	-1.7985	-1.79466	-1.8183
-1.8211	-1.81725	-1.81337	-1.80949	-1.80563	-1.80177	-1.79791	-1.79407	-1.8177
-1.82053	-1.81667	-1.81278	-1.8089	-1.80503	-1.80118	-1.79734	-1.79352	-1.85098
-1.81995	-1.81608	-1.81219	-1.8083	-1.80444	-1.80059	-1.79678	-1.79299	-1.85046
-1.8194	-1.81552	-1.81163	-1.80774	-1.80388	-1.80003	-1.79622	-1.79245	-1.8499
-1.8189	-1.81502	-1.81113	-1.80725	-1.80338	-1.79952	-1.79569	-1.7919	-1.84929
-1.8184	-1.81451	-1.81063	-1.80676	-1.80288	-1.799	-1.79515	-1.79133	-1.84867
-1.81784	-1.81395	-1.81007	-1.8062	-1.80231	-1.79843	-1.8221	-1.81821	-1.86045
-1.81727	-1.81337	-1.80949	-1.80562	-1.80174	-1.79786	-1.82151	-1.81762	-1.85983
-1.81675	-1.81286	-1.80897	-1.8051	-1.80122	-1.79734	-1.82098	-1.81709	-1.88871
-1.81628	-1.81238	-1.80849	-1.80461	-1.80073	-1.79686	-1.8205	-1.81661	-1.88819
-1.8158	-1.8119	-1.80801	-1.80413	-1.80025	-1.79639	-1.85074	-1.84675	-1.88767
-1.81533	-1.81143	-1.80753	-1.80364	-1.79977	-1.79593	-1.85025	-1.84625	-1.88714
-1.81483	-1.81093	-1.80703	-1.80313	-1.79927	-1.79543	-1.84973	-1.84572	-1.88658
-1.81425	-1.81036	-1.80646	-1.80256	-1.7987	-1.79487	-1.84913	-1.84512	-1.88596
-1.81368	-1.80979	-1.80589	-1.802	-1.79814	-1.7943	-1.84853	-1.84453	-1.88534
-1.81317	-1.80928	-1.80538	-1.80149	-1.85601	-1.852	-1.88104	-1.87689	-1.88478
-1.81269	-1.8088	-1.80491	-1.80102	-1.85551	-1.8515	-1.88052	-1.87637	-1.88425
-1.81222	-1.80833	-1.80444	-1.80056	-1.85501	-1.851	-1.88001	-1.87585	-1.88372
-1.81175	-1.80786	-1.80397	-1.80009	-1.85452	-1.85051	-1.8795	-1.87534	-1.88321
-1.81129	-1.83922	-1.83523	-1.83125	-1.85403	-1.85002	-1.87899	-1.87484	-1.8827
-1.81082	-1.83874	-1.83475	-1.83077	-1.85354	-1.84953	-1.87849	-1.87433	-1.88219
-1.81036	-1.83826	-1.83427	-1.83029	-1.85305	-1.84904	-1.87798	-1.87383	-1.88169
-1.80989	-1.83778	-1.83379	-1.8298	-1.85256	-1.84855	-1.87748	-1.87333	-1.88118
-1.80943	-1.8373	-1.83331	-1.82932	-1.85207	-1.84807	-1.87697	-1.87282	-1.88067
-1.80897	-1.83682	-1.83283	-1.82885	-1.85158	-1.84757	-1.87646	-1.87232	-1.88016
-1.80851	-1.83634	-1.83237	-1.8284	-1.85111	-1.84708	-1.87595	-1.87181	-1.87965
-1.83992	-1.83592	-1.83195	-1.82798	-1.85068	-1.84665	-1.87548	-1.87132	-1.87915
-1.83955	-1.83555	-1.83158	-1.82762	-1.8503	-1.84627	-1.87506	-1.87086	-1.87865
-1.83915	-1.83515	-1.83118	-1.82722	-1.88302	-1.87883	-1.88666	-1.88241	-1.87819
-1.83869	-1.83469	-1.83073	-1.82677	-1.88255	-1.87837	-1.88622	-1.88199	-1.8778

-1.83822	-1.83422	-1.83026	-1.8263	-1.88206	-1.8779	-1.88576	-1.88157	-1.87739
-1.83775	-1.83376	-1.82979	-1.82583	-1.88157	-1.87741	-1.88527	-1.88109	-1.87692
-1.83729	-1.83331	-1.82933	-1.82536	-1.88108	-1.87693	-1.88479	-1.8806	-1.87643
-1.83688	-1.83291	-1.82893	-1.82495	-1.88065	-1.8765	-1.88435	-1.88017	-1.87599
-1.83651	-1.83254	-1.82856	-1.82459	-1.88026	-1.87611	-1.88396	-1.87977	-1.87557
-1.8361	-1.83213	-1.82816	-1.82419	-1.87984	-1.87569	-1.88353	-1.87934	-1.87514
-1.83564	-1.83167	-1.82771	-1.82376	-1.87937	-1.87521	-1.88306	-1.87888	-1.87471
-1.83522	-1.83125	-1.82729	-1.82334	-1.87892	-1.87476	-1.88261	-1.87845	-1.87429
-1.83485	-1.83089	-1.82693	-1.82298	-1.87854	-1.87439	-1.88222	-1.87805	-1.87389
-1.83451	-1.83055	-1.82659	-1.82263	-1.87818	-1.87403	-1.88186	-1.87768	-1.87351
-1.83416	-1.8302	-1.82624	-1.82229	-1.87781	-1.87367	-1.88149	-1.8773	-1.87312
-1.83382	-1.82985	-1.82589	-1.82194	-1.87744	-1.8733	-1.88112	-1.87692	-1.87272
-1.86772	-1.88953	-1.88535	-1.88117	-1.87702	-1.87287	-1.88069	-1.8765	-1.8723
-1.86736	-1.88914	-1.88492	-1.88071	-1.87654	-1.87239	-1.88021	-1.87605	-1.87188
-1.86697	-1.88873	-1.8845	-1.88028	-1.8761	-1.87195	-1.87978	-1.87562	-1.87147
-1.86655	-1.88828	-1.88407	-1.87988	-1.87572	-1.87159	-1.87941	-1.87524	-1.87109
-1.86617	-1.88787	-1.88368	-1.87951	-1.87536	-1.87123	-1.87904	-1.87487	-1.87072
-1.86586	-1.88757	-1.88335	-1.87915	-1.87499	-1.87086	-1.87868	-1.87452	-1.87037
-1.86557	-1.88729	-1.88304	-1.87881	-1.87463	-1.87049	-1.87831	-1.87417	-1.87002
-1.86526	-1.88699	-1.88275	-1.87851	-1.87431	-1.87014	-1.87794	-1.8738	-1.86966
-1.86493	-1.88667	-1.88247	-1.87826	-1.87403	-1.86981	-1.87757	-1.87342	-1.86928
-1.86459	-1.88634	-1.88215	-1.87796	-1.87374	-1.86951	-1.87725	-1.87307	-1.86892
-1.86424	-1.88598	-1.88181	-1.87763	-1.87344	-1.86924	-1.87698	-1.87275	-1.86856
-1.86388	-1.88562	-1.88146	-1.87729	-1.87313	-1.86896	-1.8767	-1.87244	-1.86823
-1.86352	-1.88525	-1.88109	-1.87694	-1.87278	-1.86863	-1.87639	-1.87217	-1.86796
-1.86317	-1.88488	-1.88073	-1.87658	-1.87243	-1.86829	-1.87607	-1.87189	-1.86769
-1.88872	-1.88452	-1.88035	-1.87621	-1.88406	-1.8799	-1.87574	-1.87157	-1.86739
-1.88841	-1.88417	-1.87999	-1.87584	-1.8837	-1.87954	-1.87539	-1.87123	-1.86707
-1.88812	-1.88387	-1.87966	-1.87549	-1.88333	-1.87919	-1.87504	-1.87088	-1.86673
-1.88784	-1.8836	-1.87936	-1.87514	-1.88297	-1.87883	-1.87468	-1.87053	-1.86638
-1.88754	-1.88333	-1.87908	-1.87485	-1.88267	-1.87853	-1.87438	-1.87022	-1.86606
-1.88722	-1.88303	-1.87881	-1.8746	-1.88243	-1.87828	-1.87413	-1.86997	-1.8658
-1.88692	-1.88275	-1.87856	-1.87437	-1.88219	-1.87802	-1.87386	-1.8697	-1.86553
-1.88666	-1.8825	-1.87832	-1.87413	-1.88192	-1.87771	-1.87354	-1.86939	-1.86524
-1.88641	-1.88224	-1.87807	-1.87389	-1.88165	-1.8774	-1.87322	-1.86909	-1.86495
-1.88613	-1.88193	-1.87776	-1.8736	-1.88137	-1.87714	-1.87296	-1.86882	-1.86469
-1.88583	-1.8816	-1.87744	-1.87329	-1.88109	-1.87689	-1.87272	-1.86858	-1.86444
-1.88555	-1.88133	-1.87717	-1.87303	-1.88084	-1.87665	-1.87247	-1.8683	-1.86415
-1.88528	-1.88109	-1.87694	-1.87281	-1.88061	-1.87641	-1.87221	-1.868	-1.86382
-1.88504	-1.88086	-1.87672	-1.87259	-1.88039	-1.87618	-1.87196	-1.86773	-1.86353
-1.88481	-1.88064	-1.8765	-1.87236	-1.88017	-1.87597	-1.87175	-1.8675	-1.86329
-1.86902	-1.88041	-1.87625	-1.87211	-1.87992	-1.87574	-1.87152	-1.86727	-1.90546
-1.86878	-1.88016	-1.87597	-1.87179	-1.8796	-1.87544	-1.87125	-1.86703	-1.90523
-1.86855	-1.87991	-1.87568	-1.87147	-1.87927	-1.87513	-1.87097	-1.8668	-1.90501
-1.86834	-1.87968	-1.87544	-1.87121	-1.879	-1.87487	-1.87073	-1.86657	-1.90479
-1.86813	-1.87948	-1.87522	-1.87098	-1.87877	-1.87465	-1.87051	-1.86636	-1.90456
-1.86791	-1.87927	-1.87501	-1.87076	-1.87855	-1.87442	-1.87029	-1.86615	-1.90433
-1.8677	-1.87906	-1.87481	-1.87057	-1.87835	-1.87421	-1.87007	-1.86593	-1.9041
-1.86748	-1.86333	-1.85915	-1.85497	-1.87818	-1.87402	-1.90072	-1.89648	-1.90387
-1.86727	-1.86314	-1.859	-1.85486	-1.87808	-1.8739	-1.90055	-1.89627	-1.90363

-1.86707	-1.86295	-1.85885	-1.85474	-1.87797	-1.87377	-1.90038	-1.89606	-1.9034
-1.86688	-1.86274	-1.85864	-1.85456	-1.90879	-1.9045	-1.90019	-1.89586	-1.90317
-1.8667	-1.86252	-1.85842	-1.85434	-1.90858	-1.9043	-1.89999	-1.89566	-1.90297
-1.86651	-1.86231	-1.8582	-1.85413	-1.89349	-1.88926	-1.8998	-1.8955	-1.90282
-1.86631	-1.86212	-1.85799	-1.8539	-1.89325	-1.88904	-1.89962	-1.89537	-1.90272
-1.86615	-1.90576	-1.90151	-1.89728	-1.89305	-1.88883	-1.91109	-1.90684	-1.90256
-1.86602	-1.90566	-1.9014	-1.89714	-1.89288	-1.88863	-1.91087	-1.90661	-1.90234
-1.86587	-1.90552	-1.90126	-1.897	-1.89272	-1.88845	-1.91068	-1.90642	-1.90214
-1.86566	-1.90531	-1.90106	-1.89682	-1.89257	-1.88832	-1.91056	-1.90628	-1.90198
-1.86546	-1.90508	-1.90085	-1.89662	-1.8924	-1.88819	-1.91044	-1.90614	-1.90184
-1.89729	-1.90492	-1.90066	-1.89641	-1.89219	-1.88799	-1.89561	-1.89136	-1.90167
-1.89717	-1.90477	-1.90047	-1.8962	-1.89197	-1.88778	-1.8954	-1.89117	-1.90151
-1.89702	-1.90461	-1.90031	-1.89604	-1.89181	-1.88762	-1.89525	-1.89102	-1.90137
-1.89686	-1.90443	-1.90016	-1.89592	-1.89171	-1.88752	-1.89514	-1.89091	-1.90125
-1.86409	-1.87215	-1.86804	-1.86396	-1.89161	-1.88742	-1.89503	-1.89079	-1.90112
-1.86395	-1.87205	-1.86795	-1.86387	-1.89152	-1.88732	-1.89492	-1.89067	-1.90099
-1.86383	-1.87195	-1.86786	-1.86378	-1.89143	-1.88721	-1.8948	-1.89053	-1.88625
-1.86373	-1.87186	-1.86777	-1.86369	-1.89132	-1.88709	-1.89466	-1.89037	-1.88608
-1.86364	-1.87177	-1.86768	-1.86358	-1.89121	-1.88696	-1.89451	-1.8902	-1.88591
-1.86354	-1.87167	-1.86757	-1.86347	-1.90296	-1.89865	-1.89435	-1.89005	-1.88578
-1.86344	-1.87156	-1.86745	-1.86333	-1.9028	-1.89848	-1.89418	-1.88992	-1.88566
-1.86335	-1.87146	-1.86731	-1.86317	-1.90264	-1.89833	-1.89405	-1.8898	-1.88556
-1.86326	-1.87135	-1.86717	-1.86299	-1.90246	-1.89819	-1.89394	-1.88971	-1.88548
-1.86317	-1.87125	-1.86706	-1.86287	-1.90235	-1.89808	-1.89383	-1.88961	-1.88539
-1.86309	-1.87118	-1.86702	-1.86286	-1.9023	-1.89799	-1.89372	-1.88951	-1.88529
-1.863	-1.87112	-1.86699	-1.86286	-1.90228	-1.89792	-1.89364	-1.88941	-1.88519
-1.86291	-1.87103	-1.86693	-1.86282	-1.90226	-1.89792	-1.89362	-1.88934	-1.88509
-1.86283	-1.87095	-1.86685	-1.86275	-1.90224	-1.89794	-1.89362	-1.88929	-1.885
-1.86273	-1.87086	-1.86676	-1.86268	-1.90218	-1.8979	-1.8936	-1.88927	-1.88497
-1.86263	-1.87076	-1.86667	-1.86259	-1.90209	-1.89783	-1.89356	-1.88928	-1.88499
-1.86255	-1.87067	-1.86657	-1.86249	-1.902	-1.89775	-1.8935	-1.88925	-1.88498
-1.86251	-1.87057	-1.86646	-1.86239	-1.9019	-1.89765	-1.89341	-1.88918	-1.88493
-1.87471	-1.88285	-1.87869	-1.87456	-1.9018	-1.89756	-1.89332	-1.8891	-1.88486
-1.87471	-1.88285	-1.87865	-1.87448	-1.9017	-1.89747	-1.89323	-1.889	-1.88476
-1.87473	-1.88288	-1.87864	-1.87442	-1.90163	-1.89741	-1.89317	-1.88892	-1.88468
-1.87477	-1.88294	-1.87868	-1.87443	-1.90163	-1.89741	-1.89316	-1.88891	-1.88466
-1.8748	-1.88299	-1.87873	-1.87447	-1.90167	-1.89743	-1.89318	-1.88894	-1.88467
-1.86145	-1.88298	-1.87875	-1.87451	-1.90168	-1.89741	-1.89315	-1.88892	-1.88466
-1.86136	-1.88292	-1.87873	-1.87454	-1.90168	-1.89735	-1.89308	-1.88887	-1.88464
-1.86129	-1.88285	-1.8787	-1.87454	-1.90168	-1.89733	-1.89305	-1.88885	-1.88463
-1.86123	-1.88276	-1.87862	-1.87449	-1.90167	-1.89735	-1.89308	-1.88887	-1.88465
-1.86121	-1.86955	-1.86544	-1.86136	-1.87032	-1.86615	-1.89311	-1.88888	-1.88465
-1.86124	-1.86957	-1.86546	-1.86139	-1.87035	-1.86619	-1.89313	-1.88884	-1.88459
-1.86128	-1.86962	-1.86551	-1.86143	-1.87039	-1.86623	-1.89314	-1.88881	-1.88453
-1.8613	-1.86966	-1.86555	-1.86146	-1.87043	-1.86628	-1.89319	-1.88884	-1.88454
-1.8613	-1.86969	-1.86558	-1.86149	-1.87047	-1.86633	-1.89324	-1.88889	-1.88459
-1.87387	-1.86972	-1.86558	-1.86146	-1.85736	-1.85327	-1.89326	-1.88893	-1.88465
-1.8739	-1.86973	-1.86556	-1.86139	-1.85728	-1.85321	-1.89325	-1.88897	-1.88471
-1.87393	-1.86976	-1.86555	-1.86136	-1.85725	-1.85319	-1.89326	-1.889	-1.88477
-1.87398	-1.8698	-1.86558	-1.86138	-1.85727	-1.85321	-1.89329	-1.88905	-1.88483

-1.87403	-1.86985	-1.86563	-1.86143	-1.85731	-1.85325	-1.88063	-1.87643	-1.88488
-1.87408	-1.86991	-1.86569	-1.86148	-1.85736	-1.85328	-1.88067	-1.87648	-1.88493
-1.87413	-1.86997	-1.86576	-1.86156	-1.85742	-1.85334	-1.88072	-1.87652	-1.88498
-1.87418	-1.87003	-1.86585	-1.86168	-1.85755	-1.85344	-1.8808	-1.87658	-1.88502
-1.87423	-1.8701	-1.86597	-1.86183	-1.8577	-1.85357	-1.8809	-1.87664	-1.88506
-1.87429	-1.87015	-1.86604	-1.86193	-1.85781	-1.85367	-1.84953	-1.84537	-1.87245
-1.87436	-1.87019	-1.86608	-1.86198	-1.85786	-1.85373	-1.84958	-1.84542	-1.87249
-1.87443	-1.87024	-1.86612	-1.86202	-1.85791	-1.85378	-1.84965	-1.8455	-1.87258
-1.87449	-1.87029	-1.86616	-1.86206	-1.85794	-1.85383	-1.84972	-1.84562	-1.87274
-1.87457	-1.87037	-1.86623	-1.86211	-1.85799	-1.85388	-1.8498	-1.84574	-1.87289
-1.87469	-1.8705	-1.86636	-1.86222	-1.85808	-1.85394	-1.84985	-1.8458	-1.87296
-1.87483	-1.87067	-1.86651	-1.86236	-1.85819	-1.85401	-1.8499	-1.84584	-1.873
-1.87498	-1.87082	-1.86664	-1.86246	-1.85829	-1.85412	-1.85001	-1.84594	-1.87309
-1.87514	-1.87096	-1.86675	-1.86254	-1.85839	-1.85426	-1.85016	-1.84608	-1.87322
-1.87526	-1.87107	-1.86684	-1.86262	-1.85847	-1.85437	-1.85028	-1.8462	-1.87334
-1.87532	-1.87114	-1.86691	-1.86268	-1.85853	-1.85443	-1.85035	-1.84629	-1.87346
-1.87538	-1.87121	-1.86699	-1.86277	-1.85863	-1.85452	-1.85045	-1.84641	-1.8736
-1.87547	-1.87129	-1.8671	-1.86293	-1.8588	-1.8547	-1.85062	-1.84657	-1.87376
-1.87558	-1.87139	-1.86724	-1.86311	-1.85899	-1.85489	-1.85081	-1.84674	-1.87393
-1.87571	-1.87154	-1.86741	-1.8633	-1.85918	-1.85508	-1.85099	-1.84691	-1.85949
-1.87585	-1.87171	-1.86759	-1.86348	-1.85937	-1.85526	-1.85116	-1.84707	-1.85963
-1.87602	-1.87189	-1.86777	-1.86367	-1.85955	-1.85544	-1.85132	-1.84721	-1.85975
-1.8762	-1.87207	-1.86796	-1.86384	-1.85972	-1.8556	-1.85146	-1.84732	-1.85986
-1.87638	-1.87225	-1.86813	-1.86401	-1.85988	-1.85574	-1.86833	-1.86415	-1.85999
-1.87655	-1.87243	-1.8683	-1.86417	-1.86001	-1.85585	-1.86845	-1.86429	-1.86015
-1.87672	-1.8726	-1.86846	-1.86431	-1.87696	-1.87275	-1.86858	-1.86445	-1.86033
-1.8769	-1.87276	-1.86859	-1.86442	-1.87708	-1.87289	-1.86874	-1.86463	-1.86052
-1.87708	-1.87293	-1.86873	-1.86454	-1.87721	-1.87305	-1.86892	-1.86482	-1.86071
-1.87727	-1.87312	-1.86894	-1.86475	-1.87741	-1.87323	-1.86909	-1.86499	-1.89064
-1.87745	-1.87333	-1.86918	-1.86503	-1.87766	-1.87343	-1.86926	-1.86516	-1.89081
-1.87763	-1.89045	-1.88629	-1.88212	-1.8779	-1.87366	-1.86948	-1.86536	-1.89098
-1.87781	-1.89063	-1.88648	-1.88232	-1.87814	-1.87395	-1.86976	-1.86557	-1.89115
-1.87799	-1.8908	-1.88666	-1.88251	-1.87836	-1.87421	-1.87003	-1.8658	-1.89135
-1.87815	-1.89097	-1.88683	-1.88269	-1.87856	-1.87442	-1.87026	-1.86608	-1.89162
-1.87832	-1.89114	-1.887	-1.88287	-1.87874	-1.87462	-1.87048	-1.86634	-1.8919
-1.89549	-1.8913	-1.88716	-1.88304	-1.87892	-1.8748	-1.87068	-1.86655	-1.89213
-1.89572	-1.89148	-1.88733	-1.88321	-1.87909	-1.87497	-1.87085	-1.86674	-1.89234
-1.89597	-1.89172	-1.88754	-1.88339	-1.87926	-1.87515	-1.90094	-1.89673	-1.90626
-1.89624	-1.89201	-1.88778	-1.88357	-1.87943	-1.87534	-1.90112	-1.8969	-1.90644
-1.89648	-1.89228	-1.88803	-1.8838	-1.87966	-1.87557	-1.90135	-1.89712	-1.90666
-1.89668	-1.89251	-1.88829	-1.88409	-1.87995	-1.87585	-1.90162	-1.89741	-1.90694
-1.8969	-1.89275	-1.88857	-1.88439	-1.88025	-1.87612	-1.90188	-1.89768	-1.90721
-1.89718	-1.89303	-1.88886	-1.8847	-1.88052	-1.87635	-1.90209	-1.89791	-1.90746
-1.89747	-1.8933	-1.88915	-1.885	-1.8808	-1.87658	-1.90231	-1.89813	-1.9077
-1.89772	-1.89353	-1.88938	-1.88525	-1.88106	-1.87686	-1.90258	-1.89839	-1.90797
-1.89797	-1.89374	-1.88959	-1.88548	-1.88132	-1.87715	-1.90288	-1.89867	-1.90825
-1.89825	-1.89401	-1.88986	-1.88575	-1.88161	-1.87745	-1.90317	-1.89893	-1.9085
-1.89858	-1.89433	-1.89018	-1.88606	-1.88191	-1.87776	-1.90345	-1.89916	-1.90871
-1.89889	-1.89465	-1.89049	-1.88636	-1.88222	-1.87807	-1.90375	-1.89943	-1.90896
-1.89918	-1.89496	-1.89081	-1.88667	-1.88253	-1.87839	-1.90407	-1.89974	-1.90926

-1.89947	-1.89528	-1.89111	-1.88696	-1.91288	-1.90866	-1.91829	-1.91391	-1.90958
-1.89977	-1.89557	-1.89137	-1.88718	-1.91309	-1.90889	-1.91856	-1.91422	-1.90991
-1.90008	-1.89587	-1.89162	-1.88739	-1.9133	-1.90912	-1.91881	-1.91452	-1.91024
-1.9004	-1.89618	-1.89192	-1.88768	-1.91357	-1.90939	-1.9191	-1.91483	-1.91057
-1.90072	-1.89651	-1.89224	-1.888	-1.91388	-1.90969	-1.91941	-1.91516	-1.9109
-1.90105	-1.89683	-1.89257	-1.88833	-1.91419	-1.90999	-1.91972	-1.91548	-1.91123
-1.90137	-1.89716	-1.89291	-1.88866	-1.91452	-1.9103	-1.92004	-1.9158	-1.91156
-1.87224	-1.8975	-1.89326	-1.88903	-1.91488	-1.91065	-1.92038	-1.91613	-1.91188
-1.87256	-1.89784	-1.89365	-1.88946	-1.9153	-1.91106	-1.92077	-1.91647	-1.91219
-1.87289	-1.89819	-1.89403	-1.88987	-1.91572	-1.91146	-1.92115	-1.91682	-1.91251
-1.8732	-1.89851	-1.89436	-1.89021	-1.91607	-1.91182	-1.92151	-1.91716	-1.91284
-1.87351	-1.89882	-1.89467	-1.89053	-1.9164	-1.91216	-1.92186	-1.9175	-1.91318
-1.87385	-1.86973	-1.86564	-1.86156	-1.91672	-1.91249	-1.92222	-1.91788	-1.91357
-1.87421	-1.87005	-1.86595	-1.86187	-1.91703	-1.91281	-1.92257	-1.91829	-1.91401
-1.85875	-1.87043	-1.86631	-1.86222	-1.91737	-1.91314	-1.92291	-1.91866	-1.91441
-1.85915	-1.87086	-1.86674	-1.86263	-1.91775	-1.91348	-1.92324	-1.91899	-1.91474
-1.85953	-1.90228	-1.89807	-1.89385	-1.90408	-1.89982	-1.92359	-1.91934	-1.91509
-1.85985	-1.9026	-1.8984	-1.89421	-1.90447	-1.90023	-1.92402	-1.91975	-1.91548
-1.86017	-1.90293	-1.89873	-1.89455	-1.90484	-1.90064	-1.92445	-1.92017	-1.91589
-1.86056	-1.90331	-1.89908	-1.89488	-1.90517	-1.90098	-1.92481	-1.92055	-1.91628
-1.86097	-1.90371	-1.89945	-1.89521	-1.90549	-1.9013	-1.92515	-1.92091	-1.91666
-1.86136	-1.90409	-1.89983	-1.89559	-1.90588	-1.90169	-1.89751	-1.89335	-1.91707
-1.86173	-1.90446	-1.90022	-1.89601	-1.90632	-1.90213	-1.89795	-1.89378	-1.9175
-1.86212	-1.90486	-1.90064	-1.89645	-1.90677	-1.90258	-1.89839	-1.89421	-1.91792
-1.86252	-1.90528	-1.90108	-1.8969	-1.90723	-1.90303	-1.89883	-1.89464	-1.91835
-1.86293	-1.89023	-1.88608	-1.88195	-1.90769	-1.90348	-1.89927	-1.89506	-1.89084
-1.86336	-1.89067	-1.88652	-1.88239	-1.90813	-1.90391	-1.89968	-1.89544	-1.89121
-1.86379	-1.89111	-1.88696	-1.88283	-1.90857	-1.90433	-1.90008	-1.89582	-1.89159
-1.86423	-1.89155	-1.88739	-1.88324	-1.90898	-1.90472	-1.90047	-1.89622	-1.892
-1.86467	-1.89199	-1.88782	-1.88365	-1.90938	-1.9051	-1.90085	-1.89663	-1.89243
-1.89658	-1.90728	-1.90304	-1.89881	-1.90978	-1.9055	-1.90126	-1.89705	-1.89287
-1.897	-1.90769	-1.90343	-1.89917	-1.91016	-1.90592	-1.9017	-1.8975	-1.89332
-1.8974	-1.90808	-1.90382	-1.89956	-1.91057	-1.90635	-1.90214	-1.89795	-1.89377
-1.89778	-1.90845	-1.9042	-1.89998	-1.91101	-1.9068	-1.90259	-1.89841	-1.89423
-1.89819	-1.90885	-1.90461	-1.90041	-1.91146	-1.90725	-1.90306	-1.89887	-1.89469
-1.89868	-1.90936	-1.90509	-1.90086	-1.89667	-1.89252	-1.90352	-1.89932	-1.89513
-1.89921	-1.90991	-1.90561	-1.90133	-1.89714	-1.893	-1.904	-1.89979	-1.89559
-1.8997	-1.91042	-1.90614	-1.90187	-1.89767	-1.89352	-1.90453	-1.90032	-1.89611
-1.90016	-1.91092	-1.90668	-1.90246	-1.89826	-1.89407	-1.90509	-1.90089	-1.89667
-1.90062	-1.91139	-1.90719	-1.90299	-1.89879	-1.89459	-1.90561	-1.90142	-1.8972
-1.90106	-1.91184	-1.90764	-1.90345	-1.89926	-1.89506	-1.90609	-1.9019	-1.8977
-1.90152	-1.9123	-1.9081	-1.90391	-1.89972	-1.89553	-1.89136	-1.8872	-1.89817
-1.90197	-1.91278	-1.90856	-1.90436	-1.90017	-1.896	-1.89182	-1.88764	-1.89861
-1.90245	-1.91327	-1.90905	-1.90483	-1.90064	-1.89648	-1.8923	-1.8881	-1.89907
-1.91801	-1.91381	-1.90959	-1.90538	-1.90119	-1.89701	-1.89283	-1.88864	-1.89961
-1.91858	-1.91437	-1.91016	-1.90596	-1.90176	-1.89756	-1.89338	-1.88921	-1.90018
-1.905	-1.91488	-1.91068	-1.90649	-1.90228	-1.89807	-1.89388	-1.88972	-1.90071
-1.90547	-1.91535	-1.91116	-1.90697	-1.90276	-1.89854	-1.89435	-1.89021	-1.90122
-1.89107	-1.91587	-1.91168	-1.90749	-1.90327	-1.89904	-1.89486	-1.89071	-1.88655
-1.89163	-1.91644	-1.91224	-1.90805	-1.90383	-1.8996	-1.89541	-1.89124	-1.88708

-1.89219	-1.90294	-1.89876	-1.89458	-1.9044	-1.90017	-1.89596	-1.89177	-1.8876
-1.89276	-1.90352	-1.89933	-1.89515	-1.90497	-1.90075	-1.89652	-1.89227	-1.88806
-1.89331	-1.90408	-1.89989	-1.8957	-1.90552	-1.90131	-1.89706	-1.89277	-1.88853
-1.88091	-1.90458	-1.90038	-1.89618	-1.89201	-1.88784	-1.89758	-1.8933	-1.88907
-1.88137	-1.90505	-1.90084	-1.89664	-1.89247	-1.88832	-1.89809	-1.89385	-1.88965
-1.88187	-1.89064	-1.88647	-1.88231	-1.89298	-1.88883	-1.89862	-1.89441	-1.89022
-1.88242	-1.89118	-1.88703	-1.88287	-1.89354	-1.88938	-1.89918	-1.89498	-1.8908
-1.88298	-1.89174	-1.88759	-1.88344	-1.89412	-1.88994	-1.88578	-1.88162	-1.89138
-1.88354	-1.89231	-1.88816	-1.88402	-1.89469	-1.89052	-1.88635	-1.88219	-1.89196
-1.88411	-1.89289	-1.88874	-1.88459	-1.89528	-1.8911	-1.88693	-1.88276	-1.89254
-1.88468	-1.89346	-1.88931	-1.88517	-1.89585	-1.89167	-1.8875	-1.88334	-1.87919
-1.88524	-1.89403	-1.88988	-1.88574	-1.89643	-1.89225	-1.88808	-1.88391	-1.87976
-1.85267	-1.89459	-1.89045	-1.8863	-1.88216	-1.87802	-1.88865	-1.88448	-1.88033
-1.85322	-1.89515	-1.89101	-1.88687	-1.88273	-1.87859	-1.88923	-1.88505	-1.8809
-1.85378	-1.89572	-1.89158	-1.88744	-1.8833	-1.87917	-1.88981	-1.88564	-1.88148
-1.85434	-1.89628	-1.89215	-1.88802	-1.88388	-1.87974	-1.89039	-1.88623	-1.88207
-1.85493	-1.88399	-1.8799	-1.87583	-1.88449	-1.88034	-1.87621	-1.8721	-1.8827
-1.85559	-1.88465	-1.88057	-1.8765	-1.88517	-1.88101	-1.87688	-1.87278	-1.88338
-1.85624	-1.88532	-1.88123	-1.87716	-1.88583	-1.88168	-1.87755	-1.87345	-1.88406
-1.85684	-1.85281	-1.84883	-1.84485	-1.88643	-1.88229	-1.87816	-1.87405	-1.88467
-1.8574	-1.85338	-1.84939	-1.8454	-1.887	-1.88287	-1.87874	-1.87463	-1.88525
-1.85797	-1.85395	-1.84995	-1.84595	-1.88757	-1.88345	-1.87932	-1.8752	-1.87108
-1.85854	-1.85453	-1.85051	-1.84651	-1.88814	-1.88402	-1.8799	-1.87576	-1.87165
-1.85914	-1.85513	-1.85111	-1.84711	-1.88875	-1.88464	-1.88052	-1.87638	-1.87226
-1.8598	-1.85579	-1.85177	-1.84776	-1.88943	-1.88533	-1.88121	-1.87707	-1.87294
-1.86045	-1.85643	-1.85242	-1.84841	-1.89009	-1.886	-1.88188	-1.87776	-1.87364
-1.86103	-1.857	-1.853	-1.849	-1.8907	-1.88659	-1.8825	-1.87843	-1.87434
-1.86161	-1.85758	-1.85358	-1.8496	-1.8913	-1.88718	-1.8831	-1.87908	-1.87502
-1.86225	-1.85822	-1.85422	-1.85023	-1.89195	-1.88782	-1.88374	-1.8797	-1.87563
-1.86293	-1.8589	-1.85489	-1.85089	-1.89263	-1.8885	-1.88438	-1.88029	-1.87619
-1.86358	-1.85958	-1.85557	-1.85156	-1.84756	-1.84358	-1.88505	-1.88093	-1.87682
-1.86422	-1.86026	-1.85626	-1.85224	-1.84824	-1.84426	-1.88575	-1.88162	-1.8775
-1.86484	-1.86091	-1.85692	-1.85292	-1.84893	-1.84493	-1.88645	-1.88232	-1.87819
-1.86544	-1.86148	-1.85754	-1.8536	-1.84962	-1.84562	-1.88714	-1.88301	-1.87889
-1.86605	-1.86206	-1.85815	-1.85425	-1.85029	-1.8463	-1.88784	-1.88371	-1.87959
-1.8667	-1.8627	-1.85876	-1.85484	-1.8509	-1.84696	-1.88855	-1.88441	-1.88028
-1.86738	-1.86336	-1.85937	-1.85541	-1.85149	-1.84761	-1.88925	-1.88511	-1.88097
-1.86806	-1.86403	-1.86002	-1.85603	-1.85211	-1.84822	-1.88989	-1.88578	-1.88167
-1.86875	-1.86471	-1.86069	-1.8567	-1.85274	-1.84881	-1.89049	-1.88644	-1.88236
-1.86944	-1.8654	-1.86138	-1.85738	-1.85341	-1.84947	-1.8784	-1.8744	-1.88305
-1.87013	-1.8661	-1.86208	-1.85809	-1.85413	-1.8502	-1.87915	-1.8751	-1.88373
-1.87083	-1.86681	-1.8628	-1.85882	-1.85487	-1.85094	-1.87988	-1.87581	-1.88442
-1.87154	-1.86753	-1.86354	-1.85957	-1.85561	-1.85166	-1.88059	-1.8765	-1.88511
-1.87226	-1.86826	-1.86428	-1.86032	-1.85635	-1.85237	-1.88129	-1.87719	-1.88581
-1.873	-1.86901	-1.86502	-1.86104	-1.85705	-1.85305	-1.84905	-1.84505	-1.88651
-1.87375	-1.86976	-1.86576	-1.86175	-1.85774	-1.85372	-1.84972	-1.84574	-1.88722
-1.87449	-1.87049	-1.86647	-1.86244	-1.85842	-1.85441	-1.85041	-1.84643	-1.87522
-1.87523	-1.8712	-1.86716	-1.86312	-1.8591	-1.85509	-1.85111	-1.84714	-1.87595
-1.87596	-1.87191	-1.86785	-1.86381	-1.85979	-1.85579	-1.85181	-1.84785	-1.8767
-1.87667	-1.87261	-1.86855	-1.8645	-1.86048	-1.85649	-1.85253	-1.84859	-1.87746

-1.8774	-1.87334	-1.86928	-1.86523	-1.86121	-1.85722	-1.85326	-1.84933	-1.87822
-1.87815	-1.87412	-1.87007	-1.86603	-1.86201	-1.858	-1.85401	-1.85006	-1.87894
-1.87892	-1.8749	-1.87087	-1.86684	-1.86282	-1.85879	-1.85479	-1.8508	-1.87966
-1.87967	-1.87564	-1.87161	-1.86759	-1.86358	-1.85958	-1.85558	-1.85158	-1.88045
-1.88042	-1.87636	-1.87232	-1.86831	-1.86433	-1.86036	-1.85638	-1.85239	-1.88127
-1.8812	-1.87713	-1.87309	-1.86907	-1.86508	-1.86111	-1.85713	-1.85315	-1.84917
-1.88202	-1.87794	-1.87389	-1.86985	-1.86583	-1.86182	-1.85783	-1.85386	-1.8499
-1.88279	-1.87872	-1.87467	-1.87063	-1.8666	-1.86257	-1.85857	-1.8546	-1.85065
-1.8835	-1.87945	-1.87543	-1.87142	-1.8674	-1.86338	-1.85937	-1.85538	-1.85142
-1.88423	-1.88018	-1.87618	-1.8722	-1.86819	-1.86417	-1.86017	-1.85617	-1.85221
-1.885	-1.88093	-1.87691	-1.87291	-1.8689	-1.8649	-1.86091	-1.85695	-1.85299
-1.8858	-1.88171	-1.87766	-1.87362	-1.86961	-1.86562	-1.86165	-1.85772	-1.85377
-1.88658	-1.88251	-1.87844	-1.87439	-1.87037	-1.86638	-1.86243	-1.85851	-1.85457
-1.88737	-1.88333	-1.87926	-1.8752	-1.87117	-1.86718	-1.86322	-1.85931	-1.85537
-1.88817	-1.88415	-1.88008	-1.876	-1.87197	-1.86797	-1.86402	-1.86012	-1.85618
-1.88898	-1.88497	-1.88089	-1.87681	-1.87278	-1.86878	-1.86482	-1.86092	-1.85698
-1.8898	-1.88579	-1.88171	-1.87762	-1.87359	-1.86959	-1.86563	-1.86173	-1.85779
-1.89062	-1.88661	-1.88252	-1.87843	-1.87439	-1.87039	-1.86644	-1.86253	-1.85859
-1.89144	-1.88743	-1.88333	-1.87923	-1.8752	-1.8712	-1.86724	-1.86334	-1.8594
-1.87417	-1.88825	-1.88414	-1.88004	-1.876	-1.872	-1.86805	-1.86415	-1.86022
-1.87501	-1.88907	-1.88496	-1.88085	-1.8768	-1.8728	-1.86886	-1.86497	-1.86104
-1.87585	-1.87185	-1.86777	-1.86367	-1.87762	-1.87361	-1.86966	-1.86578	-1.86188
-1.8767	-1.8727	-1.86862	-1.86453	-1.87845	-1.87442	-1.87047	-1.8666	-1.86272
-1.87756	-1.87356	-1.8695	-1.86543	-1.87933	-1.87527	-1.87131	-1.86743	-1.86355
-1.87844	-1.87443	-1.87041	-1.86638	-1.88026	-1.87618	-1.87218	-1.86826	-1.86436
-1.87932	-1.87529	-1.8713	-1.8673	-1.86326	-1.85919	-1.87306	-1.8691	-1.86517
-1.88017	-1.87613	-1.87215	-1.86818	-1.86414	-1.86007	-1.8739	-1.86991	-1.86597
-1.88104	-1.87698	-1.873	-1.86904	-1.86501	-1.86095	-1.87476	-1.87074	-1.86678
-1.88196	-1.87787	-1.87387	-1.8699	-1.86589	-1.86187	-1.85785	-1.85384	-1.86766
-1.8829	-1.8788	-1.87476	-1.87076	-1.86677	-1.8628	-1.85881	-1.85479	-1.86858
-1.88381	-1.87969	-1.87563	-1.87161	-1.86763	-1.86369	-1.85973	-1.85574	-1.85175
-1.88468	-1.88057	-1.87648	-1.87244	-1.86847	-1.86455	-1.86062	-1.85668	-1.85272
-1.8531	-1.88147	-1.87738	-1.87332	-1.86934	-1.86542	-1.8615	-1.85759	-1.85366
-1.85399	-1.88243	-1.87834	-1.87427	-1.87025	-1.86628	-1.86234	-1.85843	-1.85452
-1.8356	-1.85092	-1.84691	-1.8429	-1.8712	-1.86719	-1.86322	-1.8593	-1.85539
-1.83646	-1.85178	-1.84781	-1.84385	-1.8722	-1.8682	-1.86422	-1.86025	-1.8563
-1.83732	-1.85263	-1.8487	-1.84479	-1.8732	-1.86923	-1.86524	-1.86122	-1.85723
-1.83823	-1.85352	-1.84956	-1.84564	-1.84174	-1.83785	-1.86617	-1.86217	-1.85817
-1.83916	-1.85443	-1.85042	-1.84645	-1.84254	-1.83869	-1.86705	-1.86309	-1.85912
-1.84008	-1.85536	-1.85132	-1.84731	-1.84339	-1.83952	-1.83564	-1.83176	-1.86002
-1.84098	-1.85631	-1.85226	-1.84822	-1.84426	-1.84034	-1.83644	-1.83256	-1.86088
-1.84187	-1.83792	-1.83393	-1.82993	-1.84517	-1.84122	-1.8373	-1.83341	-1.82955
-1.84273	-1.83878	-1.83482	-1.83087	-1.84614	-1.8422	-1.83827	-1.83434	-1.83044
-1.84362	-1.83965	-1.83572	-1.83181	-1.84711	-1.84319	-1.83925	-1.83529	-1.83136
-1.84459	-1.84062	-1.83667	-1.83274	-1.84803	-1.84409	-1.84015	-1.83621	-1.8323
-1.84558	-1.84162	-1.83764	-1.83367	-1.84894	-1.84497	-1.84103	-1.83713	-1.83325
-1.8465	-1.84254	-1.83857	-1.83461	-1.84989	-1.84592	-1.84198	-1.83807	-1.83417
-1.84739	-1.84342	-1.83948	-1.83555	-1.85087	-1.84692	-1.84296	-1.83901	-1.83508
-1.84833	-1.84435	-1.84041	-1.83649	-1.83257	-1.82867	-1.84391	-1.83996	-1.83602
-1.84932	-1.84535	-1.84138	-1.83742	-1.83347	-1.82955	-1.84481	-1.84089	-1.83699

-1.8503	-1.84633	-1.84234	-1.83836	-1.83439	-1.83045	-1.84574	-1.84185	-1.83797
-1.85121	-1.84723	-1.84327	-1.83932	-1.83537	-1.83143	-1.84674	-1.84286	-1.83897
-1.85212	-1.84815	-1.84421	-1.8403	-1.83637	-1.83243	-1.84774	-1.84385	-1.83995
-1.8531	-1.84913	-1.84521	-1.8413	-1.83736	-1.8334	-1.8487	-1.84478	-1.84086
-1.85411	-1.85015	-1.84623	-1.84231	-1.83834	-1.83435	-1.84963	-1.84567	-1.84175
-1.82174	-1.85112	-1.84718	-1.84325	-1.83929	-1.83532	-1.83137	-1.82746	-1.84272
-1.8227	-1.85207	-1.84809	-1.84414	-1.8402	-1.83628	-1.83237	-1.82847	-1.84375
-1.82366	-1.85304	-1.84905	-1.84509	-1.84117	-1.83728	-1.83339	-1.8295	-1.84478
-1.8246	-1.85404	-1.85007	-1.84612	-1.84221	-1.83833	-1.83443	-1.83053	-1.84581
-1.80883	-1.8217	-1.81784	-1.81398	-1.84327	-1.83938	-1.83547	-1.83155	-1.82762
-1.80985	-1.82273	-1.81887	-1.81501	-1.84432	-1.84042	-1.83649	-1.83253	-1.82858
-1.8109	-1.82376	-1.81989	-1.81603	-1.84535	-1.84145	-1.8375	-1.8335	-1.82953
-1.81193	-1.80808	-1.80421	-1.80035	-1.82829	-1.82442	-1.83843	-1.83445	-1.83051
-1.81297	-1.80909	-1.80519	-1.80128	-1.82919	-1.82529	-1.83932	-1.8354	-1.83149
-1.81398	-1.81007	-1.80615	-1.80223	-1.79835	-1.7945	-1.82237	-1.81854	-1.83252
-1.81495	-1.81103	-1.80711	-1.8032	-1.79934	-1.79551	-1.8234	-1.81958	-1.83357
-1.81591	-1.81199	-1.80808	-1.80419	-1.80035	-1.79654	-1.82446	-1.82064	-1.83464
-1.81684	-1.81295	-1.80908	-1.80521	-1.80139	-1.79759	-1.82552	-1.8217	-1.83571
-1.81778	-1.81394	-1.81009	-1.80625	-1.80244	-1.79864	-1.82659	-1.82277	-1.83678
-1.81879	-1.81496	-1.81112	-1.8073	-1.80349	-1.79969	-1.81129	-1.80749	-1.82001
-1.81983	-1.816	-1.81217	-1.80836	-1.80455	-1.80075	-1.81235	-1.80855	-1.82107
-1.82088	-1.81704	-1.81322	-1.80941	-1.8056	-1.8018	-1.79802	-1.79425	-1.80582
-1.82194	-1.8181	-1.81428	-1.81047	-1.80666	-1.80286	-1.79907	-1.7953	-1.80688
-1.823	-1.81916	-1.81534	-1.81152	-1.80771	-1.80391	-1.80013	-1.79635	-1.80794
-1.82406	-1.82022	-1.81639	-1.81258	-1.80877	-1.80497	-1.80118	-1.7974	-1.80899
-1.82512	-1.82128	-1.81745	-1.81363	-1.80982	-1.80602	-1.80223	-1.79845	-1.79467
-1.82618	-1.82233	-1.8185	-1.81468	-1.81087	-1.80707	-1.80327	-1.79949	-1.79571
-1.82724	-1.82339	-1.81956	-1.81573	-1.81192	-1.80812	-1.80432	-1.80053	-1.79675
-1.8283	-1.82445	-1.82061	-1.81678	-1.81297	-1.80917	-1.80537	-1.80157	-1.79779
-1.82935	-1.82551	-1.82167	-1.81783	-1.81402	-1.81022	-1.80642	-1.80262	-1.79883
-1.83041	-1.82657	-1.82272	-1.81889	-1.81507	-1.81126	-1.80746	-1.80366	-1.79988
-1.83147	-1.82763	-1.82378	-1.81995	-1.81612	-1.81231	-1.8085	-1.80471	-1.80092
-1.83253	-1.82869	-1.82484	-1.821	-1.81717	-1.81336	-1.80955	-1.80575	-1.80196
-1.83358	-1.82974	-1.82589	-1.82206	-1.81823	-1.81441	-1.8106	-1.8068	-1.803
-1.83464	-1.83079	-1.82695	-1.82311	-1.81928	-1.81546	-1.81165	-1.80784	-1.80405
-1.8357	-1.83183	-1.82799	-1.82416	-1.82033	-1.81651	-1.81269	-1.80889	-1.80509
-1.83676	-1.83289	-1.82905	-1.82522	-1.82139	-1.81756	-1.81374	-1.80994	-1.80614
-1.83784	-1.83396	-1.83012	-1.82629	-1.82246	-1.81863	-1.81481	-1.81101	-1.8072
-1.83895	-1.83506	-1.83121	-1.82739	-1.82356	-1.81973	-1.8159	-1.81209	-1.80829
-1.84014	-1.83625	-1.8324	-1.82857	-1.82474	-1.82091	-1.81708	-1.81327	-1.80946
-1.84143	-1.83753	-1.83367	-1.82984	-1.82601	-1.82218	-1.81836	-1.81454	-1.81072
-1.84265	-1.83875	-1.83488	-1.83104	-1.82721	-1.82339	-1.81956	-1.81574	-1.81192
-1.84377	-1.83987	-1.836	-1.83214	-1.82831	-1.82449	-1.82067	-1.81684	-1.81302
-1.84486	-1.84095	-1.83707	-1.83321	-1.82937	-1.82556	-1.82173	-1.81791	-1.81408
-1.82426	-1.84202	-1.83813	-1.83425	-1.83041	-1.8266	-1.82277	-1.81895	-1.81512
-1.8253	-1.84307	-1.83917	-1.83529	-1.83145	-1.82762	-1.8238	-1.81997	-1.81615
-1.82636	-1.84414	-1.84024	-1.83635	-1.8325	-1.82867	-1.82484	-1.82101	-1.8172
-1.82744	-1.84523	-1.84132	-1.83743	-1.83357	-1.82973	-1.8259	-1.82207	-1.81826
-1.82859	-1.82472	-1.82087	-1.81703	-1.83472	-1.83088	-1.82705	-1.82322	-1.81941
-1.82985	-1.82597	-1.82211	-1.81827	-1.83598	-1.83213	-1.82829	-1.82447	-1.82065

-1.83106	-1.82719	-1.82333	-1.81948	-1.83721	-1.83334	-1.8295	-1.82568	-1.82186
-1.83218	-1.82832	-1.82445	-1.8206	-1.83833	-1.83446	-1.83061	-1.82679	-1.82297
-1.83329	-1.82944	-1.82557	-1.82172	-1.83946	-1.83558	-1.83173	-1.8279	-1.82408
-1.83442	-1.8306	-1.82677	-1.82293	-1.81909	-1.81526	-1.83295	-1.82911	-1.82528
-1.83556	-1.83178	-1.82798	-1.82417	-1.82034	-1.81649	-1.83419	-1.83035	-1.82652
-1.83672	-1.83293	-1.82913	-1.82533	-1.8215	-1.81765	-1.83536	-1.83151	-1.82767
-1.83789	-1.83406	-1.83025	-1.82644	-1.8226	-1.81874	-1.83646	-1.8326	-1.82876
-1.83904	-1.83518	-1.83137	-1.82757	-1.82374	-1.81989	-1.81606	-1.81226	-1.82991
-1.84014	-1.83629	-1.8325	-1.82873	-1.82493	-1.82112	-1.81731	-1.81349	-1.83115
-1.84128	-1.83743	-1.83365	-1.82989	-1.82611	-1.82232	-1.81852	-1.81469	-1.83237
-1.84253	-1.83867	-1.83485	-1.83104	-1.82724	-1.82344	-1.81963	-1.81579	-1.83349
-1.8438	-1.83994	-1.83607	-1.83221	-1.82837	-1.82455	-1.82072	-1.81689	-1.8346
-1.82829	-1.84116	-1.8373	-1.83344	-1.82959	-1.82575	-1.82192	-1.81808	-1.81427
-1.82945	-1.84236	-1.83853	-1.8347	-1.83085	-1.82699	-1.82314	-1.81932	-1.81551
-1.81066	-1.84354	-1.83971	-1.83587	-1.83201	-1.82815	-1.8243	-1.82048	-1.81669
-1.81183	-1.84468	-1.84082	-1.83697	-1.8331	-1.82923	-1.82539	-1.82157	-1.81779
-1.81302	-1.82915	-1.82531	-1.82147	-1.83425	-1.83039	-1.82655	-1.82272	-1.81895
-1.81429	-1.83041	-1.82657	-1.82273	-1.83552	-1.83165	-1.82779	-1.82396	-1.82016
-1.81552	-1.83166	-1.82782	-1.82399	-1.8368	-1.83292	-1.82907	-1.82523	-1.82141
-1.81662	-1.83278	-1.82897	-1.82518	-1.82138	-1.81757	-1.83036	-1.82653	-1.8227
-1.81771	-1.83389	-1.83011	-1.82635	-1.82259	-1.81881	-1.83164	-1.82782	-1.824
-1.81889	-1.83508	-1.83128	-1.8275	-1.82373	-1.81996	-1.8162	-1.81245	-1.82523
-1.82015	-1.83633	-1.83247	-1.82864	-1.82483	-1.82104	-1.81729	-1.81358	-1.82643
-1.80233	-1.8376	-1.83371	-1.82985	-1.82601	-1.82219	-1.81843	-1.81472	-1.81103
-1.80364	-1.83892	-1.83502	-1.83113	-1.82726	-1.82342	-1.81962	-1.81586	-1.81215
-1.8049	-1.82013	-1.81629	-1.81245	-1.82854	-1.82468	-1.82085	-1.81704	-1.8133
-1.80607	-1.82135	-1.81754	-1.81374	-1.82985	-1.82597	-1.82212	-1.81828	-1.8145
-1.80721	-1.82254	-1.81877	-1.815	-1.83114	-1.82727	-1.82341	-1.81955	-1.81574
-1.80837	-1.80463	-1.80092	-1.79721	-1.83239	-1.82855	-1.8247	-1.82083	-1.817
-1.80954	-1.80576	-1.80206	-1.79838	-1.83362	-1.82982	-1.82599	-1.82212	-1.81828
-1.81077	-1.80696	-1.80323	-1.79954	-1.81611	-1.81237	-1.82725	-1.8234	-1.81956
-1.81205	-1.80822	-1.80443	-1.80068	-1.81727	-1.81357	-1.82848	-1.82467	-1.82085
-1.81333	-1.80948	-1.80566	-1.80189	-1.7982	-1.79456	-1.82973	-1.82594	-1.82214
-1.81463	-1.81077	-1.80694	-1.80316	-1.79946	-1.79581	-1.83099	-1.82722	-1.82342
-1.78012	-1.81206	-1.80823	-1.80445	-1.80073	-1.79707	-1.81361	-1.80987	-1.82471
-1.78138	-1.81335	-1.80952	-1.80573	-1.80201	-1.79835	-1.81489	-1.81115	-1.82599
-1.78265	-1.81464	-1.81081	-1.80702	-1.8033	-1.79963	-1.81619	-1.81244	-1.82726
-1.78392	-1.81593	-1.8121	-1.8083	-1.80458	-1.80091	-1.81748	-1.81372	-1.80994
-1.78519	-1.81722	-1.81338	-1.80958	-1.80586	-1.80218	-1.81877	-1.815	-1.81122
-1.78645	-1.81851	-1.81467	-1.81086	-1.80713	-1.80346	-1.79976	-1.79605	-1.81249
-1.78771	-1.8198	-1.81595	-1.81214	-1.80841	-1.80473	-1.80103	-1.79731	-1.81376
-1.78896	-1.78517	-1.78141	-1.77769	-1.80968	-1.806	-1.80229	-1.79857	-1.81503
-1.79021	-1.78642	-1.78265	-1.77893	-1.81095	-1.80726	-1.80355	-1.79982	-1.8163
-1.79146	-1.78766	-1.78389	-1.78016	-1.81221	-1.80852	-1.80481	-1.80108	-1.79733
-1.79271	-1.78891	-1.78513	-1.7814	-1.81347	-1.80978	-1.80607	-1.80234	-1.79859
-1.79395	-1.79015	-1.78637	-1.78263	-1.81474	-1.81105	-1.80733	-1.8036	-1.79985
-1.7952	-1.7914	-1.78762	-1.78388	-1.81601	-1.81231	-1.80859	-1.80485	-1.8011
-1.79645	-1.79265	-1.78888	-1.78515	-1.8173	-1.81357	-1.80984	-1.8061	-1.80235
-1.79771	-1.79391	-1.79016	-1.78645	-1.78276	-1.7791	-1.81112	-1.80737	-1.80362
-1.79898	-1.79518	-1.79147	-1.7878	-1.78411	-1.78042	-1.81244	-1.80866	-1.80488

-1.80025	-1.79645	-1.79277	-1.78911	-1.78543	-1.78172	-1.81376	-1.80995	-1.80616
-1.80152	-1.79772	-1.79402	-1.79037	-1.78668	-1.78299	-1.81506	-1.81124	-1.80746
-1.80281	-1.799	-1.79529	-1.79162	-1.78794	-1.78426	-1.81636	-1.81255	-1.80877
-1.77722	-1.80035	-1.79663	-1.79293	-1.78923	-1.78553	-1.81767	-1.81388	-1.81012
-1.77848	-1.80172	-1.79798	-1.79425	-1.79053	-1.78681	-1.81899	-1.81523	-1.81149
-1.77969	-1.80303	-1.79928	-1.79555	-1.79183	-1.78813	-1.78444	-1.78078	-1.81284
-1.78088	-1.8043	-1.80055	-1.79683	-1.79314	-1.78947	-1.78579	-1.78211	-1.81419
-1.76154	-1.77857	-1.77505	-1.77154	-1.79441	-1.79076	-1.78709	-1.78341	-1.81552
-1.76281	-1.77984	-1.77627	-1.77272	-1.79565	-1.79199	-1.78833	-1.78467	-1.81683
-1.76409	-1.7811	-1.77751	-1.77393	-1.79692	-1.79324	-1.78958	-1.78593	-1.78229
-1.76534	-1.78235	-1.77876	-1.77519	-1.79826	-1.79455	-1.79086	-1.78719	-1.78354
-1.76659	-1.7836	-1.78003	-1.77648	-1.79961	-1.79588	-1.79217	-1.78846	-1.78479
-1.76786	-1.76431	-1.76078	-1.75727	-1.80096	-1.79719	-1.79347	-1.78978	-1.78611
-1.76914	-1.76559	-1.76206	-1.75854	-1.8023	-1.79851	-1.79478	-1.79111	-1.78746
-1.77042	-1.76687	-1.76332	-1.75979	-1.77672	-1.77316	-1.79612	-1.79246	-1.78881
-1.7717	-1.76813	-1.76457	-1.76102	-1.77797	-1.77444	-1.7975	-1.79382	-1.79017
-1.77296	-1.76939	-1.76582	-1.76227	-1.75877	-1.75529	-1.79887	-1.79519	-1.79153
-1.77421	-1.77063	-1.76708	-1.76356	-1.76006	-1.75657	-1.80024	-1.79656	-1.7929
-1.77546	-1.77188	-1.76835	-1.76485	-1.76135	-1.75786	-1.80161	-1.79793	-1.79426
-1.77673	-1.77317	-1.76964	-1.76613	-1.76263	-1.75915	-1.803	-1.79929	-1.79561
-1.77801	-1.77446	-1.77093	-1.76741	-1.76392	-1.76045	-1.80438	-1.80065	-1.79695
-1.77929	-1.77575	-1.77222	-1.7687	-1.76521	-1.76173	-1.75826	-1.7548	-1.79832
-1.78057	-1.77704	-1.77351	-1.77	-1.76649	-1.763	-1.75953	-1.75608	-1.79969
-1.78187	-1.77833	-1.7748	-1.77128	-1.76777	-1.76427	-1.7608	-1.75735	-1.80106
-1.78317	-1.77963	-1.77608	-1.77255	-1.76904	-1.76554	-1.76207	-1.75861	-1.8024
-1.78447	-1.78092	-1.77737	-1.77383	-1.77031	-1.76682	-1.76334	-1.75987	-1.78371
-1.78577	-1.78221	-1.77866	-1.77513	-1.7716	-1.76808	-1.76459	-1.76114	-1.78507
-1.78707	-1.7835	-1.77995	-1.77643	-1.77289	-1.76935	-1.76585	-1.76241	-1.78643
-1.72655	-1.78479	-1.78123	-1.77769	-1.77415	-1.77062	-1.76712	-1.76366	-1.76022
-1.72782	-1.78608	-1.78251	-1.77895	-1.77541	-1.7719	-1.7684	-1.76491	-1.76146
-1.70236	-1.74441	-1.74091	-1.73743	-1.7767	-1.77318	-1.76967	-1.76618	-1.76272
-1.70362	-1.74567	-1.74219	-1.73872	-1.77801	-1.77447	-1.77095	-1.76746	-1.764
-1.70491	-1.72818	-1.72473	-1.7213	-1.73656	-1.73309	-1.77227	-1.76878	-1.7653
-1.70626	-1.72955	-1.72609	-1.72264	-1.7379	-1.73444	-1.77365	-1.77014	-1.76665
-1.68085	-1.70416	-1.70073	-1.69732	-1.73923	-1.73578	-1.73233	-1.7289	-1.768
-1.68212	-1.70545	-1.70201	-1.69858	-1.74052	-1.73707	-1.73362	-1.73018	-1.7693
-1.68337	-1.70673	-1.70328	-1.69983	-1.74179	-1.73834	-1.7349	-1.73145	-1.7706
-1.6846	-1.68124	-1.67786	-1.6745	-1.69769	-1.69431	-1.73615	-1.73272	-1.72933
-1.68582	-1.68245	-1.67909	-1.67574	-1.69894	-1.69553	-1.73739	-1.734	-1.73062
-1.63862	-1.68372	-1.68037	-1.67703	-1.67368	-1.67033	-1.71245	-1.7091	-1.73192
-1.63992	-1.68507	-1.6817	-1.67836	-1.67501	-1.67167	-1.71379	-1.71041	-1.73322
-1.64119	-1.68638	-1.68301	-1.67965	-1.67631	-1.67298	-1.69614	-1.69279	-1.73454
-1.64241	-1.68764	-1.68426	-1.6809	-1.67756	-1.67425	-1.69745	-1.69412	-1.73591
-1.64364	-1.66369	-1.66037	-1.65705	-1.67883	-1.67552	-1.67224	-1.66898	-1.71102
-1.64492	-1.66499	-1.66167	-1.65836	-1.68014	-1.67681	-1.6735	-1.67021	-1.71227
-1.6462	-1.66628	-1.66297	-1.65968	-1.68145	-1.6781	-1.67477	-1.67144	-1.7135
-1.64744	-1.66753	-1.66423	-1.66096	-1.68276	-1.67942	-1.67609	-1.67275	-1.66945
-1.64865	-1.66877	-1.66548	-1.66222	-1.68405	-1.68075	-1.67743	-1.67409	-1.67077
-1.6499	-1.64663	-1.64341	-1.64022	-1.66022	-1.65698	-1.67871	-1.67538	-1.67207
-1.65118	-1.64791	-1.64467	-1.64146	-1.66146	-1.65819	-1.67993	-1.67663	-1.67334

-1.65248	-1.6492	-1.64595	-1.64272	-1.66272	-1.65943	-1.68118	-1.6779	-1.67463
-1.6538	-1.65051	-1.64726	-1.64402	-1.66404	-1.66074	-1.68249	-1.6792	-1.67593
-1.65511	-1.65182	-1.64856	-1.64533	-1.66536	-1.66206	-1.68382	-1.68052	-1.67725
-1.63411	-1.65309	-1.64985	-1.64663	-1.66669	-1.66337	-1.6601	-1.65686	-1.67857
-1.63536	-1.65434	-1.65112	-1.64792	-1.66801	-1.66469	-1.66141	-1.65817	-1.6799
-1.61326	-1.63339	-1.63019	-1.62699	-1.66933	-1.666	-1.66272	-1.65948	-1.68122
-1.61454	-1.63468	-1.63147	-1.62829	-1.67064	-1.66731	-1.66402	-1.66077	-1.68253
-1.61584	-1.63599	-1.63278	-1.62959	-1.65011	-1.64679	-1.64351	-1.64026	-1.65886
-1.61716	-1.63733	-1.6341	-1.6309	-1.65145	-1.64812	-1.64484	-1.64159	-1.66016
-1.61848	-1.61525	-1.61207	-1.60891	-1.62895	-1.6257	-1.64616	-1.64291	-1.6397
-1.61979	-1.61655	-1.61335	-1.61019	-1.63024	-1.62698	-1.64747	-1.64422	-1.64101
-1.62109	-1.61784	-1.61464	-1.61146	-1.63153	-1.62826	-1.64878	-1.64553	-1.64232
-1.62241	-1.61916	-1.61594	-1.61275	-1.63284	-1.62956	-1.6501	-1.64685	-1.64364
-1.62375	-1.62048	-1.61726	-1.61406	-1.63415	-1.63086	-1.65142	-1.64818	-1.64497
-1.62509	-1.6218	-1.61858	-1.61537	-1.61213	-1.60887	-1.65275	-1.64949	-1.64629
-1.62643	-1.62313	-1.6199	-1.6167	-1.61344	-1.61016	-1.65408	-1.65081	-1.6476
-1.62777	-1.62445	-1.62122	-1.61802	-1.61476	-1.61149	-1.65545	-1.65216	-1.64892
-1.6291	-1.62576	-1.62253	-1.61934	-1.61612	-1.61289	-1.65689	-1.65354	-1.65026
-1.63046	-1.6271	-1.62386	-1.62066	-1.61746	-1.61427	-1.63438	-1.63108	-1.65162
-1.63189	-1.6285	-1.62522	-1.62199	-1.61879	-1.61561	-1.63577	-1.63248	-1.65306
-1.63334	-1.62992	-1.62659	-1.62332	-1.6201	-1.61694	-1.63714	-1.63389	-1.65451
-1.63475	-1.63131	-1.62794	-1.62464	-1.62141	-1.61825	-1.61508	-1.61191	-1.65589
-1.63613	-1.63269	-1.6293	-1.62596	-1.62272	-1.61955	-1.61638	-1.61321	-1.65724
-1.61268	-1.63413	-1.6307	-1.62732	-1.62405	-1.62085	-1.61767	-1.6145	-1.65859
-1.61411	-1.63561	-1.63216	-1.62872	-1.6254	-1.62215	-1.61894	-1.61579	-1.65995
-1.6155	-1.63705	-1.6336	-1.63015	-1.62679	-1.62349	-1.62025	-1.61709	-1.66131
-1.61681	-1.63841	-1.63502	-1.63164	-1.62826	-1.62489	-1.62161	-1.6184	-1.66264
-1.61811	-1.63975	-1.63643	-1.6331	-1.62972	-1.62632	-1.623	-1.61976	-1.66402
-1.61943	-1.61612	-1.61286	-1.60962	-1.63114	-1.62778	-1.62448	-1.62123	-1.618
-1.62078	-1.61743	-1.61416	-1.61094	-1.63253	-1.62925	-1.62598	-1.62273	-1.61947
-1.6222	-1.6188	-1.61549	-1.61224	-1.60901	-1.6058	-1.62737	-1.62413	-1.62088
-1.62368	-1.62021	-1.61684	-1.61354	-1.61029	-1.60709	-1.6287	-1.62547	-1.62224
-1.59739	-1.62167	-1.61826	-1.61492	-1.61164	-1.60842	-1.60521	-1.60201	-1.62357
-1.59885	-1.62319	-1.61977	-1.61639	-1.61306	-1.60978	-1.60652	-1.6033	-1.62487
-1.60027	-1.62468	-1.62125	-1.61785	-1.61449	-1.61116	-1.60788	-1.60463	-1.62742
-1.60162	-1.62607	-1.62265	-1.61926	-1.61591	-1.61259	-1.60931	-1.60608	-1.62887
-1.60297	-1.62745	-1.62404	-1.62066	-1.61732	-1.61401	-1.61075	-1.60753	-1.63033
-1.60441	-1.62888	-1.62544	-1.62204	-1.61868	-1.61536	-1.61209	-1.60888	-1.63171
-1.60589	-1.63035	-1.62687	-1.62344	-1.62005	-1.6167	-1.61342	-1.61021	-1.63307
-1.60738	-1.60394	-1.60055	-1.59719	-1.62152	-1.61815	-1.61483	-1.61157	-1.63441
-1.60889	-1.60549	-1.6021	-1.59874	-1.6231	-1.6197	-1.61633	-1.61296	-1.63573
-1.58126	-1.60697	-1.60359	-1.60023	-1.62461	-1.6212	-1.61779	-1.61437	-1.63712
-1.58263	-1.60836	-1.60499	-1.60163	-1.62604	-1.62262	-1.61921	-1.6158	-1.63858
-1.58403	-1.60977	-1.6064	-1.60306	-1.6275	-1.62406	-1.64686	-1.64342	-1.64005
-1.5855	-1.61127	-1.60791	-1.60457	-1.62904	-1.6256	-1.64838	-1.64489	-1.64147
-1.58698	-1.61279	-1.60943	-1.60609	-1.6306	-1.62715	-1.64992	-1.64637	-1.6429
-1.58839	-1.61423	-1.61087	-1.60752	-1.63207	-1.62864	-1.65143	-1.64789	-1.64441
-1.58977	-1.61564	-1.61227	-1.60891	-1.63351	-1.63009	-1.65293	-1.64943	-1.64597
-1.56634	-1.58789	-1.58457	-1.58129	-1.63383	-1.63047	-1.65447	-1.651	-1.64753
-1.56788	-1.58941	-1.58608	-1.58279	-1.63538	-1.63202	-1.65606	-1.6526	-1.64911

-1.51612	-1.56599	-1.56268	-1.55941	-1.63688	-1.63351	-1.65759	-1.65413	-1.65064
-1.5175	-1.56741	-1.56408	-1.5608	-1.63829	-1.63491	-1.65901	-1.65555	-1.65208
-1.51892	-1.56887	-1.5655	-1.56219	-1.63971	-1.63631	-1.66044	-1.65699	-1.65353
-1.49298	-1.57043	-1.56701	-1.56365	-1.61718	-1.6138	-1.66198	-1.65852	-1.65507
-1.49455	-1.57203	-1.56856	-1.56513	-1.61869	-1.61531	-1.66354	-1.66007	-1.65662
-1.49607	-1.52179	-1.51837	-1.51501	-1.59091	-1.58759	-1.61337	-1.61002	-1.65808
-1.49758	-1.52324	-1.51976	-1.51635	-1.5923	-1.58896	-1.61476	-1.6114	-1.6595
-1.46341	-1.52473	-1.52123	-1.51779	-1.5677	-1.56436	-1.61623	-1.61286	-1.63745
-1.46481	-1.52628	-1.52278	-1.51934	-1.56929	-1.5659	-1.61777	-1.6144	-1.63903
-1.39782	-1.52784	-1.52436	-1.52092	-1.51755	-1.51424	-1.59375	-1.5904	-1.64064
-1.39909	-1.52942	-1.52596	-1.52253	-1.51914	-1.5158	-1.59538	-1.59201	-1.64227
-1.40035	-1.53099	-1.52753	-1.52412	-1.52072	-1.51736	-1.59702	-1.59362	-1.64391
-1.40155	-1.53246	-1.529	-1.52558	-1.52221	-1.51889	-1.59865	-1.59521	-1.59181
-1.40272	-1.53391	-1.53043	-1.52701	-1.52368	-1.52041	-1.60026	-1.59679	-1.59336
-1.40397	-1.46861	-1.46553	-1.46251	-1.52516	-1.52188	-1.51857	-1.51525	-1.59483
-1.40528	-1.47002	-1.46692	-1.46385	-1.52667	-1.52331	-1.51995	-1.5166	-1.59625
-1.40661	-1.43459	-1.43167	-1.42878	-1.52822	-1.5248	-1.52141	-1.51803	-1.59776
-1.40798	-1.43597	-1.43304	-1.43012	-1.52984	-1.52637	-1.52295	-1.51956	-1.59937
-1.38004	-1.43736	-1.43441	-1.43148	-1.46503	-1.46196	-1.52453	-1.52113	-1.54844
-1.38138	-1.43875	-1.43578	-1.43283	-1.46646	-1.46336	-1.52612	-1.52271	-1.55005
-1.38272	-1.44013	-1.43715	-1.43419	-1.46789	-1.46478	-1.52774	-1.52432	-1.55167
-1.38401	-1.38113	-1.37826	-1.3754	-1.46936	-1.46622	-1.52937	-1.52593	-1.52252
-1.38527	-1.38242	-1.37956	-1.37671	-1.47083	-1.46767	-1.53101	-1.52754	-1.52412
-1.31622	-1.38363	-1.38078	-1.37794	-1.43529	-1.43237	-1.466	-1.46293	-1.52572
-1.31731	-1.38477	-1.38191	-1.37909	-1.43654	-1.43367	-1.46742	-1.46436	-1.52734
-1.18647	-1.38599	-1.3831	-1.38026	-1.37747	-1.37473	-1.46881	-1.46576	-1.52896
-1.18757	-1.38731	-1.38437	-1.38147	-1.37865	-1.37589	-1.47014	-1.46713	-1.53056
-1.15296	-1.31815	-1.31534	-1.31257	-1.37986	-1.37708	-1.41235	-1.40946	-1.46553
-1.15404	-1.31941	-1.31658	-1.31378	-1.38116	-1.37834	-1.4137	-1.41082	-1.46694
-1.15514	-1.32068	-1.31784	-1.31504	-1.38249	-1.37964	-1.41509	-1.41219	-1.46837
-1.1254	-1.19619	-1.19361	-1.19107	-1.31354	-1.31084	-1.37816	-1.37546	-1.46981
-1.12651	-1.19733	-1.19475	-1.19221	-1.31479	-1.31206	-1.37946	-1.37675	-1.47127
-1.04526	-1.16271	-1.16019	-1.15772	-1.25482	-1.25216	-1.38077	-1.37805	-1.41346
-1.04617	-1.16384	-1.16131	-1.15883	-1.25605	-1.25338	-1.38211	-1.37935	-1.41485
-1.04708	-1.12739	-1.12493	-1.12252	-1.15748	-1.15505	-1.25201	-1.24947	-1.41624
-1.04802	-1.12855	-1.12606	-1.12361	-1.15858	-1.15615	-1.25322	-1.25065	-1.41762
-1.04897	-1.12973	-1.1272	-1.12472	-1.1597	-1.15725	-1.25444	-1.25184	-1.41903
-0.97558	-1.04787	-1.04583	-1.04383	-1.16082	-1.15837	-1.25567	-1.25305	-1.25046
-0.97648	-1.04882	-1.04676	-1.04473	-1.16196	-1.15949	-1.25691	-1.25428	-1.25168
-0.97738	-0.97546	-0.97354	-0.97164	-1.16312	-1.16061	-1.15815	-1.15574	-1.25289
-0.97827	-0.97635	-0.97443	-0.97253	-1.16429	-1.16173	-1.15925	-1.15684	-1.25412
-0.85203	-0.97725	-0.97533	-0.97343	-1.01315	-1.01118	-1.1604	-1.15797	-1.25536
-0.85276	-0.97813	-0.97622	-0.97432	-1.01412	-1.01216	-1.16161	-1.15913	-1.25661
-0.8535	-0.89856	-0.89685	-0.89516	-0.97336	-0.97152	-1.16283	-1.1603	-1.15781
-0.85424	-0.89936	-0.89763	-0.89593	-0.97425	-0.97242	-1.16402	-1.16147	-1.15895
-0.85499	-0.90016	-0.89842	-0.89672	-0.97514	-0.97332	-1.16522	-1.16264	-1.1601
-0.81469	-0.8541	-0.85249	-0.85091	-0.89589	-0.89424	-1.05777	-1.05537	-1.16124
-0.81541	-0.85487	-0.85327	-0.8517	-0.89673	-0.89506	-1.05891	-1.05648	-1.16239
-0.81616	-0.8146	-0.81307	-0.81156	-0.8509	-0.84932	-0.9831	-0.98084	-1.13317
-0.81693	-0.81538	-0.81383	-0.81229	-0.85165	-0.85006	-0.98417	-0.98188	-1.13439

-0.81769	-0.81614	-0.81458	-0.81302	-0.81148	-0.80993	-0.89575	-0.89403	-0.98071
-0.81842	-0.81687	-0.81531	-0.81375	-0.81219	-0.81062	-0.89654	-0.89486	-0.98183
-0.81917	-0.8176	-0.81603	-0.81448	-0.8129	-0.81132	-0.80979	-0.80832	-0.98293
-0.81996	-0.81835	-0.81677	-0.8152	-0.81361	-0.81202	-0.81048	-0.809	-0.98399
-0.82075	-0.81912	-0.81751	-0.81592	-0.81432	-0.81273	-0.81119	-0.80968	-0.98505
-0.77597	-0.81988	-0.81825	-0.81664	-0.81506	-0.81349	-0.81194	-0.8104	-0.94765
-0.77667	-0.82064	-0.819	-0.81738	-0.81581	-0.81427	-0.81272	-0.81114	-0.94872
-0.73382	-0.77581	-0.77428	-0.77277	-0.81658	-0.81504	-0.81348	-0.81189	-0.81033
-0.73448	-0.7765	-0.77499	-0.7735	-0.81736	-0.8158	-0.81423	-0.81264	-0.81108
-0.73515	-0.73371	-0.7323	-0.73092	-0.81814	-0.81656	-0.81498	-0.8134	-0.81185
-0.73585	-0.73442	-0.73301	-0.7316	-0.81891	-0.8173	-0.81572	-0.81417	-0.81262
-0.73655	-0.73514	-0.73371	-0.73228	-0.81967	-0.81806	-0.81648	-0.81494	-0.81339
-0.73724	-0.73582	-0.73438	-0.73294	-0.73153	-0.73013	-0.81726	-0.81571	-0.81415
-0.73793	-0.73648	-0.73504	-0.7336	-0.7322	-0.73082	-0.81805	-0.81647	-0.81489
-0.69725	-0.73716	-0.73571	-0.73428	-0.73288	-0.7315	-0.77329	-0.7718	-0.81563
-0.69789	-0.73783	-0.7364	-0.73498	-0.73357	-0.73217	-0.77398	-0.77248	-0.81637
-0.69853	-0.73852	-0.73708	-0.73566	-0.73424	-0.73283	-0.73143	-0.73003	-0.81713
-0.69919	-0.73923	-0.73778	-0.73633	-0.7349	-0.73348	-0.73208	-0.7307	-0.81792
-0.60809	-0.69848	-0.69709	-0.69569	-0.73556	-0.73414	-0.73275	-0.73138	-0.73001
-0.60866	-0.69913	-0.69773	-0.69633	-0.73624	-0.73483	-0.73343	-0.73205	-0.73068
-0.60923	-0.69977	-0.69837	-0.69698	-0.73694	-0.73552	-0.73411	-0.73272	-0.73135
-0.6098	-0.70041	-0.69901	-0.69764	-0.69627	-0.69491	-0.73479	-0.73341	-0.73203
-0.61038	-0.70105	-0.69967	-0.6983	-0.69692	-0.69554	-0.73546	-0.73409	-0.73272
-0.56916	-0.6098	-0.60865	-0.60752	-0.69758	-0.6962	-0.69485	-0.69354	-0.7334
-0.56975	-0.61043	-0.60926	-0.6081	-0.69824	-0.69688	-0.69552	-0.69418	-0.73406
-0.52192	-0.56922	-0.5681	-0.567	-0.60755	-0.60644	-0.69619	-0.69483	-0.73474
-0.52242	-0.56977	-0.56866	-0.56756	-0.60813	-0.60701	-0.69685	-0.69549	-0.73544
-0.52292	-0.52189	-0.52088	-0.51989	-0.56705	-0.56598	-0.64984	-0.64866	-0.6948
-0.52343	-0.52241	-0.52139	-0.5204	-0.5676	-0.56653	-0.65043	-0.64924	-0.69544
-0.52395	-0.52293	-0.52191	-0.5209	-0.56816	-0.56709	-0.65101	-0.64981	-0.69609
-0.46797	-0.52345	-0.52243	-0.52142	-0.52042	-0.51944	-0.6082	-0.60708	-0.69676
-0.46843	-0.52397	-0.52295	-0.52195	-0.52094	-0.51994	-0.60879	-0.60766	-0.69744
-0.46889	-0.52449	-0.52347	-0.52246	-0.52145	-0.52045	-0.51947	-0.51851	-0.60714
-0.46935	-0.52501	-0.52398	-0.52297	-0.52197	-0.52097	-0.51999	-0.51903	-0.60775
-0.46984	-0.4689	-0.46799	-0.46709	-0.52249	-0.5215	-0.52052	-0.51955	-0.51859
-0.47036	-0.46942	-0.46849	-0.46758	-0.52304	-0.52204	-0.52105	-0.52007	-0.51909
-0.47089	-0.46993	-0.46899	-0.46806	-0.52359	-0.52258	-0.52158	-0.52058	-0.51959
-0.47139	-0.47042	-0.46947	-0.46853	-0.52412	-0.52311	-0.5221	-0.5211	-0.52011
-0.47189	-0.47091	-0.46994	-0.469	-0.52465	-0.52362	-0.52261	-0.52161	-0.52062
-0.4724	-0.47141	-0.47043	-0.46948	-0.46854	-0.46763	-0.52313	-0.52212	-0.52112
-0.47291	-0.47192	-0.47094	-0.46997	-0.46902	-0.46809	-0.52365	-0.52263	-0.52162
-0.47343	-0.47243	-0.47144	-0.47047	-0.46951	-0.46858	-0.52419	-0.52316	-0.52214
-0.47394	-0.47294	-0.47195	-0.47098	-0.47002	-0.46908	-0.52477	-0.52372	-0.52269
-0.47447	-0.47345	-0.47246	-0.47149	-0.47053	-0.46959	-0.52534	-0.52428	-0.52324
-0.47503	-0.474	-0.473	-0.47202	-0.47104	-0.47008	-0.52589	-0.52481	-0.52376
-0.47558	-0.47455	-0.47354	-0.47254	-0.47155	-0.47057	-0.52644	-0.52536	-0.5243
-0.47613	-0.47509	-0.47406	-0.47305	-0.47206	-0.47108	-0.47012	-0.46916	-0.52487
-0.47666	-0.47562	-0.47458	-0.47356	-0.47257	-0.4716	-0.47064	-0.46969	-0.52547
-0.47722	-0.47618	-0.47513	-0.4741	-0.4731	-0.47212	-0.47115	-0.47019	-0.52604
-0.4778	-0.47674	-0.47569	-0.47466	-0.47364	-0.47265	-0.47166	-0.47068	-0.52659

-0.47838	-0.4773	-0.47625	-0.47521	-0.47419	-0.47319	-0.47219	-0.4712	-0.47022
-0.47894	-0.47784	-0.47679	-0.47575	-0.47474	-0.47374	-0.47274	-0.47173	-0.47075
-0.47952	-0.4784	-0.47734	-0.4763	-0.47529	-0.47429	-0.47328	-0.47227	-0.47128
-0.48011	-0.479	-0.47792	-0.47687	-0.47583	-0.47481	-0.4738	-0.47279	-0.47181
-0.48072	-0.47962	-0.47852	-0.47744	-0.47638	-0.47534	-0.47432	-0.47332	-0.47234
-0.43364	-0.48023	-0.47913	-0.47805	-0.47697	-0.47591	-0.47487	-0.47387	-0.47288
-0.4342	-0.48084	-0.47974	-0.47866	-0.47758	-0.47649	-0.47545	-0.47443	-0.47343
-0.38794	-0.48145	-0.48035	-0.47926	-0.47816	-0.47707	-0.47602	-0.47499	-0.47398
-0.38846	-0.48206	-0.48094	-0.47983	-0.47873	-0.47764	-0.47658	-0.47556	-0.47454
-0.389	-0.43487	-0.43382	-0.43279	-0.47931	-0.47823	-0.47717	-0.47613	-0.4751
-0.38957	-0.43547	-0.43441	-0.43336	-0.47993	-0.47884	-0.47776	-0.47672	-0.47568
-0.39014	-0.43607	-0.43499	-0.43393	-0.48055	-0.47945	-0.47837	-0.47731	-0.47626
-0.39069	-0.3897	-0.38872	-0.38777	-0.48116	-0.48006	-0.47898	-0.47792	-0.47685
-0.39124	-0.39022	-0.38924	-0.38829	-0.48177	-0.48068	-0.4796	-0.47852	-0.47745
-0.34007	-0.39079	-0.38979	-0.38883	-0.43459	-0.43359	-0.48021	-0.47913	-0.47805
-0.34061	-0.39139	-0.39038	-0.38939	-0.43517	-0.43415	-0.48082	-0.47974	-0.47866
-0.34115	-0.3402	-0.33928	-0.33839	-0.38901	-0.38809	-0.48145	-0.48036	-0.47926
-0.34168	-0.34073	-0.33981	-0.33891	-0.38959	-0.38865	-0.48209	-0.48098	-0.47987
-0.34221	-0.34126	-0.34034	-0.33944	-0.39018	-0.38922	-0.43489	-0.43385	-0.48048
-0.34274	-0.34179	-0.34087	-0.33997	-0.39076	-0.38979	-0.43548	-0.43443	-0.4811
-0.34329	-0.34234	-0.34141	-0.3405	-0.39136	-0.39037	-0.43608	-0.43502	-0.48173
-0.34384	-0.34289	-0.34196	-0.34105	-0.34015	-0.33925	-0.38998	-0.389	-0.43454
-0.34441	-0.34345	-0.34252	-0.3416	-0.34069	-0.33979	-0.39057	-0.38958	-0.43512
-0.34499	-0.34402	-0.34308	-0.34215	-0.34124	-0.34033	-0.33942	-0.33852	-0.43573
-0.34556	-0.34459	-0.34364	-0.3427	-0.34178	-0.34087	-0.33997	-0.33906	-0.43636
-0.34614	-0.34517	-0.34421	-0.34326	-0.34233	-0.34142	-0.34051	-0.33959	-0.39036
-0.34673	-0.34576	-0.34479	-0.34383	-0.34289	-0.34197	-0.34105	-0.34013	-0.39097
-0.34732	-0.34634	-0.34537	-0.34441	-0.34346	-0.34252	-0.34159	-0.34068	-0.33977
-0.34791	-0.34692	-0.34595	-0.34499	-0.34403	-0.34308	-0.34214	-0.34122	-0.34031
-0.34851	-0.3475	-0.34653	-0.34557	-0.34461	-0.34365	-0.3427	-0.34177	-0.34085
-0.34913	-0.34811	-0.34712	-0.34615	-0.34519	-0.34422	-0.34327	-0.34233	-0.3414
-0.34977	-0.34873	-0.34772	-0.34674	-0.34576	-0.34481	-0.34385	-0.34289	-0.34195
-0.35039	-0.34934	-0.34832	-0.34732	-0.34635	-0.34539	-0.34443	-0.34346	-0.34251
-0.35101	-0.34995	-0.34892	-0.34792	-0.34693	-0.34597	-0.345	-0.34404	-0.34308
-0.35164	-0.35058	-0.34954	-0.34852	-0.34753	-0.34655	-0.34558	-0.34462	-0.34366
-0.3523	-0.35124	-0.35018	-0.34915	-0.34813	-0.34714	-0.34616	-0.34519	-0.34423
-0.35297	-0.3519	-0.35083	-0.34978	-0.34875	-0.34774	-0.34675	-0.34578	-0.34482
-0.31097	-0.35255	-0.35148	-0.35043	-0.34939	-0.34836	-0.34736	-0.34638	-0.3454
-0.31152	-0.3532	-0.35213	-0.35108	-0.35004	-0.349	-0.34798	-0.34699	-0.346
-0.31208	-0.35387	-0.35279	-0.35173	-0.35068	-0.34964	-0.34862	-0.34763	-0.34664
-0.31264	-0.35455	-0.35345	-0.35238	-0.35132	-0.3503	-0.34929	-0.34829	-0.3473
-0.3132	-0.31232	-0.31145	-0.3106	-0.35197	-0.35094	-0.34992	-0.34893	-0.34794
-0.31378	-0.31289	-0.312	-0.31113	-0.35261	-0.35156	-0.35054	-0.34953	-0.34854
-0.31436	-0.31347	-0.31257	-0.31168	-0.31082	-0.31	-0.35116	-0.35015	-0.34915
-0.31492	-0.31404	-0.31314	-0.31225	-0.31138	-0.31053	-0.35181	-0.35078	-0.34977
-0.31551	-0.31463	-0.31373	-0.31283	-0.31195	-0.31107	-0.35247	-0.35142	-0.3504
-0.25199	-0.31525	-0.31433	-0.31342	-0.31252	-0.31163	-0.35313	-0.35207	-0.35103
-0.25242	-0.31588	-0.31495	-0.31403	-0.31311	-0.3122	-0.35382	-0.35273	-0.35167
-0.25281	-0.31649	-0.31557	-0.31464	-0.31373	-0.31282	-0.31193	-0.31106	-0.35235
-0.25319	-0.31709	-0.31618	-0.31528	-0.31437	-0.31348	-0.31258	-0.31168	-0.35308

-0.25357	-0.253	-0.25244	-0.25187	-0.31499	-0.3141	-0.3132	-0.31229	-0.3114
-0.25394	-0.25337	-0.25281	-0.25225	-0.31556	-0.31468	-0.31378	-0.31287	-0.31198
-0.25432	-0.25375	-0.25318	-0.25262	-0.31613	-0.31525	-0.31435	-0.31345	-0.31255
-0.25471	-0.25412	-0.25356	-0.253	-0.31672	-0.31582	-0.31492	-0.31402	-0.31313
-0.2551	-0.25451	-0.25394	-0.25338	-0.31732	-0.31641	-0.3155	-0.31461	-0.31372
-0.25554	-0.25494	-0.25436	-0.25379	-0.31797	-0.31705	-0.31614	-0.31524	-0.31435
-0.256	-0.25538	-0.25479	-0.25421	-0.31864	-0.31772	-0.3168	-0.3159	-0.315
-0.25643	-0.25581	-0.2552	-0.25461	-0.25403	-0.25347	-0.31742	-0.31651	-0.31561
-0.25684	-0.25621	-0.2556	-0.25499	-0.25441	-0.25385	-0.31801	-0.31708	-0.31617
-0.25726	-0.25663	-0.256	-0.25539	-0.2548	-0.25423	-0.31863	-0.31769	-0.31678
-0.25768	-0.25705	-0.25642	-0.2558	-0.25521	-0.25463	-0.3193	-0.31837	-0.31745
-0.25811	-0.25748	-0.25685	-0.25623	-0.25563	-0.25504	-0.25448	-0.25392	-0.31811
-0.25857	-0.25794	-0.25731	-0.25668	-0.25606	-0.25546	-0.25488	-0.25431	-0.31872
-0.25903	-0.25841	-0.25778	-0.25714	-0.25651	-0.25589	-0.25528	-0.2547	-0.31932
-0.25946	-0.25884	-0.25821	-0.25758	-0.25694	-0.25631	-0.2557	-0.2551	-0.31997
-0.25986	-0.25925	-0.25863	-0.258	-0.25737	-0.25673	-0.25612	-0.25553	-0.32066
-0.26028	-0.25968	-0.25906	-0.25844	-0.25781	-0.25718	-0.25656	-0.25596	-0.25536
-0.26073	-0.26013	-0.25951	-0.2589	-0.25827	-0.25765	-0.25702	-0.2564	-0.25579
-0.19582	-0.26056	-0.25995	-0.25934	-0.25872	-0.2581	-0.25747	-0.25684	-0.25622
-0.19612	-0.26096	-0.26035	-0.25975	-0.25914	-0.25852	-0.2579	-0.25728	-0.25666
-0.19643	-0.26136	-0.26076	-0.26015	-0.25955	-0.25894	-0.25834	-0.25772	-0.25711
-0.19676	-0.26181	-0.2612	-0.26059	-0.25999	-0.25939	-0.25878	-0.25817	-0.25755
-0.1971	-0.26227	-0.26165	-0.26104	-0.26043	-0.25983	-0.25923	-0.25862	-0.258
-0.19744	-0.19695	-0.19648	-0.19602	-0.26085	-0.26025	-0.25965	-0.25905	-0.25844
-0.19778	-0.19727	-0.19678	-0.1963	-0.26124	-0.26065	-0.26006	-0.25948	-0.25888
-0.06361	-0.19762	-0.19711	-0.19662	-0.19616	-0.19571	-0.26049	-0.2599	-0.25931
-0.06371	-0.198	-0.19749	-0.19698	-0.1965	-0.19605	-0.26092	-0.26033	-0.25973
-0.06382	-0.06368	-0.06354	-0.06341	-0.19685	-0.19638	-0.26136	-0.26076	-0.26016
-0.06391	-0.06377	-0.06364	-0.0635	-0.19718	-0.19671	-0.26181	-0.26121	-0.26061
-0.064	-0.06386	-0.06373	-0.06359	-0.19752	-0.19704	-0.26226	-0.26166	-0.26106
	-0.06396	-0.06383	-0.06369	-0.13257	-0.13225	-0.19691	-0.19645	-0.26148
	-0.06406	-0.06393	-0.06379	-0.13282	-0.13248	-0.19725	-0.19676	-0.2619
				-0.06376	-0.06362	-0.19761	-0.19711	-0.19662
				-0.06386	-0.06372	-0.19799	-0.19748	-0.19698
				-0.06395	-0.06382	-0.06369	-0.06356	-0.19735
				-0.06404	-0.06392	-0.06379	-0.06366	-0.19772
						-0.06388	-0.06375	-0.13269
						-0.06397	-0.06384	-0.1329
						-0.06406	-0.06393	-0.13312
								-0.0639
								-0.064

		-0.12926
		-0.12902
-0.12949	-0.12914	-0.44066
-0.12924	-0.12888	-0.43987
-0.12899	-0.12862	-0.43908
-0.44052	-0.43941	-0.65956

						-0.43973	-0.43863	-0.65844
				-0.44126	-0.44009	-0.43895	-0.43785	-0.7108
				-0.44046	-0.43932	-0.43819	-0.43708	-0.70958
		-0.12919	-0.12881	-0.43969	-0.43856	-0.71197	-0.71017	-0.75768
		-0.12894	-0.12856	-0.43894	-0.43783	-0.71083	-0.70902	-0.75643
		-0.44046	-0.43932	-0.65967	-0.65801	-0.75903	-0.7571	-0.75519
		-0.4397	-0.43858	-0.6586	-0.65692	-0.75776	-0.75584	-0.75395
		-0.43894	-0.43784	-0.65753	-0.65582	-0.75648	-0.75458	-0.75273
-0.44042	-0.43928	-0.43817	-0.43707	-0.70975	-0.70793	-0.75523	-0.75336	-0.75152
-0.43964	-0.43851	-0.43739	-0.43629	-0.70854	-0.70677	-0.75401	-0.75215	-0.75033
-0.43887	-0.43775	-0.71093	-0.70914	-0.7565	-0.75464	-0.7528	-0.75097	-0.79752
-0.43813	-0.43702	-0.70978	-0.70799	-0.75528	-0.75343	-0.75161	-0.74982	-0.79637
-0.71221	-0.71042	-0.75781	-0.75592	-0.75407	-0.75224	-0.79885	-0.79703	-0.79521
-0.71106	-0.70925	-0.75656	-0.7547	-0.75288	-0.75108	-0.79768	-0.79586	-0.79402
-0.70991	-0.70809	-0.75533	-0.7535	-0.7517	-0.74993	-0.79651	-0.79467	-0.79282
-0.70874	-0.70695	-0.75413	-0.75233	-0.79898	-0.79716	-0.79532	-0.79346	-0.97996
-0.70757	-0.70583	-0.75295	-0.75118	-0.79781	-0.79597	-0.79412	-0.79224	-0.97842
-0.7554	-0.75358	-0.75179	-0.75002	-0.79662	-0.79477	-0.98152	-0.9792	-0.97689
-0.75422	-0.75243	-0.75064	-0.74886	-0.79542	-0.79354	-0.97999	-0.97767	-0.97537
-0.75305	-0.75127	-0.79792	-0.79608	-0.89288	-0.89074	-0.97846	-0.97615	-1.02108
-0.75192	-0.75011	-0.79672	-0.79486	-0.89148	-0.88933	-0.97692	-0.97465	-1.01954
-0.75077	-0.74894	-0.89434	-0.89223	-0.98005	-0.97772	-0.97542	-0.97318	-1.01801
-0.74958	-0.74777	-0.89298	-0.89089	-0.9786	-0.97629	-0.97401	-0.97175	-1.0165
-0.74841	-0.74661	-0.89163	-0.88956	-0.97716	-0.97488	-0.9726	-0.97034	-1.015
-0.7957	-0.79384	-0.98024	-0.97796	-0.97568	-0.9734	-1.01819	-1.01583	-1.01351
-0.79458	-0.7927	-0.97879	-0.97646	-0.97417	-0.97191	-1.01664	-1.01432	-1.01204
-0.89187	-0.88974	-0.97729	-0.97496	-1.01981	-1.01748	-1.01517	-1.01288	-1.01062
-0.89047	-0.88832	-0.97576	-0.97346	-1.01827	-1.016	-1.01375	-1.0115	-1.00926
-0.97893	-0.9766	-0.97429	-0.97202	-1.01678	-1.01454	-1.01231	-1.01009	-1.00785
-0.97751	-0.97521	-0.97292	-0.97067	-1.01535	-1.01309	-1.01085	-1.00862	-1.00638
-0.97609	-0.97381	-1.01859	-1.01623	-1.01392	-1.01165	-1.00939	-1.00716	-1.00492
-0.97461	-0.97234	-1.01707	-1.01474	-1.01244	-1.01018	-1.00794	-1.00573	-1.00351
-0.97313	-0.97089	-1.01556	-1.01326	-1.01098	-1.00873	-1.0065	-1.0043	-1.00209
-1.01879	-1.01646	-1.01416	-1.01187	-1.0096	-1.00734	-1.0051	-1.00287	-1.00064
-1.01738	-1.01509	-1.01281	-1.01053	-1.00825	-1.00598	-1.00371	-1.00144	-0.99916
-1.01592	-1.01365	-1.01138	-1.0091	-1.00682	-1.00454	-1.00226	-0.99999	-1.03791
-1.01442	-1.01216	-1.00989	-1.00761	-1.00532	-1.00303	-1.00076	-0.99852	-1.0364
-1.01298	-1.01072	-1.00844	-1.00615	-1.00386	-1.00156	-1.0396	-1.03723	-1.16865
-1.01164	-1.00937	-1.00708	-1.00478	-1.00247	-1.00015	-1.03809	-1.03571	-1.16673
-1.01028	-1.00799	-1.00569	-1.00338	-1.00106	-0.99873	-1.03658	-1.0342	-1.16483
-1.00881	-1.0065	-1.00419	-1.00186	-1.03988	-1.03742	-1.12313	-1.12035	-1.16298
-1.0073	-1.00499	-1.00265	-1.00031	-1.03826	-1.03583	-1.12124	-1.11854	-1.16116
-1.00587	-1.00354	-1.04166	-1.03918	-1.03674	-1.03435	-1.16493	-1.16212	-1.21322
-1.00447	-1.00214	-1.04017	-1.0377	-1.03529	-1.03294	-1.16319	-1.16037	-1.21139
-1.003	-1.00066	-1.03862	-1.03618	-1.17712	-1.17427	-1.21545	-1.21253	-1.20964
-1.00144	-0.99909	-1.03701	-1.03462	-1.17526	-1.17249	-1.21365	-1.21081	-1.20796
-1.04034	-1.03787	-1.17918	-1.17627	-1.21753	-1.21468	-1.21185	-1.20905	-1.24552
-1.03885	-1.03641	-1.17742	-1.17454	-1.21573	-1.21288	-1.21003	-1.20719	-1.24351
-1.03741	-1.035	-1.17573	-1.17288	-1.21399	-1.21111	-1.20823	-1.20534	-1.24151
-1.09435	-1.09186	-1.17406	-1.17126	-1.21229	-1.2094	-1.20651	-1.20361	-1.3221

-1.09288	-1.09043	-1.17241	-1.16965	-1.21061	-1.2077	-1.20481	-1.20192	-1.31996
-1.1763	-1.17346	-1.21459	-1.21171	-1.2088	-1.20589	-1.24207	-1.239	-1.31775
-1.17447	-1.17166	-1.21271	-1.20982	-1.20691	-1.20398	-1.24005	-1.23699	-1.31547
-1.17273	-1.16995	-1.2109	-1.208	-1.24434	-1.24122	-1.32038	-1.31682	-1.31332
-1.17109	-1.16833	-1.20919	-1.20629	-1.24253	-1.23944	-1.31829	-1.31477	-1.31131
-1.2133	-1.2104	-1.24692	-1.24382	-1.32341	-1.31979	-1.31624	-1.31277	-1.30935
-1.2116	-1.20869	-1.2451	-1.242	-1.32132	-1.31775	-1.31425	-1.31081	-1.30743
-1.2099	-1.20697	-1.24327	-1.24018	-1.31922	-1.31571	-1.31225	-1.30885	-1.30549
-1.24768	-1.24455	-1.28061	-1.2772	-1.31706	-1.31356	-1.31013	-1.30676	-1.30344
-1.24582	-1.24271	-1.27859	-1.27515	-1.31489	-1.31138	-1.30796	-1.30461	-1.30133
-1.28332	-1.27994	-1.31996	-1.31633	-1.31279	-1.30931	-1.30592	-1.3026	-1.33835
-1.28112	-1.27777	-1.31775	-1.31423	-1.31076	-1.30734	-1.30399	-1.30071	-1.33646
-1.32266	-1.3191	-1.31562	-1.31219	-1.30878	-1.3054	-1.3021	-1.29886	-1.33463
-1.32056	-1.31705	-1.3136	-1.31021	-1.30684	-1.3035	-1.30024	-1.29706	-1.33285
-1.31853	-1.31506	-1.31164	-1.30828	-1.30495	-1.30164	-1.33741	-1.33421	-1.33115
-1.31655	-1.31312	-1.30973	-1.30638	-1.30308	-1.29983	-1.33562	-1.33251	-1.32954
-1.31461	-1.31122	-1.30784	-1.30451	-1.30125	-1.29806	-1.33389	-1.33087	-1.32799
-1.31266	-1.3093	-1.30595	-1.30265	-1.33846	-1.33526	-1.33219	-1.32924	-1.32643
-1.31072	-1.30738	-1.30405	-1.30079	-1.33663	-1.33353	-1.33053	-1.32765	-1.32488
-1.30879	-1.30547	-1.30217	-1.29894	-1.33479	-1.33177	-1.32886	-1.32608	-1.35951
-1.30688	-1.30357	-1.30029	-1.29708	-1.33292	-1.32997	-1.32716	-1.32453	-1.35804
-1.305	-1.30172	-1.33748	-1.33423	-1.33118	-1.3283	-1.32558	-1.32305	-1.3566
-1.30315	-1.29992	-1.33573	-1.33258	-1.32962	-1.32683	-1.32417	-1.32167	-1.35519
-1.30132	-1.29816	-1.33402	-1.33099	-1.32813	-1.32542	-1.32282	-1.32033	-1.35381
-1.2995	-1.2964	-1.33231	-1.32939	-1.32663	-1.324	-1.35746	-1.35493	-1.35244
-1.29772	-1.29468	-1.33063	-1.32783	-1.32515	-1.3226	-1.35607	-1.35355	-1.35108
-1.33495	-1.3319	-1.32905	-1.32634	-1.35978	-1.35721	-1.35468	-1.35218	-1.38483
-1.33326	-1.33031	-1.32754	-1.32491	-1.35835	-1.3558	-1.35329	-1.35082	-1.38343
-1.33167	-1.3288	-1.3261	-1.32351	-1.35694	-1.35442	-1.38712	-1.38457	-1.38203
-1.33018	-1.3274	-1.32473	-1.32215	-1.35555	-1.35306	-1.38572	-1.38318	-1.38065
-1.32873	-1.32602	-1.35936	-1.35672	-1.35417	-1.3517	-1.38433	-1.38179	-1.45003
-1.32729	-1.32463	-1.35795	-1.35533	-1.3528	-1.35034	-1.38293	-1.3804	-1.44849
-1.32588	-1.32325	-1.35654	-1.35395	-1.35143	-1.34899	-1.38154	-1.37901	-1.44695
-1.36046	-1.35778	-1.35515	-1.35257	-1.38519	-1.38266	-1.38015	-1.37764	-1.44546
-1.35905	-1.35638	-1.35376	-1.35121	-1.38378	-1.38127	-1.37877	-1.37629	-1.44398
-1.35764	-1.35499	-1.38759	-1.38495	-1.38238	-1.37988	-1.44791	-1.44521	-1.4425
-1.35625	-1.35362	-1.38618	-1.38355	-1.381	-1.37851	-1.4464	-1.44372	-1.44103
-1.35486	-1.35225	-1.38477	-1.38215	-1.4144	-1.41185	-1.4449	-1.44223	-1.43956
-1.35348	-1.35089	-1.38336	-1.38075	-1.41297	-1.41043	-1.44341	-1.44076	-1.4381
-1.38728	-1.3846	-1.38196	-1.37937	-1.44725	-1.44458	-1.44193	-1.4393	-1.43666
-1.38585	-1.38319	-1.38058	-1.37801	-1.44576	-1.44312	-1.44048	-1.43787	-1.43524
-1.38444	-1.38181	-1.37922	-1.37667	-1.4443	-1.44166	-1.43904	-1.43645	-1.43384
-1.38307	-1.38048	-1.4126	-1.40996	-1.44285	-1.44021	-1.4376	-1.43502	-1.43243
-1.38172	-1.37919	-1.41126	-1.40862	-1.44143	-1.43877	-1.43615	-1.43359	-1.43101
-1.4151	-1.41253	-1.44551	-1.44272	-1.44	-1.43733	-1.43471	-1.43215	-1.42959
-1.41366	-1.41112	-1.44404	-1.44129	-1.43857	-1.43589	-1.43327	-1.43072	-1.42817
-1.41224	-1.40972	-1.44258	-1.43986	-1.43716	-1.43448	-1.43185	-1.42929	-1.50241
-1.41085	-1.40832	-1.44114	-1.43848	-1.43579	-1.4331	-1.43046	-1.42787	-1.50068
-1.40947	-1.40693	-1.4397	-1.43708	-1.43442	-1.43172	-1.42907	-1.42646	-1.49897
-1.44356	-1.44087	-1.43824	-1.43565	-1.433	-1.43031	-1.50337	-1.50028	-1.49725

-1.44209	-1.43939	-1.43678	-1.43421	-1.43157	-1.42889	-1.50167	-1.49857	-1.49556
-1.44067	-1.43796	-1.43534	-1.43276	-1.43014	-1.42751	-1.50004	-1.49696	-1.52583
-1.43929	-1.43656	-1.43391	-1.4313	-1.42871	-1.42615	-1.49848	-1.4954	-1.5242
-1.43789	-1.43516	-1.43248	-1.42985	-1.50285	-1.49987	-1.49686	-1.49382	-1.52256
-1.43644	-1.43374	-1.43105	-1.42839	-1.5011	-1.49816	-1.4952	-1.49219	-1.5209
-1.435	-1.43233	-1.50559	-1.50242	-1.49939	-1.49647	-1.52541	-1.52233	-1.51926
-1.43361	-1.43095	-1.50393	-1.50077	-1.49774	-1.49482	-1.52372	-1.52071	-1.5177
-1.43222	-1.42957	-1.50229	-1.49916	-1.49614	-1.49321	-1.52205	-1.51909	-1.51613
-1.43078	-1.42814	-1.50065	-1.49761	-1.49461	-1.49164	-1.5204	-1.51744	-1.55003
-1.42934	-1.42671	-1.49901	-1.49608	-1.49311	-1.4901	-1.51876	-1.51578	-1.54832
-1.50343	-1.50033	-1.49738	-1.49449	-1.5233	-1.52018	-1.51712	-1.51415	-1.54665
-1.50178	-1.4987	-1.49574	-1.49284	-1.5216	-1.51851	-1.51549	-1.51253	-1.545
-1.50016	-1.49711	-1.49414	-1.49121	-1.51993	-1.51689	-1.5139	-1.51096	-1.5434
-1.49856	-1.49557	-1.49259	-1.48963	-1.51831	-1.51533	-1.51237	-1.50944	-1.54184
-1.49698	-1.49402	-1.49104	-1.48808	-1.51671	-1.51377	-1.51085	-1.50794	-1.5738
-1.49542	-1.49245	-1.48949	-1.48656	-1.51514	-1.51221	-1.50932	-1.50646	-1.57227
-1.49387	-1.49086	-1.48793	-1.48505	-1.51358	-1.51065	-1.50777	-1.50498	-1.57074
-1.49225	-1.48926	-1.5179	-1.51492	-1.51197	-1.50906	-1.54025	-1.53739	-1.56912
-1.4906	-1.48765	-1.51624	-1.51327	-1.51035	-1.50746	-1.53862	-1.53576	-1.56746
-1.48899	-1.48608	-1.51463	-1.51168	-1.50878	-1.50593	-1.53706	-1.5342	-1.56585
-1.48742	-1.48454	-1.51305	-1.51014	-1.50727	-1.50446	-1.53556	-1.53271	-1.56431
-1.48588	-1.48303	-1.51151	-1.50862	-1.53978	-1.53692	-1.53407	-1.53123	-1.56278
-1.48438	-1.48153	-1.50998	-1.50714	-1.53827	-1.53541	-1.53257	-1.52976	-1.56129
-1.48289	-1.48007	-1.50848	-1.50567	-1.53676	-1.5339	-1.53108	-1.52829	-1.5598
-1.48141	-1.47863	-1.54099	-1.5381	-1.53524	-1.53242	-1.56401	-1.56114	-1.55829
-1.47994	-1.4772	-1.53946	-1.53657	-1.53373	-1.53093	-1.5625	-1.55963	-1.55679
-1.5097	-1.50687	-1.53794	-1.53507	-1.53224	-1.52944	-1.56098	-1.55813	-1.55531
-1.5082	-1.50536	-1.53643	-1.53361	-1.53078	-1.52795	-1.55945	-1.55663	-1.55385
-1.54066	-1.53777	-1.53493	-1.53212	-1.5293	-1.52648	-1.55794	-1.55515	-1.55238
-1.53912	-1.53628	-1.53344	-1.53062	-1.52782	-1.52503	-1.55648	-1.55368	-1.55091
-1.53759	-1.53478	-1.53195	-1.52912	-1.52633	-1.52358	-1.55501	-1.55221	-1.57071
-1.53608	-1.53326	-1.53044	-1.52764	-1.52486	-1.52211	-1.55352	-1.55076	-1.56927
-1.53458	-1.53174	-1.52894	-1.52616	-1.52339	-1.52065	-1.55203	-1.54931	-1.56783
-1.53308	-1.53026	-1.52745	-1.52468	-1.52193	-1.51922	-1.57192	-1.56913	-1.60909
-1.53158	-1.52879	-1.52598	-1.5232	-1.52047	-1.51781	-1.57047	-1.56767	-1.60763
-1.5301	-1.5273	-1.52451	-1.52174	-1.54089	-1.53818	-1.61184	-1.60901	-1.6062
-1.52866	-1.52579	-1.52302	-1.52031	-1.53945	-1.53674	-1.61037	-1.60758	-1.60479
-1.52723	-1.52435	-1.54351	-1.54076	-1.53802	-1.5353	-1.63088	-1.62809	-1.62532
-1.52583	-1.52299	-1.54211	-1.5393	-1.53656	-1.53388	-1.62943	-1.62664	-1.62387
-1.54644	-1.54358	-1.56378	-1.56092	-1.60132	-1.59861	-1.62799	-1.6252	-1.62243
-1.54497	-1.54217	-1.56238	-1.55955	-1.59995	-1.59719	-1.62652	-1.62376	-1.62102
-1.54349	-1.54073	-1.56097	-1.5582	-1.5986	-1.59579	-1.62506	-1.62233	-1.61961
-1.56504	-1.56226	-1.60279	-1.60002	-1.62937	-1.6265	-1.62368	-1.62092	-1.61818
-1.56358	-1.56076	-1.60133	-1.59861	-1.62797	-1.62516	-1.62235	-1.61953	-1.61675
-1.56216	-1.55933	-1.59991	-1.5972	-1.62654	-1.62378	-1.62099	-1.61817	-1.63441
-1.56078	-1.55796	-1.59854	-1.59577	-1.62508	-1.62236	-1.61962	-1.61685	-1.63311
-1.60284	-1.60002	-1.5972	-1.5944	-1.62366	-1.62095	-1.61823	-1.6155	-1.66337
-1.60152	-1.59872	-1.59591	-1.59311	-1.62231	-1.61955	-1.61681	-1.61412	-1.6619
-1.60018	-1.59741	-1.59462	-1.59184	-1.62098	-1.61817	-1.61542	-1.61273	-1.66044
-1.59878	-1.59605	-1.5933	-1.59056	-1.61968	-1.61686	-1.63319	-1.63046	-1.65902

-1.59736	-1.59466	-1.59196	-1.58928	-1.61838	-1.61556	-1.63189	-1.62914	-1.65759
-1.59597	-1.59326	-1.59059	-1.58794	-1.63624	-1.63339	-1.66198	-1.65903	-1.68419
-1.59459	-1.59185	-1.58919	-1.58657	-1.63487	-1.63207	-1.66056	-1.65755	-1.68263
-1.59326	-1.5905	-1.63897	-1.63624	-1.63348	-1.63072	-1.65912	-1.65609	-1.70999
-1.59196	-1.5892	-1.63761	-1.63483	-1.63207	-1.62933	-1.65766	-1.65465	-1.70845
-1.61044	-1.60764	-1.63627	-1.63345	-1.66202	-1.65913	-1.68556	-1.68249	-1.70692
-1.60917	-1.60636	-1.63495	-1.63213	-1.66059	-1.65769	-1.68407	-1.68101	-1.70541
-1.6079	-1.6051	-1.63365	-1.63082	-1.65917	-1.65624	-1.68256	-1.67951	-1.7039
-1.60661	-1.60383	-1.63235	-1.62949	-1.65771	-1.65473	-1.68098	-1.67796	-1.74981
-1.60531	-1.60257	-1.63105	-1.62816	-1.65624	-1.65319	-1.67936	-1.67638	-1.74818
-1.60402	-1.60129	-1.66092	-1.65786	-1.68404	-1.68087	-1.67779	-1.67481	-1.74653
-1.60273	-1.6	-1.6595	-1.65642	-1.68254	-1.67935	-1.67626	-1.67326	-1.74487
-1.63274	-1.62992	-1.68738	-1.68421	-1.68104	-1.67786	-1.74956	-1.74637	-1.74326
-1.63143	-1.62858	-1.68587	-1.6827	-1.67954	-1.6764	-1.74802	-1.74483	-1.74171
-1.66127	-1.65823	-1.68436	-1.68118	-1.67805	-1.67495	-1.7465	-1.74331	-1.74019
-1.65983	-1.65679	-1.68285	-1.67965	-1.67654	-1.67349	-1.74498	-1.7418	-1.73867
-1.65839	-1.65535	-1.68135	-1.67814	-1.67505	-1.67204	-1.74346	-1.7403	-1.73717
-1.68616	-1.68303	-1.67986	-1.67667	-1.72169	-1.71858	-1.74195	-1.7388	-1.7357
-1.68464	-1.68151	-1.67837	-1.67523	-1.7202	-1.7171	-1.74044	-1.73732	-1.73424
-1.68313	-1.68001	-1.67689	-1.67379	-1.71872	-1.71563	-1.73895	-1.73585	-1.73279
-1.68163	-1.67851	-1.67542	-1.67235	-1.71724	-1.71417	-1.73747	-1.73439	-1.73134
-1.68014	-1.67704	-1.67396	-1.67092	-1.74222	-1.7391	-1.736	-1.73294	-1.75201
-1.67865	-1.6756	-1.67254	-1.6695	-1.74073	-1.73762	-1.73454	-1.73149	-1.75059
-1.67719	-1.67417	-1.67113	-1.6681	-1.73924	-1.73615	-1.73308	-1.73005	-1.74917
-1.67575	-1.67274	-1.7176	-1.71448	-1.73777	-1.73469	-1.75383	-1.75078	-1.74776
-1.67432	-1.67131	-1.71612	-1.71303	-1.7363	-1.73324	-1.7524	-1.74936	-1.74634
-1.67289	-1.66989	-1.74108	-1.73794	-1.75712	-1.75404	-1.75098	-1.74794	-1.74492
-1.67146	-1.66847	-1.73959	-1.73648	-1.75569	-1.75262	-1.74956	-1.74653	-1.74351
-1.67004	-1.66706	-1.76046	-1.75736	-1.75427	-1.7512	-1.74815	-1.74511	-1.76623
-1.66861	-1.66565	-1.759	-1.75593	-1.75286	-1.74979	-1.74674	-1.74369	-1.76477
-1.76377	-1.76063	-1.75755	-1.75451	-1.75145	-1.74838	-1.76955	-1.76642	-1.80883
-1.76229	-1.75918	-1.75611	-1.75307	-1.75002	-1.74697	-1.76811	-1.76498	-1.80738
-1.76083	-1.75774	-1.75468	-1.75164	-1.7486	-1.74557	-1.76668	-1.76356	-1.80596
-1.75937	-1.75631	-1.75326	-1.75022	-1.74719	-1.74416	-1.76525	-1.76217	-1.80458
-1.75794	-1.7549	-1.75186	-1.74882	-1.74579	-1.74277	-1.76384	-1.7608	-1.80321
-1.75662	-1.75358	-1.75054	-1.7475	-1.76865	-1.76554	-1.76247	-1.75943	-1.80182
-1.75538	-1.75232	-1.74928	-1.74626	-1.76736	-1.76423	-1.76114	-1.75807	-1.80042
-1.75407	-1.75099	-1.77222	-1.7691	-1.76598	-1.76285	-1.75974	-1.75666	-1.79898
-1.75267	-1.74959	-1.77075	-1.76762	-1.7645	-1.76138	-1.75827	-1.75519	-1.79749
-1.75124	-1.74816	-1.76927	-1.76612	-1.76299	-1.75988	-1.75679	-1.75371	-1.796
-1.74979	-1.74672	-1.76777	-1.76461	-1.76148	-1.75837	-1.75529	-1.75222	-1.79451
-1.74832	-1.74526	-1.76628	-1.7631	-1.75997	-1.75687	-1.75379	-1.75074	-1.79303
-1.77109	-1.76794	-1.76478	-1.76162	-1.75849	-1.75539	-1.75231	-1.74927	-1.79156
-1.7696	-1.76644	-1.7633	-1.76016	-1.75704	-1.75394	-1.75086	-1.74783	-1.7901
-1.76819	-1.76503	-1.7619	-1.75878	-1.75567	-1.75259	-1.74952	-1.74648	-1.78873
-1.76687	-1.7637	-1.76058	-1.75747	-1.75439	-1.75133	-1.74828	-1.74523	-1.78746
-1.76548	-1.76232	-1.7592	-1.75611	-1.75304	-1.75001	-1.74697	-1.74391	-1.78612
-1.76398	-1.76082	-1.75771	-1.75464	-1.75159	-1.74857	-1.74553	-1.74249	-1.78468
-1.76249	-1.75934	-1.75623	-1.75316	-1.75012	-1.7471	-1.74408	-1.74104	-1.78322
-1.76109	-1.75791	-1.75479	-1.7517	-1.74865	-1.74564	-1.74262	-1.73958	-1.78174

-1.75973	-1.75653	-1.75338	-1.75028	-1.74722	-1.7442	-1.74118	-1.73814	-1.78028
-1.75834	-1.75517	-1.75205	-1.74896	-1.7459	-1.74287	-1.73984	-1.7368	-1.77892
-1.75694	-1.75384	-1.75076	-1.74771	-1.74466	-1.74161	-1.73857	-1.73551	-1.77761
-1.75553	-1.75245	-1.7494	-1.74637	-1.74333	-1.74028	-1.73723	-1.73416	-1.8005
-1.75409	-1.75101	-1.74797	-1.74495	-1.74191	-1.73886	-1.73581	-1.73274	-1.79903
-1.75269	-1.74962	-1.74658	-1.74356	-1.74053	-1.73749	-1.73443	-1.73137	-1.79762
-1.75137	-1.74833	-1.7453	-1.74227	-1.73924	-1.73619	-1.73315	-1.73009	-1.79633
-1.75006	-1.74703	-1.744	-1.74098	-1.73793	-1.73489	-1.73185	-1.72882	-1.79503
-1.74868	-1.74563	-1.7426	-1.73957	-1.73652	-1.73347	-1.75537	-1.75231	-1.79364
-1.74729	-1.74421	-1.74116	-1.73813	-1.73509	-1.73204	-1.75393	-1.75088	-1.79222
-1.74597	-1.74288	-1.73983	-1.73679	-1.73375	-1.73071	-1.75258	-1.74954	-1.79087
-1.7447	-1.74163	-1.73857	-1.73553	-1.73249	-1.72945	-1.7513	-1.74826	-1.78957
-1.74341	-1.74033	-1.73726	-1.73419	-1.7561	-1.75303	-1.74997	-1.74694	-1.78824
-1.74209	-1.73898	-1.73587	-1.73278	-1.75466	-1.75161	-1.74857	-1.74557	-1.78686
-1.74075	-1.73763	-1.7345	-1.7314	-1.75326	-1.75022	-1.79162	-1.78856	-1.7855
-1.73937	-1.73628	-1.73318	-1.7301	-1.75196	-1.74894	-1.79033	-1.78727	-1.78421
-1.73801	-1.73495	-1.73188	-1.72883	-1.75068	-1.74766	-1.78904	-1.78599	-1.78292
-1.73671	-1.73364	-1.75552	-1.7524	-1.74932	-1.7463	-1.78764	-1.7846	-1.78155
-1.73544	-1.73236	-1.75418	-1.75104	-1.74795	-1.7449	-1.78622	-1.78318	-1.78016
-1.73416	-1.73106	-1.75286	-1.74971	-1.74662	-1.74359	-1.78488	-1.78185	-1.77882
-1.73287	-1.72975	-1.75154	-1.74841	-1.74535	-1.74234	-1.78362	-1.78058	-1.77755
-1.73155	-1.72845	-1.75024	-1.74714	-1.74409	-1.7411	-1.78236	-1.7793	-1.77625
-1.73019	-1.72714	-1.74895	-1.74589	-1.74286	-1.73986	-1.78108	-1.77799	-1.7749
-1.75378	-1.75072	-1.74767	-1.74464	-1.74162	-1.7386	-1.77979	-1.77667	-1.77356
-1.75249	-1.74943	-1.7464	-1.74338	-1.74035	-1.73731	-1.77845	-1.77533	-1.77223
-1.75122	-1.74815	-1.74512	-1.74211	-1.73906	-1.73599	-1.7771	-1.77399	-1.77092
-1.74992	-1.74684	-1.74379	-1.74076	-1.73772	-1.73468	-1.77578	-1.77269	-1.78988
-1.74863	-1.7455	-1.74242	-1.73938	-1.73635	-1.73335	-1.77447	-1.77142	-1.7886
-1.74735	-1.74421	-1.74111	-1.73805	-1.73504	-1.73207	-1.77319	-1.77015	-1.78731
-1.74609	-1.74296	-1.73986	-1.73679	-1.73378	-1.73082	-1.77193	-1.76889	-1.78603
-1.74482	-1.7417	-1.73861	-1.73554	-1.73254	-1.72958	-1.7909	-1.78781	-1.78471
-1.74349	-1.74039	-1.73732	-1.73429	-1.73129	-1.72832	-1.78958	-1.78646	-1.78335
-1.74217	-1.73908	-1.73605	-1.73305	-1.73005	-1.72706	-1.78825	-1.78511	-1.78198
-1.74088	-1.73782	-1.7348	-1.73181	-1.7288	-1.72578	-1.7869	-1.78375	-1.79956
-1.7396	-1.73658	-1.73357	-1.73057	-1.72754	-1.7245	-1.78554	-1.78241	-1.79822
-1.73834	-1.73533	-1.73232	-1.72932	-1.7471	-1.74398	-1.78424	-1.78112	-1.79692
-1.73708	-1.73408	-1.73107	-1.72807	-1.74582	-1.74272	-1.78296	-1.77986	-1.79565
-1.73583	-1.73283	-1.72981	-1.7268	-1.74452	-1.74143	-1.78166	-1.77859	-1.79439
-1.73458	-1.73157	-1.72853	-1.72549	-1.74317	-1.7401	-1.78034	-1.77731	-1.79312
-1.73335	-1.73033	-1.72727	-1.7242	-1.74185	-1.73879	-1.77903	-1.77604	-1.79184
-1.73211	-1.7291	-1.72603	-1.72296	-1.74059	-1.73755	-1.77777	-1.77478	-1.79056
-1.73089	-1.72788	-1.72481	-1.72174	-1.73935	-1.73633	-1.77653	-1.77354	-1.78927
-1.72966	-1.72665	-1.7443	-1.74117	-1.73811	-1.7351	-1.77528	-1.77229	-1.788
-1.72843	-1.72543	-1.74305	-1.73992	-1.73687	-1.73388	-1.77404	-1.77105	-1.78673
-1.72721	-1.7242	-1.74181	-1.73868	-1.73564	-1.73267	-1.7728	-1.76981	-1.78546
-1.72598	-1.72298	-1.74057	-1.73747	-1.73443	-1.73145	-1.77156	-1.76857	-1.78419
-1.72475	-1.72175	-1.73933	-1.73624	-1.77641	-1.77334	-1.77031	-1.76731	-1.78292
-1.7235	-1.72051	-1.73808	-1.73501	-1.77515	-1.77208	-1.76905	-1.76605	-1.78163
-1.74292	-1.73988	-1.73683	-1.73378	-1.7739	-1.77083	-1.76778	-1.76478	-1.78034
-1.74166	-1.73863	-1.73558	-1.73254	-1.77263	-1.76957	-1.76652	-1.76352	-1.77905

-1.7404	-1.73739	-1.73435	-1.73131	-1.77137	-1.76831	-1.76526	-1.76225	-1.77776
-1.73917	-1.73616	-1.73312	-1.73009	-1.77012	-1.76705	-1.764	-1.76099	-1.77647
-1.73795	-1.73493	-1.7319	-1.72888	-1.76888	-1.76579	-1.76274	-1.75972	-1.77519
-1.73673	-1.73371	-1.7307	-1.7277	-1.76767	-1.76457	-1.76149	-1.75847	-1.80259
-1.7355	-1.73249	-1.72951	-1.72655	-1.7665	-1.76338	-1.76028	-1.75722	-1.80119
-1.73429	-1.73128	-1.72832	-1.72538	-1.76531	-1.76217	-1.75905	-1.75596	-1.81719
-1.73314	-1.73011	-1.72713	-1.72416	-1.76405	-1.76091	-1.75779	-1.75469	-1.81581
-1.73198	-1.72894	-1.72593	-1.72292	-1.76278	-1.75964	-1.75653	-1.75343	-1.81444
-1.73077	-1.72773	-1.7247	-1.72168	-1.7615	-1.75837	-1.80193	-1.79859	-1.83739
-1.72955	-1.72652	-1.72348	-1.72044	-1.76023	-1.7571	-1.80058	-1.79724	-1.83604
-1.72836	-1.72535	-1.7223	-1.71925	-1.759	-1.75589	-1.82397	-1.82059	-1.83472
-1.72718	-1.7242	-1.72115	-1.71809	-1.75782	-1.75471	-1.82271	-1.81933	-1.83342
-1.72598	-1.723	-1.76288	-1.75973	-1.8282	-1.8248	-1.82142	-1.81804	-1.83211
-1.72477	-1.72176	-1.76161	-1.75847	-1.82685	-1.82345	-1.82007	-1.8167	-1.83076
-1.72358	-1.72054	-1.80241	-1.79917	-1.86555	-1.86205	-1.87553	-1.87204	-1.86856
-1.72241	-1.71936	-1.80117	-1.79794	-1.86424	-1.86074	-1.87421	-1.87073	-1.86726
-1.72125	-1.71819	-1.79993	-1.7967	-1.86293	-1.85943	-1.87288	-1.86942	-1.86596
-1.72003	-1.71697	-1.86857	-1.86506	-1.86156	-1.85807	-1.87151	-1.86804	-1.86459
-1.71879	-1.71573	-1.86717	-1.86366	-1.86017	-1.85669	-1.87012	-1.86665	-1.8632
-1.74391	-1.7408	-1.86583	-1.86232	-1.85883	-1.85537	-1.86877	-1.86529	-1.86184
-1.74274	-1.73964	-1.86454	-1.86102	-1.85754	-1.85408	-1.86747	-1.86397	-1.8605
-1.75998	-1.75686	-1.82328	-1.81987	-1.85625	-1.85279	-1.86615	-1.86266	-1.85918
-1.75882	-1.7557	-1.82201	-1.81862	-1.85496	-1.85149	-1.86483	-1.86136	-1.85789
-1.78666	-1.7833	-1.82075	-1.81737	-1.814	-1.81062	-1.86352	-1.86006	-1.8566
-1.78542	-1.78208	-1.8195	-1.81612	-1.81274	-1.80937	-1.86222	-1.85876	-1.8553
-1.78418	-1.78085	-1.81825	-1.81486	-1.81149	-1.80812	-1.86091	-1.85744	-1.85399
-1.78294	-1.77961	-1.81699	-1.8136	-1.81022	-1.80686	-1.85958	-1.85609	-1.85262
-1.7817	-1.77836	-1.81572	-1.81233	-1.80895	-1.80558	-1.85823	-1.85472	-1.85124
-1.78046	-1.77713	-1.81446	-1.81107	-1.80768	-1.80429	-1.81809	-1.81468	-1.86613
-1.77921	-1.7759	-1.81321	-1.8098	-1.8064	-1.803	-1.81677	-1.8134	-1.8648
-1.77797	-1.77467	-1.81195	-1.80855	-1.80514	-1.80174	-1.8155	-1.81213	-1.88167
-1.77673	-1.77342	-1.81069	-1.8073	-1.80393	-1.80056	-1.8143	-1.8109	-1.88033
-1.77549	-1.77218	-1.80943	-1.80606	-1.80271	-1.79936	-1.81309	-1.80966	-1.87899
-1.77425	-1.77094	-1.80818	-1.80482	-1.80145	-1.79808	-1.81179	-1.80838	-1.87765
-1.77301	-1.76971	-1.80693	-1.80357	-1.80019	-1.79679	-1.81047	-1.80709	-1.87629
-1.77176	-1.76846	-1.80567	-1.80233	-1.79896	-1.79557	-1.84457	-1.84106	-1.87492
-1.7705	-1.7672	-1.80441	-1.80111	-1.79777	-1.7944	-1.84334	-1.83976	-1.87354
-1.76926	-1.76596	-1.80316	-1.79986	-1.79654	-1.79319	-1.84207	-1.83848	-1.8722
-1.76803	-1.76476	-1.80191	-1.79859	-1.79526	-1.79195	-1.84079	-1.83723	-1.87091
-1.76683	-1.76358	-1.8007	-1.79735	-1.79401	-1.79069	-1.83948	-1.83595	-1.86961
-1.76568	-1.76243	-1.79954	-1.79619	-1.79281	-1.78942	-1.83813	-1.83463	-1.86829
-1.76452	-1.76128	-1.79838	-1.79503	-1.79162	-1.78817	-1.83678	-1.83329	-1.86695
-1.76334	-1.76007	-1.83631	-1.83287	-1.84798	-1.84438	-1.87281	-1.86918	-1.86558
-1.76215	-1.75883	-1.83501	-1.83159	-1.84669	-1.84313	-1.87152	-1.86784	-1.8642
-1.84073	-1.83722	-1.83375	-1.83029	-1.86145	-1.85787	-1.8702	-1.86652	-1.86288
-1.83952	-1.83602	-1.8325	-1.82898	-1.86008	-1.85652	-1.86887	-1.86523	-1.8616
-1.83827	-1.83478	-1.83125	-1.82769	-1.85874	-1.85517	-1.86751	-1.86391	-1.86031
-1.83697	-1.83351	-1.83	-1.82646	-1.85745	-1.85382	-1.86613	-1.86256	-1.859
-1.83567	-1.83222	-1.84731	-1.84375	-1.85616	-1.85249	-1.86476	-1.86122	-1.85769
-1.8344	-1.83092	-1.84599	-1.84245	-1.85485	-1.8512	-1.86345	-1.85988	-1.85635

-1.83314	-1.82962	-1.84467	-1.84115	-1.85355	-1.84993	-1.86217	-1.85857	-1.85501
-1.83191	-1.82836	-1.8434	-1.83989	-1.85229	-1.84869	-1.86093	-1.85731	-1.85374
-1.8307	-1.82714	-1.84214	-1.83864	-1.85104	-1.84747	-1.8597	-1.85608	-1.8525
-1.82949	-1.8259	-1.84087	-1.83735	-1.84974	-1.84621	-1.85845	-1.85484	-1.86838
-1.82828	-1.82467	-1.83959	-1.83603	-1.84841	-1.84491	-1.85717	-1.85359	-1.86714
-1.84562	-1.84194	-1.85428	-1.85065	-1.84711	-1.84363	-1.87304	-1.86948	-1.8659
-1.84439	-1.84072	-1.85303	-1.8494	-1.84585	-1.84239	-1.8718	-1.86825	-1.86468
-1.84317	-1.8395	-1.85179	-1.84816	-1.84462	-1.84117	-1.87056	-1.86702	-1.86345
-1.84194	-1.83828	-1.85057	-1.84695	-1.86081	-1.85734	-1.86934	-1.8658	-1.86222
-1.84072	-1.83706	-1.84935	-1.84574	-1.85961	-1.85614	-1.86812	-1.86457	-1.86099
-1.83949	-1.83584	-1.84812	-1.84452	-1.8584	-1.85493	-1.86689	-1.86335	-1.87525
-1.83827	-1.83463	-1.84689	-1.8433	-1.85717	-1.8537	-1.86565	-1.86211	-1.87401
-1.83705	-1.83341	-1.87893	-1.87529	-1.87172	-1.8682	-1.87989	-1.87633	-1.87274
-1.83584	-1.83221	-1.8777	-1.87406	-1.87046	-1.86691	-1.87856	-1.875	-1.87142
-1.88385	-1.88013	-1.87647	-1.87284	-1.86923	-1.86565	-1.87728	-1.87371	-1.87013
-1.8826	-1.8789	-1.87525	-1.87163	-1.86804	-1.86449	-1.8761	-1.87253	-1.86893
-1.88136	-1.87767	-1.87403	-1.87042	-1.86686	-1.86333	-1.87495	-1.87136	-1.86774
-1.88013	-1.87644	-1.8728	-1.86919	-1.86564	-1.86213	-1.87374	-1.87014	-1.86652
-1.87889	-1.87521	-1.87157	-1.86796	-1.86441	-1.86091	-1.8725	-1.86891	-1.86528
-1.87766	-1.87399	-1.87035	-1.86674	-1.86319	-1.85968	-1.87126	-1.86766	-1.86405
-1.87643	-1.87278	-1.86914	-1.86553	-1.86197	-1.85844	-1.87	-1.86641	-1.8628
-1.87521	-1.87157	-1.86795	-1.86436	-1.86079	-1.85724	-1.86877	-1.86517	-1.86156
-1.87399	-1.87038	-1.86679	-1.86322	-1.85966	-1.85609	-1.86758	-1.86395	-1.8603
-1.8728	-1.86921	-1.86564	-1.86209	-1.85852	-1.85494	-1.86638	-1.86272	-1.85905
-1.87165	-1.86808	-1.86452	-1.86097	-1.85737	-1.85375	-1.86516	-1.86148	-1.85781
-1.87051	-1.86696	-1.86339	-1.85982	-1.8562	-1.85255	-1.86392	-1.86024	-1.85657
-1.86938	-1.86582	-1.86224	-1.85863	-1.85499	-1.85133	-1.86268	-1.85899	-1.85533
-1.86826	-1.86468	-1.86106	-1.85741	-1.85376	-1.85009	-1.86143	-1.85776	-1.85411
-1.8671	-1.8635	-1.85985	-1.85618	-1.85252	-1.84886	-1.8602	-1.85653	-1.8529
-1.86591	-1.86228	-1.85862	-1.85494	-1.85129	-1.84765	-1.85898	-1.85532	-1.8517
-1.86472	-1.86107	-1.8574	-1.85372	-1.85008	-1.84646	-1.8578	-1.85416	-1.85055
-1.86351	-1.85985	-1.85618	-1.85251	-1.8489	-1.84532	-1.85669	-1.85309	-1.84949
-1.86231	-1.85866	-1.855	-1.85134	-1.84775	-1.84419	-1.85557	-1.852	-1.8484
-1.86115	-1.85753	-1.85389	-1.85026	-1.84666	-1.84308	-1.85441	-1.85081	-1.8472
-1.86001	-1.85641	-1.8528	-1.8492	-1.84558	-1.84195	-1.85323	-1.8496	-1.84596
-1.85889	-1.85527	-1.85165	-1.84804	-1.84442	-1.84079	-1.85203	-1.84838	-1.84472
-1.85778	-1.85412	-1.85047	-1.84683	-1.84321	-1.83959	-1.85083	-1.84716	-1.84349
-1.85664	-1.85294	-1.84927	-1.84562	-1.84201	-1.83842	-1.84966	-1.84601	-1.84234
-1.85544	-1.85174	-1.84806	-1.84441	-1.84081	-1.83727	-1.84855	-1.84494	-1.8413
-1.85429	-1.85059	-1.8469	-1.84325	-1.83966	-1.83613	-1.84742	-1.84383	-1.84021
-1.85323	-1.84953	-1.84584	-1.84218	-1.83858	-1.83501	-1.84626	-1.84266	-1.83903
-1.85216	-1.84846	-1.84478	-1.84112	-1.83749	-1.83389	-1.84509	-1.84147	-1.83784
-1.851	-1.8473	-1.84362	-1.83996	-1.83633	-1.83271	-1.8439	-1.8403	-1.83669
-1.8498	-1.8461	-1.84243	-1.83878	-1.83515	-1.83153	-1.84271	-1.83913	-1.83555
-1.8487	-1.84499	-1.84133	-1.83768	-1.83405	-1.83043	-1.84159	-1.83799	-1.8344
-1.84765	-1.84395	-1.84028	-1.83664	-1.83301	-1.82939	-1.84051	-1.83687	-1.83324
-1.84654	-1.84284	-1.83918	-1.83554	-1.83191	-1.82828	-1.83938	-1.83571	-1.83206
-1.84535	-1.84166	-1.83799	-1.83435	-1.83072	-1.82709	-1.83817	-1.83449	-1.83084
-1.84419	-1.84051	-1.83685	-1.83321	-1.82957	-1.82594	-1.837	-1.83332	-1.82967
-1.84313	-1.83947	-1.83582	-1.83218	-1.82854	-1.8249	-1.83594	-1.83226	-1.82859

-1.84208	-1.83843	-1.83479	-1.83116	-1.82753	-1.82389	-1.83491	-1.83122	-1.82754
-1.84092	-1.83729	-1.83368	-1.83009	-1.82648	-1.82286	-1.83388	-1.83019	-1.8265
-1.83973	-1.8361	-1.83253	-1.82898	-1.82541	-1.82182	-1.83285	-1.82915	-1.82546
-1.83863	-1.835	-1.83142	-1.82786	-1.82429	-1.8207	-1.83171	-1.82801	-1.82432
-1.8376	-1.83396	-1.83034	-1.82674	-1.82312	-1.8195	-1.83048	-1.82678	-1.8231
-1.8366	-1.83294	-1.8293	-1.82566	-1.82202	-1.81837	-1.82932	-1.82562	-1.82194
-1.83562	-1.83195	-1.8283	-1.82465	-1.82099	-1.81733	-1.82826	-1.82457	-1.82089
-1.83464	-1.83096	-1.8273	-1.82364	-1.81998	-1.81631	-1.82723	-1.82354	-1.81987
-1.83363	-1.82996	-1.82629	-1.82263	-1.81896	-1.81529	-1.8262	-1.82251	-1.81884
-1.83259	-1.82892	-1.82525	-1.82158	-1.81791	-1.81424	-1.82515	-1.82147	-1.8178
-1.83145	-1.82778	-1.82411	-1.82043	-1.81676	-1.81309	-1.82398	-1.82031	-1.81665
-1.83024	-1.82657	-1.8229	-1.81922	-1.81555	-1.81188	-1.82276	-1.81909	-1.81543
-1.82912	-1.82546	-1.82178	-1.8181	-1.81443	-1.81076	-1.82162	-1.81796	-1.83301
-1.82809	-1.82442	-1.82075	-1.81707	-1.81339	-1.80973	-1.82058	-1.81692	-1.83196
-1.82708	-1.82341	-1.81973	-1.81605	-1.81238	-1.80872	-1.81956	-1.8159	-1.83093
-1.82608	-1.82241	-1.81873	-1.81505	-1.81138	-1.80772	-1.81856	-1.8149	-1.82991
-1.82509	-1.82142	-1.81773	-1.81405	-1.81039	-1.80673	-1.81756	-1.8139	-1.82889
-1.82409	-1.82042	-1.81673	-1.81305	-1.80939	-1.80574	-1.81656	-1.81289	-1.82787
-1.8231	-1.81942	-1.81574	-1.81206	-1.80839	-1.80475	-1.81555	-1.81188	-1.82685
-1.82211	-1.81842	-1.81473	-1.81105	-1.80739	-1.80374	-1.83324	-1.82952	-1.8258
-1.82111	-1.81742	-1.81373	-1.81004	-1.80637	-1.80272	-1.83219	-1.82847	-1.82475
-1.82011	-1.81642	-1.81273	-1.80903	-1.80534	-1.80167	-1.83109	-1.82736	-1.82362
-1.81911	-1.81542	-1.81172	-1.80802	-1.8043	-1.80057	-1.82992	-1.82616	-1.82241
-1.81811	-1.81442	-1.81072	-1.80701	-1.80327	-1.7995	-1.82879	-1.825	-1.82125
-1.8171	-1.81341	-1.80971	-1.80601	-1.80226	-1.79848	-1.82774	-1.82394	-1.82018
-1.81609	-1.8124	-1.80871	-1.80501	-1.80126	-1.79749	-1.82672	-1.82291	-1.81915
-1.81508	-1.81139	-1.8077	-1.804	-1.81919	-1.81536	-1.82569	-1.82189	-1.81813
-1.81407	-1.81038	-1.8067	-1.803	-1.81818	-1.81436	-1.82467	-1.82087	-1.81711
-1.81306	-1.80937	-1.80569	-1.80201	-1.81717	-1.81335	-1.82366	-1.81985	-1.81609
-1.81205	-1.80836	-1.80469	-1.80102	-1.81617	-1.81236	-1.82265	-1.81883	-1.81505
-1.81104	-1.80736	-1.80369	-1.80003	-1.81519	-1.8114	-1.82168	-1.81785	-1.81406
-1.81003	-1.80635	-1.8027	-1.79904	-1.81422	-1.81048	-1.82078	-1.81694	-1.81313
-1.80903	-1.80535	-1.82064	-1.81694	-1.81325	-1.80955	-1.81986	-1.81604	-1.81221
-1.80802	-1.80435	-1.81963	-1.81594	-1.81225	-1.80857	-1.8189	-1.81512	-1.81132
-1.80701	-1.80335	-1.81861	-1.81492	-1.81124	-1.80757	-1.81792	-1.81418	-1.81042
-1.806	-1.80234	-1.8176	-1.81391	-1.81023	-1.80656	-1.81691	-1.8132	-1.80946
-1.80498	-1.80134	-1.81658	-1.8129	-1.80922	-1.80555	-1.81589	-1.81218	-1.80847
-1.80399	-1.80033	-1.81556	-1.81188	-1.8082	-1.80453	-1.81486	-1.81116	-1.80745
-1.80302	-1.79932	-1.81452	-1.81086	-1.80719	-1.80351	-1.81383	-1.81013	-1.80643
-1.82102	-1.81724	-1.81352	-1.80985	-1.80617	-1.8025	-1.8128	-1.8091	-1.8054
-1.82013	-1.81632	-1.81257	-1.80885	-1.80515	-1.80147	-1.81176	-1.80806	-1.80436
-1.81924	-1.81542	-1.81164	-1.80789	-1.80417	-1.80047	-1.81074	-1.80703	-1.80334
-1.81832	-1.81454	-1.81078	-1.80703	-1.80329	-1.79957	-1.80978	-1.80604	-1.80231
-1.81738	-1.81365	-1.80993	-1.80621	-1.80246	-1.7987	-1.80886	-1.80507	-1.80131
-1.81638	-1.81268	-1.80898	-1.80528	-1.80156	-1.7978	-1.80797	-1.80418	-1.80041
-1.81536	-1.81166	-1.80797	-1.8043	-1.8006	-1.7969	-1.8071	-1.80335	-1.79959
-1.8144	-1.81069	-1.80699	-1.80331	-1.79963	-1.79595	-1.80616	-1.80244	-1.7987
-1.81354	-1.80977	-1.80603	-1.8023	-1.7986	-1.79493	-1.80514	-1.80144	-1.79773
-1.81266	-1.80887	-1.8051	-1.80135	-1.79764	-1.79395	-1.80414	-1.80044	-1.79674
-1.81173	-1.80799	-1.80425	-1.80051	-1.79679	-1.79306	-1.8032	-1.79945	-1.79572

-1.81079	-1.80709	-1.80339	-1.79968	-1.79595	-1.79219	-1.80228	-1.79849	-1.79473
-1.80984	-1.80613	-1.80244	-1.79875	-1.79504	-1.79129	-1.80139	-1.79761	-1.79385
-1.80889	-1.80515	-1.80146	-1.7978	-1.7941	-1.79039	-1.80051	-1.79677	-1.79302
-1.808	-1.80427	-1.80058	-1.79692	-1.79323	-1.78951	-1.79961	-1.79587	-1.79211
-1.80716	-1.80346	-1.79978	-1.79611	-1.79239	-1.78864	-1.7987	-1.79491	-1.79115
-1.80628	-1.80259	-1.79892	-1.79524	-1.79152	-1.78776	-1.79779	-1.794	-1.79023
-1.80534	-1.80163	-1.79795	-1.79428	-1.79058	-1.78685	-1.79691	-1.79315	-1.78939
-1.80442	-1.80069	-1.79701	-1.79334	-1.78965	-1.78595	-1.79601	-1.79228	-1.78853
-1.80357	-1.79985	-1.79617	-1.7925	-1.7888	-1.78506	-1.79509	-1.79133	-1.78757
-1.80274	-1.79902	-1.79535	-1.79167	-1.78795	-1.78418	-1.79417	-1.79038	-1.78661
-1.80189	-1.79816	-1.79446	-1.79076	-1.78703	-1.78329	-1.79328	-1.7895	-1.79236
-1.80104	-1.79728	-1.79354	-1.78981	-1.78609	-1.78239	-1.79241	-1.78865	-1.79154
-1.80018	-1.79641	-1.79266	-1.78892	-1.78522	-1.78154	-1.79156	-1.7878	-1.7907
-1.79931	-1.79554	-1.7918	-1.78809	-1.7844	-1.78073	-1.79073	-1.78695	-1.78985
-1.79845	-1.79468	-1.79095	-1.78725	-1.78356	-1.77987	-1.79658	-1.79279	-1.789
-1.79761	-1.79386	-1.79012	-1.78638	-1.78265	-1.77893	-1.79565	-1.7919	-1.78815
-1.79677	-1.79303	-1.78927	-1.78551	-1.78175	-1.77801	-1.79474	-1.79103	-1.78731
-1.79593	-1.79217	-1.78841	-1.78464	-1.78089	-1.77717	-1.79393	-1.79022	-1.7865
-1.79508	-1.7913	-1.78754	-1.78378	-1.78006	-1.77637	-1.79315	-1.78944	-1.7857
-1.79422	-1.79044	-1.78669	-1.78296	-1.78622	-1.78254	-1.79236	-1.78862	-1.78487
-1.79335	-1.78958	-1.78586	-1.78217	-1.78547	-1.78178	-1.79157	-1.7878	-1.78402
-1.7925	-1.78876	-1.78506	-1.78137	-1.7847	-1.78099	-1.79075	-1.78696	-1.81092
-1.79168	-1.78797	-1.78427	-1.78058	-1.78391	-1.78017	-1.7899	-1.78611	-1.81006
-1.79088	-1.78719	-1.79056	-1.78685	-1.78311	-1.77934	-1.78906	-1.78528	-1.80923
-1.7901	-1.78641	-1.78977	-1.78602	-1.78226	-1.7785	-1.78823	-1.78448	-1.80845
-1.78934	-1.78563	-1.78899	-1.7852	-1.78143	-1.77767	-1.78741	-1.7837	-1.80767
-1.78856	-1.78485	-1.78823	-1.78443	-1.78066	-1.7769	-1.78664	-1.78294	-1.80689
-1.78779	-1.78407	-1.78747	-1.78369	-1.77992	-1.77616	-1.78589	-1.78219	-1.80611
-1.79417	-1.79043	-1.78667	-1.7829	-1.77915	-1.77541	-1.78514	-1.78144	-1.83914
-1.79337	-1.78959	-1.78582	-1.78207	-1.77835	-1.77466	-1.7844	-1.78069	-1.83834
-1.79259	-1.78879	-1.78502	-1.78127	-1.77757	-1.7739	-1.81137	-1.80757	-1.8375
-1.79184	-1.78803	-1.78426	-1.78052	-1.77682	-1.77316	-1.81058	-1.80674	-1.8366
-1.7911	-1.78729	-1.78352	-1.77977	-1.77607	-1.77241	-1.8098	-1.80592	-1.85461
-1.79036	-1.78655	-1.78278	-1.77903	-1.77533	-1.77167	-1.80903	-1.80515	-1.85378
-1.78963	-1.78582	-1.78205	-1.7783	-1.7746	-1.77094	-1.80827	-1.80439	-1.85298
-1.78889	-1.78508	-1.78131	-1.77757	-1.77387	-1.77021	-1.84139	-1.83735	-1.85218
-1.78816	-1.78435	-1.78058	-1.77684	-1.77314	-1.76948	-1.8406	-1.83657	-1.85138
-1.78744	-1.78363	-1.77986	-1.77611	-1.77241	-1.76875	-1.85874	-1.85466	-1.85059
-1.78672	-1.78292	-1.77914	-1.77538	-1.77167	-1.76802	-1.85794	-1.85386	-1.84979
-1.78603	-1.78224	-1.77846	-1.77468	-1.79106	-1.78734	-1.85715	-1.85307	-1.849
-1.78536	-1.78162	-1.77784	-1.77405	-1.79039	-1.78663	-1.85636	-1.85228	-1.84822
-1.78469	-1.78098	-1.77721	-1.77341	-1.78972	-1.78593	-1.85558	-1.85152	-1.84746
-1.78398	-1.78029	-1.79668	-1.79283	-1.78901	-1.78521	-1.85483	-1.8508	-1.84678
-1.78325	-1.77957	-1.79595	-1.7921	-1.78829	-1.78449	-1.85408	-1.8501	-1.84611
-1.78256	-1.77886	-1.79522	-1.79138	-1.86135	-1.85731	-1.8533	-1.84935	-1.84538
-1.78188	-1.77815	-1.79449	-1.79066	-1.86057	-1.85652	-1.85251	-1.84857	-1.84461
-1.8014	-1.79759	-1.79379	-1.78999	-1.85985	-1.85578	-1.85177	-1.84781	-1.84385
-1.80068	-1.79687	-1.79311	-1.78936	-1.85919	-1.85511	-1.85107	-1.84708	-1.84309
-1.8	-1.79618	-1.79244	-1.78871	-1.85851	-1.85444	-1.85039	-1.84636	-1.84235
-1.79937	-1.79554	-1.79176	-1.78801	-1.85778	-1.85376	-1.84973	-1.84568	-1.84164

-1.79875	-1.79492	-1.79111	-1.78731	-1.85704	-1.85307	-1.84906	-1.84499	-1.84094
-1.7981	-1.79431	-1.79049	-1.78666	-1.85632	-1.85232	-1.8483	-1.84425	-1.8402
-1.79744	-1.79369	-1.78988	-1.78604	-1.85561	-1.85157	-1.84753	-1.84348	-1.83945
-1.79676	-1.79302	-1.78923	-1.78542	-1.85493	-1.85087	-1.84682	-1.84278	-1.83875
-1.79608	-1.7923	-1.78855	-1.7848	-1.85429	-1.85022	-1.84617	-1.84212	-1.83808
-1.79542	-1.79162	-1.78787	-1.78415	-1.85361	-1.84956	-1.84551	-1.84147	-1.83743
-1.7948	-1.79098	-1.7872	-1.78344	-1.85287	-1.84888	-1.84487	-1.84082	-1.83678
-1.79419	-1.79036	-1.84775	-1.84375	-1.85214	-1.84818	-1.84419	-1.84015	-1.83611
-1.79358	-1.78975	-1.84708	-1.84307	-1.85143	-1.84744	-1.84343	-1.83941	-1.83538
-1.79298	-1.78914	-1.84643	-1.84241	-1.85073	-1.8467	-1.84268	-1.83866	-1.83465
-1.79238	-1.78854	-1.84578	-1.84175	-1.85006	-1.84602	-1.84199	-1.83798	-1.83398
-1.79178	-1.78794	-1.84514	-1.8411	-1.84941	-1.84537	-1.84135	-1.83733	-1.83334
-1.79118	-1.78734	-1.84451	-1.84047	-1.84877	-1.84473	-1.8407	-1.83669	-1.83269
-1.79058	-1.78674	-1.84387	-1.83984	-1.84813	-1.84409	-1.84006	-1.83604	-1.83203
-1.78998	-1.78614	-1.84323	-1.83921	-1.8475	-1.84345	-1.83942	-1.83539	-1.83138
-1.78938	-1.78555	-1.8426	-1.83857	-1.84685	-1.84282	-1.83878	-1.83475	-1.83072
-1.78878	-1.78496	-1.84197	-1.83793	-1.84622	-1.84219	-1.83815	-1.83411	-1.83008
-1.78817	-1.78435	-1.84133	-1.83731	-1.84559	-1.84156	-1.83752	-1.83348	-1.82944
-1.78757	-1.78375	-1.8407	-1.83669	-1.84497	-1.84094	-1.83691	-1.83286	-1.82883
-1.81439	-1.8105	-1.85242	-1.84839	-1.84437	-1.84037	-1.83635	-1.83231	-1.85706
-1.81379	-1.8099	-1.8518	-1.84778	-1.84379	-1.83982	-1.83583	-1.8318	-1.85653
-1.84693	-1.84289	-1.85122	-1.8472	-1.84321	-1.83925	-1.86419	-1.86007	-1.85595
-1.84642	-1.84238	-1.85069	-1.84665	-1.84264	-1.83865	-1.86356	-1.85943	-1.85531
-1.84587	-1.84183	-1.85013	-1.84608	-1.87112	-1.86703	-1.86292	-1.85879	-1.85467
-1.84525	-1.84122	-1.84952	-1.84548	-1.8705	-1.86639	-1.86227	-1.85814	-1.85402
-1.84462	-1.84058	-1.8489	-1.84487	-1.86988	-1.86576	-1.86164	-1.85752	-1.8534
-1.85637	-1.85231	-1.87747	-1.87337	-1.86927	-1.86519	-1.86108	-1.85697	-1.85285
-1.85575	-1.85169	-1.87684	-1.87275	-1.86867	-1.86463	-1.86055	-1.85644	-1.85232
-1.88454	-1.88038	-1.87626	-1.87217	-1.86809	-1.86404	-1.85996	-1.85585	-1.85173
-1.88402	-1.87986	-1.87573	-1.87162	-1.86752	-1.86343	-1.85933	-1.85522	-1.85111
-1.88349	-1.87934	-1.8752	-1.87108	-1.86696	-1.86285	-1.85873	-1.85463	-1.85052
-1.88298	-1.87883	-1.87468	-1.87055	-1.86643	-1.86231	-1.8582	-1.85409	-1.84999
-1.88243	-1.87828	-1.87414	-1.87	-1.86587	-1.86176	-1.85764	-1.85354	-1.84944
-1.88181	-1.87766	-1.87352	-1.86938	-1.86525	-1.86114	-1.85703	-1.85292	-1.84883
-1.88118	-1.87704	-1.87289	-1.86876	-1.86464	-1.86053	-1.85642	-1.85231	-1.84822
-1.88062	-1.87648	-1.87234	-1.8682	-1.86408	-1.85998	-1.85587	-1.85176	-1.84767
-1.8801	-1.87596	-1.87182	-1.86768	-1.86356	-1.85946	-1.85535	-1.85125	-1.84715
-1.87958	-1.87544	-1.8713	-1.86717	-1.86305	-1.85894	-1.85484	-1.85074	-1.84664
-1.87907	-1.87493	-1.87079	-1.86666	-1.86254	-1.85844	-1.85434	-1.85023	-1.84614
-1.87856	-1.87443	-1.87029	-1.86615	-1.86203	-1.85793	-1.85383	-1.84973	-1.84564
-1.87805	-1.87392	-1.86978	-1.86564	-1.86152	-1.85743	-1.85333	-1.84923	-1.84513
-1.87754	-1.8734	-1.86926	-1.86513	-1.86102	-1.85692	-1.85282	-1.84872	-1.84463
-1.87703	-1.8729	-1.86875	-1.86462	-1.86051	-1.85641	-1.85232	-1.84822	-1.84413
-1.87653	-1.87239	-1.86825	-1.86411	-1.86	-1.85591	-1.85181	-1.84772	-1.84363
-1.87602	-1.87188	-1.86774	-1.8636	-1.85949	-1.8554	-1.85131	-1.84722	-1.84313
-1.8755	-1.87137	-1.86723	-1.8631	-1.85899	-1.8549	-1.85081	-1.84671	-1.84262
-1.875	-1.87086	-1.86673	-1.8626	-1.85849	-1.8544	-1.85031	-1.84622	-1.84213
-1.87449	-1.87036	-1.86623	-1.86212	-1.85801	-1.85391	-1.84982	-1.84573	-1.84164
-1.87403	-1.86989	-1.86577	-1.86166	-1.85756	-1.85345	-1.84936	-1.84528	-1.85304
-1.87364	-1.8695	-1.86538	-1.86127	-1.85716	-1.85306	-1.84897	-1.84489	-1.85264

-1.87324	-1.8691	-1.86498	-1.86087	-1.85676	-1.85266	-1.84857	-1.84449	-1.85224
-1.87277	-1.86863	-1.86451	-1.86041	-1.8563	-1.8522	-1.84811	-1.84403	-1.85178
-1.87228	-1.86814	-1.86402	-1.85992	-1.85581	-1.85171	-1.84762	-1.84354	-1.85129
-1.87181	-1.86765	-1.86353	-1.85942	-1.85532	-1.85122	-1.84713	-1.84306	-1.85079
-1.87136	-1.86717	-1.86303	-1.85892	-1.85482	-1.85074	-1.84665	-1.84257	-1.85029
-1.87093	-1.86673	-1.86259	-1.85848	-1.85438	-1.8503	-1.84621	-1.84212	-1.84984
-1.87051	-1.86634	-1.86221	-1.85811	-1.85401	-1.84991	-1.84582	-1.84173	-1.84944
-1.87012	-1.86596	-1.86185	-1.85775	-1.85365	-1.84953	-1.84543	-1.84133	-1.84903
-1.86974	-1.8656	-1.86149	-1.85739	-1.85328	-1.84915	-1.84503	-1.8409	-1.84857
-1.86936	-1.86522	-1.86111	-1.85701	-1.85289	-1.84877	-1.84463	-1.84048	-1.84812
-1.86894	-1.86478	-1.86066	-1.85656	-1.85245	-1.84834	-1.85606	-1.85187	-1.8477
-1.86851	-1.86432	-1.86018	-1.85608	-1.85198	-1.84789	-1.85563	-1.85147	-1.84732
-1.86809	-1.8639	-1.85977	-1.85567	-1.85157	-1.84748	-1.85523	-1.85108	-1.84695
-1.86769	-1.86352	-1.8594	-1.85531	-1.85121	-1.84711	-1.85484	-1.85072	-1.8466
-1.8673	-1.86315	-1.85904	-1.85495	-1.86275	-1.8586	-1.85447	-1.85036	-1.84624
-1.86694	-1.86281	-1.85869	-1.85459	-1.86238	-1.85825	-1.85412	-1.84999	-1.84586
-1.86659	-1.86246	-1.85834	-1.85423	-1.86202	-1.8579	-1.85376	-1.84963	-1.84548
-1.86623	-1.8621	-1.85799	-1.85388	-1.86166	-1.85753	-1.85339	-1.84926	-1.84511
-1.86588	-1.86174	-1.85763	-1.85354	-1.86131	-1.85716	-1.85302	-1.84889	-1.84474
-1.86552	-1.86139	-1.85727	-1.85317	-1.86094	-1.8568	-1.85266	-1.84853	-1.84438
-1.86516	-1.86104	-1.85692	-1.85281	-1.86057	-1.85644	-1.85231	-1.84817	-1.84403
-1.86479	-1.86068	-1.85656	-1.85245	-1.86021	-1.85608	-1.85195	-1.84781	-1.84366
-1.86442	-1.86031	-1.8562	-1.8521	-1.85985	-1.85571	-1.85157	-1.84743	-1.84329
-1.86407	-1.85995	-1.85584	-1.85174	-1.85949	-1.85534	-1.8512	-1.84707	-1.87281
-1.86376	-1.85959	-1.85547	-1.85137	-1.85911	-1.85498	-1.85085	-1.84672	-1.87246
-1.86346	-1.85926	-1.85511	-1.85101	-1.85875	-1.85462	-1.8505	-1.84637	-1.87211
-1.86318	-1.85898	-1.85481	-1.85066	-1.85838	-1.85426	-1.85014	-1.84602	-1.87174
-1.8629	-1.85873	-1.85453	-1.85034	-1.85803	-1.85392	-1.8498	-1.84567	-1.87137
-1.86258	-1.85843	-1.86615	-1.86192	-1.85775	-1.85363	-1.8795	-1.87528	-1.87106
-1.86224	-1.8581	-1.86586	-1.86167	-1.85751	-1.85338	-1.87923	-1.87502	-1.87079
-1.86191	-1.85777	-1.86554	-1.86138	-1.85724	-1.85309	-1.87892	-1.87472	-1.8705
-1.8616	-1.85743	-1.86519	-1.86105	-1.8569	-1.85276	-1.87857	-1.87437	-1.87017
-1.86131	-1.85711	-1.86487	-1.86074	-1.85659	-1.85244	-1.87823	-1.87403	-1.86983
-1.86104	-1.85686	-1.86462	-1.86048	-1.85633	-1.85218	-1.87794	-1.87369	-1.86946
-1.86079	-1.85663	-1.86438	-1.86023	-1.85608	-1.85193	-1.87766	-1.87337	-1.86911
-1.86054	-1.8564	-1.86411	-1.85992	-1.88582	-1.8816	-1.87736	-1.87308	-1.86883
-1.86031	-1.85616	-1.86384	-1.8596	-1.88547	-1.88127	-1.87706	-1.87281	-1.86858
-1.86002	-1.85589	-1.89377	-1.88944	-1.88518	-1.88099	-1.87679	-1.87256	-1.86833
-1.8597	-1.85558	-1.89348	-1.88919	-1.88495	-1.88076	-1.87655	-1.87231	-1.86807
-1.85942	-1.85531	-1.89322	-1.88895	-1.88472	-1.88052	-1.8763	-1.87207	-1.86782
-1.85918	-1.85509	-1.89298	-1.88872	-1.88449	-1.88028	-1.87606	-1.87184	-1.8676
-1.90122	-1.897	-1.89275	-1.88848	-1.88424	-1.88002	-1.8758	-1.87159	-1.86736
-1.901	-1.89678	-1.89251	-1.88824	-1.88397	-1.8797	-1.87546	-1.87126	-1.86705
-1.90078	-1.89655	-1.89228	-1.88799	-1.88369	-1.87938	-1.87512	-1.87093	-1.86674
-1.90056	-1.89632	-1.89205	-1.88776	-1.88345	-1.87911	-1.87484	-1.87065	-1.86647
-1.90033	-1.8961	-1.89183	-1.88754	-1.88322	-1.87888	-1.8746	-1.87041	-1.86623
-1.9001	-1.89587	-1.8916	-1.88731	-1.88299	-1.87864	-1.87437	-1.87017	-1.866
-1.89987	-1.89563	-1.89136	-1.88707	-1.88275	-1.87841	-1.87413	-1.86994	-1.86577
-1.89964	-1.8954	-1.89113	-1.88684	-1.88252	-1.87818	-1.87391	-1.86972	-1.86554
-1.8994	-1.89517	-1.8909	-1.88661	-1.8823	-1.87797	-1.87369	-1.86948	-1.8653

-1.89917	-1.89494	-1.89068	-1.88639	-1.88209	-1.87777	-1.8735	-1.86928	-1.86508
-1.89893	-1.8947	-1.89044	-1.88618	-1.8819	-1.87762	-1.87337	-1.86914	-1.86491
-1.89871	-1.89447	-1.89021	-1.88596	-1.88172	-1.87749	-1.87325	-1.86901	-1.86477
-1.89855	-1.89428	-1.89001	-1.88574	-1.88151	-1.8773	-1.87307	-1.86883	-1.86458
-1.89843	-1.89413	-1.88982	-1.88551	-1.88127	-1.87707	-1.87285	-1.86862	-1.86437
-1.89826	-1.89395	-1.88962	-1.88529	-1.88103	-1.87684	-1.87263	-1.8684	-1.86416
-1.89805	-1.89374	-1.88941	-1.88507	-1.88081	-1.8766	-1.87239	-1.86817	-1.86396
-1.89784	-1.89354	-1.88921	-1.88488	-1.88062	-1.8764	-1.87218	-1.86796	-1.86375
-1.89767	-1.89335	-1.88904	-1.88475	-1.88049	-1.87626	-1.87202	-1.86777	-1.86354
-1.8975	-1.89318	-1.88889	-1.88463	-1.88037	-1.87613	-1.87187	-1.8676	-1.86335
-1.89735	-1.89304	-1.88877	-1.88451	-1.88024	-1.87597	-1.87171	-1.86745	-1.86321
-1.89721	-1.89292	-1.88865	-1.88439	-1.88011	-1.87581	-1.87155	-1.86732	-1.8631
-1.89708	-1.89279	-1.88851	-1.88424	-1.87995	-1.87567	-1.87142	-1.86719	-1.86297
-1.89696	-1.89266	-1.88836	-1.88406	-1.87979	-1.87554	-1.8713	-1.86708	-1.86285
-1.89682	-1.89251	-1.88821	-1.88391	-1.87965	-1.87541	-1.87118	-1.86695	-1.86271
-1.89666	-1.89234	-1.88805	-1.88378	-1.87952	-1.87529	-1.87105	-1.86679	-1.86254
-1.88196	-1.87767	-1.88791	-1.88365	-1.8794	-1.87516	-1.87091	-1.86664	-1.86239
-1.8818	-1.87754	-1.88778	-1.88353	-1.87927	-1.875	-1.87075	-1.8665	-1.86227
-1.88165	-1.87741	-1.88766	-1.8834	-1.87913	-1.87484	-1.87058	-1.86636	-1.86216
-1.88153	-1.87729	-1.88752	-1.88324	-1.87897	-1.8747	-1.87046	-1.86626	-1.86207
-1.88142	-1.87718	-1.88739	-1.88307	-1.8788	-1.87457	-1.87036	-1.86618	-1.86199
-1.88133	-1.87709	-1.8873	-1.88298	-1.87871	-1.87448	-1.87027	-1.86609	-1.8619
-1.88125	-1.87701	-1.88724	-1.88295	-1.87867	-1.87441	-1.87018	-1.86599	-1.86181
-1.88116	-1.87693	-1.87268	-1.86844	-1.8786	-1.87433	-1.87009	-1.8659	-1.86171
-1.88106	-1.87682	-1.87255	-1.86828	-1.87846	-1.87421	-1.86999	-1.8658	-1.86162
-1.88096	-1.87671	-1.87243	-1.86815	-1.87833	-1.8741	-1.86989	-1.86571	-1.86153
-1.88086	-1.87664	-1.87238	-1.86811	-1.87828	-1.87402	-1.86979	-1.86561	-1.86143
-1.88078	-1.87657	-1.87234	-1.8681	-1.87827	-1.87396	-1.8697	-1.86551	-1.86133
-1.88072	-1.87648	-1.87227	-1.86806	-1.87824	-1.87394	-1.86967	-1.86544	-1.86123
-1.88068	-1.87639	-1.87217	-1.86799	-1.8782	-1.87394	-1.86967	-1.86539	-1.86114
-1.88065	-1.87634	-1.87211	-1.86791	-1.86371	-1.85952	-1.86965	-1.86536	-1.86109
-1.88065	-1.87636	-1.87209	-1.86783	-1.86362	-1.85944	-1.86961	-1.86535	-1.86109
-1.88062	-1.87636	-1.87207	-1.86777	-1.86354	-1.85937	-1.86954	-1.86532	-1.88916
-1.88054	-1.87631	-1.87205	-1.86778	-1.86353	-1.85929	-1.86945	-1.86525	-1.88911
-1.88046	-1.87625	-1.87202	-1.86778	-1.86351	-1.85923	-1.86937	-1.86517	-1.88905
-1.8804	-1.87616	-1.87194	-1.86773	-1.86348	-1.85921	-1.86934	-1.86511	-1.88895
-1.88036	-1.87606	-1.87184	-1.86765	-1.86344	-1.85921	-1.86934	-1.86506	-1.88885
-1.88034	-1.87603	-1.87179	-1.86758	-1.86337	-1.85918	-1.85496	-1.8507	-1.8888
-1.88035	-1.87605	-1.87177	-1.8675	-1.86329	-1.85913	-1.85494	-1.8507	-1.8888
-1.88037	-1.87609	-1.87178	-1.86747	-1.86326	-1.85911	-1.85493	-1.85072	-1.88882
-1.88041	-1.87614	-1.87183	-1.86751	-1.86328	-1.85912	-1.85495	-1.85076	-1.88887
-1.88042	-1.87618	-1.87187	-1.86755	-1.86331	-1.85912	-1.85495	-1.85078	-1.88891
-1.88037	-1.87615	-1.87187	-1.86757	-1.86331	-1.85908	-1.85488	-1.85073	-1.88888
-1.88032	-1.87612	-1.87187	-1.86759	-1.86332	-1.85904	-1.85482	-1.85067	-1.88884
-1.88034	-1.87614	-1.8719	-1.86764	-1.86336	-1.85905	-1.85482	-1.85068	-1.87487
-1.88038	-1.87619	-1.87195	-1.86769	-1.86341	-1.8591	-1.85487	-1.85072	-1.8749
-1.88044	-1.87624	-1.872	-1.86775	-1.86346	-1.85915	-1.88343	-1.87918	-1.87493
-1.8805	-1.8763	-1.87206	-1.86781	-1.86352	-1.85921	-1.88348	-1.87922	-1.87498
-1.88057	-1.87636	-1.87212	-1.86786	-1.86357	-1.85926	-1.88352	-1.87927	-1.87502
-1.88062	-1.87641	-1.87217	-1.86791	-1.86363	-1.85931	-1.88357	-1.87931	-1.87506

-1.88068	-1.87647	-1.87223	-1.86797	-1.86368	-1.85936	-1.88361	-1.87936	-1.88798
-1.88073	-1.87652	-1.87228	-1.86802	-1.86373	-1.85941	-1.88366	-1.8794	-1.88803
-1.88078	-1.87657	-1.87233	-1.86807	-1.86378	-1.85947	-1.88371	-1.87945	-1.88808
-1.88082	-1.87662	-1.87238	-1.86812	-1.86383	-1.85952	-1.88375	-1.8795	-1.88814
-1.88086	-1.87666	-1.87243	-1.86817	-1.86388	-1.85956	-1.88379	-1.87954	-1.88819
-1.86827	-1.8641	-1.87247	-1.86821	-1.86393	-1.85962	-1.88384	-1.87959	-1.90348
-1.86831	-1.86414	-1.87251	-1.86826	-1.86398	-1.85968	-1.8839	-1.87963	-1.90352
-1.86839	-1.8642	-1.87257	-1.86832	-1.86405	-1.85977	-1.88399	-1.87971	-1.90359
-1.86853	-1.86432	-1.87267	-1.8684	-1.86415	-1.85991	-1.88415	-1.87986	-1.90372
-1.86867	-1.86445	-1.87277	-1.86848	-1.86424	-1.86006	-1.88431	-1.88	-1.90385
-1.86875	-1.86453	-1.86027	-1.856	-1.89296	-1.88868	-1.9127	-1.90833	-1.90394
-1.8688	-1.86459	-1.86033	-1.85607	-1.89302	-1.88874	-1.91276	-1.9084	-1.90402
-1.86888	-1.86466	-1.86043	-1.85619	-1.90869	-1.90436	-1.91286	-1.90849	-1.9041
-1.86898	-1.86475	-1.86054	-1.85634	-1.90885	-1.90451	-1.913	-1.90859	-1.9042
-1.8691	-1.86488	-1.86068	-1.8565	-1.89697	-1.89265	-1.91313	-1.90872	-1.90433
-1.86925	-1.86504	-1.86085	-1.85667	-1.89712	-1.89278	-1.91326	-1.90887	-1.90449
-1.8694	-1.86521	-1.87717	-1.87294	-1.89727	-1.89291	-1.9134	-1.90903	-1.90467
-1.86957	-1.86537	-1.87731	-1.87306	-1.89739	-1.89306	-1.91357	-1.9092	-1.90483
-1.86972	-1.86552	-1.87745	-1.87318	-1.89752	-1.89321	-1.91374	-1.90937	-1.90499
-1.85536	-1.85123	-1.87757	-1.87332	-1.89766	-1.89336	-1.90207	-1.89771	-1.90512
-1.85548	-1.85133	-1.8777	-1.87348	-1.89782	-1.89351	-1.90222	-1.89784	-1.90524
-1.8556	-1.85146	-1.90659	-1.90228	-1.91107	-1.90672	-1.90235	-1.89797	-1.90538
-1.85573	-1.85161	-1.90675	-1.90244	-1.91122	-1.90684	-1.90247	-1.89812	-1.90555
-1.85587	-1.85177	-1.9069	-1.90258	-1.91136	-1.90697	-1.90261	-1.89828	-1.89396
-1.85605	-1.85194	-1.90704	-1.90268	-1.91147	-1.90711	-1.90278	-1.89846	-1.89416
-1.85623	-1.85212	-1.9072	-1.90282	-1.91161	-1.90728	-1.90296	-1.89866	-1.89436
-1.85642	-1.85231	-1.90741	-1.90304	-1.91184	-1.90748	-1.90315	-1.89885	-1.89455
-1.85661	-1.8525	-1.90763	-1.90329	-1.91208	-1.9077	-1.90334	-1.89904	-1.89474
-1.88643	-1.88222	-1.92103	-1.91664	-1.91225	-1.90788	-1.90353	-1.89923	-1.89493
-1.8866	-1.88239	-1.92118	-1.91676	-1.91238	-1.90804	-1.90372	-1.89942	-1.89513
-1.88678	-1.88257	-1.92137	-1.91695	-1.91257	-1.90823	-1.9039	-1.89961	-1.89532
-1.88695	-1.88277	-1.92161	-1.91722	-1.91283	-1.90843	-1.90408	-1.89979	-1.8955
-1.88715	-1.88296	-1.89235	-1.88811	-1.91308	-1.90867	-1.9043	-1.89999	-1.89569
-1.88736	-1.88313	-1.89253	-1.88832	-1.91334	-1.90895	-1.90458	-1.90022	-1.89587
-1.8876	-1.88332	-1.89272	-1.88852	-1.91358	-1.90923	-1.90487	-1.90046	-1.89608
-1.88785	-1.88358	-1.89295	-1.88871	-1.91377	-1.90946	-1.90512	-1.90073	-1.89635
-1.8881	-1.88387	-1.89321	-1.88891	-1.91396	-1.90966	-1.90535	-1.90101	-1.89665
-1.90202	-1.89776	-1.89347	-1.88916	-1.9142	-1.90987	-1.90555	-1.90124	-1.8969
-1.90221	-1.89798	-1.89372	-1.88946	-1.91447	-1.91008	-1.90573	-1.90143	-1.89713
-1.90241	-1.89817	-1.89394	-1.88971	-1.91474	-1.91032	-1.90596	-1.90165	-1.89735
-1.90263	-1.89834	-1.89412	-1.88991	-1.91498	-1.91061	-1.90624	-1.90188	-1.89754
-1.90287	-1.89855	-1.89431	-1.89011	-1.91522	-1.91089	-1.90653	-1.90214	-1.89777
-1.90313	-1.89881	-1.89455	-1.8903	-1.91542	-1.91112	-1.90679	-1.90242	-1.89807
-1.90341	-1.89911	-1.89481	-1.89052	-1.91563	-1.91135	-1.90705	-1.90271	-1.89838
-1.90371	-1.89943	-1.89511	-1.8908	-1.91591	-1.91163	-1.90734	-1.90301	-1.89869
-1.90401	-1.89976	-1.89544	-1.89112	-1.91623	-1.91195	-1.90765	-1.90333	-1.89899
-1.90427	-1.90004	-1.89574	-1.89142	-1.88719	-1.88302	-1.90796	-1.90365	-1.89931
-1.90448	-1.90026	-1.896	-1.89172	-1.8875	-1.88333	-1.90827	-1.90397	-1.89964
-1.90473	-1.90052	-1.89627	-1.89202	-1.8878	-1.8836	-1.90855	-1.90425	-1.89993
-1.90503	-1.90081	-1.89657	-1.89232	-1.88808	-1.88383	-1.90875	-1.90447	-1.90017

-1.90534	-1.90112	-1.89688	-1.89263	-1.88836	-1.88407	-1.90898	-1.9047	-1.90042
-1.90567	-1.90144	-1.89719	-1.89295	-1.88866	-1.88436	-1.90926	-1.90499	-1.90072
-1.906	-1.90177	-1.89752	-1.89327	-1.88898	-1.88467	-1.90958	-1.9053	-1.90103
-1.90633	-1.9021	-1.89785	-1.89359	-1.88929	-1.88498	-1.9099	-1.90562	-1.90135
-1.90667	-1.90243	-1.89817	-1.8939	-1.88961	-1.8853	-1.91022	-1.90595	-1.90168
-1.907	-1.90275	-1.8985	-1.89423	-1.88993	-1.88562	-1.91056	-1.90627	-1.902
-1.90732	-1.90308	-1.89882	-1.89455	-1.89027	-1.88596	-1.9109	-1.9066	-1.90231
-1.90764	-1.9034	-1.89915	-1.89489	-1.89061	-1.88632	-1.91127	-1.90696	-1.90266
-1.90795	-1.90372	-1.89947	-1.89523	-1.89098	-1.88673	-1.91172	-1.90739	-1.90306
-1.90827	-1.90404	-1.8998	-1.89556	-1.89134	-1.88713	-1.91215	-1.90782	-1.90347
-1.90858	-1.90435	-1.90012	-1.89589	-1.89168	-1.88748	-1.88328	-1.87908	-1.90384
-1.90892	-1.90468	-1.90044	-1.89621	-1.892	-1.8878	-1.88361	-1.87941	-1.9042
-1.90931	-1.90506	-1.9008	-1.89655	-1.89232	-1.88812	-1.88393	-1.87974	-1.90455
-1.90975	-1.90549	-1.90119	-1.8969	-1.89265	-1.88844	-1.88424	-1.88006	-1.9049
-1.91015	-1.90588	-1.90159	-1.89728	-1.89303	-1.88881	-1.8846	-1.8804	-1.90524
-1.9105	-1.90625	-1.90199	-1.89772	-1.89347	-1.88924	-1.885	-1.88076	-1.90558
-1.91084	-1.9066	-1.90237	-1.89813	-1.89389	-1.88966	-1.88541	-1.88115	-1.90596
-1.91121	-1.90694	-1.9027	-1.89848	-1.89426	-1.89004	-1.88581	-1.88156	-1.90639
-1.91159	-1.90729	-1.90305	-1.89883	-1.89461	-1.89042	-1.88621	-1.88198	-1.90683
-1.91198	-1.9077	-1.90345	-1.89923	-1.89502	-1.89083	-1.88663	-1.88241	-1.90727
-1.91239	-1.90813	-1.90388	-1.89965	-1.89545	-1.89126	-1.88706	-1.88284	-1.9077
-1.91281	-1.90855	-1.90429	-1.90005	-1.89583	-1.89163	-1.88743	-1.88322	-1.9081
-1.91324	-1.90898	-1.90469	-1.90041	-1.89617	-1.89196	-1.88776	-1.88357	-1.90849
-1.91366	-1.90938	-1.90509	-1.9008	-1.89655	-1.89233	-1.88813	-1.88396	-1.87977
-1.91405	-1.90976	-1.90548	-1.90122	-1.89698	-1.89277	-1.88856	-1.88438	-1.88018
-1.88659	-1.88235	-1.90589	-1.90166	-1.89743	-1.89322	-1.88901	-1.88481	-1.88061
-1.88698	-1.88277	-1.90633	-1.90211	-1.89789	-1.89367	-1.88946	-1.88526	-1.88104
-1.88738	-1.8832	-1.90678	-1.90256	-1.89835	-1.89413	-1.88992	-1.88571	-1.88149
-1.88781	-1.88364	-1.90723	-1.90302	-1.8988	-1.89459	-1.89038	-1.88617	-1.88194
-1.88826	-1.8841	-1.90769	-1.90347	-1.89926	-1.89505	-1.89084	-1.88663	-1.88241
-1.8887	-1.88455	-1.90815	-1.90393	-1.89971	-1.89551	-1.89129	-1.88708	-1.88286
-1.88915	-1.885	-1.90861	-1.90439	-1.90017	-1.89596	-1.89174	-1.88753	-1.8833
-1.88961	-1.88545	-1.88129	-1.87713	-1.90063	-1.89641	-1.8922	-1.88799	-1.88376
-1.89006	-1.8859	-1.88174	-1.87759	-1.90109	-1.89687	-1.89266	-1.88845	-1.88423
-1.89051	-1.88635	-1.88219	-1.87804	-1.90155	-1.89733	-1.89311	-1.88891	-1.8847
-1.89096	-1.8868	-1.88264	-1.87849	-1.902	-1.89778	-1.89357	-1.88936	-1.88516
-1.89141	-1.88725	-1.88309	-1.87894	-1.90246	-1.89823	-1.89402	-1.88982	-1.88562
-1.8919	-1.8877	-1.88354	-1.87939	-1.87524	-1.87107	-1.89447	-1.89026	-1.88607
-1.89241	-1.88816	-1.88399	-1.87986	-1.8757	-1.87152	-1.89492	-1.89071	-1.88652
-1.89293	-1.88867	-1.8845	-1.88037	-1.8762	-1.87203	-1.89542	-1.89119	-1.88697
-1.89347	-1.88925	-1.88508	-1.88092	-1.87675	-1.87259	-1.89597	-1.89169	-1.88743
-1.89398	-1.8898	-1.88562	-1.88145	-1.87729	-1.87314	-1.86894	-1.86469	-1.88792
-1.89444	-1.89027	-1.8861	-1.88193	-1.87777	-1.87362	-1.86944	-1.86523	-1.88849
-1.89489	-1.89073	-1.88656	-1.8824	-1.87824	-1.87408	-1.86992	-1.86576	-1.88906
-1.89538	-1.89118	-1.88701	-1.88286	-1.8787	-1.87453	-1.87038	-1.86624	-1.8621
-1.8959	-1.89163	-1.88746	-1.88333	-1.87917	-1.87499	-1.87083	-1.8667	-1.86258
-1.89643	-1.89216	-1.88799	-1.88385	-1.87969	-1.87551	-1.87136	-1.86723	-1.8631
-1.89698	-1.89275	-1.88857	-1.88441	-1.88025	-1.87609	-1.87194	-1.8678	-1.86366
-1.88239	-1.87823	-1.88915	-1.88498	-1.88082	-1.87667	-1.87252	-1.86837	-1.86423
-1.88294	-1.8788	-1.88973	-1.88555	-1.88139	-1.87725	-1.87311	-1.86895	-1.8648

-1.88347	-1.87935	-1.89028	-1.8861	-1.88194	-1.87781	-1.87366	-1.8695	-1.86535
-1.88394	-1.87983	-1.89077	-1.88658	-1.88243	-1.87829	-1.87415	-1.86999	-1.86583
-1.8844	-1.8803	-1.89125	-1.88706	-1.88291	-1.87877	-1.87463	-1.87046	-1.86631
-1.88493	-1.88082	-1.89178	-1.88761	-1.88345	-1.87931	-1.87516	-1.871	-1.86686
-1.8855	-1.88137	-1.89235	-1.88819	-1.88403	-1.87988	-1.87573	-1.87158	-1.86743
-1.88607	-1.88194	-1.87781	-1.87369	-1.88461	-1.88046	-1.87631	-1.87216	-1.86802
-1.88665	-1.88251	-1.87838	-1.87426	-1.88519	-1.88105	-1.8769	-1.87275	-1.86861
-1.88723	-1.88309	-1.87896	-1.87484	-1.88578	-1.88163	-1.87749	-1.87334	-1.8692
-1.88781	-1.88367	-1.87954	-1.87541	-1.88636	-1.88222	-1.87807	-1.87393	-1.86979
-1.88839	-1.88425	-1.88012	-1.87599	-1.88695	-1.8828	-1.87866	-1.87452	-1.87037
-1.87507	-1.87096	-1.88069	-1.87657	-1.88753	-1.88339	-1.87924	-1.8751	-1.87096
-1.87564	-1.87153	-1.88127	-1.87715	-1.88812	-1.88396	-1.87982	-1.87567	-1.87153
-1.87621	-1.8721	-1.86799	-1.8639	-1.8736	-1.86949	-1.88039	-1.87625	-1.87211
-1.87678	-1.87266	-1.86856	-1.86446	-1.87417	-1.87005	-1.88097	-1.87682	-1.87268
-1.87735	-1.87323	-1.86913	-1.86503	-1.87474	-1.87063	-1.88155	-1.8774	-1.87326
-1.87793	-1.8738	-1.8697	-1.86561	-1.87532	-1.8712	-1.88213	-1.87798	-1.87385
-1.87854	-1.8744	-1.8703	-1.86622	-1.86212	-1.85802	-1.86769	-1.86359	-1.87447
-1.87922	-1.87507	-1.87097	-1.86689	-1.86279	-1.85868	-1.86835	-1.86426	-1.87515
-1.8799	-1.87574	-1.87164	-1.86755	-1.86345	-1.85934	-1.86902	-1.86493	-1.87583
-1.88051	-1.87636	-1.87225	-1.86816	-1.86406	-1.85996	-1.85587	-1.85179	-1.87644
-1.88109	-1.87695	-1.87283	-1.86874	-1.86464	-1.86055	-1.85647	-1.85238	-1.87703
-1.86697	-1.86288	-1.87342	-1.86932	-1.86524	-1.86118	-1.85711	-1.85301	-1.86412
-1.86754	-1.86346	-1.874	-1.8699	-1.86584	-1.86184	-1.85779	-1.85368	-1.8648
-1.86816	-1.86408	-1.87463	-1.87052	-1.86647	-1.86247	-1.85842	-1.85432	-1.86545
-1.86884	-1.86475	-1.8753	-1.87119	-1.86711	-1.86306	-1.85899	-1.85491	-1.86605
-1.86953	-1.86543	-1.86134	-1.85726	-1.86777	-1.86368	-1.85959	-1.85551	-1.86668
-1.87021	-1.8661	-1.86202	-1.85796	-1.86847	-1.86436	-1.86026	-1.85618	-1.86736
-1.87088	-1.86675	-1.86268	-1.85864	-1.86916	-1.86506	-1.86096	-1.85687	-1.86805
-1.8715	-1.86737	-1.86329	-1.85924	-1.8698	-1.86574	-1.86166	-1.85756	-1.85346
-1.87208	-1.86797	-1.86388	-1.85981	-1.8704	-1.8664	-1.86235	-1.85824	-1.85413
-1.87271	-1.86861	-1.86451	-1.86043	-1.85642	-1.85246	-1.86298	-1.85887	-1.85476
-1.87338	-1.86928	-1.86518	-1.86109	-1.85704	-1.85303	-1.86354	-1.85944	-1.85536
-1.87407	-1.86996	-1.86586	-1.86177	-1.8577	-1.85365	-1.86414	-1.86006	-1.85598
-1.87476	-1.87065	-1.86655	-1.86246	-1.85838	-1.85432	-1.86481	-1.86072	-1.85664
-1.87546	-1.87134	-1.86724	-1.86315	-1.85908	-1.85501	-1.8655	-1.86141	-1.85732
-1.87615	-1.87204	-1.86793	-1.86384	-1.85976	-1.8557	-1.85164	-1.84759	-1.85803
-1.87685	-1.87274	-1.86863	-1.86453	-1.86045	-1.85639	-1.85234	-1.84829	-1.85874
-1.87755	-1.87343	-1.86932	-1.86522	-1.86114	-1.85708	-1.85303	-1.84899	-1.84496
-1.87824	-1.87411	-1.87001	-1.86591	-1.86183	-1.85777	-1.85372	-1.84968	-1.84567
-1.87893	-1.87481	-1.8707	-1.8666	-1.86253	-1.85846	-1.85442	-1.85038	-1.84637
-1.87962	-1.8755	-1.87139	-1.8673	-1.86323	-1.85918	-1.85513	-1.85107	-1.84704
-1.8803	-1.87619	-1.87209	-1.868	-1.86394	-1.85991	-1.85586	-1.8518	-1.84775
-1.88099	-1.87688	-1.87279	-1.86872	-1.86468	-1.86067	-1.85665	-1.8526	-1.84856
-1.88169	-1.87759	-1.87351	-1.86945	-1.86543	-1.86144	-1.85743	-1.8534	-1.84936
-1.8824	-1.87831	-1.87425	-1.87021	-1.86619	-1.86218	-1.85816	-1.85413	-1.85009
-1.88312	-1.87904	-1.875	-1.87098	-1.86694	-1.8629	-1.85886	-1.85482	-1.85078
-1.87118	-1.86715	-1.87575	-1.87172	-1.86768	-1.86362	-1.85957	-1.85551	-1.85147
-1.87193	-1.86792	-1.87651	-1.87246	-1.8684	-1.86434	-1.86028	-1.85621	-1.85216
-1.87268	-1.86867	-1.87725	-1.87319	-1.86913	-1.86508	-1.86103	-1.85696	-1.85291
-1.87342	-1.86939	-1.87798	-1.87391	-1.86987	-1.86585	-1.86182	-1.85778	-1.85374

-1.87416	-1.8701	-1.8787	-1.87465	-1.87062	-1.86661	-1.8626	-1.85857	-1.85455
-1.87487	-1.87082	-1.87944	-1.87541	-1.87137	-1.86735	-1.86333	-1.85931	-1.85529
-1.87559	-1.87155	-1.88019	-1.87616	-1.87212	-1.86808	-1.86404	-1.86003	-1.85602
-1.87637	-1.87231	-1.88094	-1.87689	-1.87284	-1.8688	-1.86478	-1.86078	-1.85679
-1.87718	-1.87309	-1.88169	-1.87761	-1.87355	-1.86952	-1.86552	-1.86155	-1.85757
-1.84519	-1.84122	-1.88249	-1.87839	-1.87432	-1.87029	-1.86628	-1.86233	-1.85836
-1.84596	-1.84203	-1.88333	-1.87921	-1.87513	-1.87108	-1.86708	-1.86312	-1.85916
-1.84674	-1.84284	-1.87148	-1.8674	-1.87595	-1.87189	-1.86787	-1.8639	-1.85992
-1.84753	-1.84363	-1.8723	-1.86823	-1.87678	-1.8727	-1.86865	-1.86463	-1.86062
-1.8483	-1.8444	-1.87309	-1.86903	-1.87759	-1.87351	-1.86944	-1.86538	-1.86133
-1.84905	-1.84511	-1.87381	-1.86976	-1.87835	-1.87431	-1.87025	-1.86618	-1.86212
-1.84979	-1.84581	-1.87452	-1.87048	-1.87911	-1.87511	-1.87107	-1.867	-1.86294
-1.85057	-1.84657	-1.84261	-1.83868	-1.8799	-1.87592	-1.8719	-1.86782	-1.86375
-1.85137	-1.84737	-1.8434	-1.83946	-1.8807	-1.87673	-1.87272	-1.86864	-1.86457
-1.85217	-1.84817	-1.8442	-1.84026	-1.88152	-1.87756	-1.87354	-1.86946	-1.86539
-1.85298	-1.84897	-1.845	-1.84107	-1.88235	-1.87838	-1.87436	-1.87028	-1.86621
-1.85378	-1.84978	-1.84581	-1.84187	-1.87052	-1.86659	-1.87519	-1.87111	-1.86704
-1.85459	-1.85058	-1.84661	-1.84268	-1.87134	-1.86741	-1.87602	-1.87195	-1.86788
-1.8554	-1.85139	-1.84742	-1.84348	-1.87217	-1.86824	-1.87686	-1.8728	-1.86873
-1.85622	-1.85221	-1.84824	-1.84429	-1.87299	-1.86907	-1.87772	-1.87369	-1.86964
-1.85705	-1.85305	-1.84906	-1.8451	-1.87381	-1.8699	-1.87857	-1.87459	-1.87057
-1.85791	-1.85393	-1.84994	-1.84596	-1.87467	-1.87073	-1.87941	-1.87544	-1.87145
-1.8588	-1.85485	-1.85086	-1.84687	-1.87556	-1.87157	-1.88023	-1.87627	-1.87228
-1.85965	-1.85573	-1.85175	-1.84775	-1.84379	-1.83987	-1.86842	-1.86451	-1.87312
-1.86047	-1.85657	-1.8526	-1.8486	-1.84462	-1.84067	-1.86923	-1.86533	-1.87397
-1.86129	-1.8574	-1.85344	-1.84946	-1.84548	-1.84151	-1.87007	-1.86616	-1.87481
-1.8621	-1.85823	-1.85431	-1.85037	-1.8464	-1.84242	-1.87096	-1.867	-1.87562
-1.86292	-1.85906	-1.85518	-1.85128	-1.84733	-1.84335	-1.87189	-1.86787	-1.87645
-1.86375	-1.85988	-1.856	-1.85213	-1.84822	-1.84428	-1.87285	-1.86879	-1.87734
-1.86461	-1.86069	-1.85681	-1.85296	-1.84909	-1.8452	-1.87382	-1.86974	-1.87827
-1.84777	-1.84382	-1.85765	-1.85378	-1.84992	-1.84606	-1.87473	-1.87068	-1.86662
-1.84873	-1.84475	-1.85852	-1.85459	-1.85072	-1.84688	-1.8756	-1.8716	-1.86759
-1.84968	-1.8457	-1.84173	-1.83778	-1.85155	-1.84771	-1.87645	-1.87249	-1.8685
-1.8506	-1.84666	-1.84269	-1.83872	-1.85244	-1.84854	-1.87728	-1.87332	-1.86935
-1.8515	-1.8476	-1.84364	-1.83967	-1.83573	-1.83181	-1.87814	-1.87417	-1.8702
-1.85238	-1.84846	-1.84454	-1.84062	-1.83671	-1.8328	-1.87912	-1.8751	-1.8711
-1.85325	-1.84931	-1.84542	-1.84156	-1.83769	-1.8338	-1.88012	-1.87607	-1.87202
-1.85418	-1.85021	-1.84631	-1.84244	-1.83857	-1.83469	-1.83081	-1.8269	-1.87295
-1.85513	-1.85115	-1.84721	-1.84329	-1.8394	-1.83554	-1.83167	-1.82781	-1.87391
-1.85606	-1.85209	-1.84815	-1.84422	-1.84032	-1.83644	-1.83256	-1.8287	-1.85778
-1.85696	-1.85305	-1.84915	-1.84524	-1.84132	-1.83739	-1.83348	-1.82958	-1.85865
-1.82572	-1.82189	-1.8501	-1.84621	-1.84229	-1.83835	-1.83442	-1.83049	-1.85957
-1.82657	-1.82273	-1.85096	-1.8471	-1.8432	-1.83929	-1.83538	-1.83148	-1.8606
-1.82745	-1.82358	-1.85184	-1.84799	-1.84412	-1.84023	-1.83634	-1.83247	-1.86162
-1.82841	-1.82455	-1.82073	-1.81694	-1.8451	-1.84118	-1.83728	-1.83338	-1.86254
-1.82939	-1.82554	-1.82173	-1.81793	-1.84609	-1.84214	-1.83821	-1.83428	-1.86344
-1.8303	-1.82645	-1.82263	-1.81883	-1.81498	-1.8111	-1.83915	-1.83523	-1.83133
-1.83117	-1.82729	-1.82347	-1.81968	-1.81586	-1.81201	-1.84011	-1.83622	-1.83233
-1.8321	-1.82821	-1.82438	-1.82059	-1.81677	-1.81292	-1.80909	-1.80526	-1.83328
-1.83308	-1.8292	-1.82537	-1.82157	-1.81772	-1.81385	-1.80998	-1.80613	-1.83416

-1.83407	-1.83018	-1.82635	-1.82253	-1.81867	-1.81477	-1.8109	-1.80703	-1.8032
-1.83504	-1.83112	-1.82726	-1.82341	-1.81955	-1.8157	-1.81186	-1.80801	-1.80419
-1.83601	-1.83207	-1.82817	-1.82429	-1.82045	-1.81665	-1.81284	-1.80901	-1.8052
-1.83694	-1.83303	-1.82914	-1.82526	-1.82143	-1.81763	-1.81383	-1.81002	-1.80621
-1.83787	-1.83401	-1.83014	-1.82628	-1.82245	-1.81864	-1.81483	-1.81103	-1.80721
-1.83885	-1.83501	-1.83115	-1.8273	-1.82345	-1.81961	-1.81578	-1.81196	-1.80813
-1.83988	-1.83602	-1.83217	-1.82832	-1.82445	-1.82056	-1.81669	-1.81283	-1.80899
-1.84089	-1.837	-1.83314	-1.82928	-1.8254	-1.8215	-1.81762	-1.81375	-1.80991
-1.84187	-1.83794	-1.83404	-1.83015	-1.82628	-1.82243	-1.81858	-1.81474	-1.81092
-1.8237	-1.81979	-1.83495	-1.83105	-1.82719	-1.82338	-1.81957	-1.81575	-1.81195
-1.82465	-1.82074	-1.83593	-1.83204	-1.82819	-1.82439	-1.82058	-1.81678	-1.81299
-1.8256	-1.82171	-1.83693	-1.83307	-1.82924	-1.82542	-1.82162	-1.81783	-1.81404
-1.82659	-1.82271	-1.83797	-1.83412	-1.83029	-1.82646	-1.82266	-1.81887	-1.81508
-1.82761	-1.82375	-1.83903	-1.83519	-1.83135	-1.82752	-1.8237	-1.81991	-1.81611
-1.82865	-1.8248	-1.8401	-1.83625	-1.83241	-1.82857	-1.82474	-1.82093	-1.81712
-1.82971	-1.82586	-1.84117	-1.83732	-1.83346	-1.82961	-1.82577	-1.82195	-1.81812
-1.83077	-1.82692	-1.8231	-1.81928	-1.83451	-1.83064	-1.82679	-1.82294	-1.81909
-1.83184	-1.82798	-1.82415	-1.82032	-1.83555	-1.83167	-1.82779	-1.8239	-1.82002
-1.83291	-1.82904	-1.8252	-1.82136	-1.83659	-1.83268	-1.82877	-1.82486	-1.82096
-1.81618	-1.81236	-1.82625	-1.8224	-1.8376	-1.83365	-1.82972	-1.8258	-1.8219
-1.81724	-1.81342	-1.82731	-1.82343	-1.83862	-1.83463	-1.83067	-1.82674	-1.82285
-1.80202	-1.79822	-1.81065	-1.80682	-1.82055	-1.81658	-1.83168	-1.82774	-1.82385
-1.80308	-1.79928	-1.81171	-1.80787	-1.82161	-1.81763	-1.83273	-1.82877	-1.82487
-1.80414	-1.80034	-1.79653	-1.79271	-1.80503	-1.80111	-1.83378	-1.82981	-1.82591
-1.8052	-1.80141	-1.79759	-1.79376	-1.80607	-1.80214	-1.83483	-1.83086	-1.82695
-1.79091	-1.78714	-1.79865	-1.79482	-1.80713	-1.8032	-1.79929	-1.79541	-1.828
-1.79195	-1.78819	-1.79971	-1.79587	-1.80819	-1.80426	-1.80036	-1.79647	-1.82906
-1.79299	-1.78923	-1.80076	-1.79694	-1.80926	-1.80535	-1.80145	-1.79756	-1.83015
-1.79403	-1.79027	-1.80182	-1.798	-1.79417	-1.79032	-1.80261	-1.79875	-1.83136
-1.79507	-1.79131	-1.80287	-1.79907	-1.79527	-1.79147	-1.8038	-1.79997	-1.83262
-1.79611	-1.79234	-1.78858	-1.78481	-1.79634	-1.79256	-1.78878	-1.78499	-1.81659
-1.79714	-1.79337	-1.78961	-1.78585	-1.79739	-1.79361	-1.78983	-1.78606	-1.8177
-1.79818	-1.79441	-1.79064	-1.78688	-1.79843	-1.79465	-1.79088	-1.78712	-1.79947
-1.79921	-1.79544	-1.79167	-1.78791	-1.79948	-1.7957	-1.79192	-1.78816	-1.80051
-1.80025	-1.79647	-1.7927	-1.78894	-1.78519	-1.78143	-1.79296	-1.7892	-1.78545
-1.80129	-1.7975	-1.79373	-1.78997	-1.78622	-1.78246	-1.794	-1.79024	-1.78648
-1.80233	-1.79854	-1.79477	-1.79101	-1.78725	-1.78349	-1.79504	-1.79128	-1.78753
-1.80339	-1.7996	-1.79582	-1.79206	-1.7883	-1.78454	-1.78079	-1.77706	-1.78858
-1.80448	-1.80068	-1.7969	-1.79313	-1.78936	-1.78561	-1.78186	-1.77812	-1.78965
-1.80565	-1.80185	-1.79806	-1.79428	-1.79051	-1.78676	-1.78301	-1.77927	-1.79081
-1.80691	-1.8031	-1.79931	-1.79552	-1.79175	-1.788	-1.78425	-1.78051	-1.79206
-1.80811	-1.8043	-1.8005	-1.7967	-1.79293	-1.78917	-1.78542	-1.78168	-1.77794
-1.8092	-1.80539	-1.80158	-1.79778	-1.794	-1.79024	-1.78649	-1.78275	-1.77901
-1.81026	-1.80645	-1.80263	-1.79883	-1.79505	-1.79129	-1.78754	-1.7838	-1.78006
-1.81131	-1.80749	-1.80369	-1.79989	-1.79611	-1.79235	-1.78859	-1.78485	-1.78111
-1.81234	-1.80854	-1.80474	-1.80096	-1.79718	-1.79341	-1.78966	-1.78591	-1.78218
-1.81339	-1.8096	-1.80583	-1.80208	-1.79832	-1.79457	-1.79083	-1.78709	-1.78335
-1.81445	-1.81067	-1.80693	-1.80322	-1.79951	-1.79579	-1.79206	-1.78832	-1.78458
-1.8156	-1.81181	-1.80807	-1.80436	-1.80065	-1.79694	-1.79321	-1.78947	-1.78574
-1.81683	-1.81303	-1.80925	-1.80549	-1.80174	-1.798	-1.79426	-1.79053	-1.7868

-1.81804	-1.81422	-1.81041	-1.80661	-1.80283	-1.79906	-1.79531	-1.79157	-1.78785
-1.81914	-1.81533	-1.81151	-1.8077	-1.8039	-1.80011	-1.79635	-1.79261	-1.7889
-1.82025	-1.81644	-1.81262	-1.80881	-1.805	-1.8012	-1.79742	-1.79368	-1.78998
-1.82146	-1.81764	-1.81382	-1.81001	-1.8062	-1.80239	-1.79862	-1.79488	-1.79117
-1.82269	-1.81887	-1.81505	-1.81123	-1.80743	-1.80364	-1.79987	-1.79613	-1.79241
-1.82384	-1.82002	-1.8162	-1.81238	-1.80858	-1.80479	-1.80104	-1.79731	-1.79361
-1.82493	-1.82111	-1.81729	-1.81348	-1.80968	-1.80589	-1.80215	-1.79846	-1.79478
-1.82608	-1.82226	-1.81844	-1.81463	-1.81083	-1.80705	-1.80331	-1.79962	-1.79595
-1.82732	-1.8235	-1.81968	-1.81587	-1.81208	-1.8083	-1.80454	-1.80081	-1.79709
-1.82853	-1.8247	-1.82089	-1.81708	-1.8133	-1.80954	-1.80578	-1.80201	-1.79826
-1.82964	-1.82581	-1.822	-1.8182	-1.81444	-1.81071	-1.80698	-1.80324	-1.79951
-1.83075	-1.82692	-1.8231	-1.81931	-1.81556	-1.81187	-1.80817	-1.80446	-1.80075
-1.81049	-1.80672	-1.82432	-1.82052	-1.81676	-1.81303	-1.80932	-1.8056	-1.80189
-1.81174	-1.80798	-1.8256	-1.82178	-1.81799	-1.81421	-1.81045	-1.80671	-1.80297
-1.81293	-1.80919	-1.82685	-1.82305	-1.81925	-1.81545	-1.81166	-1.8079	-1.80415
-1.81407	-1.81037	-1.82809	-1.82433	-1.82054	-1.81674	-1.81295	-1.80918	-1.80541
-1.81523	-1.81153	-1.80783	-1.80414	-1.82177	-1.81799	-1.81421	-1.81044	-1.80668
-1.8164	-1.81266	-1.80894	-1.80524	-1.8229	-1.81916	-1.81542	-1.81169	-1.80796
-1.81761	-1.81382	-1.81007	-1.80635	-1.82403	-1.82031	-1.8166	-1.81291	-1.8092
-1.81888	-1.81507	-1.8113	-1.80755	-1.82523	-1.82148	-1.81774	-1.81403	-1.81032
-1.82017	-1.81636	-1.81257	-1.80879	-1.82645	-1.82266	-1.81888	-1.81513	-1.81141
-1.82144	-1.81764	-1.81385	-1.81006	-1.80628	-1.80252	-1.82011	-1.81633	-1.81259
-1.82269	-1.81894	-1.81514	-1.81134	-1.80756	-1.8038	-1.82139	-1.81759	-1.81382
-1.80736	-1.80368	-1.81641	-1.81262	-1.80884	-1.80507	-1.82268	-1.81886	-1.81508
-1.8085	-1.80485	-1.81764	-1.81389	-1.81013	-1.80635	-1.82397	-1.82015	-1.81636
-1.80964	-1.80601	-1.81883	-1.81513	-1.81139	-1.80761	-1.80385	-1.8001	-1.81765
-1.81079	-1.80712	-1.81997	-1.8163	-1.8126	-1.80887	-1.80513	-1.80137	-1.81894
-1.81197	-1.80825	-1.82111	-1.81746	-1.81379	-1.8101	-1.80639	-1.80264	-1.82023
-1.8132	-1.80945	-1.80576	-1.80211	-1.81494	-1.81129	-1.80761	-1.80389	-1.82152
-1.81447	-1.81069	-1.80695	-1.80325	-1.81609	-1.81247	-1.80882	-1.80513	-1.82281
-1.81573	-1.81194	-1.80818	-1.80446	-1.80081	-1.79723	-1.81005	-1.80639	-1.8027
-1.81701	-1.8132	-1.80944	-1.80572	-1.80206	-1.79847	-1.8113	-1.80764	-1.80396
-1.81829	-1.81447	-1.8107	-1.80697	-1.80331	-1.79971	-1.7961	-1.79249	-1.80523
-1.81957	-1.81574	-1.81196	-1.80822	-1.80455	-1.80094	-1.79733	-1.79373	-1.80649
-1.82085	-1.81701	-1.81322	-1.80947	-1.80579	-1.80217	-1.79855	-1.79495	-1.80774
-1.82213	-1.81828	-1.81448	-1.81072	-1.80701	-1.80335	-1.79972	-1.79612	-1.80893
-1.82341	-1.81956	-1.81575	-1.81198	-1.80825	-1.80456	-1.8009	-1.79729	-1.81012
-1.80611	-1.80229	-1.81702	-1.81325	-1.80952	-1.80585	-1.8022	-1.79857	-1.79494
-1.80739	-1.80357	-1.81829	-1.81452	-1.81082	-1.80719	-1.80355	-1.79989	-1.79623
-1.80866	-1.80484	-1.81956	-1.81578	-1.8121	-1.80848	-1.80485	-1.80118	-1.79751
-1.80993	-1.8061	-1.82082	-1.81704	-1.81335	-1.80974	-1.80611	-1.80245	-1.79878
-1.81119	-1.80737	-1.80358	-1.79982	-1.8146	-1.81098	-1.80735	-1.8037	-1.80003
-1.81246	-1.80863	-1.80483	-1.80108	-1.81585	-1.81222	-1.80859	-1.80494	-1.80128
-1.79354	-1.78977	-1.8061	-1.80234	-1.8171	-1.81347	-1.80983	-1.80618	-1.80252
-1.7948	-1.79102	-1.80737	-1.80362	-1.81837	-1.81471	-1.81107	-1.80743	-1.80377
-1.79606	-1.79228	-1.80866	-1.80491	-1.81965	-1.81597	-1.81232	-1.80868	-1.80503
-1.79732	-1.79355	-1.80996	-1.80625	-1.80255	-1.79887	-1.8136	-1.80994	-1.80629
-1.79857	-1.79482	-1.81129	-1.80761	-1.80391	-1.80021	-1.8149	-1.81121	-1.80755
-1.79983	-1.79609	-1.79244	-1.78883	-1.80523	-1.80151	-1.79781	-1.79413	-1.8088
-1.8011	-1.79735	-1.79369	-1.79007	-1.80649	-1.80279	-1.79909	-1.79539	-1.81004

-1.80239	-1.79865	-1.79497	-1.79133	-1.80777	-1.80408	-1.80038	-1.79667	-1.81131
-1.80371	-1.79999	-1.79631	-1.79265	-1.80908	-1.80537	-1.80168	-1.79798	-1.81263
-1.80505	-1.80135	-1.79766	-1.79397	-1.8104	-1.80668	-1.80299	-1.79931	-1.81396
-1.8064	-1.8027	-1.79899	-1.79528	-1.7916	-1.78795	-1.80432	-1.80065	-1.79698
-1.80776	-1.80404	-1.80031	-1.79659	-1.79292	-1.78928	-1.80568	-1.80199	-1.79831
-1.8091	-1.80537	-1.80164	-1.79792	-1.79425	-1.79061	-1.80702	-1.80332	-1.79963
-1.81043	-1.80668	-1.80297	-1.79927	-1.7956	-1.79194	-1.80835	-1.80464	-1.80095
-1.81176	-1.80802	-1.80431	-1.80062	-1.79693	-1.79326	-1.80968	-1.80597	-1.80228
-1.8131	-1.80938	-1.80567	-1.80197	-1.79826	-1.79456	-1.81099	-1.8073	-1.80362
-1.77867	-1.77505	-1.80701	-1.8033	-1.79959	-1.79588	-1.7922	-1.78857	-1.80496
-1.77991	-1.7763	-1.8083	-1.80461	-1.80091	-1.79721	-1.79354	-1.7899	-1.80629
-1.78115	-1.77754	-1.80958	-1.80591	-1.80224	-1.79855	-1.79488	-1.79122	-1.80761
-1.78246	-1.77885	-1.81091	-1.80725	-1.80357	-1.79989	-1.79621	-1.79253	-1.80893
-1.78381	-1.78019	-1.81228	-1.8086	-1.80492	-1.80123	-1.79754	-1.79384	-1.81025
-1.78517	-1.78154	-1.77793	-1.77433	-1.80626	-1.80255	-1.79886	-1.79516	-1.81159
-1.78654	-1.78292	-1.77929	-1.77567	-1.8076	-1.80386	-1.80015	-1.79648	-1.81295
-1.78791	-1.78428	-1.78065	-1.77701	-1.80896	-1.80521	-1.8015	-1.79783	-1.79419
-1.78926	-1.78563	-1.782	-1.77837	-1.81037	-1.80664	-1.80292	-1.79922	-1.79555
-1.79061	-1.78698	-1.78335	-1.77972	-1.7761	-1.77247	-1.80433	-1.8006	-1.79691
-1.79197	-1.78834	-1.7847	-1.78107	-1.77742	-1.77377	-1.80565	-1.80195	-1.79828
-1.79332	-1.7897	-1.78606	-1.78241	-1.77874	-1.77506	-1.80697	-1.8033	-1.79965
-1.79467	-1.79104	-1.7874	-1.78376	-1.7801	-1.77643	-1.80836	-1.80468	-1.80101
-1.79601	-1.79236	-1.78874	-1.78513	-1.78149	-1.77784	-1.80981	-1.80607	-1.80237
-1.79737	-1.7937	-1.79008	-1.78648	-1.78286	-1.77923	-1.81124	-1.8075	-1.80379
-1.79872	-1.79507	-1.79143	-1.78782	-1.7842	-1.7806	-1.81268	-1.80897	-1.80527
-1.78007	-1.77645	-1.79278	-1.78915	-1.78555	-1.78195	-1.77836	-1.77477	-1.80672
-1.78141	-1.77777	-1.79411	-1.79049	-1.78688	-1.78328	-1.77969	-1.77611	-1.80812
-1.78275	-1.7791	-1.79544	-1.79183	-1.78822	-1.78461	-1.78101	-1.77744	-1.80949
-1.75679	-1.75338	-1.79679	-1.79316	-1.78954	-1.78595	-1.78236	-1.77878	-1.81086
-1.75805	-1.75466	-1.79814	-1.79449	-1.79087	-1.78729	-1.78371	-1.78012	-1.81223
-1.7593	-1.7559	-1.77952	-1.77591	-1.79221	-1.78862	-1.80556	-1.80191	-1.83346
-1.76056	-1.75714	-1.78085	-1.77725	-1.79355	-1.78994	-1.80691	-1.80328	-1.83489
-1.76185	-1.75843	-1.78222	-1.77863	-1.79493	-1.79131	-1.8083	-1.80467	-1.80106
-1.76321	-1.75979	-1.78366	-1.78005	-1.79636	-1.79274	-1.80975	-1.8061	-1.80247
-1.76455	-1.76113	-1.75772	-1.75432	-1.77785	-1.77427	-1.81118	-1.80751	-1.80386
-1.76584	-1.76241	-1.75899	-1.75558	-1.7792	-1.77562	-1.81256	-1.80887	-1.80521
-1.76713	-1.76369	-1.76026	-1.75684	-1.78054	-1.77696	-1.81392	-1.81024	-1.80657
-1.72595	-1.7226	-1.76158	-1.75817	-1.78195	-1.77835	-1.81535	-1.81166	-1.808
-1.72727	-1.72392	-1.76293	-1.75952	-1.78337	-1.77975	-1.81678	-1.81311	-1.80946
-1.72855	-1.72519	-1.72184	-1.71849	-1.75741	-1.754	-1.81818	-1.81452	-1.81086
-1.72982	-1.72644	-1.72308	-1.71973	-1.75866	-1.75525	-1.81955	-1.81588	-1.81223
-1.73113	-1.72773	-1.72436	-1.721	-1.71765	-1.7143	-1.80134	-1.7977	-1.81362
-1.7325	-1.72909	-1.7257	-1.72232	-1.71896	-1.71563	-1.80281	-1.79914	-1.81504
-1.70767	-1.70432	-1.72704	-1.72365	-1.72029	-1.71696	-1.73577	-1.73237	-1.81647
-1.70892	-1.70559	-1.72835	-1.72498	-1.72163	-1.7183	-1.73713	-1.73372	-1.81794
-1.71016	-1.70684	-1.72964	-1.72631	-1.72297	-1.71963	-1.7385	-1.73508	-1.81942
-1.66619	-1.66296	-1.7048	-1.70151	-1.72425	-1.72092	-1.73983	-1.73644	-1.7806
-1.66749	-1.66423	-1.70607	-1.70275	-1.7255	-1.72218	-1.74115	-1.73779	-1.78208
-1.66878	-1.66551	-1.66226	-1.65903	-1.72679	-1.72347	-1.74248	-1.73913	-1.75784
-1.67007	-1.66681	-1.66356	-1.66033	-1.72812	-1.72478	-1.74381	-1.74048	-1.7592

-1.67137	-1.66813	-1.66488	-1.66163	-1.68461	-1.68135	-1.74517	-1.74183	-1.76057
-1.67269	-1.66946	-1.6662	-1.66295	-1.68594	-1.68268	-1.74655	-1.74321	-1.76195
-1.67402	-1.67079	-1.66753	-1.66427	-1.68728	-1.68402	-1.74795	-1.7446	-1.76334
-1.67534	-1.67212	-1.66885	-1.66558	-1.66235	-1.65916	-1.7085	-1.70526	-1.74273
-1.67667	-1.67344	-1.67017	-1.66688	-1.66365	-1.66047	-1.70984	-1.70659	-1.74408
-1.67799	-1.67476	-1.67148	-1.66819	-1.66496	-1.66177	-1.70858	-1.70535	-1.72741
-1.6793	-1.67607	-1.67279	-1.66949	-1.66625	-1.66306	-1.7099	-1.70667	-1.72875
-1.65568	-1.65251	-1.6741	-1.6708	-1.66756	-1.66436	-1.68475	-1.68158	-1.73011
-1.65698	-1.65381	-1.67541	-1.67211	-1.66887	-1.66567	-1.68606	-1.6829	-1.73147
-1.63653	-1.63337	-1.67673	-1.67343	-1.67018	-1.66696	-1.68737	-1.68421	-1.70746
-1.63784	-1.63468	-1.67803	-1.67473	-1.67147	-1.66825	-1.68866	-1.68552	-1.70881
-1.63916	-1.63599	-1.67934	-1.67605	-1.67278	-1.66954	-1.68997	-1.68683	-1.71015
-1.64048	-1.63733	-1.63413	-1.63092	-1.67417	-1.6709	-1.69131	-1.68815	-1.685
-1.64181	-1.63867	-1.63551	-1.63234	-1.67559	-1.6723	-1.69269	-1.68948	-1.68631
-1.64314	-1.64	-1.63686	-1.63371	-1.6305	-1.62726	-1.69404	-1.6908	-1.6876
-1.64445	-1.64132	-1.63818	-1.63503	-1.63183	-1.6286	-1.69538	-1.69211	-1.68888
-1.64576	-1.64263	-1.63949	-1.63635	-1.63317	-1.62995	-1.65111	-1.64788	-1.69019
-1.64707	-1.64392	-1.64078	-1.63766	-1.63451	-1.63135	-1.65254	-1.64927	-1.69154
-1.6484	-1.64522	-1.64208	-1.63896	-1.63584	-1.63272	-1.65395	-1.65067	-1.64742
-1.64979	-1.64656	-1.64339	-1.64026	-1.63715	-1.63405	-1.65532	-1.65209	-1.64887
-1.65119	-1.64791	-1.64471	-1.64156	-1.63844	-1.63535	-1.65666	-1.6535	-1.65032
-1.65255	-1.64925	-1.64603	-1.64286	-1.66425	-1.66111	-1.65797	-1.65484	-1.65169
-1.6539	-1.65059	-1.64735	-1.64415	-1.66554	-1.66239	-1.65926	-1.65614	-1.65302
-1.65527	-1.65197	-1.64873	-1.64553	-1.6669	-1.66373	-1.66057	-1.65745	-1.65433
-1.65666	-1.65339	-1.65017	-1.64698	-1.66834	-1.66511	-1.66191	-1.65875	-1.65561
-1.65803	-1.65478	-1.67622	-1.673	-1.66976	-1.6665	-1.66328	-1.66009	-1.65693
-1.65936	-1.65612	-1.67757	-1.67436	-1.67114	-1.66793	-1.66471	-1.66148	-1.65828
-1.66072	-1.65746	-1.67891	-1.67569	-1.6725	-1.66933	-1.66613	-1.66288	-1.65965
-1.61478	-1.6116	-1.68025	-1.677	-1.6738	-1.67065	-1.66748	-1.66427	-1.66108
-1.6162	-1.61295	-1.68161	-1.67831	-1.67508	-1.67193	-1.66879	-1.66565	-1.66251
-1.61761	-1.61435	-1.68303	-1.67967	-1.67641	-1.67323	-1.67008	-1.66696	-1.66384
-1.61901	-1.61578	-1.6845	-1.68107	-1.67776	-1.67453	-1.67135	-1.66823	-1.66512
-1.62037	-1.61716	-1.68596	-1.68252	-1.67918	-1.67592	-1.67271	-1.66958	-1.66646
-1.62167	-1.61849	-1.68741	-1.68403	-1.6807	-1.6774	-1.67417	-1.67102	-1.66789
-1.62421	-1.62103	-1.66617	-1.66289	-1.68218	-1.67886	-1.67561	-1.67245	-1.66929
-1.62561	-1.62238	-1.66755	-1.66427	-1.68357	-1.68025	-1.67699	-1.67379	-1.67061
-1.62703	-1.62375	-1.66895	-1.66565	-1.68497	-1.68166	-1.67838	-1.67514	-1.67194
-1.62844	-1.62519	-1.64646	-1.64318	-1.68647	-1.68316	-1.67986	-1.67659	-1.67335
-1.62985	-1.62664	-1.64796	-1.64467	-1.68802	-1.68469	-1.68138	-1.67807	-1.6748
-1.63119	-1.628	-1.6248	-1.62162	-1.68949	-1.68615	-1.68283	-1.6795	-1.67621
-1.63248	-1.62928	-1.6261	-1.62294	-1.69091	-1.68757	-1.68423	-1.6809	-1.67759
-1.63385	-1.63064	-1.62747	-1.62432	-1.64564	-1.64241	-1.6857	-1.68235	-1.67903
-1.63531	-1.6321	-1.62892	-1.62578	-1.64713	-1.6439	-1.68725	-1.6839	-1.68056
-1.63677	-1.63354	-1.63036	-1.62721	-1.6486	-1.64538	-1.66515	-1.66184	-1.68207
-1.63815	-1.63489	-1.6317	-1.62856	-1.64998	-1.64677	-1.66656	-1.66325	-1.68351
-1.63953	-1.63624	-1.63303	-1.62989	-1.65136	-1.64815	-1.66796	-1.66466	-1.68495
-1.64101	-1.63768	-1.63445	-1.6313	-1.6528	-1.64958	-1.66943	-1.66613	-1.66284
-1.64253	-1.63917	-1.63592	-1.63274	-1.65425	-1.65104	-1.67092	-1.66763	-1.66435
-1.64405	-1.64063	-1.63733	-1.63412	-1.65565	-1.65243	-1.64922	-1.646	-1.66578
-1.64557	-1.64207	-1.63871	-1.63543	-1.65697	-1.65376	-1.65054	-1.64733	-1.66714

-1.64708	-1.64355	-1.64014	-1.63681	-1.63358	-1.63043	-1.65192	-1.64871	-1.66854
-1.64857	-1.64508	-1.64165	-1.63828	-1.63502	-1.63186	-1.65338	-1.65017	-1.67003
-1.65007	-1.64662	-1.64318	-1.63979	-1.63651	-1.63332	-1.65486	-1.65165	-1.67153
-1.65162	-1.64818	-1.64475	-1.64135	-1.63804	-1.63482	-1.65636	-1.65314	-1.64993
-1.65317	-1.64974	-1.64632	-1.64292	-1.63959	-1.63631	-1.65786	-1.65462	-1.6514
-1.65464	-1.65121	-1.64779	-1.64438	-1.64102	-1.6377	-1.65925	-1.65599	-1.65277
-1.65606	-1.65263	-1.6492	-1.64579	-1.64239	-1.63902	-1.66058	-1.6573	-1.65407
-1.63403	-1.63062	-1.65069	-1.64726	-1.64385	-1.64044	-1.63713	-1.6339	-1.65545
-1.6356	-1.63219	-1.65225	-1.64881	-1.64539	-1.64197	-1.63863	-1.63536	-1.65691
-1.6372	-1.63378	-1.63035	-1.62693	-1.64696	-1.64354	-1.64018	-1.63687	-1.65841
-1.63882	-1.63538	-1.63195	-1.62854	-1.64856	-1.64515	-1.64176	-1.63841	-1.65995
-1.64043	-1.63698	-1.63355	-1.63015	-1.65018	-1.64676	-1.64337	-1.63998	-1.66153
-1.58846	-1.58513	-1.63516	-1.63175	-1.62835	-1.62496	-1.64498	-1.64158	-1.66315
-1.59	-1.58667	-1.63674	-1.63333	-1.62993	-1.62656	-1.64658	-1.64319	-1.66479
-1.59146	-1.58812	-1.61316	-1.60977	-1.63142	-1.62804	-1.62469	-1.62135	-1.64138
-1.59285	-1.5895	-1.61456	-1.61117	-1.63282	-1.62944	-1.6261	-1.62282	-1.6429
-1.59433	-1.59095	-1.61604	-1.61264	-1.60926	-1.60591	-1.62756	-1.62428	-1.62102
-1.5959	-1.59249	-1.61761	-1.61421	-1.61081	-1.60744	-1.62906	-1.62572	-1.62243
-1.54506	-1.54173	-1.5907	-1.58738	-1.61239	-1.609	-1.60563	-1.60227	-1.62386
-1.54664	-1.54329	-1.5923	-1.58896	-1.614	-1.61058	-1.60718	-1.6038	-1.62536
-1.54825	-1.54488	-1.59393	-1.59056	-1.61562	-1.61217	-1.60876	-1.60536	-1.6269
-1.51916	-1.51584	-1.59556	-1.59216	-1.61724	-1.61378	-1.61035	-1.60694	-1.60358
-1.52074	-1.51741	-1.59721	-1.59377	-1.61887	-1.61541	-1.61197	-1.60854	-1.60516
-1.52234	-1.51899	-1.5463	-1.54294	-1.6205	-1.61703	-1.61358	-1.61014	-1.60675
-1.52394	-1.52056	-1.54787	-1.54449	-1.62213	-1.61865	-1.61519	-1.61175	-1.60835
-1.52554	-1.52214	-1.54947	-1.54605	-1.62379	-1.62028	-1.61681	-1.61336	-1.60996
-1.52714	-1.52373	-1.55108	-1.54765	-1.62548	-1.62193	-1.61843	-1.61498	-1.61157
-1.46252	-1.45951	-1.55269	-1.54925	-1.54585	-1.5425	-1.62007	-1.61661	-1.61319
-1.46392	-1.46089	-1.55429	-1.55084	-1.54744	-1.54407	-1.62173	-1.61825	-1.61481
-1.46532	-1.46228	-1.5559	-1.55244	-1.54902	-1.54564	-1.62341	-1.6199	-1.61643
-1.46676	-1.4637	-1.52667	-1.52325	-1.55061	-1.54723	-1.6251	-1.62156	-1.61806
-1.46821	-1.46514	-1.5283	-1.52484	-1.55221	-1.54882	-1.6268	-1.62322	-1.6197
-1.41047	-1.40749	-1.4635	-1.46044	-1.55382	-1.55042	-1.57667	-1.57319	-1.62134
-1.41186	-1.40887	-1.46494	-1.46185	-1.55545	-1.55202	-1.57831	-1.5748	-1.623
-1.41325	-1.41026	-1.40724	-1.40423	-1.55709	-1.55363	-1.5502	-1.54678	-1.62467
-1.41462	-1.41163	-1.40861	-1.4056	-1.55874	-1.55524	-1.55178	-1.54836	-1.62638
-1.41601	-1.41301	-1.40998	-1.40696	-1.56041	-1.55686	-1.55337	-1.54995	-1.6281
-1.24791	-1.24537	-1.41135	-1.40833	-1.46445	-1.46139	-1.55497	-1.55153	-1.57786
-1.24908	-1.24651	-1.41272	-1.4097	-1.46587	-1.46278	-1.55658	-1.55312	-1.57951
-1.25028	-1.24768	-1.28574	-1.28297	-1.40807	-1.40506	-1.55825	-1.55479	-1.58122
-1.25149	-1.24887	-1.28703	-1.28424	-1.40945	-1.40645	-1.55999	-1.55651	-1.58298
-1.25271	-1.25008	-1.24748	-1.24491	-1.28277	-1.28004	-1.43791	-1.43493	-1.58473
-1.25394	-1.25129	-1.24867	-1.24609	-1.28408	-1.28139	-1.43943	-1.4364	-1.58648
-1.15536	-1.15293	-1.24988	-1.24728	-1.28539	-1.28272	-1.31526	-1.31243	-1.55819
-1.15647	-1.15403	-1.25108	-1.24847	-1.28669	-1.284	-1.31657	-1.31375	-1.55991
-1.15759	-1.15513	-1.2523	-1.24968	-1.288	-1.28528	-1.31788	-1.31507	-1.56164
-1.15872	-1.15624	-1.25355	-1.25095	-1.24837	-1.24584	-1.28385	-1.28112	-1.3136
-1.15985	-1.15736	-1.25482	-1.25225	-1.24965	-1.24706	-1.28515	-1.28241	-1.31493
-1.13063	-1.12814	-1.1561	-1.15374	-1.25092	-1.2483	-1.28649	-1.28374	-1.3163
-1.13183	-1.12933	-1.15726	-1.15487	-1.25217	-1.24956	-1.28787	-1.28512	-1.31771

-0.97853	-0.9764	-1.12808	-1.12568	-1.219	-1.21645	-1.28922	-1.28646	-1.31909
-0.9796	-0.97742	-1.12926	-1.12681	-1.22019	-1.21763	-1.29054	-1.28776	-1.32042
-0.98068	-0.97847	-1.01614	-1.01397	-1.19217	-1.18961	-1.21628	-1.21379	-1.28631
-0.98175	-0.97954	-1.01717	-1.01493	-1.19339	-1.19085	-1.2175	-1.21498	-1.28763
-0.98282	-0.98062	-1.01822	-1.01592	-1.19464	-1.1921	-1.21874	-1.2162	-1.28897
-0.94547	-0.94333	-0.97943	-0.97721	-1.05542	-1.05319	-1.19079	-1.18826	-1.29033
-0.9465	-0.94433	-0.9805	-0.97831	-1.05655	-1.05424	-1.19201	-1.18949	-1.29173
-0.80881	-0.80732	-0.94322	-0.94109	-1.01694	-1.01467	-1.19327	-1.19074	-1.18824
-0.80956	-0.80806	-0.94427	-0.94213	-1.01801	-1.01573	-1.19458	-1.19202	-1.1895
-0.81031	-0.8088	-0.94534	-0.94317	-0.94102	-0.93888	-1.01459	-1.01245	-1.19076
-0.81107	-0.80953	-0.94642	-0.94423	-0.94207	-0.93994	-1.01569	-1.01348	-1.19201
-0.81182	-0.81026	-0.94753	-0.9453	-0.94313	-0.941	-1.01681	-1.01453	-1.1933
-0.81256	-0.81099	-0.80946	-0.80797	-0.9442	-0.94205	-0.98029	-0.97809	-1.12357
-0.8133	-0.81173	-0.81021	-0.80871	-0.94528	-0.94311	-0.98136	-0.97918	-1.12486
-0.81405	-0.81249	-0.81096	-0.80945	-0.94638	-0.94421	-0.94206	-0.93995	-0.97809
-0.81481	-0.81326	-0.81172	-0.81019	-0.9475	-0.94534	-0.94318	-0.94101	-0.97913
-0.81557	-0.81403	-0.81248	-0.81095	-0.80946	-0.808	-0.94427	-0.94209	-0.98022
-0.81634	-0.81477	-0.81324	-0.81173	-0.81023	-0.80873	-0.94533	-0.94319	-0.98135
-0.72863	-0.72727	-0.81401	-0.81251	-0.81099	-0.80947	-0.80798	-0.80652	-0.98247
-0.72932	-0.72796	-0.81479	-0.81326	-0.81174	-0.81023	-0.80874	-0.80725	-0.98352
-0.73	-0.72865	-0.81557	-0.81401	-0.81249	-0.811	-0.8095	-0.80798	-0.98458
-0.73068	-0.72932	-0.72796	-0.7266	-0.81324	-0.81174	-0.81023	-0.80873	-0.94538
-0.73135	-0.72998	-0.72862	-0.72727	-0.814	-0.81247	-0.81095	-0.80947	-0.94658
-0.73202	-0.73065	-0.72928	-0.72792	-0.7728	-0.77135	-0.81169	-0.81021	-0.80873
-0.7327	-0.73134	-0.72995	-0.72857	-0.7735	-0.77207	-0.81244	-0.81093	-0.80944
-0.73337	-0.73201	-0.73062	-0.72923	-0.72787	-0.72653	-0.81319	-0.81167	-0.81016
-0.73405	-0.73267	-0.73129	-0.72992	-0.72855	-0.72718	-0.81392	-0.81241	-0.81092
-0.69346	-0.69214	-0.73197	-0.73061	-0.72923	-0.72784	-0.72648	-0.72517	-0.81166
-0.6941	-0.69278	-0.73264	-0.73128	-0.7299	-0.72851	-0.72715	-0.72581	-0.81238
-0.69474	-0.69342	-0.73332	-0.73193	-0.73056	-0.72919	-0.72782	-0.72646	-0.8131
-0.6954	-0.69406	-0.69274	-0.69144	-0.73123	-0.72985	-0.72848	-0.72711	-0.77203
-0.69606	-0.6947	-0.69338	-0.69208	-0.73192	-0.73052	-0.72913	-0.72777	-0.77277
-0.60601	-0.60489	-0.69405	-0.69274	-0.73262	-0.73122	-0.72983	-0.72846	-0.72712
-0.60662	-0.6055	-0.69474	-0.69341	-0.73335	-0.73196	-0.73058	-0.7292	-0.72783
-0.51763	-0.51667	-0.64806	-0.64688	-0.69276	-0.69147	-0.73131	-0.72991	-0.72853
-0.51812	-0.51716	-0.64863	-0.64745	-0.6934	-0.69211	-0.732	-0.7306	-0.72921
-0.51862	-0.51765	-0.64921	-0.64802	-0.69404	-0.69274	-0.73268	-0.73128	-0.72989
-0.51912	-0.51815	-0.51719	-0.51623	-0.64743	-0.64627	-0.69205	-0.69074	-0.73058
-0.51963	-0.51865	-0.51768	-0.51673	-0.64801	-0.64683	-0.69268	-0.69138	-0.73128
-0.52013	-0.51915	-0.51819	-0.51724	-0.56099	-0.55997	-0.64626	-0.64512	-0.69076
-0.52064	-0.51967	-0.51871	-0.51776	-0.56151	-0.56049	-0.64687	-0.64572	-0.69143
-0.52116	-0.52019	-0.51922	-0.51827	-0.51731	-0.51636	-0.55999	-0.55901	-0.64517
-0.52169	-0.52071	-0.51973	-0.51876	-0.5178	-0.51685	-0.56049	-0.55951	-0.64578
-0.52223	-0.52123	-0.52024	-0.51926	-0.5183	-0.51734	-0.56099	-0.56001	-0.55902
-0.52274	-0.52173	-0.52074	-0.51977	-0.51881	-0.51785	-0.5615	-0.56049	-0.5595
-0.52326	-0.52224	-0.52125	-0.52028	-0.51932	-0.51837	-0.56201	-0.56099	-0.55997
-0.52382	-0.52278	-0.52178	-0.52081	-0.51985	-0.5189	-0.51795	-0.51698	-0.56048
-0.5244	-0.52334	-0.52233	-0.52135	-0.52038	-0.51944	-0.51848	-0.51751	-0.56101
-0.52496	-0.5239	-0.52288	-0.52189	-0.52091	-0.51995	-0.51899	-0.51801	-0.56152
-0.52552	-0.52446	-0.52343	-0.52243	-0.52144	-0.52046	-0.51948	-0.51851	-0.56202

-0.46927	-0.46834	-0.524	-0.52298	-0.52198	-0.52098	-0.51999	-0.51902	-0.51806
-0.46979	-0.46886	-0.52458	-0.52355	-0.52253	-0.52152	-0.52053	-0.51956	-0.5186
-0.47032	-0.46937	-0.52516	-0.52411	-0.52309	-0.52207	-0.52107	-0.5201	-0.51914
-0.47084	-0.46989	-0.52573	-0.52467	-0.52363	-0.5226	-0.5216	-0.52063	-0.51967
-0.47137	-0.47041	-0.52631	-0.52523	-0.52417	-0.52314	-0.52213	-0.52117	-0.52021
-0.4719	-0.47093	-0.46996	-0.469	-0.52475	-0.5237	-0.52268	-0.52171	-0.52074
-0.47244	-0.47146	-0.47049	-0.46952	-0.52533	-0.52427	-0.52325	-0.52226	-0.52128
-0.47298	-0.47199	-0.471	-0.47003	-0.46908	-0.46815	-0.52382	-0.52282	-0.52182
-0.47351	-0.4725	-0.47151	-0.47054	-0.46959	-0.46867	-0.52441	-0.52339	-0.52237
-0.47406	-0.47303	-0.47203	-0.47105	-0.4701	-0.46918	-0.525	-0.52396	-0.52293
-0.47463	-0.47359	-0.47257	-0.47157	-0.47062	-0.46969	-0.52559	-0.52454	-0.52351
-0.47521	-0.47416	-0.47313	-0.47211	-0.47114	-0.47021	-0.52617	-0.52513	-0.52409
-0.47578	-0.47473	-0.47369	-0.47268	-0.4717	-0.47074	-0.4698	-0.46888	-0.52467
-0.47637	-0.4753	-0.47426	-0.47326	-0.47226	-0.47128	-0.47033	-0.4694	-0.52527
-0.47695	-0.47587	-0.47483	-0.47382	-0.47282	-0.47183	-0.47087	-0.46993	-0.46899
-0.47754	-0.47645	-0.4754	-0.47438	-0.47338	-0.4724	-0.47142	-0.47046	-0.46951
-0.47814	-0.47704	-0.47598	-0.47495	-0.47394	-0.47296	-0.47198	-0.471	-0.47003
-0.47874	-0.47764	-0.47656	-0.47552	-0.47451	-0.47353	-0.47254	-0.47154	-0.47056
-0.47935	-0.47824	-0.47716	-0.4761	-0.47508	-0.47409	-0.4731	-0.4721	-0.4711
-0.47996	-0.47884	-0.47775	-0.47669	-0.47566	-0.47466	-0.47366	-0.47266	-0.47168
-0.48058	-0.47944	-0.47835	-0.47728	-0.47624	-0.47522	-0.47422	-0.47323	-0.47225
-0.43346	-0.43241	-0.47895	-0.47788	-0.47683	-0.4758	-0.47478	-0.47379	-0.47281
-0.43403	-0.43297	-0.47955	-0.47848	-0.47741	-0.47637	-0.47535	-0.47435	-0.47336
-0.43462	-0.43354	-0.48017	-0.47908	-0.47801	-0.47696	-0.47593	-0.47491	-0.47391
-0.43523	-0.43414	-0.4808	-0.47968	-0.4786	-0.47755	-0.47651	-0.47549	-0.47448
-0.38934	-0.38834	-0.43368	-0.43264	-0.47921	-0.47815	-0.4771	-0.47607	-0.47504
-0.38995	-0.38895	-0.4343	-0.43323	-0.47984	-0.47876	-0.47769	-0.47665	-0.47562
-0.33885	-0.33795	-0.43493	-0.43383	-0.43278	-0.43177	-0.47829	-0.47724	-0.4762
-0.33939	-0.33848	-0.43555	-0.43443	-0.43336	-0.43233	-0.47888	-0.47783	-0.47679
-0.33992	-0.33901	-0.43617	-0.43504	-0.43395	-0.4329	-0.4795	-0.47843	-0.47738
-0.34047	-0.33955	-0.33864	-0.33773	-0.4346	-0.43353	-0.48017	-0.47907	-0.47799
-0.34102	-0.34011	-0.3392	-0.33829	-0.43527	-0.43419	-0.48087	-0.47972	-0.47861
-0.34158	-0.34066	-0.33974	-0.33884	-0.43592	-0.43483	-0.43375	-0.43268	-0.47926
-0.34213	-0.3412	-0.34027	-0.33937	-0.43655	-0.43544	-0.43436	-0.4333	-0.47995
-0.3427	-0.34175	-0.34082	-0.3399	-0.339	-0.3381	-0.43498	-0.43392	-0.43288
-0.34327	-0.34231	-0.34138	-0.34046	-0.33954	-0.33863	-0.4356	-0.43452	-0.43346
-0.34385	-0.34289	-0.34195	-0.34102	-0.34009	-0.33917	-0.43623	-0.43513	-0.43405
-0.34443	-0.34347	-0.34252	-0.34157	-0.34064	-0.33972	-0.33881	-0.33791	-0.43467
-0.34503	-0.34407	-0.3431	-0.34213	-0.34119	-0.34028	-0.33937	-0.33846	-0.4353
-0.34567	-0.3447	-0.34371	-0.34272	-0.34177	-0.34083	-0.33991	-0.339	-0.43593
-0.34632	-0.34534	-0.34434	-0.34334	-0.34236	-0.3414	-0.34046	-0.33954	-0.43657
-0.34695	-0.34597	-0.34497	-0.34397	-0.34298	-0.342	-0.34104	-0.34012	-0.33921
-0.34755	-0.34657	-0.34559	-0.3446	-0.34362	-0.34264	-0.34168	-0.34074	-0.33982
-0.34816	-0.34717	-0.3462	-0.34523	-0.34425	-0.34328	-0.34231	-0.34136	-0.34041
-0.34877	-0.34778	-0.3468	-0.34582	-0.34485	-0.34387	-0.3429	-0.34193	-0.34097
-0.34939	-0.34839	-0.3474	-0.34642	-0.34544	-0.34446	-0.34349	-0.3425	-0.34154
-0.35001	-0.349	-0.34801	-0.34703	-0.34605	-0.34507	-0.34409	-0.34311	-0.34213
-0.35064	-0.34963	-0.34863	-0.34764	-0.34666	-0.34569	-0.34471	-0.34373	-0.34275
-0.35131	-0.35029	-0.34928	-0.34829	-0.3473	-0.34633	-0.34535	-0.34437	-0.34339
-0.35201	-0.35097	-0.34996	-0.34896	-0.34797	-0.34699	-0.34601	-0.34503	-0.34405

-0.31055	-0.30972	-0.35061	-0.34961	-0.34861	-0.34763	-0.34664	-0.34566	-0.34468
-0.3111	-0.31025	-0.35123	-0.3502	-0.3492	-0.34822	-0.34723	-0.34625	-0.34527
-0.31165	-0.31078	-0.35186	-0.35082	-0.34981	-0.34882	-0.34784	-0.34686	-0.34588
-0.31222	-0.31133	-0.35254	-0.35148	-0.35047	-0.34949	-0.34851	-0.34753	-0.34655
-0.31281	-0.31191	-0.35326	-0.35217	-0.35114	-0.35016	-0.34918	-0.3482	-0.34722
-0.31344	-0.31254	-0.31165	-0.31079	-0.35184	-0.35082	-0.34982	-0.34883	-0.34784
-0.3141	-0.3132	-0.31229	-0.3114	-0.35254	-0.35148	-0.35045	-0.34945	-0.34845
-0.31471	-0.31381	-0.3129	-0.31199	-0.35325	-0.35216	-0.35111	-0.3501	-0.3491
-0.31528	-0.31439	-0.31348	-0.31257	-0.35396	-0.35285	-0.35179	-0.35078	-0.34978
-0.31588	-0.31499	-0.31408	-0.31318	-0.31228	-0.31139	-0.3525	-0.35147	-0.35046
-0.31653	-0.31563	-0.31473	-0.31383	-0.31293	-0.31203	-0.35324	-0.35217	-0.35112
-0.31718	-0.31626	-0.31536	-0.31448	-0.31358	-0.31267	-0.35399	-0.35288	-0.3518
-0.31778	-0.31686	-0.31595	-0.31506	-0.31416	-0.31326	-0.35472	-0.35362	-0.35253
-0.31838	-0.31746	-0.31654	-0.31563	-0.31473	-0.31385	-0.35546	-0.35437	-0.35329
-0.31903	-0.31811	-0.31718	-0.31626	-0.31536	-0.31447	-0.31359	-0.31272	-0.35402
-0.31972	-0.31879	-0.31785	-0.31693	-0.31602	-0.31512	-0.31423	-0.31333	-0.35474
-0.25479	-0.25422	-0.31849	-0.31756	-0.31664	-0.31574	-0.31484	-0.31395	-0.31306
-0.25519	-0.2546	-0.31909	-0.31815	-0.31722	-0.31631	-0.31542	-0.31456	-0.3137
-0.2556	-0.255	-0.31972	-0.31877	-0.31784	-0.31691	-0.31602	-0.31517	-0.31432
-0.25605	-0.25544	-0.32041	-0.31946	-0.31851	-0.31758	-0.31667	-0.31578	-0.3149
-0.25649	-0.25588	-0.32111	-0.32015	-0.3192	-0.31826	-0.31733	-0.3164	-0.31549
-0.25693	-0.2563	-0.25569	-0.25509	-0.31983	-0.31891	-0.31799	-0.31706	-0.31614
-0.25736	-0.25672	-0.25611	-0.2555	-0.32047	-0.31957	-0.31866	-0.31773	-0.3168
-0.25781	-0.25718	-0.25655	-0.25594	-0.32116	-0.32023	-0.31929	-0.31836	-0.31744
-0.25827	-0.25765	-0.25703	-0.2564	-0.32189	-0.32089	-0.31991	-0.31897	-0.31805
-0.25871	-0.2581	-0.25748	-0.25685	-0.25623	-0.25562	-0.32058	-0.31962	-0.31869
-0.25913	-0.25853	-0.25791	-0.2573	-0.25668	-0.25607	-0.32131	-0.32033	-0.31937
-0.25956	-0.25895	-0.25835	-0.25774	-0.25713	-0.25652	-0.25591	-0.25531	-0.32006
-0.26001	-0.2594	-0.25879	-0.25818	-0.25756	-0.25694	-0.25633	-0.25573	-0.32074
-0.26046	-0.25986	-0.25924	-0.25862	-0.258	-0.25737	-0.25676	-0.25616	-0.32144
-0.26089	-0.2603	-0.25969	-0.25908	-0.25846	-0.25783	-0.25721	-0.2566	-0.32216
-0.26131	-0.26073	-0.26014	-0.25954	-0.25893	-0.2583	-0.25768	-0.25706	-0.3229
-0.19617	-0.19573	-0.26057	-0.25999	-0.25939	-0.25877	-0.25815	-0.25752	-0.25689
-0.1965	-0.19604	-0.261	-0.26042	-0.25983	-0.25923	-0.25861	-0.25798	-0.25736
-0.19685	-0.19637	-0.26143	-0.26085	-0.26027	-0.25968	-0.25907	-0.25845	-0.25782
-0.19722	-0.19674	-0.26188	-0.26128	-0.26069	-0.26011	-0.25952	-0.25891	-0.25829
-0.13236	-0.13204	-0.19661	-0.19614	-0.26113	-0.26055	-0.25996	-0.25936	-0.25875
-0.13258	-0.13227	-0.19696	-0.19649	-0.26157	-0.26098	-0.26039	-0.2598	-0.2592
-0.1328	-0.13249	-0.19731	-0.19684	-0.26203	-0.26142	-0.26082	-0.26023	-0.25964
-0.06377	-0.06364	-0.1324	-0.1321	-0.19672	-0.19624	-0.26126	-0.26067	-0.26008
-0.06386	-0.06373	-0.13262	-0.13232	-0.19706	-0.19659	-0.26171	-0.26111	-0.26051
		-0.0637	-0.06358	-0.13224	-0.13193	-0.19647	-0.19599	-0.26095
		-0.0638	-0.06367	-0.13247	-0.13216	-0.19683	-0.19634	-0.2614
				-0.1327	-0.13239	-0.19718	-0.19669	-0.26186
				-0.13292	-0.13261	-0.19753	-0.19705	-0.26232
				-0.13314	-0.13283	-0.19787	-0.1974	-0.26279
				-0.06393	-0.0638	-0.13274	-0.13244	-0.19728
				-0.06403	-0.0639	-0.13296	-0.13266	-0.19762
						-0.13319	-0.13289	-0.13259
						-0.13343	-0.13313	-0.13282

-0.13306
-0.13329
-0.13353
-0.13377
-0.13401

						-0.12904	-0.12868	-0.12832
						-0.12877	-0.1284	-0.12803
				-0.12923	-0.12887	-0.4398	-0.43864	-0.4375
				-0.12898	-0.12861	-0.43899	-0.43784	-0.4367
				-0.12872	-0.12835	-0.4382	-0.43705	-0.43591
				-0.19513	-0.19458	-0.65776	-0.65607	-0.6544
				-0.19473	-0.19418	-0.65665	-0.65496	-0.65329
		-0.12867	-0.1283	-0.49068	-0.48948	-0.75849	-0.75657	-0.75468
		-0.12841	-0.12803	-0.48985	-0.48865	-0.75719	-0.75529	-0.75342
-0.12889	-0.12852	-0.43874	-0.43759	-0.65663	-0.65497	-0.75589	-0.75402	-0.75217
-0.12864	-0.12826	-0.43796	-0.43681	-0.65551	-0.65387	-0.75461	-0.75276	-0.75093
-0.4395	-0.43833	-0.65779	-0.65608	-0.75708	-0.7552	-0.75335	-0.75151	-0.74971
-0.43872	-0.43756	-0.65669	-0.65497	-0.75578	-0.75392	-0.75209	-0.75028	-0.74851
-0.43794	-0.43679	-0.65559	-0.65389	-0.75452	-0.75267	-0.75086	-0.74908	-0.74731
-0.65787	-0.65619	-0.75711	-0.75519	-0.75332	-0.75148	-0.74967	-0.74789	-0.74613

-0.65676	-0.65509	-0.75586	-0.754	-0.75215	-0.75032	-0.74851	-0.74672	-0.74496
-0.709	-0.70721	-0.7546	-0.75278	-0.75096	-0.74915	-0.79556	-0.79371	-0.79188
-0.70779	-0.70602	-0.75334	-0.75154	-0.74975	-0.74796	-0.79437	-0.79252	-0.79068
-0.75579	-0.75392	-0.7521	-0.75032	-0.79681	-0.79499	-0.9383	-0.93613	-0.93396
-0.75454	-0.75268	-0.75088	-0.74911	-0.79559	-0.79379	-0.93688	-0.93474	-0.93259
-0.75332	-0.75147	-0.798	-0.7962	-0.79439	-0.79258	-0.93544	-0.93333	-0.9312
-0.75212	-0.75033	-0.79684	-0.79502	-0.7932	-0.79136	-0.93399	-0.93187	-0.92976
-0.75095	-0.74919	-0.79569	-0.79386	-0.79201	-0.79015	-0.93252	-0.9304	-0.9283
-0.74976	-0.74802	-0.79449	-0.79266	-0.93545	-0.93324	-0.93107	-0.92894	-0.92684
-0.74856	-0.74682	-0.79327	-0.79144	-0.934	-0.93179	-0.92962	-0.92749	-0.9254
-0.7957	-0.79389	-0.9369	-0.93473	-0.97607	-0.97377	-1.01865	-1.01632	-1.01402
-0.79454	-0.79271	-0.93548	-0.93328	-0.97455	-0.97231	-1.01713	-1.01481	-1.01252
-0.79337	-0.79151	-0.9776	-0.9753	-1.02024	-1.01791	-1.0156	-1.0133	-1.01103
-0.79216	-0.79029	-0.97607	-0.97376	-1.01862	-1.01632	-1.01404	-1.01178	-1.00954
-0.79094	-0.78906	-0.97455	-0.97225	-1.01704	-1.01476	-1.0125	-1.01027	-1.00805
-0.97764	-0.97533	-0.97305	-0.9708	-1.01554	-1.01326	-1.01101	-1.00877	-1.00656
-0.97611	-0.97381	-0.97157	-0.96938	-1.01407	-1.0118	-1.00955	-1.0073	-1.00507
-0.9746	-0.97234	-1.01714	-1.01486	-1.01259	-1.01034	-1.00809	-1.00586	-1.00364
-0.97312	-0.97089	-1.01564	-1.01337	-1.01111	-1.00888	-1.00666	-1.00445	-1.00225
-1.01874	-1.01643	-1.01415	-1.01189	-1.00965	-1.00743	-1.00523	-1.00303	-1.00082
-1.01721	-1.01492	-1.01266	-1.01042	-1.0082	-1.00601	-1.00381	-1.00159	-0.99936
-1.01571	-1.01343	-1.01118	-1.00896	-1.00676	-1.00457	-1.00237	-1.00013	-0.99788
-1.01421	-1.01196	-1.00973	-1.00754	-1.00534	-1.00313	-1.00091	-0.99864	-0.99638
-1.01273	-1.0105	-1.0083	-1.00612	-1.00391	-1.00168	-0.99943	-0.99714	-0.99486
-1.01127	-1.00907	-1.00687	-1.00468	-1.00246	-1.0002	-1.04635	-1.0439	-1.04148
-1.00983	-1.00766	-1.00545	-1.00324	-1.00099	-0.9987	-1.04472	-1.04228	-1.03988
-1.00842	-1.00622	-1.004	-1.00177	-0.9995	-0.99719	-1.0431	-1.0407	-1.03834
-1.00702	-1.00478	-1.00253	-1.00026	-0.99798	-0.99567	-1.04148	-1.03914	-1.03685
-1.00559	-1.00332	-1.00105	-0.99878	-1.04477	-1.04232	-1.16505	-1.16218	-1.15937
-1.00411	-1.00183	-0.99957	-0.99733	-1.04325	-1.04085	-1.16325	-1.16039	-1.15757
-1.00264	-1.00036	-0.99811	-0.99588	-1.16725	-1.16434	-1.16145	-1.15859	-1.15577
-1.00124	-0.99897	-0.9967	-0.99445	-1.16532	-1.16242	-1.15956	-1.15673	-1.15394
-0.99984	-0.99758	-0.9953	-0.99301	-1.16339	-1.16051	-1.15767	-1.15488	-1.15214
-0.99837	-0.99609	-1.08098	-1.07843	-1.16146	-1.15866	-1.15589	-1.15315	-1.15043
-0.99687	-0.99457	-1.07929	-1.07678	-1.15956	-1.15684	-1.15415	-1.15147	-1.14878
-1.03549	-1.03308	-1.16349	-1.16058	-1.15777	-1.15507	-1.20567	-1.20288	-1.20008
-1.03403	-1.03168	-1.16178	-1.15891	-1.1561	-1.15335	-1.20387	-1.20103	-1.19821
-1.16575	-1.16288	-1.16002	-1.15719	-1.20785	-1.20494	-1.20205	-1.19921	-1.19638
-1.16383	-1.16098	-1.15815	-1.15534	-1.20598	-1.20308	-1.20022	-1.19741	-1.19463
-1.16195	-1.15911	-1.15631	-1.15353	-1.20413	-1.20125	-1.19842	-1.19564	-1.19288
-1.16017	-1.1574	-1.2082	-1.20531	-1.20242	-1.19954	-1.31687	-1.31337	-1.30993
-1.15843	-1.15572	-1.20651	-1.20362	-1.20073	-1.19784	-1.31474	-1.31124	-1.30779
-1.21038	-1.20755	-1.20468	-1.2018	-1.31968	-1.31609	-1.31255	-1.30907	-1.30565
-1.20853	-1.20567	-1.20279	-1.19991	-1.3174	-1.31385	-1.31035	-1.30689	-1.30351
-1.20675	-1.20388	-1.23994	-1.23687	-1.31521	-1.31171	-1.30825	-1.30482	-1.30148
-1.20507	-1.20218	-1.23814	-1.23509	-1.31313	-1.30967	-1.30625	-1.30287	-1.29955
-1.24244	-1.23934	-1.31806	-1.31452	-1.31103	-1.3076	-1.30423	-1.30088	-1.2976
-1.24044	-1.23736	-1.3158	-1.31229	-1.30884	-1.30544	-1.3021	-1.2988	-1.29557
-1.23845	-1.2354	-1.31356	-1.31009	-1.30668	-1.30332	-1.30002	-1.29675	-1.29358
-1.31851	-1.31496	-1.31148	-1.30805	-1.30468	-1.30135	-1.29808	-1.29485	-1.29173

-1.31641	-1.31291	-1.30947	-1.30608	-1.30274	-1.29945	-1.29623	-1.29304	-1.28998
-1.31423	-1.31077	-1.30736	-1.30401	-1.30073	-1.29751	-1.33317	-1.33001	-1.32697
-1.312	-1.30858	-1.3052	-1.30189	-1.29867	-1.29555	-1.33128	-1.32828	-1.32539
-1.30988	-1.3065	-1.30316	-1.29987	-1.33557	-1.33244	-1.32943	-1.32655	-1.32378
-1.30791	-1.30457	-1.30125	-1.29798	-1.33366	-1.33056	-1.32761	-1.32481	-1.32213
-1.30599	-1.30267	-1.29938	-1.29614	-1.33182	-1.32877	-1.32587	-1.32315	-1.32055
-1.30408	-1.30079	-1.29754	-1.29436	-1.33007	-1.3271	-1.32428	-1.32166	-1.31914
-1.30217	-1.29891	-1.29572	-1.29261	-1.32837	-1.32549	-1.32277	-1.32023	-1.31777
-1.30015	-1.29693	-1.33266	-1.32959	-1.32667	-1.3239	-1.32126	-1.31876	-1.31633
-1.2981	-1.29495	-1.33073	-1.32779	-1.325	-1.32235	-1.31979	-1.31731	-1.31488
-1.33509	-1.33195	-1.32897	-1.32614	-1.32345	-1.32089	-1.35448	-1.35195	-1.34946
-1.33329	-1.33025	-1.32736	-1.32463	-1.322	-1.3195	-1.35309	-1.35059	-1.34811
-1.33155	-1.32861	-1.32582	-1.32317	-1.35675	-1.35421	-1.35171	-1.34923	-1.34676
-1.32987	-1.32702	-1.32433	-1.32179	-1.35536	-1.35284	-1.35034	-1.34787	-1.34543
-1.32825	-1.3255	-1.3229	-1.32043	-1.35399	-1.35146	-1.34896	-1.34649	-1.34406
-1.32675	-1.3241	-1.32155	-1.31909	-1.3526	-1.35006	-1.34754	-1.34505	-1.34259
-1.3253	-1.32273	-1.32021	-1.31775	-1.3512	-1.34865	-1.34611	-1.3436	-1.34112
-1.32379	-1.32126	-1.35479	-1.35227	-1.34975	-1.34723	-1.38031	-1.37773	-1.37519
-1.32226	-1.31974	-1.35323	-1.35075	-1.34827	-1.3458	-1.37888	-1.37635	-1.37385
-1.35686	-1.35429	-1.35177	-1.3493	-1.34685	-1.34442	-1.41174	-1.40915	-1.40659
-1.35544	-1.35289	-1.35039	-1.34793	-1.34549	-1.34306	-1.41029	-1.40773	-1.4052
-1.35405	-1.35152	-1.34904	-1.34659	-1.41403	-1.41144	-1.44449	-1.44179	-1.43913
-1.35268	-1.35018	-1.34772	-1.34528	-1.41262	-1.41005	-1.44303	-1.44033	-1.43766
-1.35132	-1.34886	-1.34641	-1.34397	-1.41122	-1.40866	-1.44156	-1.43885	-1.43618
-1.34997	-1.34752	-1.38003	-1.37752	-1.44548	-1.44277	-1.44007	-1.43736	-1.43468
-1.34862	-1.34618	-1.37865	-1.37614	-1.44397	-1.44126	-1.43855	-1.43585	-1.43318
-1.3823	-1.37978	-1.41211	-1.40953	-1.44245	-1.43973	-1.43702	-1.43434	-1.43168
-1.3809	-1.37838	-1.41066	-1.40809	-1.44092	-1.43818	-1.43547	-1.43282	-1.4302
-1.37951	-1.377	-1.44485	-1.44212	-1.43939	-1.43665	-1.47239	-1.46964	-1.46693
-1.37814	-1.37563	-1.44332	-1.44056	-1.43784	-1.43514	-1.47084	-1.46812	-1.46545
-1.44729	-1.44455	-1.44178	-1.43902	-1.43631	-1.43365	-1.46931	-1.46664	-1.464
-1.44572	-1.44296	-1.44021	-1.43748	-1.43481	-1.4322	-1.46782	-1.46517	-1.46256
-1.44416	-1.44138	-1.43865	-1.43596	-1.43334	-1.43077	-1.46636	-1.46373	-1.46113
-1.44267	-1.43989	-1.43717	-1.43451	-1.4319	-1.42936	-1.46491	-1.46229	-1.45971
-1.44121	-1.43845	-1.43575	-1.43309	-1.4305	-1.42798	-1.46347	-1.46087	-1.45829
-1.43975	-1.43701	-1.43432	-1.43168	-1.46727	-1.46463	-1.5364	-1.53333	-1.5303
-1.43829	-1.43557	-1.4329	-1.43028	-1.4658	-1.46317	-1.53463	-1.53158	-1.52858
-1.43685	-1.43414	-1.43149	-1.42888	-1.46434	-1.46171	-1.53288	-1.52986	-1.52688
-1.43541	-1.43273	-1.43009	-1.42748	-1.46289	-1.46027	-1.53115	-1.52815	-1.52519
-1.43399	-1.43132	-1.42869	-1.42608	-1.53552	-1.53246	-1.56078	-1.55769	-1.55463
-1.43258	-1.42992	-1.42729	-1.42468	-1.53376	-1.53073	-1.55901	-1.55593	-1.55288
-1.43118	-1.42852	-1.42589	-1.42329	-1.53201	-1.52901	-1.55725	-1.55419	-1.55114
-1.42978	-1.42712	-1.49987	-1.49679	-1.53028	-1.52731	-1.5555	-1.55246	-1.54943
-1.42838	-1.42573	-1.49818	-1.49513	-1.52857	-1.52561	-1.55375	-1.55074	-1.54773
-1.42699	-1.42438	-1.53313	-1.52996	-1.55812	-1.55505	-1.55202	-1.54902	-1.54605
-1.42562	-1.42305	-1.53149	-1.52833	-1.55642	-1.55332	-1.55028	-1.54731	-1.54436
-1.49941	-1.49643	-1.52985	-1.52669	-1.55471	-1.5516	-1.54857	-1.54562	-1.54271
-1.49772	-1.49479	-1.52816	-1.52504	-1.553	-1.54989	-1.54687	-1.54396	-1.54109
-1.49603	-1.49313	-1.52648	-1.52338	-1.5513	-1.5482	-1.54519	-1.54232	-1.53949
-1.49435	-1.49148	-1.55591	-1.55272	-1.54959	-1.54652	-1.54354	-1.54069	-1.5379

-1.49268	-1.48983	-1.55416	-1.55099	-1.5479	-1.54487	-1.54192	-1.53909	-1.53632
-1.52282	-1.51987	-1.55246	-1.54935	-1.5463	-1.54328	-1.54035	-1.53753	-1.53477
-1.52117	-1.51819	-1.55078	-1.54777	-1.54476	-1.54175	-1.53882	-1.536	-1.53324
-1.5195	-1.51651	-1.54909	-1.54615	-1.54319	-1.5402	-1.57081	-1.56792	-1.5651
-1.51782	-1.51481	-1.54738	-1.5445	-1.54158	-1.53864	-1.56923	-1.56634	-1.56351
-1.51619	-1.51316	-1.5457	-1.54285	-1.53998	-1.5371	-1.56767	-1.56479	-1.56195
-1.51464	-1.51162	-1.5441	-1.54124	-1.5384	-1.53559	-1.56617	-1.56328	-1.56042
-1.51311	-1.5101	-1.54253	-1.53965	-1.53683	-1.53409	-1.56467	-1.56178	-1.5589
-1.54696	-1.54392	-1.54096	-1.53807	-1.56869	-1.56592	-1.56312	-1.56023	-1.55735
-1.54531	-1.54233	-1.5394	-1.5365	-1.56708	-1.56432	-1.56154	-1.55867	-1.55581
-1.54369	-1.54078	-1.57135	-1.56839	-1.56552	-1.56274	-1.55996	-1.55714	-1.55432
-1.54211	-1.53927	-1.56982	-1.56687	-1.56399	-1.56118	-1.55839	-1.55563	-1.55287
-1.54054	-1.53773	-1.56827	-1.56536	-1.56248	-1.55963	-1.55684	-1.5541	-1.55139
-1.53897	-1.53615	-1.5667	-1.56387	-1.56101	-1.55813	-1.5553	-1.55256	-1.54984
-1.57085	-1.56795	-1.56512	-1.56235	-1.55952	-1.55662	-1.55378	-1.55102	-1.54831
-1.56932	-1.5664	-1.56356	-1.56077	-1.55794	-1.55508	-1.55226	-1.54951	-1.5468
-1.5678	-1.56488	-1.56202	-1.55918	-1.55636	-1.55353	-1.55075	-1.54802	-1.54533
-1.56624	-1.56338	-1.56051	-1.55765	-1.55484	-1.55206	-1.54931	-1.54659	-1.5439
-1.56465	-1.56187	-1.55903	-1.55616	-1.55336	-1.55063	-1.54791	-1.5452	-1.54251
-1.56307	-1.56031	-1.55748	-1.55462	-1.55184	-1.54914	-1.54645	-1.54378	-1.54113
-1.56147	-1.55867	-1.55585	-1.55305	-1.5503	-1.5476	-1.54494	-1.54235	-1.53976
-1.55992	-1.55708	-1.55429	-1.55152	-1.54879	-1.54609	-1.54345	-1.5409	-1.53835
-1.55842	-1.55559	-1.55281	-1.55007	-1.54735	-1.54465	-1.542	-1.53942	-1.53685
-1.55695	-1.55412	-1.55136	-1.54864	-1.54593	-1.54323	-1.54056	-1.53795	-1.53535
-1.55547	-1.55267	-1.54992	-1.5472	-1.5445	-1.54182	-1.5821	-1.57943	-1.57678
-1.554	-1.55124	-1.54849	-1.54577	-1.54308	-1.54043	-1.58075	-1.57806	-1.57539
-1.55254	-1.54979	-1.54706	-1.54435	-1.58472	-1.58204	-1.65166	-1.64886	-1.64607
-1.55109	-1.54835	-1.54563	-1.54294	-1.58333	-1.58065	-1.65024	-1.64746	-1.64469
-1.54964	-1.54691	-1.56532	-1.56259	-1.6023	-1.59955	-1.64883	-1.64606	-1.6433
-1.54818	-1.54547	-1.56389	-1.56115	-1.60089	-1.59816	-1.64743	-1.64465	-1.64188
-1.56794	-1.56519	-1.605	-1.60223	-1.59948	-1.59676	-1.69237	-1.68948	-1.68661
-1.56649	-1.56373	-1.60358	-1.60083	-1.59808	-1.59535	-1.6909	-1.68798	-1.6851
-1.56504	-1.56228	-1.60215	-1.59942	-1.59668	-1.59393	-1.68941	-1.68649	-1.68359
-1.60628	-1.60349	-1.60074	-1.598	-1.61722	-1.61445	-1.6879	-1.68501	-1.68215
-1.60485	-1.60209	-1.59933	-1.59658	-1.61577	-1.61298	-1.6864	-1.68355	-1.68074
-1.60342	-1.60067	-1.61988	-1.61713	-1.66571	-1.66285	-1.74295	-1.73985	-1.7368
-1.602	-1.59923	-1.61844	-1.61572	-1.66432	-1.6615	-1.74142	-1.7383	-1.73523
-1.62253	-1.61976	-1.61702	-1.61431	-1.66291	-1.6601	-1.75697	-1.75382	-1.75072
-1.62111	-1.61836	-1.61562	-1.6129	-1.66147	-1.65865	-1.75535	-1.75225	-1.74918
-1.61969	-1.61696	-1.61422	-1.61149	-1.70881	-1.70577	-1.75377	-1.75069	-1.74765
-1.61828	-1.61554	-1.61282	-1.61011	-1.70735	-1.70434	-1.75228	-1.74916	-1.74608
-1.61687	-1.61413	-1.61142	-1.60874	-1.7059	-1.70294	-1.75082	-1.74766	-1.74453
-1.61545	-1.61274	-1.62895	-1.62622	-1.7317	-1.72867	-1.82273	-1.81942	-1.81613
-1.61404	-1.61135	-1.62756	-1.62482	-1.73015	-1.72714	-1.82111	-1.81785	-1.8146
-1.63164	-1.62891	-1.65742	-1.65453	-1.72861	-1.72559	-1.81945	-1.81623	-1.81303
-1.6303	-1.6275	-1.65592	-1.65306	-1.72709	-1.72403	-1.81774	-1.81458	-1.81144
-1.66036	-1.65736	-1.68251	-1.67958	-1.82266	-1.81934	-1.81609	-1.81295	-1.80984
-1.65893	-1.65596	-1.68106	-1.67812	-1.82107	-1.81779	-1.81454	-1.81135	-1.8082
-1.65751	-1.65457	-1.67962	-1.67665	-1.81947	-1.81623	-1.81301	-1.80978	-1.80659
-1.6561	-1.65318	-1.70701	-1.7039	-1.8178	-1.81461	-1.81143	-1.80823	-1.80506

-1.65469	-1.65177	-1.70548	-1.70233	-1.81612	-1.81297	-1.80984	-1.80669	-1.80355
-1.68121	-1.67824	-1.75153	-1.74829	-1.81452	-1.81135	-1.80822	-1.80511	-1.80201
-1.67965	-1.6767	-1.74994	-1.74676	-1.81298	-1.80975	-1.80658	-1.8035	-1.80045
-1.70692	-1.70388	-1.74831	-1.74519	-1.81143	-1.80818	-1.805	-1.80192	-1.79889
-1.70533	-1.70225	-1.74664	-1.74359	-1.80987	-1.80667	-1.8035	-1.80038	-1.79731
-1.70378	-1.70067	-1.745	-1.74198	-1.80828	-1.80513	-1.80199	-1.79886	-1.79576
-1.70227	-1.69917	-1.74341	-1.74035	-1.80665	-1.80355	-1.80045	-1.79736	-1.79428
-1.70078	-1.69769	-1.74184	-1.73874	-1.80502	-1.80195	-1.7989	-1.79586	-1.79282
-1.74663	-1.74347	-1.74032	-1.7372	-1.80345	-1.80037	-1.79733	-1.79432	-1.79131
-1.74505	-1.74195	-1.73882	-1.73569	-1.80192	-1.7988	-1.79575	-1.79277	-1.7898
-1.74345	-1.74039	-1.7373	-1.7342	-1.80042	-1.79729	-1.79423	-1.79126	-1.78831
-1.74182	-1.73881	-1.73577	-1.73273	-1.79897	-1.79583	-1.79276	-1.78979	-1.78684
-1.74023	-1.73725	-1.73426	-1.73127	-1.79751	-1.79438	-1.81487	-1.8118	-1.80875
-1.73869	-1.73573	-1.73276	-1.72981	-1.79606	-1.79293	-1.81336	-1.81024	-1.80716
-1.73717	-1.7342	-1.77808	-1.77511	-1.7946	-1.79148	-1.83245	-1.82927	-1.82613
-1.73563	-1.73265	-1.77653	-1.77359	-1.7931	-1.79003	-1.831	-1.8278	-1.82464
-1.73411	-1.7311	-1.77498	-1.77206	-1.7916	-1.78857	-1.82955	-1.82636	-1.82319
-1.73263	-1.72962	-1.77349	-1.77059	-1.81111	-1.80809	-1.8281	-1.82491	-1.82173
-1.73118	-1.72817	-1.77204	-1.76914	-1.80968	-1.80667	-1.82664	-1.82347	-1.8203
-1.72974	-1.72673	-1.79196	-1.78904	-1.83138	-1.8283	-1.82519	-1.82202	-1.81885
-1.72831	-1.72531	-1.79054	-1.78761	-1.82992	-1.82685	-1.82374	-1.82058	-1.81741
-1.74896	-1.74595	-1.78912	-1.78619	-1.82845	-1.82539	-1.84647	-1.84325	-1.84003
-1.74755	-1.74454	-1.78771	-1.78477	-1.82699	-1.82393	-1.845	-1.8418	-1.83859
-1.74614	-1.74314	-1.78629	-1.78335	-1.82553	-1.82247	-1.84353	-1.84035	-1.83715
-1.74473	-1.74173	-1.80829	-1.80527	-1.82407	-1.82101	-1.84206	-1.83889	-1.83571
-1.74332	-1.74033	-1.80684	-1.80382	-1.82262	-1.81955	-1.84058	-1.83742	-1.83426
-1.74191	-1.73892	-1.80538	-1.80237	-1.82116	-1.81809	-1.8391	-1.83595	-1.8328
-1.74049	-1.7375	-1.80392	-1.80091	-1.8197	-1.81663	-1.83763	-1.83448	-1.83133
-1.76314	-1.76009	-1.80246	-1.79944	-1.81824	-1.81518	-1.83616	-1.83301	-1.82987
-1.76169	-1.75864	-1.80099	-1.79798	-1.81678	-1.81373	-1.83471	-1.83155	-1.8284
-1.8057	-1.80261	-1.79956	-1.79654	-1.81535	-1.8123	-1.83324	-1.83009	-1.82693
-1.80429	-1.80123	-1.79819	-1.79516	-1.81395	-1.81087	-1.83177	-1.82861	-1.82546
-1.80289	-1.79985	-1.79681	-1.79378	-1.81255	-1.80944	-1.8303	-1.82714	-1.82399
-1.8015	-1.79845	-1.79541	-1.79236	-1.8352	-1.83202	-1.82884	-1.82567	-1.82252
-1.80012	-1.79705	-1.79398	-1.79092	-1.83373	-1.83056	-1.82739	-1.82421	-1.82105
-1.79872	-1.79563	-1.79254	-1.78947	-1.83225	-1.82909	-1.82592	-1.82274	-1.81958
-1.7973	-1.7942	-1.79109	-1.788	-1.83075	-1.8276	-1.82444	-1.82127	-1.81811
-1.79586	-1.79275	-1.78964	-1.78654	-1.82926	-1.8261	-1.82295	-1.81979	-1.81664
-1.79439	-1.7913	-1.7882	-1.78509	-1.82778	-1.8246	-1.82144	-1.81829	-1.81517
-1.79291	-1.78983	-1.78674	-1.78363	-1.8263	-1.82311	-1.8395	-1.83634	-1.8332
-1.79142	-1.78834	-1.78526	-1.78216	-1.82482	-1.82163	-1.838	-1.83484	-1.83171
-1.78994	-1.78685	-1.78377	-1.78069	-1.82335	-1.82017	-1.83654	-1.83338	-1.83026
-1.78846	-1.78538	-1.7823	-1.77923	-1.82189	-1.81875	-1.83515	-1.83203	-1.82893
-1.78701	-1.78392	-1.78084	-1.77777	-1.82044	-1.81737	-1.83381	-1.83072	-1.82766
-1.78564	-1.78255	-1.80373	-1.8006	-1.79752	-1.7945	-1.83243	-1.82936	-1.82631
-1.78435	-1.78126	-1.80242	-1.79927	-1.79618	-1.79314	-1.83104	-1.82796	-1.82489
-1.78301	-1.77991	-1.80104	-1.7979	-1.7948	-1.79174	-1.82962	-1.82654	-1.82347
-1.78156	-1.77845	-1.79957	-1.79644	-1.79335	-1.7903	-1.82816	-1.82509	-1.82203
-1.7801	-1.77698	-1.79808	-1.79497	-1.79189	-1.78885	-1.8267	-1.82365	-1.8206
-1.77863	-1.77552	-1.79661	-1.79351	-1.79044	-1.78741	-1.82525	-1.82219	-1.81915

-1.77718	-1.77408	-1.79517	-1.79208	-1.78902	-1.78599	-1.82382	-1.82077	-1.81773
-1.77583	-1.77276	-1.79384	-1.79076	-1.80741	-1.80435	-1.82251	-1.81946	-1.81642
-1.77454	-1.7715	-1.79258	-1.78952	-1.80616	-1.80311	-1.82128	-1.81822	-1.81517
-1.79738	-1.79429	-1.79123	-1.78819	-1.80482	-1.80178	-1.81995	-1.81689	-1.81384
-1.79591	-1.79283	-1.78978	-1.78676	-1.80339	-1.80035	-1.81853	-1.81548	-1.81243
-1.79449	-1.7914	-1.78835	-1.78533	-1.80194	-1.79891	-1.8171	-1.81405	-1.81101
-1.79317	-1.79004	-1.78695	-1.7839	-1.80048	-1.79746	-1.81564	-1.8126	-1.80957
-1.79186	-1.7887	-1.78559	-1.78251	-1.79905	-1.79604	-1.81422	-1.81118	-1.80815
-1.7905	-1.78738	-1.78429	-1.78123	-1.79776	-1.79474	-1.81292	-1.80988	-1.80684
-1.78914	-1.78607	-1.78302	-1.77999	-1.79651	-1.79348	-1.81165	-1.80861	-1.80557
-1.78779	-1.78473	-1.78168	-1.77865	-1.79515	-1.79212	-1.81029	-1.80724	-1.8042
-1.78647	-1.78337	-1.7803	-1.77725	-1.79372	-1.7907	-1.80887	-1.80582	-1.80278
-1.78513	-1.78203	-1.77895	-1.7759	-1.79235	-1.78933	-1.80634	-1.80328	-1.80022
-1.78377	-1.7807	-1.77765	-1.77462	-1.79106	-1.78805	-1.80504	-1.80198	-1.79892
-1.78243	-1.77937	-1.77633	-1.77332	-1.78974	-1.78673	-1.8037	-1.80064	-1.79759
-1.78112	-1.77804	-1.77498	-1.77194	-1.78832	-1.78531	-1.80225	-1.79919	-1.79616
-1.77982	-1.77671	-1.77363	-1.77056	-1.78691	-1.78388	-1.8008	-1.79775	-1.79472
-1.77847	-1.77539	-1.79174	-1.78865	-1.80557	-1.80251	-1.79946	-1.79643	-1.79341
-1.77711	-1.77407	-1.79044	-1.78736	-1.80428	-1.80122	-1.79818	-1.79515	-1.79214
-1.7758	-1.77278	-1.78912	-1.78604	-1.80292	-1.79984	-1.7968	-1.79379	-1.79079
-1.77452	-1.7715	-1.7878	-1.78468	-1.8015	-1.7984	-1.79534	-1.79234	-1.78936
-1.77321	-1.77018	-1.80642	-1.80325	-1.80012	-1.79702	-1.79396	-1.79096	-1.78798
-1.77185	-1.7688	-1.80501	-1.80189	-1.79879	-1.79572	-1.79268	-1.78968	-1.78669
-1.77049	-1.76744	-1.80361	-1.80054	-1.79748	-1.79445	-1.79142	-1.78841	-1.7854
-1.76918	-1.76615	-1.80229	-1.79923	-1.79619	-1.79318	-1.79015	-1.78711	-1.78406
-1.76789	-1.76488	-1.80099	-1.79793	-1.7949	-1.7919	-1.78887	-1.7858	-1.78271
-1.78682	-1.78377	-1.79967	-1.79659	-1.79354	-1.79052	-1.78749	-1.78443	-1.78137
-1.78553	-1.78248	-1.79833	-1.79522	-1.79214	-1.7891	-1.78606	-1.78305	-1.78003
-1.78424	-1.78117	-1.79699	-1.79388	-1.7908	-1.78774	-1.78471	-1.78171	-1.77871
-1.78292	-1.77983	-1.79563	-1.79255	-1.78949	-1.78646	-1.78343	-1.78042	-1.77741
-1.7816	-1.77849	-1.79429	-1.79124	-1.7882	-1.78518	-1.78215	-1.77912	-1.77609
-1.78025	-1.77717	-1.79297	-1.78995	-1.78692	-1.7839	-1.78086	-1.77779	-1.77471
-1.7789	-1.77586	-1.79167	-1.78866	-1.78564	-1.78261	-1.77955	-1.77644	-1.77333
-1.79647	-1.79342	-1.79039	-1.78738	-1.78434	-1.78128	-1.7782	-1.77508	-1.77198
-1.79516	-1.79214	-1.78911	-1.78609	-1.78303	-1.77993	-1.77683	-1.77372	-1.77064
-1.79388	-1.79087	-1.78782	-1.78477	-1.78169	-1.77858	-1.80436	-1.80103	-1.79773
-1.79262	-1.7896	-1.78653	-1.78342	-1.78033	-1.77723	-1.80292	-1.79962	-1.79635
-1.79136	-1.78833	-1.78524	-1.78211	-1.779	-1.7759	-1.80151	-1.79823	-1.79497
-1.79009	-1.78706	-1.78397	-1.78083	-1.77772	-1.77462	-1.80011	-1.79685	-1.7936
-1.78881	-1.78576	-1.78267	-1.77954	-1.80537	-1.80203	-1.82293	-1.81964	-1.81636
-1.78749	-1.78441	-1.78131	-1.7782	-1.80394	-1.80062	-1.82155	-1.81828	-1.81502
-1.78617	-1.78305	-1.77994	-1.77685	-1.80251	-1.79922	-1.82018	-1.81692	-1.81366
-1.78487	-1.78173	-1.77862	-1.77553	-1.80109	-1.79782	-1.8188	-1.81552	-1.81225
-1.78359	-1.78044	-1.77733	-1.77423	-1.79969	-1.79643	-1.81742	-1.81411	-1.81081
-1.78232	-1.77916	-1.77604	-1.77294	-1.7983	-1.79506	-1.81606	-1.81273	-1.80942
-1.78105	-1.77788	-1.77475	-1.77165	-1.79692	-1.79369	-1.81471	-1.81139	-1.80806
-1.77977	-1.7766	-1.80215	-1.79881	-1.81988	-1.81663	-1.81337	-1.81004	-1.80672
-1.77848	-1.77532	-1.80076	-1.79744	-1.81851	-1.81529	-1.81203	-1.8087	-1.80537
-1.77719	-1.77403	-1.79938	-1.79606	-1.81716	-1.81394	-1.84474	-1.84135	-1.83796
-1.7759	-1.77274	-1.798	-1.7947	-1.81581	-1.81259	-1.84336	-1.83998	-1.83659

-1.77461	-1.77145	-1.79662	-1.79334	-1.81446	-1.81124	-1.84199	-1.83861	-1.83521
-1.77332	-1.77016	-1.79525	-1.79198	-1.83016	-1.82693	-1.84062	-1.83724	-1.83384
-1.77204	-1.76887	-1.79387	-1.79062	-1.82882	-1.82558	-1.83925	-1.83587	-1.83247
-1.7992	-1.79581	-1.83403	-1.83074	-1.82747	-1.82423	-1.83788	-1.83449	-1.8311
-1.79781	-1.79444	-1.83267	-1.82938	-1.82611	-1.82288	-1.83651	-1.83312	-1.82972
-1.8138	-1.81044	-1.83132	-1.82802	-1.84172	-1.83844	-1.83513	-1.83174	-1.82834
-1.81244	-1.80909	-1.82997	-1.82666	-1.84033	-1.83706	-1.83375	-1.83036	-1.82696
-1.81109	-1.80777	-1.82865	-1.82532	-1.83896	-1.83568	-1.83237	-1.82898	-1.82559
-1.83405	-1.83072	-1.82737	-1.82401	-1.83761	-1.83431	-1.83099	-1.8276	-1.82421
-1.83274	-1.82945	-1.8261	-1.82272	-1.83628	-1.83295	-1.82961	-1.82622	-1.82283
-1.83142	-1.82814	-1.82479	-1.82141	-1.83493	-1.83158	-1.82823	-1.82487	-1.82151
-1.8301	-1.82679	-1.82344	-1.82007	-1.83357	-1.83021	-1.82687	-1.82355	-1.82023
-1.82876	-1.82544	-1.861	-1.85756	-1.87047	-1.86699	-1.86353	-1.86013	-1.85672
-1.8274	-1.82407	-1.85963	-1.85624	-1.86914	-1.86564	-1.86216	-1.85873	-1.85529
-1.8651	-1.86166	-1.85827	-1.8549	-1.86779	-1.86428	-1.879	-1.87549	-1.87199
-1.86379	-1.86033	-1.85691	-1.85353	-1.86638	-1.86288	-1.87758	-1.87405	-1.87053
-1.86249	-1.85901	-1.85557	-1.85215	-1.86498	-1.86148	-1.87616	-1.87262	-1.86909
-1.86115	-1.85771	-1.8706	-1.8671	-1.86361	-1.86012	-1.87478	-1.87124	-1.8677
-1.8598	-1.85641	-1.86928	-1.86576	-1.86226	-1.85878	-1.87342	-1.86987	-1.86633
-1.85845	-1.85506	-1.86791	-1.86438	-1.86088	-1.85739	-1.87201	-1.86846	-1.86492
-1.85708	-1.85367	-1.86649	-1.86298	-1.85948	-1.85597	-1.87056	-1.86702	-1.86349
-1.85574	-1.85231	-1.86511	-1.86161	-1.87626	-1.8727	-1.86916	-1.86562	-1.8621
-1.85444	-1.85099	-1.86378	-1.86028	-1.87491	-1.87135	-1.8678	-1.86427	-1.86076
-1.85314	-1.84969	-1.86245	-1.85896	-1.87357	-1.87	-1.86646	-1.86293	-1.85942
-1.85185	-1.8484	-1.86113	-1.85762	-1.87221	-1.86866	-1.86512	-1.86158	-1.85807
-1.85055	-1.84711	-1.8598	-1.85627	-1.87083	-1.86729	-1.86376	-1.86022	-1.85671
-1.84918	-1.84574	-1.87655	-1.87297	-1.86941	-1.86587	-1.86234	-1.85882	-1.85531
-1.84778	-1.84434	-1.8751	-1.87153	-1.86797	-1.86441	-1.86088	-1.85738	-1.8539
-1.86262	-1.85912	-1.87369	-1.87012	-1.86656	-1.863	-1.85948	-1.85599	-1.85252
-1.86128	-1.85776	-1.87232	-1.86875	-1.86519	-1.86164	-1.85811	-1.85463	-1.85117
-1.8781	-1.87453	-1.87096	-1.86739	-1.86383	-1.86029	-1.85677	-1.85329	-1.84983
-1.87676	-1.87318	-1.86961	-1.86604	-1.86249	-1.85896	-1.85545	-1.85197	-1.8485
-1.87541	-1.87184	-1.86826	-1.86469	-1.86114	-1.85763	-1.85414	-1.85065	-1.84717
-1.87406	-1.87048	-1.8669	-1.86333	-1.8598	-1.85629	-1.8528	-1.84932	-1.84584
-1.8727	-1.86911	-1.86554	-1.86198	-1.85845	-1.85495	-1.85147	-1.84799	-1.84451
-1.87133	-1.86775	-1.86418	-1.86063	-1.85711	-1.85362	-1.85013	-1.84665	-1.84317
-1.86995	-1.8664	-1.86284	-1.85929	-1.85577	-1.85228	-1.8488	-1.84531	-1.84182
-1.8686	-1.86503	-1.86147	-1.85794	-1.85443	-1.85095	-1.84746	-1.84396	-1.84047
-1.86726	-1.86364	-1.86009	-1.8566	-1.8531	-1.84961	-1.84612	-1.84263	-1.83914
-1.86594	-1.86229	-1.85874	-1.85525	-1.85176	-1.84827	-1.84478	-1.84129	-1.8378
-1.86465	-1.86101	-1.85744	-1.8539	-1.85039	-1.84691	-1.84344	-1.83994	-1.83645
-1.86335	-1.85975	-1.85615	-1.85257	-1.84904	-1.84557	-1.84209	-1.8386	-1.8351
-1.86201	-1.85845	-1.85488	-1.8513	-1.84776	-1.84424	-1.84073	-1.83724	-1.83375
-1.86065	-1.85714	-1.8536	-1.85006	-1.84651	-1.84292	-1.83937	-1.83588	-1.8324
-1.85932	-1.8558	-1.85229	-1.84878	-1.84523	-1.84164	-1.85508	-1.85154	-1.84802
-1.85801	-1.85445	-1.85094	-1.84746	-1.84395	-1.84039	-1.85384	-1.85025	-1.84669
-1.85672	-1.85314	-1.84962	-1.84614	-1.85967	-1.85613	-1.85258	-1.84898	-1.8454
-1.85544	-1.85189	-1.84834	-1.8448	-1.85832	-1.85481	-1.85129	-1.84772	-1.84416
-1.85416	-1.85063	-1.84707	-1.84349	-1.857	-1.85351	-1.85	-1.84646	-1.84293
-1.85284	-1.84935	-1.8458	-1.84224	-1.85575	-1.85225	-1.84875	-1.84522	-1.84169

-1.85153	-1.84807	-1.84455	-1.841	-1.85451	-1.851	-1.84748	-1.84397	-1.84044
-1.85026	-1.84681	-1.86037	-1.85682	-1.85327	-1.84971	-1.84617	-1.84267	-1.83916
-1.84902	-1.84556	-1.85912	-1.85558	-1.85201	-1.84841	-1.84484	-1.84134	-1.83785
-1.86485	-1.86134	-1.85782	-1.8543	-1.85074	-1.84712	-1.84354	-1.84002	-1.83653
-1.86356	-1.86	-1.85648	-1.85298	-1.84943	-1.84584	-1.84226	-1.83871	-1.83518
-1.8623	-1.8587	-1.85517	-1.85167	-1.84814	-1.84458	-1.84101	-1.83743	-1.83386
-1.86106	-1.85744	-1.8539	-1.8504	-1.84688	-1.84334	-1.83978	-1.83619	-1.8326
-1.85983	-1.8562	-1.85264	-1.84913	-1.84561	-1.84209	-1.83855	-1.83495	-1.83135
-1.85859	-1.85494	-1.85136	-1.84781	-1.84429	-1.84079	-1.85259	-1.849	-1.84539
-1.85735	-1.85369	-1.85008	-1.84649	-1.84296	-1.83948	-1.8513	-1.84773	-1.84415
-1.87157	-1.86788	-1.86423	-1.8606	-1.85704	-1.85354	-1.85004	-1.84649	-1.84292
-1.87033	-1.86663	-1.86298	-1.85935	-1.85579	-1.8523	-1.8488	-1.84526	-1.8417
-1.86907	-1.86539	-1.86173	-1.8581	-1.85454	-1.85105	-1.84755	-1.84403	-1.84047
-1.86779	-1.86414	-1.8605	-1.85686	-1.85329	-1.8498	-1.84631	-1.84279	-1.83925
-1.86653	-1.8629	-1.85926	-1.85562	-1.85204	-1.84853	-1.84503	-1.84152	-1.83799
-1.8653	-1.86166	-1.85802	-1.85437	-1.85077	-1.84721	-1.84368	-1.84018	-1.83666
-1.86409	-1.86043	-1.85677	-1.85313	-1.84951	-1.84591	-1.84235	-1.83885	-1.83534
-1.86286	-1.85919	-1.85554	-1.8519	-1.84827	-1.84467	-1.84109	-1.83757	-1.83406
-1.86162	-1.85795	-1.8543	-1.85067	-1.84706	-1.84346	-1.83988	-1.83634	-1.8328
-1.86038	-1.85671	-1.85307	-1.84945	-1.84585	-1.84228	-1.83871	-1.83516	-1.8316
-1.85914	-1.85547	-1.85183	-1.84822	-1.84465	-1.84113	-1.83758	-1.83402	-1.83043
-1.8579	-1.85423	-1.8506	-1.84699	-1.84343	-1.83993	-1.83641	-1.83284	-1.82924
-1.85665	-1.85299	-1.84936	-1.84575	-1.84219	-1.83869	-1.83516	-1.8316	-1.828
-1.8554	-1.85176	-1.84812	-1.84451	-1.84094	-1.83743	-1.83391	-1.83035	-1.82675
-1.85416	-1.85052	-1.84689	-1.84328	-1.83971	-1.83618	-1.83264	-1.82908	-1.82549
-1.85292	-1.84929	-1.84567	-1.84207	-1.83849	-1.83493	-1.83137	-1.82781	-1.82423
-1.85169	-1.84808	-1.84448	-1.84089	-1.83731	-1.83374	-1.83016	-1.82657	-1.82296
-1.85048	-1.84688	-1.8433	-1.83974	-1.83616	-1.83258	-1.82897	-1.82534	-1.8217
-1.84929	-1.8457	-1.84213	-1.83858	-1.83499	-1.83138	-1.82776	-1.8241	-1.82045
-1.84811	-1.84455	-1.84099	-1.83741	-1.83381	-1.83016	-1.82651	-1.82285	-1.81921
-1.84697	-1.8434	-1.83982	-1.83622	-1.83259	-1.82892	-1.82526	-1.82161	-1.81799
-1.84588	-1.84226	-1.83863	-1.83498	-1.83133	-1.82766	-1.82401	-1.82038	-1.81678
-1.84475	-1.84109	-1.83742	-1.83374	-1.83007	-1.82641	-1.82277	-1.81917	-1.8156
-1.84354	-1.83987	-1.83619	-1.8325	-1.82883	-1.82516	-1.82154	-1.81798	-1.81445
-1.8423	-1.83863	-1.83495	-1.83127	-1.82761	-1.82395	-1.82035	-1.81682	-1.81331
-1.84106	-1.83739	-1.83374	-1.83009	-1.82646	-1.82283	-1.81923	-1.81569	-1.81217
-1.83982	-1.83616	-1.83254	-1.82895	-1.82535	-1.82176	-1.81817	-1.81459	-1.81101
-1.83867	-1.83501	-1.83139	-1.8278	-1.82421	-1.82063	-1.81703	-1.81343	-1.80983
-1.83763	-1.83394	-1.83029	-1.82666	-1.82304	-1.81943	-1.81583	-1.81223	-1.80863
-1.83653	-1.83284	-1.82916	-1.82549	-1.82185	-1.81823	-1.81462	-1.81104	-1.80745
-1.83534	-1.83164	-1.82795	-1.82428	-1.82063	-1.81701	-1.81342	-1.80986	-1.80632
-1.83415	-1.83045	-1.82676	-1.82309	-1.81944	-1.81582	-1.81224	-1.80871	-1.80519
-1.83303	-1.82936	-1.82569	-1.82201	-1.81836	-1.81474	-1.82981	-1.82621	-1.82262
-1.83194	-1.8283	-1.82464	-1.82096	-1.81731	-1.81369	-1.82873	-1.82509	-1.82145
-1.83079	-1.82716	-1.8235	-1.81983	-1.81618	-1.81255	-1.82757	-1.82391	-1.82025
-1.82961	-1.82597	-1.8223	-1.81863	-1.81498	-1.81135	-1.82636	-1.82269	-1.81903
-1.82842	-1.82479	-1.82114	-1.81749	-1.81385	-1.81022	-1.82521	-1.82154	-1.81788
-1.82723	-1.82363	-1.82003	-1.81643	-1.81281	-1.80918	-1.82415	-1.82049	-1.81683
-1.82607	-1.82249	-1.81891	-1.81534	-1.83042	-1.82674	-1.82306	-1.8194	-1.81574
-1.82496	-1.82135	-1.81775	-1.81416	-1.82921	-1.82554	-1.82186	-1.81821	-1.81455

-1.82388	-1.82023	-1.8166	-1.81298	-1.828	-1.82433	-1.82066	-1.81701	-1.81335
-1.82283	-1.81917	-1.81552	-1.8119	-1.8269	-1.82323	-1.81956	-1.8159	-1.81225
-1.82179	-1.81813	-1.81448	-1.81084	-1.82583	-1.82216	-1.81849	-1.81483	-1.81118
-1.82065	-1.81699	-1.81335	-1.80971	-1.82468	-1.821	-1.81734	-1.81368	-1.81002
-1.81943	-1.81578	-1.81213	-1.8085	-1.82345	-1.81978	-1.81611	-1.81245	-1.8088
-1.81828	-1.81463	-1.82965	-1.82597	-1.8223	-1.81862	-1.81496	-1.8113	-1.80764
-1.81724	-1.81359	-1.8286	-1.82492	-1.82124	-1.81757	-1.81391	-1.81025	-1.80659
-1.81621	-1.81257	-1.82756	-1.82388	-1.82021	-1.81654	-1.81287	-1.80922	-1.80556
-1.81519	-1.81155	-1.82653	-1.82285	-1.81917	-1.81551	-1.81184	-1.80819	-1.80453
-1.81415	-1.8105	-1.82547	-1.82179	-1.81812	-1.81445	-1.81079	-1.80713	-1.80348
-1.81299	-1.80935	-1.82431	-1.82063	-1.81695	-1.81328	-1.80962	-1.80597	-1.80231
-1.81178	-1.80813	-1.82307	-1.81939	-1.81572	-1.81205	-1.80839	-1.80473	-1.80108
-1.82931	-1.82562	-1.82194	-1.81826	-1.81458	-1.81092	-1.80725	-1.8036	-1.79994
-1.82826	-1.82457	-1.82089	-1.81721	-1.81354	-1.80987	-1.8062	-1.80255	-1.79889
-1.82723	-1.82354	-1.81986	-1.81618	-1.81251	-1.80884	-1.80518	-1.80152	-1.79786
-1.82622	-1.82253	-1.81885	-1.81517	-1.8115	-1.80784	-1.80417	-1.8005	-1.79683
-1.8252	-1.82151	-1.81783	-1.81416	-1.81049	-1.80683	-1.80316	-1.79948	-1.7958
-1.82417	-1.82048	-1.8168	-1.81313	-1.80946	-1.8058	-1.80212	-1.79845	-1.79476
-1.82314	-1.81943	-1.81574	-1.81207	-1.8084	-1.80473	-1.80106	-1.79739	-1.79369
-1.82207	-1.81833	-1.81462	-1.81092	-1.80724	-1.80357	-1.7999	-1.79623	-1.79254
-1.82098	-1.8172	-1.81344	-1.8097	-1.80599	-1.80233	-1.79866	-1.79499	-1.79132
-1.81985	-1.81607	-1.8123	-1.80855	-1.80484	-1.80117	-1.79751	-1.79384	-1.79018
-1.81867	-1.81494	-1.81121	-1.80749	-1.80379	-1.80012	-1.79645	-1.79279	-1.78912
-1.81754	-1.81385	-1.81015	-1.80645	-1.80277	-1.79909	-1.79542	-1.79176	-1.78809
-1.81648	-1.8128	-1.80911	-1.80543	-1.80175	-1.79807	-1.7944	-1.79073	-1.78707
-1.81545	-1.81178	-1.80809	-1.80441	-1.80074	-1.79706	-1.79338	-1.78972	-1.78606
-1.81443	-1.81075	-1.80707	-1.80339	-1.79972	-1.79604	-1.79236	-1.7887	-1.78504
-1.81341	-1.80974	-1.80605	-1.80237	-1.79869	-1.79502	-1.79134	-1.78768	-1.78402
-1.81239	-1.80871	-1.80503	-1.80135	-1.79767	-1.79399	-1.79032	-1.78666	-1.78299
-1.81135	-1.80769	-1.80401	-1.80032	-1.79664	-1.79297	-1.78929	-1.78563	-1.78197
-1.81035	-1.80666	-1.80298	-1.7993	-1.79562	-1.79194	-1.78827	-1.7846	-1.78094
-1.80936	-1.80563	-1.80193	-1.79826	-1.79459	-1.79091	-1.78724	-1.78358	-1.77992
-1.80841	-1.80463	-1.80091	-1.79724	-1.79356	-1.78989	-1.78622	-1.78255	-1.77889
-1.8075	-1.80369	-1.79994	-1.79622	-1.79252	-1.78885	-1.78519	-1.78152	-1.77786
-1.80661	-1.80278	-1.79899	-1.79521	-1.79149	-1.78782	-1.78416	-1.7805	-1.77683
-1.80568	-1.80188	-1.79807	-1.79427	-1.79052	-1.78681	-1.78313	-1.77946	-1.7758
-1.80473	-1.80098	-1.79718	-1.79336	-1.78958	-1.78582	-1.78209	-1.77843	-1.77477
-1.80375	-1.80003	-1.79626	-1.79246	-1.78866	-1.78487	-1.78111	-1.77741	-1.77374
-1.80273	-1.79903	-1.7953	-1.79155	-1.78777	-1.78396	-1.78018	-1.77643	-1.77271
-1.80171	-1.79802	-1.79432	-1.79061	-1.78686	-1.78305	-1.77926	-1.77547	-1.77172
-1.80068	-1.79699	-1.7933	-1.78961	-1.78589	-1.78214	-1.77837	-1.77458	-1.77079
-1.79965	-1.79596	-1.79228	-1.7886	-1.7849	-1.7812	-1.77746	-1.77368	-1.76989
-1.79861	-1.79492	-1.79124	-1.78757	-1.78389	-1.7802	-1.77649	-1.77275	-1.76901
-1.79759	-1.7939	-1.79021	-1.78654	-1.78286	-1.77918	-1.7755	-1.77181	-1.76811
-1.79667	-1.79295	-1.78923	-1.78553	-1.78183	-1.77815	-1.7808	-1.77713	-1.77346
-1.79582	-1.79205	-1.78829	-1.78453	-1.78081	-1.77712	-1.77978	-1.77612	-1.77246
-1.79493	-1.79115	-1.78738	-1.78361	-1.77987	-1.77615	-1.77881	-1.77513	-1.77146
-1.794	-1.79026	-1.78652	-1.78278	-1.77903	-1.77527	-1.7779	-1.77417	-1.77045
-1.79304	-1.78934	-1.78563	-1.78191	-1.77816	-1.77438	-1.77701	-1.77325	-1.7695
-1.79201	-1.78832	-1.78463	-1.78093	-1.77721	-1.77347	-1.77614	-1.77241	-1.76867

-1.791	-1.78731	-1.78361	-1.77992	-1.77623	-1.77253	-1.77527	-1.77156	-1.76785
-1.7901	-1.78637	-1.78264	-1.77893	-1.78168	-1.778	-1.80195	-1.79818	-1.7944
-1.78925	-1.78546	-1.7817	-1.77794	-1.7807	-1.77701	-1.80095	-1.79718	-1.79341
-1.78834	-1.78456	-1.78079	-1.77704	-1.77982	-1.77613	-1.80004	-1.79625	-1.79246
-1.7874	-1.78366	-1.77993	-1.77621	-1.77902	-1.77534	-1.7992	-1.79538	-1.79154
-1.78649	-1.78276	-1.78558	-1.78186	-1.77816	-1.77448	-1.79831	-1.79448	-1.79064
-1.78562	-1.78186	-1.78466	-1.78091	-1.77719	-1.77351	-1.79734	-1.79355	-1.78975
-1.78475	-1.78096	-1.78376	-1.77998	-1.80391	-1.80014	-1.82993	-1.82602	-1.82212
-1.78381	-1.78005	-1.7829	-1.77915	-1.80304	-1.79923	-1.82895	-1.82502	-1.8211
-1.78287	-1.77914	-1.78204	-1.77832	-1.80219	-1.79835	-1.82798	-1.82402	-1.82008
-1.78861	-1.78488	-1.78115	-1.77742	-1.80128	-1.79745	-1.82706	-1.82311	-1.81918
-1.78777	-1.784	-1.78024	-1.77649	-1.80034	-1.79656	-1.82618	-1.82225	-1.81833
-1.78692	-1.78313	-1.77936	-1.77562	-1.79946	-1.7957	-1.8253	-1.82137	-1.81745
-1.78606	-1.78227	-1.77852	-1.7748	-1.79865	-1.79489	-1.82445	-1.82049	-1.81652
-1.78521	-1.78144	-1.80536	-1.80158	-1.83138	-1.82747	-1.82355	-1.81958	-1.81561
-1.78439	-1.78063	-1.80453	-1.80071	-1.83044	-1.8265	-1.82257	-1.81864	-1.8147
-1.78358	-1.77983	-1.80369	-1.79984	-1.8295	-1.82554	-1.8216	-1.81771	-1.81381
-1.78276	-1.77899	-1.80282	-1.79897	-1.8286	-1.82465	-1.82073	-1.81684	-1.81295
-1.78193	-1.77815	-1.80195	-1.7981	-1.82771	-1.82379	-1.81988	-1.81599	-1.81209
-1.78108	-1.7773	-1.80109	-1.79726	-1.82684	-1.8229	-1.81897	-1.81506	-1.81115
-1.78023	-1.77644	-1.80024	-1.79644	-1.82597	-1.822	-1.81803	-1.81409	-1.81015
-1.80707	-1.80323	-1.83302	-1.82906	-1.82509	-1.8211	-1.81712	-1.81317	-1.80924
-1.80625	-1.80244	-1.83217	-1.82817	-1.82418	-1.82019	-1.81623	-1.81231	-1.8084
-1.80545	-1.80166	-1.83133	-1.8273	-1.8233	-1.81932	-1.83404	-1.83009	-1.82615
-1.80467	-1.80088	-1.83053	-1.8265	-1.82249	-1.81851	-1.83321	-1.82926	-1.82531
-1.80389	-1.8001	-1.82972	-1.82569	-1.82169	-1.81771	-1.83239	-1.82843	-1.82447
-1.80308	-1.79926	-1.82884	-1.82484	-1.83958	-1.83556	-1.83156	-1.82757	-1.82358
-1.80226	-1.7984	-1.82795	-1.82396	-1.83871	-1.83473	-1.83073	-1.8267	-1.82267
-1.83513	-1.8311	-1.82711	-1.82313	-1.83788	-1.83392	-1.82993	-1.82588	-1.82183
-1.83433	-1.83031	-1.82631	-1.82234	-1.83708	-1.83313	-1.82915	-1.82509	-1.82103
-1.83349	-1.82948	-1.84428	-1.84026	-1.83628	-1.83233	-1.82835	-1.8243	-1.82024
-1.8326	-1.82861	-1.84342	-1.83945	-1.83549	-1.83154	-1.82755	-1.82351	-1.81947
-1.85057	-1.84655	-1.84258	-1.83864	-1.83468	-1.83072	-1.82673	-1.8227	-1.81868
-1.84974	-1.84573	-1.84176	-1.83783	-1.83385	-1.82985	-1.82584	-1.82183	-1.81783
-1.84894	-1.84493	-1.84096	-1.83701	-1.83302	-1.82898	-1.82495	-1.82094	-1.81696
-1.84814	-1.84413	-1.84017	-1.83622	-1.83222	-1.82816	-1.82412	-1.82012	-1.81615
-1.84735	-1.84334	-1.83938	-1.83543	-1.83143	-1.82737	-1.82332	-1.81933	-1.81536
-1.84656	-1.84255	-1.83859	-1.83465	-1.83064	-1.82658	-1.82254	-1.81854	-1.81458
-1.84576	-1.84176	-1.83779	-1.83386	-1.82986	-1.82579	-1.82176	-1.81776	-1.8138
-1.84497	-1.84096	-1.837	-1.83307	-1.82908	-1.82502	-1.82099	-1.81699	-1.81302
-1.84418	-1.84016	-1.83621	-1.83228	-1.8283	-1.82425	-1.82022	-1.81622	-1.81224
-1.84342	-1.83939	-1.83543	-1.83151	-1.82753	-1.8235	-1.81948	-1.81547	-1.81148
-1.84272	-1.83868	-1.8347	-1.83075	-1.82679	-1.8228	-1.8188	-1.81478	-1.81078
-1.84205	-1.83799	-1.83398	-1.83001	-1.82605	-1.8221	-1.81813	-1.81411	-1.81009
-1.84132	-1.83726	-1.83324	-1.82924	-1.82528	-1.82136	-1.81741	-1.81339	-1.80936
-1.84057	-1.83651	-1.83248	-1.82847	-1.8245	-1.82059	-1.81665	-1.81262	-1.8086
-1.83983	-1.8358	-1.83176	-1.82773	-1.82375	-1.81983	-1.81588	-1.81186	-1.80784
-1.83911	-1.83512	-1.83109	-1.82705	-1.82304	-1.81908	-1.81511	-1.81109	-1.80708
-1.83839	-1.83442	-1.8304	-1.82635	-1.82232	-1.81833	-1.81434	-1.81034	-1.80635
-1.83766	-1.83368	-1.82965	-1.8256	-1.82157	-1.81757	-1.81358	-1.80963	-1.80569

-1.83693	-1.83292	-1.8289	-1.82486	-1.82084	-1.81682	-1.81285	-1.80894	-1.80502
-1.83617	-1.83216	-1.82816	-1.82417	-1.82017	-1.81614	-1.84064	-1.83663	-1.83263
-1.83542	-1.8314	-1.82744	-1.8235	-1.81952	-1.81549	-1.83996	-1.83591	-1.83188
-1.83471	-1.8307	-1.82673	-1.82279	-1.81882	-1.81482	-1.83928	-1.83521	-1.83116
-1.83405	-1.83003	-1.82603	-1.82205	-1.81809	-1.81414	-1.83861	-1.83453	-1.83045
-1.83339	-1.82937	-1.82535	-1.82135	-1.81737	-1.81344	-1.8379	-1.83381	-1.82973
-1.83275	-1.82873	-1.82471	-1.82068	-1.81668	-1.81271	-1.83713	-1.83305	-1.82897
-1.83209	-1.82807	-1.82405	-1.82003	-1.84456	-1.84047	-1.84829	-1.84418	-1.84007
-1.83136	-1.82735	-1.82336	-1.81937	-1.8439	-1.8398	-1.84761	-1.84349	-1.83937
-1.83064	-1.82664	-1.82267	-1.81872	-1.84325	-1.83915	-1.84695	-1.84282	-1.8387
-1.83	-1.82603	-1.82206	-1.81809	-1.8426	-1.8385	-1.84628	-1.84216	-1.83805
-1.82939	-1.82546	-1.82148	-1.81747	-1.84195	-1.83784	-1.84562	-1.84151	-1.83741
-1.82874	-1.8248	-1.82083	-1.81684	-1.8413	-1.8372	-1.84497	-1.84086	-1.83675
-1.82805	-1.82408	-1.82013	-1.81618	-1.84066	-1.83656	-1.84432	-1.8402	-1.83609
-1.82737	-1.82338	-1.84804	-1.84403	-1.83998	-1.83591	-1.84367	-1.83955	-1.83544
-1.82671	-1.8227	-1.84733	-1.8433	-1.83926	-1.83523	-1.84301	-1.8389	-1.83479
-1.82606	-1.82206	-1.84664	-1.8426	-1.83856	-1.83456	-1.84236	-1.83825	-1.83414
-1.82543	-1.82143	-1.84601	-1.84198	-1.83795	-1.83393	-1.84171	-1.8376	-1.83348
-1.82482	-1.82084	-1.84542	-1.8414	-1.83736	-1.83331	-1.84106	-1.83694	-1.83282
-1.85296	-1.84887	-1.84482	-1.8408	-1.84862	-1.84451	-1.8404	-1.83628	-1.83216
-1.85242	-1.84831	-1.84424	-1.84019	-1.84799	-1.84386	-1.83974	-1.83563	-1.83152
-1.85183	-1.84772	-1.84363	-1.83956	-1.84734	-1.84321	-1.83909	-1.83498	-1.83088
-1.8512	-1.8471	-1.843	-1.83891	-1.84668	-1.84256	-1.83845	-1.83435	-1.83024
-1.85057	-1.84647	-1.84236	-1.83826	-1.84602	-1.84192	-1.83783	-1.83373	-1.82963
-1.84992	-1.84583	-1.84173	-1.83762	-1.84538	-1.84129	-1.83721	-1.83315	-1.82908
-1.8493	-1.84521	-1.84111	-1.83702	-1.84476	-1.84067	-1.8366	-1.83257	-1.82853
-1.84875	-1.84465	-1.84056	-1.83647	-1.84421	-1.8401	-1.83602	-1.83197	-1.82793
-1.84822	-1.84412	-1.84003	-1.83595	-1.84367	-1.83955	-1.83544	-1.83137	-1.8273
-1.84763	-1.84353	-1.83944	-1.83537	-1.84309	-1.83895	-1.83483	-1.83075	-1.82667
-1.84701	-1.84291	-1.83883	-1.83475	-1.84246	-1.83833	-1.83421	-1.83011	-1.82602
-1.84643	-1.84233	-1.83825	-1.83417	-1.84188	-1.83775	-1.83363	-1.82953	-1.82544
-1.8459	-1.84181	-1.83773	-1.83366	-1.84135	-1.83722	-1.83311	-1.82901	-1.82491
-1.84536	-1.84128	-1.83721	-1.83314	-1.84083	-1.83671	-1.83259	-1.82849	-1.8244
-1.84475	-1.84069	-1.83665	-1.83261	-1.84031	-1.8362	-1.83208	-1.82798	-1.82388
-1.84415	-1.8401	-1.83608	-1.83207	-1.83979	-1.83567	-1.83156	-1.82745	-1.82335
-1.8436	-1.83954	-1.84733	-1.84327	-1.83919	-1.83508	-1.83097	-1.82686	-1.82276
-1.84307	-1.839	-1.84675	-1.84266	-1.83856	-1.83446	-1.83036	-1.82625	-1.82215
-1.84256	-1.83848	-1.84621	-1.8421	-1.838	-1.8339	-1.8298	-1.82569	-1.82159
-1.84206	-1.83798	-1.8457	-1.84159	-1.83748	-1.83337	-1.82927	-1.82517	-1.82107
-1.84156	-1.83749	-1.8452	-1.84108	-1.83697	-1.83286	-1.82876	-1.82466	-1.82057
-1.84106	-1.83699	-1.84469	-1.84057	-1.83646	-1.83236	-1.82826	-1.82416	-1.82007
-1.84056	-1.83649	-1.84419	-1.84007	-1.83596	-1.83186	-1.82777	-1.82367	-1.81958
-1.84006	-1.83599	-1.84368	-1.83956	-1.83546	-1.83136	-1.82727	-1.82317	-1.81908
-1.83956	-1.83549	-1.84317	-1.83906	-1.83495	-1.83086	-1.82676	-1.82267	-1.81858
-1.83906	-1.83499	-1.84267	-1.83855	-1.83445	-1.83035	-1.82626	-1.82216	-1.81808
-1.83855	-1.83449	-1.84216	-1.83805	-1.83395	-1.82985	-1.82575	-1.82166	-1.81757
-1.83806	-1.83399	-1.84165	-1.83754	-1.83344	-1.82934	-1.82525	-1.82115	-1.81707
-1.83756	-1.83349	-1.84114	-1.83703	-1.83293	-1.82883	-1.82474	-1.82065	-1.81656
-1.84891	-1.84478	-1.84066	-1.83653	-1.83242	-1.82833	-1.82424	-1.82015	-1.81607
-1.84851	-1.84437	-1.84021	-1.83605	-1.83192	-1.82782	-1.82373	-1.81965	-1.81558

-1.8481	-1.84395	-1.83978	-1.8356	-1.83145	-1.82734	-1.82325	-1.81919	-1.81513
-1.84765	-1.8435	-1.83934	-1.83518	-1.83104	-1.82693	-1.82285	-1.81878	-1.81472
-1.84716	-1.84304	-1.8389	-1.83477	-1.83065	-1.82655	-1.82247	-1.8184	-1.81433
-1.84668	-1.84256	-1.83844	-1.83432	-1.83021	-1.82612	-1.82203	-1.81795	-1.81388
-1.84618	-1.84207	-1.83795	-1.83384	-1.82974	-1.82565	-1.82157	-1.81748	-1.8134
-1.84573	-1.84162	-1.83751	-1.8334	-1.8293	-1.82522	-1.82113	-1.81704	-1.81295
-1.84533	-1.84123	-1.83712	-1.83301	-1.82891	-1.82483	-1.82075	-1.81665	-1.81255
-1.84492	-1.84082	-1.83671	-1.8326	-1.8285	-1.82442	-1.82034	-1.81625	-1.81215
-1.84445	-1.84035	-1.83624	-1.83214	-1.82805	-1.82396	-1.81988	-1.81581	-1.81172
-1.84398	-1.83987	-1.83577	-1.83168	-1.82759	-1.8235	-1.81942	-1.81536	-1.8113
-1.84358	-1.83947	-1.83537	-1.83127	-1.82718	-1.8231	-1.84848	-1.84433	-1.84018
-1.84321	-1.83911	-1.835	-1.8309	-1.8268	-1.82271	-1.84808	-1.84393	-1.83978
-1.84284	-1.83874	-1.83463	-1.83051	-1.8264	-1.8223	-1.84763	-1.84348	-1.83933
-1.84248	-1.83837	-1.83425	-1.83013	-1.826	-1.82186	-1.84715	-1.84299	-1.83884
-1.84212	-1.83799	-1.83386	-1.82973	-1.85516	-1.85092	-1.8467	-1.84254	-1.8384
-1.84173	-1.8376	-1.83345	-1.8293	-1.85471	-1.8505	-1.84631	-1.84216	-1.83801
-1.84134	-1.8372	-1.83304	-1.82888	-1.85428	-1.8501	-1.84594	-1.84178	-1.83764
-1.84094	-1.83676	-1.83261	-1.82847	-1.85389	-1.84973	-1.84557	-1.84141	-1.83726
-1.84054	-1.83634	-1.83219	-1.82808	-1.85351	-1.84936	-1.8452	-1.84104	-1.83689
-1.84019	-1.836	-1.86152	-1.85731	-1.85313	-1.84898	-1.84483	-1.84068	-1.83653
-1.83986	-1.8357	-1.8612	-1.85696	-1.85276	-1.8486	-1.84445	-1.84031	-1.83617
-1.8395	-1.83534	-1.86083	-1.85659	-1.85239	-1.84823	-1.84408	-1.83994	-1.83581
-1.8391	-1.83492	-1.8604	-1.85619	-1.85201	-1.84786	-1.84372	-1.83957	-1.83543
-1.86853	-1.86425	-1.86002	-1.85581	-1.85164	-1.8475	-1.84336	-1.8392	-1.83505
-1.8682	-1.86394	-1.8597	-1.85546	-1.85127	-1.84713	-1.843	-1.83884	-1.83469
-1.86788	-1.86365	-1.8594	-1.85513	-1.85093	-1.84679	-1.84266	-1.8385	-1.83435
-1.86753	-1.86333	-1.8591	-1.85486	-1.85067	-1.84652	-1.84237	-1.83822	-1.83407
-1.86718	-1.86299	-1.8588	-1.8546	-1.85042	-1.84626	-1.84211	-1.83797	-1.83381
-1.86685	-1.86264	-1.85845	-1.85428	-1.85011	-1.84595	-1.8418	-1.83766	-1.8335
-1.86653	-1.86227	-1.85807	-1.85391	-1.84975	-1.84561	-1.84146	-1.83732	-1.83317
-1.86622	-1.86195	-1.85773	-1.85355	-1.84939	-1.84525	-1.84111	-1.83697	-1.83282
-1.86592	-1.86167	-1.85743	-1.8532	-1.84902	-1.84488	-1.84075	-1.83661	-1.83246
-1.86561	-1.86139	-1.85714	-1.85288	-1.84868	-1.84455	-1.84042	-1.83627	-1.83213
-1.86527	-1.86108	-1.85685	-1.85261	-1.84842	-1.84428	-1.84014	-1.836	-1.83185
-1.86493	-1.86077	-1.85657	-1.85235	-1.84817	-1.84402	-1.83988	-1.83573	-1.83158
-1.86465	-1.86049	-1.8563	-1.8521	-1.84791	-1.84372	-1.83955	-1.83541	-1.83127
-1.8644	-1.86022	-1.85604	-1.85185	-1.84764	-1.8434	-1.8392	-1.83507	-1.83095
-1.86411	-1.85991	-1.85573	-1.85155	-1.84735	-1.84311	-1.83891	-1.83479	-1.83067
-1.86381	-1.85957	-1.85538	-1.85123	-1.84705	-1.84284	-1.83867	-1.83455	-1.83043
-1.86354	-1.85926	-1.85507	-1.85093	-1.84677	-1.84259	-1.83843	-1.83431	-1.83019
-1.8633	-1.85901	-1.85482	-1.85068	-1.84652	-1.84235	-1.8382	-1.83407	-1.82994
-1.86307	-1.85878	-1.85458	-1.85044	-1.84628	-1.84212	-1.83796	-1.83381	-1.82966
-1.8628	-1.85854	-1.85436	-1.85021	-1.84605	-1.84187	-1.83769	-1.83351	-1.82933
-1.86252	-1.85831	-1.85414	-1.84999	-1.84582	-1.84163	-1.83743	-1.8332	-1.829
-1.86228	-1.85808	-1.85391	-1.84976	-1.84559	-1.84139	-1.83718	-1.83294	-1.82872
-1.86204	-1.85785	-1.85368	-1.84953	-1.84535	-1.84116	-1.83695	-1.8327	-1.82848
-1.86181	-1.85762	-1.85345	-1.8493	-1.84512	-1.84093	-1.83671	-1.83246	-1.82823
-1.86158	-1.85739	-1.85322	-1.84906	-1.84489	-1.8407	-1.83648	-1.83223	-1.828
-1.86134	-1.85716	-1.85299	-1.84883	-1.84465	-1.84046	-1.83625	-1.832	-1.82776
-1.8611	-1.85692	-1.85276	-1.8486	-1.84442	-1.84023	-1.83602	-1.83176	-1.82753

-1.86088	-1.85669	-1.85252	-1.84837	-1.84419	-1.83999	-1.83578	-1.83153	-1.82731
-1.86068	-1.85645	-1.85228	-1.84813	-1.84396	-1.83977	-1.83556	-1.83131	-1.82709
-1.86049	-1.85623	-1.85204	-1.84789	-1.84373	-1.83954	-1.83534	-1.8311	-1.82688
-1.86029	-1.85601	-1.85181	-1.84766	-1.84349	-1.83932	-1.86281	-1.85851	-1.85421
-1.86008	-1.8558	-1.85159	-1.84743	-1.84326	-1.83909	-1.8626	-1.85834	-1.85408
-1.8599	-1.85564	-1.85143	-1.84724	-1.84306	-1.83889	-1.86238	-1.85814	-1.85391
-1.85974	-1.85552	-1.85132	-1.84712	-1.84292	-1.8387	-1.86215	-1.85791	-1.85367
-1.85956	-1.85538	-1.85118	-1.84698	-1.84276	-1.83853	-1.86196	-1.8577	-1.85346
-1.85935	-1.85517	-1.85098	-1.84679	-1.8426	-1.83839	-1.86183	-1.85755	-1.85329
-1.85914	-1.85496	-1.85077	-1.84659	-1.84242	-1.83825	-1.8617	-1.85742	-1.85314
-1.859	-1.85481	-1.8506	-1.8464	-1.87002	-1.86576	-1.8615	-1.85724	-1.85297
-1.85889	-1.85468	-1.85045	-1.84621	-1.8698	-1.86554	-1.86129	-1.85704	-1.8528
-1.85875	-1.85453	-1.85029	-1.84605	-1.86963	-1.86537	-1.86112	-1.85689	-1.85266
-1.8586	-1.85436	-1.85014	-1.84593	-1.86951	-1.86524	-1.86098	-1.85676	-1.85253
-1.85846	-1.85421	-1.85	-1.8458	-1.86937	-1.86509	-1.86082	-1.85659	-1.85236
-1.85831	-1.85408	-1.84988	-1.84568	-1.86922	-1.86491	-1.86062	-1.85637	-1.85214
-1.85817	-1.85397	-1.84976	-1.84555	-1.86907	-1.86473	-1.86043	-1.85617	-1.85194
-1.85806	-1.85386	-1.84964	-1.84539	-1.86889	-1.86458	-1.86029	-1.85604	-1.85181
-1.85797	-1.85377	-1.84952	-1.84524	-1.86873	-1.86444	-1.86017	-1.85592	-1.8517
-1.85788	-1.85368	-1.87734	-1.87298	-1.86865	-1.86434	-1.87237	-1.86808	-1.86381
-1.8578	-1.8536	-1.87727	-1.87294	-1.8686	-1.86427	-1.87228	-1.86798	-1.86371
-1.85771	-1.85351	-1.87717	-1.87283	-1.8685	-1.86417	-1.87218	-1.86788	-1.8636
-1.85761	-1.85341	-1.87704	-1.87268	-1.86835	-1.86404	-1.87207	-1.86778	-1.86349
-1.85752	-1.85332	-1.87693	-1.87256	-1.86823	-1.86393	-1.87198	-1.86769	-1.8634
-1.85743	-1.85323	-1.87686	-1.87251	-1.86818	-1.86386	-1.87189	-1.86761	-1.86332
-1.85734	-1.85314	-1.87678	-1.87244	-1.86811	-1.86378	-1.87181	-1.86752	-1.86325
-1.85724	-1.85304	-1.87665	-1.8723	-1.86797	-1.86366	-1.87171	-1.86743	-1.86317
-1.85714	-1.85293	-1.87652	-1.87215	-1.86782	-1.86354	-1.87161	-1.86734	-1.86309
-1.85704	-1.85284	-1.87643	-1.87208	-1.86775	-1.86345	-1.87151	-1.86725	-1.86299
-1.85695	-1.85277	-1.87638	-1.87206	-1.86773	-1.86338	-1.87142	-1.86714	-1.86288
-1.85688	-1.85269	-1.87631	-1.87202	-1.8677	-1.86335	-1.87137	-1.86707	-1.86279
-1.85684	-1.8526	-1.87621	-1.87195	-1.86766	-1.86334	-1.87138	-1.86703	-1.8627
-1.88479	-1.88043	-1.87613	-1.87188	-1.88003	-1.87572	-1.87138	-1.86701	-1.86265
-1.88477	-1.88043	-1.8761	-1.87179	-1.87994	-1.87565	-1.87134	-1.86699	-1.86265
-1.88474	-1.88043	-1.87608	-1.87172	-1.87985	-1.87557	-1.87128	-1.86697	-1.86266
-1.88466	-1.88038	-1.87605	-1.8717	-1.87983	-1.87551	-1.8712	-1.86692	-1.86263
-1.88457	-1.88031	-1.87602	-1.87171	-1.87984	-1.87547	-1.87113	-1.86687	-1.86261
-1.8845	-1.88023	-1.87595	-1.87168	-1.87983	-1.87545	-1.87112	-1.86686	-1.86261
-1.88445	-1.88013	-1.87586	-1.87163	-1.87981	-1.87546	-1.87115	-1.86688	-1.86262
-1.88444	-1.88009	-1.87582	-1.8716	-1.8798	-1.87549	-1.87118	-1.86688	-1.8626
-1.88449	-1.88011	-1.87583	-1.8716	-1.87982	-1.87552	-1.8712	-1.86687	-1.86254
-1.88452	-1.88014	-1.87584	-1.8716	-1.87982	-1.87553	-1.87122	-1.86685	-1.8625
-1.88452	-1.88015	-1.87584	-1.87155	-1.87975	-1.87548	-1.87119	-1.86685	-1.86251
-1.88452	-1.88017	-1.87583	-1.87149	-1.87969	-1.87543	-1.87116	-1.86685	-1.86254
-1.8706	-1.8663	-1.88842	-1.88402	-1.87969	-1.87543	-1.87117	-1.86687	-1.86256
-1.87063	-1.86634	-1.88846	-1.88405	-1.87972	-1.87546	-1.87119	-1.86689	-1.86258
-1.87067	-1.86639	-1.88851	-1.8841	-1.87976	-1.8755	-1.8861	-1.88177	-1.87743
-1.87071	-1.86643	-1.88857	-1.88415	-1.87981	-1.87555	-1.88615	-1.88182	-1.87748
-1.87076	-1.86648	-1.88862	-1.88421	-1.8948	-1.89051	-1.8862	-1.88187	-1.87753
-1.8708	-1.86652	-1.88867	-1.88425	-1.89484	-1.89055	-1.88625	-1.88192	-1.87757

-1.88367	-1.87935	-1.87499	-1.87061	-1.89489	-1.8906	-1.88629	-1.88196	-1.87761
-1.88372	-1.8794	-1.87504	-1.87066	-1.89494	-1.89065	-1.88634	-1.88201	-1.87766
-1.88377	-1.87945	-1.87509	-1.87071	-1.89499	-1.8907	-1.88639	-1.88206	-1.87771
-1.88383	-1.8795	-1.8903	-1.88588	-1.89504	-1.89074	-1.88644	-1.88211	-1.87777
-1.88388	-1.87955	-1.89035	-1.88593	-1.89508	-1.89079	-1.88649	-1.88216	-1.87782
-1.89914	-1.89479	-1.8904	-1.88598	-1.89514	-1.89084	-1.88653	-1.88221	-1.87788
-1.89918	-1.89484	-1.89045	-1.88604	-1.89519	-1.89088	-1.88657	-1.88226	-1.87795
-1.89924	-1.8949	-1.89052	-1.88613	-1.88178	-1.87749	-1.88664	-1.88233	-1.87802
-1.89934	-1.89497	-1.89061	-1.88627	-1.88194	-1.87764	-1.88677	-1.88243	-1.87809
-1.89944	-1.89505	-1.89071	-1.88641	-1.8821	-1.87779	-1.88691	-1.88254	-1.87818
-1.89952	-1.89511	-1.89078	-1.88648	-1.88218	-1.87789	-1.88702	-1.88267	-1.87832
-1.8996	-1.89519	-1.89084	-1.88654	-1.88224	-1.87796	-1.88712	-1.8828	-1.87847
-1.8997	-1.89531	-1.89097	-1.88665	-1.88235	-1.87804	-1.8872	-1.88289	-1.87858
[truncated: 917,572 more chars]
