# Supplementary material for: Artefact-free topography based scleral-asymmetry
Source: PLoS One. 2019 Jul 26;14(7):e0219789. doi: 10.1371/journal.pone.0219789 (PMC6660081; doi:10.1371/journal.pone.0219789)
Supplement: S2 Table — (ZIP) [file Eye mean OS.pdf › Eye mean OS.pdf]

					-0.05777
					-0.05771
					-0.05765
					-0.0576
					-0.11536
					-0.11521
					-0.11507
					-0.11492
					-0.11477
		-0.05802	-0.05778	-0.05753	-0.31156
		-0.05798	-0.05772	-0.05747	-0.31123
		-0.05792	-0.05766	-0.0574	-0.31091
		-0.05786	-0.0576	-0.05735	-0.3106
		-0.05779	-0.05755	-0.0573	-0.31029
		-0.05774	-0.05749	-0.05724	-0.30998
		-0.05769	-0.05744	-0.05718	-0.30968
		-0.15941	-0.15889	-0.15837	-0.41379
		-0.15929	-0.15878	-0.15825	-0.41339
		-0.15918	-0.15866	-0.15814	-0.45717
		-0.15907	-0.15855	-0.15803	-0.45678
-0.05791	-0.05766	-0.25916	-0.25828	-0.25739	-0.4564
-0.05786	-0.05761	-0.25897	-0.25808	-0.25719	-0.45602
-0.05781	-0.05756	-0.25878	-0.25789	-0.257	-0.45565
-0.05776	-0.05751	-0.25859	-0.25769	-0.25681	-0.54166
-0.05771	-0.05746	-0.2584	-0.2575	-0.25661	-0.54123
-0.15946	-0.15894	-0.3651	-0.36378	-0.36248	-0.5408
-0.15934	-0.15882	-0.3648	-0.36349	-0.36218	-0.54036
-0.15923	-0.15871	-0.45263	-0.451	-0.44939	-0.53994
-0.15912	-0.15859	-0.45229	-0.45066	-0.44906	-0.53952
-0.159	-0.15848	-0.45195	-0.45033	-0.44874	-0.53911
-0.15889	-0.15836	-0.45161	-0.45	-0.44842	-0.53872

					-0.05777
					-0.05771
					-0.05765
					-0.0576
					-0.11536
					-0.11521
					-0.11507
					-0.11492
					-0.11477
		-0.05802	-0.05778	-0.05753	-0.31156
		-0.05798	-0.05772	-0.05747	-0.31123
		-0.05792	-0.05766	-0.0574	-0.31091
		-0.05786	-0.0576	-0.05735	-0.3106
		-0.05779	-0.05755	-0.0573	-0.31029
		-0.05774	-0.05749	-0.05724	-0.30998
		-0.05769	-0.05744	-0.05718	-0.30968
		-0.15941	-0.15889	-0.15837	-0.41379
		-0.15929	-0.15878	-0.15825	-0.41339
		-0.15918	-0.15866	-0.15814	-0.45717
		-0.15907	-0.15855	-0.15803	-0.45678
-0.05791	-0.05766	-0.25916	-0.25828	-0.25739	-0.4564
-0.05786	-0.05761	-0.25897	-0.25808	-0.25719	-0.45602
-0.05781	-0.05756	-0.25878	-0.25789	-0.257	-0.45565
-0.05776	-0.05751	-0.25859	-0.25769	-0.25681	-0.54166
-0.05771	-0.05746	-0.2584	-0.2575	-0.25661	-0.54123
-0.15946	-0.15894	-0.3651	-0.36378	-0.36248	-0.5408
-0.15934	-0.15882	-0.3648	-0.36349	-0.36218	-0.54036
-0.15923	-0.15871	-0.45263	-0.451	-0.44939	-0.53994
-0.15912	-0.15859	-0.45229	-0.45066	-0.44906	-0.53952
-0.159	-0.15848	-0.45195	-0.45033	-0.44874	-0.53911
-0.15889	-0.15836	-0.45161	-0.45	-0.44842	-0.53872

		-0.10914
		-0.10897
		-0.10879
		-0.10858
		-0.16332
		-0.16311
		-0.1629
		-0.16269
		-0.16249
-0.10891	-0.10828	-0.32111
-0.10873	-0.10814	-0.32077
-0.16357	-0.16276	-0.42551
-0.16339	-0.1626	-0.42512
-0.16321	-0.16243	-0.42471
-0.16303	-0.16223	-0.42425

						-0.16284	-0.16204	-0.42377
						-0.16267	-0.16185	-0.4233
						-0.16251	-0.16167	-0.42282
			-0.05771	-0.05746	-0.05721	-0.31422	-0.31293	-0.4667
			-0.05764	-0.05739	-0.05714	-0.31394	-0.31264	-0.46626
			-0.11513	-0.11443	-0.11376	-0.31362	-0.31233	-0.4658
			-0.11497	-0.11427	-0.1136	-0.31327	-0.31199	-0.46531
			-0.16428	-0.16335	-0.16245	-0.41324	-0.41159	-0.55619
			-0.1641	-0.16316	-0.16225	-0.41282	-0.41116	-0.55561
			-0.16393	-0.16298	-0.16206	-0.4124	-0.41075	-0.55505
			-0.16375	-0.16279	-0.16186	-0.412	-0.41036	-0.55455
			-0.16356	-0.16259	-0.16166	-0.41159	-0.40998	-0.5541
	-0.05768	-0.05743	-0.36212	-0.36061	-0.35914	-0.45788	-0.45611	-0.62434
	-0.05761	-0.05736	-0.36178	-0.36026	-0.35879	-0.45742	-0.45568	-0.62381
	-0.05754	-0.0573	-0.36143	-0.35991	-0.35845	-0.45697	-0.45526	-0.62329
	-0.05749	-0.05724	-0.36106	-0.35955	-0.35809	-0.45653	-0.45486	-0.62281
	-0.05744	-0.05719	-0.3607	-0.35919	-0.35774	-0.45609	-0.45446	-0.62233
	-0.05739	-0.05713	-0.36035	-0.35885	-0.35741	-0.45566	-0.45405	-0.62181
	-0.05733	-0.05707	-0.36	-0.35851	-0.35709	-0.45523	-0.45363	-0.62128
-0.05753	-0.25877	-0.25786	-0.46032	-0.45838	-0.45652	-0.45483	-0.45324	-0.62079
-0.05746	-0.25856	-0.25766	-0.45986	-0.45796	-0.45614	-0.45446	-0.45286	-0.62033
-0.0574	-0.25835	-0.25746	-0.4594	-0.45754	-0.45576	-0.5284	-0.52658	-0.65705
-0.05735	-0.25814	-0.25725	-0.45896	-0.45713	-0.45538	-0.52801	-0.52618	-0.65654
-0.11462	-0.36128	-0.35978	-0.51266	-0.51062	-0.50865	-0.62327	-0.62108	-0.65603
-0.11447	-0.36095	-0.35944	-0.51216	-0.51015	-0.50821	-0.62279	-0.62062	-0.65553
-0.11433	-0.36062	-0.3591	-0.51165	-0.50967	-0.50776	-0.62231	-0.62016	-0.65504
-0.11417	-0.36029	-0.35877	-0.55309	-0.55102	-0.54902	-0.69212	-0.68972	-0.72872
-0.11401	-0.35995	-0.35844	-0.55257	-0.55054	-0.54858	-0.69162	-0.68924	-0.72819
-0.31017	-0.46013	-0.45816	-0.55209	-0.55009	-0.54815	-0.69112	-0.68873	-0.72765
-0.30986	-0.45966	-0.45775	-0.55165	-0.54966	-0.54772	-0.69061	-0.68821	-0.72709
-0.30954	-0.50231	-0.50031	-0.62087	-0.61861	-0.61641	-0.73173	-0.72916	-0.92532
-0.30923	-0.50187	-0.49991	-0.62042	-0.61815	-0.61593	-0.73118	-0.72861	-0.92455
-0.30892	-0.50142	-0.49951	-0.61997	-0.6177	-0.61547	-0.73065	-0.72807	-0.92381
-0.30863	-0.50098	-0.49911	-0.61951	-0.61726	-0.61503	-0.73014	-0.72755	-0.92306
-0.30834	-0.50054	-0.49869	-0.61904	-0.61681	-0.6146	-0.72965	-0.72704	-0.92232
-0.41205	-0.50009	-0.49827	-0.61856	-0.61635	-0.61415	-0.72916	-0.72655	-0.92156
-0.41165	-0.49964	-0.49785	-0.61806	-0.61588	-0.61369	-0.72866	-0.72606	-0.92081
-0.45529	-0.53951	-0.53755	-0.61759	-0.61542	-0.61325	-0.84433	-0.84139	-0.92006
-0.4549	-0.53906	-0.53712	-0.61713	-0.61497	-0.61281	-0.84375	-0.84079	-0.91931
-0.45453	-0.53863	-0.53671	-0.61668	-0.61453	-0.61237	-0.84315	-0.84016	-1.02304
-0.45418	-0.53824	-0.53632	-0.61625	-0.6141	-0.61192	-0.84254	-0.8395	-1.02212
-0.45384	-0.53786	-0.53594	-0.61582	-0.61366	-0.61148	-0.84193	-0.83885	-1.02124
-0.53951	-0.61314	-0.61098	-0.65849	-0.6562	-0.65389	-0.88274	-0.87947	-1.02045
-0.53911	-0.61273	-0.61057	-0.65804	-0.65575	-0.65342	-0.88213	-0.87887	-1.01971
-0.53871	-0.61232	-0.61017	-0.65759	-0.65529	-0.65295	-0.88152	-0.87827	-1.01897
-0.53831	-0.61191	-0.60976	-0.65715	-0.65483	-0.65248	-0.88091	-0.87767	-1.01823
-0.53792	-0.6115	-0.60935	-0.77747	-0.7748	-0.7721	-0.8803	-0.87708	-1.01751
-0.53753	-0.6111	-0.60895	-0.77694	-0.77426	-0.77155	-0.8797	-0.87649	-1.01678
-0.53715	-0.61069	-0.60854	-0.77642	-0.77373	-0.77101	-0.98556	-0.98204	-1.01606
-0.53676	-0.61028	-0.60813	-0.77589	-0.77319	-0.77047	-0.9849	-0.98139	-1.01536

		-0.10914
		-0.10897
		-0.10879
		-0.10858
		-0.16332
		-0.16311
		-0.1629
		-0.16269
		-0.16249
-0.10891	-0.10828	-0.32111
-0.10873	-0.10814	-0.32077
-0.16357	-0.16276	-0.42551
-0.16339	-0.1626	-0.42512
-0.16321	-0.16243	-0.42471
-0.16303	-0.16223	-0.42425

						-0.16284	-0.16204	-0.42377
						-0.16267	-0.16185	-0.4233
						-0.16251	-0.16167	-0.42282
			-0.05771	-0.05746	-0.05721	-0.31422	-0.31293	-0.4667
			-0.05764	-0.05739	-0.05714	-0.31394	-0.31264	-0.46626
			-0.11513	-0.11443	-0.11376	-0.31362	-0.31233	-0.4658
			-0.11497	-0.11427	-0.1136	-0.31327	-0.31199	-0.46531
			-0.16428	-0.16335	-0.16245	-0.41324	-0.41159	-0.55619
			-0.1641	-0.16316	-0.16225	-0.41282	-0.41116	-0.55561
			-0.16393	-0.16298	-0.16206	-0.4124	-0.41075	-0.55505
			-0.16375	-0.16279	-0.16186	-0.412	-0.41036	-0.55455
			-0.16356	-0.16259	-0.16166	-0.41159	-0.40998	-0.5541
	-0.05768	-0.05743	-0.36212	-0.36061	-0.35914	-0.45788	-0.45611	-0.62434
	-0.05761	-0.05736	-0.36178	-0.36026	-0.35879	-0.45742	-0.45568	-0.62381
	-0.05754	-0.0573	-0.36143	-0.35991	-0.35845	-0.45697	-0.45526	-0.62329
	-0.05749	-0.05724	-0.36106	-0.35955	-0.35809	-0.45653	-0.45486	-0.62281
	-0.05744	-0.05719	-0.3607	-0.35919	-0.35774	-0.45609	-0.45446	-0.62233
	-0.05739	-0.05713	-0.36035	-0.35885	-0.35741	-0.45566	-0.45405	-0.62181
	-0.05733	-0.05707	-0.36	-0.35851	-0.35709	-0.45523	-0.45363	-0.62128
-0.05753	-0.25877	-0.25786	-0.46032	-0.45838	-0.45652	-0.45483	-0.45324	-0.62079
-0.05746	-0.25856	-0.25766	-0.45986	-0.45796	-0.45614	-0.45446	-0.45286	-0.62033
-0.0574	-0.25835	-0.25746	-0.4594	-0.45754	-0.45576	-0.5284	-0.52658	-0.65705
-0.05735	-0.25814	-0.25725	-0.45896	-0.45713	-0.45538	-0.52801	-0.52618	-0.65654
-0.11462	-0.36128	-0.35978	-0.51266	-0.51062	-0.50865	-0.62327	-0.62108	-0.65603
-0.11447	-0.36095	-0.35944	-0.51216	-0.51015	-0.50821	-0.62279	-0.62062	-0.65553
-0.11433	-0.36062	-0.3591	-0.51165	-0.50967	-0.50776	-0.62231	-0.62016	-0.65504
-0.11417	-0.36029	-0.35877	-0.55309	-0.55102	-0.54902	-0.69212	-0.68972	-0.72872
-0.11401	-0.35995	-0.35844	-0.55257	-0.55054	-0.54858	-0.69162	-0.68924	-0.72819
-0.31017	-0.46013	-0.45816	-0.55209	-0.55009	-0.54815	-0.69112	-0.68873	-0.72765
-0.30986	-0.45966	-0.45775	-0.55165	-0.54966	-0.54772	-0.69061	-0.68821	-0.72709
-0.30954	-0.50231	-0.50031	-0.62087	-0.61861	-0.61641	-0.73173	-0.72916	-0.92532
-0.30923	-0.50187	-0.49991	-0.62042	-0.61815	-0.61593	-0.73118	-0.72861	-0.92455
-0.30892	-0.50142	-0.49951	-0.61997	-0.6177	-0.61547	-0.73065	-0.72807	-0.92381
-0.30863	-0.50098	-0.49911	-0.61951	-0.61726	-0.61503	-0.73014	-0.72755	-0.92306
-0.30834	-0.50054	-0.49869	-0.61904	-0.61681	-0.6146	-0.72965	-0.72704	-0.92232
-0.41205	-0.50009	-0.49827	-0.61856	-0.61635	-0.61415	-0.72916	-0.72655	-0.92156
-0.41165	-0.49964	-0.49785	-0.61806	-0.61588	-0.61369	-0.72866	-0.72606	-0.92081
-0.45529	-0.53951	-0.53755	-0.61759	-0.61542	-0.61325	-0.84433	-0.84139	-0.92006
-0.4549	-0.53906	-0.53712	-0.61713	-0.61497	-0.61281	-0.84375	-0.84079	-0.91931
-0.45453	-0.53863	-0.53671	-0.61668	-0.61453	-0.61237	-0.84315	-0.84016	-1.02304
-0.45418	-0.53824	-0.53632	-0.61625	-0.6141	-0.61192	-0.84254	-0.8395	-1.02212
-0.45384	-0.53786	-0.53594	-0.61582	-0.61366	-0.61148	-0.84193	-0.83885	-1.02124
-0.53951	-0.61314	-0.61098	-0.65849	-0.6562	-0.65389	-0.88274	-0.87947	-1.02045
-0.53911	-0.61273	-0.61057	-0.65804	-0.65575	-0.65342	-0.88213	-0.87887	-1.01971
-0.53871	-0.61232	-0.61017	-0.65759	-0.65529	-0.65295	-0.88152	-0.87827	-1.01897
-0.53831	-0.61191	-0.60976	-0.65715	-0.65483	-0.65248	-0.88091	-0.87767	-1.01823
-0.53792	-0.6115	-0.60935	-0.77747	-0.7748	-0.7721	-0.8803	-0.87708	-1.01751
-0.53753	-0.6111	-0.60895	-0.77694	-0.77426	-0.77155	-0.8797	-0.87649	-1.01678
-0.53715	-0.61069	-0.60854	-0.77642	-0.77373	-0.77101	-0.98556	-0.98204	-1.01606
-0.53676	-0.61028	-0.60813	-0.77589	-0.77319	-0.77047	-0.9849	-0.98139	-1.01536

								-0.05885
								-0.05867
								-0.17218
								-0.17188
								-0.17158
								-0.17127
								-0.17097
								-0.17065
								-0.17035
						-0.05846	-0.05794	-0.17009
						-0.05832	-0.05783	-0.16986
						-0.17154	-0.17056	-0.32042
						-0.17126	-0.17032	-0.32008
						-0.17098	-0.17008	-0.31973
						-0.17073	-0.16985	-0.31938
						-0.17048	-0.16962	-0.31902
						-0.17024	-0.16939	-0.31864
						-0.17001	-0.16915	-0.31825
			-0.10944	-0.1087	-0.10802	-0.32057	-0.31922	-0.5069
			-0.10916	-0.10846	-0.10783	-0.32014	-0.31878	-0.50625
			-0.10891	-0.10825	-0.10765	-0.31974	-0.31838	-0.50563
			-0.10869	-0.10806	-0.10749	-0.31939	-0.31802	-0.50506
			-0.1714	-0.17049	-0.16965	-0.37015	-0.36863	-0.54918
			-0.17115	-0.17026	-0.16942	-0.36975	-0.36823	-0.54858
			-0.17093	-0.17004	-0.1692	-0.36934	-0.36783	-0.54799
			-0.17071	-0.16982	-0.16898	-0.36894	-0.36742	-0.5474
			-0.17048	-0.16961	-0.16876	-0.36852	-0.36702	-0.54683
	-0.109	-0.10834	-0.32135	-0.31997	-0.31862	-0.50581	-0.50379	-0.54625
	-0.10878	-0.10816	-0.32099	-0.31961	-0.31825	-0.50524	-0.50323	-0.54567
	-0.10857	-0.10799	-0.37183	-0.3703	-0.36878	-0.50468	-0.50267	-0.70019
	-0.1084	-0.10784	-0.37141	-0.36989	-0.36837	-0.50412	-0.50211	-0.6995
	-0.10824	-0.10769	-0.37099	-0.36947	-0.36796	-0.50357	-0.50156	-0.73611
	-0.10809	-0.10754	-0.37057	-0.36906	-0.36755	-0.50302	-0.50101	-0.73541
	-0.10795	-0.1074	-0.37014	-0.36864	-0.36714	-0.50247	-0.50047	-0.73471
-0.10844	-0.3215	-0.32013	-0.36972	-0.36821	-0.36672	-0.50192	-0.49993	-0.73403
-0.1083	-0.32119	-0.31978	-0.36929	-0.36779	-0.3663	-0.50138	-0.4994	-0.73335
-0.10816	-0.37202	-0.37045	-0.47055	-0.46864	-0.46675	-0.6554	-0.65302	-0.73268
-0.10801	-0.37167	-0.3701	-0.47007	-0.46814	-0.46624	-0.65478	-0.6524	-0.732
-0.16255	-0.3713	-0.36974	-0.4696	-0.46766	-0.46575	-0.65419	-0.65181	-0.73134
-0.16234	-0.3709	-0.36937	-0.46915	-0.46722	-0.46531	-0.65366	-0.65125	-0.73071
-0.16213	-0.37048	-0.36899	-0.46868	-0.46677	-0.46487	-0.65313	-0.65071	-0.7301
-0.16192	-0.42397	-0.42223	-0.5469	-0.54477	-0.54265	-0.69477	-0.69225	-0.84769
-0.16171	-0.42346	-0.42173	-0.54631	-0.54419	-0.54209	-0.69415	-0.69167	-0.84701
-0.31976	-0.42296	-0.42123	-0.54573	-0.54362	-0.54154	-0.69352	-0.69107	-0.88207
-0.3194	-0.42245	-0.42073	-0.54514	-0.54304	-0.54098	-0.69287	-0.69044	-0.88133
-0.42373	-0.51315	-0.51115	-0.65731	-0.65488	-0.65249	-0.69222	-0.6898	-0.88056
-0.42331	-0.51264	-0.51062	-0.65668	-0.65424	-0.65185	-0.69157	-0.68917	-0.87978
-0.4229	-0.51214	-0.51011	-0.65608	-0.65364	-0.65125	-0.69094	-0.68855	-0.91641
-0.42246	-0.51167	-0.50965	-0.65555	-0.6531	-0.65069	-0.69034	-0.68795	-0.9156

-0.42202	-0.51119	-0.50919	-0.65503	-0.65257	-0.65016	-0.68977	-0.68737	-0.91481
-0.42155	-0.51065	-0.50869	-0.65445	-0.65203	-0.64965	-0.68924	-0.68683	-0.95181
-0.42106	-0.5101	-0.50816	-0.65385	-0.65148	-0.64915	-0.68873	-0.68631	-0.95107
-0.46482	-0.62466	-0.62238	-0.65326	-0.65092	-0.64861	-0.68818	-0.68578	-0.98093
-0.46438	-0.62412	-0.62183	-0.65269	-0.65035	-0.64805	-0.68761	-0.68524	-0.98019
-0.46394	-0.62359	-0.62131	-0.65216	-0.64981	-0.64751	-0.88487	-0.88186	-1.04933
-0.46349	-0.62308	-0.62083	-0.65169	-0.64934	-0.647	-0.88414	-0.8811	-1.04832
-0.55411	-0.65588	-0.65355	-0.65122	-0.64885	-0.64651	-0.91533	-0.9121	-1.04733
-0.55357	-0.6553	-0.65298	-0.65067	-0.64834	-0.64602	-0.91464	-0.91141	-1.04647
-0.55303	-0.65473	-0.65241	-0.6501	-0.6478	-0.64552	-0.91395	-0.91072	-1.04562
-0.55255	-0.69648	-0.69404	-0.73131	-0.72876	-0.72622	-0.94836	-0.94502	-1.14689
-0.55211	-0.69598	-0.69352	-0.73073	-0.72816	-0.72559	-0.94749	-0.94413	-1.1458
-0.62216	-0.69545	-0.69299	-0.76499	-0.76227	-0.75954	-0.94669	-0.94331	-1.14473
-0.62164	-0.69489	-0.69245	-0.7644	-0.76169	-0.75896	-0.94596	-0.94256	-1.14369
-0.62113	-0.69436	-0.69192	-0.95537	-0.952	-0.94862	-1.01615	-1.01221	-1.20267
-0.62066	-0.69386	-0.69139	-0.95459	-0.95119	-0.94779	-1.01523	-1.0114	-1.20174
-0.62019	-0.69335	-0.69086	-0.95381	-0.95038	-0.94698	-1.01432	-1.0106	-1.20084
-0.61967	-0.72844	-0.72582	-0.95306	-0.94963	-0.94624	-1.01349	-1.0098	-1.26154
-0.61915	-0.72785	-0.72524	-0.95231	-0.9489	-0.94552	-1.01268	-1.00902	-1.26054
-0.61867	-0.7273	-0.72467	-0.95153	-0.94811	-0.94474	-1.01181	-1.00823	-1.28715
-0.61821	-0.72677	-0.72412	-0.95075	-0.9473	-0.94391	-1.01089	-1.00743	-1.28619
-0.65478	-0.9215	-0.91815	-1.02155	-1.01754	-1.01364	-1.10551	-1.10172	-1.28521
-0.65423	-0.92073	-0.91743	-1.02073	-1.01675	-1.01288	-1.10465	-1.10086	-1.28419
-0.6537	-0.91999	-0.91672	-1.01991	-1.01597	-1.01212	-1.19789	-1.19378	-1.31733
-0.65319	-0.9193	-0.91604	-1.0191	-1.01518	-1.01135	-1.19697	-1.19292	-1.31637
-0.65269	-0.91861	-0.91534	-1.01825	-1.01437	-1.01059	-1.19609	-1.19208	-1.31544
-0.72608	-0.91787	-0.91458	-1.05402	-1.05011	-1.04632	-1.19523	-1.19125	-1.3145
-0.72552	-0.91711	-0.91378	-1.05301	-1.04922	-1.04556	-1.19439	-1.19042	-1.31355
-0.72497	-0.9164	-0.91306	-1.08359	-1.07977	-1.07607	-1.22585	-1.22178	-1.39677
-0.72442	-0.91572	-0.9124	-1.08275	-1.07898	-1.07531	-1.22495	-1.22085	-1.39568
-0.92167	-0.98776	-0.9839	-1.1402	-1.13613	-1.1322	-1.22404	-1.21994	-1.42112
-0.92093	-0.98698	-0.98313	-1.13928	-1.13522	-1.13131	-1.2231	-1.21906	-1.42009
-0.92019	-1.0557	-1.05165	-1.20227	-1.19803	-1.19395	-1.25737	-1.2532	-1.41908
-0.91945	-1.05488	-1.05084	-1.20132	-1.19713	-1.19309	-1.25649	-1.25234	-1.41808
-0.9187	-1.05405	-1.05002	-1.2004	-1.19625	-1.19227	-1.25563	-1.25148	-1.41708
-0.9179	-1.05316	-1.0492	-1.19951	-1.19541	-1.19146	-1.28358	-1.27928	-1.41602
-0.91708	-1.05224	-1.04839	-1.19866	-1.1946	-1.19066	-1.28268	-1.27834	-1.41493
-0.91631	-1.0514	-1.04762	-1.19782	-1.1938	-1.18987	-1.28175	-1.27735	-1.4138
-0.91558	-1.05062	-1.04689	-1.197	-1.19301	-1.18908	-1.2808	-1.2763	-1.41262
-1.01904	-1.04984	-1.04616	-1.22626	-1.22218	-1.21812	-1.27987	-1.27529	-1.51228
-1.01818	-1.04902	-1.04542	-1.22542	-1.22135	-1.21728	-1.27896	-1.27438	-1.51116
-1.01735	-1.04823	-1.0447	-1.22459	-1.2205	-1.21641	-1.27803	-1.27346	-1.51007
-1.01659	-1.04749	-1.044	-1.22375	-1.21961	-1.21547	-1.27702	-1.2725	-1.50896
-1.01588	-1.04678	-1.04332	-1.22292	-1.21872	-1.21451	-1.276	-1.27153	-1.50785
-1.01518	-1.14357	-1.13975	-1.25743	-1.25297	-1.24851	-1.33669	-1.33203	-1.50676
-1.0145	-1.14282	-1.139	-1.25657	-1.25211	-1.24764	-1.33574	-1.33106	-1.50569
-1.01382	-1.14208	-1.13826	-1.25572	-1.25125	-1.24677	-1.33479	-1.3301	-1.50464
-1.01315	-1.14134	-1.13753	-1.25487	-1.25039	-1.24589	-1.33385	-1.32916	-1.5036
-1.01248	-1.17163	-1.1677	-1.25401	-1.24952	-1.24502	-1.41295	-1.40801	-1.50256
-1.01182	-1.17085	-1.16692	-1.25314	-1.24865	-1.24414	-1.41194	-1.40701	-1.50154

								-0.05885
								-0.05867
								-0.17218
								-0.17188
								-0.17158
								-0.17127
								-0.17097
								-0.17065
								-0.17035
						-0.05846	-0.05794	-0.17009
						-0.05832	-0.05783	-0.16986
						-0.17154	-0.17056	-0.32042
						-0.17126	-0.17032	-0.32008
						-0.17098	-0.17008	-0.31973
						-0.17073	-0.16985	-0.31938
						-0.17048	-0.16962	-0.31902
						-0.17024	-0.16939	-0.31864
						-0.17001	-0.16915	-0.31825
			-0.10944	-0.1087	-0.10802	-0.32057	-0.31922	-0.5069
			-0.10916	-0.10846	-0.10783	-0.32014	-0.31878	-0.50625
			-0.10891	-0.10825	-0.10765	-0.31974	-0.31838	-0.50563
			-0.10869	-0.10806	-0.10749	-0.31939	-0.31802	-0.50506
			-0.1714	-0.17049	-0.16965	-0.37015	-0.36863	-0.54918
			-0.17115	-0.17026	-0.16942	-0.36975	-0.36823	-0.54858
			-0.17093	-0.17004	-0.1692	-0.36934	-0.36783	-0.54799
			-0.17071	-0.16982	-0.16898	-0.36894	-0.36742	-0.5474
			-0.17048	-0.16961	-0.16876	-0.36852	-0.36702	-0.54683
	-0.109	-0.10834	-0.32135	-0.31997	-0.31862	-0.50581	-0.50379	-0.54625
	-0.10878	-0.10816	-0.32099	-0.31961	-0.31825	-0.50524	-0.50323	-0.54567
	-0.10857	-0.10799	-0.37183	-0.3703	-0.36878	-0.50468	-0.50267	-0.70019
	-0.1084	-0.10784	-0.37141	-0.36989	-0.36837	-0.50412	-0.50211	-0.6995
	-0.10824	-0.10769	-0.37099	-0.36947	-0.36796	-0.50357	-0.50156	-0.73611
	-0.10809	-0.10754	-0.37057	-0.36906	-0.36755	-0.50302	-0.50101	-0.73541
	-0.10795	-0.1074	-0.37014	-0.36864	-0.36714	-0.50247	-0.50047	-0.73471
-0.10844	-0.3215	-0.32013	-0.36972	-0.36821	-0.36672	-0.50192	-0.49993	-0.73403
-0.1083	-0.32119	-0.31978	-0.36929	-0.36779	-0.3663	-0.50138	-0.4994	-0.73335
-0.10816	-0.37202	-0.37045	-0.47055	-0.46864	-0.46675	-0.6554	-0.65302	-0.73268
-0.10801	-0.37167	-0.3701	-0.47007	-0.46814	-0.46624	-0.65478	-0.6524	-0.732
-0.16255	-0.3713	-0.36974	-0.4696	-0.46766	-0.46575	-0.65419	-0.65181	-0.73134
-0.16234	-0.3709	-0.36937	-0.46915	-0.46722	-0.46531	-0.65366	-0.65125	-0.73071
-0.16213	-0.37048	-0.36899	-0.46868	-0.46677	-0.46487	-0.65313	-0.65071	-0.7301
-0.16192	-0.42397	-0.42223	-0.5469	-0.54477	-0.54265	-0.69477	-0.69225	-0.84769
-0.16171	-0.42346	-0.42173	-0.54631	-0.54419	-0.54209	-0.69415	-0.69167	-0.84701
-0.31976	-0.42296	-0.42123	-0.54573	-0.54362	-0.54154	-0.69352	-0.69107	-0.88207
-0.3194	-0.42245	-0.42073	-0.54514	-0.54304	-0.54098	-0.69287	-0.69044	-0.88133
-0.42373	-0.51315	-0.51115	-0.65731	-0.65488	-0.65249	-0.69222	-0.6898	-0.88056
-0.42331	-0.51264	-0.51062	-0.65668	-0.65424	-0.65185	-0.69157	-0.68917	-0.87978
-0.4229	-0.51214	-0.51011	-0.65608	-0.65364	-0.65125	-0.69094	-0.68855	-0.91641
-0.42246	-0.51167	-0.50965	-0.65555	-0.6531	-0.65069	-0.69034	-0.68795	-0.9156

-0.42202	-0.51119	-0.50919	-0.65503	-0.65257	-0.65016	-0.68977	-0.68737	-0.91481
-0.42155	-0.51065	-0.50869	-0.65445	-0.65203	-0.64965	-0.68924	-0.68683	-0.95181
-0.42106	-0.5101	-0.50816	-0.65385	-0.65148	-0.64915	-0.68873	-0.68631	-0.95107
-0.46482	-0.62466	-0.62238	-0.65326	-0.65092	-0.64861	-0.68818	-0.68578	-0.98093
-0.46438	-0.62412	-0.62183	-0.65269	-0.65035	-0.64805	-0.68761	-0.68524	-0.98019
-0.46394	-0.62359	-0.62131	-0.65216	-0.64981	-0.64751	-0.88487	-0.88186	-1.04933
-0.46349	-0.62308	-0.62083	-0.65169	-0.64934	-0.647	-0.88414	-0.8811	-1.04832
-0.55411	-0.65588	-0.65355	-0.65122	-0.64885	-0.64651	-0.91533	-0.9121	-1.04733
-0.55357	-0.6553	-0.65298	-0.65067	-0.64834	-0.64602	-0.91464	-0.91141	-1.04647
-0.55303	-0.65473	-0.65241	-0.6501	-0.6478	-0.64552	-0.91395	-0.91072	-1.04562
-0.55255	-0.69648	-0.69404	-0.73131	-0.72876	-0.72622	-0.94836	-0.94502	-1.14689
-0.55211	-0.69598	-0.69352	-0.73073	-0.72816	-0.72559	-0.94749	-0.94413	-1.1458
-0.62216	-0.69545	-0.69299	-0.76499	-0.76227	-0.75954	-0.94669	-0.94331	-1.14473
-0.62164	-0.69489	-0.69245	-0.7644	-0.76169	-0.75896	-0.94596	-0.94256	-1.14369
-0.62113	-0.69436	-0.69192	-0.95537	-0.952	-0.94862	-1.01615	-1.01221	-1.20267
-0.62066	-0.69386	-0.69139	-0.95459	-0.95119	-0.94779	-1.01523	-1.0114	-1.20174
-0.62019	-0.69335	-0.69086	-0.95381	-0.95038	-0.94698	-1.01432	-1.0106	-1.20084
-0.61967	-0.72844	-0.72582	-0.95306	-0.94963	-0.94624	-1.01349	-1.0098	-1.26154
-0.61915	-0.72785	-0.72524	-0.95231	-0.9489	-0.94552	-1.01268	-1.00902	-1.26054
-0.61867	-0.7273	-0.72467	-0.95153	-0.94811	-0.94474	-1.01181	-1.00823	-1.28715
-0.61821	-0.72677	-0.72412	-0.95075	-0.9473	-0.94391	-1.01089	-1.00743	-1.28619
-0.65478	-0.9215	-0.91815	-1.02155	-1.01754	-1.01364	-1.10551	-1.10172	-1.28521
-0.65423	-0.92073	-0.91743	-1.02073	-1.01675	-1.01288	-1.10465	-1.10086	-1.28419
-0.6537	-0.91999	-0.91672	-1.01991	-1.01597	-1.01212	-1.19789	-1.19378	-1.31733
-0.65319	-0.9193	-0.91604	-1.0191	-1.01518	-1.01135	-1.19697	-1.19292	-1.31637
-0.65269	-0.91861	-0.91534	-1.01825	-1.01437	-1.01059	-1.19609	-1.19208	-1.31544
-0.72608	-0.91787	-0.91458	-1.05402	-1.05011	-1.04632	-1.19523	-1.19125	-1.3145
-0.72552	-0.91711	-0.91378	-1.05301	-1.04922	-1.04556	-1.19439	-1.19042	-1.31355
-0.72497	-0.9164	-0.91306	-1.08359	-1.07977	-1.07607	-1.22585	-1.22178	-1.39677
-0.72442	-0.91572	-0.9124	-1.08275	-1.07898	-1.07531	-1.22495	-1.22085	-1.39568
-0.92167	-0.98776	-0.9839	-1.1402	-1.13613	-1.1322	-1.22404	-1.21994	-1.42112
-0.92093	-0.98698	-0.98313	-1.13928	-1.13522	-1.13131	-1.2231	-1.21906	-1.42009
-0.92019	-1.0557	-1.05165	-1.20227	-1.19803	-1.19395	-1.25737	-1.2532	-1.41908
-0.91945	-1.05488	-1.05084	-1.20132	-1.19713	-1.19309	-1.25649	-1.25234	-1.41808
-0.9187	-1.05405	-1.05002	-1.2004	-1.19625	-1.19227	-1.25563	-1.25148	-1.41708
-0.9179	-1.05316	-1.0492	-1.19951	-1.19541	-1.19146	-1.28358	-1.27928	-1.41602
-0.91708	-1.05224	-1.04839	-1.19866	-1.1946	-1.19066	-1.28268	-1.27834	-1.41493
-0.91631	-1.0514	-1.04762	-1.19782	-1.1938	-1.18987	-1.28175	-1.27735	-1.4138
-0.91558	-1.05062	-1.04689	-1.197	-1.19301	-1.18908	-1.2808	-1.2763	-1.41262
-1.01904	-1.04984	-1.04616	-1.22626	-1.22218	-1.21812	-1.27987	-1.27529	-1.51228
-1.01818	-1.04902	-1.04542	-1.22542	-1.22135	-1.21728	-1.27896	-1.27438	-1.51116
-1.01735	-1.04823	-1.0447	-1.22459	-1.2205	-1.21641	-1.27803	-1.27346	-1.51007
-1.01659	-1.04749	-1.044	-1.22375	-1.21961	-1.21547	-1.27702	-1.2725	-1.50896
-1.01588	-1.04678	-1.04332	-1.22292	-1.21872	-1.21451	-1.276	-1.27153	-1.50785
-1.01518	-1.14357	-1.13975	-1.25743	-1.25297	-1.24851	-1.33669	-1.33203	-1.50676
-1.0145	-1.14282	-1.139	-1.25657	-1.25211	-1.24764	-1.33574	-1.33106	-1.50569
-1.01382	-1.14208	-1.13826	-1.25572	-1.25125	-1.24677	-1.33479	-1.3301	-1.50464
-1.01315	-1.14134	-1.13753	-1.25487	-1.25039	-1.24589	-1.33385	-1.32916	-1.5036
-1.01248	-1.17163	-1.1677	-1.25401	-1.24952	-1.24502	-1.41295	-1.40801	-1.50256
-1.01182	-1.17085	-1.16692	-1.25314	-1.24865	-1.24414	-1.41194	-1.40701	-1.50154

							-0.0588
							-0.05862
							-0.05844
							-0.05827
							-0.05809
					-0.05893	-0.05841	-0.17082
					-0.05875	-0.05823	-0.17048
					-0.05858	-0.05807	-0.21829
					-0.05845	-0.05793	-0.21798
					-0.05831	-0.0578	-0.21766
					-0.05814	-0.05765	-0.21731
					-0.05796	-0.05748	-0.21694
			-0.05882	-0.05828	-0.17062	-0.16964	-0.21658
			-0.05863	-0.0581	-0.17028	-0.16931	-0.21623
			-0.05847	-0.05794	-0.21806	-0.21698	-0.40773
			-0.05834	-0.05782	-0.21777	-0.2167	-0.40726
			-0.17148	-0.17044	-0.21746	-0.21639	-0.40678
			-0.17114	-0.17012	-0.21711	-0.21605	-0.40626
			-0.17079	-0.1698	-0.21676	-0.21571	-0.40573
			-0.17045	-0.16948	-0.21642	-0.2154	-0.54896
			-0.17012	-0.16917	-0.2161	-0.21509	-0.54828
-0.05891	-0.05835	-0.05783	-0.16983	-0.16889	-0.21579	-0.21478	-0.59202
-0.05876	-0.05822	-0.05771	-0.16958	-0.16864	-0.21549	-0.21447	-0.59128
-0.17234	-0.17129	-0.17027	-0.21727	-0.21623	-0.50588	-0.50384	-0.63455
-0.17198	-0.17095	-0.16996	-0.21693	-0.21589	-0.5052	-0.50323	-0.63383
-0.17163	-0.17063	-0.16966	-0.2166	-0.21557	-0.55002	-0.54791	-0.63311
-0.17132	-0.17034	-0.1694	-0.21632	-0.21529	-0.54936	-0.54726	-0.63236

	-0.17103	-0.17008	-0.16916	-0.21606	-0.21503	-0.54873	-0.54662	-0.63162
-0.05829	-0.17075	-0.16982	-0.16891	-0.21579	-0.21476	-0.54809	-0.54599	-0.65958
-0.05816	-0.17049	-0.16957	-0.16866	-0.21551	-0.21448	-0.54746	-0.54538	-0.65888
-0.17117	-0.21827	-0.21725	-0.21624	-0.5512	-0.54895	-0.63417	-0.63183	-0.65814
-0.17088	-0.21794	-0.21692	-0.21591	-0.55048	-0.54826	-0.6334	-0.63106	-0.65735
-0.17059	-0.21763	-0.21661	-0.21561	-0.54978	-0.5476	-0.66142	-0.659	-0.77807
-0.17032	-0.21735	-0.21634	-0.21534	-0.54913	-0.54698	-0.66072	-0.65833	-0.77732
-0.17005	-0.21707	-0.21606	-0.21506	-0.54848	-0.54636	-0.66002	-0.65765	-0.77656
-0.16975	-0.31924	-0.31786	-0.31649	-0.54777	-0.54567	-0.65925	-0.65692	-0.77576
-0.16945	-0.3188	-0.31743	-0.31608	-0.54706	-0.54497	-0.65847	-0.65617	-0.77495
-0.1692	-0.31842	-0.31706	-0.31571	-0.54643	-0.54435	-0.65774	-0.65543	-0.93394
-0.16897	-0.31807	-0.31672	-0.31537	-0.54585	-0.54378	-0.65705	-0.65469	-0.93291
-0.31906	-0.55177	-0.54956	-0.54739	-0.63241	-0.63006	-0.65639	-0.65402	-0.93195
-0.31871	-0.55116	-0.54897	-0.54681	-0.63176	-0.62942	-0.65575	-0.6534	-0.93107
-0.31836	-0.55054	-0.54836	-0.54622	-0.65987	-0.65747	-0.85531	-0.85237	-1.0267
-0.31799	-0.5499	-0.54774	-0.54562	-0.65918	-0.65679	-0.85448	-0.85156	-1.02569
-0.31762	-0.54926	-0.5471	-0.54499	-0.65846	-0.65609	-0.85362	-0.85071	-1.02467
-0.31724	-0.54859	-0.54643	-0.54432	-0.65769	-0.65533	-0.85269	-0.84981	-1.0236
-0.31684	-0.54792	-0.54575	-0.54364	-0.6569	-0.65456	-0.85174	-0.84888	-1.0225
-0.50481	-0.54729	-0.54513	-0.54303	-0.6562	-0.65387	-0.88492	-0.88198	-1.02144
-0.5042	-0.54668	-0.54455	-0.54247	-0.65557	-0.65325	-0.88409	-0.88115	-1.02042
-0.50361	-0.66217	-0.6597	-0.65729	-0.81455	-0.81176	-0.91806	-0.91504	-1.01943
-0.50304	-0.66147	-0.65904	-0.65665	-0.81379	-0.81101	-0.91724	-0.91422	-1.01848
-0.54701	-0.66079	-0.65838	-0.65601	-0.84789	-0.84501	-0.91643	-0.91341	-1.11984
-0.54642	-0.66012	-0.65772	-0.65538	-0.84711	-0.84425	-0.91562	-0.91259	-1.1187
-0.54584	-0.65945	-0.65707	-0.65474	-0.84633	-0.84348	-0.91481	-0.91177	-1.11756
-0.54527	-0.69789	-0.69544	-0.69304	-0.91782	-0.91459	-1.02183	-1.01822	-1.11641
-0.5447	-0.69722	-0.69478	-0.69239	-0.91695	-0.91373	-1.02088	-1.01727	-1.11527
-0.54414	-0.73373	-0.7312	-0.72871	-0.91609	-0.91288	-1.01993	-1.01632	-1.20259
-0.54357	-0.73305	-0.73054	-0.72808	-0.91524	-0.91202	-1.01898	-1.01537	-1.20133
-0.6977	-0.85108	-0.84819	-0.84533	-0.9486	-0.94531	-1.01803	-1.01442	-1.20011
-0.69702	-0.85029	-0.84742	-0.84457	-0.94774	-0.94444	-1.01707	-1.01349	-1.19892
-0.73355	-0.84951	-0.84665	-0.84381	-0.94688	-0.94357	-1.11806	-1.11385	-1.29311
-0.73285	-0.84872	-0.84588	-0.84305	-0.94601	-0.9427	-1.11692	-1.11276	-1.29187
-0.73217	-0.84793	-0.8451	-0.84229	-0.94514	-0.94184	-1.11578	-1.11167	-1.29064
-0.73149	-0.84714	-0.84433	-0.84152	-0.94427	-0.94097	-1.11464	-1.11058	-1.34065
-0.73082	-0.84637	-0.84356	-0.84075	-0.9434	-0.94011	-1.11351	-1.1095	-1.33936
-0.73016	-0.8456	-0.84279	-0.83998	-0.94253	-0.93926	-1.14611	-1.14204	-1.33808
-0.7295	-0.84483	-0.84202	-0.83921	-0.94167	-0.93842	-1.14501	-1.14095	-1.33681
-0.72885	-0.84407	-0.84125	-0.83844	-1.11854	-1.11432	-1.26073	-1.25625	-1.36603
-0.7282	-0.8433	-0.84047	-0.83766	-1.1174	-1.11324	-1.25952	-1.25507	-1.36477
-0.72758	-0.84256	-0.83971	-0.83689	-1.11627	-1.11216	-1.25832	-1.2539	-1.36351
-0.84476	-0.84186	-0.83897	-0.83613	-1.14891	-1.14475	-1.25711	-1.25273	-1.36226
-0.8441	-0.84118	-0.83826	-0.83538	-1.14775	-1.14365	-1.2559	-1.25156	-1.361
-0.87909	-0.9514	-0.94813	-0.9449	-1.20269	-1.19843	-1.36921	-1.36448	-1.35974
-0.8784	-0.9507	-0.94742	-0.94417	-1.20157	-1.19731	-1.36794	-1.36321	-1.35847
-0.87767	-0.98696	-0.98359	-0.98023	-1.20053	-1.19628	-1.36675	-1.36201	-1.41294
-0.87689	-0.98611	-0.98281	-0.97952	-1.1996	-1.19535	-1.36567	-1.36086	-1.41168
-0.91339	-1.18481	-1.18047	-1.17619	-1.29137	-1.28681	-1.36459	-1.35974	-1.41046
-0.91257	-1.18361	-1.17933	-1.17513	-1.29023	-1.28572	-1.36347	-1.35865	-1.40932

-0.91176	-1.18241	-1.17818	-1.17403	-1.28905	-1.2846	-1.36232	-1.35755	-1.40818
-0.94861	-1.18122	-1.17702	-1.17293	-1.28785	-1.28344	-1.41686	-1.41196	-1.4572
-0.94783	-1.18005	-1.17588	-1.17182	-1.28665	-1.28228	-1.41557	-1.41066	-1.4559
-0.97752	-1.17899	-1.17482	-1.17077	-1.2855	-1.28114	-1.4143	-1.40936	-1.47979
-0.9768	-1.17801	-1.17384	-1.16978	-1.28438	-1.28002	-1.41303	-1.40805	-1.4784
-1.04543	-1.23602	-1.23165	-1.22741	-1.28333	-1.27898	-1.43649	-1.43143	-1.47706
-1.04447	-1.23495	-1.23065	-1.22648	-1.28238	-1.27803	-1.43538	-1.43025	-1.47576
-1.04352	-1.23388	-1.22966	-1.22555	-1.33983	-1.33532	-1.45988	-1.45464	-1.47452
-1.04265	-1.23282	-1.22863	-1.22454	-1.33869	-1.33418	-1.45865	-1.45345	-1.47334
-1.04181	-1.23179	-1.22762	-1.22354	-1.33754	-1.333	-1.45738	-1.45225	-1.47218
-1.14274	-1.26617	-1.26187	-1.25764	-1.41535	-1.41037	-1.45612	-1.45098	-1.47091
-1.14177	-1.2652	-1.26097	-1.25675	-1.41424	-1.40918	-1.45486	-1.44968	-1.4696
-1.14078	-1.32367	-1.31933	-1.31498	-1.43844	-1.4333	-1.47882	-1.47355	-1.51756
-1.13977	-1.32251	-1.31821	-1.31389	-1.43728	-1.4322	-1.4777	-1.47242	-1.51639
-1.19856	-1.34791	-1.34351	-1.33911	-1.43609	-1.43104	-1.47651	-1.47123	-1.51518
-1.19768	-1.34691	-1.34246	-1.338	-1.43485	-1.42978	-1.47518	-1.46989	-1.51385
-1.19682	-1.34592	-1.34143	-1.33692	-1.43363	-1.42853	-1.47386	-1.46856	-1.51253
-1.25739	-1.34486	-1.34038	-1.33588	-1.45803	-1.45285	-1.52186	-1.51643	-1.51126
-1.25641	-1.34379	-1.33934	-1.33485	-1.4569	-1.45172	-1.52065	-1.5152	-1.51004
-1.28293	-1.34278	-1.33829	-1.33376	-1.45569	-1.4505	-1.51942	-1.51404	-1.50892
-1.28202	-1.34181	-1.33724	-1.33264	-1.45443	-1.44923	-1.51818	-1.51294	-1.50789
-1.28107	-1.3408	-1.33621	-1.33156	-1.47833	-1.47305	-1.51697	-1.51185	-1.50686
-1.28007	-1.33975	-1.33518	-1.33056	-1.4772	-1.47189	-1.51581	-1.51077	-1.50582
-1.31305	-1.33872	-1.33417	-1.32957	-1.52526	-1.51982	-1.51464	-1.50967	-1.50476
-1.31209	-1.33774	-1.33319	-1.32858	-1.52406	-1.51858	-1.51342	-1.5085	-1.50364
-1.31114	-1.33675	-1.33219	-1.32756	-1.52285	-1.51735	-1.51221	-1.50733	-1.50251
-1.31014	-1.3629	-1.35821	-1.35348	-1.52161	-1.51618	-1.51108	-1.50623	-1.50143
-1.30911	-1.36176	-1.35704	-1.3523	-1.52037	-1.51504	-1.50999	-1.50517	-1.50037
-1.39191	-1.46424	-1.45908	-1.45394	-1.51921	-1.51394	-1.50892	-1.50409	-1.4993
-1.39084	-1.46314	-1.45798	-1.45284	-1.5181	-1.51288	-1.50786	-1.50302	-1.49822
-1.41619	-1.46205	-1.45688	-1.45174	-1.517	-1.51181	-1.50681	-1.50197	-1.49718
-1.41516	-1.46099	-1.45581	-1.45066	-1.51588	-1.51072	-1.50575	-1.50094	-1.49617
-1.41414	-1.50979	-1.50447	-1.49922	-1.51475	-1.50963	-1.5047	-1.49994	-1.49517
-1.41312	-1.50868	-1.50329	-1.498	-1.51359	-1.50857	-1.50369	-1.49894	-1.49416
-1.41209	-1.50754	-1.50209	-1.49677	-1.51244	-1.50753	-1.50269	-1.49794	-1.49314
-1.41097	-1.50634	-1.5009	-1.49561	-1.51133	-1.5065	-1.50168	-1.49691	-1.49209
-1.40981	-1.5051	-1.49971	-1.49449	-1.51027	-1.5055	-1.50068	-1.49586	-1.49102
-1.40867	-1.50392	-1.49859	-1.49343	-1.48864	-1.48402	-1.47932	-1.47459	-1.49001
-1.40753	-1.50279	-1.49754	-1.49245	-1.48767	-1.48305	-1.47836	-1.47364	-1.48905
-1.50694	-1.50168	-1.4965	-1.49147	-1.48669	-1.48205	-1.47736	-1.47266	-1.48808
-1.50582	-1.50059	-1.49547	-1.49049	-1.48569	-1.481	-1.47631	-1.47164	-1.48708
-1.50473	-1.49951	-1.49444	-1.48949	-1.48467	-1.47995	-1.47526	-1.47062	-1.48608
-1.50364	-1.49844	-1.49338	-1.48844	-1.48364	-1.47894	-1.47426	-1.46962	-1.5323
-1.50257	-1.49739	-1.49232	-1.48738	-1.48261	-1.47795	-1.47329	-1.46864	-1.53127
-1.5015	-1.49635	-1.4913	-1.48638	-1.48163	-1.47698	-1.47232	-1.46767	-1.53024
-1.50046	-1.49533	-1.49033	-1.48544	-1.48069	-1.47603	-1.47137	-1.46672	-1.52922
-1.49942	-1.49432	-1.48935	-1.48449	-1.47976	-1.4751	-1.47043	-1.46577	-1.50874
-1.4984	-1.49333	-1.48839	-1.48355	-1.47883	-1.47417	-1.46949	-1.46482	-1.50775
-1.4974	-1.49236	-1.48744	-1.48262	-1.4779	-1.47324	-1.46856	-1.46388	-1.50677
-1.49642	-1.49141	-1.48651	-1.48169	-1.47697	-1.47231	-1.46762	-1.46293	-1.50579

							-0.0588
							-0.05862
							-0.05844
							-0.05827
							-0.05809
					-0.05893	-0.05841	-0.17082
					-0.05875	-0.05823	-0.17048
					-0.05858	-0.05807	-0.21829
					-0.05845	-0.05793	-0.21798
					-0.05831	-0.0578	-0.21766
					-0.05814	-0.05765	-0.21731
					-0.05796	-0.05748	-0.21694
			-0.05882	-0.05828	-0.17062	-0.16964	-0.21658
			-0.05863	-0.0581	-0.17028	-0.16931	-0.21623
			-0.05847	-0.05794	-0.21806	-0.21698	-0.40773
			-0.05834	-0.05782	-0.21777	-0.2167	-0.40726
			-0.17148	-0.17044	-0.21746	-0.21639	-0.40678
			-0.17114	-0.17012	-0.21711	-0.21605	-0.40626
			-0.17079	-0.1698	-0.21676	-0.21571	-0.40573
			-0.17045	-0.16948	-0.21642	-0.2154	-0.54896
			-0.17012	-0.16917	-0.2161	-0.21509	-0.54828
-0.05891	-0.05835	-0.05783	-0.16983	-0.16889	-0.21579	-0.21478	-0.59202
-0.05876	-0.05822	-0.05771	-0.16958	-0.16864	-0.21549	-0.21447	-0.59128
-0.17234	-0.17129	-0.17027	-0.21727	-0.21623	-0.50588	-0.50384	-0.63455
-0.17198	-0.17095	-0.16996	-0.21693	-0.21589	-0.5052	-0.50323	-0.63383
-0.17163	-0.17063	-0.16966	-0.2166	-0.21557	-0.55002	-0.54791	-0.63311
-0.17132	-0.17034	-0.1694	-0.21632	-0.21529	-0.54936	-0.54726	-0.63236

	-0.17103	-0.17008	-0.16916	-0.21606	-0.21503	-0.54873	-0.54662	-0.63162
-0.05829	-0.17075	-0.16982	-0.16891	-0.21579	-0.21476	-0.54809	-0.54599	-0.65958
-0.05816	-0.17049	-0.16957	-0.16866	-0.21551	-0.21448	-0.54746	-0.54538	-0.65888
-0.17117	-0.21827	-0.21725	-0.21624	-0.5512	-0.54895	-0.63417	-0.63183	-0.65814
-0.17088	-0.21794	-0.21692	-0.21591	-0.55048	-0.54826	-0.6334	-0.63106	-0.65735
-0.17059	-0.21763	-0.21661	-0.21561	-0.54978	-0.5476	-0.66142	-0.659	-0.77807
-0.17032	-0.21735	-0.21634	-0.21534	-0.54913	-0.54698	-0.66072	-0.65833	-0.77732
-0.17005	-0.21707	-0.21606	-0.21506	-0.54848	-0.54636	-0.66002	-0.65765	-0.77656
-0.16975	-0.31924	-0.31786	-0.31649	-0.54777	-0.54567	-0.65925	-0.65692	-0.77576
-0.16945	-0.3188	-0.31743	-0.31608	-0.54706	-0.54497	-0.65847	-0.65617	-0.77495
-0.1692	-0.31842	-0.31706	-0.31571	-0.54643	-0.54435	-0.65774	-0.65543	-0.93394
-0.16897	-0.31807	-0.31672	-0.31537	-0.54585	-0.54378	-0.65705	-0.65469	-0.93291
-0.31906	-0.55177	-0.54956	-0.54739	-0.63241	-0.63006	-0.65639	-0.65402	-0.93195
-0.31871	-0.55116	-0.54897	-0.54681	-0.63176	-0.62942	-0.65575	-0.6534	-0.93107
-0.31836	-0.55054	-0.54836	-0.54622	-0.65987	-0.65747	-0.85531	-0.85237	-1.0267
-0.31799	-0.5499	-0.54774	-0.54562	-0.65918	-0.65679	-0.85448	-0.85156	-1.02569
-0.31762	-0.54926	-0.5471	-0.54499	-0.65846	-0.65609	-0.85362	-0.85071	-1.02467
-0.31724	-0.54859	-0.54643	-0.54432	-0.65769	-0.65533	-0.85269	-0.84981	-1.0236
-0.31684	-0.54792	-0.54575	-0.54364	-0.6569	-0.65456	-0.85174	-0.84888	-1.0225
-0.50481	-0.54729	-0.54513	-0.54303	-0.6562	-0.65387	-0.88492	-0.88198	-1.02144
-0.5042	-0.54668	-0.54455	-0.54247	-0.65557	-0.65325	-0.88409	-0.88115	-1.02042
-0.50361	-0.66217	-0.6597	-0.65729	-0.81455	-0.81176	-0.91806	-0.91504	-1.01943
-0.50304	-0.66147	-0.65904	-0.65665	-0.81379	-0.81101	-0.91724	-0.91422	-1.01848
-0.54701	-0.66079	-0.65838	-0.65601	-0.84789	-0.84501	-0.91643	-0.91341	-1.11984
-0.54642	-0.66012	-0.65772	-0.65538	-0.84711	-0.84425	-0.91562	-0.91259	-1.1187
-0.54584	-0.65945	-0.65707	-0.65474	-0.84633	-0.84348	-0.91481	-0.91177	-1.11756
-0.54527	-0.69789	-0.69544	-0.69304	-0.91782	-0.91459	-1.02183	-1.01822	-1.11641
-0.5447	-0.69722	-0.69478	-0.69239	-0.91695	-0.91373	-1.02088	-1.01727	-1.11527
-0.54414	-0.73373	-0.7312	-0.72871	-0.91609	-0.91288	-1.01993	-1.01632	-1.20259
-0.54357	-0.73305	-0.73054	-0.72808	-0.91524	-0.91202	-1.01898	-1.01537	-1.20133
-0.6977	-0.85108	-0.84819	-0.84533	-0.9486	-0.94531	-1.01803	-1.01442	-1.20011
-0.69702	-0.85029	-0.84742	-0.84457	-0.94774	-0.94444	-1.01707	-1.01349	-1.19892
-0.73355	-0.84951	-0.84665	-0.84381	-0.94688	-0.94357	-1.11806	-1.11385	-1.29311
-0.73285	-0.84872	-0.84588	-0.84305	-0.94601	-0.9427	-1.11692	-1.11276	-1.29187
-0.73217	-0.84793	-0.8451	-0.84229	-0.94514	-0.94184	-1.11578	-1.11167	-1.29064
-0.73149	-0.84714	-0.84433	-0.84152	-0.94427	-0.94097	-1.11464	-1.11058	-1.34065
-0.73082	-0.84637	-0.84356	-0.84075	-0.9434	-0.94011	-1.11351	-1.1095	-1.33936
-0.73016	-0.8456	-0.84279	-0.83998	-0.94253	-0.93926	-1.14611	-1.14204	-1.33808
-0.7295	-0.84483	-0.84202	-0.83921	-0.94167	-0.93842	-1.14501	-1.14095	-1.33681
-0.72885	-0.84407	-0.84125	-0.83844	-1.11854	-1.11432	-1.26073	-1.25625	-1.36603
-0.7282	-0.8433	-0.84047	-0.83766	-1.1174	-1.11324	-1.25952	-1.25507	-1.36477
-0.72758	-0.84256	-0.83971	-0.83689	-1.11627	-1.11216	-1.25832	-1.2539	-1.36351
-0.84476	-0.84186	-0.83897	-0.83613	-1.14891	-1.14475	-1.25711	-1.25273	-1.36226
-0.8441	-0.84118	-0.83826	-0.83538	-1.14775	-1.14365	-1.2559	-1.25156	-1.361
-0.87909	-0.9514	-0.94813	-0.9449	-1.20269	-1.19843	-1.36921	-1.36448	-1.35974
-0.8784	-0.9507	-0.94742	-0.94417	-1.20157	-1.19731	-1.36794	-1.36321	-1.35847
-0.87767	-0.98696	-0.98359	-0.98023	-1.20053	-1.19628	-1.36675	-1.36201	-1.41294
-0.87689	-0.98611	-0.98281	-0.97952	-1.1996	-1.19535	-1.36567	-1.36086	-1.41168
-0.91339	-1.18481	-1.18047	-1.17619	-1.29137	-1.28681	-1.36459	-1.35974	-1.41046
-0.91257	-1.18361	-1.17933	-1.17513	-1.29023	-1.28572	-1.36347	-1.35865	-1.40932

-0.91176	-1.18241	-1.17818	-1.17403	-1.28905	-1.2846	-1.36232	-1.35755	-1.40818
-0.94861	-1.18122	-1.17702	-1.17293	-1.28785	-1.28344	-1.41686	-1.41196	-1.4572
-0.94783	-1.18005	-1.17588	-1.17182	-1.28665	-1.28228	-1.41557	-1.41066	-1.4559
-0.97752	-1.17899	-1.17482	-1.17077	-1.2855	-1.28114	-1.4143	-1.40936	-1.47979
-0.9768	-1.17801	-1.17384	-1.16978	-1.28438	-1.28002	-1.41303	-1.40805	-1.4784
-1.04543	-1.23602	-1.23165	-1.22741	-1.28333	-1.27898	-1.43649	-1.43143	-1.47706
-1.04447	-1.23495	-1.23065	-1.22648	-1.28238	-1.27803	-1.43538	-1.43025	-1.47576
-1.04352	-1.23388	-1.22966	-1.22555	-1.33983	-1.33532	-1.45988	-1.45464	-1.47452
-1.04265	-1.23282	-1.22863	-1.22454	-1.33869	-1.33418	-1.45865	-1.45345	-1.47334
-1.04181	-1.23179	-1.22762	-1.22354	-1.33754	-1.333	-1.45738	-1.45225	-1.47218
-1.14274	-1.26617	-1.26187	-1.25764	-1.41535	-1.41037	-1.45612	-1.45098	-1.47091
-1.14177	-1.2652	-1.26097	-1.25675	-1.41424	-1.40918	-1.45486	-1.44968	-1.4696
-1.14078	-1.32367	-1.31933	-1.31498	-1.43844	-1.4333	-1.47882	-1.47355	-1.51756
-1.13977	-1.32251	-1.31821	-1.31389	-1.43728	-1.4322	-1.4777	-1.47242	-1.51639
-1.19856	-1.34791	-1.34351	-1.33911	-1.43609	-1.43104	-1.47651	-1.47123	-1.51518
-1.19768	-1.34691	-1.34246	-1.338	-1.43485	-1.42978	-1.47518	-1.46989	-1.51385
-1.19682	-1.34592	-1.34143	-1.33692	-1.43363	-1.42853	-1.47386	-1.46856	-1.51253
-1.25739	-1.34486	-1.34038	-1.33588	-1.45803	-1.45285	-1.52186	-1.51643	-1.51126
-1.25641	-1.34379	-1.33934	-1.33485	-1.4569	-1.45172	-1.52065	-1.5152	-1.51004
-1.28293	-1.34278	-1.33829	-1.33376	-1.45569	-1.4505	-1.51942	-1.51404	-1.50892
-1.28202	-1.34181	-1.33724	-1.33264	-1.45443	-1.44923	-1.51818	-1.51294	-1.50789
-1.28107	-1.3408	-1.33621	-1.33156	-1.47833	-1.47305	-1.51697	-1.51185	-1.50686
-1.28007	-1.33975	-1.33518	-1.33056	-1.4772	-1.47189	-1.51581	-1.51077	-1.50582
-1.31305	-1.33872	-1.33417	-1.32957	-1.52526	-1.51982	-1.51464	-1.50967	-1.50476
-1.31209	-1.33774	-1.33319	-1.32858	-1.52406	-1.51858	-1.51342	-1.5085	-1.50364
-1.31114	-1.33675	-1.33219	-1.32756	-1.52285	-1.51735	-1.51221	-1.50733	-1.50251
-1.31014	-1.3629	-1.35821	-1.35348	-1.52161	-1.51618	-1.51108	-1.50623	-1.50143
-1.30911	-1.36176	-1.35704	-1.3523	-1.52037	-1.51504	-1.50999	-1.50517	-1.50037
-1.39191	-1.46424	-1.45908	-1.45394	-1.51921	-1.51394	-1.50892	-1.50409	-1.4993
-1.39084	-1.46314	-1.45798	-1.45284	-1.5181	-1.51288	-1.50786	-1.50302	-1.49822
-1.41619	-1.46205	-1.45688	-1.45174	-1.517	-1.51181	-1.50681	-1.50197	-1.49718
-1.41516	-1.46099	-1.45581	-1.45066	-1.51588	-1.51072	-1.50575	-1.50094	-1.49617
-1.41414	-1.50979	-1.50447	-1.49922	-1.51475	-1.50963	-1.5047	-1.49994	-1.49517
-1.41312	-1.50868	-1.50329	-1.498	-1.51359	-1.50857	-1.50369	-1.49894	-1.49416
-1.41209	-1.50754	-1.50209	-1.49677	-1.51244	-1.50753	-1.50269	-1.49794	-1.49314
-1.41097	-1.50634	-1.5009	-1.49561	-1.51133	-1.5065	-1.50168	-1.49691	-1.49209
-1.40981	-1.5051	-1.49971	-1.49449	-1.51027	-1.5055	-1.50068	-1.49586	-1.49102
-1.40867	-1.50392	-1.49859	-1.49343	-1.48864	-1.48402	-1.47932	-1.47459	-1.49001
-1.40753	-1.50279	-1.49754	-1.49245	-1.48767	-1.48305	-1.47836	-1.47364	-1.48905
-1.50694	-1.50168	-1.4965	-1.49147	-1.48669	-1.48205	-1.47736	-1.47266	-1.48808
-1.50582	-1.50059	-1.49547	-1.49049	-1.48569	-1.481	-1.47631	-1.47164	-1.48708
-1.50473	-1.49951	-1.49444	-1.48949	-1.48467	-1.47995	-1.47526	-1.47062	-1.48608
-1.50364	-1.49844	-1.49338	-1.48844	-1.48364	-1.47894	-1.47426	-1.46962	-1.5323
-1.50257	-1.49739	-1.49232	-1.48738	-1.48261	-1.47795	-1.47329	-1.46864	-1.53127
-1.5015	-1.49635	-1.4913	-1.48638	-1.48163	-1.47698	-1.47232	-1.46767	-1.53024
-1.50046	-1.49533	-1.49033	-1.48544	-1.48069	-1.47603	-1.47137	-1.46672	-1.52922
-1.49942	-1.49432	-1.48935	-1.48449	-1.47976	-1.4751	-1.47043	-1.46577	-1.50874
-1.4984	-1.49333	-1.48839	-1.48355	-1.47883	-1.47417	-1.46949	-1.46482	-1.50775
-1.4974	-1.49236	-1.48744	-1.48262	-1.4779	-1.47324	-1.46856	-1.46388	-1.50677
-1.49642	-1.49141	-1.48651	-1.48169	-1.47697	-1.47231	-1.46762	-1.46293	-1.50579

-0.10768	-0.10719	-0.10673	-0.10629	-0.15253
-0.10783	-0.10735	-0.10689	-0.10644	-0.15271
-0.108	-0.10752	-0.10705	-0.10659	-0.1529
-0.05297	-0.05271	-0.10722	-0.10676	-0.15309
-0.05307	-0.05282	-0.1074	-0.10692	-0.15328
		-0.10757	-0.10709	-0.10663
		-0.10773	-0.10725	-0.10679
		-0.05281	-0.05255	-0.10696
		-0.0529	-0.05264	-0.10713
				-0.1073
				-0.10748
				-0.10765
				-0.05276
				-0.05284

-0.05899
-0.05883
-0.05866
-0.0585

								-0.05834
								-0.05815
								-0.05795
						-0.05873	-0.05824	-0.2094
						-0.05856	-0.05807	-0.20905
						-0.0584	-0.0579	-0.20872
						-0.05823	-0.05774	-0.20838
						-0.05806	-0.05757	-0.20804
						-0.10797	-0.10726	-0.30662
						-0.10772	-0.10703	-0.30615
						-0.16989	-0.1689	-0.35552
						-0.16955	-0.16857	-0.355
				-0.10844	-0.10772	-0.30753	-0.30613	-0.35448
				-0.10819	-0.10749	-0.30706	-0.30567	-0.35397
				-0.17055	-0.16955	-0.3565	-0.35497	-0.54582
				-0.17021	-0.16921	-0.35598	-0.35445	-0.5451
				-0.16986	-0.16888	-0.35546	-0.35393	-0.54439
				-0.16952	-0.16855	-0.35494	-0.35341	-0.54367
				-0.16919	-0.16822	-0.35442	-0.35288	-0.54296
	-0.1719	-0.17087	-0.16985	-0.30703	-0.30565	-0.35391	-0.35239	-0.54227
	-0.17155	-0.17053	-0.16952	-0.30656	-0.30519	-0.35342	-0.35193	-0.54162
	-0.1712	-0.17018	-0.16918	-0.35591	-0.3544	-0.54491	-0.5429	-0.76709
	-0.17083	-0.16983	-0.16885	-0.35539	-0.35388	-0.5442	-0.54223	-0.76614
-0.05828	-0.21865	-0.21753	-0.21644	-0.35488	-0.35337	-0.54351	-0.54158	-0.76521
-0.05811	-0.21827	-0.21716	-0.21608	-0.35437	-0.35287	-0.54282	-0.54095	-0.76431
-0.05794	-0.2179	-0.21679	-0.21571	-0.35386	-0.35239	-0.54216	-0.54033	-0.76341
-0.05776	-0.21753	-0.21643	-0.21536	-0.45185	-0.45013	-0.63593	-0.63375	-0.76249
-0.05759	-0.21717	-0.21608	-0.21502	-0.45128	-0.44961	-0.6352	-0.633	-0.76158
-0.16983	-0.30705	-0.30568	-0.30433	-0.45074	-0.44909	-0.63444	-0.63222	-0.76066
-0.16953	-0.30665	-0.30529	-0.30395	-0.45022	-0.44857	-0.63366	-0.63143	-0.75974
-0.21723	-0.4549	-0.45312	-0.45137	-0.63746	-0.63514	-0.76416	-0.76147	-0.75883
-0.2169	-0.45431	-0.45252	-0.45076	-0.63663	-0.63434	-0.76322	-0.76055	-0.75792
-0.21657	-0.45372	-0.45192	-0.45016	-0.6358	-0.63354	-0.7623	-0.75965	-0.75703
-0.21622	-0.45312	-0.45135	-0.44962	-0.635	-0.63275	-0.76137	-0.75877	-0.90556
-0.21585	-0.45252	-0.45079	-0.4491	-0.63424	-0.63198	-0.76047	-0.75791	-0.90456
-0.2155	-0.45193	-0.45023	-0.44859	-0.67581	-0.6734	-0.75961	-0.75705	-0.96135
-0.21515	-0.45134	-0.44968	-0.44808	-0.67506	-0.67266	-0.75879	-0.7562	-0.96024
-0.40603	-0.59303	-0.59087	-0.58877	-0.76323	-0.76057	-0.7842	-0.78152	-0.95912
-0.40557	-0.59234	-0.59016	-0.58805	-0.76231	-0.75968	-0.7833	-0.78064	-0.958
-0.40511	-0.63511	-0.63275	-0.63044	-0.7614	-0.75878	-0.96354	-0.96023	-1.08962
-0.40461	-0.63434	-0.63199	-0.62969	-0.7605	-0.75789	-0.96241	-0.95913	-1.08834
-0.40413	-0.63359	-0.63125	-0.62895	-0.75963	-0.75701	-0.9613	-0.95804	-1.08705
-0.54686	-0.67758	-0.67512	-0.67269	-0.78512	-0.78243	-0.96024	-0.95695	-1.08571
-0.54623	-0.67684	-0.67438	-0.67196	-0.7843	-0.78161	-0.95921	-0.95587	-1.08435
-0.58982	-0.67605	-0.67361	-0.67119	-0.85792	-0.85501	-1.0212	-1.01749	-1.20675
-0.58909	-0.67524	-0.6728	-0.67039	-0.85698	-0.85407	-1.02004	-1.01633	-1.20523
-0.63223	-0.78998	-0.78719	-0.78443	-0.85606	-0.85314	-1.01889	-1.01518	-1.20376
-0.63153	-0.78911	-0.78634	-0.7836	-0.85516	-0.85224	-1.01777	-1.01408	-1.20234
-0.63082	-0.86303	-0.8601	-0.85718	-1.02405	-1.02034	-1.08613	-1.082	-1.23201
-0.63008	-0.86209	-0.85917	-0.85626	-1.0229	-1.0192	-1.08483	-1.08072	-1.23056

-0.62933	-0.86116	-0.85824	-0.85533	-1.02175	-1.01805	-1.08354	-1.07945	-1.22913
-0.65719	-0.86026	-0.85734	-0.85443	-1.02063	-1.01693	-1.17485	-1.17032	-1.29079
-0.65649	-0.85937	-0.85645	-0.85353	-1.01952	-1.01583	-1.17347	-1.16901	-1.28939
-0.65575	-0.85845	-0.85553	-0.85261	-1.0184	-1.01471	-1.2037	-1.19919	-1.28793
-0.65498	-0.85751	-0.8546	-0.85168	-1.01725	-1.01356	-1.20221	-1.19777	-1.28643
-0.77539	-0.99746	-0.99391	-0.99038	-1.08427	-1.08014	-1.2008	-1.19641	-1.28499
-0.77463	-0.99642	-0.99287	-0.98935	-1.08307	-1.07895	-1.19949	-1.19514	-1.28364
-0.77389	-0.9954	-0.99186	-0.98834	-1.08189	-1.07779	-1.19823	-1.1939	-1.28231
-0.77311	-0.99441	-0.99087	-0.98735	-1.17689	-1.17231	-1.261	-1.25639	-1.31138
-0.77232	-0.99341	-0.98988	-0.98636	-1.17557	-1.17106	-1.2597	-1.2551	-1.31004
-0.93083	-1.05279	-1.04902	-1.04528	-1.20337	-1.19886	-1.2583	-1.25371	-1.30861
-0.92981	-1.05161	-1.04784	-1.0441	-1.20188	-1.19746	-1.25684	-1.25228	-1.30714
-0.92885	-1.05048	-1.04671	-1.04297	-1.22907	-1.22457	-1.3151	-1.31041	-1.33661
-0.92795	-1.04939	-1.04563	-1.04191	-1.22773	-1.22328	-1.31374	-1.30908	-1.33528
-1.02308	-1.08565	-1.08148	-1.07739	-1.22643	-1.22201	-1.3124	-1.30775	-1.33395
-1.02208	-1.08448	-1.08035	-1.07629	-1.22518	-1.22077	-1.31106	-1.30642	-1.3326
-1.02106	-1.08329	-1.0792	-1.07519	-1.22392	-1.21952	-1.30971	-1.30506	-1.33122
-1.01998	-1.2076	-1.20291	-1.19834	-1.28896	-1.28433	-1.33917	-1.33447	-1.35321
-1.01888	-1.20607	-1.20144	-1.19695	-1.2875	-1.2829	-1.3377	-1.33299	-1.35169
-1.01783	-1.20464	-1.20007	-1.19564	-1.28613	-1.28155	-1.33632	-1.3316	-1.37586
-1.01681	-1.20329	-1.19877	-1.19438	-1.28483	-1.28027	-1.335	-1.33029	-1.3745
-1.01583	-1.20201	-1.19752	-1.19317	-1.28356	-1.27902	-1.35722	-1.35242	-1.42802
-1.01489	-1.20081	-1.19634	-1.192	-1.28232	-1.27779	-1.35594	-1.35114	-1.42667
-1.11562	-1.29511	-1.29033	-1.2857	-1.31249	-1.30791	-1.38029	-1.37543	-1.42533
-1.11451	-1.29385	-1.28909	-1.28448	-1.31126	-1.30667	-1.37898	-1.37412	-1.42397
-1.11341	-1.29259	-1.28787	-1.28327	-1.31004	-1.30544	-1.37767	-1.37281	-1.42261
-1.1123	-1.29132	-1.28665	-1.28207	-1.33277	-1.32809	-1.43124	-1.42624	-1.47239
-1.11119	-1.29006	-1.28543	-1.28088	-1.33154	-1.32685	-1.42989	-1.42487	-1.47097
-1.1981	-1.28879	-1.28421	-1.27968	-1.38346	-1.37862	-1.42853	-1.42347	-1.49287
-1.19688	-1.28753	-1.28298	-1.27849	-1.38219	-1.37734	-1.42717	-1.42207	-1.49138
-1.19569	-1.33747	-1.33281	-1.3282	-1.38089	-1.37604	-1.42577	-1.42061	-1.48984
-1.19453	-1.3362	-1.33155	-1.32695	-1.37958	-1.37469	-1.42433	-1.4191	-1.48824
-1.28843	-1.36543	-1.36074	-1.35607	-1.37825	-1.37333	-1.44703	-1.44171	-1.48667
-1.28722	-1.36418	-1.35949	-1.35479	-1.3769	-1.37191	-1.44552	-1.44023	-1.48519
-1.28602	-1.36294	-1.35824	-1.35352	-1.37556	-1.37051	-1.44404	-1.4388	-1.48376
-1.33593	-1.36169	-1.35698	-1.35224	-1.42895	-1.42376	-1.49297	-1.48759	-1.50676
-1.33468	-1.36044	-1.35572	-1.35097	-1.42764	-1.42251	-1.49169	-1.4863	-1.50545
-1.33343	-1.3592	-1.35446	-1.34969	-1.45048	-1.44534	-1.49034	-1.48499	-1.50414
-1.33219	-1.35795	-1.3532	-1.34841	-1.4491	-1.44395	-1.48892	-1.48365	-1.50283
-1.36137	-1.35669	-1.35193	-1.34713	-1.44771	-1.44255	-1.5119	-1.50663	-1.50154
-1.36009	-1.3554	-1.35064	-1.34584	-1.44632	-1.44116	-1.51046	-1.50526	-1.50023
-1.35882	-1.35411	-1.34934	-1.34454	-1.44494	-1.43979	-1.50904	-1.50393	-1.49896
-1.35755	-1.45876	-1.45372	-1.44866	-1.44356	-1.43846	-1.5077	-1.50269	-1.49778
-1.35628	-1.45738	-1.45232	-1.44725	-1.44219	-1.43716	-1.5064	-1.50149	-1.49665
-1.355	-1.456	-1.45092	-1.44585	-1.46595	-1.46091	-1.50508	-1.50025	-1.49546
-1.35372	-1.45462	-1.44952	-1.44444	-1.46455	-1.45959	-1.50376	-1.49897	-1.49423
-1.408	-1.45324	-1.44812	-1.44303	-1.51238	-1.50733	-1.50244	-1.49769	-1.49299
-1.40667	-1.45184	-1.4467	-1.44162	-1.51093	-1.50596	-1.50111	-1.49639	-1.49172
-1.40539	-1.4757	-1.47047	-1.46532	-1.50951	-1.50459	-1.49978	-1.4951	-1.49045
-1.40418	-1.47437	-1.46912	-1.46396	-1.50812	-1.50323	-1.49846	-1.4938	-1.48917

-1.403	-1.4731	-1.46783	-1.46267	-1.5068	-1.50192	-1.49715	-1.49252	-1.48789
-1.45198	-1.4719	-1.46667	-1.46154	-1.50562	-1.50071	-1.49592	-1.49125	-1.4866
-1.45076	-1.47074	-1.46558	-1.46049	-1.50452	-1.49957	-1.49473	-1.49001	-1.48532
-1.4746	-1.46946	-1.46437	-1.45935	-1.50339	-1.49847	-1.49362	-1.48884	-1.48411
-1.4732	-1.46808	-1.46307	-1.45816	-1.50224	-1.49741	-1.49257	-1.48774	-1.48295
-1.47183	-1.46671	-1.46176	-1.45693	-1.50104	-1.49628	-1.49148	-1.48665	-1.48184
-1.47049	-1.46536	-1.46043	-1.45565	-1.49977	-1.49506	-1.49032	-1.48557	-1.48081
-1.46921	-1.51321	-1.50818	-1.50329	-1.49852	-1.49384	-1.48915	-1.48445	-1.47973
-1.46806	-1.51203	-1.50699	-1.5021	-1.49733	-1.49265	-1.48796	-1.48325	-1.47853
-1.46694	-1.51089	-1.50586	-1.50096	-1.49618	-1.4915	-1.48678	-1.48203	-1.47728
-1.46572	-1.50969	-1.50471	-1.49986	-1.4951	-1.49042	-1.48568	-1.48088	-1.4761
-1.46446	-1.50847	-1.50357	-1.49879	-1.49407	-1.4894	-1.48464	-1.47978	-1.47495
-1.51235	-1.50728	-1.50243	-1.49768	-1.49297	-1.48829	-1.48352	-1.47867	-1.47383
-1.51119	-1.50613	-1.50127	-1.49652	-1.49179	-1.4871	-1.48235	-1.47754	-1.47276
-1.51002	-1.505	-1.50016	-1.49541	-1.49065	-1.48592	-1.5305	-1.52556	-1.52064
-1.50882	-1.5039	-1.49911	-1.49438	-1.4896	-1.4848	-1.5293	-1.52434	-1.51942
-1.50761	-1.5028	-1.49807	-1.49336	-1.48855	-1.48369	-1.52813	-1.52314	-1.51821
-1.50639	-1.50163	-1.49694	-1.49226	-1.48746	-1.48261	-1.52701	-1.52203	-1.5171
-1.50519	-1.50045	-1.49579	-1.49114	-1.48637	-1.48153	-1.52592	-1.52094	-1.51602
-1.50409	-1.49935	-1.49471	-1.49007	-1.48528	-1.48042	-1.52476	-1.51977	-1.51487
-1.50306	-1.49832	-1.49367	-1.48903	-1.4842	-1.47929	-1.52355	-1.51854	-1.51366
-1.50202	-1.49726	-1.4926	-1.48793	-1.48309	-1.47817	-1.52239	-1.51738	-1.5578
-1.50098	-1.49619	-1.49148	-1.48677	-1.48194	-1.47706	-1.52127	-1.5163	-1.5567
-1.49992	-1.49513	-1.49038	-1.48563	-1.4808	-1.47594	-1.52014	-1.51521	-1.55561
-1.49885	-1.49408	-1.48932	-1.48455	-1.4797	-1.4748	-1.51898	-1.51409	-1.55449
-1.49777	-1.49303	-1.48827	-1.48348	-1.4786	-1.47368	-1.51782	-1.51297	-1.55339
-1.49668	-1.49193	-1.48714	-1.48233	-1.47747	-1.47259	-1.51461	-1.50984	-1.50519
-1.49559	-1.49081	-1.48599	-1.48116	-1.47633	-1.47152	-1.51357	-1.50882	-1.50419
-1.49453	-1.48976	-1.48492	-1.48008	-1.47526	-1.47047	-1.5125	-1.50778	-1.50316
-1.4935	-1.48875	-1.48391	-1.47906	-1.47422	-1.46942	-1.51142	-1.50671	-1.5021
-1.49247	-1.48773	-1.4829	-1.47804	-1.52012	-1.51516	-1.51034	-1.50566	-1.50108
-1.49144	-1.48668	-1.48187	-1.47703	-1.51905	-1.51405	-1.50925	-1.50464	-1.5001
-1.4904	-1.48561	-1.4808	-1.47598	-1.51797	-1.51296	-1.50818	-1.50363	-1.5264
-1.48933	-1.4845	-1.47966	-1.47484	-1.51685	-1.51193	-1.5072	-1.50267	-1.52542
-1.48826	-1.48338	-1.47851	-1.47369	-1.51573	-1.51092	-1.50625	-1.50171	-1.52445
-1.48719	-1.52946	-1.52446	-1.51952	-1.51467	-1.50992	-1.50526	-1.50071	-1.52344
-1.48614	-1.5284	-1.52341	-1.51847	-1.51365	-1.50892	-1.50427	-1.49968	-1.52241
-1.48512	-1.52735	-1.52236	-1.51742	-1.51261	-1.5079	-1.50326	-1.49869	-1.52144
-1.48414	-1.52632	-1.52132	-1.51638	-1.51157	-1.50686	-1.50225	-1.49771	-1.5205
-1.48317	-1.52531	-1.52028	-1.51532	-1.51052	-1.50584	-1.50126	-1.49676	-1.51958
-1.4822	-1.52431	-1.51922	-1.5142	-1.50943	-1.50483	-1.5003	-1.49583	-1.51868
-1.48123	-1.52332	-1.51818	-1.51312	-1.50838	-1.50386	-1.49937	-1.49493	-1.51779
-1.52733	-1.52232	-1.5172	-1.51217	-1.50745	-1.50294	-1.49847	-1.49406	-1.51694
-1.52629	-1.5213	-1.51624	-1.51127	-1.50656	-1.50205	-1.4976	-1.49322	-1.51611
-1.52522	-1.52021	-1.51522	-1.51033	-1.50566	-1.50116	-1.49675	-1.49241	-1.51532
-1.52412	-1.51908	-1.51417	-1.50938	-1.50476	-1.50029	-1.49591	-1.49162	-1.51457
-1.50367	-1.51801	-1.51315	-1.50844	-1.50385	-1.49941	-1.49507	-1.49082	-1.5138
-1.50267	-1.51701	-1.51219	-1.5075	-1.50295	-1.49853	-1.49422	-1.49	-1.51302
-1.50169	-1.51604	-1.51124	-1.50659	-1.50206	-1.49766	-1.49338	-1.48921	-1.51227
-1.50072	-1.51508	-1.51032	-1.50571	-1.50119	-1.4968	-1.49257	-1.48848	-1.51158

-0.05899
-0.05883
-0.05866
-0.0585

								-0.05834
								-0.05815
								-0.05795
						-0.05873	-0.05824	-0.2094
						-0.05856	-0.05807	-0.20905
						-0.0584	-0.0579	-0.20872
						-0.05823	-0.05774	-0.20838
						-0.05806	-0.05757	-0.20804
						-0.10797	-0.10726	-0.30662
						-0.10772	-0.10703	-0.30615
						-0.16989	-0.1689	-0.35552
						-0.16955	-0.16857	-0.355
				-0.10844	-0.10772	-0.30753	-0.30613	-0.35448
				-0.10819	-0.10749	-0.30706	-0.30567	-0.35397
				-0.17055	-0.16955	-0.3565	-0.35497	-0.54582
				-0.17021	-0.16921	-0.35598	-0.35445	-0.5451
				-0.16986	-0.16888	-0.35546	-0.35393	-0.54439
				-0.16952	-0.16855	-0.35494	-0.35341	-0.54367
				-0.16919	-0.16822	-0.35442	-0.35288	-0.54296
	-0.1719	-0.17087	-0.16985	-0.30703	-0.30565	-0.35391	-0.35239	-0.54227
	-0.17155	-0.17053	-0.16952	-0.30656	-0.30519	-0.35342	-0.35193	-0.54162
	-0.1712	-0.17018	-0.16918	-0.35591	-0.3544	-0.54491	-0.5429	-0.76709
	-0.17083	-0.16983	-0.16885	-0.35539	-0.35388	-0.5442	-0.54223	-0.76614
-0.05828	-0.21865	-0.21753	-0.21644	-0.35488	-0.35337	-0.54351	-0.54158	-0.76521
-0.05811	-0.21827	-0.21716	-0.21608	-0.35437	-0.35287	-0.54282	-0.54095	-0.76431
-0.05794	-0.2179	-0.21679	-0.21571	-0.35386	-0.35239	-0.54216	-0.54033	-0.76341
-0.05776	-0.21753	-0.21643	-0.21536	-0.45185	-0.45013	-0.63593	-0.63375	-0.76249
-0.05759	-0.21717	-0.21608	-0.21502	-0.45128	-0.44961	-0.6352	-0.633	-0.76158
-0.16983	-0.30705	-0.30568	-0.30433	-0.45074	-0.44909	-0.63444	-0.63222	-0.76066
-0.16953	-0.30665	-0.30529	-0.30395	-0.45022	-0.44857	-0.63366	-0.63143	-0.75974
-0.21723	-0.4549	-0.45312	-0.45137	-0.63746	-0.63514	-0.76416	-0.76147	-0.75883
-0.2169	-0.45431	-0.45252	-0.45076	-0.63663	-0.63434	-0.76322	-0.76055	-0.75792
-0.21657	-0.45372	-0.45192	-0.45016	-0.6358	-0.63354	-0.7623	-0.75965	-0.75703
-0.21622	-0.45312	-0.45135	-0.44962	-0.635	-0.63275	-0.76137	-0.75877	-0.90556
-0.21585	-0.45252	-0.45079	-0.4491	-0.63424	-0.63198	-0.76047	-0.75791	-0.90456
-0.2155	-0.45193	-0.45023	-0.44859	-0.67581	-0.6734	-0.75961	-0.75705	-0.96135
-0.21515	-0.45134	-0.44968	-0.44808	-0.67506	-0.67266	-0.75879	-0.7562	-0.96024
-0.40603	-0.59303	-0.59087	-0.58877	-0.76323	-0.76057	-0.7842	-0.78152	-0.95912
-0.40557	-0.59234	-0.59016	-0.58805	-0.76231	-0.75968	-0.7833	-0.78064	-0.958
-0.40511	-0.63511	-0.63275	-0.63044	-0.7614	-0.75878	-0.96354	-0.96023	-1.08962
-0.40461	-0.63434	-0.63199	-0.62969	-0.7605	-0.75789	-0.96241	-0.95913	-1.08834
-0.40413	-0.63359	-0.63125	-0.62895	-0.75963	-0.75701	-0.9613	-0.95804	-1.08705
-0.54686	-0.67758	-0.67512	-0.67269	-0.78512	-0.78243	-0.96024	-0.95695	-1.08571
-0.54623	-0.67684	-0.67438	-0.67196	-0.7843	-0.78161	-0.95921	-0.95587	-1.08435
-0.58982	-0.67605	-0.67361	-0.67119	-0.85792	-0.85501	-1.0212	-1.01749	-1.20675
-0.58909	-0.67524	-0.6728	-0.67039	-0.85698	-0.85407	-1.02004	-1.01633	-1.20523
-0.63223	-0.78998	-0.78719	-0.78443	-0.85606	-0.85314	-1.01889	-1.01518	-1.20376
-0.63153	-0.78911	-0.78634	-0.7836	-0.85516	-0.85224	-1.01777	-1.01408	-1.20234
-0.63082	-0.86303	-0.8601	-0.85718	-1.02405	-1.02034	-1.08613	-1.082	-1.23201
-0.63008	-0.86209	-0.85917	-0.85626	-1.0229	-1.0192	-1.08483	-1.08072	-1.23056

-0.62933	-0.86116	-0.85824	-0.85533	-1.02175	-1.01805	-1.08354	-1.07945	-1.22913
-0.65719	-0.86026	-0.85734	-0.85443	-1.02063	-1.01693	-1.17485	-1.17032	-1.29079
-0.65649	-0.85937	-0.85645	-0.85353	-1.01952	-1.01583	-1.17347	-1.16901	-1.28939
-0.65575	-0.85845	-0.85553	-0.85261	-1.0184	-1.01471	-1.2037	-1.19919	-1.28793
-0.65498	-0.85751	-0.8546	-0.85168	-1.01725	-1.01356	-1.20221	-1.19777	-1.28643
-0.77539	-0.99746	-0.99391	-0.99038	-1.08427	-1.08014	-1.2008	-1.19641	-1.28499
-0.77463	-0.99642	-0.99287	-0.98935	-1.08307	-1.07895	-1.19949	-1.19514	-1.28364
-0.77389	-0.9954	-0.99186	-0.98834	-1.08189	-1.07779	-1.19823	-1.1939	-1.28231
-0.77311	-0.99441	-0.99087	-0.98735	-1.17689	-1.17231	-1.261	-1.25639	-1.31138
-0.77232	-0.99341	-0.98988	-0.98636	-1.17557	-1.17106	-1.2597	-1.2551	-1.31004
-0.93083	-1.05279	-1.04902	-1.04528	-1.20337	-1.19886	-1.2583	-1.25371	-1.30861
-0.92981	-1.05161	-1.04784	-1.0441	-1.20188	-1.19746	-1.25684	-1.25228	-1.30714
-0.92885	-1.05048	-1.04671	-1.04297	-1.22907	-1.22457	-1.3151	-1.31041	-1.33661
-0.92795	-1.04939	-1.04563	-1.04191	-1.22773	-1.22328	-1.31374	-1.30908	-1.33528
-1.02308	-1.08565	-1.08148	-1.07739	-1.22643	-1.22201	-1.3124	-1.30775	-1.33395
-1.02208	-1.08448	-1.08035	-1.07629	-1.22518	-1.22077	-1.31106	-1.30642	-1.3326
-1.02106	-1.08329	-1.0792	-1.07519	-1.22392	-1.21952	-1.30971	-1.30506	-1.33122
-1.01998	-1.2076	-1.20291	-1.19834	-1.28896	-1.28433	-1.33917	-1.33447	-1.35321
-1.01888	-1.20607	-1.20144	-1.19695	-1.2875	-1.2829	-1.3377	-1.33299	-1.35169
-1.01783	-1.20464	-1.20007	-1.19564	-1.28613	-1.28155	-1.33632	-1.3316	-1.37586
-1.01681	-1.20329	-1.19877	-1.19438	-1.28483	-1.28027	-1.335	-1.33029	-1.3745
-1.01583	-1.20201	-1.19752	-1.19317	-1.28356	-1.27902	-1.35722	-1.35242	-1.42802
-1.01489	-1.20081	-1.19634	-1.192	-1.28232	-1.27779	-1.35594	-1.35114	-1.42667
-1.11562	-1.29511	-1.29033	-1.2857	-1.31249	-1.30791	-1.38029	-1.37543	-1.42533
-1.11451	-1.29385	-1.28909	-1.28448	-1.31126	-1.30667	-1.37898	-1.37412	-1.42397
-1.11341	-1.29259	-1.28787	-1.28327	-1.31004	-1.30544	-1.37767	-1.37281	-1.42261
-1.1123	-1.29132	-1.28665	-1.28207	-1.33277	-1.32809	-1.43124	-1.42624	-1.47239
-1.11119	-1.29006	-1.28543	-1.28088	-1.33154	-1.32685	-1.42989	-1.42487	-1.47097
-1.1981	-1.28879	-1.28421	-1.27968	-1.38346	-1.37862	-1.42853	-1.42347	-1.49287
-1.19688	-1.28753	-1.28298	-1.27849	-1.38219	-1.37734	-1.42717	-1.42207	-1.49138
-1.19569	-1.33747	-1.33281	-1.3282	-1.38089	-1.37604	-1.42577	-1.42061	-1.48984
-1.19453	-1.3362	-1.33155	-1.32695	-1.37958	-1.37469	-1.42433	-1.4191	-1.48824
-1.28843	-1.36543	-1.36074	-1.35607	-1.37825	-1.37333	-1.44703	-1.44171	-1.48667
-1.28722	-1.36418	-1.35949	-1.35479	-1.3769	-1.37191	-1.44552	-1.44023	-1.48519
-1.28602	-1.36294	-1.35824	-1.35352	-1.37556	-1.37051	-1.44404	-1.4388	-1.48376
-1.33593	-1.36169	-1.35698	-1.35224	-1.42895	-1.42376	-1.49297	-1.48759	-1.50676
-1.33468	-1.36044	-1.35572	-1.35097	-1.42764	-1.42251	-1.49169	-1.4863	-1.50545
-1.33343	-1.3592	-1.35446	-1.34969	-1.45048	-1.44534	-1.49034	-1.48499	-1.50414
-1.33219	-1.35795	-1.3532	-1.34841	-1.4491	-1.44395	-1.48892	-1.48365	-1.50283
-1.36137	-1.35669	-1.35193	-1.34713	-1.44771	-1.44255	-1.5119	-1.50663	-1.50154
-1.36009	-1.3554	-1.35064	-1.34584	-1.44632	-1.44116	-1.51046	-1.50526	-1.50023
-1.35882	-1.35411	-1.34934	-1.34454	-1.44494	-1.43979	-1.50904	-1.50393	-1.49896
-1.35755	-1.45876	-1.45372	-1.44866	-1.44356	-1.43846	-1.5077	-1.50269	-1.49778
-1.35628	-1.45738	-1.45232	-1.44725	-1.44219	-1.43716	-1.5064	-1.50149	-1.49665
-1.355	-1.456	-1.45092	-1.44585	-1.46595	-1.46091	-1.50508	-1.50025	-1.49546
-1.35372	-1.45462	-1.44952	-1.44444	-1.46455	-1.45959	-1.50376	-1.49897	-1.49423
-1.408	-1.45324	-1.44812	-1.44303	-1.51238	-1.50733	-1.50244	-1.49769	-1.49299
-1.40667	-1.45184	-1.4467	-1.44162	-1.51093	-1.50596	-1.50111	-1.49639	-1.49172
-1.40539	-1.4757	-1.47047	-1.46532	-1.50951	-1.50459	-1.49978	-1.4951	-1.49045
-1.40418	-1.47437	-1.46912	-1.46396	-1.50812	-1.50323	-1.49846	-1.4938	-1.48917

-1.403	-1.4731	-1.46783	-1.46267	-1.5068	-1.50192	-1.49715	-1.49252	-1.48789
-1.45198	-1.4719	-1.46667	-1.46154	-1.50562	-1.50071	-1.49592	-1.49125	-1.4866
-1.45076	-1.47074	-1.46558	-1.46049	-1.50452	-1.49957	-1.49473	-1.49001	-1.48532
-1.4746	-1.46946	-1.46437	-1.45935	-1.50339	-1.49847	-1.49362	-1.48884	-1.48411
-1.4732	-1.46808	-1.46307	-1.45816	-1.50224	-1.49741	-1.49257	-1.48774	-1.48295
-1.47183	-1.46671	-1.46176	-1.45693	-1.50104	-1.49628	-1.49148	-1.48665	-1.48184
-1.47049	-1.46536	-1.46043	-1.45565	-1.49977	-1.49506	-1.49032	-1.48557	-1.48081
-1.46921	-1.51321	-1.50818	-1.50329	-1.49852	-1.49384	-1.48915	-1.48445	-1.47973
-1.46806	-1.51203	-1.50699	-1.5021	-1.49733	-1.49265	-1.48796	-1.48325	-1.47853
-1.46694	-1.51089	-1.50586	-1.50096	-1.49618	-1.4915	-1.48678	-1.48203	-1.47728
-1.46572	-1.50969	-1.50471	-1.49986	-1.4951	-1.49042	-1.48568	-1.48088	-1.4761
-1.46446	-1.50847	-1.50357	-1.49879	-1.49407	-1.4894	-1.48464	-1.47978	-1.47495
-1.51235	-1.50728	-1.50243	-1.49768	-1.49297	-1.48829	-1.48352	-1.47867	-1.47383
-1.51119	-1.50613	-1.50127	-1.49652	-1.49179	-1.4871	-1.48235	-1.47754	-1.47276
-1.51002	-1.505	-1.50016	-1.49541	-1.49065	-1.48592	-1.5305	-1.52556	-1.52064
-1.50882	-1.5039	-1.49911	-1.49438	-1.4896	-1.4848	-1.5293	-1.52434	-1.51942
-1.50761	-1.5028	-1.49807	-1.49336	-1.48855	-1.48369	-1.52813	-1.52314	-1.51821
-1.50639	-1.50163	-1.49694	-1.49226	-1.48746	-1.48261	-1.52701	-1.52203	-1.5171
-1.50519	-1.50045	-1.49579	-1.49114	-1.48637	-1.48153	-1.52592	-1.52094	-1.51602
-1.50409	-1.49935	-1.49471	-1.49007	-1.48528	-1.48042	-1.52476	-1.51977	-1.51487
-1.50306	-1.49832	-1.49367	-1.48903	-1.4842	-1.47929	-1.52355	-1.51854	-1.51366
-1.50202	-1.49726	-1.4926	-1.48793	-1.48309	-1.47817	-1.52239	-1.51738	-1.5578
-1.50098	-1.49619	-1.49148	-1.48677	-1.48194	-1.47706	-1.52127	-1.5163	-1.5567
-1.49992	-1.49513	-1.49038	-1.48563	-1.4808	-1.47594	-1.52014	-1.51521	-1.55561
-1.49885	-1.49408	-1.48932	-1.48455	-1.4797	-1.4748	-1.51898	-1.51409	-1.55449
-1.49777	-1.49303	-1.48827	-1.48348	-1.4786	-1.47368	-1.51782	-1.51297	-1.55339
-1.49668	-1.49193	-1.48714	-1.48233	-1.47747	-1.47259	-1.51461	-1.50984	-1.50519
-1.49559	-1.49081	-1.48599	-1.48116	-1.47633	-1.47152	-1.51357	-1.50882	-1.50419
-1.49453	-1.48976	-1.48492	-1.48008	-1.47526	-1.47047	-1.5125	-1.50778	-1.50316
-1.4935	-1.48875	-1.48391	-1.47906	-1.47422	-1.46942	-1.51142	-1.50671	-1.5021
-1.49247	-1.48773	-1.4829	-1.47804	-1.52012	-1.51516	-1.51034	-1.50566	-1.50108
-1.49144	-1.48668	-1.48187	-1.47703	-1.51905	-1.51405	-1.50925	-1.50464	-1.5001
-1.4904	-1.48561	-1.4808	-1.47598	-1.51797	-1.51296	-1.50818	-1.50363	-1.5264
-1.48933	-1.4845	-1.47966	-1.47484	-1.51685	-1.51193	-1.5072	-1.50267	-1.52542
-1.48826	-1.48338	-1.47851	-1.47369	-1.51573	-1.51092	-1.50625	-1.50171	-1.52445
-1.48719	-1.52946	-1.52446	-1.51952	-1.51467	-1.50992	-1.50526	-1.50071	-1.52344
-1.48614	-1.5284	-1.52341	-1.51847	-1.51365	-1.50892	-1.50427	-1.49968	-1.52241
-1.48512	-1.52735	-1.52236	-1.51742	-1.51261	-1.5079	-1.50326	-1.49869	-1.52144
-1.48414	-1.52632	-1.52132	-1.51638	-1.51157	-1.50686	-1.50225	-1.49771	-1.5205
-1.48317	-1.52531	-1.52028	-1.51532	-1.51052	-1.50584	-1.50126	-1.49676	-1.51958
-1.4822	-1.52431	-1.51922	-1.5142	-1.50943	-1.50483	-1.5003	-1.49583	-1.51868
-1.48123	-1.52332	-1.51818	-1.51312	-1.50838	-1.50386	-1.49937	-1.49493	-1.51779
-1.52733	-1.52232	-1.5172	-1.51217	-1.50745	-1.50294	-1.49847	-1.49406	-1.51694
-1.52629	-1.5213	-1.51624	-1.51127	-1.50656	-1.50205	-1.4976	-1.49322	-1.51611
-1.52522	-1.52021	-1.51522	-1.51033	-1.50566	-1.50116	-1.49675	-1.49241	-1.51532
-1.52412	-1.51908	-1.51417	-1.50938	-1.50476	-1.50029	-1.49591	-1.49162	-1.51457
-1.50367	-1.51801	-1.51315	-1.50844	-1.50385	-1.49941	-1.49507	-1.49082	-1.5138
-1.50267	-1.51701	-1.51219	-1.5075	-1.50295	-1.49853	-1.49422	-1.49	-1.51302
-1.50169	-1.51604	-1.51124	-1.50659	-1.50206	-1.49766	-1.49338	-1.48921	-1.51227
-1.50072	-1.51508	-1.51032	-1.50571	-1.50119	-1.4968	-1.49257	-1.48848	-1.51158

-0.152	-0.15148	-0.15096	-0.15043	-0.14988	-0.14932	-0.25035	-0.24938	-0.24846
-0.15218	-0.15166	-0.15114	-0.15062	-0.15008	-0.14952	-0.25066	-0.24968	-0.24875
-0.15237	-0.15184	-0.15133	-0.15081	-0.15027	-0.14972	-0.25096	-0.24999	-0.24905
-0.15254	-0.15201	-0.15149	-0.15098	-0.15044	-0.1499	-0.19908	-0.19834	-0.24934
-0.15271	-0.15217	-0.15165	-0.15114	-0.15061	-0.15007	-0.19929	-0.19856	-0.24964
-0.10619	-0.15235	-0.15183	-0.15131	-0.15078	-0.15025	-0.19951	-0.19879	-0.24995
-0.10635	-0.15255	-0.15202	-0.1515	-0.15097	-0.15043	-0.19973	-0.19903	-0.25027
-0.10651	-0.15274	-0.1522	-0.15167	-0.15114	-0.15061	-0.15008	-0.14955	-0.25058
-0.10667	-0.15292	-0.15237	-0.15184	-0.1513	-0.15077	-0.15025	-0.14973	-0.25088
-0.10682	-0.15311	-0.15255	-0.15201	-0.15147	-0.15094	-0.15042	-0.14991	-0.25117
-0.10699	-0.15331	-0.15275	-0.1522	-0.15166	-0.15114	-0.15061	-0.15009	-0.25145
-0.10716	-0.15352	-0.15295	-0.1524	-0.15186	-0.15133	-0.1508	-0.15027	-0.25174
-0.05249	-0.15371	-0.15314	-0.15259	-0.15205	-0.15152	-0.15099	-0.15046	-0.19965
-0.05256	-0.1539	-0.15333	-0.15277	-0.15223	-0.15171	-0.15118	-0.15065	-0.19988
	-0.10718	-0.10673	-0.1063	-0.15241	-0.15188	-0.15135	-0.15083	-0.2001
	-0.10737	-0.10691	-0.10645	-0.15259	-0.15205	-0.15152	-0.151	-0.20031
	-0.05259	-0.05234	-0.05209	-0.15279	-0.15223	-0.15169	-0.15118	-0.20053
	-0.0527	-0.05243	-0.05217	-0.15299	-0.15243	-0.15189	-0.15136	-0.20076
	-0.0528	-0.05253	-0.05226	-0.15321	-0.15264	-0.15209	-0.15155	-0.201
	-0.05289	-0.05262	-0.05236	-0.15343	-0.15286	-0.1523	-0.15175	-0.20125
	-0.05298	-0.05272	-0.05246	-0.15365	-0.15307	-0.15251	-0.15196	-0.20151
				-0.05229	-0.05205	-0.15272	-0.15217	-0.20176
				-0.05237	-0.05213	-0.15291	-0.15237	-0.20202
				-0.05246	-0.05221	-0.15311	-0.15257	-0.20226
				-0.05256	-0.05229	-0.1533	-0.15276	-0.20248
				-0.05266	-0.05238	-0.09995	-0.0996	-0.15241
				-0.05276	-0.05248	-0.10009	-0.09972	-0.1526
				-0.05287	-0.05259	-0.10023	-0.09985	-0.1528
				-0.05296	-0.05269	-0.10037	-0.09998	-0.15301
				-0.05306	-0.05279	-0.10052	-0.10013	-0.15323
						-0.05263	-0.05237	-0.09988
						-0.05274	-0.05246	-0.10001
						-0.05284	-0.05256	-0.10015
						-0.05294	-0.05266	-0.10029
						-0.05304	-0.05277	-0.10044
						-0.05314	-0.05287	-0.10058
						-0.05323	-0.05297	-0.10073
								-0.05281
								-0.05292
								-0.05302
								-0.05312
								-0.05321
								-0.0533

								-0.05843
								-0.05822
								-0.05803
								-0.05786
						-0.0586	-0.05814	-0.24956
						-0.05842	-0.05794	-0.24913
						-0.05824	-0.05775	-0.24871
						-0.05807	-0.05758	-0.24831
						-0.0579	-0.05741	-0.24792
						-0.05771	-0.05723	-0.24752
						-0.05751	-0.05705	-0.24711
				-0.05827	-0.05779	-0.24881	-0.24775	-0.29989
				-0.05807	-0.05761	-0.24839	-0.24732	-0.29939
				-0.05788	-0.05743	-0.24797	-0.2469	-0.39802
				-0.05769	-0.05723	-0.24754	-0.24649	-0.39746
				-0.05751	-0.05704	-0.24711	-0.24608	-0.3969
		-0.0583	-0.05782	-0.20855	-0.20759	-0.29988	-0.2987	-0.49001
		-0.05811	-0.05765	-0.20821	-0.20725	-0.29943	-0.29824	-0.4893
		-0.05793	-0.05748	-0.20786	-0.20691	-0.34882	-0.34749	-0.7506
		-0.05776	-0.05731	-0.20751	-0.20657	-0.34833	-0.347	-0.74951
-0.05851	-0.05804	-0.209	-0.20808	-0.2473	-0.24622	-0.40352	-0.40196	-0.74846
-0.05835	-0.05787	-0.20865	-0.2077	-0.24686	-0.2458	-0.40292	-0.40141	-0.74748
-0.05818	-0.0577	-0.20829	-0.20733	-0.34942	-0.34809	-0.68116	-0.6787	-0.90056
-0.05802	-0.05753	-0.20796	-0.20699	-0.3489	-0.34756	-0.68014	-0.67776	-0.89941

-0.05785	-0.05736	-0.20763	-0.20666	-0.3484	-0.34704	-0.67915	-0.67684	-0.89826
-0.05767	-0.05719	-0.2073	-0.20634	-0.40364	-0.40204	-0.67823	-0.67594	-0.89711
-0.05749	-0.05702	-0.20697	-0.20601	-0.4031	-0.40154	-0.67736	-0.67506	-0.89597
-0.20848	-0.20757	-0.30501	-0.30366	-0.40256	-0.40104	-0.67651	-0.6742	-0.89484
-0.20813	-0.20722	-0.30454	-0.30319	-0.40202	-0.40054	-0.67569	-0.67337	-0.89371
-0.20778	-0.20687	-0.40457	-0.40299	-0.67975	-0.67728	-0.86717	-0.86425	-0.9753
-0.20743	-0.2065	-0.40396	-0.40242	-0.67885	-0.67643	-0.86612	-0.86322	-0.97409
-0.20708	-0.20615	-0.40336	-0.40186	-0.67795	-0.67558	-0.86509	-0.8622	-0.9729
-0.30522	-0.30388	-0.40281	-0.40134	-0.67707	-0.67475	-0.86407	-0.86119	-1.00454
-0.30476	-0.30343	-0.40227	-0.40085	-0.67622	-0.67392	-0.86305	-0.86019	-1.00332
-0.35399	-0.35252	-0.54273	-0.54091	-0.75149	-0.74898	-0.97589	-0.97259	-1.00208
-0.35346	-0.352	-0.54203	-0.54024	-0.75059	-0.74809	-0.97471	-0.9714	-1.00084
-0.35296	-0.35151	-0.54134	-0.53958	-0.79559	-0.79292	-1.00646	-1.00304	-1.02872
-0.35248	-0.35106	-0.54068	-0.53892	-0.7946	-0.79195	-1.00525	-1.00182	-1.02739
-0.54379	-0.54184	-0.71886	-0.71647	-0.93758	-0.93454	-1.00404	-1.0006	-1.02607
-0.5431	-0.54117	-0.71798	-0.7156	-0.93645	-0.93342	-1.00283	-0.99937	-1.02474
-0.54242	-0.54052	-0.71711	-0.71475	-0.93533	-0.9323	-1.00161	-0.99813	-1.02341
-0.54174	-0.53987	-0.76286	-0.76031	-1.00709	-1.00377	-1.02965	-1.02586	-1.20461
-0.54107	-0.53924	-0.76193	-0.75937	-1.00587	-1.00254	-1.02832	-1.02454	-1.20297
-0.54043	-0.53863	-0.76103	-0.75846	-1.00467	-1.00131	-1.02699	-1.02321	-1.20132
-0.53982	-0.53805	-0.76015	-0.75758	-1.00349	-1.00008	-1.02566	-1.02188	-1.19968
-0.76443	-0.76185	-0.75927	-0.7567	-1.00229	-0.99885	-1.07751	-1.07357	-1.19806
-0.76349	-0.76092	-0.75836	-0.75583	-1.00109	-0.99761	-1.07612	-1.0722	-1.19646
-0.76257	-0.75999	-0.90708	-0.90402	-1.02923	-1.02542	-1.20406	-1.19941	-1.22245
-0.76167	-0.75908	-0.90598	-0.90289	-1.02789	-1.02409	-1.20242	-1.19784	-1.22092
-0.76077	-0.75818	-0.90487	-0.90174	-1.02655	-1.02276	-1.20081	-1.1963	-1.21944
-0.75987	-0.75728	-0.90376	-0.9006	-1.07847	-1.07454	-1.19922	-1.19482	-1.3102
-0.75897	-0.75639	-0.90265	-0.89946	-1.0771	-1.07319	-1.19766	-1.19337	-1.30868
-0.75806	-0.75549	-0.97158	-0.96817	-1.1432	-1.139	-1.22372	-1.21943	-1.30712
-0.75715	-0.75457	-0.97038	-0.96698	-1.14176	-1.13762	-1.22226	-1.21798	-1.30551
-0.75623	-0.75366	-1.02454	-1.02098	-1.14034	-1.13626	-1.28387	-1.27923	-1.3039
-0.75532	-0.75274	-1.02331	-1.01979	-1.13898	-1.13491	-1.28227	-1.27766	-1.3023
-0.75442	-0.75184	-1.02211	-1.01861	-1.13762	-1.13356	-1.28068	-1.27608	-1.3007
-0.9024	-0.89923	-1.11818	-1.11406	-1.22516	-1.22072	-1.27907	-1.27448	-1.29909
-0.9014	-0.89823	-1.11685	-1.11267	-1.22359	-1.21921	-1.27747	-1.27289	-1.29748
-0.95802	-0.95467	-1.11547	-1.1113	-1.28522	-1.28053	-1.30518	-1.3005	-1.2959
-0.95689	-0.95353	-1.11408	-1.10993	-1.28359	-1.27896	-1.30361	-1.29894	-1.29436
-0.95576	-0.95239	-1.11269	-1.10857	-1.25825	-1.25373	-1.27887	-1.27428	-1.32263
-0.95461	-0.95124	-1.11129	-1.10723	-1.25675	-1.25231	-1.27748	-1.27291	-1.32122
-1.08547	-1.08138	-1.20034	-1.19591	-1.28508	-1.28057	-1.27606	-1.27152	-1.31979
-1.08422	-1.08014	-1.19896	-1.19459	-1.28363	-1.27908	-1.27456	-1.27005	-1.31829
-1.08296	-1.07892	-1.19761	-1.19329	-1.28219	-1.2776	-1.27305	-1.26855	-1.31677
-1.08165	-1.07764	-1.2602	-1.2556	-1.28073	-1.27612	-1.30177	-1.29719	-1.34552
-1.0803	-1.07633	-1.25866	-1.25413	-1.27925	-1.27465	-1.30027	-1.29569	-1.34397
-1.20213	-1.19765	-1.287	-1.28243	-1.27783	-1.27324	-1.29885	-1.29427	-1.34249
-1.20065	-1.19622	-1.28549	-1.28099	-1.27644	-1.27187	-1.29747	-1.2929	-1.34107
-1.19923	-1.19485	-1.28401	-1.27953	-1.30528	-1.30067	-1.32684	-1.32219	-1.31756
-1.19788	-1.19355	-1.28257	-1.27805	-1.30378	-1.29922	-1.32538	-1.32072	-1.31609
-1.22749	-1.22308	-1.28112	-1.27656	-1.30228	-1.29776	-1.32392	-1.31925	-1.44054
-1.22608	-1.2217	-1.27964	-1.27507	-1.30078	-1.29631	-1.32248	-1.31783	-1.43902

-1.22468	-1.22033	-1.27818	-1.27361	-1.29931	-1.29487	-1.32104	-1.31641	-1.43751
-1.28605	-1.28141	-1.3071	-1.30247	-1.3287	-1.32415	-1.31958	-1.31495	-1.43594
-1.28467	-1.28005	-1.30575	-1.30115	-1.32734	-1.32272	-1.31811	-1.31347	-1.43435
-1.28324	-1.27864	-1.30436	-1.2998	-1.32597	-1.32132	-1.41836	-1.41345	-1.53762
-1.28177	-1.27719	-1.30294	-1.29843	-1.3246	-1.31996	-1.41692	-1.41203	-1.53612
-1.28035	-1.27579	-1.30153	-1.29704	-1.32319	-1.31856	-1.41543	-1.41058	-1.53458
-1.27903	-1.27447	-1.30016	-1.29561	-1.32171	-1.31707	-1.41386	-1.40906	-1.53296
-1.27772	-1.27317	-1.2988	-1.2942	-1.32024	-1.31559	-1.41229	-1.40756	-1.53136
-1.30672	-1.30208	-1.32822	-1.32353	-1.34215	-1.33743	-1.4108	-1.40613	-1.57872
-1.30537	-1.30073	-1.32685	-1.32217	-1.34076	-1.33603	-1.40934	-1.40471	-1.57718
-1.30395	-1.29931	-1.3254	-1.32071	-1.41758	-1.41262	-1.48445	-1.47961	-1.59848
-1.30248	-1.29785	-1.3239	-1.31921	-1.41598	-1.41106	-1.48285	-1.47804	-1.59679
-1.33189	-1.32719	-1.34582	-1.34106	-1.41448	-1.40961	-1.5318	-1.52687	-1.59519
-1.33056	-1.32585	-1.34445	-1.3397	-1.41308	-1.40827	-1.53037	-1.52545	-1.5937
-1.32923	-1.32452	-1.36859	-1.36376	-1.46462	-1.45974	-1.55761	-1.55261	-1.59222
-1.32789	-1.32318	-1.36721	-1.36238	-1.46317	-1.45835	-1.55616	-1.55117	-1.59076
-1.32651	-1.32181	-1.36579	-1.36097	-1.4617	-1.45693	-1.55467	-1.54969	-1.58926
-1.34841	-1.34363	-1.41898	-1.41406	-1.46014	-1.45542	-1.55305	-1.54809	-1.58762
-1.34688	-1.34209	-1.41738	-1.4125	-1.45856	-1.45388	-1.5514	-1.54645	-1.58594
-1.37097	-1.36612	-1.46693	-1.46193	-1.53121	-1.52626	-1.57306	-1.56804	-1.58437
-1.3696	-1.36476	-1.46551	-1.46057	-1.52979	-1.52486	-1.5716	-1.56658	-1.58289
-1.42299	-1.41804	-1.46412	-1.45923	-1.5284	-1.52349	-1.57018	-1.56516	-1.58146
-1.42164	-1.41668	-1.46275	-1.45791	-1.52704	-1.52215	-1.56881	-1.56381	-1.58011
-1.42029	-1.41533	-1.48465	-1.47978	-1.52568	-1.52081	-1.56744	-1.56246	-1.57875
-1.41893	-1.41399	-1.48328	-1.47845	-1.52433	-1.51947	-1.56606	-1.56108	-1.57736
-1.41758	-1.41266	-1.48191	-1.47712	-1.52297	-1.51813	-1.56466	-1.55968	-1.57594
-1.46722	-1.46215	-1.48054	-1.47578	-1.54511	-1.54022	-1.56324	-1.55823	-1.57444
-1.46582	-1.46078	-1.47916	-1.47444	-1.54369	-1.53881	-1.56179	-1.55675	-1.5729
-1.48767	-1.48258	-1.47773	-1.47303	-1.54219	-1.53732	-1.56029	-1.55526	-1.57141
-1.48617	-1.48109	-1.47625	-1.47156	-1.54061	-1.53576	-1.55873	-1.55376	-1.56996
-1.48466	-1.47962	-1.54898	-1.54401	-1.5391	-1.53426	-1.55723	-1.55231	-1.56853
-1.48314	-1.47818	-1.54752	-1.54258	-1.53769	-1.53287	-1.55582	-1.55091	-1.56714
-1.48164	-1.47678	-1.54608	-1.54119	-1.53633	-1.53151	-1.55445	-1.54954	-1.56576
-1.48022	-1.47542	-1.54468	-1.53983	-1.53499	-1.53019	-1.5531	-1.54818	-1.5644
-1.47885	-1.4741	-1.54331	-1.53849	-1.53368	-1.52888	-1.55176	-1.54684	-1.56304
-1.50181	-1.49702	-1.54196	-1.53716	-1.53236	-1.52756	-1.55041	-1.54549	-1.56168
-1.50052	-1.49575	-1.54064	-1.53584	-1.53103	-1.52623	-1.54906	-1.54414	-1.56031
-1.49923	-1.49448	-1.53932	-1.53452	-1.52971	-1.5249	-1.54771	-1.54279	-1.55897
-1.49796	-1.49322	-1.538	-1.53321	-1.52839	-1.52357	-1.54636	-1.54145	-1.55763
-1.49669	-1.49196	-1.53669	-1.53189	-1.52707	-1.52223	-1.545	-1.54012	-1.55631
-1.4954	-1.49069	-1.53536	-1.53057	-1.52574	-1.5209	-1.54366	-1.53881	-1.555
-1.49415	-1.48944	-1.53405	-1.52926	-1.52442	-1.51957	-1.54232	-1.53749	-1.55369
-1.49298	-1.48825	-1.53278	-1.52794	-1.52308	-1.51822	-1.54096	-1.53617	-1.55238
-1.49185	-1.48709	-1.53153	-1.52664	-1.52175	-1.51688	-1.53961	-1.53484	-1.55107
-1.49071	-1.48597	-1.53034	-1.52539	-1.52046	-1.51555	-1.53825	-1.53351	-1.54976
-1.48956	-1.48487	-1.52919	-1.52418	-1.5192	-1.51424	-1.5369	-1.53218	-1.54843
-1.48836	-1.4837	-1.52801	-1.52299	-1.51799	-1.51301	-1.53563	-1.53091	-1.54716
-1.4871	-1.48245	-1.52679	-1.52184	-1.51687	-1.51187	-1.53445	-1.52971	-1.54592
-1.48582	-1.48118	-1.52552	-1.52064	-1.54326	-1.53821	-1.53331	-1.52855	-1.54475
-1.48454	-1.47989	-1.52421	-1.51934	-1.542	-1.53705	-1.53221	-1.52748	-1.54368

-1.48325	-1.4786	-1.52287	-1.518	-1.54071	-1.53586	-1.53109	-1.52639	-1.54263
-1.48195	-1.4773	-1.52152	-1.51666	-1.53938	-1.5346	-1.52988	-1.5252	-1.54148
-1.48066	-1.47599	-1.52017	-1.51531	-1.53803	-1.53328	-1.5286	-1.52395	-1.54028
-1.47941	-1.47473	-1.51885	-1.51398	-1.5367	-1.53198	-1.52732	-1.52269	-1.53906
-1.47821	-1.47349	-1.51756	-1.51267	-1.53537	-1.53067	-1.52603	-1.5214	-1.53781
-1.47708	-1.47233	-1.51634	-1.51145	-1.53412	-1.52942	-1.5248	-1.52018	-1.59195
-1.47604	-1.47128	-1.51525	-1.51034	-1.53296	-1.52825	-1.52362	-1.51903	-1.59076
-1.47497	-1.47021	-1.51417	-1.50926	-1.53186	-1.52713	-1.5666	-1.5619	-1.58967
-1.47377	-1.46903	-1.51303	-1.5082	-1.53082	-1.52611	-1.56557	-1.56093	-1.58871
-1.47253	-1.46782	-1.51186	-1.50713	-1.52979	-1.5251	-1.56458	-1.56002	-1.58784
-1.47132	-1.4666	-1.51063	-1.50597	-1.52864	-1.52399	-1.56351	-1.55909	-1.58701
-1.47013	-1.46539	-1.50938	-1.50475	-1.52743	-1.52281	-1.56239	-1.55816	-1.58621
-1.46901	-1.46427	-1.50823	-1.50359	-1.57043	-1.56569	-1.56126	-1.55716	-1.58533
-1.46797	-1.46325	-1.50717	-1.50251	-1.56926	-1.5645	-1.56011	-1.55611	-1.58435
-1.51572	-1.51086	-1.53349	-1.52875	-1.56815	-1.5634	-1.55904	-1.55512	-1.5834
-1.51452	-1.50971	-1.53238	-1.52771	-1.56714	-1.56244	-1.55812	-1.55423	-1.5825
-1.51334	-1.50856	-1.53127	-1.52666	-1.56614	-1.56151	-1.55726	-1.5534	-1.58166
-1.51224	-1.50747	-1.57443	-1.56969	-1.57771	-1.57304	-1.56873	-1.56479	-1.58092
-1.51117	-1.50641	-1.57328	-1.56854	-1.57662	-1.57208	-1.56788	-1.56403	-1.58022
-1.51006	-1.50536	-1.5722	-1.56747	-1.57559	-1.57117	-1.56706	-1.56327	-1.57949
-1.50894	-1.50432	-1.57115	-1.56645	-1.57461	-1.57029	-1.56626	-1.56252	-1.57876
-1.55298	-1.54826	-1.57006	-1.56538	-1.57359	-1.56937	-1.56542	-1.56176	-1.57805
-1.55187	-1.54714	-1.56891	-1.56424	-1.5725	-1.56839	-1.56454	-1.56099	-1.57737
-1.55078	-1.54605	-1.56781	-1.56316	-1.57146	-1.56744	-1.56369	-1.56024	-1.57667
-1.54972	-1.54505	-1.56682	-1.5622	-1.57053	-1.56659	-1.5629	-1.55948	-1.57594
-1.54868	-1.54407	-1.56587	-1.56129	-1.56965	-1.56577	-1.56213	-1.55875	-1.57521
-1.50067	-1.49625	-1.56491	-1.5604	-1.5688	-1.56497	-1.56139	-1.55806	-1.57457
-1.49968	-1.49528	-1.56396	-1.55953	-1.56798	-1.5642	-1.56067	-1.55741	-1.57396
-1.49865	-1.49427	-1.56298	-1.55867	-1.56719	-1.56347	-1.55999	-1.55676	-1.57332
-1.49757	-1.49321	-1.56197	-1.55783	-1.56644	-1.5628	-1.55935	-1.55612	-1.57265
-1.49654	-1.49219	-1.56099	-1.55699	-1.56569	-1.56211	-1.55871	-1.55549	-1.57202
-1.49556	-1.49124	-1.56007	-1.55616	-1.56491	-1.56139	-1.55804	-1.55488	-1.57144
-1.52177	-1.51736	-1.55915	-1.55533	-1.56414	-1.56069	-1.55739	-1.55428	-1.57086
-1.5208	-1.51641	-1.55821	-1.55447	-1.56336	-1.56001	-1.55679	-1.5537	-1.57028
-1.51984	-1.51548	-1.5573	-1.55363	-1.5626	-1.55935	-1.5562	-1.55313	-1.5697
-1.51891	-1.51465	-1.55651	-1.55289	-1.56191	-1.55872	-1.55559	-1.55252	-1.56908
-1.51801	-1.51387	-1.55579	-1.55223	-1.56128	-1.5581	-1.55498	-1.5519	-1.56845
-1.51714	-1.51311	-1.55508	-1.55159	-1.56066	-1.55749	-1.55437	-1.5513	-1.56784
-1.5163	-1.51237	-1.5544	-1.55096	-1.56007	-1.55688	-1.55375	-1.55071	-1.56726
-1.51548	-1.51164	-1.50813	-1.50487	-1.55946	-1.55627	-1.55316	-1.55013	-1.56669
-1.51466	-1.51091	-1.50744	-1.50421	-1.55883	-1.55568	-1.5526	-1.54959	-1.56614
-1.51385	-1.51017	-1.50675	-1.50355	-1.5582	-1.5551	-1.55205	-1.54905	-1.56559
-1.51303	-1.5094	-1.50604	-1.50291	-1.55759	-1.55452	-1.55149	-1.5485	-1.56505
-1.51223	-1.50863	-1.50534	-1.50229	-1.557	-1.55395	-1.55093	-1.54795	-1.56449
-1.51149	-1.50794	-1.5047	-1.50169	-1.55641	-1.55337	-1.55036	-1.54736	-1.56389
-1.5108	-1.5073	-1.5041	-1.50111	-1.55582	-1.5528	-1.54978	-1.54675	-1.56325
-1.51011	-1.50668	-1.50351	-1.50054	-1.55524	-1.55221	-1.54918	-1.54615	-1.56724
-1.50942	-1.50607	-1.50295	-1.5	-1.55467	-1.55159	-1.54855	-1.54555	-1.56666
-1.50875	-1.50548	-1.50239	-1.49947	-1.50943	-1.50643	-1.54796	-1.54498	-1.5661
-1.50812	-1.5049	-1.50185	-1.49895	-1.50891	-1.5059	-1.54741	-1.54443	-1.56553

								-0.05843
								-0.05822
								-0.05803
								-0.05786
						-0.0586	-0.05814	-0.24956
						-0.05842	-0.05794	-0.24913
						-0.05824	-0.05775	-0.24871
						-0.05807	-0.05758	-0.24831
						-0.0579	-0.05741	-0.24792
						-0.05771	-0.05723	-0.24752
						-0.05751	-0.05705	-0.24711
				-0.05827	-0.05779	-0.24881	-0.24775	-0.29989
				-0.05807	-0.05761	-0.24839	-0.24732	-0.29939
				-0.05788	-0.05743	-0.24797	-0.2469	-0.39802
				-0.05769	-0.05723	-0.24754	-0.24649	-0.39746
				-0.05751	-0.05704	-0.24711	-0.24608	-0.3969
		-0.0583	-0.05782	-0.20855	-0.20759	-0.29988	-0.2987	-0.49001
		-0.05811	-0.05765	-0.20821	-0.20725	-0.29943	-0.29824	-0.4893
		-0.05793	-0.05748	-0.20786	-0.20691	-0.34882	-0.34749	-0.7506
		-0.05776	-0.05731	-0.20751	-0.20657	-0.34833	-0.347	-0.74951
-0.05851	-0.05804	-0.209	-0.20808	-0.2473	-0.24622	-0.40352	-0.40196	-0.74846
-0.05835	-0.05787	-0.20865	-0.2077	-0.24686	-0.2458	-0.40292	-0.40141	-0.74748
-0.05818	-0.0577	-0.20829	-0.20733	-0.34942	-0.34809	-0.68116	-0.6787	-0.90056
-0.05802	-0.05753	-0.20796	-0.20699	-0.3489	-0.34756	-0.68014	-0.67776	-0.89941

-0.05785	-0.05736	-0.20763	-0.20666	-0.3484	-0.34704	-0.67915	-0.67684	-0.89826
-0.05767	-0.05719	-0.2073	-0.20634	-0.40364	-0.40204	-0.67823	-0.67594	-0.89711
-0.05749	-0.05702	-0.20697	-0.20601	-0.4031	-0.40154	-0.67736	-0.67506	-0.89597
-0.20848	-0.20757	-0.30501	-0.30366	-0.40256	-0.40104	-0.67651	-0.6742	-0.89484
-0.20813	-0.20722	-0.30454	-0.30319	-0.40202	-0.40054	-0.67569	-0.67337	-0.89371
-0.20778	-0.20687	-0.40457	-0.40299	-0.67975	-0.67728	-0.86717	-0.86425	-0.9753
-0.20743	-0.2065	-0.40396	-0.40242	-0.67885	-0.67643	-0.86612	-0.86322	-0.97409
-0.20708	-0.20615	-0.40336	-0.40186	-0.67795	-0.67558	-0.86509	-0.8622	-0.9729
-0.30522	-0.30388	-0.40281	-0.40134	-0.67707	-0.67475	-0.86407	-0.86119	-1.00454
-0.30476	-0.30343	-0.40227	-0.40085	-0.67622	-0.67392	-0.86305	-0.86019	-1.00332
-0.35399	-0.35252	-0.54273	-0.54091	-0.75149	-0.74898	-0.97589	-0.97259	-1.00208
-0.35346	-0.352	-0.54203	-0.54024	-0.75059	-0.74809	-0.97471	-0.9714	-1.00084
-0.35296	-0.35151	-0.54134	-0.53958	-0.79559	-0.79292	-1.00646	-1.00304	-1.02872
-0.35248	-0.35106	-0.54068	-0.53892	-0.7946	-0.79195	-1.00525	-1.00182	-1.02739
-0.54379	-0.54184	-0.71886	-0.71647	-0.93758	-0.93454	-1.00404	-1.0006	-1.02607
-0.5431	-0.54117	-0.71798	-0.7156	-0.93645	-0.93342	-1.00283	-0.99937	-1.02474
-0.54242	-0.54052	-0.71711	-0.71475	-0.93533	-0.9323	-1.00161	-0.99813	-1.02341
-0.54174	-0.53987	-0.76286	-0.76031	-1.00709	-1.00377	-1.02965	-1.02586	-1.20461
-0.54107	-0.53924	-0.76193	-0.75937	-1.00587	-1.00254	-1.02832	-1.02454	-1.20297
-0.54043	-0.53863	-0.76103	-0.75846	-1.00467	-1.00131	-1.02699	-1.02321	-1.20132
-0.53982	-0.53805	-0.76015	-0.75758	-1.00349	-1.00008	-1.02566	-1.02188	-1.19968
-0.76443	-0.76185	-0.75927	-0.7567	-1.00229	-0.99885	-1.07751	-1.07357	-1.19806
-0.76349	-0.76092	-0.75836	-0.75583	-1.00109	-0.99761	-1.07612	-1.0722	-1.19646
-0.76257	-0.75999	-0.90708	-0.90402	-1.02923	-1.02542	-1.20406	-1.19941	-1.22245
-0.76167	-0.75908	-0.90598	-0.90289	-1.02789	-1.02409	-1.20242	-1.19784	-1.22092
-0.76077	-0.75818	-0.90487	-0.90174	-1.02655	-1.02276	-1.20081	-1.1963	-1.21944
-0.75987	-0.75728	-0.90376	-0.9006	-1.07847	-1.07454	-1.19922	-1.19482	-1.3102
-0.75897	-0.75639	-0.90265	-0.89946	-1.0771	-1.07319	-1.19766	-1.19337	-1.30868
-0.75806	-0.75549	-0.97158	-0.96817	-1.1432	-1.139	-1.22372	-1.21943	-1.30712
-0.75715	-0.75457	-0.97038	-0.96698	-1.14176	-1.13762	-1.22226	-1.21798	-1.30551
-0.75623	-0.75366	-1.02454	-1.02098	-1.14034	-1.13626	-1.28387	-1.27923	-1.3039
-0.75532	-0.75274	-1.02331	-1.01979	-1.13898	-1.13491	-1.28227	-1.27766	-1.3023
-0.75442	-0.75184	-1.02211	-1.01861	-1.13762	-1.13356	-1.28068	-1.27608	-1.3007
-0.9024	-0.89923	-1.11818	-1.11406	-1.22516	-1.22072	-1.27907	-1.27448	-1.29909
-0.9014	-0.89823	-1.11685	-1.11267	-1.22359	-1.21921	-1.27747	-1.27289	-1.29748
-0.95802	-0.95467	-1.11547	-1.1113	-1.28522	-1.28053	-1.30518	-1.3005	-1.2959
-0.95689	-0.95353	-1.11408	-1.10993	-1.28359	-1.27896	-1.30361	-1.29894	-1.29436
-0.95576	-0.95239	-1.11269	-1.10857	-1.25825	-1.25373	-1.27887	-1.27428	-1.32263
-0.95461	-0.95124	-1.11129	-1.10723	-1.25675	-1.25231	-1.27748	-1.27291	-1.32122
-1.08547	-1.08138	-1.20034	-1.19591	-1.28508	-1.28057	-1.27606	-1.27152	-1.31979
-1.08422	-1.08014	-1.19896	-1.19459	-1.28363	-1.27908	-1.27456	-1.27005	-1.31829
-1.08296	-1.07892	-1.19761	-1.19329	-1.28219	-1.2776	-1.27305	-1.26855	-1.31677
-1.08165	-1.07764	-1.2602	-1.2556	-1.28073	-1.27612	-1.30177	-1.29719	-1.34552
-1.0803	-1.07633	-1.25866	-1.25413	-1.27925	-1.27465	-1.30027	-1.29569	-1.34397
-1.20213	-1.19765	-1.287	-1.28243	-1.27783	-1.27324	-1.29885	-1.29427	-1.34249
-1.20065	-1.19622	-1.28549	-1.28099	-1.27644	-1.27187	-1.29747	-1.2929	-1.34107
-1.19923	-1.19485	-1.28401	-1.27953	-1.30528	-1.30067	-1.32684	-1.32219	-1.31756
-1.19788	-1.19355	-1.28257	-1.27805	-1.30378	-1.29922	-1.32538	-1.32072	-1.31609
-1.22749	-1.22308	-1.28112	-1.27656	-1.30228	-1.29776	-1.32392	-1.31925	-1.44054
-1.22608	-1.2217	-1.27964	-1.27507	-1.30078	-1.29631	-1.32248	-1.31783	-1.43902

-1.22468	-1.22033	-1.27818	-1.27361	-1.29931	-1.29487	-1.32104	-1.31641	-1.43751
-1.28605	-1.28141	-1.3071	-1.30247	-1.3287	-1.32415	-1.31958	-1.31495	-1.43594
-1.28467	-1.28005	-1.30575	-1.30115	-1.32734	-1.32272	-1.31811	-1.31347	-1.43435
-1.28324	-1.27864	-1.30436	-1.2998	-1.32597	-1.32132	-1.41836	-1.41345	-1.53762
-1.28177	-1.27719	-1.30294	-1.29843	-1.3246	-1.31996	-1.41692	-1.41203	-1.53612
-1.28035	-1.27579	-1.30153	-1.29704	-1.32319	-1.31856	-1.41543	-1.41058	-1.53458
-1.27903	-1.27447	-1.30016	-1.29561	-1.32171	-1.31707	-1.41386	-1.40906	-1.53296
-1.27772	-1.27317	-1.2988	-1.2942	-1.32024	-1.31559	-1.41229	-1.40756	-1.53136
-1.30672	-1.30208	-1.32822	-1.32353	-1.34215	-1.33743	-1.4108	-1.40613	-1.57872
-1.30537	-1.30073	-1.32685	-1.32217	-1.34076	-1.33603	-1.40934	-1.40471	-1.57718
-1.30395	-1.29931	-1.3254	-1.32071	-1.41758	-1.41262	-1.48445	-1.47961	-1.59848
-1.30248	-1.29785	-1.3239	-1.31921	-1.41598	-1.41106	-1.48285	-1.47804	-1.59679
-1.33189	-1.32719	-1.34582	-1.34106	-1.41448	-1.40961	-1.5318	-1.52687	-1.59519
-1.33056	-1.32585	-1.34445	-1.3397	-1.41308	-1.40827	-1.53037	-1.52545	-1.5937
-1.32923	-1.32452	-1.36859	-1.36376	-1.46462	-1.45974	-1.55761	-1.55261	-1.59222
-1.32789	-1.32318	-1.36721	-1.36238	-1.46317	-1.45835	-1.55616	-1.55117	-1.59076
-1.32651	-1.32181	-1.36579	-1.36097	-1.4617	-1.45693	-1.55467	-1.54969	-1.58926
-1.34841	-1.34363	-1.41898	-1.41406	-1.46014	-1.45542	-1.55305	-1.54809	-1.58762
-1.34688	-1.34209	-1.41738	-1.4125	-1.45856	-1.45388	-1.5514	-1.54645	-1.58594
-1.37097	-1.36612	-1.46693	-1.46193	-1.53121	-1.52626	-1.57306	-1.56804	-1.58437
-1.3696	-1.36476	-1.46551	-1.46057	-1.52979	-1.52486	-1.5716	-1.56658	-1.58289
-1.42299	-1.41804	-1.46412	-1.45923	-1.5284	-1.52349	-1.57018	-1.56516	-1.58146
-1.42164	-1.41668	-1.46275	-1.45791	-1.52704	-1.52215	-1.56881	-1.56381	-1.58011
-1.42029	-1.41533	-1.48465	-1.47978	-1.52568	-1.52081	-1.56744	-1.56246	-1.57875
-1.41893	-1.41399	-1.48328	-1.47845	-1.52433	-1.51947	-1.56606	-1.56108	-1.57736
-1.41758	-1.41266	-1.48191	-1.47712	-1.52297	-1.51813	-1.56466	-1.55968	-1.57594
-1.46722	-1.46215	-1.48054	-1.47578	-1.54511	-1.54022	-1.56324	-1.55823	-1.57444
-1.46582	-1.46078	-1.47916	-1.47444	-1.54369	-1.53881	-1.56179	-1.55675	-1.5729
-1.48767	-1.48258	-1.47773	-1.47303	-1.54219	-1.53732	-1.56029	-1.55526	-1.57141
-1.48617	-1.48109	-1.47625	-1.47156	-1.54061	-1.53576	-1.55873	-1.55376	-1.56996
-1.48466	-1.47962	-1.54898	-1.54401	-1.5391	-1.53426	-1.55723	-1.55231	-1.56853
-1.48314	-1.47818	-1.54752	-1.54258	-1.53769	-1.53287	-1.55582	-1.55091	-1.56714
-1.48164	-1.47678	-1.54608	-1.54119	-1.53633	-1.53151	-1.55445	-1.54954	-1.56576
-1.48022	-1.47542	-1.54468	-1.53983	-1.53499	-1.53019	-1.5531	-1.54818	-1.5644
-1.47885	-1.4741	-1.54331	-1.53849	-1.53368	-1.52888	-1.55176	-1.54684	-1.56304
-1.50181	-1.49702	-1.54196	-1.53716	-1.53236	-1.52756	-1.55041	-1.54549	-1.56168
-1.50052	-1.49575	-1.54064	-1.53584	-1.53103	-1.52623	-1.54906	-1.54414	-1.56031
-1.49923	-1.49448	-1.53932	-1.53452	-1.52971	-1.5249	-1.54771	-1.54279	-1.55897
-1.49796	-1.49322	-1.538	-1.53321	-1.52839	-1.52357	-1.54636	-1.54145	-1.55763
-1.49669	-1.49196	-1.53669	-1.53189	-1.52707	-1.52223	-1.545	-1.54012	-1.55631
-1.4954	-1.49069	-1.53536	-1.53057	-1.52574	-1.5209	-1.54366	-1.53881	-1.555
-1.49415	-1.48944	-1.53405	-1.52926	-1.52442	-1.51957	-1.54232	-1.53749	-1.55369
-1.49298	-1.48825	-1.53278	-1.52794	-1.52308	-1.51822	-1.54096	-1.53617	-1.55238
-1.49185	-1.48709	-1.53153	-1.52664	-1.52175	-1.51688	-1.53961	-1.53484	-1.55107
-1.49071	-1.48597	-1.53034	-1.52539	-1.52046	-1.51555	-1.53825	-1.53351	-1.54976
-1.48956	-1.48487	-1.52919	-1.52418	-1.5192	-1.51424	-1.5369	-1.53218	-1.54843
-1.48836	-1.4837	-1.52801	-1.52299	-1.51799	-1.51301	-1.53563	-1.53091	-1.54716
-1.4871	-1.48245	-1.52679	-1.52184	-1.51687	-1.51187	-1.53445	-1.52971	-1.54592
-1.48582	-1.48118	-1.52552	-1.52064	-1.54326	-1.53821	-1.53331	-1.52855	-1.54475
-1.48454	-1.47989	-1.52421	-1.51934	-1.542	-1.53705	-1.53221	-1.52748	-1.54368

-1.48325	-1.4786	-1.52287	-1.518	-1.54071	-1.53586	-1.53109	-1.52639	-1.54263
-1.48195	-1.4773	-1.52152	-1.51666	-1.53938	-1.5346	-1.52988	-1.5252	-1.54148
-1.48066	-1.47599	-1.52017	-1.51531	-1.53803	-1.53328	-1.5286	-1.52395	-1.54028
-1.47941	-1.47473	-1.51885	-1.51398	-1.5367	-1.53198	-1.52732	-1.52269	-1.53906
-1.47821	-1.47349	-1.51756	-1.51267	-1.53537	-1.53067	-1.52603	-1.5214	-1.53781
-1.47708	-1.47233	-1.51634	-1.51145	-1.53412	-1.52942	-1.5248	-1.52018	-1.59195
-1.47604	-1.47128	-1.51525	-1.51034	-1.53296	-1.52825	-1.52362	-1.51903	-1.59076
-1.47497	-1.47021	-1.51417	-1.50926	-1.53186	-1.52713	-1.5666	-1.5619	-1.58967
-1.47377	-1.46903	-1.51303	-1.5082	-1.53082	-1.52611	-1.56557	-1.56093	-1.58871
-1.47253	-1.46782	-1.51186	-1.50713	-1.52979	-1.5251	-1.56458	-1.56002	-1.58784
-1.47132	-1.4666	-1.51063	-1.50597	-1.52864	-1.52399	-1.56351	-1.55909	-1.58701
-1.47013	-1.46539	-1.50938	-1.50475	-1.52743	-1.52281	-1.56239	-1.55816	-1.58621
-1.46901	-1.46427	-1.50823	-1.50359	-1.57043	-1.56569	-1.56126	-1.55716	-1.58533
-1.46797	-1.46325	-1.50717	-1.50251	-1.56926	-1.5645	-1.56011	-1.55611	-1.58435
-1.51572	-1.51086	-1.53349	-1.52875	-1.56815	-1.5634	-1.55904	-1.55512	-1.5834
-1.51452	-1.50971	-1.53238	-1.52771	-1.56714	-1.56244	-1.55812	-1.55423	-1.5825
-1.51334	-1.50856	-1.53127	-1.52666	-1.56614	-1.56151	-1.55726	-1.5534	-1.58166
-1.51224	-1.50747	-1.57443	-1.56969	-1.57771	-1.57304	-1.56873	-1.56479	-1.58092
-1.51117	-1.50641	-1.57328	-1.56854	-1.57662	-1.57208	-1.56788	-1.56403	-1.58022
-1.51006	-1.50536	-1.5722	-1.56747	-1.57559	-1.57117	-1.56706	-1.56327	-1.57949
-1.50894	-1.50432	-1.57115	-1.56645	-1.57461	-1.57029	-1.56626	-1.56252	-1.57876
-1.55298	-1.54826	-1.57006	-1.56538	-1.57359	-1.56937	-1.56542	-1.56176	-1.57805
-1.55187	-1.54714	-1.56891	-1.56424	-1.5725	-1.56839	-1.56454	-1.56099	-1.57737
-1.55078	-1.54605	-1.56781	-1.56316	-1.57146	-1.56744	-1.56369	-1.56024	-1.57667
-1.54972	-1.54505	-1.56682	-1.5622	-1.57053	-1.56659	-1.5629	-1.55948	-1.57594
-1.54868	-1.54407	-1.56587	-1.56129	-1.56965	-1.56577	-1.56213	-1.55875	-1.57521
-1.50067	-1.49625	-1.56491	-1.5604	-1.5688	-1.56497	-1.56139	-1.55806	-1.57457
-1.49968	-1.49528	-1.56396	-1.55953	-1.56798	-1.5642	-1.56067	-1.55741	-1.57396
-1.49865	-1.49427	-1.56298	-1.55867	-1.56719	-1.56347	-1.55999	-1.55676	-1.57332
-1.49757	-1.49321	-1.56197	-1.55783	-1.56644	-1.5628	-1.55935	-1.55612	-1.57265
-1.49654	-1.49219	-1.56099	-1.55699	-1.56569	-1.56211	-1.55871	-1.55549	-1.57202
-1.49556	-1.49124	-1.56007	-1.55616	-1.56491	-1.56139	-1.55804	-1.55488	-1.57144
-1.52177	-1.51736	-1.55915	-1.55533	-1.56414	-1.56069	-1.55739	-1.55428	-1.57086
-1.5208	-1.51641	-1.55821	-1.55447	-1.56336	-1.56001	-1.55679	-1.5537	-1.57028
-1.51984	-1.51548	-1.5573	-1.55363	-1.5626	-1.55935	-1.5562	-1.55313	-1.5697
-1.51891	-1.51465	-1.55651	-1.55289	-1.56191	-1.55872	-1.55559	-1.55252	-1.56908
-1.51801	-1.51387	-1.55579	-1.55223	-1.56128	-1.5581	-1.55498	-1.5519	-1.56845
-1.51714	-1.51311	-1.55508	-1.55159	-1.56066	-1.55749	-1.55437	-1.5513	-1.56784
-1.5163	-1.51237	-1.5544	-1.55096	-1.56007	-1.55688	-1.55375	-1.55071	-1.56726
-1.51548	-1.51164	-1.50813	-1.50487	-1.55946	-1.55627	-1.55316	-1.55013	-1.56669
-1.51466	-1.51091	-1.50744	-1.50421	-1.55883	-1.55568	-1.5526	-1.54959	-1.56614
-1.51385	-1.51017	-1.50675	-1.50355	-1.5582	-1.5551	-1.55205	-1.54905	-1.56559
-1.51303	-1.5094	-1.50604	-1.50291	-1.55759	-1.55452	-1.55149	-1.5485	-1.56505
-1.51223	-1.50863	-1.50534	-1.50229	-1.557	-1.55395	-1.55093	-1.54795	-1.56449
-1.51149	-1.50794	-1.5047	-1.50169	-1.55641	-1.55337	-1.55036	-1.54736	-1.56389
-1.5108	-1.5073	-1.5041	-1.50111	-1.55582	-1.5528	-1.54978	-1.54675	-1.56325
-1.51011	-1.50668	-1.50351	-1.50054	-1.55524	-1.55221	-1.54918	-1.54615	-1.56724
-1.50942	-1.50607	-1.50295	-1.5	-1.55467	-1.55159	-1.54855	-1.54555	-1.56666
-1.50875	-1.50548	-1.50239	-1.49947	-1.50943	-1.50643	-1.54796	-1.54498	-1.5661
-1.50812	-1.5049	-1.50185	-1.49895	-1.50891	-1.5059	-1.54741	-1.54443	-1.56553

-0.24762	-0.24682	-0.24606	-0.24532	-0.31983	-0.31886	-0.4927	-0.49108	-0.609
-0.24789	-0.24706	-0.2463	-0.24557	-0.32006	-0.31909	-0.49305	-0.49145	-0.60949
-0.24816	-0.24732	-0.24655	-0.24582	-0.32028	-0.31931	-0.49339	-0.49181	-0.60996
-0.24843	-0.24757	-0.24679	-0.24605	-0.32049	-0.31952	-0.49376	-0.49219	-0.49061
-0.24872	-0.24784	-0.24704	-0.24629	-0.32069	-0.31974	-0.49413	-0.49257	-0.491
-0.24902	-0.24812	-0.2473	-0.24653	-0.3209	-0.31995	-0.41284	-0.41154	-0.49137
-0.24933	-0.24842	-0.24758	-0.24678	-0.32112	-0.32016	-0.41312	-0.41182	-0.49172
-0.24964	-0.24872	-0.24786	-0.24703	-0.32135	-0.32039	-0.41341	-0.41212	-0.49208
-0.24994	-0.24902	-0.24814	-0.2473	-0.32159	-0.32063	-0.41373	-0.41244	-0.49247
-0.25024	-0.24932	-0.24843	-0.24757	-0.24676	-0.24599	-0.3199	-0.3189	-0.49287
-0.25053	-0.24961	-0.24871	-0.24783	-0.24701	-0.24624	-0.32012	-0.31912	-0.49325
-0.25082	-0.2499	-0.249	-0.24811	-0.24728	-0.24648	-0.32033	-0.31934	-0.49364
-0.19895	-0.19826	-0.24929	-0.2484	-0.24755	-0.24674	-0.32056	-0.31957	-0.49399
-0.19919	-0.1985	-0.2496	-0.2487	-0.24783	-0.247	-0.3208	-0.3198	-0.49434
-0.19941	-0.19873	-0.24991	-0.249	-0.24813	-0.24728	-0.24645	-0.24565	-0.49473
-0.19963	-0.19895	-0.25022	-0.24932	-0.24844	-0.24757	-0.24673	-0.24593	-0.49515
-0.19984	-0.19916	-0.25052	-0.24962	-0.24874	-0.24787	-0.24702	-0.24621	-0.31958
-0.20005	-0.19936	-0.2508	-0.24991	-0.24903	-0.24817	-0.24732	-0.2465	-0.31985
-0.20028	-0.19957	-0.1989	-0.19824	-0.24932	-0.24846	-0.24762	-0.24679	-0.24597
-0.20052	-0.1998	-0.19912	-0.19845	-0.2496	-0.24874	-0.24789	-0.24707	-0.24625
-0.20077	-0.20005	-0.19935	-0.19868	-0.24988	-0.24901	-0.24816	-0.24734	-0.24653
-0.20102	-0.20029	-0.19959	-0.19891	-0.25018	-0.24929	-0.24843	-0.2476	-0.24679
-0.20128	-0.20055	-0.19984	-0.19916	-0.2505	-0.24958	-0.2487	-0.24786	-0.24704
-0.20153	-0.2008	-0.2001	-0.1994	-0.19871	-0.19802	-0.249	-0.24813	-0.2473
-0.20177	-0.20106	-0.20035	-0.19964	-0.19895	-0.19826	-0.24931	-0.24842	-0.24757
-0.15188	-0.15135	-0.2006	-0.19988	-0.19919	-0.1985	-0.24963	-0.24872	-0.24785
-0.15207	-0.15155	-0.20084	-0.20012	-0.19942	-0.19874	-0.24995	-0.24903	-0.24815
-0.15227	-0.15175	-0.20108	-0.20036	-0.19967	-0.19898	-0.25027	-0.24935	-0.24846
-0.15247	-0.15194	-0.20131	-0.20061	-0.19991	-0.19922	-0.19853	-0.19785	-0.24877
-0.15268	-0.15214	-0.20155	-0.20086	-0.20016	-0.19947	-0.19877	-0.19809	-0.24909
-0.09952	-0.09917	-0.20178	-0.2011	-0.20041	-0.19971	-0.19901	-0.19832	-0.2494
-0.09964	-0.09928	-0.20201	-0.20134	-0.20064	-0.19995	-0.19925	-0.19856	-0.24972
-0.09977	-0.0994	-0.20225	-0.20158	-0.20088	-0.20019	-0.19949	-0.1988	-0.25004
-0.09991	-0.09954	-0.2025	-0.20182	-0.20112	-0.20042	-0.19973	-0.19903	-0.25036
-0.10005	-0.09968	-0.20276	-0.20207	-0.20137	-0.20066	-0.19996	-0.19927	-0.19858
-0.10019	-0.09981	-0.20301	-0.20231	-0.20161	-0.2009	-0.2002	-0.19951	-0.19881
-0.10033	-0.09994	-0.20326	-0.20256	-0.20185	-0.20115	-0.20044	-0.19975	-0.19905
-0.05254	-0.05228	-0.09971	-0.09936	-0.20209	-0.20139	-0.20069	-0.19998	-0.19929
-0.05264	-0.05237	-0.09984	-0.09948	-0.20234	-0.20163	-0.20093	-0.20022	-0.19952
-0.05274	-0.05246	-0.09997	-0.09961	-0.20258	-0.20188	-0.20117	-0.20046	-0.19976
-0.05284	-0.05257	-0.10011	-0.09975	-0.20283	-0.20212	-0.20141	-0.2007	-0.2
-0.05294	-0.05267	-0.10025	-0.09988	-0.09952	-0.09918	-0.20165	-0.20094	-0.20024
-0.05304	-0.05277	-0.10039	-0.10001	-0.09965	-0.0993	-0.20189	-0.20117	-0.20048
		-0.10053	-0.10014	-0.09977	-0.09942	-0.20213	-0.20141	-0.20072
		-0.10069	-0.10029	-0.09992	-0.09955	-0.20238	-0.20167	-0.20097
		-0.10085	-0.10045	-0.10006	-0.09969	-0.20262	-0.20192	-0.20123
		-0.05295	-0.05268	-0.10021	-0.09982	-0.09945	-0.09911	-0.20149
		-0.05305	-0.05278	-0.10035	-0.09996	-0.09958	-0.09924	-0.20175
				-0.10049	-0.10009	-0.09971	-0.09936	-0.20199
				-0.10063	-0.10022	-0.09983	-0.09948	-0.20222

-0.05282	-0.05254	-0.09997	-0.09961	-0.15013
-0.05293	-0.05265	-0.10013	-0.09976	-0.1503
-0.05304	-0.05276	-0.10029	-0.09991	-0.15049
-0.05315	-0.05288	-0.10044	-0.10006	-0.09968
-0.05325	-0.05299	-0.1006	-0.1002	-0.09981
		-0.05284	-0.05256	-0.09995
		-0.05294	-0.05267	-0.1001
		-0.05304	-0.05277	-0.10025
		-0.05314	-0.05288	-0.10039
				-0.05272
				-0.05283
				-0.05295
				-0.05306
				-0.05317

						-0.05822	-0.05773	-0.05724
						-0.05802	-0.05755	-0.05708
						-0.05781	-0.05736	-0.05691
						-0.05759	-0.05715	-0.05672
				-0.05837	-0.05786	-0.24973	-0.24871	-0.24771
				-0.05818	-0.05768	-0.24932	-0.24828	-0.24725
				-0.05797	-0.05751	-0.24891	-0.24785	-0.2468
				-0.05776	-0.05731	-0.34369	-0.34237	-0.34107
				-0.05755	-0.05712	-0.34311	-0.34181	-0.34052
				-0.05736	-0.05692	-0.34255	-0.34126	-0.33999
				-0.05719	-0.05674	-0.342	-0.34072	-0.33947
		-0.05794	-0.05747	-0.34407	-0.34276	-0.43241	-0.43081	-0.42924
		-0.05775	-0.05728	-0.34352	-0.34223	-0.43177	-0.43017	-0.42861
		-0.05757	-0.0571	-0.34298	-0.34171	-0.52275	-0.52087	-0.51904
		-0.05738	-0.05692	-0.34245	-0.34117	-0.52197	-0.52012	-0.51831
		-0.05719	-0.05675	-0.34193	-0.34062	-0.52119	-0.51937	-0.51759
-0.05794	-0.05746	-0.24806	-0.24702	-0.43231	-0.43068	-0.56583	-0.56392	-0.56204
-0.05776	-0.05729	-0.24765	-0.2466	-0.43163	-0.43002	-0.565	-0.56312	-0.56128
-0.05757	-0.05711	-0.24723	-0.24619	-0.43097	-0.42938	-0.7426	-0.74012	-0.73769
-0.05738	-0.05692	-0.24681	-0.24578	-0.43033	-0.42876	-0.74156	-0.7391	-0.73668
-0.24849	-0.24743	-0.34183	-0.34055	-0.47696	-0.47528	-0.78726	-0.7846	-0.78197
-0.24806	-0.24701	-0.34129	-0.33999	-0.47622	-0.4746	-0.78617	-0.78353	-0.78091
-0.24765	-0.2466	-0.34076	-0.33944	-0.4755	-0.47392	-0.78509	-0.78246	-0.77985
-0.24724	-0.24618	-0.43085	-0.42924	-0.74243	-0.73996	-0.89767	-0.89474	-0.89183
-0.24683	-0.24576	-0.43019	-0.42862	-0.74138	-0.73894	-0.89643	-0.89352	-0.89062
-0.24643	-0.24536	-0.47681	-0.47512	-0.78706	-0.78442	-0.96936	-0.96621	-0.96305
-0.24604	-0.24498	-0.47616	-0.47447	-0.78595	-0.78333	-0.96808	-0.96492	-0.96175
-0.29868	-0.29748	-0.5279	-0.52599	-0.78489	-0.78227	-0.96679	-0.96362	-0.96046
-0.29819	-0.29702	-0.52714	-0.5253	-0.78388	-0.78122	-0.96548	-0.96232	-0.95916
-0.39656	-0.39515	-0.78821	-0.78554	-0.89637	-0.89338	-0.9642	-0.96103	-0.95786
-0.39601	-0.39461	-0.78709	-0.78446	-0.8952	-0.89226	-0.96301	-0.95977	-0.95654
-0.39548	-0.3941	-0.78598	-0.78338	-0.89402	-0.89114	-0.96182	-0.95853	-0.95525
-0.48828	-0.48659	-0.78494	-0.78231	-0.96689	-0.96376	-1.03075	-1.02703	-1.02332
-0.48763	-0.48598	-0.78393	-0.78126	-0.9656	-0.96248	-1.02933	-1.02566	-1.02198
-0.74798	-0.74544	-0.89638	-0.89342	-0.96436	-0.9612	-1.02786	-1.02422	-1.02059
-0.74692	-0.74442	-0.89523	-0.89232	-0.96317	-0.95993	-1.02634	-1.02273	-1.01916
-0.74588	-0.7434	-0.89405	-0.8912	-1.0032	-0.99974	-1.04633	-1.04266	-1.03906
-0.74488	-0.74237	-0.89285	-0.89003	-1.00189	-0.99846	-1.04494	-1.04121	-1.03756
-0.89753	-0.89456	-0.93224	-0.92924	-1.00057	-0.99717	-1.04357	-1.03979	-1.03611
-0.89642	-0.89347	-0.93105	-0.92798	-0.99919	-0.99583	-1.04219	-1.03845	-1.03479

-0.89532	-0.8924	-0.92988	-0.92674	-0.99781	-0.99449	-1.0408	-1.03714	-1.03354
-0.89423	-0.89135	-1.00334	-0.99988	-1.04662	-1.043	-1.14053	-1.13624	-1.132
-0.89314	-0.8903	-1.00212	-0.99863	-1.04525	-1.04159	-1.13885	-1.13466	-1.13053
-0.89203	-0.88922	-1.00088	-0.99738	-1.04389	-1.0402	-1.20387	-1.19947	-1.19515
-0.89091	-0.88811	-0.99962	-0.99614	-1.04255	-1.03882	-1.20205	-1.19775	-1.19355
-0.97215	-0.96901	-0.99836	-0.99489	-1.04123	-1.03748	-1.22897	-1.22469	-1.2205
-0.97093	-0.96777	-0.9971	-0.99363	-1.03992	-1.0362	-1.22733	-1.22305	-1.21887
-0.96971	-0.96652	-0.99583	-0.99238	-1.03862	-1.03495	-1.22573	-1.22143	-1.21722
-1.00124	-0.99795	-1.0447	-1.04099	-1.19682	-1.19238	-1.22413	-1.21978	-1.21554
-0.99999	-0.99666	-1.04334	-1.03966	-1.19525	-1.19086	-1.22256	-1.21814	-1.21386
-0.99871	-0.99534	-1.04194	-1.03832	-1.23	-1.22546	-1.278	-1.27337	-1.26885
-0.99741	-0.99399	-1.04053	-1.03697	-1.22835	-1.22387	-1.27638	-1.27175	-1.26722
-1.02495	-1.02123	-1.19906	-1.19479	-1.22672	-1.22229	-1.30071	-1.29604	-1.29145
-1.02362	-1.0199	-1.19742	-1.19321	-1.2251	-1.22071	-1.29909	-1.29444	-1.28987
-1.0223	-1.01859	-1.19582	-1.19165	-1.28069	-1.27611	-1.33142	-1.32668	-1.32201
-1.02097	-1.01728	-1.19424	-1.19011	-1.27905	-1.27448	-1.32974	-1.32505	-1.3204
-1.01966	-1.01598	-1.19269	-1.1886	-1.27741	-1.27286	-1.32807	-1.32341	-1.31879
-1.19995	-1.19542	-1.19117	-1.1871	-1.30175	-1.29714	-1.32639	-1.32177	-1.31718
-1.19836	-1.19388	-1.18966	-1.18562	-1.30011	-1.29551	-1.32472	-1.32013	-1.31558
-1.19676	-1.19234	-1.28147	-1.27705	-1.29848	-1.2939	-1.37923	-1.37447	-1.36974
-1.19517	-1.1908	-1.27982	-1.27543	-1.29687	-1.29233	-1.37757	-1.3728	-1.36807
-1.1936	-1.18929	-1.30418	-1.29973	-1.29525	-1.29076	-1.37591	-1.37114	-1.36641
-1.19207	-1.18782	-1.30255	-1.29809	-1.29363	-1.28917	-1.37425	-1.36948	-1.36476
-1.21806	-1.2138	-1.30096	-1.29648	-1.29202	-1.28758	-1.37258	-1.36783	-1.36312
-1.21662	-1.21243	-1.29944	-1.29492	-1.29044	-1.28601	-1.37091	-1.36617	-1.36148
-1.21521	-1.21107	-1.29795	-1.29337	-1.28887	-1.28444	-1.36925	-1.36451	-1.35985
-1.30555	-1.30099	-1.29641	-1.29184	-1.31816	-1.31362	-1.39567	-1.39089	-1.38622
-1.30402	-1.29942	-1.29485	-1.29032	-1.3166	-1.31203	-1.39398	-1.38923	-1.3846
-1.30246	-1.29784	-1.29328	-1.28877	-1.31502	-1.31044	-1.52041	-1.51532	-1.51036
-1.30086	-1.29625	-1.2917	-1.2872	-1.31343	-1.30884	-1.5186	-1.51357	-1.50867
-1.29926	-1.29466	-1.29011	-1.28562	-1.39855	-1.39373	-1.51682	-1.51186	-1.50702
-1.29765	-1.29305	-1.28851	-1.28404	-1.39686	-1.39207	-1.51506	-1.51021	-1.50545
-1.29606	-1.29146	-1.28694	-1.28247	-1.39519	-1.39045	-1.51335	-1.50858	-1.50388
-1.29449	-1.28993	-1.31501	-1.31046	-1.39357	-1.3889	-1.51173	-1.50697	-1.50226
-1.29293	-1.28844	-1.3135	-1.30894	-1.39198	-1.3874	-1.51014	-1.50536	-1.50063
-1.29142	-1.28698	-1.34227	-1.33763	-1.39047	-1.3859	-1.53081	-1.52597	-1.52118
-1.28994	-1.28556	-1.34084	-1.33621	-1.389	-1.38441	-1.52915	-1.5243	-1.51949
-1.31812	-1.31367	-1.33934	-1.33473	-1.51505	-1.51009	-1.54738	-1.54246	-1.53758
-1.31667	-1.31215	-1.33777	-1.33317	-1.51332	-1.50839	-1.54567	-1.54076	-1.53588
-1.3152	-1.31065	-1.33622	-1.33163	-1.55392	-1.54892	-1.56647	-1.56149	-1.55656
-1.31371	-1.30916	-1.33471	-1.33013	-1.55219	-1.54721	-1.56476	-1.55983	-1.55496
-1.31221	-1.30769	-1.33323	-1.32868	-1.55052	-1.54555	-1.56309	-1.5582	-1.55337
-1.34091	-1.33635	-1.33182	-1.32733	-1.54898	-1.54402	-1.56152	-1.55661	-1.55175
-1.3394	-1.33489	-1.33042	-1.32602	-1.5475	-1.54255	-1.56	-1.55503	-1.55012
-1.3379	-1.33339	-1.53349	-1.52856	-1.54589	-1.54095	-1.55838	-1.55341	-1.5485
-1.33643	-1.33186	-1.53178	-1.52688	-1.5442	-1.53927	-1.55668	-1.55177	-1.5469
-1.31296	-1.30843	-1.5301	-1.52521	-1.54252	-1.53759	-1.55498	-1.55013	-1.54532
-1.31151	-1.30701	-1.52845	-1.52356	-1.54083	-1.53589	-1.55327	-1.5485	-1.54377
-1.43564	-1.43081	-1.54912	-1.54416	-1.53921	-1.53426	-1.62108	-1.61618	-1.61133
-1.43416	-1.42939	-1.5476	-1.54265	-1.53771	-1.53277	-1.61948	-1.61454	-1.60965

-1.43271	-1.42799	-1.54611	-1.54117	-1.53624	-1.53131	-1.61792	-1.61292	-1.60797
-1.43118	-1.42651	-1.54452	-1.53959	-1.5579	-1.55295	-1.61628	-1.61125	-1.6063
-1.42963	-1.42502	-1.5429	-1.53797	-1.55628	-1.55135	-1.61461	-1.60959	-1.60465
-1.53261	-1.52767	-1.54134	-1.53642	-1.60192	-1.59689	-1.61301	-1.60799	-1.60306
-1.53112	-1.52619	-1.53985	-1.53493	-1.60037	-1.59534	-1.61146	-1.60646	-1.60153
-1.5296	-1.52468	-1.59128	-1.58623	-1.59878	-1.59377	-1.60988	-1.6049	-1.59998
-1.528	-1.52309	-1.58963	-1.58459	-1.59712	-1.59212	-1.60824	-1.60327	-1.59837
-1.52641	-1.52152	-1.58799	-1.58296	-1.59548	-1.59049	-1.60663	-1.60168	-1.5968
-1.57366	-1.56865	-1.58644	-1.58142	-1.59394	-1.58896	-1.60511	-1.6002	-1.59535
-1.57213	-1.56713	-1.58492	-1.5799	-1.59241	-1.58744	-1.60361	-1.59876	-1.59396
-1.59338	-1.58832	-1.5833	-1.5783	-1.59081	-1.58584	-1.60203	-1.59725	-1.59252
-1.59169	-1.58664	-1.58163	-1.57666	-1.58916	-1.5842	-1.60042	-1.5957	-1.59105
-1.5901	-1.58505	-1.58006	-1.57511	-1.58761	-1.58266	-1.57782	-1.57316	-1.56856
-1.58861	-1.58358	-1.57859	-1.57366	-1.58615	-1.58122	-1.57638	-1.57168	-1.56704
-1.58715	-1.58212	-1.57714	-1.57221	-1.56732	-1.56245	-1.57497	-1.57023	-1.56555
-1.5857	-1.58068	-1.57571	-1.57078	-1.5659	-1.56105	-1.57357	-1.56884	-1.56416
-1.58421	-1.57921	-1.57424	-1.56932	-1.56445	-1.55963	-1.57215	-1.56742	-1.56275
-1.5826	-1.57761	-1.57266	-1.56775	-1.5629	-1.5581	-1.57062	-1.56589	-1.56124
-1.58094	-1.57598	-1.57105	-1.56614	-1.56131	-1.55654	-1.56906	-1.56432	-1.55969
-1.5794	-1.57446	-1.56954	-1.56464	-1.55982	-1.55507	-1.56759	-1.56285	-1.55824
-1.57793	-1.57301	-1.5681	-1.56322	-1.55842	-1.55368	-1.56619	-1.56144	-1.55686
-1.57651	-1.5716	-1.56671	-1.56185	-1.55705	-1.55232	-1.54764	-1.54293	-1.53842
-1.57516	-1.57025	-1.56536	-1.56052	-1.55574	-1.55102	-1.54634	-1.54161	-1.53712
-1.5738	-1.56888	-1.56401	-1.55918	-1.55441	-1.5497	-1.54504	-1.54032	-1.53587
-1.57241	-1.56749	-1.56263	-1.55782	-1.55307	-1.54837	-1.54374	-1.53906	-1.53467
-1.57098	-1.56606	-1.56121	-1.55642	-1.55169	-1.547	-1.5424	-1.5378	-1.5335
-1.56945	-1.56452	-1.55968	-1.55492	-1.5502	-1.54552	-1.54097	-1.5365	-1.53235
-1.56789	-1.56294	-1.5581	-1.55336	-1.54865	-1.54397	-1.53949	-1.53519	-1.53122
-1.5664	-1.56145	-1.55663	-1.5519	-1.54721	-1.54252	-1.5381	-1.53396	-1.53016
-1.56497	-1.56004	-1.55524	-1.55053	-1.54584	-1.54115	-1.53678	-1.5328	-1.52916
-1.56358	-1.55868	-1.55389	-1.54919	-1.54452	-1.53984	-1.54765	-1.54366	-1.54003
-1.56222	-1.55736	-1.55259	-1.54791	-1.54325	-1.53858	-1.54643	-1.54257	-1.53907
-1.56088	-1.55605	-1.5513	-1.54663	-1.542	-1.53739	-1.54529	-1.54154	-1.53814
-1.55954	-1.55473	-1.55	-1.54534	-1.54078	-1.53628	-1.54427	-1.5406	-1.53727
-1.5582	-1.55342	-1.54869	-1.54404	-1.53956	-1.53522	-1.54331	-1.53972	-1.53645
-1.55685	-1.5521	-1.54738	-1.54274	-1.53835	-1.53416	-1.54235	-1.53885	-1.53565
-1.55551	-1.55078	-1.54607	-1.54144	-1.53713	-1.53311	-1.54141	-1.53802	-1.53488
-1.55418	-1.54947	-1.54478	-1.54017	-1.53594	-1.53206	-1.54047	-1.53717	-1.53411
-1.55286	-1.54817	-1.54349	-1.53891	-1.53476	-1.53102	-1.53953	-1.53632	-1.53333
-1.55155	-1.54688	-1.54224	-1.5377	-1.54581	-1.54205	-1.5386	-1.53548	-1.53256
-1.55025	-1.5456	-1.54101	-1.53655	-1.54473	-1.54107	-1.53771	-1.53465	-1.53177
-1.54896	-1.54433	-1.53981	-1.53544	-1.5437	-1.54013	-1.53684	-1.53384	-1.531
-1.54765	-1.54305	-1.53862	-1.53439	-1.54272	-1.53924	-1.53601	-1.53305	-1.53024
-1.54635	-1.54177	-1.53745	-1.53336	-1.54178	-1.53838	-1.53521	-1.53228	-1.52949
-1.54504	-1.54049	-1.53627	-1.53234	-1.54084	-1.53753	-1.53442	-1.53152	-1.52874
-1.54373	-1.53921	-1.53509	-1.53131	-1.53991	-1.53669	-1.53364	-1.53077	-1.52801
-1.54245	-1.53796	-1.54624	-1.54246	-1.53899	-1.53585	-1.57504	-1.57205	-1.56916
-1.54121	-1.53674	-1.54509	-1.54143	-1.53807	-1.53501	-1.57423	-1.57127	-1.56838
-1.54005	-1.53562	-1.54401	-1.54045	-1.57954	-1.57642	-1.57341	-1.57048	-1.56761
-1.53904	-1.53466	-1.54307	-1.53955	-1.57864	-1.57556	-1.57259	-1.56969	-1.56685

-1.53807	-1.53377	-1.54221	-1.5387	-1.57777	-1.57473	-1.57178	-1.56891	-1.56609
-1.53706	-1.53291	-1.58392	-1.58031	-1.57697	-1.57393	-1.6221	-1.61898	-1.61593
-1.53603	-1.53206	-1.58312	-1.57955	-1.57622	-1.57317	-1.62128	-1.61817	-1.61512
-1.53496	-1.53114	-1.58228	-1.57879	-1.57551	-1.57245	-1.62049	-1.61738	-1.61433
-1.53383	-1.53017	-1.5814	-1.57805	-1.57483	-1.57176	-1.61973	-1.61661	-1.61356
-1.58781	-1.58401	-1.5805	-1.57727	-1.57412	-1.57108	-1.61902	-1.6159	-1.61284
-1.58671	-1.58301	-1.57959	-1.57645	-1.57338	-1.57041	-1.61837	-1.61526	-1.61219
-1.58568	-1.58205	-1.5787	-1.57562	-1.57262	-1.56972	-1.61769	-1.6146	-1.61154
-1.58477	-1.58118	-1.57786	-1.57481	-1.57184	-1.56897	-1.61692	-1.61386	-1.61082
-1.58393	-1.58037	-1.57707	-1.57402	-1.57106	-1.5682	-1.61611	-1.61308	-1.61007
-1.58317	-1.57965	-1.57636	-1.5733	-1.57033	-1.56747	-1.61532	-1.61228	-1.60929
-1.58247	-1.579	-1.57572	-1.57263	-1.56964	-1.56676	-1.61453	-1.61148	-1.60849
-1.58168	-1.57829	-1.57505	-1.57197	-1.56899	-1.56609	-1.6138	-1.61074	-1.60773
-1.58081	-1.57752	-1.57435	-1.57133	-1.56838	-1.56549	-1.61314	-1.61005	-1.60702
-1.57994	-1.57673	-1.57362	-1.57065	-1.56772	-1.56485	-1.61246	-1.60937	-1.60633
-1.57909	-1.57591	-1.57284	-1.56989	-1.56698	-1.56413	-1.61173	-1.60869	-1.60569
-1.57828	-1.57513	-1.57207	-1.56912	-1.56623	-1.5634	-1.61097	-1.60799	-1.60505
-1.57757	-1.57443	-1.57137	-1.5684	-1.5655	-1.56267	-1.61019	-1.60723	-1.60432
-1.5769	-1.57378	-1.5707	-1.56771	-1.56479	-1.56194	-1.60942	-1.60647	-1.60358
-1.5762	-1.57309	-1.57003	-1.56705	-1.61483	-1.61174	-1.62901	-1.62602	-1.62309
-1.57549	-1.57239	-1.56936	-1.56641	-1.61418	-1.61109	-1.62835	-1.62538	-1.62245
-1.57481	-1.57172	-1.5687	-1.56576	-1.61349	-1.61041	-1.62767	-1.6247	-1.62178
-1.57416	-1.57109	-1.56805	-1.56508	-1.61275	-1.60969	-1.62695	-1.62398	-1.62106
-1.5735	-1.57044	-1.56741	-1.56443	-1.61204	-1.609	-1.62625	-1.62327	-1.62036
-1.5728	-1.56976	-1.56676	-1.56381	-1.6114	-1.60837	-1.62561	-1.62261	-1.61967
-1.5721	-1.56909	-1.56612	-1.5632	-1.61077	-1.60775	-1.62497	-1.62196	-1.61901
-1.57148	-1.56848	-1.5655	-1.56257	-1.61008	-1.60708	-1.6243	-1.62131	-1.61838
-1.57089	-1.5679	-1.56491	-1.56194	-1.60938	-1.60639	-1.62362	-1.62067	-1.61778
-1.57026	-1.56728	-1.5643	-1.56135	-1.60875	-1.60576	-1.62297	-1.62002	-1.61714
-1.5696	-1.56663	-1.56368	-1.56079	-1.60817	-1.60518	-1.62237	-1.61938	-1.61647
-1.56897	-1.566	-1.56309	-1.56023	-1.62781	-1.62476	-1.6442	-1.64109	-1.63807
-1.56839	-1.56542	-1.56252	-1.55967	-1.62719	-1.62412	-1.64353	-1.64046	-1.63747
-1.56782	-1.56484	-1.56194	-1.5591	-1.62656	-1.62348	-1.64287	-1.63984	-1.63688
-1.56723	-1.56423	-1.56132	-1.55848	-1.6259	-1.62286	-1.64226	-1.63925	-1.6363
-1.56664	-1.56363	-1.56071	-1.55787	-1.62524	-1.62225	-1.64166	-1.63866	-1.63572
-1.56604	-1.56304	-1.56013	-1.55729	-1.64716	-1.64408	-1.64105	-1.63805	-1.6351
-1.56543	-1.56247	-1.55957	-1.55673	-1.64653	-1.64346	-1.64043	-1.63742	-1.63447
-1.56484	-1.56189	-1.55899	-1.55615	-1.64586	-1.64279	-1.66297	-1.65975	-1.65663
-1.56426	-1.5613	-1.55839	-1.55553	-1.64514	-1.64206	-1.66218	-1.65905	-1.656
-1.56368	-1.56071	-1.60197	-1.59894	-1.64446	-1.64138	-1.66144	-1.65837	-1.65538
-1.56311	-1.56012	-1.60134	-1.59835	-1.64385	-1.64077	-1.66078	-1.65773	-1.65474
-1.56255	-1.55955	-1.60074	-1.59778	-1.64327	-1.64019	-1.66015	-1.6571	-1.6541
-1.56201	-1.55902	-1.60017	-1.59721	-1.64268	-1.63962	-1.65955	-1.65645	-1.65343
-1.56147	-1.5585	-1.59963	-1.59666	-1.64209	-1.63905	-1.65895	-1.65581	-1.65275
-1.5609	-1.55796	-1.59907	-1.59611	-1.6415	-1.63844	-1.65829	-1.65516	-1.65211
-1.5603	-1.5574	-1.5985	-1.59557	-1.6409	-1.6378	-1.65761	-1.65451	-1.6515
-1.56426	-1.56133	-1.57916	-1.57627	-1.64031	-1.6372	-1.65697	-1.65389	-1.6509
-1.5637	-1.56078	-1.5786	-1.57571	-1.63973	-1.63663	-1.65638	-1.65331	-1.65031
-1.56314	-1.56023	-1.57803	-1.57514	-1.66225	-1.65898	-1.68004	-1.67683	-1.67368
-1.56259	-1.55969	-1.57746	-1.57451	-1.66151	-1.6583	-1.6794	-1.6762	-1.67307

						-0.05822	-0.05773	-0.05724
						-0.05802	-0.05755	-0.05708
						-0.05781	-0.05736	-0.05691
						-0.05759	-0.05715	-0.05672
				-0.05837	-0.05786	-0.24973	-0.24871	-0.24771
				-0.05818	-0.05768	-0.24932	-0.24828	-0.24725
				-0.05797	-0.05751	-0.24891	-0.24785	-0.2468
				-0.05776	-0.05731	-0.34369	-0.34237	-0.34107
				-0.05755	-0.05712	-0.34311	-0.34181	-0.34052
				-0.05736	-0.05692	-0.34255	-0.34126	-0.33999
				-0.05719	-0.05674	-0.342	-0.34072	-0.33947
		-0.05794	-0.05747	-0.34407	-0.34276	-0.43241	-0.43081	-0.42924
		-0.05775	-0.05728	-0.34352	-0.34223	-0.43177	-0.43017	-0.42861
		-0.05757	-0.0571	-0.34298	-0.34171	-0.52275	-0.52087	-0.51904
		-0.05738	-0.05692	-0.34245	-0.34117	-0.52197	-0.52012	-0.51831
		-0.05719	-0.05675	-0.34193	-0.34062	-0.52119	-0.51937	-0.51759
-0.05794	-0.05746	-0.24806	-0.24702	-0.43231	-0.43068	-0.56583	-0.56392	-0.56204
-0.05776	-0.05729	-0.24765	-0.2466	-0.43163	-0.43002	-0.565	-0.56312	-0.56128
-0.05757	-0.05711	-0.24723	-0.24619	-0.43097	-0.42938	-0.7426	-0.74012	-0.73769
-0.05738	-0.05692	-0.24681	-0.24578	-0.43033	-0.42876	-0.74156	-0.7391	-0.73668
-0.24849	-0.24743	-0.34183	-0.34055	-0.47696	-0.47528	-0.78726	-0.7846	-0.78197
-0.24806	-0.24701	-0.34129	-0.33999	-0.47622	-0.4746	-0.78617	-0.78353	-0.78091
-0.24765	-0.2466	-0.34076	-0.33944	-0.4755	-0.47392	-0.78509	-0.78246	-0.77985
-0.24724	-0.24618	-0.43085	-0.42924	-0.74243	-0.73996	-0.89767	-0.89474	-0.89183
-0.24683	-0.24576	-0.43019	-0.42862	-0.74138	-0.73894	-0.89643	-0.89352	-0.89062
-0.24643	-0.24536	-0.47681	-0.47512	-0.78706	-0.78442	-0.96936	-0.96621	-0.96305
-0.24604	-0.24498	-0.47616	-0.47447	-0.78595	-0.78333	-0.96808	-0.96492	-0.96175
-0.29868	-0.29748	-0.5279	-0.52599	-0.78489	-0.78227	-0.96679	-0.96362	-0.96046
-0.29819	-0.29702	-0.52714	-0.5253	-0.78388	-0.78122	-0.96548	-0.96232	-0.95916
-0.39656	-0.39515	-0.78821	-0.78554	-0.89637	-0.89338	-0.9642	-0.96103	-0.95786
-0.39601	-0.39461	-0.78709	-0.78446	-0.8952	-0.89226	-0.96301	-0.95977	-0.95654
-0.39548	-0.3941	-0.78598	-0.78338	-0.89402	-0.89114	-0.96182	-0.95853	-0.95525
-0.48828	-0.48659	-0.78494	-0.78231	-0.96689	-0.96376	-1.03075	-1.02703	-1.02332
-0.48763	-0.48598	-0.78393	-0.78126	-0.9656	-0.96248	-1.02933	-1.02566	-1.02198
-0.74798	-0.74544	-0.89638	-0.89342	-0.96436	-0.9612	-1.02786	-1.02422	-1.02059
-0.74692	-0.74442	-0.89523	-0.89232	-0.96317	-0.95993	-1.02634	-1.02273	-1.01916
-0.74588	-0.7434	-0.89405	-0.8912	-1.0032	-0.99974	-1.04633	-1.04266	-1.03906
-0.74488	-0.74237	-0.89285	-0.89003	-1.00189	-0.99846	-1.04494	-1.04121	-1.03756
-0.89753	-0.89456	-0.93224	-0.92924	-1.00057	-0.99717	-1.04357	-1.03979	-1.03611
-0.89642	-0.89347	-0.93105	-0.92798	-0.99919	-0.99583	-1.04219	-1.03845	-1.03479

-0.89532	-0.8924	-0.92988	-0.92674	-0.99781	-0.99449	-1.0408	-1.03714	-1.03354
-0.89423	-0.89135	-1.00334	-0.99988	-1.04662	-1.043	-1.14053	-1.13624	-1.132
-0.89314	-0.8903	-1.00212	-0.99863	-1.04525	-1.04159	-1.13885	-1.13466	-1.13053
-0.89203	-0.88922	-1.00088	-0.99738	-1.04389	-1.0402	-1.20387	-1.19947	-1.19515
-0.89091	-0.88811	-0.99962	-0.99614	-1.04255	-1.03882	-1.20205	-1.19775	-1.19355
-0.97215	-0.96901	-0.99836	-0.99489	-1.04123	-1.03748	-1.22897	-1.22469	-1.2205
-0.97093	-0.96777	-0.9971	-0.99363	-1.03992	-1.0362	-1.22733	-1.22305	-1.21887
-0.96971	-0.96652	-0.99583	-0.99238	-1.03862	-1.03495	-1.22573	-1.22143	-1.21722
-1.00124	-0.99795	-1.0447	-1.04099	-1.19682	-1.19238	-1.22413	-1.21978	-1.21554
-0.99999	-0.99666	-1.04334	-1.03966	-1.19525	-1.19086	-1.22256	-1.21814	-1.21386
-0.99871	-0.99534	-1.04194	-1.03832	-1.23	-1.22546	-1.278	-1.27337	-1.26885
-0.99741	-0.99399	-1.04053	-1.03697	-1.22835	-1.22387	-1.27638	-1.27175	-1.26722
-1.02495	-1.02123	-1.19906	-1.19479	-1.22672	-1.22229	-1.30071	-1.29604	-1.29145
-1.02362	-1.0199	-1.19742	-1.19321	-1.2251	-1.22071	-1.29909	-1.29444	-1.28987
-1.0223	-1.01859	-1.19582	-1.19165	-1.28069	-1.27611	-1.33142	-1.32668	-1.32201
-1.02097	-1.01728	-1.19424	-1.19011	-1.27905	-1.27448	-1.32974	-1.32505	-1.3204
-1.01966	-1.01598	-1.19269	-1.1886	-1.27741	-1.27286	-1.32807	-1.32341	-1.31879
-1.19995	-1.19542	-1.19117	-1.1871	-1.30175	-1.29714	-1.32639	-1.32177	-1.31718
-1.19836	-1.19388	-1.18966	-1.18562	-1.30011	-1.29551	-1.32472	-1.32013	-1.31558
-1.19676	-1.19234	-1.28147	-1.27705	-1.29848	-1.2939	-1.37923	-1.37447	-1.36974
-1.19517	-1.1908	-1.27982	-1.27543	-1.29687	-1.29233	-1.37757	-1.3728	-1.36807
-1.1936	-1.18929	-1.30418	-1.29973	-1.29525	-1.29076	-1.37591	-1.37114	-1.36641
-1.19207	-1.18782	-1.30255	-1.29809	-1.29363	-1.28917	-1.37425	-1.36948	-1.36476
-1.21806	-1.2138	-1.30096	-1.29648	-1.29202	-1.28758	-1.37258	-1.36783	-1.36312
-1.21662	-1.21243	-1.29944	-1.29492	-1.29044	-1.28601	-1.37091	-1.36617	-1.36148
-1.21521	-1.21107	-1.29795	-1.29337	-1.28887	-1.28444	-1.36925	-1.36451	-1.35985
-1.30555	-1.30099	-1.29641	-1.29184	-1.31816	-1.31362	-1.39567	-1.39089	-1.38622
-1.30402	-1.29942	-1.29485	-1.29032	-1.3166	-1.31203	-1.39398	-1.38923	-1.3846
-1.30246	-1.29784	-1.29328	-1.28877	-1.31502	-1.31044	-1.52041	-1.51532	-1.51036
-1.30086	-1.29625	-1.2917	-1.2872	-1.31343	-1.30884	-1.5186	-1.51357	-1.50867
-1.29926	-1.29466	-1.29011	-1.28562	-1.39855	-1.39373	-1.51682	-1.51186	-1.50702
-1.29765	-1.29305	-1.28851	-1.28404	-1.39686	-1.39207	-1.51506	-1.51021	-1.50545
-1.29606	-1.29146	-1.28694	-1.28247	-1.39519	-1.39045	-1.51335	-1.50858	-1.50388
-1.29449	-1.28993	-1.31501	-1.31046	-1.39357	-1.3889	-1.51173	-1.50697	-1.50226
-1.29293	-1.28844	-1.3135	-1.30894	-1.39198	-1.3874	-1.51014	-1.50536	-1.50063
-1.29142	-1.28698	-1.34227	-1.33763	-1.39047	-1.3859	-1.53081	-1.52597	-1.52118
-1.28994	-1.28556	-1.34084	-1.33621	-1.389	-1.38441	-1.52915	-1.5243	-1.51949
-1.31812	-1.31367	-1.33934	-1.33473	-1.51505	-1.51009	-1.54738	-1.54246	-1.53758
-1.31667	-1.31215	-1.33777	-1.33317	-1.51332	-1.50839	-1.54567	-1.54076	-1.53588
-1.3152	-1.31065	-1.33622	-1.33163	-1.55392	-1.54892	-1.56647	-1.56149	-1.55656
-1.31371	-1.30916	-1.33471	-1.33013	-1.55219	-1.54721	-1.56476	-1.55983	-1.55496
-1.31221	-1.30769	-1.33323	-1.32868	-1.55052	-1.54555	-1.56309	-1.5582	-1.55337
-1.34091	-1.33635	-1.33182	-1.32733	-1.54898	-1.54402	-1.56152	-1.55661	-1.55175
-1.3394	-1.33489	-1.33042	-1.32602	-1.5475	-1.54255	-1.56	-1.55503	-1.55012
-1.3379	-1.33339	-1.53349	-1.52856	-1.54589	-1.54095	-1.55838	-1.55341	-1.5485
-1.33643	-1.33186	-1.53178	-1.52688	-1.5442	-1.53927	-1.55668	-1.55177	-1.5469
-1.31296	-1.30843	-1.5301	-1.52521	-1.54252	-1.53759	-1.55498	-1.55013	-1.54532
-1.31151	-1.30701	-1.52845	-1.52356	-1.54083	-1.53589	-1.55327	-1.5485	-1.54377
-1.43564	-1.43081	-1.54912	-1.54416	-1.53921	-1.53426	-1.62108	-1.61618	-1.61133
-1.43416	-1.42939	-1.5476	-1.54265	-1.53771	-1.53277	-1.61948	-1.61454	-1.60965

-1.43271	-1.42799	-1.54611	-1.54117	-1.53624	-1.53131	-1.61792	-1.61292	-1.60797
-1.43118	-1.42651	-1.54452	-1.53959	-1.5579	-1.55295	-1.61628	-1.61125	-1.6063
-1.42963	-1.42502	-1.5429	-1.53797	-1.55628	-1.55135	-1.61461	-1.60959	-1.60465
-1.53261	-1.52767	-1.54134	-1.53642	-1.60192	-1.59689	-1.61301	-1.60799	-1.60306
-1.53112	-1.52619	-1.53985	-1.53493	-1.60037	-1.59534	-1.61146	-1.60646	-1.60153
-1.5296	-1.52468	-1.59128	-1.58623	-1.59878	-1.59377	-1.60988	-1.6049	-1.59998
-1.528	-1.52309	-1.58963	-1.58459	-1.59712	-1.59212	-1.60824	-1.60327	-1.59837
-1.52641	-1.52152	-1.58799	-1.58296	-1.59548	-1.59049	-1.60663	-1.60168	-1.5968
-1.57366	-1.56865	-1.58644	-1.58142	-1.59394	-1.58896	-1.60511	-1.6002	-1.59535
-1.57213	-1.56713	-1.58492	-1.5799	-1.59241	-1.58744	-1.60361	-1.59876	-1.59396
-1.59338	-1.58832	-1.5833	-1.5783	-1.59081	-1.58584	-1.60203	-1.59725	-1.59252
-1.59169	-1.58664	-1.58163	-1.57666	-1.58916	-1.5842	-1.60042	-1.5957	-1.59105
-1.5901	-1.58505	-1.58006	-1.57511	-1.58761	-1.58266	-1.57782	-1.57316	-1.56856
-1.58861	-1.58358	-1.57859	-1.57366	-1.58615	-1.58122	-1.57638	-1.57168	-1.56704
-1.58715	-1.58212	-1.57714	-1.57221	-1.56732	-1.56245	-1.57497	-1.57023	-1.56555
-1.5857	-1.58068	-1.57571	-1.57078	-1.5659	-1.56105	-1.57357	-1.56884	-1.56416
-1.58421	-1.57921	-1.57424	-1.56932	-1.56445	-1.55963	-1.57215	-1.56742	-1.56275
-1.5826	-1.57761	-1.57266	-1.56775	-1.5629	-1.5581	-1.57062	-1.56589	-1.56124
-1.58094	-1.57598	-1.57105	-1.56614	-1.56131	-1.55654	-1.56906	-1.56432	-1.55969
-1.5794	-1.57446	-1.56954	-1.56464	-1.55982	-1.55507	-1.56759	-1.56285	-1.55824
-1.57793	-1.57301	-1.5681	-1.56322	-1.55842	-1.55368	-1.56619	-1.56144	-1.55686
-1.57651	-1.5716	-1.56671	-1.56185	-1.55705	-1.55232	-1.54764	-1.54293	-1.53842
-1.57516	-1.57025	-1.56536	-1.56052	-1.55574	-1.55102	-1.54634	-1.54161	-1.53712
-1.5738	-1.56888	-1.56401	-1.55918	-1.55441	-1.5497	-1.54504	-1.54032	-1.53587
-1.57241	-1.56749	-1.56263	-1.55782	-1.55307	-1.54837	-1.54374	-1.53906	-1.53467
-1.57098	-1.56606	-1.56121	-1.55642	-1.55169	-1.547	-1.5424	-1.5378	-1.5335
-1.56945	-1.56452	-1.55968	-1.55492	-1.5502	-1.54552	-1.54097	-1.5365	-1.53235
-1.56789	-1.56294	-1.5581	-1.55336	-1.54865	-1.54397	-1.53949	-1.53519	-1.53122
-1.5664	-1.56145	-1.55663	-1.5519	-1.54721	-1.54252	-1.5381	-1.53396	-1.53016
-1.56497	-1.56004	-1.55524	-1.55053	-1.54584	-1.54115	-1.53678	-1.5328	-1.52916
-1.56358	-1.55868	-1.55389	-1.54919	-1.54452	-1.53984	-1.54765	-1.54366	-1.54003
-1.56222	-1.55736	-1.55259	-1.54791	-1.54325	-1.53858	-1.54643	-1.54257	-1.53907
-1.56088	-1.55605	-1.5513	-1.54663	-1.542	-1.53739	-1.54529	-1.54154	-1.53814
-1.55954	-1.55473	-1.55	-1.54534	-1.54078	-1.53628	-1.54427	-1.5406	-1.53727
-1.5582	-1.55342	-1.54869	-1.54404	-1.53956	-1.53522	-1.54331	-1.53972	-1.53645
-1.55685	-1.5521	-1.54738	-1.54274	-1.53835	-1.53416	-1.54235	-1.53885	-1.53565
-1.55551	-1.55078	-1.54607	-1.54144	-1.53713	-1.53311	-1.54141	-1.53802	-1.53488
-1.55418	-1.54947	-1.54478	-1.54017	-1.53594	-1.53206	-1.54047	-1.53717	-1.53411
-1.55286	-1.54817	-1.54349	-1.53891	-1.53476	-1.53102	-1.53953	-1.53632	-1.53333
-1.55155	-1.54688	-1.54224	-1.5377	-1.54581	-1.54205	-1.5386	-1.53548	-1.53256
-1.55025	-1.5456	-1.54101	-1.53655	-1.54473	-1.54107	-1.53771	-1.53465	-1.53177
-1.54896	-1.54433	-1.53981	-1.53544	-1.5437	-1.54013	-1.53684	-1.53384	-1.531
-1.54765	-1.54305	-1.53862	-1.53439	-1.54272	-1.53924	-1.53601	-1.53305	-1.53024
-1.54635	-1.54177	-1.53745	-1.53336	-1.54178	-1.53838	-1.53521	-1.53228	-1.52949
-1.54504	-1.54049	-1.53627	-1.53234	-1.54084	-1.53753	-1.53442	-1.53152	-1.52874
-1.54373	-1.53921	-1.53509	-1.53131	-1.53991	-1.53669	-1.53364	-1.53077	-1.52801
-1.54245	-1.53796	-1.54624	-1.54246	-1.53899	-1.53585	-1.57504	-1.57205	-1.56916
-1.54121	-1.53674	-1.54509	-1.54143	-1.53807	-1.53501	-1.57423	-1.57127	-1.56838
-1.54005	-1.53562	-1.54401	-1.54045	-1.57954	-1.57642	-1.57341	-1.57048	-1.56761
-1.53904	-1.53466	-1.54307	-1.53955	-1.57864	-1.57556	-1.57259	-1.56969	-1.56685

-1.53807	-1.53377	-1.54221	-1.5387	-1.57777	-1.57473	-1.57178	-1.56891	-1.56609
-1.53706	-1.53291	-1.58392	-1.58031	-1.57697	-1.57393	-1.6221	-1.61898	-1.61593
-1.53603	-1.53206	-1.58312	-1.57955	-1.57622	-1.57317	-1.62128	-1.61817	-1.61512
-1.53496	-1.53114	-1.58228	-1.57879	-1.57551	-1.57245	-1.62049	-1.61738	-1.61433
-1.53383	-1.53017	-1.5814	-1.57805	-1.57483	-1.57176	-1.61973	-1.61661	-1.61356
-1.58781	-1.58401	-1.5805	-1.57727	-1.57412	-1.57108	-1.61902	-1.6159	-1.61284
-1.58671	-1.58301	-1.57959	-1.57645	-1.57338	-1.57041	-1.61837	-1.61526	-1.61219
-1.58568	-1.58205	-1.5787	-1.57562	-1.57262	-1.56972	-1.61769	-1.6146	-1.61154
-1.58477	-1.58118	-1.57786	-1.57481	-1.57184	-1.56897	-1.61692	-1.61386	-1.61082
-1.58393	-1.58037	-1.57707	-1.57402	-1.57106	-1.5682	-1.61611	-1.61308	-1.61007
-1.58317	-1.57965	-1.57636	-1.5733	-1.57033	-1.56747	-1.61532	-1.61228	-1.60929
-1.58247	-1.579	-1.57572	-1.57263	-1.56964	-1.56676	-1.61453	-1.61148	-1.60849
-1.58168	-1.57829	-1.57505	-1.57197	-1.56899	-1.56609	-1.6138	-1.61074	-1.60773
-1.58081	-1.57752	-1.57435	-1.57133	-1.56838	-1.56549	-1.61314	-1.61005	-1.60702
-1.57994	-1.57673	-1.57362	-1.57065	-1.56772	-1.56485	-1.61246	-1.60937	-1.60633
-1.57909	-1.57591	-1.57284	-1.56989	-1.56698	-1.56413	-1.61173	-1.60869	-1.60569
-1.57828	-1.57513	-1.57207	-1.56912	-1.56623	-1.5634	-1.61097	-1.60799	-1.60505
-1.57757	-1.57443	-1.57137	-1.5684	-1.5655	-1.56267	-1.61019	-1.60723	-1.60432
-1.5769	-1.57378	-1.5707	-1.56771	-1.56479	-1.56194	-1.60942	-1.60647	-1.60358
-1.5762	-1.57309	-1.57003	-1.56705	-1.61483	-1.61174	-1.62901	-1.62602	-1.62309
-1.57549	-1.57239	-1.56936	-1.56641	-1.61418	-1.61109	-1.62835	-1.62538	-1.62245
-1.57481	-1.57172	-1.5687	-1.56576	-1.61349	-1.61041	-1.62767	-1.6247	-1.62178
-1.57416	-1.57109	-1.56805	-1.56508	-1.61275	-1.60969	-1.62695	-1.62398	-1.62106
-1.5735	-1.57044	-1.56741	-1.56443	-1.61204	-1.609	-1.62625	-1.62327	-1.62036
-1.5728	-1.56976	-1.56676	-1.56381	-1.6114	-1.60837	-1.62561	-1.62261	-1.61967
-1.5721	-1.56909	-1.56612	-1.5632	-1.61077	-1.60775	-1.62497	-1.62196	-1.61901
-1.57148	-1.56848	-1.5655	-1.56257	-1.61008	-1.60708	-1.6243	-1.62131	-1.61838
-1.57089	-1.5679	-1.56491	-1.56194	-1.60938	-1.60639	-1.62362	-1.62067	-1.61778
-1.57026	-1.56728	-1.5643	-1.56135	-1.60875	-1.60576	-1.62297	-1.62002	-1.61714
-1.5696	-1.56663	-1.56368	-1.56079	-1.60817	-1.60518	-1.62237	-1.61938	-1.61647
-1.56897	-1.566	-1.56309	-1.56023	-1.62781	-1.62476	-1.6442	-1.64109	-1.63807
-1.56839	-1.56542	-1.56252	-1.55967	-1.62719	-1.62412	-1.64353	-1.64046	-1.63747
-1.56782	-1.56484	-1.56194	-1.5591	-1.62656	-1.62348	-1.64287	-1.63984	-1.63688
-1.56723	-1.56423	-1.56132	-1.55848	-1.6259	-1.62286	-1.64226	-1.63925	-1.6363
-1.56664	-1.56363	-1.56071	-1.55787	-1.62524	-1.62225	-1.64166	-1.63866	-1.63572
-1.56604	-1.56304	-1.56013	-1.55729	-1.64716	-1.64408	-1.64105	-1.63805	-1.6351
-1.56543	-1.56247	-1.55957	-1.55673	-1.64653	-1.64346	-1.64043	-1.63742	-1.63447
-1.56484	-1.56189	-1.55899	-1.55615	-1.64586	-1.64279	-1.66297	-1.65975	-1.65663
-1.56426	-1.5613	-1.55839	-1.55553	-1.64514	-1.64206	-1.66218	-1.65905	-1.656
-1.56368	-1.56071	-1.60197	-1.59894	-1.64446	-1.64138	-1.66144	-1.65837	-1.65538
-1.56311	-1.56012	-1.60134	-1.59835	-1.64385	-1.64077	-1.66078	-1.65773	-1.65474
-1.56255	-1.55955	-1.60074	-1.59778	-1.64327	-1.64019	-1.66015	-1.6571	-1.6541
-1.56201	-1.55902	-1.60017	-1.59721	-1.64268	-1.63962	-1.65955	-1.65645	-1.65343
-1.56147	-1.5585	-1.59963	-1.59666	-1.64209	-1.63905	-1.65895	-1.65581	-1.65275
-1.5609	-1.55796	-1.59907	-1.59611	-1.6415	-1.63844	-1.65829	-1.65516	-1.65211
-1.5603	-1.5574	-1.5985	-1.59557	-1.6409	-1.6378	-1.65761	-1.65451	-1.6515
-1.56426	-1.56133	-1.57916	-1.57627	-1.64031	-1.6372	-1.65697	-1.65389	-1.6509
-1.5637	-1.56078	-1.5786	-1.57571	-1.63973	-1.63663	-1.65638	-1.65331	-1.65031
-1.56314	-1.56023	-1.57803	-1.57514	-1.66225	-1.65898	-1.68004	-1.67683	-1.67368
-1.56259	-1.55969	-1.57746	-1.57451	-1.66151	-1.6583	-1.6794	-1.6762	-1.67307

-0.60709	-0.60522	-0.60345	-0.60176	-0.60008	-0.59842	-0.59674	-0.59503	-0.59329
-0.60757	-0.60568	-0.60388	-0.60216	-0.60045	-0.59879	-0.59712	-0.59544	-0.59374
-0.60805	-0.60615	-0.60433	-0.60257	-0.60085	-0.59917	-0.59751	-0.59585	-0.59418
-0.48902	-0.48743	-0.60483	-0.60305	-0.60131	-0.59962	-0.59795	-0.59628	-0.5946
-0.48943	-0.48786	-0.60536	-0.60355	-0.6018	-0.6001	-0.59841	-0.59672	-0.59502
-0.48982	-0.48827	-0.60586	-0.60405	-0.60228	-0.60057	-0.59887	-0.59716	-0.59546
-0.49019	-0.48866	-0.60636	-0.60454	-0.60277	-0.60104	-0.59932	-0.59761	-0.59591
-0.49056	-0.48904	-0.48751	-0.48599	-0.60324	-0.60149	-0.59976	-0.59805	-0.59635
-0.49093	-0.4894	-0.48787	-0.48634	-0.60369	-0.60192	-0.60017	-0.59846	-0.59677
-0.49131	-0.48977	-0.48823	-0.48671	-0.60415	-0.60236	-0.6006	-0.59889	-0.59719
-0.49171	-0.49017	-0.48864	-0.48711	-0.60465	-0.60285	-0.60108	-0.59934	-0.59763
-0.49211	-0.49058	-0.48905	-0.48751	-0.60516	-0.60335	-0.60157	-0.59981	-0.59808
-0.49247	-0.49096	-0.48944	-0.48791	-0.60566	-0.60385	-0.60206	-0.6003	-0.59856
-0.49283	-0.49132	-0.48981	-0.4883	-0.60615	-0.60435	-0.60257	-0.6008	-0.59905
-0.4932	-0.49168	-0.49017	-0.48866	-0.48716	-0.48567	-0.60305	-0.60129	-0.59954
-0.49358	-0.49203	-0.4905	-0.489	-0.4875	-0.48602	-0.60351	-0.60177	-0.60002
-0.31856	-0.31754	-0.49087	-0.48936	-0.48786	-0.48637	-0.60397	-0.60223	-0.6005
-0.31882	-0.3178	-0.49127	-0.48975	-0.48823	-0.48672	-0.60442	-0.60268	-0.60095
-0.24515	-0.24434	-0.49168	-0.49015	-0.48862	-0.48709	-0.48559	-0.48414	-0.4827
-0.24543	-0.24462	-0.4921	-0.49057	-0.48903	-0.4875	-0.48599	-0.4845	-0.48304
-0.24571	-0.24491	-0.49253	-0.49099	-0.48945	-0.48792	-0.48639	-0.48488	-0.48339
-0.24599	-0.24519	-0.38057	-0.37929	-0.48985	-0.48833	-0.4868	-0.48528	-0.48378
-0.24625	-0.24547	-0.38098	-0.37969	-0.49025	-0.48873	-0.48721	-0.48569	-0.48419
-0.24651	-0.24574	-0.38139	-0.38009	-0.49065	-0.48913	-0.48762	-0.4861	-0.4846
-0.24677	-0.24599	-0.38178	-0.38049	-0.49106	-0.48953	-0.48801	-0.4865	-0.48499
-0.24703	-0.24624	-0.24545	-0.24467	-0.37961	-0.37836	-0.48841	-0.4869	-0.48539
-0.2473	-0.24649	-0.24571	-0.24494	-0.38002	-0.37876	-0.48882	-0.48731	-0.48579
-0.24759	-0.24676	-0.24597	-0.24521	-0.38042	-0.37916	-0.48924	-0.48771	-0.48619
-0.24789	-0.24703	-0.24624	-0.24548	-0.38083	-0.37956	-0.42346	-0.42211	-0.42077
-0.24819	-0.24733	-0.24652	-0.24576	-0.38124	-0.37996	-0.42388	-0.42253	-0.42119
-0.2485	-0.24762	-0.24681	-0.24604	-0.38165	-0.38037	-0.42431	-0.42295	-0.42161
-0.24881	-0.24792	-0.2471	-0.24632	-0.38208	-0.38079	-0.42474	-0.42338	-0.42204
-0.24912	-0.24822	-0.2474	-0.24661	-0.24582	-0.24502	-0.37993	-0.37867	-0.37742
-0.24943	-0.24853	-0.24769	-0.24689	-0.2461	-0.24531	-0.38037	-0.3791	-0.37784
-0.19788	-0.1972	-0.24799	-0.24718	-0.24638	-0.24559	-0.3808	-0.37952	-0.37826
-0.19812	-0.19743	-0.24829	-0.24746	-0.24665	-0.24587	-0.38124	-0.37995	-0.37867
-0.19835	-0.19767	-0.24859	-0.24775	-0.24693	-0.24615	-0.38167	-0.38037	-0.37908
-0.1986	-0.19792	-0.24891	-0.24805	-0.24722	-0.24643	-0.29564	-0.29463	-0.29362
-0.19885	-0.19817	-0.24924	-0.24836	-0.24751	-0.24671	-0.29598	-0.29496	-0.29396
-0.19909	-0.19843	-0.24958	-0.24868	-0.24782	-0.247	-0.29632	-0.2953	-0.2943
-0.19934	-0.19868	-0.24992	-0.249	-0.24813	-0.24729	-0.29665	-0.29565	-0.29466
-0.19958	-0.19893	-0.25025	-0.24933	-0.24845	-0.24758	-0.24675	-0.24596	-0.24518
-0.19982	-0.19917	-0.25058	-0.24967	-0.24877	-0.24787	-0.24702	-0.24624	-0.24548
-0.20006	-0.1994	-0.19873	-0.19804	-0.24909	-0.24818	-0.24731	-0.24653	-0.24577
-0.2003	-0.19964	-0.19896	-0.19828	-0.24941	-0.24849	-0.24762	-0.24683	-0.24605
-0.20055	-0.19988	-0.1992	-0.19852	-0.24973	-0.24882	-0.24795	-0.24712	-0.24633
-0.20081	-0.20012	-0.19944	-0.19875	-0.25005	-0.24915	-0.24828	-0.24743	-0.24661
-0.20106	-0.20037	-0.19968	-0.19899	-0.25037	-0.24949	-0.24861	-0.24774	-0.2469
-0.2013	-0.20062	-0.19993	-0.19924	-0.2507	-0.24982	-0.24894	-0.24805	-0.24719
-0.20153	-0.20085	-0.20017	-0.19949	-0.25104	-0.25014	-0.24925	-0.24836	-0.24749

-0.14964	-0.14917	-0.20042	-0.19975	-0.19907	-0.19839	-0.24957	-0.24867	-0.2478
-0.14981	-0.14933	-0.20067	-0.2	-0.19932	-0.19864	-0.24989	-0.249	-0.24812
-0.14998	-0.14949	-0.20093	-0.20025	-0.19957	-0.19889	-0.25023	-0.24933	-0.24845
-0.09932	-0.09898	-0.20118	-0.2005	-0.19982	-0.19914	-0.25057	-0.24967	-0.24878
-0.09946	-0.09912	-0.20142	-0.20074	-0.20006	-0.19939	-0.25092	-0.25002	-0.24912
-0.09959	-0.09925	-0.14949	-0.149	-0.20032	-0.19964	-0.19897	-0.19829	-0.1976
-0.09974	-0.09938	-0.14965	-0.14917	-0.20057	-0.1999	-0.19922	-0.19853	-0.19785
-0.09988	-0.09952	-0.14982	-0.14933	-0.14884	-0.14836	-0.19947	-0.19878	-0.1981
-0.10002	-0.09965	-0.15	-0.1495	-0.14901	-0.14852	-0.19971	-0.19903	-0.19836
-0.05247	-0.05224	-0.15018	-0.14967	-0.14917	-0.14868	-0.19996	-0.19929	-0.19861
-0.05258	-0.05233	-0.15035	-0.14983	-0.14933	-0.14885	-0.20022	-0.19954	-0.19886
-0.05269	-0.05243	-0.15054	-0.15001	-0.1495	-0.14901	-0.20047	-0.19979	-0.19912
-0.0528	-0.05254	-0.09985	-0.09949	-0.14967	-0.14918	-0.14869	-0.14819	-0.1477
-0.05291	-0.05265	-0.1	-0.09963	-0.14984	-0.14935	-0.14885	-0.14837	-0.14788
		-0.0525	-0.05226	-0.15002	-0.14952	-0.14902	-0.14854	-0.14805
		-0.0526	-0.05236	-0.1502	-0.14969	-0.1492	-0.1487	-0.14821
		-0.05271	-0.05246	-0.10399	-0.10362	-0.14937	-0.14887	-0.14838
		-0.05282	-0.05256	-0.10415	-0.10375	-0.14953	-0.14904	-0.14856
		-0.05293	-0.05266	-0.05242	-0.0522	-0.1497	-0.14921	-0.14873
		-0.05303	-0.05277	-0.05253	-0.0523	-0.14989	-0.14939	-0.14889
		-0.05314	-0.05288	-0.05264	-0.0524	-0.15008	-0.14956	-0.14906
				-0.05274	-0.0525	-0.1039	-0.1035	-0.10313
				-0.05285	-0.05259	-0.10403	-0.10363	-0.10326
				-0.05295	-0.0527	-0.10418	-0.10377	-0.10338
				-0.05306	-0.0528	-0.10434	-0.10391	-0.1035
				-0.05317	-0.05291	-0.1045	-0.10406	-0.10364
				-0.05326	-0.05302	-0.10465	-0.10421	-0.10379
				-0.05336	-0.05312	-0.10481	-0.10437	-0.10394
				-0.05346	-0.05322	-0.05298	-0.05273	-0.05249
				-0.05355	-0.05332	-0.05309	-0.05284	-0.05259
						-0.05319	-0.05294	-0.05269
						-0.05329	-0.05304	-0.0528
						-0.05339	-0.05315	-0.0529
						-0.05349	-0.05326	-0.05302
						-0.05359	-0.05337	-0.05313
						-0.05369	-0.05347	-0.05323
						-0.05378	-0.05356	-0.05334

				-0.10643	-0.10583	-0.10525
				-0.10615	-0.10556	-0.10499
				-0.10588	-0.10529	-0.10472
				-0.1056	-0.10502	-0.10446
				-0.10532	-0.10475	-0.1042
		-0.10632	-0.10568	-0.30694	-0.30565	-0.30439
		-0.10605	-0.10541	-0.30636	-0.30508	-0.30382
		-0.10577	-0.10514	-0.34403	-0.34263	-0.34127
		-0.10549	-0.10487	-0.34338	-0.342	-0.34065
-0.10643	-0.10582	-0.30726	-0.30592	-0.38692	-0.38543	-0.38399
-0.10615	-0.10555	-0.30667	-0.30534	-0.38624	-0.38475	-0.38332

		-0.10586	-0.10528	-0.30609	-0.30477	-0.38558	-0.38409	-0.38265
		-0.10558	-0.105	-0.34373	-0.34229	-0.52558	-0.52368	-0.52184
		-0.1053	-0.10473	-0.34309	-0.34167	-0.52477	-0.5229	-0.52108
		-0.10502	-0.10447	-0.39046	-0.38893	-0.56897	-0.56699	-0.56505
		-0.10475	-0.1042	-0.38979	-0.38829	-0.56814	-0.56618	-0.56426
-0.1057	-0.10507	-0.344	-0.34261	-0.43224	-0.43068	-0.5673	-0.56538	-0.56348
-0.10545	-0.10484	-0.34343	-0.34203	-0.43154	-0.42999	-0.56646	-0.56456	-0.56269
-0.10519	-0.10461	-0.34285	-0.34144	-0.43084	-0.42931	-0.56561	-0.56375	-0.56191
-0.10491	-0.10436	-0.39019	-0.38864	-0.56864	-0.56668	-0.65788	-0.65569	-0.65356
-0.10464	-0.10409	-0.38951	-0.38798	-0.5678	-0.56585	-0.6569	-0.65475	-0.65264
-0.24842	-0.2474	-0.43195	-0.43036	-0.56697	-0.56504	-0.6985	-0.69624	-0.69403
-0.248	-0.24695	-0.43128	-0.42971	-0.56615	-0.56423	-0.6975	-0.69525	-0.69304
-0.34259	-0.34128	-0.52203	-0.52019	-0.65852	-0.65626	-0.8137	-0.81112	-0.80857
-0.34203	-0.34072	-0.52126	-0.51942	-0.65755	-0.65532	-0.81257	-0.80997	-0.80742
-0.43239	-0.43079	-0.52049	-0.51866	-0.73981	-0.73736	-0.8907	-0.8878	-0.88494
-0.4317	-0.43013	-0.51971	-0.5179	-0.73875	-0.73632	-0.88948	-0.88657	-0.8837
-0.43102	-0.42947	-0.51894	-0.51714	-0.73769	-0.73528	-0.88826	-0.88536	-0.88248
-0.52179	-0.51996	-0.65823	-0.65598	-0.81329	-0.81068	-0.96424	-0.96106	-0.95792
-0.52101	-0.51917	-0.65724	-0.65504	-0.81215	-0.80954	-0.96285	-0.9597	-0.95658
-0.52024	-0.51842	-0.73944	-0.73699	-0.8903	-0.88741	-0.99083	-0.98745	-0.9841
-0.51948	-0.51771	-0.73842	-0.73596	-0.88909	-0.88621	-0.98943	-0.98607	-0.98273
-0.56404	-0.56217	-0.78384	-0.78118	-0.96522	-0.96206	-0.98805	-0.98469	-0.98135
-0.56325	-0.56138	-0.78275	-0.78011	-0.96393	-0.96078	-0.98669	-0.98332	-0.97996
-0.74028	-0.73783	-0.89516	-0.89222	-0.96264	-0.95949	-1.02595	-1.0224	-1.01889
-0.73926	-0.73682	-0.89394	-0.89103	-0.96137	-0.9582	-1.02447	-1.02094	-1.01745
-0.73826	-0.73583	-0.89273	-0.88984	-0.96009	-0.9569	-1.02297	-1.01947	-1.01602
-0.78368	-0.78106	-0.96513	-0.96195	-0.98797	-0.98451	-1.18244	-1.17811	-1.17383
-0.7826	-0.77999	-0.96383	-0.96062	-0.98653	-0.98308	-1.18057	-1.17622	-1.17193
-0.89495	-0.89202	-0.96251	-0.95929	-1.02564	-1.02204	-1.17873	-1.17438	-1.1701
-0.89372	-0.89078	-0.96117	-0.95795	-1.02414	-1.02056	-1.17693	-1.17259	-1.16832
-0.96628	-0.96306	-0.98918	-0.98571	-1.14602	-1.14173	-1.17513	-1.17082	-1.16658
-0.96496	-0.96171	-0.98775	-0.98429	-1.14426	-1.13997	-1.17333	-1.16907	-1.16489
-0.96366	-0.96039	-0.98634	-0.98289	-1.14251	-1.13822	-1.17155	-1.16735	-1.16322
-0.96239	-0.95914	-1.02546	-1.02182	-1.14071	-1.13649	-1.20657	-1.20217	-1.19786
-0.96113	-0.95791	-1.02404	-1.02035	-1.13892	-1.13478	-1.20482	-1.20041	-1.19607
-0.98928	-0.9858	-1.14602	-1.14156	-1.1749	-1.17063	-1.203	-1.19863	-1.19434
-0.98789	-0.98444	-1.14435	-1.13996	-1.17327	-1.16892	-1.20116	-1.19686	-1.19265
-1.02701	-1.02342	-1.14264	-1.13832	-1.20843	-1.20387	-1.22983	-1.22547	-1.22121
-1.02554	-1.02193	-1.14087	-1.13665	-1.20671	-1.20223	-1.2282	-1.22377	-1.21944
-1.02407	-1.02045	-1.13912	-1.13496	-1.20497	-1.20057	-1.30807	-1.30338	-1.29875
-1.02257	-1.019	-1.13741	-1.13326	-1.20318	-1.19885	-1.30632	-1.3017	-1.29713
-1.0211	-1.01756	-1.13571	-1.13157	-1.20138	-1.19712	-1.30455	-1.30003	-1.29553
-1.14248	-1.13817	-1.17156	-1.16737	-1.25606	-1.25167	-1.30271	-1.29826	-1.29385
-1.14082	-1.13644	-1.1698	-1.16569	-1.25431	-1.24989	-1.30085	-1.29646	-1.29214
-1.13918	-1.13483	-1.20477	-1.20047	-1.30813	-1.30355	-1.38512	-1.3805	-1.37595
-1.13755	-1.13331	-1.20313	-1.19872	-1.30624	-1.30175	-1.38328	-1.37865	-1.37408
-1.13588	-1.13175	-1.25792	-1.25337	-1.30444	-1.29997	-1.40677	-1.40207	-1.39742
-1.13415	-1.13013	-1.25623	-1.25174	-1.30274	-1.29823	-1.40485	-1.40021	-1.39564
-1.19882	-1.19461	-1.31025	-1.30564	-1.33193	-1.3273	-1.4266	-1.42197	-1.41743
-1.1971	-1.19286	-1.30841	-1.30389	-1.33022	-1.32561	-1.42481	-1.42019	-1.41566

-1.19545	-1.19114	-1.30658	-1.30214	-1.32851	-1.32395	-1.42307	-1.41843	-1.41389
-1.25815	-1.25363	-1.30485	-1.30044	-1.32682	-1.3223	-1.42134	-1.41667	-1.41209
-1.25655	-1.25203	-1.30318	-1.29877	-1.32513	-1.32065	-1.41962	-1.41491	-1.41029
-1.31076	-1.30606	-1.33236	-1.32786	-1.42725	-1.42253	-1.46901	-1.46423	-1.45952
-1.30909	-1.3044	-1.33066	-1.32609	-1.42534	-1.42067	-1.46718	-1.46248	-1.45784
-1.3074	-1.30275	-1.35386	-1.34921	-1.42348	-1.41882	-1.51759	-1.51276	-1.50799
-1.30571	-1.30111	-1.3522	-1.34754	-1.42169	-1.41697	-1.51556	-1.51082	-1.50618
-1.304	-1.29947	-1.35055	-1.34589	-1.41994	-1.41516	-1.51357	-1.50891	-1.50436
-1.32763	-1.32314	-1.3489	-1.34425	-1.46938	-1.46451	-1.51174	-1.5071	-1.50257
-1.32586	-1.32147	-1.34724	-1.34262	-1.46766	-1.46282	-1.50999	-1.50532	-1.50077
-1.3545	-1.35008	-1.37121	-1.36653	-1.46594	-1.46114	-1.50825	-1.50354	-1.49894
-1.35278	-1.34835	-1.36947	-1.36485	-1.46422	-1.45949	-1.50654	-1.50177	-1.49709
-1.35106	-1.3466	-1.47197	-1.46721	-1.51455	-1.50966	-1.52699	-1.52218	-1.51743
-1.34935	-1.34481	-1.47006	-1.46538	-1.51269	-1.50787	-1.52526	-1.52045	-1.51569
-1.37334	-1.36865	-1.46821	-1.46359	-1.51086	-1.50611	-1.54637	-1.54148	-1.53662
-1.37166	-1.36696	-1.46644	-1.46185	-1.50908	-1.50436	-1.54462	-1.53973	-1.53485
-1.37	-1.3653	-1.46471	-1.46015	-1.50732	-1.50264	-1.54287	-1.53798	-1.5331
-1.36833	-1.36364	-1.51502	-1.5103	-1.50559	-1.50092	-1.54111	-1.53624	-1.53136
-1.36668	-1.36199	-1.51323	-1.50855	-1.50388	-1.49921	-1.53936	-1.5345	-1.52963
-1.36502	-1.36036	-1.51145	-1.50681	-1.50216	-1.49749	-1.53761	-1.53275	-1.5279
-1.36336	-1.35874	-1.50967	-1.50507	-1.50044	-1.49577	-1.53585	-1.53101	-1.52618
-1.51776	-1.51272	-1.50793	-1.50334	-1.52089	-1.51619	-1.5341	-1.52927	-1.52446
-1.51597	-1.51097	-1.5062	-1.50163	-1.51918	-1.51448	-1.53235	-1.52754	-1.52275
-1.5142	-1.50925	-1.5045	-1.49993	-1.5402	-1.53541	-1.53062	-1.52582	-1.52106
-1.51244	-1.50754	-1.50281	-1.49824	-1.53846	-1.53368	-1.52891	-1.52412	-1.51937
-1.5107	-1.50587	-1.50116	-1.49656	-1.53673	-1.53197	-1.52721	-1.52244	-1.5177
-1.509	-1.50426	-1.52175	-1.51708	-1.53503	-1.53027	-1.54475	-1.53995	-1.5352
-1.50732	-1.50268	-1.52018	-1.51546	-1.53335	-1.52857	-1.54303	-1.53826	-1.53354
-1.50565	-1.50105	-1.51855	-1.51381	-1.551	-1.54613	-1.54132	-1.53657	-1.53189
-1.50397	-1.49937	-1.51687	-1.51214	-1.54927	-1.54442	-1.53961	-1.53489	-1.53024
-1.52454	-1.51989	-1.55729	-1.55241	-1.54756	-1.54271	-1.59412	-1.58908	-1.58413
-1.52295	-1.51824	-1.55557	-1.55069	-1.54584	-1.54101	-1.59235	-1.58736	-1.58245
-1.52135	-1.5166	-1.55385	-1.54896	-1.54412	-1.53932	-1.59062	-1.58567	-1.58082
-1.53952	-1.53466	-1.55211	-1.54723	-1.54241	-1.53765	-1.58891	-1.58404	-1.57926
-1.53783	-1.53296	-1.55037	-1.54551	-1.54072	-1.536	-1.58724	-1.58245	-1.57773
-1.53612	-1.53124	-1.54864	-1.5438	-1.53906	-1.53442	-1.60758	-1.60273	-1.59795
-1.53439	-1.52952	-1.54692	-1.54212	-1.53744	-1.53287	-1.60597	-1.60111	-1.59629
-1.53269	-1.52783	-1.54525	-1.54051	-1.55864	-1.55402	-1.60434	-1.59947	-1.59463
-1.53101	-1.52617	-1.54362	-1.53896	-1.55709	-1.55245	-1.60266	-1.59779	-1.59295
-1.55165	-1.54678	-1.54204	-1.53742	-1.61091	-1.60592	-1.64549	-1.6405	-1.63558
-1.5501	-1.54528	-1.54054	-1.53587	-1.6092	-1.60422	-1.64375	-1.63874	-1.63382
-1.54856	-1.54377	-1.53902	-1.53431	-1.60748	-1.60251	-1.64201	-1.63699	-1.63208
-1.54694	-1.54217	-1.56018	-1.55538	-1.65036	-1.6453	-1.64029	-1.63525	-1.63035
-1.54529	-1.54052	-1.55852	-1.55374	-1.6486	-1.64357	-1.63859	-1.63354	-1.62867
-1.5665	-1.56167	-1.60303	-1.59813	-1.64688	-1.6419	-1.63698	-1.63196	-1.62713
-1.56488	-1.56003	-1.60134	-1.59646	-1.64518	-1.64028	-1.63544	-1.63048	-1.62571
-1.6096	-1.60463	-1.59973	-1.59487	-1.64355	-1.63867	-1.63387	-1.62899	-1.62432
-1.60802	-1.60309	-1.59822	-1.5934	-1.64201	-1.63706	-1.63224	-1.62748	-1.62297
-1.60643	-1.60154	-1.59671	-1.59192	-1.64046	-1.63544	-1.63061	-1.62599	-1.62165
-1.60477	-1.59992	-1.59511	-1.59035	-1.63882	-1.63378	-1.62897	-1.62449	-1.62035

-1.6031	-1.59829	-1.59351	-1.58878	-1.63719	-1.63214	-1.62737	-1.62304	-1.61908
-1.60147	-1.59672	-1.64565	-1.64065	-1.63567	-1.63062	-1.6259	-1.62171	-1.6179
-1.59986	-1.59517	-1.64411	-1.63914	-1.63419	-1.62918	-1.62452	-1.62045	-1.61678
-1.59828	-1.59361	-1.6425	-1.63756	-1.63266	-1.62774	-1.6338	-1.62973	-1.62606
-1.59673	-1.59203	-1.64083	-1.6359	-1.63108	-1.6263	-1.63248	-1.62852	-1.62494
-1.59517	-1.59044	-1.63917	-1.63427	-1.62953	-1.62492	-1.63124	-1.62739	-1.62389
-1.59356	-1.58884	-1.63752	-1.63266	-1.62803	-1.62362	-1.63009	-1.62635	-1.62293
-1.592	-1.58729	-1.63591	-1.63107	-1.62654	-1.62232	-1.62894	-1.62532	-1.62198
-1.59058	-1.58587	-1.63439	-1.6295	-1.63573	-1.63156	-1.62772	-1.62423	-1.62098
-1.58921	-1.58451	-1.63293	-1.62798	-1.63421	-1.6302	-1.62651	-1.62313	-1.61998
-1.5878	-1.58313	-1.63152	-1.62662	-1.63292	-1.629	-1.62541	-1.62212	-1.61904
-1.58638	-1.58174	-1.63015	-1.62539	-1.63178	-1.62792	-1.62439	-1.62116	-1.61813
-1.56393	-1.55935	-1.6287	-1.6241	-1.6306	-1.62684	-1.62338	-1.6202	-1.61721
-1.56237	-1.55778	-1.62715	-1.62276	-1.6294	-1.62575	-1.62237	-1.61923	-1.61626
-1.56085	-1.55627	-1.63651	-1.63219	-1.62824	-1.6247	-1.6214	-1.6183	-1.61536
-1.55944	-1.55485	-1.63509	-1.63094	-1.62713	-1.62371	-1.6205	-1.61745	-1.61452
-1.55805	-1.55349	-1.63373	-1.62972	-1.62605	-1.62273	-1.6196	-1.61659	-1.61369
-1.5566	-1.55213	-1.63238	-1.6285	-1.62493	-1.62169	-1.61863	-1.61569	-1.61283
-1.55515	-1.55081	-1.63108	-1.62732	-1.62384	-1.62066	-1.61765	-1.61476	-1.61196
-1.60742	-1.60294	-1.62991	-1.62625	-1.62285	-1.61972	-1.61674	-1.61386	-1.61105
-1.60611	-1.6018	-1.62886	-1.62529	-1.62195	-1.61886	-1.61589	-1.61297	-1.61012
-1.60482	-1.60068	-1.62785	-1.62437	-1.62109	-1.61803	-1.61506	-1.61213	-1.60925
-1.60357	-1.5996	-1.62687	-1.62349	-1.62027	-1.61723	-1.61427	-1.61134	-1.60846
-1.59728	-1.59333	-1.6259	-1.62261	-1.61945	-1.61643	-1.61348	-1.61056	-1.60768
-1.59609	-1.59227	-1.62492	-1.6217	-1.6186	-1.6156	-1.61266	-1.60975	-1.60688
-1.59494	-1.59121	-1.62395	-1.62079	-1.61772	-1.61474	-1.61182	-1.60892	-1.60605
-1.59379	-1.59015	-1.58677	-1.58368	-1.61678	-1.61381	-1.63147	-1.62851	-1.6256
-1.59266	-1.5891	-1.58577	-1.58271	-1.61582	-1.61286	-1.63049	-1.62756	-1.62466
-1.5916	-1.58813	-1.58486	-1.58181	-1.59909	-1.59615	-1.61405	-1.61114	-1.60827
-1.59062	-1.58724	-1.58402	-1.58099	-1.59828	-1.59534	-1.61322	-1.61033	-1.60747
-1.58966	-1.58637	-1.5832	-1.58019	-1.59749	-1.59456	-1.65147	-1.64845	-1.64547
-1.58873	-1.58552	-1.5824	-1.5794	-1.59671	-1.5938	-1.65066	-1.64765	-1.64467
-1.58783	-1.58468	-1.5816	-1.57862	-1.59594	-1.59304	-1.64986	-1.64685	-1.64388
-1.58694	-1.58383	-1.58078	-1.57782	-1.59516	-1.59227	-1.64904	-1.64604	-1.64309
-1.58608	-1.583	-1.57997	-1.57703	-1.59438	-1.59149	-1.64822	-1.64524	-1.6423
-1.58526	-1.58219	-1.57918	-1.57624	-1.61362	-1.61061	-1.6474	-1.64443	-1.6415
-1.58446	-1.5814	-1.5784	-1.57546	-1.6128	-1.6098	-1.64659	-1.64362	-1.6407
-1.58365	-1.58061	-1.57762	-1.57469	-1.63244	-1.62939	-1.64579	-1.64282	-1.63989
-1.58285	-1.57982	-1.57684	-1.57391	-1.63162	-1.62858	-1.64499	-1.64202	-1.63908
-1.58203	-1.57902	-1.61646	-1.6134	-1.63079	-1.62777	-1.6442	-1.64121	-1.63826
-1.58122	-1.57822	-1.61563	-1.61258	-1.62996	-1.62695	-1.64339	-1.64038	-1.63741
-1.5804	-1.57743	-1.61481	-1.61176	-1.62913	-1.62613	-1.64258	-1.63956	-1.63658
-1.57961	-1.57665	-1.61401	-1.61096	-1.62831	-1.62533	-1.64179	-1.63877	-1.63581
-1.57882	-1.57588	-1.61321	-1.61016	-1.6275	-1.62452	-1.641	-1.638	-1.63506
-1.61854	-1.61546	-1.61241	-1.60938	-1.62672	-1.62374	-1.62079	-1.61782	-1.61492
-1.61772	-1.61465	-1.61162	-1.60861	-1.62595	-1.62297	-1.62003	-1.61708	-1.6142
-1.61691	-1.61385	-1.61082	-1.60784	-1.62518	-1.62221	-1.61928	-1.61638	-1.61353
-1.61609	-1.61304	-1.61004	-1.60707	-1.62441	-1.62146	-1.61856	-1.61572	-1.61293
-1.61528	-1.61224	-1.62961	-1.6266	-1.62364	-1.62071	-1.64022	-1.63732	-1.63449
-1.61448	-1.61144	-1.6288	-1.62581	-1.62286	-1.61996	-1.63946	-1.63661	-1.63382

-1.61368	-1.61065	-1.628	-1.62502	-1.62209	-1.61921	-1.63871	-1.63589	-1.63314
-1.61289	-1.60987	-1.62721	-1.62423	-1.62132	-1.61845	-1.66112	-1.65811	-1.6552
-1.61209	-1.6091	-1.62642	-1.62345	-1.62054	-1.6177	-1.66027	-1.6573	-1.65445
-1.61131	-1.60833	-1.62565	-1.62268	-1.64222	-1.63928	-1.65942	-1.6565	-1.65369
-1.61054	-1.60756	-1.62487	-1.62192	-1.64144	-1.63851	-1.65859	-1.65571	-1.65294
-1.60981	-1.60682	-1.62411	-1.62117	-1.64067	-1.63776	-1.65779	-1.65494	-1.65219
-1.60913	-1.60611	-1.62338	-1.62043	-1.63991	-1.63703	-1.65704	-1.6542	-1.65144
-1.6288	-1.62569	-1.64521	-1.64214	-1.63917	-1.63632	-1.65632	-1.65347	-1.6507
-1.6281	-1.62504	-1.64453	-1.64143	-1.63845	-1.63561	-1.65558	-1.65273	-1.64994
-1.62739	-1.62438	-1.64387	-1.64075	-1.63776	-1.63493	-1.65487	-1.65201	-1.64919
-1.62661	-1.62364	-1.64315	-1.64008	-1.66017	-1.65716	-1.65423	-1.65134	-1.64849
-1.6258	-1.62286	-1.64238	-1.63941	-1.6595	-1.65654	-1.65363	-1.65071	-1.64781
-1.62503	-1.62209	-1.64162	-1.63869	-1.65875	-1.65584	-1.65297	-1.65006	-1.64716
-1.62428	-1.62134	-1.64083	-1.63792	-1.65793	-1.65507	-1.65225	-1.6494	-1.64655
-1.62358	-1.62064	-1.6401	-1.63718	-1.65715	-1.65431	-1.65152	-1.6487	-1.64588
-1.62296	-1.62003	-1.63947	-1.63653	-1.65644	-1.65359	-1.65078	-1.64796	-1.64513
-1.62233	-1.61942	-1.63884	-1.63589	-1.65577	-1.65289	-1.65006	-1.64722	-1.64438
-1.62161	-1.61872	-1.66129	-1.65816	-1.65514	-1.65228	-1.67337	-1.67035	-1.66732
-1.62086	-1.61799	-1.6605	-1.65748	-1.65454	-1.65168	-1.67274	-1.66967	-1.6666
-1.64259	-1.6396	-1.6597	-1.65675	-1.65385	-1.651	-1.67203	-1.66897	-1.66591
-1.6419	-1.63888	-1.65891	-1.65599	-1.65312	-1.65027	-1.67127	-1.66827	-1.66526
-1.64121	-1.6382	-1.65818	-1.6553	-1.65243	-1.64957	-1.67053	-1.66754	-1.66455
-1.64052	-1.63757	-1.65754	-1.65468	-1.6518	-1.64891	-1.66979	-1.66678	-1.66377
-1.63982	-1.63693	-1.65689	-1.65403	-1.65116	-1.64825	-1.66908	-1.66605	-1.66303
-1.63911	-1.63624	-1.65617	-1.65332	-1.65046	-1.64759	-1.66842	-1.6654	-1.66237
-1.63842	-1.63556	-1.65546	-1.6526	-1.64975	-1.64692	-1.66776	-1.66475	-1.66171
-1.66085	-1.65779	-1.6548	-1.65191	-1.67296	-1.66999	-1.68604	-1.68298	-1.67989
-1.6602	-1.65717	-1.65418	-1.65124	-1.67221	-1.66922	-1.68524	-1.68218	-1.67911
-1.65949	-1.6565	-1.65353	-1.65059	-1.67154	-1.66853	-1.68451	-1.68144	-1.67837
-1.65874	-1.6558	-1.65287	-1.64998	-1.67092	-1.66789	-1.68383	-1.68075	-1.67768
-1.65803	-1.65512	-1.65222	-1.64936	-1.67028	-1.66723	-1.68312	-1.68004	-1.67696
-1.6574	-1.6545	-1.65161	-1.64875	-1.66963	-1.66655	-1.68238	-1.67927	-1.67618
-1.65679	-1.65387	-1.67499	-1.672	-1.68796	-1.6848	-1.68164	-1.67852	-1.67542
-1.65615	-1.65319	-1.67426	-1.67126	-1.68721	-1.68409	-1.68096	-1.67783	-1.67472
-1.65551	-1.65252	-1.67353	-1.67051	-1.68646	-1.68338	-1.68029	-1.67716	-1.67404
-1.65487	-1.65188	-1.67286	-1.66984	-1.68575	-1.6827	-1.67962	-1.67647	-1.67332
-1.65423	-1.65128	-1.67224	-1.6692	-1.68508	-1.68202	-1.67894	-1.67576	-1.67259
-1.65361	-1.65067	-1.67159	-1.66852	-1.68436	-1.68129	-1.69576	-1.69252	-1.68929
-1.65301	-1.65005	-1.67092	-1.66781	-1.68359	-1.68051	-1.69496	-1.69178	-1.68862
-1.65238	-1.64941	-1.67024	-1.66712	-1.68287	-1.67976	-1.69419	-1.69105	-1.68794
-1.65173	-1.64873	-1.66953	-1.66645	-1.6822	-1.67909	-1.69349	-1.69037	-1.68727
-1.65107	-1.64805	-1.66884	-1.6658	-1.68155	-1.67844	-1.69281	-1.68969	-1.68657
-1.6744	-1.67127	-1.68708	-1.684	-1.69849	-1.69531	-1.69214	-1.68898	-1.68585
-1.67372	-1.67063	-1.68644	-1.68337	-1.69783	-1.69465	-1.69146	-1.68827	-1.68511
-1.67306	-1.67	-1.6858	-1.68273	-1.69716	-1.69394	-1.69074	-1.68756	-1.68441
-1.67244	-1.66938	-1.68516	-1.68209	-1.69648	-1.69322	-1.68999	-1.68684	-1.68375
-1.67182	-1.66877	-1.68451	-1.68142	-1.69577	-1.69251	-1.68928	-1.68616	-1.68309
-1.67122	-1.66817	-1.68387	-1.68072	-1.69502	-1.69181	-1.68863	-1.68552	-1.68244
-1.68947	-1.68638	-1.68323	-1.68003	-1.6943	-1.69113	-1.68799	-1.68488	-1.6818
-1.68884	-1.68575	-1.6826	-1.6794	-1.69365	-1.69048	-1.68735	-1.68425	-1.68117

				-0.10643	-0.10583	-0.10525
				-0.10615	-0.10556	-0.10499
				-0.10588	-0.10529	-0.10472
				-0.1056	-0.10502	-0.10446
				-0.10532	-0.10475	-0.1042
		-0.10632	-0.10568	-0.30694	-0.30565	-0.30439
		-0.10605	-0.10541	-0.30636	-0.30508	-0.30382
		-0.10577	-0.10514	-0.34403	-0.34263	-0.34127
		-0.10549	-0.10487	-0.34338	-0.342	-0.34065
-0.10643	-0.10582	-0.30726	-0.30592	-0.38692	-0.38543	-0.38399
-0.10615	-0.10555	-0.30667	-0.30534	-0.38624	-0.38475	-0.38332

		-0.10586	-0.10528	-0.30609	-0.30477	-0.38558	-0.38409	-0.38265
		-0.10558	-0.105	-0.34373	-0.34229	-0.52558	-0.52368	-0.52184
		-0.1053	-0.10473	-0.34309	-0.34167	-0.52477	-0.5229	-0.52108
		-0.10502	-0.10447	-0.39046	-0.38893	-0.56897	-0.56699	-0.56505
		-0.10475	-0.1042	-0.38979	-0.38829	-0.56814	-0.56618	-0.56426
-0.1057	-0.10507	-0.344	-0.34261	-0.43224	-0.43068	-0.5673	-0.56538	-0.56348
-0.10545	-0.10484	-0.34343	-0.34203	-0.43154	-0.42999	-0.56646	-0.56456	-0.56269
-0.10519	-0.10461	-0.34285	-0.34144	-0.43084	-0.42931	-0.56561	-0.56375	-0.56191
-0.10491	-0.10436	-0.39019	-0.38864	-0.56864	-0.56668	-0.65788	-0.65569	-0.65356
-0.10464	-0.10409	-0.38951	-0.38798	-0.5678	-0.56585	-0.6569	-0.65475	-0.65264
-0.24842	-0.2474	-0.43195	-0.43036	-0.56697	-0.56504	-0.6985	-0.69624	-0.69403
-0.248	-0.24695	-0.43128	-0.42971	-0.56615	-0.56423	-0.6975	-0.69525	-0.69304
-0.34259	-0.34128	-0.52203	-0.52019	-0.65852	-0.65626	-0.8137	-0.81112	-0.80857
-0.34203	-0.34072	-0.52126	-0.51942	-0.65755	-0.65532	-0.81257	-0.80997	-0.80742
-0.43239	-0.43079	-0.52049	-0.51866	-0.73981	-0.73736	-0.8907	-0.8878	-0.88494
-0.4317	-0.43013	-0.51971	-0.5179	-0.73875	-0.73632	-0.88948	-0.88657	-0.8837
-0.43102	-0.42947	-0.51894	-0.51714	-0.73769	-0.73528	-0.88826	-0.88536	-0.88248
-0.52179	-0.51996	-0.65823	-0.65598	-0.81329	-0.81068	-0.96424	-0.96106	-0.95792
-0.52101	-0.51917	-0.65724	-0.65504	-0.81215	-0.80954	-0.96285	-0.9597	-0.95658
-0.52024	-0.51842	-0.73944	-0.73699	-0.8903	-0.88741	-0.99083	-0.98745	-0.9841
-0.51948	-0.51771	-0.73842	-0.73596	-0.88909	-0.88621	-0.98943	-0.98607	-0.98273
-0.56404	-0.56217	-0.78384	-0.78118	-0.96522	-0.96206	-0.98805	-0.98469	-0.98135
-0.56325	-0.56138	-0.78275	-0.78011	-0.96393	-0.96078	-0.98669	-0.98332	-0.97996
-0.74028	-0.73783	-0.89516	-0.89222	-0.96264	-0.95949	-1.02595	-1.0224	-1.01889
-0.73926	-0.73682	-0.89394	-0.89103	-0.96137	-0.9582	-1.02447	-1.02094	-1.01745
-0.73826	-0.73583	-0.89273	-0.88984	-0.96009	-0.9569	-1.02297	-1.01947	-1.01602
-0.78368	-0.78106	-0.96513	-0.96195	-0.98797	-0.98451	-1.18244	-1.17811	-1.17383
-0.7826	-0.77999	-0.96383	-0.96062	-0.98653	-0.98308	-1.18057	-1.17622	-1.17193
-0.89495	-0.89202	-0.96251	-0.95929	-1.02564	-1.02204	-1.17873	-1.17438	-1.1701
-0.89372	-0.89078	-0.96117	-0.95795	-1.02414	-1.02056	-1.17693	-1.17259	-1.16832
-0.96628	-0.96306	-0.98918	-0.98571	-1.14602	-1.14173	-1.17513	-1.17082	-1.16658
-0.96496	-0.96171	-0.98775	-0.98429	-1.14426	-1.13997	-1.17333	-1.16907	-1.16489
-0.96366	-0.96039	-0.98634	-0.98289	-1.14251	-1.13822	-1.17155	-1.16735	-1.16322
-0.96239	-0.95914	-1.02546	-1.02182	-1.14071	-1.13649	-1.20657	-1.20217	-1.19786
-0.96113	-0.95791	-1.02404	-1.02035	-1.13892	-1.13478	-1.20482	-1.20041	-1.19607
-0.98928	-0.9858	-1.14602	-1.14156	-1.1749	-1.17063	-1.203	-1.19863	-1.19434
-0.98789	-0.98444	-1.14435	-1.13996	-1.17327	-1.16892	-1.20116	-1.19686	-1.19265
-1.02701	-1.02342	-1.14264	-1.13832	-1.20843	-1.20387	-1.22983	-1.22547	-1.22121
-1.02554	-1.02193	-1.14087	-1.13665	-1.20671	-1.20223	-1.2282	-1.22377	-1.21944
-1.02407	-1.02045	-1.13912	-1.13496	-1.20497	-1.20057	-1.30807	-1.30338	-1.29875
-1.02257	-1.019	-1.13741	-1.13326	-1.20318	-1.19885	-1.30632	-1.3017	-1.29713
-1.0211	-1.01756	-1.13571	-1.13157	-1.20138	-1.19712	-1.30455	-1.30003	-1.29553
-1.14248	-1.13817	-1.17156	-1.16737	-1.25606	-1.25167	-1.30271	-1.29826	-1.29385
-1.14082	-1.13644	-1.1698	-1.16569	-1.25431	-1.24989	-1.30085	-1.29646	-1.29214
-1.13918	-1.13483	-1.20477	-1.20047	-1.30813	-1.30355	-1.38512	-1.3805	-1.37595
-1.13755	-1.13331	-1.20313	-1.19872	-1.30624	-1.30175	-1.38328	-1.37865	-1.37408
-1.13588	-1.13175	-1.25792	-1.25337	-1.30444	-1.29997	-1.40677	-1.40207	-1.39742
-1.13415	-1.13013	-1.25623	-1.25174	-1.30274	-1.29823	-1.40485	-1.40021	-1.39564
-1.19882	-1.19461	-1.31025	-1.30564	-1.33193	-1.3273	-1.4266	-1.42197	-1.41743
-1.1971	-1.19286	-1.30841	-1.30389	-1.33022	-1.32561	-1.42481	-1.42019	-1.41566

-1.19545	-1.19114	-1.30658	-1.30214	-1.32851	-1.32395	-1.42307	-1.41843	-1.41389
-1.25815	-1.25363	-1.30485	-1.30044	-1.32682	-1.3223	-1.42134	-1.41667	-1.41209
-1.25655	-1.25203	-1.30318	-1.29877	-1.32513	-1.32065	-1.41962	-1.41491	-1.41029
-1.31076	-1.30606	-1.33236	-1.32786	-1.42725	-1.42253	-1.46901	-1.46423	-1.45952
-1.30909	-1.3044	-1.33066	-1.32609	-1.42534	-1.42067	-1.46718	-1.46248	-1.45784
-1.3074	-1.30275	-1.35386	-1.34921	-1.42348	-1.41882	-1.51759	-1.51276	-1.50799
-1.30571	-1.30111	-1.3522	-1.34754	-1.42169	-1.41697	-1.51556	-1.51082	-1.50618
-1.304	-1.29947	-1.35055	-1.34589	-1.41994	-1.41516	-1.51357	-1.50891	-1.50436
-1.32763	-1.32314	-1.3489	-1.34425	-1.46938	-1.46451	-1.51174	-1.5071	-1.50257
-1.32586	-1.32147	-1.34724	-1.34262	-1.46766	-1.46282	-1.50999	-1.50532	-1.50077
-1.3545	-1.35008	-1.37121	-1.36653	-1.46594	-1.46114	-1.50825	-1.50354	-1.49894
-1.35278	-1.34835	-1.36947	-1.36485	-1.46422	-1.45949	-1.50654	-1.50177	-1.49709
-1.35106	-1.3466	-1.47197	-1.46721	-1.51455	-1.50966	-1.52699	-1.52218	-1.51743
-1.34935	-1.34481	-1.47006	-1.46538	-1.51269	-1.50787	-1.52526	-1.52045	-1.51569
-1.37334	-1.36865	-1.46821	-1.46359	-1.51086	-1.50611	-1.54637	-1.54148	-1.53662
-1.37166	-1.36696	-1.46644	-1.46185	-1.50908	-1.50436	-1.54462	-1.53973	-1.53485
-1.37	-1.3653	-1.46471	-1.46015	-1.50732	-1.50264	-1.54287	-1.53798	-1.5331
-1.36833	-1.36364	-1.51502	-1.5103	-1.50559	-1.50092	-1.54111	-1.53624	-1.53136
-1.36668	-1.36199	-1.51323	-1.50855	-1.50388	-1.49921	-1.53936	-1.5345	-1.52963
-1.36502	-1.36036	-1.51145	-1.50681	-1.50216	-1.49749	-1.53761	-1.53275	-1.5279
-1.36336	-1.35874	-1.50967	-1.50507	-1.50044	-1.49577	-1.53585	-1.53101	-1.52618
-1.51776	-1.51272	-1.50793	-1.50334	-1.52089	-1.51619	-1.5341	-1.52927	-1.52446
-1.51597	-1.51097	-1.5062	-1.50163	-1.51918	-1.51448	-1.53235	-1.52754	-1.52275
-1.5142	-1.50925	-1.5045	-1.49993	-1.5402	-1.53541	-1.53062	-1.52582	-1.52106
-1.51244	-1.50754	-1.50281	-1.49824	-1.53846	-1.53368	-1.52891	-1.52412	-1.51937
-1.5107	-1.50587	-1.50116	-1.49656	-1.53673	-1.53197	-1.52721	-1.52244	-1.5177
-1.509	-1.50426	-1.52175	-1.51708	-1.53503	-1.53027	-1.54475	-1.53995	-1.5352
-1.50732	-1.50268	-1.52018	-1.51546	-1.53335	-1.52857	-1.54303	-1.53826	-1.53354
-1.50565	-1.50105	-1.51855	-1.51381	-1.551	-1.54613	-1.54132	-1.53657	-1.53189
-1.50397	-1.49937	-1.51687	-1.51214	-1.54927	-1.54442	-1.53961	-1.53489	-1.53024
-1.52454	-1.51989	-1.55729	-1.55241	-1.54756	-1.54271	-1.59412	-1.58908	-1.58413
-1.52295	-1.51824	-1.55557	-1.55069	-1.54584	-1.54101	-1.59235	-1.58736	-1.58245
-1.52135	-1.5166	-1.55385	-1.54896	-1.54412	-1.53932	-1.59062	-1.58567	-1.58082
-1.53952	-1.53466	-1.55211	-1.54723	-1.54241	-1.53765	-1.58891	-1.58404	-1.57926
-1.53783	-1.53296	-1.55037	-1.54551	-1.54072	-1.536	-1.58724	-1.58245	-1.57773
-1.53612	-1.53124	-1.54864	-1.5438	-1.53906	-1.53442	-1.60758	-1.60273	-1.59795
-1.53439	-1.52952	-1.54692	-1.54212	-1.53744	-1.53287	-1.60597	-1.60111	-1.59629
-1.53269	-1.52783	-1.54525	-1.54051	-1.55864	-1.55402	-1.60434	-1.59947	-1.59463
-1.53101	-1.52617	-1.54362	-1.53896	-1.55709	-1.55245	-1.60266	-1.59779	-1.59295
-1.55165	-1.54678	-1.54204	-1.53742	-1.61091	-1.60592	-1.64549	-1.6405	-1.63558
-1.5501	-1.54528	-1.54054	-1.53587	-1.6092	-1.60422	-1.64375	-1.63874	-1.63382
-1.54856	-1.54377	-1.53902	-1.53431	-1.60748	-1.60251	-1.64201	-1.63699	-1.63208
-1.54694	-1.54217	-1.56018	-1.55538	-1.65036	-1.6453	-1.64029	-1.63525	-1.63035
-1.54529	-1.54052	-1.55852	-1.55374	-1.6486	-1.64357	-1.63859	-1.63354	-1.62867
-1.5665	-1.56167	-1.60303	-1.59813	-1.64688	-1.6419	-1.63698	-1.63196	-1.62713
-1.56488	-1.56003	-1.60134	-1.59646	-1.64518	-1.64028	-1.63544	-1.63048	-1.62571
-1.6096	-1.60463	-1.59973	-1.59487	-1.64355	-1.63867	-1.63387	-1.62899	-1.62432
-1.60802	-1.60309	-1.59822	-1.5934	-1.64201	-1.63706	-1.63224	-1.62748	-1.62297
-1.60643	-1.60154	-1.59671	-1.59192	-1.64046	-1.63544	-1.63061	-1.62599	-1.62165
-1.60477	-1.59992	-1.59511	-1.59035	-1.63882	-1.63378	-1.62897	-1.62449	-1.62035

-1.6031	-1.59829	-1.59351	-1.58878	-1.63719	-1.63214	-1.62737	-1.62304	-1.61908
-1.60147	-1.59672	-1.64565	-1.64065	-1.63567	-1.63062	-1.6259	-1.62171	-1.6179
-1.59986	-1.59517	-1.64411	-1.63914	-1.63419	-1.62918	-1.62452	-1.62045	-1.61678
-1.59828	-1.59361	-1.6425	-1.63756	-1.63266	-1.62774	-1.6338	-1.62973	-1.62606
-1.59673	-1.59203	-1.64083	-1.6359	-1.63108	-1.6263	-1.63248	-1.62852	-1.62494
-1.59517	-1.59044	-1.63917	-1.63427	-1.62953	-1.62492	-1.63124	-1.62739	-1.62389
-1.59356	-1.58884	-1.63752	-1.63266	-1.62803	-1.62362	-1.63009	-1.62635	-1.62293
-1.592	-1.58729	-1.63591	-1.63107	-1.62654	-1.62232	-1.62894	-1.62532	-1.62198
-1.59058	-1.58587	-1.63439	-1.6295	-1.63573	-1.63156	-1.62772	-1.62423	-1.62098
-1.58921	-1.58451	-1.63293	-1.62798	-1.63421	-1.6302	-1.62651	-1.62313	-1.61998
-1.5878	-1.58313	-1.63152	-1.62662	-1.63292	-1.629	-1.62541	-1.62212	-1.61904
-1.58638	-1.58174	-1.63015	-1.62539	-1.63178	-1.62792	-1.62439	-1.62116	-1.61813
-1.56393	-1.55935	-1.6287	-1.6241	-1.6306	-1.62684	-1.62338	-1.6202	-1.61721
-1.56237	-1.55778	-1.62715	-1.62276	-1.6294	-1.62575	-1.62237	-1.61923	-1.61626
-1.56085	-1.55627	-1.63651	-1.63219	-1.62824	-1.6247	-1.6214	-1.6183	-1.61536
-1.55944	-1.55485	-1.63509	-1.63094	-1.62713	-1.62371	-1.6205	-1.61745	-1.61452
-1.55805	-1.55349	-1.63373	-1.62972	-1.62605	-1.62273	-1.6196	-1.61659	-1.61369
-1.5566	-1.55213	-1.63238	-1.6285	-1.62493	-1.62169	-1.61863	-1.61569	-1.61283
-1.55515	-1.55081	-1.63108	-1.62732	-1.62384	-1.62066	-1.61765	-1.61476	-1.61196
-1.60742	-1.60294	-1.62991	-1.62625	-1.62285	-1.61972	-1.61674	-1.61386	-1.61105
-1.60611	-1.6018	-1.62886	-1.62529	-1.62195	-1.61886	-1.61589	-1.61297	-1.61012
-1.60482	-1.60068	-1.62785	-1.62437	-1.62109	-1.61803	-1.61506	-1.61213	-1.60925
-1.60357	-1.5996	-1.62687	-1.62349	-1.62027	-1.61723	-1.61427	-1.61134	-1.60846
-1.59728	-1.59333	-1.6259	-1.62261	-1.61945	-1.61643	-1.61348	-1.61056	-1.60768
-1.59609	-1.59227	-1.62492	-1.6217	-1.6186	-1.6156	-1.61266	-1.60975	-1.60688
-1.59494	-1.59121	-1.62395	-1.62079	-1.61772	-1.61474	-1.61182	-1.60892	-1.60605
-1.59379	-1.59015	-1.58677	-1.58368	-1.61678	-1.61381	-1.63147	-1.62851	-1.6256
-1.59266	-1.5891	-1.58577	-1.58271	-1.61582	-1.61286	-1.63049	-1.62756	-1.62466
-1.5916	-1.58813	-1.58486	-1.58181	-1.59909	-1.59615	-1.61405	-1.61114	-1.60827
-1.59062	-1.58724	-1.58402	-1.58099	-1.59828	-1.59534	-1.61322	-1.61033	-1.60747
-1.58966	-1.58637	-1.5832	-1.58019	-1.59749	-1.59456	-1.65147	-1.64845	-1.64547
-1.58873	-1.58552	-1.5824	-1.5794	-1.59671	-1.5938	-1.65066	-1.64765	-1.64467
-1.58783	-1.58468	-1.5816	-1.57862	-1.59594	-1.59304	-1.64986	-1.64685	-1.64388
-1.58694	-1.58383	-1.58078	-1.57782	-1.59516	-1.59227	-1.64904	-1.64604	-1.64309
-1.58608	-1.583	-1.57997	-1.57703	-1.59438	-1.59149	-1.64822	-1.64524	-1.6423
-1.58526	-1.58219	-1.57918	-1.57624	-1.61362	-1.61061	-1.6474	-1.64443	-1.6415
-1.58446	-1.5814	-1.5784	-1.57546	-1.6128	-1.6098	-1.64659	-1.64362	-1.6407
-1.58365	-1.58061	-1.57762	-1.57469	-1.63244	-1.62939	-1.64579	-1.64282	-1.63989
-1.58285	-1.57982	-1.57684	-1.57391	-1.63162	-1.62858	-1.64499	-1.64202	-1.63908
-1.58203	-1.57902	-1.61646	-1.6134	-1.63079	-1.62777	-1.6442	-1.64121	-1.63826
-1.58122	-1.57822	-1.61563	-1.61258	-1.62996	-1.62695	-1.64339	-1.64038	-1.63741
-1.5804	-1.57743	-1.61481	-1.61176	-1.62913	-1.62613	-1.64258	-1.63956	-1.63658
-1.57961	-1.57665	-1.61401	-1.61096	-1.62831	-1.62533	-1.64179	-1.63877	-1.63581
-1.57882	-1.57588	-1.61321	-1.61016	-1.6275	-1.62452	-1.641	-1.638	-1.63506
-1.61854	-1.61546	-1.61241	-1.60938	-1.62672	-1.62374	-1.62079	-1.61782	-1.61492
-1.61772	-1.61465	-1.61162	-1.60861	-1.62595	-1.62297	-1.62003	-1.61708	-1.6142
-1.61691	-1.61385	-1.61082	-1.60784	-1.62518	-1.62221	-1.61928	-1.61638	-1.61353
-1.61609	-1.61304	-1.61004	-1.60707	-1.62441	-1.62146	-1.61856	-1.61572	-1.61293
-1.61528	-1.61224	-1.62961	-1.6266	-1.62364	-1.62071	-1.64022	-1.63732	-1.63449
-1.61448	-1.61144	-1.6288	-1.62581	-1.62286	-1.61996	-1.63946	-1.63661	-1.63382

-1.61368	-1.61065	-1.628	-1.62502	-1.62209	-1.61921	-1.63871	-1.63589	-1.63314
-1.61289	-1.60987	-1.62721	-1.62423	-1.62132	-1.61845	-1.66112	-1.65811	-1.6552
-1.61209	-1.6091	-1.62642	-1.62345	-1.62054	-1.6177	-1.66027	-1.6573	-1.65445
-1.61131	-1.60833	-1.62565	-1.62268	-1.64222	-1.63928	-1.65942	-1.6565	-1.65369
-1.61054	-1.60756	-1.62487	-1.62192	-1.64144	-1.63851	-1.65859	-1.65571	-1.65294
-1.60981	-1.60682	-1.62411	-1.62117	-1.64067	-1.63776	-1.65779	-1.65494	-1.65219
-1.60913	-1.60611	-1.62338	-1.62043	-1.63991	-1.63703	-1.65704	-1.6542	-1.65144
-1.6288	-1.62569	-1.64521	-1.64214	-1.63917	-1.63632	-1.65632	-1.65347	-1.6507
-1.6281	-1.62504	-1.64453	-1.64143	-1.63845	-1.63561	-1.65558	-1.65273	-1.64994
-1.62739	-1.62438	-1.64387	-1.64075	-1.63776	-1.63493	-1.65487	-1.65201	-1.64919
-1.62661	-1.62364	-1.64315	-1.64008	-1.66017	-1.65716	-1.65423	-1.65134	-1.64849
-1.6258	-1.62286	-1.64238	-1.63941	-1.6595	-1.65654	-1.65363	-1.65071	-1.64781
-1.62503	-1.62209	-1.64162	-1.63869	-1.65875	-1.65584	-1.65297	-1.65006	-1.64716
-1.62428	-1.62134	-1.64083	-1.63792	-1.65793	-1.65507	-1.65225	-1.6494	-1.64655
-1.62358	-1.62064	-1.6401	-1.63718	-1.65715	-1.65431	-1.65152	-1.6487	-1.64588
-1.62296	-1.62003	-1.63947	-1.63653	-1.65644	-1.65359	-1.65078	-1.64796	-1.64513
-1.62233	-1.61942	-1.63884	-1.63589	-1.65577	-1.65289	-1.65006	-1.64722	-1.64438
-1.62161	-1.61872	-1.66129	-1.65816	-1.65514	-1.65228	-1.67337	-1.67035	-1.66732
-1.62086	-1.61799	-1.6605	-1.65748	-1.65454	-1.65168	-1.67274	-1.66967	-1.6666
-1.64259	-1.6396	-1.6597	-1.65675	-1.65385	-1.651	-1.67203	-1.66897	-1.66591
-1.6419	-1.63888	-1.65891	-1.65599	-1.65312	-1.65027	-1.67127	-1.66827	-1.66526
-1.64121	-1.6382	-1.65818	-1.6553	-1.65243	-1.64957	-1.67053	-1.66754	-1.66455
-1.64052	-1.63757	-1.65754	-1.65468	-1.6518	-1.64891	-1.66979	-1.66678	-1.66377
-1.63982	-1.63693	-1.65689	-1.65403	-1.65116	-1.64825	-1.66908	-1.66605	-1.66303
-1.63911	-1.63624	-1.65617	-1.65332	-1.65046	-1.64759	-1.66842	-1.6654	-1.66237
-1.63842	-1.63556	-1.65546	-1.6526	-1.64975	-1.64692	-1.66776	-1.66475	-1.66171
-1.66085	-1.65779	-1.6548	-1.65191	-1.67296	-1.66999	-1.68604	-1.68298	-1.67989
-1.6602	-1.65717	-1.65418	-1.65124	-1.67221	-1.66922	-1.68524	-1.68218	-1.67911
-1.65949	-1.6565	-1.65353	-1.65059	-1.67154	-1.66853	-1.68451	-1.68144	-1.67837
-1.65874	-1.6558	-1.65287	-1.64998	-1.67092	-1.66789	-1.68383	-1.68075	-1.67768
-1.65803	-1.65512	-1.65222	-1.64936	-1.67028	-1.66723	-1.68312	-1.68004	-1.67696
-1.6574	-1.6545	-1.65161	-1.64875	-1.66963	-1.66655	-1.68238	-1.67927	-1.67618
-1.65679	-1.65387	-1.67499	-1.672	-1.68796	-1.6848	-1.68164	-1.67852	-1.67542
-1.65615	-1.65319	-1.67426	-1.67126	-1.68721	-1.68409	-1.68096	-1.67783	-1.67472
-1.65551	-1.65252	-1.67353	-1.67051	-1.68646	-1.68338	-1.68029	-1.67716	-1.67404
-1.65487	-1.65188	-1.67286	-1.66984	-1.68575	-1.6827	-1.67962	-1.67647	-1.67332
-1.65423	-1.65128	-1.67224	-1.6692	-1.68508	-1.68202	-1.67894	-1.67576	-1.67259
-1.65361	-1.65067	-1.67159	-1.66852	-1.68436	-1.68129	-1.69576	-1.69252	-1.68929
-1.65301	-1.65005	-1.67092	-1.66781	-1.68359	-1.68051	-1.69496	-1.69178	-1.68862
-1.65238	-1.64941	-1.67024	-1.66712	-1.68287	-1.67976	-1.69419	-1.69105	-1.68794
-1.65173	-1.64873	-1.66953	-1.66645	-1.6822	-1.67909	-1.69349	-1.69037	-1.68727
-1.65107	-1.64805	-1.66884	-1.6658	-1.68155	-1.67844	-1.69281	-1.68969	-1.68657
-1.6744	-1.67127	-1.68708	-1.684	-1.69849	-1.69531	-1.69214	-1.68898	-1.68585
-1.67372	-1.67063	-1.68644	-1.68337	-1.69783	-1.69465	-1.69146	-1.68827	-1.68511
-1.67306	-1.67	-1.6858	-1.68273	-1.69716	-1.69394	-1.69074	-1.68756	-1.68441
-1.67244	-1.66938	-1.68516	-1.68209	-1.69648	-1.69322	-1.68999	-1.68684	-1.68375
-1.67182	-1.66877	-1.68451	-1.68142	-1.69577	-1.69251	-1.68928	-1.68616	-1.68309
-1.67122	-1.66817	-1.68387	-1.68072	-1.69502	-1.69181	-1.68863	-1.68552	-1.68244
-1.68947	-1.68638	-1.68323	-1.68003	-1.6943	-1.69113	-1.68799	-1.68488	-1.6818
-1.68884	-1.68575	-1.6826	-1.6794	-1.69365	-1.69048	-1.68735	-1.68425	-1.68117

-0.59152	-0.58975	-0.63205	-0.63021	-0.6284	-0.62661	-0.62488	-0.62319	-0.62161
-0.59199	-0.59023	-0.63259	-0.63072	-0.62889	-0.6271	-0.62536	-0.62362	-0.62197
-0.59245	-0.5907	-0.63313	-0.63123	-0.62939	-0.62761	-0.62585	-0.62407	-0.62235
-0.59288	-0.59114	-0.63362	-0.63171	-0.62986	-0.62809	-0.62633	-0.62452	-0.62277
-0.5933	-0.59157	-0.63411	-0.63219	-0.63034	-0.62857	-0.62681	-0.62499	-0.62322
-0.59374	-0.59203	-0.63462	-0.6327	-0.63084	-0.62905	-0.62728	-0.62546	-0.62367
-0.5942	-0.59249	-0.63517	-0.63323	-0.63135	-0.62954	-0.62774	-0.62592	-0.62414
-0.59465	-0.59295	-0.63572	-0.63378	-0.63188	-0.63004	-0.62823	-0.62641	-0.62462
-0.59508	-0.59339	-0.63628	-0.63433	-0.63243	-0.63058	-0.62875	-0.62692	-0.62512
-0.5955	-0.59383	-0.63683	-0.63487	-0.63296	-0.63111	-0.62927	-0.62744	-0.62563
-0.59592	-0.59423	-0.63734	-0.63537	-0.63346	-0.63162	-0.62979	-0.62797	-0.62615
-0.59635	-0.59464	-0.63784	-0.63587	-0.63396	-0.63212	-0.63031	-0.6285	-0.62668
-0.59681	-0.59508	-0.63836	-0.63637	-0.63445	-0.63262	-0.63081	-0.629	-0.62719
-0.59729	-0.59554	-0.63889	-0.63688	-0.63495	-0.63311	-0.63129	-0.62949	-0.62769
-0.59777	-0.59601	-0.63944	-0.63743	-0.63548	-0.63361	-0.63177	-0.62997	-0.62817
-0.59826	-0.5965	-0.64003	-0.63799	-0.63602	-0.63411	-0.63225	-0.63044	-0.62866
-0.59874	-0.59698	-0.59525	-0.59354	-0.63657	-0.63463	-0.63274	-0.63092	-0.62913
-0.5992	-0.59746	-0.59573	-0.59402	-0.63714	-0.63517	-0.63325	-0.6314	-0.6296
-0.59967	-0.59794	-0.59621	-0.59449	-0.63771	-0.63572	-0.63377	-0.63191	-0.63008
-0.60013	-0.59841	-0.59669	-0.59496	-0.63828	-0.63627	-0.63431	-0.63243	-0.63059
-0.60059	-0.59888	-0.59716	-0.59543	-0.63886	-0.63684	-0.63487	-0.63297	-0.63112
-0.60108	-0.59936	-0.59764	-0.59591	-0.63944	-0.63741	-0.63543	-0.63352	-0.63165
-0.60158	-0.59984	-0.59811	-0.59639	-0.64002	-0.638	-0.63601	-0.63408	-0.63219
-0.48311	-0.48164	-0.59858	-0.59687	-0.64059	-0.63857	-0.63657	-0.63463	-0.63272
-0.48349	-0.48199	-0.59904	-0.59733	-0.64115	-0.63913	-0.63713	-0.63517	-0.63324
-0.48387	-0.48236	-0.59951	-0.5978	-0.64172	-0.6397	-0.6377	-0.63572	-0.63377
-0.48427	-0.48276	-0.60001	-0.59829	-0.64229	-0.64027	-0.63826	-0.63628	-0.63432
-0.48467	-0.48315	-0.60052	-0.59879	-0.64288	-0.64085	-0.63884	-0.63685	-0.63489
-0.48507	-0.48356	-0.60103	-0.59929	-0.59757	-0.59584	-0.63944	-0.63744	-0.63547
-0.48547	-0.48396	-0.60155	-0.59981	-0.59808	-0.59636	-0.64006	-0.63804	-0.63605
-0.48587	-0.48437	-0.4829	-0.48146	-0.59859	-0.59687	-0.64067	-0.63864	-0.63664
-0.48628	-0.48478	-0.4833	-0.48186	-0.59911	-0.59739	-0.64128	-0.63924	-0.63722
-0.42114	-0.41981	-0.48371	-0.48227	-0.59962	-0.5979	-0.59618	-0.59444	-0.5927
-0.42157	-0.42024	-0.48412	-0.48267	-0.60014	-0.59842	-0.59669	-0.59493	-0.59318
-0.42201	-0.42068	-0.41938	-0.41812	-0.56016	-0.55852	-0.5972	-0.59544	-0.59368
-0.42244	-0.42111	-0.41982	-0.41855	-0.56064	-0.55899	-0.59771	-0.59594	-0.59418
-0.42288	-0.42156	-0.42026	-0.41899	-0.56113	-0.55947	-0.59822	-0.59645	-0.59469
-0.37826	-0.37706	-0.42073	-0.41944	-0.49974	-0.49823	-0.59873	-0.59696	-0.59521
-0.37869	-0.37751	-0.42121	-0.4199	-0.50027	-0.49874	-0.59925	-0.59748	-0.59574
-0.37912	-0.37794	-0.42166	-0.42035	-0.41903	-0.4177	-0.55922	-0.55757	-0.55594
-0.37956	-0.37836	-0.42211	-0.4208	-0.41948	-0.41814	-0.55968	-0.55805	-0.55645
-0.29405	-0.29308	-0.37757	-0.37636	-0.41992	-0.41857	-0.49873	-0.49725	-0.49578
-0.29441	-0.29344	-0.37799	-0.37676	-0.42035	-0.41901	-0.49924	-0.49775	-0.49628
-0.29477	-0.29379	-0.37841	-0.37717	-0.42078	-0.41945	-0.41814	-0.41685	-0.41558
-0.2951	-0.29413	-0.37884	-0.3776	-0.42123	-0.41991	-0.4186	-0.4173	-0.41601
-0.29544	-0.29448	-0.37927	-0.37803	-0.42169	-0.42038	-0.41907	-0.41775	-0.41644
-0.29577	-0.29481	-0.3797	-0.37847	-0.37725	-0.37603	-0.41953	-0.4182	-0.41688
-0.29612	-0.29514	-0.38013	-0.37892	-0.37769	-0.37646	-0.41997	-0.41865	-0.41733
-0.24638	-0.24561	-0.38055	-0.37934	-0.37812	-0.37687	-0.42041	-0.41909	-0.41779
-0.24668	-0.2459	-0.38097	-0.37975	-0.37852	-0.37728	-0.42085	-0.41955	-0.41826

-0.24698	-0.24619	-0.3814	-0.38016	-0.37893	-0.37769	-0.37646	-0.37527	-0.37408
-0.24729	-0.24649	-0.38185	-0.3806	-0.37936	-0.37812	-0.37689	-0.37568	-0.37448
-0.2476	-0.24678	-0.38229	-0.38104	-0.37979	-0.37855	-0.37732	-0.3761	-0.37488
-0.24791	-0.24707	-0.29626	-0.2953	-0.38023	-0.37899	-0.37776	-0.37653	-0.37531
-0.24823	-0.24736	-0.29661	-0.29565	-0.38067	-0.37944	-0.37821	-0.37698	-0.37575
-0.24855	-0.24768	-0.29698	-0.29601	-0.38112	-0.37988	-0.37866	-0.37743	-0.3762
-0.24889	-0.24801	-0.29736	-0.29638	-0.38157	-0.38033	-0.3791	-0.37788	-0.37667
-0.24922	-0.24834	-0.2475	-0.2467	-0.38202	-0.38078	-0.37955	-0.37834	-0.37713
-0.24957	-0.24867	-0.24782	-0.247	-0.38246	-0.38124	-0.38002	-0.3788	-0.37757
-0.19793	-0.19725	-0.24815	-0.24731	-0.3829	-0.38169	-0.38048	-0.37924	-0.378
-0.19819	-0.19752	-0.24848	-0.24763	-0.38337	-0.38213	-0.3809	-0.37967	-0.37843
-0.19845	-0.19778	-0.24882	-0.24795	-0.38383	-0.38257	-0.38132	-0.38009	-0.37886
-0.19871	-0.19804	-0.24916	-0.24827	-0.38427	-0.38299	-0.38175	-0.38052	-0.37931
-0.19897	-0.19829	-0.2495	-0.24859	-0.3847	-0.38342	-0.38217	-0.38096	-0.37976
-0.14756	-0.14707	-0.24984	-0.24892	-0.33679	-0.33568	-0.38261	-0.3814	-0.3802
-0.14772	-0.14723	-0.25016	-0.24926	-0.33722	-0.33608	-0.38306	-0.38183	-0.38064
-0.14789	-0.14739	-0.19834	-0.19766	-0.24871	-0.24783	-0.38351	-0.38228	-0.38109
-0.14806	-0.14756	-0.19859	-0.19791	-0.24904	-0.24815	-0.38397	-0.38274	-0.38155
-0.14823	-0.14773	-0.14723	-0.14673	-0.24938	-0.24848	-0.33619	-0.33514	-0.33413
-0.1484	-0.14791	-0.14741	-0.1469	-0.24971	-0.24882	-0.33663	-0.33554	-0.3345
-0.14857	-0.1481	-0.1476	-0.14709	-0.25006	-0.24916	-0.33707	-0.33596	-0.33488
-0.14874	-0.14827	-0.14777	-0.14727	-0.20002	-0.19929	-0.33751	-0.33637	-0.33528
-0.14891	-0.14843	-0.14794	-0.14746	-0.20031	-0.19956	-0.33795	-0.3368	-0.3357
-0.14908	-0.1486	-0.14811	-0.14763	-0.20058	-0.19984	-0.33842	-0.33726	-0.33613
-0.14925	-0.14877	-0.14828	-0.1478	-0.20084	-0.20012	-0.33892	-0.33773	-0.33657
-0.10326	-0.10291	-0.14845	-0.14796	-0.14747	-0.147	-0.19969	-0.19897	-0.19825
-0.1034	-0.10303	-0.14861	-0.14813	-0.14765	-0.14716	-0.19996	-0.19924	-0.19852
-0.10354	-0.10315	-0.14878	-0.1483	-0.14782	-0.14733	-0.20022	-0.19951	-0.19879
-0.10367	-0.10328	-0.14894	-0.14846	-0.14798	-0.1475	-0.20048	-0.19978	-0.19906
-0.10381	-0.10341	-0.14912	-0.14863	-0.14815	-0.14767	-0.20073	-0.20004	-0.19932
-0.10396	-0.10355	-0.10316	-0.10279	-0.14831	-0.14784	-0.20099	-0.20029	-0.19958
-0.10411	-0.10369	-0.10329	-0.10292	-0.14848	-0.14802	-0.20124	-0.20055	-0.19984
-0.10427	-0.10383	-0.10343	-0.10305	-0.14866	-0.14819	-0.14772	-0.14723	-0.14674
-0.10444	-0.10399	-0.10357	-0.10318	-0.14884	-0.14836	-0.14789	-0.14741	-0.14693
-0.05289	-0.05264	-0.10372	-0.10333	-0.10295	-0.10259	-0.14807	-0.14759	-0.14712
-0.05299	-0.05275	-0.10388	-0.10347	-0.10309	-0.10272	-0.14824	-0.14777	-0.1473
-0.0531	-0.05285	-0.10404	-0.10362	-0.10323	-0.10285	-0.14841	-0.14794	-0.14747
-0.05321	-0.05296	-0.1042	-0.10377	-0.10337	-0.10299	-0.14859	-0.14812	-0.14766
-0.05331	-0.05308	-0.10437	-0.10393	-0.10351	-0.10313	-0.14878	-0.1483	-0.14784
-0.05341	-0.05318	-0.10453	-0.10408	-0.10366	-0.10327	-0.10289	-0.10253	-0.10218
-0.05351	-0.05328	-0.10469	-0.10424	-0.10381	-0.10341	-0.10302	-0.10266	-0.10231
		-0.05315	-0.05291	-0.10397	-0.10355	-0.10316	-0.10279	-0.10243
		-0.05326	-0.05302	-0.10414	-0.10371	-0.10331	-0.10293	-0.10256
		-0.05336	-0.05313	-0.1043	-0.10387	-0.10346	-0.10307	-0.10269
		-0.05346	-0.05323	-0.10446	-0.10403	-0.10361	-0.10321	-0.10284
		-0.05355	-0.05333	-0.10461	-0.10418	-0.10376	-0.10336	-0.10298
				-0.0532	-0.05298	-0.10391	-0.1035	-0.10311
				-0.05331	-0.05308	-0.10406	-0.10365	-0.10324
				-0.05341	-0.05319	-0.10422	-0.1038	-0.10339
				-0.05352	-0.05329	-0.10439	-0.10397	-0.10355

-0.10456	-0.10413	-0.10372
-0.10472	-0.1043	-0.10388
-0.10487	-0.10446	-0.10404
-0.05348	-0.05326	-0.05304
-0.05359	-0.05337	-0.05315

							-0.04961	-0.04949
							-0.04954	-0.04941
							-0.10561	-0.10503
							-0.10532	-0.10473
							-0.10503	-0.10443
				-0.04962	-0.0495	-0.04937	-0.15403	-0.15333
				-0.04954	-0.04941	-0.04928	-0.15368	-0.153
				-0.10588	-0.10531	-0.10474	-0.15335	-0.15268
				-0.10561	-0.10504	-0.10448	-0.15304	-0.15237
		-0.04958	-0.04945	-0.15474	-0.15406	-0.15339	-0.34863	-0.34728
		-0.04951	-0.04938	-0.15442	-0.15374	-0.15307	-0.348	-0.34665
		-0.10595	-0.10537	-0.35146	-0.35009	-0.34873	-0.38449	-0.38302
		-0.10568	-0.10511	-0.35082	-0.34945	-0.3481	-0.3838	-0.38234
		-0.10541	-0.10484	-0.35018	-0.34881	-0.34746	-0.38311	-0.38168
-0.04948	-0.04935	-0.15449	-0.15382	-0.34955	-0.34815	-0.34679	-0.42803	-0.42653
-0.04941	-0.04928	-0.15417	-0.1535	-0.34891	-0.34749	-0.34612	-0.42729	-0.42586
-0.10576	-0.10517	-0.351	-0.34965	-0.38548	-0.38393	-0.38244	-0.64896	-0.64696
-0.10548	-0.10491	-0.35037	-0.34901	-0.38479	-0.38328	-0.38183	-0.64804	-0.64604
-0.10521	-0.10465	-0.34973	-0.34836	-0.47922	-0.47746	-0.47577	-0.64711	-0.64513
-0.10495	-0.10438	-0.34909	-0.34769	-0.47836	-0.47667	-0.47505	-0.64619	-0.64422
-0.35119	-0.34983	-0.38565	-0.38411	-0.56746	-0.5656	-0.56382	-0.64526	-0.64331
-0.35055	-0.34921	-0.38498	-0.38345	-0.56658	-0.56476	-0.563	-0.64434	-0.64239
-0.34993	-0.34859	-0.38432	-0.38281	-0.56575	-0.56394	-0.56219	-0.64342	-0.64146
-0.3493	-0.34797	-0.47882	-0.47705	-0.6485	-0.64646	-0.64446	-0.73132	-0.72909
-0.34867	-0.34735	-0.47805	-0.47632	-0.64758	-0.64554	-0.64354	-0.73027	-0.72804
-0.38521	-0.38378	-0.56709	-0.56517	-0.64666	-0.64462	-0.64262	-0.77583	-0.77342
-0.38454	-0.38311	-0.56622	-0.56434	-0.64574	-0.6437	-0.64169	-0.7747	-0.77229
-0.52417	-0.52235	-0.56537	-0.56352	-0.734	-0.73167	-0.72938	-0.84829	-0.84566
-0.52335	-0.52157	-0.56456	-0.56271	-0.73293	-0.73061	-0.72833	-0.84707	-0.84444
-0.56744	-0.5656	-0.65662	-0.65444	-0.77277	-0.77033	-0.76793	-0.91317	-0.91017
-0.56656	-0.56477	-0.65566	-0.65349	-0.77165	-0.76922	-0.76682	-0.91179	-0.9088

-0.5657	-0.56394	-0.65471	-0.65255	-0.77054	-0.76811	-0.76572	-0.91041	-0.90743
-0.56485	-0.56311	-0.73349	-0.73108	-0.84328	-0.84063	-0.83802	-0.98912	-0.98584
-0.56403	-0.56229	-0.73242	-0.73002	-0.84208	-0.83943	-0.83681	-0.9876	-0.98433
-0.656	-0.65393	-0.77218	-0.7697	-0.91544	-0.91234	-0.90927	-1.08748	-1.08371
-0.65507	-0.65299	-0.77107	-0.76863	-0.9141	-0.91099	-0.90791	-1.08574	-1.08198
-0.69663	-0.69441	-0.8439	-0.84125	-1.02882	-1.02528	-1.02178	-1.12298	-1.11897
-0.69566	-0.69342	-0.84272	-0.8401	-1.02735	-1.0238	-1.02027	-1.12117	-1.11722
-0.6947	-0.69244	-0.84154	-0.83895	-1.02588	-1.02231	-1.01877	-1.11938	-1.11548
-0.81054	-0.80793	-0.96079	-0.95778	-1.02437	-1.02079	-1.01725	-1.15344	-1.14927
-0.80943	-0.80681	-0.95944	-0.95642	-1.02283	-1.01926	-1.01573	-1.15152	-1.14739
-0.84344	-0.84074	-1.0282	-1.02476	-1.12442	-1.12016	-1.11594	-1.20536	-1.20105
-0.84228	-0.8396	-1.0267	-1.02322	-1.12255	-1.11835	-1.11418	-1.20342	-1.19909
-0.96164	-0.95853	-1.02522	-1.0217	-1.159	-1.1546	-1.15027	-1.20155	-1.19719
-0.96027	-0.95723	-1.02377	-1.0202	-1.15705	-1.15276	-1.14854	-1.19974	-1.19534
-0.98796	-0.98472	-1.12564	-1.12132	-1.21105	-1.20666	-1.20234	-1.19792	-1.19352
-0.9865	-0.98326	-1.12382	-1.11955	-1.20912	-1.20473	-1.20041	-1.19603	-1.19171
-0.98504	-0.98179	-1.12199	-1.11779	-1.20724	-1.20282	-1.19847	-1.19416	-1.18993
-0.98359	-0.9803	-1.17726	-1.17299	-1.2054	-1.20095	-1.19659	-1.25073	-1.24645
-0.98215	-0.97881	-1.17533	-1.17117	-1.2036	-1.19912	-1.19473	-1.2489	-1.24473
-1.1248	-1.12059	-1.17345	-1.16933	-1.20171	-1.19726	-1.1929	-1.24706	-1.24295
-1.12309	-1.11885	-1.17161	-1.16746	-1.19976	-1.19538	-1.1911	-1.24521	-1.24111
-1.1785	-1.17411	-1.20654	-1.20217	-1.25643	-1.25199	-1.24762	-1.27512	-1.27094
-1.1767	-1.17239	-1.20476	-1.20034	-1.25453	-1.25017	-1.24589	-1.27337	-1.26917
-1.17491	-1.17065	-1.20298	-1.19855	-1.25268	-1.24838	-1.24416	-1.34906	-1.34457
-1.17313	-1.16885	-1.20112	-1.19678	-1.25092	-1.24661	-1.24239	-1.3472	-1.34277
-1.17135	-1.16703	-1.19925	-1.19501	-1.24918	-1.24485	-1.24061	-1.34533	-1.34098
-1.16952	-1.16523	-1.19739	-1.19318	-1.27362	-1.26921	-1.26487	-1.36887	-1.36445
-1.16767	-1.16344	-1.19554	-1.19132	-1.27166	-1.26733	-1.26306	-1.36692	-1.36247
-1.20241	-1.19805	-1.2522	-1.24786	-1.35347	-1.34899	-1.34455	-1.41828	-1.41371
-1.20059	-1.19629	-1.25044	-1.24611	-1.3516	-1.34715	-1.34274	-1.41638	-1.4118
-1.1988	-1.19454	-1.27504	-1.27062	-1.34977	-1.34532	-1.34092	-1.4145	-1.40995
-1.19706	-1.19278	-1.2732	-1.26886	-1.34798	-1.3435	-1.33907	-1.41263	-1.40817
-1.19532	-1.19104	-1.27138	-1.26711	-1.34619	-1.34169	-1.33725	-1.41078	-1.40641
-1.22388	-1.2196	-1.35327	-1.3488	-1.39843	-1.39386	-1.38936	-1.40895	-1.4046
-1.22212	-1.21793	-1.35147	-1.34696	-1.39651	-1.392	-1.38756	-1.40714	-1.40279
-1.30156	-1.29714	-1.40385	-1.39922	-1.41878	-1.41421	-1.40973	-1.40534	-1.40102
-1.29977	-1.29536	-1.40192	-1.39735	-1.41692	-1.41237	-1.4079	-1.40355	-1.39928
-1.35501	-1.35046	-1.40002	-1.39547	-1.41504	-1.41053	-1.4061	-1.50457	-1.50001
-1.35311	-1.34864	-1.39816	-1.39358	-1.41312	-1.40871	-1.40437	-1.50267	-1.49806
-1.40551	-1.4009	-1.3963	-1.39171	-1.41125	-1.40691	-1.40262	-1.52236	-1.51767
-1.40371	-1.39902	-1.39442	-1.38989	-1.40946	-1.40511	-1.40082	-1.52042	-1.51577
-1.40194	-1.39718	-1.39256	-1.3881	-1.4077	-1.40334	-1.39903	-1.51849	-1.51388
-1.40017	-1.39542	-1.39082	-1.38634	-1.53052	-1.52585	-1.52123	-1.5393	-1.53458
-1.39839	-1.39372	-1.38912	-1.3846	-1.5286	-1.52397	-1.51937	-1.53738	-1.53261
-1.39656	-1.39196	-1.46195	-1.45732	-1.52677	-1.52211	-1.51747	-1.53545	-1.5307
-1.39469	-1.39017	-1.46019	-1.45564	-1.52502	-1.52027	-1.51555	-1.53351	-1.52882
-1.41641	-1.4119	-1.53262	-1.52794	-1.52324	-1.51845	-1.51369	-1.53162	-1.52694
-1.41462	-1.41008	-1.53067	-1.52606	-1.52141	-1.51667	-1.51192	-1.52977	-1.52504
-1.46391	-1.45927	-1.52876	-1.52418	-1.54233	-1.53755	-1.53276	-1.58444	-1.57929
-1.46215	-1.45757	-1.52697	-1.52231	-1.54039	-1.53565	-1.53092	-1.58255	-1.57744

-1.4604	-1.4559	-1.52522	-1.52046	-1.53844	-1.53373	-1.52905	-1.58067	-1.57562
-1.51059	-1.50597	-1.52342	-1.51867	-1.53661	-1.53185	-1.52715	-1.5787	-1.57374
-1.50865	-1.50409	-1.5216	-1.51691	-1.53484	-1.53	-1.52522	-1.5767	-1.57184
-1.50679	-1.50222	-1.54251	-1.53778	-1.53302	-1.52818	-1.52339	-1.57476	-1.56992
-1.505	-1.50033	-1.54056	-1.53587	-1.53116	-1.52639	-1.52165	-1.57288	-1.56796
-1.52538	-1.52063	-1.53865	-1.53397	-1.52928	-1.52459	-1.51991	-1.57104	-1.56609
-1.52362	-1.51888	-1.53684	-1.53207	-1.52736	-1.52273	-1.51815	-1.56925	-1.56434
-1.52186	-1.51715	-1.53506	-1.53021	-1.52546	-1.52089	-1.51638	-1.56748	-1.56263
-1.54286	-1.5381	-1.53329	-1.52842	-1.58025	-1.57534	-1.57052	-1.56571	-1.56095
-1.54104	-1.53632	-1.53154	-1.52668	-1.57841	-1.57349	-1.56867	-1.56394	-1.55928
-1.53918	-1.53451	-1.52976	-1.52493	-1.57657	-1.57161	-1.56678	-1.5621	-1.55753
-1.5373	-1.53267	-1.52797	-1.52319	-1.57474	-1.56973	-1.56485	-1.56022	-1.55573
-1.53545	-1.53084	-1.52618	-1.52146	-1.57294	-1.56793	-1.56304	-1.57689	-1.57238
-1.53366	-1.52903	-1.52438	-1.51973	-1.57118	-1.56621	-1.56135	-1.57513	-1.57054
-1.53187	-1.5272	-1.57928	-1.57432	-1.58791	-1.583	-1.57816	-1.5734	-1.56874
-1.53006	-1.52534	-1.57731	-1.57244	-1.5861	-1.58128	-1.5765	-1.57172	-1.56702
-1.52828	-1.52351	-1.57537	-1.57057	-1.5843	-1.57956	-1.57484	-1.57006	-1.56533
-1.52653	-1.52175	-1.57353	-1.56876	-1.58253	-1.57784	-1.57316	-1.56837	-1.56362
-1.52481	-1.52005	-1.57174	-1.567	-1.58079	-1.57613	-1.57147	-1.56667	-1.56193
-1.5231	-1.51836	-1.58847	-1.58375	-1.57908	-1.57444	-1.5698	-1.58683	-1.58205
-1.5214	-1.51669	-1.58674	-1.58207	-1.57742	-1.57277	-1.56812	-1.58516	-1.58043
-1.5389	-1.5342	-1.58502	-1.58038	-1.57576	-1.57109	-1.56644	-1.58353	-1.57891
-1.5372	-1.53253	-1.58328	-1.57869	-1.57408	-1.56938	-1.56474	-1.58193	-1.57751
-1.59132	-1.58634	-1.58156	-1.57699	-1.59428	-1.58949	-1.58481	-1.58036	-1.57615
-1.58956	-1.58462	-1.57986	-1.5753	-1.59257	-1.58775	-1.58309	-1.57879	-1.57479
-1.58782	-1.5829	-1.57816	-1.57361	-1.59085	-1.58604	-1.58141	-1.57726	-1.57346
-1.58609	-1.5812	-1.57647	-1.57191	-1.58914	-1.58437	-1.57983	-1.58653	-1.58276
-1.58437	-1.57952	-1.5748	-1.57022	-1.58744	-1.58276	-1.57832	-1.58512	-1.58147
-1.58266	-1.57784	-1.59497	-1.5903	-1.58574	-1.58119	-1.57692	-1.62742	-1.62381
-1.58096	-1.57617	-1.59327	-1.58856	-1.58404	-1.57968	-1.57561	-1.62616	-1.62265
-1.6012	-1.59639	-1.59162	-1.58688	-1.62664	-1.62237	-1.61841	-1.62493	-1.62152
-1.59955	-1.59481	-1.59006	-1.58525	-1.62499	-1.62088	-1.6171	-1.62373	-1.62043
-1.59793	-1.59323	-1.5885	-1.58369	-1.62342	-1.61945	-1.61583	-1.62255	-1.61936
-1.59634	-1.59162	-1.63121	-1.62638	-1.63234	-1.62836	-1.62474	-1.62139	-1.61829
-1.59476	-1.58998	-1.62957	-1.62487	-1.63094	-1.62706	-1.62353	-1.62026	-1.61724
-1.63751	-1.63261	-1.62791	-1.62339	-1.62958	-1.62582	-1.62238	-1.63998	-1.63695
-1.63579	-1.63086	-1.62625	-1.62193	-1.62827	-1.62463	-1.62129	-1.63893	-1.63593
-1.63406	-1.62913	-1.62461	-1.62049	-1.62697	-1.62347	-1.62022	-1.63791	-1.63495
-1.63233	-1.6274	-1.62298	-1.61906	-1.62569	-1.62232	-1.61919	-1.63693	-1.63402
-1.63063	-1.62577	-1.62147	-1.61771	-1.62446	-1.62122	-1.61818	-1.63596	-1.63307
-1.62898	-1.62428	-1.62012	-1.61648	-1.6233	-1.62018	-1.61723	-1.635	-1.6321
-1.62737	-1.62288	-1.61887	-1.61534	-1.62222	-1.61918	-1.61629	-1.63404	-1.63111
-1.62581	-1.62156	-1.62806	-1.62453	-1.62124	-1.61822	-1.61532	-1.63306	-1.63012
-1.6243	-1.62031	-1.62696	-1.62355	-1.62033	-1.61729	-1.61436	-1.63206	-1.62912
-1.62287	-1.61905	-1.62581	-1.62251	-1.64014	-1.63706	-1.63405	-1.63107	-1.62814
-1.62154	-1.61781	-1.62463	-1.62143	-1.63911	-1.63605	-1.63306	-1.63009	-1.62717
-1.62025	-1.61663	-1.6235	-1.62037	-1.63808	-1.63504	-1.63206	-1.62912	-1.62623
-1.61902	-1.6155	-1.62242	-1.61932	-1.63704	-1.63401	-1.63106	-1.62817	-1.62534
-1.62824	-1.6247	-1.62139	-1.61832	-1.63604	-1.63303	-1.63009	-1.62724	-1.62445
-1.62706	-1.62367	-1.62044	-1.61742	-1.63514	-1.63214	-1.6292	-1.62633	-1.62353

-1.62591	-1.62265	-1.61952	-1.61654	-1.63427	-1.63128	-1.62834	-1.62544	-1.62261
-1.62481	-1.62162	-1.61854	-1.61559	-1.63333	-1.63036	-1.62743	-1.62455	-1.62172
-1.62374	-1.62059	-1.61754	-1.61462	-1.63235	-1.62941	-1.62651	-1.62366	-1.62086
-1.62267	-1.61957	-1.61655	-1.61364	-1.63138	-1.62848	-1.62561	-1.6228	-1.62002
-1.62162	-1.61856	-1.61557	-1.61266	-1.6304	-1.62755	-1.62474	-1.62197	-1.61922
-1.62062	-1.6176	-1.63531	-1.63235	-1.62946	-1.62663	-1.62386	-1.62111	-1.6184
-1.61972	-1.61673	-1.63444	-1.63148	-1.62858	-1.62573	-1.62294	-1.62021	-1.61752
-1.61883	-1.61586	-1.63357	-1.63061	-1.62771	-1.62484	-1.62203	-1.61931	-1.61664
-1.61788	-1.61494	-1.63264	-1.6297	-1.6268	-1.62394	-1.62114	-1.61844	-1.61581
-1.61694	-1.61401	-1.63171	-1.62878	-1.6259	-1.62305	-1.62027	-1.61759	-1.61498
-1.61603	-1.61313	-1.63083	-1.62791	-1.62504	-1.62221	-1.61945	-1.61677	-1.61415
-1.61516	-1.61228	-1.62997	-1.62706	-1.6242	-1.6214	-1.61864	-1.61595	-1.61332
-1.61426	-1.61139	-1.62907	-1.62617	-1.62332	-1.62053	-1.61779	-1.63748	-1.6348
-1.61333	-1.61045	-1.62812	-1.62523	-1.62239	-1.61961	-1.61688	-1.63659	-1.63396
-1.61243	-1.60956	-1.62721	-1.62433	-1.6215	-1.61872	-1.61601	-1.63572	-1.63311
-1.61161	-1.60874	-1.62638	-1.62351	-1.6207	-1.61792	-1.6152	-1.63487	-1.63223
-1.6108	-1.60793	-1.62556	-1.62272	-1.61991	-1.61714	-1.61442	-1.63404	-1.63137
-1.63053	-1.62762	-1.62475	-1.62191	-1.61912	-1.61635	-1.61364	-1.63325	-1.63059
-1.62967	-1.62678	-1.62393	-1.6211	-1.61831	-1.61556	-1.61285	-1.63247	-1.62984
-1.62875	-1.62588	-1.62304	-1.62022	-1.61745	-1.6147	-1.61201	-1.66867	-1.66593
-1.62779	-1.62493	-1.6221	-1.61929	-1.61652	-1.61378	-1.61111	-1.66773	-1.66504
-1.62689	-1.62404	-1.62121	-1.61841	-1.65357	-1.6507	-1.64791	-1.66685	-1.66419
-1.62608	-1.62323	-1.6204	-1.6176	-1.65273	-1.64987	-1.64708	-1.66603	-1.6634
-1.62528	-1.62243	-1.6196	-1.6168	-1.67372	-1.67078	-1.66793	-1.66523	-1.66263
-1.62447	-1.62162	-1.6188	-1.616	-1.67288	-1.66997	-1.66714	-1.66445	-1.66185
-1.62363	-1.62079	-1.61798	-1.61519	-1.67203	-1.66914	-1.66634	-1.66366	-1.66107
-1.62273	-1.6199	-1.65513	-1.65221	-1.67109	-1.66825	-1.66548	-1.66281	-1.66022
-1.6218	-1.61898	-1.65417	-1.65125	-1.67012	-1.66732	-1.66459	-1.66194	-1.65936
-1.65917	-1.65621	-1.65328	-1.65037	-1.66924	-1.66647	-1.66378	-1.68314	-1.68043
-1.65833	-1.65538	-1.65246	-1.64956	-1.66843	-1.66569	-1.66302	-1.68237	-1.67966
-1.64252	-1.63959	-1.65166	-1.64879	-1.66765	-1.66494	-1.66229	-1.68162	-1.67891
-1.64172	-1.6388	-1.65089	-1.64804	-1.66691	-1.66422	-1.6616	-1.68089	-1.67818
-1.64094	-1.63803	-1.63518	-1.63238	-1.66619	-1.66352	-1.66091	-1.68017	-1.67745
-1.64016	-1.63726	-1.63443	-1.63168	-1.66548	-1.66282	-1.66021	-1.67942	-1.6767
-1.63938	-1.63649	-1.63369	-1.63098	-1.66478	-1.66211	-1.65949	-1.67865	-1.67592
-1.63859	-1.63572	-1.65489	-1.65213	-1.67181	-1.669	-1.66624	-1.67782	-1.67507
-1.6378	-1.63495	-1.65412	-1.65139	-1.67104	-1.66821	-1.66541	-1.67695	-1.67417
-1.63699	-1.63414	-1.6533	-1.6506	-1.67024	-1.66741	-1.66461	-1.68523	-1.68232
-1.63617	-1.6333	-1.65243	-1.64977	-1.66939	-1.66661	-1.66383	-1.68442	-1.6815
-1.63535	-1.63249	-1.67408	-1.67131	-1.66857	-1.66582	-1.66307	-1.68362	-1.6807
-1.63454	-1.63174	-1.6733	-1.67054	-1.66781	-1.66507	-1.66232	-1.68282	-1.67991
-1.63375	-1.63102	-1.67257	-1.66981	-1.66707	-1.66432	-1.66157	-1.68203	-1.67912
-1.633	-1.63032	-1.67183	-1.66907	-1.66633	-1.66358	-1.66082	-1.68125	-1.67834
-1.63229	-1.62964	-1.67111	-1.66835	-1.6656	-1.66283	-1.66006	-1.68046	-1.67756
-1.67606	-1.6732	-1.67039	-1.66763	-1.68845	-1.68551	-1.68259	-1.67968	-1.67679
-1.67528	-1.67246	-1.66967	-1.66692	-1.68768	-1.68473	-1.6818	-1.67889	-1.676
-1.67455	-1.67174	-1.66895	-1.66619	-1.6869	-1.68394	-1.681	-1.6781	-1.67523
-1.67389	-1.67105	-1.66823	-1.66545	-1.6861	-1.68314	-1.68021	-1.67731	-1.67445
-1.67323	-1.67036	-1.66751	-1.66471	-1.6853	-1.68234	-1.67941	-1.67652	-1.67367
-1.67255	-1.66966	-1.6668	-1.66397	-1.68449	-1.68153	-1.67861	-1.67572	-1.67289

-1.67185	-1.66897	-1.66609	-1.66323	-1.68369	-1.68073	-1.6778	-1.67493	-1.67211
-1.67116	-1.66831	-1.689	-1.68594	-1.68292	-1.67994	-1.67701	-1.67415	-1.67134
-1.67046	-1.66766	-1.68832	-1.68523	-1.68217	-1.67917	-1.67623	-1.67337	-1.67059
-1.65089	-1.64812	-1.68759	-1.68451	-1.68146	-1.67844	-1.67548	-1.69134	-1.68851
-1.65015	-1.64737	-1.68682	-1.68381	-1.68079	-1.67774	-1.67475	-1.69058	-1.68775
-1.6494	-1.64661	-1.68603	-1.68306	-1.68008	-1.67704	-1.67404	-1.68985	-1.68702
-1.64865	-1.64586	-1.68523	-1.68226	-1.67929	-1.67631	-1.67337	-1.68917	-1.68632
-1.6479	-1.6451	-1.68442	-1.68144	-1.67849	-1.67557	-1.67268	-1.68848	-1.68563
-1.64713	-1.64433	-1.6836	-1.68063	-1.67769	-1.67478	-1.67192	-1.68775	-1.68497
-1.64637	-1.64355	-1.68279	-1.67983	-1.67689	-1.67398	-1.67114	-1.687	-1.68431
-1.6694	-1.66642	-1.68199	-1.67902	-1.67609	-1.6732	-1.67038	-1.68626	-1.68358
-1.66865	-1.66565	-1.68121	-1.67823	-1.6753	-1.67244	-1.66965	-1.6855	-1.68282
-1.66796	-1.66495	-1.66195	-1.65894	-1.69322	-1.69032	-1.68749	-1.70198	-1.69923
-1.66733	-1.66432	-1.66129	-1.65824	-1.69247	-1.68957	-1.68675	-1.7012	-1.69843
-1.66666	-1.66365	-1.66061	-1.65755	-1.69175	-1.68886	-1.68605	-1.70045	-1.69766
-1.66588	-1.66288	-1.65988	-1.65686	-1.69108	-1.68823	-1.68542	-1.69976	-1.69691
-1.66508	-1.66209	-1.65912	-1.65616	-1.6904	-1.68757	-1.68478	-1.69908	-1.69619
-1.66431	-1.66131	-1.67728	-1.67427	-1.68963	-1.68682	-1.68405	-1.69836	-1.69549
-1.66356	-1.66055	-1.67649	-1.67346	-1.68882	-1.68603	-1.68329	-1.69761	-1.6948
-1.66288	-1.65986	-1.67577	-1.67273	-1.70531	-1.70245	-1.69964	-1.69683	-1.69403
-1.66224	-1.65923	-1.6751	-1.67204	-1.70457	-1.70168	-1.69885	-1.69602	-1.69321
-1.68051	-1.67744	-1.67439	-1.67135	-1.68581	-1.68292	-1.68008	-1.69526	-1.69242
-1.6797	-1.67663	-1.67361	-1.67065	-1.68514	-1.68226	-1.67941	-1.69456	-1.69167
-1.67892	-1.67583	-1.67283	-1.66993	-1.68444	-1.68157	-1.67871	-1.71431	-1.71131
-1.6782	-1.67506	-1.67205	-1.66915	-1.68365	-1.68078	-1.67793	-1.71354	-1.71058
-1.6775	-1.67432	-1.67128	-1.66839	-1.68287	-1.67999	-1.67714	-1.71275	-1.70984
-1.67676	-1.6736	-1.67059	-1.6677	-1.68214	-1.67924	-1.67636	-1.71194	-1.70904
-1.67601	-1.67291	-1.66992	-1.66704	-1.68145	-1.67851	-1.6756	-1.71113	-1.70822
-1.67529	-1.6722	-1.68658	-1.68364	-1.70156	-1.69853	-1.69554	-1.71038	-1.70746
-1.67458	-1.67148	-1.68582	-1.68287	-1.70078	-1.69778	-1.69482	-1.70967	-1.70676
-1.67386	-1.67077	-1.68509	-1.68214	-1.70002	-1.69704	-1.69408	-1.69116	-1.68825
-1.67312	-1.67009	-1.68443	-1.68148	-1.69932	-1.69629	-1.6933	-1.69037	-1.68747
-1.68982	-1.68675	-1.68374	-1.6808	-1.6986	-1.69556	-1.69255	-1.68961	-1.68671
-1.68908	-1.686	-1.68299	-1.68005	-1.69784	-1.69483	-1.69185	-1.68891	-1.68601
-1.68835	-1.68525	-1.68222	-1.67928	-1.69707	-1.69411	-1.69118	-1.68824	-1.68532
-1.68762	-1.68454	-1.70238	-1.69934	-1.69634	-1.6934	-1.69048	-1.70362	-1.7006
-1.68689	-1.68386	-1.70169	-1.69864	-1.69564	-1.69271	-1.68978	-1.70287	-1.69983
-1.68619	-1.68318	-1.70098	-1.69792	-1.6949	-1.69196	-1.68904	-1.70212	-1.69908
-1.68554	-1.68252	-1.70026	-1.69715	-1.69411	-1.69117	-1.68826	-1.70136	-1.69837
-1.68487	-1.68183	-1.69953	-1.69641	-1.70957	-1.70657	-1.7036	-1.70063	-1.69766
-1.68417	-1.68109	-1.69877	-1.6957	-1.70887	-1.70587	-1.7029	-1.69993	-1.69696
-1.68346	-1.68036	-1.69803	-1.695	-1.70818	-1.70519	-1.70222	-1.69923	-1.69625
-1.68274	-1.67966	-1.69733	-1.69432	-1.7075	-1.7045	-1.70153	-1.69852	-1.69551
-1.68202	-1.67898	-1.69666	-1.69366	-1.70682	-1.70382	-1.70083	-1.6978	-1.69477
-1.70215	-1.69904	-1.71222	-1.70917	-1.70613	-1.70311	-1.70009	-1.71569	-1.71262
-1.70148	-1.69837	-1.71154	-1.70848	-1.70544	-1.70238	-1.69934	-1.71494	-1.71192
-1.70081	-1.6977	-1.71084	-1.70778	-1.70472	-1.70166	-1.69861	-1.71423	-1.71122
-1.70013	-1.69703	-1.71014	-1.70704	-1.70398	-1.70094	-1.69793	-1.71354	-1.71054
-1.69946	-1.69634	-1.70941	-1.70631	-1.70325	-1.70024	-1.69725	-1.71286	-1.70987
-1.69877	-1.69561	-1.70866	-1.70559	-1.70256	-1.69956	-1.69658	-1.71219	-1.7092

							-0.04961	-0.04949
							-0.04954	-0.04941
							-0.10561	-0.10503
							-0.10532	-0.10473
							-0.10503	-0.10443
				-0.04962	-0.0495	-0.04937	-0.15403	-0.15333
				-0.04954	-0.04941	-0.04928	-0.15368	-0.153
				-0.10588	-0.10531	-0.10474	-0.15335	-0.15268
				-0.10561	-0.10504	-0.10448	-0.15304	-0.15237
		-0.04958	-0.04945	-0.15474	-0.15406	-0.15339	-0.34863	-0.34728
		-0.04951	-0.04938	-0.15442	-0.15374	-0.15307	-0.348	-0.34665
		-0.10595	-0.10537	-0.35146	-0.35009	-0.34873	-0.38449	-0.38302
		-0.10568	-0.10511	-0.35082	-0.34945	-0.3481	-0.3838	-0.38234
		-0.10541	-0.10484	-0.35018	-0.34881	-0.34746	-0.38311	-0.38168
-0.04948	-0.04935	-0.15449	-0.15382	-0.34955	-0.34815	-0.34679	-0.42803	-0.42653
-0.04941	-0.04928	-0.15417	-0.1535	-0.34891	-0.34749	-0.34612	-0.42729	-0.42586
-0.10576	-0.10517	-0.351	-0.34965	-0.38548	-0.38393	-0.38244	-0.64896	-0.64696
-0.10548	-0.10491	-0.35037	-0.34901	-0.38479	-0.38328	-0.38183	-0.64804	-0.64604
-0.10521	-0.10465	-0.34973	-0.34836	-0.47922	-0.47746	-0.47577	-0.64711	-0.64513
-0.10495	-0.10438	-0.34909	-0.34769	-0.47836	-0.47667	-0.47505	-0.64619	-0.64422
-0.35119	-0.34983	-0.38565	-0.38411	-0.56746	-0.5656	-0.56382	-0.64526	-0.64331
-0.35055	-0.34921	-0.38498	-0.38345	-0.56658	-0.56476	-0.563	-0.64434	-0.64239
-0.34993	-0.34859	-0.38432	-0.38281	-0.56575	-0.56394	-0.56219	-0.64342	-0.64146
-0.3493	-0.34797	-0.47882	-0.47705	-0.6485	-0.64646	-0.64446	-0.73132	-0.72909
-0.34867	-0.34735	-0.47805	-0.47632	-0.64758	-0.64554	-0.64354	-0.73027	-0.72804
-0.38521	-0.38378	-0.56709	-0.56517	-0.64666	-0.64462	-0.64262	-0.77583	-0.77342
-0.38454	-0.38311	-0.56622	-0.56434	-0.64574	-0.6437	-0.64169	-0.7747	-0.77229
-0.52417	-0.52235	-0.56537	-0.56352	-0.734	-0.73167	-0.72938	-0.84829	-0.84566
-0.52335	-0.52157	-0.56456	-0.56271	-0.73293	-0.73061	-0.72833	-0.84707	-0.84444
-0.56744	-0.5656	-0.65662	-0.65444	-0.77277	-0.77033	-0.76793	-0.91317	-0.91017
-0.56656	-0.56477	-0.65566	-0.65349	-0.77165	-0.76922	-0.76682	-0.91179	-0.9088

-0.5657	-0.56394	-0.65471	-0.65255	-0.77054	-0.76811	-0.76572	-0.91041	-0.90743
-0.56485	-0.56311	-0.73349	-0.73108	-0.84328	-0.84063	-0.83802	-0.98912	-0.98584
-0.56403	-0.56229	-0.73242	-0.73002	-0.84208	-0.83943	-0.83681	-0.9876	-0.98433
-0.656	-0.65393	-0.77218	-0.7697	-0.91544	-0.91234	-0.90927	-1.08748	-1.08371
-0.65507	-0.65299	-0.77107	-0.76863	-0.9141	-0.91099	-0.90791	-1.08574	-1.08198
-0.69663	-0.69441	-0.8439	-0.84125	-1.02882	-1.02528	-1.02178	-1.12298	-1.11897
-0.69566	-0.69342	-0.84272	-0.8401	-1.02735	-1.0238	-1.02027	-1.12117	-1.11722
-0.6947	-0.69244	-0.84154	-0.83895	-1.02588	-1.02231	-1.01877	-1.11938	-1.11548
-0.81054	-0.80793	-0.96079	-0.95778	-1.02437	-1.02079	-1.01725	-1.15344	-1.14927
-0.80943	-0.80681	-0.95944	-0.95642	-1.02283	-1.01926	-1.01573	-1.15152	-1.14739
-0.84344	-0.84074	-1.0282	-1.02476	-1.12442	-1.12016	-1.11594	-1.20536	-1.20105
-0.84228	-0.8396	-1.0267	-1.02322	-1.12255	-1.11835	-1.11418	-1.20342	-1.19909
-0.96164	-0.95853	-1.02522	-1.0217	-1.159	-1.1546	-1.15027	-1.20155	-1.19719
-0.96027	-0.95723	-1.02377	-1.0202	-1.15705	-1.15276	-1.14854	-1.19974	-1.19534
-0.98796	-0.98472	-1.12564	-1.12132	-1.21105	-1.20666	-1.20234	-1.19792	-1.19352
-0.9865	-0.98326	-1.12382	-1.11955	-1.20912	-1.20473	-1.20041	-1.19603	-1.19171
-0.98504	-0.98179	-1.12199	-1.11779	-1.20724	-1.20282	-1.19847	-1.19416	-1.18993
-0.98359	-0.9803	-1.17726	-1.17299	-1.2054	-1.20095	-1.19659	-1.25073	-1.24645
-0.98215	-0.97881	-1.17533	-1.17117	-1.2036	-1.19912	-1.19473	-1.2489	-1.24473
-1.1248	-1.12059	-1.17345	-1.16933	-1.20171	-1.19726	-1.1929	-1.24706	-1.24295
-1.12309	-1.11885	-1.17161	-1.16746	-1.19976	-1.19538	-1.1911	-1.24521	-1.24111
-1.1785	-1.17411	-1.20654	-1.20217	-1.25643	-1.25199	-1.24762	-1.27512	-1.27094
-1.1767	-1.17239	-1.20476	-1.20034	-1.25453	-1.25017	-1.24589	-1.27337	-1.26917
-1.17491	-1.17065	-1.20298	-1.19855	-1.25268	-1.24838	-1.24416	-1.34906	-1.34457
-1.17313	-1.16885	-1.20112	-1.19678	-1.25092	-1.24661	-1.24239	-1.3472	-1.34277
-1.17135	-1.16703	-1.19925	-1.19501	-1.24918	-1.24485	-1.24061	-1.34533	-1.34098
-1.16952	-1.16523	-1.19739	-1.19318	-1.27362	-1.26921	-1.26487	-1.36887	-1.36445
-1.16767	-1.16344	-1.19554	-1.19132	-1.27166	-1.26733	-1.26306	-1.36692	-1.36247
-1.20241	-1.19805	-1.2522	-1.24786	-1.35347	-1.34899	-1.34455	-1.41828	-1.41371
-1.20059	-1.19629	-1.25044	-1.24611	-1.3516	-1.34715	-1.34274	-1.41638	-1.4118
-1.1988	-1.19454	-1.27504	-1.27062	-1.34977	-1.34532	-1.34092	-1.4145	-1.40995
-1.19706	-1.19278	-1.2732	-1.26886	-1.34798	-1.3435	-1.33907	-1.41263	-1.40817
-1.19532	-1.19104	-1.27138	-1.26711	-1.34619	-1.34169	-1.33725	-1.41078	-1.40641
-1.22388	-1.2196	-1.35327	-1.3488	-1.39843	-1.39386	-1.38936	-1.40895	-1.4046
-1.22212	-1.21793	-1.35147	-1.34696	-1.39651	-1.392	-1.38756	-1.40714	-1.40279
-1.30156	-1.29714	-1.40385	-1.39922	-1.41878	-1.41421	-1.40973	-1.40534	-1.40102
-1.29977	-1.29536	-1.40192	-1.39735	-1.41692	-1.41237	-1.4079	-1.40355	-1.39928
-1.35501	-1.35046	-1.40002	-1.39547	-1.41504	-1.41053	-1.4061	-1.50457	-1.50001
-1.35311	-1.34864	-1.39816	-1.39358	-1.41312	-1.40871	-1.40437	-1.50267	-1.49806
-1.40551	-1.4009	-1.3963	-1.39171	-1.41125	-1.40691	-1.40262	-1.52236	-1.51767
-1.40371	-1.39902	-1.39442	-1.38989	-1.40946	-1.40511	-1.40082	-1.52042	-1.51577
-1.40194	-1.39718	-1.39256	-1.3881	-1.4077	-1.40334	-1.39903	-1.51849	-1.51388
-1.40017	-1.39542	-1.39082	-1.38634	-1.53052	-1.52585	-1.52123	-1.5393	-1.53458
-1.39839	-1.39372	-1.38912	-1.3846	-1.5286	-1.52397	-1.51937	-1.53738	-1.53261
-1.39656	-1.39196	-1.46195	-1.45732	-1.52677	-1.52211	-1.51747	-1.53545	-1.5307
-1.39469	-1.39017	-1.46019	-1.45564	-1.52502	-1.52027	-1.51555	-1.53351	-1.52882
-1.41641	-1.4119	-1.53262	-1.52794	-1.52324	-1.51845	-1.51369	-1.53162	-1.52694
-1.41462	-1.41008	-1.53067	-1.52606	-1.52141	-1.51667	-1.51192	-1.52977	-1.52504
-1.46391	-1.45927	-1.52876	-1.52418	-1.54233	-1.53755	-1.53276	-1.58444	-1.57929
-1.46215	-1.45757	-1.52697	-1.52231	-1.54039	-1.53565	-1.53092	-1.58255	-1.57744

-1.4604	-1.4559	-1.52522	-1.52046	-1.53844	-1.53373	-1.52905	-1.58067	-1.57562
-1.51059	-1.50597	-1.52342	-1.51867	-1.53661	-1.53185	-1.52715	-1.5787	-1.57374
-1.50865	-1.50409	-1.5216	-1.51691	-1.53484	-1.53	-1.52522	-1.5767	-1.57184
-1.50679	-1.50222	-1.54251	-1.53778	-1.53302	-1.52818	-1.52339	-1.57476	-1.56992
-1.505	-1.50033	-1.54056	-1.53587	-1.53116	-1.52639	-1.52165	-1.57288	-1.56796
-1.52538	-1.52063	-1.53865	-1.53397	-1.52928	-1.52459	-1.51991	-1.57104	-1.56609
-1.52362	-1.51888	-1.53684	-1.53207	-1.52736	-1.52273	-1.51815	-1.56925	-1.56434
-1.52186	-1.51715	-1.53506	-1.53021	-1.52546	-1.52089	-1.51638	-1.56748	-1.56263
-1.54286	-1.5381	-1.53329	-1.52842	-1.58025	-1.57534	-1.57052	-1.56571	-1.56095
-1.54104	-1.53632	-1.53154	-1.52668	-1.57841	-1.57349	-1.56867	-1.56394	-1.55928
-1.53918	-1.53451	-1.52976	-1.52493	-1.57657	-1.57161	-1.56678	-1.5621	-1.55753
-1.5373	-1.53267	-1.52797	-1.52319	-1.57474	-1.56973	-1.56485	-1.56022	-1.55573
-1.53545	-1.53084	-1.52618	-1.52146	-1.57294	-1.56793	-1.56304	-1.57689	-1.57238
-1.53366	-1.52903	-1.52438	-1.51973	-1.57118	-1.56621	-1.56135	-1.57513	-1.57054
-1.53187	-1.5272	-1.57928	-1.57432	-1.58791	-1.583	-1.57816	-1.5734	-1.56874
-1.53006	-1.52534	-1.57731	-1.57244	-1.5861	-1.58128	-1.5765	-1.57172	-1.56702
-1.52828	-1.52351	-1.57537	-1.57057	-1.5843	-1.57956	-1.57484	-1.57006	-1.56533
-1.52653	-1.52175	-1.57353	-1.56876	-1.58253	-1.57784	-1.57316	-1.56837	-1.56362
-1.52481	-1.52005	-1.57174	-1.567	-1.58079	-1.57613	-1.57147	-1.56667	-1.56193
-1.5231	-1.51836	-1.58847	-1.58375	-1.57908	-1.57444	-1.5698	-1.58683	-1.58205
-1.5214	-1.51669	-1.58674	-1.58207	-1.57742	-1.57277	-1.56812	-1.58516	-1.58043
-1.5389	-1.5342	-1.58502	-1.58038	-1.57576	-1.57109	-1.56644	-1.58353	-1.57891
-1.5372	-1.53253	-1.58328	-1.57869	-1.57408	-1.56938	-1.56474	-1.58193	-1.57751
-1.59132	-1.58634	-1.58156	-1.57699	-1.59428	-1.58949	-1.58481	-1.58036	-1.57615
-1.58956	-1.58462	-1.57986	-1.5753	-1.59257	-1.58775	-1.58309	-1.57879	-1.57479
-1.58782	-1.5829	-1.57816	-1.57361	-1.59085	-1.58604	-1.58141	-1.57726	-1.57346
-1.58609	-1.5812	-1.57647	-1.57191	-1.58914	-1.58437	-1.57983	-1.58653	-1.58276
-1.58437	-1.57952	-1.5748	-1.57022	-1.58744	-1.58276	-1.57832	-1.58512	-1.58147
-1.58266	-1.57784	-1.59497	-1.5903	-1.58574	-1.58119	-1.57692	-1.62742	-1.62381
-1.58096	-1.57617	-1.59327	-1.58856	-1.58404	-1.57968	-1.57561	-1.62616	-1.62265
-1.6012	-1.59639	-1.59162	-1.58688	-1.62664	-1.62237	-1.61841	-1.62493	-1.62152
-1.59955	-1.59481	-1.59006	-1.58525	-1.62499	-1.62088	-1.6171	-1.62373	-1.62043
-1.59793	-1.59323	-1.5885	-1.58369	-1.62342	-1.61945	-1.61583	-1.62255	-1.61936
-1.59634	-1.59162	-1.63121	-1.62638	-1.63234	-1.62836	-1.62474	-1.62139	-1.61829
-1.59476	-1.58998	-1.62957	-1.62487	-1.63094	-1.62706	-1.62353	-1.62026	-1.61724
-1.63751	-1.63261	-1.62791	-1.62339	-1.62958	-1.62582	-1.62238	-1.63998	-1.63695
-1.63579	-1.63086	-1.62625	-1.62193	-1.62827	-1.62463	-1.62129	-1.63893	-1.63593
-1.63406	-1.62913	-1.62461	-1.62049	-1.62697	-1.62347	-1.62022	-1.63791	-1.63495
-1.63233	-1.6274	-1.62298	-1.61906	-1.62569	-1.62232	-1.61919	-1.63693	-1.63402
-1.63063	-1.62577	-1.62147	-1.61771	-1.62446	-1.62122	-1.61818	-1.63596	-1.63307
-1.62898	-1.62428	-1.62012	-1.61648	-1.6233	-1.62018	-1.61723	-1.635	-1.6321
-1.62737	-1.62288	-1.61887	-1.61534	-1.62222	-1.61918	-1.61629	-1.63404	-1.63111
-1.62581	-1.62156	-1.62806	-1.62453	-1.62124	-1.61822	-1.61532	-1.63306	-1.63012
-1.6243	-1.62031	-1.62696	-1.62355	-1.62033	-1.61729	-1.61436	-1.63206	-1.62912
-1.62287	-1.61905	-1.62581	-1.62251	-1.64014	-1.63706	-1.63405	-1.63107	-1.62814
-1.62154	-1.61781	-1.62463	-1.62143	-1.63911	-1.63605	-1.63306	-1.63009	-1.62717
-1.62025	-1.61663	-1.6235	-1.62037	-1.63808	-1.63504	-1.63206	-1.62912	-1.62623
-1.61902	-1.6155	-1.62242	-1.61932	-1.63704	-1.63401	-1.63106	-1.62817	-1.62534
-1.62824	-1.6247	-1.62139	-1.61832	-1.63604	-1.63303	-1.63009	-1.62724	-1.62445
-1.62706	-1.62367	-1.62044	-1.61742	-1.63514	-1.63214	-1.6292	-1.62633	-1.62353

-1.62591	-1.62265	-1.61952	-1.61654	-1.63427	-1.63128	-1.62834	-1.62544	-1.62261
-1.62481	-1.62162	-1.61854	-1.61559	-1.63333	-1.63036	-1.62743	-1.62455	-1.62172
-1.62374	-1.62059	-1.61754	-1.61462	-1.63235	-1.62941	-1.62651	-1.62366	-1.62086
-1.62267	-1.61957	-1.61655	-1.61364	-1.63138	-1.62848	-1.62561	-1.6228	-1.62002
-1.62162	-1.61856	-1.61557	-1.61266	-1.6304	-1.62755	-1.62474	-1.62197	-1.61922
-1.62062	-1.6176	-1.63531	-1.63235	-1.62946	-1.62663	-1.62386	-1.62111	-1.6184
-1.61972	-1.61673	-1.63444	-1.63148	-1.62858	-1.62573	-1.62294	-1.62021	-1.61752
-1.61883	-1.61586	-1.63357	-1.63061	-1.62771	-1.62484	-1.62203	-1.61931	-1.61664
-1.61788	-1.61494	-1.63264	-1.6297	-1.6268	-1.62394	-1.62114	-1.61844	-1.61581
-1.61694	-1.61401	-1.63171	-1.62878	-1.6259	-1.62305	-1.62027	-1.61759	-1.61498
-1.61603	-1.61313	-1.63083	-1.62791	-1.62504	-1.62221	-1.61945	-1.61677	-1.61415
-1.61516	-1.61228	-1.62997	-1.62706	-1.6242	-1.6214	-1.61864	-1.61595	-1.61332
-1.61426	-1.61139	-1.62907	-1.62617	-1.62332	-1.62053	-1.61779	-1.63748	-1.6348
-1.61333	-1.61045	-1.62812	-1.62523	-1.62239	-1.61961	-1.61688	-1.63659	-1.63396
-1.61243	-1.60956	-1.62721	-1.62433	-1.6215	-1.61872	-1.61601	-1.63572	-1.63311
-1.61161	-1.60874	-1.62638	-1.62351	-1.6207	-1.61792	-1.6152	-1.63487	-1.63223
-1.6108	-1.60793	-1.62556	-1.62272	-1.61991	-1.61714	-1.61442	-1.63404	-1.63137
-1.63053	-1.62762	-1.62475	-1.62191	-1.61912	-1.61635	-1.61364	-1.63325	-1.63059
-1.62967	-1.62678	-1.62393	-1.6211	-1.61831	-1.61556	-1.61285	-1.63247	-1.62984
-1.62875	-1.62588	-1.62304	-1.62022	-1.61745	-1.6147	-1.61201	-1.66867	-1.66593
-1.62779	-1.62493	-1.6221	-1.61929	-1.61652	-1.61378	-1.61111	-1.66773	-1.66504
-1.62689	-1.62404	-1.62121	-1.61841	-1.65357	-1.6507	-1.64791	-1.66685	-1.66419
-1.62608	-1.62323	-1.6204	-1.6176	-1.65273	-1.64987	-1.64708	-1.66603	-1.6634
-1.62528	-1.62243	-1.6196	-1.6168	-1.67372	-1.67078	-1.66793	-1.66523	-1.66263
-1.62447	-1.62162	-1.6188	-1.616	-1.67288	-1.66997	-1.66714	-1.66445	-1.66185
-1.62363	-1.62079	-1.61798	-1.61519	-1.67203	-1.66914	-1.66634	-1.66366	-1.66107
-1.62273	-1.6199	-1.65513	-1.65221	-1.67109	-1.66825	-1.66548	-1.66281	-1.66022
-1.6218	-1.61898	-1.65417	-1.65125	-1.67012	-1.66732	-1.66459	-1.66194	-1.65936
-1.65917	-1.65621	-1.65328	-1.65037	-1.66924	-1.66647	-1.66378	-1.68314	-1.68043
-1.65833	-1.65538	-1.65246	-1.64956	-1.66843	-1.66569	-1.66302	-1.68237	-1.67966
-1.64252	-1.63959	-1.65166	-1.64879	-1.66765	-1.66494	-1.66229	-1.68162	-1.67891
-1.64172	-1.6388	-1.65089	-1.64804	-1.66691	-1.66422	-1.6616	-1.68089	-1.67818
-1.64094	-1.63803	-1.63518	-1.63238	-1.66619	-1.66352	-1.66091	-1.68017	-1.67745
-1.64016	-1.63726	-1.63443	-1.63168	-1.66548	-1.66282	-1.66021	-1.67942	-1.6767
-1.63938	-1.63649	-1.63369	-1.63098	-1.66478	-1.66211	-1.65949	-1.67865	-1.67592
-1.63859	-1.63572	-1.65489	-1.65213	-1.67181	-1.669	-1.66624	-1.67782	-1.67507
-1.6378	-1.63495	-1.65412	-1.65139	-1.67104	-1.66821	-1.66541	-1.67695	-1.67417
-1.63699	-1.63414	-1.6533	-1.6506	-1.67024	-1.66741	-1.66461	-1.68523	-1.68232
-1.63617	-1.6333	-1.65243	-1.64977	-1.66939	-1.66661	-1.66383	-1.68442	-1.6815
-1.63535	-1.63249	-1.67408	-1.67131	-1.66857	-1.66582	-1.66307	-1.68362	-1.6807
-1.63454	-1.63174	-1.6733	-1.67054	-1.66781	-1.66507	-1.66232	-1.68282	-1.67991
-1.63375	-1.63102	-1.67257	-1.66981	-1.66707	-1.66432	-1.66157	-1.68203	-1.67912
-1.633	-1.63032	-1.67183	-1.66907	-1.66633	-1.66358	-1.66082	-1.68125	-1.67834
-1.63229	-1.62964	-1.67111	-1.66835	-1.6656	-1.66283	-1.66006	-1.68046	-1.67756
-1.67606	-1.6732	-1.67039	-1.66763	-1.68845	-1.68551	-1.68259	-1.67968	-1.67679
-1.67528	-1.67246	-1.66967	-1.66692	-1.68768	-1.68473	-1.6818	-1.67889	-1.676
-1.67455	-1.67174	-1.66895	-1.66619	-1.6869	-1.68394	-1.681	-1.6781	-1.67523
-1.67389	-1.67105	-1.66823	-1.66545	-1.6861	-1.68314	-1.68021	-1.67731	-1.67445
-1.67323	-1.67036	-1.66751	-1.66471	-1.6853	-1.68234	-1.67941	-1.67652	-1.67367
-1.67255	-1.66966	-1.6668	-1.66397	-1.68449	-1.68153	-1.67861	-1.67572	-1.67289

-1.67185	-1.66897	-1.66609	-1.66323	-1.68369	-1.68073	-1.6778	-1.67493	-1.67211
-1.67116	-1.66831	-1.689	-1.68594	-1.68292	-1.67994	-1.67701	-1.67415	-1.67134
-1.67046	-1.66766	-1.68832	-1.68523	-1.68217	-1.67917	-1.67623	-1.67337	-1.67059
-1.65089	-1.64812	-1.68759	-1.68451	-1.68146	-1.67844	-1.67548	-1.69134	-1.68851
-1.65015	-1.64737	-1.68682	-1.68381	-1.68079	-1.67774	-1.67475	-1.69058	-1.68775
-1.6494	-1.64661	-1.68603	-1.68306	-1.68008	-1.67704	-1.67404	-1.68985	-1.68702
-1.64865	-1.64586	-1.68523	-1.68226	-1.67929	-1.67631	-1.67337	-1.68917	-1.68632
-1.6479	-1.6451	-1.68442	-1.68144	-1.67849	-1.67557	-1.67268	-1.68848	-1.68563
-1.64713	-1.64433	-1.6836	-1.68063	-1.67769	-1.67478	-1.67192	-1.68775	-1.68497
-1.64637	-1.64355	-1.68279	-1.67983	-1.67689	-1.67398	-1.67114	-1.687	-1.68431
-1.6694	-1.66642	-1.68199	-1.67902	-1.67609	-1.6732	-1.67038	-1.68626	-1.68358
-1.66865	-1.66565	-1.68121	-1.67823	-1.6753	-1.67244	-1.66965	-1.6855	-1.68282
-1.66796	-1.66495	-1.66195	-1.65894	-1.69322	-1.69032	-1.68749	-1.70198	-1.69923
-1.66733	-1.66432	-1.66129	-1.65824	-1.69247	-1.68957	-1.68675	-1.7012	-1.69843
-1.66666	-1.66365	-1.66061	-1.65755	-1.69175	-1.68886	-1.68605	-1.70045	-1.69766
-1.66588	-1.66288	-1.65988	-1.65686	-1.69108	-1.68823	-1.68542	-1.69976	-1.69691
-1.66508	-1.66209	-1.65912	-1.65616	-1.6904	-1.68757	-1.68478	-1.69908	-1.69619
-1.66431	-1.66131	-1.67728	-1.67427	-1.68963	-1.68682	-1.68405	-1.69836	-1.69549
-1.66356	-1.66055	-1.67649	-1.67346	-1.68882	-1.68603	-1.68329	-1.69761	-1.6948
-1.66288	-1.65986	-1.67577	-1.67273	-1.70531	-1.70245	-1.69964	-1.69683	-1.69403
-1.66224	-1.65923	-1.6751	-1.67204	-1.70457	-1.70168	-1.69885	-1.69602	-1.69321
-1.68051	-1.67744	-1.67439	-1.67135	-1.68581	-1.68292	-1.68008	-1.69526	-1.69242
-1.6797	-1.67663	-1.67361	-1.67065	-1.68514	-1.68226	-1.67941	-1.69456	-1.69167
-1.67892	-1.67583	-1.67283	-1.66993	-1.68444	-1.68157	-1.67871	-1.71431	-1.71131
-1.6782	-1.67506	-1.67205	-1.66915	-1.68365	-1.68078	-1.67793	-1.71354	-1.71058
-1.6775	-1.67432	-1.67128	-1.66839	-1.68287	-1.67999	-1.67714	-1.71275	-1.70984
-1.67676	-1.6736	-1.67059	-1.6677	-1.68214	-1.67924	-1.67636	-1.71194	-1.70904
-1.67601	-1.67291	-1.66992	-1.66704	-1.68145	-1.67851	-1.6756	-1.71113	-1.70822
-1.67529	-1.6722	-1.68658	-1.68364	-1.70156	-1.69853	-1.69554	-1.71038	-1.70746
-1.67458	-1.67148	-1.68582	-1.68287	-1.70078	-1.69778	-1.69482	-1.70967	-1.70676
-1.67386	-1.67077	-1.68509	-1.68214	-1.70002	-1.69704	-1.69408	-1.69116	-1.68825
-1.67312	-1.67009	-1.68443	-1.68148	-1.69932	-1.69629	-1.6933	-1.69037	-1.68747
-1.68982	-1.68675	-1.68374	-1.6808	-1.6986	-1.69556	-1.69255	-1.68961	-1.68671
-1.68908	-1.686	-1.68299	-1.68005	-1.69784	-1.69483	-1.69185	-1.68891	-1.68601
-1.68835	-1.68525	-1.68222	-1.67928	-1.69707	-1.69411	-1.69118	-1.68824	-1.68532
-1.68762	-1.68454	-1.70238	-1.69934	-1.69634	-1.6934	-1.69048	-1.70362	-1.7006
-1.68689	-1.68386	-1.70169	-1.69864	-1.69564	-1.69271	-1.68978	-1.70287	-1.69983
-1.68619	-1.68318	-1.70098	-1.69792	-1.6949	-1.69196	-1.68904	-1.70212	-1.69908
-1.68554	-1.68252	-1.70026	-1.69715	-1.69411	-1.69117	-1.68826	-1.70136	-1.69837
-1.68487	-1.68183	-1.69953	-1.69641	-1.70957	-1.70657	-1.7036	-1.70063	-1.69766
-1.68417	-1.68109	-1.69877	-1.6957	-1.70887	-1.70587	-1.7029	-1.69993	-1.69696
-1.68346	-1.68036	-1.69803	-1.695	-1.70818	-1.70519	-1.70222	-1.69923	-1.69625
-1.68274	-1.67966	-1.69733	-1.69432	-1.7075	-1.7045	-1.70153	-1.69852	-1.69551
-1.68202	-1.67898	-1.69666	-1.69366	-1.70682	-1.70382	-1.70083	-1.6978	-1.69477
-1.70215	-1.69904	-1.71222	-1.70917	-1.70613	-1.70311	-1.70009	-1.71569	-1.71262
-1.70148	-1.69837	-1.71154	-1.70848	-1.70544	-1.70238	-1.69934	-1.71494	-1.71192
-1.70081	-1.6977	-1.71084	-1.70778	-1.70472	-1.70166	-1.69861	-1.71423	-1.71122
-1.70013	-1.69703	-1.71014	-1.70704	-1.70398	-1.70094	-1.69793	-1.71354	-1.71054
-1.69946	-1.69634	-1.70941	-1.70631	-1.70325	-1.70024	-1.69725	-1.71286	-1.70987
-1.69877	-1.69561	-1.70866	-1.70559	-1.70256	-1.69956	-1.69658	-1.71219	-1.7092

-0.62024	-0.61903	-0.61789	-0.61684	-0.61583	-0.61484	-0.61386	-0.61285	-0.61183
-0.62053	-0.61925	-0.61808	-0.61703	-0.61601	-0.61503	-0.61405	-0.61306	-0.61206
-0.62084	-0.61949	-0.61828	-0.61722	-0.6162	-0.61521	-0.61424	-0.61326	-0.61228
-0.6212	-0.61979	-0.61853	-0.61745	-0.61642	-0.61542	-0.61445	-0.61347	-0.61248
-0.6216	-0.62011	-0.61881	-0.6177	-0.61665	-0.61564	-0.61465	-0.61367	-0.61269
-0.62201	-0.62046	-0.6191	-0.61795	-0.61687	-0.61585	-0.61486	-0.61388	-0.6129
-0.62243	-0.62082	-0.61941	-0.61821	-0.61709	-0.61605	-0.61506	-0.61409	-0.61313
-0.62287	-0.62119	-0.61972	-0.61846	-0.6173	-0.61625	-0.61526	-0.61429	-0.61333
-0.62332	-0.62156	-0.62002	-0.61869	-0.6175	-0.61644	-0.61546	-0.61448	-0.61353
-0.62379	-0.62196	-0.62036	-0.61896	-0.61771	-0.61664	-0.61565	-0.61467	-0.61371
-0.62429	-0.62243	-0.62076	-0.61927	-0.61796	-0.61685	-0.61584	-0.61485	-0.6139
-0.62481	-0.62293	-0.6212	-0.61963	-0.61824	-0.61708	-0.61604	-0.61504	-0.61408
-0.62533	-0.62345	-0.62169	-0.62004	-0.61857	-0.61735	-0.61627	-0.61523	-0.61426
-0.62585	-0.62399	-0.62221	-0.62048	-0.61893	-0.61765	-0.61651	-0.61543	-0.61444
-0.62635	-0.62452	-0.62272	-0.62092	-0.61932	-0.61796	-0.61675	-0.61565	-0.61465
-0.62685	-0.62503	-0.62322	-0.62138	-0.61972	-0.61828	-0.617	-0.61587	-0.61487
-0.62733	-0.62553	-0.62371	-0.62185	-0.62014	-0.61863	-0.61728	-0.61612	-0.6151
-0.6278	-0.62601	-0.62419	-0.62232	-0.62058	-0.61901	-0.61759	-0.61638	-0.61532
-0.62827	-0.62648	-0.62467	-0.62281	-0.62105	-0.61941	-0.61793	-0.61667	-0.61556
-0.62877	-0.62696	-0.62514	-0.6233	-0.62154	-0.61984	-0.61828	-0.61696	-0.61581
-0.62927	-0.62744	-0.62562	-0.62381	-0.62205	-0.62029	-0.61866	-0.61728	-0.61608
-0.62979	-0.62795	-0.62614	-0.62434	-0.62257	-0.62076	-0.61907	-0.61763	-0.61636
-0.63033	-0.62849	-0.62668	-0.62489	-0.6231	-0.62125	-0.6195	-0.61799	-0.61665
-0.63086	-0.62902	-0.62721	-0.62542	-0.62363	-0.62176	-0.61998	-0.6184	-0.61698
-0.63138	-0.62955	-0.62774	-0.62595	-0.62415	-0.62229	-0.62049	-0.61885	-0.61734
-0.6319	-0.63008	-0.62827	-0.62648	-0.62467	-0.62282	-0.62102	-0.61931	-0.61772
-0.63244	-0.63061	-0.6288	-0.627	-0.6252	-0.62336	-0.62156	-0.61979	-0.61811
-0.63299	-0.63114	-0.62932	-0.62753	-0.62573	-0.62391	-0.6221	-0.62028	-0.61853
-0.63355	-0.63169	-0.62986	-0.62807	-0.62628	-0.62446	-0.62264	-0.62079	-0.61898
-0.63412	-0.63224	-0.6304	-0.62861	-0.62682	-0.62501	-0.62319	-0.62131	-0.61945
-0.63469	-0.63279	-0.63094	-0.62915	-0.62736	-0.62556	-0.62373	-0.62184	-0.61997
-0.63526	-0.63334	-0.63148	-0.6297	-0.62791	-0.6261	-0.62427	-0.62239	-0.62052
-0.63583	-0.63391	-0.63204	-0.63025	-0.62846	-0.62664	-0.62481	-0.62293	-0.62107
-0.63642	-0.6345	-0.63263	-0.63082	-0.62901	-0.62717	-0.62533	-0.62347	-0.62162
-0.63701	-0.6351	-0.63323	-0.6314	-0.62957	-0.62772	-0.62587	-0.62401	-0.62216
-0.63763	-0.63572	-0.63384	-0.63199	-0.63014	-0.62828	-0.62641	-0.62456	-0.62271
-0.63826	-0.63634	-0.63444	-0.63258	-0.63072	-0.62884	-0.62697	-0.62512	-0.62327
-0.59353	-0.59188	-0.63503	-0.63315	-0.63128	-0.62941	-0.62755	-0.6257	-0.62386
-0.59404	-0.59237	-0.63561	-0.63371	-0.63184	-0.62998	-0.62814	-0.6263	-0.62446
-0.59457	-0.59289	-0.6362	-0.63429	-0.63241	-0.63056	-0.62873	-0.62689	-0.62505
-0.59512	-0.59342	-0.63681	-0.63489	-0.63301	-0.63116	-0.62932	-0.62748	-0.62564
-0.59565	-0.59394	-0.63742	-0.63549	-0.6336	-0.63175	-0.62991	-0.62806	-0.62621
-0.59616	-0.59445	-0.63802	-0.63609	-0.63418	-0.63232	-0.63047	-0.62862	-0.62678
-0.53712	-0.53554	-0.59328	-0.59161	-0.63478	-0.6329	-0.63104	-0.62919	-0.62735
-0.53766	-0.53608	-0.5938	-0.59213	-0.63539	-0.63349	-0.63162	-0.62976	-0.62793
-0.53821	-0.53663	-0.59432	-0.59266	-0.63601	-0.6341	-0.63221	-0.63035	-0.6285
-0.49682	-0.49535	-0.53564	-0.5341	-0.57911	-0.57732	-0.57556	-0.63095	-0.6291
-0.49735	-0.49587	-0.53621	-0.53465	-0.57975	-0.57797	-0.57621	-0.63156	-0.6297
-0.4165	-0.41523	-0.53677	-0.53521	-0.58039	-0.57861	-0.57685	-0.5751	-0.57337
-0.41696	-0.41565	-0.53731	-0.53575	-0.58105	-0.57927	-0.5775	-0.57573	-0.57397

-0.4174	-0.41608	-0.4148	-0.41356	-0.5817	-0.57991	-0.57814	-0.57636	-0.57459
-0.41783	-0.41651	-0.41524	-0.414	-0.58237	-0.58055	-0.57876	-0.57698	-0.57522
-0.41826	-0.41695	-0.41568	-0.41444	-0.58303	-0.58119	-0.57938	-0.57761	-0.57586
-0.41871	-0.41741	-0.41613	-0.41488	-0.53642	-0.53489	-0.53336	-0.57825	-0.5765
-0.41918	-0.41787	-0.41658	-0.4153	-0.53695	-0.53541	-0.5339	-0.57889	-0.57716
-0.37498	-0.37375	-0.41703	-0.41572	-0.41445	-0.41323	-0.41202	-0.57954	-0.5778
-0.37543	-0.37418	-0.41746	-0.41614	-0.41487	-0.41365	-0.41246	-0.58018	-0.57844
-0.37588	-0.37461	-0.41791	-0.41658	-0.41529	-0.41408	-0.41289	-0.46109	-0.45961
-0.37631	-0.37505	-0.41837	-0.41703	-0.41574	-0.41452	-0.41332	-0.46164	-0.46016
-0.37675	-0.37548	-0.41883	-0.41749	-0.41619	-0.41496	-0.41375	-0.41255	-0.41137
-0.37718	-0.37593	-0.41931	-0.41796	-0.41665	-0.41541	-0.41419	-0.41299	-0.4118
-0.37762	-0.37638	-0.41979	-0.41844	-0.41712	-0.41586	-0.41463	-0.41342	-0.41223
-0.37808	-0.37684	-0.37559	-0.37435	-0.41761	-0.41632	-0.41507	-0.41385	-0.41267
-0.37853	-0.3773	-0.37606	-0.37482	-0.41811	-0.41679	-0.4155	-0.41429	-0.41312
-0.37899	-0.37777	-0.37653	-0.37529	-0.4186	-0.41727	-0.41597	-0.41474	-0.41355
-0.37944	-0.37824	-0.37701	-0.37575	-0.41908	-0.41775	-0.41645	-0.41519	-0.41397
-0.37989	-0.3787	-0.37747	-0.37621	-0.37496	-0.37373	-0.37252	-0.41565	-0.41441
-0.38035	-0.37914	-0.37792	-0.37668	-0.37544	-0.3742	-0.37297	-0.41611	-0.41485
-0.3808	-0.37959	-0.37837	-0.37715	-0.37592	-0.37467	-0.37343	-0.41658	-0.4153
-0.38123	-0.38004	-0.37884	-0.37762	-0.37639	-0.37514	-0.3739	-0.41706	-0.41575
-0.38167	-0.3805	-0.3793	-0.37808	-0.37685	-0.37562	-0.37438	-0.41755	-0.41621
-0.38213	-0.38094	-0.37974	-0.37854	-0.37733	-0.37609	-0.37484	-0.37359	-0.37236
-0.3826	-0.38138	-0.38018	-0.37901	-0.37781	-0.37655	-0.37529	-0.37406	-0.37285
-0.33505	-0.33401	-0.38064	-0.37948	-0.37829	-0.37704	-0.37578	-0.37455	-0.37333
-0.33548	-0.33443	-0.38112	-0.37995	-0.37876	-0.37754	-0.37631	-0.37507	-0.37383
-0.3359	-0.33484	-0.3816	-0.38041	-0.37922	-0.37803	-0.37682	-0.37557	-0.37431
-0.33633	-0.33524	-0.38205	-0.38086	-0.37967	-0.37849	-0.3773	-0.37605	-0.37478
-0.33677	-0.33565	-0.3825	-0.3813	-0.38012	-0.37894	-0.37776	-0.37652	-0.37525
-0.28909	-0.2881	-0.38296	-0.38177	-0.38058	-0.37939	-0.37819	-0.37697	-0.37574
-0.28945	-0.28844	-0.38343	-0.38224	-0.38105	-0.37984	-0.37863	-0.37743	-0.37623
-0.28981	-0.2888	-0.33581	-0.33478	-0.38151	-0.3803	-0.37909	-0.3779	-0.37672
-0.29017	-0.28918	-0.33628	-0.3352	-0.38198	-0.38078	-0.37958	-0.37839	-0.3772
-0.19941	-0.19871	-0.2886	-0.28765	-0.38245	-0.38125	-0.38006	-0.37886	-0.37766
-0.1997	-0.19899	-0.28898	-0.28799	-0.38292	-0.38172	-0.38054	-0.37932	-0.3781
-0.19999	-0.19927	-0.28936	-0.28836	-0.33642	-0.33535	-0.33428	-0.37978	-0.37855
-0.20026	-0.19955	-0.28975	-0.28875	-0.33688	-0.33577	-0.33469	-0.38025	-0.37903
-0.20052	-0.19983	-0.29015	-0.28914	-0.33734	-0.3362	-0.33509	-0.38072	-0.37952
-0.14718	-0.14669	-0.29052	-0.28953	-0.33779	-0.33662	-0.33548	-0.33438	-0.33332
-0.14736	-0.14688	-0.29089	-0.28991	-0.33825	-0.33705	-0.33587	-0.33477	-0.33372
-0.14754	-0.14707	-0.29127	-0.2903	-0.28931	-0.28829	-0.28728	-0.33519	-0.33413
-0.14772	-0.14726	-0.29166	-0.29069	-0.2897	-0.28868	-0.28767	-0.33564	-0.33455
-0.1479	-0.14744	-0.14696	-0.14647	-0.29009	-0.28908	-0.28807	-0.33609	-0.33497
-0.14806	-0.14761	-0.14714	-0.14665	-0.29049	-0.28948	-0.28847	-0.33655	-0.3354
-0.10233	-0.10199	-0.14731	-0.14683	-0.29087	-0.28987	-0.28887	-0.33701	-0.33585
-0.10247	-0.10212	-0.14749	-0.14701	-0.29123	-0.29024	-0.28925	-0.33747	-0.33632
-0.10262	-0.10226	-0.14767	-0.1472	-0.29158	-0.29061	-0.28963	-0.33794	-0.33679
-0.10275	-0.1024	-0.10204	-0.10168	-0.29193	-0.29097	-0.29001	-0.28906	-0.2881
-0.10288	-0.10253	-0.10217	-0.10181	-0.29229	-0.29134	-0.29039	-0.28944	-0.28849
-0.10302	-0.10267	-0.10231	-0.10194	-0.14726	-0.14678	-0.14629	-0.28984	-0.28889
-0.10317	-0.10281	-0.10244	-0.10208	-0.14744	-0.14697	-0.14649	-0.29024	-0.28928

-0.10332	-0.10295	-0.10258	-0.10221	-0.10185	-0.1015	-0.10116	-0.29063	-0.28967
-0.10348	-0.10309	-0.10272	-0.10235	-0.10199	-0.10164	-0.10129	-0.291	-0.29006
-0.10364	-0.10324	-0.10286	-0.10249	-0.10212	-0.10177	-0.10142	-0.29138	-0.29045
-0.1038	-0.1034	-0.10301	-0.10263	-0.10226	-0.10191	-0.10156	-0.24022	-0.23949
-0.10396	-0.10356	-0.10317	-0.10278	-0.10241	-0.10205	-0.10169	-0.24052	-0.23978
-0.10411	-0.10371	-0.10332	-0.10293	-0.10255	-0.10218	-0.10183	-0.19714	-0.1965
-0.10427	-0.10387	-0.10348	-0.10307	-0.10269	-0.10232	-0.10196	-0.1974	-0.19674
-0.05233	-0.05213	-0.10363	-0.10322	-0.10283	-0.10246	-0.1021	-0.10175	-0.1014
-0.05239	-0.0522	-0.10379	-0.10338	-0.10297	-0.1026	-0.10224	-0.10189	-0.10154
-0.05245	-0.05226	-0.10395	-0.10353	-0.10313	-0.10275	-0.10238	-0.10202	-0.10167
-0.0525	-0.05231	-0.10411	-0.1037	-0.10329	-0.1029	-0.10252	-0.10216	-0.10181
-0.05256	-0.05237	-0.10426	-0.10386	-0.10346	-0.10306	-0.10266	-0.1023	-0.10195
		-0.05223	-0.05204	-0.10363	-0.10321	-0.10281	-0.10244	-0.10208
		-0.05229	-0.05209	-0.10379	-0.10337	-0.10297	-0.10259	-0.10222
		-0.05234	-0.05214	-0.10394	-0.10353	-0.10312	-0.10274	-0.10236
		-0.05238	-0.05219	-0.10408	-0.10368	-0.10328	-0.10289	-0.1025
		-0.05243	-0.05224	-0.05205	-0.05185	-0.05166	-0.10305	-0.10265
		-0.05247	-0.05228	-0.05209	-0.05191	-0.05172	-0.10321	-0.10281
				-0.05214	-0.05196	-0.05177	-0.10336	-0.10297
				-0.0522	-0.05201	-0.05182	-0.10351	-0.10312
				-0.05225	-0.05206	-0.05187	-0.10365	-0.10327
				-0.0523	-0.05211	-0.05192	-0.05173	-0.05153
				-0.05234	-0.05215	-0.05196	-0.05177	-0.05158
							-0.05183	-0.05163
							-0.05188	-0.05168
							-0.05193	-0.05173
							-0.05199	-0.05179

							-0.10613	-0.10553
							-0.10581	-0.10522
							-0.10549	-0.10492
							-0.10517	-0.10461
				-0.04994	-0.0498	-0.04967	-0.15425	-0.15358
				-0.04985	-0.04972	-0.04959	-0.15393	-0.15326
				-0.04977	-0.04964	-0.04951	-0.15361	-0.15295
				-0.15537	-0.15465	-0.15395	-0.15326	-0.1526
				-0.15503	-0.1543	-0.15359	-0.15291	-0.15224
		-0.0498	-0.04967	-0.15467	-0.15396	-0.15326	-0.35067	-0.34939
		-0.04972	-0.04958	-0.15431	-0.15362	-0.15293	-0.35005	-0.34878
		-0.15536	-0.15466	-0.15397	-0.15328	-0.1526	-0.47536	-0.47366
		-0.15502	-0.15433	-0.15365	-0.15295	-0.15226	-0.47449	-0.47283
-0.04974	-0.04962	-0.15468	-0.154	-0.25474	-0.25366	-0.2526	-0.60578	-0.60378
-0.04966	-0.04953	-0.15432	-0.15364	-0.25421	-0.25315	-0.25212	-0.6048	-0.60284
-0.04957	-0.04945	-0.15396	-0.15328	-0.25368	-0.25265	-0.25166	-0.60384	-0.60191
-0.155	-0.15431	-0.15363	-0.15295	-0.38485	-0.38339	-0.38197	-0.60291	-0.60099
-0.15468	-0.15399	-0.15332	-0.15264	-0.38414	-0.38273	-0.38137	-0.602	-0.60009
-0.15435	-0.15368	-0.25427	-0.25321	-0.60694	-0.60494	-0.60301	-0.60108	-0.59919
-0.15403	-0.15336	-0.25377	-0.2527	-0.60589	-0.60392	-0.60203	-0.60015	-0.59829
-0.1537	-0.15302	-0.38497	-0.38342	-0.6049	-0.60295	-0.60108	-0.64868	-0.64666
-0.15333	-0.15266	-0.38421	-0.38273	-0.60398	-0.60205	-0.60017	-0.64769	-0.64568
-0.15297	-0.15231	-0.38346	-0.38207	-0.60308	-0.60116	-0.59928	-0.6467	-0.64468
-0.34847	-0.34709	-0.42832	-0.42683	-0.60217	-0.60026	-0.59837	-0.68976	-0.68762
-0.34782	-0.34646	-0.4276	-0.42615	-0.60125	-0.59935	-0.59746	-0.6887	-0.68652
-0.38431	-0.38285	-0.60417	-0.60224	-0.64553	-0.64352	-0.64153	-0.77423	-0.77179
-0.38364	-0.38223	-0.60324	-0.60133	-0.64456	-0.64256	-0.64058	-0.77308	-0.77064
-0.42859	-0.42706	-0.64763	-0.64561	-0.69213	-0.68995	-0.68779	-0.81083	-0.80826
-0.42785	-0.42636	-0.64666	-0.64466	-0.69111	-0.68895	-0.68679	-0.80964	-0.80705
-0.64978	-0.64772	-0.6457	-0.64372	-0.77699	-0.77455	-0.77211	-0.80843	-0.80582
-0.64877	-0.64675	-0.64475	-0.6428	-0.77587	-0.77341	-0.77095	-0.80719	-0.80455
-0.64779	-0.6458	-0.64382	-0.64187	-0.77474	-0.77226	-0.76977	-0.80594	-0.80329
-0.64684	-0.64487	-0.69138	-0.68923	-0.77358	-0.77109	-0.76858	-0.90684	-0.90375
-0.64591	-0.64397	-0.69039	-0.6882	-0.77242	-0.7699	-0.76739	-0.90538	-0.90233
-0.64499	-0.64306	-0.77623	-0.77374	-0.87635	-0.87335	-0.87036	-1.04467	-1.04104
-0.64408	-0.64215	-0.77512	-0.77258	-0.87493	-0.87192	-0.86895	-1.04295	-1.0394
-0.69168	-0.68956	-0.77398	-0.77141	-0.91032	-0.90719	-0.90411	-1.11872	-1.11488
-0.69068	-0.68852	-0.77281	-0.77025	-0.90891	-0.90577	-0.9027	-1.11686	-1.11314
-0.77659	-0.77413	-0.87686	-0.87383	-1.04894	-1.04516	-1.04147	-1.15083	-1.14684
-0.77545	-0.77294	-0.87544	-0.87243	-1.0472	-1.04349	-1.03986	-1.14894	-1.14497
-0.77431	-0.77176	-0.87402	-0.87103	-1.04547	-1.04184	-1.0383	-1.14709	-1.14309
-0.84785	-0.84507	-0.90908	-0.90596	-1.12147	-1.11752	-1.11369	-1.17729	-1.17316
-0.84662	-0.84383	-0.90768	-0.90455	-1.11956	-1.11572	-1.11199	-1.17538	-1.1712
-0.87605	-0.87299	-1.05331	-1.04949	-1.18599	-1.18179	-1.17769	-1.1735	-1.16931
-0.87471	-0.87166	-1.05161	-1.04784	-1.18405	-1.17988	-1.17581	-1.17165	-1.16748
-0.90984	-0.90666	-1.12302	-1.11895	-1.18213	-1.178	-1.17395	-1.2345	-1.23012
-0.90845	-0.90528	-1.12116	-1.11717	-1.18024	-1.17614	-1.17211	-1.23258	-1.22826
-1.08854	-1.0845	-1.15533	-1.1511	-1.17835	-1.17428	-1.17028	-1.30671	-1.30225
-1.0868	-1.08279	-1.1534	-1.1492	-1.17646	-1.17242	-1.16845	-1.30474	-1.30033

-1.08507	-1.0811	-1.1515	-1.14733	-1.17458	-1.17057	-1.16663	-1.30279	-1.29841
-1.12226	-1.11807	-1.18103	-1.17681	-1.25545	-1.25123	-1.24709	-1.30085	-1.2965
-1.12042	-1.11632	-1.17914	-1.17494	-1.25347	-1.2493	-1.2452	-1.29893	-1.29459
-1.15454	-1.15015	-1.2135	-1.2092	-1.28331	-1.27912	-1.27501	-1.37735	-1.37284
-1.15259	-1.14825	-1.21154	-1.20727	-1.28134	-1.2772	-1.27313	-1.37537	-1.37083
-1.15066	-1.14636	-1.25619	-1.25184	-1.27938	-1.27529	-1.27125	-1.39844	-1.39385
-1.14877	-1.14448	-1.25418	-1.24989	-1.27746	-1.27339	-1.26937	-1.39643	-1.39182
-1.14691	-1.14263	-1.25221	-1.24797	-1.27555	-1.27151	-1.2675	-1.3944	-1.38979
-1.20059	-1.19619	-1.28208	-1.27782	-1.35635	-1.35213	-1.34792	-1.41548	-1.41079
-1.19868	-1.19434	-1.28019	-1.27595	-1.3544	-1.35016	-1.34593	-1.41342	-1.40876
-1.19677	-1.19254	-1.27837	-1.27412	-1.37795	-1.37363	-1.36935	-1.4356	-1.43092
-1.19488	-1.19078	-1.27661	-1.27234	-1.376	-1.37165	-1.36735	-1.43354	-1.42895
-1.25142	-1.24728	-1.27482	-1.27055	-1.42244	-1.41787	-1.41336	-1.43151	-1.42702
-1.24953	-1.24543	-1.27299	-1.26875	-1.42044	-1.41585	-1.41132	-1.42951	-1.42515
-1.27942	-1.27529	-1.37914	-1.3747	-1.44125	-1.43656	-1.43195	-1.42755	-1.42328
-1.27754	-1.2734	-1.37715	-1.37279	-1.4393	-1.43462	-1.43	-1.42561	-1.42137
-1.27569	-1.27153	-1.37517	-1.37087	-1.43734	-1.43269	-1.42809	-1.4237	-1.41945
-1.27393	-1.26972	-1.42157	-1.41708	-1.43531	-1.43072	-1.42618	-1.4218	-1.41754
-1.2722	-1.26794	-1.41954	-1.41504	-1.43326	-1.42875	-1.42429	-1.41993	-1.41565
-1.34769	-1.34315	-1.41753	-1.41303	-1.43124	-1.42681	-1.42244	-1.41809	-1.41377
-1.34574	-1.34125	-1.41554	-1.41105	-1.42925	-1.42491	-1.42063	-1.41628	-1.41192
-1.36931	-1.3648	-1.4136	-1.40912	-1.42732	-1.42302	-1.41878	-1.5381	-1.53328
-1.36741	-1.36295	-1.41173	-1.40726	-1.42544	-1.42114	-1.41689	-1.53602	-1.53126
-1.4189	-1.41435	-1.43254	-1.42804	-1.42361	-1.41928	-1.415	-1.53395	-1.52926
-1.41702	-1.41245	-1.43062	-1.4262	-1.42182	-1.41745	-1.41312	-1.53189	-1.5273
-1.41514	-1.41054	-1.4287	-1.42436	-1.42004	-1.41563	-1.41125	-1.52985	-1.52534
-1.41318	-1.40863	-1.4268	-1.42247	-1.54212	-1.53727	-1.53244	-1.58346	-1.5786
-1.41118	-1.40673	-1.42492	-1.42056	-1.53997	-1.53517	-1.53041	-1.58132	-1.57647
-1.40925	-1.40488	-1.42307	-1.4187	-1.53788	-1.53313	-1.52844	-1.57926	-1.5744
-1.40737	-1.40306	-1.42127	-1.41688	-1.53585	-1.53115	-1.52653	-1.57727	-1.57241
-1.40554	-1.40127	-1.52066	-1.51592	-1.53386	-1.5292	-1.52462	-1.57528	-1.57044
-1.40379	-1.39947	-1.51867	-1.51398	-1.53193	-1.52728	-1.5227	-1.57329	-1.56852
-1.40204	-1.3977	-1.5167	-1.51204	-1.53	-1.52537	-1.5208	-1.57132	-1.56661
-1.40026	-1.39594	-1.53748	-1.53272	-1.584	-1.57905	-1.57417	-1.58935	-1.58461
-1.39847	-1.39419	-1.53554	-1.53075	-1.5819	-1.57702	-1.57223	-1.5874	-1.58263
-1.49919	-1.49458	-1.53355	-1.52876	-1.59982	-1.59493	-1.59015	-1.58538	-1.58064
-1.4973	-1.49264	-1.53154	-1.52676	-1.5977	-1.59281	-1.58803	-1.58331	-1.57864
-1.53963	-1.53484	-1.60588	-1.60072	-1.59566	-1.59076	-1.58598	-1.58129	-1.57669
-1.53765	-1.53291	-1.60389	-1.59877	-1.59373	-1.58881	-1.58399	-1.57933	-1.57478
-1.53568	-1.53099	-1.60188	-1.5968	-1.59179	-1.58687	-1.58206	-1.57741	-1.57288
-1.53376	-1.52905	-1.5998	-1.59473	-1.58975	-1.58493	-1.58021	-1.57556	-1.57099
-1.53184	-1.52713	-1.59774	-1.59268	-1.58774	-1.583	-1.57837	-1.57372	-1.56909
-1.58631	-1.58128	-1.59578	-1.59076	-1.58585	-1.58111	-1.57647	-1.57182	-1.56722
-1.58425	-1.5793	-1.59387	-1.58891	-1.58403	-1.57925	-1.57456	-1.56992	-1.56535
-1.58224	-1.57732	-1.59191	-1.58703	-1.58221	-1.57745	-1.57276	-1.56809	-1.56349
-1.58028	-1.57533	-1.58992	-1.58514	-1.58041	-1.57571	-1.57104	-1.56632	-1.56163
-1.57831	-1.57337	-1.58797	-1.58324	-1.57858	-1.57393	-1.5693	-1.5646	-1.55991
-1.57629	-1.57144	-1.58607	-1.58136	-1.57671	-1.57209	-1.56753	-1.56293	-1.55839
-1.57433	-1.56952	-1.58417	-1.57948	-1.57484	-1.57024	-1.56571	-1.56126	-1.55692
-1.57246	-1.5676	-1.58223	-1.5776	-1.57301	-1.56837	-1.56383	-1.55953	-1.55546

-1.57064	-1.56572	-1.58031	-1.57573	-1.57119	-1.56651	-1.56194	-1.55781	-1.55402
-1.56883	-1.56395	-1.57853	-1.57392	-1.56935	-1.56467	-1.56016	-1.57416	-1.57052
-1.56702	-1.56226	-1.57684	-1.57214	-1.56751	-1.56287	-1.55845	-1.57255	-1.56906
-1.56516	-1.56049	-1.57511	-1.57037	-1.58387	-1.57926	-1.57489	-1.59292	-1.58951
-1.56323	-1.55867	-1.57335	-1.56863	-1.58215	-1.57766	-1.57345	-1.59158	-1.5883
-1.56137	-1.55685	-1.58973	-1.585	-1.58044	-1.57611	-1.57206	-1.61137	-1.60812
-1.5596	-1.55503	-1.58786	-1.58317	-1.5787	-1.57456	-1.57074	-1.61014	-1.607
-1.55789	-1.55324	-1.58602	-1.58138	-1.57702	-1.57307	-1.56946	-1.60894	-1.60589
-1.55623	-1.55155	-1.5843	-1.57972	-1.59693	-1.59306	-1.58955	-1.61766	-1.61455
-1.55459	-1.54991	-1.58264	-1.57812	-1.59539	-1.59167	-1.5883	-1.61643	-1.61337
-1.57134	-1.56661	-1.58096	-1.57656	-1.59393	-1.59034	-1.5871	-1.61526	-1.61225
-1.56957	-1.56488	-1.57928	-1.57504	-1.59253	-1.58907	-1.58593	-1.61411	-1.61118
-1.56778	-1.56313	-1.59911	-1.59497	-1.62279	-1.61925	-1.61603	-1.61297	-1.61009
-1.56594	-1.56135	-1.59739	-1.59346	-1.6214	-1.61796	-1.6148	-1.61181	-1.60899
-1.58602	-1.58146	-1.59578	-1.59203	-1.62007	-1.61672	-1.61362	-1.61068	-1.6079
-1.58432	-1.5799	-1.59434	-1.59072	-1.6188	-1.61555	-1.61251	-1.6096	-1.60682
-1.58268	-1.57842	-1.59297	-1.58947	-1.61758	-1.61442	-1.61144	-1.60854	-1.60574
-1.58103	-1.57696	-1.60203	-1.59853	-1.61644	-1.61333	-1.61038	-1.60747	-1.60465
-1.57942	-1.57553	-1.60072	-1.59735	-1.61534	-1.61227	-1.60933	-1.60641	-1.60355
-1.57784	-1.57411	-1.6206	-1.61733	-1.61423	-1.61121	-1.60829	-1.60538	-1.60251
-1.5763	-1.57268	-1.61921	-1.61609	-1.61312	-1.61015	-1.60725	-1.60436	-1.6015
-1.58556	-1.5819	-1.61791	-1.6149	-1.61201	-1.6091	-1.60622	-1.64543	-1.64252
-1.58425	-1.58068	-1.61673	-1.61377	-1.61093	-1.60804	-1.60519	-1.64438	-1.64148
-1.58299	-1.57951	-1.65796	-1.65497	-1.65208	-1.64915	-1.64624	-1.64334	-1.64046
-1.58174	-1.57838	-1.65684	-1.65388	-1.651	-1.64809	-1.64519	-1.64231	-1.63946
-1.58051	-1.57728	-1.65574	-1.6528	-1.64994	-1.64703	-1.64415	-1.6413	-1.63848
-1.62285	-1.61965	-1.65464	-1.65172	-1.64887	-1.64598	-1.64312	-1.6403	-1.63752
-1.62164	-1.61853	-1.65355	-1.65065	-1.6478	-1.64493	-1.64209	-1.63931	-1.63658
-1.64131	-1.63821	-1.65248	-1.64959	-1.64675	-1.64389	-1.64107	-1.63832	-1.63564
-1.64019	-1.63713	-1.65141	-1.64853	-1.6457	-1.64286	-1.64006	-1.63734	-1.6347
-1.63911	-1.63608	-1.65037	-1.6475	-1.64467	-1.64185	-1.63907	-1.63638	-1.63376
-1.63806	-1.63506	-1.64935	-1.64648	-1.64367	-1.64086	-1.63811	-1.63544	-1.63284
-1.63704	-1.63406	-1.64836	-1.64549	-1.64268	-1.6399	-1.63717	-1.63452	-1.63193
-1.63602	-1.6331	-1.64741	-1.64453	-1.64171	-1.63895	-1.63625	-1.63362	-1.63104
-1.63501	-1.63215	-1.64648	-1.64359	-1.64076	-1.63802	-1.63535	-1.63272	-1.63016
-1.634	-1.63116	-1.6455	-1.64262	-1.6398	-1.63708	-1.63442	-1.63182	-1.62928
-1.633	-1.63015	-1.64448	-1.64163	-1.63884	-1.63613	-1.6335	-1.63092	-1.6284
-1.63203	-1.62917	-1.64349	-1.64066	-1.63789	-1.6352	-1.63258	-1.65206	-1.6495
-1.63111	-1.62823	-1.64253	-1.63971	-1.63696	-1.63428	-1.63167	-1.65114	-1.6486
-1.64736	-1.64443	-1.64157	-1.63876	-1.63603	-1.63337	-1.63078	-1.65025	-1.64774
-1.64637	-1.64345	-1.6406	-1.63782	-1.63511	-1.63247	-1.6299	-1.64939	-1.64692
-1.64537	-1.64247	-1.63964	-1.63688	-1.63419	-1.63158	-1.62905	-1.64855	-1.64612
-1.64437	-1.64147	-1.63865	-1.63593	-1.63328	-1.63072	-1.62822	-1.64772	-1.64529
-1.64336	-1.64047	-1.63767	-1.63498	-1.63238	-1.62987	-1.62742	-1.64691	-1.64446
-1.64238	-1.63951	-1.63674	-1.63409	-1.63152	-1.62902	-1.62658	-1.64606	-1.6436
-1.64142	-1.63858	-1.63584	-1.63323	-1.63069	-1.62818	-1.62573	-1.64518	-1.64273
-1.6405	-1.63769	-1.63498	-1.63238	-1.65188	-1.64931	-1.64678	-1.6443	-1.64185
-1.63965	-1.63688	-1.63418	-1.63155	-1.651	-1.64843	-1.6459	-1.64341	-1.64096
-1.63879	-1.63605	-1.63336	-1.63071	-1.65012	-1.64755	-1.64504	-1.66482	-1.66219
-1.63787	-1.63515	-1.63249	-1.62986	-1.64926	-1.64669	-1.64418	-1.66391	-1.66127

-1.63694	-1.63425	-1.6316	-1.629	-1.6484	-1.64585	-1.64335	-1.66303	-1.66039
-1.63605	-1.63335	-1.63072	-1.62816	-1.64758	-1.64505	-1.64257	-1.66221	-1.65958
-1.63518	-1.63247	-1.62985	-1.62732	-1.64677	-1.64428	-1.64182	-1.66143	-1.6588
-1.63435	-1.63164	-1.65104	-1.64846	-1.64595	-1.64346	-1.64101	-1.66058	-1.65796
-1.63356	-1.63087	-1.65025	-1.64765	-1.64511	-1.64262	-1.64016	-1.65967	-1.65706
-1.63275	-1.63007	-1.64944	-1.64683	-1.6667	-1.66402	-1.66139	-1.65876	-1.65616
-1.63188	-1.62922	-1.64857	-1.64599	-1.66581	-1.66313	-1.66049	-1.65787	-1.65527
-1.63101	-1.62837	-1.64772	-1.64515	-1.66495	-1.66227	-1.65962	-1.657	-1.6544
-1.65223	-1.64954	-1.64692	-1.64437	-1.66413	-1.66146	-1.65882	-1.69155	-1.68885
-1.6514	-1.64874	-1.64613	-1.6436	-1.66332	-1.66067	-1.65803	-1.69074	-1.68804
-1.65055	-1.64791	-1.64532	-1.64279	-1.69807	-1.69532	-1.69259	-1.7079	-1.70515
-1.64969	-1.64707	-1.64449	-1.64195	-1.69715	-1.69441	-1.69168	-1.70699	-1.70424
-1.64886	-1.64626	-1.66608	-1.66339	-1.69628	-1.69354	-1.69082	-1.70612	-1.70337
-1.64806	-1.64548	-1.66528	-1.6626	-1.69547	-1.69273	-1.69001	-1.7053	-1.70255
-1.64723	-1.64468	-1.70016	-1.69738	-1.69463	-1.69189	-1.68917	-1.72685	-1.72399
-1.64633	-1.64381	-1.69923	-1.69647	-1.69372	-1.69099	-1.68826	-1.7259	-1.72305
-1.64546	-1.64295	-1.69831	-1.69556	-1.69281	-1.69008	-1.68735	-1.72496	-1.72211
-1.68104	-1.67843	-1.69745	-1.69471	-1.69197	-1.68923	-1.68649	-1.72407	-1.72123
-1.68026	-1.67763	-1.6966	-1.69387	-1.69114	-1.6884	-1.68566	-1.7232	-1.72037
-1.70124	-1.69846	-1.69571	-1.69298	-1.69026	-1.68751	-1.68477	-1.72228	-1.71946
-1.70031	-1.69753	-1.69478	-1.69205	-1.68932	-1.68658	-1.68384	-1.72133	-1.7185
-1.69943	-1.69666	-1.69391	-1.69118	-1.71118	-1.70833	-1.7055	-1.72043	-1.71762
-1.69862	-1.69587	-1.69312	-1.69037	-1.71034	-1.70749	-1.70467	-1.71961	-1.71682
-1.69782	-1.69508	-1.69233	-1.68958	-1.7095	-1.70666	-1.70385	-1.7188	-1.71603
-1.697	-1.69428	-1.69154	-1.68878	-1.70867	-1.70582	-1.70301	-1.71798	-1.71524
-1.69617	-1.69345	-1.69072	-1.68795	-1.70781	-1.70497	-1.70216	-1.71714	-1.71442
-1.69528	-1.69255	-1.7126	-1.70973	-1.70688	-1.70404	-1.70124	-1.73437	-1.73161
-1.69435	-1.69162	-1.71162	-1.70875	-1.70591	-1.70308	-1.70029	-1.7334	-1.73066
-1.69351	-1.69076	-1.71072	-1.70786	-1.70502	-1.7022	-1.69943	-1.73253	-1.72978
-1.69273	-1.68997	-1.70989	-1.70702	-1.70419	-1.7014	-1.69865	-1.73172	-1.72897
-1.71487	-1.71196	-1.70908	-1.70621	-1.72185	-1.71902	-1.71622	-1.73093	-1.72818
-1.71408	-1.71117	-1.70829	-1.70542	-1.72105	-1.71822	-1.71545	-1.73016	-1.72741
-1.71329	-1.71038	-1.7075	-1.70463	-1.72024	-1.71743	-1.71467	-1.72938	-1.72663
-1.7125	-1.70958	-1.70669	-1.70383	-1.71942	-1.71663	-1.71389	-1.7286	-1.72583
-1.71167	-1.70875	-1.70586	-1.70301	-1.71859	-1.71582	-1.71309	-1.72779	-1.72502
-1.71077	-1.70785	-1.70497	-1.70213	-1.71769	-1.71494	-1.71222	-1.72691	-1.72413
-1.70982	-1.70691	-1.70404	-1.70122	-1.71677	-1.71402	-1.7113	-1.72599	-1.7232
-1.70894	-1.70603	-1.72159	-1.71873	-1.71591	-1.71316	-1.71044	-1.72513	-1.72234
-1.70811	-1.70522	-1.72077	-1.71793	-1.71513	-1.71238	-1.70964	-1.72432	-1.72153
-1.6935	-1.69063	-1.7064	-1.7036	-1.71436	-1.7116	-1.70886	-1.72353	-1.72073
-1.6927	-1.68984	-1.7056	-1.70283	-1.7136	-1.71084	-1.70809	-1.72275	-1.71994
-1.69191	-1.68906	-1.70481	-1.70206	-1.71283	-1.71007	-1.70732	-1.72197	-1.71916
-1.69114	-1.68829	-1.70403	-1.70129	-1.71631	-1.71352	-1.71073	-1.73782	-1.73494
-1.69037	-1.68753	-1.70326	-1.70052	-1.71555	-1.71275	-1.70995	-1.73701	-1.73413
-1.6896	-1.68678	-1.7025	-1.69976	-1.73173	-1.72888	-1.72602	-1.74324	-1.74026
-1.68883	-1.68604	-1.70175	-1.69901	-1.73095	-1.72808	-1.72521	-1.74239	-1.7394
-1.70661	-1.70378	-1.701	-1.69825	-1.73015	-1.72728	-1.7244	-1.74154	-1.73855
-1.70582	-1.70302	-1.70024	-1.69749	-1.72936	-1.72648	-1.72359	-1.74069	-1.7377
-1.70503	-1.70225	-1.71667	-1.71386	-1.74883	-1.74585	-1.74285	-1.7581	-1.75505
-1.70423	-1.70148	-1.71588	-1.71307	-1.74799	-1.745	-1.742	-1.75724	-1.75421

-1.70344	-1.70071	-1.71509	-1.71228	-1.74714	-1.74414	-1.74114	-1.75639	-1.75338
-1.68728	-1.68459	-1.7143	-1.71148	-1.74629	-1.74329	-1.74029	-1.75553	-1.75254
-1.68651	-1.68383	-1.71349	-1.71068	-1.74544	-1.74243	-1.73944	-1.75467	-1.75171
-1.70311	-1.70037	-1.71269	-1.70987	-1.74458	-1.74158	-1.73859	-1.75382	-1.75087
-1.70232	-1.69958	-1.71188	-1.70906	-1.74373	-1.74073	-1.73774	-1.75297	-1.75004
-1.70155	-1.6988	-1.71108	-1.70825	-1.74287	-1.73988	-1.73691	-1.75213	-1.74922
-1.70081	-1.69804	-1.71029	-1.70745	-1.74202	-1.73904	-1.73608	-1.75131	-1.74841
-1.7001	-1.6973	-1.72991	-1.72695	-1.74119	-1.73821	-1.73527	-1.75049	-1.74762
-1.69944	-1.69665	-1.72919	-1.72619	-1.74039	-1.7374	-1.73445	-1.74967	-1.74682
-1.69879	-1.696	-1.72848	-1.72545	-1.73961	-1.7366	-1.73365	-1.74887	-1.74604
-1.69805	-1.69527	-1.72773	-1.72471	-1.7571	-1.75401	-1.75098	-1.7633	-1.76043
-1.69726	-1.69449	-1.72694	-1.72397	-1.75637	-1.75327	-1.75021	-1.7625	-1.75965
-1.69647	-1.6937	-1.72613	-1.72319	-1.75559	-1.75251	-1.74947	-1.76176	-1.75892
-1.69566	-1.69289	-1.72529	-1.72236	-1.75478	-1.75176	-1.74877	-1.76107	-1.75825
-1.69487	-1.69208	-1.72444	-1.72152	-1.76927	-1.76622	-1.76323	-1.76034	-1.75753
-1.69408	-1.69127	-1.72358	-1.72066	-1.76838	-1.76538	-1.76242	-1.75955	-1.75676
-1.69333	-1.69048	-1.72273	-1.71982	-1.7675	-1.76452	-1.76158	-1.75874	-1.75595
-1.69262	-1.68973	-1.72191	-1.71899	-1.76663	-1.76366	-1.76074	-1.75789	-1.7551
-1.69192	-1.689	-1.72112	-1.71818	-1.76577	-1.7628	-1.75989	-1.75704	-1.75423
-1.71157	-1.70858	-1.72037	-1.71744	-1.76498	-1.762	-1.75908	-1.75621	-1.75339
-1.71076	-1.70783	-1.71966	-1.71674	-1.76423	-1.76123	-1.75828	-1.75539	-1.75255
-1.70995	-1.70705	-1.73461	-1.73163	-1.76344	-1.76045	-1.7575	-1.75461	-1.75175
-1.70914	-1.70624	-1.73377	-1.7308	-1.76261	-1.75966	-1.75676	-1.75388	-1.75102
-1.70836	-1.70545	-1.73295	-1.72998	-1.76178	-1.75886	-1.75599	-1.75312	-1.75026
-1.70765	-1.70474	-1.7322	-1.72921	-1.76096	-1.75803	-1.75516	-1.75229	-1.74943
-1.70693	-1.70403	-1.73146	-1.72845	-1.76016	-1.75722	-1.75433	-1.75145	-1.74859
-1.70614	-1.70324	-1.7488	-1.74576	-1.7594	-1.75646	-1.75357	-1.76511	-1.76218
-1.70531	-1.70241	-1.74796	-1.74498	-1.75866	-1.75573	-1.75283	-1.76433	-1.76137
-1.72042	-1.71743	-1.74712	-1.74417	-1.75786	-1.75494	-1.75204	-1.76354	-1.76058
-1.71967	-1.71663	-1.74627	-1.74332	-1.75702	-1.75411	-1.75122	-1.76273	-1.75981
-1.71891	-1.71586	-1.74548	-1.74253	-1.77072	-1.76777	-1.76485	-1.76193	-1.75903
-1.71813	-1.71513	-1.74475	-1.74181	-1.76998	-1.76702	-1.76409	-1.76116	-1.75823
-1.71738	-1.71442	-1.74405	-1.7411	-1.76923	-1.76627	-1.76333	-1.76038	-1.75744
-1.71667	-1.71373	-1.74334	-1.74036	-1.76844	-1.76547	-1.76253	-1.7596	-1.75669
-1.71596	-1.71303	-1.74263	-1.73962	-1.76765	-1.76467	-1.76173	-1.75883	-1.75595
-1.7152	-1.71228	-1.74187	-1.73888	-1.7669	-1.76392	-1.76099	-1.75807	-1.75519
-1.71442	-1.7115	-1.7411	-1.73814	-1.76616	-1.7632	-1.76027	-1.75733	-1.75442
-1.73202	-1.72905	-1.74035	-1.73738	-1.76539	-1.76244	-1.75951	-1.75659	-1.75368
-1.73131	-1.72834	-1.73961	-1.73662	-1.76458	-1.76163	-1.75873	-1.75584	-1.75297
-1.71328	-1.7103	-1.73888	-1.73587	-1.76379	-1.76085	-1.75797	-1.7551	-1.75226
-1.71255	-1.70955	-1.73812	-1.73513	-1.76306	-1.76013	-1.75725	-1.75439	-1.75156
-1.71181	-1.7088	-1.73737	-1.73441	-1.76234	-1.75941	-1.75653	-1.75367	-1.75084
-1.71107	-1.70808	-1.75126	-1.74827	-1.76161	-1.75867	-1.75578	-1.75291	-1.75007
-1.71033	-1.70736	-1.75054	-1.74755	-1.76087	-1.75792	-1.75501	-1.75214	-1.7493
-1.70961	-1.70665	-1.74982	-1.74682	-1.76013	-1.75718	-1.75428	-1.75142	-1.74858
-1.70892	-1.70595	-1.7491	-1.74608	-1.75937	-1.75645	-1.75359	-1.75073	-1.7479
-1.70822	-1.70524	-1.73146	-1.72845	-1.75865	-1.75575	-1.7529	-1.75005	-1.74723
-1.70753	-1.7045	-1.73069	-1.72773	-1.75796	-1.75508	-1.75224	-1.74939	-1.74656
-1.70684	-1.70378	-1.72996	-1.72703	-1.75728	-1.75441	-1.75157	-1.74873	-1.74589
-1.70616	-1.7031	-1.72927	-1.72635	-1.75662	-1.75375	-1.7509	-1.74804	-1.74518

							-0.10613	-0.10553
							-0.10581	-0.10522
							-0.10549	-0.10492
							-0.10517	-0.10461
				-0.04994	-0.0498	-0.04967	-0.15425	-0.15358
				-0.04985	-0.04972	-0.04959	-0.15393	-0.15326
				-0.04977	-0.04964	-0.04951	-0.15361	-0.15295
				-0.15537	-0.15465	-0.15395	-0.15326	-0.1526
				-0.15503	-0.1543	-0.15359	-0.15291	-0.15224
		-0.0498	-0.04967	-0.15467	-0.15396	-0.15326	-0.35067	-0.34939
		-0.04972	-0.04958	-0.15431	-0.15362	-0.15293	-0.35005	-0.34878
		-0.15536	-0.15466	-0.15397	-0.15328	-0.1526	-0.47536	-0.47366
		-0.15502	-0.15433	-0.15365	-0.15295	-0.15226	-0.47449	-0.47283
-0.04974	-0.04962	-0.15468	-0.154	-0.25474	-0.25366	-0.2526	-0.60578	-0.60378
-0.04966	-0.04953	-0.15432	-0.15364	-0.25421	-0.25315	-0.25212	-0.6048	-0.60284
-0.04957	-0.04945	-0.15396	-0.15328	-0.25368	-0.25265	-0.25166	-0.60384	-0.60191
-0.155	-0.15431	-0.15363	-0.15295	-0.38485	-0.38339	-0.38197	-0.60291	-0.60099
-0.15468	-0.15399	-0.15332	-0.15264	-0.38414	-0.38273	-0.38137	-0.602	-0.60009
-0.15435	-0.15368	-0.25427	-0.25321	-0.60694	-0.60494	-0.60301	-0.60108	-0.59919
-0.15403	-0.15336	-0.25377	-0.2527	-0.60589	-0.60392	-0.60203	-0.60015	-0.59829
-0.1537	-0.15302	-0.38497	-0.38342	-0.6049	-0.60295	-0.60108	-0.64868	-0.64666
-0.15333	-0.15266	-0.38421	-0.38273	-0.60398	-0.60205	-0.60017	-0.64769	-0.64568
-0.15297	-0.15231	-0.38346	-0.38207	-0.60308	-0.60116	-0.59928	-0.6467	-0.64468
-0.34847	-0.34709	-0.42832	-0.42683	-0.60217	-0.60026	-0.59837	-0.68976	-0.68762
-0.34782	-0.34646	-0.4276	-0.42615	-0.60125	-0.59935	-0.59746	-0.6887	-0.68652
-0.38431	-0.38285	-0.60417	-0.60224	-0.64553	-0.64352	-0.64153	-0.77423	-0.77179
-0.38364	-0.38223	-0.60324	-0.60133	-0.64456	-0.64256	-0.64058	-0.77308	-0.77064
-0.42859	-0.42706	-0.64763	-0.64561	-0.69213	-0.68995	-0.68779	-0.81083	-0.80826
-0.42785	-0.42636	-0.64666	-0.64466	-0.69111	-0.68895	-0.68679	-0.80964	-0.80705
-0.64978	-0.64772	-0.6457	-0.64372	-0.77699	-0.77455	-0.77211	-0.80843	-0.80582
-0.64877	-0.64675	-0.64475	-0.6428	-0.77587	-0.77341	-0.77095	-0.80719	-0.80455
-0.64779	-0.6458	-0.64382	-0.64187	-0.77474	-0.77226	-0.76977	-0.80594	-0.80329
-0.64684	-0.64487	-0.69138	-0.68923	-0.77358	-0.77109	-0.76858	-0.90684	-0.90375
-0.64591	-0.64397	-0.69039	-0.6882	-0.77242	-0.7699	-0.76739	-0.90538	-0.90233
-0.64499	-0.64306	-0.77623	-0.77374	-0.87635	-0.87335	-0.87036	-1.04467	-1.04104
-0.64408	-0.64215	-0.77512	-0.77258	-0.87493	-0.87192	-0.86895	-1.04295	-1.0394
-0.69168	-0.68956	-0.77398	-0.77141	-0.91032	-0.90719	-0.90411	-1.11872	-1.11488
-0.69068	-0.68852	-0.77281	-0.77025	-0.90891	-0.90577	-0.9027	-1.11686	-1.11314
-0.77659	-0.77413	-0.87686	-0.87383	-1.04894	-1.04516	-1.04147	-1.15083	-1.14684
-0.77545	-0.77294	-0.87544	-0.87243	-1.0472	-1.04349	-1.03986	-1.14894	-1.14497
-0.77431	-0.77176	-0.87402	-0.87103	-1.04547	-1.04184	-1.0383	-1.14709	-1.14309
-0.84785	-0.84507	-0.90908	-0.90596	-1.12147	-1.11752	-1.11369	-1.17729	-1.17316
-0.84662	-0.84383	-0.90768	-0.90455	-1.11956	-1.11572	-1.11199	-1.17538	-1.1712
-0.87605	-0.87299	-1.05331	-1.04949	-1.18599	-1.18179	-1.17769	-1.1735	-1.16931
-0.87471	-0.87166	-1.05161	-1.04784	-1.18405	-1.17988	-1.17581	-1.17165	-1.16748
-0.90984	-0.90666	-1.12302	-1.11895	-1.18213	-1.178	-1.17395	-1.2345	-1.23012
-0.90845	-0.90528	-1.12116	-1.11717	-1.18024	-1.17614	-1.17211	-1.23258	-1.22826
-1.08854	-1.0845	-1.15533	-1.1511	-1.17835	-1.17428	-1.17028	-1.30671	-1.30225
-1.0868	-1.08279	-1.1534	-1.1492	-1.17646	-1.17242	-1.16845	-1.30474	-1.30033

-1.08507	-1.0811	-1.1515	-1.14733	-1.17458	-1.17057	-1.16663	-1.30279	-1.29841
-1.12226	-1.11807	-1.18103	-1.17681	-1.25545	-1.25123	-1.24709	-1.30085	-1.2965
-1.12042	-1.11632	-1.17914	-1.17494	-1.25347	-1.2493	-1.2452	-1.29893	-1.29459
-1.15454	-1.15015	-1.2135	-1.2092	-1.28331	-1.27912	-1.27501	-1.37735	-1.37284
-1.15259	-1.14825	-1.21154	-1.20727	-1.28134	-1.2772	-1.27313	-1.37537	-1.37083
-1.15066	-1.14636	-1.25619	-1.25184	-1.27938	-1.27529	-1.27125	-1.39844	-1.39385
-1.14877	-1.14448	-1.25418	-1.24989	-1.27746	-1.27339	-1.26937	-1.39643	-1.39182
-1.14691	-1.14263	-1.25221	-1.24797	-1.27555	-1.27151	-1.2675	-1.3944	-1.38979
-1.20059	-1.19619	-1.28208	-1.27782	-1.35635	-1.35213	-1.34792	-1.41548	-1.41079
-1.19868	-1.19434	-1.28019	-1.27595	-1.3544	-1.35016	-1.34593	-1.41342	-1.40876
-1.19677	-1.19254	-1.27837	-1.27412	-1.37795	-1.37363	-1.36935	-1.4356	-1.43092
-1.19488	-1.19078	-1.27661	-1.27234	-1.376	-1.37165	-1.36735	-1.43354	-1.42895
-1.25142	-1.24728	-1.27482	-1.27055	-1.42244	-1.41787	-1.41336	-1.43151	-1.42702
-1.24953	-1.24543	-1.27299	-1.26875	-1.42044	-1.41585	-1.41132	-1.42951	-1.42515
-1.27942	-1.27529	-1.37914	-1.3747	-1.44125	-1.43656	-1.43195	-1.42755	-1.42328
-1.27754	-1.2734	-1.37715	-1.37279	-1.4393	-1.43462	-1.43	-1.42561	-1.42137
-1.27569	-1.27153	-1.37517	-1.37087	-1.43734	-1.43269	-1.42809	-1.4237	-1.41945
-1.27393	-1.26972	-1.42157	-1.41708	-1.43531	-1.43072	-1.42618	-1.4218	-1.41754
-1.2722	-1.26794	-1.41954	-1.41504	-1.43326	-1.42875	-1.42429	-1.41993	-1.41565
-1.34769	-1.34315	-1.41753	-1.41303	-1.43124	-1.42681	-1.42244	-1.41809	-1.41377
-1.34574	-1.34125	-1.41554	-1.41105	-1.42925	-1.42491	-1.42063	-1.41628	-1.41192
-1.36931	-1.3648	-1.4136	-1.40912	-1.42732	-1.42302	-1.41878	-1.5381	-1.53328
-1.36741	-1.36295	-1.41173	-1.40726	-1.42544	-1.42114	-1.41689	-1.53602	-1.53126
-1.4189	-1.41435	-1.43254	-1.42804	-1.42361	-1.41928	-1.415	-1.53395	-1.52926
-1.41702	-1.41245	-1.43062	-1.4262	-1.42182	-1.41745	-1.41312	-1.53189	-1.5273
-1.41514	-1.41054	-1.4287	-1.42436	-1.42004	-1.41563	-1.41125	-1.52985	-1.52534
-1.41318	-1.40863	-1.4268	-1.42247	-1.54212	-1.53727	-1.53244	-1.58346	-1.5786
-1.41118	-1.40673	-1.42492	-1.42056	-1.53997	-1.53517	-1.53041	-1.58132	-1.57647
-1.40925	-1.40488	-1.42307	-1.4187	-1.53788	-1.53313	-1.52844	-1.57926	-1.5744
-1.40737	-1.40306	-1.42127	-1.41688	-1.53585	-1.53115	-1.52653	-1.57727	-1.57241
-1.40554	-1.40127	-1.52066	-1.51592	-1.53386	-1.5292	-1.52462	-1.57528	-1.57044
-1.40379	-1.39947	-1.51867	-1.51398	-1.53193	-1.52728	-1.5227	-1.57329	-1.56852
-1.40204	-1.3977	-1.5167	-1.51204	-1.53	-1.52537	-1.5208	-1.57132	-1.56661
-1.40026	-1.39594	-1.53748	-1.53272	-1.584	-1.57905	-1.57417	-1.58935	-1.58461
-1.39847	-1.39419	-1.53554	-1.53075	-1.5819	-1.57702	-1.57223	-1.5874	-1.58263
-1.49919	-1.49458	-1.53355	-1.52876	-1.59982	-1.59493	-1.59015	-1.58538	-1.58064
-1.4973	-1.49264	-1.53154	-1.52676	-1.5977	-1.59281	-1.58803	-1.58331	-1.57864
-1.53963	-1.53484	-1.60588	-1.60072	-1.59566	-1.59076	-1.58598	-1.58129	-1.57669
-1.53765	-1.53291	-1.60389	-1.59877	-1.59373	-1.58881	-1.58399	-1.57933	-1.57478
-1.53568	-1.53099	-1.60188	-1.5968	-1.59179	-1.58687	-1.58206	-1.57741	-1.57288
-1.53376	-1.52905	-1.5998	-1.59473	-1.58975	-1.58493	-1.58021	-1.57556	-1.57099
-1.53184	-1.52713	-1.59774	-1.59268	-1.58774	-1.583	-1.57837	-1.57372	-1.56909
-1.58631	-1.58128	-1.59578	-1.59076	-1.58585	-1.58111	-1.57647	-1.57182	-1.56722
-1.58425	-1.5793	-1.59387	-1.58891	-1.58403	-1.57925	-1.57456	-1.56992	-1.56535
-1.58224	-1.57732	-1.59191	-1.58703	-1.58221	-1.57745	-1.57276	-1.56809	-1.56349
-1.58028	-1.57533	-1.58992	-1.58514	-1.58041	-1.57571	-1.57104	-1.56632	-1.56163
-1.57831	-1.57337	-1.58797	-1.58324	-1.57858	-1.57393	-1.5693	-1.5646	-1.55991
-1.57629	-1.57144	-1.58607	-1.58136	-1.57671	-1.57209	-1.56753	-1.56293	-1.55839
-1.57433	-1.56952	-1.58417	-1.57948	-1.57484	-1.57024	-1.56571	-1.56126	-1.55692
-1.57246	-1.5676	-1.58223	-1.5776	-1.57301	-1.56837	-1.56383	-1.55953	-1.55546

-1.57064	-1.56572	-1.58031	-1.57573	-1.57119	-1.56651	-1.56194	-1.55781	-1.55402
-1.56883	-1.56395	-1.57853	-1.57392	-1.56935	-1.56467	-1.56016	-1.57416	-1.57052
-1.56702	-1.56226	-1.57684	-1.57214	-1.56751	-1.56287	-1.55845	-1.57255	-1.56906
-1.56516	-1.56049	-1.57511	-1.57037	-1.58387	-1.57926	-1.57489	-1.59292	-1.58951
-1.56323	-1.55867	-1.57335	-1.56863	-1.58215	-1.57766	-1.57345	-1.59158	-1.5883
-1.56137	-1.55685	-1.58973	-1.585	-1.58044	-1.57611	-1.57206	-1.61137	-1.60812
-1.5596	-1.55503	-1.58786	-1.58317	-1.5787	-1.57456	-1.57074	-1.61014	-1.607
-1.55789	-1.55324	-1.58602	-1.58138	-1.57702	-1.57307	-1.56946	-1.60894	-1.60589
-1.55623	-1.55155	-1.5843	-1.57972	-1.59693	-1.59306	-1.58955	-1.61766	-1.61455
-1.55459	-1.54991	-1.58264	-1.57812	-1.59539	-1.59167	-1.5883	-1.61643	-1.61337
-1.57134	-1.56661	-1.58096	-1.57656	-1.59393	-1.59034	-1.5871	-1.61526	-1.61225
-1.56957	-1.56488	-1.57928	-1.57504	-1.59253	-1.58907	-1.58593	-1.61411	-1.61118
-1.56778	-1.56313	-1.59911	-1.59497	-1.62279	-1.61925	-1.61603	-1.61297	-1.61009
-1.56594	-1.56135	-1.59739	-1.59346	-1.6214	-1.61796	-1.6148	-1.61181	-1.60899
-1.58602	-1.58146	-1.59578	-1.59203	-1.62007	-1.61672	-1.61362	-1.61068	-1.6079
-1.58432	-1.5799	-1.59434	-1.59072	-1.6188	-1.61555	-1.61251	-1.6096	-1.60682
-1.58268	-1.57842	-1.59297	-1.58947	-1.61758	-1.61442	-1.61144	-1.60854	-1.60574
-1.58103	-1.57696	-1.60203	-1.59853	-1.61644	-1.61333	-1.61038	-1.60747	-1.60465
-1.57942	-1.57553	-1.60072	-1.59735	-1.61534	-1.61227	-1.60933	-1.60641	-1.60355
-1.57784	-1.57411	-1.6206	-1.61733	-1.61423	-1.61121	-1.60829	-1.60538	-1.60251
-1.5763	-1.57268	-1.61921	-1.61609	-1.61312	-1.61015	-1.60725	-1.60436	-1.6015
-1.58556	-1.5819	-1.61791	-1.6149	-1.61201	-1.6091	-1.60622	-1.64543	-1.64252
-1.58425	-1.58068	-1.61673	-1.61377	-1.61093	-1.60804	-1.60519	-1.64438	-1.64148
-1.58299	-1.57951	-1.65796	-1.65497	-1.65208	-1.64915	-1.64624	-1.64334	-1.64046
-1.58174	-1.57838	-1.65684	-1.65388	-1.651	-1.64809	-1.64519	-1.64231	-1.63946
-1.58051	-1.57728	-1.65574	-1.6528	-1.64994	-1.64703	-1.64415	-1.6413	-1.63848
-1.62285	-1.61965	-1.65464	-1.65172	-1.64887	-1.64598	-1.64312	-1.6403	-1.63752
-1.62164	-1.61853	-1.65355	-1.65065	-1.6478	-1.64493	-1.64209	-1.63931	-1.63658
-1.64131	-1.63821	-1.65248	-1.64959	-1.64675	-1.64389	-1.64107	-1.63832	-1.63564
-1.64019	-1.63713	-1.65141	-1.64853	-1.6457	-1.64286	-1.64006	-1.63734	-1.6347
-1.63911	-1.63608	-1.65037	-1.6475	-1.64467	-1.64185	-1.63907	-1.63638	-1.63376
-1.63806	-1.63506	-1.64935	-1.64648	-1.64367	-1.64086	-1.63811	-1.63544	-1.63284
-1.63704	-1.63406	-1.64836	-1.64549	-1.64268	-1.6399	-1.63717	-1.63452	-1.63193
-1.63602	-1.6331	-1.64741	-1.64453	-1.64171	-1.63895	-1.63625	-1.63362	-1.63104
-1.63501	-1.63215	-1.64648	-1.64359	-1.64076	-1.63802	-1.63535	-1.63272	-1.63016
-1.634	-1.63116	-1.6455	-1.64262	-1.6398	-1.63708	-1.63442	-1.63182	-1.62928
-1.633	-1.63015	-1.64448	-1.64163	-1.63884	-1.63613	-1.6335	-1.63092	-1.6284
-1.63203	-1.62917	-1.64349	-1.64066	-1.63789	-1.6352	-1.63258	-1.65206	-1.6495
-1.63111	-1.62823	-1.64253	-1.63971	-1.63696	-1.63428	-1.63167	-1.65114	-1.6486
-1.64736	-1.64443	-1.64157	-1.63876	-1.63603	-1.63337	-1.63078	-1.65025	-1.64774
-1.64637	-1.64345	-1.6406	-1.63782	-1.63511	-1.63247	-1.6299	-1.64939	-1.64692
-1.64537	-1.64247	-1.63964	-1.63688	-1.63419	-1.63158	-1.62905	-1.64855	-1.64612
-1.64437	-1.64147	-1.63865	-1.63593	-1.63328	-1.63072	-1.62822	-1.64772	-1.64529
-1.64336	-1.64047	-1.63767	-1.63498	-1.63238	-1.62987	-1.62742	-1.64691	-1.64446
-1.64238	-1.63951	-1.63674	-1.63409	-1.63152	-1.62902	-1.62658	-1.64606	-1.6436
-1.64142	-1.63858	-1.63584	-1.63323	-1.63069	-1.62818	-1.62573	-1.64518	-1.64273
-1.6405	-1.63769	-1.63498	-1.63238	-1.65188	-1.64931	-1.64678	-1.6443	-1.64185
-1.63965	-1.63688	-1.63418	-1.63155	-1.651	-1.64843	-1.6459	-1.64341	-1.64096
-1.63879	-1.63605	-1.63336	-1.63071	-1.65012	-1.64755	-1.64504	-1.66482	-1.66219
-1.63787	-1.63515	-1.63249	-1.62986	-1.64926	-1.64669	-1.64418	-1.66391	-1.66127

-1.63694	-1.63425	-1.6316	-1.629	-1.6484	-1.64585	-1.64335	-1.66303	-1.66039
-1.63605	-1.63335	-1.63072	-1.62816	-1.64758	-1.64505	-1.64257	-1.66221	-1.65958
-1.63518	-1.63247	-1.62985	-1.62732	-1.64677	-1.64428	-1.64182	-1.66143	-1.6588
-1.63435	-1.63164	-1.65104	-1.64846	-1.64595	-1.64346	-1.64101	-1.66058	-1.65796
-1.63356	-1.63087	-1.65025	-1.64765	-1.64511	-1.64262	-1.64016	-1.65967	-1.65706
-1.63275	-1.63007	-1.64944	-1.64683	-1.6667	-1.66402	-1.66139	-1.65876	-1.65616
-1.63188	-1.62922	-1.64857	-1.64599	-1.66581	-1.66313	-1.66049	-1.65787	-1.65527
-1.63101	-1.62837	-1.64772	-1.64515	-1.66495	-1.66227	-1.65962	-1.657	-1.6544
-1.65223	-1.64954	-1.64692	-1.64437	-1.66413	-1.66146	-1.65882	-1.69155	-1.68885
-1.6514	-1.64874	-1.64613	-1.6436	-1.66332	-1.66067	-1.65803	-1.69074	-1.68804
-1.65055	-1.64791	-1.64532	-1.64279	-1.69807	-1.69532	-1.69259	-1.7079	-1.70515
-1.64969	-1.64707	-1.64449	-1.64195	-1.69715	-1.69441	-1.69168	-1.70699	-1.70424
-1.64886	-1.64626	-1.66608	-1.66339	-1.69628	-1.69354	-1.69082	-1.70612	-1.70337
-1.64806	-1.64548	-1.66528	-1.6626	-1.69547	-1.69273	-1.69001	-1.7053	-1.70255
-1.64723	-1.64468	-1.70016	-1.69738	-1.69463	-1.69189	-1.68917	-1.72685	-1.72399
-1.64633	-1.64381	-1.69923	-1.69647	-1.69372	-1.69099	-1.68826	-1.7259	-1.72305
-1.64546	-1.64295	-1.69831	-1.69556	-1.69281	-1.69008	-1.68735	-1.72496	-1.72211
-1.68104	-1.67843	-1.69745	-1.69471	-1.69197	-1.68923	-1.68649	-1.72407	-1.72123
-1.68026	-1.67763	-1.6966	-1.69387	-1.69114	-1.6884	-1.68566	-1.7232	-1.72037
-1.70124	-1.69846	-1.69571	-1.69298	-1.69026	-1.68751	-1.68477	-1.72228	-1.71946
-1.70031	-1.69753	-1.69478	-1.69205	-1.68932	-1.68658	-1.68384	-1.72133	-1.7185
-1.69943	-1.69666	-1.69391	-1.69118	-1.71118	-1.70833	-1.7055	-1.72043	-1.71762
-1.69862	-1.69587	-1.69312	-1.69037	-1.71034	-1.70749	-1.70467	-1.71961	-1.71682
-1.69782	-1.69508	-1.69233	-1.68958	-1.7095	-1.70666	-1.70385	-1.7188	-1.71603
-1.697	-1.69428	-1.69154	-1.68878	-1.70867	-1.70582	-1.70301	-1.71798	-1.71524
-1.69617	-1.69345	-1.69072	-1.68795	-1.70781	-1.70497	-1.70216	-1.71714	-1.71442
-1.69528	-1.69255	-1.7126	-1.70973	-1.70688	-1.70404	-1.70124	-1.73437	-1.73161
-1.69435	-1.69162	-1.71162	-1.70875	-1.70591	-1.70308	-1.70029	-1.7334	-1.73066
-1.69351	-1.69076	-1.71072	-1.70786	-1.70502	-1.7022	-1.69943	-1.73253	-1.72978
-1.69273	-1.68997	-1.70989	-1.70702	-1.70419	-1.7014	-1.69865	-1.73172	-1.72897
-1.71487	-1.71196	-1.70908	-1.70621	-1.72185	-1.71902	-1.71622	-1.73093	-1.72818
-1.71408	-1.71117	-1.70829	-1.70542	-1.72105	-1.71822	-1.71545	-1.73016	-1.72741
-1.71329	-1.71038	-1.7075	-1.70463	-1.72024	-1.71743	-1.71467	-1.72938	-1.72663
-1.7125	-1.70958	-1.70669	-1.70383	-1.71942	-1.71663	-1.71389	-1.7286	-1.72583
-1.71167	-1.70875	-1.70586	-1.70301	-1.71859	-1.71582	-1.71309	-1.72779	-1.72502
-1.71077	-1.70785	-1.70497	-1.70213	-1.71769	-1.71494	-1.71222	-1.72691	-1.72413
-1.70982	-1.70691	-1.70404	-1.70122	-1.71677	-1.71402	-1.7113	-1.72599	-1.7232
-1.70894	-1.70603	-1.72159	-1.71873	-1.71591	-1.71316	-1.71044	-1.72513	-1.72234
-1.70811	-1.70522	-1.72077	-1.71793	-1.71513	-1.71238	-1.70964	-1.72432	-1.72153
-1.6935	-1.69063	-1.7064	-1.7036	-1.71436	-1.7116	-1.70886	-1.72353	-1.72073
-1.6927	-1.68984	-1.7056	-1.70283	-1.7136	-1.71084	-1.70809	-1.72275	-1.71994
-1.69191	-1.68906	-1.70481	-1.70206	-1.71283	-1.71007	-1.70732	-1.72197	-1.71916
-1.69114	-1.68829	-1.70403	-1.70129	-1.71631	-1.71352	-1.71073	-1.73782	-1.73494
-1.69037	-1.68753	-1.70326	-1.70052	-1.71555	-1.71275	-1.70995	-1.73701	-1.73413
-1.6896	-1.68678	-1.7025	-1.69976	-1.73173	-1.72888	-1.72602	-1.74324	-1.74026
-1.68883	-1.68604	-1.70175	-1.69901	-1.73095	-1.72808	-1.72521	-1.74239	-1.7394
-1.70661	-1.70378	-1.701	-1.69825	-1.73015	-1.72728	-1.7244	-1.74154	-1.73855
-1.70582	-1.70302	-1.70024	-1.69749	-1.72936	-1.72648	-1.72359	-1.74069	-1.7377
-1.70503	-1.70225	-1.71667	-1.71386	-1.74883	-1.74585	-1.74285	-1.7581	-1.75505
-1.70423	-1.70148	-1.71588	-1.71307	-1.74799	-1.745	-1.742	-1.75724	-1.75421

-1.70344	-1.70071	-1.71509	-1.71228	-1.74714	-1.74414	-1.74114	-1.75639	-1.75338
-1.68728	-1.68459	-1.7143	-1.71148	-1.74629	-1.74329	-1.74029	-1.75553	-1.75254
-1.68651	-1.68383	-1.71349	-1.71068	-1.74544	-1.74243	-1.73944	-1.75467	-1.75171
-1.70311	-1.70037	-1.71269	-1.70987	-1.74458	-1.74158	-1.73859	-1.75382	-1.75087
-1.70232	-1.69958	-1.71188	-1.70906	-1.74373	-1.74073	-1.73774	-1.75297	-1.75004
-1.70155	-1.6988	-1.71108	-1.70825	-1.74287	-1.73988	-1.73691	-1.75213	-1.74922
-1.70081	-1.69804	-1.71029	-1.70745	-1.74202	-1.73904	-1.73608	-1.75131	-1.74841
-1.7001	-1.6973	-1.72991	-1.72695	-1.74119	-1.73821	-1.73527	-1.75049	-1.74762
-1.69944	-1.69665	-1.72919	-1.72619	-1.74039	-1.7374	-1.73445	-1.74967	-1.74682
-1.69879	-1.696	-1.72848	-1.72545	-1.73961	-1.7366	-1.73365	-1.74887	-1.74604
-1.69805	-1.69527	-1.72773	-1.72471	-1.7571	-1.75401	-1.75098	-1.7633	-1.76043
-1.69726	-1.69449	-1.72694	-1.72397	-1.75637	-1.75327	-1.75021	-1.7625	-1.75965
-1.69647	-1.6937	-1.72613	-1.72319	-1.75559	-1.75251	-1.74947	-1.76176	-1.75892
-1.69566	-1.69289	-1.72529	-1.72236	-1.75478	-1.75176	-1.74877	-1.76107	-1.75825
-1.69487	-1.69208	-1.72444	-1.72152	-1.76927	-1.76622	-1.76323	-1.76034	-1.75753
-1.69408	-1.69127	-1.72358	-1.72066	-1.76838	-1.76538	-1.76242	-1.75955	-1.75676
-1.69333	-1.69048	-1.72273	-1.71982	-1.7675	-1.76452	-1.76158	-1.75874	-1.75595
-1.69262	-1.68973	-1.72191	-1.71899	-1.76663	-1.76366	-1.76074	-1.75789	-1.7551
-1.69192	-1.689	-1.72112	-1.71818	-1.76577	-1.7628	-1.75989	-1.75704	-1.75423
-1.71157	-1.70858	-1.72037	-1.71744	-1.76498	-1.762	-1.75908	-1.75621	-1.75339
-1.71076	-1.70783	-1.71966	-1.71674	-1.76423	-1.76123	-1.75828	-1.75539	-1.75255
-1.70995	-1.70705	-1.73461	-1.73163	-1.76344	-1.76045	-1.7575	-1.75461	-1.75175
-1.70914	-1.70624	-1.73377	-1.7308	-1.76261	-1.75966	-1.75676	-1.75388	-1.75102
-1.70836	-1.70545	-1.73295	-1.72998	-1.76178	-1.75886	-1.75599	-1.75312	-1.75026
-1.70765	-1.70474	-1.7322	-1.72921	-1.76096	-1.75803	-1.75516	-1.75229	-1.74943
-1.70693	-1.70403	-1.73146	-1.72845	-1.76016	-1.75722	-1.75433	-1.75145	-1.74859
-1.70614	-1.70324	-1.7488	-1.74576	-1.7594	-1.75646	-1.75357	-1.76511	-1.76218
-1.70531	-1.70241	-1.74796	-1.74498	-1.75866	-1.75573	-1.75283	-1.76433	-1.76137
-1.72042	-1.71743	-1.74712	-1.74417	-1.75786	-1.75494	-1.75204	-1.76354	-1.76058
-1.71967	-1.71663	-1.74627	-1.74332	-1.75702	-1.75411	-1.75122	-1.76273	-1.75981
-1.71891	-1.71586	-1.74548	-1.74253	-1.77072	-1.76777	-1.76485	-1.76193	-1.75903
-1.71813	-1.71513	-1.74475	-1.74181	-1.76998	-1.76702	-1.76409	-1.76116	-1.75823
-1.71738	-1.71442	-1.74405	-1.7411	-1.76923	-1.76627	-1.76333	-1.76038	-1.75744
-1.71667	-1.71373	-1.74334	-1.74036	-1.76844	-1.76547	-1.76253	-1.7596	-1.75669
-1.71596	-1.71303	-1.74263	-1.73962	-1.76765	-1.76467	-1.76173	-1.75883	-1.75595
-1.7152	-1.71228	-1.74187	-1.73888	-1.7669	-1.76392	-1.76099	-1.75807	-1.75519
-1.71442	-1.7115	-1.7411	-1.73814	-1.76616	-1.7632	-1.76027	-1.75733	-1.75442
-1.73202	-1.72905	-1.74035	-1.73738	-1.76539	-1.76244	-1.75951	-1.75659	-1.75368
-1.73131	-1.72834	-1.73961	-1.73662	-1.76458	-1.76163	-1.75873	-1.75584	-1.75297
-1.71328	-1.7103	-1.73888	-1.73587	-1.76379	-1.76085	-1.75797	-1.7551	-1.75226
-1.71255	-1.70955	-1.73812	-1.73513	-1.76306	-1.76013	-1.75725	-1.75439	-1.75156
-1.71181	-1.7088	-1.73737	-1.73441	-1.76234	-1.75941	-1.75653	-1.75367	-1.75084
-1.71107	-1.70808	-1.75126	-1.74827	-1.76161	-1.75867	-1.75578	-1.75291	-1.75007
-1.71033	-1.70736	-1.75054	-1.74755	-1.76087	-1.75792	-1.75501	-1.75214	-1.7493
-1.70961	-1.70665	-1.74982	-1.74682	-1.76013	-1.75718	-1.75428	-1.75142	-1.74858
-1.70892	-1.70595	-1.7491	-1.74608	-1.75937	-1.75645	-1.75359	-1.75073	-1.7479
-1.70822	-1.70524	-1.73146	-1.72845	-1.75865	-1.75575	-1.7529	-1.75005	-1.74723
-1.70753	-1.7045	-1.73069	-1.72773	-1.75796	-1.75508	-1.75224	-1.74939	-1.74656
-1.70684	-1.70378	-1.72996	-1.72703	-1.75728	-1.75441	-1.75157	-1.74873	-1.74589
-1.70616	-1.7031	-1.72927	-1.72635	-1.75662	-1.75375	-1.7509	-1.74804	-1.74518

-0.61081	-0.60977	-0.60875	-0.60774	-0.60676	-0.60582	-0.60491	-0.60399	-0.60307
-0.61104	-0.61001	-0.60898	-0.60796	-0.60697	-0.60603	-0.60511	-0.60419	-0.60327
-0.61127	-0.61024	-0.60921	-0.60818	-0.60718	-0.60623	-0.60531	-0.60439	-0.60348
-0.61148	-0.61045	-0.60942	-0.60839	-0.60738	-0.60643	-0.6055	-0.60459	-0.60368
-0.61168	-0.61066	-0.60963	-0.60859	-0.60758	-0.60662	-0.60569	-0.60478	-0.60388
-0.6119	-0.61087	-0.60983	-0.60879	-0.60778	-0.60681	-0.60588	-0.60496	-0.60407
-0.61212	-0.61108	-0.61004	-0.609	-0.60798	-0.60701	-0.60607	-0.60515	-0.60425
-0.61233	-0.61129	-0.61026	-0.60921	-0.60819	-0.60721	-0.60626	-0.60533	-0.60444
-0.61253	-0.61151	-0.61049	-0.60944	-0.60841	-0.60741	-0.60644	-0.60552	-0.60463
-0.61273	-0.61173	-0.61072	-0.60967	-0.60864	-0.60762	-0.60663	-0.6057	-0.60481
-0.61292	-0.61194	-0.61094	-0.6099	-0.60886	-0.60783	-0.60683	-0.60588	-0.60498
-0.61311	-0.61214	-0.61115	-0.61012	-0.60909	-0.60805	-0.60703	-0.60607	-0.60515
-0.61329	-0.61234	-0.61136	-0.61033	-0.6093	-0.60827	-0.60725	-0.60627	-0.60534
-0.61347	-0.61253	-0.61155	-0.61054	-0.60952	-0.60849	-0.60747	-0.60649	-0.60554
-0.61366	-0.61272	-0.61175	-0.61075	-0.60974	-0.60871	-0.60769	-0.6067	-0.60574
-0.61387	-0.61291	-0.61194	-0.61095	-0.60996	-0.60894	-0.60791	-0.60692	-0.60595
-0.61408	-0.6131	-0.61212	-0.61115	-0.61017	-0.60915	-0.60813	-0.60713	-0.60615
-0.61428	-0.61329	-0.61231	-0.61134	-0.61037	-0.60936	-0.60834	-0.60733	-0.60634
-0.61449	-0.61348	-0.6125	-0.61154	-0.61057	-0.60957	-0.60856	-0.60755	-0.60654
-0.61472	-0.6137	-0.61271	-0.61175	-0.61078	-0.60979	-0.60878	-0.60777	-0.60677
-0.61495	-0.61393	-0.61293	-0.61197	-0.611	-0.61001	-0.60901	-0.608	-0.607
-0.6152	-0.61416	-0.61315	-0.61218	-0.61121	-0.61023	-0.60924	-0.60824	-0.60724
-0.61545	-0.6144	-0.61339	-0.6124	-0.61143	-0.61046	-0.60949	-0.60849	-0.60747
-0.61573	-0.61466	-0.61363	-0.61263	-0.61165	-0.61069	-0.60973	-0.60873	-0.6077
-0.61604	-0.61494	-0.61389	-0.61287	-0.61188	-0.61092	-0.60996	-0.60896	-0.60794
-0.61636	-0.61522	-0.61414	-0.61311	-0.61212	-0.61115	-0.61019	-0.60919	-0.60818
-0.61669	-0.6155	-0.61438	-0.61335	-0.61236	-0.61139	-0.61043	-0.60943	-0.60842
-0.61705	-0.61579	-0.61464	-0.6136	-0.6126	-0.61163	-0.61066	-0.60967	-0.60866
-0.61743	-0.61611	-0.61491	-0.61386	-0.61286	-0.61187	-0.61089	-0.6099	-0.60891
-0.61785	-0.61645	-0.61521	-0.61414	-0.61312	-0.61211	-0.61111	-0.61013	-0.60916
-0.61831	-0.61684	-0.61553	-0.61443	-0.6134	-0.61236	-0.61135	-0.61036	-0.60939
-0.61882	-0.61726	-0.61589	-0.61475	-0.61368	-0.61262	-0.61159	-0.61059	-0.60962
-0.61933	-0.61769	-0.61625	-0.61506	-0.61395	-0.61287	-0.61184	-0.61083	-0.60986
-0.61984	-0.61812	-0.61662	-0.61536	-0.61422	-0.61312	-0.61209	-0.61109	-0.61012
-0.62035	-0.61858	-0.61701	-0.61569	-0.61449	-0.61338	-0.61235	-0.61134	-0.61037
-0.62088	-0.61907	-0.61745	-0.61605	-0.61479	-0.61366	-0.61262	-0.6116	-0.61062
-0.62143	-0.61959	-0.61793	-0.61644	-0.61511	-0.61395	-0.6129	-0.61186	-0.61086
-0.62201	-0.62016	-0.61845	-0.61688	-0.61547	-0.61427	-0.6132	-0.61214	-0.61113
-0.62261	-0.62076	-0.61901	-0.61735	-0.61586	-0.61462	-0.61352	-0.61244	-0.6114
-0.6232	-0.62134	-0.61956	-0.61783	-0.61627	-0.61498	-0.61383	-0.61272	-0.61168
-0.62378	-0.62192	-0.62012	-0.61834	-0.61671	-0.61534	-0.61413	-0.613	-0.61196
-0.62436	-0.6225	-0.62068	-0.61886	-0.61719	-0.61574	-0.61446	-0.6133	-0.61226
-0.62493	-0.62307	-0.62123	-0.6194	-0.61769	-0.61618	-0.61482	-0.61362	-0.61257
-0.6255	-0.62364	-0.6218	-0.61996	-0.61822	-0.61664	-0.61521	-0.61396	-0.61287
-0.62608	-0.62423	-0.62239	-0.62054	-0.61877	-0.61712	-0.6156	-0.61429	-0.61317
-0.62667	-0.62483	-0.623	-0.62114	-0.61934	-0.61762	-0.61601	-0.61464	-0.61347
-0.62725	-0.62541	-0.62357	-0.62173	-0.61992	-0.61814	-0.61646	-0.61503	-0.6138
-0.62784	-0.62598	-0.62414	-0.62231	-0.6205	-0.61868	-0.61695	-0.61546	-0.61415
-0.62843	-0.62657	-0.62473	-0.6229	-0.62109	-0.61926	-0.61749	-0.61594	-0.61455
-0.62901	-0.62717	-0.62533	-0.6235	-0.62168	-0.61986	-0.61809	-0.61647	-0.61499

-0.57285	-0.57113	-0.62594	-0.6241	-0.62228	-0.62046	-0.61869	-0.61702	-0.61545
-0.57348	-0.57176	-0.62654	-0.6247	-0.62288	-0.62107	-0.6193	-0.61758	-0.61592
-0.57412	-0.5724	-0.62714	-0.62531	-0.62348	-0.62168	-0.6199	-0.61814	-0.61641
-0.57477	-0.57304	-0.57132	-0.56959	-0.56789	-0.56621	-0.56456	-0.61869	-0.61693
-0.57542	-0.57368	-0.57196	-0.57024	-0.56854	-0.56683	-0.56515	-0.61925	-0.61747
-0.57606	-0.57433	-0.57261	-0.57088	-0.56916	-0.56744	-0.56574	-0.56407	-0.56244
-0.57671	-0.57499	-0.57326	-0.57151	-0.56977	-0.56804	-0.56634	-0.56467	-0.56304
-0.57735	-0.57563	-0.5739	-0.57214	-0.57039	-0.56865	-0.56694	-0.56527	-0.56364
-0.578	-0.57627	-0.57453	-0.57277	-0.57103	-0.56928	-0.56755	-0.56587	-0.56424
-0.50161	-0.50004	-0.57517	-0.57342	-0.57167	-0.56991	-0.56817	-0.56649	-0.56484
-0.50219	-0.50064	-0.57584	-0.57408	-0.57232	-0.57056	-0.56881	-0.56712	-0.56547
-0.50278	-0.50124	-0.57651	-0.57475	-0.57298	-0.57121	-0.56946	-0.56776	-0.5661
-0.46083	-0.45937	-0.57714	-0.57538	-0.57363	-0.57187	-0.57012	-0.56841	-0.56672
-0.46137	-0.45988	-0.57776	-0.57602	-0.57428	-0.57253	-0.57079	-0.56906	-0.56735
-0.41235	-0.41116	-0.50138	-0.49981	-0.57495	-0.57321	-0.57148	-0.56974	-0.56801
-0.41277	-0.4116	-0.502	-0.50044	-0.57565	-0.57391	-0.57218	-0.57043	-0.56868
-0.4132	-0.41203	-0.46005	-0.4586	-0.4995	-0.49794	-0.49637	-0.57111	-0.56936
-0.41363	-0.41245	-0.46058	-0.45913	-0.50009	-0.49853	-0.49696	-0.57178	-0.57004
-0.41407	-0.41288	-0.4117	-0.41054	-0.50068	-0.49912	-0.49755	-0.49599	-0.49443
-0.41451	-0.41332	-0.41214	-0.41097	-0.50125	-0.4997	-0.49815	-0.49659	-0.49502
-0.41495	-0.41376	-0.41257	-0.41139	-0.50183	-0.50028	-0.49875	-0.49718	-0.4956
-0.41543	-0.41419	-0.41299	-0.41182	-0.45981	-0.45837	-0.45693	-0.49777	-0.4962
-0.41592	-0.41463	-0.4134	-0.41225	-0.46036	-0.4589	-0.45746	-0.49836	-0.49681
-0.41642	-0.4151	-0.41385	-0.4127	-0.46092	-0.45946	-0.45801	-0.45656	-0.45511
-0.41693	-0.4156	-0.41434	-0.41317	-0.4615	-0.46002	-0.45858	-0.45713	-0.45568
-0.41744	-0.41611	-0.41484	-0.41363	-0.41245	-0.41128	-0.41012	-0.45769	-0.45623
-0.41793	-0.41661	-0.41532	-0.41409	-0.41288	-0.41172	-0.41058	-0.45823	-0.45678
-0.41843	-0.4171	-0.41581	-0.41455	-0.41332	-0.41217	-0.41103	-0.45878	-0.45732
-0.37449	-0.37324	-0.41628	-0.41501	-0.41378	-0.41262	-0.41148	-0.45933	-0.45788
-0.37499	-0.37371	-0.41677	-0.41549	-0.41426	-0.41308	-0.41193	-0.45989	-0.45845
-0.37548	-0.3742	-0.41728	-0.416	-0.41475	-0.41355	-0.41237	-0.4112	-0.41003
-0.37597	-0.37471	-0.41782	-0.41653	-0.41526	-0.41402	-0.41281	-0.41163	-0.41048
-0.37644	-0.3752	-0.37397	-0.37276	-0.41577	-0.41451	-0.41327	-0.41209	-0.41094
-0.37688	-0.37568	-0.37447	-0.37326	-0.4163	-0.41502	-0.41376	-0.41256	-0.41141
-0.37735	-0.37616	-0.37496	-0.37376	-0.41681	-0.41552	-0.41425	-0.41305	-0.41188
-0.37784	-0.37666	-0.37546	-0.37423	-0.4173	-0.41601	-0.41474	-0.41353	-0.41235
-0.37833	-0.37715	-0.37595	-0.37471	-0.41779	-0.4165	-0.41524	-0.41402	-0.41282
-0.37881	-0.37763	-0.37644	-0.3752	-0.37397	-0.37277	-0.37159	-0.41451	-0.41328
-0.37928	-0.3781	-0.37691	-0.37569	-0.37448	-0.37328	-0.37208	-0.415	-0.41373
-0.37975	-0.37856	-0.37737	-0.37617	-0.37497	-0.37378	-0.37258	-0.37137	-0.37017
-0.38022	-0.37901	-0.37782	-0.37664	-0.37546	-0.37427	-0.37308	-0.37187	-0.37065
-0.33387	-0.33279	-0.37828	-0.37711	-0.37594	-0.37476	-0.37358	-0.37236	-0.37113
-0.33429	-0.33321	-0.37877	-0.37758	-0.37641	-0.37524	-0.37408	-0.37286	-0.37162
-0.33473	-0.33364	-0.37926	-0.37806	-0.37688	-0.37573	-0.37457	-0.37336	-0.37212
-0.33518	-0.33407	-0.37975	-0.37855	-0.37737	-0.37622	-0.37507	-0.37386	-0.37262
-0.33565	-0.33451	-0.38025	-0.37905	-0.37787	-0.37672	-0.37557	-0.37437	-0.37312
-0.33611	-0.33495	-0.33383	-0.33276	-0.37836	-0.37721	-0.37607	-0.37486	-0.37362
-0.33657	-0.33538	-0.33425	-0.33318	-0.37886	-0.37771	-0.37656	-0.37535	-0.37412
-0.33703	-0.33583	-0.33468	-0.3336	-0.37936	-0.3782	-0.37704	-0.37585	-0.37462
-0.33749	-0.33628	-0.33513	-0.33403	-0.37985	-0.37868	-0.37753	-0.37634	-0.37512

-0.28868	-0.28766	-0.33558	-0.33446	-0.33339	-0.33236	-0.33135	-0.37683	-0.37562
-0.28908	-0.28805	-0.33603	-0.33489	-0.3338	-0.33277	-0.33176	-0.37732	-0.37612
-0.28947	-0.28845	-0.33649	-0.33533	-0.33422	-0.33318	-0.33218	-0.37781	-0.37661
-0.28986	-0.28884	-0.33695	-0.33577	-0.33464	-0.3336	-0.33259	-0.3783	-0.37709
-0.29026	-0.28924	-0.33742	-0.33623	-0.33508	-0.33403	-0.33301	-0.37879	-0.37757
-0.29064	-0.28962	-0.28862	-0.28762	-0.33553	-0.33446	-0.33342	-0.33237	-0.33132
-0.29101	-0.29001	-0.28901	-0.28802	-0.33599	-0.33489	-0.33383	-0.33278	-0.33174
-0.23987	-0.23908	-0.28941	-0.28841	-0.33646	-0.33534	-0.33425	-0.33319	-0.33216
-0.24017	-0.23938	-0.28981	-0.28881	-0.33693	-0.33579	-0.33467	-0.33361	-0.33259
-0.19683	-0.19616	-0.29022	-0.28922	-0.33741	-0.33624	-0.33511	-0.33404	-0.33302
-0.1971	-0.19643	-0.29064	-0.28963	-0.3379	-0.33672	-0.33556	-0.33447	-0.33343
-0.19738	-0.19671	-0.29105	-0.29005	-0.33839	-0.3372	-0.33603	-0.33491	-0.33384
-0.10173	-0.10139	-0.1963	-0.19563	-0.28945	-0.28846	-0.28748	-0.33536	-0.33425
-0.10186	-0.10152	-0.19655	-0.19588	-0.28987	-0.28887	-0.28787	-0.33581	-0.33468
-0.102	-0.10165	-0.19682	-0.19615	-0.29029	-0.28929	-0.28828	-0.33629	-0.33512
-0.10213	-0.10178	-0.19709	-0.19643	-0.29071	-0.28971	-0.2887	-0.33678	-0.33558
-0.10228	-0.10192	-0.10156	-0.10121	-0.19603	-0.19537	-0.19471	-0.28812	-0.28711
-0.10243	-0.10205	-0.10169	-0.10134	-0.19628	-0.19563	-0.19498	-0.28856	-0.28754
-0.10258	-0.1022	-0.10182	-0.10147	-0.19654	-0.19589	-0.19525	-0.19459	-0.19393
-0.10273	-0.10235	-0.10197	-0.1016	-0.1968	-0.19615	-0.19551	-0.19486	-0.1942
-0.10289	-0.1025	-0.10212	-0.10174	-0.19707	-0.19641	-0.19576	-0.19512	-0.19448
-0.10303	-0.10265	-0.10227	-0.10189	-0.10152	-0.10116	-0.10081	-0.19537	-0.19473
-0.10317	-0.10279	-0.10241	-0.10204	-0.10167	-0.1013	-0.10094	-0.19562	-0.19498
-0.10331	-0.10293	-0.10255	-0.10219	-0.10182	-0.10145	-0.10108	-0.19589	-0.19524
-0.10345	-0.10307	-0.1027	-0.10233	-0.10197	-0.1016	-0.10124	-0.19616	-0.19551
-0.05154	-0.05135	-0.10284	-0.10248	-0.10211	-0.10175	-0.10138	-0.10102	-0.10067
-0.0516	-0.05141	-0.103	-0.10261	-0.10224	-0.10188	-0.10152	-0.10117	-0.10081
-0.05166	-0.05148	-0.10315	-0.10276	-0.10237	-0.10201	-0.10166	-0.10131	-0.10095
-0.05172	-0.05153	-0.10331	-0.10292	-0.10253	-0.10216	-0.1018	-0.10144	-0.10109
-0.05177	-0.05158	-0.10347	-0.10308	-0.1027	-0.10232	-0.10195	-0.10158	-0.10122
-0.05182	-0.05163	-0.10362	-0.10323	-0.10285	-0.10247	-0.1021	-0.10173	-0.10137
-0.05186	-0.05167	-0.10375	-0.10338	-0.103	-0.10262	-0.10225	-0.10188	-0.10152
-0.05192	-0.05172	-0.05154	-0.05135	-0.10315	-0.10277	-0.1024	-0.10203	-0.10167
-0.05198	-0.05178	-0.05159	-0.05141	-0.1033	-0.10292	-0.10254	-0.10218	-0.10182
		-0.05165	-0.05146	-0.10345	-0.10307	-0.10269	-0.10232	-0.10197
		-0.0517	-0.05152	-0.10362	-0.10324	-0.10286	-0.10248	-0.10212
		-0.05175	-0.05157	-0.10378	-0.1034	-0.10303	-0.10264	-0.10226
		-0.05181	-0.05163	-0.05144	-0.05125	-0.05107	-0.1028	-0.10242
		-0.05188	-0.05169	-0.0515	-0.0513	-0.05111	-0.10294	-0.10258
				-0.05155	-0.05136	-0.05116	-0.10309	-0.10273
				-0.0516	-0.05141	-0.05122	-0.10325	-0.10288
				-0.05165	-0.05146	-0.05127	-0.05109	-0.05091
				-0.05171	-0.05151	-0.05131	-0.05113	-0.05095
							-0.05117	-0.05099
							-0.05124	-0.05105
							-0.05131	-0.05112

							-0.05017	-0.05004
							-0.05008	-0.04996
							-0.10569	-0.1051
							-0.10539	-0.10481
					-0.05009	-0.04996	-0.15459	-0.15392
					-0.05001	-0.04988	-0.15423	-0.15357
					-0.10565	-0.10507	-0.30927	-0.3082
					-0.10534	-0.1048	-0.30874	-0.30768
					-0.10505	-0.10453	-0.30821	-0.30716
		-0.10649	-0.10589	-0.1053	-0.15417	-0.15355	-0.35118	-0.34993
		-0.10621	-0.10562	-0.10504	-0.15384	-0.15319	-0.35049	-0.34925
		-0.10591	-0.10533	-0.10476	-0.30859	-0.30753	-0.39557	-0.39417
		-0.10559	-0.10501	-0.10446	-0.30801	-0.30696	-0.39484	-0.39348
-0.10646	-0.10585	-0.15476	-0.15408	-0.15342	-0.35095	-0.3497	-0.39412	-0.3928
-0.10613	-0.10553	-0.15439	-0.15371	-0.15305	-0.35027	-0.34902	-0.39343	-0.39214
-0.10581	-0.10523	-0.15403	-0.15336	-0.1527	-0.34961	-0.34838	-0.39277	-0.39151
-0.10553	-0.10495	-0.30894	-0.30787	-0.30681	-0.39484	-0.39347	-0.6031	-0.60123

-0.10525	-0.10469	-0.30841	-0.30734	-0.30629	-0.3942	-0.39286	-0.60218	-0.60032
-0.15436	-0.15369	-0.35149	-0.35022	-0.34897	-0.60504	-0.60309	-0.65073	-0.64868
-0.15399	-0.15332	-0.3508	-0.34953	-0.3483	-0.60393	-0.60204	-0.64961	-0.64757
-0.30882	-0.30774	-0.39623	-0.3948	-0.39342	-0.60288	-0.60103	-0.69022	-0.68804
-0.30823	-0.30716	-0.39551	-0.39412	-0.39278	-0.60191	-0.60006	-0.68908	-0.68691
-0.35132	-0.35004	-0.6068	-0.6048	-0.60285	-0.65049	-0.64845	-0.68797	-0.68582
-0.3507	-0.34942	-0.60577	-0.60381	-0.60191	-0.64949	-0.64746	-0.68692	-0.68478
-0.35008	-0.34882	-0.60477	-0.60285	-0.60098	-0.64849	-0.64648	-0.68587	-0.68374
-0.47542	-0.47373	-0.60379	-0.60189	-0.60004	-0.68909	-0.68693	-0.68477	-0.68263
-0.47451	-0.47289	-0.60282	-0.60095	-0.59911	-0.68799	-0.68583	-0.68366	-0.68149
-0.60576	-0.6038	-0.60189	-0.60002	-0.59819	-0.68692	-0.68476	-0.72573	-0.72345
-0.60476	-0.60285	-0.60097	-0.59912	-0.59729	-0.68588	-0.68373	-0.72464	-0.72233
-0.60377	-0.60189	-0.69131	-0.68913	-0.68696	-0.72804	-0.7258	-0.8078	-0.80522
-0.6028	-0.60094	-0.69019	-0.68801	-0.68585	-0.72688	-0.72465	-0.80654	-0.80396
-0.60185	-0.6	-0.68909	-0.68691	-0.68475	-0.72573	-0.7235	-0.90875	-0.9057
-0.60094	-0.5991	-0.68803	-0.68586	-0.6837	-0.72463	-0.72239	-0.90729	-0.90424
-0.60003	-0.5982	-0.68697	-0.68481	-0.68265	-0.72352	-0.72128	-0.90582	-0.90279
-0.64853	-0.6465	-0.72912	-0.72687	-0.72463	-0.80659	-0.80408	-0.97374	-0.97034
-0.64752	-0.64547	-0.72795	-0.7257	-0.72346	-0.80532	-0.8028	-0.97207	-0.96873
-0.69068	-0.68852	-0.8116	-0.80908	-0.80658	-0.87478	-0.87188	-1.17608	-1.17188
-0.68967	-0.68749	-0.81036	-0.80784	-0.80533	-0.87335	-0.87048	-1.17406	-1.16983
-0.72925	-0.72692	-0.80913	-0.80659	-0.80408	-1.00989	-1.00648	-1.20163	-1.1973
-0.72813	-0.7258	-0.80789	-0.80532	-0.80279	-1.00817	-1.00481	-1.19956	-1.19533
-0.72701	-0.72467	-0.80665	-0.80407	-0.80153	-1.00647	-1.00315	-1.19751	-1.19337
-0.81059	-0.80801	-0.87643	-0.87346	-0.87053	-1.17423	-1.17012	-1.26051	-1.25625
-0.80933	-0.80677	-0.87504	-0.87209	-0.86918	-1.17227	-1.16815	-1.25842	-1.25421
-0.8081	-0.80553	-1.01193	-1.00845	-1.00502	-1.26505	-1.26061	-1.31497	-1.31068
-0.80689	-0.80429	-1.01021	-1.0068	-1.00344	-1.26297	-1.2585	-1.3128	-1.30852
-0.90792	-0.90487	-1.11987	-1.11604	-1.11228	-1.26087	-1.25644	-1.33967	-1.33534
-0.9065	-0.90345	-1.11789	-1.11408	-1.11038	-1.25873	-1.25445	-1.33766	-1.33341
-1.01225	-1.00866	-1.26968	-1.26524	-1.26091	-1.28662	-1.28235	-1.36857	-1.36427
-1.01052	-1.00702	-1.2676	-1.26319	-1.25889	-1.28457	-1.28032	-1.36648	-1.36227
-1.00882	-1.00539	-1.26555	-1.2612	-1.25692	-1.28256	-1.27831	-1.36439	-1.36023
-1.11823	-1.11442	-1.26351	-1.2592	-1.25496	-1.3381	-1.33377	-1.36228	-1.35808
-1.11636	-1.11262	-1.26148	-1.25722	-1.25301	-1.33608	-1.33175	-1.36018	-1.3559
-1.1503	-1.14625	-1.28949	-1.28514	-1.28084	-1.36686	-1.36248	-1.35813	-1.35382
-1.1484	-1.14438	-1.28746	-1.28307	-1.27875	-1.36473	-1.36044	-1.35612	-1.35179
-1.17863	-1.17451	-1.2854	-1.28103	-1.27674	-1.36266	-1.35842	-1.37011	-1.36575
-1.17672	-1.17256	-1.28332	-1.27904	-1.27484	-1.36066	-1.3564	-1.36808	-1.36375
-1.29015	-1.28566	-1.31522	-1.3109	-1.30668	-1.35868	-1.35438	-1.38942	-1.38503
-1.28814	-1.28367	-1.31321	-1.30894	-1.30474	-1.35669	-1.35237	-1.38736	-1.38299
-1.28611	-1.28171	-1.31124	-1.30699	-1.30281	-1.35469	-1.35035	-1.38529	-1.38095
-1.28405	-1.27974	-1.36564	-1.36132	-1.35702	-1.36865	-1.36427	-1.40776	-1.40337
-1.28197	-1.27776	-1.36364	-1.35932	-1.35501	-1.36661	-1.36222	-1.40565	-1.40125
-1.31383	-1.30958	-1.37767	-1.37332	-1.36897	-1.38792	-1.38342	-1.42786	-1.42344
-1.31184	-1.30761	-1.37565	-1.37129	-1.36693	-1.38582	-1.38131	-1.42574	-1.4214
-1.32665	-1.3224	-1.37363	-1.36927	-1.36491	-1.40833	-1.40379	-1.44751	-1.44318
-1.32471	-1.32046	-1.37162	-1.36726	-1.36289	-1.4063	-1.4018	-1.44548	-1.44118
-1.37834	-1.37396	-1.41774	-1.41325	-1.40878	-1.42853	-1.42402	-1.44349	-1.43922
-1.37635	-1.37195	-1.41567	-1.41121	-1.40678	-1.42653	-1.42206	-1.44153	-1.43727

-1.37437	-1.36994	-1.4136	-1.40918	-1.4048	-1.42456	-1.42012	-1.43958	-1.43534
-1.37237	-1.36793	-1.41154	-1.40716	-1.40283	-1.44645	-1.44198	-1.57098	-1.56616
-1.37037	-1.36591	-1.40949	-1.40515	-1.40087	-1.44447	-1.44003	-1.56882	-1.56403
-1.41649	-1.41193	-1.43169	-1.42735	-1.42306	-1.4425	-1.43809	-1.56666	-1.5619
-1.41443	-1.4099	-1.42966	-1.42536	-1.4211	-1.44054	-1.43615	-1.56449	-1.55975
-1.43666	-1.43209	-1.42765	-1.42338	-1.41917	-1.57221	-1.5672	-1.58422	-1.57944
-1.43457	-1.43005	-1.42566	-1.42143	-1.41725	-1.57004	-1.56505	-1.58205	-1.57726
-1.43249	-1.42803	-1.42369	-1.41949	-1.41534	-1.56787	-1.56291	-1.57989	-1.57509
-1.43043	-1.42603	-1.42173	-1.41757	-1.41344	-1.58762	-1.58262	-1.57773	-1.57293
-1.4284	-1.42404	-1.41978	-1.41565	-1.41154	-1.58542	-1.58044	-1.57556	-1.57076
-1.42642	-1.42208	-1.5759	-1.57107	-1.56627	-1.58323	-1.57826	-1.5734	-1.5686
-1.42449	-1.42016	-1.57369	-1.56888	-1.5641	-1.58104	-1.57608	-1.57122	-1.56645
-1.42261	-1.41828	-1.59349	-1.58863	-1.5838	-1.57886	-1.5739	-1.56906	-1.56432
-1.4208	-1.41645	-1.59132	-1.58645	-1.58162	-1.57668	-1.57173	-1.56691	-1.5622
-1.41897	-1.41461	-1.58915	-1.58427	-1.57944	-1.57451	-1.56958	-1.56479	-1.56012
-1.41708	-1.41273	-1.58696	-1.58209	-1.57728	-1.57237	-1.56746	-1.5627	-1.55807
-1.41517	-1.41085	-1.58478	-1.57992	-1.57512	-1.57025	-1.5654	-1.56067	-1.55605
-1.59275	-1.58764	-1.58263	-1.57777	-1.57297	-1.56818	-1.56342	-1.55873	-1.5541
-1.59053	-1.58548	-1.58051	-1.57564	-1.57085	-1.56614	-1.56151	-1.55685	-1.55219
-1.58834	-1.58336	-1.57844	-1.57356	-1.56876	-1.56411	-1.55954	-1.55492	-1.55026
-1.58618	-1.5813	-1.57644	-1.57154	-1.56671	-1.56207	-1.55754	-1.55294	-1.54832
-1.58404	-1.57921	-1.5744	-1.56951	-1.56468	-1.56005	-1.55553	-1.57365	-1.56902
-1.58194	-1.5771	-1.57229	-1.56745	-1.56268	-1.55805	-1.55352	-1.57165	-1.5671
-1.57986	-1.57501	-1.5702	-1.56543	-1.56072	-1.55609	-1.55154	-1.58923	-1.58472
-1.57784	-1.57296	-1.56816	-1.56348	-1.55885	-1.55422	-1.54963	-1.58729	-1.5828
-1.57582	-1.57093	-1.56615	-1.56155	-1.557	-1.55237	-1.54776	-1.58539	-1.58094
-1.57375	-1.56889	-1.56415	-1.55957	-1.55505	-1.57034	-1.56574	-1.58357	-1.57925
-1.57165	-1.56687	-1.56217	-1.55758	-1.55305	-1.56838	-1.56386	-1.58182	-1.57769
-1.56962	-1.56491	-1.58018	-1.57555	-1.57098	-1.56641	-1.56194	-1.58002	-1.57611
-1.56765	-1.56302	-1.57834	-1.57367	-1.56904	-1.56444	-1.55996	-1.57815	-1.5745
-1.58567	-1.58106	-1.57645	-1.57177	-1.56713	-1.58482	-1.58034	-1.57642	-1.57299
-1.58377	-1.57912	-1.57449	-1.56984	-1.56527	-1.58304	-1.57869	-1.57491	-1.57162
-1.58185	-1.57716	-1.57252	-1.5679	-1.5634	-1.58129	-1.57713	-1.57348	-1.57031
-1.57988	-1.57518	-1.57053	-1.56589	-1.56142	-1.57947	-1.57556	-1.57208	-1.56902
-1.5779	-1.57319	-1.56855	-1.56389	-1.55946	-1.57768	-1.57404	-1.57073	-1.56776
-1.57594	-1.57126	-1.56665	-1.56202	-1.55767	-1.57604	-1.57261	-1.56944	-1.56655
-1.57399	-1.56935	-1.5648	-1.56026	-1.55602	-1.57452	-1.57126	-1.5682	-1.56539
-1.57207	-1.56743	-1.5852	-1.58077	-1.57665	-1.57311	-1.56997	-1.567	-1.56426
-1.57017	-1.56548	-1.58327	-1.57909	-1.57523	-1.57181	-1.56873	-1.56585	-1.56317
-1.56828	-1.56357	-1.58141	-1.57745	-1.57384	-1.57055	-1.56755	-1.60231	-1.59963
-1.56639	-1.56172	-1.57963	-1.57585	-1.57242	-1.56928	-1.56641	-1.60121	-1.59852
-1.56452	-1.55995	-1.57796	-1.57432	-1.57104	-1.56805	-1.5653	-1.60013	-1.59742
-1.56272	-1.5583	-1.57644	-1.57295	-1.56979	-1.5845	-1.58179	-1.62908	-1.62627
-1.56097	-1.55675	-1.57504	-1.57167	-1.56861	-1.58336	-1.58067	-1.62793	-1.62516
-1.5592	-1.5552	-1.57367	-1.57042	-1.56744	-1.59216	-1.58942	-1.6268	-1.62403
-1.55741	-1.55368	-1.57234	-1.56919	-1.56628	-1.59102	-1.58832	-1.62569	-1.62289
-1.57795	-1.57434	-1.58866	-1.58562	-1.58277	-1.61062	-1.60789	-1.62457	-1.62176
-1.57652	-1.57298	-1.58736	-1.58443	-1.58169	-1.60951	-1.60674	-1.62342	-1.62066
-1.59286	-1.58934	-1.617	-1.61403	-1.61123	-1.6279	-1.62507	-1.62229	-1.61956
-1.59154	-1.58816	-1.61584	-1.61288	-1.61008	-1.62677	-1.62396	-1.62119	-1.61844

-1.59026	-1.58703	-1.61475	-1.61177	-1.60893	-1.62564	-1.62287	-1.62009	-1.61733
-1.61	-1.60686	-1.61365	-1.6107	-1.60785	-1.62454	-1.62175	-1.61899	-1.61627
-1.60864	-1.60567	-1.61255	-1.60966	-1.60682	-1.62348	-1.62063	-1.61789	-1.61524
-1.61726	-1.61429	-1.61142	-1.60857	-1.60576	-1.62242	-1.61957	-1.61684	-1.61422
-1.61604	-1.6131	-1.61024	-1.60744	-1.60468	-1.62137	-1.61855	-1.61583	-1.6132
-1.61488	-1.61196	-1.60912	-1.60632	-1.60357	-1.6203	-1.61753	-1.61484	-1.61221
-1.61379	-1.6109	-1.60806	-1.60522	-1.60243	-1.61919	-1.61648	-1.61384	-1.61126
-1.61271	-1.60985	-1.60702	-1.60414	-1.60132	-1.61809	-1.61543	-1.61284	-1.61033
-1.61157	-1.60875	-1.60594	-1.60309	-1.60029	-1.61706	-1.6144	-1.61184	-1.60937
-1.61043	-1.60763	-1.60486	-1.60205	-1.59929	-1.61606	-1.61339	-1.61085	-1.60842
-1.60933	-1.60651	-1.62332	-1.62052	-1.61776	-1.61506	-1.61243	-1.63227	-1.62978
-1.60827	-1.60539	-1.62217	-1.6194	-1.6167	-1.61407	-1.6115	-1.63133	-1.62881
-1.6072	-1.60431	-1.62107	-1.61832	-1.61565	-1.61307	-1.61056	-1.67107	-1.66849
-1.60615	-1.60328	-1.62005	-1.61728	-1.6146	-1.61206	-1.60961	-1.67012	-1.66758
-1.60509	-1.60226	-1.61905	-1.61628	-1.6136	-1.65241	-1.64994	-1.66918	-1.66668
-1.60403	-1.60124	-1.61806	-1.61532	-1.61267	-1.65146	-1.64901	-1.66826	-1.6658
-1.60297	-1.60022	-1.61708	-1.61439	-1.61177	-1.65054	-1.64808	-1.66734	-1.66492
-1.60188	-1.59916	-1.65749	-1.65478	-1.65214	-1.6496	-1.64713	-1.66638	-1.664
-1.60078	-1.59811	-1.65644	-1.65379	-1.6512	-1.64866	-1.64618	-1.66542	-1.66307
-1.64177	-1.63907	-1.65545	-1.65284	-1.65028	-1.64775	-1.64528	-1.66449	-1.66213
-1.64075	-1.63808	-1.65449	-1.6519	-1.64937	-1.64688	-1.64442	-1.66359	-1.66118
-1.63973	-1.63708	-1.65351	-1.65096	-1.64847	-1.66767	-1.66518	-1.66271	-1.66026
-1.63871	-1.63606	-1.6525	-1.65	-1.64756	-1.66678	-1.66431	-1.66185	-1.65939
-1.6377	-1.63506	-1.65152	-1.64906	-1.64666	-1.66589	-1.66344	-1.66099	-1.65853
-1.63673	-1.6341	-1.65058	-1.64815	-1.64578	-1.665	-1.66257	-1.66012	-1.65767
-1.63577	-1.63317	-1.64967	-1.64726	-1.6449	-1.66412	-1.66169	-1.65926	-1.65681
-1.63484	-1.63226	-1.67051	-1.66806	-1.66566	-1.66324	-1.66082	-1.65839	-1.65595
-1.63393	-1.63137	-1.66961	-1.66718	-1.66479	-1.66237	-1.65995	-1.65752	-1.65509
-1.63303	-1.63048	-1.66872	-1.66629	-1.66392	-1.6615	-1.65908	-1.67824	-1.67561
-1.63212	-1.6296	-1.66783	-1.66542	-1.66305	-1.66063	-1.65822	-1.67731	-1.67469
-1.65327	-1.65072	-1.66695	-1.66455	-1.66218	-1.65977	-1.65735	-1.67639	-1.67378
-1.65235	-1.64982	-1.66606	-1.66367	-1.66131	-1.6589	-1.65649	-1.67547	-1.67288
-1.65143	-1.64892	-1.66518	-1.66279	-1.66043	-1.65803	-1.65563	-1.67455	-1.67197
-1.65053	-1.64803	-1.66429	-1.6619	-1.65955	-1.6788	-1.6762	-1.69675	-1.69406
-1.64964	-1.64714	-1.66341	-1.66102	-1.65866	-1.67786	-1.67526	-1.69576	-1.6931
-1.64875	-1.64627	-1.66253	-1.66015	-1.65779	-1.67692	-1.67434	-1.6948	-1.69216
-1.64787	-1.6454	-1.66167	-1.65929	-1.65692	-1.676	-1.67342	-1.69385	-1.69121
-1.647	-1.64456	-1.66085	-1.65845	-1.65607	-1.67508	-1.67252	-1.69291	-1.69028
-1.64613	-1.64375	-1.66006	-1.65764	-1.65524	-1.67418	-1.67161	-1.69198	-1.68936
-1.64529	-1.64293	-1.68109	-1.67847	-1.67586	-1.69638	-1.6937	-1.69104	-1.68843
-1.6445	-1.64211	-1.68019	-1.67756	-1.67494	-1.69541	-1.69275	-1.69011	-1.68749
-1.6437	-1.64129	-1.67927	-1.67664	-1.67402	-1.69445	-1.6918	-1.68917	-1.68656
-1.66163	-1.65916	-1.67836	-1.67572	-1.67311	-1.6935	-1.69086	-1.68824	-1.68563
-1.66078	-1.65831	-1.67744	-1.67481	-1.67221	-1.69256	-1.68992	-1.68731	-1.68472
-1.68181	-1.67915	-1.67651	-1.67389	-1.6713	-1.69163	-1.68899	-1.7202	-1.71754
-1.68088	-1.67822	-1.67558	-1.67297	-1.67039	-1.69069	-1.68807	-1.71928	-1.71667
-1.67995	-1.67729	-1.67466	-1.67206	-1.66949	-1.68978	-1.68718	-1.7397	-1.73695
-1.679	-1.67636	-1.67375	-1.67116	-1.6686	-1.68889	-1.68633	-1.73878	-1.73598
-1.67806	-1.67546	-1.69592	-1.69323	-1.69059	-1.72191	-1.71927	-1.73784	-1.735
-1.67717	-1.6746	-1.69507	-1.6924	-1.68976	-1.72103	-1.71836	-1.73685	-1.734

-1.6763	-1.67377	-1.69423	-1.69157	-1.68894	-1.72015	-1.71744	-1.73584	-1.73299
-1.67547	-1.67293	-1.72746	-1.7247	-1.72196	-1.71922	-1.7165	-1.75277	-1.74984
-1.67468	-1.67209	-1.72652	-1.72375	-1.721	-1.71826	-1.71554	-1.75173	-1.7488
-1.70864	-1.70593	-1.72558	-1.7228	-1.72004	-1.71731	-1.71459	-1.73004	-1.72731
-1.70771	-1.70501	-1.72463	-1.72185	-1.71909	-1.71635	-1.71364	-1.7291	-1.72641
-1.72927	-1.72648	-1.72371	-1.72093	-1.71818	-1.71545	-1.71274	-1.72818	-1.7255
-1.72832	-1.72558	-1.72285	-1.72009	-1.71734	-1.71462	-1.71192	-1.7273	-1.72457
-1.72741	-1.72469	-1.72197	-1.71923	-1.7165	-1.71379	-1.71109	-1.72642	-1.72364
-1.72653	-1.72378	-1.72104	-1.71831	-1.71559	-1.73103	-1.72826	-1.72548	-1.72271
-1.72566	-1.72286	-1.72009	-1.71737	-1.71467	-1.73009	-1.72732	-1.72455	-1.72178
-1.72474	-1.72192	-1.71915	-1.71646	-1.7138	-1.7292	-1.72644	-1.72368	-1.72092
-1.72379	-1.72097	-1.71822	-1.71557	-1.71295	-1.72834	-1.72559	-1.72284	-1.72009
-1.72289	-1.72007	-1.71732	-1.71468	-1.71207	-1.72744	-1.7247	-1.72196	-1.71922
-1.72204	-1.71923	-1.71648	-1.71381	-1.71115	-1.72649	-1.72375	-1.72102	-1.7183
-1.72117	-1.71837	-1.71562	-1.71291	-1.71024	-1.72555	-1.72282	-1.72011	-1.7174
-1.72023	-1.71745	-1.7147	-1.71198	-1.7093	-1.72461	-1.72193	-1.71924	-1.71655
-1.7193	-1.71653	-1.71379	-1.71107	-1.7084	-1.7237	-1.72104	-1.71837	-1.7157
-1.71844	-1.71568	-1.73106	-1.72829	-1.72555	-1.72284	-1.72015	-1.73597	-1.73324
-1.71759	-1.71486	-1.73023	-1.72748	-1.72474	-1.722	-1.71927	-1.73505	-1.73232
-1.71669	-1.71398	-1.72934	-1.72661	-1.7239	-1.72116	-1.71843	-1.73419	-1.73145
-1.71574	-1.71304	-1.72841	-1.72572	-1.72304	-1.72033	-1.71761	-1.73336	-1.73063
-1.733	-1.73024	-1.72752	-1.72483	-1.72216	-1.71945	-1.71675	-1.73249	-1.72976
-1.73219	-1.72942	-1.72668	-1.72396	-1.72124	-1.71853	-1.71582	-1.73155	-1.72883
-1.73138	-1.72862	-1.72586	-1.7231	-1.72035	-1.73614	-1.73337	-1.73063	-1.72792
-1.73058	-1.72781	-1.72505	-1.72228	-1.71952	-1.73529	-1.73252	-1.72978	-1.72707
-1.72975	-1.72698	-1.72422	-1.72145	-1.71869	-1.73444	-1.73167	-1.72894	-1.72624
-1.72884	-1.72608	-1.72332	-1.72055	-1.71779	-1.73353	-1.73076	-1.72804	-1.72536
-1.7279	-1.72514	-1.72238	-1.71962	-1.71686	-1.73258	-1.72983	-1.72713	-1.72448
-1.72703	-1.72427	-1.72151	-1.71874	-1.71599	-1.7317	-1.72896	-1.74578	-1.74308
-1.72622	-1.72345	-1.72068	-1.71792	-1.71518	-1.73087	-1.72813	-1.74494	-1.74227
-1.72542	-1.72265	-1.71988	-1.71711	-1.71437	-1.74966	-1.74685	-1.78334	-1.78051
-1.72465	-1.72187	-1.7191	-1.71632	-1.71357	-1.74881	-1.74601	-1.78246	-1.77965
-1.72386	-1.72109	-1.73685	-1.73399	-1.73117	-1.76402	-1.76118	-1.78155	-1.77876
-1.72306	-1.72029	-1.73602	-1.73314	-1.73029	-1.76308	-1.76026	-1.7806	-1.77781
-1.72224	-1.71946	-1.73517	-1.73226	-1.72939	-1.76214	-1.75933	-1.77963	-1.77684
-1.72135	-1.71856	-1.77013	-1.7671	-1.76412	-1.76125	-1.75847	-1.79321	-1.79038
-1.72042	-1.71763	-1.76913	-1.76616	-1.76323	-1.76041	-1.75765	-1.79235	-1.78952
-1.7362	-1.73335	-1.76819	-1.76525	-1.76236	-1.75957	-1.75684	-1.79151	-1.78867
-1.73536	-1.73251	-1.76729	-1.76437	-1.7615	-1.75874	-1.75604	-1.79068	-1.78784
-1.77242	-1.7694	-1.76643	-1.76351	-1.76066	-1.75792	-1.75525	-1.78985	-1.787
-1.77156	-1.76854	-1.76558	-1.76269	-1.75986	-1.75713	-1.75446	-1.78902	-1.78616
-1.7707	-1.76769	-1.76475	-1.76187	-1.75906	-1.75634	-1.75367	-1.78818	-1.78532
-1.76983	-1.76684	-1.76391	-1.76106	-1.75827	-1.77857	-1.77579	-1.78735	-1.78448
-1.76897	-1.76598	-1.76307	-1.76024	-1.75748	-1.77776	-1.77497	-1.78651	-1.78363
-1.7681	-1.76512	-1.76223	-1.75943	-1.75669	-1.77694	-1.77414	-1.8001	-1.79717
-1.76722	-1.76427	-1.76139	-1.75862	-1.7559	-1.77612	-1.77331	-1.79924	-1.7963
-1.76636	-1.76341	-1.76056	-1.75781	-1.75511	-1.80419	-1.80129	-1.79837	-1.79542
-1.76549	-1.76256	-1.75972	-1.757	-1.75432	-1.80334	-1.80043	-1.79749	-1.79453
-1.75208	-1.74919	-1.78208	-1.77926	-1.77649	-1.80249	-1.79956	-1.7966	-1.79361
-1.75127	-1.7484	-1.78126	-1.77845	-1.77567	-1.80163	-1.79869	-1.79569	-1.79265

-1.75046	-1.74762	-1.78046	-1.77764	-1.77485	-1.80077	-1.79781	-1.79478	-1.79169
-1.74964	-1.74683	-1.79441	-1.79154	-1.78868	-1.79991	-1.79694	-1.7939	-1.79079
-1.74883	-1.74605	-1.79358	-1.79071	-1.78783	-1.79905	-1.79607	-1.79303	-1.78993
-1.74802	-1.74526	-1.78068	-1.77782	-1.77495	-1.79818	-1.79521	-1.79219	-1.78912
-1.7472	-1.74448	-1.77986	-1.77698	-1.7741	-1.79731	-1.79435	-1.79136	-1.78834
-1.76161	-1.75884	-1.77903	-1.77615	-1.77326	-1.79644	-1.79347	-1.79051	-1.78754
-1.7608	-1.75804	-1.7782	-1.77531	-1.77242	-1.79557	-1.79259	-1.78963	-1.78668
-1.75999	-1.75724	-1.77737	-1.77448	-1.77157	-1.78288	-1.77991	-1.78875	-1.78581
-1.75919	-1.75643	-1.77652	-1.77363	-1.77072	-1.782	-1.77903	-1.78786	-1.78493
-1.75839	-1.75562	-1.77568	-1.77278	-1.76987	-1.78113	-1.77815	-1.78698	-1.78406
-1.7576	-1.75482	-1.77483	-1.77192	-1.76901	-1.78025	-1.77728	-1.7861	-1.78319
-1.75682	-1.75402	-1.77399	-1.77107	-1.76815	-1.77937	-1.7764	-1.78523	-1.78234
-1.75609	-1.75325	-1.78743	-1.78445	-1.78148	-1.77849	-1.77552	-1.77262	-1.76977
-1.7554	-1.75252	-1.78661	-1.7836	-1.7806	-1.77761	-1.77464	-1.77175	-1.76893
-1.75468	-1.75179	-1.78581	-1.78277	-1.77973	-1.77673	-1.77377	-1.77089	-1.76809
-1.75393	-1.75106	-1.78507	-1.78198	-1.77889	-1.77587	-1.7729	-1.77003	-1.76725
-1.75314	-1.75031	-1.7843	-1.78119	-1.77807	-1.77503	-1.77206	-1.76919	-1.76642
-1.7523	-1.74949	-1.78345	-1.78037	-1.77729	-1.77426	-1.77128	-1.7684	-1.76563
-1.75143	-1.74863	-1.78256	-1.77953	-1.7765	-1.7735	-1.77052	-1.76765	-1.76486
-1.75057	-1.74777	-1.78165	-1.77864	-1.77565	-1.77267	-1.76973	-1.76688	-1.7641
-1.74972	-1.7469	-1.78071	-1.77772	-1.77474	-1.77181	-1.76892	-1.7661	-1.76336
-1.76334	-1.76046	-1.7798	-1.77681	-1.77385	-1.77094	-1.76809	-1.7653	-1.76258
-1.76257	-1.75965	-1.77892	-1.77591	-1.77295	-1.77006	-1.76723	-1.76446	-1.76175
-1.76179	-1.75884	-1.77806	-1.77505	-1.77209	-1.76921	-1.76639	-1.76363	-1.76093
-1.76096	-1.75805	-1.77727	-1.77427	-1.77132	-1.76843	-1.76559	-1.76282	-1.76011
-1.76012	-1.75726	-1.77649	-1.7735	-1.77055	-1.76766	-1.76482	-1.76203	-1.7593
-1.75929	-1.75643	-1.77563	-1.77266	-1.76973	-1.76687	-1.76406	-1.76129	-1.75856
-1.75845	-1.75559	-1.77476	-1.77179	-1.76888	-1.76607	-1.76332	-1.76058	-1.75786
-1.77991	-1.77691	-1.77394	-1.77098	-1.76807	-1.76527	-1.76254	-1.78244	-1.77951
-1.77914	-1.77615	-1.77318	-1.7702	-1.76727	-1.76447	-1.76173	-1.78158	-1.77867
-1.77834	-1.77536	-1.77239	-1.76942	-1.76651	-1.7637	-1.76096	-1.78076	-1.77786
-1.77749	-1.77451	-1.77157	-1.76865	-1.76578	-1.76299	-1.76025	-1.78001	-1.77711
-1.77666	-1.77368	-1.77076	-1.76788	-1.76505	-1.76227	-1.75953	-1.77925	-1.77634
-1.7759	-1.77293	-1.77	-1.76712	-1.76428	-1.7615	-1.75875	-1.77842	-1.77551
-1.77515	-1.77219	-1.76926	-1.76637	-1.76352	-1.76072	-1.75797	-1.77758	-1.77467
-1.77435	-1.77139	-1.76848	-1.76561	-1.76278	-1.75999	-1.75724	-1.77678	-1.77385
-1.77353	-1.77058	-1.76768	-1.76486	-1.76206	-1.75929	-1.75653	-1.77601	-1.77305
-1.77276	-1.76982	-1.76693	-1.76411	-1.76131	-1.75854	-1.75578	-1.77522	-1.77228
-1.77205	-1.76912	-1.76622	-1.76336	-1.76054	-1.75775	-1.755	-1.77443	-1.77152
-1.77134	-1.7684	-1.7655	-1.76262	-1.75978	-1.757	-1.75425	-1.77365	-1.77077
-1.77061	-1.76766	-1.76475	-1.7619	-1.75908	-1.75629	-1.75355	-1.77292	-1.77004
-1.76987	-1.76692	-1.76402	-1.76118	-1.75838	-1.7556	-1.75285	-1.77217	-1.7693
-1.76908	-1.76618	-1.76331	-1.76049	-1.75769	-1.75489	-1.75211	-1.77139	-1.76852
-1.7683	-1.76545	-1.76262	-1.7598	-1.75699	-1.75418	-1.75137	-1.77059	-1.76772
-1.76756	-1.76473	-1.7848	-1.78176	-1.77874	-1.77573	-1.77276	-1.76985	-1.76698
-1.76686	-1.76403	-1.78403	-1.78097	-1.77794	-1.77496	-1.77203	-1.76913	-1.76627
-1.76616	-1.76331	-1.78325	-1.78019	-1.77716	-1.77421	-1.77131	-1.76843	-1.76557
-1.76546	-1.76257	-1.78244	-1.7794	-1.77641	-1.77349	-1.77061	-1.76773	-1.76487
-1.76475	-1.76184	-1.78166	-1.77865	-1.77568	-1.77277	-1.7699	-1.78477	-1.78171
-1.76402	-1.76113	-1.78093	-1.77793	-1.77498	-1.77207	-1.7692	-1.784	-1.78092

							-0.05017	-0.05004
							-0.05008	-0.04996
							-0.10569	-0.1051
							-0.10539	-0.10481
					-0.05009	-0.04996	-0.15459	-0.15392
					-0.05001	-0.04988	-0.15423	-0.15357
					-0.10565	-0.10507	-0.30927	-0.3082
					-0.10534	-0.1048	-0.30874	-0.30768
					-0.10505	-0.10453	-0.30821	-0.30716
		-0.10649	-0.10589	-0.1053	-0.15417	-0.15355	-0.35118	-0.34993
		-0.10621	-0.10562	-0.10504	-0.15384	-0.15319	-0.35049	-0.34925
		-0.10591	-0.10533	-0.10476	-0.30859	-0.30753	-0.39557	-0.39417
		-0.10559	-0.10501	-0.10446	-0.30801	-0.30696	-0.39484	-0.39348
-0.10646	-0.10585	-0.15476	-0.15408	-0.15342	-0.35095	-0.3497	-0.39412	-0.3928
-0.10613	-0.10553	-0.15439	-0.15371	-0.15305	-0.35027	-0.34902	-0.39343	-0.39214
-0.10581	-0.10523	-0.15403	-0.15336	-0.1527	-0.34961	-0.34838	-0.39277	-0.39151
-0.10553	-0.10495	-0.30894	-0.30787	-0.30681	-0.39484	-0.39347	-0.6031	-0.60123

-0.10525	-0.10469	-0.30841	-0.30734	-0.30629	-0.3942	-0.39286	-0.60218	-0.60032
-0.15436	-0.15369	-0.35149	-0.35022	-0.34897	-0.60504	-0.60309	-0.65073	-0.64868
-0.15399	-0.15332	-0.3508	-0.34953	-0.3483	-0.60393	-0.60204	-0.64961	-0.64757
-0.30882	-0.30774	-0.39623	-0.3948	-0.39342	-0.60288	-0.60103	-0.69022	-0.68804
-0.30823	-0.30716	-0.39551	-0.39412	-0.39278	-0.60191	-0.60006	-0.68908	-0.68691
-0.35132	-0.35004	-0.6068	-0.6048	-0.60285	-0.65049	-0.64845	-0.68797	-0.68582
-0.3507	-0.34942	-0.60577	-0.60381	-0.60191	-0.64949	-0.64746	-0.68692	-0.68478
-0.35008	-0.34882	-0.60477	-0.60285	-0.60098	-0.64849	-0.64648	-0.68587	-0.68374
-0.47542	-0.47373	-0.60379	-0.60189	-0.60004	-0.68909	-0.68693	-0.68477	-0.68263
-0.47451	-0.47289	-0.60282	-0.60095	-0.59911	-0.68799	-0.68583	-0.68366	-0.68149
-0.60576	-0.6038	-0.60189	-0.60002	-0.59819	-0.68692	-0.68476	-0.72573	-0.72345
-0.60476	-0.60285	-0.60097	-0.59912	-0.59729	-0.68588	-0.68373	-0.72464	-0.72233
-0.60377	-0.60189	-0.69131	-0.68913	-0.68696	-0.72804	-0.7258	-0.8078	-0.80522
-0.6028	-0.60094	-0.69019	-0.68801	-0.68585	-0.72688	-0.72465	-0.80654	-0.80396
-0.60185	-0.6	-0.68909	-0.68691	-0.68475	-0.72573	-0.7235	-0.90875	-0.9057
-0.60094	-0.5991	-0.68803	-0.68586	-0.6837	-0.72463	-0.72239	-0.90729	-0.90424
-0.60003	-0.5982	-0.68697	-0.68481	-0.68265	-0.72352	-0.72128	-0.90582	-0.90279
-0.64853	-0.6465	-0.72912	-0.72687	-0.72463	-0.80659	-0.80408	-0.97374	-0.97034
-0.64752	-0.64547	-0.72795	-0.7257	-0.72346	-0.80532	-0.8028	-0.97207	-0.96873
-0.69068	-0.68852	-0.8116	-0.80908	-0.80658	-0.87478	-0.87188	-1.17608	-1.17188
-0.68967	-0.68749	-0.81036	-0.80784	-0.80533	-0.87335	-0.87048	-1.17406	-1.16983
-0.72925	-0.72692	-0.80913	-0.80659	-0.80408	-1.00989	-1.00648	-1.20163	-1.1973
-0.72813	-0.7258	-0.80789	-0.80532	-0.80279	-1.00817	-1.00481	-1.19956	-1.19533
-0.72701	-0.72467	-0.80665	-0.80407	-0.80153	-1.00647	-1.00315	-1.19751	-1.19337
-0.81059	-0.80801	-0.87643	-0.87346	-0.87053	-1.17423	-1.17012	-1.26051	-1.25625
-0.80933	-0.80677	-0.87504	-0.87209	-0.86918	-1.17227	-1.16815	-1.25842	-1.25421
-0.8081	-0.80553	-1.01193	-1.00845	-1.00502	-1.26505	-1.26061	-1.31497	-1.31068
-0.80689	-0.80429	-1.01021	-1.0068	-1.00344	-1.26297	-1.2585	-1.3128	-1.30852
-0.90792	-0.90487	-1.11987	-1.11604	-1.11228	-1.26087	-1.25644	-1.33967	-1.33534
-0.9065	-0.90345	-1.11789	-1.11408	-1.11038	-1.25873	-1.25445	-1.33766	-1.33341
-1.01225	-1.00866	-1.26968	-1.26524	-1.26091	-1.28662	-1.28235	-1.36857	-1.36427
-1.01052	-1.00702	-1.2676	-1.26319	-1.25889	-1.28457	-1.28032	-1.36648	-1.36227
-1.00882	-1.00539	-1.26555	-1.2612	-1.25692	-1.28256	-1.27831	-1.36439	-1.36023
-1.11823	-1.11442	-1.26351	-1.2592	-1.25496	-1.3381	-1.33377	-1.36228	-1.35808
-1.11636	-1.11262	-1.26148	-1.25722	-1.25301	-1.33608	-1.33175	-1.36018	-1.3559
-1.1503	-1.14625	-1.28949	-1.28514	-1.28084	-1.36686	-1.36248	-1.35813	-1.35382
-1.1484	-1.14438	-1.28746	-1.28307	-1.27875	-1.36473	-1.36044	-1.35612	-1.35179
-1.17863	-1.17451	-1.2854	-1.28103	-1.27674	-1.36266	-1.35842	-1.37011	-1.36575
-1.17672	-1.17256	-1.28332	-1.27904	-1.27484	-1.36066	-1.3564	-1.36808	-1.36375
-1.29015	-1.28566	-1.31522	-1.3109	-1.30668	-1.35868	-1.35438	-1.38942	-1.38503
-1.28814	-1.28367	-1.31321	-1.30894	-1.30474	-1.35669	-1.35237	-1.38736	-1.38299
-1.28611	-1.28171	-1.31124	-1.30699	-1.30281	-1.35469	-1.35035	-1.38529	-1.38095
-1.28405	-1.27974	-1.36564	-1.36132	-1.35702	-1.36865	-1.36427	-1.40776	-1.40337
-1.28197	-1.27776	-1.36364	-1.35932	-1.35501	-1.36661	-1.36222	-1.40565	-1.40125
-1.31383	-1.30958	-1.37767	-1.37332	-1.36897	-1.38792	-1.38342	-1.42786	-1.42344
-1.31184	-1.30761	-1.37565	-1.37129	-1.36693	-1.38582	-1.38131	-1.42574	-1.4214
-1.32665	-1.3224	-1.37363	-1.36927	-1.36491	-1.40833	-1.40379	-1.44751	-1.44318
-1.32471	-1.32046	-1.37162	-1.36726	-1.36289	-1.4063	-1.4018	-1.44548	-1.44118
-1.37834	-1.37396	-1.41774	-1.41325	-1.40878	-1.42853	-1.42402	-1.44349	-1.43922
-1.37635	-1.37195	-1.41567	-1.41121	-1.40678	-1.42653	-1.42206	-1.44153	-1.43727

-1.37437	-1.36994	-1.4136	-1.40918	-1.4048	-1.42456	-1.42012	-1.43958	-1.43534
-1.37237	-1.36793	-1.41154	-1.40716	-1.40283	-1.44645	-1.44198	-1.57098	-1.56616
-1.37037	-1.36591	-1.40949	-1.40515	-1.40087	-1.44447	-1.44003	-1.56882	-1.56403
-1.41649	-1.41193	-1.43169	-1.42735	-1.42306	-1.4425	-1.43809	-1.56666	-1.5619
-1.41443	-1.4099	-1.42966	-1.42536	-1.4211	-1.44054	-1.43615	-1.56449	-1.55975
-1.43666	-1.43209	-1.42765	-1.42338	-1.41917	-1.57221	-1.5672	-1.58422	-1.57944
-1.43457	-1.43005	-1.42566	-1.42143	-1.41725	-1.57004	-1.56505	-1.58205	-1.57726
-1.43249	-1.42803	-1.42369	-1.41949	-1.41534	-1.56787	-1.56291	-1.57989	-1.57509
-1.43043	-1.42603	-1.42173	-1.41757	-1.41344	-1.58762	-1.58262	-1.57773	-1.57293
-1.4284	-1.42404	-1.41978	-1.41565	-1.41154	-1.58542	-1.58044	-1.57556	-1.57076
-1.42642	-1.42208	-1.5759	-1.57107	-1.56627	-1.58323	-1.57826	-1.5734	-1.5686
-1.42449	-1.42016	-1.57369	-1.56888	-1.5641	-1.58104	-1.57608	-1.57122	-1.56645
-1.42261	-1.41828	-1.59349	-1.58863	-1.5838	-1.57886	-1.5739	-1.56906	-1.56432
-1.4208	-1.41645	-1.59132	-1.58645	-1.58162	-1.57668	-1.57173	-1.56691	-1.5622
-1.41897	-1.41461	-1.58915	-1.58427	-1.57944	-1.57451	-1.56958	-1.56479	-1.56012
-1.41708	-1.41273	-1.58696	-1.58209	-1.57728	-1.57237	-1.56746	-1.5627	-1.55807
-1.41517	-1.41085	-1.58478	-1.57992	-1.57512	-1.57025	-1.5654	-1.56067	-1.55605
-1.59275	-1.58764	-1.58263	-1.57777	-1.57297	-1.56818	-1.56342	-1.55873	-1.5541
-1.59053	-1.58548	-1.58051	-1.57564	-1.57085	-1.56614	-1.56151	-1.55685	-1.55219
-1.58834	-1.58336	-1.57844	-1.57356	-1.56876	-1.56411	-1.55954	-1.55492	-1.55026
-1.58618	-1.5813	-1.57644	-1.57154	-1.56671	-1.56207	-1.55754	-1.55294	-1.54832
-1.58404	-1.57921	-1.5744	-1.56951	-1.56468	-1.56005	-1.55553	-1.57365	-1.56902
-1.58194	-1.5771	-1.57229	-1.56745	-1.56268	-1.55805	-1.55352	-1.57165	-1.5671
-1.57986	-1.57501	-1.5702	-1.56543	-1.56072	-1.55609	-1.55154	-1.58923	-1.58472
-1.57784	-1.57296	-1.56816	-1.56348	-1.55885	-1.55422	-1.54963	-1.58729	-1.5828
-1.57582	-1.57093	-1.56615	-1.56155	-1.557	-1.55237	-1.54776	-1.58539	-1.58094
-1.57375	-1.56889	-1.56415	-1.55957	-1.55505	-1.57034	-1.56574	-1.58357	-1.57925
-1.57165	-1.56687	-1.56217	-1.55758	-1.55305	-1.56838	-1.56386	-1.58182	-1.57769
-1.56962	-1.56491	-1.58018	-1.57555	-1.57098	-1.56641	-1.56194	-1.58002	-1.57611
-1.56765	-1.56302	-1.57834	-1.57367	-1.56904	-1.56444	-1.55996	-1.57815	-1.5745
-1.58567	-1.58106	-1.57645	-1.57177	-1.56713	-1.58482	-1.58034	-1.57642	-1.57299
-1.58377	-1.57912	-1.57449	-1.56984	-1.56527	-1.58304	-1.57869	-1.57491	-1.57162
-1.58185	-1.57716	-1.57252	-1.5679	-1.5634	-1.58129	-1.57713	-1.57348	-1.57031
-1.57988	-1.57518	-1.57053	-1.56589	-1.56142	-1.57947	-1.57556	-1.57208	-1.56902
-1.5779	-1.57319	-1.56855	-1.56389	-1.55946	-1.57768	-1.57404	-1.57073	-1.56776
-1.57594	-1.57126	-1.56665	-1.56202	-1.55767	-1.57604	-1.57261	-1.56944	-1.56655
-1.57399	-1.56935	-1.5648	-1.56026	-1.55602	-1.57452	-1.57126	-1.5682	-1.56539
-1.57207	-1.56743	-1.5852	-1.58077	-1.57665	-1.57311	-1.56997	-1.567	-1.56426
-1.57017	-1.56548	-1.58327	-1.57909	-1.57523	-1.57181	-1.56873	-1.56585	-1.56317
-1.56828	-1.56357	-1.58141	-1.57745	-1.57384	-1.57055	-1.56755	-1.60231	-1.59963
-1.56639	-1.56172	-1.57963	-1.57585	-1.57242	-1.56928	-1.56641	-1.60121	-1.59852
-1.56452	-1.55995	-1.57796	-1.57432	-1.57104	-1.56805	-1.5653	-1.60013	-1.59742
-1.56272	-1.5583	-1.57644	-1.57295	-1.56979	-1.5845	-1.58179	-1.62908	-1.62627
-1.56097	-1.55675	-1.57504	-1.57167	-1.56861	-1.58336	-1.58067	-1.62793	-1.62516
-1.5592	-1.5552	-1.57367	-1.57042	-1.56744	-1.59216	-1.58942	-1.6268	-1.62403
-1.55741	-1.55368	-1.57234	-1.56919	-1.56628	-1.59102	-1.58832	-1.62569	-1.62289
-1.57795	-1.57434	-1.58866	-1.58562	-1.58277	-1.61062	-1.60789	-1.62457	-1.62176
-1.57652	-1.57298	-1.58736	-1.58443	-1.58169	-1.60951	-1.60674	-1.62342	-1.62066
-1.59286	-1.58934	-1.617	-1.61403	-1.61123	-1.6279	-1.62507	-1.62229	-1.61956
-1.59154	-1.58816	-1.61584	-1.61288	-1.61008	-1.62677	-1.62396	-1.62119	-1.61844

-1.59026	-1.58703	-1.61475	-1.61177	-1.60893	-1.62564	-1.62287	-1.62009	-1.61733
-1.61	-1.60686	-1.61365	-1.6107	-1.60785	-1.62454	-1.62175	-1.61899	-1.61627
-1.60864	-1.60567	-1.61255	-1.60966	-1.60682	-1.62348	-1.62063	-1.61789	-1.61524
-1.61726	-1.61429	-1.61142	-1.60857	-1.60576	-1.62242	-1.61957	-1.61684	-1.61422
-1.61604	-1.6131	-1.61024	-1.60744	-1.60468	-1.62137	-1.61855	-1.61583	-1.6132
-1.61488	-1.61196	-1.60912	-1.60632	-1.60357	-1.6203	-1.61753	-1.61484	-1.61221
-1.61379	-1.6109	-1.60806	-1.60522	-1.60243	-1.61919	-1.61648	-1.61384	-1.61126
-1.61271	-1.60985	-1.60702	-1.60414	-1.60132	-1.61809	-1.61543	-1.61284	-1.61033
-1.61157	-1.60875	-1.60594	-1.60309	-1.60029	-1.61706	-1.6144	-1.61184	-1.60937
-1.61043	-1.60763	-1.60486	-1.60205	-1.59929	-1.61606	-1.61339	-1.61085	-1.60842
-1.60933	-1.60651	-1.62332	-1.62052	-1.61776	-1.61506	-1.61243	-1.63227	-1.62978
-1.60827	-1.60539	-1.62217	-1.6194	-1.6167	-1.61407	-1.6115	-1.63133	-1.62881
-1.6072	-1.60431	-1.62107	-1.61832	-1.61565	-1.61307	-1.61056	-1.67107	-1.66849
-1.60615	-1.60328	-1.62005	-1.61728	-1.6146	-1.61206	-1.60961	-1.67012	-1.66758
-1.60509	-1.60226	-1.61905	-1.61628	-1.6136	-1.65241	-1.64994	-1.66918	-1.66668
-1.60403	-1.60124	-1.61806	-1.61532	-1.61267	-1.65146	-1.64901	-1.66826	-1.6658
-1.60297	-1.60022	-1.61708	-1.61439	-1.61177	-1.65054	-1.64808	-1.66734	-1.66492
-1.60188	-1.59916	-1.65749	-1.65478	-1.65214	-1.6496	-1.64713	-1.66638	-1.664
-1.60078	-1.59811	-1.65644	-1.65379	-1.6512	-1.64866	-1.64618	-1.66542	-1.66307
-1.64177	-1.63907	-1.65545	-1.65284	-1.65028	-1.64775	-1.64528	-1.66449	-1.66213
-1.64075	-1.63808	-1.65449	-1.6519	-1.64937	-1.64688	-1.64442	-1.66359	-1.66118
-1.63973	-1.63708	-1.65351	-1.65096	-1.64847	-1.66767	-1.66518	-1.66271	-1.66026
-1.63871	-1.63606	-1.6525	-1.65	-1.64756	-1.66678	-1.66431	-1.66185	-1.65939
-1.6377	-1.63506	-1.65152	-1.64906	-1.64666	-1.66589	-1.66344	-1.66099	-1.65853
-1.63673	-1.6341	-1.65058	-1.64815	-1.64578	-1.665	-1.66257	-1.66012	-1.65767
-1.63577	-1.63317	-1.64967	-1.64726	-1.6449	-1.66412	-1.66169	-1.65926	-1.65681
-1.63484	-1.63226	-1.67051	-1.66806	-1.66566	-1.66324	-1.66082	-1.65839	-1.65595
-1.63393	-1.63137	-1.66961	-1.66718	-1.66479	-1.66237	-1.65995	-1.65752	-1.65509
-1.63303	-1.63048	-1.66872	-1.66629	-1.66392	-1.6615	-1.65908	-1.67824	-1.67561
-1.63212	-1.6296	-1.66783	-1.66542	-1.66305	-1.66063	-1.65822	-1.67731	-1.67469
-1.65327	-1.65072	-1.66695	-1.66455	-1.66218	-1.65977	-1.65735	-1.67639	-1.67378
-1.65235	-1.64982	-1.66606	-1.66367	-1.66131	-1.6589	-1.65649	-1.67547	-1.67288
-1.65143	-1.64892	-1.66518	-1.66279	-1.66043	-1.65803	-1.65563	-1.67455	-1.67197
-1.65053	-1.64803	-1.66429	-1.6619	-1.65955	-1.6788	-1.6762	-1.69675	-1.69406
-1.64964	-1.64714	-1.66341	-1.66102	-1.65866	-1.67786	-1.67526	-1.69576	-1.6931
-1.64875	-1.64627	-1.66253	-1.66015	-1.65779	-1.67692	-1.67434	-1.6948	-1.69216
-1.64787	-1.6454	-1.66167	-1.65929	-1.65692	-1.676	-1.67342	-1.69385	-1.69121
-1.647	-1.64456	-1.66085	-1.65845	-1.65607	-1.67508	-1.67252	-1.69291	-1.69028
-1.64613	-1.64375	-1.66006	-1.65764	-1.65524	-1.67418	-1.67161	-1.69198	-1.68936
-1.64529	-1.64293	-1.68109	-1.67847	-1.67586	-1.69638	-1.6937	-1.69104	-1.68843
-1.6445	-1.64211	-1.68019	-1.67756	-1.67494	-1.69541	-1.69275	-1.69011	-1.68749
-1.6437	-1.64129	-1.67927	-1.67664	-1.67402	-1.69445	-1.6918	-1.68917	-1.68656
-1.66163	-1.65916	-1.67836	-1.67572	-1.67311	-1.6935	-1.69086	-1.68824	-1.68563
-1.66078	-1.65831	-1.67744	-1.67481	-1.67221	-1.69256	-1.68992	-1.68731	-1.68472
-1.68181	-1.67915	-1.67651	-1.67389	-1.6713	-1.69163	-1.68899	-1.7202	-1.71754
-1.68088	-1.67822	-1.67558	-1.67297	-1.67039	-1.69069	-1.68807	-1.71928	-1.71667
-1.67995	-1.67729	-1.67466	-1.67206	-1.66949	-1.68978	-1.68718	-1.7397	-1.73695
-1.679	-1.67636	-1.67375	-1.67116	-1.6686	-1.68889	-1.68633	-1.73878	-1.73598
-1.67806	-1.67546	-1.69592	-1.69323	-1.69059	-1.72191	-1.71927	-1.73784	-1.735
-1.67717	-1.6746	-1.69507	-1.6924	-1.68976	-1.72103	-1.71836	-1.73685	-1.734

-1.6763	-1.67377	-1.69423	-1.69157	-1.68894	-1.72015	-1.71744	-1.73584	-1.73299
-1.67547	-1.67293	-1.72746	-1.7247	-1.72196	-1.71922	-1.7165	-1.75277	-1.74984
-1.67468	-1.67209	-1.72652	-1.72375	-1.721	-1.71826	-1.71554	-1.75173	-1.7488
-1.70864	-1.70593	-1.72558	-1.7228	-1.72004	-1.71731	-1.71459	-1.73004	-1.72731
-1.70771	-1.70501	-1.72463	-1.72185	-1.71909	-1.71635	-1.71364	-1.7291	-1.72641
-1.72927	-1.72648	-1.72371	-1.72093	-1.71818	-1.71545	-1.71274	-1.72818	-1.7255
-1.72832	-1.72558	-1.72285	-1.72009	-1.71734	-1.71462	-1.71192	-1.7273	-1.72457
-1.72741	-1.72469	-1.72197	-1.71923	-1.7165	-1.71379	-1.71109	-1.72642	-1.72364
-1.72653	-1.72378	-1.72104	-1.71831	-1.71559	-1.73103	-1.72826	-1.72548	-1.72271
-1.72566	-1.72286	-1.72009	-1.71737	-1.71467	-1.73009	-1.72732	-1.72455	-1.72178
-1.72474	-1.72192	-1.71915	-1.71646	-1.7138	-1.7292	-1.72644	-1.72368	-1.72092
-1.72379	-1.72097	-1.71822	-1.71557	-1.71295	-1.72834	-1.72559	-1.72284	-1.72009
-1.72289	-1.72007	-1.71732	-1.71468	-1.71207	-1.72744	-1.7247	-1.72196	-1.71922
-1.72204	-1.71923	-1.71648	-1.71381	-1.71115	-1.72649	-1.72375	-1.72102	-1.7183
-1.72117	-1.71837	-1.71562	-1.71291	-1.71024	-1.72555	-1.72282	-1.72011	-1.7174
-1.72023	-1.71745	-1.7147	-1.71198	-1.7093	-1.72461	-1.72193	-1.71924	-1.71655
-1.7193	-1.71653	-1.71379	-1.71107	-1.7084	-1.7237	-1.72104	-1.71837	-1.7157
-1.71844	-1.71568	-1.73106	-1.72829	-1.72555	-1.72284	-1.72015	-1.73597	-1.73324
-1.71759	-1.71486	-1.73023	-1.72748	-1.72474	-1.722	-1.71927	-1.73505	-1.73232
-1.71669	-1.71398	-1.72934	-1.72661	-1.7239	-1.72116	-1.71843	-1.73419	-1.73145
-1.71574	-1.71304	-1.72841	-1.72572	-1.72304	-1.72033	-1.71761	-1.73336	-1.73063
-1.733	-1.73024	-1.72752	-1.72483	-1.72216	-1.71945	-1.71675	-1.73249	-1.72976
-1.73219	-1.72942	-1.72668	-1.72396	-1.72124	-1.71853	-1.71582	-1.73155	-1.72883
-1.73138	-1.72862	-1.72586	-1.7231	-1.72035	-1.73614	-1.73337	-1.73063	-1.72792
-1.73058	-1.72781	-1.72505	-1.72228	-1.71952	-1.73529	-1.73252	-1.72978	-1.72707
-1.72975	-1.72698	-1.72422	-1.72145	-1.71869	-1.73444	-1.73167	-1.72894	-1.72624
-1.72884	-1.72608	-1.72332	-1.72055	-1.71779	-1.73353	-1.73076	-1.72804	-1.72536
-1.7279	-1.72514	-1.72238	-1.71962	-1.71686	-1.73258	-1.72983	-1.72713	-1.72448
-1.72703	-1.72427	-1.72151	-1.71874	-1.71599	-1.7317	-1.72896	-1.74578	-1.74308
-1.72622	-1.72345	-1.72068	-1.71792	-1.71518	-1.73087	-1.72813	-1.74494	-1.74227
-1.72542	-1.72265	-1.71988	-1.71711	-1.71437	-1.74966	-1.74685	-1.78334	-1.78051
-1.72465	-1.72187	-1.7191	-1.71632	-1.71357	-1.74881	-1.74601	-1.78246	-1.77965
-1.72386	-1.72109	-1.73685	-1.73399	-1.73117	-1.76402	-1.76118	-1.78155	-1.77876
-1.72306	-1.72029	-1.73602	-1.73314	-1.73029	-1.76308	-1.76026	-1.7806	-1.77781
-1.72224	-1.71946	-1.73517	-1.73226	-1.72939	-1.76214	-1.75933	-1.77963	-1.77684
-1.72135	-1.71856	-1.77013	-1.7671	-1.76412	-1.76125	-1.75847	-1.79321	-1.79038
-1.72042	-1.71763	-1.76913	-1.76616	-1.76323	-1.76041	-1.75765	-1.79235	-1.78952
-1.7362	-1.73335	-1.76819	-1.76525	-1.76236	-1.75957	-1.75684	-1.79151	-1.78867
-1.73536	-1.73251	-1.76729	-1.76437	-1.7615	-1.75874	-1.75604	-1.79068	-1.78784
-1.77242	-1.7694	-1.76643	-1.76351	-1.76066	-1.75792	-1.75525	-1.78985	-1.787
-1.77156	-1.76854	-1.76558	-1.76269	-1.75986	-1.75713	-1.75446	-1.78902	-1.78616
-1.7707	-1.76769	-1.76475	-1.76187	-1.75906	-1.75634	-1.75367	-1.78818	-1.78532
-1.76983	-1.76684	-1.76391	-1.76106	-1.75827	-1.77857	-1.77579	-1.78735	-1.78448
-1.76897	-1.76598	-1.76307	-1.76024	-1.75748	-1.77776	-1.77497	-1.78651	-1.78363
-1.7681	-1.76512	-1.76223	-1.75943	-1.75669	-1.77694	-1.77414	-1.8001	-1.79717
-1.76722	-1.76427	-1.76139	-1.75862	-1.7559	-1.77612	-1.77331	-1.79924	-1.7963
-1.76636	-1.76341	-1.76056	-1.75781	-1.75511	-1.80419	-1.80129	-1.79837	-1.79542
-1.76549	-1.76256	-1.75972	-1.757	-1.75432	-1.80334	-1.80043	-1.79749	-1.79453
-1.75208	-1.74919	-1.78208	-1.77926	-1.77649	-1.80249	-1.79956	-1.7966	-1.79361
-1.75127	-1.7484	-1.78126	-1.77845	-1.77567	-1.80163	-1.79869	-1.79569	-1.79265

-1.75046	-1.74762	-1.78046	-1.77764	-1.77485	-1.80077	-1.79781	-1.79478	-1.79169
-1.74964	-1.74683	-1.79441	-1.79154	-1.78868	-1.79991	-1.79694	-1.7939	-1.79079
-1.74883	-1.74605	-1.79358	-1.79071	-1.78783	-1.79905	-1.79607	-1.79303	-1.78993
-1.74802	-1.74526	-1.78068	-1.77782	-1.77495	-1.79818	-1.79521	-1.79219	-1.78912
-1.7472	-1.74448	-1.77986	-1.77698	-1.7741	-1.79731	-1.79435	-1.79136	-1.78834
-1.76161	-1.75884	-1.77903	-1.77615	-1.77326	-1.79644	-1.79347	-1.79051	-1.78754
-1.7608	-1.75804	-1.7782	-1.77531	-1.77242	-1.79557	-1.79259	-1.78963	-1.78668
-1.75999	-1.75724	-1.77737	-1.77448	-1.77157	-1.78288	-1.77991	-1.78875	-1.78581
-1.75919	-1.75643	-1.77652	-1.77363	-1.77072	-1.782	-1.77903	-1.78786	-1.78493
-1.75839	-1.75562	-1.77568	-1.77278	-1.76987	-1.78113	-1.77815	-1.78698	-1.78406
-1.7576	-1.75482	-1.77483	-1.77192	-1.76901	-1.78025	-1.77728	-1.7861	-1.78319
-1.75682	-1.75402	-1.77399	-1.77107	-1.76815	-1.77937	-1.7764	-1.78523	-1.78234
-1.75609	-1.75325	-1.78743	-1.78445	-1.78148	-1.77849	-1.77552	-1.77262	-1.76977
-1.7554	-1.75252	-1.78661	-1.7836	-1.7806	-1.77761	-1.77464	-1.77175	-1.76893
-1.75468	-1.75179	-1.78581	-1.78277	-1.77973	-1.77673	-1.77377	-1.77089	-1.76809
-1.75393	-1.75106	-1.78507	-1.78198	-1.77889	-1.77587	-1.7729	-1.77003	-1.76725
-1.75314	-1.75031	-1.7843	-1.78119	-1.77807	-1.77503	-1.77206	-1.76919	-1.76642
-1.7523	-1.74949	-1.78345	-1.78037	-1.77729	-1.77426	-1.77128	-1.7684	-1.76563
-1.75143	-1.74863	-1.78256	-1.77953	-1.7765	-1.7735	-1.77052	-1.76765	-1.76486
-1.75057	-1.74777	-1.78165	-1.77864	-1.77565	-1.77267	-1.76973	-1.76688	-1.7641
-1.74972	-1.7469	-1.78071	-1.77772	-1.77474	-1.77181	-1.76892	-1.7661	-1.76336
-1.76334	-1.76046	-1.7798	-1.77681	-1.77385	-1.77094	-1.76809	-1.7653	-1.76258
-1.76257	-1.75965	-1.77892	-1.77591	-1.77295	-1.77006	-1.76723	-1.76446	-1.76175
-1.76179	-1.75884	-1.77806	-1.77505	-1.77209	-1.76921	-1.76639	-1.76363	-1.76093
-1.76096	-1.75805	-1.77727	-1.77427	-1.77132	-1.76843	-1.76559	-1.76282	-1.76011
-1.76012	-1.75726	-1.77649	-1.7735	-1.77055	-1.76766	-1.76482	-1.76203	-1.7593
-1.75929	-1.75643	-1.77563	-1.77266	-1.76973	-1.76687	-1.76406	-1.76129	-1.75856
-1.75845	-1.75559	-1.77476	-1.77179	-1.76888	-1.76607	-1.76332	-1.76058	-1.75786
-1.77991	-1.77691	-1.77394	-1.77098	-1.76807	-1.76527	-1.76254	-1.78244	-1.77951
-1.77914	-1.77615	-1.77318	-1.7702	-1.76727	-1.76447	-1.76173	-1.78158	-1.77867
-1.77834	-1.77536	-1.77239	-1.76942	-1.76651	-1.7637	-1.76096	-1.78076	-1.77786
-1.77749	-1.77451	-1.77157	-1.76865	-1.76578	-1.76299	-1.76025	-1.78001	-1.77711
-1.77666	-1.77368	-1.77076	-1.76788	-1.76505	-1.76227	-1.75953	-1.77925	-1.77634
-1.7759	-1.77293	-1.77	-1.76712	-1.76428	-1.7615	-1.75875	-1.77842	-1.77551
-1.77515	-1.77219	-1.76926	-1.76637	-1.76352	-1.76072	-1.75797	-1.77758	-1.77467
-1.77435	-1.77139	-1.76848	-1.76561	-1.76278	-1.75999	-1.75724	-1.77678	-1.77385
-1.77353	-1.77058	-1.76768	-1.76486	-1.76206	-1.75929	-1.75653	-1.77601	-1.77305
-1.77276	-1.76982	-1.76693	-1.76411	-1.76131	-1.75854	-1.75578	-1.77522	-1.77228
-1.77205	-1.76912	-1.76622	-1.76336	-1.76054	-1.75775	-1.755	-1.77443	-1.77152
-1.77134	-1.7684	-1.7655	-1.76262	-1.75978	-1.757	-1.75425	-1.77365	-1.77077
-1.77061	-1.76766	-1.76475	-1.7619	-1.75908	-1.75629	-1.75355	-1.77292	-1.77004
-1.76987	-1.76692	-1.76402	-1.76118	-1.75838	-1.7556	-1.75285	-1.77217	-1.7693
-1.76908	-1.76618	-1.76331	-1.76049	-1.75769	-1.75489	-1.75211	-1.77139	-1.76852
-1.7683	-1.76545	-1.76262	-1.7598	-1.75699	-1.75418	-1.75137	-1.77059	-1.76772
-1.76756	-1.76473	-1.7848	-1.78176	-1.77874	-1.77573	-1.77276	-1.76985	-1.76698
-1.76686	-1.76403	-1.78403	-1.78097	-1.77794	-1.77496	-1.77203	-1.76913	-1.76627
-1.76616	-1.76331	-1.78325	-1.78019	-1.77716	-1.77421	-1.77131	-1.76843	-1.76557
-1.76546	-1.76257	-1.78244	-1.7794	-1.77641	-1.77349	-1.77061	-1.76773	-1.76487
-1.76475	-1.76184	-1.78166	-1.77865	-1.77568	-1.77277	-1.7699	-1.78477	-1.78171
-1.76402	-1.76113	-1.78093	-1.77793	-1.77498	-1.77207	-1.7692	-1.784	-1.78092

-0.63544	-0.63442	-0.6677	-0.66659	-0.66551	-0.69903	-0.6979	-0.69679	-0.6957
-0.63567	-0.63464	-0.66795	-0.66684	-0.66575	-0.6993	-0.69817	-0.69706	-0.69596
-0.63591	-0.63486	-0.6682	-0.66709	-0.666	-0.69957	-0.69844	-0.69732	-0.69622
-0.60276	-0.60181	-0.66847	-0.66735	-0.66626	-0.6652	-0.66417	-0.69757	-0.69647
-0.60297	-0.60202	-0.66875	-0.66763	-0.66652	-0.66545	-0.66439	-0.69781	-0.69672
-0.60316	-0.60223	-0.63456	-0.63352	-0.63249	-0.6657	-0.66463	-0.69806	-0.69697
-0.60335	-0.60244	-0.63482	-0.63378	-0.63274	-0.66597	-0.66487	-0.69832	-0.69721
-0.60354	-0.60264	-0.63507	-0.63403	-0.63299	-0.63195	-0.63091	-0.66408	-0.66307
-0.60373	-0.60284	-0.63532	-0.63429	-0.63325	-0.63221	-0.63117	-0.66436	-0.66332
-0.60392	-0.60303	-0.63556	-0.63454	-0.6335	-0.63247	-0.63143	-0.66464	-0.66358
-0.60409	-0.60321	-0.63579	-0.63477	-0.63374	-0.63272	-0.63169	-0.66492	-0.66384
-0.60425	-0.60339	-0.63601	-0.635	-0.63398	-0.63296	-0.63195	-0.66521	-0.66411
-0.60443	-0.60356	-0.60268	-0.60178	-0.60088	-0.6332	-0.63219	-0.63116	-0.63013
-0.60461	-0.60372	-0.60284	-0.60196	-0.60108	-0.63344	-0.63242	-0.6314	-0.63037
-0.6048	-0.60391	-0.60302	-0.60215	-0.60128	-0.63368	-0.63266	-0.63164	-0.63061
-0.60501	-0.6041	-0.60321	-0.60235	-0.60148	-0.63393	-0.63291	-0.63189	-0.63086
-0.6052	-0.60429	-0.60341	-0.60254	-0.60168	-0.63417	-0.63315	-0.63213	-0.63111
-0.60539	-0.60449	-0.6036	-0.60273	-0.60187	-0.63441	-0.6334	-0.63238	-0.63135
-0.60559	-0.60468	-0.6038	-0.60293	-0.60206	-0.60118	-0.60029	-0.63262	-0.63159
-0.60581	-0.60489	-0.60399	-0.60312	-0.60225	-0.60138	-0.60049	-0.63287	-0.63183
-0.60604	-0.6051	-0.60419	-0.60331	-0.60245	-0.60157	-0.6007	-0.63312	-0.63208
-0.60626	-0.60532	-0.6044	-0.60351	-0.60264	-0.60177	-0.6009	-0.63336	-0.63232
-0.60648	-0.60553	-0.6046	-0.60371	-0.60284	-0.60197	-0.60109	-0.6336	-0.63256
-0.60671	-0.60575	-0.60481	-0.60392	-0.60304	-0.60216	-0.60129	-0.60039	-0.59946
-0.60694	-0.60597	-0.60502	-0.60412	-0.60324	-0.60236	-0.60148	-0.60058	-0.59968
-0.60718	-0.60619	-0.60524	-0.60433	-0.60345	-0.60256	-0.60167	-0.60078	-0.59989
-0.60742	-0.60642	-0.60546	-0.60455	-0.60366	-0.60277	-0.60187	-0.60098	-0.60009
-0.60766	-0.60666	-0.6057	-0.60478	-0.60388	-0.60298	-0.60208	-0.60118	-0.60029
-0.60792	-0.60692	-0.60594	-0.60501	-0.60409	-0.60318	-0.60228	-0.60138	-0.60048
-0.60817	-0.60718	-0.6062	-0.60524	-0.60431	-0.60339	-0.60249	-0.60159	-0.60068
-0.60842	-0.60743	-0.60645	-0.60548	-0.60453	-0.60361	-0.60271	-0.6018	-0.6009
-0.60865	-0.60767	-0.6067	-0.60572	-0.60475	-0.60382	-0.60293	-0.60203	-0.60113
-0.60889	-0.60792	-0.60695	-0.60596	-0.60498	-0.60405	-0.60315	-0.60224	-0.60135
-0.60915	-0.60818	-0.6072	-0.60621	-0.60523	-0.60428	-0.60336	-0.60246	-0.60156
-0.6094	-0.60843	-0.60745	-0.60646	-0.60547	-0.60452	-0.60359	-0.60267	-0.60178
-0.60964	-0.60867	-0.60769	-0.6067	-0.60571	-0.60475	-0.60381	-0.60289	-0.60198
-0.60988	-0.60891	-0.60794	-0.60694	-0.60594	-0.60498	-0.60403	-0.6031	-0.60219
-0.61013	-0.60916	-0.60819	-0.60719	-0.6062	-0.60522	-0.60426	-0.60332	-0.60241
-0.61039	-0.60941	-0.60844	-0.60745	-0.60647	-0.60547	-0.60449	-0.60354	-0.60263
-0.61066	-0.60968	-0.6087	-0.60772	-0.60673	-0.60573	-0.60472	-0.60377	-0.60285
-0.61095	-0.60996	-0.60898	-0.60798	-0.60698	-0.60598	-0.60497	-0.60401	-0.60308
-0.61124	-0.61024	-0.60925	-0.60825	-0.60724	-0.60623	-0.60523	-0.60426	-0.60331
-0.61152	-0.61051	-0.60951	-0.60851	-0.60752	-0.60651	-0.6055	-0.60451	-0.60354
-0.61181	-0.61078	-0.60977	-0.60878	-0.60779	-0.60678	-0.60576	-0.60476	-0.60377
-0.61208	-0.61105	-0.61004	-0.60905	-0.60806	-0.60705	-0.60603	-0.60501	-0.60401
-0.61236	-0.61133	-0.61032	-0.60933	-0.60834	-0.60732	-0.60629	-0.60527	-0.60426
-0.61266	-0.61162	-0.61061	-0.60961	-0.60861	-0.60759	-0.60656	-0.60554	-0.60452
-0.61297	-0.61193	-0.61092	-0.6099	-0.60888	-0.60786	-0.60684	-0.60581	-0.60479
-0.61331	-0.61224	-0.61121	-0.61018	-0.60916	-0.60814	-0.60712	-0.6061	-0.60506
-0.61368	-0.61256	-0.61149	-0.61045	-0.60944	-0.60842	-0.60741	-0.60639	-0.60535

-0.61407	-0.61289	-0.61178	-0.61073	-0.60972	-0.60871	-0.6077	-0.60668	-0.60565
-0.61446	-0.61322	-0.61207	-0.61102	-0.61001	-0.609	-0.60799	-0.60698	-0.60595
-0.61489	-0.61358	-0.61238	-0.61132	-0.61031	-0.60929	-0.60829	-0.60727	-0.60626
-0.61535	-0.61396	-0.61271	-0.61162	-0.61061	-0.60959	-0.60859	-0.60758	-0.60656
-0.61585	-0.61438	-0.61306	-0.61194	-0.61091	-0.60989	-0.6089	-0.60789	-0.60686
-0.56091	-0.55949	-0.61344	-0.61228	-0.61122	-0.61019	-0.6092	-0.60819	-0.60717
-0.56149	-0.56001	-0.61385	-0.61264	-0.61154	-0.61049	-0.60949	-0.60848	-0.60747
-0.56206	-0.56053	-0.55913	-0.5579	-0.55677	-0.5557	-0.55468	-0.60877	-0.60777
-0.56264	-0.56107	-0.55962	-0.55834	-0.55717	-0.55608	-0.55506	-0.60907	-0.60807
-0.56323	-0.56163	-0.56014	-0.55881	-0.55758	-0.55647	-0.55544	-0.55441	-0.55338
-0.56384	-0.56222	-0.5607	-0.55931	-0.55803	-0.55688	-0.55583	-0.55478	-0.55376
-0.56446	-0.56283	-0.56129	-0.55984	-0.5585	-0.55731	-0.55622	-0.55516	-0.55414
-0.56507	-0.56344	-0.56188	-0.56038	-0.55898	-0.55774	-0.55662	-0.55554	-0.55452
-0.56568	-0.56406	-0.56249	-0.56093	-0.55947	-0.55819	-0.55703	-0.55593	-0.5549
-0.56632	-0.56469	-0.56309	-0.5615	-0.56	-0.55867	-0.55747	-0.55633	-0.55528
-0.56698	-0.56532	-0.5637	-0.56209	-0.56057	-0.55918	-0.55791	-0.55674	-0.55568
-0.56765	-0.56596	-0.56432	-0.5627	-0.56116	-0.55973	-0.55839	-0.55718	-0.55609
-0.56832	-0.56663	-0.56497	-0.56335	-0.56179	-0.5603	-0.5589	-0.55764	-0.55652
-0.56899	-0.56728	-0.56562	-0.56399	-0.56242	-0.56089	-0.55942	-0.55812	-0.55696
-0.56964	-0.56792	-0.56624	-0.56461	-0.56302	-0.56145	-0.55995	-0.5586	-0.55739
-0.57028	-0.56856	-0.56687	-0.56522	-0.56361	-0.56203	-0.5605	-0.55911	-0.55784
-0.49463	-0.49307	-0.5675	-0.56585	-0.56423	-0.56264	-0.56109	-0.55966	-0.55833
-0.49524	-0.49365	-0.56814	-0.56649	-0.56487	-0.56327	-0.56172	-0.56024	-0.55884
-0.49585	-0.49426	-0.56881	-0.56714	-0.56551	-0.5639	-0.56233	-0.56082	-0.55936
-0.49648	-0.4949	-0.56952	-0.56781	-0.56614	-0.56451	-0.56293	-0.56138	-0.55988
-0.45477	-0.45331	-0.49398	-0.49242	-0.49089	-0.56514	-0.56354	-0.56197	-0.56043
-0.45532	-0.45387	-0.49459	-0.49302	-0.49147	-0.56578	-0.56417	-0.56259	-0.56104
-0.45587	-0.45443	-0.49521	-0.49362	-0.49206	-0.56643	-0.56481	-0.56322	-0.56165
-0.45643	-0.45499	-0.49582	-0.49425	-0.49269	-0.49118	-0.48971	-0.56384	-0.56226
-0.457	-0.45555	-0.49644	-0.49488	-0.49333	-0.4918	-0.4903	-0.56448	-0.56288
-0.45755	-0.4561	-0.45465	-0.4532	-0.45175	-0.49243	-0.4909	-0.56514	-0.56351
-0.4581	-0.45664	-0.45519	-0.45376	-0.45233	-0.49305	-0.49151	-0.5658	-0.56417
-0.45865	-0.45719	-0.45574	-0.45432	-0.4529	-0.45145	-0.44999	-0.49061	-0.48913
-0.45922	-0.45776	-0.45631	-0.45489	-0.45346	-0.452	-0.45053	-0.4912	-0.48972
-0.4107	-0.4095	-0.45688	-0.45546	-0.45403	-0.45256	-0.45108	-0.44965	-0.44826
-0.41117	-0.40997	-0.45746	-0.45604	-0.45461	-0.45315	-0.45167	-0.45023	-0.44881
-0.41163	-0.41044	-0.45804	-0.45662	-0.4552	-0.45374	-0.45226	-0.45081	-0.44938
-0.41207	-0.4109	-0.45861	-0.45719	-0.45578	-0.45432	-0.45285	-0.4514	-0.44996
-0.41251	-0.41134	-0.45918	-0.45776	-0.45635	-0.4549	-0.45344	-0.45199	-0.45054
-0.41298	-0.41181	-0.45976	-0.45834	-0.45693	-0.45548	-0.45403	-0.45258	-0.45113
-0.41348	-0.41228	-0.46036	-0.45892	-0.45751	-0.45606	-0.45461	-0.45317	-0.45173
-0.41398	-0.41277	-0.41159	-0.41045	-0.40931	-0.45664	-0.4552	-0.45375	-0.45232
-0.41449	-0.41326	-0.41206	-0.41092	-0.40979	-0.45723	-0.45577	-0.45434	-0.45291
-0.37092	-0.36977	-0.41255	-0.4114	-0.41026	-0.45781	-0.45635	-0.45492	-0.4535
-0.37141	-0.37024	-0.41303	-0.41188	-0.41074	-0.45839	-0.45693	-0.45549	-0.45408
-0.37191	-0.37072	-0.41353	-0.41236	-0.41121	-0.45897	-0.4575	-0.45607	-0.45466
-0.37241	-0.37121	-0.41403	-0.41285	-0.41168	-0.41048	-0.40928	-0.45664	-0.45524
-0.37291	-0.37171	-0.41455	-0.41334	-0.41215	-0.41095	-0.40975	-0.45722	-0.45582
-0.37341	-0.37221	-0.37103	-0.36989	-0.36877	-0.41142	-0.41022	-0.4578	-0.4564
-0.37392	-0.37271	-0.37153	-0.37037	-0.36923	-0.4119	-0.41069	-0.45838	-0.45696

-0.37442	-0.37322	-0.37203	-0.37086	-0.3697	-0.41239	-0.41116	-0.40997	-0.40881
-0.37492	-0.37372	-0.37253	-0.37136	-0.37019	-0.41288	-0.41163	-0.41043	-0.40928
-0.37541	-0.37422	-0.37304	-0.37186	-0.37068	-0.41338	-0.41211	-0.4109	-0.40974
-0.3759	-0.37472	-0.37354	-0.37236	-0.37118	-0.36998	-0.36877	-0.41138	-0.41021
-0.37638	-0.37522	-0.37405	-0.37287	-0.37169	-0.37047	-0.36926	-0.41188	-0.41069
-0.37688	-0.37571	-0.37455	-0.37337	-0.37219	-0.37098	-0.36977	-0.3686	-0.36747
-0.37738	-0.37621	-0.37504	-0.37387	-0.37269	-0.37149	-0.37029	-0.36911	-0.36795
-0.33113	-0.33011	-0.37553	-0.37436	-0.37319	-0.372	-0.37081	-0.36962	-0.36845
-0.33157	-0.33053	-0.37602	-0.37486	-0.37369	-0.37251	-0.37131	-0.37013	-0.36895
-0.33199	-0.33095	-0.32991	-0.32889	-0.32786	-0.37301	-0.37181	-0.37063	-0.36945
-0.33239	-0.33135	-0.33032	-0.32931	-0.32828	-0.3735	-0.37231	-0.37112	-0.36994
-0.33278	-0.33176	-0.33074	-0.32972	-0.32871	-0.37399	-0.37281	-0.37162	-0.37043
-0.33319	-0.33218	-0.33117	-0.33015	-0.32912	-0.37449	-0.37331	-0.37212	-0.37093
-0.33361	-0.3326	-0.3316	-0.33058	-0.32955	-0.375	-0.37382	-0.37263	-0.37143
-0.33403	-0.33301	-0.33201	-0.331	-0.32998	-0.32895	-0.32791	-0.37315	-0.37195
-0.33446	-0.33342	-0.3324	-0.33142	-0.33044	-0.32941	-0.32837	-0.37367	-0.37247
-0.33492	-0.33384	-0.3328	-0.33183	-0.33086	-0.32986	-0.32882	-0.32777	-0.32669
-0.3354	-0.33427	-0.3332	-0.33222	-0.33126	-0.33028	-0.32928	-0.32825	-0.32719
-0.33591	-0.33473	-0.33362	-0.33262	-0.33165	-0.33068	-0.32971	-0.32871	-0.32767
-0.33645	-0.33523	-0.33408	-0.33304	-0.33204	-0.33107	-0.33011	-0.32912	-0.32811
-0.337	-0.33575	-0.33456	-0.33348	-0.33245	-0.33146	-0.3305	-0.32952	-0.32853
-0.28829	-0.28727	-0.33508	-0.33396	-0.33289	-0.33188	-0.3309	-0.32992	-0.32895
-0.2887	-0.2877	-0.33562	-0.33447	-0.33336	-0.33231	-0.33132	-0.33033	-0.32935
-0.1946	-0.19395	-0.33613	-0.33496	-0.33382	-0.33275	-0.33174	-0.33075	-0.32978
-0.19486	-0.1942	-0.33662	-0.33543	-0.33427	-0.3332	-0.33218	-0.33119	-0.33023
-0.19513	-0.19446	-0.33711	-0.33591	-0.33474	-0.33365	-0.33261	-0.33162	-0.33066
-0.19542	-0.19474	-0.33763	-0.33641	-0.33522	-0.3341	-0.33303	-0.33202	-0.33106
-0.19571	-0.19502	-0.19434	-0.19368	-0.19302	-0.33457	-0.33346	-0.33243	-0.33146
-0.19598	-0.1953	-0.19463	-0.19396	-0.1933	-0.33509	-0.33395	-0.33288	-0.33188
-0.19624	-0.19558	-0.19492	-0.19425	-0.19359	-0.33563	-0.33446	-0.33336	-0.33232
-0.10101	-0.10065	-0.19519	-0.19453	-0.19387	-0.33616	-0.33497	-0.33384	-0.33277
-0.10116	-0.1008	-0.19546	-0.1948	-0.19414	-0.33669	-0.33549	-0.33434	-0.33324
-0.10131	-0.10095	-0.19575	-0.19509	-0.19443	-0.19376	-0.1931	-0.33486	-0.33371
-0.10146	-0.1011	-0.19605	-0.19539	-0.19473	-0.19407	-0.1934	-0.3354	-0.3342
-0.10161	-0.10126	-0.1009	-0.10055	-0.1002	-0.19436	-0.19369	-0.33594	-0.3347
-0.10175	-0.1014	-0.10105	-0.10069	-0.10034	-0.19464	-0.19396	-0.33646	-0.33523
-0.1019	-0.10154	-0.10119	-0.10083	-0.10048	-0.19491	-0.19424	-0.33699	-0.33577
-0.10205	-0.1017	-0.10134	-0.10098	-0.10063	-0.19521	-0.19453	-0.19385	-0.19315
-0.10222	-0.10186	-0.1015	-0.10114	-0.10078	-0.19551	-0.19484	-0.19415	-0.19344
-0.10237	-0.10201	-0.10164	-0.10129	-0.10093	-0.10057	-0.10022	-0.19444	-0.19373
-0.10251	-0.10215	-0.10179	-0.10143	-0.10107	-0.10072	-0.10036	-0.19473	-0.19402
-0.10266	-0.1023	-0.10194	-0.10158	-0.10122	-0.10086	-0.1005	-0.19502	-0.19433
-0.10281	-0.10245	-0.1021	-0.10174	-0.10138	-0.10102	-0.10066	-0.19533	-0.19464
-0.10297	-0.10261	-0.10225	-0.10189	-0.10153	-0.10118	-0.10082	-0.10047	-0.10011
-0.10311	-0.10276	-0.1024	-0.10204	-0.10168	-0.10132	-0.10097	-0.10061	-0.10025
-0.10326	-0.10291	-0.10255	-0.10219	-0.10183	-0.10147	-0.10111	-0.10075	-0.10039
-0.10343	-0.10307	-0.10271	-0.10235	-0.10199	-0.10163	-0.10127	-0.10091	-0.10055
-0.1036	-0.10323	-0.10287	-0.10251	-0.10216	-0.1018	-0.10143	-0.10107	-0.10071
-0.0511	-0.05092	-0.10302	-0.10266	-0.10231	-0.10195	-0.10159	-0.10123	-0.10087
-0.05115	-0.05096	-0.10317	-0.10281	-0.10246	-0.1021	-0.10173	-0.10137	-0.10102

-0.10332	-0.10295	-0.1026	-0.10224	-0.10188	-0.10152	-0.10117
-0.10347	-0.10309	-0.10273	-0.10237	-0.10202	-0.10166	-0.10131
-0.05093	-0.05075	-0.05057	-0.10252	-0.10217	-0.10181	-0.10145
-0.051	-0.05082	-0.05063	-0.10268	-0.10233	-0.10197	-0.10161
-0.05107	-0.05088	-0.0507	-0.10284	-0.10249	-0.10214	-0.10177
			-0.10301	-0.10265	-0.1023	-0.10194
			-0.10317	-0.10281	-0.10245	-0.1021
			-0.05066	-0.0505	-0.1026	-0.10225
			-0.05072	-0.05054	-0.10274	-0.10239
					-0.10288	-0.10253
					-0.10304	-0.10268
					-0.1032	-0.10282
					-0.05057	-0.05039
					-0.05064	-0.05045

							-0.05042	-0.05029
							-0.05032	-0.0502
							-0.15489	-0.15426
							-0.15449	-0.15391
					-0.0503	-0.05017	-0.15412	-0.15357
					-0.05021	-0.05008	-0.15379	-0.15324
					-0.05013	-0.05	-0.15347	-0.15292
					-0.1544	-0.15376	-0.30828	-0.30732
					-0.15405	-0.15344	-0.30773	-0.30675
		-0.10606	-0.10543	-0.10483	-0.3092	-0.30819	-0.35054	-0.34936
		-0.10573	-0.10513	-0.10456	-0.30866	-0.30765	-0.3499	-0.34872
		-0.15505	-0.15436	-0.15371	-0.35164	-0.35045	-0.3944	-0.39306
		-0.15468	-0.15401	-0.15338	-0.351	-0.3498	-0.3937	-0.39237
-0.10602	-0.10541	-0.31013	-0.30906	-0.30803	-0.35035	-0.34916	-0.48641	-0.48479
-0.10571	-0.10511	-0.30956	-0.3085	-0.30748	-0.34971	-0.34852	-0.48557	-0.48397
-0.1054	-0.10483	-0.309	-0.30795	-0.30693	-0.34906	-0.34789	-0.48474	-0.48317
-0.15464	-0.15397	-0.35206	-0.35082	-0.3496	-0.3936	-0.39228	-0.58111	-0.57927
-0.15429	-0.15363	-0.35139	-0.35016	-0.34895	-0.3929	-0.3916	-0.58011	-0.5783
-0.30941	-0.30835	-0.35073	-0.34951	-0.34831	-0.43909	-0.43766	-0.72747	-0.72524
-0.30884	-0.30778	-0.35006	-0.34887	-0.3477	-0.43838	-0.43696	-0.72625	-0.724
-0.35189	-0.35063	-0.39486	-0.39354	-0.39227	-0.57511	-0.5732	-0.72507	-0.72281
-0.35123	-0.34997	-0.39416	-0.39289	-0.39166	-0.57417	-0.57229	-0.72396	-0.72167
-0.35058	-0.34933	-0.44047	-0.43908	-0.43773	-0.69106	-0.68885	-0.72283	-0.72054
-0.34997	-0.34875	-0.43977	-0.43838	-0.43702	-0.6899	-0.6877	-0.72164	-0.71936
-0.34937	-0.34817	-0.43908	-0.43768	-0.43631	-0.68873	-0.68652	-0.72043	-0.71817
-0.39425	-0.39293	-0.65223	-0.65015	-0.64809	-0.7238	-0.7215	-0.76173	-0.75939
-0.39353	-0.39224	-0.6511	-0.64902	-0.64697	-0.72257	-0.72029	-0.76048	-0.75815
-0.60433	-0.60242	-0.69178	-0.68956	-0.68736	-0.72134	-0.71909	-0.75923	-0.7569
-0.60328	-0.60139	-0.69058	-0.68836	-0.68617	-0.72012	-0.71787	-0.75798	-0.75566
-0.65187	-0.6498	-0.68939	-0.6872	-0.68501	-0.7614	-0.75907	-0.78797	-0.78541
-0.65078	-0.64872	-0.68823	-0.68605	-0.68389	-0.76019	-0.75786	-0.78661	-0.78407
-0.64971	-0.64766	-0.6871	-0.68494	-0.6828	-0.759	-0.75667	-0.7853	-0.78279
-0.69042	-0.68822	-0.7223	-0.72004	-0.7178	-0.75788	-0.75555	-1.08166	-1.078

-0.68935	-0.68716	-0.72121	-0.71896	-0.71673	-0.75676	-0.75444	-1.07988	-1.07628
-0.68821	-0.68604	-0.76255	-0.76022	-0.75789	-0.78664	-0.7841	-1.07803	-1.07447
-0.68703	-0.68488	-0.76132	-0.75898	-0.75665	-0.78528	-0.78276	-1.07613	-1.0726
-0.68588	-0.68375	-0.76009	-0.75776	-0.75543	-1.08154	-1.07787	-1.1106	-1.10673
-0.68476	-0.68263	-0.75888	-0.75655	-0.75422	-1.07964	-1.07603	-1.10859	-1.10473
-0.72689	-0.72467	-0.91009	-0.90713	-0.9042	-1.0778	-1.07422	-1.19726	-1.19317
-0.72581	-0.72359	-0.90863	-0.90568	-0.90278	-1.07603	-1.07248	-1.19526	-1.19117
-0.72473	-0.7225	-0.90717	-0.90425	-0.90138	-1.07427	-1.07073	-1.19325	-1.18919
-0.75889	-0.75655	-1.08339	-1.07967	-1.07603	-1.10859	-1.10472	-1.2521	-1.24792
-0.75764	-0.7553	-1.08143	-1.07776	-1.07416	-1.10655	-1.10269	-1.24987	-1.24574
-0.90854	-0.90557	-1.07951	-1.07587	-1.07231	-1.19502	-1.19093	-1.27542	-1.27129
-0.90703	-0.90406	-1.07761	-1.074	-1.07047	-1.19287	-1.1888	-1.27322	-1.26921
-0.97512	-0.97176	-1.17356	-1.16947	-1.16545	-1.25168	-1.2475	-1.33268	-1.32856
-0.97348	-0.97017	-1.17159	-1.16752	-1.16351	-1.24965	-1.24552	-1.33062	-1.32655
-1.17787	-1.17371	-1.19916	-1.19502	-1.19095	-1.31032	-1.30607	-1.32859	-1.32456
-1.1758	-1.17165	-1.19707	-1.19294	-1.1889	-1.30816	-1.304	-1.32653	-1.3225
-1.17373	-1.1696	-1.19498	-1.19088	-1.18686	-1.30602	-1.30196	-1.3245	-1.32046
-1.17173	-1.16762	-1.31675	-1.31237	-1.3081	-1.33063	-1.32657	-1.38295	-1.37874
-1.16976	-1.16567	-1.31463	-1.3103	-1.30607	-1.32861	-1.3246	-1.38091	-1.37672
-1.26236	-1.25802	-1.31246	-1.30819	-1.30403	-1.35487	-1.35078	-1.37879	-1.37461
-1.26014	-1.25582	-1.31024	-1.30607	-1.30199	-1.35277	-1.34866	-1.37661	-1.37244
-1.31679	-1.31238	-1.33699	-1.3328	-1.32871	-1.3829	-1.37868	-1.37448	-1.37031
-1.31471	-1.31032	-1.33491	-1.33077	-1.32673	-1.38085	-1.37662	-1.37243	-1.36828
-1.31266	-1.3083	-1.33287	-1.32875	-1.32473	-1.37877	-1.37455	-1.37037	-1.36623
-1.31061	-1.30629	-1.36364	-1.35939	-1.35523	-1.3766	-1.3724	-1.393	-1.38882
-1.30855	-1.3043	-1.36158	-1.3573	-1.35309	-1.37442	-1.37024	-1.39083	-1.38666
-1.33531	-1.33107	-1.38521	-1.38087	-1.37657	-1.37234	-1.36816	-1.40402	-1.39985
-1.33316	-1.32904	-1.3832	-1.37888	-1.37457	-1.37032	-1.36612	-1.40196	-1.39783
-1.36392	-1.35973	-1.38115	-1.37685	-1.37256	-1.39309	-1.38882	-1.42208	-1.41787
-1.36195	-1.35772	-1.37907	-1.37481	-1.37056	-1.39106	-1.38674	-1.41992	-1.41571
-1.35999	-1.35569	-1.37697	-1.37273	-1.36851	-1.42658	-1.42214	-1.44426	-1.43997
-1.358	-1.35363	-1.37482	-1.37056	-1.36635	-1.42441	-1.42007	-1.44221	-1.43793
-1.35597	-1.35156	-1.37268	-1.3684	-1.36421	-1.42226	-1.41803	-1.44019	-1.43592
-1.35383	-1.34947	-1.43324	-1.42882	-1.42447	-1.4202	-1.416	-1.54614	-1.54134
-1.35164	-1.34738	-1.43114	-1.42676	-1.42243	-1.41818	-1.41399	-1.54388	-1.5391
-1.39069	-1.3864	-1.42906	-1.42472	-1.42042	-1.49522	-1.4908	-1.56347	-1.5587
-1.38863	-1.38437	-1.42701	-1.4227	-1.41843	-1.49311	-1.48865	-1.56117	-1.55647
-1.43366	-1.42928	-1.42496	-1.42067	-1.41641	-1.56857	-1.5637	-1.55893	-1.55427
-1.4316	-1.42724	-1.42292	-1.41861	-1.41434	-1.56626	-1.56148	-1.55676	-1.55212
-1.42955	-1.42519	-1.52279	-1.51822	-1.51371	-1.56397	-1.55929	-1.55462	-1.54997
-1.42749	-1.42311	-1.52056	-1.51603	-1.51158	-1.56176	-1.55712	-1.55247	-1.54782
-1.42541	-1.42103	-1.51834	-1.51387	-1.50948	-1.55959	-1.55496	-1.55032	-1.54567
-1.4233	-1.41896	-1.57161	-1.56682	-1.56211	-1.55743	-1.5528	-1.56989	-1.56515
-1.42117	-1.41692	-1.56937	-1.56464	-1.55996	-1.55529	-1.55063	-1.56766	-1.56289
-1.52079	-1.51635	-1.56716	-1.56248	-1.55783	-1.57494	-1.57016	-1.5654	-1.56065
-1.51867	-1.51427	-1.56497	-1.56034	-1.55572	-1.57274	-1.56786	-1.56309	-1.55844
-1.57225	-1.5675	-1.56282	-1.5582	-1.55358	-1.57054	-1.56561	-1.56086	-1.55627
-1.57005	-1.56535	-1.56069	-1.55605	-1.55141	-1.56835	-1.56345	-1.55873	-1.55417
-1.56788	-1.56321	-1.58043	-1.57569	-1.57094	-1.56614	-1.56131	-1.57942	-1.57484
-1.56571	-1.56107	-1.57826	-1.57345	-1.56863	-1.56386	-1.55914	-1.5773	-1.57278

-1.56356	-1.55893	-1.57608	-1.5712	-1.56631	-1.56159	-1.55699	-1.57521	-1.57074
-1.58331	-1.57863	-1.57391	-1.56901	-1.5641	-1.55941	-1.55487	-1.57312	-1.56869
-1.58116	-1.57646	-1.57173	-1.56685	-1.56197	-1.55731	-1.5528	-1.57106	-1.56666
-1.57901	-1.5743	-1.56957	-1.56471	-1.55988	-1.55524	-1.55076	-1.56904	-1.5647
-1.57685	-1.57214	-1.56742	-1.5626	-1.55782	-1.55323	-1.54878	-1.56708	-1.56281
-1.57469	-1.56997	-1.56527	-1.5605	-1.55578	-1.57392	-1.56944	-1.56515	-1.561
-1.57252	-1.56781	-1.56312	-1.5584	-1.55372	-1.57186	-1.5674	-1.56322	-1.55931
-1.57035	-1.56566	-1.561	-1.55632	-1.55169	-1.56983	-1.56539	-1.56134	-1.55766
-1.5682	-1.56354	-1.55892	-1.55428	-1.54968	-1.56783	-1.56343	-1.55949	-1.55601
-1.56605	-1.56145	-1.55688	-1.55226	-1.5477	-1.56588	-1.56155	-1.55771	-1.5544
-1.56392	-1.55936	-1.57757	-1.57292	-1.56834	-1.56398	-1.55982	-1.55611	-1.55289
-1.56179	-1.55728	-1.57551	-1.57087	-1.56632	-1.56213	-1.55822	-1.55466	-1.55147
-1.55969	-1.55522	-1.57347	-1.56885	-1.56435	-1.56032	-1.55665	-1.55324	-1.55013
-1.55762	-1.55318	-1.57145	-1.56687	-1.56242	-1.55853	-1.55507	-1.55186	-1.54891
-1.57831	-1.57385	-1.56945	-1.56494	-1.56059	-1.55685	-1.55356	-1.56986	-1.56704
-1.57625	-1.5718	-1.56745	-1.56309	-1.55893	-1.55532	-1.55213	-1.56852	-1.56578
-1.57423	-1.56977	-1.56547	-1.56129	-1.55735	-1.55386	-1.55075	-1.5672	-1.56453
-1.57225	-1.56778	-1.56352	-1.55953	-1.5558	-1.57183	-1.56879	-1.58591	-1.58326
-1.5703	-1.56582	-1.56162	-1.55781	-1.55431	-1.57046	-1.5675	-1.58469	-1.58207
-1.56837	-1.56394	-1.57929	-1.57561	-1.57227	-1.56914	-1.56626	-1.58349	-1.5809
-1.56647	-1.56213	-1.57758	-1.57408	-1.57092	-1.5679	-1.56506	-1.58232	-1.57974
-1.58412	-1.57988	-1.57599	-1.57263	-1.5696	-1.56666	-1.56388	-1.58117	-1.57859
-1.5823	-1.57824	-1.57451	-1.57126	-1.5683	-1.56543	-1.5627	-1.58002	-1.57745
-1.58051	-1.57664	-1.57308	-1.56992	-1.56703	-1.58424	-1.58152	-1.57887	-1.57631
-1.57869	-1.57501	-1.57164	-1.5686	-1.56579	-1.58306	-1.58037	-1.57774	-1.57518
-1.57693	-1.57344	-1.57025	-1.56732	-1.56458	-1.58189	-1.57923	-1.57661	-1.57405
-1.57538	-1.57204	-1.56897	-1.56611	-1.56341	-1.58076	-1.57813	-1.57553	-1.57295
-1.57398	-1.57076	-1.56777	-1.56494	-1.56226	-1.57964	-1.57706	-1.57447	-1.57186
-1.57257	-1.56947	-1.58668	-1.58385	-1.58115	-1.57852	-1.57596	-1.60009	-1.59742
-1.57117	-1.56819	-1.58547	-1.5827	-1.58002	-1.5774	-1.57483	-1.59893	-1.5963
-1.56982	-1.56694	-1.58429	-1.58156	-1.57891	-1.58592	-1.58327	-1.61814	-1.61552
-1.56856	-1.56573	-1.58311	-1.58045	-1.57785	-1.58483	-1.58217	-1.61703	-1.61446
-1.56733	-1.56454	-1.58196	-1.57933	-1.57677	-1.58374	-1.58108	-1.61595	-1.61341
-1.58627	-1.58349	-1.58081	-1.57819	-1.57563	-1.59975	-1.59714	-1.61488	-1.61234
-1.58508	-1.58234	-1.57968	-1.57706	-1.57448	-1.5986	-1.59606	-1.61382	-1.61128
-1.58393	-1.58123	-1.60546	-1.60274	-1.60006	-1.6178	-1.61526	-1.63514	-1.63254
-1.58281	-1.58015	-1.60436	-1.60163	-1.59895	-1.61668	-1.61415	-1.63402	-1.63145
-1.60869	-1.60595	-1.62362	-1.62089	-1.61819	-1.61559	-1.61306	-1.63292	-1.63039
-1.6076	-1.60483	-1.62248	-1.61977	-1.61712	-1.61453	-1.612	-1.63184	-1.62936
-1.62691	-1.62411	-1.62135	-1.61867	-1.61605	-1.63592	-1.63332	-1.63079	-1.62834
-1.62577	-1.623	-1.62027	-1.6176	-1.61498	-1.63484	-1.63229	-1.62978	-1.62731
-1.62462	-1.62189	-1.6192	-1.61653	-1.61392	-1.63377	-1.63127	-1.62878	-1.62629
-1.62351	-1.62077	-1.61809	-1.61546	-1.61288	-1.63274	-1.63024	-1.62775	-1.62528
-1.62241	-1.61965	-1.61696	-1.61438	-1.61186	-1.63171	-1.62919	-1.62672	-1.62428
-1.62128	-1.61854	-1.63839	-1.63576	-1.6332	-1.63066	-1.62815	-1.6257	-1.62331
-1.62014	-1.61744	-1.63729	-1.63467	-1.63212	-1.62959	-1.6271	-1.62469	-1.62235
-1.61902	-1.61636	-1.63621	-1.6336	-1.63105	-1.62854	-1.62609	-1.6237	-1.6214
-1.61795	-1.6153	-1.63514	-1.63253	-1.62999	-1.62752	-1.62512	-1.62276	-1.62045
-1.63946	-1.63674	-1.63408	-1.63149	-1.62897	-1.62653	-1.62417	-1.62183	-1.61951
-1.6383	-1.63565	-1.63307	-1.63052	-1.62802	-1.62559	-1.62321	-1.62088	-1.61859

-1.63716	-1.63458	-1.63207	-1.62957	-1.62711	-1.62468	-1.62228	-1.61995	-1.61769
-1.6361	-1.63354	-1.63105	-1.6286	-1.62618	-1.62378	-1.6214	-1.61907	-1.61678
-1.6351	-1.63253	-1.63003	-1.62762	-1.62525	-1.62289	-1.62054	-1.61821	-1.61589
-1.63408	-1.63153	-1.62905	-1.62666	-1.62432	-1.62199	-1.61967	-1.65776	-1.65536
-1.63304	-1.63055	-1.62812	-1.62572	-1.62338	-1.62107	-1.61879	-1.65687	-1.6545
-1.63204	-1.62958	-1.62717	-1.62479	-1.62245	-1.66062	-1.65829	-1.65596	-1.65363
-1.6311	-1.62861	-1.62619	-1.62385	-1.62155	-1.65969	-1.65734	-1.65503	-1.65274
-1.63018	-1.62767	-1.62524	-1.62292	-1.62066	-1.65875	-1.6564	-1.6541	-1.65186
-1.62925	-1.62678	-1.66492	-1.66251	-1.66015	-1.6578	-1.65547	-1.65319	-1.65095
-1.62831	-1.6259	-1.66403	-1.66159	-1.65919	-1.65685	-1.65456	-1.65229	-1.65005
-1.66798	-1.66554	-1.66313	-1.6607	-1.65829	-1.65594	-1.65364	-1.69552	-1.69303
-1.66696	-1.66458	-1.66222	-1.65982	-1.65743	-1.65506	-1.65273	-1.69448	-1.69207
-1.666	-1.66363	-1.66129	-1.65892	-1.65656	-1.6542	-1.65186	-1.69351	-1.69113
-1.6651	-1.66269	-1.66032	-1.65798	-1.65567	-1.65335	-1.65104	-1.69262	-1.69023
-1.66421	-1.66177	-1.65938	-1.65706	-1.65479	-1.67367	-1.67121	-1.69173	-1.68931
-1.66335	-1.6609	-1.6585	-1.65619	-1.65392	-1.67275	-1.67033	-1.69082	-1.68836
-1.66249	-1.66005	-1.65764	-1.65532	-1.65305	-1.67183	-1.66945	-1.6899	-1.68741
-1.66161	-1.65918	-1.65679	-1.65445	-1.65216	-1.67089	-1.66854	-1.68897	-1.68649
-1.66071	-1.65832	-1.65594	-1.65358	-1.65127	-1.66994	-1.66761	-1.68803	-1.68557
-1.65978	-1.65742	-1.67647	-1.67393	-1.67143	-1.69193	-1.68949	-1.72755	-1.72486
-1.65881	-1.65649	-1.67552	-1.67302	-1.67054	-1.69099	-1.6885	-1.72644	-1.72379
-1.65788	-1.65559	-1.67458	-1.67211	-1.66966	-1.69005	-1.68753	-1.72537	-1.72274
-1.65701	-1.65472	-1.67366	-1.67121	-1.66877	-1.68914	-1.68661	-1.72434	-1.7217
-1.67767	-1.67518	-1.69581	-1.69326	-1.69075	-1.68822	-1.68569	-1.72333	-1.72065
-1.67674	-1.67427	-1.69487	-1.69233	-1.68983	-1.6873	-1.68477	-1.7223	-1.71956
-1.67581	-1.67335	-1.69392	-1.6914	-1.6889	-1.68638	-1.68384	-1.72127	-1.71847
-1.67488	-1.6724	-1.69292	-1.69042	-1.68795	-1.70721	-1.70449	-1.72025	-1.71742
-1.67395	-1.67144	-1.69189	-1.68942	-1.68699	-1.70621	-1.7035	-1.71922	-1.7164
-1.69612	-1.69347	-1.69091	-1.68846	-1.68604	-1.70521	-1.70251	-1.71821	-1.71537
-1.69516	-1.69253	-1.68997	-1.68752	-1.6851	-1.70422	-1.70152	-1.71719	-1.71435
-1.69422	-1.69159	-1.68904	-1.68659	-1.68417	-1.72174	-1.71897	-1.71617	-1.71333
-1.69329	-1.69066	-1.68811	-1.68566	-1.68324	-1.72073	-1.71795	-1.71515	-1.71229
-1.69236	-1.68974	-1.68718	-1.68473	-1.68231	-1.71971	-1.71693	-1.71412	-1.71127
-1.69142	-1.68881	-1.68626	-1.6838	-1.68138	-1.71869	-1.71591	-1.71311	-1.71028
-1.69048	-1.68788	-1.68533	-1.68288	-1.68044	-1.71767	-1.71488	-1.7121	-1.7093
-1.68954	-1.68695	-1.70618	-1.70355	-1.70095	-1.71665	-1.71386	-1.74329	-1.74044
-1.68861	-1.68602	-1.7052	-1.70257	-1.69996	-1.71563	-1.71283	-1.74226	-1.73944
-1.68768	-1.6851	-1.70422	-1.70159	-1.69897	-1.74695	-1.74405	-1.78351	-1.78054
-1.68676	-1.68419	-1.70324	-1.7006	-1.69798	-1.74589	-1.74298	-1.78239	-1.77946
-1.68584	-1.68328	-1.73533	-1.73258	-1.72984	-1.74484	-1.74194	-1.78129	-1.7784
-1.68492	-1.68241	-1.73441	-1.73163	-1.72885	-1.74382	-1.74095	-1.78025	-1.77737
-1.68401	-1.68154	-1.7335	-1.73069	-1.72786	-1.74281	-1.73997	-1.77922	-1.77634
-1.71675	-1.71419	-1.75042	-1.74753	-1.74463	-1.74178	-1.73897	-1.77819	-1.7753
-1.71581	-1.71324	-1.74937	-1.74648	-1.74358	-1.74075	-1.73798	-1.77715	-1.77428
-1.71491	-1.71232	-1.74833	-1.74543	-1.74253	-1.78196	-1.77902	-1.79104	-1.78814
-1.71405	-1.71142	-1.7473	-1.74439	-1.74149	-1.78086	-1.77795	-1.78999	-1.78711
-1.7521	-1.74919	-1.74627	-1.74335	-1.74047	-1.77979	-1.77691	-1.78896	-1.78611
-1.75107	-1.74815	-1.74523	-1.74234	-1.73948	-1.77875	-1.77588	-1.78795	-1.78515
-1.75004	-1.7471	-1.74419	-1.74132	-1.73851	-1.77774	-1.77488	-1.78696	-1.78421
-1.74899	-1.74604	-1.74313	-1.7403	-1.73753	-1.77675	-1.77394	-1.78602	-1.78325

-1.74794	-1.74499	-1.74209	-1.73929	-1.73656	-1.77577	-1.77301	-1.78509	-1.78229
-1.7469	-1.74395	-1.74106	-1.73832	-1.73565	-1.77482	-1.77204	-1.7841	-1.78129
-1.74588	-1.74293	-1.74007	-1.73738	-1.73476	-1.77387	-1.77106	-1.78308	-1.78028
-1.72458	-1.72183	-1.7814	-1.77852	-1.77572	-1.78776	-1.78489	-1.78206	-1.77927
-1.72372	-1.72099	-1.7805	-1.77759	-1.77473	-1.78673	-1.78386	-1.78103	-1.77824
-1.72282	-1.72012	-1.77957	-1.77663	-1.77373	-1.78571	-1.78284	-1.78002	-1.77723
-1.72187	-1.71918	-1.77855	-1.77562	-1.77273	-1.7847	-1.78184	-1.77902	-1.77623
-1.72091	-1.71823	-1.77753	-1.77462	-1.77175	-1.78372	-1.78088	-1.77806	-1.77527
-1.71997	-1.71729	-1.77651	-1.77364	-1.77082	-1.78281	-1.77999	-1.77718	-1.77439
-1.71905	-1.71637	-1.7755	-1.77267	-1.7699	-1.78191	-1.77912	-1.77633	-1.77354
-1.73667	-1.73394	-1.77456	-1.77172	-1.76894	-1.78095	-1.77817	-1.79395	-1.79109
-1.73584	-1.73311	-1.77367	-1.77079	-1.76796	-1.77994	-1.77718	-1.79294	-1.79009
-1.73496	-1.73223	-1.75349	-1.75071	-1.74797	-1.77896	-1.77619	-1.79194	-1.78912
-1.73404	-1.73132	-1.75253	-1.74977	-1.74705	-1.77798	-1.77521	-1.79095	-1.78818
-1.75727	-1.75441	-1.76664	-1.76386	-1.76112	-1.79565	-1.7928	-1.78999	-1.78724
-1.75639	-1.75354	-1.76575	-1.76299	-1.76028	-1.79476	-1.79191	-1.78908	-1.78628
-1.7555	-1.75266	-1.76487	-1.76213	-1.75944	-1.79387	-1.79101	-1.78816	-1.78532
-1.75455	-1.75172	-1.76393	-1.7612	-1.75852	-1.79291	-1.79005	-1.78719	-1.78434
-1.75358	-1.75076	-1.76298	-1.76026	-1.75759	-1.79192	-1.78906	-1.78621	-1.78335
-1.75268	-1.74988	-1.78103	-1.77824	-1.7755	-1.79099	-1.78813	-1.78527	-1.78242
-1.75183	-1.74905	-1.78018	-1.77739	-1.77464	-1.79011	-1.78724	-1.78438	-1.78153
-1.75093	-1.74817	-1.77928	-1.7765	-1.77373	-1.78918	-1.78631	-1.79859	-1.79571
-1.74996	-1.74723	-1.77832	-1.77554	-1.77277	-1.78818	-1.78531	-1.79758	-1.79469
-1.76822	-1.76544	-1.77738	-1.7746	-1.77183	-1.80239	-1.79948	-1.79659	-1.79371
-1.76732	-1.76456	-1.77651	-1.77373	-1.77096	-1.80147	-1.79857	-1.79569	-1.79282
-1.76643	-1.76369	-1.77564	-1.77286	-1.77009	-1.80055	-1.79768	-1.79482	-1.79196
-1.78022	-1.77745	-1.79016	-1.78732	-1.7845	-1.7817	-1.77893	-1.79392	-1.79108
-1.77927	-1.77649	-1.78916	-1.78632	-1.7835	-1.78074	-1.77801	-1.79302	-1.7902
-1.79389	-1.79106	-1.78823	-1.78539	-1.78257	-1.7798	-1.77708	-1.79207	-1.78927
-1.79302	-1.79019	-1.78736	-1.78452	-1.78169	-1.77889	-1.77612	-1.79108	-1.78829
-1.79216	-1.78932	-1.78649	-1.78365	-1.78081	-1.778	-1.7752	-1.80419	-1.80136
-1.7913	-1.78846	-1.78563	-1.78279	-1.77995	-1.77714	-1.77434	-1.80329	-1.80048
-1.79041	-1.78757	-1.78473	-1.78189	-1.77906	-1.79056	-1.78772	-1.80239	-1.7996
-1.78944	-1.78659	-1.78375	-1.78091	-1.77808	-1.78957	-1.78674	-1.8014	-1.79864
-1.78846	-1.7856	-1.78275	-1.77991	-1.77708	-1.78856	-1.78574	-1.80041	-1.79767
-1.78754	-1.78469	-1.78184	-1.779	-1.77617	-1.78764	-1.78483	-1.79949	-1.79677
-1.78668	-1.78383	-1.78098	-1.77814	-1.77532	-1.78677	-1.78398	-1.79864	-1.79592
-1.78582	-1.78297	-1.79449	-1.7916	-1.78873	-1.78592	-1.78314	-1.82026	-1.81733
-1.78499	-1.78213	-1.79363	-1.79075	-1.78789	-1.78509	-1.78232	-1.81939	-1.81646
-1.79858	-1.79567	-1.79277	-1.78989	-1.78705	-1.78425	-1.78151	-1.81853	-1.81559
-1.79771	-1.7948	-1.7919	-1.78903	-1.7862	-1.78342	-1.78069	-1.81766	-1.81473
-1.79685	-1.79393	-1.79104	-1.78817	-1.78535	-1.78258	-1.77986	-1.81679	-1.81385
-1.79598	-1.79306	-1.79017	-1.78731	-1.7845	-1.78174	-1.77903	-1.79875	-1.79584
-1.79511	-1.79219	-1.7893	-1.78645	-1.78364	-1.78089	-1.77818	-1.79783	-1.79493
-1.79424	-1.79132	-1.78843	-1.78559	-1.78279	-1.80275	-1.7998	-1.79687	-1.79395
-1.79338	-1.79044	-1.78755	-1.78472	-1.78193	-1.80181	-1.79883	-1.79587	-1.79293
-1.79248	-1.78954	-1.78663	-1.78381	-1.78103	-1.80085	-1.79787	-1.7949	-1.79196
-1.79156	-1.78858	-1.78565	-1.78284	-1.78008	-1.79988	-1.79693	-1.79399	-1.79105
-1.79062	-1.78763	-1.78469	-1.78189	-1.77916	-1.79893	-1.79601	-1.79309	-1.79015
-1.78964	-1.78668	-1.78378	-1.78101	-1.77829	-1.79802	-1.79512	-1.7922	-1.78926

-1.78868	-1.78575	-1.7829	-1.78015	-1.77745	-1.79713	-1.79423	-1.79132	-1.78839
-1.78778	-1.78487	-1.78204	-1.7793	-1.77663	-1.79625	-1.79336	-1.79044	-1.78751
-1.78692	-1.78402	-1.7812	-1.77848	-1.77581	-1.79538	-1.79248	-1.78957	-1.78664
-1.78612	-1.78321	-1.80326	-1.8003	-1.79741	-1.7945	-1.7916	-1.78869	-1.78576
-1.78537	-1.78245	-1.8024	-1.79943	-1.79653	-1.79362	-1.79071	-1.7878	-1.78487
-1.78459	-1.78166	-1.80154	-1.79856	-1.79565	-1.79273	-1.78982	-1.78691	-1.78398
-1.78376	-1.78084	-1.80068	-1.79769	-1.79476	-1.79183	-1.78892	-1.78601	-1.78309
-1.78291	-1.78003	-1.79983	-1.79683	-1.79388	-1.79094	-1.78802	-1.78511	-1.7822
-1.78206	-1.77925	-1.79903	-1.79602	-1.79303	-1.79007	-1.78713	-1.78421	-1.7813
-1.78121	-1.77846	-1.79824	-1.79522	-1.7922	-1.78921	-1.78625	-1.78332	-1.78041
-1.78037	-1.77765	-1.79741	-1.79441	-1.7914	-1.78839	-1.78539	-1.79996	-1.79687
-1.77953	-1.77682	-1.79656	-1.7936	-1.79062	-1.7876	-1.78456	-1.79905	-1.79596
-1.80153	-1.7986	-1.79568	-1.79275	-1.7898	-1.78679	-1.78375	-1.79819	-1.79507
-1.80064	-1.7977	-1.79479	-1.79187	-1.78894	-1.78596	-1.78296	-1.79737	-1.79422
-1.79974	-1.79681	-1.79389	-1.79097	-1.78806	-1.78511	-1.78215	-1.79654	-1.79339
-1.79884	-1.79591	-1.79299	-1.79007	-1.78715	-1.78423	-1.7813	-1.79569	-1.79259
-1.79795	-1.79502	-1.7921	-1.78917	-1.78624	-1.78333	-1.78043	-1.79482	-1.79178
-1.78564	-1.78271	-1.7912	-1.78827	-1.78534	-1.78243	-1.77954	-1.79391	-1.79092
-1.78479	-1.78185	-1.79032	-1.78737	-1.78444	-1.78153	-1.77864	-1.79299	-1.79003
-1.78398	-1.78101	-1.78945	-1.78648	-1.78354	-1.79812	-1.79507	-1.79207	-1.78914
-1.78319	-1.78019	-1.78859	-1.78561	-1.78266	-1.79719	-1.79414	-1.79115	-1.78823
-1.78238	-1.77938	-1.7764	-1.77344	-1.7705	-1.79631	-1.79325	-1.79026	-1.78734
-1.78154	-1.77859	-1.77564	-1.7727	-1.76976	-1.79551	-1.79241	-1.78939	-1.78647
-1.78068	-1.77776	-1.77485	-1.77192	-1.769	-1.7947	-1.79159	-1.78857	-1.78564
-1.7798	-1.77688	-1.77398	-1.77107	-1.76818	-1.79388	-1.79082	-1.78783	-1.7849
-1.77893	-1.776	-1.77309	-1.77021	-1.76734	-1.79304	-1.79005	-1.78709	-1.78416
-1.77813	-1.77518	-1.77225	-1.76936	-1.7665	-1.78107	-1.77812	-1.78626	-1.78334
-1.77737	-1.77438	-1.77142	-1.76852	-1.76566	-1.78019	-1.77725	-1.78539	-1.78249
-1.77657	-1.77359	-1.77064	-1.76774	-1.76488	-1.77936	-1.77641	-1.7735	-1.77061
-1.77575	-1.77281	-1.76989	-1.76701	-1.76415	-1.77859	-1.77561	-1.77267	-1.76977
-1.77495	-1.77203	-1.78688	-1.78383	-1.78081	-1.7778	-1.77482	-1.77188	-1.76898
-1.77419	-1.77125	-1.78604	-1.78299	-1.77998	-1.777	-1.77406	-1.77114	-1.76825
-1.77342	-1.77047	-1.7852	-1.78217	-1.77917	-1.77621	-1.77329	-1.77039	-1.76751
-1.77261	-1.76969	-1.78443	-1.78141	-1.77842	-1.77545	-1.7725	-1.76958	-1.7667
-1.77178	-1.76891	-1.78366	-1.78066	-1.77768	-1.77469	-1.7717	-1.76877	-1.76589
-1.77096	-1.7681	-1.78282	-1.77984	-1.77688	-1.7739	-1.77094	-1.76801	-1.76513
-1.77014	-1.76727	-1.78195	-1.77899	-1.77605	-1.77311	-1.77018	-1.76728	-1.76441
-1.76936	-1.7665	-1.78114	-1.77818	-1.77526	-1.77233	-1.7694	-1.7665	-1.76364
-1.76863	-1.76577	-1.78038	-1.77743	-1.7745	-1.77155	-1.76859	-1.76568	-1.76281
-1.76791	-1.76504	-1.77961	-1.77665	-1.77372	-1.77076	-1.7678	-1.76488	-1.76201
-1.76717	-1.76428	-1.77878	-1.77582	-1.7729	-1.76997	-1.76703	-1.76413	-1.76127
-1.76643	-1.76353	-1.77797	-1.77501	-1.77209	-1.76917	-1.76627	-1.76338	-1.76052
-1.78327	-1.78022	-1.77721	-1.77425	-1.77132	-1.76839	-1.76546	-1.76257	-1.75971
-1.78245	-1.77945	-1.77647	-1.77351	-1.77056	-1.76761	-1.76466	-1.76175	-1.75889
-1.78168	-1.7787	-1.77573	-1.77276	-1.76979	-1.76684	-1.7639	-1.761	-1.75814
-1.78094	-1.77795	-1.77498	-1.77199	-1.76902	-1.76608	-1.76317	-1.76029	-1.75744
-1.78019	-1.7772	-1.77421	-1.77122	-1.76826	-1.76533	-1.76244	-1.75957	-1.75673
-1.77944	-1.77642	-1.77342	-1.77045	-1.76751	-1.7646	-1.76172	-1.75886	-1.75602
-1.77867	-1.77564	-1.77264	-1.76969	-1.76677	-1.76386	-1.76097	-1.75811	-1.75528
-1.77787	-1.77487	-1.77189	-1.76895	-1.76602	-1.7631	-1.76018	-1.75731	-1.75447

							-0.05042	-0.05029
							-0.05032	-0.0502
							-0.15489	-0.15426
							-0.15449	-0.15391
					-0.0503	-0.05017	-0.15412	-0.15357
					-0.05021	-0.05008	-0.15379	-0.15324
					-0.05013	-0.05	-0.15347	-0.15292
					-0.1544	-0.15376	-0.30828	-0.30732
					-0.15405	-0.15344	-0.30773	-0.30675
		-0.10606	-0.10543	-0.10483	-0.3092	-0.30819	-0.35054	-0.34936
		-0.10573	-0.10513	-0.10456	-0.30866	-0.30765	-0.3499	-0.34872
		-0.15505	-0.15436	-0.15371	-0.35164	-0.35045	-0.3944	-0.39306
		-0.15468	-0.15401	-0.15338	-0.351	-0.3498	-0.3937	-0.39237
-0.10602	-0.10541	-0.31013	-0.30906	-0.30803	-0.35035	-0.34916	-0.48641	-0.48479
-0.10571	-0.10511	-0.30956	-0.3085	-0.30748	-0.34971	-0.34852	-0.48557	-0.48397
-0.1054	-0.10483	-0.309	-0.30795	-0.30693	-0.34906	-0.34789	-0.48474	-0.48317
-0.15464	-0.15397	-0.35206	-0.35082	-0.3496	-0.3936	-0.39228	-0.58111	-0.57927
-0.15429	-0.15363	-0.35139	-0.35016	-0.34895	-0.3929	-0.3916	-0.58011	-0.5783
-0.30941	-0.30835	-0.35073	-0.34951	-0.34831	-0.43909	-0.43766	-0.72747	-0.72524
-0.30884	-0.30778	-0.35006	-0.34887	-0.3477	-0.43838	-0.43696	-0.72625	-0.724
-0.35189	-0.35063	-0.39486	-0.39354	-0.39227	-0.57511	-0.5732	-0.72507	-0.72281
-0.35123	-0.34997	-0.39416	-0.39289	-0.39166	-0.57417	-0.57229	-0.72396	-0.72167
-0.35058	-0.34933	-0.44047	-0.43908	-0.43773	-0.69106	-0.68885	-0.72283	-0.72054
-0.34997	-0.34875	-0.43977	-0.43838	-0.43702	-0.6899	-0.6877	-0.72164	-0.71936
-0.34937	-0.34817	-0.43908	-0.43768	-0.43631	-0.68873	-0.68652	-0.72043	-0.71817
-0.39425	-0.39293	-0.65223	-0.65015	-0.64809	-0.7238	-0.7215	-0.76173	-0.75939
-0.39353	-0.39224	-0.6511	-0.64902	-0.64697	-0.72257	-0.72029	-0.76048	-0.75815
-0.60433	-0.60242	-0.69178	-0.68956	-0.68736	-0.72134	-0.71909	-0.75923	-0.7569
-0.60328	-0.60139	-0.69058	-0.68836	-0.68617	-0.72012	-0.71787	-0.75798	-0.75566
-0.65187	-0.6498	-0.68939	-0.6872	-0.68501	-0.7614	-0.75907	-0.78797	-0.78541
-0.65078	-0.64872	-0.68823	-0.68605	-0.68389	-0.76019	-0.75786	-0.78661	-0.78407
-0.64971	-0.64766	-0.6871	-0.68494	-0.6828	-0.759	-0.75667	-0.7853	-0.78279
-0.69042	-0.68822	-0.7223	-0.72004	-0.7178	-0.75788	-0.75555	-1.08166	-1.078

-0.68935	-0.68716	-0.72121	-0.71896	-0.71673	-0.75676	-0.75444	-1.07988	-1.07628
-0.68821	-0.68604	-0.76255	-0.76022	-0.75789	-0.78664	-0.7841	-1.07803	-1.07447
-0.68703	-0.68488	-0.76132	-0.75898	-0.75665	-0.78528	-0.78276	-1.07613	-1.0726
-0.68588	-0.68375	-0.76009	-0.75776	-0.75543	-1.08154	-1.07787	-1.1106	-1.10673
-0.68476	-0.68263	-0.75888	-0.75655	-0.75422	-1.07964	-1.07603	-1.10859	-1.10473
-0.72689	-0.72467	-0.91009	-0.90713	-0.9042	-1.0778	-1.07422	-1.19726	-1.19317
-0.72581	-0.72359	-0.90863	-0.90568	-0.90278	-1.07603	-1.07248	-1.19526	-1.19117
-0.72473	-0.7225	-0.90717	-0.90425	-0.90138	-1.07427	-1.07073	-1.19325	-1.18919
-0.75889	-0.75655	-1.08339	-1.07967	-1.07603	-1.10859	-1.10472	-1.2521	-1.24792
-0.75764	-0.7553	-1.08143	-1.07776	-1.07416	-1.10655	-1.10269	-1.24987	-1.24574
-0.90854	-0.90557	-1.07951	-1.07587	-1.07231	-1.19502	-1.19093	-1.27542	-1.27129
-0.90703	-0.90406	-1.07761	-1.074	-1.07047	-1.19287	-1.1888	-1.27322	-1.26921
-0.97512	-0.97176	-1.17356	-1.16947	-1.16545	-1.25168	-1.2475	-1.33268	-1.32856
-0.97348	-0.97017	-1.17159	-1.16752	-1.16351	-1.24965	-1.24552	-1.33062	-1.32655
-1.17787	-1.17371	-1.19916	-1.19502	-1.19095	-1.31032	-1.30607	-1.32859	-1.32456
-1.1758	-1.17165	-1.19707	-1.19294	-1.1889	-1.30816	-1.304	-1.32653	-1.3225
-1.17373	-1.1696	-1.19498	-1.19088	-1.18686	-1.30602	-1.30196	-1.3245	-1.32046
-1.17173	-1.16762	-1.31675	-1.31237	-1.3081	-1.33063	-1.32657	-1.38295	-1.37874
-1.16976	-1.16567	-1.31463	-1.3103	-1.30607	-1.32861	-1.3246	-1.38091	-1.37672
-1.26236	-1.25802	-1.31246	-1.30819	-1.30403	-1.35487	-1.35078	-1.37879	-1.37461
-1.26014	-1.25582	-1.31024	-1.30607	-1.30199	-1.35277	-1.34866	-1.37661	-1.37244
-1.31679	-1.31238	-1.33699	-1.3328	-1.32871	-1.3829	-1.37868	-1.37448	-1.37031
-1.31471	-1.31032	-1.33491	-1.33077	-1.32673	-1.38085	-1.37662	-1.37243	-1.36828
-1.31266	-1.3083	-1.33287	-1.32875	-1.32473	-1.37877	-1.37455	-1.37037	-1.36623
-1.31061	-1.30629	-1.36364	-1.35939	-1.35523	-1.3766	-1.3724	-1.393	-1.38882
-1.30855	-1.3043	-1.36158	-1.3573	-1.35309	-1.37442	-1.37024	-1.39083	-1.38666
-1.33531	-1.33107	-1.38521	-1.38087	-1.37657	-1.37234	-1.36816	-1.40402	-1.39985
-1.33316	-1.32904	-1.3832	-1.37888	-1.37457	-1.37032	-1.36612	-1.40196	-1.39783
-1.36392	-1.35973	-1.38115	-1.37685	-1.37256	-1.39309	-1.38882	-1.42208	-1.41787
-1.36195	-1.35772	-1.37907	-1.37481	-1.37056	-1.39106	-1.38674	-1.41992	-1.41571
-1.35999	-1.35569	-1.37697	-1.37273	-1.36851	-1.42658	-1.42214	-1.44426	-1.43997
-1.358	-1.35363	-1.37482	-1.37056	-1.36635	-1.42441	-1.42007	-1.44221	-1.43793
-1.35597	-1.35156	-1.37268	-1.3684	-1.36421	-1.42226	-1.41803	-1.44019	-1.43592
-1.35383	-1.34947	-1.43324	-1.42882	-1.42447	-1.4202	-1.416	-1.54614	-1.54134
-1.35164	-1.34738	-1.43114	-1.42676	-1.42243	-1.41818	-1.41399	-1.54388	-1.5391
-1.39069	-1.3864	-1.42906	-1.42472	-1.42042	-1.49522	-1.4908	-1.56347	-1.5587
-1.38863	-1.38437	-1.42701	-1.4227	-1.41843	-1.49311	-1.48865	-1.56117	-1.55647
-1.43366	-1.42928	-1.42496	-1.42067	-1.41641	-1.56857	-1.5637	-1.55893	-1.55427
-1.4316	-1.42724	-1.42292	-1.41861	-1.41434	-1.56626	-1.56148	-1.55676	-1.55212
-1.42955	-1.42519	-1.52279	-1.51822	-1.51371	-1.56397	-1.55929	-1.55462	-1.54997
-1.42749	-1.42311	-1.52056	-1.51603	-1.51158	-1.56176	-1.55712	-1.55247	-1.54782
-1.42541	-1.42103	-1.51834	-1.51387	-1.50948	-1.55959	-1.55496	-1.55032	-1.54567
-1.4233	-1.41896	-1.57161	-1.56682	-1.56211	-1.55743	-1.5528	-1.56989	-1.56515
-1.42117	-1.41692	-1.56937	-1.56464	-1.55996	-1.55529	-1.55063	-1.56766	-1.56289
-1.52079	-1.51635	-1.56716	-1.56248	-1.55783	-1.57494	-1.57016	-1.5654	-1.56065
-1.51867	-1.51427	-1.56497	-1.56034	-1.55572	-1.57274	-1.56786	-1.56309	-1.55844
-1.57225	-1.5675	-1.56282	-1.5582	-1.55358	-1.57054	-1.56561	-1.56086	-1.55627
-1.57005	-1.56535	-1.56069	-1.55605	-1.55141	-1.56835	-1.56345	-1.55873	-1.55417
-1.56788	-1.56321	-1.58043	-1.57569	-1.57094	-1.56614	-1.56131	-1.57942	-1.57484
-1.56571	-1.56107	-1.57826	-1.57345	-1.56863	-1.56386	-1.55914	-1.5773	-1.57278

-1.56356	-1.55893	-1.57608	-1.5712	-1.56631	-1.56159	-1.55699	-1.57521	-1.57074
-1.58331	-1.57863	-1.57391	-1.56901	-1.5641	-1.55941	-1.55487	-1.57312	-1.56869
-1.58116	-1.57646	-1.57173	-1.56685	-1.56197	-1.55731	-1.5528	-1.57106	-1.56666
-1.57901	-1.5743	-1.56957	-1.56471	-1.55988	-1.55524	-1.55076	-1.56904	-1.5647
-1.57685	-1.57214	-1.56742	-1.5626	-1.55782	-1.55323	-1.54878	-1.56708	-1.56281
-1.57469	-1.56997	-1.56527	-1.5605	-1.55578	-1.57392	-1.56944	-1.56515	-1.561
-1.57252	-1.56781	-1.56312	-1.5584	-1.55372	-1.57186	-1.5674	-1.56322	-1.55931
-1.57035	-1.56566	-1.561	-1.55632	-1.55169	-1.56983	-1.56539	-1.56134	-1.55766
-1.5682	-1.56354	-1.55892	-1.55428	-1.54968	-1.56783	-1.56343	-1.55949	-1.55601
-1.56605	-1.56145	-1.55688	-1.55226	-1.5477	-1.56588	-1.56155	-1.55771	-1.5544
-1.56392	-1.55936	-1.57757	-1.57292	-1.56834	-1.56398	-1.55982	-1.55611	-1.55289
-1.56179	-1.55728	-1.57551	-1.57087	-1.56632	-1.56213	-1.55822	-1.55466	-1.55147
-1.55969	-1.55522	-1.57347	-1.56885	-1.56435	-1.56032	-1.55665	-1.55324	-1.55013
-1.55762	-1.55318	-1.57145	-1.56687	-1.56242	-1.55853	-1.55507	-1.55186	-1.54891
-1.57831	-1.57385	-1.56945	-1.56494	-1.56059	-1.55685	-1.55356	-1.56986	-1.56704
-1.57625	-1.5718	-1.56745	-1.56309	-1.55893	-1.55532	-1.55213	-1.56852	-1.56578
-1.57423	-1.56977	-1.56547	-1.56129	-1.55735	-1.55386	-1.55075	-1.5672	-1.56453
-1.57225	-1.56778	-1.56352	-1.55953	-1.5558	-1.57183	-1.56879	-1.58591	-1.58326
-1.5703	-1.56582	-1.56162	-1.55781	-1.55431	-1.57046	-1.5675	-1.58469	-1.58207
-1.56837	-1.56394	-1.57929	-1.57561	-1.57227	-1.56914	-1.56626	-1.58349	-1.5809
-1.56647	-1.56213	-1.57758	-1.57408	-1.57092	-1.5679	-1.56506	-1.58232	-1.57974
-1.58412	-1.57988	-1.57599	-1.57263	-1.5696	-1.56666	-1.56388	-1.58117	-1.57859
-1.5823	-1.57824	-1.57451	-1.57126	-1.5683	-1.56543	-1.5627	-1.58002	-1.57745
-1.58051	-1.57664	-1.57308	-1.56992	-1.56703	-1.58424	-1.58152	-1.57887	-1.57631
-1.57869	-1.57501	-1.57164	-1.5686	-1.56579	-1.58306	-1.58037	-1.57774	-1.57518
-1.57693	-1.57344	-1.57025	-1.56732	-1.56458	-1.58189	-1.57923	-1.57661	-1.57405
-1.57538	-1.57204	-1.56897	-1.56611	-1.56341	-1.58076	-1.57813	-1.57553	-1.57295
-1.57398	-1.57076	-1.56777	-1.56494	-1.56226	-1.57964	-1.57706	-1.57447	-1.57186
-1.57257	-1.56947	-1.58668	-1.58385	-1.58115	-1.57852	-1.57596	-1.60009	-1.59742
-1.57117	-1.56819	-1.58547	-1.5827	-1.58002	-1.5774	-1.57483	-1.59893	-1.5963
-1.56982	-1.56694	-1.58429	-1.58156	-1.57891	-1.58592	-1.58327	-1.61814	-1.61552
-1.56856	-1.56573	-1.58311	-1.58045	-1.57785	-1.58483	-1.58217	-1.61703	-1.61446
-1.56733	-1.56454	-1.58196	-1.57933	-1.57677	-1.58374	-1.58108	-1.61595	-1.61341
-1.58627	-1.58349	-1.58081	-1.57819	-1.57563	-1.59975	-1.59714	-1.61488	-1.61234
-1.58508	-1.58234	-1.57968	-1.57706	-1.57448	-1.5986	-1.59606	-1.61382	-1.61128
-1.58393	-1.58123	-1.60546	-1.60274	-1.60006	-1.6178	-1.61526	-1.63514	-1.63254
-1.58281	-1.58015	-1.60436	-1.60163	-1.59895	-1.61668	-1.61415	-1.63402	-1.63145
-1.60869	-1.60595	-1.62362	-1.62089	-1.61819	-1.61559	-1.61306	-1.63292	-1.63039
-1.6076	-1.60483	-1.62248	-1.61977	-1.61712	-1.61453	-1.612	-1.63184	-1.62936
-1.62691	-1.62411	-1.62135	-1.61867	-1.61605	-1.63592	-1.63332	-1.63079	-1.62834
-1.62577	-1.623	-1.62027	-1.6176	-1.61498	-1.63484	-1.63229	-1.62978	-1.62731
-1.62462	-1.62189	-1.6192	-1.61653	-1.61392	-1.63377	-1.63127	-1.62878	-1.62629
-1.62351	-1.62077	-1.61809	-1.61546	-1.61288	-1.63274	-1.63024	-1.62775	-1.62528
-1.62241	-1.61965	-1.61696	-1.61438	-1.61186	-1.63171	-1.62919	-1.62672	-1.62428
-1.62128	-1.61854	-1.63839	-1.63576	-1.6332	-1.63066	-1.62815	-1.6257	-1.62331
-1.62014	-1.61744	-1.63729	-1.63467	-1.63212	-1.62959	-1.6271	-1.62469	-1.62235
-1.61902	-1.61636	-1.63621	-1.6336	-1.63105	-1.62854	-1.62609	-1.6237	-1.6214
-1.61795	-1.6153	-1.63514	-1.63253	-1.62999	-1.62752	-1.62512	-1.62276	-1.62045
-1.63946	-1.63674	-1.63408	-1.63149	-1.62897	-1.62653	-1.62417	-1.62183	-1.61951
-1.6383	-1.63565	-1.63307	-1.63052	-1.62802	-1.62559	-1.62321	-1.62088	-1.61859

-1.63716	-1.63458	-1.63207	-1.62957	-1.62711	-1.62468	-1.62228	-1.61995	-1.61769
-1.6361	-1.63354	-1.63105	-1.6286	-1.62618	-1.62378	-1.6214	-1.61907	-1.61678
-1.6351	-1.63253	-1.63003	-1.62762	-1.62525	-1.62289	-1.62054	-1.61821	-1.61589
-1.63408	-1.63153	-1.62905	-1.62666	-1.62432	-1.62199	-1.61967	-1.65776	-1.65536
-1.63304	-1.63055	-1.62812	-1.62572	-1.62338	-1.62107	-1.61879	-1.65687	-1.6545
-1.63204	-1.62958	-1.62717	-1.62479	-1.62245	-1.66062	-1.65829	-1.65596	-1.65363
-1.6311	-1.62861	-1.62619	-1.62385	-1.62155	-1.65969	-1.65734	-1.65503	-1.65274
-1.63018	-1.62767	-1.62524	-1.62292	-1.62066	-1.65875	-1.6564	-1.6541	-1.65186
-1.62925	-1.62678	-1.66492	-1.66251	-1.66015	-1.6578	-1.65547	-1.65319	-1.65095
-1.62831	-1.6259	-1.66403	-1.66159	-1.65919	-1.65685	-1.65456	-1.65229	-1.65005
-1.66798	-1.66554	-1.66313	-1.6607	-1.65829	-1.65594	-1.65364	-1.69552	-1.69303
-1.66696	-1.66458	-1.66222	-1.65982	-1.65743	-1.65506	-1.65273	-1.69448	-1.69207
-1.666	-1.66363	-1.66129	-1.65892	-1.65656	-1.6542	-1.65186	-1.69351	-1.69113
-1.6651	-1.66269	-1.66032	-1.65798	-1.65567	-1.65335	-1.65104	-1.69262	-1.69023
-1.66421	-1.66177	-1.65938	-1.65706	-1.65479	-1.67367	-1.67121	-1.69173	-1.68931
-1.66335	-1.6609	-1.6585	-1.65619	-1.65392	-1.67275	-1.67033	-1.69082	-1.68836
-1.66249	-1.66005	-1.65764	-1.65532	-1.65305	-1.67183	-1.66945	-1.6899	-1.68741
-1.66161	-1.65918	-1.65679	-1.65445	-1.65216	-1.67089	-1.66854	-1.68897	-1.68649
-1.66071	-1.65832	-1.65594	-1.65358	-1.65127	-1.66994	-1.66761	-1.68803	-1.68557
-1.65978	-1.65742	-1.67647	-1.67393	-1.67143	-1.69193	-1.68949	-1.72755	-1.72486
-1.65881	-1.65649	-1.67552	-1.67302	-1.67054	-1.69099	-1.6885	-1.72644	-1.72379
-1.65788	-1.65559	-1.67458	-1.67211	-1.66966	-1.69005	-1.68753	-1.72537	-1.72274
-1.65701	-1.65472	-1.67366	-1.67121	-1.66877	-1.68914	-1.68661	-1.72434	-1.7217
-1.67767	-1.67518	-1.69581	-1.69326	-1.69075	-1.68822	-1.68569	-1.72333	-1.72065
-1.67674	-1.67427	-1.69487	-1.69233	-1.68983	-1.6873	-1.68477	-1.7223	-1.71956
-1.67581	-1.67335	-1.69392	-1.6914	-1.6889	-1.68638	-1.68384	-1.72127	-1.71847
-1.67488	-1.6724	-1.69292	-1.69042	-1.68795	-1.70721	-1.70449	-1.72025	-1.71742
-1.67395	-1.67144	-1.69189	-1.68942	-1.68699	-1.70621	-1.7035	-1.71922	-1.7164
-1.69612	-1.69347	-1.69091	-1.68846	-1.68604	-1.70521	-1.70251	-1.71821	-1.71537
-1.69516	-1.69253	-1.68997	-1.68752	-1.6851	-1.70422	-1.70152	-1.71719	-1.71435
-1.69422	-1.69159	-1.68904	-1.68659	-1.68417	-1.72174	-1.71897	-1.71617	-1.71333
-1.69329	-1.69066	-1.68811	-1.68566	-1.68324	-1.72073	-1.71795	-1.71515	-1.71229
-1.69236	-1.68974	-1.68718	-1.68473	-1.68231	-1.71971	-1.71693	-1.71412	-1.71127
-1.69142	-1.68881	-1.68626	-1.6838	-1.68138	-1.71869	-1.71591	-1.71311	-1.71028
-1.69048	-1.68788	-1.68533	-1.68288	-1.68044	-1.71767	-1.71488	-1.7121	-1.7093
-1.68954	-1.68695	-1.70618	-1.70355	-1.70095	-1.71665	-1.71386	-1.74329	-1.74044
-1.68861	-1.68602	-1.7052	-1.70257	-1.69996	-1.71563	-1.71283	-1.74226	-1.73944
-1.68768	-1.6851	-1.70422	-1.70159	-1.69897	-1.74695	-1.74405	-1.78351	-1.78054
-1.68676	-1.68419	-1.70324	-1.7006	-1.69798	-1.74589	-1.74298	-1.78239	-1.77946
-1.68584	-1.68328	-1.73533	-1.73258	-1.72984	-1.74484	-1.74194	-1.78129	-1.7784
-1.68492	-1.68241	-1.73441	-1.73163	-1.72885	-1.74382	-1.74095	-1.78025	-1.77737
-1.68401	-1.68154	-1.7335	-1.73069	-1.72786	-1.74281	-1.73997	-1.77922	-1.77634
-1.71675	-1.71419	-1.75042	-1.74753	-1.74463	-1.74178	-1.73897	-1.77819	-1.7753
-1.71581	-1.71324	-1.74937	-1.74648	-1.74358	-1.74075	-1.73798	-1.77715	-1.77428
-1.71491	-1.71232	-1.74833	-1.74543	-1.74253	-1.78196	-1.77902	-1.79104	-1.78814
-1.71405	-1.71142	-1.7473	-1.74439	-1.74149	-1.78086	-1.77795	-1.78999	-1.78711
-1.7521	-1.74919	-1.74627	-1.74335	-1.74047	-1.77979	-1.77691	-1.78896	-1.78611
-1.75107	-1.74815	-1.74523	-1.74234	-1.73948	-1.77875	-1.77588	-1.78795	-1.78515
-1.75004	-1.7471	-1.74419	-1.74132	-1.73851	-1.77774	-1.77488	-1.78696	-1.78421
-1.74899	-1.74604	-1.74313	-1.7403	-1.73753	-1.77675	-1.77394	-1.78602	-1.78325

-1.74794	-1.74499	-1.74209	-1.73929	-1.73656	-1.77577	-1.77301	-1.78509	-1.78229
-1.7469	-1.74395	-1.74106	-1.73832	-1.73565	-1.77482	-1.77204	-1.7841	-1.78129
-1.74588	-1.74293	-1.74007	-1.73738	-1.73476	-1.77387	-1.77106	-1.78308	-1.78028
-1.72458	-1.72183	-1.7814	-1.77852	-1.77572	-1.78776	-1.78489	-1.78206	-1.77927
-1.72372	-1.72099	-1.7805	-1.77759	-1.77473	-1.78673	-1.78386	-1.78103	-1.77824
-1.72282	-1.72012	-1.77957	-1.77663	-1.77373	-1.78571	-1.78284	-1.78002	-1.77723
-1.72187	-1.71918	-1.77855	-1.77562	-1.77273	-1.7847	-1.78184	-1.77902	-1.77623
-1.72091	-1.71823	-1.77753	-1.77462	-1.77175	-1.78372	-1.78088	-1.77806	-1.77527
-1.71997	-1.71729	-1.77651	-1.77364	-1.77082	-1.78281	-1.77999	-1.77718	-1.77439
-1.71905	-1.71637	-1.7755	-1.77267	-1.7699	-1.78191	-1.77912	-1.77633	-1.77354
-1.73667	-1.73394	-1.77456	-1.77172	-1.76894	-1.78095	-1.77817	-1.79395	-1.79109
-1.73584	-1.73311	-1.77367	-1.77079	-1.76796	-1.77994	-1.77718	-1.79294	-1.79009
-1.73496	-1.73223	-1.75349	-1.75071	-1.74797	-1.77896	-1.77619	-1.79194	-1.78912
-1.73404	-1.73132	-1.75253	-1.74977	-1.74705	-1.77798	-1.77521	-1.79095	-1.78818
-1.75727	-1.75441	-1.76664	-1.76386	-1.76112	-1.79565	-1.7928	-1.78999	-1.78724
-1.75639	-1.75354	-1.76575	-1.76299	-1.76028	-1.79476	-1.79191	-1.78908	-1.78628
-1.7555	-1.75266	-1.76487	-1.76213	-1.75944	-1.79387	-1.79101	-1.78816	-1.78532
-1.75455	-1.75172	-1.76393	-1.7612	-1.75852	-1.79291	-1.79005	-1.78719	-1.78434
-1.75358	-1.75076	-1.76298	-1.76026	-1.75759	-1.79192	-1.78906	-1.78621	-1.78335
-1.75268	-1.74988	-1.78103	-1.77824	-1.7755	-1.79099	-1.78813	-1.78527	-1.78242
-1.75183	-1.74905	-1.78018	-1.77739	-1.77464	-1.79011	-1.78724	-1.78438	-1.78153
-1.75093	-1.74817	-1.77928	-1.7765	-1.77373	-1.78918	-1.78631	-1.79859	-1.79571
-1.74996	-1.74723	-1.77832	-1.77554	-1.77277	-1.78818	-1.78531	-1.79758	-1.79469
-1.76822	-1.76544	-1.77738	-1.7746	-1.77183	-1.80239	-1.79948	-1.79659	-1.79371
-1.76732	-1.76456	-1.77651	-1.77373	-1.77096	-1.80147	-1.79857	-1.79569	-1.79282
-1.76643	-1.76369	-1.77564	-1.77286	-1.77009	-1.80055	-1.79768	-1.79482	-1.79196
-1.78022	-1.77745	-1.79016	-1.78732	-1.7845	-1.7817	-1.77893	-1.79392	-1.79108
-1.77927	-1.77649	-1.78916	-1.78632	-1.7835	-1.78074	-1.77801	-1.79302	-1.7902
-1.79389	-1.79106	-1.78823	-1.78539	-1.78257	-1.7798	-1.77708	-1.79207	-1.78927
-1.79302	-1.79019	-1.78736	-1.78452	-1.78169	-1.77889	-1.77612	-1.79108	-1.78829
-1.79216	-1.78932	-1.78649	-1.78365	-1.78081	-1.778	-1.7752	-1.80419	-1.80136
-1.7913	-1.78846	-1.78563	-1.78279	-1.77995	-1.77714	-1.77434	-1.80329	-1.80048
-1.79041	-1.78757	-1.78473	-1.78189	-1.77906	-1.79056	-1.78772	-1.80239	-1.7996
-1.78944	-1.78659	-1.78375	-1.78091	-1.77808	-1.78957	-1.78674	-1.8014	-1.79864
-1.78846	-1.7856	-1.78275	-1.77991	-1.77708	-1.78856	-1.78574	-1.80041	-1.79767
-1.78754	-1.78469	-1.78184	-1.779	-1.77617	-1.78764	-1.78483	-1.79949	-1.79677
-1.78668	-1.78383	-1.78098	-1.77814	-1.77532	-1.78677	-1.78398	-1.79864	-1.79592
-1.78582	-1.78297	-1.79449	-1.7916	-1.78873	-1.78592	-1.78314	-1.82026	-1.81733
-1.78499	-1.78213	-1.79363	-1.79075	-1.78789	-1.78509	-1.78232	-1.81939	-1.81646
-1.79858	-1.79567	-1.79277	-1.78989	-1.78705	-1.78425	-1.78151	-1.81853	-1.81559
-1.79771	-1.7948	-1.7919	-1.78903	-1.7862	-1.78342	-1.78069	-1.81766	-1.81473
-1.79685	-1.79393	-1.79104	-1.78817	-1.78535	-1.78258	-1.77986	-1.81679	-1.81385
-1.79598	-1.79306	-1.79017	-1.78731	-1.7845	-1.78174	-1.77903	-1.79875	-1.79584
-1.79511	-1.79219	-1.7893	-1.78645	-1.78364	-1.78089	-1.77818	-1.79783	-1.79493
-1.79424	-1.79132	-1.78843	-1.78559	-1.78279	-1.80275	-1.7998	-1.79687	-1.79395
-1.79338	-1.79044	-1.78755	-1.78472	-1.78193	-1.80181	-1.79883	-1.79587	-1.79293
-1.79248	-1.78954	-1.78663	-1.78381	-1.78103	-1.80085	-1.79787	-1.7949	-1.79196
-1.79156	-1.78858	-1.78565	-1.78284	-1.78008	-1.79988	-1.79693	-1.79399	-1.79105
-1.79062	-1.78763	-1.78469	-1.78189	-1.77916	-1.79893	-1.79601	-1.79309	-1.79015
-1.78964	-1.78668	-1.78378	-1.78101	-1.77829	-1.79802	-1.79512	-1.7922	-1.78926

-1.78868	-1.78575	-1.7829	-1.78015	-1.77745	-1.79713	-1.79423	-1.79132	-1.78839
-1.78778	-1.78487	-1.78204	-1.7793	-1.77663	-1.79625	-1.79336	-1.79044	-1.78751
-1.78692	-1.78402	-1.7812	-1.77848	-1.77581	-1.79538	-1.79248	-1.78957	-1.78664
-1.78612	-1.78321	-1.80326	-1.8003	-1.79741	-1.7945	-1.7916	-1.78869	-1.78576
-1.78537	-1.78245	-1.8024	-1.79943	-1.79653	-1.79362	-1.79071	-1.7878	-1.78487
-1.78459	-1.78166	-1.80154	-1.79856	-1.79565	-1.79273	-1.78982	-1.78691	-1.78398
-1.78376	-1.78084	-1.80068	-1.79769	-1.79476	-1.79183	-1.78892	-1.78601	-1.78309
-1.78291	-1.78003	-1.79983	-1.79683	-1.79388	-1.79094	-1.78802	-1.78511	-1.7822
-1.78206	-1.77925	-1.79903	-1.79602	-1.79303	-1.79007	-1.78713	-1.78421	-1.7813
-1.78121	-1.77846	-1.79824	-1.79522	-1.7922	-1.78921	-1.78625	-1.78332	-1.78041
-1.78037	-1.77765	-1.79741	-1.79441	-1.7914	-1.78839	-1.78539	-1.79996	-1.79687
-1.77953	-1.77682	-1.79656	-1.7936	-1.79062	-1.7876	-1.78456	-1.79905	-1.79596
-1.80153	-1.7986	-1.79568	-1.79275	-1.7898	-1.78679	-1.78375	-1.79819	-1.79507
-1.80064	-1.7977	-1.79479	-1.79187	-1.78894	-1.78596	-1.78296	-1.79737	-1.79422
-1.79974	-1.79681	-1.79389	-1.79097	-1.78806	-1.78511	-1.78215	-1.79654	-1.79339
-1.79884	-1.79591	-1.79299	-1.79007	-1.78715	-1.78423	-1.7813	-1.79569	-1.79259
-1.79795	-1.79502	-1.7921	-1.78917	-1.78624	-1.78333	-1.78043	-1.79482	-1.79178
-1.78564	-1.78271	-1.7912	-1.78827	-1.78534	-1.78243	-1.77954	-1.79391	-1.79092
-1.78479	-1.78185	-1.79032	-1.78737	-1.78444	-1.78153	-1.77864	-1.79299	-1.79003
-1.78398	-1.78101	-1.78945	-1.78648	-1.78354	-1.79812	-1.79507	-1.79207	-1.78914
-1.78319	-1.78019	-1.78859	-1.78561	-1.78266	-1.79719	-1.79414	-1.79115	-1.78823
-1.78238	-1.77938	-1.7764	-1.77344	-1.7705	-1.79631	-1.79325	-1.79026	-1.78734
-1.78154	-1.77859	-1.77564	-1.7727	-1.76976	-1.79551	-1.79241	-1.78939	-1.78647
-1.78068	-1.77776	-1.77485	-1.77192	-1.769	-1.7947	-1.79159	-1.78857	-1.78564
-1.7798	-1.77688	-1.77398	-1.77107	-1.76818	-1.79388	-1.79082	-1.78783	-1.7849
-1.77893	-1.776	-1.77309	-1.77021	-1.76734	-1.79304	-1.79005	-1.78709	-1.78416
-1.77813	-1.77518	-1.77225	-1.76936	-1.7665	-1.78107	-1.77812	-1.78626	-1.78334
-1.77737	-1.77438	-1.77142	-1.76852	-1.76566	-1.78019	-1.77725	-1.78539	-1.78249
-1.77657	-1.77359	-1.77064	-1.76774	-1.76488	-1.77936	-1.77641	-1.7735	-1.77061
-1.77575	-1.77281	-1.76989	-1.76701	-1.76415	-1.77859	-1.77561	-1.77267	-1.76977
-1.77495	-1.77203	-1.78688	-1.78383	-1.78081	-1.7778	-1.77482	-1.77188	-1.76898
-1.77419	-1.77125	-1.78604	-1.78299	-1.77998	-1.777	-1.77406	-1.77114	-1.76825
-1.77342	-1.77047	-1.7852	-1.78217	-1.77917	-1.77621	-1.77329	-1.77039	-1.76751
-1.77261	-1.76969	-1.78443	-1.78141	-1.77842	-1.77545	-1.7725	-1.76958	-1.7667
-1.77178	-1.76891	-1.78366	-1.78066	-1.77768	-1.77469	-1.7717	-1.76877	-1.76589
-1.77096	-1.7681	-1.78282	-1.77984	-1.77688	-1.7739	-1.77094	-1.76801	-1.76513
-1.77014	-1.76727	-1.78195	-1.77899	-1.77605	-1.77311	-1.77018	-1.76728	-1.76441
-1.76936	-1.7665	-1.78114	-1.77818	-1.77526	-1.77233	-1.7694	-1.7665	-1.76364
-1.76863	-1.76577	-1.78038	-1.77743	-1.7745	-1.77155	-1.76859	-1.76568	-1.76281
-1.76791	-1.76504	-1.77961	-1.77665	-1.77372	-1.77076	-1.7678	-1.76488	-1.76201
-1.76717	-1.76428	-1.77878	-1.77582	-1.7729	-1.76997	-1.76703	-1.76413	-1.76127
-1.76643	-1.76353	-1.77797	-1.77501	-1.77209	-1.76917	-1.76627	-1.76338	-1.76052
-1.78327	-1.78022	-1.77721	-1.77425	-1.77132	-1.76839	-1.76546	-1.76257	-1.75971
-1.78245	-1.77945	-1.77647	-1.77351	-1.77056	-1.76761	-1.76466	-1.76175	-1.75889
-1.78168	-1.7787	-1.77573	-1.77276	-1.76979	-1.76684	-1.7639	-1.761	-1.75814
-1.78094	-1.77795	-1.77498	-1.77199	-1.76902	-1.76608	-1.76317	-1.76029	-1.75744
-1.78019	-1.7772	-1.77421	-1.77122	-1.76826	-1.76533	-1.76244	-1.75957	-1.75673
-1.77944	-1.77642	-1.77342	-1.77045	-1.76751	-1.7646	-1.76172	-1.75886	-1.75602
-1.77867	-1.77564	-1.77264	-1.76969	-1.76677	-1.76386	-1.76097	-1.75811	-1.75528
-1.77787	-1.77487	-1.77189	-1.76895	-1.76602	-1.7631	-1.76018	-1.75731	-1.75447

-0.7311	-0.72992	-0.72877	-0.72764	-0.72653	-0.72544	-0.72437	-0.74369	-0.74259
-0.7314	-0.73022	-0.72906	-0.72792	-0.72679	-0.72569	-0.72461	-0.7439	-0.7428
-0.73169	-0.73052	-0.72936	-0.7282	-0.72706	-0.72595	-0.72486	-0.74411	-0.743
-0.69538	-0.6943	-0.72963	-0.72848	-0.72734	-0.72622	-0.72512	-0.74432	-0.74322
-0.69563	-0.69454	-0.7299	-0.72875	-0.72762	-0.7265	-0.72539	-0.74455	-0.74344
-0.69588	-0.69478	-0.6937	-0.69265	-0.69162	-0.72678	-0.72566	-0.74477	-0.74366
-0.69612	-0.69503	-0.69396	-0.6929	-0.69186	-0.72705	-0.72593	-0.745	-0.74389
-0.69637	-0.69529	-0.69422	-0.69315	-0.6921	-0.72732	-0.72621	-0.74523	-0.74412
-0.69664	-0.69556	-0.69449	-0.69341	-0.69234	-0.7276	-0.72649	-0.74547	-0.74436
-0.69692	-0.69583	-0.69475	-0.69367	-0.69259	-0.69154	-0.69052	-0.74571	-0.74459
-0.69719	-0.69609	-0.695	-0.69391	-0.69283	-0.69178	-0.69075	-0.74594	-0.74481
-0.69747	-0.69635	-0.69525	-0.69416	-0.69308	-0.69202	-0.69098	-0.74617	-0.74504
-0.66331	-0.66229	-0.69551	-0.69442	-0.69335	-0.69228	-0.69123	-0.71067	-0.70963
-0.66357	-0.66254	-0.69578	-0.6947	-0.69363	-0.69255	-0.69148	-0.71089	-0.70984
-0.62961	-0.62863	-0.69606	-0.69497	-0.6939	-0.69283	-0.69175	-0.71111	-0.71006
-0.62986	-0.62887	-0.69634	-0.69525	-0.69418	-0.6931	-0.69202	-0.71134	-0.71027
-0.6301	-0.6291	-0.66228	-0.66129	-0.66033	-0.69337	-0.69229	-0.71157	-0.71048
-0.63033	-0.62933	-0.66254	-0.66154	-0.66057	-0.69364	-0.69257	-0.71182	-0.7107
-0.63057	-0.62956	-0.62858	-0.62765	-0.62674	-0.69391	-0.69284	-0.71207	-0.71093
-0.6308	-0.62979	-0.6288	-0.62786	-0.62695	-0.69418	-0.6931	-0.7123	-0.71117
-0.63104	-0.63002	-0.62903	-0.62808	-0.62717	-0.69445	-0.69336	-0.71254	-0.71141
-0.63129	-0.63026	-0.62926	-0.62831	-0.62739	-0.69473	-0.69363	-0.71277	-0.71165
-0.63153	-0.6305	-0.6295	-0.62855	-0.62762	-0.69501	-0.6939	-0.71301	-0.71189
-0.63179	-0.63076	-0.62975	-0.62879	-0.62785	-0.66178	-0.66074	-0.71325	-0.71213
-0.63205	-0.63103	-0.63002	-0.62903	-0.62807	-0.66203	-0.66099	-0.7135	-0.71237
-0.63231	-0.63129	-0.63028	-0.62928	-0.62829	-0.62734	-0.62643	-0.71375	-0.71263
-0.63256	-0.63154	-0.63053	-0.62952	-0.62852	-0.62757	-0.62665	-0.71401	-0.71288
-0.6328	-0.63179	-0.63078	-0.62976	-0.62875	-0.62779	-0.62686	-0.71427	-0.71314
-0.59959	-0.59869	-0.63104	-0.63001	-0.62899	-0.62802	-0.62708	-0.6815	-0.68046
-0.59979	-0.5989	-0.63129	-0.63026	-0.62923	-0.62825	-0.62729	-0.68172	-0.68068
-0.6	-0.59911	-0.63154	-0.63052	-0.62949	-0.6285	-0.62752	-0.68195	-0.68092
-0.60023	-0.59933	-0.63179	-0.63078	-0.62977	-0.62876	-0.62776	-0.68221	-0.68116
-0.60045	-0.59954	-0.63205	-0.63104	-0.63004	-0.62903	-0.62802	-0.64805	-0.64711
-0.60067	-0.59976	-0.63231	-0.63131	-0.63031	-0.6293	-0.6283	-0.64829	-0.64734
-0.60088	-0.59997	-0.59907	-0.59818	-0.59728	-0.62957	-0.62857	-0.64854	-0.64757
-0.60108	-0.60017	-0.59927	-0.59838	-0.59749	-0.62984	-0.62885	-0.64879	-0.6478
-0.60128	-0.60037	-0.59947	-0.59858	-0.5977	-0.6301	-0.62912	-0.64904	-0.64803
-0.6015	-0.60059	-0.59969	-0.5988	-0.59791	-0.63035	-0.62938	-0.64928	-0.64825
-0.60172	-0.60082	-0.59993	-0.59902	-0.59812	-0.6306	-0.62963	-0.64951	-0.64848
-0.60195	-0.60104	-0.60014	-0.59924	-0.59834	-0.63086	-0.62989	-0.64975	-0.64872
-0.60217	-0.60126	-0.60035	-0.59945	-0.59856	-0.63113	-0.63015	-0.65	-0.64898
-0.60239	-0.60147	-0.60057	-0.59967	-0.59878	-0.5979	-0.59702	-0.65025	-0.64924
-0.6026	-0.60169	-0.60079	-0.5999	-0.599	-0.59812	-0.59724	-0.65051	-0.64951
-0.60281	-0.60191	-0.60102	-0.60012	-0.59923	-0.59834	-0.59746	-0.65076	-0.64977
-0.60305	-0.60214	-0.60124	-0.60034	-0.59945	-0.59857	-0.59769	-0.65101	-0.65001
-0.60329	-0.60237	-0.60146	-0.60056	-0.59968	-0.5988	-0.59793	-0.65126	-0.65024
-0.60354	-0.6026	-0.60168	-0.60079	-0.59991	-0.59903	-0.59815	-0.65151	-0.65047
-0.60379	-0.60283	-0.60191	-0.60102	-0.60016	-0.59927	-0.59837	-0.65176	-0.65071
-0.60406	-0.60309	-0.60215	-0.60126	-0.60039	-0.59949	-0.59859	-0.61889	-0.61796
-0.60434	-0.60337	-0.60242	-0.6015	-0.6006	-0.5997	-0.59881	-0.6191	-0.61816

-0.60463	-0.60365	-0.60268	-0.60175	-0.60083	-0.59993	-0.59904	-0.61932	-0.61838
-0.60494	-0.60393	-0.60294	-0.602	-0.60109	-0.60018	-0.59928	-0.61954	-0.61859
-0.60524	-0.60421	-0.60321	-0.60227	-0.60135	-0.60043	-0.59952	-0.61977	-0.61882
-0.60554	-0.60452	-0.60351	-0.60255	-0.60161	-0.60069	-0.59978	-0.62001	-0.61905
-0.60584	-0.60483	-0.60383	-0.60284	-0.60187	-0.60095	-0.60004	-0.62026	-0.61929
-0.60615	-0.60513	-0.60412	-0.60313	-0.60216	-0.60122	-0.60031	-0.5994	-0.59848
-0.60646	-0.60543	-0.60442	-0.60343	-0.60246	-0.60151	-0.60058	-0.59966	-0.59874
-0.60676	-0.60573	-0.60472	-0.60372	-0.60275	-0.60179	-0.60085	-0.59992	-0.599
-0.60706	-0.60603	-0.60502	-0.60402	-0.60303	-0.60207	-0.60113	-0.6002	-0.59928
-0.55237	-0.55135	-0.60532	-0.60431	-0.60332	-0.60235	-0.60141	-0.60048	-0.59955
-0.55274	-0.55172	-0.60562	-0.60461	-0.60361	-0.60263	-0.60168	-0.60074	-0.59982
-0.55312	-0.55209	-0.60591	-0.6049	-0.60391	-0.60292	-0.60195	-0.60101	-0.60009
-0.55349	-0.55246	-0.55144	-0.55044	-0.54946	-0.54849	-0.54754	-0.60129	-0.60036
-0.55387	-0.55284	-0.55182	-0.55081	-0.54981	-0.54884	-0.5479	-0.60157	-0.60063
-0.55425	-0.55322	-0.5522	-0.55118	-0.55017	-0.5492	-0.54826	-0.54732	-0.54638
-0.55463	-0.5536	-0.55258	-0.55155	-0.55055	-0.54957	-0.54863	-0.54768	-0.54675
-0.55503	-0.55399	-0.55297	-0.55194	-0.55092	-0.54994	-0.54899	-0.54805	-0.54711
-0.55544	-0.5544	-0.55337	-0.55233	-0.5513	-0.55031	-0.54935	-0.5484	-0.54747
-0.55585	-0.5548	-0.55377	-0.55273	-0.55169	-0.55069	-0.54971	-0.54876	-0.54782
-0.55625	-0.5552	-0.55417	-0.55312	-0.55209	-0.55108	-0.55008	-0.54911	-0.54817
-0.55667	-0.55559	-0.55456	-0.55352	-0.55249	-0.55147	-0.55046	-0.54948	-0.54853
-0.55711	-0.55601	-0.55497	-0.55393	-0.5529	-0.55188	-0.55085	-0.54986	-0.5489
-0.55756	-0.55645	-0.55538	-0.55434	-0.55332	-0.55229	-0.55126	-0.55026	-0.54928
-0.55803	-0.55688	-0.55579	-0.55474	-0.55372	-0.55269	-0.55165	-0.55065	-0.54967
-0.55851	-0.55731	-0.55619	-0.55513	-0.55411	-0.55308	-0.55205	-0.55104	-0.55005
-0.55903	-0.55777	-0.5566	-0.55553	-0.55451	-0.55348	-0.55245	-0.55144	-0.55044
-0.55959	-0.55827	-0.55705	-0.55596	-0.55493	-0.5539	-0.55287	-0.55185	-0.55083
-0.56018	-0.55879	-0.55751	-0.55639	-0.55536	-0.55432	-0.5533	-0.55227	-0.55123
-0.56075	-0.55931	-0.55798	-0.55683	-0.55578	-0.55473	-0.55371	-0.55269	-0.55165
-0.56134	-0.55985	-0.55847	-0.55728	-0.5562	-0.55514	-0.55412	-0.5531	-0.55207
-0.56195	-0.56042	-0.559	-0.55775	-0.55662	-0.55554	-0.55453	-0.55351	-0.55249
-0.56257	-0.56102	-0.55956	-0.55824	-0.55704	-0.55594	-0.55493	-0.55391	-0.5529
-0.56321	-0.56163	-0.56014	-0.55877	-0.55749	-0.55636	-0.55534	-0.55432	-0.55331
-0.56385	-0.56227	-0.56076	-0.55932	-0.55797	-0.5568	-0.55575	-0.55473	-0.55372
-0.48886	-0.48745	-0.56139	-0.55989	-0.55848	-0.55726	-0.55618	-0.55513	-0.55413
-0.48945	-0.48803	-0.56202	-0.56049	-0.55903	-0.55775	-0.55661	-0.55554	-0.55454
-0.49005	-0.48862	-0.56266	-0.5611	-0.5596	-0.55827	-0.55706	-0.55595	-0.55494
-0.44856	-0.4472	-0.56332	-0.56174	-0.56021	-0.55882	-0.55753	-0.55637	-0.55534
-0.44913	-0.44776	-0.56399	-0.5624	-0.56085	-0.55939	-0.55802	-0.5568	-0.55575
-0.44972	-0.44833	-0.44697	-0.44561	-0.44429	-0.55998	-0.55855	-0.55728	-0.55619
-0.45031	-0.44891	-0.44753	-0.44615	-0.44481	-0.56058	-0.55911	-0.5578	-0.55666
-0.4509	-0.44949	-0.44809	-0.4467	-0.44533	-0.44403	-0.44278	-0.55835	-0.55714
-0.45149	-0.45007	-0.44866	-0.44725	-0.44587	-0.44456	-0.4433	-0.55893	-0.55765
-0.45208	-0.45065	-0.44924	-0.44781	-0.44642	-0.44509	-0.44382	-0.51931	-0.51802
-0.45267	-0.45125	-0.44982	-0.44839	-0.44697	-0.44563	-0.44434	-0.51986	-0.51852
-0.45326	-0.45184	-0.45042	-0.44897	-0.44753	-0.44617	-0.44487	-0.52043	-0.51905
-0.45384	-0.45243	-0.45101	-0.44955	-0.4481	-0.44672	-0.4454	-0.44412	-0.44287
-0.45442	-0.45302	-0.4516	-0.45013	-0.44867	-0.44728	-0.44594	-0.44464	-0.44338
-0.45499	-0.4536	-0.45219	-0.45072	-0.44926	-0.44785	-0.44648	-0.44517	-0.4439
-0.45556	-0.45418	-0.45278	-0.45132	-0.44986	-0.44843	-0.44704	-0.4457	-0.44443

-0.45614	-0.45476	-0.45337	-0.45193	-0.45048	-0.44904	-0.44761	-0.44625	-0.44497
-0.45672	-0.45535	-0.45397	-0.45255	-0.45112	-0.44966	-0.44821	-0.44682	-0.4455
-0.4573	-0.45593	-0.45456	-0.45316	-0.45175	-0.45029	-0.44881	-0.4474	-0.44605
-0.45787	-0.4565	-0.45514	-0.45375	-0.45235	-0.4509	-0.44942	-0.44798	-0.4466
-0.45845	-0.45707	-0.45571	-0.45433	-0.45295	-0.45151	-0.45003	-0.44859	-0.44716
-0.45905	-0.45766	-0.45628	-0.45492	-0.45355	-0.45212	-0.45066	-0.44921	-0.44776
-0.45968	-0.45826	-0.45687	-0.45551	-0.45415	-0.45274	-0.4513	-0.44984	-0.44837
-0.41101	-0.40985	-0.45747	-0.4561	-0.45475	-0.45335	-0.45193	-0.45048	-0.449
-0.41154	-0.41037	-0.45808	-0.45669	-0.45533	-0.45394	-0.45254	-0.45111	-0.44964
-0.36831	-0.36722	-0.45871	-0.4573	-0.45592	-0.45453	-0.45315	-0.45173	-0.45028
-0.36879	-0.3677	-0.45936	-0.45793	-0.45652	-0.45513	-0.45375	-0.45233	-0.45089
-0.36928	-0.36818	-0.46001	-0.45856	-0.45714	-0.45574	-0.45435	-0.45294	-0.4515
-0.36976	-0.36864	-0.36755	-0.3665	-0.36547	-0.45636	-0.45497	-0.45355	-0.45211
-0.37026	-0.36911	-0.368	-0.36696	-0.36594	-0.457	-0.45559	-0.45417	-0.45272
-0.37076	-0.36958	-0.36845	-0.3674	-0.36638	-0.45761	-0.45622	-0.4548	-0.45336
-0.37127	-0.37007	-0.36891	-0.36783	-0.3668	-0.45822	-0.45685	-0.45544	-0.454
-0.37181	-0.37058	-0.3694	-0.36829	-0.36723	-0.36622	-0.36524	-0.45606	-0.45465
-0.37237	-0.37113	-0.36991	-0.36877	-0.36767	-0.36663	-0.36565	-0.45666	-0.45529
-0.32659	-0.32546	-0.37045	-0.36927	-0.36813	-0.36707	-0.36607	-0.41473	-0.41347
-0.32706	-0.32595	-0.37101	-0.36981	-0.36864	-0.36755	-0.36651	-0.41528	-0.41402
-0.3275	-0.32642	-0.37157	-0.37036	-0.36917	-0.36804	-0.36696	-0.41583	-0.41457
-0.32793	-0.32687	-0.32579	-0.32469	-0.32361	-0.36853	-0.36743	-0.36639	-0.36541
-0.32835	-0.3273	-0.32623	-0.32514	-0.32405	-0.36903	-0.36791	-0.36686	-0.36588
-0.32878	-0.32774	-0.32668	-0.3256	-0.3245	-0.36953	-0.36839	-0.36732	-0.36632
-0.32924	-0.32821	-0.32715	-0.32606	-0.32496	-0.37004	-0.36887	-0.36777	-0.36675
-0.32968	-0.32866	-0.32762	-0.32653	-0.32543	-0.32434	-0.32327	-0.36826	-0.36721
-0.33009	-0.32911	-0.3281	-0.32703	-0.32594	-0.32485	-0.32376	-0.36879	-0.36771
-0.3305	-0.32955	-0.32856	-0.32752	-0.32644	-0.32536	-0.32426	-0.32322	-0.32223
-0.33091	-0.32996	-0.32899	-0.32797	-0.32692	-0.32584	-0.32475	-0.32368	-0.32265
-0.33132	-0.33036	-0.3294	-0.32841	-0.32739	-0.32632	-0.32523	-0.32415	-0.32309
-0.33175	-0.33079	-0.32984	-0.32886	-0.32787	-0.32682	-0.32573	-0.32465	-0.32356
-0.3322	-0.33123	-0.33028	-0.32933	-0.32836	-0.32733	-0.32625	-0.32516	-0.32406
-0.33264	-0.33166	-0.33071	-0.32977	-0.32881	-0.3278	-0.32674	-0.32566	-0.32455
-0.33308	-0.33208	-0.33111	-0.33017	-0.32923	-0.32824	-0.3272	-0.32613	-0.32503
-0.33355	-0.33251	-0.33152	-0.33058	-0.32965	-0.32867	-0.32767	-0.32661	-0.32552
-0.33406	-0.33298	-0.33196	-0.33101	-0.33009	-0.32913	-0.32815	-0.32712	-0.32603
-0.33459	-0.33347	-0.33241	-0.33144	-0.33051	-0.32957	-0.32862	-0.32761	-0.32654
-0.3351	-0.33394	-0.33284	-0.33185	-0.33091	-0.32998	-0.32905	-0.32806	-0.327
-0.33561	-0.33441	-0.33328	-0.33226	-0.33131	-0.33037	-0.32945	-0.32848	-0.32745
-0.33615	-0.33493	-0.33377	-0.33272	-0.33174	-0.33079	-0.32987	-0.32891	-0.32792
-0.33672	-0.33549	-0.3343	-0.33321	-0.33218	-0.33121	-0.3303	-0.32935	-0.32839
-0.29607	-0.29494	-0.33486	-0.33373	-0.33265	-0.33166	-0.33073	-0.3298	-0.32886
-0.2966	-0.29549	-0.33545	-0.33428	-0.33315	-0.33213	-0.33118	-0.33024	-0.32932
-0.19427	-0.19359	-0.33603	-0.33483	-0.33366	-0.3326	-0.33163	-0.33068	-0.32976
-0.19457	-0.19388	-0.33656	-0.33535	-0.33416	-0.33307	-0.33205	-0.33108	-0.33015
-0.19485	-0.19416	-0.33709	-0.33588	-0.33468	-0.33356	-0.33249	-0.33148	-0.33055
-0.19515	-0.19447	-0.2964	-0.2953	-0.2942	-0.33408	-0.33296	-0.33192	-0.33097
-0.19546	-0.19478	-0.29692	-0.29583	-0.29473	-0.33463	-0.33347	-0.33239	-0.33142
-0.10051	-0.10015	-0.19442	-0.19374	-0.19306	-0.29416	-0.29307	-0.3329	-0.33188
-0.10066	-0.1003	-0.19474	-0.19406	-0.19337	-0.2947	-0.2936	-0.33345	-0.33238

-0.10081	-0.10045	-0.19504	-0.19436	-0.19367	-0.29521	-0.29411	-0.33398	-0.33287
-0.10095	-0.10059	-0.1953	-0.19463	-0.19395	-0.29569	-0.29459	-0.33449	-0.33333
-0.10109	-0.10073	-0.10038	-0.10002	-0.09967	-0.19356	-0.19288	-0.29399	-0.2929
-0.10125	-0.1009	-0.10054	-0.10018	-0.09983	-0.19388	-0.1932	-0.2945	-0.2934
-0.10142	-0.10106	-0.10071	-0.10035	-0.1	-0.19421	-0.19352	-0.29503	-0.29393
-0.10158	-0.10123	-0.10087	-0.10051	-0.10016	-0.19452	-0.19384	-0.29557	-0.29447
-0.10174	-0.10138	-0.10103	-0.10067	-0.10032	-0.19483	-0.19416	-0.29612	-0.29502
-0.1019	-0.10154	-0.10118	-0.10083	-0.10048	-0.19514	-0.19448	-0.24524	-0.24435
-0.10204	-0.10168	-0.10132	-0.10098	-0.10063	-0.19545	-0.1948	-0.24566	-0.24478
-0.10218	-0.10182	-0.10147	-0.10112	-0.10078	-0.10043	-0.10008	-0.19445	-0.19378
-0.10232	-0.10196	-0.10161	-0.10126	-0.10092	-0.10057	-0.10023	-0.19476	-0.19409
-0.10246	-0.1021	-0.10175	-0.1014	-0.10106	-0.10071	-0.10037	-0.19505	-0.19441
-0.10262	-0.10226	-0.10191	-0.10156	-0.10121	-0.10086	-0.10052	-0.19534	-0.19471
-0.10279	-0.10243	-0.10207	-0.10172	-0.10136	-0.10101	-0.10066	-0.19561	-0.19501
-0.10296	-0.10259	-0.10223	-0.10187	-0.10152	-0.10117	-0.10082	-0.19591	-0.19531
-0.10313	-0.10275	-0.10239	-0.10203	-0.10168	-0.10133	-0.10097	-0.19621	-0.19561
-0.05043	-0.05025	-0.10254	-0.10218	-0.10184	-0.10148	-0.10113	-0.10078	-0.10044
-0.05049	-0.0503	-0.1027	-0.10234	-0.10199	-0.10164	-0.10128	-0.10093	-0.10058
		-0.10287	-0.1025	-0.10214	-0.10179	-0.10144	-0.10109	-0.10074
		-0.10306	-0.10267	-0.1023	-0.10194	-0.10159	-0.10124	-0.10089
		-0.10325	-0.10284	-0.10246	-0.10209	-0.10175	-0.1014	-0.10104
		-0.05036	-0.05016	-0.04997	-0.10225	-0.10191	-0.10156	-0.1012
		-0.05042	-0.05022	-0.05003	-0.10242	-0.10207	-0.10172	-0.10136
					-0.10259	-0.10223	-0.10187	-0.10152
					-0.10276	-0.10238	-0.10202	-0.10168
					-0.05001	-0.04983	-0.10218	-0.10184
					-0.05007	-0.04988	-0.10234	-0.102
							-0.04975	-0.0496
							-0.04982	-0.04965
							-0.04988	-0.04971
							-0.04995	-0.04977
							-0.05002	-0.04983

							-0.05073	-0.05061
							-0.05065	-0.05052
					-0.05081	-0.05069	-0.15986	-0.15909
					-0.05071	-0.05059	-0.1594	-0.15869
					-0.05062	-0.0505	-0.20754	-0.20677
					-0.05053	-0.05041	-0.20707	-0.20633
					-0.05044	-0.05031	-0.20662	-0.20589
			-0.0506	-0.05047	-0.15918	-0.15849	-0.254	-0.25311
			-0.05051	-0.05039	-0.15878	-0.15813	-0.25353	-0.25263
			-0.10523	-0.10464	-0.20686	-0.2061	-0.44312	-0.44169
			-0.10488	-0.10438	-0.20645	-0.20569	-0.44234	-0.44088
-0.05061	-0.05048	-0.05036	-0.15417	-0.1536	-0.40045	-0.39917	-0.44156	-0.44007
-0.05052	-0.05039	-0.05027	-0.15381	-0.15325	-0.39967	-0.39843	-0.44078	-0.43929
-0.1057	-0.10508	-0.1045	-0.20661	-0.20586	-0.44278	-0.44141	-0.62231	-0.6203
-0.10534	-0.10477	-0.10424	-0.20619	-0.20544	-0.44198	-0.4406	-0.62121	-0.61922
-0.10501	-0.10448	-0.104	-0.20579	-0.20503	-0.44118	-0.43978	-0.62008	-0.61811
-0.15431	-0.1537	-0.15312	-0.39931	-0.398	-0.44039	-0.43894	-0.6575	-0.65544
-0.15398	-0.15338	-0.1528	-0.39859	-0.39728	-0.43959	-0.4381	-0.65629	-0.65422
-0.30917	-0.30818	-0.3072	-0.44178	-0.4403	-0.62073	-0.61866	-0.69116	-0.6889
-0.30862	-0.30764	-0.30666	-0.441	-0.43951	-0.61965	-0.61758	-0.68993	-0.68763
-0.35155	-0.35041	-0.34926	-0.48627	-0.48465	-0.61857	-0.6165	-0.73224	-0.72983
-0.3509	-0.34977	-0.34863	-0.48542	-0.48379	-0.61748	-0.61541	-0.73098	-0.72861
-0.35024	-0.34912	-0.34799	-0.48457	-0.48293	-0.6164	-0.61432	-0.72973	-0.72743
-0.44219	-0.44077	-0.43935	-0.61946	-0.61738	-0.65194	-0.64972	-0.79841	-0.79597
-0.44135	-0.43995	-0.43854	-0.61835	-0.61629	-0.65078	-0.64858	-0.7971	-0.7947
-0.4866	-0.48508	-0.48357	-0.61726	-0.6152	-0.73076	-0.72841	-0.79582	-0.79345
-0.48575	-0.48423	-0.48271	-0.61617	-0.61412	-0.72953	-0.72723	-0.79458	-0.79221
-0.58237	-0.58054	-0.57872	-0.65167	-0.64949	-0.79816	-0.79573	-0.83396	-0.8315
-0.58133	-0.57951	-0.5777	-0.65053	-0.64839	-0.79688	-0.79447	-0.83267	-0.83021
-0.65568	-0.65358	-0.65151	-0.72222	-0.71994	-0.79561	-0.79322	-0.83138	-0.82893
-0.65455	-0.65244	-0.65036	-0.72099	-0.71876	-0.79435	-0.79198	-0.83009	-0.82765
-0.65345	-0.65132	-0.64923	-0.71978	-0.71759	-0.7931	-0.79074	-0.82881	-0.82638
-0.72538	-0.72309	-0.72082	-0.79659	-0.79422	-0.83244	-0.82997	-1.00681	-1.00339
-0.72422	-0.72194	-0.71967	-0.79534	-0.79296	-0.83113	-0.82867	-1.00497	-1.00159
-0.72301	-0.72076	-0.71851	-0.7941	-0.79172	-0.82984	-0.82739	-1.07446	-1.07065
-0.72177	-0.71955	-0.71735	-0.79285	-0.79047	-0.82855	-0.82612	-1.07245	-1.06865
-0.76314	-0.76085	-0.75858	-0.8322	-0.82972	-1.0064	-1.00299	-1.14031	-1.13633
-0.76194	-0.75964	-0.75736	-0.8309	-0.82842	-1.00456	-1.0012	-1.13821	-1.13424
-0.76074	-0.75843	-0.75614	-0.85928	-0.8566	-1.11164	-1.10779	-1.19556	-1.19148
-0.75953	-0.75721	-0.75492	-0.85786	-0.85521	-1.10961	-1.10575	-1.1934	-1.18931
-0.75831	-0.75599	-0.7537	-0.85646	-0.85383	-1.10758	-1.10373	-1.19125	-1.18718
-0.83315	-0.83065	-0.82817	-1.00416	-1.00082	-1.13616	-1.13224	-1.18911	-1.18509
-0.83183	-0.82934	-0.82688	-1.00235	-0.99906	-1.13408	-1.13016	-1.18698	-1.18305
-0.86023	-0.85753	-0.85488	-1.10913	-1.10531	-1.19291	-1.18885	-1.18489	-1.18104
-0.85878	-0.85612	-0.85349	-1.1071	-1.10328	-1.19077	-1.18676	-1.18285	-1.17907
-1.07986	-1.07628	-1.07276	-1.13566	-1.13178	-1.18867	-1.18473	-1.29709	-1.29307
-1.07797	-1.07442	-1.07093	-1.13361	-1.12973	-1.1866	-1.1828	-1.29508	-1.29112
-1.07614	-1.07262	-1.06915	-1.1316	-1.12773	-1.18457	-1.18088	-1.29309	-1.28918
-1.11076	-1.1069	-1.10308	-1.1297	-1.12583	-1.29892	-1.29496	-1.32375	-1.31972

-1.1089	-1.10505	-1.10123	-1.12783	-1.12398	-1.29684	-1.29288	-1.32161	-1.31759
-1.13753	-1.1336	-1.12972	-1.27409	-1.26994	-1.3276	-1.32351	-1.31946	-1.31547
-1.13545	-1.13153	-1.12766	-1.27189	-1.26785	-1.32543	-1.32135	-1.31732	-1.31335
-1.19332	-1.18927	-1.18529	-1.26974	-1.26578	-1.32329	-1.31922	-1.36787	-1.36379
-1.1912	-1.18717	-1.18323	-1.26761	-1.26373	-1.32116	-1.31712	-1.36572	-1.36165
-1.27782	-1.27362	-1.26951	-1.29889	-1.29492	-1.34654	-1.34246	-1.42188	-1.41766
-1.27574	-1.27162	-1.26759	-1.29693	-1.29298	-1.34455	-1.3405	-1.41981	-1.41559
-1.27367	-1.26964	-1.26569	-1.295	-1.29104	-1.34256	-1.33854	-1.41774	-1.41352
-1.30503	-1.30095	-1.29694	-1.3486	-1.34452	-1.42402	-1.41977	-1.41554	-1.41133
-1.30281	-1.29878	-1.29483	-1.34644	-1.34237	-1.42175	-1.4175	-1.41328	-1.40908
-1.32867	-1.32462	-1.32063	-1.3443	-1.34024	-1.41951	-1.41526	-1.41103	-1.40683
-1.32655	-1.32251	-1.31852	-1.34216	-1.33813	-1.41727	-1.41301	-1.40878	-1.40456
-1.3245	-1.32047	-1.31649	-1.42361	-1.41935	-1.41509	-1.41085	-1.43336	-1.42906
-1.32254	-1.31854	-1.31456	-1.42153	-1.41726	-1.41302	-1.40879	-1.43128	-1.42695
-1.38093	-1.37676	-1.37261	-1.41941	-1.41514	-1.41091	-1.4067	-1.49992	-1.49519
-1.37876	-1.37459	-1.37043	-1.41716	-1.4129	-1.40868	-1.40448	-1.49755	-1.49297
-1.3766	-1.3724	-1.36823	-1.41489	-1.41063	-1.40642	-1.40225	-1.49517	-1.49075
-1.37453	-1.37029	-1.36608	-1.41265	-1.4084	-1.44555	-1.4413	-1.56353	-1.55895
-1.3725	-1.36822	-1.36397	-1.41044	-1.4062	-1.44331	-1.43911	-1.56112	-1.55657
-1.39518	-1.39086	-1.38656	-1.42328	-1.41902	-1.56822	-1.56348	-1.55883	-1.5543
-1.39301	-1.38874	-1.38451	-1.4212	-1.41696	-1.56594	-1.56127	-1.55668	-1.55216
-1.39088	-1.38665	-1.38245	-1.46677	-1.46241	-1.56363	-1.55902	-1.55447	-1.54996
-1.38883	-1.38457	-1.38036	-1.46456	-1.46024	-1.56125	-1.55668	-1.55216	-1.54766
-1.38677	-1.38251	-1.37829	-1.46238	-1.4581	-1.55891	-1.55437	-1.54986	-1.54538
-1.42209	-1.41776	-1.41348	-1.56608	-1.56134	-1.5567	-1.55217	-1.54767	-1.5432
-1.4199	-1.41563	-1.41143	-1.56379	-1.55913	-1.55452	-1.54999	-1.5455	-1.54105
-1.44424	-1.43992	-1.43565	-1.56144	-1.55682	-1.55224	-1.54772	-1.54323	-1.53879
-1.44217	-1.43784	-1.43356	-1.55907	-1.55446	-1.54989	-1.54538	-1.54091	-1.53649
-1.54811	-1.54325	-1.53846	-1.55681	-1.5522	-1.56908	-1.56454	-1.60392	-1.59938
-1.54569	-1.54097	-1.53631	-1.55467	-1.55006	-1.56691	-1.56238	-1.60172	-1.59719
-1.54334	-1.53872	-1.53416	-1.57399	-1.56934	-1.56475	-1.5602	-1.59949	-1.59496
-1.5411	-1.53652	-1.532	-1.57182	-1.5672	-1.56259	-1.55799	-1.5972	-1.59263
-1.53888	-1.53431	-1.5298	-1.5696	-1.56502	-1.56041	-1.55579	-1.59492	-1.59033
-1.55846	-1.55384	-1.54928	-1.56728	-1.56273	-1.60198	-1.59732	-1.59273	-1.58816
-1.55619	-1.55154	-1.54695	-1.56492	-1.56041	-1.59967	-1.5951	-1.59059	-1.58606
-1.55398	-1.54932	-1.54473	-1.58403	-1.57948	-1.59742	-1.59291	-1.58848	-1.58405
-1.55181	-1.54718	-1.5426	-1.58184	-1.5773	-1.59523	-1.59073	-1.58638	-1.58213
-1.57143	-1.56676	-1.56214	-1.57966	-1.57511	-1.59301	-1.58853	-1.58428	-1.58024
-1.56926	-1.56462	-1.56001	-1.57748	-1.57288	-1.59074	-1.58625	-1.58212	-1.57839
-1.56708	-1.56245	-1.55787	-1.59773	-1.59307	-1.58854	-1.58411	-1.58012	-1.57665
-1.5649	-1.56025	-1.55566	-1.59548	-1.59089	-1.58647	-1.58222	-1.57839	-1.57508
-1.56271	-1.55804	-1.55345	-1.59326	-1.58875	-1.58449	-1.58046	-1.5768	-1.57359
-1.56046	-1.55584	-1.55131	-1.5911	-1.58666	-1.58255	-1.57875	-1.57527	-1.57215
-1.5582	-1.55365	-1.54921	-1.589	-1.58462	-1.58067	-1.57712	-1.57381	-1.57076
-1.5788	-1.57425	-1.56982	-1.58698	-1.58271	-1.5789	-1.57551	-1.57234	-1.56938
-1.57664	-1.57213	-1.56776	-1.58502	-1.58092	-1.57724	-1.57394	-1.57087	-1.56803
-1.57452	-1.57005	-1.56574	-1.58312	-1.57919	-1.57564	-1.57245	-1.58921	-1.58642
-1.57244	-1.568	-1.56375	-1.58124	-1.57751	-1.57411	-1.57104	-1.58789	-1.58517
-1.57039	-1.566	-1.56183	-1.57945	-1.5759	-1.57265	-1.56969	-1.58662	-1.58394
-1.56839	-1.56409	-1.56003	-1.57776	-1.57438	-1.57125	-1.56838	-1.58537	-1.58273

-1.56642	-1.56224	-1.5583	-1.57616	-1.57292	-1.5699	-1.56711	-1.58415	-1.58154
-1.56445	-1.56041	-1.55665	-1.57462	-1.57151	-1.58837	-1.58561	-1.58294	-1.58036
-1.5625	-1.55864	-1.55506	-1.57315	-1.57015	-1.58709	-1.58438	-1.58175	-1.57918
-1.56064	-1.55694	-1.55353	-1.55042	-1.54756	-1.58583	-1.58317	-1.58056	-1.57798
-1.55885	-1.55531	-1.55206	-1.54907	-1.5463	-1.58461	-1.58199	-1.57938	-1.57676
-1.55718	-1.55378	-1.55066	-1.54776	-1.54507	-1.5834	-1.58081	-1.59698	-1.59433
-1.55568	-1.55238	-1.54933	-1.54649	-1.54386	-1.5822	-1.57964	-1.59582	-1.59316
-1.55424	-1.55103	-1.54804	-1.54527	-1.54267	-1.58101	-1.57847	-1.59466	-1.59201
-1.55277	-1.54969	-1.5468	-1.5642	-1.56158	-1.5986	-1.59606	-1.59349	-1.59086
-1.55131	-1.54837	-1.54558	-1.56302	-1.56043	-1.59743	-1.59488	-1.59232	-1.58971
-1.54988	-1.54704	-1.54434	-1.58088	-1.57832	-1.59624	-1.59367	-1.59111	-1.58854
-1.54849	-1.54572	-1.54309	-1.57968	-1.57715	-1.59505	-1.59243	-1.58986	-1.58736
-1.5865	-1.58373	-1.58109	-1.57852	-1.576	-1.57344	-1.57086	-1.58867	-1.5862
-1.58536	-1.58261	-1.57996	-1.57738	-1.57487	-1.57231	-1.56973	-1.58753	-1.58508
-1.58423	-1.5815	-1.57884	-1.57626	-1.57374	-1.57119	-1.56862	-1.59557	-1.59309
-1.58304	-1.58034	-1.5777	-1.57513	-1.57261	-1.57008	-1.56754	-1.59448	-1.59201
-1.58183	-1.57918	-1.57657	-1.57401	-1.57149	-1.56899	-1.56649	-1.59341	-1.59095
-1.58065	-1.57802	-1.57542	-1.57287	-1.57036	-1.57728	-1.57481	-1.63491	-1.63235
-1.57948	-1.57686	-1.57427	-1.57173	-1.56922	-1.57617	-1.57377	-1.63387	-1.63131
-1.57832	-1.57571	-1.57313	-1.5706	-1.56811	-1.57507	-1.57272	-1.64917	-1.64663
-1.57717	-1.57457	-1.572	-1.56949	-1.56703	-1.57399	-1.57165	-1.64805	-1.64555
-1.57603	-1.57343	-1.57087	-1.5778	-1.57532	-1.63268	-1.63025	-1.64694	-1.64447
-1.57489	-1.5723	-1.56974	-1.57667	-1.57422	-1.63155	-1.62915	-1.64585	-1.6434
-1.5833	-1.58067	-1.57808	-1.61304	-1.61056	-1.63044	-1.62806	-1.64476	-1.64234
-1.58215	-1.57957	-1.57704	-1.61198	-1.6095	-1.62934	-1.62697	-1.64367	-1.64129
-1.58101	-1.5785	-1.57602	-1.61095	-1.60845	-1.62827	-1.6259	-1.6426	-1.64024
-1.59706	-1.59456	-1.59211	-1.63221	-1.62966	-1.62721	-1.62485	-1.64155	-1.63924
-1.59593	-1.59345	-1.59102	-1.63111	-1.62859	-1.62617	-1.62382	-1.64052	-1.63825
-1.61514	-1.61263	-1.61018	-1.63002	-1.62755	-1.62515	-1.62281	-1.62053	-1.61834
-1.61403	-1.61152	-1.60908	-1.62894	-1.62654	-1.62417	-1.62183	-1.61955	-1.61734
-1.63536	-1.63279	-1.63029	-1.62787	-1.62552	-1.62317	-1.62084	-1.61856	-1.61636
-1.63431	-1.63174	-1.62923	-1.62682	-1.62447	-1.62214	-1.61984	-1.61758	-1.61536
-1.63327	-1.6307	-1.6282	-1.62578	-1.62344	-1.62113	-1.61885	-1.61661	-1.61439
-1.63218	-1.62966	-1.6272	-1.62479	-1.62244	-1.62015	-1.6179	-1.61568	-1.61345
-1.63109	-1.62862	-1.6262	-1.62381	-1.62147	-1.61918	-1.61697	-1.61476	-1.61253
-1.63001	-1.62756	-1.62517	-1.6228	-1.62047	-1.61821	-1.61601	-1.6138	-1.61159
-1.62894	-1.62649	-1.6241	-1.62176	-1.61947	-1.61723	-1.61503	-1.61284	-1.61065
-1.6279	-1.62546	-1.62308	-1.62077	-1.61851	-1.61628	-1.61407	-1.63342	-1.63111
-1.62691	-1.62449	-1.62212	-1.61983	-1.61759	-1.61536	-1.61315	-1.63243	-1.63017
-1.62592	-1.62353	-1.62118	-1.6189	-1.61668	-1.61445	-1.61225	-1.6551	-1.65277
-1.6249	-1.62256	-1.62026	-1.61799	-1.61575	-1.61354	-1.61137	-1.65416	-1.65181
-1.62388	-1.62159	-1.61934	-1.61707	-1.61481	-1.61262	-1.6105	-1.65324	-1.65086
-1.62289	-1.62061	-1.61837	-1.61611	-1.61386	-1.61168	-1.60958	-1.65224	-1.64988
-1.62191	-1.61963	-1.61738	-1.61513	-1.6129	-1.61074	-1.60864	-1.65122	-1.64889
-1.62097	-1.61868	-1.61642	-1.6142	-1.612	-1.65486	-1.65254	-1.67262	-1.67011
-1.62004	-1.61775	-1.61549	-1.61329	-1.61114	-1.65393	-1.65158	-1.67156	-1.6691
-1.61912	-1.61683	-1.61458	-1.6124	-1.61027	-1.65299	-1.65064	-1.75351	-1.75075
-1.61817	-1.61592	-1.6137	-1.61153	-1.60939	-1.65204	-1.64973	-1.75241	-1.74959
-1.61724	-1.61502	-1.61283	-1.69494	-1.69247	-1.71197	-1.70944	-1.7513	-1.74843
-1.61634	-1.61412	-1.61193	-1.69391	-1.6915	-1.71095	-1.70839	-1.75014	-1.74728

-1.61545	-1.61322	-1.61103	-1.69289	-1.69054	-1.70993	-1.70733	-1.74896	-1.74614
-1.65489	-1.65262	-1.65039	-1.69191	-1.68955	-1.70887	-1.70629	-1.74783	-1.74497
-1.65394	-1.6517	-1.64951	-1.69096	-1.68856	-1.7078	-1.70526	-1.74671	-1.7438
-1.69736	-1.69484	-1.69241	-1.71193	-1.70933	-1.70675	-1.70419	-1.74555	-1.74263
-1.69636	-1.69384	-1.6914	-1.71088	-1.70833	-1.70574	-1.7031	-1.74433	-1.74146
-1.6954	-1.69288	-1.69044	-1.70985	-1.70731	-1.74894	-1.74603	-1.74314	-1.74029
-1.69447	-1.69198	-1.68954	-1.70884	-1.70625	-1.74776	-1.74488	-1.742	-1.7391
-1.69354	-1.69108	-1.68865	-1.70785	-1.70519	-1.74659	-1.74374	-1.74086	-1.73792
-1.69255	-1.69014	-1.68773	-1.70687	-1.70419	-1.74547	-1.74256	-1.73966	-1.73675
-1.69155	-1.68917	-1.6868	-1.70588	-1.7032	-1.74437	-1.74138	-1.73846	-1.73561
-1.71263	-1.71005	-1.70748	-1.72344	-1.72072	-1.74327	-1.74028	-1.73735	-1.73451
-1.71164	-1.70902	-1.7064	-1.72234	-1.71965	-1.74218	-1.73921	-1.7363	-1.73345
-1.71066	-1.70802	-1.70536	-1.72126	-1.71856	-1.74104	-1.73811	-1.75351	-1.75065
-1.70968	-1.70704	-1.70438	-1.7202	-1.71743	-1.73984	-1.73695	-1.7524	-1.74961
-1.72735	-1.72466	-1.72194	-1.71915	-1.71634	-1.73867	-1.73581	-1.75129	-1.74856
-1.72627	-1.72359	-1.7209	-1.71812	-1.71531	-1.73757	-1.73469	-1.75016	-1.74749
-1.72518	-1.72251	-1.71983	-1.71708	-1.71429	-1.73651	-1.7336	-1.74905	-1.74641
-1.72416	-1.72144	-1.71872	-1.74122	-1.73832	-1.75373	-1.75082	-1.76343	-1.76073
-1.72315	-1.72037	-1.71759	-1.74004	-1.73718	-1.75264	-1.7498	-1.76242	-1.75966
-1.72213	-1.71933	-1.71653	-1.7389	-1.73602	-1.75151	-1.74875	-1.76139	-1.75863
-1.7211	-1.71832	-1.71552	-1.73778	-1.73484	-1.75033	-1.74767	-1.76037	-1.75763
-1.72006	-1.7173	-1.7145	-1.75504	-1.75203	-1.76465	-1.76201	-1.75935	-1.75662
-1.71902	-1.71626	-1.71349	-1.75398	-1.75101	-1.76364	-1.76099	-1.75832	-1.75561
-1.71795	-1.71521	-1.71246	-1.75293	-1.75001	-1.76263	-1.75995	-1.75727	-1.75458
-1.71683	-1.71411	-1.7114	-1.75185	-1.74898	-1.76159	-1.75887	-1.75617	-1.75352
-1.71571	-1.71301	-1.71034	-1.75077	-1.74796	-1.76055	-1.75778	-1.75507	-1.75244
-1.71465	-1.71195	-1.7093	-1.74971	-1.74694	-1.75953	-1.75675	-1.78427	-1.78153
-1.71362	-1.71092	-1.70828	-1.74864	-1.74592	-1.75852	-1.75574	-1.78322	-1.78043
-1.75607	-1.75316	-1.75031	-1.76296	-1.76025	-1.78788	-1.78502	-1.78219	-1.77937
-1.75497	-1.75204	-1.7492	-1.76186	-1.7592	-1.78683	-1.784	-1.78118	-1.77836
-1.75387	-1.75095	-1.74812	-1.79128	-1.78857	-1.7858	-1.78299	-1.78018	-1.77736
-1.75277	-1.74988	-1.74709	-1.79023	-1.78753	-1.78477	-1.78197	-1.77917	-1.77635
-1.75169	-1.74884	-1.74608	-1.7892	-1.78649	-1.78374	-1.78096	-1.77817	-1.77535
-1.78173	-1.77881	-1.77598	-1.78816	-1.78546	-1.78272	-1.77995	-1.77716	-1.77435
-1.7807	-1.77779	-1.77496	-1.78714	-1.78444	-1.7817	-1.77894	-1.77616	-1.77335
-1.77966	-1.77677	-1.77395	-1.78611	-1.78343	-1.78069	-1.77793	-1.77515	-1.77235
-1.77864	-1.77575	-1.77294	-1.7851	-1.78242	-1.77969	-1.77693	-1.77415	-1.77134
-1.79257	-1.78966	-1.78682	-1.78408	-1.78141	-1.77869	-1.77593	-1.77316	-1.77035
-1.79153	-1.78863	-1.78579	-1.78307	-1.78041	-1.77769	-1.77493	-1.77216	-1.76938
-1.79048	-1.7876	-1.78477	-1.78205	-1.7794	-1.77668	-1.77393	-1.81748	-1.81432
-1.78943	-1.78656	-1.78373	-1.78103	-1.77838	-1.77567	-1.77292	-1.81637	-1.81324
-1.78839	-1.78552	-1.7827	-1.78	-1.77737	-1.77467	-1.77192	-1.81525	-1.81216
-1.78735	-1.78449	-1.78169	-1.77899	-1.77635	-1.77365	-1.7709	-1.81413	-1.81106
-1.78631	-1.78348	-1.78069	-1.778	-1.77534	-1.77264	-1.76989	-1.81301	-1.80997
-1.78529	-1.7825	-1.77975	-1.77704	-1.77436	-1.79011	-1.78729	-1.81191	-1.80888
-1.78428	-1.78155	-1.77884	-1.77611	-1.77338	-1.78909	-1.78628	-1.81083	-1.8078
-1.78331	-1.7806	-1.7779	-1.77516	-1.7724	-1.78807	-1.78527	-1.80975	-1.80672
-1.78239	-1.77966	-1.77693	-1.77417	-1.7714	-1.78704	-1.78425	-1.80868	-1.80565
-1.78146	-1.7787	-1.77594	-1.79168	-1.78883	-1.78602	-1.78323	-1.8076	-1.80458
-1.78048	-1.77772	-1.77495	-1.79065	-1.7878	-1.78498	-1.7822	-1.80652	-1.8035

-1.77949	-1.77672	-1.77394	-1.78961	-1.78676	-1.78396	-1.78119	-1.80545	-1.80245
-1.7785	-1.77571	-1.77294	-1.78859	-1.78575	-1.81041	-1.80739	-1.8486	-1.84533
-1.77749	-1.7747	-1.77193	-1.78757	-1.78476	-1.80937	-1.80635	-1.8475	-1.84429
-1.79508	-1.79221	-1.78937	-1.78657	-1.78381	-1.80838	-1.80537	-1.84645	-1.84326
-1.79403	-1.79117	-1.78835	-1.7856	-1.7829	-1.80745	-1.80444	-1.84545	-1.84223
-1.793	-1.79016	-1.78737	-1.78465	-1.78197	-1.80648	-1.80348	-1.8444	-1.84117
-1.79199	-1.7892	-1.78644	-1.78371	-1.78101	-1.80544	-1.80243	-1.84328	-1.84007
-1.79101	-1.78825	-1.78551	-1.78277	-1.78003	-1.80438	-1.80137	-1.84215	-1.83896
-1.7901	-1.78732	-1.78455	-1.78179	-1.77903	-1.80331	-1.80032	-1.86301	-1.85961
-1.7892	-1.78638	-1.78358	-1.78079	-1.77803	-1.80224	-1.79928	-1.86185	-1.85846
-1.78824	-1.78541	-1.7826	-1.7798	-1.77703	-1.80121	-1.7983	-1.87487	-1.87149
-1.78725	-1.78443	-1.78162	-1.77882	-1.77605	-1.8002	-1.79736	-1.87384	-1.87048
-1.7863	-1.78349	-1.78068	-1.77789	-1.77512	-1.81401	-1.81116	-1.87275	-1.8694
-1.7854	-1.7826	-1.7798	-1.77701	-1.77424	-1.81304	-1.81017	-1.87158	-1.86824
-1.78448	-1.78168	-1.77889	-1.79127	-1.78847	-1.83434	-1.83123	-1.87041	-1.86706
-1.78349	-1.78069	-1.77791	-1.79028	-1.7875	-1.83324	-1.83013	-1.86924	-1.86589
-1.7825	-1.77968	-1.7769	-1.78928	-1.78654	-1.83217	-1.82907	-1.86811	-1.86475
-1.80925	-1.80617	-1.80314	-1.81492	-1.81199	-1.83117	-1.82808	-1.87995	-1.87655
-1.80819	-1.80511	-1.80209	-1.8139	-1.81105	-1.8302	-1.82713	-1.87892	-1.87553
-1.82197	-1.81887	-1.81582	-1.81291	-1.81009	-1.84254	-1.83943	-1.87782	-1.87443
-1.82102	-1.81791	-1.81487	-1.81195	-1.8091	-1.84146	-1.83835	-1.87667	-1.87329
-1.82001	-1.81692	-1.81389	-1.83321	-1.83015	-1.84042	-1.83732	-1.87556	-1.87219
-1.81893	-1.81586	-1.81287	-1.83216	-1.82915	-1.83944	-1.83635	-1.87452	-1.87117
-1.81789	-1.81485	-1.81188	-1.84453	-1.8415	-1.83844	-1.83535	-1.87346	-1.87012
-1.81696	-1.81394	-1.81098	-1.84352	-1.84046	-1.83738	-1.83429	-1.87232	-1.86899
-1.81606	-1.81306	-1.81011	-1.84253	-1.83943	-1.83633	-1.83323	-1.87119	-1.86786
-1.82885	-1.82582	-1.82285	-1.84157	-1.83846	-1.83535	-1.83224	-1.87014	-1.86681
-1.82791	-1.82492	-1.82197	-1.84063	-1.83751	-1.83439	-1.83127	-1.86912	-1.8658
-1.84909	-1.8459	-1.84275	-1.83961	-1.83649	-1.83336	-1.83024	-1.86803	-1.86471
-1.84797	-1.8448	-1.84166	-1.83853	-1.8354	-1.83228	-1.82914	-1.86688	-1.86357
-1.84691	-1.84375	-1.84062	-1.83749	-1.83437	-1.83124	-1.8281	-1.88208	-1.87861
-1.84595	-1.8428	-1.83968	-1.83655	-1.83342	-1.83028	-1.82715	-1.88103	-1.87756
-1.84498	-1.84185	-1.83874	-1.83561	-1.83248	-1.82934	-1.82621	-1.88	-1.87654
-1.84393	-1.84085	-1.83778	-1.83466	-1.83153	-1.8284	-1.82526	-1.87896	-1.87552
-1.84287	-1.83983	-1.83679	-1.83369	-1.83056	-1.82743	-1.8243	-1.87791	-1.87449
-1.84186	-1.83881	-1.83575	-1.83264	-1.8295	-1.86735	-1.864	-1.87677	-1.87337
-1.8409	-1.83779	-1.83468	-1.83154	-1.82839	-1.86618	-1.86284	-1.87558	-1.8722
-1.83996	-1.83682	-1.83368	-1.83052	-1.82737	-1.8651	-1.86177	-1.87447	-1.87111
-1.83905	-1.8359	-1.83275	-1.82959	-1.82642	-1.86409	-1.86076	-1.87343	-1.8701
-1.83814	-1.83499	-1.83183	-1.82866	-1.82549	-1.87932	-1.87583	-1.87242	-1.8691
-1.83724	-1.83407	-1.83091	-1.82773	-1.82457	-1.8783	-1.87482	-1.87142	-1.86812
-1.83632	-1.83315	-1.82997	-1.8268	-1.82364	-1.87728	-1.8738	-1.87041	-1.86711
-1.83538	-1.83221	-1.82904	-1.82587	-1.82271	-1.87624	-1.87274	-1.86932	-1.86602
-1.83442	-1.83125	-1.82808	-1.82492	-1.82177	-1.87519	-1.87165	-1.86822	-1.86492
-1.80809	-1.80513	-1.80218	-1.79925	-1.79633	-1.8741	-1.8706	-1.8672	-1.86389
-1.80705	-1.80409	-1.80114	-1.79822	-1.79532	-1.87298	-1.86958	-1.86623	-1.86294
-1.80607	-1.8031	-1.80016	-1.81429	-1.81124	-1.87191	-1.86857	-1.86527	-1.86199
-1.80515	-1.80218	-1.79924	-1.81334	-1.81031	-1.87088	-1.86759	-1.86431	-1.86105
-1.80425	-1.80129	-1.79836	-1.81242	-1.8094	-1.86989	-1.86662	-1.86336	-1.86011
-1.80336	-1.80042	-1.7975	-1.81153	-1.80852	-1.86895	-1.86568	-1.86242	-1.85918

-1.80249	-1.79955	-1.79664	-1.81065	-1.80766	-1.86802	-1.86474	-1.86149	-1.85824
-1.80161	-1.79868	-1.79579	-1.80977	-1.80678	-1.86708	-1.86381	-1.86055	-1.85732
-1.80074	-1.79782	-1.79493	-1.80888	-1.80591	-1.86614	-1.86287	-1.85963	-1.8564
-1.81712	-1.81403	-1.81099	-1.80799	-1.80503	-1.86521	-1.86194	-1.8587	-1.85548
-1.81619	-1.81312	-1.81009	-1.8071	-1.80416	-1.86428	-1.86101	-1.85777	-1.85456
-1.81525	-1.8122	-1.80918	-1.80621	-1.80328	-1.86334	-1.86008	-1.85684	-1.85364
-1.81432	-1.81128	-1.80827	-1.80531	-1.80239	-1.86239	-1.85914	-1.85591	-1.85271
-1.81339	-1.81036	-1.80737	-1.80442	-1.8015	-1.86144	-1.8582	-1.85498	-1.85179
-1.81246	-1.80945	-1.80647	-1.80352	-1.80061	-1.86049	-1.85725	-1.85404	-1.85085
-1.81153	-1.80853	-1.80557	-1.80263	-1.79972	-1.85954	-1.85631	-1.8531	-1.84991
-1.79385	-1.79089	-1.78798	-1.80174	-1.79883	-1.83586	-1.83277	-1.85216	-1.84898
-1.79293	-1.78999	-1.78708	-1.80085	-1.79795	-1.83493	-1.83185	-1.85123	-1.84804
-1.79203	-1.78909	-1.7862	-1.79996	-1.79706	-1.834	-1.83093	-1.85029	-1.84711
-1.79115	-1.7882	-1.78531	-1.79907	-1.79617	-1.83307	-1.83	-1.84935	-1.84617
-1.79031	-1.78735	-1.78445	-1.7982	-1.7953	-1.83215	-1.82908	-1.84841	-1.84523
-1.78954	-1.78655	-1.78361	-1.79734	-1.79443	-1.83123	-1.82816	-1.84747	-1.84429
-1.78877	-1.78577	-1.78279	-1.79652	-1.7936	-1.83034	-1.82725	-1.84653	-1.84336
-1.78795	-1.78498	-1.78203	-1.79577	-1.79284	-1.82952	-1.82639	-1.84562	-1.84243
-1.7871	-1.78418	-1.78128	-1.79504	-1.79212	-1.82873	-1.82555	-1.84473	-1.84151
-1.78622	-1.78334	-1.78046	-1.79423	-1.79133	-1.82791	-1.82473	-1.84389	-1.84065
-1.78533	-1.78245	-1.77958	-1.79338	-1.79049	-1.82706	-1.82394	-1.84311	-1.83983
-1.78445	-1.78157	-1.7787	-1.79251	-1.78964	-1.82618	-1.8231	-1.84228	-1.83902
-1.78357	-1.78069	-1.77782	-1.79163	-1.78876	-1.82526	-1.8222	-1.84142	-1.83823
-1.78274	-1.77983	-1.77695	-1.79076	-1.7879	-1.82435	-1.8213	-1.84053	-1.8374
-1.78198	-1.77904	-1.77612	-1.78991	-1.78704	-1.82344	-1.82039	-1.83962	-1.83652
-1.78123	-1.77826	-1.77532	-1.7891	-1.78622	-1.82256	-1.8195	-1.83871	-1.83562
-1.78042	-1.77748	-1.77456	-1.77169	-1.76886	-1.82175	-1.81865	-1.83782	-1.83474
-1.77959	-1.7767	-1.77382	-1.77098	-1.76814	-1.82099	-1.81785	-1.83697	-1.83388
-1.77875	-1.77589	-1.77303	-1.7702	-1.76737	-1.82019	-1.81707	-1.81401	-1.81104
-1.7779	-1.77504	-1.7722	-1.76937	-1.76656	-1.81938	-1.81631	-1.81329	-1.81034
-1.7771	-1.77424	-1.7714	-1.76857	-1.76575	-1.81855	-1.81553	-1.81254	-1.80958
-1.77637	-1.77352	-1.77067	-1.76782	-1.76496	-1.8177	-1.81469	-1.8117	-1.80875
-1.76465	-1.76182	-1.75899	-1.76706	-1.76419	-1.81687	-1.81387	-1.81088	-1.80793
-1.76385	-1.76102	-1.7582	-1.76629	-1.76345	-1.81613	-1.81313	-1.81013	-1.80714
-1.76303	-1.7602	-1.75739	-1.7655	-1.7627	-1.81539	-1.8124	-1.8094	-1.80635
-1.76227	-1.75942	-1.75659	-1.75379	-1.75103	-1.81458	-1.8116	-1.80861	-1.80558
-1.76154	-1.75865	-1.75578	-1.75297	-1.7502	-1.81372	-1.81075	-1.80778	-1.8048
-1.76077	-1.75788	-1.75502	-1.75221	-1.74943	-1.79665	-1.7937	-1.80695	-1.80399
-1.75996	-1.75711	-1.75429	-1.75149	-1.74871	-1.79586	-1.79286	-1.80609	-1.80312
-1.75917	-1.75634	-1.75353	-1.75074	-1.74796	-1.78462	-1.78162	-1.80527	-1.80229
-1.75842	-1.75556	-1.75273	-1.74993	-1.74716	-1.78381	-1.78084	-1.8045	-1.80153
-1.75766	-1.75478	-1.75193	-1.74913	-1.74637	-1.783	-1.78006	-1.80374	-1.80078
-1.75686	-1.75401	-1.75118	-1.74839	-1.74564	-1.78223	-1.77931	-1.80298	-1.79998
-1.75606	-1.75325	-1.75045	-1.74768	-1.74492	-1.78148	-1.77855	-1.80221	-1.79918
-1.75532	-1.75251	-1.74972	-1.74693	-1.74415	-1.78066	-1.77773	-1.79118	-1.78819
-1.75461	-1.7518	-1.749	-1.74618	-1.74336	-1.77981	-1.77687	-1.79034	-1.7874
-1.7539	-1.75107	-1.74825	-1.74542	-1.74258	-1.77899	-1.77605	-1.77313	-1.77025
-1.75318	-1.75032	-1.74747	-1.74465	-1.74184	-1.77822	-1.77527	-1.77236	-1.76949
-1.75243	-1.74957	-1.74671	-1.74389	-1.7411	-1.77746	-1.7745	-1.77157	-1.76871
-1.75165	-1.74881	-1.74598	-1.74317	-1.74038	-1.77669	-1.77369	-1.77074	-1.76787

							-0.05073	-0.05061
							-0.05065	-0.05052
					-0.05081	-0.05069	-0.15986	-0.15909
					-0.05071	-0.05059	-0.1594	-0.15869
					-0.05062	-0.0505	-0.20754	-0.20677
					-0.05053	-0.05041	-0.20707	-0.20633
					-0.05044	-0.05031	-0.20662	-0.20589
			-0.0506	-0.05047	-0.15918	-0.15849	-0.254	-0.25311
			-0.05051	-0.05039	-0.15878	-0.15813	-0.25353	-0.25263
			-0.10523	-0.10464	-0.20686	-0.2061	-0.44312	-0.44169
			-0.10488	-0.10438	-0.20645	-0.20569	-0.44234	-0.44088
-0.05061	-0.05048	-0.05036	-0.15417	-0.1536	-0.40045	-0.39917	-0.44156	-0.44007
-0.05052	-0.05039	-0.05027	-0.15381	-0.15325	-0.39967	-0.39843	-0.44078	-0.43929
-0.1057	-0.10508	-0.1045	-0.20661	-0.20586	-0.44278	-0.44141	-0.62231	-0.6203
-0.10534	-0.10477	-0.10424	-0.20619	-0.20544	-0.44198	-0.4406	-0.62121	-0.61922
-0.10501	-0.10448	-0.104	-0.20579	-0.20503	-0.44118	-0.43978	-0.62008	-0.61811
-0.15431	-0.1537	-0.15312	-0.39931	-0.398	-0.44039	-0.43894	-0.6575	-0.65544
-0.15398	-0.15338	-0.1528	-0.39859	-0.39728	-0.43959	-0.4381	-0.65629	-0.65422
-0.30917	-0.30818	-0.3072	-0.44178	-0.4403	-0.62073	-0.61866	-0.69116	-0.6889
-0.30862	-0.30764	-0.30666	-0.441	-0.43951	-0.61965	-0.61758	-0.68993	-0.68763
-0.35155	-0.35041	-0.34926	-0.48627	-0.48465	-0.61857	-0.6165	-0.73224	-0.72983
-0.3509	-0.34977	-0.34863	-0.48542	-0.48379	-0.61748	-0.61541	-0.73098	-0.72861
-0.35024	-0.34912	-0.34799	-0.48457	-0.48293	-0.6164	-0.61432	-0.72973	-0.72743
-0.44219	-0.44077	-0.43935	-0.61946	-0.61738	-0.65194	-0.64972	-0.79841	-0.79597
-0.44135	-0.43995	-0.43854	-0.61835	-0.61629	-0.65078	-0.64858	-0.7971	-0.7947
-0.4866	-0.48508	-0.48357	-0.61726	-0.6152	-0.73076	-0.72841	-0.79582	-0.79345
-0.48575	-0.48423	-0.48271	-0.61617	-0.61412	-0.72953	-0.72723	-0.79458	-0.79221
-0.58237	-0.58054	-0.57872	-0.65167	-0.64949	-0.79816	-0.79573	-0.83396	-0.8315
-0.58133	-0.57951	-0.5777	-0.65053	-0.64839	-0.79688	-0.79447	-0.83267	-0.83021
-0.65568	-0.65358	-0.65151	-0.72222	-0.71994	-0.79561	-0.79322	-0.83138	-0.82893
-0.65455	-0.65244	-0.65036	-0.72099	-0.71876	-0.79435	-0.79198	-0.83009	-0.82765
-0.65345	-0.65132	-0.64923	-0.71978	-0.71759	-0.7931	-0.79074	-0.82881	-0.82638
-0.72538	-0.72309	-0.72082	-0.79659	-0.79422	-0.83244	-0.82997	-1.00681	-1.00339
-0.72422	-0.72194	-0.71967	-0.79534	-0.79296	-0.83113	-0.82867	-1.00497	-1.00159
-0.72301	-0.72076	-0.71851	-0.7941	-0.79172	-0.82984	-0.82739	-1.07446	-1.07065
-0.72177	-0.71955	-0.71735	-0.79285	-0.79047	-0.82855	-0.82612	-1.07245	-1.06865
-0.76314	-0.76085	-0.75858	-0.8322	-0.82972	-1.0064	-1.00299	-1.14031	-1.13633
-0.76194	-0.75964	-0.75736	-0.8309	-0.82842	-1.00456	-1.0012	-1.13821	-1.13424
-0.76074	-0.75843	-0.75614	-0.85928	-0.8566	-1.11164	-1.10779	-1.19556	-1.19148
-0.75953	-0.75721	-0.75492	-0.85786	-0.85521	-1.10961	-1.10575	-1.1934	-1.18931
-0.75831	-0.75599	-0.7537	-0.85646	-0.85383	-1.10758	-1.10373	-1.19125	-1.18718
-0.83315	-0.83065	-0.82817	-1.00416	-1.00082	-1.13616	-1.13224	-1.18911	-1.18509
-0.83183	-0.82934	-0.82688	-1.00235	-0.99906	-1.13408	-1.13016	-1.18698	-1.18305
-0.86023	-0.85753	-0.85488	-1.10913	-1.10531	-1.19291	-1.18885	-1.18489	-1.18104
-0.85878	-0.85612	-0.85349	-1.1071	-1.10328	-1.19077	-1.18676	-1.18285	-1.17907
-1.07986	-1.07628	-1.07276	-1.13566	-1.13178	-1.18867	-1.18473	-1.29709	-1.29307
-1.07797	-1.07442	-1.07093	-1.13361	-1.12973	-1.1866	-1.1828	-1.29508	-1.29112
-1.07614	-1.07262	-1.06915	-1.1316	-1.12773	-1.18457	-1.18088	-1.29309	-1.28918
-1.11076	-1.1069	-1.10308	-1.1297	-1.12583	-1.29892	-1.29496	-1.32375	-1.31972

-1.1089	-1.10505	-1.10123	-1.12783	-1.12398	-1.29684	-1.29288	-1.32161	-1.31759
-1.13753	-1.1336	-1.12972	-1.27409	-1.26994	-1.3276	-1.32351	-1.31946	-1.31547
-1.13545	-1.13153	-1.12766	-1.27189	-1.26785	-1.32543	-1.32135	-1.31732	-1.31335
-1.19332	-1.18927	-1.18529	-1.26974	-1.26578	-1.32329	-1.31922	-1.36787	-1.36379
-1.1912	-1.18717	-1.18323	-1.26761	-1.26373	-1.32116	-1.31712	-1.36572	-1.36165
-1.27782	-1.27362	-1.26951	-1.29889	-1.29492	-1.34654	-1.34246	-1.42188	-1.41766
-1.27574	-1.27162	-1.26759	-1.29693	-1.29298	-1.34455	-1.3405	-1.41981	-1.41559
-1.27367	-1.26964	-1.26569	-1.295	-1.29104	-1.34256	-1.33854	-1.41774	-1.41352
-1.30503	-1.30095	-1.29694	-1.3486	-1.34452	-1.42402	-1.41977	-1.41554	-1.41133
-1.30281	-1.29878	-1.29483	-1.34644	-1.34237	-1.42175	-1.4175	-1.41328	-1.40908
-1.32867	-1.32462	-1.32063	-1.3443	-1.34024	-1.41951	-1.41526	-1.41103	-1.40683
-1.32655	-1.32251	-1.31852	-1.34216	-1.33813	-1.41727	-1.41301	-1.40878	-1.40456
-1.3245	-1.32047	-1.31649	-1.42361	-1.41935	-1.41509	-1.41085	-1.43336	-1.42906
-1.32254	-1.31854	-1.31456	-1.42153	-1.41726	-1.41302	-1.40879	-1.43128	-1.42695
-1.38093	-1.37676	-1.37261	-1.41941	-1.41514	-1.41091	-1.4067	-1.49992	-1.49519
-1.37876	-1.37459	-1.37043	-1.41716	-1.4129	-1.40868	-1.40448	-1.49755	-1.49297
-1.3766	-1.3724	-1.36823	-1.41489	-1.41063	-1.40642	-1.40225	-1.49517	-1.49075
-1.37453	-1.37029	-1.36608	-1.41265	-1.4084	-1.44555	-1.4413	-1.56353	-1.55895
-1.3725	-1.36822	-1.36397	-1.41044	-1.4062	-1.44331	-1.43911	-1.56112	-1.55657
-1.39518	-1.39086	-1.38656	-1.42328	-1.41902	-1.56822	-1.56348	-1.55883	-1.5543
-1.39301	-1.38874	-1.38451	-1.4212	-1.41696	-1.56594	-1.56127	-1.55668	-1.55216
-1.39088	-1.38665	-1.38245	-1.46677	-1.46241	-1.56363	-1.55902	-1.55447	-1.54996
-1.38883	-1.38457	-1.38036	-1.46456	-1.46024	-1.56125	-1.55668	-1.55216	-1.54766
-1.38677	-1.38251	-1.37829	-1.46238	-1.4581	-1.55891	-1.55437	-1.54986	-1.54538
-1.42209	-1.41776	-1.41348	-1.56608	-1.56134	-1.5567	-1.55217	-1.54767	-1.5432
-1.4199	-1.41563	-1.41143	-1.56379	-1.55913	-1.55452	-1.54999	-1.5455	-1.54105
-1.44424	-1.43992	-1.43565	-1.56144	-1.55682	-1.55224	-1.54772	-1.54323	-1.53879
-1.44217	-1.43784	-1.43356	-1.55907	-1.55446	-1.54989	-1.54538	-1.54091	-1.53649
-1.54811	-1.54325	-1.53846	-1.55681	-1.5522	-1.56908	-1.56454	-1.60392	-1.59938
-1.54569	-1.54097	-1.53631	-1.55467	-1.55006	-1.56691	-1.56238	-1.60172	-1.59719
-1.54334	-1.53872	-1.53416	-1.57399	-1.56934	-1.56475	-1.5602	-1.59949	-1.59496
-1.5411	-1.53652	-1.532	-1.57182	-1.5672	-1.56259	-1.55799	-1.5972	-1.59263
-1.53888	-1.53431	-1.5298	-1.5696	-1.56502	-1.56041	-1.55579	-1.59492	-1.59033
-1.55846	-1.55384	-1.54928	-1.56728	-1.56273	-1.60198	-1.59732	-1.59273	-1.58816
-1.55619	-1.55154	-1.54695	-1.56492	-1.56041	-1.59967	-1.5951	-1.59059	-1.58606
-1.55398	-1.54932	-1.54473	-1.58403	-1.57948	-1.59742	-1.59291	-1.58848	-1.58405
-1.55181	-1.54718	-1.5426	-1.58184	-1.5773	-1.59523	-1.59073	-1.58638	-1.58213
-1.57143	-1.56676	-1.56214	-1.57966	-1.57511	-1.59301	-1.58853	-1.58428	-1.58024
-1.56926	-1.56462	-1.56001	-1.57748	-1.57288	-1.59074	-1.58625	-1.58212	-1.57839
-1.56708	-1.56245	-1.55787	-1.59773	-1.59307	-1.58854	-1.58411	-1.58012	-1.57665
-1.5649	-1.56025	-1.55566	-1.59548	-1.59089	-1.58647	-1.58222	-1.57839	-1.57508
-1.56271	-1.55804	-1.55345	-1.59326	-1.58875	-1.58449	-1.58046	-1.5768	-1.57359
-1.56046	-1.55584	-1.55131	-1.5911	-1.58666	-1.58255	-1.57875	-1.57527	-1.57215
-1.5582	-1.55365	-1.54921	-1.589	-1.58462	-1.58067	-1.57712	-1.57381	-1.57076
-1.5788	-1.57425	-1.56982	-1.58698	-1.58271	-1.5789	-1.57551	-1.57234	-1.56938
-1.57664	-1.57213	-1.56776	-1.58502	-1.58092	-1.57724	-1.57394	-1.57087	-1.56803
-1.57452	-1.57005	-1.56574	-1.58312	-1.57919	-1.57564	-1.57245	-1.58921	-1.58642
-1.57244	-1.568	-1.56375	-1.58124	-1.57751	-1.57411	-1.57104	-1.58789	-1.58517
-1.57039	-1.566	-1.56183	-1.57945	-1.5759	-1.57265	-1.56969	-1.58662	-1.58394
-1.56839	-1.56409	-1.56003	-1.57776	-1.57438	-1.57125	-1.56838	-1.58537	-1.58273

-1.56642	-1.56224	-1.5583	-1.57616	-1.57292	-1.5699	-1.56711	-1.58415	-1.58154
-1.56445	-1.56041	-1.55665	-1.57462	-1.57151	-1.58837	-1.58561	-1.58294	-1.58036
-1.5625	-1.55864	-1.55506	-1.57315	-1.57015	-1.58709	-1.58438	-1.58175	-1.57918
-1.56064	-1.55694	-1.55353	-1.55042	-1.54756	-1.58583	-1.58317	-1.58056	-1.57798
-1.55885	-1.55531	-1.55206	-1.54907	-1.5463	-1.58461	-1.58199	-1.57938	-1.57676
-1.55718	-1.55378	-1.55066	-1.54776	-1.54507	-1.5834	-1.58081	-1.59698	-1.59433
-1.55568	-1.55238	-1.54933	-1.54649	-1.54386	-1.5822	-1.57964	-1.59582	-1.59316
-1.55424	-1.55103	-1.54804	-1.54527	-1.54267	-1.58101	-1.57847	-1.59466	-1.59201
-1.55277	-1.54969	-1.5468	-1.5642	-1.56158	-1.5986	-1.59606	-1.59349	-1.59086
-1.55131	-1.54837	-1.54558	-1.56302	-1.56043	-1.59743	-1.59488	-1.59232	-1.58971
-1.54988	-1.54704	-1.54434	-1.58088	-1.57832	-1.59624	-1.59367	-1.59111	-1.58854
-1.54849	-1.54572	-1.54309	-1.57968	-1.57715	-1.59505	-1.59243	-1.58986	-1.58736
-1.5865	-1.58373	-1.58109	-1.57852	-1.576	-1.57344	-1.57086	-1.58867	-1.5862
-1.58536	-1.58261	-1.57996	-1.57738	-1.57487	-1.57231	-1.56973	-1.58753	-1.58508
-1.58423	-1.5815	-1.57884	-1.57626	-1.57374	-1.57119	-1.56862	-1.59557	-1.59309
-1.58304	-1.58034	-1.5777	-1.57513	-1.57261	-1.57008	-1.56754	-1.59448	-1.59201
-1.58183	-1.57918	-1.57657	-1.57401	-1.57149	-1.56899	-1.56649	-1.59341	-1.59095
-1.58065	-1.57802	-1.57542	-1.57287	-1.57036	-1.57728	-1.57481	-1.63491	-1.63235
-1.57948	-1.57686	-1.57427	-1.57173	-1.56922	-1.57617	-1.57377	-1.63387	-1.63131
-1.57832	-1.57571	-1.57313	-1.5706	-1.56811	-1.57507	-1.57272	-1.64917	-1.64663
-1.57717	-1.57457	-1.572	-1.56949	-1.56703	-1.57399	-1.57165	-1.64805	-1.64555
-1.57603	-1.57343	-1.57087	-1.5778	-1.57532	-1.63268	-1.63025	-1.64694	-1.64447
-1.57489	-1.5723	-1.56974	-1.57667	-1.57422	-1.63155	-1.62915	-1.64585	-1.6434
-1.5833	-1.58067	-1.57808	-1.61304	-1.61056	-1.63044	-1.62806	-1.64476	-1.64234
-1.58215	-1.57957	-1.57704	-1.61198	-1.6095	-1.62934	-1.62697	-1.64367	-1.64129
-1.58101	-1.5785	-1.57602	-1.61095	-1.60845	-1.62827	-1.6259	-1.6426	-1.64024
-1.59706	-1.59456	-1.59211	-1.63221	-1.62966	-1.62721	-1.62485	-1.64155	-1.63924
-1.59593	-1.59345	-1.59102	-1.63111	-1.62859	-1.62617	-1.62382	-1.64052	-1.63825
-1.61514	-1.61263	-1.61018	-1.63002	-1.62755	-1.62515	-1.62281	-1.62053	-1.61834
-1.61403	-1.61152	-1.60908	-1.62894	-1.62654	-1.62417	-1.62183	-1.61955	-1.61734
-1.63536	-1.63279	-1.63029	-1.62787	-1.62552	-1.62317	-1.62084	-1.61856	-1.61636
-1.63431	-1.63174	-1.62923	-1.62682	-1.62447	-1.62214	-1.61984	-1.61758	-1.61536
-1.63327	-1.6307	-1.6282	-1.62578	-1.62344	-1.62113	-1.61885	-1.61661	-1.61439
-1.63218	-1.62966	-1.6272	-1.62479	-1.62244	-1.62015	-1.6179	-1.61568	-1.61345
-1.63109	-1.62862	-1.6262	-1.62381	-1.62147	-1.61918	-1.61697	-1.61476	-1.61253
-1.63001	-1.62756	-1.62517	-1.6228	-1.62047	-1.61821	-1.61601	-1.6138	-1.61159
-1.62894	-1.62649	-1.6241	-1.62176	-1.61947	-1.61723	-1.61503	-1.61284	-1.61065
-1.6279	-1.62546	-1.62308	-1.62077	-1.61851	-1.61628	-1.61407	-1.63342	-1.63111
-1.62691	-1.62449	-1.62212	-1.61983	-1.61759	-1.61536	-1.61315	-1.63243	-1.63017
-1.62592	-1.62353	-1.62118	-1.6189	-1.61668	-1.61445	-1.61225	-1.6551	-1.65277
-1.6249	-1.62256	-1.62026	-1.61799	-1.61575	-1.61354	-1.61137	-1.65416	-1.65181
-1.62388	-1.62159	-1.61934	-1.61707	-1.61481	-1.61262	-1.6105	-1.65324	-1.65086
-1.62289	-1.62061	-1.61837	-1.61611	-1.61386	-1.61168	-1.60958	-1.65224	-1.64988
-1.62191	-1.61963	-1.61738	-1.61513	-1.6129	-1.61074	-1.60864	-1.65122	-1.64889
-1.62097	-1.61868	-1.61642	-1.6142	-1.612	-1.65486	-1.65254	-1.67262	-1.67011
-1.62004	-1.61775	-1.61549	-1.61329	-1.61114	-1.65393	-1.65158	-1.67156	-1.6691
-1.61912	-1.61683	-1.61458	-1.6124	-1.61027	-1.65299	-1.65064	-1.75351	-1.75075
-1.61817	-1.61592	-1.6137	-1.61153	-1.60939	-1.65204	-1.64973	-1.75241	-1.74959
-1.61724	-1.61502	-1.61283	-1.69494	-1.69247	-1.71197	-1.70944	-1.7513	-1.74843
-1.61634	-1.61412	-1.61193	-1.69391	-1.6915	-1.71095	-1.70839	-1.75014	-1.74728

-1.61545	-1.61322	-1.61103	-1.69289	-1.69054	-1.70993	-1.70733	-1.74896	-1.74614
-1.65489	-1.65262	-1.65039	-1.69191	-1.68955	-1.70887	-1.70629	-1.74783	-1.74497
-1.65394	-1.6517	-1.64951	-1.69096	-1.68856	-1.7078	-1.70526	-1.74671	-1.7438
-1.69736	-1.69484	-1.69241	-1.71193	-1.70933	-1.70675	-1.70419	-1.74555	-1.74263
-1.69636	-1.69384	-1.6914	-1.71088	-1.70833	-1.70574	-1.7031	-1.74433	-1.74146
-1.6954	-1.69288	-1.69044	-1.70985	-1.70731	-1.74894	-1.74603	-1.74314	-1.74029
-1.69447	-1.69198	-1.68954	-1.70884	-1.70625	-1.74776	-1.74488	-1.742	-1.7391
-1.69354	-1.69108	-1.68865	-1.70785	-1.70519	-1.74659	-1.74374	-1.74086	-1.73792
-1.69255	-1.69014	-1.68773	-1.70687	-1.70419	-1.74547	-1.74256	-1.73966	-1.73675
-1.69155	-1.68917	-1.6868	-1.70588	-1.7032	-1.74437	-1.74138	-1.73846	-1.73561
-1.71263	-1.71005	-1.70748	-1.72344	-1.72072	-1.74327	-1.74028	-1.73735	-1.73451
-1.71164	-1.70902	-1.7064	-1.72234	-1.71965	-1.74218	-1.73921	-1.7363	-1.73345
-1.71066	-1.70802	-1.70536	-1.72126	-1.71856	-1.74104	-1.73811	-1.75351	-1.75065
-1.70968	-1.70704	-1.70438	-1.7202	-1.71743	-1.73984	-1.73695	-1.7524	-1.74961
-1.72735	-1.72466	-1.72194	-1.71915	-1.71634	-1.73867	-1.73581	-1.75129	-1.74856
-1.72627	-1.72359	-1.7209	-1.71812	-1.71531	-1.73757	-1.73469	-1.75016	-1.74749
-1.72518	-1.72251	-1.71983	-1.71708	-1.71429	-1.73651	-1.7336	-1.74905	-1.74641
-1.72416	-1.72144	-1.71872	-1.74122	-1.73832	-1.75373	-1.75082	-1.76343	-1.76073
-1.72315	-1.72037	-1.71759	-1.74004	-1.73718	-1.75264	-1.7498	-1.76242	-1.75966
-1.72213	-1.71933	-1.71653	-1.7389	-1.73602	-1.75151	-1.74875	-1.76139	-1.75863
-1.7211	-1.71832	-1.71552	-1.73778	-1.73484	-1.75033	-1.74767	-1.76037	-1.75763
-1.72006	-1.7173	-1.7145	-1.75504	-1.75203	-1.76465	-1.76201	-1.75935	-1.75662
-1.71902	-1.71626	-1.71349	-1.75398	-1.75101	-1.76364	-1.76099	-1.75832	-1.75561
-1.71795	-1.71521	-1.71246	-1.75293	-1.75001	-1.76263	-1.75995	-1.75727	-1.75458
-1.71683	-1.71411	-1.7114	-1.75185	-1.74898	-1.76159	-1.75887	-1.75617	-1.75352
-1.71571	-1.71301	-1.71034	-1.75077	-1.74796	-1.76055	-1.75778	-1.75507	-1.75244
-1.71465	-1.71195	-1.7093	-1.74971	-1.74694	-1.75953	-1.75675	-1.78427	-1.78153
-1.71362	-1.71092	-1.70828	-1.74864	-1.74592	-1.75852	-1.75574	-1.78322	-1.78043
-1.75607	-1.75316	-1.75031	-1.76296	-1.76025	-1.78788	-1.78502	-1.78219	-1.77937
-1.75497	-1.75204	-1.7492	-1.76186	-1.7592	-1.78683	-1.784	-1.78118	-1.77836
-1.75387	-1.75095	-1.74812	-1.79128	-1.78857	-1.7858	-1.78299	-1.78018	-1.77736
-1.75277	-1.74988	-1.74709	-1.79023	-1.78753	-1.78477	-1.78197	-1.77917	-1.77635
-1.75169	-1.74884	-1.74608	-1.7892	-1.78649	-1.78374	-1.78096	-1.77817	-1.77535
-1.78173	-1.77881	-1.77598	-1.78816	-1.78546	-1.78272	-1.77995	-1.77716	-1.77435
-1.7807	-1.77779	-1.77496	-1.78714	-1.78444	-1.7817	-1.77894	-1.77616	-1.77335
-1.77966	-1.77677	-1.77395	-1.78611	-1.78343	-1.78069	-1.77793	-1.77515	-1.77235
-1.77864	-1.77575	-1.77294	-1.7851	-1.78242	-1.77969	-1.77693	-1.77415	-1.77134
-1.79257	-1.78966	-1.78682	-1.78408	-1.78141	-1.77869	-1.77593	-1.77316	-1.77035
-1.79153	-1.78863	-1.78579	-1.78307	-1.78041	-1.77769	-1.77493	-1.77216	-1.76938
-1.79048	-1.7876	-1.78477	-1.78205	-1.7794	-1.77668	-1.77393	-1.81748	-1.81432
-1.78943	-1.78656	-1.78373	-1.78103	-1.77838	-1.77567	-1.77292	-1.81637	-1.81324
-1.78839	-1.78552	-1.7827	-1.78	-1.77737	-1.77467	-1.77192	-1.81525	-1.81216
-1.78735	-1.78449	-1.78169	-1.77899	-1.77635	-1.77365	-1.7709	-1.81413	-1.81106
-1.78631	-1.78348	-1.78069	-1.778	-1.77534	-1.77264	-1.76989	-1.81301	-1.80997
-1.78529	-1.7825	-1.77975	-1.77704	-1.77436	-1.79011	-1.78729	-1.81191	-1.80888
-1.78428	-1.78155	-1.77884	-1.77611	-1.77338	-1.78909	-1.78628	-1.81083	-1.8078
-1.78331	-1.7806	-1.7779	-1.77516	-1.7724	-1.78807	-1.78527	-1.80975	-1.80672
-1.78239	-1.77966	-1.77693	-1.77417	-1.7714	-1.78704	-1.78425	-1.80868	-1.80565
-1.78146	-1.7787	-1.77594	-1.79168	-1.78883	-1.78602	-1.78323	-1.8076	-1.80458
-1.78048	-1.77772	-1.77495	-1.79065	-1.7878	-1.78498	-1.7822	-1.80652	-1.8035

-1.77949	-1.77672	-1.77394	-1.78961	-1.78676	-1.78396	-1.78119	-1.80545	-1.80245
-1.7785	-1.77571	-1.77294	-1.78859	-1.78575	-1.81041	-1.80739	-1.8486	-1.84533
-1.77749	-1.7747	-1.77193	-1.78757	-1.78476	-1.80937	-1.80635	-1.8475	-1.84429
-1.79508	-1.79221	-1.78937	-1.78657	-1.78381	-1.80838	-1.80537	-1.84645	-1.84326
-1.79403	-1.79117	-1.78835	-1.7856	-1.7829	-1.80745	-1.80444	-1.84545	-1.84223
-1.793	-1.79016	-1.78737	-1.78465	-1.78197	-1.80648	-1.80348	-1.8444	-1.84117
-1.79199	-1.7892	-1.78644	-1.78371	-1.78101	-1.80544	-1.80243	-1.84328	-1.84007
-1.79101	-1.78825	-1.78551	-1.78277	-1.78003	-1.80438	-1.80137	-1.84215	-1.83896
-1.7901	-1.78732	-1.78455	-1.78179	-1.77903	-1.80331	-1.80032	-1.86301	-1.85961
-1.7892	-1.78638	-1.78358	-1.78079	-1.77803	-1.80224	-1.79928	-1.86185	-1.85846
-1.78824	-1.78541	-1.7826	-1.7798	-1.77703	-1.80121	-1.7983	-1.87487	-1.87149
-1.78725	-1.78443	-1.78162	-1.77882	-1.77605	-1.8002	-1.79736	-1.87384	-1.87048
-1.7863	-1.78349	-1.78068	-1.77789	-1.77512	-1.81401	-1.81116	-1.87275	-1.8694
-1.7854	-1.7826	-1.7798	-1.77701	-1.77424	-1.81304	-1.81017	-1.87158	-1.86824
-1.78448	-1.78168	-1.77889	-1.79127	-1.78847	-1.83434	-1.83123	-1.87041	-1.86706
-1.78349	-1.78069	-1.77791	-1.79028	-1.7875	-1.83324	-1.83013	-1.86924	-1.86589
-1.7825	-1.77968	-1.7769	-1.78928	-1.78654	-1.83217	-1.82907	-1.86811	-1.86475
-1.80925	-1.80617	-1.80314	-1.81492	-1.81199	-1.83117	-1.82808	-1.87995	-1.87655
-1.80819	-1.80511	-1.80209	-1.8139	-1.81105	-1.8302	-1.82713	-1.87892	-1.87553
-1.82197	-1.81887	-1.81582	-1.81291	-1.81009	-1.84254	-1.83943	-1.87782	-1.87443
-1.82102	-1.81791	-1.81487	-1.81195	-1.8091	-1.84146	-1.83835	-1.87667	-1.87329
-1.82001	-1.81692	-1.81389	-1.83321	-1.83015	-1.84042	-1.83732	-1.87556	-1.87219
-1.81893	-1.81586	-1.81287	-1.83216	-1.82915	-1.83944	-1.83635	-1.87452	-1.87117
-1.81789	-1.81485	-1.81188	-1.84453	-1.8415	-1.83844	-1.83535	-1.87346	-1.87012
-1.81696	-1.81394	-1.81098	-1.84352	-1.84046	-1.83738	-1.83429	-1.87232	-1.86899
-1.81606	-1.81306	-1.81011	-1.84253	-1.83943	-1.83633	-1.83323	-1.87119	-1.86786
-1.82885	-1.82582	-1.82285	-1.84157	-1.83846	-1.83535	-1.83224	-1.87014	-1.86681
-1.82791	-1.82492	-1.82197	-1.84063	-1.83751	-1.83439	-1.83127	-1.86912	-1.8658
-1.84909	-1.8459	-1.84275	-1.83961	-1.83649	-1.83336	-1.83024	-1.86803	-1.86471
-1.84797	-1.8448	-1.84166	-1.83853	-1.8354	-1.83228	-1.82914	-1.86688	-1.86357
-1.84691	-1.84375	-1.84062	-1.83749	-1.83437	-1.83124	-1.8281	-1.88208	-1.87861
-1.84595	-1.8428	-1.83968	-1.83655	-1.83342	-1.83028	-1.82715	-1.88103	-1.87756
-1.84498	-1.84185	-1.83874	-1.83561	-1.83248	-1.82934	-1.82621	-1.88	-1.87654
-1.84393	-1.84085	-1.83778	-1.83466	-1.83153	-1.8284	-1.82526	-1.87896	-1.87552
-1.84287	-1.83983	-1.83679	-1.83369	-1.83056	-1.82743	-1.8243	-1.87791	-1.87449
-1.84186	-1.83881	-1.83575	-1.83264	-1.8295	-1.86735	-1.864	-1.87677	-1.87337
-1.8409	-1.83779	-1.83468	-1.83154	-1.82839	-1.86618	-1.86284	-1.87558	-1.8722
-1.83996	-1.83682	-1.83368	-1.83052	-1.82737	-1.8651	-1.86177	-1.87447	-1.87111
-1.83905	-1.8359	-1.83275	-1.82959	-1.82642	-1.86409	-1.86076	-1.87343	-1.8701
-1.83814	-1.83499	-1.83183	-1.82866	-1.82549	-1.87932	-1.87583	-1.87242	-1.8691
-1.83724	-1.83407	-1.83091	-1.82773	-1.82457	-1.8783	-1.87482	-1.87142	-1.86812
-1.83632	-1.83315	-1.82997	-1.8268	-1.82364	-1.87728	-1.8738	-1.87041	-1.86711
-1.83538	-1.83221	-1.82904	-1.82587	-1.82271	-1.87624	-1.87274	-1.86932	-1.86602
-1.83442	-1.83125	-1.82808	-1.82492	-1.82177	-1.87519	-1.87165	-1.86822	-1.86492
-1.80809	-1.80513	-1.80218	-1.79925	-1.79633	-1.8741	-1.8706	-1.8672	-1.86389
-1.80705	-1.80409	-1.80114	-1.79822	-1.79532	-1.87298	-1.86958	-1.86623	-1.86294
-1.80607	-1.8031	-1.80016	-1.81429	-1.81124	-1.87191	-1.86857	-1.86527	-1.86199
-1.80515	-1.80218	-1.79924	-1.81334	-1.81031	-1.87088	-1.86759	-1.86431	-1.86105
-1.80425	-1.80129	-1.79836	-1.81242	-1.8094	-1.86989	-1.86662	-1.86336	-1.86011
-1.80336	-1.80042	-1.7975	-1.81153	-1.80852	-1.86895	-1.86568	-1.86242	-1.85918

-1.80249	-1.79955	-1.79664	-1.81065	-1.80766	-1.86802	-1.86474	-1.86149	-1.85824
-1.80161	-1.79868	-1.79579	-1.80977	-1.80678	-1.86708	-1.86381	-1.86055	-1.85732
-1.80074	-1.79782	-1.79493	-1.80888	-1.80591	-1.86614	-1.86287	-1.85963	-1.8564
-1.81712	-1.81403	-1.81099	-1.80799	-1.80503	-1.86521	-1.86194	-1.8587	-1.85548
-1.81619	-1.81312	-1.81009	-1.8071	-1.80416	-1.86428	-1.86101	-1.85777	-1.85456
-1.81525	-1.8122	-1.80918	-1.80621	-1.80328	-1.86334	-1.86008	-1.85684	-1.85364
-1.81432	-1.81128	-1.80827	-1.80531	-1.80239	-1.86239	-1.85914	-1.85591	-1.85271
-1.81339	-1.81036	-1.80737	-1.80442	-1.8015	-1.86144	-1.8582	-1.85498	-1.85179
-1.81246	-1.80945	-1.80647	-1.80352	-1.80061	-1.86049	-1.85725	-1.85404	-1.85085
-1.81153	-1.80853	-1.80557	-1.80263	-1.79972	-1.85954	-1.85631	-1.8531	-1.84991
-1.79385	-1.79089	-1.78798	-1.80174	-1.79883	-1.83586	-1.83277	-1.85216	-1.84898
-1.79293	-1.78999	-1.78708	-1.80085	-1.79795	-1.83493	-1.83185	-1.85123	-1.84804
-1.79203	-1.78909	-1.7862	-1.79996	-1.79706	-1.834	-1.83093	-1.85029	-1.84711
-1.79115	-1.7882	-1.78531	-1.79907	-1.79617	-1.83307	-1.83	-1.84935	-1.84617
-1.79031	-1.78735	-1.78445	-1.7982	-1.7953	-1.83215	-1.82908	-1.84841	-1.84523
-1.78954	-1.78655	-1.78361	-1.79734	-1.79443	-1.83123	-1.82816	-1.84747	-1.84429
-1.78877	-1.78577	-1.78279	-1.79652	-1.7936	-1.83034	-1.82725	-1.84653	-1.84336
-1.78795	-1.78498	-1.78203	-1.79577	-1.79284	-1.82952	-1.82639	-1.84562	-1.84243
-1.7871	-1.78418	-1.78128	-1.79504	-1.79212	-1.82873	-1.82555	-1.84473	-1.84151
-1.78622	-1.78334	-1.78046	-1.79423	-1.79133	-1.82791	-1.82473	-1.84389	-1.84065
-1.78533	-1.78245	-1.77958	-1.79338	-1.79049	-1.82706	-1.82394	-1.84311	-1.83983
-1.78445	-1.78157	-1.7787	-1.79251	-1.78964	-1.82618	-1.8231	-1.84228	-1.83902
-1.78357	-1.78069	-1.77782	-1.79163	-1.78876	-1.82526	-1.8222	-1.84142	-1.83823
-1.78274	-1.77983	-1.77695	-1.79076	-1.7879	-1.82435	-1.8213	-1.84053	-1.8374
-1.78198	-1.77904	-1.77612	-1.78991	-1.78704	-1.82344	-1.82039	-1.83962	-1.83652
-1.78123	-1.77826	-1.77532	-1.7891	-1.78622	-1.82256	-1.8195	-1.83871	-1.83562
-1.78042	-1.77748	-1.77456	-1.77169	-1.76886	-1.82175	-1.81865	-1.83782	-1.83474
-1.77959	-1.7767	-1.77382	-1.77098	-1.76814	-1.82099	-1.81785	-1.83697	-1.83388
-1.77875	-1.77589	-1.77303	-1.7702	-1.76737	-1.82019	-1.81707	-1.81401	-1.81104
-1.7779	-1.77504	-1.7722	-1.76937	-1.76656	-1.81938	-1.81631	-1.81329	-1.81034
-1.7771	-1.77424	-1.7714	-1.76857	-1.76575	-1.81855	-1.81553	-1.81254	-1.80958
-1.77637	-1.77352	-1.77067	-1.76782	-1.76496	-1.8177	-1.81469	-1.8117	-1.80875
-1.76465	-1.76182	-1.75899	-1.76706	-1.76419	-1.81687	-1.81387	-1.81088	-1.80793
-1.76385	-1.76102	-1.7582	-1.76629	-1.76345	-1.81613	-1.81313	-1.81013	-1.80714
-1.76303	-1.7602	-1.75739	-1.7655	-1.7627	-1.81539	-1.8124	-1.8094	-1.80635
-1.76227	-1.75942	-1.75659	-1.75379	-1.75103	-1.81458	-1.8116	-1.80861	-1.80558
-1.76154	-1.75865	-1.75578	-1.75297	-1.7502	-1.81372	-1.81075	-1.80778	-1.8048
-1.76077	-1.75788	-1.75502	-1.75221	-1.74943	-1.79665	-1.7937	-1.80695	-1.80399
-1.75996	-1.75711	-1.75429	-1.75149	-1.74871	-1.79586	-1.79286	-1.80609	-1.80312
-1.75917	-1.75634	-1.75353	-1.75074	-1.74796	-1.78462	-1.78162	-1.80527	-1.80229
-1.75842	-1.75556	-1.75273	-1.74993	-1.74716	-1.78381	-1.78084	-1.8045	-1.80153
-1.75766	-1.75478	-1.75193	-1.74913	-1.74637	-1.783	-1.78006	-1.80374	-1.80078
-1.75686	-1.75401	-1.75118	-1.74839	-1.74564	-1.78223	-1.77931	-1.80298	-1.79998
-1.75606	-1.75325	-1.75045	-1.74768	-1.74492	-1.78148	-1.77855	-1.80221	-1.79918
-1.75532	-1.75251	-1.74972	-1.74693	-1.74415	-1.78066	-1.77773	-1.79118	-1.78819
-1.75461	-1.7518	-1.749	-1.74618	-1.74336	-1.77981	-1.77687	-1.79034	-1.7874
-1.7539	-1.75107	-1.74825	-1.74542	-1.74258	-1.77899	-1.77605	-1.77313	-1.77025
-1.75318	-1.75032	-1.74747	-1.74465	-1.74184	-1.77822	-1.77527	-1.77236	-1.76949
-1.75243	-1.74957	-1.74671	-1.74389	-1.7411	-1.77746	-1.7745	-1.77157	-1.76871
-1.75165	-1.74881	-1.74598	-1.74317	-1.74038	-1.77669	-1.77369	-1.77074	-1.76787

-0.80909	-0.80775	-0.80641	-0.80508	-0.80377	-0.80247	-0.8012	-0.83359	-0.83219
-0.80937	-0.80804	-0.80671	-0.80538	-0.80405	-0.80275	-0.80148	-0.83392	-0.83252
-0.80965	-0.80833	-0.80701	-0.80568	-0.80434	-0.80304	-0.80176	-0.83426	-0.83285
-0.80992	-0.80861	-0.8073	-0.80597	-0.80464	-0.80334	-0.80205	-0.80077	-0.79951
-0.8102	-0.80889	-0.80759	-0.80627	-0.80495	-0.80363	-0.80233	-0.80105	-0.79979
-0.74257	-0.74149	-0.74042	-0.80654	-0.80523	-0.80392	-0.80262	-0.80134	-0.80008
-0.74279	-0.74169	-0.74061	-0.80679	-0.8055	-0.80421	-0.80291	-0.80163	-0.80037
-0.74302	-0.74191	-0.74082	-0.80706	-0.80578	-0.80449	-0.8032	-0.80191	-0.80065
-0.74325	-0.74215	-0.74106	-0.80736	-0.80608	-0.80478	-0.80348	-0.8022	-0.80093
-0.74348	-0.74238	-0.7413	-0.80767	-0.80638	-0.80508	-0.80377	-0.80248	-0.8012
-0.74371	-0.74261	-0.74153	-0.80799	-0.8067	-0.80539	-0.80407	-0.80277	-0.80149
-0.74393	-0.74284	-0.74175	-0.8083	-0.80702	-0.80571	-0.80438	-0.80306	-0.80177
-0.74417	-0.74307	-0.74198	-0.74093	-0.73989	-0.80601	-0.80468	-0.80337	-0.80206
-0.74442	-0.74331	-0.74221	-0.74115	-0.74011	-0.80631	-0.805	-0.80368	-0.80236
-0.70902	-0.70799	-0.70698	-0.74138	-0.74033	-0.80662	-0.80531	-0.80399	-0.80265
-0.70922	-0.7082	-0.70719	-0.74162	-0.74056	-0.80692	-0.80562	-0.8043	-0.80295
-0.70942	-0.7084	-0.7074	-0.74186	-0.74079	-0.73973	-0.73868	-0.80461	-0.80326
-0.70963	-0.7086	-0.70761	-0.7421	-0.74104	-0.73997	-0.73891	-0.80492	-0.80357
-0.70984	-0.70881	-0.70781	-0.70683	-0.70586	-0.74021	-0.73914	-0.80522	-0.80388
-0.71008	-0.70904	-0.70803	-0.70704	-0.70608	-0.74046	-0.73938	-0.80552	-0.80419
-0.71032	-0.70927	-0.70825	-0.70726	-0.7063	-0.74071	-0.73961	-0.80582	-0.80449
-0.71056	-0.7095	-0.70847	-0.70748	-0.70651	-0.70551	-0.7045	-0.73876	-0.73767
-0.7108	-0.70974	-0.70871	-0.7077	-0.70672	-0.70572	-0.70472	-0.739	-0.73791
-0.71104	-0.70998	-0.70894	-0.70793	-0.70693	-0.70593	-0.70493	-0.70392	-0.70292
-0.71128	-0.71022	-0.70919	-0.70816	-0.70714	-0.70613	-0.70513	-0.70413	-0.70315
-0.71153	-0.71047	-0.70943	-0.70839	-0.70736	-0.70634	-0.70534	-0.70435	-0.70338
-0.71178	-0.71072	-0.70967	-0.70862	-0.70759	-0.70657	-0.70556	-0.70457	-0.7036
-0.71204	-0.71097	-0.70991	-0.70886	-0.70782	-0.7068	-0.70579	-0.7048	-0.70382
-0.7123	-0.71121	-0.71014	-0.70909	-0.70805	-0.70703	-0.70603	-0.70504	-0.70405
-0.71256	-0.71146	-0.71038	-0.70932	-0.70828	-0.70726	-0.70627	-0.70528	-0.70427
-0.67989	-0.67886	-0.67784	-0.70956	-0.70852	-0.70751	-0.70651	-0.70551	-0.7045
-0.68013	-0.67909	-0.67806	-0.70982	-0.70878	-0.70776	-0.70676	-0.70575	-0.70473
-0.68037	-0.67933	-0.67831	-0.71009	-0.70904	-0.70801	-0.707	-0.70599	-0.70497
-0.68062	-0.67959	-0.67857	-0.71037	-0.7093	-0.70825	-0.70723	-0.70622	-0.70522
-0.68088	-0.67985	-0.67884	-0.67781	-0.67679	-0.7085	-0.70747	-0.70646	-0.70547
-0.68114	-0.6801	-0.67909	-0.67806	-0.67703	-0.70876	-0.70772	-0.7067	-0.70571
-0.6814	-0.68036	-0.67933	-0.6783	-0.67727	-0.70903	-0.70799	-0.70696	-0.70595
-0.64727	-0.64633	-0.64542	-0.67854	-0.67753	-0.67652	-0.67553	-0.70721	-0.7062
-0.64748	-0.64654	-0.64562	-0.67878	-0.67779	-0.67678	-0.67577	-0.70747	-0.70644
-0.64772	-0.64676	-0.64583	-0.67903	-0.67804	-0.67703	-0.67601	-0.70773	-0.7067
-0.64798	-0.64701	-0.64606	-0.67929	-0.67828	-0.67726	-0.67624	-0.70799	-0.70696
-0.64824	-0.64726	-0.64629	-0.64536	-0.64445	-0.67749	-0.67648	-0.67549	-0.67452
-0.64851	-0.6475	-0.64651	-0.64556	-0.64465	-0.67773	-0.67673	-0.67574	-0.67476
-0.64877	-0.64774	-0.64673	-0.64578	-0.64486	-0.67797	-0.67698	-0.676	-0.675
-0.649	-0.64798	-0.64697	-0.64601	-0.64508	-0.67822	-0.67722	-0.67623	-0.67524
-0.64922	-0.64821	-0.64721	-0.64625	-0.6453	-0.67847	-0.67745	-0.67645	-0.67547
-0.64945	-0.64845	-0.64746	-0.64649	-0.64553	-0.64459	-0.64368	-0.67669	-0.6757
-0.64969	-0.64869	-0.64771	-0.64673	-0.64575	-0.64481	-0.6439	-0.67694	-0.67593
-0.64993	-0.64893	-0.64796	-0.64697	-0.64599	-0.64504	-0.64412	-0.67718	-0.67616
-0.65018	-0.64918	-0.6482	-0.64721	-0.64623	-0.64527	-0.64435	-0.67743	-0.67641

-0.65044	-0.64943	-0.64844	-0.64745	-0.64647	-0.64551	-0.64458	-0.64365	-0.64274
-0.65071	-0.64969	-0.64869	-0.6477	-0.64672	-0.64576	-0.64482	-0.64388	-0.64296
-0.65098	-0.64995	-0.64894	-0.64795	-0.64697	-0.64601	-0.64505	-0.64411	-0.64319
-0.6181	-0.61717	-0.61625	-0.64819	-0.64721	-0.64624	-0.64528	-0.64433	-0.64341
-0.61833	-0.61739	-0.61645	-0.64844	-0.64745	-0.64647	-0.6455	-0.64455	-0.64363
-0.61857	-0.61761	-0.61667	-0.64869	-0.64769	-0.64671	-0.64573	-0.64478	-0.64386
-0.61881	-0.61785	-0.6169	-0.64896	-0.64794	-0.64695	-0.64598	-0.64503	-0.6441
-0.61906	-0.6181	-0.61714	-0.6162	-0.61526	-0.64721	-0.64624	-0.64528	-0.64434
-0.61933	-0.61836	-0.6174	-0.61645	-0.61551	-0.6475	-0.64651	-0.64555	-0.6446
-0.61959	-0.61862	-0.61765	-0.6167	-0.61576	-0.64778	-0.64679	-0.64581	-0.64485
-0.61985	-0.61886	-0.61789	-0.61693	-0.61599	-0.64805	-0.64705	-0.64606	-0.64509
-0.6201	-0.61911	-0.61813	-0.61717	-0.61622	-0.64833	-0.64731	-0.64631	-0.64533
-0.62035	-0.61936	-0.61838	-0.61741	-0.61646	-0.64861	-0.64759	-0.64658	-0.64559
-0.62061	-0.61962	-0.61865	-0.61767	-0.6167	-0.64891	-0.64788	-0.64686	-0.64586
-0.56726	-0.56626	-0.56526	-0.61794	-0.61697	-0.61602	-0.61509	-0.64716	-0.64615
-0.56762	-0.56663	-0.56564	-0.61823	-0.61726	-0.6163	-0.61536	-0.64747	-0.64645
-0.54618	-0.54524	-0.54431	-0.56501	-0.56402	-0.61658	-0.61563	-0.64778	-0.64675
-0.54654	-0.5456	-0.54466	-0.56535	-0.56437	-0.61685	-0.61588	-0.64807	-0.64705
-0.54689	-0.54595	-0.54502	-0.5657	-0.56472	-0.56374	-0.56276	-0.56181	-0.56088
-0.54724	-0.54631	-0.54539	-0.56606	-0.56509	-0.56411	-0.56312	-0.56216	-0.56122
-0.54759	-0.54667	-0.54576	-0.56642	-0.56545	-0.56447	-0.56349	-0.56252	-0.56157
-0.54796	-0.54704	-0.54613	-0.54521	-0.54429	-0.56483	-0.56385	-0.56288	-0.56192
-0.54833	-0.54741	-0.54649	-0.54557	-0.54466	-0.56519	-0.56421	-0.56324	-0.56227
-0.54871	-0.54778	-0.54686	-0.54594	-0.54503	-0.56554	-0.56457	-0.5636	-0.56263
-0.54909	-0.54815	-0.54723	-0.54631	-0.5454	-0.5659	-0.56493	-0.56396	-0.56298
-0.54947	-0.54853	-0.5476	-0.54668	-0.54577	-0.54485	-0.54394	-0.56432	-0.56334
-0.54984	-0.54889	-0.54796	-0.54704	-0.54613	-0.54522	-0.54432	-0.5647	-0.56372
-0.55023	-0.54926	-0.54832	-0.5474	-0.54649	-0.54559	-0.54469	-0.56507	-0.56409
-0.55064	-0.54966	-0.5487	-0.54777	-0.54686	-0.54596	-0.54506	-0.56544	-0.56447
-0.55106	-0.55007	-0.54909	-0.54815	-0.54723	-0.54632	-0.54543	-0.5658	-0.56483
-0.55148	-0.55047	-0.54948	-0.54852	-0.5476	-0.54669	-0.5458	-0.56616	-0.56519
-0.55189	-0.55087	-0.54987	-0.5489	-0.54796	-0.54705	-0.54615	-0.56651	-0.56555
-0.5523	-0.55128	-0.55026	-0.54929	-0.54834	-0.54741	-0.54651	-0.56686	-0.56589
-0.55272	-0.55169	-0.55068	-0.54968	-0.54871	-0.54777	-0.54685	-0.5672	-0.56623
-0.55313	-0.55211	-0.55109	-0.55008	-0.54909	-0.54813	-0.5472	-0.56754	-0.56657
-0.55354	-0.55251	-0.55148	-0.55047	-0.54947	-0.5485	-0.54757	-0.5679	-0.56693
-0.55394	-0.55291	-0.55188	-0.55086	-0.54985	-0.54888	-0.54794	-0.56826	-0.56728
-0.55434	-0.55331	-0.55228	-0.55126	-0.55024	-0.54926	-0.54831	-0.54738	-0.54647
-0.55473	-0.55371	-0.55269	-0.55166	-0.55064	-0.54965	-0.54869	-0.54776	-0.54684
-0.55515	-0.55412	-0.5531	-0.55207	-0.55105	-0.55006	-0.54909	-0.54814	-0.54722
-0.55558	-0.55454	-0.55353	-0.5525	-0.55148	-0.55048	-0.5495	-0.54854	-0.5476
-0.55602	-0.55497	-0.55395	-0.55292	-0.5519	-0.55089	-0.5499	-0.54893	-0.54799
-0.55646	-0.5554	-0.55438	-0.55334	-0.55231	-0.55129	-0.55029	-0.54932	-0.54837
-0.55693	-0.55584	-0.55481	-0.55376	-0.55272	-0.5517	-0.55069	-0.54971	-0.54875
-0.55742	-0.55629	-0.55523	-0.55417	-0.55313	-0.55211	-0.5511	-0.55011	-0.54913
-0.55794	-0.55675	-0.55565	-0.55458	-0.55355	-0.55253	-0.55151	-0.55051	-0.54953
-0.51829	-0.51711	-0.51602	-0.555	-0.55398	-0.55296	-0.55193	-0.55093	-0.54993
-0.51885	-0.51759	-0.51643	-0.55544	-0.55442	-0.55339	-0.55237	-0.55136	-0.55036
-0.4427	-0.44155	-0.44049	-0.51581	-0.51485	-0.55382	-0.55279	-0.55178	-0.55078
-0.44321	-0.44201	-0.4409	-0.51625	-0.51525	-0.55425	-0.55322	-0.5522	-0.55121

-0.44372	-0.44249	-0.44133	-0.44033	-0.43944	-0.55467	-0.55364	-0.55263	-0.55163
-0.44423	-0.44298	-0.4418	-0.44074	-0.43978	-0.5551	-0.55407	-0.55305	-0.55204
-0.44475	-0.44348	-0.44228	-0.44117	-0.44014	-0.55555	-0.55451	-0.55348	-0.55246
-0.44527	-0.444	-0.44278	-0.44163	-0.44054	-0.51587	-0.51488	-0.55391	-0.55289
-0.4458	-0.44452	-0.4433	-0.4421	-0.44096	-0.51632	-0.51529	-0.55435	-0.55332
-0.44636	-0.44506	-0.44381	-0.44258	-0.44141	-0.44037	-0.43945	-0.51471	-0.51374
-0.44695	-0.4456	-0.44432	-0.44307	-0.44189	-0.44081	-0.43983	-0.51512	-0.51414
-0.44756	-0.44618	-0.44485	-0.44358	-0.44238	-0.44126	-0.44022	-0.43928	-0.43845
-0.44819	-0.44678	-0.44541	-0.44411	-0.44288	-0.44172	-0.44062	-0.43964	-0.4388
-0.44883	-0.44739	-0.44599	-0.44466	-0.4434	-0.4422	-0.44105	-0.44002	-0.43916
-0.44945	-0.448	-0.44658	-0.44523	-0.44393	-0.44269	-0.4415	-0.44043	-0.4395
-0.45006	-0.44862	-0.44719	-0.44582	-0.44449	-0.44321	-0.44199	-0.44088	-0.43988
-0.45067	-0.44922	-0.44779	-0.44641	-0.44506	-0.44378	-0.44254	-0.44138	-0.4403
-0.45128	-0.44982	-0.44839	-0.447	-0.44565	-0.44436	-0.44311	-0.44192	-0.44076
-0.45191	-0.45045	-0.449	-0.44759	-0.44623	-0.44492	-0.44366	-0.44244	-0.44124
-0.45255	-0.45108	-0.44961	-0.44818	-0.44679	-0.44546	-0.44419	-0.44296	-0.44175
-0.45321	-0.45174	-0.45027	-0.44882	-0.44739	-0.44603	-0.44473	-0.44348	-0.44226
-0.45389	-0.45244	-0.45097	-0.4495	-0.44805	-0.44665	-0.4453	-0.44401	-0.44278
-0.45454	-0.45311	-0.45166	-0.45018	-0.44871	-0.44727	-0.44588	-0.44456	-0.44332
-0.45514	-0.45373	-0.45229	-0.45083	-0.44935	-0.4479	-0.44649	-0.44515	-0.4439
-0.45571	-0.45433	-0.45291	-0.45146	-0.44998	-0.44854	-0.44711	-0.44575	-0.44448
-0.45632	-0.45494	-0.45354	-0.45209	-0.45061	-0.44915	-0.44771	-0.44633	-0.44504
-0.45694	-0.45556	-0.45416	-0.45271	-0.45123	-0.44977	-0.44831	-0.44692	-0.44559
-0.41503	-0.41374	-0.41244	-0.45336	-0.4519	-0.45044	-0.44897	-0.44755	-0.4462
-0.4156	-0.41433	-0.41305	-0.45403	-0.45261	-0.45116	-0.44968	-0.44824	-0.44685
-0.3662	-0.36523	-0.36425	-0.45468	-0.45328	-0.45184	-0.45037	-0.44892	-0.44749
-0.36667	-0.36567	-0.36469	-0.45528	-0.45391	-0.45249	-0.45102	-0.44956	-0.4481
-0.36714	-0.36612	-0.36513	-0.41349	-0.41222	-0.45312	-0.45167	-0.45021	-0.44874
-0.36761	-0.36657	-0.36558	-0.41408	-0.41283	-0.45379	-0.45236	-0.45091	-0.44943
-0.36808	-0.36703	-0.36603	-0.41468	-0.41344	-0.45445	-0.45305	-0.45161	-0.45013
-0.3686	-0.36751	-0.36647	-0.36549	-0.36453	-0.45506	-0.45368	-0.45227	-0.45079
-0.36915	-0.36801	-0.36692	-0.36591	-0.36495	-0.45563	-0.45429	-0.4529	-0.45143
-0.32347	-0.32243	-0.32143	-0.36636	-0.3654	-0.41385	-0.41261	-0.45354	-0.4521
-0.32394	-0.32287	-0.32185	-0.36684	-0.36586	-0.41445	-0.41323	-0.45421	-0.45279
-0.32442	-0.32334	-0.32229	-0.32134	-0.32045	-0.36535	-0.36441	-0.41257	-0.41124
-0.32494	-0.32383	-0.32276	-0.32178	-0.32087	-0.36581	-0.36488	-0.41316	-0.41185
-0.32545	-0.32433	-0.32324	-0.32223	-0.32128	-0.36628	-0.36534	-0.41374	-0.41245
-0.32593	-0.32481	-0.32371	-0.32267	-0.32168	-0.32077	-0.31992	-0.41431	-0.41304
-0.3264	-0.32529	-0.32419	-0.32312	-0.32209	-0.32114	-0.32027	-0.41487	-0.41364
-0.32689	-0.3258	-0.3247	-0.32361	-0.32254	-0.32157	-0.32067	-0.3198	-0.31897
-0.32739	-0.32632	-0.32522	-0.32412	-0.32304	-0.32203	-0.32109	-0.3202	-0.31936
-0.32788	-0.32683	-0.32575	-0.32465	-0.32355	-0.32252	-0.32153	-0.32061	-0.31975
-0.32836	-0.32734	-0.32628	-0.3252	-0.3241	-0.32304	-0.322	-0.32104	-0.32017
-0.32881	-0.32782	-0.32679	-0.32572	-0.32463	-0.32355	-0.32248	-0.32148	-0.32058
-0.32922	-0.32825	-0.32725	-0.3262	-0.32511	-0.32402	-0.32293	-0.32191	-0.32098
-0.32961	-0.32867	-0.3277	-0.32666	-0.32558	-0.3245	-0.3234	-0.32236	-0.32139
-0.33003	-0.3291	-0.32815	-0.32713	-0.32608	-0.325	-0.3239	-0.32285	-0.32183
-0.33047	-0.32954	-0.3286	-0.32761	-0.32658	-0.32552	-0.32443	-0.32336	-0.3223
-0.33091	-0.32998	-0.32905	-0.32807	-0.32707	-0.32603	-0.32495	-0.32388	-0.3228
-0.33136	-0.33042	-0.32949	-0.32854	-0.32757	-0.32655	-0.32549	-0.32441	-0.32333

-0.33181	-0.33084	-0.32991	-0.32897	-0.32803	-0.32703	-0.32599	-0.32493	-0.32385
-0.33224	-0.33125	-0.3303	-0.32936	-0.32843	-0.32746	-0.32647	-0.32544	-0.32438
-0.33269	-0.33167	-0.3307	-0.32976	-0.32883	-0.32789	-0.32693	-0.32594	-0.32489
-0.33322	-0.33216	-0.33115	-0.33019	-0.32926	-0.32832	-0.32738	-0.32639	-0.32535
-0.33378	-0.33267	-0.33161	-0.33064	-0.32971	-0.32877	-0.32782	-0.32684	-0.32581
-0.2934	-0.29235	-0.29135	-0.3311	-0.33016	-0.32922	-0.32828	-0.32731	-0.32629
-0.29393	-0.29285	-0.29181	-0.33159	-0.33063	-0.32967	-0.32874	-0.32779	-0.32679
-0.29447	-0.29337	-0.29231	-0.29135	-0.29046	-0.33014	-0.32921	-0.32826	-0.32728
-0.29501	-0.29392	-0.29285	-0.29185	-0.2909	-0.33061	-0.32967	-0.32872	-0.32776
-0.29555	-0.29446	-0.29339	-0.29235	-0.29136	-0.33109	-0.33014	-0.32919	-0.32824
-0.29608	-0.29501	-0.29393	-0.29287	-0.29184	-0.3316	-0.33062	-0.32965	-0.32871
-0.2966	-0.29554	-0.29446	-0.29339	-0.29233	-0.33212	-0.33111	-0.33012	-0.32917
-0.24558	-0.24471	-0.24384	-0.29392	-0.29285	-0.29185	-0.2909	-0.3306	-0.32964
-0.24598	-0.24512	-0.24425	-0.29445	-0.29339	-0.29236	-0.29137	-0.33109	-0.33012
-0.1947	-0.19405	-0.19339	-0.24381	-0.24295	-0.29288	-0.29185	-0.29089	-0.29
-0.195	-0.19436	-0.19371	-0.24424	-0.24337	-0.2934	-0.29236	-0.29137	-0.29046
-0.19529	-0.19466	-0.19403	-0.24466	-0.24379	-0.29393	-0.29288	-0.29187	-0.29092
-0.19556	-0.19495	-0.19433	-0.24506	-0.2442	-0.29445	-0.2934	-0.29238	-0.29139
-0.19585	-0.19524	-0.19463	-0.194	-0.19335	-0.24376	-0.24292	-0.29289	-0.29188
-0.19615	-0.19554	-0.19494	-0.19431	-0.19366	-0.24417	-0.24333	-0.29341	-0.29238
-0.19645	-0.19585	-0.19525	-0.19462	-0.19397	-0.24458	-0.24373	-0.29392	-0.29288
-0.10086	-0.10053	-0.1002	-0.19493	-0.19428	-0.24499	-0.24413	-0.24329	-0.24245
-0.10102	-0.10069	-0.10036	-0.19524	-0.19459	-0.24539	-0.24453	-0.24369	-0.24285
-0.10118	-0.10085	-0.10052	-0.19555	-0.1949	-0.19425	-0.1936	-0.24409	-0.24326
-0.10134	-0.101	-0.10067	-0.19586	-0.19522	-0.19457	-0.19391	-0.2445	-0.24366
-0.10151	-0.10116	-0.10082	-0.10048	-0.10015	-0.19488	-0.19422	-0.2449	-0.24406
-0.10167	-0.10133	-0.10098	-0.10064	-0.1003	-0.19519	-0.19453	-0.24531	-0.24446
-0.10183	-0.10149	-0.10115	-0.1008	-0.10045	-0.1955	-0.19484	-0.19418	-0.19353
-0.10199	-0.10164	-0.1013	-0.10095	-0.1006	-0.19581	-0.19514	-0.19449	-0.19383
-0.10215	-0.1018	-0.10146	-0.10111	-0.10076	-0.19612	-0.19546	-0.1948	-0.19414
-0.10231	-0.10196	-0.10162	-0.10127	-0.10092	-0.10057	-0.10023	-0.19512	-0.19447
-0.10249	-0.10213	-0.10178	-0.10143	-0.10108	-0.10074	-0.1004	-0.19546	-0.19481
-0.0497	-0.04953	-0.04936	-0.10159	-0.10124	-0.10089	-0.10056	-0.10022	-0.09988
-0.04976	-0.04958	-0.0494	-0.10174	-0.10139	-0.10105	-0.10072	-0.10038	-0.10005
-0.04982	-0.04963	-0.04945	-0.04928	-0.04911	-0.10121	-0.10087	-0.10054	-0.10021
-0.04988	-0.04969	-0.0495	-0.04933	-0.04916	-0.10137	-0.10103	-0.1007	-0.10036
-0.04994	-0.04974	-0.04955	-0.04938	-0.04922	-0.10153	-0.10119	-0.10085	-0.10051
			-0.04945	-0.04928	-0.1017	-0.10135	-0.10101	-0.10067
			-0.04952	-0.04934	-0.10187	-0.10152	-0.10118	-0.10083
			-0.04959	-0.0494	-0.04921	-0.04904	-0.10134	-0.101
			-0.04965	-0.04945	-0.04927	-0.04909	-0.1015	-0.10117
					-0.04933	-0.04915	-0.10167	-0.10134
					-0.04939	-0.04922	-0.10185	-0.10151
							-0.04911	-0.04894
							-0.04916	-0.04899
							-0.04922	-0.04904
							-0.04929	-0.04911
							-0.04936	-0.04919

							-0.10735	-0.10701
							-0.10713	-0.10679
							-0.1069	-0.10657
							-0.10667	-0.10636
					-0.10712	-0.10678	-0.25553	-0.25452
					-0.1069	-0.10657	-0.25496	-0.25396
					-0.10667	-0.10636	-0.25443	-0.25342
					-0.10646	-0.10615	-0.3516	-0.35034
					-0.10627	-0.10594	-0.3509	-0.34967
			-0.10668	-0.10637	-0.25471	-0.25373	-0.39414	-0.39279
			-0.10647	-0.10615	-0.25415	-0.25319	-0.39341	-0.39207
-0.05096	-0.05084	-0.05071	-0.20782	-0.20698	-0.30741	-0.30633	-0.39268	-0.39136
-0.05086	-0.05074	-0.05062	-0.20735	-0.20654	-0.30681	-0.3057	-0.39193	-0.39066
-0.05076	-0.05065	-0.05053	-0.20688	-0.2061	-0.30622	-0.3051	-0.3912	-0.38997
-0.10661	-0.10629	-0.10598	-0.25398	-0.25303	-0.34933	-0.3481	-0.61558	-0.61363

-0.10641	-0.10609	-0.10576	-0.25344	-0.2525	-0.34869	-0.3475	-0.61457	-0.61258
-0.20783	-0.20702	-0.20626	-0.30657	-0.3055	-0.52233	-0.52068	-0.65669	-0.65454
-0.20733	-0.20656	-0.20583	-0.30597	-0.3049	-0.52139	-0.51976	-0.65554	-0.65342
-0.25468	-0.25373	-0.25281	-0.48254	-0.48098	-0.61523	-0.6133	-0.697	-0.69481
-0.25418	-0.25322	-0.25228	-0.48165	-0.48013	-0.61419	-0.61221	-0.69572	-0.69353
-0.3074	-0.3063	-0.30521	-0.52193	-0.52034	-0.65668	-0.65457	-0.77245	-0.77
-0.30683	-0.30574	-0.30465	-0.52105	-0.51945	-0.65555	-0.65346	-0.77112	-0.76865
-0.30626	-0.30519	-0.30411	-0.52017	-0.51854	-0.6544	-0.65234	-0.7698	-0.76733
-0.48302	-0.48145	-0.47986	-0.61383	-0.61185	-0.73278	-0.73048	-0.76844	-0.76603
-0.48217	-0.48061	-0.47904	-0.61278	-0.61074	-0.73144	-0.72914	-0.76706	-0.76474
-0.52252	-0.52087	-0.51921	-0.65514	-0.65298	-0.7705	-0.76807	-0.84591	-0.84345
-0.52157	-0.51993	-0.51829	-0.65398	-0.65185	-0.76917	-0.76674	-0.84448	-0.84204
-0.659	-0.65693	-0.65488	-0.73227	-0.72993	-0.76784	-0.76544	-0.90673	-0.9041
-0.65788	-0.65576	-0.65366	-0.7309	-0.72861	-0.76651	-0.7642	-0.9053	-0.90264
-0.69298	-0.69065	-0.68833	-0.72955	-0.72731	-0.8735	-0.87099	-1.01299	-1.0096
-0.69177	-0.68943	-0.68709	-0.72826	-0.72604	-0.87201	-0.86954	-1.01112	-1.00779
-0.69056	-0.68822	-0.68589	-0.72702	-0.72481	-0.87053	-0.86809	-1.00922	-1.00599
-0.73285	-0.73047	-0.72811	-0.79565	-0.79328	-0.86913	-0.86666	-1.0073	-1.00413
-0.73149	-0.7292	-0.72694	-0.79442	-0.79206	-0.86778	-0.86526	-1.00538	-1.00225
-0.73014	-0.72792	-0.72575	-0.87158	-0.86899	-1.0102	-1.00681	-1.10456	-1.10091
-0.72879	-0.72663	-0.72455	-0.87019	-0.86763	-1.0084	-1.00504	-1.1025	-1.09881
-0.7974	-0.7951	-0.79289	-0.8688	-0.86628	-1.00662	-1.00328	-1.131	-1.1272
-0.79611	-0.79382	-0.7916	-0.86741	-0.86493	-1.00485	-1.00153	-1.12889	-1.12502
-0.79486	-0.79256	-0.79032	-0.86601	-0.86357	-1.00309	-0.99978	-1.12679	-1.12288
-0.83421	-0.83179	-0.82941	-0.97447	-0.9714	-1.10197	-1.09811	-1.12472	-1.12084
-0.83291	-0.83046	-0.82806	-0.97263	-0.96961	-1.0999	-1.09606	-1.12266	-1.11885
-0.83162	-0.82917	-0.82676	-1.07374	-1.0702	-1.12832	-1.12445	-1.18768	-1.18371
-0.83033	-0.8279	-0.82549	-1.07172	-1.06817	-1.12622	-1.12238	-1.18555	-1.18168
-0.97706	-0.97378	-0.97054	-1.10098	-1.09737	-1.19137	-1.18738	-1.18347	-1.17968
-0.97533	-0.97205	-0.96881	-1.09891	-1.09529	-1.18918	-1.18526	-1.18143	-1.17771
-1.07698	-1.07312	-1.06931	-1.12731	-1.12359	-1.18697	-1.18316	-1.17942	-1.17577
-1.07494	-1.07109	-1.0673	-1.12518	-1.1214	-1.18471	-1.18106	-1.17746	-1.17385
-1.07291	-1.06908	-1.06529	-1.12308	-1.11926	-1.1825	-1.179	-1.17552	-1.17195
-1.07089	-1.06708	-1.06331	-1.18816	-1.18417	-1.18044	-1.17701	-1.23415	-1.2305
-1.06887	-1.06508	-1.06134	-1.18606	-1.18215	-1.17848	-1.17506	-1.23213	-1.22852
-1.19606	-1.19196	-1.18791	-1.18398	-1.18016	-1.17653	-1.17313	-1.28243	-1.27872
-1.19389	-1.1898	-1.18577	-1.18191	-1.17819	-1.17461	-1.17122	-1.28037	-1.27667
-1.19174	-1.18768	-1.18369	-1.17988	-1.17623	-1.25895	-1.25538	-1.33916	-1.33528
-1.18959	-1.1856	-1.18169	-1.17792	-1.17427	-1.2569	-1.25336	-1.33699	-1.33312
-1.18746	-1.18354	-1.17971	-1.26238	-1.25856	-1.34244	-1.33865	-1.33484	-1.33098
-1.18531	-1.18147	-1.17773	-1.26033	-1.25654	-1.34029	-1.3365	-1.33271	-1.32885
-1.1832	-1.17943	-1.17577	-1.25831	-1.25456	-1.33817	-1.33439	-1.33059	-1.32673
-1.26784	-1.2639	-1.26008	-1.31829	-1.31439	-1.33613	-1.33232	-1.32849	-1.32463
-1.26575	-1.26185	-1.25807	-1.31622	-1.31241	-1.33414	-1.33028	-1.32642	-1.32254
-1.29324	-1.28933	-1.28554	-1.33983	-1.33598	-1.33212	-1.32823	-1.35606	-1.35208
-1.29117	-1.28734	-1.28361	-1.33781	-1.33394	-1.33006	-1.32617	-1.35394	-1.34995
-1.3218	-1.31787	-1.31402	-1.33576	-1.33188	-1.38615	-1.38208	-1.48698	-1.48238
-1.31981	-1.31587	-1.31199	-1.33368	-1.32981	-1.38397	-1.3799	-1.48457	-1.48002
-1.31781	-1.31385	-1.30994	-1.33159	-1.32772	-1.38179	-1.37771	-1.48217	-1.47769
-1.34141	-1.33741	-1.33344	-1.35634	-1.35238	-1.40606	-1.40192	-1.47978	-1.47535

-1.33928	-1.3353	-1.33136	-1.35421	-1.35024	-1.40382	-1.3997	-1.47741	-1.47302
-1.36408	-1.36005	-1.35605	-1.40985	-1.4057	-1.4841	-1.47955	-1.47508	-1.47072
-1.36189	-1.35787	-1.35388	-1.40758	-1.40344	-1.48168	-1.4772	-1.47279	-1.46845
-1.41785	-1.41366	-1.40949	-1.40535	-1.40122	-1.4793	-1.47488	-1.49096	-1.48656
-1.41563	-1.41144	-1.40728	-1.40315	-1.39905	-1.47696	-1.4726	-1.48867	-1.4843
-1.41346	-1.40928	-1.40513	-1.48371	-1.47917	-1.49524	-1.49083	-1.48647	-1.48212
-1.4114	-1.40723	-1.40308	-1.48153	-1.47706	-1.49314	-1.48875	-1.4844	-1.48009
-1.40933	-1.40517	-1.40104	-1.47937	-1.47497	-1.49106	-1.48668	-1.48234	-1.47806
-1.40715	-1.40301	-1.39891	-1.4771	-1.47276	-1.48885	-1.48449	-1.56701	-1.56257
-1.40492	-1.4008	-1.39674	-1.47479	-1.47049	-1.48658	-1.48224	-1.56466	-1.56024
-1.48586	-1.48133	-1.47687	-1.493	-1.48864	-1.54793	-1.5435	-1.56231	-1.55789
-1.4834	-1.47894	-1.47457	-1.4907	-1.48634	-1.54555	-1.54114	-1.55994	-1.55552
-1.51542	-1.5109	-1.50649	-1.55212	-1.54765	-1.56646	-1.56201	-1.55759	-1.55317
-1.51311	-1.5086	-1.50421	-1.54977	-1.54533	-1.56412	-1.55968	-1.55526	-1.55082
-1.56117	-1.55651	-1.55198	-1.5475	-1.54307	-1.56185	-1.55741	-1.553	-1.54855
-1.55895	-1.55434	-1.54982	-1.54536	-1.54094	-1.55971	-1.55527	-1.55087	-1.54642
-1.55674	-1.55219	-1.54769	-1.54324	-1.53883	-1.55757	-1.55313	-1.54875	-1.54436
-1.55443	-1.54993	-1.54546	-1.56422	-1.55976	-1.5553	-1.55084	-1.5465	-1.54227
-1.55208	-1.54761	-1.54318	-1.56192	-1.55745	-1.55298	-1.54848	-1.5442	-1.5402
-1.54983	-1.54539	-1.54098	-1.5597	-1.55523	-1.55076	-1.54623	-1.56328	-1.55947
-1.54769	-1.54325	-1.53884	-1.55756	-1.5531	-1.54863	-1.54406	-1.56115	-1.55754
-1.54549	-1.54105	-1.53665	-1.55534	-1.55089	-1.58837	-1.58384	-1.57972	-1.57626
-1.5432	-1.53876	-1.53435	-1.553	-1.54855	-1.58611	-1.58187	-1.57802	-1.57471
-1.54092	-1.53649	-1.53207	-1.55069	-1.54623	-1.58389	-1.57997	-1.5764	-1.57323
-1.56193	-1.55746	-1.55301	-1.5904	-1.58584	-1.58173	-1.57807	-1.57473	-1.57171
-1.55977	-1.55531	-1.55088	-1.58823	-1.58368	-1.57968	-1.57624	-1.5731	-1.57023
-1.59952	-1.595	-1.59052	-1.58608	-1.58172	-1.57789	-1.57462	-1.57161	-1.56885
-1.59716	-1.59263	-1.58818	-1.58395	-1.57991	-1.5763	-1.57315	-1.57023	-1.56754
-1.59489	-1.59037	-1.58597	-1.58191	-1.57814	-1.57472	-1.57169	-1.56886	-1.56622
-1.59272	-1.58823	-1.58388	-1.57997	-1.5764	-1.57315	-1.57023	-1.56749	-1.56489
-1.59052	-1.58611	-1.58188	-1.57813	-1.57476	-1.57165	-1.56884	-1.56616	-1.56359
-1.58822	-1.58397	-1.57996	-1.5764	-1.57322	-1.57025	-1.56752	-1.5649	-1.56235
-1.58597	-1.58191	-1.57815	-1.57478	-1.57176	-1.5689	-1.56624	-1.56365	-1.56111
-1.58385	-1.57999	-1.57647	-1.57327	-1.57038	-1.5676	-1.56495	-1.58184	-1.57926
-1.58184	-1.57818	-1.57489	-1.57184	-1.56906	-1.56633	-1.56367	-1.58054	-1.57795
-1.57996	-1.57645	-1.57331	-1.57038	-1.56769	-1.56503	-1.5624	-1.57928	-1.57669
-1.5782	-1.5748	-1.57176	-1.56892	-1.5663	-1.5637	-1.56114	-1.57805	-1.57546
-1.57654	-1.57327	-1.57032	-1.56755	-1.56497	-1.58194	-1.57938	-1.57682	-1.57423
-1.57498	-1.57187	-1.56902	-1.5663	-1.56373	-1.58071	-1.57815	-1.57559	-1.57298
-1.57348	-1.57052	-1.56776	-1.58467	-1.58206	-1.57947	-1.57691	-1.57435	-1.57174
-1.57203	-1.56919	-1.56651	-1.58343	-1.58079	-1.57818	-1.57561	-1.57306	-1.57052
-1.57063	-1.56787	-1.56525	-1.58219	-1.57953	-1.5769	-1.57432	-1.57179	-1.56932
-1.58894	-1.58618	-1.58356	-1.58091	-1.57828	-1.57568	-1.5731	-1.57058	-1.56816
-1.58758	-1.58484	-1.58222	-1.57962	-1.57705	-1.57448	-1.57192	-1.56943	-1.56703
-1.58626	-1.58356	-1.58096	-1.57838	-1.57584	-1.5733	-1.57077	-1.5683	-1.56591
-1.58499	-1.58234	-1.57976	-1.57719	-1.57466	-1.57214	-1.56965	-1.56721	-1.56479
-1.58375	-1.58113	-1.57858	-1.57602	-1.57348	-1.57098	-1.56852	-1.58918	-1.58668
-1.58254	-1.57995	-1.5774	-1.57485	-1.57232	-1.56982	-1.56736	-1.58799	-1.58553
-1.58134	-1.57877	-1.57623	-1.57369	-1.57115	-1.56866	-1.56621	-1.60522	-1.6028
-1.58016	-1.5776	-1.57506	-1.57252	-1.56997	-1.5675	-1.56508	-1.60409	-1.60171

-1.57898	-1.57642	-1.57388	-1.57134	-1.5688	-1.56634	-1.56397	-1.60299	-1.60064
-1.5778	-1.57523	-1.57266	-1.58888	-1.58637	-1.58394	-1.58159	-1.61064	-1.60828
-1.57662	-1.57402	-1.57143	-1.58766	-1.58522	-1.58285	-1.58054	-1.60956	-1.60724
-1.59417	-1.59156	-1.58897	-1.5865	-1.58411	-1.58177	-1.57949	-1.60849	-1.60619
-1.59293	-1.59035	-1.58782	-1.58538	-1.58301	-1.5807	-1.57845	-1.60742	-1.60513
-1.59171	-1.58917	-1.58669	-1.58428	-1.58194	-1.61105	-1.60868	-1.62627	-1.62396
-1.59054	-1.58803	-1.58558	-1.5832	-1.58089	-1.60996	-1.60758	-1.62516	-1.62289
-1.5894	-1.58691	-1.58448	-1.58213	-1.57985	-1.60889	-1.60651	-1.62408	-1.62183
-1.58828	-1.5858	-1.5834	-1.5901	-1.58779	-1.62774	-1.62536	-1.66549	-1.66306
-1.58716	-1.58471	-1.58233	-1.58903	-1.58675	-1.62668	-1.62433	-1.66439	-1.66195
-1.59517	-1.5927	-1.59031	-1.63033	-1.62797	-1.62563	-1.6233	-1.67916	-1.67671
-1.59403	-1.5916	-1.58923	-1.62923	-1.6269	-1.62458	-1.62227	-1.67805	-1.6756
-1.5929	-1.59051	-1.58817	-1.62814	-1.62581	-1.63989	-1.63761	-1.67693	-1.67449
-1.59178	-1.58942	-1.58712	-1.62705	-1.62471	-1.63879	-1.63654	-1.67579	-1.67338
-1.63315	-1.63072	-1.62834	-1.64236	-1.64001	-1.66205	-1.65965	-1.69549	-1.69291
-1.63203	-1.62962	-1.62727	-1.6413	-1.63897	-1.66094	-1.65852	-1.69424	-1.69173
-1.63093	-1.62854	-1.62622	-1.64026	-1.63794	-1.65984	-1.65741	-1.69302	-1.69057
-1.64625	-1.64387	-1.64156	-1.63923	-1.63691	-1.65874	-1.65632	-1.69183	-1.68943
-1.6452	-1.64283	-1.64053	-1.6382	-1.63588	-1.65764	-1.65523	-1.69069	-1.68831
-1.64415	-1.6418	-1.6395	-1.63718	-1.63486	-1.6739	-1.67147	-1.68957	-1.68722
-1.6431	-1.64076	-1.63848	-1.63616	-1.63384	-1.67279	-1.6704	-1.68848	-1.68614
-1.64205	-1.63972	-1.63745	-1.63515	-1.63285	-1.67172	-1.66934	-1.6874	-1.68507
-1.64101	-1.63869	-1.63641	-1.63414	-1.63189	-1.6707	-1.66832	-1.68633	-1.68401
-1.63997	-1.63766	-1.63539	-1.65714	-1.65476	-1.69016	-1.68768	-1.73325	-1.7306
-1.63894	-1.63663	-1.63436	-1.65606	-1.65372	-1.68909	-1.68662	-1.73205	-1.72939
-1.63792	-1.63562	-1.63334	-1.65498	-1.65267	-1.68802	-1.68557	-1.73086	-1.72818
-1.63694	-1.63463	-1.63235	-1.65391	-1.65163	-1.68695	-1.68452	-1.74811	-1.74535
-1.63599	-1.63367	-1.63137	-1.65285	-1.6506	-1.68588	-1.68347	-1.74688	-1.74409
-1.63501	-1.6327	-1.6304	-1.67254	-1.67021	-1.68482	-1.68242	-1.74565	-1.74284
-1.634	-1.63172	-1.62945	-1.67152	-1.66917	-1.68376	-1.68137	-1.74443	-1.74159
-1.65696	-1.65456	-1.65219	-1.67052	-1.66815	-1.74887	-1.74606	-1.74323	-1.74036
-1.65587	-1.65353	-1.65123	-1.66955	-1.66716	-1.74768	-1.7449	-1.74208	-1.73916
-1.6548	-1.65251	-1.65027	-1.66857	-1.66617	-1.74649	-1.74372	-1.7409	-1.73795
-1.67463	-1.67223	-1.66992	-1.66755	-1.66517	-1.74528	-1.7425	-1.73966	-1.7367
-1.67358	-1.67118	-1.66887	-1.66652	-1.66416	-1.74408	-1.74125	-1.73839	-1.73544
-1.67253	-1.67014	-1.66783	-1.74854	-1.74574	-1.7429	-1.74003	-1.73714	-1.73422
-1.6715	-1.66912	-1.66679	-1.74733	-1.74459	-1.74175	-1.73882	-1.73591	-1.73304
-1.6705	-1.66812	-1.66578	-1.74612	-1.74339	-1.76051	-1.75744	-1.80762	-1.80458
-1.66954	-1.66716	-1.66479	-1.7449	-1.74214	-1.75922	-1.75615	-1.8063	-1.80332
-1.73607	-1.73325	-1.73042	-1.7437	-1.7409	-1.79359	-1.79049	-1.80502	-1.80209
-1.73489	-1.7321	-1.72932	-1.74256	-1.73968	-1.79229	-1.78924	-1.8038	-1.8009
-1.73369	-1.73094	-1.72821	-1.74142	-1.73848	-1.79099	-1.788	-1.8026	-1.79972
-1.73251	-1.72975	-1.72701	-1.77637	-1.77335	-1.80729	-1.80429	-1.80136	-1.79852
-1.73133	-1.72855	-1.72578	-1.77508	-1.77208	-1.806	-1.80302	-1.80012	-1.79732
-1.76693	-1.76406	-1.76117	-1.77383	-1.77086	-1.80476	-1.8018	-1.79893	-1.79617
-1.76581	-1.76289	-1.75995	-1.7726	-1.76969	-1.80357	-1.80064	-1.79779	-1.79506
-1.74797	-1.74507	-1.74215	-1.7714	-1.76851	-1.80237	-1.79948	-1.79667	-1.79396
-1.74676	-1.74389	-1.74102	-1.77023	-1.76729	-1.80113	-1.79834	-1.7956	-1.79286
-1.74556	-1.74272	-1.73988	-1.7869	-1.7839	-1.79993	-1.79721	-1.79451	-1.79176
-1.74442	-1.74155	-1.73868	-1.78571	-1.7828	-1.79883	-1.7961	-1.79338	-1.79065

-1.7433	-1.74038	-1.73748	-1.78452	-1.78172	-1.79777	-1.79499	-1.79224	-1.78953
-1.74211	-1.7392	-1.73632	-1.78336	-1.78061	-1.79665	-1.79388	-1.79114	-1.78841
-1.74089	-1.73802	-1.73519	-1.78221	-1.77947	-1.79551	-1.79279	-1.79006	-1.78729
-1.73972	-1.73687	-1.73407	-1.78108	-1.77837	-1.79439	-1.79167	-1.78893	-1.78614
-1.73861	-1.73575	-1.73295	-1.77995	-1.7773	-1.7933	-1.79052	-1.78775	-1.78495
-1.75576	-1.75289	-1.75007	-1.77884	-1.77622	-1.79218	-1.78937	-1.78657	-1.78379
-1.75454	-1.75174	-1.74902	-1.77778	-1.77512	-1.79102	-1.78819	-1.7854	-1.78266
-1.75333	-1.7506	-1.74796	-1.77672	-1.77403	-1.78988	-1.78705	-1.78426	-1.78155
-1.75218	-1.74949	-1.74688	-1.77563	-1.77295	-1.7888	-1.78598	-1.7832	-1.78046
-1.75108	-1.74839	-1.74578	-1.77452	-1.77188	-1.78773	-1.78495	-1.78218	-1.77939
-1.74998	-1.74731	-1.74472	-1.77344	-1.77079	-1.78662	-1.78388	-1.82774	-1.82459
-1.74891	-1.74626	-1.74368	-1.77236	-1.76967	-1.78549	-1.78279	-1.82659	-1.82347
-1.76329	-1.76061	-1.75799	-1.77127	-1.76858	-1.78437	-1.78169	-1.82539	-1.82231
-1.76228	-1.75956	-1.75689	-1.77015	-1.7675	-1.78329	-1.78056	-1.82412	-1.8211
-1.76126	-1.75853	-1.75581	-1.75317	-1.75057	-1.78219	-1.77944	-1.85093	-1.84789
-1.76023	-1.75751	-1.7548	-1.75212	-1.74948	-1.78105	-1.77833	-1.84972	-1.84667
-1.75917	-1.75648	-1.75379	-1.75109	-1.74839	-1.77992	-1.77722	-1.84852	-1.84545
-1.75807	-1.75541	-1.75276	-1.75007	-1.74736	-1.83605	-1.83298	-1.85607	-1.85282
-1.75696	-1.75432	-1.75171	-1.74904	-1.74636	-1.83489	-1.83175	-1.8547	-1.85154
-1.7559	-1.75325	-1.75062	-1.77798	-1.77526	-1.83375	-1.8306	-1.87076	-1.86757
-1.7549	-1.75219	-1.74951	-1.77686	-1.7742	-1.83263	-1.82951	-1.8696	-1.8664
-1.75389	-1.75116	-1.74844	-1.77578	-1.77315	-1.85788	-1.85448	-1.86843	-1.86521
-1.75289	-1.75015	-1.74743	-1.77474	-1.7721	-1.85664	-1.85331	-1.86725	-1.86398
-1.78204	-1.77923	-1.77643	-1.77371	-1.77105	-1.87271	-1.86937	-1.86606	-1.86276
-1.78099	-1.7782	-1.77541	-1.77267	-1.76995	-1.87137	-1.86811	-1.86487	-1.86158
-1.77993	-1.77716	-1.77439	-1.77162	-1.76886	-1.87003	-1.86685	-1.86367	-1.86041
-1.77881	-1.77608	-1.77335	-1.8438	-1.84036	-1.86878	-1.86558	-1.86241	-1.85921
-1.77768	-1.77498	-1.77231	-1.84262	-1.83922	-1.8676	-1.86433	-1.86112	-1.858
-1.7766	-1.77392	-1.77127	-1.85894	-1.85553	-1.86644	-1.86313	-1.85992	-1.85683
-1.77558	-1.77289	-1.77023	-1.85771	-1.85438	-1.8653	-1.862	-1.85878	-1.8557
-1.77457	-1.77186	-1.76918	-1.85647	-1.85321	-1.86416	-1.86086	-1.85764	-1.85454
-1.77356	-1.77081	-1.76811	-1.85519	-1.85202	-1.86301	-1.85971	-1.85647	-1.85334
-1.77256	-1.76978	-1.76706	-1.85395	-1.85084	-1.86186	-1.85857	-1.85532	-1.85216
-1.81777	-1.81461	-1.81152	-1.85278	-1.84968	-1.84651	-1.84328	-1.8542	-1.85103
-1.81666	-1.81353	-1.81047	-1.85165	-1.84853	-1.84537	-1.84217	-1.8531	-1.84994
-1.8605	-1.85707	-1.85374	-1.85051	-1.84739	-1.84424	-1.84106	-1.87366	-1.8703
-1.85928	-1.85589	-1.85259	-1.84938	-1.84625	-1.84311	-1.83996	-1.8725	-1.86913
-1.8581	-1.85473	-1.85144	-1.84824	-1.84511	-1.84199	-1.83886	-1.87133	-1.86796
-1.85696	-1.85359	-1.8503	-1.8471	-1.84398	-1.84086	-1.83776	-1.87015	-1.8668
-1.85582	-1.85245	-1.84916	-1.84596	-1.84285	-1.83975	-1.83666	-1.86898	-1.86563
-1.85467	-1.85131	-1.84802	-1.84484	-1.84175	-1.83865	-1.83556	-1.86781	-1.86447
-1.85352	-1.85017	-1.84688	-1.84372	-1.84065	-1.83757	-1.83448	-1.86664	-1.86332
-1.85237	-1.84902	-1.84573	-1.84258	-1.83954	-1.85825	-1.85496	-1.86549	-1.86217
-1.85121	-1.84787	-1.84457	-1.84144	-1.83844	-1.85709	-1.85381	-1.86434	-1.86101
-1.85005	-1.84672	-1.84343	-1.84032	-1.83734	-1.85593	-1.85266	-1.86318	-1.85986
-1.84888	-1.84557	-1.8423	-1.83921	-1.83624	-1.85476	-1.85149	-1.86201	-1.85869
-1.84773	-1.84445	-1.84124	-1.83815	-1.83517	-1.8536	-1.85033	-1.86084	-1.85753
-1.84658	-1.84339	-1.84026	-1.83717	-1.83413	-1.85245	-1.84918	-1.85968	-1.85636
-1.84544	-1.84231	-1.83926	-1.85788	-1.85458	-1.8513	-1.84802	-1.87253	-1.8692
-1.84429	-1.8412	-1.83818	-1.85673	-1.85344	-1.85015	-1.84687	-1.87135	-1.86802

-1.84317	-1.84009	-1.83708	-1.85557	-1.85228	-1.84899	-1.84571	-1.87016	-1.86684
-1.84213	-1.83904	-1.83601	-1.85441	-1.85112	-1.86201	-1.8587	-1.88525	-1.88176
-1.84114	-1.83802	-1.83494	-1.85325	-1.84997	-1.86083	-1.85753	-1.88401	-1.88056
-1.86205	-1.85871	-1.85539	-1.86625	-1.86295	-1.85966	-1.85636	-1.89524	-1.89178
-1.86091	-1.85756	-1.85425	-1.86508	-1.86178	-1.85849	-1.85521	-1.89404	-1.89063
-1.85976	-1.85641	-1.8531	-1.86392	-1.86061	-1.87014	-1.86685	-1.89287	-1.88949
-1.8586	-1.85525	-1.85194	-1.86275	-1.85946	-1.869	-1.86576	-1.89175	-1.88836
-1.85744	-1.8541	-1.85079	-1.86159	-1.85833	-1.86788	-1.86467	-1.89063	-1.88721
-1.87037	-1.86703	-1.86373	-1.86047	-1.85725	-1.86679	-1.86356	-1.88945	-1.88602
-1.8692	-1.86587	-1.86259	-1.85936	-1.85618	-1.86571	-1.86244	-1.88824	-1.88482
-1.86813	-1.86481	-1.86152	-1.87107	-1.86783	-1.88075	-1.8773	-1.88703	-1.88361
-1.86714	-1.86381	-1.86049	-1.86998	-1.8667	-1.87953	-1.87608	-1.88581	-1.88241
-1.87895	-1.87557	-1.8722	-1.86887	-1.86556	-1.87832	-1.87488	-1.88462	-1.88126
-1.87778	-1.87441	-1.87105	-1.86772	-1.86441	-1.87713	-1.87371	-1.88347	-1.88018
-1.87659	-1.87323	-1.86989	-1.86658	-1.8633	-1.87599	-1.87259	-1.88237	-1.8791
-1.8754	-1.87205	-1.86873	-1.86546	-1.86224	-1.87493	-1.87155	-1.88133	-1.87803
-1.87423	-1.87089	-1.86759	-1.86436	-1.86119	-1.87387	-1.87052	-1.88029	-1.87696
-1.87317	-1.86983	-1.86653	-1.87942	-1.87607	-1.87273	-1.8694	-1.86608	-1.86278
-1.87216	-1.86881	-1.8655	-1.87831	-1.87492	-1.87156	-1.86824	-1.86494	-1.86167
-1.87107	-1.86773	-1.86442	-1.87717	-1.87377	-1.87041	-1.8671	-1.86383	-1.86059
-1.86993	-1.86661	-1.86331	-1.87602	-1.87262	-1.86927	-1.86597	-1.86272	-1.85954
-1.86884	-1.86553	-1.86225	-1.87492	-1.87153	-1.86819	-1.8649	-1.86166	-1.8585
-1.86783	-1.86453	-1.86126	-1.8739	-1.87054	-1.86721	-1.86391	-1.86066	-1.85748
-1.88313	-1.87967	-1.87624	-1.87286	-1.86953	-1.86621	-1.86291	-1.85965	-1.85644
-1.88196	-1.87851	-1.8751	-1.87174	-1.86842	-1.86512	-1.86183	-1.85858	-1.85537
-1.88078	-1.87735	-1.87395	-1.87061	-1.8673	-1.86401	-1.86074	-1.85751	-1.85432
-1.87969	-1.87627	-1.87289	-1.86956	-1.86627	-1.86299	-1.85974	-1.85652	-1.85334
-1.87864	-1.87523	-1.87187	-1.86855	-1.86527	-1.86201	-1.85876	-1.85555	-1.8524
-1.87751	-1.87412	-1.87078	-1.86747	-1.86419	-1.86094	-1.85771	-1.85453	-1.85142
-1.87633	-1.87295	-1.86962	-1.86632	-1.86305	-1.85981	-1.85661	-1.85346	-1.8504
-1.8752	-1.87184	-1.86853	-1.86523	-1.86197	-1.85874	-1.85555	-1.85242	-1.84938
-1.87415	-1.87082	-1.86752	-1.86424	-1.86099	-1.85776	-1.85458	-1.85144	-1.84836
-1.87314	-1.86981	-1.86654	-1.86327	-1.86002	-1.8568	-1.85362	-1.85047	-1.84736
-1.87213	-1.86882	-1.86555	-1.86229	-1.85905	-1.85585	-1.85268	-1.84953	-1.8464
-1.87112	-1.86781	-1.86455	-1.8613	-1.85807	-1.85487	-1.85172	-1.84858	-1.84545
-1.87001	-1.86672	-1.86346	-1.86022	-1.857	-1.85382	-1.85067	-1.84754	-1.8444
-1.86886	-1.86558	-1.86233	-1.8591	-1.85589	-1.85271	-1.84957	-1.84644	-1.84331
-1.86779	-1.86451	-1.86127	-1.85805	-1.85485	-1.85169	-1.84855	-1.84542	-1.8423
-1.8668	-1.86353	-1.86029	-1.85709	-1.8539	-1.85074	-1.84761	-1.84448	-1.84136
-1.86583	-1.86256	-1.85933	-1.85613	-1.85296	-1.84981	-1.84667	-1.84354	-1.84043
-1.86485	-1.8616	-1.85837	-1.85518	-1.85202	-1.84887	-1.84573	-1.84261	-1.83951
-1.86386	-1.86061	-1.85739	-1.8542	-1.85105	-1.8479	-1.84477	-1.84165	-1.83856
-1.86277	-1.85953	-1.85632	-1.85314	-1.84999	-1.84685	-1.84371	-1.84061	-1.83753
-1.86166	-1.85842	-1.85522	-1.85205	-1.84891	-1.84577	-1.84263	-1.83953	-1.83647
-1.86064	-1.85741	-1.85421	-1.85105	-1.84791	-1.84477	-1.84164	-1.83854	-1.83549
-1.85969	-1.85647	-1.85328	-1.85011	-1.84697	-1.84384	-1.84071	-1.83761	-1.83457
-1.85874	-1.85553	-1.85235	-1.84919	-1.84605	-1.84291	-1.83979	-1.83671	-1.83367
-1.85781	-1.85461	-1.85144	-1.84827	-1.84513	-1.84201	-1.8389	-1.83582	-1.83279
-1.85689	-1.85369	-1.85052	-1.84736	-1.84422	-1.8411	-1.83801	-1.83494	-1.83191
-1.85595	-1.85277	-1.84961	-1.84645	-1.84331	-1.8402	-1.83712	-1.83406	-1.83104

-1.85503	-1.85185	-1.8487	-1.84555	-1.8424	-1.8393	-1.83622	-1.83318	-1.83016
-1.85411	-1.85093	-1.84778	-1.84463	-1.84149	-1.83839	-1.83533	-1.8323	-1.82928
-1.8532	-1.85002	-1.84686	-1.84371	-1.84057	-1.83748	-1.83443	-1.83141	-1.8284
-1.85228	-1.84911	-1.84595	-1.8428	-1.83967	-1.83658	-1.83354	-1.83053	-1.82752
-1.85137	-1.84819	-1.84504	-1.8419	-1.83878	-1.8357	-1.83266	-1.82964	-1.82664
-1.85045	-1.84728	-1.84413	-1.841	-1.83789	-1.83481	-1.83177	-1.82876	-1.82576
-1.84953	-1.84636	-1.84322	-1.84009	-1.837	-1.83393	-1.8309	-1.82789	-1.82489
-1.84861	-1.84545	-1.8423	-1.83918	-1.8361	-1.83304	-1.83002	-1.82702	-1.82403
-1.84768	-1.84451	-1.84137	-1.83827	-1.8352	-1.83215	-1.82914	-1.82614	-1.82316
-1.84674	-1.84358	-1.84044	-1.83735	-1.83429	-1.83126	-1.82824	-1.82525	-1.82229
-1.84581	-1.84265	-1.83951	-1.83643	-1.83338	-1.83036	-1.82735	-1.82437	-1.82142
-1.84488	-1.84173	-1.83859	-1.83551	-1.83248	-1.82946	-1.82646	-1.82348	-1.82054
-1.84395	-1.8408	-1.83768	-1.83461	-1.83158	-1.82857	-1.82557	-1.8226	-1.81967
-1.84301	-1.83988	-1.83677	-1.83371	-1.83069	-1.82768	-1.8247	-1.82173	-1.81881
-1.84208	-1.83896	-1.83587	-1.83281	-1.8298	-1.8268	-1.82382	-1.82086	-1.81795
-1.84115	-1.83804	-1.83496	-1.83192	-1.82891	-1.82591	-1.82295	-1.82	-1.81709
-1.84022	-1.83712	-1.83406	-1.83102	-1.82802	-1.82503	-1.82207	-1.81914	-1.81623
-1.83928	-1.8362	-1.83315	-1.83013	-1.82713	-1.82415	-1.8212	-1.81827	-1.81537
-1.83835	-1.83528	-1.83225	-1.82923	-1.82624	-1.82327	-1.82033	-1.81741	-1.8145
-1.83747	-1.83438	-1.83136	-1.82834	-1.82535	-1.82239	-1.81946	-1.81655	-1.81364
-1.83661	-1.83352	-1.83049	-1.82746	-1.82447	-1.8215	-1.81858	-1.81568	-1.81277
-1.83581	-1.83271	-1.82968	-1.82664	-1.82362	-1.82064	-1.81772	-1.81481	-1.8119
-1.83507	-1.83199	-1.82895	-1.82588	-1.82281	-1.81981	-1.81686	-1.81394	-1.81103
-1.8343	-1.83124	-1.82819	-1.82512	-1.82203	-1.819	-1.81603	-1.8131	-1.81018
-1.83345	-1.8304	-1.82738	-1.82433	-1.82129	-1.81826	-1.81525	-1.81227	-1.80935
-1.83256	-1.82954	-1.82653	-1.82353	-1.82053	-1.81752	-1.81448	-1.81148	-1.80855
-1.8317	-1.82867	-1.82567	-1.82268	-1.81971	-1.81673	-1.81372	-1.81074	-1.80783
-1.83084	-1.8278	-1.82479	-1.82181	-1.81886	-1.81591	-1.81295	-1.81002	-1.80713
-1.83005	-1.82699	-1.82394	-1.82095	-1.81801	-1.81507	-1.81214	-1.80924	-1.80636
-1.82932	-1.82621	-1.82312	-1.82011	-1.81716	-1.81422	-1.81129	-1.80839	-1.80554
-1.82856	-1.82544	-1.82234	-1.81932	-1.81636	-1.81341	-1.81047	-1.80756	-1.80471
-1.82774	-1.82467	-1.82161	-1.81861	-1.81564	-1.81267	-1.80968	-1.80674	-1.80388
-1.82691	-1.82388	-1.82086	-1.81788	-1.81491	-1.81193	-1.80891	-1.80595	-1.80309
-1.82607	-1.82304	-1.82004	-1.81706	-1.81411	-1.81114	-1.80816	-1.80523	-1.80238
-1.82525	-1.82221	-1.81921	-1.81623	-1.81328	-1.81034	-1.80742	-1.80453	-1.80168
-1.82449	-1.82145	-1.81845	-1.81545	-1.81247	-1.80953	-1.80662	-1.80375	-1.80091
-1.82375	-1.82073	-1.81772	-1.8147	-1.81167	-1.80871	-1.80581	-1.80294	-1.8001
-1.82295	-1.81995	-1.81695	-1.81393	-1.8109	-1.80794	-1.80505	-1.80218	-1.79933
-1.82209	-1.8191	-1.81614	-1.81315	-1.81017	-1.80723	-1.80435	-1.80147	-1.79857
-1.79935	-1.79644	-1.79355	-1.81237	-1.80941	-1.80648	-1.80361	-1.80073	-1.79782
-1.79859	-1.79568	-1.79279	-1.81159	-1.8086	-1.80566	-1.8028	-1.79995	-1.79707
-1.79783	-1.79492	-1.79202	-1.8108	-1.80779	-1.80485	-1.80199	-1.79916	-1.79631
-1.79701	-1.79409	-1.79119	-1.81	-1.80701	-1.80409	-1.80125	-1.79841	-1.79554
-1.79617	-1.79324	-1.79035	-1.80919	-1.80625	-1.80336	-1.80051	-1.79766	-1.79477
-1.79539	-1.79246	-1.78955	-1.78666	-1.78377	-1.80257	-1.79973	-1.79689	-1.79401
-1.79463	-1.79171	-1.78879	-1.78587	-1.78296	-1.80175	-1.79892	-1.79609	-1.79326
-1.78373	-1.78082	-1.77791	-1.78509	-1.78217	-1.80098	-1.79815	-1.79533	-1.79253
-1.78298	-1.78004	-1.77711	-1.7843	-1.78142	-1.80027	-1.79743	-1.79462	-1.79182
-1.7822	-1.77926	-1.77632	-1.78352	-1.78068	-1.77785	-1.77504	-1.79388	-1.79109
-1.78137	-1.77846	-1.77556	-1.78277	-1.77994	-1.77711	-1.77426	-1.7931	-1.7903

							-0.10735	-0.10701
							-0.10713	-0.10679
							-0.1069	-0.10657
							-0.10667	-0.10636
					-0.10712	-0.10678	-0.25553	-0.25452
					-0.1069	-0.10657	-0.25496	-0.25396
					-0.10667	-0.10636	-0.25443	-0.25342
					-0.10646	-0.10615	-0.3516	-0.35034
					-0.10627	-0.10594	-0.3509	-0.34967
			-0.10668	-0.10637	-0.25471	-0.25373	-0.39414	-0.39279
			-0.10647	-0.10615	-0.25415	-0.25319	-0.39341	-0.39207
-0.05096	-0.05084	-0.05071	-0.20782	-0.20698	-0.30741	-0.30633	-0.39268	-0.39136
-0.05086	-0.05074	-0.05062	-0.20735	-0.20654	-0.30681	-0.3057	-0.39193	-0.39066
-0.05076	-0.05065	-0.05053	-0.20688	-0.2061	-0.30622	-0.3051	-0.3912	-0.38997
-0.10661	-0.10629	-0.10598	-0.25398	-0.25303	-0.34933	-0.3481	-0.61558	-0.61363

-0.10641	-0.10609	-0.10576	-0.25344	-0.2525	-0.34869	-0.3475	-0.61457	-0.61258
-0.20783	-0.20702	-0.20626	-0.30657	-0.3055	-0.52233	-0.52068	-0.65669	-0.65454
-0.20733	-0.20656	-0.20583	-0.30597	-0.3049	-0.52139	-0.51976	-0.65554	-0.65342
-0.25468	-0.25373	-0.25281	-0.48254	-0.48098	-0.61523	-0.6133	-0.697	-0.69481
-0.25418	-0.25322	-0.25228	-0.48165	-0.48013	-0.61419	-0.61221	-0.69572	-0.69353
-0.3074	-0.3063	-0.30521	-0.52193	-0.52034	-0.65668	-0.65457	-0.77245	-0.77
-0.30683	-0.30574	-0.30465	-0.52105	-0.51945	-0.65555	-0.65346	-0.77112	-0.76865
-0.30626	-0.30519	-0.30411	-0.52017	-0.51854	-0.6544	-0.65234	-0.7698	-0.76733
-0.48302	-0.48145	-0.47986	-0.61383	-0.61185	-0.73278	-0.73048	-0.76844	-0.76603
-0.48217	-0.48061	-0.47904	-0.61278	-0.61074	-0.73144	-0.72914	-0.76706	-0.76474
-0.52252	-0.52087	-0.51921	-0.65514	-0.65298	-0.7705	-0.76807	-0.84591	-0.84345
-0.52157	-0.51993	-0.51829	-0.65398	-0.65185	-0.76917	-0.76674	-0.84448	-0.84204
-0.659	-0.65693	-0.65488	-0.73227	-0.72993	-0.76784	-0.76544	-0.90673	-0.9041
-0.65788	-0.65576	-0.65366	-0.7309	-0.72861	-0.76651	-0.7642	-0.9053	-0.90264
-0.69298	-0.69065	-0.68833	-0.72955	-0.72731	-0.8735	-0.87099	-1.01299	-1.0096
-0.69177	-0.68943	-0.68709	-0.72826	-0.72604	-0.87201	-0.86954	-1.01112	-1.00779
-0.69056	-0.68822	-0.68589	-0.72702	-0.72481	-0.87053	-0.86809	-1.00922	-1.00599
-0.73285	-0.73047	-0.72811	-0.79565	-0.79328	-0.86913	-0.86666	-1.0073	-1.00413
-0.73149	-0.7292	-0.72694	-0.79442	-0.79206	-0.86778	-0.86526	-1.00538	-1.00225
-0.73014	-0.72792	-0.72575	-0.87158	-0.86899	-1.0102	-1.00681	-1.10456	-1.10091
-0.72879	-0.72663	-0.72455	-0.87019	-0.86763	-1.0084	-1.00504	-1.1025	-1.09881
-0.7974	-0.7951	-0.79289	-0.8688	-0.86628	-1.00662	-1.00328	-1.131	-1.1272
-0.79611	-0.79382	-0.7916	-0.86741	-0.86493	-1.00485	-1.00153	-1.12889	-1.12502
-0.79486	-0.79256	-0.79032	-0.86601	-0.86357	-1.00309	-0.99978	-1.12679	-1.12288
-0.83421	-0.83179	-0.82941	-0.97447	-0.9714	-1.10197	-1.09811	-1.12472	-1.12084
-0.83291	-0.83046	-0.82806	-0.97263	-0.96961	-1.0999	-1.09606	-1.12266	-1.11885
-0.83162	-0.82917	-0.82676	-1.07374	-1.0702	-1.12832	-1.12445	-1.18768	-1.18371
-0.83033	-0.8279	-0.82549	-1.07172	-1.06817	-1.12622	-1.12238	-1.18555	-1.18168
-0.97706	-0.97378	-0.97054	-1.10098	-1.09737	-1.19137	-1.18738	-1.18347	-1.17968
-0.97533	-0.97205	-0.96881	-1.09891	-1.09529	-1.18918	-1.18526	-1.18143	-1.17771
-1.07698	-1.07312	-1.06931	-1.12731	-1.12359	-1.18697	-1.18316	-1.17942	-1.17577
-1.07494	-1.07109	-1.0673	-1.12518	-1.1214	-1.18471	-1.18106	-1.17746	-1.17385
-1.07291	-1.06908	-1.06529	-1.12308	-1.11926	-1.1825	-1.179	-1.17552	-1.17195
-1.07089	-1.06708	-1.06331	-1.18816	-1.18417	-1.18044	-1.17701	-1.23415	-1.2305
-1.06887	-1.06508	-1.06134	-1.18606	-1.18215	-1.17848	-1.17506	-1.23213	-1.22852
-1.19606	-1.19196	-1.18791	-1.18398	-1.18016	-1.17653	-1.17313	-1.28243	-1.27872
-1.19389	-1.1898	-1.18577	-1.18191	-1.17819	-1.17461	-1.17122	-1.28037	-1.27667
-1.19174	-1.18768	-1.18369	-1.17988	-1.17623	-1.25895	-1.25538	-1.33916	-1.33528
-1.18959	-1.1856	-1.18169	-1.17792	-1.17427	-1.2569	-1.25336	-1.33699	-1.33312
-1.18746	-1.18354	-1.17971	-1.26238	-1.25856	-1.34244	-1.33865	-1.33484	-1.33098
-1.18531	-1.18147	-1.17773	-1.26033	-1.25654	-1.34029	-1.3365	-1.33271	-1.32885
-1.1832	-1.17943	-1.17577	-1.25831	-1.25456	-1.33817	-1.33439	-1.33059	-1.32673
-1.26784	-1.2639	-1.26008	-1.31829	-1.31439	-1.33613	-1.33232	-1.32849	-1.32463
-1.26575	-1.26185	-1.25807	-1.31622	-1.31241	-1.33414	-1.33028	-1.32642	-1.32254
-1.29324	-1.28933	-1.28554	-1.33983	-1.33598	-1.33212	-1.32823	-1.35606	-1.35208
-1.29117	-1.28734	-1.28361	-1.33781	-1.33394	-1.33006	-1.32617	-1.35394	-1.34995
-1.3218	-1.31787	-1.31402	-1.33576	-1.33188	-1.38615	-1.38208	-1.48698	-1.48238
-1.31981	-1.31587	-1.31199	-1.33368	-1.32981	-1.38397	-1.3799	-1.48457	-1.48002
-1.31781	-1.31385	-1.30994	-1.33159	-1.32772	-1.38179	-1.37771	-1.48217	-1.47769
-1.34141	-1.33741	-1.33344	-1.35634	-1.35238	-1.40606	-1.40192	-1.47978	-1.47535

-1.33928	-1.3353	-1.33136	-1.35421	-1.35024	-1.40382	-1.3997	-1.47741	-1.47302
-1.36408	-1.36005	-1.35605	-1.40985	-1.4057	-1.4841	-1.47955	-1.47508	-1.47072
-1.36189	-1.35787	-1.35388	-1.40758	-1.40344	-1.48168	-1.4772	-1.47279	-1.46845
-1.41785	-1.41366	-1.40949	-1.40535	-1.40122	-1.4793	-1.47488	-1.49096	-1.48656
-1.41563	-1.41144	-1.40728	-1.40315	-1.39905	-1.47696	-1.4726	-1.48867	-1.4843
-1.41346	-1.40928	-1.40513	-1.48371	-1.47917	-1.49524	-1.49083	-1.48647	-1.48212
-1.4114	-1.40723	-1.40308	-1.48153	-1.47706	-1.49314	-1.48875	-1.4844	-1.48009
-1.40933	-1.40517	-1.40104	-1.47937	-1.47497	-1.49106	-1.48668	-1.48234	-1.47806
-1.40715	-1.40301	-1.39891	-1.4771	-1.47276	-1.48885	-1.48449	-1.56701	-1.56257
-1.40492	-1.4008	-1.39674	-1.47479	-1.47049	-1.48658	-1.48224	-1.56466	-1.56024
-1.48586	-1.48133	-1.47687	-1.493	-1.48864	-1.54793	-1.5435	-1.56231	-1.55789
-1.4834	-1.47894	-1.47457	-1.4907	-1.48634	-1.54555	-1.54114	-1.55994	-1.55552
-1.51542	-1.5109	-1.50649	-1.55212	-1.54765	-1.56646	-1.56201	-1.55759	-1.55317
-1.51311	-1.5086	-1.50421	-1.54977	-1.54533	-1.56412	-1.55968	-1.55526	-1.55082
-1.56117	-1.55651	-1.55198	-1.5475	-1.54307	-1.56185	-1.55741	-1.553	-1.54855
-1.55895	-1.55434	-1.54982	-1.54536	-1.54094	-1.55971	-1.55527	-1.55087	-1.54642
-1.55674	-1.55219	-1.54769	-1.54324	-1.53883	-1.55757	-1.55313	-1.54875	-1.54436
-1.55443	-1.54993	-1.54546	-1.56422	-1.55976	-1.5553	-1.55084	-1.5465	-1.54227
-1.55208	-1.54761	-1.54318	-1.56192	-1.55745	-1.55298	-1.54848	-1.5442	-1.5402
-1.54983	-1.54539	-1.54098	-1.5597	-1.55523	-1.55076	-1.54623	-1.56328	-1.55947
-1.54769	-1.54325	-1.53884	-1.55756	-1.5531	-1.54863	-1.54406	-1.56115	-1.55754
-1.54549	-1.54105	-1.53665	-1.55534	-1.55089	-1.58837	-1.58384	-1.57972	-1.57626
-1.5432	-1.53876	-1.53435	-1.553	-1.54855	-1.58611	-1.58187	-1.57802	-1.57471
-1.54092	-1.53649	-1.53207	-1.55069	-1.54623	-1.58389	-1.57997	-1.5764	-1.57323
-1.56193	-1.55746	-1.55301	-1.5904	-1.58584	-1.58173	-1.57807	-1.57473	-1.57171
-1.55977	-1.55531	-1.55088	-1.58823	-1.58368	-1.57968	-1.57624	-1.5731	-1.57023
-1.59952	-1.595	-1.59052	-1.58608	-1.58172	-1.57789	-1.57462	-1.57161	-1.56885
-1.59716	-1.59263	-1.58818	-1.58395	-1.57991	-1.5763	-1.57315	-1.57023	-1.56754
-1.59489	-1.59037	-1.58597	-1.58191	-1.57814	-1.57472	-1.57169	-1.56886	-1.56622
-1.59272	-1.58823	-1.58388	-1.57997	-1.5764	-1.57315	-1.57023	-1.56749	-1.56489
-1.59052	-1.58611	-1.58188	-1.57813	-1.57476	-1.57165	-1.56884	-1.56616	-1.56359
-1.58822	-1.58397	-1.57996	-1.5764	-1.57322	-1.57025	-1.56752	-1.5649	-1.56235
-1.58597	-1.58191	-1.57815	-1.57478	-1.57176	-1.5689	-1.56624	-1.56365	-1.56111
-1.58385	-1.57999	-1.57647	-1.57327	-1.57038	-1.5676	-1.56495	-1.58184	-1.57926
-1.58184	-1.57818	-1.57489	-1.57184	-1.56906	-1.56633	-1.56367	-1.58054	-1.57795
-1.57996	-1.57645	-1.57331	-1.57038	-1.56769	-1.56503	-1.5624	-1.57928	-1.57669
-1.5782	-1.5748	-1.57176	-1.56892	-1.5663	-1.5637	-1.56114	-1.57805	-1.57546
-1.57654	-1.57327	-1.57032	-1.56755	-1.56497	-1.58194	-1.57938	-1.57682	-1.57423
-1.57498	-1.57187	-1.56902	-1.5663	-1.56373	-1.58071	-1.57815	-1.57559	-1.57298
-1.57348	-1.57052	-1.56776	-1.58467	-1.58206	-1.57947	-1.57691	-1.57435	-1.57174
-1.57203	-1.56919	-1.56651	-1.58343	-1.58079	-1.57818	-1.57561	-1.57306	-1.57052
-1.57063	-1.56787	-1.56525	-1.58219	-1.57953	-1.5769	-1.57432	-1.57179	-1.56932
-1.58894	-1.58618	-1.58356	-1.58091	-1.57828	-1.57568	-1.5731	-1.57058	-1.56816
-1.58758	-1.58484	-1.58222	-1.57962	-1.57705	-1.57448	-1.57192	-1.56943	-1.56703
-1.58626	-1.58356	-1.58096	-1.57838	-1.57584	-1.5733	-1.57077	-1.5683	-1.56591
-1.58499	-1.58234	-1.57976	-1.57719	-1.57466	-1.57214	-1.56965	-1.56721	-1.56479
-1.58375	-1.58113	-1.57858	-1.57602	-1.57348	-1.57098	-1.56852	-1.58918	-1.58668
-1.58254	-1.57995	-1.5774	-1.57485	-1.57232	-1.56982	-1.56736	-1.58799	-1.58553
-1.58134	-1.57877	-1.57623	-1.57369	-1.57115	-1.56866	-1.56621	-1.60522	-1.6028
-1.58016	-1.5776	-1.57506	-1.57252	-1.56997	-1.5675	-1.56508	-1.60409	-1.60171

-1.57898	-1.57642	-1.57388	-1.57134	-1.5688	-1.56634	-1.56397	-1.60299	-1.60064
-1.5778	-1.57523	-1.57266	-1.58888	-1.58637	-1.58394	-1.58159	-1.61064	-1.60828
-1.57662	-1.57402	-1.57143	-1.58766	-1.58522	-1.58285	-1.58054	-1.60956	-1.60724
-1.59417	-1.59156	-1.58897	-1.5865	-1.58411	-1.58177	-1.57949	-1.60849	-1.60619
-1.59293	-1.59035	-1.58782	-1.58538	-1.58301	-1.5807	-1.57845	-1.60742	-1.60513
-1.59171	-1.58917	-1.58669	-1.58428	-1.58194	-1.61105	-1.60868	-1.62627	-1.62396
-1.59054	-1.58803	-1.58558	-1.5832	-1.58089	-1.60996	-1.60758	-1.62516	-1.62289
-1.5894	-1.58691	-1.58448	-1.58213	-1.57985	-1.60889	-1.60651	-1.62408	-1.62183
-1.58828	-1.5858	-1.5834	-1.5901	-1.58779	-1.62774	-1.62536	-1.66549	-1.66306
-1.58716	-1.58471	-1.58233	-1.58903	-1.58675	-1.62668	-1.62433	-1.66439	-1.66195
-1.59517	-1.5927	-1.59031	-1.63033	-1.62797	-1.62563	-1.6233	-1.67916	-1.67671
-1.59403	-1.5916	-1.58923	-1.62923	-1.6269	-1.62458	-1.62227	-1.67805	-1.6756
-1.5929	-1.59051	-1.58817	-1.62814	-1.62581	-1.63989	-1.63761	-1.67693	-1.67449
-1.59178	-1.58942	-1.58712	-1.62705	-1.62471	-1.63879	-1.63654	-1.67579	-1.67338
-1.63315	-1.63072	-1.62834	-1.64236	-1.64001	-1.66205	-1.65965	-1.69549	-1.69291
-1.63203	-1.62962	-1.62727	-1.6413	-1.63897	-1.66094	-1.65852	-1.69424	-1.69173
-1.63093	-1.62854	-1.62622	-1.64026	-1.63794	-1.65984	-1.65741	-1.69302	-1.69057
-1.64625	-1.64387	-1.64156	-1.63923	-1.63691	-1.65874	-1.65632	-1.69183	-1.68943
-1.6452	-1.64283	-1.64053	-1.6382	-1.63588	-1.65764	-1.65523	-1.69069	-1.68831
-1.64415	-1.6418	-1.6395	-1.63718	-1.63486	-1.6739	-1.67147	-1.68957	-1.68722
-1.6431	-1.64076	-1.63848	-1.63616	-1.63384	-1.67279	-1.6704	-1.68848	-1.68614
-1.64205	-1.63972	-1.63745	-1.63515	-1.63285	-1.67172	-1.66934	-1.6874	-1.68507
-1.64101	-1.63869	-1.63641	-1.63414	-1.63189	-1.6707	-1.66832	-1.68633	-1.68401
-1.63997	-1.63766	-1.63539	-1.65714	-1.65476	-1.69016	-1.68768	-1.73325	-1.7306
-1.63894	-1.63663	-1.63436	-1.65606	-1.65372	-1.68909	-1.68662	-1.73205	-1.72939
-1.63792	-1.63562	-1.63334	-1.65498	-1.65267	-1.68802	-1.68557	-1.73086	-1.72818
-1.63694	-1.63463	-1.63235	-1.65391	-1.65163	-1.68695	-1.68452	-1.74811	-1.74535
-1.63599	-1.63367	-1.63137	-1.65285	-1.6506	-1.68588	-1.68347	-1.74688	-1.74409
-1.63501	-1.6327	-1.6304	-1.67254	-1.67021	-1.68482	-1.68242	-1.74565	-1.74284
-1.634	-1.63172	-1.62945	-1.67152	-1.66917	-1.68376	-1.68137	-1.74443	-1.74159
-1.65696	-1.65456	-1.65219	-1.67052	-1.66815	-1.74887	-1.74606	-1.74323	-1.74036
-1.65587	-1.65353	-1.65123	-1.66955	-1.66716	-1.74768	-1.7449	-1.74208	-1.73916
-1.6548	-1.65251	-1.65027	-1.66857	-1.66617	-1.74649	-1.74372	-1.7409	-1.73795
-1.67463	-1.67223	-1.66992	-1.66755	-1.66517	-1.74528	-1.7425	-1.73966	-1.7367
-1.67358	-1.67118	-1.66887	-1.66652	-1.66416	-1.74408	-1.74125	-1.73839	-1.73544
-1.67253	-1.67014	-1.66783	-1.74854	-1.74574	-1.7429	-1.74003	-1.73714	-1.73422
-1.6715	-1.66912	-1.66679	-1.74733	-1.74459	-1.74175	-1.73882	-1.73591	-1.73304
-1.6705	-1.66812	-1.66578	-1.74612	-1.74339	-1.76051	-1.75744	-1.80762	-1.80458
-1.66954	-1.66716	-1.66479	-1.7449	-1.74214	-1.75922	-1.75615	-1.8063	-1.80332
-1.73607	-1.73325	-1.73042	-1.7437	-1.7409	-1.79359	-1.79049	-1.80502	-1.80209
-1.73489	-1.7321	-1.72932	-1.74256	-1.73968	-1.79229	-1.78924	-1.8038	-1.8009
-1.73369	-1.73094	-1.72821	-1.74142	-1.73848	-1.79099	-1.788	-1.8026	-1.79972
-1.73251	-1.72975	-1.72701	-1.77637	-1.77335	-1.80729	-1.80429	-1.80136	-1.79852
-1.73133	-1.72855	-1.72578	-1.77508	-1.77208	-1.806	-1.80302	-1.80012	-1.79732
-1.76693	-1.76406	-1.76117	-1.77383	-1.77086	-1.80476	-1.8018	-1.79893	-1.79617
-1.76581	-1.76289	-1.75995	-1.7726	-1.76969	-1.80357	-1.80064	-1.79779	-1.79506
-1.74797	-1.74507	-1.74215	-1.7714	-1.76851	-1.80237	-1.79948	-1.79667	-1.79396
-1.74676	-1.74389	-1.74102	-1.77023	-1.76729	-1.80113	-1.79834	-1.7956	-1.79286
-1.74556	-1.74272	-1.73988	-1.7869	-1.7839	-1.79993	-1.79721	-1.79451	-1.79176
-1.74442	-1.74155	-1.73868	-1.78571	-1.7828	-1.79883	-1.7961	-1.79338	-1.79065

-1.7433	-1.74038	-1.73748	-1.78452	-1.78172	-1.79777	-1.79499	-1.79224	-1.78953
-1.74211	-1.7392	-1.73632	-1.78336	-1.78061	-1.79665	-1.79388	-1.79114	-1.78841
-1.74089	-1.73802	-1.73519	-1.78221	-1.77947	-1.79551	-1.79279	-1.79006	-1.78729
-1.73972	-1.73687	-1.73407	-1.78108	-1.77837	-1.79439	-1.79167	-1.78893	-1.78614
-1.73861	-1.73575	-1.73295	-1.77995	-1.7773	-1.7933	-1.79052	-1.78775	-1.78495
-1.75576	-1.75289	-1.75007	-1.77884	-1.77622	-1.79218	-1.78937	-1.78657	-1.78379
-1.75454	-1.75174	-1.74902	-1.77778	-1.77512	-1.79102	-1.78819	-1.7854	-1.78266
-1.75333	-1.7506	-1.74796	-1.77672	-1.77403	-1.78988	-1.78705	-1.78426	-1.78155
-1.75218	-1.74949	-1.74688	-1.77563	-1.77295	-1.7888	-1.78598	-1.7832	-1.78046
-1.75108	-1.74839	-1.74578	-1.77452	-1.77188	-1.78773	-1.78495	-1.78218	-1.77939
-1.74998	-1.74731	-1.74472	-1.77344	-1.77079	-1.78662	-1.78388	-1.82774	-1.82459
-1.74891	-1.74626	-1.74368	-1.77236	-1.76967	-1.78549	-1.78279	-1.82659	-1.82347
-1.76329	-1.76061	-1.75799	-1.77127	-1.76858	-1.78437	-1.78169	-1.82539	-1.82231
-1.76228	-1.75956	-1.75689	-1.77015	-1.7675	-1.78329	-1.78056	-1.82412	-1.8211
-1.76126	-1.75853	-1.75581	-1.75317	-1.75057	-1.78219	-1.77944	-1.85093	-1.84789
-1.76023	-1.75751	-1.7548	-1.75212	-1.74948	-1.78105	-1.77833	-1.84972	-1.84667
-1.75917	-1.75648	-1.75379	-1.75109	-1.74839	-1.77992	-1.77722	-1.84852	-1.84545
-1.75807	-1.75541	-1.75276	-1.75007	-1.74736	-1.83605	-1.83298	-1.85607	-1.85282
-1.75696	-1.75432	-1.75171	-1.74904	-1.74636	-1.83489	-1.83175	-1.8547	-1.85154
-1.7559	-1.75325	-1.75062	-1.77798	-1.77526	-1.83375	-1.8306	-1.87076	-1.86757
-1.7549	-1.75219	-1.74951	-1.77686	-1.7742	-1.83263	-1.82951	-1.8696	-1.8664
-1.75389	-1.75116	-1.74844	-1.77578	-1.77315	-1.85788	-1.85448	-1.86843	-1.86521
-1.75289	-1.75015	-1.74743	-1.77474	-1.7721	-1.85664	-1.85331	-1.86725	-1.86398
-1.78204	-1.77923	-1.77643	-1.77371	-1.77105	-1.87271	-1.86937	-1.86606	-1.86276
-1.78099	-1.7782	-1.77541	-1.77267	-1.76995	-1.87137	-1.86811	-1.86487	-1.86158
-1.77993	-1.77716	-1.77439	-1.77162	-1.76886	-1.87003	-1.86685	-1.86367	-1.86041
-1.77881	-1.77608	-1.77335	-1.8438	-1.84036	-1.86878	-1.86558	-1.86241	-1.85921
-1.77768	-1.77498	-1.77231	-1.84262	-1.83922	-1.8676	-1.86433	-1.86112	-1.858
-1.7766	-1.77392	-1.77127	-1.85894	-1.85553	-1.86644	-1.86313	-1.85992	-1.85683
-1.77558	-1.77289	-1.77023	-1.85771	-1.85438	-1.8653	-1.862	-1.85878	-1.8557
-1.77457	-1.77186	-1.76918	-1.85647	-1.85321	-1.86416	-1.86086	-1.85764	-1.85454
-1.77356	-1.77081	-1.76811	-1.85519	-1.85202	-1.86301	-1.85971	-1.85647	-1.85334
-1.77256	-1.76978	-1.76706	-1.85395	-1.85084	-1.86186	-1.85857	-1.85532	-1.85216
-1.81777	-1.81461	-1.81152	-1.85278	-1.84968	-1.84651	-1.84328	-1.8542	-1.85103
-1.81666	-1.81353	-1.81047	-1.85165	-1.84853	-1.84537	-1.84217	-1.8531	-1.84994
-1.8605	-1.85707	-1.85374	-1.85051	-1.84739	-1.84424	-1.84106	-1.87366	-1.8703
-1.85928	-1.85589	-1.85259	-1.84938	-1.84625	-1.84311	-1.83996	-1.8725	-1.86913
-1.8581	-1.85473	-1.85144	-1.84824	-1.84511	-1.84199	-1.83886	-1.87133	-1.86796
-1.85696	-1.85359	-1.8503	-1.8471	-1.84398	-1.84086	-1.83776	-1.87015	-1.8668
-1.85582	-1.85245	-1.84916	-1.84596	-1.84285	-1.83975	-1.83666	-1.86898	-1.86563
-1.85467	-1.85131	-1.84802	-1.84484	-1.84175	-1.83865	-1.83556	-1.86781	-1.86447
-1.85352	-1.85017	-1.84688	-1.84372	-1.84065	-1.83757	-1.83448	-1.86664	-1.86332
-1.85237	-1.84902	-1.84573	-1.84258	-1.83954	-1.85825	-1.85496	-1.86549	-1.86217
-1.85121	-1.84787	-1.84457	-1.84144	-1.83844	-1.85709	-1.85381	-1.86434	-1.86101
-1.85005	-1.84672	-1.84343	-1.84032	-1.83734	-1.85593	-1.85266	-1.86318	-1.85986
-1.84888	-1.84557	-1.8423	-1.83921	-1.83624	-1.85476	-1.85149	-1.86201	-1.85869
-1.84773	-1.84445	-1.84124	-1.83815	-1.83517	-1.8536	-1.85033	-1.86084	-1.85753
-1.84658	-1.84339	-1.84026	-1.83717	-1.83413	-1.85245	-1.84918	-1.85968	-1.85636
-1.84544	-1.84231	-1.83926	-1.85788	-1.85458	-1.8513	-1.84802	-1.87253	-1.8692
-1.84429	-1.8412	-1.83818	-1.85673	-1.85344	-1.85015	-1.84687	-1.87135	-1.86802

-1.84317	-1.84009	-1.83708	-1.85557	-1.85228	-1.84899	-1.84571	-1.87016	-1.86684
-1.84213	-1.83904	-1.83601	-1.85441	-1.85112	-1.86201	-1.8587	-1.88525	-1.88176
-1.84114	-1.83802	-1.83494	-1.85325	-1.84997	-1.86083	-1.85753	-1.88401	-1.88056
-1.86205	-1.85871	-1.85539	-1.86625	-1.86295	-1.85966	-1.85636	-1.89524	-1.89178
-1.86091	-1.85756	-1.85425	-1.86508	-1.86178	-1.85849	-1.85521	-1.89404	-1.89063
-1.85976	-1.85641	-1.8531	-1.86392	-1.86061	-1.87014	-1.86685	-1.89287	-1.88949
-1.8586	-1.85525	-1.85194	-1.86275	-1.85946	-1.869	-1.86576	-1.89175	-1.88836
-1.85744	-1.8541	-1.85079	-1.86159	-1.85833	-1.86788	-1.86467	-1.89063	-1.88721
-1.87037	-1.86703	-1.86373	-1.86047	-1.85725	-1.86679	-1.86356	-1.88945	-1.88602
-1.8692	-1.86587	-1.86259	-1.85936	-1.85618	-1.86571	-1.86244	-1.88824	-1.88482
-1.86813	-1.86481	-1.86152	-1.87107	-1.86783	-1.88075	-1.8773	-1.88703	-1.88361
-1.86714	-1.86381	-1.86049	-1.86998	-1.8667	-1.87953	-1.87608	-1.88581	-1.88241
-1.87895	-1.87557	-1.8722	-1.86887	-1.86556	-1.87832	-1.87488	-1.88462	-1.88126
-1.87778	-1.87441	-1.87105	-1.86772	-1.86441	-1.87713	-1.87371	-1.88347	-1.88018
-1.87659	-1.87323	-1.86989	-1.86658	-1.8633	-1.87599	-1.87259	-1.88237	-1.8791
-1.8754	-1.87205	-1.86873	-1.86546	-1.86224	-1.87493	-1.87155	-1.88133	-1.87803
-1.87423	-1.87089	-1.86759	-1.86436	-1.86119	-1.87387	-1.87052	-1.88029	-1.87696
-1.87317	-1.86983	-1.86653	-1.87942	-1.87607	-1.87273	-1.8694	-1.86608	-1.86278
-1.87216	-1.86881	-1.8655	-1.87831	-1.87492	-1.87156	-1.86824	-1.86494	-1.86167
-1.87107	-1.86773	-1.86442	-1.87717	-1.87377	-1.87041	-1.8671	-1.86383	-1.86059
-1.86993	-1.86661	-1.86331	-1.87602	-1.87262	-1.86927	-1.86597	-1.86272	-1.85954
-1.86884	-1.86553	-1.86225	-1.87492	-1.87153	-1.86819	-1.8649	-1.86166	-1.8585
-1.86783	-1.86453	-1.86126	-1.8739	-1.87054	-1.86721	-1.86391	-1.86066	-1.85748
-1.88313	-1.87967	-1.87624	-1.87286	-1.86953	-1.86621	-1.86291	-1.85965	-1.85644
-1.88196	-1.87851	-1.8751	-1.87174	-1.86842	-1.86512	-1.86183	-1.85858	-1.85537
-1.88078	-1.87735	-1.87395	-1.87061	-1.8673	-1.86401	-1.86074	-1.85751	-1.85432
-1.87969	-1.87627	-1.87289	-1.86956	-1.86627	-1.86299	-1.85974	-1.85652	-1.85334
-1.87864	-1.87523	-1.87187	-1.86855	-1.86527	-1.86201	-1.85876	-1.85555	-1.8524
-1.87751	-1.87412	-1.87078	-1.86747	-1.86419	-1.86094	-1.85771	-1.85453	-1.85142
-1.87633	-1.87295	-1.86962	-1.86632	-1.86305	-1.85981	-1.85661	-1.85346	-1.8504
-1.8752	-1.87184	-1.86853	-1.86523	-1.86197	-1.85874	-1.85555	-1.85242	-1.84938
-1.87415	-1.87082	-1.86752	-1.86424	-1.86099	-1.85776	-1.85458	-1.85144	-1.84836
-1.87314	-1.86981	-1.86654	-1.86327	-1.86002	-1.8568	-1.85362	-1.85047	-1.84736
-1.87213	-1.86882	-1.86555	-1.86229	-1.85905	-1.85585	-1.85268	-1.84953	-1.8464
-1.87112	-1.86781	-1.86455	-1.8613	-1.85807	-1.85487	-1.85172	-1.84858	-1.84545
-1.87001	-1.86672	-1.86346	-1.86022	-1.857	-1.85382	-1.85067	-1.84754	-1.8444
-1.86886	-1.86558	-1.86233	-1.8591	-1.85589	-1.85271	-1.84957	-1.84644	-1.84331
-1.86779	-1.86451	-1.86127	-1.85805	-1.85485	-1.85169	-1.84855	-1.84542	-1.8423
-1.8668	-1.86353	-1.86029	-1.85709	-1.8539	-1.85074	-1.84761	-1.84448	-1.84136
-1.86583	-1.86256	-1.85933	-1.85613	-1.85296	-1.84981	-1.84667	-1.84354	-1.84043
-1.86485	-1.8616	-1.85837	-1.85518	-1.85202	-1.84887	-1.84573	-1.84261	-1.83951
-1.86386	-1.86061	-1.85739	-1.8542	-1.85105	-1.8479	-1.84477	-1.84165	-1.83856
-1.86277	-1.85953	-1.85632	-1.85314	-1.84999	-1.84685	-1.84371	-1.84061	-1.83753
-1.86166	-1.85842	-1.85522	-1.85205	-1.84891	-1.84577	-1.84263	-1.83953	-1.83647
-1.86064	-1.85741	-1.85421	-1.85105	-1.84791	-1.84477	-1.84164	-1.83854	-1.83549
-1.85969	-1.85647	-1.85328	-1.85011	-1.84697	-1.84384	-1.84071	-1.83761	-1.83457
-1.85874	-1.85553	-1.85235	-1.84919	-1.84605	-1.84291	-1.83979	-1.83671	-1.83367
-1.85781	-1.85461	-1.85144	-1.84827	-1.84513	-1.84201	-1.8389	-1.83582	-1.83279
-1.85689	-1.85369	-1.85052	-1.84736	-1.84422	-1.8411	-1.83801	-1.83494	-1.83191
-1.85595	-1.85277	-1.84961	-1.84645	-1.84331	-1.8402	-1.83712	-1.83406	-1.83104

-1.85503	-1.85185	-1.8487	-1.84555	-1.8424	-1.8393	-1.83622	-1.83318	-1.83016
-1.85411	-1.85093	-1.84778	-1.84463	-1.84149	-1.83839	-1.83533	-1.8323	-1.82928
-1.8532	-1.85002	-1.84686	-1.84371	-1.84057	-1.83748	-1.83443	-1.83141	-1.8284
-1.85228	-1.84911	-1.84595	-1.8428	-1.83967	-1.83658	-1.83354	-1.83053	-1.82752
-1.85137	-1.84819	-1.84504	-1.8419	-1.83878	-1.8357	-1.83266	-1.82964	-1.82664
-1.85045	-1.84728	-1.84413	-1.841	-1.83789	-1.83481	-1.83177	-1.82876	-1.82576
-1.84953	-1.84636	-1.84322	-1.84009	-1.837	-1.83393	-1.8309	-1.82789	-1.82489
-1.84861	-1.84545	-1.8423	-1.83918	-1.8361	-1.83304	-1.83002	-1.82702	-1.82403
-1.84768	-1.84451	-1.84137	-1.83827	-1.8352	-1.83215	-1.82914	-1.82614	-1.82316
-1.84674	-1.84358	-1.84044	-1.83735	-1.83429	-1.83126	-1.82824	-1.82525	-1.82229
-1.84581	-1.84265	-1.83951	-1.83643	-1.83338	-1.83036	-1.82735	-1.82437	-1.82142
-1.84488	-1.84173	-1.83859	-1.83551	-1.83248	-1.82946	-1.82646	-1.82348	-1.82054
-1.84395	-1.8408	-1.83768	-1.83461	-1.83158	-1.82857	-1.82557	-1.8226	-1.81967
-1.84301	-1.83988	-1.83677	-1.83371	-1.83069	-1.82768	-1.8247	-1.82173	-1.81881
-1.84208	-1.83896	-1.83587	-1.83281	-1.8298	-1.8268	-1.82382	-1.82086	-1.81795
-1.84115	-1.83804	-1.83496	-1.83192	-1.82891	-1.82591	-1.82295	-1.82	-1.81709
-1.84022	-1.83712	-1.83406	-1.83102	-1.82802	-1.82503	-1.82207	-1.81914	-1.81623
-1.83928	-1.8362	-1.83315	-1.83013	-1.82713	-1.82415	-1.8212	-1.81827	-1.81537
-1.83835	-1.83528	-1.83225	-1.82923	-1.82624	-1.82327	-1.82033	-1.81741	-1.8145
-1.83747	-1.83438	-1.83136	-1.82834	-1.82535	-1.82239	-1.81946	-1.81655	-1.81364
-1.83661	-1.83352	-1.83049	-1.82746	-1.82447	-1.8215	-1.81858	-1.81568	-1.81277
-1.83581	-1.83271	-1.82968	-1.82664	-1.82362	-1.82064	-1.81772	-1.81481	-1.8119
-1.83507	-1.83199	-1.82895	-1.82588	-1.82281	-1.81981	-1.81686	-1.81394	-1.81103
-1.8343	-1.83124	-1.82819	-1.82512	-1.82203	-1.819	-1.81603	-1.8131	-1.81018
-1.83345	-1.8304	-1.82738	-1.82433	-1.82129	-1.81826	-1.81525	-1.81227	-1.80935
-1.83256	-1.82954	-1.82653	-1.82353	-1.82053	-1.81752	-1.81448	-1.81148	-1.80855
-1.8317	-1.82867	-1.82567	-1.82268	-1.81971	-1.81673	-1.81372	-1.81074	-1.80783
-1.83084	-1.8278	-1.82479	-1.82181	-1.81886	-1.81591	-1.81295	-1.81002	-1.80713
-1.83005	-1.82699	-1.82394	-1.82095	-1.81801	-1.81507	-1.81214	-1.80924	-1.80636
-1.82932	-1.82621	-1.82312	-1.82011	-1.81716	-1.81422	-1.81129	-1.80839	-1.80554
-1.82856	-1.82544	-1.82234	-1.81932	-1.81636	-1.81341	-1.81047	-1.80756	-1.80471
-1.82774	-1.82467	-1.82161	-1.81861	-1.81564	-1.81267	-1.80968	-1.80674	-1.80388
-1.82691	-1.82388	-1.82086	-1.81788	-1.81491	-1.81193	-1.80891	-1.80595	-1.80309
-1.82607	-1.82304	-1.82004	-1.81706	-1.81411	-1.81114	-1.80816	-1.80523	-1.80238
-1.82525	-1.82221	-1.81921	-1.81623	-1.81328	-1.81034	-1.80742	-1.80453	-1.80168
-1.82449	-1.82145	-1.81845	-1.81545	-1.81247	-1.80953	-1.80662	-1.80375	-1.80091
-1.82375	-1.82073	-1.81772	-1.8147	-1.81167	-1.80871	-1.80581	-1.80294	-1.8001
-1.82295	-1.81995	-1.81695	-1.81393	-1.8109	-1.80794	-1.80505	-1.80218	-1.79933
-1.82209	-1.8191	-1.81614	-1.81315	-1.81017	-1.80723	-1.80435	-1.80147	-1.79857
-1.79935	-1.79644	-1.79355	-1.81237	-1.80941	-1.80648	-1.80361	-1.80073	-1.79782
-1.79859	-1.79568	-1.79279	-1.81159	-1.8086	-1.80566	-1.8028	-1.79995	-1.79707
-1.79783	-1.79492	-1.79202	-1.8108	-1.80779	-1.80485	-1.80199	-1.79916	-1.79631
-1.79701	-1.79409	-1.79119	-1.81	-1.80701	-1.80409	-1.80125	-1.79841	-1.79554
-1.79617	-1.79324	-1.79035	-1.80919	-1.80625	-1.80336	-1.80051	-1.79766	-1.79477
-1.79539	-1.79246	-1.78955	-1.78666	-1.78377	-1.80257	-1.79973	-1.79689	-1.79401
-1.79463	-1.79171	-1.78879	-1.78587	-1.78296	-1.80175	-1.79892	-1.79609	-1.79326
-1.78373	-1.78082	-1.77791	-1.78509	-1.78217	-1.80098	-1.79815	-1.79533	-1.79253
-1.78298	-1.78004	-1.77711	-1.7843	-1.78142	-1.80027	-1.79743	-1.79462	-1.79182
-1.7822	-1.77926	-1.77632	-1.78352	-1.78068	-1.77785	-1.77504	-1.79388	-1.79109
-1.78137	-1.77846	-1.77556	-1.78277	-1.77994	-1.77711	-1.77426	-1.7931	-1.7903

-0.86223	-0.86088	-0.85955	-0.85817	-0.85677	-0.85538	-0.85399	-0.85261	-0.85122
-0.86258	-0.86121	-0.85987	-0.8585	-0.85712	-0.85573	-0.85435	-0.85296	-0.85157
-0.86293	-0.86155	-0.86019	-0.85883	-0.85746	-0.85608	-0.85469	-0.85331	-0.85193
-0.8318	-0.83044	-0.8291	-0.85912	-0.85778	-0.85642	-0.85504	-0.85365	-0.85227
-0.83214	-0.83074	-0.82937	-0.85942	-0.8581	-0.85675	-0.85537	-0.85399	-0.85261
-0.79883	-0.79757	-0.79632	-0.82836	-0.82709	-0.85708	-0.8557	-0.85432	-0.85294
-0.79911	-0.79786	-0.79661	-0.82869	-0.8274	-0.8574	-0.85604	-0.85466	-0.85328
-0.79939	-0.79814	-0.79689	-0.79567	-0.79447	-0.82641	-0.82509	-0.85499	-0.85361
-0.79967	-0.79841	-0.79716	-0.79595	-0.79475	-0.82673	-0.8254	-0.85532	-0.85393
-0.79994	-0.79868	-0.79743	-0.79622	-0.79503	-0.82705	-0.82572	-0.82438	-0.82304
-0.80022	-0.79895	-0.79769	-0.79648	-0.79528	-0.82735	-0.82603	-0.8247	-0.82335
-0.8005	-0.79922	-0.79796	-0.79673	-0.79553	-0.82765	-0.82634	-0.82502	-0.82367
-0.80077	-0.7995	-0.79824	-0.79701	-0.7958	-0.79459	-0.79338	-0.82533	-0.82398
-0.80105	-0.79978	-0.79852	-0.79729	-0.79607	-0.79485	-0.79364	-0.82562	-0.82429
-0.80133	-0.80005	-0.7988	-0.79756	-0.79635	-0.79512	-0.7939	-0.79267	-0.79144
-0.80162	-0.80033	-0.79906	-0.79783	-0.79662	-0.7954	-0.79416	-0.79293	-0.7917
-0.80192	-0.80061	-0.79933	-0.7981	-0.7969	-0.79568	-0.79443	-0.79319	-0.79196
-0.80223	-0.80091	-0.79961	-0.79837	-0.79716	-0.79594	-0.7947	-0.79346	-0.79223
-0.80255	-0.80122	-0.79991	-0.79865	-0.79744	-0.79621	-0.79497	-0.79374	-0.7925
-0.80286	-0.80154	-0.80024	-0.79897	-0.79773	-0.79648	-0.79524	-0.79401	-0.79279
-0.80317	-0.80187	-0.80058	-0.7993	-0.79802	-0.79676	-0.79551	-0.79428	-0.79308
-0.80347	-0.80218	-0.8009	-0.79961	-0.79832	-0.79705	-0.79579	-0.79455	-0.79336
-0.80378	-0.80248	-0.8012	-0.79991	-0.79862	-0.79734	-0.79607	-0.79483	-0.79363
-0.80412	-0.80281	-0.80152	-0.80022	-0.79892	-0.79764	-0.79638	-0.79513	-0.79393
-0.8045	-0.80317	-0.80184	-0.80053	-0.79922	-0.79795	-0.79671	-0.79547	-0.79424
-0.77087	-0.76961	-0.76834	-0.80085	-0.79954	-0.79827	-0.79704	-0.79581	-0.79456
-0.77117	-0.76991	-0.76865	-0.8012	-0.79988	-0.79861	-0.79737	-0.79613	-0.79487
-0.77147	-0.77021	-0.76896	-0.80154	-0.80023	-0.79895	-0.79769	-0.79644	-0.79519
-0.70306	-0.70207	-0.70108	-0.80188	-0.80057	-0.79928	-0.79801	-0.79675	-0.79551
-0.70327	-0.70228	-0.7013	-0.80221	-0.80091	-0.79962	-0.79833	-0.79707	-0.79584
-0.70349	-0.7025	-0.70153	-0.76864	-0.76741	-0.79995	-0.79864	-0.79737	-0.79615
-0.70372	-0.70273	-0.70176	-0.76896	-0.76772	-0.80028	-0.79895	-0.79766	-0.79644
-0.70396	-0.70297	-0.70198	-0.701	-0.70002	-0.76679	-0.76554	-0.79799	-0.79676
-0.70422	-0.70321	-0.7022	-0.70121	-0.70024	-0.76709	-0.76587	-0.79836	-0.7971
-0.70448	-0.70345	-0.70243	-0.70143	-0.70046	-0.7674	-0.76619	-0.79873	-0.79745
-0.70472	-0.70369	-0.70267	-0.70167	-0.70069	-0.76771	-0.7665	-0.79907	-0.7978
-0.70495	-0.70394	-0.70292	-0.70192	-0.70093	-0.76803	-0.76681	-0.79941	-0.79816
-0.70519	-0.70418	-0.70318	-0.70217	-0.70117	-0.70018	-0.69922	-0.76593	-0.76474
-0.70544	-0.70444	-0.70344	-0.70243	-0.70142	-0.70043	-0.69946	-0.76626	-0.76504
-0.70568	-0.70468	-0.70369	-0.70269	-0.70167	-0.70068	-0.69971	-0.76657	-0.76534
-0.70594	-0.70493	-0.70393	-0.70293	-0.70194	-0.70095	-0.69995	-0.76687	-0.76563
-0.70619	-0.70518	-0.70418	-0.70318	-0.7022	-0.7012	-0.7002	-0.6992	-0.69822
-0.70646	-0.70544	-0.70444	-0.70344	-0.70243	-0.70143	-0.70044	-0.69945	-0.69846
-0.67401	-0.67307	-0.67214	-0.70369	-0.70268	-0.70167	-0.70068	-0.69969	-0.69871
-0.67426	-0.6733	-0.67236	-0.70395	-0.70294	-0.70193	-0.70093	-0.69994	-0.69896
-0.67451	-0.67354	-0.67257	-0.7042	-0.70321	-0.7022	-0.70119	-0.70019	-0.69922
-0.67473	-0.67376	-0.6728	-0.67186	-0.67092	-0.70246	-0.70145	-0.70045	-0.69947
-0.67494	-0.67398	-0.67304	-0.67209	-0.67114	-0.70272	-0.70172	-0.70073	-0.69974
-0.67516	-0.6742	-0.67326	-0.67231	-0.67137	-0.70298	-0.70198	-0.70099	-0.7
-0.67541	-0.67443	-0.67347	-0.67253	-0.6716	-0.70325	-0.70224	-0.70124	-0.70025

-0.67566	-0.67467	-0.67369	-0.67275	-0.67183	-0.6709	-0.66995	-0.7015	-0.70051
-0.6759	-0.6749	-0.67393	-0.67297	-0.67204	-0.67111	-0.67018	-0.70177	-0.70077
-0.67615	-0.67515	-0.67416	-0.6732	-0.67225	-0.67132	-0.67041	-0.70203	-0.70103
-0.67641	-0.67539	-0.67439	-0.67342	-0.67246	-0.67153	-0.67062	-0.66971	-0.66878
-0.67667	-0.67564	-0.67462	-0.67364	-0.67268	-0.67174	-0.67083	-0.66992	-0.669
-0.64295	-0.64203	-0.64112	-0.67388	-0.6729	-0.67195	-0.67103	-0.67012	-0.66922
-0.64318	-0.64226	-0.64135	-0.67413	-0.67313	-0.67216	-0.67124	-0.67033	-0.66942
-0.64342	-0.6425	-0.64159	-0.6744	-0.67338	-0.6724	-0.67146	-0.67054	-0.66964
-0.64366	-0.64274	-0.64183	-0.67468	-0.67365	-0.67265	-0.6717	-0.67077	-0.66985
-0.6439	-0.64299	-0.64208	-0.64116	-0.64024	-0.67292	-0.67195	-0.671	-0.67007
-0.64414	-0.64322	-0.64231	-0.64139	-0.64048	-0.67318	-0.67219	-0.67123	-0.67029
-0.64437	-0.64345	-0.64254	-0.64162	-0.64071	-0.67346	-0.67245	-0.67146	-0.67052
-0.64462	-0.64369	-0.64278	-0.64186	-0.64096	-0.67374	-0.67271	-0.67171	-0.67076
-0.64489	-0.64394	-0.64302	-0.64211	-0.64121	-0.67402	-0.67298	-0.67197	-0.67101
-0.64516	-0.64421	-0.64327	-0.64236	-0.64145	-0.64054	-0.63961	-0.67224	-0.67126
-0.64545	-0.64448	-0.64353	-0.6426	-0.6417	-0.64078	-0.63985	-0.67252	-0.67152
-0.64575	-0.64476	-0.6438	-0.64286	-0.64195	-0.64103	-0.6401	-0.6728	-0.67178
-0.64604	-0.64505	-0.64408	-0.64313	-0.64219	-0.64127	-0.64035	-0.6731	-0.67206
-0.64634	-0.64535	-0.64437	-0.6434	-0.64244	-0.64151	-0.6406	-0.63969	-0.63875
-0.64664	-0.64564	-0.64465	-0.64367	-0.6427	-0.64175	-0.64083	-0.63991	-0.63898
-0.64695	-0.64594	-0.64494	-0.64394	-0.64296	-0.642	-0.64106	-0.64013	-0.63922
-0.59522	-0.59418	-0.59316	-0.64423	-0.64324	-0.64227	-0.64132	-0.64038	-0.63946
-0.59561	-0.59457	-0.59353	-0.64452	-0.64354	-0.64256	-0.64159	-0.64064	-0.63971
-0.56166	-0.56071	-0.55977	-0.59289	-0.59189	-0.64286	-0.64188	-0.64092	-0.63998
-0.562	-0.56105	-0.56011	-0.59327	-0.59228	-0.64316	-0.64217	-0.6412	-0.64026
-0.56236	-0.5614	-0.56045	-0.59366	-0.59266	-0.59166	-0.59065	-0.64149	-0.64055
-0.56274	-0.56177	-0.56081	-0.59405	-0.59304	-0.59204	-0.59102	-0.64179	-0.64084
-0.56312	-0.56214	-0.56116	-0.59445	-0.59343	-0.59241	-0.5914	-0.64209	-0.64114
-0.56349	-0.5625	-0.56152	-0.59484	-0.59381	-0.59278	-0.59178	-0.59078	-0.58982
-0.56386	-0.56287	-0.56188	-0.59524	-0.59418	-0.59315	-0.59215	-0.59116	-0.59019
-0.56422	-0.56324	-0.56225	-0.56128	-0.56031	-0.59353	-0.59253	-0.59154	-0.59057
-0.56458	-0.5636	-0.56263	-0.56165	-0.56067	-0.59393	-0.59292	-0.59192	-0.59095
-0.56493	-0.56396	-0.56299	-0.56201	-0.56103	-0.59432	-0.59331	-0.59231	-0.59133
-0.56527	-0.56431	-0.56335	-0.56238	-0.56139	-0.59473	-0.5937	-0.59269	-0.59172
-0.56562	-0.56466	-0.56371	-0.56274	-0.56176	-0.56079	-0.55985	-0.59309	-0.5921
-0.56596	-0.56502	-0.56407	-0.56311	-0.56213	-0.56117	-0.56021	-0.59349	-0.5925
-0.56632	-0.56537	-0.56443	-0.56348	-0.56251	-0.56155	-0.56058	-0.59389	-0.5929
-0.56669	-0.56574	-0.5648	-0.56385	-0.56289	-0.56193	-0.56096	-0.5943	-0.5933
-0.56706	-0.5661	-0.56516	-0.56422	-0.56328	-0.56232	-0.56134	-0.59472	-0.5937
-0.56743	-0.56647	-0.56553	-0.56459	-0.56365	-0.5627	-0.56171	-0.56074	-0.5598
-0.56781	-0.56685	-0.56589	-0.56495	-0.56401	-0.56306	-0.56208	-0.56112	-0.56016
-0.56819	-0.56721	-0.56625	-0.5653	-0.56437	-0.56342	-0.56245	-0.56149	-0.56052
-0.56856	-0.56758	-0.56661	-0.56565	-0.56471	-0.56376	-0.56281	-0.56185	-0.56088
-0.56894	-0.56795	-0.56697	-0.56601	-0.56506	-0.56412	-0.56317	-0.56222	-0.56125
-0.56931	-0.56832	-0.56734	-0.56638	-0.56543	-0.56448	-0.56354	-0.56259	-0.56163
-0.56969	-0.56869	-0.56772	-0.56675	-0.5658	-0.56485	-0.56392	-0.56297	-0.56201
-0.57009	-0.56909	-0.56811	-0.56713	-0.56617	-0.56522	-0.56428	-0.56334	-0.56239
-0.57051	-0.5695	-0.5685	-0.56752	-0.56655	-0.5656	-0.56465	-0.56371	-0.56277
-0.5498	-0.54884	-0.5479	-0.56791	-0.56694	-0.56597	-0.56502	-0.56408	-0.56315
-0.55022	-0.54926	-0.54831	-0.56832	-0.56733	-0.56636	-0.5654	-0.56446	-0.56352

-0.55064	-0.54967	-0.54872	-0.56872	-0.56773	-0.56675	-0.56579	-0.56483	-0.56389
-0.55105	-0.55008	-0.54912	-0.56913	-0.56814	-0.56715	-0.56618	-0.56521	-0.56426
-0.55146	-0.55048	-0.54952	-0.56953	-0.56854	-0.56756	-0.56657	-0.56559	-0.56463
-0.55189	-0.5509	-0.54993	-0.56993	-0.56894	-0.56795	-0.56697	-0.56599	-0.56502
-0.55231	-0.55132	-0.55033	-0.57032	-0.56933	-0.56834	-0.56736	-0.56639	-0.56541
-0.55274	-0.55174	-0.55075	-0.57073	-0.56973	-0.56874	-0.56776	-0.56678	-0.56581
-0.55318	-0.55217	-0.55118	-0.57115	-0.57013	-0.56913	-0.56814	-0.56717	-0.56621
-0.55362	-0.55261	-0.55162	-0.55063	-0.54966	-0.56953	-0.56854	-0.56756	-0.5666
-0.55408	-0.55306	-0.55206	-0.55107	-0.55009	-0.56995	-0.56895	-0.56796	-0.56699
-0.43834	-0.43755	-0.43677	-0.55151	-0.55052	-0.57037	-0.56936	-0.56837	-0.56738
-0.43865	-0.43785	-0.43709	-0.55194	-0.55096	-0.57081	-0.56979	-0.56878	-0.56779
-0.43897	-0.43816	-0.43741	-0.55237	-0.5514	-0.57125	-0.57022	-0.5692	-0.56821
-0.43932	-0.43847	-0.43771	-0.43694	-0.43619	-0.57167	-0.57064	-0.56963	-0.56863
-0.4397	-0.43881	-0.43801	-0.43723	-0.43648	-0.57209	-0.57107	-0.57006	-0.56905
-0.44014	-0.4392	-0.43836	-0.43756	-0.4368	-0.43605	-0.4353	-0.57048	-0.56947
-0.44062	-0.43964	-0.43874	-0.43791	-0.43715	-0.43638	-0.43562	-0.57091	-0.56988
-0.44112	-0.44008	-0.43912	-0.43826	-0.43748	-0.43671	-0.43593	-0.53202	-0.53106
-0.44162	-0.44052	-0.4395	-0.43861	-0.43781	-0.43703	-0.43626	-0.53244	-0.53147
-0.44213	-0.44099	-0.43992	-0.43898	-0.43815	-0.43735	-0.43659	-0.43584	-0.4351
-0.44269	-0.44151	-0.44039	-0.43939	-0.4385	-0.43767	-0.4369	-0.43615	-0.4354
-0.44326	-0.44205	-0.44089	-0.43984	-0.43888	-0.438	-0.43721	-0.43646	-0.4357
-0.4438	-0.44258	-0.44141	-0.44031	-0.43929	-0.43837	-0.43757	-0.4368	-0.43604
-0.44432	-0.44311	-0.44195	-0.44082	-0.43973	-0.43877	-0.43794	-0.43716	-0.43639
-0.4449	-0.44367	-0.44249	-0.44133	-0.44019	-0.43918	-0.43831	-0.4375	-0.43672
-0.44551	-0.44425	-0.44305	-0.44185	-0.44066	-0.43961	-0.43868	-0.43782	-0.43703
-0.44611	-0.44483	-0.4436	-0.44238	-0.44117	-0.44007	-0.43908	-0.43817	-0.43737
-0.4467	-0.44539	-0.44414	-0.44292	-0.44173	-0.4406	-0.43954	-0.43857	-0.43774
-0.44731	-0.44597	-0.44469	-0.44347	-0.44229	-0.44115	-0.44002	-0.43899	-0.43812
-0.44798	-0.4466	-0.44528	-0.44403	-0.44285	-0.44168	-0.4405	-0.43942	-0.43849
-0.44867	-0.44725	-0.44588	-0.4446	-0.4434	-0.4422	-0.441	-0.43987	-0.43888
-0.44932	-0.44788	-0.44647	-0.44517	-0.44394	-0.44273	-0.44152	-0.44039	-0.43933
-0.44996	-0.4485	-0.44707	-0.44573	-0.44448	-0.44326	-0.44208	-0.44094	-0.43982
-0.45063	-0.44916	-0.44771	-0.44635	-0.44506	-0.44382	-0.44264	-0.44149	-0.44034
-0.45134	-0.44987	-0.4484	-0.44701	-0.44567	-0.4444	-0.44321	-0.44205	-0.44087
-0.45203	-0.45056	-0.44909	-0.44766	-0.44628	-0.44498	-0.44378	-0.4426	-0.44141
-0.45268	-0.45121	-0.44973	-0.44829	-0.44688	-0.44555	-0.44431	-0.44311	-0.44192
-0.45333	-0.45187	-0.45039	-0.44893	-0.4475	-0.44614	-0.44487	-0.44364	-0.44245
-0.45399	-0.45255	-0.45109	-0.44963	-0.44818	-0.44679	-0.44547	-0.44421	-0.44302
-0.45465	-0.45324	-0.45181	-0.45035	-0.44889	-0.44748	-0.44612	-0.44482	-0.44361
-0.36831	-0.36712	-0.3659	-0.45108	-0.44961	-0.44818	-0.44678	-0.44545	-0.44422
-0.36884	-0.36768	-0.3665	-0.45181	-0.45034	-0.4489	-0.44748	-0.44611	-0.44484
-0.31891	-0.31806	-0.31719	-0.36587	-0.36462	-0.44962	-0.44818	-0.44679	-0.44548
-0.31932	-0.31848	-0.31763	-0.36647	-0.36523	-0.45035	-0.44889	-0.44747	-0.44612
-0.31972	-0.31887	-0.31804	-0.36703	-0.36581	-0.40752	-0.40618	-0.44815	-0.44676
-0.32009	-0.31924	-0.3184	-0.36753	-0.36634	-0.40809	-0.40675	-0.44879	-0.44738
-0.32046	-0.3196	-0.31876	-0.36802	-0.36686	-0.40866	-0.40732	-0.44943	-0.448
-0.32087	-0.31999	-0.31914	-0.31829	-0.31743	-0.36621	-0.36497	-0.40661	-0.40529
-0.3213	-0.32039	-0.31954	-0.31868	-0.31784	-0.36678	-0.36557	-0.40726	-0.40593
-0.32177	-0.32083	-0.31995	-0.31909	-0.31825	-0.36735	-0.36615	-0.36494	-0.36369
-0.32227	-0.3213	-0.32037	-0.31949	-0.31865	-0.3679	-0.36674	-0.36555	-0.36431

-0.32279	-0.32178	-0.32081	-0.31991	-0.31906	-0.31821	-0.31735	-0.36614	-0.36491
-0.32331	-0.32227	-0.32127	-0.32034	-0.31948	-0.31862	-0.31777	-0.3667	-0.3655
-0.32383	-0.32277	-0.32174	-0.32079	-0.31991	-0.31904	-0.31819	-0.36727	-0.36608
-0.3243	-0.32326	-0.32223	-0.32126	-0.32035	-0.31946	-0.31861	-0.36782	-0.36665
-0.32477	-0.32374	-0.32272	-0.32175	-0.32081	-0.3199	-0.31903	-0.36838	-0.36722
-0.32526	-0.32423	-0.32321	-0.32222	-0.32125	-0.32033	-0.31946	-0.31859	-0.31772
-0.32577	-0.32473	-0.32369	-0.32268	-0.3217	-0.32077	-0.31989	-0.31902	-0.31815
-0.32628	-0.32524	-0.32419	-0.32316	-0.32216	-0.32121	-0.32032	-0.31945	-0.31857
-0.32678	-0.32575	-0.32471	-0.32367	-0.32264	-0.32167	-0.32077	-0.31988	-0.319
-0.32728	-0.32626	-0.32522	-0.32418	-0.32313	-0.32215	-0.32122	-0.32032	-0.31944
-0.32775	-0.32675	-0.32573	-0.3247	-0.32365	-0.32265	-0.3217	-0.32078	-0.31989
-0.32822	-0.32723	-0.32623	-0.32521	-0.32417	-0.32316	-0.32219	-0.32125	-0.32035
-0.32869	-0.32771	-0.32673	-0.32571	-0.32467	-0.32367	-0.32269	-0.32174	-0.32082
-0.32916	-0.32819	-0.32721	-0.3262	-0.32517	-0.32417	-0.32321	-0.32225	-0.3213
-0.32964	-0.32866	-0.32769	-0.32669	-0.32567	-0.32468	-0.32371	-0.32275	-0.32179
-0.33012	-0.32914	-0.32817	-0.32717	-0.32617	-0.32518	-0.32422	-0.32325	-0.32228
-0.29001	-0.28913	-0.28828	-0.32766	-0.32666	-0.32568	-0.32471	-0.32375	-0.32277
-0.29045	-0.28956	-0.2887	-0.32815	-0.32716	-0.32618	-0.3252	-0.32424	-0.32326
-0.2909	-0.29	-0.28913	-0.28827	-0.28742	-0.32668	-0.3257	-0.32473	-0.32376
-0.29138	-0.29045	-0.28956	-0.2887	-0.28786	-0.32721	-0.32623	-0.32524	-0.32426
-0.29187	-0.29091	-0.28999	-0.28913	-0.28831	-0.32774	-0.32675	-0.32576	-0.32477
-0.29237	-0.29139	-0.29044	-0.28956	-0.28874	-0.32824	-0.32725	-0.32626	-0.32527
-0.29288	-0.29187	-0.2909	-0.29	-0.28917	-0.32873	-0.32775	-0.32676	-0.32576
-0.29339	-0.29237	-0.29137	-0.29046	-0.28961	-0.28876	-0.2879	-0.32726	-0.32625
-0.2939	-0.29288	-0.29187	-0.29094	-0.29005	-0.28918	-0.28833	-0.32775	-0.32674
-0.24324	-0.24242	-0.24161	-0.29144	-0.29052	-0.28963	-0.28876	-0.32825	-0.32723
-0.24363	-0.24283	-0.24205	-0.29197	-0.29102	-0.29009	-0.2892	-0.32874	-0.32772
-0.24403	-0.24324	-0.24248	-0.2925	-0.29153	-0.29058	-0.28966	-0.28875	-0.28786
-0.24443	-0.24364	-0.24287	-0.29301	-0.29203	-0.29107	-0.29012	-0.2892	-0.28832
-0.24483	-0.24403	-0.24326	-0.29351	-0.29254	-0.29157	-0.29059	-0.28966	-0.28879
-0.19383	-0.19321	-0.19261	-0.24287	-0.24209	-0.29206	-0.29107	-0.29013	-0.28925
-0.19416	-0.19353	-0.19291	-0.24326	-0.24247	-0.29255	-0.29156	-0.29061	-0.28971
-0.1945	-0.19386	-0.19321	-0.24365	-0.24286	-0.29304	-0.29207	-0.29111	-0.2902
-0.19484	-0.19418	-0.19351	-0.24405	-0.24324	-0.29353	-0.29258	-0.29164	-0.2907
-0.19517	-0.1945	-0.19382	-0.19318	-0.19257	-0.24285	-0.2421	-0.29216	-0.2912
-0.19549	-0.19481	-0.19414	-0.1935	-0.19289	-0.24326	-0.24251	-0.29267	-0.2917
-0.19582	-0.19514	-0.19447	-0.19383	-0.19321	-0.24368	-0.24291	-0.29317	-0.29221
-0.10033	-0.1	-0.09966	-0.19417	-0.19353	-0.2441	-0.24331	-0.29368	-0.29273
-0.10049	-0.10016	-0.09982	-0.19452	-0.19385	-0.24452	-0.24371	-0.29418	-0.29325
-0.10067	-0.10032	-0.09998	-0.09965	-0.09932	-0.19354	-0.19292	-0.24334	-0.24258
-0.10084	-0.10049	-0.10015	-0.09981	-0.09948	-0.19387	-0.19325	-0.24376	-0.24299
-0.10101	-0.10066	-0.10031	-0.09997	-0.09965	-0.09931	-0.09898	-0.24419	-0.2434
-0.10117	-0.10082	-0.10048	-0.10014	-0.09982	-0.09948	-0.09915	-0.24463	-0.24381
-0.10133	-0.10099	-0.10064	-0.10031	-0.09998	-0.09965	-0.09931	-0.19362	-0.19299
-0.1015	-0.10116	-0.10082	-0.10048	-0.10014	-0.0998	-0.09947	-0.19397	-0.19333
-0.10168	-0.10133	-0.10099	-0.10064	-0.10029	-0.09996	-0.09964	-0.19432	-0.19367
-0.10187	-0.10152	-0.10117	-0.10082	-0.10047	-0.10013	-0.0998	-0.09948	-0.09914
-0.10208	-0.10171	-0.10136	-0.10101	-0.10066	-0.10031	-0.09997	-0.09963	-0.0993
-0.04908	-0.04891	-0.04874	-0.10119	-0.10084	-0.1005	-0.10015	-0.0998	-0.09947
-0.04914	-0.04897	-0.0488	-0.10138	-0.10103	-0.10068	-0.10033	-0.09998	-0.09963

-0.10157	-0.10122	-0.10086	-0.10051	-0.10015	-0.0998
-0.10176	-0.1014	-0.10104	-0.10068	-0.10033	-0.09998
-0.04881	-0.04863	-0.10122	-0.10086	-0.10051	-0.10015
-0.04887	-0.0487	-0.1014	-0.10104	-0.10069	-0.10033
-0.04894	-0.04876	-0.10159	-0.10123	-0.10087	-0.10052
		-0.10179	-0.10142	-0.10106	-0.1007
		-0.10199	-0.10163	-0.10127	-0.10089
				-0.10146	-0.10108
				-0.10164	-0.10128
				-0.04855	-0.04839
				-0.04861	-0.04844
				-0.04868	-0.0485

								-0.10719
								-0.10696
								-0.10673
					-0.1075	-0.10715	-0.10682	-0.15857
					-0.10727	-0.10693	-0.1066	-0.1581
					-0.10704	-0.10671	-0.10639	-0.30692
					-0.10681	-0.10651	-0.1062	-0.30627
			-0.10726	-0.10691	-0.15893	-0.15824	-0.15756	-0.39268
			-0.10703	-0.10669	-0.15847	-0.15779	-0.15712	-0.39189
	-0.10745	-0.10713	-0.15956	-0.15878	-0.39518	-0.3938	-0.39246	-0.48538
	-0.10723	-0.1069	-0.15908	-0.15835	-0.39443	-0.39303	-0.39166	-0.48445
	-0.10702	-0.10668	-0.15862	-0.15794	-0.39369	-0.39227	-0.3909	-0.48353
	-0.1068	-0.10648	-0.35167	-0.35041	-0.39291	-0.39153	-0.39018	-0.48267
	-0.10658	-0.10628	-0.35099	-0.34969	-0.39212	-0.39079	-0.38951	-0.48184
-0.10667	-0.15879	-0.15812	-0.39414	-0.39271	-0.48558	-0.48402	-0.48251	-0.60545
-0.10645	-0.15834	-0.15769	-0.39334	-0.39193	-0.48464	-0.48312	-0.48164	-0.60438
-0.10624	-0.3513	-0.35007	-0.39256	-0.39118	-0.48376	-0.48225	-0.48079	-0.65227
-0.10604	-0.35061	-0.34935	-0.3918	-0.39048	-0.48294	-0.48144	-0.47996	-0.65107
-0.25355	-0.39378	-0.39239	-0.48519	-0.48365	-0.65604	-0.65397	-0.65191	-0.68582
-0.25299	-0.39303	-0.39166	-0.48432	-0.48281	-0.65488	-0.65282	-0.65077	-0.68456
-0.25243	-0.39228	-0.39097	-0.48349	-0.48198	-0.65371	-0.65167	-0.64964	-0.68332
-0.3491	-0.39157	-0.39028	-0.60731	-0.60545	-0.65256	-0.65051	-0.64847	-0.84398
-0.34845	-0.39088	-0.3896	-0.60626	-0.60441	-0.65143	-0.64935	-0.64729	-0.8425
-0.39148	-0.48405	-0.48253	-0.65434	-0.65231	-0.72799	-0.72565	-0.72334	-0.87934
-0.39076	-0.48321	-0.48173	-0.6532	-0.65113	-0.72664	-0.72434	-0.72206	-0.87785
-0.39007	-0.65614	-0.65411	-0.65205	-0.64996	-0.84576	-0.84321	-0.84069	-0.91188
-0.38942	-0.65499	-0.65293	-0.65086	-0.6488	-0.84428	-0.84174	-0.83924	-0.91031
-0.38876	-0.65383	-0.65174	-0.64968	-0.64765	-0.84282	-0.84029	-0.83779	-0.90876
-0.6117	-0.65264	-0.65056	-0.76856	-0.76619	-0.91529	-0.91256	-0.90987	-0.90724

-0.6106	-0.65144	-0.64939	-0.76717	-0.76482	-0.9137	-0.911	-0.90835	-0.90575
-0.65239	-0.7706	-0.76817	-0.84591	-0.84341	-0.91214	-0.90946	-0.90683	-1.07584
-0.65129	-0.76927	-0.7668	-0.84444	-0.84197	-0.9106	-0.90794	-0.90533	-1.07381
-0.69261	-0.76794	-0.76548	-0.9144	-0.91172	-1.04273	-1.03932	-1.03599	-1.07182
-0.69135	-0.76659	-0.76422	-0.91293	-0.9102	-1.04071	-1.0374	-1.03416	-1.06987
-0.76759	-0.84536	-0.84289	-0.93841	-0.93557	-1.07821	-1.07473	-1.07133	-1.0928
-0.76623	-0.84393	-0.8415	-0.93689	-0.9341	-1.07626	-1.07275	-1.06932	-1.0907
-0.76491	-0.84252	-0.84011	-0.93536	-0.93266	-1.07437	-1.07081	-1.06733	-1.08863
-0.76366	-0.90473	-0.90209	-1.03998	-1.03668	-1.07245	-1.06893	-1.06545	-1.12088
-0.76245	-0.90328	-0.90059	-1.03801	-1.03478	-1.07052	-1.06707	-1.06363	-1.11883
-0.84102	-1.01045	-1.00706	-1.0754	-1.07195	-1.128	-1.12425	-1.12053	-1.18018
-0.83965	-1.00863	-1.00533	-1.0735	-1.06996	-1.12575	-1.12207	-1.11845	-1.17812
-0.90152	-1.03922	-1.03591	-1.09669	-1.0929	-1.12359	-1.11993	-1.11635	-1.24039
-0.90002	-1.03728	-1.03404	-1.09467	-1.09094	-1.12155	-1.11784	-1.11423	-1.23813
-1.00626	-1.07457	-1.07116	-1.1271	-1.12328	-1.1829	-1.17905	-1.17531	-1.23592
-1.00449	-1.0726	-1.06912	-1.12485	-1.12112	-1.18073	-1.17699	-1.17334	-1.2338
-1.00275	-1.07067	-1.06709	-1.1226	-1.11894	-1.17856	-1.17497	-1.17142	-1.23173
-1.00096	-1.12821	-1.12427	-1.12048	-1.11683	-1.24084	-1.23711	-1.23343	-1.25663
-0.99915	-1.12611	-1.12221	-1.11844	-1.11478	-1.23858	-1.23493	-1.23133	-1.2546
-1.0973	-1.124	-1.12016	-1.24428	-1.24029	-1.23644	-1.2328	-1.22921	-1.25252
-1.09517	-1.12187	-1.11811	-1.24211	-1.23822	-1.23442	-1.23071	-1.22707	-1.25039
-1.12346	-1.18673	-1.18283	-1.23998	-1.23618	-1.2594	-1.25561	-1.25188	-1.24828
-1.12124	-1.18448	-1.18073	-1.23788	-1.23416	-1.2574	-1.25363	-1.24989	-1.24623
-1.11908	-1.18227	-1.17864	-1.23579	-1.23213	-1.25541	-1.25167	-1.24794	-1.2442
-1.11706	-1.24107	-1.23733	-1.23367	-1.23008	-1.2534	-1.24969	-1.24597	-1.27482
-1.11513	-1.23893	-1.23518	-1.23155	-1.22804	-1.25138	-1.2477	-1.244	-1.27281
-1.17982	-1.23687	-1.23312	-1.25647	-1.25293	-1.24935	-1.2457	-1.24202	-1.29984
-1.17789	-1.23489	-1.23114	-1.25446	-1.2509	-1.24732	-1.24369	-1.24004	-1.29779
-1.17597	-1.23292	-1.22918	-1.25245	-1.24885	-1.27793	-1.27424	-1.27054	-1.34743
-1.17406	-1.23096	-1.22724	-1.25045	-1.24676	-1.27576	-1.27212	-1.2685	-1.34532
-1.17216	-1.25598	-1.25221	-1.3059	-1.30199	-1.3546	-1.35081	-1.34705	-1.43014
-1.17026	-1.254	-1.25024	-1.30384	-1.29991	-1.35242	-1.34862	-1.34486	-1.42785
-1.16838	-1.25202	-1.24827	-1.30178	-1.29786	-1.35028	-1.34644	-1.34269	-1.42557
-1.22683	-1.27521	-1.2714	-1.32795	-1.32396	-1.40563	-1.40138	-1.39724	-1.42326
-1.22488	-1.2732	-1.26938	-1.32584	-1.32188	-1.40335	-1.39907	-1.39492	-1.42096
-1.27498	-1.33169	-1.3277	-1.35187	-1.34785	-1.43122	-1.42689	-1.4227	-1.41873
-1.27294	-1.32956	-1.32558	-1.34971	-1.34571	-1.42892	-1.42467	-1.42054	-1.41656
-1.33138	-1.32743	-1.32346	-1.43548	-1.43101	-1.42667	-1.42248	-1.41839	-1.46396
-1.32923	-1.3253	-1.32136	-1.43317	-1.42878	-1.42449	-1.42033	-1.41625	-1.46169
-1.32709	-1.35487	-1.35086	-1.43088	-1.42656	-1.47212	-1.46786	-1.46364	-1.48409
-1.32497	-1.3527	-1.34871	-1.42857	-1.42434	-1.46988	-1.46563	-1.46141	-1.48184
-1.32286	-1.35055	-1.34658	-1.42629	-1.42213	-1.46764	-1.4634	-1.45918	-1.4796
-1.32077	-1.43276	-1.42835	-1.47389	-1.46964	-1.46541	-1.46117	-1.45697	-1.47736
-1.31869	-1.43048	-1.42615	-1.47166	-1.4674	-1.46317	-1.45895	-1.45476	-1.47512
-1.34813	-1.47813	-1.47374	-1.46941	-1.46516	-1.48559	-1.48134	-1.47712	-1.54228
-1.34601	-1.47578	-1.47145	-1.46716	-1.46291	-1.48333	-1.4791	-1.4749	-1.53993
-1.47784	-1.47346	-1.46918	-1.46492	-1.46068	-1.50093	-1.49664	-1.4924	-1.5376
-1.47555	-1.47119	-1.46692	-1.46267	-1.45845	-1.49865	-1.49438	-1.49015	-1.53527
-1.47327	-1.46894	-1.46467	-1.46043	-1.45623	-1.49639	-1.49213	-1.48794	-1.53301
-1.47099	-1.46667	-1.46242	-1.50268	-1.4984	-1.54375	-1.53937	-1.53508	-1.53087

-1.46871	-1.46442	-1.46017	-1.5004	-1.49614	-1.54144	-1.53708	-1.53286	-1.52882
-1.46643	-1.48245	-1.47816	-1.54782	-1.54347	-1.55204	-1.54771	-1.54356	-1.5397
-1.46416	-1.48017	-1.47592	-1.54551	-1.54118	-1.54979	-1.54552	-1.54149	-1.53783
-1.48221	-1.50218	-1.49792	-1.5561	-1.5518	-1.54759	-1.54341	-1.5395	-1.53601
-1.47998	-1.49995	-1.49572	-1.55384	-1.54959	-1.54546	-1.54138	-1.53758	-1.53425
-1.47783	-1.56026	-1.55591	-1.55162	-1.5474	-1.54334	-1.53941	-1.53577	-1.53258
-1.47582	-1.55815	-1.5538	-1.54949	-1.5452	-1.54119	-1.53749	-1.53409	-1.53103
-1.47382	-1.55604	-1.55168	-1.54736	-1.54304	-1.53911	-1.53565	-1.53248	-1.52954
-1.55817	-1.55377	-1.54941	-1.54515	-1.54097	-1.53719	-1.5339	-1.53088	-1.54934
-1.55585	-1.55144	-1.54707	-1.54292	-1.53897	-1.53538	-1.53223	-1.52933	-1.54789
-1.55351	-1.5491	-1.54476	-1.54075	-1.53704	-1.55493	-1.55191	-1.54911	-1.54647
-1.55115	-1.54676	-1.54246	-1.53861	-1.53518	-1.55327	-1.55039	-1.54768	-1.54509
-1.54881	-1.5445	-1.54033	-1.55794	-1.5547	-1.55169	-1.54892	-1.54629	-1.54373
-1.54648	-1.54233	-1.53839	-1.55616	-1.55306	-1.55016	-1.54749	-1.54493	-1.54239
-1.54425	-1.56157	-1.55786	-1.55451	-1.55153	-1.54874	-1.54614	-1.54362	-1.5411
-1.54218	-1.55967	-1.5562	-1.55303	-1.55017	-1.54745	-1.54489	-1.54239	-1.53988
-1.54022	-1.55787	-1.55463	-1.55162	-1.54886	-1.54621	-1.54367	-1.54117	-1.53867
-1.53832	-1.55613	-1.55304	-1.55015	-1.54748	-1.56533	-1.5628	-1.5603	-1.5578
-1.53651	-1.55445	-1.55148	-1.54868	-1.54609	-1.56398	-1.56146	-1.55896	-1.55646
-1.55599	-1.5733	-1.57044	-1.56771	-1.56516	-1.56264	-1.56013	-1.55763	-1.57434
-1.55429	-1.57175	-1.569	-1.56634	-1.56381	-1.5613	-1.55879	-1.55631	-1.573
-1.57316	-1.57028	-1.56763	-1.56503	-1.56251	-1.56	-1.55751	-1.55503	-1.57173
-1.57172	-1.56894	-1.56635	-1.56379	-1.56128	-1.55879	-1.5563	-1.55383	-1.57055
-1.57033	-1.56762	-1.56509	-1.56256	-1.56006	-1.55757	-1.55508	-1.55262	-1.56937
-1.56892	-1.56628	-1.56376	-1.56125	-1.55876	-1.57555	-1.57302	-1.57053	-1.58747
-1.56755	-1.56495	-1.56244	-1.55994	-1.55745	-1.57424	-1.57172	-1.56924	-1.58617
-1.56625	-1.56368	-1.56118	-1.55868	-1.55621	-1.573	-1.57051	-1.56804	-1.60768
-1.56499	-1.56245	-1.55995	-1.55747	-1.555	-1.5718	-1.56935	-1.56692	-1.60648
-1.5637	-1.56118	-1.55869	-1.57553	-1.57302	-1.57055	-1.56813	-1.56574	-1.60524
-1.56238	-1.55987	-1.55738	-1.57421	-1.57169	-1.56924	-1.56685	-1.56449	-1.60399
-1.56109	-1.57796	-1.57544	-1.57292	-1.57042	-1.5911	-1.58863	-1.58619	-1.60274
-1.55985	-1.57673	-1.57421	-1.57172	-1.56925	-1.58991	-1.5874	-1.58495	-1.60148
-1.55862	-1.57549	-1.57298	-1.57051	-1.56809	-1.58872	-1.5862	-1.58373	-1.60026
-1.57672	-1.57419	-1.57168	-1.56924	-1.56685	-1.60647	-1.60395	-1.60149	-1.67051
-1.5754	-1.57287	-1.57037	-1.56795	-1.56561	-1.60519	-1.60274	-1.60035	-1.66921
-1.57414	-1.57162	-1.56916	-1.58986	-1.58743	-1.60398	-1.60154	-1.59918	-1.66785
-1.57292	-1.57044	-1.56801	-1.5887	-1.58628	-1.60282	-1.60037	-1.598	-1.66645
-1.57168	-1.56922	-1.56684	-1.5875	-1.5851	-1.62735	-1.62482	-1.62235	-1.66511
-1.5704	-1.56796	-1.5656	-1.58624	-1.58387	-1.62608	-1.62364	-1.62125	-1.66386
-1.56917	-1.58984	-1.58741	-1.58502	-1.58267	-1.62485	-1.62249	-1.62018	-1.66261
-1.56802	-1.58868	-1.58626	-1.58388	-1.58155	-1.62368	-1.62138	-1.6191	-1.66133
-1.56691	-1.58756	-1.58515	-1.58279	-1.58047	-1.62255	-1.62028	-1.61802	-1.66005
-1.5658	-1.58644	-1.58406	-1.62624	-1.62378	-1.68404	-1.68137	-1.67872	-1.70326
-1.56469	-1.58532	-1.58298	-1.62511	-1.62266	-1.68271	-1.68002	-1.67737	-1.70193
-1.56357	-1.58417	-1.58185	-1.64175	-1.63935	-1.68911	-1.68638	-1.6837	-1.71907
-1.56244	-1.58299	-1.58067	-1.64054	-1.63822	-1.68786	-1.68513	-1.68245	-1.71781
-1.58423	-1.60025	-1.59795	-1.64755	-1.64524	-1.70534	-1.70256	-1.69984	-1.71653
-1.58314	-1.59921	-1.59696	-1.64647	-1.64416	-1.70406	-1.70125	-1.69851	-1.71519
-1.60045	-1.65019	-1.64778	-1.70828	-1.70553	-1.70276	-1.69995	-1.69722	-1.71389
-1.59939	-1.64907	-1.6467	-1.707	-1.70419	-1.70143	-1.69869	-1.69604	-1.7127

-1.59835	-1.64797	-1.64561	-1.7057	-1.70285	-1.7001	-1.69746	-1.6949	-1.71155
-1.60597	-1.66619	-1.66378	-1.70437	-1.70154	-1.71813	-1.7155	-1.71293	-1.71039
-1.60497	-1.66506	-1.66262	-1.70303	-1.70025	-1.71686	-1.71428	-1.71175	-1.70923
-1.60394	-1.66392	-1.66148	-1.70174	-1.69901	-1.71564	-1.71309	-1.71058	-1.72318
-1.60289	-1.66276	-1.66035	-1.70049	-1.69783	-1.71447	-1.71193	-1.70941	-1.72198
-1.62169	-1.66162	-1.65923	-1.71463	-1.71203	-1.72844	-1.72588	-1.72334	-1.76673
-1.62065	-1.6605	-1.65812	-1.7134	-1.71087	-1.72727	-1.72469	-1.72211	-1.76535
-1.61963	-1.65939	-1.65702	-1.71219	-1.70972	-1.72611	-1.7235	-1.7209	-1.76399
-1.66067	-1.71627	-1.71359	-1.71103	-1.70858	-1.72495	-1.72234	-1.71974	-1.76268
-1.65956	-1.71504	-1.71243	-1.70989	-1.70744	-1.72378	-1.7212	-1.71861	-1.76139
-1.67431	-1.71382	-1.71127	-1.72768	-1.72512	-1.76886	-1.76593	-1.76301	-1.77817
-1.67319	-1.71261	-1.71012	-1.7265	-1.7239	-1.76744	-1.76456	-1.76168	-1.77685
-1.67209	-1.71142	-1.70896	-1.72532	-1.72271	-1.76609	-1.76321	-1.76036	-1.77552
-1.67101	-1.71026	-1.70781	-1.72415	-1.72156	-1.7648	-1.7619	-1.75903	-1.77419
-1.69041	-1.7091	-1.70665	-1.76936	-1.76642	-1.7635	-1.76059	-1.75771	-1.77285
-1.6893	-1.70794	-1.70545	-1.76796	-1.76506	-1.76217	-1.75927	-1.75639	-1.77151
-1.68819	-1.70678	-1.70426	-1.76656	-1.7637	-1.76083	-1.75795	-1.75507	-1.77017
-1.68709	-1.72448	-1.72188	-1.76524	-1.76237	-1.77757	-1.77465	-1.77174	-1.76883
-1.68599	-1.72334	-1.72074	-1.76395	-1.76105	-1.77621	-1.7733	-1.7704	-1.7675
-1.68491	-1.76855	-1.76559	-1.76265	-1.75973	-1.77487	-1.77195	-1.76907	-1.7662
-1.68383	-1.76725	-1.76428	-1.76134	-1.75841	-1.77353	-1.7706	-1.76773	-1.76492
-1.68275	-1.76597	-1.76298	-1.76003	-1.75709	-1.77219	-1.76928	-1.76644	-1.76367
-1.68169	-1.76469	-1.76168	-1.75871	-1.75576	-1.77087	-1.768	-1.76519	-1.76246
-1.72794	-1.76342	-1.7604	-1.75742	-1.75445	-1.76955	-1.76673	-1.76397	-1.76127
-1.72673	-1.76217	-1.75919	-1.75619	-1.75317	-1.76823	-1.76547	-1.76278	-1.7601
-1.7255	-1.76092	-1.75798	-1.75498	-1.7519	-1.76693	-1.76424	-1.76161	-1.75894
-1.7426	-1.75962	-1.7567	-1.7717	-1.76859	-1.76567	-1.76303	-1.76044	-1.75778
-1.74132	-1.75829	-1.75536	-1.77038	-1.76733	-1.76445	-1.76184	-1.75927	-1.75664
-1.74004	-1.75696	-1.75403	-1.76908	-1.76609	-1.76327	-1.76067	-1.75812	-1.79043
-1.73875	-1.75562	-1.75271	-1.76778	-1.76489	-1.76212	-1.75953	-1.75698	-1.78929
-1.73749	-1.75431	-1.75141	-1.76651	-1.7637	-1.796	-1.79336	-1.79075	-1.78813
-1.73624	-1.75301	-1.75012	-1.76525	-1.76252	-1.79483	-1.79219	-1.78957	-1.78694
-1.73501	-1.75174	-1.74887	-1.76404	-1.76138	-1.79369	-1.79104	-1.7884	-1.78575
-1.73376	-1.76848	-1.76563	-1.79793	-1.79526	-1.7926	-1.78991	-1.78723	-1.78455
-1.73253	-1.76726	-1.76449	-1.7968	-1.79418	-1.79152	-1.78879	-1.78606	-1.78335
-1.73136	-1.80113	-1.79835	-1.79565	-1.79305	-1.79039	-1.78764	-1.78487	-1.82825
-1.73023	-1.79995	-1.79718	-1.79449	-1.7919	-1.78923	-1.78646	-1.78368	-1.82693
-1.80162	-1.79878	-1.79602	-1.79333	-1.79073	-1.78806	-1.7853	-1.78253	-1.82564
-1.80042	-1.79761	-1.79486	-1.79217	-1.78954	-1.78688	-1.78416	-1.78143	-1.8244
-1.79924	-1.79645	-1.79373	-1.79104	-1.78837	-1.83237	-1.82934	-1.8263	-1.82315
-1.79807	-1.79533	-1.79266	-1.78995	-1.78723	-1.83104	-1.82802	-1.825	-1.82186
-1.79691	-1.79421	-1.79158	-1.78887	-1.7861	-1.82975	-1.82671	-1.82368	-1.82057
-1.79574	-1.79306	-1.79042	-1.78773	-1.78498	-1.82854	-1.82544	-1.82238	-1.81934
-1.79457	-1.79188	-1.78924	-1.78656	-1.78388	-1.82735	-1.8242	-1.8211	-1.81814
-1.79345	-1.79075	-1.78806	-1.83216	-1.82915	-1.82609	-1.82293	-1.81982	-1.81691
-1.79237	-1.78964	-1.7869	-1.83082	-1.82781	-1.82477	-1.82163	-1.81855	-1.81567
-1.79127	-1.78853	-1.78577	-1.82953	-1.82651	-1.82347	-1.82037	-1.81734	-1.83962
-1.79014	-1.78741	-1.78468	-1.82833	-1.82525	-1.8222	-1.81918	-1.81622	-1.83838
-1.78901	-1.78629	-1.78358	-1.82713	-1.82401	-1.82096	-1.81801	-1.81509	-1.83716
-1.78791	-1.78518	-1.78243	-1.82587	-1.8228	-1.81978	-1.81683	-1.81392	-1.83593

-1.78682	-1.78405	-1.78127	-1.8246	-1.82159	-1.81861	-1.81564	-1.81272	-1.8347
-1.78568	-1.82951	-1.82637	-1.82331	-1.82034	-1.84304	-1.83976	-1.83654	-1.85073
-1.78451	-1.8282	-1.82507	-1.82202	-1.81906	-1.84167	-1.83843	-1.83523	-1.8494
-1.78334	-1.82693	-1.82382	-1.82079	-1.81783	-1.84036	-1.83716	-1.834	-1.84813
-1.78217	-1.82568	-1.82263	-1.81962	-1.81664	-1.83909	-1.83595	-1.83283	-1.84691
-1.78103	-1.82445	-1.82143	-1.84436	-1.84107	-1.85526	-1.85208	-1.84891	-1.84569
-1.77994	-1.82323	-1.82018	-1.84304	-1.83986	-1.85407	-1.85084	-1.84764	-1.84446
-1.77886	-1.82203	-1.81896	-1.84175	-1.83865	-1.85287	-1.8496	-1.84637	-1.84325
-1.77775	-1.82083	-1.81783	-1.84055	-1.83742	-1.87819	-1.87487	-1.8716	-1.86843
-1.77664	-1.81964	-1.81673	-1.83939	-1.83619	-1.87689	-1.87363	-1.87041	-1.86722
-1.82147	-1.8446	-1.84138	-1.88229	-1.87895	-1.87565	-1.8724	-1.86919	-1.86602
-1.82037	-1.84338	-1.84012	-1.881	-1.87772	-1.87446	-1.87118	-1.86796	-1.86483
-1.81925	-1.8694	-1.86605	-1.87974	-1.87647	-1.87322	-1.86997	-1.86676	-1.88489
-1.81811	-1.86819	-1.86487	-1.87851	-1.8752	-1.87194	-1.86876	-1.86561	-1.88363
-1.84488	-1.884	-1.88065	-1.87731	-1.87396	-1.87069	-1.86755	-1.86444	-1.88236
-1.84365	-1.88271	-1.87944	-1.87612	-1.87278	-1.8695	-1.86633	-1.86319	-1.88106
-1.84243	-1.88141	-1.8782	-1.87493	-1.87161	-1.86834	-1.86512	-1.86195	-1.87977
-1.84963	-1.88014	-1.8769	-1.87366	-1.87042	-1.86719	-1.86397	-1.86078	-1.8785
-1.84845	-1.87889	-1.87558	-1.87236	-1.86921	-1.86605	-1.86284	-1.85965	-1.87726
-1.86445	-1.87767	-1.87434	-1.87111	-1.86798	-1.88612	-1.88275	-1.8794	-1.87605
-1.86326	-1.87648	-1.87316	-1.86991	-1.86672	-1.88478	-1.88149	-1.8782	-1.87488
-1.86204	-1.87526	-1.87198	-1.86874	-1.86552	-1.88349	-1.88022	-1.87695	-1.87366
-1.86077	-1.874	-1.87081	-1.8676	-1.8644	-1.88226	-1.87893	-1.87563	-1.87239
-1.85951	-1.87276	-1.86962	-1.88781	-1.88442	-1.88104	-1.87766	-1.87433	-1.87112
-1.85832	-1.87154	-1.86838	-1.88652	-1.88318	-1.87984	-1.87646	-1.87312	-1.86988
-1.85716	-1.87034	-1.86714	-1.88521	-1.88192	-1.87862	-1.87527	-1.87194	-1.86865
-1.85602	-1.86919	-1.86597	-1.88394	-1.88064	-1.87734	-1.87405	-1.87076	-1.86747
-1.8549	-1.86807	-1.86485	-1.8827	-1.87934	-1.87603	-1.87281	-1.86959	-1.86632
-1.85377	-1.88831	-1.88489	-1.88149	-1.87811	-1.8748	-1.87161	-1.86842	-1.86515
-1.85265	-1.88712	-1.88371	-1.88032	-1.87694	-1.87363	-1.87043	-1.86725	-1.86399
-1.85149	-1.88589	-1.88252	-1.87915	-1.87577	-1.87246	-1.86924	-1.86604	-1.87851
-1.85026	-1.8846	-1.88131	-1.87797	-1.87459	-1.87126	-1.86799	-1.86477	-1.87724
-1.84905	-1.88333	-1.88009	-1.87678	-1.87341	-1.87007	-1.86676	-1.86351	-1.87596
-1.84792	-1.8821	-1.87888	-1.87558	-1.87223	-1.8689	-1.86558	-1.86231	-1.87469
-1.84682	-1.88089	-1.87765	-1.87437	-1.87104	-1.86773	-1.86442	-1.86115	-1.87344
-1.86698	-1.87966	-1.87639	-1.87311	-1.86984	-1.88249	-1.87901	-1.87558	-1.87223
-1.86579	-1.87841	-1.8751	-1.87183	-1.86863	-1.88127	-1.8778	-1.87437	-1.87104
-1.86462	-1.8772	-1.87386	-1.8706	-1.86743	-1.88005	-1.87661	-1.87319	-1.86987
-1.86346	-1.87602	-1.87268	-1.86943	-1.86626	-1.87884	-1.87544	-1.87205	-1.86872
-1.8623	-1.87485	-1.87152	-1.88432	-1.88098	-1.87764	-1.87427	-1.87091	-1.87987
-1.86115	-1.87369	-1.87035	-1.8831	-1.87978	-1.87645	-1.8731	-1.86975	-1.8787
-1.86	-1.87252	-1.86918	-1.88189	-1.87858	-1.87526	-1.87192	-1.86859	-1.87753
-1.85885	-1.87136	-1.86802	-1.88068	-1.87737	-1.89995	-1.89657	-1.8932	-1.88984
-1.8577	-1.87019	-1.86687	-1.87948	-1.87617	-1.89872	-1.89536	-1.892	-1.88865
-1.85655	-1.88514	-1.88166	-1.89194	-1.88863	-1.89751	-1.89416	-1.89081	-1.88747
-1.85539	-1.88392	-1.88044	-1.89072	-1.88744	-1.89631	-1.89296	-1.88962	-1.88628
-1.85423	-1.89634	-1.89286	-1.9017	-1.89842	-1.89511	-1.89177	-1.88843	-1.8851
-1.85306	-1.89509	-1.89161	-1.90045	-1.89719	-1.89391	-1.89057	-1.88724	-1.88393
-1.86588	-1.90609	-1.90258	-1.89921	-1.89597	-1.8927	-1.88937	-1.88605	-1.88276
-1.86471	-1.90483	-1.90135	-1.89799	-1.89474	-1.89148	-1.88817	-1.88486	-1.88157

-1.86354	-1.90361	-1.90014	-1.89679	-1.89353	-1.89027	-1.88696	-1.88367	-1.88039
-1.87833	-1.90242	-1.89901	-1.89565	-1.89237	-1.88908	-1.88577	-1.88248	-1.87922
-1.87715	-1.90126	-1.89791	-1.89456	-1.89122	-1.8879	-1.88459	-1.88131	-1.87805
-1.88838	-1.90012	-1.89677	-1.89342	-1.89006	-1.88672	-1.88342	-1.88014	-1.87689
-1.88727	-1.89898	-1.89561	-1.89224	-1.88887	-1.88554	-1.88224	-1.87898	-1.87574
-1.88614	-1.89782	-1.89443	-1.89104	-1.88768	-1.88435	-1.88107	-1.87782	-1.8746
-1.88498	-1.89663	-1.89321	-1.88983	-1.88647	-1.88316	-1.87989	-1.87666	-1.87345
-1.88381	-1.89542	-1.892	-1.88862	-1.88528	-1.88198	-1.87873	-1.87552	-1.87232
-1.88262	-1.89421	-1.8908	-1.88743	-1.8841	-1.88082	-1.87759	-1.87439	-1.87123
-1.88142	-1.893	-1.8896	-1.88625	-1.88295	-1.87969	-1.87647	-1.87329	-1.87018
-1.88023	-1.8918	-1.88842	-1.88511	-1.88187	-1.87864	-1.87543	-1.87226	-1.86914
-1.87905	-1.89061	-1.88725	-1.88399	-1.88082	-1.87765	-1.87445	-1.87127	-1.86811
-1.87794	-1.8895	-1.88615	-1.88291	-1.87976	-1.8766	-1.87341	-1.87022	-1.86705
-1.87692	-1.88848	-1.88513	-1.88187	-1.87869	-1.8755	-1.8723	-1.86911	-1.86595
-1.87587	-1.88743	-1.88409	-1.88082	-1.8776	-1.8744	-1.87119	-1.868	-1.86485
-1.87477	-1.8863	-1.88299	-1.87972	-1.8765	-1.87329	-1.87008	-1.8669	-1.86375
-1.87365	-1.88517	-1.88188	-1.87862	-1.87539	-1.87219	-1.86899	-1.86582	-1.86268
-1.85952	-1.86928	-1.86607	-1.87751	-1.87429	-1.87111	-1.86796	-1.86483	-1.86171
-1.85843	-1.86819	-1.86498	-1.87641	-1.87321	-1.87005	-1.86695	-1.86387	-1.86076
-1.85739	-1.86717	-1.86398	-1.8754	-1.87219	-1.86903	-1.86593	-1.86285	-1.85974
-1.85639	-1.86621	-1.86304	-1.87445	-1.87123	-1.86804	-1.86491	-1.86179	-1.85866
-1.85538	-1.86521	-1.86205	-1.87345	-1.87022	-1.86702	-1.86386	-1.86072	-1.85759
-1.85433	-1.86414	-1.86099	-1.87238	-1.86916	-1.86596	-1.86279	-1.85963	-1.8565
-1.85328	-1.86308	-1.85994	-1.87132	-1.86812	-1.86492	-1.86174	-1.85859	-1.85544
-1.85222	-1.86205	-1.85896	-1.87035	-1.86715	-1.86396	-1.86078	-1.85762	-1.85447
-1.85117	-1.86103	-1.85799	-1.86939	-1.86619	-1.863	-1.85983	-1.85667	-1.85351
-1.85021	-1.84714	-1.84411	-1.86835	-1.86516	-1.86198	-1.8588	-1.85564	-1.85249
-1.84928	-1.84618	-1.84309	-1.86728	-1.8641	-1.86092	-1.85773	-1.85457	-1.85145
-1.84833	-1.84522	-1.84211	-1.85362	-1.85048	-1.85991	-1.85673	-1.85357	-1.85046
-1.84736	-1.84428	-1.84118	-1.85267	-1.84953	-1.85895	-1.85577	-1.85262	-1.84953
-1.84636	-1.84329	-1.84019	-1.85167	-1.84854	-1.84541	-1.84228	-1.83918	-1.84856
-1.8453	-1.84222	-1.83912	-1.85059	-1.84747	-1.84435	-1.84121	-1.83813	-1.84753
-1.84426	-1.84116	-1.83807	-1.84953	-1.84641	-1.8433	-1.84018	-1.8371	-1.83413
-1.84329	-1.84019	-1.8371	-1.84855	-1.84543	-1.84233	-1.83923	-1.83618	-1.83321
-1.84233	-1.83923	-1.83615	-1.84759	-1.84447	-1.84139	-1.83831	-1.83528	-1.83232
-1.84129	-1.83819	-1.83512	-1.84655	-1.84345	-1.84037	-1.83733	-1.83432	-1.83136
-1.8402	-1.83712	-1.83405	-1.84548	-1.84239	-1.83933	-1.83631	-1.83332	-1.83037
-1.83919	-1.83611	-1.83306	-1.84448	-1.8414	-1.83836	-1.83536	-1.83239	-1.82945
-1.83826	-1.83519	-1.83215	-1.84357	-1.84049	-1.83745	-1.83448	-1.83153	-1.82859
-1.83734	-1.83428	-1.83124	-1.84266	-1.83959	-1.83657	-1.83361	-1.83068	-1.82773
-1.83643	-1.83338	-1.83035	-1.84176	-1.83871	-1.8357	-1.83275	-1.82982	-1.82688
-1.8355	-1.83245	-1.82943	-1.84084	-1.83781	-1.83481	-1.83186	-1.82893	-1.82599
-1.83448	-1.83145	-1.82844	-1.83984	-1.83683	-1.83384	-1.8309	-1.82797	-1.82504
-1.83343	-1.83041	-1.82742	-1.83882	-1.83582	-1.83285	-1.82991	-1.82698	-1.82406
-1.83247	-1.82946	-1.82647	-1.83787	-1.83489	-1.83193	-1.82899	-1.82607	-1.82315
-1.83156	-1.82855	-1.82557	-1.83697	-1.83401	-1.83106	-1.82813	-1.8252	-1.82229
-1.83066	-1.82766	-1.82469	-1.83609	-1.83314	-1.8302	-1.82726	-1.82433	-1.82143
-1.82978	-1.82679	-1.82383	-1.83521	-1.83227	-1.82933	-1.82639	-1.82347	-1.82058
-1.82891	-1.82593	-1.82297	-1.83435	-1.83141	-1.82847	-1.82553	-1.82261	-1.81973
-1.82804	-1.82507	-1.82212	-1.83348	-1.83054	-1.82761	-1.82467	-1.82175	-1.81887

-1.82717	-1.8242	-1.82127	-1.83261	-1.82967	-1.82674	-1.82381	-1.8209	-1.81802
-1.8263	-1.82334	-1.82041	-1.83174	-1.8288	-1.82586	-1.82294	-1.82003	-1.81716
-1.82542	-1.82247	-1.81955	-1.83086	-1.82791	-1.82497	-1.82205	-1.81916	-1.8163
-1.82454	-1.8216	-1.81868	-1.82996	-1.82697	-1.82402	-1.82111	-1.81822	-1.81537
-1.82366	-1.82072	-1.8178	-1.82904	-1.826	-1.82301	-1.8201	-1.81723	-1.81438
-1.82278	-1.81983	-1.8169	-1.8281	-1.82504	-1.82205	-1.81914	-1.81628	-1.81345
-1.82191	-1.81893	-1.81595	-1.82713	-1.82411	-1.82114	-1.81825	-1.8154	-1.81258
-1.82104	-1.81803	-1.81502	-1.82618	-1.8232	-1.82026	-1.81739	-1.81455	-1.81173
-1.82018	-1.81716	-1.81414	-1.82529	-1.82232	-1.8194	-1.81654	-1.81371	-1.8109
-1.81932	-1.81631	-1.81328	-1.82441	-1.82146	-1.81855	-1.8157	-1.81288	-1.81008
-1.81846	-1.81545	-1.81241	-1.82354	-1.8206	-1.8177	-1.81486	-1.81205	-1.80926
-1.8176	-1.81458	-1.81155	-1.82267	-1.81974	-1.81686	-1.81403	-1.81123	-1.80844
-1.81674	-1.81373	-1.81069	-1.8218	-1.81889	-1.81602	-1.81319	-1.8104	-1.80762
-1.81588	-1.81287	-1.80984	-1.82093	-1.81803	-1.81517	-1.81235	-1.80956	-1.80678
-1.81502	-1.81203	-1.80902	-1.80609	-1.80324	-1.81433	-1.81151	-1.80872	-1.80595
-1.81418	-1.81123	-1.80826	-1.80534	-1.80246	-1.81351	-1.81067	-1.80788	-1.80511
-1.81334	-1.81042	-1.80751	-1.8046	-1.80169	-1.81271	-1.80986	-1.80705	-1.80428
-1.81248	-1.80959	-1.80671	-1.80383	-1.80096	-1.81197	-1.80909	-1.80625	-1.80346
-1.81161	-1.80874	-1.80589	-1.80305	-1.80023	-1.81125	-1.80834	-1.80547	-1.80265
-1.81075	-1.80789	-1.80506	-1.80224	-1.79945	-1.81048	-1.8076	-1.80472	-1.80189
-1.80988	-1.80704	-1.80422	-1.80142	-1.79864	-1.80968	-1.80685	-1.80401	-1.80116
-1.80902	-1.80618	-1.80337	-1.80059	-1.79782	-1.80887	-1.80607	-1.80326	-1.80042
-1.80815	-1.80532	-1.80253	-1.79975	-1.79699	-1.80803	-1.80525	-1.80247	-1.79967
-1.8073	-1.80448	-1.80169	-1.79891	-1.79615	-1.80718	-1.80442	-1.80166	-1.79889
-1.80647	-1.80364	-1.80085	-1.79807	-1.79531	-1.80633	-1.80358	-1.80083	-1.79808
-1.80567	-1.80283	-1.80003	-1.79725	-1.79448	-1.80549	-1.80274	-1.8	-1.79725
-1.80494	-1.80208	-1.79925	-1.79645	-1.79367	-1.80467	-1.80191	-1.79916	-1.79642
-1.80425	-1.80137	-1.79849	-1.79567	-1.79289	-1.80386	-1.80109	-1.79833	-1.79559
-1.8035	-1.80063	-1.79776	-1.79494	-1.79215	-1.80311	-1.80031	-1.79753	-1.79478
-1.80271	-1.79987	-1.79705	-1.79425	-1.79147	-1.80241	-1.79957	-1.79675	-1.79398
-1.8019	-1.79909	-1.79629	-1.79351	-1.79074	-1.80167	-1.79883	-1.796	-1.79322
-1.80107	-1.79826	-1.79547	-1.7927	-1.78995	-1.80089	-1.79809	-1.7953	-1.79253
-1.80027	-1.79745	-1.79465	-1.79188	-1.78914	-1.80009	-1.79733	-1.79457	-1.79181
-1.79955	-1.79671	-1.79387	-1.79108	-1.78833	-1.79927	-1.79652	-1.79377	-1.79101
-1.79885	-1.79598	-1.7931	-1.79029	-1.78754	-1.79846	-1.7957	-1.79295	-1.79018
-1.79808	-1.79523	-1.79237	-1.78957	-1.78681	-1.79772	-1.79493	-1.79215	-1.78937
-1.79728	-1.79447	-1.79166	-1.78888	-1.78613	-1.79701	-1.79418	-1.79136	-1.78856
-1.79649	-1.79369	-1.7909	-1.78814	-1.7854	-1.79626	-1.79344	-1.79061	-1.7878
-1.7957	-1.79288	-1.7901	-1.78735	-1.78461	-1.79548	-1.79269	-1.7899	-1.7871
-1.79493	-1.79211	-1.78934	-1.78658	-1.78383	-1.79469	-1.79192	-1.78915	-1.78636
-1.79421	-1.7914	-1.78863	-1.78586	-1.78308	-1.7939	-1.79112	-1.78834	-1.78555
-1.79348	-1.79069	-1.78792	-1.78514	-1.78233	-1.79312	-1.79033	-1.78754	-1.78475
-1.79269	-1.7899	-1.78714	-1.78437	-1.78159	-1.79238	-1.78959	-1.7868	-1.78401
-1.79189	-1.7891	-1.78635	-1.7836	-1.78086	-1.79165	-1.78887	-1.78607	-1.78329
-1.79115	-1.78836	-1.78561	-1.78286	-1.7801	-1.79087	-1.78808	-1.78528	-1.78249
-1.79045	-1.78766	-1.7849	-1.78212	-1.77933	-1.79006	-1.78725	-1.78445	-1.78166
-1.78974	-1.78695	-1.78417	-1.78138	-1.77858	-1.77579	-1.77303	-1.77028	-1.78088
-1.78903	-1.78623	-1.78341	-1.78062	-1.77785	-1.77508	-1.77232	-1.76957	-1.78014
-1.78831	-1.78549	-1.78267	-1.77988	-1.77713	-1.77437	-1.7716	-1.76884	-1.7794
-1.78753	-1.78473	-1.78195	-1.77918	-1.77643	-1.77366	-1.77086	-1.76806	-1.7786

								-0.10719
								-0.10696
								-0.10673
					-0.1075	-0.10715	-0.10682	-0.15857
					-0.10727	-0.10693	-0.1066	-0.1581
					-0.10704	-0.10671	-0.10639	-0.30692
					-0.10681	-0.10651	-0.1062	-0.30627
			-0.10726	-0.10691	-0.15893	-0.15824	-0.15756	-0.39268
			-0.10703	-0.10669	-0.15847	-0.15779	-0.15712	-0.39189
	-0.10745	-0.10713	-0.15956	-0.15878	-0.39518	-0.3938	-0.39246	-0.48538
	-0.10723	-0.1069	-0.15908	-0.15835	-0.39443	-0.39303	-0.39166	-0.48445
	-0.10702	-0.10668	-0.15862	-0.15794	-0.39369	-0.39227	-0.3909	-0.48353
	-0.1068	-0.10648	-0.35167	-0.35041	-0.39291	-0.39153	-0.39018	-0.48267
	-0.10658	-0.10628	-0.35099	-0.34969	-0.39212	-0.39079	-0.38951	-0.48184
-0.10667	-0.15879	-0.15812	-0.39414	-0.39271	-0.48558	-0.48402	-0.48251	-0.60545
-0.10645	-0.15834	-0.15769	-0.39334	-0.39193	-0.48464	-0.48312	-0.48164	-0.60438
-0.10624	-0.3513	-0.35007	-0.39256	-0.39118	-0.48376	-0.48225	-0.48079	-0.65227
-0.10604	-0.35061	-0.34935	-0.3918	-0.39048	-0.48294	-0.48144	-0.47996	-0.65107
-0.25355	-0.39378	-0.39239	-0.48519	-0.48365	-0.65604	-0.65397	-0.65191	-0.68582
-0.25299	-0.39303	-0.39166	-0.48432	-0.48281	-0.65488	-0.65282	-0.65077	-0.68456
-0.25243	-0.39228	-0.39097	-0.48349	-0.48198	-0.65371	-0.65167	-0.64964	-0.68332
-0.3491	-0.39157	-0.39028	-0.60731	-0.60545	-0.65256	-0.65051	-0.64847	-0.84398
-0.34845	-0.39088	-0.3896	-0.60626	-0.60441	-0.65143	-0.64935	-0.64729	-0.8425
-0.39148	-0.48405	-0.48253	-0.65434	-0.65231	-0.72799	-0.72565	-0.72334	-0.87934
-0.39076	-0.48321	-0.48173	-0.6532	-0.65113	-0.72664	-0.72434	-0.72206	-0.87785
-0.39007	-0.65614	-0.65411	-0.65205	-0.64996	-0.84576	-0.84321	-0.84069	-0.91188
-0.38942	-0.65499	-0.65293	-0.65086	-0.6488	-0.84428	-0.84174	-0.83924	-0.91031
-0.38876	-0.65383	-0.65174	-0.64968	-0.64765	-0.84282	-0.84029	-0.83779	-0.90876
-0.6117	-0.65264	-0.65056	-0.76856	-0.76619	-0.91529	-0.91256	-0.90987	-0.90724

-0.6106	-0.65144	-0.64939	-0.76717	-0.76482	-0.9137	-0.911	-0.90835	-0.90575
-0.65239	-0.7706	-0.76817	-0.84591	-0.84341	-0.91214	-0.90946	-0.90683	-1.07584
-0.65129	-0.76927	-0.7668	-0.84444	-0.84197	-0.9106	-0.90794	-0.90533	-1.07381
-0.69261	-0.76794	-0.76548	-0.9144	-0.91172	-1.04273	-1.03932	-1.03599	-1.07182
-0.69135	-0.76659	-0.76422	-0.91293	-0.9102	-1.04071	-1.0374	-1.03416	-1.06987
-0.76759	-0.84536	-0.84289	-0.93841	-0.93557	-1.07821	-1.07473	-1.07133	-1.0928
-0.76623	-0.84393	-0.8415	-0.93689	-0.9341	-1.07626	-1.07275	-1.06932	-1.0907
-0.76491	-0.84252	-0.84011	-0.93536	-0.93266	-1.07437	-1.07081	-1.06733	-1.08863
-0.76366	-0.90473	-0.90209	-1.03998	-1.03668	-1.07245	-1.06893	-1.06545	-1.12088
-0.76245	-0.90328	-0.90059	-1.03801	-1.03478	-1.07052	-1.06707	-1.06363	-1.11883
-0.84102	-1.01045	-1.00706	-1.0754	-1.07195	-1.128	-1.12425	-1.12053	-1.18018
-0.83965	-1.00863	-1.00533	-1.0735	-1.06996	-1.12575	-1.12207	-1.11845	-1.17812
-0.90152	-1.03922	-1.03591	-1.09669	-1.0929	-1.12359	-1.11993	-1.11635	-1.24039
-0.90002	-1.03728	-1.03404	-1.09467	-1.09094	-1.12155	-1.11784	-1.11423	-1.23813
-1.00626	-1.07457	-1.07116	-1.1271	-1.12328	-1.1829	-1.17905	-1.17531	-1.23592
-1.00449	-1.0726	-1.06912	-1.12485	-1.12112	-1.18073	-1.17699	-1.17334	-1.2338
-1.00275	-1.07067	-1.06709	-1.1226	-1.11894	-1.17856	-1.17497	-1.17142	-1.23173
-1.00096	-1.12821	-1.12427	-1.12048	-1.11683	-1.24084	-1.23711	-1.23343	-1.25663
-0.99915	-1.12611	-1.12221	-1.11844	-1.11478	-1.23858	-1.23493	-1.23133	-1.2546
-1.0973	-1.124	-1.12016	-1.24428	-1.24029	-1.23644	-1.2328	-1.22921	-1.25252
-1.09517	-1.12187	-1.11811	-1.24211	-1.23822	-1.23442	-1.23071	-1.22707	-1.25039
-1.12346	-1.18673	-1.18283	-1.23998	-1.23618	-1.2594	-1.25561	-1.25188	-1.24828
-1.12124	-1.18448	-1.18073	-1.23788	-1.23416	-1.2574	-1.25363	-1.24989	-1.24623
-1.11908	-1.18227	-1.17864	-1.23579	-1.23213	-1.25541	-1.25167	-1.24794	-1.2442
-1.11706	-1.24107	-1.23733	-1.23367	-1.23008	-1.2534	-1.24969	-1.24597	-1.27482
-1.11513	-1.23893	-1.23518	-1.23155	-1.22804	-1.25138	-1.2477	-1.244	-1.27281
-1.17982	-1.23687	-1.23312	-1.25647	-1.25293	-1.24935	-1.2457	-1.24202	-1.29984
-1.17789	-1.23489	-1.23114	-1.25446	-1.2509	-1.24732	-1.24369	-1.24004	-1.29779
-1.17597	-1.23292	-1.22918	-1.25245	-1.24885	-1.27793	-1.27424	-1.27054	-1.34743
-1.17406	-1.23096	-1.22724	-1.25045	-1.24676	-1.27576	-1.27212	-1.2685	-1.34532
-1.17216	-1.25598	-1.25221	-1.3059	-1.30199	-1.3546	-1.35081	-1.34705	-1.43014
-1.17026	-1.254	-1.25024	-1.30384	-1.29991	-1.35242	-1.34862	-1.34486	-1.42785
-1.16838	-1.25202	-1.24827	-1.30178	-1.29786	-1.35028	-1.34644	-1.34269	-1.42557
-1.22683	-1.27521	-1.2714	-1.32795	-1.32396	-1.40563	-1.40138	-1.39724	-1.42326
-1.22488	-1.2732	-1.26938	-1.32584	-1.32188	-1.40335	-1.39907	-1.39492	-1.42096
-1.27498	-1.33169	-1.3277	-1.35187	-1.34785	-1.43122	-1.42689	-1.4227	-1.41873
-1.27294	-1.32956	-1.32558	-1.34971	-1.34571	-1.42892	-1.42467	-1.42054	-1.41656
-1.33138	-1.32743	-1.32346	-1.43548	-1.43101	-1.42667	-1.42248	-1.41839	-1.46396
-1.32923	-1.3253	-1.32136	-1.43317	-1.42878	-1.42449	-1.42033	-1.41625	-1.46169
-1.32709	-1.35487	-1.35086	-1.43088	-1.42656	-1.47212	-1.46786	-1.46364	-1.48409
-1.32497	-1.3527	-1.34871	-1.42857	-1.42434	-1.46988	-1.46563	-1.46141	-1.48184
-1.32286	-1.35055	-1.34658	-1.42629	-1.42213	-1.46764	-1.4634	-1.45918	-1.4796
-1.32077	-1.43276	-1.42835	-1.47389	-1.46964	-1.46541	-1.46117	-1.45697	-1.47736
-1.31869	-1.43048	-1.42615	-1.47166	-1.4674	-1.46317	-1.45895	-1.45476	-1.47512
-1.34813	-1.47813	-1.47374	-1.46941	-1.46516	-1.48559	-1.48134	-1.47712	-1.54228
-1.34601	-1.47578	-1.47145	-1.46716	-1.46291	-1.48333	-1.4791	-1.4749	-1.53993
-1.47784	-1.47346	-1.46918	-1.46492	-1.46068	-1.50093	-1.49664	-1.4924	-1.5376
-1.47555	-1.47119	-1.46692	-1.46267	-1.45845	-1.49865	-1.49438	-1.49015	-1.53527
-1.47327	-1.46894	-1.46467	-1.46043	-1.45623	-1.49639	-1.49213	-1.48794	-1.53301
-1.47099	-1.46667	-1.46242	-1.50268	-1.4984	-1.54375	-1.53937	-1.53508	-1.53087

-1.46871	-1.46442	-1.46017	-1.5004	-1.49614	-1.54144	-1.53708	-1.53286	-1.52882
-1.46643	-1.48245	-1.47816	-1.54782	-1.54347	-1.55204	-1.54771	-1.54356	-1.5397
-1.46416	-1.48017	-1.47592	-1.54551	-1.54118	-1.54979	-1.54552	-1.54149	-1.53783
-1.48221	-1.50218	-1.49792	-1.5561	-1.5518	-1.54759	-1.54341	-1.5395	-1.53601
-1.47998	-1.49995	-1.49572	-1.55384	-1.54959	-1.54546	-1.54138	-1.53758	-1.53425
-1.47783	-1.56026	-1.55591	-1.55162	-1.5474	-1.54334	-1.53941	-1.53577	-1.53258
-1.47582	-1.55815	-1.5538	-1.54949	-1.5452	-1.54119	-1.53749	-1.53409	-1.53103
-1.47382	-1.55604	-1.55168	-1.54736	-1.54304	-1.53911	-1.53565	-1.53248	-1.52954
-1.55817	-1.55377	-1.54941	-1.54515	-1.54097	-1.53719	-1.5339	-1.53088	-1.54934
-1.55585	-1.55144	-1.54707	-1.54292	-1.53897	-1.53538	-1.53223	-1.52933	-1.54789
-1.55351	-1.5491	-1.54476	-1.54075	-1.53704	-1.55493	-1.55191	-1.54911	-1.54647
-1.55115	-1.54676	-1.54246	-1.53861	-1.53518	-1.55327	-1.55039	-1.54768	-1.54509
-1.54881	-1.5445	-1.54033	-1.55794	-1.5547	-1.55169	-1.54892	-1.54629	-1.54373
-1.54648	-1.54233	-1.53839	-1.55616	-1.55306	-1.55016	-1.54749	-1.54493	-1.54239
-1.54425	-1.56157	-1.55786	-1.55451	-1.55153	-1.54874	-1.54614	-1.54362	-1.5411
-1.54218	-1.55967	-1.5562	-1.55303	-1.55017	-1.54745	-1.54489	-1.54239	-1.53988
-1.54022	-1.55787	-1.55463	-1.55162	-1.54886	-1.54621	-1.54367	-1.54117	-1.53867
-1.53832	-1.55613	-1.55304	-1.55015	-1.54748	-1.56533	-1.5628	-1.5603	-1.5578
-1.53651	-1.55445	-1.55148	-1.54868	-1.54609	-1.56398	-1.56146	-1.55896	-1.55646
-1.55599	-1.5733	-1.57044	-1.56771	-1.56516	-1.56264	-1.56013	-1.55763	-1.57434
-1.55429	-1.57175	-1.569	-1.56634	-1.56381	-1.5613	-1.55879	-1.55631	-1.573
-1.57316	-1.57028	-1.56763	-1.56503	-1.56251	-1.56	-1.55751	-1.55503	-1.57173
-1.57172	-1.56894	-1.56635	-1.56379	-1.56128	-1.55879	-1.5563	-1.55383	-1.57055
-1.57033	-1.56762	-1.56509	-1.56256	-1.56006	-1.55757	-1.55508	-1.55262	-1.56937
-1.56892	-1.56628	-1.56376	-1.56125	-1.55876	-1.57555	-1.57302	-1.57053	-1.58747
-1.56755	-1.56495	-1.56244	-1.55994	-1.55745	-1.57424	-1.57172	-1.56924	-1.58617
-1.56625	-1.56368	-1.56118	-1.55868	-1.55621	-1.573	-1.57051	-1.56804	-1.60768
-1.56499	-1.56245	-1.55995	-1.55747	-1.555	-1.5718	-1.56935	-1.56692	-1.60648
-1.5637	-1.56118	-1.55869	-1.57553	-1.57302	-1.57055	-1.56813	-1.56574	-1.60524
-1.56238	-1.55987	-1.55738	-1.57421	-1.57169	-1.56924	-1.56685	-1.56449	-1.60399
-1.56109	-1.57796	-1.57544	-1.57292	-1.57042	-1.5911	-1.58863	-1.58619	-1.60274
-1.55985	-1.57673	-1.57421	-1.57172	-1.56925	-1.58991	-1.5874	-1.58495	-1.60148
-1.55862	-1.57549	-1.57298	-1.57051	-1.56809	-1.58872	-1.5862	-1.58373	-1.60026
-1.57672	-1.57419	-1.57168	-1.56924	-1.56685	-1.60647	-1.60395	-1.60149	-1.67051
-1.5754	-1.57287	-1.57037	-1.56795	-1.56561	-1.60519	-1.60274	-1.60035	-1.66921
-1.57414	-1.57162	-1.56916	-1.58986	-1.58743	-1.60398	-1.60154	-1.59918	-1.66785
-1.57292	-1.57044	-1.56801	-1.5887	-1.58628	-1.60282	-1.60037	-1.598	-1.66645
-1.57168	-1.56922	-1.56684	-1.5875	-1.5851	-1.62735	-1.62482	-1.62235	-1.66511
-1.5704	-1.56796	-1.5656	-1.58624	-1.58387	-1.62608	-1.62364	-1.62125	-1.66386
-1.56917	-1.58984	-1.58741	-1.58502	-1.58267	-1.62485	-1.62249	-1.62018	-1.66261
-1.56802	-1.58868	-1.58626	-1.58388	-1.58155	-1.62368	-1.62138	-1.6191	-1.66133
-1.56691	-1.58756	-1.58515	-1.58279	-1.58047	-1.62255	-1.62028	-1.61802	-1.66005
-1.5658	-1.58644	-1.58406	-1.62624	-1.62378	-1.68404	-1.68137	-1.67872	-1.70326
-1.56469	-1.58532	-1.58298	-1.62511	-1.62266	-1.68271	-1.68002	-1.67737	-1.70193
-1.56357	-1.58417	-1.58185	-1.64175	-1.63935	-1.68911	-1.68638	-1.6837	-1.71907
-1.56244	-1.58299	-1.58067	-1.64054	-1.63822	-1.68786	-1.68513	-1.68245	-1.71781
-1.58423	-1.60025	-1.59795	-1.64755	-1.64524	-1.70534	-1.70256	-1.69984	-1.71653
-1.58314	-1.59921	-1.59696	-1.64647	-1.64416	-1.70406	-1.70125	-1.69851	-1.71519
-1.60045	-1.65019	-1.64778	-1.70828	-1.70553	-1.70276	-1.69995	-1.69722	-1.71389
-1.59939	-1.64907	-1.6467	-1.707	-1.70419	-1.70143	-1.69869	-1.69604	-1.7127

-1.59835	-1.64797	-1.64561	-1.7057	-1.70285	-1.7001	-1.69746	-1.6949	-1.71155
-1.60597	-1.66619	-1.66378	-1.70437	-1.70154	-1.71813	-1.7155	-1.71293	-1.71039
-1.60497	-1.66506	-1.66262	-1.70303	-1.70025	-1.71686	-1.71428	-1.71175	-1.70923
-1.60394	-1.66392	-1.66148	-1.70174	-1.69901	-1.71564	-1.71309	-1.71058	-1.72318
-1.60289	-1.66276	-1.66035	-1.70049	-1.69783	-1.71447	-1.71193	-1.70941	-1.72198
-1.62169	-1.66162	-1.65923	-1.71463	-1.71203	-1.72844	-1.72588	-1.72334	-1.76673
-1.62065	-1.6605	-1.65812	-1.7134	-1.71087	-1.72727	-1.72469	-1.72211	-1.76535
-1.61963	-1.65939	-1.65702	-1.71219	-1.70972	-1.72611	-1.7235	-1.7209	-1.76399
-1.66067	-1.71627	-1.71359	-1.71103	-1.70858	-1.72495	-1.72234	-1.71974	-1.76268
-1.65956	-1.71504	-1.71243	-1.70989	-1.70744	-1.72378	-1.7212	-1.71861	-1.76139
-1.67431	-1.71382	-1.71127	-1.72768	-1.72512	-1.76886	-1.76593	-1.76301	-1.77817
-1.67319	-1.71261	-1.71012	-1.7265	-1.7239	-1.76744	-1.76456	-1.76168	-1.77685
-1.67209	-1.71142	-1.70896	-1.72532	-1.72271	-1.76609	-1.76321	-1.76036	-1.77552
-1.67101	-1.71026	-1.70781	-1.72415	-1.72156	-1.7648	-1.7619	-1.75903	-1.77419
-1.69041	-1.7091	-1.70665	-1.76936	-1.76642	-1.7635	-1.76059	-1.75771	-1.77285
-1.6893	-1.70794	-1.70545	-1.76796	-1.76506	-1.76217	-1.75927	-1.75639	-1.77151
-1.68819	-1.70678	-1.70426	-1.76656	-1.7637	-1.76083	-1.75795	-1.75507	-1.77017
-1.68709	-1.72448	-1.72188	-1.76524	-1.76237	-1.77757	-1.77465	-1.77174	-1.76883
-1.68599	-1.72334	-1.72074	-1.76395	-1.76105	-1.77621	-1.7733	-1.7704	-1.7675
-1.68491	-1.76855	-1.76559	-1.76265	-1.75973	-1.77487	-1.77195	-1.76907	-1.7662
-1.68383	-1.76725	-1.76428	-1.76134	-1.75841	-1.77353	-1.7706	-1.76773	-1.76492
-1.68275	-1.76597	-1.76298	-1.76003	-1.75709	-1.77219	-1.76928	-1.76644	-1.76367
-1.68169	-1.76469	-1.76168	-1.75871	-1.75576	-1.77087	-1.768	-1.76519	-1.76246
-1.72794	-1.76342	-1.7604	-1.75742	-1.75445	-1.76955	-1.76673	-1.76397	-1.76127
-1.72673	-1.76217	-1.75919	-1.75619	-1.75317	-1.76823	-1.76547	-1.76278	-1.7601
-1.7255	-1.76092	-1.75798	-1.75498	-1.7519	-1.76693	-1.76424	-1.76161	-1.75894
-1.7426	-1.75962	-1.7567	-1.7717	-1.76859	-1.76567	-1.76303	-1.76044	-1.75778
-1.74132	-1.75829	-1.75536	-1.77038	-1.76733	-1.76445	-1.76184	-1.75927	-1.75664
-1.74004	-1.75696	-1.75403	-1.76908	-1.76609	-1.76327	-1.76067	-1.75812	-1.79043
-1.73875	-1.75562	-1.75271	-1.76778	-1.76489	-1.76212	-1.75953	-1.75698	-1.78929
-1.73749	-1.75431	-1.75141	-1.76651	-1.7637	-1.796	-1.79336	-1.79075	-1.78813
-1.73624	-1.75301	-1.75012	-1.76525	-1.76252	-1.79483	-1.79219	-1.78957	-1.78694
-1.73501	-1.75174	-1.74887	-1.76404	-1.76138	-1.79369	-1.79104	-1.7884	-1.78575
-1.73376	-1.76848	-1.76563	-1.79793	-1.79526	-1.7926	-1.78991	-1.78723	-1.78455
-1.73253	-1.76726	-1.76449	-1.7968	-1.79418	-1.79152	-1.78879	-1.78606	-1.78335
-1.73136	-1.80113	-1.79835	-1.79565	-1.79305	-1.79039	-1.78764	-1.78487	-1.82825
-1.73023	-1.79995	-1.79718	-1.79449	-1.7919	-1.78923	-1.78646	-1.78368	-1.82693
-1.80162	-1.79878	-1.79602	-1.79333	-1.79073	-1.78806	-1.7853	-1.78253	-1.82564
-1.80042	-1.79761	-1.79486	-1.79217	-1.78954	-1.78688	-1.78416	-1.78143	-1.8244
-1.79924	-1.79645	-1.79373	-1.79104	-1.78837	-1.83237	-1.82934	-1.8263	-1.82315
-1.79807	-1.79533	-1.79266	-1.78995	-1.78723	-1.83104	-1.82802	-1.825	-1.82186
-1.79691	-1.79421	-1.79158	-1.78887	-1.7861	-1.82975	-1.82671	-1.82368	-1.82057
-1.79574	-1.79306	-1.79042	-1.78773	-1.78498	-1.82854	-1.82544	-1.82238	-1.81934
-1.79457	-1.79188	-1.78924	-1.78656	-1.78388	-1.82735	-1.8242	-1.8211	-1.81814
-1.79345	-1.79075	-1.78806	-1.83216	-1.82915	-1.82609	-1.82293	-1.81982	-1.81691
-1.79237	-1.78964	-1.7869	-1.83082	-1.82781	-1.82477	-1.82163	-1.81855	-1.81567
-1.79127	-1.78853	-1.78577	-1.82953	-1.82651	-1.82347	-1.82037	-1.81734	-1.83962
-1.79014	-1.78741	-1.78468	-1.82833	-1.82525	-1.8222	-1.81918	-1.81622	-1.83838
-1.78901	-1.78629	-1.78358	-1.82713	-1.82401	-1.82096	-1.81801	-1.81509	-1.83716
-1.78791	-1.78518	-1.78243	-1.82587	-1.8228	-1.81978	-1.81683	-1.81392	-1.83593

-1.78682	-1.78405	-1.78127	-1.8246	-1.82159	-1.81861	-1.81564	-1.81272	-1.8347
-1.78568	-1.82951	-1.82637	-1.82331	-1.82034	-1.84304	-1.83976	-1.83654	-1.85073
-1.78451	-1.8282	-1.82507	-1.82202	-1.81906	-1.84167	-1.83843	-1.83523	-1.8494
-1.78334	-1.82693	-1.82382	-1.82079	-1.81783	-1.84036	-1.83716	-1.834	-1.84813
-1.78217	-1.82568	-1.82263	-1.81962	-1.81664	-1.83909	-1.83595	-1.83283	-1.84691
-1.78103	-1.82445	-1.82143	-1.84436	-1.84107	-1.85526	-1.85208	-1.84891	-1.84569
-1.77994	-1.82323	-1.82018	-1.84304	-1.83986	-1.85407	-1.85084	-1.84764	-1.84446
-1.77886	-1.82203	-1.81896	-1.84175	-1.83865	-1.85287	-1.8496	-1.84637	-1.84325
-1.77775	-1.82083	-1.81783	-1.84055	-1.83742	-1.87819	-1.87487	-1.8716	-1.86843
-1.77664	-1.81964	-1.81673	-1.83939	-1.83619	-1.87689	-1.87363	-1.87041	-1.86722
-1.82147	-1.8446	-1.84138	-1.88229	-1.87895	-1.87565	-1.8724	-1.86919	-1.86602
-1.82037	-1.84338	-1.84012	-1.881	-1.87772	-1.87446	-1.87118	-1.86796	-1.86483
-1.81925	-1.8694	-1.86605	-1.87974	-1.87647	-1.87322	-1.86997	-1.86676	-1.88489
-1.81811	-1.86819	-1.86487	-1.87851	-1.8752	-1.87194	-1.86876	-1.86561	-1.88363
-1.84488	-1.884	-1.88065	-1.87731	-1.87396	-1.87069	-1.86755	-1.86444	-1.88236
-1.84365	-1.88271	-1.87944	-1.87612	-1.87278	-1.8695	-1.86633	-1.86319	-1.88106
-1.84243	-1.88141	-1.8782	-1.87493	-1.87161	-1.86834	-1.86512	-1.86195	-1.87977
-1.84963	-1.88014	-1.8769	-1.87366	-1.87042	-1.86719	-1.86397	-1.86078	-1.8785
-1.84845	-1.87889	-1.87558	-1.87236	-1.86921	-1.86605	-1.86284	-1.85965	-1.87726
-1.86445	-1.87767	-1.87434	-1.87111	-1.86798	-1.88612	-1.88275	-1.8794	-1.87605
-1.86326	-1.87648	-1.87316	-1.86991	-1.86672	-1.88478	-1.88149	-1.8782	-1.87488
-1.86204	-1.87526	-1.87198	-1.86874	-1.86552	-1.88349	-1.88022	-1.87695	-1.87366
-1.86077	-1.874	-1.87081	-1.8676	-1.8644	-1.88226	-1.87893	-1.87563	-1.87239
-1.85951	-1.87276	-1.86962	-1.88781	-1.88442	-1.88104	-1.87766	-1.87433	-1.87112
-1.85832	-1.87154	-1.86838	-1.88652	-1.88318	-1.87984	-1.87646	-1.87312	-1.86988
-1.85716	-1.87034	-1.86714	-1.88521	-1.88192	-1.87862	-1.87527	-1.87194	-1.86865
-1.85602	-1.86919	-1.86597	-1.88394	-1.88064	-1.87734	-1.87405	-1.87076	-1.86747
-1.8549	-1.86807	-1.86485	-1.8827	-1.87934	-1.87603	-1.87281	-1.86959	-1.86632
-1.85377	-1.88831	-1.88489	-1.88149	-1.87811	-1.8748	-1.87161	-1.86842	-1.86515
-1.85265	-1.88712	-1.88371	-1.88032	-1.87694	-1.87363	-1.87043	-1.86725	-1.86399
-1.85149	-1.88589	-1.88252	-1.87915	-1.87577	-1.87246	-1.86924	-1.86604	-1.87851
-1.85026	-1.8846	-1.88131	-1.87797	-1.87459	-1.87126	-1.86799	-1.86477	-1.87724
-1.84905	-1.88333	-1.88009	-1.87678	-1.87341	-1.87007	-1.86676	-1.86351	-1.87596
-1.84792	-1.8821	-1.87888	-1.87558	-1.87223	-1.8689	-1.86558	-1.86231	-1.87469
-1.84682	-1.88089	-1.87765	-1.87437	-1.87104	-1.86773	-1.86442	-1.86115	-1.87344
-1.86698	-1.87966	-1.87639	-1.87311	-1.86984	-1.88249	-1.87901	-1.87558	-1.87223
-1.86579	-1.87841	-1.8751	-1.87183	-1.86863	-1.88127	-1.8778	-1.87437	-1.87104
-1.86462	-1.8772	-1.87386	-1.8706	-1.86743	-1.88005	-1.87661	-1.87319	-1.86987
-1.86346	-1.87602	-1.87268	-1.86943	-1.86626	-1.87884	-1.87544	-1.87205	-1.86872
-1.8623	-1.87485	-1.87152	-1.88432	-1.88098	-1.87764	-1.87427	-1.87091	-1.87987
-1.86115	-1.87369	-1.87035	-1.8831	-1.87978	-1.87645	-1.8731	-1.86975	-1.8787
-1.86	-1.87252	-1.86918	-1.88189	-1.87858	-1.87526	-1.87192	-1.86859	-1.87753
-1.85885	-1.87136	-1.86802	-1.88068	-1.87737	-1.89995	-1.89657	-1.8932	-1.88984
-1.8577	-1.87019	-1.86687	-1.87948	-1.87617	-1.89872	-1.89536	-1.892	-1.88865
-1.85655	-1.88514	-1.88166	-1.89194	-1.88863	-1.89751	-1.89416	-1.89081	-1.88747
-1.85539	-1.88392	-1.88044	-1.89072	-1.88744	-1.89631	-1.89296	-1.88962	-1.88628
-1.85423	-1.89634	-1.89286	-1.9017	-1.89842	-1.89511	-1.89177	-1.88843	-1.8851
-1.85306	-1.89509	-1.89161	-1.90045	-1.89719	-1.89391	-1.89057	-1.88724	-1.88393
-1.86588	-1.90609	-1.90258	-1.89921	-1.89597	-1.8927	-1.88937	-1.88605	-1.88276
-1.86471	-1.90483	-1.90135	-1.89799	-1.89474	-1.89148	-1.88817	-1.88486	-1.88157

-1.86354	-1.90361	-1.90014	-1.89679	-1.89353	-1.89027	-1.88696	-1.88367	-1.88039
-1.87833	-1.90242	-1.89901	-1.89565	-1.89237	-1.88908	-1.88577	-1.88248	-1.87922
-1.87715	-1.90126	-1.89791	-1.89456	-1.89122	-1.8879	-1.88459	-1.88131	-1.87805
-1.88838	-1.90012	-1.89677	-1.89342	-1.89006	-1.88672	-1.88342	-1.88014	-1.87689
-1.88727	-1.89898	-1.89561	-1.89224	-1.88887	-1.88554	-1.88224	-1.87898	-1.87574
-1.88614	-1.89782	-1.89443	-1.89104	-1.88768	-1.88435	-1.88107	-1.87782	-1.8746
-1.88498	-1.89663	-1.89321	-1.88983	-1.88647	-1.88316	-1.87989	-1.87666	-1.87345
-1.88381	-1.89542	-1.892	-1.88862	-1.88528	-1.88198	-1.87873	-1.87552	-1.87232
-1.88262	-1.89421	-1.8908	-1.88743	-1.8841	-1.88082	-1.87759	-1.87439	-1.87123
-1.88142	-1.893	-1.8896	-1.88625	-1.88295	-1.87969	-1.87647	-1.87329	-1.87018
-1.88023	-1.8918	-1.88842	-1.88511	-1.88187	-1.87864	-1.87543	-1.87226	-1.86914
-1.87905	-1.89061	-1.88725	-1.88399	-1.88082	-1.87765	-1.87445	-1.87127	-1.86811
-1.87794	-1.8895	-1.88615	-1.88291	-1.87976	-1.8766	-1.87341	-1.87022	-1.86705
-1.87692	-1.88848	-1.88513	-1.88187	-1.87869	-1.8755	-1.8723	-1.86911	-1.86595
-1.87587	-1.88743	-1.88409	-1.88082	-1.8776	-1.8744	-1.87119	-1.868	-1.86485
-1.87477	-1.8863	-1.88299	-1.87972	-1.8765	-1.87329	-1.87008	-1.8669	-1.86375
-1.87365	-1.88517	-1.88188	-1.87862	-1.87539	-1.87219	-1.86899	-1.86582	-1.86268
-1.85952	-1.86928	-1.86607	-1.87751	-1.87429	-1.87111	-1.86796	-1.86483	-1.86171
-1.85843	-1.86819	-1.86498	-1.87641	-1.87321	-1.87005	-1.86695	-1.86387	-1.86076
-1.85739	-1.86717	-1.86398	-1.8754	-1.87219	-1.86903	-1.86593	-1.86285	-1.85974
-1.85639	-1.86621	-1.86304	-1.87445	-1.87123	-1.86804	-1.86491	-1.86179	-1.85866
-1.85538	-1.86521	-1.86205	-1.87345	-1.87022	-1.86702	-1.86386	-1.86072	-1.85759
-1.85433	-1.86414	-1.86099	-1.87238	-1.86916	-1.86596	-1.86279	-1.85963	-1.8565
-1.85328	-1.86308	-1.85994	-1.87132	-1.86812	-1.86492	-1.86174	-1.85859	-1.85544
-1.85222	-1.86205	-1.85896	-1.87035	-1.86715	-1.86396	-1.86078	-1.85762	-1.85447
-1.85117	-1.86103	-1.85799	-1.86939	-1.86619	-1.863	-1.85983	-1.85667	-1.85351
-1.85021	-1.84714	-1.84411	-1.86835	-1.86516	-1.86198	-1.8588	-1.85564	-1.85249
-1.84928	-1.84618	-1.84309	-1.86728	-1.8641	-1.86092	-1.85773	-1.85457	-1.85145
-1.84833	-1.84522	-1.84211	-1.85362	-1.85048	-1.85991	-1.85673	-1.85357	-1.85046
-1.84736	-1.84428	-1.84118	-1.85267	-1.84953	-1.85895	-1.85577	-1.85262	-1.84953
-1.84636	-1.84329	-1.84019	-1.85167	-1.84854	-1.84541	-1.84228	-1.83918	-1.84856
-1.8453	-1.84222	-1.83912	-1.85059	-1.84747	-1.84435	-1.84121	-1.83813	-1.84753
-1.84426	-1.84116	-1.83807	-1.84953	-1.84641	-1.8433	-1.84018	-1.8371	-1.83413
-1.84329	-1.84019	-1.8371	-1.84855	-1.84543	-1.84233	-1.83923	-1.83618	-1.83321
-1.84233	-1.83923	-1.83615	-1.84759	-1.84447	-1.84139	-1.83831	-1.83528	-1.83232
-1.84129	-1.83819	-1.83512	-1.84655	-1.84345	-1.84037	-1.83733	-1.83432	-1.83136
-1.8402	-1.83712	-1.83405	-1.84548	-1.84239	-1.83933	-1.83631	-1.83332	-1.83037
-1.83919	-1.83611	-1.83306	-1.84448	-1.8414	-1.83836	-1.83536	-1.83239	-1.82945
-1.83826	-1.83519	-1.83215	-1.84357	-1.84049	-1.83745	-1.83448	-1.83153	-1.82859
-1.83734	-1.83428	-1.83124	-1.84266	-1.83959	-1.83657	-1.83361	-1.83068	-1.82773
-1.83643	-1.83338	-1.83035	-1.84176	-1.83871	-1.8357	-1.83275	-1.82982	-1.82688
-1.8355	-1.83245	-1.82943	-1.84084	-1.83781	-1.83481	-1.83186	-1.82893	-1.82599
-1.83448	-1.83145	-1.82844	-1.83984	-1.83683	-1.83384	-1.8309	-1.82797	-1.82504
-1.83343	-1.83041	-1.82742	-1.83882	-1.83582	-1.83285	-1.82991	-1.82698	-1.82406
-1.83247	-1.82946	-1.82647	-1.83787	-1.83489	-1.83193	-1.82899	-1.82607	-1.82315
-1.83156	-1.82855	-1.82557	-1.83697	-1.83401	-1.83106	-1.82813	-1.8252	-1.82229
-1.83066	-1.82766	-1.82469	-1.83609	-1.83314	-1.8302	-1.82726	-1.82433	-1.82143
-1.82978	-1.82679	-1.82383	-1.83521	-1.83227	-1.82933	-1.82639	-1.82347	-1.82058
-1.82891	-1.82593	-1.82297	-1.83435	-1.83141	-1.82847	-1.82553	-1.82261	-1.81973
-1.82804	-1.82507	-1.82212	-1.83348	-1.83054	-1.82761	-1.82467	-1.82175	-1.81887

-1.82717	-1.8242	-1.82127	-1.83261	-1.82967	-1.82674	-1.82381	-1.8209	-1.81802
-1.8263	-1.82334	-1.82041	-1.83174	-1.8288	-1.82586	-1.82294	-1.82003	-1.81716
-1.82542	-1.82247	-1.81955	-1.83086	-1.82791	-1.82497	-1.82205	-1.81916	-1.8163
-1.82454	-1.8216	-1.81868	-1.82996	-1.82697	-1.82402	-1.82111	-1.81822	-1.81537
-1.82366	-1.82072	-1.8178	-1.82904	-1.826	-1.82301	-1.8201	-1.81723	-1.81438
-1.82278	-1.81983	-1.8169	-1.8281	-1.82504	-1.82205	-1.81914	-1.81628	-1.81345
-1.82191	-1.81893	-1.81595	-1.82713	-1.82411	-1.82114	-1.81825	-1.8154	-1.81258
-1.82104	-1.81803	-1.81502	-1.82618	-1.8232	-1.82026	-1.81739	-1.81455	-1.81173
-1.82018	-1.81716	-1.81414	-1.82529	-1.82232	-1.8194	-1.81654	-1.81371	-1.8109
-1.81932	-1.81631	-1.81328	-1.82441	-1.82146	-1.81855	-1.8157	-1.81288	-1.81008
-1.81846	-1.81545	-1.81241	-1.82354	-1.8206	-1.8177	-1.81486	-1.81205	-1.80926
-1.8176	-1.81458	-1.81155	-1.82267	-1.81974	-1.81686	-1.81403	-1.81123	-1.80844
-1.81674	-1.81373	-1.81069	-1.8218	-1.81889	-1.81602	-1.81319	-1.8104	-1.80762
-1.81588	-1.81287	-1.80984	-1.82093	-1.81803	-1.81517	-1.81235	-1.80956	-1.80678
-1.81502	-1.81203	-1.80902	-1.80609	-1.80324	-1.81433	-1.81151	-1.80872	-1.80595
-1.81418	-1.81123	-1.80826	-1.80534	-1.80246	-1.81351	-1.81067	-1.80788	-1.80511
-1.81334	-1.81042	-1.80751	-1.8046	-1.80169	-1.81271	-1.80986	-1.80705	-1.80428
-1.81248	-1.80959	-1.80671	-1.80383	-1.80096	-1.81197	-1.80909	-1.80625	-1.80346
-1.81161	-1.80874	-1.80589	-1.80305	-1.80023	-1.81125	-1.80834	-1.80547	-1.80265
-1.81075	-1.80789	-1.80506	-1.80224	-1.79945	-1.81048	-1.8076	-1.80472	-1.80189
-1.80988	-1.80704	-1.80422	-1.80142	-1.79864	-1.80968	-1.80685	-1.80401	-1.80116
-1.80902	-1.80618	-1.80337	-1.80059	-1.79782	-1.80887	-1.80607	-1.80326	-1.80042
-1.80815	-1.80532	-1.80253	-1.79975	-1.79699	-1.80803	-1.80525	-1.80247	-1.79967
-1.8073	-1.80448	-1.80169	-1.79891	-1.79615	-1.80718	-1.80442	-1.80166	-1.79889
-1.80647	-1.80364	-1.80085	-1.79807	-1.79531	-1.80633	-1.80358	-1.80083	-1.79808
-1.80567	-1.80283	-1.80003	-1.79725	-1.79448	-1.80549	-1.80274	-1.8	-1.79725
-1.80494	-1.80208	-1.79925	-1.79645	-1.79367	-1.80467	-1.80191	-1.79916	-1.79642
-1.80425	-1.80137	-1.79849	-1.79567	-1.79289	-1.80386	-1.80109	-1.79833	-1.79559
-1.8035	-1.80063	-1.79776	-1.79494	-1.79215	-1.80311	-1.80031	-1.79753	-1.79478
-1.80271	-1.79987	-1.79705	-1.79425	-1.79147	-1.80241	-1.79957	-1.79675	-1.79398
-1.8019	-1.79909	-1.79629	-1.79351	-1.79074	-1.80167	-1.79883	-1.796	-1.79322
-1.80107	-1.79826	-1.79547	-1.7927	-1.78995	-1.80089	-1.79809	-1.7953	-1.79253
-1.80027	-1.79745	-1.79465	-1.79188	-1.78914	-1.80009	-1.79733	-1.79457	-1.79181
-1.79955	-1.79671	-1.79387	-1.79108	-1.78833	-1.79927	-1.79652	-1.79377	-1.79101
-1.79885	-1.79598	-1.7931	-1.79029	-1.78754	-1.79846	-1.7957	-1.79295	-1.79018
-1.79808	-1.79523	-1.79237	-1.78957	-1.78681	-1.79772	-1.79493	-1.79215	-1.78937
-1.79728	-1.79447	-1.79166	-1.78888	-1.78613	-1.79701	-1.79418	-1.79136	-1.78856
-1.79649	-1.79369	-1.7909	-1.78814	-1.7854	-1.79626	-1.79344	-1.79061	-1.7878
-1.7957	-1.79288	-1.7901	-1.78735	-1.78461	-1.79548	-1.79269	-1.7899	-1.7871
-1.79493	-1.79211	-1.78934	-1.78658	-1.78383	-1.79469	-1.79192	-1.78915	-1.78636
-1.79421	-1.7914	-1.78863	-1.78586	-1.78308	-1.7939	-1.79112	-1.78834	-1.78555
-1.79348	-1.79069	-1.78792	-1.78514	-1.78233	-1.79312	-1.79033	-1.78754	-1.78475
-1.79269	-1.7899	-1.78714	-1.78437	-1.78159	-1.79238	-1.78959	-1.7868	-1.78401
-1.79189	-1.7891	-1.78635	-1.7836	-1.78086	-1.79165	-1.78887	-1.78607	-1.78329
-1.79115	-1.78836	-1.78561	-1.78286	-1.7801	-1.79087	-1.78808	-1.78528	-1.78249
-1.79045	-1.78766	-1.7849	-1.78212	-1.77933	-1.79006	-1.78725	-1.78445	-1.78166
-1.78974	-1.78695	-1.78417	-1.78138	-1.77858	-1.77579	-1.77303	-1.77028	-1.78088
-1.78903	-1.78623	-1.78341	-1.78062	-1.77785	-1.77508	-1.77232	-1.76957	-1.78014
-1.78831	-1.78549	-1.78267	-1.77988	-1.77713	-1.77437	-1.7716	-1.76884	-1.7794
-1.78753	-1.78473	-1.78195	-1.77918	-1.77643	-1.77366	-1.77086	-1.76806	-1.7786

-0.84983	-0.84846	-0.84712	-0.84583	-0.84461	-0.88024	-0.87891	-0.87759	-0.87625
-0.85019	-0.8488	-0.84743	-0.84614	-0.84492	-0.88055	-0.87923	-0.87791	-0.87658
-0.85054	-0.84915	-0.84777	-0.84646	-0.84523	-0.88087	-0.87955	-0.87823	-0.8769
-0.85089	-0.8495	-0.84811	-0.8468	-0.84554	-0.88118	-0.87986	-0.87854	-0.87721
-0.85123	-0.84984	-0.84847	-0.84714	-0.84586	-0.88149	-0.88017	-0.87885	-0.87752
-0.85156	-0.85018	-0.84881	-0.84747	-0.84618	-0.88181	-0.88048	-0.87916	-0.87783
-0.85189	-0.85051	-0.84913	-0.8478	-0.84649	-0.88213	-0.8808	-0.87947	-0.87813
-0.85222	-0.85084	-0.84946	-0.84812	-0.84681	-0.88246	-0.88111	-0.87978	-0.87844
-0.85254	-0.85116	-0.84978	-0.84844	-0.84713	-0.88278	-0.88144	-0.8801	-0.87875
-0.8217	-0.85148	-0.85011	-0.84876	-0.84744	-0.88311	-0.88176	-0.88041	-0.87906
-0.822	-0.8518	-0.85043	-0.84908	-0.84777	-0.88344	-0.88207	-0.88071	-0.87936
-0.82231	-0.85213	-0.85075	-0.84941	-0.84809	-0.88376	-0.88238	-0.88101	-0.87965
-0.82263	-0.82128	-0.81995	-0.84973	-0.84842	-0.88409	-0.88269	-0.8813	-0.87995
-0.82295	-0.82161	-0.82027	-0.85007	-0.84875	-0.88442	-0.883	-0.8816	-0.88024
-0.79021	-0.82193	-0.82061	-0.8193	-0.818	-0.88476	-0.88332	-0.88191	-0.88055
-0.79047	-0.82227	-0.82097	-0.81966	-0.81834	-0.88511	-0.88366	-0.88224	-0.88088
-0.79074	-0.82259	-0.82132	-0.82001	-0.81867	-0.85498	-0.85356	-0.85219	-0.88121
-0.791	-0.8229	-0.82163	-0.82033	-0.819	-0.85532	-0.8539	-0.85251	-0.88155
-0.79127	-0.79008	-0.78889	-0.82064	-0.81932	-0.85566	-0.85424	-0.85285	-0.85151
-0.79158	-0.79037	-0.78917	-0.82096	-0.81964	-0.85599	-0.8546	-0.85322	-0.85185
-0.79189	-0.79068	-0.78946	-0.82128	-0.81995	-0.85631	-0.85495	-0.85359	-0.8522
-0.79218	-0.79097	-0.78974	-0.78852	-0.78729	-0.85664	-0.85528	-0.85392	-0.85253
-0.79245	-0.79124	-0.79003	-0.7888	-0.78756	-0.85697	-0.8556	-0.85424	-0.85285
-0.79273	-0.79152	-0.79031	-0.78909	-0.78786	-0.85732	-0.85594	-0.85456	-0.85317
-0.79301	-0.7918	-0.79059	-0.78938	-0.78818	-0.85771	-0.85629	-0.85489	-0.8535
-0.79331	-0.79209	-0.79088	-0.78968	-0.7885	-0.82563	-0.8243	-0.82299	-0.85383
-0.79362	-0.7924	-0.79119	-0.78999	-0.78879	-0.82593	-0.82461	-0.8233	-0.8542
-0.79395	-0.79272	-0.79151	-0.7903	-0.78909	-0.82622	-0.82492	-0.82362	-0.85457
-0.79428	-0.79305	-0.79183	-0.7906	-0.78938	-0.82652	-0.82522	-0.82393	-0.82265
-0.79462	-0.79338	-0.79214	-0.7909	-0.78967	-0.82683	-0.82553	-0.82425	-0.82296
-0.79493	-0.7937	-0.79245	-0.79122	-0.78999	-0.82715	-0.82585	-0.82455	-0.82325
-0.79523	-0.794	-0.79276	-0.79154	-0.79033	-0.82749	-0.82616	-0.82484	-0.82354
-0.79554	-0.79431	-0.79309	-0.79187	-0.79065	-0.82783	-0.82648	-0.82515	-0.82384
-0.79585	-0.79463	-0.79342	-0.7922	-0.79097	-0.82815	-0.8268	-0.82547	-0.82416
-0.79619	-0.79496	-0.79375	-0.79252	-0.79128	-0.82847	-0.82713	-0.8258	-0.82448
-0.79655	-0.7953	-0.79406	-0.79282	-0.79158	-0.8288	-0.82746	-0.82613	-0.82482
-0.79691	-0.79565	-0.79438	-0.79312	-0.79188	-0.82913	-0.82779	-0.82647	-0.82516
-0.76355	-0.79597	-0.7947	-0.79345	-0.79221	-0.82948	-0.82812	-0.82679	-0.82549
-0.76383	-0.79628	-0.79503	-0.79378	-0.79255	-0.82984	-0.82847	-0.82712	-0.82583
-0.76412	-0.76293	-0.76177	-0.79412	-0.79288	-0.83021	-0.82883	-0.82748	-0.82617
-0.76441	-0.76323	-0.76207	-0.79446	-0.79322	-0.83059	-0.82921	-0.82786	-0.82654
-0.69725	-0.76353	-0.76237	-0.79479	-0.79356	-0.83096	-0.82959	-0.82824	-0.8269
-0.69749	-0.76385	-0.76265	-0.7951	-0.79389	-0.83133	-0.82997	-0.82861	-0.82727
-0.69774	-0.76417	-0.76295	-0.76178	-0.76065	-0.8317	-0.83034	-0.82898	-0.82764
-0.698	-0.76451	-0.76329	-0.76211	-0.76095	-0.83204	-0.83069	-0.82934	-0.828
-0.69825	-0.76485	-0.76364	-0.76244	-0.76125	-0.83239	-0.83104	-0.8297	-0.82836
-0.69851	-0.69753	-0.69657	-0.76277	-0.76158	-0.79982	-0.79852	-0.79724	-0.82872
-0.69875	-0.69778	-0.69682	-0.7631	-0.76191	-0.80019	-0.79887	-0.79757	-0.82908
-0.69901	-0.69804	-0.69708	-0.76344	-0.76226	-0.80057	-0.79924	-0.79792	-0.82946
-0.69927	-0.69829	-0.69733	-0.76379	-0.76261	-0.80097	-0.79963	-0.7983	-0.82986

-0.69953	-0.69855	-0.69758	-0.69662	-0.69568	-0.80135	-0.80001	-0.79868	-0.79738
-0.69978	-0.6988	-0.69783	-0.69687	-0.69592	-0.8017	-0.80036	-0.79903	-0.79772
-0.70004	-0.69906	-0.69809	-0.69712	-0.69615	-0.80205	-0.8007	-0.79938	-0.79807
-0.66785	-0.69932	-0.69834	-0.69737	-0.6964	-0.80242	-0.80105	-0.79971	-0.79841
-0.66809	-0.69958	-0.69859	-0.69761	-0.69665	-0.8028	-0.8014	-0.80005	-0.79875
-0.66831	-0.66739	-0.66646	-0.69788	-0.69692	-0.8032	-0.80179	-0.80042	-0.79912
-0.66852	-0.66761	-0.6667	-0.69815	-0.69719	-0.80361	-0.80221	-0.80083	-0.7995
-0.66874	-0.66783	-0.66693	-0.69842	-0.69746	-0.73773	-0.73658	-0.73543	-0.79988
-0.66895	-0.66806	-0.66717	-0.69869	-0.69771	-0.73804	-0.7369	-0.73575	-0.80027
-0.66917	-0.66827	-0.66739	-0.66649	-0.66558	-0.697	-0.69604	-0.69509	-0.7612
-0.66938	-0.66848	-0.66759	-0.6667	-0.6658	-0.69727	-0.69631	-0.69536	-0.76155
-0.6696	-0.66869	-0.66779	-0.6669	-0.66602	-0.69753	-0.69658	-0.69563	-0.76191
-0.66984	-0.66891	-0.668	-0.66711	-0.66623	-0.66536	-0.66448	-0.6636	-0.69493
-0.67008	-0.66914	-0.66822	-0.66733	-0.66645	-0.66557	-0.6647	-0.66382	-0.69519
-0.67031	-0.66937	-0.66845	-0.66755	-0.66667	-0.66579	-0.66491	-0.66403	-0.69545
-0.67054	-0.6696	-0.66868	-0.66778	-0.6669	-0.66602	-0.66512	-0.66424	-0.69571
-0.67078	-0.66983	-0.66892	-0.66802	-0.66713	-0.66623	-0.66533	-0.66445	-0.66359
-0.67104	-0.67008	-0.66916	-0.66824	-0.66734	-0.66644	-0.66555	-0.66466	-0.66381
-0.63783	-0.67034	-0.6694	-0.66848	-0.66756	-0.66666	-0.66577	-0.66489	-0.66403
-0.63807	-0.67061	-0.66966	-0.66872	-0.6678	-0.66689	-0.666	-0.66512	-0.66426
-0.63832	-0.67089	-0.66992	-0.66897	-0.66804	-0.66713	-0.66624	-0.66535	-0.66448
-0.63856	-0.67116	-0.67019	-0.66924	-0.6683	-0.66738	-0.66648	-0.66559	-0.66471
-0.6388	-0.67144	-0.67047	-0.66951	-0.66855	-0.66762	-0.66673	-0.66584	-0.66494
-0.63906	-0.63816	-0.63729	-0.66978	-0.66882	-0.66788	-0.66698	-0.66608	-0.66518
-0.63934	-0.63843	-0.63753	-0.67005	-0.66908	-0.66814	-0.66723	-0.66634	-0.66544
-0.63962	-0.6387	-0.63779	-0.67033	-0.66935	-0.6684	-0.66749	-0.6666	-0.66569
-0.63991	-0.63897	-0.63805	-0.6706	-0.66961	-0.66866	-0.66775	-0.66685	-0.66594
-0.6402	-0.63926	-0.63832	-0.67088	-0.66988	-0.66892	-0.66801	-0.66711	-0.6662
-0.58886	-0.63954	-0.6386	-0.63768	-0.63676	-0.6692	-0.66827	-0.66736	-0.66645
-0.58923	-0.63983	-0.63889	-0.63796	-0.63704	-0.66949	-0.66854	-0.66762	-0.6667
-0.58961	-0.58864	-0.58768	-0.58674	-0.5858	-0.63642	-0.63556	-0.63472	-0.66696
-0.58999	-0.58902	-0.58806	-0.58711	-0.58617	-0.63671	-0.63583	-0.63497	-0.66722
-0.59037	-0.5894	-0.58844	-0.58749	-0.58655	-0.58563	-0.58472	-0.58383	-0.6675
-0.59075	-0.58978	-0.58882	-0.58787	-0.58693	-0.58601	-0.58508	-0.58418	-0.6678
-0.59114	-0.59017	-0.58921	-0.58825	-0.58732	-0.58639	-0.58546	-0.58454	-0.58366
-0.59153	-0.59056	-0.5896	-0.58865	-0.58771	-0.58677	-0.58583	-0.58492	-0.58403
-0.59192	-0.59095	-0.59	-0.58904	-0.58809	-0.58715	-0.58622	-0.58529	-0.58439
-0.59231	-0.59134	-0.59039	-0.58943	-0.58848	-0.58753	-0.5866	-0.58567	-0.58476
-0.5927	-0.59173	-0.59077	-0.58981	-0.58886	-0.58791	-0.58697	-0.58605	-0.58512
-0.55889	-0.59211	-0.59116	-0.5902	-0.58924	-0.58829	-0.58735	-0.58642	-0.58549
-0.55923	-0.59249	-0.59154	-0.59058	-0.58963	-0.58868	-0.58773	-0.58679	-0.58585
-0.55958	-0.59288	-0.59192	-0.59096	-0.59001	-0.58906	-0.58811	-0.58716	-0.58623
-0.55993	-0.59327	-0.5923	-0.59134	-0.59039	-0.58944	-0.58849	-0.58754	-0.5866
-0.56029	-0.55937	-0.55848	-0.59172	-0.59078	-0.58983	-0.58887	-0.58791	-0.58697
-0.56067	-0.55974	-0.55884	-0.59211	-0.59116	-0.59021	-0.58925	-0.5883	-0.58735
-0.56105	-0.56012	-0.5592	-0.59251	-0.59156	-0.5906	-0.58964	-0.58869	-0.58774
-0.56144	-0.5605	-0.55956	-0.5929	-0.59194	-0.59099	-0.59003	-0.58908	-0.58813
-0.56184	-0.56088	-0.55992	-0.5933	-0.59234	-0.59138	-0.59043	-0.58948	-0.58853
-0.56221	-0.56126	-0.5603	-0.55938	-0.55849	-0.59177	-0.59082	-0.58987	-0.58892
-0.56258	-0.56163	-0.56069	-0.55976	-0.55885	-0.59216	-0.59121	-0.59026	-0.58931

-0.56295	-0.56201	-0.56107	-0.56014	-0.55922	-0.59256	-0.59161	-0.59066	-0.5897
-0.56331	-0.56238	-0.56144	-0.56051	-0.55958	-0.59297	-0.592	-0.59105	-0.59009
-0.56368	-0.56274	-0.56181	-0.56088	-0.55995	-0.59339	-0.59241	-0.59144	-0.59049
-0.56406	-0.56311	-0.56217	-0.56124	-0.56031	-0.55941	-0.55852	-0.55765	-0.5909
-0.56444	-0.56349	-0.56254	-0.5616	-0.56068	-0.55978	-0.55889	-0.55801	-0.59131
-0.56484	-0.56388	-0.56293	-0.56198	-0.56105	-0.56014	-0.55924	-0.55836	-0.5575
-0.56525	-0.56429	-0.56333	-0.56238	-0.56143	-0.5605	-0.55959	-0.5587	-0.55784
-0.56564	-0.56468	-0.56373	-0.56277	-0.56182	-0.56088	-0.55996	-0.55906	-0.55819
-0.56602	-0.56506	-0.56411	-0.56316	-0.56221	-0.56127	-0.56034	-0.55942	-0.55854
-0.56641	-0.56545	-0.56449	-0.56354	-0.56259	-0.56165	-0.56072	-0.5598	-0.55889
-0.56681	-0.56584	-0.56487	-0.56392	-0.56297	-0.56203	-0.5611	-0.56017	-0.55926
-0.56722	-0.56624	-0.56526	-0.5643	-0.56335	-0.56241	-0.56147	-0.56055	-0.55963
-0.56764	-0.56665	-0.56568	-0.56471	-0.56376	-0.5628	-0.56186	-0.56092	-0.55999
-0.56806	-0.56708	-0.5661	-0.56513	-0.56417	-0.56321	-0.56224	-0.56129	-0.56036
-0.56847	-0.56748	-0.56651	-0.56554	-0.56457	-0.56361	-0.56264	-0.56168	-0.56074
-0.56887	-0.56788	-0.56691	-0.56593	-0.56497	-0.564	-0.56304	-0.56209	-0.56115
-0.53011	-0.52919	-0.52828	-0.56634	-0.56537	-0.56441	-0.56345	-0.56249	-0.56154
-0.53052	-0.5296	-0.52868	-0.56678	-0.5658	-0.56482	-0.56384	-0.56287	-0.56192
-0.43436	-0.53	-0.52908	-0.52816	-0.52725	-0.56524	-0.56424	-0.56326	-0.5623
-0.43466	-0.53037	-0.52944	-0.52852	-0.52761	-0.56565	-0.56466	-0.56368	-0.56272
-0.43497	-0.53075	-0.52981	-0.52889	-0.52798	-0.56606	-0.56508	-0.56411	-0.56314
-0.4353	-0.45692	-0.45613	-0.52929	-0.52837	-0.52746	-0.52655	-0.52564	-0.56355
-0.43564	-0.45727	-0.45648	-0.52971	-0.52879	-0.52787	-0.52695	-0.52603	-0.56396
-0.43597	-0.43523	-0.43449	-0.45602	-0.45525	-0.52825	-0.52733	-0.52641	-0.52548
-0.43628	-0.43553	-0.43479	-0.45633	-0.45555	-0.52862	-0.5277	-0.52678	-0.52585
-0.43661	-0.43585	-0.43511	-0.45665	-0.45587	-0.45509	-0.45431	-0.45353	-0.52624
-0.43697	-0.43619	-0.43544	-0.457	-0.45621	-0.45543	-0.45465	-0.45388	-0.52666
-0.43732	-0.43653	-0.43578	-0.43504	-0.43432	-0.45577	-0.45499	-0.45422	-0.52707
-0.43764	-0.43684	-0.43609	-0.43535	-0.43462	-0.45607	-0.4553	-0.45452	-0.52745
-0.43797	-0.43715	-0.4364	-0.43565	-0.43492	-0.45638	-0.4556	-0.45482	-0.52782
-0.43835	-0.4375	-0.43673	-0.43597	-0.43523	-0.45671	-0.45592	-0.45515	-0.45437
-0.43878	-0.43789	-0.43709	-0.43631	-0.43557	-0.45705	-0.45627	-0.45549	-0.45471
-0.43925	-0.4383	-0.43746	-0.43666	-0.43591	-0.43518	-0.43446	-0.43375	-0.45506
-0.43975	-0.43876	-0.43785	-0.43702	-0.43626	-0.43552	-0.43479	-0.43408	-0.45541
-0.44027	-0.43922	-0.43825	-0.43738	-0.4366	-0.43585	-0.43512	-0.4344	-0.45574
-0.44077	-0.43968	-0.43864	-0.43772	-0.43692	-0.43615	-0.43541	-0.43469	-0.45604
-0.4413	-0.44015	-0.43906	-0.43809	-0.43724	-0.43645	-0.4357	-0.43498	-0.45635
-0.44185	-0.44068	-0.43954	-0.43853	-0.43762	-0.43679	-0.43603	-0.4353	-0.43458
-0.44243	-0.44124	-0.44008	-0.43901	-0.43804	-0.43715	-0.43637	-0.43564	-0.43491
-0.44302	-0.44182	-0.44064	-0.43954	-0.43849	-0.43755	-0.43674	-0.43598	-0.43524
-0.44362	-0.44241	-0.44124	-0.4401	-0.43898	-0.43798	-0.43712	-0.43632	-0.43557
-0.44422	-0.44301	-0.44184	-0.44067	-0.4395	-0.43845	-0.43753	-0.43669	-0.43591
-0.44483	-0.4436	-0.44242	-0.44124	-0.44006	-0.43897	-0.43797	-0.43706	-0.43626
-0.44543	-0.44418	-0.443	-0.44181	-0.44062	-0.4395	-0.43844	-0.43747	-0.43663
-0.44601	-0.44474	-0.44353	-0.44234	-0.44118	-0.44005	-0.43894	-0.43791	-0.43702
-0.44661	-0.44531	-0.44407	-0.44288	-0.44174	-0.4406	-0.43946	-0.43838	-0.43744
-0.404	-0.44593	-0.44466	-0.44344	-0.44229	-0.44115	-0.43999	-0.43889	-0.43789
-0.40462	-0.44659	-0.44528	-0.44403	-0.44285	-0.44169	-0.44053	-0.43941	-0.43837
-0.36246	-0.40397	-0.40274	-0.40157	-0.40047	-0.44225	-0.44108	-0.43995	-0.43886
-0.36306	-0.40461	-0.40335	-0.40215	-0.40102	-0.44284	-0.44165	-0.4405	-0.43936

-0.36367	-0.36245	-0.36125	-0.40274	-0.40157	-0.40044	-0.39934	-0.39827	-0.43989
-0.36427	-0.36305	-0.36184	-0.40334	-0.40214	-0.40098	-0.39987	-0.39879	-0.44045
-0.36487	-0.36365	-0.36242	-0.40395	-0.40272	-0.40154	-0.40042	-0.39932	-0.39821
-0.36546	-0.36424	-0.36301	-0.40457	-0.40332	-0.40212	-0.40098	-0.39986	-0.39874
-0.36605	-0.36484	-0.36361	-0.40519	-0.40393	-0.40272	-0.40155	-0.40041	-0.39929
-0.31684	-0.36542	-0.3642	-0.363	-0.3618	-0.40331	-0.40212	-0.40096	-0.39984
-0.31727	-0.36599	-0.36479	-0.36359	-0.36238	-0.40391	-0.4027	-0.40152	-0.40039
-0.3177	-0.36656	-0.36537	-0.36417	-0.36296	-0.36177	-0.36062	-0.35951	-0.40095
-0.31813	-0.36713	-0.36595	-0.36475	-0.36354	-0.36235	-0.36119	-0.36006	-0.40152
-0.31856	-0.31767	-0.31677	-0.36534	-0.36412	-0.36293	-0.36176	-0.36061	-0.35951
-0.31901	-0.31812	-0.31722	-0.36591	-0.36471	-0.36351	-0.36232	-0.36116	-0.36005
-0.31946	-0.31856	-0.31767	-0.36649	-0.36529	-0.36409	-0.3629	-0.36172	-0.3606
-0.31991	-0.31901	-0.31811	-0.36707	-0.36587	-0.36468	-0.36348	-0.3623	-0.36118
-0.32037	-0.31946	-0.31856	-0.36765	-0.36645	-0.36526	-0.36406	-0.36289	-0.36177
-0.32084	-0.31992	-0.31901	-0.31809	-0.31718	-0.36584	-0.36465	-0.36348	-0.36236
-0.32132	-0.32038	-0.31946	-0.31854	-0.31763	-0.36643	-0.36524	-0.36407	-0.36294
-0.3218	-0.32085	-0.31992	-0.31899	-0.31808	-0.36702	-0.36584	-0.36468	-0.36354
-0.3223	-0.32133	-0.32038	-0.31944	-0.31852	-0.36764	-0.36646	-0.3653	-0.36415
-0.32279	-0.32182	-0.32085	-0.3199	-0.31898	-0.31807	-0.31718	-0.3163	-0.36475
-0.32328	-0.3223	-0.32132	-0.32037	-0.31944	-0.31853	-0.31764	-0.31676	-0.36534
-0.32378	-0.32279	-0.32181	-0.32085	-0.31991	-0.31899	-0.31811	-0.31722	-0.36593
-0.32427	-0.32328	-0.32229	-0.32134	-0.3204	-0.31949	-0.31859	-0.31769	-0.31678
-0.32476	-0.32377	-0.32278	-0.32183	-0.32091	-0.31999	-0.31908	-0.31817	-0.31724
-0.32525	-0.32425	-0.32327	-0.32232	-0.3214	-0.32048	-0.31957	-0.31865	-0.31771
-0.32573	-0.32473	-0.32375	-0.3228	-0.32189	-0.32097	-0.32005	-0.31913	-0.31818
-0.32622	-0.32522	-0.32424	-0.3233	-0.32238	-0.32146	-0.32054	-0.31961	-0.31866
-0.32671	-0.32572	-0.32476	-0.32381	-0.32288	-0.32194	-0.32102	-0.32008	-0.31914
-0.28698	-0.32623	-0.32527	-0.32432	-0.32337	-0.32243	-0.32149	-0.32056	-0.31962
-0.28745	-0.32676	-0.32578	-0.32481	-0.32385	-0.32291	-0.32197	-0.32103	-0.3201
-0.28794	-0.3273	-0.32628	-0.32529	-0.32433	-0.32338	-0.32244	-0.32151	-0.3206
-0.28839	-0.32782	-0.3268	-0.3258	-0.32482	-0.32386	-0.32292	-0.322	-0.32109
-0.28884	-0.32834	-0.32732	-0.32632	-0.32531	-0.32434	-0.32341	-0.3225	-0.32159
-0.2893	-0.2884	-0.28751	-0.32683	-0.32582	-0.32484	-0.32391	-0.323	-0.32209
-0.28977	-0.28886	-0.28796	-0.32735	-0.32634	-0.32537	-0.32442	-0.32349	-0.32257
-0.29025	-0.28933	-0.28843	-0.28754	-0.28666	-0.3259	-0.32493	-0.32399	-0.32306
-0.29075	-0.28983	-0.28892	-0.28803	-0.28714	-0.32643	-0.32545	-0.32448	-0.32356
-0.29126	-0.29033	-0.28942	-0.28852	-0.28762	-0.32697	-0.32597	-0.32499	-0.32406
-0.29179	-0.29085	-0.28992	-0.289	-0.28809	-0.32751	-0.32651	-0.32552	-0.32457
-0.29232	-0.29137	-0.29042	-0.28949	-0.28857	-0.32805	-0.32705	-0.32606	-0.3251
-0.24185	-0.29188	-0.29092	-0.28999	-0.28906	-0.28816	-0.28727	-0.28639	-0.32562
-0.24224	-0.29238	-0.29143	-0.29049	-0.28957	-0.28866	-0.28776	-0.28687	-0.32614
-0.24263	-0.29288	-0.29194	-0.29101	-0.29008	-0.28916	-0.28824	-0.28734	-0.28644
-0.24302	-0.2934	-0.29246	-0.29153	-0.29058	-0.28965	-0.28873	-0.28782	-0.28692
-0.19239	-0.24266	-0.24193	-0.29204	-0.29109	-0.29014	-0.28922	-0.2883	-0.28741
-0.19271	-0.24308	-0.24232	-0.29254	-0.29159	-0.29065	-0.28971	-0.2888	-0.2879
-0.19304	-0.2435	-0.24272	-0.29304	-0.29209	-0.29115	-0.29022	-0.2893	-0.28839
-0.09881	-0.24393	-0.24313	-0.29355	-0.29261	-0.29167	-0.29073	-0.2898	-0.2889
-0.09897	-0.24437	-0.24355	-0.29408	-0.29314	-0.2922	-0.29125	-0.29031	-0.2894
-0.09913	-0.0988	-0.09847	-0.24318	-0.24243	-0.29272	-0.29177	-0.29084	-0.28992
-0.0993	-0.09896	-0.09863	-0.24361	-0.24284	-0.29323	-0.29231	-0.29139	-0.29044

-0.09946	-0.09913	-0.09879	-0.1518	-0.15127	-0.24249	-0.24176	-0.24102	-0.29097
-0.09963	-0.09929	-0.09897	-0.15209	-0.15155	-0.24291	-0.24216	-0.24142	-0.2915
-0.0998	-0.09947	-0.09914	-0.09881	-0.09848	-0.1513	-0.1508	-0.1503	-0.29202
-0.09998	-0.09964	-0.09931	-0.09898	-0.09865	-0.15159	-0.15107	-0.15056	-0.29255
-0.10017	-0.09982	-0.09948	-0.09915	-0.09883	-0.15189	-0.15135	-0.15082	-0.29308
-0.10034	-0.09999	-0.09965	-0.09931	-0.09899	-0.15218	-0.15163	-0.1511	-0.24233
-0.10052	-0.10016	-0.09982	-0.09948	-0.09915	-0.15247	-0.15192	-0.15139	-0.24274
-0.10071	-0.10035	-0.1	-0.09965	-0.09931	-0.09898	-0.09866	-0.09835	-0.15114
-0.10091	-0.10055	-0.10018	-0.09982	-0.09948	-0.09915	-0.09882	-0.0985	-0.15143
-0.04822	-0.10074	-0.10037	-0.10001	-0.09965	-0.09931	-0.09898	-0.09866	-0.15172
-0.04827	-0.10092	-0.10056	-0.10019	-0.09983	-0.09948	-0.09915	-0.09882	-0.152
-0.04832	-0.10111	-0.10075	-0.10038	-0.10001	-0.09966	-0.09932	-0.09899	-0.1523
	-0.1013	-0.10094	-0.10057	-0.1002	-0.09984	-0.09949	-0.09916	-0.09884
	-0.1015	-0.10114	-0.10077	-0.10039	-0.10003	-0.09967	-0.09933	-0.09901
	-0.04836	-0.04818	-0.10096	-0.10059	-0.10022	-0.09986	-0.09951	-0.09918
	-0.04843	-0.04824	-0.10117	-0.1008	-0.10043	-0.10005	-0.09969	-0.09935
			-0.04813	-0.04796	-0.10062	-0.10025	-0.09988	-0.09953
			-0.0482	-0.04802	-0.10082	-0.10045	-0.10008	-0.09972
			-0.04827	-0.04808	-0.0479	-0.04774	-0.04757	-0.09991
			-0.04834	-0.04815	-0.04796	-0.0478	-0.04764	-0.10011
			-0.04841	-0.04822	-0.04803	-0.04786	-0.0477	-0.1003
					-0.04811	-0.04793	-0.04775	-0.04758
					-0.04819	-0.04799	-0.0478	-0.04763
								-0.0477
								-0.04778
								-0.04785
								-0.04792

								-0.10789
								-0.10767
								-0.10745
								-0.10723
								-0.107
					-0.10769	-0.10738	-0.10708	-0.25351
					-0.10747	-0.10716	-0.10686	-0.25291
					-0.10725	-0.10694	-0.10663	-0.25231
					-0.10705	-0.10673	-0.10641	-0.25171
			-0.10747	-0.10716	-0.25424	-0.25317	-0.25211	-0.35486
			-0.10724	-0.10694	-0.25363	-0.25257	-0.2515	-0.35406
			-0.10702	-0.10671	-0.25301	-0.25195	-0.25089	-0.35326
	-0.10741	-0.1071	-0.25438	-0.2534	-0.30637	-0.30517	-0.30398	-0.48288
	-0.10718	-0.10688	-0.25376	-0.25277	-0.30565	-0.30446	-0.30329	-0.48196
	-0.10697	-0.10666	-0.25314	-0.25215	-0.48598	-0.48429	-0.48263	-0.52041
	-0.10676	-0.10644	-0.25252	-0.25153	-0.48499	-0.48338	-0.4818	-0.51949
-0.10687	-0.25399	-0.25293	-0.39261	-0.39125	-0.48402	-0.48248	-0.48097	-0.60634
-0.10665	-0.25338	-0.25233	-0.39181	-0.39045	-0.48305	-0.48157	-0.48012	-0.60529
-0.10643	-0.25277	-0.25174	-0.39104	-0.38967	-0.48212	-0.48067	-0.47926	-0.60424
-0.15789	-0.39305	-0.39166	-0.48444	-0.48281	-0.52054	-0.51894	-0.51739	-0.68483
-0.15743	-0.39226	-0.3909	-0.48356	-0.48197	-0.51963	-0.51801	-0.51644	-0.68366
-0.30579	-0.48583	-0.48423	-0.48267	-0.48114	-0.68848	-0.68643	-0.68441	-0.71764
-0.30511	-0.48488	-0.4833	-0.48177	-0.48032	-0.68736	-0.68529	-0.68324	-0.71635
-0.39127	-0.48396	-0.48239	-0.5201	-0.51853	-0.72167	-0.71943	-0.71719	-0.80105
-0.39053	-0.48308	-0.48153	-0.51914	-0.51758	-0.72035	-0.71813	-0.71592	-0.79961
-0.48379	-0.48222	-0.48068	-0.68773	-0.68576	-0.7652	-0.76285	-0.76051	-0.87746
-0.4829	-0.48136	-0.47984	-0.68655	-0.68456	-0.76377	-0.76148	-0.7592	-0.87596
-0.48202	-0.48051	-0.47902	-0.68538	-0.68338	-0.76236	-0.76012	-0.75789	-0.87446
-0.48117	-0.60384	-0.60199	-0.76569	-0.76332	-0.80108	-0.79873	-0.79639	-0.87291
-0.48035	-0.60279	-0.60097	-0.76436	-0.76199	-0.79968	-0.79729	-0.79492	-0.87135
-0.6036	-0.68663	-0.68443	-0.80316	-0.80072	-0.87754	-0.87494	-0.87237	-0.86985
-0.60257	-0.68538	-0.68314	-0.8017	-0.79932	-0.87606	-0.87346	-0.87089	-0.86842
-0.65031	-0.80524	-0.8027	-0.8796	-0.87707	-0.87454	-0.87198	-0.86945	-1.04839
-0.64908	-0.80373	-0.80124	-0.87805	-0.87548	-0.87295	-0.87048	-0.86805	-1.04644
-0.68363	-0.8433	-0.84078	-0.87651	-0.87391	-1.05462	-1.05122	-1.04788	-1.04449
-0.68236	-0.84182	-0.83932	-0.87501	-0.87242	-1.05248	-1.04912	-1.04584	-1.04249
-0.68113	-0.84036	-0.83788	-0.87352	-0.87097	-1.05041	-1.04707	-1.0438	-1.0405
-0.84143	-0.87716	-0.87459	-0.90739	-0.90476	-1.04845	-1.04511	-1.04184	-1.129
-0.84	-0.87568	-0.87311	-0.90585	-0.90331	-1.04658	-1.04323	-1.03994	-1.12699
-0.87677	-0.90962	-0.90695	-1.05138	-1.048	-1.06923	-1.06588	-1.06256	-1.14847
-0.87527	-0.90807	-0.90545	-1.04941	-1.04605	-1.06727	-1.06396	-1.06068	-1.14643
-0.90917	-1.05437	-1.05088	-1.07203	-1.06866	-1.15504	-1.15146	-1.14793	-1.17734
-0.90764	-1.05234	-1.0489	-1.07007	-1.06675	-1.15296	-1.14937	-1.14582	-1.17521
-0.90614	-1.05035	-1.04695	-1.06813	-1.06485	-1.15089	-1.14729	-1.14373	-1.17309
-0.90466	-1.07293	-1.06957	-1.06623	-1.06292	-1.18186	-1.17819	-1.17457	-1.171

-0.9032	-1.07095	-1.06766	-1.06434	-1.06098	-1.17966	-1.17607	-1.17252	-1.16891
-1.07238	-1.069	-1.06572	-1.15176	-1.14815	-1.17754	-1.17398	-1.17044	-1.22564
-1.07045	-1.06709	-1.06375	-1.14961	-1.14608	-1.17546	-1.17189	-1.16834	-1.22347
-1.0685	-1.08987	-1.08635	-1.18053	-1.17693	-1.17336	-1.16981	-1.16625	-1.28301
-1.06652	-1.08779	-1.08433	-1.17838	-1.1748	-1.17124	-1.16771	-1.16419	-1.28086
-1.08921	-1.18352	-1.17986	-1.17624	-1.17268	-1.29004	-1.28624	-1.28248	-1.27872
-1.08719	-1.18135	-1.1777	-1.17411	-1.1706	-1.28781	-1.28402	-1.28026	-1.27653
-1.08518	-1.17921	-1.17557	-1.17201	-1.16852	-1.28561	-1.28183	-1.27808	-1.27437
-1.11727	-1.17702	-1.17347	-1.2609	-1.25718	-1.28337	-1.27966	-1.27598	-1.27232
-1.11515	-1.17485	-1.17139	-1.25874	-1.25499	-1.28114	-1.27751	-1.27392	-1.27031
-1.1764	-1.23696	-1.23329	-1.28646	-1.28268	-1.27898	-1.27537	-1.27182	-1.26824
-1.17445	-1.23487	-1.23112	-1.28421	-1.28052	-1.27687	-1.27326	-1.26967	-1.26613
-1.2366	-1.25976	-1.25593	-1.28205	-1.27839	-1.27476	-1.27114	-1.26756	-1.26403
-1.23446	-1.25768	-1.2539	-1.27999	-1.27628	-1.27262	-1.26903	-1.26548	-1.26195
-1.23231	-1.25558	-1.25188	-1.27796	-1.27421	-1.27052	-1.26694	-1.2634	-1.34913
-1.23014	-1.25342	-1.24981	-1.27591	-1.27218	-1.2685	-1.26486	-1.26128	-1.34681
-1.22801	-1.25127	-1.24773	-1.27384	-1.27017	-1.2665	-1.26281	-1.25917	-1.34452
-1.25288	-1.24922	-1.24563	-1.27171	-1.26808	-1.29639	-1.29265	-1.28894	-1.34232
-1.25091	-1.24722	-1.24354	-1.26954	-1.26596	-1.29428	-1.29062	-1.28697	-1.34018
-1.24889	-1.27491	-1.27113	-1.29944	-1.29576	-1.34993	-1.34596	-1.34201	-1.3876
-1.24683	-1.27286	-1.26912	-1.29736	-1.29359	-1.34755	-1.34367	-1.33986	-1.38545
-1.24475	-1.30285	-1.29908	-1.2953	-1.29149	-1.39503	-1.39108	-1.3872	-1.4131
-1.24261	-1.30064	-1.29695	-1.29325	-1.28949	-1.39285	-1.38884	-1.3849	-1.4108
-1.24051	-1.29844	-1.29483	-1.2912	-1.28753	-1.39073	-1.38666	-1.38266	-1.40851
-1.27101	-1.29633	-1.29272	-1.39676	-1.39269	-1.41856	-1.41441	-1.4103	-1.43177
-1.269	-1.29427	-1.29063	-1.39449	-1.39052	-1.41643	-1.4123	-1.4082	-1.42958
-1.29596	-1.40039	-1.39625	-1.42222	-1.41824	-1.41424	-1.41017	-1.40609	-1.47601
-1.29393	-1.39813	-1.39398	-1.41991	-1.41596	-1.41202	-1.40801	-1.404	-1.47384
-1.34349	-1.42599	-1.42177	-1.41766	-1.4137	-1.48402	-1.47991	-1.4758	-1.47165
-1.34142	-1.42379	-1.41961	-1.41549	-1.41143	-1.48159	-1.47754	-1.47352	-1.46943
-1.42583	-1.42161	-1.41747	-1.46296	-1.45872	-1.47923	-1.47519	-1.47122	-1.46719
-1.42365	-1.41947	-1.41533	-1.46076	-1.45653	-1.477	-1.47289	-1.46887	-1.46488
-1.42147	-1.41734	-1.4132	-1.45857	-1.45437	-1.47482	-1.47063	-1.46655	-1.46263
-1.41927	-1.46484	-1.46061	-1.48106	-1.47682	-1.47262	-1.46841	-1.46433	-1.48003
-1.41706	-1.46262	-1.45842	-1.47886	-1.47464	-1.47045	-1.46623	-1.46219	-1.478
-1.41486	-1.48507	-1.48088	-1.47668	-1.47246	-1.48792	-1.48366	-1.47961	-1.52528
-1.41266	-1.48279	-1.47866	-1.47449	-1.47028	-1.48574	-1.48153	-1.47755	-1.52327
-1.4599	-1.48049	-1.47641	-1.47229	-1.46809	-1.53306	-1.52883	-1.52486	-1.54255
-1.45757	-1.47813	-1.47412	-1.47005	-1.46587	-1.53079	-1.5268	-1.52309	-1.54091
-1.47988	-1.47581	-1.47184	-1.53704	-1.53269	-1.52858	-1.52484	-1.52141	-1.53935
-1.47763	-1.47356	-1.4696	-1.53469	-1.5304	-1.52641	-1.52292	-1.51973	-1.53782
-1.47541	-1.47135	-1.46739	-1.53239	-1.5282	-1.52436	-1.52109	-1.51812	-1.53634
-1.47318	-1.53835	-1.53419	-1.53015	-1.5262	-1.52258	-1.51946	-1.51661	-1.54758
-1.47096	-1.53601	-1.53187	-1.52798	-1.52434	-1.52099	-1.51797	-1.51518	-1.54615
-1.53792	-1.53371	-1.52963	-1.52589	-1.52252	-1.53231	-1.52941	-1.52669	-1.54476
-1.53558	-1.53143	-1.52745	-1.52388	-1.52074	-1.53071	-1.52795	-1.52532	-1.5434
-1.53329	-1.52924	-1.52544	-1.53492	-1.53196	-1.52918	-1.52652	-1.52396	-1.54205
-1.53101	-1.52714	-1.52362	-1.53328	-1.53042	-1.52772	-1.52512	-1.5226	-1.54069
-1.52884	-1.52517	-1.52191	-1.53173	-1.52895	-1.5263	-1.52374	-1.52125	-1.53933
-1.52686	-1.5362	-1.5331	-1.5302	-1.52752	-1.54559	-1.54302	-1.54049	-1.55892

-1.52502	-1.5345	-1.53152	-1.52872	-1.52612	-1.54422	-1.54166	-1.53914	-1.55756
-1.5361	-1.53289	-1.53002	-1.5273	-1.52474	-1.56381	-1.56125	-1.55872	-1.6035
-1.53446	-1.53138	-1.52859	-1.52592	-1.52339	-1.56243	-1.55988	-1.55736	-1.60204
-1.53284	-1.52989	-1.52717	-1.54583	-1.5433	-1.56106	-1.55852	-1.556	-1.60059
-1.53123	-1.5284	-1.52578	-1.54448	-1.54196	-1.55968	-1.55715	-1.55464	-1.59915
-1.52969	-1.54824	-1.54568	-1.54314	-1.54062	-1.55831	-1.55579	-1.55328	-1.66454
-1.52823	-1.54684	-1.54431	-1.54179	-1.53928	-1.55695	-1.55442	-1.55192	-1.66296
-1.52682	-1.54546	-1.54295	-1.54045	-1.53794	-1.55558	-1.55306	-1.55058	-1.6614
-1.5467	-1.54411	-1.54161	-1.55929	-1.55674	-1.57329	-1.57075	-1.56825	-1.65988
-1.54533	-1.54279	-1.54028	-1.55793	-1.55538	-1.57192	-1.56941	-1.56696	-1.6584
-1.54397	-1.54145	-1.53894	-1.5757	-1.5731	-1.66551	-1.66258	-1.65972	-1.65695
-1.5426	-1.5401	-1.53761	-1.57433	-1.57174	-1.66394	-1.66104	-1.65824	-1.65555
-1.54125	-1.53877	-1.53631	-1.57302	-1.57046	-1.68112	-1.67826	-1.67549	-1.69551
-1.53991	-1.53746	-1.53505	-1.57178	-1.56927	-1.67977	-1.67694	-1.67418	-1.6941
-1.53861	-1.59531	-1.59284	-1.59038	-1.58792	-1.67841	-1.6756	-1.67286	-1.69268
-1.53739	-1.59403	-1.59152	-1.58906	-1.58664	-1.67696	-1.67418	-1.67146	-1.69122
-1.53618	-1.59276	-1.59021	-1.58774	-1.58535	-1.67549	-1.67274	-1.67005	-1.68975
-1.55531	-1.59144	-1.5889	-1.65882	-1.65608	-1.69679	-1.69389	-1.69105	-1.68827
-1.55398	-1.5901	-1.58761	-1.65737	-1.65464	-1.69524	-1.69236	-1.68954	-1.68679
-1.57182	-1.58877	-1.58632	-1.65593	-1.65321	-1.69373	-1.69088	-1.68808	-1.68535
-1.57049	-1.58744	-1.58504	-1.65449	-1.65181	-1.69224	-1.68944	-1.68668	-1.68395
-1.56923	-1.60893	-1.60646	-1.69655	-1.69366	-1.69082	-1.68805	-1.68532	-1.6826
-1.56809	-1.60775	-1.60529	-1.69517	-1.69229	-1.68948	-1.68674	-1.68403	-1.68132
-1.56695	-1.60657	-1.60412	-1.69378	-1.69093	-1.68814	-1.68541	-1.68273	-1.68003
-1.58505	-1.6053	-1.60286	-1.69232	-1.6895	-1.68673	-1.68402	-1.68133	-1.67864
-1.58379	-1.604	-1.60159	-1.69083	-1.68805	-1.68531	-1.68259	-1.67991	-1.67724
-1.60518	-1.67472	-1.6719	-1.68936	-1.68661	-1.68389	-1.68118	-1.6785	-1.67586
-1.60394	-1.67325	-1.67044	-1.68791	-1.68518	-1.68248	-1.67978	-1.67711	-1.67451
-1.60272	-1.67186	-1.66907	-1.68653	-1.68383	-1.68114	-1.67844	-1.67579	-1.67323
-1.60154	-1.67054	-1.66779	-1.68525	-1.68255	-1.67987	-1.67719	-1.67455	-1.67202
-1.60035	-1.6692	-1.66648	-1.68394	-1.68124	-1.67857	-1.67592	-1.67331	-1.67081
-1.59912	-1.66777	-1.66509	-1.68256	-1.67986	-1.6772	-1.67459	-1.67204	-1.66955
-1.59792	-1.66637	-1.66371	-1.68118	-1.67848	-1.67585	-1.67329	-1.67078	-1.66829
-1.66777	-1.68525	-1.68256	-1.67988	-1.6772	-1.67458	-1.67206	-1.66959	-1.67457
-1.6665	-1.68397	-1.68128	-1.67861	-1.67594	-1.67335	-1.67087	-1.66842	-1.67338
-1.66516	-1.68263	-1.67994	-1.67728	-1.67466	-1.69647	-1.69398	-1.69152	-1.7102
-1.66378	-1.68123	-1.67855	-1.67592	-1.67336	-1.6952	-1.69272	-1.69026	-1.70888
-1.66245	-1.67989	-1.67722	-1.69901	-1.69647	-1.69397	-1.6915	-1.68904	-1.76926
-1.6612	-1.67862	-1.67596	-1.69776	-1.69526	-1.69279	-1.69034	-1.68788	-1.76787
-1.65993	-1.70176	-1.69909	-1.69652	-1.69405	-1.73105	-1.72839	-1.72572	-1.76647
-1.65862	-1.70043	-1.69781	-1.69527	-1.6928	-1.72969	-1.72703	-1.72436	-1.76503
-1.65732	-1.69911	-1.69654	-1.69402	-1.69156	-1.72833	-1.72566	-1.72299	-1.76359
-1.70049	-1.71624	-1.71367	-1.71113	-1.70864	-1.7709	-1.768	-1.76511	-1.76218
-1.69924	-1.715	-1.71246	-1.70994	-1.70745	-1.76954	-1.76665	-1.76375	-1.76079
-1.71639	-1.71378	-1.71123	-1.7298	-1.72714	-1.76812	-1.76523	-1.76233	-1.77383
-1.71518	-1.71257	-1.70999	-1.72845	-1.72577	-1.76664	-1.76376	-1.76087	-1.77241
-1.71395	-1.71135	-1.70876	-1.77101	-1.76811	-1.77964	-1.77676	-1.77388	-1.77103
-1.71265	-1.71011	-1.70757	-1.76968	-1.76676	-1.77829	-1.7754	-1.77253	-1.76969
-1.71138	-1.70888	-1.70639	-1.78277	-1.77986	-1.77695	-1.77404	-1.77117	-1.76836
-1.71019	-1.70769	-1.7052	-1.78143	-1.77853	-1.77562	-1.77267	-1.76975	-1.76698

-1.70903	-1.70652	-1.70401	-1.78008	-1.77718	-1.77427	-1.77128	-1.76835	-1.76561
-1.70787	-1.76634	-1.76345	-1.77866	-1.77576	-1.77286	-1.76991	-1.76702	-1.76433
-1.70672	-1.76501	-1.76204	-1.7772	-1.77428	-1.77141	-1.76854	-1.76574	-1.76311
-1.72065	-1.78177	-1.77876	-1.7758	-1.77288	-1.77003	-1.76724	-1.76452	-1.76191
-1.71942	-1.78036	-1.77739	-1.77446	-1.77155	-1.76872	-1.766	-1.76334	-1.76075
-1.76379	-1.77897	-1.77604	-1.77314	-1.77025	-1.76744	-1.76477	-1.76216	-1.77124
-1.76244	-1.77762	-1.77471	-1.77182	-1.76895	-1.76617	-1.76355	-1.76098	-1.76999
-1.76112	-1.7763	-1.77339	-1.77053	-1.76768	-1.76492	-1.76233	-1.75977	-1.76872
-1.7598	-1.77497	-1.77208	-1.76924	-1.76645	-1.76373	-1.7611	-1.75851	-1.76741
-1.7585	-1.77365	-1.77077	-1.76797	-1.76524	-1.76255	-1.75988	-1.75724	-1.7661
-1.77524	-1.77233	-1.76946	-1.76666	-1.76397	-1.7613	-1.75864	-1.756	-1.76483
-1.77393	-1.77103	-1.76814	-1.76535	-1.76266	-1.76002	-1.7574	-1.75479	-1.76358
-1.77262	-1.76972	-1.76684	-1.76406	-1.76139	-1.75876	-1.75617	-1.75359	-1.76233
-1.7713	-1.76842	-1.76556	-1.7628	-1.76015	-1.75754	-1.75496	-1.75238	-1.76109
-1.76998	-1.76712	-1.76429	-1.76156	-1.75894	-1.76793	-1.76523	-1.76254	-1.80624
-1.76863	-1.76581	-1.76305	-1.76036	-1.75775	-1.7667	-1.764	-1.7613	-1.80482
-1.76728	-1.76451	-1.76181	-1.75916	-1.75657	-1.76548	-1.76278	-1.76007	-1.80341
-1.76596	-1.76321	-1.76057	-1.75796	-1.75538	-1.76425	-1.76155	-1.75883	-1.80203
-1.76465	-1.76194	-1.75934	-1.75675	-1.75418	-1.76302	-1.76032	-1.7576	-1.80066
-1.76339	-1.76071	-1.75813	-1.75555	-1.75299	-1.76178	-1.75906	-1.75632	-1.79924
-1.76218	-1.75952	-1.75695	-1.75437	-1.7518	-1.76053	-1.75776	-1.75499	-1.79779
-1.76098	-1.75835	-1.75577	-1.75319	-1.75061	-1.80577	-1.8026	-1.79945	-1.82972
-1.75979	-1.75717	-1.7546	-1.75202	-1.74942	-1.80441	-1.80123	-1.79808	-1.82836
-1.75861	-1.756	-1.75343	-1.75084	-1.74823	-1.83647	-1.83323	-1.83003	-1.84473
-1.75744	-1.75482	-1.75224	-1.74964	-1.74704	-1.83508	-1.83183	-1.82864	-1.84337
-1.75628	-1.75365	-1.75105	-1.74845	-1.74585	-1.83369	-1.83046	-1.82728	-1.84204
-1.75513	-1.78738	-1.7847	-1.82791	-1.82491	-1.83231	-1.82912	-1.826	-1.86427
-1.75399	-1.78618	-1.78347	-1.82653	-1.82354	-1.83094	-1.82782	-1.82477	-1.86296
-1.78774	-1.78502	-1.78227	-1.82517	-1.82218	-1.82957	-1.82652	-1.82354	-1.86165
-1.78663	-1.78389	-1.78109	-1.82382	-1.82082	-1.82821	-1.82522	-1.8223	-1.86034
-1.78549	-1.82878	-1.82558	-1.82249	-1.8195	-1.85134	-1.8481	-1.84495	-1.85902
-1.78429	-1.82746	-1.82425	-1.82117	-1.81821	-1.84997	-1.84677	-1.84365	-1.85769
-1.78307	-1.82611	-1.82292	-1.81986	-1.81694	-1.84864	-1.84547	-1.84238	-1.85639
-1.78186	-1.82477	-1.82159	-1.81856	-1.81569	-1.86458	-1.86141	-1.85828	-1.85513
-1.78064	-1.82342	-1.82026	-1.81726	-1.81446	-1.86329	-1.86017	-1.85707	-1.85389
-1.82521	-1.8221	-1.81897	-1.816	-1.81324	-1.86197	-1.85887	-1.85577	-1.85259
-1.82389	-1.8208	-1.81771	-1.81478	-1.81203	-1.86063	-1.85753	-1.85443	-1.85125
-1.82258	-1.81953	-1.81651	-1.83831	-1.83528	-1.8593	-1.8562	-1.85309	-1.84992
-1.82129	-1.81829	-1.81538	-1.83715	-1.83405	-1.85797	-1.85486	-1.85176	-1.84859
-1.82001	-1.81706	-1.81424	-1.85334	-1.85014	-1.85665	-1.85353	-1.85043	-1.86894
-1.81873	-1.81582	-1.81305	-1.85207	-1.84884	-1.85532	-1.8522	-1.84912	-1.86759
-1.81749	-1.8146	-1.81185	-1.85077	-1.84755	-1.854	-1.85088	-1.84781	-1.86624
-1.81634	-1.84842	-1.84527	-1.85923	-1.85596	-1.85275	-1.84961	-1.84652	-1.86487
-1.81525	-1.84723	-1.84399	-1.85791	-1.8547	-1.85152	-1.84835	-1.84523	-1.8635
-1.81408	-1.84596	-1.84269	-1.85657	-1.8534	-1.87204	-1.86868	-1.86535	-1.86213
-1.81286	-1.84464	-1.84137	-1.85523	-1.85208	-1.87065	-1.86729	-1.86398	-1.86076
-1.8365	-1.86048	-1.85716	-1.85392	-1.85078	-1.86929	-1.86596	-1.86266	-1.85945
-1.83522	-1.85916	-1.85588	-1.85267	-1.84952	-1.86795	-1.86468	-1.86144	-1.85821
-1.83397	-1.85787	-1.85463	-1.87328	-1.86994	-1.86664	-1.86341	-1.86021	-1.88229
-1.83278	-1.85665	-1.85339	-1.87198	-1.86866	-1.86538	-1.86214	-1.85892	-1.88104

-1.8316	-1.85543	-1.85216	-1.87067	-1.86739	-1.86413	-1.86086	-1.85762	-1.87977
-1.84759	-1.85414	-1.85089	-1.86933	-1.86607	-1.88829	-1.88496	-1.88167	-1.87845
-1.84626	-1.85282	-1.8496	-1.86797	-1.86473	-1.88694	-1.88362	-1.88032	-1.87711
-1.84497	-1.85154	-1.84836	-1.8922	-1.8889	-1.88561	-1.88231	-1.87904	-1.87583
-1.8437	-1.85029	-1.84717	-1.89094	-1.88759	-1.88429	-1.88104	-1.87782	-1.87461
-1.84248	-1.89633	-1.893	-1.88967	-1.88632	-1.88301	-1.87978	-1.87659	-1.88893
-1.84133	-1.89507	-1.89171	-1.88838	-1.88507	-1.88179	-1.87855	-1.87534	-1.88763
-1.84017	-1.89382	-1.89042	-1.88709	-1.88383	-1.88058	-1.87733	-1.8741	-1.88634
-1.8653	-1.89253	-1.88917	-1.88585	-1.88257	-1.87932	-1.87609	-1.8729	-1.88508
-1.86408	-1.89123	-1.88792	-1.88462	-1.88131	-1.87805	-1.87486	-1.8717	-1.88381
-1.8629	-1.88996	-1.88664	-1.88334	-1.88006	-1.89257	-1.88919	-1.88585	-1.88251
-1.86175	-1.8887	-1.88534	-1.88204	-1.87883	-1.8913	-1.8879	-1.88452	-1.88119
-1.88162	-1.88743	-1.88406	-1.88078	-1.87759	-1.89001	-1.88662	-1.88324	-1.87993
-1.88032	-1.88613	-1.88282	-1.87956	-1.87634	-1.88868	-1.88535	-1.88204	-1.87875
-1.87902	-1.88484	-1.88158	-1.89417	-1.89075	-1.88738	-1.88409	-1.88082	-1.87754
-1.87777	-1.88358	-1.88032	-1.89286	-1.88949	-1.88615	-1.88283	-1.87954	-1.87625
-1.87651	-1.88233	-1.87906	-1.89155	-1.88822	-1.88491	-1.88158	-1.87826	-1.87497
-1.87524	-1.88105	-1.87782	-1.89025	-1.88692	-1.88361	-1.88032	-1.87705	-1.87377
-1.87396	-1.87978	-1.87659	-1.88896	-1.88559	-1.88228	-1.87906	-1.87586	-1.87261
-1.87274	-1.87854	-1.87533	-1.88764	-1.88429	-1.881	-1.87779	-1.87462	-1.8714
-1.87156	-1.87732	-1.87407	-1.88631	-1.88301	-1.87975	-1.87653	-1.87333	-1.87015
-1.87037	-1.88298	-1.87956	-1.87623	-1.87299	-1.8785	-1.87527	-1.87207	-1.86891
-1.86917	-1.88176	-1.87834	-1.875	-1.87173	-1.87721	-1.87401	-1.87085	-1.86768
-1.86795	-1.88051	-1.87712	-1.87378	-1.87049	-1.87596	-1.87277	-1.86962	-1.86645
-1.86668	-1.87921	-1.87588	-1.87257	-1.86928	-1.87475	-1.87153	-1.86836	-1.86522
-1.86541	-1.87789	-1.87462	-1.87136	-1.8681	-1.87356	-1.87032	-1.86712	-1.864
-1.86421	-1.87662	-1.87335	-1.87011	-1.8669	-1.8724	-1.86916	-1.86594	-1.87491
-1.86304	-1.87537	-1.87207	-1.86885	-1.8657	-1.87126	-1.86803	-1.86482	-1.87376
-1.86188	-1.87416	-1.87086	-1.86764	-1.86453	-1.87011	-1.86689	-1.86369	-1.8726
-1.86074	-1.87298	-1.8697	-1.86649	-1.86338	-1.86897	-1.86576	-1.86256	-1.87143
-1.87513	-1.87181	-1.86854	-1.86534	-1.8622	-1.87989	-1.87667	-1.87346	-1.87026
-1.87394	-1.87065	-1.86738	-1.86415	-1.86096	-1.87862	-1.87545	-1.8723	-1.86912
-1.87272	-1.86946	-1.86621	-1.86297	-1.85974	-1.87736	-1.87423	-1.87113	-1.86797
-1.87145	-1.86822	-1.86502	-1.87405	-1.87078	-1.87617	-1.87301	-1.86989	-1.89221
-1.87016	-1.86696	-1.86383	-1.87288	-1.86962	-1.875	-1.8718	-1.86864	-1.89097
-1.86895	-1.87805	-1.8749	-1.87171	-1.86846	-1.87877	-1.87556	-1.8724	-1.88979
-1.86777	-1.87686	-1.87372	-1.87053	-1.8673	-1.87761	-1.8744	-1.87125	-1.88864
-1.8666	-1.87566	-1.87249	-1.88283	-1.87963	-1.87644	-1.87325	-1.87009	-1.88747
-1.86543	-1.87443	-1.87121	-1.88154	-1.87841	-1.87527	-1.8721	-1.86894	-1.88629
-1.87652	-1.88673	-1.88346	-1.88028	-1.8772	-1.8862	-1.88304	-1.87988	-1.88512
-1.87535	-1.88555	-1.88228	-1.8791	-1.87602	-1.88503	-1.88187	-1.87873	-1.88399
-1.87419	-1.88438	-1.88112	-1.87794	-1.87486	-1.88386	-1.88072	-1.87759	-1.88287
-1.8865	-1.88321	-1.87995	-1.87678	-1.87371	-1.88271	-1.87958	-1.87647	-1.88175
-1.88532	-1.88204	-1.87879	-1.87563	-1.87256	-1.88157	-1.87846	-1.87536	-1.88065
-1.88414	-1.88087	-1.87763	-1.87447	-1.87142	-1.88042	-1.87734	-1.87425	-1.87118
-1.88296	-1.87969	-1.87647	-1.87332	-1.87027	-1.87928	-1.87621	-1.87314	-1.87008
-1.88179	-1.87853	-1.87531	-1.87217	-1.86913	-1.87814	-1.87508	-1.87203	-1.86898
-1.88064	-1.87738	-1.87416	-1.87103	-1.86799	-1.877	-1.87396	-1.87093	-1.86788
-1.87948	-1.88827	-1.88505	-1.88192	-1.87889	-1.87587	-1.87285	-1.86983	-1.86678
-1.87831	-1.88711	-1.8839	-1.88078	-1.87777	-1.87477	-1.87174	-1.86872	-1.86569

-1.87714	-1.88594	-1.88274	-1.87964	-1.87665	-1.87367	-1.87064	-1.86762	-1.86459
-1.87598	-1.88479	-1.8816	-1.8785	-1.87553	-1.87256	-1.86954	-1.86652	-1.86351
-1.87483	-1.88365	-1.88046	-1.87738	-1.87442	-1.87146	-1.86845	-1.86543	-1.86242
-1.87368	-1.88253	-1.87938	-1.87631	-1.87334	-1.87037	-1.86735	-1.86433	-1.86133
-1.87255	-1.88144	-1.87836	-1.87531	-1.8723	-1.86928	-1.86625	-1.86322	-1.86023
-1.87142	-1.88034	-1.87731	-1.87427	-1.87124	-1.8682	-1.86515	-1.86212	-1.85914
-1.87027	-1.87922	-1.87622	-1.87319	-1.87015	-1.8671	-1.86406	-1.86104	-1.85807
-1.86916	-1.87811	-1.87512	-1.8721	-1.86905	-1.86601	-1.86298	-1.85997	-1.85701
-1.86812	-1.87706	-1.87404	-1.87101	-1.86795	-1.86491	-1.86189	-1.85889	-1.85594
-1.8671	-1.87604	-1.87297	-1.86991	-1.86685	-1.86381	-1.8608	-1.85782	-1.85488
-1.86605	-1.87496	-1.87188	-1.86881	-1.86574	-1.86271	-1.85972	-1.85676	-1.85386
-1.86497	-1.87387	-1.87078	-1.86771	-1.86463	-1.8616	-1.85864	-1.85573	-1.85286
-1.86389	-1.87278	-1.86969	-1.86661	-1.86354	-1.86053	-1.8576	-1.85472	-1.85187
-1.8628	-1.87168	-1.86859	-1.86552	-1.86247	-1.85949	-1.8566	-1.85375	-1.85089
-1.86172	-1.87061	-1.86753	-1.86448	-1.86145	-1.85848	-1.85561	-1.85278	-1.8499
-1.86064	-1.86956	-1.86653	-1.86352	-1.8605	-1.85754	-1.85464	-1.85177	-1.84887
-1.85958	-1.86853	-1.86554	-1.86255	-1.85956	-1.85659	-1.85366	-1.85075	-1.84784
-1.8586	-1.86752	-1.8645	-1.86151	-1.85853	-1.85558	-1.85264	-1.84971	-1.8468
-1.85764	-1.86653	-1.86345	-1.86043	-1.85748	-1.85454	-1.8516	-1.84867	-1.84576
-1.85662	-1.85352	-1.85043	-1.84742	-1.84447	-1.8535	-1.85056	-1.84764	-1.84476
-1.85555	-1.85246	-1.8494	-1.84639	-1.84344	-1.85246	-1.84953	-1.84662	-1.84378
-1.85447	-1.85141	-1.84839	-1.84541	-1.84246	-1.85148	-1.84855	-1.84565	-1.84282
-1.85339	-1.85038	-1.84743	-1.84449	-1.84155	-1.85058	-1.84765	-1.84474	-1.84188
-1.85234	-1.84935	-1.84645	-1.84354	-1.84062	-1.84965	-1.84672	-1.84381	-1.84093
-1.85135	-1.84835	-1.84545	-1.84254	-1.83961	-1.84864	-1.84573	-1.84283	-1.83995
-1.85038	-1.84736	-1.84443	-1.84151	-1.83858	-1.84761	-1.84472	-1.84185	-1.83898
-1.84937	-1.84635	-1.84341	-1.84048	-1.83755	-1.84659	-1.84374	-1.84091	-1.83806
-1.84836	-1.84534	-1.84239	-1.83946	-1.83653	-1.84558	-1.84277	-1.83999	-1.83717
-1.8474	-1.84439	-1.84144	-1.8385	-1.83557	-1.84462	-1.84182	-1.83904	-1.83623
-1.84648	-1.84349	-1.84054	-1.8376	-1.83467	-1.84371	-1.84088	-1.83806	-1.83525
-1.84554	-1.84255	-1.83961	-1.83667	-1.83375	-1.84278	-1.83992	-1.83709	-1.83429
-1.84454	-1.84157	-1.83861	-1.83568	-1.83276	-1.8418	-1.83894	-1.83611	-1.83335
-1.8312	-1.84059	-1.83763	-1.8347	-1.8318	-1.84085	-1.83799	-1.83517	-1.83243
-1.83029	-1.83969	-1.83673	-1.83381	-1.83092	-1.83997	-1.83712	-1.8343	-1.83154
-1.8294	-1.83879	-1.83584	-1.83293	-1.83006	-1.83912	-1.83628	-1.83346	-1.83067
-1.82844	-1.82553	-1.82262	-1.832	-1.82916	-1.83826	-1.83543	-1.83262	-1.82982
-1.82745	-1.82454	-1.82165	-1.83104	-1.82825	-1.83738	-1.83457	-1.83177	-1.82898
-1.82653	-1.82362	-1.82074	-1.81792	-1.81516	-1.83647	-1.83367	-1.83088	-1.82809
-1.82566	-1.82276	-1.81988	-1.81705	-1.81426	-1.83552	-1.83272	-1.82993	-1.82715
-1.82481	-1.82191	-1.81903	-1.81619	-1.81337	-1.82263	-1.81986	-1.81711	-1.82622
-1.82395	-1.82106	-1.81819	-1.81535	-1.81252	-1.82177	-1.819	-1.81625	-1.82536
-1.82307	-1.82018	-1.81732	-1.81448	-1.81166	-1.82091	-1.81814	-1.81539	-1.82449
-1.82212	-1.81923	-1.81637	-1.81354	-1.81072	-1.81997	-1.81721	-1.81446	-1.81171
-1.82114	-1.81826	-1.8154	-1.81257	-1.80976	-1.81902	-1.81626	-1.81351	-1.81077
-1.82024	-1.81736	-1.81451	-1.81168	-1.80888	-1.81814	-1.81538	-1.81264	-1.8099
-1.81939	-1.81652	-1.81368	-1.81086	-1.80806	-1.81731	-1.81456	-1.81182	-1.80907
-1.81855	-1.81569	-1.81285	-1.81004	-1.80725	-1.8165	-1.81375	-1.81101	-1.80826
-1.81771	-1.81486	-1.81203	-1.80923	-1.80645	-1.81572	-1.81296	-1.81022	-1.80748
-1.81687	-1.81403	-1.8112	-1.80841	-1.80565	-1.81491	-1.81216	-1.80942	-1.80668
-1.81602	-1.81318	-1.81037	-1.80757	-1.80482	-1.81409	-1.81134	-1.8086	-1.80586

-1.81517	-1.81233	-1.80951	-1.80671	-1.80396	-1.81323	-1.81048	-1.80774	-1.80501
-1.81431	-1.81146	-1.80861	-1.8058	-1.80304	-1.81231	-1.80956	-1.80682	-1.80408
-1.81345	-1.81057	-1.80769	-1.80486	-1.80209	-1.81135	-1.8086	-1.80586	-1.80312
-1.81253	-1.80967	-1.80679	-1.80397	-1.8012	-1.79844	-1.79569	-1.79296	-1.80223
-1.81156	-1.80874	-1.80592	-1.80312	-1.80036	-1.7976	-1.79485	-1.79211	-1.80138
-1.81064	-1.80784	-1.80506	-1.80229	-1.79953	-1.79678	-1.79403	-1.79128	-1.80056
-1.80978	-1.80699	-1.80423	-1.80147	-1.79871	-1.79596	-1.79321	-1.79047	-1.79974
-1.80894	-1.80616	-1.80341	-1.80065	-1.7979	-1.79515	-1.7924	-1.78966	-1.79893
-1.80811	-1.80534	-1.80259	-1.79984	-1.79708	-1.79433	-1.79158	-1.78884	-1.79811
-1.8073	-1.80453	-1.80178	-1.79902	-1.79627	-1.79352	-1.79077	-1.78802	-1.79729
-1.80648	-1.80372	-1.80097	-1.79821	-1.79546	-1.7927	-1.78995	-1.7872	-1.79647
-1.80567	-1.80291	-1.80015	-1.7974	-1.79464	-1.79188	-1.78913	-1.78638	-1.79565
-1.80485	-1.80209	-1.79934	-1.79658	-1.79382	-1.79107	-1.78831	-1.78556	-1.79483
-1.80402	-1.80126	-1.79851	-1.79576	-1.793	-1.79025	-1.78749	-1.78474	-1.79401
-1.80319	-1.80044	-1.79768	-1.79493	-1.79218	-1.78942	-1.78667	-1.78392	-1.79319
-1.80236	-1.79961	-1.79685	-1.7941	-1.79135	-1.7886	-1.78585	-1.7831	-1.79237
-1.80153	-1.79877	-1.79602	-1.79327	-1.79052	-1.78777	-1.78502	-1.78228	-1.79155
-1.8007	-1.79794	-1.79519	-1.79244	-1.78969	-1.78694	-1.78419	-1.78145	-1.79073
-1.79988	-1.79711	-1.79436	-1.79161	-1.78886	-1.78611	-1.78336	-1.78062	-1.78991
-1.79908	-1.7963	-1.79354	-1.79079	-1.78803	-1.78528	-1.78253	-1.77979	-1.78908
-1.79831	-1.7955	-1.79272	-1.78996	-1.7872	-1.78445	-1.7817	-1.77896	-1.78825
-1.79756	-1.79474	-1.79193	-1.78915	-1.78639	-1.78363	-1.78087	-1.77813	-1.78742
-1.79685	-1.79402	-1.79117	-1.78836	-1.78558	-1.78282	-1.78005	-1.77731	-1.78659
-1.79611	-1.79329	-1.79043	-1.7876	-1.78482	-1.78204	-1.77926	-1.7765	-1.78578
-1.79532	-1.79253	-1.78971	-1.7869	-1.78412	-1.78133	-1.77852	-1.77572	-1.78497
-1.79451	-1.79175	-1.78898	-1.7862	-1.78342	-1.78063	-1.77779	-1.77496	-1.78418
-1.79369	-1.79094	-1.78819	-1.78542	-1.78265	-1.77987	-1.77705	-1.77423	-1.78343
-1.79286	-1.79012	-1.78736	-1.7846	-1.78184	-1.77908	-1.7763	-1.77351	-1.7827
-1.79204	-1.78929	-1.78653	-1.78378	-1.78102	-1.77826	-1.7755	-1.77274	-1.78194
-1.79123	-1.78847	-1.7857	-1.78294	-1.78017	-1.77741	-1.77467	-1.77192	-1.78116
-1.79046	-1.78768	-1.78489	-1.78211	-1.77933	-1.77657	-1.77383	-1.77109	-1.78036
-1.78975	-1.78694	-1.7841	-1.78129	-1.7785	-1.77573	-1.77298	-1.77025	-1.77952
-1.78903	-1.7862	-1.78333	-1.7805	-1.7777	-1.77492	-1.77216	-1.76942	-1.7787
-1.78824	-1.78543	-1.78259	-1.77977	-1.77698	-1.7742	-1.77141	-1.76863	-1.77789
-1.78742	-1.78463	-1.78184	-1.77905	-1.77627	-1.77349	-1.77067	-1.76787	-1.77712
-1.78659	-1.78381	-1.78103	-1.77825	-1.77549	-1.77272	-1.76992	-1.76714	-1.7764
-1.78577	-1.78297	-1.78018	-1.77741	-1.77466	-1.77192	-1.76917	-1.76643	-1.77571
-1.785	-1.78218	-1.77937	-1.77658	-1.77384	-1.77111	-1.76838	-1.76566	-1.77496
-1.78429	-1.78145	-1.77858	-1.77577	-1.77302	-1.77028	-1.76755	-1.76484	-1.77415
-1.78355	-1.7807	-1.77782	-1.77499	-1.77224	-1.7695	-1.76676	-1.76404	-1.77334
-1.78275	-1.77993	-1.77708	-1.77428	-1.77153	-1.76878	-1.76601	-1.76326	-1.77255
-1.78196	-1.77916	-1.77635	-1.77357	-1.77082	-1.76807	-1.76528	-1.7625	-1.77178
-1.78122	-1.77841	-1.77559	-1.7728	-1.77006	-1.76731	-1.76454	-1.76179	-1.77108
-1.78049	-1.77766	-1.77482	-1.77202	-1.76928	-1.76654	-1.76381	-1.76109	-1.77039
-1.7797	-1.77689	-1.77407	-1.77129	-1.76855	-1.76582	-1.76308	-1.76035	-1.76965
-1.77888	-1.7761	-1.77333	-1.77058	-1.76785	-1.76512	-1.76235	-1.75959	-1.76887
-1.7781	-1.77533	-1.77257	-1.76983	-1.76711	-1.76438	-1.76161	-1.75885	-1.76813
-1.77736	-1.77458	-1.77179	-1.76904	-1.76632	-1.7636	-1.76087	-1.75814	-1.76743
-1.77662	-1.77382	-1.77103	-1.76827	-1.76555	-1.76284	-1.76013	-1.75742	-1.75472
-1.77583	-1.77306	-1.7703	-1.76756	-1.76485	-1.76213	-1.75939	-1.75665	-1.75395

								-0.10789
								-0.10767
								-0.10745
								-0.10723
								-0.107
					-0.10769	-0.10738	-0.10708	-0.25351
					-0.10747	-0.10716	-0.10686	-0.25291
					-0.10725	-0.10694	-0.10663	-0.25231
					-0.10705	-0.10673	-0.10641	-0.25171
			-0.10747	-0.10716	-0.25424	-0.25317	-0.25211	-0.35486
			-0.10724	-0.10694	-0.25363	-0.25257	-0.2515	-0.35406
			-0.10702	-0.10671	-0.25301	-0.25195	-0.25089	-0.35326
	-0.10741	-0.1071	-0.25438	-0.2534	-0.30637	-0.30517	-0.30398	-0.48288
	-0.10718	-0.10688	-0.25376	-0.25277	-0.30565	-0.30446	-0.30329	-0.48196
	-0.10697	-0.10666	-0.25314	-0.25215	-0.48598	-0.48429	-0.48263	-0.52041
	-0.10676	-0.10644	-0.25252	-0.25153	-0.48499	-0.48338	-0.4818	-0.51949
-0.10687	-0.25399	-0.25293	-0.39261	-0.39125	-0.48402	-0.48248	-0.48097	-0.60634
-0.10665	-0.25338	-0.25233	-0.39181	-0.39045	-0.48305	-0.48157	-0.48012	-0.60529
-0.10643	-0.25277	-0.25174	-0.39104	-0.38967	-0.48212	-0.48067	-0.47926	-0.60424
-0.15789	-0.39305	-0.39166	-0.48444	-0.48281	-0.52054	-0.51894	-0.51739	-0.68483
-0.15743	-0.39226	-0.3909	-0.48356	-0.48197	-0.51963	-0.51801	-0.51644	-0.68366
-0.30579	-0.48583	-0.48423	-0.48267	-0.48114	-0.68848	-0.68643	-0.68441	-0.71764
-0.30511	-0.48488	-0.4833	-0.48177	-0.48032	-0.68736	-0.68529	-0.68324	-0.71635
-0.39127	-0.48396	-0.48239	-0.5201	-0.51853	-0.72167	-0.71943	-0.71719	-0.80105
-0.39053	-0.48308	-0.48153	-0.51914	-0.51758	-0.72035	-0.71813	-0.71592	-0.79961
-0.48379	-0.48222	-0.48068	-0.68773	-0.68576	-0.7652	-0.76285	-0.76051	-0.87746
-0.4829	-0.48136	-0.47984	-0.68655	-0.68456	-0.76377	-0.76148	-0.7592	-0.87596
-0.48202	-0.48051	-0.47902	-0.68538	-0.68338	-0.76236	-0.76012	-0.75789	-0.87446
-0.48117	-0.60384	-0.60199	-0.76569	-0.76332	-0.80108	-0.79873	-0.79639	-0.87291
-0.48035	-0.60279	-0.60097	-0.76436	-0.76199	-0.79968	-0.79729	-0.79492	-0.87135
-0.6036	-0.68663	-0.68443	-0.80316	-0.80072	-0.87754	-0.87494	-0.87237	-0.86985
-0.60257	-0.68538	-0.68314	-0.8017	-0.79932	-0.87606	-0.87346	-0.87089	-0.86842
-0.65031	-0.80524	-0.8027	-0.8796	-0.87707	-0.87454	-0.87198	-0.86945	-1.04839
-0.64908	-0.80373	-0.80124	-0.87805	-0.87548	-0.87295	-0.87048	-0.86805	-1.04644
-0.68363	-0.8433	-0.84078	-0.87651	-0.87391	-1.05462	-1.05122	-1.04788	-1.04449
-0.68236	-0.84182	-0.83932	-0.87501	-0.87242	-1.05248	-1.04912	-1.04584	-1.04249
-0.68113	-0.84036	-0.83788	-0.87352	-0.87097	-1.05041	-1.04707	-1.0438	-1.0405
-0.84143	-0.87716	-0.87459	-0.90739	-0.90476	-1.04845	-1.04511	-1.04184	-1.129
-0.84	-0.87568	-0.87311	-0.90585	-0.90331	-1.04658	-1.04323	-1.03994	-1.12699
-0.87677	-0.90962	-0.90695	-1.05138	-1.048	-1.06923	-1.06588	-1.06256	-1.14847
-0.87527	-0.90807	-0.90545	-1.04941	-1.04605	-1.06727	-1.06396	-1.06068	-1.14643
-0.90917	-1.05437	-1.05088	-1.07203	-1.06866	-1.15504	-1.15146	-1.14793	-1.17734
-0.90764	-1.05234	-1.0489	-1.07007	-1.06675	-1.15296	-1.14937	-1.14582	-1.17521
-0.90614	-1.05035	-1.04695	-1.06813	-1.06485	-1.15089	-1.14729	-1.14373	-1.17309
-0.90466	-1.07293	-1.06957	-1.06623	-1.06292	-1.18186	-1.17819	-1.17457	-1.171

-0.9032	-1.07095	-1.06766	-1.06434	-1.06098	-1.17966	-1.17607	-1.17252	-1.16891
-1.07238	-1.069	-1.06572	-1.15176	-1.14815	-1.17754	-1.17398	-1.17044	-1.22564
-1.07045	-1.06709	-1.06375	-1.14961	-1.14608	-1.17546	-1.17189	-1.16834	-1.22347
-1.0685	-1.08987	-1.08635	-1.18053	-1.17693	-1.17336	-1.16981	-1.16625	-1.28301
-1.06652	-1.08779	-1.08433	-1.17838	-1.1748	-1.17124	-1.16771	-1.16419	-1.28086
-1.08921	-1.18352	-1.17986	-1.17624	-1.17268	-1.29004	-1.28624	-1.28248	-1.27872
-1.08719	-1.18135	-1.1777	-1.17411	-1.1706	-1.28781	-1.28402	-1.28026	-1.27653
-1.08518	-1.17921	-1.17557	-1.17201	-1.16852	-1.28561	-1.28183	-1.27808	-1.27437
-1.11727	-1.17702	-1.17347	-1.2609	-1.25718	-1.28337	-1.27966	-1.27598	-1.27232
-1.11515	-1.17485	-1.17139	-1.25874	-1.25499	-1.28114	-1.27751	-1.27392	-1.27031
-1.1764	-1.23696	-1.23329	-1.28646	-1.28268	-1.27898	-1.27537	-1.27182	-1.26824
-1.17445	-1.23487	-1.23112	-1.28421	-1.28052	-1.27687	-1.27326	-1.26967	-1.26613
-1.2366	-1.25976	-1.25593	-1.28205	-1.27839	-1.27476	-1.27114	-1.26756	-1.26403
-1.23446	-1.25768	-1.2539	-1.27999	-1.27628	-1.27262	-1.26903	-1.26548	-1.26195
-1.23231	-1.25558	-1.25188	-1.27796	-1.27421	-1.27052	-1.26694	-1.2634	-1.34913
-1.23014	-1.25342	-1.24981	-1.27591	-1.27218	-1.2685	-1.26486	-1.26128	-1.34681
-1.22801	-1.25127	-1.24773	-1.27384	-1.27017	-1.2665	-1.26281	-1.25917	-1.34452
-1.25288	-1.24922	-1.24563	-1.27171	-1.26808	-1.29639	-1.29265	-1.28894	-1.34232
-1.25091	-1.24722	-1.24354	-1.26954	-1.26596	-1.29428	-1.29062	-1.28697	-1.34018
-1.24889	-1.27491	-1.27113	-1.29944	-1.29576	-1.34993	-1.34596	-1.34201	-1.3876
-1.24683	-1.27286	-1.26912	-1.29736	-1.29359	-1.34755	-1.34367	-1.33986	-1.38545
-1.24475	-1.30285	-1.29908	-1.2953	-1.29149	-1.39503	-1.39108	-1.3872	-1.4131
-1.24261	-1.30064	-1.29695	-1.29325	-1.28949	-1.39285	-1.38884	-1.3849	-1.4108
-1.24051	-1.29844	-1.29483	-1.2912	-1.28753	-1.39073	-1.38666	-1.38266	-1.40851
-1.27101	-1.29633	-1.29272	-1.39676	-1.39269	-1.41856	-1.41441	-1.4103	-1.43177
-1.269	-1.29427	-1.29063	-1.39449	-1.39052	-1.41643	-1.4123	-1.4082	-1.42958
-1.29596	-1.40039	-1.39625	-1.42222	-1.41824	-1.41424	-1.41017	-1.40609	-1.47601
-1.29393	-1.39813	-1.39398	-1.41991	-1.41596	-1.41202	-1.40801	-1.404	-1.47384
-1.34349	-1.42599	-1.42177	-1.41766	-1.4137	-1.48402	-1.47991	-1.4758	-1.47165
-1.34142	-1.42379	-1.41961	-1.41549	-1.41143	-1.48159	-1.47754	-1.47352	-1.46943
-1.42583	-1.42161	-1.41747	-1.46296	-1.45872	-1.47923	-1.47519	-1.47122	-1.46719
-1.42365	-1.41947	-1.41533	-1.46076	-1.45653	-1.477	-1.47289	-1.46887	-1.46488
-1.42147	-1.41734	-1.4132	-1.45857	-1.45437	-1.47482	-1.47063	-1.46655	-1.46263
-1.41927	-1.46484	-1.46061	-1.48106	-1.47682	-1.47262	-1.46841	-1.46433	-1.48003
-1.41706	-1.46262	-1.45842	-1.47886	-1.47464	-1.47045	-1.46623	-1.46219	-1.478
-1.41486	-1.48507	-1.48088	-1.47668	-1.47246	-1.48792	-1.48366	-1.47961	-1.52528
-1.41266	-1.48279	-1.47866	-1.47449	-1.47028	-1.48574	-1.48153	-1.47755	-1.52327
-1.4599	-1.48049	-1.47641	-1.47229	-1.46809	-1.53306	-1.52883	-1.52486	-1.54255
-1.45757	-1.47813	-1.47412	-1.47005	-1.46587	-1.53079	-1.5268	-1.52309	-1.54091
-1.47988	-1.47581	-1.47184	-1.53704	-1.53269	-1.52858	-1.52484	-1.52141	-1.53935
-1.47763	-1.47356	-1.4696	-1.53469	-1.5304	-1.52641	-1.52292	-1.51973	-1.53782
-1.47541	-1.47135	-1.46739	-1.53239	-1.5282	-1.52436	-1.52109	-1.51812	-1.53634
-1.47318	-1.53835	-1.53419	-1.53015	-1.5262	-1.52258	-1.51946	-1.51661	-1.54758
-1.47096	-1.53601	-1.53187	-1.52798	-1.52434	-1.52099	-1.51797	-1.51518	-1.54615
-1.53792	-1.53371	-1.52963	-1.52589	-1.52252	-1.53231	-1.52941	-1.52669	-1.54476
-1.53558	-1.53143	-1.52745	-1.52388	-1.52074	-1.53071	-1.52795	-1.52532	-1.5434
-1.53329	-1.52924	-1.52544	-1.53492	-1.53196	-1.52918	-1.52652	-1.52396	-1.54205
-1.53101	-1.52714	-1.52362	-1.53328	-1.53042	-1.52772	-1.52512	-1.5226	-1.54069
-1.52884	-1.52517	-1.52191	-1.53173	-1.52895	-1.5263	-1.52374	-1.52125	-1.53933
-1.52686	-1.5362	-1.5331	-1.5302	-1.52752	-1.54559	-1.54302	-1.54049	-1.55892

-1.52502	-1.5345	-1.53152	-1.52872	-1.52612	-1.54422	-1.54166	-1.53914	-1.55756
-1.5361	-1.53289	-1.53002	-1.5273	-1.52474	-1.56381	-1.56125	-1.55872	-1.6035
-1.53446	-1.53138	-1.52859	-1.52592	-1.52339	-1.56243	-1.55988	-1.55736	-1.60204
-1.53284	-1.52989	-1.52717	-1.54583	-1.5433	-1.56106	-1.55852	-1.556	-1.60059
-1.53123	-1.5284	-1.52578	-1.54448	-1.54196	-1.55968	-1.55715	-1.55464	-1.59915
-1.52969	-1.54824	-1.54568	-1.54314	-1.54062	-1.55831	-1.55579	-1.55328	-1.66454
-1.52823	-1.54684	-1.54431	-1.54179	-1.53928	-1.55695	-1.55442	-1.55192	-1.66296
-1.52682	-1.54546	-1.54295	-1.54045	-1.53794	-1.55558	-1.55306	-1.55058	-1.6614
-1.5467	-1.54411	-1.54161	-1.55929	-1.55674	-1.57329	-1.57075	-1.56825	-1.65988
-1.54533	-1.54279	-1.54028	-1.55793	-1.55538	-1.57192	-1.56941	-1.56696	-1.6584
-1.54397	-1.54145	-1.53894	-1.5757	-1.5731	-1.66551	-1.66258	-1.65972	-1.65695
-1.5426	-1.5401	-1.53761	-1.57433	-1.57174	-1.66394	-1.66104	-1.65824	-1.65555
-1.54125	-1.53877	-1.53631	-1.57302	-1.57046	-1.68112	-1.67826	-1.67549	-1.69551
-1.53991	-1.53746	-1.53505	-1.57178	-1.56927	-1.67977	-1.67694	-1.67418	-1.6941
-1.53861	-1.59531	-1.59284	-1.59038	-1.58792	-1.67841	-1.6756	-1.67286	-1.69268
-1.53739	-1.59403	-1.59152	-1.58906	-1.58664	-1.67696	-1.67418	-1.67146	-1.69122
-1.53618	-1.59276	-1.59021	-1.58774	-1.58535	-1.67549	-1.67274	-1.67005	-1.68975
-1.55531	-1.59144	-1.5889	-1.65882	-1.65608	-1.69679	-1.69389	-1.69105	-1.68827
-1.55398	-1.5901	-1.58761	-1.65737	-1.65464	-1.69524	-1.69236	-1.68954	-1.68679
-1.57182	-1.58877	-1.58632	-1.65593	-1.65321	-1.69373	-1.69088	-1.68808	-1.68535
-1.57049	-1.58744	-1.58504	-1.65449	-1.65181	-1.69224	-1.68944	-1.68668	-1.68395
-1.56923	-1.60893	-1.60646	-1.69655	-1.69366	-1.69082	-1.68805	-1.68532	-1.6826
-1.56809	-1.60775	-1.60529	-1.69517	-1.69229	-1.68948	-1.68674	-1.68403	-1.68132
-1.56695	-1.60657	-1.60412	-1.69378	-1.69093	-1.68814	-1.68541	-1.68273	-1.68003
-1.58505	-1.6053	-1.60286	-1.69232	-1.6895	-1.68673	-1.68402	-1.68133	-1.67864
-1.58379	-1.604	-1.60159	-1.69083	-1.68805	-1.68531	-1.68259	-1.67991	-1.67724
-1.60518	-1.67472	-1.6719	-1.68936	-1.68661	-1.68389	-1.68118	-1.6785	-1.67586
-1.60394	-1.67325	-1.67044	-1.68791	-1.68518	-1.68248	-1.67978	-1.67711	-1.67451
-1.60272	-1.67186	-1.66907	-1.68653	-1.68383	-1.68114	-1.67844	-1.67579	-1.67323
-1.60154	-1.67054	-1.66779	-1.68525	-1.68255	-1.67987	-1.67719	-1.67455	-1.67202
-1.60035	-1.6692	-1.66648	-1.68394	-1.68124	-1.67857	-1.67592	-1.67331	-1.67081
-1.59912	-1.66777	-1.66509	-1.68256	-1.67986	-1.6772	-1.67459	-1.67204	-1.66955
-1.59792	-1.66637	-1.66371	-1.68118	-1.67848	-1.67585	-1.67329	-1.67078	-1.66829
-1.66777	-1.68525	-1.68256	-1.67988	-1.6772	-1.67458	-1.67206	-1.66959	-1.67457
-1.6665	-1.68397	-1.68128	-1.67861	-1.67594	-1.67335	-1.67087	-1.66842	-1.67338
-1.66516	-1.68263	-1.67994	-1.67728	-1.67466	-1.69647	-1.69398	-1.69152	-1.7102
-1.66378	-1.68123	-1.67855	-1.67592	-1.67336	-1.6952	-1.69272	-1.69026	-1.70888
-1.66245	-1.67989	-1.67722	-1.69901	-1.69647	-1.69397	-1.6915	-1.68904	-1.76926
-1.6612	-1.67862	-1.67596	-1.69776	-1.69526	-1.69279	-1.69034	-1.68788	-1.76787
-1.65993	-1.70176	-1.69909	-1.69652	-1.69405	-1.73105	-1.72839	-1.72572	-1.76647
-1.65862	-1.70043	-1.69781	-1.69527	-1.6928	-1.72969	-1.72703	-1.72436	-1.76503
-1.65732	-1.69911	-1.69654	-1.69402	-1.69156	-1.72833	-1.72566	-1.72299	-1.76359
-1.70049	-1.71624	-1.71367	-1.71113	-1.70864	-1.7709	-1.768	-1.76511	-1.76218
-1.69924	-1.715	-1.71246	-1.70994	-1.70745	-1.76954	-1.76665	-1.76375	-1.76079
-1.71639	-1.71378	-1.71123	-1.7298	-1.72714	-1.76812	-1.76523	-1.76233	-1.77383
-1.71518	-1.71257	-1.70999	-1.72845	-1.72577	-1.76664	-1.76376	-1.76087	-1.77241
-1.71395	-1.71135	-1.70876	-1.77101	-1.76811	-1.77964	-1.77676	-1.77388	-1.77103
-1.71265	-1.71011	-1.70757	-1.76968	-1.76676	-1.77829	-1.7754	-1.77253	-1.76969
-1.71138	-1.70888	-1.70639	-1.78277	-1.77986	-1.77695	-1.77404	-1.77117	-1.76836
-1.71019	-1.70769	-1.7052	-1.78143	-1.77853	-1.77562	-1.77267	-1.76975	-1.76698

-1.70903	-1.70652	-1.70401	-1.78008	-1.77718	-1.77427	-1.77128	-1.76835	-1.76561
-1.70787	-1.76634	-1.76345	-1.77866	-1.77576	-1.77286	-1.76991	-1.76702	-1.76433
-1.70672	-1.76501	-1.76204	-1.7772	-1.77428	-1.77141	-1.76854	-1.76574	-1.76311
-1.72065	-1.78177	-1.77876	-1.7758	-1.77288	-1.77003	-1.76724	-1.76452	-1.76191
-1.71942	-1.78036	-1.77739	-1.77446	-1.77155	-1.76872	-1.766	-1.76334	-1.76075
-1.76379	-1.77897	-1.77604	-1.77314	-1.77025	-1.76744	-1.76477	-1.76216	-1.77124
-1.76244	-1.77762	-1.77471	-1.77182	-1.76895	-1.76617	-1.76355	-1.76098	-1.76999
-1.76112	-1.7763	-1.77339	-1.77053	-1.76768	-1.76492	-1.76233	-1.75977	-1.76872
-1.7598	-1.77497	-1.77208	-1.76924	-1.76645	-1.76373	-1.7611	-1.75851	-1.76741
-1.7585	-1.77365	-1.77077	-1.76797	-1.76524	-1.76255	-1.75988	-1.75724	-1.7661
-1.77524	-1.77233	-1.76946	-1.76666	-1.76397	-1.7613	-1.75864	-1.756	-1.76483
-1.77393	-1.77103	-1.76814	-1.76535	-1.76266	-1.76002	-1.7574	-1.75479	-1.76358
-1.77262	-1.76972	-1.76684	-1.76406	-1.76139	-1.75876	-1.75617	-1.75359	-1.76233
-1.7713	-1.76842	-1.76556	-1.7628	-1.76015	-1.75754	-1.75496	-1.75238	-1.76109
-1.76998	-1.76712	-1.76429	-1.76156	-1.75894	-1.76793	-1.76523	-1.76254	-1.80624
-1.76863	-1.76581	-1.76305	-1.76036	-1.75775	-1.7667	-1.764	-1.7613	-1.80482
-1.76728	-1.76451	-1.76181	-1.75916	-1.75657	-1.76548	-1.76278	-1.76007	-1.80341
-1.76596	-1.76321	-1.76057	-1.75796	-1.75538	-1.76425	-1.76155	-1.75883	-1.80203
-1.76465	-1.76194	-1.75934	-1.75675	-1.75418	-1.76302	-1.76032	-1.7576	-1.80066
-1.76339	-1.76071	-1.75813	-1.75555	-1.75299	-1.76178	-1.75906	-1.75632	-1.79924
-1.76218	-1.75952	-1.75695	-1.75437	-1.7518	-1.76053	-1.75776	-1.75499	-1.79779
-1.76098	-1.75835	-1.75577	-1.75319	-1.75061	-1.80577	-1.8026	-1.79945	-1.82972
-1.75979	-1.75717	-1.7546	-1.75202	-1.74942	-1.80441	-1.80123	-1.79808	-1.82836
-1.75861	-1.756	-1.75343	-1.75084	-1.74823	-1.83647	-1.83323	-1.83003	-1.84473
-1.75744	-1.75482	-1.75224	-1.74964	-1.74704	-1.83508	-1.83183	-1.82864	-1.84337
-1.75628	-1.75365	-1.75105	-1.74845	-1.74585	-1.83369	-1.83046	-1.82728	-1.84204
-1.75513	-1.78738	-1.7847	-1.82791	-1.82491	-1.83231	-1.82912	-1.826	-1.86427
-1.75399	-1.78618	-1.78347	-1.82653	-1.82354	-1.83094	-1.82782	-1.82477	-1.86296
-1.78774	-1.78502	-1.78227	-1.82517	-1.82218	-1.82957	-1.82652	-1.82354	-1.86165
-1.78663	-1.78389	-1.78109	-1.82382	-1.82082	-1.82821	-1.82522	-1.8223	-1.86034
-1.78549	-1.82878	-1.82558	-1.82249	-1.8195	-1.85134	-1.8481	-1.84495	-1.85902
-1.78429	-1.82746	-1.82425	-1.82117	-1.81821	-1.84997	-1.84677	-1.84365	-1.85769
-1.78307	-1.82611	-1.82292	-1.81986	-1.81694	-1.84864	-1.84547	-1.84238	-1.85639
-1.78186	-1.82477	-1.82159	-1.81856	-1.81569	-1.86458	-1.86141	-1.85828	-1.85513
-1.78064	-1.82342	-1.82026	-1.81726	-1.81446	-1.86329	-1.86017	-1.85707	-1.85389
-1.82521	-1.8221	-1.81897	-1.816	-1.81324	-1.86197	-1.85887	-1.85577	-1.85259
-1.82389	-1.8208	-1.81771	-1.81478	-1.81203	-1.86063	-1.85753	-1.85443	-1.85125
-1.82258	-1.81953	-1.81651	-1.83831	-1.83528	-1.8593	-1.8562	-1.85309	-1.84992
-1.82129	-1.81829	-1.81538	-1.83715	-1.83405	-1.85797	-1.85486	-1.85176	-1.84859
-1.82001	-1.81706	-1.81424	-1.85334	-1.85014	-1.85665	-1.85353	-1.85043	-1.86894
-1.81873	-1.81582	-1.81305	-1.85207	-1.84884	-1.85532	-1.8522	-1.84912	-1.86759
-1.81749	-1.8146	-1.81185	-1.85077	-1.84755	-1.854	-1.85088	-1.84781	-1.86624
-1.81634	-1.84842	-1.84527	-1.85923	-1.85596	-1.85275	-1.84961	-1.84652	-1.86487
-1.81525	-1.84723	-1.84399	-1.85791	-1.8547	-1.85152	-1.84835	-1.84523	-1.8635
-1.81408	-1.84596	-1.84269	-1.85657	-1.8534	-1.87204	-1.86868	-1.86535	-1.86213
-1.81286	-1.84464	-1.84137	-1.85523	-1.85208	-1.87065	-1.86729	-1.86398	-1.86076
-1.8365	-1.86048	-1.85716	-1.85392	-1.85078	-1.86929	-1.86596	-1.86266	-1.85945
-1.83522	-1.85916	-1.85588	-1.85267	-1.84952	-1.86795	-1.86468	-1.86144	-1.85821
-1.83397	-1.85787	-1.85463	-1.87328	-1.86994	-1.86664	-1.86341	-1.86021	-1.88229
-1.83278	-1.85665	-1.85339	-1.87198	-1.86866	-1.86538	-1.86214	-1.85892	-1.88104

-1.8316	-1.85543	-1.85216	-1.87067	-1.86739	-1.86413	-1.86086	-1.85762	-1.87977
-1.84759	-1.85414	-1.85089	-1.86933	-1.86607	-1.88829	-1.88496	-1.88167	-1.87845
-1.84626	-1.85282	-1.8496	-1.86797	-1.86473	-1.88694	-1.88362	-1.88032	-1.87711
-1.84497	-1.85154	-1.84836	-1.8922	-1.8889	-1.88561	-1.88231	-1.87904	-1.87583
-1.8437	-1.85029	-1.84717	-1.89094	-1.88759	-1.88429	-1.88104	-1.87782	-1.87461
-1.84248	-1.89633	-1.893	-1.88967	-1.88632	-1.88301	-1.87978	-1.87659	-1.88893
-1.84133	-1.89507	-1.89171	-1.88838	-1.88507	-1.88179	-1.87855	-1.87534	-1.88763
-1.84017	-1.89382	-1.89042	-1.88709	-1.88383	-1.88058	-1.87733	-1.8741	-1.88634
-1.8653	-1.89253	-1.88917	-1.88585	-1.88257	-1.87932	-1.87609	-1.8729	-1.88508
-1.86408	-1.89123	-1.88792	-1.88462	-1.88131	-1.87805	-1.87486	-1.8717	-1.88381
-1.8629	-1.88996	-1.88664	-1.88334	-1.88006	-1.89257	-1.88919	-1.88585	-1.88251
-1.86175	-1.8887	-1.88534	-1.88204	-1.87883	-1.8913	-1.8879	-1.88452	-1.88119
-1.88162	-1.88743	-1.88406	-1.88078	-1.87759	-1.89001	-1.88662	-1.88324	-1.87993
-1.88032	-1.88613	-1.88282	-1.87956	-1.87634	-1.88868	-1.88535	-1.88204	-1.87875
-1.87902	-1.88484	-1.88158	-1.89417	-1.89075	-1.88738	-1.88409	-1.88082	-1.87754
-1.87777	-1.88358	-1.88032	-1.89286	-1.88949	-1.88615	-1.88283	-1.87954	-1.87625
-1.87651	-1.88233	-1.87906	-1.89155	-1.88822	-1.88491	-1.88158	-1.87826	-1.87497
-1.87524	-1.88105	-1.87782	-1.89025	-1.88692	-1.88361	-1.88032	-1.87705	-1.87377
-1.87396	-1.87978	-1.87659	-1.88896	-1.88559	-1.88228	-1.87906	-1.87586	-1.87261
-1.87274	-1.87854	-1.87533	-1.88764	-1.88429	-1.881	-1.87779	-1.87462	-1.8714
-1.87156	-1.87732	-1.87407	-1.88631	-1.88301	-1.87975	-1.87653	-1.87333	-1.87015
-1.87037	-1.88298	-1.87956	-1.87623	-1.87299	-1.8785	-1.87527	-1.87207	-1.86891
-1.86917	-1.88176	-1.87834	-1.875	-1.87173	-1.87721	-1.87401	-1.87085	-1.86768
-1.86795	-1.88051	-1.87712	-1.87378	-1.87049	-1.87596	-1.87277	-1.86962	-1.86645
-1.86668	-1.87921	-1.87588	-1.87257	-1.86928	-1.87475	-1.87153	-1.86836	-1.86522
-1.86541	-1.87789	-1.87462	-1.87136	-1.8681	-1.87356	-1.87032	-1.86712	-1.864
-1.86421	-1.87662	-1.87335	-1.87011	-1.8669	-1.8724	-1.86916	-1.86594	-1.87491
-1.86304	-1.87537	-1.87207	-1.86885	-1.8657	-1.87126	-1.86803	-1.86482	-1.87376
-1.86188	-1.87416	-1.87086	-1.86764	-1.86453	-1.87011	-1.86689	-1.86369	-1.8726
-1.86074	-1.87298	-1.8697	-1.86649	-1.86338	-1.86897	-1.86576	-1.86256	-1.87143
-1.87513	-1.87181	-1.86854	-1.86534	-1.8622	-1.87989	-1.87667	-1.87346	-1.87026
-1.87394	-1.87065	-1.86738	-1.86415	-1.86096	-1.87862	-1.87545	-1.8723	-1.86912
-1.87272	-1.86946	-1.86621	-1.86297	-1.85974	-1.87736	-1.87423	-1.87113	-1.86797
-1.87145	-1.86822	-1.86502	-1.87405	-1.87078	-1.87617	-1.87301	-1.86989	-1.89221
-1.87016	-1.86696	-1.86383	-1.87288	-1.86962	-1.875	-1.8718	-1.86864	-1.89097
-1.86895	-1.87805	-1.8749	-1.87171	-1.86846	-1.87877	-1.87556	-1.8724	-1.88979
-1.86777	-1.87686	-1.87372	-1.87053	-1.8673	-1.87761	-1.8744	-1.87125	-1.88864
-1.8666	-1.87566	-1.87249	-1.88283	-1.87963	-1.87644	-1.87325	-1.87009	-1.88747
-1.86543	-1.87443	-1.87121	-1.88154	-1.87841	-1.87527	-1.8721	-1.86894	-1.88629
-1.87652	-1.88673	-1.88346	-1.88028	-1.8772	-1.8862	-1.88304	-1.87988	-1.88512
-1.87535	-1.88555	-1.88228	-1.8791	-1.87602	-1.88503	-1.88187	-1.87873	-1.88399
-1.87419	-1.88438	-1.88112	-1.87794	-1.87486	-1.88386	-1.88072	-1.87759	-1.88287
-1.8865	-1.88321	-1.87995	-1.87678	-1.87371	-1.88271	-1.87958	-1.87647	-1.88175
-1.88532	-1.88204	-1.87879	-1.87563	-1.87256	-1.88157	-1.87846	-1.87536	-1.88065
-1.88414	-1.88087	-1.87763	-1.87447	-1.87142	-1.88042	-1.87734	-1.87425	-1.87118
-1.88296	-1.87969	-1.87647	-1.87332	-1.87027	-1.87928	-1.87621	-1.87314	-1.87008
-1.88179	-1.87853	-1.87531	-1.87217	-1.86913	-1.87814	-1.87508	-1.87203	-1.86898
-1.88064	-1.87738	-1.87416	-1.87103	-1.86799	-1.877	-1.87396	-1.87093	-1.86788
-1.87948	-1.88827	-1.88505	-1.88192	-1.87889	-1.87587	-1.87285	-1.86983	-1.86678
-1.87831	-1.88711	-1.8839	-1.88078	-1.87777	-1.87477	-1.87174	-1.86872	-1.86569

-1.87714	-1.88594	-1.88274	-1.87964	-1.87665	-1.87367	-1.87064	-1.86762	-1.86459
-1.87598	-1.88479	-1.8816	-1.8785	-1.87553	-1.87256	-1.86954	-1.86652	-1.86351
-1.87483	-1.88365	-1.88046	-1.87738	-1.87442	-1.87146	-1.86845	-1.86543	-1.86242
-1.87368	-1.88253	-1.87938	-1.87631	-1.87334	-1.87037	-1.86735	-1.86433	-1.86133
-1.87255	-1.88144	-1.87836	-1.87531	-1.8723	-1.86928	-1.86625	-1.86322	-1.86023
-1.87142	-1.88034	-1.87731	-1.87427	-1.87124	-1.8682	-1.86515	-1.86212	-1.85914
-1.87027	-1.87922	-1.87622	-1.87319	-1.87015	-1.8671	-1.86406	-1.86104	-1.85807
-1.86916	-1.87811	-1.87512	-1.8721	-1.86905	-1.86601	-1.86298	-1.85997	-1.85701
-1.86812	-1.87706	-1.87404	-1.87101	-1.86795	-1.86491	-1.86189	-1.85889	-1.85594
-1.8671	-1.87604	-1.87297	-1.86991	-1.86685	-1.86381	-1.8608	-1.85782	-1.85488
-1.86605	-1.87496	-1.87188	-1.86881	-1.86574	-1.86271	-1.85972	-1.85676	-1.85386
-1.86497	-1.87387	-1.87078	-1.86771	-1.86463	-1.8616	-1.85864	-1.85573	-1.85286
-1.86389	-1.87278	-1.86969	-1.86661	-1.86354	-1.86053	-1.8576	-1.85472	-1.85187
-1.8628	-1.87168	-1.86859	-1.86552	-1.86247	-1.85949	-1.8566	-1.85375	-1.85089
-1.86172	-1.87061	-1.86753	-1.86448	-1.86145	-1.85848	-1.85561	-1.85278	-1.8499
-1.86064	-1.86956	-1.86653	-1.86352	-1.8605	-1.85754	-1.85464	-1.85177	-1.84887
-1.85958	-1.86853	-1.86554	-1.86255	-1.85956	-1.85659	-1.85366	-1.85075	-1.84784
-1.8586	-1.86752	-1.8645	-1.86151	-1.85853	-1.85558	-1.85264	-1.84971	-1.8468
-1.85764	-1.86653	-1.86345	-1.86043	-1.85748	-1.85454	-1.8516	-1.84867	-1.84576
-1.85662	-1.85352	-1.85043	-1.84742	-1.84447	-1.8535	-1.85056	-1.84764	-1.84476
-1.85555	-1.85246	-1.8494	-1.84639	-1.84344	-1.85246	-1.84953	-1.84662	-1.84378
-1.85447	-1.85141	-1.84839	-1.84541	-1.84246	-1.85148	-1.84855	-1.84565	-1.84282
-1.85339	-1.85038	-1.84743	-1.84449	-1.84155	-1.85058	-1.84765	-1.84474	-1.84188
-1.85234	-1.84935	-1.84645	-1.84354	-1.84062	-1.84965	-1.84672	-1.84381	-1.84093
-1.85135	-1.84835	-1.84545	-1.84254	-1.83961	-1.84864	-1.84573	-1.84283	-1.83995
-1.85038	-1.84736	-1.84443	-1.84151	-1.83858	-1.84761	-1.84472	-1.84185	-1.83898
-1.84937	-1.84635	-1.84341	-1.84048	-1.83755	-1.84659	-1.84374	-1.84091	-1.83806
-1.84836	-1.84534	-1.84239	-1.83946	-1.83653	-1.84558	-1.84277	-1.83999	-1.83717
-1.8474	-1.84439	-1.84144	-1.8385	-1.83557	-1.84462	-1.84182	-1.83904	-1.83623
-1.84648	-1.84349	-1.84054	-1.8376	-1.83467	-1.84371	-1.84088	-1.83806	-1.83525
-1.84554	-1.84255	-1.83961	-1.83667	-1.83375	-1.84278	-1.83992	-1.83709	-1.83429
-1.84454	-1.84157	-1.83861	-1.83568	-1.83276	-1.8418	-1.83894	-1.83611	-1.83335
-1.8312	-1.84059	-1.83763	-1.8347	-1.8318	-1.84085	-1.83799	-1.83517	-1.83243
-1.83029	-1.83969	-1.83673	-1.83381	-1.83092	-1.83997	-1.83712	-1.8343	-1.83154
-1.8294	-1.83879	-1.83584	-1.83293	-1.83006	-1.83912	-1.83628	-1.83346	-1.83067
-1.82844	-1.82553	-1.82262	-1.832	-1.82916	-1.83826	-1.83543	-1.83262	-1.82982
-1.82745	-1.82454	-1.82165	-1.83104	-1.82825	-1.83738	-1.83457	-1.83177	-1.82898
-1.82653	-1.82362	-1.82074	-1.81792	-1.81516	-1.83647	-1.83367	-1.83088	-1.82809
-1.82566	-1.82276	-1.81988	-1.81705	-1.81426	-1.83552	-1.83272	-1.82993	-1.82715
-1.82481	-1.82191	-1.81903	-1.81619	-1.81337	-1.82263	-1.81986	-1.81711	-1.82622
-1.82395	-1.82106	-1.81819	-1.81535	-1.81252	-1.82177	-1.819	-1.81625	-1.82536
-1.82307	-1.82018	-1.81732	-1.81448	-1.81166	-1.82091	-1.81814	-1.81539	-1.82449
-1.82212	-1.81923	-1.81637	-1.81354	-1.81072	-1.81997	-1.81721	-1.81446	-1.81171
-1.82114	-1.81826	-1.8154	-1.81257	-1.80976	-1.81902	-1.81626	-1.81351	-1.81077
-1.82024	-1.81736	-1.81451	-1.81168	-1.80888	-1.81814	-1.81538	-1.81264	-1.8099
-1.81939	-1.81652	-1.81368	-1.81086	-1.80806	-1.81731	-1.81456	-1.81182	-1.80907
-1.81855	-1.81569	-1.81285	-1.81004	-1.80725	-1.8165	-1.81375	-1.81101	-1.80826
-1.81771	-1.81486	-1.81203	-1.80923	-1.80645	-1.81572	-1.81296	-1.81022	-1.80748
-1.81687	-1.81403	-1.8112	-1.80841	-1.80565	-1.81491	-1.81216	-1.80942	-1.80668
-1.81602	-1.81318	-1.81037	-1.80757	-1.80482	-1.81409	-1.81134	-1.8086	-1.80586

-1.81517	-1.81233	-1.80951	-1.80671	-1.80396	-1.81323	-1.81048	-1.80774	-1.80501
-1.81431	-1.81146	-1.80861	-1.8058	-1.80304	-1.81231	-1.80956	-1.80682	-1.80408
-1.81345	-1.81057	-1.80769	-1.80486	-1.80209	-1.81135	-1.8086	-1.80586	-1.80312
-1.81253	-1.80967	-1.80679	-1.80397	-1.8012	-1.79844	-1.79569	-1.79296	-1.80223
-1.81156	-1.80874	-1.80592	-1.80312	-1.80036	-1.7976	-1.79485	-1.79211	-1.80138
-1.81064	-1.80784	-1.80506	-1.80229	-1.79953	-1.79678	-1.79403	-1.79128	-1.80056
-1.80978	-1.80699	-1.80423	-1.80147	-1.79871	-1.79596	-1.79321	-1.79047	-1.79974
-1.80894	-1.80616	-1.80341	-1.80065	-1.7979	-1.79515	-1.7924	-1.78966	-1.79893
-1.80811	-1.80534	-1.80259	-1.79984	-1.79708	-1.79433	-1.79158	-1.78884	-1.79811
-1.8073	-1.80453	-1.80178	-1.79902	-1.79627	-1.79352	-1.79077	-1.78802	-1.79729
-1.80648	-1.80372	-1.80097	-1.79821	-1.79546	-1.7927	-1.78995	-1.7872	-1.79647
-1.80567	-1.80291	-1.80015	-1.7974	-1.79464	-1.79188	-1.78913	-1.78638	-1.79565
-1.80485	-1.80209	-1.79934	-1.79658	-1.79382	-1.79107	-1.78831	-1.78556	-1.79483
-1.80402	-1.80126	-1.79851	-1.79576	-1.793	-1.79025	-1.78749	-1.78474	-1.79401
-1.80319	-1.80044	-1.79768	-1.79493	-1.79218	-1.78942	-1.78667	-1.78392	-1.79319
-1.80236	-1.79961	-1.79685	-1.7941	-1.79135	-1.7886	-1.78585	-1.7831	-1.79237
-1.80153	-1.79877	-1.79602	-1.79327	-1.79052	-1.78777	-1.78502	-1.78228	-1.79155
-1.8007	-1.79794	-1.79519	-1.79244	-1.78969	-1.78694	-1.78419	-1.78145	-1.79073
-1.79988	-1.79711	-1.79436	-1.79161	-1.78886	-1.78611	-1.78336	-1.78062	-1.78991
-1.79908	-1.7963	-1.79354	-1.79079	-1.78803	-1.78528	-1.78253	-1.77979	-1.78908
-1.79831	-1.7955	-1.79272	-1.78996	-1.7872	-1.78445	-1.7817	-1.77896	-1.78825
-1.79756	-1.79474	-1.79193	-1.78915	-1.78639	-1.78363	-1.78087	-1.77813	-1.78742
-1.79685	-1.79402	-1.79117	-1.78836	-1.78558	-1.78282	-1.78005	-1.77731	-1.78659
-1.79611	-1.79329	-1.79043	-1.7876	-1.78482	-1.78204	-1.77926	-1.7765	-1.78578
-1.79532	-1.79253	-1.78971	-1.7869	-1.78412	-1.78133	-1.77852	-1.77572	-1.78497
-1.79451	-1.79175	-1.78898	-1.7862	-1.78342	-1.78063	-1.77779	-1.77496	-1.78418
-1.79369	-1.79094	-1.78819	-1.78542	-1.78265	-1.77987	-1.77705	-1.77423	-1.78343
-1.79286	-1.79012	-1.78736	-1.7846	-1.78184	-1.77908	-1.7763	-1.77351	-1.7827
-1.79204	-1.78929	-1.78653	-1.78378	-1.78102	-1.77826	-1.7755	-1.77274	-1.78194
-1.79123	-1.78847	-1.7857	-1.78294	-1.78017	-1.77741	-1.77467	-1.77192	-1.78116
-1.79046	-1.78768	-1.78489	-1.78211	-1.77933	-1.77657	-1.77383	-1.77109	-1.78036
-1.78975	-1.78694	-1.7841	-1.78129	-1.7785	-1.77573	-1.77298	-1.77025	-1.77952
-1.78903	-1.7862	-1.78333	-1.7805	-1.7777	-1.77492	-1.77216	-1.76942	-1.7787
-1.78824	-1.78543	-1.78259	-1.77977	-1.77698	-1.7742	-1.77141	-1.76863	-1.77789
-1.78742	-1.78463	-1.78184	-1.77905	-1.77627	-1.77349	-1.77067	-1.76787	-1.77712
-1.78659	-1.78381	-1.78103	-1.77825	-1.77549	-1.77272	-1.76992	-1.76714	-1.7764
-1.78577	-1.78297	-1.78018	-1.77741	-1.77466	-1.77192	-1.76917	-1.76643	-1.77571
-1.785	-1.78218	-1.77937	-1.77658	-1.77384	-1.77111	-1.76838	-1.76566	-1.77496
-1.78429	-1.78145	-1.77858	-1.77577	-1.77302	-1.77028	-1.76755	-1.76484	-1.77415
-1.78355	-1.7807	-1.77782	-1.77499	-1.77224	-1.7695	-1.76676	-1.76404	-1.77334
-1.78275	-1.77993	-1.77708	-1.77428	-1.77153	-1.76878	-1.76601	-1.76326	-1.77255
-1.78196	-1.77916	-1.77635	-1.77357	-1.77082	-1.76807	-1.76528	-1.7625	-1.77178
-1.78122	-1.77841	-1.77559	-1.7728	-1.77006	-1.76731	-1.76454	-1.76179	-1.77108
-1.78049	-1.77766	-1.77482	-1.77202	-1.76928	-1.76654	-1.76381	-1.76109	-1.77039
-1.7797	-1.77689	-1.77407	-1.77129	-1.76855	-1.76582	-1.76308	-1.76035	-1.76965
-1.77888	-1.7761	-1.77333	-1.77058	-1.76785	-1.76512	-1.76235	-1.75959	-1.76887
-1.7781	-1.77533	-1.77257	-1.76983	-1.76711	-1.76438	-1.76161	-1.75885	-1.76813
-1.77736	-1.77458	-1.77179	-1.76904	-1.76632	-1.7636	-1.76087	-1.75814	-1.76743
-1.77662	-1.77382	-1.77103	-1.76827	-1.76555	-1.76284	-1.76013	-1.75742	-1.75472
-1.77583	-1.77306	-1.7703	-1.76756	-1.76485	-1.76213	-1.75939	-1.75665	-1.75395

-0.87491	-0.89752	-0.89611	-0.89475	-0.89344	-1.02033	-1.01879	-1.01724	-1.01567
-0.87523	-0.89787	-0.89646	-0.8951	-0.89378	-1.02073	-1.01918	-1.01764	-1.01607
-0.87556	-0.89821	-0.89681	-0.89544	-0.89411	-1.02113	-1.01958	-1.01803	-1.01646
-0.87588	-0.87452	-0.87316	-0.89577	-0.89444	-0.94651	-0.94508	-0.94364	-1.01686
-0.87619	-0.87484	-0.87347	-0.8961	-0.89477	-0.94688	-0.94544	-0.944	-1.01725
-0.87649	-0.87515	-0.87379	-0.87247	-0.87117	-0.89379	-0.89247	-0.89116	-0.97163
-0.8768	-0.87546	-0.87412	-0.8728	-0.8715	-0.89412	-0.8928	-0.89148	-0.97201
-0.8771	-0.87576	-0.87443	-0.87311	-0.87182	-0.87052	-0.86923	-0.86794	-0.89048
-0.8774	-0.87606	-0.87473	-0.87342	-0.87214	-0.87085	-0.86955	-0.86824	-0.8908
-0.8777	-0.87636	-0.87503	-0.87373	-0.87246	-0.87118	-0.86986	-0.86855	-0.86726
-0.87801	-0.87667	-0.87535	-0.87404	-0.87277	-0.87149	-0.87017	-0.86886	-0.86758
-0.87832	-0.87699	-0.87567	-0.87437	-0.87309	-0.8718	-0.87049	-0.86918	-0.8679
-0.87861	-0.8773	-0.87599	-0.8747	-0.87341	-0.87211	-0.8708	-0.86949	-0.86821
-0.87891	-0.87761	-0.87632	-0.87503	-0.87374	-0.87243	-0.87111	-0.8698	-0.86853
-0.87922	-0.87792	-0.87665	-0.87535	-0.87405	-0.87274	-0.87142	-0.87011	-0.86884
-0.87955	-0.87824	-0.87696	-0.87566	-0.87435	-0.87304	-0.87173	-0.87043	-0.86916
-0.87988	-0.87857	-0.87728	-0.87597	-0.87465	-0.87334	-0.87205	-0.87076	-0.86947
-0.88022	-0.8789	-0.8776	-0.8763	-0.87497	-0.87366	-0.87237	-0.87109	-0.8698
-0.85021	-0.87922	-0.87792	-0.87662	-0.8753	-0.87399	-0.8727	-0.87142	-0.87012
-0.85051	-0.87953	-0.87823	-0.87692	-0.87563	-0.87433	-0.87303	-0.87173	-0.87043
-0.85082	-0.87984	-0.87852	-0.87723	-0.87595	-0.87467	-0.87336	-0.87204	-0.87073
-0.85114	-0.88016	-0.87883	-0.87752	-0.87626	-0.87498	-0.87366	-0.87234	-0.87104
-0.85147	-0.8805	-0.87914	-0.87782	-0.87655	-0.87527	-0.87396	-0.87264	-0.87136
-0.8518	-0.85045	-0.84913	-0.87814	-0.87686	-0.87557	-0.87426	-0.87295	-0.87166
-0.85212	-0.85078	-0.84946	-0.87849	-0.87718	-0.87588	-0.87456	-0.87324	-0.87196
-0.85246	-0.85111	-0.84979	-0.8485	-0.84723	-0.87619	-0.87487	-0.87356	-0.87227
-0.85282	-0.85146	-0.85011	-0.8488	-0.84753	-0.87651	-0.8752	-0.87391	-0.87261
-0.85319	-0.85181	-0.85044	-0.84911	-0.84783	-0.87683	-0.87554	-0.87426	-0.87295
-0.82137	-0.85216	-0.85077	-0.84943	-0.84813	-0.87716	-0.87587	-0.87458	-0.87329
-0.82167	-0.8525	-0.85111	-0.84976	-0.84845	-0.87749	-0.87619	-0.87491	-0.87363
-0.82196	-0.85285	-0.85147	-0.85012	-0.84878	-0.84749	-0.84624	-0.84503	-0.87397
-0.82225	-0.8532	-0.85184	-0.85049	-0.84913	-0.84782	-0.84657	-0.84535	-0.8743
-0.82255	-0.82128	-0.82005	-0.85085	-0.84949	-0.84817	-0.84691	-0.84568	-0.87464
-0.82286	-0.82159	-0.82034	-0.8512	-0.84986	-0.84854	-0.84726	-0.846	-0.87498
-0.82318	-0.82191	-0.82065	-0.81941	-0.81819	-0.84891	-0.84761	-0.84633	-0.84507
-0.82352	-0.82224	-0.82098	-0.81973	-0.81849	-0.84925	-0.84795	-0.84667	-0.84541
-0.82387	-0.82259	-0.82131	-0.82005	-0.8188	-0.84961	-0.8483	-0.84702	-0.84576
-0.82421	-0.82293	-0.82165	-0.82039	-0.81914	-0.8179	-0.81669	-0.81549	-0.84609
-0.82456	-0.82328	-0.822	-0.82073	-0.81949	-0.81825	-0.81701	-0.81579	-0.84642
-0.8249	-0.82361	-0.82234	-0.82108	-0.81983	-0.81859	-0.81734	-0.81611	-0.8149
-0.82523	-0.82395	-0.82268	-0.82142	-0.82017	-0.81892	-0.81769	-0.81646	-0.81524
-0.82558	-0.82429	-0.82302	-0.82176	-0.8205	-0.81926	-0.81802	-0.8168	-0.81558
-0.82595	-0.82465	-0.82336	-0.82209	-0.82083	-0.81958	-0.81834	-0.81711	-0.8159
-0.82632	-0.82501	-0.82371	-0.82243	-0.82117	-0.81991	-0.81866	-0.81743	-0.81622
-0.82668	-0.82536	-0.82407	-0.82278	-0.82151	-0.82024	-0.81899	-0.81775	-0.81653
-0.82703	-0.82572	-0.82444	-0.82315	-0.82185	-0.82057	-0.81931	-0.81807	-0.81684
-0.82739	-0.82609	-0.82479	-0.8235	-0.82221	-0.82093	-0.81966	-0.81841	-0.81717
-0.82776	-0.82645	-0.82515	-0.82386	-0.82259	-0.82131	-0.82003	-0.81875	-0.8175
-0.82814	-0.82682	-0.82551	-0.82422	-0.82296	-0.82169	-0.82039	-0.81911	-0.81786
-0.82852	-0.8272	-0.82589	-0.82459	-0.82333	-0.82206	-0.82077	-0.81949	-0.81824

-0.7961	-0.82757	-0.82625	-0.82495	-0.82368	-0.82241	-0.82113	-0.81985	-0.81859
-0.79643	-0.82792	-0.8266	-0.82529	-0.824	-0.82272	-0.82145	-0.82019	-0.81892
-0.79677	-0.82828	-0.82695	-0.82563	-0.82433	-0.82304	-0.82177	-0.82052	-0.81924
-0.79712	-0.79584	-0.79456	-0.82598	-0.82468	-0.82339	-0.82211	-0.82084	-0.81957
-0.79749	-0.7962	-0.7949	-0.82635	-0.82504	-0.82375	-0.82246	-0.82117	-0.8199
-0.79784	-0.79655	-0.79525	-0.79398	-0.79274	-0.82412	-0.82282	-0.82153	-0.82026
-0.79819	-0.7969	-0.79562	-0.79436	-0.7931	-0.8245	-0.8232	-0.82191	-0.82064
-0.79854	-0.79725	-0.79598	-0.79471	-0.79344	-0.79219	-0.79095	-0.78973	-0.82102
-0.7989	-0.79759	-0.79633	-0.79505	-0.79377	-0.7925	-0.79128	-0.79007	-0.8214
-0.76	-0.79795	-0.79668	-0.7954	-0.7941	-0.79283	-0.79161	-0.79041	-0.78921
-0.76035	-0.79835	-0.79705	-0.79575	-0.79445	-0.79317	-0.79194	-0.79074	-0.78952
-0.76071	-0.79875	-0.79742	-0.79611	-0.7948	-0.79352	-0.79229	-0.79106	-0.78984
-0.69397	-0.79914	-0.79779	-0.79646	-0.79516	-0.79389	-0.79264	-0.79141	-0.79017
-0.69423	-0.79954	-0.79816	-0.79682	-0.79554	-0.79427	-0.79301	-0.79177	-0.79052
-0.69451	-0.79994	-0.79856	-0.79722	-0.79592	-0.79465	-0.79339	-0.79214	-0.79088
-0.69478	-0.80037	-0.79898	-0.79763	-0.79632	-0.79503	-0.79377	-0.79251	-0.79124
-0.66275	-0.7349	-0.73374	-0.79804	-0.79672	-0.79542	-0.79414	-0.79287	-0.7916
-0.66297	-0.73522	-0.73406	-0.79845	-0.79713	-0.79582	-0.79451	-0.79322	-0.79194
-0.66318	-0.70389	-0.7028	-0.79884	-0.79752	-0.79621	-0.79488	-0.79357	-0.79229
-0.6634	-0.70415	-0.70307	-0.79922	-0.7979	-0.79658	-0.79525	-0.79393	-0.79265
-0.66362	-0.70441	-0.70333	-0.79959	-0.79827	-0.79696	-0.79562	-0.79429	-0.79302
-0.66384	-0.66296	-0.66208	-0.73414	-0.73303	-0.79733	-0.79598	-0.79465	-0.79337
-0.66406	-0.66318	-0.66231	-0.73445	-0.73334	-0.79771	-0.79634	-0.79499	-0.79371
-0.66429	-0.66341	-0.66254	-0.7031	-0.70205	-0.7981	-0.79672	-0.79537	-0.79408
-0.66454	-0.66365	-0.66278	-0.70339	-0.70234	-0.7985	-0.79713	-0.79576	-0.79446
-0.66479	-0.6639	-0.66302	-0.66215	-0.66129	-0.70158	-0.70049	-0.69941	-0.79484
-0.66504	-0.66415	-0.66327	-0.6624	-0.66154	-0.70187	-0.70078	-0.6997	-0.79523
-0.6653	-0.6644	-0.66352	-0.66264	-0.66178	-0.70217	-0.70107	-0.69999	-0.79562
-0.66555	-0.66466	-0.66377	-0.66289	-0.66203	-0.70246	-0.70137	-0.70029	-0.79602
-0.66581	-0.66491	-0.66402	-0.66314	-0.66228	-0.70276	-0.70168	-0.70059	-0.79644
-0.66605	-0.66516	-0.66427	-0.6634	-0.66253	-0.66166	-0.66077	-0.65988	-0.69983
-0.6663	-0.6654	-0.66452	-0.66365	-0.66278	-0.66191	-0.66103	-0.66015	-0.70015
-0.66656	-0.66566	-0.66478	-0.6639	-0.66303	-0.66216	-0.66129	-0.66041	-0.70046
-0.66685	-0.66593	-0.66503	-0.66414	-0.66327	-0.6624	-0.66153	-0.66067	-0.70076
-0.58281	-0.66621	-0.66529	-0.66439	-0.66351	-0.66264	-0.66178	-0.66091	-0.70105
-0.58316	-0.6665	-0.66556	-0.66465	-0.66377	-0.66289	-0.66202	-0.66114	-0.70135
-0.58351	-0.6668	-0.66585	-0.66492	-0.66404	-0.66315	-0.66227	-0.66138	-0.70165
-0.58386	-0.61717	-0.61621	-0.61526	-0.61434	-0.66342	-0.66253	-0.66164	-0.66076
-0.5842	-0.61755	-0.61659	-0.61565	-0.61471	-0.6637	-0.6628	-0.66192	-0.66103
-0.58456	-0.58368	-0.58282	-0.61603	-0.61509	-0.61416	-0.61324	-0.61232	-0.61141
-0.58493	-0.58404	-0.58317	-0.61641	-0.61548	-0.61454	-0.61361	-0.61268	-0.61176
-0.5853	-0.5844	-0.58352	-0.61679	-0.61585	-0.61492	-0.61398	-0.61305	-0.61212
-0.58567	-0.58475	-0.58385	-0.61715	-0.61622	-0.61529	-0.61436	-0.61343	-0.61249
-0.58604	-0.58511	-0.5842	-0.58332	-0.58248	-0.61565	-0.61473	-0.61381	-0.61286
-0.58642	-0.58548	-0.58456	-0.58367	-0.58282	-0.61602	-0.61509	-0.61417	-0.61323
-0.5868	-0.58586	-0.58494	-0.58404	-0.58316	-0.61638	-0.61545	-0.61454	-0.61361
-0.58719	-0.58625	-0.58531	-0.5844	-0.58351	-0.61676	-0.61582	-0.6149	-0.61397
-0.58758	-0.58663	-0.58569	-0.58477	-0.58387	-0.61714	-0.6162	-0.61527	-0.61434
-0.58797	-0.58702	-0.58608	-0.58515	-0.58424	-0.58335	-0.5825	-0.58167	-0.61471
-0.58835	-0.58741	-0.58647	-0.58555	-0.58463	-0.58373	-0.58286	-0.58202	-0.61508

-0.58874	-0.5878	-0.58687	-0.58594	-0.58501	-0.58411	-0.58323	-0.58236	-0.61547
-0.58914	-0.58819	-0.58725	-0.58632	-0.58539	-0.58448	-0.58359	-0.58272	-0.61586
-0.58954	-0.58859	-0.58764	-0.5867	-0.58577	-0.58486	-0.58396	-0.58307	-0.61626
-0.58995	-0.589	-0.58803	-0.58708	-0.58615	-0.58523	-0.58432	-0.58342	-0.58256
-0.59037	-0.58941	-0.58843	-0.58748	-0.58654	-0.58561	-0.58469	-0.58378	-0.58291
-0.55665	-0.58981	-0.58884	-0.58788	-0.58693	-0.586	-0.58507	-0.58415	-0.58326
-0.557	-0.59022	-0.58925	-0.58829	-0.58734	-0.5864	-0.58546	-0.58453	-0.58361
-0.55734	-0.55647	-0.5556	-0.58869	-0.58773	-0.58679	-0.58585	-0.58491	-0.58398
-0.55766	-0.55679	-0.55593	-0.58907	-0.58811	-0.58715	-0.58622	-0.58529	-0.58436
-0.558	-0.55713	-0.55627	-0.58946	-0.5885	-0.58754	-0.58661	-0.58568	-0.58475
-0.55836	-0.55749	-0.55663	-0.58988	-0.58891	-0.58796	-0.58701	-0.58607	-0.58513
-0.55873	-0.55785	-0.55699	-0.59029	-0.58933	-0.58838	-0.58742	-0.58647	-0.58552
-0.55908	-0.5582	-0.55734	-0.55648	-0.55562	-0.58878	-0.58782	-0.58686	-0.58592
-0.55944	-0.55855	-0.55768	-0.55682	-0.55596	-0.58919	-0.58822	-0.58726	-0.58631
-0.55982	-0.55893	-0.55805	-0.55718	-0.55632	-0.55546	-0.55459	-0.55371	-0.58671
-0.56022	-0.55931	-0.55843	-0.55756	-0.5567	-0.55584	-0.55496	-0.55408	-0.58711
-0.5606	-0.55969	-0.5588	-0.55792	-0.55706	-0.5562	-0.55532	-0.55444	-0.55356
-0.56097	-0.56006	-0.55916	-0.55827	-0.55741	-0.55654	-0.55567	-0.5548	-0.55392
-0.56136	-0.56043	-0.55952	-0.55863	-0.55776	-0.55689	-0.55603	-0.55517	-0.55429
-0.56177	-0.56084	-0.55992	-0.55902	-0.55814	-0.55727	-0.5564	-0.55554	-0.55466
-0.56219	-0.56124	-0.56031	-0.55941	-0.55852	-0.55765	-0.55678	-0.55591	-0.55503
-0.56258	-0.56163	-0.5607	-0.55978	-0.55889	-0.55801	-0.55714	-0.55627	-0.55539
-0.56297	-0.56201	-0.56108	-0.56016	-0.55925	-0.55836	-0.55748	-0.55661	-0.55574
-0.52455	-0.56242	-0.56148	-0.56055	-0.55964	-0.55874	-0.55786	-0.55699	-0.55611
-0.52494	-0.56285	-0.5619	-0.56097	-0.56005	-0.55915	-0.55827	-0.55739	-0.55651
-0.52533	-0.52443	-0.52355	-0.56139	-0.56047	-0.55956	-0.55867	-0.55779	-0.55691
-0.52574	-0.52484	-0.52394	-0.56182	-0.56089	-0.55998	-0.55908	-0.55819	-0.5573
-0.52615	-0.52524	-0.52432	-0.56223	-0.5613	-0.56039	-0.55948	-0.55858	-0.55769
-0.52653	-0.52561	-0.52469	-0.56263	-0.56169	-0.56077	-0.55985	-0.55895	-0.55805
-0.5269	-0.52598	-0.52505	-0.56304	-0.56209	-0.56115	-0.56022	-0.55931	-0.55842
-0.45359	-0.52638	-0.52545	-0.52454	-0.52364	-0.56156	-0.56063	-0.55971	-0.55881
-0.45394	-0.5268	-0.52588	-0.52496	-0.52404	-0.562	-0.56106	-0.56014	-0.55923
-0.45428	-0.4535	-0.45272	-0.52538	-0.52446	-0.52355	-0.52267	-0.5218	-0.55965
-0.45463	-0.45385	-0.45308	-0.52581	-0.52488	-0.52397	-0.52308	-0.5222	-0.56008
-0.45497	-0.45419	-0.45342	-0.52623	-0.52531	-0.5244	-0.52349	-0.52259	-0.52172
-0.45527	-0.45451	-0.45376	-0.52665	-0.52573	-0.52482	-0.5239	-0.52299	-0.5221
-0.45558	-0.45482	-0.45408	-0.52705	-0.52614	-0.52523	-0.5243	-0.52338	-0.52249
-0.43389	-0.45514	-0.4544	-0.45364	-0.45286	-0.52561	-0.52468	-0.52376	-0.52288
-0.4342	-0.45547	-0.4547	-0.45393	-0.45316	-0.52598	-0.52506	-0.52415	-0.52326
-0.43453	-0.4558	-0.45503	-0.45426	-0.45348	-0.4527	-0.45193	-0.45117	-0.52366
-0.43485	-0.45615	-0.45537	-0.45459	-0.45382	-0.45305	-0.45227	-0.4515	-0.52406
-0.43519	-0.43447	-0.43376	-0.45493	-0.45416	-0.45339	-0.45262	-0.45184	-0.45107
-0.43553	-0.4348	-0.43408	-0.45527	-0.4545	-0.45373	-0.45296	-0.45219	-0.45142
-0.43587	-0.43513	-0.43441	-0.45561	-0.45484	-0.45407	-0.4533	-0.45253	-0.45177
-0.43623	-0.43546	-0.43473	-0.45595	-0.45517	-0.4544	-0.45364	-0.45288	-0.45212
-0.43659	-0.4358	-0.43507	-0.4563	-0.45551	-0.45474	-0.45398	-0.45322	-0.45246
-0.43698	-0.43616	-0.43541	-0.43468	-0.43396	-0.45507	-0.45431	-0.45355	-0.4528
-0.43739	-0.43653	-0.43576	-0.43501	-0.43429	-0.45541	-0.45464	-0.45388	-0.45313
-0.43782	-0.43692	-0.43612	-0.43535	-0.43462	-0.4339	-0.43321	-0.43253	-0.45346
-0.43828	-0.43733	-0.43649	-0.4357	-0.43495	-0.43423	-0.43352	-0.43283	-0.45379

-0.43876	-0.43777	-0.43688	-0.43605	-0.43529	-0.43456	-0.43384	-0.43315	-0.45412
-0.43929	-0.43824	-0.43728	-0.43641	-0.43564	-0.4349	-0.43417	-0.43346	-0.45445
-0.39712	-0.43875	-0.43771	-0.4368	-0.436	-0.43525	-0.4345	-0.43378	-0.43308
-0.39765	-0.4393	-0.43821	-0.43723	-0.43639	-0.43559	-0.43484	-0.43411	-0.4334
-0.3982	-0.43989	-0.43874	-0.4377	-0.43679	-0.43595	-0.43518	-0.43445	-0.43373
-0.39874	-0.39765	-0.39657	-0.39558	-0.39468	-0.43634	-0.43553	-0.43479	-0.43405
-0.3993	-0.3982	-0.39711	-0.39608	-0.3951	-0.43674	-0.43591	-0.43514	-0.43439
-0.39985	-0.39875	-0.39766	-0.3966	-0.39555	-0.3946	-0.3938	-0.39308	-0.43473
-0.4004	-0.3993	-0.39822	-0.39714	-0.39604	-0.39503	-0.39417	-0.3934	-0.43509
-0.35844	-0.39985	-0.39877	-0.39768	-0.39655	-0.3955	-0.39457	-0.39374	-0.39301
-0.35897	-0.40041	-0.39933	-0.39824	-0.39711	-0.39603	-0.39503	-0.39411	-0.39334
-0.35951	-0.40098	-0.3999	-0.3988	-0.39768	-0.39658	-0.39551	-0.39451	-0.39369
-0.36009	-0.40158	-0.40048	-0.39936	-0.39824	-0.39713	-0.396	-0.39495	-0.39407
-0.36068	-0.4022	-0.40106	-0.39993	-0.39881	-0.39768	-0.39652	-0.39542	-0.39449
-0.36126	-0.36017	-0.35909	-0.40051	-0.39937	-0.39824	-0.39707	-0.39595	-0.39495
-0.36183	-0.36073	-0.35965	-0.40109	-0.39995	-0.39881	-0.39765	-0.39652	-0.39547
-0.36241	-0.3613	-0.3602	-0.40167	-0.40051	-0.39937	-0.39822	-0.39709	-0.396
-0.363	-0.36187	-0.36076	-0.40225	-0.40108	-0.39992	-0.39878	-0.39766	-0.39654
-0.36359	-0.36245	-0.36132	-0.36021	-0.3591	-0.40049	-0.39936	-0.39823	-0.39711
-0.36418	-0.36302	-0.36187	-0.36075	-0.35966	-0.40109	-0.39996	-0.39884	-0.3977
-0.36476	-0.3636	-0.36243	-0.36131	-0.36022	-0.4017	-0.40057	-0.39945	-0.39831
-0.31586	-0.36417	-0.36301	-0.36188	-0.3608	-0.40231	-0.40118	-0.40005	-0.39891
-0.31631	-0.36475	-0.36359	-0.36247	-0.36138	-0.40292	-0.40178	-0.40065	-0.39951
-0.31677	-0.36534	-0.36418	-0.36306	-0.36196	-0.36087	-0.35979	-0.35872	-0.4001
-0.31724	-0.36594	-0.36478	-0.36365	-0.36253	-0.36143	-0.36035	-0.35927	-0.40068
-0.31771	-0.31679	-0.31588	-0.36424	-0.36311	-0.36199	-0.3609	-0.35982	-0.35875
-0.31819	-0.31726	-0.31635	-0.36482	-0.36368	-0.36256	-0.36146	-0.36038	-0.35931
-0.31868	-0.31775	-0.31683	-0.31591	-0.31501	-0.36313	-0.36202	-0.36094	-0.35988
-0.31918	-0.31825	-0.31732	-0.3164	-0.31547	-0.36369	-0.36259	-0.36151	-0.36044
-0.31968	-0.31876	-0.31783	-0.31689	-0.31593	-0.36428	-0.36317	-0.36209	-0.36101
-0.32018	-0.31926	-0.31833	-0.31739	-0.31643	-0.3649	-0.36377	-0.36267	-0.36158
-0.32068	-0.31976	-0.31882	-0.31789	-0.31696	-0.36556	-0.3644	-0.36326	-0.36215
-0.32117	-0.32025	-0.31932	-0.31839	-0.31747	-0.31655	-0.31562	-0.3147	-0.36274
-0.32165	-0.32074	-0.31982	-0.3189	-0.31798	-0.31705	-0.3161	-0.31517	-0.36333
-0.32214	-0.32123	-0.32032	-0.31941	-0.31848	-0.31754	-0.31659	-0.31566	-0.36394
-0.32264	-0.32173	-0.32082	-0.3199	-0.31897	-0.31803	-0.3171	-0.31617	-0.36457
-0.32315	-0.32223	-0.32132	-0.32039	-0.31946	-0.31853	-0.31761	-0.31669	-0.36521
-0.32365	-0.32272	-0.32181	-0.32088	-0.31996	-0.31904	-0.31812	-0.3172	-0.31628
-0.32414	-0.32321	-0.32229	-0.32138	-0.32047	-0.31955	-0.31863	-0.3177	-0.31679
-0.32464	-0.3237	-0.32279	-0.32187	-0.32097	-0.32006	-0.31914	-0.31822	-0.3173
-0.32514	-0.32419	-0.32328	-0.32237	-0.32147	-0.32056	-0.31966	-0.31875	-0.31782
-0.28556	-0.32471	-0.32378	-0.32287	-0.32197	-0.32107	-0.32017	-0.31927	-0.31834
-0.28604	-0.32524	-0.32429	-0.32337	-0.32248	-0.32158	-0.32068	-0.31978	-0.31884
-0.28653	-0.32579	-0.32482	-0.32388	-0.32298	-0.32209	-0.32119	-0.32029	-0.31935
-0.28701	-0.32634	-0.32536	-0.32441	-0.32348	-0.32258	-0.32169	-0.3208	-0.31987
-0.2875	-0.32689	-0.32591	-0.32494	-0.32399	-0.32307	-0.32218	-0.32131	-0.32039
-0.28801	-0.28712	-0.28624	-0.32548	-0.32452	-0.32359	-0.32269	-0.3218	-0.32089
-0.28851	-0.28762	-0.28673	-0.32603	-0.32507	-0.32413	-0.32321	-0.32229	-0.32138
-0.28901	-0.28812	-0.28723	-0.28635	-0.28549	-0.32466	-0.32372	-0.3228	-0.32189
-0.28951	-0.28861	-0.28773	-0.28686	-0.28597	-0.32518	-0.32425	-0.32333	-0.32242

-0.29002	-0.28912	-0.28824	-0.28736	-0.28646	-0.28558	-0.28474	-0.28392	-0.32294
-0.29057	-0.28966	-0.28875	-0.28785	-0.28697	-0.28609	-0.28522	-0.28437	-0.32344
-0.29112	-0.29019	-0.28926	-0.28835	-0.28747	-0.2866	-0.28571	-0.28484	-0.32396
-0.29163	-0.29071	-0.28979	-0.28888	-0.28798	-0.2871	-0.28621	-0.28533	-0.3245
-0.29214	-0.29122	-0.29032	-0.28941	-0.2885	-0.28759	-0.28671	-0.28583	-0.32505
-0.24159	-0.29175	-0.29084	-0.28993	-0.28901	-0.2881	-0.28721	-0.28633	-0.28545
-0.242	-0.29228	-0.29135	-0.29044	-0.28953	-0.28862	-0.28772	-0.28683	-0.28594
-0.15062	-0.2928	-0.29188	-0.29096	-0.29004	-0.28914	-0.28824	-0.28734	-0.28645
-0.1509	-0.29333	-0.29242	-0.2915	-0.29057	-0.28965	-0.28875	-0.28787	-0.28696
-0.15119	-0.15068	-0.15018	-0.29203	-0.29109	-0.29018	-0.28928	-0.28839	-0.28748
-0.15147	-0.15095	-0.15044	-0.29253	-0.29162	-0.29072	-0.28982	-0.28892	-0.28802
-0.15176	-0.15123	-0.15071	-0.29305	-0.29216	-0.29127	-0.29036	-0.28946	-0.28855
-0.09852	-0.15153	-0.151	-0.29359	-0.29269	-0.2918	-0.29089	-0.28999	-0.28908
-0.0987	-0.15184	-0.1513	-0.29415	-0.29323	-0.29233	-0.29142	-0.29052	-0.28961
-0.09886	-0.15214	-0.15159	-0.15106	-0.15055	-0.29285	-0.29195	-0.29105	-0.29014
-0.09903	-0.15243	-0.15188	-0.15135	-0.15082	-0.29337	-0.29247	-0.29158	-0.29066
-0.09919	-0.09886	-0.09854	-0.15164	-0.1511	-0.15058	-0.1501	-0.14962	-0.29119
-0.09936	-0.09903	-0.0987	-0.15192	-0.15138	-0.15086	-0.15036	-0.14988	-0.29172
-0.09954	-0.0992	-0.09887	-0.09855	-0.09824	-0.15115	-0.15064	-0.15014	-0.29225
-0.09974	-0.09939	-0.09905	-0.09872	-0.09841	-0.15146	-0.15093	-0.15042	-0.29279
-0.09994	-0.09958	-0.09923	-0.0989	-0.09858	-0.15177	-0.15123	-0.15071	-0.29332
-0.04741	-0.09977	-0.09942	-0.09908	-0.09876	-0.15208	-0.15154	-0.151	-0.1505
-0.04748	-0.09997	-0.09961	-0.09927	-0.09894	-0.15239	-0.15184	-0.15131	-0.15079
-0.04754	-0.10016	-0.0998	-0.09945	-0.09912	-0.09879	-0.09847	-0.09815	-0.15108
-0.04761	-0.10036	-0.09999	-0.09964	-0.09929	-0.09896	-0.09864	-0.09833	-0.15138
-0.04768	-0.0475	-0.04732	-0.09982	-0.09947	-0.09913	-0.09881	-0.0985	-0.09818
-0.04774	-0.04757	-0.04739	-0.10002	-0.09966	-0.09931	-0.09898	-0.09867	-0.09835
	-0.04763	-0.04746	-0.04729	-0.04712	-0.0995	-0.09917	-0.09884	-0.09852
	-0.04769	-0.04752	-0.04735	-0.04719	-0.0997	-0.09936	-0.09903	-0.0987
	-0.04775	-0.04758	-0.04741	-0.04726	-0.09991	-0.09956	-0.09921	-0.09888
			-0.04748	-0.04732	-0.10011	-0.09975	-0.09939	-0.09905
			-0.04755	-0.04738	-0.1003	-0.09994	-0.09958	-0.09923
			-0.04762	-0.04745	-0.04728	-0.04711	-0.04695	-0.09942
			-0.0477	-0.04752	-0.04734	-0.04717	-0.047	-0.09961
					-0.04741	-0.04723	-0.04707	-0.04691
					-0.04747	-0.0473	-0.04713	-0.04698
					-0.04754	-0.04737	-0.0472	-0.04705
								-0.04711
								-0.04717
								-0.04725
								-0.04734

					-0.10806
					-0.10784
					-0.10762
					-0.1074
			-0.10777	-0.10748	-0.25722
			-0.10755	-0.10725	-0.25659
			-0.10733	-0.10703	-0.25596
-0.108	-0.10771	-0.10741	-0.10711	-0.1068	-0.30219

			-0.10778	-0.10749	-0.10719	-0.10689	-0.10657	-0.30149
	-0.10816	-0.10786	-0.10757	-0.10727	-0.10697	-0.3031	-0.30197	-0.39522
	-0.10795	-0.10765	-0.10735	-0.10704	-0.10673	-0.30242	-0.30128	-0.39439
	-0.10773	-0.10743	-0.25376	-0.25278	-0.25182	-0.39635	-0.39495	-0.47614
	-0.10751	-0.10721	-0.25316	-0.2522	-0.25124	-0.39548	-0.39403	-0.4751
-0.10759	-0.10729	-0.10698	-0.3038	-0.30265	-0.3015	-0.47742	-0.47569	-0.56212
-0.10737	-0.10706	-0.10676	-0.30311	-0.30195	-0.3008	-0.47644	-0.47474	-0.56105
-0.10715	-0.10684	-0.10653	-0.30241	-0.30124	-0.30009	-0.47546	-0.47382	-0.56001
-0.10692	-0.25274	-0.25177	-0.39634	-0.39491	-0.39351	-0.51968	-0.51802	-0.55897
-0.1067	-0.25216	-0.25118	-0.39549	-0.39404	-0.39263	-0.51862	-0.51703	-0.55794
-0.25254	-0.35549	-0.3542	-0.52282	-0.52102	-0.51926	-0.56026	-0.55858	-0.60171
-0.25194	-0.35469	-0.3534	-0.52178	-0.52003	-0.51833	-0.55924	-0.55755	-0.60063
-0.25133	-0.4382	-0.4367	-0.56356	-0.56177	-0.56	-0.60316	-0.60134	-0.59958
-0.25073	-0.4373	-0.43583	-0.5625	-0.56077	-0.55905	-0.60217	-0.60032	-0.59856
-0.35357	-0.52209	-0.52043	-0.6066	-0.60482	-0.60303	-0.60117	-0.59931	-0.71372
-0.35277	-0.52106	-0.51945	-0.60553	-0.60376	-0.60199	-0.60015	-0.59831	-0.71248
-0.352	-0.52004	-0.51847	-0.60446	-0.60271	-0.60094	-0.59912	-0.5973	-0.71125
-0.48133	-0.56169	-0.56004	-0.6034	-0.60166	-0.59991	-0.71429	-0.7121	-0.83623
-0.48047	-0.56068	-0.55903	-0.60234	-0.60063	-0.5989	-0.71302	-0.71083	-0.83476
-0.51877	-0.60476	-0.60301	-0.71807	-0.71601	-0.71392	-0.71175	-0.70957	-0.87168
-0.51783	-0.6037	-0.60196	-0.71677	-0.71471	-0.71263	-0.71048	-0.70831	-0.87014
-0.60445	-0.71967	-0.71755	-0.71547	-0.71341	-0.71135	-0.87368	-0.87111	-1.01075
-0.60342	-0.71838	-0.71626	-0.71417	-0.71213	-0.71008	-0.87214	-0.86959	-1.00874
-0.6024	-0.71712	-0.71498	-0.71288	-0.71085	-0.70881	-0.87061	-0.8681	-1.0068
-0.68284	-0.7159	-0.71372	-0.87648	-0.87401	-0.87157	-1.01145	-1.00817	-1.11172
-0.68173	-0.71469	-0.71248	-0.87494	-0.87245	-0.87001	-1.00949	-1.00635	-1.10972
-0.71558	-0.79925	-0.79679	-0.87342	-0.87092	-0.86848	-1.11456	-1.11116	-1.10764
-0.71428	-0.7978	-0.79537	-0.87191	-0.8694	-0.86695	-1.11234	-1.109	-1.10554
-0.79871	-0.8755	-0.87295	-1.0132	-1.00987	-1.00664	-1.11017	-1.10686	-1.10345
-0.79724	-0.87393	-0.87145	-1.01129	-1.00798	-1.00475	-1.10806	-1.10473	-1.10139
-0.87488	-0.87238	-0.86996	-1.11664	-1.11299	-1.10941	-1.10597	-1.10263	-1.09935
-0.87334	-0.87084	-0.86845	-1.11445	-1.11087	-1.10734	-1.1039	-1.10054	-1.0973
-0.87182	-0.86932	-0.86695	-1.11227	-1.10877	-1.10531	-1.10187	-1.0985	-1.09527
-0.87031	-1.04952	-1.04619	-1.11013	-1.10672	-1.10334	-1.09993	-1.09656	-1.1785
-0.86881	-1.04744	-1.04414	-1.10802	-1.1047	-1.10139	-1.09804	-1.09469	-1.17642
-0.86738	-1.11281	-1.10936	-1.10598	-1.10266	-1.09938	-1.11959	-1.11626	-1.19648
-0.86601	-1.11086	-1.10739	-1.10398	-1.10062	-1.09731	-1.11758	-1.11436	-1.19444
-1.04506	-1.10887	-1.10543	-1.12557	-1.12215	-1.1188	-1.19934	-1.19591	-1.2234
-1.04308	-1.10682	-1.10346	-1.12363	-1.12023	-1.11687	-1.19719	-1.19373	-1.22118
-1.04111	-1.12833	-1.125	-1.17802	-1.17442	-1.17085	-1.19507	-1.19155	-1.21898
-1.03914	-1.12625	-1.12291	-1.17581	-1.17231	-1.16881	-1.19297	-1.1894	-1.21682
-1.0372	-1.12418	-1.12081	-1.17359	-1.17018	-1.16676	-1.19088	-1.18727	-1.2147
-1.12557	-1.17855	-1.17496	-1.19925	-1.19578	-1.19231	-1.21968	-1.21605	-1.21259
-1.12358	-1.17645	-1.17286	-1.19711	-1.19358	-1.19009	-1.21747	-1.21395	-1.2105
-1.14497	-1.17434	-1.17078	-1.22605	-1.22242	-1.21884	-1.21534	-1.21189	-1.20846
-1.14291	-1.17225	-1.16873	-1.22395	-1.2203	-1.21671	-1.21327	-1.20989	-1.20648
-1.17372	-1.198	-1.19445	-1.22185	-1.21822	-1.21465	-1.21123	-1.20789	-1.26529
-1.17167	-1.19594	-1.19235	-1.21971	-1.21617	-1.21268	-1.20925	-1.20586	-1.2632
-1.16959	-1.19386	-1.19025	-1.21758	-1.21413	-1.21071	-1.20728	-1.20386	-1.26113
-1.16746	-1.22267	-1.21907	-1.21554	-1.21208	-1.20866	-1.26616	-1.26263	-1.28561

-1.1653	-1.22048	-1.21698	-1.21351	-1.21002	-1.20658	-1.26406	-1.2606	-1.28356
-1.22189	-1.27982	-1.27623	-1.27264	-1.26901	-1.26542	-1.26194	-1.25851	-1.33758
-1.21971	-1.27758	-1.27401	-1.27044	-1.26686	-1.26331	-1.25982	-1.25637	-1.33528
-1.27914	-1.27542	-1.27184	-1.26829	-1.26475	-1.26123	-1.31515	-1.31133	-1.39034
-1.27706	-1.27336	-1.26975	-1.26619	-1.26268	-1.2592	-1.31298	-1.30916	-1.38805
-1.27498	-1.27131	-1.26768	-1.26412	-1.26064	-1.25718	-1.34156	-1.33764	-1.41182
-1.27284	-1.26921	-1.26564	-1.26211	-1.25861	-1.25514	-1.33934	-1.33548	-1.40956
-1.27071	-1.26713	-1.26361	-1.2601	-1.25659	-1.25312	-1.33714	-1.33332	-1.4073
-1.26868	-1.26509	-1.26155	-1.31582	-1.31198	-1.30817	-1.3599	-1.35602	-1.40502
-1.2667	-1.26309	-1.25948	-1.31357	-1.3098	-1.30606	-1.35767	-1.35375	-1.40274
-1.26467	-1.31933	-1.31533	-1.34215	-1.33827	-1.33442	-1.38182	-1.37788	-1.40051
-1.26262	-1.31719	-1.31326	-1.34003	-1.33607	-1.33217	-1.37954	-1.37569	-1.39831
-1.26054	-1.34582	-1.34186	-1.33791	-1.33393	-1.33	-1.37733	-1.37352	-1.47115
-1.25844	-1.34352	-1.33966	-1.33578	-1.33184	-1.32794	-1.37519	-1.37135	-1.46885
-1.34513	-1.34124	-1.33744	-1.38492	-1.38095	-1.377	-1.44942	-1.44537	-1.46661
-1.3429	-1.33903	-1.33521	-1.38263	-1.37875	-1.37489	-1.44726	-1.44325	-1.46444
-1.3407	-1.33685	-1.333	-1.38035	-1.37654	-1.37277	-1.4451	-1.44116	-1.46233
-1.33848	-1.33465	-1.33082	-1.4547	-1.45072	-1.4468	-1.46808	-1.46413	-1.46025
-1.33627	-1.33243	-1.32868	-1.45242	-1.44844	-1.4445	-1.46578	-1.46191	-1.45822
-1.38355	-1.43488	-1.43089	-1.45013	-1.44616	-1.44226	-1.46353	-1.45972	-1.50279
-1.38144	-1.43269	-1.42864	-1.44782	-1.44391	-1.44007	-1.46132	-1.45754	-1.50073
-1.40907	-1.4305	-1.42643	-1.4933	-1.48929	-1.48538	-1.50585	-1.50208	-1.51754
-1.40686	-1.42832	-1.42429	-1.49107	-1.48698	-1.48306	-1.50372	-1.50027	-1.51586
-1.40464	-1.42613	-1.42217	-1.48889	-1.48474	-1.48084	-1.50171	-1.49857	-1.51427
-1.42781	-1.49477	-1.49072	-1.48671	-1.48263	-1.47882	-1.49983	-1.49687	-1.51269
-1.42557	-1.49242	-1.48846	-1.48456	-1.48062	-1.47696	-1.49808	-1.49525	-1.51116
-1.47184	-1.49016	-1.48624	-1.50686	-1.50308	-1.49957	-1.51517	-1.51237	-1.5573
-1.46967	-1.48797	-1.48405	-1.50474	-1.50128	-1.49807	-1.51375	-1.51099	-1.55585
-1.4675	-1.4858	-1.48192	-1.5215	-1.51822	-1.5152	-1.56001	-1.55717	-1.57837
-1.46533	-1.48367	-1.47985	-1.51951	-1.51646	-1.51364	-1.55849	-1.55577	-1.57699
-1.46317	-1.50076	-1.49702	-1.56554	-1.56251	-1.55971	-1.55698	-1.55435	-1.57559
-1.46099	-1.4987	-1.49524	-1.56382	-1.56086	-1.55809	-1.55545	-1.55291	-1.57418
-1.45889	-1.49674	-1.49356	-1.56221	-1.55929	-1.55653	-1.55394	-1.55146	-1.57273
-1.4764	-1.54346	-1.54035	-1.56063	-1.5578	-1.55509	-1.5525	-1.54999	-1.60845
-1.47457	-1.54167	-1.53872	-1.55912	-1.55637	-1.55371	-1.55109	-1.54853	-1.60683
-1.52192	-1.53994	-1.53715	-1.55764	-1.55496	-1.55234	-1.61086	-1.60803	-1.63237
-1.52011	-1.53828	-1.53564	-1.55621	-1.55358	-1.55098	-1.60938	-1.60656	-1.63076
-1.53952	-1.55995	-1.55736	-1.56728	-1.56469	-1.56212	-1.60791	-1.6051	-1.62919
-1.53797	-1.55845	-1.55591	-1.56585	-1.5633	-1.56076	-1.60645	-1.60365	-1.62767
-1.53649	-1.56947	-1.56693	-1.58999	-1.58731	-1.58464	-1.63203	-1.62907	-1.64584
-1.53507	-1.56804	-1.56548	-1.58843	-1.58581	-1.58319	-1.63046	-1.62752	-1.64432
-1.53369	-1.56665	-1.56404	-1.58689	-1.58431	-1.58175	-1.6289	-1.62599	-1.64283
-1.54498	-1.56525	-1.56265	-1.58542	-1.58285	-1.58031	-1.64704	-1.64417	-1.64138
-1.54359	-1.56387	-1.56128	-1.58398	-1.58142	-1.57888	-1.64552	-1.64272	-1.63995
-1.54222	-1.58794	-1.58521	-1.65237	-1.64951	-1.64671	-1.64396	-1.64124	-1.66179
-1.54087	-1.58649	-1.58378	-1.65079	-1.64789	-1.64506	-1.64237	-1.63973	-1.66023
-1.53952	-1.60536	-1.60266	-1.64921	-1.6463	-1.64347	-1.64081	-1.63824	-1.67596
-1.53817	-1.60391	-1.60121	-1.64764	-1.64476	-1.64197	-1.63933	-1.63676	-1.67441
-1.53682	-1.60246	-1.59977	-1.64608	-1.64325	-1.64049	-1.63786	-1.63531	-1.67287
-1.55641	-1.60101	-1.59834	-1.64454	-1.64173	-1.63901	-1.67688	-1.67412	-1.6917

-1.55506	-1.59957	-1.59692	-1.64301	-1.64023	-1.63753	-1.67531	-1.67259	-1.69019
-1.6008	-1.6472	-1.64432	-1.65919	-1.65639	-1.65369	-1.67378	-1.67109	-1.68871
-1.59934	-1.64563	-1.64279	-1.65769	-1.65492	-1.65223	-1.67226	-1.66962	-1.68724
-1.59791	-1.66182	-1.65899	-1.67926	-1.67631	-1.67346	-1.67077	-1.66818	-1.6858
-1.59648	-1.66029	-1.65748	-1.67769	-1.67476	-1.67194	-1.6693	-1.66676	-1.68438
-1.66162	-1.65878	-1.656	-1.67613	-1.67324	-1.67046	-1.66786	-1.66535	-1.68297
-1.66009	-1.65728	-1.65453	-1.67461	-1.67177	-1.66903	-1.66645	-1.66395	-1.68154
-1.65857	-1.6558	-1.65308	-1.67311	-1.67034	-1.66765	-1.66507	-1.66255	-1.68012
-1.65709	-1.67731	-1.67442	-1.67163	-1.66895	-1.66633	-1.66374	-1.66118	-1.69675
-1.65565	-1.67581	-1.67294	-1.67019	-1.66759	-1.66503	-1.66243	-1.65982	-1.69535
-1.65427	-1.69275	-1.68992	-1.68718	-1.68458	-1.682	-1.69926	-1.69657	-1.69394
-1.65295	-1.69141	-1.68864	-1.68591	-1.68326	-1.68062	-1.69784	-1.69515	-1.69254
-1.69276	-1.69002	-1.68729	-1.68458	-1.68189	-1.67923	-1.69642	-1.69375	-1.69116
-1.69131	-1.68857	-1.68588	-1.68319	-1.68049	-1.67782	-1.69501	-1.69238	-1.6898
-1.68987	-1.68712	-1.68444	-1.68177	-1.67907	-1.6764	-1.69361	-1.69102	-1.68846
-1.68843	-1.68569	-1.68301	-1.68034	-1.67764	-1.67499	-1.69221	-1.68965	-1.68713
-1.687	-1.68427	-1.68158	-1.6789	-1.67622	-1.67359	-1.69082	-1.6883	-1.6858
-1.68555	-1.68284	-1.68014	-1.67748	-1.67483	-1.67224	-1.68946	-1.68696	-1.70573
-1.6841	-1.6814	-1.67871	-1.67606	-1.67347	-1.67092	-1.68814	-1.68564	-1.70432
-1.68267	-1.67999	-1.6773	-1.67467	-1.67212	-1.66962	-1.68683	-1.68434	-1.72865
-1.68127	-1.67859	-1.67591	-1.67331	-1.6708	-1.66833	-1.68554	-1.68306	-1.7272
-1.67991	-1.67724	-1.6746	-1.6918	-1.68927	-1.68678	-1.73145	-1.72861	-1.7258
-1.67863	-1.67599	-1.67339	-1.6906	-1.68809	-1.6856	-1.73012	-1.72729	-1.7245
-1.67735	-1.67473	-1.67219	-1.68941	-1.6869	-1.68441	-1.72879	-1.72596	-1.72318
-1.67598	-1.67339	-1.67089	-1.68812	-1.68562	-1.68315	-1.72736	-1.72455	-1.72179
-1.67459	-1.67204	-1.66957	-1.68681	-1.68433	-1.68186	-1.72591	-1.72313	-1.72037
-1.67326	-1.67074	-1.66829	-1.73293	-1.73008	-1.72726	-1.72447	-1.7217	-1.72565
-1.67197	-1.66948	-1.66703	-1.73147	-1.72863	-1.72581	-1.72304	-1.72029	-1.72424
-1.67073	-1.66826	-1.66582	-1.73691	-1.73404	-1.73121	-1.72841	-1.72564	-1.72289
-1.66956	-1.66711	-1.66468	-1.73559	-1.73274	-1.72992	-1.72713	-1.72437	-1.72162
-1.66837	-1.73994	-1.73708	-1.73423	-1.7314	-1.72859	-1.74189	-1.73912	-1.7364
-1.6671	-1.73848	-1.73561	-1.73277	-1.72996	-1.72717	-1.74046	-1.73771	-1.73502
-1.66584	-1.73699	-1.73413	-1.7313	-1.7285	-1.72572	-1.73902	-1.73628	-1.73363
-1.67206	-1.7516	-1.74874	-1.74592	-1.74312	-1.74036	-1.75487	-1.75213	-1.74949
-1.67084	-1.75015	-1.7473	-1.74448	-1.74169	-1.73893	-1.75346	-1.75075	-1.74814
-1.70747	-1.74877	-1.74593	-1.76045	-1.75762	-1.75484	-1.75212	-1.74947	-1.75852
-1.7062	-1.74744	-1.7446	-1.75911	-1.75629	-1.75352	-1.75086	-1.74825	-1.75728
-1.76632	-1.76342	-1.76055	-1.75772	-1.75492	-1.75219	-1.74956	-1.747	-1.7703
-1.76489	-1.76197	-1.7591	-1.75628	-1.75351	-1.75081	-1.74821	-1.74569	-1.76897
-1.76347	-1.76054	-1.75767	-1.75487	-1.75213	-1.74947	-1.77292	-1.77029	-1.76765
-1.76208	-1.75917	-1.75631	-1.75354	-1.75085	-1.74824	-1.77165	-1.76903	-1.76639
-1.76069	-1.75782	-1.75497	-1.75222	-1.74959	-1.74702	-1.7704	-1.76777	-1.76513
-1.75926	-1.75639	-1.75358	-1.76532	-1.76274	-1.76021	-1.76908	-1.76646	-1.76381
-1.75782	-1.75495	-1.75217	-1.76395	-1.76141	-1.7589	-1.76775	-1.76512	-1.76247
-1.77088	-1.76805	-1.76532	-1.77436	-1.77171	-1.76909	-1.76646	-1.76382	-1.76117
-1.76953	-1.76676	-1.76409	-1.77311	-1.77046	-1.76784	-1.7652	-1.76255	-1.7599
-1.7682	-1.76548	-1.76285	-1.77185	-1.7692	-1.76656	-1.76391	-1.76126	-1.80446
-1.7669	-1.7642	-1.76158	-1.77054	-1.76788	-1.76524	-1.76258	-1.75992	-1.80297
-1.76561	-1.77469	-1.77195	-1.76925	-1.76658	-1.76393	-1.76126	-1.75859	-1.80149
-1.7643	-1.77338	-1.77068	-1.768	-1.76533	-1.76267	-1.76	-1.75733	-1.80009

-1.763	-1.77208	-1.76942	-1.76677	-1.7641	-1.76144	-1.75876	-1.75608	-1.79871
-1.76176	-1.77083	-1.76818	-1.76553	-1.76287	-1.7602	-1.80332	-1.80028	-1.79728
-1.76057	-1.7696	-1.76695	-1.76429	-1.76163	-1.75895	-1.80191	-1.79882	-1.79582
-1.75938	-1.76836	-1.76568	-1.763	-1.76034	-1.75766	-1.80047	-1.79739	-1.79442
-1.75821	-1.76711	-1.76438	-1.76166	-1.75898	-1.7563	-1.799	-1.79598	-1.79307
-1.76856	-1.76584	-1.76307	-1.80661	-1.80358	-1.80058	-1.79757	-1.7946	-1.79174
-1.76726	-1.76453	-1.76178	-1.80519	-1.80216	-1.79917	-1.79619	-1.79326	-1.79045
-1.76596	-1.76322	-1.7605	-1.8038	-1.80078	-1.79779	-1.79483	-1.79193	-1.78915
-1.76467	-1.76195	-1.75925	-1.80241	-1.7994	-1.79642	-1.79348	-1.79059	-1.829
-1.76339	-1.76069	-1.758	-1.80104	-1.79803	-1.79505	-1.79213	-1.78926	-1.82753
-1.76213	-1.8057	-1.80268	-1.79966	-1.79663	-1.79365	-1.79077	-1.78797	-1.82613
-1.76088	-1.80431	-1.8013	-1.79829	-1.79523	-1.79224	-1.78943	-1.78671	-1.8248
-1.75963	-1.80292	-1.79989	-1.79687	-1.79384	-1.7909	-1.82953	-1.82652	-1.82349
-1.75839	-1.80151	-1.79842	-1.7954	-1.79248	-1.78965	-1.82822	-1.82522	-1.82222
-1.80318	-1.8001	-1.79697	-1.81211	-1.80922	-1.80642	-1.85904	-1.856	-1.85295
-1.80172	-1.79864	-1.79556	-1.81073	-1.80791	-1.80517	-1.85771	-1.85467	-1.8516
-1.80026	-1.79719	-1.79419	-1.8094	-1.80664	-1.80394	-1.85637	-1.85333	-1.85023
-1.79886	-1.79582	-1.7929	-1.86412	-1.86108	-1.85807	-1.855	-1.85193	-1.84882
-1.7975	-1.7945	-1.79165	-1.8628	-1.85976	-1.85674	-1.85363	-1.8505	-1.8474
-1.79613	-1.84417	-1.84129	-1.86147	-1.85845	-1.85542	-1.85228	-1.84912	-1.86772
-1.79475	-1.8428	-1.83999	-1.86015	-1.85715	-1.85412	-1.85097	-1.84778	-1.86628
-1.8267	-1.86485	-1.86183	-1.85883	-1.85584	-1.8528	-1.84964	-1.84645	-1.86486
-1.82542	-1.8635	-1.8605	-1.8575	-1.8545	-1.85147	-1.8483	-1.84511	-1.86343
-1.8418	-1.86218	-1.85917	-1.85616	-1.85315	-1.8501	-1.86866	-1.86529	-1.86202
-1.84052	-1.86086	-1.85785	-1.85481	-1.85173	-1.84864	-1.86715	-1.86384	-1.8606
-1.83925	-1.85954	-1.85652	-1.85346	-1.85031	-1.84717	-1.86563	-1.86239	-1.85919
-1.86126	-1.85823	-1.8552	-1.85213	-1.84895	-1.84579	-1.86416	-1.86095	-1.85779
-1.85994	-1.85691	-1.85389	-1.8508	-1.84761	-1.84444	-1.86273	-1.85954	-1.85641
-1.85862	-1.85558	-1.85256	-1.87131	-1.86793	-1.86457	-1.86132	-1.85813	-1.85503
-1.85729	-1.85425	-1.85122	-1.86988	-1.8665	-1.86315	-1.85991	-1.85674	-1.85367
-1.85597	-1.85291	-1.84988	-1.86845	-1.86507	-1.86174	-1.85852	-1.85537	-1.85233
-1.85463	-1.85157	-1.84854	-1.86702	-1.86365	-1.86033	-1.85715	-1.85405	-1.85102
-1.8533	-1.85023	-1.8472	-1.8656	-1.86224	-1.85894	-1.85579	-1.85273	-1.84972
-1.85199	-1.87069	-1.86745	-1.86418	-1.86083	-1.85755	-1.85443	-1.8514	-1.84841
-1.85069	-1.86928	-1.86603	-1.86277	-1.85944	-1.85618	-1.85309	-1.85009	-1.8471
-1.84937	-1.86787	-1.86464	-1.8614	-1.85813	-1.85491	-1.85182	-1.8488	-1.8458
-1.84804	-1.86647	-1.86325	-1.86006	-1.85688	-1.85372	-1.85061	-1.84753	-1.84451
-1.84673	-1.86507	-1.86187	-1.8587	-1.85559	-1.85248	-1.84936	-1.84625	-1.85881
-1.84541	-1.86367	-1.86047	-1.85732	-1.85425	-1.85119	-1.84807	-1.84496	-1.85746
-1.86559	-1.86232	-1.85911	-1.85597	-1.85292	-1.84988	-1.84678	-1.84367	-1.88062
-1.8643	-1.86104	-1.8578	-1.85465	-1.8516	-1.84857	-1.84547	-1.84236	-1.87926
-1.86302	-1.85977	-1.85652	-1.85334	-1.8503	-1.84727	-1.84417	-1.84108	-1.87792
-1.86168	-1.85845	-1.8552	-1.85204	-1.849	-1.84598	-1.8831	-1.87985	-1.87667
-1.86032	-1.85711	-1.85389	-1.85075	-1.8477	-1.8447	-1.88182	-1.87864	-1.87547
-1.85896	-1.85579	-1.85263	-1.87459	-1.87146	-1.86838	-1.8805	-1.87737	-1.87421
-1.85761	-1.85449	-1.85141	-1.8734	-1.87022	-1.86709	-1.87915	-1.87606	-1.87291
-1.8563	-1.87839	-1.87529	-1.88764	-1.88428	-1.88097	-1.87782	-1.87474	-1.87161
-1.85502	-1.8771	-1.87399	-1.88628	-1.88293	-1.87963	-1.8765	-1.87343	-1.87031
-1.87902	-1.87582	-1.8727	-1.88491	-1.88159	-1.87834	-1.87521	-1.87214	-1.86903
-1.8778	-1.87459	-1.87143	-1.88357	-1.88032	-1.87712	-1.87398	-1.87089	-1.8678

-1.87658	-1.87337	-1.87016	-1.88224	-1.87906	-1.87592	-1.87277	-1.86965	-1.86658
-1.87529	-1.88756	-1.88417	-1.88088	-1.87776	-1.87467	-1.87153	-1.8684	-1.86534
-1.87396	-1.88616	-1.88278	-1.87952	-1.87645	-1.8734	-1.87027	-1.86715	-1.86408
-1.87267	-1.88483	-1.88149	-1.87826	-1.87519	-1.87214	-1.86904	-1.86594	-1.86286
-1.87142	-1.88355	-1.8803	-1.8771	-1.87398	-1.8709	-1.86783	-1.86478	-1.86168
-1.88557	-1.88229	-1.87909	-1.87593	-1.87278	-1.86967	-1.86662	-1.86358	-1.86048
-1.88431	-1.88105	-1.87786	-1.8747	-1.87158	-1.86849	-1.8654	-1.86232	-1.85924
-1.88305	-1.87981	-1.87662	-1.87347	-1.87037	-1.86729	-1.86417	-1.86105	-1.85798
-1.88178	-1.87855	-1.8754	-1.87227	-1.86915	-1.86604	-1.86289	-1.85975	-1.85669
-1.88049	-1.87728	-1.87417	-1.87106	-1.86792	-1.86477	-1.86161	-1.85846	-1.8554
-1.8792	-1.876	-1.8729	-1.86979	-1.86664	-1.86349	-1.86034	-1.85722	-1.86656
-1.87791	-1.87471	-1.87161	-1.86849	-1.86534	-1.8622	-1.8591	-1.85602	-1.86536
-1.87666	-1.87346	-1.87033	-1.8672	-1.86406	-1.86095	-1.85787	-1.85481	-1.86416
-1.87547	-1.87225	-1.86907	-1.86593	-1.86283	-1.85974	-1.85666	-1.8536	-1.86297
-1.87426	-1.87102	-1.86783	-1.86468	-1.8616	-1.85854	-1.86785	-1.86479	-1.8736
-1.87298	-1.86976	-1.8666	-1.86347	-1.86037	-1.8573	-1.86664	-1.86362	-1.87241
-1.8717	-1.86851	-1.86538	-1.86227	-1.85915	-1.85607	-1.86543	-1.86244	-1.87123
-1.87051	-1.8673	-1.86415	-1.86103	-1.85794	-1.85488	-1.87606	-1.87302	-1.87002
-1.86935	-1.86613	-1.86293	-1.8598	-1.85674	-1.85372	-1.87486	-1.8718	-1.86882
-1.86818	-1.86497	-1.86176	-1.87099	-1.86792	-1.86489	-1.87366	-1.87062	-1.86764
-1.86699	-1.86381	-1.86062	-1.86984	-1.86674	-1.86368	-1.87246	-1.86947	-1.86649
-1.86578	-1.86263	-1.85947	-1.88057	-1.87741	-1.8743	-1.87128	-1.8683	-1.86532
-1.86452	-1.8614	-1.85831	-1.87944	-1.87628	-1.87315	-1.87011	-1.8671	-1.86413
-1.86329	-1.87225	-1.86916	-1.87828	-1.87514	-1.87202	-1.86895	-1.86593	-1.86295
-1.8621	-1.87105	-1.86794	-1.87705	-1.87397	-1.8709	-1.86784	-1.8648	-1.86181
-1.86093	-1.86986	-1.86672	-1.87582	-1.87279	-1.86977	-1.86673	-1.8637	-1.86069
-1.87183	-1.8809	-1.87775	-1.87464	-1.87161	-1.8686	-1.8656	-1.8626	-1.87291
-1.87068	-1.87975	-1.87661	-1.8735	-1.87044	-1.86741	-1.86444	-1.86149	-1.87182
-1.8695	-1.87857	-1.87546	-1.87236	-1.8693	-1.86626	-1.87658	-1.87363	-1.87069
-1.86828	-1.87736	-1.8743	-1.87125	-1.86819	-1.86515	-1.87541	-1.87242	-1.86951
-1.86709	-1.87617	-1.87313	-1.8834	-1.88035	-1.87731	-1.87426	-1.87125	-1.86835
-1.86594	-1.875	-1.87193	-1.88218	-1.87918	-1.87619	-1.87316	-1.87015	-1.86724
-1.86481	-1.87384	-1.87075	-1.88097	-1.878	-1.87505	-1.87206	-1.86907	-1.86613
-1.88909	-1.88599	-1.88288	-1.87983	-1.87683	-1.87386	-1.8709	-1.86796	-1.86505
-1.88793	-1.88486	-1.88178	-1.87871	-1.87566	-1.87266	-1.86973	-1.86685	-1.86396
-1.88678	-1.88374	-1.88067	-1.87761	-1.87454	-1.87152	-1.86859	-1.86571	-1.86284
-1.88564	-1.88261	-1.87956	-1.87651	-1.87345	-1.87042	-1.86746	-1.86456	-1.8617
-1.88447	-1.88145	-1.87844	-1.87541	-1.87236	-1.86933	-1.86636	-1.86344	-1.86059
-1.88325	-1.88024	-1.87728	-1.8743	-1.87127	-1.86826	-1.86529	-1.86236	-1.85951
-1.88205	-1.87906	-1.87613	-1.87319	-1.87019	-1.86719	-1.86423	-1.8613	-1.85844
-1.88092	-1.87793	-1.87501	-1.87208	-1.8691	-1.86613	-1.86318	-1.86025	-1.85737
-1.87981	-1.87681	-1.87388	-1.87095	-1.86801	-1.86506	-1.86212	-1.8592	-1.85631
-1.87868	-1.87567	-1.87271	-1.86978	-1.86688	-1.86399	-1.86107	-1.85815	-1.85527
-1.87757	-1.87453	-1.87153	-1.86859	-1.86575	-1.86291	-1.86001	-1.85709	-1.85423
-1.86812	-1.87342	-1.87041	-1.86747	-1.86465	-1.86184	-1.85896	-1.85606	-1.8532
-1.86703	-1.87234	-1.86933	-1.8664	-1.86358	-1.86078	-1.85791	-1.85503	-1.85218
-1.86594	-1.87126	-1.86826	-1.86534	-1.86253	-1.85973	-1.85687	-1.85401	-1.85117
-1.86483	-1.87018	-1.8672	-1.86429	-1.86149	-1.8587	-1.85586	-1.853	-1.85017
-1.86374	-1.8691	-1.86613	-1.86324	-1.86045	-1.85767	-1.85484	-1.85199	-1.84917
-1.86265	-1.86803	-1.86508	-1.8622	-1.85941	-1.85663	-1.85381	-1.85098	-1.84817

-1.86157	-1.86697	-1.86403	-1.86116	-1.85837	-1.85559	-1.85279	-1.84997	-1.84717
-1.8605	-1.86591	-1.86298	-1.86012	-1.85734	-1.85457	-1.85177	-1.84896	-1.84616
-1.85943	-1.86486	-1.86194	-1.85908	-1.85632	-1.85356	-1.85076	-1.84795	-1.84514
-1.85836	-1.85542	-1.8525	-1.85804	-1.85528	-1.85254	-1.84974	-1.84693	-1.84413
-1.85728	-1.85435	-1.85144	-1.85699	-1.85424	-1.8515	-1.84872	-1.84592	-1.84313
-1.8562	-1.85328	-1.85038	-1.85595	-1.8532	-1.85047	-1.84769	-1.8449	-1.84212
-1.85513	-1.85222	-1.84932	-1.8549	-1.85216	-1.84944	-1.84666	-1.84387	-1.8411
-1.85408	-1.85117	-1.84828	-1.85389	-1.85115	-1.84842	-1.84563	-1.84284	-1.84008
-1.85303	-1.85015	-1.8473	-1.85294	-1.85017	-1.8474	-1.84461	-1.84182	-1.83906
-1.85199	-1.84914	-1.84635	-1.85201	-1.8492	-1.84639	-1.84359	-1.84079	-1.83804
-1.851	-1.84817	-1.84537	-1.85103	-1.8482	-1.84538	-1.84257	-1.83978	-1.83703
-1.85004	-1.84721	-1.84437	-1.85003	-1.84719	-1.84437	-1.84156	-1.83878	-1.83602
-1.84905	-1.84621	-1.84335	-1.8405	-1.83766	-1.83483	-1.84055	-1.83777	-1.83503
-1.84804	-1.84518	-1.84232	-1.83946	-1.83661	-1.83378	-1.83953	-1.83677	-1.83403
-1.84701	-1.84414	-1.84127	-1.83842	-1.83557	-1.83275	-1.83851	-1.83577	-1.83305
-1.84597	-1.84309	-1.84022	-1.83737	-1.83452	-1.83172	-1.8375	-1.83477	-1.83208
-1.84494	-1.84205	-1.83917	-1.83633	-1.8335	-1.83072	-1.83653	-1.83381	-1.83114
-1.8439	-1.84102	-1.83815	-1.83532	-1.83254	-1.82979	-1.83562	-1.83291	-1.83023
-1.84288	-1.84	-1.83714	-1.83433	-1.8316	-1.82889	-1.83475	-1.83203	-1.82933
-1.84191	-1.83906	-1.8362	-1.8334	-1.83067	-1.82796	-1.82525	-1.82253	-1.82839
-1.84098	-1.83816	-1.83531	-1.8325	-1.82975	-1.82702	-1.82428	-1.82156	-1.82742
-1.84003	-1.83722	-1.83438	-1.83157	-1.82881	-1.82606	-1.82332	-1.82059	-1.82646
-1.83906	-1.83623	-1.8334	-1.83059	-1.82783	-1.82508	-1.82234	-1.81962	-1.8255
-1.83807	-1.83523	-1.83241	-1.82961	-1.82685	-1.82411	-1.82138	-1.81867	-1.81597
-1.83709	-1.83425	-1.83144	-1.82865	-1.82588	-1.82315	-1.82045	-1.81778	-1.81509
-1.83612	-1.83329	-1.83048	-1.8277	-1.82494	-1.82221	-1.81953	-1.81689	-1.81422
-1.83522	-1.8324	-1.8296	-1.82682	-1.82405	-1.82132	-1.81863	-1.81597	-1.8133
-1.83435	-1.83154	-1.82875	-1.82597	-1.8232	-1.82046	-1.81774	-1.81503	-1.81235
-1.83343	-1.83063	-1.82785	-1.82507	-1.82231	-1.81955	-1.81681	-1.81409	-1.8114
-1.83246	-1.82967	-1.8269	-1.82413	-1.82136	-1.81861	-1.81587	-1.81315	-1.81045
-1.83152	-1.82875	-1.82599	-1.82322	-1.82045	-1.81769	-1.81495	-1.81223	-1.80953
-1.83063	-1.82788	-1.82512	-1.82236	-1.81959	-1.81683	-1.8141	-1.81138	-1.80868
-1.82974	-1.82701	-1.82425	-1.82149	-1.81872	-1.81597	-1.81323	-1.81052	-1.80782
-1.82882	-1.82608	-1.82332	-1.82056	-1.81779	-1.81504	-1.81231	-1.8096	-1.8069
-1.82791	-1.82515	-1.82238	-1.81962	-1.81685	-1.8141	-1.81137	-1.80867	-1.80597
-1.82705	-1.82427	-1.8215	-1.81874	-1.81597	-1.81322	-1.8105	-1.80779	-1.8051
-1.82621	-1.82343	-1.82065	-1.81788	-1.81513	-1.81238	-1.80966	-1.80695	-1.80426
-1.82532	-1.82254	-1.81975	-1.81698	-1.81423	-1.81149	-1.80877	-1.80607	-1.80338
-1.82437	-1.82159	-1.8188	-1.81603	-1.81329	-1.81055	-1.80783	-1.80513	-1.80244
-1.82344	-1.82066	-1.81788	-1.81512	-1.81237	-1.80964	-1.80693	-1.80423	-1.80155
-1.82258	-1.8198	-1.81703	-1.81427	-1.81153	-1.80881	-1.8061	-1.8034	-1.80072
-1.82171	-1.81893	-1.81617	-1.81342	-1.8107	-1.80799	-1.80528	-1.80259	-1.79991
-1.80897	-1.818	-1.81524	-1.81251	-1.80982	-1.80715	-1.80446	-1.80178	-1.7991
-1.80803	-1.81705	-1.81429	-1.81158	-1.80894	-1.8063	-1.80363	-1.80095	-1.79828
-1.80716	-1.80443	-1.80171	-1.8107	-1.80805	-1.80541	-1.80274	-1.80007	-1.7974
-1.80633	-1.8036	-1.80089	-1.80985	-1.80716	-1.80448	-1.80181	-1.79914	-1.79647
-1.80552	-1.8028	-1.80008	-1.79738	-1.79471	-1.79204	-1.80093	-1.79826	-1.79559
-1.80474	-1.80201	-1.7993	-1.7966	-1.79391	-1.79124	-1.80011	-1.79744	-1.79477
-1.80394	-1.80122	-1.7985	-1.7958	-1.79311	-1.79044	-1.7993	-1.79663	-1.79395
-1.80313	-1.80041	-1.79769	-1.79499	-1.7923	-1.78963	-1.79849	-1.79581	-1.79314

-1.80228	-1.79956	-1.79685	-1.79416	-1.79147	-1.7888	-1.79765	-1.79497	-1.7923
-1.80135	-1.79864	-1.79593	-1.79324	-1.79056	-1.7879	-1.79674	-1.79406	-1.79139
-1.80039	-1.79768	-1.79497	-1.79229	-1.78962	-1.78696	-1.79579	-1.79311	-1.79044
-1.7995	-1.79678	-1.79408	-1.7914	-1.78873	-1.78608	-1.7949	-1.79223	-1.78955
-1.79865	-1.79594	-1.79323	-1.79055	-1.78789	-1.78525	-1.79406	-1.79139	-1.78871
-1.79783	-1.79511	-1.7924	-1.78972	-1.78707	-1.78443	-1.79323	-1.79056	-1.78789
-1.79701	-1.7943	-1.79159	-1.78891	-1.78626	-1.78362	-1.79241	-1.78974	-1.78707
-1.7962	-1.79348	-1.79078	-1.78811	-1.78545	-1.78281	-1.7916	-1.78893	-1.78626
-1.79538	-1.79267	-1.78998	-1.7873	-1.78465	-1.78201	-1.79079	-1.78812	-1.78545
-1.79456	-1.79186	-1.78917	-1.7865	-1.78384	-1.7812	-1.78997	-1.78731	-1.78463
-1.79374	-1.79104	-1.78836	-1.78569	-1.78304	-1.7804	-1.78916	-1.78649	-1.78382
-1.79292	-1.79022	-1.78754	-1.78488	-1.78223	-1.77959	-1.78834	-1.78567	-1.783
-1.7921	-1.7894	-1.78673	-1.78408	-1.78143	-1.77879	-1.78753	-1.78485	-1.78218
-1.79128	-1.78859	-1.78592	-1.78327	-1.78062	-1.77798	-1.78672	-1.78404	-1.78136
-1.79046	-1.78777	-1.78511	-1.78246	-1.77982	-1.77718	-1.7859	-1.78322	-1.78055
-1.78965	-1.78696	-1.7843	-1.78165	-1.77901	-1.77637	-1.78509	-1.78241	-1.77973
-1.78884	-1.78615	-1.78349	-1.78084	-1.7782	-1.77556	-1.78427	-1.78159	-1.77891
-1.78802	-1.78534	-1.78267	-1.78002	-1.77738	-1.77474	-1.78344	-1.78076	-1.77808
-1.7872	-1.78452	-1.78185	-1.7792	-1.77656	-1.77392	-1.78261	-1.77993	-1.77725
-1.78638	-1.7837	-1.78103	-1.77837	-1.77573	-1.77309	-1.78177	-1.77909	-1.77641
-1.78556	-1.78287	-1.78021	-1.77755	-1.7749	-1.77226	-1.78093	-1.77825	-1.77557
-1.78473	-1.78205	-1.77938	-1.77672	-1.77407	-1.77143	-1.78009	-1.77741	-1.77473
-1.7839	-1.78122	-1.77855	-1.77589	-1.77324	-1.77059	-1.77925	-1.77657	-1.7739
-1.78308	-1.78039	-1.77771	-1.77505	-1.7724	-1.76976	-1.77841	-1.77573	-1.77306
-1.78225	-1.77956	-1.77688	-1.77422	-1.77157	-1.76893	-1.77757	-1.77489	-1.77221
-1.78144	-1.77873	-1.77605	-1.77338	-1.77074	-1.7681	-1.77673	-1.77405	-1.77137
-1.78065	-1.77792	-1.77522	-1.77255	-1.7699	-1.76726	-1.77589	-1.77321	-1.77053
-1.77988	-1.77712	-1.77441	-1.77172	-1.76907	-1.76643	-1.77504	-1.77237	-1.76969
-1.77913	-1.77636	-1.77362	-1.77092	-1.76826	-1.76561	-1.77421	-1.77152	-1.76884
-1.7784	-1.77563	-1.77287	-1.77014	-1.76746	-1.7648	-1.77338	-1.77068	-1.76799
-1.77763	-1.77488	-1.77212	-1.76939	-1.7667	-1.76403	-1.77258	-1.76987	-1.76717
-1.77681	-1.7741	-1.77139	-1.76869	-1.76601	-1.76333	-1.77184	-1.76908	-1.76636
-1.77599	-1.77331	-1.77064	-1.76797	-1.7653	-1.76261	-1.77111	-1.76832	-1.76559
-1.77519	-1.7725	-1.76984	-1.76718	-1.76452	-1.76184	-1.77035	-1.7676	-1.76487
-1.7744	-1.77171	-1.76904	-1.76637	-1.76371	-1.76105	-1.76959	-1.76688	-1.76417
-1.77368	-1.77098	-1.76827	-1.76558	-1.76291	-1.76025	-1.76879	-1.76608	-1.76338
-1.773	-1.77028	-1.76753	-1.76481	-1.76212	-1.75945	-1.76797	-1.76526	-1.76256
-1.77226	-1.76954	-1.76679	-1.77537	-1.77264	-1.76992	-1.76719	-1.76446	-1.78929
-1.77147	-1.76878	-1.76607	-1.77467	-1.77195	-1.76922	-1.76645	-1.76368	-1.78847
-1.77067	-1.768	-1.76533	-1.77394	-1.77123	-1.76849	-1.76571	-1.76292	-1.78768
-1.76987	-1.7672	-1.76453	-1.77314	-1.77043	-1.7677	-1.76495	-1.76219	-1.78696
-1.7691	-1.76642	-1.76375	-1.77235	-1.76963	-1.7669	-1.76418	-1.76146	-1.78624
-1.76839	-1.76571	-1.76304	-1.77163	-1.76887	-1.76611	-1.79096	-1.7882	-1.78543
-1.76771	-1.76503	-1.76235	-1.77092	-1.76812	-1.76533	-1.79015	-1.78738	-1.78461
-1.76696	-1.76428	-1.7616	-1.77016	-1.76737	-1.76459	-1.78939	-1.78663	-1.78384
-1.76617	-1.76348	-1.7608	-1.76936	-1.76662	-1.76387	-1.78868	-1.78591	-1.7831
-1.76543	-1.77404	-1.7713	-1.79622	-1.79345	-1.79069	-1.78792	-1.78515	-1.78234
-1.76473	-1.77332	-1.77054	-1.79541	-1.79263	-1.78986	-1.7871	-1.78434	-1.78155
-1.75202	-1.77259	-1.76978	-1.79463	-1.79184	-1.78907	-1.78631	-1.78355	-1.78078
-1.75125	-1.77183	-1.76905	-1.79391	-1.79112	-1.78835	-1.78558	-1.78282	-1.78003

					-0.10806
					-0.10784
					-0.10762
					-0.1074
			-0.10777	-0.10748	-0.25722
			-0.10755	-0.10725	-0.25659
			-0.10733	-0.10703	-0.25596
-0.108	-0.10771	-0.10741	-0.10711	-0.1068	-0.30219

			-0.10778	-0.10749	-0.10719	-0.10689	-0.10657	-0.30149
	-0.10816	-0.10786	-0.10757	-0.10727	-0.10697	-0.3031	-0.30197	-0.39522
	-0.10795	-0.10765	-0.10735	-0.10704	-0.10673	-0.30242	-0.30128	-0.39439
	-0.10773	-0.10743	-0.25376	-0.25278	-0.25182	-0.39635	-0.39495	-0.47614
	-0.10751	-0.10721	-0.25316	-0.2522	-0.25124	-0.39548	-0.39403	-0.4751
-0.10759	-0.10729	-0.10698	-0.3038	-0.30265	-0.3015	-0.47742	-0.47569	-0.56212
-0.10737	-0.10706	-0.10676	-0.30311	-0.30195	-0.3008	-0.47644	-0.47474	-0.56105
-0.10715	-0.10684	-0.10653	-0.30241	-0.30124	-0.30009	-0.47546	-0.47382	-0.56001
-0.10692	-0.25274	-0.25177	-0.39634	-0.39491	-0.39351	-0.51968	-0.51802	-0.55897
-0.1067	-0.25216	-0.25118	-0.39549	-0.39404	-0.39263	-0.51862	-0.51703	-0.55794
-0.25254	-0.35549	-0.3542	-0.52282	-0.52102	-0.51926	-0.56026	-0.55858	-0.60171
-0.25194	-0.35469	-0.3534	-0.52178	-0.52003	-0.51833	-0.55924	-0.55755	-0.60063
-0.25133	-0.4382	-0.4367	-0.56356	-0.56177	-0.56	-0.60316	-0.60134	-0.59958
-0.25073	-0.4373	-0.43583	-0.5625	-0.56077	-0.55905	-0.60217	-0.60032	-0.59856
-0.35357	-0.52209	-0.52043	-0.6066	-0.60482	-0.60303	-0.60117	-0.59931	-0.71372
-0.35277	-0.52106	-0.51945	-0.60553	-0.60376	-0.60199	-0.60015	-0.59831	-0.71248
-0.352	-0.52004	-0.51847	-0.60446	-0.60271	-0.60094	-0.59912	-0.5973	-0.71125
-0.48133	-0.56169	-0.56004	-0.6034	-0.60166	-0.59991	-0.71429	-0.7121	-0.83623
-0.48047	-0.56068	-0.55903	-0.60234	-0.60063	-0.5989	-0.71302	-0.71083	-0.83476
-0.51877	-0.60476	-0.60301	-0.71807	-0.71601	-0.71392	-0.71175	-0.70957	-0.87168
-0.51783	-0.6037	-0.60196	-0.71677	-0.71471	-0.71263	-0.71048	-0.70831	-0.87014
-0.60445	-0.71967	-0.71755	-0.71547	-0.71341	-0.71135	-0.87368	-0.87111	-1.01075
-0.60342	-0.71838	-0.71626	-0.71417	-0.71213	-0.71008	-0.87214	-0.86959	-1.00874
-0.6024	-0.71712	-0.71498	-0.71288	-0.71085	-0.70881	-0.87061	-0.8681	-1.0068
-0.68284	-0.7159	-0.71372	-0.87648	-0.87401	-0.87157	-1.01145	-1.00817	-1.11172
-0.68173	-0.71469	-0.71248	-0.87494	-0.87245	-0.87001	-1.00949	-1.00635	-1.10972
-0.71558	-0.79925	-0.79679	-0.87342	-0.87092	-0.86848	-1.11456	-1.11116	-1.10764
-0.71428	-0.7978	-0.79537	-0.87191	-0.8694	-0.86695	-1.11234	-1.109	-1.10554
-0.79871	-0.8755	-0.87295	-1.0132	-1.00987	-1.00664	-1.11017	-1.10686	-1.10345
-0.79724	-0.87393	-0.87145	-1.01129	-1.00798	-1.00475	-1.10806	-1.10473	-1.10139
-0.87488	-0.87238	-0.86996	-1.11664	-1.11299	-1.10941	-1.10597	-1.10263	-1.09935
-0.87334	-0.87084	-0.86845	-1.11445	-1.11087	-1.10734	-1.1039	-1.10054	-1.0973
-0.87182	-0.86932	-0.86695	-1.11227	-1.10877	-1.10531	-1.10187	-1.0985	-1.09527
-0.87031	-1.04952	-1.04619	-1.11013	-1.10672	-1.10334	-1.09993	-1.09656	-1.1785
-0.86881	-1.04744	-1.04414	-1.10802	-1.1047	-1.10139	-1.09804	-1.09469	-1.17642
-0.86738	-1.11281	-1.10936	-1.10598	-1.10266	-1.09938	-1.11959	-1.11626	-1.19648
-0.86601	-1.11086	-1.10739	-1.10398	-1.10062	-1.09731	-1.11758	-1.11436	-1.19444
-1.04506	-1.10887	-1.10543	-1.12557	-1.12215	-1.1188	-1.19934	-1.19591	-1.2234
-1.04308	-1.10682	-1.10346	-1.12363	-1.12023	-1.11687	-1.19719	-1.19373	-1.22118
-1.04111	-1.12833	-1.125	-1.17802	-1.17442	-1.17085	-1.19507	-1.19155	-1.21898
-1.03914	-1.12625	-1.12291	-1.17581	-1.17231	-1.16881	-1.19297	-1.1894	-1.21682
-1.0372	-1.12418	-1.12081	-1.17359	-1.17018	-1.16676	-1.19088	-1.18727	-1.2147
-1.12557	-1.17855	-1.17496	-1.19925	-1.19578	-1.19231	-1.21968	-1.21605	-1.21259
-1.12358	-1.17645	-1.17286	-1.19711	-1.19358	-1.19009	-1.21747	-1.21395	-1.2105
-1.14497	-1.17434	-1.17078	-1.22605	-1.22242	-1.21884	-1.21534	-1.21189	-1.20846
-1.14291	-1.17225	-1.16873	-1.22395	-1.2203	-1.21671	-1.21327	-1.20989	-1.20648
-1.17372	-1.198	-1.19445	-1.22185	-1.21822	-1.21465	-1.21123	-1.20789	-1.26529
-1.17167	-1.19594	-1.19235	-1.21971	-1.21617	-1.21268	-1.20925	-1.20586	-1.2632
-1.16959	-1.19386	-1.19025	-1.21758	-1.21413	-1.21071	-1.20728	-1.20386	-1.26113
-1.16746	-1.22267	-1.21907	-1.21554	-1.21208	-1.20866	-1.26616	-1.26263	-1.28561

-1.1653	-1.22048	-1.21698	-1.21351	-1.21002	-1.20658	-1.26406	-1.2606	-1.28356
-1.22189	-1.27982	-1.27623	-1.27264	-1.26901	-1.26542	-1.26194	-1.25851	-1.33758
-1.21971	-1.27758	-1.27401	-1.27044	-1.26686	-1.26331	-1.25982	-1.25637	-1.33528
-1.27914	-1.27542	-1.27184	-1.26829	-1.26475	-1.26123	-1.31515	-1.31133	-1.39034
-1.27706	-1.27336	-1.26975	-1.26619	-1.26268	-1.2592	-1.31298	-1.30916	-1.38805
-1.27498	-1.27131	-1.26768	-1.26412	-1.26064	-1.25718	-1.34156	-1.33764	-1.41182
-1.27284	-1.26921	-1.26564	-1.26211	-1.25861	-1.25514	-1.33934	-1.33548	-1.40956
-1.27071	-1.26713	-1.26361	-1.2601	-1.25659	-1.25312	-1.33714	-1.33332	-1.4073
-1.26868	-1.26509	-1.26155	-1.31582	-1.31198	-1.30817	-1.3599	-1.35602	-1.40502
-1.2667	-1.26309	-1.25948	-1.31357	-1.3098	-1.30606	-1.35767	-1.35375	-1.40274
-1.26467	-1.31933	-1.31533	-1.34215	-1.33827	-1.33442	-1.38182	-1.37788	-1.40051
-1.26262	-1.31719	-1.31326	-1.34003	-1.33607	-1.33217	-1.37954	-1.37569	-1.39831
-1.26054	-1.34582	-1.34186	-1.33791	-1.33393	-1.33	-1.37733	-1.37352	-1.47115
-1.25844	-1.34352	-1.33966	-1.33578	-1.33184	-1.32794	-1.37519	-1.37135	-1.46885
-1.34513	-1.34124	-1.33744	-1.38492	-1.38095	-1.377	-1.44942	-1.44537	-1.46661
-1.3429	-1.33903	-1.33521	-1.38263	-1.37875	-1.37489	-1.44726	-1.44325	-1.46444
-1.3407	-1.33685	-1.333	-1.38035	-1.37654	-1.37277	-1.4451	-1.44116	-1.46233
-1.33848	-1.33465	-1.33082	-1.4547	-1.45072	-1.4468	-1.46808	-1.46413	-1.46025
-1.33627	-1.33243	-1.32868	-1.45242	-1.44844	-1.4445	-1.46578	-1.46191	-1.45822
-1.38355	-1.43488	-1.43089	-1.45013	-1.44616	-1.44226	-1.46353	-1.45972	-1.50279
-1.38144	-1.43269	-1.42864	-1.44782	-1.44391	-1.44007	-1.46132	-1.45754	-1.50073
-1.40907	-1.4305	-1.42643	-1.4933	-1.48929	-1.48538	-1.50585	-1.50208	-1.51754
-1.40686	-1.42832	-1.42429	-1.49107	-1.48698	-1.48306	-1.50372	-1.50027	-1.51586
-1.40464	-1.42613	-1.42217	-1.48889	-1.48474	-1.48084	-1.50171	-1.49857	-1.51427
-1.42781	-1.49477	-1.49072	-1.48671	-1.48263	-1.47882	-1.49983	-1.49687	-1.51269
-1.42557	-1.49242	-1.48846	-1.48456	-1.48062	-1.47696	-1.49808	-1.49525	-1.51116
-1.47184	-1.49016	-1.48624	-1.50686	-1.50308	-1.49957	-1.51517	-1.51237	-1.5573
-1.46967	-1.48797	-1.48405	-1.50474	-1.50128	-1.49807	-1.51375	-1.51099	-1.55585
-1.4675	-1.4858	-1.48192	-1.5215	-1.51822	-1.5152	-1.56001	-1.55717	-1.57837
-1.46533	-1.48367	-1.47985	-1.51951	-1.51646	-1.51364	-1.55849	-1.55577	-1.57699
-1.46317	-1.50076	-1.49702	-1.56554	-1.56251	-1.55971	-1.55698	-1.55435	-1.57559
-1.46099	-1.4987	-1.49524	-1.56382	-1.56086	-1.55809	-1.55545	-1.55291	-1.57418
-1.45889	-1.49674	-1.49356	-1.56221	-1.55929	-1.55653	-1.55394	-1.55146	-1.57273
-1.4764	-1.54346	-1.54035	-1.56063	-1.5578	-1.55509	-1.5525	-1.54999	-1.60845
-1.47457	-1.54167	-1.53872	-1.55912	-1.55637	-1.55371	-1.55109	-1.54853	-1.60683
-1.52192	-1.53994	-1.53715	-1.55764	-1.55496	-1.55234	-1.61086	-1.60803	-1.63237
-1.52011	-1.53828	-1.53564	-1.55621	-1.55358	-1.55098	-1.60938	-1.60656	-1.63076
-1.53952	-1.55995	-1.55736	-1.56728	-1.56469	-1.56212	-1.60791	-1.6051	-1.62919
-1.53797	-1.55845	-1.55591	-1.56585	-1.5633	-1.56076	-1.60645	-1.60365	-1.62767
-1.53649	-1.56947	-1.56693	-1.58999	-1.58731	-1.58464	-1.63203	-1.62907	-1.64584
-1.53507	-1.56804	-1.56548	-1.58843	-1.58581	-1.58319	-1.63046	-1.62752	-1.64432
-1.53369	-1.56665	-1.56404	-1.58689	-1.58431	-1.58175	-1.6289	-1.62599	-1.64283
-1.54498	-1.56525	-1.56265	-1.58542	-1.58285	-1.58031	-1.64704	-1.64417	-1.64138
-1.54359	-1.56387	-1.56128	-1.58398	-1.58142	-1.57888	-1.64552	-1.64272	-1.63995
-1.54222	-1.58794	-1.58521	-1.65237	-1.64951	-1.64671	-1.64396	-1.64124	-1.66179
-1.54087	-1.58649	-1.58378	-1.65079	-1.64789	-1.64506	-1.64237	-1.63973	-1.66023
-1.53952	-1.60536	-1.60266	-1.64921	-1.6463	-1.64347	-1.64081	-1.63824	-1.67596
-1.53817	-1.60391	-1.60121	-1.64764	-1.64476	-1.64197	-1.63933	-1.63676	-1.67441
-1.53682	-1.60246	-1.59977	-1.64608	-1.64325	-1.64049	-1.63786	-1.63531	-1.67287
-1.55641	-1.60101	-1.59834	-1.64454	-1.64173	-1.63901	-1.67688	-1.67412	-1.6917

-1.55506	-1.59957	-1.59692	-1.64301	-1.64023	-1.63753	-1.67531	-1.67259	-1.69019
-1.6008	-1.6472	-1.64432	-1.65919	-1.65639	-1.65369	-1.67378	-1.67109	-1.68871
-1.59934	-1.64563	-1.64279	-1.65769	-1.65492	-1.65223	-1.67226	-1.66962	-1.68724
-1.59791	-1.66182	-1.65899	-1.67926	-1.67631	-1.67346	-1.67077	-1.66818	-1.6858
-1.59648	-1.66029	-1.65748	-1.67769	-1.67476	-1.67194	-1.6693	-1.66676	-1.68438
-1.66162	-1.65878	-1.656	-1.67613	-1.67324	-1.67046	-1.66786	-1.66535	-1.68297
-1.66009	-1.65728	-1.65453	-1.67461	-1.67177	-1.66903	-1.66645	-1.66395	-1.68154
-1.65857	-1.6558	-1.65308	-1.67311	-1.67034	-1.66765	-1.66507	-1.66255	-1.68012
-1.65709	-1.67731	-1.67442	-1.67163	-1.66895	-1.66633	-1.66374	-1.66118	-1.69675
-1.65565	-1.67581	-1.67294	-1.67019	-1.66759	-1.66503	-1.66243	-1.65982	-1.69535
-1.65427	-1.69275	-1.68992	-1.68718	-1.68458	-1.682	-1.69926	-1.69657	-1.69394
-1.65295	-1.69141	-1.68864	-1.68591	-1.68326	-1.68062	-1.69784	-1.69515	-1.69254
-1.69276	-1.69002	-1.68729	-1.68458	-1.68189	-1.67923	-1.69642	-1.69375	-1.69116
-1.69131	-1.68857	-1.68588	-1.68319	-1.68049	-1.67782	-1.69501	-1.69238	-1.6898
-1.68987	-1.68712	-1.68444	-1.68177	-1.67907	-1.6764	-1.69361	-1.69102	-1.68846
-1.68843	-1.68569	-1.68301	-1.68034	-1.67764	-1.67499	-1.69221	-1.68965	-1.68713
-1.687	-1.68427	-1.68158	-1.6789	-1.67622	-1.67359	-1.69082	-1.6883	-1.6858
-1.68555	-1.68284	-1.68014	-1.67748	-1.67483	-1.67224	-1.68946	-1.68696	-1.70573
-1.6841	-1.6814	-1.67871	-1.67606	-1.67347	-1.67092	-1.68814	-1.68564	-1.70432
-1.68267	-1.67999	-1.6773	-1.67467	-1.67212	-1.66962	-1.68683	-1.68434	-1.72865
-1.68127	-1.67859	-1.67591	-1.67331	-1.6708	-1.66833	-1.68554	-1.68306	-1.7272
-1.67991	-1.67724	-1.6746	-1.6918	-1.68927	-1.68678	-1.73145	-1.72861	-1.7258
-1.67863	-1.67599	-1.67339	-1.6906	-1.68809	-1.6856	-1.73012	-1.72729	-1.7245
-1.67735	-1.67473	-1.67219	-1.68941	-1.6869	-1.68441	-1.72879	-1.72596	-1.72318
-1.67598	-1.67339	-1.67089	-1.68812	-1.68562	-1.68315	-1.72736	-1.72455	-1.72179
-1.67459	-1.67204	-1.66957	-1.68681	-1.68433	-1.68186	-1.72591	-1.72313	-1.72037
-1.67326	-1.67074	-1.66829	-1.73293	-1.73008	-1.72726	-1.72447	-1.7217	-1.72565
-1.67197	-1.66948	-1.66703	-1.73147	-1.72863	-1.72581	-1.72304	-1.72029	-1.72424
-1.67073	-1.66826	-1.66582	-1.73691	-1.73404	-1.73121	-1.72841	-1.72564	-1.72289
-1.66956	-1.66711	-1.66468	-1.73559	-1.73274	-1.72992	-1.72713	-1.72437	-1.72162
-1.66837	-1.73994	-1.73708	-1.73423	-1.7314	-1.72859	-1.74189	-1.73912	-1.7364
-1.6671	-1.73848	-1.73561	-1.73277	-1.72996	-1.72717	-1.74046	-1.73771	-1.73502
-1.66584	-1.73699	-1.73413	-1.7313	-1.7285	-1.72572	-1.73902	-1.73628	-1.73363
-1.67206	-1.7516	-1.74874	-1.74592	-1.74312	-1.74036	-1.75487	-1.75213	-1.74949
-1.67084	-1.75015	-1.7473	-1.74448	-1.74169	-1.73893	-1.75346	-1.75075	-1.74814
-1.70747	-1.74877	-1.74593	-1.76045	-1.75762	-1.75484	-1.75212	-1.74947	-1.75852
-1.7062	-1.74744	-1.7446	-1.75911	-1.75629	-1.75352	-1.75086	-1.74825	-1.75728
-1.76632	-1.76342	-1.76055	-1.75772	-1.75492	-1.75219	-1.74956	-1.747	-1.7703
-1.76489	-1.76197	-1.7591	-1.75628	-1.75351	-1.75081	-1.74821	-1.74569	-1.76897
-1.76347	-1.76054	-1.75767	-1.75487	-1.75213	-1.74947	-1.77292	-1.77029	-1.76765
-1.76208	-1.75917	-1.75631	-1.75354	-1.75085	-1.74824	-1.77165	-1.76903	-1.76639
-1.76069	-1.75782	-1.75497	-1.75222	-1.74959	-1.74702	-1.7704	-1.76777	-1.76513
-1.75926	-1.75639	-1.75358	-1.76532	-1.76274	-1.76021	-1.76908	-1.76646	-1.76381
-1.75782	-1.75495	-1.75217	-1.76395	-1.76141	-1.7589	-1.76775	-1.76512	-1.76247
-1.77088	-1.76805	-1.76532	-1.77436	-1.77171	-1.76909	-1.76646	-1.76382	-1.76117
-1.76953	-1.76676	-1.76409	-1.77311	-1.77046	-1.76784	-1.7652	-1.76255	-1.7599
-1.7682	-1.76548	-1.76285	-1.77185	-1.7692	-1.76656	-1.76391	-1.76126	-1.80446
-1.7669	-1.7642	-1.76158	-1.77054	-1.76788	-1.76524	-1.76258	-1.75992	-1.80297
-1.76561	-1.77469	-1.77195	-1.76925	-1.76658	-1.76393	-1.76126	-1.75859	-1.80149
-1.7643	-1.77338	-1.77068	-1.768	-1.76533	-1.76267	-1.76	-1.75733	-1.80009

-1.763	-1.77208	-1.76942	-1.76677	-1.7641	-1.76144	-1.75876	-1.75608	-1.79871
-1.76176	-1.77083	-1.76818	-1.76553	-1.76287	-1.7602	-1.80332	-1.80028	-1.79728
-1.76057	-1.7696	-1.76695	-1.76429	-1.76163	-1.75895	-1.80191	-1.79882	-1.79582
-1.75938	-1.76836	-1.76568	-1.763	-1.76034	-1.75766	-1.80047	-1.79739	-1.79442
-1.75821	-1.76711	-1.76438	-1.76166	-1.75898	-1.7563	-1.799	-1.79598	-1.79307
-1.76856	-1.76584	-1.76307	-1.80661	-1.80358	-1.80058	-1.79757	-1.7946	-1.79174
-1.76726	-1.76453	-1.76178	-1.80519	-1.80216	-1.79917	-1.79619	-1.79326	-1.79045
-1.76596	-1.76322	-1.7605	-1.8038	-1.80078	-1.79779	-1.79483	-1.79193	-1.78915
-1.76467	-1.76195	-1.75925	-1.80241	-1.7994	-1.79642	-1.79348	-1.79059	-1.829
-1.76339	-1.76069	-1.758	-1.80104	-1.79803	-1.79505	-1.79213	-1.78926	-1.82753
-1.76213	-1.8057	-1.80268	-1.79966	-1.79663	-1.79365	-1.79077	-1.78797	-1.82613
-1.76088	-1.80431	-1.8013	-1.79829	-1.79523	-1.79224	-1.78943	-1.78671	-1.8248
-1.75963	-1.80292	-1.79989	-1.79687	-1.79384	-1.7909	-1.82953	-1.82652	-1.82349
-1.75839	-1.80151	-1.79842	-1.7954	-1.79248	-1.78965	-1.82822	-1.82522	-1.82222
-1.80318	-1.8001	-1.79697	-1.81211	-1.80922	-1.80642	-1.85904	-1.856	-1.85295
-1.80172	-1.79864	-1.79556	-1.81073	-1.80791	-1.80517	-1.85771	-1.85467	-1.8516
-1.80026	-1.79719	-1.79419	-1.8094	-1.80664	-1.80394	-1.85637	-1.85333	-1.85023
-1.79886	-1.79582	-1.7929	-1.86412	-1.86108	-1.85807	-1.855	-1.85193	-1.84882
-1.7975	-1.7945	-1.79165	-1.8628	-1.85976	-1.85674	-1.85363	-1.8505	-1.8474
-1.79613	-1.84417	-1.84129	-1.86147	-1.85845	-1.85542	-1.85228	-1.84912	-1.86772
-1.79475	-1.8428	-1.83999	-1.86015	-1.85715	-1.85412	-1.85097	-1.84778	-1.86628
-1.8267	-1.86485	-1.86183	-1.85883	-1.85584	-1.8528	-1.84964	-1.84645	-1.86486
-1.82542	-1.8635	-1.8605	-1.8575	-1.8545	-1.85147	-1.8483	-1.84511	-1.86343
-1.8418	-1.86218	-1.85917	-1.85616	-1.85315	-1.8501	-1.86866	-1.86529	-1.86202
-1.84052	-1.86086	-1.85785	-1.85481	-1.85173	-1.84864	-1.86715	-1.86384	-1.8606
-1.83925	-1.85954	-1.85652	-1.85346	-1.85031	-1.84717	-1.86563	-1.86239	-1.85919
-1.86126	-1.85823	-1.8552	-1.85213	-1.84895	-1.84579	-1.86416	-1.86095	-1.85779
-1.85994	-1.85691	-1.85389	-1.8508	-1.84761	-1.84444	-1.86273	-1.85954	-1.85641
-1.85862	-1.85558	-1.85256	-1.87131	-1.86793	-1.86457	-1.86132	-1.85813	-1.85503
-1.85729	-1.85425	-1.85122	-1.86988	-1.8665	-1.86315	-1.85991	-1.85674	-1.85367
-1.85597	-1.85291	-1.84988	-1.86845	-1.86507	-1.86174	-1.85852	-1.85537	-1.85233
-1.85463	-1.85157	-1.84854	-1.86702	-1.86365	-1.86033	-1.85715	-1.85405	-1.85102
-1.8533	-1.85023	-1.8472	-1.8656	-1.86224	-1.85894	-1.85579	-1.85273	-1.84972
-1.85199	-1.87069	-1.86745	-1.86418	-1.86083	-1.85755	-1.85443	-1.8514	-1.84841
-1.85069	-1.86928	-1.86603	-1.86277	-1.85944	-1.85618	-1.85309	-1.85009	-1.8471
-1.84937	-1.86787	-1.86464	-1.8614	-1.85813	-1.85491	-1.85182	-1.8488	-1.8458
-1.84804	-1.86647	-1.86325	-1.86006	-1.85688	-1.85372	-1.85061	-1.84753	-1.84451
-1.84673	-1.86507	-1.86187	-1.8587	-1.85559	-1.85248	-1.84936	-1.84625	-1.85881
-1.84541	-1.86367	-1.86047	-1.85732	-1.85425	-1.85119	-1.84807	-1.84496	-1.85746
-1.86559	-1.86232	-1.85911	-1.85597	-1.85292	-1.84988	-1.84678	-1.84367	-1.88062
-1.8643	-1.86104	-1.8578	-1.85465	-1.8516	-1.84857	-1.84547	-1.84236	-1.87926
-1.86302	-1.85977	-1.85652	-1.85334	-1.8503	-1.84727	-1.84417	-1.84108	-1.87792
-1.86168	-1.85845	-1.8552	-1.85204	-1.849	-1.84598	-1.8831	-1.87985	-1.87667
-1.86032	-1.85711	-1.85389	-1.85075	-1.8477	-1.8447	-1.88182	-1.87864	-1.87547
-1.85896	-1.85579	-1.85263	-1.87459	-1.87146	-1.86838	-1.8805	-1.87737	-1.87421
-1.85761	-1.85449	-1.85141	-1.8734	-1.87022	-1.86709	-1.87915	-1.87606	-1.87291
-1.8563	-1.87839	-1.87529	-1.88764	-1.88428	-1.88097	-1.87782	-1.87474	-1.87161
-1.85502	-1.8771	-1.87399	-1.88628	-1.88293	-1.87963	-1.8765	-1.87343	-1.87031
-1.87902	-1.87582	-1.8727	-1.88491	-1.88159	-1.87834	-1.87521	-1.87214	-1.86903
-1.8778	-1.87459	-1.87143	-1.88357	-1.88032	-1.87712	-1.87398	-1.87089	-1.8678

-1.87658	-1.87337	-1.87016	-1.88224	-1.87906	-1.87592	-1.87277	-1.86965	-1.86658
-1.87529	-1.88756	-1.88417	-1.88088	-1.87776	-1.87467	-1.87153	-1.8684	-1.86534
-1.87396	-1.88616	-1.88278	-1.87952	-1.87645	-1.8734	-1.87027	-1.86715	-1.86408
-1.87267	-1.88483	-1.88149	-1.87826	-1.87519	-1.87214	-1.86904	-1.86594	-1.86286
-1.87142	-1.88355	-1.8803	-1.8771	-1.87398	-1.8709	-1.86783	-1.86478	-1.86168
-1.88557	-1.88229	-1.87909	-1.87593	-1.87278	-1.86967	-1.86662	-1.86358	-1.86048
-1.88431	-1.88105	-1.87786	-1.8747	-1.87158	-1.86849	-1.8654	-1.86232	-1.85924
-1.88305	-1.87981	-1.87662	-1.87347	-1.87037	-1.86729	-1.86417	-1.86105	-1.85798
-1.88178	-1.87855	-1.8754	-1.87227	-1.86915	-1.86604	-1.86289	-1.85975	-1.85669
-1.88049	-1.87728	-1.87417	-1.87106	-1.86792	-1.86477	-1.86161	-1.85846	-1.8554
-1.8792	-1.876	-1.8729	-1.86979	-1.86664	-1.86349	-1.86034	-1.85722	-1.86656
-1.87791	-1.87471	-1.87161	-1.86849	-1.86534	-1.8622	-1.8591	-1.85602	-1.86536
-1.87666	-1.87346	-1.87033	-1.8672	-1.86406	-1.86095	-1.85787	-1.85481	-1.86416
-1.87547	-1.87225	-1.86907	-1.86593	-1.86283	-1.85974	-1.85666	-1.8536	-1.86297
-1.87426	-1.87102	-1.86783	-1.86468	-1.8616	-1.85854	-1.86785	-1.86479	-1.8736
-1.87298	-1.86976	-1.8666	-1.86347	-1.86037	-1.8573	-1.86664	-1.86362	-1.87241
-1.8717	-1.86851	-1.86538	-1.86227	-1.85915	-1.85607	-1.86543	-1.86244	-1.87123
-1.87051	-1.8673	-1.86415	-1.86103	-1.85794	-1.85488	-1.87606	-1.87302	-1.87002
-1.86935	-1.86613	-1.86293	-1.8598	-1.85674	-1.85372	-1.87486	-1.8718	-1.86882
-1.86818	-1.86497	-1.86176	-1.87099	-1.86792	-1.86489	-1.87366	-1.87062	-1.86764
-1.86699	-1.86381	-1.86062	-1.86984	-1.86674	-1.86368	-1.87246	-1.86947	-1.86649
-1.86578	-1.86263	-1.85947	-1.88057	-1.87741	-1.8743	-1.87128	-1.8683	-1.86532
-1.86452	-1.8614	-1.85831	-1.87944	-1.87628	-1.87315	-1.87011	-1.8671	-1.86413
-1.86329	-1.87225	-1.86916	-1.87828	-1.87514	-1.87202	-1.86895	-1.86593	-1.86295
-1.8621	-1.87105	-1.86794	-1.87705	-1.87397	-1.8709	-1.86784	-1.8648	-1.86181
-1.86093	-1.86986	-1.86672	-1.87582	-1.87279	-1.86977	-1.86673	-1.8637	-1.86069
-1.87183	-1.8809	-1.87775	-1.87464	-1.87161	-1.8686	-1.8656	-1.8626	-1.87291
-1.87068	-1.87975	-1.87661	-1.8735	-1.87044	-1.86741	-1.86444	-1.86149	-1.87182
-1.8695	-1.87857	-1.87546	-1.87236	-1.8693	-1.86626	-1.87658	-1.87363	-1.87069
-1.86828	-1.87736	-1.8743	-1.87125	-1.86819	-1.86515	-1.87541	-1.87242	-1.86951
-1.86709	-1.87617	-1.87313	-1.8834	-1.88035	-1.87731	-1.87426	-1.87125	-1.86835
-1.86594	-1.875	-1.87193	-1.88218	-1.87918	-1.87619	-1.87316	-1.87015	-1.86724
-1.86481	-1.87384	-1.87075	-1.88097	-1.878	-1.87505	-1.87206	-1.86907	-1.86613
-1.88909	-1.88599	-1.88288	-1.87983	-1.87683	-1.87386	-1.8709	-1.86796	-1.86505
-1.88793	-1.88486	-1.88178	-1.87871	-1.87566	-1.87266	-1.86973	-1.86685	-1.86396
-1.88678	-1.88374	-1.88067	-1.87761	-1.87454	-1.87152	-1.86859	-1.86571	-1.86284
-1.88564	-1.88261	-1.87956	-1.87651	-1.87345	-1.87042	-1.86746	-1.86456	-1.8617
-1.88447	-1.88145	-1.87844	-1.87541	-1.87236	-1.86933	-1.86636	-1.86344	-1.86059
-1.88325	-1.88024	-1.87728	-1.8743	-1.87127	-1.86826	-1.86529	-1.86236	-1.85951
-1.88205	-1.87906	-1.87613	-1.87319	-1.87019	-1.86719	-1.86423	-1.8613	-1.85844
-1.88092	-1.87793	-1.87501	-1.87208	-1.8691	-1.86613	-1.86318	-1.86025	-1.85737
-1.87981	-1.87681	-1.87388	-1.87095	-1.86801	-1.86506	-1.86212	-1.8592	-1.85631
-1.87868	-1.87567	-1.87271	-1.86978	-1.86688	-1.86399	-1.86107	-1.85815	-1.85527
-1.87757	-1.87453	-1.87153	-1.86859	-1.86575	-1.86291	-1.86001	-1.85709	-1.85423
-1.86812	-1.87342	-1.87041	-1.86747	-1.86465	-1.86184	-1.85896	-1.85606	-1.8532
-1.86703	-1.87234	-1.86933	-1.8664	-1.86358	-1.86078	-1.85791	-1.85503	-1.85218
-1.86594	-1.87126	-1.86826	-1.86534	-1.86253	-1.85973	-1.85687	-1.85401	-1.85117
-1.86483	-1.87018	-1.8672	-1.86429	-1.86149	-1.8587	-1.85586	-1.853	-1.85017
-1.86374	-1.8691	-1.86613	-1.86324	-1.86045	-1.85767	-1.85484	-1.85199	-1.84917
-1.86265	-1.86803	-1.86508	-1.8622	-1.85941	-1.85663	-1.85381	-1.85098	-1.84817

-1.86157	-1.86697	-1.86403	-1.86116	-1.85837	-1.85559	-1.85279	-1.84997	-1.84717
-1.8605	-1.86591	-1.86298	-1.86012	-1.85734	-1.85457	-1.85177	-1.84896	-1.84616
-1.85943	-1.86486	-1.86194	-1.85908	-1.85632	-1.85356	-1.85076	-1.84795	-1.84514
-1.85836	-1.85542	-1.8525	-1.85804	-1.85528	-1.85254	-1.84974	-1.84693	-1.84413
-1.85728	-1.85435	-1.85144	-1.85699	-1.85424	-1.8515	-1.84872	-1.84592	-1.84313
-1.8562	-1.85328	-1.85038	-1.85595	-1.8532	-1.85047	-1.84769	-1.8449	-1.84212
-1.85513	-1.85222	-1.84932	-1.8549	-1.85216	-1.84944	-1.84666	-1.84387	-1.8411
-1.85408	-1.85117	-1.84828	-1.85389	-1.85115	-1.84842	-1.84563	-1.84284	-1.84008
-1.85303	-1.85015	-1.8473	-1.85294	-1.85017	-1.8474	-1.84461	-1.84182	-1.83906
-1.85199	-1.84914	-1.84635	-1.85201	-1.8492	-1.84639	-1.84359	-1.84079	-1.83804
-1.851	-1.84817	-1.84537	-1.85103	-1.8482	-1.84538	-1.84257	-1.83978	-1.83703
-1.85004	-1.84721	-1.84437	-1.85003	-1.84719	-1.84437	-1.84156	-1.83878	-1.83602
-1.84905	-1.84621	-1.84335	-1.8405	-1.83766	-1.83483	-1.84055	-1.83777	-1.83503
-1.84804	-1.84518	-1.84232	-1.83946	-1.83661	-1.83378	-1.83953	-1.83677	-1.83403
-1.84701	-1.84414	-1.84127	-1.83842	-1.83557	-1.83275	-1.83851	-1.83577	-1.83305
-1.84597	-1.84309	-1.84022	-1.83737	-1.83452	-1.83172	-1.8375	-1.83477	-1.83208
-1.84494	-1.84205	-1.83917	-1.83633	-1.8335	-1.83072	-1.83653	-1.83381	-1.83114
-1.8439	-1.84102	-1.83815	-1.83532	-1.83254	-1.82979	-1.83562	-1.83291	-1.83023
-1.84288	-1.84	-1.83714	-1.83433	-1.8316	-1.82889	-1.83475	-1.83203	-1.82933
-1.84191	-1.83906	-1.8362	-1.8334	-1.83067	-1.82796	-1.82525	-1.82253	-1.82839
-1.84098	-1.83816	-1.83531	-1.8325	-1.82975	-1.82702	-1.82428	-1.82156	-1.82742
-1.84003	-1.83722	-1.83438	-1.83157	-1.82881	-1.82606	-1.82332	-1.82059	-1.82646
-1.83906	-1.83623	-1.8334	-1.83059	-1.82783	-1.82508	-1.82234	-1.81962	-1.8255
-1.83807	-1.83523	-1.83241	-1.82961	-1.82685	-1.82411	-1.82138	-1.81867	-1.81597
-1.83709	-1.83425	-1.83144	-1.82865	-1.82588	-1.82315	-1.82045	-1.81778	-1.81509
-1.83612	-1.83329	-1.83048	-1.8277	-1.82494	-1.82221	-1.81953	-1.81689	-1.81422
-1.83522	-1.8324	-1.8296	-1.82682	-1.82405	-1.82132	-1.81863	-1.81597	-1.8133
-1.83435	-1.83154	-1.82875	-1.82597	-1.8232	-1.82046	-1.81774	-1.81503	-1.81235
-1.83343	-1.83063	-1.82785	-1.82507	-1.82231	-1.81955	-1.81681	-1.81409	-1.8114
-1.83246	-1.82967	-1.8269	-1.82413	-1.82136	-1.81861	-1.81587	-1.81315	-1.81045
-1.83152	-1.82875	-1.82599	-1.82322	-1.82045	-1.81769	-1.81495	-1.81223	-1.80953
-1.83063	-1.82788	-1.82512	-1.82236	-1.81959	-1.81683	-1.8141	-1.81138	-1.80868
-1.82974	-1.82701	-1.82425	-1.82149	-1.81872	-1.81597	-1.81323	-1.81052	-1.80782
-1.82882	-1.82608	-1.82332	-1.82056	-1.81779	-1.81504	-1.81231	-1.8096	-1.8069
-1.82791	-1.82515	-1.82238	-1.81962	-1.81685	-1.8141	-1.81137	-1.80867	-1.80597
-1.82705	-1.82427	-1.8215	-1.81874	-1.81597	-1.81322	-1.8105	-1.80779	-1.8051
-1.82621	-1.82343	-1.82065	-1.81788	-1.81513	-1.81238	-1.80966	-1.80695	-1.80426
-1.82532	-1.82254	-1.81975	-1.81698	-1.81423	-1.81149	-1.80877	-1.80607	-1.80338
-1.82437	-1.82159	-1.8188	-1.81603	-1.81329	-1.81055	-1.80783	-1.80513	-1.80244
-1.82344	-1.82066	-1.81788	-1.81512	-1.81237	-1.80964	-1.80693	-1.80423	-1.80155
-1.82258	-1.8198	-1.81703	-1.81427	-1.81153	-1.80881	-1.8061	-1.8034	-1.80072
-1.82171	-1.81893	-1.81617	-1.81342	-1.8107	-1.80799	-1.80528	-1.80259	-1.79991
-1.80897	-1.818	-1.81524	-1.81251	-1.80982	-1.80715	-1.80446	-1.80178	-1.7991
-1.80803	-1.81705	-1.81429	-1.81158	-1.80894	-1.8063	-1.80363	-1.80095	-1.79828
-1.80716	-1.80443	-1.80171	-1.8107	-1.80805	-1.80541	-1.80274	-1.80007	-1.7974
-1.80633	-1.8036	-1.80089	-1.80985	-1.80716	-1.80448	-1.80181	-1.79914	-1.79647
-1.80552	-1.8028	-1.80008	-1.79738	-1.79471	-1.79204	-1.80093	-1.79826	-1.79559
-1.80474	-1.80201	-1.7993	-1.7966	-1.79391	-1.79124	-1.80011	-1.79744	-1.79477
-1.80394	-1.80122	-1.7985	-1.7958	-1.79311	-1.79044	-1.7993	-1.79663	-1.79395
-1.80313	-1.80041	-1.79769	-1.79499	-1.7923	-1.78963	-1.79849	-1.79581	-1.79314

-1.80228	-1.79956	-1.79685	-1.79416	-1.79147	-1.7888	-1.79765	-1.79497	-1.7923
-1.80135	-1.79864	-1.79593	-1.79324	-1.79056	-1.7879	-1.79674	-1.79406	-1.79139
-1.80039	-1.79768	-1.79497	-1.79229	-1.78962	-1.78696	-1.79579	-1.79311	-1.79044
-1.7995	-1.79678	-1.79408	-1.7914	-1.78873	-1.78608	-1.7949	-1.79223	-1.78955
-1.79865	-1.79594	-1.79323	-1.79055	-1.78789	-1.78525	-1.79406	-1.79139	-1.78871
-1.79783	-1.79511	-1.7924	-1.78972	-1.78707	-1.78443	-1.79323	-1.79056	-1.78789
-1.79701	-1.7943	-1.79159	-1.78891	-1.78626	-1.78362	-1.79241	-1.78974	-1.78707
-1.7962	-1.79348	-1.79078	-1.78811	-1.78545	-1.78281	-1.7916	-1.78893	-1.78626
-1.79538	-1.79267	-1.78998	-1.7873	-1.78465	-1.78201	-1.79079	-1.78812	-1.78545
-1.79456	-1.79186	-1.78917	-1.7865	-1.78384	-1.7812	-1.78997	-1.78731	-1.78463
-1.79374	-1.79104	-1.78836	-1.78569	-1.78304	-1.7804	-1.78916	-1.78649	-1.78382
-1.79292	-1.79022	-1.78754	-1.78488	-1.78223	-1.77959	-1.78834	-1.78567	-1.783
-1.7921	-1.7894	-1.78673	-1.78408	-1.78143	-1.77879	-1.78753	-1.78485	-1.78218
-1.79128	-1.78859	-1.78592	-1.78327	-1.78062	-1.77798	-1.78672	-1.78404	-1.78136
-1.79046	-1.78777	-1.78511	-1.78246	-1.77982	-1.77718	-1.7859	-1.78322	-1.78055
-1.78965	-1.78696	-1.7843	-1.78165	-1.77901	-1.77637	-1.78509	-1.78241	-1.77973
-1.78884	-1.78615	-1.78349	-1.78084	-1.7782	-1.77556	-1.78427	-1.78159	-1.77891
-1.78802	-1.78534	-1.78267	-1.78002	-1.77738	-1.77474	-1.78344	-1.78076	-1.77808
-1.7872	-1.78452	-1.78185	-1.7792	-1.77656	-1.77392	-1.78261	-1.77993	-1.77725
-1.78638	-1.7837	-1.78103	-1.77837	-1.77573	-1.77309	-1.78177	-1.77909	-1.77641
-1.78556	-1.78287	-1.78021	-1.77755	-1.7749	-1.77226	-1.78093	-1.77825	-1.77557
-1.78473	-1.78205	-1.77938	-1.77672	-1.77407	-1.77143	-1.78009	-1.77741	-1.77473
-1.7839	-1.78122	-1.77855	-1.77589	-1.77324	-1.77059	-1.77925	-1.77657	-1.7739
-1.78308	-1.78039	-1.77771	-1.77505	-1.7724	-1.76976	-1.77841	-1.77573	-1.77306
-1.78225	-1.77956	-1.77688	-1.77422	-1.77157	-1.76893	-1.77757	-1.77489	-1.77221
-1.78144	-1.77873	-1.77605	-1.77338	-1.77074	-1.7681	-1.77673	-1.77405	-1.77137
-1.78065	-1.77792	-1.77522	-1.77255	-1.7699	-1.76726	-1.77589	-1.77321	-1.77053
-1.77988	-1.77712	-1.77441	-1.77172	-1.76907	-1.76643	-1.77504	-1.77237	-1.76969
-1.77913	-1.77636	-1.77362	-1.77092	-1.76826	-1.76561	-1.77421	-1.77152	-1.76884
-1.7784	-1.77563	-1.77287	-1.77014	-1.76746	-1.7648	-1.77338	-1.77068	-1.76799
-1.77763	-1.77488	-1.77212	-1.76939	-1.7667	-1.76403	-1.77258	-1.76987	-1.76717
-1.77681	-1.7741	-1.77139	-1.76869	-1.76601	-1.76333	-1.77184	-1.76908	-1.76636
-1.77599	-1.77331	-1.77064	-1.76797	-1.7653	-1.76261	-1.77111	-1.76832	-1.76559
-1.77519	-1.7725	-1.76984	-1.76718	-1.76452	-1.76184	-1.77035	-1.7676	-1.76487
-1.7744	-1.77171	-1.76904	-1.76637	-1.76371	-1.76105	-1.76959	-1.76688	-1.76417
-1.77368	-1.77098	-1.76827	-1.76558	-1.76291	-1.76025	-1.76879	-1.76608	-1.76338
-1.773	-1.77028	-1.76753	-1.76481	-1.76212	-1.75945	-1.76797	-1.76526	-1.76256
-1.77226	-1.76954	-1.76679	-1.77537	-1.77264	-1.76992	-1.76719	-1.76446	-1.78929
-1.77147	-1.76878	-1.76607	-1.77467	-1.77195	-1.76922	-1.76645	-1.76368	-1.78847
-1.77067	-1.768	-1.76533	-1.77394	-1.77123	-1.76849	-1.76571	-1.76292	-1.78768
-1.76987	-1.7672	-1.76453	-1.77314	-1.77043	-1.7677	-1.76495	-1.76219	-1.78696
-1.7691	-1.76642	-1.76375	-1.77235	-1.76963	-1.7669	-1.76418	-1.76146	-1.78624
-1.76839	-1.76571	-1.76304	-1.77163	-1.76887	-1.76611	-1.79096	-1.7882	-1.78543
-1.76771	-1.76503	-1.76235	-1.77092	-1.76812	-1.76533	-1.79015	-1.78738	-1.78461
-1.76696	-1.76428	-1.7616	-1.77016	-1.76737	-1.76459	-1.78939	-1.78663	-1.78384
-1.76617	-1.76348	-1.7608	-1.76936	-1.76662	-1.76387	-1.78868	-1.78591	-1.7831
-1.76543	-1.77404	-1.7713	-1.79622	-1.79345	-1.79069	-1.78792	-1.78515	-1.78234
-1.76473	-1.77332	-1.77054	-1.79541	-1.79263	-1.78986	-1.7871	-1.78434	-1.78155
-1.75202	-1.77259	-1.76978	-1.79463	-1.79184	-1.78907	-1.78631	-1.78355	-1.78078
-1.75125	-1.77183	-1.76905	-1.79391	-1.79112	-1.78835	-1.78558	-1.78282	-1.78003

-1.01409	-1.01255	-1.01103	-1.02895	-1.02746	-1.02597	-1.02447	-1.02298	-1.02149
-1.01449	-1.01294	-1.01141	-1.02934	-1.02784	-1.02636	-1.02486	-1.02336	-1.02187
-1.01489	-1.01333	-1.01178	-1.02972	-1.02823	-1.02674	-1.02524	-1.02374	-1.02224
-1.01528	-1.01372	-1.01216	-1.03011	-1.02861	-1.02713	-1.02561	-1.0241	-1.02261
-1.01567	-1.01411	-1.01255	-1.0305	-1.029	-1.02751	-1.02598	-1.02446	-1.02297
-0.97012	-1.01449	-1.01293	-1.0114	-1.00993	-1.00846	-1.02637	-1.02484	-1.02335
-0.9705	-1.01488	-1.01331	-1.01178	-1.0103	-1.00884	-1.02676	-1.02524	-1.02373
-0.88918	-0.96938	-0.96787	-1.01218	-1.0107	-1.00922	-1.02715	-1.02563	-1.02412
-0.8895	-0.96978	-0.96829	-1.01262	-1.01111	-1.00961	-1.02755	-1.02602	-1.02451
-0.866	-0.97018	-0.96871	-0.96725	-0.96579	-0.96433	-1.00848	-1.00698	-1.02489
-0.86631	-0.97058	-0.96911	-0.96763	-0.96616	-0.9647	-1.00886	-1.00735	-1.02528
-0.86663	-0.97098	-0.9695	-0.96802	-0.96653	-0.96506	-1.00923	-1.00773	-1.02567
-0.86696	-0.86571	-0.86446	-0.96843	-0.96691	-0.96542	-0.96396	-0.96253	-1.02606
-0.86729	-0.86605	-0.8648	-0.96884	-0.9673	-0.96578	-0.96432	-0.96289	-1.02645
-0.8676	-0.86635	-0.8651	-0.86384	-0.86254	-0.86125	-0.9647	-0.96328	-0.96186
-0.86789	-0.86663	-0.86538	-0.86412	-0.86284	-0.86157	-0.96511	-0.96368	-0.96225
-0.86819	-0.86692	-0.86567	-0.86441	-0.86314	-0.86188	-0.86065	-0.85943	-0.96264
-0.86851	-0.86723	-0.86596	-0.8647	-0.86344	-0.86219	-0.86095	-0.85971	-0.96301
-0.86883	-0.86754	-0.86626	-0.865	-0.86375	-0.8625	-0.86125	-0.86	-0.94099
-0.86913	-0.86785	-0.86657	-0.86531	-0.86406	-0.86281	-0.86155	-0.86029	-0.94136
-0.86944	-0.86816	-0.86688	-0.86562	-0.86436	-0.86311	-0.86185	-0.8606	-0.94174
-0.86976	-0.86847	-0.86718	-0.86591	-0.86465	-0.8634	-0.86215	-0.86091	-0.85969
-0.87009	-0.86879	-0.86748	-0.86619	-0.86493	-0.86369	-0.86246	-0.86123	-0.86001
-0.87039	-0.8691	-0.86778	-0.86649	-0.86523	-0.86399	-0.86276	-0.86153	-0.8603
-0.87068	-0.8694	-0.8681	-0.86682	-0.86555	-0.8643	-0.86305	-0.8618	-0.86057
-0.87099	-0.8697	-0.86842	-0.86715	-0.86587	-0.8646	-0.86334	-0.86209	-0.86085
-0.87131	-0.87003	-0.86875	-0.86747	-0.86617	-0.86488	-0.86362	-0.86238	-0.86116
-0.87165	-0.87036	-0.86909	-0.8678	-0.86647	-0.86517	-0.86391	-0.86269	-0.86147
-0.872	-0.87071	-0.86941	-0.86811	-0.86678	-0.86547	-0.86422	-0.86299	-0.86177
-0.87236	-0.87106	-0.86973	-0.86841	-0.86709	-0.86579	-0.86453	-0.8633	-0.86209
-0.87269	-0.87138	-0.87006	-0.86874	-0.86743	-0.86613	-0.86487	-0.86362	-0.8624
-0.873	-0.87169	-0.87038	-0.86907	-0.86778	-0.8665	-0.86522	-0.86396	-0.86273
-0.87332	-0.87201	-0.87071	-0.86941	-0.86813	-0.86686	-0.86557	-0.8643	-0.86307
-0.87366	-0.87235	-0.87105	-0.86975	-0.86847	-0.8672	-0.86593	-0.86467	-0.86343
-0.84381	-0.8727	-0.8714	-0.8701	-0.86882	-0.86754	-0.86628	-0.86503	-0.86379
-0.84416	-0.87308	-0.87176	-0.87046	-0.86917	-0.8679	-0.86663	-0.86537	-0.86413
-0.84451	-0.87346	-0.87214	-0.87083	-0.86954	-0.86826	-0.86699	-0.86572	-0.86447
-0.84484	-0.87381	-0.87251	-0.8712	-0.86991	-0.86862	-0.86734	-0.86607	-0.86481
-0.84516	-0.87417	-0.87288	-0.87159	-0.87029	-0.86899	-0.86771	-0.86643	-0.86516
-0.81371	-0.84426	-0.84304	-0.87197	-0.87066	-0.86936	-0.86807	-0.86679	-0.86552
-0.81404	-0.84461	-0.84339	-0.87235	-0.87104	-0.86974	-0.86844	-0.86715	-0.86588
-0.81437	-0.81317	-0.812	-0.87273	-0.87142	-0.87012	-0.86881	-0.86751	-0.86625
-0.8147	-0.8135	-0.81232	-0.8731	-0.8718	-0.8705	-0.86919	-0.86789	-0.86662
-0.81502	-0.81383	-0.81264	-0.81145	-0.81027	-0.80909	-0.86956	-0.86826	-0.86699
-0.81532	-0.81413	-0.81295	-0.81177	-0.81059	-0.80941	-0.86994	-0.86863	-0.86735
-0.81563	-0.81443	-0.81325	-0.81208	-0.8109	-0.80972	-0.8703	-0.869	-0.86771
-0.81595	-0.81474	-0.81355	-0.81236	-0.81119	-0.81002	-0.83954	-0.83829	-0.86806
-0.81628	-0.81506	-0.81384	-0.81264	-0.81148	-0.81032	-0.83986	-0.8386	-0.86841
-0.81663	-0.8154	-0.81416	-0.81295	-0.81179	-0.81063	-0.80943	-0.80824	-0.86877
-0.817	-0.81575	-0.81451	-0.81329	-0.81211	-0.81094	-0.80974	-0.80855	-0.86914

-0.81734	-0.8161	-0.81486	-0.81364	-0.81244	-0.81125	-0.81006	-0.80887	-0.83838
-0.81766	-0.81642	-0.8152	-0.81398	-0.81276	-0.81156	-0.81037	-0.80919	-0.83871
-0.81797	-0.81673	-0.81553	-0.81432	-0.81309	-0.81187	-0.81068	-0.80951	-0.83906
-0.8183	-0.81706	-0.81586	-0.81465	-0.81341	-0.81219	-0.81101	-0.80985	-0.80866
-0.81864	-0.8174	-0.81619	-0.81497	-0.81374	-0.81252	-0.81135	-0.81019	-0.809
-0.819	-0.81776	-0.81652	-0.81529	-0.81406	-0.81285	-0.81167	-0.81051	-0.80933
-0.81939	-0.81813	-0.81686	-0.81561	-0.81438	-0.81317	-0.81199	-0.81082	-0.80964
-0.81976	-0.81849	-0.81721	-0.81595	-0.81472	-0.81351	-0.81231	-0.81112	-0.80994
-0.82014	-0.81887	-0.81758	-0.81632	-0.81509	-0.81387	-0.81264	-0.81142	-0.81023
-0.78801	-0.81923	-0.81795	-0.81669	-0.81545	-0.81423	-0.81298	-0.81174	-0.81054
-0.78832	-0.81957	-0.81831	-0.81706	-0.81583	-0.8146	-0.81335	-0.8121	-0.81089
-0.78862	-0.81991	-0.81867	-0.81744	-0.8162	-0.81497	-0.81372	-0.81247	-0.81125
-0.78894	-0.78774	-0.78657	-0.8178	-0.81657	-0.81533	-0.81408	-0.81283	-0.81161
-0.78928	-0.78807	-0.78689	-0.81815	-0.81692	-0.81568	-0.81443	-0.81319	-0.81197
-0.78962	-0.7884	-0.78722	-0.78605	-0.78486	-0.78368	-0.81477	-0.81353	-0.81232
-0.78997	-0.78875	-0.78756	-0.78637	-0.78516	-0.78396	-0.81509	-0.81387	-0.81266
-0.79033	-0.7891	-0.7879	-0.7867	-0.78548	-0.78427	-0.81543	-0.81421	-0.81301
-0.79067	-0.78944	-0.78825	-0.78705	-0.78582	-0.78461	-0.8158	-0.81458	-0.81337
-0.79102	-0.7898	-0.7886	-0.78739	-0.78617	-0.78496	-0.78377	-0.7826	-0.81374
-0.7914	-0.79017	-0.78896	-0.78775	-0.78652	-0.78531	-0.78411	-0.78294	-0.81411
-0.79177	-0.79054	-0.78932	-0.7881	-0.78687	-0.78566	-0.78446	-0.78328	-0.81448
-0.79213	-0.7909	-0.78967	-0.78845	-0.78721	-0.78599	-0.7848	-0.78362	-0.78247
-0.79248	-0.79124	-0.79002	-0.78879	-0.78755	-0.78632	-0.78514	-0.78397	-0.78281
-0.79283	-0.79159	-0.79037	-0.78914	-0.78789	-0.78666	-0.78547	-0.7843	-0.78315
-0.79319	-0.79194	-0.79072	-0.78948	-0.78823	-0.78699	-0.7858	-0.78463	-0.78347
-0.79356	-0.7923	-0.79107	-0.78984	-0.78859	-0.78735	-0.78615	-0.78497	-0.7838
-0.79393	-0.79267	-0.79145	-0.79022	-0.78898	-0.78775	-0.78653	-0.78532	-0.78413
-0.79431	-0.79304	-0.79182	-0.7906	-0.78937	-0.78814	-0.7869	-0.78567	-0.78447
-0.7947	-0.79342	-0.79219	-0.79097	-0.78975	-0.78852	-0.78727	-0.78602	-0.78481
-0.7951	-0.79381	-0.79257	-0.79133	-0.79012	-0.78889	-0.78764	-0.78638	-0.78516
-0.69876	-0.79421	-0.79294	-0.7917	-0.79048	-0.78926	-0.78801	-0.78674	-0.78552
-0.69907	-0.79461	-0.79332	-0.79207	-0.79085	-0.78963	-0.78837	-0.78711	-0.78589
-0.69937	-0.76465	-0.7634	-0.79244	-0.79122	-0.79	-0.78875	-0.7875	-0.78627
-0.69967	-0.76502	-0.76375	-0.7928	-0.79158	-0.79037	-0.78914	-0.78791	-0.78667
-0.69997	-0.6989	-0.69783	-0.79319	-0.79195	-0.79074	-0.78952	-0.7883	-0.78706
-0.70027	-0.6992	-0.69813	-0.79358	-0.79232	-0.79109	-0.78988	-0.78867	-0.78744
-0.70058	-0.6995	-0.69843	-0.79399	-0.7927	-0.79145	-0.79023	-0.78902	-0.7878
-0.65988	-0.69982	-0.69875	-0.76401	-0.76276	-0.76153	-0.79059	-0.78938	-0.78815
-0.66015	-0.70014	-0.69908	-0.76444	-0.76315	-0.76189	-0.79096	-0.78973	-0.7885
-0.6105	-0.70046	-0.6994	-0.7281	-0.72698	-0.72588	-0.76106	-0.75986	-0.78887
-0.61084	-0.70078	-0.69971	-0.72843	-0.72732	-0.72622	-0.76145	-0.76023	-0.78926
-0.61119	-0.65256	-0.65146	-0.68118	-0.68005	-0.67894	-0.76184	-0.7606	-0.78967
-0.61155	-0.65298	-0.65186	-0.68159	-0.68045	-0.67934	-0.76224	-0.76099	-0.79009
-0.61191	-0.61098	-0.61006	-0.65115	-0.65008	-0.64902	-0.67863	-0.67754	-0.71377
-0.61229	-0.61135	-0.61042	-0.65156	-0.65048	-0.64941	-0.67904	-0.67793	-0.71421
-0.61267	-0.61173	-0.61077	-0.65198	-0.65089	-0.64982	-0.67945	-0.67833	-0.71465
-0.61304	-0.6121	-0.61114	-0.65242	-0.65131	-0.65023	-0.67988	-0.67875	-0.67765
-0.6134	-0.61246	-0.61152	-0.65287	-0.65175	-0.65064	-0.68032	-0.67919	-0.67807
-0.61377	-0.61283	-0.61189	-0.61096	-0.61005	-0.60914	-0.68076	-0.67962	-0.6785
-0.61415	-0.61321	-0.61226	-0.61133	-0.6104	-0.60948	-0.68119	-0.68006	-0.67893

-0.61454	-0.61359	-0.61264	-0.61169	-0.61076	-0.60983	-0.65077	-0.6497	-0.67936
-0.61493	-0.61398	-0.61302	-0.61206	-0.61112	-0.61019	-0.65119	-0.6501	-0.67978
-0.61532	-0.61437	-0.6134	-0.61244	-0.61148	-0.61055	-0.65161	-0.6505	-0.6802
-0.58172	-0.61474	-0.61378	-0.61282	-0.61186	-0.61092	-0.61	-0.6091	-0.68065
-0.58206	-0.61511	-0.61415	-0.6132	-0.61225	-0.6113	-0.61038	-0.60947	-0.6811
-0.58238	-0.61545	-0.6145	-0.61356	-0.61261	-0.61167	-0.61074	-0.60983	-0.65068
-0.5827	-0.61579	-0.61485	-0.61391	-0.61297	-0.61204	-0.61111	-0.61019	-0.65112
-0.58305	-0.58217	-0.58132	-0.61427	-0.61334	-0.61241	-0.61148	-0.61056	-0.60966
-0.58343	-0.58253	-0.58167	-0.61465	-0.61372	-0.6128	-0.61187	-0.61096	-0.61004
-0.58381	-0.5829	-0.58202	-0.61504	-0.6141	-0.61318	-0.61226	-0.61134	-0.61043
-0.58419	-0.58327	-0.58237	-0.61541	-0.61446	-0.61354	-0.61262	-0.6117	-0.61079
-0.58458	-0.58365	-0.58273	-0.61579	-0.61483	-0.6139	-0.61298	-0.61206	-0.61115
-0.58498	-0.58404	-0.58311	-0.58221	-0.58134	-0.5805	-0.61335	-0.61244	-0.61153
-0.58538	-0.58444	-0.58349	-0.58258	-0.58171	-0.58085	-0.61374	-0.61282	-0.61191
-0.58577	-0.58482	-0.58388	-0.58296	-0.58207	-0.58119	-0.61411	-0.61319	-0.61227
-0.58615	-0.5852	-0.58427	-0.58334	-0.58242	-0.58153	-0.61447	-0.61354	-0.61263
-0.55268	-0.58559	-0.58466	-0.58373	-0.5828	-0.5819	-0.58104	-0.5802	-0.61301
-0.55303	-0.586	-0.58507	-0.58414	-0.58321	-0.58229	-0.58142	-0.58058	-0.61341
-0.5534	-0.55253	-0.55169	-0.58455	-0.58362	-0.5827	-0.58182	-0.58095	-0.61382
-0.55378	-0.55291	-0.55206	-0.58496	-0.58403	-0.58312	-0.58221	-0.58133	-0.61423
-0.55415	-0.55328	-0.55242	-0.58537	-0.58445	-0.58353	-0.58261	-0.58171	-0.61463
-0.55451	-0.55364	-0.55276	-0.5519	-0.55106	-0.55023	-0.58299	-0.58207	-0.58121
-0.55486	-0.55398	-0.55309	-0.55223	-0.55138	-0.55055	-0.58336	-0.58244	-0.58156
-0.55524	-0.55435	-0.55346	-0.55258	-0.55173	-0.5509	-0.55006	-0.54923	-0.58194
-0.55563	-0.55475	-0.55386	-0.55298	-0.55211	-0.55126	-0.55042	-0.5496	-0.58234
-0.55603	-0.55514	-0.55425	-0.55337	-0.55249	-0.55163	-0.55079	-0.54996	-0.54914
-0.55642	-0.55554	-0.55465	-0.55376	-0.55287	-0.55199	-0.55115	-0.55032	-0.54949
-0.55681	-0.55592	-0.55504	-0.55415	-0.55325	-0.55236	-0.5515	-0.55066	-0.54983
-0.55717	-0.55629	-0.5554	-0.5545	-0.5536	-0.55271	-0.55184	-0.55099	-0.55016
-0.55753	-0.55664	-0.55575	-0.55486	-0.55396	-0.55306	-0.55218	-0.55132	-0.55048
-0.55792	-0.55703	-0.55614	-0.55524	-0.55434	-0.55345	-0.55256	-0.55168	-0.55083
-0.55833	-0.55744	-0.55655	-0.55565	-0.55475	-0.55386	-0.55295	-0.55206	-0.55119
-0.55875	-0.55785	-0.55696	-0.55606	-0.55517	-0.55426	-0.55336	-0.55245	-0.55158
-0.55917	-0.55826	-0.55738	-0.55648	-0.55558	-0.55468	-0.55377	-0.55286	-0.55198
-0.52086	-0.55868	-0.55779	-0.5569	-0.556	-0.55509	-0.55419	-0.55328	-0.5524
-0.52124	-0.5591	-0.55821	-0.55731	-0.55642	-0.55552	-0.55461	-0.55371	-0.55282
-0.52162	-0.55952	-0.55862	-0.55773	-0.55684	-0.55594	-0.55504	-0.55414	-0.55324
-0.52201	-0.52115	-0.52031	-0.55814	-0.55725	-0.55636	-0.55547	-0.55458	-0.55367
-0.52239	-0.52153	-0.52069	-0.55856	-0.55767	-0.55678	-0.55589	-0.555	-0.55411
-0.52277	-0.52191	-0.52106	-0.52022	-0.5194	-0.51859	-0.55631	-0.55542	-0.55453
-0.52315	-0.52227	-0.52142	-0.52058	-0.51976	-0.51895	-0.55672	-0.55584	-0.55495
-0.45029	-0.52267	-0.5218	-0.52096	-0.52013	-0.51931	-0.5185	-0.51769	-0.51688
-0.45065	-0.52309	-0.52221	-0.52134	-0.5205	-0.51968	-0.51887	-0.51807	-0.51725
-0.451	-0.45024	-0.44949	-0.52174	-0.52089	-0.52005	-0.51924	-0.51844	-0.51763
-0.45136	-0.45059	-0.44983	-0.52215	-0.52129	-0.52045	-0.51963	-0.51882	-0.51801
-0.4517	-0.45094	-0.45017	-0.52257	-0.5217	-0.52085	-0.52003	-0.51921	-0.5184
-0.45204	-0.45128	-0.45052	-0.44977	-0.44904	-0.44833	-0.52043	-0.51961	-0.51879
-0.45238	-0.45162	-0.45087	-0.45012	-0.44938	-0.44866	-0.52083	-0.52001	-0.51919
-0.45271	-0.45196	-0.45121	-0.45047	-0.44972	-0.44899	-0.4483	-0.44762	-0.51959
-0.45304	-0.4523	-0.45155	-0.45081	-0.45006	-0.44933	-0.44863	-0.44795	-0.51999

-0.45337	-0.45263	-0.45189	-0.45115	-0.4504	-0.44967	-0.44897	-0.44828	-0.44758
-0.4537	-0.45295	-0.45221	-0.45148	-0.45074	-0.45001	-0.4493	-0.4486	-0.4479
-0.43241	-0.45327	-0.45254	-0.4518	-0.45108	-0.45035	-0.44964	-0.44893	-0.44823
-0.43271	-0.4536	-0.45286	-0.45213	-0.4514	-0.45069	-0.44998	-0.44927	-0.44857
-0.43303	-0.45392	-0.45318	-0.45245	-0.45174	-0.45103	-0.45032	-0.44962	-0.44891
-0.43334	-0.43266	-0.432	-0.45279	-0.45208	-0.45138	-0.45068	-0.44997	-0.44926
-0.43366	-0.43297	-0.43231	-0.45314	-0.45244	-0.45175	-0.45104	-0.45033	-0.44961
-0.434	-0.43331	-0.43264	-0.45348	-0.45279	-0.4521	-0.45139	-0.45068	-0.44995
-0.43436	-0.43366	-0.43298	-0.45383	-0.45313	-0.45244	-0.45173	-0.45102	-0.45029
-0.39236	-0.39172	-0.39111	-0.43266	-0.43202	-0.43139	-0.45207	-0.45136	-0.45064
-0.39268	-0.39203	-0.39141	-0.43301	-0.43236	-0.43172	-0.45241	-0.45169	-0.45098
-0.393	-0.39234	-0.39172	-0.43337	-0.4327	-0.43204	-0.45275	-0.45203	-0.45132
-0.39333	-0.39264	-0.39202	-0.39141	-0.39081	-0.39023	-0.43172	-0.43108	-0.45165
-0.39367	-0.39294	-0.39232	-0.39171	-0.3911	-0.39051	-0.43205	-0.43139	-0.45199
-0.39406	-0.39329	-0.39264	-0.39201	-0.39139	-0.39079	-0.3902	-0.38962	-0.45234
-0.3945	-0.39367	-0.39297	-0.39232	-0.39168	-0.39107	-0.39047	-0.38989	-0.4527
-0.39497	-0.39408	-0.39333	-0.39263	-0.39198	-0.39137	-0.39076	-0.39017	-0.3896
-0.39547	-0.39452	-0.39369	-0.39294	-0.39229	-0.39167	-0.39106	-0.39047	-0.38989
-0.396	-0.395	-0.39409	-0.39328	-0.39261	-0.39199	-0.39137	-0.39077	-0.39018
-0.39657	-0.39552	-0.39453	-0.39365	-0.39294	-0.3923	-0.39167	-0.39107	-0.39048
-0.39717	-0.39607	-0.39501	-0.39407	-0.3933	-0.39261	-0.39197	-0.39138	-0.39078
-0.39776	-0.39664	-0.39554	-0.39455	-0.3937	-0.39294	-0.39228	-0.39168	-0.39107
-0.39836	-0.39723	-0.39611	-0.39508	-0.39413	-0.39329	-0.39259	-0.39197	-0.39135
-0.39895	-0.39782	-0.39668	-0.39561	-0.39459	-0.39367	-0.39292	-0.39227	-0.39163
-0.39953	-0.39839	-0.39726	-0.39616	-0.39507	-0.39408	-0.39327	-0.39257	-0.39192
-0.35768	-0.39899	-0.39785	-0.39673	-0.39559	-0.39455	-0.39367	-0.3929	-0.39222
-0.35826	-0.39961	-0.39847	-0.39733	-0.39617	-0.39509	-0.39413	-0.39327	-0.39254
-0.35884	-0.40023	-0.39908	-0.39794	-0.39678	-0.39566	-0.39463	-0.39368	-0.3929
-0.3594	-0.40082	-0.39968	-0.39854	-0.39739	-0.39626	-0.39517	-0.39415	-0.3933
-0.35995	-0.40141	-0.40028	-0.39914	-0.39801	-0.39688	-0.39575	-0.39466	-0.39375
-0.36051	-0.35945	-0.35839	-0.39976	-0.39863	-0.3975	-0.39636	-0.39525	-0.39426
-0.36108	-0.36002	-0.35897	-0.40038	-0.39926	-0.39813	-0.397	-0.39588	-0.39482
-0.36165	-0.36059	-0.35955	-0.3585	-0.35745	-0.35639	-0.39763	-0.3965	-0.39539
-0.36223	-0.36116	-0.36012	-0.35907	-0.35803	-0.35698	-0.39825	-0.39711	-0.39597
-0.36283	-0.36175	-0.36069	-0.35964	-0.35861	-0.35756	-0.39886	-0.39771	-0.39656
-0.36344	-0.36234	-0.36128	-0.36022	-0.35917	-0.35812	-0.39946	-0.39832	-0.39717
-0.36406	-0.36295	-0.36187	-0.3608	-0.35974	-0.35868	-0.40006	-0.39892	-0.39778
-0.31536	-0.36357	-0.36247	-0.36138	-0.3603	-0.35924	-0.35818	-0.35712	-0.39841
-0.31588	-0.36419	-0.36306	-0.36195	-0.36087	-0.3598	-0.35875	-0.35771	-0.39904
-0.31638	-0.36481	-0.36366	-0.36253	-0.36144	-0.36037	-0.35932	-0.35828	-0.35723
-0.31689	-0.36543	-0.36425	-0.36311	-0.36202	-0.36095	-0.3599	-0.35886	-0.35781
-0.31739	-0.31645	-0.31551	-0.36372	-0.36262	-0.36154	-0.36048	-0.35944	-0.35839
-0.3179	-0.31696	-0.31602	-0.36435	-0.36323	-0.36214	-0.36107	-0.36003	-0.35898
-0.31841	-0.31747	-0.31654	-0.31562	-0.31472	-0.31384	-0.36167	-0.36062	-0.35957
-0.31893	-0.31799	-0.31706	-0.31613	-0.31522	-0.31433	-0.36226	-0.3612	-0.36016
-0.31946	-0.31852	-0.31758	-0.31665	-0.31573	-0.31483	-0.36287	-0.36179	-0.36074
-0.31997	-0.31904	-0.31812	-0.31719	-0.31626	-0.31534	-0.36349	-0.36239	-0.36133
-0.32047	-0.31956	-0.31865	-0.31773	-0.31679	-0.31586	-0.36413	-0.36301	-0.36192
-0.32098	-0.32008	-0.31919	-0.31828	-0.31734	-0.3164	-0.31549	-0.3146	-0.36255
-0.32151	-0.32061	-0.31972	-0.31883	-0.3179	-0.31697	-0.31605	-0.31514	-0.36321

-0.32203	-0.32113	-0.32025	-0.31936	-0.31845	-0.31753	-0.3166	-0.31568	-0.31477
-0.32255	-0.32165	-0.32077	-0.31987	-0.31897	-0.31807	-0.31714	-0.31621	-0.31529
-0.32307	-0.32217	-0.32128	-0.32038	-0.3195	-0.3186	-0.31767	-0.31674	-0.31581
-0.32358	-0.32267	-0.32179	-0.3209	-0.32001	-0.31912	-0.3182	-0.31728	-0.31635
-0.32409	-0.32317	-0.3223	-0.32142	-0.32053	-0.31963	-0.31873	-0.31782	-0.31689
-0.28459	-0.32369	-0.32279	-0.32191	-0.32103	-0.32015	-0.31925	-0.31834	-0.31741
-0.28507	-0.32421	-0.32329	-0.32239	-0.32154	-0.32067	-0.31977	-0.31884	-0.31793
-0.28556	-0.28469	-0.28385	-0.3229	-0.32205	-0.32119	-0.32029	-0.31937	-0.31847
-0.28605	-0.28518	-0.28433	-0.32343	-0.32256	-0.3217	-0.32082	-0.31993	-0.31903
-0.28657	-0.28569	-0.28483	-0.284	-0.28319	-0.2824	-0.32134	-0.32047	-0.31958
-0.28711	-0.28622	-0.28535	-0.28449	-0.28366	-0.28285	-0.32184	-0.32098	-0.32009
-0.28765	-0.28676	-0.28587	-0.28499	-0.28414	-0.28331	-0.32234	-0.32149	-0.32061
-0.28818	-0.28728	-0.28637	-0.28549	-0.28463	-0.28379	-0.28299	-0.28221	-0.32112
-0.2887	-0.28779	-0.28688	-0.28599	-0.28512	-0.28427	-0.28347	-0.28268	-0.32164
-0.28922	-0.28831	-0.2874	-0.28651	-0.28564	-0.28478	-0.28395	-0.28315	-0.28234
-0.28973	-0.28882	-0.28793	-0.28705	-0.28616	-0.28529	-0.28445	-0.28361	-0.2828
-0.29026	-0.28935	-0.28846	-0.28758	-0.28669	-0.28581	-0.28494	-0.28409	-0.28327
-0.2908	-0.2899	-0.289	-0.2881	-0.2872	-0.28631	-0.28543	-0.28457	-0.28375
-0.29135	-0.29044	-0.28954	-0.28863	-0.28773	-0.28683	-0.28594	-0.28507	-0.28424
-0.29189	-0.29099	-0.29009	-0.28918	-0.28826	-0.28736	-0.28647	-0.2856	-0.28476
-0.29243	-0.29153	-0.29064	-0.28973	-0.28881	-0.28789	-0.287	-0.28614	-0.28528
-0.15001	-0.29207	-0.29117	-0.29026	-0.28933	-0.28842	-0.28754	-0.28667	-0.2858
-0.15029	-0.29261	-0.29169	-0.29078	-0.28986	-0.28896	-0.28808	-0.28721	-0.28633
-0.15057	-0.15007	-0.14958	-0.29131	-0.2904	-0.28951	-0.28862	-0.28774	-0.28685
-0.15085	-0.15034	-0.14985	-0.29186	-0.29096	-0.29006	-0.28916	-0.28827	-0.28738
-0.09786	-0.15063	-0.15013	-0.29241	-0.29151	-0.29061	-0.28971	-0.2888	-0.2879
-0.09803	-0.15092	-0.15041	-0.29296	-0.29206	-0.29117	-0.29025	-0.28933	-0.28842
-0.09821	-0.15122	-0.15069	-0.15019	-0.14971	-0.14924	-0.2908	-0.28987	-0.28895
-0.09838	-0.15152	-0.15099	-0.15048	-0.15	-0.14952	-0.29136	-0.29042	-0.2895
-0.09855	-0.15182	-0.15129	-0.15078	-0.15029	-0.14981	-0.29192	-0.29098	-0.29005
-0.09872	-0.09841	-0.0981	-0.15108	-0.15059	-0.1501	-0.14959	-0.14909	-0.29061
-0.0989	-0.09858	-0.09827	-0.15138	-0.15088	-0.15038	-0.14988	-0.14938	-0.29118
-0.09908	-0.09876	-0.09844	-0.09813	-0.09783	-0.09753	-0.15017	-0.14966	-0.2023
-0.09927	-0.09894	-0.09862	-0.09831	-0.09801	-0.0977	-0.15046	-0.14995	-0.20271
-0.04676	-0.09913	-0.09881	-0.09849	-0.09818	-0.09787	-0.15075	-0.15023	-0.14973
-0.04682	-0.09933	-0.099	-0.09867	-0.09836	-0.09804	-0.15105	-0.15052	-0.15001
-0.04689	-0.09954	-0.09919	-0.09885	-0.09853	-0.09822	-0.15134	-0.15082	-0.1503
-0.04696	-0.09974	-0.09939	-0.09905	-0.09871	-0.09838	-0.09806	-0.09774	-0.1506
-0.04702	-0.09994	-0.0996	-0.09926	-0.0989	-0.09855	-0.09823	-0.09792	-0.15089
-0.04709	-0.04693	-0.04678	-0.09946	-0.0991	-0.09874	-0.09841	-0.09809	-0.09778
-0.04716	-0.04699	-0.04684	-0.09966	-0.09931	-0.09896	-0.09861	-0.09827	-0.09795
	-0.04706	-0.0469	-0.04675	-0.04659	-0.04643	-0.09881	-0.09846	-0.09813
	-0.04715	-0.04698	-0.04682	-0.04666	-0.04649	-0.09902	-0.09866	-0.09833
			-0.04689	-0.04673	-0.04656	-0.09923	-0.09887	-0.09852
			-0.04696	-0.0468	-0.04664	-0.09945	-0.09908	-0.09872
			-0.04704	-0.04687	-0.04671	-0.09966	-0.09929	-0.09893
			-0.04712	-0.04695	-0.04678	-0.04661	-0.04645	-0.09915
			-0.04721	-0.04703	-0.04685	-0.04669	-0.04652	-0.09938
						-0.04676	-0.0466	-0.04642
						-0.04684	-0.04667	-0.0465

-0.04658
-0.04666
-0.04674
-0.04682
-0.0469

								-0.10814
								-0.1079
								-0.10765
								-0.25616
								-0.25553
						-0.20472	-0.20385	-0.25492
						-0.20416	-0.20334	-0.25433
			-0.20636	-0.20539	-0.20446	-0.25567	-0.2547	-0.38536
			-0.2057	-0.20478	-0.20391	-0.25501	-0.25404	-0.38447
			-0.20508	-0.20421	-0.20339	-0.25438	-0.2534	-0.3836
	-0.10793	-0.10762	-0.25679	-0.25575	-0.25477	-0.38543	-0.3841	-0.42482
	-0.1077	-0.10739	-0.25617	-0.25516	-0.25418	-0.38463	-0.3833	-0.42397
-0.10776	-0.20526	-0.20438	-0.30257	-0.30148	-0.3004	-0.38382	-0.3825	-0.5165
-0.10754	-0.20471	-0.20387	-0.3019	-0.30081	-0.29973	-0.38302	-0.38171	-0.5155
-0.10732	-0.30342	-0.30231	-0.3862	-0.38485	-0.38352	-0.47394	-0.47241	-0.55871
-0.1071	-0.30274	-0.30164	-0.38538	-0.38403	-0.38269	-0.47297	-0.47146	-0.55763
-0.25619	-0.34468	-0.34345	-0.47669	-0.47509	-0.47351	-0.5599	-0.55822	-0.6011
-0.25557	-0.34394	-0.34271	-0.4757	-0.4741	-0.47252	-0.55876	-0.55707	-0.5999
-0.25494	-0.34318	-0.34196	-0.4747	-0.47311	-0.47153	-0.55763	-0.55593	-0.59872
-0.30104	-0.47683	-0.47525	-0.51742	-0.51575	-0.5141	-0.60116	-0.59937	-0.59763

-0.3003	-0.47578	-0.47422	-0.51633	-0.51471	-0.5131	-0.60007	-0.59829	-0.59658
-0.39376	-0.47478	-0.47324	-0.55952	-0.55783	-0.55615	-0.59899	-0.59725	-0.64379
-0.39295	-0.47383	-0.47231	-0.55846	-0.55678	-0.55512	-0.59793	-0.59623	-0.64269
-0.47449	-0.56081	-0.5591	-0.60213	-0.60035	-0.5986	-0.59689	-0.59522	-0.7608
-0.47352	-0.55976	-0.55805	-0.60104	-0.59927	-0.59754	-0.59586	-0.59423	-0.75951
-0.56041	-0.60354	-0.60173	-0.59996	-0.59821	-0.59651	-0.64299	-0.64122	-0.75822
-0.55935	-0.60244	-0.60065	-0.5989	-0.59719	-0.59551	-0.64191	-0.64014	-0.75694
-0.55831	-0.60136	-0.59959	-0.59786	-0.59618	-0.59453	-0.64083	-0.63906	-0.75565
-0.55729	-0.6003	-0.59856	-0.64514	-0.64332	-0.64153	-0.7585	-0.75643	-1.01266
-0.55627	-0.59926	-0.59755	-0.64405	-0.64224	-0.64044	-0.7572	-0.75512	-1.01052
-0.59995	-0.59823	-0.59654	-0.76218	-0.76008	-0.75799	-1.01514	-1.01172	-1.04329
-0.5989	-0.5972	-0.59553	-0.76087	-0.75878	-0.75669	-1.013	-1.00957	-1.04111
-0.59787	-0.67994	-0.67798	-0.83417	-0.8319	-0.82962	-1.04586	-1.04235	-1.10635
-0.59686	-0.67873	-0.6768	-0.83273	-0.83046	-0.82819	-1.04377	-1.0403	-1.10421
-0.71167	-0.83587	-0.83357	-1.05189	-1.0485	-1.04513	-1.10913	-1.10556	-1.13492
-0.71044	-0.83442	-0.83212	-1.04971	-1.04638	-1.04307	-1.10699	-1.10343	-1.13279
-0.70921	-0.83297	-0.83067	-1.04755	-1.04428	-1.04103	-1.10487	-1.10131	-1.13068
-0.83386	-1.01239	-1.0092	-1.113	-1.10961	-1.10625	-1.10275	-1.09921	-1.12859
-0.83239	-1.01037	-1.00722	-1.11084	-1.10747	-1.10412	-1.10064	-1.09713	-1.12652
-0.86922	-1.04794	-1.04463	-1.10869	-1.10534	-1.102	-1.09857	-1.09511	-1.15314
-0.8677	-1.04581	-1.04255	-1.10656	-1.10322	-1.09989	-1.09651	-1.09315	-1.15116
-1.00754	-1.11112	-1.10776	-1.10443	-1.10111	-1.09781	-1.09449	-1.0912	-1.14919
-1.00558	-1.10897	-1.10561	-1.1023	-1.09902	-1.09576	-1.0925	-1.08925	-1.14721
-1.00366	-1.10685	-1.10348	-1.10018	-1.09695	-1.09376	-1.09054	-1.08733	-1.14525
-1.1082	-1.10475	-1.10138	-1.09809	-1.09493	-1.09181	-1.1499	-1.14657	-1.19756
-1.10614	-1.10267	-1.0993	-1.09604	-1.09294	-1.08988	-1.14795	-1.14466	-1.1955
-1.10407	-1.10062	-1.09728	-1.12325	-1.12012	-1.11705	-1.20044	-1.19696	-1.27293
-1.10201	-1.0986	-1.09531	-1.12128	-1.11815	-1.11509	-1.19835	-1.19494	-1.27081
-1.10001	-1.12591	-1.12257	-1.17512	-1.17178	-1.16849	-1.24838	-1.24491	-1.26866
-1.09807	-1.124	-1.12065	-1.17304	-1.16967	-1.16637	-1.24615	-1.24272	-1.26645
-1.09614	-1.17805	-1.17447	-1.25448	-1.25089	-1.24737	-1.24392	-1.24052	-1.26424
-1.09415	-1.17594	-1.1724	-1.25229	-1.24868	-1.24515	-1.24171	-1.23833	-1.26204
-1.09217	-1.17381	-1.17033	-1.25012	-1.24651	-1.24295	-1.23951	-1.23615	-1.25986
-1.1751	-1.19379	-1.19032	-1.248	-1.24438	-1.2408	-1.26455	-1.26114	-1.31428
-1.17298	-1.19163	-1.18824	-1.24589	-1.24227	-1.23868	-1.26238	-1.25895	-1.31197
-1.19294	-1.22049	-1.21707	-1.24375	-1.24015	-1.23658	-1.26023	-1.25678	-1.30965
-1.19083	-1.2183	-1.2149	-1.24157	-1.23802	-1.23449	-1.25809	-1.25462	-1.30733
-1.2197	-1.21614	-1.21274	-1.26661	-1.26306	-1.25952	-1.31263	-1.30877	-1.39153
-1.21754	-1.21401	-1.21061	-1.26441	-1.26094	-1.25749	-1.31047	-1.30662	-1.38923
-1.21542	-1.24206	-1.23856	-1.26224	-1.25882	-1.25544	-1.365	-1.36105	-1.38699
-1.21338	-1.24003	-1.23651	-1.26012	-1.25669	-1.25331	-1.36273	-1.35891	-1.38482
-1.21135	-1.23803	-1.23449	-1.25805	-1.25458	-1.25118	-1.36046	-1.35679	-1.38267
-1.20926	-1.26304	-1.25952	-1.3698	-1.36582	-1.36193	-1.38797	-1.38426	-1.40649
-1.20713	-1.26086	-1.25744	-1.36757	-1.36359	-1.35968	-1.38567	-1.38197	-1.40419
-1.20506	-1.3175	-1.31397	-1.36529	-1.36135	-1.35747	-1.38339	-1.37968	-1.45017
-1.20306	-1.31532	-1.31178	-1.36295	-1.35909	-1.35529	-1.38114	-1.37739	-1.4478
-1.26171	-1.36843	-1.36452	-1.39048	-1.38658	-1.38273	-1.4049	-1.40107	-1.44544
-1.25968	-1.36621	-1.36231	-1.38823	-1.38437	-1.38055	-1.40269	-1.39883	-1.44311
-1.25766	-1.36401	-1.36014	-1.38601	-1.38217	-1.37836	-1.40048	-1.39663	-1.4408
-1.28207	-1.39162	-1.3877	-1.40987	-1.40595	-1.40206	-1.39823	-1.39446	-1.46683

-1.27999	-1.38933	-1.38549	-1.40766	-1.40369	-1.39975	-1.39598	-1.3923	-1.46458
-1.33369	-1.38703	-1.38319	-1.40535	-1.4014	-1.39749	-1.42283	-1.41909	-1.46228
-1.33145	-1.38472	-1.38085	-1.40295	-1.39908	-1.39524	-1.42052	-1.41674	-1.45994
-1.38637	-1.40845	-1.40451	-1.40063	-1.39681	-1.39304	-1.41826	-1.41447	-1.45771
-1.38412	-1.40619	-1.40225	-1.39838	-1.39461	-1.39088	-1.41608	-1.41228	-1.45559
-1.40786	-1.40393	-1.40002	-1.42532	-1.42149	-1.41773	-1.41394	-1.41024	-1.5229
-1.40557	-1.40165	-1.39783	-1.42313	-1.41926	-1.41549	-1.41186	-1.40839	-1.52108
-1.40329	-1.3994	-1.39565	-1.42096	-1.41707	-1.4133	-1.40986	-1.40667	-1.51941
-1.40108	-1.42638	-1.42254	-1.46453	-1.46061	-1.45685	-1.49883	-1.49568	-1.5178
-1.39891	-1.42421	-1.42032	-1.46226	-1.45845	-1.45485	-1.49695	-1.49403	-1.51629
-1.39672	-1.46784	-1.46389	-1.46008	-1.45643	-1.45302	-1.49524	-1.4925	-1.55907
-1.39451	-1.46555	-1.46166	-1.45798	-1.45453	-1.45134	-1.4937	-1.49109	-1.55763
-1.46718	-1.46331	-1.45951	-1.45595	-1.45272	-1.44975	-1.5615	-1.55883	-1.57381
-1.46493	-1.46113	-1.45741	-1.45398	-1.45098	-1.44824	-1.55999	-1.55736	-1.57238
-1.46273	-1.45901	-1.45542	-1.49863	-1.49572	-1.49307	-1.57614	-1.57352	-1.59643
-1.46051	-1.45689	-1.45357	-1.49695	-1.49415	-1.49156	-1.57464	-1.5721	-1.59494
-1.45838	-1.45487	-1.45183	-1.49539	-1.49266	-1.49011	-1.57316	-1.57069	-1.59345
-1.45646	-1.49958	-1.49659	-1.51241	-1.50974	-1.50722	-1.57175	-1.56927	-1.63656
-1.45469	-1.49793	-1.495	-1.51085	-1.5083	-1.50587	-1.57037	-1.56786	-1.63495
-1.49944	-1.51498	-1.5121	-1.53404	-1.53147	-1.529	-1.63895	-1.63615	-1.63339
-1.49769	-1.51344	-1.5107	-1.53269	-1.5301	-1.52759	-1.63735	-1.63462	-1.63187
-1.51466	-1.51192	-1.50931	-1.57816	-1.57544	-1.57277	-1.63578	-1.63307	-1.65692
-1.51305	-1.51039	-1.5079	-1.57671	-1.57404	-1.57139	-1.63426	-1.63149	-1.65525
-1.51152	-1.50892	-1.5065	-1.57526	-1.57264	-1.57002	-1.63276	-1.62994	-1.6536
-1.51007	-1.57895	-1.57634	-1.59908	-1.59634	-1.5936	-1.63128	-1.62847	-1.67311
-1.50867	-1.57749	-1.57485	-1.59748	-1.59478	-1.59209	-1.62981	-1.62704	-1.67156
-1.55471	-1.576	-1.57339	-1.59594	-1.59323	-1.59055	-1.65471	-1.65184	-1.68123
-1.55323	-1.5745	-1.57195	-1.59446	-1.59171	-1.58899	-1.65309	-1.65035	-1.67978
-1.57567	-1.59833	-1.59566	-1.62037	-1.61743	-1.61452	-1.66287	-1.6602	-1.67829
-1.57428	-1.59686	-1.59419	-1.61881	-1.6159	-1.61303	-1.66135	-1.65866	-1.67675
-1.5729	-1.60747	-1.60477	-1.629	-1.62614	-1.62333	-1.65986	-1.65717	-1.7161
-1.57151	-1.60598	-1.60323	-1.62732	-1.62455	-1.62185	-1.65843	-1.65576	-1.71453
-1.57011	-1.60449	-1.6017	-1.62567	-1.62297	-1.62037	-1.65699	-1.65438	-1.71299
-1.60573	-1.62995	-1.627	-1.62415	-1.62146	-1.61886	-1.69503	-1.6924	-1.71146
-1.60417	-1.62835	-1.62549	-1.62269	-1.61998	-1.61735	-1.69348	-1.69092	-1.70993
-1.62952	-1.62672	-1.62396	-1.6409	-1.6382	-1.63555	-1.71386	-1.7111	-1.70838
-1.62784	-1.62504	-1.6224	-1.63944	-1.63679	-1.63417	-1.7123	-1.70951	-1.7068
-1.62625	-1.64314	-1.64055	-1.66119	-1.65835	-1.65553	-1.71077	-1.70798	-1.70528
-1.62478	-1.64169	-1.6391	-1.65963	-1.65682	-1.65404	-1.70928	-1.70652	-1.70382
-1.64299	-1.64025	-1.63765	-1.6787	-1.67588	-1.67309	-1.70781	-1.70509	-1.71866
-1.64152	-1.6388	-1.63616	-1.67704	-1.67429	-1.6716	-1.70636	-1.70366	-1.71723
-1.64007	-1.63736	-1.63467	-1.6754	-1.67272	-1.6701	-1.7049	-1.70223	-1.71582
-1.63863	-1.65899	-1.65609	-1.67385	-1.67118	-1.66858	-1.71972	-1.71707	-1.7144
-1.63721	-1.65747	-1.6546	-1.67236	-1.66966	-1.66705	-1.7182	-1.71561	-1.71299
-1.65885	-1.69353	-1.69063	-1.6878	-1.68507	-1.68241	-1.71673	-1.71416	-1.71158
-1.65732	-1.69202	-1.68918	-1.6864	-1.68367	-1.681	-1.7153	-1.71273	-1.71017
-1.67305	-1.69052	-1.68774	-1.68499	-1.68227	-1.6796	-1.71388	-1.7113	-1.70876
-1.67155	-1.68904	-1.6863	-1.68359	-1.68088	-1.67821	-1.71246	-1.70986	-1.70733
-1.67007	-1.68758	-1.68487	-1.68219	-1.67949	-1.67682	-1.71105	-1.70844	-1.70592
-1.68891	-1.68616	-1.68347	-1.68079	-1.67811	-1.67545	-1.70967	-1.70707	-1.70455

-1.68747	-1.68476	-1.68207	-1.6794	-1.67673	-1.67409	-1.7083	-1.70571	-1.7032
-1.68603	-1.68335	-1.68066	-1.678	-1.67536	-1.67275	-1.70695	-1.70437	-1.72252
-1.68459	-1.68193	-1.67925	-1.6766	-1.67399	-1.67141	-1.70562	-1.70304	-1.72108
-1.68317	-1.68052	-1.67785	-1.67521	-1.67263	-1.67008	-1.70427	-1.7017	-1.74491
-1.68174	-1.6791	-1.67645	-1.67383	-1.67128	-1.66874	-1.70293	-1.70036	-1.74341
-1.68032	-1.67769	-1.67506	-1.67247	-1.66993	-1.66741	-1.73949	-1.73672	-1.75884
-1.6789	-1.67628	-1.67369	-1.67112	-1.66859	-1.66608	-1.73806	-1.73532	-1.75739
-1.67748	-1.67488	-1.67232	-1.66978	-1.66726	-1.66476	-1.73664	-1.73394	-1.75595
-1.69411	-1.69151	-1.68895	-1.70769	-1.70496	-1.70225	-1.76011	-1.75729	-1.75452
-1.69273	-1.69014	-1.6876	-1.70626	-1.70354	-1.70084	-1.75862	-1.75585	-1.75309
-1.69135	-1.68879	-1.68626	-1.70485	-1.70214	-1.69946	-1.75716	-1.75441	-1.75167
-1.68998	-1.68744	-1.68493	-1.70344	-1.70076	-1.6981	-1.75572	-1.75298	-1.75025
-1.68862	-1.74318	-1.74043	-1.76276	-1.7599	-1.75707	-1.75429	-1.75156	-1.74884
-1.68728	-1.74174	-1.73899	-1.76124	-1.75842	-1.75563	-1.75287	-1.75014	-1.74745
-1.68595	-1.76554	-1.76262	-1.75975	-1.75695	-1.7542	-1.75145	-1.74873	-1.7578
-1.68463	-1.76403	-1.76114	-1.7583	-1.75552	-1.75278	-1.75004	-1.74733	-1.75638
-1.68331	-1.76254	-1.75969	-1.75687	-1.7541	-1.75136	-1.74865	-1.74597	-1.755
-1.70306	-1.76105	-1.75824	-1.75546	-1.75269	-1.74996	-1.74729	-1.74469	-1.75988
-1.70166	-1.75958	-1.75682	-1.75407	-1.75129	-1.74856	-1.74597	-1.74347	-1.75862
-1.72582	-1.75815	-1.75543	-1.75272	-1.74996	-1.74724	-1.76254	-1.75993	-1.75732
-1.7244	-1.75675	-1.75408	-1.75142	-1.74869	-1.74599	-1.76122	-1.75861	-1.75599
-1.72303	-1.75538	-1.75273	-1.7564	-1.7537	-1.75103	-1.75989	-1.75727	-1.75465
-1.72175	-1.75408	-1.75137	-1.75501	-1.75234	-1.74972	-1.75854	-1.75592	-1.75329
-1.72045	-1.75276	-1.75	-1.75362	-1.75098	-1.74839	-1.75718	-1.75456	-1.75192
-1.71906	-1.7577	-1.75493	-1.75223	-1.74962	-1.74707	-1.75581	-1.75319	-1.75055
-1.71764	-1.75627	-1.75353	-1.75085	-1.74827	-1.74575	-1.75446	-1.75181	-1.74917
-1.7229	-1.75486	-1.75216	-1.76108	-1.7584	-1.75576	-1.76983	-1.76716	-1.76448
-1.72148	-1.75346	-1.75082	-1.75973	-1.75706	-1.75442	-1.76848	-1.7658	-1.76312
-1.72015	-1.75213	-1.74954	-1.79044	-1.78774	-1.78507	-1.78237	-1.77966	-1.82196
-1.7189	-1.75089	-1.74833	-1.78919	-1.7865	-1.78382	-1.78111	-1.7784	-1.82055
-1.7337	-1.79328	-1.79058	-1.78791	-1.78522	-1.78253	-1.77982	-1.77711	-1.83261
-1.73238	-1.79192	-1.78923	-1.78655	-1.78386	-1.78117	-1.77847	-1.77576	-1.8311
-1.73105	-1.79055	-1.78786	-1.78518	-1.78249	-1.77979	-1.77709	-1.77439	-1.82959
-1.74693	-1.78918	-1.78649	-1.79773	-1.79504	-1.79234	-1.83408	-1.83107	-1.82807
-1.7456	-1.78782	-1.78513	-1.79636	-1.79367	-1.79097	-1.83254	-1.82954	-1.82656
-1.75588	-1.80043	-1.79774	-1.79505	-1.79236	-1.78966	-1.83109	-1.82809	-1.82514
-1.75464	-1.79918	-1.79649	-1.79379	-1.7911	-1.78841	-1.82969	-1.8267	-1.82379
-1.76767	-1.79788	-1.79518	-1.83729	-1.83426	-1.83125	-1.82825	-1.82528	-1.8224
-1.76633	-1.79652	-1.79381	-1.83576	-1.83273	-1.82973	-1.82676	-1.82382	-1.82097
-1.76501	-1.79517	-1.79245	-1.83424	-1.8312	-1.82821	-1.82526	-1.82237	-1.81956
-1.76374	-1.79388	-1.79111	-1.83272	-1.82967	-1.82667	-1.82377	-1.82093	-1.81816
-1.76248	-1.79258	-1.78979	-1.83122	-1.82816	-1.82517	-1.82231	-1.81953	-1.81679
-1.76115	-1.83599	-1.83287	-1.82979	-1.82675	-1.82379	-1.82095	-1.81821	-1.81549
-1.7598	-1.8345	-1.83142	-1.82838	-1.82539	-1.82247	-1.81966	-1.81694	-1.81423
-1.7585	-1.83304	-1.82996	-1.82694	-1.82399	-1.82112	-1.81833	-1.81562	-1.85145
-1.75725	-1.83163	-1.8285	-1.82545	-1.82256	-1.81974	-1.81698	-1.81428	-1.84998
-1.80148	-1.83019	-1.82706	-1.82403	-1.82117	-1.8184	-1.81566	-1.81297	-1.84856
-1.79998	-1.82871	-1.82565	-1.82268	-1.81986	-1.81712	-1.81439	-1.81171	-1.84721
-1.7985	-1.82724	-1.82426	-1.82135	-1.81856	-1.81583	-1.85185	-1.84883	-1.84586
-1.7971	-1.82584	-1.82288	-1.82	-1.81721	-1.81449	-1.85039	-1.84739	-1.84445

-1.79573	-1.82447	-1.82153	-1.81865	-1.81588	-1.81315	-1.84893	-1.84596	-1.84304
-1.79432	-1.82308	-1.8202	-1.85681	-1.85366	-1.85059	-1.84758	-1.84463	-1.86338
-1.79289	-1.82169	-1.81889	-1.85545	-1.85233	-1.84927	-1.84628	-1.84335	-1.86201
-1.79153	-1.82037	-1.81762	-1.85409	-1.85101	-1.84796	-1.84497	-1.84203	-1.86059
-1.79025	-1.81911	-1.81637	-1.85275	-1.84969	-1.84666	-1.84366	-1.84068	-1.85912
-1.78898	-1.85757	-1.85445	-1.85137	-1.84834	-1.84534	-1.86399	-1.86081	-1.88842
-1.78774	-1.85619	-1.85303	-1.84993	-1.84692	-1.84396	-1.86255	-1.85941	-1.88701
-1.78648	-1.8548	-1.85162	-1.84851	-1.84552	-1.84258	-1.86112	-1.85801	-1.8856
-1.82595	-1.85337	-1.85024	-1.84717	-1.84419	-1.84126	-1.89045	-1.88731	-1.88417
-1.82449	-1.85193	-1.84888	-1.84586	-1.84289	-1.83995	-1.88901	-1.88587	-1.88272
-1.82311	-1.85057	-1.84755	-1.89709	-1.89387	-1.89067	-1.88751	-1.88439	-1.88126
-1.82179	-1.84925	-1.84624	-1.89565	-1.89238	-1.88913	-1.88597	-1.88287	-1.87979
-1.82049	-1.87919	-1.87613	-1.89418	-1.89089	-1.88764	-1.88449	-1.88142	-1.89388
-1.81922	-1.87785	-1.87474	-1.89267	-1.88941	-1.8862	-1.88308	-1.88003	-1.89245
-1.84991	-1.8978	-1.89447	-1.89118	-1.88795	-1.88478	-1.88169	-1.87866	-1.89103
-1.84851	-1.89629	-1.89298	-1.88973	-1.88653	-1.88339	-1.88033	-1.87733	-1.88964
-1.84709	-1.89477	-1.89151	-1.88829	-1.88513	-1.88202	-1.87898	-1.87599	-1.88824
-1.8457	-1.8933	-1.89005	-1.88686	-1.88374	-1.88067	-1.89311	-1.88991	-1.88675
-1.84432	-1.89185	-1.88862	-1.88545	-1.88237	-1.87932	-1.89166	-1.88841	-1.88523
-1.86447	-1.89041	-1.8872	-1.88405	-1.881	-1.87799	-1.89024	-1.88697	-1.88379
-1.86304	-1.88898	-1.88579	-1.88267	-1.87966	-1.87666	-1.88886	-1.88559	-1.88241
-1.86162	-1.88756	-1.88441	-1.89704	-1.89384	-1.89067	-1.88744	-1.8842	-1.88104
-1.8602	-1.88615	-1.88305	-1.89562	-1.89239	-1.88918	-1.88598	-1.88279	-1.87966
-1.8588	-1.88476	-1.8817	-1.89421	-1.89095	-1.88771	-1.91879	-1.91538	-1.912
-1.85741	-1.88339	-1.88036	-1.89283	-1.88956	-1.88631	-1.91728	-1.91389	-1.91052
-1.85604	-1.88203	-1.87903	-1.89144	-1.88818	-1.88494	-1.9158	-1.91242	-1.90905
-1.85468	-1.88067	-1.87765	-1.88997	-1.88675	-1.88354	-1.9143	-1.91093	-1.90758
-1.85334	-1.87933	-1.87626	-1.88847	-1.88528	-1.88213	-1.91279	-1.90943	-1.9061
-1.85201	-1.89353	-1.89025	-1.88702	-1.88386	-1.88074	-1.91129	-1.90794	-1.90462
-1.85069	-1.89214	-1.88886	-1.88562	-1.88247	-1.87936	-1.9098	-1.90645	-1.90312
-1.84937	-1.89075	-1.88747	-1.88423	-1.88109	-1.878	-1.90832	-1.90499	-1.90166
-1.84806	-1.88936	-1.88608	-1.88285	-1.87972	-1.87664	-1.90686	-1.90354	-1.90023
-1.84675	-1.88797	-1.8847	-1.88148	-1.87836	-1.87529	-1.90541	-1.90212	-1.89882
-1.84545	-1.8866	-1.88334	-1.88013	-1.87702	-1.87395	-1.90398	-1.9007	-1.92027
-1.84415	-1.88523	-1.88199	-1.87879	-1.87568	-1.87263	-1.90256	-1.8993	-1.91888
-1.84285	-1.88385	-1.88062	-1.87744	-1.87435	-1.87131	-1.92407	-1.92079	-1.9175
-1.84154	-1.88246	-1.87926	-1.8761	-1.87302	-1.87	-1.92266	-1.91939	-1.91612
-1.85567	-1.9049	-1.90166	-1.89848	-1.89537	-1.89232	-1.92125	-1.918	-1.91473
-1.85434	-1.90356	-1.90033	-1.89714	-1.89405	-1.891	-1.91983	-1.91659	-1.91334
-1.87748	-1.90224	-1.89902	-1.89585	-1.89276	-1.88971	-1.91843	-1.9152	-1.92383
-1.87615	-1.90093	-1.89778	-1.89464	-1.89153	-1.88846	-1.91707	-1.91384	-1.92246
-1.87484	-1.89964	-1.89655	-1.89345	-1.89032	-1.88721	-1.91571	-1.91249	-1.92112
-1.87356	-1.89835	-1.89527	-1.89219	-1.88905	-1.88593	-1.9262	-1.92299	-1.91981
-1.8723	-1.89706	-1.89398	-1.89091	-1.88777	-1.88465	-1.92482	-1.92165	-1.91852
-1.87103	-1.86792	-1.86489	-1.86185	-1.85874	-1.85565	-1.92344	-1.92029	-1.91718
-1.86974	-1.86664	-1.86361	-1.86057	-1.85747	-1.85439	-1.92206	-1.91891	-1.91582
-1.86846	-1.86536	-1.86233	-1.87177	-1.8687	-1.86564	-1.92073	-1.91757	-1.91449
-1.86717	-1.86407	-1.86104	-1.8705	-1.86748	-1.86448	-1.91948	-1.9163	-1.91321
-1.8659	-1.86282	-1.85978	-1.86924	-1.86627	-1.86332	-1.91822	-1.91504	-1.91194
-1.86473	-1.86165	-1.85857	-1.86799	-1.86502	-1.86209	-1.91691	-1.91374	-1.91066

-1.86356	-1.8605	-1.85738	-1.86676	-1.86378	-1.86084	-1.91558	-1.91245	-1.90939
-1.86233	-1.85929	-1.8562	-1.86559	-1.86259	-1.85964	-1.9143	-1.91121	-1.91927
-1.86106	-1.85804	-1.85502	-1.86445	-1.86143	-1.85845	-1.91304	-1.90999	-1.91803
-1.85981	-1.85678	-1.8538	-1.86325	-1.86024	-1.85727	-1.91177	-1.90875	-1.91677
-1.85856	-1.85551	-1.85253	-1.86199	-1.85902	-1.85609	-1.9105	-1.90747	-1.91548
-1.85734	-1.8667	-1.8637	-1.86074	-1.85782	-1.85493	-1.92036	-1.91728	-1.91419
-1.85616	-1.86553	-1.86249	-1.85952	-1.85664	-1.85381	-1.91911	-1.91601	-1.91293
-1.85496	-1.86435	-1.86129	-1.85831	-1.85547	-1.85268	-1.91786	-1.91473	-1.91166
-1.8537	-1.8631	-1.86006	-1.86886	-1.866	-1.86318	-1.91654	-1.91341	-1.91035
-1.85241	-1.86182	-1.85883	-1.86764	-1.86478	-1.86195	-1.91521	-1.91209	-1.90905
-1.86355	-1.87238	-1.8694	-1.86647	-1.86359	-1.86074	-1.91392	-1.91083	-1.90779
-1.86231	-1.87113	-1.86821	-1.86533	-1.86243	-1.85955	-1.91266	-1.90961	-1.90657
-1.86112	-1.86992	-1.86704	-1.86417	-1.86126	-1.85837	-1.91141	-1.90837	-1.90535
-1.85998	-1.86879	-1.86587	-1.86298	-1.86009	-1.85722	-1.91016	-1.90713	-1.90413
-1.87061	-1.86765	-1.86471	-1.8618	-1.85892	-1.85607	-1.90892	-1.9059	-1.90292
-1.86942	-1.86647	-1.86355	-1.86066	-1.85777	-1.85491	-1.9077	-1.90471	-1.90174
-1.86824	-1.86529	-1.86239	-1.85951	-1.85662	-1.85376	-1.90648	-1.90352	-1.90057
-1.86706	-1.86412	-1.86121	-1.85832	-1.85547	-1.85265	-1.90529	-1.90233	-1.89939
-1.86589	-1.86297	-1.86003	-1.85713	-1.85432	-1.85155	-1.9041	-1.90114	-1.89821
-1.86471	-1.86179	-1.85885	-1.85597	-1.85318	-1.85042	-1.90291	-1.89997	-1.89705
-1.86352	-1.86059	-1.85769	-1.85484	-1.85204	-1.84928	-1.90172	-1.89881	-1.89591
-1.86234	-1.85941	-1.85654	-1.85371	-1.85092	-1.84816	-1.91325	-1.91036	-1.90746
-1.86117	-1.85826	-1.85539	-1.85257	-1.84981	-1.84709	-1.91208	-1.90917	-1.90628
-1.86001	-1.85712	-1.85425	-1.86477	-1.86203	-1.85932	-1.91091	-1.90799	-1.90511
-1.85885	-1.85595	-1.85313	-1.86367	-1.86091	-1.85818	-1.90972	-1.90683	-1.90395
-1.8577	-1.8548	-1.85203	-1.86257	-1.85979	-1.85703	-1.90853	-1.90567	-1.90279
-1.86991	-1.867	-1.8642	-1.86143	-1.85866	-1.85592	-1.90735	-1.90449	-1.90162
-1.86884	-1.86592	-1.86308	-1.86029	-1.85754	-1.85482	-1.90618	-1.9033	-1.90043
-1.86775	-1.86485	-1.86199	-1.85918	-1.85642	-1.8537	-1.905	-1.90212	-1.89926
-1.86666	-1.8638	-1.86094	-1.85811	-1.85532	-1.85256	-1.9038	-1.90095	-1.8981
-1.86554	-1.86271	-1.85987	-1.85704	-1.85423	-1.85145	-1.87498	-1.87227	-1.86957
-1.86439	-1.86156	-1.85875	-1.85596	-1.85316	-1.85039	-1.87389	-1.87116	-1.86846
-1.86324	-1.8604	-1.85763	-1.85486	-1.8521	-1.84935	-1.87282	-1.87007	-1.86738
-1.86215	-1.8593	-1.8565	-1.85374	-1.85101	-1.8483	-1.87177	-1.869	-1.86633
-1.86108	-1.85822	-1.85539	-1.85261	-1.84992	-1.84726	-1.87073	-1.86797	-1.8653
-1.85999	-1.85715	-1.85431	-1.85153	-1.84886	-1.84622	-1.8697	-1.86695	-1.86428
-1.85889	-1.85608	-1.85326	-1.8505	-1.84783	-1.8452	-1.86868	-1.86595	-1.86326
-1.8578	-1.85501	-1.85222	-1.84946	-1.84679	-1.84414	-1.86763	-1.86493	-1.86225
-1.85672	-1.85395	-1.85118	-1.84844	-1.84572	-1.84304	-1.86653	-1.86388	-1.86123
-1.85564	-1.85287	-1.85014	-1.84742	-1.84467	-1.84196	-1.86545	-1.86282	-1.86019
-1.85454	-1.85178	-1.84908	-1.84639	-1.84365	-1.84093	-1.86438	-1.86175	-1.85913
-1.85346	-1.85069	-1.84802	-1.84536	-1.84264	-1.83993	-1.86334	-1.86068	-1.85807
-1.85242	-1.84966	-1.847	-1.84435	-1.84164	-1.83894	-1.86233	-1.85965	-1.85704
-1.8514	-1.84865	-1.84599	-1.84333	-1.84065	-1.83796	-1.86134	-1.85865	-1.85604
-1.85038	-1.84763	-1.84494	-1.84227	-1.83961	-1.83697	-1.86034	-1.85765	-1.85503
-1.84937	-1.8466	-1.84385	-1.84116	-1.83855	-1.83596	-1.85934	-1.85667	-1.85402
-1.84836	-1.84558	-1.84281	-1.84011	-1.83751	-1.83496	-1.85834	-1.85567	-1.85301
-1.84736	-1.84457	-1.84181	-1.83911	-1.83652	-1.83396	-1.85734	-1.85467	-1.852
-1.84636	-1.84358	-1.84082	-1.83812	-1.83553	-1.83298	-1.85634	-1.85367	-1.851
-1.84536	-1.84258	-1.83983	-1.83714	-1.83455	-1.832	-1.85535	-1.85268	-1.85

-1.84436	-1.84159	-1.83884	-1.83616	-1.83357	-1.83102	-1.85436	-1.85169	-1.849
-1.84335	-1.84059	-1.83785	-1.83517	-1.83259	-1.83004	-1.85336	-1.85069	-1.848
-1.84234	-1.83958	-1.83686	-1.83419	-1.83161	-1.82905	-1.85235	-1.84968	-1.84699
-1.84134	-1.83858	-1.83587	-1.83321	-1.83062	-1.82806	-1.85135	-1.84867	-1.84598
-1.84034	-1.8376	-1.83488	-1.83221	-1.82963	-1.82707	-1.85034	-1.84766	-1.84497
-1.83935	-1.8366	-1.83389	-1.83122	-1.82864	-1.82607	-1.84933	-1.84664	-1.84395
-1.83834	-1.8356	-1.83289	-1.83023	-1.82764	-1.82507	-1.84831	-1.84563	-1.84293
-1.83733	-1.8346	-1.83189	-1.82923	-1.82664	-1.82407	-1.84729	-1.84461	-1.84192
-1.83632	-1.8336	-1.83089	-1.82822	-1.82564	-1.82308	-1.84629	-1.84361	-1.84092
-1.83531	-1.83259	-1.82988	-1.82722	-1.82465	-1.82209	-1.84529	-1.84261	-1.83992
-1.8343	-1.83159	-1.82888	-1.82622	-1.82365	-1.8211	-1.84429	-1.84161	-1.83893
-1.8333	-1.83059	-1.82789	-1.82524	-1.82266	-1.82011	-1.84328	-1.84061	-1.83794
-1.83231	-1.82961	-1.82693	-1.82429	-1.82171	-1.81913	-1.84229	-1.83962	-1.83695
-1.83132	-1.82864	-1.82601	-1.82339	-1.82079	-1.81818	-1.8413	-1.83862	-1.83595
-1.83035	-1.8277	-1.82509	-1.82248	-1.81985	-1.81722	-1.84032	-1.83763	-1.83496
-1.82943	-1.82679	-1.82416	-1.82153	-1.8189	-1.81626	-1.83933	-1.83665	-1.83398
-1.82852	-1.82588	-1.82323	-1.82057	-1.81793	-1.81529	-1.83835	-1.83568	-1.83302
-1.82758	-1.85085	-1.84814	-1.84543	-1.84273	-1.84005	-1.83737	-1.8347	-1.83206
-1.82663	-1.84986	-1.84715	-1.84444	-1.84174	-1.83906	-1.83639	-1.83374	-1.83112
-1.82567	-1.84888	-1.84616	-1.84346	-1.84076	-1.83809	-1.83543	-1.83281	-1.8302
-1.82471	-1.84791	-1.84519	-1.84249	-1.83979	-1.83712	-1.8345	-1.83191	-1.82931
-1.82375	-1.84694	-1.84424	-1.84156	-1.83887	-1.8362	-1.83358	-1.83099	-1.82838
-1.82279	-1.84599	-1.84334	-1.84069	-1.83801	-1.83534	-1.83269	-1.83007	-1.82743
-1.81327	-1.84507	-1.84244	-1.8398	-1.83713	-1.83446	-1.83179	-1.82913	-1.82647
-1.81241	-1.84419	-1.84153	-1.83887	-1.83619	-1.83352	-1.83084	-1.82817	-1.82551
-1.81155	-1.84331	-1.84061	-1.83792	-1.83524	-1.83256	-1.82989	-1.82721	-1.82456
-1.81063	-1.81658	-1.81393	-1.81128	-1.80864	-1.80601	-1.82893	-1.82626	-1.82362
-1.80967	-1.81565	-1.81299	-1.81035	-1.80771	-1.80508	-1.82798	-1.82531	-1.82269
-1.80872	-1.80607	-1.80345	-1.80946	-1.80683	-1.8042	-1.82708	-1.82442	-1.82179
-1.80777	-1.80514	-1.80256	-1.80862	-1.806	-1.80337	-1.82624	-1.82357	-1.82092
-1.80686	-1.80423	-1.80166	-1.80775	-1.80514	-1.80252	-1.82537	-1.8227	-1.82003
-1.806	-1.80336	-1.80076	-1.80684	-1.80422	-1.80161	-1.82445	-1.82177	-1.8191
-1.80514	-1.80248	-1.79985	-1.79723	-1.79462	-1.79202	-1.82353	-1.82086	-1.81819
-1.80422	-1.80156	-1.79891	-1.79629	-1.79372	-1.79115	-1.82267	-1.82	-1.81733
-1.80329	-1.80063	-1.79799	-1.79537	-1.79282	-1.79028	-1.82181	-1.81915	-1.81648
-1.80242	-1.79976	-1.79711	-1.7945	-1.79194	-1.78939	-1.8209	-1.81824	-1.81556
-1.80158	-1.79892	-1.79628	-1.79365	-1.79106	-1.78848	-1.81997	-1.8173	-1.81462
-1.8007	-1.79804	-1.7954	-1.79277	-1.79018	-1.78759	-1.78499	-1.78237	-1.81372
-1.79977	-1.79712	-1.79447	-1.79186	-1.78929	-1.78674	-1.78414	-1.78153	-1.81287
-1.79888	-1.79623	-1.79358	-1.79097	-1.78841	-1.78586	-1.78327	-1.78066	-1.81199
-1.79806	-1.7954	-1.79276	-1.79013	-1.78754	-1.78496	-1.78235	-1.77974	-1.81105
-1.79725	-1.79459	-1.79195	-1.78931	-1.78668	-1.78406	-1.78144	-1.77881	-1.81011
-1.79644	-1.79378	-1.79114	-1.78849	-1.78585	-1.78321	-1.78058	-1.77794	-1.80072
-1.79562	-1.79296	-1.79031	-1.78767	-1.78502	-1.78238	-1.77973	-1.77709	-1.79985
-1.79473	-1.79208	-1.78943	-1.78678	-1.78413	-1.78148	-1.77883	-1.77619	-1.79893
-1.7938	-1.79114	-1.78849	-1.78584	-1.78319	-1.78054	-1.77789	-1.77524	-1.79797
-1.79292	-1.79026	-1.7876	-1.78495	-1.7823	-1.77965	-1.77699	-1.77434	-1.77169
-1.7921	-1.78944	-1.78678	-1.78412	-1.78147	-1.77881	-1.77616	-1.77351	-1.77085
-1.79128	-1.78862	-1.78596	-1.7833	-1.78064	-1.77799	-1.77533	-1.77267	-1.77002
-1.79047	-1.7878	-1.78514	-1.78248	-1.77982	-1.77717	-1.77451	-1.77184	-1.76918

-1.78963	-1.78697	-1.7843	-1.78164	-1.77898	-1.77632	-1.77366	-1.77099	-1.76833
-1.78872	-1.78605	-1.78339	-1.78072	-1.77806	-1.7754	-1.77273	-1.77006	-1.76739
-1.78777	-1.7851	-1.78244	-1.77977	-1.77711	-1.77445	-1.77177	-1.7691	-1.76643
-1.78688	-1.78422	-1.78155	-1.77888	-1.77622	-1.77355	-1.77088	-1.7682	-1.76553
-1.78604	-1.78338	-1.78071	-1.77804	-1.77538	-1.77271	-1.77003	-1.76735	-1.76468
-1.78522	-1.78255	-1.77988	-1.77722	-1.77455	-1.77187	-1.7692	-1.76652	-1.76384
-1.7844	-1.78173	-1.77907	-1.7764	-1.77373	-1.77105	-1.76838	-1.7657	-1.76302
-1.78359	-1.78092	-1.77826	-1.77559	-1.77291	-1.77024	-1.76756	-1.76488	-1.76219
-1.78278	-1.78011	-1.77744	-1.77477	-1.7721	-1.76942	-1.76674	-1.76405	-1.76137
-1.78196	-1.77929	-1.77662	-1.77395	-1.77128	-1.7686	-1.76591	-1.76323	-1.76053
-1.78115	-1.77847	-1.77581	-1.77313	-1.77045	-1.76776	-1.76508	-1.76239	-1.75967
-1.78033	-1.77766	-1.77498	-1.77231	-1.76962	-1.76692	-1.76423	-1.76154	-1.7588
-1.77951	-1.77683	-1.77416	-1.77147	-1.76875	-1.76603	-1.76333	-1.76064	-1.75789
-1.77869	-1.77601	-1.77332	-1.77061	-1.76786	-1.7651	-1.76238	-1.75967	-1.75695
-1.77787	-1.77518	-1.77247	-1.76974	-1.76696	-1.76419	-1.76145	-1.75874	-1.78345
-1.77705	-1.77433	-1.77158	-1.76882	-1.76606	-1.76331	-1.76058	-1.75787	-1.78256
-1.77622	-1.77349	-1.7707	-1.76791	-1.76517	-1.76245	-1.75973	-1.75702	-1.7817
-1.7754	-1.77265	-1.76985	-1.76705	-1.76432	-1.76161	-1.75889	-1.75617	-1.78084
-1.77456	-1.77182	-1.76901	-1.76622	-1.76348	-1.76077	-1.75805	-1.75534	-1.77999
-1.77373	-1.77099	-1.76818	-1.76539	-1.76265	-1.75993	-1.75721	-1.75449	-1.77913
-1.77289	-1.77016	-1.76735	-1.76456	-1.76181	-1.75908	-1.75636	-1.75365	-1.77828
-1.77206	-1.76933	-1.76655	-1.76377	-1.761	-1.75826	-1.78294	-1.78018	-1.77742
-1.77122	-1.76852	-1.76578	-1.76302	-1.76024	-1.75745	-1.7821	-1.77933	-1.77656
-1.77038	-1.7677	-1.76499	-1.76226	-1.75947	-1.75667	-1.78129	-1.77849	-1.77571
-1.76954	-1.76686	-1.76417	-1.76146	-1.75871	-1.75593	-1.78052	-1.77768	-1.77488
-1.76869	-1.76601	-1.76333	-1.76064	-1.75793	-1.75519	-1.77976	-1.77689	-1.77406
-1.76785	-1.76517	-1.76249	-1.75981	-1.75711	-1.75439	-1.77897	-1.77612	-1.77329
-1.767	-1.76432	-1.76165	-1.75896	-1.75627	-1.75356	-1.77817	-1.77537	-1.77255
-1.76616	-1.76348	-1.7608	-1.75812	-1.75542	-1.75271	-1.77733	-1.77456	-1.77177
-1.76531	-1.76263	-1.75995	-1.75727	-1.75457	-1.75186	-1.77647	-1.77372	-1.77096
-1.76448	-1.7618	-1.75911	-1.78386	-1.78111	-1.77836	-1.77561	-1.77287	-1.77013
-1.76366	-1.76097	-1.75827	-1.783	-1.78025	-1.7775	-1.77475	-1.77201	-1.76928
-1.76288	-1.76017	-1.75745	-1.78215	-1.7794	-1.77665	-1.7739	-1.77115	-1.76843
-1.76217	-1.75943	-1.75668	-1.78133	-1.77856	-1.7758	-1.77305	-1.7703	-1.76758
-1.76146	-1.75872	-1.75592	-1.78053	-1.77775	-1.77498	-1.77222	-1.76947	-1.76673
-1.76068	-1.78543	-1.7826	-1.77978	-1.777	-1.77423	-1.77144	-1.76866	-1.7659
-1.75986	-1.78461	-1.78184	-1.77906	-1.77629	-1.77352	-1.7707	-1.76788	-1.76509
-1.78654	-1.78379	-1.78103	-1.77828	-1.77552	-1.77274	-1.76994	-1.76711	-1.76431
-1.7857	-1.78295	-1.78019	-1.77744	-1.77468	-1.77192	-1.76915	-1.76637	-1.76358
-1.78491	-1.78214	-1.77937	-1.7766	-1.77384	-1.77109	-1.76835	-1.7656	-1.76282
-1.7842	-1.78141	-1.77859	-1.77579	-1.77301	-1.77025	-1.76751	-1.76478	-1.76202
-1.78348	-1.78068	-1.77783	-1.775	-1.77221	-1.76944	-1.76669	-1.76395	-1.76121
-1.78267	-1.77989	-1.77706	-1.77425	-1.77147	-1.76871	-1.76593	-1.76316	-1.7604
-1.78185	-1.77908	-1.7763	-1.77352	-1.77076	-1.76799	-1.76519	-1.76238	-1.7596
-1.78105	-1.77827	-1.7755	-1.77273	-1.76997	-1.76721	-1.76442	-1.76162	-1.75886
-1.78026	-1.77745	-1.77467	-1.7719	-1.76914	-1.76639	-1.76364	-1.76089	-1.75814
-1.7795	-1.77668	-1.7739	-1.77112	-1.76835	-1.76558	-1.76284	-1.76011	-1.75738
-1.77875	-1.77596	-1.77318	-1.7704	-1.76759	-1.76479	-1.76203	-1.75929	-1.75656
-1.778	-1.77522	-1.77244	-1.76965	-1.76683	-1.76401	-1.76124	-1.7585	-1.75575
-1.77721	-1.77441	-1.77164	-1.76886	-1.76606	-1.76327	-1.76051	-1.75777	-1.755

								-0.10814
								-0.1079
								-0.10765
								-0.25616
								-0.25553
						-0.20472	-0.20385	-0.25492
						-0.20416	-0.20334	-0.25433
			-0.20636	-0.20539	-0.20446	-0.25567	-0.2547	-0.38536
			-0.2057	-0.20478	-0.20391	-0.25501	-0.25404	-0.38447
			-0.20508	-0.20421	-0.20339	-0.25438	-0.2534	-0.3836
	-0.10793	-0.10762	-0.25679	-0.25575	-0.25477	-0.38543	-0.3841	-0.42482
	-0.1077	-0.10739	-0.25617	-0.25516	-0.25418	-0.38463	-0.3833	-0.42397
-0.10776	-0.20526	-0.20438	-0.30257	-0.30148	-0.3004	-0.38382	-0.3825	-0.5165
-0.10754	-0.20471	-0.20387	-0.3019	-0.30081	-0.29973	-0.38302	-0.38171	-0.5155
-0.10732	-0.30342	-0.30231	-0.3862	-0.38485	-0.38352	-0.47394	-0.47241	-0.55871
-0.1071	-0.30274	-0.30164	-0.38538	-0.38403	-0.38269	-0.47297	-0.47146	-0.55763
-0.25619	-0.34468	-0.34345	-0.47669	-0.47509	-0.47351	-0.5599	-0.55822	-0.6011
-0.25557	-0.34394	-0.34271	-0.4757	-0.4741	-0.47252	-0.55876	-0.55707	-0.5999
-0.25494	-0.34318	-0.34196	-0.4747	-0.47311	-0.47153	-0.55763	-0.55593	-0.59872
-0.30104	-0.47683	-0.47525	-0.51742	-0.51575	-0.5141	-0.60116	-0.59937	-0.59763

-0.3003	-0.47578	-0.47422	-0.51633	-0.51471	-0.5131	-0.60007	-0.59829	-0.59658
-0.39376	-0.47478	-0.47324	-0.55952	-0.55783	-0.55615	-0.59899	-0.59725	-0.64379
-0.39295	-0.47383	-0.47231	-0.55846	-0.55678	-0.55512	-0.59793	-0.59623	-0.64269
-0.47449	-0.56081	-0.5591	-0.60213	-0.60035	-0.5986	-0.59689	-0.59522	-0.7608
-0.47352	-0.55976	-0.55805	-0.60104	-0.59927	-0.59754	-0.59586	-0.59423	-0.75951
-0.56041	-0.60354	-0.60173	-0.59996	-0.59821	-0.59651	-0.64299	-0.64122	-0.75822
-0.55935	-0.60244	-0.60065	-0.5989	-0.59719	-0.59551	-0.64191	-0.64014	-0.75694
-0.55831	-0.60136	-0.59959	-0.59786	-0.59618	-0.59453	-0.64083	-0.63906	-0.75565
-0.55729	-0.6003	-0.59856	-0.64514	-0.64332	-0.64153	-0.7585	-0.75643	-1.01266
-0.55627	-0.59926	-0.59755	-0.64405	-0.64224	-0.64044	-0.7572	-0.75512	-1.01052
-0.59995	-0.59823	-0.59654	-0.76218	-0.76008	-0.75799	-1.01514	-1.01172	-1.04329
-0.5989	-0.5972	-0.59553	-0.76087	-0.75878	-0.75669	-1.013	-1.00957	-1.04111
-0.59787	-0.67994	-0.67798	-0.83417	-0.8319	-0.82962	-1.04586	-1.04235	-1.10635
-0.59686	-0.67873	-0.6768	-0.83273	-0.83046	-0.82819	-1.04377	-1.0403	-1.10421
-0.71167	-0.83587	-0.83357	-1.05189	-1.0485	-1.04513	-1.10913	-1.10556	-1.13492
-0.71044	-0.83442	-0.83212	-1.04971	-1.04638	-1.04307	-1.10699	-1.10343	-1.13279
-0.70921	-0.83297	-0.83067	-1.04755	-1.04428	-1.04103	-1.10487	-1.10131	-1.13068
-0.83386	-1.01239	-1.0092	-1.113	-1.10961	-1.10625	-1.10275	-1.09921	-1.12859
-0.83239	-1.01037	-1.00722	-1.11084	-1.10747	-1.10412	-1.10064	-1.09713	-1.12652
-0.86922	-1.04794	-1.04463	-1.10869	-1.10534	-1.102	-1.09857	-1.09511	-1.15314
-0.8677	-1.04581	-1.04255	-1.10656	-1.10322	-1.09989	-1.09651	-1.09315	-1.15116
-1.00754	-1.11112	-1.10776	-1.10443	-1.10111	-1.09781	-1.09449	-1.0912	-1.14919
-1.00558	-1.10897	-1.10561	-1.1023	-1.09902	-1.09576	-1.0925	-1.08925	-1.14721
-1.00366	-1.10685	-1.10348	-1.10018	-1.09695	-1.09376	-1.09054	-1.08733	-1.14525
-1.1082	-1.10475	-1.10138	-1.09809	-1.09493	-1.09181	-1.1499	-1.14657	-1.19756
-1.10614	-1.10267	-1.0993	-1.09604	-1.09294	-1.08988	-1.14795	-1.14466	-1.1955
-1.10407	-1.10062	-1.09728	-1.12325	-1.12012	-1.11705	-1.20044	-1.19696	-1.27293
-1.10201	-1.0986	-1.09531	-1.12128	-1.11815	-1.11509	-1.19835	-1.19494	-1.27081
-1.10001	-1.12591	-1.12257	-1.17512	-1.17178	-1.16849	-1.24838	-1.24491	-1.26866
-1.09807	-1.124	-1.12065	-1.17304	-1.16967	-1.16637	-1.24615	-1.24272	-1.26645
-1.09614	-1.17805	-1.17447	-1.25448	-1.25089	-1.24737	-1.24392	-1.24052	-1.26424
-1.09415	-1.17594	-1.1724	-1.25229	-1.24868	-1.24515	-1.24171	-1.23833	-1.26204
-1.09217	-1.17381	-1.17033	-1.25012	-1.24651	-1.24295	-1.23951	-1.23615	-1.25986
-1.1751	-1.19379	-1.19032	-1.248	-1.24438	-1.2408	-1.26455	-1.26114	-1.31428
-1.17298	-1.19163	-1.18824	-1.24589	-1.24227	-1.23868	-1.26238	-1.25895	-1.31197
-1.19294	-1.22049	-1.21707	-1.24375	-1.24015	-1.23658	-1.26023	-1.25678	-1.30965
-1.19083	-1.2183	-1.2149	-1.24157	-1.23802	-1.23449	-1.25809	-1.25462	-1.30733
-1.2197	-1.21614	-1.21274	-1.26661	-1.26306	-1.25952	-1.31263	-1.30877	-1.39153
-1.21754	-1.21401	-1.21061	-1.26441	-1.26094	-1.25749	-1.31047	-1.30662	-1.38923
-1.21542	-1.24206	-1.23856	-1.26224	-1.25882	-1.25544	-1.365	-1.36105	-1.38699
-1.21338	-1.24003	-1.23651	-1.26012	-1.25669	-1.25331	-1.36273	-1.35891	-1.38482
-1.21135	-1.23803	-1.23449	-1.25805	-1.25458	-1.25118	-1.36046	-1.35679	-1.38267
-1.20926	-1.26304	-1.25952	-1.3698	-1.36582	-1.36193	-1.38797	-1.38426	-1.40649
-1.20713	-1.26086	-1.25744	-1.36757	-1.36359	-1.35968	-1.38567	-1.38197	-1.40419
-1.20506	-1.3175	-1.31397	-1.36529	-1.36135	-1.35747	-1.38339	-1.37968	-1.45017
-1.20306	-1.31532	-1.31178	-1.36295	-1.35909	-1.35529	-1.38114	-1.37739	-1.4478
-1.26171	-1.36843	-1.36452	-1.39048	-1.38658	-1.38273	-1.4049	-1.40107	-1.44544
-1.25968	-1.36621	-1.36231	-1.38823	-1.38437	-1.38055	-1.40269	-1.39883	-1.44311
-1.25766	-1.36401	-1.36014	-1.38601	-1.38217	-1.37836	-1.40048	-1.39663	-1.4408
-1.28207	-1.39162	-1.3877	-1.40987	-1.40595	-1.40206	-1.39823	-1.39446	-1.46683

-1.27999	-1.38933	-1.38549	-1.40766	-1.40369	-1.39975	-1.39598	-1.3923	-1.46458
-1.33369	-1.38703	-1.38319	-1.40535	-1.4014	-1.39749	-1.42283	-1.41909	-1.46228
-1.33145	-1.38472	-1.38085	-1.40295	-1.39908	-1.39524	-1.42052	-1.41674	-1.45994
-1.38637	-1.40845	-1.40451	-1.40063	-1.39681	-1.39304	-1.41826	-1.41447	-1.45771
-1.38412	-1.40619	-1.40225	-1.39838	-1.39461	-1.39088	-1.41608	-1.41228	-1.45559
-1.40786	-1.40393	-1.40002	-1.42532	-1.42149	-1.41773	-1.41394	-1.41024	-1.5229
-1.40557	-1.40165	-1.39783	-1.42313	-1.41926	-1.41549	-1.41186	-1.40839	-1.52108
-1.40329	-1.3994	-1.39565	-1.42096	-1.41707	-1.4133	-1.40986	-1.40667	-1.51941
-1.40108	-1.42638	-1.42254	-1.46453	-1.46061	-1.45685	-1.49883	-1.49568	-1.5178
-1.39891	-1.42421	-1.42032	-1.46226	-1.45845	-1.45485	-1.49695	-1.49403	-1.51629
-1.39672	-1.46784	-1.46389	-1.46008	-1.45643	-1.45302	-1.49524	-1.4925	-1.55907
-1.39451	-1.46555	-1.46166	-1.45798	-1.45453	-1.45134	-1.4937	-1.49109	-1.55763
-1.46718	-1.46331	-1.45951	-1.45595	-1.45272	-1.44975	-1.5615	-1.55883	-1.57381
-1.46493	-1.46113	-1.45741	-1.45398	-1.45098	-1.44824	-1.55999	-1.55736	-1.57238
-1.46273	-1.45901	-1.45542	-1.49863	-1.49572	-1.49307	-1.57614	-1.57352	-1.59643
-1.46051	-1.45689	-1.45357	-1.49695	-1.49415	-1.49156	-1.57464	-1.5721	-1.59494
-1.45838	-1.45487	-1.45183	-1.49539	-1.49266	-1.49011	-1.57316	-1.57069	-1.59345
-1.45646	-1.49958	-1.49659	-1.51241	-1.50974	-1.50722	-1.57175	-1.56927	-1.63656
-1.45469	-1.49793	-1.495	-1.51085	-1.5083	-1.50587	-1.57037	-1.56786	-1.63495
-1.49944	-1.51498	-1.5121	-1.53404	-1.53147	-1.529	-1.63895	-1.63615	-1.63339
-1.49769	-1.51344	-1.5107	-1.53269	-1.5301	-1.52759	-1.63735	-1.63462	-1.63187
-1.51466	-1.51192	-1.50931	-1.57816	-1.57544	-1.57277	-1.63578	-1.63307	-1.65692
-1.51305	-1.51039	-1.5079	-1.57671	-1.57404	-1.57139	-1.63426	-1.63149	-1.65525
-1.51152	-1.50892	-1.5065	-1.57526	-1.57264	-1.57002	-1.63276	-1.62994	-1.6536
-1.51007	-1.57895	-1.57634	-1.59908	-1.59634	-1.5936	-1.63128	-1.62847	-1.67311
-1.50867	-1.57749	-1.57485	-1.59748	-1.59478	-1.59209	-1.62981	-1.62704	-1.67156
-1.55471	-1.576	-1.57339	-1.59594	-1.59323	-1.59055	-1.65471	-1.65184	-1.68123
-1.55323	-1.5745	-1.57195	-1.59446	-1.59171	-1.58899	-1.65309	-1.65035	-1.67978
-1.57567	-1.59833	-1.59566	-1.62037	-1.61743	-1.61452	-1.66287	-1.6602	-1.67829
-1.57428	-1.59686	-1.59419	-1.61881	-1.6159	-1.61303	-1.66135	-1.65866	-1.67675
-1.5729	-1.60747	-1.60477	-1.629	-1.62614	-1.62333	-1.65986	-1.65717	-1.7161
-1.57151	-1.60598	-1.60323	-1.62732	-1.62455	-1.62185	-1.65843	-1.65576	-1.71453
-1.57011	-1.60449	-1.6017	-1.62567	-1.62297	-1.62037	-1.65699	-1.65438	-1.71299
-1.60573	-1.62995	-1.627	-1.62415	-1.62146	-1.61886	-1.69503	-1.6924	-1.71146
-1.60417	-1.62835	-1.62549	-1.62269	-1.61998	-1.61735	-1.69348	-1.69092	-1.70993
-1.62952	-1.62672	-1.62396	-1.6409	-1.6382	-1.63555	-1.71386	-1.7111	-1.70838
-1.62784	-1.62504	-1.6224	-1.63944	-1.63679	-1.63417	-1.7123	-1.70951	-1.7068
-1.62625	-1.64314	-1.64055	-1.66119	-1.65835	-1.65553	-1.71077	-1.70798	-1.70528
-1.62478	-1.64169	-1.6391	-1.65963	-1.65682	-1.65404	-1.70928	-1.70652	-1.70382
-1.64299	-1.64025	-1.63765	-1.6787	-1.67588	-1.67309	-1.70781	-1.70509	-1.71866
-1.64152	-1.6388	-1.63616	-1.67704	-1.67429	-1.6716	-1.70636	-1.70366	-1.71723
-1.64007	-1.63736	-1.63467	-1.6754	-1.67272	-1.6701	-1.7049	-1.70223	-1.71582
-1.63863	-1.65899	-1.65609	-1.67385	-1.67118	-1.66858	-1.71972	-1.71707	-1.7144
-1.63721	-1.65747	-1.6546	-1.67236	-1.66966	-1.66705	-1.7182	-1.71561	-1.71299
-1.65885	-1.69353	-1.69063	-1.6878	-1.68507	-1.68241	-1.71673	-1.71416	-1.71158
-1.65732	-1.69202	-1.68918	-1.6864	-1.68367	-1.681	-1.7153	-1.71273	-1.71017
-1.67305	-1.69052	-1.68774	-1.68499	-1.68227	-1.6796	-1.71388	-1.7113	-1.70876
-1.67155	-1.68904	-1.6863	-1.68359	-1.68088	-1.67821	-1.71246	-1.70986	-1.70733
-1.67007	-1.68758	-1.68487	-1.68219	-1.67949	-1.67682	-1.71105	-1.70844	-1.70592
-1.68891	-1.68616	-1.68347	-1.68079	-1.67811	-1.67545	-1.70967	-1.70707	-1.70455

-1.68747	-1.68476	-1.68207	-1.6794	-1.67673	-1.67409	-1.7083	-1.70571	-1.7032
-1.68603	-1.68335	-1.68066	-1.678	-1.67536	-1.67275	-1.70695	-1.70437	-1.72252
-1.68459	-1.68193	-1.67925	-1.6766	-1.67399	-1.67141	-1.70562	-1.70304	-1.72108
-1.68317	-1.68052	-1.67785	-1.67521	-1.67263	-1.67008	-1.70427	-1.7017	-1.74491
-1.68174	-1.6791	-1.67645	-1.67383	-1.67128	-1.66874	-1.70293	-1.70036	-1.74341
-1.68032	-1.67769	-1.67506	-1.67247	-1.66993	-1.66741	-1.73949	-1.73672	-1.75884
-1.6789	-1.67628	-1.67369	-1.67112	-1.66859	-1.66608	-1.73806	-1.73532	-1.75739
-1.67748	-1.67488	-1.67232	-1.66978	-1.66726	-1.66476	-1.73664	-1.73394	-1.75595
-1.69411	-1.69151	-1.68895	-1.70769	-1.70496	-1.70225	-1.76011	-1.75729	-1.75452
-1.69273	-1.69014	-1.6876	-1.70626	-1.70354	-1.70084	-1.75862	-1.75585	-1.75309
-1.69135	-1.68879	-1.68626	-1.70485	-1.70214	-1.69946	-1.75716	-1.75441	-1.75167
-1.68998	-1.68744	-1.68493	-1.70344	-1.70076	-1.6981	-1.75572	-1.75298	-1.75025
-1.68862	-1.74318	-1.74043	-1.76276	-1.7599	-1.75707	-1.75429	-1.75156	-1.74884
-1.68728	-1.74174	-1.73899	-1.76124	-1.75842	-1.75563	-1.75287	-1.75014	-1.74745
-1.68595	-1.76554	-1.76262	-1.75975	-1.75695	-1.7542	-1.75145	-1.74873	-1.7578
-1.68463	-1.76403	-1.76114	-1.7583	-1.75552	-1.75278	-1.75004	-1.74733	-1.75638
-1.68331	-1.76254	-1.75969	-1.75687	-1.7541	-1.75136	-1.74865	-1.74597	-1.755
-1.70306	-1.76105	-1.75824	-1.75546	-1.75269	-1.74996	-1.74729	-1.74469	-1.75988
-1.70166	-1.75958	-1.75682	-1.75407	-1.75129	-1.74856	-1.74597	-1.74347	-1.75862
-1.72582	-1.75815	-1.75543	-1.75272	-1.74996	-1.74724	-1.76254	-1.75993	-1.75732
-1.7244	-1.75675	-1.75408	-1.75142	-1.74869	-1.74599	-1.76122	-1.75861	-1.75599
-1.72303	-1.75538	-1.75273	-1.7564	-1.7537	-1.75103	-1.75989	-1.75727	-1.75465
-1.72175	-1.75408	-1.75137	-1.75501	-1.75234	-1.74972	-1.75854	-1.75592	-1.75329
-1.72045	-1.75276	-1.75	-1.75362	-1.75098	-1.74839	-1.75718	-1.75456	-1.75192
-1.71906	-1.7577	-1.75493	-1.75223	-1.74962	-1.74707	-1.75581	-1.75319	-1.75055
-1.71764	-1.75627	-1.75353	-1.75085	-1.74827	-1.74575	-1.75446	-1.75181	-1.74917
-1.7229	-1.75486	-1.75216	-1.76108	-1.7584	-1.75576	-1.76983	-1.76716	-1.76448
-1.72148	-1.75346	-1.75082	-1.75973	-1.75706	-1.75442	-1.76848	-1.7658	-1.76312
-1.72015	-1.75213	-1.74954	-1.79044	-1.78774	-1.78507	-1.78237	-1.77966	-1.82196
-1.7189	-1.75089	-1.74833	-1.78919	-1.7865	-1.78382	-1.78111	-1.7784	-1.82055
-1.7337	-1.79328	-1.79058	-1.78791	-1.78522	-1.78253	-1.77982	-1.77711	-1.83261
-1.73238	-1.79192	-1.78923	-1.78655	-1.78386	-1.78117	-1.77847	-1.77576	-1.8311
-1.73105	-1.79055	-1.78786	-1.78518	-1.78249	-1.77979	-1.77709	-1.77439	-1.82959
-1.74693	-1.78918	-1.78649	-1.79773	-1.79504	-1.79234	-1.83408	-1.83107	-1.82807
-1.7456	-1.78782	-1.78513	-1.79636	-1.79367	-1.79097	-1.83254	-1.82954	-1.82656
-1.75588	-1.80043	-1.79774	-1.79505	-1.79236	-1.78966	-1.83109	-1.82809	-1.82514
-1.75464	-1.79918	-1.79649	-1.79379	-1.7911	-1.78841	-1.82969	-1.8267	-1.82379
-1.76767	-1.79788	-1.79518	-1.83729	-1.83426	-1.83125	-1.82825	-1.82528	-1.8224
-1.76633	-1.79652	-1.79381	-1.83576	-1.83273	-1.82973	-1.82676	-1.82382	-1.82097
-1.76501	-1.79517	-1.79245	-1.83424	-1.8312	-1.82821	-1.82526	-1.82237	-1.81956
-1.76374	-1.79388	-1.79111	-1.83272	-1.82967	-1.82667	-1.82377	-1.82093	-1.81816
-1.76248	-1.79258	-1.78979	-1.83122	-1.82816	-1.82517	-1.82231	-1.81953	-1.81679
-1.76115	-1.83599	-1.83287	-1.82979	-1.82675	-1.82379	-1.82095	-1.81821	-1.81549
-1.7598	-1.8345	-1.83142	-1.82838	-1.82539	-1.82247	-1.81966	-1.81694	-1.81423
-1.7585	-1.83304	-1.82996	-1.82694	-1.82399	-1.82112	-1.81833	-1.81562	-1.85145
-1.75725	-1.83163	-1.8285	-1.82545	-1.82256	-1.81974	-1.81698	-1.81428	-1.84998
-1.80148	-1.83019	-1.82706	-1.82403	-1.82117	-1.8184	-1.81566	-1.81297	-1.84856
-1.79998	-1.82871	-1.82565	-1.82268	-1.81986	-1.81712	-1.81439	-1.81171	-1.84721
-1.7985	-1.82724	-1.82426	-1.82135	-1.81856	-1.81583	-1.85185	-1.84883	-1.84586
-1.7971	-1.82584	-1.82288	-1.82	-1.81721	-1.81449	-1.85039	-1.84739	-1.84445

-1.79573	-1.82447	-1.82153	-1.81865	-1.81588	-1.81315	-1.84893	-1.84596	-1.84304
-1.79432	-1.82308	-1.8202	-1.85681	-1.85366	-1.85059	-1.84758	-1.84463	-1.86338
-1.79289	-1.82169	-1.81889	-1.85545	-1.85233	-1.84927	-1.84628	-1.84335	-1.86201
-1.79153	-1.82037	-1.81762	-1.85409	-1.85101	-1.84796	-1.84497	-1.84203	-1.86059
-1.79025	-1.81911	-1.81637	-1.85275	-1.84969	-1.84666	-1.84366	-1.84068	-1.85912
-1.78898	-1.85757	-1.85445	-1.85137	-1.84834	-1.84534	-1.86399	-1.86081	-1.88842
-1.78774	-1.85619	-1.85303	-1.84993	-1.84692	-1.84396	-1.86255	-1.85941	-1.88701
-1.78648	-1.8548	-1.85162	-1.84851	-1.84552	-1.84258	-1.86112	-1.85801	-1.8856
-1.82595	-1.85337	-1.85024	-1.84717	-1.84419	-1.84126	-1.89045	-1.88731	-1.88417
-1.82449	-1.85193	-1.84888	-1.84586	-1.84289	-1.83995	-1.88901	-1.88587	-1.88272
-1.82311	-1.85057	-1.84755	-1.89709	-1.89387	-1.89067	-1.88751	-1.88439	-1.88126
-1.82179	-1.84925	-1.84624	-1.89565	-1.89238	-1.88913	-1.88597	-1.88287	-1.87979
-1.82049	-1.87919	-1.87613	-1.89418	-1.89089	-1.88764	-1.88449	-1.88142	-1.89388
-1.81922	-1.87785	-1.87474	-1.89267	-1.88941	-1.8862	-1.88308	-1.88003	-1.89245
-1.84991	-1.8978	-1.89447	-1.89118	-1.88795	-1.88478	-1.88169	-1.87866	-1.89103
-1.84851	-1.89629	-1.89298	-1.88973	-1.88653	-1.88339	-1.88033	-1.87733	-1.88964
-1.84709	-1.89477	-1.89151	-1.88829	-1.88513	-1.88202	-1.87898	-1.87599	-1.88824
-1.8457	-1.8933	-1.89005	-1.88686	-1.88374	-1.88067	-1.89311	-1.88991	-1.88675
-1.84432	-1.89185	-1.88862	-1.88545	-1.88237	-1.87932	-1.89166	-1.88841	-1.88523
-1.86447	-1.89041	-1.8872	-1.88405	-1.881	-1.87799	-1.89024	-1.88697	-1.88379
-1.86304	-1.88898	-1.88579	-1.88267	-1.87966	-1.87666	-1.88886	-1.88559	-1.88241
-1.86162	-1.88756	-1.88441	-1.89704	-1.89384	-1.89067	-1.88744	-1.8842	-1.88104
-1.8602	-1.88615	-1.88305	-1.89562	-1.89239	-1.88918	-1.88598	-1.88279	-1.87966
-1.8588	-1.88476	-1.8817	-1.89421	-1.89095	-1.88771	-1.91879	-1.91538	-1.912
-1.85741	-1.88339	-1.88036	-1.89283	-1.88956	-1.88631	-1.91728	-1.91389	-1.91052
-1.85604	-1.88203	-1.87903	-1.89144	-1.88818	-1.88494	-1.9158	-1.91242	-1.90905
-1.85468	-1.88067	-1.87765	-1.88997	-1.88675	-1.88354	-1.9143	-1.91093	-1.90758
-1.85334	-1.87933	-1.87626	-1.88847	-1.88528	-1.88213	-1.91279	-1.90943	-1.9061
-1.85201	-1.89353	-1.89025	-1.88702	-1.88386	-1.88074	-1.91129	-1.90794	-1.90462
-1.85069	-1.89214	-1.88886	-1.88562	-1.88247	-1.87936	-1.9098	-1.90645	-1.90312
-1.84937	-1.89075	-1.88747	-1.88423	-1.88109	-1.878	-1.90832	-1.90499	-1.90166
-1.84806	-1.88936	-1.88608	-1.88285	-1.87972	-1.87664	-1.90686	-1.90354	-1.90023
-1.84675	-1.88797	-1.8847	-1.88148	-1.87836	-1.87529	-1.90541	-1.90212	-1.89882
-1.84545	-1.8866	-1.88334	-1.88013	-1.87702	-1.87395	-1.90398	-1.9007	-1.92027
-1.84415	-1.88523	-1.88199	-1.87879	-1.87568	-1.87263	-1.90256	-1.8993	-1.91888
-1.84285	-1.88385	-1.88062	-1.87744	-1.87435	-1.87131	-1.92407	-1.92079	-1.9175
-1.84154	-1.88246	-1.87926	-1.8761	-1.87302	-1.87	-1.92266	-1.91939	-1.91612
-1.85567	-1.9049	-1.90166	-1.89848	-1.89537	-1.89232	-1.92125	-1.918	-1.91473
-1.85434	-1.90356	-1.90033	-1.89714	-1.89405	-1.891	-1.91983	-1.91659	-1.91334
-1.87748	-1.90224	-1.89902	-1.89585	-1.89276	-1.88971	-1.91843	-1.9152	-1.92383
-1.87615	-1.90093	-1.89778	-1.89464	-1.89153	-1.88846	-1.91707	-1.91384	-1.92246
-1.87484	-1.89964	-1.89655	-1.89345	-1.89032	-1.88721	-1.91571	-1.91249	-1.92112
-1.87356	-1.89835	-1.89527	-1.89219	-1.88905	-1.88593	-1.9262	-1.92299	-1.91981
-1.8723	-1.89706	-1.89398	-1.89091	-1.88777	-1.88465	-1.92482	-1.92165	-1.91852
-1.87103	-1.86792	-1.86489	-1.86185	-1.85874	-1.85565	-1.92344	-1.92029	-1.91718
-1.86974	-1.86664	-1.86361	-1.86057	-1.85747	-1.85439	-1.92206	-1.91891	-1.91582
-1.86846	-1.86536	-1.86233	-1.87177	-1.8687	-1.86564	-1.92073	-1.91757	-1.91449
-1.86717	-1.86407	-1.86104	-1.8705	-1.86748	-1.86448	-1.91948	-1.9163	-1.91321
-1.8659	-1.86282	-1.85978	-1.86924	-1.86627	-1.86332	-1.91822	-1.91504	-1.91194
-1.86473	-1.86165	-1.85857	-1.86799	-1.86502	-1.86209	-1.91691	-1.91374	-1.91066

-1.86356	-1.8605	-1.85738	-1.86676	-1.86378	-1.86084	-1.91558	-1.91245	-1.90939
-1.86233	-1.85929	-1.8562	-1.86559	-1.86259	-1.85964	-1.9143	-1.91121	-1.91927
-1.86106	-1.85804	-1.85502	-1.86445	-1.86143	-1.85845	-1.91304	-1.90999	-1.91803
-1.85981	-1.85678	-1.8538	-1.86325	-1.86024	-1.85727	-1.91177	-1.90875	-1.91677
-1.85856	-1.85551	-1.85253	-1.86199	-1.85902	-1.85609	-1.9105	-1.90747	-1.91548
-1.85734	-1.8667	-1.8637	-1.86074	-1.85782	-1.85493	-1.92036	-1.91728	-1.91419
-1.85616	-1.86553	-1.86249	-1.85952	-1.85664	-1.85381	-1.91911	-1.91601	-1.91293
-1.85496	-1.86435	-1.86129	-1.85831	-1.85547	-1.85268	-1.91786	-1.91473	-1.91166
-1.8537	-1.8631	-1.86006	-1.86886	-1.866	-1.86318	-1.91654	-1.91341	-1.91035
-1.85241	-1.86182	-1.85883	-1.86764	-1.86478	-1.86195	-1.91521	-1.91209	-1.90905
-1.86355	-1.87238	-1.8694	-1.86647	-1.86359	-1.86074	-1.91392	-1.91083	-1.90779
-1.86231	-1.87113	-1.86821	-1.86533	-1.86243	-1.85955	-1.91266	-1.90961	-1.90657
-1.86112	-1.86992	-1.86704	-1.86417	-1.86126	-1.85837	-1.91141	-1.90837	-1.90535
-1.85998	-1.86879	-1.86587	-1.86298	-1.86009	-1.85722	-1.91016	-1.90713	-1.90413
-1.87061	-1.86765	-1.86471	-1.8618	-1.85892	-1.85607	-1.90892	-1.9059	-1.90292
-1.86942	-1.86647	-1.86355	-1.86066	-1.85777	-1.85491	-1.9077	-1.90471	-1.90174
-1.86824	-1.86529	-1.86239	-1.85951	-1.85662	-1.85376	-1.90648	-1.90352	-1.90057
-1.86706	-1.86412	-1.86121	-1.85832	-1.85547	-1.85265	-1.90529	-1.90233	-1.89939
-1.86589	-1.86297	-1.86003	-1.85713	-1.85432	-1.85155	-1.9041	-1.90114	-1.89821
-1.86471	-1.86179	-1.85885	-1.85597	-1.85318	-1.85042	-1.90291	-1.89997	-1.89705
-1.86352	-1.86059	-1.85769	-1.85484	-1.85204	-1.84928	-1.90172	-1.89881	-1.89591
-1.86234	-1.85941	-1.85654	-1.85371	-1.85092	-1.84816	-1.91325	-1.91036	-1.90746
-1.86117	-1.85826	-1.85539	-1.85257	-1.84981	-1.84709	-1.91208	-1.90917	-1.90628
-1.86001	-1.85712	-1.85425	-1.86477	-1.86203	-1.85932	-1.91091	-1.90799	-1.90511
-1.85885	-1.85595	-1.85313	-1.86367	-1.86091	-1.85818	-1.90972	-1.90683	-1.90395
-1.8577	-1.8548	-1.85203	-1.86257	-1.85979	-1.85703	-1.90853	-1.90567	-1.90279
-1.86991	-1.867	-1.8642	-1.86143	-1.85866	-1.85592	-1.90735	-1.90449	-1.90162
-1.86884	-1.86592	-1.86308	-1.86029	-1.85754	-1.85482	-1.90618	-1.9033	-1.90043
-1.86775	-1.86485	-1.86199	-1.85918	-1.85642	-1.8537	-1.905	-1.90212	-1.89926
-1.86666	-1.8638	-1.86094	-1.85811	-1.85532	-1.85256	-1.9038	-1.90095	-1.8981
-1.86554	-1.86271	-1.85987	-1.85704	-1.85423	-1.85145	-1.87498	-1.87227	-1.86957
-1.86439	-1.86156	-1.85875	-1.85596	-1.85316	-1.85039	-1.87389	-1.87116	-1.86846
-1.86324	-1.8604	-1.85763	-1.85486	-1.8521	-1.84935	-1.87282	-1.87007	-1.86738
-1.86215	-1.8593	-1.8565	-1.85374	-1.85101	-1.8483	-1.87177	-1.869	-1.86633
-1.86108	-1.85822	-1.85539	-1.85261	-1.84992	-1.84726	-1.87073	-1.86797	-1.8653
-1.85999	-1.85715	-1.85431	-1.85153	-1.84886	-1.84622	-1.8697	-1.86695	-1.86428
-1.85889	-1.85608	-1.85326	-1.8505	-1.84783	-1.8452	-1.86868	-1.86595	-1.86326
-1.8578	-1.85501	-1.85222	-1.84946	-1.84679	-1.84414	-1.86763	-1.86493	-1.86225
-1.85672	-1.85395	-1.85118	-1.84844	-1.84572	-1.84304	-1.86653	-1.86388	-1.86123
-1.85564	-1.85287	-1.85014	-1.84742	-1.84467	-1.84196	-1.86545	-1.86282	-1.86019
-1.85454	-1.85178	-1.84908	-1.84639	-1.84365	-1.84093	-1.86438	-1.86175	-1.85913
-1.85346	-1.85069	-1.84802	-1.84536	-1.84264	-1.83993	-1.86334	-1.86068	-1.85807
-1.85242	-1.84966	-1.847	-1.84435	-1.84164	-1.83894	-1.86233	-1.85965	-1.85704
-1.8514	-1.84865	-1.84599	-1.84333	-1.84065	-1.83796	-1.86134	-1.85865	-1.85604
-1.85038	-1.84763	-1.84494	-1.84227	-1.83961	-1.83697	-1.86034	-1.85765	-1.85503
-1.84937	-1.8466	-1.84385	-1.84116	-1.83855	-1.83596	-1.85934	-1.85667	-1.85402
-1.84836	-1.84558	-1.84281	-1.84011	-1.83751	-1.83496	-1.85834	-1.85567	-1.85301
-1.84736	-1.84457	-1.84181	-1.83911	-1.83652	-1.83396	-1.85734	-1.85467	-1.852
-1.84636	-1.84358	-1.84082	-1.83812	-1.83553	-1.83298	-1.85634	-1.85367	-1.851
-1.84536	-1.84258	-1.83983	-1.83714	-1.83455	-1.832	-1.85535	-1.85268	-1.85

-1.84436	-1.84159	-1.83884	-1.83616	-1.83357	-1.83102	-1.85436	-1.85169	-1.849
-1.84335	-1.84059	-1.83785	-1.83517	-1.83259	-1.83004	-1.85336	-1.85069	-1.848
-1.84234	-1.83958	-1.83686	-1.83419	-1.83161	-1.82905	-1.85235	-1.84968	-1.84699
-1.84134	-1.83858	-1.83587	-1.83321	-1.83062	-1.82806	-1.85135	-1.84867	-1.84598
-1.84034	-1.8376	-1.83488	-1.83221	-1.82963	-1.82707	-1.85034	-1.84766	-1.84497
-1.83935	-1.8366	-1.83389	-1.83122	-1.82864	-1.82607	-1.84933	-1.84664	-1.84395
-1.83834	-1.8356	-1.83289	-1.83023	-1.82764	-1.82507	-1.84831	-1.84563	-1.84293
-1.83733	-1.8346	-1.83189	-1.82923	-1.82664	-1.82407	-1.84729	-1.84461	-1.84192
-1.83632	-1.8336	-1.83089	-1.82822	-1.82564	-1.82308	-1.84629	-1.84361	-1.84092
-1.83531	-1.83259	-1.82988	-1.82722	-1.82465	-1.82209	-1.84529	-1.84261	-1.83992
-1.8343	-1.83159	-1.82888	-1.82622	-1.82365	-1.8211	-1.84429	-1.84161	-1.83893
-1.8333	-1.83059	-1.82789	-1.82524	-1.82266	-1.82011	-1.84328	-1.84061	-1.83794
-1.83231	-1.82961	-1.82693	-1.82429	-1.82171	-1.81913	-1.84229	-1.83962	-1.83695
-1.83132	-1.82864	-1.82601	-1.82339	-1.82079	-1.81818	-1.8413	-1.83862	-1.83595
-1.83035	-1.8277	-1.82509	-1.82248	-1.81985	-1.81722	-1.84032	-1.83763	-1.83496
-1.82943	-1.82679	-1.82416	-1.82153	-1.8189	-1.81626	-1.83933	-1.83665	-1.83398
-1.82852	-1.82588	-1.82323	-1.82057	-1.81793	-1.81529	-1.83835	-1.83568	-1.83302
-1.82758	-1.85085	-1.84814	-1.84543	-1.84273	-1.84005	-1.83737	-1.8347	-1.83206
-1.82663	-1.84986	-1.84715	-1.84444	-1.84174	-1.83906	-1.83639	-1.83374	-1.83112
-1.82567	-1.84888	-1.84616	-1.84346	-1.84076	-1.83809	-1.83543	-1.83281	-1.8302
-1.82471	-1.84791	-1.84519	-1.84249	-1.83979	-1.83712	-1.8345	-1.83191	-1.82931
-1.82375	-1.84694	-1.84424	-1.84156	-1.83887	-1.8362	-1.83358	-1.83099	-1.82838
-1.82279	-1.84599	-1.84334	-1.84069	-1.83801	-1.83534	-1.83269	-1.83007	-1.82743
-1.81327	-1.84507	-1.84244	-1.8398	-1.83713	-1.83446	-1.83179	-1.82913	-1.82647
-1.81241	-1.84419	-1.84153	-1.83887	-1.83619	-1.83352	-1.83084	-1.82817	-1.82551
-1.81155	-1.84331	-1.84061	-1.83792	-1.83524	-1.83256	-1.82989	-1.82721	-1.82456
-1.81063	-1.81658	-1.81393	-1.81128	-1.80864	-1.80601	-1.82893	-1.82626	-1.82362
-1.80967	-1.81565	-1.81299	-1.81035	-1.80771	-1.80508	-1.82798	-1.82531	-1.82269
-1.80872	-1.80607	-1.80345	-1.80946	-1.80683	-1.8042	-1.82708	-1.82442	-1.82179
-1.80777	-1.80514	-1.80256	-1.80862	-1.806	-1.80337	-1.82624	-1.82357	-1.82092
-1.80686	-1.80423	-1.80166	-1.80775	-1.80514	-1.80252	-1.82537	-1.8227	-1.82003
-1.806	-1.80336	-1.80076	-1.80684	-1.80422	-1.80161	-1.82445	-1.82177	-1.8191
-1.80514	-1.80248	-1.79985	-1.79723	-1.79462	-1.79202	-1.82353	-1.82086	-1.81819
-1.80422	-1.80156	-1.79891	-1.79629	-1.79372	-1.79115	-1.82267	-1.82	-1.81733
-1.80329	-1.80063	-1.79799	-1.79537	-1.79282	-1.79028	-1.82181	-1.81915	-1.81648
-1.80242	-1.79976	-1.79711	-1.7945	-1.79194	-1.78939	-1.8209	-1.81824	-1.81556
-1.80158	-1.79892	-1.79628	-1.79365	-1.79106	-1.78848	-1.81997	-1.8173	-1.81462
-1.8007	-1.79804	-1.7954	-1.79277	-1.79018	-1.78759	-1.78499	-1.78237	-1.81372
-1.79977	-1.79712	-1.79447	-1.79186	-1.78929	-1.78674	-1.78414	-1.78153	-1.81287
-1.79888	-1.79623	-1.79358	-1.79097	-1.78841	-1.78586	-1.78327	-1.78066	-1.81199
-1.79806	-1.7954	-1.79276	-1.79013	-1.78754	-1.78496	-1.78235	-1.77974	-1.81105
-1.79725	-1.79459	-1.79195	-1.78931	-1.78668	-1.78406	-1.78144	-1.77881	-1.81011
-1.79644	-1.79378	-1.79114	-1.78849	-1.78585	-1.78321	-1.78058	-1.77794	-1.80072
-1.79562	-1.79296	-1.79031	-1.78767	-1.78502	-1.78238	-1.77973	-1.77709	-1.79985
-1.79473	-1.79208	-1.78943	-1.78678	-1.78413	-1.78148	-1.77883	-1.77619	-1.79893
-1.7938	-1.79114	-1.78849	-1.78584	-1.78319	-1.78054	-1.77789	-1.77524	-1.79797
-1.79292	-1.79026	-1.7876	-1.78495	-1.7823	-1.77965	-1.77699	-1.77434	-1.77169
-1.7921	-1.78944	-1.78678	-1.78412	-1.78147	-1.77881	-1.77616	-1.77351	-1.77085
-1.79128	-1.78862	-1.78596	-1.7833	-1.78064	-1.77799	-1.77533	-1.77267	-1.77002
-1.79047	-1.7878	-1.78514	-1.78248	-1.77982	-1.77717	-1.77451	-1.77184	-1.76918

-1.78963	-1.78697	-1.7843	-1.78164	-1.77898	-1.77632	-1.77366	-1.77099	-1.76833
-1.78872	-1.78605	-1.78339	-1.78072	-1.77806	-1.7754	-1.77273	-1.77006	-1.76739
-1.78777	-1.7851	-1.78244	-1.77977	-1.77711	-1.77445	-1.77177	-1.7691	-1.76643
-1.78688	-1.78422	-1.78155	-1.77888	-1.77622	-1.77355	-1.77088	-1.7682	-1.76553
-1.78604	-1.78338	-1.78071	-1.77804	-1.77538	-1.77271	-1.77003	-1.76735	-1.76468
-1.78522	-1.78255	-1.77988	-1.77722	-1.77455	-1.77187	-1.7692	-1.76652	-1.76384
-1.7844	-1.78173	-1.77907	-1.7764	-1.77373	-1.77105	-1.76838	-1.7657	-1.76302
-1.78359	-1.78092	-1.77826	-1.77559	-1.77291	-1.77024	-1.76756	-1.76488	-1.76219
-1.78278	-1.78011	-1.77744	-1.77477	-1.7721	-1.76942	-1.76674	-1.76405	-1.76137
-1.78196	-1.77929	-1.77662	-1.77395	-1.77128	-1.7686	-1.76591	-1.76323	-1.76053
-1.78115	-1.77847	-1.77581	-1.77313	-1.77045	-1.76776	-1.76508	-1.76239	-1.75967
-1.78033	-1.77766	-1.77498	-1.77231	-1.76962	-1.76692	-1.76423	-1.76154	-1.7588
-1.77951	-1.77683	-1.77416	-1.77147	-1.76875	-1.76603	-1.76333	-1.76064	-1.75789
-1.77869	-1.77601	-1.77332	-1.77061	-1.76786	-1.7651	-1.76238	-1.75967	-1.75695
-1.77787	-1.77518	-1.77247	-1.76974	-1.76696	-1.76419	-1.76145	-1.75874	-1.78345
-1.77705	-1.77433	-1.77158	-1.76882	-1.76606	-1.76331	-1.76058	-1.75787	-1.78256
-1.77622	-1.77349	-1.7707	-1.76791	-1.76517	-1.76245	-1.75973	-1.75702	-1.7817
-1.7754	-1.77265	-1.76985	-1.76705	-1.76432	-1.76161	-1.75889	-1.75617	-1.78084
-1.77456	-1.77182	-1.76901	-1.76622	-1.76348	-1.76077	-1.75805	-1.75534	-1.77999
-1.77373	-1.77099	-1.76818	-1.76539	-1.76265	-1.75993	-1.75721	-1.75449	-1.77913
-1.77289	-1.77016	-1.76735	-1.76456	-1.76181	-1.75908	-1.75636	-1.75365	-1.77828
-1.77206	-1.76933	-1.76655	-1.76377	-1.761	-1.75826	-1.78294	-1.78018	-1.77742
-1.77122	-1.76852	-1.76578	-1.76302	-1.76024	-1.75745	-1.7821	-1.77933	-1.77656
-1.77038	-1.7677	-1.76499	-1.76226	-1.75947	-1.75667	-1.78129	-1.77849	-1.77571
-1.76954	-1.76686	-1.76417	-1.76146	-1.75871	-1.75593	-1.78052	-1.77768	-1.77488
-1.76869	-1.76601	-1.76333	-1.76064	-1.75793	-1.75519	-1.77976	-1.77689	-1.77406
-1.76785	-1.76517	-1.76249	-1.75981	-1.75711	-1.75439	-1.77897	-1.77612	-1.77329
-1.767	-1.76432	-1.76165	-1.75896	-1.75627	-1.75356	-1.77817	-1.77537	-1.77255
-1.76616	-1.76348	-1.7608	-1.75812	-1.75542	-1.75271	-1.77733	-1.77456	-1.77177
-1.76531	-1.76263	-1.75995	-1.75727	-1.75457	-1.75186	-1.77647	-1.77372	-1.77096
-1.76448	-1.7618	-1.75911	-1.78386	-1.78111	-1.77836	-1.77561	-1.77287	-1.77013
-1.76366	-1.76097	-1.75827	-1.783	-1.78025	-1.7775	-1.77475	-1.77201	-1.76928
-1.76288	-1.76017	-1.75745	-1.78215	-1.7794	-1.77665	-1.7739	-1.77115	-1.76843
-1.76217	-1.75943	-1.75668	-1.78133	-1.77856	-1.7758	-1.77305	-1.7703	-1.76758
-1.76146	-1.75872	-1.75592	-1.78053	-1.77775	-1.77498	-1.77222	-1.76947	-1.76673
-1.76068	-1.78543	-1.7826	-1.77978	-1.777	-1.77423	-1.77144	-1.76866	-1.7659
-1.75986	-1.78461	-1.78184	-1.77906	-1.77629	-1.77352	-1.7707	-1.76788	-1.76509
-1.78654	-1.78379	-1.78103	-1.77828	-1.77552	-1.77274	-1.76994	-1.76711	-1.76431
-1.7857	-1.78295	-1.78019	-1.77744	-1.77468	-1.77192	-1.76915	-1.76637	-1.76358
-1.78491	-1.78214	-1.77937	-1.7766	-1.77384	-1.77109	-1.76835	-1.7656	-1.76282
-1.7842	-1.78141	-1.77859	-1.77579	-1.77301	-1.77025	-1.76751	-1.76478	-1.76202
-1.78348	-1.78068	-1.77783	-1.775	-1.77221	-1.76944	-1.76669	-1.76395	-1.76121
-1.78267	-1.77989	-1.77706	-1.77425	-1.77147	-1.76871	-1.76593	-1.76316	-1.7604
-1.78185	-1.77908	-1.7763	-1.77352	-1.77076	-1.76799	-1.76519	-1.76238	-1.7596
-1.78105	-1.77827	-1.7755	-1.77273	-1.76997	-1.76721	-1.76442	-1.76162	-1.75886
-1.78026	-1.77745	-1.77467	-1.7719	-1.76914	-1.76639	-1.76364	-1.76089	-1.75814
-1.7795	-1.77668	-1.7739	-1.77112	-1.76835	-1.76558	-1.76284	-1.76011	-1.75738
-1.77875	-1.77596	-1.77318	-1.7704	-1.76759	-1.76479	-1.76203	-1.75929	-1.75656
-1.778	-1.77522	-1.77244	-1.76965	-1.76683	-1.76401	-1.76124	-1.7585	-1.75575
-1.77721	-1.77441	-1.77164	-1.76886	-1.76606	-1.76327	-1.76051	-1.75777	-1.755

-1.02002	-1.01856	-1.01711	-1.01569	-1.01428	-1.01289	-1.01149	-1.01008	-1.00866
-1.02039	-1.01893	-1.01748	-1.01605	-1.01465	-1.01325	-1.01185	-1.01044	-1.00901
-1.02076	-1.01929	-1.01785	-1.01642	-1.01502	-1.01362	-1.01221	-1.0108	-1.00937
-1.02113	-1.01967	-1.01822	-1.01679	-1.01538	-1.01398	-1.01258	-1.01116	-1.00974
-1.0215	-1.02004	-1.0186	-1.01717	-1.01576	-1.01435	-1.01294	-1.01153	-1.01011
-1.02187	-1.02041	-1.01898	-1.01756	-1.01615	-1.01474	-1.01332	-1.0119	-1.01048
-1.02225	-1.02079	-1.01938	-1.01797	-1.01656	-1.01513	-1.0137	-1.01227	-1.01085
-1.02262	-1.02117	-1.01977	-1.01837	-1.01694	-1.01552	-1.01408	-1.01264	-1.01123
-1.02301	-1.02155	-1.02014	-1.01874	-1.01731	-1.01588	-1.01444	-1.01301	-1.0116
-1.02339	-1.02193	-1.02052	-1.01911	-1.01768	-1.01624	-1.01481	-1.01339	-1.01197
-1.02378	-1.02232	-1.0209	-1.01949	-1.01806	-1.01662	-1.0152	-1.01378	-1.01236
-1.02417	-1.02271	-1.02129	-1.01987	-1.01844	-1.01701	-1.01559	-1.01417	-1.01274
-1.02456	-1.0231	-1.02166	-1.02024	-1.01881	-1.01738	-1.01597	-1.01455	-1.01312
-1.02496	-1.02349	-1.02204	-1.0206	-1.01918	-1.01776	-1.01634	-1.01493	-1.0135
-0.96045	-1.02388	-1.02241	-1.02096	-1.01954	-1.01813	-1.01671	-1.01529	-1.01387
-0.96082	-1.02426	-1.02279	-1.02133	-1.01991	-1.0185	-1.01708	-1.01565	-1.01422
-0.9612	-0.97993	-0.97851	-0.97712	-0.97575	-0.97439	-1.01744	-1.01601	-1.01458
-0.96159	-0.98034	-0.97892	-0.97751	-0.97612	-0.97475	-1.0178	-1.01637	-1.01495
-0.93962	-0.96058	-0.95917	-0.9779	-0.9765	-0.97511	-0.97374	-0.97237	-1.01532
-0.94	-0.96097	-0.95956	-0.97829	-0.97689	-0.97549	-0.97411	-0.97275	-1.01571
-0.94038	-0.96136	-0.95994	-0.97868	-0.97728	-0.97588	-0.9745	-0.97314	-1.01611
-0.8585	-0.93936	-0.93797	-0.97907	-0.97766	-0.97627	-0.97489	-0.97353	-0.97216
-0.85879	-0.93971	-0.93832	-0.97945	-0.97805	-0.97666	-0.97529	-0.97392	-0.97256
-0.85907	-0.85786	-0.85668	-0.93732	-0.93598	-0.93464	-0.97568	-0.97432	-0.97295
-0.85934	-0.85814	-0.85698	-0.93771	-0.93635	-0.93502	-0.97608	-0.97471	-0.97334
-0.85963	-0.85844	-0.85727	-0.90912	-0.90783	-0.90654	-0.95456	-0.95323	-0.97373
-0.85994	-0.85874	-0.85757	-0.90945	-0.90817	-0.9069	-0.95496	-0.9536	-0.97413
-0.86025	-0.85906	-0.85787	-0.90978	-0.90851	-0.90726	-0.95535	-0.95398	-0.97452
-0.86057	-0.85937	-0.85819	-0.85702	-0.85588	-0.85474	-0.93519	-0.93385	-0.95302
-0.86088	-0.85969	-0.85851	-0.85734	-0.85619	-0.85504	-0.93555	-0.93422	-0.95339
-0.8612	-0.86001	-0.85882	-0.85765	-0.8565	-0.85536	-0.90699	-0.90571	-0.95378
-0.86153	-0.86033	-0.85913	-0.85796	-0.85681	-0.85567	-0.90734	-0.90606	-0.95417
-0.86186	-0.86066	-0.85946	-0.85827	-0.85712	-0.85598	-0.85484	-0.8537	-0.9261
-0.8622	-0.86099	-0.85979	-0.85861	-0.85743	-0.85627	-0.85513	-0.85401	-0.92646
-0.86255	-0.86133	-0.86013	-0.85894	-0.85775	-0.85657	-0.85543	-0.85431	-0.90583
-0.86289	-0.86167	-0.86046	-0.85926	-0.85807	-0.85688	-0.85574	-0.85462	-0.90616
-0.86324	-0.86201	-0.86079	-0.85959	-0.85839	-0.85721	-0.85606	-0.85492	-0.90649
-0.86357	-0.86233	-0.86112	-0.85991	-0.85871	-0.85753	-0.85637	-0.85523	-0.85409
-0.86389	-0.86265	-0.86144	-0.86023	-0.85903	-0.85785	-0.85668	-0.85554	-0.8544
-0.86425	-0.863	-0.86178	-0.86057	-0.85937	-0.85818	-0.85701	-0.85584	-0.8547
-0.86463	-0.86339	-0.86215	-0.86092	-0.85972	-0.85853	-0.85733	-0.85614	-0.85499
-0.86501	-0.86376	-0.86251	-0.86128	-0.86008	-0.85889	-0.85767	-0.85645	-0.8553
-0.86538	-0.86413	-0.86288	-0.86164	-0.86044	-0.85925	-0.85803	-0.85681	-0.85563
-0.86574	-0.86449	-0.86324	-0.862	-0.86079	-0.85959	-0.85838	-0.85716	-0.85597
-0.86608	-0.86482	-0.86358	-0.86235	-0.86113	-0.85991	-0.8587	-0.8575	-0.8563
-0.86642	-0.86516	-0.86392	-0.86269	-0.86145	-0.86023	-0.85902	-0.85783	-0.85663
-0.86678	-0.86552	-0.86428	-0.86305	-0.86181	-0.86057	-0.85936	-0.85817	-0.85696
-0.86716	-0.8659	-0.86466	-0.86341	-0.86217	-0.86093	-0.85971	-0.8585	-0.85728
-0.86752	-0.86626	-0.86501	-0.86376	-0.86252	-0.86128	-0.86005	-0.85883	-0.85761
-0.86787	-0.8666	-0.86534	-0.86409	-0.86286	-0.86163	-0.8604	-0.85917	-0.85795

-0.83715	-0.86694	-0.86568	-0.86443	-0.8632	-0.86197	-0.86074	-0.85951	-0.8583
-0.83747	-0.86727	-0.86602	-0.86477	-0.86354	-0.86231	-0.86108	-0.85985	-0.85865
-0.8378	-0.86762	-0.86637	-0.86513	-0.86389	-0.86265	-0.86143	-0.86021	-0.859
-0.80746	-0.83692	-0.83571	-0.86549	-0.86424	-0.863	-0.86177	-0.86055	-0.85935
-0.8078	-0.83729	-0.83607	-0.86587	-0.8646	-0.86335	-0.86212	-0.8609	-0.8597
-0.80813	-0.83765	-0.83643	-0.86625	-0.86497	-0.86372	-0.86248	-0.86127	-0.86006
-0.80845	-0.838	-0.83678	-0.86663	-0.86536	-0.8641	-0.86287	-0.86165	-0.86044
-0.80876	-0.80759	-0.80642	-0.8359	-0.83469	-0.83349	-0.86324	-0.86202	-0.86081
-0.80906	-0.80789	-0.80673	-0.83623	-0.83502	-0.83382	-0.8636	-0.86239	-0.86117
-0.80937	-0.8082	-0.80703	-0.80587	-0.80472	-0.80358	-0.83297	-0.8318	-0.86153
-0.80971	-0.80853	-0.80735	-0.80618	-0.80503	-0.80389	-0.8333	-0.83213	-0.86189
-0.81006	-0.80887	-0.80768	-0.8065	-0.80535	-0.8042	-0.83364	-0.83247	-0.86226
-0.81042	-0.80922	-0.80803	-0.80685	-0.80568	-0.80454	-0.83399	-0.83283	-0.83164
-0.81078	-0.80958	-0.80838	-0.8072	-0.80603	-0.80488	-0.83436	-0.83319	-0.832
-0.81113	-0.80993	-0.80873	-0.80755	-0.80638	-0.80523	-0.80409	-0.80297	-0.83235
-0.81147	-0.81028	-0.80908	-0.80789	-0.80673	-0.80557	-0.80443	-0.80329	-0.8327
-0.81182	-0.81063	-0.80943	-0.80824	-0.80707	-0.80591	-0.80476	-0.80362	-0.80248
-0.81218	-0.81098	-0.80977	-0.80857	-0.8074	-0.80624	-0.80509	-0.80394	-0.80279
-0.81254	-0.81134	-0.81011	-0.8089	-0.80773	-0.80658	-0.80542	-0.80427	-0.80312
-0.81291	-0.8117	-0.81047	-0.80926	-0.80809	-0.80693	-0.80578	-0.80463	-0.80347
-0.81329	-0.81208	-0.81084	-0.80963	-0.80845	-0.80729	-0.80614	-0.80499	-0.80383
-0.78133	-0.81244	-0.81121	-0.81	-0.80882	-0.80765	-0.8065	-0.80535	-0.80419
-0.78167	-0.81281	-0.81159	-0.81037	-0.80919	-0.80802	-0.80686	-0.80572	-0.80457
-0.782	-0.81318	-0.81197	-0.81076	-0.80956	-0.80838	-0.80722	-0.80607	-0.80492
-0.78232	-0.81355	-0.81235	-0.81115	-0.80994	-0.80874	-0.80757	-0.80642	-0.80527
-0.78264	-0.7815	-0.78038	-0.81152	-0.8103	-0.80909	-0.80791	-0.80676	-0.8056
-0.78296	-0.78181	-0.78068	-0.81186	-0.81064	-0.80943	-0.80825	-0.80709	-0.80594
-0.78329	-0.78213	-0.78099	-0.8122	-0.81099	-0.80978	-0.8086	-0.80743	-0.80627
-0.78363	-0.78246	-0.78132	-0.78018	-0.77905	-0.77793	-0.80898	-0.80779	-0.80662
-0.78397	-0.7828	-0.78166	-0.78052	-0.77941	-0.77829	-0.80938	-0.80817	-0.80699
-0.78433	-0.78315	-0.782	-0.78086	-0.77975	-0.77864	-0.7775	-0.77634	-0.80735
-0.78468	-0.7835	-0.78234	-0.7812	-0.78008	-0.77897	-0.77783	-0.77669	-0.80773
-0.78505	-0.78386	-0.78269	-0.78154	-0.78042	-0.77931	-0.77817	-0.77703	-0.80812
-0.78543	-0.78423	-0.78305	-0.7819	-0.78077	-0.77964	-0.77852	-0.77739	-0.80852
-0.78582	-0.7846	-0.78342	-0.78225	-0.78111	-0.77998	-0.77885	-0.77773	-0.77662
-0.7862	-0.78498	-0.78378	-0.7826	-0.78144	-0.7803	-0.77918	-0.77806	-0.77694
-0.78657	-0.78535	-0.78414	-0.78295	-0.78178	-0.78063	-0.7795	-0.77838	-0.77726
-0.78693	-0.78571	-0.7845	-0.7833	-0.78212	-0.78096	-0.77983	-0.77871	-0.77759
-0.78728	-0.78606	-0.78485	-0.78365	-0.78247	-0.7813	-0.78016	-0.77905	-0.77793
-0.78765	-0.78644	-0.78522	-0.78402	-0.78283	-0.78166	-0.78052	-0.77941	-0.77829
-0.78805	-0.78683	-0.7856	-0.78438	-0.7832	-0.78204	-0.7809	-0.77978	-0.77866
-0.78846	-0.78723	-0.78599	-0.78476	-0.78358	-0.78242	-0.78127	-0.78015	-0.77902
-0.78887	-0.78765	-0.7864	-0.78516	-0.78396	-0.78278	-0.78163	-0.7805	-0.77938
-0.71259	-0.78805	-0.78681	-0.78557	-0.78435	-0.78316	-0.782	-0.78087	-0.77974
-0.71302	-0.78844	-0.78722	-0.786	-0.78478	-0.78357	-0.78239	-0.78124	-0.7801
-0.71344	-0.78882	-0.78762	-0.78642	-0.7852	-0.78399	-0.78279	-0.78162	-0.78047
-0.67657	-0.71268	-0.71152	-0.74151	-0.74026	-0.73902	-0.78319	-0.782	-0.78085
-0.67697	-0.71312	-0.71194	-0.74195	-0.74071	-0.73947	-0.78358	-0.78239	-0.78124
-0.67738	-0.71357	-0.71237	-0.71119	-0.71	-0.70883	-0.73869	-0.73747	-0.73627
-0.67781	-0.71405	-0.71282	-0.71161	-0.71042	-0.70925	-0.73914	-0.73791	-0.7367

-0.67823	-0.67712	-0.67603	-0.71204	-0.71085	-0.70968	-0.73961	-0.73837	-0.73716
-0.67865	-0.67753	-0.67642	-0.71249	-0.7113	-0.71014	-0.7401	-0.73887	-0.73766
-0.67907	-0.67795	-0.67683	-0.71294	-0.71175	-0.71059	-0.7406	-0.73938	-0.73816
-0.67952	-0.67839	-0.67725	-0.67614	-0.67509	-0.67406	-0.70986	-0.70871	-0.73864
-0.67997	-0.67883	-0.67768	-0.67656	-0.67549	-0.67444	-0.71029	-0.70915	-0.73911
-0.6496	-0.67927	-0.67812	-0.67699	-0.6759	-0.67483	-0.71074	-0.7096	-0.70845
-0.65002	-0.67971	-0.67856	-0.67742	-0.67632	-0.67524	-0.7112	-0.71006	-0.70891
-0.60876	-0.68016	-0.679	-0.67786	-0.67674	-0.67565	-0.6746	-0.6736	-0.70936
-0.60914	-0.68062	-0.67945	-0.6783	-0.67717	-0.67606	-0.675	-0.67397	-0.70978
-0.60951	-0.65022	-0.64911	-0.67875	-0.67761	-0.67649	-0.67541	-0.67436	-0.71022
-0.60987	-0.65066	-0.64955	-0.67922	-0.67808	-0.67695	-0.67586	-0.67479	-0.7107
-0.61024	-0.6511	-0.64999	-0.67969	-0.67855	-0.67742	-0.67632	-0.67524	-0.7112
-0.61062	-0.60971	-0.60882	-0.64934	-0.64827	-0.64721	-0.67678	-0.67569	-0.67464
-0.611	-0.6101	-0.6092	-0.64979	-0.64871	-0.64765	-0.67725	-0.67615	-0.67507
-0.61137	-0.61047	-0.60957	-0.65023	-0.64914	-0.64808	-0.67771	-0.67659	-0.6755
-0.61173	-0.61083	-0.60993	-0.65067	-0.64958	-0.6485	-0.67815	-0.67703	-0.67592
-0.61211	-0.61121	-0.61031	-0.60941	-0.60852	-0.60763	-0.64787	-0.64682	-0.67637
-0.61251	-0.61161	-0.61071	-0.60981	-0.60892	-0.60803	-0.64833	-0.64727	-0.67685
-0.61291	-0.61201	-0.61111	-0.61022	-0.60932	-0.60842	-0.6488	-0.64772	-0.64666
-0.61331	-0.6124	-0.61151	-0.61062	-0.60972	-0.60882	-0.64926	-0.64816	-0.64709
-0.61371	-0.6128	-0.6119	-0.61101	-0.61011	-0.60921	-0.64971	-0.64861	-0.64752
-0.58038	-0.61317	-0.61227	-0.61138	-0.61048	-0.60959	-0.60868	-0.60778	-0.64795
-0.58072	-0.61353	-0.61263	-0.61173	-0.61084	-0.60995	-0.60904	-0.60814	-0.64838
-0.58108	-0.58024	-0.57943	-0.61211	-0.61122	-0.61033	-0.60942	-0.60852	-0.64884
-0.58145	-0.5806	-0.57979	-0.61251	-0.61161	-0.61072	-0.60982	-0.60892	-0.64932
-0.54832	-0.58097	-0.58016	-0.6129	-0.61201	-0.61112	-0.61022	-0.60932	-0.60842
-0.54867	-0.58136	-0.58053	-0.6133	-0.61241	-0.61152	-0.61062	-0.60972	-0.60883
-0.54901	-0.54821	-0.54745	-0.58007	-0.57927	-0.57848	-0.61102	-0.61013	-0.60924
-0.54934	-0.54854	-0.54776	-0.58042	-0.57962	-0.57883	-0.6114	-0.61052	-0.60964
-0.54966	-0.54886	-0.54807	-0.58077	-0.57996	-0.57917	-0.61178	-0.61092	-0.61005
-0.55001	-0.54919	-0.54839	-0.58113	-0.58031	-0.57951	-0.61216	-0.61131	-0.61045
-0.55036	-0.54954	-0.54873	-0.58151	-0.58067	-0.57985	-0.61255	-0.6117	-0.61085
-0.55073	-0.5499	-0.54908	-0.54829	-0.54752	-0.54677	-0.57942	-0.57866	-0.61124
-0.55112	-0.55028	-0.54947	-0.54867	-0.54787	-0.5471	-0.57977	-0.57901	-0.61162
-0.55152	-0.55067	-0.54986	-0.54905	-0.54824	-0.54745	-0.58015	-0.57937	-0.612
-0.55193	-0.55107	-0.55024	-0.54943	-0.54861	-0.54782	-0.58055	-0.57974	-0.6124
-0.55234	-0.55147	-0.55063	-0.54981	-0.54899	-0.5482	-0.58097	-0.58013	-0.61281
-0.55277	-0.55189	-0.55104	-0.5502	-0.54938	-0.54858	-0.54779	-0.54702	-0.5797
-0.55321	-0.55232	-0.55145	-0.5506	-0.54977	-0.54896	-0.54816	-0.54737	-0.58009
-0.55364	-0.55275	-0.55186	-0.551	-0.55016	-0.54935	-0.54854	-0.54774	-0.58049
-0.55407	-0.55317	-0.55228	-0.5514	-0.55055	-0.54973	-0.54891	-0.54812	-0.58092
-0.51606	-0.5536	-0.5527	-0.55181	-0.55095	-0.55012	-0.5493	-0.5485	-0.54772
-0.51644	-0.55403	-0.55313	-0.55224	-0.55137	-0.55051	-0.54969	-0.54889	-0.5481
-0.51682	-0.516	-0.51517	-0.55267	-0.55178	-0.55092	-0.55008	-0.54927	-0.54847
-0.5172	-0.51638	-0.51556	-0.5531	-0.55221	-0.55133	-0.55048	-0.54966	-0.54885
-0.51759	-0.51677	-0.51594	-0.55353	-0.55263	-0.55174	-0.55088	-0.55004	-0.54923
-0.51798	-0.51716	-0.51633	-0.5155	-0.51467	-0.51385	-0.55129	-0.55044	-0.54962
-0.51837	-0.51755	-0.51672	-0.51588	-0.51505	-0.51423	-0.55171	-0.55084	-0.55001
-0.51876	-0.51794	-0.5171	-0.51627	-0.51544	-0.51462	-0.5138	-0.51299	-0.55043
-0.51916	-0.51833	-0.5175	-0.51666	-0.51583	-0.51501	-0.51419	-0.51338	-0.55086

-0.44689	-0.51873	-0.51789	-0.51706	-0.51623	-0.5154	-0.51458	-0.51377	-0.51299
-0.44721	-0.51913	-0.51829	-0.51745	-0.51662	-0.5158	-0.51498	-0.51417	-0.51338
-0.44754	-0.44685	-0.44615	-0.51785	-0.51702	-0.5162	-0.51538	-0.51458	-0.51378
-0.44787	-0.44717	-0.44647	-0.51826	-0.51743	-0.51661	-0.5158	-0.515	-0.51419
-0.44821	-0.4475	-0.4468	-0.51866	-0.51784	-0.51702	-0.51621	-0.51542	-0.51461
-0.44854	-0.44783	-0.44712	-0.51907	-0.51824	-0.51742	-0.51662	-0.51582	-0.51502
-0.44888	-0.44816	-0.44745	-0.51948	-0.51864	-0.51782	-0.51702	-0.51623	-0.51543
-0.44922	-0.44849	-0.44778	-0.44709	-0.44641	-0.44573	-0.51743	-0.51664	-0.51583
-0.44956	-0.44884	-0.44813	-0.44743	-0.44675	-0.44608	-0.51786	-0.51706	-0.51625
-0.44991	-0.44919	-0.44848	-0.44778	-0.4471	-0.44643	-0.44576	-0.44508	-0.51666
-0.45026	-0.44955	-0.44883	-0.44813	-0.44744	-0.44676	-0.44608	-0.44541	-0.51706
-0.45061	-0.4499	-0.44919	-0.44849	-0.44779	-0.4471	-0.44641	-0.44573	-0.51747
-0.45094	-0.45024	-0.44954	-0.44884	-0.44813	-0.44743	-0.44674	-0.44606	-0.44539
-0.45128	-0.45057	-0.44988	-0.44918	-0.44847	-0.44777	-0.44708	-0.4464	-0.44573
-0.45163	-0.45093	-0.45023	-0.44953	-0.44882	-0.44811	-0.44742	-0.44674	-0.44607
-0.452	-0.45129	-0.45059	-0.44988	-0.44916	-0.44845	-0.44776	-0.44709	-0.44641
-0.38906	-0.41029	-0.40966	-0.45023	-0.44952	-0.44881	-0.44812	-0.44743	-0.44675
-0.38933	-0.41059	-0.40995	-0.45059	-0.44988	-0.44918	-0.44848	-0.44777	-0.44708
-0.38961	-0.38904	-0.3885	-0.40962	-0.409	-0.40837	-0.44883	-0.44812	-0.44742
-0.3899	-0.38933	-0.38878	-0.40993	-0.4093	-0.40867	-0.44918	-0.44847	-0.44777
-0.39019	-0.38961	-0.38905	-0.41024	-0.4096	-0.40897	-0.44953	-0.44882	-0.44813
-0.39047	-0.38988	-0.38932	-0.41053	-0.40989	-0.40926	-0.40862	-0.40799	-0.40736
-0.39074	-0.39015	-0.38958	-0.41082	-0.41018	-0.40956	-0.40893	-0.4083	-0.40767
-0.39103	-0.39043	-0.38986	-0.38929	-0.38874	-0.3882	-0.40923	-0.4086	-0.40796
-0.39133	-0.39074	-0.39015	-0.38958	-0.38902	-0.38847	-0.40954	-0.4089	-0.40826
-0.39163	-0.39104	-0.39045	-0.38987	-0.38931	-0.38875	-0.38819	-0.38763	-0.40856
-0.39194	-0.39135	-0.39076	-0.39017	-0.3896	-0.38902	-0.38846	-0.3879	-0.40886
-0.39226	-0.39165	-0.39106	-0.39048	-0.38989	-0.3893	-0.38873	-0.38818	-0.40917
-0.3926	-0.39195	-0.39135	-0.39077	-0.39017	-0.38958	-0.38901	-0.38845	-0.40947
-0.39296	-0.39226	-0.39165	-0.39106	-0.39046	-0.38987	-0.3893	-0.38873	-0.40978
-0.39337	-0.3926	-0.39196	-0.39135	-0.39075	-0.39017	-0.38959	-0.38902	-0.38845
-0.39382	-0.39296	-0.39228	-0.39165	-0.39104	-0.39046	-0.38988	-0.38931	-0.38874
-0.39431	-0.39338	-0.39263	-0.39195	-0.39134	-0.39076	-0.39018	-0.3896	-0.38903
-0.39484	-0.39385	-0.39301	-0.39227	-0.39163	-0.39106	-0.39047	-0.3899	-0.38933
-0.39541	-0.39437	-0.39344	-0.39262	-0.39195	-0.39136	-0.39077	-0.3902	-0.38962
-0.39602	-0.39494	-0.39392	-0.39302	-0.3923	-0.39167	-0.39106	-0.39049	-0.38992
-0.39665	-0.39554	-0.39445	-0.39347	-0.39268	-0.39199	-0.39137	-0.3908	-0.39022
-0.39727	-0.39615	-0.39503	-0.394	-0.39312	-0.39235	-0.39168	-0.3911	-0.39052
-0.3979	-0.39678	-0.39565	-0.39459	-0.39361	-0.39273	-0.39202	-0.39142	-0.39083
-0.35617	-0.3974	-0.39627	-0.39518	-0.39413	-0.39317	-0.39239	-0.39174	-0.39112
-0.35676	-0.39804	-0.3969	-0.39579	-0.39468	-0.39366	-0.3928	-0.39208	-0.39142
-0.35735	-0.3563	-0.35523	-0.39641	-0.39527	-0.3942	-0.39327	-0.39246	-0.39175
-0.35795	-0.3569	-0.35584	-0.39705	-0.39591	-0.3948	-0.3938	-0.39288	-0.39211
-0.35854	-0.35749	-0.35644	-0.39769	-0.39655	-0.39542	-0.39436	-0.39336	-0.39251
-0.35913	-0.35808	-0.35703	-0.39832	-0.39718	-0.39606	-0.39495	-0.39388	-0.39296
-0.35971	-0.35867	-0.35762	-0.39894	-0.39782	-0.3967	-0.39557	-0.39445	-0.39347
-0.36029	-0.35925	-0.35821	-0.35717	-0.3561	-0.35504	-0.39619	-0.39507	-0.39403
-0.36086	-0.35982	-0.35881	-0.35778	-0.35671	-0.35563	-0.39682	-0.39571	-0.39464
-0.36147	-0.36042	-0.35941	-0.35838	-0.35732	-0.35626	-0.3552	-0.35415	-0.39527
-0.36212	-0.36105	-0.36002	-0.35899	-0.35795	-0.35691	-0.35585	-0.35478	-0.39591

-0.31389	-0.36167	-0.36062	-0.35958	-0.35856	-0.35754	-0.35648	-0.3554	-0.35433
-0.31439	-0.36227	-0.3612	-0.36016	-0.35914	-0.35813	-0.35708	-0.35601	-0.35494
-0.31489	-0.36288	-0.3618	-0.36075	-0.35972	-0.35871	-0.35767	-0.35661	-0.35555
-0.31541	-0.3635	-0.36241	-0.36134	-0.3603	-0.35928	-0.35825	-0.35723	-0.35618
-0.31594	-0.36415	-0.36304	-0.36196	-0.36089	-0.35985	-0.35883	-0.35784	-0.35681
-0.31647	-0.31556	-0.31467	-0.36259	-0.36151	-0.36045	-0.35943	-0.35844	-0.35741
-0.31702	-0.31611	-0.3152	-0.36324	-0.36214	-0.36108	-0.36005	-0.35903	-0.35799
-0.31757	-0.31666	-0.31574	-0.31483	-0.31395	-0.3131	-0.36066	-0.35963	-0.35859
-0.31812	-0.3172	-0.31627	-0.31536	-0.31446	-0.31359	-0.36127	-0.36024	-0.3592
-0.31866	-0.31774	-0.31681	-0.31589	-0.31499	-0.3141	-0.3619	-0.36085	-0.35981
-0.31918	-0.31827	-0.31735	-0.31643	-0.31553	-0.31463	-0.36253	-0.36147	-0.36041
-0.3197	-0.3188	-0.31789	-0.31698	-0.31607	-0.31517	-0.36318	-0.36209	-0.36102
-0.32023	-0.31933	-0.31843	-0.31753	-0.31661	-0.31569	-0.3148	-0.31391	-0.36164
-0.32076	-0.31987	-0.31898	-0.31807	-0.31715	-0.31622	-0.31531	-0.31441	-0.36227
-0.28154	-0.3204	-0.31952	-0.31862	-0.3177	-0.31676	-0.31585	-0.31494	-0.31408
-0.28201	-0.32092	-0.32006	-0.31917	-0.31825	-0.31732	-0.3164	-0.31549	-0.31462
-0.28248	-0.32145	-0.32058	-0.3197	-0.31879	-0.31787	-0.31696	-0.31605	-0.31516
-0.28296	-0.32197	-0.32109	-0.3202	-0.3193	-0.3184	-0.31751	-0.31661	-0.31572
-0.28344	-0.28264	-0.28183	-0.32071	-0.31982	-0.31894	-0.31805	-0.31717	-0.31627
-0.28394	-0.28312	-0.28231	-0.32124	-0.32035	-0.31948	-0.3186	-0.31771	-0.31681
-0.28444	-0.28361	-0.28279	-0.32177	-0.32089	-0.32002	-0.31914	-0.31826	-0.31736
-0.28494	-0.28409	-0.28327	-0.28246	-0.28166	-0.28087	-0.31968	-0.31881	-0.31791
-0.28544	-0.28458	-0.28375	-0.28294	-0.28215	-0.28136	-0.32023	-0.31937	-0.31847
-0.28595	-0.28509	-0.28425	-0.28343	-0.28263	-0.28185	-0.28106	-0.28027	-0.319
-0.28648	-0.28561	-0.28475	-0.28392	-0.28312	-0.28234	-0.28153	-0.28073	-0.3195
-0.28701	-0.28613	-0.28526	-0.28442	-0.28362	-0.28284	-0.28202	-0.2812	-0.28038
-0.28752	-0.28665	-0.28579	-0.28495	-0.28414	-0.28335	-0.28252	-0.28169	-0.28087
-0.28805	-0.28717	-0.28631	-0.28548	-0.28467	-0.28386	-0.28303	-0.28219	-0.28137
-0.28858	-0.2877	-0.28685	-0.28601	-0.28519	-0.28437	-0.28354	-0.2827	-0.28187
-0.28913	-0.28824	-0.28738	-0.28654	-0.28571	-0.28488	-0.28405	-0.28321	-0.28238
-0.28969	-0.28879	-0.28792	-0.28707	-0.28623	-0.2854	-0.28456	-0.28371	-0.28287
-0.29026	-0.28935	-0.28847	-0.2876	-0.28676	-0.28593	-0.28507	-0.2842	-0.28337
-0.20161	-0.28992	-0.28902	-0.28815	-0.2873	-0.28646	-0.28559	-0.28471	-0.28387
-0.20202	-0.29048	-0.28958	-0.2887	-0.28784	-0.28699	-0.28611	-0.28523	-0.28439
-0.14924	-0.29104	-0.29014	-0.28926	-0.28838	-0.28752	-0.28664	-0.28576	-0.2849
-0.14951	-0.2916	-0.29071	-0.28982	-0.28893	-0.28805	-0.28716	-0.28628	-0.28543
-0.1498	-0.29216	-0.29128	-0.29039	-0.28949	-0.28859	-0.2877	-0.28681	-0.28595
-0.15009	-0.1496	-0.14912	-0.29095	-0.29005	-0.28915	-0.28824	-0.28733	-0.28646
-0.15039	-0.14989	-0.1494	-0.2915	-0.29062	-0.28972	-0.2888	-0.28787	-0.28698
-0.09748	-0.15018	-0.14969	-0.20244	-0.20177	-0.20111	-0.28937	-0.28844	-0.28754
-0.09765	-0.15048	-0.14999	-0.20285	-0.20218	-0.20151	-0.28994	-0.28903	-0.28813
-0.09782	-0.15078	-0.15028	-0.14979	-0.1493	-0.14882	-0.20125	-0.20059	-0.28871
-0.09801	-0.15107	-0.15057	-0.15008	-0.14959	-0.1491	-0.20166	-0.201	-0.28928
-0.09819	-0.09787	-0.09756	-0.15037	-0.14988	-0.14939	-0.14891	-0.14844	-0.28985
-0.09838	-0.09805	-0.09774	-0.15068	-0.15018	-0.14969	-0.1492	-0.14873	-0.29043
-0.09858	-0.09824	-0.09793	-0.15099	-0.15048	-0.14998	-0.1495	-0.14902	-0.291
-0.09878	-0.09844	-0.09812	-0.09781	-0.0975	-0.09719	-0.1498	-0.14932	-0.20198
-0.099	-0.09865	-0.09831	-0.098	-0.09769	-0.09738	-0.15011	-0.14962	-0.2024
-0.04625	-0.09886	-0.09852	-0.09819	-0.09788	-0.09757	-0.15042	-0.14992	-0.14944
-0.04634	-0.09909	-0.09874	-0.09839	-0.09807	-0.09776	-0.15072	-0.15023	-0.14974

-0.04642	-0.04626	-0.04608	-0.09861	-0.09828	-0.09796	-0.09765	-0.09734	-0.15005
-0.0465	-0.04633	-0.04616	-0.09885	-0.09851	-0.09818	-0.09785	-0.09754	-0.15037
-0.04657	-0.04641	-0.04624	-0.09908	-0.09873	-0.09839	-0.09806	-0.09774	-0.15069
-0.04665	-0.04649	-0.04632	-0.09929	-0.09894	-0.0986	-0.09827	-0.09795	-0.15103
-0.04673	-0.04657	-0.0464	-0.0995	-0.09915	-0.09881	-0.09849	-0.09817	-0.15137
	-0.04664	-0.04648	-0.04631	-0.04615	-0.04599	-0.0987	-0.09837	-0.09805
	-0.04672	-0.04655	-0.04639	-0.04623	-0.04607	-0.09891	-0.09856	-0.09824
	-0.0468	-0.04664	-0.04647	-0.04631	-0.04615	-0.04598	-0.04581	-0.09844
	-0.0469	-0.04672	-0.04655	-0.04638	-0.04622	-0.04606	-0.04589	-0.09865
			-0.04663	-0.04646	-0.0463	-0.04614	-0.04598	-0.04581
			-0.04672	-0.04656	-0.04639	-0.04623	-0.04606	-0.0459
			-0.04681	-0.04665	-0.04648	-0.04632	-0.04615	-0.04598
			-0.04689	-0.04672	-0.04656	-0.04639	-0.04623	-0.04606
			-0.04696	-0.0468	-0.04663	-0.04647	-0.04631	-0.04614
						-0.04654	-0.04638	-0.04621
						-0.04661	-0.04644	-0.04627
								-0.04635
								-0.04644

								-0.10805
								-0.1078
						-0.10816	-0.10786	-0.255
						-0.10792	-0.10763	-0.25435
						-0.10768	-0.1074	-0.25372
				-0.10804	-0.10774	-0.25499	-0.25402	-0.34205
				-0.10782	-0.10752	-0.25434	-0.25335	-0.34123
				-0.20376	-0.20291	-0.34283	-0.34165	-0.4287
				-0.20316	-0.20233	-0.34203	-0.34088	-0.42778
	-0.20524	-0.20433	-0.20344	-0.25434	-0.25334	-0.38366	-0.38239	-0.47306
	-0.20462	-0.20376	-0.20293	-0.25373	-0.25274	-0.38283	-0.38154	-0.47204
-0.10785	-0.25612	-0.25512	-0.25414	-0.38467	-0.38334	-0.4278	-0.42638	-0.51099
-0.1076	-0.25543	-0.25447	-0.25353	-0.38384	-0.38251	-0.42691	-0.4255	-0.50998
-0.10735	-0.25478	-0.25384	-0.25292	-0.383	-0.38167	-0.42601	-0.42463	-0.50898
-0.25514	-0.34342	-0.34227	-0.34112	-0.38214	-0.38083	-0.5111	-0.50952	-0.59979
-0.25454	-0.3427	-0.34152	-0.34035	-0.38127	-0.37998	-0.51004	-0.50845	-0.5986
-0.25394	-0.38438	-0.38305	-0.38174	-0.46695	-0.46546	-0.55662	-0.55493	-0.59746
-0.25335	-0.38356	-0.38225	-0.38094	-0.46601	-0.46452	-0.55553	-0.55385	-0.59636
-0.38404	-0.42471	-0.42329	-0.42188	-0.5578	-0.55611	-0.59873	-0.59698	-0.59525
-0.38316	-0.42377	-0.42237	-0.42097	-0.55666	-0.55496	-0.59756	-0.59583	-0.59413
-0.38229	-0.42286	-0.42146	-0.42007	-0.55554	-0.55385	-0.59641	-0.59471	-0.59303
-0.42341	-0.51528	-0.51367	-0.51208	-0.59883	-0.59707	-0.59535	-0.59367	-0.68206
-0.42257	-0.51428	-0.51268	-0.5111	-0.59775	-0.59602	-0.59432	-0.59266	-0.68088
-0.51489	-0.60199	-0.60019	-0.59841	-0.59667	-0.59497	-0.68352	-0.68161	-0.79578
-0.5139	-0.60089	-0.59908	-0.5973	-0.59559	-0.59392	-0.68231	-0.6804	-0.79442
-0.55702	-0.59978	-0.59797	-0.5962	-0.68489	-0.68298	-0.75809	-0.756	-0.93958
-0.55594	-0.59867	-0.59687	-0.5951	-0.68361	-0.68169	-0.75666	-0.75457	-0.93753
-0.59932	-0.59755	-0.59577	-0.59403	-0.68236	-0.68044	-0.9051	-0.90214	-1.04227
-0.59815	-0.59641	-0.5947	-0.593	-0.68116	-0.67924	-0.90324	-0.90035	-1.04025
-0.597	-0.5953	-0.59364	-0.59199	-0.67999	-0.67807	-0.90144	-0.89862	-1.0383
-0.59593	-0.68451	-0.68262	-0.68073	-0.75555	-0.75347	-1.04276	-1.03954	-1.03634

-0.59491	-0.68335	-0.68145	-0.67955	-0.75427	-0.75219	-1.04074	-1.03756	-1.03438
-0.64199	-0.7592	-0.75713	-0.75505	-1.0107	-1.00748	-1.03872	-1.03554	-1.16292
-0.64091	-0.75792	-0.75585	-0.75378	-1.00865	-1.00547	-1.03668	-1.03351	-1.16072
-0.75872	-0.90692	-0.90397	-0.90106	-1.0411	-1.03783	-1.13497	-1.13161	-1.15847
-0.75744	-0.90508	-0.90217	-0.89927	-1.03902	-1.03572	-1.1327	-1.12931	-1.15615
-0.75615	-1.01231	-1.00904	-1.00579	-1.07116	-1.06777	-1.16065	-1.15723	-1.21421
-0.75487	-1.01023	-1.00695	-1.0037	-1.06903	-1.06568	-1.15849	-1.15515	-1.21204
-0.75356	-1.00814	-1.00485	-1.00161	-1.06691	-1.06363	-1.15638	-1.15313	-1.20992
-1.00934	-1.07489	-1.07149	-1.06814	-1.13083	-1.1275	-1.15431	-1.15111	-1.23483
-1.00721	-1.07272	-1.06936	-1.06606	-1.12872	-1.12546	-1.15227	-1.1491	-1.23266
-1.03995	-1.13673	-1.13328	-1.12991	-1.1567	-1.15345	-1.21026	-1.20685	-1.23049
-1.03784	-1.13454	-1.13112	-1.12779	-1.1546	-1.15142	-1.20809	-1.20469	-1.22832
-1.10298	-1.13239	-1.12902	-1.12573	-1.15255	-1.14941	-1.23301	-1.22957	-1.22617
-1.10085	-1.13025	-1.12697	-1.12376	-1.15055	-1.14742	-1.23084	-1.22741	-1.22403
-1.13149	-1.12816	-1.12495	-1.12181	-1.17663	-1.17344	-1.22868	-1.22527	-1.30231
-1.1294	-1.12612	-1.12296	-1.11985	-1.17461	-1.17143	-1.22653	-1.22319	-1.30004
-1.12735	-1.12412	-1.12098	-1.11789	-1.17259	-1.16941	-1.2244	-1.22112	-1.2978
-1.12532	-1.18016	-1.17694	-1.17376	-1.17058	-1.16741	-1.30268	-1.29914	-1.29555
-1.12332	-1.17813	-1.17492	-1.17175	-1.16858	-1.16543	-1.3004	-1.29686	-1.29331
-1.14993	-1.17611	-1.17291	-1.16974	-1.2499	-1.24646	-1.29813	-1.29457	-1.36495
-1.14796	-1.17411	-1.1709	-1.16773	-1.24771	-1.24431	-1.29588	-1.29226	-1.3624
-1.146	-1.25594	-1.25242	-1.24894	-1.30076	-1.29725	-1.31361	-1.30991	-1.35991
-1.14402	-1.25372	-1.25024	-1.24679	-1.29852	-1.29502	-1.31136	-1.30768	-1.35751
-1.14206	-1.2515	-1.24806	-1.24464	-1.2963	-1.2928	-1.30913	-1.30549	-1.35517
-1.19412	-1.2701	-1.26664	-1.26321	-1.31402	-1.3105	-1.36073	-1.35674	-1.38274
-1.19204	-1.26789	-1.26443	-1.26102	-1.31177	-1.30829	-1.35837	-1.35442	-1.38038
-1.26928	-1.26571	-1.26224	-1.25883	-1.36373	-1.3599	-1.38597	-1.38195	-1.37801
-1.26714	-1.26355	-1.26007	-1.25666	-1.36134	-1.35755	-1.38357	-1.37957	-1.37564
-1.26498	-1.26138	-1.25791	-1.25451	-1.35896	-1.35521	-1.38119	-1.37721	-1.45218
-1.26277	-1.25919	-1.25574	-1.25237	-1.3566	-1.35289	-1.37883	-1.37487	-1.44975
-1.26059	-1.31334	-1.30952	-1.30582	-1.38413	-1.38035	-1.43018	-1.42614	-1.44738
-1.25844	-1.31104	-1.30726	-1.30358	-1.38176	-1.37799	-1.42781	-1.42384	-1.4451
-1.25632	-1.30879	-1.30504	-1.30138	-1.37941	-1.37565	-1.42545	-1.42157	-1.44284
-1.31043	-1.33747	-1.33362	-1.32985	-1.43079	-1.42692	-1.44831	-1.44444	-1.44052
-1.30824	-1.3353	-1.33144	-1.32765	-1.4284	-1.42453	-1.4459	-1.44208	-1.43819
-1.30601	-1.43817	-1.43406	-1.42999	-1.45129	-1.44737	-1.44352	-1.43972	-1.43586
-1.30375	-1.43575	-1.43169	-1.42765	-1.44893	-1.44499	-1.44114	-1.43736	-1.43353
-1.38778	-1.43336	-1.42935	-1.42536	-1.4466	-1.44264	-1.43878	-1.435	-1.45755
-1.38547	-1.43099	-1.42705	-1.42312	-1.44432	-1.44033	-1.43644	-1.43263	-1.45525
-1.3832	-1.45389	-1.44997	-1.44604	-1.44204	-1.43803	-1.43413	-1.43032	-1.48067
-1.38097	-1.4515	-1.4476	-1.44371	-1.43972	-1.43573	-1.43185	-1.42808	-1.47847
-1.37876	-1.44912	-1.44523	-1.44136	-1.4374	-1.43346	-1.42965	-1.42596	-1.47641
-1.40253	-1.44678	-1.44289	-1.43903	-1.43511	-1.43125	-1.48149	-1.47784	-1.49913
-1.40028	-1.44448	-1.44056	-1.4367	-1.43285	-1.42911	-1.47947	-1.47609	-1.49754
-1.44618	-1.44222	-1.43825	-1.43438	-1.45875	-1.4551	-1.47753	-1.4744	-1.49599
-1.44392	-1.44001	-1.43598	-1.43207	-1.45652	-1.45307	-1.47567	-1.47278	-1.4945
-1.44163	-1.46602	-1.46189	-1.45794	-1.48024	-1.47693	-1.47394	-1.47126	-1.51633
-1.43928	-1.46364	-1.45957	-1.45572	-1.47819	-1.47515	-1.47238	-1.46989	-1.51499
-1.43694	-1.46129	-1.45734	-1.45367	-1.47631	-1.47351	-1.47093	-1.46857	-1.51368
-1.46283	-1.45899	-1.45531	-1.45191	-1.47468	-1.47201	-1.51268	-1.51031	-1.55765

-1.46053	-1.45678	-1.45341	-1.45034	-1.47323	-1.47063	-1.51129	-1.50895	-1.5562
-1.45833	-1.48059	-1.47739	-1.47448	-1.51501	-1.51244	-1.53361	-1.5312	-1.55473
-1.45622	-1.47865	-1.47565	-1.47292	-1.51357	-1.51114	-1.53231	-1.52988	-1.55327
-1.45425	-1.52025	-1.51731	-1.51462	-1.51214	-1.5098	-1.55703	-1.55441	-1.63843
-1.45242	-1.51855	-1.51576	-1.51316	-1.51072	-1.5084	-1.55554	-1.55299	-1.63686
-1.51982	-1.51695	-1.51428	-1.51176	-1.53587	-1.53338	-1.61347	-1.61085	-1.63526
-1.51815	-1.51542	-1.51288	-1.51045	-1.53446	-1.53193	-1.61188	-1.60927	-1.63359
-1.51659	-1.51396	-1.51152	-1.50916	-1.53308	-1.5305	-1.61031	-1.60769	-1.63192
-1.51513	-1.51259	-1.51018	-1.50783	-1.61424	-1.6115	-1.63588	-1.63305	-1.6734
-1.51375	-1.51128	-1.50887	-1.5065	-1.61271	-1.61003	-1.63431	-1.63143	-1.67172
-1.5565	-1.59709	-1.59437	-1.59168	-1.61119	-1.60853	-1.6759	-1.67295	-1.67015
-1.55507	-1.59555	-1.59288	-1.59023	-1.60969	-1.607	-1.67422	-1.6714	-1.66866
-1.57123	-1.59404	-1.59139	-1.58875	-1.6784	-1.67542	-1.67258	-1.66985	-1.66717
-1.56986	-1.59258	-1.5899	-1.58724	-1.6767	-1.67377	-1.67097	-1.66829	-1.66566
-1.59377	-1.63577	-1.63297	-1.6302	-1.67503	-1.67215	-1.66939	-1.66674	-1.68664
-1.59226	-1.63416	-1.6314	-1.62867	-1.67342	-1.67059	-1.66786	-1.6652	-1.68496
-1.59074	-1.63256	-1.62984	-1.62717	-1.67183	-1.66906	-1.66634	-1.66368	-1.6833
-1.63377	-1.67884	-1.6759	-1.67303	-1.67023	-1.6675	-1.66483	-1.66221	-1.68171
-1.63223	-1.67721	-1.67427	-1.6714	-1.66863	-1.66595	-1.66333	-1.66077	-1.68018
-1.63068	-1.67554	-1.67267	-1.66986	-1.66713	-1.66447	-1.68416	-1.68138	-1.68965
-1.62912	-1.67384	-1.67109	-1.66839	-1.66572	-1.66307	-1.68262	-1.6798	-1.68808
-1.65397	-1.67222	-1.66954	-1.66692	-1.66428	-1.66167	-1.6921	-1.68929	-1.68656
-1.65242	-1.6707	-1.66802	-1.66539	-1.6628	-1.66024	-1.6906	-1.68784	-1.68512
-1.65088	-1.66921	-1.66651	-1.66388	-1.66132	-1.6588	-1.68909	-1.68639	-1.68369
-1.67036	-1.67884	-1.67621	-1.67362	-1.69297	-1.69024	-1.68755	-1.6849	-1.70076
-1.6688	-1.6773	-1.67472	-1.67217	-1.69141	-1.68868	-1.68601	-1.6834	-1.69929
-1.6785	-1.67583	-1.67324	-1.6707	-1.68984	-1.68716	-1.70304	-1.70042	-1.69779
-1.67708	-1.67442	-1.67178	-1.6692	-1.68828	-1.68568	-1.70156	-1.6989	-1.69627
-1.67564	-1.6951	-1.69223	-1.68943	-1.70529	-1.70269	-1.70006	-1.69741	-1.6948
-1.67416	-1.69356	-1.69076	-1.688	-1.70382	-1.70116	-1.69853	-1.69594	-1.69338
-1.71331	-1.71055	-1.70782	-1.7051	-1.70237	-1.69966	-1.69704	-1.69449	-1.69198
-1.71171	-1.70895	-1.70628	-1.70363	-1.70093	-1.69823	-1.69562	-1.69306	-1.69057
-1.71013	-1.70737	-1.70475	-1.70215	-1.69949	-1.69683	-1.69422	-1.69167	-1.68918
-1.70864	-1.7059	-1.70324	-1.70063	-1.69801	-1.69541	-1.70893	-1.70637	-1.70386
-1.7072	-1.70448	-1.70176	-1.69909	-1.69651	-1.69398	-1.70756	-1.70505	-1.70254
-1.70571	-1.70303	-1.70031	-1.69763	-1.7112	-1.70866	-1.70616	-1.70369	-1.74698
-1.70419	-1.70157	-1.69888	-1.69623	-1.70978	-1.70723	-1.70474	-1.70232	-1.74548
-1.70268	-1.7163	-1.71363	-1.71098	-1.70839	-1.70584	-1.70336	-1.70094	-1.74395
-1.70116	-1.71476	-1.71216	-1.70958	-1.70703	-1.70451	-1.70201	-1.69955	-1.74234
-1.71592	-1.71325	-1.71069	-1.70817	-1.70567	-1.70319	-1.7464	-1.74353	-1.74079
-1.71449	-1.71182	-1.70925	-1.70673	-1.70427	-1.70184	-1.74492	-1.74208	-1.73933
-1.71309	-1.71042	-1.70783	-1.7053	-1.70287	-1.70048	-1.74345	-1.74067	-1.73793
-1.7117	-1.70906	-1.70647	-1.70395	-1.72206	-1.71951	-1.74198	-1.73925	-1.73654
-1.71034	-1.70773	-1.70515	-1.70263	-1.72065	-1.71809	-1.7405	-1.73783	-1.73515
-1.70897	-1.70639	-1.70383	-1.70131	-1.74441	-1.74168	-1.739	-1.73639	-1.73375
-1.70761	-1.70506	-1.70252	-1.7	-1.74291	-1.74015	-1.7375	-1.73493	-1.73233
-1.70624	-1.72448	-1.72176	-1.71906	-1.74143	-1.73867	-1.73602	-1.73346	-1.74263
-1.70486	-1.72305	-1.72035	-1.71767	-1.73998	-1.73726	-1.73458	-1.73195	-1.74107
-1.70349	-1.72162	-1.71895	-1.71629	-1.73856	-1.73586	-1.73316	-1.73048	-1.73953
-1.70213	-1.74548	-1.74267	-1.73989	-1.73716	-1.73446	-1.73176	-1.72908	-1.73807

-1.70077	-1.74396	-1.74121	-1.73848	-1.73576	-1.73306	-1.73038	-1.72772	-1.73665
-1.71988	-1.7424	-1.73972	-1.73705	-1.73436	-1.73167	-1.74072	-1.73794	-1.73526
-1.7184	-1.74082	-1.73821	-1.73561	-1.73294	-1.73027	-1.7393	-1.73657	-1.73389
-1.74206	-1.73932	-1.73674	-1.73418	-1.7484	-1.74573	-1.75453	-1.75182	-1.74912
-1.7406	-1.73789	-1.73532	-1.73277	-1.747	-1.74437	-1.75314	-1.75044	-1.74775
-1.75606	-1.75336	-1.75077	-1.74821	-1.7572	-1.75446	-1.75175	-1.74906	-1.74638
-1.75462	-1.75194	-1.74935	-1.7468	-1.75577	-1.75307	-1.75038	-1.7477	-1.74502
-1.7532	-1.75052	-1.74794	-1.74541	-1.75435	-1.75169	-1.74902	-1.74633	-1.74366
-1.75177	-1.74911	-1.74656	-1.74405	-1.75295	-1.75032	-1.74765	-1.74496	-1.7423
-1.75035	-1.74771	-1.74519	-1.74272	-1.75157	-1.74894	-1.74628	-1.74359	-1.74094
-1.74893	-1.75799	-1.75538	-1.75282	-1.7502	-1.74757	-1.74491	-1.74223	-1.74574
-1.74751	-1.75652	-1.75396	-1.75144	-1.74884	-1.74621	-1.74355	-1.74087	-1.74439
-1.74613	-1.7551	-1.75257	-1.75006	-1.74747	-1.74484	-1.74836	-1.74568	-1.78862
-1.7448	-1.75374	-1.75121	-1.74869	-1.7461	-1.74347	-1.74699	-1.74434	-1.78713
-1.75506	-1.75241	-1.74987	-1.74734	-1.75091	-1.74827	-1.74563	-1.74299	-1.78563
-1.75374	-1.75114	-1.74858	-1.74601	-1.74956	-1.74691	-1.74428	-1.74165	-1.78414
-1.75244	-1.74988	-1.74729	-1.74469	-1.74822	-1.74556	-1.74293	-1.74031	-1.78264
-1.75733	-1.75475	-1.75214	-1.74951	-1.74687	-1.74422	-1.78703	-1.78407	-1.78116
-1.75603	-1.75343	-1.7508	-1.74816	-1.74552	-1.74288	-1.78553	-1.78258	-1.77969
-1.75471	-1.7521	-1.74945	-1.7468	-1.74416	-1.74153	-1.78403	-1.78109	-1.79429
-1.75338	-1.75075	-1.74809	-1.74544	-1.7428	-1.74018	-1.78253	-1.77961	-1.79283
-1.75202	-1.74939	-1.74673	-1.74407	-1.80302	-1.80005	-1.79711	-1.79421	-1.80598
-1.75065	-1.74801	-1.74536	-1.74271	-1.80149	-1.79852	-1.79562	-1.79278	-1.80457
-1.74928	-1.74663	-1.74399	-1.74135	-1.79996	-1.797	-1.79414	-1.79136	-1.80317
-1.7479	-1.76191	-1.75924	-1.75659	-1.79843	-1.79551	-1.8208	-1.81805	-1.81533
-1.74652	-1.76052	-1.75786	-1.75521	-1.79693	-1.79404	-1.81936	-1.81665	-1.81395
-1.76181	-1.81895	-1.81593	-1.81295	-1.82356	-1.82071	-1.81795	-1.81526	-1.81259
-1.76046	-1.81742	-1.8144	-1.81144	-1.82209	-1.81931	-1.81657	-1.81389	-1.81124
-1.81895	-1.82945	-1.82646	-1.82352	-1.82071	-1.81797	-1.81525	-1.81258	-1.80994
-1.81754	-1.82806	-1.82509	-1.82219	-1.81941	-1.8167	-1.81401	-1.81133	-1.80868
-1.82961	-1.82664	-1.82371	-1.82085	-1.8181	-1.81541	-1.81273	-1.81006	-1.82457
-1.8281	-1.82515	-1.82228	-1.81948	-1.81675	-1.81407	-1.81138	-1.80872	-1.8232
-1.82658	-1.82365	-1.82085	-1.8181	-1.81538	-1.8127	-1.81002	-1.80735	-1.82182
-1.82508	-1.82219	-1.81941	-1.81669	-1.81399	-1.81131	-1.80864	-1.80598	-1.86191
-1.82361	-1.82075	-1.818	-1.81529	-1.8126	-1.80993	-1.80726	-1.80461	-1.86034
-1.82224	-1.81941	-1.81664	-1.81392	-1.81123	-1.80856	-1.84316	-1.84022	-1.85879
-1.82095	-1.81815	-1.81534	-1.81258	-1.80987	-1.80721	-1.84171	-1.83879	-1.85727
-1.81962	-1.81684	-1.81404	-1.81127	-1.82578	-1.82306	-1.862	-1.85891	-1.85581
-1.81823	-1.81549	-1.81274	-1.81002	-1.82452	-1.8218	-1.8606	-1.85752	-1.85444
-1.81684	-1.81413	-1.81143	-1.80875	-1.84355	-1.84062	-1.85919	-1.85611	-1.85305
-1.81545	-1.81276	-1.81008	-1.80742	-1.84215	-1.83924	-1.85772	-1.85462	-1.85157
-1.81409	-1.8114	-1.80874	-1.80609	-1.84076	-1.83787	-1.85625	-1.85314	-1.85009
-1.8128	-1.8482	-1.84525	-1.84234	-1.86103	-1.85796	-1.85488	-1.85178	-1.84872
-1.81155	-1.84686	-1.84394	-1.84104	-1.85965	-1.8566	-1.85353	-1.85047	-1.84741
-1.84844	-1.84548	-1.84257	-1.83969	-1.8582	-1.85515	-1.85211	-1.84908	-1.84605
-1.847	-1.84405	-1.84116	-1.83829	-1.85669	-1.85362	-1.85061	-1.84763	-1.84465
-1.8456	-1.86443	-1.86136	-1.85829	-1.8552	-1.85213	-1.84912	-1.84617	-1.85884
-1.84428	-1.86304	-1.85996	-1.85689	-1.85378	-1.85067	-1.84765	-1.84469	-1.85729
-1.84296	-1.86164	-1.85856	-1.85548	-1.85236	-1.84925	-1.86199	-1.85886	-1.85578
-1.84156	-1.86015	-1.85708	-1.85401	-1.85093	-1.84787	-1.86057	-1.85745	-1.85438

-1.84016	-1.85866	-1.85559	-1.85253	-1.8495	-1.8465	-1.85917	-1.85607	-1.85302
-1.86031	-1.85725	-1.85418	-1.85113	-1.84809	-1.84509	-1.88789	-1.88477	-1.91574
-1.85894	-1.85588	-1.85282	-1.84976	-1.8467	-1.84366	-1.88633	-1.88323	-1.91408
-1.85752	-1.85446	-1.85141	-1.84838	-1.89127	-1.88802	-1.88485	-1.88176	-1.9125
-1.85604	-1.85299	-1.84997	-1.84696	-1.8898	-1.8866	-1.88347	-1.88038	-1.91099
-1.88529	-1.8822	-1.87916	-1.87614	-1.88835	-1.8852	-1.91613	-1.91278	-1.90947
-1.88389	-1.8808	-1.87777	-1.87476	-1.88691	-1.88378	-1.91458	-1.91122	-1.90791
-1.88249	-1.87941	-1.87638	-1.87337	-1.88547	-1.88236	-1.91303	-1.90967	-1.90636
-1.88104	-1.89343	-1.89023	-1.88708	-1.88397	-1.88089	-1.91147	-1.90815	-1.90486
-1.87957	-1.89187	-1.88868	-1.88554	-1.88246	-1.87942	-1.90992	-1.90664	-1.9034
-1.87814	-1.89039	-1.8872	-1.88408	-1.91496	-1.91168	-1.90841	-1.90517	-1.90193
-1.87673	-1.88895	-1.88579	-1.88268	-1.91345	-1.91019	-1.90694	-1.90371	-1.90046
-1.8907	-1.88754	-1.8844	-1.8813	-1.91193	-1.90866	-1.90542	-1.90221	-1.89897
-1.88929	-1.88614	-1.88302	-1.87993	-1.9104	-1.90709	-1.90384	-1.90064	-1.89744
-1.88789	-1.88476	-1.88164	-1.87855	-1.90887	-1.90554	-1.90229	-1.89911	-1.89593
-1.8865	-1.88336	-1.88024	-1.87713	-1.90736	-1.90406	-1.90083	-1.89764	-1.89447
-1.88509	-1.88196	-1.87882	-1.87571	-1.90586	-1.90261	-1.89939	-1.8962	-1.89303
-1.88362	-1.91438	-1.911	-1.90765	-1.90438	-1.90116	-1.89795	-1.89476	-1.89159
-1.88212	-1.91279	-1.90946	-1.90617	-1.90292	-1.89971	-1.89651	-1.89333	-1.89015
-1.88069	-1.91126	-1.90796	-1.9047	-1.90146	-1.89825	-1.89505	-1.89187	-1.88869
-1.87931	-1.90977	-1.90649	-1.90324	-1.90001	-1.8968	-1.89359	-1.89038	-1.88722
-1.87795	-1.90829	-1.90502	-1.90178	-1.89854	-1.89533	-1.89212	-1.88892	-1.88578
-1.87659	-1.90681	-1.90354	-1.90029	-1.89705	-1.89382	-1.89064	-1.8875	-1.8844
-1.90865	-1.90533	-1.90206	-1.8988	-1.89556	-1.89234	-1.88919	-1.8861	-1.88305
-1.90717	-1.90385	-1.90056	-1.89729	-1.89408	-1.8909	-1.88779	-1.88473	-1.8817
-1.90571	-1.90238	-1.89906	-1.89579	-1.89261	-1.8895	-1.88642	-1.88338	-1.88037
-1.90425	-1.90094	-1.89763	-1.89436	-1.89121	-1.88813	-1.88507	-1.88205	-1.90161
-1.9028	-1.89951	-1.89622	-1.89298	-1.88985	-1.88678	-1.88374	-1.88073	-1.90028
-1.90132	-1.89804	-1.89479	-1.89159	-1.88848	-1.88543	-1.90505	-1.90202	-1.91087
-1.8998	-1.89654	-1.89333	-1.89018	-1.88711	-1.88408	-1.90372	-1.90071	-1.90957
-1.89832	-1.91784	-1.91465	-1.91153	-1.90844	-1.9054	-1.91428	-1.91126	-1.90822
-1.8969	-1.91644	-1.91327	-1.91016	-1.90709	-1.90405	-1.9129	-1.90985	-1.90681
-1.89551	-1.91506	-1.91191	-1.90881	-1.90574	-1.9027	-1.91153	-1.90845	-1.90542
-1.91694	-1.91369	-1.91055	-1.90747	-1.9163	-1.91327	-1.91021	-1.90713	-1.90409
-1.91557	-1.91234	-1.90921	-1.90614	-1.91498	-1.91197	-1.90892	-1.90583	-1.90281
-1.9142	-1.92287	-1.91975	-1.91669	-1.91366	-1.91066	-1.90761	-1.90453	-1.90151
-1.91284	-1.92151	-1.91841	-1.91536	-1.91234	-1.90935	-1.90631	-1.90323	-1.90023
-1.91147	-1.92015	-1.91707	-1.91403	-1.91103	-1.90804	-1.90501	-1.90194	-1.89894
-1.91009	-1.91879	-1.91573	-1.91272	-1.90972	-1.90674	-1.90371	-1.90065	-1.89766
-1.92059	-1.91744	-1.9144	-1.91141	-1.90842	-1.90544	-1.90242	-1.89937	-1.90744
-1.91924	-1.9161	-1.91308	-1.91011	-1.90712	-1.90415	-1.90114	-1.89811	-1.90617
-1.91792	-1.91479	-1.91178	-1.90881	-1.90583	-1.90286	-1.89986	-1.89685	-1.90491
-1.91665	-1.91355	-1.91052	-1.90753	-1.90454	-1.90157	-1.90964	-1.90661	-1.90364
-1.91542	-1.91234	-1.90928	-1.90625	-1.90325	-1.90028	-1.90834	-1.90532	-1.90237
-1.91413	-1.91108	-1.90801	-1.90496	-1.90197	-1.899	-1.90705	-1.90406	-1.90112
-1.91281	-1.90978	-1.90671	-1.90367	-1.90068	-1.89773	-1.90578	-1.9028	-1.89989
-1.9115	-1.90849	-1.90542	-1.90238	-1.91046	-1.90749	-1.90453	-1.90158	-1.89868
-1.91022	-1.9072	-1.90413	-1.90109	-1.90917	-1.90623	-1.9033	-1.90039	-1.89751
-1.90894	-1.91704	-1.91394	-1.91088	-1.9079	-1.90497	-1.90207	-1.8992	-1.89633
-1.90767	-1.91576	-1.9127	-1.90966	-1.90667	-1.90371	-1.90081	-1.89795	-1.8951

-1.90639	-1.91448	-1.91147	-1.90846	-1.90545	-1.90246	-1.89955	-1.8967	-1.89385
-1.91621	-1.91319	-1.91019	-1.90721	-1.9042	-1.90121	-1.89831	-1.89547	-1.89263
-1.91493	-1.91188	-1.90889	-1.90593	-1.90295	-1.89998	-1.89708	-1.89425	-1.89142
-1.91364	-1.91058	-1.90761	-1.90466	-1.9017	-1.89876	-1.89588	-1.89305	-1.89022
-1.91235	-1.90929	-1.90632	-1.90339	-1.90047	-1.89758	-1.89471	-1.89186	-1.88904
-1.91109	-1.90804	-1.90506	-1.90213	-1.89924	-1.89638	-1.89352	-1.89067	-1.88785
-1.90987	-1.90683	-1.90384	-1.90088	-1.898	-1.89516	-1.89231	-1.88945	-1.88665
-1.90864	-1.90563	-1.90262	-1.89965	-1.89677	-1.89394	-1.89109	-1.88825	-1.88545
-1.90736	-1.90438	-1.90141	-1.89846	-1.89558	-1.89274	-1.88991	-1.8871	-1.88431
-1.90607	-1.90312	-1.90019	-1.89729	-1.8944	-1.89154	-1.88873	-1.88595	-1.88318
-1.90481	-1.90186	-1.89895	-1.89607	-1.89319	-1.89033	-1.88753	-1.88477	-1.882
-1.90356	-1.9006	-1.89771	-1.89484	-1.89197	-1.88912	-1.88632	-1.88356	-1.8808
-1.90235	-1.89939	-1.8965	-1.89364	-1.89079	-1.88795	-1.88515	-1.88238	-1.87963
-1.90116	-1.89823	-1.89533	-1.89247	-1.88964	-1.88684	-1.88404	-1.88125	-1.8785
-1.89998	-1.89707	-1.89418	-1.89132	-1.88851	-1.88572	-1.88292	-1.88013	-1.87739
-1.8988	-1.8959	-1.89303	-1.89019	-1.88737	-1.88456	-1.88178	-1.87902	-1.8763
-1.89763	-1.89473	-1.89188	-1.88906	-1.88622	-1.88339	-1.88062	-1.8779	-1.87519
-1.89647	-1.89358	-1.89073	-1.88789	-1.88504	-1.8822	-1.89215	-1.88945	-1.88674
-1.89533	-1.89245	-1.88957	-1.8867	-1.88384	-1.88102	-1.89095	-1.88825	-1.88556
-1.89418	-1.90401	-1.90111	-1.89822	-1.89537	-1.89256	-1.8898	-1.88709	-1.8844
-1.89302	-1.90282	-1.89991	-1.89703	-1.89421	-1.89144	-1.88868	-1.88596	-1.88327
-1.90456	-1.90166	-1.89874	-1.89586	-1.89306	-1.89031	-1.88757	-1.88484	-1.88215
-1.90341	-1.90053	-1.89762	-1.89473	-1.89193	-1.88917	-1.88644	-1.88375	-1.88107
-1.90225	-1.89939	-1.89648	-1.8936	-1.89079	-1.88803	-1.88532	-1.88265	-1.87998
-1.90107	-1.8982	-1.8953	-1.89244	-1.88966	-1.88692	-1.88421	-1.88154	-1.87887
-1.89989	-1.89701	-1.89413	-1.89129	-1.88853	-1.88581	-1.88311	-1.88042	-1.87776
-1.89873	-1.89587	-1.89301	-1.89018	-1.88742	-1.8847	-1.882	-1.87933	-1.87667
-1.89759	-1.89475	-1.89191	-1.8891	-1.88632	-1.88358	-1.88089	-1.87825	-1.8756
-1.89643	-1.89361	-1.89081	-1.88802	-1.88525	-1.8825	-1.87981	-1.87716	-1.87451
-1.89525	-1.89245	-1.8897	-1.88697	-1.88422	-1.88147	-1.87876	-1.87607	-1.8734
-1.86687	-1.8913	-1.88858	-1.88588	-1.88316	-1.88044	-1.87772	-1.875	-1.87231
-1.8658	-1.8902	-1.88746	-1.88475	-1.88206	-1.87939	-1.87669	-1.87396	-1.87126
-1.86476	-1.88912	-1.88636	-1.88363	-1.88096	-1.87833	-1.87564	-1.87293	-1.87023
-1.86373	-1.88806	-1.88531	-1.88258	-1.87989	-1.87723	-1.87456	-1.87188	-1.86919
-1.86272	-1.88701	-1.88427	-1.88155	-1.87883	-1.87613	-1.87346	-1.87082	-1.86815
-1.86167	-1.88591	-1.88321	-1.88051	-1.87779	-1.87507	-1.87239	-1.86973	-1.86706
-1.86061	-1.88478	-1.88212	-1.87947	-1.87676	-1.87404	-1.87133	-1.86863	-1.86595
-1.85957	-1.88369	-1.88104	-1.8784	-1.87571	-1.873	-1.87028	-1.86755	-1.86486
-1.85857	-1.88264	-1.87996	-1.87729	-1.87462	-1.87195	-1.86924	-1.86651	-1.86379
-1.85756	-1.8816	-1.87889	-1.8762	-1.87354	-1.87088	-1.86818	-1.86546	-1.86274
-1.85654	-1.88058	-1.87786	-1.87514	-1.87245	-1.86977	-1.86708	-1.86439	-1.86169
-1.85551	-1.87956	-1.87683	-1.87409	-1.87137	-1.86866	-1.86597	-1.86332	-1.86064
-1.85451	-1.87853	-1.87581	-1.87307	-1.87033	-1.86759	-1.86489	-1.86222	-1.85954
-1.8535	-1.8775	-1.87479	-1.87206	-1.86931	-1.86655	-1.86382	-1.86111	-1.85843
-1.85246	-1.84988	-1.84728	-1.84466	-1.86828	-1.86551	-1.86277	-1.86004	-1.85736
-1.85139	-1.84879	-1.84621	-1.84363	-1.86723	-1.86448	-1.86174	-1.859	-1.85632
-1.85035	-1.84772	-1.84516	-1.8426	-1.86618	-1.86344	-1.8607	-1.85797	-1.85528
-1.84933	-1.8467	-1.84414	-1.84157	-1.86513	-1.8624	-1.85967	-1.85694	-1.85424
-1.84832	-1.84568	-1.8431	-1.84053	-1.86407	-1.86135	-1.85863	-1.8559	-1.8532
-1.84731	-1.84465	-1.84203	-1.83943	-1.86296	-1.86029	-1.85759	-1.85488	-1.85217

-1.8463	-1.84362	-1.84096	-1.83833	-1.86186	-1.85923	-1.85656	-1.85385	-1.85116
-1.8453	-1.8426	-1.83992	-1.83728	-1.8608	-1.85818	-1.85552	-1.85283	-1.85015
-1.84429	-1.8416	-1.83892	-1.83627	-1.85976	-1.85715	-1.85449	-1.85182	-1.84914
-1.84328	-1.84059	-1.83791	-1.83526	-1.83271	-1.83019	-1.85347	-1.85081	-1.84813
-1.84226	-1.83958	-1.8369	-1.83427	-1.83171	-1.82919	-1.85247	-1.8498	-1.84713
-1.84125	-1.83856	-1.8359	-1.83327	-1.83072	-1.8282	-1.85146	-1.84879	-1.84613
-1.84023	-1.83755	-1.83489	-1.83226	-1.82972	-1.82721	-1.85045	-1.8478	-1.84513
-1.83922	-1.83654	-1.83388	-1.83126	-1.82872	-1.82621	-1.84945	-1.8468	-1.84414
-1.83823	-1.83555	-1.83289	-1.83027	-1.82773	-1.82521	-1.84843	-1.84579	-1.84314
-1.83724	-1.83456	-1.83191	-1.82928	-1.82674	-1.82422	-1.84742	-1.84479	-1.84214
-1.83625	-1.83358	-1.83092	-1.8283	-1.82575	-1.82324	-1.82069	-1.81812	-1.84114
-1.83526	-1.83259	-1.82993	-1.82731	-1.82478	-1.82228	-1.81973	-1.81715	-1.84014
-1.83427	-1.83161	-1.82895	-1.82634	-1.8238	-1.8213	-1.81875	-1.81617	-1.83914
-1.83328	-1.83062	-1.82798	-1.82537	-1.82283	-1.82032	-1.81777	-1.81519	-1.83814
-1.8323	-1.82965	-1.82703	-1.82443	-1.82188	-1.81936	-1.8168	-1.81421	-1.83714
-1.83132	-1.8287	-1.8261	-1.82353	-1.82097	-1.81841	-1.81583	-1.81324	-1.83613
-1.83037	-1.82776	-1.82519	-1.82263	-1.82006	-1.81747	-1.81487	-1.81226	-1.83513
-1.82945	-1.82686	-1.82428	-1.82171	-1.81911	-1.81651	-1.8139	-1.81129	-1.83413
-1.82855	-1.82597	-1.82337	-1.82076	-1.81815	-1.81554	-1.81293	-1.81031	-1.83313
-1.82763	-1.82504	-1.82243	-1.8198	-1.81718	-1.81456	-1.81194	-1.80932	-1.8067
-1.8267	-1.82409	-1.82146	-1.81884	-1.81621	-1.81358	-1.81095	-1.80834	-1.80572
-1.82575	-1.82312	-1.82049	-1.81786	-1.81523	-1.8126	-1.80998	-1.80736	-1.80475
-1.82478	-1.82214	-1.81951	-1.81689	-1.81426	-1.81164	-1.80901	-1.80639	-1.80379
-1.82381	-1.82117	-1.81854	-1.81592	-1.8133	-1.81069	-1.80807	-1.80544	-1.80285
-1.82286	-1.82021	-1.81758	-1.81496	-1.81237	-1.8098	-1.80719	-1.80457	-1.80197
-1.82191	-1.81927	-1.81663	-1.81402	-1.81145	-1.80891	-1.80632	-1.8037	-1.80108
-1.821	-1.81839	-1.81575	-1.81313	-1.81055	-1.80798	-1.80538	-1.80277	-1.80014
-1.82011	-1.81752	-1.8149	-1.81227	-1.80965	-1.80703	-1.80442	-1.8018	-1.79917
-1.81921	-1.81662	-1.81399	-1.81135	-1.80872	-1.80608	-1.80345	-1.80083	-1.7982
-1.8183	-1.81568	-1.81304	-1.8104	-1.80776	-1.80513	-1.80249	-1.79985	-1.79722
-1.81738	-1.81473	-1.81208	-1.80944	-1.80682	-1.80419	-1.80156	-1.79892	-1.79628
-1.81644	-1.81378	-1.81112	-1.80848	-1.80588	-1.80329	-1.80068	-1.79804	-1.79541
-1.81551	-1.81284	-1.81017	-1.80753	-1.80495	-1.80239	-1.79979	-1.79716	-1.79452
-1.81465	-1.81197	-1.8093	-1.80664	-1.80404	-1.80145	-1.79884	-1.79621	-1.79357
-1.81379	-1.81111	-1.80844	-1.80577	-1.80313	-1.8005	-1.79787	-1.79523	-1.7926
-1.81288	-1.8102	-1.80752	-1.80485	-1.80219	-1.79954	-1.7969	-1.79426	-1.81824
-1.81193	-1.80925	-1.80657	-1.8039	-1.80124	-1.79858	-1.79593	-1.79328	-1.81725
-1.81103	-1.80835	-1.80567	-1.803	-1.80033	-1.79767	-1.79501	-1.79235	-1.8163
-1.81017	-1.80748	-1.80481	-1.80214	-1.79947	-1.7968	-1.79414	-1.79148	-1.81539
-1.80929	-1.8066	-1.80392	-1.80125	-1.79858	-1.79591	-1.79325	-1.79058	-1.81446
-1.80835	-1.80566	-1.80298	-1.80031	-1.79764	-1.79497	-1.7923	-1.78963	-1.81348
-1.80741	-1.80472	-1.80204	-1.79937	-1.7967	-1.79403	-1.79135	-1.78868	-1.81251
-1.79802	-1.80384	-1.80116	-1.79848	-1.79581	-1.79313	-1.81705	-1.81432	-1.8116
-1.79715	-1.80298	-1.80029	-1.79761	-1.79493	-1.79225	-1.81615	-1.81343	-1.81071
-1.79622	-1.80206	-1.79937	-1.79669	-1.794	-1.79132	-1.81519	-1.81247	-1.80976
-1.79526	-1.80111	-1.79842	-1.79573	-1.79304	-1.79035	-1.8142	-1.81147	-1.80878
-1.76904	-1.79164	-1.78894	-1.78624	-1.79212	-1.78943	-1.81325	-1.81051	-1.80782
-1.7682	-1.79077	-1.78807	-1.78537	-1.79126	-1.78856	-1.81236	-1.80961	-1.8069
-1.76736	-1.78992	-1.78721	-1.7845	-1.81698	-1.81423	-1.81148	-1.80872	-1.80599
-1.76652	-1.78906	-1.78635	-1.78364	-1.81611	-1.81335	-1.8106	-1.80784	-1.8051

-1.76566	-1.78819	-1.78547	-1.78275	-1.81521	-1.81246	-1.8097	-1.80694	-1.8042
-1.76473	-1.78724	-1.78452	-1.78179	-1.80586	-1.80308	-1.80872	-1.80597	-1.80323
-1.76376	-1.78625	-1.78353	-1.7808	-1.80484	-1.80206	-1.80771	-1.80497	-1.80224
-1.76285	-1.78533	-1.7826	-1.77987	-1.80388	-1.8011	-1.80676	-1.80402	-1.8013
-1.762	-1.78446	-1.78172	-1.77898	-1.80298	-1.80019	-1.80586	-1.80313	-1.80041
-1.76116	-1.81047	-1.80768	-1.80488	-1.80209	-1.7993	-1.79654	-1.79381	-1.79954
-1.76034	-1.80961	-1.80682	-1.80402	-1.80121	-1.79842	-1.79566	-1.79294	-1.79868
-1.75951	-1.80875	-1.80595	-1.80315	-1.80034	-1.79755	-1.79479	-1.79206	-1.79781
-1.75868	-1.80788	-1.80507	-1.80227	-1.79947	-1.79668	-1.79392	-1.79119	-1.79694
-1.75782	-1.80698	-1.80417	-1.80137	-1.79858	-1.79579	-1.79304	-1.79031	-1.79606
-1.75693	-1.7816	-1.77883	-1.77609	-1.79761	-1.79484	-1.79208	-1.78935	-1.79512
-1.756	-1.78062	-1.77784	-1.77509	-1.7966	-1.79384	-1.79109	-1.78836	-1.79413
-1.75509	-1.77968	-1.7769	-1.77415	-1.79566	-1.7929	-1.79016	-1.78742	-1.78469
-1.75419	-1.7788	-1.77602	-1.77327	-1.79477	-1.79202	-1.78928	-1.78654	-1.78381
-1.78068	-1.77792	-1.77516	-1.77241	-1.7939	-1.79115	-1.78841	-1.78567	-1.78294
-1.77981	-1.77706	-1.7743	-1.77156	-1.79304	-1.7903	-1.78755	-1.78482	-1.78208
-1.77895	-1.7762	-1.77346	-1.77073	-1.79219	-1.78945	-1.7867	-1.78397	-1.78123
-1.77809	-1.77535	-1.77261	-1.76989	-1.79134	-1.78859	-1.78585	-1.78311	-1.78037
-1.77724	-1.7745	-1.77177	-1.76905	-1.79049	-1.78774	-1.78499	-1.78225	-1.77951
-1.77638	-1.77365	-1.77092	-1.76821	-1.78963	-1.78688	-1.78414	-1.78139	-1.77865
-1.77553	-1.77279	-1.77007	-1.76737	-1.78878	-1.78603	-1.78328	-1.78053	-1.77779
-1.77467	-1.77193	-1.76922	-1.76652	-1.76382	-1.76113	-1.78242	-1.77967	-1.77692
-1.77381	-1.77107	-1.76837	-1.76567	-1.76297	-1.76028	-1.78155	-1.7788	-1.77606
-1.77295	-1.77022	-1.76751	-1.76482	-1.76212	-1.75942	-1.78069	-1.77794	-1.77519
-1.77211	-1.76937	-1.76666	-1.76397	-1.76127	-1.75857	-1.77982	-1.77707	-1.77433
-1.77128	-1.76853	-1.76582	-1.76312	-1.76041	-1.75772	-1.77895	-1.77621	-1.77346
-1.77048	-1.76771	-1.76498	-1.76227	-1.75956	-1.75686	-1.77809	-1.77534	-1.77259
-1.76971	-1.7669	-1.76415	-1.76143	-1.75871	-1.75601	-1.77723	-1.77447	-1.77172
-1.76894	-1.76614	-1.76336	-1.7606	-1.75787	-1.75516	-1.77636	-1.77361	-1.77085
-1.76819	-1.76541	-1.7626	-1.7598	-1.75704	-1.75431	-1.77549	-1.77274	-1.76999
-1.7674	-1.76464	-1.76183	-1.75902	-1.75624	-1.75349	-1.75076	-1.74806	-1.76913
-1.76657	-1.76383	-1.76106	-1.75828	-1.75549	-1.75269	-1.74994	-1.74723	-1.76826
-1.76572	-1.763	-1.76028	-1.75753	-1.75473	-1.75191	-1.74915	-1.74642	-1.76743
-1.76487	-1.76216	-1.75945	-1.75672	-1.75395	-1.75117	-1.74842	-1.74569	-1.76667
-1.76402	-1.76131	-1.7586	-1.75588	-1.75316	-1.75042	-1.74769	-1.74497	-1.76592
-1.76318	-1.76046	-1.75775	-1.75504	-1.75233	-1.74961	-1.7469	-1.74418	-1.76512
-1.76234	-1.75961	-1.75689	-1.75418	-1.75147	-1.74876	-1.74606	-1.74335	-1.7643
-1.76154	-1.75878	-1.75606	-1.75334	-1.75063	-1.74792	-1.74521	-1.74251	-1.76347
-1.76076	-1.75798	-1.75523	-1.75251	-1.74978	-1.74706	-1.74435	-1.74166	-1.76262
-1.76	-1.7572	-1.75445	-1.75171	-1.74897	-1.74623	-1.74352	-1.74081	-1.76176
-1.75924	-1.75648	-1.75374	-1.75099	-1.74822	-1.74544	-1.7427	-1.73999	-1.76091
-1.75848	-1.75575	-1.75302	-1.75027	-1.74747	-1.74466	-1.74191	-1.73919	-1.76009
-1.75767	-1.75494	-1.75222	-1.74947	-1.7467	-1.74392	-1.74117	-1.73846	-1.73574
-1.75686	-1.75413	-1.75139	-1.74866	-1.74592	-1.74318	-1.74046	-1.73775	-1.73502
-1.75612	-1.75337	-1.75061	-1.74785	-1.74512	-1.74239	-1.73968	-1.73698	-1.73425
-1.75541	-1.75265	-1.74985	-1.74706	-1.74431	-1.74158	-1.73886	-1.73616	-1.73344
-1.75465	-1.75189	-1.74909	-1.74629	-1.74354	-1.74081	-1.73808	-1.73535	-1.73263
-1.75383	-1.75109	-1.74833	-1.74557	-1.74283	-1.7401	-1.73735	-1.73457	-1.73182
-1.75301	-1.75028	-1.74754	-1.74482	-1.7421	-1.73937	-1.7366	-1.7338	-1.73104
-1.75222	-1.74946	-1.74673	-1.74401	-1.74129	-1.73858	-1.73583	-1.73306	-1.73031

								-0.10805
								-0.1078
						-0.10816	-0.10786	-0.255
						-0.10792	-0.10763	-0.25435
						-0.10768	-0.1074	-0.25372
				-0.10804	-0.10774	-0.25499	-0.25402	-0.34205
				-0.10782	-0.10752	-0.25434	-0.25335	-0.34123
				-0.20376	-0.20291	-0.34283	-0.34165	-0.4287
				-0.20316	-0.20233	-0.34203	-0.34088	-0.42778
	-0.20524	-0.20433	-0.20344	-0.25434	-0.25334	-0.38366	-0.38239	-0.47306
	-0.20462	-0.20376	-0.20293	-0.25373	-0.25274	-0.38283	-0.38154	-0.47204
-0.10785	-0.25612	-0.25512	-0.25414	-0.38467	-0.38334	-0.4278	-0.42638	-0.51099
-0.1076	-0.25543	-0.25447	-0.25353	-0.38384	-0.38251	-0.42691	-0.4255	-0.50998
-0.10735	-0.25478	-0.25384	-0.25292	-0.383	-0.38167	-0.42601	-0.42463	-0.50898
-0.25514	-0.34342	-0.34227	-0.34112	-0.38214	-0.38083	-0.5111	-0.50952	-0.59979
-0.25454	-0.3427	-0.34152	-0.34035	-0.38127	-0.37998	-0.51004	-0.50845	-0.5986
-0.25394	-0.38438	-0.38305	-0.38174	-0.46695	-0.46546	-0.55662	-0.55493	-0.59746
-0.25335	-0.38356	-0.38225	-0.38094	-0.46601	-0.46452	-0.55553	-0.55385	-0.59636
-0.38404	-0.42471	-0.42329	-0.42188	-0.5578	-0.55611	-0.59873	-0.59698	-0.59525
-0.38316	-0.42377	-0.42237	-0.42097	-0.55666	-0.55496	-0.59756	-0.59583	-0.59413
-0.38229	-0.42286	-0.42146	-0.42007	-0.55554	-0.55385	-0.59641	-0.59471	-0.59303
-0.42341	-0.51528	-0.51367	-0.51208	-0.59883	-0.59707	-0.59535	-0.59367	-0.68206
-0.42257	-0.51428	-0.51268	-0.5111	-0.59775	-0.59602	-0.59432	-0.59266	-0.68088
-0.51489	-0.60199	-0.60019	-0.59841	-0.59667	-0.59497	-0.68352	-0.68161	-0.79578
-0.5139	-0.60089	-0.59908	-0.5973	-0.59559	-0.59392	-0.68231	-0.6804	-0.79442
-0.55702	-0.59978	-0.59797	-0.5962	-0.68489	-0.68298	-0.75809	-0.756	-0.93958
-0.55594	-0.59867	-0.59687	-0.5951	-0.68361	-0.68169	-0.75666	-0.75457	-0.93753
-0.59932	-0.59755	-0.59577	-0.59403	-0.68236	-0.68044	-0.9051	-0.90214	-1.04227
-0.59815	-0.59641	-0.5947	-0.593	-0.68116	-0.67924	-0.90324	-0.90035	-1.04025
-0.597	-0.5953	-0.59364	-0.59199	-0.67999	-0.67807	-0.90144	-0.89862	-1.0383
-0.59593	-0.68451	-0.68262	-0.68073	-0.75555	-0.75347	-1.04276	-1.03954	-1.03634

-0.59491	-0.68335	-0.68145	-0.67955	-0.75427	-0.75219	-1.04074	-1.03756	-1.03438
-0.64199	-0.7592	-0.75713	-0.75505	-1.0107	-1.00748	-1.03872	-1.03554	-1.16292
-0.64091	-0.75792	-0.75585	-0.75378	-1.00865	-1.00547	-1.03668	-1.03351	-1.16072
-0.75872	-0.90692	-0.90397	-0.90106	-1.0411	-1.03783	-1.13497	-1.13161	-1.15847
-0.75744	-0.90508	-0.90217	-0.89927	-1.03902	-1.03572	-1.1327	-1.12931	-1.15615
-0.75615	-1.01231	-1.00904	-1.00579	-1.07116	-1.06777	-1.16065	-1.15723	-1.21421
-0.75487	-1.01023	-1.00695	-1.0037	-1.06903	-1.06568	-1.15849	-1.15515	-1.21204
-0.75356	-1.00814	-1.00485	-1.00161	-1.06691	-1.06363	-1.15638	-1.15313	-1.20992
-1.00934	-1.07489	-1.07149	-1.06814	-1.13083	-1.1275	-1.15431	-1.15111	-1.23483
-1.00721	-1.07272	-1.06936	-1.06606	-1.12872	-1.12546	-1.15227	-1.1491	-1.23266
-1.03995	-1.13673	-1.13328	-1.12991	-1.1567	-1.15345	-1.21026	-1.20685	-1.23049
-1.03784	-1.13454	-1.13112	-1.12779	-1.1546	-1.15142	-1.20809	-1.20469	-1.22832
-1.10298	-1.13239	-1.12902	-1.12573	-1.15255	-1.14941	-1.23301	-1.22957	-1.22617
-1.10085	-1.13025	-1.12697	-1.12376	-1.15055	-1.14742	-1.23084	-1.22741	-1.22403
-1.13149	-1.12816	-1.12495	-1.12181	-1.17663	-1.17344	-1.22868	-1.22527	-1.30231
-1.1294	-1.12612	-1.12296	-1.11985	-1.17461	-1.17143	-1.22653	-1.22319	-1.30004
-1.12735	-1.12412	-1.12098	-1.11789	-1.17259	-1.16941	-1.2244	-1.22112	-1.2978
-1.12532	-1.18016	-1.17694	-1.17376	-1.17058	-1.16741	-1.30268	-1.29914	-1.29555
-1.12332	-1.17813	-1.17492	-1.17175	-1.16858	-1.16543	-1.3004	-1.29686	-1.29331
-1.14993	-1.17611	-1.17291	-1.16974	-1.2499	-1.24646	-1.29813	-1.29457	-1.36495
-1.14796	-1.17411	-1.1709	-1.16773	-1.24771	-1.24431	-1.29588	-1.29226	-1.3624
-1.146	-1.25594	-1.25242	-1.24894	-1.30076	-1.29725	-1.31361	-1.30991	-1.35991
-1.14402	-1.25372	-1.25024	-1.24679	-1.29852	-1.29502	-1.31136	-1.30768	-1.35751
-1.14206	-1.2515	-1.24806	-1.24464	-1.2963	-1.2928	-1.30913	-1.30549	-1.35517
-1.19412	-1.2701	-1.26664	-1.26321	-1.31402	-1.3105	-1.36073	-1.35674	-1.38274
-1.19204	-1.26789	-1.26443	-1.26102	-1.31177	-1.30829	-1.35837	-1.35442	-1.38038
-1.26928	-1.26571	-1.26224	-1.25883	-1.36373	-1.3599	-1.38597	-1.38195	-1.37801
-1.26714	-1.26355	-1.26007	-1.25666	-1.36134	-1.35755	-1.38357	-1.37957	-1.37564
-1.26498	-1.26138	-1.25791	-1.25451	-1.35896	-1.35521	-1.38119	-1.37721	-1.45218
-1.26277	-1.25919	-1.25574	-1.25237	-1.3566	-1.35289	-1.37883	-1.37487	-1.44975
-1.26059	-1.31334	-1.30952	-1.30582	-1.38413	-1.38035	-1.43018	-1.42614	-1.44738
-1.25844	-1.31104	-1.30726	-1.30358	-1.38176	-1.37799	-1.42781	-1.42384	-1.4451
-1.25632	-1.30879	-1.30504	-1.30138	-1.37941	-1.37565	-1.42545	-1.42157	-1.44284
-1.31043	-1.33747	-1.33362	-1.32985	-1.43079	-1.42692	-1.44831	-1.44444	-1.44052
-1.30824	-1.3353	-1.33144	-1.32765	-1.4284	-1.42453	-1.4459	-1.44208	-1.43819
-1.30601	-1.43817	-1.43406	-1.42999	-1.45129	-1.44737	-1.44352	-1.43972	-1.43586
-1.30375	-1.43575	-1.43169	-1.42765	-1.44893	-1.44499	-1.44114	-1.43736	-1.43353
-1.38778	-1.43336	-1.42935	-1.42536	-1.4466	-1.44264	-1.43878	-1.435	-1.45755
-1.38547	-1.43099	-1.42705	-1.42312	-1.44432	-1.44033	-1.43644	-1.43263	-1.45525
-1.3832	-1.45389	-1.44997	-1.44604	-1.44204	-1.43803	-1.43413	-1.43032	-1.48067
-1.38097	-1.4515	-1.4476	-1.44371	-1.43972	-1.43573	-1.43185	-1.42808	-1.47847
-1.37876	-1.44912	-1.44523	-1.44136	-1.4374	-1.43346	-1.42965	-1.42596	-1.47641
-1.40253	-1.44678	-1.44289	-1.43903	-1.43511	-1.43125	-1.48149	-1.47784	-1.49913
-1.40028	-1.44448	-1.44056	-1.4367	-1.43285	-1.42911	-1.47947	-1.47609	-1.49754
-1.44618	-1.44222	-1.43825	-1.43438	-1.45875	-1.4551	-1.47753	-1.4744	-1.49599
-1.44392	-1.44001	-1.43598	-1.43207	-1.45652	-1.45307	-1.47567	-1.47278	-1.4945
-1.44163	-1.46602	-1.46189	-1.45794	-1.48024	-1.47693	-1.47394	-1.47126	-1.51633
-1.43928	-1.46364	-1.45957	-1.45572	-1.47819	-1.47515	-1.47238	-1.46989	-1.51499
-1.43694	-1.46129	-1.45734	-1.45367	-1.47631	-1.47351	-1.47093	-1.46857	-1.51368
-1.46283	-1.45899	-1.45531	-1.45191	-1.47468	-1.47201	-1.51268	-1.51031	-1.55765

-1.46053	-1.45678	-1.45341	-1.45034	-1.47323	-1.47063	-1.51129	-1.50895	-1.5562
-1.45833	-1.48059	-1.47739	-1.47448	-1.51501	-1.51244	-1.53361	-1.5312	-1.55473
-1.45622	-1.47865	-1.47565	-1.47292	-1.51357	-1.51114	-1.53231	-1.52988	-1.55327
-1.45425	-1.52025	-1.51731	-1.51462	-1.51214	-1.5098	-1.55703	-1.55441	-1.63843
-1.45242	-1.51855	-1.51576	-1.51316	-1.51072	-1.5084	-1.55554	-1.55299	-1.63686
-1.51982	-1.51695	-1.51428	-1.51176	-1.53587	-1.53338	-1.61347	-1.61085	-1.63526
-1.51815	-1.51542	-1.51288	-1.51045	-1.53446	-1.53193	-1.61188	-1.60927	-1.63359
-1.51659	-1.51396	-1.51152	-1.50916	-1.53308	-1.5305	-1.61031	-1.60769	-1.63192
-1.51513	-1.51259	-1.51018	-1.50783	-1.61424	-1.6115	-1.63588	-1.63305	-1.6734
-1.51375	-1.51128	-1.50887	-1.5065	-1.61271	-1.61003	-1.63431	-1.63143	-1.67172
-1.5565	-1.59709	-1.59437	-1.59168	-1.61119	-1.60853	-1.6759	-1.67295	-1.67015
-1.55507	-1.59555	-1.59288	-1.59023	-1.60969	-1.607	-1.67422	-1.6714	-1.66866
-1.57123	-1.59404	-1.59139	-1.58875	-1.6784	-1.67542	-1.67258	-1.66985	-1.66717
-1.56986	-1.59258	-1.5899	-1.58724	-1.6767	-1.67377	-1.67097	-1.66829	-1.66566
-1.59377	-1.63577	-1.63297	-1.6302	-1.67503	-1.67215	-1.66939	-1.66674	-1.68664
-1.59226	-1.63416	-1.6314	-1.62867	-1.67342	-1.67059	-1.66786	-1.6652	-1.68496
-1.59074	-1.63256	-1.62984	-1.62717	-1.67183	-1.66906	-1.66634	-1.66368	-1.6833
-1.63377	-1.67884	-1.6759	-1.67303	-1.67023	-1.6675	-1.66483	-1.66221	-1.68171
-1.63223	-1.67721	-1.67427	-1.6714	-1.66863	-1.66595	-1.66333	-1.66077	-1.68018
-1.63068	-1.67554	-1.67267	-1.66986	-1.66713	-1.66447	-1.68416	-1.68138	-1.68965
-1.62912	-1.67384	-1.67109	-1.66839	-1.66572	-1.66307	-1.68262	-1.6798	-1.68808
-1.65397	-1.67222	-1.66954	-1.66692	-1.66428	-1.66167	-1.6921	-1.68929	-1.68656
-1.65242	-1.6707	-1.66802	-1.66539	-1.6628	-1.66024	-1.6906	-1.68784	-1.68512
-1.65088	-1.66921	-1.66651	-1.66388	-1.66132	-1.6588	-1.68909	-1.68639	-1.68369
-1.67036	-1.67884	-1.67621	-1.67362	-1.69297	-1.69024	-1.68755	-1.6849	-1.70076
-1.6688	-1.6773	-1.67472	-1.67217	-1.69141	-1.68868	-1.68601	-1.6834	-1.69929
-1.6785	-1.67583	-1.67324	-1.6707	-1.68984	-1.68716	-1.70304	-1.70042	-1.69779
-1.67708	-1.67442	-1.67178	-1.6692	-1.68828	-1.68568	-1.70156	-1.6989	-1.69627
-1.67564	-1.6951	-1.69223	-1.68943	-1.70529	-1.70269	-1.70006	-1.69741	-1.6948
-1.67416	-1.69356	-1.69076	-1.688	-1.70382	-1.70116	-1.69853	-1.69594	-1.69338
-1.71331	-1.71055	-1.70782	-1.7051	-1.70237	-1.69966	-1.69704	-1.69449	-1.69198
-1.71171	-1.70895	-1.70628	-1.70363	-1.70093	-1.69823	-1.69562	-1.69306	-1.69057
-1.71013	-1.70737	-1.70475	-1.70215	-1.69949	-1.69683	-1.69422	-1.69167	-1.68918
-1.70864	-1.7059	-1.70324	-1.70063	-1.69801	-1.69541	-1.70893	-1.70637	-1.70386
-1.7072	-1.70448	-1.70176	-1.69909	-1.69651	-1.69398	-1.70756	-1.70505	-1.70254
-1.70571	-1.70303	-1.70031	-1.69763	-1.7112	-1.70866	-1.70616	-1.70369	-1.74698
-1.70419	-1.70157	-1.69888	-1.69623	-1.70978	-1.70723	-1.70474	-1.70232	-1.74548
-1.70268	-1.7163	-1.71363	-1.71098	-1.70839	-1.70584	-1.70336	-1.70094	-1.74395
-1.70116	-1.71476	-1.71216	-1.70958	-1.70703	-1.70451	-1.70201	-1.69955	-1.74234
-1.71592	-1.71325	-1.71069	-1.70817	-1.70567	-1.70319	-1.7464	-1.74353	-1.74079
-1.71449	-1.71182	-1.70925	-1.70673	-1.70427	-1.70184	-1.74492	-1.74208	-1.73933
-1.71309	-1.71042	-1.70783	-1.7053	-1.70287	-1.70048	-1.74345	-1.74067	-1.73793
-1.7117	-1.70906	-1.70647	-1.70395	-1.72206	-1.71951	-1.74198	-1.73925	-1.73654
-1.71034	-1.70773	-1.70515	-1.70263	-1.72065	-1.71809	-1.7405	-1.73783	-1.73515
-1.70897	-1.70639	-1.70383	-1.70131	-1.74441	-1.74168	-1.739	-1.73639	-1.73375
-1.70761	-1.70506	-1.70252	-1.7	-1.74291	-1.74015	-1.7375	-1.73493	-1.73233
-1.70624	-1.72448	-1.72176	-1.71906	-1.74143	-1.73867	-1.73602	-1.73346	-1.74263
-1.70486	-1.72305	-1.72035	-1.71767	-1.73998	-1.73726	-1.73458	-1.73195	-1.74107
-1.70349	-1.72162	-1.71895	-1.71629	-1.73856	-1.73586	-1.73316	-1.73048	-1.73953
-1.70213	-1.74548	-1.74267	-1.73989	-1.73716	-1.73446	-1.73176	-1.72908	-1.73807

-1.70077	-1.74396	-1.74121	-1.73848	-1.73576	-1.73306	-1.73038	-1.72772	-1.73665
-1.71988	-1.7424	-1.73972	-1.73705	-1.73436	-1.73167	-1.74072	-1.73794	-1.73526
-1.7184	-1.74082	-1.73821	-1.73561	-1.73294	-1.73027	-1.7393	-1.73657	-1.73389
-1.74206	-1.73932	-1.73674	-1.73418	-1.7484	-1.74573	-1.75453	-1.75182	-1.74912
-1.7406	-1.73789	-1.73532	-1.73277	-1.747	-1.74437	-1.75314	-1.75044	-1.74775
-1.75606	-1.75336	-1.75077	-1.74821	-1.7572	-1.75446	-1.75175	-1.74906	-1.74638
-1.75462	-1.75194	-1.74935	-1.7468	-1.75577	-1.75307	-1.75038	-1.7477	-1.74502
-1.7532	-1.75052	-1.74794	-1.74541	-1.75435	-1.75169	-1.74902	-1.74633	-1.74366
-1.75177	-1.74911	-1.74656	-1.74405	-1.75295	-1.75032	-1.74765	-1.74496	-1.7423
-1.75035	-1.74771	-1.74519	-1.74272	-1.75157	-1.74894	-1.74628	-1.74359	-1.74094
-1.74893	-1.75799	-1.75538	-1.75282	-1.7502	-1.74757	-1.74491	-1.74223	-1.74574
-1.74751	-1.75652	-1.75396	-1.75144	-1.74884	-1.74621	-1.74355	-1.74087	-1.74439
-1.74613	-1.7551	-1.75257	-1.75006	-1.74747	-1.74484	-1.74836	-1.74568	-1.78862
-1.7448	-1.75374	-1.75121	-1.74869	-1.7461	-1.74347	-1.74699	-1.74434	-1.78713
-1.75506	-1.75241	-1.74987	-1.74734	-1.75091	-1.74827	-1.74563	-1.74299	-1.78563
-1.75374	-1.75114	-1.74858	-1.74601	-1.74956	-1.74691	-1.74428	-1.74165	-1.78414
-1.75244	-1.74988	-1.74729	-1.74469	-1.74822	-1.74556	-1.74293	-1.74031	-1.78264
-1.75733	-1.75475	-1.75214	-1.74951	-1.74687	-1.74422	-1.78703	-1.78407	-1.78116
-1.75603	-1.75343	-1.7508	-1.74816	-1.74552	-1.74288	-1.78553	-1.78258	-1.77969
-1.75471	-1.7521	-1.74945	-1.7468	-1.74416	-1.74153	-1.78403	-1.78109	-1.79429
-1.75338	-1.75075	-1.74809	-1.74544	-1.7428	-1.74018	-1.78253	-1.77961	-1.79283
-1.75202	-1.74939	-1.74673	-1.74407	-1.80302	-1.80005	-1.79711	-1.79421	-1.80598
-1.75065	-1.74801	-1.74536	-1.74271	-1.80149	-1.79852	-1.79562	-1.79278	-1.80457
-1.74928	-1.74663	-1.74399	-1.74135	-1.79996	-1.797	-1.79414	-1.79136	-1.80317
-1.7479	-1.76191	-1.75924	-1.75659	-1.79843	-1.79551	-1.8208	-1.81805	-1.81533
-1.74652	-1.76052	-1.75786	-1.75521	-1.79693	-1.79404	-1.81936	-1.81665	-1.81395
-1.76181	-1.81895	-1.81593	-1.81295	-1.82356	-1.82071	-1.81795	-1.81526	-1.81259
-1.76046	-1.81742	-1.8144	-1.81144	-1.82209	-1.81931	-1.81657	-1.81389	-1.81124
-1.81895	-1.82945	-1.82646	-1.82352	-1.82071	-1.81797	-1.81525	-1.81258	-1.80994
-1.81754	-1.82806	-1.82509	-1.82219	-1.81941	-1.8167	-1.81401	-1.81133	-1.80868
-1.82961	-1.82664	-1.82371	-1.82085	-1.8181	-1.81541	-1.81273	-1.81006	-1.82457
-1.8281	-1.82515	-1.82228	-1.81948	-1.81675	-1.81407	-1.81138	-1.80872	-1.8232
-1.82658	-1.82365	-1.82085	-1.8181	-1.81538	-1.8127	-1.81002	-1.80735	-1.82182
-1.82508	-1.82219	-1.81941	-1.81669	-1.81399	-1.81131	-1.80864	-1.80598	-1.86191
-1.82361	-1.82075	-1.818	-1.81529	-1.8126	-1.80993	-1.80726	-1.80461	-1.86034
-1.82224	-1.81941	-1.81664	-1.81392	-1.81123	-1.80856	-1.84316	-1.84022	-1.85879
-1.82095	-1.81815	-1.81534	-1.81258	-1.80987	-1.80721	-1.84171	-1.83879	-1.85727
-1.81962	-1.81684	-1.81404	-1.81127	-1.82578	-1.82306	-1.862	-1.85891	-1.85581
-1.81823	-1.81549	-1.81274	-1.81002	-1.82452	-1.8218	-1.8606	-1.85752	-1.85444
-1.81684	-1.81413	-1.81143	-1.80875	-1.84355	-1.84062	-1.85919	-1.85611	-1.85305
-1.81545	-1.81276	-1.81008	-1.80742	-1.84215	-1.83924	-1.85772	-1.85462	-1.85157
-1.81409	-1.8114	-1.80874	-1.80609	-1.84076	-1.83787	-1.85625	-1.85314	-1.85009
-1.8128	-1.8482	-1.84525	-1.84234	-1.86103	-1.85796	-1.85488	-1.85178	-1.84872
-1.81155	-1.84686	-1.84394	-1.84104	-1.85965	-1.8566	-1.85353	-1.85047	-1.84741
-1.84844	-1.84548	-1.84257	-1.83969	-1.8582	-1.85515	-1.85211	-1.84908	-1.84605
-1.847	-1.84405	-1.84116	-1.83829	-1.85669	-1.85362	-1.85061	-1.84763	-1.84465
-1.8456	-1.86443	-1.86136	-1.85829	-1.8552	-1.85213	-1.84912	-1.84617	-1.85884
-1.84428	-1.86304	-1.85996	-1.85689	-1.85378	-1.85067	-1.84765	-1.84469	-1.85729
-1.84296	-1.86164	-1.85856	-1.85548	-1.85236	-1.84925	-1.86199	-1.85886	-1.85578
-1.84156	-1.86015	-1.85708	-1.85401	-1.85093	-1.84787	-1.86057	-1.85745	-1.85438

-1.84016	-1.85866	-1.85559	-1.85253	-1.8495	-1.8465	-1.85917	-1.85607	-1.85302
-1.86031	-1.85725	-1.85418	-1.85113	-1.84809	-1.84509	-1.88789	-1.88477	-1.91574
-1.85894	-1.85588	-1.85282	-1.84976	-1.8467	-1.84366	-1.88633	-1.88323	-1.91408
-1.85752	-1.85446	-1.85141	-1.84838	-1.89127	-1.88802	-1.88485	-1.88176	-1.9125
-1.85604	-1.85299	-1.84997	-1.84696	-1.8898	-1.8866	-1.88347	-1.88038	-1.91099
-1.88529	-1.8822	-1.87916	-1.87614	-1.88835	-1.8852	-1.91613	-1.91278	-1.90947
-1.88389	-1.8808	-1.87777	-1.87476	-1.88691	-1.88378	-1.91458	-1.91122	-1.90791
-1.88249	-1.87941	-1.87638	-1.87337	-1.88547	-1.88236	-1.91303	-1.90967	-1.90636
-1.88104	-1.89343	-1.89023	-1.88708	-1.88397	-1.88089	-1.91147	-1.90815	-1.90486
-1.87957	-1.89187	-1.88868	-1.88554	-1.88246	-1.87942	-1.90992	-1.90664	-1.9034
-1.87814	-1.89039	-1.8872	-1.88408	-1.91496	-1.91168	-1.90841	-1.90517	-1.90193
-1.87673	-1.88895	-1.88579	-1.88268	-1.91345	-1.91019	-1.90694	-1.90371	-1.90046
-1.8907	-1.88754	-1.8844	-1.8813	-1.91193	-1.90866	-1.90542	-1.90221	-1.89897
-1.88929	-1.88614	-1.88302	-1.87993	-1.9104	-1.90709	-1.90384	-1.90064	-1.89744
-1.88789	-1.88476	-1.88164	-1.87855	-1.90887	-1.90554	-1.90229	-1.89911	-1.89593
-1.8865	-1.88336	-1.88024	-1.87713	-1.90736	-1.90406	-1.90083	-1.89764	-1.89447
-1.88509	-1.88196	-1.87882	-1.87571	-1.90586	-1.90261	-1.89939	-1.8962	-1.89303
-1.88362	-1.91438	-1.911	-1.90765	-1.90438	-1.90116	-1.89795	-1.89476	-1.89159
-1.88212	-1.91279	-1.90946	-1.90617	-1.90292	-1.89971	-1.89651	-1.89333	-1.89015
-1.88069	-1.91126	-1.90796	-1.9047	-1.90146	-1.89825	-1.89505	-1.89187	-1.88869
-1.87931	-1.90977	-1.90649	-1.90324	-1.90001	-1.8968	-1.89359	-1.89038	-1.88722
-1.87795	-1.90829	-1.90502	-1.90178	-1.89854	-1.89533	-1.89212	-1.88892	-1.88578
-1.87659	-1.90681	-1.90354	-1.90029	-1.89705	-1.89382	-1.89064	-1.8875	-1.8844
-1.90865	-1.90533	-1.90206	-1.8988	-1.89556	-1.89234	-1.88919	-1.8861	-1.88305
-1.90717	-1.90385	-1.90056	-1.89729	-1.89408	-1.8909	-1.88779	-1.88473	-1.8817
-1.90571	-1.90238	-1.89906	-1.89579	-1.89261	-1.8895	-1.88642	-1.88338	-1.88037
-1.90425	-1.90094	-1.89763	-1.89436	-1.89121	-1.88813	-1.88507	-1.88205	-1.90161
-1.9028	-1.89951	-1.89622	-1.89298	-1.88985	-1.88678	-1.88374	-1.88073	-1.90028
-1.90132	-1.89804	-1.89479	-1.89159	-1.88848	-1.88543	-1.90505	-1.90202	-1.91087
-1.8998	-1.89654	-1.89333	-1.89018	-1.88711	-1.88408	-1.90372	-1.90071	-1.90957
-1.89832	-1.91784	-1.91465	-1.91153	-1.90844	-1.9054	-1.91428	-1.91126	-1.90822
-1.8969	-1.91644	-1.91327	-1.91016	-1.90709	-1.90405	-1.9129	-1.90985	-1.90681
-1.89551	-1.91506	-1.91191	-1.90881	-1.90574	-1.9027	-1.91153	-1.90845	-1.90542
-1.91694	-1.91369	-1.91055	-1.90747	-1.9163	-1.91327	-1.91021	-1.90713	-1.90409
-1.91557	-1.91234	-1.90921	-1.90614	-1.91498	-1.91197	-1.90892	-1.90583	-1.90281
-1.9142	-1.92287	-1.91975	-1.91669	-1.91366	-1.91066	-1.90761	-1.90453	-1.90151
-1.91284	-1.92151	-1.91841	-1.91536	-1.91234	-1.90935	-1.90631	-1.90323	-1.90023
-1.91147	-1.92015	-1.91707	-1.91403	-1.91103	-1.90804	-1.90501	-1.90194	-1.89894
-1.91009	-1.91879	-1.91573	-1.91272	-1.90972	-1.90674	-1.90371	-1.90065	-1.89766
-1.92059	-1.91744	-1.9144	-1.91141	-1.90842	-1.90544	-1.90242	-1.89937	-1.90744
-1.91924	-1.9161	-1.91308	-1.91011	-1.90712	-1.90415	-1.90114	-1.89811	-1.90617
-1.91792	-1.91479	-1.91178	-1.90881	-1.90583	-1.90286	-1.89986	-1.89685	-1.90491
-1.91665	-1.91355	-1.91052	-1.90753	-1.90454	-1.90157	-1.90964	-1.90661	-1.90364
-1.91542	-1.91234	-1.90928	-1.90625	-1.90325	-1.90028	-1.90834	-1.90532	-1.90237
-1.91413	-1.91108	-1.90801	-1.90496	-1.90197	-1.899	-1.90705	-1.90406	-1.90112
-1.91281	-1.90978	-1.90671	-1.90367	-1.90068	-1.89773	-1.90578	-1.9028	-1.89989
-1.9115	-1.90849	-1.90542	-1.90238	-1.91046	-1.90749	-1.90453	-1.90158	-1.89868
-1.91022	-1.9072	-1.90413	-1.90109	-1.90917	-1.90623	-1.9033	-1.90039	-1.89751
-1.90894	-1.91704	-1.91394	-1.91088	-1.9079	-1.90497	-1.90207	-1.8992	-1.89633
-1.90767	-1.91576	-1.9127	-1.90966	-1.90667	-1.90371	-1.90081	-1.89795	-1.8951

-1.90639	-1.91448	-1.91147	-1.90846	-1.90545	-1.90246	-1.89955	-1.8967	-1.89385
-1.91621	-1.91319	-1.91019	-1.90721	-1.9042	-1.90121	-1.89831	-1.89547	-1.89263
-1.91493	-1.91188	-1.90889	-1.90593	-1.90295	-1.89998	-1.89708	-1.89425	-1.89142
-1.91364	-1.91058	-1.90761	-1.90466	-1.9017	-1.89876	-1.89588	-1.89305	-1.89022
-1.91235	-1.90929	-1.90632	-1.90339	-1.90047	-1.89758	-1.89471	-1.89186	-1.88904
-1.91109	-1.90804	-1.90506	-1.90213	-1.89924	-1.89638	-1.89352	-1.89067	-1.88785
-1.90987	-1.90683	-1.90384	-1.90088	-1.898	-1.89516	-1.89231	-1.88945	-1.88665
-1.90864	-1.90563	-1.90262	-1.89965	-1.89677	-1.89394	-1.89109	-1.88825	-1.88545
-1.90736	-1.90438	-1.90141	-1.89846	-1.89558	-1.89274	-1.88991	-1.8871	-1.88431
-1.90607	-1.90312	-1.90019	-1.89729	-1.8944	-1.89154	-1.88873	-1.88595	-1.88318
-1.90481	-1.90186	-1.89895	-1.89607	-1.89319	-1.89033	-1.88753	-1.88477	-1.882
-1.90356	-1.9006	-1.89771	-1.89484	-1.89197	-1.88912	-1.88632	-1.88356	-1.8808
-1.90235	-1.89939	-1.8965	-1.89364	-1.89079	-1.88795	-1.88515	-1.88238	-1.87963
-1.90116	-1.89823	-1.89533	-1.89247	-1.88964	-1.88684	-1.88404	-1.88125	-1.8785
-1.89998	-1.89707	-1.89418	-1.89132	-1.88851	-1.88572	-1.88292	-1.88013	-1.87739
-1.8988	-1.8959	-1.89303	-1.89019	-1.88737	-1.88456	-1.88178	-1.87902	-1.8763
-1.89763	-1.89473	-1.89188	-1.88906	-1.88622	-1.88339	-1.88062	-1.8779	-1.87519
-1.89647	-1.89358	-1.89073	-1.88789	-1.88504	-1.8822	-1.89215	-1.88945	-1.88674
-1.89533	-1.89245	-1.88957	-1.8867	-1.88384	-1.88102	-1.89095	-1.88825	-1.88556
-1.89418	-1.90401	-1.90111	-1.89822	-1.89537	-1.89256	-1.8898	-1.88709	-1.8844
-1.89302	-1.90282	-1.89991	-1.89703	-1.89421	-1.89144	-1.88868	-1.88596	-1.88327
-1.90456	-1.90166	-1.89874	-1.89586	-1.89306	-1.89031	-1.88757	-1.88484	-1.88215
-1.90341	-1.90053	-1.89762	-1.89473	-1.89193	-1.88917	-1.88644	-1.88375	-1.88107
-1.90225	-1.89939	-1.89648	-1.8936	-1.89079	-1.88803	-1.88532	-1.88265	-1.87998
-1.90107	-1.8982	-1.8953	-1.89244	-1.88966	-1.88692	-1.88421	-1.88154	-1.87887
-1.89989	-1.89701	-1.89413	-1.89129	-1.88853	-1.88581	-1.88311	-1.88042	-1.87776
-1.89873	-1.89587	-1.89301	-1.89018	-1.88742	-1.8847	-1.882	-1.87933	-1.87667
-1.89759	-1.89475	-1.89191	-1.8891	-1.88632	-1.88358	-1.88089	-1.87825	-1.8756
-1.89643	-1.89361	-1.89081	-1.88802	-1.88525	-1.8825	-1.87981	-1.87716	-1.87451
-1.89525	-1.89245	-1.8897	-1.88697	-1.88422	-1.88147	-1.87876	-1.87607	-1.8734
-1.86687	-1.8913	-1.88858	-1.88588	-1.88316	-1.88044	-1.87772	-1.875	-1.87231
-1.8658	-1.8902	-1.88746	-1.88475	-1.88206	-1.87939	-1.87669	-1.87396	-1.87126
-1.86476	-1.88912	-1.88636	-1.88363	-1.88096	-1.87833	-1.87564	-1.87293	-1.87023
-1.86373	-1.88806	-1.88531	-1.88258	-1.87989	-1.87723	-1.87456	-1.87188	-1.86919
-1.86272	-1.88701	-1.88427	-1.88155	-1.87883	-1.87613	-1.87346	-1.87082	-1.86815
-1.86167	-1.88591	-1.88321	-1.88051	-1.87779	-1.87507	-1.87239	-1.86973	-1.86706
-1.86061	-1.88478	-1.88212	-1.87947	-1.87676	-1.87404	-1.87133	-1.86863	-1.86595
-1.85957	-1.88369	-1.88104	-1.8784	-1.87571	-1.873	-1.87028	-1.86755	-1.86486
-1.85857	-1.88264	-1.87996	-1.87729	-1.87462	-1.87195	-1.86924	-1.86651	-1.86379
-1.85756	-1.8816	-1.87889	-1.8762	-1.87354	-1.87088	-1.86818	-1.86546	-1.86274
-1.85654	-1.88058	-1.87786	-1.87514	-1.87245	-1.86977	-1.86708	-1.86439	-1.86169
-1.85551	-1.87956	-1.87683	-1.87409	-1.87137	-1.86866	-1.86597	-1.86332	-1.86064
-1.85451	-1.87853	-1.87581	-1.87307	-1.87033	-1.86759	-1.86489	-1.86222	-1.85954
-1.8535	-1.8775	-1.87479	-1.87206	-1.86931	-1.86655	-1.86382	-1.86111	-1.85843
-1.85246	-1.84988	-1.84728	-1.84466	-1.86828	-1.86551	-1.86277	-1.86004	-1.85736
-1.85139	-1.84879	-1.84621	-1.84363	-1.86723	-1.86448	-1.86174	-1.859	-1.85632
-1.85035	-1.84772	-1.84516	-1.8426	-1.86618	-1.86344	-1.8607	-1.85797	-1.85528
-1.84933	-1.8467	-1.84414	-1.84157	-1.86513	-1.8624	-1.85967	-1.85694	-1.85424
-1.84832	-1.84568	-1.8431	-1.84053	-1.86407	-1.86135	-1.85863	-1.8559	-1.8532
-1.84731	-1.84465	-1.84203	-1.83943	-1.86296	-1.86029	-1.85759	-1.85488	-1.85217

-1.8463	-1.84362	-1.84096	-1.83833	-1.86186	-1.85923	-1.85656	-1.85385	-1.85116
-1.8453	-1.8426	-1.83992	-1.83728	-1.8608	-1.85818	-1.85552	-1.85283	-1.85015
-1.84429	-1.8416	-1.83892	-1.83627	-1.85976	-1.85715	-1.85449	-1.85182	-1.84914
-1.84328	-1.84059	-1.83791	-1.83526	-1.83271	-1.83019	-1.85347	-1.85081	-1.84813
-1.84226	-1.83958	-1.8369	-1.83427	-1.83171	-1.82919	-1.85247	-1.8498	-1.84713
-1.84125	-1.83856	-1.8359	-1.83327	-1.83072	-1.8282	-1.85146	-1.84879	-1.84613
-1.84023	-1.83755	-1.83489	-1.83226	-1.82972	-1.82721	-1.85045	-1.8478	-1.84513
-1.83922	-1.83654	-1.83388	-1.83126	-1.82872	-1.82621	-1.84945	-1.8468	-1.84414
-1.83823	-1.83555	-1.83289	-1.83027	-1.82773	-1.82521	-1.84843	-1.84579	-1.84314
-1.83724	-1.83456	-1.83191	-1.82928	-1.82674	-1.82422	-1.84742	-1.84479	-1.84214
-1.83625	-1.83358	-1.83092	-1.8283	-1.82575	-1.82324	-1.82069	-1.81812	-1.84114
-1.83526	-1.83259	-1.82993	-1.82731	-1.82478	-1.82228	-1.81973	-1.81715	-1.84014
-1.83427	-1.83161	-1.82895	-1.82634	-1.8238	-1.8213	-1.81875	-1.81617	-1.83914
-1.83328	-1.83062	-1.82798	-1.82537	-1.82283	-1.82032	-1.81777	-1.81519	-1.83814
-1.8323	-1.82965	-1.82703	-1.82443	-1.82188	-1.81936	-1.8168	-1.81421	-1.83714
-1.83132	-1.8287	-1.8261	-1.82353	-1.82097	-1.81841	-1.81583	-1.81324	-1.83613
-1.83037	-1.82776	-1.82519	-1.82263	-1.82006	-1.81747	-1.81487	-1.81226	-1.83513
-1.82945	-1.82686	-1.82428	-1.82171	-1.81911	-1.81651	-1.8139	-1.81129	-1.83413
-1.82855	-1.82597	-1.82337	-1.82076	-1.81815	-1.81554	-1.81293	-1.81031	-1.83313
-1.82763	-1.82504	-1.82243	-1.8198	-1.81718	-1.81456	-1.81194	-1.80932	-1.8067
-1.8267	-1.82409	-1.82146	-1.81884	-1.81621	-1.81358	-1.81095	-1.80834	-1.80572
-1.82575	-1.82312	-1.82049	-1.81786	-1.81523	-1.8126	-1.80998	-1.80736	-1.80475
-1.82478	-1.82214	-1.81951	-1.81689	-1.81426	-1.81164	-1.80901	-1.80639	-1.80379
-1.82381	-1.82117	-1.81854	-1.81592	-1.8133	-1.81069	-1.80807	-1.80544	-1.80285
-1.82286	-1.82021	-1.81758	-1.81496	-1.81237	-1.8098	-1.80719	-1.80457	-1.80197
-1.82191	-1.81927	-1.81663	-1.81402	-1.81145	-1.80891	-1.80632	-1.8037	-1.80108
-1.821	-1.81839	-1.81575	-1.81313	-1.81055	-1.80798	-1.80538	-1.80277	-1.80014
-1.82011	-1.81752	-1.8149	-1.81227	-1.80965	-1.80703	-1.80442	-1.8018	-1.79917
-1.81921	-1.81662	-1.81399	-1.81135	-1.80872	-1.80608	-1.80345	-1.80083	-1.7982
-1.8183	-1.81568	-1.81304	-1.8104	-1.80776	-1.80513	-1.80249	-1.79985	-1.79722
-1.81738	-1.81473	-1.81208	-1.80944	-1.80682	-1.80419	-1.80156	-1.79892	-1.79628
-1.81644	-1.81378	-1.81112	-1.80848	-1.80588	-1.80329	-1.80068	-1.79804	-1.79541
-1.81551	-1.81284	-1.81017	-1.80753	-1.80495	-1.80239	-1.79979	-1.79716	-1.79452
-1.81465	-1.81197	-1.8093	-1.80664	-1.80404	-1.80145	-1.79884	-1.79621	-1.79357
-1.81379	-1.81111	-1.80844	-1.80577	-1.80313	-1.8005	-1.79787	-1.79523	-1.7926
-1.81288	-1.8102	-1.80752	-1.80485	-1.80219	-1.79954	-1.7969	-1.79426	-1.81824
-1.81193	-1.80925	-1.80657	-1.8039	-1.80124	-1.79858	-1.79593	-1.79328	-1.81725
-1.81103	-1.80835	-1.80567	-1.803	-1.80033	-1.79767	-1.79501	-1.79235	-1.8163
-1.81017	-1.80748	-1.80481	-1.80214	-1.79947	-1.7968	-1.79414	-1.79148	-1.81539
-1.80929	-1.8066	-1.80392	-1.80125	-1.79858	-1.79591	-1.79325	-1.79058	-1.81446
-1.80835	-1.80566	-1.80298	-1.80031	-1.79764	-1.79497	-1.7923	-1.78963	-1.81348
-1.80741	-1.80472	-1.80204	-1.79937	-1.7967	-1.79403	-1.79135	-1.78868	-1.81251
-1.79802	-1.80384	-1.80116	-1.79848	-1.79581	-1.79313	-1.81705	-1.81432	-1.8116
-1.79715	-1.80298	-1.80029	-1.79761	-1.79493	-1.79225	-1.81615	-1.81343	-1.81071
-1.79622	-1.80206	-1.79937	-1.79669	-1.794	-1.79132	-1.81519	-1.81247	-1.80976
-1.79526	-1.80111	-1.79842	-1.79573	-1.79304	-1.79035	-1.8142	-1.81147	-1.80878
-1.76904	-1.79164	-1.78894	-1.78624	-1.79212	-1.78943	-1.81325	-1.81051	-1.80782
-1.7682	-1.79077	-1.78807	-1.78537	-1.79126	-1.78856	-1.81236	-1.80961	-1.8069
-1.76736	-1.78992	-1.78721	-1.7845	-1.81698	-1.81423	-1.81148	-1.80872	-1.80599
-1.76652	-1.78906	-1.78635	-1.78364	-1.81611	-1.81335	-1.8106	-1.80784	-1.8051

-1.76566	-1.78819	-1.78547	-1.78275	-1.81521	-1.81246	-1.8097	-1.80694	-1.8042
-1.76473	-1.78724	-1.78452	-1.78179	-1.80586	-1.80308	-1.80872	-1.80597	-1.80323
-1.76376	-1.78625	-1.78353	-1.7808	-1.80484	-1.80206	-1.80771	-1.80497	-1.80224
-1.76285	-1.78533	-1.7826	-1.77987	-1.80388	-1.8011	-1.80676	-1.80402	-1.8013
-1.762	-1.78446	-1.78172	-1.77898	-1.80298	-1.80019	-1.80586	-1.80313	-1.80041
-1.76116	-1.81047	-1.80768	-1.80488	-1.80209	-1.7993	-1.79654	-1.79381	-1.79954
-1.76034	-1.80961	-1.80682	-1.80402	-1.80121	-1.79842	-1.79566	-1.79294	-1.79868
-1.75951	-1.80875	-1.80595	-1.80315	-1.80034	-1.79755	-1.79479	-1.79206	-1.79781
-1.75868	-1.80788	-1.80507	-1.80227	-1.79947	-1.79668	-1.79392	-1.79119	-1.79694
-1.75782	-1.80698	-1.80417	-1.80137	-1.79858	-1.79579	-1.79304	-1.79031	-1.79606
-1.75693	-1.7816	-1.77883	-1.77609	-1.79761	-1.79484	-1.79208	-1.78935	-1.79512
-1.756	-1.78062	-1.77784	-1.77509	-1.7966	-1.79384	-1.79109	-1.78836	-1.79413
-1.75509	-1.77968	-1.7769	-1.77415	-1.79566	-1.7929	-1.79016	-1.78742	-1.78469
-1.75419	-1.7788	-1.77602	-1.77327	-1.79477	-1.79202	-1.78928	-1.78654	-1.78381
-1.78068	-1.77792	-1.77516	-1.77241	-1.7939	-1.79115	-1.78841	-1.78567	-1.78294
-1.77981	-1.77706	-1.7743	-1.77156	-1.79304	-1.7903	-1.78755	-1.78482	-1.78208
-1.77895	-1.7762	-1.77346	-1.77073	-1.79219	-1.78945	-1.7867	-1.78397	-1.78123
-1.77809	-1.77535	-1.77261	-1.76989	-1.79134	-1.78859	-1.78585	-1.78311	-1.78037
-1.77724	-1.7745	-1.77177	-1.76905	-1.79049	-1.78774	-1.78499	-1.78225	-1.77951
-1.77638	-1.77365	-1.77092	-1.76821	-1.78963	-1.78688	-1.78414	-1.78139	-1.77865
-1.77553	-1.77279	-1.77007	-1.76737	-1.78878	-1.78603	-1.78328	-1.78053	-1.77779
-1.77467	-1.77193	-1.76922	-1.76652	-1.76382	-1.76113	-1.78242	-1.77967	-1.77692
-1.77381	-1.77107	-1.76837	-1.76567	-1.76297	-1.76028	-1.78155	-1.7788	-1.77606
-1.77295	-1.77022	-1.76751	-1.76482	-1.76212	-1.75942	-1.78069	-1.77794	-1.77519
-1.77211	-1.76937	-1.76666	-1.76397	-1.76127	-1.75857	-1.77982	-1.77707	-1.77433
-1.77128	-1.76853	-1.76582	-1.76312	-1.76041	-1.75772	-1.77895	-1.77621	-1.77346
-1.77048	-1.76771	-1.76498	-1.76227	-1.75956	-1.75686	-1.77809	-1.77534	-1.77259
-1.76971	-1.7669	-1.76415	-1.76143	-1.75871	-1.75601	-1.77723	-1.77447	-1.77172
-1.76894	-1.76614	-1.76336	-1.7606	-1.75787	-1.75516	-1.77636	-1.77361	-1.77085
-1.76819	-1.76541	-1.7626	-1.7598	-1.75704	-1.75431	-1.77549	-1.77274	-1.76999
-1.7674	-1.76464	-1.76183	-1.75902	-1.75624	-1.75349	-1.75076	-1.74806	-1.76913
-1.76657	-1.76383	-1.76106	-1.75828	-1.75549	-1.75269	-1.74994	-1.74723	-1.76826
-1.76572	-1.763	-1.76028	-1.75753	-1.75473	-1.75191	-1.74915	-1.74642	-1.76743
-1.76487	-1.76216	-1.75945	-1.75672	-1.75395	-1.75117	-1.74842	-1.74569	-1.76667
-1.76402	-1.76131	-1.7586	-1.75588	-1.75316	-1.75042	-1.74769	-1.74497	-1.76592
-1.76318	-1.76046	-1.75775	-1.75504	-1.75233	-1.74961	-1.7469	-1.74418	-1.76512
-1.76234	-1.75961	-1.75689	-1.75418	-1.75147	-1.74876	-1.74606	-1.74335	-1.7643
-1.76154	-1.75878	-1.75606	-1.75334	-1.75063	-1.74792	-1.74521	-1.74251	-1.76347
-1.76076	-1.75798	-1.75523	-1.75251	-1.74978	-1.74706	-1.74435	-1.74166	-1.76262
-1.76	-1.7572	-1.75445	-1.75171	-1.74897	-1.74623	-1.74352	-1.74081	-1.76176
-1.75924	-1.75648	-1.75374	-1.75099	-1.74822	-1.74544	-1.7427	-1.73999	-1.76091
-1.75848	-1.75575	-1.75302	-1.75027	-1.74747	-1.74466	-1.74191	-1.73919	-1.76009
-1.75767	-1.75494	-1.75222	-1.74947	-1.7467	-1.74392	-1.74117	-1.73846	-1.73574
-1.75686	-1.75413	-1.75139	-1.74866	-1.74592	-1.74318	-1.74046	-1.73775	-1.73502
-1.75612	-1.75337	-1.75061	-1.74785	-1.74512	-1.74239	-1.73968	-1.73698	-1.73425
-1.75541	-1.75265	-1.74985	-1.74706	-1.74431	-1.74158	-1.73886	-1.73616	-1.73344
-1.75465	-1.75189	-1.74909	-1.74629	-1.74354	-1.74081	-1.73808	-1.73535	-1.73263
-1.75383	-1.75109	-1.74833	-1.74557	-1.74283	-1.7401	-1.73735	-1.73457	-1.73182
-1.75301	-1.75028	-1.74754	-1.74482	-1.7421	-1.73937	-1.7366	-1.7338	-1.73104
-1.75222	-1.74946	-1.74673	-1.74401	-1.74129	-1.73858	-1.73583	-1.73306	-1.73031

-1.00721	-1.00576	-1.00432	-1.00289	-1.00149	-1.0001	-1.01529	-1.01388	-1.01247
-1.00757	-1.00613	-1.00471	-1.0033	-1.00189	-1.00048	-1.01562	-1.0142	-1.01279
-1.00793	-1.00651	-1.0051	-1.0037	-1.00228	-1.00086	-1.01594	-1.01452	-1.01311
-1.00831	-1.00689	-1.00548	-1.00408	-1.00265	-1.00122	-1.01626	-1.01483	-1.01343
-1.00869	-1.00727	-1.00586	-1.00445	-1.00302	-1.00158	-1.01657	-1.01515	-1.01375
-1.00907	-1.00766	-1.00625	-1.00484	-1.0034	-1.00195	-1.01689	-1.01547	-1.01407
-1.00945	-1.00805	-1.00665	-1.00523	-1.00378	-1.00232	-1.01722	-1.0158	-1.0144
-1.00983	-1.00843	-1.00703	-1.00561	-1.00415	-1.00269	-1.01755	-1.01613	-1.01473
-1.0102	-1.0088	-1.00739	-1.00596	-1.00451	-1.00305	-1.01788	-1.01647	-1.01508
-1.01057	-1.00916	-1.00774	-1.00631	-1.00487	-1.00342	-1.01821	-1.01681	-1.01542
-1.01094	-1.00951	-1.0081	-1.00668	-1.00525	-1.00382	-1.01856	-1.01715	-1.01574
-1.0113	-1.00987	-1.00846	-1.00705	-1.00563	-1.00421	-1.01891	-1.01748	-1.01606
-1.01168	-1.01024	-1.00883	-1.00743	-1.00601	-1.00459	-1.01925	-1.01781	-1.0164
-1.01206	-1.01063	-1.00922	-1.00781	-1.00639	-1.00495	-1.01957	-1.01814	-1.01674
-1.01243	-1.011	-1.0096	-1.00819	-1.00676	-1.00533	-1.0199	-1.01848	-1.01707
-1.0128	-1.01138	-1.00997	-1.00855	-1.00713	-1.0057	-1.02025	-1.01882	-1.01741
-1.01317	-1.01175	-1.01034	-1.00892	-1.0075	-1.00608	-1.02058	-1.01916	-1.01774
-1.01353	-1.01212	-1.0107	-1.00929	-1.00787	-1.00646	-1.02091	-1.01948	-1.01805
-1.0139	-1.01249	-1.01107	-1.00966	-1.00825	-1.00683	-1.02125	-1.0198	-1.01837
-1.01428	-1.01287	-1.01146	-1.01005	-1.00863	-1.00721	-1.02158	-1.02014	-1.01872
-1.01468	-1.01325	-1.01185	-1.01045	-1.00902	-1.00758	-1.02192	-1.02049	-1.01907
-0.97079	-1.01365	-1.01224	-1.01083	-1.00939	-1.00795	-1.02225	-1.02083	-1.01941
-0.97119	-1.01407	-1.01263	-1.0112	-1.00976	-1.00832	-1.02258	-1.02116	-1.01975
-0.97158	-0.97021	-0.96882	-0.96744	-1.01014	-1.00871	-1.02294	-1.02152	-1.02012
-0.97196	-0.97058	-0.9692	-0.96783	-1.01055	-1.00912	-1.02331	-1.0219	-1.0205
-0.97235	-0.97097	-0.96959	-0.96822	-0.96685	-0.96548	-1.00272	-1.00135	-1.02089
-0.97276	-0.97139	-0.97	-0.96861	-0.96724	-0.96587	-1.00307	-1.00172	-1.02126
-0.97317	-0.97181	-0.97041	-0.96901	-0.96763	-0.96626	-1.00342	-1.00208	-1.02163
-0.9517	-0.97219	-0.9708	-0.96941	-0.96803	-0.96666	-0.98136	-0.98002	-1.00108
-0.95206	-0.97257	-0.97118	-0.9698	-0.96843	-0.96706	-0.98172	-0.98037	-1.00143
-0.95244	-0.9511	-0.94975	-0.94841	-0.96882	-0.96746	-0.98207	-0.98073	-0.97939
-0.95283	-0.95149	-0.95014	-0.9488	-0.96922	-0.96785	-0.98241	-0.98108	-0.97975
-0.92481	-0.95187	-0.95053	-0.94919	-0.94785	-0.9465	-0.98278	-0.98145	-0.98012
-0.92516	-0.95225	-0.95092	-0.9496	-0.94826	-0.94693	-0.98318	-0.98184	-0.98051
-0.90455	-0.95262	-0.9513	-0.94999	-0.94866	-0.94734	-0.96213	-0.96082	-0.98088
-0.90488	-0.953	-0.95167	-0.95034	-0.94903	-0.94773	-0.96248	-0.96116	-0.98124
-0.90522	-0.95338	-0.95203	-0.9507	-0.9494	-0.94811	-0.96281	-0.9615	-0.98159
-0.85295	-0.92527	-0.92398	-0.92271	-0.94978	-0.94848	-0.96314	-0.96183	-0.96051
-0.85327	-0.92563	-0.92436	-0.92309	-0.95017	-0.94885	-0.96347	-0.96216	-0.96084
-0.85358	-0.926	-0.92473	-0.92346	-0.92219	-0.92092	-0.96382	-0.96251	-0.96119
-0.85388	-0.92637	-0.9251	-0.92383	-0.92256	-0.92129	-0.96419	-0.96288	-0.96156
-0.85419	-0.85308	-0.85195	-0.85082	-0.92292	-0.92165	-0.96454	-0.96323	-0.96192
-0.85451	-0.85338	-0.85224	-0.85111	-0.92326	-0.922	-0.96486	-0.96354	-0.96223
-0.85482	-0.85368	-0.85253	-0.8514	-0.87206	-0.87092	-0.93719	-0.93592	-0.96256
-0.85512	-0.85396	-0.85283	-0.8517	-0.87239	-0.87125	-0.93752	-0.93626	-0.96293
-0.85543	-0.85425	-0.85313	-0.85202	-0.87272	-0.87158	-0.93787	-0.93661	-0.96331
-0.85574	-0.85455	-0.85343	-0.85232	-0.8512	-0.85008	-0.93818	-0.93693	-0.93568
-0.85605	-0.85486	-0.85374	-0.85263	-0.8515	-0.85037	-0.93849	-0.93724	-0.93599
-0.85639	-0.8552	-0.85406	-0.85294	-0.8518	-0.85067	-0.88798	-0.88685	-0.93631
-0.85675	-0.85556	-0.8544	-0.85326	-0.85212	-0.85099	-0.88827	-0.88714	-0.93664

-0.8571	-0.85592	-0.85473	-0.85357	-0.85242	-0.8513	-0.86706	-0.86594	-0.88629
-0.85745	-0.85626	-0.85505	-0.85386	-0.85272	-0.85161	-0.86734	-0.86622	-0.88658
-0.8578	-0.8566	-0.85537	-0.85416	-0.85302	-0.85192	-0.86761	-0.86649	-0.88686
-0.85815	-0.85694	-0.85571	-0.85449	-0.85334	-0.85222	-0.86787	-0.86674	-0.88713
-0.8585	-0.85729	-0.85606	-0.85484	-0.85366	-0.85252	-0.86812	-0.86698	-0.88739
-0.85886	-0.85765	-0.85642	-0.8552	-0.85401	-0.85285	-0.86842	-0.86727	-0.88769
-0.85924	-0.85803	-0.8568	-0.85557	-0.85438	-0.85321	-0.86874	-0.86759	-0.88802
-0.8596	-0.85839	-0.85716	-0.85595	-0.85475	-0.85357	-0.86906	-0.86791	-0.86678
-0.85995	-0.85873	-0.85752	-0.85631	-0.85511	-0.85393	-0.86938	-0.86822	-0.86709
-0.8603	-0.85908	-0.85787	-0.85667	-0.85548	-0.85429	-0.8697	-0.86853	-0.86738
-0.86066	-0.85944	-0.85824	-0.85704	-0.85585	-0.85466	-0.87002	-0.86882	-0.86767
-0.86103	-0.85981	-0.85861	-0.85741	-0.85622	-0.85503	-0.87034	-0.86912	-0.86795
-0.83044	-0.86019	-0.85899	-0.8578	-0.85661	-0.85542	-0.85421	-0.85297	-0.86827
-0.8308	-0.86057	-0.85938	-0.8582	-0.85701	-0.85582	-0.85459	-0.85335	-0.8686
-0.83115	-0.82997	-0.82881	-0.82768	-0.8574	-0.85621	-0.85498	-0.85373	-0.86894
-0.83149	-0.83031	-0.82915	-0.82801	-0.85778	-0.85659	-0.85537	-0.85412	-0.86928
-0.80133	-0.83065	-0.82948	-0.82834	-0.82722	-0.82613	-0.85575	-0.8545	-0.86962
-0.80164	-0.83097	-0.82981	-0.82866	-0.82754	-0.82644	-0.85613	-0.85489	-0.86996
-0.80196	-0.83131	-0.83014	-0.829	-0.82787	-0.82676	-0.82565	-0.82454	-0.87032
-0.8023	-0.83167	-0.8305	-0.82935	-0.82822	-0.8271	-0.82599	-0.82489	-0.87072
-0.80265	-0.83205	-0.83087	-0.82971	-0.82857	-0.82745	-0.82634	-0.82525	-0.87113
-0.80303	-0.80187	-0.80073	-0.79961	-0.82892	-0.8278	-0.82669	-0.8256	-0.8411
-0.80341	-0.80225	-0.8011	-0.79996	-0.82928	-0.82815	-0.82703	-0.82594	-0.84142
-0.80377	-0.80261	-0.80144	-0.80029	-0.79918	-0.79809	-0.82738	-0.82628	-0.84175
-0.80411	-0.80295	-0.80178	-0.80063	-0.79951	-0.79841	-0.82771	-0.82661	-0.84207
-0.80445	-0.80329	-0.80212	-0.80097	-0.79984	-0.79874	-0.82806	-0.82694	-0.84239
-0.80478	-0.80363	-0.80247	-0.80132	-0.80018	-0.79907	-0.8284	-0.82728	-0.84271
-0.80513	-0.80398	-0.80282	-0.80167	-0.80053	-0.79941	-0.82876	-0.82763	-0.84303
-0.80548	-0.80433	-0.80318	-0.80204	-0.8009	-0.79977	-0.79866	-0.79758	-0.82688
-0.80584	-0.80469	-0.80354	-0.8024	-0.80127	-0.80014	-0.79903	-0.79793	-0.82725
-0.8062	-0.80506	-0.80391	-0.80277	-0.80163	-0.8005	-0.79939	-0.79829	-0.7972
-0.80657	-0.80542	-0.80427	-0.80313	-0.80199	-0.80085	-0.79974	-0.79865	-0.79756
-0.80695	-0.8058	-0.80464	-0.80349	-0.80234	-0.8012	-0.80009	-0.79899	-0.7979
-0.80735	-0.80618	-0.80502	-0.80386	-0.8027	-0.80155	-0.80042	-0.79932	-0.79823
-0.77551	-0.80655	-0.80539	-0.80422	-0.80306	-0.8019	-0.80076	-0.79964	-0.79855
-0.77583	-0.80691	-0.80575	-0.80459	-0.80342	-0.80225	-0.8011	-0.79997	-0.79887
-0.77615	-0.80727	-0.80611	-0.80495	-0.80378	-0.80261	-0.80145	-0.80032	-0.79921
-0.77648	-0.77537	-0.77427	-0.77317	-0.80415	-0.803	-0.80185	-0.80071	-0.79958
-0.77682	-0.77571	-0.7746	-0.7735	-0.80453	-0.8034	-0.80226	-0.80112	-0.79998
-0.77717	-0.77606	-0.77494	-0.77384	-0.77275	-0.77168	-0.80265	-0.80151	-0.80037
-0.77754	-0.77642	-0.7753	-0.77419	-0.7731	-0.77202	-0.80302	-0.80188	-0.80075
-0.7779	-0.77678	-0.77566	-0.77455	-0.77346	-0.77238	-0.77129	-0.7702	-0.80113
-0.77827	-0.77715	-0.77604	-0.77494	-0.77384	-0.77275	-0.77165	-0.77055	-0.8015
-0.77863	-0.77752	-0.77642	-0.77532	-0.77422	-0.77312	-0.77201	-0.7709	-0.80187
-0.77898	-0.77787	-0.77676	-0.77566	-0.77456	-0.77345	-0.77235	-0.77124	-0.80225
-0.77934	-0.77822	-0.77711	-0.776	-0.77489	-0.77378	-0.77268	-0.77158	-0.80263
-0.77972	-0.77859	-0.77747	-0.77636	-0.77524	-0.77411	-0.77301	-0.77191	-0.77083
-0.7801	-0.77898	-0.77785	-0.77672	-0.77558	-0.77444	-0.77333	-0.77224	-0.77116
-0.7351	-0.73393	-0.73276	-0.73159	-0.77594	-0.7748	-0.77368	-0.77259	-0.77151
-0.73552	-0.73435	-0.73318	-0.73202	-0.7763	-0.77518	-0.77406	-0.77296	-0.77188

-0.73597	-0.7348	-0.73363	-0.73247	-0.73131	-0.73016	-0.72901	-0.72788	-0.72676
-0.73646	-0.73528	-0.7341	-0.73293	-0.73176	-0.73059	-0.72944	-0.7283	-0.72718
-0.73695	-0.73575	-0.73458	-0.7334	-0.73222	-0.73103	-0.72987	-0.72874	-0.72761
-0.73742	-0.73621	-0.73503	-0.73386	-0.73267	-0.73149	-0.73033	-0.7292	-0.72807
-0.73788	-0.73667	-0.73549	-0.73431	-0.73313	-0.73196	-0.73081	-0.72967	-0.72854
-0.70728	-0.73716	-0.73597	-0.73479	-0.73361	-0.73244	-0.73128	-0.73014	-0.729
-0.70776	-0.73767	-0.73646	-0.73527	-0.7341	-0.73293	-0.73177	-0.73061	-0.72945
-0.70821	-0.70706	-0.70591	-0.70477	-0.73457	-0.7334	-0.73223	-0.73106	-0.7299
-0.70864	-0.7075	-0.70635	-0.7052	-0.73503	-0.73386	-0.73268	-0.7315	-0.73034
-0.70909	-0.70795	-0.70679	-0.70564	-0.70452	-0.7034	-0.73314	-0.73195	-0.73079
-0.70957	-0.70843	-0.70728	-0.70612	-0.70498	-0.70385	-0.73362	-0.73244	-0.73128
-0.71006	-0.70892	-0.70776	-0.7066	-0.70545	-0.7043	-0.7341	-0.73293	-0.73177
-0.67362	-0.70938	-0.70822	-0.70705	-0.70589	-0.70474	-0.73456	-0.7334	-0.73223
-0.67403	-0.70984	-0.70866	-0.70749	-0.70632	-0.70517	-0.73503	-0.73386	-0.73269
-0.67443	-0.71029	-0.70911	-0.70795	-0.70678	-0.70563	-0.7045	-0.70339	-0.73318
-0.67483	-0.71073	-0.70956	-0.70842	-0.70726	-0.70612	-0.70499	-0.70387	-0.7337
-0.67526	-0.67419	-0.67317	-0.67218	-0.70775	-0.70662	-0.70548	-0.70436	-0.70324
-0.67573	-0.67464	-0.67361	-0.6726	-0.70824	-0.70712	-0.70599	-0.70485	-0.70373
-0.64562	-0.67511	-0.67404	-0.67302	-0.70873	-0.70761	-0.70648	-0.70534	-0.70422
-0.64604	-0.67557	-0.67448	-0.67344	-0.70922	-0.7081	-0.70696	-0.70583	-0.7047
-0.64647	-0.67603	-0.67493	-0.67386	-0.7097	-0.70856	-0.70743	-0.7063	-0.70518
-0.64689	-0.67648	-0.67536	-0.67428	-0.67325	-0.67226	-0.70787	-0.70674	-0.70563
-0.64731	-0.67692	-0.6758	-0.6747	-0.67365	-0.67263	-0.70829	-0.70718	-0.70607
-0.64775	-0.64669	-0.64564	-0.64462	-0.67408	-0.67304	-0.67204	-0.67109	-0.70653
-0.64822	-0.64715	-0.64609	-0.64506	-0.67454	-0.67347	-0.67246	-0.67149	-0.70699
-0.60753	-0.64761	-0.64655	-0.64551	-0.64448	-0.64348	-0.6729	-0.67191	-0.70747
-0.60794	-0.64809	-0.64702	-0.64597	-0.64494	-0.64392	-0.67336	-0.67234	-0.70796
-0.60835	-0.64858	-0.6475	-0.64645	-0.6454	-0.64437	-0.67383	-0.67278	-0.67177
-0.60876	-0.64908	-0.648	-0.64693	-0.64587	-0.64483	-0.67432	-0.67324	-0.67221
-0.60918	-0.64958	-0.64849	-0.64741	-0.64635	-0.6453	-0.67482	-0.67372	-0.67266
-0.60959	-0.60872	-0.60785	-0.60698	-0.64684	-0.64578	-0.64474	-0.64371	-0.67313
-0.60999	-0.60913	-0.60827	-0.6074	-0.64734	-0.64627	-0.64521	-0.64417	-0.6736
-0.61039	-0.60953	-0.60868	-0.60782	-0.64783	-0.64676	-0.64569	-0.64463	-0.64359
-0.61078	-0.60994	-0.60908	-0.60823	-0.64832	-0.64725	-0.64617	-0.6451	-0.64404
-0.61117	-0.61033	-0.60948	-0.60863	-0.60778	-0.60692	-0.64666	-0.64558	-0.64451
-0.61155	-0.61071	-0.60987	-0.60904	-0.60819	-0.60733	-0.64715	-0.64607	-0.645
-0.61195	-0.6111	-0.61027	-0.60944	-0.6086	-0.60774	-0.64765	-0.64656	-0.64548
-0.57892	-0.61149	-0.61067	-0.60984	-0.60901	-0.60816	-0.64814	-0.64706	-0.64598
-0.57929	-0.6119	-0.61107	-0.61025	-0.60942	-0.60858	-0.64864	-0.64755	-0.64647
-0.57968	-0.61232	-0.61148	-0.61066	-0.60983	-0.60899	-0.60814	-0.60727	-0.64696
-0.58008	-0.61274	-0.6119	-0.61107	-0.61023	-0.6094	-0.60854	-0.60768	-0.64746
-0.54696	-0.57966	-0.5789	-0.57815	-0.61064	-0.6098	-0.60895	-0.60809	-0.64795
-0.54731	-0.58006	-0.57928	-0.57853	-0.61105	-0.61021	-0.60936	-0.6085	-0.64844
-0.54768	-0.54692	-0.54621	-0.54552	-0.61147	-0.61062	-0.60976	-0.6089	-0.60804
-0.54806	-0.5473	-0.54656	-0.54585	-0.61188	-0.61103	-0.61017	-0.60931	-0.60844
-0.54844	-0.54767	-0.54693	-0.5462	-0.61231	-0.61145	-0.61058	-0.60971	-0.60885
-0.54883	-0.54806	-0.5473	-0.54657	-0.54586	-0.54516	-0.61099	-0.61012	-0.60925
-0.54922	-0.54845	-0.54769	-0.54694	-0.54622	-0.54551	-0.61141	-0.61053	-0.60966
-0.54963	-0.54885	-0.54808	-0.54733	-0.54659	-0.54586	-0.61184	-0.61095	-0.61008
-0.55005	-0.54926	-0.54849	-0.54773	-0.54697	-0.54622	-0.61227	-0.61138	-0.61051

-0.51223	-0.54967	-0.5489	-0.54813	-0.54736	-0.54659	-0.54585	-0.54513	-0.61094
-0.51261	-0.55008	-0.5493	-0.54852	-0.54774	-0.54696	-0.54621	-0.54548	-0.61137
-0.51299	-0.5505	-0.5497	-0.54891	-0.54812	-0.54734	-0.54658	-0.54584	-0.57903
-0.51339	-0.55094	-0.55012	-0.54932	-0.54852	-0.54774	-0.54697	-0.54623	-0.57944
-0.5138	-0.55138	-0.55054	-0.54972	-0.54892	-0.54814	-0.54737	-0.54662	-0.57987
-0.51421	-0.51341	-0.51261	-0.51183	-0.54933	-0.54854	-0.54777	-0.54701	-0.54627
-0.51462	-0.51381	-0.51299	-0.5122	-0.54974	-0.54895	-0.54818	-0.54741	-0.54665
-0.51502	-0.51421	-0.51339	-0.51258	-0.51183	-0.51111	-0.54858	-0.54781	-0.54704
-0.51543	-0.51461	-0.51379	-0.51298	-0.51222	-0.51148	-0.54899	-0.54821	-0.54743
-0.51584	-0.51502	-0.5142	-0.51339	-0.51261	-0.51185	-0.51111	-0.51039	-0.54783
-0.51625	-0.51543	-0.51462	-0.51382	-0.51302	-0.51223	-0.51147	-0.51074	-0.54823
-0.51666	-0.51585	-0.51505	-0.51424	-0.51343	-0.51262	-0.51185	-0.51111	-0.54864
-0.44473	-0.51627	-0.51547	-0.51466	-0.51384	-0.51303	-0.51225	-0.5115	-0.51076
-0.44507	-0.5167	-0.51589	-0.51507	-0.51426	-0.51346	-0.51268	-0.5119	-0.51115
-0.44541	-0.44474	-0.44407	-0.44339	-0.51469	-0.51389	-0.5131	-0.51231	-0.51154
-0.44574	-0.44507	-0.44441	-0.44374	-0.51512	-0.51432	-0.51352	-0.51271	-0.51193
-0.44608	-0.44541	-0.44475	-0.44409	-0.44342	-0.44275	-0.51394	-0.51313	-0.51234
-0.44641	-0.44574	-0.44509	-0.44444	-0.44377	-0.44309	-0.51436	-0.51355	-0.51276
-0.44675	-0.44608	-0.44543	-0.44478	-0.44411	-0.44343	-0.44277	-0.4421	-0.51319
-0.4471	-0.44643	-0.44577	-0.44511	-0.44444	-0.44378	-0.44312	-0.44245	-0.51362
-0.44745	-0.44677	-0.4461	-0.44543	-0.44478	-0.44413	-0.44347	-0.4428	-0.51404
-0.40674	-0.4471	-0.44643	-0.44577	-0.44511	-0.44447	-0.44381	-0.44314	-0.44247
-0.40702	-0.44743	-0.44676	-0.4461	-0.44545	-0.4448	-0.44414	-0.44348	-0.44281
-0.40732	-0.40669	-0.40608	-0.40549	-0.44578	-0.44514	-0.44448	-0.44383	-0.44317
-0.40762	-0.40699	-0.40638	-0.40578	-0.44612	-0.44547	-0.44483	-0.44419	-0.44353
-0.40792	-0.40729	-0.40667	-0.40606	-0.40547	-0.40489	-0.40432	-0.40376	-0.44388
-0.40823	-0.4076	-0.40697	-0.40635	-0.40576	-0.40518	-0.40461	-0.40404	-0.44421
-0.40854	-0.40791	-0.40727	-0.40664	-0.40605	-0.40547	-0.40489	-0.40431	-0.40372
-0.40884	-0.40821	-0.40758	-0.40695	-0.40634	-0.40574	-0.40515	-0.40457	-0.40399
-0.40914	-0.40851	-0.40789	-0.40727	-0.40664	-0.40602	-0.40542	-0.40484	-0.40426
-0.3879	-0.40883	-0.4082	-0.40756	-0.40693	-0.4063	-0.4057	-0.40512	-0.40454
-0.38819	-0.40914	-0.4085	-0.40786	-0.40722	-0.40659	-0.40599	-0.40541	-0.40483
-0.38847	-0.38791	-0.38735	-0.38678	-0.40753	-0.4069	-0.40629	-0.4057	-0.40512
-0.38876	-0.38819	-0.38764	-0.38708	-0.40786	-0.40723	-0.4066	-0.40599	-0.4054
-0.38905	-0.38848	-0.38793	-0.38738	-0.40819	-0.40756	-0.40693	-0.4063	-0.4057
-0.38935	-0.38878	-0.38822	-0.38766	-0.40851	-0.40789	-0.40726	-0.40663	-0.40602
-0.38965	-0.38908	-0.38851	-0.38795	-0.40883	-0.40821	-0.40759	-0.40696	-0.40635
-0.38995	-0.38937	-0.3888	-0.38824	-0.38768	-0.38714	-0.4079	-0.40729	-0.40668
-0.39025	-0.38967	-0.3891	-0.38853	-0.38797	-0.38742	-0.40822	-0.40761	-0.407
-0.39055	-0.38998	-0.3894	-0.38883	-0.38826	-0.38771	-0.38716	-0.38663	-0.40731
-0.39085	-0.39028	-0.3897	-0.38913	-0.38856	-0.38799	-0.38744	-0.38691	-0.40761
-0.39116	-0.39059	-0.39001	-0.38943	-0.38886	-0.38829	-0.38773	-0.38718	-0.40792
-0.39148	-0.39089	-0.39031	-0.38973	-0.38915	-0.38858	-0.38801	-0.38746	-0.40823
-0.39183	-0.39119	-0.39061	-0.39004	-0.38946	-0.38888	-0.38831	-0.38775	-0.38721
-0.39219	-0.3915	-0.3909	-0.39034	-0.38976	-0.38919	-0.38862	-0.38806	-0.38751
-0.39259	-0.39183	-0.39121	-0.39065	-0.39007	-0.3895	-0.38893	-0.38836	-0.38781
-0.39306	-0.39223	-0.39156	-0.39097	-0.39038	-0.38982	-0.38924	-0.38867	-0.38811
-0.39359	-0.39268	-0.39195	-0.3913	-0.39069	-0.39013	-0.38956	-0.38898	-0.38841
-0.39417	-0.39319	-0.39237	-0.39165	-0.39101	-0.39044	-0.38986	-0.38929	-0.38872
-0.39479	-0.39376	-0.39283	-0.39201	-0.39134	-0.39076	-0.39017	-0.3896	-0.38902

-0.35326	-0.39435	-0.39333	-0.39242	-0.3917	-0.39108	-0.39048	-0.38991	-0.38934
-0.35387	-0.39496	-0.39387	-0.39288	-0.39209	-0.39141	-0.39079	-0.39022	-0.38965
-0.35448	-0.35343	-0.35235	-0.35136	-0.39253	-0.39178	-0.39112	-0.39054	-0.38997
-0.35512	-0.35406	-0.35297	-0.35194	-0.39306	-0.3922	-0.39147	-0.39086	-0.39027
-0.35576	-0.3547	-0.35362	-0.35257	-0.39365	-0.39267	-0.39187	-0.39121	-0.39058
-0.35637	-0.35531	-0.35424	-0.35319	-0.35216	-0.35119	-0.39235	-0.3916	-0.39093
-0.35695	-0.35591	-0.35486	-0.35381	-0.35277	-0.35176	-0.39289	-0.39203	-0.39129
-0.35756	-0.35652	-0.35547	-0.35443	-0.35337	-0.35234	-0.39344	-0.3925	-0.39169
-0.35817	-0.35714	-0.35609	-0.35504	-0.35398	-0.35292	-0.39401	-0.39299	-0.39212
-0.35879	-0.35775	-0.35671	-0.35566	-0.35459	-0.35353	-0.35249	-0.35148	-0.39261
-0.35939	-0.35836	-0.35733	-0.35629	-0.35523	-0.35416	-0.35312	-0.35208	-0.3932
-0.35999	-0.35896	-0.35794	-0.35691	-0.35586	-0.3548	-0.35375	-0.35271	-0.39382
-0.3606	-0.35957	-0.35853	-0.3575	-0.35646	-0.35542	-0.35437	-0.35334	-0.35233
-0.36122	-0.36017	-0.35912	-0.35808	-0.35705	-0.35602	-0.35499	-0.35396	-0.35293
-0.31327	-0.3608	-0.35974	-0.35869	-0.35766	-0.35664	-0.35561	-0.35458	-0.35355
-0.31378	-0.36145	-0.36038	-0.35932	-0.35828	-0.35726	-0.35623	-0.35521	-0.35417
-0.3143	-0.36208	-0.36101	-0.35995	-0.35891	-0.35788	-0.35685	-0.35583	-0.35479
-0.31481	-0.36271	-0.36164	-0.36058	-0.35954	-0.3585	-0.35748	-0.35645	-0.35542
-0.31533	-0.31444	-0.3136	-0.31277	-0.36017	-0.35913	-0.3581	-0.35707	-0.35604
-0.31588	-0.31498	-0.31411	-0.31326	-0.36079	-0.35975	-0.35871	-0.35767	-0.35663
-0.31644	-0.31553	-0.31463	-0.31376	-0.36143	-0.36039	-0.35933	-0.35827	-0.35722
-0.31699	-0.31608	-0.31517	-0.31429	-0.31347	-0.31268	-0.35997	-0.35889	-0.35783
-0.31754	-0.31662	-0.31573	-0.31485	-0.31401	-0.31319	-0.36062	-0.35952	-0.35845
-0.31807	-0.31716	-0.31627	-0.3154	-0.31455	-0.31372	-0.36128	-0.36017	-0.35909
-0.3186	-0.3177	-0.31682	-0.31594	-0.31509	-0.31425	-0.36196	-0.36084	-0.35974
-0.27958	-0.31824	-0.31736	-0.31648	-0.31563	-0.31478	-0.31392	-0.31305	-0.3604
-0.28006	-0.31878	-0.31789	-0.31701	-0.31616	-0.31532	-0.31445	-0.31357	-0.36107
-0.28056	-0.27976	-0.27895	-0.27817	-0.3167	-0.31585	-0.31498	-0.31409	-0.31323
-0.28106	-0.28025	-0.27945	-0.27866	-0.31724	-0.31639	-0.31551	-0.31462	-0.31375
-0.28156	-0.28075	-0.27995	-0.27917	-0.31779	-0.31692	-0.31604	-0.31515	-0.31429
-0.28206	-0.28125	-0.28045	-0.27966	-0.27887	-0.27808	-0.31658	-0.31571	-0.31484
-0.28256	-0.28176	-0.28096	-0.28016	-0.27936	-0.27857	-0.31713	-0.31627	-0.3154
-0.28306	-0.28226	-0.28146	-0.28067	-0.27986	-0.27906	-0.27828	-0.27752	-0.31595
-0.28357	-0.28277	-0.28197	-0.28117	-0.28037	-0.27957	-0.27878	-0.278	-0.31648
-0.28408	-0.28327	-0.28248	-0.28168	-0.28088	-0.28007	-0.27928	-0.27849	-0.2777
-0.2846	-0.28378	-0.28298	-0.28219	-0.28139	-0.28059	-0.27978	-0.27898	-0.27819
-0.28511	-0.28429	-0.28349	-0.2827	-0.2819	-0.2811	-0.28029	-0.27947	-0.27868
-0.28562	-0.2848	-0.284	-0.2832	-0.2824	-0.2816	-0.2808	-0.27998	-0.27918
-0.28614	-0.28531	-0.28451	-0.28371	-0.28291	-0.28211	-0.2813	-0.2805	-0.27969
-0.28667	-0.28583	-0.28502	-0.28422	-0.28342	-0.28262	-0.28182	-0.28101	-0.2802
-0.28723	-0.28636	-0.28553	-0.28472	-0.28393	-0.28314	-0.28233	-0.28152	-0.28071
-0.28779	-0.28691	-0.28606	-0.28524	-0.28444	-0.28364	-0.28284	-0.28202	-0.28121
-0.28837	-0.28748	-0.28661	-0.28577	-0.28494	-0.28414	-0.28333	-0.28252	-0.28172
-0.28895	-0.28806	-0.28718	-0.28631	-0.28547	-0.28465	-0.28383	-0.28303	-0.28222
-0.28953	-0.28863	-0.28775	-0.28687	-0.28601	-0.28517	-0.28434	-0.28354	-0.28273
-0.2901	-0.2892	-0.28832	-0.28744	-0.28657	-0.2857	-0.28487	-0.28406	-0.28325
-0.20131	-0.28978	-0.28888	-0.288	-0.28712	-0.28626	-0.28542	-0.2846	-0.28379
-0.20174	-0.29036	-0.28945	-0.28855	-0.28768	-0.28682	-0.28598	-0.28516	-0.28435
-0.14896	-0.29094	-0.29002	-0.28912	-0.28824	-0.28737	-0.28652	-0.2857	-0.28489
-0.14925	-0.29151	-0.29061	-0.2897	-0.2888	-0.2879	-0.28705	-0.28623	-0.28541

-0.14956	-0.20232	-0.20165	-0.20098	-0.28937	-0.28846	-0.28759	-0.28676	-0.28593
-0.14988	-0.20275	-0.20207	-0.20139	-0.28995	-0.28904	-0.28815	-0.28728	-0.28644
-0.1502	-0.14971	-0.14921	-0.14871	-0.29053	-0.28963	-0.28872	-0.28782	-0.28696
-0.15054	-0.15004	-0.14954	-0.14904	-0.29113	-0.29021	-0.2893	-0.28839	-0.28751
-0.15087	-0.15038	-0.14988	-0.14937	-0.29174	-0.29081	-0.28989	-0.28897	-0.28808
-0.09774	-0.1507	-0.1502	-0.1497	-0.20247	-0.20178	-0.29052	-0.2896	-0.28869
-0.09793	-0.15101	-0.15052	-0.15002	-0.20292	-0.20224	-0.29117	-0.29025	-0.28933
-0.09812	-0.15132	-0.15083	-0.15034	-0.14984	-0.14935	-0.2918	-0.29088	-0.28996
-0.09832	-0.15162	-0.15113	-0.15065	-0.15015	-0.14966	-0.2924	-0.29149	-0.29057
-0.04565	-0.09819	-0.09788	-0.09758	-0.15046	-0.14997	-0.24168	-0.24095	-0.29118
-0.04573	-0.09841	-0.09808	-0.09777	-0.15076	-0.15028	-0.24217	-0.24143	-0.29178
-0.04582	-0.04566	-0.0455	-0.04534	-0.09766	-0.09735	-0.15011	-0.14962	-0.29239
-0.0459	-0.04573	-0.04557	-0.0454	-0.09786	-0.09755	-0.15044	-0.14995	-0.29304
-0.04597	-0.0458	-0.04563	-0.04547	-0.09807	-0.09775	-0.15077	-0.15029	-0.29369
-0.04604	-0.04587	-0.04571	-0.04555	-0.09828	-0.09795	-0.15108	-0.1506	-0.24278
-0.04611	-0.04595	-0.04579	-0.04563	-0.09848	-0.09815	-0.1514	-0.15091	-0.24328
-0.04619	-0.04602	-0.04586	-0.0457	-0.04554	-0.04538	-0.09803	-0.09772	-0.15074
-0.04627	-0.0461	-0.04594	-0.04578	-0.04561	-0.04544	-0.09825	-0.09793	-0.15107
	-0.04617	-0.04601	-0.04585	-0.04568	-0.04551	-0.04536	-0.0452	-0.09782
	-0.04625	-0.04608	-0.04591	-0.04575	-0.04559	-0.04543	-0.04527	-0.09801
	-0.04633	-0.04616	-0.04598	-0.04582	-0.04566	-0.0455	-0.04534	-0.09821
	-0.04642	-0.04624	-0.04607	-0.0459	-0.04573	-0.04557	-0.04541	-0.09842
	-0.0465	-0.04633	-0.04616	-0.04598	-0.04581	-0.04565	-0.04549	-0.09863
				-0.04606	-0.04589	-0.04572	-0.04557	-0.04541
				-0.04614	-0.04596	-0.0458	-0.04565	-0.04549
						-0.04588	-0.04573	-0.04557
						-0.04597	-0.0458	-0.04563
								-0.0457
								-0.0458
								-0.04591

								-0.25476
								-0.25402
						-0.25545	-0.25436	-0.30078
						-0.25469	-0.25364	-0.30001
				-0.10823	-0.10792	-0.25398	-0.25295	-0.389
				-0.10798	-0.10769	-0.25335	-0.25232	-0.38816
				-0.10774	-0.10747	-0.25271	-0.25168	-0.38731
	-0.10839	-0.10809	-0.10779	-0.25399	-0.25302	-0.34078	-0.33955	-0.43225
	-0.10813	-0.10785	-0.10757	-0.25334	-0.25232	-0.33994	-0.33871	-0.43128
	-0.25569	-0.25466	-0.25368	-0.34156	-0.34033	-0.43312	-0.43172	-0.43037
	-0.25501	-0.25397	-0.25297	-0.34073	-0.33951	-0.43217	-0.4308	-0.42949
-0.10775	-0.30167	-0.30054	-0.29943	-0.3881	-0.38683	-0.47157	-0.47008	-0.46862
-0.1075	-0.30089	-0.29976	-0.29866	-0.38725	-0.38602	-0.4706	-0.46913	-0.46768
-0.25396	-0.34195	-0.34071	-0.3395	-0.47251	-0.47107	-0.46962	-0.46817	-0.59715
-0.25332	-0.34118	-0.33996	-0.33876	-0.4715	-0.47005	-0.46861	-0.46717	-0.59597
-0.25268	-0.34041	-0.33921	-0.33803	-0.4705	-0.46904	-0.4676	-0.46618	-0.5948
-0.34082	-0.47402	-0.47249	-0.47099	-0.4695	-0.46803	-0.50622	-0.50473	-0.63664
-0.34001	-0.47297	-0.47147	-0.46998	-0.4685	-0.46704	-0.50518	-0.50373	-0.63549
-0.42725	-0.47198	-0.47049	-0.46902	-0.50723	-0.50568	-0.63781	-0.63607	-0.63433
-0.42637	-0.47103	-0.46955	-0.46808	-0.50624	-0.5047	-0.63666	-0.63492	-0.63317
-0.47156	-0.50993	-0.50835	-0.50679	-0.59671	-0.59499	-0.63551	-0.63378	-0.71732
-0.47056	-0.50888	-0.50731	-0.50575	-0.59556	-0.59386	-0.63436	-0.63265	-0.71608
-0.50941	-0.59966	-0.59789	-0.59614	-0.59443	-0.59275	-0.71858	-0.71672	-0.90491
-0.5084	-0.59853	-0.59678	-0.59505	-0.59336	-0.59169	-0.71735	-0.71547	-0.90294
-0.5074	-0.59741	-0.59568	-0.59398	-0.5923	-0.59065	-0.71612	-0.71423	-0.90099
-0.59801	-0.59626	-0.59456	-0.59288	-0.67552	-0.67366	-0.90484	-0.90196	-1.00766
-0.59684	-0.59512	-0.59343	-0.59177	-0.67429	-0.67243	-0.90286	-0.90009	-1.00563
-0.59572	-0.63712	-0.63535	-0.63361	-0.79459	-0.79239	-0.93595	-0.93314	-1.07009
-0.59465	-0.636	-0.63424	-0.6325	-0.79322	-0.79104	-0.9341	-0.93129	-1.06788
-0.59357	-0.76503	-0.76292	-0.76083	-0.9381	-0.93516	-1.00566	-1.00259	-1.12841
-0.59246	-0.76363	-0.76151	-0.75941	-0.93611	-0.9333	-1.00371	-1.00065	-1.12625
-0.59138	-0.76226	-0.76013	-0.75804	-0.93416	-0.93145	-1.00175	-0.99869	-1.12408
-0.68014	-0.90614	-0.90317	-0.90027	-0.97245	-0.96952	-1.12855	-1.12518	-1.18587
-0.67896	-0.90432	-0.90143	-0.89859	-0.97055	-0.96755	-1.12629	-1.12292	-1.18354
-0.79359	-0.93744	-0.93451	-0.93165	-1.06675	-1.06355	-1.18818	-1.18473	-1.21253
-0.79222	-0.93555	-0.93268	-0.92987	-1.06473	-1.06154	-1.18597	-1.18256	-1.21033
-0.93659	-1.04016	-1.03697	-1.03384	-1.12651	-1.12315	-1.21495	-1.2115	-1.20808
-0.93462	-1.03801	-1.03484	-1.0317	-1.12423	-1.1209	-1.2126	-1.20917	-1.20576
-1.03908	-1.06793	-1.06471	-1.06152	-1.21721	-1.21375	-1.21031	-1.2069	-1.23046
-1.03708	-1.06589	-1.06269	-1.05953	-1.215	-1.21157	-1.20814	-1.20473	-1.22827
-1.03512	-1.0639	-1.06071	-1.05757	-1.21282	-1.20941	-1.20599	-1.20257	-1.2261
-1.03317	-1.16045	-1.1571	-1.15381	-1.21061	-1.20721	-1.23077	-1.2273	-1.2239

-1.03122	-1.15833	-1.15501	-1.15173	-1.20839	-1.20498	-1.22853	-1.22506	-1.22169
-1.15955	-1.2166	-1.21307	-1.20957	-1.23312	-1.22967	-1.22623	-1.22282	-1.24991
-1.15739	-1.21434	-1.2108	-1.20727	-1.23078	-1.2273	-1.2239	-1.22057	-1.24766
-1.1552	-1.21205	-1.20853	-1.20501	-1.22851	-1.22504	-1.25212	-1.24878	-1.27551
-1.15294	-1.20972	-1.20625	-1.20279	-1.22632	-1.2229	-1.24997	-1.24668	-1.2734
-1.21081	-1.23449	-1.23103	-1.22758	-1.22417	-1.22081	-1.27794	-1.27461	-1.29087
-1.20863	-1.23228	-1.22882	-1.2254	-1.22205	-1.21877	-1.27584	-1.27252	-1.28872
-1.2065	-1.23011	-1.22666	-1.22325	-1.21996	-1.21675	-1.27375	-1.27044	-1.28658
-1.23137	-1.22793	-1.22453	-1.22118	-1.2783	-1.27494	-1.29116	-1.28777	-1.33779
-1.22919	-1.22577	-1.22244	-1.21915	-1.27617	-1.27279	-1.28894	-1.28557	-1.33543
-1.22704	-1.25407	-1.2507	-1.24738	-1.29363	-1.29012	-1.34043	-1.33671	-1.3634
-1.22492	-1.25193	-1.24858	-1.24526	-1.29137	-1.28785	-1.33795	-1.33422	-1.36082
-1.22281	-1.29971	-1.29615	-1.29263	-1.28911	-1.28562	-1.3848	-1.38095	-1.37712
-1.22071	-1.29744	-1.29387	-1.29033	-1.28685	-1.28342	-1.38238	-1.37855	-1.37472
-1.29873	-1.29516	-1.29159	-1.28805	-1.35757	-1.35381	-1.37999	-1.37617	-1.37236
-1.29642	-1.29284	-1.28931	-1.28583	-1.35519	-1.35146	-1.37759	-1.37378	-1.37
-1.29413	-1.29053	-1.28705	-1.28364	-1.35284	-1.34914	-1.37521	-1.37141	-1.36765
-1.2919	-1.36183	-1.35799	-1.35423	-1.38041	-1.37662	-1.37283	-1.36904	-1.41776
-1.2897	-1.35947	-1.35564	-1.35189	-1.37801	-1.37424	-1.37046	-1.36668	-1.41536
-1.36099	-1.38714	-1.38325	-1.37943	-1.37562	-1.37186	-1.39417	-1.39039	-1.41303
-1.35852	-1.38468	-1.38084	-1.37704	-1.37324	-1.36946	-1.39179	-1.3881	-1.41078
-1.35609	-1.38225	-1.37844	-1.37466	-1.39697	-1.39316	-1.38945	-1.38583	-1.48572
-1.35373	-1.37983	-1.37603	-1.37227	-1.39459	-1.39083	-1.38716	-1.38357	-1.48335
-1.35139	-1.37744	-1.37364	-1.3699	-1.39223	-1.38854	-1.3849	-1.38132	-1.481
-1.37887	-1.37505	-1.37128	-1.36756	-1.3899	-1.38626	-1.48621	-1.48236	-1.47865
-1.3765	-1.37269	-1.36893	-1.36524	-1.38759	-1.384	-1.48383	-1.47999	-1.47636
-1.37414	-1.39641	-1.39264	-1.38895	-1.43754	-1.43383	-1.48146	-1.47771	-1.47421
-1.37179	-1.39404	-1.39033	-1.38669	-1.43521	-1.43149	-1.47912	-1.47551	-1.47219
-1.44815	-1.44421	-1.44038	-1.43664	-1.4843	-1.48048	-1.47687	-1.47347	-1.49748
-1.44575	-1.44184	-1.43803	-1.4343	-1.48192	-1.47816	-1.47472	-1.47159	-1.49577
-1.44339	-1.43949	-1.43568	-1.43198	-1.47959	-1.47596	-1.4999	-1.49694	-1.49414
-1.4411	-1.43719	-1.43335	-1.42966	-1.47733	-1.47392	-1.498	-1.49522	-1.49256
-1.43882	-1.43489	-1.43106	-1.42741	-1.47516	-1.47201	-1.49623	-1.49358	-1.49103
-1.4365	-1.45891	-1.45504	-1.45141	-1.47311	-1.47019	-1.49454	-1.49202	-1.48957
-1.43416	-1.45656	-1.4528	-1.44932	-1.47117	-1.46848	-1.49294	-1.49053	-1.48816
-1.43186	-1.47929	-1.47565	-1.47232	-1.49652	-1.49391	-1.49142	-1.48908	-1.48677
-1.42959	-1.47705	-1.47366	-1.4706	-1.4949	-1.49239	-1.48997	-1.48767	-1.4854
-1.45367	-1.50222	-1.49897	-1.49604	-1.49336	-1.49093	-1.48857	-1.4863	-1.51062
-1.45164	-1.50037	-1.49729	-1.49448	-1.4919	-1.48953	-1.48722	-1.48498	-1.50919
-1.4773	-1.49864	-1.4957	-1.493	-1.49049	-1.48817	-1.51259	-1.51018	-1.55909
-1.47537	-1.49694	-1.49417	-1.49158	-1.48914	-1.48684	-1.51116	-1.50879	-1.55757
-1.47356	-1.49532	-1.49271	-1.49022	-1.48783	-1.48553	-1.50975	-1.50741	-1.55607
-1.49638	-1.49381	-1.49132	-1.48893	-1.51327	-1.51077	-1.55966	-1.55711	-1.55458
-1.49488	-1.49239	-1.48999	-1.48768	-1.51194	-1.50941	-1.55814	-1.5556	-1.55309
-1.49342	-1.491	-1.48867	-1.48641	-1.53329	-1.53071	-1.57499	-1.57242	-1.59098
-1.49202	-1.48965	-1.48736	-1.48513	-1.53187	-1.52933	-1.57348	-1.5709	-1.58943
-1.51386	-1.55673	-1.5542	-1.55171	-1.57737	-1.57465	-1.59311	-1.59048	-1.62473
-1.51253	-1.55526	-1.55274	-1.55026	-1.57585	-1.57319	-1.59165	-1.58903	-1.62326
-1.51124	-1.55383	-1.5513	-1.54882	-1.57433	-1.57174	-1.59021	-1.58761	-1.62182
-1.55504	-1.58084	-1.5781	-1.57541	-1.59389	-1.5913	-1.62552	-1.62292	-1.62038

-1.55365	-1.57938	-1.57661	-1.57387	-1.59233	-1.58978	-1.62401	-1.62146	-1.61895
-1.55221	-1.63608	-1.63317	-1.63031	-1.62763	-1.62507	-1.66545	-1.66285	-1.68241
-1.55076	-1.63442	-1.63153	-1.62869	-1.62606	-1.62358	-1.66393	-1.66139	-1.68085
-1.6356	-1.63278	-1.62995	-1.62718	-1.66756	-1.66498	-1.68478	-1.68202	-1.67926
-1.63397	-1.63115	-1.62844	-1.6258	-1.66611	-1.66349	-1.68312	-1.68036	-1.67762
-1.63235	-1.6727	-1.66997	-1.66733	-1.66467	-1.66203	-1.6815	-1.67873	-1.67601
-1.63072	-1.67103	-1.66835	-1.66577	-1.66317	-1.6606	-1.67996	-1.67718	-1.67447
-1.62912	-1.6694	-1.66676	-1.6642	-1.66167	-1.65918	-1.67843	-1.67565	-1.67295
-1.6706	-1.66788	-1.66525	-1.66268	-1.68228	-1.67957	-1.67685	-1.6741	-1.67144
-1.66905	-1.66642	-1.66378	-1.66118	-1.68063	-1.67793	-1.67524	-1.67256	-1.66993
-1.66752	-1.68752	-1.68465	-1.6818	-1.67904	-1.67635	-1.67369	-1.67106	-1.67956
-1.66602	-1.68586	-1.68305	-1.68027	-1.67752	-1.67481	-1.67216	-1.6696	-1.67814
-1.66454	-1.68424	-1.68146	-1.67872	-1.67599	-1.6733	-1.68175	-1.67922	-1.6767
-1.66308	-1.68268	-1.67989	-1.67715	-1.67447	-1.67185	-1.68032	-1.67773	-1.67521
-1.68386	-1.6811	-1.67832	-1.67557	-1.684	-1.68145	-1.67888	-1.67627	-1.67376
-1.6822	-1.67947	-1.67671	-1.674	-1.68244	-1.67991	-1.67738	-1.67487	-1.6724
-1.68055	-1.67783	-1.67512	-1.67244	-1.6809	-1.67836	-1.67588	-1.67347	-1.67107
-1.67897	-1.6873	-1.68463	-1.682	-1.67941	-1.67686	-1.6744	-1.67204	-1.66966
-1.67743	-1.68576	-1.68314	-1.68056	-1.67796	-1.67539	-1.67292	-1.67058	-1.66823
-1.68694	-1.68427	-1.68166	-1.67908	-1.67652	-1.67397	-1.69	-1.68763	-1.73135
-1.68543	-1.68281	-1.68018	-1.67758	-1.67507	-1.67261	-1.68865	-1.68623	-1.72973
-1.68393	-1.69987	-1.69723	-1.69463	-1.69214	-1.68972	-1.68729	-1.68485	-1.72816
-1.68245	-1.69835	-1.69575	-1.69321	-1.69074	-1.68832	-1.6859	-1.6835	-1.72664
-1.68097	-1.69684	-1.69429	-1.6918	-1.68934	-1.68692	-1.68452	-1.68215	-1.72515
-1.69805	-1.6954	-1.69287	-1.69039	-1.68795	-1.68554	-1.72902	-1.7263	-1.7389
-1.69661	-1.69399	-1.69146	-1.68899	-1.68656	-1.68418	-1.72749	-1.72478	-1.73739
-1.69514	-1.69255	-1.69006	-1.68762	-1.68519	-1.6828	-1.74125	-1.73857	-1.73594
-1.69366	-1.69111	-1.68866	-1.68626	-1.68384	-1.68143	-1.73968	-1.7371	-1.73453
-1.69224	-1.68972	-1.68729	-1.68489	-1.74366	-1.74085	-1.73818	-1.73564	-1.73309
-1.69088	-1.6884	-1.68593	-1.68349	-1.74209	-1.7394	-1.73676	-1.73417	-1.73161
-1.68953	-1.70305	-1.70053	-1.69803	-1.74056	-1.73794	-1.73533	-1.73272	-1.73014
-1.68816	-1.7017	-1.69921	-1.69673	-1.73908	-1.73645	-1.73385	-1.73128	-1.72873
-1.68679	-1.70035	-1.69789	-1.69545	-1.73764	-1.73498	-1.73238	-1.72984	-1.72732
-1.70139	-1.74439	-1.74164	-1.73893	-1.73624	-1.73358	-1.73097	-1.7284	-1.73713
-1.70003	-1.74279	-1.74012	-1.7375	-1.73486	-1.73222	-1.72959	-1.72697	-1.73561
-1.7441	-1.74129	-1.73863	-1.73602	-1.73341	-1.73082	-1.72821	-1.7256	-1.73418
-1.74266	-1.73988	-1.73717	-1.73452	-1.73193	-1.72938	-1.72684	-1.72428	-1.73281
-1.74119	-1.73846	-1.73574	-1.73307	-1.73048	-1.72795	-1.73673	-1.73408	-1.73144
-1.73969	-1.73705	-1.73436	-1.73169	-1.72908	-1.72651	-1.73523	-1.73265	-1.73007
-1.73819	-1.73561	-1.73296	-1.73032	-1.73915	-1.7364	-1.73376	-1.73122	-1.72867
-1.7367	-1.7341	-1.7315	-1.72891	-1.73771	-1.735	-1.73236	-1.72978	-1.72722
-1.73524	-1.7326	-1.73002	-1.72749	-1.73628	-1.73363	-1.73099	-1.72835	-1.72577
-1.73382	-1.73117	-1.72859	-1.72606	-1.73483	-1.73223	-1.72961	-1.72696	-1.72436
-1.73245	-1.72978	-1.72718	-1.72464	-1.73337	-1.73082	-1.72823	-1.72559	-1.723
-1.73106	-1.73996	-1.73723	-1.73455	-1.73194	-1.72939	-1.72681	-1.72422	-1.72165
-1.72966	-1.73853	-1.73583	-1.73317	-1.73054	-1.72794	-1.72537	-1.72284	-1.72033
-1.73985	-1.73712	-1.73444	-1.7318	-1.72915	-1.72652	-1.72396	-1.72149	-1.76519
-1.73838	-1.73572	-1.73308	-1.73044	-1.72779	-1.72515	-1.7226	-1.72015	-1.76371
-1.73693	-1.73434	-1.73172	-1.72909	-1.72645	-1.7238	-1.72126	-1.71882	-1.76221
-1.73551	-1.73296	-1.73036	-1.72774	-1.72511	-1.72248	-1.76623	-1.76342	-1.77662

-1.73411	-1.73158	-1.72899	-1.72639	-1.72378	-1.72117	-1.76474	-1.76188	-1.77505
-1.73269	-1.74673	-1.74414	-1.74155	-1.73895	-1.73635	-1.77923	-1.77634	-1.77354
-1.73126	-1.74525	-1.74268	-1.74014	-1.73759	-1.73504	-1.77777	-1.77488	-1.77206
-1.74645	-1.74382	-1.74125	-1.73872	-1.78201	-1.77916	-1.77631	-1.77343	-1.77638
-1.74507	-1.74243	-1.73983	-1.73728	-1.78041	-1.77764	-1.77484	-1.772	-1.77496
-1.7437	-1.74106	-1.73844	-1.73588	-1.78462	-1.7819	-1.77913	-1.77633	-1.77357
-1.74235	-1.73971	-1.7371	-1.73454	-1.7831	-1.78038	-1.77765	-1.77492	-1.77221
-1.741	-1.73837	-1.73578	-1.73323	-1.78161	-1.77888	-1.77617	-1.77351	-1.77085
-1.73965	-1.78875	-1.78582	-1.78293	-1.78012	-1.77737	-1.77469	-1.77208	-1.76946
-1.73831	-1.78726	-1.78433	-1.78145	-1.77863	-1.77587	-1.77321	-1.77065	-1.76808
-1.74313	-1.78577	-1.78285	-1.77997	-1.77716	-1.77443	-1.77179	-1.76926	-1.76671
-1.7418	-1.78429	-1.78136	-1.77848	-1.77571	-1.77303	-1.77042	-1.7679	-1.76535
-1.7857	-1.7828	-1.7799	-1.77704	-1.7743	-1.77165	-1.76906	-1.76653	-1.76399
-1.7842	-1.78132	-1.77846	-1.77566	-1.77293	-1.77028	-1.76768	-1.76517	-1.76264
-1.78271	-1.77984	-1.77703	-1.77428	-1.77157	-1.76891	-1.76632	-1.76381	-1.81454
-1.78121	-1.77836	-1.7756	-1.7729	-1.77021	-1.76755	-1.76496	-1.76245	-1.81307
-1.77973	-1.7769	-1.77418	-1.77152	-1.76884	-1.76619	-1.76361	-1.76111	-1.8116
-1.77829	-1.77549	-1.77279	-1.77014	-1.78344	-1.78076	-1.79268	-1.79016	-1.81014
-1.77687	-1.77411	-1.77142	-1.76876	-1.78205	-1.77939	-1.7913	-1.7888	-1.80869
-1.79149	-1.78874	-1.78603	-1.78335	-1.79519	-1.79252	-1.82598	-1.8233	-1.85542
-1.79007	-1.78736	-1.78466	-1.78198	-1.79381	-1.79114	-1.82451	-1.82184	-1.85388
-1.80324	-1.81407	-1.81137	-1.80868	-1.80601	-1.80335	-1.83979	-1.83705	-1.85235
-1.80183	-1.81267	-1.80998	-1.8073	-1.80464	-1.802	-1.83831	-1.83558	-1.85083
-1.80044	-1.81129	-1.8086	-1.80594	-1.8033	-1.80068	-1.83687	-1.83412	-1.84932
-1.81261	-1.80992	-1.80724	-1.80458	-1.8243	-1.82154	-1.85377	-1.85078	-1.84783
-1.81125	-1.80857	-1.8059	-1.80326	-1.8229	-1.82017	-1.85234	-1.84933	-1.84637
-1.80993	-1.80729	-1.80463	-1.80199	-1.85692	-1.85388	-1.85087	-1.84787	-1.84493
-1.80865	-1.80604	-1.80341	-1.80077	-1.85547	-1.85239	-1.84938	-1.84642	-1.8435
-1.80736	-1.84411	-1.84123	-1.83834	-1.85399	-1.8509	-1.8479	-1.84497	-1.84208
-1.80607	-1.84267	-1.83978	-1.83689	-1.85248	-1.84941	-1.84643	-1.84353	-1.84066
-1.82187	-1.86035	-1.85719	-1.85405	-1.85097	-1.84793	-1.84497	-1.84209	-1.88999
-1.82051	-1.85879	-1.85564	-1.85251	-1.84945	-1.84645	-1.84352	-1.84067	-1.88834
-1.81913	-1.85723	-1.85409	-1.85097	-1.84795	-1.84499	-1.84209	-1.83926	-1.8867
-1.85879	-1.85568	-1.85257	-1.84949	-1.8465	-1.84357	-1.84069	-1.83786	-1.88508
-1.85722	-1.85413	-1.85106	-1.84804	-1.84508	-1.84219	-1.83932	-1.83648	-1.88347
-1.85567	-1.85259	-1.84957	-1.8466	-1.8437	-1.84085	-1.88848	-1.88521	-1.88195
-1.85414	-1.85107	-1.84809	-1.84517	-1.84233	-1.83955	-1.88701	-1.88375	-1.88049
-1.8527	-1.84965	-1.84669	-1.8438	-1.84098	-1.83822	-1.88548	-1.88222	-1.87897
-1.85137	-1.84834	-1.84539	-1.84249	-1.83965	-1.83685	-1.88388	-1.88063	-1.87738
-1.85001	-1.84702	-1.84407	-1.84116	-1.88877	-1.88551	-1.88225	-1.87901	-1.87577
-1.84857	-1.8456	-1.84267	-1.83977	-1.88715	-1.88388	-1.88062	-1.87738	-1.87414
-1.84711	-1.84417	-1.84126	-1.83837	-1.88552	-1.88225	-1.87899	-1.87575	-1.87252
-1.84571	-1.85833	-1.85526	-1.85223	-1.8839	-1.88064	-1.87739	-1.87414	-1.87093
-1.84435	-1.85684	-1.85376	-1.85074	-1.88228	-1.87904	-1.8758	-1.87257	-1.86937
-1.843	-1.89064	-1.88729	-1.88401	-1.88075	-1.87751	-1.90361	-1.90036	-1.89714
-1.84167	-1.88917	-1.88584	-1.88255	-1.87929	-1.87605	-1.90213	-1.89888	-1.89569
-1.85576	-1.88765	-1.88433	-1.88105	-1.90713	-1.90386	-1.90061	-1.89738	-1.8942
-1.85423	-1.88602	-1.88275	-1.87949	-1.90554	-1.90227	-1.89903	-1.89581	-1.89264
-1.85274	-1.91378	-1.9105	-1.90724	-1.90397	-1.9007	-1.89747	-1.89426	-1.90636
-1.85136	-1.91225	-1.90898	-1.90573	-1.90247	-1.89922	-1.896	-1.89281	-1.90487

-1.85001	-1.91075	-1.90748	-1.90423	-1.90099	-1.89777	-1.89456	-1.89137	-1.90339
-1.91244	-1.90917	-1.90591	-1.90267	-1.89944	-1.89623	-1.89304	-1.88988	-1.90188
-1.91081	-1.90756	-1.90431	-1.90108	-1.89785	-1.89465	-1.89148	-1.88834	-1.90034
-1.90924	-1.906	-1.90277	-1.89955	-1.89633	-1.89313	-1.88997	-1.88684	-1.89882
-1.90774	-1.90451	-1.90129	-1.89808	-1.89488	-1.89169	-1.88852	-1.88537	-1.89731
-1.90623	-1.903	-1.89979	-1.89659	-1.89341	-1.89023	-1.88707	-1.88391	-1.89582
-1.90466	-1.90144	-1.89822	-1.89503	-1.89186	-1.88871	-1.88558	-1.88247	-1.8944
-1.90311	-1.89989	-1.89668	-1.89348	-1.89032	-1.88719	-1.8841	-1.88104	-1.89298
-1.90163	-1.89842	-1.89522	-1.89204	-1.88889	-1.88577	-1.88268	-1.87962	-1.89152
-1.90018	-1.89698	-1.8938	-1.89064	-1.8875	-1.88438	-1.88128	-1.8782	-1.89006
-1.89869	-1.89547	-1.8923	-1.88915	-1.88603	-1.88293	-1.89482	-1.89171	-1.88867
-1.89718	-1.89393	-1.89075	-1.88761	-1.88451	-1.88146	-1.89336	-1.89032	-1.88732
-1.89569	-1.89245	-1.88927	-1.88614	-1.88306	-1.88004	-1.89194	-1.88894	-1.88596
-1.89423	-1.89104	-1.88788	-1.88475	-1.8817	-1.87869	-1.89057	-1.88758	-1.8846
-1.89277	-1.88962	-1.88649	-1.88338	-1.88033	-1.87733	-1.88919	-1.88621	-1.88323
-1.89133	-1.8882	-1.88508	-1.88199	-1.87894	-1.87592	-1.88775	-1.88478	-1.88183
-1.88988	-1.88676	-1.88367	-1.8806	-1.87755	-1.87452	-1.88632	-1.88336	-1.88044
-1.88842	-1.8853	-1.88223	-1.8792	-1.87617	-1.87317	-1.88496	-1.88201	-1.8791
-1.88696	-1.88383	-1.88078	-1.87779	-1.87481	-1.87185	-1.88363	-1.88069	-1.87778
-1.88552	-1.88241	-1.87939	-1.87642	-1.87347	-1.87054	-1.88231	-1.87938	-1.88854
-1.88409	-1.88103	-1.87804	-1.87509	-1.87216	-1.86924	-1.88099	-1.87807	-1.88723
-1.88271	-1.87968	-1.8767	-1.87376	-1.87082	-1.86791	-1.91343	-1.91051	-1.9076
-1.88137	-1.87836	-1.87539	-1.87243	-1.86947	-1.86652	-1.91201	-1.90911	-1.90624
-1.88004	-1.87705	-1.87406	-1.87109	-1.90242	-1.89942	-1.9106	-1.90773	-1.90488
-1.87871	-1.87572	-1.87272	-1.86972	-1.90106	-1.8981	-1.90927	-1.90641	-1.90358
-1.87738	-1.87438	-1.87136	-1.86835	-1.89972	-1.89681	-1.90798	-1.90512	-1.90229
-1.89856	-1.90743	-1.90438	-1.90136	-1.91252	-1.9096	-1.90671	-1.90386	-1.91188
-1.89719	-1.90604	-1.90302	-1.90005	-1.91123	-1.90833	-1.90546	-1.90261	-1.9106
-1.90777	-1.9047	-1.90171	-1.89876	-1.90994	-1.90706	-1.9042	-1.90135	-1.90933
-1.90647	-1.90341	-1.90043	-1.8975	-1.90866	-1.9058	-1.90294	-1.90009	-1.90805
-1.90515	-1.90211	-1.89915	-1.89624	-1.90738	-1.90452	-1.91251	-1.90963	-1.90676
-1.90378	-1.90078	-1.89786	-1.89497	-1.9061	-1.90324	-1.9112	-1.90831	-1.90545
-1.90242	-1.89946	-1.89657	-1.89371	-1.90481	-1.90194	-1.90988	-1.90699	-1.90414
-1.90111	-1.89818	-1.8953	-1.89245	-1.91436	-1.91143	-1.90855	-1.90569	-1.90289
-1.89984	-1.89692	-1.89404	-1.89119	-1.91303	-1.91009	-1.90722	-1.90442	-1.90165
-1.89857	-1.90671	-1.90381	-1.90094	-1.91175	-1.9088	-1.90594	-1.90316	-1.90042
-1.8973	-1.90544	-1.90256	-1.8997	-1.9105	-1.90756	-1.90471	-1.90193	-1.89919
-1.89603	-1.90417	-1.9013	-1.89845	-1.90924	-1.90633	-1.90348	-1.90071	-1.89798
-1.89476	-1.9029	-1.90004	-1.89719	-1.90798	-1.90509	-1.90226	-1.8995	-1.89677
-1.90452	-1.90164	-1.89878	-1.89595	-1.90673	-1.90386	-1.90105	-1.8983	-1.89557
-1.90326	-1.90039	-1.89755	-1.89473	-1.9055	-1.90264	-1.89984	-1.8971	-1.89438
-1.90201	-1.89915	-1.89632	-1.89352	-1.90429	-1.90143	-1.89864	-1.89591	-1.8932
-1.90075	-1.8979	-1.8951	-1.89231	-1.90307	-1.90022	-1.89744	-1.89471	-1.89201
-1.8995	-1.89666	-1.89388	-1.89111	-1.90185	-1.89901	-1.89623	-1.89351	-1.89082
-1.89826	-1.89544	-1.89267	-1.88991	-1.90064	-1.89781	-1.89504	-1.89232	-1.88963
-1.89704	-1.89424	-1.89147	-1.88872	-1.89944	-1.89661	-1.89384	-1.89113	-1.88845
-1.89584	-1.89304	-1.89028	-1.88753	-1.88476	-1.88197	-1.89265	-1.88994	-1.88726
-1.89466	-1.89184	-1.88908	-1.88634	-1.88357	-1.88079	-1.89145	-1.88874	-1.88607
-1.89347	-1.89064	-1.88788	-1.88514	-1.88237	-1.8796	-1.89025	-1.88755	-1.88489
-1.89224	-1.88942	-1.88666	-1.88393	-1.88117	-1.8784	-1.88904	-1.88635	-1.8837

-1.891	-1.88819	-1.88545	-1.88273	-1.87997	-1.87721	-1.88784	-1.88516	-1.88251
-1.88978	-1.88698	-1.88424	-1.88153	-1.8788	-1.87607	-1.88669	-1.884	-1.88134
-1.88857	-1.88577	-1.88304	-1.88034	-1.87764	-1.87496	-1.88556	-1.88286	-1.88019
-1.88739	-1.8846	-1.88186	-1.87915	-1.87648	-1.87382	-1.87116	-1.86848	-1.87904
-1.88625	-1.88348	-1.88071	-1.87798	-1.8753	-1.87267	-1.87002	-1.86735	-1.87789
-1.88509	-1.88233	-1.87956	-1.87681	-1.87414	-1.87152	-1.86888	-1.86623	-1.87676
-1.88389	-1.88114	-1.87837	-1.87563	-1.87297	-1.87037	-1.86776	-1.86516	-1.87568
-1.88269	-1.87995	-1.8772	-1.87448	-1.87183	-1.86923	-1.86664	-1.86408	-1.87459
-1.88154	-1.87879	-1.87607	-1.87338	-1.87072	-1.86811	-1.86552	-1.86296	-1.87346
-1.88039	-1.87764	-1.87495	-1.8723	-1.86964	-1.86699	-1.86439	-1.86182	-1.8723
-1.87922	-1.87648	-1.87382	-1.87118	-1.86853	-1.86587	-1.86326	-1.86069	-1.87084
-1.87804	-1.87532	-1.87267	-1.87005	-1.8674	-1.86476	-1.86214	-1.85955	-1.86969
-1.8769	-1.87419	-1.87155	-1.86893	-1.87904	-1.87639	-1.87376	-1.87114	-1.86854
-1.8758	-1.87312	-1.87047	-1.86783	-1.87793	-1.87531	-1.87268	-1.87002	-1.86739
-1.8747	-1.88477	-1.88211	-1.87946	-1.87684	-1.87422	-1.87158	-1.86891	-1.86626
-1.87359	-1.88364	-1.881	-1.87838	-1.87575	-1.87311	-1.87046	-1.86781	-1.86516
-1.87248	-1.88251	-1.87989	-1.87729	-1.87464	-1.87197	-1.86932	-1.8667	-1.86406
-1.88403	-1.88136	-1.87874	-1.87614	-1.87349	-1.87081	-1.86816	-1.86554	-1.86291
-1.88287	-1.8802	-1.87758	-1.87497	-1.87231	-1.86964	-1.86699	-1.86436	-1.86173
-1.88173	-1.87908	-1.87644	-1.87382	-1.87117	-1.86851	-1.86586	-1.86323	-1.8606
-1.88062	-1.87798	-1.87533	-1.87268	-1.87004	-1.8674	-1.86476	-1.86212	-1.85949
-1.87951	-1.87687	-1.87422	-1.87157	-1.86893	-1.8663	-1.86366	-1.86102	-1.85839
-1.87841	-1.87576	-1.87312	-1.87048	-1.86784	-1.86519	-1.86256	-1.85993	-1.85732
-1.87731	-1.87466	-1.87202	-1.86939	-1.86675	-1.86409	-1.86146	-1.85885	-1.85625
-1.87621	-1.87356	-1.87092	-1.86828	-1.86564	-1.86301	-1.86039	-1.85777	-1.85517
-1.87512	-1.87248	-1.86982	-1.86717	-1.86455	-1.86193	-1.85932	-1.8567	-1.8541
-1.87402	-1.87138	-1.86873	-1.86609	-1.86346	-1.86084	-1.85823	-1.85563	-1.85304
-1.87293	-1.87027	-1.86764	-1.86501	-1.86238	-1.85975	-1.85714	-1.85457	-1.85199
-1.87184	-1.86919	-1.86655	-1.86393	-1.8613	-1.85867	-1.85607	-1.8535	-1.85093
-1.87075	-1.86812	-1.86547	-1.86283	-1.86021	-1.8576	-1.85501	-1.85243	-1.84986
-1.86967	-1.86704	-1.86439	-1.86175	-1.85913	-1.85653	-1.85394	-1.85136	-1.84879
-1.86859	-1.86594	-1.8633	-1.86068	-1.85806	-1.85545	-1.85286	-1.8503	-1.84773
-1.86752	-1.86484	-1.86222	-1.85961	-1.85699	-1.85437	-1.85178	-1.84923	-1.84666
-1.86648	-1.86378	-1.86113	-1.8585	-1.85589	-1.8533	-1.85072	-1.84815	-1.84558
-1.86544	-1.86274	-1.86005	-1.85739	-1.85479	-1.85222	-1.84965	-1.84706	-1.84448
-1.86438	-1.8617	-1.85901	-1.85633	-1.85371	-1.85113	-1.84855	-1.84597	-1.84339
-1.86331	-1.86066	-1.85798	-1.85529	-1.85265	-1.85002	-1.84743	-1.84487	-1.84231
-1.86222	-1.85958	-1.85692	-1.85425	-1.8516	-1.84895	-1.84634	-1.84379	-1.84122
-1.86111	-1.85845	-1.85582	-1.85321	-1.85057	-1.84792	-1.8453	-1.8427	-1.84012
-1.86002	-1.85734	-1.85473	-1.85214	-1.84952	-1.84689	-1.84426	-1.84164	-1.83905
-1.85897	-1.85628	-1.85364	-1.85103	-1.84843	-1.84584	-1.84323	-1.84061	-1.83801
-1.85794	-1.85524	-1.85257	-1.84992	-1.84734	-1.84479	-1.8422	-1.83959	-1.837
-1.85687	-1.8542	-1.85153	-1.84887	-1.8463	-1.84376	-1.84118	-1.83857	-1.83597
-1.8558	-1.85317	-1.8505	-1.84785	-1.84527	-1.84274	-1.84016	-1.83755	-1.83494
-1.85475	-1.85213	-1.84948	-1.84683	-1.84423	-1.84168	-1.8391	-1.83651	-1.83391
-1.85371	-1.85109	-1.84844	-1.8458	-1.84319	-1.84059	-1.83802	-1.83546	-1.83289
-1.85265	-1.85003	-1.8474	-1.84478	-1.84215	-1.83953	-1.83695	-1.8344	-1.83183
-1.85157	-1.84894	-1.84634	-1.84376	-1.84114	-1.8385	-1.83589	-1.83331	-1.83073
-1.85051	-1.84786	-1.84529	-1.84274	-1.84012	-1.83748	-1.83486	-1.83224	-1.82965
-1.84948	-1.84683	-1.84426	-1.84172	-1.83912	-1.83648	-1.83385	-1.83122	-1.82861

-1.84846	-1.84581	-1.84324	-1.84069	-1.8381	-1.83549	-1.83286	-1.83022	-1.82759
-1.84746	-1.8448	-1.8422	-1.83962	-1.83704	-1.83446	-1.83185	-1.82921	-1.82658
-1.84645	-1.84379	-1.84115	-1.83854	-1.83596	-1.83341	-1.83083	-1.8282	-1.82557
-1.84545	-1.84278	-1.84012	-1.83749	-1.83492	-1.83239	-1.82981	-1.82719	-1.82456
-1.84445	-1.84178	-1.83912	-1.83649	-1.83391	-1.83138	-1.8288	-1.82618	-1.82355
-1.84345	-1.84079	-1.83812	-1.83548	-1.83291	-1.83037	-1.82779	-1.82517	-1.82254
-1.84246	-1.83979	-1.83712	-1.83448	-1.8319	-1.82936	-1.82678	-1.82416	-1.82153
-1.84146	-1.83879	-1.83612	-1.83348	-1.8309	-1.82836	-1.82578	-1.82315	-1.82052
-1.84046	-1.83779	-1.83512	-1.83247	-1.82989	-1.82735	-1.82476	-1.82214	-1.81951
-1.83946	-1.83679	-1.83412	-1.83147	-1.82888	-1.82634	-1.82375	-1.82113	-1.81849
-1.83846	-1.83579	-1.83312	-1.83046	-1.82788	-1.82532	-1.82274	-1.82011	-1.81748
-1.83746	-1.83479	-1.83211	-1.82946	-1.82687	-1.82432	-1.82172	-1.8191	-1.81646
-1.83646	-1.83378	-1.83111	-1.82845	-1.82586	-1.8233	-1.82071	-1.81808	-1.84142
-1.83546	-1.83278	-1.8301	-1.82744	-1.82484	-1.82229	-1.8197	-1.81707	-1.84039
-1.83446	-1.83178	-1.82909	-1.82643	-1.82383	-1.82128	-1.81868	-1.81606	-1.83936
-1.83345	-1.83077	-1.82808	-1.82542	-1.82282	-1.82027	-1.81768	-1.81505	-1.83833
-1.83244	-1.82976	-1.82707	-1.82441	-1.82182	-1.81926	-1.81667	-1.81404	-1.83729
-1.83143	-1.82875	-1.82607	-1.82341	-1.82082	-1.81826	-1.84165	-1.83896	-1.83626
-1.83043	-1.82775	-1.82507	-1.82243	-1.81983	-1.81726	-1.84063	-1.83793	-1.83523
-1.80407	-1.82676	-1.82411	-1.82148	-1.81887	-1.81628	-1.83961	-1.83691	-1.83421
-1.8031	-1.82579	-1.82317	-1.82056	-1.81793	-1.81531	-1.8386	-1.83589	-1.83318
-1.80215	-1.82485	-1.82223	-1.81962	-1.843	-1.8403	-1.83759	-1.83487	-1.83216
-1.80123	-1.82393	-1.8213	-1.81866	-1.842	-1.83929	-1.83657	-1.83385	-1.83114
-1.80031	-1.823	-1.82035	-1.81768	-1.84099	-1.83827	-1.83555	-1.83283	-1.83013
-1.79939	-1.82203	-1.81936	-1.81668	-1.83997	-1.83725	-1.83453	-1.83182	-1.82913
-1.79847	-1.82104	-1.81836	-1.81568	-1.83895	-1.83623	-1.83352	-1.83082	-1.82814
-1.79751	-1.79488	-1.79226	-1.78964	-1.83793	-1.83521	-1.83252	-1.82985	-1.82719
-1.79654	-1.79391	-1.79129	-1.78867	-1.83693	-1.83421	-1.83153	-1.82889	-1.82624
-1.79556	-1.81957	-1.81692	-1.81427	-1.83597	-1.83325	-1.83057	-1.82793	-1.82527
-1.79458	-1.81858	-1.81596	-1.81335	-1.83506	-1.83235	-1.82964	-1.82696	-1.82426
-1.79364	-1.81763	-1.81501	-1.81241	-1.80977	-1.8071	-1.82868	-1.82597	-1.82324
-1.79276	-1.81672	-1.81407	-1.81143	-1.80877	-1.8061	-1.82767	-1.82494	-1.82221
-1.79188	-1.8158	-1.81312	-1.81044	-1.80777	-1.80509	-1.82664	-1.82391	-1.82118
-1.79093	-1.81483	-1.81214	-1.80945	-1.80676	-1.80408	-1.82561	-1.82288	-1.82016
-1.78996	-1.81385	-1.81116	-1.80846	-1.80577	-1.80309	-1.8246	-1.82186	-1.81915
-1.81558	-1.81291	-1.81022	-1.80752	-1.80484	-1.80215	-1.79947	-1.79678	-1.81818
-1.81463	-1.812	-1.80932	-1.80662	-1.80394	-1.80126	-1.79858	-1.79588	-1.81724
-1.81368	-1.81104	-1.80837	-1.80568	-1.803	-1.80032	-1.79764	-1.79494	-1.81627
-1.81273	-1.81006	-1.80738	-1.8047	-1.80202	-1.79933	-1.79665	-1.79395	-1.81527
-1.81176	-1.80907	-1.8064	-1.80373	-1.80105	-1.79837	-1.79568	-1.79299	-1.79029
-1.81076	-1.80807	-1.80543	-1.8028	-1.80014	-1.79746	-1.79477	-1.79208	-1.78939
-1.80978	-1.80709	-1.80447	-1.80187	-1.79922	-1.79654	-1.79386	-1.79117	-1.78848
-1.80888	-1.80618	-1.80354	-1.80091	-1.79825	-1.79557	-1.79289	-1.7902	-1.7875
-1.80799	-1.80529	-1.80262	-1.79995	-1.79727	-1.79458	-1.7919	-1.78921	-1.78652
-1.80708	-1.80439	-1.80171	-1.79903	-1.79634	-1.79365	-1.79096	-1.78827	-1.78558
-1.80614	-1.80349	-1.80082	-1.79814	-1.79544	-1.79274	-1.79005	-1.78736	-1.78467
-1.80519	-1.80255	-1.79988	-1.7972	-1.7945	-1.7918	-1.7891	-1.78641	-1.78372
-1.80423	-1.80157	-1.79889	-1.7962	-1.7935	-1.7908	-1.7881	-1.78541	-1.78272
-1.80329	-1.8006	-1.79791	-1.79522	-1.79252	-1.78982	-1.78712	-1.78443	-1.78173
-1.8024	-1.7997	-1.797	-1.7943	-1.7916	-1.7889	-1.7862	-1.7835	-1.78081

-1.80149	-1.79879	-1.79609	-1.79338	-1.79068	-1.78798	-1.78528	-1.78258	-1.7799
-1.80052	-1.79782	-1.79511	-1.7924	-1.7897	-1.787	-1.7843	-1.7816	-1.77892
-1.79953	-1.79682	-1.79411	-1.7914	-1.7887	-1.786	-1.7833	-1.78061	-1.77792
-1.79859	-1.79588	-1.79317	-1.79047	-1.78776	-1.78506	-1.78237	-1.77967	-1.77699
-1.7977	-1.795	-1.79229	-1.78958	-1.78688	-1.78418	-1.78148	-1.77879	-1.77611
-1.79683	-1.79412	-1.79142	-1.78871	-1.786	-1.7833	-1.78061	-1.77792	-1.77524
-1.79597	-1.79326	-1.79056	-1.78785	-1.78514	-1.78244	-1.77974	-1.77706	-1.77439
-1.7951	-1.7924	-1.78969	-1.78698	-1.78428	-1.78157	-1.77888	-1.7762	-1.77353
-1.79424	-1.79153	-1.78882	-1.78611	-1.7834	-1.7807	-1.778	-1.77533	-1.77265
-1.79335	-1.79064	-1.78792	-1.78521	-1.78251	-1.7798	-1.77711	-1.77443	-1.77176
-1.7924	-1.78968	-1.78697	-1.78426	-1.78155	-1.77884	-1.77615	-1.77348	-1.7708
-1.79141	-1.78869	-1.78598	-1.78327	-1.78056	-1.77785	-1.77516	-1.77248	-1.76981
-1.78196	-1.78776	-1.78505	-1.78233	-1.77962	-1.77692	-1.77423	-1.77155	-1.76887
-1.78108	-1.78689	-1.78417	-1.78145	-1.77874	-1.77604	-1.77335	-1.77066	-1.76798
-1.78021	-1.78603	-1.78331	-1.78059	-1.77788	-1.77518	-1.77248	-1.76979	-1.76711
-1.77934	-1.78517	-1.78245	-1.77973	-1.77702	-1.77432	-1.77162	-1.76894	-1.76625
-1.77849	-1.78433	-1.7816	-1.77888	-1.77617	-1.77347	-1.77077	-1.76809	-1.7654
-1.77763	-1.77489	-1.77216	-1.76943	-1.77531	-1.77261	-1.76991	-1.76723	-1.76454
-1.77677	-1.77403	-1.7713	-1.76857	-1.77445	-1.77175	-1.76905	-1.76636	-1.76368
-1.77591	-1.77317	-1.77044	-1.76771	-1.77359	-1.77089	-1.76819	-1.7655	-1.76281
-1.77505	-1.77231	-1.76958	-1.76685	-1.77274	-1.77003	-1.76733	-1.76464	-1.76195
-1.77419	-1.77145	-1.76871	-1.76598	-1.76326	-1.76053	-1.76647	-1.76377	-1.76109
-1.77332	-1.77058	-1.76785	-1.76511	-1.76239	-1.75967	-1.76561	-1.76291	-1.76023
-1.77245	-1.76972	-1.76698	-1.76424	-1.76152	-1.7588	-1.76475	-1.76205	-1.75936
-1.77159	-1.76885	-1.76611	-1.76337	-1.76065	-1.75793	-1.76389	-1.76119	-1.7585
-1.77072	-1.76797	-1.76523	-1.7625	-1.75978	-1.75706	-1.76303	-1.76033	-1.75764
-1.76984	-1.7671	-1.76436	-1.76163	-1.75891	-1.7562	-1.75349	-1.75078	-1.75679
-1.76897	-1.76623	-1.7635	-1.76077	-1.75805	-1.75533	-1.75262	-1.74992	-1.75593
-1.7681	-1.76536	-1.76263	-1.7599	-1.75718	-1.75447	-1.75175	-1.74905	-1.75507
-1.76724	-1.7645	-1.76176	-1.75904	-1.75632	-1.7536	-1.75089	-1.74818	-1.75421
-1.76637	-1.76363	-1.7609	-1.75817	-1.75545	-1.75274	-1.75002	-1.74731	-1.7446
-1.76551	-1.76276	-1.76003	-1.75731	-1.75459	-1.75187	-1.74915	-1.74644	-1.74373
-1.76466	-1.76191	-1.75918	-1.75646	-1.75373	-1.75101	-1.74829	-1.74557	-1.74285
-1.76386	-1.76107	-1.75832	-1.75559	-1.75286	-1.75014	-1.74742	-1.7447	-1.74198
-1.76308	-1.76026	-1.75749	-1.75474	-1.752	-1.74928	-1.74655	-1.74383	-1.74111
-1.76231	-1.7595	-1.75669	-1.75391	-1.75115	-1.74842	-1.74569	-1.74297	-1.74024
-1.76154	-1.75875	-1.75593	-1.7531	-1.75033	-1.74758	-1.74484	-1.7421	-1.73937
-1.76073	-1.75797	-1.75515	-1.75232	-1.74954	-1.74679	-1.74403	-1.74127	-1.73852
-1.75988	-1.75714	-1.75436	-1.75158	-1.74881	-1.74606	-1.74328	-1.74047	-1.73768
-1.75903	-1.7563	-1.75355	-1.75079	-1.74804	-1.74529	-1.7425	-1.73968	-1.73687
-1.75817	-1.75543	-1.75269	-1.74995	-1.7472	-1.74446	-1.74169	-1.7389	-1.7361
-1.75733	-1.75457	-1.75183	-1.74909	-1.74635	-1.74361	-1.74086	-1.73811	-1.73533
-1.73299	-1.75374	-1.75098	-1.74823	-1.74548	-1.74274	-1.74	-1.73726	-1.7345
-1.73224	-1.75293	-1.75015	-1.74739	-1.74463	-1.74187	-1.73913	-1.7364	-1.73366
-1.73147	-1.75216	-1.74938	-1.74661	-1.74383	-1.74104	-1.73828	-1.73554	-1.73281
-1.73071	-1.75143	-1.74866	-1.74588	-1.74307	-1.74024	-1.73745	-1.73469	-1.73195
-1.72993	-1.75065	-1.74788	-1.7451	-1.74229	-1.73945	-1.73666	-1.7339	-1.73114
-1.7291	-1.7498	-1.74703	-1.74427	-1.74148	-1.73869	-1.73592	-1.73317	-1.7304
-1.72831	-1.74899	-1.7462	-1.74343	-1.74067	-1.73791	-1.73516	-1.73241	-1.72963
-1.72758	-1.74824	-1.74542	-1.74261	-1.73983	-1.73707	-1.73432	-1.73157	-1.72881

								-0.25476
								-0.25402
						-0.25545	-0.25436	-0.30078
						-0.25469	-0.25364	-0.30001
				-0.10823	-0.10792	-0.25398	-0.25295	-0.389
				-0.10798	-0.10769	-0.25335	-0.25232	-0.38816
				-0.10774	-0.10747	-0.25271	-0.25168	-0.38731
	-0.10839	-0.10809	-0.10779	-0.25399	-0.25302	-0.34078	-0.33955	-0.43225
	-0.10813	-0.10785	-0.10757	-0.25334	-0.25232	-0.33994	-0.33871	-0.43128
	-0.25569	-0.25466	-0.25368	-0.34156	-0.34033	-0.43312	-0.43172	-0.43037
	-0.25501	-0.25397	-0.25297	-0.34073	-0.33951	-0.43217	-0.4308	-0.42949
-0.10775	-0.30167	-0.30054	-0.29943	-0.3881	-0.38683	-0.47157	-0.47008	-0.46862
-0.1075	-0.30089	-0.29976	-0.29866	-0.38725	-0.38602	-0.4706	-0.46913	-0.46768
-0.25396	-0.34195	-0.34071	-0.3395	-0.47251	-0.47107	-0.46962	-0.46817	-0.59715
-0.25332	-0.34118	-0.33996	-0.33876	-0.4715	-0.47005	-0.46861	-0.46717	-0.59597
-0.25268	-0.34041	-0.33921	-0.33803	-0.4705	-0.46904	-0.4676	-0.46618	-0.5948
-0.34082	-0.47402	-0.47249	-0.47099	-0.4695	-0.46803	-0.50622	-0.50473	-0.63664
-0.34001	-0.47297	-0.47147	-0.46998	-0.4685	-0.46704	-0.50518	-0.50373	-0.63549
-0.42725	-0.47198	-0.47049	-0.46902	-0.50723	-0.50568	-0.63781	-0.63607	-0.63433
-0.42637	-0.47103	-0.46955	-0.46808	-0.50624	-0.5047	-0.63666	-0.63492	-0.63317
-0.47156	-0.50993	-0.50835	-0.50679	-0.59671	-0.59499	-0.63551	-0.63378	-0.71732
-0.47056	-0.50888	-0.50731	-0.50575	-0.59556	-0.59386	-0.63436	-0.63265	-0.71608
-0.50941	-0.59966	-0.59789	-0.59614	-0.59443	-0.59275	-0.71858	-0.71672	-0.90491
-0.5084	-0.59853	-0.59678	-0.59505	-0.59336	-0.59169	-0.71735	-0.71547	-0.90294
-0.5074	-0.59741	-0.59568	-0.59398	-0.5923	-0.59065	-0.71612	-0.71423	-0.90099
-0.59801	-0.59626	-0.59456	-0.59288	-0.67552	-0.67366	-0.90484	-0.90196	-1.00766
-0.59684	-0.59512	-0.59343	-0.59177	-0.67429	-0.67243	-0.90286	-0.90009	-1.00563
-0.59572	-0.63712	-0.63535	-0.63361	-0.79459	-0.79239	-0.93595	-0.93314	-1.07009
-0.59465	-0.636	-0.63424	-0.6325	-0.79322	-0.79104	-0.9341	-0.93129	-1.06788
-0.59357	-0.76503	-0.76292	-0.76083	-0.9381	-0.93516	-1.00566	-1.00259	-1.12841
-0.59246	-0.76363	-0.76151	-0.75941	-0.93611	-0.9333	-1.00371	-1.00065	-1.12625
-0.59138	-0.76226	-0.76013	-0.75804	-0.93416	-0.93145	-1.00175	-0.99869	-1.12408
-0.68014	-0.90614	-0.90317	-0.90027	-0.97245	-0.96952	-1.12855	-1.12518	-1.18587
-0.67896	-0.90432	-0.90143	-0.89859	-0.97055	-0.96755	-1.12629	-1.12292	-1.18354
-0.79359	-0.93744	-0.93451	-0.93165	-1.06675	-1.06355	-1.18818	-1.18473	-1.21253
-0.79222	-0.93555	-0.93268	-0.92987	-1.06473	-1.06154	-1.18597	-1.18256	-1.21033
-0.93659	-1.04016	-1.03697	-1.03384	-1.12651	-1.12315	-1.21495	-1.2115	-1.20808
-0.93462	-1.03801	-1.03484	-1.0317	-1.12423	-1.1209	-1.2126	-1.20917	-1.20576
-1.03908	-1.06793	-1.06471	-1.06152	-1.21721	-1.21375	-1.21031	-1.2069	-1.23046
-1.03708	-1.06589	-1.06269	-1.05953	-1.215	-1.21157	-1.20814	-1.20473	-1.22827
-1.03512	-1.0639	-1.06071	-1.05757	-1.21282	-1.20941	-1.20599	-1.20257	-1.2261
-1.03317	-1.16045	-1.1571	-1.15381	-1.21061	-1.20721	-1.23077	-1.2273	-1.2239

-1.03122	-1.15833	-1.15501	-1.15173	-1.20839	-1.20498	-1.22853	-1.22506	-1.22169
-1.15955	-1.2166	-1.21307	-1.20957	-1.23312	-1.22967	-1.22623	-1.22282	-1.24991
-1.15739	-1.21434	-1.2108	-1.20727	-1.23078	-1.2273	-1.2239	-1.22057	-1.24766
-1.1552	-1.21205	-1.20853	-1.20501	-1.22851	-1.22504	-1.25212	-1.24878	-1.27551
-1.15294	-1.20972	-1.20625	-1.20279	-1.22632	-1.2229	-1.24997	-1.24668	-1.2734
-1.21081	-1.23449	-1.23103	-1.22758	-1.22417	-1.22081	-1.27794	-1.27461	-1.29087
-1.20863	-1.23228	-1.22882	-1.2254	-1.22205	-1.21877	-1.27584	-1.27252	-1.28872
-1.2065	-1.23011	-1.22666	-1.22325	-1.21996	-1.21675	-1.27375	-1.27044	-1.28658
-1.23137	-1.22793	-1.22453	-1.22118	-1.2783	-1.27494	-1.29116	-1.28777	-1.33779
-1.22919	-1.22577	-1.22244	-1.21915	-1.27617	-1.27279	-1.28894	-1.28557	-1.33543
-1.22704	-1.25407	-1.2507	-1.24738	-1.29363	-1.29012	-1.34043	-1.33671	-1.3634
-1.22492	-1.25193	-1.24858	-1.24526	-1.29137	-1.28785	-1.33795	-1.33422	-1.36082
-1.22281	-1.29971	-1.29615	-1.29263	-1.28911	-1.28562	-1.3848	-1.38095	-1.37712
-1.22071	-1.29744	-1.29387	-1.29033	-1.28685	-1.28342	-1.38238	-1.37855	-1.37472
-1.29873	-1.29516	-1.29159	-1.28805	-1.35757	-1.35381	-1.37999	-1.37617	-1.37236
-1.29642	-1.29284	-1.28931	-1.28583	-1.35519	-1.35146	-1.37759	-1.37378	-1.37
-1.29413	-1.29053	-1.28705	-1.28364	-1.35284	-1.34914	-1.37521	-1.37141	-1.36765
-1.2919	-1.36183	-1.35799	-1.35423	-1.38041	-1.37662	-1.37283	-1.36904	-1.41776
-1.2897	-1.35947	-1.35564	-1.35189	-1.37801	-1.37424	-1.37046	-1.36668	-1.41536
-1.36099	-1.38714	-1.38325	-1.37943	-1.37562	-1.37186	-1.39417	-1.39039	-1.41303
-1.35852	-1.38468	-1.38084	-1.37704	-1.37324	-1.36946	-1.39179	-1.3881	-1.41078
-1.35609	-1.38225	-1.37844	-1.37466	-1.39697	-1.39316	-1.38945	-1.38583	-1.48572
-1.35373	-1.37983	-1.37603	-1.37227	-1.39459	-1.39083	-1.38716	-1.38357	-1.48335
-1.35139	-1.37744	-1.37364	-1.3699	-1.39223	-1.38854	-1.3849	-1.38132	-1.481
-1.37887	-1.37505	-1.37128	-1.36756	-1.3899	-1.38626	-1.48621	-1.48236	-1.47865
-1.3765	-1.37269	-1.36893	-1.36524	-1.38759	-1.384	-1.48383	-1.47999	-1.47636
-1.37414	-1.39641	-1.39264	-1.38895	-1.43754	-1.43383	-1.48146	-1.47771	-1.47421
-1.37179	-1.39404	-1.39033	-1.38669	-1.43521	-1.43149	-1.47912	-1.47551	-1.47219
-1.44815	-1.44421	-1.44038	-1.43664	-1.4843	-1.48048	-1.47687	-1.47347	-1.49748
-1.44575	-1.44184	-1.43803	-1.4343	-1.48192	-1.47816	-1.47472	-1.47159	-1.49577
-1.44339	-1.43949	-1.43568	-1.43198	-1.47959	-1.47596	-1.4999	-1.49694	-1.49414
-1.4411	-1.43719	-1.43335	-1.42966	-1.47733	-1.47392	-1.498	-1.49522	-1.49256
-1.43882	-1.43489	-1.43106	-1.42741	-1.47516	-1.47201	-1.49623	-1.49358	-1.49103
-1.4365	-1.45891	-1.45504	-1.45141	-1.47311	-1.47019	-1.49454	-1.49202	-1.48957
-1.43416	-1.45656	-1.4528	-1.44932	-1.47117	-1.46848	-1.49294	-1.49053	-1.48816
-1.43186	-1.47929	-1.47565	-1.47232	-1.49652	-1.49391	-1.49142	-1.48908	-1.48677
-1.42959	-1.47705	-1.47366	-1.4706	-1.4949	-1.49239	-1.48997	-1.48767	-1.4854
-1.45367	-1.50222	-1.49897	-1.49604	-1.49336	-1.49093	-1.48857	-1.4863	-1.51062
-1.45164	-1.50037	-1.49729	-1.49448	-1.4919	-1.48953	-1.48722	-1.48498	-1.50919
-1.4773	-1.49864	-1.4957	-1.493	-1.49049	-1.48817	-1.51259	-1.51018	-1.55909
-1.47537	-1.49694	-1.49417	-1.49158	-1.48914	-1.48684	-1.51116	-1.50879	-1.55757
-1.47356	-1.49532	-1.49271	-1.49022	-1.48783	-1.48553	-1.50975	-1.50741	-1.55607
-1.49638	-1.49381	-1.49132	-1.48893	-1.51327	-1.51077	-1.55966	-1.55711	-1.55458
-1.49488	-1.49239	-1.48999	-1.48768	-1.51194	-1.50941	-1.55814	-1.5556	-1.55309
-1.49342	-1.491	-1.48867	-1.48641	-1.53329	-1.53071	-1.57499	-1.57242	-1.59098
-1.49202	-1.48965	-1.48736	-1.48513	-1.53187	-1.52933	-1.57348	-1.5709	-1.58943
-1.51386	-1.55673	-1.5542	-1.55171	-1.57737	-1.57465	-1.59311	-1.59048	-1.62473
-1.51253	-1.55526	-1.55274	-1.55026	-1.57585	-1.57319	-1.59165	-1.58903	-1.62326
-1.51124	-1.55383	-1.5513	-1.54882	-1.57433	-1.57174	-1.59021	-1.58761	-1.62182
-1.55504	-1.58084	-1.5781	-1.57541	-1.59389	-1.5913	-1.62552	-1.62292	-1.62038

-1.55365	-1.57938	-1.57661	-1.57387	-1.59233	-1.58978	-1.62401	-1.62146	-1.61895
-1.55221	-1.63608	-1.63317	-1.63031	-1.62763	-1.62507	-1.66545	-1.66285	-1.68241
-1.55076	-1.63442	-1.63153	-1.62869	-1.62606	-1.62358	-1.66393	-1.66139	-1.68085
-1.6356	-1.63278	-1.62995	-1.62718	-1.66756	-1.66498	-1.68478	-1.68202	-1.67926
-1.63397	-1.63115	-1.62844	-1.6258	-1.66611	-1.66349	-1.68312	-1.68036	-1.67762
-1.63235	-1.6727	-1.66997	-1.66733	-1.66467	-1.66203	-1.6815	-1.67873	-1.67601
-1.63072	-1.67103	-1.66835	-1.66577	-1.66317	-1.6606	-1.67996	-1.67718	-1.67447
-1.62912	-1.6694	-1.66676	-1.6642	-1.66167	-1.65918	-1.67843	-1.67565	-1.67295
-1.6706	-1.66788	-1.66525	-1.66268	-1.68228	-1.67957	-1.67685	-1.6741	-1.67144
-1.66905	-1.66642	-1.66378	-1.66118	-1.68063	-1.67793	-1.67524	-1.67256	-1.66993
-1.66752	-1.68752	-1.68465	-1.6818	-1.67904	-1.67635	-1.67369	-1.67106	-1.67956
-1.66602	-1.68586	-1.68305	-1.68027	-1.67752	-1.67481	-1.67216	-1.6696	-1.67814
-1.66454	-1.68424	-1.68146	-1.67872	-1.67599	-1.6733	-1.68175	-1.67922	-1.6767
-1.66308	-1.68268	-1.67989	-1.67715	-1.67447	-1.67185	-1.68032	-1.67773	-1.67521
-1.68386	-1.6811	-1.67832	-1.67557	-1.684	-1.68145	-1.67888	-1.67627	-1.67376
-1.6822	-1.67947	-1.67671	-1.674	-1.68244	-1.67991	-1.67738	-1.67487	-1.6724
-1.68055	-1.67783	-1.67512	-1.67244	-1.6809	-1.67836	-1.67588	-1.67347	-1.67107
-1.67897	-1.6873	-1.68463	-1.682	-1.67941	-1.67686	-1.6744	-1.67204	-1.66966
-1.67743	-1.68576	-1.68314	-1.68056	-1.67796	-1.67539	-1.67292	-1.67058	-1.66823
-1.68694	-1.68427	-1.68166	-1.67908	-1.67652	-1.67397	-1.69	-1.68763	-1.73135
-1.68543	-1.68281	-1.68018	-1.67758	-1.67507	-1.67261	-1.68865	-1.68623	-1.72973
-1.68393	-1.69987	-1.69723	-1.69463	-1.69214	-1.68972	-1.68729	-1.68485	-1.72816
-1.68245	-1.69835	-1.69575	-1.69321	-1.69074	-1.68832	-1.6859	-1.6835	-1.72664
-1.68097	-1.69684	-1.69429	-1.6918	-1.68934	-1.68692	-1.68452	-1.68215	-1.72515
-1.69805	-1.6954	-1.69287	-1.69039	-1.68795	-1.68554	-1.72902	-1.7263	-1.7389
-1.69661	-1.69399	-1.69146	-1.68899	-1.68656	-1.68418	-1.72749	-1.72478	-1.73739
-1.69514	-1.69255	-1.69006	-1.68762	-1.68519	-1.6828	-1.74125	-1.73857	-1.73594
-1.69366	-1.69111	-1.68866	-1.68626	-1.68384	-1.68143	-1.73968	-1.7371	-1.73453
-1.69224	-1.68972	-1.68729	-1.68489	-1.74366	-1.74085	-1.73818	-1.73564	-1.73309
-1.69088	-1.6884	-1.68593	-1.68349	-1.74209	-1.7394	-1.73676	-1.73417	-1.73161
-1.68953	-1.70305	-1.70053	-1.69803	-1.74056	-1.73794	-1.73533	-1.73272	-1.73014
-1.68816	-1.7017	-1.69921	-1.69673	-1.73908	-1.73645	-1.73385	-1.73128	-1.72873
-1.68679	-1.70035	-1.69789	-1.69545	-1.73764	-1.73498	-1.73238	-1.72984	-1.72732
-1.70139	-1.74439	-1.74164	-1.73893	-1.73624	-1.73358	-1.73097	-1.7284	-1.73713
-1.70003	-1.74279	-1.74012	-1.7375	-1.73486	-1.73222	-1.72959	-1.72697	-1.73561
-1.7441	-1.74129	-1.73863	-1.73602	-1.73341	-1.73082	-1.72821	-1.7256	-1.73418
-1.74266	-1.73988	-1.73717	-1.73452	-1.73193	-1.72938	-1.72684	-1.72428	-1.73281
-1.74119	-1.73846	-1.73574	-1.73307	-1.73048	-1.72795	-1.73673	-1.73408	-1.73144
-1.73969	-1.73705	-1.73436	-1.73169	-1.72908	-1.72651	-1.73523	-1.73265	-1.73007
-1.73819	-1.73561	-1.73296	-1.73032	-1.73915	-1.7364	-1.73376	-1.73122	-1.72867
-1.7367	-1.7341	-1.7315	-1.72891	-1.73771	-1.735	-1.73236	-1.72978	-1.72722
-1.73524	-1.7326	-1.73002	-1.72749	-1.73628	-1.73363	-1.73099	-1.72835	-1.72577
-1.73382	-1.73117	-1.72859	-1.72606	-1.73483	-1.73223	-1.72961	-1.72696	-1.72436
-1.73245	-1.72978	-1.72718	-1.72464	-1.73337	-1.73082	-1.72823	-1.72559	-1.723
-1.73106	-1.73996	-1.73723	-1.73455	-1.73194	-1.72939	-1.72681	-1.72422	-1.72165
-1.72966	-1.73853	-1.73583	-1.73317	-1.73054	-1.72794	-1.72537	-1.72284	-1.72033
-1.73985	-1.73712	-1.73444	-1.7318	-1.72915	-1.72652	-1.72396	-1.72149	-1.76519
-1.73838	-1.73572	-1.73308	-1.73044	-1.72779	-1.72515	-1.7226	-1.72015	-1.76371
-1.73693	-1.73434	-1.73172	-1.72909	-1.72645	-1.7238	-1.72126	-1.71882	-1.76221
-1.73551	-1.73296	-1.73036	-1.72774	-1.72511	-1.72248	-1.76623	-1.76342	-1.77662

-1.73411	-1.73158	-1.72899	-1.72639	-1.72378	-1.72117	-1.76474	-1.76188	-1.77505
-1.73269	-1.74673	-1.74414	-1.74155	-1.73895	-1.73635	-1.77923	-1.77634	-1.77354
-1.73126	-1.74525	-1.74268	-1.74014	-1.73759	-1.73504	-1.77777	-1.77488	-1.77206
-1.74645	-1.74382	-1.74125	-1.73872	-1.78201	-1.77916	-1.77631	-1.77343	-1.77638
-1.74507	-1.74243	-1.73983	-1.73728	-1.78041	-1.77764	-1.77484	-1.772	-1.77496
-1.7437	-1.74106	-1.73844	-1.73588	-1.78462	-1.7819	-1.77913	-1.77633	-1.77357
-1.74235	-1.73971	-1.7371	-1.73454	-1.7831	-1.78038	-1.77765	-1.77492	-1.77221
-1.741	-1.73837	-1.73578	-1.73323	-1.78161	-1.77888	-1.77617	-1.77351	-1.77085
-1.73965	-1.78875	-1.78582	-1.78293	-1.78012	-1.77737	-1.77469	-1.77208	-1.76946
-1.73831	-1.78726	-1.78433	-1.78145	-1.77863	-1.77587	-1.77321	-1.77065	-1.76808
-1.74313	-1.78577	-1.78285	-1.77997	-1.77716	-1.77443	-1.77179	-1.76926	-1.76671
-1.7418	-1.78429	-1.78136	-1.77848	-1.77571	-1.77303	-1.77042	-1.7679	-1.76535
-1.7857	-1.7828	-1.7799	-1.77704	-1.7743	-1.77165	-1.76906	-1.76653	-1.76399
-1.7842	-1.78132	-1.77846	-1.77566	-1.77293	-1.77028	-1.76768	-1.76517	-1.76264
-1.78271	-1.77984	-1.77703	-1.77428	-1.77157	-1.76891	-1.76632	-1.76381	-1.81454
-1.78121	-1.77836	-1.7756	-1.7729	-1.77021	-1.76755	-1.76496	-1.76245	-1.81307
-1.77973	-1.7769	-1.77418	-1.77152	-1.76884	-1.76619	-1.76361	-1.76111	-1.8116
-1.77829	-1.77549	-1.77279	-1.77014	-1.78344	-1.78076	-1.79268	-1.79016	-1.81014
-1.77687	-1.77411	-1.77142	-1.76876	-1.78205	-1.77939	-1.7913	-1.7888	-1.80869
-1.79149	-1.78874	-1.78603	-1.78335	-1.79519	-1.79252	-1.82598	-1.8233	-1.85542
-1.79007	-1.78736	-1.78466	-1.78198	-1.79381	-1.79114	-1.82451	-1.82184	-1.85388
-1.80324	-1.81407	-1.81137	-1.80868	-1.80601	-1.80335	-1.83979	-1.83705	-1.85235
-1.80183	-1.81267	-1.80998	-1.8073	-1.80464	-1.802	-1.83831	-1.83558	-1.85083
-1.80044	-1.81129	-1.8086	-1.80594	-1.8033	-1.80068	-1.83687	-1.83412	-1.84932
-1.81261	-1.80992	-1.80724	-1.80458	-1.8243	-1.82154	-1.85377	-1.85078	-1.84783
-1.81125	-1.80857	-1.8059	-1.80326	-1.8229	-1.82017	-1.85234	-1.84933	-1.84637
-1.80993	-1.80729	-1.80463	-1.80199	-1.85692	-1.85388	-1.85087	-1.84787	-1.84493
-1.80865	-1.80604	-1.80341	-1.80077	-1.85547	-1.85239	-1.84938	-1.84642	-1.8435
-1.80736	-1.84411	-1.84123	-1.83834	-1.85399	-1.8509	-1.8479	-1.84497	-1.84208
-1.80607	-1.84267	-1.83978	-1.83689	-1.85248	-1.84941	-1.84643	-1.84353	-1.84066
-1.82187	-1.86035	-1.85719	-1.85405	-1.85097	-1.84793	-1.84497	-1.84209	-1.88999
-1.82051	-1.85879	-1.85564	-1.85251	-1.84945	-1.84645	-1.84352	-1.84067	-1.88834
-1.81913	-1.85723	-1.85409	-1.85097	-1.84795	-1.84499	-1.84209	-1.83926	-1.8867
-1.85879	-1.85568	-1.85257	-1.84949	-1.8465	-1.84357	-1.84069	-1.83786	-1.88508
-1.85722	-1.85413	-1.85106	-1.84804	-1.84508	-1.84219	-1.83932	-1.83648	-1.88347
-1.85567	-1.85259	-1.84957	-1.8466	-1.8437	-1.84085	-1.88848	-1.88521	-1.88195
-1.85414	-1.85107	-1.84809	-1.84517	-1.84233	-1.83955	-1.88701	-1.88375	-1.88049
-1.8527	-1.84965	-1.84669	-1.8438	-1.84098	-1.83822	-1.88548	-1.88222	-1.87897
-1.85137	-1.84834	-1.84539	-1.84249	-1.83965	-1.83685	-1.88388	-1.88063	-1.87738
-1.85001	-1.84702	-1.84407	-1.84116	-1.88877	-1.88551	-1.88225	-1.87901	-1.87577
-1.84857	-1.8456	-1.84267	-1.83977	-1.88715	-1.88388	-1.88062	-1.87738	-1.87414
-1.84711	-1.84417	-1.84126	-1.83837	-1.88552	-1.88225	-1.87899	-1.87575	-1.87252
-1.84571	-1.85833	-1.85526	-1.85223	-1.8839	-1.88064	-1.87739	-1.87414	-1.87093
-1.84435	-1.85684	-1.85376	-1.85074	-1.88228	-1.87904	-1.8758	-1.87257	-1.86937
-1.843	-1.89064	-1.88729	-1.88401	-1.88075	-1.87751	-1.90361	-1.90036	-1.89714
-1.84167	-1.88917	-1.88584	-1.88255	-1.87929	-1.87605	-1.90213	-1.89888	-1.89569
-1.85576	-1.88765	-1.88433	-1.88105	-1.90713	-1.90386	-1.90061	-1.89738	-1.8942
-1.85423	-1.88602	-1.88275	-1.87949	-1.90554	-1.90227	-1.89903	-1.89581	-1.89264
-1.85274	-1.91378	-1.9105	-1.90724	-1.90397	-1.9007	-1.89747	-1.89426	-1.90636
-1.85136	-1.91225	-1.90898	-1.90573	-1.90247	-1.89922	-1.896	-1.89281	-1.90487

-1.85001	-1.91075	-1.90748	-1.90423	-1.90099	-1.89777	-1.89456	-1.89137	-1.90339
-1.91244	-1.90917	-1.90591	-1.90267	-1.89944	-1.89623	-1.89304	-1.88988	-1.90188
-1.91081	-1.90756	-1.90431	-1.90108	-1.89785	-1.89465	-1.89148	-1.88834	-1.90034
-1.90924	-1.906	-1.90277	-1.89955	-1.89633	-1.89313	-1.88997	-1.88684	-1.89882
-1.90774	-1.90451	-1.90129	-1.89808	-1.89488	-1.89169	-1.88852	-1.88537	-1.89731
-1.90623	-1.903	-1.89979	-1.89659	-1.89341	-1.89023	-1.88707	-1.88391	-1.89582
-1.90466	-1.90144	-1.89822	-1.89503	-1.89186	-1.88871	-1.88558	-1.88247	-1.8944
-1.90311	-1.89989	-1.89668	-1.89348	-1.89032	-1.88719	-1.8841	-1.88104	-1.89298
-1.90163	-1.89842	-1.89522	-1.89204	-1.88889	-1.88577	-1.88268	-1.87962	-1.89152
-1.90018	-1.89698	-1.8938	-1.89064	-1.8875	-1.88438	-1.88128	-1.8782	-1.89006
-1.89869	-1.89547	-1.8923	-1.88915	-1.88603	-1.88293	-1.89482	-1.89171	-1.88867
-1.89718	-1.89393	-1.89075	-1.88761	-1.88451	-1.88146	-1.89336	-1.89032	-1.88732
-1.89569	-1.89245	-1.88927	-1.88614	-1.88306	-1.88004	-1.89194	-1.88894	-1.88596
-1.89423	-1.89104	-1.88788	-1.88475	-1.8817	-1.87869	-1.89057	-1.88758	-1.8846
-1.89277	-1.88962	-1.88649	-1.88338	-1.88033	-1.87733	-1.88919	-1.88621	-1.88323
-1.89133	-1.8882	-1.88508	-1.88199	-1.87894	-1.87592	-1.88775	-1.88478	-1.88183
-1.88988	-1.88676	-1.88367	-1.8806	-1.87755	-1.87452	-1.88632	-1.88336	-1.88044
-1.88842	-1.8853	-1.88223	-1.8792	-1.87617	-1.87317	-1.88496	-1.88201	-1.8791
-1.88696	-1.88383	-1.88078	-1.87779	-1.87481	-1.87185	-1.88363	-1.88069	-1.87778
-1.88552	-1.88241	-1.87939	-1.87642	-1.87347	-1.87054	-1.88231	-1.87938	-1.88854
-1.88409	-1.88103	-1.87804	-1.87509	-1.87216	-1.86924	-1.88099	-1.87807	-1.88723
-1.88271	-1.87968	-1.8767	-1.87376	-1.87082	-1.86791	-1.91343	-1.91051	-1.9076
-1.88137	-1.87836	-1.87539	-1.87243	-1.86947	-1.86652	-1.91201	-1.90911	-1.90624
-1.88004	-1.87705	-1.87406	-1.87109	-1.90242	-1.89942	-1.9106	-1.90773	-1.90488
-1.87871	-1.87572	-1.87272	-1.86972	-1.90106	-1.8981	-1.90927	-1.90641	-1.90358
-1.87738	-1.87438	-1.87136	-1.86835	-1.89972	-1.89681	-1.90798	-1.90512	-1.90229
-1.89856	-1.90743	-1.90438	-1.90136	-1.91252	-1.9096	-1.90671	-1.90386	-1.91188
-1.89719	-1.90604	-1.90302	-1.90005	-1.91123	-1.90833	-1.90546	-1.90261	-1.9106
-1.90777	-1.9047	-1.90171	-1.89876	-1.90994	-1.90706	-1.9042	-1.90135	-1.90933
-1.90647	-1.90341	-1.90043	-1.8975	-1.90866	-1.9058	-1.90294	-1.90009	-1.90805
-1.90515	-1.90211	-1.89915	-1.89624	-1.90738	-1.90452	-1.91251	-1.90963	-1.90676
-1.90378	-1.90078	-1.89786	-1.89497	-1.9061	-1.90324	-1.9112	-1.90831	-1.90545
-1.90242	-1.89946	-1.89657	-1.89371	-1.90481	-1.90194	-1.90988	-1.90699	-1.90414
-1.90111	-1.89818	-1.8953	-1.89245	-1.91436	-1.91143	-1.90855	-1.90569	-1.90289
-1.89984	-1.89692	-1.89404	-1.89119	-1.91303	-1.91009	-1.90722	-1.90442	-1.90165
-1.89857	-1.90671	-1.90381	-1.90094	-1.91175	-1.9088	-1.90594	-1.90316	-1.90042
-1.8973	-1.90544	-1.90256	-1.8997	-1.9105	-1.90756	-1.90471	-1.90193	-1.89919
-1.89603	-1.90417	-1.9013	-1.89845	-1.90924	-1.90633	-1.90348	-1.90071	-1.89798
-1.89476	-1.9029	-1.90004	-1.89719	-1.90798	-1.90509	-1.90226	-1.8995	-1.89677
-1.90452	-1.90164	-1.89878	-1.89595	-1.90673	-1.90386	-1.90105	-1.8983	-1.89557
-1.90326	-1.90039	-1.89755	-1.89473	-1.9055	-1.90264	-1.89984	-1.8971	-1.89438
-1.90201	-1.89915	-1.89632	-1.89352	-1.90429	-1.90143	-1.89864	-1.89591	-1.8932
-1.90075	-1.8979	-1.8951	-1.89231	-1.90307	-1.90022	-1.89744	-1.89471	-1.89201
-1.8995	-1.89666	-1.89388	-1.89111	-1.90185	-1.89901	-1.89623	-1.89351	-1.89082
-1.89826	-1.89544	-1.89267	-1.88991	-1.90064	-1.89781	-1.89504	-1.89232	-1.88963
-1.89704	-1.89424	-1.89147	-1.88872	-1.89944	-1.89661	-1.89384	-1.89113	-1.88845
-1.89584	-1.89304	-1.89028	-1.88753	-1.88476	-1.88197	-1.89265	-1.88994	-1.88726
-1.89466	-1.89184	-1.88908	-1.88634	-1.88357	-1.88079	-1.89145	-1.88874	-1.88607
-1.89347	-1.89064	-1.88788	-1.88514	-1.88237	-1.8796	-1.89025	-1.88755	-1.88489
-1.89224	-1.88942	-1.88666	-1.88393	-1.88117	-1.8784	-1.88904	-1.88635	-1.8837

-1.891	-1.88819	-1.88545	-1.88273	-1.87997	-1.87721	-1.88784	-1.88516	-1.88251
-1.88978	-1.88698	-1.88424	-1.88153	-1.8788	-1.87607	-1.88669	-1.884	-1.88134
-1.88857	-1.88577	-1.88304	-1.88034	-1.87764	-1.87496	-1.88556	-1.88286	-1.88019
-1.88739	-1.8846	-1.88186	-1.87915	-1.87648	-1.87382	-1.87116	-1.86848	-1.87904
-1.88625	-1.88348	-1.88071	-1.87798	-1.8753	-1.87267	-1.87002	-1.86735	-1.87789
-1.88509	-1.88233	-1.87956	-1.87681	-1.87414	-1.87152	-1.86888	-1.86623	-1.87676
-1.88389	-1.88114	-1.87837	-1.87563	-1.87297	-1.87037	-1.86776	-1.86516	-1.87568
-1.88269	-1.87995	-1.8772	-1.87448	-1.87183	-1.86923	-1.86664	-1.86408	-1.87459
-1.88154	-1.87879	-1.87607	-1.87338	-1.87072	-1.86811	-1.86552	-1.86296	-1.87346
-1.88039	-1.87764	-1.87495	-1.8723	-1.86964	-1.86699	-1.86439	-1.86182	-1.8723
-1.87922	-1.87648	-1.87382	-1.87118	-1.86853	-1.86587	-1.86326	-1.86069	-1.87084
-1.87804	-1.87532	-1.87267	-1.87005	-1.8674	-1.86476	-1.86214	-1.85955	-1.86969
-1.8769	-1.87419	-1.87155	-1.86893	-1.87904	-1.87639	-1.87376	-1.87114	-1.86854
-1.8758	-1.87312	-1.87047	-1.86783	-1.87793	-1.87531	-1.87268	-1.87002	-1.86739
-1.8747	-1.88477	-1.88211	-1.87946	-1.87684	-1.87422	-1.87158	-1.86891	-1.86626
-1.87359	-1.88364	-1.881	-1.87838	-1.87575	-1.87311	-1.87046	-1.86781	-1.86516
-1.87248	-1.88251	-1.87989	-1.87729	-1.87464	-1.87197	-1.86932	-1.8667	-1.86406
-1.88403	-1.88136	-1.87874	-1.87614	-1.87349	-1.87081	-1.86816	-1.86554	-1.86291
-1.88287	-1.8802	-1.87758	-1.87497	-1.87231	-1.86964	-1.86699	-1.86436	-1.86173
-1.88173	-1.87908	-1.87644	-1.87382	-1.87117	-1.86851	-1.86586	-1.86323	-1.8606
-1.88062	-1.87798	-1.87533	-1.87268	-1.87004	-1.8674	-1.86476	-1.86212	-1.85949
-1.87951	-1.87687	-1.87422	-1.87157	-1.86893	-1.8663	-1.86366	-1.86102	-1.85839
-1.87841	-1.87576	-1.87312	-1.87048	-1.86784	-1.86519	-1.86256	-1.85993	-1.85732
-1.87731	-1.87466	-1.87202	-1.86939	-1.86675	-1.86409	-1.86146	-1.85885	-1.85625
-1.87621	-1.87356	-1.87092	-1.86828	-1.86564	-1.86301	-1.86039	-1.85777	-1.85517
-1.87512	-1.87248	-1.86982	-1.86717	-1.86455	-1.86193	-1.85932	-1.8567	-1.8541
-1.87402	-1.87138	-1.86873	-1.86609	-1.86346	-1.86084	-1.85823	-1.85563	-1.85304
-1.87293	-1.87027	-1.86764	-1.86501	-1.86238	-1.85975	-1.85714	-1.85457	-1.85199
-1.87184	-1.86919	-1.86655	-1.86393	-1.8613	-1.85867	-1.85607	-1.8535	-1.85093
-1.87075	-1.86812	-1.86547	-1.86283	-1.86021	-1.8576	-1.85501	-1.85243	-1.84986
-1.86967	-1.86704	-1.86439	-1.86175	-1.85913	-1.85653	-1.85394	-1.85136	-1.84879
-1.86859	-1.86594	-1.8633	-1.86068	-1.85806	-1.85545	-1.85286	-1.8503	-1.84773
-1.86752	-1.86484	-1.86222	-1.85961	-1.85699	-1.85437	-1.85178	-1.84923	-1.84666
-1.86648	-1.86378	-1.86113	-1.8585	-1.85589	-1.8533	-1.85072	-1.84815	-1.84558
-1.86544	-1.86274	-1.86005	-1.85739	-1.85479	-1.85222	-1.84965	-1.84706	-1.84448
-1.86438	-1.8617	-1.85901	-1.85633	-1.85371	-1.85113	-1.84855	-1.84597	-1.84339
-1.86331	-1.86066	-1.85798	-1.85529	-1.85265	-1.85002	-1.84743	-1.84487	-1.84231
-1.86222	-1.85958	-1.85692	-1.85425	-1.8516	-1.84895	-1.84634	-1.84379	-1.84122
-1.86111	-1.85845	-1.85582	-1.85321	-1.85057	-1.84792	-1.8453	-1.8427	-1.84012
-1.86002	-1.85734	-1.85473	-1.85214	-1.84952	-1.84689	-1.84426	-1.84164	-1.83905
-1.85897	-1.85628	-1.85364	-1.85103	-1.84843	-1.84584	-1.84323	-1.84061	-1.83801
-1.85794	-1.85524	-1.85257	-1.84992	-1.84734	-1.84479	-1.8422	-1.83959	-1.837
-1.85687	-1.8542	-1.85153	-1.84887	-1.8463	-1.84376	-1.84118	-1.83857	-1.83597
-1.8558	-1.85317	-1.8505	-1.84785	-1.84527	-1.84274	-1.84016	-1.83755	-1.83494
-1.85475	-1.85213	-1.84948	-1.84683	-1.84423	-1.84168	-1.8391	-1.83651	-1.83391
-1.85371	-1.85109	-1.84844	-1.8458	-1.84319	-1.84059	-1.83802	-1.83546	-1.83289
-1.85265	-1.85003	-1.8474	-1.84478	-1.84215	-1.83953	-1.83695	-1.8344	-1.83183
-1.85157	-1.84894	-1.84634	-1.84376	-1.84114	-1.8385	-1.83589	-1.83331	-1.83073
-1.85051	-1.84786	-1.84529	-1.84274	-1.84012	-1.83748	-1.83486	-1.83224	-1.82965
-1.84948	-1.84683	-1.84426	-1.84172	-1.83912	-1.83648	-1.83385	-1.83122	-1.82861

-1.84846	-1.84581	-1.84324	-1.84069	-1.8381	-1.83549	-1.83286	-1.83022	-1.82759
-1.84746	-1.8448	-1.8422	-1.83962	-1.83704	-1.83446	-1.83185	-1.82921	-1.82658
-1.84645	-1.84379	-1.84115	-1.83854	-1.83596	-1.83341	-1.83083	-1.8282	-1.82557
-1.84545	-1.84278	-1.84012	-1.83749	-1.83492	-1.83239	-1.82981	-1.82719	-1.82456
-1.84445	-1.84178	-1.83912	-1.83649	-1.83391	-1.83138	-1.8288	-1.82618	-1.82355
-1.84345	-1.84079	-1.83812	-1.83548	-1.83291	-1.83037	-1.82779	-1.82517	-1.82254
-1.84246	-1.83979	-1.83712	-1.83448	-1.8319	-1.82936	-1.82678	-1.82416	-1.82153
-1.84146	-1.83879	-1.83612	-1.83348	-1.8309	-1.82836	-1.82578	-1.82315	-1.82052
-1.84046	-1.83779	-1.83512	-1.83247	-1.82989	-1.82735	-1.82476	-1.82214	-1.81951
-1.83946	-1.83679	-1.83412	-1.83147	-1.82888	-1.82634	-1.82375	-1.82113	-1.81849
-1.83846	-1.83579	-1.83312	-1.83046	-1.82788	-1.82532	-1.82274	-1.82011	-1.81748
-1.83746	-1.83479	-1.83211	-1.82946	-1.82687	-1.82432	-1.82172	-1.8191	-1.81646
-1.83646	-1.83378	-1.83111	-1.82845	-1.82586	-1.8233	-1.82071	-1.81808	-1.84142
-1.83546	-1.83278	-1.8301	-1.82744	-1.82484	-1.82229	-1.8197	-1.81707	-1.84039
-1.83446	-1.83178	-1.82909	-1.82643	-1.82383	-1.82128	-1.81868	-1.81606	-1.83936
-1.83345	-1.83077	-1.82808	-1.82542	-1.82282	-1.82027	-1.81768	-1.81505	-1.83833
-1.83244	-1.82976	-1.82707	-1.82441	-1.82182	-1.81926	-1.81667	-1.81404	-1.83729
-1.83143	-1.82875	-1.82607	-1.82341	-1.82082	-1.81826	-1.84165	-1.83896	-1.83626
-1.83043	-1.82775	-1.82507	-1.82243	-1.81983	-1.81726	-1.84063	-1.83793	-1.83523
-1.80407	-1.82676	-1.82411	-1.82148	-1.81887	-1.81628	-1.83961	-1.83691	-1.83421
-1.8031	-1.82579	-1.82317	-1.82056	-1.81793	-1.81531	-1.8386	-1.83589	-1.83318
-1.80215	-1.82485	-1.82223	-1.81962	-1.843	-1.8403	-1.83759	-1.83487	-1.83216
-1.80123	-1.82393	-1.8213	-1.81866	-1.842	-1.83929	-1.83657	-1.83385	-1.83114
-1.80031	-1.823	-1.82035	-1.81768	-1.84099	-1.83827	-1.83555	-1.83283	-1.83013
-1.79939	-1.82203	-1.81936	-1.81668	-1.83997	-1.83725	-1.83453	-1.83182	-1.82913
-1.79847	-1.82104	-1.81836	-1.81568	-1.83895	-1.83623	-1.83352	-1.83082	-1.82814
-1.79751	-1.79488	-1.79226	-1.78964	-1.83793	-1.83521	-1.83252	-1.82985	-1.82719
-1.79654	-1.79391	-1.79129	-1.78867	-1.83693	-1.83421	-1.83153	-1.82889	-1.82624
-1.79556	-1.81957	-1.81692	-1.81427	-1.83597	-1.83325	-1.83057	-1.82793	-1.82527
-1.79458	-1.81858	-1.81596	-1.81335	-1.83506	-1.83235	-1.82964	-1.82696	-1.82426
-1.79364	-1.81763	-1.81501	-1.81241	-1.80977	-1.8071	-1.82868	-1.82597	-1.82324
-1.79276	-1.81672	-1.81407	-1.81143	-1.80877	-1.8061	-1.82767	-1.82494	-1.82221
-1.79188	-1.8158	-1.81312	-1.81044	-1.80777	-1.80509	-1.82664	-1.82391	-1.82118
-1.79093	-1.81483	-1.81214	-1.80945	-1.80676	-1.80408	-1.82561	-1.82288	-1.82016
-1.78996	-1.81385	-1.81116	-1.80846	-1.80577	-1.80309	-1.8246	-1.82186	-1.81915
-1.81558	-1.81291	-1.81022	-1.80752	-1.80484	-1.80215	-1.79947	-1.79678	-1.81818
-1.81463	-1.812	-1.80932	-1.80662	-1.80394	-1.80126	-1.79858	-1.79588	-1.81724
-1.81368	-1.81104	-1.80837	-1.80568	-1.803	-1.80032	-1.79764	-1.79494	-1.81627
-1.81273	-1.81006	-1.80738	-1.8047	-1.80202	-1.79933	-1.79665	-1.79395	-1.81527
-1.81176	-1.80907	-1.8064	-1.80373	-1.80105	-1.79837	-1.79568	-1.79299	-1.79029
-1.81076	-1.80807	-1.80543	-1.8028	-1.80014	-1.79746	-1.79477	-1.79208	-1.78939
-1.80978	-1.80709	-1.80447	-1.80187	-1.79922	-1.79654	-1.79386	-1.79117	-1.78848
-1.80888	-1.80618	-1.80354	-1.80091	-1.79825	-1.79557	-1.79289	-1.7902	-1.7875
-1.80799	-1.80529	-1.80262	-1.79995	-1.79727	-1.79458	-1.7919	-1.78921	-1.78652
-1.80708	-1.80439	-1.80171	-1.79903	-1.79634	-1.79365	-1.79096	-1.78827	-1.78558
-1.80614	-1.80349	-1.80082	-1.79814	-1.79544	-1.79274	-1.79005	-1.78736	-1.78467
-1.80519	-1.80255	-1.79988	-1.7972	-1.7945	-1.7918	-1.7891	-1.78641	-1.78372
-1.80423	-1.80157	-1.79889	-1.7962	-1.7935	-1.7908	-1.7881	-1.78541	-1.78272
-1.80329	-1.8006	-1.79791	-1.79522	-1.79252	-1.78982	-1.78712	-1.78443	-1.78173
-1.8024	-1.7997	-1.797	-1.7943	-1.7916	-1.7889	-1.7862	-1.7835	-1.78081

-1.80149	-1.79879	-1.79609	-1.79338	-1.79068	-1.78798	-1.78528	-1.78258	-1.7799
-1.80052	-1.79782	-1.79511	-1.7924	-1.7897	-1.787	-1.7843	-1.7816	-1.77892
-1.79953	-1.79682	-1.79411	-1.7914	-1.7887	-1.786	-1.7833	-1.78061	-1.77792
-1.79859	-1.79588	-1.79317	-1.79047	-1.78776	-1.78506	-1.78237	-1.77967	-1.77699
-1.7977	-1.795	-1.79229	-1.78958	-1.78688	-1.78418	-1.78148	-1.77879	-1.77611
-1.79683	-1.79412	-1.79142	-1.78871	-1.786	-1.7833	-1.78061	-1.77792	-1.77524
-1.79597	-1.79326	-1.79056	-1.78785	-1.78514	-1.78244	-1.77974	-1.77706	-1.77439
-1.7951	-1.7924	-1.78969	-1.78698	-1.78428	-1.78157	-1.77888	-1.7762	-1.77353
-1.79424	-1.79153	-1.78882	-1.78611	-1.7834	-1.7807	-1.778	-1.77533	-1.77265
-1.79335	-1.79064	-1.78792	-1.78521	-1.78251	-1.7798	-1.77711	-1.77443	-1.77176
-1.7924	-1.78968	-1.78697	-1.78426	-1.78155	-1.77884	-1.77615	-1.77348	-1.7708
-1.79141	-1.78869	-1.78598	-1.78327	-1.78056	-1.77785	-1.77516	-1.77248	-1.76981
-1.78196	-1.78776	-1.78505	-1.78233	-1.77962	-1.77692	-1.77423	-1.77155	-1.76887
-1.78108	-1.78689	-1.78417	-1.78145	-1.77874	-1.77604	-1.77335	-1.77066	-1.76798
-1.78021	-1.78603	-1.78331	-1.78059	-1.77788	-1.77518	-1.77248	-1.76979	-1.76711
-1.77934	-1.78517	-1.78245	-1.77973	-1.77702	-1.77432	-1.77162	-1.76894	-1.76625
-1.77849	-1.78433	-1.7816	-1.77888	-1.77617	-1.77347	-1.77077	-1.76809	-1.7654
-1.77763	-1.77489	-1.77216	-1.76943	-1.77531	-1.77261	-1.76991	-1.76723	-1.76454
-1.77677	-1.77403	-1.7713	-1.76857	-1.77445	-1.77175	-1.76905	-1.76636	-1.76368
-1.77591	-1.77317	-1.77044	-1.76771	-1.77359	-1.77089	-1.76819	-1.7655	-1.76281
-1.77505	-1.77231	-1.76958	-1.76685	-1.77274	-1.77003	-1.76733	-1.76464	-1.76195
-1.77419	-1.77145	-1.76871	-1.76598	-1.76326	-1.76053	-1.76647	-1.76377	-1.76109
-1.77332	-1.77058	-1.76785	-1.76511	-1.76239	-1.75967	-1.76561	-1.76291	-1.76023
-1.77245	-1.76972	-1.76698	-1.76424	-1.76152	-1.7588	-1.76475	-1.76205	-1.75936
-1.77159	-1.76885	-1.76611	-1.76337	-1.76065	-1.75793	-1.76389	-1.76119	-1.7585
-1.77072	-1.76797	-1.76523	-1.7625	-1.75978	-1.75706	-1.76303	-1.76033	-1.75764
-1.76984	-1.7671	-1.76436	-1.76163	-1.75891	-1.7562	-1.75349	-1.75078	-1.75679
-1.76897	-1.76623	-1.7635	-1.76077	-1.75805	-1.75533	-1.75262	-1.74992	-1.75593
-1.7681	-1.76536	-1.76263	-1.7599	-1.75718	-1.75447	-1.75175	-1.74905	-1.75507
-1.76724	-1.7645	-1.76176	-1.75904	-1.75632	-1.7536	-1.75089	-1.74818	-1.75421
-1.76637	-1.76363	-1.7609	-1.75817	-1.75545	-1.75274	-1.75002	-1.74731	-1.7446
-1.76551	-1.76276	-1.76003	-1.75731	-1.75459	-1.75187	-1.74915	-1.74644	-1.74373
-1.76466	-1.76191	-1.75918	-1.75646	-1.75373	-1.75101	-1.74829	-1.74557	-1.74285
-1.76386	-1.76107	-1.75832	-1.75559	-1.75286	-1.75014	-1.74742	-1.7447	-1.74198
-1.76308	-1.76026	-1.75749	-1.75474	-1.752	-1.74928	-1.74655	-1.74383	-1.74111
-1.76231	-1.7595	-1.75669	-1.75391	-1.75115	-1.74842	-1.74569	-1.74297	-1.74024
-1.76154	-1.75875	-1.75593	-1.7531	-1.75033	-1.74758	-1.74484	-1.7421	-1.73937
-1.76073	-1.75797	-1.75515	-1.75232	-1.74954	-1.74679	-1.74403	-1.74127	-1.73852
-1.75988	-1.75714	-1.75436	-1.75158	-1.74881	-1.74606	-1.74328	-1.74047	-1.73768
-1.75903	-1.7563	-1.75355	-1.75079	-1.74804	-1.74529	-1.7425	-1.73968	-1.73687
-1.75817	-1.75543	-1.75269	-1.74995	-1.7472	-1.74446	-1.74169	-1.7389	-1.7361
-1.75733	-1.75457	-1.75183	-1.74909	-1.74635	-1.74361	-1.74086	-1.73811	-1.73533
-1.73299	-1.75374	-1.75098	-1.74823	-1.74548	-1.74274	-1.74	-1.73726	-1.7345
-1.73224	-1.75293	-1.75015	-1.74739	-1.74463	-1.74187	-1.73913	-1.7364	-1.73366
-1.73147	-1.75216	-1.74938	-1.74661	-1.74383	-1.74104	-1.73828	-1.73554	-1.73281
-1.73071	-1.75143	-1.74866	-1.74588	-1.74307	-1.74024	-1.73745	-1.73469	-1.73195
-1.72993	-1.75065	-1.74788	-1.7451	-1.74229	-1.73945	-1.73666	-1.7339	-1.73114
-1.7291	-1.7498	-1.74703	-1.74427	-1.74148	-1.73869	-1.73592	-1.73317	-1.7304
-1.72831	-1.74899	-1.7462	-1.74343	-1.74067	-1.73791	-1.73516	-1.73241	-1.72963
-1.72758	-1.74824	-1.74542	-1.74261	-1.73983	-1.73707	-1.73432	-1.73157	-1.72881

-1.01106	-1.00967	-1.0083	-1.00695	-1.00561	-1.00426	-1.00291	-1.00155	-1.00019
-1.01139	-1.01	-1.00864	-1.00729	-1.00594	-1.00458	-1.00323	-1.00188	-1.00053
-1.01171	-1.01034	-1.00898	-1.00762	-1.00626	-1.0049	-1.00355	-1.00221	-1.00087
-1.01205	-1.01068	-1.00931	-1.00795	-1.00659	-1.00523	-1.00389	-1.00256	-1.00123
-1.01238	-1.01102	-1.00965	-1.00828	-1.00692	-1.00556	-1.00423	-1.00292	-1.00159
-1.0127	-1.01134	-1.00997	-1.00861	-1.00725	-1.00591	-1.00458	-1.00326	-1.00194
-1.01302	-1.01165	-1.01029	-1.00893	-1.00759	-1.00626	-1.00493	-1.0036	-1.00227
-1.01335	-1.01197	-1.01061	-1.00925	-1.00791	-1.00659	-1.00526	-1.00392	-1.00259
-1.01369	-1.0123	-1.01093	-1.00956	-1.00822	-1.0069	-1.00557	-1.00423	-1.00291
-1.01402	-1.01263	-1.01125	-1.00989	-1.00854	-1.00721	-1.00588	-1.00454	-1.00322
-1.01434	-1.01295	-1.01158	-1.01022	-1.00887	-1.00753	-1.0062	-1.00487	-1.00355
-1.01466	-1.01328	-1.01191	-1.01055	-1.0092	-1.00786	-1.00652	-1.0052	-1.00388
-1.015	-1.01362	-1.01225	-1.01089	-1.00953	-1.00819	-1.00685	-1.00553	-1.00421
-1.01535	-1.01397	-1.01259	-1.01122	-1.00986	-1.00852	-1.00718	-1.00586	-1.00455
-1.01568	-1.0143	-1.01292	-1.01154	-1.01019	-1.00885	-1.00752	-1.0062	-1.00489
-1.016	-1.01461	-1.01322	-1.01185	-1.01051	-1.00918	-1.00786	-1.00655	-1.00524
-1.01632	-1.01492	-1.01354	-1.01217	-1.01083	-1.00952	-1.0082	-1.00689	-1.00559
-1.01664	-1.01524	-1.01386	-1.01251	-1.01117	-1.00985	-1.00853	-1.00723	-1.00592
-1.01696	-1.01556	-1.0142	-1.01285	-1.01151	-1.01018	-1.00886	-1.00756	-1.00626
-1.0173	-1.01591	-1.01453	-1.01318	-1.01184	-1.01051	-1.00919	-1.00789	-1.00659
-1.01765	-1.01626	-1.01488	-1.01351	-1.01217	-1.01084	-1.00952	-1.00822	-1.00692
-1.018	-1.01661	-1.01523	-1.01386	-1.01251	-1.01118	-1.00986	-1.00855	-1.00725
-1.01836	-1.01697	-1.01559	-1.01422	-1.01287	-1.01153	-1.01021	-1.0089	-1.00759
-1.01872	-1.01734	-1.01595	-1.01458	-1.01322	-1.01188	-1.01055	-1.00924	-1.00793
-1.01911	-1.01772	-1.01633	-1.01494	-1.01357	-1.01223	-1.01089	-1.00958	-1.00826
-1.01949	-1.0181	-1.0167	-1.01531	-1.01394	-1.01258	-1.01124	-1.00992	-1.0086
-1.01987	-1.01848	-1.01708	-1.0157	-1.01432	-1.01295	-1.0116	-1.01027	-1.00895
-1.02024	-1.01885	-1.01746	-1.01608	-1.0147	-1.01332	-1.01196	-1.01062	-1.00929
-0.99972	-1.01921	-1.01782	-1.01644	-1.01506	-1.01368	-1.01231	-1.01097	-1.00964
-1.00007	-1.01957	-1.01818	-1.0168	-1.01541	-1.01403	-1.01267	-1.01132	-1.00998
-0.97806	-0.99908	-0.99773	-0.99639	-1.01579	-1.01441	-1.01304	-1.01169	-1.01035
-0.97842	-0.99944	-0.99811	-0.99678	-1.01619	-1.0148	-1.01343	-1.01207	-1.01072
-0.97879	-0.97746	-0.97615	-0.97484	-0.99581	-0.99445	-0.99311	-0.99179	-1.0111
-0.97917	-0.97783	-0.97652	-0.97521	-0.99619	-0.99484	-0.9935	-0.99218	-1.01149
-0.97954	-0.9782	-0.97688	-0.97557	-0.97425	-0.97293	-0.99389	-0.99256	-0.99123
-0.9799	-0.97856	-0.97724	-0.97592	-0.97461	-0.9733	-0.99426	-0.99292	-0.99158
-0.98025	-0.97891	-0.97758	-0.97626	-0.97495	-0.97366	-0.99463	-0.99328	-0.99194
-0.9592	-0.97924	-0.97791	-0.97659	-0.97529	-0.974	-0.97269	-0.97137	-0.99231
-0.95951	-0.97956	-0.97823	-0.97692	-0.97561	-0.97432	-0.97302	-0.97173	-0.99269
-0.95986	-0.97993	-0.9786	-0.97728	-0.97597	-0.97466	-0.97336	-0.97208	-0.97079
-0.96024	-0.98033	-0.97899	-0.97766	-0.97634	-0.97502	-0.97372	-0.97244	-0.97115
-0.96061	-0.9593	-0.95799	-0.95668	-0.9767	-0.97539	-0.97408	-0.9728	-0.97151
-0.96093	-0.95963	-0.95832	-0.95701	-0.97705	-0.97574	-0.97445	-0.97317	-0.97187
-0.96126	-0.95996	-0.95865	-0.95734	-0.95605	-0.95478	-0.97481	-0.97352	-0.97223
-0.96163	-0.96032	-0.959	-0.95769	-0.9564	-0.95512	-0.97515	-0.97385	-0.97255
-0.96199	-0.96068	-0.95936	-0.95804	-0.95675	-0.95546	-0.9755	-0.97418	-0.97287
-0.93442	-0.96102	-0.9597	-0.95839	-0.9571	-0.95581	-0.97586	-0.97453	-0.97322
-0.93473	-0.96136	-0.96005	-0.95874	-0.95745	-0.95617	-0.97623	-0.9749	-0.97359
-0.93504	-0.9617	-0.96039	-0.95909	-0.95779	-0.9565	-0.95521	-0.95392	-0.97395
-0.93537	-0.96206	-0.96074	-0.95943	-0.95812	-0.95682	-0.95552	-0.95424	-0.97429

-0.88514	-0.93444	-0.93318	-0.93193	-0.95848	-0.95716	-0.95586	-0.95458	-0.97466
-0.88544	-0.93479	-0.93354	-0.93229	-0.95887	-0.95754	-0.95624	-0.95497	-0.97506
-0.88573	-0.93514	-0.9339	-0.93266	-0.95926	-0.95794	-0.95664	-0.95536	-0.97547
-0.886	-0.88489	-0.88378	-0.88267	-0.93176	-0.93049	-0.95703	-0.95575	-0.95448
-0.88627	-0.88516	-0.88407	-0.88298	-0.93211	-0.93085	-0.95742	-0.95614	-0.95487
-0.88657	-0.88547	-0.88438	-0.88329	-0.88216	-0.88103	-0.92996	-0.92874	-0.95525
-0.88689	-0.88578	-0.88469	-0.8836	-0.88247	-0.88133	-0.93031	-0.92909	-0.95563
-0.86568	-0.8861	-0.88501	-0.88391	-0.88279	-0.88165	-0.88053	-0.87941	-0.95601
-0.86598	-0.88643	-0.88533	-0.88423	-0.88311	-0.88197	-0.88085	-0.87973	-0.9564
-0.86628	-0.88675	-0.88564	-0.88455	-0.88343	-0.88229	-0.88117	-0.88005	-0.87894
-0.86657	-0.88705	-0.88595	-0.88485	-0.88373	-0.8826	-0.88148	-0.88036	-0.87925
-0.86685	-0.88735	-0.88625	-0.88516	-0.88404	-0.88291	-0.88179	-0.88067	-0.87956
-0.86716	-0.86608	-0.865	-0.86393	-0.88436	-0.88323	-0.88211	-0.881	-0.87989
-0.86749	-0.86639	-0.86532	-0.86425	-0.88469	-0.88356	-0.88244	-0.88133	-0.88023
-0.8678	-0.86669	-0.86562	-0.86455	-0.88502	-0.88389	-0.88277	-0.88167	-0.88057
-0.86811	-0.86699	-0.86591	-0.86485	-0.88533	-0.88421	-0.8831	-0.882	-0.8809
-0.86843	-0.86728	-0.8662	-0.86514	-0.86406	-0.86297	-0.88344	-0.88233	-0.88123
-0.86874	-0.86757	-0.86648	-0.86543	-0.86436	-0.86329	-0.88377	-0.88266	-0.88155
-0.86908	-0.86789	-0.86678	-0.86572	-0.86465	-0.8636	-0.86253	-0.86144	-0.88188
-0.86946	-0.86825	-0.86711	-0.86602	-0.86496	-0.86392	-0.86286	-0.86178	-0.88224
-0.86986	-0.86863	-0.86746	-0.86634	-0.86526	-0.86423	-0.86318	-0.86212	-0.88261
-0.83997	-0.86901	-0.86781	-0.86665	-0.86557	-0.86454	-0.86349	-0.86244	-0.88295
-0.84029	-0.86941	-0.86817	-0.86698	-0.86588	-0.86484	-0.8638	-0.86274	-0.88327
-0.84063	-0.86981	-0.86856	-0.86734	-0.86622	-0.86515	-0.86409	-0.86304	-0.86198
-0.84096	-0.87022	-0.86897	-0.86774	-0.86658	-0.86547	-0.86438	-0.86333	-0.86227
-0.84129	-0.84018	-0.83906	-0.83795	-0.86695	-0.8658	-0.8647	-0.86364	-0.86258
-0.8416	-0.84049	-0.83939	-0.83829	-0.86734	-0.86615	-0.86503	-0.86397	-0.86292
-0.84191	-0.84081	-0.83972	-0.83863	-0.86774	-0.86653	-0.86539	-0.86433	-0.86327
-0.82578	-0.84113	-0.84004	-0.83896	-0.83788	-0.83679	-0.86579	-0.8647	-0.86361
-0.82613	-0.84147	-0.84037	-0.83929	-0.83821	-0.83714	-0.8662	-0.86507	-0.86396
-0.79613	-0.8418	-0.8407	-0.83962	-0.83854	-0.83747	-0.86658	-0.86543	-0.8643
-0.79647	-0.84214	-0.84103	-0.83995	-0.83886	-0.83779	-0.86694	-0.86577	-0.86462
-0.79681	-0.84249	-0.84138	-0.84028	-0.83919	-0.8381	-0.83702	-0.83595	-0.86497
-0.79715	-0.84285	-0.84172	-0.84061	-0.83951	-0.83842	-0.83734	-0.83626	-0.86534
-0.79748	-0.81318	-0.81211	-0.81104	-0.83984	-0.83875	-0.83766	-0.83659	-0.83552
-0.7978	-0.81347	-0.81241	-0.81135	-0.84019	-0.8391	-0.83802	-0.83695	-0.83587
-0.79812	-0.81377	-0.81271	-0.81167	-0.84053	-0.83946	-0.83838	-0.83731	-0.83623
-0.79848	-0.8141	-0.81303	-0.81198	-0.84087	-0.83979	-0.83871	-0.83764	-0.83656
-0.79886	-0.81444	-0.81336	-0.81231	-0.84121	-0.84012	-0.83904	-0.83796	-0.83688
-0.79924	-0.79813	-0.79706	-0.79602	-0.81159	-0.81056	-0.83938	-0.83829	-0.8372
-0.79962	-0.79851	-0.79743	-0.79638	-0.81194	-0.8109	-0.83973	-0.83862	-0.83752
-0.8	-0.79889	-0.7978	-0.79673	-0.81227	-0.81123	-0.84008	-0.83896	-0.83785
-0.80036	-0.79924	-0.79814	-0.79706	-0.81259	-0.81154	-0.84041	-0.8393	-0.8382
-0.80073	-0.79961	-0.7985	-0.79741	-0.81292	-0.81186	-0.8108	-0.80975	-0.83855
-0.80111	-0.79999	-0.79888	-0.79779	-0.81328	-0.81221	-0.81114	-0.81008	-0.83889
-0.8015	-0.80038	-0.79927	-0.79817	-0.81365	-0.81257	-0.81149	-0.81042	-0.83925
-0.76976	-0.80076	-0.79964	-0.79854	-0.81401	-0.81292	-0.81185	-0.81078	-0.83963
-0.7701	-0.80114	-0.80002	-0.79891	-0.81437	-0.81328	-0.8122	-0.81114	-0.84003
-0.77045	-0.7694	-0.76834	-0.76729	-0.79821	-0.79713	-0.81256	-0.8115	-0.81044
-0.77082	-0.76977	-0.76871	-0.76766	-0.79861	-0.79751	-0.81293	-0.81185	-0.81078

-0.72567	-0.77013	-0.76907	-0.76802	-0.76697	-0.76592	-0.81328	-0.8122	-0.81112
-0.72608	-0.77046	-0.76941	-0.76836	-0.7673	-0.76624	-0.81361	-0.81252	-0.81144
-0.7265	-0.7708	-0.76974	-0.76869	-0.76762	-0.76656	-0.81396	-0.81286	-0.81177
-0.72695	-0.72585	-0.72476	-0.72368	-0.72259	-0.72151	-0.78276	-0.7817	-0.81214
-0.72741	-0.7263	-0.72519	-0.7241	-0.72302	-0.72195	-0.78311	-0.78205	-0.81251
-0.72785	-0.72672	-0.72561	-0.72452	-0.72343	-0.72237	-0.7213	-0.72023	-0.78133
-0.72828	-0.72714	-0.72602	-0.72492	-0.72384	-0.72278	-0.72171	-0.72063	-0.78165
-0.72873	-0.72758	-0.72646	-0.72536	-0.72428	-0.72321	-0.72214	-0.72106	-0.73753
-0.72918	-0.72805	-0.72693	-0.72582	-0.72474	-0.72367	-0.7226	-0.72152	-0.73798
-0.72965	-0.72852	-0.7274	-0.72629	-0.72521	-0.72413	-0.72306	-0.72198	-0.73844
-0.73013	-0.72899	-0.72787	-0.72676	-0.72567	-0.72459	-0.72352	-0.72245	-0.73891
-0.73061	-0.72946	-0.72834	-0.72723	-0.72613	-0.72505	-0.72398	-0.72291	-0.73938
-0.73107	-0.72991	-0.72878	-0.72767	-0.72658	-0.72551	-0.72443	-0.72336	-0.72228
-0.73152	-0.73036	-0.72922	-0.72811	-0.72702	-0.72596	-0.72488	-0.7238	-0.72271
-0.73201	-0.73085	-0.7297	-0.72857	-0.72748	-0.7264	-0.72531	-0.72422	-0.72313
-0.73253	-0.73137	-0.73021	-0.72906	-0.72794	-0.72684	-0.72574	-0.72464	-0.72354
-0.70213	-0.73189	-0.73072	-0.72956	-0.72842	-0.7273	-0.72618	-0.72508	-0.72398
-0.70263	-0.73241	-0.73124	-0.73007	-0.72892	-0.72778	-0.72665	-0.72554	-0.72444
-0.70312	-0.70201	-0.70089	-0.69977	-0.72942	-0.72826	-0.72713	-0.72602	-0.72492
-0.7036	-0.70249	-0.70137	-0.70025	-0.72993	-0.72877	-0.72763	-0.72651	-0.72541
-0.70407	-0.70296	-0.70185	-0.70073	-0.73044	-0.72928	-0.72813	-0.72701	-0.7259
-0.70452	-0.70342	-0.70231	-0.70121	-0.7001	-0.69899	-0.72864	-0.72752	-0.7264
-0.70497	-0.70387	-0.70277	-0.70167	-0.70057	-0.69947	-0.72916	-0.72802	-0.7269
-0.70541	-0.70431	-0.70321	-0.70212	-0.70103	-0.69994	-0.72966	-0.72853	-0.7274
-0.70586	-0.70475	-0.70364	-0.70255	-0.70147	-0.70041	-0.73016	-0.72903	-0.7279
-0.70633	-0.70521	-0.70409	-0.70299	-0.70192	-0.70087	-0.6998	-0.69872	-0.7284
-0.70682	-0.7057	-0.70457	-0.70346	-0.70238	-0.70132	-0.70025	-0.69919	-0.72891
-0.6708	-0.70619	-0.70506	-0.70394	-0.70285	-0.70177	-0.70071	-0.69966	-0.69861
-0.67122	-0.70668	-0.70555	-0.70443	-0.70333	-0.70224	-0.70118	-0.70014	-0.69909
-0.67165	-0.70717	-0.70604	-0.70493	-0.70382	-0.70272	-0.70166	-0.70063	-0.69958
-0.67208	-0.67107	-0.67011	-0.66916	-0.70431	-0.70321	-0.70214	-0.70112	-0.70007
-0.67253	-0.67149	-0.67052	-0.66958	-0.7048	-0.70369	-0.70263	-0.7016	-0.70056
-0.64256	-0.67194	-0.67095	-0.66999	-0.70529	-0.70418	-0.70311	-0.70208	-0.70104
-0.64301	-0.6724	-0.67138	-0.6704	-0.70577	-0.70467	-0.7036	-0.70256	-0.70151
-0.64347	-0.67287	-0.67183	-0.67082	-0.66985	-0.66892	-0.70408	-0.70303	-0.70198
-0.64394	-0.67337	-0.67229	-0.67125	-0.67027	-0.66933	-0.70457	-0.70351	-0.70245
-0.64442	-0.67387	-0.67276	-0.67169	-0.67069	-0.66974	-0.70505	-0.70399	-0.70293
-0.6449	-0.64384	-0.6428	-0.64177	-0.67114	-0.67016	-0.70553	-0.70447	-0.70342
-0.64539	-0.64433	-0.64327	-0.64224	-0.6716	-0.6706	-0.70602	-0.70497	-0.70391
-0.64588	-0.64481	-0.64375	-0.64271	-0.6417	-0.64073	-0.67008	-0.66917	-0.70441
-0.64638	-0.6453	-0.64424	-0.64319	-0.64217	-0.64118	-0.67055	-0.66962	-0.70492
-0.64687	-0.6458	-0.64473	-0.64367	-0.64265	-0.64166	-0.67104	-0.67009	-0.70543
-0.64736	-0.64629	-0.64522	-0.64416	-0.64314	-0.64215	-0.67156	-0.67057	-0.70595
-0.60717	-0.64678	-0.64571	-0.64466	-0.64364	-0.64265	-0.64167	-0.6407	-0.67005
-0.60758	-0.64727	-0.6462	-0.64516	-0.64414	-0.64315	-0.64216	-0.64116	-0.67053
-0.60799	-0.64776	-0.6467	-0.64566	-0.64465	-0.64365	-0.64264	-0.64163	-0.67101
-0.60839	-0.64826	-0.64722	-0.64618	-0.64516	-0.64414	-0.64313	-0.6421	-0.64111
-0.6088	-0.64877	-0.64773	-0.6467	-0.64567	-0.64464	-0.64362	-0.64258	-0.64158
-0.60923	-0.60838	-0.60755	-0.60673	-0.64618	-0.64514	-0.6441	-0.64307	-0.64206
-0.60966	-0.60882	-0.60799	-0.60715	-0.64668	-0.64563	-0.64459	-0.64356	-0.64255

-0.61009	-0.60925	-0.60841	-0.60757	-0.64717	-0.64612	-0.64508	-0.64405	-0.64304
-0.61052	-0.60968	-0.60884	-0.60799	-0.64766	-0.6466	-0.64556	-0.64454	-0.64354
-0.57824	-0.61011	-0.60926	-0.6084	-0.60755	-0.60669	-0.64605	-0.64504	-0.64403
-0.57864	-0.61054	-0.60968	-0.60882	-0.60796	-0.6071	-0.64655	-0.64553	-0.64452
-0.57905	-0.61098	-0.6101	-0.60924	-0.60837	-0.60751	-0.64705	-0.64602	-0.64501
-0.54553	-0.57863	-0.57782	-0.57703	-0.6088	-0.60795	-0.60711	-0.60628	-0.6455
-0.54591	-0.57903	-0.57821	-0.57741	-0.60924	-0.6084	-0.60756	-0.60671	-0.64601
-0.54628	-0.54553	-0.54482	-0.54412	-0.57703	-0.57627	-0.608	-0.60714	-0.64653
-0.54665	-0.5459	-0.54518	-0.54449	-0.57745	-0.57667	-0.60845	-0.60759	-0.64706
-0.54704	-0.54628	-0.54556	-0.54487	-0.57786	-0.57707	-0.57629	-0.57551	-0.60719
-0.54745	-0.54669	-0.54595	-0.54523	-0.57826	-0.57747	-0.57669	-0.57593	-0.60765
-0.54787	-0.54711	-0.54635	-0.54561	-0.57866	-0.57787	-0.5771	-0.57634	-0.60811
-0.51005	-0.54752	-0.54674	-0.54598	-0.54527	-0.54458	-0.57751	-0.57674	-0.57597
-0.51042	-0.54792	-0.54714	-0.54637	-0.54565	-0.54495	-0.57793	-0.57715	-0.57636
-0.5108	-0.51007	-0.50936	-0.50866	-0.50799	-0.50734	-0.54462	-0.54394	-0.57676
-0.51118	-0.51044	-0.50973	-0.50903	-0.50834	-0.50767	-0.54498	-0.54429	-0.57716
-0.51157	-0.51083	-0.5101	-0.5094	-0.5087	-0.50801	-0.50734	-0.50671	-0.57756
-0.51198	-0.51122	-0.51048	-0.50976	-0.50905	-0.50835	-0.50768	-0.50704	-0.57798
-0.51239	-0.51161	-0.51086	-0.51013	-0.50941	-0.50871	-0.50803	-0.50738	-0.50675
-0.51281	-0.51202	-0.51125	-0.51051	-0.50978	-0.50908	-0.50839	-0.50772	-0.50707
-0.51323	-0.51243	-0.51165	-0.5109	-0.51016	-0.50945	-0.50875	-0.50806	-0.5074
-0.4418	-0.51286	-0.51206	-0.51129	-0.51054	-0.50982	-0.50912	-0.50842	-0.50775
-0.44214	-0.51329	-0.51248	-0.51169	-0.51093	-0.5102	-0.50949	-0.5088	-0.50812
-0.4425	-0.44183	-0.44116	-0.44049	-0.51134	-0.51059	-0.50987	-0.50917	-0.50849
-0.44286	-0.44219	-0.44152	-0.44085	-0.51177	-0.51101	-0.51027	-0.50956	-0.50886
-0.44321	-0.44254	-0.44187	-0.44121	-0.44056	-0.43992	-0.51068	-0.50995	-0.50924
-0.44355	-0.44289	-0.44223	-0.44157	-0.44091	-0.44025	-0.51108	-0.51035	-0.50963
-0.40314	-0.44323	-0.44258	-0.44192	-0.44126	-0.4406	-0.43996	-0.43936	-0.51003
-0.40342	-0.44357	-0.44292	-0.44227	-0.44161	-0.44095	-0.44031	-0.43968	-0.51042
-0.4037	-0.44391	-0.44326	-0.44261	-0.44196	-0.44131	-0.44066	-0.44001	-0.51082
-0.40398	-0.40341	-0.40284	-0.40226	-0.44231	-0.44166	-0.44101	-0.44036	-0.43973
-0.40426	-0.40369	-0.40312	-0.40255	-0.44266	-0.44201	-0.44136	-0.44071	-0.44008
-0.40454	-0.40397	-0.4034	-0.40283	-0.40226	-0.40169	-0.40111	-0.40054	-0.44043
-0.40482	-0.40425	-0.40368	-0.40311	-0.40255	-0.40198	-0.40142	-0.40085	-0.4408
-0.40512	-0.40455	-0.40397	-0.4034	-0.40284	-0.40228	-0.40171	-0.40115	-0.40058
-0.40544	-0.40486	-0.40428	-0.40371	-0.40314	-0.40257	-0.402	-0.40144	-0.40087
-0.40576	-0.40518	-0.4046	-0.40402	-0.40344	-0.40287	-0.40229	-0.40172	-0.40116
-0.40607	-0.40548	-0.4049	-0.40432	-0.40374	-0.40316	-0.40258	-0.40201	-0.40144
-0.40638	-0.40578	-0.40519	-0.40461	-0.40403	-0.40345	-0.40288	-0.4023	-0.40173
-0.40669	-0.40609	-0.40549	-0.40491	-0.40432	-0.40374	-0.40316	-0.40259	-0.40201
-0.40701	-0.40641	-0.4058	-0.4052	-0.40462	-0.40404	-0.40345	-0.40286	-0.40229
-0.40732	-0.40672	-0.40611	-0.40551	-0.40492	-0.40434	-0.40375	-0.40316	-0.40258
-0.40762	-0.40702	-0.40642	-0.40582	-0.40523	-0.40465	-0.40407	-0.40348	-0.4029
-0.38668	-0.40733	-0.40673	-0.40614	-0.40555	-0.40496	-0.40438	-0.4038	-0.40322
-0.38697	-0.40765	-0.40705	-0.40646	-0.40587	-0.40527	-0.40468	-0.4041	-0.40351
-0.38727	-0.40798	-0.40738	-0.40678	-0.40618	-0.40558	-0.40498	-0.4044	-0.40381
-0.38755	-0.38701	-0.38648	-0.38597	-0.40649	-0.40589	-0.4053	-0.40471	-0.40412
-0.38784	-0.38728	-0.38675	-0.38623	-0.40679	-0.40621	-0.40562	-0.40503	-0.40445
-0.38814	-0.38758	-0.38704	-0.38652	-0.386	-0.38548	-0.40593	-0.40534	-0.40476
-0.38845	-0.38789	-0.38734	-0.38681	-0.38628	-0.38576	-0.40624	-0.40565	-0.40506

-0.38876	-0.3882	-0.38764	-0.3871	-0.38657	-0.38605	-0.38553	-0.38501	-0.38449
-0.38907	-0.3885	-0.38794	-0.38739	-0.38686	-0.38634	-0.38582	-0.3853	-0.38479
-0.38938	-0.38881	-0.38824	-0.38769	-0.38715	-0.38663	-0.38611	-0.3856	-0.38508
-0.38969	-0.38912	-0.38855	-0.38799	-0.38744	-0.38691	-0.38639	-0.38588	-0.38536
-0.39	-0.38944	-0.38886	-0.38829	-0.38774	-0.38719	-0.38666	-0.38615	-0.38564
-0.39033	-0.38976	-0.38918	-0.38861	-0.38804	-0.38748	-0.38695	-0.38643	-0.38592
-0.39067	-0.39009	-0.38951	-0.38894	-0.38836	-0.38779	-0.38724	-0.38672	-0.38621
-0.39103	-0.39042	-0.38983	-0.38926	-0.38868	-0.3881	-0.38754	-0.38701	-0.38649
-0.3914	-0.39075	-0.39014	-0.38957	-0.38899	-0.38842	-0.38785	-0.3873	-0.38677
-0.39182	-0.39111	-0.39048	-0.3899	-0.38931	-0.38873	-0.38817	-0.3876	-0.38706
-0.39231	-0.39152	-0.39084	-0.39024	-0.38964	-0.38905	-0.38848	-0.38792	-0.38737
-0.39284	-0.39196	-0.39124	-0.3906	-0.38997	-0.38938	-0.3888	-0.38824	-0.38768
-0.35133	-0.39245	-0.39166	-0.39095	-0.39031	-0.38972	-0.38914	-0.38856	-0.388
-0.35189	-0.39298	-0.39211	-0.39133	-0.39066	-0.39007	-0.38947	-0.38889	-0.38832
-0.35248	-0.35149	-0.35059	-0.34979	-0.39104	-0.39042	-0.38982	-0.38923	-0.38865
-0.35311	-0.35209	-0.35111	-0.35022	-0.39145	-0.3908	-0.39017	-0.38957	-0.38899
-0.35374	-0.3527	-0.35168	-0.35072	-0.34994	-0.34928	-0.39054	-0.38993	-0.38933
-0.35437	-0.35333	-0.3523	-0.35132	-0.35045	-0.34968	-0.39092	-0.39029	-0.38968
-0.35499	-0.35395	-0.35292	-0.35193	-0.351	-0.35014	-0.39135	-0.39068	-0.39004
-0.35558	-0.35455	-0.35354	-0.35255	-0.35159	-0.35066	-0.39184	-0.39108	-0.39039
-0.35618	-0.35515	-0.35415	-0.35317	-0.35219	-0.35122	-0.39237	-0.39153	-0.39077
-0.35679	-0.35576	-0.35476	-0.35378	-0.35279	-0.35182	-0.3509	-0.35002	-0.39119
-0.3574	-0.35637	-0.35537	-0.35438	-0.35341	-0.35244	-0.35149	-0.35053	-0.39166
-0.35803	-0.35699	-0.35598	-0.35499	-0.35401	-0.35305	-0.35208	-0.35109	-0.35019
-0.35866	-0.35761	-0.3566	-0.3556	-0.35462	-0.35365	-0.35268	-0.3517	-0.35077
-0.35931	-0.35825	-0.35722	-0.35622	-0.35523	-0.35425	-0.35328	-0.35231	-0.35137
-0.35997	-0.3589	-0.35786	-0.35684	-0.35584	-0.35485	-0.35388	-0.35292	-0.35197
-0.31239	-0.35956	-0.3585	-0.35747	-0.35645	-0.35545	-0.35448	-0.35353	-0.35258
-0.31291	-0.36023	-0.35916	-0.3581	-0.35708	-0.35607	-0.35508	-0.35412	-0.35316
-0.31344	-0.36092	-0.35983	-0.35875	-0.35771	-0.35669	-0.35569	-0.35471	-0.35374
-0.31398	-0.3616	-0.36051	-0.35942	-0.35836	-0.35732	-0.3563	-0.35531	-0.35434
-0.31452	-0.36229	-0.3612	-0.36012	-0.35903	-0.35795	-0.35691	-0.35591	-0.35494
-0.31506	-0.31419	-0.31335	-0.31253	-0.3597	-0.35861	-0.35755	-0.35654	-0.35554
-0.3156	-0.31472	-0.31388	-0.31304	-0.36038	-0.35929	-0.35822	-0.35718	-0.35616
-0.27692	-0.31527	-0.31441	-0.31357	-0.31274	-0.31192	-0.3589	-0.35783	-0.35679
-0.27741	-0.31583	-0.31496	-0.3141	-0.31327	-0.31245	-0.35959	-0.3585	-0.35743
-0.27791	-0.31639	-0.31552	-0.31465	-0.31381	-0.31299	-0.36028	-0.35917	-0.35809
-0.2784	-0.27762	-0.27685	-0.27609	-0.31436	-0.31352	-0.36096	-0.35986	-0.35877
-0.27888	-0.27809	-0.27734	-0.2766	-0.31492	-0.31406	-0.36166	-0.36057	-0.35948
-0.27938	-0.27859	-0.27783	-0.27708	-0.27631	-0.27555	-0.31377	-0.31295	-0.36022
-0.2799	-0.2791	-0.27831	-0.27754	-0.27678	-0.27605	-0.31434	-0.31352	-0.36097
-0.28041	-0.2796	-0.2788	-0.27801	-0.27726	-0.27653	-0.27579	-0.27506	-0.31325
-0.28091	-0.28011	-0.2793	-0.2785	-0.27773	-0.27698	-0.27625	-0.27552	-0.31378
-0.28142	-0.28061	-0.2798	-0.279	-0.27821	-0.27745	-0.2767	-0.27598	-0.27526
-0.28192	-0.28111	-0.2803	-0.2795	-0.2787	-0.27792	-0.27717	-0.27644	-0.27572
-0.28244	-0.28163	-0.28082	-0.28001	-0.2792	-0.27841	-0.27764	-0.2769	-0.27617
-0.28299	-0.28218	-0.28135	-0.28052	-0.27971	-0.2789	-0.27812	-0.27736	-0.27662
-0.28355	-0.28274	-0.2819	-0.28105	-0.28023	-0.27941	-0.27862	-0.27784	-0.27708
-0.28409	-0.28328	-0.28244	-0.2816	-0.28077	-0.27995	-0.27914	-0.27835	-0.27758
-0.2846	-0.28379	-0.28297	-0.28216	-0.28133	-0.28051	-0.27969	-0.27889	-0.27811

-0.28511	-0.2843	-0.2835	-0.28269	-0.28187	-0.28105	-0.28024	-0.27943	-0.27864
-0.28562	-0.28481	-0.284	-0.28319	-0.28238	-0.28157	-0.28077	-0.27996	-0.27916
-0.28614	-0.28533	-0.28451	-0.2837	-0.28289	-0.2821	-0.28129	-0.28049	-0.27968
-0.28666	-0.28584	-0.28502	-0.28421	-0.28341	-0.28261	-0.28181	-0.28101	-0.28021
-0.28721	-0.28636	-0.28554	-0.28474	-0.28394	-0.28314	-0.28234	-0.28154	-0.28074
-0.2878	-0.28693	-0.2861	-0.2853	-0.28449	-0.2837	-0.2829	-0.2821	-0.2813
-0.28842	-0.28753	-0.28669	-0.28587	-0.28506	-0.28427	-0.28348	-0.28268	-0.28188
-0.28903	-0.28813	-0.28727	-0.28643	-0.28561	-0.28482	-0.28403	-0.28324	-0.28245
-0.28965	-0.28873	-0.28784	-0.28698	-0.28616	-0.28536	-0.28457	-0.28379	-0.28299
-0.29026	-0.28934	-0.28843	-0.28754	-0.28671	-0.28591	-0.28512	-0.28434	-0.28355
-0.29086	-0.28995	-0.28902	-0.28811	-0.28727	-0.28647	-0.28568	-0.2849	-0.28412
-0.29148	-0.29057	-0.28963	-0.28871	-0.28785	-0.28704	-0.28624	-0.28546	-0.28468
-0.29213	-0.29122	-0.29029	-0.28936	-0.28847	-0.28761	-0.28679	-0.286	-0.28522
-0.29278	-0.29187	-0.29096	-0.29003	-0.28911	-0.2882	-0.28734	-0.28654	-0.28576
-0.24204	-0.29251	-0.2916	-0.29067	-0.28974	-0.28881	-0.28793	-0.28711	-0.2863
-0.24254	-0.29313	-0.29222	-0.2913	-0.29037	-0.28944	-0.28855	-0.28769	-0.28686
-0.15026	-0.24231	-0.24157	-0.24082	-0.29102	-0.29009	-0.28919	-0.2883	-0.28745
-0.15059	-0.24285	-0.24211	-0.24137	-0.2917	-0.29076	-0.28984	-0.28893	-0.28805
-0.09751	-0.24338	-0.24264	-0.2419	-0.29235	-0.29142	-0.2905	-0.28958	-0.28868
-0.09771	-0.24389	-0.24314	-0.2424	-0.29297	-0.29206	-0.29115	-0.29024	-0.28933
-0.0979	-0.2444	-0.24364	-0.2429	-0.29358	-0.29269	-0.2918	-0.2909	-0.28999
-0.0981	-0.15138	-0.15089	-0.15041	-0.24269	-0.24195	-0.29244	-0.29155	-0.29064
-0.0983	-0.15172	-0.15122	-0.15075	-0.24323	-0.24248	-0.29309	-0.29219	-0.29128
-0.04524	-0.09818	-0.09788	-0.09758	-0.24374	-0.24299	-0.24225	-0.24153	-0.29193
-0.04532	-0.0984	-0.09808	-0.09777	-0.24423	-0.24349	-0.24275	-0.24204	-0.29258
-0.04541	-0.04524	-0.04508	-0.04493	-0.09766	-0.09736	-0.24327	-0.24255	-0.24183
-0.04548	-0.04532	-0.04515	-0.04499	-0.09786	-0.09757	-0.2438	-0.24307	-0.24233
-0.04555	-0.0454	-0.04523	-0.04507	-0.04492	-0.04479	-0.24434	-0.2436	-0.24285
-0.04564	-0.04548	-0.04531	-0.04515	-0.045	-0.04485	-0.24488	-0.24413	-0.24339
-0.04573	-0.04556	-0.0454	-0.04524	-0.04508	-0.04491	-0.24541	-0.24467	-0.24393
	-0.04565	-0.04549	-0.04533	-0.04517	-0.045	-0.09806	-0.09775	-0.24443
	-0.04573	-0.04557	-0.04542	-0.04526	-0.04509	-0.09828	-0.09794	-0.24493
	-0.04582	-0.04566	-0.0455	-0.04534	-0.04518	-0.04502	-0.04484	-0.09783
	-0.04591	-0.04575	-0.04559	-0.04543	-0.04527	-0.04511	-0.04494	-0.09807
				-0.04551	-0.04536	-0.0452	-0.04503	-0.04486
				-0.0456	-0.04544	-0.04528	-0.04511	-0.04494
						-0.04536	-0.04519	-0.04502
						-0.04546	-0.04529	-0.04512
						-0.04556	-0.04539	-0.04522
						-0.04565	-0.04549	-0.04532
						-0.04574	-0.04558	-0.04542
								-0.04551
								-0.0456

		-0.10882
		-0.10859
-0.10892	-0.10864	-0.30195
-0.10866	-0.10839	-0.30115
-0.10841	-0.10813	-0.30036
-0.2539	-0.25294	-0.34064

						-0.25318	-0.25224	-0.33981
				-0.25456	-0.25349	-0.30007	-0.29904	-0.38721
				-0.25381	-0.25279	-0.2993	-0.29826	-0.38631
		-0.10835	-0.10805	-0.30072	-0.29963	-0.38793	-0.38667	-0.47728
		-0.10809	-0.10779	-0.29996	-0.29889	-0.38709	-0.38581	-0.47625
		-0.25375	-0.25275	-0.34044	-0.33927	-0.4322	-0.43081	-0.47524
		-0.25307	-0.2521	-0.33965	-0.33846	-0.43122	-0.42987	-0.47425
		-0.2524	-0.25146	-0.33886	-0.33765	-0.43025	-0.42894	-0.47329
-0.2537	-0.25268	-0.29907	-0.29804	-0.38616	-0.38487	-0.47516	-0.47373	-0.51139
-0.25299	-0.25198	-0.29829	-0.29726	-0.38527	-0.38403	-0.47421	-0.4728	-0.51034
-0.29968	-0.2986	-0.3869	-0.38565	-0.43016	-0.42886	-0.47326	-0.47188	-0.55689
-0.29893	-0.29788	-0.38606	-0.38481	-0.42925	-0.42797	-0.47234	-0.47098	-0.55581
-0.38773	-0.38647	-0.43099	-0.42966	-0.47417	-0.47279	-0.55791	-0.55632	-0.59233
-0.38687	-0.38561	-0.43007	-0.42877	-0.47324	-0.47188	-0.55682	-0.55522	-0.59117
-0.38601	-0.38476	-0.42916	-0.4279	-0.47232	-0.47098	-0.55572	-0.55412	-0.59001
-0.43089	-0.42956	-0.42829	-0.42706	-0.55781	-0.5562	-0.59221	-0.59053	-0.78982
-0.42997	-0.42869	-0.42745	-0.42623	-0.5567	-0.55508	-0.59104	-0.58936	-0.78826
-0.42909	-0.42784	-0.51122	-0.5097	-0.59325	-0.59155	-0.63295	-0.63119	-0.78673
-0.42824	-0.42701	-0.51018	-0.50865	-0.59206	-0.59038	-0.63172	-0.62998	-0.78521
-0.46722	-0.46583	-0.59432	-0.5926	-0.5909	-0.58924	-0.78803	-0.78586	-1.07583
-0.46625	-0.46484	-0.59314	-0.59146	-0.58979	-0.58812	-0.78651	-0.78437	-1.07353
-0.59539	-0.59366	-0.59199	-0.59035	-0.67347	-0.6716	-1.03965	-1.03646	-1.07124
-0.59422	-0.5925	-0.59086	-0.58926	-0.67228	-0.67042	-1.03747	-1.03429	-1.06895
-0.59307	-0.59138	-0.58976	-0.58817	-0.67109	-0.66925	-1.03532	-1.03214	-1.06668
-0.63484	-0.63307	-0.67346	-0.67166	-1.03965	-1.03638	-1.07112	-1.06775	-1.06443
-0.63372	-0.63195	-0.67226	-0.67042	-1.03736	-1.03417	-1.06885	-1.06549	-1.0622
-0.63257	-0.63082	-0.71407	-0.71215	-1.07327	-1.06996	-1.06665	-1.06331	-1.12653
-0.63141	-0.62966	-0.7128	-0.7109	-1.07096	-1.06776	-1.06451	-1.0612	-1.12432
-0.71539	-0.71347	-1.03721	-1.03396	-1.06872	-1.06555	-1.06234	-1.05906	-1.18243
-0.71416	-0.71225	-1.03509	-1.03192	-1.06657	-1.06331	-1.06009	-1.05688	-1.18012
-0.90196	-0.89904	-1.07106	-1.06775	-1.06443	-1.0611	-1.18455	-1.18118	-1.17786
-0.90005	-0.89721	-1.06883	-1.06553	-1.06222	-1.05892	-1.18222	-1.17894	-1.17569
-0.89818	-0.89542	-1.06662	-1.06332	-1.06004	-1.05678	-1.17994	-1.17673	-1.17353
-1.00454	-1.00146	-1.0645	-1.06121	-1.18454	-1.1811	-1.17776	-1.17455	-1.17134
-1.00256	-0.99951	-1.06242	-1.05915	-1.18234	-1.17896	-1.17564	-1.17238	-1.16913
-1.06681	-1.06355	-1.18696	-1.1835	-1.1801	-1.17675	-1.17344	-1.17018	-1.2555
-1.0646	-1.06135	-1.18461	-1.1812	-1.17782	-1.17447	-1.17119	-1.16797	-1.2532
-1.12502	-1.12166	-1.18233	-1.17895	-1.17559	-1.17224	-1.25762	-1.25424	-1.25091
-1.12287	-1.11953	-1.18014	-1.17679	-1.17344	-1.1701	-1.25534	-1.25196	-1.24864
-1.12073	-1.11742	-1.17796	-1.17463	-1.17128	-1.16794	-1.25306	-1.2497	-1.2464
-1.18246	-1.17907	-1.17571	-1.17237	-1.22695	-1.22351	-1.25073	-1.24746	-1.27421
-1.18017	-1.17681	-1.17345	-1.1701	-1.22463	-1.22121	-1.24841	-1.24524	-1.27204
-1.20911	-1.20571	-1.22924	-1.2258	-1.25298	-1.24955	-1.24625	-1.24309	-1.26985
-1.20691	-1.20351	-1.22701	-1.22358	-1.25076	-1.24745	-1.24419	-1.241	-1.26767
-1.20465	-1.20125	-1.22475	-1.22135	-1.24854	-1.24532	-1.27204	-1.26878	-1.26554
-1.20233	-1.19893	-1.22245	-1.21911	-1.2463	-1.24317	-1.26991	-1.26669	-1.26347
-1.227	-1.22357	-1.2507	-1.24735	-1.27411	-1.27094	-1.26776	-1.26457	-1.36331
-1.22483	-1.22144	-1.24856	-1.24527	-1.27199	-1.26878	-1.26558	-1.26239	-1.36082
-1.2227	-1.21935	-1.24647	-1.24323	-1.2699	-1.26665	-1.26342	-1.26022	-1.35833
-1.22056	-1.21729	-1.24439	-1.24117	-1.2678	-1.26456	-1.36343	-1.35967	-1.38319

-1.21844	-1.21524	-1.24231	-1.2391	-1.2657	-1.26248	-1.36108	-1.35729	-1.38075
-1.24663	-1.2434	-1.27006	-1.26679	-1.28288	-1.27955	-1.35861	-1.35482	-1.37825
-1.24441	-1.2412	-1.26784	-1.26459	-1.28061	-1.27726	-1.35607	-1.35229	-1.37571
-1.2722	-1.26893	-1.28508	-1.28174	-1.3612	-1.3574	-1.38087	-1.37704	-1.39169
-1.27012	-1.26686	-1.28294	-1.27962	-1.35886	-1.35505	-1.37848	-1.37465	-1.38929
-1.2875	-1.28415	-1.36406	-1.3603	-1.3565	-1.3527	-1.39454	-1.3907	-1.38695
-1.28537	-1.28203	-1.3617	-1.3579	-1.3541	-1.3503	-1.39211	-1.38835	-1.38468
-1.28324	-1.27991	-1.35931	-1.35547	-1.35167	-1.34789	-1.38969	-1.38601	-1.38242
-1.33411	-1.33042	-1.35688	-1.35299	-1.39479	-1.39095	-1.38721	-1.38361	-1.40557
-1.33175	-1.32806	-1.35441	-1.35049	-1.39222	-1.38842	-1.38474	-1.38118	-1.40317
-1.35962	-1.35582	-1.3707	-1.36677	-1.38976	-1.38603	-1.40793	-1.40434	-1.42737
-1.35704	-1.35326	-1.36818	-1.36434	-1.38739	-1.38377	-1.40569	-1.4021	-1.42514
-1.3733	-1.36951	-1.39255	-1.38875	-1.41064	-1.40704	-1.43003	-1.42642	-1.42305
-1.37089	-1.3671	-1.39018	-1.38649	-1.40839	-1.40478	-1.4278	-1.42433	-1.42111
-1.36852	-1.36475	-1.41345	-1.4098	-1.43278	-1.42912	-1.42564	-1.42234	-1.46919
-1.3662	-1.36248	-1.41115	-1.40755	-1.43054	-1.42693	-1.42357	-1.42049	-1.46749
-1.36392	-1.36026	-1.4089	-1.40532	-1.42834	-1.42481	-1.42161	-1.41875	-1.4659
-1.41399	-1.41028	-1.40663	-1.40307	-1.47632	-1.47288	-1.46974	-1.46692	-1.4643
-1.41166	-1.40803	-1.40438	-1.40083	-1.47414	-1.47096	-1.468	-1.46527	-1.46272
-1.40937	-1.40577	-1.479	-1.47534	-1.47205	-1.46908	-1.4663	-1.46369	-1.48817
-1.40711	-1.40351	-1.47672	-1.47316	-1.47002	-1.46724	-1.46463	-1.46219	-1.48674
-1.48187	-1.47814	-1.47455	-1.47116	-1.46817	-1.46554	-1.49004	-1.48765	-1.48534
-1.47955	-1.47591	-1.47252	-1.46938	-1.46655	-1.46402	-1.48855	-1.48622	-1.48397
-1.47728	-1.47377	-1.4706	-1.46771	-1.46503	-1.46258	-1.48712	-1.48484	-1.48262
-1.47508	-1.47175	-1.46878	-1.46607	-1.4905	-1.48806	-1.48572	-1.48349	-1.48132
-1.47298	-1.46985	-1.46705	-1.4645	-1.48899	-1.48664	-1.48436	-1.48218	-1.48004
-1.47102	-1.46808	-1.49245	-1.48995	-1.48754	-1.48525	-1.48303	-1.48088	-1.5339
-1.46919	-1.46644	-1.49089	-1.48848	-1.48614	-1.4839	-1.48172	-1.4796	-1.53241
-1.49457	-1.49188	-1.48938	-1.48704	-1.48477	-1.48257	-1.53578	-1.53334	-1.53092
-1.49299	-1.49038	-1.48795	-1.48565	-1.48342	-1.48127	-1.53428	-1.53184	-1.52942
-1.49147	-1.48893	-1.48655	-1.48429	-1.50854	-1.50625	-1.53278	-1.53035	-1.52793
-1.48998	-1.48752	-1.48519	-1.48297	-1.50713	-1.50487	-1.53129	-1.52886	-1.52644
-1.48853	-1.48614	-1.48386	-1.48167	-1.50575	-1.5035	-1.52981	-1.52739	-1.52498
-1.48714	-1.48478	-1.50899	-1.50666	-1.53316	-1.53074	-1.52833	-1.52595	-1.54567
-1.48578	-1.48346	-1.50758	-1.50528	-1.53167	-1.52925	-1.52687	-1.52452	-1.54425
-1.48443	-1.48215	-1.5351	-1.53263	-1.53019	-1.52779	-1.54757	-1.54521	-1.54283
-1.48311	-1.48086	-1.53359	-1.53113	-1.52872	-1.52636	-1.54613	-1.54378	-1.54142
-1.50816	-1.50574	-1.55434	-1.55185	-1.54942	-1.54706	-1.5447	-1.54237	-1.60227
-1.50675	-1.50435	-1.55282	-1.55035	-1.54796	-1.54563	-1.5433	-1.54096	-1.60068
-1.55644	-1.55383	-1.55132	-1.54888	-1.58573	-1.58335	-1.60439	-1.60173	-1.63504
-1.55493	-1.55234	-1.54986	-1.54746	-1.58427	-1.5819	-1.60279	-1.60017	-1.6335
-1.55345	-1.55088	-1.54844	-1.54607	-1.58283	-1.58045	-1.60121	-1.59864	-1.63199
-1.55202	-1.5495	-1.58629	-1.58383	-1.5814	-1.57901	-1.63561	-1.63306	-1.67248
-1.55062	-1.54818	-1.58492	-1.58243	-1.57998	-1.57758	-1.63404	-1.63158	-1.671
-1.58849	-1.58603	-1.58352	-1.58102	-1.63775	-1.63508	-1.67448	-1.67199	-1.66951
-1.58701	-1.5846	-1.58211	-1.57962	-1.63619	-1.63358	-1.67295	-1.67047	-1.668
-1.62228	-1.61985	-1.64011	-1.63734	-1.67667	-1.67401	-1.67143	-1.66895	-1.66649
-1.62081	-1.61839	-1.63855	-1.63584	-1.67514	-1.67251	-1.66994	-1.66744	-1.66499
-1.61936	-1.61694	-1.63699	-1.63436	-1.67364	-1.67101	-1.66845	-1.66594	-1.66349
-1.61791	-1.61549	-1.67741	-1.67476	-1.67213	-1.66951	-1.66695	-1.66443	-1.66199

-1.61648	-1.61405	-1.6758	-1.67322	-1.67063	-1.66802	-1.66547	-1.66295	-1.66051
-1.67959	-1.67683	-1.67422	-1.67169	-1.66913	-1.66656	-1.66403	-1.66152	-1.67031
-1.67802	-1.67526	-1.67266	-1.67016	-1.66764	-1.66513	-1.66263	-1.66014	-1.66895
-1.67645	-1.67371	-1.67113	-1.66863	-1.66614	-1.66367	-1.67242	-1.66997	-1.66758
-1.67487	-1.67219	-1.6696	-1.66709	-1.66463	-1.6622	-1.67099	-1.66858	-1.66621
-1.67333	-1.6707	-1.66812	-1.66559	-1.67431	-1.67193	-1.66957	-1.66721	-1.71097
-1.67186	-1.66928	-1.66668	-1.66412	-1.67284	-1.6705	-1.66818	-1.66587	-1.70951
-1.67039	-1.66786	-1.66527	-1.66269	-1.67142	-1.6691	-1.6668	-1.66453	-1.70804
-1.66888	-1.66637	-1.67498	-1.67249	-1.67008	-1.66773	-1.71169	-1.7091	-1.70651
-1.66737	-1.66486	-1.67355	-1.67117	-1.66878	-1.66639	-1.71012	-1.70753	-1.70496
-1.67701	-1.67452	-1.67213	-1.6698	-1.66743	-1.66503	-1.70856	-1.70598	-1.70344
-1.67559	-1.67309	-1.67071	-1.6684	-1.66604	-1.66366	-1.707	-1.70444	-1.70194
-1.67418	-1.6717	-1.66933	-1.66701	-1.71086	-1.70814	-1.7055	-1.70296	-1.71829
-1.67277	-1.67037	-1.66799	-1.66564	-1.70932	-1.70668	-1.70408	-1.70153	-1.71683
-1.67138	-1.66904	-1.71319	-1.71045	-1.70779	-1.70522	-1.72048	-1.71792	-1.7154
-1.67001	-1.66764	-1.71163	-1.70894	-1.70631	-1.70374	-1.71901	-1.7165	-1.71403
-1.66864	-1.66624	-1.71007	-1.70744	-1.70484	-1.70226	-1.71753	-1.71508	-1.71265
-1.66724	-1.66484	-1.72634	-1.72374	-1.72114	-1.71855	-1.71603	-1.7136	-1.72622
-1.66583	-1.66345	-1.72475	-1.72219	-1.71961	-1.71704	-1.71453	-1.71208	-1.72471
-1.72858	-1.72584	-1.72322	-1.72067	-1.71812	-1.71558	-1.72817	-1.72568	-1.73433
-1.72702	-1.72436	-1.72175	-1.71919	-1.71666	-1.71417	-1.72677	-1.72429	-1.73283
-1.72549	-1.72288	-1.73549	-1.7329	-1.73036	-1.72785	-1.72537	-1.7229	-1.73137
-1.72399	-1.72139	-1.73403	-1.73149	-1.72895	-1.72642	-1.72394	-1.72153	-1.72996
-1.72251	-1.71992	-1.73258	-1.73007	-1.72754	-1.725	-1.72253	-1.72016	-1.72855
-1.73628	-1.7337	-1.73114	-1.7286	-1.7261	-1.72362	-1.73213	-1.72961	-1.72711
-1.73483	-1.73229	-1.7297	-1.72713	-1.72465	-1.72226	-1.73072	-1.72818	-1.72567
-1.73338	-1.73084	-1.72827	-1.72572	-1.73433	-1.73181	-1.72929	-1.72676	-1.72426
-1.73194	-1.72937	-1.72684	-1.72436	-1.73288	-1.73035	-1.72784	-1.72535	-1.72288
-1.7305	-1.72793	-1.72543	-1.72298	-1.73144	-1.72892	-1.72642	-1.72394	-1.72149
-1.72905	-1.72652	-1.72403	-1.72157	-1.73	-1.7275	-1.72502	-1.72254	-1.7201
-1.72761	-1.72511	-1.73366	-1.73109	-1.72857	-1.72609	-1.72362	-1.72116	-1.7648
-1.72619	-1.72369	-1.73221	-1.72968	-1.72717	-1.72467	-1.72221	-1.71979	-1.7633
-1.72479	-1.72229	-1.73077	-1.72828	-1.72577	-1.72325	-1.7208	-1.71843	-1.76181
-1.73449	-1.73189	-1.72935	-1.72685	-1.72435	-1.72188	-1.71945	-1.71708	-1.76026
-1.73303	-1.73049	-1.72794	-1.72541	-1.72294	-1.72053	-1.71812	-1.71572	-1.75869
-1.73159	-1.72906	-1.72651	-1.724	-1.72155	-1.71916	-1.76261	-1.75986	-1.75715
-1.73018	-1.7276	-1.72509	-1.72262	-1.72019	-1.71778	-1.76103	-1.75832	-1.75564
-1.72879	-1.72619	-1.72368	-1.72124	-1.71882	-1.71641	-1.75948	-1.75678	-1.75411
-1.72743	-1.72483	-1.72231	-1.71985	-1.71743	-1.71506	-1.75796	-1.75524	-1.75257
-1.72606	-1.72348	-1.72095	-1.71848	-1.76181	-1.75913	-1.75644	-1.75372	-1.75105
-1.72466	-1.72213	-1.71963	-1.71717	-1.76029	-1.75757	-1.75488	-1.75222	-1.7496
-1.72325	-1.72077	-1.71831	-1.71587	-1.7588	-1.75602	-1.75332	-1.75074	-1.74818
-1.72186	-1.71939	-1.76284	-1.76009	-1.75733	-1.75455	-1.75186	-1.74927	-1.74671
-1.72047	-1.718	-1.76128	-1.75859	-1.75586	-1.75313	-1.75045	-1.74782	-1.74524
-1.71913	-1.71665	-1.75974	-1.75705	-1.75437	-1.75169	-1.74904	-1.74641	-1.74381
-1.71783	-1.71536	-1.75822	-1.75549	-1.75283	-1.75025	-1.74766	-1.74503	-1.74244
-1.76236	-1.75955	-1.75675	-1.75399	-1.75135	-1.74881	-1.76788	-1.76527	-1.76269
-1.7609	-1.75811	-1.75533	-1.75258	-1.74993	-1.74735	-1.7664	-1.76385	-1.76132
-1.75943	-1.75667	-1.75393	-1.75121	-1.74854	-1.74591	-1.76494	-1.76244	-1.75996
-1.77388	-1.77116	-1.76846	-1.76578	-1.76881	-1.76613	-1.76354	-1.76107	-1.78186

-1.77235	-1.76969	-1.76704	-1.76441	-1.76745	-1.76476	-1.76218	-1.75972	-1.78042
-1.77084	-1.7682	-1.77132	-1.76873	-1.76608	-1.76341	-1.76082	-1.75836	-1.79612
-1.76936	-1.76671	-1.76986	-1.7673	-1.7647	-1.76206	-1.75949	-1.75698	-1.79459
-1.77366	-1.771	-1.76841	-1.76586	-1.76329	-1.7607	-1.78132	-1.77861	-1.79308
-1.77226	-1.76961	-1.76698	-1.76439	-1.76184	-1.75933	-1.77988	-1.77718	-1.79161
-1.77089	-1.76824	-1.76559	-1.76296	-1.76043	-1.75797	-1.79565	-1.79288	-1.82303
-1.76954	-1.76688	-1.76423	-1.7616	-1.75908	-1.75664	-1.79419	-1.79144	-1.82153
-1.76819	-1.76554	-1.76288	-1.76026	-1.75774	-1.75531	-1.79273	-1.78999	-1.82003
-1.76682	-1.76418	-1.76155	-1.75893	-1.7794	-1.77674	-1.80657	-1.80385	-1.81853
-1.76545	-1.76283	-1.76022	-1.75762	-1.77794	-1.77523	-1.80502	-1.80234	-1.81703
-1.7641	-1.76149	-1.78205	-1.77926	-1.80907	-1.80624	-1.83492	-1.83205	-1.82922
-1.76275	-1.76015	-1.78062	-1.77784	-1.8076	-1.80476	-1.83336	-1.83048	-1.82766
-1.76141	-1.75882	-1.80906	-1.80625	-1.83789	-1.83483	-1.83185	-1.82897	-1.89023
-1.76007	-1.7575	-1.80761	-1.8048	-1.83638	-1.83336	-1.83041	-1.82752	-1.88852
-1.81176	-1.80897	-1.82295	-1.82009	-1.84781	-1.84484	-1.84191	-1.83904	-1.88683
-1.81029	-1.80751	-1.82148	-1.81863	-1.84632	-1.84339	-1.84049	-1.83762	-1.88514
-1.80883	-1.80606	-1.82001	-1.81718	-1.84485	-1.84195	-1.83908	-1.83621	-1.88347
-1.80737	-1.80461	-1.84933	-1.84634	-1.84341	-1.84054	-1.88843	-1.88511	-1.88185
-1.80592	-1.80316	-1.84785	-1.8449	-1.842	-1.83914	-1.88679	-1.8835	-1.88026
-1.85236	-1.84932	-1.84636	-1.84345	-1.84058	-1.83774	-1.88515	-1.88188	-1.87867
-1.85083	-1.84781	-1.84488	-1.84201	-1.83915	-1.83632	-1.88351	-1.88027	-1.87707
-1.84933	-1.84633	-1.84343	-1.84058	-1.88841	-1.88513	-1.88188	-1.87865	-1.87547
-1.84785	-1.8449	-1.84201	-1.83915	-1.88674	-1.88349	-1.88025	-1.87703	-1.87386
-1.84638	-1.84348	-1.8406	-1.83774	-1.88509	-1.88185	-1.87863	-1.87542	-1.87227
-1.84493	-1.84205	-1.83918	-1.83633	-1.88345	-1.88022	-1.87701	-1.87382	-1.87069
-1.84348	-1.84063	-1.83776	-1.83492	-1.88182	-1.87859	-1.8754	-1.87223	-1.86911
-1.84206	-1.83921	-1.88672	-1.88344	-1.88019	-1.87697	-1.87378	-1.87063	-1.86753
-1.84064	-1.83781	-1.88509	-1.88183	-1.87858	-1.87535	-1.87216	-1.86903	-1.86595
-1.83923	-1.8364	-1.88346	-1.88021	-1.87696	-1.87373	-1.87056	-1.86745	-1.8644
-1.83782	-1.835	-1.88183	-1.87858	-1.87535	-1.87213	-1.86897	-1.86589	-1.86287
-1.88671	-1.88346	-1.8802	-1.87696	-1.87374	-1.87053	-1.8674	-1.86435	-1.87706
-1.88507	-1.88182	-1.87857	-1.87533	-1.87212	-1.86894	-1.86584	-1.86283	-1.87555
-1.88344	-1.8802	-1.87695	-1.87371	-1.87052	-1.86737	-1.86431	-1.86135	-1.87407
-1.88182	-1.87858	-1.87534	-1.87211	-1.86895	-1.86584	-1.86282	-1.85991	-1.87261
-1.88022	-1.87699	-1.87375	-1.87054	-1.86741	-1.86435	-1.86137	-1.85849	-1.87118
-1.8787	-1.87547	-1.87224	-1.86905	-1.86594	-1.86291	-1.87559	-1.87265	-1.89866
-1.87724	-1.87401	-1.87079	-1.86762	-1.86453	-1.86153	-1.87418	-1.87121	-1.89715
-1.87572	-1.87249	-1.8693	-1.86614	-1.86309	-1.86011	-1.90165	-1.89863	-1.89563
-1.87413	-1.87091	-1.86774	-1.86463	-1.86159	-1.85863	-1.9001	-1.89709	-1.89409
-1.87252	-1.86931	-1.86618	-1.8631	-1.90463	-1.90157	-1.89854	-1.89554	-1.89255
-1.87091	-1.86772	-1.86461	-1.86156	-1.90303	-1.89999	-1.89697	-1.89397	-1.89099
-1.8693	-1.86614	-1.86305	-1.86003	-1.90143	-1.89841	-1.8954	-1.89241	-1.88944
-1.86774	-1.8646	-1.89078	-1.88774	-1.89987	-1.89685	-1.89385	-1.89087	-1.88792
-1.86622	-1.86311	-1.88926	-1.88623	-1.89834	-1.89532	-1.89233	-1.88936	-1.88642
-1.89399	-1.89087	-1.90301	-1.89993	-1.89688	-1.89387	-1.89088	-1.88792	-1.88499
-1.89256	-1.88947	-1.90159	-1.89852	-1.89549	-1.89249	-1.88951	-1.88655	-1.88362
-1.89108	-1.88801	-1.90011	-1.89706	-1.89405	-1.89107	-1.8881	-1.88515	-1.88222
-1.88953	-1.88647	-1.89855	-1.89552	-1.89253	-1.88957	-1.88662	-1.88368	-1.88078
-1.90318	-1.90005	-1.89698	-1.89397	-1.891	-1.88806	-1.88514	-1.88222	-1.87933
-1.90166	-1.89851	-1.89545	-1.89246	-1.8895	-1.88658	-1.88367	-1.88077	-1.8779

-1.90016	-1.897	-1.89395	-1.89098	-1.88803	-1.88512	-1.88222	-1.87934	-1.8765
-1.89868	-1.89555	-1.89252	-1.88957	-1.88664	-1.88373	-1.88085	-1.878	-1.87518
-1.89721	-1.89414	-1.89114	-1.8882	-1.88529	-1.8824	-1.87953	-1.8767	-1.8739
-1.89573	-1.8927	-1.88973	-1.8868	-1.88389	-1.88101	-1.87817	-1.87535	-1.87257
-1.89424	-1.89123	-1.88828	-1.88536	-1.88246	-1.87959	-1.87675	-1.87395	-1.87117
-1.89278	-1.8898	-1.88685	-1.88395	-1.88106	-1.8782	-1.87537	-1.87258	-1.88195
-1.89139	-1.88843	-1.8855	-1.8826	-1.87973	-1.87689	-1.87407	-1.87129	-1.88064
-1.88999	-1.88705	-1.88413	-1.88125	-1.8784	-1.87558	-1.87278	-1.86999	-1.87934
-1.88854	-1.88561	-1.88271	-1.87983	-1.877	-1.8742	-1.88353	-1.88075	-1.87799
-1.88709	-1.88416	-1.88126	-1.87841	-1.87558	-1.87279	-1.88214	-1.87937	-1.87662
-1.8857	-1.88278	-1.87989	-1.87704	-1.87423	-1.87145	-1.8808	-1.87803	-1.8753
-1.88436	-1.88144	-1.87856	-1.87572	-1.87292	-1.87015	-1.8795	-1.87674	-1.874
-1.883	-1.88007	-1.8772	-1.87438	-1.88368	-1.88091	-1.87816	-1.87541	-1.8941
-1.88162	-1.87868	-1.87581	-1.873	-1.88229	-1.87951	-1.87676	-1.87404	-1.89277
-1.88025	-1.87731	-1.88655	-1.88373	-1.88093	-1.87814	-1.89688	-1.89416	-1.90229
-1.87889	-1.87599	-1.88523	-1.88242	-1.87962	-1.87684	-1.89561	-1.8929	-1.90102
-1.87755	-1.87468	-1.88392	-1.8811	-1.87831	-1.87555	-1.89436	-1.89166	-1.89977
-1.87622	-1.87337	-1.88259	-1.87974	-1.89853	-1.89576	-1.89305	-1.89038	-1.89849
-1.87492	-1.87206	-1.88125	-1.87837	-1.89717	-1.89442	-1.89172	-1.88908	-1.89721
-1.88567	-1.88281	-1.90155	-1.89866	-1.89587	-1.89314	-1.90124	-1.89858	-1.89596
-1.88434	-1.88146	-1.90024	-1.89739	-1.89462	-1.89191	-1.90001	-1.89736	-1.89474
-1.90469	-1.9018	-1.89895	-1.89614	-1.90419	-1.90147	-1.89878	-1.89613	-1.89351
-1.90336	-1.90052	-1.8977	-1.89493	-1.90297	-1.90025	-1.89756	-1.89488	-1.89225
-1.90205	-1.89924	-1.90727	-1.90449	-1.90175	-1.89903	-1.89633	-1.89364	-1.891
-1.90075	-1.89796	-1.906	-1.90324	-1.90049	-1.89777	-1.89508	-1.89242	-1.8898
-1.89947	-1.89667	-1.90471	-1.90197	-1.89923	-1.8965	-1.89382	-1.8912	-1.88861
-1.909	-1.90617	-1.9034	-1.90067	-1.89795	-1.89524	-1.89259	-1.88999	-1.88742
-1.9077	-1.90484	-1.90207	-1.89935	-1.89666	-1.89399	-1.89136	-1.88879	-1.88624
-1.90643	-1.90357	-1.9008	-1.89809	-1.89541	-1.89277	-1.89017	-1.8876	-1.88505
-1.90518	-1.90234	-1.89958	-1.89688	-1.89421	-1.89159	-1.889	-1.88642	-1.88386
-1.90392	-1.90112	-1.89837	-1.89567	-1.89302	-1.89041	-1.88781	-1.88522	-1.88265
-1.90264	-1.89987	-1.89714	-1.89445	-1.89181	-1.8892	-1.8866	-1.884	-1.88143
-1.90136	-1.89862	-1.89592	-1.89325	-1.8906	-1.88798	-1.88537	-1.88277	-1.8802
-1.90013	-1.89741	-1.89472	-1.89206	-1.8894	-1.88675	-1.88413	-1.88155	-1.879
-1.89892	-1.89622	-1.89353	-1.89087	-1.88819	-1.88551	-1.88289	-1.88033	-1.87781
-1.8977	-1.89501	-1.89234	-1.88968	-1.88701	-1.88432	-1.8817	-1.87916	-1.87665
-1.89649	-1.89381	-1.89115	-1.8885	-1.88583	-1.88316	-1.88055	-1.87802	-1.87551
-1.89528	-1.8926	-1.88994	-1.88728	-1.88463	-1.88199	-1.8794	-1.87687	-1.87437
-1.89408	-1.8914	-1.88871	-1.88602	-1.88338	-1.88078	-1.87823	-1.87572	-1.87323
-1.89289	-1.8902	-1.88749	-1.88479	-1.88216	-1.8796	-1.87706	-1.87457	-1.87208
-1.8917	-1.88903	-1.88632	-1.88362	-1.881	-1.87844	-1.87591	-1.87342	-1.87093
-1.89052	-1.88785	-1.88516	-1.88247	-1.87985	-1.8773	-1.87477	-1.87227	-1.86977
-1.88934	-1.88667	-1.88398	-1.8813	-1.87869	-1.87614	-1.87362	-1.87112	-1.86861
-1.88815	-1.88549	-1.8828	-1.88013	-1.87753	-1.87499	-1.87247	-1.86997	-1.86746
-1.88697	-1.88432	-1.88164	-1.87898	-1.87638	-1.87384	-1.87131	-1.86881	-1.8663
-1.8858	-1.88316	-1.88049	-1.87783	-1.87523	-1.87269	-1.87016	-1.86766	-1.86514
-1.88463	-1.882	-1.87934	-1.87668	-1.87408	-1.87153	-1.869	-1.8665	-1.86398
-1.88345	-1.88083	-1.87817	-1.87552	-1.87292	-1.87037	-1.86784	-1.86534	-1.86281
-1.88227	-1.87965	-1.87701	-1.87436	-1.87176	-1.86921	-1.86667	-1.86417	-1.86165
-1.88109	-1.87848	-1.87583	-1.87318	-1.87059	-1.86804	-1.8655	-1.86299	-1.86047

-1.87991	-1.87731	-1.87466	-1.87201	-1.86942	-1.86686	-1.86433	-1.86181	-1.85929
-1.87875	-1.87615	-1.8735	-1.87085	-1.86825	-1.86569	-1.86315	-1.86064	-1.85811
-1.87759	-1.875	-1.87235	-1.8697	-1.86709	-1.86453	-1.86198	-1.85947	-1.85695
-1.87644	-1.87384	-1.87121	-1.86857	-1.86596	-1.86338	-1.86082	-1.8583	-1.85578
-1.87528	-1.87269	-1.87009	-1.86748	-1.86486	-1.86224	-1.85966	-1.85714	-1.85462
-1.87414	-1.87154	-1.86895	-1.86636	-1.86374	-1.8611	-1.85851	-1.85599	-1.86606
-1.87303	-1.8704	-1.8678	-1.86521	-1.86259	-1.85994	-1.85735	-1.85483	-1.8649
-1.87192	-1.86925	-1.86664	-1.86405	-1.86143	-1.85878	-1.85619	-1.85368	-1.86374
-1.87077	-1.8681	-1.86548	-1.86288	-1.87284	-1.8702	-1.86761	-1.86509	-1.86257
-1.86962	-1.86695	-1.86432	-1.86172	-1.87167	-1.86903	-1.86645	-1.86392	-1.86141
-1.86821	-1.86557	-1.87576	-1.87313	-1.87051	-1.86791	-1.86533	-1.86278	-1.86026
-1.86709	-1.86448	-1.87464	-1.87198	-1.86938	-1.86681	-1.86425	-1.86166	-1.8591
-1.86595	-1.86336	-1.87349	-1.87082	-1.86823	-1.86569	-1.86312	-1.86052	-1.85794
-1.86479	-1.8622	-1.87232	-1.86966	-1.86706	-1.86453	-1.86196	-1.85936	-1.85678
-1.86365	-1.86104	-1.87115	-1.86851	-1.86592	-1.86337	-1.8608	-1.85821	-1.85563
-1.86252	-1.8599	-1.87002	-1.86741	-1.86481	-1.86223	-1.85965	-1.85709	-1.85453
-1.8614	-1.85876	-1.86888	-1.8663	-1.8637	-1.86109	-1.85851	-1.85597	-1.85343
-1.86024	-1.8576	-1.86771	-1.86515	-1.86255	-1.85992	-1.85734	-1.85481	-1.85228
-1.85908	-1.85645	-1.86654	-1.86397	-1.86137	-1.85876	-1.85617	-1.85363	-1.85111
-1.85796	-1.85535	-1.85277	-1.85022	-1.86024	-1.85763	-1.85505	-1.85249	-1.84995
-1.85688	-1.85429	-1.8517	-1.84913	-1.85913	-1.85654	-1.85396	-1.85137	-1.84881
-1.8558	-1.85323	-1.85064	-1.84805	-1.85804	-1.85545	-1.85287	-1.85027	-1.8477
-1.85473	-1.85215	-1.84957	-1.847	-1.85697	-1.85437	-1.85178	-1.8492	-1.84663
-1.85366	-1.85107	-1.84851	-1.84595	-1.85589	-1.85328	-1.85069	-1.84812	-1.84556
-1.85259	-1.85002	-1.84745	-1.84488	-1.85481	-1.85221	-1.84961	-1.84703	-1.84445
-1.85154	-1.84898	-1.84639	-1.8438	-1.85372	-1.85113	-1.84853	-1.84592	-1.84334
-1.85047	-1.8479	-1.84532	-1.84274	-1.85263	-1.85003	-1.84742	-1.8448	-1.84221
-1.8494	-1.84682	-1.84425	-1.84168	-1.85154	-1.84892	-1.8463	-1.84367	-1.84107
-1.84834	-1.84576	-1.84318	-1.84061	-1.83801	-1.83539	-1.84517	-1.84255	-1.83996
-1.84729	-1.84471	-1.84212	-1.83952	-1.83691	-1.83427	-1.84403	-1.84144	-1.83887
-1.84622	-1.84365	-1.84106	-1.83845	-1.83583	-1.83318	-1.84293	-1.84035	-1.83777
-1.84515	-1.84257	-1.83999	-1.8374	-1.83479	-1.83215	-1.84187	-1.83926	-1.83667
-1.84408	-1.84148	-1.83891	-1.83634	-1.83374	-1.83111	-1.84081	-1.83818	-1.83557
-1.843	-1.84042	-1.83784	-1.83525	-1.83264	-1.83	-1.83969	-1.83708	-1.83448
-1.84192	-1.83935	-1.83676	-1.83415	-1.83152	-1.82889	-1.83857	-1.83598	-1.8334
-1.84083	-1.83826	-1.83568	-1.83308	-1.83046	-1.82782	-1.82521	-1.82263	-1.85809
-1.83973	-1.83715	-1.83459	-1.83203	-1.82943	-1.8268	-1.82417	-1.82156	-1.85696
-1.83864	-1.83606	-1.83351	-1.83095	-1.82836	-1.82575	-1.82314	-1.82051	-1.85586
-1.83756	-1.835	-1.83242	-1.82984	-1.82727	-1.8247	-1.82211	-1.81949	-1.85481
-1.8365	-1.83395	-1.83136	-1.82875	-1.82618	-1.82363	-1.82106	-1.81846	-1.84185
-1.83548	-1.83293	-1.83034	-1.82772	-1.82513	-1.82254	-1.81997	-1.8174	-1.84081
-1.83446	-1.83191	-1.82932	-1.82671	-1.82409	-1.82147	-1.81888	-1.81633	-1.83974
-1.8334	-1.83083	-1.82826	-1.82568	-1.82307	-1.82044	-1.84386	-1.84125	-1.83864
-1.83233	-1.82973	-1.82718	-1.82464	-1.82205	-1.81943	-1.84282	-1.84016	-1.83752
-1.83129	-1.82868	-1.82611	-1.82356	-1.82099	-1.81839	-1.84178	-1.8391	-1.83645
-1.83027	-1.82765	-1.82505	-1.82246	-1.81989	-1.81734	-1.84073	-1.83807	-1.83541
-1.82924	-1.82663	-1.82401	-1.82139	-1.84485	-1.84227	-1.83966	-1.83701	-1.83436
-1.82817	-1.8256	-1.82299	-1.82037	-1.84379	-1.84116	-1.83854	-1.83593	-1.83331
-1.82711	-1.82456	-1.82196	-1.81935	-1.84274	-1.84008	-1.83744	-1.83484	-1.83224
-1.82604	-1.82347	-1.8209	-1.81833	-1.84171	-1.83904	-1.83638	-1.83376	-1.83115

-1.82497	-1.82238	-1.81983	-1.8173	-1.84068	-1.83801	-1.83534	-1.83269	-1.83006
-1.82395	-1.82134	-1.8188	-1.81628	-1.83964	-1.83697	-1.83431	-1.83164	-1.82901
-1.82294	-1.82032	-1.81777	-1.81524	-1.83859	-1.83593	-1.83328	-1.83062	-1.82799
-1.82192	-1.8193	-1.81672	-1.81417	-1.83751	-1.83488	-1.83224	-1.82959	-1.82696
-1.82091	-1.81827	-1.81566	-1.81307	-1.83639	-1.8338	-1.83119	-1.82854	-1.82591
-1.81989	-1.81725	-1.84057	-1.83791	-1.8353	-1.83273	-1.83014	-1.82751	-1.82487
-1.81888	-1.81623	-1.83953	-1.83685	-1.83424	-1.83169	-1.8291	-1.82647	-1.82383
-1.81787	-1.81521	-1.83849	-1.83581	-1.8332	-1.83065	-1.82807	-1.82544	-1.8228
-1.81685	-1.8142	-1.83745	-1.83477	-1.83216	-1.82961	-1.82703	-1.82441	-1.82177
-1.81584	-1.81319	-1.83642	-1.83373	-1.83112	-1.82858	-1.826	-1.82338	-1.82073
-1.81482	-1.81217	-1.83538	-1.83269	-1.83008	-1.82753	-1.82495	-1.82234	-1.8197
-1.8138	-1.81115	-1.83434	-1.83166	-1.82904	-1.82648	-1.8239	-1.82129	-1.81866
-1.83871	-1.83601	-1.8333	-1.83062	-1.82799	-1.82541	-1.82282	-1.82023	-1.81761
-1.83768	-1.83497	-1.83227	-1.82958	-1.82693	-1.82431	-1.82171	-1.81913	-1.81654
-1.83664	-1.83393	-1.83123	-1.82855	-1.82589	-1.82325	-1.82064	-1.81807	-1.81548
-1.83561	-1.8329	-1.8302	-1.82752	-1.82488	-1.82228	-1.81967	-1.81707	-1.81445
-1.83458	-1.83187	-1.82917	-1.8265	-1.82388	-1.82131	-1.81871	-1.81607	-1.81342
-1.83355	-1.83084	-1.82815	-1.82547	-1.82286	-1.8203	-1.8177	-1.81505	-1.81239
-1.83252	-1.82982	-1.82712	-1.82444	-1.82182	-1.81926	-1.81666	-1.81402	-1.81135
-1.8315	-1.82879	-1.82609	-1.82342	-1.8208	-1.81823	-1.81563	-1.81297	-1.8103
-1.83047	-1.82776	-1.82507	-1.8224	-1.81978	-1.81719	-1.81458	-1.81192	-1.80924
-1.82945	-1.82675	-1.82407	-1.82141	-1.81878	-1.81617	-1.81354	-1.81087	-1.80819
-1.82843	-1.82575	-1.8231	-1.82046	-1.81782	-1.81517	-1.81251	-1.80982	-1.80713
-1.82744	-1.82476	-1.82212	-1.8195	-1.81684	-1.81416	-1.81148	-1.80877	-1.80607
-1.82647	-1.82381	-1.82116	-1.8185	-1.81583	-1.81313	-1.81043	-1.80772	-1.80501
-1.8255	-1.82286	-1.82019	-1.81749	-1.81479	-1.81208	-1.80937	-1.80666	-1.80395
-1.82454	-1.82188	-1.81918	-1.81646	-1.81375	-1.81103	-1.80831	-1.8056	-1.8029
-1.82357	-1.82087	-1.81815	-1.81542	-1.81269	-1.80996	-1.80725	-1.80454	-1.80185
-1.82256	-1.81984	-1.81711	-1.81438	-1.81164	-1.80891	-1.8062	-1.80349	-1.80081
-1.82153	-1.8188	-1.81606	-1.81333	-1.8106	-1.80787	-1.80515	-1.80245	-1.79978
-1.8205	-1.81776	-1.81502	-1.81229	-1.80957	-1.80686	-1.80415	-1.80145	-1.79878
-1.81947	-1.81673	-1.81399	-1.81126	-1.80856	-1.80589	-1.80321	-1.80052	-1.79784
-1.81845	-1.81572	-1.81298	-1.81025	-1.80756	-1.80492	-1.80226	-1.79957	-1.79689
-1.81746	-1.81476	-1.81203	-1.8093	-1.8066	-1.80393	-1.80126	-1.79857	-1.79589
-1.81648	-1.81382	-1.8111	-1.80837	-1.80565	-1.80294	-1.80024	-1.79755	-1.79487
-1.81551	-1.81283	-1.81012	-1.80738	-1.80466	-1.80194	-1.79923	-1.79654	-1.79385
-1.81453	-1.81182	-1.80909	-1.80636	-1.80365	-1.80094	-1.79823	-1.79553	-1.79283
-1.81354	-1.8108	-1.80807	-1.80534	-1.80264	-1.79995	-1.79726	-1.79456	-1.79185
-1.81252	-1.80978	-1.80704	-1.80432	-1.80164	-1.799	-1.79633	-1.79364	-1.79093
-1.7876	-1.78491	-1.80605	-1.80333	-1.80066	-1.79804	-1.79538	-1.7927	-1.78999
-1.78669	-1.784	-1.80513	-1.80241	-1.79972	-1.79706	-1.79439	-1.7917	-1.789
-1.78578	-1.78309	-1.80421	-1.80148	-1.79878	-1.79608	-1.79339	-1.7907	-1.788
-1.78481	-1.78212	-1.80323	-1.8005	-1.79779	-1.79508	-1.79239	-1.78972	-1.78704
-1.78382	-1.78113	-1.80223	-1.7995	-1.79679	-1.79408	-1.7914	-1.78875	-1.7861
-1.78288	-1.78019	-1.77751	-1.77484	-1.79584	-1.79312	-1.79043	-1.78778	-1.78512
-1.78198	-1.77929	-1.77661	-1.77395	-1.79493	-1.79221	-1.7895	-1.7868	-1.78411
-1.78103	-1.77835	-1.77568	-1.77301	-1.79398	-1.79125	-1.78853	-1.78581	-1.78311
-1.78003	-1.77735	-1.77468	-1.77202	-1.79297	-1.79024	-1.78752	-1.7848	-1.7821
-1.77905	-1.77638	-1.77371	-1.77105	-1.79199	-1.78926	-1.78654	-1.78382	-1.78112
-1.77814	-1.77547	-1.77281	-1.77015	-1.79109	-1.78836	-1.78563	-1.7829	-1.78019

-1.77722	-1.77456	-1.77191	-1.76927	-1.7902	-1.78747	-1.78474	-1.78201	-1.77929
-1.77625	-1.77359	-1.77097	-1.76837	-1.76573	-1.76307	-1.78384	-1.78111	-1.77839
-1.77525	-1.77261	-1.77002	-1.76745	-1.76483	-1.76217	-1.78294	-1.78021	-1.77748
-1.77432	-1.77168	-1.76908	-1.7665	-1.76388	-1.76123	-1.78197	-1.77925	-1.77652
-1.77344	-1.77079	-1.76816	-1.76554	-1.76289	-1.76023	-1.78096	-1.77824	-1.77551
-1.77257	-1.76991	-1.76726	-1.76461	-1.76194	-1.75928	-1.75661	-1.75393	-1.77453
-1.77172	-1.76906	-1.7664	-1.76373	-1.76106	-1.75839	-1.75571	-1.75304	-1.77362
-1.77086	-1.7682	-1.76553	-1.76286	-1.76018	-1.75751	-1.75483	-1.75215	-1.77272
-1.76999	-1.76733	-1.76466	-1.76198	-1.7593	-1.75663	-1.75395	-1.75126	-1.77182
-1.7691	-1.76643	-1.76376	-1.76108	-1.7584	-1.75572	-1.75304	-1.75036	-1.77091
-1.76814	-1.76547	-1.7628	-1.76011	-1.75743	-1.75476	-1.75207	-1.74939	-1.76992
-1.76714	-1.76447	-1.76179	-1.75911	-1.75643	-1.75375	-1.75106	-1.74838	-1.7689
-1.7662	-1.76353	-1.76084	-1.75816	-1.75548	-1.7528	-1.75011	-1.74743	-1.74474
-1.76531	-1.76263	-1.75995	-1.75726	-1.75458	-1.7519	-1.74922	-1.74653	-1.74384
-1.76443	-1.76176	-1.75907	-1.75639	-1.75371	-1.75103	-1.74834	-1.74565	-1.74296
-1.76357	-1.76089	-1.75821	-1.75553	-1.75285	-1.75016	-1.74748	-1.74479	-1.7421
-1.76272	-1.76003	-1.75735	-1.75467	-1.75199	-1.7493	-1.74661	-1.74392	-1.74123
-1.76185	-1.75917	-1.75649	-1.75381	-1.75113	-1.74844	-1.74575	-1.74306	-1.74036
-1.76099	-1.75831	-1.75563	-1.75295	-1.75027	-1.74757	-1.74488	-1.74218	-1.73948
-1.76013	-1.75745	-1.75477	-1.75209	-1.7494	-1.7467	-1.744	-1.7413	-1.73858
-1.75926	-1.75658	-1.7539	-1.75122	-1.74852	-1.74581	-1.7431	-1.7404	-1.73768
-1.7584	-1.75572	-1.75303	-1.75034	-1.74763	-1.7449	-1.74217	-1.73946	-1.73673
-1.75754	-1.75486	-1.75217	-1.74948	-1.74673	-1.74394	-1.74118	-1.73845	-1.73573
-1.75668	-1.754	-1.75131	-1.74861	-1.74583	-1.743	-1.74021	-1.73747	-1.73475
-1.75582	-1.75314	-1.75045	-1.74774	-1.74495	-1.74211	-1.73931	-1.73656	-1.73384
-1.75496	-1.75228	-1.74958	-1.74687	-1.74408	-1.74123	-1.73843	-1.73567	-1.73295
-1.7541	-1.75141	-1.74872	-1.746	-1.74321	-1.74035	-1.73755	-1.73479	-1.73207
-1.75324	-1.75055	-1.74785	-1.74513	-1.74233	-1.73948	-1.73668	-1.73393	-1.7312
-1.75238	-1.74969	-1.74698	-1.74426	-1.74147	-1.73862	-1.73581	-1.73306	-1.73033
-1.75152	-1.74882	-1.74612	-1.74339	-1.7406	-1.73776	-1.73496	-1.7322	-1.72946
-1.7419	-1.73918	-1.74525	-1.74252	-1.73975	-1.73693	-1.73413	-1.73136	-1.72861
-1.74102	-1.7383	-1.74438	-1.74166	-1.73891	-1.73614	-1.73336	-1.73056	-1.72778
-1.74014	-1.73742	-1.7347	-1.73198	-1.73807	-1.73534	-1.73257	-1.72976	-1.72697
-1.73926	-1.73654	-1.73382	-1.7311	-1.7372	-1.73449	-1.73175	-1.72898	-1.7262
-1.73839	-1.73566	-1.73294	-1.73021	-1.73633	-1.73362	-1.73091	-1.72818	-1.72543
-1.73751	-1.73479	-1.73206	-1.72933	-1.73546	-1.73275	-1.73005	-1.72734	-1.72462
-1.73664	-1.73392	-1.73118	-1.72845	-1.73458	-1.73188	-1.72917	-1.72648	-1.72377
-1.73578	-1.73304	-1.73031	-1.72758	-1.72485	-1.72214	-1.7283	-1.72561	-1.72291
-1.73492	-1.73217	-1.72943	-1.7267	-1.72398	-1.72127	-1.72744	-1.72474	-1.72203
-1.73408	-1.73131	-1.72856	-1.72583	-1.72311	-1.7204	-1.71769	-1.71497	-1.72116
-1.73328	-1.73048	-1.72771	-1.72497	-1.72223	-1.71952	-1.7168	-1.71409	-1.72028
-1.7325	-1.72967	-1.72689	-1.72413	-1.72139	-1.71866	-1.71593	-1.71321	-1.71941
-1.7317	-1.7289	-1.72613	-1.72337	-1.72061	-1.71784	-1.71509	-1.71235	-1.71854
-1.73091	-1.72815	-1.72539	-1.72263	-1.71985	-1.71704	-1.71426	-1.71152	-1.7177
-1.73007	-1.72733	-1.72458	-1.72183	-1.71906	-1.71626	-1.71349	-1.71074	-1.70798
-1.7292	-1.72646	-1.72372	-1.72098	-1.71824	-1.71549	-1.71274	-1.71	-1.70724
-1.72837	-1.72561	-1.72287	-1.72013	-1.7174	-1.71468	-1.71195	-1.70921	-1.70645
-1.72759	-1.72478	-1.72202	-1.71928	-1.71654	-1.71381	-1.71108	-1.70835	-1.70561
-1.7268	-1.72398	-1.7212	-1.71845	-1.7157	-1.71296	-1.71022	-1.70749	-1.70476
-1.72602	-1.72322	-1.72046	-1.7177	-1.71493	-1.71215	-1.70938	-1.70664	-1.7039

		-0.10882
		-0.10859
-0.10892	-0.10864	-0.30195
-0.10866	-0.10839	-0.30115
-0.10841	-0.10813	-0.30036
-0.2539	-0.25294	-0.34064

						-0.25318	-0.25224	-0.33981
				-0.25456	-0.25349	-0.30007	-0.29904	-0.38721
				-0.25381	-0.25279	-0.2993	-0.29826	-0.38631
		-0.10835	-0.10805	-0.30072	-0.29963	-0.38793	-0.38667	-0.47728
		-0.10809	-0.10779	-0.29996	-0.29889	-0.38709	-0.38581	-0.47625
		-0.25375	-0.25275	-0.34044	-0.33927	-0.4322	-0.43081	-0.47524
		-0.25307	-0.2521	-0.33965	-0.33846	-0.43122	-0.42987	-0.47425
		-0.2524	-0.25146	-0.33886	-0.33765	-0.43025	-0.42894	-0.47329
-0.2537	-0.25268	-0.29907	-0.29804	-0.38616	-0.38487	-0.47516	-0.47373	-0.51139
-0.25299	-0.25198	-0.29829	-0.29726	-0.38527	-0.38403	-0.47421	-0.4728	-0.51034
-0.29968	-0.2986	-0.3869	-0.38565	-0.43016	-0.42886	-0.47326	-0.47188	-0.55689
-0.29893	-0.29788	-0.38606	-0.38481	-0.42925	-0.42797	-0.47234	-0.47098	-0.55581
-0.38773	-0.38647	-0.43099	-0.42966	-0.47417	-0.47279	-0.55791	-0.55632	-0.59233
-0.38687	-0.38561	-0.43007	-0.42877	-0.47324	-0.47188	-0.55682	-0.55522	-0.59117
-0.38601	-0.38476	-0.42916	-0.4279	-0.47232	-0.47098	-0.55572	-0.55412	-0.59001
-0.43089	-0.42956	-0.42829	-0.42706	-0.55781	-0.5562	-0.59221	-0.59053	-0.78982
-0.42997	-0.42869	-0.42745	-0.42623	-0.5567	-0.55508	-0.59104	-0.58936	-0.78826
-0.42909	-0.42784	-0.51122	-0.5097	-0.59325	-0.59155	-0.63295	-0.63119	-0.78673
-0.42824	-0.42701	-0.51018	-0.50865	-0.59206	-0.59038	-0.63172	-0.62998	-0.78521
-0.46722	-0.46583	-0.59432	-0.5926	-0.5909	-0.58924	-0.78803	-0.78586	-1.07583
-0.46625	-0.46484	-0.59314	-0.59146	-0.58979	-0.58812	-0.78651	-0.78437	-1.07353
-0.59539	-0.59366	-0.59199	-0.59035	-0.67347	-0.6716	-1.03965	-1.03646	-1.07124
-0.59422	-0.5925	-0.59086	-0.58926	-0.67228	-0.67042	-1.03747	-1.03429	-1.06895
-0.59307	-0.59138	-0.58976	-0.58817	-0.67109	-0.66925	-1.03532	-1.03214	-1.06668
-0.63484	-0.63307	-0.67346	-0.67166	-1.03965	-1.03638	-1.07112	-1.06775	-1.06443
-0.63372	-0.63195	-0.67226	-0.67042	-1.03736	-1.03417	-1.06885	-1.06549	-1.0622
-0.63257	-0.63082	-0.71407	-0.71215	-1.07327	-1.06996	-1.06665	-1.06331	-1.12653
-0.63141	-0.62966	-0.7128	-0.7109	-1.07096	-1.06776	-1.06451	-1.0612	-1.12432
-0.71539	-0.71347	-1.03721	-1.03396	-1.06872	-1.06555	-1.06234	-1.05906	-1.18243
-0.71416	-0.71225	-1.03509	-1.03192	-1.06657	-1.06331	-1.06009	-1.05688	-1.18012
-0.90196	-0.89904	-1.07106	-1.06775	-1.06443	-1.0611	-1.18455	-1.18118	-1.17786
-0.90005	-0.89721	-1.06883	-1.06553	-1.06222	-1.05892	-1.18222	-1.17894	-1.17569
-0.89818	-0.89542	-1.06662	-1.06332	-1.06004	-1.05678	-1.17994	-1.17673	-1.17353
-1.00454	-1.00146	-1.0645	-1.06121	-1.18454	-1.1811	-1.17776	-1.17455	-1.17134
-1.00256	-0.99951	-1.06242	-1.05915	-1.18234	-1.17896	-1.17564	-1.17238	-1.16913
-1.06681	-1.06355	-1.18696	-1.1835	-1.1801	-1.17675	-1.17344	-1.17018	-1.2555
-1.0646	-1.06135	-1.18461	-1.1812	-1.17782	-1.17447	-1.17119	-1.16797	-1.2532
-1.12502	-1.12166	-1.18233	-1.17895	-1.17559	-1.17224	-1.25762	-1.25424	-1.25091
-1.12287	-1.11953	-1.18014	-1.17679	-1.17344	-1.1701	-1.25534	-1.25196	-1.24864
-1.12073	-1.11742	-1.17796	-1.17463	-1.17128	-1.16794	-1.25306	-1.2497	-1.2464
-1.18246	-1.17907	-1.17571	-1.17237	-1.22695	-1.22351	-1.25073	-1.24746	-1.27421
-1.18017	-1.17681	-1.17345	-1.1701	-1.22463	-1.22121	-1.24841	-1.24524	-1.27204
-1.20911	-1.20571	-1.22924	-1.2258	-1.25298	-1.24955	-1.24625	-1.24309	-1.26985
-1.20691	-1.20351	-1.22701	-1.22358	-1.25076	-1.24745	-1.24419	-1.241	-1.26767
-1.20465	-1.20125	-1.22475	-1.22135	-1.24854	-1.24532	-1.27204	-1.26878	-1.26554
-1.20233	-1.19893	-1.22245	-1.21911	-1.2463	-1.24317	-1.26991	-1.26669	-1.26347
-1.227	-1.22357	-1.2507	-1.24735	-1.27411	-1.27094	-1.26776	-1.26457	-1.36331
-1.22483	-1.22144	-1.24856	-1.24527	-1.27199	-1.26878	-1.26558	-1.26239	-1.36082
-1.2227	-1.21935	-1.24647	-1.24323	-1.2699	-1.26665	-1.26342	-1.26022	-1.35833
-1.22056	-1.21729	-1.24439	-1.24117	-1.2678	-1.26456	-1.36343	-1.35967	-1.38319

-1.21844	-1.21524	-1.24231	-1.2391	-1.2657	-1.26248	-1.36108	-1.35729	-1.38075
-1.24663	-1.2434	-1.27006	-1.26679	-1.28288	-1.27955	-1.35861	-1.35482	-1.37825
-1.24441	-1.2412	-1.26784	-1.26459	-1.28061	-1.27726	-1.35607	-1.35229	-1.37571
-1.2722	-1.26893	-1.28508	-1.28174	-1.3612	-1.3574	-1.38087	-1.37704	-1.39169
-1.27012	-1.26686	-1.28294	-1.27962	-1.35886	-1.35505	-1.37848	-1.37465	-1.38929
-1.2875	-1.28415	-1.36406	-1.3603	-1.3565	-1.3527	-1.39454	-1.3907	-1.38695
-1.28537	-1.28203	-1.3617	-1.3579	-1.3541	-1.3503	-1.39211	-1.38835	-1.38468
-1.28324	-1.27991	-1.35931	-1.35547	-1.35167	-1.34789	-1.38969	-1.38601	-1.38242
-1.33411	-1.33042	-1.35688	-1.35299	-1.39479	-1.39095	-1.38721	-1.38361	-1.40557
-1.33175	-1.32806	-1.35441	-1.35049	-1.39222	-1.38842	-1.38474	-1.38118	-1.40317
-1.35962	-1.35582	-1.3707	-1.36677	-1.38976	-1.38603	-1.40793	-1.40434	-1.42737
-1.35704	-1.35326	-1.36818	-1.36434	-1.38739	-1.38377	-1.40569	-1.4021	-1.42514
-1.3733	-1.36951	-1.39255	-1.38875	-1.41064	-1.40704	-1.43003	-1.42642	-1.42305
-1.37089	-1.3671	-1.39018	-1.38649	-1.40839	-1.40478	-1.4278	-1.42433	-1.42111
-1.36852	-1.36475	-1.41345	-1.4098	-1.43278	-1.42912	-1.42564	-1.42234	-1.46919
-1.3662	-1.36248	-1.41115	-1.40755	-1.43054	-1.42693	-1.42357	-1.42049	-1.46749
-1.36392	-1.36026	-1.4089	-1.40532	-1.42834	-1.42481	-1.42161	-1.41875	-1.4659
-1.41399	-1.41028	-1.40663	-1.40307	-1.47632	-1.47288	-1.46974	-1.46692	-1.4643
-1.41166	-1.40803	-1.40438	-1.40083	-1.47414	-1.47096	-1.468	-1.46527	-1.46272
-1.40937	-1.40577	-1.479	-1.47534	-1.47205	-1.46908	-1.4663	-1.46369	-1.48817
-1.40711	-1.40351	-1.47672	-1.47316	-1.47002	-1.46724	-1.46463	-1.46219	-1.48674
-1.48187	-1.47814	-1.47455	-1.47116	-1.46817	-1.46554	-1.49004	-1.48765	-1.48534
-1.47955	-1.47591	-1.47252	-1.46938	-1.46655	-1.46402	-1.48855	-1.48622	-1.48397
-1.47728	-1.47377	-1.4706	-1.46771	-1.46503	-1.46258	-1.48712	-1.48484	-1.48262
-1.47508	-1.47175	-1.46878	-1.46607	-1.4905	-1.48806	-1.48572	-1.48349	-1.48132
-1.47298	-1.46985	-1.46705	-1.4645	-1.48899	-1.48664	-1.48436	-1.48218	-1.48004
-1.47102	-1.46808	-1.49245	-1.48995	-1.48754	-1.48525	-1.48303	-1.48088	-1.5339
-1.46919	-1.46644	-1.49089	-1.48848	-1.48614	-1.4839	-1.48172	-1.4796	-1.53241
-1.49457	-1.49188	-1.48938	-1.48704	-1.48477	-1.48257	-1.53578	-1.53334	-1.53092
-1.49299	-1.49038	-1.48795	-1.48565	-1.48342	-1.48127	-1.53428	-1.53184	-1.52942
-1.49147	-1.48893	-1.48655	-1.48429	-1.50854	-1.50625	-1.53278	-1.53035	-1.52793
-1.48998	-1.48752	-1.48519	-1.48297	-1.50713	-1.50487	-1.53129	-1.52886	-1.52644
-1.48853	-1.48614	-1.48386	-1.48167	-1.50575	-1.5035	-1.52981	-1.52739	-1.52498
-1.48714	-1.48478	-1.50899	-1.50666	-1.53316	-1.53074	-1.52833	-1.52595	-1.54567
-1.48578	-1.48346	-1.50758	-1.50528	-1.53167	-1.52925	-1.52687	-1.52452	-1.54425
-1.48443	-1.48215	-1.5351	-1.53263	-1.53019	-1.52779	-1.54757	-1.54521	-1.54283
-1.48311	-1.48086	-1.53359	-1.53113	-1.52872	-1.52636	-1.54613	-1.54378	-1.54142
-1.50816	-1.50574	-1.55434	-1.55185	-1.54942	-1.54706	-1.5447	-1.54237	-1.60227
-1.50675	-1.50435	-1.55282	-1.55035	-1.54796	-1.54563	-1.5433	-1.54096	-1.60068
-1.55644	-1.55383	-1.55132	-1.54888	-1.58573	-1.58335	-1.60439	-1.60173	-1.63504
-1.55493	-1.55234	-1.54986	-1.54746	-1.58427	-1.5819	-1.60279	-1.60017	-1.6335
-1.55345	-1.55088	-1.54844	-1.54607	-1.58283	-1.58045	-1.60121	-1.59864	-1.63199
-1.55202	-1.5495	-1.58629	-1.58383	-1.5814	-1.57901	-1.63561	-1.63306	-1.67248
-1.55062	-1.54818	-1.58492	-1.58243	-1.57998	-1.57758	-1.63404	-1.63158	-1.671
-1.58849	-1.58603	-1.58352	-1.58102	-1.63775	-1.63508	-1.67448	-1.67199	-1.66951
-1.58701	-1.5846	-1.58211	-1.57962	-1.63619	-1.63358	-1.67295	-1.67047	-1.668
-1.62228	-1.61985	-1.64011	-1.63734	-1.67667	-1.67401	-1.67143	-1.66895	-1.66649
-1.62081	-1.61839	-1.63855	-1.63584	-1.67514	-1.67251	-1.66994	-1.66744	-1.66499
-1.61936	-1.61694	-1.63699	-1.63436	-1.67364	-1.67101	-1.66845	-1.66594	-1.66349
-1.61791	-1.61549	-1.67741	-1.67476	-1.67213	-1.66951	-1.66695	-1.66443	-1.66199

-1.61648	-1.61405	-1.6758	-1.67322	-1.67063	-1.66802	-1.66547	-1.66295	-1.66051
-1.67959	-1.67683	-1.67422	-1.67169	-1.66913	-1.66656	-1.66403	-1.66152	-1.67031
-1.67802	-1.67526	-1.67266	-1.67016	-1.66764	-1.66513	-1.66263	-1.66014	-1.66895
-1.67645	-1.67371	-1.67113	-1.66863	-1.66614	-1.66367	-1.67242	-1.66997	-1.66758
-1.67487	-1.67219	-1.6696	-1.66709	-1.66463	-1.6622	-1.67099	-1.66858	-1.66621
-1.67333	-1.6707	-1.66812	-1.66559	-1.67431	-1.67193	-1.66957	-1.66721	-1.71097
-1.67186	-1.66928	-1.66668	-1.66412	-1.67284	-1.6705	-1.66818	-1.66587	-1.70951
-1.67039	-1.66786	-1.66527	-1.66269	-1.67142	-1.6691	-1.6668	-1.66453	-1.70804
-1.66888	-1.66637	-1.67498	-1.67249	-1.67008	-1.66773	-1.71169	-1.7091	-1.70651
-1.66737	-1.66486	-1.67355	-1.67117	-1.66878	-1.66639	-1.71012	-1.70753	-1.70496
-1.67701	-1.67452	-1.67213	-1.6698	-1.66743	-1.66503	-1.70856	-1.70598	-1.70344
-1.67559	-1.67309	-1.67071	-1.6684	-1.66604	-1.66366	-1.707	-1.70444	-1.70194
-1.67418	-1.6717	-1.66933	-1.66701	-1.71086	-1.70814	-1.7055	-1.70296	-1.71829
-1.67277	-1.67037	-1.66799	-1.66564	-1.70932	-1.70668	-1.70408	-1.70153	-1.71683
-1.67138	-1.66904	-1.71319	-1.71045	-1.70779	-1.70522	-1.72048	-1.71792	-1.7154
-1.67001	-1.66764	-1.71163	-1.70894	-1.70631	-1.70374	-1.71901	-1.7165	-1.71403
-1.66864	-1.66624	-1.71007	-1.70744	-1.70484	-1.70226	-1.71753	-1.71508	-1.71265
-1.66724	-1.66484	-1.72634	-1.72374	-1.72114	-1.71855	-1.71603	-1.7136	-1.72622
-1.66583	-1.66345	-1.72475	-1.72219	-1.71961	-1.71704	-1.71453	-1.71208	-1.72471
-1.72858	-1.72584	-1.72322	-1.72067	-1.71812	-1.71558	-1.72817	-1.72568	-1.73433
-1.72702	-1.72436	-1.72175	-1.71919	-1.71666	-1.71417	-1.72677	-1.72429	-1.73283
-1.72549	-1.72288	-1.73549	-1.7329	-1.73036	-1.72785	-1.72537	-1.7229	-1.73137
-1.72399	-1.72139	-1.73403	-1.73149	-1.72895	-1.72642	-1.72394	-1.72153	-1.72996
-1.72251	-1.71992	-1.73258	-1.73007	-1.72754	-1.725	-1.72253	-1.72016	-1.72855
-1.73628	-1.7337	-1.73114	-1.7286	-1.7261	-1.72362	-1.73213	-1.72961	-1.72711
-1.73483	-1.73229	-1.7297	-1.72713	-1.72465	-1.72226	-1.73072	-1.72818	-1.72567
-1.73338	-1.73084	-1.72827	-1.72572	-1.73433	-1.73181	-1.72929	-1.72676	-1.72426
-1.73194	-1.72937	-1.72684	-1.72436	-1.73288	-1.73035	-1.72784	-1.72535	-1.72288
-1.7305	-1.72793	-1.72543	-1.72298	-1.73144	-1.72892	-1.72642	-1.72394	-1.72149
-1.72905	-1.72652	-1.72403	-1.72157	-1.73	-1.7275	-1.72502	-1.72254	-1.7201
-1.72761	-1.72511	-1.73366	-1.73109	-1.72857	-1.72609	-1.72362	-1.72116	-1.7648
-1.72619	-1.72369	-1.73221	-1.72968	-1.72717	-1.72467	-1.72221	-1.71979	-1.7633
-1.72479	-1.72229	-1.73077	-1.72828	-1.72577	-1.72325	-1.7208	-1.71843	-1.76181
-1.73449	-1.73189	-1.72935	-1.72685	-1.72435	-1.72188	-1.71945	-1.71708	-1.76026
-1.73303	-1.73049	-1.72794	-1.72541	-1.72294	-1.72053	-1.71812	-1.71572	-1.75869
-1.73159	-1.72906	-1.72651	-1.724	-1.72155	-1.71916	-1.76261	-1.75986	-1.75715
-1.73018	-1.7276	-1.72509	-1.72262	-1.72019	-1.71778	-1.76103	-1.75832	-1.75564
-1.72879	-1.72619	-1.72368	-1.72124	-1.71882	-1.71641	-1.75948	-1.75678	-1.75411
-1.72743	-1.72483	-1.72231	-1.71985	-1.71743	-1.71506	-1.75796	-1.75524	-1.75257
-1.72606	-1.72348	-1.72095	-1.71848	-1.76181	-1.75913	-1.75644	-1.75372	-1.75105
-1.72466	-1.72213	-1.71963	-1.71717	-1.76029	-1.75757	-1.75488	-1.75222	-1.7496
-1.72325	-1.72077	-1.71831	-1.71587	-1.7588	-1.75602	-1.75332	-1.75074	-1.74818
-1.72186	-1.71939	-1.76284	-1.76009	-1.75733	-1.75455	-1.75186	-1.74927	-1.74671
-1.72047	-1.718	-1.76128	-1.75859	-1.75586	-1.75313	-1.75045	-1.74782	-1.74524
-1.71913	-1.71665	-1.75974	-1.75705	-1.75437	-1.75169	-1.74904	-1.74641	-1.74381
-1.71783	-1.71536	-1.75822	-1.75549	-1.75283	-1.75025	-1.74766	-1.74503	-1.74244
-1.76236	-1.75955	-1.75675	-1.75399	-1.75135	-1.74881	-1.76788	-1.76527	-1.76269
-1.7609	-1.75811	-1.75533	-1.75258	-1.74993	-1.74735	-1.7664	-1.76385	-1.76132
-1.75943	-1.75667	-1.75393	-1.75121	-1.74854	-1.74591	-1.76494	-1.76244	-1.75996
-1.77388	-1.77116	-1.76846	-1.76578	-1.76881	-1.76613	-1.76354	-1.76107	-1.78186

-1.77235	-1.76969	-1.76704	-1.76441	-1.76745	-1.76476	-1.76218	-1.75972	-1.78042
-1.77084	-1.7682	-1.77132	-1.76873	-1.76608	-1.76341	-1.76082	-1.75836	-1.79612
-1.76936	-1.76671	-1.76986	-1.7673	-1.7647	-1.76206	-1.75949	-1.75698	-1.79459
-1.77366	-1.771	-1.76841	-1.76586	-1.76329	-1.7607	-1.78132	-1.77861	-1.79308
-1.77226	-1.76961	-1.76698	-1.76439	-1.76184	-1.75933	-1.77988	-1.77718	-1.79161
-1.77089	-1.76824	-1.76559	-1.76296	-1.76043	-1.75797	-1.79565	-1.79288	-1.82303
-1.76954	-1.76688	-1.76423	-1.7616	-1.75908	-1.75664	-1.79419	-1.79144	-1.82153
-1.76819	-1.76554	-1.76288	-1.76026	-1.75774	-1.75531	-1.79273	-1.78999	-1.82003
-1.76682	-1.76418	-1.76155	-1.75893	-1.7794	-1.77674	-1.80657	-1.80385	-1.81853
-1.76545	-1.76283	-1.76022	-1.75762	-1.77794	-1.77523	-1.80502	-1.80234	-1.81703
-1.7641	-1.76149	-1.78205	-1.77926	-1.80907	-1.80624	-1.83492	-1.83205	-1.82922
-1.76275	-1.76015	-1.78062	-1.77784	-1.8076	-1.80476	-1.83336	-1.83048	-1.82766
-1.76141	-1.75882	-1.80906	-1.80625	-1.83789	-1.83483	-1.83185	-1.82897	-1.89023
-1.76007	-1.7575	-1.80761	-1.8048	-1.83638	-1.83336	-1.83041	-1.82752	-1.88852
-1.81176	-1.80897	-1.82295	-1.82009	-1.84781	-1.84484	-1.84191	-1.83904	-1.88683
-1.81029	-1.80751	-1.82148	-1.81863	-1.84632	-1.84339	-1.84049	-1.83762	-1.88514
-1.80883	-1.80606	-1.82001	-1.81718	-1.84485	-1.84195	-1.83908	-1.83621	-1.88347
-1.80737	-1.80461	-1.84933	-1.84634	-1.84341	-1.84054	-1.88843	-1.88511	-1.88185
-1.80592	-1.80316	-1.84785	-1.8449	-1.842	-1.83914	-1.88679	-1.8835	-1.88026
-1.85236	-1.84932	-1.84636	-1.84345	-1.84058	-1.83774	-1.88515	-1.88188	-1.87867
-1.85083	-1.84781	-1.84488	-1.84201	-1.83915	-1.83632	-1.88351	-1.88027	-1.87707
-1.84933	-1.84633	-1.84343	-1.84058	-1.88841	-1.88513	-1.88188	-1.87865	-1.87547
-1.84785	-1.8449	-1.84201	-1.83915	-1.88674	-1.88349	-1.88025	-1.87703	-1.87386
-1.84638	-1.84348	-1.8406	-1.83774	-1.88509	-1.88185	-1.87863	-1.87542	-1.87227
-1.84493	-1.84205	-1.83918	-1.83633	-1.88345	-1.88022	-1.87701	-1.87382	-1.87069
-1.84348	-1.84063	-1.83776	-1.83492	-1.88182	-1.87859	-1.8754	-1.87223	-1.86911
-1.84206	-1.83921	-1.88672	-1.88344	-1.88019	-1.87697	-1.87378	-1.87063	-1.86753
-1.84064	-1.83781	-1.88509	-1.88183	-1.87858	-1.87535	-1.87216	-1.86903	-1.86595
-1.83923	-1.8364	-1.88346	-1.88021	-1.87696	-1.87373	-1.87056	-1.86745	-1.8644
-1.83782	-1.835	-1.88183	-1.87858	-1.87535	-1.87213	-1.86897	-1.86589	-1.86287
-1.88671	-1.88346	-1.8802	-1.87696	-1.87374	-1.87053	-1.8674	-1.86435	-1.87706
-1.88507	-1.88182	-1.87857	-1.87533	-1.87212	-1.86894	-1.86584	-1.86283	-1.87555
-1.88344	-1.8802	-1.87695	-1.87371	-1.87052	-1.86737	-1.86431	-1.86135	-1.87407
-1.88182	-1.87858	-1.87534	-1.87211	-1.86895	-1.86584	-1.86282	-1.85991	-1.87261
-1.88022	-1.87699	-1.87375	-1.87054	-1.86741	-1.86435	-1.86137	-1.85849	-1.87118
-1.8787	-1.87547	-1.87224	-1.86905	-1.86594	-1.86291	-1.87559	-1.87265	-1.89866
-1.87724	-1.87401	-1.87079	-1.86762	-1.86453	-1.86153	-1.87418	-1.87121	-1.89715
-1.87572	-1.87249	-1.8693	-1.86614	-1.86309	-1.86011	-1.90165	-1.89863	-1.89563
-1.87413	-1.87091	-1.86774	-1.86463	-1.86159	-1.85863	-1.9001	-1.89709	-1.89409
-1.87252	-1.86931	-1.86618	-1.8631	-1.90463	-1.90157	-1.89854	-1.89554	-1.89255
-1.87091	-1.86772	-1.86461	-1.86156	-1.90303	-1.89999	-1.89697	-1.89397	-1.89099
-1.8693	-1.86614	-1.86305	-1.86003	-1.90143	-1.89841	-1.8954	-1.89241	-1.88944
-1.86774	-1.8646	-1.89078	-1.88774	-1.89987	-1.89685	-1.89385	-1.89087	-1.88792
-1.86622	-1.86311	-1.88926	-1.88623	-1.89834	-1.89532	-1.89233	-1.88936	-1.88642
-1.89399	-1.89087	-1.90301	-1.89993	-1.89688	-1.89387	-1.89088	-1.88792	-1.88499
-1.89256	-1.88947	-1.90159	-1.89852	-1.89549	-1.89249	-1.88951	-1.88655	-1.88362
-1.89108	-1.88801	-1.90011	-1.89706	-1.89405	-1.89107	-1.8881	-1.88515	-1.88222
-1.88953	-1.88647	-1.89855	-1.89552	-1.89253	-1.88957	-1.88662	-1.88368	-1.88078
-1.90318	-1.90005	-1.89698	-1.89397	-1.891	-1.88806	-1.88514	-1.88222	-1.87933
-1.90166	-1.89851	-1.89545	-1.89246	-1.8895	-1.88658	-1.88367	-1.88077	-1.8779

-1.90016	-1.897	-1.89395	-1.89098	-1.88803	-1.88512	-1.88222	-1.87934	-1.8765
-1.89868	-1.89555	-1.89252	-1.88957	-1.88664	-1.88373	-1.88085	-1.878	-1.87518
-1.89721	-1.89414	-1.89114	-1.8882	-1.88529	-1.8824	-1.87953	-1.8767	-1.8739
-1.89573	-1.8927	-1.88973	-1.8868	-1.88389	-1.88101	-1.87817	-1.87535	-1.87257
-1.89424	-1.89123	-1.88828	-1.88536	-1.88246	-1.87959	-1.87675	-1.87395	-1.87117
-1.89278	-1.8898	-1.88685	-1.88395	-1.88106	-1.8782	-1.87537	-1.87258	-1.88195
-1.89139	-1.88843	-1.8855	-1.8826	-1.87973	-1.87689	-1.87407	-1.87129	-1.88064
-1.88999	-1.88705	-1.88413	-1.88125	-1.8784	-1.87558	-1.87278	-1.86999	-1.87934
-1.88854	-1.88561	-1.88271	-1.87983	-1.877	-1.8742	-1.88353	-1.88075	-1.87799
-1.88709	-1.88416	-1.88126	-1.87841	-1.87558	-1.87279	-1.88214	-1.87937	-1.87662
-1.8857	-1.88278	-1.87989	-1.87704	-1.87423	-1.87145	-1.8808	-1.87803	-1.8753
-1.88436	-1.88144	-1.87856	-1.87572	-1.87292	-1.87015	-1.8795	-1.87674	-1.874
-1.883	-1.88007	-1.8772	-1.87438	-1.88368	-1.88091	-1.87816	-1.87541	-1.8941
-1.88162	-1.87868	-1.87581	-1.873	-1.88229	-1.87951	-1.87676	-1.87404	-1.89277
-1.88025	-1.87731	-1.88655	-1.88373	-1.88093	-1.87814	-1.89688	-1.89416	-1.90229
-1.87889	-1.87599	-1.88523	-1.88242	-1.87962	-1.87684	-1.89561	-1.8929	-1.90102
-1.87755	-1.87468	-1.88392	-1.8811	-1.87831	-1.87555	-1.89436	-1.89166	-1.89977
-1.87622	-1.87337	-1.88259	-1.87974	-1.89853	-1.89576	-1.89305	-1.89038	-1.89849
-1.87492	-1.87206	-1.88125	-1.87837	-1.89717	-1.89442	-1.89172	-1.88908	-1.89721
-1.88567	-1.88281	-1.90155	-1.89866	-1.89587	-1.89314	-1.90124	-1.89858	-1.89596
-1.88434	-1.88146	-1.90024	-1.89739	-1.89462	-1.89191	-1.90001	-1.89736	-1.89474
-1.90469	-1.9018	-1.89895	-1.89614	-1.90419	-1.90147	-1.89878	-1.89613	-1.89351
-1.90336	-1.90052	-1.8977	-1.89493	-1.90297	-1.90025	-1.89756	-1.89488	-1.89225
-1.90205	-1.89924	-1.90727	-1.90449	-1.90175	-1.89903	-1.89633	-1.89364	-1.891
-1.90075	-1.89796	-1.906	-1.90324	-1.90049	-1.89777	-1.89508	-1.89242	-1.8898
-1.89947	-1.89667	-1.90471	-1.90197	-1.89923	-1.8965	-1.89382	-1.8912	-1.88861
-1.909	-1.90617	-1.9034	-1.90067	-1.89795	-1.89524	-1.89259	-1.88999	-1.88742
-1.9077	-1.90484	-1.90207	-1.89935	-1.89666	-1.89399	-1.89136	-1.88879	-1.88624
-1.90643	-1.90357	-1.9008	-1.89809	-1.89541	-1.89277	-1.89017	-1.8876	-1.88505
-1.90518	-1.90234	-1.89958	-1.89688	-1.89421	-1.89159	-1.889	-1.88642	-1.88386
-1.90392	-1.90112	-1.89837	-1.89567	-1.89302	-1.89041	-1.88781	-1.88522	-1.88265
-1.90264	-1.89987	-1.89714	-1.89445	-1.89181	-1.8892	-1.8866	-1.884	-1.88143
-1.90136	-1.89862	-1.89592	-1.89325	-1.8906	-1.88798	-1.88537	-1.88277	-1.8802
-1.90013	-1.89741	-1.89472	-1.89206	-1.8894	-1.88675	-1.88413	-1.88155	-1.879
-1.89892	-1.89622	-1.89353	-1.89087	-1.88819	-1.88551	-1.88289	-1.88033	-1.87781
-1.8977	-1.89501	-1.89234	-1.88968	-1.88701	-1.88432	-1.8817	-1.87916	-1.87665
-1.89649	-1.89381	-1.89115	-1.8885	-1.88583	-1.88316	-1.88055	-1.87802	-1.87551
-1.89528	-1.8926	-1.88994	-1.88728	-1.88463	-1.88199	-1.8794	-1.87687	-1.87437
-1.89408	-1.8914	-1.88871	-1.88602	-1.88338	-1.88078	-1.87823	-1.87572	-1.87323
-1.89289	-1.8902	-1.88749	-1.88479	-1.88216	-1.8796	-1.87706	-1.87457	-1.87208
-1.8917	-1.88903	-1.88632	-1.88362	-1.881	-1.87844	-1.87591	-1.87342	-1.87093
-1.89052	-1.88785	-1.88516	-1.88247	-1.87985	-1.8773	-1.87477	-1.87227	-1.86977
-1.88934	-1.88667	-1.88398	-1.8813	-1.87869	-1.87614	-1.87362	-1.87112	-1.86861
-1.88815	-1.88549	-1.8828	-1.88013	-1.87753	-1.87499	-1.87247	-1.86997	-1.86746
-1.88697	-1.88432	-1.88164	-1.87898	-1.87638	-1.87384	-1.87131	-1.86881	-1.8663
-1.8858	-1.88316	-1.88049	-1.87783	-1.87523	-1.87269	-1.87016	-1.86766	-1.86514
-1.88463	-1.882	-1.87934	-1.87668	-1.87408	-1.87153	-1.869	-1.8665	-1.86398
-1.88345	-1.88083	-1.87817	-1.87552	-1.87292	-1.87037	-1.86784	-1.86534	-1.86281
-1.88227	-1.87965	-1.87701	-1.87436	-1.87176	-1.86921	-1.86667	-1.86417	-1.86165
-1.88109	-1.87848	-1.87583	-1.87318	-1.87059	-1.86804	-1.8655	-1.86299	-1.86047

-1.87991	-1.87731	-1.87466	-1.87201	-1.86942	-1.86686	-1.86433	-1.86181	-1.85929
-1.87875	-1.87615	-1.8735	-1.87085	-1.86825	-1.86569	-1.86315	-1.86064	-1.85811
-1.87759	-1.875	-1.87235	-1.8697	-1.86709	-1.86453	-1.86198	-1.85947	-1.85695
-1.87644	-1.87384	-1.87121	-1.86857	-1.86596	-1.86338	-1.86082	-1.8583	-1.85578
-1.87528	-1.87269	-1.87009	-1.86748	-1.86486	-1.86224	-1.85966	-1.85714	-1.85462
-1.87414	-1.87154	-1.86895	-1.86636	-1.86374	-1.8611	-1.85851	-1.85599	-1.86606
-1.87303	-1.8704	-1.8678	-1.86521	-1.86259	-1.85994	-1.85735	-1.85483	-1.8649
-1.87192	-1.86925	-1.86664	-1.86405	-1.86143	-1.85878	-1.85619	-1.85368	-1.86374
-1.87077	-1.8681	-1.86548	-1.86288	-1.87284	-1.8702	-1.86761	-1.86509	-1.86257
-1.86962	-1.86695	-1.86432	-1.86172	-1.87167	-1.86903	-1.86645	-1.86392	-1.86141
-1.86821	-1.86557	-1.87576	-1.87313	-1.87051	-1.86791	-1.86533	-1.86278	-1.86026
-1.86709	-1.86448	-1.87464	-1.87198	-1.86938	-1.86681	-1.86425	-1.86166	-1.8591
-1.86595	-1.86336	-1.87349	-1.87082	-1.86823	-1.86569	-1.86312	-1.86052	-1.85794
-1.86479	-1.8622	-1.87232	-1.86966	-1.86706	-1.86453	-1.86196	-1.85936	-1.85678
-1.86365	-1.86104	-1.87115	-1.86851	-1.86592	-1.86337	-1.8608	-1.85821	-1.85563
-1.86252	-1.8599	-1.87002	-1.86741	-1.86481	-1.86223	-1.85965	-1.85709	-1.85453
-1.8614	-1.85876	-1.86888	-1.8663	-1.8637	-1.86109	-1.85851	-1.85597	-1.85343
-1.86024	-1.8576	-1.86771	-1.86515	-1.86255	-1.85992	-1.85734	-1.85481	-1.85228
-1.85908	-1.85645	-1.86654	-1.86397	-1.86137	-1.85876	-1.85617	-1.85363	-1.85111
-1.85796	-1.85535	-1.85277	-1.85022	-1.86024	-1.85763	-1.85505	-1.85249	-1.84995
-1.85688	-1.85429	-1.8517	-1.84913	-1.85913	-1.85654	-1.85396	-1.85137	-1.84881
-1.8558	-1.85323	-1.85064	-1.84805	-1.85804	-1.85545	-1.85287	-1.85027	-1.8477
-1.85473	-1.85215	-1.84957	-1.847	-1.85697	-1.85437	-1.85178	-1.8492	-1.84663
-1.85366	-1.85107	-1.84851	-1.84595	-1.85589	-1.85328	-1.85069	-1.84812	-1.84556
-1.85259	-1.85002	-1.84745	-1.84488	-1.85481	-1.85221	-1.84961	-1.84703	-1.84445
-1.85154	-1.84898	-1.84639	-1.8438	-1.85372	-1.85113	-1.84853	-1.84592	-1.84334
-1.85047	-1.8479	-1.84532	-1.84274	-1.85263	-1.85003	-1.84742	-1.8448	-1.84221
-1.8494	-1.84682	-1.84425	-1.84168	-1.85154	-1.84892	-1.8463	-1.84367	-1.84107
-1.84834	-1.84576	-1.84318	-1.84061	-1.83801	-1.83539	-1.84517	-1.84255	-1.83996
-1.84729	-1.84471	-1.84212	-1.83952	-1.83691	-1.83427	-1.84403	-1.84144	-1.83887
-1.84622	-1.84365	-1.84106	-1.83845	-1.83583	-1.83318	-1.84293	-1.84035	-1.83777
-1.84515	-1.84257	-1.83999	-1.8374	-1.83479	-1.83215	-1.84187	-1.83926	-1.83667
-1.84408	-1.84148	-1.83891	-1.83634	-1.83374	-1.83111	-1.84081	-1.83818	-1.83557
-1.843	-1.84042	-1.83784	-1.83525	-1.83264	-1.83	-1.83969	-1.83708	-1.83448
-1.84192	-1.83935	-1.83676	-1.83415	-1.83152	-1.82889	-1.83857	-1.83598	-1.8334
-1.84083	-1.83826	-1.83568	-1.83308	-1.83046	-1.82782	-1.82521	-1.82263	-1.85809
-1.83973	-1.83715	-1.83459	-1.83203	-1.82943	-1.8268	-1.82417	-1.82156	-1.85696
-1.83864	-1.83606	-1.83351	-1.83095	-1.82836	-1.82575	-1.82314	-1.82051	-1.85586
-1.83756	-1.835	-1.83242	-1.82984	-1.82727	-1.8247	-1.82211	-1.81949	-1.85481
-1.8365	-1.83395	-1.83136	-1.82875	-1.82618	-1.82363	-1.82106	-1.81846	-1.84185
-1.83548	-1.83293	-1.83034	-1.82772	-1.82513	-1.82254	-1.81997	-1.8174	-1.84081
-1.83446	-1.83191	-1.82932	-1.82671	-1.82409	-1.82147	-1.81888	-1.81633	-1.83974
-1.8334	-1.83083	-1.82826	-1.82568	-1.82307	-1.82044	-1.84386	-1.84125	-1.83864
-1.83233	-1.82973	-1.82718	-1.82464	-1.82205	-1.81943	-1.84282	-1.84016	-1.83752
-1.83129	-1.82868	-1.82611	-1.82356	-1.82099	-1.81839	-1.84178	-1.8391	-1.83645
-1.83027	-1.82765	-1.82505	-1.82246	-1.81989	-1.81734	-1.84073	-1.83807	-1.83541
-1.82924	-1.82663	-1.82401	-1.82139	-1.84485	-1.84227	-1.83966	-1.83701	-1.83436
-1.82817	-1.8256	-1.82299	-1.82037	-1.84379	-1.84116	-1.83854	-1.83593	-1.83331
-1.82711	-1.82456	-1.82196	-1.81935	-1.84274	-1.84008	-1.83744	-1.83484	-1.83224
-1.82604	-1.82347	-1.8209	-1.81833	-1.84171	-1.83904	-1.83638	-1.83376	-1.83115

-1.82497	-1.82238	-1.81983	-1.8173	-1.84068	-1.83801	-1.83534	-1.83269	-1.83006
-1.82395	-1.82134	-1.8188	-1.81628	-1.83964	-1.83697	-1.83431	-1.83164	-1.82901
-1.82294	-1.82032	-1.81777	-1.81524	-1.83859	-1.83593	-1.83328	-1.83062	-1.82799
-1.82192	-1.8193	-1.81672	-1.81417	-1.83751	-1.83488	-1.83224	-1.82959	-1.82696
-1.82091	-1.81827	-1.81566	-1.81307	-1.83639	-1.8338	-1.83119	-1.82854	-1.82591
-1.81989	-1.81725	-1.84057	-1.83791	-1.8353	-1.83273	-1.83014	-1.82751	-1.82487
-1.81888	-1.81623	-1.83953	-1.83685	-1.83424	-1.83169	-1.8291	-1.82647	-1.82383
-1.81787	-1.81521	-1.83849	-1.83581	-1.8332	-1.83065	-1.82807	-1.82544	-1.8228
-1.81685	-1.8142	-1.83745	-1.83477	-1.83216	-1.82961	-1.82703	-1.82441	-1.82177
-1.81584	-1.81319	-1.83642	-1.83373	-1.83112	-1.82858	-1.826	-1.82338	-1.82073
-1.81482	-1.81217	-1.83538	-1.83269	-1.83008	-1.82753	-1.82495	-1.82234	-1.8197
-1.8138	-1.81115	-1.83434	-1.83166	-1.82904	-1.82648	-1.8239	-1.82129	-1.81866
-1.83871	-1.83601	-1.8333	-1.83062	-1.82799	-1.82541	-1.82282	-1.82023	-1.81761
-1.83768	-1.83497	-1.83227	-1.82958	-1.82693	-1.82431	-1.82171	-1.81913	-1.81654
-1.83664	-1.83393	-1.83123	-1.82855	-1.82589	-1.82325	-1.82064	-1.81807	-1.81548
-1.83561	-1.8329	-1.8302	-1.82752	-1.82488	-1.82228	-1.81967	-1.81707	-1.81445
-1.83458	-1.83187	-1.82917	-1.8265	-1.82388	-1.82131	-1.81871	-1.81607	-1.81342
-1.83355	-1.83084	-1.82815	-1.82547	-1.82286	-1.8203	-1.8177	-1.81505	-1.81239
-1.83252	-1.82982	-1.82712	-1.82444	-1.82182	-1.81926	-1.81666	-1.81402	-1.81135
-1.8315	-1.82879	-1.82609	-1.82342	-1.8208	-1.81823	-1.81563	-1.81297	-1.8103
-1.83047	-1.82776	-1.82507	-1.8224	-1.81978	-1.81719	-1.81458	-1.81192	-1.80924
-1.82945	-1.82675	-1.82407	-1.82141	-1.81878	-1.81617	-1.81354	-1.81087	-1.80819
-1.82843	-1.82575	-1.8231	-1.82046	-1.81782	-1.81517	-1.81251	-1.80982	-1.80713
-1.82744	-1.82476	-1.82212	-1.8195	-1.81684	-1.81416	-1.81148	-1.80877	-1.80607
-1.82647	-1.82381	-1.82116	-1.8185	-1.81583	-1.81313	-1.81043	-1.80772	-1.80501
-1.8255	-1.82286	-1.82019	-1.81749	-1.81479	-1.81208	-1.80937	-1.80666	-1.80395
-1.82454	-1.82188	-1.81918	-1.81646	-1.81375	-1.81103	-1.80831	-1.8056	-1.8029
-1.82357	-1.82087	-1.81815	-1.81542	-1.81269	-1.80996	-1.80725	-1.80454	-1.80185
-1.82256	-1.81984	-1.81711	-1.81438	-1.81164	-1.80891	-1.8062	-1.80349	-1.80081
-1.82153	-1.8188	-1.81606	-1.81333	-1.8106	-1.80787	-1.80515	-1.80245	-1.79978
-1.8205	-1.81776	-1.81502	-1.81229	-1.80957	-1.80686	-1.80415	-1.80145	-1.79878
-1.81947	-1.81673	-1.81399	-1.81126	-1.80856	-1.80589	-1.80321	-1.80052	-1.79784
-1.81845	-1.81572	-1.81298	-1.81025	-1.80756	-1.80492	-1.80226	-1.79957	-1.79689
-1.81746	-1.81476	-1.81203	-1.8093	-1.8066	-1.80393	-1.80126	-1.79857	-1.79589
-1.81648	-1.81382	-1.8111	-1.80837	-1.80565	-1.80294	-1.80024	-1.79755	-1.79487
-1.81551	-1.81283	-1.81012	-1.80738	-1.80466	-1.80194	-1.79923	-1.79654	-1.79385
-1.81453	-1.81182	-1.80909	-1.80636	-1.80365	-1.80094	-1.79823	-1.79553	-1.79283
-1.81354	-1.8108	-1.80807	-1.80534	-1.80264	-1.79995	-1.79726	-1.79456	-1.79185
-1.81252	-1.80978	-1.80704	-1.80432	-1.80164	-1.799	-1.79633	-1.79364	-1.79093
-1.7876	-1.78491	-1.80605	-1.80333	-1.80066	-1.79804	-1.79538	-1.7927	-1.78999
-1.78669	-1.784	-1.80513	-1.80241	-1.79972	-1.79706	-1.79439	-1.7917	-1.789
-1.78578	-1.78309	-1.80421	-1.80148	-1.79878	-1.79608	-1.79339	-1.7907	-1.788
-1.78481	-1.78212	-1.80323	-1.8005	-1.79779	-1.79508	-1.79239	-1.78972	-1.78704
-1.78382	-1.78113	-1.80223	-1.7995	-1.79679	-1.79408	-1.7914	-1.78875	-1.7861
-1.78288	-1.78019	-1.77751	-1.77484	-1.79584	-1.79312	-1.79043	-1.78778	-1.78512
-1.78198	-1.77929	-1.77661	-1.77395	-1.79493	-1.79221	-1.7895	-1.7868	-1.78411
-1.78103	-1.77835	-1.77568	-1.77301	-1.79398	-1.79125	-1.78853	-1.78581	-1.78311
-1.78003	-1.77735	-1.77468	-1.77202	-1.79297	-1.79024	-1.78752	-1.7848	-1.7821
-1.77905	-1.77638	-1.77371	-1.77105	-1.79199	-1.78926	-1.78654	-1.78382	-1.78112
-1.77814	-1.77547	-1.77281	-1.77015	-1.79109	-1.78836	-1.78563	-1.7829	-1.78019

-1.77722	-1.77456	-1.77191	-1.76927	-1.7902	-1.78747	-1.78474	-1.78201	-1.77929
-1.77625	-1.77359	-1.77097	-1.76837	-1.76573	-1.76307	-1.78384	-1.78111	-1.77839
-1.77525	-1.77261	-1.77002	-1.76745	-1.76483	-1.76217	-1.78294	-1.78021	-1.77748
-1.77432	-1.77168	-1.76908	-1.7665	-1.76388	-1.76123	-1.78197	-1.77925	-1.77652
-1.77344	-1.77079	-1.76816	-1.76554	-1.76289	-1.76023	-1.78096	-1.77824	-1.77551
-1.77257	-1.76991	-1.76726	-1.76461	-1.76194	-1.75928	-1.75661	-1.75393	-1.77453
-1.77172	-1.76906	-1.7664	-1.76373	-1.76106	-1.75839	-1.75571	-1.75304	-1.77362
-1.77086	-1.7682	-1.76553	-1.76286	-1.76018	-1.75751	-1.75483	-1.75215	-1.77272
-1.76999	-1.76733	-1.76466	-1.76198	-1.7593	-1.75663	-1.75395	-1.75126	-1.77182
-1.7691	-1.76643	-1.76376	-1.76108	-1.7584	-1.75572	-1.75304	-1.75036	-1.77091
-1.76814	-1.76547	-1.7628	-1.76011	-1.75743	-1.75476	-1.75207	-1.74939	-1.76992
-1.76714	-1.76447	-1.76179	-1.75911	-1.75643	-1.75375	-1.75106	-1.74838	-1.7689
-1.7662	-1.76353	-1.76084	-1.75816	-1.75548	-1.7528	-1.75011	-1.74743	-1.74474
-1.76531	-1.76263	-1.75995	-1.75726	-1.75458	-1.7519	-1.74922	-1.74653	-1.74384
-1.76443	-1.76176	-1.75907	-1.75639	-1.75371	-1.75103	-1.74834	-1.74565	-1.74296
-1.76357	-1.76089	-1.75821	-1.75553	-1.75285	-1.75016	-1.74748	-1.74479	-1.7421
-1.76272	-1.76003	-1.75735	-1.75467	-1.75199	-1.7493	-1.74661	-1.74392	-1.74123
-1.76185	-1.75917	-1.75649	-1.75381	-1.75113	-1.74844	-1.74575	-1.74306	-1.74036
-1.76099	-1.75831	-1.75563	-1.75295	-1.75027	-1.74757	-1.74488	-1.74218	-1.73948
-1.76013	-1.75745	-1.75477	-1.75209	-1.7494	-1.7467	-1.744	-1.7413	-1.73858
-1.75926	-1.75658	-1.7539	-1.75122	-1.74852	-1.74581	-1.7431	-1.7404	-1.73768
-1.7584	-1.75572	-1.75303	-1.75034	-1.74763	-1.7449	-1.74217	-1.73946	-1.73673
-1.75754	-1.75486	-1.75217	-1.74948	-1.74673	-1.74394	-1.74118	-1.73845	-1.73573
-1.75668	-1.754	-1.75131	-1.74861	-1.74583	-1.743	-1.74021	-1.73747	-1.73475
-1.75582	-1.75314	-1.75045	-1.74774	-1.74495	-1.74211	-1.73931	-1.73656	-1.73384
-1.75496	-1.75228	-1.74958	-1.74687	-1.74408	-1.74123	-1.73843	-1.73567	-1.73295
-1.7541	-1.75141	-1.74872	-1.746	-1.74321	-1.74035	-1.73755	-1.73479	-1.73207
-1.75324	-1.75055	-1.74785	-1.74513	-1.74233	-1.73948	-1.73668	-1.73393	-1.7312
-1.75238	-1.74969	-1.74698	-1.74426	-1.74147	-1.73862	-1.73581	-1.73306	-1.73033
-1.75152	-1.74882	-1.74612	-1.74339	-1.7406	-1.73776	-1.73496	-1.7322	-1.72946
-1.7419	-1.73918	-1.74525	-1.74252	-1.73975	-1.73693	-1.73413	-1.73136	-1.72861
-1.74102	-1.7383	-1.74438	-1.74166	-1.73891	-1.73614	-1.73336	-1.73056	-1.72778
-1.74014	-1.73742	-1.7347	-1.73198	-1.73807	-1.73534	-1.73257	-1.72976	-1.72697
-1.73926	-1.73654	-1.73382	-1.7311	-1.7372	-1.73449	-1.73175	-1.72898	-1.7262
-1.73839	-1.73566	-1.73294	-1.73021	-1.73633	-1.73362	-1.73091	-1.72818	-1.72543
-1.73751	-1.73479	-1.73206	-1.72933	-1.73546	-1.73275	-1.73005	-1.72734	-1.72462
-1.73664	-1.73392	-1.73118	-1.72845	-1.73458	-1.73188	-1.72917	-1.72648	-1.72377
-1.73578	-1.73304	-1.73031	-1.72758	-1.72485	-1.72214	-1.7283	-1.72561	-1.72291
-1.73492	-1.73217	-1.72943	-1.7267	-1.72398	-1.72127	-1.72744	-1.72474	-1.72203
-1.73408	-1.73131	-1.72856	-1.72583	-1.72311	-1.7204	-1.71769	-1.71497	-1.72116
-1.73328	-1.73048	-1.72771	-1.72497	-1.72223	-1.71952	-1.7168	-1.71409	-1.72028
-1.7325	-1.72967	-1.72689	-1.72413	-1.72139	-1.71866	-1.71593	-1.71321	-1.71941
-1.7317	-1.7289	-1.72613	-1.72337	-1.72061	-1.71784	-1.71509	-1.71235	-1.71854
-1.73091	-1.72815	-1.72539	-1.72263	-1.71985	-1.71704	-1.71426	-1.71152	-1.7177
-1.73007	-1.72733	-1.72458	-1.72183	-1.71906	-1.71626	-1.71349	-1.71074	-1.70798
-1.7292	-1.72646	-1.72372	-1.72098	-1.71824	-1.71549	-1.71274	-1.71	-1.70724
-1.72837	-1.72561	-1.72287	-1.72013	-1.7174	-1.71468	-1.71195	-1.70921	-1.70645
-1.72759	-1.72478	-1.72202	-1.71928	-1.71654	-1.71381	-1.71108	-1.70835	-1.70561
-1.7268	-1.72398	-1.7212	-1.71845	-1.7157	-1.71296	-1.71022	-1.70749	-1.70476
-1.72602	-1.72322	-1.72046	-1.7177	-1.71493	-1.71215	-1.70938	-1.70664	-1.7039

-0.99884	-0.99749	-0.99615	-0.9948	-0.99345	-0.9921	-0.99076	-0.98944	-1.00893
-0.99919	-0.99785	-0.9965	-0.99515	-0.9938	-0.99245	-0.99111	-0.9898	-1.00931
-0.99954	-0.9982	-0.99685	-0.9955	-0.99415	-0.99281	-0.99147	-0.99016	-1.0097
-0.99989	-0.99855	-0.9972	-0.99585	-0.9945	-0.99316	-0.99183	-0.9905	-0.98918
-1.00025	-0.9989	-0.99755	-0.9962	-0.99486	-0.99352	-0.99218	-0.99084	-0.98952
-1.00059	-0.99924	-0.99789	-0.99655	-0.99521	-0.99387	-0.99253	-0.99119	-0.98986
-1.00092	-0.99958	-0.99824	-0.9969	-0.99556	-0.99421	-0.99288	-0.99155	-0.99023
-1.00126	-0.99993	-0.99859	-0.99725	-0.9959	-0.99456	-0.99322	-0.9919	-0.99058
-1.00159	-1.00028	-0.99894	-0.9976	-0.99625	-0.9949	-0.99356	-0.99224	-0.99092
-1.00193	-1.00063	-0.99929	-0.99794	-0.99659	-0.99525	-0.99391	-0.99258	-0.99126
-1.00225	-1.00095	-0.99962	-0.99828	-0.99694	-0.99559	-0.99425	-0.99292	-0.99159
-1.00258	-1.00127	-0.99995	-0.99861	-0.99728	-0.99594	-0.9946	-0.99325	-0.99192
-1.00291	-1.00161	-1.00029	-0.99897	-0.99764	-0.99629	-0.99495	-0.9936	-0.99226
-1.00325	-1.00195	-1.00065	-0.99934	-0.998	-0.99665	-0.99529	-0.99395	-0.99262
-1.00359	-1.0023	-1.001	-0.99969	-0.99836	-0.99699	-0.99564	-0.9943	-0.99297
-1.00395	-1.00266	-1.00136	-1.00004	-0.9987	-0.99734	-0.99599	-0.99464	-0.99331
-1.0043	-1.00301	-1.0017	-1.00039	-0.99905	-0.99769	-0.99634	-0.99499	-0.99366
-1.00463	-1.00333	-1.00203	-1.00073	-0.99941	-0.99806	-0.99671	-0.99537	-0.99403
-1.00495	-1.00365	-1.00237	-1.00108	-0.99976	-0.99842	-0.99708	-0.99573	-0.9944
-1.00529	-1.004	-1.00271	-1.00141	-1.0001	-0.99876	-0.99742	-0.99608	-0.99475
-1.00563	-1.00434	-1.00304	-1.00175	-1.00043	-0.9991	-0.99777	-0.99643	-0.9951
-1.00596	-1.00466	-1.00337	-1.00208	-1.00077	-0.99945	-0.99812	-0.99679	-0.99546
-1.00629	-1.00499	-1.00369	-1.00241	-1.00111	-0.9998	-0.99848	-0.99715	-0.99583
-1.00662	-1.00532	-1.00403	-1.00274	-1.00145	-1.00015	-0.99884	-0.99752	-0.9962
-1.00696	-1.00566	-1.00437	-1.00308	-1.00179	-1.00051	-0.99921	-0.9979	-0.99659
-1.0073	-1.006	-1.0047	-1.00341	-1.00213	-1.00085	-0.99956	-0.99826	-0.99695
-1.00763	-1.00632	-1.00502	-1.00373	-1.00245	-1.00117	-0.99988	-0.99859	-0.9973
-1.00796	-1.00664	-1.00534	-1.00406	-1.00277	-1.00148	-1.0002	-0.99892	-0.99763
-1.00831	-1.00699	-1.00568	-1.00439	-1.0031	-1.00182	-1.00054	-0.99926	-0.99798
-1.00866	-1.00734	-1.00603	-1.00473	-1.00344	-1.00216	-1.00088	-0.9996	-0.99833
-1.00901	-1.00768	-1.00637	-1.00506	-1.00377	-1.00249	-1.00121	-0.99994	-0.99867
-1.00937	-1.00802	-1.0067	-1.00539	-1.00409	-1.00281	-1.00153	-1.00027	-0.999
-1.00974	-1.00838	-1.00706	-1.00575	-1.00444	-1.00315	-1.00187	-1.00061	-0.99935
-1.01013	-1.00878	-1.00745	-1.00613	-1.00483	-1.00354	-1.00226	-1.00098	-0.99971
-0.9899	-0.98858	-1.00783	-1.00651	-1.00521	-1.00392	-1.00263	-1.00135	-1.00007
-0.99026	-0.98894	-1.00819	-1.00686	-1.00556	-1.00427	-1.00298	-1.00169	-1.0004
-0.99062	-0.9893	-1.00855	-1.00721	-1.0059	-1.00461	-1.00332	-1.00203	-1.00074
-0.99099	-0.98968	-0.98836	-0.98706	-0.98577	-0.98451	-1.0037	-1.0024	-1.00111
-0.99137	-0.99005	-0.98874	-0.98744	-0.98616	-0.98489	-1.00408	-1.00279	-1.0015
-0.96948	-0.96818	-0.96689	-0.96561	-0.98652	-0.98524	-0.98398	-0.98273	-1.00187
-0.96983	-0.96851	-0.96722	-0.96595	-0.98685	-0.98558	-0.98431	-0.98307	-1.00223
-0.97018	-0.96887	-0.96757	-0.9663	-0.96503	-0.96377	-0.98466	-0.98343	-0.98219
-0.97056	-0.96925	-0.96795	-0.96667	-0.9654	-0.96415	-0.98505	-0.98381	-0.98257
-0.97092	-0.96962	-0.96833	-0.96704	-0.96577	-0.96452	-0.96327	-0.96205	-0.98294
-0.97126	-0.96997	-0.96868	-0.96739	-0.96612	-0.96486	-0.96362	-0.96241	-0.9833
-0.97159	-0.97031	-0.96902	-0.96773	-0.96646	-0.9652	-0.96397	-0.96275	-0.98366
-0.97194	-0.97067	-0.96937	-0.96808	-0.96681	-0.96555	-0.96431	-0.96311	-0.96189
-0.97231	-0.97103	-0.96973	-0.96843	-0.96715	-0.96589	-0.96466	-0.96346	-0.96226
-0.97267	-0.97139	-0.97009	-0.96879	-0.96751	-0.96624	-0.96501	-0.96381	-0.96261
-0.97301	-0.97173	-0.97044	-0.96915	-0.96787	-0.9666	-0.96536	-0.96415	-0.96295

-0.97338	-0.9721	-0.97081	-0.96952	-0.96824	-0.96697	-0.96573	-0.96451	-0.9633
-0.97378	-0.9725	-0.97121	-0.96992	-0.96864	-0.96737	-0.96613	-0.9649	-0.96369
-0.97418	-0.9729	-0.97161	-0.97032	-0.96905	-0.96778	-0.96653	-0.9653	-0.96407
-0.95323	-0.95198	-0.97201	-0.97071	-0.96944	-0.96817	-0.96692	-0.96568	-0.96445
-0.95362	-0.95236	-0.9724	-0.9711	-0.96982	-0.96856	-0.96731	-0.96606	-0.96482
-0.954	-0.95274	-0.97278	-0.97147	-0.97019	-0.96894	-0.96768	-0.96644	-0.96519
-0.95437	-0.9531	-0.97316	-0.97184	-0.97056	-0.9693	-0.96806	-0.96681	-0.96556
-0.95475	-0.95348	-0.97355	-0.97222	-0.97093	-0.96968	-0.96843	-0.96719	-0.96594
-0.95514	-0.95387	-0.97395	-0.97262	-0.97133	-0.97007	-0.96881	-0.96757	-0.96633
-0.87785	-0.87675	-0.95297	-0.95166	-0.97173	-0.97045	-0.96919	-0.96796	-0.96672
-0.87815	-0.87705	-0.95335	-0.95204	-0.97211	-0.97082	-0.96956	-0.96833	-0.96709
-0.87847	-0.87737	-0.95373	-0.95243	-0.97251	-0.97121	-0.96993	-0.9687	-0.96747
-0.8788	-0.8777	-0.87658	-0.87546	-0.97295	-0.97163	-0.97035	-0.9691	-0.96786
-0.87914	-0.87804	-0.87694	-0.87583	-0.97341	-0.97208	-0.97078	-0.96951	-0.96826
-0.87947	-0.87838	-0.87728	-0.87618	-0.90367	-0.90248	-0.9712	-0.96991	-0.96864
-0.8798	-0.8787	-0.87761	-0.87651	-0.90404	-0.90285	-0.97162	-0.97031	-0.96902
-0.88013	-0.87903	-0.87793	-0.87684	-0.87573	-0.8746	-0.90204	-0.90085	-0.96943
-0.88044	-0.87934	-0.87825	-0.87716	-0.87605	-0.87494	-0.90242	-0.90124	-0.96986
-0.88077	-0.87967	-0.87857	-0.87748	-0.87638	-0.87528	-0.9028	-0.90162	-0.90046
-0.88113	-0.88002	-0.87892	-0.87782	-0.87672	-0.87563	-0.90318	-0.90202	-0.90085
-0.88149	-0.88037	-0.87926	-0.87816	-0.87706	-0.87597	-0.90356	-0.9024	-0.90125
-0.88183	-0.88071	-0.8796	-0.8785	-0.8774	-0.87631	-0.87521	-0.87411	-0.90162
-0.88215	-0.88104	-0.87994	-0.87884	-0.87774	-0.87664	-0.87554	-0.87444	-0.90197
-0.8609	-0.85982	-0.88028	-0.87919	-0.87809	-0.87699	-0.87588	-0.87478	-0.90234
-0.86121	-0.86014	-0.88063	-0.87954	-0.87845	-0.87734	-0.87623	-0.87513	-0.90272
-0.86152	-0.86047	-0.88097	-0.87989	-0.8788	-0.87769	-0.87659	-0.87548	-0.87438
-0.86186	-0.86079	-0.88131	-0.88023	-0.87914	-0.87805	-0.87695	-0.87584	-0.87474
-0.86219	-0.86112	-0.88165	-0.88057	-0.87948	-0.87839	-0.8773	-0.87619	-0.87509
-0.86253	-0.86146	-0.86039	-0.85932	-0.87981	-0.87872	-0.87763	-0.87653	-0.87544
-0.86287	-0.86179	-0.86071	-0.85963	-0.88013	-0.87904	-0.87795	-0.87687	-0.87578
-0.86321	-0.86212	-0.86103	-0.85994	-0.85887	-0.85781	-0.87828	-0.8772	-0.87613
-0.86353	-0.86246	-0.86135	-0.86025	-0.85918	-0.85812	-0.8786	-0.87753	-0.87646
-0.86387	-0.86279	-0.86169	-0.86059	-0.85951	-0.85845	-0.87895	-0.87788	-0.87681
-0.86422	-0.86312	-0.86203	-0.86095	-0.85987	-0.85881	-0.87933	-0.87825	-0.87718
-0.83446	-0.8334	-0.86237	-0.8613	-0.86023	-0.85916	-0.8581	-0.85705	-0.87753
-0.8348	-0.83373	-0.86271	-0.86164	-0.86056	-0.85949	-0.85843	-0.85737	-0.87787
-0.83514	-0.83406	-0.86306	-0.86198	-0.8609	-0.85982	-0.85876	-0.85769	-0.87821
-0.83548	-0.8344	-0.83333	-0.83227	-0.86124	-0.86017	-0.8591	-0.85803	-0.85697
-0.8358	-0.83473	-0.83366	-0.8326	-0.8616	-0.86052	-0.85945	-0.85837	-0.8573
-0.83613	-0.83505	-0.83399	-0.83293	-0.86194	-0.86087	-0.85979	-0.85872	-0.85765
-0.83644	-0.83536	-0.8343	-0.83324	-0.86228	-0.86121	-0.86014	-0.85907	-0.858
-0.83677	-0.83569	-0.83463	-0.83357	-0.83252	-0.83148	-0.8605	-0.85942	-0.85835
-0.83712	-0.83605	-0.83498	-0.83393	-0.83288	-0.83184	-0.86087	-0.85979	-0.8587
-0.83747	-0.8364	-0.83534	-0.83428	-0.83323	-0.83219	-0.83114	-0.83008	-0.85905
-0.83782	-0.83675	-0.83568	-0.83461	-0.83356	-0.83251	-0.83146	-0.8304	-0.8594
-0.83817	-0.83709	-0.83601	-0.83494	-0.83388	-0.83283	-0.83177	-0.83072	-0.85975
-0.83853	-0.83744	-0.83636	-0.83528	-0.83421	-0.83316	-0.83211	-0.83106	-0.86012
-0.8389	-0.83779	-0.83671	-0.83563	-0.83456	-0.8335	-0.83244	-0.83139	-0.86049
-0.80936	-0.8083	-0.83705	-0.83597	-0.8349	-0.83383	-0.83278	-0.83173	-0.83068
-0.80969	-0.80862	-0.83739	-0.8363	-0.83523	-0.83416	-0.8331	-0.83206	-0.83101

-0.81003	-0.80895	-0.8079	-0.80686	-0.83557	-0.83451	-0.83345	-0.8324	-0.83135
-0.81036	-0.8093	-0.80825	-0.80721	-0.83595	-0.8349	-0.83384	-0.83278	-0.83172
-0.81071	-0.80966	-0.80861	-0.80757	-0.83634	-0.83529	-0.83423	-0.83316	-0.83209
-0.81107	-0.81002	-0.80897	-0.80794	-0.80692	-0.80591	-0.8346	-0.83352	-0.83245
-0.81144	-0.81038	-0.80934	-0.80831	-0.80728	-0.80625	-0.83496	-0.83388	-0.83281
-0.78032	-0.77932	-0.80968	-0.80864	-0.8076	-0.80657	-0.83529	-0.83422	-0.83315
-0.78063	-0.77962	-0.81	-0.80895	-0.80791	-0.80686	-0.83561	-0.83454	-0.83348
-0.73647	-0.73542	-0.76676	-0.76568	-0.80824	-0.80719	-0.80616	-0.80515	-0.83384
-0.7369	-0.73583	-0.7672	-0.76611	-0.80859	-0.80755	-0.80652	-0.80551	-0.83422
-0.73734	-0.73626	-0.7352	-0.73417	-0.76547	-0.7644	-0.80689	-0.80587	-0.80486
-0.7378	-0.7367	-0.73563	-0.73459	-0.76593	-0.76485	-0.80726	-0.80624	-0.80522
-0.73825	-0.73714	-0.73607	-0.73502	-0.76639	-0.76531	-0.80764	-0.80661	-0.80559
-0.72119	-0.72012	-0.7365	-0.73543	-0.7344	-0.73338	-0.76468	-0.76361	-0.80595
-0.72163	-0.72056	-0.73693	-0.73585	-0.7348	-0.73378	-0.76512	-0.76405	-0.80631
-0.72206	-0.72098	-0.73735	-0.73625	-0.73519	-0.73417	-0.73315	-0.73216	-0.76342
-0.72247	-0.72139	-0.73775	-0.73665	-0.73558	-0.73454	-0.73352	-0.73253	-0.76384
-0.7229	-0.72183	-0.73819	-0.73707	-0.736	-0.73494	-0.73391	-0.73291	-0.76426
-0.72336	-0.72228	-0.73865	-0.73754	-0.73645	-0.73537	-0.73432	-0.73331	-0.76468
-0.72383	-0.72276	-0.72168	-0.72061	-0.73692	-0.73582	-0.73475	-0.73372	-0.7327
-0.72432	-0.72323	-0.72216	-0.72109	-0.7374	-0.73628	-0.7352	-0.73415	-0.73312
-0.72481	-0.72372	-0.72265	-0.72158	-0.73788	-0.73676	-0.73566	-0.73459	-0.73355
-0.7253	-0.72421	-0.72313	-0.72206	-0.73836	-0.73724	-0.73613	-0.73505	-0.73399
-0.7258	-0.7247	-0.72362	-0.72254	-0.73885	-0.73772	-0.73661	-0.73551	-0.73444
-0.7263	-0.7252	-0.7241	-0.72302	-0.73933	-0.73821	-0.73709	-0.73599	-0.7349
-0.72679	-0.72569	-0.72459	-0.72349	-0.73981	-0.73869	-0.73758	-0.73646	-0.73537
-0.72729	-0.72619	-0.72508	-0.72397	-0.74029	-0.73918	-0.73806	-0.73695	-0.73585
-0.72779	-0.72668	-0.72557	-0.72446	-0.74079	-0.73967	-0.73855	-0.73744	-0.73633
-0.69754	-0.69647	-0.72606	-0.72495	-0.72386	-0.72278	-0.73904	-0.73793	-0.73682
-0.69801	-0.69693	-0.72655	-0.72544	-0.72435	-0.72326	-0.73953	-0.73842	-0.7373
-0.69849	-0.6974	-0.72705	-0.72594	-0.72484	-0.72375	-0.74002	-0.7389	-0.73779
-0.69898	-0.69788	-0.72756	-0.72644	-0.72533	-0.72424	-0.74051	-0.73939	-0.73828
-0.69947	-0.69838	-0.72807	-0.72694	-0.72583	-0.72473	-0.741	-0.73988	-0.73876
-0.69996	-0.69887	-0.69778	-0.69669	-0.72633	-0.72522	-0.72413	-0.72304	-0.73924
-0.70044	-0.69937	-0.69827	-0.69717	-0.72683	-0.72572	-0.72462	-0.72352	-0.73972
-0.70092	-0.69986	-0.69876	-0.69765	-0.69658	-0.69552	-0.72512	-0.724	-0.74021
-0.7014	-0.70034	-0.69925	-0.69815	-0.69706	-0.69599	-0.72561	-0.7245	-0.74073
-0.70188	-0.70081	-0.69973	-0.69864	-0.69755	-0.69647	-0.72612	-0.725	-0.74125
-0.70236	-0.70129	-0.70021	-0.69913	-0.69804	-0.69694	-0.69586	-0.6948	-0.72441
-0.70284	-0.70177	-0.7007	-0.69963	-0.69853	-0.69742	-0.69634	-0.69527	-0.72492
-0.70334	-0.70226	-0.70119	-0.70011	-0.69902	-0.69792	-0.69683	-0.69577	-0.72545
-0.70384	-0.70275	-0.70167	-0.70059	-0.69951	-0.69842	-0.69734	-0.69628	-0.72599
-0.70434	-0.70326	-0.70217	-0.70108	-0.7	-0.69893	-0.69786	-0.69679	-0.69574
-0.70485	-0.70376	-0.70266	-0.70157	-0.7005	-0.69945	-0.69838	-0.6973	-0.69623
-0.6691	-0.66815	-0.70316	-0.70206	-0.701	-0.69996	-0.6989	-0.69781	-0.69672
-0.66955	-0.66859	-0.70367	-0.70257	-0.7015	-0.70045	-0.69939	-0.6983	-0.69722
-0.67001	-0.66903	-0.70418	-0.70309	-0.70201	-0.70095	-0.69987	-0.69879	-0.69772
-0.64014	-0.6392	-0.70471	-0.70363	-0.70254	-0.70146	-0.70038	-0.6993	-0.69822
-0.6406	-0.63964	-0.70525	-0.70418	-0.70309	-0.70198	-0.70088	-0.6998	-0.69873
-0.64107	-0.64011	-0.66949	-0.66856	-0.70362	-0.7025	-0.7014	-0.70032	-0.69925
-0.64157	-0.64061	-0.67	-0.66903	-0.70414	-0.70302	-0.70192	-0.70085	-0.69978

-0.64206	-0.64109	-0.64014	-0.63919	-0.70467	-0.70354	-0.70244	-0.70137	-0.70031
-0.64255	-0.64158	-0.6406	-0.63963	-0.70519	-0.70406	-0.70296	-0.70189	-0.70083
-0.64304	-0.64205	-0.64107	-0.64008	-0.63912	-0.63818	-0.70348	-0.70241	-0.70135
-0.64352	-0.64252	-0.64153	-0.64055	-0.63959	-0.63865	-0.70403	-0.70294	-0.70186
-0.644	-0.643	-0.64201	-0.64103	-0.64007	-0.63912	-0.70459	-0.70348	-0.70238
-0.64449	-0.64348	-0.6425	-0.64152	-0.64055	-0.63958	-0.67544	-0.67436	-0.70291
-0.64499	-0.64398	-0.643	-0.64203	-0.64104	-0.64005	-0.67596	-0.67487	-0.70344
-0.6455	-0.64449	-0.6435	-0.64252	-0.64153	-0.64054	-0.63956	-0.63861	-0.67433
-0.64603	-0.64501	-0.64401	-0.64301	-0.64202	-0.64103	-0.64006	-0.63909	-0.67487
-0.60636	-0.60553	-0.64451	-0.64351	-0.64251	-0.64153	-0.64056	-0.63958	-0.63862
-0.6068	-0.60596	-0.64502	-0.64401	-0.64301	-0.64204	-0.64105	-0.64007	-0.6391
-0.60725	-0.60639	-0.64553	-0.64452	-0.64352	-0.64254	-0.64156	-0.64056	-0.63959
-0.57518	-0.57439	-0.60599	-0.60517	-0.64403	-0.64305	-0.64206	-0.64107	-0.6401
-0.57557	-0.57479	-0.60644	-0.60561	-0.64455	-0.64356	-0.64258	-0.64159	-0.64061
-0.57597	-0.5752	-0.57443	-0.57368	-0.64507	-0.64408	-0.64309	-0.6421	-0.64112
-0.57639	-0.57562	-0.57484	-0.57407	-0.64561	-0.64461	-0.64361	-0.64261	-0.64162
-0.5768	-0.57603	-0.57525	-0.57448	-0.57372	-0.57299	-0.64413	-0.64313	-0.64214
-0.5772	-0.57643	-0.57567	-0.57491	-0.57414	-0.57338	-0.64465	-0.64365	-0.64266
-0.50612	-0.50551	-0.57608	-0.57533	-0.57456	-0.57378	-0.57302	-0.57228	-0.64318
-0.50645	-0.50584	-0.5765	-0.57573	-0.57497	-0.5742	-0.57344	-0.57269	-0.64371
-0.50678	-0.50618	-0.57692	-0.57613	-0.57537	-0.57463	-0.57387	-0.57309	-0.64424
-0.50712	-0.5065	-0.50588	-0.50527	-0.57579	-0.57504	-0.57428	-0.57351	-0.64478
-0.50746	-0.50682	-0.5062	-0.5056	-0.57622	-0.57545	-0.57468	-0.57393	-0.64533
-0.50782	-0.50716	-0.50654	-0.50593	-0.53943	-0.53872	-0.5751	-0.57435	-0.57359
-0.50818	-0.50752	-0.50688	-0.50626	-0.5398	-0.5391	-0.57553	-0.57476	-0.57399
-0.50855	-0.50788	-0.50723	-0.50659	-0.50598	-0.50538	-0.53878	-0.53806	-0.5744
-0.50893	-0.50825	-0.50759	-0.50694	-0.50631	-0.5057	-0.53914	-0.53844	-0.57484
-0.50932	-0.50862	-0.50795	-0.5073	-0.50666	-0.50603	-0.50542	-0.50482	-0.53812
-0.50971	-0.50901	-0.50832	-0.50765	-0.507	-0.50637	-0.50575	-0.50515	-0.53849
-0.5101	-0.5094	-0.5087	-0.50801	-0.50735	-0.50672	-0.5061	-0.50548	-0.53885
-0.43913	-0.43854	-0.50908	-0.50839	-0.50771	-0.50706	-0.50643	-0.50581	-0.53924
-0.43945	-0.43885	-0.50947	-0.50878	-0.50809	-0.50741	-0.50676	-0.50615	-0.53963
-0.43979	-0.43917	-0.43859	-0.43804	-0.50848	-0.50779	-0.50712	-0.5065	-0.50589
-0.44015	-0.43951	-0.43893	-0.43836	-0.50889	-0.50819	-0.50752	-0.50687	-0.50625
-0.4	-0.39942	-0.43926	-0.43868	-0.43811	-0.43755	-0.50791	-0.50725	-0.5066
-0.40029	-0.39973	-0.4396	-0.439	-0.43842	-0.43786	-0.50829	-0.50761	-0.50694
-0.40059	-0.40002	-0.43994	-0.43931	-0.43872	-0.43816	-0.50868	-0.50798	-0.5073
-0.40088	-0.40031	-0.39974	-0.39917	-0.43904	-0.43847	-0.4379	-0.43734	-0.50768
-0.40116	-0.40059	-0.40002	-0.39945	-0.43937	-0.43878	-0.4382	-0.43765	-0.50808
-0.40144	-0.40088	-0.40031	-0.39975	-0.3992	-0.39867	-0.43852	-0.43797	-0.43743
-0.40173	-0.40117	-0.40062	-0.40006	-0.39951	-0.39895	-0.43885	-0.43829	-0.43774
-0.40202	-0.40147	-0.40092	-0.40037	-0.39981	-0.39924	-0.39871	-0.39821	-0.39772
-0.40233	-0.40177	-0.40121	-0.40065	-0.4001	-0.39954	-0.399	-0.39849	-0.39799
-0.40264	-0.40206	-0.4015	-0.40094	-0.40039	-0.39984	-0.3993	-0.39877	-0.39826
-0.40293	-0.40235	-0.40179	-0.40124	-0.40068	-0.40013	-0.39959	-0.39904	-0.39851
-0.40322	-0.40264	-0.40208	-0.40154	-0.40098	-0.40043	-0.39988	-0.39932	-0.39877
-0.40354	-0.40295	-0.40239	-0.40184	-0.40129	-0.40074	-0.40019	-0.39962	-0.39907
-0.40386	-0.40328	-0.40271	-0.40215	-0.40159	-0.40105	-0.4005	-0.39993	-0.39938
-0.40417	-0.40359	-0.40302	-0.40245	-0.40189	-0.40135	-0.40079	-0.40024	-0.39968
-0.40448	-0.4039	-0.40332	-0.40275	-0.40219	-0.40163	-0.40108	-0.40053	-0.39998

-0.38398	-0.38348	-0.40363	-0.40306	-0.40249	-0.40193	-0.40137	-0.40083	-0.40028
-0.38427	-0.38375	-0.40395	-0.40338	-0.40281	-0.40224	-0.40168	-0.40113	-0.40058
-0.38455	-0.38403	-0.38353	-0.38304	-0.40313	-0.40255	-0.40199	-0.40143	-0.40089
-0.38483	-0.38431	-0.38381	-0.38331	-0.40343	-0.40285	-0.40229	-0.40174	-0.40119
-0.38512	-0.3846	-0.38408	-0.38357	-0.40372	-0.40315	-0.40259	-0.40204	-0.4015
-0.38541	-0.38489	-0.38437	-0.38385	-0.38334	-0.38284	-0.4029	-0.40235	-0.4018
-0.3857	-0.38519	-0.38467	-0.38414	-0.38363	-0.38312	-0.40321	-0.40265	-0.4021
-0.38598	-0.38548	-0.38495	-0.38443	-0.38391	-0.38341	-0.38291	-0.38242	-0.4024
-0.38625	-0.38575	-0.38523	-0.38471	-0.3842	-0.38369	-0.38319	-0.3827	-0.40271
-0.38654	-0.38602	-0.38551	-0.385	-0.38449	-0.38398	-0.38348	-0.38298	-0.38249
-0.38683	-0.38631	-0.3858	-0.38529	-0.38478	-0.38428	-0.38377	-0.38328	-0.38278
-0.38714	-0.3866	-0.38609	-0.38558	-0.38508	-0.38457	-0.38407	-0.38357	-0.38307
-0.38744	-0.3869	-0.38639	-0.38588	-0.38538	-0.38488	-0.38437	-0.38387	-0.38336
-0.38776	-0.38721	-0.38669	-0.38618	-0.38568	-0.38518	-0.38468	-0.38416	-0.38365
-0.38808	-0.38753	-0.387	-0.38648	-0.38597	-0.38548	-0.38498	-0.38446	-0.38394
-0.38842	-0.38786	-0.38731	-0.38678	-0.38627	-0.38578	-0.38528	-0.38476	-0.38424
-0.38875	-0.38818	-0.38763	-0.38709	-0.38657	-0.38608	-0.38558	-0.38506	-0.38455
-0.38908	-0.3885	-0.38794	-0.3874	-0.38688	-0.38638	-0.38588	-0.38537	-0.38485
-0.38942	-0.38883	-0.38826	-0.38771	-0.38719	-0.38668	-0.38618	-0.38567	-0.38516
-0.38976	-0.38917	-0.38859	-0.38803	-0.38749	-0.38698	-0.38647	-0.38598	-0.38547
-0.39012	-0.38952	-0.38892	-0.38835	-0.3878	-0.38728	-0.38677	-0.38628	-0.38578
-0.3905	-0.38987	-0.38926	-0.38868	-0.38812	-0.3876	-0.38708	-0.38658	-0.38608
-0.39091	-0.39024	-0.3896	-0.38901	-0.38845	-0.38792	-0.38739	-0.38688	-0.38638
-0.34942	-0.34874	-0.38996	-0.38936	-0.38878	-0.38824	-0.38771	-0.38719	-0.38668
-0.3499	-0.34912	-0.39034	-0.38971	-0.38912	-0.38856	-0.38802	-0.3875	-0.38699
-0.35043	-0.34957	-0.34886	-0.34826	-0.38946	-0.38889	-0.38834	-0.38781	-0.38729
-0.351	-0.35009	-0.34931	-0.34862	-0.38982	-0.38923	-0.38866	-0.38812	-0.3876
-0.35159	-0.35065	-0.34979	-0.34902	-0.34836	-0.34781	-0.389	-0.38844	-0.38791
-0.35219	-0.35124	-0.35032	-0.34946	-0.34873	-0.34813	-0.38934	-0.38877	-0.38823
-0.35279	-0.35185	-0.35089	-0.34994	-0.34915	-0.34849	-0.3897	-0.38911	-0.38856
-0.35339	-0.35245	-0.35147	-0.35052	-0.34967	-0.34892	-0.34825	-0.34769	-0.3889
-0.35398	-0.35303	-0.35208	-0.35114	-0.35025	-0.34939	-0.34865	-0.34803	-0.38925
-0.35457	-0.35362	-0.35267	-0.35175	-0.35082	-0.3499	-0.3491	-0.34842	-0.34782
-0.35517	-0.35419	-0.35325	-0.35233	-0.35139	-0.35045	-0.3496	-0.34886	-0.34819
-0.35578	-0.35479	-0.35384	-0.35291	-0.35196	-0.35102	-0.35013	-0.34932	-0.34859
-0.3564	-0.3554	-0.35443	-0.35348	-0.35254	-0.3516	-0.3507	-0.3498	-0.349
-0.35705	-0.35603	-0.35503	-0.35406	-0.35311	-0.35219	-0.35127	-0.35032	-0.34946
-0.3577	-0.35665	-0.35564	-0.35465	-0.35369	-0.35277	-0.35183	-0.35088	-0.34999
-0.35838	-0.3573	-0.35626	-0.35526	-0.35429	-0.35334	-0.3524	-0.35147	-0.35057
-0.3591	-0.35801	-0.35694	-0.3559	-0.35489	-0.35392	-0.35297	-0.35204	-0.35113
-0.35987	-0.35877	-0.35766	-0.35656	-0.35552	-0.35453	-0.35356	-0.35262	-0.35171
-0.31244	-0.31162	-0.35838	-0.35726	-0.3562	-0.35517	-0.35419	-0.35324	-0.35231
-0.31297	-0.31216	-0.35912	-0.35801	-0.35693	-0.35587	-0.35486	-0.35389	-0.35295
-0.27455	-0.27382	-0.35984	-0.35874	-0.35765	-0.35657	-0.35553	-0.35454	-0.35358
-0.275	-0.27428	-0.36054	-0.35944	-0.35834	-0.35724	-0.35618	-0.35517	-0.35419
-0.27545	-0.27473	-0.36122	-0.36013	-0.35903	-0.35791	-0.35683	-0.3558	-0.3548
-0.2759	-0.27518	-0.27446	-0.27374	-0.35972	-0.35861	-0.35753	-0.35646	-0.35543
-0.27635	-0.27564	-0.27492	-0.27419	-0.36041	-0.35932	-0.35824	-0.35715	-0.35609
-0.27684	-0.27611	-0.27539	-0.27468	-0.27396	-0.27325	-0.35898	-0.35788	-0.3568
-0.27735	-0.27661	-0.27589	-0.27518	-0.27447	-0.27376	-0.35974	-0.35865	-0.35757

-0.27786	-0.27711	-0.27638	-0.27567	-0.27496	-0.27425	-0.27353	-0.27281	-0.35831
-0.27837	-0.2776	-0.27686	-0.27614	-0.27543	-0.27473	-0.27401	-0.27329	-0.35902
-0.27888	-0.2781	-0.27734	-0.27661	-0.2759	-0.2752	-0.2745	-0.27378	-0.35974
-0.2794	-0.2786	-0.27783	-0.27708	-0.27637	-0.27568	-0.27499	-0.27429	-0.36049
-0.27993	-0.27913	-0.27834	-0.27757	-0.27685	-0.27617	-0.27548	-0.27479	-0.36125
-0.2805	-0.27969	-0.27889	-0.2781	-0.27736	-0.27666	-0.27596	-0.27527	-0.27457
-0.28108	-0.28028	-0.27946	-0.27865	-0.27789	-0.27715	-0.27643	-0.27574	-0.27504
-0.28165	-0.28084	-0.28003	-0.27921	-0.27842	-0.27765	-0.27692	-0.27622	-0.27553
-0.2822	-0.28139	-0.28058	-0.27977	-0.27896	-0.27816	-0.2774	-0.27671	-0.27602
-0.28275	-0.28195	-0.28115	-0.28033	-0.27952	-0.27869	-0.27792	-0.27721	-0.27651
-0.28333	-0.28254	-0.28173	-0.28092	-0.2801	-0.27927	-0.27848	-0.27773	-0.27699
-0.2839	-0.28312	-0.28232	-0.2815	-0.28068	-0.27986	-0.27905	-0.27826	-0.27749
-0.28444	-0.28366	-0.28287	-0.28207	-0.28125	-0.28042	-0.27961	-0.27881	-0.27804
-0.28497	-0.28419	-0.28341	-0.28263	-0.28182	-0.28099	-0.28017	-0.27938	-0.2786
-0.28552	-0.28474	-0.28397	-0.28319	-0.28239	-0.28157	-0.28076	-0.27996	-0.27916
-0.28608	-0.28531	-0.28453	-0.28376	-0.28297	-0.28217	-0.28136	-0.28054	-0.27972
-0.28664	-0.28586	-0.28508	-0.2843	-0.28352	-0.28274	-0.28194	-0.28112	-0.2803
-0.2872	-0.28638	-0.2856	-0.28483	-0.28406	-0.28328	-0.28249	-0.2817	-0.2809
-0.28779	-0.28694	-0.28614	-0.28537	-0.28459	-0.28382	-0.28304	-0.28228	-0.28149
-0.28842	-0.28754	-0.28671	-0.28591	-0.28513	-0.28437	-0.28361	-0.28284	-0.28205
-0.28906	-0.28815	-0.28729	-0.28646	-0.28568	-0.28492	-0.28416	-0.28339	-0.2826
-0.2897	-0.28877	-0.28788	-0.28701	-0.28621	-0.28546	-0.28471	-0.28395	-0.28317
-0.29034	-0.2894	-0.28848	-0.28757	-0.28675	-0.286	-0.28525	-0.2845	-0.28374
-0.291	-0.29006	-0.28912	-0.28819	-0.28734	-0.28655	-0.28577	-0.28502	-0.28427
-0.29166	-0.29073	-0.28979	-0.28885	-0.28796	-0.28711	-0.28629	-0.28553	-0.28478
-0.24109	-0.24034	-0.29045	-0.28951	-0.28859	-0.2877	-0.28685	-0.28606	-0.2853
-0.24158	-0.24083	-0.2911	-0.29018	-0.28926	-0.28833	-0.28746	-0.28663	-0.28584
-0.24209	-0.24134	-0.29174	-0.29084	-0.28992	-0.28897	-0.28808	-0.28722	-0.2864
-0.24263	-0.24188	-0.29239	-0.29147	-0.29055	-0.28961	-0.2887	-0.28781	-0.28697
-0.24318	-0.24243	-0.29303	-0.29211	-0.29118	-0.29025	-0.28933	-0.28842	-0.28755
-0.2437	-0.24297	-0.24222	-0.24145	-0.29185	-0.29091	-0.28997	-0.28905	-0.28816
-0.24421	-0.2435	-0.24276	-0.24201	-0.29254	-0.29158	-0.29064	-0.2897	-0.28879
-0.09753	-0.09724	-0.24328	-0.24255	-0.2418	-0.24102	-0.29132	-0.29037	-0.28944
-0.09774	-0.09743	-0.24379	-0.24308	-0.24234	-0.24158	-0.29203	-0.29107	-0.29012
-0.04469	-0.04453	-0.24431	-0.24361	-0.24288	-0.24213	-0.24137	-0.24059	-0.29081
-0.04478	-0.04462	-0.24485	-0.24411	-0.24338	-0.24266	-0.24192	-0.24116	-0.29151
-0.04487	-0.04471	-0.04455	-0.04438	-0.2439	-0.24319	-0.24246	-0.24171	-0.24096
-0.04495	-0.04479	-0.04462	-0.04446	-0.24444	-0.24371	-0.24298	-0.24225	-0.24151
-0.04504	-0.04487	-0.0447	-0.04455	-0.245	-0.24424	-0.2435	-0.24278	-0.24206
-0.04514	-0.04496	-0.0448	-0.04464	-0.04448	-0.04432	-0.24404	-0.24331	-0.24259
-0.04524	-0.04506	-0.0449	-0.04474	-0.04457	-0.0444	-0.24459	-0.24385	-0.24311
-0.04534	-0.04517	-0.045	-0.04483	-0.04466	-0.04449	-0.04433	-0.04418	-0.24365
-0.04544	-0.04527	-0.0451	-0.04492	-0.04475	-0.04459	-0.04443	-0.04426	-0.24421
		-0.0452	-0.04502	-0.04485	-0.04469	-0.04453	-0.04435	-0.24477
		-0.04529	-0.04512	-0.04495	-0.04478	-0.04461	-0.04444	-0.24534
		-0.04538	-0.04522	-0.04505	-0.04487	-0.04469	-0.04454	-0.24592
				-0.04514	-0.04497	-0.0448	-0.04463	-0.04447
				-0.04523	-0.04507	-0.0449	-0.04473	-0.04457
				-0.04533	-0.04516	-0.045	-0.04484	-0.04467
				-0.04542	-0.04525	-0.04509	-0.04494	-0.04477

-0.04519	-0.04503	-0.04488
-0.04528	-0.04513	-0.04497
		-0.04507
		-0.04515
		-0.04523

						-0.1091	-0.10882	-0.10854
						-0.10885	-0.10855	-0.10826
				-0.10915	-0.10887	-0.25543	-0.25464	-0.25386
				-0.10889	-0.10862	-0.25484	-0.25405	-0.25327
				-0.10864	-0.10836	-0.25425	-0.25347	-0.25269
				-0.16198	-0.16151	-0.33983	-0.33862	-0.33742
				-0.16158	-0.16112	-0.33899	-0.33778	-0.3366
		-0.10846	-0.10816	-0.29958	-0.29856	-0.43208	-0.43064	-0.42922
		-0.10821	-0.1079	-0.29879	-0.29777	-0.43109	-0.42967	-0.42828
-0.10853	-0.10825	-0.30015	-0.29905	-0.33875	-0.33764	-0.47629	-0.47479	-0.47332
-0.1083	-0.108	-0.29936	-0.29826	-0.33792	-0.33682	-0.47524	-0.47377	-0.47232
-0.30082	-0.2997	-0.33947	-0.33825	-0.47705	-0.47563	-0.47421	-0.47275	-0.47132
-0.30003	-0.29892	-0.33864	-0.33743	-0.47601	-0.47461	-0.4732	-0.47175	-0.47033
-0.29925	-0.29814	-0.33783	-0.33664	-0.475	-0.4736	-0.4722	-0.47076	-0.46935
-0.33943	-0.33821	-0.47695	-0.47549	-0.47405	-0.47264	-0.51027	-0.50867	-0.50712

-0.3386	-0.3374	-0.47594	-0.47454	-0.47312	-0.47169	-0.50919	-0.5076	-0.50607
-0.38591	-0.38463	-0.47493	-0.47357	-0.51129	-0.50969	-0.64123	-0.6392	-0.63725
-0.38503	-0.38377	-0.47392	-0.47258	-0.5102	-0.50862	-0.63981	-0.63786	-0.63599
-0.47578	-0.47432	-0.47294	-0.47161	-0.55677	-0.5551	-0.67904	-0.67706	-0.67515
-0.47478	-0.47336	-0.47198	-0.47064	-0.5556	-0.55397	-0.67762	-0.67573	-0.67388
-0.47381	-0.47242	-0.55767	-0.55606	-0.63958	-0.63764	-0.75	-0.74802	-0.74607
-0.47287	-0.47152	-0.55657	-0.55494	-0.6382	-0.63632	-0.74851	-0.74657	-0.74463
-0.47195	-0.47062	-0.55547	-0.55384	-0.63686	-0.63502	-0.74705	-0.74511	-0.74318
-0.5099	-0.50843	-0.55436	-0.55275	-0.7497	-0.74762	-0.7854	-0.78337	-0.78135
-0.50885	-0.50737	-0.55324	-0.55167	-0.74826	-0.74619	-0.78389	-0.78186	-0.77982
-0.55528	-0.5537	-0.7123	-0.71028	-0.78665	-0.78452	-0.93294	-0.93001	-0.9271
-0.55422	-0.55263	-0.71087	-0.70885	-0.78511	-0.78304	-0.93091	-0.92797	-0.92502
-0.59064	-0.58896	-0.78787	-0.78569	-0.89398	-0.8913	-1.03503	-1.03179	-1.02856
-0.58949	-0.58781	-0.78634	-0.78417	-0.89199	-0.88933	-1.03272	-1.02947	-1.02625
-0.58833	-0.58668	-0.78482	-0.78268	-0.89004	-0.88737	-1.03042	-1.02717	-1.02397
-0.78762	-0.78545	-0.78332	-0.78121	-1.03452	-1.03133	-1.06181	-1.0585	-1.05527
-0.78609	-0.78395	-0.78184	-0.77976	-1.03234	-1.02909	-1.05952	-1.05626	-1.05309
-0.78459	-0.78247	-1.03652	-1.03332	-1.06385	-1.06053	-1.05728	-1.05411	-1.05103
-0.7831	-0.78101	-1.03429	-1.03105	-1.06155	-1.05827	-1.05509	-1.05204	-1.04907
-1.07249	-1.0692	-1.06588	-1.06257	-1.0593	-1.05608	-1.11933	-1.11623	-1.11319
-1.07019	-1.06689	-1.06359	-1.0603	-1.0571	-1.05398	-1.1172	-1.11412	-1.11106
-1.0679	-1.06461	-1.06133	-1.05809	-1.12134	-1.11819	-1.11508	-1.11199	-1.10893
-1.06563	-1.06235	-1.05912	-1.05596	-1.11916	-1.11604	-1.11295	-1.10986	-1.10678
-1.06338	-1.06013	-1.05696	-1.05387	-1.11702	-1.11391	-1.11082	-1.10772	-1.10464
-1.06117	-1.05798	-1.12119	-1.11802	-1.11488	-1.11177	-1.2271	-1.22374	-1.22042
-1.05901	-1.05588	-1.11903	-1.11588	-1.11275	-1.10963	-1.22476	-1.22142	-1.21814
-1.12325	-1.12003	-1.11687	-1.11374	-1.17045	-1.16717	-1.22243	-1.21915	-1.21593
-1.12106	-1.11786	-1.11471	-1.11161	-1.16821	-1.16493	-1.22012	-1.21693	-1.21378
-1.17905	-1.17573	-1.17247	-1.16925	-1.22457	-1.22118	-1.21789	-1.21473	-1.21163
-1.17678	-1.17348	-1.17025	-1.16708	-1.22234	-1.21902	-1.21575	-1.21257	-1.20945
-1.17455	-1.17127	-1.22667	-1.22339	-1.25007	-1.24675	-1.27341	-1.2701	-1.26684
-1.1724	-1.16914	-1.22443	-1.22111	-1.24775	-1.24449	-1.27115	-1.26786	-1.26461
-1.17027	-1.16701	-1.22219	-1.21883	-1.24544	-1.24224	-1.26891	-1.26564	-1.26242
-1.16807	-1.1648	-1.24985	-1.24646	-1.27314	-1.26993	-1.29588	-1.29258	-1.28932
-1.16584	-1.16257	-1.24752	-1.2442	-1.27089	-1.26774	-1.2937	-1.29045	-1.28722
-1.25207	-1.24865	-1.24531	-1.24204	-1.26871	-1.26557	-1.32273	-1.31942	-1.31611
-1.24984	-1.2465	-1.24322	-1.23997	-1.26661	-1.26341	-1.32044	-1.31716	-1.31387
-1.24761	-1.24434	-1.271	-1.26773	-1.29359	-1.29031	-1.36839	-1.36481	-1.36125
-1.24536	-1.24213	-1.26878	-1.26554	-1.29138	-1.28811	-1.36599	-1.36243	-1.35888
-1.24316	-1.23996	-1.26659	-1.26336	-1.28919	-1.28593	-1.36361	-1.36005	-1.3565
-1.27094	-1.26771	-1.26448	-1.26128	-1.36859	-1.3649	-1.37884	-1.37514	-1.37147
-1.26882	-1.26561	-1.2624	-1.25921	-1.36628	-1.36257	-1.3764	-1.37264	-1.36894
-1.26665	-1.26345	-1.34357	-1.33993	-1.38151	-1.3777	-1.37391	-1.37015	-1.36647
-1.26446	-1.26127	-1.34114	-1.33752	-1.379	-1.37517	-1.3714	-1.36767	-1.36404
-1.26233	-1.25914	-1.38411	-1.38032	-1.37653	-1.37272	-1.36897	-1.36529	-1.36172
-1.26027	-1.25707	-1.38171	-1.3779	-1.37411	-1.37033	-1.36663	-1.36302	-1.35952
-1.35956	-1.35582	-1.37927	-1.37544	-1.37167	-1.36794	-1.3643	-1.36076	-1.35731
-1.35706	-1.3533	-1.37671	-1.37289	-1.36916	-1.36551	-1.36194	-1.35842	-1.35503
-1.35455	-1.35078	-1.37417	-1.37038	-1.3667	-1.36313	-1.35963	-1.35615	-1.35283
-1.37935	-1.37552	-1.37175	-1.36802	-1.36441	-1.3609	-1.37585	-1.3724	-1.36914

-1.37691	-1.3731	-1.36938	-1.36574	-1.36219	-1.35874	-1.37374	-1.37038	-1.36725
-1.37442	-1.37064	-1.36698	-1.36341	-1.37832	-1.37492	-1.4234	-1.42011	-1.41706
-1.37191	-1.36817	-1.36457	-1.36107	-1.37603	-1.37271	-1.42128	-1.41828	-1.41549
-1.38792	-1.38422	-1.38063	-1.37715	-1.42564	-1.42234	-1.41927	-1.4165	-1.41393
-1.38562	-1.38202	-1.37844	-1.37493	-1.42343	-1.42029	-1.41739	-1.41477	-1.41234
-1.38335	-1.37984	-1.40175	-1.39821	-1.42134	-1.41838	-1.41564	-1.41316	-1.41083
-1.38111	-1.37763	-1.39959	-1.39616	-1.41941	-1.41664	-1.41407	-1.41171	-1.40948
-1.37888	-1.37543	-1.39747	-1.39422	-1.41761	-1.41502	-1.4126	-1.41034	-1.40819
-1.40202	-1.39858	-1.42176	-1.4187	-1.41593	-1.41347	-1.46063	-1.45834	-1.45615
-1.39967	-1.39633	-1.41969	-1.41694	-1.41437	-1.412	-1.45918	-1.45694	-1.45477
-1.42394	-1.42072	-1.41786	-1.41531	-1.46245	-1.46008	-1.45781	-1.45562	-1.45349
-1.42193	-1.41895	-1.41626	-1.41381	-1.461	-1.45872	-1.45652	-1.45438	-1.45229
-1.42004	-1.41728	-1.46439	-1.46195	-1.45961	-1.45739	-1.48204	-1.47987	-1.47775
-1.41827	-1.4157	-1.46289	-1.46055	-1.45828	-1.4561	-1.48071	-1.47858	-1.47648
-1.46641	-1.46386	-1.46146	-1.4592	-1.48382	-1.48157	-1.53493	-1.53249	-1.53008
-1.46485	-1.4624	-1.46007	-1.45785	-1.48245	-1.48022	-1.53335	-1.53089	-1.52848
-1.46336	-1.46099	-1.45871	-1.45652	-1.48109	-1.47888	-1.53177	-1.5293	-1.52688
-1.46185	-1.45954	-1.48414	-1.48193	-1.47973	-1.47755	-1.53024	-1.52778	-1.52537
-1.46034	-1.45809	-1.48269	-1.48053	-1.47838	-1.47624	-1.52875	-1.52631	-1.52391
-1.48579	-1.48352	-1.48132	-1.47919	-1.53211	-1.52967	-1.52726	-1.52485	-1.52248
-1.48442	-1.48218	-1.48001	-1.4779	-1.53061	-1.52817	-1.52577	-1.5234	-1.52107
-1.48308	-1.48088	-1.53399	-1.53156	-1.52913	-1.52669	-1.52429	-1.52195	-1.51965
-1.48176	-1.47961	-1.53251	-1.53008	-1.52765	-1.52523	-1.52285	-1.52051	-1.51821
-1.48046	-1.47835	-1.53104	-1.5286	-1.52618	-1.52378	-1.52141	-1.51907	-1.51677
-1.47918	-1.47708	-1.52957	-1.52713	-1.52472	-1.52234	-1.51997	-1.51761	-1.5153
-1.47792	-1.47582	-1.52809	-1.52565	-1.52325	-1.52088	-1.51853	-1.51615	-1.51384
-1.53146	-1.52903	-1.5266	-1.52418	-1.52178	-1.5194	-1.54119	-1.53863	-1.53615
-1.52996	-1.52753	-1.52511	-1.52272	-1.52031	-1.51789	-1.53953	-1.53707	-1.53472
-1.52847	-1.52604	-1.52363	-1.52123	-1.51883	-1.51642	-1.55961	-1.55722	-1.55493
-1.52699	-1.52457	-1.52215	-1.51972	-1.51734	-1.51498	-1.55807	-1.55578	-1.55358
-1.52551	-1.5231	-1.52067	-1.51824	-1.56157	-1.55902	-1.55661	-1.5544	-1.55225
-1.52401	-1.5216	-1.51919	-1.5168	-1.56003	-1.55759	-1.55526	-1.55306	-1.55092
-1.52254	-1.52011	-1.51773	-1.51541	-1.55854	-1.55622	-1.55396	-1.55176	-1.54959
-1.54319	-1.54073	-1.56204	-1.5595	-1.5571	-1.55486	-1.55265	-1.55045	-1.54828
-1.54178	-1.53932	-1.56053	-1.55805	-1.55571	-1.55351	-1.55133	-1.54914	-1.54698
-1.54037	-1.53793	-1.55905	-1.55666	-1.59246	-1.5902	-1.62362	-1.62133	-1.61906
-1.53898	-1.53656	-1.5576	-1.55531	-1.59111	-1.58883	-1.62222	-1.61995	-1.61772
-1.59952	-1.59682	-1.59436	-1.59205	-1.62546	-1.62312	-1.66248	-1.66014	-1.65785
-1.59801	-1.59541	-1.59298	-1.59067	-1.62405	-1.62172	-1.66101	-1.65871	-1.65646
-1.63241	-1.62983	-1.66925	-1.66678	-1.66433	-1.66191	-1.65956	-1.6573	-1.65507
-1.63092	-1.6284	-1.66776	-1.66528	-1.66286	-1.66048	-1.65816	-1.65592	-1.65369
-1.62946	-1.62698	-1.66628	-1.66379	-1.66139	-1.65907	-1.65679	-1.65455	-1.65231
-1.66989	-1.66733	-1.6648	-1.66232	-1.65994	-1.65766	-1.66671	-1.66449	-1.66227
-1.66842	-1.66585	-1.66334	-1.66087	-1.65851	-1.65626	-1.66533	-1.66312	-1.66092
-1.66695	-1.66441	-1.66191	-1.65947	-1.6684	-1.66616	-1.70975	-1.70724	-1.70474
-1.66549	-1.66299	-1.66053	-1.65811	-1.66705	-1.66479	-1.70819	-1.70571	-1.70323
-1.66403	-1.66158	-1.65915	-1.65675	-1.66569	-1.66342	-1.70665	-1.70419	-1.70174
-1.66256	-1.66015	-1.65775	-1.65537	-1.66432	-1.66205	-1.70512	-1.70268	-1.70026
-1.66109	-1.65873	-1.65636	-1.65399	-1.66295	-1.66069	-1.70361	-1.70119	-1.69878
-1.65964	-1.65733	-1.66623	-1.66387	-1.70713	-1.70459	-1.70211	-1.6997	-1.6973

-1.6582	-1.65594	-1.66486	-1.66251	-1.7056	-1.70309	-1.70063	-1.69821	-1.69582
-1.66803	-1.66579	-1.70936	-1.70672	-1.70414	-1.70163	-1.69917	-1.69674	-1.69436
-1.66665	-1.66441	-1.70785	-1.70528	-1.70274	-1.70021	-1.69772	-1.69529	-1.6929
-1.66527	-1.66302	-1.70632	-1.70382	-1.70131	-1.69877	-1.69628	-1.69384	-1.69147
-1.6639	-1.66163	-1.70477	-1.70233	-1.69985	-1.69733	-1.69485	-1.69242	-1.69005
-1.70832	-1.70572	-1.70324	-1.70083	-1.69839	-1.69589	-1.70502	-1.70244	-1.69994
-1.70684	-1.70423	-1.70175	-1.69936	-1.69694	-1.69449	-1.70356	-1.70101	-1.69852
-1.70538	-1.70276	-1.70028	-1.69789	-1.69549	-1.69309	-1.70211	-1.6996	-1.69713
-1.70389	-1.70129	-1.69882	-1.69643	-1.69405	-1.69169	-1.71819	-1.71567	-1.71319
-1.70238	-1.69984	-1.69738	-1.69498	-1.69261	-1.6903	-1.71673	-1.71423	-1.71175
-1.70093	-1.69845	-1.71377	-1.71132	-1.72019	-1.71772	-1.71526	-1.71279	-1.71034
-1.69951	-1.69711	-1.71241	-1.70992	-1.71869	-1.71623	-1.71379	-1.71137	-1.70896
-1.71588	-1.71349	-1.71103	-1.70855	-1.71723	-1.71476	-1.72701	-1.72459	-1.72218
-1.7144	-1.71201	-1.70961	-1.70722	-1.71584	-1.71333	-1.72554	-1.72312	-1.72071
-1.71295	-1.71055	-1.7342	-1.7317	-1.72916	-1.72659	-1.72408	-1.72165	-1.71926
-1.71156	-1.70913	-1.7327	-1.73022	-1.72769	-1.72511	-1.7226	-1.72019	-1.71783
-1.71019	-1.70775	-1.73121	-1.72873	-1.7262	-1.72364	-1.72114	-1.71875	-1.71642
-1.72378	-1.72135	-1.72976	-1.72725	-1.72473	-1.72222	-1.71977	-1.71739	-1.71506
-1.72235	-1.72001	-1.72833	-1.72577	-1.72327	-1.72084	-1.71845	-1.71607	-1.71373
-1.73186	-1.72941	-1.72686	-1.72429	-1.72181	-1.71944	-1.76282	-1.76014	-1.7575
-1.73035	-1.72789	-1.72535	-1.7228	-1.72035	-1.71802	-1.76126	-1.75866	-1.75607
-1.72887	-1.72639	-1.72387	-1.72136	-1.76494	-1.7623	-1.75971	-1.75715	-1.7546
-1.72742	-1.72491	-1.72244	-1.72	-1.76343	-1.76077	-1.75815	-1.75559	-1.75304
-1.726	-1.72348	-1.72104	-1.71866	-1.76195	-1.75926	-1.75661	-1.75404	-1.75148
-1.72459	-1.72209	-1.71967	-1.71731	-1.76044	-1.75777	-1.75513	-1.75253	-1.74995
-1.72318	-1.72074	-1.71834	-1.71598	-1.75893	-1.75629	-1.75366	-1.75105	-1.74845
-1.7218	-1.71938	-1.76278	-1.7601	-1.75744	-1.75479	-1.75217	-1.74957	-1.747
-1.72044	-1.71802	-1.76127	-1.75863	-1.75597	-1.75329	-1.75066	-1.74811	-1.74558
-1.71908	-1.71669	-1.75976	-1.75712	-1.75446	-1.75179	-1.74918	-1.74664	-1.74414
-1.71773	-1.71538	-1.75827	-1.75558	-1.75293	-1.75032	-1.74774	-1.74518	-1.74265
-1.76212	-1.75947	-1.75677	-1.75405	-1.75141	-1.74884	-1.74629	-1.74371	-1.74117
-1.7606	-1.75792	-1.75524	-1.75257	-1.74994	-1.74736	-1.7448	-1.74221	-1.73968
-1.75908	-1.75637	-1.75371	-1.75109	-1.74848	-1.74588	-1.74331	-1.74073	-1.73822
-1.75755	-1.75485	-1.75221	-1.74961	-1.747	-1.74439	-1.74182	-1.73931	-1.73685
-1.756	-1.75334	-1.75072	-1.74813	-1.74552	-1.7429	-1.74035	-1.73792	-1.73553
-1.75447	-1.75183	-1.74923	-1.74666	-1.74408	-1.74148	-1.73896	-1.73655	-1.73419
-1.75296	-1.75031	-1.74774	-1.74522	-1.74268	-1.74013	-1.73763	-1.73521	-1.73283
-1.75146	-1.74883	-1.74628	-1.74377	-1.74126	-1.73874	-1.75996	-1.75736	-1.7548
-1.74997	-1.74741	-1.74485	-1.74231	-1.7398	-1.73731	-1.75845	-1.75589	-1.75335
-1.74849	-1.74598	-1.74343	-1.74088	-1.76221	-1.75956	-1.77439	-1.77179	-1.7692
-1.74704	-1.74451	-1.74199	-1.73948	-1.76074	-1.75812	-1.77292	-1.77029	-1.76768
-1.7456	-1.74304	-1.74055	-1.7381	-1.7593	-1.75669	-1.77147	-1.76879	-1.76615
-1.74415	-1.74161	-1.73913	-1.7367	-1.78084	-1.77823	-1.7756	-1.77293	-1.77029
-1.7427	-1.7402	-1.73773	-1.7353	-1.77933	-1.77675	-1.77416	-1.77151	-1.76887
-1.74128	-1.73879	-1.7657	-1.76311	-1.79309	-1.79049	-1.78789	-1.78526	-1.78264
-1.73988	-1.73738	-1.76421	-1.76166	-1.79159	-1.78896	-1.78635	-1.78377	-1.78122
-1.76014	-1.75763	-1.79536	-1.79275	-1.79012	-1.78746	-1.8001	-1.79753	-1.795
-1.75879	-1.7563	-1.7939	-1.7913	-1.78868	-1.78602	-1.79865	-1.79606	-1.79351
-1.75746	-1.75499	-1.79245	-1.78985	-1.78723	-1.78459	-1.79722	-1.79461	-1.79205
-1.77919	-1.77655	-1.79099	-1.78834	-1.78573	-1.78314	-1.81216	-1.80937	-1.80661

-1.77777	-1.77513	-1.78951	-1.78682	-1.78421	-1.78169	-1.81072	-1.80796	-1.80521
-1.79343	-1.79075	-1.78805	-1.78536	-1.81469	-1.81195	-1.86113	-1.85793	-1.85477
-1.79192	-1.78927	-1.7866	-1.78393	-1.81321	-1.81043	-1.85933	-1.85621	-1.85312
-1.79041	-1.78777	-1.81747	-1.81459	-1.81175	-1.80896	-1.85759	-1.8545	-1.85145
-1.78891	-1.78625	-1.81592	-1.81312	-1.81033	-1.80754	-1.85588	-1.85279	-1.84974
-1.82009	-1.81719	-1.81438	-1.81164	-1.86064	-1.8574	-1.8673	-1.86417	-1.8611
-1.81859	-1.81569	-1.81288	-1.81012	-1.85888	-1.85572	-1.86566	-1.86253	-1.85945
-1.81711	-1.81423	-1.8114	-1.80862	-1.85713	-1.85404	-1.86403	-1.86091	-1.85783
-1.81565	-1.81279	-1.87495	-1.8717	-1.86852	-1.86542	-1.86235	-1.85929	-1.85625
-1.8142	-1.81138	-1.87328	-1.87002	-1.86683	-1.86372	-1.86066	-1.85767	-1.85471
-1.82642	-1.82364	-1.87162	-1.86838	-1.87763	-1.87452	-1.87147	-1.86853	-1.86561
-1.82491	-1.8222	-1.86999	-1.86677	-1.87604	-1.87292	-1.86987	-1.86695	-1.86405
-1.88706	-1.88394	-1.88077	-1.87759	-1.87445	-1.87133	-1.86828	-1.86536	-1.86247
-1.88537	-1.88227	-1.87914	-1.87599	-1.87287	-1.86975	-1.8667	-1.86375	-1.86086
-1.88369	-1.88061	-1.8775	-1.87439	-1.87129	-1.86819	-1.88099	-1.87795	-1.87497
-1.88199	-1.87891	-1.87583	-1.87277	-1.86971	-1.86665	-1.87943	-1.87638	-1.87338
-1.88031	-1.87722	-1.87417	-1.87116	-1.86815	-1.86512	-1.87789	-1.87483	-1.87182
-1.87868	-1.87558	-1.87255	-1.86958	-1.8666	-1.8636	-1.87635	-1.8733	-1.87028
-1.87709	-1.87398	-1.87097	-1.86803	-1.86507	-1.8621	-1.87482	-1.87178	-1.86877
-1.8755	-1.87239	-1.8694	-1.86648	-1.87933	-1.87631	-1.87329	-1.87027	-1.86726
-1.87391	-1.87082	-1.86784	-1.86494	-1.87777	-1.87478	-1.87177	-1.86875	-1.86575
-1.87233	-1.86926	-1.86629	-1.86341	-1.87622	-1.87324	-1.87025	-1.86724	-1.86425
-1.87075	-1.86769	-1.86475	-1.8619	-1.87468	-1.87172	-1.86874	-1.86575	-1.86278
-1.86917	-1.86614	-1.86322	-1.86038	-1.87314	-1.87019	-1.86723	-1.86426	-1.86131
-1.86761	-1.8646	-1.86169	-1.85887	-1.8716	-1.86866	-1.86571	-1.86277	-1.85984
-1.86606	-1.86307	-1.86018	-1.85736	-1.87006	-1.86713	-1.8642	-1.86127	-1.85837
-1.86451	-1.86155	-1.87431	-1.87143	-1.86853	-1.8656	-1.86269	-1.85979	-1.8569
-1.86296	-1.86003	-1.87276	-1.86989	-1.867	-1.86409	-1.86119	-1.8583	-1.85543
-1.86143	-1.85853	-1.87124	-1.86837	-1.86549	-1.86259	-1.88865	-1.88573	-1.88284
-1.85993	-1.85704	-1.86973	-1.86687	-1.86399	-1.86109	-1.88713	-1.88422	-1.88135
-1.87408	-1.87115	-1.86825	-1.86538	-1.89144	-1.88851	-1.8856	-1.88271	-1.87986
-1.87261	-1.86971	-1.86681	-1.86392	-1.88993	-1.88698	-1.88407	-1.8812	-1.87837
-1.87116	-1.86828	-1.86537	-1.86246	-1.88842	-1.88546	-1.88255	-1.87969	-1.87689
-1.86971	-1.86682	-1.89281	-1.88985	-1.8869	-1.88396	-1.88106	-1.87822	-1.87544
-1.86826	-1.86535	-1.89129	-1.88833	-1.88539	-1.88246	-1.87959	-1.87677	-1.874
-1.89569	-1.89273	-1.88976	-1.8868	-1.88386	-1.88095	-1.8781	-1.87532	-1.87259
-1.89417	-1.89119	-1.88821	-1.88525	-1.88233	-1.87943	-1.87661	-1.87387	-1.87119
-1.89263	-1.88965	-1.88667	-1.88372	-1.88081	-1.87794	-1.87515	-1.87245	-1.86979
-1.89109	-1.8881	-1.88514	-1.88219	-1.8793	-1.87648	-1.87372	-1.87104	-1.86839
-1.88955	-1.88657	-1.88361	-1.88068	-1.87783	-1.87504	-1.87231	-1.86965	-1.86701
-1.888	-1.88503	-1.8821	-1.87919	-1.87637	-1.87362	-1.87093	-1.86827	-1.86564
-1.88647	-1.88352	-1.88061	-1.87773	-1.87494	-1.87224	-1.86957	-1.86692	-1.86429
-1.88496	-1.88203	-1.87915	-1.87632	-1.87357	-1.87091	-1.86827	-1.86563	-1.86301
-1.88348	-1.88058	-1.87773	-1.87494	-1.87223	-1.86961	-1.867	-1.86437	-1.86176
-1.88207	-1.87919	-1.87637	-1.87362	-1.87092	-1.8683	-1.86568	-1.86306	-1.86045
-1.88072	-1.87786	-1.87507	-1.87234	-1.86964	-1.86698	-1.86434	-1.86171	-1.8591
-1.87934	-1.8765	-1.87373	-1.87102	-1.86832	-1.86564	-1.86298	-1.86035	-1.85775
-1.87791	-1.87509	-1.87234	-1.86964	-1.86695	-1.86427	-1.86162	-1.85899	-1.8564
-1.87649	-1.87369	-1.87095	-1.86825	-1.86557	-1.8629	-1.86026	-1.85765	-1.85507
-1.87509	-1.87231	-1.86957	-1.86687	-1.86419	-1.86154	-1.85892	-1.85633	-1.85378

-1.87371	-1.87095	-1.86821	-1.86551	-1.86284	-1.86021	-1.85761	-1.85504	-1.85251
-1.8724	-1.86966	-1.86693	-1.86422	-1.86155	-1.85894	-1.8685	-1.86595	-1.86344
-1.87114	-1.86841	-1.86569	-1.86298	-1.86032	-1.85771	-1.86728	-1.86475	-1.86226
-1.86982	-1.86709	-1.87652	-1.87383	-1.87118	-1.86858	-1.86602	-1.8635	-1.86103
-1.86842	-1.8657	-1.87514	-1.87248	-1.86985	-1.86727	-1.86472	-1.86222	-1.85975
-1.87918	-1.87645	-1.87377	-1.87113	-1.86853	-1.86596	-1.88454	-1.88202	-1.87953
-1.87785	-1.87509	-1.8724	-1.86978	-1.8672	-1.86466	-1.88325	-1.88075	-1.87828
-1.87653	-1.87375	-1.87107	-1.86846	-1.86589	-1.86338	-1.88199	-1.87951	-1.87705
-1.87521	-1.87247	-1.86981	-1.86721	-1.88584	-1.88329	-1.89149	-1.88898	-1.88651
-1.8739	-1.87121	-1.86858	-1.86599	-1.88464	-1.8821	-1.89028	-1.88779	-1.88534
-1.87259	-1.86992	-1.88859	-1.88598	-1.89414	-1.89158	-1.88905	-1.88657	-1.88411
-1.87129	-1.86862	-1.8873	-1.8847	-1.89286	-1.89032	-1.88781	-1.88532	-1.88286
-1.89137	-1.88867	-1.8968	-1.89419	-1.89162	-1.8891	-1.8866	-1.88412	-1.88166
-1.89008	-1.88742	-1.89556	-1.89298	-1.89043	-1.88792	-1.88543	-1.88297	-1.88052
-1.89959	-1.89693	-1.89432	-1.89175	-1.88922	-1.88672	-1.88425	-1.8818	-1.87937
-1.89832	-1.89565	-1.89304	-1.89048	-1.88796	-1.88547	-1.88301	-1.88057	-1.87814
-1.89706	-1.89439	-1.89178	-1.88922	-1.88671	-1.88423	-1.88177	-1.87934	-1.8769
-1.89581	-1.89317	-1.89057	-1.88803	-1.88552	-1.88305	-1.88059	-1.87815	-1.87572
-1.89458	-1.89197	-1.8894	-1.88686	-1.88436	-1.88189	-1.87944	-1.87699	-1.87454
-1.89336	-1.89078	-1.88821	-1.88567	-1.88316	-1.8807	-1.87824	-1.87579	-1.87334
-1.89216	-1.88959	-1.88702	-1.88445	-1.88194	-1.87947	-1.87701	-1.87456	-1.87211
-1.89093	-1.88838	-1.88581	-1.88325	-1.88074	-1.87827	-1.87581	-1.87335	-1.87089
-1.88967	-1.88713	-1.88459	-1.88208	-1.87959	-1.87711	-1.87465	-1.87218	-1.86972
-1.88843	-1.88589	-1.88339	-1.88091	-1.87844	-1.87596	-1.87349	-1.87101	-1.86854
-1.88723	-1.88471	-1.88222	-1.87975	-1.87728	-1.87481	-1.87233	-1.86982	-1.86732
-1.88606	-1.88355	-1.88106	-1.87859	-1.87612	-1.87365	-1.87115	-1.86863	-1.8661
-1.88489	-1.88237	-1.87987	-1.87738	-1.87489	-1.87242	-1.86994	-1.86743	-1.86492
-1.88371	-1.88119	-1.87866	-1.87613	-1.87363	-1.87116	-1.86869	-1.86622	-1.86375
-1.88252	-1.87999	-1.87745	-1.87492	-1.87241	-1.86995	-1.86749	-1.86503	-1.86257
-1.88131	-1.87877	-1.87624	-1.87372	-1.87123	-1.86877	-1.86631	-1.86385	-1.86139
-1.8801	-1.87756	-1.87504	-1.87254	-1.87006	-1.8676	-1.86514	-1.86266	-1.86019
-1.87888	-1.87636	-1.87387	-1.87139	-1.86892	-1.86645	-1.86397	-1.86146	-1.85896
-1.87768	-1.87518	-1.8727	-1.87024	-1.86777	-1.86529	-1.86279	-1.86026	-1.85773
-1.87649	-1.87401	-1.87154	-1.86908	-1.86659	-1.86409	-1.86158	-1.85906	-1.85655
-1.87532	-1.87285	-1.87037	-1.8679	-1.8654	-1.86287	-1.86036	-1.85786	-1.85539
-1.87416	-1.87169	-1.86921	-1.86671	-1.8642	-1.86168	-1.85917	-1.85669	-1.85423
-1.87303	-1.87055	-1.86804	-1.86551	-1.86299	-1.86049	-1.858	-1.85554	-1.85308
-1.87189	-1.86941	-1.86688	-1.86433	-1.86181	-1.85932	-1.85685	-1.85439	-1.85194
-1.87075	-1.86827	-1.86574	-1.86318	-1.86066	-1.85818	-1.85571	-1.85326	-1.8508
-1.8696	-1.86711	-1.86458	-1.86203	-1.85951	-1.85704	-1.85458	-1.85213	-1.84966
-1.86841	-1.8659	-1.86337	-1.86084	-1.85835	-1.85589	-1.85343	-1.85098	-1.84851
-1.86723	-1.86468	-1.86216	-1.85966	-1.85718	-1.85473	-1.85228	-1.84982	-1.84735
-1.86606	-1.8635	-1.86098	-1.85849	-1.85602	-1.85357	-1.85112	-1.84863	-1.84614
-1.86489	-1.86233	-1.85981	-1.85733	-1.85486	-1.85241	-1.84994	-1.84743	-1.84491
-1.86373	-1.86115	-1.85864	-1.85616	-1.8537	-1.85125	-1.84878	-1.84625	-1.84372
-1.86256	-1.85998	-1.85747	-1.855	-1.85253	-1.85009	-1.84761	-1.84508	-1.84255
-1.86139	-1.85881	-1.8563	-1.85383	-1.85137	-1.84893	-1.84645	-1.84391	-1.84138
-1.86023	-1.85765	-1.85514	-1.85267	-1.85021	-1.84777	-1.8453	-1.84276	-1.84022
-1.85907	-1.85649	-1.85398	-1.85151	-1.84905	-1.84661	-1.84414	-1.8416	-1.83907
-1.8579	-1.85533	-1.85282	-1.85034	-1.84788	-1.84544	-1.84297	-1.84044	-1.83791

-1.85672	-1.85416	-1.85165	-1.84918	-1.84672	-1.84428	-1.84181	-1.83928	-1.83675
-1.85555	-1.853	-1.85049	-1.84802	-1.85817	-1.85572	-1.85324	-1.8507	-1.84816
-1.85439	-1.85183	-1.84933	-1.84686	-1.857	-1.85455	-1.85206	-1.84952	-1.84698
-1.85322	-1.85067	-1.86076	-1.85829	-1.85583	-1.85337	-1.85089	-1.84834	-1.84581
-1.85206	-1.84951	-1.85959	-1.85712	-1.85465	-1.8522	-1.84971	-1.84717	-1.84463
-1.86349	-1.86093	-1.85842	-1.85595	-1.85348	-1.85102	-1.84854	-1.84599	-1.84345
-1.86233	-1.85976	-1.85725	-1.85477	-1.8523	-1.84984	-1.84736	-1.84481	-1.84227
-1.86118	-1.85862	-1.8561	-1.8536	-1.85112	-1.84867	-1.84618	-1.84363	-1.84109
-1.86004	-1.85751	-1.85498	-1.85245	-1.84995	-1.84749	-1.87102	-1.86845	-1.86588
-1.85891	-1.85641	-1.85386	-1.8513	-1.84879	-1.84632	-1.86983	-1.86727	-1.8647
-1.85776	-1.85527	-1.85272	-1.85015	-1.84762	-1.84515	-1.86865	-1.86611	-1.86357
-1.8566	-1.8541	-1.85156	-1.84898	-1.84646	-1.84398	-1.86747	-1.86497	-1.86246
-1.85543	-1.85293	-1.8504	-1.84785	-1.84532	-1.84283	-1.86629	-1.86381	-1.86131
-1.85425	-1.85175	-1.84924	-1.84674	-1.84422	-1.84169	-1.86511	-1.86262	-1.86012
-1.85309	-1.85058	-1.84809	-1.84562	-1.86908	-1.86649	-1.86393	-1.86143	-1.85892
-1.85198	-1.84943	-1.84693	-1.84446	-1.86791	-1.86532	-1.86276	-1.86024	-1.85772
-1.85086	-1.8483	-1.84578	-1.8433	-1.86673	-1.86416	-1.86159	-1.85906	-1.85652
-1.84974	-1.84719	-1.84467	-1.84215	-1.86557	-1.86302	-1.86047	-1.8579	-1.85534
-1.8486	-1.8461	-1.84357	-1.84101	-1.86441	-1.86189	-1.85935	-1.85676	-1.85416
-1.84746	-1.84497	-1.84243	-1.83986	-1.86324	-1.86073	-1.85819	-1.85558	-1.85298
-1.8463	-1.8438	-1.84126	-1.83871	-1.86207	-1.85954	-1.85699	-1.85439	-1.85179
-1.84517	-1.84265	-1.84012	-1.83758	-1.86092	-1.85837	-1.85581	-1.85322	-1.85063
-1.84408	-1.84154	-1.83901	-1.83649	-1.85981	-1.85723	-1.85465	-1.85208	-1.84951
-1.843	-1.84045	-1.83791	-1.83539	-1.8587	-1.85609	-1.8535	-1.85094	-1.84838
-1.8419	-1.83936	-1.83682	-1.83427	-1.85755	-1.85497	-1.85239	-1.84981	-1.84723
-1.8408	-1.83828	-1.83572	-1.83314	-1.85639	-1.85385	-1.85128	-1.84867	-1.84606
-1.83967	-1.83714	-1.83458	-1.832	-1.85522	-1.85268	-1.85011	-1.8475	-1.84489
-1.83852	-1.83598	-1.83342	-1.83085	-1.85405	-1.85149	-1.84892	-1.84631	-1.84372
-1.8374	-1.83485	-1.83229	-1.82973	-1.8529	-1.85032	-1.84775	-1.84516	-1.84258
-1.83629	-1.83372	-1.83117	-1.82863	-1.85178	-1.84918	-1.84659	-1.84402	-1.84146
-1.83519	-1.83262	-1.85583	-1.85325	-1.85066	-1.84805	-1.84546	-1.8429	-1.84034
-1.83409	-1.83153	-1.85472	-1.85212	-1.84953	-1.84694	-1.84435	-1.84177	-1.83921
-1.833	-1.83045	-1.85361	-1.851	-1.8484	-1.84582	-1.84324	-1.84066	-1.83808
-1.83191	-1.82934	-1.85249	-1.84989	-1.84729	-1.8447	-1.84212	-1.83954	-1.83696
-1.83081	-1.82823	-1.85136	-1.84877	-1.84618	-1.84358	-1.84099	-1.83842	-1.83586
-1.85546	-1.85285	-1.85024	-1.84765	-1.84505	-1.84246	-1.83987	-1.8373	-1.83473
-1.85434	-1.85174	-1.84913	-1.84651	-1.84391	-1.84134	-1.83876	-1.83618	-1.8336
-1.85324	-1.85063	-1.84801	-1.84539	-1.8428	-1.84022	-1.83764	-1.83505	-1.83247
-1.85215	-1.84951	-1.84689	-1.84429	-1.8417	-1.8391	-1.83651	-1.83393	-1.83135
-1.83919	-1.83653	-1.84578	-1.84319	-1.8406	-1.83799	-1.83539	-1.83281	-1.83024
-1.83814	-1.83548	-1.8447	-1.84208	-1.83948	-1.83689	-1.8343	-1.83171	-1.82911
-1.8371	-1.83444	-1.84363	-1.84098	-1.83836	-1.83579	-1.83321	-1.8306	-1.82799
-1.83602	-1.83339	-1.84257	-1.83991	-1.83728	-1.83468	-1.83209	-1.82949	-1.82689
-1.83493	-1.83234	-1.84152	-1.83887	-1.83622	-1.83358	-1.83096	-1.82838	-1.82579
-1.83384	-1.83125	-1.82865	-1.82604	-1.83517	-1.83251	-1.82988	-1.82728	-1.82468
-1.83277	-1.83014	-1.82755	-1.82499	-1.83412	-1.83147	-1.82882	-1.82619	-1.82356
-1.83171	-1.82906	-1.82647	-1.82392	-1.83306	-1.83042	-1.82778	-1.82512	-1.82247
-1.83066	-1.82802	-1.82541	-1.82283	-1.83195	-1.82936	-1.82674	-1.82408	-1.82143
-1.82962	-1.82699	-1.82437	-1.82175	-1.81917	-1.81662	-1.8257	-1.82305	-1.82039
-1.82855	-1.82596	-1.82334	-1.82071	-1.81812	-1.81558	-1.82466	-1.82201	-1.81935

-1.82749	-1.82493	-1.82232	-1.81969	-1.81709	-1.81455	-1.82361	-1.82097	-1.8183
-1.82645	-1.8239	-1.8213	-1.81867	-1.81606	-1.81348	-1.82251	-1.81988	-1.81724
-1.82543	-1.82288	-1.82028	-1.81765	-1.81502	-1.81239	-1.82139	-1.81878	-1.81616
-1.82438	-1.82181	-1.81922	-1.81662	-1.81399	-1.81133	-1.80869	-1.80608	-1.80348
-1.8233	-1.8207	-1.81813	-1.81557	-1.81295	-1.81029	-1.80763	-1.80498	-1.80235
-1.82223	-1.81961	-1.81704	-1.81451	-1.81191	-1.80924	-1.80657	-1.8039	-1.80124
-1.82119	-1.81855	-1.81598	-1.81344	-1.81084	-1.80819	-1.80553	-1.80284	-1.80016
-1.82015	-1.8175	-1.81491	-1.81235	-1.80976	-1.80713	-1.80447	-1.80178	-1.79909
-1.81911	-1.81645	-1.81383	-1.81123	-1.80863	-1.80603	-1.8034	-1.80072	-1.79803
-1.81807	-1.8154	-1.81275	-1.8101	-1.80749	-1.80492	-1.80232	-1.79966	-1.79697
-1.81703	-1.81436	-1.81169	-1.80902	-1.8064	-1.80384	-1.80125	-1.79859	-1.79591
-1.816	-1.81332	-1.81064	-1.80796	-1.80534	-1.80277	-1.80018	-1.79753	-1.79485
-1.81495	-1.81227	-1.80959	-1.80691	-1.80428	-1.80171	-1.79911	-1.79646	-1.79379
-1.81389	-1.81122	-1.80854	-1.80586	-1.80323	-1.80065	-1.79805	-1.7954	-1.79273
-1.81283	-1.81016	-1.80748	-1.8048	-1.80216	-1.79957	-1.79696	-1.79432	-1.79167
-1.81179	-1.80911	-1.80642	-1.80374	-1.80108	-1.79844	-1.79582	-1.79322	-1.7906
-1.81074	-1.80806	-1.80537	-1.80267	-1.79999	-1.79733	-1.79469	-1.79211	-1.78953
-1.8097	-1.80701	-1.80432	-1.80162	-1.79894	-1.79625	-1.79361	-1.79104	-1.78846
-1.80866	-1.80596	-1.80327	-1.80057	-1.79789	-1.79521	-1.79257	-1.78999	-1.7874
-1.80761	-1.80491	-1.80222	-1.79953	-1.79685	-1.7942	-1.79157	-1.78897	-1.78636
-1.80655	-1.80386	-1.80117	-1.79848	-1.79583	-1.79322	-1.7906	-1.78798	-1.78533
-1.80549	-1.8028	-1.80011	-1.79743	-1.7948	-1.79221	-1.78961	-1.78696	-1.7843
-1.80443	-1.80173	-1.79905	-1.79637	-1.79374	-1.79117	-1.78857	-1.78592	-1.78325
-1.80337	-1.80067	-1.79799	-1.79531	-1.79269	-1.79012	-1.78752	-1.78487	-1.78219
-1.80231	-1.79961	-1.79693	-1.79426	-1.79164	-1.78907	-1.78647	-1.78381	-1.78113
-1.80125	-1.79856	-1.79588	-1.79322	-1.7906	-1.78802	-1.78541	-1.78275	-1.78007
-1.8002	-1.79752	-1.79486	-1.79223	-1.7896	-1.787	-1.78437	-1.7817	-1.77901
-1.79916	-1.79649	-1.79386	-1.79126	-1.78863	-1.78599	-1.78333	-1.78065	-1.77795
-1.79814	-1.7955	-1.79288	-1.79027	-1.78763	-1.78496	-1.78228	-1.77959	-1.77688
-1.79715	-1.79454	-1.79191	-1.78926	-1.7866	-1.78391	-1.78122	-1.77852	-1.77582
-1.79617	-1.79356	-1.79091	-1.78824	-1.78556	-1.78286	-1.78016	-1.77746	-1.77476
-1.79519	-1.79255	-1.78988	-1.7872	-1.78451	-1.78181	-1.7791	-1.7764	-1.7737
-1.79421	-1.79153	-1.78884	-1.78616	-1.78346	-1.78076	-1.77805	-1.77535	-1.77267
-1.79319	-1.7905	-1.78781	-1.78512	-1.78242	-1.77971	-1.77701	-1.77434	-1.77168
-1.79217	-1.78947	-1.78678	-1.78409	-1.78139	-1.77868	-1.77599	-1.77334	-1.77069
-1.79114	-1.78844	-1.78576	-1.7831	-1.78041	-1.77771	-1.77501	-1.77235	-1.76968
-1.79012	-1.78742	-1.78476	-1.78214	-1.77947	-1.77678	-1.77407	-1.77136	-1.76865
-1.78914	-1.78644	-1.78379	-1.78116	-1.77849	-1.7758	-1.77309	-1.77036	-1.76762
-1.78822	-1.78551	-1.78283	-1.78016	-1.77747	-1.77477	-1.77206	-1.76933	-1.7666
-1.78728	-1.78456	-1.78186	-1.77915	-1.77645	-1.77374	-1.77103	-1.76831	-1.76559
-1.78628	-1.78356	-1.78085	-1.77813	-1.77541	-1.7727	-1.76999	-1.76732	-1.76464
-1.78529	-1.78257	-1.77985	-1.77713	-1.77441	-1.77168	-1.76898	-1.76633	-1.76367
-1.78434	-1.78163	-1.77891	-1.77619	-1.77346	-1.77073	-1.76802	-1.76534	-1.76267
-1.78341	-1.7807	-1.77799	-1.77527	-1.77254	-1.7698	-1.76707	-1.76436	-1.76165
-1.78243	-1.77972	-1.77701	-1.7743	-1.77157	-1.76882	-1.76608	-1.76335	-1.76062
-1.78142	-1.77872	-1.77601	-1.77329	-1.77056	-1.76781	-1.76507	-1.76233	-1.75959
-1.78042	-1.77774	-1.77503	-1.77232	-1.76958	-1.76684	-1.76409	-1.76135	-1.75861
-1.77944	-1.77679	-1.7741	-1.77139	-1.76866	-1.76591	-1.76317	-1.76042	-1.75768
-1.77848	-1.77584	-1.77316	-1.77045	-1.76772	-1.76497	-1.76222	-1.75948	-1.75674
-1.77752	-1.77486	-1.77216	-1.76945	-1.76671	-1.76397	-1.76122	-1.75847	-1.75573

-1.77658	-1.77388	-1.77116	-1.76844	-1.76571	-1.76296	-1.76021	-1.75746	-1.75472
-1.77566	-1.77294	-1.77021	-1.76749	-1.76475	-1.762	-1.75926	-1.75651	-1.75377
-1.77475	-1.77202	-1.76929	-1.76655	-1.76382	-1.76107	-1.75833	-1.75558	-1.75284
-1.77379	-1.77105	-1.76831	-1.76557	-1.76283	-1.76009	-1.75735	-1.75461	-1.75186
-1.77277	-1.77003	-1.76729	-1.76455	-1.76181	-1.75907	-1.75633	-1.75359	-1.75085
-1.7718	-1.76906	-1.76632	-1.76357	-1.76083	-1.75809	-1.75536	-1.75261	-1.74987
-1.77089	-1.76815	-1.76541	-1.76266	-1.75992	-1.75718	-1.75444	-1.7517	-1.74896
-1.76999	-1.76725	-1.76451	-1.76176	-1.75901	-1.75628	-1.75354	-1.7508	-1.74806
-1.76909	-1.76635	-1.7636	-1.76085	-1.75811	-1.75537	-1.75264	-1.7499	-1.74716
-1.76817	-1.76543	-1.76268	-1.75993	-1.75719	-1.75446	-1.75172	-1.74898	-1.74624
-1.76718	-1.76445	-1.7617	-1.75895	-1.75621	-1.75348	-1.75074	-1.748	-1.74526
-1.76616	-1.76342	-1.76068	-1.75793	-1.75519	-1.75246	-1.74972	-1.74699	-1.74425
-1.74205	-1.73937	-1.75971	-1.75697	-1.75423	-1.7515	-1.74877	-1.74603	-1.74329
-1.74115	-1.73847	-1.7588	-1.75606	-1.75333	-1.7506	-1.74787	-1.74513	-1.74239
-1.74027	-1.73758	-1.75791	-1.75517	-1.75243	-1.74971	-1.74698	-1.74424	-1.7415
-1.73941	-1.73672	-1.75704	-1.75429	-1.75155	-1.74882	-1.74609	-1.74336	-1.74061
-1.73854	-1.73585	-1.75616	-1.75341	-1.75067	-1.74794	-1.7452	-1.74247	-1.73972
-1.73766	-1.73496	-1.73227	-1.72958	-1.74978	-1.74704	-1.7443	-1.74157	-1.73881
-1.73677	-1.73405	-1.73136	-1.72868	-1.74887	-1.74613	-1.74339	-1.74065	-1.73789
-1.73585	-1.73311	-1.7304	-1.72772	-1.74789	-1.74515	-1.74241	-1.73967	-1.73691
-1.7349	-1.73213	-1.7294	-1.72671	-1.74686	-1.74412	-1.74138	-1.73864	-1.73589
-1.73395	-1.73117	-1.72844	-1.72575	-1.72305	-1.72036	-1.7404	-1.73766	-1.73491
-1.73298	-1.73024	-1.72753	-1.72484	-1.72215	-1.71945	-1.73948	-1.73673	-1.73399
-1.73204	-1.72933	-1.72663	-1.72395	-1.72126	-1.71856	-1.73857	-1.73583	-1.73308
-1.73113	-1.72844	-1.72575	-1.72307	-1.72038	-1.71768	-1.73768	-1.73493	-1.73219
-1.73024	-1.72755	-1.72487	-1.72219	-1.7195	-1.71681	-1.73679	-1.73404	-1.7313
-1.72936	-1.72667	-1.72399	-1.72132	-1.71863	-1.71593	-1.7359	-1.73315	-1.73041
-1.72849	-1.7258	-1.72312	-1.72044	-1.71775	-1.71505	-1.73501	-1.73226	-1.72951
-1.72762	-1.72493	-1.72224	-1.71957	-1.71688	-1.71418	-1.71148	-1.70879	-1.70609
-1.72675	-1.72406	-1.72137	-1.71869	-1.716	-1.7133	-1.7106	-1.70791	-1.70521
-1.72589	-1.7232	-1.7205	-1.71781	-1.71512	-1.71242	-1.70972	-1.70703	-1.70433
-1.72504	-1.72233	-1.71963	-1.71694	-1.71424	-1.71154	-1.70884	-1.70614	-1.70345
-1.72421	-1.72147	-1.71876	-1.71607	-1.71337	-1.71066	-1.70796	-1.70526	-1.70257
-1.72342	-1.72064	-1.71791	-1.7152	-1.71249	-1.70978	-1.70708	-1.70438	-1.70169
-1.72264	-1.71983	-1.71707	-1.71434	-1.71162	-1.70891	-1.7062	-1.7035	-1.70081
-1.72184	-1.71905	-1.71627	-1.7135	-1.71076	-1.70803	-1.70532	-1.70262	-1.69993
-1.72104	-1.71828	-1.7155	-1.71269	-1.70992	-1.70717	-1.70444	-1.70174	-1.69905
-1.7202	-1.71747	-1.7147	-1.71189	-1.70911	-1.70633	-1.70358	-1.70087	-1.69818
-1.71933	-1.71661	-1.71387	-1.71112	-1.70833	-1.70552	-1.70274	-1.70001	-1.6973
-1.71845	-1.71575	-1.71303	-1.71031	-1.70754	-1.70472	-1.70192	-1.69917	-1.69644
-1.71757	-1.71487	-1.71216	-1.70945	-1.7067	-1.70393	-1.70115	-1.69837	-1.6956
-1.7167	-1.71398	-1.71128	-1.70857	-1.70585	-1.70312	-1.70037	-1.69757	-1.69478
-1.71582	-1.7131	-1.7104	-1.70769	-1.70498	-1.70227	-1.69954	-1.69677	-1.694
-1.71496	-1.71223	-1.70952	-1.70681	-1.7041	-1.7014	-1.69868	-1.69595	-1.69322
-1.70521	-1.70244	-1.70866	-1.70594	-1.70323	-1.70052	-1.69782	-1.6951	-1.69239
-1.70443	-1.70161	-1.70782	-1.70509	-1.70237	-1.69965	-1.69694	-1.69423	-1.69152
-1.70364	-1.70082	-1.70702	-1.70429	-1.70155	-1.69881	-1.69608	-1.69337	-1.69066
-1.70284	-1.70006	-1.70628	-1.70355	-1.70079	-1.69801	-1.69524	-1.69251	-1.68979
-1.70202	-1.69927	-1.69653	-1.69379	-1.70002	-1.69722	-1.69444	-1.69169	-1.68896
-1.70115	-1.69841	-1.69568	-1.69295	-1.6992	-1.69644	-1.69368	-1.69094	-1.68821

						-0.1091	-0.10882	-0.10854
						-0.10885	-0.10855	-0.10826
				-0.10915	-0.10887	-0.25543	-0.25464	-0.25386
				-0.10889	-0.10862	-0.25484	-0.25405	-0.25327
				-0.10864	-0.10836	-0.25425	-0.25347	-0.25269
				-0.16198	-0.16151	-0.33983	-0.33862	-0.33742
				-0.16158	-0.16112	-0.33899	-0.33778	-0.3366
		-0.10846	-0.10816	-0.29958	-0.29856	-0.43208	-0.43064	-0.42922
		-0.10821	-0.1079	-0.29879	-0.29777	-0.43109	-0.42967	-0.42828
-0.10853	-0.10825	-0.30015	-0.29905	-0.33875	-0.33764	-0.47629	-0.47479	-0.47332
-0.1083	-0.108	-0.29936	-0.29826	-0.33792	-0.33682	-0.47524	-0.47377	-0.47232
-0.30082	-0.2997	-0.33947	-0.33825	-0.47705	-0.47563	-0.47421	-0.47275	-0.47132
-0.30003	-0.29892	-0.33864	-0.33743	-0.47601	-0.47461	-0.4732	-0.47175	-0.47033
-0.29925	-0.29814	-0.33783	-0.33664	-0.475	-0.4736	-0.4722	-0.47076	-0.46935
-0.33943	-0.33821	-0.47695	-0.47549	-0.47405	-0.47264	-0.51027	-0.50867	-0.50712

-0.3386	-0.3374	-0.47594	-0.47454	-0.47312	-0.47169	-0.50919	-0.5076	-0.50607
-0.38591	-0.38463	-0.47493	-0.47357	-0.51129	-0.50969	-0.64123	-0.6392	-0.63725
-0.38503	-0.38377	-0.47392	-0.47258	-0.5102	-0.50862	-0.63981	-0.63786	-0.63599
-0.47578	-0.47432	-0.47294	-0.47161	-0.55677	-0.5551	-0.67904	-0.67706	-0.67515
-0.47478	-0.47336	-0.47198	-0.47064	-0.5556	-0.55397	-0.67762	-0.67573	-0.67388
-0.47381	-0.47242	-0.55767	-0.55606	-0.63958	-0.63764	-0.75	-0.74802	-0.74607
-0.47287	-0.47152	-0.55657	-0.55494	-0.6382	-0.63632	-0.74851	-0.74657	-0.74463
-0.47195	-0.47062	-0.55547	-0.55384	-0.63686	-0.63502	-0.74705	-0.74511	-0.74318
-0.5099	-0.50843	-0.55436	-0.55275	-0.7497	-0.74762	-0.7854	-0.78337	-0.78135
-0.50885	-0.50737	-0.55324	-0.55167	-0.74826	-0.74619	-0.78389	-0.78186	-0.77982
-0.55528	-0.5537	-0.7123	-0.71028	-0.78665	-0.78452	-0.93294	-0.93001	-0.9271
-0.55422	-0.55263	-0.71087	-0.70885	-0.78511	-0.78304	-0.93091	-0.92797	-0.92502
-0.59064	-0.58896	-0.78787	-0.78569	-0.89398	-0.8913	-1.03503	-1.03179	-1.02856
-0.58949	-0.58781	-0.78634	-0.78417	-0.89199	-0.88933	-1.03272	-1.02947	-1.02625
-0.58833	-0.58668	-0.78482	-0.78268	-0.89004	-0.88737	-1.03042	-1.02717	-1.02397
-0.78762	-0.78545	-0.78332	-0.78121	-1.03452	-1.03133	-1.06181	-1.0585	-1.05527
-0.78609	-0.78395	-0.78184	-0.77976	-1.03234	-1.02909	-1.05952	-1.05626	-1.05309
-0.78459	-0.78247	-1.03652	-1.03332	-1.06385	-1.06053	-1.05728	-1.05411	-1.05103
-0.7831	-0.78101	-1.03429	-1.03105	-1.06155	-1.05827	-1.05509	-1.05204	-1.04907
-1.07249	-1.0692	-1.06588	-1.06257	-1.0593	-1.05608	-1.11933	-1.11623	-1.11319
-1.07019	-1.06689	-1.06359	-1.0603	-1.0571	-1.05398	-1.1172	-1.11412	-1.11106
-1.0679	-1.06461	-1.06133	-1.05809	-1.12134	-1.11819	-1.11508	-1.11199	-1.10893
-1.06563	-1.06235	-1.05912	-1.05596	-1.11916	-1.11604	-1.11295	-1.10986	-1.10678
-1.06338	-1.06013	-1.05696	-1.05387	-1.11702	-1.11391	-1.11082	-1.10772	-1.10464
-1.06117	-1.05798	-1.12119	-1.11802	-1.11488	-1.11177	-1.2271	-1.22374	-1.22042
-1.05901	-1.05588	-1.11903	-1.11588	-1.11275	-1.10963	-1.22476	-1.22142	-1.21814
-1.12325	-1.12003	-1.11687	-1.11374	-1.17045	-1.16717	-1.22243	-1.21915	-1.21593
-1.12106	-1.11786	-1.11471	-1.11161	-1.16821	-1.16493	-1.22012	-1.21693	-1.21378
-1.17905	-1.17573	-1.17247	-1.16925	-1.22457	-1.22118	-1.21789	-1.21473	-1.21163
-1.17678	-1.17348	-1.17025	-1.16708	-1.22234	-1.21902	-1.21575	-1.21257	-1.20945
-1.17455	-1.17127	-1.22667	-1.22339	-1.25007	-1.24675	-1.27341	-1.2701	-1.26684
-1.1724	-1.16914	-1.22443	-1.22111	-1.24775	-1.24449	-1.27115	-1.26786	-1.26461
-1.17027	-1.16701	-1.22219	-1.21883	-1.24544	-1.24224	-1.26891	-1.26564	-1.26242
-1.16807	-1.1648	-1.24985	-1.24646	-1.27314	-1.26993	-1.29588	-1.29258	-1.28932
-1.16584	-1.16257	-1.24752	-1.2442	-1.27089	-1.26774	-1.2937	-1.29045	-1.28722
-1.25207	-1.24865	-1.24531	-1.24204	-1.26871	-1.26557	-1.32273	-1.31942	-1.31611
-1.24984	-1.2465	-1.24322	-1.23997	-1.26661	-1.26341	-1.32044	-1.31716	-1.31387
-1.24761	-1.24434	-1.271	-1.26773	-1.29359	-1.29031	-1.36839	-1.36481	-1.36125
-1.24536	-1.24213	-1.26878	-1.26554	-1.29138	-1.28811	-1.36599	-1.36243	-1.35888
-1.24316	-1.23996	-1.26659	-1.26336	-1.28919	-1.28593	-1.36361	-1.36005	-1.3565
-1.27094	-1.26771	-1.26448	-1.26128	-1.36859	-1.3649	-1.37884	-1.37514	-1.37147
-1.26882	-1.26561	-1.2624	-1.25921	-1.36628	-1.36257	-1.3764	-1.37264	-1.36894
-1.26665	-1.26345	-1.34357	-1.33993	-1.38151	-1.3777	-1.37391	-1.37015	-1.36647
-1.26446	-1.26127	-1.34114	-1.33752	-1.379	-1.37517	-1.3714	-1.36767	-1.36404
-1.26233	-1.25914	-1.38411	-1.38032	-1.37653	-1.37272	-1.36897	-1.36529	-1.36172
-1.26027	-1.25707	-1.38171	-1.3779	-1.37411	-1.37033	-1.36663	-1.36302	-1.35952
-1.35956	-1.35582	-1.37927	-1.37544	-1.37167	-1.36794	-1.3643	-1.36076	-1.35731
-1.35706	-1.3533	-1.37671	-1.37289	-1.36916	-1.36551	-1.36194	-1.35842	-1.35503
-1.35455	-1.35078	-1.37417	-1.37038	-1.3667	-1.36313	-1.35963	-1.35615	-1.35283
-1.37935	-1.37552	-1.37175	-1.36802	-1.36441	-1.3609	-1.37585	-1.3724	-1.36914

-1.37691	-1.3731	-1.36938	-1.36574	-1.36219	-1.35874	-1.37374	-1.37038	-1.36725
-1.37442	-1.37064	-1.36698	-1.36341	-1.37832	-1.37492	-1.4234	-1.42011	-1.41706
-1.37191	-1.36817	-1.36457	-1.36107	-1.37603	-1.37271	-1.42128	-1.41828	-1.41549
-1.38792	-1.38422	-1.38063	-1.37715	-1.42564	-1.42234	-1.41927	-1.4165	-1.41393
-1.38562	-1.38202	-1.37844	-1.37493	-1.42343	-1.42029	-1.41739	-1.41477	-1.41234
-1.38335	-1.37984	-1.40175	-1.39821	-1.42134	-1.41838	-1.41564	-1.41316	-1.41083
-1.38111	-1.37763	-1.39959	-1.39616	-1.41941	-1.41664	-1.41407	-1.41171	-1.40948
-1.37888	-1.37543	-1.39747	-1.39422	-1.41761	-1.41502	-1.4126	-1.41034	-1.40819
-1.40202	-1.39858	-1.42176	-1.4187	-1.41593	-1.41347	-1.46063	-1.45834	-1.45615
-1.39967	-1.39633	-1.41969	-1.41694	-1.41437	-1.412	-1.45918	-1.45694	-1.45477
-1.42394	-1.42072	-1.41786	-1.41531	-1.46245	-1.46008	-1.45781	-1.45562	-1.45349
-1.42193	-1.41895	-1.41626	-1.41381	-1.461	-1.45872	-1.45652	-1.45438	-1.45229
-1.42004	-1.41728	-1.46439	-1.46195	-1.45961	-1.45739	-1.48204	-1.47987	-1.47775
-1.41827	-1.4157	-1.46289	-1.46055	-1.45828	-1.4561	-1.48071	-1.47858	-1.47648
-1.46641	-1.46386	-1.46146	-1.4592	-1.48382	-1.48157	-1.53493	-1.53249	-1.53008
-1.46485	-1.4624	-1.46007	-1.45785	-1.48245	-1.48022	-1.53335	-1.53089	-1.52848
-1.46336	-1.46099	-1.45871	-1.45652	-1.48109	-1.47888	-1.53177	-1.5293	-1.52688
-1.46185	-1.45954	-1.48414	-1.48193	-1.47973	-1.47755	-1.53024	-1.52778	-1.52537
-1.46034	-1.45809	-1.48269	-1.48053	-1.47838	-1.47624	-1.52875	-1.52631	-1.52391
-1.48579	-1.48352	-1.48132	-1.47919	-1.53211	-1.52967	-1.52726	-1.52485	-1.52248
-1.48442	-1.48218	-1.48001	-1.4779	-1.53061	-1.52817	-1.52577	-1.5234	-1.52107
-1.48308	-1.48088	-1.53399	-1.53156	-1.52913	-1.52669	-1.52429	-1.52195	-1.51965
-1.48176	-1.47961	-1.53251	-1.53008	-1.52765	-1.52523	-1.52285	-1.52051	-1.51821
-1.48046	-1.47835	-1.53104	-1.5286	-1.52618	-1.52378	-1.52141	-1.51907	-1.51677
-1.47918	-1.47708	-1.52957	-1.52713	-1.52472	-1.52234	-1.51997	-1.51761	-1.5153
-1.47792	-1.47582	-1.52809	-1.52565	-1.52325	-1.52088	-1.51853	-1.51615	-1.51384
-1.53146	-1.52903	-1.5266	-1.52418	-1.52178	-1.5194	-1.54119	-1.53863	-1.53615
-1.52996	-1.52753	-1.52511	-1.52272	-1.52031	-1.51789	-1.53953	-1.53707	-1.53472
-1.52847	-1.52604	-1.52363	-1.52123	-1.51883	-1.51642	-1.55961	-1.55722	-1.55493
-1.52699	-1.52457	-1.52215	-1.51972	-1.51734	-1.51498	-1.55807	-1.55578	-1.55358
-1.52551	-1.5231	-1.52067	-1.51824	-1.56157	-1.55902	-1.55661	-1.5544	-1.55225
-1.52401	-1.5216	-1.51919	-1.5168	-1.56003	-1.55759	-1.55526	-1.55306	-1.55092
-1.52254	-1.52011	-1.51773	-1.51541	-1.55854	-1.55622	-1.55396	-1.55176	-1.54959
-1.54319	-1.54073	-1.56204	-1.5595	-1.5571	-1.55486	-1.55265	-1.55045	-1.54828
-1.54178	-1.53932	-1.56053	-1.55805	-1.55571	-1.55351	-1.55133	-1.54914	-1.54698
-1.54037	-1.53793	-1.55905	-1.55666	-1.59246	-1.5902	-1.62362	-1.62133	-1.61906
-1.53898	-1.53656	-1.5576	-1.55531	-1.59111	-1.58883	-1.62222	-1.61995	-1.61772
-1.59952	-1.59682	-1.59436	-1.59205	-1.62546	-1.62312	-1.66248	-1.66014	-1.65785
-1.59801	-1.59541	-1.59298	-1.59067	-1.62405	-1.62172	-1.66101	-1.65871	-1.65646
-1.63241	-1.62983	-1.66925	-1.66678	-1.66433	-1.66191	-1.65956	-1.6573	-1.65507
-1.63092	-1.6284	-1.66776	-1.66528	-1.66286	-1.66048	-1.65816	-1.65592	-1.65369
-1.62946	-1.62698	-1.66628	-1.66379	-1.66139	-1.65907	-1.65679	-1.65455	-1.65231
-1.66989	-1.66733	-1.6648	-1.66232	-1.65994	-1.65766	-1.66671	-1.66449	-1.66227
-1.66842	-1.66585	-1.66334	-1.66087	-1.65851	-1.65626	-1.66533	-1.66312	-1.66092
-1.66695	-1.66441	-1.66191	-1.65947	-1.6684	-1.66616	-1.70975	-1.70724	-1.70474
-1.66549	-1.66299	-1.66053	-1.65811	-1.66705	-1.66479	-1.70819	-1.70571	-1.70323
-1.66403	-1.66158	-1.65915	-1.65675	-1.66569	-1.66342	-1.70665	-1.70419	-1.70174
-1.66256	-1.66015	-1.65775	-1.65537	-1.66432	-1.66205	-1.70512	-1.70268	-1.70026
-1.66109	-1.65873	-1.65636	-1.65399	-1.66295	-1.66069	-1.70361	-1.70119	-1.69878
-1.65964	-1.65733	-1.66623	-1.66387	-1.70713	-1.70459	-1.70211	-1.6997	-1.6973

-1.6582	-1.65594	-1.66486	-1.66251	-1.7056	-1.70309	-1.70063	-1.69821	-1.69582
-1.66803	-1.66579	-1.70936	-1.70672	-1.70414	-1.70163	-1.69917	-1.69674	-1.69436
-1.66665	-1.66441	-1.70785	-1.70528	-1.70274	-1.70021	-1.69772	-1.69529	-1.6929
-1.66527	-1.66302	-1.70632	-1.70382	-1.70131	-1.69877	-1.69628	-1.69384	-1.69147
-1.6639	-1.66163	-1.70477	-1.70233	-1.69985	-1.69733	-1.69485	-1.69242	-1.69005
-1.70832	-1.70572	-1.70324	-1.70083	-1.69839	-1.69589	-1.70502	-1.70244	-1.69994
-1.70684	-1.70423	-1.70175	-1.69936	-1.69694	-1.69449	-1.70356	-1.70101	-1.69852
-1.70538	-1.70276	-1.70028	-1.69789	-1.69549	-1.69309	-1.70211	-1.6996	-1.69713
-1.70389	-1.70129	-1.69882	-1.69643	-1.69405	-1.69169	-1.71819	-1.71567	-1.71319
-1.70238	-1.69984	-1.69738	-1.69498	-1.69261	-1.6903	-1.71673	-1.71423	-1.71175
-1.70093	-1.69845	-1.71377	-1.71132	-1.72019	-1.71772	-1.71526	-1.71279	-1.71034
-1.69951	-1.69711	-1.71241	-1.70992	-1.71869	-1.71623	-1.71379	-1.71137	-1.70896
-1.71588	-1.71349	-1.71103	-1.70855	-1.71723	-1.71476	-1.72701	-1.72459	-1.72218
-1.7144	-1.71201	-1.70961	-1.70722	-1.71584	-1.71333	-1.72554	-1.72312	-1.72071
-1.71295	-1.71055	-1.7342	-1.7317	-1.72916	-1.72659	-1.72408	-1.72165	-1.71926
-1.71156	-1.70913	-1.7327	-1.73022	-1.72769	-1.72511	-1.7226	-1.72019	-1.71783
-1.71019	-1.70775	-1.73121	-1.72873	-1.7262	-1.72364	-1.72114	-1.71875	-1.71642
-1.72378	-1.72135	-1.72976	-1.72725	-1.72473	-1.72222	-1.71977	-1.71739	-1.71506
-1.72235	-1.72001	-1.72833	-1.72577	-1.72327	-1.72084	-1.71845	-1.71607	-1.71373
-1.73186	-1.72941	-1.72686	-1.72429	-1.72181	-1.71944	-1.76282	-1.76014	-1.7575
-1.73035	-1.72789	-1.72535	-1.7228	-1.72035	-1.71802	-1.76126	-1.75866	-1.75607
-1.72887	-1.72639	-1.72387	-1.72136	-1.76494	-1.7623	-1.75971	-1.75715	-1.7546
-1.72742	-1.72491	-1.72244	-1.72	-1.76343	-1.76077	-1.75815	-1.75559	-1.75304
-1.726	-1.72348	-1.72104	-1.71866	-1.76195	-1.75926	-1.75661	-1.75404	-1.75148
-1.72459	-1.72209	-1.71967	-1.71731	-1.76044	-1.75777	-1.75513	-1.75253	-1.74995
-1.72318	-1.72074	-1.71834	-1.71598	-1.75893	-1.75629	-1.75366	-1.75105	-1.74845
-1.7218	-1.71938	-1.76278	-1.7601	-1.75744	-1.75479	-1.75217	-1.74957	-1.747
-1.72044	-1.71802	-1.76127	-1.75863	-1.75597	-1.75329	-1.75066	-1.74811	-1.74558
-1.71908	-1.71669	-1.75976	-1.75712	-1.75446	-1.75179	-1.74918	-1.74664	-1.74414
-1.71773	-1.71538	-1.75827	-1.75558	-1.75293	-1.75032	-1.74774	-1.74518	-1.74265
-1.76212	-1.75947	-1.75677	-1.75405	-1.75141	-1.74884	-1.74629	-1.74371	-1.74117
-1.7606	-1.75792	-1.75524	-1.75257	-1.74994	-1.74736	-1.7448	-1.74221	-1.73968
-1.75908	-1.75637	-1.75371	-1.75109	-1.74848	-1.74588	-1.74331	-1.74073	-1.73822
-1.75755	-1.75485	-1.75221	-1.74961	-1.747	-1.74439	-1.74182	-1.73931	-1.73685
-1.756	-1.75334	-1.75072	-1.74813	-1.74552	-1.7429	-1.74035	-1.73792	-1.73553
-1.75447	-1.75183	-1.74923	-1.74666	-1.74408	-1.74148	-1.73896	-1.73655	-1.73419
-1.75296	-1.75031	-1.74774	-1.74522	-1.74268	-1.74013	-1.73763	-1.73521	-1.73283
-1.75146	-1.74883	-1.74628	-1.74377	-1.74126	-1.73874	-1.75996	-1.75736	-1.7548
-1.74997	-1.74741	-1.74485	-1.74231	-1.7398	-1.73731	-1.75845	-1.75589	-1.75335
-1.74849	-1.74598	-1.74343	-1.74088	-1.76221	-1.75956	-1.77439	-1.77179	-1.7692
-1.74704	-1.74451	-1.74199	-1.73948	-1.76074	-1.75812	-1.77292	-1.77029	-1.76768
-1.7456	-1.74304	-1.74055	-1.7381	-1.7593	-1.75669	-1.77147	-1.76879	-1.76615
-1.74415	-1.74161	-1.73913	-1.7367	-1.78084	-1.77823	-1.7756	-1.77293	-1.77029
-1.7427	-1.7402	-1.73773	-1.7353	-1.77933	-1.77675	-1.77416	-1.77151	-1.76887
-1.74128	-1.73879	-1.7657	-1.76311	-1.79309	-1.79049	-1.78789	-1.78526	-1.78264
-1.73988	-1.73738	-1.76421	-1.76166	-1.79159	-1.78896	-1.78635	-1.78377	-1.78122
-1.76014	-1.75763	-1.79536	-1.79275	-1.79012	-1.78746	-1.8001	-1.79753	-1.795
-1.75879	-1.7563	-1.7939	-1.7913	-1.78868	-1.78602	-1.79865	-1.79606	-1.79351
-1.75746	-1.75499	-1.79245	-1.78985	-1.78723	-1.78459	-1.79722	-1.79461	-1.79205
-1.77919	-1.77655	-1.79099	-1.78834	-1.78573	-1.78314	-1.81216	-1.80937	-1.80661

-1.77777	-1.77513	-1.78951	-1.78682	-1.78421	-1.78169	-1.81072	-1.80796	-1.80521
-1.79343	-1.79075	-1.78805	-1.78536	-1.81469	-1.81195	-1.86113	-1.85793	-1.85477
-1.79192	-1.78927	-1.7866	-1.78393	-1.81321	-1.81043	-1.85933	-1.85621	-1.85312
-1.79041	-1.78777	-1.81747	-1.81459	-1.81175	-1.80896	-1.85759	-1.8545	-1.85145
-1.78891	-1.78625	-1.81592	-1.81312	-1.81033	-1.80754	-1.85588	-1.85279	-1.84974
-1.82009	-1.81719	-1.81438	-1.81164	-1.86064	-1.8574	-1.8673	-1.86417	-1.8611
-1.81859	-1.81569	-1.81288	-1.81012	-1.85888	-1.85572	-1.86566	-1.86253	-1.85945
-1.81711	-1.81423	-1.8114	-1.80862	-1.85713	-1.85404	-1.86403	-1.86091	-1.85783
-1.81565	-1.81279	-1.87495	-1.8717	-1.86852	-1.86542	-1.86235	-1.85929	-1.85625
-1.8142	-1.81138	-1.87328	-1.87002	-1.86683	-1.86372	-1.86066	-1.85767	-1.85471
-1.82642	-1.82364	-1.87162	-1.86838	-1.87763	-1.87452	-1.87147	-1.86853	-1.86561
-1.82491	-1.8222	-1.86999	-1.86677	-1.87604	-1.87292	-1.86987	-1.86695	-1.86405
-1.88706	-1.88394	-1.88077	-1.87759	-1.87445	-1.87133	-1.86828	-1.86536	-1.86247
-1.88537	-1.88227	-1.87914	-1.87599	-1.87287	-1.86975	-1.8667	-1.86375	-1.86086
-1.88369	-1.88061	-1.8775	-1.87439	-1.87129	-1.86819	-1.88099	-1.87795	-1.87497
-1.88199	-1.87891	-1.87583	-1.87277	-1.86971	-1.86665	-1.87943	-1.87638	-1.87338
-1.88031	-1.87722	-1.87417	-1.87116	-1.86815	-1.86512	-1.87789	-1.87483	-1.87182
-1.87868	-1.87558	-1.87255	-1.86958	-1.8666	-1.8636	-1.87635	-1.8733	-1.87028
-1.87709	-1.87398	-1.87097	-1.86803	-1.86507	-1.8621	-1.87482	-1.87178	-1.86877
-1.8755	-1.87239	-1.8694	-1.86648	-1.87933	-1.87631	-1.87329	-1.87027	-1.86726
-1.87391	-1.87082	-1.86784	-1.86494	-1.87777	-1.87478	-1.87177	-1.86875	-1.86575
-1.87233	-1.86926	-1.86629	-1.86341	-1.87622	-1.87324	-1.87025	-1.86724	-1.86425
-1.87075	-1.86769	-1.86475	-1.8619	-1.87468	-1.87172	-1.86874	-1.86575	-1.86278
-1.86917	-1.86614	-1.86322	-1.86038	-1.87314	-1.87019	-1.86723	-1.86426	-1.86131
-1.86761	-1.8646	-1.86169	-1.85887	-1.8716	-1.86866	-1.86571	-1.86277	-1.85984
-1.86606	-1.86307	-1.86018	-1.85736	-1.87006	-1.86713	-1.8642	-1.86127	-1.85837
-1.86451	-1.86155	-1.87431	-1.87143	-1.86853	-1.8656	-1.86269	-1.85979	-1.8569
-1.86296	-1.86003	-1.87276	-1.86989	-1.867	-1.86409	-1.86119	-1.8583	-1.85543
-1.86143	-1.85853	-1.87124	-1.86837	-1.86549	-1.86259	-1.88865	-1.88573	-1.88284
-1.85993	-1.85704	-1.86973	-1.86687	-1.86399	-1.86109	-1.88713	-1.88422	-1.88135
-1.87408	-1.87115	-1.86825	-1.86538	-1.89144	-1.88851	-1.8856	-1.88271	-1.87986
-1.87261	-1.86971	-1.86681	-1.86392	-1.88993	-1.88698	-1.88407	-1.8812	-1.87837
-1.87116	-1.86828	-1.86537	-1.86246	-1.88842	-1.88546	-1.88255	-1.87969	-1.87689
-1.86971	-1.86682	-1.89281	-1.88985	-1.8869	-1.88396	-1.88106	-1.87822	-1.87544
-1.86826	-1.86535	-1.89129	-1.88833	-1.88539	-1.88246	-1.87959	-1.87677	-1.874
-1.89569	-1.89273	-1.88976	-1.8868	-1.88386	-1.88095	-1.8781	-1.87532	-1.87259
-1.89417	-1.89119	-1.88821	-1.88525	-1.88233	-1.87943	-1.87661	-1.87387	-1.87119
-1.89263	-1.88965	-1.88667	-1.88372	-1.88081	-1.87794	-1.87515	-1.87245	-1.86979
-1.89109	-1.8881	-1.88514	-1.88219	-1.8793	-1.87648	-1.87372	-1.87104	-1.86839
-1.88955	-1.88657	-1.88361	-1.88068	-1.87783	-1.87504	-1.87231	-1.86965	-1.86701
-1.888	-1.88503	-1.8821	-1.87919	-1.87637	-1.87362	-1.87093	-1.86827	-1.86564
-1.88647	-1.88352	-1.88061	-1.87773	-1.87494	-1.87224	-1.86957	-1.86692	-1.86429
-1.88496	-1.88203	-1.87915	-1.87632	-1.87357	-1.87091	-1.86827	-1.86563	-1.86301
-1.88348	-1.88058	-1.87773	-1.87494	-1.87223	-1.86961	-1.867	-1.86437	-1.86176
-1.88207	-1.87919	-1.87637	-1.87362	-1.87092	-1.8683	-1.86568	-1.86306	-1.86045
-1.88072	-1.87786	-1.87507	-1.87234	-1.86964	-1.86698	-1.86434	-1.86171	-1.8591
-1.87934	-1.8765	-1.87373	-1.87102	-1.86832	-1.86564	-1.86298	-1.86035	-1.85775
-1.87791	-1.87509	-1.87234	-1.86964	-1.86695	-1.86427	-1.86162	-1.85899	-1.8564
-1.87649	-1.87369	-1.87095	-1.86825	-1.86557	-1.8629	-1.86026	-1.85765	-1.85507
-1.87509	-1.87231	-1.86957	-1.86687	-1.86419	-1.86154	-1.85892	-1.85633	-1.85378

-1.87371	-1.87095	-1.86821	-1.86551	-1.86284	-1.86021	-1.85761	-1.85504	-1.85251
-1.8724	-1.86966	-1.86693	-1.86422	-1.86155	-1.85894	-1.8685	-1.86595	-1.86344
-1.87114	-1.86841	-1.86569	-1.86298	-1.86032	-1.85771	-1.86728	-1.86475	-1.86226
-1.86982	-1.86709	-1.87652	-1.87383	-1.87118	-1.86858	-1.86602	-1.8635	-1.86103
-1.86842	-1.8657	-1.87514	-1.87248	-1.86985	-1.86727	-1.86472	-1.86222	-1.85975
-1.87918	-1.87645	-1.87377	-1.87113	-1.86853	-1.86596	-1.88454	-1.88202	-1.87953
-1.87785	-1.87509	-1.8724	-1.86978	-1.8672	-1.86466	-1.88325	-1.88075	-1.87828
-1.87653	-1.87375	-1.87107	-1.86846	-1.86589	-1.86338	-1.88199	-1.87951	-1.87705
-1.87521	-1.87247	-1.86981	-1.86721	-1.88584	-1.88329	-1.89149	-1.88898	-1.88651
-1.8739	-1.87121	-1.86858	-1.86599	-1.88464	-1.8821	-1.89028	-1.88779	-1.88534
-1.87259	-1.86992	-1.88859	-1.88598	-1.89414	-1.89158	-1.88905	-1.88657	-1.88411
-1.87129	-1.86862	-1.8873	-1.8847	-1.89286	-1.89032	-1.88781	-1.88532	-1.88286
-1.89137	-1.88867	-1.8968	-1.89419	-1.89162	-1.8891	-1.8866	-1.88412	-1.88166
-1.89008	-1.88742	-1.89556	-1.89298	-1.89043	-1.88792	-1.88543	-1.88297	-1.88052
-1.89959	-1.89693	-1.89432	-1.89175	-1.88922	-1.88672	-1.88425	-1.8818	-1.87937
-1.89832	-1.89565	-1.89304	-1.89048	-1.88796	-1.88547	-1.88301	-1.88057	-1.87814
-1.89706	-1.89439	-1.89178	-1.88922	-1.88671	-1.88423	-1.88177	-1.87934	-1.8769
-1.89581	-1.89317	-1.89057	-1.88803	-1.88552	-1.88305	-1.88059	-1.87815	-1.87572
-1.89458	-1.89197	-1.8894	-1.88686	-1.88436	-1.88189	-1.87944	-1.87699	-1.87454
-1.89336	-1.89078	-1.88821	-1.88567	-1.88316	-1.8807	-1.87824	-1.87579	-1.87334
-1.89216	-1.88959	-1.88702	-1.88445	-1.88194	-1.87947	-1.87701	-1.87456	-1.87211
-1.89093	-1.88838	-1.88581	-1.88325	-1.88074	-1.87827	-1.87581	-1.87335	-1.87089
-1.88967	-1.88713	-1.88459	-1.88208	-1.87959	-1.87711	-1.87465	-1.87218	-1.86972
-1.88843	-1.88589	-1.88339	-1.88091	-1.87844	-1.87596	-1.87349	-1.87101	-1.86854
-1.88723	-1.88471	-1.88222	-1.87975	-1.87728	-1.87481	-1.87233	-1.86982	-1.86732
-1.88606	-1.88355	-1.88106	-1.87859	-1.87612	-1.87365	-1.87115	-1.86863	-1.8661
-1.88489	-1.88237	-1.87987	-1.87738	-1.87489	-1.87242	-1.86994	-1.86743	-1.86492
-1.88371	-1.88119	-1.87866	-1.87613	-1.87363	-1.87116	-1.86869	-1.86622	-1.86375
-1.88252	-1.87999	-1.87745	-1.87492	-1.87241	-1.86995	-1.86749	-1.86503	-1.86257
-1.88131	-1.87877	-1.87624	-1.87372	-1.87123	-1.86877	-1.86631	-1.86385	-1.86139
-1.8801	-1.87756	-1.87504	-1.87254	-1.87006	-1.8676	-1.86514	-1.86266	-1.86019
-1.87888	-1.87636	-1.87387	-1.87139	-1.86892	-1.86645	-1.86397	-1.86146	-1.85896
-1.87768	-1.87518	-1.8727	-1.87024	-1.86777	-1.86529	-1.86279	-1.86026	-1.85773
-1.87649	-1.87401	-1.87154	-1.86908	-1.86659	-1.86409	-1.86158	-1.85906	-1.85655
-1.87532	-1.87285	-1.87037	-1.8679	-1.8654	-1.86287	-1.86036	-1.85786	-1.85539
-1.87416	-1.87169	-1.86921	-1.86671	-1.8642	-1.86168	-1.85917	-1.85669	-1.85423
-1.87303	-1.87055	-1.86804	-1.86551	-1.86299	-1.86049	-1.858	-1.85554	-1.85308
-1.87189	-1.86941	-1.86688	-1.86433	-1.86181	-1.85932	-1.85685	-1.85439	-1.85194
-1.87075	-1.86827	-1.86574	-1.86318	-1.86066	-1.85818	-1.85571	-1.85326	-1.8508
-1.8696	-1.86711	-1.86458	-1.86203	-1.85951	-1.85704	-1.85458	-1.85213	-1.84966
-1.86841	-1.8659	-1.86337	-1.86084	-1.85835	-1.85589	-1.85343	-1.85098	-1.84851
-1.86723	-1.86468	-1.86216	-1.85966	-1.85718	-1.85473	-1.85228	-1.84982	-1.84735
-1.86606	-1.8635	-1.86098	-1.85849	-1.85602	-1.85357	-1.85112	-1.84863	-1.84614
-1.86489	-1.86233	-1.85981	-1.85733	-1.85486	-1.85241	-1.84994	-1.84743	-1.84491
-1.86373	-1.86115	-1.85864	-1.85616	-1.8537	-1.85125	-1.84878	-1.84625	-1.84372
-1.86256	-1.85998	-1.85747	-1.855	-1.85253	-1.85009	-1.84761	-1.84508	-1.84255
-1.86139	-1.85881	-1.8563	-1.85383	-1.85137	-1.84893	-1.84645	-1.84391	-1.84138
-1.86023	-1.85765	-1.85514	-1.85267	-1.85021	-1.84777	-1.8453	-1.84276	-1.84022
-1.85907	-1.85649	-1.85398	-1.85151	-1.84905	-1.84661	-1.84414	-1.8416	-1.83907
-1.8579	-1.85533	-1.85282	-1.85034	-1.84788	-1.84544	-1.84297	-1.84044	-1.83791

-1.85672	-1.85416	-1.85165	-1.84918	-1.84672	-1.84428	-1.84181	-1.83928	-1.83675
-1.85555	-1.853	-1.85049	-1.84802	-1.85817	-1.85572	-1.85324	-1.8507	-1.84816
-1.85439	-1.85183	-1.84933	-1.84686	-1.857	-1.85455	-1.85206	-1.84952	-1.84698
-1.85322	-1.85067	-1.86076	-1.85829	-1.85583	-1.85337	-1.85089	-1.84834	-1.84581
-1.85206	-1.84951	-1.85959	-1.85712	-1.85465	-1.8522	-1.84971	-1.84717	-1.84463
-1.86349	-1.86093	-1.85842	-1.85595	-1.85348	-1.85102	-1.84854	-1.84599	-1.84345
-1.86233	-1.85976	-1.85725	-1.85477	-1.8523	-1.84984	-1.84736	-1.84481	-1.84227
-1.86118	-1.85862	-1.8561	-1.8536	-1.85112	-1.84867	-1.84618	-1.84363	-1.84109
-1.86004	-1.85751	-1.85498	-1.85245	-1.84995	-1.84749	-1.87102	-1.86845	-1.86588
-1.85891	-1.85641	-1.85386	-1.8513	-1.84879	-1.84632	-1.86983	-1.86727	-1.8647
-1.85776	-1.85527	-1.85272	-1.85015	-1.84762	-1.84515	-1.86865	-1.86611	-1.86357
-1.8566	-1.8541	-1.85156	-1.84898	-1.84646	-1.84398	-1.86747	-1.86497	-1.86246
-1.85543	-1.85293	-1.8504	-1.84785	-1.84532	-1.84283	-1.86629	-1.86381	-1.86131
-1.85425	-1.85175	-1.84924	-1.84674	-1.84422	-1.84169	-1.86511	-1.86262	-1.86012
-1.85309	-1.85058	-1.84809	-1.84562	-1.86908	-1.86649	-1.86393	-1.86143	-1.85892
-1.85198	-1.84943	-1.84693	-1.84446	-1.86791	-1.86532	-1.86276	-1.86024	-1.85772
-1.85086	-1.8483	-1.84578	-1.8433	-1.86673	-1.86416	-1.86159	-1.85906	-1.85652
-1.84974	-1.84719	-1.84467	-1.84215	-1.86557	-1.86302	-1.86047	-1.8579	-1.85534
-1.8486	-1.8461	-1.84357	-1.84101	-1.86441	-1.86189	-1.85935	-1.85676	-1.85416
-1.84746	-1.84497	-1.84243	-1.83986	-1.86324	-1.86073	-1.85819	-1.85558	-1.85298
-1.8463	-1.8438	-1.84126	-1.83871	-1.86207	-1.85954	-1.85699	-1.85439	-1.85179
-1.84517	-1.84265	-1.84012	-1.83758	-1.86092	-1.85837	-1.85581	-1.85322	-1.85063
-1.84408	-1.84154	-1.83901	-1.83649	-1.85981	-1.85723	-1.85465	-1.85208	-1.84951
-1.843	-1.84045	-1.83791	-1.83539	-1.8587	-1.85609	-1.8535	-1.85094	-1.84838
-1.8419	-1.83936	-1.83682	-1.83427	-1.85755	-1.85497	-1.85239	-1.84981	-1.84723
-1.8408	-1.83828	-1.83572	-1.83314	-1.85639	-1.85385	-1.85128	-1.84867	-1.84606
-1.83967	-1.83714	-1.83458	-1.832	-1.85522	-1.85268	-1.85011	-1.8475	-1.84489
-1.83852	-1.83598	-1.83342	-1.83085	-1.85405	-1.85149	-1.84892	-1.84631	-1.84372
-1.8374	-1.83485	-1.83229	-1.82973	-1.8529	-1.85032	-1.84775	-1.84516	-1.84258
-1.83629	-1.83372	-1.83117	-1.82863	-1.85178	-1.84918	-1.84659	-1.84402	-1.84146
-1.83519	-1.83262	-1.85583	-1.85325	-1.85066	-1.84805	-1.84546	-1.8429	-1.84034
-1.83409	-1.83153	-1.85472	-1.85212	-1.84953	-1.84694	-1.84435	-1.84177	-1.83921
-1.833	-1.83045	-1.85361	-1.851	-1.8484	-1.84582	-1.84324	-1.84066	-1.83808
-1.83191	-1.82934	-1.85249	-1.84989	-1.84729	-1.8447	-1.84212	-1.83954	-1.83696
-1.83081	-1.82823	-1.85136	-1.84877	-1.84618	-1.84358	-1.84099	-1.83842	-1.83586
-1.85546	-1.85285	-1.85024	-1.84765	-1.84505	-1.84246	-1.83987	-1.8373	-1.83473
-1.85434	-1.85174	-1.84913	-1.84651	-1.84391	-1.84134	-1.83876	-1.83618	-1.8336
-1.85324	-1.85063	-1.84801	-1.84539	-1.8428	-1.84022	-1.83764	-1.83505	-1.83247
-1.85215	-1.84951	-1.84689	-1.84429	-1.8417	-1.8391	-1.83651	-1.83393	-1.83135
-1.83919	-1.83653	-1.84578	-1.84319	-1.8406	-1.83799	-1.83539	-1.83281	-1.83024
-1.83814	-1.83548	-1.8447	-1.84208	-1.83948	-1.83689	-1.8343	-1.83171	-1.82911
-1.8371	-1.83444	-1.84363	-1.84098	-1.83836	-1.83579	-1.83321	-1.8306	-1.82799
-1.83602	-1.83339	-1.84257	-1.83991	-1.83728	-1.83468	-1.83209	-1.82949	-1.82689
-1.83493	-1.83234	-1.84152	-1.83887	-1.83622	-1.83358	-1.83096	-1.82838	-1.82579
-1.83384	-1.83125	-1.82865	-1.82604	-1.83517	-1.83251	-1.82988	-1.82728	-1.82468
-1.83277	-1.83014	-1.82755	-1.82499	-1.83412	-1.83147	-1.82882	-1.82619	-1.82356
-1.83171	-1.82906	-1.82647	-1.82392	-1.83306	-1.83042	-1.82778	-1.82512	-1.82247
-1.83066	-1.82802	-1.82541	-1.82283	-1.83195	-1.82936	-1.82674	-1.82408	-1.82143
-1.82962	-1.82699	-1.82437	-1.82175	-1.81917	-1.81662	-1.8257	-1.82305	-1.82039
-1.82855	-1.82596	-1.82334	-1.82071	-1.81812	-1.81558	-1.82466	-1.82201	-1.81935

-1.82749	-1.82493	-1.82232	-1.81969	-1.81709	-1.81455	-1.82361	-1.82097	-1.8183
-1.82645	-1.8239	-1.8213	-1.81867	-1.81606	-1.81348	-1.82251	-1.81988	-1.81724
-1.82543	-1.82288	-1.82028	-1.81765	-1.81502	-1.81239	-1.82139	-1.81878	-1.81616
-1.82438	-1.82181	-1.81922	-1.81662	-1.81399	-1.81133	-1.80869	-1.80608	-1.80348
-1.8233	-1.8207	-1.81813	-1.81557	-1.81295	-1.81029	-1.80763	-1.80498	-1.80235
-1.82223	-1.81961	-1.81704	-1.81451	-1.81191	-1.80924	-1.80657	-1.8039	-1.80124
-1.82119	-1.81855	-1.81598	-1.81344	-1.81084	-1.80819	-1.80553	-1.80284	-1.80016
-1.82015	-1.8175	-1.81491	-1.81235	-1.80976	-1.80713	-1.80447	-1.80178	-1.79909
-1.81911	-1.81645	-1.81383	-1.81123	-1.80863	-1.80603	-1.8034	-1.80072	-1.79803
-1.81807	-1.8154	-1.81275	-1.8101	-1.80749	-1.80492	-1.80232	-1.79966	-1.79697
-1.81703	-1.81436	-1.81169	-1.80902	-1.8064	-1.80384	-1.80125	-1.79859	-1.79591
-1.816	-1.81332	-1.81064	-1.80796	-1.80534	-1.80277	-1.80018	-1.79753	-1.79485
-1.81495	-1.81227	-1.80959	-1.80691	-1.80428	-1.80171	-1.79911	-1.79646	-1.79379
-1.81389	-1.81122	-1.80854	-1.80586	-1.80323	-1.80065	-1.79805	-1.7954	-1.79273
-1.81283	-1.81016	-1.80748	-1.8048	-1.80216	-1.79957	-1.79696	-1.79432	-1.79167
-1.81179	-1.80911	-1.80642	-1.80374	-1.80108	-1.79844	-1.79582	-1.79322	-1.7906
-1.81074	-1.80806	-1.80537	-1.80267	-1.79999	-1.79733	-1.79469	-1.79211	-1.78953
-1.8097	-1.80701	-1.80432	-1.80162	-1.79894	-1.79625	-1.79361	-1.79104	-1.78846
-1.80866	-1.80596	-1.80327	-1.80057	-1.79789	-1.79521	-1.79257	-1.78999	-1.7874
-1.80761	-1.80491	-1.80222	-1.79953	-1.79685	-1.7942	-1.79157	-1.78897	-1.78636
-1.80655	-1.80386	-1.80117	-1.79848	-1.79583	-1.79322	-1.7906	-1.78798	-1.78533
-1.80549	-1.8028	-1.80011	-1.79743	-1.7948	-1.79221	-1.78961	-1.78696	-1.7843
-1.80443	-1.80173	-1.79905	-1.79637	-1.79374	-1.79117	-1.78857	-1.78592	-1.78325
-1.80337	-1.80067	-1.79799	-1.79531	-1.79269	-1.79012	-1.78752	-1.78487	-1.78219
-1.80231	-1.79961	-1.79693	-1.79426	-1.79164	-1.78907	-1.78647	-1.78381	-1.78113
-1.80125	-1.79856	-1.79588	-1.79322	-1.7906	-1.78802	-1.78541	-1.78275	-1.78007
-1.8002	-1.79752	-1.79486	-1.79223	-1.7896	-1.787	-1.78437	-1.7817	-1.77901
-1.79916	-1.79649	-1.79386	-1.79126	-1.78863	-1.78599	-1.78333	-1.78065	-1.77795
-1.79814	-1.7955	-1.79288	-1.79027	-1.78763	-1.78496	-1.78228	-1.77959	-1.77688
-1.79715	-1.79454	-1.79191	-1.78926	-1.7866	-1.78391	-1.78122	-1.77852	-1.77582
-1.79617	-1.79356	-1.79091	-1.78824	-1.78556	-1.78286	-1.78016	-1.77746	-1.77476
-1.79519	-1.79255	-1.78988	-1.7872	-1.78451	-1.78181	-1.7791	-1.7764	-1.7737
-1.79421	-1.79153	-1.78884	-1.78616	-1.78346	-1.78076	-1.77805	-1.77535	-1.77267
-1.79319	-1.7905	-1.78781	-1.78512	-1.78242	-1.77971	-1.77701	-1.77434	-1.77168
-1.79217	-1.78947	-1.78678	-1.78409	-1.78139	-1.77868	-1.77599	-1.77334	-1.77069
-1.79114	-1.78844	-1.78576	-1.7831	-1.78041	-1.77771	-1.77501	-1.77235	-1.76968
-1.79012	-1.78742	-1.78476	-1.78214	-1.77947	-1.77678	-1.77407	-1.77136	-1.76865
-1.78914	-1.78644	-1.78379	-1.78116	-1.77849	-1.7758	-1.77309	-1.77036	-1.76762
-1.78822	-1.78551	-1.78283	-1.78016	-1.77747	-1.77477	-1.77206	-1.76933	-1.7666
-1.78728	-1.78456	-1.78186	-1.77915	-1.77645	-1.77374	-1.77103	-1.76831	-1.76559
-1.78628	-1.78356	-1.78085	-1.77813	-1.77541	-1.7727	-1.76999	-1.76732	-1.76464
-1.78529	-1.78257	-1.77985	-1.77713	-1.77441	-1.77168	-1.76898	-1.76633	-1.76367
-1.78434	-1.78163	-1.77891	-1.77619	-1.77346	-1.77073	-1.76802	-1.76534	-1.76267
-1.78341	-1.7807	-1.77799	-1.77527	-1.77254	-1.7698	-1.76707	-1.76436	-1.76165
-1.78243	-1.77972	-1.77701	-1.7743	-1.77157	-1.76882	-1.76608	-1.76335	-1.76062
-1.78142	-1.77872	-1.77601	-1.77329	-1.77056	-1.76781	-1.76507	-1.76233	-1.75959
-1.78042	-1.77774	-1.77503	-1.77232	-1.76958	-1.76684	-1.76409	-1.76135	-1.75861
-1.77944	-1.77679	-1.7741	-1.77139	-1.76866	-1.76591	-1.76317	-1.76042	-1.75768
-1.77848	-1.77584	-1.77316	-1.77045	-1.76772	-1.76497	-1.76222	-1.75948	-1.75674
-1.77752	-1.77486	-1.77216	-1.76945	-1.76671	-1.76397	-1.76122	-1.75847	-1.75573

-1.77658	-1.77388	-1.77116	-1.76844	-1.76571	-1.76296	-1.76021	-1.75746	-1.75472
-1.77566	-1.77294	-1.77021	-1.76749	-1.76475	-1.762	-1.75926	-1.75651	-1.75377
-1.77475	-1.77202	-1.76929	-1.76655	-1.76382	-1.76107	-1.75833	-1.75558	-1.75284
-1.77379	-1.77105	-1.76831	-1.76557	-1.76283	-1.76009	-1.75735	-1.75461	-1.75186
-1.77277	-1.77003	-1.76729	-1.76455	-1.76181	-1.75907	-1.75633	-1.75359	-1.75085
-1.7718	-1.76906	-1.76632	-1.76357	-1.76083	-1.75809	-1.75536	-1.75261	-1.74987
-1.77089	-1.76815	-1.76541	-1.76266	-1.75992	-1.75718	-1.75444	-1.7517	-1.74896
-1.76999	-1.76725	-1.76451	-1.76176	-1.75901	-1.75628	-1.75354	-1.7508	-1.74806
-1.76909	-1.76635	-1.7636	-1.76085	-1.75811	-1.75537	-1.75264	-1.7499	-1.74716
-1.76817	-1.76543	-1.76268	-1.75993	-1.75719	-1.75446	-1.75172	-1.74898	-1.74624
-1.76718	-1.76445	-1.7617	-1.75895	-1.75621	-1.75348	-1.75074	-1.748	-1.74526
-1.76616	-1.76342	-1.76068	-1.75793	-1.75519	-1.75246	-1.74972	-1.74699	-1.74425
-1.74205	-1.73937	-1.75971	-1.75697	-1.75423	-1.7515	-1.74877	-1.74603	-1.74329
-1.74115	-1.73847	-1.7588	-1.75606	-1.75333	-1.7506	-1.74787	-1.74513	-1.74239
-1.74027	-1.73758	-1.75791	-1.75517	-1.75243	-1.74971	-1.74698	-1.74424	-1.7415
-1.73941	-1.73672	-1.75704	-1.75429	-1.75155	-1.74882	-1.74609	-1.74336	-1.74061
-1.73854	-1.73585	-1.75616	-1.75341	-1.75067	-1.74794	-1.7452	-1.74247	-1.73972
-1.73766	-1.73496	-1.73227	-1.72958	-1.74978	-1.74704	-1.7443	-1.74157	-1.73881
-1.73677	-1.73405	-1.73136	-1.72868	-1.74887	-1.74613	-1.74339	-1.74065	-1.73789
-1.73585	-1.73311	-1.7304	-1.72772	-1.74789	-1.74515	-1.74241	-1.73967	-1.73691
-1.7349	-1.73213	-1.7294	-1.72671	-1.74686	-1.74412	-1.74138	-1.73864	-1.73589
-1.73395	-1.73117	-1.72844	-1.72575	-1.72305	-1.72036	-1.7404	-1.73766	-1.73491
-1.73298	-1.73024	-1.72753	-1.72484	-1.72215	-1.71945	-1.73948	-1.73673	-1.73399
-1.73204	-1.72933	-1.72663	-1.72395	-1.72126	-1.71856	-1.73857	-1.73583	-1.73308
-1.73113	-1.72844	-1.72575	-1.72307	-1.72038	-1.71768	-1.73768	-1.73493	-1.73219
-1.73024	-1.72755	-1.72487	-1.72219	-1.7195	-1.71681	-1.73679	-1.73404	-1.7313
-1.72936	-1.72667	-1.72399	-1.72132	-1.71863	-1.71593	-1.7359	-1.73315	-1.73041
-1.72849	-1.7258	-1.72312	-1.72044	-1.71775	-1.71505	-1.73501	-1.73226	-1.72951
-1.72762	-1.72493	-1.72224	-1.71957	-1.71688	-1.71418	-1.71148	-1.70879	-1.70609
-1.72675	-1.72406	-1.72137	-1.71869	-1.716	-1.7133	-1.7106	-1.70791	-1.70521
-1.72589	-1.7232	-1.7205	-1.71781	-1.71512	-1.71242	-1.70972	-1.70703	-1.70433
-1.72504	-1.72233	-1.71963	-1.71694	-1.71424	-1.71154	-1.70884	-1.70614	-1.70345
-1.72421	-1.72147	-1.71876	-1.71607	-1.71337	-1.71066	-1.70796	-1.70526	-1.70257
-1.72342	-1.72064	-1.71791	-1.7152	-1.71249	-1.70978	-1.70708	-1.70438	-1.70169
-1.72264	-1.71983	-1.71707	-1.71434	-1.71162	-1.70891	-1.7062	-1.7035	-1.70081
-1.72184	-1.71905	-1.71627	-1.7135	-1.71076	-1.70803	-1.70532	-1.70262	-1.69993
-1.72104	-1.71828	-1.7155	-1.71269	-1.70992	-1.70717	-1.70444	-1.70174	-1.69905
-1.7202	-1.71747	-1.7147	-1.71189	-1.70911	-1.70633	-1.70358	-1.70087	-1.69818
-1.71933	-1.71661	-1.71387	-1.71112	-1.70833	-1.70552	-1.70274	-1.70001	-1.6973
-1.71845	-1.71575	-1.71303	-1.71031	-1.70754	-1.70472	-1.70192	-1.69917	-1.69644
-1.71757	-1.71487	-1.71216	-1.70945	-1.7067	-1.70393	-1.70115	-1.69837	-1.6956
-1.7167	-1.71398	-1.71128	-1.70857	-1.70585	-1.70312	-1.70037	-1.69757	-1.69478
-1.71582	-1.7131	-1.7104	-1.70769	-1.70498	-1.70227	-1.69954	-1.69677	-1.694
-1.71496	-1.71223	-1.70952	-1.70681	-1.7041	-1.7014	-1.69868	-1.69595	-1.69322
-1.70521	-1.70244	-1.70866	-1.70594	-1.70323	-1.70052	-1.69782	-1.6951	-1.69239
-1.70443	-1.70161	-1.70782	-1.70509	-1.70237	-1.69965	-1.69694	-1.69423	-1.69152
-1.70364	-1.70082	-1.70702	-1.70429	-1.70155	-1.69881	-1.69608	-1.69337	-1.69066
-1.70284	-1.70006	-1.70628	-1.70355	-1.70079	-1.69801	-1.69524	-1.69251	-1.68979
-1.70202	-1.69927	-1.69653	-1.69379	-1.70002	-1.69722	-1.69444	-1.69169	-1.68896
-1.70115	-1.69841	-1.69568	-1.69295	-1.6992	-1.69644	-1.69368	-1.69094	-1.68821

-1.00759	-1.00625	-1.05311	-1.05165	-1.05021	-1.0488	-1.10688	-1.10529	-1.1037
-1.00796	-1.00661	-1.05351	-1.05205	-1.05061	-1.0492	-1.10735	-1.10576	-1.10418
-1.00833	-1.00697	-1.05391	-1.05246	-1.05102	-1.04961	-1.1078	-1.10622	-1.10465
-0.98786	-0.98655	-1.05434	-1.0529	-1.05146	-1.05002	-1.0874	-1.08587	-1.08434
-0.9882	-0.9869	-1.05478	-1.05335	-1.0519	-1.05045	-1.08784	-1.0863	-1.08477
-0.98856	-0.98726	-0.98597	-0.98468	-1.05232	-1.05086	-1.0494	-1.04795	-1.0465
-0.98892	-0.98762	-0.98632	-0.98501	-1.05271	-1.05126	-1.04981	-1.04837	-1.04693
-0.98927	-0.98797	-0.98666	-0.98534	-1.05311	-1.05168	-1.05024	-1.04881	-1.04737
-0.98961	-0.9883	-0.98699	-0.98568	-1.05352	-1.0521	-1.05068	-1.04925	-1.04782
-0.98994	-0.98863	-0.98732	-0.98602	-0.98473	-0.98347	-1.05112	-1.0497	-1.04826
-0.99028	-0.98897	-0.98767	-0.98638	-0.9851	-0.98384	-1.05155	-1.05013	-1.04871
-0.99061	-0.98932	-0.98803	-0.98675	-0.98547	-0.9842	-1.05198	-1.05057	-1.04915
-0.99096	-0.98967	-0.98838	-0.9871	-0.98582	-0.98455	-0.98328	-0.98201	-0.98074
-0.99131	-0.99001	-0.98872	-0.98744	-0.98616	-0.9849	-0.98363	-0.98236	-0.98109
-0.99166	-0.99036	-0.98907	-0.98779	-0.98651	-0.98524	-0.98398	-0.98271	-0.98145
-0.99199	-0.99069	-0.98941	-0.98813	-0.98686	-0.98559	-0.98433	-0.98308	-0.98182
-0.99235	-0.99104	-0.98976	-0.98848	-0.98721	-0.98595	-0.98469	-0.98343	-0.98217
-0.99272	-0.99141	-0.99012	-0.98884	-0.98757	-0.9863	-0.98504	-0.98378	-0.98251
-0.99308	-0.99178	-0.99048	-0.9892	-0.98792	-0.98665	-0.98539	-0.98412	-0.98285
-0.99343	-0.99212	-0.99083	-0.98954	-0.98826	-0.98699	-0.98572	-0.98445	-0.98319
-0.99378	-0.99246	-0.99117	-0.98988	-0.9886	-0.98733	-0.98606	-0.9848	-0.98353
-0.99415	-0.99283	-0.99153	-0.99024	-0.98895	-0.98769	-0.98642	-0.98516	-0.98389
-0.99452	-0.99322	-0.99191	-0.9906	-0.98932	-0.98806	-0.9868	-0.98553	-0.98426
-0.9949	-0.9936	-0.99229	-0.99098	-0.98969	-0.98843	-0.98717	-0.9859	-0.98463
-0.99528	-0.99397	-0.99266	-0.99135	-0.99007	-0.9888	-0.98753	-0.98627	-0.98501
-0.99564	-0.99433	-0.99302	-0.99173	-0.99044	-0.98916	-0.98789	-0.98663	-0.98537
-0.99599	-0.99467	-0.99337	-0.99208	-0.99079	-0.98951	-0.98824	-0.98698	-0.98573
-0.99633	-0.99502	-0.99373	-0.99244	-0.99115	-0.98986	-0.98858	-0.98733	-0.98608
-0.99669	-0.99539	-0.99409	-0.99279	-0.9915	-0.9902	-0.98892	-0.98766	-0.9864
-0.99705	-0.99576	-0.99446	-0.99314	-0.99184	-0.99055	-0.98926	-0.98799	-0.98672
-0.9974	-0.99612	-0.99482	-0.9935	-0.9922	-0.9909	-0.98961	-0.98833	-0.98706
-0.99774	-0.99647	-0.99517	-0.99386	-0.99256	-0.99126	-0.98997	-0.98868	-0.98741
-0.99808	-0.99681	-0.99553	-0.99423	-0.99293	-0.99162	-0.99033	-0.98904	-0.98777
-0.99844	-0.99716	-0.99588	-0.9946	-0.9933	-0.99199	-0.99069	-0.9894	-0.98813
-0.99879	-0.99751	-0.99624	-0.99496	-0.99366	-0.99235	-0.99105	-0.98977	-0.98849
-0.99912	-0.99785	-0.99658	-0.9953	-0.99401	-0.99271	-0.99141	-0.99013	-0.98886
-0.99946	-0.9982	-0.99692	-0.99565	-0.99436	-0.99307	-0.99177	-0.9905	-0.98922
-0.99984	-0.99857	-0.99729	-0.99601	-0.99472	-0.99343	-0.99215	-0.99087	-0.9896
-1.00022	-0.99895	-0.99766	-0.99637	-0.99508	-0.9938	-0.99252	-0.99125	-0.98997
-1.00059	-0.99931	-0.99802	-0.99673	-0.99544	-0.99416	-0.99288	-0.9916	-0.99032
-1.00095	-0.99967	-0.99838	-0.99708	-0.9958	-0.99452	-0.99324	-0.99194	-0.99065
-0.98094	-0.97969	-0.97843	-0.97716	-0.99616	-0.99488	-0.9936	-0.9923	-0.991
-0.98131	-0.98005	-0.97879	-0.97753	-0.99653	-0.99526	-0.99398	-0.99268	-0.99139
-0.98168	-0.98041	-0.97915	-0.97789	-0.97665	-0.97541	-0.99437	-0.99307	-0.99178
-0.98204	-0.98077	-0.97951	-0.97825	-0.97701	-0.97577	-0.99474	-0.99344	-0.99215
-0.9824	-0.98114	-0.97988	-0.97862	-0.97737	-0.97614	-0.99511	-0.99381	-0.99253
-0.96066	-0.95942	-0.98026	-0.979	-0.97776	-0.97652	-0.97528	-0.97402	-0.97276
-0.96103	-0.9598	-0.98064	-0.9794	-0.97815	-0.97691	-0.97567	-0.97441	-0.97315
-0.96138	-0.96015	-0.95893	-0.9577	-0.97853	-0.97729	-0.97605	-0.97479	-0.97353
-0.96173	-0.9605	-0.95928	-0.95806	-0.9789	-0.97766	-0.97641	-0.97515	-0.9739

-0.96208	-0.96085	-0.95963	-0.95842	-0.9572	-0.95598	-0.97679	-0.97553	-0.97427
-0.96245	-0.96122	-0.96	-0.95879	-0.95757	-0.95636	-0.97718	-0.97591	-0.97465
-0.96283	-0.96159	-0.96037	-0.95916	-0.95795	-0.95674	-0.97756	-0.9763	-0.97504
-0.96319	-0.96195	-0.96072	-0.95951	-0.9583	-0.9571	-0.95588	-0.95463	-0.95339
-0.96356	-0.96231	-0.96108	-0.95986	-0.95865	-0.95745	-0.95623	-0.95499	-0.95375
-0.96393	-0.96267	-0.96144	-0.96023	-0.95902	-0.95782	-0.9566	-0.95536	-0.95412
-0.9643	-0.96305	-0.96182	-0.9606	-0.95939	-0.95819	-0.95697	-0.95574	-0.95451
-0.96468	-0.96343	-0.96219	-0.96098	-0.95977	-0.95856	-0.95735	-0.95613	-0.9549
-0.96507	-0.96381	-0.96258	-0.96135	-0.96014	-0.95893	-0.95772	-0.95651	-0.95529
-0.96546	-0.9642	-0.96296	-0.96173	-0.96051	-0.9593	-0.95809	-0.95689	-0.95568
-0.96585	-0.9646	-0.96336	-0.96211	-0.96089	-0.95968	-0.95847	-0.95727	-0.95607
-0.96624	-0.965	-0.96375	-0.9625	-0.96126	-0.96006	-0.95885	-0.95766	-0.95646
-0.96663	-0.96539	-0.96414	-0.96289	-0.96165	-0.96044	-0.95923	-0.95804	-0.95684
-0.96702	-0.96579	-0.96454	-0.96329	-0.96205	-0.96082	-0.95961	-0.95842	-0.95723
-0.9674	-0.96617	-0.96493	-0.96368	-0.96244	-0.96121	-0.96	-0.9588	-0.9576
-0.96778	-0.96656	-0.96532	-0.96408	-0.96284	-0.96161	-0.96038	-0.95917	-0.95797
-0.96818	-0.96695	-0.96572	-0.96449	-0.96325	-0.96201	-0.96078	-0.95956	-0.95835
-0.9686	-0.96736	-0.96613	-0.9649	-0.96367	-0.96243	-0.9612	-0.95998	-0.95876
-0.89932	-0.89819	-0.96654	-0.96531	-0.96408	-0.96285	-0.96162	-0.96039	-0.95916
-0.8997	-0.89856	-0.96695	-0.96572	-0.96448	-0.96324	-0.96201	-0.96079	-0.95957
-0.90009	-0.89894	-0.96736	-0.96612	-0.96488	-0.96363	-0.9624	-0.96119	-0.95998
-0.90046	-0.8993	-0.89818	-0.89706	-0.96528	-0.96404	-0.96281	-0.96159	-0.96038
-0.90082	-0.89967	-0.89854	-0.89742	-0.96569	-0.96445	-0.96322	-0.96199	-0.96078
-0.90119	-0.90004	-0.89891	-0.89779	-0.89668	-0.89559	-0.96365	-0.96241	-0.96118
-0.90157	-0.90042	-0.89929	-0.89817	-0.89706	-0.89598	-0.9641	-0.96285	-0.9616
-0.87329	-0.8722	-0.89966	-0.89853	-0.89743	-0.89635	-0.89526	-0.89415	-0.89303
-0.87364	-0.87255	-0.90003	-0.89888	-0.89777	-0.89668	-0.8956	-0.8945	-0.8934
-0.87399	-0.8729	-0.9004	-0.89924	-0.89811	-0.89702	-0.89594	-0.89485	-0.89376
-0.87434	-0.87325	-0.87215	-0.87104	-0.89849	-0.89738	-0.89629	-0.89521	-0.89412
-0.8747	-0.87361	-0.8725	-0.8714	-0.89887	-0.89775	-0.89665	-0.89556	-0.89449
-0.87505	-0.87396	-0.87286	-0.87176	-0.87067	-0.86961	-0.89702	-0.89594	-0.89486
-0.87539	-0.87432	-0.87322	-0.87212	-0.87104	-0.86997	-0.89742	-0.89633	-0.89525
-0.87574	-0.87467	-0.87357	-0.87248	-0.87139	-0.87032	-0.89779	-0.8967	-0.89562
-0.87609	-0.87501	-0.87391	-0.87282	-0.87173	-0.87065	-0.89816	-0.89706	-0.89597
-0.87644	-0.87535	-0.87426	-0.87317	-0.87208	-0.871	-0.86994	-0.86889	-0.86785
-0.87678	-0.87569	-0.87461	-0.87353	-0.87246	-0.87138	-0.87031	-0.86925	-0.8682
-0.87712	-0.87604	-0.87497	-0.8739	-0.87283	-0.87176	-0.87069	-0.86962	-0.86856
-0.85592	-0.85487	-0.87532	-0.87425	-0.87318	-0.87211	-0.87104	-0.86997	-0.8689
-0.85625	-0.8552	-0.87567	-0.8746	-0.87353	-0.87246	-0.87139	-0.87031	-0.86925
-0.85658	-0.85553	-0.87602	-0.87496	-0.87389	-0.87282	-0.87175	-0.87067	-0.8696
-0.85693	-0.85586	-0.87638	-0.87531	-0.87425	-0.87318	-0.87212	-0.87104	-0.86997
-0.85727	-0.85619	-0.85515	-0.85412	-0.87459	-0.87353	-0.87247	-0.8714	-0.87034
-0.8576	-0.85651	-0.85545	-0.85442	-0.87492	-0.87386	-0.8728	-0.87174	-0.87068
-0.85794	-0.85684	-0.85577	-0.85474	-0.87526	-0.8742	-0.87315	-0.87209	-0.87104
-0.8583	-0.8572	-0.85613	-0.85509	-0.87563	-0.87458	-0.87353	-0.87248	-0.87143
-0.85866	-0.85757	-0.85651	-0.85546	-0.87602	-0.87496	-0.87391	-0.87287	-0.87182
-0.85903	-0.85795	-0.85688	-0.85582	-0.85478	-0.85376	-0.87429	-0.87325	-0.8722
-0.85941	-0.85833	-0.85725	-0.85617	-0.85512	-0.8541	-0.87466	-0.87361	-0.87257
-0.82964	-0.8286	-0.85761	-0.85652	-0.85546	-0.85443	-0.875	-0.87396	-0.87292
-0.82996	-0.82891	-0.85795	-0.85685	-0.85578	-0.85474	-0.87532	-0.87429	-0.87325

-0.8303	-0.82925	-0.85831	-0.85721	-0.85613	-0.85507	-0.85404	-0.85303	-0.85204
-0.83066	-0.8296	-0.85872	-0.85761	-0.85652	-0.85545	-0.8544	-0.85339	-0.85239
-0.83103	-0.82997	-0.85912	-0.85802	-0.85692	-0.85583	-0.85477	-0.85375	-0.85274
-0.83139	-0.83033	-0.82927	-0.82822	-0.85732	-0.85623	-0.85515	-0.85411	-0.85309
-0.83174	-0.83068	-0.82963	-0.82858	-0.85773	-0.85662	-0.85554	-0.85448	-0.85344
-0.83208	-0.83102	-0.82997	-0.82893	-0.85812	-0.85702	-0.85592	-0.85484	-0.85379
-0.83241	-0.83135	-0.8303	-0.82926	-0.8585	-0.8574	-0.85631	-0.8552	-0.85413
-0.83277	-0.83171	-0.83066	-0.82961	-0.82857	-0.82752	-0.85669	-0.85557	-0.85448
-0.83316	-0.8321	-0.83104	-0.82998	-0.82892	-0.82786	-0.85707	-0.85594	-0.85483
-0.80385	-0.80285	-0.83142	-0.83035	-0.82928	-0.82821	-0.82716	-0.82611	-0.82507
-0.80421	-0.8032	-0.8318	-0.83072	-0.82965	-0.82858	-0.82752	-0.82647	-0.82543
-0.80457	-0.80356	-0.83218	-0.8311	-0.83002	-0.82895	-0.82789	-0.82684	-0.8258
-0.80493	-0.80392	-0.83256	-0.83148	-0.8304	-0.82933	-0.82827	-0.82721	-0.82616
-0.80529	-0.80427	-0.83294	-0.83187	-0.83079	-0.82972	-0.82865	-0.82758	-0.82653
-0.76236	-0.76131	-0.8036	-0.80259	-0.83119	-0.83011	-0.82903	-0.82796	-0.8269
-0.76278	-0.76173	-0.80396	-0.80296	-0.83158	-0.8305	-0.82941	-0.82833	-0.82726
-0.76321	-0.76216	-0.76112	-0.76009	-0.78962	-0.7885	-0.8298	-0.82871	-0.82763
-0.76363	-0.7626	-0.76156	-0.76052	-0.79008	-0.78896	-0.83019	-0.82909	-0.82801
-0.73172	-0.73075	-0.76199	-0.76095	-0.75991	-0.75885	-0.78831	-0.78719	-0.78607
-0.73212	-0.73114	-0.76241	-0.76138	-0.76034	-0.75928	-0.78877	-0.78765	-0.78654
-0.73253	-0.73153	-0.76284	-0.76181	-0.76077	-0.75972	-0.78924	-0.78813	-0.78702
-0.73296	-0.73195	-0.73096	-0.72998	-0.76121	-0.76016	-0.78971	-0.7886	-0.78749
-0.7334	-0.73238	-0.73138	-0.7304	-0.76166	-0.76061	-0.79018	-0.78907	-0.78796
-0.73385	-0.73281	-0.73181	-0.73082	-0.76211	-0.76106	-0.76001	-0.75895	-0.75789
-0.7343	-0.73325	-0.73224	-0.73125	-0.76258	-0.76152	-0.76046	-0.7594	-0.75834
-0.73476	-0.7337	-0.73267	-0.73168	-0.73069	-0.72968	-0.76092	-0.75986	-0.75879
-0.73522	-0.73414	-0.73311	-0.73212	-0.73112	-0.73012	-0.76138	-0.76031	-0.75924
-0.7357	-0.7346	-0.73356	-0.73256	-0.73155	-0.73055	-0.72954	-0.72853	-0.72751
-0.73618	-0.73507	-0.73402	-0.733	-0.73199	-0.73099	-0.72998	-0.72896	-0.72793
-0.73667	-0.73555	-0.73449	-0.73346	-0.73244	-0.73144	-0.73043	-0.72939	-0.72837
-0.73715	-0.73603	-0.73496	-0.73392	-0.7329	-0.73189	-0.73088	-0.72984	-0.72881
-0.73763	-0.73652	-0.73544	-0.7344	-0.73336	-0.73235	-0.73133	-0.73029	-0.72926
-0.73811	-0.73701	-0.73593	-0.73488	-0.73384	-0.73281	-0.73178	-0.73074	-0.72971
-0.7386	-0.7375	-0.73643	-0.73538	-0.73432	-0.73327	-0.73223	-0.73119	-0.73016
-0.7391	-0.73802	-0.73694	-0.73588	-0.73481	-0.73374	-0.73268	-0.73164	-0.73061
-0.73963	-0.73854	-0.73747	-0.73639	-0.73531	-0.73422	-0.73314	-0.7321	-0.73108
-0.74015	-0.73908	-0.73799	-0.73691	-0.73581	-0.7347	-0.73362	-0.73257	-0.73155
-0.72337	-0.72234	-0.73851	-0.73742	-0.73631	-0.73519	-0.7341	-0.73305	-0.73203
-0.72388	-0.72284	-0.73902	-0.73792	-0.73681	-0.73569	-0.7346	-0.73355	-0.73252
-0.72439	-0.72335	-0.72229	-0.72123	-0.73731	-0.73619	-0.73509	-0.73404	-0.733
-0.72492	-0.72386	-0.7228	-0.72173	-0.73782	-0.73669	-0.73559	-0.73453	-0.73349
-0.69471	-0.6937	-0.7233	-0.72222	-0.73834	-0.73722	-0.73611	-0.73504	-0.73398
-0.69519	-0.69417	-0.7238	-0.72273	-0.73886	-0.73775	-0.73665	-0.73556	-0.73449
-0.69567	-0.69464	-0.69362	-0.69261	-0.72218	-0.72112	-0.7372	-0.73609	-0.735
-0.69615	-0.6951	-0.69409	-0.6931	-0.72271	-0.72164	-0.73773	-0.73662	-0.73552
-0.69664	-0.69558	-0.69457	-0.69359	-0.72324	-0.72216	-0.73826	-0.73715	-0.73606
-0.69715	-0.69609	-0.69507	-0.69407	-0.69307	-0.69206	-0.7388	-0.7377	-0.7366
-0.69767	-0.69662	-0.69558	-0.69455	-0.69353	-0.69254	-0.73935	-0.73826	-0.73716
-0.69819	-0.69714	-0.69609	-0.69504	-0.69402	-0.69303	-0.69205	-0.69107	-0.69008
-0.69872	-0.69767	-0.6966	-0.69554	-0.69452	-0.69354	-0.69256	-0.69157	-0.69056

-0.69924	-0.69818	-0.69712	-0.69605	-0.69503	-0.69405	-0.69307	-0.69206	-0.69105
-0.69976	-0.69869	-0.69763	-0.69658	-0.69555	-0.69455	-0.69355	-0.69255	-0.69155
-0.70027	-0.6992	-0.69815	-0.69711	-0.69608	-0.69506	-0.69404	-0.69305	-0.69205
-0.70078	-0.6997	-0.69865	-0.69762	-0.69659	-0.69555	-0.69453	-0.69355	-0.69257
-0.70129	-0.70021	-0.69916	-0.69813	-0.6971	-0.69605	-0.69503	-0.69406	-0.69308
-0.70182	-0.70074	-0.69969	-0.69865	-0.69761	-0.69658	-0.69556	-0.69457	-0.69358
-0.70236	-0.7013	-0.70023	-0.69916	-0.69812	-0.69711	-0.69611	-0.69509	-0.69407
-0.67329	-0.67226	-0.70076	-0.69969	-0.69865	-0.69764	-0.69663	-0.6956	-0.69457
-0.67381	-0.67277	-0.7013	-0.70024	-0.69919	-0.69816	-0.69713	-0.69609	-0.69507
-0.63769	-0.63679	-0.67226	-0.67123	-0.69972	-0.69867	-0.69763	-0.6966	-0.69558
-0.63817	-0.63726	-0.67278	-0.67174	-0.70024	-0.69918	-0.69814	-0.69712	-0.6961
-0.63865	-0.63773	-0.67331	-0.67225	-0.70076	-0.69969	-0.69865	-0.69764	-0.69663
-0.63914	-0.63819	-0.67384	-0.67278	-0.67174	-0.6707	-0.69917	-0.69815	-0.69714
-0.63963	-0.63866	-0.67437	-0.67333	-0.67228	-0.67122	-0.69971	-0.69867	-0.69764
-0.64013	-0.63915	-0.63821	-0.63729	-0.6728	-0.67173	-0.70024	-0.69919	-0.69816
-0.64064	-0.63967	-0.63871	-0.63776	-0.67332	-0.67225	-0.70077	-0.69971	-0.69868
-0.64115	-0.64018	-0.63921	-0.63824	-0.6373	-0.63637	-0.67172	-0.6707	-0.6697
-0.64167	-0.64069	-0.63971	-0.63874	-0.63778	-0.63684	-0.67225	-0.67122	-0.67021
-0.64219	-0.64121	-0.64022	-0.63924	-0.63828	-0.63732	-0.6728	-0.67175	-0.67072
-0.64271	-0.64173	-0.64074	-0.63976	-0.63878	-0.63781	-0.67336	-0.6723	-0.67126
-0.64324	-0.64225	-0.64126	-0.64028	-0.63929	-0.63832	-0.67393	-0.67285	-0.67181
-0.64377	-0.64278	-0.64179	-0.6408	-0.63981	-0.63882	-0.63785	-0.63692	-0.636
-0.64431	-0.6433	-0.64231	-0.64133	-0.64033	-0.63932	-0.63834	-0.6374	-0.63648
-0.57282	-0.57205	-0.64283	-0.64185	-0.64085	-0.63984	-0.63885	-0.63789	-0.63695
-0.57323	-0.57247	-0.64336	-0.64237	-0.64137	-0.64037	-0.63937	-0.63838	-0.63742
-0.57364	-0.57288	-0.57212	-0.57136	-0.6419	-0.6409	-0.6399	-0.63889	-0.63791
-0.57406	-0.57328	-0.57252	-0.57178	-0.64242	-0.64142	-0.64043	-0.63943	-0.63844
-0.53739	-0.53666	-0.57293	-0.57219	-0.64295	-0.64196	-0.64096	-0.63997	-0.63899
-0.53777	-0.53705	-0.57336	-0.5726	-0.64349	-0.6425	-0.64151	-0.64052	-0.63953
-0.53815	-0.53745	-0.57379	-0.573	-0.64404	-0.64305	-0.64207	-0.64108	-0.64008
-0.53854	-0.53785	-0.53713	-0.53639	-0.57267	-0.57194	-0.64262	-0.64162	-0.64062
-0.53894	-0.53825	-0.53753	-0.5368	-0.57312	-0.57237	-0.64316	-0.64216	-0.64116
-0.50529	-0.5047	-0.53793	-0.5372	-0.53648	-0.53577	-0.57203	-0.57127	-0.57052
-0.50563	-0.50502	-0.5383	-0.53759	-0.53688	-0.53615	-0.57245	-0.57169	-0.57095
-0.50597	-0.50536	-0.50475	-0.50415	-0.53727	-0.53654	-0.53582	-0.53513	-0.53446
-0.50631	-0.5057	-0.5051	-0.5045	-0.53768	-0.53695	-0.53623	-0.53554	-0.53485
-0.50666	-0.50605	-0.50544	-0.50484	-0.53808	-0.53735	-0.53664	-0.53594	-0.53525
-0.50703	-0.5064	-0.50578	-0.50518	-0.53846	-0.53775	-0.53704	-0.53635	-0.53566
-0.50741	-0.50676	-0.50612	-0.50551	-0.53883	-0.53814	-0.53744	-0.53676	-0.53607
-0.43688	-0.43633	-0.50648	-0.50585	-0.50524	-0.50465	-0.53784	-0.53715	-0.53646
-0.43718	-0.43663	-0.50684	-0.5062	-0.50559	-0.50499	-0.53823	-0.53753	-0.53684
-0.39721	-0.39672	-0.50722	-0.50657	-0.50594	-0.50534	-0.50474	-0.50414	-0.50354
-0.39749	-0.39699	-0.50761	-0.50694	-0.5063	-0.5057	-0.50509	-0.50449	-0.50388
-0.39775	-0.39726	-0.39677	-0.39629	-0.46803	-0.46748	-0.50544	-0.50483	-0.50423
-0.39801	-0.39752	-0.39703	-0.39655	-0.46836	-0.46778	-0.50578	-0.50517	-0.50456
-0.39827	-0.39779	-0.3973	-0.39682	-0.4687	-0.4681	-0.50613	-0.5055	-0.5049
-0.39855	-0.39805	-0.39756	-0.39708	-0.3966	-0.39611	-0.46783	-0.46725	-0.4667
-0.39884	-0.39832	-0.39782	-0.39735	-0.39687	-0.39638	-0.46816	-0.46757	-0.467
-0.39913	-0.3986	-0.39809	-0.39761	-0.39713	-0.39663	-0.39615	-0.39568	-0.39523
-0.39943	-0.39888	-0.39837	-0.39787	-0.39738	-0.39689	-0.39641	-0.39593	-0.39547

-0.39973	-0.39918	-0.39865	-0.39813	-0.39763	-0.39715	-0.39667	-0.39619	-0.39572
-0.40003	-0.39949	-0.39894	-0.3984	-0.39789	-0.39741	-0.39693	-0.39645	-0.39599
-0.40034	-0.39979	-0.39924	-0.39869	-0.39817	-0.39767	-0.39719	-0.39672	-0.39625
-0.40065	-0.40011	-0.39955	-0.39899	-0.39846	-0.39795	-0.39746	-0.39698	-0.39652
-0.40096	-0.40041	-0.39986	-0.3993	-0.39876	-0.39823	-0.39773	-0.39725	-0.39678
-0.40125	-0.40071	-0.40016	-0.3996	-0.39906	-0.39853	-0.39801	-0.39753	-0.39705
-0.40154	-0.40099	-0.40044	-0.3999	-0.39936	-0.39883	-0.39831	-0.39781	-0.39732
-0.40184	-0.40129	-0.40074	-0.4002	-0.39966	-0.39913	-0.39861	-0.39809	-0.39759
-0.40215	-0.4016	-0.40105	-0.40051	-0.39997	-0.39944	-0.39892	-0.39838	-0.39786
-0.38201	-0.38153	-0.40136	-0.40082	-0.40028	-0.39975	-0.39922	-0.39868	-0.39815
-0.38229	-0.3818	-0.40167	-0.40113	-0.40059	-0.40006	-0.39953	-0.399	-0.39847
-0.38258	-0.38209	-0.40198	-0.40144	-0.4009	-0.40037	-0.39984	-0.39932	-0.3988
-0.38286	-0.38237	-0.38188	-0.3814	-0.4012	-0.40068	-0.40015	-0.39963	-0.3991
-0.38315	-0.38265	-0.38216	-0.38168	-0.40151	-0.40099	-0.40046	-0.39994	-0.39941
-0.38344	-0.38294	-0.38244	-0.38195	-0.38148	-0.38103	-0.40077	-0.40024	-0.39971
-0.38373	-0.38322	-0.38272	-0.38223	-0.38176	-0.3813	-0.40108	-0.40054	-0.40001
-0.38403	-0.38351	-0.38301	-0.38251	-0.38204	-0.38158	-0.38112	-0.38066	-0.38021
-0.38432	-0.3838	-0.38329	-0.3828	-0.38232	-0.38185	-0.38139	-0.38094	-0.38049
-0.38463	-0.3841	-0.38359	-0.38309	-0.3826	-0.38213	-0.38166	-0.38121	-0.38076
-0.38494	-0.38442	-0.38389	-0.38338	-0.38289	-0.38241	-0.38194	-0.38148	-0.38103
-0.38526	-0.38474	-0.38421	-0.38368	-0.38317	-0.38269	-0.38222	-0.38176	-0.3813
-0.38557	-0.38506	-0.38453	-0.384	-0.38349	-0.38299	-0.3825	-0.38203	-0.38158
-0.38588	-0.38538	-0.38486	-0.38433	-0.38381	-0.3833	-0.3828	-0.38232	-0.38185
-0.38619	-0.38569	-0.38518	-0.38466	-0.38414	-0.38361	-0.3831	-0.38261	-0.38213
-0.38649	-0.38599	-0.38549	-0.38498	-0.38446	-0.38394	-0.38342	-0.38291	-0.38242
-0.38679	-0.38629	-0.38579	-0.38529	-0.38478	-0.38426	-0.38374	-0.38322	-0.38272
-0.38709	-0.38658	-0.38609	-0.38559	-0.38509	-0.38457	-0.38406	-0.38355	-0.38304
-0.38739	-0.38688	-0.38638	-0.38589	-0.38539	-0.38488	-0.38437	-0.38387	-0.38337
-0.3877	-0.38719	-0.38668	-0.38619	-0.38569	-0.38519	-0.38469	-0.38418	-0.38368
-0.38802	-0.38749	-0.38699	-0.38649	-0.38599	-0.3855	-0.385	-0.38449	-0.38398
-0.38834	-0.3878	-0.38728	-0.38678	-0.38629	-0.3858	-0.3853	-0.38479	-0.38428
-0.38866	-0.38811	-0.38758	-0.38707	-0.38658	-0.38609	-0.38561	-0.3851	-0.38459
-0.34727	-0.34677	-0.34629	-0.34584	-0.38688	-0.38639	-0.3859	-0.3854	-0.38489
-0.34761	-0.3471	-0.3466	-0.34613	-0.3872	-0.38669	-0.38618	-0.38569	-0.3852
-0.34797	-0.34743	-0.34691	-0.34642	-0.34595	-0.34548	-0.38649	-0.386	-0.38551
-0.34834	-0.34776	-0.34722	-0.34672	-0.34624	-0.34577	-0.38682	-0.38633	-0.38584
-0.34874	-0.34811	-0.34754	-0.34702	-0.34653	-0.34607	-0.38716	-0.38666	-0.38617
-0.3492	-0.34848	-0.34786	-0.34733	-0.34682	-0.34634	-0.34588	-0.34544	-0.345
-0.34969	-0.34889	-0.34822	-0.34765	-0.34711	-0.34661	-0.34614	-0.34569	-0.34525
-0.35022	-0.34935	-0.34862	-0.34799	-0.34741	-0.34689	-0.3464	-0.34594	-0.3455
-0.35077	-0.34988	-0.34907	-0.34834	-0.34771	-0.34717	-0.34667	-0.34619	-0.34574
-0.35137	-0.35045	-0.34959	-0.34878	-0.34808	-0.34751	-0.34698	-0.34649	-0.34602
-0.35201	-0.3511	-0.35018	-0.34929	-0.34853	-0.3479	-0.34734	-0.34682	-0.34634
-0.35265	-0.35173	-0.35078	-0.34984	-0.34902	-0.34833	-0.34771	-0.34716	-0.34666
-0.35324	-0.3523	-0.35136	-0.35043	-0.34958	-0.34879	-0.34809	-0.3475	-0.34697
-0.35383	-0.35288	-0.35195	-0.35104	-0.35015	-0.34928	-0.34851	-0.34787	-0.3473
-0.35444	-0.35347	-0.35254	-0.35163	-0.35074	-0.34984	-0.34903	-0.34832	-0.34768
-0.35507	-0.35409	-0.35314	-0.35223	-0.35133	-0.35045	-0.34961	-0.34881	-0.34809
-0.35576	-0.35476	-0.35379	-0.35286	-0.35195	-0.35106	-0.35019	-0.34933	-0.34854
-0.35651	-0.35547	-0.35448	-0.35352	-0.35259	-0.35168	-0.35078	-0.34987	-0.34902

-0.35723	-0.35617	-0.35516	-0.35417	-0.35322	-0.35229	-0.35137	-0.35044	-0.34956
-0.35794	-0.35686	-0.35582	-0.35481	-0.35383	-0.35289	-0.35197	-0.35104	-0.35015
-0.35865	-0.35757	-0.35651	-0.35547	-0.35448	-0.35352	-0.35258	-0.35166	-0.35076
-0.35941	-0.35833	-0.35726	-0.3562	-0.35518	-0.35419	-0.35323	-0.3523	-0.35138
-0.36018	-0.3591	-0.35802	-0.35695	-0.3559	-0.35488	-0.35389	-0.35294	-0.35202
-0.27385	-0.27313	-0.35874	-0.35766	-0.3566	-0.35556	-0.35455	-0.35358	-0.35265
-0.27434	-0.27362	-0.35945	-0.35837	-0.3573	-0.35623	-0.35521	-0.35423	-0.35328
-0.27483	-0.27412	-0.2734	-0.27265	-0.35803	-0.35695	-0.35591	-0.35491	-0.35394
-0.27533	-0.27462	-0.27391	-0.27318	-0.35879	-0.35771	-0.35666	-0.35562	-0.35462
-0.27581	-0.27512	-0.27441	-0.2737	-0.27296	-0.27221	-0.3574	-0.35635	-0.35532
-0.27629	-0.2756	-0.27489	-0.27418	-0.27346	-0.27271	-0.35812	-0.35709	-0.35607
-0.27677	-0.27608	-0.27537	-0.27467	-0.27395	-0.27321	-0.31125	-0.31045	-0.30965
-0.2773	-0.27658	-0.27586	-0.27516	-0.27445	-0.27374	-0.31185	-0.31104	-0.31022
-0.27783	-0.27708	-0.27636	-0.27566	-0.27496	-0.27427	-0.31246	-0.31163	-0.31079
-0.27837	-0.27759	-0.27685	-0.27614	-0.27545	-0.27477	-0.27408	-0.27335	-0.2726
-0.2789	-0.2781	-0.27734	-0.27661	-0.27592	-0.27525	-0.27458	-0.27388	-0.27316
-0.27947	-0.27865	-0.27787	-0.27711	-0.2764	-0.27573	-0.27506	-0.27438	-0.27368
-0.28007	-0.27924	-0.27843	-0.27764	-0.2769	-0.27621	-0.27553	-0.27484	-0.27415
-0.28066	-0.27982	-0.279	-0.27819	-0.27742	-0.27671	-0.27601	-0.27532	-0.27463
-0.28123	-0.2804	-0.27958	-0.27876	-0.27798	-0.27723	-0.27651	-0.27582	-0.27513
-0.28179	-0.28097	-0.28016	-0.27935	-0.27856	-0.27777	-0.27702	-0.27632	-0.27564
-0.28237	-0.28156	-0.28074	-0.27993	-0.27911	-0.2783	-0.27753	-0.27681	-0.27612
-0.28295	-0.28215	-0.28134	-0.2805	-0.27967	-0.27884	-0.27805	-0.27731	-0.27661
-0.28351	-0.28273	-0.28193	-0.2811	-0.28027	-0.27942	-0.27861	-0.27784	-0.2771
-0.28404	-0.2833	-0.28252	-0.28171	-0.28089	-0.28003	-0.2792	-0.27838	-0.2776
-0.28457	-0.28384	-0.28309	-0.28231	-0.28149	-0.28065	-0.2798	-0.27896	-0.27814
-0.28509	-0.28435	-0.28362	-0.28288	-0.2821	-0.28127	-0.28043	-0.27958	-0.27874
-0.28562	-0.28487	-0.28414	-0.28343	-0.28268	-0.28187	-0.28105	-0.2802	-0.27935
-0.28618	-0.28542	-0.28468	-0.28395	-0.28321	-0.28245	-0.28166	-0.28082	-0.27997
-0.28675	-0.28598	-0.28522	-0.28447	-0.28373	-0.28301	-0.28225	-0.28143	-0.28059
-0.28733	-0.28654	-0.28576	-0.28501	-0.28427	-0.28354	-0.28279	-0.28201	-0.2812
-0.28792	-0.28709	-0.28631	-0.28557	-0.28482	-0.28406	-0.28331	-0.28255	-0.28178
-0.28854	-0.28768	-0.28688	-0.28612	-0.28536	-0.2846	-0.28385	-0.28311	-0.28235
-0.28919	-0.28829	-0.28746	-0.28667	-0.2859	-0.28516	-0.28441	-0.28366	-0.2829
-0.28986	-0.28894	-0.28807	-0.28724	-0.28645	-0.28571	-0.28497	-0.28421	-0.28345
-0.29056	-0.28962	-0.28871	-0.28783	-0.28701	-0.28626	-0.28551	-0.28476	-0.28401
-0.24019	-0.23941	-0.28938	-0.28845	-0.2876	-0.28683	-0.28607	-0.28532	-0.28458
-0.24075	-0.23998	-0.29007	-0.28913	-0.28825	-0.28743	-0.28664	-0.28588	-0.28514
-0.24131	-0.24055	-0.29078	-0.28983	-0.28892	-0.28805	-0.28722	-0.28645	-0.2857
-0.24186	-0.24112	-0.29149	-0.29053	-0.2896	-0.2887	-0.28784	-0.28704	-0.28627
-0.24239	-0.24168	-0.29219	-0.29124	-0.2903	-0.28936	-0.28847	-0.28764	-0.28685
-0.24294	-0.24223	-0.24149	-0.24072	-0.291	-0.29005	-0.28914	-0.28828	-0.28746
-0.24349	-0.24279	-0.24205	-0.2413	-0.29172	-0.29076	-0.28983	-0.28895	-0.2881
-0.24404	-0.24333	-0.2426	-0.24186	-0.24111	-0.24032	-0.29053	-0.28964	-0.28877
-0.24459	-0.24386	-0.24313	-0.24241	-0.24167	-0.24089	-0.29124	-0.29034	-0.28946
-0.24515	-0.24439	-0.24366	-0.24296	-0.24223	-0.24147	-0.29195	-0.29105	-0.29015
-0.0443	-0.04414	-0.24421	-0.24352	-0.24279	-0.24204	-0.29265	-0.29173	-0.29082
-0.04439	-0.04422	-0.24478	-0.24408	-0.24335	-0.24261	-0.29334	-0.29241	-0.29148
-0.04449	-0.04432	-0.04416	-0.04401	-0.24391	-0.24317	-0.24243	-0.24165	-0.24088
-0.0446	-0.04442	-0.04424	-0.04408	-0.24445	-0.24373	-0.243	-0.24223	-0.24146

-0.0447	-0.04452	-0.04434	-0.04416	-0.04401	-0.04388	-0.24355	-0.24279	-0.24203
-0.0448	-0.04462	-0.04444	-0.04426	-0.0441	-0.04395	-0.24409	-0.24335	-0.2426
-0.0449	-0.04473	-0.04455	-0.04436	-0.04419	-0.04404	-0.04389	-0.04375	-0.04362
-0.04499	-0.04483	-0.04466	-0.04447	-0.0443	-0.04413	-0.04398	-0.04383	-0.04369
-0.04508	-0.04493	-0.04476	-0.04458	-0.0444	-0.04423	-0.04407	-0.04391	-0.04376
		-0.04486	-0.04468	-0.0445	-0.04433	-0.04417	-0.044	-0.04384
		-0.04494	-0.04477	-0.04461	-0.04444	-0.04427	-0.04409	-0.04392
				-0.0447	-0.04454	-0.04437	-0.04419	-0.04401
				-0.04479	-0.04464	-0.04447	-0.04429	-0.04412
						-0.04457	-0.04439	-0.04422
						-0.04466	-0.04449	-0.04431
						-0.04475	-0.04458	-0.04441
						-0.04485	-0.04468	-0.04451
						-0.04495	-0.04478	-0.04462

						-0.10822	-0.10792	-0.10761
						-0.10795	-0.10766	-0.10736
						-0.30503	-0.30412	-0.3032
						-0.30434	-0.30339	-0.30244
				-0.16248	-0.16201	-0.30362	-0.30267	-0.30171
				-0.16207	-0.16161	-0.30289	-0.30195	-0.30102
		-0.1626	-0.16213	-0.30399	-0.30308	-0.43168	-0.43023	-0.4288
		-0.1622	-0.16173	-0.30329	-0.30235	-0.43058	-0.42917	-0.42781
		-0.1618	-0.16134	-0.30259	-0.30163	-0.42949	-0.42814	-0.42685
-0.1624	-0.16189	-0.3037	-0.30281	-0.43139	-0.42991	-0.56548	-0.5637	-0.56196
-0.162	-0.16149	-0.30301	-0.30212	-0.43037	-0.42893	-0.56416	-0.56241	-0.5607
-0.25435	-0.25358	-0.43213	-0.43075	-0.47554	-0.47404	-0.6006	-0.59874	-0.59691
-0.25372	-0.25298	-0.4311	-0.42972	-0.47446	-0.47302	-0.59919	-0.59733	-0.5955
-0.3865	-0.38523	-0.47629	-0.47483	-0.60161	-0.59968	-0.64392	-0.64198	-0.64006
-0.38557	-0.38431	-0.47522	-0.47376	-0.60014	-0.59823	-0.64242	-0.64051	-0.63864
-0.38466	-0.38341	-0.47417	-0.47272	-0.59869	-0.5968	-0.64093	-0.63907	-0.63725
-0.476	-0.47459	-0.47316	-0.47171	-0.59727	-0.5954	-0.63947	-0.63765	-0.63588
-0.47497	-0.47359	-0.47217	-0.47073	-0.59587	-0.59403	-0.63805	-0.63626	-0.63452
-0.47395	-0.47259	-0.59832	-0.59637	-0.6404	-0.63851	-0.67733	-0.67545	-0.6736
-0.47294	-0.47159	-0.59691	-0.59498	-0.63896	-0.63713	-0.6759	-0.67401	-0.67214
-0.51113	-0.50966	-0.64146	-0.63946	-0.67824	-0.67635	-0.71395	-0.71199	-0.71004
-0.51002	-0.50855	-0.63999	-0.63805	-0.67679	-0.67494	-0.71251	-0.71056	-0.70861
-0.59647	-0.5946	-0.63856	-0.63668	-0.71483	-0.71295	-0.89565	-0.8928	-0.88996
-0.59507	-0.59325	-0.63717	-0.63535	-0.71339	-0.71153	-0.8936	-0.89078	-0.88797
-0.59371	-0.59195	-0.63583	-0.63406	-0.71197	-0.71012	-0.89157	-0.88878	-0.886
-0.63812	-0.63632	-0.67499	-0.67313	-0.74469	-0.74276	-0.92974	-0.92675	-0.92377

-0.63677	-0.63505	-0.67366	-0.6718	-0.74324	-0.7413	-0.9276	-0.92463	-0.92168
-0.67594	-0.67417	-0.74579	-0.74377	-0.89091	-0.88825	-0.92549	-0.92254	-0.91959
-0.67462	-0.67285	-0.74435	-0.74232	-0.8889	-0.88628	-0.92341	-0.92045	-0.9175
-0.74685	-0.74491	-0.89252	-0.88968	-0.92694	-0.92414	-0.9983	-0.99521	-0.99214
-0.74547	-0.74348	-0.89054	-0.88774	-0.92489	-0.92207	-0.9961	-0.99304	-0.99001
-0.89419	-0.89135	-0.92873	-0.92578	-0.99993	-0.99692	-1.09021	-1.08695	-1.08375
-0.89219	-0.88936	-0.92665	-0.92378	-0.99785	-0.9948	-1.08789	-1.08469	-1.08157
-0.89017	-0.88737	-0.92458	-0.92179	-0.99577	-0.99272	-1.08562	-1.08248	-1.07943
-0.92836	-0.92536	-0.99951	-0.99659	-1.0898	-1.08657	-1.0834	-1.08032	-1.07732
-0.92623	-0.92325	-0.9973	-0.99439	-1.08744	-1.08431	-1.08122	-1.0782	-1.07524
-1.00122	-0.99811	-1.02356	-1.02057	-1.08515	-1.08209	-1.13935	-1.13627	-1.13324
-0.99903	-0.99593	-1.02132	-1.01838	-1.08292	-1.07991	-1.13716	-1.13415	-1.13117
-1.02535	-1.02218	-1.11903	-1.11595	-1.11289	-1.10984	-1.13498	-1.13201	-1.12907
-1.02312	-1.02008	-1.11686	-1.1138	-1.11075	-1.10768	-1.13279	-1.12983	-1.12692
-1.02094	-1.01801	-1.11472	-1.11168	-1.10863	-1.10554	-1.13062	-1.12767	-1.12477
-1.11865	-1.11561	-1.11257	-1.10953	-1.13465	-1.13153	-1.192	-1.18894	-1.18592
-1.11642	-1.11344	-1.11042	-1.10738	-1.13249	-1.1294	-1.18978	-1.18672	-1.1837
-1.11429	-1.1113	-1.13647	-1.13338	-1.22022	-1.217	-1.27399	-1.27077	-1.2676
-1.11222	-1.10919	-1.13431	-1.13119	-1.21789	-1.21478	-1.27176	-1.26854	-1.26533
-1.11012	-1.10706	-1.22212	-1.2188	-1.24583	-1.24271	-1.30144	-1.29811	-1.29476
-1.10798	-1.10491	-1.2198	-1.21653	-1.24355	-1.24042	-1.29904	-1.2957	-1.29238
-1.22409	-1.22077	-1.21751	-1.21431	-1.27121	-1.26803	-1.32493	-1.32156	-1.31822
-1.22176	-1.21847	-1.21527	-1.21214	-1.26899	-1.26577	-1.32253	-1.3192	-1.31592
-1.21945	-1.21622	-1.21307	-1.20999	-1.26678	-1.26354	-1.32016	-1.31688	-1.31366
-1.2172	-1.21403	-1.27094	-1.26774	-1.32458	-1.32118	-1.31784	-1.31462	-1.31145
-1.21498	-1.21188	-1.26872	-1.26549	-1.3222	-1.31886	-1.31557	-1.3124	-1.30927
-1.24321	-1.24008	-1.32662	-1.32322	-1.31988	-1.3166	-1.36248	-1.359	-1.35555
-1.24102	-1.23786	-1.32425	-1.32089	-1.3176	-1.3144	-1.36019	-1.35667	-1.35315
-1.29725	-1.29396	-1.32192	-1.31861	-1.3648	-1.36132	-1.35784	-1.35429	-1.35074
-1.29496	-1.2917	-1.31964	-1.31642	-1.36249	-1.35895	-1.35541	-1.35185	-1.34831
-1.32397	-1.32064	-1.36715	-1.36367	-1.36015	-1.35655	-1.35297	-1.34942	-1.34591
-1.32168	-1.31842	-1.36479	-1.36127	-1.35772	-1.35412	-1.35054	-1.34701	-1.34354
-1.31943	-1.31623	-1.36244	-1.35886	-1.35528	-1.35168	-1.34812	-1.34464	-1.34122
-1.36714	-1.36361	-1.36004	-1.35644	-1.35286	-1.34926	-1.36303	-1.35953	-1.3561
-1.36478	-1.3612	-1.35761	-1.35402	-1.35045	-1.34688	-1.36062	-1.35719	-1.35385
-1.36239	-1.35877	-1.35519	-1.35164	-1.36544	-1.36183	-1.35831	-1.3549	-1.35161
-1.35998	-1.35633	-1.35277	-1.34929	-1.3631	-1.35956	-1.35608	-1.35266	-1.34938
-1.37513	-1.37138	-1.36776	-1.36425	-1.36076	-1.35726	-1.35385	-1.35049	-1.34731
-1.37271	-1.36898	-1.36537	-1.36185	-1.35837	-1.35489	-1.35154	-1.34838	-1.34544
-1.37029	-1.3666	-1.363	-1.35949	-1.35603	-1.35259	-1.34936	-1.34641	-1.34373
-1.36781	-1.3642	-1.36064	-1.35714	-1.35376	-1.35045	-1.34738	-1.34466	-1.34219
-1.36535	-1.36182	-1.35832	-1.35486	-1.35157	-1.34844	-1.34557	-1.34306	-1.34079
-1.36294	-1.35951	-1.35609	-1.3527	-1.3765	-1.37351	-1.41367	-1.41117	-1.40887
-1.36059	-1.35726	-1.35394	-1.35065	-1.37457	-1.37182	-1.41213	-1.40973	-1.4075
-1.3583	-1.35502	-1.37884	-1.37566	-1.41568	-1.41305	-1.41062	-1.40833	-1.40618
-1.35609	-1.35278	-1.3767	-1.37378	-1.41395	-1.41145	-1.40913	-1.40696	-1.40492
-1.39907	-1.39573	-1.41765	-1.4149	-1.41233	-1.40995	-1.40772	-1.40564	-1.40367
-1.39688	-1.39376	-1.41584	-1.41327	-1.41085	-1.40857	-1.40641	-1.40437	-1.40241
-1.3948	-1.39193	-1.41416	-1.41174	-1.40943	-1.40725	-1.40515	-1.40312	-1.40115
-1.4178	-1.41509	-1.41257	-1.41025	-1.40803	-1.4059	-1.40384	-1.40185	-1.39993

-1.416	-1.41348	-1.41108	-1.40883	-1.40666	-1.40457	-1.40255	-1.4006	-1.39872
-1.41439	-1.41198	-1.40967	-1.40749	-1.40538	-1.40333	-1.50656	-1.50428	-1.50206
-1.41295	-1.4106	-1.40835	-1.40622	-1.40416	-1.40217	-1.50521	-1.50293	-1.5007
-1.4115	-1.40922	-1.40703	-1.40495	-1.47876	-1.47663	-1.50382	-1.50156	-1.49932
-1.41002	-1.40783	-1.40571	-1.40367	-1.47741	-1.47531	-1.50238	-1.50013	-1.49791
-1.45798	-1.45575	-1.48038	-1.47822	-1.50548	-1.50321	-1.52661	-1.52422	-1.52187
-1.45662	-1.45444	-1.47906	-1.47694	-1.50411	-1.50185	-1.52515	-1.5228	-1.52048
-1.45531	-1.45315	-1.47776	-1.47567	-1.50275	-1.5005	-1.52373	-1.52141	-1.51913
-1.45398	-1.45186	-1.50581	-1.50355	-1.52693	-1.5246	-1.5223	-1.52002	-1.51776
-1.45266	-1.45059	-1.50437	-1.50208	-1.52537	-1.52312	-1.52088	-1.51863	-1.51639
-1.47809	-1.47598	-1.52867	-1.52624	-1.52389	-1.52165	-1.51943	-1.5172	-1.51498
-1.47687	-1.47476	-1.52725	-1.52483	-1.52248	-1.52019	-1.51795	-1.51573	-1.51354
-1.53064	-1.52822	-1.52582	-1.52343	-1.52108	-1.51878	-1.54068	-1.53825	-1.53588
-1.52917	-1.52676	-1.52438	-1.52204	-1.51972	-1.51743	-1.53921	-1.53679	-1.53443
-1.52767	-1.52527	-1.52293	-1.52062	-1.51834	-1.51608	-1.53774	-1.53534	-1.53301
-1.52609	-1.52373	-1.52141	-1.51913	-1.51687	-1.51464	-1.5362	-1.53388	-1.53162
-1.52451	-1.52219	-1.51989	-1.51762	-1.51539	-1.51319	-1.53468	-1.53244	-1.53026
-1.52302	-1.52072	-1.51844	-1.51619	-1.53788	-1.53553	-1.53326	-1.53107	-1.52894
-1.52158	-1.5193	-1.51704	-1.51481	-1.53641	-1.53412	-1.5319	-1.52976	-1.52765
-1.52016	-1.5179	-1.5397	-1.53729	-1.53496	-1.53273	-1.53056	-1.52844	-1.52635
-1.51877	-1.5165	-1.5382	-1.53583	-1.53355	-1.53137	-1.52924	-1.52712	-1.52503
-1.51736	-1.51512	-1.53672	-1.53441	-1.53217	-1.53001	-1.54942	-1.54726	-1.54514
-1.51595	-1.51374	-1.53526	-1.53303	-1.53083	-1.52865	-1.548	-1.54584	-1.54372
-1.51454	-1.51237	-1.53381	-1.53165	-1.52948	-1.52728	-1.54659	-1.54444	-1.54234
-1.53688	-1.53457	-1.53236	-1.53023	-1.54961	-1.54738	-1.54521	-1.54311	-1.54108
-1.53532	-1.5331	-1.53093	-1.52881	-1.54817	-1.54599	-1.54386	-1.54184	-1.53987
-1.55548	-1.55327	-1.55109	-1.54894	-1.54681	-1.54467	-1.54259	-1.5406	-1.53866
-1.55408	-1.55191	-1.54974	-1.5476	-1.54549	-1.5434	-1.54137	-1.5394	-1.53746
-1.55271	-1.55056	-1.54842	-1.54628	-1.56064	-1.55858	-1.61315	-1.61105	-1.60898
-1.55139	-1.54924	-1.5471	-1.54498	-1.55935	-1.55734	-1.61187	-1.60976	-1.60767
-1.55008	-1.54793	-1.61917	-1.61695	-1.65629	-1.6541	-1.6833	-1.68092	-1.67856
-1.54875	-1.54661	-1.61781	-1.61563	-1.65492	-1.65274	-1.68185	-1.67946	-1.67708
-1.54744	-1.54531	-1.61647	-1.61432	-1.65357	-1.65137	-1.68039	-1.67799	-1.6756
-1.61954	-1.61731	-1.65662	-1.65441	-1.66358	-1.66137	-1.704	-1.70148	-1.69899
-1.61819	-1.61599	-1.65524	-1.65305	-1.66221	-1.65996	-1.70236	-1.69992	-1.69754
-1.65837	-1.6561	-1.66522	-1.66302	-1.70595	-1.70335	-1.70081	-1.69842	-1.69609
-1.65698	-1.65474	-1.66384	-1.66159	-1.70431	-1.7018	-1.69935	-1.69698	-1.69466
-1.66693	-1.66471	-1.7079	-1.70527	-1.70273	-1.70028	-1.69789	-1.69554	-1.69321
-1.66555	-1.66334	-1.70636	-1.70374	-1.70122	-1.69881	-1.69644	-1.69408	-1.69176
-1.66417	-1.66196	-1.70481	-1.70223	-1.69973	-1.69734	-1.69499	-1.69264	-1.69031
-1.66277	-1.66054	-1.70321	-1.70069	-1.69825	-1.69588	-1.69354	-1.69121	-1.6889
-1.66138	-1.65912	-1.70161	-1.69917	-1.69677	-1.69442	-1.6921	-1.68979	-1.68751
-1.70519	-1.70259	-1.7001	-1.69768	-1.6953	-1.69295	-1.7021	-1.69969	-1.69732
-1.70369	-1.70112	-1.69864	-1.69622	-1.69384	-1.69147	-1.70054	-1.69819	-1.69588
-1.70219	-1.69964	-1.69717	-1.69476	-1.69239	-1.69005	-1.69904	-1.69672	-1.69445
-1.7007	-1.69816	-1.6957	-1.6933	-1.69096	-1.68867	-1.6976	-1.6953	-1.69303
-1.69921	-1.69668	-1.69424	-1.69186	-1.70091	-1.69852	-1.69617	-1.69388	-1.69161
-1.69775	-1.69522	-1.69279	-1.69043	-1.69942	-1.69706	-1.69475	-1.69246	-1.69019
-1.69629	-1.69379	-1.69138	-1.68904	-1.69795	-1.69562	-1.69333	-1.69105	-1.68878
-1.69486	-1.69241	-1.7014	-1.69892	-1.69652	-1.69421	-1.69192	-1.68964	-1.68737

-1.69343	-1.69105	-1.69998	-1.69752	-1.69512	-1.69281	-1.69052	-1.68823	-1.68596
-1.69201	-1.68969	-1.69856	-1.69611	-1.71123	-1.70889	-1.70657	-1.70426	-1.70197
-1.6906	-1.68833	-1.69714	-1.69471	-1.70981	-1.70747	-1.70515	-1.70285	-1.70056
-1.7005	-1.69814	-1.71324	-1.71079	-1.70839	-1.70605	-1.70373	-1.70143	-1.69916
-1.69903	-1.69671	-1.71181	-1.70936	-1.70696	-1.70462	-1.70231	-1.70002	-1.69775
-1.71509	-1.71277	-1.71038	-1.70795	-1.70556	-1.70321	-1.76077	-1.75814	-1.75556
-1.71365	-1.71131	-1.70895	-1.70657	-1.7042	-1.70183	-1.75916	-1.75654	-1.75397
-1.71221	-1.70986	-1.70751	-1.7052	-1.70285	-1.70044	-1.75756	-1.75495	-1.75239
-1.71076	-1.70839	-1.72063	-1.71832	-1.76146	-1.75869	-1.75598	-1.75338	-1.75085
-1.70932	-1.70693	-1.71917	-1.71687	-1.75986	-1.75711	-1.75441	-1.75183	-1.74932
-1.72252	-1.72008	-1.71773	-1.71546	-1.75827	-1.75553	-1.75285	-1.7503	-1.74782
-1.72113	-1.71868	-1.71632	-1.71405	-1.75669	-1.75396	-1.75129	-1.74878	-1.74635
-1.71972	-1.71728	-1.7603	-1.75773	-1.75512	-1.75243	-1.7498	-1.74731	-1.74489
-1.71829	-1.71588	-1.75874	-1.75616	-1.75358	-1.75098	-1.74842	-1.74592	-1.74347
-1.76253	-1.75984	-1.7572	-1.75461	-1.75205	-1.74953	-1.74703	-1.74454	-1.74206
-1.76097	-1.75836	-1.75572	-1.75309	-1.75052	-1.74804	-1.74559	-1.74312	-1.74067
-1.75945	-1.75689	-1.75426	-1.75158	-1.749	-1.74655	-1.74413	-1.7417	-1.73928
-1.75791	-1.75537	-1.75276	-1.75008	-1.7475	-1.74505	-1.74264	-1.74024	-1.73786
-1.75638	-1.75384	-1.75124	-1.7486	-1.74603	-1.74356	-1.74114	-1.73876	-1.73641
-1.7549	-1.75233	-1.74974	-1.74714	-1.7446	-1.7421	-1.73966	-1.7373	-1.73498
-1.75345	-1.75084	-1.74827	-1.74573	-1.7432	-1.74067	-1.7382	-1.73586	-1.73357
-1.75198	-1.74935	-1.74679	-1.74429	-1.74178	-1.73926	-1.7368	-1.73445	-1.73218
-1.75044	-1.74783	-1.74528	-1.74279	-1.74032	-1.73788	-1.73547	-1.73312	-1.7308
-1.7489	-1.74632	-1.74379	-1.74129	-1.73885	-1.7365	-1.73416	-1.73179	-1.72944
-1.74739	-1.74485	-1.74233	-1.73982	-1.7374	-1.73509	-1.77356	-1.77096	-1.76838
-1.74591	-1.74341	-1.7409	-1.73839	-1.73598	-1.73368	-1.77203	-1.76945	-1.76688
-1.74446	-1.74195	-1.73946	-1.73701	-1.75223	-1.74987	-1.77052	-1.76796	-1.76541
-1.74303	-1.74049	-1.73804	-1.73567	-1.7509	-1.74851	-1.76904	-1.76649	-1.76396
-1.7416	-1.73907	-1.73665	-1.73432	-1.7727	-1.77012	-1.76756	-1.76502	-1.7625
-1.74016	-1.73771	-1.73531	-1.73296	-1.7712	-1.76865	-1.7661	-1.76355	-1.76102
-1.73872	-1.73634	-1.77487	-1.77227	-1.76969	-1.76715	-1.76462	-1.76207	-1.75953
-1.73727	-1.73493	-1.77334	-1.77074	-1.76816	-1.76562	-1.76309	-1.76055	-1.75803
-1.73584	-1.73353	-1.77181	-1.76921	-1.76664	-1.76409	-1.76156	-1.75904	-1.75654
-1.73447	-1.73215	-1.7703	-1.76771	-1.76515	-1.7626	-1.76007	-1.75758	-1.75511
-1.73315	-1.73079	-1.7688	-1.76624	-1.76368	-1.76112	-1.7586	-1.75614	-1.75372
-1.77251	-1.76991	-1.76733	-1.76476	-1.76221	-1.75967	-1.81698	-1.81407	-1.8112
-1.77103	-1.76845	-1.76587	-1.76328	-1.76074	-1.75824	-1.8153	-1.8124	-1.80954
-1.76955	-1.76698	-1.76998	-1.76742	-1.76489	-1.76242	-1.82833	-1.82543	-1.82258
-1.76808	-1.76548	-1.76851	-1.76598	-1.76348	-1.76101	-1.82666	-1.8238	-1.82098
-1.77218	-1.76958	-1.76704	-1.76455	-1.83085	-1.82791	-1.85333	-1.8503	-1.84731
-1.77068	-1.76811	-1.76559	-1.76311	-1.82915	-1.82624	-1.85167	-1.84865	-1.84567
-1.76918	-1.76666	-1.76416	-1.76168	-1.82746	-1.82459	-1.85001	-1.847	-1.84403
-1.78291	-1.78037	-1.79304	-1.79055	-1.85442	-1.85136	-1.84834	-1.84536	-1.8424
-1.78146	-1.7789	-1.79158	-1.78914	-1.85273	-1.84968	-1.84667	-1.84371	-1.84079
-1.79525	-1.79266	-1.85714	-1.85407	-1.85103	-1.848	-1.845	-1.84206	-1.83916
-1.79386	-1.79129	-1.85545	-1.85237	-1.84933	-1.84632	-1.84335	-1.84041	-1.83752
-1.80832	-1.80558	-1.85378	-1.85069	-1.84765	-1.84465	-1.8417	-1.83878	-1.8359
-1.80684	-1.80416	-1.85215	-1.84906	-1.84601	-1.843	-1.84004	-1.83716	-1.83432
-1.80536	-1.80274	-1.85052	-1.84744	-1.8444	-1.84137	-1.83842	-1.83556	-1.83275
-1.85496	-1.85189	-1.84885	-1.84582	-1.84282	-1.83981	-1.85302	-1.85007	-1.84717

-1.85328	-1.85019	-1.84717	-1.84421	-1.84125	-1.83826	-1.85145	-1.84848	-1.84556
-1.85163	-1.84853	-1.84551	-1.84257	-1.83964	-1.83671	-1.84989	-1.84692	-1.84399
-1.85001	-1.84691	-1.84388	-1.84092	-1.838	-1.83514	-1.84833	-1.84539	-1.84246
-1.84837	-1.8453	-1.8553	-1.85231	-1.86536	-1.86243	-1.87183	-1.8689	-1.86599
-1.84671	-1.8437	-1.85373	-1.85075	-1.86375	-1.8608	-1.8702	-1.86731	-1.86445
-1.85809	-1.85512	-1.86462	-1.86166	-1.87447	-1.87151	-1.86859	-1.86573	-1.8629
-1.85643	-1.85346	-1.863	-1.86011	-1.87291	-1.86995	-1.86703	-1.86415	-1.86131
-1.8548	-1.85182	-1.86138	-1.85855	-1.87134	-1.8684	-1.86549	-1.86259	-1.85974
-1.8657	-1.86271	-1.87555	-1.87263	-1.86973	-1.86683	-1.86395	-1.86107	-1.85822
-1.86417	-1.86118	-1.87395	-1.87101	-1.8681	-1.86525	-1.86241	-1.85956	-1.85674
-1.87844	-1.8754	-1.8724	-1.86943	-1.86651	-1.86367	-1.86086	-1.85806	-1.85527
-1.87688	-1.87388	-1.87088	-1.86789	-1.86496	-1.86211	-1.85931	-1.85655	-1.85382
-1.87529	-1.87233	-1.86935	-1.86636	-1.86343	-1.86058	-1.85778	-1.85504	-1.85235
-1.87367	-1.87076	-1.86781	-1.86484	-1.86192	-1.85908	-1.85629	-1.85355	-1.85085
-1.87205	-1.86918	-1.86626	-1.86332	-1.86044	-1.85761	-1.85482	-1.85208	-1.84938
-1.87045	-1.86757	-1.86469	-1.86183	-1.85898	-1.85615	-1.85337	-1.85063	-1.84795
-1.86887	-1.86597	-1.86312	-1.86033	-1.85754	-1.85472	-1.85194	-1.84922	-1.84655
-1.86733	-1.86442	-1.8616	-1.85884	-1.85608	-1.85329	-1.85053	-1.84782	-1.84517
-1.86582	-1.86291	-1.86009	-1.85735	-1.85462	-1.85186	-1.84914	-1.84645	-1.84381
-1.86431	-1.86141	-1.85859	-1.85585	-1.85313	-1.85042	-1.84774	-1.84508	-1.84246
-1.86281	-1.85992	-1.8571	-1.85433	-1.85162	-1.84895	-1.84632	-1.84371	-1.84113
-1.86133	-1.85845	-1.85562	-1.85285	-1.87909	-1.87644	-1.87382	-1.87121	-1.86861
-1.85986	-1.85698	-1.85416	-1.85141	-1.87763	-1.87501	-1.87242	-1.86982	-1.86723
-1.85839	-1.85553	-1.85272	-1.84999	-1.87619	-1.87359	-1.87101	-1.86843	-1.86585
-1.85694	-1.85409	-1.88021	-1.87744	-1.87474	-1.87214	-1.86957	-1.867	-1.86446
-1.8555	-1.85266	-1.87875	-1.876	-1.87331	-1.87069	-1.86812	-1.86557	-1.86306
-1.88296	-1.8801	-1.87731	-1.87458	-1.8719	-1.86929	-1.86672	-1.86419	-1.8617
-1.88147	-1.87864	-1.87587	-1.87317	-1.87052	-1.86791	-1.86535	-1.86284	-1.86036
-1.87999	-1.87719	-1.87445	-1.87177	-1.86913	-1.86653	-1.86397	-1.86147	-1.85901
-1.87853	-1.87575	-1.87303	-1.87036	-1.86773	-1.86513	-1.86258	-1.86009	-1.85765
-1.87707	-1.87432	-1.87162	-1.86896	-1.86634	-1.86373	-1.86118	-1.85871	-1.85628
-1.87561	-1.8729	-1.87023	-1.86758	-1.86495	-1.86234	-1.85978	-1.85729	-1.85486
-1.87416	-1.8715	-1.86884	-1.8662	-1.86357	-1.86096	-1.8584	-1.8559	-1.85347
-1.87273	-1.87009	-1.86744	-1.86481	-1.8622	-1.8596	-1.85705	-1.85457	-1.85215
-1.87132	-1.86868	-1.86605	-1.86342	-1.86083	-1.85825	-1.85572	-1.85327	-1.85087
-1.86991	-1.86728	-1.86465	-1.86204	-1.85946	-1.8569	-1.8544	-1.85199	-1.84963
-1.86852	-1.86588	-1.86326	-1.86066	-1.8581	-1.85556	-1.85309	-1.85073	-1.84842
-1.86713	-1.86449	-1.86188	-1.85929	-1.85675	-1.85424	-1.8518	-1.84947	-1.8472
-1.86575	-1.86312	-1.86052	-1.85794	-1.85542	-1.85295	-1.85054	-1.84822	-1.84596
-1.86438	-1.86176	-1.85918	-1.85662	-1.85412	-1.85168	-1.8493	-1.84699	-1.84472
-1.86301	-1.86041	-1.85786	-1.85535	-1.85289	-1.85047	-1.8481	-1.84578	-1.8435
-1.86168	-1.85909	-1.85657	-1.8541	-1.85167	-1.84927	-1.84691	-1.84458	-1.84227
-1.8604	-1.85782	-1.8553	-1.85284	-1.85041	-1.84803	-1.84567	-1.84335	-1.84104
-1.85916	-1.85658	-1.85405	-1.85158	-1.84915	-1.84677	-1.84442	-1.8421	-1.83979
-1.85786	-1.8553	-1.85278	-1.85031	-1.84789	-1.84552	-1.85537	-1.85304	-1.85073
-1.85652	-1.85398	-1.85149	-1.84904	-1.84663	-1.84426	-1.85411	-1.85178	-1.84946
-1.85519	-1.85267	-1.85019	-1.84777	-1.85755	-1.85519	-1.8844	-1.882	-1.87961
-1.85386	-1.85135	-1.8489	-1.8465	-1.85629	-1.85394	-1.88313	-1.88074	-1.87835
-1.86471	-1.86222	-1.85979	-1.8574	-1.88665	-1.88425	-1.88186	-1.87947	-1.87708
-1.86343	-1.86096	-1.85854	-1.85615	-1.88538	-1.88299	-1.8806	-1.87821	-1.87582

-1.86218	-1.85973	-1.85731	-1.85493	-1.88414	-1.88175	-1.87935	-1.87696	-1.87457
-1.86097	-1.85854	-1.88781	-1.88538	-1.88296	-1.88056	-1.87817	-1.87577	-1.87337
-1.85981	-1.85739	-1.88665	-1.88423	-1.88182	-1.87942	-1.87702	-1.8746	-1.87219
-1.87962	-1.8772	-1.88543	-1.88302	-1.88062	-1.87821	-1.87581	-1.87339	-1.87097
-1.87835	-1.87593	-1.88415	-1.88176	-1.87936	-1.87695	-1.87454	-1.87212	-1.86971
-1.88775	-1.8853	-1.88288	-1.88048	-1.87809	-1.87568	-1.87327	-1.87086	-1.86845
-1.88648	-1.88404	-1.88161	-1.87921	-1.87681	-1.8744	-1.87199	-1.86959	-1.86719
-1.88524	-1.8828	-1.88038	-1.87796	-1.87555	-1.87314	-1.87074	-1.86834	-1.86594
-1.88406	-1.88163	-1.8792	-1.87678	-1.87437	-1.87195	-1.86954	-1.86714	-1.86476
-1.8829	-1.88048	-1.87805	-1.87563	-1.8732	-1.87078	-1.86836	-1.86597	-1.86359
-1.88168	-1.87926	-1.87683	-1.8744	-1.87198	-1.86955	-1.86714	-1.86474	-1.86236
-1.88043	-1.878	-1.87557	-1.87313	-1.87071	-1.8683	-1.86589	-1.86349	-1.8611
-1.87922	-1.87679	-1.87434	-1.87189	-1.86945	-1.86704	-1.86464	-1.86224	-1.85985
-1.87808	-1.87563	-1.87316	-1.87066	-1.8682	-1.86578	-1.86337	-1.86098	-1.8586
-1.87692	-1.87446	-1.87197	-1.86945	-1.86698	-1.86454	-1.86213	-1.85974	-1.85737
-1.87569	-1.87324	-1.87076	-1.86827	-1.8658	-1.86336	-1.86095	-1.85857	-1.8562
-1.87446	-1.87201	-1.86955	-1.86708	-1.86463	-1.8622	-1.85978	-1.8574	-1.85504
-1.87327	-1.87083	-1.86836	-1.86589	-1.86343	-1.86099	-1.85857	-1.85618	-1.85382
-1.8721	-1.86965	-1.86718	-1.86468	-1.86221	-1.85976	-1.85734	-1.85495	-1.85258
-1.87089	-1.86844	-1.86597	-1.86347	-1.861	-1.85856	-1.85614	-1.85375	-1.85138
-1.86965	-1.8672	-1.86474	-1.86226	-1.8598	-1.85738	-1.85498	-1.85259	-1.85022
-1.86844	-1.86599	-1.86352	-1.86104	-1.85859	-1.85618	-1.85378	-1.8514	-1.84903
-1.86727	-1.86482	-1.86234	-1.85983	-1.85736	-1.85493	-1.85253	-1.85015	-1.84778
-1.86609	-1.86363	-1.86114	-1.85862	-1.85614	-1.8537	-1.87813	-1.87571	-1.87331
-1.86485	-1.86239	-1.85991	-1.85742	-1.85496	-1.85253	-1.87694	-1.87452	-1.87211
-1.86361	-1.86114	-1.85868	-1.85624	-1.8538	-1.85139	-1.87577	-1.87334	-1.87092
-1.86243	-1.85995	-1.8575	-1.85506	-1.85264	-1.85023	-1.87458	-1.87213	-1.86968
-1.86127	-1.8588	-1.85634	-1.85389	-1.85147	-1.84906	-1.87339	-1.87091	-1.86843
-1.86011	-1.85764	-1.85517	-1.85271	-1.85028	-1.84787	-1.87217	-1.86969	-1.8672
-1.85893	-1.85647	-1.854	-1.85151	-1.84906	-1.84665	-1.87092	-1.86846	-1.86599
-1.85774	-1.85529	-1.85281	-1.85031	-1.84786	-1.84545	-1.86969	-1.86724	-1.86479
-1.85649	-1.85405	-1.85159	-1.84913	-1.84669	-1.84428	-1.86849	-1.86605	-1.8636
-1.85526	-1.85281	-1.85037	-1.84795	-1.84553	-1.84312	-1.8673	-1.86485	-1.86239
-1.85408	-1.85164	-1.84921	-1.84679	-1.84438	-1.84197	-1.86612	-1.86364	-1.86116
-1.85293	-1.8505	-1.84806	-1.84564	-1.84322	-1.84081	-1.86493	-1.86243	-1.85991
-1.85178	-1.84934	-1.84689	-1.84444	-1.8686	-1.86615	-1.86369	-1.8612	-1.8587
-1.85063	-1.84818	-1.84571	-1.84322	-1.86733	-1.86487	-1.86242	-1.85996	-1.8575
-1.84947	-1.847	-1.84452	-1.84202	-1.86609	-1.86363	-1.86119	-1.85874	-1.85631
-1.84831	-1.8458	-1.84331	-1.84084	-1.8649	-1.86244	-1.86	-1.85756	-1.85513
-1.84714	-1.84461	-1.84212	-1.83968	-1.86372	-1.86126	-1.85882	-1.85638	-1.85394
-1.84599	-1.84345	-1.84096	-1.83852	-1.86254	-1.8601	-1.85765	-1.85521	-1.85276
-1.84483	-1.84229	-1.83981	-1.83737	-1.86138	-1.85893	-1.85649	-1.85403	-1.85156
-1.84363	-1.84113	-1.86514	-1.86267	-1.86021	-1.85776	-1.86764	-1.86514	-1.86264
-1.84243	-1.83996	-1.86396	-1.86151	-1.85905	-1.85658	-1.86642	-1.8639	-1.86137
-1.84124	-1.83879	-1.86278	-1.86033	-1.87019	-1.86769	-1.86519	-1.86266	-1.86013
-1.84007	-1.83763	-1.8616	-1.85915	-1.86898	-1.86645	-1.86392	-1.86141	-1.85892
-1.83891	-1.83647	-1.87273	-1.87027	-1.86778	-1.86522	-1.86268	-1.86018	-1.85771
-1.83775	-1.83531	-1.87154	-1.86908	-1.86658	-1.86401	-1.86146	-1.85897	-1.8565
-1.87531	-1.87282	-1.87035	-1.86789	-1.86538	-1.86281	-1.86025	-1.85776	-1.8553
-1.87411	-1.87163	-1.86915	-1.8667	-1.86419	-1.86162	-1.85906	-1.85656	-1.8541

-1.87292	-1.87043	-1.86796	-1.8655	-1.863	-1.86043	-1.85787	-1.85537	-1.8529
-1.87173	-1.86924	-1.86677	-1.86431	-1.8618	-1.85923	-1.85667	-1.85417	-1.8517
-1.87053	-1.86805	-1.86557	-1.86311	-1.8606	-1.85803	-1.85546	-1.85297	-1.8505
-1.86934	-1.86685	-1.86438	-1.86191	-1.85941	-1.85683	-1.85426	-1.85176	-1.84929
-1.86814	-1.86566	-1.86318	-1.86072	-1.85821	-1.85563	-1.85306	-1.85056	-1.84808
-1.86695	-1.86446	-1.86198	-1.85952	-1.857	-1.85442	-1.85186	-1.84935	-1.84687
-1.86575	-1.86327	-1.86079	-1.85831	-1.85579	-1.85321	-1.85064	-1.84814	-1.84566
-1.86455	-1.86207	-1.85959	-1.85711	-1.85458	-1.852	-1.84943	-1.84692	-1.84444
-1.86336	-1.86088	-1.85839	-1.8559	-1.85337	-1.85078	-1.84821	-1.84571	-1.84324
-1.86218	-1.85968	-1.85718	-1.85469	-1.85216	-1.84957	-1.847	-1.8445	-1.84203
-1.86103	-1.8585	-1.85598	-1.85348	-1.85095	-1.84835	-1.84578	-1.84329	-1.84082
-1.8599	-1.85733	-1.85479	-1.85227	-1.84973	-1.84714	-1.84457	-1.84208	-1.83961
-1.85874	-1.85614	-1.85359	-1.85107	-1.84852	-1.84593	-1.84337	-1.84088	-1.83841
-1.85754	-1.85493	-1.85238	-1.84986	-1.84731	-1.84473	-1.84218	-1.83968	-1.83721
-1.85634	-1.85373	-1.85117	-1.84865	-1.84612	-1.84355	-1.84101	-1.8385	-1.83601
-1.85513	-1.85253	-1.84997	-1.84745	-1.84493	-1.84241	-1.83988	-1.83735	-1.83483
-1.85393	-1.85134	-1.84878	-1.84626	-1.84375	-1.84126	-1.83876	-1.83621	-1.83365
-1.85276	-1.85019	-1.84763	-1.84509	-1.84257	-1.84009	-1.83759	-1.83503	-1.83247
-1.85161	-1.84907	-1.8465	-1.84392	-1.84138	-1.8389	-1.8364	-1.83385	-1.83129
-1.85043	-1.8479	-1.84533	-1.84274	-1.8402	-1.8377	-1.83521	-1.83268	-1.83014
-1.84924	-1.8467	-1.84414	-1.84156	-1.83901	-1.8365	-1.83401	-1.83151	-1.82901
-1.84807	-1.84553	-1.84297	-1.8404	-1.83785	-1.83533	-1.83282	-1.83034	-1.82786
-1.84694	-1.84437	-1.84182	-1.83928	-1.83673	-1.83418	-1.83165	-1.82916	-1.82667
-1.8458	-1.84323	-1.84068	-1.83815	-1.83561	-1.83304	-1.83049	-1.82799	-1.82548
-1.84466	-1.84211	-1.83955	-1.837	-1.83445	-1.83191	-1.82938	-1.82684	-1.82431
-1.84351	-1.84099	-1.83843	-1.83584	-1.83329	-1.83078	-1.82826	-1.8257	-1.82314
-1.84235	-1.83982	-1.83726	-1.83467	-1.83211	-1.8296	-1.82709	-1.82452	-1.82195
-1.84117	-1.83864	-1.83607	-1.83348	-1.83092	-1.82841	-1.82588	-1.82332	-1.82076
-1.84002	-1.83747	-1.83491	-1.83234	-1.82978	-1.82724	-1.82471	-1.82216	-1.8196
-1.8389	-1.83633	-1.83377	-1.83122	-1.82866	-1.82611	-1.82356	-1.82102	-1.81848
-1.83777	-1.8352	-1.83264	-1.8301	-1.82754	-1.82498	-1.82242	-1.81988	-1.81735
-1.83664	-1.83408	-1.83152	-1.82896	-1.82641	-1.82386	-1.8213	-1.81875	-1.8162
-1.83552	-1.83296	-1.8304	-1.82783	-1.82527	-1.82272	-1.82018	-1.81761	-1.81505
-1.8344	-1.83183	-1.82926	-1.8267	-1.82414	-1.82157	-1.81901	-1.81646	-1.81391
-1.83328	-1.83069	-1.82813	-1.82557	-1.82301	-1.82041	-1.81783	-1.8153	-1.81278
-1.83215	-1.82957	-1.827	-1.82444	-1.82185	-1.81924	-1.81664	-1.81411	-1.81159
-1.83103	-1.82846	-1.82588	-1.82329	-1.82069	-1.81805	-1.81544	-1.8129	-1.81038
-1.8299	-1.82733	-1.82475	-1.82216	-1.81954	-1.81691	-1.81429	-1.81173	-1.80919
-1.82877	-1.82619	-1.8236	-1.82103	-1.81843	-1.81581	-1.8132	-1.8106	-1.80802
-1.82764	-1.82505	-1.82247	-1.81989	-1.81731	-1.81471	-1.81211	-1.80949	-1.80687
-1.82652	-1.82393	-1.82134	-1.81875	-1.81616	-1.81359	-1.811	-1.80839	-1.80576
-1.8254	-1.82282	-1.82022	-1.8176	-1.81501	-1.81246	-1.80989	-1.80728	-1.80465
-1.82429	-1.82169	-1.81909	-1.81649	-1.8139	-1.81133	-1.80874	-1.80612	-1.80348
-1.82318	-1.82056	-1.81797	-1.81539	-1.8128	-1.8102	-1.80758	-1.80494	-1.8023
-1.82207	-1.81946	-1.81686	-1.81427	-1.81168	-1.80908	-1.80647	-1.80382	-1.80118
-1.82096	-1.81837	-1.81576	-1.81314	-1.81054	-1.80797	-1.80538	-1.80275	-1.8001
-1.81988	-1.8173	-1.81468	-1.81205	-1.80943	-1.80685	-1.80426	-1.80164	-1.79901
-1.81883	-1.81625	-1.81363	-1.81099	-1.80835	-1.80573	-1.80311	-1.80051	-1.79791
-1.81778	-1.81519	-1.81256	-1.80992	-1.80727	-1.80462	-1.80197	-1.79938	-1.79679
-1.81671	-1.81408	-1.81145	-1.80884	-1.8062	-1.80354	-1.80088	-1.79825	-1.79564

-1.81563	-1.81296	-1.81033	-1.80774	-1.80512	-1.80246	-1.7998	-1.79714	-1.79448
-1.81456	-1.81188	-1.80923	-1.80661	-1.80399	-1.80136	-1.79871	-1.79605	-1.79338
-1.81349	-1.81081	-1.80814	-1.80548	-1.80284	-1.80024	-1.79762	-1.79497	-1.7923
-1.81239	-1.80973	-1.80706	-1.80437	-1.80172	-1.79911	-1.7965	-1.79386	-1.79121
-1.81127	-1.80864	-1.80597	-1.80329	-1.80062	-1.79797	-1.79534	-1.79272	-1.79011
-1.81014	-1.80752	-1.80487	-1.8022	-1.79953	-1.79686	-1.7942	-1.79159	-1.78899
-1.80902	-1.80637	-1.80373	-1.80111	-1.79846	-1.79578	-1.79311	-1.79046	-1.78783
-1.80792	-1.80523	-1.8026	-1.80001	-1.79738	-1.79471	-1.79202	-1.78934	-1.78668
-1.80684	-1.80414	-1.80151	-1.79892	-1.7963	-1.79363	-1.79094	-1.78825	-1.78558
-1.80577	-1.80307	-1.80043	-1.79783	-1.7952	-1.79254	-1.78987	-1.78718	-1.7845
-1.79323	-1.79054	-1.79933	-1.79671	-1.79408	-1.79144	-1.78878	-1.78609	-1.78341
-1.79217	-1.78948	-1.79823	-1.79557	-1.79293	-1.79031	-1.78769	-1.78501	-1.78232
-1.79111	-1.78841	-1.79715	-1.79446	-1.79181	-1.78921	-1.7866	-1.78393	-1.78123
-1.79005	-1.78736	-1.79607	-1.79338	-1.79072	-1.78813	-1.78552	-1.78285	-1.78015
-1.78899	-1.7863	-1.78362	-1.78093	-1.78964	-1.78705	-1.78444	-1.78177	-1.77907
-1.78794	-1.78525	-1.78256	-1.77987	-1.78856	-1.78596	-1.78335	-1.78068	-1.778
-1.78688	-1.78419	-1.7815	-1.77881	-1.78748	-1.78486	-1.78224	-1.77958	-1.77691
-1.78582	-1.78314	-1.78045	-1.77775	-1.78638	-1.78373	-1.78108	-1.77844	-1.7758
-1.78476	-1.78209	-1.7794	-1.77668	-1.78528	-1.78258	-1.77991	-1.77729	-1.77467
-1.78371	-1.78104	-1.77834	-1.77562	-1.78419	-1.78147	-1.77878	-1.77616	-1.77356
-1.78267	-1.77998	-1.77728	-1.77456	-1.78311	-1.78038	-1.77768	-1.77506	-1.77246
-1.78162	-1.77893	-1.77622	-1.7735	-1.77078	-1.76807	-1.7766	-1.77398	-1.77137
-1.78056	-1.77787	-1.77516	-1.77244	-1.76973	-1.76701	-1.77553	-1.7729	-1.77028
-1.7795	-1.7768	-1.77409	-1.77138	-1.76868	-1.76597	-1.77449	-1.77184	-1.7692
-1.77844	-1.77573	-1.77302	-1.77031	-1.76762	-1.76496	-1.77349	-1.77082	-1.76814
-1.77737	-1.77465	-1.77194	-1.76923	-1.76656	-1.76395	-1.77249	-1.7698	-1.76709
-1.7763	-1.77357	-1.77086	-1.76816	-1.7655	-1.7629	-1.76028	-1.75761	-1.75491
-1.77523	-1.7725	-1.76979	-1.7671	-1.76444	-1.76183	-1.75921	-1.75654	-1.75385
-1.77417	-1.77145	-1.76875	-1.76607	-1.76341	-1.76079	-1.75815	-1.75548	-1.75278
-1.7731	-1.7704	-1.76772	-1.76508	-1.76242	-1.75977	-1.7571	-1.75441	-1.7517
-1.77206	-1.76937	-1.76671	-1.76407	-1.76141	-1.75873	-1.75604	-1.75334	-1.75063
-1.77104	-1.76839	-1.76572	-1.76305	-1.76037	-1.75767	-1.75497	-1.75226	-1.74955
-1.77003	-1.76739	-1.76472	-1.76202	-1.75932	-1.75661	-1.76491	-1.76216	-1.75941
-1.76902	-1.76637	-1.76368	-1.76097	-1.75826	-1.75554	-1.76382	-1.76107	-1.75832
-1.76802	-1.76533	-1.76263	-1.75991	-1.75719	-1.75447	-1.76273	-1.75998	-1.75724
-1.76699	-1.76428	-1.76157	-1.75885	-1.75613	-1.75341	-1.76166	-1.75891	-1.75616
-1.76593	-1.76322	-1.7605	-1.75779	-1.75508	-1.75236	-1.7606	-1.75785	-1.75511
-1.76489	-1.76217	-1.75945	-1.75674	-1.75404	-1.75134	-1.75958	-1.75683	-1.7541
-1.76386	-1.76113	-1.75841	-1.7557	-1.75302	-1.75036	-1.75862	-1.75588	-1.75314
-1.76286	-1.76013	-1.75741	-1.7547	-1.75202	-1.74937	-1.75763	-1.7549	-1.75216
-1.76192	-1.75919	-1.75647	-1.75375	-1.75105	-1.74839	-1.75661	-1.75387	-1.75112
-1.76097	-1.75825	-1.75553	-1.7528	-1.75009	-1.74739	-1.75557	-1.75282	-1.75007
-1.75996	-1.75724	-1.75453	-1.7518	-1.74909	-1.74637	-1.75453	-1.75177	-1.74902
-1.75893	-1.75621	-1.7535	-1.75079	-1.74808	-1.74536	-1.75349	-1.75073	-1.74797
-1.7579	-1.75518	-1.75248	-1.74979	-1.7471	-1.74439	-1.7525	-1.74974	-1.74697
-1.75686	-1.75414	-1.75146	-1.74882	-1.74615	-1.74345	-1.75155	-1.74879	-1.74602
-1.75587	-1.75315	-1.75047	-1.74783	-1.74516	-1.74246	-1.75056	-1.7478	-1.74504
-1.75494	-1.75222	-1.74952	-1.74683	-1.74414	-1.74143	-1.74952	-1.74676	-1.74401
-1.754	-1.75127	-1.74854	-1.74582	-1.7431	-1.74039	-1.74847	-1.74573	-1.74298
-1.75299	-1.75026	-1.74753	-1.74479	-1.74206	-1.73934	-1.74741	-1.7447	-1.74199

-1.75199	-1.74925	-1.74652	-1.74377	-1.74104	-1.73831	-1.74637	-1.74368	-1.74101
-1.75103	-1.7483	-1.74556	-1.74282	-1.74009	-1.73736	-1.74539	-1.74269	-1.74
-1.75011	-1.74737	-1.74464	-1.7419	-1.73916	-1.73644	-1.74446	-1.74172	-1.73899
-1.74913	-1.7464	-1.74366	-1.74092	-1.7382	-1.7355	-1.74352	-1.74077	-1.73802
-1.74811	-1.74538	-1.74264	-1.73991	-1.7372	-1.73454	-1.74258	-1.73984	-1.73709
-1.74713	-1.7444	-1.74167	-1.73893	-1.73622	-1.73357	-1.74161	-1.73888	-1.73612
-1.74622	-1.74349	-1.74075	-1.73801	-1.73529	-1.7326	-1.7406	-1.73785	-1.7351
-1.74532	-1.74259	-1.73985	-1.7371	-1.73436	-1.73164	-1.73959	-1.73684	-1.73408
-1.74442	-1.74169	-1.73894	-1.7362	-1.73345	-1.73072	-1.73865	-1.73589	-1.73312
-1.74351	-1.74077	-1.73803	-1.73528	-1.73254	-1.7298	-1.73771	-1.73494	-1.73217
-1.74253	-1.73979	-1.73705	-1.7343	-1.74227	-1.73949	-1.73671	-1.73394	-1.73117
-1.74151	-1.73877	-1.73603	-1.73329	-1.74124	-1.73847	-1.73569	-1.73292	-1.73014
-1.74055	-1.73781	-1.73507	-1.73233	-1.74027	-1.73749	-1.73472	-1.73194	-1.72917
-1.73965	-1.73691	-1.73417	-1.73143	-1.73935	-1.73658	-1.7338	-1.73102	-1.72825
-1.73876	-1.73602	-1.73327	-1.73053	-1.73844	-1.73566	-1.73288	-1.73011	-1.72733
-1.73787	-1.73511	-1.73237	-1.72962	-1.73753	-1.73475	-1.73197	-1.7292	-1.72642
-1.73696	-1.73419	-1.73144	-1.7287	-1.73659	-1.73381	-1.73104	-1.72826	-1.72548
-1.73602	-1.73322	-1.74114	-1.73835	-1.73557	-1.7328	-1.73002	-1.72725	-1.72447
-1.73507	-1.73222	-1.74011	-1.73732	-1.73454	-1.73176	-1.72898	-1.72621	-1.72343
-1.73409	-1.73126	-1.73914	-1.73635	-1.73356	-1.73078	-1.72801	-1.72523	-1.72246
-1.73311	-1.73032	-1.7382	-1.73542	-1.73264	-1.72985	-1.72707	-1.7243	-1.72152
-1.73215	-1.72939	-1.73727	-1.7345	-1.73172	-1.72893	-1.72615	-1.72338	-1.7206
-1.73124	-1.72848	-1.73636	-1.73359	-1.73081	-1.72803	-1.72524	-1.72247	-1.7197
-1.73033	-1.72758	-1.73545	-1.73268	-1.72991	-1.72712	-1.72434	-1.72157	-1.71879
-1.72944	-1.72669	-1.73455	-1.73178	-1.729	-1.72622	-1.72343	-1.72066	-1.71789
-1.72855	-1.7258	-1.73365	-1.73087	-1.7281	-1.72531	-1.72253	-1.71976	-1.71699
-1.72765	-1.7249	-1.73274	-1.72997	-1.72719	-1.72441	-1.72162	-1.71885	-1.71608
-1.72676	-1.72401	-1.73184	-1.72907	-1.72629	-1.7235	-1.72072	-1.71795	-1.71517
-1.72587	-1.72312	-1.73093	-1.72816	-1.72538	-1.7226	-1.71982	-1.71704	-1.71426
-1.72498	-1.72223	-1.73003	-1.72725	-1.72447	-1.72169	-1.71891	-1.71613	-1.71335
-1.72408	-1.72134	-1.72912	-1.72634	-1.72356	-1.72079	-1.71801	-1.71523	-1.71244
-1.72319	-1.72045	-1.72822	-1.72543	-1.72265	-1.71988	-1.71711	-1.71433	-1.71154
-1.7223	-1.71956	-1.72731	-1.72452	-1.72174	-1.71898	-1.71621	-1.71343	-1.71064
-1.72141	-1.71866	-1.7264	-1.72361	-1.72083	-1.71807	-1.7153	-1.71252	-1.70974
-1.72052	-1.71777	-1.7255	-1.72271	-1.71993	-1.71716	-1.7144	-1.71162	-1.70883
-1.69723	-1.69453	-1.72459	-1.7218	-1.71902	-1.71626	-1.71349	-1.71071	-1.70792
-1.69635	-1.69365	-1.72369	-1.7209	-1.71812	-1.71535	-1.71258	-1.7098	-1.70702
-1.69547	-1.69277	-1.72279	-1.72	-1.71722	-1.71445	-1.71167	-1.70889	-1.70611
-1.69459	-1.69188	-1.72189	-1.7191	-1.71632	-1.71354	-1.71077	-1.70798	-1.7052
-1.70446	-1.70172	-1.69897	-1.69624	-1.71542	-1.71264	-1.70986	-1.70708	-1.7043
-1.7036	-1.70084	-1.69809	-1.69535	-1.71452	-1.71174	-1.70896	-1.70617	-1.70339
-1.70276	-1.69999	-1.69723	-1.69447	-1.71362	-1.71084	-1.70806	-1.70527	-1.70249
-1.70197	-1.69921	-1.69642	-1.69363	-1.71275	-1.70995	-1.70715	-1.70437	-1.70159
-1.7012	-1.69844	-1.69564	-1.69282	-1.71189	-1.70906	-1.70626	-1.70347	-1.70069
-1.70037	-1.69762	-1.69483	-1.69202	-1.68921	-1.68642	-1.70539	-1.70259	-1.6998
-1.6995	-1.69676	-1.694	-1.69123	-1.68843	-1.68561	-1.70454	-1.70172	-1.69892
-1.69862	-1.69589	-1.69315	-1.6904	-1.68762	-1.6848	-1.70372	-1.7009	-1.69809
-1.69773	-1.695	-1.69226	-1.68953	-1.68677	-1.68399	-1.70294	-1.70013	-1.69732
-1.69687	-1.69413	-1.69139	-1.68865	-1.68591	-1.68317	-1.68042	-1.67768	-1.67493
-1.69608	-1.69329	-1.69052	-1.68778	-1.68503	-1.68229	-1.67956	-1.67683	-1.67409

						-0.10822	-0.10792	-0.10761
						-0.10795	-0.10766	-0.10736
						-0.30503	-0.30412	-0.3032
						-0.30434	-0.30339	-0.30244
				-0.16248	-0.16201	-0.30362	-0.30267	-0.30171
				-0.16207	-0.16161	-0.30289	-0.30195	-0.30102
		-0.1626	-0.16213	-0.30399	-0.30308	-0.43168	-0.43023	-0.4288
		-0.1622	-0.16173	-0.30329	-0.30235	-0.43058	-0.42917	-0.42781
		-0.1618	-0.16134	-0.30259	-0.30163	-0.42949	-0.42814	-0.42685
-0.1624	-0.16189	-0.3037	-0.30281	-0.43139	-0.42991	-0.56548	-0.5637	-0.56196
-0.162	-0.16149	-0.30301	-0.30212	-0.43037	-0.42893	-0.56416	-0.56241	-0.5607
-0.25435	-0.25358	-0.43213	-0.43075	-0.47554	-0.47404	-0.6006	-0.59874	-0.59691
-0.25372	-0.25298	-0.4311	-0.42972	-0.47446	-0.47302	-0.59919	-0.59733	-0.5955
-0.3865	-0.38523	-0.47629	-0.47483	-0.60161	-0.59968	-0.64392	-0.64198	-0.64006
-0.38557	-0.38431	-0.47522	-0.47376	-0.60014	-0.59823	-0.64242	-0.64051	-0.63864
-0.38466	-0.38341	-0.47417	-0.47272	-0.59869	-0.5968	-0.64093	-0.63907	-0.63725
-0.476	-0.47459	-0.47316	-0.47171	-0.59727	-0.5954	-0.63947	-0.63765	-0.63588
-0.47497	-0.47359	-0.47217	-0.47073	-0.59587	-0.59403	-0.63805	-0.63626	-0.63452
-0.47395	-0.47259	-0.59832	-0.59637	-0.6404	-0.63851	-0.67733	-0.67545	-0.6736
-0.47294	-0.47159	-0.59691	-0.59498	-0.63896	-0.63713	-0.6759	-0.67401	-0.67214
-0.51113	-0.50966	-0.64146	-0.63946	-0.67824	-0.67635	-0.71395	-0.71199	-0.71004
-0.51002	-0.50855	-0.63999	-0.63805	-0.67679	-0.67494	-0.71251	-0.71056	-0.70861
-0.59647	-0.5946	-0.63856	-0.63668	-0.71483	-0.71295	-0.89565	-0.8928	-0.88996
-0.59507	-0.59325	-0.63717	-0.63535	-0.71339	-0.71153	-0.8936	-0.89078	-0.88797
-0.59371	-0.59195	-0.63583	-0.63406	-0.71197	-0.71012	-0.89157	-0.88878	-0.886
-0.63812	-0.63632	-0.67499	-0.67313	-0.74469	-0.74276	-0.92974	-0.92675	-0.92377

-0.63677	-0.63505	-0.67366	-0.6718	-0.74324	-0.7413	-0.9276	-0.92463	-0.92168
-0.67594	-0.67417	-0.74579	-0.74377	-0.89091	-0.88825	-0.92549	-0.92254	-0.91959
-0.67462	-0.67285	-0.74435	-0.74232	-0.8889	-0.88628	-0.92341	-0.92045	-0.9175
-0.74685	-0.74491	-0.89252	-0.88968	-0.92694	-0.92414	-0.9983	-0.99521	-0.99214
-0.74547	-0.74348	-0.89054	-0.88774	-0.92489	-0.92207	-0.9961	-0.99304	-0.99001
-0.89419	-0.89135	-0.92873	-0.92578	-0.99993	-0.99692	-1.09021	-1.08695	-1.08375
-0.89219	-0.88936	-0.92665	-0.92378	-0.99785	-0.9948	-1.08789	-1.08469	-1.08157
-0.89017	-0.88737	-0.92458	-0.92179	-0.99577	-0.99272	-1.08562	-1.08248	-1.07943
-0.92836	-0.92536	-0.99951	-0.99659	-1.0898	-1.08657	-1.0834	-1.08032	-1.07732
-0.92623	-0.92325	-0.9973	-0.99439	-1.08744	-1.08431	-1.08122	-1.0782	-1.07524
-1.00122	-0.99811	-1.02356	-1.02057	-1.08515	-1.08209	-1.13935	-1.13627	-1.13324
-0.99903	-0.99593	-1.02132	-1.01838	-1.08292	-1.07991	-1.13716	-1.13415	-1.13117
-1.02535	-1.02218	-1.11903	-1.11595	-1.11289	-1.10984	-1.13498	-1.13201	-1.12907
-1.02312	-1.02008	-1.11686	-1.1138	-1.11075	-1.10768	-1.13279	-1.12983	-1.12692
-1.02094	-1.01801	-1.11472	-1.11168	-1.10863	-1.10554	-1.13062	-1.12767	-1.12477
-1.11865	-1.11561	-1.11257	-1.10953	-1.13465	-1.13153	-1.192	-1.18894	-1.18592
-1.11642	-1.11344	-1.11042	-1.10738	-1.13249	-1.1294	-1.18978	-1.18672	-1.1837
-1.11429	-1.1113	-1.13647	-1.13338	-1.22022	-1.217	-1.27399	-1.27077	-1.2676
-1.11222	-1.10919	-1.13431	-1.13119	-1.21789	-1.21478	-1.27176	-1.26854	-1.26533
-1.11012	-1.10706	-1.22212	-1.2188	-1.24583	-1.24271	-1.30144	-1.29811	-1.29476
-1.10798	-1.10491	-1.2198	-1.21653	-1.24355	-1.24042	-1.29904	-1.2957	-1.29238
-1.22409	-1.22077	-1.21751	-1.21431	-1.27121	-1.26803	-1.32493	-1.32156	-1.31822
-1.22176	-1.21847	-1.21527	-1.21214	-1.26899	-1.26577	-1.32253	-1.3192	-1.31592
-1.21945	-1.21622	-1.21307	-1.20999	-1.26678	-1.26354	-1.32016	-1.31688	-1.31366
-1.2172	-1.21403	-1.27094	-1.26774	-1.32458	-1.32118	-1.31784	-1.31462	-1.31145
-1.21498	-1.21188	-1.26872	-1.26549	-1.3222	-1.31886	-1.31557	-1.3124	-1.30927
-1.24321	-1.24008	-1.32662	-1.32322	-1.31988	-1.3166	-1.36248	-1.359	-1.35555
-1.24102	-1.23786	-1.32425	-1.32089	-1.3176	-1.3144	-1.36019	-1.35667	-1.35315
-1.29725	-1.29396	-1.32192	-1.31861	-1.3648	-1.36132	-1.35784	-1.35429	-1.35074
-1.29496	-1.2917	-1.31964	-1.31642	-1.36249	-1.35895	-1.35541	-1.35185	-1.34831
-1.32397	-1.32064	-1.36715	-1.36367	-1.36015	-1.35655	-1.35297	-1.34942	-1.34591
-1.32168	-1.31842	-1.36479	-1.36127	-1.35772	-1.35412	-1.35054	-1.34701	-1.34354
-1.31943	-1.31623	-1.36244	-1.35886	-1.35528	-1.35168	-1.34812	-1.34464	-1.34122
-1.36714	-1.36361	-1.36004	-1.35644	-1.35286	-1.34926	-1.36303	-1.35953	-1.3561
-1.36478	-1.3612	-1.35761	-1.35402	-1.35045	-1.34688	-1.36062	-1.35719	-1.35385
-1.36239	-1.35877	-1.35519	-1.35164	-1.36544	-1.36183	-1.35831	-1.3549	-1.35161
-1.35998	-1.35633	-1.35277	-1.34929	-1.3631	-1.35956	-1.35608	-1.35266	-1.34938
-1.37513	-1.37138	-1.36776	-1.36425	-1.36076	-1.35726	-1.35385	-1.35049	-1.34731
-1.37271	-1.36898	-1.36537	-1.36185	-1.35837	-1.35489	-1.35154	-1.34838	-1.34544
-1.37029	-1.3666	-1.363	-1.35949	-1.35603	-1.35259	-1.34936	-1.34641	-1.34373
-1.36781	-1.3642	-1.36064	-1.35714	-1.35376	-1.35045	-1.34738	-1.34466	-1.34219
-1.36535	-1.36182	-1.35832	-1.35486	-1.35157	-1.34844	-1.34557	-1.34306	-1.34079
-1.36294	-1.35951	-1.35609	-1.3527	-1.3765	-1.37351	-1.41367	-1.41117	-1.40887
-1.36059	-1.35726	-1.35394	-1.35065	-1.37457	-1.37182	-1.41213	-1.40973	-1.4075
-1.3583	-1.35502	-1.37884	-1.37566	-1.41568	-1.41305	-1.41062	-1.40833	-1.40618
-1.35609	-1.35278	-1.3767	-1.37378	-1.41395	-1.41145	-1.40913	-1.40696	-1.40492
-1.39907	-1.39573	-1.41765	-1.4149	-1.41233	-1.40995	-1.40772	-1.40564	-1.40367
-1.39688	-1.39376	-1.41584	-1.41327	-1.41085	-1.40857	-1.40641	-1.40437	-1.40241
-1.3948	-1.39193	-1.41416	-1.41174	-1.40943	-1.40725	-1.40515	-1.40312	-1.40115
-1.4178	-1.41509	-1.41257	-1.41025	-1.40803	-1.4059	-1.40384	-1.40185	-1.39993

-1.416	-1.41348	-1.41108	-1.40883	-1.40666	-1.40457	-1.40255	-1.4006	-1.39872
-1.41439	-1.41198	-1.40967	-1.40749	-1.40538	-1.40333	-1.50656	-1.50428	-1.50206
-1.41295	-1.4106	-1.40835	-1.40622	-1.40416	-1.40217	-1.50521	-1.50293	-1.5007
-1.4115	-1.40922	-1.40703	-1.40495	-1.47876	-1.47663	-1.50382	-1.50156	-1.49932
-1.41002	-1.40783	-1.40571	-1.40367	-1.47741	-1.47531	-1.50238	-1.50013	-1.49791
-1.45798	-1.45575	-1.48038	-1.47822	-1.50548	-1.50321	-1.52661	-1.52422	-1.52187
-1.45662	-1.45444	-1.47906	-1.47694	-1.50411	-1.50185	-1.52515	-1.5228	-1.52048
-1.45531	-1.45315	-1.47776	-1.47567	-1.50275	-1.5005	-1.52373	-1.52141	-1.51913
-1.45398	-1.45186	-1.50581	-1.50355	-1.52693	-1.5246	-1.5223	-1.52002	-1.51776
-1.45266	-1.45059	-1.50437	-1.50208	-1.52537	-1.52312	-1.52088	-1.51863	-1.51639
-1.47809	-1.47598	-1.52867	-1.52624	-1.52389	-1.52165	-1.51943	-1.5172	-1.51498
-1.47687	-1.47476	-1.52725	-1.52483	-1.52248	-1.52019	-1.51795	-1.51573	-1.51354
-1.53064	-1.52822	-1.52582	-1.52343	-1.52108	-1.51878	-1.54068	-1.53825	-1.53588
-1.52917	-1.52676	-1.52438	-1.52204	-1.51972	-1.51743	-1.53921	-1.53679	-1.53443
-1.52767	-1.52527	-1.52293	-1.52062	-1.51834	-1.51608	-1.53774	-1.53534	-1.53301
-1.52609	-1.52373	-1.52141	-1.51913	-1.51687	-1.51464	-1.5362	-1.53388	-1.53162
-1.52451	-1.52219	-1.51989	-1.51762	-1.51539	-1.51319	-1.53468	-1.53244	-1.53026
-1.52302	-1.52072	-1.51844	-1.51619	-1.53788	-1.53553	-1.53326	-1.53107	-1.52894
-1.52158	-1.5193	-1.51704	-1.51481	-1.53641	-1.53412	-1.5319	-1.52976	-1.52765
-1.52016	-1.5179	-1.5397	-1.53729	-1.53496	-1.53273	-1.53056	-1.52844	-1.52635
-1.51877	-1.5165	-1.5382	-1.53583	-1.53355	-1.53137	-1.52924	-1.52712	-1.52503
-1.51736	-1.51512	-1.53672	-1.53441	-1.53217	-1.53001	-1.54942	-1.54726	-1.54514
-1.51595	-1.51374	-1.53526	-1.53303	-1.53083	-1.52865	-1.548	-1.54584	-1.54372
-1.51454	-1.51237	-1.53381	-1.53165	-1.52948	-1.52728	-1.54659	-1.54444	-1.54234
-1.53688	-1.53457	-1.53236	-1.53023	-1.54961	-1.54738	-1.54521	-1.54311	-1.54108
-1.53532	-1.5331	-1.53093	-1.52881	-1.54817	-1.54599	-1.54386	-1.54184	-1.53987
-1.55548	-1.55327	-1.55109	-1.54894	-1.54681	-1.54467	-1.54259	-1.5406	-1.53866
-1.55408	-1.55191	-1.54974	-1.5476	-1.54549	-1.5434	-1.54137	-1.5394	-1.53746
-1.55271	-1.55056	-1.54842	-1.54628	-1.56064	-1.55858	-1.61315	-1.61105	-1.60898
-1.55139	-1.54924	-1.5471	-1.54498	-1.55935	-1.55734	-1.61187	-1.60976	-1.60767
-1.55008	-1.54793	-1.61917	-1.61695	-1.65629	-1.6541	-1.6833	-1.68092	-1.67856
-1.54875	-1.54661	-1.61781	-1.61563	-1.65492	-1.65274	-1.68185	-1.67946	-1.67708
-1.54744	-1.54531	-1.61647	-1.61432	-1.65357	-1.65137	-1.68039	-1.67799	-1.6756
-1.61954	-1.61731	-1.65662	-1.65441	-1.66358	-1.66137	-1.704	-1.70148	-1.69899
-1.61819	-1.61599	-1.65524	-1.65305	-1.66221	-1.65996	-1.70236	-1.69992	-1.69754
-1.65837	-1.6561	-1.66522	-1.66302	-1.70595	-1.70335	-1.70081	-1.69842	-1.69609
-1.65698	-1.65474	-1.66384	-1.66159	-1.70431	-1.7018	-1.69935	-1.69698	-1.69466
-1.66693	-1.66471	-1.7079	-1.70527	-1.70273	-1.70028	-1.69789	-1.69554	-1.69321
-1.66555	-1.66334	-1.70636	-1.70374	-1.70122	-1.69881	-1.69644	-1.69408	-1.69176
-1.66417	-1.66196	-1.70481	-1.70223	-1.69973	-1.69734	-1.69499	-1.69264	-1.69031
-1.66277	-1.66054	-1.70321	-1.70069	-1.69825	-1.69588	-1.69354	-1.69121	-1.6889
-1.66138	-1.65912	-1.70161	-1.69917	-1.69677	-1.69442	-1.6921	-1.68979	-1.68751
-1.70519	-1.70259	-1.7001	-1.69768	-1.6953	-1.69295	-1.7021	-1.69969	-1.69732
-1.70369	-1.70112	-1.69864	-1.69622	-1.69384	-1.69147	-1.70054	-1.69819	-1.69588
-1.70219	-1.69964	-1.69717	-1.69476	-1.69239	-1.69005	-1.69904	-1.69672	-1.69445
-1.7007	-1.69816	-1.6957	-1.6933	-1.69096	-1.68867	-1.6976	-1.6953	-1.69303
-1.69921	-1.69668	-1.69424	-1.69186	-1.70091	-1.69852	-1.69617	-1.69388	-1.69161
-1.69775	-1.69522	-1.69279	-1.69043	-1.69942	-1.69706	-1.69475	-1.69246	-1.69019
-1.69629	-1.69379	-1.69138	-1.68904	-1.69795	-1.69562	-1.69333	-1.69105	-1.68878
-1.69486	-1.69241	-1.7014	-1.69892	-1.69652	-1.69421	-1.69192	-1.68964	-1.68737

-1.69343	-1.69105	-1.69998	-1.69752	-1.69512	-1.69281	-1.69052	-1.68823	-1.68596
-1.69201	-1.68969	-1.69856	-1.69611	-1.71123	-1.70889	-1.70657	-1.70426	-1.70197
-1.6906	-1.68833	-1.69714	-1.69471	-1.70981	-1.70747	-1.70515	-1.70285	-1.70056
-1.7005	-1.69814	-1.71324	-1.71079	-1.70839	-1.70605	-1.70373	-1.70143	-1.69916
-1.69903	-1.69671	-1.71181	-1.70936	-1.70696	-1.70462	-1.70231	-1.70002	-1.69775
-1.71509	-1.71277	-1.71038	-1.70795	-1.70556	-1.70321	-1.76077	-1.75814	-1.75556
-1.71365	-1.71131	-1.70895	-1.70657	-1.7042	-1.70183	-1.75916	-1.75654	-1.75397
-1.71221	-1.70986	-1.70751	-1.7052	-1.70285	-1.70044	-1.75756	-1.75495	-1.75239
-1.71076	-1.70839	-1.72063	-1.71832	-1.76146	-1.75869	-1.75598	-1.75338	-1.75085
-1.70932	-1.70693	-1.71917	-1.71687	-1.75986	-1.75711	-1.75441	-1.75183	-1.74932
-1.72252	-1.72008	-1.71773	-1.71546	-1.75827	-1.75553	-1.75285	-1.7503	-1.74782
-1.72113	-1.71868	-1.71632	-1.71405	-1.75669	-1.75396	-1.75129	-1.74878	-1.74635
-1.71972	-1.71728	-1.7603	-1.75773	-1.75512	-1.75243	-1.7498	-1.74731	-1.74489
-1.71829	-1.71588	-1.75874	-1.75616	-1.75358	-1.75098	-1.74842	-1.74592	-1.74347
-1.76253	-1.75984	-1.7572	-1.75461	-1.75205	-1.74953	-1.74703	-1.74454	-1.74206
-1.76097	-1.75836	-1.75572	-1.75309	-1.75052	-1.74804	-1.74559	-1.74312	-1.74067
-1.75945	-1.75689	-1.75426	-1.75158	-1.749	-1.74655	-1.74413	-1.7417	-1.73928
-1.75791	-1.75537	-1.75276	-1.75008	-1.7475	-1.74505	-1.74264	-1.74024	-1.73786
-1.75638	-1.75384	-1.75124	-1.7486	-1.74603	-1.74356	-1.74114	-1.73876	-1.73641
-1.7549	-1.75233	-1.74974	-1.74714	-1.7446	-1.7421	-1.73966	-1.7373	-1.73498
-1.75345	-1.75084	-1.74827	-1.74573	-1.7432	-1.74067	-1.7382	-1.73586	-1.73357
-1.75198	-1.74935	-1.74679	-1.74429	-1.74178	-1.73926	-1.7368	-1.73445	-1.73218
-1.75044	-1.74783	-1.74528	-1.74279	-1.74032	-1.73788	-1.73547	-1.73312	-1.7308
-1.7489	-1.74632	-1.74379	-1.74129	-1.73885	-1.7365	-1.73416	-1.73179	-1.72944
-1.74739	-1.74485	-1.74233	-1.73982	-1.7374	-1.73509	-1.77356	-1.77096	-1.76838
-1.74591	-1.74341	-1.7409	-1.73839	-1.73598	-1.73368	-1.77203	-1.76945	-1.76688
-1.74446	-1.74195	-1.73946	-1.73701	-1.75223	-1.74987	-1.77052	-1.76796	-1.76541
-1.74303	-1.74049	-1.73804	-1.73567	-1.7509	-1.74851	-1.76904	-1.76649	-1.76396
-1.7416	-1.73907	-1.73665	-1.73432	-1.7727	-1.77012	-1.76756	-1.76502	-1.7625
-1.74016	-1.73771	-1.73531	-1.73296	-1.7712	-1.76865	-1.7661	-1.76355	-1.76102
-1.73872	-1.73634	-1.77487	-1.77227	-1.76969	-1.76715	-1.76462	-1.76207	-1.75953
-1.73727	-1.73493	-1.77334	-1.77074	-1.76816	-1.76562	-1.76309	-1.76055	-1.75803
-1.73584	-1.73353	-1.77181	-1.76921	-1.76664	-1.76409	-1.76156	-1.75904	-1.75654
-1.73447	-1.73215	-1.7703	-1.76771	-1.76515	-1.7626	-1.76007	-1.75758	-1.75511
-1.73315	-1.73079	-1.7688	-1.76624	-1.76368	-1.76112	-1.7586	-1.75614	-1.75372
-1.77251	-1.76991	-1.76733	-1.76476	-1.76221	-1.75967	-1.81698	-1.81407	-1.8112
-1.77103	-1.76845	-1.76587	-1.76328	-1.76074	-1.75824	-1.8153	-1.8124	-1.80954
-1.76955	-1.76698	-1.76998	-1.76742	-1.76489	-1.76242	-1.82833	-1.82543	-1.82258
-1.76808	-1.76548	-1.76851	-1.76598	-1.76348	-1.76101	-1.82666	-1.8238	-1.82098
-1.77218	-1.76958	-1.76704	-1.76455	-1.83085	-1.82791	-1.85333	-1.8503	-1.84731
-1.77068	-1.76811	-1.76559	-1.76311	-1.82915	-1.82624	-1.85167	-1.84865	-1.84567
-1.76918	-1.76666	-1.76416	-1.76168	-1.82746	-1.82459	-1.85001	-1.847	-1.84403
-1.78291	-1.78037	-1.79304	-1.79055	-1.85442	-1.85136	-1.84834	-1.84536	-1.8424
-1.78146	-1.7789	-1.79158	-1.78914	-1.85273	-1.84968	-1.84667	-1.84371	-1.84079
-1.79525	-1.79266	-1.85714	-1.85407	-1.85103	-1.848	-1.845	-1.84206	-1.83916
-1.79386	-1.79129	-1.85545	-1.85237	-1.84933	-1.84632	-1.84335	-1.84041	-1.83752
-1.80832	-1.80558	-1.85378	-1.85069	-1.84765	-1.84465	-1.8417	-1.83878	-1.8359
-1.80684	-1.80416	-1.85215	-1.84906	-1.84601	-1.843	-1.84004	-1.83716	-1.83432
-1.80536	-1.80274	-1.85052	-1.84744	-1.8444	-1.84137	-1.83842	-1.83556	-1.83275
-1.85496	-1.85189	-1.84885	-1.84582	-1.84282	-1.83981	-1.85302	-1.85007	-1.84717

-1.85328	-1.85019	-1.84717	-1.84421	-1.84125	-1.83826	-1.85145	-1.84848	-1.84556
-1.85163	-1.84853	-1.84551	-1.84257	-1.83964	-1.83671	-1.84989	-1.84692	-1.84399
-1.85001	-1.84691	-1.84388	-1.84092	-1.838	-1.83514	-1.84833	-1.84539	-1.84246
-1.84837	-1.8453	-1.8553	-1.85231	-1.86536	-1.86243	-1.87183	-1.8689	-1.86599
-1.84671	-1.8437	-1.85373	-1.85075	-1.86375	-1.8608	-1.8702	-1.86731	-1.86445
-1.85809	-1.85512	-1.86462	-1.86166	-1.87447	-1.87151	-1.86859	-1.86573	-1.8629
-1.85643	-1.85346	-1.863	-1.86011	-1.87291	-1.86995	-1.86703	-1.86415	-1.86131
-1.8548	-1.85182	-1.86138	-1.85855	-1.87134	-1.8684	-1.86549	-1.86259	-1.85974
-1.8657	-1.86271	-1.87555	-1.87263	-1.86973	-1.86683	-1.86395	-1.86107	-1.85822
-1.86417	-1.86118	-1.87395	-1.87101	-1.8681	-1.86525	-1.86241	-1.85956	-1.85674
-1.87844	-1.8754	-1.8724	-1.86943	-1.86651	-1.86367	-1.86086	-1.85806	-1.85527
-1.87688	-1.87388	-1.87088	-1.86789	-1.86496	-1.86211	-1.85931	-1.85655	-1.85382
-1.87529	-1.87233	-1.86935	-1.86636	-1.86343	-1.86058	-1.85778	-1.85504	-1.85235
-1.87367	-1.87076	-1.86781	-1.86484	-1.86192	-1.85908	-1.85629	-1.85355	-1.85085
-1.87205	-1.86918	-1.86626	-1.86332	-1.86044	-1.85761	-1.85482	-1.85208	-1.84938
-1.87045	-1.86757	-1.86469	-1.86183	-1.85898	-1.85615	-1.85337	-1.85063	-1.84795
-1.86887	-1.86597	-1.86312	-1.86033	-1.85754	-1.85472	-1.85194	-1.84922	-1.84655
-1.86733	-1.86442	-1.8616	-1.85884	-1.85608	-1.85329	-1.85053	-1.84782	-1.84517
-1.86582	-1.86291	-1.86009	-1.85735	-1.85462	-1.85186	-1.84914	-1.84645	-1.84381
-1.86431	-1.86141	-1.85859	-1.85585	-1.85313	-1.85042	-1.84774	-1.84508	-1.84246
-1.86281	-1.85992	-1.8571	-1.85433	-1.85162	-1.84895	-1.84632	-1.84371	-1.84113
-1.86133	-1.85845	-1.85562	-1.85285	-1.87909	-1.87644	-1.87382	-1.87121	-1.86861
-1.85986	-1.85698	-1.85416	-1.85141	-1.87763	-1.87501	-1.87242	-1.86982	-1.86723
-1.85839	-1.85553	-1.85272	-1.84999	-1.87619	-1.87359	-1.87101	-1.86843	-1.86585
-1.85694	-1.85409	-1.88021	-1.87744	-1.87474	-1.87214	-1.86957	-1.867	-1.86446
-1.8555	-1.85266	-1.87875	-1.876	-1.87331	-1.87069	-1.86812	-1.86557	-1.86306
-1.88296	-1.8801	-1.87731	-1.87458	-1.8719	-1.86929	-1.86672	-1.86419	-1.8617
-1.88147	-1.87864	-1.87587	-1.87317	-1.87052	-1.86791	-1.86535	-1.86284	-1.86036
-1.87999	-1.87719	-1.87445	-1.87177	-1.86913	-1.86653	-1.86397	-1.86147	-1.85901
-1.87853	-1.87575	-1.87303	-1.87036	-1.86773	-1.86513	-1.86258	-1.86009	-1.85765
-1.87707	-1.87432	-1.87162	-1.86896	-1.86634	-1.86373	-1.86118	-1.85871	-1.85628
-1.87561	-1.8729	-1.87023	-1.86758	-1.86495	-1.86234	-1.85978	-1.85729	-1.85486
-1.87416	-1.8715	-1.86884	-1.8662	-1.86357	-1.86096	-1.8584	-1.8559	-1.85347
-1.87273	-1.87009	-1.86744	-1.86481	-1.8622	-1.8596	-1.85705	-1.85457	-1.85215
-1.87132	-1.86868	-1.86605	-1.86342	-1.86083	-1.85825	-1.85572	-1.85327	-1.85087
-1.86991	-1.86728	-1.86465	-1.86204	-1.85946	-1.8569	-1.8544	-1.85199	-1.84963
-1.86852	-1.86588	-1.86326	-1.86066	-1.8581	-1.85556	-1.85309	-1.85073	-1.84842
-1.86713	-1.86449	-1.86188	-1.85929	-1.85675	-1.85424	-1.8518	-1.84947	-1.8472
-1.86575	-1.86312	-1.86052	-1.85794	-1.85542	-1.85295	-1.85054	-1.84822	-1.84596
-1.86438	-1.86176	-1.85918	-1.85662	-1.85412	-1.85168	-1.8493	-1.84699	-1.84472
-1.86301	-1.86041	-1.85786	-1.85535	-1.85289	-1.85047	-1.8481	-1.84578	-1.8435
-1.86168	-1.85909	-1.85657	-1.8541	-1.85167	-1.84927	-1.84691	-1.84458	-1.84227
-1.8604	-1.85782	-1.8553	-1.85284	-1.85041	-1.84803	-1.84567	-1.84335	-1.84104
-1.85916	-1.85658	-1.85405	-1.85158	-1.84915	-1.84677	-1.84442	-1.8421	-1.83979
-1.85786	-1.8553	-1.85278	-1.85031	-1.84789	-1.84552	-1.85537	-1.85304	-1.85073
-1.85652	-1.85398	-1.85149	-1.84904	-1.84663	-1.84426	-1.85411	-1.85178	-1.84946
-1.85519	-1.85267	-1.85019	-1.84777	-1.85755	-1.85519	-1.8844	-1.882	-1.87961
-1.85386	-1.85135	-1.8489	-1.8465	-1.85629	-1.85394	-1.88313	-1.88074	-1.87835
-1.86471	-1.86222	-1.85979	-1.8574	-1.88665	-1.88425	-1.88186	-1.87947	-1.87708
-1.86343	-1.86096	-1.85854	-1.85615	-1.88538	-1.88299	-1.8806	-1.87821	-1.87582

-1.86218	-1.85973	-1.85731	-1.85493	-1.88414	-1.88175	-1.87935	-1.87696	-1.87457
-1.86097	-1.85854	-1.88781	-1.88538	-1.88296	-1.88056	-1.87817	-1.87577	-1.87337
-1.85981	-1.85739	-1.88665	-1.88423	-1.88182	-1.87942	-1.87702	-1.8746	-1.87219
-1.87962	-1.8772	-1.88543	-1.88302	-1.88062	-1.87821	-1.87581	-1.87339	-1.87097
-1.87835	-1.87593	-1.88415	-1.88176	-1.87936	-1.87695	-1.87454	-1.87212	-1.86971
-1.88775	-1.8853	-1.88288	-1.88048	-1.87809	-1.87568	-1.87327	-1.87086	-1.86845
-1.88648	-1.88404	-1.88161	-1.87921	-1.87681	-1.8744	-1.87199	-1.86959	-1.86719
-1.88524	-1.8828	-1.88038	-1.87796	-1.87555	-1.87314	-1.87074	-1.86834	-1.86594
-1.88406	-1.88163	-1.8792	-1.87678	-1.87437	-1.87195	-1.86954	-1.86714	-1.86476
-1.8829	-1.88048	-1.87805	-1.87563	-1.8732	-1.87078	-1.86836	-1.86597	-1.86359
-1.88168	-1.87926	-1.87683	-1.8744	-1.87198	-1.86955	-1.86714	-1.86474	-1.86236
-1.88043	-1.878	-1.87557	-1.87313	-1.87071	-1.8683	-1.86589	-1.86349	-1.8611
-1.87922	-1.87679	-1.87434	-1.87189	-1.86945	-1.86704	-1.86464	-1.86224	-1.85985
-1.87808	-1.87563	-1.87316	-1.87066	-1.8682	-1.86578	-1.86337	-1.86098	-1.8586
-1.87692	-1.87446	-1.87197	-1.86945	-1.86698	-1.86454	-1.86213	-1.85974	-1.85737
-1.87569	-1.87324	-1.87076	-1.86827	-1.8658	-1.86336	-1.86095	-1.85857	-1.8562
-1.87446	-1.87201	-1.86955	-1.86708	-1.86463	-1.8622	-1.85978	-1.8574	-1.85504
-1.87327	-1.87083	-1.86836	-1.86589	-1.86343	-1.86099	-1.85857	-1.85618	-1.85382
-1.8721	-1.86965	-1.86718	-1.86468	-1.86221	-1.85976	-1.85734	-1.85495	-1.85258
-1.87089	-1.86844	-1.86597	-1.86347	-1.861	-1.85856	-1.85614	-1.85375	-1.85138
-1.86965	-1.8672	-1.86474	-1.86226	-1.8598	-1.85738	-1.85498	-1.85259	-1.85022
-1.86844	-1.86599	-1.86352	-1.86104	-1.85859	-1.85618	-1.85378	-1.8514	-1.84903
-1.86727	-1.86482	-1.86234	-1.85983	-1.85736	-1.85493	-1.85253	-1.85015	-1.84778
-1.86609	-1.86363	-1.86114	-1.85862	-1.85614	-1.8537	-1.87813	-1.87571	-1.87331
-1.86485	-1.86239	-1.85991	-1.85742	-1.85496	-1.85253	-1.87694	-1.87452	-1.87211
-1.86361	-1.86114	-1.85868	-1.85624	-1.8538	-1.85139	-1.87577	-1.87334	-1.87092
-1.86243	-1.85995	-1.8575	-1.85506	-1.85264	-1.85023	-1.87458	-1.87213	-1.86968
-1.86127	-1.8588	-1.85634	-1.85389	-1.85147	-1.84906	-1.87339	-1.87091	-1.86843
-1.86011	-1.85764	-1.85517	-1.85271	-1.85028	-1.84787	-1.87217	-1.86969	-1.8672
-1.85893	-1.85647	-1.854	-1.85151	-1.84906	-1.84665	-1.87092	-1.86846	-1.86599
-1.85774	-1.85529	-1.85281	-1.85031	-1.84786	-1.84545	-1.86969	-1.86724	-1.86479
-1.85649	-1.85405	-1.85159	-1.84913	-1.84669	-1.84428	-1.86849	-1.86605	-1.8636
-1.85526	-1.85281	-1.85037	-1.84795	-1.84553	-1.84312	-1.8673	-1.86485	-1.86239
-1.85408	-1.85164	-1.84921	-1.84679	-1.84438	-1.84197	-1.86612	-1.86364	-1.86116
-1.85293	-1.8505	-1.84806	-1.84564	-1.84322	-1.84081	-1.86493	-1.86243	-1.85991
-1.85178	-1.84934	-1.84689	-1.84444	-1.8686	-1.86615	-1.86369	-1.8612	-1.8587
-1.85063	-1.84818	-1.84571	-1.84322	-1.86733	-1.86487	-1.86242	-1.85996	-1.8575
-1.84947	-1.847	-1.84452	-1.84202	-1.86609	-1.86363	-1.86119	-1.85874	-1.85631
-1.84831	-1.8458	-1.84331	-1.84084	-1.8649	-1.86244	-1.86	-1.85756	-1.85513
-1.84714	-1.84461	-1.84212	-1.83968	-1.86372	-1.86126	-1.85882	-1.85638	-1.85394
-1.84599	-1.84345	-1.84096	-1.83852	-1.86254	-1.8601	-1.85765	-1.85521	-1.85276
-1.84483	-1.84229	-1.83981	-1.83737	-1.86138	-1.85893	-1.85649	-1.85403	-1.85156
-1.84363	-1.84113	-1.86514	-1.86267	-1.86021	-1.85776	-1.86764	-1.86514	-1.86264
-1.84243	-1.83996	-1.86396	-1.86151	-1.85905	-1.85658	-1.86642	-1.8639	-1.86137
-1.84124	-1.83879	-1.86278	-1.86033	-1.87019	-1.86769	-1.86519	-1.86266	-1.86013
-1.84007	-1.83763	-1.8616	-1.85915	-1.86898	-1.86645	-1.86392	-1.86141	-1.85892
-1.83891	-1.83647	-1.87273	-1.87027	-1.86778	-1.86522	-1.86268	-1.86018	-1.85771
-1.83775	-1.83531	-1.87154	-1.86908	-1.86658	-1.86401	-1.86146	-1.85897	-1.8565
-1.87531	-1.87282	-1.87035	-1.86789	-1.86538	-1.86281	-1.86025	-1.85776	-1.8553
-1.87411	-1.87163	-1.86915	-1.8667	-1.86419	-1.86162	-1.85906	-1.85656	-1.8541

-1.87292	-1.87043	-1.86796	-1.8655	-1.863	-1.86043	-1.85787	-1.85537	-1.8529
-1.87173	-1.86924	-1.86677	-1.86431	-1.8618	-1.85923	-1.85667	-1.85417	-1.8517
-1.87053	-1.86805	-1.86557	-1.86311	-1.8606	-1.85803	-1.85546	-1.85297	-1.8505
-1.86934	-1.86685	-1.86438	-1.86191	-1.85941	-1.85683	-1.85426	-1.85176	-1.84929
-1.86814	-1.86566	-1.86318	-1.86072	-1.85821	-1.85563	-1.85306	-1.85056	-1.84808
-1.86695	-1.86446	-1.86198	-1.85952	-1.857	-1.85442	-1.85186	-1.84935	-1.84687
-1.86575	-1.86327	-1.86079	-1.85831	-1.85579	-1.85321	-1.85064	-1.84814	-1.84566
-1.86455	-1.86207	-1.85959	-1.85711	-1.85458	-1.852	-1.84943	-1.84692	-1.84444
-1.86336	-1.86088	-1.85839	-1.8559	-1.85337	-1.85078	-1.84821	-1.84571	-1.84324
-1.86218	-1.85968	-1.85718	-1.85469	-1.85216	-1.84957	-1.847	-1.8445	-1.84203
-1.86103	-1.8585	-1.85598	-1.85348	-1.85095	-1.84835	-1.84578	-1.84329	-1.84082
-1.8599	-1.85733	-1.85479	-1.85227	-1.84973	-1.84714	-1.84457	-1.84208	-1.83961
-1.85874	-1.85614	-1.85359	-1.85107	-1.84852	-1.84593	-1.84337	-1.84088	-1.83841
-1.85754	-1.85493	-1.85238	-1.84986	-1.84731	-1.84473	-1.84218	-1.83968	-1.83721
-1.85634	-1.85373	-1.85117	-1.84865	-1.84612	-1.84355	-1.84101	-1.8385	-1.83601
-1.85513	-1.85253	-1.84997	-1.84745	-1.84493	-1.84241	-1.83988	-1.83735	-1.83483
-1.85393	-1.85134	-1.84878	-1.84626	-1.84375	-1.84126	-1.83876	-1.83621	-1.83365
-1.85276	-1.85019	-1.84763	-1.84509	-1.84257	-1.84009	-1.83759	-1.83503	-1.83247
-1.85161	-1.84907	-1.8465	-1.84392	-1.84138	-1.8389	-1.8364	-1.83385	-1.83129
-1.85043	-1.8479	-1.84533	-1.84274	-1.8402	-1.8377	-1.83521	-1.83268	-1.83014
-1.84924	-1.8467	-1.84414	-1.84156	-1.83901	-1.8365	-1.83401	-1.83151	-1.82901
-1.84807	-1.84553	-1.84297	-1.8404	-1.83785	-1.83533	-1.83282	-1.83034	-1.82786
-1.84694	-1.84437	-1.84182	-1.83928	-1.83673	-1.83418	-1.83165	-1.82916	-1.82667
-1.8458	-1.84323	-1.84068	-1.83815	-1.83561	-1.83304	-1.83049	-1.82799	-1.82548
-1.84466	-1.84211	-1.83955	-1.837	-1.83445	-1.83191	-1.82938	-1.82684	-1.82431
-1.84351	-1.84099	-1.83843	-1.83584	-1.83329	-1.83078	-1.82826	-1.8257	-1.82314
-1.84235	-1.83982	-1.83726	-1.83467	-1.83211	-1.8296	-1.82709	-1.82452	-1.82195
-1.84117	-1.83864	-1.83607	-1.83348	-1.83092	-1.82841	-1.82588	-1.82332	-1.82076
-1.84002	-1.83747	-1.83491	-1.83234	-1.82978	-1.82724	-1.82471	-1.82216	-1.8196
-1.8389	-1.83633	-1.83377	-1.83122	-1.82866	-1.82611	-1.82356	-1.82102	-1.81848
-1.83777	-1.8352	-1.83264	-1.8301	-1.82754	-1.82498	-1.82242	-1.81988	-1.81735
-1.83664	-1.83408	-1.83152	-1.82896	-1.82641	-1.82386	-1.8213	-1.81875	-1.8162
-1.83552	-1.83296	-1.8304	-1.82783	-1.82527	-1.82272	-1.82018	-1.81761	-1.81505
-1.8344	-1.83183	-1.82926	-1.8267	-1.82414	-1.82157	-1.81901	-1.81646	-1.81391
-1.83328	-1.83069	-1.82813	-1.82557	-1.82301	-1.82041	-1.81783	-1.8153	-1.81278
-1.83215	-1.82957	-1.827	-1.82444	-1.82185	-1.81924	-1.81664	-1.81411	-1.81159
-1.83103	-1.82846	-1.82588	-1.82329	-1.82069	-1.81805	-1.81544	-1.8129	-1.81038
-1.8299	-1.82733	-1.82475	-1.82216	-1.81954	-1.81691	-1.81429	-1.81173	-1.80919
-1.82877	-1.82619	-1.8236	-1.82103	-1.81843	-1.81581	-1.8132	-1.8106	-1.80802
-1.82764	-1.82505	-1.82247	-1.81989	-1.81731	-1.81471	-1.81211	-1.80949	-1.80687
-1.82652	-1.82393	-1.82134	-1.81875	-1.81616	-1.81359	-1.811	-1.80839	-1.80576
-1.8254	-1.82282	-1.82022	-1.8176	-1.81501	-1.81246	-1.80989	-1.80728	-1.80465
-1.82429	-1.82169	-1.81909	-1.81649	-1.8139	-1.81133	-1.80874	-1.80612	-1.80348
-1.82318	-1.82056	-1.81797	-1.81539	-1.8128	-1.8102	-1.80758	-1.80494	-1.8023
-1.82207	-1.81946	-1.81686	-1.81427	-1.81168	-1.80908	-1.80647	-1.80382	-1.80118
-1.82096	-1.81837	-1.81576	-1.81314	-1.81054	-1.80797	-1.80538	-1.80275	-1.8001
-1.81988	-1.8173	-1.81468	-1.81205	-1.80943	-1.80685	-1.80426	-1.80164	-1.79901
-1.81883	-1.81625	-1.81363	-1.81099	-1.80835	-1.80573	-1.80311	-1.80051	-1.79791
-1.81778	-1.81519	-1.81256	-1.80992	-1.80727	-1.80462	-1.80197	-1.79938	-1.79679
-1.81671	-1.81408	-1.81145	-1.80884	-1.8062	-1.80354	-1.80088	-1.79825	-1.79564

-1.81563	-1.81296	-1.81033	-1.80774	-1.80512	-1.80246	-1.7998	-1.79714	-1.79448
-1.81456	-1.81188	-1.80923	-1.80661	-1.80399	-1.80136	-1.79871	-1.79605	-1.79338
-1.81349	-1.81081	-1.80814	-1.80548	-1.80284	-1.80024	-1.79762	-1.79497	-1.7923
-1.81239	-1.80973	-1.80706	-1.80437	-1.80172	-1.79911	-1.7965	-1.79386	-1.79121
-1.81127	-1.80864	-1.80597	-1.80329	-1.80062	-1.79797	-1.79534	-1.79272	-1.79011
-1.81014	-1.80752	-1.80487	-1.8022	-1.79953	-1.79686	-1.7942	-1.79159	-1.78899
-1.80902	-1.80637	-1.80373	-1.80111	-1.79846	-1.79578	-1.79311	-1.79046	-1.78783
-1.80792	-1.80523	-1.8026	-1.80001	-1.79738	-1.79471	-1.79202	-1.78934	-1.78668
-1.80684	-1.80414	-1.80151	-1.79892	-1.7963	-1.79363	-1.79094	-1.78825	-1.78558
-1.80577	-1.80307	-1.80043	-1.79783	-1.7952	-1.79254	-1.78987	-1.78718	-1.7845
-1.79323	-1.79054	-1.79933	-1.79671	-1.79408	-1.79144	-1.78878	-1.78609	-1.78341
-1.79217	-1.78948	-1.79823	-1.79557	-1.79293	-1.79031	-1.78769	-1.78501	-1.78232
-1.79111	-1.78841	-1.79715	-1.79446	-1.79181	-1.78921	-1.7866	-1.78393	-1.78123
-1.79005	-1.78736	-1.79607	-1.79338	-1.79072	-1.78813	-1.78552	-1.78285	-1.78015
-1.78899	-1.7863	-1.78362	-1.78093	-1.78964	-1.78705	-1.78444	-1.78177	-1.77907
-1.78794	-1.78525	-1.78256	-1.77987	-1.78856	-1.78596	-1.78335	-1.78068	-1.778
-1.78688	-1.78419	-1.7815	-1.77881	-1.78748	-1.78486	-1.78224	-1.77958	-1.77691
-1.78582	-1.78314	-1.78045	-1.77775	-1.78638	-1.78373	-1.78108	-1.77844	-1.7758
-1.78476	-1.78209	-1.7794	-1.77668	-1.78528	-1.78258	-1.77991	-1.77729	-1.77467
-1.78371	-1.78104	-1.77834	-1.77562	-1.78419	-1.78147	-1.77878	-1.77616	-1.77356
-1.78267	-1.77998	-1.77728	-1.77456	-1.78311	-1.78038	-1.77768	-1.77506	-1.77246
-1.78162	-1.77893	-1.77622	-1.7735	-1.77078	-1.76807	-1.7766	-1.77398	-1.77137
-1.78056	-1.77787	-1.77516	-1.77244	-1.76973	-1.76701	-1.77553	-1.7729	-1.77028
-1.7795	-1.7768	-1.77409	-1.77138	-1.76868	-1.76597	-1.77449	-1.77184	-1.7692
-1.77844	-1.77573	-1.77302	-1.77031	-1.76762	-1.76496	-1.77349	-1.77082	-1.76814
-1.77737	-1.77465	-1.77194	-1.76923	-1.76656	-1.76395	-1.77249	-1.7698	-1.76709
-1.7763	-1.77357	-1.77086	-1.76816	-1.7655	-1.7629	-1.76028	-1.75761	-1.75491
-1.77523	-1.7725	-1.76979	-1.7671	-1.76444	-1.76183	-1.75921	-1.75654	-1.75385
-1.77417	-1.77145	-1.76875	-1.76607	-1.76341	-1.76079	-1.75815	-1.75548	-1.75278
-1.7731	-1.7704	-1.76772	-1.76508	-1.76242	-1.75977	-1.7571	-1.75441	-1.7517
-1.77206	-1.76937	-1.76671	-1.76407	-1.76141	-1.75873	-1.75604	-1.75334	-1.75063
-1.77104	-1.76839	-1.76572	-1.76305	-1.76037	-1.75767	-1.75497	-1.75226	-1.74955
-1.77003	-1.76739	-1.76472	-1.76202	-1.75932	-1.75661	-1.76491	-1.76216	-1.75941
-1.76902	-1.76637	-1.76368	-1.76097	-1.75826	-1.75554	-1.76382	-1.76107	-1.75832
-1.76802	-1.76533	-1.76263	-1.75991	-1.75719	-1.75447	-1.76273	-1.75998	-1.75724
-1.76699	-1.76428	-1.76157	-1.75885	-1.75613	-1.75341	-1.76166	-1.75891	-1.75616
-1.76593	-1.76322	-1.7605	-1.75779	-1.75508	-1.75236	-1.7606	-1.75785	-1.75511
-1.76489	-1.76217	-1.75945	-1.75674	-1.75404	-1.75134	-1.75958	-1.75683	-1.7541
-1.76386	-1.76113	-1.75841	-1.7557	-1.75302	-1.75036	-1.75862	-1.75588	-1.75314
-1.76286	-1.76013	-1.75741	-1.7547	-1.75202	-1.74937	-1.75763	-1.7549	-1.75216
-1.76192	-1.75919	-1.75647	-1.75375	-1.75105	-1.74839	-1.75661	-1.75387	-1.75112
-1.76097	-1.75825	-1.75553	-1.7528	-1.75009	-1.74739	-1.75557	-1.75282	-1.75007
-1.75996	-1.75724	-1.75453	-1.7518	-1.74909	-1.74637	-1.75453	-1.75177	-1.74902
-1.75893	-1.75621	-1.7535	-1.75079	-1.74808	-1.74536	-1.75349	-1.75073	-1.74797
-1.7579	-1.75518	-1.75248	-1.74979	-1.7471	-1.74439	-1.7525	-1.74974	-1.74697
-1.75686	-1.75414	-1.75146	-1.74882	-1.74615	-1.74345	-1.75155	-1.74879	-1.74602
-1.75587	-1.75315	-1.75047	-1.74783	-1.74516	-1.74246	-1.75056	-1.7478	-1.74504
-1.75494	-1.75222	-1.74952	-1.74683	-1.74414	-1.74143	-1.74952	-1.74676	-1.74401
-1.754	-1.75127	-1.74854	-1.74582	-1.7431	-1.74039	-1.74847	-1.74573	-1.74298
-1.75299	-1.75026	-1.74753	-1.74479	-1.74206	-1.73934	-1.74741	-1.7447	-1.74199

-1.75199	-1.74925	-1.74652	-1.74377	-1.74104	-1.73831	-1.74637	-1.74368	-1.74101
-1.75103	-1.7483	-1.74556	-1.74282	-1.74009	-1.73736	-1.74539	-1.74269	-1.74
-1.75011	-1.74737	-1.74464	-1.7419	-1.73916	-1.73644	-1.74446	-1.74172	-1.73899
-1.74913	-1.7464	-1.74366	-1.74092	-1.7382	-1.7355	-1.74352	-1.74077	-1.73802
-1.74811	-1.74538	-1.74264	-1.73991	-1.7372	-1.73454	-1.74258	-1.73984	-1.73709
-1.74713	-1.7444	-1.74167	-1.73893	-1.73622	-1.73357	-1.74161	-1.73888	-1.73612
-1.74622	-1.74349	-1.74075	-1.73801	-1.73529	-1.7326	-1.7406	-1.73785	-1.7351
-1.74532	-1.74259	-1.73985	-1.7371	-1.73436	-1.73164	-1.73959	-1.73684	-1.73408
-1.74442	-1.74169	-1.73894	-1.7362	-1.73345	-1.73072	-1.73865	-1.73589	-1.73312
-1.74351	-1.74077	-1.73803	-1.73528	-1.73254	-1.7298	-1.73771	-1.73494	-1.73217
-1.74253	-1.73979	-1.73705	-1.7343	-1.74227	-1.73949	-1.73671	-1.73394	-1.73117
-1.74151	-1.73877	-1.73603	-1.73329	-1.74124	-1.73847	-1.73569	-1.73292	-1.73014
-1.74055	-1.73781	-1.73507	-1.73233	-1.74027	-1.73749	-1.73472	-1.73194	-1.72917
-1.73965	-1.73691	-1.73417	-1.73143	-1.73935	-1.73658	-1.7338	-1.73102	-1.72825
-1.73876	-1.73602	-1.73327	-1.73053	-1.73844	-1.73566	-1.73288	-1.73011	-1.72733
-1.73787	-1.73511	-1.73237	-1.72962	-1.73753	-1.73475	-1.73197	-1.7292	-1.72642
-1.73696	-1.73419	-1.73144	-1.7287	-1.73659	-1.73381	-1.73104	-1.72826	-1.72548
-1.73602	-1.73322	-1.74114	-1.73835	-1.73557	-1.7328	-1.73002	-1.72725	-1.72447
-1.73507	-1.73222	-1.74011	-1.73732	-1.73454	-1.73176	-1.72898	-1.72621	-1.72343
-1.73409	-1.73126	-1.73914	-1.73635	-1.73356	-1.73078	-1.72801	-1.72523	-1.72246
-1.73311	-1.73032	-1.7382	-1.73542	-1.73264	-1.72985	-1.72707	-1.7243	-1.72152
-1.73215	-1.72939	-1.73727	-1.7345	-1.73172	-1.72893	-1.72615	-1.72338	-1.7206
-1.73124	-1.72848	-1.73636	-1.73359	-1.73081	-1.72803	-1.72524	-1.72247	-1.7197
-1.73033	-1.72758	-1.73545	-1.73268	-1.72991	-1.72712	-1.72434	-1.72157	-1.71879
-1.72944	-1.72669	-1.73455	-1.73178	-1.729	-1.72622	-1.72343	-1.72066	-1.71789
-1.72855	-1.7258	-1.73365	-1.73087	-1.7281	-1.72531	-1.72253	-1.71976	-1.71699
-1.72765	-1.7249	-1.73274	-1.72997	-1.72719	-1.72441	-1.72162	-1.71885	-1.71608
-1.72676	-1.72401	-1.73184	-1.72907	-1.72629	-1.7235	-1.72072	-1.71795	-1.71517
-1.72587	-1.72312	-1.73093	-1.72816	-1.72538	-1.7226	-1.71982	-1.71704	-1.71426
-1.72498	-1.72223	-1.73003	-1.72725	-1.72447	-1.72169	-1.71891	-1.71613	-1.71335
-1.72408	-1.72134	-1.72912	-1.72634	-1.72356	-1.72079	-1.71801	-1.71523	-1.71244
-1.72319	-1.72045	-1.72822	-1.72543	-1.72265	-1.71988	-1.71711	-1.71433	-1.71154
-1.7223	-1.71956	-1.72731	-1.72452	-1.72174	-1.71898	-1.71621	-1.71343	-1.71064
-1.72141	-1.71866	-1.7264	-1.72361	-1.72083	-1.71807	-1.7153	-1.71252	-1.70974
-1.72052	-1.71777	-1.7255	-1.72271	-1.71993	-1.71716	-1.7144	-1.71162	-1.70883
-1.69723	-1.69453	-1.72459	-1.7218	-1.71902	-1.71626	-1.71349	-1.71071	-1.70792
-1.69635	-1.69365	-1.72369	-1.7209	-1.71812	-1.71535	-1.71258	-1.7098	-1.70702
-1.69547	-1.69277	-1.72279	-1.72	-1.71722	-1.71445	-1.71167	-1.70889	-1.70611
-1.69459	-1.69188	-1.72189	-1.7191	-1.71632	-1.71354	-1.71077	-1.70798	-1.7052
-1.70446	-1.70172	-1.69897	-1.69624	-1.71542	-1.71264	-1.70986	-1.70708	-1.7043
-1.7036	-1.70084	-1.69809	-1.69535	-1.71452	-1.71174	-1.70896	-1.70617	-1.70339
-1.70276	-1.69999	-1.69723	-1.69447	-1.71362	-1.71084	-1.70806	-1.70527	-1.70249
-1.70197	-1.69921	-1.69642	-1.69363	-1.71275	-1.70995	-1.70715	-1.70437	-1.70159
-1.7012	-1.69844	-1.69564	-1.69282	-1.71189	-1.70906	-1.70626	-1.70347	-1.70069
-1.70037	-1.69762	-1.69483	-1.69202	-1.68921	-1.68642	-1.70539	-1.70259	-1.6998
-1.6995	-1.69676	-1.694	-1.69123	-1.68843	-1.68561	-1.70454	-1.70172	-1.69892
-1.69862	-1.69589	-1.69315	-1.6904	-1.68762	-1.6848	-1.70372	-1.7009	-1.69809
-1.69773	-1.695	-1.69226	-1.68953	-1.68677	-1.68399	-1.70294	-1.70013	-1.69732
-1.69687	-1.69413	-1.69139	-1.68865	-1.68591	-1.68317	-1.68042	-1.67768	-1.67493
-1.69608	-1.69329	-1.69052	-1.68778	-1.68503	-1.68229	-1.67956	-1.67683	-1.67409

-1.14427	-1.1426	-1.14094	-1.13928	-1.13762	-1.13594	-1.20611	-1.20434	-1.20258
-1.1448	-1.14314	-1.14147	-1.1398	-1.13813	-1.13645	-1.20666	-1.2049	-1.20315
-1.14532	-1.14367	-1.142	-1.14031	-1.13863	-1.13695	-1.20722	-1.20546	-1.20371
-1.10355	-1.102	-1.1425	-1.14082	-1.13915	-1.13747	-1.15235	-1.15067	-1.14901
-1.104	-1.10244	-1.14299	-1.14133	-1.13967	-1.13801	-1.15289	-1.15119	-1.14951
-1.10447	-1.10291	-1.10136	-1.09983	-1.14021	-1.13855	-1.13689	-1.13522	-1.13356
-1.10497	-1.1034	-1.10185	-1.10033	-1.14077	-1.1391	-1.13743	-1.13576	-1.13409
-1.04592	-1.04447	-1.10236	-1.10083	-1.0993	-1.09775	-1.13798	-1.1363	-1.13463
-1.04638	-1.04494	-1.10288	-1.10133	-1.09978	-1.09825	-1.13855	-1.13686	-1.13517
-1.04684	-1.0454	-1.04396	-1.04252	-1.10027	-1.09874	-1.0972	-1.09562	-1.09404
-1.04728	-1.04583	-1.04439	-1.04295	-1.10076	-1.09922	-1.09766	-1.09609	-1.09451
-1.04771	-1.04626	-1.04482	-1.04338	-1.10125	-1.09968	-1.09812	-1.09655	-1.09498
-1.04814	-1.04669	-1.04525	-1.04381	-1.04236	-1.04091	-1.09859	-1.09704	-1.09549
-1.04856	-1.04713	-1.04569	-1.04423	-1.04278	-1.04134	-1.09908	-1.09754	-1.09602
-1.049	-1.04757	-1.04613	-1.04468	-1.04323	-1.04179	-1.06172	-1.06027	-1.05882
-1.04944	-1.04801	-1.04658	-1.04515	-1.04371	-1.04226	-1.06222	-1.06076	-1.05931
-0.9809	-0.97963	-1.04702	-1.04559	-1.04416	-1.04272	-1.04128	-1.03987	-1.03846
-0.98125	-0.97998	-1.04745	-1.04602	-1.04458	-1.04315	-1.04172	-1.0403	-1.03888
-0.98159	-0.98032	-1.04787	-1.04644	-1.04501	-1.04358	-1.04216	-1.04074	-1.03931
-0.98192	-0.98066	-1.04829	-1.04687	-1.04545	-1.04403	-1.0426	-1.04118	-1.03976
-0.98227	-0.981	-1.0487	-1.04731	-1.0459	-1.04447	-1.04304	-1.04163	-1.04022
-0.98263	-0.98137	-0.98011	-0.97885	-1.04633	-1.04491	-1.04349	-1.04206	-1.04064
-0.98301	-0.98176	-0.98049	-0.97922	-1.04676	-1.04535	-1.04393	-1.04249	-1.04106
-0.98338	-0.98213	-0.98087	-0.97959	-1.02413	-1.02276	-1.04437	-1.04293	-1.04149
-0.98375	-0.9825	-0.98123	-0.97996	-1.02455	-1.02318	-1.04481	-1.04338	-1.04195
-0.98411	-0.98285	-0.98159	-0.98032	-0.97905	-0.97778	-1.04525	-1.04383	-1.04241
-0.98447	-0.98321	-0.98195	-0.98068	-0.97942	-0.97814	-1.04568	-1.04427	-1.04286
-0.98482	-0.98356	-0.9823	-0.98104	-0.97977	-0.97849	-1.0461	-1.04471	-1.04332
-0.98515	-0.9839	-0.98264	-0.98137	-0.9801	-0.97884	-1.02343	-1.02208	-1.02074
-0.98547	-0.98423	-0.98297	-0.9817	-0.98043	-0.97917	-1.02382	-1.02248	-1.02113
-0.98581	-0.98458	-0.98332	-0.98204	-0.98078	-0.97953	-0.99924	-0.99796	-0.99667
-0.98616	-0.98492	-0.98367	-0.9824	-0.98115	-0.97989	-0.99963	-0.99833	-0.99703
-0.98652	-0.98527	-0.98403	-0.98277	-0.98152	-0.98027	-0.97901	-0.97774	-0.97648
-0.98687	-0.98562	-0.98438	-0.98315	-0.9819	-0.98065	-0.9794	-0.97813	-0.97686
-0.98723	-0.98598	-0.98474	-0.98351	-0.98228	-0.98103	-0.97977	-0.9785	-0.97724
-0.98759	-0.98633	-0.98509	-0.98387	-0.98263	-0.98138	-0.98011	-0.97885	-0.9776
-0.98795	-0.98669	-0.98545	-0.98423	-0.98299	-0.98172	-0.98046	-0.9792	-0.97795
-0.98832	-0.98706	-0.98582	-0.9846	-0.98336	-0.9821	-0.98084	-0.97959	-0.97834
-0.9887	-0.98743	-0.98619	-0.98498	-0.98375	-0.98249	-0.98124	-0.97998	-0.97874
-0.98905	-0.98778	-0.98655	-0.98533	-0.98411	-0.98286	-0.98162	-0.98037	-0.97912
-0.98938	-0.98812	-0.98689	-0.98568	-0.98446	-0.98322	-0.98198	-0.98074	-0.9795
-0.98973	-0.98848	-0.98725	-0.98603	-0.98482	-0.98359	-0.98235	-0.98112	-0.97989
-0.99012	-0.98886	-0.98763	-0.98641	-0.98519	-0.98396	-0.98274	-0.98151	-0.98029
-0.9905	-0.98925	-0.98801	-0.98679	-0.98557	-0.98434	-0.98312	-0.9819	-0.98068
-0.99088	-0.98962	-0.98838	-0.98716	-0.98594	-0.9847	-0.98348	-0.98226	-0.98104
-0.99126	-0.99	-0.98876	-0.98754	-0.98631	-0.98507	-0.98383	-0.98262	-0.9814
-0.97152	-0.9703	-0.98915	-0.98793	-0.98669	-0.98545	-0.98421	-0.98299	-0.98177
-0.97192	-0.97069	-0.98955	-0.98832	-0.98709	-0.98583	-0.98459	-0.98336	-0.98214
-0.9723	-0.97107	-0.96986	-0.96866	-0.98747	-0.98621	-0.98496	-0.98373	-0.98252
-0.97266	-0.97144	-0.97023	-0.96902	-0.98784	-0.98658	-0.98533	-0.9841	-0.98289

-0.97303	-0.97181	-0.9706	-0.9694	-0.96818	-0.96695	-0.96573	-0.96453	-0.96334
-0.97342	-0.9722	-0.97098	-0.96978	-0.96857	-0.96734	-0.96611	-0.9649	-0.9637
-0.9738	-0.97258	-0.97137	-0.97017	-0.96896	-0.96773	-0.9665	-0.96528	-0.96407
-0.97417	-0.97294	-0.97173	-0.97054	-0.96933	-0.9681	-0.96687	-0.96565	-0.96444
-0.97453	-0.9733	-0.97209	-0.9709	-0.96969	-0.96847	-0.96725	-0.96602	-0.96481
-0.9529	-0.9517	-0.97247	-0.97128	-0.97007	-0.96886	-0.96764	-0.96642	-0.9652
-0.95329	-0.95208	-0.97286	-0.97166	-0.97046	-0.96926	-0.96806	-0.96683	-0.9656
-0.95368	-0.95246	-0.95126	-0.95007	-0.97084	-0.96965	-0.96845	-0.96723	-0.96601
-0.95406	-0.95283	-0.95162	-0.95043	-0.9712	-0.97001	-0.96882	-0.96762	-0.96641
-0.95445	-0.95321	-0.95199	-0.95079	-0.94961	-0.94844	-0.94727	-0.94609	-0.94491
-0.95484	-0.9536	-0.95238	-0.95117	-0.94998	-0.9488	-0.94763	-0.94646	-0.94528
-0.95523	-0.95399	-0.95277	-0.95155	-0.95035	-0.94917	-0.948	-0.94682	-0.94565
-0.95562	-0.95439	-0.95317	-0.95194	-0.95073	-0.94954	-0.94837	-0.94719	-0.94602
-0.95602	-0.9548	-0.95357	-0.95233	-0.95112	-0.94992	-0.94874	-0.94756	-0.94639
-0.9564	-0.95519	-0.95397	-0.95274	-0.95152	-0.95032	-0.94913	-0.94795	-0.94677
-0.95677	-0.95557	-0.95436	-0.95315	-0.95194	-0.95075	-0.94955	-0.94836	-0.94717
-0.95715	-0.95596	-0.95476	-0.95355	-0.95235	-0.95115	-0.94996	-0.94876	-0.94757
-0.95756	-0.95636	-0.95515	-0.95393	-0.95272	-0.95152	-0.95033	-0.94914	-0.94795
-0.95796	-0.95676	-0.95555	-0.95433	-0.95311	-0.9519	-0.9507	-0.94951	-0.94833
-0.95837	-0.95717	-0.95596	-0.95473	-0.95351	-0.9523	-0.95109	-0.9499	-0.94871
-0.95878	-0.95758	-0.95637	-0.95515	-0.95393	-0.9527	-0.95149	-0.95029	-0.9491
-0.95918	-0.95798	-0.95678	-0.95557	-0.95435	-0.95313	-0.95191	-0.9507	-0.9495
-0.95957	-0.95837	-0.95718	-0.95599	-0.95479	-0.95357	-0.95234	-0.95111	-0.9499
-0.95997	-0.95876	-0.95757	-0.95639	-0.95519	-0.95398	-0.95275	-0.95153	-0.95031
-0.96037	-0.95915	-0.95795	-0.95676	-0.95556	-0.95436	-0.95316	-0.95195	-0.95074
-0.96078	-0.95956	-0.95834	-0.95714	-0.95594	-0.95475	-0.95356	-0.95236	-0.95117
-0.96121	-0.95998	-0.95876	-0.95754	-0.95634	-0.95515	-0.95397	-0.95277	-0.95157
-0.96164	-0.96041	-0.95919	-0.95796	-0.95675	-0.95556	-0.95438	-0.95318	-0.95198
-0.89304	-0.89194	-0.9596	-0.95838	-0.95717	-0.95598	-0.95479	-0.9536	-0.95242
-0.8934	-0.89231	-0.96002	-0.9588	-0.95759	-0.95639	-0.95521	-0.95403	-0.95286
-0.89378	-0.89269	-0.93921	-0.93801	-0.958	-0.9568	-0.95562	-0.95445	-0.95328
-0.89416	-0.89307	-0.93962	-0.93841	-0.95841	-0.95721	-0.95602	-0.95485	-0.95368
-0.89453	-0.89344	-0.89234	-0.89123	-0.93762	-0.93643	-0.95643	-0.95525	-0.95409
-0.89489	-0.89382	-0.89272	-0.89161	-0.93803	-0.93684	-0.95685	-0.95567	-0.95451
-0.89526	-0.89419	-0.8931	-0.89199	-0.89089	-0.88979	-0.93608	-0.93492	-0.93378
-0.89564	-0.89457	-0.89348	-0.89236	-0.89125	-0.89016	-0.93646	-0.93531	-0.93416
-0.89603	-0.89495	-0.89385	-0.89273	-0.89163	-0.89053	-0.93686	-0.9357	-0.93455
-0.86786	-0.86683	-0.89421	-0.89311	-0.89201	-0.89091	-0.88982	-0.88876	-0.8877
-0.86819	-0.86715	-0.89457	-0.89349	-0.8924	-0.89131	-0.89022	-0.88915	-0.8881
-0.86855	-0.8675	-0.86647	-0.86545	-0.89279	-0.8917	-0.89061	-0.88955	-0.88849
-0.86891	-0.86786	-0.86683	-0.8658	-0.89318	-0.89209	-0.89101	-0.88994	-0.88887
-0.86927	-0.86822	-0.86717	-0.86614	-0.89355	-0.89247	-0.89139	-0.89032	-0.88925
-0.86962	-0.86856	-0.86751	-0.86647	-0.8939	-0.89282	-0.89175	-0.89068	-0.88961
-0.86998	-0.86891	-0.86786	-0.86681	-0.86577	-0.86476	-0.89212	-0.89105	-0.88998
-0.87037	-0.8693	-0.86824	-0.86718	-0.86614	-0.86513	-0.89252	-0.89145	-0.89038
-0.87076	-0.8697	-0.86863	-0.86757	-0.86653	-0.86551	-0.89294	-0.89186	-0.89079
-0.87115	-0.87008	-0.86902	-0.86796	-0.86691	-0.86588	-0.86486	-0.86384	-0.86283
-0.87152	-0.87047	-0.86941	-0.86836	-0.8673	-0.86625	-0.86521	-0.86419	-0.86318
-0.87187	-0.87083	-0.86978	-0.86872	-0.86767	-0.8666	-0.86555	-0.86453	-0.86352
-0.87221	-0.87117	-0.87012	-0.86907	-0.86801	-0.86694	-0.86588	-0.86485	-0.86384

-0.87256	-0.87152	-0.87048	-0.86942	-0.86836	-0.8673	-0.86624	-0.8652	-0.86418
-0.87293	-0.8719	-0.87085	-0.86981	-0.86875	-0.86768	-0.86662	-0.86558	-0.86454
-0.87331	-0.87228	-0.87124	-0.8702	-0.86915	-0.86809	-0.86702	-0.86596	-0.86491
-0.87368	-0.87266	-0.87163	-0.87059	-0.86955	-0.86849	-0.86743	-0.86636	-0.8653
-0.87405	-0.87303	-0.87201	-0.87099	-0.86996	-0.8689	-0.86783	-0.86676	-0.86569
-0.85278	-0.85179	-0.87238	-0.87137	-0.87034	-0.86929	-0.86823	-0.86716	-0.8661
-0.85311	-0.85213	-0.87274	-0.87174	-0.87072	-0.86968	-0.86863	-0.86757	-0.8665
-0.85345	-0.85246	-0.87309	-0.87209	-0.87107	-0.87005	-0.86902	-0.86797	-0.86691
-0.85378	-0.85277	-0.87342	-0.87242	-0.87142	-0.87041	-0.86939	-0.86836	-0.86731
-0.85414	-0.8531	-0.85209	-0.85113	-0.87176	-0.87076	-0.86975	-0.86873	-0.8677
-0.85452	-0.85345	-0.85242	-0.85145	-0.87209	-0.87109	-0.8701	-0.86909	-0.86808
-0.85492	-0.85381	-0.85277	-0.85178	-0.87243	-0.87143	-0.87044	-0.86945	-0.86846
-0.85533	-0.85421	-0.85314	-0.85213	-0.87278	-0.87179	-0.8708	-0.86982	-0.86883
-0.85575	-0.85463	-0.85354	-0.85249	-0.87315	-0.87215	-0.87117	-0.87018	-0.86919
-0.82584	-0.8248	-0.85394	-0.85286	-0.85183	-0.85086	-0.87153	-0.87054	-0.86955
-0.82621	-0.82516	-0.85435	-0.85325	-0.8522	-0.85121	-0.87189	-0.87089	-0.86989
-0.82657	-0.82553	-0.8245	-0.82348	-0.85258	-0.85157	-0.87225	-0.87124	-0.87025
-0.82695	-0.8259	-0.82486	-0.82384	-0.85298	-0.85193	-0.87261	-0.8716	-0.87062
-0.82733	-0.82628	-0.82523	-0.8242	-0.85339	-0.85231	-0.85127	-0.85029	-0.84935
-0.82772	-0.82666	-0.8256	-0.82455	-0.8538	-0.85269	-0.85164	-0.85066	-0.84973
-0.82812	-0.82705	-0.82598	-0.82492	-0.85421	-0.85309	-0.85202	-0.85105	-0.85012
-0.78638	-0.78527	-0.78417	-0.78308	-0.82424	-0.82323	-0.85244	-0.85144	-0.85049
-0.78684	-0.78572	-0.78462	-0.78352	-0.82462	-0.8236	-0.85288	-0.85185	-0.85086
-0.78731	-0.78618	-0.78507	-0.78398	-0.78291	-0.78186	-0.78083	-0.77984	-0.77886
-0.78778	-0.78665	-0.78554	-0.78444	-0.78336	-0.78232	-0.78129	-0.78029	-0.7793
-0.75772	-0.75664	-0.78601	-0.78491	-0.78383	-0.78279	-0.78177	-0.78075	-0.77975
-0.75817	-0.7571	-0.78649	-0.78539	-0.78432	-0.78328	-0.78226	-0.78123	-0.78022
-0.75862	-0.75756	-0.78699	-0.78588	-0.78481	-0.78377	-0.78274	-0.78171	-0.78068
-0.75908	-0.75802	-0.78748	-0.78638	-0.78531	-0.78426	-0.78322	-0.78218	-0.78115
-0.75955	-0.75849	-0.78798	-0.78689	-0.78581	-0.78474	-0.78369	-0.78265	-0.78161
-0.72779	-0.72679	-0.75791	-0.75689	-0.78633	-0.78525	-0.78418	-0.78312	-0.78208
-0.72824	-0.72723	-0.75839	-0.75738	-0.78686	-0.78577	-0.78468	-0.78361	-0.78256
-0.72869	-0.72768	-0.7267	-0.72574	-0.75685	-0.7558	-0.78518	-0.7841	-0.78303
-0.72914	-0.72813	-0.72715	-0.72619	-0.75732	-0.75628	-0.78568	-0.78459	-0.78351
-0.7296	-0.72859	-0.72761	-0.72664	-0.72566	-0.72467	-0.78618	-0.78508	-0.784
-0.73007	-0.72908	-0.72808	-0.72709	-0.72609	-0.7251	-0.78667	-0.78558	-0.78449
-0.73055	-0.72957	-0.72856	-0.72754	-0.72653	-0.72554	-0.78717	-0.78608	-0.785
-0.73103	-0.73004	-0.72903	-0.728	-0.72698	-0.72599	-0.725	-0.72402	-0.72305
-0.73151	-0.73051	-0.72949	-0.72846	-0.72744	-0.72644	-0.72545	-0.72448	-0.72352
-0.73198	-0.73097	-0.72995	-0.72892	-0.72791	-0.72691	-0.72592	-0.72494	-0.72396
-0.73245	-0.73142	-0.7304	-0.72938	-0.72837	-0.72738	-0.72639	-0.7254	-0.72441
-0.73293	-0.73188	-0.73086	-0.72985	-0.72884	-0.72785	-0.72686	-0.72586	-0.72486
-0.73341	-0.73235	-0.73133	-0.73033	-0.72933	-0.72833	-0.72732	-0.72632	-0.72532
-0.73391	-0.73284	-0.73181	-0.73081	-0.72981	-0.7288	-0.72779	-0.72678	-0.72578
-0.73443	-0.73336	-0.73232	-0.7313	-0.73029	-0.72928	-0.72827	-0.72726	-0.72626
-0.73497	-0.73389	-0.73283	-0.73179	-0.73076	-0.72976	-0.72875	-0.72775	-0.72675
-0.73551	-0.73442	-0.73335	-0.73229	-0.73126	-0.73025	-0.72925	-0.72823	-0.72722
-0.73605	-0.73495	-0.73386	-0.73279	-0.73176	-0.73075	-0.72975	-0.72872	-0.72769
-0.7366	-0.73548	-0.73439	-0.73332	-0.73227	-0.73125	-0.73024	-0.7292	-0.72817
-0.73713	-0.73601	-0.73492	-0.73385	-0.7328	-0.73175	-0.73071	-0.72968	-0.72865

-0.70758	-0.70652	-0.73546	-0.73439	-0.73332	-0.73225	-0.73119	-0.73016	-0.72915
-0.70811	-0.70705	-0.73602	-0.73492	-0.73384	-0.73275	-0.73169	-0.73066	-0.72965
-0.69105	-0.69004	-0.70652	-0.70547	-0.73436	-0.73326	-0.73219	-0.73116	-0.73015
-0.69156	-0.69054	-0.70703	-0.70597	-0.73489	-0.7338	-0.73272	-0.73168	-0.73066
-0.69207	-0.69103	-0.70754	-0.70648	-0.73544	-0.73434	-0.73327	-0.73221	-0.73117
-0.69256	-0.69153	-0.70807	-0.70701	-0.70595	-0.70491	-0.73381	-0.73274	-0.73169
-0.69305	-0.69203	-0.7086	-0.70754	-0.70648	-0.70542	-0.73435	-0.73328	-0.73223
-0.69355	-0.69254	-0.69154	-0.69053	-0.70701	-0.70595	-0.7049	-0.70388	-0.70288
-0.69406	-0.69306	-0.69206	-0.69104	-0.70755	-0.70649	-0.70544	-0.7044	-0.70339
-0.69457	-0.69357	-0.69257	-0.69156	-0.69055	-0.68954	-0.70597	-0.70493	-0.7039
-0.69509	-0.69407	-0.69307	-0.69208	-0.69108	-0.69006	-0.7065	-0.70546	-0.70444
-0.6956	-0.69457	-0.69358	-0.6926	-0.69161	-0.69057	-0.70703	-0.706	-0.70498
-0.69612	-0.69509	-0.69409	-0.69311	-0.69211	-0.6911	-0.69009	-0.68909	-0.6881
-0.69663	-0.69562	-0.69461	-0.69361	-0.69261	-0.69162	-0.69063	-0.68961	-0.6886
-0.69714	-0.69613	-0.69512	-0.69412	-0.69312	-0.69214	-0.69115	-0.69013	-0.68912
-0.69765	-0.69663	-0.69562	-0.69464	-0.69364	-0.69264	-0.69164	-0.69065	-0.68966
-0.69817	-0.69714	-0.69614	-0.69515	-0.69416	-0.69315	-0.69215	-0.69117	-0.69019
-0.6987	-0.69767	-0.69666	-0.69566	-0.69466	-0.69366	-0.69267	-0.69169	-0.69071
-0.69924	-0.69821	-0.69719	-0.69618	-0.69517	-0.69419	-0.6932	-0.69221	-0.69123
-0.6998	-0.69876	-0.69774	-0.69671	-0.69571	-0.69472	-0.69374	-0.69275	-0.69176
-0.70036	-0.69932	-0.69828	-0.69726	-0.69625	-0.69526	-0.69428	-0.69328	-0.69229
-0.67132	-0.6703	-0.69881	-0.69779	-0.69678	-0.69578	-0.69479	-0.6938	-0.69281
-0.67185	-0.67081	-0.69934	-0.69832	-0.69731	-0.6963	-0.69529	-0.69431	-0.69333
-0.67238	-0.67133	-0.69988	-0.69885	-0.69783	-0.69681	-0.69581	-0.69482	-0.69384
-0.6729	-0.67187	-0.70044	-0.69937	-0.69834	-0.69734	-0.69634	-0.69533	-0.69433
-0.63697	-0.63607	-0.67137	-0.67034	-0.69887	-0.69787	-0.69688	-0.69585	-0.69484
-0.63748	-0.63655	-0.67191	-0.67088	-0.69943	-0.69842	-0.69742	-0.69639	-0.69538
-0.63801	-0.63704	-0.63611	-0.63522	-0.70001	-0.69898	-0.69796	-0.69694	-0.69592
-0.63854	-0.63755	-0.63661	-0.6357	-0.70059	-0.69954	-0.6985	-0.69748	-0.69647
-0.63908	-0.63807	-0.63712	-0.6362	-0.70118	-0.7001	-0.69905	-0.69803	-0.69703
-0.63962	-0.63862	-0.63765	-0.6367	-0.63577	-0.63488	-0.69962	-0.69859	-0.69759
-0.64017	-0.63918	-0.63819	-0.6372	-0.63626	-0.63536	-0.70021	-0.69918	-0.69816
-0.64072	-0.63974	-0.63874	-0.63773	-0.63677	-0.63586	-0.70082	-0.69977	-0.69874
-0.64128	-0.6403	-0.6393	-0.63828	-0.63731	-0.63639	-0.70144	-0.70039	-0.69935
-0.61023	-0.60932	-0.60838	-0.60744	-0.63786	-0.63691	-0.636	-0.63512	-0.63425
-0.61076	-0.60982	-0.60889	-0.60797	-0.63842	-0.63744	-0.63649	-0.63559	-0.63472
-0.61128	-0.61034	-0.60941	-0.60849	-0.63899	-0.63798	-0.637	-0.63608	-0.63519
-0.53496	-0.53426	-0.57379	-0.57293	-0.6081	-0.60713	-0.63754	-0.63659	-0.63568
-0.53537	-0.53467	-0.57428	-0.57343	-0.60863	-0.60766	-0.63809	-0.63712	-0.63618
-0.53576	-0.53506	-0.53437	-0.5337	-0.57304	-0.57213	-0.60723	-0.60631	-0.60542
-0.53614	-0.53545	-0.53477	-0.5341	-0.57354	-0.57263	-0.60777	-0.60683	-0.60592
-0.53653	-0.53584	-0.53517	-0.5345	-0.57402	-0.57313	-0.57223	-0.57134	-0.57047
-0.53692	-0.53624	-0.53557	-0.5349	-0.57451	-0.57362	-0.57273	-0.57184	-0.57097
-0.53732	-0.53664	-0.53597	-0.5353	-0.53462	-0.53392	-0.57323	-0.57235	-0.57147
-0.53771	-0.53704	-0.53636	-0.53568	-0.535	-0.53431	-0.57372	-0.57285	-0.57198
-0.5381	-0.53744	-0.53675	-0.53606	-0.53537	-0.5347	-0.57421	-0.57335	-0.57248
-0.46617	-0.46566	-0.53715	-0.53645	-0.53577	-0.53509	-0.53441	-0.53373	-0.53305
-0.46647	-0.46595	-0.53754	-0.53685	-0.53616	-0.53548	-0.5348	-0.53412	-0.53345
-0.46677	-0.46625	-0.46572	-0.4652	-0.53656	-0.53587	-0.53519	-0.53452	-0.53385
-0.46708	-0.46655	-0.46602	-0.46549	-0.53695	-0.53627	-0.53559	-0.53492	-0.53425

-0.39528	-0.39486	-0.46632	-0.46579	-0.49959	-0.49897	-0.49835	-0.49774	-0.49712
-0.39553	-0.39508	-0.46661	-0.46608	-0.49993	-0.49931	-0.4987	-0.49809	-0.49748
-0.39578	-0.39531	-0.39488	-0.39447	-0.46585	-0.46532	-0.49905	-0.49844	-0.49783
-0.39604	-0.39556	-0.39511	-0.3947	-0.46615	-0.46563	-0.4994	-0.49879	-0.49818
-0.3963	-0.39582	-0.39536	-0.39493	-0.46645	-0.46593	-0.49975	-0.49914	-0.49854
-0.39656	-0.39608	-0.39562	-0.39516	-0.39473	-0.39434	-0.46572	-0.46521	-0.46469
-0.39683	-0.39636	-0.39588	-0.3954	-0.39496	-0.39457	-0.46604	-0.46552	-0.465
-0.3971	-0.39662	-0.39614	-0.39565	-0.39521	-0.3948	-0.39441	-0.39402	-0.39363
-0.39736	-0.39687	-0.39639	-0.39592	-0.39546	-0.39504	-0.39463	-0.39424	-0.39385
-0.39764	-0.39714	-0.39666	-0.39618	-0.39573	-0.39529	-0.39487	-0.39446	-0.39407
-0.39795	-0.39744	-0.39694	-0.39646	-0.396	-0.39554	-0.3951	-0.3947	-0.3943
-0.39826	-0.39773	-0.39723	-0.39674	-0.39627	-0.3958	-0.39535	-0.39493	-0.39453
-0.39857	-0.39803	-0.39751	-0.39702	-0.39653	-0.39606	-0.3956	-0.39517	-0.39475
-0.39887	-0.39833	-0.3978	-0.39729	-0.3968	-0.39632	-0.39586	-0.39541	-0.39498
-0.39918	-0.39864	-0.39811	-0.39758	-0.39707	-0.39659	-0.39612	-0.39566	-0.39522
-0.39949	-0.39897	-0.39843	-0.39788	-0.39736	-0.39687	-0.39639	-0.39592	-0.39547
-0.3998	-0.39929	-0.39875	-0.3982	-0.39767	-0.39716	-0.39667	-0.3962	-0.39574
-0.40011	-0.39959	-0.39907	-0.39853	-0.398	-0.39748	-0.39697	-0.39648	-0.39601
-0.38031	-0.37986	-0.39938	-0.39886	-0.39833	-0.3978	-0.39728	-0.39678	-0.39629
-0.38058	-0.38013	-0.39969	-0.39917	-0.39865	-0.39811	-0.39759	-0.39709	-0.3966
-0.38085	-0.3804	-0.4	-0.39949	-0.39896	-0.39843	-0.3979	-0.3974	-0.39691
-0.38112	-0.38068	-0.38023	-0.37978	-0.37933	-0.37886	-0.39822	-0.3977	-0.39719
-0.3814	-0.38096	-0.38051	-0.38005	-0.3796	-0.37914	-0.39854	-0.39801	-0.39748
-0.38167	-0.38123	-0.38078	-0.38032	-0.37987	-0.37942	-0.37897	-0.37849	-0.37803
-0.38195	-0.38149	-0.38104	-0.38059	-0.38014	-0.3797	-0.37925	-0.37878	-0.37831
-0.38224	-0.38178	-0.38132	-0.38087	-0.38043	-0.37998	-0.37953	-0.37906	-0.3786
-0.38256	-0.38209	-0.38163	-0.38118	-0.38072	-0.38027	-0.37981	-0.37934	-0.37888
-0.38288	-0.3824	-0.38194	-0.38148	-0.38102	-0.38056	-0.38009	-0.37963	-0.37917
-0.38318	-0.3827	-0.38223	-0.38177	-0.38132	-0.38086	-0.3804	-0.37994	-0.37948
-0.38348	-0.38299	-0.38252	-0.38206	-0.3816	-0.38115	-0.3807	-0.38025	-0.37979
-0.38378	-0.38329	-0.3828	-0.38234	-0.38188	-0.38143	-0.38099	-0.38054	-0.38009
-0.38408	-0.38358	-0.38309	-0.38261	-0.38215	-0.3817	-0.38126	-0.38082	-0.38037
-0.38439	-0.38388	-0.38338	-0.38289	-0.38242	-0.38197	-0.38153	-0.38108	-0.38064
-0.38469	-0.38418	-0.38368	-0.38318	-0.3827	-0.38224	-0.38179	-0.38134	-0.38089
-0.385	-0.38449	-0.38399	-0.38349	-0.383	-0.38252	-0.38206	-0.38161	-0.38117
-0.38534	-0.38484	-0.38434	-0.38383	-0.38333	-0.38284	-0.38236	-0.38191	-0.38148
-0.38568	-0.38519	-0.38469	-0.38418	-0.38367	-0.38316	-0.38267	-0.38222	-0.38178
-0.38598	-0.3855	-0.385	-0.38449	-0.38398	-0.38347	-0.38298	-0.38251	-0.38206
-0.38628	-0.3858	-0.3853	-0.38479	-0.38429	-0.38379	-0.38329	-0.38281	-0.38233
-0.34507	-0.34464	-0.38559	-0.3851	-0.38461	-0.38411	-0.38362	-0.38313	-0.38264
-0.3453	-0.34487	-0.38586	-0.3854	-0.38493	-0.38444	-0.38395	-0.38346	-0.38298
-0.34557	-0.34513	-0.34471	-0.34431	-0.38524	-0.38476	-0.38427	-0.38379	-0.3833
-0.34587	-0.34543	-0.345	-0.34458	-0.38555	-0.38507	-0.38459	-0.38411	-0.38361
-0.34618	-0.34573	-0.34528	-0.34485	-0.34442	-0.344	-0.3849	-0.38442	-0.38392
-0.34647	-0.346	-0.34555	-0.34511	-0.34468	-0.34426	-0.3852	-0.38471	-0.38422
-0.34677	-0.34629	-0.34582	-0.34537	-0.34494	-0.34451	-0.38549	-0.38501	-0.38453
-0.34711	-0.34661	-0.34613	-0.34567	-0.34522	-0.34479	-0.34436	-0.34393	-0.34351
-0.34749	-0.34696	-0.34646	-0.34598	-0.34552	-0.34507	-0.34463	-0.34421	-0.3438
-0.34788	-0.34731	-0.34677	-0.34627	-0.3458	-0.34534	-0.3449	-0.34448	-0.34407
-0.34829	-0.34764	-0.34707	-0.34656	-0.34608	-0.34562	-0.34517	-0.34474	-0.34432

-0.34876	-0.34804	-0.34742	-0.34689	-0.34639	-0.34591	-0.34545	-0.34501	-0.34458
-0.3493	-0.3485	-0.34783	-0.34726	-0.34674	-0.34624	-0.34577	-0.34531	-0.34486
-0.34987	-0.34901	-0.34829	-0.34767	-0.3471	-0.34658	-0.34609	-0.34561	-0.34516
-0.35047	-0.34959	-0.34879	-0.34808	-0.34744	-0.34691	-0.34641	-0.34593	-0.34547
-0.3511	-0.3502	-0.34934	-0.34853	-0.34783	-0.34726	-0.34675	-0.34626	-0.34579
-0.35173	-0.35083	-0.34994	-0.34908	-0.34831	-0.34767	-0.34709	-0.34658	-0.3461
-0.35236	-0.35147	-0.35058	-0.34968	-0.34886	-0.34812	-0.34745	-0.3469	-0.34641
-0.35301	-0.35211	-0.3512	-0.35029	-0.34943	-0.34861	-0.34788	-0.34728	-0.34676
-0.35366	-0.35273	-0.35181	-0.3509	-0.35002	-0.34915	-0.34836	-0.34771	-0.34714
-0.35434	-0.35338	-0.35244	-0.35153	-0.35063	-0.34972	-0.34889	-0.34818	-0.34755
-0.35505	-0.35406	-0.3531	-0.35218	-0.35126	-0.35034	-0.34947	-0.34868	-0.34797
-0.35578	-0.35476	-0.35378	-0.35284	-0.35191	-0.35098	-0.35008	-0.34922	-0.34843
-0.35649	-0.35546	-0.35447	-0.35351	-0.35257	-0.35163	-0.35072	-0.34983	-0.34898
-0.3572	-0.35618	-0.35518	-0.35419	-0.35323	-0.3523	-0.35138	-0.35046	-0.34958
-0.35794	-0.35691	-0.35589	-0.35488	-0.3539	-0.35295	-0.35202	-0.35109	-0.35019
-0.35871	-0.35766	-0.35661	-0.35558	-0.35458	-0.35361	-0.35266	-0.35173	-0.35083
-0.31179	-0.31095	-0.35736	-0.35631	-0.35529	-0.35429	-0.35332	-0.35238	-0.35147
-0.31235	-0.31156	-0.35813	-0.35709	-0.35605	-0.35501	-0.354	-0.35304	-0.35211
-0.27395	-0.27327	-0.31134	-0.31052	-0.35681	-0.35575	-0.35471	-0.35373	-0.35277
-0.27445	-0.27376	-0.31191	-0.31112	-0.35757	-0.35653	-0.35549	-0.35446	-0.35345
-0.27494	-0.27425	-0.31248	-0.31171	-0.35832	-0.3573	-0.35627	-0.35521	-0.35416
-0.27544	-0.27475	-0.31305	-0.31228	-0.35903	-0.35803	-0.35701	-0.35597	-0.35492
-0.27593	-0.27526	-0.31363	-0.31285	-0.35972	-0.35874	-0.35774	-0.35673	-0.3557
-0.27642	-0.27576	-0.27508	-0.27439	-0.31263	-0.31185	-0.35846	-0.35747	-0.35646
-0.2769	-0.27624	-0.27557	-0.2749	-0.31322	-0.31243	-0.35917	-0.35818	-0.35719
-0.27741	-0.27672	-0.27605	-0.27539	-0.27471	-0.27401	-0.31221	-0.31141	-0.31061
-0.27796	-0.27721	-0.27652	-0.27586	-0.2752	-0.27452	-0.31279	-0.312	-0.31119
-0.27853	-0.27773	-0.27701	-0.27634	-0.27568	-0.27501	-0.27433	-0.27363	-0.27291
-0.27913	-0.2783	-0.27754	-0.27683	-0.27615	-0.2755	-0.27484	-0.27415	-0.27344
-0.27975	-0.2789	-0.2781	-0.27734	-0.27663	-0.27598	-0.27534	-0.27466	-0.27397
-0.28036	-0.27951	-0.27869	-0.27788	-0.27714	-0.27648	-0.27583	-0.27517	-0.27449
-0.28097	-0.28014	-0.2793	-0.27846	-0.27768	-0.27699	-0.27633	-0.27567	-0.27501
-0.28156	-0.28074	-0.27991	-0.27906	-0.27826	-0.27752	-0.27682	-0.27615	-0.2755
-0.28212	-0.28133	-0.28052	-0.27968	-0.27886	-0.27806	-0.2773	-0.27663	-0.27599
-0.28269	-0.28193	-0.28113	-0.2803	-0.27947	-0.27862	-0.27783	-0.27714	-0.27648
-0.28327	-0.28252	-0.28173	-0.2809	-0.28007	-0.27923	-0.27842	-0.27769	-0.27699
-0.28384	-0.28311	-0.28232	-0.28151	-0.28068	-0.27984	-0.27903	-0.27826	-0.27753
-0.2844	-0.28365	-0.28289	-0.2821	-0.2813	-0.28045	-0.27962	-0.27884	-0.27809
-0.28494	-0.28419	-0.28344	-0.2827	-0.28191	-0.28106	-0.28023	-0.27944	-0.27868
-0.2855	-0.28474	-0.284	-0.28328	-0.28251	-0.28168	-0.28085	-0.28004	-0.27924
-0.28607	-0.2853	-0.28456	-0.28385	-0.28309	-0.28229	-0.28147	-0.28064	-0.27981
-0.28666	-0.28588	-0.28513	-0.28441	-0.28367	-0.28289	-0.28209	-0.28124	-0.2804
-0.28727	-0.28646	-0.2857	-0.28498	-0.28424	-0.28348	-0.28269	-0.28186	-0.28101
-0.28791	-0.28707	-0.28628	-0.28554	-0.2848	-0.28405	-0.28329	-0.28247	-0.28163
-0.28857	-0.2877	-0.28688	-0.2861	-0.28534	-0.28461	-0.28387	-0.28307	-0.28226
-0.28924	-0.28835	-0.28749	-0.28666	-0.28588	-0.28516	-0.28444	-0.28367	-0.28288
-0.28991	-0.28902	-0.28813	-0.28726	-0.28645	-0.28573	-0.28501	-0.28425	-0.28348
-0.29059	-0.28971	-0.2888	-0.28788	-0.28704	-0.28631	-0.28558	-0.28483	-0.28408
-0.29128	-0.29039	-0.28948	-0.28854	-0.28768	-0.28691	-0.28616	-0.28541	-0.28466
-0.29197	-0.29108	-0.29017	-0.28924	-0.28836	-0.28753	-0.28674	-0.28598	-0.28525

-0.24129	-0.24057	-0.29087	-0.28995	-0.28905	-0.28819	-0.28736	-0.28658	-0.28583
-0.24187	-0.24114	-0.29158	-0.29065	-0.28975	-0.28888	-0.28803	-0.28721	-0.28641
-0.24244	-0.24172	-0.29229	-0.29136	-0.29046	-0.28958	-0.28871	-0.28785	-0.28702
-0.24301	-0.2423	-0.29299	-0.29207	-0.29116	-0.29028	-0.28941	-0.28852	-0.28765
-0.24358	-0.24287	-0.29368	-0.29277	-0.29188	-0.29099	-0.29011	-0.2892	-0.28831
-0.04371	-0.04358	-0.24271	-0.24195	-0.29258	-0.2917	-0.29081	-0.2899	-0.289
-0.04378	-0.04365	-0.24327	-0.24253	-0.29328	-0.2924	-0.29151	-0.2906	-0.2897
-0.04386	-0.04373	-0.19382	-0.19322	-0.29397	-0.2931	-0.29222	-0.29132	-0.29043
-0.04395	-0.0438	-0.19424	-0.19366	-0.29465	-0.2938	-0.29293	-0.29205	-0.29117
-0.04405	-0.04388	-0.04373	-0.0436	-0.19351	-0.19294	-0.29363	-0.29277	-0.2919
-0.04415	-0.04398	-0.04383	-0.04368	-0.19393	-0.19337	-0.29431	-0.29348	-0.29263
-0.04425	-0.04409	-0.04392	-0.04376	-0.04361	-0.04348	-0.24609	-0.24541	-0.2447
-0.04435	-0.04418	-0.04402	-0.04386	-0.0437	-0.04356	-0.24664	-0.24596	-0.24527
-0.04445	-0.04428	-0.04412	-0.04396	-0.0438	-0.04364	-0.24719	-0.2465	-0.24582
-0.04455	-0.04437	-0.04421	-0.04406	-0.0439	-0.04373	-0.04357	-0.04343	-0.04331
-0.04464	-0.04446	-0.0443	-0.04415	-0.04399	-0.04382	-0.04366	-0.04351	-0.04338
		-0.04439	-0.04424	-0.04408	-0.04392	-0.04376	-0.0436	-0.04346
		-0.04449	-0.04433	-0.04417	-0.04402	-0.04387	-0.04371	-0.04355
				-0.04427	-0.04412	-0.04397	-0.04381	-0.04364
				-0.04437	-0.04422	-0.04406	-0.0439	-0.04374
				-0.04447	-0.04431	-0.04416	-0.044	-0.04384
						-0.04425	-0.0441	-0.04394
						-0.04435	-0.0442	-0.04405
						-0.04446	-0.0443	-0.04414
						-0.04457	-0.0444	-0.04424

							-0.2526	-0.25186
							-0.25203	-0.25128
				-0.25366	-0.25293	-0.25219	-0.25145	-0.2507
				-0.25309	-0.25235	-0.25161	-0.25086	-0.25012
		-0.25398	-0.25324	-0.25251	-0.25176	-0.25102	-0.52814	-0.52658
		-0.2534	-0.25266	-0.25192	-0.25116	-0.25042	-0.52691	-0.52541
-0.1081	-0.10779	-0.25282	-0.25207	-0.53036	-0.52876	-0.52719	-0.56405	-0.56233
-0.10784	-0.10754	-0.25222	-0.25147	-0.52908	-0.52753	-0.52601	-0.5627	-0.561
-0.10757	-0.10728	-0.25163	-0.25087	-0.52782	-0.52632	-0.52484	-0.56137	-0.55968
-0.25253	-0.25178	-0.3891	-0.38797	-0.56523	-0.56348	-0.56177	-0.59773	-0.59589
-0.25194	-0.25118	-0.38822	-0.38713	-0.56385	-0.56215	-0.56047	-0.5963	-0.59442
-0.38959	-0.38846	-0.56604	-0.56427	-0.60038	-0.59855	-0.59672	-0.59487	-0.59302
-0.38871	-0.38762	-0.56468	-0.56297	-0.59901	-0.59713	-0.59526	-0.59344	-0.59167
-0.56689	-0.56509	-0.60123	-0.59943	-0.59761	-0.59571	-0.59384	-0.63798	-0.63617
-0.56551	-0.56376	-0.59982	-0.59801	-0.59619	-0.59431	-0.59247	-0.63657	-0.63479
-0.56415	-0.56245	-0.59842	-0.59658	-0.64081	-0.63887	-0.63697	-0.71557	-0.71366
-0.56283	-0.56115	-0.59699	-0.59513	-0.63927	-0.6374	-0.63558	-0.71405	-0.71215
-0.56153	-0.55985	-0.59556	-0.59369	-0.63776	-0.63596	-0.6342	-0.71254	-0.71064
-0.59781	-0.59596	-0.64011	-0.63819	-0.6768	-0.67498	-0.67319	-0.71102	-0.70908
-0.59641	-0.59451	-0.63859	-0.63675	-0.67536	-0.67358	-0.6718	-0.7095	-0.70751
-0.64103	-0.63904	-0.63715	-0.63536	-0.71373	-0.71186	-0.70998	-0.708	-0.70599
-0.63958	-0.63764	-0.63578	-0.63401	-0.71224	-0.71037	-0.70848	-0.70652	-0.70453
-0.63815	-0.63627	-0.71459	-0.71267	-0.71077	-0.70887	-0.70697	-0.92635	-0.92338
-0.63677	-0.63492	-0.71311	-0.71121	-0.70931	-0.70737	-0.70543	-0.92412	-0.92129
-0.63541	-0.63359	-0.71164	-0.70976	-0.70786	-0.70588	-0.70392	-0.92193	-0.91923
-0.71411	-0.71212	-0.71018	-0.7083	-0.92866	-0.92562	-0.92264	-0.91985	-0.91717
-0.71263	-0.71065	-0.70872	-0.70684	-0.92647	-0.92352	-0.92061	-0.91783	-0.91513
-0.71112	-0.70918	-0.93011	-0.92722	-0.92435	-0.92143	-0.91856	-0.98482	-0.98197
-0.70961	-0.70771	-0.92791	-0.92509	-0.92227	-0.91936	-0.9165	-0.98266	-0.97985
-0.89127	-0.88858	-0.92575	-0.92294	-0.9894	-0.98636	-0.98337	-1.01671	-1.01389
-0.88925	-0.88661	-0.92364	-0.92076	-0.98703	-0.98407	-0.9812	-1.01458	-1.01183
-0.92721	-0.92442	-0.9616	-0.95857	-1.021	-1.01807	-1.01522	-1.07998	-1.07715
-0.9251	-0.92233	-0.95944	-0.95643	-1.01878	-1.01591	-1.01312	-1.07778	-1.07495
-0.92302	-0.92025	-0.95732	-0.95435	-1.01661	-1.01379	-1.01104	-1.07559	-1.07275
-0.96094	-0.95808	-1.02051	-1.01745	-1.08208	-1.07915	-1.07628	-1.07339	-1.0705

-0.95879	-0.95595	-1.0183	-1.01531	-1.07988	-1.07699	-1.07413	-1.07119	-1.06825
-1.02203	-1.01907	-1.08375	-1.08069	-1.0777	-1.07483	-1.07198	-1.13295	-1.12988
-1.01979	-1.01689	-1.0815	-1.0785	-1.07555	-1.07269	-1.06984	-1.13077	-1.12773
-1.08526	-1.08228	-1.07931	-1.07634	-1.10199	-1.09909	-1.09622	-1.15607	-1.15304
-1.08299	-1.0801	-1.07716	-1.0742	-1.09983	-1.09695	-1.0941	-1.1539	-1.15089
-1.08077	-1.07794	-1.10365	-1.10064	-1.1607	-1.15771	-1.15475	-1.26982	-1.26663
-1.07863	-1.07581	-1.1015	-1.0985	-1.15849	-1.15553	-1.1526	-1.26751	-1.26435
-1.07652	-1.0737	-1.09937	-1.09637	-1.1563	-1.15338	-1.15048	-1.26523	-1.26208
-1.10303	-1.10016	-1.1603	-1.15718	-1.18587	-1.1829	-1.17997	-1.29467	-1.29139
-1.10093	-1.09803	-1.1581	-1.15503	-1.18368	-1.18073	-1.17781	-1.29232	-1.28904
-1.16203	-1.15899	-1.18767	-1.18456	-1.29957	-1.29638	-1.29322	-1.28998	-1.28673
-1.15993	-1.15687	-1.18549	-1.18239	-1.29721	-1.29402	-1.29086	-1.28765	-1.28444
-1.18953	-1.18641	-1.3015	-1.29818	-1.2949	-1.2917	-1.28853	-1.28534	-1.28216
-1.1873	-1.18419	-1.29913	-1.29588	-1.29266	-1.28945	-1.28625	-1.28306	-1.27988
-1.18507	-1.18198	-1.29676	-1.29358	-1.2904	-1.2872	-1.284	-1.28079	-1.27761
-1.26902	-1.2658	-1.29438	-1.29124	-1.28809	-1.28492	-1.28173	-1.30655	-1.30331
-1.26669	-1.26349	-1.292	-1.28888	-1.28577	-1.28263	-1.27947	-1.30427	-1.30105
-1.29616	-1.29283	-1.28963	-1.28656	-1.31157	-1.30838	-1.30518	-1.34936	-1.3459
-1.29379	-1.29046	-1.28728	-1.28426	-1.30925	-1.30605	-1.30286	-1.34697	-1.34362
-1.31966	-1.31631	-1.3131	-1.31003	-1.35491	-1.3514	-1.34792	-1.34458	-1.3413
-1.31729	-1.31402	-1.31083	-1.30773	-1.35243	-1.34894	-1.34549	-1.34217	-1.33893
-1.31497	-1.31178	-1.35687	-1.35342	-1.34998	-1.34651	-1.34309	-1.3398	-1.33658
-1.31271	-1.30957	-1.35454	-1.35106	-1.34759	-1.34413	-1.34073	-1.33744	-1.33424
-1.3105	-1.30737	-1.35222	-1.34871	-1.34523	-1.34179	-1.33842	-1.33515	-1.33197
-1.35676	-1.35331	-1.34983	-1.34631	-1.34285	-1.33949	-1.3362	-1.33295	-1.3298
-1.35443	-1.35092	-1.34742	-1.3439	-1.34049	-1.33721	-1.33403	-1.33083	-1.32775
-1.35205	-1.34853	-1.34503	-1.34154	-1.33817	-1.33493	-1.33182	-1.34575	-1.3428
-1.34964	-1.34613	-1.34266	-1.33921	-1.33588	-1.33265	-1.3296	-1.34372	-1.3411
-1.34723	-1.34375	-1.34032	-1.33693	-1.35072	-1.3475	-1.3445	-1.34187	-1.33951
-1.34483	-1.34139	-1.33801	-1.33466	-1.34849	-1.34546	-1.34266	-1.34021	-1.33802
-1.34246	-1.33908	-1.35284	-1.34949	-1.34638	-1.34355	-1.34098	-1.33869	-1.33664
-1.34015	-1.33683	-1.3506	-1.34735	-1.34437	-1.34177	-1.33942	-1.3373	-1.33538
-1.33788	-1.33463	-1.34845	-1.34533	-1.34252	-1.34013	-1.33799	-1.33601	-1.33419
-1.35273	-1.34944	-1.34637	-1.3435	-1.34091	-1.33867	-1.33664	-1.36015	-1.35833
-1.35054	-1.3473	-1.3444	-1.34184	-1.33949	-1.33736	-1.33539	-1.3589	-1.3571
-1.34842	-1.34537	-1.34267	-1.34031	-1.36361	-1.36152	-1.35957	-1.38367	-1.38182
-1.34639	-1.34365	-1.34116	-1.33892	-1.36228	-1.36029	-1.3584	-1.3825	-1.38066
-1.34453	-1.34206	-1.39152	-1.38924	-1.40484	-1.40284	-1.40093	-1.39905	-1.39723
-1.34287	-1.3406	-1.39013	-1.38796	-1.40362	-1.40164	-1.39974	-1.39787	-1.39605
-1.34135	-1.33923	-1.38879	-1.38671	-1.40241	-1.40045	-1.39855	-1.3967	-1.39489
-1.39172	-1.38956	-1.40517	-1.40314	-1.40117	-1.39924	-1.39736	-1.42592	-1.42398
-1.39034	-1.38822	-1.40386	-1.40188	-1.39994	-1.39804	-1.39619	-1.42465	-1.42274
-1.40667	-1.40458	-1.40257	-1.40067	-1.42945	-1.42739	-1.42537	-1.4234	-1.42148
-1.40533	-1.40327	-1.40132	-1.3995	-1.42822	-1.42619	-1.42417	-1.42217	-1.42019
-1.40407	-1.40204	-1.40012	-1.39833	-1.45386	-1.45178	-1.4497	-1.49448	-1.49234
-1.40289	-1.40092	-1.39901	-1.39717	-1.45248	-1.45041	-1.44835	-1.49311	-1.491
-1.40171	-1.39978	-1.50252	-1.50031	-1.49813	-1.49597	-1.49385	-1.51692	-1.51472
-1.40047	-1.39857	-1.50111	-1.4989	-1.49672	-1.49457	-1.49246	-1.51547	-1.51333
-1.39924	-1.39737	-1.4997	-1.49751	-1.49535	-1.4932	-1.49111	-1.51404	-1.51195
-1.50268	-1.50051	-1.49834	-1.49617	-1.51929	-1.51703	-1.51482	-1.51266	-1.51056

-1.50128	-1.49914	-1.497	-1.49485	-1.5179	-1.51567	-1.51348	-1.51131	-1.50919
-1.49989	-1.49777	-1.52099	-1.51872	-1.51647	-1.51426	-1.51209	-1.53354	-1.53129
-1.49853	-1.49639	-1.51951	-1.51726	-1.51503	-1.51281	-1.51066	-1.53208	-1.52996
-1.52257	-1.52031	-1.51807	-1.51584	-1.53756	-1.53515	-1.53281	-1.53066	-1.52861
-1.52108	-1.51888	-1.51668	-1.51448	-1.5361	-1.53373	-1.53144	-1.5293	-1.52725
-1.51963	-1.51746	-1.53936	-1.53697	-1.53463	-1.53233	-1.5301	-1.52796	-1.52588
-1.51824	-1.51604	-1.53779	-1.53541	-1.53309	-1.53089	-1.52874	-1.52662	-1.52453
-1.51687	-1.51465	-1.53625	-1.53387	-1.53159	-1.52947	-1.5274	-1.52529	-1.52319
-1.51552	-1.5133	-1.5348	-1.53249	-1.53026	-1.52815	-1.52609	-1.52398	-1.5219
-1.51417	-1.51199	-1.53341	-1.53118	-1.52901	-1.52688	-1.52479	-1.52269	-1.52063
-1.53661	-1.53426	-1.532	-1.52983	-1.52769	-1.52556	-1.52346	-1.52139	-1.51935
-1.53507	-1.53277	-1.53056	-1.52844	-1.52633	-1.5242	-1.52211	-1.52006	-1.51807
-1.53359	-1.53136	-1.52919	-1.52708	-1.52499	-1.52288	-1.52081	-1.5188	-1.51685
-1.53218	-1.53003	-1.5279	-1.52578	-1.52369	-1.52162	-1.51959	-1.51762	-1.5157
-1.53081	-1.52872	-1.52661	-1.52448	-1.52239	-1.52036	-1.51838	-1.5378	-1.53587
-1.52945	-1.52736	-1.52525	-1.52313	-1.52106	-1.51907	-1.51713	-1.53656	-1.53467
-1.52811	-1.526	-1.52389	-1.52179	-1.51975	-1.51779	-1.51589	-1.53532	-1.53347
-1.5268	-1.52468	-1.54404	-1.54196	-1.53992	-1.53793	-1.53599	-1.53411	-1.53225
-1.52552	-1.52339	-1.54275	-1.54074	-1.53876	-1.53679	-1.53484	-1.53292	-1.53102
-1.5457	-1.54355	-1.54149	-1.53952	-1.53757	-1.53562	-1.53368	-1.5688	-1.56668
-1.54439	-1.5423	-1.54027	-1.53832	-1.53638	-1.53445	-1.53253	-1.56755	-1.56546
-1.54306	-1.54103	-1.53904	-1.53709	-1.5726	-1.57047	-1.56836	-1.61208	-1.60982
-1.54169	-1.53971	-1.53776	-1.53582	-1.57121	-1.56909	-1.567	-1.61061	-1.60838
-1.54034	-1.53841	-1.53648	-1.53456	-1.56983	-1.56774	-1.56567	-1.60916	-1.60695
-1.5391	-1.53717	-1.57278	-1.57064	-1.61453	-1.61226	-1.61001	-1.6934	-1.69109
-1.53791	-1.53597	-1.57147	-1.56936	-1.61315	-1.6109	-1.60867	-1.69199	-1.68969
-1.57309	-1.57109	-1.69138	-1.68895	-1.69756	-1.69521	-1.69289	-1.69057	-1.68828
-1.57187	-1.56987	-1.68987	-1.68748	-1.69612	-1.69378	-1.69146	-1.68915	-1.68687
-1.69333	-1.69083	-1.69939	-1.69702	-1.69467	-1.69232	-1.68999	-1.69911	-1.6967
-1.6918	-1.68936	-1.69795	-1.6956	-1.69324	-1.69084	-1.68848	-1.69751	-1.69515
-1.70123	-1.69885	-1.69648	-1.69413	-1.69177	-1.68937	-1.687	-1.69595	-1.69364
-1.69964	-1.69728	-1.69493	-1.69258	-1.69024	-1.6879	-1.6856	-1.69449	-1.6922
-1.69807	-1.69572	-1.69337	-1.69103	-1.68872	-1.68646	-1.68423	-1.69305	-1.69078
-1.69658	-1.69423	-1.6919	-1.68958	-1.69864	-1.69627	-1.69393	-1.69163	-1.68936
-1.69515	-1.69279	-1.69047	-1.68819	-1.69717	-1.69483	-1.69252	-1.69022	-1.68795
-1.69372	-1.69136	-1.70047	-1.69808	-1.69572	-1.69341	-1.69112	-1.68883	-1.68655
-1.6923	-1.68995	-1.69898	-1.69662	-1.6943	-1.69201	-1.68973	-1.68744	-1.68517
-1.69088	-1.68856	-1.69752	-1.69519	-1.69288	-1.69059	-1.68832	-1.68605	-1.68379
-1.68947	-1.68721	-1.6961	-1.69377	-1.69146	-1.68916	-1.68689	-1.68464	-1.68242
-1.69938	-1.69703	-1.69469	-1.69236	-1.69004	-1.68773	-1.68545	-1.68323	-1.68104
-1.6979	-1.69559	-1.69327	-1.69094	-1.68862	-1.68629	-1.68399	-1.68177	-1.67961
-1.69644	-1.69415	-1.69184	-1.68952	-1.6872	-1.68485	-1.68254	-1.68033	-1.67817
-1.69499	-1.6927	-1.69041	-1.68811	-1.6858	-1.68346	-1.68115	-1.67894	-1.67679
-1.69357	-1.69126	-1.68897	-1.6867	-1.68441	-1.68209	-1.6798	-1.6776	-1.67545
-1.69215	-1.68983	-1.68754	-1.68527	-1.68299	-1.6807	-1.67844	-1.72153	-1.7191
-1.69073	-1.68841	-1.68611	-1.68381	-1.68154	-1.67929	-1.67707	-1.72001	-1.7176
-1.68931	-1.68701	-1.6847	-1.68239	-1.74283	-1.74027	-1.73778	-1.73533	-1.73293
-1.68791	-1.68563	-1.68332	-1.681	-1.74124	-1.7387	-1.73623	-1.73382	-1.73146
-1.68652	-1.68425	-1.68196	-1.67963	-1.73967	-1.73715	-1.7347	-1.73232	-1.73
-1.70254	-1.70028	-1.7434	-1.74072	-1.73812	-1.73563	-1.73322	-1.74463	-1.7423

-1.70113	-1.69889	-1.74185	-1.73918	-1.73659	-1.73414	-1.73175	-1.74318	-1.74086
-1.69972	-1.6975	-1.7403	-1.73764	-1.74891	-1.74647	-1.74409	-1.74174	-1.73941
-1.69832	-1.69611	-1.73875	-1.73612	-1.74741	-1.745	-1.74265	-1.7403	-1.73797
-1.75617	-1.75364	-1.75107	-1.74846	-1.74593	-1.74353	-1.74119	-1.73886	-1.73655
-1.75458	-1.75205	-1.74951	-1.74696	-1.74446	-1.74207	-1.73973	-1.73742	-1.73515
-1.75301	-1.75049	-1.74798	-1.74547	-1.74301	-1.74062	-1.73828	-1.736	-1.73376
-1.75144	-1.74897	-1.74649	-1.74401	-1.74157	-1.73918	-1.73685	-1.73459	-1.73238
-1.7499	-1.74746	-1.74502	-1.74256	-1.74014	-1.73776	-1.73543	-1.73319	-1.73101
-1.74839	-1.74599	-1.74356	-1.74111	-1.7387	-1.73635	-1.73404	-1.7554	-1.75303
-1.74689	-1.74452	-1.74212	-1.73968	-1.73728	-1.73495	-1.73267	-1.75391	-1.75154
-1.74541	-1.74306	-1.74069	-1.73828	-1.73591	-1.73359	-1.73131	-1.75241	-1.75003
-1.74395	-1.7416	-1.73926	-1.73692	-1.73459	-1.73226	-1.72995	-1.75091	-1.74852
-1.7425	-1.74014	-1.73782	-1.73554	-1.75688	-1.75436	-1.75186	-1.74941	-1.74701
-1.74105	-1.73867	-1.73635	-1.73412	-1.75537	-1.75286	-1.75036	-1.74792	-1.74552
-1.73962	-1.73723	-1.75869	-1.75628	-1.75385	-1.75135	-1.74886	-1.74643	-1.74403
-1.73824	-1.73584	-1.75719	-1.75477	-1.75233	-1.74984	-1.74736	-1.74493	-1.74255
-1.73687	-1.73448	-1.7557	-1.75327	-1.75083	-1.74834	-1.74586	-1.74345	-1.74107
-1.73547	-1.7331	-1.7542	-1.75176	-1.74933	-1.74687	-1.74443	-1.75889	-1.75645
-1.73406	-1.73172	-1.75271	-1.75026	-1.74784	-1.74543	-1.74303	-1.75746	-1.755
-1.75621	-1.75372	-1.75124	-1.74878	-1.76336	-1.76091	-1.75848	-1.75602	-1.75356
-1.75473	-1.75228	-1.74981	-1.74732	-1.76185	-1.75942	-1.75701	-1.75456	-1.75212
-1.77042	-1.76794	-1.76543	-1.76286	-1.76035	-1.75793	-1.75554	-1.80705	-1.80417
-1.7689	-1.76643	-1.76392	-1.76135	-1.75885	-1.75644	-1.75408	-1.80536	-1.80254
-1.76738	-1.76491	-1.76241	-1.75986	-1.75737	-1.75498	-1.75263	-1.80368	-1.80091
-1.76586	-1.76339	-1.76091	-1.75842	-1.81044	-1.80757	-1.80475	-1.80198	-1.79926
-1.76436	-1.76188	-1.75942	-1.757	-1.80877	-1.80588	-1.80303	-1.80029	-1.79761
-1.76288	-1.76039	-1.75794	-1.75555	-1.80707	-1.80421	-1.80139	-1.81188	-1.80903
-1.76143	-1.75892	-1.75647	-1.75409	-1.80537	-1.80257	-1.79979	-1.81026	-1.80737
-1.75998	-1.75748	-1.80931	-1.80647	-1.80367	-1.8009	-1.79816	-1.82308	-1.82015
-1.75853	-1.75609	-1.80764	-1.80477	-1.80195	-1.79921	-1.79649	-1.82142	-1.81851
-1.81175	-1.80885	-1.80597	-1.80309	-1.82843	-1.82552	-1.82266	-1.82476	-1.82189
-1.80996	-1.8071	-1.80426	-1.80145	-1.8268	-1.8239	-1.82102	-1.82316	-1.82032
-1.80818	-1.80535	-1.80255	-1.7998	-1.82519	-1.82228	-1.81938	-1.82155	-1.81875
-1.80648	-1.80364	-1.83423	-1.83135	-1.82848	-1.82559	-1.82272	-1.81991	-1.81713
-1.80481	-1.80196	-1.83253	-1.82966	-1.82679	-1.82391	-1.82106	-1.81826	-1.81549
-1.83686	-1.83384	-1.83089	-1.82801	-1.82515	-1.82229	-1.81945	-1.83286	-1.83001
-1.8352	-1.83223	-1.82929	-1.8264	-1.82354	-1.82071	-1.81789	-1.83124	-1.82837
-1.83356	-1.83061	-1.82769	-1.82481	-1.83594	-1.83311	-1.8303	-1.84341	-1.84053
-1.83194	-1.82897	-1.82606	-1.82321	-1.83436	-1.83154	-1.82873	-1.84182	-1.83897
-1.84432	-1.84135	-1.83844	-1.83561	-1.84886	-1.84597	-1.8431	-1.84025	-1.83742
-1.84271	-1.83977	-1.83687	-1.83403	-1.84724	-1.84437	-1.84152	-1.83868	-1.83586
-1.84109	-1.8382	-1.83532	-1.83246	-1.84563	-1.84278	-1.83996	-1.83713	-1.83432
-1.83949	-1.8366	-1.84983	-1.84692	-1.84404	-1.8412	-1.83839	-1.83558	-1.8328
-1.83788	-1.835	-1.84821	-1.84533	-1.84248	-1.83964	-1.83683	-1.83405	-1.8313
-1.83628	-1.83342	-1.84661	-1.84375	-1.84091	-1.83808	-1.83529	-1.83252	-1.82979
-1.83467	-1.83186	-1.84502	-1.84217	-1.83934	-1.83654	-1.83376	-1.831	-1.82827
-1.84915	-1.84629	-1.84344	-1.84059	-1.83777	-1.83499	-1.83224	-1.82949	-1.82677
-1.84753	-1.84468	-1.84184	-1.83901	-1.83621	-1.83344	-1.8307	-1.82797	-1.82528
-1.84593	-1.84308	-1.84025	-1.83744	-1.83466	-1.8319	-1.82917	-1.82646	-1.82379
-1.84431	-1.84149	-1.83868	-1.83588	-1.84581	-1.84306	-1.84034	-1.88041	-1.87767

-1.84271	-1.83991	-1.83711	-1.83432	-1.84425	-1.84153	-1.83883	-1.87887	-1.87613
-1.85388	-1.85106	-1.86057	-1.85779	-1.85503	-1.85232	-1.84964	-1.87737	-1.87469
-1.85232	-1.84947	-1.85898	-1.85623	-1.85351	-1.85081	-1.84815	-1.87591	-1.8733
-1.8631	-1.86024	-1.85744	-1.8547	-1.85199	-1.84931	-1.84668	-1.87447	-1.87191
-1.86159	-1.85875	-1.85594	-1.85318	-1.85047	-1.84782	-1.84524	-1.87303	-1.87051
-1.86007	-1.85726	-1.85447	-1.85169	-1.84898	-1.84637	-1.84382	-1.87161	-1.86911
-1.85852	-1.85576	-1.85301	-1.85024	-1.84754	-1.84494	-1.84241	-1.87019	-1.86772
-1.85697	-1.85427	-1.85155	-1.84882	-1.84614	-1.84355	-1.84102	-1.86878	-1.86632
-1.85546	-1.85276	-1.85008	-1.8474	-1.84477	-1.8422	-1.83967	-1.89575	-1.89322
-1.85398	-1.85126	-1.8486	-1.846	-1.84342	-1.84086	-1.83834	-1.89433	-1.89177
-1.85252	-1.84981	-1.84716	-1.84459	-1.87234	-1.86976	-1.8672	-1.89293	-1.89037
-1.85109	-1.84838	-1.84574	-1.84317	-1.8709	-1.86836	-1.86584	-1.89156	-1.88901
-1.84965	-1.84697	-1.84435	-1.84179	-1.8978	-1.89523	-1.89269	-1.89016	-1.88764
-1.8482	-1.84558	-1.84299	-1.84044	-1.89637	-1.89377	-1.89121	-1.88871	-1.88626
-1.84676	-1.8442	-1.84165	-1.8391	-1.89497	-1.89234	-1.88976	-1.88729	-1.88488
-1.84536	-1.84283	-1.8403	-1.83777	-1.89359	-1.89096	-1.88838	-1.88589	-1.88347
-1.84397	-1.84146	-1.83895	-1.83644	-1.89221	-1.88961	-1.88704	-1.88453	-1.88207
-1.84259	-1.84007	-1.86644	-1.86392	-1.8908	-1.88824	-1.8857	-1.8832	-1.88074
-1.84122	-1.83868	-1.865	-1.8625	-1.88938	-1.88686	-1.88436	-1.88189	-1.87945
-1.86873	-1.86613	-1.86359	-1.86109	-1.88795	-1.88547	-1.88302	-1.88058	-1.87816
-1.86736	-1.86475	-1.86219	-1.85967	-1.8865	-1.88407	-1.88167	-1.87927	-1.87689
-1.86599	-1.86338	-1.86081	-1.85829	-1.88509	-1.88268	-1.88031	-1.87795	-1.87561
-1.86463	-1.86203	-1.85947	-1.85694	-1.88371	-1.8813	-1.87894	-1.87661	-1.87432
-1.86327	-1.86069	-1.85814	-1.85562	-1.88237	-1.87995	-1.87759	-1.87529	-1.87302
-1.8619	-1.85936	-1.85684	-1.85433	-1.88106	-1.87864	-1.87628	-1.87399	-1.87174
-1.86054	-1.85803	-1.85554	-1.85307	-1.87979	-1.87737	-1.875	-1.87271	-1.87045
-1.85919	-1.8567	-1.85423	-1.85179	-1.8785	-1.87609	-1.87372	-1.87141	-1.86915
-1.85786	-1.85537	-1.85293	-1.85053	-1.87723	-1.87483	-1.87245	-1.87012	-1.86783
-1.85653	-1.85407	-1.85165	-1.84927	-1.87596	-1.87357	-1.8712	-1.86885	-1.86654
-1.85521	-1.85279	-1.85039	-1.84803	-1.87471	-1.87232	-1.86995	-1.8676	-1.86528
-1.85387	-1.85149	-1.84913	-1.84679	-1.87345	-1.87108	-1.86871	-1.86636	-1.86403
-1.85249	-1.85017	-1.84785	-1.84553	-1.87218	-1.86983	-1.86748	-1.86513	-1.86278
-1.85113	-1.84886	-1.84657	-1.84427	-1.87091	-1.86858	-1.86624	-1.86388	-1.86153
-1.84983	-1.84758	-1.84531	-1.84302	-1.86964	-1.86731	-1.86498	-1.86262	-1.86026
-1.84857	-1.84632	-1.84406	-1.84177	-1.86836	-1.86604	-1.8637	-1.86135	-1.85898
-1.84733	-1.84508	-1.84281	-1.84053	-1.86708	-1.86476	-1.86242	-1.86006	-1.8577
-1.84613	-1.84386	-1.84158	-1.83929	-1.86581	-1.86347	-1.86112	-1.85876	-1.8564
-1.84491	-1.84263	-1.84034	-1.83805	-1.86453	-1.86218	-1.85982	-1.89953	-1.89714
-1.84367	-1.84138	-1.8391	-1.83681	-1.86326	-1.86089	-1.85853	-1.89823	-1.89585
-1.84243	-1.84014	-1.83785	-1.83556	-1.90417	-1.90175	-1.89934	-1.89694	-1.89455
-1.8412	-1.8389	-1.8366	-1.83429	-1.90286	-1.90045	-1.89804	-1.89565	-1.89327
-1.83997	-1.83766	-1.83535	-1.83302	-1.90155	-1.89914	-1.89674	-1.89436	-1.89198
-1.85093	-1.84862	-1.9051	-1.90266	-1.90024	-1.89783	-1.89544	-1.89306	-1.8907
-1.84968	-1.84737	-1.90381	-1.90137	-1.89894	-1.89653	-1.89413	-1.89176	-1.8894
-1.87978	-1.87741	-1.9025	-1.90006	-1.89763	-1.89522	-1.89283	-1.89046	-1.88811
-1.8785	-1.87612	-1.90118	-1.89874	-1.89632	-1.89391	-1.89153	-1.88916	-1.88682
-1.87722	-1.87484	-1.89986	-1.89743	-1.89501	-1.89262	-1.89024	-1.88788	-1.88554
-1.87595	-1.87355	-1.89854	-1.89612	-1.89372	-1.89133	-1.88896	-1.88661	-1.88426
-1.87468	-1.87227	-1.89723	-1.89482	-1.89243	-1.89007	-1.88772	-1.88536	-1.88302
-1.87342	-1.871	-1.89593	-1.89353	-1.89115	-1.88883	-1.88651	-1.88418	-1.88184

-1.87217	-1.86977	-1.89466	-1.89225	-1.88989	-1.8876	-1.88532	-1.883	-1.88067
-1.87097	-1.86857	-1.89345	-1.89104	-1.88868	-1.88637	-1.88408	-1.88177	-1.87944
-1.8698	-1.86741	-1.89227	-1.88987	-1.88749	-1.88515	-1.88282	-1.88049	-1.87817
-1.86858	-1.8662	-1.89103	-1.88864	-1.88627	-1.8839	-1.88155	-1.87921	-1.87688
-1.86731	-1.86493	-1.88974	-1.88736	-1.88499	-1.88263	-1.88027	-1.87792	-1.87558
-1.86605	-1.86367	-1.88845	-1.88608	-1.88372	-1.88136	-1.879	-1.87664	-1.87429
-1.86479	-1.86241	-1.88717	-1.8848	-1.88243	-1.88008	-1.87773	-1.87537	-1.87302
-1.86356	-1.86119	-1.88592	-1.88354	-1.88117	-1.87882	-1.87647	-1.87412	-1.87177
-1.88951	-1.88711	-1.88473	-1.88235	-1.87998	-1.87763	-1.87527	-1.87292	-1.87057
-1.88833	-1.88594	-1.88355	-1.88118	-1.87881	-1.87645	-1.87409	-1.87174	-1.86939
-1.88708	-1.88469	-1.88232	-1.87995	-1.87758	-1.87521	-1.87285	-1.8705	-1.86815
-1.88579	-1.8834	-1.88103	-1.87867	-1.8763	-1.87394	-1.87158	-1.86922	-1.86687
-1.88451	-1.88213	-1.87975	-1.87739	-1.87502	-1.87266	-1.8703	-1.86794	-1.86559
-1.88323	-1.88085	-1.87848	-1.8761	-1.87373	-1.87137	-1.86901	-1.86666	-1.86431
-1.88199	-1.87961	-1.87723	-1.87485	-1.87247	-1.87011	-1.86775	-1.8654	-1.86305
-1.8808	-1.87842	-1.87604	-1.87366	-1.87128	-1.86892	-1.86656	-1.86421	-1.86185
-1.87961	-1.87723	-1.87485	-1.87247	-1.8701	-1.86774	-1.86538	-1.86302	-1.86065
-1.87836	-1.87597	-1.87359	-1.87121	-1.86884	-1.86648	-1.86411	-1.86176	-1.8594
-1.87708	-1.8747	-1.87231	-1.86993	-1.86755	-1.86518	-1.86282	-1.86046	-1.8581
-1.87586	-1.87347	-1.87108	-1.8687	-1.86631	-1.86392	-1.86153	-1.85916	-1.8568
-1.87468	-1.87229	-1.8699	-1.86751	-1.86511	-1.86268	-1.86025	-1.85786	-1.85549
-1.87346	-1.87107	-1.86868	-1.86629	-1.86388	-1.86144	-1.859	-1.85659	-1.85421
-1.87218	-1.86979	-1.8674	-1.86502	-1.86262	-1.8602	-1.85778	-1.85538	-1.85299
-1.87091	-1.86852	-1.86613	-1.86375	-1.86136	-1.85895	-1.85655	-1.85415	-1.85177
-1.86971	-1.8673	-1.86491	-1.86253	-1.86013	-1.8577	-1.85527	-1.85288	-1.85049
-1.86851	-1.8661	-1.8637	-1.86131	-1.8589	-1.85645	-1.854	-1.85159	-1.84922
-1.86726	-1.86485	-1.86245	-1.86005	-1.85764	-1.85519	-1.85275	-1.85035	-1.84798
-1.86599	-1.86358	-1.86117	-1.85877	-1.85637	-1.85395	-1.85153	-1.84914	-1.84675
-1.86475	-1.86233	-1.85992	-1.85752	-1.85512	-1.85272	-1.85032	-1.84791	-1.84551
-1.86355	-1.86111	-1.85869	-1.8563	-1.8539	-1.85151	-1.84911	-1.84669	-1.84425
-1.86234	-1.85989	-1.85746	-1.85506	-1.85266	-1.85027	-1.84788	-1.84544	-1.843
-1.86113	-1.85864	-1.85619	-1.85377	-1.85137	-1.84898	-1.84659	-1.84418	-1.84175
-1.85991	-1.8574	-1.85493	-1.8525	-1.85009	-1.8477	-1.84531	-1.84292	-1.84052
-1.85867	-1.85618	-1.85372	-1.85129	-1.84888	-1.84648	-1.84409	-1.84169	-1.8393
-1.85743	-1.85497	-1.85252	-1.8501	-1.84769	-1.84528	-1.84287	-1.84048	-1.83808
-1.85623	-1.85378	-1.85134	-1.84892	-1.8465	-1.84406	-1.84163	-1.83922	-1.83683
-1.85505	-1.85261	-1.85018	-1.84775	-1.84531	-1.84283	-1.84037	-1.83794	-1.83554
-1.85387	-1.85144	-1.84899	-1.84655	-1.85641	-1.85391	-1.85143	-1.84899	-1.84658
-1.85269	-1.85024	-1.84779	-1.84531	-1.85515	-1.85268	-1.85022	-1.84779	-1.84537
-1.85149	-1.84904	-1.85888	-1.85638	-1.8539	-1.85145	-1.84901	-1.84659	-1.84417
-1.85029	-1.8478	-1.85763	-1.85514	-1.85267	-1.85024	-1.84782	-1.84539	-1.84297
-1.84907	-1.84656	-1.85637	-1.85391	-1.85146	-1.84904	-1.84662	-1.84419	-1.84175
-1.86013	-1.85762	-1.85515	-1.8527	-1.85026	-1.84784	-1.84541	-1.84296	-1.84049
-1.85887	-1.8564	-1.85394	-1.85151	-1.84908	-1.84664	-1.8442	-1.84172	-1.83923
-1.85764	-1.85519	-1.85275	-1.85032	-1.84788	-1.84542	-1.84295	-1.84046	-1.83797
-1.85645	-1.85401	-1.85157	-1.84914	-1.84669	-1.84418	-1.84168	-1.83919	-1.83673
-1.85526	-1.85282	-1.85038	-1.84795	-1.84549	-1.84296	-1.84043	-1.83795	-1.8355
-1.85405	-1.85163	-1.84919	-1.84677	-1.8443	-1.84176	-1.83923	-1.83675	-1.8343
-1.85285	-1.85043	-1.84799	-1.84556	-1.84308	-1.84055	-1.83802	-1.83555	-1.8331
-1.85165	-1.84922	-1.84677	-1.84429	-1.8418	-1.83929	-1.83679	-1.83433	-1.8319

-1.85045	-1.84801	-1.84553	-1.84301	-1.84051	-1.83802	-1.83555	-1.83311	-1.83069
-1.84925	-1.84681	-1.84432	-1.84178	-1.83926	-1.83679	-1.83433	-1.8319	-1.82948
-1.84805	-1.84561	-1.84312	-1.84057	-1.83805	-1.83557	-1.83312	-1.83068	-1.82826
-1.84684	-1.8444	-1.84191	-1.83936	-1.83683	-1.83436	-1.8319	-1.82947	-1.82704
-1.84563	-1.84319	-1.8407	-1.83815	-1.83563	-1.83315	-1.8307	-1.82826	-1.82583
-1.84442	-1.84198	-1.83949	-1.83694	-1.83442	-1.83194	-1.82949	-1.82705	-1.82462
-1.8432	-1.84077	-1.83828	-1.83574	-1.83321	-1.83074	-1.82828	-1.82584	-1.8234
-1.84199	-1.83956	-1.83708	-1.83453	-1.832	-1.82953	-1.82707	-1.82463	-1.82219
-1.84079	-1.83835	-1.83586	-1.83331	-1.83079	-1.82831	-1.82585	-1.82341	-1.82096
-1.83958	-1.83714	-1.83465	-1.83209	-1.82957	-1.82709	-1.82463	-1.82218	-1.81972
-1.83837	-1.83593	-1.83343	-1.83088	-1.82834	-1.82587	-1.8234	-1.82095	-1.81849
-1.83716	-1.83472	-1.83222	-1.82966	-1.82712	-1.82464	-1.82217	-1.81972	-1.81726
-1.83595	-1.8335	-1.831	-1.82844	-1.8259	-1.82341	-1.82094	-1.81849	-1.81603
-1.83475	-1.83229	-1.82978	-1.82721	-1.82467	-1.82218	-1.81971	-1.81725	-1.81479
-1.83354	-1.83108	-1.82857	-1.82599	-1.82345	-1.82095	-1.81847	-1.81601	-1.81355
-1.83235	-1.82988	-1.82736	-1.82478	-1.82223	-1.81973	-1.81725	-1.81478	-1.81232
-1.83115	-1.82868	-1.82616	-1.82359	-1.82104	-1.81853	-1.81603	-1.81356	-1.8111
-1.82996	-1.82748	-1.82497	-1.82243	-1.81989	-1.81736	-1.81484	-1.81236	-1.80988
-1.82877	-1.82628	-1.82379	-1.82128	-1.81876	-1.81621	-1.81365	-1.81116	-1.80868
-1.82761	-1.8251	-1.82259	-1.82011	-1.8176	-1.81503	-1.81247	-1.80996	-1.80747
-1.82647	-1.82392	-1.8214	-1.81891	-1.8164	-1.81384	-1.81128	-1.80876	-1.80626
-1.82531	-1.82274	-1.8202	-1.81771	-1.81521	-1.81266	-1.81011	-1.80759	-1.80506
-1.82412	-1.82154	-1.81901	-1.81651	-1.81402	-1.81151	-1.80898	-1.80644	-1.80388
-1.82294	-1.82037	-1.81783	-1.81532	-1.81283	-1.81034	-1.80784	-1.80528	-1.8027
-1.82177	-1.81923	-1.8167	-1.81416	-1.81164	-1.80915	-1.80666	-1.8041	-1.80151
-1.82061	-1.8181	-1.81557	-1.813	-1.81045	-1.80796	-1.80546	-1.8029	-1.80032
-1.81943	-1.81692	-1.81439	-1.81181	-1.80925	-1.80675	-1.80425	-1.80172	-1.79916
-1.81823	-1.81572	-1.81318	-1.81061	-1.80806	-1.80554	-1.80303	-1.80052	-1.79801
-1.81707	-1.81454	-1.812	-1.80945	-1.8069	-1.80436	-1.80183	-1.79933	-1.79683
-1.81594	-1.81339	-1.81085	-1.80832	-1.80577	-1.80321	-1.80065	-1.79812	-1.79561
-1.8148	-1.81225	-1.80971	-1.80717	-1.80463	-1.80205	-1.79948	-1.79694	-1.7944
-1.81366	-1.81112	-1.80857	-1.80601	-1.80345	-1.8009	-1.79835	-1.79578	-1.7932
-1.81251	-1.80998	-1.80743	-1.80484	-1.80227	-1.79973	-1.7972	-1.79461	-1.792
-1.81136	-1.80881	-1.80623	-1.80363	-1.80105	-1.79852	-1.79598	-1.79339	-1.79078
-1.81021	-1.80762	-1.80503	-1.80242	-1.79983	-1.79729	-1.79474	-1.79215	-1.78955
-1.80903	-1.80643	-1.80383	-1.80124	-1.79865	-1.79609	-1.79353	-1.79095	-1.78836
-1.80782	-1.80523	-1.80265	-1.80008	-1.7975	-1.79492	-1.79233	-1.78976	-1.78719
-1.80662	-1.80405	-1.80148	-1.79891	-1.79633	-1.79375	-1.79116	-1.78859	-1.78602
-1.80545	-1.80289	-1.80032	-1.79774	-1.79516	-1.79258	-1.79	-1.78742	-1.78484
-1.80429	-1.80174	-1.79917	-1.79657	-1.79399	-1.79142	-1.78884	-1.79746	-1.79483
-1.80316	-1.80057	-1.79799	-1.79541	-1.79283	-1.79025	-1.78766	-1.79627	-1.79365
-1.80203	-1.79941	-1.79681	-1.79424	-1.79167	-1.78907	-1.78648	-1.79507	-1.79247
-1.80087	-1.79826	-1.79566	-1.79307	-1.79049	-1.7879	-1.78531	-1.79388	-1.79127
-1.7997	-1.79711	-1.79451	-1.7919	-1.7893	-1.78673	-1.78415	-1.7927	-1.79007
-1.79856	-1.79596	-1.79335	-1.79074	-1.78814	-1.78556	-1.78298	-1.79151	-1.78888
-1.79745	-1.7948	-1.79218	-1.78959	-1.787	-1.7844	-1.7818	-1.79032	-1.78771
-1.79635	-1.79368	-1.79104	-1.78845	-1.78585	-1.78324	-1.78064	-1.78914	-1.78653
-1.79527	-1.7926	-1.78994	-1.78731	-1.78469	-1.78209	-1.77951	-1.78798	-1.78535
-1.79416	-1.79151	-1.78885	-1.78618	-1.78354	-1.78095	-1.77837	-1.78682	-1.78418
-1.79302	-1.7904	-1.78776	-1.78509	-1.78244	-1.77981	-1.7772	-1.78564	-1.78302

-1.79187	-1.78928	-1.78666	-1.784	-1.78135	-1.77869	-1.77604	-1.78446	-1.78185
-1.79074	-1.78813	-1.78551	-1.78288	-1.79136	-1.78866	-1.78596	-1.78331	-1.78068
-1.78963	-1.78697	-1.78434	-1.78175	-1.79023	-1.78755	-1.78486	-1.78217	-1.7795
-1.78854	-1.78585	-1.78321	-1.78061	-1.78908	-1.78641	-1.78373	-1.78105	-1.77836
-1.78745	-1.78477	-1.78211	-1.77946	-1.7879	-1.78525	-1.7826	-1.77993	-1.77724
-1.78635	-1.78368	-1.78102	-1.77834	-1.78674	-1.78409	-1.78146	-1.77879	-1.77613
-1.7852	-1.78258	-1.77993	-1.77726	-1.78562	-1.78294	-1.78028	-1.77763	-1.77499
-1.78406	-1.78148	-1.77885	-1.77618	-1.78452	-1.78181	-1.77912	-1.77648	-1.77386
-1.78296	-1.78039	-1.77777	-1.7751	-1.78342	-1.7807	-1.778	-1.77537	-1.77276
-1.78188	-1.77929	-1.77667	-1.77402	-1.78233	-1.77961	-1.7769	-1.77426	-1.77164
-1.78077	-1.77815	-1.78657	-1.7839	-1.78122	-1.77851	-1.77579	-1.77313	-1.77048
-1.77965	-1.77699	-1.78538	-1.78276	-1.7801	-1.77739	-1.77468	-1.77198	-1.76928
-1.77854	-1.77586	-1.78422	-1.78161	-1.77898	-1.77628	-1.77356	-1.77084	-1.76812
-1.77745	-1.77476	-1.7831	-1.78048	-1.77784	-1.77515	-1.77244	-1.76972	-1.76698
-1.77637	-1.77366	-1.78197	-1.77933	-1.77669	-1.77401	-1.77131	-1.76859	-1.76585
-1.77529	-1.77257	-1.78084	-1.77816	-1.77549	-1.77283	-1.77017	-1.76746	-1.76472
-1.77421	-1.77149	-1.77972	-1.77699	-1.77429	-1.77166	-1.76902	-1.76633	-1.7636
-1.77311	-1.7704	-1.77861	-1.77586	-1.77315	-1.77052	-1.76789	-1.7652	-1.76247
-1.772	-1.7693	-1.77751	-1.77475	-1.77204	-1.7694	-1.76676	-1.76407	-1.76135
-1.78187	-1.77914	-1.7764	-1.77364	-1.77092	-1.76827	-1.76563	-1.76294	-1.76022
-1.78076	-1.77802	-1.77528	-1.77253	-1.76981	-1.76715	-1.7645	-1.76181	-1.75909
-1.77964	-1.7769	-1.77416	-1.77141	-1.76868	-1.76601	-1.76334	-1.76066	-1.75796
-1.77853	-1.77579	-1.77304	-1.77028	-1.76754	-1.76483	-1.76214	-1.75947	-1.75681
-1.77743	-1.77468	-1.77193	-1.76916	-1.76641	-1.76367	-1.76096	-1.7583	-1.75566
-1.77633	-1.77358	-1.77082	-1.76805	-1.76529	-1.76254	-1.75982	-1.75717	-1.75454
-1.77525	-1.77248	-1.76971	-1.76694	-1.76418	-1.76144	-1.75872	-1.75606	-1.75343
-1.77415	-1.77138	-1.7686	-1.76583	-1.76309	-1.76037	-1.75768	-1.755	-1.75234
-1.77306	-1.77028	-1.7675	-1.76473	-1.762	-1.75933	-1.75666	-1.75397	-1.75127
-1.77196	-1.76917	-1.7664	-1.76363	-1.76091	-1.75826	-1.75561	-1.75291	-1.75019
-1.77086	-1.76807	-1.7653	-1.76253	-1.75981	-1.75716	-1.75452	-1.75183	-1.74911
-1.75884	-1.75607	-1.7642	-1.76144	-1.75872	-1.75607	-1.75344	-1.75074	-1.74801
-1.75775	-1.75499	-1.76311	-1.76035	-1.75763	-1.75499	-1.75234	-1.74964	-1.74692
-1.75666	-1.75391	-1.76203	-1.75929	-1.75658	-1.75391	-1.75125	-1.74855	-1.74582
-1.75558	-1.75284	-1.76097	-1.75826	-1.75556	-1.75287	-1.75018	-1.74746	-1.74473
-1.7545	-1.75178	-1.75993	-1.75725	-1.75456	-1.75184	-1.74912	-1.74638	-1.74363
-1.75345	-1.75076	-1.74808	-1.74542	-1.75352	-1.75079	-1.74804	-1.74529	-1.74254
-1.75242	-1.74977	-1.74709	-1.7444	-1.75247	-1.74972	-1.74696	-1.7442	-1.74144
-1.75141	-1.74876	-1.74607	-1.74336	-1.7514	-1.74864	-1.74587	-1.74311	-1.74035
-1.75043	-1.74774	-1.74503	-1.7423	-1.75033	-1.74756	-1.74479	-1.74202	-1.73928
-1.74943	-1.7467	-1.74397	-1.74124	-1.7385	-1.73574	-1.73299	-1.74095	-1.73822
-1.74839	-1.74565	-1.74291	-1.74018	-1.73744	-1.73468	-1.73193	-1.7399	-1.7372
-1.74733	-1.74459	-1.74186	-1.73913	-1.73639	-1.73363	-1.73088	-1.73887	-1.73618
-1.74627	-1.74353	-1.74081	-1.73811	-1.73539	-1.73263	-1.72988	-1.73784	-1.73514
-1.74522	-1.74248	-1.73977	-1.7371	-1.73441	-1.73166	-1.7289	-1.73682	-1.73408
-1.74422	-1.74148	-1.73876	-1.73608	-1.73338	-1.73063	-1.72787	-1.72513	-1.72238
-1.74327	-1.74051	-1.73778	-1.73506	-1.73233	-1.72957	-1.72681	-1.72407	-1.72133
-1.74228	-1.73952	-1.73677	-1.73402	-1.73127	-1.72851	-1.72575	-1.72302	-1.72029
-1.74125	-1.73849	-1.73572	-1.73296	-1.7302	-1.72743	-1.72468	-1.72198	-1.71929
-1.74023	-1.73747	-1.7347	-1.73193	-1.72916	-1.72639	-1.72364	-1.72095	-1.71828
-1.73925	-1.7365	-1.73373	-1.73096	-1.72819	-1.72541	-1.72266	-1.71994	-1.71725

-1.73828	-1.73553	-1.73277	-1.73	-1.72722	-1.72445	-1.72168	-1.71893	-1.7162
-1.73727	-1.73451	-1.73175	-1.72897	-1.7262	-1.72342	-1.72065	-1.71789	-1.71514
-1.73624	-1.73347	-1.7307	-1.72793	-1.72516	-1.72238	-1.71961	-1.71684	-1.71408
-1.73526	-1.73248	-1.72971	-1.72693	-1.72416	-1.72139	-1.71861	-1.71584	-1.71308
-1.73432	-1.73154	-1.72876	-1.72598	-1.7232	-1.72043	-1.71765	-1.71488	-1.71212
-1.73335	-1.73056	-1.72778	-1.725	-1.72222	-1.71944	-1.71667	-1.71389	-1.71113
-1.73232	-1.72953	-1.72675	-1.72397	-1.7212	-1.71842	-1.71564	-1.71286	-1.71008
-1.7313	-1.72852	-1.72574	-1.72296	-1.72018	-1.7174	-1.71462	-1.71184	-1.70906
-1.73034	-1.72756	-1.72478	-1.722	-1.71922	-1.71644	-1.71366	-1.71088	-1.7081
-1.72939	-1.7266	-1.72382	-1.72105	-1.71827	-1.71549	-1.71271	-1.70993	-1.70716
-1.72839	-1.7256	-1.72282	-1.72004	-1.71727	-1.71448	-1.7117	-1.70893	-1.70616
-1.72736	-1.72458	-1.72179	-1.71902	-1.71624	-1.71345	-1.71067	-1.70789	-1.70513
-1.72639	-1.7236	-1.72082	-1.71804	-1.71526	-1.71247	-1.70969	-1.70691	-1.70415
-1.72547	-1.72268	-1.7199	-1.71712	-1.71434	-1.71155	-1.70876	-1.70599	-1.70322
-1.72455	-1.72176	-1.71898	-1.7162	-1.71342	-1.71063	-1.70784	-1.70506	-1.7023
-1.72363	-1.72085	-1.71806	-1.71528	-1.7125	-1.70971	-1.70693	-1.70415	-1.70138
-1.72269	-1.71991	-1.71712	-1.71434	-1.71156	-1.70878	-1.70599	-1.70322	-1.70044
-1.72168	-1.7189	-1.71611	-1.71333	-1.71055	-1.70777	-1.70499	-1.70221	-1.69944
-1.72065	-1.71786	-1.71508	-1.7123	-1.70952	-1.70674	-1.70396	-1.70118	-1.69841
-1.71968	-1.71689	-1.7141	-1.71132	-1.70855	-1.70577	-1.70299	-1.70021	-1.69744
-1.71874	-1.71595	-1.71317	-1.71039	-1.70762	-1.70484	-1.70206	-1.69929	-1.69651
-1.71782	-1.71503	-1.71225	-1.70947	-1.7067	-1.70392	-1.70115	-1.69837	-1.69559
-1.71691	-1.71413	-1.71135	-1.70857	-1.70579	-1.70302	-1.70024	-1.69747	-1.69469
-1.71601	-1.71322	-1.71044	-1.70767	-1.70489	-1.70212	-1.69934	-1.69656	-1.69378
-1.71511	-1.71232	-1.70954	-1.70677	-1.70399	-1.70122	-1.69844	-1.69566	-1.69288
-1.7142	-1.71142	-1.70864	-1.70586	-1.70309	-1.70032	-1.69754	-1.69476	-1.69197
-1.7133	-1.71051	-1.70773	-1.70496	-1.70219	-1.69941	-1.69663	-1.69384	-1.69105
-1.71239	-1.7096	-1.70682	-1.70405	-1.70128	-1.6985	-1.69572	-1.69292	-1.69011
-1.71148	-1.7087	-1.70592	-1.70314	-1.70036	-1.69758	-1.69479	-1.69197	-1.68913
-1.71057	-1.70779	-1.70501	-1.70223	-1.69944	-1.69666	-1.69385	-1.69099	-1.68811
-1.70966	-1.70688	-1.7041	-1.70132	-1.69852	-1.69572	-1.69289	-1.69002	-1.68712
-1.70876	-1.70598	-1.7032	-1.70041	-1.6976	-1.69476	-1.69189	-1.68903	-1.68616
-1.70786	-1.70508	-1.7023	-1.69951	-1.69669	-1.6938	-1.69091	-1.68805	-1.68521
-1.70696	-1.70418	-1.7014	-1.69861	-1.69579	-1.69289	-1.68998	-1.68713	-1.6843
-1.70605	-1.70328	-1.7005	-1.69772	-1.69489	-1.69199	-1.68908	-1.68622	-1.6834
-1.70515	-1.70238	-1.6996	-1.69682	-1.69399	-1.69109	-1.68818	-1.68532	-1.68249
-1.70424	-1.70148	-1.6987	-1.69591	-1.69309	-1.69019	-1.68729	-1.68443	-1.68159
-1.70334	-1.70057	-1.69779	-1.69501	-1.69219	-1.68932	-1.68644	-1.68357	-1.68072
-1.70243	-1.69967	-1.69689	-1.69411	-1.69131	-1.68847	-1.68562	-1.68274	-1.67986
-1.70153	-1.69876	-1.69598	-1.69321	-1.69041	-1.68761	-1.68478	-1.68191	-1.67902
-1.70062	-1.69785	-1.69507	-1.69229	-1.68951	-1.68671	-1.68391	-1.68107	-1.67822
-1.69971	-1.69694	-1.69416	-1.69138	-1.68859	-1.68581	-1.68302	-1.68022	-1.6774
-1.69881	-1.69603	-1.69325	-1.69047	-1.68768	-1.6849	-1.68212	-1.67933	-1.67654
-1.69791	-1.69513	-1.69234	-1.68956	-1.68677	-1.68399	-1.6812	-1.67843	-1.67565
-1.69701	-1.69422	-1.69143	-1.68865	-1.68586	-1.68308	-1.6803	-1.67752	-1.67475
-1.69612	-1.69331	-1.69052	-1.68774	-1.68496	-1.68218	-1.6794	-1.67662	-1.67383
-1.69527	-1.69244	-1.68963	-1.68685	-1.68406	-1.68128	-1.67849	-1.67571	-1.67293
-1.69447	-1.6916	-1.68876	-1.68596	-1.68317	-1.68038	-1.67759	-1.6748	-1.67202
-1.69367	-1.69078	-1.68793	-1.68512	-1.68231	-1.67951	-1.6767	-1.67391	-1.67113
-1.69284	-1.68999	-1.68715	-1.68434	-1.68152	-1.67868	-1.67584	-1.67304	-1.67025

							-0.2526	-0.25186
							-0.25203	-0.25128
				-0.25366	-0.25293	-0.25219	-0.25145	-0.2507
				-0.25309	-0.25235	-0.25161	-0.25086	-0.25012
		-0.25398	-0.25324	-0.25251	-0.25176	-0.25102	-0.52814	-0.52658
		-0.2534	-0.25266	-0.25192	-0.25116	-0.25042	-0.52691	-0.52541
-0.1081	-0.10779	-0.25282	-0.25207	-0.53036	-0.52876	-0.52719	-0.56405	-0.56233
-0.10784	-0.10754	-0.25222	-0.25147	-0.52908	-0.52753	-0.52601	-0.5627	-0.561
-0.10757	-0.10728	-0.25163	-0.25087	-0.52782	-0.52632	-0.52484	-0.56137	-0.55968
-0.25253	-0.25178	-0.3891	-0.38797	-0.56523	-0.56348	-0.56177	-0.59773	-0.59589
-0.25194	-0.25118	-0.38822	-0.38713	-0.56385	-0.56215	-0.56047	-0.5963	-0.59442
-0.38959	-0.38846	-0.56604	-0.56427	-0.60038	-0.59855	-0.59672	-0.59487	-0.59302
-0.38871	-0.38762	-0.56468	-0.56297	-0.59901	-0.59713	-0.59526	-0.59344	-0.59167
-0.56689	-0.56509	-0.60123	-0.59943	-0.59761	-0.59571	-0.59384	-0.63798	-0.63617
-0.56551	-0.56376	-0.59982	-0.59801	-0.59619	-0.59431	-0.59247	-0.63657	-0.63479
-0.56415	-0.56245	-0.59842	-0.59658	-0.64081	-0.63887	-0.63697	-0.71557	-0.71366
-0.56283	-0.56115	-0.59699	-0.59513	-0.63927	-0.6374	-0.63558	-0.71405	-0.71215
-0.56153	-0.55985	-0.59556	-0.59369	-0.63776	-0.63596	-0.6342	-0.71254	-0.71064
-0.59781	-0.59596	-0.64011	-0.63819	-0.6768	-0.67498	-0.67319	-0.71102	-0.70908
-0.59641	-0.59451	-0.63859	-0.63675	-0.67536	-0.67358	-0.6718	-0.7095	-0.70751
-0.64103	-0.63904	-0.63715	-0.63536	-0.71373	-0.71186	-0.70998	-0.708	-0.70599
-0.63958	-0.63764	-0.63578	-0.63401	-0.71224	-0.71037	-0.70848	-0.70652	-0.70453
-0.63815	-0.63627	-0.71459	-0.71267	-0.71077	-0.70887	-0.70697	-0.92635	-0.92338
-0.63677	-0.63492	-0.71311	-0.71121	-0.70931	-0.70737	-0.70543	-0.92412	-0.92129
-0.63541	-0.63359	-0.71164	-0.70976	-0.70786	-0.70588	-0.70392	-0.92193	-0.91923
-0.71411	-0.71212	-0.71018	-0.7083	-0.92866	-0.92562	-0.92264	-0.91985	-0.91717
-0.71263	-0.71065	-0.70872	-0.70684	-0.92647	-0.92352	-0.92061	-0.91783	-0.91513
-0.71112	-0.70918	-0.93011	-0.92722	-0.92435	-0.92143	-0.91856	-0.98482	-0.98197
-0.70961	-0.70771	-0.92791	-0.92509	-0.92227	-0.91936	-0.9165	-0.98266	-0.97985
-0.89127	-0.88858	-0.92575	-0.92294	-0.9894	-0.98636	-0.98337	-1.01671	-1.01389
-0.88925	-0.88661	-0.92364	-0.92076	-0.98703	-0.98407	-0.9812	-1.01458	-1.01183
-0.92721	-0.92442	-0.9616	-0.95857	-1.021	-1.01807	-1.01522	-1.07998	-1.07715
-0.9251	-0.92233	-0.95944	-0.95643	-1.01878	-1.01591	-1.01312	-1.07778	-1.07495
-0.92302	-0.92025	-0.95732	-0.95435	-1.01661	-1.01379	-1.01104	-1.07559	-1.07275
-0.96094	-0.95808	-1.02051	-1.01745	-1.08208	-1.07915	-1.07628	-1.07339	-1.0705

-0.95879	-0.95595	-1.0183	-1.01531	-1.07988	-1.07699	-1.07413	-1.07119	-1.06825
-1.02203	-1.01907	-1.08375	-1.08069	-1.0777	-1.07483	-1.07198	-1.13295	-1.12988
-1.01979	-1.01689	-1.0815	-1.0785	-1.07555	-1.07269	-1.06984	-1.13077	-1.12773
-1.08526	-1.08228	-1.07931	-1.07634	-1.10199	-1.09909	-1.09622	-1.15607	-1.15304
-1.08299	-1.0801	-1.07716	-1.0742	-1.09983	-1.09695	-1.0941	-1.1539	-1.15089
-1.08077	-1.07794	-1.10365	-1.10064	-1.1607	-1.15771	-1.15475	-1.26982	-1.26663
-1.07863	-1.07581	-1.1015	-1.0985	-1.15849	-1.15553	-1.1526	-1.26751	-1.26435
-1.07652	-1.0737	-1.09937	-1.09637	-1.1563	-1.15338	-1.15048	-1.26523	-1.26208
-1.10303	-1.10016	-1.1603	-1.15718	-1.18587	-1.1829	-1.17997	-1.29467	-1.29139
-1.10093	-1.09803	-1.1581	-1.15503	-1.18368	-1.18073	-1.17781	-1.29232	-1.28904
-1.16203	-1.15899	-1.18767	-1.18456	-1.29957	-1.29638	-1.29322	-1.28998	-1.28673
-1.15993	-1.15687	-1.18549	-1.18239	-1.29721	-1.29402	-1.29086	-1.28765	-1.28444
-1.18953	-1.18641	-1.3015	-1.29818	-1.2949	-1.2917	-1.28853	-1.28534	-1.28216
-1.1873	-1.18419	-1.29913	-1.29588	-1.29266	-1.28945	-1.28625	-1.28306	-1.27988
-1.18507	-1.18198	-1.29676	-1.29358	-1.2904	-1.2872	-1.284	-1.28079	-1.27761
-1.26902	-1.2658	-1.29438	-1.29124	-1.28809	-1.28492	-1.28173	-1.30655	-1.30331
-1.26669	-1.26349	-1.292	-1.28888	-1.28577	-1.28263	-1.27947	-1.30427	-1.30105
-1.29616	-1.29283	-1.28963	-1.28656	-1.31157	-1.30838	-1.30518	-1.34936	-1.3459
-1.29379	-1.29046	-1.28728	-1.28426	-1.30925	-1.30605	-1.30286	-1.34697	-1.34362
-1.31966	-1.31631	-1.3131	-1.31003	-1.35491	-1.3514	-1.34792	-1.34458	-1.3413
-1.31729	-1.31402	-1.31083	-1.30773	-1.35243	-1.34894	-1.34549	-1.34217	-1.33893
-1.31497	-1.31178	-1.35687	-1.35342	-1.34998	-1.34651	-1.34309	-1.3398	-1.33658
-1.31271	-1.30957	-1.35454	-1.35106	-1.34759	-1.34413	-1.34073	-1.33744	-1.33424
-1.3105	-1.30737	-1.35222	-1.34871	-1.34523	-1.34179	-1.33842	-1.33515	-1.33197
-1.35676	-1.35331	-1.34983	-1.34631	-1.34285	-1.33949	-1.3362	-1.33295	-1.3298
-1.35443	-1.35092	-1.34742	-1.3439	-1.34049	-1.33721	-1.33403	-1.33083	-1.32775
-1.35205	-1.34853	-1.34503	-1.34154	-1.33817	-1.33493	-1.33182	-1.34575	-1.3428
-1.34964	-1.34613	-1.34266	-1.33921	-1.33588	-1.33265	-1.3296	-1.34372	-1.3411
-1.34723	-1.34375	-1.34032	-1.33693	-1.35072	-1.3475	-1.3445	-1.34187	-1.33951
-1.34483	-1.34139	-1.33801	-1.33466	-1.34849	-1.34546	-1.34266	-1.34021	-1.33802
-1.34246	-1.33908	-1.35284	-1.34949	-1.34638	-1.34355	-1.34098	-1.33869	-1.33664
-1.34015	-1.33683	-1.3506	-1.34735	-1.34437	-1.34177	-1.33942	-1.3373	-1.33538
-1.33788	-1.33463	-1.34845	-1.34533	-1.34252	-1.34013	-1.33799	-1.33601	-1.33419
-1.35273	-1.34944	-1.34637	-1.3435	-1.34091	-1.33867	-1.33664	-1.36015	-1.35833
-1.35054	-1.3473	-1.3444	-1.34184	-1.33949	-1.33736	-1.33539	-1.3589	-1.3571
-1.34842	-1.34537	-1.34267	-1.34031	-1.36361	-1.36152	-1.35957	-1.38367	-1.38182
-1.34639	-1.34365	-1.34116	-1.33892	-1.36228	-1.36029	-1.3584	-1.3825	-1.38066
-1.34453	-1.34206	-1.39152	-1.38924	-1.40484	-1.40284	-1.40093	-1.39905	-1.39723
-1.34287	-1.3406	-1.39013	-1.38796	-1.40362	-1.40164	-1.39974	-1.39787	-1.39605
-1.34135	-1.33923	-1.38879	-1.38671	-1.40241	-1.40045	-1.39855	-1.3967	-1.39489
-1.39172	-1.38956	-1.40517	-1.40314	-1.40117	-1.39924	-1.39736	-1.42592	-1.42398
-1.39034	-1.38822	-1.40386	-1.40188	-1.39994	-1.39804	-1.39619	-1.42465	-1.42274
-1.40667	-1.40458	-1.40257	-1.40067	-1.42945	-1.42739	-1.42537	-1.4234	-1.42148
-1.40533	-1.40327	-1.40132	-1.3995	-1.42822	-1.42619	-1.42417	-1.42217	-1.42019
-1.40407	-1.40204	-1.40012	-1.39833	-1.45386	-1.45178	-1.4497	-1.49448	-1.49234
-1.40289	-1.40092	-1.39901	-1.39717	-1.45248	-1.45041	-1.44835	-1.49311	-1.491
-1.40171	-1.39978	-1.50252	-1.50031	-1.49813	-1.49597	-1.49385	-1.51692	-1.51472
-1.40047	-1.39857	-1.50111	-1.4989	-1.49672	-1.49457	-1.49246	-1.51547	-1.51333
-1.39924	-1.39737	-1.4997	-1.49751	-1.49535	-1.4932	-1.49111	-1.51404	-1.51195
-1.50268	-1.50051	-1.49834	-1.49617	-1.51929	-1.51703	-1.51482	-1.51266	-1.51056

-1.50128	-1.49914	-1.497	-1.49485	-1.5179	-1.51567	-1.51348	-1.51131	-1.50919
-1.49989	-1.49777	-1.52099	-1.51872	-1.51647	-1.51426	-1.51209	-1.53354	-1.53129
-1.49853	-1.49639	-1.51951	-1.51726	-1.51503	-1.51281	-1.51066	-1.53208	-1.52996
-1.52257	-1.52031	-1.51807	-1.51584	-1.53756	-1.53515	-1.53281	-1.53066	-1.52861
-1.52108	-1.51888	-1.51668	-1.51448	-1.5361	-1.53373	-1.53144	-1.5293	-1.52725
-1.51963	-1.51746	-1.53936	-1.53697	-1.53463	-1.53233	-1.5301	-1.52796	-1.52588
-1.51824	-1.51604	-1.53779	-1.53541	-1.53309	-1.53089	-1.52874	-1.52662	-1.52453
-1.51687	-1.51465	-1.53625	-1.53387	-1.53159	-1.52947	-1.5274	-1.52529	-1.52319
-1.51552	-1.5133	-1.5348	-1.53249	-1.53026	-1.52815	-1.52609	-1.52398	-1.5219
-1.51417	-1.51199	-1.53341	-1.53118	-1.52901	-1.52688	-1.52479	-1.52269	-1.52063
-1.53661	-1.53426	-1.532	-1.52983	-1.52769	-1.52556	-1.52346	-1.52139	-1.51935
-1.53507	-1.53277	-1.53056	-1.52844	-1.52633	-1.5242	-1.52211	-1.52006	-1.51807
-1.53359	-1.53136	-1.52919	-1.52708	-1.52499	-1.52288	-1.52081	-1.5188	-1.51685
-1.53218	-1.53003	-1.5279	-1.52578	-1.52369	-1.52162	-1.51959	-1.51762	-1.5157
-1.53081	-1.52872	-1.52661	-1.52448	-1.52239	-1.52036	-1.51838	-1.5378	-1.53587
-1.52945	-1.52736	-1.52525	-1.52313	-1.52106	-1.51907	-1.51713	-1.53656	-1.53467
-1.52811	-1.526	-1.52389	-1.52179	-1.51975	-1.51779	-1.51589	-1.53532	-1.53347
-1.5268	-1.52468	-1.54404	-1.54196	-1.53992	-1.53793	-1.53599	-1.53411	-1.53225
-1.52552	-1.52339	-1.54275	-1.54074	-1.53876	-1.53679	-1.53484	-1.53292	-1.53102
-1.5457	-1.54355	-1.54149	-1.53952	-1.53757	-1.53562	-1.53368	-1.5688	-1.56668
-1.54439	-1.5423	-1.54027	-1.53832	-1.53638	-1.53445	-1.53253	-1.56755	-1.56546
-1.54306	-1.54103	-1.53904	-1.53709	-1.5726	-1.57047	-1.56836	-1.61208	-1.60982
-1.54169	-1.53971	-1.53776	-1.53582	-1.57121	-1.56909	-1.567	-1.61061	-1.60838
-1.54034	-1.53841	-1.53648	-1.53456	-1.56983	-1.56774	-1.56567	-1.60916	-1.60695
-1.5391	-1.53717	-1.57278	-1.57064	-1.61453	-1.61226	-1.61001	-1.6934	-1.69109
-1.53791	-1.53597	-1.57147	-1.56936	-1.61315	-1.6109	-1.60867	-1.69199	-1.68969
-1.57309	-1.57109	-1.69138	-1.68895	-1.69756	-1.69521	-1.69289	-1.69057	-1.68828
-1.57187	-1.56987	-1.68987	-1.68748	-1.69612	-1.69378	-1.69146	-1.68915	-1.68687
-1.69333	-1.69083	-1.69939	-1.69702	-1.69467	-1.69232	-1.68999	-1.69911	-1.6967
-1.6918	-1.68936	-1.69795	-1.6956	-1.69324	-1.69084	-1.68848	-1.69751	-1.69515
-1.70123	-1.69885	-1.69648	-1.69413	-1.69177	-1.68937	-1.687	-1.69595	-1.69364
-1.69964	-1.69728	-1.69493	-1.69258	-1.69024	-1.6879	-1.6856	-1.69449	-1.6922
-1.69807	-1.69572	-1.69337	-1.69103	-1.68872	-1.68646	-1.68423	-1.69305	-1.69078
-1.69658	-1.69423	-1.6919	-1.68958	-1.69864	-1.69627	-1.69393	-1.69163	-1.68936
-1.69515	-1.69279	-1.69047	-1.68819	-1.69717	-1.69483	-1.69252	-1.69022	-1.68795
-1.69372	-1.69136	-1.70047	-1.69808	-1.69572	-1.69341	-1.69112	-1.68883	-1.68655
-1.6923	-1.68995	-1.69898	-1.69662	-1.6943	-1.69201	-1.68973	-1.68744	-1.68517
-1.69088	-1.68856	-1.69752	-1.69519	-1.69288	-1.69059	-1.68832	-1.68605	-1.68379
-1.68947	-1.68721	-1.6961	-1.69377	-1.69146	-1.68916	-1.68689	-1.68464	-1.68242
-1.69938	-1.69703	-1.69469	-1.69236	-1.69004	-1.68773	-1.68545	-1.68323	-1.68104
-1.6979	-1.69559	-1.69327	-1.69094	-1.68862	-1.68629	-1.68399	-1.68177	-1.67961
-1.69644	-1.69415	-1.69184	-1.68952	-1.6872	-1.68485	-1.68254	-1.68033	-1.67817
-1.69499	-1.6927	-1.69041	-1.68811	-1.6858	-1.68346	-1.68115	-1.67894	-1.67679
-1.69357	-1.69126	-1.68897	-1.6867	-1.68441	-1.68209	-1.6798	-1.6776	-1.67545
-1.69215	-1.68983	-1.68754	-1.68527	-1.68299	-1.6807	-1.67844	-1.72153	-1.7191
-1.69073	-1.68841	-1.68611	-1.68381	-1.68154	-1.67929	-1.67707	-1.72001	-1.7176
-1.68931	-1.68701	-1.6847	-1.68239	-1.74283	-1.74027	-1.73778	-1.73533	-1.73293
-1.68791	-1.68563	-1.68332	-1.681	-1.74124	-1.7387	-1.73623	-1.73382	-1.73146
-1.68652	-1.68425	-1.68196	-1.67963	-1.73967	-1.73715	-1.7347	-1.73232	-1.73
-1.70254	-1.70028	-1.7434	-1.74072	-1.73812	-1.73563	-1.73322	-1.74463	-1.7423

-1.70113	-1.69889	-1.74185	-1.73918	-1.73659	-1.73414	-1.73175	-1.74318	-1.74086
-1.69972	-1.6975	-1.7403	-1.73764	-1.74891	-1.74647	-1.74409	-1.74174	-1.73941
-1.69832	-1.69611	-1.73875	-1.73612	-1.74741	-1.745	-1.74265	-1.7403	-1.73797
-1.75617	-1.75364	-1.75107	-1.74846	-1.74593	-1.74353	-1.74119	-1.73886	-1.73655
-1.75458	-1.75205	-1.74951	-1.74696	-1.74446	-1.74207	-1.73973	-1.73742	-1.73515
-1.75301	-1.75049	-1.74798	-1.74547	-1.74301	-1.74062	-1.73828	-1.736	-1.73376
-1.75144	-1.74897	-1.74649	-1.74401	-1.74157	-1.73918	-1.73685	-1.73459	-1.73238
-1.7499	-1.74746	-1.74502	-1.74256	-1.74014	-1.73776	-1.73543	-1.73319	-1.73101
-1.74839	-1.74599	-1.74356	-1.74111	-1.7387	-1.73635	-1.73404	-1.7554	-1.75303
-1.74689	-1.74452	-1.74212	-1.73968	-1.73728	-1.73495	-1.73267	-1.75391	-1.75154
-1.74541	-1.74306	-1.74069	-1.73828	-1.73591	-1.73359	-1.73131	-1.75241	-1.75003
-1.74395	-1.7416	-1.73926	-1.73692	-1.73459	-1.73226	-1.72995	-1.75091	-1.74852
-1.7425	-1.74014	-1.73782	-1.73554	-1.75688	-1.75436	-1.75186	-1.74941	-1.74701
-1.74105	-1.73867	-1.73635	-1.73412	-1.75537	-1.75286	-1.75036	-1.74792	-1.74552
-1.73962	-1.73723	-1.75869	-1.75628	-1.75385	-1.75135	-1.74886	-1.74643	-1.74403
-1.73824	-1.73584	-1.75719	-1.75477	-1.75233	-1.74984	-1.74736	-1.74493	-1.74255
-1.73687	-1.73448	-1.7557	-1.75327	-1.75083	-1.74834	-1.74586	-1.74345	-1.74107
-1.73547	-1.7331	-1.7542	-1.75176	-1.74933	-1.74687	-1.74443	-1.75889	-1.75645
-1.73406	-1.73172	-1.75271	-1.75026	-1.74784	-1.74543	-1.74303	-1.75746	-1.755
-1.75621	-1.75372	-1.75124	-1.74878	-1.76336	-1.76091	-1.75848	-1.75602	-1.75356
-1.75473	-1.75228	-1.74981	-1.74732	-1.76185	-1.75942	-1.75701	-1.75456	-1.75212
-1.77042	-1.76794	-1.76543	-1.76286	-1.76035	-1.75793	-1.75554	-1.80705	-1.80417
-1.7689	-1.76643	-1.76392	-1.76135	-1.75885	-1.75644	-1.75408	-1.80536	-1.80254
-1.76738	-1.76491	-1.76241	-1.75986	-1.75737	-1.75498	-1.75263	-1.80368	-1.80091
-1.76586	-1.76339	-1.76091	-1.75842	-1.81044	-1.80757	-1.80475	-1.80198	-1.79926
-1.76436	-1.76188	-1.75942	-1.757	-1.80877	-1.80588	-1.80303	-1.80029	-1.79761
-1.76288	-1.76039	-1.75794	-1.75555	-1.80707	-1.80421	-1.80139	-1.81188	-1.80903
-1.76143	-1.75892	-1.75647	-1.75409	-1.80537	-1.80257	-1.79979	-1.81026	-1.80737
-1.75998	-1.75748	-1.80931	-1.80647	-1.80367	-1.8009	-1.79816	-1.82308	-1.82015
-1.75853	-1.75609	-1.80764	-1.80477	-1.80195	-1.79921	-1.79649	-1.82142	-1.81851
-1.81175	-1.80885	-1.80597	-1.80309	-1.82843	-1.82552	-1.82266	-1.82476	-1.82189
-1.80996	-1.8071	-1.80426	-1.80145	-1.8268	-1.8239	-1.82102	-1.82316	-1.82032
-1.80818	-1.80535	-1.80255	-1.7998	-1.82519	-1.82228	-1.81938	-1.82155	-1.81875
-1.80648	-1.80364	-1.83423	-1.83135	-1.82848	-1.82559	-1.82272	-1.81991	-1.81713
-1.80481	-1.80196	-1.83253	-1.82966	-1.82679	-1.82391	-1.82106	-1.81826	-1.81549
-1.83686	-1.83384	-1.83089	-1.82801	-1.82515	-1.82229	-1.81945	-1.83286	-1.83001
-1.8352	-1.83223	-1.82929	-1.8264	-1.82354	-1.82071	-1.81789	-1.83124	-1.82837
-1.83356	-1.83061	-1.82769	-1.82481	-1.83594	-1.83311	-1.8303	-1.84341	-1.84053
-1.83194	-1.82897	-1.82606	-1.82321	-1.83436	-1.83154	-1.82873	-1.84182	-1.83897
-1.84432	-1.84135	-1.83844	-1.83561	-1.84886	-1.84597	-1.8431	-1.84025	-1.83742
-1.84271	-1.83977	-1.83687	-1.83403	-1.84724	-1.84437	-1.84152	-1.83868	-1.83586
-1.84109	-1.8382	-1.83532	-1.83246	-1.84563	-1.84278	-1.83996	-1.83713	-1.83432
-1.83949	-1.8366	-1.84983	-1.84692	-1.84404	-1.8412	-1.83839	-1.83558	-1.8328
-1.83788	-1.835	-1.84821	-1.84533	-1.84248	-1.83964	-1.83683	-1.83405	-1.8313
-1.83628	-1.83342	-1.84661	-1.84375	-1.84091	-1.83808	-1.83529	-1.83252	-1.82979
-1.83467	-1.83186	-1.84502	-1.84217	-1.83934	-1.83654	-1.83376	-1.831	-1.82827
-1.84915	-1.84629	-1.84344	-1.84059	-1.83777	-1.83499	-1.83224	-1.82949	-1.82677
-1.84753	-1.84468	-1.84184	-1.83901	-1.83621	-1.83344	-1.8307	-1.82797	-1.82528
-1.84593	-1.84308	-1.84025	-1.83744	-1.83466	-1.8319	-1.82917	-1.82646	-1.82379
-1.84431	-1.84149	-1.83868	-1.83588	-1.84581	-1.84306	-1.84034	-1.88041	-1.87767

-1.84271	-1.83991	-1.83711	-1.83432	-1.84425	-1.84153	-1.83883	-1.87887	-1.87613
-1.85388	-1.85106	-1.86057	-1.85779	-1.85503	-1.85232	-1.84964	-1.87737	-1.87469
-1.85232	-1.84947	-1.85898	-1.85623	-1.85351	-1.85081	-1.84815	-1.87591	-1.8733
-1.8631	-1.86024	-1.85744	-1.8547	-1.85199	-1.84931	-1.84668	-1.87447	-1.87191
-1.86159	-1.85875	-1.85594	-1.85318	-1.85047	-1.84782	-1.84524	-1.87303	-1.87051
-1.86007	-1.85726	-1.85447	-1.85169	-1.84898	-1.84637	-1.84382	-1.87161	-1.86911
-1.85852	-1.85576	-1.85301	-1.85024	-1.84754	-1.84494	-1.84241	-1.87019	-1.86772
-1.85697	-1.85427	-1.85155	-1.84882	-1.84614	-1.84355	-1.84102	-1.86878	-1.86632
-1.85546	-1.85276	-1.85008	-1.8474	-1.84477	-1.8422	-1.83967	-1.89575	-1.89322
-1.85398	-1.85126	-1.8486	-1.846	-1.84342	-1.84086	-1.83834	-1.89433	-1.89177
-1.85252	-1.84981	-1.84716	-1.84459	-1.87234	-1.86976	-1.8672	-1.89293	-1.89037
-1.85109	-1.84838	-1.84574	-1.84317	-1.8709	-1.86836	-1.86584	-1.89156	-1.88901
-1.84965	-1.84697	-1.84435	-1.84179	-1.8978	-1.89523	-1.89269	-1.89016	-1.88764
-1.8482	-1.84558	-1.84299	-1.84044	-1.89637	-1.89377	-1.89121	-1.88871	-1.88626
-1.84676	-1.8442	-1.84165	-1.8391	-1.89497	-1.89234	-1.88976	-1.88729	-1.88488
-1.84536	-1.84283	-1.8403	-1.83777	-1.89359	-1.89096	-1.88838	-1.88589	-1.88347
-1.84397	-1.84146	-1.83895	-1.83644	-1.89221	-1.88961	-1.88704	-1.88453	-1.88207
-1.84259	-1.84007	-1.86644	-1.86392	-1.8908	-1.88824	-1.8857	-1.8832	-1.88074
-1.84122	-1.83868	-1.865	-1.8625	-1.88938	-1.88686	-1.88436	-1.88189	-1.87945
-1.86873	-1.86613	-1.86359	-1.86109	-1.88795	-1.88547	-1.88302	-1.88058	-1.87816
-1.86736	-1.86475	-1.86219	-1.85967	-1.8865	-1.88407	-1.88167	-1.87927	-1.87689
-1.86599	-1.86338	-1.86081	-1.85829	-1.88509	-1.88268	-1.88031	-1.87795	-1.87561
-1.86463	-1.86203	-1.85947	-1.85694	-1.88371	-1.8813	-1.87894	-1.87661	-1.87432
-1.86327	-1.86069	-1.85814	-1.85562	-1.88237	-1.87995	-1.87759	-1.87529	-1.87302
-1.8619	-1.85936	-1.85684	-1.85433	-1.88106	-1.87864	-1.87628	-1.87399	-1.87174
-1.86054	-1.85803	-1.85554	-1.85307	-1.87979	-1.87737	-1.875	-1.87271	-1.87045
-1.85919	-1.8567	-1.85423	-1.85179	-1.8785	-1.87609	-1.87372	-1.87141	-1.86915
-1.85786	-1.85537	-1.85293	-1.85053	-1.87723	-1.87483	-1.87245	-1.87012	-1.86783
-1.85653	-1.85407	-1.85165	-1.84927	-1.87596	-1.87357	-1.8712	-1.86885	-1.86654
-1.85521	-1.85279	-1.85039	-1.84803	-1.87471	-1.87232	-1.86995	-1.8676	-1.86528
-1.85387	-1.85149	-1.84913	-1.84679	-1.87345	-1.87108	-1.86871	-1.86636	-1.86403
-1.85249	-1.85017	-1.84785	-1.84553	-1.87218	-1.86983	-1.86748	-1.86513	-1.86278
-1.85113	-1.84886	-1.84657	-1.84427	-1.87091	-1.86858	-1.86624	-1.86388	-1.86153
-1.84983	-1.84758	-1.84531	-1.84302	-1.86964	-1.86731	-1.86498	-1.86262	-1.86026
-1.84857	-1.84632	-1.84406	-1.84177	-1.86836	-1.86604	-1.8637	-1.86135	-1.85898
-1.84733	-1.84508	-1.84281	-1.84053	-1.86708	-1.86476	-1.86242	-1.86006	-1.8577
-1.84613	-1.84386	-1.84158	-1.83929	-1.86581	-1.86347	-1.86112	-1.85876	-1.8564
-1.84491	-1.84263	-1.84034	-1.83805	-1.86453	-1.86218	-1.85982	-1.89953	-1.89714
-1.84367	-1.84138	-1.8391	-1.83681	-1.86326	-1.86089	-1.85853	-1.89823	-1.89585
-1.84243	-1.84014	-1.83785	-1.83556	-1.90417	-1.90175	-1.89934	-1.89694	-1.89455
-1.8412	-1.8389	-1.8366	-1.83429	-1.90286	-1.90045	-1.89804	-1.89565	-1.89327
-1.83997	-1.83766	-1.83535	-1.83302	-1.90155	-1.89914	-1.89674	-1.89436	-1.89198
-1.85093	-1.84862	-1.9051	-1.90266	-1.90024	-1.89783	-1.89544	-1.89306	-1.8907
-1.84968	-1.84737	-1.90381	-1.90137	-1.89894	-1.89653	-1.89413	-1.89176	-1.8894
-1.87978	-1.87741	-1.9025	-1.90006	-1.89763	-1.89522	-1.89283	-1.89046	-1.88811
-1.8785	-1.87612	-1.90118	-1.89874	-1.89632	-1.89391	-1.89153	-1.88916	-1.88682
-1.87722	-1.87484	-1.89986	-1.89743	-1.89501	-1.89262	-1.89024	-1.88788	-1.88554
-1.87595	-1.87355	-1.89854	-1.89612	-1.89372	-1.89133	-1.88896	-1.88661	-1.88426
-1.87468	-1.87227	-1.89723	-1.89482	-1.89243	-1.89007	-1.88772	-1.88536	-1.88302
-1.87342	-1.871	-1.89593	-1.89353	-1.89115	-1.88883	-1.88651	-1.88418	-1.88184

-1.87217	-1.86977	-1.89466	-1.89225	-1.88989	-1.8876	-1.88532	-1.883	-1.88067
-1.87097	-1.86857	-1.89345	-1.89104	-1.88868	-1.88637	-1.88408	-1.88177	-1.87944
-1.8698	-1.86741	-1.89227	-1.88987	-1.88749	-1.88515	-1.88282	-1.88049	-1.87817
-1.86858	-1.8662	-1.89103	-1.88864	-1.88627	-1.8839	-1.88155	-1.87921	-1.87688
-1.86731	-1.86493	-1.88974	-1.88736	-1.88499	-1.88263	-1.88027	-1.87792	-1.87558
-1.86605	-1.86367	-1.88845	-1.88608	-1.88372	-1.88136	-1.879	-1.87664	-1.87429
-1.86479	-1.86241	-1.88717	-1.8848	-1.88243	-1.88008	-1.87773	-1.87537	-1.87302
-1.86356	-1.86119	-1.88592	-1.88354	-1.88117	-1.87882	-1.87647	-1.87412	-1.87177
-1.88951	-1.88711	-1.88473	-1.88235	-1.87998	-1.87763	-1.87527	-1.87292	-1.87057
-1.88833	-1.88594	-1.88355	-1.88118	-1.87881	-1.87645	-1.87409	-1.87174	-1.86939
-1.88708	-1.88469	-1.88232	-1.87995	-1.87758	-1.87521	-1.87285	-1.8705	-1.86815
-1.88579	-1.8834	-1.88103	-1.87867	-1.8763	-1.87394	-1.87158	-1.86922	-1.86687
-1.88451	-1.88213	-1.87975	-1.87739	-1.87502	-1.87266	-1.8703	-1.86794	-1.86559
-1.88323	-1.88085	-1.87848	-1.8761	-1.87373	-1.87137	-1.86901	-1.86666	-1.86431
-1.88199	-1.87961	-1.87723	-1.87485	-1.87247	-1.87011	-1.86775	-1.8654	-1.86305
-1.8808	-1.87842	-1.87604	-1.87366	-1.87128	-1.86892	-1.86656	-1.86421	-1.86185
-1.87961	-1.87723	-1.87485	-1.87247	-1.8701	-1.86774	-1.86538	-1.86302	-1.86065
-1.87836	-1.87597	-1.87359	-1.87121	-1.86884	-1.86648	-1.86411	-1.86176	-1.8594
-1.87708	-1.8747	-1.87231	-1.86993	-1.86755	-1.86518	-1.86282	-1.86046	-1.8581
-1.87586	-1.87347	-1.87108	-1.8687	-1.86631	-1.86392	-1.86153	-1.85916	-1.8568
-1.87468	-1.87229	-1.8699	-1.86751	-1.86511	-1.86268	-1.86025	-1.85786	-1.85549
-1.87346	-1.87107	-1.86868	-1.86629	-1.86388	-1.86144	-1.859	-1.85659	-1.85421
-1.87218	-1.86979	-1.8674	-1.86502	-1.86262	-1.8602	-1.85778	-1.85538	-1.85299
-1.87091	-1.86852	-1.86613	-1.86375	-1.86136	-1.85895	-1.85655	-1.85415	-1.85177
-1.86971	-1.8673	-1.86491	-1.86253	-1.86013	-1.8577	-1.85527	-1.85288	-1.85049
-1.86851	-1.8661	-1.8637	-1.86131	-1.8589	-1.85645	-1.854	-1.85159	-1.84922
-1.86726	-1.86485	-1.86245	-1.86005	-1.85764	-1.85519	-1.85275	-1.85035	-1.84798
-1.86599	-1.86358	-1.86117	-1.85877	-1.85637	-1.85395	-1.85153	-1.84914	-1.84675
-1.86475	-1.86233	-1.85992	-1.85752	-1.85512	-1.85272	-1.85032	-1.84791	-1.84551
-1.86355	-1.86111	-1.85869	-1.8563	-1.8539	-1.85151	-1.84911	-1.84669	-1.84425
-1.86234	-1.85989	-1.85746	-1.85506	-1.85266	-1.85027	-1.84788	-1.84544	-1.843
-1.86113	-1.85864	-1.85619	-1.85377	-1.85137	-1.84898	-1.84659	-1.84418	-1.84175
-1.85991	-1.8574	-1.85493	-1.8525	-1.85009	-1.8477	-1.84531	-1.84292	-1.84052
-1.85867	-1.85618	-1.85372	-1.85129	-1.84888	-1.84648	-1.84409	-1.84169	-1.8393
-1.85743	-1.85497	-1.85252	-1.8501	-1.84769	-1.84528	-1.84287	-1.84048	-1.83808
-1.85623	-1.85378	-1.85134	-1.84892	-1.8465	-1.84406	-1.84163	-1.83922	-1.83683
-1.85505	-1.85261	-1.85018	-1.84775	-1.84531	-1.84283	-1.84037	-1.83794	-1.83554
-1.85387	-1.85144	-1.84899	-1.84655	-1.85641	-1.85391	-1.85143	-1.84899	-1.84658
-1.85269	-1.85024	-1.84779	-1.84531	-1.85515	-1.85268	-1.85022	-1.84779	-1.84537
-1.85149	-1.84904	-1.85888	-1.85638	-1.8539	-1.85145	-1.84901	-1.84659	-1.84417
-1.85029	-1.8478	-1.85763	-1.85514	-1.85267	-1.85024	-1.84782	-1.84539	-1.84297
-1.84907	-1.84656	-1.85637	-1.85391	-1.85146	-1.84904	-1.84662	-1.84419	-1.84175
-1.86013	-1.85762	-1.85515	-1.8527	-1.85026	-1.84784	-1.84541	-1.84296	-1.84049
-1.85887	-1.8564	-1.85394	-1.85151	-1.84908	-1.84664	-1.8442	-1.84172	-1.83923
-1.85764	-1.85519	-1.85275	-1.85032	-1.84788	-1.84542	-1.84295	-1.84046	-1.83797
-1.85645	-1.85401	-1.85157	-1.84914	-1.84669	-1.84418	-1.84168	-1.83919	-1.83673
-1.85526	-1.85282	-1.85038	-1.84795	-1.84549	-1.84296	-1.84043	-1.83795	-1.8355
-1.85405	-1.85163	-1.84919	-1.84677	-1.8443	-1.84176	-1.83923	-1.83675	-1.8343
-1.85285	-1.85043	-1.84799	-1.84556	-1.84308	-1.84055	-1.83802	-1.83555	-1.8331
-1.85165	-1.84922	-1.84677	-1.84429	-1.8418	-1.83929	-1.83679	-1.83433	-1.8319

-1.85045	-1.84801	-1.84553	-1.84301	-1.84051	-1.83802	-1.83555	-1.83311	-1.83069
-1.84925	-1.84681	-1.84432	-1.84178	-1.83926	-1.83679	-1.83433	-1.8319	-1.82948
-1.84805	-1.84561	-1.84312	-1.84057	-1.83805	-1.83557	-1.83312	-1.83068	-1.82826
-1.84684	-1.8444	-1.84191	-1.83936	-1.83683	-1.83436	-1.8319	-1.82947	-1.82704
-1.84563	-1.84319	-1.8407	-1.83815	-1.83563	-1.83315	-1.8307	-1.82826	-1.82583
-1.84442	-1.84198	-1.83949	-1.83694	-1.83442	-1.83194	-1.82949	-1.82705	-1.82462
-1.8432	-1.84077	-1.83828	-1.83574	-1.83321	-1.83074	-1.82828	-1.82584	-1.8234
-1.84199	-1.83956	-1.83708	-1.83453	-1.832	-1.82953	-1.82707	-1.82463	-1.82219
-1.84079	-1.83835	-1.83586	-1.83331	-1.83079	-1.82831	-1.82585	-1.82341	-1.82096
-1.83958	-1.83714	-1.83465	-1.83209	-1.82957	-1.82709	-1.82463	-1.82218	-1.81972
-1.83837	-1.83593	-1.83343	-1.83088	-1.82834	-1.82587	-1.8234	-1.82095	-1.81849
-1.83716	-1.83472	-1.83222	-1.82966	-1.82712	-1.82464	-1.82217	-1.81972	-1.81726
-1.83595	-1.8335	-1.831	-1.82844	-1.8259	-1.82341	-1.82094	-1.81849	-1.81603
-1.83475	-1.83229	-1.82978	-1.82721	-1.82467	-1.82218	-1.81971	-1.81725	-1.81479
-1.83354	-1.83108	-1.82857	-1.82599	-1.82345	-1.82095	-1.81847	-1.81601	-1.81355
-1.83235	-1.82988	-1.82736	-1.82478	-1.82223	-1.81973	-1.81725	-1.81478	-1.81232
-1.83115	-1.82868	-1.82616	-1.82359	-1.82104	-1.81853	-1.81603	-1.81356	-1.8111
-1.82996	-1.82748	-1.82497	-1.82243	-1.81989	-1.81736	-1.81484	-1.81236	-1.80988
-1.82877	-1.82628	-1.82379	-1.82128	-1.81876	-1.81621	-1.81365	-1.81116	-1.80868
-1.82761	-1.8251	-1.82259	-1.82011	-1.8176	-1.81503	-1.81247	-1.80996	-1.80747
-1.82647	-1.82392	-1.8214	-1.81891	-1.8164	-1.81384	-1.81128	-1.80876	-1.80626
-1.82531	-1.82274	-1.8202	-1.81771	-1.81521	-1.81266	-1.81011	-1.80759	-1.80506
-1.82412	-1.82154	-1.81901	-1.81651	-1.81402	-1.81151	-1.80898	-1.80644	-1.80388
-1.82294	-1.82037	-1.81783	-1.81532	-1.81283	-1.81034	-1.80784	-1.80528	-1.8027
-1.82177	-1.81923	-1.8167	-1.81416	-1.81164	-1.80915	-1.80666	-1.8041	-1.80151
-1.82061	-1.8181	-1.81557	-1.813	-1.81045	-1.80796	-1.80546	-1.8029	-1.80032
-1.81943	-1.81692	-1.81439	-1.81181	-1.80925	-1.80675	-1.80425	-1.80172	-1.79916
-1.81823	-1.81572	-1.81318	-1.81061	-1.80806	-1.80554	-1.80303	-1.80052	-1.79801
-1.81707	-1.81454	-1.812	-1.80945	-1.8069	-1.80436	-1.80183	-1.79933	-1.79683
-1.81594	-1.81339	-1.81085	-1.80832	-1.80577	-1.80321	-1.80065	-1.79812	-1.79561
-1.8148	-1.81225	-1.80971	-1.80717	-1.80463	-1.80205	-1.79948	-1.79694	-1.7944
-1.81366	-1.81112	-1.80857	-1.80601	-1.80345	-1.8009	-1.79835	-1.79578	-1.7932
-1.81251	-1.80998	-1.80743	-1.80484	-1.80227	-1.79973	-1.7972	-1.79461	-1.792
-1.81136	-1.80881	-1.80623	-1.80363	-1.80105	-1.79852	-1.79598	-1.79339	-1.79078
-1.81021	-1.80762	-1.80503	-1.80242	-1.79983	-1.79729	-1.79474	-1.79215	-1.78955
-1.80903	-1.80643	-1.80383	-1.80124	-1.79865	-1.79609	-1.79353	-1.79095	-1.78836
-1.80782	-1.80523	-1.80265	-1.80008	-1.7975	-1.79492	-1.79233	-1.78976	-1.78719
-1.80662	-1.80405	-1.80148	-1.79891	-1.79633	-1.79375	-1.79116	-1.78859	-1.78602
-1.80545	-1.80289	-1.80032	-1.79774	-1.79516	-1.79258	-1.79	-1.78742	-1.78484
-1.80429	-1.80174	-1.79917	-1.79657	-1.79399	-1.79142	-1.78884	-1.79746	-1.79483
-1.80316	-1.80057	-1.79799	-1.79541	-1.79283	-1.79025	-1.78766	-1.79627	-1.79365
-1.80203	-1.79941	-1.79681	-1.79424	-1.79167	-1.78907	-1.78648	-1.79507	-1.79247
-1.80087	-1.79826	-1.79566	-1.79307	-1.79049	-1.7879	-1.78531	-1.79388	-1.79127
-1.7997	-1.79711	-1.79451	-1.7919	-1.7893	-1.78673	-1.78415	-1.7927	-1.79007
-1.79856	-1.79596	-1.79335	-1.79074	-1.78814	-1.78556	-1.78298	-1.79151	-1.78888
-1.79745	-1.7948	-1.79218	-1.78959	-1.787	-1.7844	-1.7818	-1.79032	-1.78771
-1.79635	-1.79368	-1.79104	-1.78845	-1.78585	-1.78324	-1.78064	-1.78914	-1.78653
-1.79527	-1.7926	-1.78994	-1.78731	-1.78469	-1.78209	-1.77951	-1.78798	-1.78535
-1.79416	-1.79151	-1.78885	-1.78618	-1.78354	-1.78095	-1.77837	-1.78682	-1.78418
-1.79302	-1.7904	-1.78776	-1.78509	-1.78244	-1.77981	-1.7772	-1.78564	-1.78302

-1.79187	-1.78928	-1.78666	-1.784	-1.78135	-1.77869	-1.77604	-1.78446	-1.78185
-1.79074	-1.78813	-1.78551	-1.78288	-1.79136	-1.78866	-1.78596	-1.78331	-1.78068
-1.78963	-1.78697	-1.78434	-1.78175	-1.79023	-1.78755	-1.78486	-1.78217	-1.7795
-1.78854	-1.78585	-1.78321	-1.78061	-1.78908	-1.78641	-1.78373	-1.78105	-1.77836
-1.78745	-1.78477	-1.78211	-1.77946	-1.7879	-1.78525	-1.7826	-1.77993	-1.77724
-1.78635	-1.78368	-1.78102	-1.77834	-1.78674	-1.78409	-1.78146	-1.77879	-1.77613
-1.7852	-1.78258	-1.77993	-1.77726	-1.78562	-1.78294	-1.78028	-1.77763	-1.77499
-1.78406	-1.78148	-1.77885	-1.77618	-1.78452	-1.78181	-1.77912	-1.77648	-1.77386
-1.78296	-1.78039	-1.77777	-1.7751	-1.78342	-1.7807	-1.778	-1.77537	-1.77276
-1.78188	-1.77929	-1.77667	-1.77402	-1.78233	-1.77961	-1.7769	-1.77426	-1.77164
-1.78077	-1.77815	-1.78657	-1.7839	-1.78122	-1.77851	-1.77579	-1.77313	-1.77048
-1.77965	-1.77699	-1.78538	-1.78276	-1.7801	-1.77739	-1.77468	-1.77198	-1.76928
-1.77854	-1.77586	-1.78422	-1.78161	-1.77898	-1.77628	-1.77356	-1.77084	-1.76812
-1.77745	-1.77476	-1.7831	-1.78048	-1.77784	-1.77515	-1.77244	-1.76972	-1.76698
-1.77637	-1.77366	-1.78197	-1.77933	-1.77669	-1.77401	-1.77131	-1.76859	-1.76585
-1.77529	-1.77257	-1.78084	-1.77816	-1.77549	-1.77283	-1.77017	-1.76746	-1.76472
-1.77421	-1.77149	-1.77972	-1.77699	-1.77429	-1.77166	-1.76902	-1.76633	-1.7636
-1.77311	-1.7704	-1.77861	-1.77586	-1.77315	-1.77052	-1.76789	-1.7652	-1.76247
-1.772	-1.7693	-1.77751	-1.77475	-1.77204	-1.7694	-1.76676	-1.76407	-1.76135
-1.78187	-1.77914	-1.7764	-1.77364	-1.77092	-1.76827	-1.76563	-1.76294	-1.76022
-1.78076	-1.77802	-1.77528	-1.77253	-1.76981	-1.76715	-1.7645	-1.76181	-1.75909
-1.77964	-1.7769	-1.77416	-1.77141	-1.76868	-1.76601	-1.76334	-1.76066	-1.75796
-1.77853	-1.77579	-1.77304	-1.77028	-1.76754	-1.76483	-1.76214	-1.75947	-1.75681
-1.77743	-1.77468	-1.77193	-1.76916	-1.76641	-1.76367	-1.76096	-1.7583	-1.75566
-1.77633	-1.77358	-1.77082	-1.76805	-1.76529	-1.76254	-1.75982	-1.75717	-1.75454
-1.77525	-1.77248	-1.76971	-1.76694	-1.76418	-1.76144	-1.75872	-1.75606	-1.75343
-1.77415	-1.77138	-1.7686	-1.76583	-1.76309	-1.76037	-1.75768	-1.755	-1.75234
-1.77306	-1.77028	-1.7675	-1.76473	-1.762	-1.75933	-1.75666	-1.75397	-1.75127
-1.77196	-1.76917	-1.7664	-1.76363	-1.76091	-1.75826	-1.75561	-1.75291	-1.75019
-1.77086	-1.76807	-1.7653	-1.76253	-1.75981	-1.75716	-1.75452	-1.75183	-1.74911
-1.75884	-1.75607	-1.7642	-1.76144	-1.75872	-1.75607	-1.75344	-1.75074	-1.74801
-1.75775	-1.75499	-1.76311	-1.76035	-1.75763	-1.75499	-1.75234	-1.74964	-1.74692
-1.75666	-1.75391	-1.76203	-1.75929	-1.75658	-1.75391	-1.75125	-1.74855	-1.74582
-1.75558	-1.75284	-1.76097	-1.75826	-1.75556	-1.75287	-1.75018	-1.74746	-1.74473
-1.7545	-1.75178	-1.75993	-1.75725	-1.75456	-1.75184	-1.74912	-1.74638	-1.74363
-1.75345	-1.75076	-1.74808	-1.74542	-1.75352	-1.75079	-1.74804	-1.74529	-1.74254
-1.75242	-1.74977	-1.74709	-1.7444	-1.75247	-1.74972	-1.74696	-1.7442	-1.74144
-1.75141	-1.74876	-1.74607	-1.74336	-1.7514	-1.74864	-1.74587	-1.74311	-1.74035
-1.75043	-1.74774	-1.74503	-1.7423	-1.75033	-1.74756	-1.74479	-1.74202	-1.73928
-1.74943	-1.7467	-1.74397	-1.74124	-1.7385	-1.73574	-1.73299	-1.74095	-1.73822
-1.74839	-1.74565	-1.74291	-1.74018	-1.73744	-1.73468	-1.73193	-1.7399	-1.7372
-1.74733	-1.74459	-1.74186	-1.73913	-1.73639	-1.73363	-1.73088	-1.73887	-1.73618
-1.74627	-1.74353	-1.74081	-1.73811	-1.73539	-1.73263	-1.72988	-1.73784	-1.73514
-1.74522	-1.74248	-1.73977	-1.7371	-1.73441	-1.73166	-1.7289	-1.73682	-1.73408
-1.74422	-1.74148	-1.73876	-1.73608	-1.73338	-1.73063	-1.72787	-1.72513	-1.72238
-1.74327	-1.74051	-1.73778	-1.73506	-1.73233	-1.72957	-1.72681	-1.72407	-1.72133
-1.74228	-1.73952	-1.73677	-1.73402	-1.73127	-1.72851	-1.72575	-1.72302	-1.72029
-1.74125	-1.73849	-1.73572	-1.73296	-1.7302	-1.72743	-1.72468	-1.72198	-1.71929
-1.74023	-1.73747	-1.7347	-1.73193	-1.72916	-1.72639	-1.72364	-1.72095	-1.71828
-1.73925	-1.7365	-1.73373	-1.73096	-1.72819	-1.72541	-1.72266	-1.71994	-1.71725

-1.73828	-1.73553	-1.73277	-1.73	-1.72722	-1.72445	-1.72168	-1.71893	-1.7162
-1.73727	-1.73451	-1.73175	-1.72897	-1.7262	-1.72342	-1.72065	-1.71789	-1.71514
-1.73624	-1.73347	-1.7307	-1.72793	-1.72516	-1.72238	-1.71961	-1.71684	-1.71408
-1.73526	-1.73248	-1.72971	-1.72693	-1.72416	-1.72139	-1.71861	-1.71584	-1.71308
-1.73432	-1.73154	-1.72876	-1.72598	-1.7232	-1.72043	-1.71765	-1.71488	-1.71212
-1.73335	-1.73056	-1.72778	-1.725	-1.72222	-1.71944	-1.71667	-1.71389	-1.71113
-1.73232	-1.72953	-1.72675	-1.72397	-1.7212	-1.71842	-1.71564	-1.71286	-1.71008
-1.7313	-1.72852	-1.72574	-1.72296	-1.72018	-1.7174	-1.71462	-1.71184	-1.70906
-1.73034	-1.72756	-1.72478	-1.722	-1.71922	-1.71644	-1.71366	-1.71088	-1.7081
-1.72939	-1.7266	-1.72382	-1.72105	-1.71827	-1.71549	-1.71271	-1.70993	-1.70716
-1.72839	-1.7256	-1.72282	-1.72004	-1.71727	-1.71448	-1.7117	-1.70893	-1.70616
-1.72736	-1.72458	-1.72179	-1.71902	-1.71624	-1.71345	-1.71067	-1.70789	-1.70513
-1.72639	-1.7236	-1.72082	-1.71804	-1.71526	-1.71247	-1.70969	-1.70691	-1.70415
-1.72547	-1.72268	-1.7199	-1.71712	-1.71434	-1.71155	-1.70876	-1.70599	-1.70322
-1.72455	-1.72176	-1.71898	-1.7162	-1.71342	-1.71063	-1.70784	-1.70506	-1.7023
-1.72363	-1.72085	-1.71806	-1.71528	-1.7125	-1.70971	-1.70693	-1.70415	-1.70138
-1.72269	-1.71991	-1.71712	-1.71434	-1.71156	-1.70878	-1.70599	-1.70322	-1.70044
-1.72168	-1.7189	-1.71611	-1.71333	-1.71055	-1.70777	-1.70499	-1.70221	-1.69944
-1.72065	-1.71786	-1.71508	-1.7123	-1.70952	-1.70674	-1.70396	-1.70118	-1.69841
-1.71968	-1.71689	-1.7141	-1.71132	-1.70855	-1.70577	-1.70299	-1.70021	-1.69744
-1.71874	-1.71595	-1.71317	-1.71039	-1.70762	-1.70484	-1.70206	-1.69929	-1.69651
-1.71782	-1.71503	-1.71225	-1.70947	-1.7067	-1.70392	-1.70115	-1.69837	-1.69559
-1.71691	-1.71413	-1.71135	-1.70857	-1.70579	-1.70302	-1.70024	-1.69747	-1.69469
-1.71601	-1.71322	-1.71044	-1.70767	-1.70489	-1.70212	-1.69934	-1.69656	-1.69378
-1.71511	-1.71232	-1.70954	-1.70677	-1.70399	-1.70122	-1.69844	-1.69566	-1.69288
-1.7142	-1.71142	-1.70864	-1.70586	-1.70309	-1.70032	-1.69754	-1.69476	-1.69197
-1.7133	-1.71051	-1.70773	-1.70496	-1.70219	-1.69941	-1.69663	-1.69384	-1.69105
-1.71239	-1.7096	-1.70682	-1.70405	-1.70128	-1.6985	-1.69572	-1.69292	-1.69011
-1.71148	-1.7087	-1.70592	-1.70314	-1.70036	-1.69758	-1.69479	-1.69197	-1.68913
-1.71057	-1.70779	-1.70501	-1.70223	-1.69944	-1.69666	-1.69385	-1.69099	-1.68811
-1.70966	-1.70688	-1.7041	-1.70132	-1.69852	-1.69572	-1.69289	-1.69002	-1.68712
-1.70876	-1.70598	-1.7032	-1.70041	-1.6976	-1.69476	-1.69189	-1.68903	-1.68616
-1.70786	-1.70508	-1.7023	-1.69951	-1.69669	-1.6938	-1.69091	-1.68805	-1.68521
-1.70696	-1.70418	-1.7014	-1.69861	-1.69579	-1.69289	-1.68998	-1.68713	-1.6843
-1.70605	-1.70328	-1.7005	-1.69772	-1.69489	-1.69199	-1.68908	-1.68622	-1.6834
-1.70515	-1.70238	-1.6996	-1.69682	-1.69399	-1.69109	-1.68818	-1.68532	-1.68249
-1.70424	-1.70148	-1.6987	-1.69591	-1.69309	-1.69019	-1.68729	-1.68443	-1.68159
-1.70334	-1.70057	-1.69779	-1.69501	-1.69219	-1.68932	-1.68644	-1.68357	-1.68072
-1.70243	-1.69967	-1.69689	-1.69411	-1.69131	-1.68847	-1.68562	-1.68274	-1.67986
-1.70153	-1.69876	-1.69598	-1.69321	-1.69041	-1.68761	-1.68478	-1.68191	-1.67902
-1.70062	-1.69785	-1.69507	-1.69229	-1.68951	-1.68671	-1.68391	-1.68107	-1.67822
-1.69971	-1.69694	-1.69416	-1.69138	-1.68859	-1.68581	-1.68302	-1.68022	-1.6774
-1.69881	-1.69603	-1.69325	-1.69047	-1.68768	-1.6849	-1.68212	-1.67933	-1.67654
-1.69791	-1.69513	-1.69234	-1.68956	-1.68677	-1.68399	-1.6812	-1.67843	-1.67565
-1.69701	-1.69422	-1.69143	-1.68865	-1.68586	-1.68308	-1.6803	-1.67752	-1.67475
-1.69612	-1.69331	-1.69052	-1.68774	-1.68496	-1.68218	-1.6794	-1.67662	-1.67383
-1.69527	-1.69244	-1.68963	-1.68685	-1.68406	-1.68128	-1.67849	-1.67571	-1.67293
-1.69447	-1.6916	-1.68876	-1.68596	-1.68317	-1.68038	-1.67759	-1.6748	-1.67202
-1.69367	-1.69078	-1.68793	-1.68512	-1.68231	-1.67951	-1.6767	-1.67391	-1.67113
-1.69284	-1.68999	-1.68715	-1.68434	-1.68152	-1.67868	-1.67584	-1.67304	-1.67025

-1.20083	-1.19909	-1.21667	-1.21485	-1.22861	-1.22676	-1.22492	-1.2231	-1.22128
-1.20139	-1.19964	-1.21724	-1.21542	-1.2292	-1.22736	-1.22554	-1.22372	-1.2219
-1.20195	-1.20019	-1.2178	-1.21599	-1.2298	-1.22798	-1.22616	-1.22434	-1.22253
-1.2025	-1.20074	-1.19898	-1.19722	-1.23041	-1.2286	-1.22678	-1.22497	-1.22316
-1.20304	-1.2013	-1.19955	-1.19781	-1.23103	-1.22922	-1.22741	-1.2256	-1.2238
-1.14836	-1.14672	-1.20012	-1.19838	-1.19664	-1.19488	-1.19312	-1.22621	-1.22441
-1.14889	-1.14725	-1.20069	-1.19895	-1.1972	-1.19546	-1.1937	-1.22681	-1.225
-1.13299	-1.13137	-1.14612	-1.14448	-1.19777	-1.19603	-1.19428	-1.20834	-1.20656
-1.13351	-1.13188	-1.14664	-1.145	-1.19834	-1.19659	-1.19484	-1.20893	-1.20715
-1.13404	-1.1324	-1.13077	-1.12917	-1.14388	-1.14224	-1.14059	-1.19367	-1.19193
-1.13456	-1.13292	-1.1313	-1.12969	-1.1444	-1.14276	-1.14112	-1.19424	-1.1925
-1.13509	-1.13346	-1.13183	-1.13021	-1.14492	-1.14328	-1.14165	-1.19482	-1.19308
-1.09395	-1.09241	-1.13237	-1.13075	-1.14546	-1.14382	-1.14218	-1.14054	-1.1389
-1.09447	-1.09292	-1.13292	-1.1313	-1.14601	-1.14436	-1.14271	-1.14108	-1.13945
-1.09499	-1.09344	-1.09189	-1.09037	-1.13022	-1.12861	-1.12699	-1.14162	-1.13998
-1.09552	-1.09396	-1.09241	-1.09086	-1.13074	-1.12914	-1.12754	-1.14215	-1.1405
-1.05832	-1.05684	-1.09293	-1.09136	-1.08982	-1.08832	-1.08682	-1.12646	-1.12483
-1.05878	-1.05731	-1.09344	-1.09188	-1.09035	-1.08884	-1.08734	-1.12702	-1.12539
-1.0379	-1.0365	-1.05632	-1.05485	-1.09087	-1.08936	-1.08785	-1.08634	-1.08483
-1.03835	-1.03694	-1.05679	-1.05533	-1.09139	-1.08987	-1.08836	-1.08685	-1.08536
-1.0388	-1.03738	-1.05726	-1.05581	-1.0919	-1.09038	-1.08886	-1.08737	-1.08588
-1.03923	-1.03782	-1.05773	-1.05627	-1.05482	-1.05337	-1.05194	-1.08788	-1.0864
-1.03965	-1.03826	-1.05819	-1.05672	-1.05526	-1.05383	-1.05241	-1.08839	-1.08691
-1.04009	-1.0387	-1.03729	-1.03587	-1.05573	-1.05431	-1.05289	-1.05147	-1.05005
-1.04054	-1.03914	-1.03774	-1.03632	-1.05622	-1.0548	-1.05337	-1.05195	-1.05052
-1.04099	-1.03959	-1.03818	-1.03678	-1.05672	-1.05529	-1.05387	-1.05243	-1.05099
-1.04145	-1.04003	-1.03863	-1.03724	-1.05722	-1.05579	-1.05437	-1.05294	-1.0515
-1.0419	-1.04047	-1.03907	-1.0377	-1.05772	-1.05629	-1.05486	-1.05343	-1.052
-1.04233	-1.04091	-1.03952	-1.03815	-1.03677	-1.03539	-1.034	-1.0539	-1.05247
-1.04276	-1.04135	-1.03996	-1.03859	-1.03721	-1.03581	-1.03442	-1.05435	-1.05293
-1.02017	-1.01882	-1.0404	-1.03903	-1.03765	-1.03625	-1.03485	-1.05482	-1.0534
-1.02056	-1.01923	-1.04084	-1.03947	-1.03809	-1.0367	-1.03531	-1.05531	-1.05389
-1.02097	-1.01965	-1.04128	-1.0399	-1.03853	-1.03714	-1.03576	-1.0344	-1.03304
-1.0214	-1.02008	-1.04173	-1.04034	-1.03896	-1.03759	-1.03623	-1.03487	-1.03352
-0.99695	-0.99569	-1.01918	-1.01783	-1.0394	-1.03804	-1.03669	-1.03534	-1.03399
-0.99733	-0.99608	-1.01959	-1.01824	-1.03984	-1.03848	-1.03713	-1.03579	-1.03445
-0.99772	-0.99647	-1.02001	-1.01866	-1.04029	-1.03893	-1.03758	-1.03624	-1.0349
-0.99813	-0.99688	-0.99562	-0.99434	-1.01777	-1.01646	-1.01515	-1.0367	-1.03537
-0.99855	-0.9973	-0.99604	-0.99477	-1.01822	-1.0169	-1.01559	-1.03717	-1.03583
-0.97789	-0.97667	-0.99645	-0.99518	-1.01866	-1.01734	-1.01603	-1.03763	-1.0363
-0.97827	-0.97704	-0.99685	-0.99557	-1.01908	-1.01777	-1.01647	-1.0381	-1.03678
-0.97866	-0.97743	-0.99725	-0.99597	-0.99471	-0.99347	-0.99224	-1.01563	-1.01435
-0.97906	-0.97782	-0.99767	-0.99638	-0.99511	-0.99387	-0.99264	-1.01605	-1.01478
-0.97945	-0.97822	-0.97697	-0.97572	-0.99551	-0.99427	-0.99304	-1.01648	-1.01521
-0.97982	-0.9786	-0.97736	-0.97611	-0.99593	-0.99469	-0.99346	-1.01692	-1.01565
-0.98019	-0.97898	-0.97775	-0.9765	-0.99635	-0.99511	-0.99388	-1.01737	-1.0161
-0.98056	-0.97936	-0.97813	-0.97688	-0.99676	-0.99551	-0.99428	-0.99306	-0.99186
-0.98094	-0.97973	-0.9785	-0.97725	-0.99716	-0.99591	-0.99467	-0.99345	-0.99225
-0.98131	-0.98011	-0.97889	-0.97764	-0.9764	-0.97519	-0.97398	-0.99386	-0.99265
-0.98168	-0.98049	-0.97928	-0.97804	-0.97681	-0.97559	-0.97438	-0.99428	-0.99306

-0.98206	-0.98087	-0.97966	-0.97844	-0.97722	-0.97599	-0.97478	-0.97358	-0.97239
-0.98242	-0.98123	-0.98003	-0.97883	-0.97762	-0.97639	-0.97517	-0.97396	-0.97278
-0.98279	-0.9816	-0.98041	-0.97922	-0.97801	-0.97678	-0.97556	-0.97435	-0.97316
-0.96326	-0.9621	-0.98079	-0.9796	-0.97841	-0.97719	-0.97596	-0.97475	-0.97355
-0.96363	-0.96247	-0.98117	-0.97999	-0.9788	-0.97759	-0.97637	-0.97516	-0.97394
-0.96401	-0.96284	-0.96168	-0.96053	-0.97917	-0.97797	-0.97677	-0.97556	-0.97434
-0.9644	-0.96321	-0.96204	-0.96088	-0.97953	-0.97835	-0.97716	-0.97596	-0.97475
-0.9648	-0.96359	-0.96241	-0.96125	-0.96009	-0.95895	-0.9578	-0.97635	-0.97515
-0.9652	-0.96399	-0.9628	-0.96163	-0.96047	-0.95932	-0.95817	-0.97675	-0.97556
-0.9656	-0.96438	-0.96319	-0.96202	-0.96085	-0.9597	-0.95855	-0.9574	-0.95626
-0.96598	-0.96478	-0.96359	-0.9624	-0.96122	-0.96006	-0.95891	-0.95777	-0.95663
-0.96637	-0.96518	-0.96398	-0.96278	-0.9616	-0.96043	-0.95928	-0.95814	-0.95701
-0.94484	-0.94367	-0.96437	-0.96318	-0.96199	-0.96082	-0.95966	-0.95852	-0.95739
-0.94521	-0.94404	-0.96475	-0.96357	-0.96239	-0.96122	-0.96005	-0.95891	-0.95777
-0.94559	-0.94442	-0.94324	-0.94208	-0.94092	-0.93977	-0.93863	-0.95929	-0.95816
-0.94598	-0.94479	-0.94361	-0.94244	-0.94128	-0.94014	-0.93901	-0.95968	-0.95854
-0.94637	-0.94516	-0.94398	-0.94281	-0.94165	-0.94051	-0.93938	-0.93826	-0.93713
-0.94675	-0.94555	-0.94436	-0.94319	-0.94203	-0.94089	-0.93975	-0.93862	-0.9375
-0.94713	-0.94594	-0.94475	-0.94358	-0.94242	-0.94127	-0.94013	-0.939	-0.93788
-0.94753	-0.94634	-0.94516	-0.94399	-0.94283	-0.94168	-0.94053	-0.93939	-0.93826
-0.94792	-0.94675	-0.94558	-0.94441	-0.94325	-0.94209	-0.94094	-0.93979	-0.93865
-0.94831	-0.94714	-0.94598	-0.94481	-0.94365	-0.94249	-0.94134	-0.94018	-0.93904
-0.94871	-0.94754	-0.94637	-0.94521	-0.94405	-0.94289	-0.94173	-0.94058	-0.93943
-0.94912	-0.94795	-0.94679	-0.94562	-0.94446	-0.94329	-0.94212	-0.94096	-0.93982
-0.94956	-0.94839	-0.94722	-0.94605	-0.94488	-0.94369	-0.94251	-0.94135	-0.94021
-0.94998	-0.94881	-0.94764	-0.94647	-0.9453	-0.9441	-0.94292	-0.94175	-0.94061
-0.95038	-0.94921	-0.94804	-0.94688	-0.9457	-0.94452	-0.94334	-0.94217	-0.94103
-0.95079	-0.94961	-0.94844	-0.94728	-0.94612	-0.94494	-0.94376	-0.9426	-0.94144
-0.95123	-0.95005	-0.94888	-0.94772	-0.94654	-0.94535	-0.94417	-0.94299	-0.94183
-0.95168	-0.9505	-0.94932	-0.94815	-0.94697	-0.94577	-0.94457	-0.94339	-0.94222
-0.9521	-0.95092	-0.94974	-0.94857	-0.94739	-0.94619	-0.945	-0.94382	-0.94265
-0.9525	-0.95132	-0.95014	-0.94897	-0.94779	-0.94662	-0.94545	-0.94427	-0.9431
-0.95292	-0.95174	-0.95056	-0.94938	-0.94821	-0.94705	-0.94589	-0.94472	-0.94354
-0.95334	-0.95217	-0.951	-0.94983	-0.94866	-0.94749	-0.94633	-0.94516	-0.94399
-0.95376	-0.9526	-0.95143	-0.95026	-0.94909	-0.94793	-0.94677	-0.9456	-0.94442
-0.95415	-0.95299	-0.95183	-0.95065	-0.94948	-0.94832	-0.94717	-0.94601	-0.94484
-0.95455	-0.95339	-0.95222	-0.95106	-0.94989	-0.94873	-0.94757	-0.94642	-0.94527
-0.95499	-0.95383	-0.95266	-0.9515	-0.95034	-0.94918	-0.94802	-0.94687	-0.94573
-0.95545	-0.95428	-0.95312	-0.95196	-0.95081	-0.94964	-0.94848	-0.94734	-0.94619
-0.88744	-0.88639	-0.95357	-0.95241	-0.95126	-0.95009	-0.94893	-0.94779	-0.94664
-0.88782	-0.88678	-0.95402	-0.95286	-0.9517	-0.95054	-0.94938	-0.94823	-0.94708
-0.88819	-0.88715	-0.90804	-0.90697	-0.95213	-0.95096	-0.9498	-0.94865	-0.94751
-0.88855	-0.8875	-0.90841	-0.90734	-0.95252	-0.95136	-0.95021	-0.94905	-0.9479
-0.88892	-0.88786	-0.88681	-0.88577	-0.90664	-0.90558	-0.90451	-0.94946	-0.94831
-0.88931	-0.88825	-0.8872	-0.88616	-0.90705	-0.90599	-0.90492	-0.94991	-0.94875
-0.88972	-0.88865	-0.8876	-0.88656	-0.90747	-0.90641	-0.90534	-0.95036	-0.9492
-0.89011	-0.88905	-0.88799	-0.88695	-0.90788	-0.90682	-0.90575	-0.90469	-0.90362
-0.8905	-0.88944	-0.88838	-0.88733	-0.90829	-0.90722	-0.90617	-0.90511	-0.90405
-0.89087	-0.88981	-0.88875	-0.88769	-0.88665	-0.88563	-0.88461	-0.90552	-0.90447
-0.89122	-0.89015	-0.88909	-0.88804	-0.887	-0.88599	-0.88498	-0.90591	-0.90487

-0.86316	-0.86216	-0.88945	-0.8884	-0.88737	-0.88635	-0.88535	-0.88433	-0.88331
-0.86352	-0.86251	-0.88985	-0.88879	-0.88775	-0.88672	-0.88571	-0.88468	-0.88366
-0.86389	-0.86288	-0.89025	-0.88919	-0.88815	-0.8871	-0.88607	-0.88504	-0.88402
-0.86427	-0.86325	-0.89066	-0.88961	-0.88856	-0.8875	-0.88646	-0.88542	-0.8844
-0.86465	-0.86364	-0.89108	-0.89002	-0.88897	-0.88791	-0.88686	-0.88582	-0.88479
-0.86505	-0.86402	-0.86301	-0.86202	-0.88939	-0.88833	-0.88727	-0.88623	-0.8852
-0.86545	-0.86442	-0.8634	-0.8624	-0.8898	-0.88874	-0.88769	-0.88664	-0.88561
-0.86586	-0.86481	-0.86379	-0.86278	-0.89021	-0.88915	-0.8881	-0.88706	-0.88602
-0.86626	-0.86521	-0.86418	-0.86316	-0.89062	-0.88957	-0.88852	-0.88747	-0.88642
-0.86666	-0.86561	-0.86457	-0.86355	-0.86254	-0.86155	-0.86056	-0.88787	-0.88682
-0.86705	-0.866	-0.86496	-0.86393	-0.86291	-0.86192	-0.86093	-0.88828	-0.88723
-0.86743	-0.86639	-0.86535	-0.86432	-0.86329	-0.86229	-0.8613	-0.88869	-0.88763
-0.86781	-0.86678	-0.86575	-0.86471	-0.86369	-0.86268	-0.86168	-0.8891	-0.88804
-0.86819	-0.86717	-0.86614	-0.86511	-0.86408	-0.86306	-0.86206	-0.88951	-0.88846
-0.86855	-0.86755	-0.86654	-0.8655	-0.86447	-0.86345	-0.86243	-0.86144	-0.86045
-0.86891	-0.86793	-0.86693	-0.86589	-0.86486	-0.86383	-0.86281	-0.86182	-0.86084
-0.86927	-0.86831	-0.86731	-0.86628	-0.86525	-0.86422	-0.8632	-0.8622	-0.86122
-0.86965	-0.86868	-0.86769	-0.86667	-0.86564	-0.86461	-0.86359	-0.86259	-0.8616
-0.87003	-0.86906	-0.86807	-0.86706	-0.86604	-0.86501	-0.86399	-0.86298	-0.86199
-0.87042	-0.86945	-0.86846	-0.86745	-0.86644	-0.86541	-0.86438	-0.86337	-0.86237
-0.87082	-0.86984	-0.86884	-0.86785	-0.86684	-0.86581	-0.86478	-0.86376	-0.86276
-0.87121	-0.87023	-0.86924	-0.86825	-0.86724	-0.86621	-0.86518	-0.86416	-0.86316
-0.8716	-0.87062	-0.86964	-0.86864	-0.86764	-0.86661	-0.86558	-0.86457	-0.86357
-0.80891	-0.80793	-0.87003	-0.86903	-0.86803	-0.86701	-0.86599	-0.86498	-0.86399
-0.80939	-0.80837	-0.87042	-0.86942	-0.86841	-0.8674	-0.8664	-0.86541	-0.86443
-0.77875	-0.77776	-0.83026	-0.82922	-0.82819	-0.82716	-0.82613	-0.82513	-0.82413
-0.77921	-0.77821	-0.83073	-0.82968	-0.82865	-0.82761	-0.82659	-0.8256	-0.82461
-0.77967	-0.77865	-0.80873	-0.8077	-0.82911	-0.82808	-0.82707	-0.82607	-0.82508
-0.78012	-0.7791	-0.80921	-0.80817	-0.82959	-0.82857	-0.82756	-0.82656	-0.82555
-0.78058	-0.77955	-0.80971	-0.80865	-0.83009	-0.82907	-0.82807	-0.82705	-0.82603
-0.78104	-0.78001	-0.77899	-0.77798	-0.83061	-0.82958	-0.82857	-0.82753	-0.82649
-0.78152	-0.78049	-0.77947	-0.77846	-0.83115	-0.8301	-0.82906	-0.82801	-0.82696
-0.78199	-0.78096	-0.77994	-0.77894	-0.77794	-0.77694	-0.77594	-0.82848	-0.82743
-0.78246	-0.78144	-0.78042	-0.77941	-0.7784	-0.77739	-0.77637	-0.82896	-0.82791
-0.78295	-0.78192	-0.7809	-0.77988	-0.77886	-0.77783	-0.77681	-0.82945	-0.82841
-0.78344	-0.7824	-0.78137	-0.78034	-0.77932	-0.77829	-0.77726	-0.82997	-0.82893
-0.78393	-0.78289	-0.78184	-0.78081	-0.77978	-0.77875	-0.77773	-0.8305	-0.82946
-0.78444	-0.78337	-0.78232	-0.78129	-0.78026	-0.77923	-0.77822	-0.77722	-0.77623
-0.78496	-0.78386	-0.7828	-0.78177	-0.78075	-0.77973	-0.77871	-0.7777	-0.77669
-0.754	-0.75296	-0.7833	-0.78227	-0.78125	-0.78022	-0.7792	-0.77818	-0.77717
-0.75447	-0.75345	-0.78383	-0.78278	-0.78174	-0.78071	-0.77968	-0.77866	-0.77765
-0.72388	-0.72292	-0.75293	-0.75194	-0.78224	-0.78119	-0.78016	-0.77915	-0.77814
-0.72434	-0.72339	-0.75343	-0.7524	-0.78272	-0.78168	-0.78066	-0.77964	-0.77864
-0.72481	-0.72386	-0.75392	-0.75287	-0.78322	-0.78218	-0.78115	-0.78014	-0.77914
-0.72528	-0.72431	-0.7544	-0.75336	-0.78374	-0.78269	-0.78166	-0.78064	-0.77963
-0.72575	-0.72477	-0.75488	-0.75385	-0.78426	-0.78321	-0.78217	-0.78114	-0.78013
-0.72622	-0.72523	-0.72425	-0.72328	-0.75333	-0.75233	-0.75134	-0.78165	-0.78063
-0.72668	-0.72569	-0.72471	-0.72374	-0.75382	-0.75282	-0.75183	-0.78217	-0.78114
-0.72716	-0.72616	-0.72518	-0.72421	-0.75432	-0.75332	-0.75232	-0.75134	-0.75036
-0.72765	-0.72665	-0.72566	-0.72467	-0.75481	-0.75382	-0.75283	-0.75183	-0.75083

-0.72814	-0.72714	-0.72614	-0.72515	-0.72417	-0.72324	-0.72231	-0.75232	-0.75131
-0.72863	-0.72763	-0.72663	-0.72563	-0.72465	-0.7237	-0.72276	-0.75282	-0.75182
-0.72914	-0.72813	-0.72712	-0.72612	-0.72513	-0.72417	-0.72323	-0.72229	-0.72136
-0.72964	-0.72864	-0.72763	-0.72661	-0.72562	-0.72465	-0.72371	-0.72276	-0.72183
-0.73015	-0.72916	-0.72815	-0.72712	-0.72611	-0.72514	-0.72419	-0.72324	-0.72229
-0.73067	-0.72967	-0.72866	-0.72764	-0.72663	-0.72564	-0.72468	-0.72372	-0.72277
-0.7312	-0.73018	-0.72917	-0.72816	-0.72716	-0.72615	-0.72517	-0.7242	-0.72326
-0.73173	-0.7307	-0.72969	-0.72868	-0.72768	-0.72667	-0.72567	-0.7247	-0.72374
-0.73227	-0.73124	-0.73022	-0.7292	-0.72819	-0.72719	-0.7262	-0.72521	-0.72423
-0.7029	-0.70192	-0.73074	-0.72972	-0.72871	-0.72772	-0.72673	-0.72572	-0.72472
-0.70342	-0.70241	-0.73126	-0.73025	-0.72924	-0.72824	-0.72724	-0.72624	-0.72524
-0.70394	-0.70291	-0.73178	-0.73078	-0.72977	-0.72877	-0.72776	-0.72676	-0.72576
-0.70446	-0.70343	-0.70243	-0.70145	-0.73029	-0.72929	-0.72829	-0.72728	-0.72627
-0.70499	-0.70397	-0.70296	-0.70195	-0.7308	-0.72981	-0.72882	-0.72781	-0.72679
-0.70553	-0.7045	-0.70348	-0.70246	-0.70148	-0.70053	-0.69959	-0.72834	-0.72732
-0.70608	-0.70502	-0.70399	-0.70299	-0.702	-0.70103	-0.70007	-0.72887	-0.72787
-0.68919	-0.68816	-0.70453	-0.70353	-0.70254	-0.70155	-0.70057	-0.69962	-0.69869
-0.68971	-0.6887	-0.70509	-0.70409	-0.70309	-0.70208	-0.70109	-0.70013	-0.6992
-0.69024	-0.68924	-0.68826	-0.6873	-0.70363	-0.70262	-0.70162	-0.70065	-0.6997
-0.69078	-0.68981	-0.68882	-0.68782	-0.70416	-0.70315	-0.70215	-0.70116	-0.70019
-0.69132	-0.69037	-0.68937	-0.68835	-0.70469	-0.70368	-0.70269	-0.70168	-0.70068
-0.69184	-0.69088	-0.68989	-0.68887	-0.68788	-0.68692	-0.68596	-0.70222	-0.70121
-0.69235	-0.69137	-0.69039	-0.68941	-0.68843	-0.68746	-0.6865	-0.70277	-0.70174
-0.69286	-0.69188	-0.69091	-0.68995	-0.68898	-0.688	-0.68702	-0.70332	-0.70229
-0.69335	-0.6924	-0.69144	-0.6905	-0.68953	-0.68853	-0.68753	-0.70386	-0.70285
-0.69387	-0.69292	-0.69198	-0.69103	-0.69007	-0.68907	-0.68806	-0.68709	-0.68614
-0.69441	-0.69346	-0.69251	-0.69155	-0.69058	-0.6896	-0.68863	-0.68765	-0.68668
-0.69495	-0.694	-0.69304	-0.69206	-0.69109	-0.69014	-0.68919	-0.68821	-0.68722
-0.6955	-0.69454	-0.69358	-0.69259	-0.69162	-0.69068	-0.68973	-0.68875	-0.68775
-0.69605	-0.69508	-0.69411	-0.69312	-0.69215	-0.69121	-0.69027	-0.68929	-0.68829
-0.6966	-0.69562	-0.69464	-0.69365	-0.69268	-0.69175	-0.69081	-0.68983	-0.68883
-0.69715	-0.69615	-0.69516	-0.69418	-0.69322	-0.69228	-0.69134	-0.69037	-0.68939
-0.69771	-0.69669	-0.69569	-0.69472	-0.69375	-0.6928	-0.69186	-0.69089	-0.68992
-0.6983	-0.69726	-0.69624	-0.69526	-0.69429	-0.69332	-0.69236	-0.6914	-0.69045
-0.69888	-0.69782	-0.6968	-0.69582	-0.69484	-0.69385	-0.69288	-0.69192	-0.69098
-0.69945	-0.6984	-0.69737	-0.69639	-0.6954	-0.6944	-0.69342	-0.69246	-0.69151
-0.70002	-0.69898	-0.69796	-0.69696	-0.69597	-0.69496	-0.69397	-0.693	-0.69205
-0.66511	-0.6642	-0.69854	-0.69754	-0.69654	-0.69552	-0.69452	-0.69354	-0.69259
-0.66565	-0.66473	-0.69914	-0.69813	-0.69711	-0.69609	-0.69507	-0.6941	-0.69314
-0.60458	-0.60377	-0.66435	-0.66345	-0.69768	-0.69664	-0.69563	-0.69465	-0.69369
-0.60506	-0.60423	-0.66489	-0.66397	-0.69824	-0.6972	-0.69618	-0.6952	-0.69425
-0.60556	-0.60471	-0.60388	-0.60306	-0.66357	-0.66267	-0.66178	-0.69576	-0.69481
-0.60607	-0.60519	-0.60434	-0.60352	-0.6641	-0.66319	-0.66229	-0.69634	-0.69537
-0.57061	-0.56976	-0.60483	-0.60399	-0.63409	-0.63322	-0.63238	-0.69692	-0.69594
-0.57111	-0.57025	-0.60535	-0.60447	-0.6346	-0.63373	-0.63288	-0.69752	-0.69653
-0.57162	-0.57076	-0.60588	-0.60497	-0.63512	-0.63424	-0.63339	-0.69813	-0.69712
-0.57211	-0.57126	-0.5704	-0.56953	-0.60459	-0.60376	-0.60296	-0.63305	-0.63223
-0.57261	-0.57176	-0.5709	-0.57001	-0.60509	-0.60425	-0.60343	-0.63356	-0.63272
-0.5731	-0.57226	-0.57139	-0.57049	-0.60561	-0.60474	-0.60391	-0.6031	-0.60231
-0.57359	-0.57275	-0.57188	-0.57099	-0.60614	-0.60524	-0.60438	-0.60356	-0.60277

-0.53398	-0.53332	-0.57237	-0.57148	-0.5706	-0.56975	-0.56892	-0.60404	-0.60324
-0.53438	-0.53371	-0.57286	-0.57197	-0.5711	-0.57024	-0.5694	-0.60454	-0.60372
-0.49722	-0.4966	-0.53343	-0.53274	-0.57159	-0.57073	-0.56989	-0.56907	-0.56829
-0.49757	-0.49695	-0.53382	-0.53313	-0.57209	-0.57122	-0.57038	-0.56955	-0.56874
-0.49792	-0.49729	-0.53421	-0.53352	-0.57258	-0.57172	-0.57087	-0.57003	-0.56921
-0.49827	-0.49765	-0.49702	-0.49639	-0.53634	-0.53553	-0.53472	-0.57051	-0.56969
-0.49863	-0.49801	-0.49739	-0.49676	-0.5368	-0.53597	-0.53516	-0.57099	-0.57017
-0.49899	-0.49837	-0.49775	-0.49712	-0.4965	-0.49586	-0.49523	-0.53481	-0.53403
-0.49934	-0.49873	-0.49811	-0.49748	-0.49684	-0.49621	-0.49558	-0.53526	-0.53448
-0.39369	-0.3933	-0.49847	-0.49784	-0.4972	-0.49656	-0.49593	-0.49532	-0.49472
-0.39391	-0.39352	-0.49883	-0.4982	-0.49757	-0.49694	-0.49631	-0.4957	-0.4951
-0.39414	-0.39374	-0.49919	-0.49857	-0.49794	-0.49732	-0.49669	-0.49608	-0.49548
-0.39435	-0.39396	-0.39357	-0.39318	-0.49831	-0.49769	-0.49707	-0.49645	-0.49584
-0.39457	-0.39418	-0.39379	-0.39341	-0.49869	-0.49806	-0.49744	-0.49682	-0.49621
-0.3948	-0.3944	-0.39401	-0.39363	-0.39324	-0.39284	-0.39244	-0.49719	-0.49657
-0.39505	-0.39464	-0.39424	-0.39385	-0.39346	-0.39307	-0.39267	-0.49755	-0.49694
-0.3953	-0.39487	-0.39446	-0.39407	-0.39368	-0.39329	-0.3929	-0.3925	-0.3921
-0.39555	-0.39511	-0.39469	-0.39429	-0.39389	-0.39351	-0.39313	-0.39273	-0.39234
-0.39582	-0.39537	-0.39494	-0.39452	-0.39412	-0.39373	-0.39335	-0.39296	-0.39257
-0.39612	-0.39567	-0.39522	-0.39478	-0.39436	-0.39396	-0.39357	-0.39318	-0.39279
-0.39643	-0.39597	-0.39551	-0.39505	-0.39461	-0.3942	-0.3938	-0.3934	-0.39301
-0.39671	-0.39624	-0.39578	-0.39532	-0.39488	-0.39445	-0.39404	-0.39364	-0.39325
-0.39698	-0.39651	-0.39605	-0.3956	-0.39516	-0.39472	-0.39429	-0.39389	-0.3935
-0.37759	-0.37717	-0.39632	-0.39587	-0.39542	-0.39497	-0.39454	-0.39413	-0.39374
-0.37786	-0.37743	-0.3966	-0.39614	-0.39568	-0.39523	-0.39479	-0.39437	-0.39397
-0.37814	-0.37768	-0.37726	-0.37685	-0.37646	-0.37607	-0.37569	-0.39461	-0.39419
-0.37841	-0.37795	-0.3775	-0.37708	-0.37668	-0.37629	-0.37591	-0.39486	-0.39442
-0.3787	-0.37823	-0.37777	-0.37734	-0.37692	-0.37653	-0.37615	-0.37576	-0.37537
-0.37901	-0.37854	-0.37808	-0.37763	-0.37719	-0.37679	-0.3764	-0.37601	-0.37562
-0.37933	-0.37886	-0.3784	-0.37793	-0.37748	-0.37706	-0.37666	-0.37626	-0.37588
-0.37963	-0.37917	-0.3787	-0.37823	-0.37777	-0.37733	-0.37691	-0.37651	-0.37612
-0.37991	-0.37946	-0.37899	-0.37852	-0.37806	-0.37759	-0.37715	-0.37674	-0.37636
-0.38019	-0.37974	-0.37928	-0.37881	-0.37834	-0.37787	-0.37742	-0.377	-0.37661
-0.38045	-0.38	-0.37955	-0.37908	-0.37861	-0.37815	-0.37771	-0.37728	-0.37687
-0.38073	-0.38028	-0.37983	-0.37936	-0.3789	-0.37845	-0.378	-0.37756	-0.37713
-0.38103	-0.38058	-0.38013	-0.37968	-0.37923	-0.37877	-0.37831	-0.37785	-0.3774
-0.38133	-0.38088	-0.38044	-0.38	-0.37955	-0.37908	-0.37862	-0.37815	-0.37768
-0.38161	-0.38116	-0.38072	-0.38028	-0.37983	-0.37936	-0.3789	-0.37844	-0.37798
-0.38187	-0.38144	-0.38099	-0.38055	-0.3801	-0.37964	-0.37918	-0.37873	-0.37829
-0.38218	-0.38173	-0.38129	-0.38084	-0.38039	-0.37994	-0.37949	-0.37904	-0.37859
-0.38251	-0.38204	-0.38159	-0.38114	-0.38069	-0.38026	-0.37982	-0.37936	-0.3789
-0.38282	-0.38234	-0.38188	-0.38143	-0.38098	-0.38055	-0.38011	-0.37966	-0.37919
-0.38311	-0.38261	-0.38214	-0.3817	-0.38126	-0.38082	-0.38037	-0.37993	-0.37948
-0.38342	-0.38291	-0.38243	-0.38199	-0.38154	-0.38109	-0.38064	-0.3802	-0.37977
-0.38373	-0.38324	-0.38277	-0.3823	-0.38184	-0.38139	-0.38094	-0.3805	-0.38006
-0.38405	-0.38358	-0.38311	-0.38262	-0.38215	-0.38169	-0.38124	-0.3808	-0.38036
-0.38437	-0.3839	-0.38341	-0.38292	-0.38244	-0.38198	-0.38153	-0.38109	-0.38066
-0.3847	-0.3842	-0.38371	-0.38321	-0.38273	-0.38227	-0.38182	-0.38139	-0.38096
-0.34363	-0.34319	-0.38403	-0.38354	-0.38306	-0.38259	-0.38213	-0.38168	-0.38125
-0.34389	-0.34347	-0.38436	-0.38389	-0.38341	-0.38292	-0.38243	-0.38197	-0.38153

-0.34416	-0.34375	-0.34333	-0.34291	-0.38374	-0.38324	-0.38275	-0.38228	-0.38183
-0.34443	-0.34402	-0.34359	-0.34316	-0.38403	-0.38355	-0.38308	-0.38261	-0.38214
-0.34472	-0.34429	-0.34386	-0.34343	-0.343	-0.34259	-0.34218	-0.38293	-0.38246
-0.34502	-0.34458	-0.34415	-0.34372	-0.34329	-0.34287	-0.34245	-0.38324	-0.38277
-0.34533	-0.34488	-0.34444	-0.34402	-0.34359	-0.34315	-0.34272	-0.38355	-0.38309
-0.34563	-0.34518	-0.34473	-0.34431	-0.34388	-0.34344	-0.34301	-0.34259	-0.34217
-0.34593	-0.34548	-0.34503	-0.34459	-0.34416	-0.34373	-0.34331	-0.34289	-0.34246
-0.34626	-0.34579	-0.34533	-0.34488	-0.34444	-0.34402	-0.3436	-0.34317	-0.34275
-0.34661	-0.34613	-0.34565	-0.34518	-0.34472	-0.34429	-0.34387	-0.34345	-0.34303
-0.34698	-0.34648	-0.34598	-0.34549	-0.34502	-0.34458	-0.34415	-0.34373	-0.34332
-0.34736	-0.34684	-0.34633	-0.34584	-0.34536	-0.34489	-0.34443	-0.344	-0.34359
-0.34778	-0.34722	-0.34669	-0.3462	-0.34571	-0.34521	-0.34473	-0.34429	-0.34387
-0.34825	-0.34761	-0.34703	-0.34653	-0.34604	-0.34554	-0.34506	-0.3446	-0.34416
-0.34877	-0.34802	-0.34738	-0.34686	-0.34637	-0.34588	-0.34541	-0.34493	-0.34445
-0.34933	-0.34852	-0.34782	-0.34724	-0.3467	-0.34621	-0.34574	-0.34525	-0.34477
-0.34994	-0.34908	-0.34832	-0.34765	-0.34705	-0.34655	-0.34607	-0.34559	-0.34511
-0.35056	-0.34968	-0.34886	-0.34811	-0.34745	-0.34691	-0.34642	-0.34593	-0.34545
-0.3512	-0.3503	-0.34945	-0.34863	-0.3479	-0.3473	-0.34677	-0.34628	-0.34581
-0.35184	-0.35094	-0.35006	-0.34918	-0.3484	-0.34774	-0.34715	-0.34664	-0.34617
-0.3525	-0.35158	-0.35068	-0.3498	-0.34897	-0.34823	-0.34756	-0.347	-0.34651
-0.35318	-0.35224	-0.35133	-0.35043	-0.34958	-0.34876	-0.34801	-0.34739	-0.34686
-0.35391	-0.35293	-0.35198	-0.35106	-0.35018	-0.34932	-0.34853	-0.34784	-0.34724
-0.35467	-0.35364	-0.35265	-0.3517	-0.3508	-0.34993	-0.3491	-0.34834	-0.34764
-0.35542	-0.35437	-0.35337	-0.35239	-0.35146	-0.35056	-0.34971	-0.34888	-0.34812
-0.35617	-0.35514	-0.35412	-0.35313	-0.35216	-0.35123	-0.35034	-0.34947	-0.34865
-0.35691	-0.3559	-0.35488	-0.35386	-0.35287	-0.35191	-0.35099	-0.35009	-0.34923
-0.35764	-0.35665	-0.35563	-0.35459	-0.35358	-0.35261	-0.35166	-0.35073	-0.34984
-0.35835	-0.35738	-0.35638	-0.35533	-0.35431	-0.35332	-0.35235	-0.35141	-0.35049
-0.35905	-0.35808	-0.35709	-0.35609	-0.35508	-0.35406	-0.35306	-0.35211	-0.35118
-0.35976	-0.35878	-0.3578	-0.35684	-0.35586	-0.35482	-0.35379	-0.35283	-0.35189
-0.31276	-0.31194	-0.35853	-0.35758	-0.35661	-0.35559	-0.35456	-0.35358	-0.35261
-0.31336	-0.31255	-0.35926	-0.35831	-0.35734	-0.35636	-0.35536	-0.35435	-0.35335
-0.27482	-0.27413	-0.31237	-0.31159	-0.35808	-0.35711	-0.35612	-0.3551	-0.35408
-0.27532	-0.27464	-0.31297	-0.3122	-0.35882	-0.35784	-0.35685	-0.35584	-0.35482
-0.27582	-0.27515	-0.27448	-0.27382	-0.35955	-0.35857	-0.35759	-0.35658	-0.35556
-0.27631	-0.27565	-0.27499	-0.27434	-0.36027	-0.3593	-0.35833	-0.35734	-0.35633
-0.27682	-0.27615	-0.27549	-0.27485	-0.27418	-0.2735	-0.27278	-0.35808	-0.35708
-0.27737	-0.27667	-0.27599	-0.27533	-0.27467	-0.27401	-0.27332	-0.35881	-0.35782
-0.27793	-0.2772	-0.27649	-0.27581	-0.27514	-0.2745	-0.27385	-0.35952	-0.35855
-0.27847	-0.27773	-0.27701	-0.27631	-0.27564	-0.275	-0.27436	-0.27366	-0.27293
-0.27902	-0.27827	-0.27754	-0.27683	-0.27614	-0.2755	-0.27486	-0.27418	-0.27348
-0.2796	-0.27884	-0.27809	-0.27736	-0.27666	-0.27601	-0.27537	-0.2747	-0.27402
-0.28021	-0.27944	-0.27867	-0.2779	-0.27717	-0.27652	-0.27589	-0.27522	-0.27455
-0.28083	-0.28006	-0.27927	-0.27847	-0.27771	-0.27705	-0.2764	-0.27574	-0.27507
-0.28146	-0.28067	-0.27988	-0.27907	-0.2783	-0.27759	-0.27691	-0.27624	-0.27559
-0.28209	-0.28129	-0.2805	-0.27969	-0.27891	-0.27816	-0.27744	-0.27676	-0.2761
-0.28271	-0.28192	-0.28113	-0.28031	-0.27952	-0.27876	-0.27802	-0.27731	-0.27662
-0.28332	-0.28255	-0.28176	-0.28094	-0.28014	-0.27937	-0.27861	-0.27787	-0.27714
-0.28392	-0.28317	-0.28239	-0.28157	-0.28076	-0.27998	-0.27922	-0.27845	-0.2777
-0.28451	-0.28377	-0.283	-0.28219	-0.28139	-0.28061	-0.27983	-0.27905	-0.27828

-0.28509	-0.28437	-0.28361	-0.28281	-0.28202	-0.28123	-0.28045	-0.27966	-0.27888
-0.28567	-0.28495	-0.28421	-0.28343	-0.28264	-0.28186	-0.28107	-0.28029	-0.27952
-0.28625	-0.28554	-0.2848	-0.28404	-0.28327	-0.28248	-0.28169	-0.28093	-0.28017
-0.28686	-0.28612	-0.28538	-0.28463	-0.28387	-0.2831	-0.28233	-0.28156	-0.2808
-0.28749	-0.28672	-0.28596	-0.28522	-0.28448	-0.28372	-0.28296	-0.2822	-0.28143
-0.28815	-0.28733	-0.28656	-0.28583	-0.28509	-0.28433	-0.28358	-0.28282	-0.28207
-0.28883	-0.28797	-0.28718	-0.28644	-0.2857	-0.28494	-0.28418	-0.28344	-0.2827
-0.28953	-0.28865	-0.28782	-0.28706	-0.28631	-0.28554	-0.28479	-0.28405	-0.28332
-0.29027	-0.28936	-0.2885	-0.28769	-0.2869	-0.28614	-0.28539	-0.28465	-0.28391
-0.291	-0.29008	-0.28919	-0.28834	-0.28753	-0.28675	-0.28599	-0.28525	-0.28451
-0.29172	-0.29078	-0.28988	-0.28902	-0.28819	-0.28738	-0.28659	-0.28584	-0.2851
-0.29244	-0.29149	-0.29059	-0.28972	-0.28887	-0.28803	-0.28722	-0.28645	-0.28571
-0.29314	-0.29221	-0.2913	-0.29042	-0.28956	-0.28872	-0.28789	-0.2871	-0.28633
-0.29383	-0.29292	-0.29202	-0.29113	-0.29026	-0.28942	-0.2886	-0.28777	-0.28696
-0.24564	-0.24488	-0.29273	-0.29184	-0.29097	-0.29012	-0.28929	-0.28845	-0.28761
-0.24619	-0.24546	-0.29345	-0.29255	-0.29167	-0.29083	-0.28999	-0.28913	-0.28827
-0.04333	-0.04321	-0.24531	-0.24455	-0.29239	-0.29153	-0.29069	-0.28982	-0.28896
-0.04341	-0.04328	-0.2459	-0.24515	-0.29311	-0.29224	-0.29138	-0.29053	-0.28969
-0.04349	-0.04336	-0.1955	-0.19499	-0.29385	-0.29296	-0.29208	-0.29123	-0.2904
-0.04359	-0.04344	-0.1959	-0.19541	-0.29459	-0.29369	-0.2928	-0.29194	-0.29109
-0.04369	-0.04353	-0.19631	-0.19582	-0.29533	-0.29443	-0.29352	-0.29264	-0.29178
-0.04379	-0.04363	-0.04348	-0.04334	-0.19573	-0.1952	-0.19466	-0.29335	-0.29248
-0.04389	-0.04373	-0.04358	-0.04343	-0.19615	-0.19561	-0.19508	-0.29407	-0.29318
-0.04399	-0.04383	-0.04368	-0.04352	-0.04338	-0.04324	-0.0431	-0.19498	-0.19444
-0.04408	-0.04393	-0.04378	-0.04362	-0.04347	-0.04333	-0.04319	-0.19542	-0.19487
-0.04418	-0.04403	-0.04388	-0.04372	-0.04357	-0.04342	-0.04327	-0.19585	-0.1953
-0.04429	-0.04413	-0.04397	-0.04382	-0.04367	-0.04352	-0.04337	-0.19629	-0.19576
		-0.04407	-0.04392	-0.04377	-0.04362	-0.04346	-0.04332	-0.04317
		-0.04417	-0.04402	-0.04387	-0.04372	-0.04356	-0.04341	-0.04326
		-0.04428	-0.04412	-0.04396	-0.04381	-0.04367	-0.04351	-0.04336
		-0.04438	-0.04422	-0.04406	-0.04391	-0.04376	-0.04361	-0.04346
		-0.04448	-0.04432	-0.04416	-0.04401	-0.04386	-0.04371	-0.04356
				-0.04427	-0.04411	-0.04396	-0.0438	-0.04366
				-0.04439	-0.04422	-0.04406	-0.0439	-0.04375
							-0.04401	-0.04386
							-0.04413	-0.04397

							-0.21611	-0.21536
							-0.21545	-0.21476
				-0.2171	-0.21628	-0.21553	-0.40304	-0.40181
				-0.21638	-0.21563	-0.21493	-0.40198	-0.40076
				-0.21571	-0.21501	-0.21437	-0.40095	-0.39973
		-0.10729	-0.10701	-0.4036	-0.40235	-0.40114	-0.48274	-0.48133
		-0.10706	-0.10676	-0.40257	-0.40132	-0.40011	-0.48159	-0.48019
-0.10741	-0.10711	-0.35473	-0.35361	-0.48466	-0.48324	-0.48183	-0.5201	-0.51861
-0.10717	-0.10686	-0.3538	-0.35271	-0.4835	-0.48207	-0.48066	-0.51884	-0.51736
-0.25289	-0.25213	-0.48518	-0.48375	-0.48234	-0.48092	-0.47951	-0.5617	-0.56016
-0.2523	-0.25156	-0.48406	-0.48262	-0.4812	-0.47979	-0.4784	-0.56042	-0.55887
-0.25171	-0.25099	-0.48294	-0.4815	-0.48007	-0.47867	-0.47729	-0.55914	-0.5576
-0.40139	-0.40016	-0.52694	-0.52541	-0.56257	-0.56098	-0.55941	-0.6405	-0.63865
-0.40038	-0.39914	-0.52572	-0.52421	-0.56127	-0.55968	-0.55813	-0.63898	-0.63715
-0.52752	-0.52602	-0.60052	-0.59865	-0.59681	-0.59501	-0.59325	-0.67827	-0.67639
-0.52627	-0.5248	-0.59904	-0.59723	-0.59544	-0.59365	-0.59189	-0.67677	-0.67491
-0.56326	-0.56155	-0.59761	-0.59582	-0.64018	-0.63832	-0.63647	-0.67528	-0.67343
-0.56194	-0.56026	-0.59622	-0.59442	-0.63869	-0.63686	-0.63505	-0.6738	-0.67194
-0.5984	-0.59661	-0.59483	-0.59302	-0.67789	-0.67602	-0.67418	-0.71215	-0.71018
-0.59698	-0.59519	-0.59341	-0.59162	-0.67634	-0.6745	-0.67269	-0.71059	-0.70864
-0.59555	-0.59377	-0.592	-0.59023	-0.67481	-0.67299	-0.67121	-0.70903	-0.70713
-0.59409	-0.59233	-0.6771	-0.67522	-0.71322	-0.71129	-0.70939	-0.7075	-0.70562
-0.59263	-0.59091	-0.67557	-0.67376	-0.71171	-0.70979	-0.70786	-0.70598	-0.70411
-0.67776	-0.67592	-0.71398	-0.71209	-0.71019	-0.70825	-0.70631	-0.85392	-0.8514
-0.6763	-0.67449	-0.71247	-0.71057	-0.70865	-0.70669	-0.70475	-0.85184	-0.84941
-0.71477	-0.71287	-0.71096	-0.70903	-0.85776	-0.85502	-0.85235	-0.92001	-0.91739
-0.71325	-0.71135	-0.70943	-0.70746	-0.85556	-0.85296	-0.85046	-0.91801	-0.91539
-0.71174	-0.70982	-0.859	-0.85617	-0.92406	-0.92131	-0.91869	-0.98524	-0.98246
-0.71022	-0.70826	-0.85682	-0.85413	-0.92198	-0.91928	-0.91667	-0.9831	-0.98033
-0.70869	-0.70669	-0.85468	-0.85217	-0.91999	-0.91731	-0.91467	-0.98097	-0.9782
-0.85807	-0.85532	-0.92333	-0.92063	-0.98726	-0.98442	-0.98161	-0.97882	-0.97605
-0.85593	-0.85335	-0.92129	-0.91862	-0.98511	-0.98228	-0.97947	-0.97667	-0.97389
-0.92471	-0.92196	-0.98861	-0.98576	-0.98295	-0.98012	-0.97731	-0.97452	-0.97175
-0.92262	-0.91991	-0.98643	-0.9836	-0.98079	-0.97795	-0.97513	-0.97237	-0.96965
-0.92056	-0.91789	-0.98426	-0.98143	-0.97862	-0.97579	-0.97299	-1.00623	-1.00349
-0.91854	-0.91588	-0.98212	-0.97924	-0.9764	-0.97363	-0.9709	-1.00413	-1.00141
-0.91654	-0.91387	-0.97998	-0.97708	-0.97423	-0.9715	-0.96884	-1.00206	-0.99935
-0.98347	-0.98067	-1.01397	-1.01106	-1.0756	-1.0727	-1.06986	-1.09966	-1.09681
-0.9813	-0.97852	-1.01182	-1.00896	-1.07345	-1.07055	-1.06769	-1.09746	-1.09464
-1.0153	-1.01247	-1.0771	-1.07416	-1.07126	-1.06838	-1.06554	-1.09528	-1.09246
-1.01314	-1.0103	-1.07482	-1.07192	-1.06906	-1.06622	-1.06341	-1.09311	-1.09029
-1.07852	-1.07553	-1.0726	-1.06972	-1.09952	-1.09664	-1.09379	-1.23699	-1.23396
-1.07637	-1.07337	-1.07043	-1.06756	-1.09733	-1.09446	-1.09163	-1.2347	-1.23174
-1.07422	-1.07122	-1.13203	-1.12905	-1.2416	-1.23849	-1.23544	-1.26268	-1.25969
-1.07203	-1.06904	-1.12981	-1.12691	-1.23936	-1.23628	-1.23324	-1.26043	-1.25744
-1.06984	-1.06688	-1.12762	-1.12479	-1.23716	-1.23411	-1.23107	-1.2582	-1.25519
-1.13141	-1.12839	-1.24088	-1.2379	-1.26516	-1.26207	-1.25897	-1.2874	-1.28431

-1.12911	-1.12619	-1.2386	-1.23564	-1.26287	-1.25978	-1.25669	-1.28506	-1.28198
-1.15435	-1.15146	-1.26657	-1.26355	-1.29214	-1.28897	-1.28581	-1.28271	-1.27965
-1.15222	-1.14935	-1.26428	-1.2612	-1.28967	-1.28654	-1.28343	-1.28035	-1.2773
-1.26811	-1.26507	-1.29367	-1.29045	-1.28726	-1.28414	-1.28106	-1.278	-1.27496
-1.26582	-1.2628	-1.29133	-1.28811	-1.28492	-1.2818	-1.27872	-1.27565	-1.2726
-1.29526	-1.29215	-1.289	-1.28579	-1.2826	-1.27948	-1.2764	-1.27332	-1.27025
-1.29291	-1.2898	-1.28666	-1.28347	-1.28029	-1.27717	-1.27408	-1.27099	-1.26792
-1.29057	-1.28747	-1.28434	-1.28115	-1.27798	-1.27487	-1.27178	-1.2687	-1.26563
-1.28823	-1.28516	-1.28204	-1.27884	-1.27567	-1.27258	-1.26953	-1.34035	-1.33697
-1.2859	-1.28286	-1.27976	-1.27654	-1.27336	-1.27031	-1.26731	-1.33801	-1.33469
-1.2836	-1.28057	-1.30548	-1.30221	-1.34561	-1.34226	-1.33899	-1.33569	-1.3324
-1.28132	-1.27828	-1.30315	-1.29991	-1.34319	-1.33988	-1.33665	-1.33337	-1.33011
-1.30708	-1.30398	-1.34777	-1.34426	-1.34081	-1.33754	-1.33435	-1.33112	-1.32792
-1.30476	-1.30166	-1.34532	-1.34186	-1.33847	-1.33523	-1.33208	-1.32893	-1.32584
-1.30245	-1.29936	-1.34291	-1.3395	-1.33616	-1.33296	-1.32987	-1.32684	-1.32392
-1.34722	-1.34387	-1.34052	-1.33715	-1.33387	-1.33071	-1.32771	-1.32489	-1.32227
-1.34482	-1.34149	-1.33817	-1.33483	-1.33161	-1.32854	-1.32566	-1.32309	-1.32078
-1.34251	-1.33917	-1.33587	-1.33255	-1.3294	-1.3265	-1.32384	-1.33835	-1.33618
-1.34026	-1.33691	-1.33361	-1.33031	-1.32726	-1.3246	-1.32222	-1.33685	-1.33482
-1.33797	-1.33462	-1.33138	-1.32821	-1.34229	-1.33977	-1.33754	-1.33544	-1.33351
-1.33563	-1.33229	-1.32916	-1.32624	-1.3405	-1.33818	-1.33611	-1.33413	-1.33226
-1.33333	-1.33006	-1.34408	-1.34134	-1.33887	-1.33672	-1.33477	-1.33287	-1.33105
-1.33108	-1.32795	-1.3421	-1.33965	-1.33742	-1.33538	-1.3335	-1.33166	-1.32989
-1.32892	-1.32599	-1.34029	-1.33809	-1.33608	-1.33413	-1.33227	-1.33048	-1.32876
-1.34385	-1.34112	-1.3387	-1.33667	-1.33477	-1.33289	-1.33108	-1.35461	-1.35289
-1.34192	-1.33949	-1.33729	-1.33535	-1.33351	-1.33168	-1.32992	-1.35346	-1.35179
-1.34022	-1.33798	-1.33592	-1.33406	-1.35763	-1.35579	-1.35401	-1.35231	-1.35068
-1.33873	-1.33661	-1.33463	-1.33282	-1.35638	-1.35459	-1.35283	-1.35116	-1.34956
-1.33731	-1.3353	-1.35878	-1.35695	-1.35518	-1.35342	-1.35169	-1.4064	-1.40457
-1.33597	-1.33407	-1.35759	-1.35579	-1.35404	-1.35229	-1.35058	-1.40511	-1.40327
-1.36011	-1.35824	-1.38231	-1.38047	-1.40965	-1.40768	-1.40574	-1.42124	-1.41939
-1.35889	-1.35705	-1.3811	-1.37927	-1.40834	-1.40641	-1.40451	-1.42001	-1.41816
-1.35772	-1.35589	-1.37991	-1.37809	-1.40704	-1.40515	-1.40329	-1.4188	-1.41694
-1.38242	-1.38055	-1.39647	-1.39466	-1.42313	-1.42125	-1.41941	-1.41755	-1.41569
-1.38118	-1.37935	-1.39528	-1.3935	-1.42184	-1.41997	-1.41814	-1.41628	-1.41443
-1.39773	-1.39591	-1.42445	-1.4225	-1.42058	-1.41872	-1.41689	-1.41503	-1.41318
-1.39657	-1.39476	-1.42317	-1.42125	-1.41936	-1.4175	-1.41565	-1.41379	-1.41194
-1.42583	-1.42386	-1.42192	-1.42001	-1.44481	-1.44288	-1.44096	-1.51032	-1.50819
-1.42455	-1.42262	-1.4207	-1.41879	-1.44353	-1.44158	-1.43966	-1.50887	-1.50678
-1.42328	-1.42138	-1.41948	-1.41757	-1.44225	-1.44029	-1.43837	-1.50747	-1.50544
-1.42204	-1.42013	-1.44488	-1.4429	-1.51243	-1.51026	-1.50814	-1.52936	-1.52725
-1.42081	-1.41888	-1.44356	-1.44159	-1.51098	-1.50884	-1.50676	-1.52795	-1.52596
-1.493	-1.49092	-1.51391	-1.51171	-1.53314	-1.53086	-1.52867	-1.52661	-1.52466
-1.49162	-1.48958	-1.51249	-1.5103	-1.53161	-1.52944	-1.52736	-1.52533	-1.52335
-1.51539	-1.51325	-1.53483	-1.53246	-1.53018	-1.52807	-1.52605	-1.52403	-1.52204
-1.51397	-1.51185	-1.53335	-1.53106	-1.52885	-1.52675	-1.52473	-1.52271	-1.52072
-1.53642	-1.53413	-1.53188	-1.52967	-1.52752	-1.52544	-1.5234	-1.52139	-1.51942
-1.53492	-1.53261	-1.53038	-1.52825	-1.52617	-1.5241	-1.52207	-1.5201	-1.51818
-1.53343	-1.53112	-1.52892	-1.52685	-1.52482	-1.52276	-1.52075	-1.51882	-1.51695
-1.53194	-1.5297	-1.52755	-1.52549	-1.52347	-1.52145	-1.51947	-1.51756	-1.51571

-1.53047	-1.52833	-1.52623	-1.52416	-1.52214	-1.52016	-1.51822	-1.51633	-1.51446
-1.52911	-1.52702	-1.52494	-1.52287	-1.52084	-1.51889	-1.51698	-1.51509	-1.51323
-1.52785	-1.52576	-1.52369	-1.52161	-1.51958	-1.51764	-1.51575	-1.51387	-1.51202
-1.52654	-1.52447	-1.52241	-1.52034	-1.51832	-1.51639	-1.51451	-1.51265	-1.51082
-1.52519	-1.52314	-1.5211	-1.51906	-1.51707	-1.51514	-1.51326	-1.51143	-1.50964
-1.52383	-1.52181	-1.5198	-1.51781	-1.51585	-1.51393	-1.51204	-1.53143	-1.52951
-1.52249	-1.5205	-1.51854	-1.5166	-1.51469	-1.51278	-1.51089	-1.53018	-1.52823
-1.52117	-1.51921	-1.51729	-1.51541	-1.51353	-1.51164	-1.50975	-1.52895	-1.52696
-1.51989	-1.51796	-1.51606	-1.51419	-1.51232	-1.51045	-1.50858	-1.52769	-1.52569
-1.51865	-1.51674	-1.51485	-1.51296	-1.51109	-1.50924	-1.50739	-1.52644	-1.52444
-1.51741	-1.51554	-1.51367	-1.51179	-1.53119	-1.52919	-1.5272	-1.57252	-1.57035
-1.51617	-1.51435	-1.51252	-1.51065	-1.52996	-1.52797	-1.52599	-1.57121	-1.56906
-1.51497	-1.51317	-1.55371	-1.55164	-1.57635	-1.57417	-1.57201	-1.58542	-1.58326
-1.51382	-1.51198	-1.55235	-1.55032	-1.57496	-1.57278	-1.57061	-1.584	-1.58184
-1.55518	-1.55308	-1.57789	-1.57573	-1.58918	-1.58698	-1.58479	-1.61435	-1.612
-1.55392	-1.55182	-1.57655	-1.57438	-1.58783	-1.58564	-1.58346	-1.61291	-1.61062
-1.55265	-1.55059	-1.57523	-1.57305	-1.58648	-1.5843	-1.58215	-1.61151	-1.60928
-1.5939	-1.59168	-1.58948	-1.58727	-1.60425	-1.60205	-1.5999	-1.69428	-1.692
-1.59242	-1.59025	-1.58807	-1.58586	-1.60281	-1.60064	-1.59853	-1.69276	-1.69052
-1.61028	-1.60808	-1.60587	-1.60364	-1.69831	-1.69591	-1.69357	-1.6913	-1.6891
-1.60894	-1.60671	-1.6045	-1.6023	-1.69682	-1.69447	-1.69218	-1.68993	-1.68772
-1.69322	-1.69087	-1.70012	-1.6977	-1.69534	-1.69303	-1.69077	-1.68854	-1.68634
-1.6917	-1.68934	-1.69851	-1.69615	-1.69384	-1.69155	-1.6893	-1.68711	-1.68495
-1.6902	-1.68786	-1.69694	-1.69463	-1.69236	-1.69009	-1.68785	-1.68567	-1.68356
-1.68879	-1.68649	-1.69549	-1.6932	-1.69094	-1.68868	-1.68645	-1.68428	-1.68215
-1.68741	-1.68517	-1.6941	-1.69181	-1.68955	-1.68731	-1.68509	-1.68291	-1.68075
-1.69735	-1.69501	-1.6927	-1.69042	-1.68817	-1.68594	-1.68373	-1.68154	-1.67938
-1.69587	-1.69359	-1.69132	-1.68905	-1.6868	-1.68457	-1.68236	-1.68019	-1.67805
-1.69437	-1.69212	-1.68987	-1.68761	-1.68537	-1.68315	-1.68096	-1.67879	-1.67666
-1.69284	-1.69058	-1.68833	-1.68609	-1.68388	-1.68168	-1.6795	-1.67734	-1.67519
-1.69134	-1.68906	-1.68682	-1.68461	-1.68241	-1.68024	-1.67808	-1.72096	-1.71849
-1.68991	-1.68763	-1.68538	-1.68319	-1.68101	-1.67886	-1.67672	-1.71943	-1.71698
-1.6885	-1.68623	-1.68399	-1.6818	-1.67965	-1.67751	-1.67539	-1.71793	-1.71552
-1.6871	-1.68485	-1.68264	-1.68046	-1.72373	-1.72127	-1.71884	-1.71645	-1.71409
-1.68571	-1.68351	-1.68132	-1.67914	-1.72223	-1.71975	-1.71732	-1.71497	-1.71269
-1.68433	-1.68215	-1.67998	-1.67781	-1.72071	-1.71824	-1.71581	-1.71348	-1.71124
-1.68295	-1.68079	-1.67863	-1.67646	-1.7192	-1.71673	-1.7143	-1.71198	-1.70975
-1.68158	-1.6794	-1.72258	-1.72012	-1.71767	-1.71523	-1.71282	-1.71052	-1.70829
-1.6802	-1.67798	-1.72095	-1.71851	-1.71611	-1.71372	-1.71138	-1.70911	-1.70689
-1.72441	-1.72183	-1.71933	-1.71693	-1.71457	-1.71225	-1.70996	-1.70772	-1.7055
-1.72281	-1.72026	-1.7178	-1.71544	-1.71312	-1.71083	-1.70856	-1.70633	-1.70412
-1.72121	-1.71872	-1.71631	-1.71399	-1.7117	-1.70943	-1.70717	-1.70495	-1.70275
-1.71968	-1.71724	-1.71486	-1.71256	-1.72708	-1.7248	-1.72255	-1.72033	-1.71814
-1.71819	-1.7158	-1.71345	-1.71115	-1.72566	-1.7234	-1.72117	-1.71897	-1.7168
-1.73353	-1.73117	-1.72884	-1.72652	-1.72424	-1.722	-1.7198	-1.75485	-1.75247
-1.73205	-1.72973	-1.72743	-1.72512	-1.72284	-1.72063	-1.71844	-1.75337	-1.75099
-1.73058	-1.7283	-1.7397	-1.73738	-1.73509	-1.73287	-1.73069	-1.75188	-1.74948
-1.72914	-1.72686	-1.73827	-1.73597	-1.7337	-1.73151	-1.72933	-1.75036	-1.74793
-1.7277	-1.72542	-1.73684	-1.73457	-1.73232	-1.73013	-1.72795	-1.74884	-1.74638
-1.73999	-1.73769	-1.73542	-1.73318	-1.75451	-1.7521	-1.7497	-1.74728	-1.74486

-1.73855	-1.73626	-1.734	-1.73179	-1.753	-1.75055	-1.74811	-1.74571	-1.74335
-1.73712	-1.73485	-1.75629	-1.75387	-1.75145	-1.749	-1.74656	-1.74419	-1.74186
-1.7357	-1.73346	-1.75477	-1.75232	-1.74988	-1.74744	-1.74503	-1.74269	-1.7404
-1.73429	-1.73208	-1.75326	-1.75079	-1.74834	-1.74592	-1.74353	-1.74121	-1.73894
-1.73291	-1.73071	-1.75177	-1.74929	-1.74683	-1.74442	-1.74205	-1.73975	-1.7375
-1.75511	-1.75272	-1.75028	-1.7478	-1.74534	-1.74295	-1.7406	-1.73831	-1.73607
-1.75362	-1.75123	-1.7488	-1.74632	-1.74387	-1.74149	-1.73916	-1.73688	-1.73465
-1.75213	-1.74974	-1.74731	-1.74484	-1.74241	-1.74004	-1.73773	-1.73547	-1.73325
-1.75064	-1.74824	-1.74582	-1.74336	-1.74094	-1.7386	-1.73631	-1.80423	-1.80152
-1.74915	-1.74674	-1.74433	-1.74188	-1.73948	-1.73717	-1.7349	-1.80252	-1.79984
-1.74765	-1.74526	-1.74285	-1.74042	-1.73803	-1.73574	-1.7335	-1.83459	-1.83189
-1.74614	-1.74378	-1.7414	-1.73898	-1.7366	-1.73432	-1.73211	-1.83288	-1.83019
-1.74464	-1.74231	-1.73995	-1.73755	-1.8056	-1.80283	-1.80013	-1.83118	-1.82851
-1.74317	-1.74086	-1.73852	-1.73614	-1.80389	-1.80114	-1.79846	-1.82949	-1.82684
-1.7417	-1.73941	-1.80793	-1.80505	-1.80222	-1.79948	-1.7968	-1.83919	-1.83637
-1.74023	-1.73796	-1.80622	-1.80339	-1.8006	-1.79787	-1.79518	-1.83751	-1.83469
-1.73877	-1.73652	-1.80452	-1.80175	-1.799	-1.79627	-1.79356	-1.83583	-1.83302
-1.80829	-1.80554	-1.8028	-1.80007	-1.80977	-1.80686	-1.80397	-1.84809	-1.84528
-1.80653	-1.80379	-1.80107	-1.79837	-1.80806	-1.80517	-1.80231	-1.84643	-1.84364
-1.80479	-1.80207	-1.81209	-1.80922	-1.82072	-1.81784	-1.81499	-1.84478	-1.84201
-1.80307	-1.80036	-1.81037	-1.80753	-1.81905	-1.81618	-1.81335	-1.84313	-1.84038
-1.80139	-1.79868	-1.82306	-1.82022	-1.81739	-1.81455	-1.81173	-1.85743	-1.85461
-1.79977	-1.79704	-1.82138	-1.81856	-1.81576	-1.81295	-1.81017	-1.8558	-1.85297
-1.79816	-1.79543	-1.81974	-1.81692	-1.81413	-1.81136	-1.80861	-1.85418	-1.85134
-1.82392	-1.82103	-1.81816	-1.81532	-1.84508	-1.84229	-1.83952	-1.85716	-1.85432
-1.82228	-1.81945	-1.8166	-1.81373	-1.84344	-1.84065	-1.83789	-1.85551	-1.85269
-1.82063	-1.81782	-1.815	-1.81212	-1.86233	-1.85948	-1.85668	-1.85388	-1.85111
-1.81896	-1.81617	-1.81336	-1.8105	-1.86066	-1.85781	-1.85502	-1.85228	-1.84957
-1.8223	-1.81954	-1.83298	-1.83007	-1.85902	-1.85617	-1.85338	-1.85067	-1.84802
-1.82068	-1.81791	-1.83132	-1.82846	-1.85742	-1.85456	-1.85175	-1.84905	-1.84644
-1.81907	-1.8163	-1.82967	-1.82685	-1.8558	-1.85295	-1.85014	-1.84746	-1.84487
-1.81751	-1.81471	-1.82803	-1.8252	-1.85415	-1.85133	-1.84856	-1.8459	-1.84333
-1.81594	-1.81313	-1.8264	-1.82357	-1.8525	-1.84973	-1.84701	-1.84437	-1.84181
-1.8305	-1.82764	-1.85657	-1.85371	-1.86427	-1.86153	-1.85885	-1.85621	-1.85362
-1.82884	-1.82604	-1.85499	-1.85214	-1.86271	-1.86001	-1.85738	-1.85475	-1.85214
-1.84096	-1.83817	-1.86674	-1.86391	-1.86114	-1.85847	-1.85587	-1.85327	-1.85068
-1.83931	-1.83652	-1.86508	-1.8623	-1.85957	-1.85693	-1.85434	-1.85178	-1.84925
-1.8377	-1.83492	-1.86348	-1.86073	-1.85804	-1.85541	-1.85283	-1.85029	-1.84779
-1.83616	-1.83339	-1.86194	-1.85922	-1.85655	-1.85391	-1.85132	-1.84878	-1.84629
-1.86596	-1.86317	-1.86043	-1.85773	-1.85507	-1.85244	-1.84984	-1.8473	-1.84481
-1.8644	-1.86165	-1.85893	-1.85624	-1.85359	-1.85098	-1.84841	-1.84588	-1.8434
-1.86285	-1.86013	-1.85744	-1.85475	-1.85211	-1.84954	-1.847	-1.84449	-1.84201
-1.86132	-1.85861	-1.85593	-1.85327	-1.85065	-1.8481	-1.84559	-1.84311	-1.84065
-1.85981	-1.8571	-1.85442	-1.85178	-1.84919	-1.84667	-1.84419	-1.84173	-1.8393
-1.85829	-1.85558	-1.85293	-1.85031	-1.85989	-1.85739	-1.85493	-1.85249	-1.85006
-1.85675	-1.85407	-1.85144	-1.84884	-1.85844	-1.85598	-1.85355	-1.85111	-1.84868
-1.86737	-1.8647	-1.87426	-1.87167	-1.86915	-1.86669	-1.86427	-1.89035	-1.88788
-1.86586	-1.8632	-1.87275	-1.8702	-1.86769	-1.86525	-1.86283	-1.88889	-1.88644
-1.86436	-1.86171	-1.87128	-1.86875	-1.86626	-1.86382	-1.86141	-1.88744	-1.88502
-1.87501	-1.87239	-1.86984	-1.86734	-1.89338	-1.89091	-1.88847	-1.88604	-1.88364

-1.8735	-1.87095	-1.86843	-1.86596	-1.89198	-1.88952	-1.88709	-1.88467	-1.88229
-1.87209	-1.86956	-1.89556	-1.89305	-1.89057	-1.88813	-1.88571	-1.88332	-1.88094
-1.87074	-1.86824	-1.89419	-1.89165	-1.88916	-1.88674	-1.88436	-1.88197	-1.87961
-1.89788	-1.89534	-1.89282	-1.89028	-1.88779	-1.88537	-1.88299	-1.88063	-1.87829
-1.89646	-1.89395	-1.89145	-1.88894	-1.88647	-1.88402	-1.88162	-1.87928	-1.87698
-1.89503	-1.89254	-1.89006	-1.88759	-1.88514	-1.88269	-1.88028	-1.87795	-1.87568
-1.89361	-1.89111	-1.88864	-1.88621	-1.88379	-1.88138	-1.87899	-1.87665	-1.87435
-1.89217	-1.88968	-1.88722	-1.88482	-1.88244	-1.88007	-1.87771	-1.87536	-1.87304
-1.89073	-1.88825	-1.88582	-1.88343	-1.88107	-1.87873	-1.87641	-1.87408	-1.87176
-1.88928	-1.88684	-1.88443	-1.88205	-1.87969	-1.87738	-1.8751	-1.8728	-1.8705
-1.88787	-1.88543	-1.88303	-1.88067	-1.87835	-1.87605	-1.87378	-1.8715	-1.86923
-1.8865	-1.88403	-1.88163	-1.87931	-1.87702	-1.87473	-1.87245	-1.87019	-1.86794
-1.88515	-1.88267	-1.88027	-1.87797	-1.87568	-1.8734	-1.87112	-1.86887	-1.86664
-1.88382	-1.88138	-1.87898	-1.87664	-1.87433	-1.87206	-1.86981	-1.86757	-1.86533
-1.88247	-1.88008	-1.8777	-1.87534	-1.87301	-1.87074	-1.8685	-1.86625	-1.86402
-1.88108	-1.87875	-1.87641	-1.87405	-1.87172	-1.86944	-1.86718	-1.86494	-1.86271
-1.8797	-1.87741	-1.8751	-1.87278	-1.87046	-1.86816	-1.86587	-1.86363	-1.86141
-1.87837	-1.87607	-1.87378	-1.87148	-1.86919	-1.86689	-1.8646	-1.86235	-1.86011
-1.87707	-1.87474	-1.87245	-1.87018	-1.86792	-1.86564	-1.86336	-1.86109	-1.85883
-1.87579	-1.87345	-1.87115	-1.8689	-1.86666	-1.86439	-1.86212	-1.85984	-1.85756
-1.87452	-1.87218	-1.86988	-1.86764	-1.8654	-1.86315	-1.86088	-1.8586	-1.85632
-1.87326	-1.87091	-1.86861	-1.86636	-1.86413	-1.86188	-1.85962	-1.85735	-1.85508
-1.87199	-1.86965	-1.86734	-1.86506	-1.86281	-1.86057	-1.85834	-1.85609	-1.85384
-1.87072	-1.86839	-1.86607	-1.86377	-1.86149	-1.85926	-1.85704	-1.85481	-1.85259
-1.86945	-1.86713	-1.86482	-1.86251	-1.86022	-1.85796	-1.85572	-1.8535	-1.8513
-1.86817	-1.86588	-1.86358	-1.86127	-1.85896	-1.85667	-1.85439	-1.85218	-1.85
-1.86688	-1.86461	-1.86233	-1.86001	-1.8577	-1.85539	-1.85311	-1.85088	-1.84869
-1.86556	-1.86333	-1.86107	-1.85875	-1.85643	-1.85414	-1.85186	-1.8496	-1.84736
-1.86427	-1.86206	-1.8598	-1.85749	-1.85518	-1.85289	-1.85061	-1.87839	-1.87607
-1.86301	-1.86078	-1.85853	-1.85624	-1.85394	-1.85165	-1.84936	-1.87714	-1.87482
-1.86174	-1.85949	-1.85724	-1.85497	-1.88293	-1.88059	-1.87824	-1.88752	-1.88519
-1.86046	-1.85816	-1.85589	-1.85366	-1.88166	-1.87933	-1.87699	-1.88626	-1.88393
-1.85918	-1.85684	-1.85456	-1.85235	-1.88039	-1.87807	-1.87574	-1.88501	-1.88267
-1.85791	-1.85556	-1.8952	-1.89297	-1.89073	-1.88842	-1.88609	-1.88375	-1.88141
-1.85664	-1.8543	-1.89395	-1.89172	-1.88948	-1.88717	-1.88483	-1.88249	-1.88015
-1.89735	-1.89499	-1.89268	-1.89045	-1.8882	-1.8859	-1.88357	-1.88124	-1.87889
-1.89607	-1.89372	-1.89141	-1.88917	-1.88692	-1.88462	-1.8823	-1.87998	-1.87765
-1.89478	-1.89243	-1.89012	-1.88785	-1.88559	-1.88331	-1.88102	-1.87872	-1.8764
-1.89348	-1.89114	-1.88882	-1.88651	-1.88422	-1.88196	-1.87971	-1.87744	-1.87515
-1.89219	-1.88985	-1.88751	-1.88517	-1.88286	-1.88062	-1.8784	-1.87615	-1.87389
-1.8909	-1.88855	-1.88621	-1.88387	-1.88156	-1.87933	-1.87712	-1.87488	-1.87263
-1.88962	-1.88726	-1.88492	-1.88259	-1.8803	-1.87806	-1.87585	-1.87361	-1.87137
-1.88833	-1.88598	-1.88365	-1.88135	-1.87908	-1.87683	-1.8746	-1.87235	-1.8701
-1.88705	-1.88469	-1.88238	-1.88013	-1.87789	-1.87563	-1.87337	-1.8711	-1.86883
-1.88576	-1.88342	-1.88112	-1.87888	-1.87665	-1.87439	-1.87212	-1.86985	-1.86757
-1.88448	-1.88215	-1.87985	-1.87762	-1.87539	-1.87313	-1.87086	-1.86859	-1.8663
-1.88321	-1.8809	-1.87861	-1.87637	-1.87412	-1.87187	-1.8696	-1.86732	-1.86503
-1.88196	-1.87969	-1.87742	-1.87515	-1.87288	-1.8706	-1.86832	-1.86603	-1.86375
-1.88072	-1.87848	-1.87621	-1.87392	-1.87163	-1.86933	-1.86703	-1.86475	-1.86246
-1.87953	-1.87725	-1.87496	-1.87266	-1.87036	-1.86806	-1.86576	-1.86346	-1.86117

-1.87834	-1.87602	-1.8737	-1.87139	-1.86909	-1.86678	-1.86448	-1.86217	-1.85987
-1.8771	-1.87476	-1.87243	-1.87011	-1.8678	-1.8655	-1.86319	-1.86088	-1.85857
-1.87583	-1.87349	-1.87115	-1.86883	-1.86651	-1.8642	-1.86189	-1.85959	-1.85728
-1.87454	-1.8722	-1.86987	-1.86754	-1.86521	-1.8629	-1.86059	-1.85828	-1.85597
-1.87324	-1.8709	-1.86857	-1.86625	-1.86393	-1.8616	-1.85928	-1.85697	-1.85466
-1.87195	-1.86962	-1.86729	-1.86497	-1.86264	-1.86032	-1.85799	-1.85567	-1.85336
-1.87067	-1.86834	-1.86601	-1.86369	-1.86136	-1.85904	-1.85672	-1.85439	-1.85206
-1.86942	-1.86708	-1.86475	-1.86243	-1.86011	-1.85779	-1.85546	-1.85313	-1.8508
-1.86823	-1.8659	-1.86357	-1.86124	-1.85891	-1.85658	-1.85426	-1.85192	-1.84959
-1.86706	-1.86473	-1.8624	-1.86007	-1.85773	-1.85539	-1.85306	-1.85073	-1.8484
-1.86582	-1.86349	-1.86116	-1.85882	-1.85649	-1.85414	-1.8518	-1.84947	-1.84715
-1.86453	-1.8622	-1.85987	-1.85753	-1.8552	-1.85285	-1.85051	-1.84818	-1.84584
-1.86325	-1.8609	-1.85856	-1.85623	-1.85389	-1.85155	-1.84921	-1.84687	-1.84454
-1.86196	-1.8596	-1.85725	-1.8549	-1.85256	-1.85022	-1.84788	-1.84555	-1.84322
-1.86069	-1.85832	-1.85595	-1.85359	-1.85124	-1.8489	-1.84657	-1.84424	-1.84191
-1.85947	-1.85707	-1.85468	-1.8523	-1.84994	-1.8476	-1.84527	-1.84294	-1.84061
-1.85826	-1.85583	-1.85342	-1.85104	-1.84867	-1.84632	-1.84399	-1.84165	-1.83933
-1.857	-1.85458	-1.85219	-1.84981	-1.84745	-1.84511	-1.84277	-1.84044	-1.8381
-1.85573	-1.85334	-1.85096	-1.8486	-1.84625	-1.84392	-1.84159	-1.83925	-1.8369
-1.85444	-1.85207	-1.84971	-1.84736	-1.84501	-1.84268	-1.84035	-1.83801	-1.83565
-1.85313	-1.85077	-1.84842	-1.84608	-1.84373	-1.8414	-1.83907	-1.83672	-1.83436
-1.85185	-1.8495	-1.84716	-1.84482	-1.84248	-1.84014	-1.8378	-1.83545	-1.8331
-1.85063	-1.84828	-1.84594	-1.84361	-1.84127	-1.83893	-1.83658	-1.83423	-1.83188
-1.8494	-1.84705	-1.84471	-1.84238	-1.84004	-1.8377	-1.83535	-1.83299	-1.83064
-1.84813	-1.84577	-1.84342	-1.84109	-1.83875	-1.8364	-1.83404	-1.83169	-1.82933
-1.84685	-1.84449	-1.84214	-1.83979	-1.83744	-1.83509	-1.83273	-1.83037	-1.828
-1.84561	-1.84325	-1.84089	-1.83854	-1.83619	-1.83383	-1.83147	-1.82909	-1.8267
-1.84438	-1.84203	-1.83967	-1.83732	-1.83496	-1.8326	-1.83023	-1.82783	-1.82541
-1.84313	-1.84077	-1.83841	-1.83605	-1.83369	-1.83133	-1.82896	-1.82656	-1.82413
-1.84185	-1.83947	-1.83709	-1.83473	-1.83237	-1.83001	-1.82765	-1.82527	-1.82288
-1.84058	-1.83819	-1.83581	-1.83344	-1.84338	-1.84101	-1.83864	-1.83626	-1.83386
-1.83934	-1.83695	-1.83457	-1.83221	-1.84213	-1.83977	-1.83739	-1.83499	-1.83256
-1.83812	-1.83571	-1.83333	-1.83097	-1.84089	-1.83852	-1.83614	-1.83372	-1.83127
-1.83688	-1.83445	-1.84434	-1.84196	-1.83959	-1.83722	-1.83484	-1.83244	-1.83001
-1.83565	-1.83318	-1.84303	-1.84064	-1.83827	-1.8359	-1.83354	-1.83116	-1.82876
-1.84669	-1.84422	-1.84178	-1.83939	-1.83702	-1.83464	-1.83227	-1.8299	-1.82752
-1.84541	-1.84299	-1.84058	-1.83819	-1.83581	-1.83342	-1.83103	-1.82865	-1.82628
-1.84416	-1.84176	-1.83937	-1.83698	-1.83459	-1.83218	-1.82977	-1.82738	-1.825
-1.84296	-1.84055	-1.83815	-1.83577	-1.83337	-1.83093	-1.82849	-1.82607	-1.82368
-1.84175	-1.83933	-1.83692	-1.83453	-1.83212	-1.82967	-1.82721	-1.82478	-1.82238
-1.84052	-1.83807	-1.83563	-1.83323	-1.83083	-1.8284	-1.82597	-1.82355	-1.82113
-1.83928	-1.8368	-1.83435	-1.83194	-1.82954	-1.82714	-1.82473	-1.82232	-1.81991
-1.83802	-1.83555	-1.8331	-1.83069	-1.82829	-1.82589	-1.8235	-1.82109	-1.81867
-1.83675	-1.8343	-1.83187	-1.82947	-1.82707	-1.82466	-1.82225	-1.81984	-1.81743
-1.83551	-1.83307	-1.83065	-1.82825	-1.82584	-1.82341	-1.82098	-1.83025	-1.82779
-1.83428	-1.83186	-1.82944	-1.82704	-1.82462	-1.82215	-1.81967	-1.8289	-1.82643
-1.83307	-1.83065	-1.82823	-1.82581	-1.82336	-1.82087	-1.81838	-1.82758	-1.8251
-1.83187	-1.82946	-1.82702	-1.82455	-1.82207	-1.81959	-1.81712	-1.8263	-1.82382
-1.83067	-1.82826	-1.82581	-1.8233	-1.82079	-1.81832	-1.81587	-1.82503	-1.82256
-1.82948	-1.82706	-1.82461	-1.82208	-1.81956	-1.81708	-1.81463	-1.82377	-1.8213

-1.82827	-1.82585	-1.82339	-1.82086	-1.81834	-1.81586	-1.8134	-1.82252	-1.82005
-1.82704	-1.8246	-1.82212	-1.81961	-1.8171	-1.81462	-1.81216	-1.82126	-1.81879
-1.8258	-1.82331	-1.82082	-1.81833	-1.81585	-1.81338	-1.81092	-1.82	-1.81752
-1.82457	-1.82205	-1.81955	-1.81707	-1.82624	-1.82373	-1.82123	-1.81873	-1.81623
-1.82334	-1.82081	-1.8183	-1.81582	-1.82497	-1.82247	-1.81996	-1.81744	-1.8149
-1.82212	-1.81958	-1.81706	-1.81458	-1.82371	-1.8212	-1.8187	-1.81616	-1.8136
-1.8209	-1.81835	-1.81582	-1.81334	-1.82244	-1.81994	-1.81744	-1.8149	-1.81233
-1.81968	-1.81712	-1.81459	-1.8121	-1.82118	-1.81868	-1.81619	-1.81365	-1.81107
-1.81845	-1.81588	-1.81335	-1.81086	-1.81992	-1.81743	-1.81495	-1.8124	-1.80982
-1.81721	-1.81465	-1.81211	-1.80962	-1.81866	-1.81618	-1.8137	-1.81116	-1.80857
-1.81598	-1.81341	-1.81088	-1.80838	-1.8174	-1.81492	-1.81244	-1.80989	-1.80731
-1.81475	-1.81218	-1.80964	-1.80715	-1.81615	-1.81366	-1.81117	-1.80862	-1.80604
-1.81351	-1.81095	-1.81994	-1.81741	-1.81489	-1.81238	-1.80987	-1.80732	-1.80475
-1.81227	-1.80971	-1.81869	-1.81616	-1.81363	-1.81109	-1.80854	-1.80599	-1.80344
-1.81104	-1.80847	-1.81743	-1.81491	-1.81237	-1.80981	-1.80725	-1.8047	-1.80215
-1.8098	-1.80724	-1.81618	-1.81366	-1.81113	-1.8086	-1.80605	-1.80348	-1.80091
-1.80857	-1.80601	-1.81493	-1.81241	-1.8099	-1.80738	-1.80486	-1.80228	-1.79968
-1.80736	-1.80478	-1.81368	-1.81116	-1.80864	-1.80614	-1.80362	-1.80104	-1.79842
-1.80614	-1.80356	-1.81244	-1.80991	-1.80739	-1.80488	-1.80235	-1.79977	-1.79716
-1.80493	-1.80235	-1.8112	-1.80866	-1.80613	-1.80362	-1.80109	-1.79851	-1.79589
-1.80371	-1.80113	-1.80996	-1.80741	-1.80488	-1.80235	-1.79983	-1.79724	-1.79462
-1.80251	-1.79995	-1.80876	-1.80619	-1.80363	-1.8011	-1.79857	-1.79599	-1.79336
-1.80134	-1.7988	-1.80759	-1.80499	-1.8024	-1.79986	-1.79733	-1.79474	-1.79212
-1.80016	-1.79764	-1.80642	-1.80379	-1.80117	-1.79862	-1.79608	-1.7935	-1.79089
-1.79896	-1.79644	-1.80521	-1.80256	-1.79994	-1.79738	-1.79484	-1.79227	-1.7897
-1.79777	-1.79524	-1.80398	-1.80134	-1.79871	-1.79614	-1.79359	-1.79104	-1.7885
-1.80796	-1.80536	-1.80276	-1.80014	-1.79753	-1.79493	-1.79235	-1.7898	-1.78727
-1.80679	-1.80415	-1.80153	-1.79894	-1.79635	-1.79373	-1.79111	-1.78855	-1.78602
-1.80558	-1.80292	-1.8003	-1.79773	-1.79515	-1.79251	-1.78987	-1.7873	-1.78477
-1.80435	-1.80169	-1.79907	-1.79649	-1.7939	-1.79126	-1.78862	-1.78605	-1.78351
-1.80311	-1.80047	-1.79785	-1.79526	-1.79266	-1.79003	-1.7874	-1.78482	-1.78226
-1.8019	-1.79929	-1.79668	-1.79406	-1.79144	-1.78883	-1.78623	-1.78364	-1.78105
-1.80068	-1.7981	-1.79549	-1.79284	-1.79021	-1.78762	-1.78505	-1.78246	-1.77985
-1.79943	-1.79686	-1.79425	-1.7916	-1.78896	-1.78638	-1.78382	-1.78124	-1.77866
-1.79819	-1.7956	-1.793	-1.79035	-1.78772	-1.78513	-1.78257	-1.78002	-1.77747
-1.79698	-1.79437	-1.79176	-1.78913	-1.78651	-1.78392	-1.78134	-1.77879	-1.77625
-1.79579	-1.79316	-1.79055	-1.78793	-1.78533	-1.78272	-1.78012	-1.77755	-1.775
-1.7946	-1.79197	-1.78935	-1.78674	-1.78414	-1.78153	-1.77892	-1.77634	-1.77377
-1.79341	-1.79079	-1.78818	-1.78556	-1.78295	-1.78035	-1.77776	-1.77516	-1.77256
-1.79222	-1.78961	-1.787	-1.78438	-1.78177	-1.77917	-1.77658	-1.77398	-1.77136
-1.79103	-1.78842	-1.7858	-1.7832	-1.78059	-1.77799	-1.77538	-1.77278	-1.77017
-1.78985	-1.78722	-1.7846	-1.78201	-1.77941	-1.7768	-1.77418	-1.77158	-1.76898
-1.78865	-1.78603	-1.78342	-1.78082	-1.77821	-1.7756	-1.77299	-1.77038	-1.76776
-1.78745	-1.78485	-1.78224	-1.77962	-1.77701	-1.77441	-1.77181	-1.76917	-1.76652
-1.78627	-1.78366	-1.78105	-1.77843	-1.77581	-1.77321	-1.7706	-1.76795	-1.76528
-1.78509	-1.78246	-1.77985	-1.77724	-1.77464	-1.77201	-1.76937	-1.76671	-1.76402
-1.78391	-1.78128	-1.77866	-1.77606	-1.77345	-1.77082	-1.76817	-1.7655	-1.76281
-1.78273	-1.78011	-1.77749	-1.77487	-1.77224	-1.76963	-1.767	-1.76434	-1.76166
-1.78155	-1.77894	-1.77632	-1.77368	-1.77105	-1.76844	-1.76583	-1.76318	-1.76051
-1.78039	-1.77776	-1.77513	-1.7725	-1.76988	-1.76725	-1.76463	-1.76199	-1.75935

-1.77922	-1.77658	-1.77394	-1.77133	-1.76871	-1.76607	-1.76342	-1.7608	-1.75818
-1.77804	-1.77541	-1.77278	-1.77015	-1.76752	-1.76488	-1.76224	-1.75961	-1.75698
-1.77686	-1.77426	-1.77163	-1.76897	-1.76631	-1.76369	-1.76107	-1.75842	-1.75576
-1.77572	-1.77312	-1.77049	-1.76782	-1.76514	-1.7625	-1.75987	-1.75722	-1.75457
-1.77461	-1.77201	-1.76938	-1.7667	-1.76401	-1.76133	-1.75866	-1.75601	-1.75339
-1.77349	-1.77087	-1.76823	-1.76556	-1.76287	-1.76017	-1.75747	-1.75482	-1.7522
-1.77234	-1.76969	-1.76703	-1.76439	-1.76173	-1.75903	-1.75633	-1.75365	-1.75099
-1.7712	-1.76851	-1.76584	-1.76321	-1.76057	-1.75789	-1.75518	-1.75249	-1.7498
-1.77009	-1.76738	-1.76469	-1.76203	-1.75936	-1.7567	-1.75403	-1.75134	-1.74864
-1.76897	-1.76627	-1.76356	-1.76084	-1.75815	-1.7555	-1.75286	-1.75019	-1.7475
-1.76781	-1.76513	-1.76242	-1.75969	-1.75698	-1.75432	-1.75167	-1.74901	-1.74634
-1.76662	-1.76397	-1.76129	-1.75856	-1.75584	-1.75314	-1.75045	-1.7478	-1.74517
-1.76544	-1.76279	-1.76012	-1.75742	-1.75471	-1.75199	-1.74928	-1.74662	-1.74398
-1.76427	-1.76158	-1.75891	-1.75625	-1.75358	-1.75087	-1.74815	-1.74545	-1.74278
-1.76312	-1.7604	-1.75772	-1.75509	-1.75245	-1.74975	-1.74703	-1.74431	-1.7416
-1.76199	-1.75925	-1.75657	-1.75394	-1.75131	-1.74862	-1.74591	-1.7432	-1.74048
-1.76087	-1.75812	-1.75542	-1.75279	-1.75016	-1.74748	-1.74479	-1.74209	-1.73936
-1.75974	-1.75699	-1.75428	-1.75161	-1.74896	-1.74631	-1.74365	-1.74096	-1.73825
-1.75861	-1.75586	-1.75314	-1.75043	-1.74775	-1.74512	-1.7425	-1.73983	-1.73713
-1.75749	-1.75474	-1.75201	-1.74928	-1.74659	-1.74397	-1.74137	-1.73871	-1.73601
-1.75636	-1.75362	-1.75089	-1.74816	-1.74546	-1.74284	-1.74024	-1.73758	-1.73489
-1.75524	-1.7525	-1.74976	-1.74703	-1.74434	-1.74171	-1.73911	-1.73646	-1.73377
-1.7541	-1.75137	-1.74864	-1.74591	-1.74321	-1.74059	-1.73798	-1.73533	-1.73265
-1.75298	-1.75025	-1.74752	-1.74479	-1.74209	-1.73945	-1.73683	-1.73418	-1.73152
-1.75186	-1.74913	-1.74641	-1.74367	-1.74096	-1.73829	-1.73564	-1.733	-1.73037
-1.75074	-1.74802	-1.74529	-1.74255	-1.73983	-1.73712	-1.73444	-1.73181	-1.72921
-1.74964	-1.74691	-1.74418	-1.74144	-1.7387	-1.73598	-1.73329	-1.73066	-1.72808
-1.74855	-1.74581	-1.74307	-1.74033	-1.73759	-1.73486	-1.73216	-1.72954	-1.72696
-1.74746	-1.74471	-1.74197	-1.73922	-1.73648	-1.73376	-1.73106	-1.72843	-1.72584
-1.74637	-1.74362	-1.74087	-1.73812	-1.73538	-1.73266	-1.72997	-1.72733	-1.72474
-1.74528	-1.74252	-1.73977	-1.73702	-1.73429	-1.73159	-1.7289	-1.72626	-1.72365
-1.74418	-1.74143	-1.73868	-1.73593	-1.7332	-1.73053	-1.72789	-1.72523	-1.72258
-1.74308	-1.74033	-1.73758	-1.73483	-1.73212	-1.72948	-1.72686	-1.7242	-1.72151
-1.74198	-1.73923	-1.73648	-1.73374	-1.73103	-1.7284	-1.72578	-1.72311	-1.72041
-1.74088	-1.73813	-1.73539	-1.73266	-1.72996	-1.72731	-1.72469	-1.72201	-1.71931
-1.73979	-1.73704	-1.73431	-1.73161	-1.72892	-1.72626	-1.7236	-1.72091	-1.7182
-1.73869	-1.73596	-1.73326	-1.73058	-1.72791	-1.72522	-1.72252	-1.71981	-1.71708
-1.73762	-1.73491	-1.73222	-1.72955	-1.72687	-1.72416	-1.72143	-1.7187	-1.71596
-1.73657	-1.73389	-1.73121	-1.72851	-1.7258	-1.72307	-1.72033	-1.71758	-1.71483
-1.73553	-1.73287	-1.73018	-1.72746	-1.72472	-1.72197	-1.71922	-1.71647	-1.71372
-1.73451	-1.73182	-1.72912	-1.72638	-1.72363	-1.72087	-1.71811	-1.71535	-1.7126
-1.73348	-1.73077	-1.72804	-1.72528	-1.72252	-1.71976	-1.717	-1.71425	-1.71151
-1.73242	-1.72969	-1.72694	-1.72418	-1.72142	-1.71866	-1.7159	-1.71317	-1.71046
-1.73133	-1.72859	-1.72584	-1.72308	-1.72032	-1.71756	-1.71481	-1.7121	-1.70943
-1.73026	-1.72751	-1.72475	-1.72199	-1.71923	-1.71649	-1.71376	-1.71105	-1.70837
-1.72919	-1.72643	-1.72367	-1.7209	-1.71815	-1.71543	-1.71273	-1.71002	-1.7073
-1.72815	-1.72539	-1.72262	-1.71985	-1.7171	-1.71439	-1.71168	-1.70896	-1.70623
-1.72716	-1.7244	-1.72164	-1.71887	-1.7161	-1.71336	-1.71062	-1.70788	-1.70514
-1.71557	-1.71284	-1.72064	-1.71786	-1.71509	-1.71232	-1.70956	-1.7068	-1.70406
-1.71453	-1.71179	-1.71957	-1.7168	-1.71403	-1.71125	-1.70848	-1.70572	-1.70297

-1.71346	-1.71072	-1.71849	-1.71573	-1.71297	-1.71019	-1.70741	-1.70465	-1.70191
-1.71239	-1.70964	-1.71742	-1.71468	-1.71194	-1.70918	-1.70641	-1.70365	-1.70089
-1.71133	-1.70857	-1.71635	-1.71364	-1.71093	-1.70819	-1.70543	-1.70267	-1.69991
-1.71032	-1.70756	-1.70483	-1.70215	-1.70991	-1.70716	-1.70441	-1.70165	-1.69889
-1.70936	-1.7066	-1.70386	-1.70114	-1.70887	-1.70612	-1.70336	-1.70059	-1.69783
-1.70837	-1.70561	-1.70287	-1.70014	-1.70785	-1.70509	-1.70233	-1.69957	-1.6968
-1.70732	-1.70456	-1.70183	-1.69913	-1.70685	-1.70411	-1.70135	-1.69859	-1.69582
-1.70629	-1.70354	-1.70081	-1.69813	-1.69544	-1.69271	-1.68998	-1.6976	-1.69482
-1.70534	-1.70259	-1.69985	-1.69714	-1.69442	-1.69169	-1.68894	-1.69655	-1.69377
-1.7044	-1.70166	-1.69891	-1.69616	-1.69341	-1.69066	-1.6879	-1.69549	-1.69271
-1.70342	-1.7007	-1.69797	-1.69521	-1.69244	-1.68968	-1.68692	-1.69449	-1.6917
-1.70241	-1.69973	-1.69702	-1.69427	-1.6915	-1.68873	-1.68596	-1.69352	-1.69073
-1.70143	-1.69875	-1.69604	-1.69328	-1.69051	-1.68774	-1.68496	-1.68218	-1.67941
-1.70048	-1.69776	-1.69502	-1.69225	-1.68947	-1.6867	-1.68391	-1.68113	-1.67835
-1.69953	-1.69678	-1.69401	-1.69124	-1.68846	-1.68568	-1.68289	-1.68011	-1.67733
-1.69861	-1.69584	-1.69306	-1.69028	-1.6875	-1.68471	-1.68193	-1.67915	-1.67638
-1.69767	-1.69488	-1.6921	-1.68931	-1.68653	-1.68374	-1.68096	-1.6782	-1.67544
-1.69665	-1.69386	-1.69107	-1.68828	-1.68549	-1.68271	-1.67994	-1.67719	-1.67446
-1.69562	-1.69283	-1.69003	-1.68723	-1.68445	-1.68166	-1.67889	-1.67617	-1.67348
-1.69465	-1.69185	-1.68905	-1.68625	-1.68346	-1.68068	-1.67791	-1.67518	-1.67248
-1.69372	-1.69092	-1.68812	-1.68532	-1.68252	-1.67974	-1.67696	-1.6742	-1.67146
-1.6928	-1.69	-1.68719	-1.68439	-1.6816	-1.67881	-1.67603	-1.67325	-1.67048
-1.69189	-1.68909	-1.68628	-1.68348	-1.68069	-1.6779	-1.67511	-1.67233	-1.66955
-1.69099	-1.68818	-1.68538	-1.68258	-1.67978	-1.67699	-1.6742	-1.67142	-1.66863
-1.69008	-1.68728	-1.68447	-1.68167	-1.67888	-1.67608	-1.67329	-1.6705	-1.66771
-1.68917	-1.68637	-1.68356	-1.68076	-1.67797	-1.67517	-1.67238	-1.66958	-1.66678
-1.68825	-1.68544	-1.68264	-1.67984	-1.67705	-1.67426	-1.67146	-1.66864	-1.6658
-1.6873	-1.68449	-1.68169	-1.6789	-1.67611	-1.67332	-1.67052	-1.66768	-1.66481
-1.68631	-1.6835	-1.6807	-1.67791	-1.67512	-1.67233	-1.66953	-1.6667	-1.66383
-1.68527	-1.68245	-1.67965	-1.67686	-1.67407	-1.67129	-1.6685	-1.66569	-1.66287
-1.68427	-1.68145	-1.67865	-1.67585	-1.67307	-1.67029	-1.66751	-1.66472	-1.66193
-1.68332	-1.68051	-1.67771	-1.67492	-1.67213	-1.66935	-1.66657	-1.66379	-1.661
-1.68239	-1.67959	-1.67679	-1.674	-1.67122	-1.66843	-1.66566	-1.66287	-1.66009
-1.68148	-1.67868	-1.67589	-1.6731	-1.67031	-1.66753	-1.66475	-1.66197	-1.65919
-1.68058	-1.67779	-1.675	-1.67221	-1.66942	-1.66664	-1.66386	-1.66108	-1.6583
-1.67968	-1.67689	-1.6741	-1.67132	-1.66853	-1.66575	-1.66297	-1.66019	-1.6574
-1.67878	-1.676	-1.67321	-1.67042	-1.66764	-1.66486	-1.66208	-1.65929	-1.6565
-1.67789	-1.6751	-1.67231	-1.66953	-1.66674	-1.66396	-1.66118	-1.65839	-1.6556
-1.67701	-1.6742	-1.6714	-1.66862	-1.66584	-1.66306	-1.66027	-1.65748	-1.6547
-1.67616	-1.67332	-1.67051	-1.66772	-1.66494	-1.66215	-1.65936	-1.65658	-1.65379
-1.67535	-1.67248	-1.66963	-1.66683	-1.66404	-1.66125	-1.65846	-1.65567	-1.65289
-1.67454	-1.67165	-1.66878	-1.66596	-1.66315	-1.66035	-1.65756	-1.65477	-1.65198
-1.6737	-1.67084	-1.66797	-1.66512	-1.66227	-1.65946	-1.65666	-1.65386	-1.65106
-1.67285	-1.67003	-1.66718	-1.6643	-1.66142	-1.65858	-1.65576	-1.65295	-1.65015
-1.67196	-1.66917	-1.66634	-1.66347	-1.6606	-1.65774	-1.65489	-1.65206	-1.64924
-1.67106	-1.66828	-1.66548	-1.66265	-1.6598	-1.65692	-1.65403	-1.65118	-1.64834
-1.67015	-1.66738	-1.6646	-1.6618	-1.65897	-1.65609	-1.6532	-1.65033	-1.64746
-1.66925	-1.66648	-1.6637	-1.66091	-1.6581	-1.65526	-1.6524	-1.64952	-1.64661
-1.66835	-1.66558	-1.6628	-1.66001	-1.65721	-1.65441	-1.65158	-1.6487	-1.64578
-1.66746	-1.66468	-1.6619	-1.65911	-1.65632	-1.65352	-1.6507	-1.64785	-1.64497

							-0.21611	-0.21536
							-0.21545	-0.21476
				-0.2171	-0.21628	-0.21553	-0.40304	-0.40181
				-0.21638	-0.21563	-0.21493	-0.40198	-0.40076
				-0.21571	-0.21501	-0.21437	-0.40095	-0.39973
		-0.10729	-0.10701	-0.4036	-0.40235	-0.40114	-0.48274	-0.48133
		-0.10706	-0.10676	-0.40257	-0.40132	-0.40011	-0.48159	-0.48019
-0.10741	-0.10711	-0.35473	-0.35361	-0.48466	-0.48324	-0.48183	-0.5201	-0.51861
-0.10717	-0.10686	-0.3538	-0.35271	-0.4835	-0.48207	-0.48066	-0.51884	-0.51736
-0.25289	-0.25213	-0.48518	-0.48375	-0.48234	-0.48092	-0.47951	-0.5617	-0.56016
-0.2523	-0.25156	-0.48406	-0.48262	-0.4812	-0.47979	-0.4784	-0.56042	-0.55887
-0.25171	-0.25099	-0.48294	-0.4815	-0.48007	-0.47867	-0.47729	-0.55914	-0.5576
-0.40139	-0.40016	-0.52694	-0.52541	-0.56257	-0.56098	-0.55941	-0.6405	-0.63865
-0.40038	-0.39914	-0.52572	-0.52421	-0.56127	-0.55968	-0.55813	-0.63898	-0.63715
-0.52752	-0.52602	-0.60052	-0.59865	-0.59681	-0.59501	-0.59325	-0.67827	-0.67639
-0.52627	-0.5248	-0.59904	-0.59723	-0.59544	-0.59365	-0.59189	-0.67677	-0.67491
-0.56326	-0.56155	-0.59761	-0.59582	-0.64018	-0.63832	-0.63647	-0.67528	-0.67343
-0.56194	-0.56026	-0.59622	-0.59442	-0.63869	-0.63686	-0.63505	-0.6738	-0.67194
-0.5984	-0.59661	-0.59483	-0.59302	-0.67789	-0.67602	-0.67418	-0.71215	-0.71018
-0.59698	-0.59519	-0.59341	-0.59162	-0.67634	-0.6745	-0.67269	-0.71059	-0.70864
-0.59555	-0.59377	-0.592	-0.59023	-0.67481	-0.67299	-0.67121	-0.70903	-0.70713
-0.59409	-0.59233	-0.6771	-0.67522	-0.71322	-0.71129	-0.70939	-0.7075	-0.70562
-0.59263	-0.59091	-0.67557	-0.67376	-0.71171	-0.70979	-0.70786	-0.70598	-0.70411
-0.67776	-0.67592	-0.71398	-0.71209	-0.71019	-0.70825	-0.70631	-0.85392	-0.8514
-0.6763	-0.67449	-0.71247	-0.71057	-0.70865	-0.70669	-0.70475	-0.85184	-0.84941
-0.71477	-0.71287	-0.71096	-0.70903	-0.85776	-0.85502	-0.85235	-0.92001	-0.91739
-0.71325	-0.71135	-0.70943	-0.70746	-0.85556	-0.85296	-0.85046	-0.91801	-0.91539
-0.71174	-0.70982	-0.859	-0.85617	-0.92406	-0.92131	-0.91869	-0.98524	-0.98246
-0.71022	-0.70826	-0.85682	-0.85413	-0.92198	-0.91928	-0.91667	-0.9831	-0.98033
-0.70869	-0.70669	-0.85468	-0.85217	-0.91999	-0.91731	-0.91467	-0.98097	-0.9782
-0.85807	-0.85532	-0.92333	-0.92063	-0.98726	-0.98442	-0.98161	-0.97882	-0.97605
-0.85593	-0.85335	-0.92129	-0.91862	-0.98511	-0.98228	-0.97947	-0.97667	-0.97389
-0.92471	-0.92196	-0.98861	-0.98576	-0.98295	-0.98012	-0.97731	-0.97452	-0.97175
-0.92262	-0.91991	-0.98643	-0.9836	-0.98079	-0.97795	-0.97513	-0.97237	-0.96965
-0.92056	-0.91789	-0.98426	-0.98143	-0.97862	-0.97579	-0.97299	-1.00623	-1.00349
-0.91854	-0.91588	-0.98212	-0.97924	-0.9764	-0.97363	-0.9709	-1.00413	-1.00141
-0.91654	-0.91387	-0.97998	-0.97708	-0.97423	-0.9715	-0.96884	-1.00206	-0.99935
-0.98347	-0.98067	-1.01397	-1.01106	-1.0756	-1.0727	-1.06986	-1.09966	-1.09681
-0.9813	-0.97852	-1.01182	-1.00896	-1.07345	-1.07055	-1.06769	-1.09746	-1.09464
-1.0153	-1.01247	-1.0771	-1.07416	-1.07126	-1.06838	-1.06554	-1.09528	-1.09246
-1.01314	-1.0103	-1.07482	-1.07192	-1.06906	-1.06622	-1.06341	-1.09311	-1.09029
-1.07852	-1.07553	-1.0726	-1.06972	-1.09952	-1.09664	-1.09379	-1.23699	-1.23396
-1.07637	-1.07337	-1.07043	-1.06756	-1.09733	-1.09446	-1.09163	-1.2347	-1.23174
-1.07422	-1.07122	-1.13203	-1.12905	-1.2416	-1.23849	-1.23544	-1.26268	-1.25969
-1.07203	-1.06904	-1.12981	-1.12691	-1.23936	-1.23628	-1.23324	-1.26043	-1.25744
-1.06984	-1.06688	-1.12762	-1.12479	-1.23716	-1.23411	-1.23107	-1.2582	-1.25519
-1.13141	-1.12839	-1.24088	-1.2379	-1.26516	-1.26207	-1.25897	-1.2874	-1.28431

-1.12911	-1.12619	-1.2386	-1.23564	-1.26287	-1.25978	-1.25669	-1.28506	-1.28198
-1.15435	-1.15146	-1.26657	-1.26355	-1.29214	-1.28897	-1.28581	-1.28271	-1.27965
-1.15222	-1.14935	-1.26428	-1.2612	-1.28967	-1.28654	-1.28343	-1.28035	-1.2773
-1.26811	-1.26507	-1.29367	-1.29045	-1.28726	-1.28414	-1.28106	-1.278	-1.27496
-1.26582	-1.2628	-1.29133	-1.28811	-1.28492	-1.2818	-1.27872	-1.27565	-1.2726
-1.29526	-1.29215	-1.289	-1.28579	-1.2826	-1.27948	-1.2764	-1.27332	-1.27025
-1.29291	-1.2898	-1.28666	-1.28347	-1.28029	-1.27717	-1.27408	-1.27099	-1.26792
-1.29057	-1.28747	-1.28434	-1.28115	-1.27798	-1.27487	-1.27178	-1.2687	-1.26563
-1.28823	-1.28516	-1.28204	-1.27884	-1.27567	-1.27258	-1.26953	-1.34035	-1.33697
-1.2859	-1.28286	-1.27976	-1.27654	-1.27336	-1.27031	-1.26731	-1.33801	-1.33469
-1.2836	-1.28057	-1.30548	-1.30221	-1.34561	-1.34226	-1.33899	-1.33569	-1.3324
-1.28132	-1.27828	-1.30315	-1.29991	-1.34319	-1.33988	-1.33665	-1.33337	-1.33011
-1.30708	-1.30398	-1.34777	-1.34426	-1.34081	-1.33754	-1.33435	-1.33112	-1.32792
-1.30476	-1.30166	-1.34532	-1.34186	-1.33847	-1.33523	-1.33208	-1.32893	-1.32584
-1.30245	-1.29936	-1.34291	-1.3395	-1.33616	-1.33296	-1.32987	-1.32684	-1.32392
-1.34722	-1.34387	-1.34052	-1.33715	-1.33387	-1.33071	-1.32771	-1.32489	-1.32227
-1.34482	-1.34149	-1.33817	-1.33483	-1.33161	-1.32854	-1.32566	-1.32309	-1.32078
-1.34251	-1.33917	-1.33587	-1.33255	-1.3294	-1.3265	-1.32384	-1.33835	-1.33618
-1.34026	-1.33691	-1.33361	-1.33031	-1.32726	-1.3246	-1.32222	-1.33685	-1.33482
-1.33797	-1.33462	-1.33138	-1.32821	-1.34229	-1.33977	-1.33754	-1.33544	-1.33351
-1.33563	-1.33229	-1.32916	-1.32624	-1.3405	-1.33818	-1.33611	-1.33413	-1.33226
-1.33333	-1.33006	-1.34408	-1.34134	-1.33887	-1.33672	-1.33477	-1.33287	-1.33105
-1.33108	-1.32795	-1.3421	-1.33965	-1.33742	-1.33538	-1.3335	-1.33166	-1.32989
-1.32892	-1.32599	-1.34029	-1.33809	-1.33608	-1.33413	-1.33227	-1.33048	-1.32876
-1.34385	-1.34112	-1.3387	-1.33667	-1.33477	-1.33289	-1.33108	-1.35461	-1.35289
-1.34192	-1.33949	-1.33729	-1.33535	-1.33351	-1.33168	-1.32992	-1.35346	-1.35179
-1.34022	-1.33798	-1.33592	-1.33406	-1.35763	-1.35579	-1.35401	-1.35231	-1.35068
-1.33873	-1.33661	-1.33463	-1.33282	-1.35638	-1.35459	-1.35283	-1.35116	-1.34956
-1.33731	-1.3353	-1.35878	-1.35695	-1.35518	-1.35342	-1.35169	-1.4064	-1.40457
-1.33597	-1.33407	-1.35759	-1.35579	-1.35404	-1.35229	-1.35058	-1.40511	-1.40327
-1.36011	-1.35824	-1.38231	-1.38047	-1.40965	-1.40768	-1.40574	-1.42124	-1.41939
-1.35889	-1.35705	-1.3811	-1.37927	-1.40834	-1.40641	-1.40451	-1.42001	-1.41816
-1.35772	-1.35589	-1.37991	-1.37809	-1.40704	-1.40515	-1.40329	-1.4188	-1.41694
-1.38242	-1.38055	-1.39647	-1.39466	-1.42313	-1.42125	-1.41941	-1.41755	-1.41569
-1.38118	-1.37935	-1.39528	-1.3935	-1.42184	-1.41997	-1.41814	-1.41628	-1.41443
-1.39773	-1.39591	-1.42445	-1.4225	-1.42058	-1.41872	-1.41689	-1.41503	-1.41318
-1.39657	-1.39476	-1.42317	-1.42125	-1.41936	-1.4175	-1.41565	-1.41379	-1.41194
-1.42583	-1.42386	-1.42192	-1.42001	-1.44481	-1.44288	-1.44096	-1.51032	-1.50819
-1.42455	-1.42262	-1.4207	-1.41879	-1.44353	-1.44158	-1.43966	-1.50887	-1.50678
-1.42328	-1.42138	-1.41948	-1.41757	-1.44225	-1.44029	-1.43837	-1.50747	-1.50544
-1.42204	-1.42013	-1.44488	-1.4429	-1.51243	-1.51026	-1.50814	-1.52936	-1.52725
-1.42081	-1.41888	-1.44356	-1.44159	-1.51098	-1.50884	-1.50676	-1.52795	-1.52596
-1.493	-1.49092	-1.51391	-1.51171	-1.53314	-1.53086	-1.52867	-1.52661	-1.52466
-1.49162	-1.48958	-1.51249	-1.5103	-1.53161	-1.52944	-1.52736	-1.52533	-1.52335
-1.51539	-1.51325	-1.53483	-1.53246	-1.53018	-1.52807	-1.52605	-1.52403	-1.52204
-1.51397	-1.51185	-1.53335	-1.53106	-1.52885	-1.52675	-1.52473	-1.52271	-1.52072
-1.53642	-1.53413	-1.53188	-1.52967	-1.52752	-1.52544	-1.5234	-1.52139	-1.51942
-1.53492	-1.53261	-1.53038	-1.52825	-1.52617	-1.5241	-1.52207	-1.5201	-1.51818
-1.53343	-1.53112	-1.52892	-1.52685	-1.52482	-1.52276	-1.52075	-1.51882	-1.51695
-1.53194	-1.5297	-1.52755	-1.52549	-1.52347	-1.52145	-1.51947	-1.51756	-1.51571

-1.53047	-1.52833	-1.52623	-1.52416	-1.52214	-1.52016	-1.51822	-1.51633	-1.51446
-1.52911	-1.52702	-1.52494	-1.52287	-1.52084	-1.51889	-1.51698	-1.51509	-1.51323
-1.52785	-1.52576	-1.52369	-1.52161	-1.51958	-1.51764	-1.51575	-1.51387	-1.51202
-1.52654	-1.52447	-1.52241	-1.52034	-1.51832	-1.51639	-1.51451	-1.51265	-1.51082
-1.52519	-1.52314	-1.5211	-1.51906	-1.51707	-1.51514	-1.51326	-1.51143	-1.50964
-1.52383	-1.52181	-1.5198	-1.51781	-1.51585	-1.51393	-1.51204	-1.53143	-1.52951
-1.52249	-1.5205	-1.51854	-1.5166	-1.51469	-1.51278	-1.51089	-1.53018	-1.52823
-1.52117	-1.51921	-1.51729	-1.51541	-1.51353	-1.51164	-1.50975	-1.52895	-1.52696
-1.51989	-1.51796	-1.51606	-1.51419	-1.51232	-1.51045	-1.50858	-1.52769	-1.52569
-1.51865	-1.51674	-1.51485	-1.51296	-1.51109	-1.50924	-1.50739	-1.52644	-1.52444
-1.51741	-1.51554	-1.51367	-1.51179	-1.53119	-1.52919	-1.5272	-1.57252	-1.57035
-1.51617	-1.51435	-1.51252	-1.51065	-1.52996	-1.52797	-1.52599	-1.57121	-1.56906
-1.51497	-1.51317	-1.55371	-1.55164	-1.57635	-1.57417	-1.57201	-1.58542	-1.58326
-1.51382	-1.51198	-1.55235	-1.55032	-1.57496	-1.57278	-1.57061	-1.584	-1.58184
-1.55518	-1.55308	-1.57789	-1.57573	-1.58918	-1.58698	-1.58479	-1.61435	-1.612
-1.55392	-1.55182	-1.57655	-1.57438	-1.58783	-1.58564	-1.58346	-1.61291	-1.61062
-1.55265	-1.55059	-1.57523	-1.57305	-1.58648	-1.5843	-1.58215	-1.61151	-1.60928
-1.5939	-1.59168	-1.58948	-1.58727	-1.60425	-1.60205	-1.5999	-1.69428	-1.692
-1.59242	-1.59025	-1.58807	-1.58586	-1.60281	-1.60064	-1.59853	-1.69276	-1.69052
-1.61028	-1.60808	-1.60587	-1.60364	-1.69831	-1.69591	-1.69357	-1.6913	-1.6891
-1.60894	-1.60671	-1.6045	-1.6023	-1.69682	-1.69447	-1.69218	-1.68993	-1.68772
-1.69322	-1.69087	-1.70012	-1.6977	-1.69534	-1.69303	-1.69077	-1.68854	-1.68634
-1.6917	-1.68934	-1.69851	-1.69615	-1.69384	-1.69155	-1.6893	-1.68711	-1.68495
-1.6902	-1.68786	-1.69694	-1.69463	-1.69236	-1.69009	-1.68785	-1.68567	-1.68356
-1.68879	-1.68649	-1.69549	-1.6932	-1.69094	-1.68868	-1.68645	-1.68428	-1.68215
-1.68741	-1.68517	-1.6941	-1.69181	-1.68955	-1.68731	-1.68509	-1.68291	-1.68075
-1.69735	-1.69501	-1.6927	-1.69042	-1.68817	-1.68594	-1.68373	-1.68154	-1.67938
-1.69587	-1.69359	-1.69132	-1.68905	-1.6868	-1.68457	-1.68236	-1.68019	-1.67805
-1.69437	-1.69212	-1.68987	-1.68761	-1.68537	-1.68315	-1.68096	-1.67879	-1.67666
-1.69284	-1.69058	-1.68833	-1.68609	-1.68388	-1.68168	-1.6795	-1.67734	-1.67519
-1.69134	-1.68906	-1.68682	-1.68461	-1.68241	-1.68024	-1.67808	-1.72096	-1.71849
-1.68991	-1.68763	-1.68538	-1.68319	-1.68101	-1.67886	-1.67672	-1.71943	-1.71698
-1.6885	-1.68623	-1.68399	-1.6818	-1.67965	-1.67751	-1.67539	-1.71793	-1.71552
-1.6871	-1.68485	-1.68264	-1.68046	-1.72373	-1.72127	-1.71884	-1.71645	-1.71409
-1.68571	-1.68351	-1.68132	-1.67914	-1.72223	-1.71975	-1.71732	-1.71497	-1.71269
-1.68433	-1.68215	-1.67998	-1.67781	-1.72071	-1.71824	-1.71581	-1.71348	-1.71124
-1.68295	-1.68079	-1.67863	-1.67646	-1.7192	-1.71673	-1.7143	-1.71198	-1.70975
-1.68158	-1.6794	-1.72258	-1.72012	-1.71767	-1.71523	-1.71282	-1.71052	-1.70829
-1.6802	-1.67798	-1.72095	-1.71851	-1.71611	-1.71372	-1.71138	-1.70911	-1.70689
-1.72441	-1.72183	-1.71933	-1.71693	-1.71457	-1.71225	-1.70996	-1.70772	-1.7055
-1.72281	-1.72026	-1.7178	-1.71544	-1.71312	-1.71083	-1.70856	-1.70633	-1.70412
-1.72121	-1.71872	-1.71631	-1.71399	-1.7117	-1.70943	-1.70717	-1.70495	-1.70275
-1.71968	-1.71724	-1.71486	-1.71256	-1.72708	-1.7248	-1.72255	-1.72033	-1.71814
-1.71819	-1.7158	-1.71345	-1.71115	-1.72566	-1.7234	-1.72117	-1.71897	-1.7168
-1.73353	-1.73117	-1.72884	-1.72652	-1.72424	-1.722	-1.7198	-1.75485	-1.75247
-1.73205	-1.72973	-1.72743	-1.72512	-1.72284	-1.72063	-1.71844	-1.75337	-1.75099
-1.73058	-1.7283	-1.7397	-1.73738	-1.73509	-1.73287	-1.73069	-1.75188	-1.74948
-1.72914	-1.72686	-1.73827	-1.73597	-1.7337	-1.73151	-1.72933	-1.75036	-1.74793
-1.7277	-1.72542	-1.73684	-1.73457	-1.73232	-1.73013	-1.72795	-1.74884	-1.74638
-1.73999	-1.73769	-1.73542	-1.73318	-1.75451	-1.7521	-1.7497	-1.74728	-1.74486

-1.73855	-1.73626	-1.734	-1.73179	-1.753	-1.75055	-1.74811	-1.74571	-1.74335
-1.73712	-1.73485	-1.75629	-1.75387	-1.75145	-1.749	-1.74656	-1.74419	-1.74186
-1.7357	-1.73346	-1.75477	-1.75232	-1.74988	-1.74744	-1.74503	-1.74269	-1.7404
-1.73429	-1.73208	-1.75326	-1.75079	-1.74834	-1.74592	-1.74353	-1.74121	-1.73894
-1.73291	-1.73071	-1.75177	-1.74929	-1.74683	-1.74442	-1.74205	-1.73975	-1.7375
-1.75511	-1.75272	-1.75028	-1.7478	-1.74534	-1.74295	-1.7406	-1.73831	-1.73607
-1.75362	-1.75123	-1.7488	-1.74632	-1.74387	-1.74149	-1.73916	-1.73688	-1.73465
-1.75213	-1.74974	-1.74731	-1.74484	-1.74241	-1.74004	-1.73773	-1.73547	-1.73325
-1.75064	-1.74824	-1.74582	-1.74336	-1.74094	-1.7386	-1.73631	-1.80423	-1.80152
-1.74915	-1.74674	-1.74433	-1.74188	-1.73948	-1.73717	-1.7349	-1.80252	-1.79984
-1.74765	-1.74526	-1.74285	-1.74042	-1.73803	-1.73574	-1.7335	-1.83459	-1.83189
-1.74614	-1.74378	-1.7414	-1.73898	-1.7366	-1.73432	-1.73211	-1.83288	-1.83019
-1.74464	-1.74231	-1.73995	-1.73755	-1.8056	-1.80283	-1.80013	-1.83118	-1.82851
-1.74317	-1.74086	-1.73852	-1.73614	-1.80389	-1.80114	-1.79846	-1.82949	-1.82684
-1.7417	-1.73941	-1.80793	-1.80505	-1.80222	-1.79948	-1.7968	-1.83919	-1.83637
-1.74023	-1.73796	-1.80622	-1.80339	-1.8006	-1.79787	-1.79518	-1.83751	-1.83469
-1.73877	-1.73652	-1.80452	-1.80175	-1.799	-1.79627	-1.79356	-1.83583	-1.83302
-1.80829	-1.80554	-1.8028	-1.80007	-1.80977	-1.80686	-1.80397	-1.84809	-1.84528
-1.80653	-1.80379	-1.80107	-1.79837	-1.80806	-1.80517	-1.80231	-1.84643	-1.84364
-1.80479	-1.80207	-1.81209	-1.80922	-1.82072	-1.81784	-1.81499	-1.84478	-1.84201
-1.80307	-1.80036	-1.81037	-1.80753	-1.81905	-1.81618	-1.81335	-1.84313	-1.84038
-1.80139	-1.79868	-1.82306	-1.82022	-1.81739	-1.81455	-1.81173	-1.85743	-1.85461
-1.79977	-1.79704	-1.82138	-1.81856	-1.81576	-1.81295	-1.81017	-1.8558	-1.85297
-1.79816	-1.79543	-1.81974	-1.81692	-1.81413	-1.81136	-1.80861	-1.85418	-1.85134
-1.82392	-1.82103	-1.81816	-1.81532	-1.84508	-1.84229	-1.83952	-1.85716	-1.85432
-1.82228	-1.81945	-1.8166	-1.81373	-1.84344	-1.84065	-1.83789	-1.85551	-1.85269
-1.82063	-1.81782	-1.815	-1.81212	-1.86233	-1.85948	-1.85668	-1.85388	-1.85111
-1.81896	-1.81617	-1.81336	-1.8105	-1.86066	-1.85781	-1.85502	-1.85228	-1.84957
-1.8223	-1.81954	-1.83298	-1.83007	-1.85902	-1.85617	-1.85338	-1.85067	-1.84802
-1.82068	-1.81791	-1.83132	-1.82846	-1.85742	-1.85456	-1.85175	-1.84905	-1.84644
-1.81907	-1.8163	-1.82967	-1.82685	-1.8558	-1.85295	-1.85014	-1.84746	-1.84487
-1.81751	-1.81471	-1.82803	-1.8252	-1.85415	-1.85133	-1.84856	-1.8459	-1.84333
-1.81594	-1.81313	-1.8264	-1.82357	-1.8525	-1.84973	-1.84701	-1.84437	-1.84181
-1.8305	-1.82764	-1.85657	-1.85371	-1.86427	-1.86153	-1.85885	-1.85621	-1.85362
-1.82884	-1.82604	-1.85499	-1.85214	-1.86271	-1.86001	-1.85738	-1.85475	-1.85214
-1.84096	-1.83817	-1.86674	-1.86391	-1.86114	-1.85847	-1.85587	-1.85327	-1.85068
-1.83931	-1.83652	-1.86508	-1.8623	-1.85957	-1.85693	-1.85434	-1.85178	-1.84925
-1.8377	-1.83492	-1.86348	-1.86073	-1.85804	-1.85541	-1.85283	-1.85029	-1.84779
-1.83616	-1.83339	-1.86194	-1.85922	-1.85655	-1.85391	-1.85132	-1.84878	-1.84629
-1.86596	-1.86317	-1.86043	-1.85773	-1.85507	-1.85244	-1.84984	-1.8473	-1.84481
-1.8644	-1.86165	-1.85893	-1.85624	-1.85359	-1.85098	-1.84841	-1.84588	-1.8434
-1.86285	-1.86013	-1.85744	-1.85475	-1.85211	-1.84954	-1.847	-1.84449	-1.84201
-1.86132	-1.85861	-1.85593	-1.85327	-1.85065	-1.8481	-1.84559	-1.84311	-1.84065
-1.85981	-1.8571	-1.85442	-1.85178	-1.84919	-1.84667	-1.84419	-1.84173	-1.8393
-1.85829	-1.85558	-1.85293	-1.85031	-1.85989	-1.85739	-1.85493	-1.85249	-1.85006
-1.85675	-1.85407	-1.85144	-1.84884	-1.85844	-1.85598	-1.85355	-1.85111	-1.84868
-1.86737	-1.8647	-1.87426	-1.87167	-1.86915	-1.86669	-1.86427	-1.89035	-1.88788
-1.86586	-1.8632	-1.87275	-1.8702	-1.86769	-1.86525	-1.86283	-1.88889	-1.88644
-1.86436	-1.86171	-1.87128	-1.86875	-1.86626	-1.86382	-1.86141	-1.88744	-1.88502
-1.87501	-1.87239	-1.86984	-1.86734	-1.89338	-1.89091	-1.88847	-1.88604	-1.88364

-1.8735	-1.87095	-1.86843	-1.86596	-1.89198	-1.88952	-1.88709	-1.88467	-1.88229
-1.87209	-1.86956	-1.89556	-1.89305	-1.89057	-1.88813	-1.88571	-1.88332	-1.88094
-1.87074	-1.86824	-1.89419	-1.89165	-1.88916	-1.88674	-1.88436	-1.88197	-1.87961
-1.89788	-1.89534	-1.89282	-1.89028	-1.88779	-1.88537	-1.88299	-1.88063	-1.87829
-1.89646	-1.89395	-1.89145	-1.88894	-1.88647	-1.88402	-1.88162	-1.87928	-1.87698
-1.89503	-1.89254	-1.89006	-1.88759	-1.88514	-1.88269	-1.88028	-1.87795	-1.87568
-1.89361	-1.89111	-1.88864	-1.88621	-1.88379	-1.88138	-1.87899	-1.87665	-1.87435
-1.89217	-1.88968	-1.88722	-1.88482	-1.88244	-1.88007	-1.87771	-1.87536	-1.87304
-1.89073	-1.88825	-1.88582	-1.88343	-1.88107	-1.87873	-1.87641	-1.87408	-1.87176
-1.88928	-1.88684	-1.88443	-1.88205	-1.87969	-1.87738	-1.8751	-1.8728	-1.8705
-1.88787	-1.88543	-1.88303	-1.88067	-1.87835	-1.87605	-1.87378	-1.8715	-1.86923
-1.8865	-1.88403	-1.88163	-1.87931	-1.87702	-1.87473	-1.87245	-1.87019	-1.86794
-1.88515	-1.88267	-1.88027	-1.87797	-1.87568	-1.8734	-1.87112	-1.86887	-1.86664
-1.88382	-1.88138	-1.87898	-1.87664	-1.87433	-1.87206	-1.86981	-1.86757	-1.86533
-1.88247	-1.88008	-1.8777	-1.87534	-1.87301	-1.87074	-1.8685	-1.86625	-1.86402
-1.88108	-1.87875	-1.87641	-1.87405	-1.87172	-1.86944	-1.86718	-1.86494	-1.86271
-1.8797	-1.87741	-1.8751	-1.87278	-1.87046	-1.86816	-1.86587	-1.86363	-1.86141
-1.87837	-1.87607	-1.87378	-1.87148	-1.86919	-1.86689	-1.8646	-1.86235	-1.86011
-1.87707	-1.87474	-1.87245	-1.87018	-1.86792	-1.86564	-1.86336	-1.86109	-1.85883
-1.87579	-1.87345	-1.87115	-1.8689	-1.86666	-1.86439	-1.86212	-1.85984	-1.85756
-1.87452	-1.87218	-1.86988	-1.86764	-1.8654	-1.86315	-1.86088	-1.8586	-1.85632
-1.87326	-1.87091	-1.86861	-1.86636	-1.86413	-1.86188	-1.85962	-1.85735	-1.85508
-1.87199	-1.86965	-1.86734	-1.86506	-1.86281	-1.86057	-1.85834	-1.85609	-1.85384
-1.87072	-1.86839	-1.86607	-1.86377	-1.86149	-1.85926	-1.85704	-1.85481	-1.85259
-1.86945	-1.86713	-1.86482	-1.86251	-1.86022	-1.85796	-1.85572	-1.8535	-1.8513
-1.86817	-1.86588	-1.86358	-1.86127	-1.85896	-1.85667	-1.85439	-1.85218	-1.85
-1.86688	-1.86461	-1.86233	-1.86001	-1.8577	-1.85539	-1.85311	-1.85088	-1.84869
-1.86556	-1.86333	-1.86107	-1.85875	-1.85643	-1.85414	-1.85186	-1.8496	-1.84736
-1.86427	-1.86206	-1.8598	-1.85749	-1.85518	-1.85289	-1.85061	-1.87839	-1.87607
-1.86301	-1.86078	-1.85853	-1.85624	-1.85394	-1.85165	-1.84936	-1.87714	-1.87482
-1.86174	-1.85949	-1.85724	-1.85497	-1.88293	-1.88059	-1.87824	-1.88752	-1.88519
-1.86046	-1.85816	-1.85589	-1.85366	-1.88166	-1.87933	-1.87699	-1.88626	-1.88393
-1.85918	-1.85684	-1.85456	-1.85235	-1.88039	-1.87807	-1.87574	-1.88501	-1.88267
-1.85791	-1.85556	-1.8952	-1.89297	-1.89073	-1.88842	-1.88609	-1.88375	-1.88141
-1.85664	-1.8543	-1.89395	-1.89172	-1.88948	-1.88717	-1.88483	-1.88249	-1.88015
-1.89735	-1.89499	-1.89268	-1.89045	-1.8882	-1.8859	-1.88357	-1.88124	-1.87889
-1.89607	-1.89372	-1.89141	-1.88917	-1.88692	-1.88462	-1.8823	-1.87998	-1.87765
-1.89478	-1.89243	-1.89012	-1.88785	-1.88559	-1.88331	-1.88102	-1.87872	-1.8764
-1.89348	-1.89114	-1.88882	-1.88651	-1.88422	-1.88196	-1.87971	-1.87744	-1.87515
-1.89219	-1.88985	-1.88751	-1.88517	-1.88286	-1.88062	-1.8784	-1.87615	-1.87389
-1.8909	-1.88855	-1.88621	-1.88387	-1.88156	-1.87933	-1.87712	-1.87488	-1.87263
-1.88962	-1.88726	-1.88492	-1.88259	-1.8803	-1.87806	-1.87585	-1.87361	-1.87137
-1.88833	-1.88598	-1.88365	-1.88135	-1.87908	-1.87683	-1.8746	-1.87235	-1.8701
-1.88705	-1.88469	-1.88238	-1.88013	-1.87789	-1.87563	-1.87337	-1.8711	-1.86883
-1.88576	-1.88342	-1.88112	-1.87888	-1.87665	-1.87439	-1.87212	-1.86985	-1.86757
-1.88448	-1.88215	-1.87985	-1.87762	-1.87539	-1.87313	-1.87086	-1.86859	-1.8663
-1.88321	-1.8809	-1.87861	-1.87637	-1.87412	-1.87187	-1.8696	-1.86732	-1.86503
-1.88196	-1.87969	-1.87742	-1.87515	-1.87288	-1.8706	-1.86832	-1.86603	-1.86375
-1.88072	-1.87848	-1.87621	-1.87392	-1.87163	-1.86933	-1.86703	-1.86475	-1.86246
-1.87953	-1.87725	-1.87496	-1.87266	-1.87036	-1.86806	-1.86576	-1.86346	-1.86117

-1.87834	-1.87602	-1.8737	-1.87139	-1.86909	-1.86678	-1.86448	-1.86217	-1.85987
-1.8771	-1.87476	-1.87243	-1.87011	-1.8678	-1.8655	-1.86319	-1.86088	-1.85857
-1.87583	-1.87349	-1.87115	-1.86883	-1.86651	-1.8642	-1.86189	-1.85959	-1.85728
-1.87454	-1.8722	-1.86987	-1.86754	-1.86521	-1.8629	-1.86059	-1.85828	-1.85597
-1.87324	-1.8709	-1.86857	-1.86625	-1.86393	-1.8616	-1.85928	-1.85697	-1.85466
-1.87195	-1.86962	-1.86729	-1.86497	-1.86264	-1.86032	-1.85799	-1.85567	-1.85336
-1.87067	-1.86834	-1.86601	-1.86369	-1.86136	-1.85904	-1.85672	-1.85439	-1.85206
-1.86942	-1.86708	-1.86475	-1.86243	-1.86011	-1.85779	-1.85546	-1.85313	-1.8508
-1.86823	-1.8659	-1.86357	-1.86124	-1.85891	-1.85658	-1.85426	-1.85192	-1.84959
-1.86706	-1.86473	-1.8624	-1.86007	-1.85773	-1.85539	-1.85306	-1.85073	-1.8484
-1.86582	-1.86349	-1.86116	-1.85882	-1.85649	-1.85414	-1.8518	-1.84947	-1.84715
-1.86453	-1.8622	-1.85987	-1.85753	-1.8552	-1.85285	-1.85051	-1.84818	-1.84584
-1.86325	-1.8609	-1.85856	-1.85623	-1.85389	-1.85155	-1.84921	-1.84687	-1.84454
-1.86196	-1.8596	-1.85725	-1.8549	-1.85256	-1.85022	-1.84788	-1.84555	-1.84322
-1.86069	-1.85832	-1.85595	-1.85359	-1.85124	-1.8489	-1.84657	-1.84424	-1.84191
-1.85947	-1.85707	-1.85468	-1.8523	-1.84994	-1.8476	-1.84527	-1.84294	-1.84061
-1.85826	-1.85583	-1.85342	-1.85104	-1.84867	-1.84632	-1.84399	-1.84165	-1.83933
-1.857	-1.85458	-1.85219	-1.84981	-1.84745	-1.84511	-1.84277	-1.84044	-1.8381
-1.85573	-1.85334	-1.85096	-1.8486	-1.84625	-1.84392	-1.84159	-1.83925	-1.8369
-1.85444	-1.85207	-1.84971	-1.84736	-1.84501	-1.84268	-1.84035	-1.83801	-1.83565
-1.85313	-1.85077	-1.84842	-1.84608	-1.84373	-1.8414	-1.83907	-1.83672	-1.83436
-1.85185	-1.8495	-1.84716	-1.84482	-1.84248	-1.84014	-1.8378	-1.83545	-1.8331
-1.85063	-1.84828	-1.84594	-1.84361	-1.84127	-1.83893	-1.83658	-1.83423	-1.83188
-1.8494	-1.84705	-1.84471	-1.84238	-1.84004	-1.8377	-1.83535	-1.83299	-1.83064
-1.84813	-1.84577	-1.84342	-1.84109	-1.83875	-1.8364	-1.83404	-1.83169	-1.82933
-1.84685	-1.84449	-1.84214	-1.83979	-1.83744	-1.83509	-1.83273	-1.83037	-1.828
-1.84561	-1.84325	-1.84089	-1.83854	-1.83619	-1.83383	-1.83147	-1.82909	-1.8267
-1.84438	-1.84203	-1.83967	-1.83732	-1.83496	-1.8326	-1.83023	-1.82783	-1.82541
-1.84313	-1.84077	-1.83841	-1.83605	-1.83369	-1.83133	-1.82896	-1.82656	-1.82413
-1.84185	-1.83947	-1.83709	-1.83473	-1.83237	-1.83001	-1.82765	-1.82527	-1.82288
-1.84058	-1.83819	-1.83581	-1.83344	-1.84338	-1.84101	-1.83864	-1.83626	-1.83386
-1.83934	-1.83695	-1.83457	-1.83221	-1.84213	-1.83977	-1.83739	-1.83499	-1.83256
-1.83812	-1.83571	-1.83333	-1.83097	-1.84089	-1.83852	-1.83614	-1.83372	-1.83127
-1.83688	-1.83445	-1.84434	-1.84196	-1.83959	-1.83722	-1.83484	-1.83244	-1.83001
-1.83565	-1.83318	-1.84303	-1.84064	-1.83827	-1.8359	-1.83354	-1.83116	-1.82876
-1.84669	-1.84422	-1.84178	-1.83939	-1.83702	-1.83464	-1.83227	-1.8299	-1.82752
-1.84541	-1.84299	-1.84058	-1.83819	-1.83581	-1.83342	-1.83103	-1.82865	-1.82628
-1.84416	-1.84176	-1.83937	-1.83698	-1.83459	-1.83218	-1.82977	-1.82738	-1.825
-1.84296	-1.84055	-1.83815	-1.83577	-1.83337	-1.83093	-1.82849	-1.82607	-1.82368
-1.84175	-1.83933	-1.83692	-1.83453	-1.83212	-1.82967	-1.82721	-1.82478	-1.82238
-1.84052	-1.83807	-1.83563	-1.83323	-1.83083	-1.8284	-1.82597	-1.82355	-1.82113
-1.83928	-1.8368	-1.83435	-1.83194	-1.82954	-1.82714	-1.82473	-1.82232	-1.81991
-1.83802	-1.83555	-1.8331	-1.83069	-1.82829	-1.82589	-1.8235	-1.82109	-1.81867
-1.83675	-1.8343	-1.83187	-1.82947	-1.82707	-1.82466	-1.82225	-1.81984	-1.81743
-1.83551	-1.83307	-1.83065	-1.82825	-1.82584	-1.82341	-1.82098	-1.83025	-1.82779
-1.83428	-1.83186	-1.82944	-1.82704	-1.82462	-1.82215	-1.81967	-1.8289	-1.82643
-1.83307	-1.83065	-1.82823	-1.82581	-1.82336	-1.82087	-1.81838	-1.82758	-1.8251
-1.83187	-1.82946	-1.82702	-1.82455	-1.82207	-1.81959	-1.81712	-1.8263	-1.82382
-1.83067	-1.82826	-1.82581	-1.8233	-1.82079	-1.81832	-1.81587	-1.82503	-1.82256
-1.82948	-1.82706	-1.82461	-1.82208	-1.81956	-1.81708	-1.81463	-1.82377	-1.8213

-1.82827	-1.82585	-1.82339	-1.82086	-1.81834	-1.81586	-1.8134	-1.82252	-1.82005
-1.82704	-1.8246	-1.82212	-1.81961	-1.8171	-1.81462	-1.81216	-1.82126	-1.81879
-1.8258	-1.82331	-1.82082	-1.81833	-1.81585	-1.81338	-1.81092	-1.82	-1.81752
-1.82457	-1.82205	-1.81955	-1.81707	-1.82624	-1.82373	-1.82123	-1.81873	-1.81623
-1.82334	-1.82081	-1.8183	-1.81582	-1.82497	-1.82247	-1.81996	-1.81744	-1.8149
-1.82212	-1.81958	-1.81706	-1.81458	-1.82371	-1.8212	-1.8187	-1.81616	-1.8136
-1.8209	-1.81835	-1.81582	-1.81334	-1.82244	-1.81994	-1.81744	-1.8149	-1.81233
-1.81968	-1.81712	-1.81459	-1.8121	-1.82118	-1.81868	-1.81619	-1.81365	-1.81107
-1.81845	-1.81588	-1.81335	-1.81086	-1.81992	-1.81743	-1.81495	-1.8124	-1.80982
-1.81721	-1.81465	-1.81211	-1.80962	-1.81866	-1.81618	-1.8137	-1.81116	-1.80857
-1.81598	-1.81341	-1.81088	-1.80838	-1.8174	-1.81492	-1.81244	-1.80989	-1.80731
-1.81475	-1.81218	-1.80964	-1.80715	-1.81615	-1.81366	-1.81117	-1.80862	-1.80604
-1.81351	-1.81095	-1.81994	-1.81741	-1.81489	-1.81238	-1.80987	-1.80732	-1.80475
-1.81227	-1.80971	-1.81869	-1.81616	-1.81363	-1.81109	-1.80854	-1.80599	-1.80344
-1.81104	-1.80847	-1.81743	-1.81491	-1.81237	-1.80981	-1.80725	-1.8047	-1.80215
-1.8098	-1.80724	-1.81618	-1.81366	-1.81113	-1.8086	-1.80605	-1.80348	-1.80091
-1.80857	-1.80601	-1.81493	-1.81241	-1.8099	-1.80738	-1.80486	-1.80228	-1.79968
-1.80736	-1.80478	-1.81368	-1.81116	-1.80864	-1.80614	-1.80362	-1.80104	-1.79842
-1.80614	-1.80356	-1.81244	-1.80991	-1.80739	-1.80488	-1.80235	-1.79977	-1.79716
-1.80493	-1.80235	-1.8112	-1.80866	-1.80613	-1.80362	-1.80109	-1.79851	-1.79589
-1.80371	-1.80113	-1.80996	-1.80741	-1.80488	-1.80235	-1.79983	-1.79724	-1.79462
-1.80251	-1.79995	-1.80876	-1.80619	-1.80363	-1.8011	-1.79857	-1.79599	-1.79336
-1.80134	-1.7988	-1.80759	-1.80499	-1.8024	-1.79986	-1.79733	-1.79474	-1.79212
-1.80016	-1.79764	-1.80642	-1.80379	-1.80117	-1.79862	-1.79608	-1.7935	-1.79089
-1.79896	-1.79644	-1.80521	-1.80256	-1.79994	-1.79738	-1.79484	-1.79227	-1.7897
-1.79777	-1.79524	-1.80398	-1.80134	-1.79871	-1.79614	-1.79359	-1.79104	-1.7885
-1.80796	-1.80536	-1.80276	-1.80014	-1.79753	-1.79493	-1.79235	-1.7898	-1.78727
-1.80679	-1.80415	-1.80153	-1.79894	-1.79635	-1.79373	-1.79111	-1.78855	-1.78602
-1.80558	-1.80292	-1.8003	-1.79773	-1.79515	-1.79251	-1.78987	-1.7873	-1.78477
-1.80435	-1.80169	-1.79907	-1.79649	-1.7939	-1.79126	-1.78862	-1.78605	-1.78351
-1.80311	-1.80047	-1.79785	-1.79526	-1.79266	-1.79003	-1.7874	-1.78482	-1.78226
-1.8019	-1.79929	-1.79668	-1.79406	-1.79144	-1.78883	-1.78623	-1.78364	-1.78105
-1.80068	-1.7981	-1.79549	-1.79284	-1.79021	-1.78762	-1.78505	-1.78246	-1.77985
-1.79943	-1.79686	-1.79425	-1.7916	-1.78896	-1.78638	-1.78382	-1.78124	-1.77866
-1.79819	-1.7956	-1.793	-1.79035	-1.78772	-1.78513	-1.78257	-1.78002	-1.77747
-1.79698	-1.79437	-1.79176	-1.78913	-1.78651	-1.78392	-1.78134	-1.77879	-1.77625
-1.79579	-1.79316	-1.79055	-1.78793	-1.78533	-1.78272	-1.78012	-1.77755	-1.775
-1.7946	-1.79197	-1.78935	-1.78674	-1.78414	-1.78153	-1.77892	-1.77634	-1.77377
-1.79341	-1.79079	-1.78818	-1.78556	-1.78295	-1.78035	-1.77776	-1.77516	-1.77256
-1.79222	-1.78961	-1.787	-1.78438	-1.78177	-1.77917	-1.77658	-1.77398	-1.77136
-1.79103	-1.78842	-1.7858	-1.7832	-1.78059	-1.77799	-1.77538	-1.77278	-1.77017
-1.78985	-1.78722	-1.7846	-1.78201	-1.77941	-1.7768	-1.77418	-1.77158	-1.76898
-1.78865	-1.78603	-1.78342	-1.78082	-1.77821	-1.7756	-1.77299	-1.77038	-1.76776
-1.78745	-1.78485	-1.78224	-1.77962	-1.77701	-1.77441	-1.77181	-1.76917	-1.76652
-1.78627	-1.78366	-1.78105	-1.77843	-1.77581	-1.77321	-1.7706	-1.76795	-1.76528
-1.78509	-1.78246	-1.77985	-1.77724	-1.77464	-1.77201	-1.76937	-1.76671	-1.76402
-1.78391	-1.78128	-1.77866	-1.77606	-1.77345	-1.77082	-1.76817	-1.7655	-1.76281
-1.78273	-1.78011	-1.77749	-1.77487	-1.77224	-1.76963	-1.767	-1.76434	-1.76166
-1.78155	-1.77894	-1.77632	-1.77368	-1.77105	-1.76844	-1.76583	-1.76318	-1.76051
-1.78039	-1.77776	-1.77513	-1.7725	-1.76988	-1.76725	-1.76463	-1.76199	-1.75935

-1.77922	-1.77658	-1.77394	-1.77133	-1.76871	-1.76607	-1.76342	-1.7608	-1.75818
-1.77804	-1.77541	-1.77278	-1.77015	-1.76752	-1.76488	-1.76224	-1.75961	-1.75698
-1.77686	-1.77426	-1.77163	-1.76897	-1.76631	-1.76369	-1.76107	-1.75842	-1.75576
-1.77572	-1.77312	-1.77049	-1.76782	-1.76514	-1.7625	-1.75987	-1.75722	-1.75457
-1.77461	-1.77201	-1.76938	-1.7667	-1.76401	-1.76133	-1.75866	-1.75601	-1.75339
-1.77349	-1.77087	-1.76823	-1.76556	-1.76287	-1.76017	-1.75747	-1.75482	-1.7522
-1.77234	-1.76969	-1.76703	-1.76439	-1.76173	-1.75903	-1.75633	-1.75365	-1.75099
-1.7712	-1.76851	-1.76584	-1.76321	-1.76057	-1.75789	-1.75518	-1.75249	-1.7498
-1.77009	-1.76738	-1.76469	-1.76203	-1.75936	-1.7567	-1.75403	-1.75134	-1.74864
-1.76897	-1.76627	-1.76356	-1.76084	-1.75815	-1.7555	-1.75286	-1.75019	-1.7475
-1.76781	-1.76513	-1.76242	-1.75969	-1.75698	-1.75432	-1.75167	-1.74901	-1.74634
-1.76662	-1.76397	-1.76129	-1.75856	-1.75584	-1.75314	-1.75045	-1.7478	-1.74517
-1.76544	-1.76279	-1.76012	-1.75742	-1.75471	-1.75199	-1.74928	-1.74662	-1.74398
-1.76427	-1.76158	-1.75891	-1.75625	-1.75358	-1.75087	-1.74815	-1.74545	-1.74278
-1.76312	-1.7604	-1.75772	-1.75509	-1.75245	-1.74975	-1.74703	-1.74431	-1.7416
-1.76199	-1.75925	-1.75657	-1.75394	-1.75131	-1.74862	-1.74591	-1.7432	-1.74048
-1.76087	-1.75812	-1.75542	-1.75279	-1.75016	-1.74748	-1.74479	-1.74209	-1.73936
-1.75974	-1.75699	-1.75428	-1.75161	-1.74896	-1.74631	-1.74365	-1.74096	-1.73825
-1.75861	-1.75586	-1.75314	-1.75043	-1.74775	-1.74512	-1.7425	-1.73983	-1.73713
-1.75749	-1.75474	-1.75201	-1.74928	-1.74659	-1.74397	-1.74137	-1.73871	-1.73601
-1.75636	-1.75362	-1.75089	-1.74816	-1.74546	-1.74284	-1.74024	-1.73758	-1.73489
-1.75524	-1.7525	-1.74976	-1.74703	-1.74434	-1.74171	-1.73911	-1.73646	-1.73377
-1.7541	-1.75137	-1.74864	-1.74591	-1.74321	-1.74059	-1.73798	-1.73533	-1.73265
-1.75298	-1.75025	-1.74752	-1.74479	-1.74209	-1.73945	-1.73683	-1.73418	-1.73152
-1.75186	-1.74913	-1.74641	-1.74367	-1.74096	-1.73829	-1.73564	-1.733	-1.73037
-1.75074	-1.74802	-1.74529	-1.74255	-1.73983	-1.73712	-1.73444	-1.73181	-1.72921
-1.74964	-1.74691	-1.74418	-1.74144	-1.7387	-1.73598	-1.73329	-1.73066	-1.72808
-1.74855	-1.74581	-1.74307	-1.74033	-1.73759	-1.73486	-1.73216	-1.72954	-1.72696
-1.74746	-1.74471	-1.74197	-1.73922	-1.73648	-1.73376	-1.73106	-1.72843	-1.72584
-1.74637	-1.74362	-1.74087	-1.73812	-1.73538	-1.73266	-1.72997	-1.72733	-1.72474
-1.74528	-1.74252	-1.73977	-1.73702	-1.73429	-1.73159	-1.7289	-1.72626	-1.72365
-1.74418	-1.74143	-1.73868	-1.73593	-1.7332	-1.73053	-1.72789	-1.72523	-1.72258
-1.74308	-1.74033	-1.73758	-1.73483	-1.73212	-1.72948	-1.72686	-1.7242	-1.72151
-1.74198	-1.73923	-1.73648	-1.73374	-1.73103	-1.7284	-1.72578	-1.72311	-1.72041
-1.74088	-1.73813	-1.73539	-1.73266	-1.72996	-1.72731	-1.72469	-1.72201	-1.71931
-1.73979	-1.73704	-1.73431	-1.73161	-1.72892	-1.72626	-1.7236	-1.72091	-1.7182
-1.73869	-1.73596	-1.73326	-1.73058	-1.72791	-1.72522	-1.72252	-1.71981	-1.71708
-1.73762	-1.73491	-1.73222	-1.72955	-1.72687	-1.72416	-1.72143	-1.7187	-1.71596
-1.73657	-1.73389	-1.73121	-1.72851	-1.7258	-1.72307	-1.72033	-1.71758	-1.71483
-1.73553	-1.73287	-1.73018	-1.72746	-1.72472	-1.72197	-1.71922	-1.71647	-1.71372
-1.73451	-1.73182	-1.72912	-1.72638	-1.72363	-1.72087	-1.71811	-1.71535	-1.7126
-1.73348	-1.73077	-1.72804	-1.72528	-1.72252	-1.71976	-1.717	-1.71425	-1.71151
-1.73242	-1.72969	-1.72694	-1.72418	-1.72142	-1.71866	-1.7159	-1.71317	-1.71046
-1.73133	-1.72859	-1.72584	-1.72308	-1.72032	-1.71756	-1.71481	-1.7121	-1.70943
-1.73026	-1.72751	-1.72475	-1.72199	-1.71923	-1.71649	-1.71376	-1.71105	-1.70837
-1.72919	-1.72643	-1.72367	-1.7209	-1.71815	-1.71543	-1.71273	-1.71002	-1.7073
-1.72815	-1.72539	-1.72262	-1.71985	-1.7171	-1.71439	-1.71168	-1.70896	-1.70623
-1.72716	-1.7244	-1.72164	-1.71887	-1.7161	-1.71336	-1.71062	-1.70788	-1.70514
-1.71557	-1.71284	-1.72064	-1.71786	-1.71509	-1.71232	-1.70956	-1.7068	-1.70406
-1.71453	-1.71179	-1.71957	-1.7168	-1.71403	-1.71125	-1.70848	-1.70572	-1.70297

-1.71346	-1.71072	-1.71849	-1.71573	-1.71297	-1.71019	-1.70741	-1.70465	-1.70191
-1.71239	-1.70964	-1.71742	-1.71468	-1.71194	-1.70918	-1.70641	-1.70365	-1.70089
-1.71133	-1.70857	-1.71635	-1.71364	-1.71093	-1.70819	-1.70543	-1.70267	-1.69991
-1.71032	-1.70756	-1.70483	-1.70215	-1.70991	-1.70716	-1.70441	-1.70165	-1.69889
-1.70936	-1.7066	-1.70386	-1.70114	-1.70887	-1.70612	-1.70336	-1.70059	-1.69783
-1.70837	-1.70561	-1.70287	-1.70014	-1.70785	-1.70509	-1.70233	-1.69957	-1.6968
-1.70732	-1.70456	-1.70183	-1.69913	-1.70685	-1.70411	-1.70135	-1.69859	-1.69582
-1.70629	-1.70354	-1.70081	-1.69813	-1.69544	-1.69271	-1.68998	-1.6976	-1.69482
-1.70534	-1.70259	-1.69985	-1.69714	-1.69442	-1.69169	-1.68894	-1.69655	-1.69377
-1.7044	-1.70166	-1.69891	-1.69616	-1.69341	-1.69066	-1.6879	-1.69549	-1.69271
-1.70342	-1.7007	-1.69797	-1.69521	-1.69244	-1.68968	-1.68692	-1.69449	-1.6917
-1.70241	-1.69973	-1.69702	-1.69427	-1.6915	-1.68873	-1.68596	-1.69352	-1.69073
-1.70143	-1.69875	-1.69604	-1.69328	-1.69051	-1.68774	-1.68496	-1.68218	-1.67941
-1.70048	-1.69776	-1.69502	-1.69225	-1.68947	-1.6867	-1.68391	-1.68113	-1.67835
-1.69953	-1.69678	-1.69401	-1.69124	-1.68846	-1.68568	-1.68289	-1.68011	-1.67733
-1.69861	-1.69584	-1.69306	-1.69028	-1.6875	-1.68471	-1.68193	-1.67915	-1.67638
-1.69767	-1.69488	-1.6921	-1.68931	-1.68653	-1.68374	-1.68096	-1.6782	-1.67544
-1.69665	-1.69386	-1.69107	-1.68828	-1.68549	-1.68271	-1.67994	-1.67719	-1.67446
-1.69562	-1.69283	-1.69003	-1.68723	-1.68445	-1.68166	-1.67889	-1.67617	-1.67348
-1.69465	-1.69185	-1.68905	-1.68625	-1.68346	-1.68068	-1.67791	-1.67518	-1.67248
-1.69372	-1.69092	-1.68812	-1.68532	-1.68252	-1.67974	-1.67696	-1.6742	-1.67146
-1.6928	-1.69	-1.68719	-1.68439	-1.6816	-1.67881	-1.67603	-1.67325	-1.67048
-1.69189	-1.68909	-1.68628	-1.68348	-1.68069	-1.6779	-1.67511	-1.67233	-1.66955
-1.69099	-1.68818	-1.68538	-1.68258	-1.67978	-1.67699	-1.6742	-1.67142	-1.66863
-1.69008	-1.68728	-1.68447	-1.68167	-1.67888	-1.67608	-1.67329	-1.6705	-1.66771
-1.68917	-1.68637	-1.68356	-1.68076	-1.67797	-1.67517	-1.67238	-1.66958	-1.66678
-1.68825	-1.68544	-1.68264	-1.67984	-1.67705	-1.67426	-1.67146	-1.66864	-1.6658
-1.6873	-1.68449	-1.68169	-1.6789	-1.67611	-1.67332	-1.67052	-1.66768	-1.66481
-1.68631	-1.6835	-1.6807	-1.67791	-1.67512	-1.67233	-1.66953	-1.6667	-1.66383
-1.68527	-1.68245	-1.67965	-1.67686	-1.67407	-1.67129	-1.6685	-1.66569	-1.66287
-1.68427	-1.68145	-1.67865	-1.67585	-1.67307	-1.67029	-1.66751	-1.66472	-1.66193
-1.68332	-1.68051	-1.67771	-1.67492	-1.67213	-1.66935	-1.66657	-1.66379	-1.661
-1.68239	-1.67959	-1.67679	-1.674	-1.67122	-1.66843	-1.66566	-1.66287	-1.66009
-1.68148	-1.67868	-1.67589	-1.6731	-1.67031	-1.66753	-1.66475	-1.66197	-1.65919
-1.68058	-1.67779	-1.675	-1.67221	-1.66942	-1.66664	-1.66386	-1.66108	-1.6583
-1.67968	-1.67689	-1.6741	-1.67132	-1.66853	-1.66575	-1.66297	-1.66019	-1.6574
-1.67878	-1.676	-1.67321	-1.67042	-1.66764	-1.66486	-1.66208	-1.65929	-1.6565
-1.67789	-1.6751	-1.67231	-1.66953	-1.66674	-1.66396	-1.66118	-1.65839	-1.6556
-1.67701	-1.6742	-1.6714	-1.66862	-1.66584	-1.66306	-1.66027	-1.65748	-1.6547
-1.67616	-1.67332	-1.67051	-1.66772	-1.66494	-1.66215	-1.65936	-1.65658	-1.65379
-1.67535	-1.67248	-1.66963	-1.66683	-1.66404	-1.66125	-1.65846	-1.65567	-1.65289
-1.67454	-1.67165	-1.66878	-1.66596	-1.66315	-1.66035	-1.65756	-1.65477	-1.65198
-1.6737	-1.67084	-1.66797	-1.66512	-1.66227	-1.65946	-1.65666	-1.65386	-1.65106
-1.67285	-1.67003	-1.66718	-1.6643	-1.66142	-1.65858	-1.65576	-1.65295	-1.65015
-1.67196	-1.66917	-1.66634	-1.66347	-1.6606	-1.65774	-1.65489	-1.65206	-1.64924
-1.67106	-1.66828	-1.66548	-1.66265	-1.6598	-1.65692	-1.65403	-1.65118	-1.64834
-1.67015	-1.66738	-1.6646	-1.6618	-1.65897	-1.65609	-1.6532	-1.65033	-1.64746
-1.66925	-1.66648	-1.6637	-1.66091	-1.6581	-1.65526	-1.6524	-1.64952	-1.64661
-1.66835	-1.66558	-1.6628	-1.66001	-1.65721	-1.65441	-1.65158	-1.6487	-1.64578
-1.66746	-1.66468	-1.6619	-1.65911	-1.65632	-1.65352	-1.6507	-1.64785	-1.64497

-1.21946	-1.21764	-1.25097	-1.24911	-1.2786	-1.27668	-1.27475	-1.27284	-1.27094
-1.22009	-1.21828	-1.25163	-1.24976	-1.27927	-1.27735	-1.27543	-1.27351	-1.27158
-1.22072	-1.21892	-1.25229	-1.25041	-1.27994	-1.27802	-1.27611	-1.27417	-1.27222
-1.22135	-1.21955	-1.21774	-1.21592	-1.24918	-1.24731	-1.24544	-1.27481	-1.27285
-1.22198	-1.22017	-1.21835	-1.21654	-1.24981	-1.24794	-1.24605	-1.27544	-1.27348
-1.2226	-1.22078	-1.21897	-1.21716	-1.25044	-1.24856	-1.24668	-1.24479	-1.2429
-1.2232	-1.2214	-1.21959	-1.21777	-1.25106	-1.24919	-1.24733	-1.24545	-1.24356
-1.2238	-1.22201	-1.2202	-1.21839	-1.21657	-1.21477	-1.21296	-1.24609	-1.24421
-1.22441	-1.22261	-1.22081	-1.219	-1.21719	-1.21538	-1.21357	-1.24671	-1.24486
-1.22501	-1.22321	-1.22141	-1.21961	-1.21781	-1.216	-1.21418	-1.24734	-1.2455
-1.22561	-1.22382	-1.22203	-1.22023	-1.21842	-1.21661	-1.2148	-1.24797	-1.24612
-1.22621	-1.22443	-1.22264	-1.22084	-1.21904	-1.21723	-1.21543	-1.24861	-1.24674
-1.20775	-1.20599	-1.22326	-1.22146	-1.21967	-1.21786	-1.21606	-1.21425	-1.21246
-1.20836	-1.20659	-1.22387	-1.22209	-1.22031	-1.2185	-1.21669	-1.21489	-1.21311
-1.13835	-1.13672	-1.20543	-1.20368	-1.22092	-1.21913	-1.21733	-1.21554	-1.21377
-1.13887	-1.13725	-1.20604	-1.20428	-1.22153	-1.21975	-1.21797	-1.21619	-1.21441
-1.1394	-1.1378	-1.15275	-1.15108	-1.20312	-1.20138	-1.19963	-1.21678	-1.21501
-1.13997	-1.13836	-1.15332	-1.15166	-1.20375	-1.202	-1.20026	-1.21738	-1.2156
-1.12436	-1.12278	-1.13731	-1.13568	-1.15058	-1.14893	-1.14727	-1.21798	-1.21619
-1.12494	-1.12334	-1.13786	-1.13625	-1.15117	-1.14951	-1.14784	-1.21858	-1.21681
-1.12551	-1.12389	-1.13842	-1.13682	-1.15177	-1.15009	-1.14841	-1.21919	-1.21743
-1.0849	-1.08339	-1.12286	-1.12129	-1.15264	-1.15098	-1.14932	-1.16418	-1.16253
-1.08541	-1.08391	-1.12342	-1.12185	-1.15319	-1.15154	-1.14988	-1.16476	-1.16311
-1.08592	-1.08443	-1.08293	-1.08144	-1.15372	-1.15208	-1.15044	-1.14881	-1.14719
-1.0864	-1.08493	-1.08344	-1.08193	-1.15424	-1.15261	-1.15098	-1.14936	-1.14773
-1.08691	-1.08544	-1.08471	-1.08321	-1.08171	-1.08024	-1.07878	-1.14991	-1.14827
-1.08744	-1.08597	-1.08522	-1.08372	-1.08222	-1.08075	-1.07929	-1.15046	-1.14881
-1.08798	-1.0865	-1.08572	-1.08423	-1.08274	-1.08127	-1.0798	-1.15103	-1.14935
-1.05105	-1.04962	-1.08619	-1.08472	-1.08325	-1.08179	-1.08033	-1.09607	-1.09452
-1.0515	-1.05007	-1.08665	-1.0852	-1.08376	-1.08231	-1.08086	-1.09663	-1.09507
-1.05198	-1.05054	-1.05009	-1.04871	-1.08426	-1.08282	-1.08138	-1.09717	-1.09562
-1.05247	-1.05104	-1.05055	-1.04917	-1.08475	-1.08332	-1.08189	-1.0977	-1.09615
-1.05296	-1.05153	-1.05103	-1.04964	-1.04826	-1.0469	-1.04554	-1.08092	-1.07941
-1.05348	-1.05204	-1.05151	-1.05012	-1.04874	-1.04737	-1.046	-1.08141	-1.07992
-1.03365	-1.03227	-1.052	-1.0506	-1.04922	-1.04784	-1.04646	-1.04506	-1.04365
-1.03409	-1.03271	-1.05247	-1.05108	-1.04969	-1.04831	-1.04692	-1.04553	-1.04414
-1.03453	-1.03315	-1.05294	-1.05155	-1.05017	-1.04878	-1.04739	-1.04601	-1.04462
-1.034	-1.03261	-1.05341	-1.05203	-1.05065	-1.04927	-1.04789	-1.04651	-1.04512
-1.03447	-1.03308	-1.05389	-1.05251	-1.05113	-1.04977	-1.0484	-1.04702	-1.04561
-1.03494	-1.03355	-1.03314	-1.0318	-1.05162	-1.05026	-1.0489	-1.04751	-1.0461
-1.03542	-1.03404	-1.03361	-1.03227	-1.05211	-1.05075	-1.04939	-1.04799	-1.04658
-1.03589	-1.03451	-1.03314	-1.0318	-1.0526	-1.05124	-1.04987	-1.04848	-1.04707
-1.03634	-1.03497	-1.0336	-1.03225	-1.05308	-1.05172	-1.05036	-1.04896	-1.04756
-1.0368	-1.03542	-1.03406	-1.03271	-1.03229	-1.03098	-1.02967	-1.04945	-1.04804
-1.03726	-1.03589	-1.03452	-1.03317	-1.03273	-1.03142	-1.03011	-1.04993	-1.04853
-1.03773	-1.03635	-1.03499	-1.03364	-1.03318	-1.03187	-1.03056	-1.05041	-1.04901
-1.01523	-1.01391	-1.03546	-1.03411	-1.03277	-1.03145	-1.03013	-1.05019	-1.0488
-1.01565	-1.01435	-1.03593	-1.03459	-1.03326	-1.03194	-1.03062	-1.05071	-1.04933
-0.99143	-0.99021	-1.03641	-1.03507	-1.03373	-1.03241	-1.03109	-1.02977	-1.02845
-0.99185	-0.99064	-1.0369	-1.03555	-1.0342	-1.03287	-1.03154	-1.03022	-1.02891

-0.99227	-0.99107	-1.01442	-1.01312	-1.03467	-1.03333	-1.032	-1.03068	-1.02938
-0.99268	-0.99148	-1.01487	-1.01358	-1.03516	-1.03382	-1.03247	-1.03114	-1.02983
-0.99309	-0.9919	-1.01532	-1.01404	-1.03565	-1.0343	-1.03295	-1.03161	-1.03029
-0.97237	-0.9712	-0.99111	-0.98991	-1.03614	-1.03479	-1.03344	-1.03211	-1.03078
-0.97276	-0.97159	-0.99152	-0.99032	-1.03662	-1.03528	-1.03394	-1.03261	-1.03128
-0.97315	-0.97198	-0.97081	-0.96964	-0.98954	-0.98833	-0.98711	-1.03312	-1.03179
-0.97356	-0.97238	-0.97121	-0.97004	-0.98996	-0.98877	-0.98757	-1.03363	-1.03231
-0.97396	-0.97278	-0.9716	-0.97043	-0.96927	-0.96811	-0.96695	-1.03413	-1.03281
-0.97437	-0.97318	-0.97199	-0.97083	-0.96967	-0.96851	-0.96735	-1.03461	-1.0333
-0.97477	-0.97358	-0.9724	-0.97123	-0.97008	-0.96892	-0.96775	-0.96659	-0.96541
-0.97517	-0.974	-0.97283	-0.97166	-0.9705	-0.96933	-0.96816	-0.96699	-0.96582
-0.97556	-0.97441	-0.97325	-0.97209	-0.97092	-0.96975	-0.96857	-0.9674	-0.96623
-0.95626	-0.95513	-0.97364	-0.97247	-0.97131	-0.97015	-0.96898	-0.96782	-0.96665
-0.95664	-0.9555	-0.97401	-0.97284	-0.97169	-0.97054	-0.9694	-0.96825	-0.96708
-0.95702	-0.95588	-0.95475	-0.95361	-0.97207	-0.97093	-0.9698	-0.96865	-0.96749
-0.95741	-0.95627	-0.95514	-0.954	-0.97247	-0.97132	-0.97019	-0.96904	-0.96789
-0.95779	-0.95666	-0.95553	-0.9544	-0.95327	-0.95216	-0.95105	-0.94993	-0.9488
-0.95818	-0.95706	-0.95593	-0.95481	-0.95369	-0.95258	-0.95147	-0.95034	-0.9492
-0.93677	-0.93567	-0.95633	-0.95522	-0.95411	-0.95299	-0.95188	-0.95075	-0.94961
-0.93715	-0.93605	-0.95672	-0.95561	-0.9545	-0.95339	-0.95228	-0.95115	-0.95002
-0.93752	-0.93642	-0.95711	-0.956	-0.9549	-0.95379	-0.95268	-0.95156	-0.95043
-0.93791	-0.93681	-0.95751	-0.95641	-0.95531	-0.9542	-0.95309	-0.95197	-0.95084
-0.93831	-0.93721	-0.95792	-0.95682	-0.95572	-0.95462	-0.95351	-0.95238	-0.95124
-0.9387	-0.9376	-0.93651	-0.93543	-0.95612	-0.95502	-0.95392	-0.95279	-0.95165
-0.93909	-0.93799	-0.93689	-0.9358	-0.9565	-0.95541	-0.95431	-0.95319	-0.95207
-0.93948	-0.93838	-0.93728	-0.93618	-0.9351	-0.93402	-0.93294	-0.9536	-0.95249
-0.93989	-0.93878	-0.93768	-0.93659	-0.93551	-0.93443	-0.93335	-0.95402	-0.95291
-0.9403	-0.93918	-0.93808	-0.93699	-0.93592	-0.93484	-0.93375	-0.95444	-0.95333
-0.94069	-0.93956	-0.93845	-0.93737	-0.9363	-0.93522	-0.93413	-0.93305	-0.93197
-0.94107	-0.93994	-0.93883	-0.93775	-0.93668	-0.93559	-0.93451	-0.93343	-0.93236
-0.94149	-0.94035	-0.93923	-0.93814	-0.93706	-0.93598	-0.9349	-0.93383	-0.93276
-0.94193	-0.94078	-0.93965	-0.93855	-0.93746	-0.93638	-0.9353	-0.93423	-0.93316
-0.94237	-0.94121	-0.94006	-0.93895	-0.93785	-0.93676	-0.93569	-0.93462	-0.93355
-0.94281	-0.94163	-0.94047	-0.93935	-0.93824	-0.93714	-0.93606	-0.93498	-0.93391
-0.94324	-0.94206	-0.9409	-0.93976	-0.93864	-0.93754	-0.93645	-0.93537	-0.93429
-0.94368	-0.9425	-0.94134	-0.9402	-0.93907	-0.93796	-0.93687	-0.93579	-0.93471
-0.94411	-0.94295	-0.9418	-0.94065	-0.93951	-0.9384	-0.93731	-0.93622	-0.93514
-0.94458	-0.94342	-0.94227	-0.94112	-0.93998	-0.93886	-0.93775	-0.93666	-0.93557
-0.94505	-0.94389	-0.94274	-0.94159	-0.94045	-0.93933	-0.93821	-0.93711	-0.93601
-0.9455	-0.94435	-0.9432	-0.94206	-0.94091	-0.93978	-0.93866	-0.93754	-0.93644
-0.94594	-0.9448	-0.94365	-0.94251	-0.94137	-0.94023	-0.93909	-0.93797	-0.93687
-0.94636	-0.94522	-0.94408	-0.94294	-0.9418	-0.94066	-0.93951	-0.93839	-0.93728
-0.94676	-0.94562	-0.94448	-0.94335	-0.94221	-0.94106	-0.93991	-0.93879	-0.93767
-0.94717	-0.94603	-0.94489	-0.94376	-0.94262	-0.94147	-0.94033	-0.9392	-0.93808
-0.9476	-0.94647	-0.94534	-0.94421	-0.94307	-0.94192	-0.94077	-0.93964	-0.93851
-0.94806	-0.94692	-0.94579	-0.94466	-0.94353	-0.94238	-0.94124	-0.94009	-0.93895
-0.94851	-0.94738	-0.94624	-0.94511	-0.94398	-0.94284	-0.9417	-0.94055	-0.93941
-0.94898	-0.94783	-0.94669	-0.94556	-0.94443	-0.94329	-0.94216	-0.94102	-0.93987
-0.94943	-0.94828	-0.94714	-0.946	-0.94487	-0.94374	-0.94262	-0.94148	-0.94033
-0.94987	-0.94872	-0.94758	-0.94645	-0.94532	-0.94419	-0.94306	-0.94192	-0.94078

-0.9042	-0.90315	-0.94801	-0.94689	-0.94576	-0.94463	-0.9435	-0.94237	-0.94123
-0.90458	-0.90354	-0.94844	-0.94732	-0.9462	-0.94507	-0.94394	-0.94281	-0.94168
-0.90496	-0.90393	-0.94887	-0.94775	-0.94663	-0.9455	-0.94438	-0.94325	-0.94213
-0.88338	-0.88237	-0.94929	-0.94817	-0.94706	-0.94594	-0.94482	-0.9437	-0.94257
-0.88377	-0.88275	-0.94972	-0.9486	-0.94748	-0.94637	-0.94526	-0.94414	-0.94301
-0.88417	-0.88315	-0.88213	-0.88113	-0.94793	-0.94682	-0.9457	-0.94458	-0.94345
-0.88458	-0.88355	-0.88253	-0.88152	-0.94838	-0.94726	-0.94614	-0.94502	-0.94388
-0.88498	-0.88395	-0.88292	-0.88191	-0.92761	-0.92651	-0.92542	-0.94545	-0.94432
-0.88538	-0.88434	-0.88331	-0.88229	-0.92801	-0.92691	-0.92582	-0.94588	-0.94476
-0.88578	-0.88474	-0.8837	-0.88268	-0.88166	-0.88066	-0.87965	-0.92515	-0.92406
-0.88618	-0.88514	-0.88411	-0.88308	-0.88206	-0.88106	-0.88006	-0.92558	-0.9245
-0.88659	-0.88555	-0.88451	-0.88349	-0.88247	-0.88147	-0.88047	-0.92604	-0.92494
-0.887	-0.88596	-0.88492	-0.88391	-0.8829	-0.8819	-0.8809	-0.8799	-0.87889
-0.88741	-0.88637	-0.88534	-0.88433	-0.88333	-0.88233	-0.88133	-0.88032	-0.8793
-0.88783	-0.88678	-0.88575	-0.88475	-0.88375	-0.88276	-0.88175	-0.88074	-0.87972
-0.88825	-0.88719	-0.88616	-0.88517	-0.88417	-0.88318	-0.88217	-0.88116	-0.88013
-0.86024	-0.85925	-0.88659	-0.88559	-0.8846	-0.8836	-0.88259	-0.88157	-0.88055
-0.86062	-0.85965	-0.88703	-0.88603	-0.88504	-0.88402	-0.88301	-0.88199	-0.88097
-0.86101	-0.86005	-0.8591	-0.85817	-0.88547	-0.88445	-0.88343	-0.88241	-0.88139
-0.8614	-0.86046	-0.85952	-0.85858	-0.88591	-0.88489	-0.88386	-0.88283	-0.88181
-0.8618	-0.86087	-0.85993	-0.85898	-0.88635	-0.88532	-0.88429	-0.88326	-0.88223
-0.8622	-0.86127	-0.86033	-0.85938	-0.88678	-0.88575	-0.88472	-0.88368	-0.88265
-0.8626	-0.86166	-0.86071	-0.85976	-0.8872	-0.88617	-0.88514	-0.88411	-0.88308
-0.86302	-0.86206	-0.8611	-0.86015	-0.85919	-0.85823	-0.85727	-0.88454	-0.88352
-0.86345	-0.86246	-0.86149	-0.86054	-0.85958	-0.85863	-0.85767	-0.88499	-0.88398
-0.82312	-0.82209	-0.86189	-0.86093	-0.85998	-0.85902	-0.85807	-0.85713	-0.85619
-0.82359	-0.82256	-0.86229	-0.86133	-0.86038	-0.85943	-0.85849	-0.85754	-0.8566
-0.82407	-0.82303	-0.82202	-0.82103	-0.82005	-0.81908	-0.81812	-0.81716	-0.8162
-0.82454	-0.8235	-0.82249	-0.8215	-0.82052	-0.81955	-0.81858	-0.81762	-0.81666
-0.825	-0.82398	-0.82296	-0.82197	-0.82099	-0.82001	-0.81904	-0.81808	-0.81712
-0.82546	-0.82444	-0.82343	-0.82244	-0.82145	-0.82047	-0.81949	-0.81853	-0.81757
-0.82592	-0.82491	-0.8239	-0.8229	-0.8219	-0.82092	-0.81994	-0.81897	-0.81802
-0.8264	-0.82538	-0.82438	-0.82337	-0.82237	-0.82138	-0.8204	-0.81943	-0.81848
-0.82688	-0.82588	-0.82487	-0.82386	-0.82286	-0.82186	-0.82088	-0.81991	-0.81895
-0.82738	-0.82637	-0.82537	-0.82435	-0.82334	-0.82235	-0.82136	-0.82039	-0.81943
-0.8279	-0.82687	-0.82586	-0.82484	-0.82384	-0.82285	-0.82186	-0.82089	-0.81991
-0.82842	-0.82738	-0.82635	-0.82534	-0.82434	-0.82335	-0.82237	-0.82139	-0.8204
-0.82893	-0.82788	-0.82685	-0.82584	-0.82483	-0.82385	-0.82287	-0.82189	-0.82091
-0.82944	-0.82839	-0.82736	-0.82634	-0.82534	-0.82435	-0.82337	-0.82239	-0.82141
-0.82998	-0.82892	-0.82788	-0.82686	-0.82585	-0.82485	-0.82387	-0.82289	-0.82191
-0.83053	-0.82946	-0.82841	-0.82738	-0.82637	-0.82536	-0.82437	-0.82338	-0.82239
-0.77714	-0.77614	-0.82895	-0.82792	-0.8269	-0.82588	-0.82487	-0.82387	-0.82288
-0.77763	-0.77663	-0.8295	-0.82846	-0.82744	-0.8264	-0.82536	-0.82436	-0.82337
-0.77813	-0.77712	-0.83006	-0.82901	-0.82797	-0.82691	-0.82586	-0.82485	-0.82386
-0.77862	-0.77762	-0.83064	-0.82956	-0.82849	-0.82742	-0.82637	-0.82535	-0.82435
-0.77912	-0.77812	-0.83122	-0.8301	-0.829	-0.82794	-0.82689	-0.82586	-0.82485
-0.77962	-0.77861	-0.7776	-0.77658	-0.82954	-0.82847	-0.82742	-0.82639	-0.82537
-0.78011	-0.77909	-0.77807	-0.77705	-0.83009	-0.82901	-0.82797	-0.82692	-0.82589
-0.78061	-0.77958	-0.77856	-0.77753	-0.77653	-0.77555	-0.77458	-0.82746	-0.82643
-0.78111	-0.78008	-0.77906	-0.77804	-0.77703	-0.77604	-0.77506	-0.82801	-0.82698

-0.75033	-0.74937	-0.77957	-0.77855	-0.77754	-0.77654	-0.77556	-0.79761	-0.79662
-0.75084	-0.74987	-0.7801	-0.77907	-0.77805	-0.77705	-0.77607	-0.79813	-0.79713
-0.75135	-0.75037	-0.7494	-0.74843	-0.77857	-0.77758	-0.7766	-0.77561	-0.77462
-0.75184	-0.75087	-0.7499	-0.74893	-0.77911	-0.77811	-0.77713	-0.77613	-0.77514
-0.75234	-0.75137	-0.7504	-0.74943	-0.77965	-0.77864	-0.77766	-0.77666	-0.77566
-0.72184	-0.72093	-0.7509	-0.74992	-0.78018	-0.77917	-0.77817	-0.77717	-0.77617
-0.72232	-0.7214	-0.75139	-0.75042	-0.78071	-0.77969	-0.77868	-0.77768	-0.77668
-0.7228	-0.72187	-0.7519	-0.75091	-0.74994	-0.74898	-0.74803	-0.77819	-0.77719
-0.72328	-0.72235	-0.75241	-0.75142	-0.75043	-0.74947	-0.74852	-0.7787	-0.77769
-0.72376	-0.72284	-0.72191	-0.72096	-0.75093	-0.74996	-0.74901	-0.74805	-0.74709
-0.72426	-0.72332	-0.72238	-0.72144	-0.75144	-0.75046	-0.74948	-0.74852	-0.74757
-0.72477	-0.72381	-0.72285	-0.72192	-0.75196	-0.75096	-0.74997	-0.74901	-0.74807
-0.72528	-0.72431	-0.72335	-0.7224	-0.72147	-0.72053	-0.7196	-0.74952	-0.74857
-0.72579	-0.72482	-0.72385	-0.72289	-0.72195	-0.72101	-0.72009	-0.75004	-0.74908
-0.72633	-0.72535	-0.72438	-0.7234	-0.72244	-0.7215	-0.72058	-0.71966	-0.71875
-0.72689	-0.7259	-0.72492	-0.72393	-0.72295	-0.72201	-0.72108	-0.72015	-0.71923
-0.72743	-0.72644	-0.72545	-0.72445	-0.72347	-0.72251	-0.72157	-0.72064	-0.71971
-0.72797	-0.72697	-0.72597	-0.72497	-0.72398	-0.72301	-0.72206	-0.72112	-0.72021
-0.69875	-0.6978	-0.72649	-0.7255	-0.72451	-0.72352	-0.72255	-0.72161	-0.72071
-0.69924	-0.6983	-0.72704	-0.72604	-0.72505	-0.72405	-0.72306	-0.72212	-0.72121
-0.69973	-0.6988	-0.72758	-0.72659	-0.72559	-0.72457	-0.72358	-0.72263	-0.72172
-0.70024	-0.69932	-0.72813	-0.72712	-0.72612	-0.72511	-0.7241	-0.72315	-0.72223
-0.70077	-0.69984	-0.72867	-0.72766	-0.72665	-0.72564	-0.72464	-0.72368	-0.72274
-0.70131	-0.70036	-0.69941	-0.69844	-0.72719	-0.72618	-0.72519	-0.72421	-0.72325
-0.70186	-0.70089	-0.69992	-0.69895	-0.72774	-0.72673	-0.72574	-0.72474	-0.72375
-0.70242	-0.70142	-0.70044	-0.69947	-0.69851	-0.69756	-0.69662	-0.72528	-0.72428
-0.70297	-0.70196	-0.70096	-0.69999	-0.69902	-0.69808	-0.69713	-0.72583	-0.72482
-0.68624	-0.68526	-0.70149	-0.70051	-0.69954	-0.69859	-0.69765	-0.72639	-0.72537
-0.68677	-0.6858	-0.70205	-0.70105	-0.70007	-0.69911	-0.69816	-0.72693	-0.72591
-0.68731	-0.68634	-0.70261	-0.7016	-0.70061	-0.69963	-0.69866	-0.72747	-0.72646
-0.68785	-0.68689	-0.70318	-0.70217	-0.70117	-0.70018	-0.69919	-0.69823	-0.69727
-0.68841	-0.68744	-0.70376	-0.70276	-0.70176	-0.70074	-0.69974	-0.69876	-0.6978
-0.68896	-0.688	-0.68704	-0.68609	-0.70234	-0.70131	-0.70029	-0.6993	-0.69834
-0.6895	-0.68856	-0.68761	-0.68665	-0.70292	-0.70189	-0.70086	-0.69986	-0.69888
-0.69004	-0.68911	-0.68817	-0.68721	-0.68623	-0.68524	-0.68425	-0.70042	-0.69942
-0.69058	-0.68966	-0.68872	-0.68774	-0.68676	-0.68578	-0.6848	-0.70098	-0.69997
-0.69111	-0.6902	-0.68926	-0.68827	-0.68728	-0.68631	-0.68535	-0.70155	-0.70053
-0.69166	-0.69074	-0.68979	-0.68881	-0.68783	-0.68686	-0.6859	-0.68493	-0.68397
-0.6922	-0.69127	-0.69033	-0.68936	-0.68838	-0.68742	-0.68645	-0.68549	-0.68453
-0.69275	-0.69181	-0.69086	-0.68989	-0.68893	-0.68797	-0.68701	-0.68605	-0.68509
-0.69329	-0.69235	-0.6914	-0.69043	-0.68947	-0.68852	-0.68757	-0.68661	-0.68566
-0.69385	-0.69289	-0.69194	-0.69097	-0.69001	-0.68906	-0.68812	-0.68717	-0.68621
-0.69441	-0.69345	-0.69248	-0.69151	-0.69054	-0.6896	-0.68866	-0.68772	-0.68677
-0.69497	-0.694	-0.69303	-0.69205	-0.69108	-0.69013	-0.6892	-0.68826	-0.68732
-0.69554	-0.69457	-0.69359	-0.6926	-0.69162	-0.69066	-0.68972	-0.68879	-0.68787
-0.69612	-0.69514	-0.69415	-0.69315	-0.69216	-0.69119	-0.69025	-0.68932	-0.68842
-0.69671	-0.69572	-0.69472	-0.69371	-0.69271	-0.69174	-0.69078	-0.68986	-0.68895
-0.69731	-0.69631	-0.6953	-0.69428	-0.69328	-0.6923	-0.69133	-0.6904	-0.68948
-0.6324	-0.63158	-0.69589	-0.69488	-0.69387	-0.69287	-0.69189	-0.69095	-0.69002
-0.6329	-0.63208	-0.6965	-0.69549	-0.69448	-0.69347	-0.69247	-0.69151	-0.69057

-0.6334	-0.63257	-0.66726	-0.66632	-0.69509	-0.69408	-0.69307	-0.69209	-0.69113
-0.63391	-0.63308	-0.66784	-0.66687	-0.69569	-0.69468	-0.69369	-0.6927	-0.69171
-0.60339	-0.60261	-0.63275	-0.63192	-0.66647	-0.66554	-0.66462	-0.69331	-0.69231
-0.60387	-0.60308	-0.63326	-0.63242	-0.66703	-0.6661	-0.66518	-0.69393	-0.69293
-0.60437	-0.60356	-0.63377	-0.63292	-0.66761	-0.66667	-0.66574	-0.69454	-0.69356
-0.56888	-0.5681	-0.60322	-0.60243	-0.6326	-0.63179	-0.63099	-0.66538	-0.66447
-0.56935	-0.56854	-0.60369	-0.6029	-0.63312	-0.6323	-0.63148	-0.66594	-0.66502
-0.56983	-0.569	-0.56822	-0.56748	-0.63363	-0.6328	-0.63198	-0.66651	-0.66559
-0.57032	-0.56948	-0.56868	-0.56792	-0.63414	-0.6333	-0.63249	-0.66709	-0.66616
-0.53415	-0.53336	-0.56916	-0.56838	-0.56761	-0.56687	-0.56615	-0.63218	-0.63138
-0.53461	-0.53383	-0.56967	-0.56886	-0.56807	-0.56732	-0.56658	-0.63269	-0.63188
-0.53507	-0.5343	-0.57017	-0.56935	-0.56855	-0.56777	-0.56702	-0.6332	-0.63238
-0.49524	-0.49464	-0.53399	-0.53321	-0.53245	-0.5317	-0.53097	-0.56672	-0.566
-0.49561	-0.49502	-0.53445	-0.53368	-0.53291	-0.53214	-0.53138	-0.56716	-0.56644
-0.49597	-0.49538	-0.5349	-0.53413	-0.53336	-0.53258	-0.53182	-0.5311	-0.53041
-0.49633	-0.49573	-0.53533	-0.53456	-0.5338	-0.53303	-0.53228	-0.53154	-0.53083
-0.49669	-0.49609	-0.49549	-0.4949	-0.53423	-0.53347	-0.53272	-0.53198	-0.53124
-0.49706	-0.49644	-0.49584	-0.49525	-0.53466	-0.5339	-0.53315	-0.5324	-0.53165
-0.39217	-0.39177	-0.49619	-0.4956	-0.49501	-0.49443	-0.49385	-0.53282	-0.53207
-0.39239	-0.392	-0.49656	-0.49596	-0.49536	-0.49477	-0.49419	-0.53327	-0.53251
-0.39262	-0.39223	-0.49694	-0.49633	-0.49573	-0.49513	-0.49454	-0.53372	-0.53297
-0.39287	-0.39248	-0.39209	-0.3917	-0.49612	-0.49552	-0.49493	-0.49434	-0.49376
-0.39312	-0.39274	-0.39235	-0.39195	-0.49652	-0.49592	-0.49532	-0.49473	-0.49415
-0.39335	-0.39297	-0.39258	-0.39219	-0.3918	-0.39142	-0.39103	-0.4951	-0.49451
-0.39357	-0.39319	-0.39281	-0.39242	-0.39204	-0.39165	-0.39125	-0.49545	-0.49485
-0.39379	-0.3934	-0.39302	-0.39265	-0.39227	-0.39188	-0.39148	-0.42612	-0.42565
-0.394	-0.39361	-0.39323	-0.39286	-0.39248	-0.39209	-0.3917	-0.42639	-0.42593
-0.39424	-0.39384	-0.39345	-0.39308	-0.3927	-0.39232	-0.39193	-0.39155	-0.39118
-0.39452	-0.3941	-0.3937	-0.39332	-0.39294	-0.39256	-0.39218	-0.3918	-0.39142
-0.39481	-0.39437	-0.39396	-0.39357	-0.39319	-0.3928	-0.39243	-0.39204	-0.39166
-0.37574	-0.37536	-0.375	-0.37466	-0.39341	-0.39303	-0.39266	-0.39227	-0.39189
-0.37597	-0.37559	-0.37521	-0.37486	-0.39363	-0.39325	-0.39288	-0.3925	-0.39212
-0.37622	-0.37583	-0.37545	-0.37508	-0.37473	-0.3744	-0.37409	-0.39274	-0.39235
-0.37647	-0.37609	-0.37571	-0.37533	-0.37497	-0.37463	-0.3743	-0.39298	-0.3926
-0.37673	-0.37634	-0.37596	-0.37558	-0.3752	-0.37485	-0.37451	-0.37418	-0.37386
-0.37698	-0.37659	-0.3762	-0.37581	-0.37543	-0.37506	-0.37469	-0.37436	-0.37405
-0.37725	-0.37684	-0.37644	-0.37605	-0.37567	-0.37528	-0.3749	-0.37456	-0.37425
-0.37753	-0.3771	-0.37669	-0.3763	-0.37592	-0.37553	-0.37515	-0.37479	-0.37446
-0.37784	-0.37738	-0.37695	-0.37656	-0.37617	-0.37579	-0.37541	-0.37504	-0.37468
-0.37813	-0.37766	-0.37722	-0.37682	-0.37642	-0.37603	-0.37565	-0.37528	-0.3749
-0.37843	-0.37796	-0.37751	-0.37708	-0.37667	-0.37627	-0.37588	-0.3755	-0.37513
-0.37873	-0.37827	-0.37781	-0.37737	-0.37694	-0.37653	-0.37613	-0.37575	-0.37537
-0.37903	-0.37859	-0.37814	-0.37768	-0.37724	-0.37681	-0.3764	-0.37602	-0.37564
-0.37933	-0.37889	-0.37845	-0.37799	-0.37753	-0.3771	-0.37668	-0.37629	-0.37591
-0.37962	-0.37918	-0.37873	-0.37827	-0.37782	-0.37738	-0.37695	-0.37655	-0.37618
-0.37991	-0.37946	-0.37901	-0.37856	-0.37811	-0.37767	-0.37723	-0.37682	-0.37644
-0.38022	-0.37977	-0.37933	-0.37888	-0.37843	-0.37798	-0.37753	-0.3771	-0.3767
-0.38052	-0.38009	-0.37965	-0.37921	-0.37876	-0.3783	-0.37784	-0.37739	-0.37696
-0.38081	-0.38038	-0.37995	-0.37951	-0.37907	-0.37861	-0.37816	-0.3777	-0.37725
-0.38109	-0.38066	-0.38023	-0.37979	-0.37935	-0.37891	-0.37847	-0.37802	-0.37758

-0.38138	-0.38095	-0.38051	-0.38008	-0.37965	-0.37921	-0.37878	-0.37834	-0.3779
-0.38169	-0.38125	-0.38081	-0.38037	-0.37994	-0.37951	-0.37908	-0.37865	-0.37822
-0.382	-0.38155	-0.38111	-0.38067	-0.38024	-0.37981	-0.37938	-0.37895	-0.37852
-0.38231	-0.38187	-0.38143	-0.38099	-0.38055	-0.38012	-0.37968	-0.37925	-0.37882
-0.38263	-0.38219	-0.38175	-0.38131	-0.38087	-0.38042	-0.37998	-0.37954	-0.37912
-0.38295	-0.3825	-0.38205	-0.3816	-0.38116	-0.38072	-0.38029	-0.37985	-0.37942
-0.38327	-0.38279	-0.38233	-0.38188	-0.38145	-0.38102	-0.3806	-0.38017	-0.37973
-0.34232	-0.3419	-0.38264	-0.38219	-0.38174	-0.38132	-0.3809	-0.38047	-0.38003
-0.34261	-0.34219	-0.38298	-0.38251	-0.38205	-0.38161	-0.38118	-0.38075	-0.38033
-0.3429	-0.34248	-0.34207	-0.34165	-0.38237	-0.38192	-0.38148	-0.38105	-0.38063
-0.34318	-0.34277	-0.34235	-0.34193	-0.3827	-0.38224	-0.38179	-0.38136	-0.38093
-0.34346	-0.34305	-0.34264	-0.34222	-0.34181	-0.3414	-0.34101	-0.38167	-0.38124
-0.34373	-0.34332	-0.34291	-0.3425	-0.3421	-0.34169	-0.34129	-0.38199	-0.38156
-0.34401	-0.34359	-0.34318	-0.34279	-0.3424	-0.34199	-0.34157	-0.38233	-0.38188
-0.34431	-0.34388	-0.34346	-0.34306	-0.34267	-0.34226	-0.34185	-0.34146	-0.34107
-0.34464	-0.34418	-0.34375	-0.34334	-0.34293	-0.34253	-0.34213	-0.34174	-0.34135
-0.34498	-0.34451	-0.34406	-0.34363	-0.34321	-0.34281	-0.34242	-0.34202	-0.34163
-0.34533	-0.34484	-0.34438	-0.34394	-0.34351	-0.34311	-0.34271	-0.34231	-0.34191
-0.34568	-0.34519	-0.34471	-0.34426	-0.34382	-0.3434	-0.343	-0.3426	-0.34219
-0.34602	-0.34554	-0.34507	-0.34459	-0.34413	-0.34369	-0.34327	-0.34287	-0.34247
-0.34636	-0.3459	-0.34542	-0.34493	-0.34445	-0.34399	-0.34355	-0.34314	-0.34275
-0.3467	-0.34623	-0.34576	-0.34526	-0.34478	-0.34432	-0.34387	-0.34344	-0.34303
-0.34705	-0.34656	-0.34608	-0.3456	-0.34513	-0.34466	-0.34421	-0.34376	-0.34332
-0.34747	-0.34693	-0.34642	-0.34594	-0.34548	-0.34501	-0.34455	-0.34409	-0.34364
-0.34794	-0.34731	-0.34676	-0.34629	-0.34584	-0.34536	-0.34489	-0.34442	-0.34397
-0.34845	-0.34774	-0.34714	-0.34665	-0.34619	-0.34571	-0.34524	-0.34477	-0.34431
-0.34901	-0.34822	-0.34755	-0.34701	-0.34652	-0.34605	-0.34559	-0.34512	-0.34464
-0.34961	-0.34876	-0.34803	-0.34742	-0.34687	-0.34639	-0.34594	-0.34546	-0.34497
-0.35027	-0.34938	-0.34857	-0.34786	-0.34724	-0.34673	-0.34627	-0.34579	-0.34532
-0.35096	-0.35003	-0.34917	-0.34836	-0.34765	-0.34709	-0.3466	-0.34612	-0.34567
-0.35166	-0.35071	-0.3498	-0.34893	-0.34815	-0.34751	-0.34696	-0.34646	-0.34602
-0.35236	-0.3514	-0.35047	-0.34956	-0.34872	-0.34799	-0.34735	-0.34681	-0.34636
-0.35309	-0.35211	-0.35116	-0.35021	-0.34933	-0.34853	-0.34781	-0.34721	-0.34671
-0.35382	-0.35284	-0.35187	-0.3509	-0.34998	-0.34913	-0.34835	-0.34766	-0.34707
-0.35456	-0.35358	-0.35259	-0.35161	-0.35067	-0.34977	-0.34892	-0.34816	-0.34748
-0.35532	-0.35432	-0.35334	-0.35235	-0.35139	-0.35045	-0.34955	-0.34872	-0.34796
-0.35608	-0.35508	-0.35409	-0.35311	-0.35213	-0.35116	-0.35021	-0.34933	-0.3485
-0.35684	-0.35586	-0.35487	-0.35386	-0.35286	-0.35189	-0.35094	-0.35002	-0.34913
-0.35759	-0.35664	-0.35566	-0.35463	-0.35361	-0.35264	-0.35169	-0.35075	-0.34981
-0.35832	-0.35738	-0.35641	-0.35539	-0.35438	-0.3534	-0.35244	-0.35147	-0.35051
-0.35905	-0.3581	-0.35714	-0.35616	-0.35518	-0.35418	-0.35318	-0.3522	-0.35124
-0.31227	-0.31148	-0.35787	-0.35692	-0.35594	-0.35494	-0.35394	-0.35295	-0.35198
-0.31288	-0.3121	-0.35861	-0.35765	-0.35668	-0.3557	-0.35471	-0.35373	-0.35274
-0.2744	-0.27372	-0.31193	-0.31111	-0.35742	-0.35645	-0.35549	-0.3545	-0.35351
-0.27492	-0.27426	-0.31255	-0.31173	-0.35815	-0.3572	-0.35625	-0.35528	-0.35431
-0.27544	-0.27479	-0.31316	-0.31236	-0.35889	-0.35795	-0.35701	-0.35606	-0.3551
-0.27595	-0.2753	-0.27463	-0.27393	-0.31218	-0.31137	-0.31058	-0.35681	-0.35587
-0.27646	-0.27581	-0.27515	-0.27448	-0.31281	-0.31201	-0.3112	-0.35755	-0.35663
-0.27699	-0.27634	-0.27568	-0.27502	-0.31345	-0.31265	-0.31184	-0.31106	-0.3103
-0.27755	-0.27687	-0.2762	-0.27556	-0.31409	-0.3133	-0.3125	-0.31171	-0.31092

-0.27814	-0.27742	-0.27674	-0.2761	-0.27545	-0.27477	-0.27406	-0.31234	-0.31155
-0.27876	-0.278	-0.27728	-0.27663	-0.27598	-0.2753	-0.27461	-0.31297	-0.31218
-0.27939	-0.27859	-0.27785	-0.27718	-0.27651	-0.27583	-0.27515	-0.27447	-0.27378
-0.28001	-0.2792	-0.27844	-0.27773	-0.27704	-0.27636	-0.27569	-0.27501	-0.27434
-0.28064	-0.27982	-0.27904	-0.27829	-0.27757	-0.27689	-0.27624	-0.27557	-0.27489
-0.28127	-0.28045	-0.27965	-0.27889	-0.27815	-0.27746	-0.2768	-0.27612	-0.27545
-0.28191	-0.28108	-0.28028	-0.2795	-0.27876	-0.27805	-0.27737	-0.27669	-0.27602
-0.28254	-0.28173	-0.28093	-0.28014	-0.27938	-0.27866	-0.27796	-0.27727	-0.2766
-0.28316	-0.28239	-0.28161	-0.2808	-0.28001	-0.27927	-0.27857	-0.27787	-0.27719
-0.28377	-0.28304	-0.28227	-0.28145	-0.28065	-0.2799	-0.27917	-0.27847	-0.27778
-0.28438	-0.28365	-0.28289	-0.28209	-0.28129	-0.28052	-0.27978	-0.27906	-0.27837
-0.28498	-0.28426	-0.28351	-0.28272	-0.28193	-0.28116	-0.28039	-0.27967	-0.27897
-0.28558	-0.28486	-0.28413	-0.28336	-0.28259	-0.2818	-0.28101	-0.28027	-0.27956
-0.2862	-0.28547	-0.28474	-0.284	-0.28324	-0.28245	-0.28165	-0.28089	-0.28016
-0.28682	-0.28608	-0.28535	-0.28462	-0.28387	-0.28309	-0.2823	-0.28154	-0.28078
-0.28746	-0.2867	-0.28595	-0.28523	-0.28449	-0.28374	-0.28298	-0.2822	-0.28141
-0.28814	-0.28734	-0.28658	-0.28584	-0.28511	-0.28437	-0.28362	-0.28285	-0.28205
-0.28885	-0.28801	-0.28721	-0.28646	-0.28572	-0.28499	-0.28425	-0.28348	-0.28271
-0.28956	-0.2887	-0.28787	-0.2871	-0.28635	-0.2856	-0.28487	-0.28412	-0.28336
-0.29025	-0.28941	-0.28859	-0.28777	-0.28698	-0.28622	-0.28549	-0.28474	-0.28399
-0.29094	-0.29013	-0.28931	-0.28846	-0.28763	-0.28686	-0.28611	-0.28536	-0.28462
-0.29163	-0.29081	-0.28999	-0.28916	-0.28834	-0.28754	-0.28676	-0.28599	-0.28524
-0.29232	-0.29148	-0.29066	-0.28986	-0.28906	-0.28823	-0.28742	-0.28664	-0.28588
-0.29303	-0.29217	-0.29134	-0.29054	-0.28974	-0.28892	-0.2881	-0.28732	-0.28656
-0.29375	-0.29288	-0.29203	-0.29121	-0.2904	-0.2896	-0.28881	-0.28804	-0.28727
-0.19477	-0.19424	-0.29275	-0.2919	-0.29107	-0.29028	-0.2895	-0.28873	-0.28796
-0.19523	-0.1947	-0.29352	-0.29263	-0.29176	-0.29095	-0.29017	-0.28939	-0.28863
-0.19569	-0.19515	-0.1946	-0.19403	-0.29248	-0.29165	-0.29085	-0.29007	-0.2893
-0.19613	-0.19559	-0.19505	-0.1945	-0.29325	-0.2924	-0.29157	-0.29076	-0.28997
-0.19657	-0.19604	-0.1955	-0.19496	-0.29403	-0.29316	-0.2923	-0.29147	-0.29065
-0.04331	-0.04316	-0.19595	-0.19541	-0.19487	-0.19432	-0.19377	-0.29218	-0.29135
-0.04341	-0.04326	-0.1964	-0.19585	-0.19531	-0.19476	-0.19422	-0.2929	-0.29206
-0.04351	-0.04336	-0.1423	-0.1419	-0.19576	-0.19521	-0.19467	-0.19412	-0.19358
-0.0436	-0.04345	-0.14264	-0.14223	-0.19621	-0.19566	-0.19512	-0.19457	-0.19402
-0.04371	-0.04355	-0.0434	-0.04325	-0.14216	-0.14176	-0.14137	-0.14098	-0.14059
-0.04382	-0.04365	-0.0435	-0.04334	-0.1425	-0.1421	-0.14171	-0.14131	-0.14092
-0.04393	-0.04376	-0.0436	-0.04344	-0.14286	-0.14245	-0.14205	-0.14165	-0.14126
-0.04404	-0.04388	-0.04372	-0.04356	-0.14323	-0.14281	-0.1424	-0.14199	-0.14159
-0.04415	-0.04399	-0.04384	-0.04368	-0.1436	-0.14318	-0.14276	-0.14234	-0.14193
		-0.04394	-0.04379	-0.04363	-0.04347	-0.04331	-0.14271	-0.14229
		-0.04405	-0.04389	-0.04374	-0.04358	-0.04341	-0.14309	-0.14267
				-0.04385	-0.04369	-0.04353	-0.14346	-0.14304
				-0.04397	-0.04381	-0.04365	-0.14382	-0.14341
							-0.04361	-0.04345
							-0.04372	-0.04356
							-0.04383	-0.04367

					-0.05392	-0.05383
					-0.05381	-0.05371
			-0.05391	-0.0538	-0.40355	-0.40232
			-0.05379	-0.05368	-0.40245	-0.40123
-0.05399	-0.05388	-0.05378	-0.40385	-0.40261	-0.48408	-0.48267
-0.05387	-0.05376	-0.05366	-0.40277	-0.40154	-0.48288	-0.48147
-0.05375	-0.05364	-0.05353	-0.40172	-0.4005	-0.4817	-0.4803
-0.40444	-0.40316	-0.40191	-0.48335	-0.48195	-0.52033	-0.51882

		-0.40335	-0.4021	-0.40087	-0.4822	-0.4808	-0.51909	-0.5176
-0.21589	-0.21517	-0.40228	-0.40104	-0.39983	-0.52085	-0.51934	-0.5661	-0.56453
-0.21526	-0.21458	-0.40123	-0.4	-0.39879	-0.51957	-0.51808	-0.56477	-0.56322
-0.40264	-0.40141	-0.4829	-0.48149	-0.4801	-0.51831	-0.51684	-0.60655	-0.60494
-0.40161	-0.40038	-0.48175	-0.48036	-0.47898	-0.51709	-0.51562	-0.60519	-0.60359
-0.48341	-0.482	-0.52034	-0.51884	-0.51735	-0.60707	-0.60544	-0.60383	-0.60223
-0.48224	-0.48084	-0.51908	-0.51758	-0.5161	-0.60567	-0.60404	-0.60243	-0.60085
-0.48108	-0.47969	-0.51783	-0.51633	-0.51484	-0.60427	-0.60263	-0.60104	-0.59948
-0.51959	-0.5181	-0.60787	-0.6062	-0.60453	-0.60289	-0.60127	-0.67521	-0.67341
-0.51836	-0.51688	-0.60652	-0.60484	-0.60316	-0.60153	-0.59993	-0.67367	-0.67191
-0.60843	-0.60679	-0.60515	-0.60348	-0.60182	-0.67583	-0.67398	-0.67218	-0.67044
-0.60705	-0.60541	-0.60378	-0.60213	-0.6005	-0.67434	-0.67253	-0.67076	-0.66901
-0.64134	-0.63948	-0.67848	-0.67657	-0.67468	-0.67286	-0.67108	-0.90213	-0.89928
-0.63983	-0.63799	-0.67694	-0.67505	-0.67319	-0.67138	-0.6696	-0.89975	-0.89694
-0.63833	-0.63651	-0.67542	-0.67355	-0.67171	-0.66991	-0.66814	-0.8974	-0.89467
-0.67761	-0.67575	-0.67391	-0.67207	-0.67025	-0.8586	-0.8559	-0.89518	-0.89256
-0.67606	-0.67423	-0.67242	-0.67061	-0.66881	-0.85644	-0.8538	-0.89304	-0.89054
-0.67455	-0.67275	-0.71073	-0.7088	-0.70689	-0.8962	-0.89351	-0.9598	-0.95709
-0.67309	-0.67131	-0.70919	-0.70724	-0.70532	-0.89395	-0.89139	-0.95752	-0.95483
-0.71142	-0.70953	-0.85815	-0.85537	-0.85267	-0.93077	-0.92811	-0.9553	-0.95262
-0.70982	-0.70794	-0.85593	-0.85331	-0.85077	-0.92867	-0.92601	-0.95315	-0.95048
-0.85911	-0.8564	-0.93486	-0.93204	-0.92931	-0.95646	-0.9537	-0.97996	-0.97725
-0.85699	-0.85434	-0.93258	-0.92986	-0.9272	-0.95428	-0.9515	-0.97772	-0.97502
-0.85491	-0.85234	-0.93038	-0.92772	-0.9251	-0.95211	-0.94931	-0.97549	-0.9728
-0.85287	-0.85035	-0.98721	-0.98443	-0.9817	-0.9789	-0.9761	-0.97336	-0.97066
-0.85088	-0.84839	-0.98492	-0.98214	-0.97942	-0.97669	-0.97398	-0.97126	-0.96855
-0.95901	-0.95622	-0.98271	-0.97994	-0.97722	-0.97453	-0.97187	-0.96915	-0.9664
-0.95683	-0.9541	-0.98057	-0.9778	-0.97507	-0.97241	-0.96976	-0.96703	-0.96422
-0.98393	-0.98118	-0.97844	-0.97567	-0.97293	-0.97026	-0.96761	-1.06798	-1.06505
-0.9818	-0.97906	-0.97632	-0.97353	-0.97077	-0.96806	-0.96539	-1.06567	-1.06285
-0.97969	-0.97695	-0.97419	-0.9714	-0.96863	-0.96589	-0.9632	-1.09588	-1.09309
-0.97756	-0.9748	-0.97204	-0.96928	-0.96653	-0.96381	-0.96112	-1.09367	-1.09093
-0.97543	-0.97266	-0.9699	-0.96717	-0.96446	-0.96176	-0.9591	-1.09151	-1.0888
-0.97329	-0.97053	-0.96779	-0.96505	-0.96235	-1.09495	-1.09215	-1.14901	-1.14618
-0.97113	-0.96842	-0.9657	-0.96295	-0.96025	-1.09275	-1.09004	-1.14684	-1.14398
-1.00496	-1.00221	-1.09915	-1.09621	-1.09331	-1.15025	-1.14748	-1.23462	-1.23163
-1.00285	-1.00009	-1.09691	-1.09399	-1.09113	-1.148	-1.14524	-1.23229	-1.22936
-1.1005	-1.09758	-1.15455	-1.15156	-1.14863	-1.23581	-1.23291	-1.23	-1.22709
-1.09829	-1.09536	-1.15226	-1.14934	-1.14648	-1.23357	-1.23067	-1.22775	-1.22483
-1.0961	-1.09318	-1.15002	-1.14716	-1.14437	-1.23135	-1.22844	-1.2255	-1.22254
-1.15379	-1.15079	-1.20527	-1.20239	-1.19954	-1.22911	-1.22618	-1.25317	-1.2501
-1.15157	-1.14864	-1.2031	-1.20023	-1.19738	-1.22687	-1.2239	-1.2508	-1.24768
-1.20677	-1.20383	-1.23348	-1.23052	-1.22756	-1.28212	-1.279	-1.27587	-1.27271
-1.20449	-1.20162	-1.23124	-1.22827	-1.22528	-1.27972	-1.27655	-1.27342	-1.27031
-1.29283	-1.28979	-1.28672	-1.28361	-1.28047	-1.2773	-1.27413	-1.27103	-1.26799
-1.29051	-1.28748	-1.2844	-1.28121	-1.27801	-1.27485	-1.27171	-1.26868	-1.26575
-1.2882	-1.28516	-1.28205	-1.27882	-1.27559	-1.27244	-1.26936	-1.2664	-1.26355
-1.28587	-1.28281	-1.27969	-1.27645	-1.27321	-1.27011	-1.26708	-1.26418	-1.2614
-1.28354	-1.28044	-1.27731	-1.27407	-1.27087	-1.26782	-1.26487	-1.26202	-1.25927
-1.28119	-1.27802	-1.27485	-1.27167	-1.26855	-1.26559	-1.26272	-1.33242	-1.32933

-1.27883	-1.27558	-1.27239	-1.26928	-1.26627	-1.2634	-1.26062	-1.33017	-1.32701
-1.27649	-1.27321	-1.27003	-1.267	-1.26407	-1.33409	-1.33105	-1.32796	-1.32485
-1.27416	-1.27091	-1.26776	-1.2648	-1.26194	-1.33183	-1.3288	-1.3258	-1.32281
-1.27184	-1.26864	-1.33901	-1.33578	-1.33267	-1.32959	-1.3266	-1.32373	-1.32097
-1.26953	-1.26642	-1.33669	-1.33351	-1.33042	-1.32737	-1.32442	-1.32175	-1.31932
-1.341	-1.33766	-1.33441	-1.33127	-1.32823	-1.32526	-1.32241	-1.31995	-1.31782
-1.33851	-1.33527	-1.33213	-1.32905	-1.3261	-1.32331	-1.3207	-1.31842	-1.31646
-1.33607	-1.33292	-1.32988	-1.3269	-1.32408	-1.32151	-1.31916	-1.31705	-1.3152
-1.33374	-1.33063	-1.32767	-1.32485	-1.32222	-1.31986	-1.31773	-1.31577	-1.314
-1.33149	-1.3284	-1.32552	-1.32291	-1.32052	-1.31836	-1.31642	-1.31458	-1.31287
-1.32928	-1.32629	-1.32356	-1.32114	-1.31895	-1.31696	-1.31514	-1.33009	-1.32839
-1.32709	-1.3243	-1.32178	-1.31955	-1.31753	-1.31565	-1.31391	-1.32888	-1.32723
-1.32504	-1.32248	-1.32016	-1.31809	-1.3162	-1.33111	-1.32938	-1.3277	-1.32608
-1.32314	-1.32084	-1.31874	-1.3168	-1.31498	-1.3299	-1.32821	-1.32655	-1.32493
-1.32142	-1.31936	-1.31743	-1.31558	-1.31383	-1.32874	-1.32707	-1.32542	-1.3238
-1.33682	-1.3348	-1.33291	-1.33107	-1.3293	-1.32761	-1.32597	-1.32433	-1.3227
-1.33548	-1.33353	-1.33168	-1.32989	-1.32817	-1.32651	-1.3249	-1.32327	-1.32163
-1.33416	-1.33229	-1.33049	-1.32875	-1.32705	-1.35056	-1.34891	-1.37823	-1.37635
-1.3329	-1.3311	-1.32935	-1.32763	-1.32595	-1.34944	-1.34779	-1.37699	-1.37512
-1.33167	-1.32993	-1.35347	-1.35172	-1.35	-1.37942	-1.3776	-1.37577	-1.37392
-1.33047	-1.32877	-1.35231	-1.35059	-1.3489	-1.3782	-1.37638	-1.37456	-1.37275
-1.35458	-1.35287	-1.35117	-1.34948	-1.34781	-1.40203	-1.40017	-1.39831	-1.39646
-1.35342	-1.35173	-1.35006	-1.34838	-1.34671	-1.40078	-1.39895	-1.39711	-1.39528
-1.35228	-1.35062	-1.34896	-1.34728	-1.34561	-1.39955	-1.39774	-1.39592	-1.39411
-1.35119	-1.34952	-1.40395	-1.40204	-1.40016	-1.39834	-1.39656	-1.4122	-1.41039
-1.35012	-1.34843	-1.40268	-1.4008	-1.39894	-1.39715	-1.39539	-1.41104	-1.40922
-1.40526	-1.40334	-1.40143	-1.39956	-1.39772	-1.41337	-1.41163	-1.40987	-1.40809
-1.40399	-1.40208	-1.40019	-1.39833	-1.3965	-1.41215	-1.41041	-1.40869	-1.40698
-1.42013	-1.41823	-1.41635	-1.41452	-1.41273	-1.41096	-1.40923	-1.43212	-1.43027
-1.41882	-1.41695	-1.41512	-1.41333	-1.41157	-1.40981	-1.40808	-1.43085	-1.42906
-1.41754	-1.41569	-1.41388	-1.41214	-1.41041	-1.43339	-1.43145	-1.45481	-1.45296
-1.4163	-1.41445	-1.41264	-1.4109	-1.4092	-1.43209	-1.43024	-1.45357	-1.45176
-1.41508	-1.41322	-1.41142	-1.40968	-1.40801	-1.4308	-1.42905	-1.45234	-1.45057
-1.41384	-1.41199	-1.43506	-1.43317	-1.43135	-1.4802	-1.4783	-1.52087	-1.51897
-1.4126	-1.41076	-1.43372	-1.43189	-1.43013	-1.47887	-1.47697	-1.51951	-1.51763
-1.46338	-1.4614	-1.52809	-1.52604	-1.52405	-1.52206	-1.52009	-1.51818	-1.51632
-1.46202	-1.46013	-1.52672	-1.5247	-1.52271	-1.52073	-1.51877	-1.51687	-1.51504
-1.52944	-1.52739	-1.52537	-1.52336	-1.52138	-1.51942	-1.51748	-1.51561	-1.51379
-1.52795	-1.52598	-1.52402	-1.52203	-1.52005	-1.51813	-1.51625	-1.5144	-1.51257
-1.52652	-1.5246	-1.52268	-1.5207	-1.51874	-1.51686	-1.51503	-1.5132	-1.51137
-1.52522	-1.52328	-1.52135	-1.51938	-1.51745	-1.5156	-1.5138	-1.51198	-1.51016
-1.52398	-1.52199	-1.52003	-1.51808	-1.51618	-1.51435	-1.51257	-1.51076	-1.50895
-1.52268	-1.52068	-1.51873	-1.51683	-1.51497	-1.51315	-1.51136	-1.50955	-1.50773
-1.52137	-1.51938	-1.51745	-1.51561	-1.5138	-1.51198	-1.51016	-1.50833	-1.50651
-1.52007	-1.51812	-1.51622	-1.51441	-1.51262	-1.51079	-1.50895	-1.52773	-1.52574
-1.51878	-1.51689	-1.51503	-1.51322	-1.51141	-1.50957	-1.50773	-1.52641	-1.52446
-1.51751	-1.51567	-1.51384	-1.51202	-1.5102	-1.52914	-1.5271	-1.52513	-1.52322
-1.5163	-1.51445	-1.51262	-1.51078	-1.50895	-1.52782	-1.52583	-1.5239	-1.52202
-1.5151	-1.51324	-1.5114	-1.50955	-1.50772	-1.52651	-1.52458	-1.52269	-1.52083
-1.51386	-1.51203	-1.53124	-1.5292	-1.52718	-1.52524	-1.52335	-1.52147	-1.5196

-1.51262	-1.51081	-1.52995	-1.52792	-1.52591	-1.524	-1.52214	-1.52026	-1.51836
-1.5114	-1.50961	-1.52867	-1.52668	-1.5247	-1.52279	-1.52092	-1.54543	-1.5434
-1.5102	-1.50842	-1.52742	-1.52548	-1.52355	-1.52161	-1.51969	-1.54411	-1.54212
-1.53004	-1.52809	-1.52616	-1.52424	-1.52233	-1.5469	-1.54482	-1.56317	-1.56121
-1.52881	-1.52685	-1.5249	-1.52297	-1.52105	-1.54553	-1.5435	-1.56188	-1.56
-1.52757	-1.52561	-1.55039	-1.54829	-1.54621	-1.56455	-1.56254	-1.58858	-1.5866
-1.52629	-1.52434	-1.54904	-1.54692	-1.54485	-1.56322	-1.5613	-1.58724	-1.58526
-1.525	-1.52308	-1.54771	-1.54559	-1.54353	-1.56194	-1.56009	-1.58593	-1.58394
-1.57093	-1.56884	-1.58226	-1.58015	-1.57808	-1.58863	-1.58663	-1.58462	-1.58262
-1.56956	-1.56749	-1.58094	-1.57891	-1.57692	-1.58735	-1.58533	-1.58332	-1.58132
-1.58375	-1.58167	-1.59232	-1.59018	-1.58809	-1.60478	-1.60272	-1.61236	-1.61033
-1.58246	-1.58037	-1.59091	-1.5888	-1.58674	-1.60342	-1.60139	-1.61104	-1.60906
-1.61279	-1.61054	-1.60835	-1.60624	-1.60416	-1.68591	-1.68376	-1.68165	-1.67959
-1.61127	-1.60914	-1.60705	-1.60497	-1.60291	-1.68457	-1.68241	-1.68029	-1.67822
-1.6098	-1.60776	-1.68974	-1.68758	-1.68541	-1.68323	-1.68106	-1.67893	-1.67683
-1.60844	-1.60641	-1.6883	-1.68615	-1.684	-1.68184	-1.67967	-1.67753	-1.6754
-1.60714	-1.60507	-1.68686	-1.68472	-1.68258	-1.68043	-1.67828	-1.67613	-1.67398
-1.68978	-1.68761	-1.68546	-1.68333	-1.6812	-1.67906	-1.67692	-1.71949	-1.71701
-1.68834	-1.68622	-1.6841	-1.68196	-1.67983	-1.67771	-1.67559	-1.71797	-1.7155
-1.68693	-1.68482	-1.68271	-1.68057	-1.67844	-1.72128	-1.71884	-1.71641	-1.71399
-1.68555	-1.68342	-1.68129	-1.67915	-1.67701	-1.71963	-1.71717	-1.71479	-1.71247
-1.68418	-1.68204	-1.6799	-1.67776	-1.67561	-1.71802	-1.71557	-1.71323	-1.71099
-1.68281	-1.68069	-1.67856	-1.67641	-1.67427	-1.71651	-1.7141	-1.7118	-1.70959
-1.68144	-1.67933	-1.67721	-1.67506	-1.67293	-1.71502	-1.71267	-1.7104	-1.70821
-1.68002	-1.6779	-1.72073	-1.71828	-1.71585	-1.7135	-1.71121	-1.70897	-1.70678
-1.6786	-1.67645	-1.71907	-1.71666	-1.71428	-1.71199	-1.70976	-1.70754	-1.70535
-1.72242	-1.71993	-1.71749	-1.71512	-1.7128	-1.71055	-1.70836	-1.70617	-1.70398
-1.72089	-1.71839	-1.71596	-1.71364	-1.71138	-1.70917	-1.70701	-1.70484	-1.70265
-1.71932	-1.71685	-1.71446	-1.71217	-1.70995	-1.70776	-1.70561	-1.74085	-1.73867
-1.71768	-1.7153	-1.71297	-1.71072	-1.7085	-1.70631	-1.70414	-1.73939	-1.73728
-1.7161	-1.7138	-1.71153	-1.7093	-1.70709	-1.70489	-1.70271	-1.73795	-1.73588
-1.71462	-1.71238	-1.71016	-1.70794	-1.70574	-1.70355	-1.70137	-1.73656	-1.73446
-1.7132	-1.711	-1.70881	-1.70661	-1.70442	-1.70223	-1.70006	-1.73521	-1.73304
-1.71181	-1.70962	-1.70744	-1.70526	-1.70309	-1.73827	-1.73607	-1.75722	-1.75484
-1.71044	-1.70824	-1.70605	-1.7039	-1.70175	-1.73693	-1.73474	-1.75576	-1.75337
-1.70901	-1.7068	-1.70462	-1.70247	-1.70034	-1.75897	-1.75661	-1.75424	-1.75186
-1.70753	-1.70531	-1.70313	-1.70099	-1.69887	-1.75735	-1.75501	-1.75266	-1.75031
-1.70608	-1.70388	-1.72601	-1.72368	-1.72137	-1.77179	-1.76945	-1.76712	-1.76478
-1.70469	-1.7025	-1.72452	-1.72219	-1.71988	-1.77027	-1.76794	-1.76562	-1.76332
-1.72007	-1.71789	-1.77583	-1.77347	-1.77111	-1.76877	-1.76644	-1.77692	-1.77462
-1.7187	-1.71655	-1.77436	-1.77198	-1.76962	-1.76728	-1.76497	-1.77545	-1.77319
-1.71734	-1.71521	-1.77289	-1.77051	-1.76814	-1.76581	-1.7635	-1.774	-1.77175
-1.73977	-1.73745	-1.78425	-1.78186	-1.77948	-1.77714	-1.77483	-1.77255	-1.77029
-1.73829	-1.73596	-1.78273	-1.78036	-1.778	-1.77568	-1.77339	-1.77109	-1.76881
-1.75009	-1.74772	-1.78117	-1.77881	-1.77647	-1.77417	-1.7719	-1.76962	-1.76733
-1.74859	-1.74617	-1.77956	-1.77721	-1.7749	-1.77262	-1.77036	-1.76811	-1.76586
-1.74707	-1.74463	-1.778	-1.77566	-1.77337	-1.7711	-1.76886	-1.83426	-1.83157
-1.74552	-1.74311	-1.77649	-1.77418	-1.77191	-1.76965	-1.76742	-1.83253	-1.82987
-1.74398	-1.74162	-1.77502	-1.77273	-1.77048	-1.76823	-1.766	-1.83083	-1.82819
-1.77824	-1.77588	-1.77356	-1.77129	-1.76904	-1.83451	-1.83182	-1.82915	-1.82651

-1.77675	-1.77442	-1.77212	-1.76985	-1.76762	-1.83281	-1.83014	-1.82748	-1.82484
-1.77527	-1.77296	-1.77068	-1.76843	-1.7662	-1.83111	-1.82846	-1.82581	-1.82317
-1.77381	-1.77152	-1.76925	-1.76701	-1.76478	-1.82941	-1.82677	-1.82413	-1.82149
-1.77235	-1.77008	-1.83578	-1.83306	-1.83038	-1.82772	-1.82509	-1.82245	-1.81981
-1.77089	-1.76864	-1.83406	-1.83136	-1.82868	-1.82603	-1.8234	-1.82076	-1.81811
-1.83777	-1.83505	-1.83234	-1.82966	-1.82699	-1.82434	-1.82171	-1.83555	-1.83285
-1.83603	-1.83333	-1.83065	-1.82796	-1.82529	-1.82264	-1.82	-1.83382	-1.83115
-1.83431	-1.83163	-1.82896	-1.82628	-1.82361	-1.82095	-1.8183	-1.8321	-1.82947
-1.8326	-1.82994	-1.82728	-1.82462	-1.82196	-1.84587	-1.84299	-1.84018	-1.83742
-1.8309	-1.82826	-1.82562	-1.82297	-1.82032	-1.84417	-1.8413	-1.8385	-1.83578
-1.82922	-1.82659	-1.83476	-1.83196	-1.82917	-1.84249	-1.83963	-1.83685	-1.83415
-1.82754	-1.82493	-1.8331	-1.83032	-1.82754	-1.84083	-1.83798	-1.83522	-1.83254
-1.83694	-1.8342	-1.84769	-1.84487	-1.84204	-1.83919	-1.83635	-1.84721	-1.84453
-1.83527	-1.83253	-1.846	-1.84319	-1.84038	-1.83756	-1.83474	-1.84562	-1.84298
-1.83359	-1.83087	-1.85799	-1.85519	-1.8524	-1.84957	-1.84676	-1.84405	-1.84143
-1.83192	-1.82921	-1.85631	-1.85354	-1.85077	-1.84797	-1.84517	-1.84248	-1.83989
-1.83026	-1.82757	-1.85463	-1.85189	-1.84915	-1.84637	-1.8436	-1.84093	-1.83835
-1.85849	-1.85572	-1.85298	-1.85026	-1.84754	-1.84479	-1.84204	-1.83939	-1.83683
-1.85682	-1.85407	-1.85135	-1.84864	-1.84594	-1.84321	-1.84049	-1.83786	-1.83531
-1.85516	-1.85243	-1.84972	-1.84704	-1.84436	-1.84626	-1.84355	-1.84093	-1.83839
-1.8535	-1.85079	-1.8481	-1.84544	-1.84279	-1.8447	-1.84201	-1.8394	-1.83687
-1.85185	-1.84915	-1.8511	-1.84847	-1.84583	-1.84316	-1.84048	-1.83789	-1.83538
-1.85021	-1.84752	-1.84948	-1.84687	-1.84427	-1.84162	-1.83897	-1.83641	-1.83392
-1.84857	-1.84589	-1.84787	-1.84529	-1.84271	-1.8401	-1.83748	-1.83495	-1.8325
-1.85157	-1.8489	-1.84628	-1.84372	-1.84118	-1.8386	-1.83604	-1.83354	-1.8311
-1.84996	-1.8473	-1.8447	-1.84216	-1.83965	-1.83712	-1.83461	-1.83214	-1.82971
-1.8484	-1.84574	-1.84314	-1.84061	-1.83811	-1.83562	-1.83316	-1.83071	-1.8283
-1.84689	-1.84421	-1.84159	-1.83906	-1.83658	-1.83412	-1.83169	-1.82928	-1.82687
-1.84536	-1.84268	-1.84007	-1.83754	-1.83507	-1.8459	-1.8435	-1.84109	-1.83868
-1.84381	-1.84115	-1.83856	-1.83605	-1.83359	-1.84443	-1.84205	-1.83966	-1.83726
-1.85558	-1.85294	-1.85036	-1.84785	-1.84539	-1.84298	-1.84061	-1.83824	-1.83586
-1.85406	-1.85147	-1.84892	-1.84642	-1.84396	-1.84155	-1.83919	-1.83684	-1.83451
-1.85256	-1.85001	-1.8475	-1.84501	-1.84255	-1.84014	-1.83778	-1.83545	-1.83317
-1.85106	-1.84854	-1.84606	-1.8436	-1.84116	-1.83875	-1.83637	-1.83406	-1.8318
-1.84958	-1.84708	-1.84461	-1.84219	-1.83979	-1.83738	-1.83498	-1.83267	-1.83043
-1.84814	-1.84563	-1.84317	-1.84077	-1.83839	-1.83599	-1.8336	-1.8313	-1.82907
-1.84673	-1.8442	-1.84173	-1.83932	-1.83696	-1.83459	-1.83224	-1.82995	-1.82773
-1.84529	-1.84276	-1.84029	-1.8379	-1.83555	-1.83321	-1.8309	-1.82863	-1.82641
-1.8438	-1.84129	-1.83885	-1.83648	-1.83416	-1.83186	-1.8296	-1.82736	-1.82514
-1.84233	-1.83986	-1.83744	-1.83509	-1.83279	-1.84266	-1.84042	-1.86693	-1.86465
-1.84093	-1.83849	-1.8361	-1.83375	-1.83145	-1.84132	-1.8391	-1.86559	-1.86335
-1.83957	-1.83716	-1.83478	-1.83244	-1.83013	-1.84	-1.83779	-1.86425	-1.86204
-1.85035	-1.84796	-1.8456	-1.84327	-1.84096	-1.8791	-1.87681	-1.87456	-1.87234
-1.84902	-1.84665	-1.8443	-1.84199	-1.8397	-1.87778	-1.87547	-1.87321	-1.87098
-1.85976	-1.8574	-1.88349	-1.88113	-1.87878	-1.87646	-1.87416	-1.87189	-1.86964
-1.8584	-1.85607	-1.88214	-1.87978	-1.87744	-1.87515	-1.87288	-1.87061	-1.86833
-1.88546	-1.88311	-1.88077	-1.87842	-1.87608	-1.87381	-1.87158	-1.86931	-1.86701
-1.88404	-1.88171	-1.87938	-1.87704	-1.87472	-1.87245	-1.87021	-1.86796	-1.86569
-1.88264	-1.88031	-1.87799	-1.87566	-1.87336	-1.8711	-1.86886	-1.86662	-1.86439
-1.88128	-1.87895	-1.87663	-1.87433	-1.87205	-1.86979	-1.86755	-1.86532	-1.86312

-1.87993	-1.87761	-1.8753	-1.87302	-1.87076	-1.8685	-1.86626	-1.86405	-1.86186
-1.8786	-1.87629	-1.874	-1.87172	-1.86946	-1.86721	-1.86499	-1.86279	-1.8606
-1.87728	-1.87499	-1.87271	-1.87042	-1.86816	-1.86593	-1.86374	-1.86154	-1.85934
-1.87597	-1.87369	-1.87142	-1.86914	-1.86689	-1.86467	-1.86248	-1.86029	-1.85809
-1.87469	-1.8724	-1.87013	-1.86788	-1.86565	-1.86343	-1.86122	-1.85903	-1.85685
-1.8734	-1.87111	-1.86884	-1.86662	-1.8644	-1.86218	-1.85996	-1.85777	-1.8556
-1.87208	-1.86982	-1.86758	-1.86535	-1.86314	-1.86092	-1.85871	-1.85651	-1.85434
-1.87076	-1.86853	-1.8663	-1.86408	-1.86187	-1.85966	-1.85745	-1.85526	-1.85307
-1.86948	-1.86723	-1.865	-1.86279	-1.86059	-1.85839	-1.85618	-1.85398	-1.8518
-1.86822	-1.86594	-1.86369	-1.86149	-1.85931	-1.85711	-1.85489	-1.8527	-1.85053
-1.86695	-1.86467	-1.86242	-1.86021	-1.85801	-1.85581	-1.85361	-1.85142	-1.84924
-1.86569	-1.86344	-1.86119	-1.85893	-1.8567	-1.85451	-1.85234	-1.85015	-1.84794
-1.86441	-1.86218	-1.85994	-1.85767	-1.85542	-1.85322	-1.85105	-1.84885	-1.84664
-1.86311	-1.8609	-1.85868	-1.85643	-1.85418	-1.85195	-1.84974	-1.84753	-1.84534
-1.8618	-1.8596	-1.85739	-1.85517	-1.85294	-1.85069	-1.84844	-1.84623	-1.84404
-1.86049	-1.85828	-1.85608	-1.85388	-1.85168	-1.84944	-1.84719	-1.84496	-1.84273
-1.85919	-1.85697	-1.85476	-1.85258	-1.8504	-1.84818	-1.84595	-1.8437	-1.84144
-1.85789	-1.85568	-1.85348	-1.85129	-1.8491	-1.8469	-1.84468	-1.85293	-1.85065
-1.8566	-1.85441	-1.85222	-1.85001	-1.84779	-1.8456	-1.8434	-1.85166	-1.84941
-1.85533	-1.85315	-1.85097	-1.84874	-1.84652	-1.84431	-1.84211	-1.86947	-1.86724
-1.85409	-1.85192	-1.84973	-1.84751	-1.84528	-1.84304	-1.84081	-1.86818	-1.86598
-1.85285	-1.85066	-1.84847	-1.84626	-1.84403	-1.87144	-1.86915	-1.8669	-1.86471
-1.85161	-1.84939	-1.84718	-1.84498	-1.84278	-1.87022	-1.86794	-1.86568	-1.86343
-1.85035	-1.84811	-1.84588	-1.84369	-1.84151	-1.86898	-1.86672	-1.86445	-1.86216
-1.84908	-1.84684	-1.87444	-1.87218	-1.86994	-1.86771	-1.86548	-1.87488	-1.87259
-1.8478	-1.84557	-1.87317	-1.87089	-1.86863	-1.86641	-1.86421	-1.87363	-1.87135
-1.87642	-1.87417	-1.87191	-1.86963	-1.86736	-1.87678	-1.87456	-1.87233	-1.87008
-1.8751	-1.87288	-1.87065	-1.86838	-1.8661	-1.87548	-1.87323	-1.871	-1.8688
-1.88544	-1.88324	-1.88102	-1.87876	-1.87649	-1.8742	-1.87193	-1.86969	-1.86749
-1.88417	-1.88196	-1.87974	-1.8775	-1.87523	-1.87295	-1.87066	-1.8684	-1.86617
-1.88291	-1.88068	-1.87845	-1.87622	-1.87397	-1.87169	-1.8694	-1.86713	-1.86487
-1.88163	-1.87937	-1.87712	-1.87491	-1.87269	-1.87043	-1.86816	-1.86589	-1.86362
-1.88036	-1.87806	-1.8758	-1.87359	-1.87139	-1.86916	-1.86691	-1.86465	-1.86238
-1.87909	-1.87678	-1.87451	-1.87228	-1.87006	-1.86785	-1.86565	-1.86341	-1.86114
-1.87782	-1.87552	-1.87324	-1.87097	-1.86873	-1.86654	-1.86437	-1.86215	-1.8599
-1.87657	-1.87427	-1.87198	-1.86971	-1.86745	-1.86527	-1.86311	-1.8609	-1.85866
-1.87534	-1.87303	-1.87075	-1.86847	-1.86621	-1.86401	-1.86184	-1.85964	-1.85742
-1.8741	-1.8718	-1.86951	-1.86723	-1.86498	-1.86276	-1.86056	-1.85836	-1.85616
-1.87286	-1.87056	-1.86828	-1.866	-1.86374	-1.86149	-1.85927	-1.85706	-1.85488
-1.87161	-1.86932	-1.86704	-1.86477	-1.8625	-1.86023	-1.85798	-1.85577	-1.8536
-1.87037	-1.86809	-1.86581	-1.86353	-1.86126	-1.85899	-1.85673	-1.85451	-1.85235
-1.86911	-1.86685	-1.86457	-1.86229	-1.86001	-1.85774	-1.85548	-1.85326	-1.85109
-1.86785	-1.86558	-1.86331	-1.86103	-1.85875	-1.85648	-1.85422	-1.852	-1.84983
-1.86657	-1.8643	-1.86204	-1.85976	-1.85748	-1.85521	-1.85295	-1.85074	-1.84856
-1.86529	-1.86302	-1.86075	-1.85848	-1.85621	-1.85393	-1.85167	-1.84944	-1.84724
-1.86402	-1.86174	-1.85946	-1.85719	-1.85492	-1.85264	-1.85036	-1.84811	-1.84588
-1.86274	-1.86045	-1.85817	-1.8559	-1.85362	-1.85134	-1.84906	-1.84678	-1.84453
-1.86146	-1.85917	-1.85688	-1.8546	-1.85232	-1.85003	-1.84775	-1.84547	-1.84319
-1.86017	-1.85788	-1.85559	-1.8533	-1.85101	-1.84872	-1.84643	-1.84415	-1.84187
-1.85888	-1.85659	-1.85429	-1.852	-1.8497	-1.84741	-1.84512	-1.84284	-1.84056

-1.85757	-1.85528	-1.85299	-1.85069	-1.84839	-1.8461	-1.84381	-1.84153	-1.83925
-1.85627	-1.85398	-1.85168	-1.84938	-1.84708	-1.84479	-1.8425	-1.84023	-1.83798
-1.85497	-1.85267	-1.85036	-1.84806	-1.84577	-1.84348	-1.84121	-1.83896	-1.83673
-1.85366	-1.85135	-1.84905	-1.84675	-1.84447	-1.8422	-1.83994	-1.83769	-1.83546
-1.85235	-1.85004	-1.84774	-1.84545	-1.84317	-1.84092	-1.83869	-1.83644	-1.83418
-1.85105	-1.84873	-1.84643	-1.84415	-1.84189	-1.83966	-1.83743	-1.83517	-1.83289
-1.84974	-1.84743	-1.84513	-1.84288	-1.84064	-1.8384	-1.83615	-1.83387	-1.83157
-1.84847	-1.84615	-1.84386	-1.84162	-1.83939	-1.83713	-1.83484	-1.83255	-1.83025
-1.84726	-1.84494	-1.84264	-1.84037	-1.83811	-1.83582	-1.83352	-1.83121	-1.8289
-1.84608	-1.84375	-1.84143	-1.83912	-1.83681	-1.8345	-1.83218	-1.82987	-1.82755
-1.84482	-1.8425	-1.84017	-1.83784	-1.83551	-1.83318	-1.83085	-1.84065	-1.83829
-1.84352	-1.8412	-1.83887	-1.83654	-1.8342	-1.83186	-1.82952	-1.83929	-1.83694
-1.84221	-1.83988	-1.83756	-1.83522	-1.83287	-1.83053	-1.82819	-1.83794	-1.83558
-1.84089	-1.83855	-1.83622	-1.83387	-1.83153	-1.8292	-1.82686	-1.83659	-1.83423
-1.83958	-1.83723	-1.83489	-1.83254	-1.83021	-1.82787	-1.82554	-1.83525	-1.83289
-1.83827	-1.83593	-1.83358	-1.83123	-1.82889	-1.82655	-1.82422	-1.83391	-1.83156
-1.83699	-1.83465	-1.8323	-1.82995	-1.8276	-1.82526	-1.82293	-1.83259	-1.83024
-1.83576	-1.83341	-1.83106	-1.82872	-1.82637	-1.82403	-1.8217	-1.83133	-1.82898
-1.83455	-1.8322	-1.82985	-1.82751	-1.82517	-1.82283	-1.82049	-1.8301	-1.82773
-1.83329	-1.83093	-1.82858	-1.82624	-1.8239	-1.83357	-1.83119	-1.82881	-1.82644
-1.832	-1.82963	-1.82727	-1.82492	-1.82257	-1.83222	-1.82985	-1.82748	-1.8251
-1.83073	-1.82834	-1.82596	-1.8236	-1.82125	-1.83088	-1.82851	-1.82614	-1.82376
-1.82949	-1.82707	-1.82467	-1.8223	-1.81994	-1.82955	-1.82717	-1.82479	-1.8224
-1.82824	-1.82581	-1.82339	-1.82101	-1.81865	-1.84035	-1.83795	-1.83555	-1.83314
-1.82695	-1.82454	-1.82214	-1.81977	-1.81742	-1.83908	-1.83669	-1.83428	-1.83187
-1.82563	-1.82326	-1.82089	-1.81854	-1.81619	-1.83783	-1.83543	-1.83303	-1.83061
-1.82432	-1.82196	-1.83185	-1.82949	-1.82713	-1.83652	-1.83411	-1.8317	-1.82929
-1.82301	-1.82064	-1.83052	-1.82817	-1.82581	-1.83517	-1.83276	-1.83035	-1.82793
-1.83399	-1.83161	-1.82923	-1.82687	-1.82452	-1.83386	-1.83145	-1.82903	-1.82661
-1.83274	-1.83036	-1.82799	-1.82562	-1.82326	-1.83259	-1.83017	-1.82776	-1.82533
-1.83147	-1.82909	-1.83851	-1.8361	-1.8337	-1.83129	-1.82887	-1.82645	-1.82403
-1.83016	-1.82778	-1.83718	-1.83477	-1.83236	-1.82994	-1.82752	-1.82509	-1.82267
-1.82885	-1.82647	-1.83585	-1.83343	-1.83102	-1.82859	-1.82616	-1.82374	-1.82131
-1.8276	-1.82521	-1.83456	-1.83214	-1.82972	-1.82729	-1.82487	-1.82244	-1.82002
-1.82636	-1.82396	-1.83328	-1.83085	-1.82843	-1.82601	-1.82358	-1.82116	-1.81874
-1.82511	-1.82268	-1.83196	-1.82953	-1.82711	-1.82468	-1.82225	-1.81982	-1.8174
-1.82386	-1.8214	-1.83063	-1.82819	-1.82575	-1.82331	-1.82088	-1.81845	-1.81603
-1.83432	-1.83182	-1.82933	-1.82687	-1.82442	-1.82198	-1.81954	-1.81712	-1.8147
-1.83299	-1.83052	-1.82805	-1.82558	-1.82312	-1.82068	-1.81826	-1.81583	-1.81341
-1.83168	-1.82923	-1.82677	-1.82429	-1.82182	-1.81938	-1.81695	-1.81453	-1.81211
-1.83041	-1.82796	-1.8255	-1.82299	-1.8205	-1.81804	-1.8156	-1.81317	-1.81075
-1.82915	-1.82669	-1.82421	-1.82169	-1.81917	-1.8167	-1.81426	-1.81182	-1.80939
-1.82789	-1.8254	-1.8229	-1.82038	-1.81788	-1.81541	-1.81296	-1.81052	-1.80808
-1.82661	-1.82409	-1.82157	-1.81908	-1.8166	-1.81414	-1.8117	-1.80925	-1.80679
-1.8253	-1.82278	-1.82027	-1.81779	-1.81533	-1.81288	-1.81043	-1.80796	-1.80547
-1.82395	-1.82146	-1.81898	-1.81653	-1.81408	-1.81162	-1.80916	-1.80666	-1.80413
-1.82263	-1.82017	-1.81771	-1.81527	-1.81282	-1.81035	-1.80786	-1.80535	-1.8028
-1.82135	-1.8189	-1.81645	-1.814	-1.81154	-1.80904	-1.80653	-1.804	-1.80148
-1.82009	-1.81764	-1.81519	-1.81273	-1.81025	-1.80773	-1.80519	-1.80267	-1.80017
-1.81884	-1.81638	-1.81391	-1.81143	-1.80892	-1.8064	-1.80387	-1.80137	-1.79888

-1.81758	-1.81511	-1.81262	-1.81011	-1.80759	-1.80508	-1.80257	-1.80008	-1.79759
-1.81631	-1.81381	-1.81131	-1.80879	-1.80627	-1.80377	-1.80128	-1.79879	-1.7963
-1.81502	-1.8125	-1.80998	-1.80747	-1.80498	-1.80249	-1.8	-1.7975	-1.795
-1.81371	-1.81118	-1.80867	-1.80617	-1.80369	-1.8012	-1.79871	-1.7962	-1.79367
-1.81238	-1.80987	-1.80737	-1.80489	-1.80241	-1.79992	-1.79742	-1.79489	-1.79233
-1.81107	-1.80858	-1.80609	-1.80361	-1.80113	-1.79862	-1.79611	-1.79356	-1.79099
-1.8098	-1.8073	-1.80482	-1.80234	-1.79985	-1.79731	-1.79476	-1.79221	-1.78966
-1.80853	-1.80603	-1.80354	-1.80106	-1.79857	-1.79601	-1.79342	-1.79087	-1.78834
-1.80727	-1.80477	-1.80228	-1.7998	-1.79729	-1.79472	-1.79211	-1.78956	-1.78705
-1.80602	-1.80351	-1.80101	-1.79852	-1.79602	-1.79344	-1.79082	-1.78827	-1.78576
-1.80476	-1.80224	-1.79974	-1.79724	-1.79473	-1.79215	-1.78954	-1.78699	-1.78449
-1.80349	-1.80097	-1.79845	-1.79595	-1.79344	-1.79087	-1.78827	-1.78573	-1.78322
-1.8022	-1.79969	-1.79717	-1.79465	-1.79212	-1.78955	-1.78698	-1.78445	-1.78195
-1.80091	-1.7984	-1.79588	-1.79332	-1.79076	-1.78821	-1.78567	-1.78315	-1.78066
-1.79963	-1.79712	-1.79459	-1.79201	-1.78942	-1.78688	-1.78436	-1.78186	-1.77936
-1.79837	-1.79585	-1.79332	-1.79072	-1.78812	-1.78558	-1.78307	-1.78057	-1.77807
-1.79711	-1.79459	-1.79205	-1.78944	-1.78684	-1.7843	-1.78179	-1.77928	-1.77678
-1.79585	-1.79332	-1.79078	-1.78817	-1.78557	-1.78302	-1.7805	-1.77799	-1.7755
-1.79459	-1.79206	-1.78951	-1.78692	-1.78432	-1.78176	-1.77923	-1.77671	-1.77422
-1.79332	-1.79079	-1.78825	-1.78568	-1.78311	-1.78054	-1.77798	-1.77544	-1.77294
-1.79205	-1.78952	-1.787	-1.78447	-1.78192	-1.77934	-1.77674	-1.77419	-1.77167
-1.79079	-1.78826	-1.78574	-1.78323	-1.7807	-1.77811	-1.77549	-1.77292	-1.77039
-1.78954	-1.787	-1.78447	-1.78196	-1.77944	-1.77684	-1.77421	-1.77164	-1.76911
-1.78831	-1.78576	-1.78321	-1.7807	-1.77816	-1.77556	-1.77293	-1.77035	-1.76783
-1.78712	-1.78453	-1.78196	-1.77943	-1.77689	-1.77428	-1.77165	-1.76907	-1.76654
-1.78592	-1.7833	-1.7807	-1.77816	-1.77561	-1.773	-1.77037	-1.76779	-1.76526
-1.7847	-1.78207	-1.77946	-1.7769	-1.77434	-1.77173	-1.7691	-1.76652	-1.76398
-1.78345	-1.78082	-1.77822	-1.77565	-1.77308	-1.77047	-1.76785	-1.76527	-1.76272
-1.7822	-1.7796	-1.77701	-1.77442	-1.77183	-1.76923	-1.76663	-1.76404	-1.76147
-1.78096	-1.77841	-1.77583	-1.77322	-1.7706	-1.76802	-1.76544	-1.76284	-1.76023
-1.77972	-1.77719	-1.77463	-1.772	-1.76937	-1.76679	-1.76423	-1.76163	-1.75898
-1.77848	-1.77595	-1.77338	-1.77075	-1.76812	-1.76554	-1.76298	-1.76037	-1.75772
-1.77726	-1.7747	-1.77213	-1.7695	-1.76688	-1.76429	-1.76172	-1.75911	-1.75647
-1.77607	-1.77348	-1.77088	-1.76828	-1.76568	-1.76307	-1.76047	-1.75786	-1.75525
-1.77489	-1.77226	-1.76964	-1.76707	-1.76448	-1.76186	-1.75922	-1.75661	-1.75404
-1.77366	-1.77102	-1.7684	-1.76582	-1.76325	-1.76061	-1.75796	-1.75536	-1.75279
-1.77241	-1.76977	-1.76715	-1.76457	-1.76198	-1.75935	-1.75671	-1.7541	-1.75152
-1.77117	-1.76855	-1.76593	-1.76334	-1.76074	-1.75812	-1.75549	-1.75288	-1.75028
-1.76996	-1.76736	-1.76475	-1.76213	-1.75951	-1.7569	-1.7543	-1.75169	-1.74907
-1.76876	-1.76616	-1.76355	-1.76092	-1.75829	-1.75569	-1.7531	-1.75049	-1.74787
-1.76756	-1.76494	-1.76232	-1.75971	-1.7571	-1.75448	-1.75187	-1.74926	-1.74666
-1.76636	-1.76371	-1.76107	-1.75848	-1.75589	-1.75326	-1.75062	-1.74802	-1.74545
-1.76512	-1.76245	-1.75981	-1.75722	-1.75463	-1.752	-1.74936	-1.74675	-1.74418
-1.76386	-1.76119	-1.75854	-1.75595	-1.75335	-1.75073	-1.74809	-1.74548	-1.7429
-1.76261	-1.75995	-1.75731	-1.7547	-1.7521	-1.74948	-1.74686	-1.74424	-1.74165
-1.76136	-1.75874	-1.75611	-1.75348	-1.75086	-1.74825	-1.74565	-1.74303	-1.74041
-1.76015	-1.75753	-1.7549	-1.75227	-1.74964	-1.74704	-1.74444	-1.74182	-1.73919
-1.75899	-1.75633	-1.75368	-1.75106	-1.74845	-1.74583	-1.74322	-1.7406	-1.73798
-1.75784	-1.75514	-1.75247	-1.74986	-1.74725	-1.74463	-1.742	-1.73939	-1.73678
-1.75669	-1.75399	-1.75131	-1.74867	-1.74604	-1.74342	-1.7408	-1.73818	-1.73556

-1.75553	-1.75283	-1.75015	-1.74748	-1.74483	-1.74221	-1.7396	-1.73698	-1.73435
-1.75432	-1.75162	-1.74894	-1.74628	-1.74364	-1.74101	-1.7384	-1.73578	-1.73315
-1.75309	-1.75039	-1.74771	-1.74508	-1.74246	-1.73983	-1.7372	-1.73457	-1.73195
-1.75189	-1.7492	-1.74652	-1.74389	-1.74126	-1.73863	-1.736	-1.73337	-1.73075
-1.75073	-1.74805	-1.74537	-1.7427	-1.74004	-1.73741	-1.7348	-1.73217	-1.72953
-1.74955	-1.74689	-1.74421	-1.74152	-1.73885	-1.73622	-1.7336	-1.73097	-1.72832
-1.74834	-1.74571	-1.74306	-1.74038	-1.7377	-1.73504	-1.73239	-1.72976	-1.72713
-1.74714	-1.74452	-1.74189	-1.73923	-1.73656	-1.73388	-1.7312	-1.72856	-1.72596
-1.74597	-1.74332	-1.74068	-1.73804	-1.7354	-1.73273	-1.73004	-1.7274	-1.72481
-1.74481	-1.74212	-1.73946	-1.73684	-1.73423	-1.73157	-1.7289	-1.72627	-1.72366
-1.74366	-1.74096	-1.73829	-1.73565	-1.73303	-1.73038	-1.72773	-1.72509	-1.72247
-1.74251	-1.73982	-1.73714	-1.73447	-1.73181	-1.72917	-1.72654	-1.72391	-1.72126
-1.74133	-1.73867	-1.736	-1.7333	-1.73062	-1.72798	-1.72535	-1.72271	-1.72006
-1.74013	-1.73751	-1.73486	-1.73217	-1.72947	-1.7268	-1.72414	-1.7215	-1.71887
-1.73895	-1.73635	-1.73373	-1.73104	-1.72834	-1.72564	-1.72295	-1.72031	-1.71771
-1.73781	-1.73522	-1.73259	-1.72992	-1.72722	-1.72452	-1.72181	-1.71917	-1.71657
-1.73669	-1.73408	-1.73145	-1.72878	-1.7261	-1.7234	-1.72069	-1.71803	-1.71542
-1.73556	-1.73291	-1.73027	-1.72762	-1.72497	-1.72227	-1.71956	-1.71689	-1.71424
-1.73444	-1.73174	-1.72907	-1.72646	-1.72383	-1.72115	-1.71843	-1.71574	-1.71305
-1.73331	-1.7306	-1.72792	-1.72531	-1.72269	-1.72002	-1.71731	-1.7146	-1.71189
-1.73219	-1.72948	-1.7268	-1.72417	-1.72155	-1.71888	-1.71619	-1.71348	-1.71076
-1.73107	-1.72836	-1.72567	-1.72302	-1.72039	-1.71773	-1.71505	-1.71235	-1.70963
-1.72996	-1.72724	-1.72454	-1.72185	-1.71918	-1.71654	-1.7139	-1.71122	-1.70849
-1.72884	-1.72612	-1.72341	-1.7207	-1.718	-1.71536	-1.71275	-1.71008	-1.70736
-1.7277	-1.725	-1.72229	-1.71957	-1.71686	-1.71422	-1.7116	-1.70893	-1.70623
-1.72657	-1.72387	-1.72117	-1.71845	-1.71574	-1.71308	-1.71044	-1.70778	-1.70509
-1.72544	-1.72276	-1.72005	-1.71733	-1.71461	-1.71192	-1.70925	-1.70658	-1.70392
-1.72432	-1.72164	-1.71894	-1.71621	-1.71348	-1.71076	-1.70804	-1.70537	-1.70274
-1.72321	-1.72053	-1.71782	-1.7151	-1.71236	-1.70961	-1.70687	-1.7042	-1.70158
-1.7221	-1.71941	-1.71671	-1.71398	-1.71124	-1.70848	-1.70574	-1.70306	-1.70044
-1.72099	-1.7183	-1.71559	-1.71286	-1.71011	-1.70736	-1.7046	-1.70192	-1.6993
-1.7199	-1.7172	-1.71448	-1.71174	-1.70899	-1.70623	-1.70347	-1.70079	-1.69817
-1.71881	-1.71609	-1.71336	-1.71061	-1.70786	-1.7051	-1.70235	-1.69967	-1.69704
-1.7177	-1.71497	-1.71223	-1.70949	-1.70674	-1.70398	-1.70123	-1.69855	-1.69592
-1.71659	-1.71385	-1.71111	-1.70836	-1.70562	-1.70287	-1.70013	-1.69744	-1.6948
-1.71547	-1.71272	-1.70998	-1.70723	-1.70449	-1.70177	-1.69906	-1.69638	-1.69371
-1.71434	-1.71159	-1.70885	-1.7061	-1.70337	-1.70069	-1.69802	-1.69534	-1.69263
-1.71321	-1.71046	-1.70772	-1.70498	-1.70226	-1.69959	-1.69695	-1.69426	-1.69154
-1.71208	-1.70933	-1.70659	-1.70386	-1.70115	-1.69848	-1.69584	-1.69315	-1.69042
-1.71097	-1.70822	-1.70548	-1.70277	-1.70007	-1.69739	-1.69473	-1.69203	-1.6893
-1.70986	-1.70711	-1.70439	-1.7017	-1.69903	-1.69634	-1.69364	-1.69092	-1.68818
-1.70877	-1.70603	-1.70332	-1.70065	-1.69799	-1.69528	-1.69254	-1.6898	-1.68706
-1.70774	-1.70501	-1.70229	-1.69961	-1.69692	-1.69419	-1.69144	-1.68868	-1.68593
-1.70673	-1.704	-1.70128	-1.69856	-1.69583	-1.69308	-1.69032	-1.68756	-1.6848
-1.70567	-1.70295	-1.70021	-1.69747	-1.69472	-1.69197	-1.6892	-1.68644	-1.68368
-1.70458	-1.70185	-1.69911	-1.69636	-1.6936	-1.69084	-1.68808	-1.68532	-1.68257
-1.70349	-1.70076	-1.69801	-1.69525	-1.69249	-1.68972	-1.68696	-1.68422	-1.68149
-1.7024	-1.69967	-1.69692	-1.69415	-1.69138	-1.68861	-1.68586	-1.68313	-1.68044
-1.70132	-1.6986	-1.69586	-1.69309	-1.69031	-1.68754	-1.68478	-1.68207	-1.67939
-1.70025	-1.69756	-1.69484	-1.69208	-1.68931	-1.68654	-1.68377	-1.68104	-1.67833

-1.69919	-1.69652	-1.69383	-1.69108	-1.68831	-1.68554	-1.68277	-1.68001	-1.67727
-1.69817	-1.69548	-1.69277	-1.69002	-1.68725	-1.68449	-1.68172	-1.67896	-1.6762
-1.69716	-1.69443	-1.69169	-1.68893	-1.68616	-1.68341	-1.68065	-1.67789	-1.67513
-1.69612	-1.69336	-1.6906	-1.68783	-1.68508	-1.68234	-1.67961	-1.67686	-1.6741
-1.69506	-1.69228	-1.6895	-1.68673	-1.68398	-1.68127	-1.67858	-1.67585	-1.6731
-1.69402	-1.69123	-1.68845	-1.68568	-1.68292	-1.68022	-1.67753	-1.67482	-1.67207
-1.69304	-1.69024	-1.68746	-1.68468	-1.68192	-1.67919	-1.67647	-1.67374	-1.67099
-1.69204	-1.68925	-1.68646	-1.68368	-1.68091	-1.67815	-1.67541	-1.67265	-1.66989
-1.69098	-1.68819	-1.68541	-1.68263	-1.67985	-1.67709	-1.67433	-1.67156	-1.6688
-1.68992	-1.68713	-1.68435	-1.68157	-1.67879	-1.67602	-1.67326	-1.67049	-1.66772
-1.68892	-1.68613	-1.68334	-1.68056	-1.67778	-1.67501	-1.67224	-1.66947	-1.6667
-1.68794	-1.68515	-1.68236	-1.67957	-1.67679	-1.67402	-1.67126	-1.66849	-1.66572
-1.68691	-1.68412	-1.68134	-1.67855	-1.67577	-1.673	-1.67023	-1.66746	-1.66469
-1.68585	-1.68306	-1.68028	-1.67749	-1.67471	-1.67193	-1.66916	-1.66639	-1.66362
-1.68482	-1.68203	-1.67925	-1.67647	-1.67369	-1.67091	-1.66813	-1.66535	-1.66258
-1.68386	-1.68107	-1.67829	-1.67551	-1.67273	-1.66995	-1.66717	-1.66439	-1.66161
-1.68292	-1.68013	-1.67734	-1.67456	-1.67179	-1.66901	-1.66622	-1.66344	-1.66065
-1.67172	-1.66896	-1.6764	-1.67361	-1.67083	-1.66805	-1.66527	-1.66249	-1.6597
-1.67076	-1.668	-1.67544	-1.67265	-1.66987	-1.66709	-1.66431	-1.66153	-1.65874
-1.66975	-1.667	-1.67442	-1.67164	-1.66885	-1.66607	-1.66329	-1.6605	-1.65772
-1.66871	-1.66595	-1.67336	-1.67058	-1.66779	-1.66501	-1.66222	-1.65944	-1.65665
-1.66772	-1.66495	-1.66219	-1.65941	-1.65664	-1.66399	-1.66121	-1.65842	-1.65562
-1.66678	-1.66401	-1.66124	-1.65847	-1.65569	-1.66304	-1.66025	-1.65746	-1.65466
-1.66585	-1.66308	-1.66031	-1.65753	-1.65475	-1.65198	-1.6492	-1.6565	-1.6537
-1.66492	-1.66214	-1.65937	-1.65659	-1.65381	-1.65104	-1.64826	-1.65555	-1.65274
-1.66398	-1.66119	-1.65841	-1.65563	-1.65286	-1.65008	-1.6473	-1.65457	-1.65177
-1.66298	-1.66019	-1.6574	-1.65462	-1.65184	-1.64906	-1.64627	-1.65354	-1.65073
-1.66197	-1.65916	-1.65636	-1.65358	-1.6508	-1.64801	-1.64521	-1.65247	-1.64967
-1.66099	-1.65818	-1.65539	-1.6526	-1.64982	-1.64702	-1.64423	-1.64143	-1.63864
-1.66006	-1.65725	-1.65446	-1.65167	-1.64888	-1.64609	-1.64329	-1.64049	-1.6377
-1.65913	-1.65634	-1.65354	-1.65075	-1.64796	-1.64516	-1.64236	-1.63956	-1.63677
-1.65822	-1.65543	-1.65264	-1.64984	-1.64705	-1.64425	-1.64145	-1.63865	-1.63585
-1.65731	-1.65453	-1.65174	-1.64894	-1.64614	-1.64334	-1.64054	-1.63774	-1.63494
-1.65641	-1.65363	-1.65084	-1.64804	-1.64523	-1.64243	-1.63963	-1.63682	-1.63402
-1.65551	-1.65273	-1.64994	-1.64713	-1.64432	-1.64152	-1.63872	-1.63591	-1.63309
-1.65461	-1.65183	-1.64904	-1.64623	-1.64342	-1.64061	-1.6378	-1.63499	-1.63218
-1.65372	-1.65093	-1.64813	-1.64532	-1.64251	-1.6397	-1.63689	-1.63408	-1.63126
-1.65281	-1.65002	-1.64722	-1.64441	-1.64159	-1.63878	-1.63597	-1.63316	-1.63034
-1.65191	-1.64911	-1.6463	-1.64349	-1.64068	-1.63786	-1.63505	-1.63223	-1.62942
-1.651	-1.64819	-1.64538	-1.64258	-1.63976	-1.63694	-1.63412	-1.63131	-1.6285
-1.65009	-1.64728	-1.64447	-1.64166	-1.63885	-1.63603	-1.6332	-1.63039	-1.62758
-1.64918	-1.64636	-1.64355	-1.64074	-1.63793	-1.63511	-1.63229	-1.62947	-1.62666
-1.64826	-1.64545	-1.64263	-1.63982	-1.637	-1.63419	-1.63137	-1.62855	-1.62573
-1.64734	-1.64453	-1.64172	-1.6389	-1.63608	-1.63326	-1.63045	-1.62763	-1.62481
-1.64643	-1.64361	-1.6408	-1.63798	-1.63516	-1.63235	-1.62953	-1.62672	-1.62389
-1.64551	-1.6427	-1.63988	-1.63706	-1.63425	-1.63143	-1.62862	-1.6258	-1.62297
-1.64462	-1.64179	-1.63897	-1.63615	-1.63333	-1.63052	-1.6277	-1.62488	-1.62206
-1.64374	-1.6409	-1.63807	-1.63524	-1.63242	-1.6296	-1.62678	-1.62396	-1.62114
-1.6429	-1.64005	-1.6372	-1.63436	-1.63152	-1.62869	-1.62587	-1.62305	-1.62023
-1.64211	-1.63926	-1.6364	-1.63352	-1.63065	-1.6278	-1.62496	-1.62213	-1.61931

					-0.05392	-0.05383
					-0.05381	-0.05371
			-0.05391	-0.0538	-0.40355	-0.40232
			-0.05379	-0.05368	-0.40245	-0.40123
-0.05399	-0.05388	-0.05378	-0.40385	-0.40261	-0.48408	-0.48267
-0.05387	-0.05376	-0.05366	-0.40277	-0.40154	-0.48288	-0.48147
-0.05375	-0.05364	-0.05353	-0.40172	-0.4005	-0.4817	-0.4803
-0.40444	-0.40316	-0.40191	-0.48335	-0.48195	-0.52033	-0.51882

		-0.40335	-0.4021	-0.40087	-0.4822	-0.4808	-0.51909	-0.5176
-0.21589	-0.21517	-0.40228	-0.40104	-0.39983	-0.52085	-0.51934	-0.5661	-0.56453
-0.21526	-0.21458	-0.40123	-0.4	-0.39879	-0.51957	-0.51808	-0.56477	-0.56322
-0.40264	-0.40141	-0.4829	-0.48149	-0.4801	-0.51831	-0.51684	-0.60655	-0.60494
-0.40161	-0.40038	-0.48175	-0.48036	-0.47898	-0.51709	-0.51562	-0.60519	-0.60359
-0.48341	-0.482	-0.52034	-0.51884	-0.51735	-0.60707	-0.60544	-0.60383	-0.60223
-0.48224	-0.48084	-0.51908	-0.51758	-0.5161	-0.60567	-0.60404	-0.60243	-0.60085
-0.48108	-0.47969	-0.51783	-0.51633	-0.51484	-0.60427	-0.60263	-0.60104	-0.59948
-0.51959	-0.5181	-0.60787	-0.6062	-0.60453	-0.60289	-0.60127	-0.67521	-0.67341
-0.51836	-0.51688	-0.60652	-0.60484	-0.60316	-0.60153	-0.59993	-0.67367	-0.67191
-0.60843	-0.60679	-0.60515	-0.60348	-0.60182	-0.67583	-0.67398	-0.67218	-0.67044
-0.60705	-0.60541	-0.60378	-0.60213	-0.6005	-0.67434	-0.67253	-0.67076	-0.66901
-0.64134	-0.63948	-0.67848	-0.67657	-0.67468	-0.67286	-0.67108	-0.90213	-0.89928
-0.63983	-0.63799	-0.67694	-0.67505	-0.67319	-0.67138	-0.6696	-0.89975	-0.89694
-0.63833	-0.63651	-0.67542	-0.67355	-0.67171	-0.66991	-0.66814	-0.8974	-0.89467
-0.67761	-0.67575	-0.67391	-0.67207	-0.67025	-0.8586	-0.8559	-0.89518	-0.89256
-0.67606	-0.67423	-0.67242	-0.67061	-0.66881	-0.85644	-0.8538	-0.89304	-0.89054
-0.67455	-0.67275	-0.71073	-0.7088	-0.70689	-0.8962	-0.89351	-0.9598	-0.95709
-0.67309	-0.67131	-0.70919	-0.70724	-0.70532	-0.89395	-0.89139	-0.95752	-0.95483
-0.71142	-0.70953	-0.85815	-0.85537	-0.85267	-0.93077	-0.92811	-0.9553	-0.95262
-0.70982	-0.70794	-0.85593	-0.85331	-0.85077	-0.92867	-0.92601	-0.95315	-0.95048
-0.85911	-0.8564	-0.93486	-0.93204	-0.92931	-0.95646	-0.9537	-0.97996	-0.97725
-0.85699	-0.85434	-0.93258	-0.92986	-0.9272	-0.95428	-0.9515	-0.97772	-0.97502
-0.85491	-0.85234	-0.93038	-0.92772	-0.9251	-0.95211	-0.94931	-0.97549	-0.9728
-0.85287	-0.85035	-0.98721	-0.98443	-0.9817	-0.9789	-0.9761	-0.97336	-0.97066
-0.85088	-0.84839	-0.98492	-0.98214	-0.97942	-0.97669	-0.97398	-0.97126	-0.96855
-0.95901	-0.95622	-0.98271	-0.97994	-0.97722	-0.97453	-0.97187	-0.96915	-0.9664
-0.95683	-0.9541	-0.98057	-0.9778	-0.97507	-0.97241	-0.96976	-0.96703	-0.96422
-0.98393	-0.98118	-0.97844	-0.97567	-0.97293	-0.97026	-0.96761	-1.06798	-1.06505
-0.9818	-0.97906	-0.97632	-0.97353	-0.97077	-0.96806	-0.96539	-1.06567	-1.06285
-0.97969	-0.97695	-0.97419	-0.9714	-0.96863	-0.96589	-0.9632	-1.09588	-1.09309
-0.97756	-0.9748	-0.97204	-0.96928	-0.96653	-0.96381	-0.96112	-1.09367	-1.09093
-0.97543	-0.97266	-0.9699	-0.96717	-0.96446	-0.96176	-0.9591	-1.09151	-1.0888
-0.97329	-0.97053	-0.96779	-0.96505	-0.96235	-1.09495	-1.09215	-1.14901	-1.14618
-0.97113	-0.96842	-0.9657	-0.96295	-0.96025	-1.09275	-1.09004	-1.14684	-1.14398
-1.00496	-1.00221	-1.09915	-1.09621	-1.09331	-1.15025	-1.14748	-1.23462	-1.23163
-1.00285	-1.00009	-1.09691	-1.09399	-1.09113	-1.148	-1.14524	-1.23229	-1.22936
-1.1005	-1.09758	-1.15455	-1.15156	-1.14863	-1.23581	-1.23291	-1.23	-1.22709
-1.09829	-1.09536	-1.15226	-1.14934	-1.14648	-1.23357	-1.23067	-1.22775	-1.22483
-1.0961	-1.09318	-1.15002	-1.14716	-1.14437	-1.23135	-1.22844	-1.2255	-1.22254
-1.15379	-1.15079	-1.20527	-1.20239	-1.19954	-1.22911	-1.22618	-1.25317	-1.2501
-1.15157	-1.14864	-1.2031	-1.20023	-1.19738	-1.22687	-1.2239	-1.2508	-1.24768
-1.20677	-1.20383	-1.23348	-1.23052	-1.22756	-1.28212	-1.279	-1.27587	-1.27271
-1.20449	-1.20162	-1.23124	-1.22827	-1.22528	-1.27972	-1.27655	-1.27342	-1.27031
-1.29283	-1.28979	-1.28672	-1.28361	-1.28047	-1.2773	-1.27413	-1.27103	-1.26799
-1.29051	-1.28748	-1.2844	-1.28121	-1.27801	-1.27485	-1.27171	-1.26868	-1.26575
-1.2882	-1.28516	-1.28205	-1.27882	-1.27559	-1.27244	-1.26936	-1.2664	-1.26355
-1.28587	-1.28281	-1.27969	-1.27645	-1.27321	-1.27011	-1.26708	-1.26418	-1.2614
-1.28354	-1.28044	-1.27731	-1.27407	-1.27087	-1.26782	-1.26487	-1.26202	-1.25927
-1.28119	-1.27802	-1.27485	-1.27167	-1.26855	-1.26559	-1.26272	-1.33242	-1.32933

-1.27883	-1.27558	-1.27239	-1.26928	-1.26627	-1.2634	-1.26062	-1.33017	-1.32701
-1.27649	-1.27321	-1.27003	-1.267	-1.26407	-1.33409	-1.33105	-1.32796	-1.32485
-1.27416	-1.27091	-1.26776	-1.2648	-1.26194	-1.33183	-1.3288	-1.3258	-1.32281
-1.27184	-1.26864	-1.33901	-1.33578	-1.33267	-1.32959	-1.3266	-1.32373	-1.32097
-1.26953	-1.26642	-1.33669	-1.33351	-1.33042	-1.32737	-1.32442	-1.32175	-1.31932
-1.341	-1.33766	-1.33441	-1.33127	-1.32823	-1.32526	-1.32241	-1.31995	-1.31782
-1.33851	-1.33527	-1.33213	-1.32905	-1.3261	-1.32331	-1.3207	-1.31842	-1.31646
-1.33607	-1.33292	-1.32988	-1.3269	-1.32408	-1.32151	-1.31916	-1.31705	-1.3152
-1.33374	-1.33063	-1.32767	-1.32485	-1.32222	-1.31986	-1.31773	-1.31577	-1.314
-1.33149	-1.3284	-1.32552	-1.32291	-1.32052	-1.31836	-1.31642	-1.31458	-1.31287
-1.32928	-1.32629	-1.32356	-1.32114	-1.31895	-1.31696	-1.31514	-1.33009	-1.32839
-1.32709	-1.3243	-1.32178	-1.31955	-1.31753	-1.31565	-1.31391	-1.32888	-1.32723
-1.32504	-1.32248	-1.32016	-1.31809	-1.3162	-1.33111	-1.32938	-1.3277	-1.32608
-1.32314	-1.32084	-1.31874	-1.3168	-1.31498	-1.3299	-1.32821	-1.32655	-1.32493
-1.32142	-1.31936	-1.31743	-1.31558	-1.31383	-1.32874	-1.32707	-1.32542	-1.3238
-1.33682	-1.3348	-1.33291	-1.33107	-1.3293	-1.32761	-1.32597	-1.32433	-1.3227
-1.33548	-1.33353	-1.33168	-1.32989	-1.32817	-1.32651	-1.3249	-1.32327	-1.32163
-1.33416	-1.33229	-1.33049	-1.32875	-1.32705	-1.35056	-1.34891	-1.37823	-1.37635
-1.3329	-1.3311	-1.32935	-1.32763	-1.32595	-1.34944	-1.34779	-1.37699	-1.37512
-1.33167	-1.32993	-1.35347	-1.35172	-1.35	-1.37942	-1.3776	-1.37577	-1.37392
-1.33047	-1.32877	-1.35231	-1.35059	-1.3489	-1.3782	-1.37638	-1.37456	-1.37275
-1.35458	-1.35287	-1.35117	-1.34948	-1.34781	-1.40203	-1.40017	-1.39831	-1.39646
-1.35342	-1.35173	-1.35006	-1.34838	-1.34671	-1.40078	-1.39895	-1.39711	-1.39528
-1.35228	-1.35062	-1.34896	-1.34728	-1.34561	-1.39955	-1.39774	-1.39592	-1.39411
-1.35119	-1.34952	-1.40395	-1.40204	-1.40016	-1.39834	-1.39656	-1.4122	-1.41039
-1.35012	-1.34843	-1.40268	-1.4008	-1.39894	-1.39715	-1.39539	-1.41104	-1.40922
-1.40526	-1.40334	-1.40143	-1.39956	-1.39772	-1.41337	-1.41163	-1.40987	-1.40809
-1.40399	-1.40208	-1.40019	-1.39833	-1.3965	-1.41215	-1.41041	-1.40869	-1.40698
-1.42013	-1.41823	-1.41635	-1.41452	-1.41273	-1.41096	-1.40923	-1.43212	-1.43027
-1.41882	-1.41695	-1.41512	-1.41333	-1.41157	-1.40981	-1.40808	-1.43085	-1.42906
-1.41754	-1.41569	-1.41388	-1.41214	-1.41041	-1.43339	-1.43145	-1.45481	-1.45296
-1.4163	-1.41445	-1.41264	-1.4109	-1.4092	-1.43209	-1.43024	-1.45357	-1.45176
-1.41508	-1.41322	-1.41142	-1.40968	-1.40801	-1.4308	-1.42905	-1.45234	-1.45057
-1.41384	-1.41199	-1.43506	-1.43317	-1.43135	-1.4802	-1.4783	-1.52087	-1.51897
-1.4126	-1.41076	-1.43372	-1.43189	-1.43013	-1.47887	-1.47697	-1.51951	-1.51763
-1.46338	-1.4614	-1.52809	-1.52604	-1.52405	-1.52206	-1.52009	-1.51818	-1.51632
-1.46202	-1.46013	-1.52672	-1.5247	-1.52271	-1.52073	-1.51877	-1.51687	-1.51504
-1.52944	-1.52739	-1.52537	-1.52336	-1.52138	-1.51942	-1.51748	-1.51561	-1.51379
-1.52795	-1.52598	-1.52402	-1.52203	-1.52005	-1.51813	-1.51625	-1.5144	-1.51257
-1.52652	-1.5246	-1.52268	-1.5207	-1.51874	-1.51686	-1.51503	-1.5132	-1.51137
-1.52522	-1.52328	-1.52135	-1.51938	-1.51745	-1.5156	-1.5138	-1.51198	-1.51016
-1.52398	-1.52199	-1.52003	-1.51808	-1.51618	-1.51435	-1.51257	-1.51076	-1.50895
-1.52268	-1.52068	-1.51873	-1.51683	-1.51497	-1.51315	-1.51136	-1.50955	-1.50773
-1.52137	-1.51938	-1.51745	-1.51561	-1.5138	-1.51198	-1.51016	-1.50833	-1.50651
-1.52007	-1.51812	-1.51622	-1.51441	-1.51262	-1.51079	-1.50895	-1.52773	-1.52574
-1.51878	-1.51689	-1.51503	-1.51322	-1.51141	-1.50957	-1.50773	-1.52641	-1.52446
-1.51751	-1.51567	-1.51384	-1.51202	-1.5102	-1.52914	-1.5271	-1.52513	-1.52322
-1.5163	-1.51445	-1.51262	-1.51078	-1.50895	-1.52782	-1.52583	-1.5239	-1.52202
-1.5151	-1.51324	-1.5114	-1.50955	-1.50772	-1.52651	-1.52458	-1.52269	-1.52083
-1.51386	-1.51203	-1.53124	-1.5292	-1.52718	-1.52524	-1.52335	-1.52147	-1.5196

-1.51262	-1.51081	-1.52995	-1.52792	-1.52591	-1.524	-1.52214	-1.52026	-1.51836
-1.5114	-1.50961	-1.52867	-1.52668	-1.5247	-1.52279	-1.52092	-1.54543	-1.5434
-1.5102	-1.50842	-1.52742	-1.52548	-1.52355	-1.52161	-1.51969	-1.54411	-1.54212
-1.53004	-1.52809	-1.52616	-1.52424	-1.52233	-1.5469	-1.54482	-1.56317	-1.56121
-1.52881	-1.52685	-1.5249	-1.52297	-1.52105	-1.54553	-1.5435	-1.56188	-1.56
-1.52757	-1.52561	-1.55039	-1.54829	-1.54621	-1.56455	-1.56254	-1.58858	-1.5866
-1.52629	-1.52434	-1.54904	-1.54692	-1.54485	-1.56322	-1.5613	-1.58724	-1.58526
-1.525	-1.52308	-1.54771	-1.54559	-1.54353	-1.56194	-1.56009	-1.58593	-1.58394
-1.57093	-1.56884	-1.58226	-1.58015	-1.57808	-1.58863	-1.58663	-1.58462	-1.58262
-1.56956	-1.56749	-1.58094	-1.57891	-1.57692	-1.58735	-1.58533	-1.58332	-1.58132
-1.58375	-1.58167	-1.59232	-1.59018	-1.58809	-1.60478	-1.60272	-1.61236	-1.61033
-1.58246	-1.58037	-1.59091	-1.5888	-1.58674	-1.60342	-1.60139	-1.61104	-1.60906
-1.61279	-1.61054	-1.60835	-1.60624	-1.60416	-1.68591	-1.68376	-1.68165	-1.67959
-1.61127	-1.60914	-1.60705	-1.60497	-1.60291	-1.68457	-1.68241	-1.68029	-1.67822
-1.6098	-1.60776	-1.68974	-1.68758	-1.68541	-1.68323	-1.68106	-1.67893	-1.67683
-1.60844	-1.60641	-1.6883	-1.68615	-1.684	-1.68184	-1.67967	-1.67753	-1.6754
-1.60714	-1.60507	-1.68686	-1.68472	-1.68258	-1.68043	-1.67828	-1.67613	-1.67398
-1.68978	-1.68761	-1.68546	-1.68333	-1.6812	-1.67906	-1.67692	-1.71949	-1.71701
-1.68834	-1.68622	-1.6841	-1.68196	-1.67983	-1.67771	-1.67559	-1.71797	-1.7155
-1.68693	-1.68482	-1.68271	-1.68057	-1.67844	-1.72128	-1.71884	-1.71641	-1.71399
-1.68555	-1.68342	-1.68129	-1.67915	-1.67701	-1.71963	-1.71717	-1.71479	-1.71247
-1.68418	-1.68204	-1.6799	-1.67776	-1.67561	-1.71802	-1.71557	-1.71323	-1.71099
-1.68281	-1.68069	-1.67856	-1.67641	-1.67427	-1.71651	-1.7141	-1.7118	-1.70959
-1.68144	-1.67933	-1.67721	-1.67506	-1.67293	-1.71502	-1.71267	-1.7104	-1.70821
-1.68002	-1.6779	-1.72073	-1.71828	-1.71585	-1.7135	-1.71121	-1.70897	-1.70678
-1.6786	-1.67645	-1.71907	-1.71666	-1.71428	-1.71199	-1.70976	-1.70754	-1.70535
-1.72242	-1.71993	-1.71749	-1.71512	-1.7128	-1.71055	-1.70836	-1.70617	-1.70398
-1.72089	-1.71839	-1.71596	-1.71364	-1.71138	-1.70917	-1.70701	-1.70484	-1.70265
-1.71932	-1.71685	-1.71446	-1.71217	-1.70995	-1.70776	-1.70561	-1.74085	-1.73867
-1.71768	-1.7153	-1.71297	-1.71072	-1.7085	-1.70631	-1.70414	-1.73939	-1.73728
-1.7161	-1.7138	-1.71153	-1.7093	-1.70709	-1.70489	-1.70271	-1.73795	-1.73588
-1.71462	-1.71238	-1.71016	-1.70794	-1.70574	-1.70355	-1.70137	-1.73656	-1.73446
-1.7132	-1.711	-1.70881	-1.70661	-1.70442	-1.70223	-1.70006	-1.73521	-1.73304
-1.71181	-1.70962	-1.70744	-1.70526	-1.70309	-1.73827	-1.73607	-1.75722	-1.75484
-1.71044	-1.70824	-1.70605	-1.7039	-1.70175	-1.73693	-1.73474	-1.75576	-1.75337
-1.70901	-1.7068	-1.70462	-1.70247	-1.70034	-1.75897	-1.75661	-1.75424	-1.75186
-1.70753	-1.70531	-1.70313	-1.70099	-1.69887	-1.75735	-1.75501	-1.75266	-1.75031
-1.70608	-1.70388	-1.72601	-1.72368	-1.72137	-1.77179	-1.76945	-1.76712	-1.76478
-1.70469	-1.7025	-1.72452	-1.72219	-1.71988	-1.77027	-1.76794	-1.76562	-1.76332
-1.72007	-1.71789	-1.77583	-1.77347	-1.77111	-1.76877	-1.76644	-1.77692	-1.77462
-1.7187	-1.71655	-1.77436	-1.77198	-1.76962	-1.76728	-1.76497	-1.77545	-1.77319
-1.71734	-1.71521	-1.77289	-1.77051	-1.76814	-1.76581	-1.7635	-1.774	-1.77175
-1.73977	-1.73745	-1.78425	-1.78186	-1.77948	-1.77714	-1.77483	-1.77255	-1.77029
-1.73829	-1.73596	-1.78273	-1.78036	-1.778	-1.77568	-1.77339	-1.77109	-1.76881
-1.75009	-1.74772	-1.78117	-1.77881	-1.77647	-1.77417	-1.7719	-1.76962	-1.76733
-1.74859	-1.74617	-1.77956	-1.77721	-1.7749	-1.77262	-1.77036	-1.76811	-1.76586
-1.74707	-1.74463	-1.778	-1.77566	-1.77337	-1.7711	-1.76886	-1.83426	-1.83157
-1.74552	-1.74311	-1.77649	-1.77418	-1.77191	-1.76965	-1.76742	-1.83253	-1.82987
-1.74398	-1.74162	-1.77502	-1.77273	-1.77048	-1.76823	-1.766	-1.83083	-1.82819
-1.77824	-1.77588	-1.77356	-1.77129	-1.76904	-1.83451	-1.83182	-1.82915	-1.82651

-1.77675	-1.77442	-1.77212	-1.76985	-1.76762	-1.83281	-1.83014	-1.82748	-1.82484
-1.77527	-1.77296	-1.77068	-1.76843	-1.7662	-1.83111	-1.82846	-1.82581	-1.82317
-1.77381	-1.77152	-1.76925	-1.76701	-1.76478	-1.82941	-1.82677	-1.82413	-1.82149
-1.77235	-1.77008	-1.83578	-1.83306	-1.83038	-1.82772	-1.82509	-1.82245	-1.81981
-1.77089	-1.76864	-1.83406	-1.83136	-1.82868	-1.82603	-1.8234	-1.82076	-1.81811
-1.83777	-1.83505	-1.83234	-1.82966	-1.82699	-1.82434	-1.82171	-1.83555	-1.83285
-1.83603	-1.83333	-1.83065	-1.82796	-1.82529	-1.82264	-1.82	-1.83382	-1.83115
-1.83431	-1.83163	-1.82896	-1.82628	-1.82361	-1.82095	-1.8183	-1.8321	-1.82947
-1.8326	-1.82994	-1.82728	-1.82462	-1.82196	-1.84587	-1.84299	-1.84018	-1.83742
-1.8309	-1.82826	-1.82562	-1.82297	-1.82032	-1.84417	-1.8413	-1.8385	-1.83578
-1.82922	-1.82659	-1.83476	-1.83196	-1.82917	-1.84249	-1.83963	-1.83685	-1.83415
-1.82754	-1.82493	-1.8331	-1.83032	-1.82754	-1.84083	-1.83798	-1.83522	-1.83254
-1.83694	-1.8342	-1.84769	-1.84487	-1.84204	-1.83919	-1.83635	-1.84721	-1.84453
-1.83527	-1.83253	-1.846	-1.84319	-1.84038	-1.83756	-1.83474	-1.84562	-1.84298
-1.83359	-1.83087	-1.85799	-1.85519	-1.8524	-1.84957	-1.84676	-1.84405	-1.84143
-1.83192	-1.82921	-1.85631	-1.85354	-1.85077	-1.84797	-1.84517	-1.84248	-1.83989
-1.83026	-1.82757	-1.85463	-1.85189	-1.84915	-1.84637	-1.8436	-1.84093	-1.83835
-1.85849	-1.85572	-1.85298	-1.85026	-1.84754	-1.84479	-1.84204	-1.83939	-1.83683
-1.85682	-1.85407	-1.85135	-1.84864	-1.84594	-1.84321	-1.84049	-1.83786	-1.83531
-1.85516	-1.85243	-1.84972	-1.84704	-1.84436	-1.84626	-1.84355	-1.84093	-1.83839
-1.8535	-1.85079	-1.8481	-1.84544	-1.84279	-1.8447	-1.84201	-1.8394	-1.83687
-1.85185	-1.84915	-1.8511	-1.84847	-1.84583	-1.84316	-1.84048	-1.83789	-1.83538
-1.85021	-1.84752	-1.84948	-1.84687	-1.84427	-1.84162	-1.83897	-1.83641	-1.83392
-1.84857	-1.84589	-1.84787	-1.84529	-1.84271	-1.8401	-1.83748	-1.83495	-1.8325
-1.85157	-1.8489	-1.84628	-1.84372	-1.84118	-1.8386	-1.83604	-1.83354	-1.8311
-1.84996	-1.8473	-1.8447	-1.84216	-1.83965	-1.83712	-1.83461	-1.83214	-1.82971
-1.8484	-1.84574	-1.84314	-1.84061	-1.83811	-1.83562	-1.83316	-1.83071	-1.8283
-1.84689	-1.84421	-1.84159	-1.83906	-1.83658	-1.83412	-1.83169	-1.82928	-1.82687
-1.84536	-1.84268	-1.84007	-1.83754	-1.83507	-1.8459	-1.8435	-1.84109	-1.83868
-1.84381	-1.84115	-1.83856	-1.83605	-1.83359	-1.84443	-1.84205	-1.83966	-1.83726
-1.85558	-1.85294	-1.85036	-1.84785	-1.84539	-1.84298	-1.84061	-1.83824	-1.83586
-1.85406	-1.85147	-1.84892	-1.84642	-1.84396	-1.84155	-1.83919	-1.83684	-1.83451
-1.85256	-1.85001	-1.8475	-1.84501	-1.84255	-1.84014	-1.83778	-1.83545	-1.83317
-1.85106	-1.84854	-1.84606	-1.8436	-1.84116	-1.83875	-1.83637	-1.83406	-1.8318
-1.84958	-1.84708	-1.84461	-1.84219	-1.83979	-1.83738	-1.83498	-1.83267	-1.83043
-1.84814	-1.84563	-1.84317	-1.84077	-1.83839	-1.83599	-1.8336	-1.8313	-1.82907
-1.84673	-1.8442	-1.84173	-1.83932	-1.83696	-1.83459	-1.83224	-1.82995	-1.82773
-1.84529	-1.84276	-1.84029	-1.8379	-1.83555	-1.83321	-1.8309	-1.82863	-1.82641
-1.8438	-1.84129	-1.83885	-1.83648	-1.83416	-1.83186	-1.8296	-1.82736	-1.82514
-1.84233	-1.83986	-1.83744	-1.83509	-1.83279	-1.84266	-1.84042	-1.86693	-1.86465
-1.84093	-1.83849	-1.8361	-1.83375	-1.83145	-1.84132	-1.8391	-1.86559	-1.86335
-1.83957	-1.83716	-1.83478	-1.83244	-1.83013	-1.84	-1.83779	-1.86425	-1.86204
-1.85035	-1.84796	-1.8456	-1.84327	-1.84096	-1.8791	-1.87681	-1.87456	-1.87234
-1.84902	-1.84665	-1.8443	-1.84199	-1.8397	-1.87778	-1.87547	-1.87321	-1.87098
-1.85976	-1.8574	-1.88349	-1.88113	-1.87878	-1.87646	-1.87416	-1.87189	-1.86964
-1.8584	-1.85607	-1.88214	-1.87978	-1.87744	-1.87515	-1.87288	-1.87061	-1.86833
-1.88546	-1.88311	-1.88077	-1.87842	-1.87608	-1.87381	-1.87158	-1.86931	-1.86701
-1.88404	-1.88171	-1.87938	-1.87704	-1.87472	-1.87245	-1.87021	-1.86796	-1.86569
-1.88264	-1.88031	-1.87799	-1.87566	-1.87336	-1.8711	-1.86886	-1.86662	-1.86439
-1.88128	-1.87895	-1.87663	-1.87433	-1.87205	-1.86979	-1.86755	-1.86532	-1.86312

-1.87993	-1.87761	-1.8753	-1.87302	-1.87076	-1.8685	-1.86626	-1.86405	-1.86186
-1.8786	-1.87629	-1.874	-1.87172	-1.86946	-1.86721	-1.86499	-1.86279	-1.8606
-1.87728	-1.87499	-1.87271	-1.87042	-1.86816	-1.86593	-1.86374	-1.86154	-1.85934
-1.87597	-1.87369	-1.87142	-1.86914	-1.86689	-1.86467	-1.86248	-1.86029	-1.85809
-1.87469	-1.8724	-1.87013	-1.86788	-1.86565	-1.86343	-1.86122	-1.85903	-1.85685
-1.8734	-1.87111	-1.86884	-1.86662	-1.8644	-1.86218	-1.85996	-1.85777	-1.8556
-1.87208	-1.86982	-1.86758	-1.86535	-1.86314	-1.86092	-1.85871	-1.85651	-1.85434
-1.87076	-1.86853	-1.8663	-1.86408	-1.86187	-1.85966	-1.85745	-1.85526	-1.85307
-1.86948	-1.86723	-1.865	-1.86279	-1.86059	-1.85839	-1.85618	-1.85398	-1.8518
-1.86822	-1.86594	-1.86369	-1.86149	-1.85931	-1.85711	-1.85489	-1.8527	-1.85053
-1.86695	-1.86467	-1.86242	-1.86021	-1.85801	-1.85581	-1.85361	-1.85142	-1.84924
-1.86569	-1.86344	-1.86119	-1.85893	-1.8567	-1.85451	-1.85234	-1.85015	-1.84794
-1.86441	-1.86218	-1.85994	-1.85767	-1.85542	-1.85322	-1.85105	-1.84885	-1.84664
-1.86311	-1.8609	-1.85868	-1.85643	-1.85418	-1.85195	-1.84974	-1.84753	-1.84534
-1.8618	-1.8596	-1.85739	-1.85517	-1.85294	-1.85069	-1.84844	-1.84623	-1.84404
-1.86049	-1.85828	-1.85608	-1.85388	-1.85168	-1.84944	-1.84719	-1.84496	-1.84273
-1.85919	-1.85697	-1.85476	-1.85258	-1.8504	-1.84818	-1.84595	-1.8437	-1.84144
-1.85789	-1.85568	-1.85348	-1.85129	-1.8491	-1.8469	-1.84468	-1.85293	-1.85065
-1.8566	-1.85441	-1.85222	-1.85001	-1.84779	-1.8456	-1.8434	-1.85166	-1.84941
-1.85533	-1.85315	-1.85097	-1.84874	-1.84652	-1.84431	-1.84211	-1.86947	-1.86724
-1.85409	-1.85192	-1.84973	-1.84751	-1.84528	-1.84304	-1.84081	-1.86818	-1.86598
-1.85285	-1.85066	-1.84847	-1.84626	-1.84403	-1.87144	-1.86915	-1.8669	-1.86471
-1.85161	-1.84939	-1.84718	-1.84498	-1.84278	-1.87022	-1.86794	-1.86568	-1.86343
-1.85035	-1.84811	-1.84588	-1.84369	-1.84151	-1.86898	-1.86672	-1.86445	-1.86216
-1.84908	-1.84684	-1.87444	-1.87218	-1.86994	-1.86771	-1.86548	-1.87488	-1.87259
-1.8478	-1.84557	-1.87317	-1.87089	-1.86863	-1.86641	-1.86421	-1.87363	-1.87135
-1.87642	-1.87417	-1.87191	-1.86963	-1.86736	-1.87678	-1.87456	-1.87233	-1.87008
-1.8751	-1.87288	-1.87065	-1.86838	-1.8661	-1.87548	-1.87323	-1.871	-1.8688
-1.88544	-1.88324	-1.88102	-1.87876	-1.87649	-1.8742	-1.87193	-1.86969	-1.86749
-1.88417	-1.88196	-1.87974	-1.8775	-1.87523	-1.87295	-1.87066	-1.8684	-1.86617
-1.88291	-1.88068	-1.87845	-1.87622	-1.87397	-1.87169	-1.8694	-1.86713	-1.86487
-1.88163	-1.87937	-1.87712	-1.87491	-1.87269	-1.87043	-1.86816	-1.86589	-1.86362
-1.88036	-1.87806	-1.8758	-1.87359	-1.87139	-1.86916	-1.86691	-1.86465	-1.86238
-1.87909	-1.87678	-1.87451	-1.87228	-1.87006	-1.86785	-1.86565	-1.86341	-1.86114
-1.87782	-1.87552	-1.87324	-1.87097	-1.86873	-1.86654	-1.86437	-1.86215	-1.8599
-1.87657	-1.87427	-1.87198	-1.86971	-1.86745	-1.86527	-1.86311	-1.8609	-1.85866
-1.87534	-1.87303	-1.87075	-1.86847	-1.86621	-1.86401	-1.86184	-1.85964	-1.85742
-1.8741	-1.8718	-1.86951	-1.86723	-1.86498	-1.86276	-1.86056	-1.85836	-1.85616
-1.87286	-1.87056	-1.86828	-1.866	-1.86374	-1.86149	-1.85927	-1.85706	-1.85488
-1.87161	-1.86932	-1.86704	-1.86477	-1.8625	-1.86023	-1.85798	-1.85577	-1.8536
-1.87037	-1.86809	-1.86581	-1.86353	-1.86126	-1.85899	-1.85673	-1.85451	-1.85235
-1.86911	-1.86685	-1.86457	-1.86229	-1.86001	-1.85774	-1.85548	-1.85326	-1.85109
-1.86785	-1.86558	-1.86331	-1.86103	-1.85875	-1.85648	-1.85422	-1.852	-1.84983
-1.86657	-1.8643	-1.86204	-1.85976	-1.85748	-1.85521	-1.85295	-1.85074	-1.84856
-1.86529	-1.86302	-1.86075	-1.85848	-1.85621	-1.85393	-1.85167	-1.84944	-1.84724
-1.86402	-1.86174	-1.85946	-1.85719	-1.85492	-1.85264	-1.85036	-1.84811	-1.84588
-1.86274	-1.86045	-1.85817	-1.8559	-1.85362	-1.85134	-1.84906	-1.84678	-1.84453
-1.86146	-1.85917	-1.85688	-1.8546	-1.85232	-1.85003	-1.84775	-1.84547	-1.84319
-1.86017	-1.85788	-1.85559	-1.8533	-1.85101	-1.84872	-1.84643	-1.84415	-1.84187
-1.85888	-1.85659	-1.85429	-1.852	-1.8497	-1.84741	-1.84512	-1.84284	-1.84056

-1.85757	-1.85528	-1.85299	-1.85069	-1.84839	-1.8461	-1.84381	-1.84153	-1.83925
-1.85627	-1.85398	-1.85168	-1.84938	-1.84708	-1.84479	-1.8425	-1.84023	-1.83798
-1.85497	-1.85267	-1.85036	-1.84806	-1.84577	-1.84348	-1.84121	-1.83896	-1.83673
-1.85366	-1.85135	-1.84905	-1.84675	-1.84447	-1.8422	-1.83994	-1.83769	-1.83546
-1.85235	-1.85004	-1.84774	-1.84545	-1.84317	-1.84092	-1.83869	-1.83644	-1.83418
-1.85105	-1.84873	-1.84643	-1.84415	-1.84189	-1.83966	-1.83743	-1.83517	-1.83289
-1.84974	-1.84743	-1.84513	-1.84288	-1.84064	-1.8384	-1.83615	-1.83387	-1.83157
-1.84847	-1.84615	-1.84386	-1.84162	-1.83939	-1.83713	-1.83484	-1.83255	-1.83025
-1.84726	-1.84494	-1.84264	-1.84037	-1.83811	-1.83582	-1.83352	-1.83121	-1.8289
-1.84608	-1.84375	-1.84143	-1.83912	-1.83681	-1.8345	-1.83218	-1.82987	-1.82755
-1.84482	-1.8425	-1.84017	-1.83784	-1.83551	-1.83318	-1.83085	-1.84065	-1.83829
-1.84352	-1.8412	-1.83887	-1.83654	-1.8342	-1.83186	-1.82952	-1.83929	-1.83694
-1.84221	-1.83988	-1.83756	-1.83522	-1.83287	-1.83053	-1.82819	-1.83794	-1.83558
-1.84089	-1.83855	-1.83622	-1.83387	-1.83153	-1.8292	-1.82686	-1.83659	-1.83423
-1.83958	-1.83723	-1.83489	-1.83254	-1.83021	-1.82787	-1.82554	-1.83525	-1.83289
-1.83827	-1.83593	-1.83358	-1.83123	-1.82889	-1.82655	-1.82422	-1.83391	-1.83156
-1.83699	-1.83465	-1.8323	-1.82995	-1.8276	-1.82526	-1.82293	-1.83259	-1.83024
-1.83576	-1.83341	-1.83106	-1.82872	-1.82637	-1.82403	-1.8217	-1.83133	-1.82898
-1.83455	-1.8322	-1.82985	-1.82751	-1.82517	-1.82283	-1.82049	-1.8301	-1.82773
-1.83329	-1.83093	-1.82858	-1.82624	-1.8239	-1.83357	-1.83119	-1.82881	-1.82644
-1.832	-1.82963	-1.82727	-1.82492	-1.82257	-1.83222	-1.82985	-1.82748	-1.8251
-1.83073	-1.82834	-1.82596	-1.8236	-1.82125	-1.83088	-1.82851	-1.82614	-1.82376
-1.82949	-1.82707	-1.82467	-1.8223	-1.81994	-1.82955	-1.82717	-1.82479	-1.8224
-1.82824	-1.82581	-1.82339	-1.82101	-1.81865	-1.84035	-1.83795	-1.83555	-1.83314
-1.82695	-1.82454	-1.82214	-1.81977	-1.81742	-1.83908	-1.83669	-1.83428	-1.83187
-1.82563	-1.82326	-1.82089	-1.81854	-1.81619	-1.83783	-1.83543	-1.83303	-1.83061
-1.82432	-1.82196	-1.83185	-1.82949	-1.82713	-1.83652	-1.83411	-1.8317	-1.82929
-1.82301	-1.82064	-1.83052	-1.82817	-1.82581	-1.83517	-1.83276	-1.83035	-1.82793
-1.83399	-1.83161	-1.82923	-1.82687	-1.82452	-1.83386	-1.83145	-1.82903	-1.82661
-1.83274	-1.83036	-1.82799	-1.82562	-1.82326	-1.83259	-1.83017	-1.82776	-1.82533
-1.83147	-1.82909	-1.83851	-1.8361	-1.8337	-1.83129	-1.82887	-1.82645	-1.82403
-1.83016	-1.82778	-1.83718	-1.83477	-1.83236	-1.82994	-1.82752	-1.82509	-1.82267
-1.82885	-1.82647	-1.83585	-1.83343	-1.83102	-1.82859	-1.82616	-1.82374	-1.82131
-1.8276	-1.82521	-1.83456	-1.83214	-1.82972	-1.82729	-1.82487	-1.82244	-1.82002
-1.82636	-1.82396	-1.83328	-1.83085	-1.82843	-1.82601	-1.82358	-1.82116	-1.81874
-1.82511	-1.82268	-1.83196	-1.82953	-1.82711	-1.82468	-1.82225	-1.81982	-1.8174
-1.82386	-1.8214	-1.83063	-1.82819	-1.82575	-1.82331	-1.82088	-1.81845	-1.81603
-1.83432	-1.83182	-1.82933	-1.82687	-1.82442	-1.82198	-1.81954	-1.81712	-1.8147
-1.83299	-1.83052	-1.82805	-1.82558	-1.82312	-1.82068	-1.81826	-1.81583	-1.81341
-1.83168	-1.82923	-1.82677	-1.82429	-1.82182	-1.81938	-1.81695	-1.81453	-1.81211
-1.83041	-1.82796	-1.8255	-1.82299	-1.8205	-1.81804	-1.8156	-1.81317	-1.81075
-1.82915	-1.82669	-1.82421	-1.82169	-1.81917	-1.8167	-1.81426	-1.81182	-1.80939
-1.82789	-1.8254	-1.8229	-1.82038	-1.81788	-1.81541	-1.81296	-1.81052	-1.80808
-1.82661	-1.82409	-1.82157	-1.81908	-1.8166	-1.81414	-1.8117	-1.80925	-1.80679
-1.8253	-1.82278	-1.82027	-1.81779	-1.81533	-1.81288	-1.81043	-1.80796	-1.80547
-1.82395	-1.82146	-1.81898	-1.81653	-1.81408	-1.81162	-1.80916	-1.80666	-1.80413
-1.82263	-1.82017	-1.81771	-1.81527	-1.81282	-1.81035	-1.80786	-1.80535	-1.8028
-1.82135	-1.8189	-1.81645	-1.814	-1.81154	-1.80904	-1.80653	-1.804	-1.80148
-1.82009	-1.81764	-1.81519	-1.81273	-1.81025	-1.80773	-1.80519	-1.80267	-1.80017
-1.81884	-1.81638	-1.81391	-1.81143	-1.80892	-1.8064	-1.80387	-1.80137	-1.79888

-1.81758	-1.81511	-1.81262	-1.81011	-1.80759	-1.80508	-1.80257	-1.80008	-1.79759
-1.81631	-1.81381	-1.81131	-1.80879	-1.80627	-1.80377	-1.80128	-1.79879	-1.7963
-1.81502	-1.8125	-1.80998	-1.80747	-1.80498	-1.80249	-1.8	-1.7975	-1.795
-1.81371	-1.81118	-1.80867	-1.80617	-1.80369	-1.8012	-1.79871	-1.7962	-1.79367
-1.81238	-1.80987	-1.80737	-1.80489	-1.80241	-1.79992	-1.79742	-1.79489	-1.79233
-1.81107	-1.80858	-1.80609	-1.80361	-1.80113	-1.79862	-1.79611	-1.79356	-1.79099
-1.8098	-1.8073	-1.80482	-1.80234	-1.79985	-1.79731	-1.79476	-1.79221	-1.78966
-1.80853	-1.80603	-1.80354	-1.80106	-1.79857	-1.79601	-1.79342	-1.79087	-1.78834
-1.80727	-1.80477	-1.80228	-1.7998	-1.79729	-1.79472	-1.79211	-1.78956	-1.78705
-1.80602	-1.80351	-1.80101	-1.79852	-1.79602	-1.79344	-1.79082	-1.78827	-1.78576
-1.80476	-1.80224	-1.79974	-1.79724	-1.79473	-1.79215	-1.78954	-1.78699	-1.78449
-1.80349	-1.80097	-1.79845	-1.79595	-1.79344	-1.79087	-1.78827	-1.78573	-1.78322
-1.8022	-1.79969	-1.79717	-1.79465	-1.79212	-1.78955	-1.78698	-1.78445	-1.78195
-1.80091	-1.7984	-1.79588	-1.79332	-1.79076	-1.78821	-1.78567	-1.78315	-1.78066
-1.79963	-1.79712	-1.79459	-1.79201	-1.78942	-1.78688	-1.78436	-1.78186	-1.77936
-1.79837	-1.79585	-1.79332	-1.79072	-1.78812	-1.78558	-1.78307	-1.78057	-1.77807
-1.79711	-1.79459	-1.79205	-1.78944	-1.78684	-1.7843	-1.78179	-1.77928	-1.77678
-1.79585	-1.79332	-1.79078	-1.78817	-1.78557	-1.78302	-1.7805	-1.77799	-1.7755
-1.79459	-1.79206	-1.78951	-1.78692	-1.78432	-1.78176	-1.77923	-1.77671	-1.77422
-1.79332	-1.79079	-1.78825	-1.78568	-1.78311	-1.78054	-1.77798	-1.77544	-1.77294
-1.79205	-1.78952	-1.787	-1.78447	-1.78192	-1.77934	-1.77674	-1.77419	-1.77167
-1.79079	-1.78826	-1.78574	-1.78323	-1.7807	-1.77811	-1.77549	-1.77292	-1.77039
-1.78954	-1.787	-1.78447	-1.78196	-1.77944	-1.77684	-1.77421	-1.77164	-1.76911
-1.78831	-1.78576	-1.78321	-1.7807	-1.77816	-1.77556	-1.77293	-1.77035	-1.76783
-1.78712	-1.78453	-1.78196	-1.77943	-1.77689	-1.77428	-1.77165	-1.76907	-1.76654
-1.78592	-1.7833	-1.7807	-1.77816	-1.77561	-1.773	-1.77037	-1.76779	-1.76526
-1.7847	-1.78207	-1.77946	-1.7769	-1.77434	-1.77173	-1.7691	-1.76652	-1.76398
-1.78345	-1.78082	-1.77822	-1.77565	-1.77308	-1.77047	-1.76785	-1.76527	-1.76272
-1.7822	-1.7796	-1.77701	-1.77442	-1.77183	-1.76923	-1.76663	-1.76404	-1.76147
-1.78096	-1.77841	-1.77583	-1.77322	-1.7706	-1.76802	-1.76544	-1.76284	-1.76023
-1.77972	-1.77719	-1.77463	-1.772	-1.76937	-1.76679	-1.76423	-1.76163	-1.75898
-1.77848	-1.77595	-1.77338	-1.77075	-1.76812	-1.76554	-1.76298	-1.76037	-1.75772
-1.77726	-1.7747	-1.77213	-1.7695	-1.76688	-1.76429	-1.76172	-1.75911	-1.75647
-1.77607	-1.77348	-1.77088	-1.76828	-1.76568	-1.76307	-1.76047	-1.75786	-1.75525
-1.77489	-1.77226	-1.76964	-1.76707	-1.76448	-1.76186	-1.75922	-1.75661	-1.75404
-1.77366	-1.77102	-1.7684	-1.76582	-1.76325	-1.76061	-1.75796	-1.75536	-1.75279
-1.77241	-1.76977	-1.76715	-1.76457	-1.76198	-1.75935	-1.75671	-1.7541	-1.75152
-1.77117	-1.76855	-1.76593	-1.76334	-1.76074	-1.75812	-1.75549	-1.75288	-1.75028
-1.76996	-1.76736	-1.76475	-1.76213	-1.75951	-1.7569	-1.7543	-1.75169	-1.74907
-1.76876	-1.76616	-1.76355	-1.76092	-1.75829	-1.75569	-1.7531	-1.75049	-1.74787
-1.76756	-1.76494	-1.76232	-1.75971	-1.7571	-1.75448	-1.75187	-1.74926	-1.74666
-1.76636	-1.76371	-1.76107	-1.75848	-1.75589	-1.75326	-1.75062	-1.74802	-1.74545
-1.76512	-1.76245	-1.75981	-1.75722	-1.75463	-1.752	-1.74936	-1.74675	-1.74418
-1.76386	-1.76119	-1.75854	-1.75595	-1.75335	-1.75073	-1.74809	-1.74548	-1.7429
-1.76261	-1.75995	-1.75731	-1.7547	-1.7521	-1.74948	-1.74686	-1.74424	-1.74165
-1.76136	-1.75874	-1.75611	-1.75348	-1.75086	-1.74825	-1.74565	-1.74303	-1.74041
-1.76015	-1.75753	-1.7549	-1.75227	-1.74964	-1.74704	-1.74444	-1.74182	-1.73919
-1.75899	-1.75633	-1.75368	-1.75106	-1.74845	-1.74583	-1.74322	-1.7406	-1.73798
-1.75784	-1.75514	-1.75247	-1.74986	-1.74725	-1.74463	-1.742	-1.73939	-1.73678
-1.75669	-1.75399	-1.75131	-1.74867	-1.74604	-1.74342	-1.7408	-1.73818	-1.73556

-1.75553	-1.75283	-1.75015	-1.74748	-1.74483	-1.74221	-1.7396	-1.73698	-1.73435
-1.75432	-1.75162	-1.74894	-1.74628	-1.74364	-1.74101	-1.7384	-1.73578	-1.73315
-1.75309	-1.75039	-1.74771	-1.74508	-1.74246	-1.73983	-1.7372	-1.73457	-1.73195
-1.75189	-1.7492	-1.74652	-1.74389	-1.74126	-1.73863	-1.736	-1.73337	-1.73075
-1.75073	-1.74805	-1.74537	-1.7427	-1.74004	-1.73741	-1.7348	-1.73217	-1.72953
-1.74955	-1.74689	-1.74421	-1.74152	-1.73885	-1.73622	-1.7336	-1.73097	-1.72832
-1.74834	-1.74571	-1.74306	-1.74038	-1.7377	-1.73504	-1.73239	-1.72976	-1.72713
-1.74714	-1.74452	-1.74189	-1.73923	-1.73656	-1.73388	-1.7312	-1.72856	-1.72596
-1.74597	-1.74332	-1.74068	-1.73804	-1.7354	-1.73273	-1.73004	-1.7274	-1.72481
-1.74481	-1.74212	-1.73946	-1.73684	-1.73423	-1.73157	-1.7289	-1.72627	-1.72366
-1.74366	-1.74096	-1.73829	-1.73565	-1.73303	-1.73038	-1.72773	-1.72509	-1.72247
-1.74251	-1.73982	-1.73714	-1.73447	-1.73181	-1.72917	-1.72654	-1.72391	-1.72126
-1.74133	-1.73867	-1.736	-1.7333	-1.73062	-1.72798	-1.72535	-1.72271	-1.72006
-1.74013	-1.73751	-1.73486	-1.73217	-1.72947	-1.7268	-1.72414	-1.7215	-1.71887
-1.73895	-1.73635	-1.73373	-1.73104	-1.72834	-1.72564	-1.72295	-1.72031	-1.71771
-1.73781	-1.73522	-1.73259	-1.72992	-1.72722	-1.72452	-1.72181	-1.71917	-1.71657
-1.73669	-1.73408	-1.73145	-1.72878	-1.7261	-1.7234	-1.72069	-1.71803	-1.71542
-1.73556	-1.73291	-1.73027	-1.72762	-1.72497	-1.72227	-1.71956	-1.71689	-1.71424
-1.73444	-1.73174	-1.72907	-1.72646	-1.72383	-1.72115	-1.71843	-1.71574	-1.71305
-1.73331	-1.7306	-1.72792	-1.72531	-1.72269	-1.72002	-1.71731	-1.7146	-1.71189
-1.73219	-1.72948	-1.7268	-1.72417	-1.72155	-1.71888	-1.71619	-1.71348	-1.71076
-1.73107	-1.72836	-1.72567	-1.72302	-1.72039	-1.71773	-1.71505	-1.71235	-1.70963
-1.72996	-1.72724	-1.72454	-1.72185	-1.71918	-1.71654	-1.7139	-1.71122	-1.70849
-1.72884	-1.72612	-1.72341	-1.7207	-1.718	-1.71536	-1.71275	-1.71008	-1.70736
-1.7277	-1.725	-1.72229	-1.71957	-1.71686	-1.71422	-1.7116	-1.70893	-1.70623
-1.72657	-1.72387	-1.72117	-1.71845	-1.71574	-1.71308	-1.71044	-1.70778	-1.70509
-1.72544	-1.72276	-1.72005	-1.71733	-1.71461	-1.71192	-1.70925	-1.70658	-1.70392
-1.72432	-1.72164	-1.71894	-1.71621	-1.71348	-1.71076	-1.70804	-1.70537	-1.70274
-1.72321	-1.72053	-1.71782	-1.7151	-1.71236	-1.70961	-1.70687	-1.7042	-1.70158
-1.7221	-1.71941	-1.71671	-1.71398	-1.71124	-1.70848	-1.70574	-1.70306	-1.70044
-1.72099	-1.7183	-1.71559	-1.71286	-1.71011	-1.70736	-1.7046	-1.70192	-1.6993
-1.7199	-1.7172	-1.71448	-1.71174	-1.70899	-1.70623	-1.70347	-1.70079	-1.69817
-1.71881	-1.71609	-1.71336	-1.71061	-1.70786	-1.7051	-1.70235	-1.69967	-1.69704
-1.7177	-1.71497	-1.71223	-1.70949	-1.70674	-1.70398	-1.70123	-1.69855	-1.69592
-1.71659	-1.71385	-1.71111	-1.70836	-1.70562	-1.70287	-1.70013	-1.69744	-1.6948
-1.71547	-1.71272	-1.70998	-1.70723	-1.70449	-1.70177	-1.69906	-1.69638	-1.69371
-1.71434	-1.71159	-1.70885	-1.7061	-1.70337	-1.70069	-1.69802	-1.69534	-1.69263
-1.71321	-1.71046	-1.70772	-1.70498	-1.70226	-1.69959	-1.69695	-1.69426	-1.69154
-1.71208	-1.70933	-1.70659	-1.70386	-1.70115	-1.69848	-1.69584	-1.69315	-1.69042
-1.71097	-1.70822	-1.70548	-1.70277	-1.70007	-1.69739	-1.69473	-1.69203	-1.6893
-1.70986	-1.70711	-1.70439	-1.7017	-1.69903	-1.69634	-1.69364	-1.69092	-1.68818
-1.70877	-1.70603	-1.70332	-1.70065	-1.69799	-1.69528	-1.69254	-1.6898	-1.68706
-1.70774	-1.70501	-1.70229	-1.69961	-1.69692	-1.69419	-1.69144	-1.68868	-1.68593
-1.70673	-1.704	-1.70128	-1.69856	-1.69583	-1.69308	-1.69032	-1.68756	-1.6848
-1.70567	-1.70295	-1.70021	-1.69747	-1.69472	-1.69197	-1.6892	-1.68644	-1.68368
-1.70458	-1.70185	-1.69911	-1.69636	-1.6936	-1.69084	-1.68808	-1.68532	-1.68257
-1.70349	-1.70076	-1.69801	-1.69525	-1.69249	-1.68972	-1.68696	-1.68422	-1.68149
-1.7024	-1.69967	-1.69692	-1.69415	-1.69138	-1.68861	-1.68586	-1.68313	-1.68044
-1.70132	-1.6986	-1.69586	-1.69309	-1.69031	-1.68754	-1.68478	-1.68207	-1.67939
-1.70025	-1.69756	-1.69484	-1.69208	-1.68931	-1.68654	-1.68377	-1.68104	-1.67833

-1.69919	-1.69652	-1.69383	-1.69108	-1.68831	-1.68554	-1.68277	-1.68001	-1.67727
-1.69817	-1.69548	-1.69277	-1.69002	-1.68725	-1.68449	-1.68172	-1.67896	-1.6762
-1.69716	-1.69443	-1.69169	-1.68893	-1.68616	-1.68341	-1.68065	-1.67789	-1.67513
-1.69612	-1.69336	-1.6906	-1.68783	-1.68508	-1.68234	-1.67961	-1.67686	-1.6741
-1.69506	-1.69228	-1.6895	-1.68673	-1.68398	-1.68127	-1.67858	-1.67585	-1.6731
-1.69402	-1.69123	-1.68845	-1.68568	-1.68292	-1.68022	-1.67753	-1.67482	-1.67207
-1.69304	-1.69024	-1.68746	-1.68468	-1.68192	-1.67919	-1.67647	-1.67374	-1.67099
-1.69204	-1.68925	-1.68646	-1.68368	-1.68091	-1.67815	-1.67541	-1.67265	-1.66989
-1.69098	-1.68819	-1.68541	-1.68263	-1.67985	-1.67709	-1.67433	-1.67156	-1.6688
-1.68992	-1.68713	-1.68435	-1.68157	-1.67879	-1.67602	-1.67326	-1.67049	-1.66772
-1.68892	-1.68613	-1.68334	-1.68056	-1.67778	-1.67501	-1.67224	-1.66947	-1.6667
-1.68794	-1.68515	-1.68236	-1.67957	-1.67679	-1.67402	-1.67126	-1.66849	-1.66572
-1.68691	-1.68412	-1.68134	-1.67855	-1.67577	-1.673	-1.67023	-1.66746	-1.66469
-1.68585	-1.68306	-1.68028	-1.67749	-1.67471	-1.67193	-1.66916	-1.66639	-1.66362
-1.68482	-1.68203	-1.67925	-1.67647	-1.67369	-1.67091	-1.66813	-1.66535	-1.66258
-1.68386	-1.68107	-1.67829	-1.67551	-1.67273	-1.66995	-1.66717	-1.66439	-1.66161
-1.68292	-1.68013	-1.67734	-1.67456	-1.67179	-1.66901	-1.66622	-1.66344	-1.66065
-1.67172	-1.66896	-1.6764	-1.67361	-1.67083	-1.66805	-1.66527	-1.66249	-1.6597
-1.67076	-1.668	-1.67544	-1.67265	-1.66987	-1.66709	-1.66431	-1.66153	-1.65874
-1.66975	-1.667	-1.67442	-1.67164	-1.66885	-1.66607	-1.66329	-1.6605	-1.65772
-1.66871	-1.66595	-1.67336	-1.67058	-1.66779	-1.66501	-1.66222	-1.65944	-1.65665
-1.66772	-1.66495	-1.66219	-1.65941	-1.65664	-1.66399	-1.66121	-1.65842	-1.65562
-1.66678	-1.66401	-1.66124	-1.65847	-1.65569	-1.66304	-1.66025	-1.65746	-1.65466
-1.66585	-1.66308	-1.66031	-1.65753	-1.65475	-1.65198	-1.6492	-1.6565	-1.6537
-1.66492	-1.66214	-1.65937	-1.65659	-1.65381	-1.65104	-1.64826	-1.65555	-1.65274
-1.66398	-1.66119	-1.65841	-1.65563	-1.65286	-1.65008	-1.6473	-1.65457	-1.65177
-1.66298	-1.66019	-1.6574	-1.65462	-1.65184	-1.64906	-1.64627	-1.65354	-1.65073
-1.66197	-1.65916	-1.65636	-1.65358	-1.6508	-1.64801	-1.64521	-1.65247	-1.64967
-1.66099	-1.65818	-1.65539	-1.6526	-1.64982	-1.64702	-1.64423	-1.64143	-1.63864
-1.66006	-1.65725	-1.65446	-1.65167	-1.64888	-1.64609	-1.64329	-1.64049	-1.6377
-1.65913	-1.65634	-1.65354	-1.65075	-1.64796	-1.64516	-1.64236	-1.63956	-1.63677
-1.65822	-1.65543	-1.65264	-1.64984	-1.64705	-1.64425	-1.64145	-1.63865	-1.63585
-1.65731	-1.65453	-1.65174	-1.64894	-1.64614	-1.64334	-1.64054	-1.63774	-1.63494
-1.65641	-1.65363	-1.65084	-1.64804	-1.64523	-1.64243	-1.63963	-1.63682	-1.63402
-1.65551	-1.65273	-1.64994	-1.64713	-1.64432	-1.64152	-1.63872	-1.63591	-1.63309
-1.65461	-1.65183	-1.64904	-1.64623	-1.64342	-1.64061	-1.6378	-1.63499	-1.63218
-1.65372	-1.65093	-1.64813	-1.64532	-1.64251	-1.6397	-1.63689	-1.63408	-1.63126
-1.65281	-1.65002	-1.64722	-1.64441	-1.64159	-1.63878	-1.63597	-1.63316	-1.63034
-1.65191	-1.64911	-1.6463	-1.64349	-1.64068	-1.63786	-1.63505	-1.63223	-1.62942
-1.651	-1.64819	-1.64538	-1.64258	-1.63976	-1.63694	-1.63412	-1.63131	-1.6285
-1.65009	-1.64728	-1.64447	-1.64166	-1.63885	-1.63603	-1.6332	-1.63039	-1.62758
-1.64918	-1.64636	-1.64355	-1.64074	-1.63793	-1.63511	-1.63229	-1.62947	-1.62666
-1.64826	-1.64545	-1.64263	-1.63982	-1.637	-1.63419	-1.63137	-1.62855	-1.62573
-1.64734	-1.64453	-1.64172	-1.6389	-1.63608	-1.63326	-1.63045	-1.62763	-1.62481
-1.64643	-1.64361	-1.6408	-1.63798	-1.63516	-1.63235	-1.62953	-1.62672	-1.62389
-1.64551	-1.6427	-1.63988	-1.63706	-1.63425	-1.63143	-1.62862	-1.6258	-1.62297
-1.64462	-1.64179	-1.63897	-1.63615	-1.63333	-1.63052	-1.6277	-1.62488	-1.62206
-1.64374	-1.6409	-1.63807	-1.63524	-1.63242	-1.6296	-1.62678	-1.62396	-1.62114
-1.6429	-1.64005	-1.6372	-1.63436	-1.63152	-1.62869	-1.62587	-1.62305	-1.62023
-1.64211	-1.63926	-1.6364	-1.63352	-1.63065	-1.6278	-1.62496	-1.62213	-1.61931

-1.26902	-1.26708	-1.26515	-1.26323	-1.26132	-1.29164	-1.28971	-1.28768	-1.28576
-1.26965	-1.26772	-1.26579	-1.26387	-1.26197	-1.29231	-1.29036	-1.2883	-1.2864
-1.27028	-1.26835	-1.26643	-1.26452	-1.26262	-1.29297	-1.29101	-1.28893	-1.28704
-1.27091	-1.26899	-1.26707	-1.26516	-1.26326	-1.26136	-1.25949	-1.28956	-1.28766
-1.27154	-1.26963	-1.26772	-1.2658	-1.26389	-1.26197	-1.26011	-1.29019	-1.28828
-1.2722	-1.27028	-1.26837	-1.26646	-1.26455	-1.2626	-1.26072	-1.29082	-1.28892
-1.27288	-1.27094	-1.26902	-1.26712	-1.26523	-1.26324	-1.26134	-1.29146	-1.28957
-1.24233	-1.24044	-1.26951	-1.26765	-1.26579	-1.26389	-1.26198	-1.26012	-1.25829
-1.24299	-1.24111	-1.27018	-1.26831	-1.26645	-1.26456	-1.26265	-1.26078	-1.25894
-1.24364	-1.24177	-1.23989	-1.23808	-1.23626	-1.26521	-1.26332	-1.26144	-1.25958
-1.24426	-1.24241	-1.24052	-1.23869	-1.23687	-1.26585	-1.26397	-1.26209	-1.26021
-1.24489	-1.24305	-1.24113	-1.23931	-1.23748	-1.26649	-1.26462	-1.26273	-1.26084
-1.24538	-1.24357	-1.24175	-1.23994	-1.23812	-1.2363	-1.23447	-1.26338	-1.2615
-1.24603	-1.24419	-1.24236	-1.24057	-1.23877	-1.23694	-1.2351	-1.26404	-1.26218
-1.21201	-1.21022	-1.24299	-1.2412	-1.23942	-1.23759	-1.23574	-1.23392	-1.23211
-1.21263	-1.21084	-1.24363	-1.24184	-1.24005	-1.23823	-1.23639	-1.23458	-1.23277
-1.21324	-1.21146	-1.2097	-1.20795	-1.20621	-1.23885	-1.23704	-1.23522	-1.23341
-1.21383	-1.21207	-1.21031	-1.20855	-1.20679	-1.23945	-1.23766	-1.23585	-1.23403
-1.21443	-1.21268	-1.21093	-1.20915	-1.20738	-1.24006	-1.23828	-1.23647	-1.23464
-1.21505	-1.2133	-1.21154	-1.20977	-1.20799	-1.24068	-1.2389	-1.2371	-1.23527
-1.21568	-1.21392	-1.21216	-1.21039	-1.20862	-1.24132	-1.23952	-1.23772	-1.23591
-1.21631	-1.21454	-1.21277	-1.21101	-1.20925	-1.2075	-1.20575	-1.23833	-1.23653
-1.21694	-1.21515	-1.21338	-1.21163	-1.20989	-1.20812	-1.20634	-1.23893	-1.23715
-1.18143	-1.17971	-1.214	-1.21226	-1.21052	-1.20874	-1.20696	-1.20522	-1.20352
-1.182	-1.18029	-1.21462	-1.21289	-1.21115	-1.20938	-1.2076	-1.20586	-1.20416
-1.14663	-1.14499	-1.17919	-1.17753	-1.17588	-1.21002	-1.20825	-1.20651	-1.2048
-1.14716	-1.14553	-1.17976	-1.17812	-1.17647	-1.21066	-1.20889	-1.20715	-1.20544
-1.1477	-1.14607	-1.18034	-1.1787	-1.17706	-1.21129	-1.20952	-1.20778	-1.20607
-1.14828	-1.14666	-1.14504	-1.14345	-1.14185	-1.17597	-1.17428	-1.20842	-1.2067
-1.14888	-1.14725	-1.14563	-1.14402	-1.1424	-1.17655	-1.17487	-1.20905	-1.20733
-1.09408	-1.09257	-1.14621	-1.14458	-1.14295	-1.14133	-1.13971	-1.1738	-1.17216
-1.09461	-1.0931	-1.14678	-1.14513	-1.14349	-1.14187	-1.14027	-1.1744	-1.17276
-1.09514	-1.09363	-1.09211	-1.09057	-1.08904	-1.14243	-1.14084	-1.13925	-1.13767
-1.09567	-1.09416	-1.09264	-1.0911	-1.08956	-1.14299	-1.14141	-1.13982	-1.13825
-1.0962	-1.09469	-1.09318	-1.09164	-1.0901	-1.08861	-1.08713	-1.10162	-1.10015
-1.09673	-1.09523	-1.09372	-1.09219	-1.09066	-1.08915	-1.08767	-1.10216	-1.10068
-1.09727	-1.09577	-1.09427	-1.09275	-1.09122	-1.08971	-1.08821	-1.1027	-1.10122
-1.04373	-1.04235	-1.05873	-1.0573	-1.05586	-1.0903	-1.08878	-1.08729	-1.08582
-1.04422	-1.04285	-1.05926	-1.05785	-1.05642	-1.09089	-1.08935	-1.08784	-1.08637
-1.04471	-1.04334	-1.05977	-1.05837	-1.05695	-1.0555	-1.05402	-1.0884	-1.08691
-1.04519	-1.04381	-1.06026	-1.05886	-1.05746	-1.05601	-1.05454	-1.08895	-1.08746
-1.04568	-1.0443	-1.04293	-1.04157	-1.0402	-1.05652	-1.05506	-1.05362	-1.0522
-1.04617	-1.04479	-1.04342	-1.04206	-1.04069	-1.05703	-1.05558	-1.05414	-1.05271
-1.04666	-1.04528	-1.04392	-1.04255	-1.04118	-1.05754	-1.0561	-1.05466	-1.05323
-1.04714	-1.04576	-1.04439	-1.04304	-1.04167	-1.05806	-1.05663	-1.0552	-1.05377
-1.04763	-1.04624	-1.04487	-1.04352	-1.04216	-1.05858	-1.05717	-1.05575	-1.05431
-1.04813	-1.04674	-1.04536	-1.044	-1.04264	-1.04129	-1.03993	-1.05628	-1.05486
-1.04866	-1.04726	-1.04587	-1.04449	-1.04312	-1.04176	-1.04042	-1.0568	-1.05541
-1.04847	-1.04708	-1.04568	-1.04429	-1.0429	-1.04154	-1.04018	-1.0573	-1.05592
-1.04898	-1.04761	-1.04623	-1.04483	-1.04343	-1.04205	-1.04068	-1.05779	-1.05641

-1.04948	-1.04813	-1.04676	-1.04536	-1.04396	-1.04257	-1.04119	-1.05773	-1.05633
-1.04997	-1.04862	-1.04726	-1.04588	-1.0445	-1.04311	-1.04172	-1.05827	-1.05686
-1.05046	-1.0491	-1.04775	-1.04639	-1.04503	-1.04365	-1.04225	-1.05881	-1.05739
-1.02946	-1.02814	-1.04824	-1.04689	-1.04553	-1.04416	-1.04278	-1.04139	-1.04
-1.02995	-1.02862	-1.04874	-1.04738	-1.04603	-1.04466	-1.04329	-1.04192	-1.04053
-1.03046	-1.02912	-1.04926	-1.04789	-1.04653	-1.04517	-1.0438	-1.04243	-1.04106
-1.03097	-1.02962	-1.04979	-1.04841	-1.04704	-1.04567	-1.0443	-1.04294	-1.04158
-1.03147	-1.03012	-1.02878	-1.02744	-1.02611	-1.04618	-1.04481	-1.04345	-1.0421
-1.03197	-1.03063	-1.02929	-1.02794	-1.02659	-1.04669	-1.04533	-1.04397	-1.04262
-1.03246	-1.03113	-1.02979	-1.02843	-1.02709	-1.02576	-1.02445	-1.04451	-1.04316
-1.03294	-1.03161	-1.03027	-1.02894	-1.0276	-1.02629	-1.02498	-1.04507	-1.04371
-1.03342	-1.03209	-1.03076	-1.02944	-1.02812	-1.02681	-1.02551	-1.04563	-1.04427
-0.96547	-0.96427	-1.03125	-1.02993	-1.02862	-1.02731	-1.02601	-1.04617	-1.04481
-0.9659	-0.9647	-1.03175	-1.03042	-1.0291	-1.0278	-1.02651	-1.0467	-1.04535
-0.96632	-0.96513	-0.98757	-0.98633	-0.98509	-1.02831	-1.02702	-1.02572	-1.02442
-0.96673	-0.96557	-0.98805	-0.98682	-0.98558	-1.02885	-1.02755	-1.02625	-1.02495
-0.96713	-0.96599	-0.96483	-0.96365	-0.96247	-1.00904	-1.00777	-1.0065	-1.00522
-0.96754	-0.96639	-0.96524	-0.96407	-0.9629	-1.00951	-1.00825	-1.00698	-1.0057
-0.94848	-0.94736	-0.96564	-0.96449	-0.96333	-0.98576	-0.98455	-1.00746	-1.00619
-0.94889	-0.94778	-0.96608	-0.96493	-0.96378	-0.98624	-0.98503	-1.00796	-1.00669
-0.94931	-0.9482	-0.96651	-0.96537	-0.96423	-0.98671	-0.9855	-1.00846	-1.00719
-0.94972	-0.9486	-0.94749	-0.94639	-0.94528	-0.94416	-0.94304	-0.98475	-0.98354
-0.95011	-0.94899	-0.94788	-0.94678	-0.94568	-0.94457	-0.94346	-0.98521	-0.98399
-0.95052	-0.94941	-0.94829	-0.94719	-0.94609	-0.94499	-0.94388	-0.94276	-0.94162
-0.95095	-0.94984	-0.94873	-0.94762	-0.94651	-0.94541	-0.94431	-0.94319	-0.94206
-0.95138	-0.95026	-0.94915	-0.94803	-0.94692	-0.94582	-0.94473	-0.94362	-0.94249
-0.95179	-0.95067	-0.94955	-0.94843	-0.94732	-0.94622	-0.94513	-0.94402	-0.9429
-0.95221	-0.95108	-0.94995	-0.94883	-0.94772	-0.94662	-0.94553	-0.94443	-0.94331
-0.95262	-0.9515	-0.95038	-0.94927	-0.94816	-0.94706	-0.94596	-0.94486	-0.94376
-0.95303	-0.95193	-0.95082	-0.94971	-0.94861	-0.94751	-0.94641	-0.94531	-0.94421
-0.93168	-0.9306	-0.95125	-0.95015	-0.94905	-0.94795	-0.94685	-0.94575	-0.94465
-0.93209	-0.93101	-0.95168	-0.95059	-0.9495	-0.9484	-0.94729	-0.94619	-0.94509
-0.93248	-0.9314	-0.93033	-0.92927	-0.9282	-0.94882	-0.94771	-0.94661	-0.94551
-0.93284	-0.93177	-0.9307	-0.92964	-0.92858	-0.94921	-0.94811	-0.94701	-0.94591
-0.93321	-0.93215	-0.93108	-0.93003	-0.92897	-0.9279	-0.92683	-0.94743	-0.94633
-0.93363	-0.93256	-0.93149	-0.93043	-0.92938	-0.92832	-0.92725	-0.94786	-0.94676
-0.93406	-0.93298	-0.93191	-0.93085	-0.92979	-0.92874	-0.92768	-0.94831	-0.94721
-0.93449	-0.93341	-0.93233	-0.93127	-0.93022	-0.92916	-0.92811	-0.92704	-0.92596
-0.93492	-0.93384	-0.93276	-0.9317	-0.93064	-0.92959	-0.92853	-0.92747	-0.92641
-0.93535	-0.93426	-0.93319	-0.93212	-0.93106	-0.93001	-0.92895	-0.9279	-0.92684
-0.93577	-0.93468	-0.93361	-0.93253	-0.93147	-0.93042	-0.92937	-0.92831	-0.92726
-0.93618	-0.93509	-0.93401	-0.93294	-0.93187	-0.93082	-0.92977	-0.92872	-0.92767
-0.93657	-0.93547	-0.93439	-0.93332	-0.93226	-0.93121	-0.93017	-0.92913	-0.92809
-0.93697	-0.93586	-0.93478	-0.93371	-0.93265	-0.9316	-0.93056	-0.92953	-0.9285
-0.93739	-0.93628	-0.93518	-0.9341	-0.93304	-0.932	-0.93097	-0.92993	-0.9289
-0.93782	-0.9367	-0.9356	-0.93451	-0.93344	-0.9324	-0.93137	-0.93034	-0.92931
-0.93827	-0.93715	-0.93603	-0.93494	-0.93386	-0.93281	-0.93177	-0.93073	-0.9297
-0.93873	-0.9376	-0.93649	-0.93539	-0.9343	-0.93322	-0.93216	-0.93112	-0.93009
-0.93919	-0.93806	-0.93694	-0.93583	-0.93474	-0.93365	-0.93257	-0.93152	-0.93048
-0.93964	-0.93851	-0.93739	-0.93628	-0.93517	-0.93408	-0.933	-0.93193	-0.93088

-0.9401	-0.93897	-0.93784	-0.93673	-0.93562	-0.93452	-0.93343	-0.93235	-0.93128
-0.94055	-0.93942	-0.93829	-0.93717	-0.93606	-0.93496	-0.93387	-0.93278	-0.9317
-0.941	-0.93987	-0.93874	-0.93762	-0.93651	-0.93541	-0.93431	-0.93322	-0.93214
-0.94145	-0.94031	-0.93919	-0.93807	-0.93695	-0.93585	-0.93475	-0.93367	-0.93258
-0.94188	-0.94076	-0.93963	-0.93851	-0.9374	-0.9363	-0.9352	-0.93411	-0.93304
-0.94232	-0.9412	-0.94008	-0.93896	-0.93785	-0.93674	-0.93564	-0.93456	-0.9335
-0.94276	-0.94164	-0.94052	-0.9394	-0.93829	-0.93718	-0.93609	-0.93501	-0.93396
-0.9432	-0.94208	-0.94096	-0.93984	-0.93873	-0.93762	-0.93653	-0.93546	-0.93441
-0.94364	-0.94252	-0.9414	-0.94028	-0.93916	-0.93806	-0.93697	-0.9359	-0.93485
-0.94409	-0.94296	-0.94184	-0.94072	-0.93961	-0.93851	-0.93742	-0.93635	-0.93529
-0.94454	-0.94341	-0.94228	-0.94116	-0.94006	-0.93896	-0.93787	-0.9368	-0.93575
-0.945	-0.94386	-0.94273	-0.94162	-0.94051	-0.93942	-0.93833	-0.93726	-0.9362
-0.92428	-0.92318	-0.9432	-0.94208	-0.94097	-0.93988	-0.9388	-0.93773	-0.93667
-0.92473	-0.92363	-0.94367	-0.94255	-0.94143	-0.94035	-0.93928	-0.93821	-0.93713
-0.8787	-0.87769	-0.92298	-0.92188	-0.92081	-0.94082	-0.93975	-0.93867	-0.93758
-0.87912	-0.8781	-0.92342	-0.92233	-0.92125	-0.94128	-0.94022	-0.93913	-0.93804
-0.87953	-0.87852	-0.87751	-0.87651	-0.87553	-0.92066	-0.91962	-0.91857	-0.91751
-0.87995	-0.87894	-0.87794	-0.87695	-0.87597	-0.92113	-0.92007	-0.91901	-0.91795
-0.88038	-0.87937	-0.87837	-0.87739	-0.87642	-0.87543	-0.87445	-0.91945	-0.91839
-0.8808	-0.87981	-0.87882	-0.87783	-0.87685	-0.87586	-0.87486	-0.91989	-0.91883
-0.88123	-0.88024	-0.87926	-0.87827	-0.87728	-0.87628	-0.87527	-0.92034	-0.91927
-0.88165	-0.88067	-0.87969	-0.8787	-0.8777	-0.8767	-0.87569	-0.8747	-0.87373
-0.88207	-0.88109	-0.88011	-0.87912	-0.87812	-0.87712	-0.87612	-0.87514	-0.87416
-0.88251	-0.88153	-0.88054	-0.87954	-0.87855	-0.87755	-0.87656	-0.87557	-0.87459
-0.88298	-0.88198	-0.88098	-0.87998	-0.87898	-0.87799	-0.877	-0.87602	-0.87503
-0.88343	-0.88242	-0.88142	-0.88042	-0.87942	-0.87843	-0.87745	-0.87646	-0.87546
-0.88387	-0.88286	-0.88186	-0.88086	-0.87987	-0.87888	-0.87789	-0.8769	-0.8759
-0.81524	-0.81428	-0.88229	-0.88131	-0.88032	-0.87932	-0.87833	-0.87733	-0.87635
-0.8157	-0.81474	-0.88274	-0.88175	-0.88076	-0.87976	-0.87876	-0.87777	-0.87679
-0.81616	-0.81521	-0.88319	-0.88219	-0.8812	-0.8802	-0.8792	-0.87822	-0.87724
-0.81662	-0.81567	-0.81472	-0.81376	-0.81281	-0.84075	-0.83972	-0.83871	-0.83771
-0.81707	-0.81612	-0.81517	-0.81423	-0.81328	-0.84126	-0.84024	-0.83923	-0.83821
-0.81753	-0.81658	-0.81564	-0.8147	-0.81375	-0.84177	-0.84075	-0.83973	-0.83872
-0.818	-0.81706	-0.81611	-0.81517	-0.81422	-0.84227	-0.84126	-0.84024	-0.83921
-0.81847	-0.81753	-0.81659	-0.81565	-0.8147	-0.81374	-0.81278	-0.84074	-0.83972
-0.81896	-0.81802	-0.81708	-0.81612	-0.81516	-0.81421	-0.81325	-0.84125	-0.84023
-0.81944	-0.81851	-0.81756	-0.8166	-0.81563	-0.81467	-0.81373	-0.84176	-0.84074
-0.81994	-0.81899	-0.81803	-0.81706	-0.8161	-0.81514	-0.8142	-0.81325	-0.8123
-0.82044	-0.81946	-0.81849	-0.81752	-0.81657	-0.81562	-0.81467	-0.81373	-0.81278
-0.82092	-0.81993	-0.81895	-0.81799	-0.81704	-0.81609	-0.81514	-0.8142	-0.81326
-0.8214	-0.8204	-0.81942	-0.81846	-0.81751	-0.81656	-0.81561	-0.81467	-0.81374
-0.82188	-0.82089	-0.81991	-0.81895	-0.818	-0.81704	-0.81609	-0.81516	-0.81423
-0.82238	-0.82139	-0.8204	-0.81944	-0.81849	-0.81753	-0.81659	-0.81565	-0.81471
-0.82287	-0.82189	-0.82091	-0.81994	-0.81898	-0.81803	-0.81708	-0.81614	-0.8152
-0.82337	-0.82239	-0.82142	-0.82045	-0.81949	-0.81853	-0.81757	-0.81663	-0.8157
-0.82386	-0.82289	-0.82192	-0.82096	-0.82	-0.81903	-0.81807	-0.81713	-0.81619
-0.82437	-0.8234	-0.82244	-0.82147	-0.8205	-0.81953	-0.81858	-0.81762	-0.81668
-0.82489	-0.82392	-0.82295	-0.82197	-0.821	-0.82004	-0.81908	-0.81813	-0.81717
-0.82542	-0.82444	-0.82346	-0.82249	-0.82151	-0.82055	-0.81959	-0.81863	-0.81767
-0.82597	-0.82496	-0.82397	-0.823	-0.82204	-0.82107	-0.8201	-0.81913	-0.81818

-0.82651	-0.8255	-0.8245	-0.82352	-0.82256	-0.82159	-0.82062	-0.81965	-0.81869
-0.82706	-0.82605	-0.82505	-0.82406	-0.82307	-0.82211	-0.82114	-0.82018	-0.8192
-0.82762	-0.82661	-0.82561	-0.8246	-0.8236	-0.82263	-0.82168	-0.82071	-0.81972
-0.8282	-0.82719	-0.82618	-0.82516	-0.82415	-0.82318	-0.82222	-0.82125	-0.82026
-0.8288	-0.82777	-0.82675	-0.82573	-0.82471	-0.82373	-0.82276	-0.82179	-0.8208
-0.7752	-0.77425	-0.8273	-0.82628	-0.82527	-0.82427	-0.82329	-0.82231	-0.82134
-0.7757	-0.77473	-0.82784	-0.82682	-0.82581	-0.8248	-0.8238	-0.82282	-0.82187
-0.7762	-0.77522	-0.7973	-0.79632	-0.79536	-0.82534	-0.82433	-0.82335	-0.82241
-0.77669	-0.77571	-0.79781	-0.79682	-0.79584	-0.82588	-0.82489	-0.82391	-0.82295
-0.77719	-0.7762	-0.77523	-0.77427	-0.77332	-0.79539	-0.79445	-0.82447	-0.8235
-0.77771	-0.7767	-0.77572	-0.77476	-0.77382	-0.7959	-0.79496	-0.82502	-0.82404
-0.77823	-0.77721	-0.77622	-0.77526	-0.77432	-0.79643	-0.79547	-0.82557	-0.82458
-0.74762	-0.74666	-0.77674	-0.77578	-0.77484	-0.79696	-0.79599	-0.82612	-0.82514
-0.74812	-0.74716	-0.77728	-0.77632	-0.77536	-0.7975	-0.79651	-0.82668	-0.8257
-0.74862	-0.74767	-0.77783	-0.77686	-0.7759	-0.77493	-0.77396	-0.79608	-0.79514
-0.74913	-0.74818	-0.77838	-0.7774	-0.77643	-0.77546	-0.77448	-0.79662	-0.79568
-0.74964	-0.74869	-0.74775	-0.74683	-0.74592	-0.77598	-0.77501	-0.79717	-0.79621
-0.75017	-0.74921	-0.74826	-0.74732	-0.7464	-0.7765	-0.77554	-0.79773	-0.79675
-0.71979	-0.71887	-0.74876	-0.74782	-0.74689	-0.74598	-0.74508	-0.77512	-0.77417
-0.72029	-0.71936	-0.74928	-0.74833	-0.7474	-0.74648	-0.74558	-0.77564	-0.77468
-0.7208	-0.71986	-0.7498	-0.74885	-0.74792	-0.74699	-0.74608	-0.77617	-0.7752
-0.7213	-0.72036	-0.71943	-0.71853	-0.71765	-0.74751	-0.7466	-0.77672	-0.77574
-0.7218	-0.72086	-0.71994	-0.71904	-0.71815	-0.74804	-0.74712	-0.77727	-0.77629
-0.7223	-0.72136	-0.72044	-0.71954	-0.71865	-0.74857	-0.74764	-0.74671	-0.74579
-0.72279	-0.72185	-0.72094	-0.72004	-0.71915	-0.7491	-0.74816	-0.74723	-0.7463
-0.7233	-0.72236	-0.72144	-0.72055	-0.71966	-0.71876	-0.71786	-0.74773	-0.7468
-0.72384	-0.72288	-0.72195	-0.72105	-0.72017	-0.71926	-0.71834	-0.74824	-0.7473
-0.72438	-0.72341	-0.72246	-0.72156	-0.72067	-0.71976	-0.71883	-0.71793	-0.71705
-0.72492	-0.72394	-0.72299	-0.72209	-0.72119	-0.72027	-0.71934	-0.71843	-0.71754
-0.72547	-0.72449	-0.72353	-0.72262	-0.72171	-0.72078	-0.71985	-0.71894	-0.71804
-0.72603	-0.72504	-0.72408	-0.72315	-0.72224	-0.7213	-0.72037	-0.71946	-0.71855
-0.72659	-0.72561	-0.72464	-0.7237	-0.72277	-0.72183	-0.7209	-0.71998	-0.71908
-0.69739	-0.69646	-0.7252	-0.72426	-0.72332	-0.72237	-0.72143	-0.7205	-0.7196
-0.69792	-0.69699	-0.72578	-0.72482	-0.72387	-0.72291	-0.72196	-0.72103	-0.72013
-0.69846	-0.69753	-0.69661	-0.6957	-0.6948	-0.72346	-0.7225	-0.72156	-0.72066
-0.699	-0.69807	-0.69714	-0.69623	-0.69532	-0.72402	-0.72304	-0.7221	-0.72119
-0.69956	-0.69861	-0.69768	-0.69676	-0.69584	-0.72457	-0.72359	-0.72264	-0.72172
-0.70013	-0.69917	-0.69822	-0.69729	-0.69637	-0.72513	-0.72414	-0.72318	-0.72226
-0.70071	-0.69973	-0.69876	-0.69782	-0.69689	-0.72568	-0.72469	-0.72373	-0.72279
-0.68414	-0.68319	-0.69932	-0.69836	-0.69742	-0.69647	-0.69552	-0.72428	-0.72333
-0.6847	-0.68375	-0.69989	-0.69892	-0.69795	-0.69699	-0.69604	-0.72483	-0.72388
-0.68526	-0.68431	-0.68336	-0.68241	-0.68146	-0.69752	-0.69657	-0.69565	-0.69475
-0.68582	-0.68488	-0.68393	-0.68296	-0.682	-0.69807	-0.6971	-0.69617	-0.69526
-0.68638	-0.68544	-0.6845	-0.68352	-0.68255	-0.68159	-0.68065	-0.69671	-0.69579
-0.68694	-0.686	-0.68506	-0.68409	-0.68312	-0.68216	-0.68121	-0.69727	-0.69633
-0.6875	-0.68656	-0.68562	-0.68466	-0.6837	-0.68274	-0.68178	-0.69784	-0.69688
-0.68804	-0.68711	-0.68617	-0.68522	-0.68427	-0.68331	-0.68235	-0.6814	-0.68046
-0.68857	-0.68765	-0.68673	-0.68579	-0.68484	-0.68388	-0.68293	-0.68198	-0.68103
-0.6891	-0.68819	-0.68728	-0.68634	-0.6854	-0.68446	-0.68351	-0.68256	-0.6816
-0.68964	-0.68873	-0.68782	-0.6869	-0.68597	-0.68504	-0.6841	-0.68314	-0.68218

-0.69019	-0.68928	-0.68836	-0.68745	-0.68654	-0.68561	-0.68468	-0.68372	-0.68275
-0.69076	-0.68983	-0.68891	-0.68801	-0.68711	-0.68618	-0.68524	-0.68429	-0.68333
-0.69134	-0.6904	-0.68947	-0.68857	-0.68767	-0.68675	-0.68581	-0.68487	-0.68392
-0.69195	-0.69099	-0.69004	-0.68913	-0.68823	-0.68731	-0.68638	-0.68545	-0.68451
-0.69258	-0.69159	-0.69063	-0.6897	-0.68879	-0.68788	-0.68696	-0.68603	-0.6851
-0.69319	-0.69221	-0.69125	-0.69031	-0.68938	-0.68847	-0.68756	-0.68663	-0.68569
-0.69381	-0.69284	-0.69188	-0.69093	-0.68999	-0.68908	-0.68817	-0.68723	-0.68628
-0.66467	-0.66377	-0.6925	-0.69154	-0.69059	-0.68967	-0.68875	-0.68782	-0.68688
-0.66524	-0.66433	-0.6931	-0.69213	-0.69117	-0.69024	-0.68932	-0.6884	-0.68748
-0.6658	-0.66489	-0.66398	-0.66306	-0.66215	-0.69082	-0.68988	-0.68897	-0.68806
-0.66637	-0.66545	-0.66453	-0.66361	-0.6627	-0.6914	-0.69046	-0.68953	-0.68862
-0.66694	-0.66601	-0.66509	-0.66417	-0.66326	-0.69199	-0.69104	-0.6901	-0.68918
-0.66753	-0.66659	-0.66566	-0.66474	-0.66384	-0.66293	-0.66202	-0.69069	-0.68976
-0.66813	-0.66717	-0.66624	-0.66532	-0.66442	-0.66351	-0.6626	-0.6913	-0.69035
-0.59787	-0.59712	-0.66685	-0.66594	-0.66503	-0.66411	-0.6632	-0.66229	-0.66138
-0.59837	-0.59762	-0.66749	-0.66657	-0.66565	-0.66473	-0.66382	-0.66291	-0.662
-0.53054	-0.52989	-0.59737	-0.59663	-0.59589	-0.66532	-0.6644	-0.66349	-0.66259
-0.53094	-0.53027	-0.59781	-0.59707	-0.59634	-0.66587	-0.66496	-0.66405	-0.66315
-0.53134	-0.53066	-0.52999	-0.52933	-0.52868	-0.63194	-0.63106	-0.66461	-0.66372
-0.53178	-0.53106	-0.53037	-0.5297	-0.52905	-0.63247	-0.63159	-0.66519	-0.66432
-0.53223	-0.53149	-0.53077	-0.53009	-0.52944	-0.63301	-0.63214	-0.66578	-0.66492
-0.53269	-0.53194	-0.53121	-0.53052	-0.52985	-0.52919	-0.52855	-0.66638	-0.6655
-0.53316	-0.53241	-0.53167	-0.53096	-0.53027	-0.52961	-0.52896	-0.66699	-0.66607
-0.53361	-0.53286	-0.53213	-0.5314	-0.5307	-0.53002	-0.52936	-0.56111	-0.56043
-0.53404	-0.53331	-0.53258	-0.53185	-0.53112	-0.53043	-0.52975	-0.56153	-0.56084
-0.49462	-0.49405	-0.53303	-0.53229	-0.53156	-0.53085	-0.53016	-0.52948	-0.52883
-0.495	-0.49443	-0.53348	-0.53275	-0.53202	-0.53129	-0.53058	-0.5299	-0.52924
-0.42576	-0.42531	-0.49423	-0.49366	-0.49309	-0.53173	-0.53101	-0.53032	-0.52966
-0.42604	-0.42558	-0.49458	-0.49401	-0.49344	-0.53217	-0.53145	-0.53075	-0.53006
-0.42632	-0.42585	-0.49493	-0.49435	-0.49378	-0.5326	-0.53189	-0.53118	-0.53047
-0.39151	-0.39112	-0.42566	-0.42521	-0.42476	-0.53305	-0.53233	-0.53162	-0.5309
-0.39173	-0.39135	-0.42595	-0.4255	-0.42505	-0.5335	-0.53278	-0.53206	-0.53135
-0.39197	-0.39159	-0.42624	-0.42578	-0.42532	-0.42488	-0.42444	-0.5325	-0.53178
-0.39222	-0.39184	-0.42653	-0.42605	-0.42558	-0.42512	-0.42468	-0.53292	-0.53221
-0.37354	-0.37321	-0.3917	-0.39131	-0.39092	-0.42539	-0.42494	-0.4245	-0.42408
-0.37374	-0.37342	-0.39195	-0.39156	-0.39118	-0.4257	-0.42524	-0.42479	-0.42436
-0.37394	-0.37363	-0.3922	-0.39182	-0.39143	-0.42601	-0.42554	-0.42508	-0.42465
-0.37414	-0.37383	-0.37352	-0.3732	-0.37288	-0.39129	-0.39091	-0.42536	-0.42493
-0.37434	-0.37402	-0.37371	-0.3734	-0.37309	-0.39153	-0.39114	-0.42564	-0.4252
-0.37456	-0.37424	-0.37393	-0.37362	-0.37331	-0.37298	-0.37264	-0.37232	-0.372
-0.37478	-0.37446	-0.37415	-0.37384	-0.37353	-0.3732	-0.37287	-0.37254	-0.3722
-0.37502	-0.37469	-0.37436	-0.37405	-0.37374	-0.37342	-0.37309	-0.37276	-0.37243
-0.37527	-0.37491	-0.37456	-0.37424	-0.37393	-0.37362	-0.37331	-0.37299	-0.37266
-0.37553	-0.37515	-0.37478	-0.37445	-0.37413	-0.37383	-0.37353	-0.37322	-0.3729
-0.3758	-0.37541	-0.37504	-0.37469	-0.37436	-0.37404	-0.37373	-0.37342	-0.37311
-0.37606	-0.37569	-0.37532	-0.37495	-0.37459	-0.37426	-0.37394	-0.37363	-0.37333
-0.37631	-0.37595	-0.37559	-0.37521	-0.37485	-0.3745	-0.37416	-0.37384	-0.37353
-0.37656	-0.37621	-0.37586	-0.37549	-0.37511	-0.37475	-0.37439	-0.37406	-0.37374
-0.37684	-0.37648	-0.37611	-0.37574	-0.37537	-0.375	-0.37464	-0.37429	-0.37396
-0.37715	-0.37674	-0.37634	-0.37597	-0.37561	-0.37525	-0.37489	-0.37454	-0.37421

-0.37746	-0.37702	-0.3766	-0.37622	-0.37586	-0.3755	-0.37514	-0.37479	-0.37444
-0.37777	-0.37732	-0.37689	-0.3765	-0.37613	-0.37575	-0.37539	-0.37503	-0.37468
-0.37808	-0.37763	-0.37719	-0.37679	-0.37639	-0.37601	-0.37565	-0.37528	-0.37492
-0.37839	-0.37795	-0.37752	-0.37708	-0.37666	-0.37627	-0.3759	-0.37554	-0.37518
-0.37869	-0.37827	-0.37784	-0.37739	-0.37694	-0.37655	-0.37617	-0.3758	-0.37544
-0.37899	-0.37857	-0.37814	-0.37769	-0.37726	-0.37684	-0.37645	-0.37607	-0.37571
-0.37929	-0.37886	-0.37843	-0.378	-0.37758	-0.37716	-0.37674	-0.37635	-0.37598
-0.37959	-0.37916	-0.37873	-0.37831	-0.37789	-0.37746	-0.37703	-0.37663	-0.37626
-0.3799	-0.37947	-0.37904	-0.37861	-0.37819	-0.37776	-0.37734	-0.37693	-0.37653
-0.38021	-0.37978	-0.37935	-0.37892	-0.37849	-0.37807	-0.37765	-0.37723	-0.37682
-0.38051	-0.38008	-0.37966	-0.37923	-0.3788	-0.37839	-0.37797	-0.37755	-0.37713
-0.38081	-0.38039	-0.37996	-0.37954	-0.37912	-0.3787	-0.37829	-0.37787	-0.37745
-0.38113	-0.3807	-0.38028	-0.37986	-0.37943	-0.37901	-0.3786	-0.37818	-0.37776
-0.38145	-0.38103	-0.3806	-0.38017	-0.37975	-0.37932	-0.37889	-0.37848	-0.37806
-0.38177	-0.38134	-0.38091	-0.38048	-0.38005	-0.37963	-0.3792	-0.37878	-0.37837
-0.3821	-0.38166	-0.38122	-0.38079	-0.38036	-0.37993	-0.37951	-0.3791	-0.37869
-0.34124	-0.34086	-0.34048	-0.34011	-0.33974	-0.38025	-0.37983	-0.37941	-0.379
-0.34152	-0.34113	-0.34075	-0.34038	-0.34001	-0.38058	-0.38015	-0.37973	-0.37931
-0.3418	-0.34141	-0.34103	-0.34066	-0.34029	-0.33992	-0.33955	-0.38005	-0.37962
-0.34208	-0.3417	-0.34132	-0.34094	-0.34057	-0.34019	-0.33981	-0.38035	-0.37992
-0.34236	-0.34198	-0.3416	-0.34123	-0.34085	-0.34047	-0.34008	-0.38067	-0.38023
-0.34263	-0.34225	-0.34187	-0.34149	-0.34112	-0.34074	-0.34036	-0.33998	-0.3396
-0.34291	-0.34251	-0.34213	-0.34175	-0.34138	-0.34101	-0.34063	-0.34026	-0.33988
-0.34321	-0.3428	-0.34241	-0.34202	-0.34164	-0.34127	-0.3409	-0.34053	-0.34015
-0.34353	-0.34311	-0.34269	-0.3423	-0.34192	-0.34154	-0.34117	-0.3408	-0.34043
-0.34385	-0.34341	-0.34298	-0.34258	-0.34219	-0.34181	-0.34144	-0.34107	-0.34071
-0.34417	-0.34371	-0.34327	-0.34286	-0.34247	-0.34209	-0.34171	-0.34135	-0.34099
-0.3445	-0.34403	-0.34358	-0.34315	-0.34275	-0.34236	-0.34199	-0.34162	-0.34126
-0.34485	-0.34437	-0.34391	-0.34346	-0.34304	-0.34264	-0.34226	-0.34189	-0.34153
-0.34521	-0.34472	-0.34425	-0.34378	-0.34334	-0.34292	-0.34253	-0.34215	-0.3418
-0.34555	-0.34507	-0.34459	-0.34412	-0.34367	-0.34323	-0.34281	-0.34242	-0.34206
-0.34589	-0.34541	-0.34494	-0.34447	-0.34401	-0.34355	-0.3431	-0.34269	-0.34233
-0.34623	-0.34576	-0.3453	-0.34483	-0.34436	-0.34388	-0.34341	-0.34299	-0.3426
-0.34655	-0.34611	-0.34567	-0.34519	-0.3447	-0.34422	-0.34374	-0.3433	-0.34288
-0.34691	-0.34646	-0.34602	-0.34554	-0.34506	-0.34457	-0.34409	-0.34363	-0.34318
-0.34731	-0.34679	-0.34631	-0.34587	-0.34543	-0.34494	-0.34444	-0.34397	-0.3435
-0.34778	-0.34717	-0.34664	-0.3462	-0.34578	-0.34531	-0.34481	-0.34432	-0.34385
-0.34833	-0.34763	-0.34702	-0.34653	-0.34609	-0.34563	-0.34517	-0.3447	-0.34422
-0.34895	-0.34815	-0.34746	-0.34689	-0.34641	-0.34596	-0.34554	-0.34509	-0.34459
-0.34961	-0.34875	-0.34799	-0.34735	-0.34679	-0.34631	-0.34589	-0.34543	-0.34495
-0.35032	-0.34942	-0.3486	-0.34787	-0.34722	-0.34669	-0.34623	-0.34576	-0.3453
-0.35104	-0.35011	-0.34924	-0.34844	-0.34772	-0.34711	-0.34659	-0.3461	-0.34565
-0.35177	-0.35083	-0.34992	-0.34907	-0.34828	-0.34758	-0.34696	-0.34644	-0.346
-0.35254	-0.35157	-0.35063	-0.34973	-0.34888	-0.3481	-0.3474	-0.34682	-0.34635
-0.35333	-0.35234	-0.35138	-0.35044	-0.34954	-0.34869	-0.34791	-0.34725	-0.3467
-0.35413	-0.35313	-0.35214	-0.35117	-0.35024	-0.34934	-0.34849	-0.34775	-0.34709
-0.3549	-0.35389	-0.3529	-0.35193	-0.35099	-0.35006	-0.34916	-0.34834	-0.34759
-0.35567	-0.35466	-0.35366	-0.35271	-0.35178	-0.35083	-0.3499	-0.34901	-0.34816
-0.35642	-0.35542	-0.35444	-0.35348	-0.35254	-0.35159	-0.35064	-0.34972	-0.34882
-0.35715	-0.35619	-0.35523	-0.35425	-0.35329	-0.35234	-0.35142	-0.35048	-0.34954

-0.31077	-0.30998	-0.35601	-0.35503	-0.35405	-0.35311	-0.3522	-0.35125	-0.3503
-0.3114	-0.31063	-0.35677	-0.3558	-0.35484	-0.3539	-0.35298	-0.35204	-0.35109
-0.31204	-0.31127	-0.35753	-0.35657	-0.35561	-0.35468	-0.35376	-0.35282	-0.35188
-0.31267	-0.31191	-0.35828	-0.35732	-0.35636	-0.35543	-0.35451	-0.35358	-0.35266
-0.31331	-0.31254	-0.35904	-0.35807	-0.35711	-0.35617	-0.35525	-0.35434	-0.35343
-0.27477	-0.2741	-0.31241	-0.31162	-0.31084	-0.35694	-0.356	-0.35508	-0.35418
-0.27534	-0.27466	-0.31305	-0.31228	-0.3115	-0.35772	-0.35676	-0.35583	-0.35492
-0.27591	-0.27523	-0.27455	-0.27387	-0.27318	-0.31137	-0.31059	-0.3566	-0.35568
-0.2765	-0.27581	-0.27512	-0.27443	-0.27375	-0.31202	-0.31125	-0.35738	-0.35643
-0.27709	-0.27638	-0.27569	-0.275	-0.27431	-0.27363	-0.27295	-0.35817	-0.3572
-0.27767	-0.27696	-0.27625	-0.27556	-0.27487	-0.27419	-0.27351	-0.35896	-0.35799
-0.27826	-0.27754	-0.27683	-0.27613	-0.27544	-0.27475	-0.27407	-0.2734	-0.27272
-0.27885	-0.27814	-0.27744	-0.27673	-0.27602	-0.27533	-0.27464	-0.27396	-0.27327
-0.27945	-0.27875	-0.27805	-0.27734	-0.27662	-0.27592	-0.27523	-0.27453	-0.27382
-0.28005	-0.27935	-0.27865	-0.27794	-0.27723	-0.27654	-0.27584	-0.27513	-0.27442
-0.28066	-0.27994	-0.27924	-0.27855	-0.27786	-0.27717	-0.27648	-0.27577	-0.27504
-0.28128	-0.28055	-0.27984	-0.27914	-0.27846	-0.27778	-0.27709	-0.27639	-0.27566
-0.28194	-0.28117	-0.28043	-0.27972	-0.27904	-0.27836	-0.27769	-0.277	-0.2763
-0.28259	-0.2818	-0.28104	-0.28032	-0.27962	-0.27894	-0.27828	-0.2776	-0.27691
-0.28322	-0.28245	-0.28168	-0.28094	-0.28022	-0.27953	-0.27886	-0.27818	-0.27751
-0.28386	-0.28309	-0.28233	-0.28157	-0.28083	-0.28012	-0.27944	-0.27876	-0.27809
-0.28449	-0.28373	-0.28297	-0.2822	-0.28146	-0.28075	-0.28006	-0.27937	-0.27869
-0.28512	-0.28436	-0.28361	-0.28284	-0.28209	-0.28138	-0.28069	-0.27999	-0.27929
-0.28579	-0.28502	-0.28425	-0.28348	-0.28273	-0.282	-0.2813	-0.28059	-0.2799
-0.28649	-0.2857	-0.28491	-0.28413	-0.28337	-0.28262	-0.28189	-0.28118	-0.2805
-0.28718	-0.28638	-0.28559	-0.28481	-0.28403	-0.28326	-0.2825	-0.28178	-0.2811
-0.28786	-0.28708	-0.2863	-0.28552	-0.28473	-0.28393	-0.28314	-0.28239	-0.28168
-0.28853	-0.28777	-0.287	-0.28622	-0.28543	-0.28462	-0.2838	-0.28303	-0.28229
-0.2892	-0.28844	-0.28767	-0.2869	-0.28611	-0.28531	-0.28451	-0.28372	-0.28294
-0.28987	-0.2891	-0.28834	-0.28757	-0.28679	-0.28601	-0.28522	-0.28443	-0.28362
-0.29056	-0.28979	-0.28903	-0.28826	-0.28748	-0.2867	-0.28591	-0.28511	-0.2843
-0.29126	-0.29049	-0.28973	-0.28895	-0.28816	-0.28738	-0.28659	-0.28579	-0.285
-0.19305	-0.19255	-0.2904	-0.28963	-0.28885	-0.28807	-0.28729	-0.2865	-0.28571
-0.19349	-0.19298	-0.29106	-0.2903	-0.28954	-0.28878	-0.28802	-0.28724	-0.28645
-0.19393	-0.19341	-0.1929	-0.19239	-0.19189	-0.28946	-0.28872	-0.28795	-0.28716
-0.1944	-0.19386	-0.19333	-0.19281	-0.19231	-0.29011	-0.28937	-0.28862	-0.28787
-0.14087	-0.14047	-0.19377	-0.19324	-0.19272	-0.19223	-0.19175	-0.28928	-0.28856
-0.14119	-0.1408	-0.19423	-0.19368	-0.19314	-0.19265	-0.19217	-0.28996	-0.28924
-0.14153	-0.14113	-0.1947	-0.19413	-0.19358	-0.19307	-0.1926	-0.29064	-0.28991
-0.14188	-0.14148	-0.14107	-0.14065	-0.14024	-0.19351	-0.19301	-0.19253	-0.19206
-0.14225	-0.14183	-0.14142	-0.14099	-0.14057	-0.19395	-0.19343	-0.19294	-0.19247
-0.14262	-0.1422	-0.14177	-0.14134	-0.14091	-0.14052	-0.14014	-0.19337	-0.19289
-0.14299	-0.14256	-0.14214	-0.1417	-0.14127	-0.14087	-0.14048	-0.19382	-0.19332
-0.14335	-0.14293	-0.14251	-0.14207	-0.14164	-0.14123	-0.14083	-0.14044	-0.14007
-0.14372	-0.1433	-0.14288	-0.14245	-0.14201	-0.14159	-0.14118	-0.14079	-0.1404
-0.14408	-0.14367	-0.14325	-0.14282	-0.14239	-0.14196	-0.14154	-0.14114	-0.14074
-0.04363	-0.04346	-0.14361	-0.14318	-0.14276	-0.14233	-0.1419	-0.14148	-0.14108
-0.04375	-0.04358	-0.14395	-0.14354	-0.14313	-0.14269	-0.14225	-0.14183	-0.14142
		-0.04354	-0.04337	-0.0432	-0.14306	-0.14263	-0.1422	-0.14177
		-0.04365	-0.04348	-0.04331	-0.14343	-0.14301	-0.14258	-0.14213

-0.04376	-0.0436	-0.04343	-0.04326	-0.04309	-0.04292	-0.04274
-0.04387	-0.04371	-0.04356	-0.04339	-0.04322	-0.04305	-0.04287
			-0.04351	-0.04335	-0.04318	-0.043
			-0.04364	-0.04348	-0.04331	-0.04313
			-0.04375	-0.0436	-0.04344	-0.04326
					-0.04355	-0.04339
					-0.04365	-0.04351

							-0.30615	-0.30524
							-0.30531	-0.30446
					-0.30632	-0.3054	-0.30451	-0.30367
					-0.30548	-0.30462	-0.30375	-0.30289
		-0.05408	-0.05397	-0.05386	-0.40257	-0.40145	-0.52263	-0.5211
		-0.05396	-0.05385	-0.05375	-0.40153	-0.40037	-0.52126	-0.51974
		-0.05384	-0.05374	-0.05364	-0.4005	-0.3993	-0.51989	-0.51839
-0.05395	-0.05384	-0.40296	-0.40177	-0.4006	-0.48163	-0.48024	-0.51855	-0.51707
-0.05384	-0.05373	-0.40193	-0.40071	-0.39951	-0.48039	-0.47901	-0.51723	-0.51577
-0.40328	-0.40206	-0.48336	-0.48195	-0.48055	-0.51887	-0.5174	-0.56415	-0.5626
-0.40219	-0.40096	-0.48212	-0.48072	-0.47934	-0.51756	-0.5161	-0.56278	-0.56126
-0.4837	-0.48229	-0.52074	-0.51924	-0.51776	-0.56451	-0.56296	-0.60445	-0.6029
-0.48248	-0.48109	-0.51944	-0.51797	-0.51651	-0.56319	-0.56165	-0.6031	-0.60158
-0.52114	-0.51965	-0.56651	-0.56495	-0.56342	-0.60491	-0.60332	-0.60176	-0.60025
-0.51984	-0.51839	-0.56519	-0.56365	-0.56212	-0.60357	-0.60198	-0.60043	-0.59891
-0.51858	-0.51715	-0.56388	-0.56235	-0.56083	-0.60223	-0.60065	-0.5991	-0.59757
-0.56558	-0.56406	-0.6056	-0.60402	-0.60243	-0.60086	-0.5993	-0.68161	-0.67993

-0.56428	-0.56274	-0.60421	-0.60262	-0.60105	-0.59949	-0.59795	-0.68012	-0.67844
-0.60605	-0.60444	-0.60284	-0.60126	-0.5997	-0.68202	-0.68033	-0.71118	-0.70932
-0.60469	-0.60309	-0.6015	-0.59994	-0.59839	-0.68056	-0.67888	-0.7096	-0.70775
-0.60334	-0.60175	-0.64176	-0.64014	-0.63853	-0.71175	-0.7099	-0.89336	-0.89053
-0.602	-0.60042	-0.64038	-0.63878	-0.63719	-0.71023	-0.70839	-0.8911	-0.88837
-0.6763	-0.67447	-0.71425	-0.71239	-0.71055	-0.86276	-0.86005	-0.92418	-0.92146
-0.67472	-0.67292	-0.71265	-0.7108	-0.70897	-0.86049	-0.85785	-0.92187	-0.91923
-0.67315	-0.67138	-0.71105	-0.70922	-0.7074	-0.85824	-0.85569	-0.91959	-0.91701
-0.71315	-0.7113	-0.90008	-0.8973	-0.89457	-0.92268	-0.92002	-0.91741	-0.91487
-0.71159	-0.70975	-0.89772	-0.895	-0.89235	-0.92043	-0.91786	-0.9153	-0.91277
-0.90105	-0.89822	-0.92634	-0.92358	-0.9209	-0.95619	-0.95349	-0.95079	-0.9481
-0.89877	-0.89601	-0.9241	-0.92143	-0.91884	-0.95406	-0.95137	-0.94868	-0.94599
-0.89652	-0.89383	-0.9601	-0.95732	-0.95462	-0.95192	-0.94922	-0.94654	-0.94386
-0.89425	-0.89169	-0.95781	-0.95508	-0.9524	-0.94971	-0.94702	-0.94434	-0.94168
-0.89206	-0.8896	-0.95558	-0.95288	-0.9502	-0.94751	-0.94483	-0.94216	-0.93951
-0.95877	-0.95608	-0.95342	-0.95073	-0.94804	-0.97421	-0.97151	-0.9688	-0.9661
-0.95662	-0.95395	-0.95129	-0.9486	-0.94591	-0.97205	-0.96938	-0.96669	-0.96397
-0.9544	-0.95174	-0.97799	-0.97527	-0.97255	-0.96985	-0.96718	-1.06763	-1.06477
-0.95216	-0.94949	-0.97572	-0.97302	-0.97033	-0.96763	-0.96492	-1.06525	-1.06241
-0.9789	-0.97619	-0.9735	-0.97082	-0.96814	-1.06863	-1.06578	-1.09536	-1.0925
-0.97673	-0.97404	-0.97136	-0.96867	-0.96598	-1.06637	-1.06356	-1.09313	-1.09033
-0.97456	-0.97188	-1.07259	-1.06974	-1.0669	-1.09654	-1.09373	-1.11852	-1.11573
-0.97233	-0.96964	-1.07022	-1.06738	-1.06457	-1.09422	-1.09147	-1.11631	-1.11358
-0.97012	-0.96742	-1.06786	-1.06505	-1.06229	-1.09194	-1.08926	-1.11413	-1.11145
-0.96797	-0.96526	-1.09808	-1.09526	-1.09249	-1.11738	-1.1147	-1.17736	-1.17449
-0.96585	-0.96315	-1.09583	-1.09306	-1.09035	-1.11526	-1.11261	-1.17514	-1.17222
-1.09927	-1.09638	-1.12124	-1.11848	-1.11577	-1.17852	-1.17572	-1.17286	-1.16995
-1.09692	-1.09408	-1.11897	-1.11624	-1.11356	-1.17618	-1.17338	-1.17055	-1.16768
-1.09464	-1.09185	-1.1824	-1.17953	-1.17671	-1.1739	-1.1711	-1.25459	-1.25156
-1.09247	-1.08971	-1.18013	-1.17728	-1.17447	-1.17167	-1.16889	-1.25221	-1.24914
-1.14998	-1.14713	-1.23431	-1.23141	-1.22854	-1.25578	-1.25283	-1.24982	-1.24675
-1.14776	-1.14492	-1.23197	-1.22905	-1.22616	-1.25333	-1.25039	-1.24741	-1.24436
-1.14557	-1.14273	-1.22964	-1.22669	-1.22378	-1.25087	-1.24794	-1.24499	-1.242
-1.23327	-1.2303	-1.22735	-1.22442	-1.22149	-1.24851	-1.24555	-1.24261	-1.23969
-1.23095	-1.22802	-1.2251	-1.22218	-1.21925	-1.24619	-1.24318	-1.24026	-1.23742
-1.22867	-1.22574	-1.25282	-1.24983	-1.24685	-1.24384	-1.24083	-1.2651	-1.26228
-1.22642	-1.22347	-1.25046	-1.24746	-1.24448	-1.24148	-1.23849	-1.26278	-1.26001
-1.25417	-1.25112	-1.24808	-1.24507	-1.24209	-1.26637	-1.2634	-1.26054	-1.25781
-1.25181	-1.24871	-1.24563	-1.24262	-1.23968	-1.26402	-1.2612	-1.25842	-1.2557
-1.24944	-1.2463	-1.24321	-1.24023	-1.23733	-1.26175	-1.25904	-1.25635	-1.25364
-1.27444	-1.27129	-1.2682	-1.26524	-1.26238	-1.2596	-1.25692	-1.25427	-1.25161
-1.272	-1.26892	-1.26592	-1.26306	-1.26028	-1.25754	-1.25485	-1.25224	-1.24968
-1.26963	-1.26663	-1.26372	-1.26091	-1.25819	-1.25548	-1.25281	-1.32124	-1.31861
-1.2673	-1.2644	-1.26158	-1.25881	-1.25611	-1.25343	-1.25081	-1.31925	-1.31694
-1.26505	-1.26222	-1.25946	-1.25672	-1.25406	-1.32268	-1.31992	-1.3175	-1.31543
-1.26288	-1.26009	-1.25736	-1.25463	-1.252	-1.32067	-1.31819	-1.31599	-1.31406
-1.26075	-1.25798	-1.32706	-1.32411	-1.3213	-1.31882	-1.31661	-1.31459	-1.31278
-1.25864	-1.25585	-1.32481	-1.32203	-1.31944	-1.31718	-1.31517	-1.31329	-1.31154
-1.25654	-1.25378	-1.32265	-1.32008	-1.31776	-1.31569	-1.31384	-1.31206	-1.31036
-1.32634	-1.32341	-1.3207	-1.31836	-1.31625	-1.31433	-1.31258	-1.31087	-1.30922

-1.32408	-1.32138	-1.31893	-1.3168	-1.3149	-1.31308	-1.31138	-1.30973	-1.30813
-1.32204	-1.31958	-1.31736	-1.31539	-1.3136	-1.31186	-1.31021	-1.30861	-1.30705
-1.32018	-1.31799	-1.31601	-1.31413	-1.31237	-1.31069	-1.30909	-1.30752	-1.30599
-1.31854	-1.31655	-1.31472	-1.31292	-1.3112	-1.30956	-1.30799	-1.323	-1.32144
-1.31715	-1.31527	-1.31351	-1.31177	-1.31009	-1.30848	-1.30694	-1.32191	-1.32037
-1.31586	-1.31406	-1.32904	-1.3273	-1.32562	-1.32399	-1.32241	-1.35222	-1.3505
-1.3146	-1.31285	-1.32782	-1.32615	-1.32453	-1.32292	-1.32134	-1.35101	-1.34923
-1.31341	-1.31168	-1.32664	-1.32502	-1.32344	-1.32186	-1.32028	-1.3498	-1.34797
-1.32895	-1.32721	-1.32552	-1.32391	-1.32235	-1.35213	-1.35038	-1.37306	-1.37118
-1.32783	-1.32611	-1.32445	-1.32284	-1.32126	-1.3509	-1.34915	-1.37182	-1.36999
-1.3267	-1.32501	-1.35499	-1.35321	-1.35145	-1.37418	-1.37237	-1.37057	-1.36878
-1.32558	-1.32392	-1.35377	-1.352	-1.35024	-1.37293	-1.3711	-1.36931	-1.36757
-1.35619	-1.35436	-1.37713	-1.37532	-1.37353	-1.3717	-1.36986	-1.36809	-1.36638
-1.35491	-1.35311	-1.37587	-1.37408	-1.3723	-1.37049	-1.36867	-1.36691	-1.36522
-1.35364	-1.35187	-1.37462	-1.37285	-1.37109	-1.3693	-1.3675	-1.36577	-1.3641
-1.37698	-1.37516	-1.37337	-1.37163	-1.36991	-1.36815	-1.36636	-1.3893	-1.38763
-1.37574	-1.37392	-1.37214	-1.37043	-1.36875	-1.36701	-1.36525	-1.3882	-1.3866
-1.37451	-1.37271	-1.3958	-1.39403	-1.39228	-1.39052	-1.38876	-1.41183	-1.41011
-1.37331	-1.37155	-1.39462	-1.39281	-1.39102	-1.38929	-1.38762	-1.41062	-1.40898
-1.39701	-1.39522	-1.39344	-1.39162	-1.38983	-1.41295	-1.41117	-1.42668	-1.42506
-1.39582	-1.39402	-1.39225	-1.39047	-1.38872	-1.41175	-1.41007	-1.42559	-1.42394
-1.39464	-1.39285	-1.43341	-1.43144	-1.42953	-1.42779	-1.42616	-1.42451	-1.42283
-1.39348	-1.3917	-1.43212	-1.43022	-1.42839	-1.42668	-1.42506	-1.42341	-1.42171
-1.39232	-1.39058	-1.43087	-1.42904	-1.42728	-1.42559	-1.42396	-1.42231	-1.42062
-1.40863	-1.40693	-1.42966	-1.42789	-1.42617	-1.4245	-1.42286	-1.446	-1.44424
-1.40748	-1.40584	-1.42849	-1.42676	-1.42507	-1.42341	-1.42177	-1.44483	-1.44311
-1.43085	-1.42908	-1.45249	-1.45066	-1.44886	-1.4471	-1.44537	-1.48828	-1.48649
-1.42967	-1.42794	-1.45128	-1.44944	-1.44763	-1.44588	-1.4442	-1.48709	-1.48532
-1.45364	-1.45184	-1.45006	-1.44823	-1.44642	-1.51391	-1.51211	-1.51032	-1.50852
-1.45238	-1.45059	-1.44883	-1.44702	-1.44524	-1.51268	-1.51091	-1.50914	-1.50735
-1.47653	-1.47469	-1.51708	-1.51516	-1.51327	-1.51146	-1.50971	-1.50796	-1.50619
-1.47531	-1.47346	-1.51578	-1.51389	-1.51203	-1.51025	-1.50851	-1.50678	-1.50504
-1.47409	-1.47223	-1.51451	-1.51264	-1.51081	-1.50904	-1.50732	-1.50561	-1.50389
-1.51708	-1.51517	-1.51328	-1.51142	-1.5096	-1.50784	-1.50614	-1.52452	-1.52267
-1.51576	-1.51391	-1.51207	-1.51023	-1.50841	-1.50666	-1.50496	-1.52326	-1.52146
-1.51448	-1.51265	-1.51085	-1.50903	-1.50724	-1.52573	-1.52387	-1.52204	-1.52025
-1.51322	-1.51141	-1.50961	-1.50785	-1.5061	-1.52452	-1.52267	-1.52085	-1.51906
-1.51198	-1.51017	-1.52897	-1.52705	-1.52517	-1.52331	-1.52148	-1.51966	-1.51786
-1.51076	-1.50895	-1.52764	-1.52577	-1.52394	-1.5221	-1.52028	-1.51846	-1.51665
-1.50955	-1.50774	-1.52634	-1.52449	-1.5227	-1.52088	-1.51907	-1.51726	-1.51545
-1.52899	-1.52699	-1.52504	-1.52322	-1.52145	-1.51965	-1.51784	-1.54218	-1.54028
-1.52769	-1.52571	-1.52378	-1.52197	-1.5202	-1.51841	-1.51661	-1.54089	-1.53907
-1.52639	-1.52447	-1.52258	-1.52076	-1.51898	-1.5434	-1.54146	-1.5396	-1.53782
-1.5251	-1.52325	-1.52143	-1.51959	-1.51777	-1.54208	-1.54015	-1.5383	-1.53655
-1.52382	-1.52202	-1.52023	-1.51839	-1.51655	-1.54079	-1.53888	-1.55009	-1.54824
-1.52257	-1.52076	-1.51896	-1.51714	-1.51534	-1.53952	-1.53765	-1.54877	-1.54692
-1.52134	-1.51951	-1.544	-1.54206	-1.54015	-1.53828	-1.53645	-1.59058	-1.58868
-1.52016	-1.5183	-1.54267	-1.54076	-1.53889	-1.53707	-1.5353	-1.58933	-1.5874
-1.51898	-1.51711	-1.54138	-1.5395	-1.53767	-1.53589	-1.53415	-1.58808	-1.58613
-1.54405	-1.54208	-1.54015	-1.53831	-1.53651	-1.58254	-1.58061	-1.62062	-1.61862

-1.54272	-1.54082	-1.53896	-1.53716	-1.53538	-1.58127	-1.57929	-1.61926	-1.61729
-1.54145	-1.53959	-1.58584	-1.58388	-1.58195	-1.62197	-1.61994	-1.61795	-1.61598
-1.54021	-1.53841	-1.58455	-1.58259	-1.58064	-1.62064	-1.61864	-1.61665	-1.61469
-1.58717	-1.5852	-1.58326	-1.58129	-1.57933	-1.61931	-1.61732	-1.67302	-1.67076
-1.5859	-1.58392	-1.58195	-1.57997	-1.57801	-1.61796	-1.61598	-1.6715	-1.66932
-1.58462	-1.58263	-1.58064	-1.57866	-1.5767	-1.67451	-1.67222	-1.72425	-1.72202
-1.58329	-1.58131	-1.57934	-1.57737	-1.57541	-1.67299	-1.67076	-1.72273	-1.7205
-1.58196	-1.57999	-1.57803	-1.57608	-1.57414	-1.67151	-1.66931	-1.72124	-1.71899
-1.58064	-1.57867	-1.64632	-1.6443	-1.6423	-1.75458	-1.75225	-1.74996	-1.74769
-1.57933	-1.57735	-1.64493	-1.64295	-1.641	-1.75305	-1.7507	-1.74841	-1.74618
-1.68017	-1.67803	-1.72078	-1.71845	-1.71617	-1.75148	-1.74916	-1.74691	-1.74472
-1.67885	-1.67673	-1.71929	-1.71691	-1.71458	-1.74988	-1.74763	-1.74544	-1.74331
-1.72261	-1.72018	-1.75542	-1.75298	-1.75059	-1.74832	-1.74614	-1.74399	-1.74188
-1.72103	-1.71859	-1.75381	-1.7514	-1.74905	-1.74682	-1.74469	-1.74256	-1.74042
-1.71943	-1.717	-1.75221	-1.74985	-1.74755	-1.74536	-1.74326	-1.74112	-1.73896
-1.71779	-1.71542	-1.75067	-1.74839	-1.74615	-1.74397	-1.74183	-1.73968	-1.73751
-1.71617	-1.71387	-1.74917	-1.74695	-1.74477	-1.74258	-1.7404	-1.73822	-1.73607
-1.71462	-1.71237	-1.74768	-1.74549	-1.74332	-1.74114	-1.73895	-1.73678	-1.73465
-1.71314	-1.71092	-1.74623	-1.74402	-1.74184	-1.73966	-1.73748	-1.73534	-1.73325
-1.71168	-1.7095	-1.74481	-1.74261	-1.74041	-1.73823	-1.73606	-1.75746	-1.75518
-1.71023	-1.70811	-1.74343	-1.74124	-1.73905	-1.73686	-1.73469	-1.75594	-1.75364
-1.70882	-1.70672	-1.74204	-1.73986	-1.73767	-1.73548	-1.7333	-1.7544	-1.75209
-1.70743	-1.70533	-1.74061	-1.73843	-1.73624	-1.73405	-1.73186	-1.75282	-1.75049
-1.70605	-1.70393	-1.73917	-1.737	-1.73482	-1.73263	-1.73043	-1.75126	-1.74892
-1.74204	-1.7399	-1.73777	-1.73561	-1.73345	-1.7545	-1.75211	-1.74976	-1.74744
-1.74059	-1.73847	-1.73637	-1.73423	-1.73209	-1.75302	-1.75063	-1.7483	-1.74601
-1.73919	-1.73707	-1.75848	-1.75616	-1.75382	-1.75148	-1.74912	-1.74682	-1.74455
-1.73784	-1.73568	-1.75692	-1.75457	-1.75223	-1.7499	-1.7476	-1.74533	-1.7431
-1.76013	-1.75776	-1.7554	-1.75304	-1.75069	-1.74839	-1.74612	-1.74389	-1.74168
-1.75866	-1.75629	-1.75393	-1.75156	-1.74922	-1.74694	-1.74471	-1.74251	-1.74033
-1.75715	-1.7548	-1.75243	-1.75008	-1.74775	-1.74549	-1.74329	-1.74111	-1.73896
-1.75557	-1.75322	-1.75088	-1.74855	-1.74625	-1.74401	-1.74182	-1.73966	-1.73753
-1.75399	-1.75165	-1.74933	-1.74703	-1.74476	-1.74254	-1.74036	-1.73821	-1.7361
-1.75249	-1.75017	-1.74787	-1.74559	-1.74335	-1.74114	-1.73898	-1.82089	-1.81828
-1.75102	-1.74872	-1.74644	-1.74419	-1.74197	-1.73978	-1.73762	-1.81923	-1.81664
-1.74952	-1.74723	-1.76094	-1.7587	-1.7565	-1.82276	-1.82013	-1.8294	-1.82682
-1.74799	-1.74571	-1.75943	-1.75722	-1.75504	-1.82099	-1.81837	-1.82767	-1.82512
-1.76247	-1.7602	-1.77067	-1.76846	-1.76628	-1.83116	-1.82854	-1.82596	-1.82343
-1.76105	-1.75881	-1.76929	-1.7671	-1.76492	-1.82951	-1.82688	-1.82429	-1.82173
-1.77236	-1.77013	-1.83582	-1.83314	-1.8305	-1.82786	-1.82523	-1.82263	-1.82006
-1.77094	-1.76872	-1.8341	-1.83145	-1.82882	-1.82621	-1.82361	-1.82102	-1.81845
-1.76952	-1.7673	-1.83237	-1.82973	-1.82712	-1.82454	-1.82197	-1.81941	-1.81684
-1.83596	-1.83325	-1.83058	-1.82795	-1.82535	-1.82278	-1.82024	-1.8177	-1.81516
-1.83412	-1.83142	-1.82876	-1.82614	-1.82355	-1.82099	-1.81845	-1.81593	-1.81344
-1.83234	-1.82966	-1.82702	-1.82441	-1.82183	-1.81927	-1.81675	-1.83905	-1.83636
-1.83062	-1.82797	-1.82535	-1.82275	-1.82018	-1.81764	-1.81513	-1.83739	-1.83471
-1.82892	-1.8263	-1.8237	-1.82112	-1.81856	-1.8412	-1.83846	-1.83576	-1.83309
-1.82724	-1.82465	-1.82207	-1.8195	-1.81695	-1.83955	-1.83681	-1.83412	-1.83147
-1.82558	-1.823	-1.82044	-1.81789	-1.81535	-1.8379	-1.83517	-1.83248	-1.82985
-1.8239	-1.82133	-1.83528	-1.83268	-1.8301	-1.83625	-1.83353	-1.83084	-1.82819

-1.82223	-1.81965	-1.83355	-1.83098	-1.82843	-1.83459	-1.83188	-1.8292	-1.82654
-1.82055	-1.81797	-1.841	-1.83827	-1.83558	-1.83288	-1.8302	-1.82755	-1.82493
-1.81887	-1.81627	-1.83921	-1.83648	-1.83379	-1.83113	-1.82849	-1.82591	-1.82336
-1.84315	-1.84029	-1.83748	-1.83476	-1.83209	-1.82946	-1.82687	-1.82432	-1.82183
-1.84139	-1.83858	-1.83581	-1.83312	-1.83048	-1.82788	-1.82533	-1.82281	-1.82034
-1.83967	-1.8369	-1.83418	-1.83152	-1.82891	-1.82634	-1.82382	-1.83479	-1.83231
-1.83799	-1.83525	-1.83257	-1.82995	-1.82738	-1.82484	-1.82233	-1.83331	-1.83083
-1.83633	-1.83363	-1.83098	-1.8284	-1.82587	-1.82335	-1.82085	-1.83184	-1.82936
-1.8347	-1.83202	-1.8294	-1.82684	-1.82433	-1.83533	-1.83284	-1.83037	-1.82791
-1.83309	-1.83044	-1.82783	-1.82529	-1.8228	-1.83382	-1.83136	-1.82891	-1.82647
-1.83149	-1.82887	-1.83983	-1.83729	-1.8348	-1.83232	-1.82987	-1.82743	-1.825
-1.82991	-1.82732	-1.83831	-1.83579	-1.83331	-1.83084	-1.82839	-1.82595	-1.8235
-1.84191	-1.83934	-1.8368	-1.83429	-1.83181	-1.82936	-1.82693	-1.8245	-1.82205
-1.84037	-1.83781	-1.83528	-1.83278	-1.8303	-1.82788	-1.82548	-1.82308	-1.82065
-1.83885	-1.83629	-1.83377	-1.83128	-1.82881	-1.83099	-1.82862	-1.82623	-1.82383
-1.83732	-1.83478	-1.83227	-1.82979	-1.82735	-1.82952	-1.82714	-1.82478	-1.82241
-1.83581	-1.83328	-1.83079	-1.82833	-1.8259	-1.82808	-1.82568	-1.82333	-1.821
-1.83889	-1.83638	-1.83391	-1.83147	-1.82907	-1.82667	-1.82428	-1.82194	-1.81964
-1.83739	-1.83489	-1.83244	-1.83004	-1.82767	-1.82529	-1.82292	-1.82059	-1.81832
-1.83589	-1.83343	-1.831	-1.82863	-1.82628	-1.82392	-1.82156	-1.81925	-1.817
-1.83439	-1.83197	-1.82959	-1.82723	-1.8249	-1.82255	-1.82022	-1.81793	-1.81569
-1.83293	-1.83054	-1.82819	-1.82585	-1.82352	-1.8212	-1.81888	-1.81661	-1.81438
-1.8315	-1.82913	-1.82679	-1.82446	-1.82215	-1.81985	-1.81755	-1.81529	-1.81308
-1.83009	-1.82773	-1.82541	-1.82309	-1.82079	-1.8185	-1.81622	-1.81398	-1.81179
-1.8287	-1.82635	-1.82403	-1.82173	-1.81945	-1.83035	-1.82805	-1.82581	-1.82362
-1.82732	-1.82498	-1.82267	-1.82039	-1.81812	-1.82902	-1.82672	-1.82449	-1.82232
-1.83915	-1.83682	-1.83451	-1.83223	-1.82997	-1.82769	-1.82542	-1.8232	-1.82104
-1.83773	-1.83541	-1.83313	-1.83087	-1.82862	-1.82638	-1.82413	-1.82193	-1.81977
-1.83632	-1.83401	-1.83174	-1.8295	-1.82728	-1.82507	-1.82287	-1.82069	-1.81853
-1.83491	-1.83261	-1.83035	-1.82813	-1.82594	-1.82378	-1.82163	-1.81948	-1.81731
-1.83353	-1.83123	-1.82897	-1.82677	-1.82461	-1.82248	-1.82038	-1.84733	-1.84511
-1.8322	-1.82988	-1.82761	-1.82542	-1.82329	-1.82117	-1.81909	-1.84601	-1.84381
-1.83088	-1.82856	-1.82628	-1.8241	-1.82197	-1.81987	-1.8178	-1.84468	-1.8425
-1.82954	-1.82723	-1.82497	-1.82279	-1.82067	-1.8476	-1.84549	-1.84336	-1.84119
-1.82818	-1.82591	-1.82368	-1.82151	-1.81938	-1.84627	-1.84416	-1.84203	-1.83989
-1.82684	-1.82462	-1.85147	-1.84925	-1.84707	-1.85668	-1.85457	-1.86401	-1.86187
-1.82552	-1.82335	-1.85019	-1.84796	-1.84575	-1.85535	-1.85325	-1.86271	-1.8606
-1.86506	-1.86285	-1.86065	-1.8584	-1.85617	-1.8656	-1.86349	-1.8614	-1.85932
-1.86372	-1.86151	-1.85931	-1.85708	-1.85486	-1.86428	-1.86216	-1.86007	-1.858
-1.87404	-1.8718	-1.86958	-1.86735	-1.86515	-1.86298	-1.86085	-1.85875	-1.85668
-1.87274	-1.87049	-1.86825	-1.86606	-1.86389	-1.86172	-1.85957	-1.85745	-1.85537
-1.87145	-1.86917	-1.86693	-1.86477	-1.86263	-1.86048	-1.85831	-1.85617	-1.85407
-1.87011	-1.86784	-1.8656	-1.86345	-1.86134	-1.8592	-1.85706	-1.85493	-1.8528
-1.86875	-1.86649	-1.86427	-1.86213	-1.86002	-1.85792	-1.85582	-1.8537	-1.85154
-1.86741	-1.86518	-1.86299	-1.86084	-1.85872	-1.85663	-1.85455	-1.85243	-1.85027
-1.86609	-1.86391	-1.86174	-1.85959	-1.85745	-1.85533	-1.85324	-1.85113	-1.84897
-1.86478	-1.86263	-1.86049	-1.85833	-1.85618	-1.85406	-1.85195	-1.84983	-1.84769
-1.86348	-1.86133	-1.8592	-1.85707	-1.85494	-1.85281	-1.85067	-1.84854	-1.84643
-1.86219	-1.86005	-1.85792	-1.85581	-1.8537	-1.85155	-1.84938	-1.84725	-1.84516
-1.86094	-1.85878	-1.85663	-1.85451	-1.8524	-1.85026	-1.84809	-1.84595	-1.84385

-1.85969	-1.85751	-1.85535	-1.85322	-1.85109	-1.84895	-1.8468	-1.84466	-1.84253
-1.85843	-1.85626	-1.8541	-1.85195	-1.84981	-1.84766	-1.84551	-1.84337	-1.84123
-1.85716	-1.85501	-1.85286	-1.8507	-1.84853	-1.84638	-1.84424	-1.8421	-1.83995
-1.85591	-1.85376	-1.85161	-1.84943	-1.84726	-1.8451	-1.84296	-1.84082	-1.83868
-1.85467	-1.8525	-1.85033	-1.84816	-1.84599	-1.84383	-1.84167	-1.83953	-1.83741
-1.85342	-1.85123	-1.84905	-1.84688	-1.84472	-1.84255	-1.84039	-1.83826	-1.83615
-1.85216	-1.84997	-1.84779	-1.84561	-1.84343	-1.84127	-1.83912	-1.83699	-1.83487
-1.85088	-1.8487	-1.84652	-1.84433	-1.84214	-1.83998	-1.83785	-1.83572	-1.8336
-1.84961	-1.84742	-1.84523	-1.84304	-1.84086	-1.83871	-1.83658	-1.84487	-1.84273
-1.84834	-1.84612	-1.84392	-1.84174	-1.83959	-1.83744	-1.8353	-1.84358	-1.84145
-1.84705	-1.84483	-1.84263	-1.84046	-1.8383	-1.84659	-1.84444	-1.84229	-1.84016
-1.84574	-1.84354	-1.84136	-1.83918	-1.83701	-1.84528	-1.84314	-1.841	-1.83885
-1.84444	-1.84225	-1.85054	-1.84834	-1.84615	-1.84399	-1.84185	-1.85844	-1.85626
-1.84314	-1.84095	-1.84922	-1.84705	-1.84488	-1.84272	-1.84057	-1.85716	-1.85495
-1.85233	-1.85011	-1.84791	-1.84575	-1.8436	-1.86027	-1.8581	-1.8559	-1.85367
-1.851	-1.84881	-1.84663	-1.84446	-1.8423	-1.859	-1.85684	-1.85466	-1.85244
-1.84968	-1.84751	-1.84535	-1.84317	-1.84099	-1.85772	-1.85558	-1.85342	-1.85123
-1.84841	-1.84622	-1.86301	-1.86081	-1.85863	-1.85646	-1.85431	-1.85215	-1.84999
-1.84717	-1.84494	-1.86172	-1.85954	-1.85738	-1.85521	-1.85303	-1.85087	-1.84874
-1.86499	-1.86272	-1.86047	-1.85828	-1.85611	-1.85393	-1.85176	-1.86129	-1.85915
-1.86376	-1.8615	-1.85924	-1.85702	-1.85482	-1.85264	-1.85048	-1.86001	-1.85785
-1.8625	-1.86026	-1.86971	-1.86746	-1.86523	-1.86305	-1.86089	-1.85872	-1.85655
-1.8612	-1.859	-1.86847	-1.86624	-1.864	-1.86179	-1.8596	-1.85742	-1.85526
-1.85991	-1.85773	-1.86722	-1.865	-1.86277	-1.86054	-1.85832	-1.85613	-1.85397
-1.87033	-1.86812	-1.86591	-1.86371	-1.86151	-1.8593	-1.85707	-1.85487	-1.85268
-1.86909	-1.86683	-1.8646	-1.86242	-1.86024	-1.85805	-1.85583	-1.85361	-1.85139
-1.86784	-1.86558	-1.86334	-1.86114	-1.85896	-1.85676	-1.85457	-1.85236	-1.85013
-1.86658	-1.86434	-1.8621	-1.85987	-1.85765	-1.85547	-1.8533	-1.8511	-1.84889
-1.86529	-1.86307	-1.86084	-1.85861	-1.85638	-1.85418	-1.85201	-1.84982	-1.84763
-1.86396	-1.86177	-1.85957	-1.85735	-1.85512	-1.85291	-1.8507	-1.84851	-1.84634
-1.86265	-1.86047	-1.85829	-1.85608	-1.85387	-1.85164	-1.84941	-1.85961	-1.8574
-1.86138	-1.85917	-1.85698	-1.85479	-1.85261	-1.85039	-1.84816	-1.85831	-1.85607
-1.86013	-1.85789	-1.85567	-1.8535	-1.85134	-1.84914	-1.84691	-1.85703	-1.85474
-1.85888	-1.85663	-1.85441	-1.85224	-1.85009	-1.84789	-1.84567	-1.85575	-1.85343
-1.85764	-1.85539	-1.85317	-1.85099	-1.84883	-1.84663	-1.84441	-1.85446	-1.85213
-1.85641	-1.85415	-1.85191	-1.84971	-1.84753	-1.84533	-1.84313	-1.85316	-1.85083
-1.85518	-1.85291	-1.85065	-1.84842	-1.8462	-1.844	-1.84181	-1.85183	-1.84952
-1.85393	-1.85166	-1.84939	-1.84714	-1.84489	-1.85499	-1.85275	-1.85048	-1.8482
-1.85267	-1.85042	-1.84815	-1.84588	-1.84361	-1.85365	-1.85137	-1.8491	-1.84684
-1.85141	-1.84917	-1.8469	-1.84462	-1.84233	-1.85232	-1.85	-1.84773	-1.84549
-1.85015	-1.84791	-1.84565	-1.84337	-1.84107	-1.85102	-1.84868	-1.8464	-1.84417
-1.84889	-1.84665	-1.84439	-1.84211	-1.8398	-1.84972	-1.84738	-1.84508	-1.84284
-1.84763	-1.84539	-1.84312	-1.84083	-1.83853	-1.84842	-1.84606	-1.84375	-1.84147
-1.84636	-1.84411	-1.84184	-1.83955	-1.83724	-1.84711	-1.84475	-1.84241	-1.8401
-1.84503	-1.8428	-1.85284	-1.85051	-1.84816	-1.8458	-1.84344	-1.84109	-1.83875
-1.84366	-1.84146	-1.85151	-1.84919	-1.84685	-1.8445	-1.84214	-1.83978	-1.83742
-1.8423	-1.84012	-1.85018	-1.84787	-1.84553	-1.84318	-1.84083	-1.83847	-1.8361
-1.84096	-1.83879	-1.84883	-1.84653	-1.8442	-1.84186	-1.83951	-1.83715	-1.83479
-1.83963	-1.83746	-1.84748	-1.84518	-1.84286	-1.84053	-1.83818	-1.83583	-1.83347
-1.83832	-1.83613	-1.84612	-1.84383	-1.84151	-1.83918	-1.83684	-1.83449	-1.83214

-1.83701	-1.83481	-1.84476	-1.84247	-1.84016	-1.83783	-1.83548	-1.83313	-1.83079
-1.84796	-1.8457	-1.84342	-1.84112	-1.8388	-1.83647	-1.83413	-1.83178	-1.82944
-1.84669	-1.8444	-1.84209	-1.83977	-1.83744	-1.83511	-1.83277	-1.83043	-1.82808
-1.84539	-1.84308	-1.84075	-1.83842	-1.83608	-1.83374	-1.83141	-1.82907	-1.82672
-1.84407	-1.84174	-1.8394	-1.83706	-1.83472	-1.83239	-1.83004	-1.8277	-1.82535
-1.84274	-1.84039	-1.83804	-1.8357	-1.83337	-1.83102	-1.82868	-1.82633	-1.82397
-1.84139	-1.83904	-1.83669	-1.83434	-1.832	-1.82966	-1.82732	-1.82496	-1.82259
-1.84004	-1.83768	-1.83533	-1.83298	-1.83064	-1.8283	-1.82595	-1.82359	-1.82121
-1.83867	-1.83632	-1.83397	-1.83163	-1.82928	-1.82693	-1.82457	-1.82221	-1.81983
-1.8373	-1.83495	-1.83261	-1.83026	-1.82792	-1.82556	-1.82319	-1.82081	-1.81844
-1.83594	-1.83359	-1.83124	-1.82889	-1.82654	-1.82418	-1.8218	-1.81943	-1.81704
-1.83458	-1.83222	-1.82986	-1.82752	-1.82517	-1.8228	-1.82042	-1.81804	-1.81565
-1.83323	-1.83087	-1.82851	-1.82616	-1.8238	-1.82143	-1.81905	-1.81666	-1.81427
-1.83188	-1.82954	-1.82719	-1.82482	-1.82245	-1.82006	-1.81767	-1.81528	-1.81289
-1.83054	-1.82822	-1.82588	-1.82351	-1.82113	-1.81873	-1.81633	-1.81394	-1.81155
-1.82922	-1.82692	-1.8246	-1.82224	-1.81986	-1.81747	-1.81507	-1.81267	-1.81027
-1.82792	-1.82563	-1.82333	-1.82098	-1.8186	-1.81621	-1.81381	-1.81141	-1.809
-1.82664	-1.82433	-1.82201	-1.81965	-1.81727	-1.81488	-1.81247	-1.82211	-1.81969
-1.82537	-1.82303	-1.82068	-1.8183	-1.8159	-1.81351	-1.8111	-1.82073	-1.81832
-1.82407	-1.8217	-1.8314	-1.829	-1.82658	-1.82417	-1.82175	-1.81934	-1.81694
-1.82272	-1.82033	-1.83001	-1.8276	-1.82519	-1.82277	-1.82036	-1.81795	-1.81554
-1.83346	-1.83104	-1.82863	-1.82621	-1.8238	-1.82139	-1.81898	-1.81657	-1.81416
-1.83208	-1.82967	-1.82726	-1.82484	-1.82242	-1.82001	-1.8176	-1.81519	-1.81279
-1.83074	-1.82833	-1.82592	-1.8235	-1.82108	-1.81867	-1.81626	-1.81385	-1.81145
-1.82946	-1.82705	-1.82464	-1.82222	-1.8198	-1.81739	-1.81498	-1.81257	-1.81016
-1.8282	-1.82578	-1.82336	-1.82094	-1.81853	-1.81612	-1.81371	-1.8113	-1.80888
-1.82687	-1.82445	-1.82203	-1.81961	-1.8172	-1.81478	-1.81237	-1.80996	-1.80753
-1.82551	-1.82309	-1.82066	-1.81824	-1.81583	-1.81341	-1.81099	-1.80857	-1.80614
-1.82418	-1.82174	-1.8193	-1.81688	-1.81446	-1.81204	-1.80962	-1.80719	-1.80475
-1.82289	-1.82041	-1.81794	-1.81551	-1.81309	-1.81066	-1.80824	-1.8058	-1.80335
-1.82158	-1.81908	-1.8166	-1.81416	-1.81174	-1.80931	-1.80688	-1.80444	-1.80198
-1.82023	-1.81776	-1.8153	-1.81287	-1.81044	-1.80801	-1.80558	-1.80313	-1.80067
-1.81888	-1.81644	-1.81399	-1.81156	-1.80914	-1.8067	-1.80426	-1.80181	-1.79935
-1.81758	-1.81511	-1.81265	-1.81021	-1.80778	-1.80533	-1.80289	-1.80043	-1.79797
-1.81629	-1.81379	-1.8113	-1.80885	-1.8064	-1.80395	-1.8015	-1.79904	-1.79657
-1.81495	-1.81247	-1.80998	-1.80752	-1.80507	-1.80261	-1.80015	-1.79769	-1.79523
-1.8136	-1.81114	-1.80867	-1.80622	-1.80376	-1.8013	-1.79884	-1.79637	-1.79392
-1.81227	-1.80981	-1.80734	-1.80488	-1.80242	-1.79996	-1.79749	-1.79503	-1.79257
-1.81096	-1.80847	-1.80597	-1.8035	-1.80103	-1.79857	-1.7961	-1.79364	-1.79118
-1.80965	-1.80713	-1.80462	-1.80213	-1.79966	-1.79719	-1.79473	-1.79227	-1.78981
-1.80829	-1.8058	-1.8033	-1.80081	-1.79834	-1.79587	-1.7934	-1.79094	-1.78849
-1.80694	-1.80447	-1.80199	-1.79951	-1.79701	-1.79454	-1.79207	-1.78962	-1.78717
-1.80563	-1.80317	-1.80069	-1.79818	-1.79566	-1.79317	-1.7907	-1.78824	-1.78579
-1.80433	-1.80186	-1.79938	-1.79684	-1.79429	-1.79179	-1.78931	-1.78685	-1.78439
-1.80299	-1.80052	-1.79803	-1.7955	-1.79296	-1.79046	-1.78798	-1.7855	-1.78304
-1.80162	-1.79913	-1.79665	-1.79415	-1.79165	-1.78916	-1.78668	-1.7842	-1.78172
-1.80028	-1.79778	-1.7953	-1.79282	-1.79034	-1.78786	-1.78538	-1.78289	-1.78039
-1.79897	-1.79648	-1.794	-1.79152	-1.78904	-1.78657	-1.78409	-1.78158	-1.77904
-1.79767	-1.79519	-1.7927	-1.79021	-1.78772	-1.78524	-1.78277	-1.78025	-1.7777
-1.79639	-1.7939	-1.7914	-1.78888	-1.78636	-1.78387	-1.78139	-1.77889	-1.77635

-1.7951	-1.7926	-1.79009	-1.78754	-1.78499	-1.78248	-1.78	-1.77751	-1.77502
-1.79379	-1.79127	-1.78875	-1.7862	-1.78366	-1.78115	-1.77867	-1.77618	-1.77369
-1.79247	-1.78993	-1.78739	-1.78486	-1.78235	-1.77985	-1.77737	-1.77488	-1.77238
-1.79114	-1.78859	-1.78605	-1.78354	-1.78105	-1.77856	-1.77607	-1.77357	-1.77105
-1.78978	-1.78725	-1.78474	-1.78224	-1.77976	-1.77727	-1.77477	-1.77225	-1.76969
-1.78844	-1.78593	-1.78344	-1.78095	-1.77847	-1.77597	-1.77345	-1.77092	-1.76835
-1.78713	-1.78464	-1.78216	-1.77968	-1.77719	-1.77466	-1.77211	-1.76955	-1.767
-1.78584	-1.78336	-1.78088	-1.7784	-1.77591	-1.77336	-1.77077	-1.7682	-1.76568
-1.78455	-1.78208	-1.77961	-1.77713	-1.77463	-1.77207	-1.76947	-1.7669	-1.76437
-1.78328	-1.78081	-1.77833	-1.77585	-1.77334	-1.77078	-1.76818	-1.76561	-1.76308
-1.782	-1.77953	-1.77705	-1.77454	-1.772	-1.76944	-1.76687	-1.76432	-1.76179
-1.78073	-1.77826	-1.77576	-1.77321	-1.77063	-1.76808	-1.76554	-1.76301	-1.7605
-1.77945	-1.77697	-1.77447	-1.77189	-1.76929	-1.76674	-1.76423	-1.76171	-1.7592
-1.77816	-1.77567	-1.77316	-1.77058	-1.76798	-1.76544	-1.76293	-1.76041	-1.75791
-1.77686	-1.77436	-1.77183	-1.76926	-1.76668	-1.76414	-1.76162	-1.75911	-1.75661
-1.77556	-1.77301	-1.77046	-1.7679	-1.76535	-1.76283	-1.76032	-1.75781	-1.75529
-1.77425	-1.77167	-1.76909	-1.76655	-1.76403	-1.76152	-1.75901	-1.75649	-1.75397
-1.77296	-1.77036	-1.76777	-1.76524	-1.76272	-1.76021	-1.7577	-1.75516	-1.75261
-1.77168	-1.76907	-1.76648	-1.76394	-1.76142	-1.75891	-1.75639	-1.75383	-1.75123
-1.7704	-1.76779	-1.76519	-1.76265	-1.76013	-1.7576	-1.75508	-1.75252	-1.74989
-1.76912	-1.76652	-1.76392	-1.76136	-1.75883	-1.7563	-1.75378	-1.75121	-1.74859
-1.76785	-1.76526	-1.76267	-1.7601	-1.75754	-1.75501	-1.75248	-1.74991	-1.74728
-1.76658	-1.76403	-1.76147	-1.75888	-1.75628	-1.75372	-1.75119	-1.74861	-1.74598
-1.76531	-1.76279	-1.76025	-1.75764	-1.75501	-1.75244	-1.7499	-1.74731	-1.74468
-1.76402	-1.76152	-1.75899	-1.75637	-1.75374	-1.75115	-1.7486	-1.74601	-1.74338
-1.76274	-1.76023	-1.7577	-1.75509	-1.75245	-1.74986	-1.7473	-1.74472	-1.74209
-1.76146	-1.75895	-1.75641	-1.75379	-1.75115	-1.74856	-1.746	-1.74343	-1.74084
-1.76018	-1.75766	-1.75511	-1.75249	-1.74984	-1.74726	-1.7447	-1.74215	-1.7396
-1.75891	-1.75638	-1.75382	-1.75119	-1.74855	-1.74596	-1.74341	-1.74086	-1.73833
-1.75764	-1.7551	-1.75254	-1.74991	-1.74727	-1.74467	-1.74211	-1.73956	-1.73703
-1.75638	-1.75382	-1.75126	-1.74864	-1.74601	-1.74341	-1.74083	-1.73827	-1.73574
-1.75511	-1.75254	-1.74998	-1.74739	-1.7448	-1.74219	-1.73957	-1.73699	-1.73445
-1.75385	-1.75127	-1.7487	-1.74614	-1.74358	-1.74096	-1.73832	-1.73571	-1.73316
-1.75264	-1.75003	-1.74744	-1.74488	-1.74232	-1.7397	-1.73705	-1.73444	-1.73187
-1.75144	-1.7488	-1.74618	-1.74361	-1.74105	-1.73843	-1.73579	-1.73317	-1.73059
-1.7502	-1.74755	-1.74491	-1.74234	-1.73977	-1.73717	-1.73455	-1.73193	-1.72932
-1.74892	-1.74629	-1.74366	-1.74107	-1.73849	-1.73591	-1.73333	-1.73072	-1.72806
-1.74768	-1.74505	-1.74243	-1.73983	-1.73723	-1.73465	-1.73208	-1.72946	-1.72678
-1.74646	-1.74385	-1.74124	-1.73861	-1.73598	-1.73338	-1.73079	-1.72817	-1.72549
-1.74525	-1.74264	-1.74003	-1.73739	-1.73474	-1.73212	-1.72951	-1.72688	-1.72422
-1.74405	-1.74143	-1.73879	-1.73617	-1.73354	-1.73089	-1.72824	-1.7256	-1.72297
-1.74284	-1.74019	-1.73754	-1.73493	-1.73232	-1.72966	-1.72697	-1.72432	-1.72172
-1.74158	-1.73892	-1.73627	-1.73365	-1.73104	-1.72838	-1.72569	-1.72304	-1.72044
-1.74029	-1.73764	-1.73499	-1.73237	-1.72974	-1.72708	-1.72441	-1.72176	-1.71915
-1.73903	-1.73639	-1.73375	-1.73111	-1.72847	-1.72582	-1.72316	-1.72052	-1.71789
-1.73778	-1.73516	-1.73253	-1.72988	-1.72722	-1.72458	-1.72194	-1.7193	-1.71666
-1.73656	-1.73394	-1.73131	-1.72866	-1.726	-1.72336	-1.72073	-1.71809	-1.71544
-1.73536	-1.73272	-1.73008	-1.72744	-1.7248	-1.72216	-1.71951	-1.71688	-1.71424
-1.73415	-1.73151	-1.72886	-1.72623	-1.7236	-1.72096	-1.71831	-1.71567	-1.71305
-1.73293	-1.7303	-1.72765	-1.72502	-1.72238	-1.71975	-1.71711	-1.71448	-1.71185

-1.73172	-1.72909	-1.72645	-1.72381	-1.72117	-1.71854	-1.71592	-1.71329	-1.71065
-1.73052	-1.72788	-1.72524	-1.72261	-1.71997	-1.71734	-1.71471	-1.71209	-1.70945
-1.72932	-1.72668	-1.72403	-1.7214	-1.71878	-1.71614	-1.7135	-1.71087	-1.70825
-1.72812	-1.72547	-1.72283	-1.7202	-1.71757	-1.71494	-1.7123	-1.70966	-1.70705
-1.72689	-1.72427	-1.72164	-1.719	-1.71636	-1.71373	-1.71111	-1.70847	-1.70584
-1.72569	-1.72306	-1.72044	-1.71779	-1.71515	-1.71252	-1.70991	-1.70728	-1.70463
-1.7245	-1.72186	-1.71921	-1.71658	-1.71396	-1.71133	-1.7087	-1.70607	-1.70345
-1.72333	-1.72066	-1.718	-1.71538	-1.71276	-1.71013	-1.70749	-1.70487	-1.70226
-1.72218	-1.71951	-1.71684	-1.71419	-1.71155	-1.70892	-1.70629	-1.70367	-1.70104
-1.72103	-1.71836	-1.71569	-1.71301	-1.71033	-1.7077	-1.70509	-1.70247	-1.69982
-1.71984	-1.71719	-1.71453	-1.71185	-1.70916	-1.70651	-1.70388	-1.70125	-1.6986
-1.71862	-1.716	-1.71337	-1.7107	-1.70802	-1.70534	-1.70267	-1.70002	-1.69739
-1.71742	-1.71481	-1.71218	-1.70953	-1.70686	-1.70418	-1.70148	-1.69882	-1.69618
-1.71624	-1.7136	-1.71095	-1.70832	-1.70569	-1.70302	-1.70033	-1.69765	-1.69497
-1.71508	-1.71241	-1.70975	-1.70712	-1.70451	-1.70185	-1.69917	-1.69648	-1.69377
-1.71394	-1.71126	-1.70859	-1.70593	-1.70328	-1.70064	-1.69799	-1.69531	-1.69261
-1.71279	-1.71012	-1.70744	-1.70475	-1.70207	-1.69942	-1.69679	-1.69414	-1.69145
-1.7116	-1.70895	-1.70628	-1.70359	-1.70089	-1.69823	-1.69558	-1.69292	-1.69026
-1.71038	-1.70776	-1.70512	-1.70244	-1.69974	-1.69705	-1.69436	-1.69169	-1.68905
-1.70921	-1.70657	-1.70392	-1.70126	-1.69858	-1.69589	-1.69317	-1.69049	-1.68785
-1.70805	-1.70537	-1.7027	-1.70006	-1.69742	-1.69474	-1.69202	-1.68932	-1.68663
-1.70691	-1.70419	-1.70151	-1.69888	-1.69626	-1.69359	-1.69087	-1.68816	-1.68545
-1.70577	-1.70305	-1.70035	-1.69772	-1.6951	-1.69243	-1.68973	-1.68702	-1.6843
-1.70464	-1.70191	-1.69921	-1.69656	-1.69393	-1.69126	-1.68858	-1.68588	-1.68315
-1.70351	-1.70078	-1.69806	-1.69538	-1.69271	-1.69006	-1.68741	-1.68472	-1.682
-1.70238	-1.69964	-1.69691	-1.6942	-1.6915	-1.68884	-1.68623	-1.68356	-1.68084
-1.70123	-1.6985	-1.69577	-1.69304	-1.69033	-1.68768	-1.68507	-1.68241	-1.67969
-1.70008	-1.69736	-1.69464	-1.6919	-1.68918	-1.68653	-1.68392	-1.68126	-1.67854
-1.69893	-1.69622	-1.6935	-1.69076	-1.68804	-1.68538	-1.68277	-1.6801	-1.67739
-1.69778	-1.69508	-1.69236	-1.68962	-1.6869	-1.68423	-1.68161	-1.67894	-1.67623
-1.69665	-1.69394	-1.69122	-1.68849	-1.68576	-1.68307	-1.68042	-1.67775	-1.67506
-1.69552	-1.69281	-1.69009	-1.68735	-1.68461	-1.68189	-1.6792	-1.67651	-1.67385
-1.69439	-1.69168	-1.68896	-1.68621	-1.68346	-1.68072	-1.67798	-1.67529	-1.67265
-1.69326	-1.69055	-1.68783	-1.68508	-1.68232	-1.67956	-1.67681	-1.67411	-1.67148
-1.69213	-1.68943	-1.6867	-1.68395	-1.68118	-1.67842	-1.67566	-1.67295	-1.67032
-1.69102	-1.6883	-1.68557	-1.68281	-1.68005	-1.67728	-1.67452	-1.67181	-1.66917
-1.68992	-1.68719	-1.68444	-1.68168	-1.67891	-1.67615	-1.6734	-1.67069	-1.66804
-1.6888	-1.68606	-1.68331	-1.68055	-1.67778	-1.67504	-1.67231	-1.6696	-1.66693
-1.68768	-1.68493	-1.68218	-1.67941	-1.67665	-1.67394	-1.67125	-1.66856	-1.66586
-1.68656	-1.6838	-1.68104	-1.67828	-1.67553	-1.67283	-1.67018	-1.66749	-1.66476
-1.68543	-1.68267	-1.67991	-1.67715	-1.67441	-1.67172	-1.66907	-1.66638	-1.66364
-1.6843	-1.68154	-1.67878	-1.67603	-1.6733	-1.67061	-1.66795	-1.66525	-1.66251
-1.68317	-1.68041	-1.67767	-1.67495	-1.67225	-1.66955	-1.66685	-1.66413	-1.66139
-1.68205	-1.6793	-1.67658	-1.67389	-1.67121	-1.66849	-1.66576	-1.66301	-1.66026
-1.68094	-1.67822	-1.67551	-1.67283	-1.67014	-1.66741	-1.66465	-1.66189	-1.65913
-1.67985	-1.67717	-1.67448	-1.67177	-1.66905	-1.66631	-1.66354	-1.66077	-1.658
-1.67878	-1.67611	-1.67342	-1.6707	-1.66795	-1.66519	-1.66242	-1.65965	-1.65688
-1.67774	-1.67505	-1.67233	-1.66959	-1.66682	-1.66406	-1.66129	-1.65853	-1.65577
-1.67669	-1.67397	-1.67124	-1.66848	-1.6657	-1.66294	-1.66018	-1.65742	-1.65468
-1.67562	-1.67288	-1.67013	-1.66737	-1.6646	-1.66183	-1.65907	-1.65633	-1.65362

-1.67453	-1.67178	-1.66903	-1.66627	-1.66351	-1.66074	-1.65798	-1.65525	-1.65257
-1.67345	-1.67068	-1.66793	-1.66519	-1.66246	-1.65971	-1.65695	-1.65422	-1.65151
-1.67237	-1.66959	-1.66684	-1.66413	-1.66144	-1.6587	-1.65595	-1.6532	-1.65045
-1.67133	-1.66855	-1.66579	-1.66308	-1.66038	-1.65765	-1.65489	-1.65213	-1.64936
-1.67034	-1.66756	-1.66479	-1.66204	-1.6593	-1.65655	-1.6538	-1.65103	-1.64825
-1.66931	-1.66654	-1.66376	-1.66099	-1.65822	-1.65546	-1.6527	-1.64992	-1.64714
-1.66823	-1.66546	-1.66269	-1.65991	-1.65714	-1.65436	-1.65159	-1.64881	-1.64602
-1.66713	-1.66438	-1.66163	-1.65885	-1.65607	-1.65329	-1.65052	-1.64773	-1.64492
-1.66605	-1.66333	-1.6606	-1.65784	-1.65506	-1.65228	-1.6495	-1.64671	-1.6439
-1.66498	-1.66228	-1.65957	-1.65683	-1.65406	-1.65128	-1.6485	-1.6457	-1.64289
-1.66395	-1.66124	-1.65853	-1.65578	-1.65301	-1.65023	-1.64744	-1.64464	-1.64184
[truncated: 629,404 more chars]
