# Supplementary material for: Artefact-free topography based scleral-asymmetry
Source: PLoS One. 2019 Jul 26;14(7):e0219789. doi: 10.1371/journal.pone.0219789 (PMC6660081; doi:10.1371/journal.pone.0219789)
Supplement: S3 Table — (ZIP) [file Eye std OD.pdf › Eye std OD.pdf]

0.615377	0.613736
0.615183	0.613576
0.615016	0.613428
0.614864	0.613289
0.614722	0.613152
0.614591	0.613021
0.614456	0.612885
0.614311	0.612741
0.614162	0.612591
0.614005	0.612435
0.61385	0.612279
0.613737	0.612143
0.613659	0.612024
0.613565	0.611899
0.613449	0.611765
0.613319	0.611625
0.613169	0.611479
0.613009	0.611328
0.612849	0.611165
0.612689	0.610997
0.612544	0.610865
0.612415	0.610768
0.612282	0.610662
0.612131	0.610536

				0.611996	0.610412
				0.611912	0.610303
				0.611845	0.610198
				0.611745	0.610079
				0.611626	0.609953
				0.611504	0.609844
				0.611375	0.609752
				0.611266	0.609666
				0.611195	0.609585
				0.611129	0.609508
0.617527	0.615863	0.859091	0.856875	0.854679	0.85251
0.617424	0.615786	0.858989	0.856761	0.854563	0.852407
0.617323	0.615691	0.858876	0.856654	0.854456	0.852298
0.617226	0.615583	0.858756	0.856551	0.854357	0.852185
0.617133	0.615488	0.858642	0.856441	0.854241	0.852047
0.617047	0.61541	0.858534	0.856318	0.854098	0.85187
0.616959	0.615328	0.858427	0.856202	0.853968	0.85172
0.616867	0.61523	0.858315	0.856103	0.85388	0.851641
0.616774	0.615132	0.858203	0.856007	0.853801	0.851582
0.616686	0.61505	0.858098	0.855897	0.853697	0.851501
0.616601	0.614977	0.857996	0.855781	0.853582	0.85141
0.61651	0.61489	0.857888	0.855674	0.853475	0.851305
0.616413	0.61479	0.857775	0.855575	0.853377	0.851186
0.616332	0.614699	0.857673	0.855471	0.853269	0.85107
0.616276	0.614619	0.857583	0.855353	0.853142	0.850959
0.616224	0.614552	0.857505	0.855249	0.853026	0.85085
0.616168	0.614517	0.85745	0.855187	0.852947	0.85074
0.616105	0.614485	0.857397	0.855137	0.852884	0.850638
0.616018	0.614407	1.051018	1.048258	1.045483	1.042688
0.615915	0.614299	1.050924	1.048201	1.045457	1.042685
0.615839	0.614209	1.050856	1.048129	1.045393	1.042647
0.615791	0.614137	1.050813	1.048033	1.045282	1.042566
0.615739	0.614078	1.050784	1.04797	1.045208	1.042515
0.615671	0.614036	1.05077	1.047963	1.04521	1.042529
0.615605	0.614002	1.050771	1.04798	1.045232	1.042546
0.615557	0.613974	1.050802	1.048012	1.045245	1.042511
0.615514	0.613943	1.050836	1.048047	1.045256	1.042459
0.615448	0.613872	1.050796	1.048029	1.045246	1.042436
0.615365	0.613771	1.050699	1.047971	1.045218	1.042433
		0.872887	0.870612	1.045213	1.042443
		0.872934	0.870659	1.045238	1.042469
		0.872989	0.870711	1.045266	1.042495
		0.873036	0.870761	1.045299	1.042526
		0.873078	0.870799	1.045312	1.042542
		0.873104	0.87079	1.045251	1.042504
		0.873123	0.87076	1.045154	1.042438
		0.873157	0.870773	1.045116	1.042417
		0.873205	0.870825	1.045127	1.042435
		0.873253	0.870876	1.045147	1.042457
		0.873301	0.870923	1.04517	1.04248

0.873344	0.870967	1.045194	1.042502
0.87338	0.871002	1.04521	1.042518
0.873411	0.871033	1.045224	1.042533
0.873442	0.871063	1.045234	1.042543
0.873475	0.871094	1.045245	1.042554
0.873532	0.871174	1.045323	1.042616
0.873619	0.87131	1.045475	1.042736
0.87369	0.871414	1.045592	1.042828
0.873725	0.871452	1.045631	1.042857
0.873752	0.871474	1.045647	1.042869
0.873779	0.8715	1.045662	1.042884
0.873814	0.871533	1.045675	1.042898
0.873908	0.871615	1.045717	1.04292
0.874046	0.871735	1.045781	1.042948
0.874132	0.871813	1.045834	1.043009
0.874148	0.871837	1.045875	1.043114
0.874178	0.871864	1.045909	1.0432
0.874258	0.871909	1.045919	1.043224
0.874352	0.871972	1.045948	1.043247
0.874429	0.872073	1.046058	1.043336
0.874506	0.872197	1.04621	1.043453
0.874615	0.872315	1.046322	1.043526
0.874752	0.872431	1.046404	1.043563
0.874864	0.872516	1.046468	1.043621
0.874935	0.872548	1.046498	1.043711
0.875003	0.872598	1.046556	1.043817
0.875086	0.872716	1.046693	1.043937
0.875179	0.872854	1.046854	1.044066
0.875294	0.872967	1.046971	1.044187
0.875425	0.87307	1.047069	1.044307
0.87555	0.873189	1.04719	1.044425
0.875668	0.873325	1.047337	1.044547
0.875792	0.873455	1.047463	1.04464
0.875929	0.873572	1.047542	1.044672
0.876062	0.873687	1.047632	1.044729
0.876169	0.873811	1.047785	1.044898
0.876268	0.873934	1.047959	1.045109
0.876378	0.874043	1.048087	1.045271
0.876496	0.874141	1.048187	1.045403
0.876608	0.874252	1.048305	1.04553
0.876707	0.874378	1.048449	1.045649

							0.615439	0.613739
							0.615193	0.613492
							0.614913	0.613224
							0.61459	0.612929
							0.859477	0.857241
							0.859096	0.856876
							0.858736	0.856527
							0.858385	0.856182
							0.858044	0.855844
					0.616308	0.614614	0.857702	0.855505
					0.616058	0.614366	0.857359	0.855166
					0.61581	0.61412	0.857019	0.854828
					0.615565	0.613877	0.85668	0.854489
					0.615322	0.613635	0.856341	0.854151
					0.61508	0.613394	0.856002	0.853813
					0.614839	0.613154	0.855664	0.853477
					0.614596	0.61291	0.855323	0.853137
					0.614354	0.612665	0.854984	0.852799
					0.614123	0.61245	0.854689	0.852494
					0.613905	0.612268	0.854443	0.852227
			0.61695	0.615307	0.85863	0.856404	1.039587	1.03683
			0.616737	0.615072	0.858295	0.856083	1.039264	1.036499
			0.616512	0.614833	0.857951	0.855747	1.03892	1.036152
			0.616271	0.614585	0.857605	0.855404	1.038575	1.035809
			0.616026	0.614341	0.857266	0.855062	1.038233	1.035473
			0.615802	0.614138	0.856981	0.854761	1.037934	1.035192
			0.615594	0.613966	0.856739	0.854491	1.037665	1.034953
			0.615372	0.613775	0.856456	0.85419	1.037364	1.034672
			0.615131	0.613559	0.856123	0.853845	1.037012	1.034331
			0.614903	0.613339	0.855792	0.853515	1.036695	1.034012
			0.614713	0.613122	0.85549	0.853237	1.03647	1.033766
			0.614527	0.6129	0.855185	0.85296	1.036251	1.033522
			0.61431	0.612661	0.854841	0.852635	1.035947	1.033207
			0.614081	0.612417	0.854485	0.852291	1.035607	1.032863
			0.61386	0.612209	0.854177	0.851977	1.035305	1.032574
			0.613644	0.612031	0.853912	0.851687	1.035033	1.032331
			0.613445	0.611848	0.853642	0.851408	1.034778	1.03209
			0.613268	0.611653	0.853358	0.85114	1.03455	1.031853
0.617925	0.616312	0.614713	0.85762	0.855327	1.039746	1.037027	1.034314	1.031598
0.61774	0.616122	0.614517	0.857344	0.855058	1.039507	1.03678	1.034049	1.031295
0.617569	0.61594	0.61432	0.857064	0.854789	1.039269	1.03652	1.033765	1.030981
0.617376	0.615753	0.614138	0.856788	0.854505	1.038998	1.036219	1.033451	1.030692
0.617175	0.615564	0.613964	0.856513	0.85421	1.038706	1.035891	1.03312	1.03042
0.616988	0.615375	0.613772	0.856219	0.853895	1.038397	1.035596	1.032844	1.030165
0.616815	0.615186	0.613558	0.855902	0.853554	1.038061	1.035332	1.032626	1.029929
0.616635	0.615001	0.613363	0.855614	0.853259	1.037783	1.035086	1.032401	1.029695
0.616438	0.614823	0.613208	0.855379	0.853043	1.037605	1.034859	1.032143	1.029457

0.616244	0.614645	0.613053	0.85515	0.852844	1.037447	1.034652	1.031901	1.029226
0.616066	0.614463	0.612866	0.854894	0.852624	1.037265	1.034476	1.031718	1.029008
0.615893	0.614278	0.61267	0.85463	0.852396	1.037071	1.034313	1.031559	1.028803
0.615705	0.614095	0.612494	0.854372	0.852149	1.036843	1.034118	1.031385	1.028626
0.615508	0.613913	0.612329	0.854117	0.851885	1.036588	1.033901	1.031202	1.02847
0.615321	0.613731	0.612153	0.853857	0.851628	1.036348	1.033671	1.030987	1.02828
0.615144	0.613548	0.611961	0.853587	0.851379	1.036128	1.033425	1.030731	1.02805
0.614985	0.613379	0.611777	0.85333	0.851131	1.035898	1.033181	1.030481	1.027814
0.614864	0.613239	0.611608	0.853097	0.850875	1.035636	1.032943	1.030259	1.027581
0.61474	0.613104	0.611452	0.852877	0.850629	1.035384	1.03271	1.030039	1.027346
0.614565	0.612951	0.611325	1.040736	1.037944	1.035193	1.032484	1.029788	1.027102
0.614366	0.612789	0.611209	1.040554	1.037793	1.035032	1.032266	1.029533	1.026862
0.614183	0.612617	0.61105	1.040334	1.037605	1.034855	1.032077	1.029337	1.026677
0.614009	0.612433	0.610856	1.04008	1.03739	1.034671	1.031914	1.029191	1.026541
0.613858	0.612268	0.610673	1.039845	1.037158	1.034453	1.031725	1.029019	1.026365
0.613743	0.612131	0.610504	1.03964	1.036899	1.034182	1.031489	1.028804	1.026122
0.613612	0.61199	0.610347	1.039445	1.03666	1.033932	1.031267	1.028595	1.025888
0.613426	0.611824	0.610207	1.039255	1.036475	1.033754	1.0311	1.028432	1.025718
0.613228	0.611653	0.610075	1.039075	1.036314	1.0336	1.030939	1.028263	1.025552
0.61306	0.611501	0.609946	1.03892	1.036163	1.033429	1.03072	1.028016	1.025317
0.612907	0.611357	0.609816	1.038775	1.036018	1.033253	1.030472	1.027726	1.025048
0.612759	0.611198	0.60965	1.038567	1.035829	1.033062	1.030253	1.027493	1.02483
0.612617	0.611023	0.609447	1.038292	1.035592	1.032852	1.03006	1.027314	1.02466
0.61248	0.610859	0.609257	1.038053	1.03538	1.032659	1.029881	1.027144	1.024495
0.612351	0.61071	0.609093	1.037878	1.035213	1.032496	1.029716	1.026979	1.024336
0.612223	0.610571	0.608942	1.037725	1.03506	1.032341	1.029557	1.026815	1.024162
1.045835	1.043055	1.040284	1.037576	1.034906	1.032182	1.029394	1.026637	1.023944
1.045624	1.042877	1.040133	1.037432	1.034755	1.032024	1.029232	1.026457	1.023714
1.045449	1.042715	1.039979	1.037278	1.034596	1.031862	1.029069	1.026286	1.023521
1.045299	1.042563	1.039822	1.037119	1.034434	1.031698	1.028905	1.026121	1.023353
1.045148	1.042409	1.039665	1.036959	1.034273	1.031536	1.028742	1.025958	1.023188
1.044996	1.042255	1.039507	1.036801	1.034115	1.031378	1.028581	1.025796	1.023026
1.044839	1.042095	1.039345	1.036638	1.033953	1.031216	1.02842	1.025637	1.022868
1.044673	1.041928	1.039175	1.036467	1.033782	1.031048	1.02826	1.025482	1.022714
1.044504	1.041758	1.039004	1.036295	1.033609	1.030878	1.028098	1.025327	1.022561
1.044333	1.041589	1.038836	1.036125	1.033437	1.030707	1.027932	1.025164	1.022403
1.044165	1.041422	1.038668	1.035956	1.033266	1.030536	1.027763	1.024999	1.022241
1.044025	1.041267	1.038502	1.035786	1.033096	1.030368	1.027596	1.024834	1.022077
1.043908	1.041124	1.038338	1.035615	1.032927	1.030201	1.027431	1.024669	1.021909
1.043769	1.040968	1.038171	1.035443	1.032758	1.030034	1.027266	1.024511	1.021771
1.043597	1.040789	1.037987	1.035261	1.032582	1.029864	1.0271	1.024364	1.021678
1.043423	1.040627	1.037838	1.035112	1.032427	1.029706	1.026939	1.024216	1.021575
1.043263	1.040512	1.03777	1.035042	1.032324	1.029575	1.026783	1.024048	1.021421
1.043104	1.040404	1.037715	1.034984	1.032228	1.029451	1.026641	1.023894	1.021268
1.042932	1.04025	1.037586	1.034853	1.032081	1.029312	1.026537	1.023812	1.021175
1.04276	1.040078	1.03742	1.034685	1.031908	1.029165	1.026456	1.023769	1.021113
1.04265	1.039961	1.037288	1.034537	1.031742	1.029006	1.026331	1.023666	1.020998
1.042603	1.039899	1.037188	1.034404	1.031579	1.028831	1.026165	1.023507	1.020835
1.04252	1.039803	1.037065	1.034274	1.031451	1.028701	1.026029	1.023363	1.020678
1.042367	1.039643	1.036901	1.034148	1.031387	1.028655	1.025954	1.023254	1.020534

1.04221	1.039475	1.036731	1.034013	1.031311	1.028596	1.025869	1.023143	1.020405
1.042091	1.039328	1.036559	1.033843	1.031158	1.028449	1.025714	1.022995	1.020299
1.04198	1.039191	1.036401	1.033673	1.030985	1.028277	1.025544	1.022846	1.02021
1.041837	1.039067	1.036305	1.033572	1.030859	1.028148	1.025435	1.022757	1.020139
1.041678	1.038957	1.036256	1.033518	1.030763	1.028046	1.025365	1.022707	1.02008
1.041575	1.038873	1.036188	1.033449	1.030682	1.027962	1.025292	1.022635	1.019987
1.041538	1.038819	1.0361	1.033363	1.030618	1.027903	1.02522	1.022542	1.019854
1.041491	1.038763	1.036021	1.033285	1.03055	1.02783	1.025125	1.022429	1.019728
1.041402	1.03869	1.035969	1.033229	1.030475	1.027729	1.024983	1.022274	1.019622
1.041312	1.038615	1.035913	1.033162	1.03038	1.027609	1.024839	1.022132	1.019531
1.041257	1.038552	1.035831	1.033049	1.030228	1.027457	1.024733	1.022061	1.019469
1.041212	1.038486	1.03573	1.032912	1.030054	1.027298	1.024646	1.022022	1.019417
1.041136	1.038379	1.035591	1.032773	1.029937	1.027199	1.024565	1.021945	1.019318
1.041036	1.038237	1.035416	1.032628	1.029861	1.027147	1.024492	1.021843	1.019181
1.040912	1.038106	1.03529	1.03252	1.02978	1.027079	1.02442	1.021766	1.019098
1.040748	1.037988	1.03523	1.032459	1.029688	1.026982	1.024349	1.021726	1.019096
1.040624	1.037893	1.035171	1.032398	1.029602	1.026893	1.024278	1.021675	1.01907
1.040599	1.037836	1.035075	1.032305	1.029534	1.026831	1.024201	1.021579	1.018948
1.040603	1.037799	1.034986	1.032215	1.029472	1.026774	1.024126	1.021482	1.01882
1.040579	1.03778	1.034962	1.032173	1.029404	1.026698	1.024063	1.021431	1.018778
1.04054	1.037768	1.034965	1.032152	1.029333	1.026615	1.024006	1.021403	1.01878
1.040462	1.037684	1.034878	1.032068	1.029259	1.026546	1.023938	1.021343	1.018744
1.040343	1.037531	1.034703	1.031925	1.029181	1.026491	1.02386	1.021253	1.018679
1.040247	1.037424	1.034588	1.031823	1.029104	1.026424	1.023785	1.021175	1.018608
1.040198	1.037399	1.034578	1.031789	1.029023	1.02633	1.023716	1.021122	1.018535
1.040145	1.037376	1.034581	1.031774	1.028962	1.026252	1.023652	1.021064	1.01846
1.040046	1.037304	1.034552	1.031763	1.028956	1.02623	1.023587	1.020971	1.018375
1.039945	1.037221	1.034506	1.031747	1.028966	1.026229	1.023533	1.020883	1.018301
1.039905	1.037157	1.034423	1.031678	1.028932	1.026212	1.023513	1.020862	1.018292
1.039903	1.037105	1.034315	1.031577	1.028875	1.02619	1.023519	1.020887	1.018325
1.039882	1.037069	1.034258	1.03153	1.028856	1.026191	1.023534	1.020903	1.01832
1.039839	1.037048	1.034255	1.031542	1.028881	1.026221	1.023566	1.020916	1.018271
1.039815	1.037043	1.034264	1.031549	1.028875	1.026213	1.023567	1.020913	1.018231
1.039837	1.037069	1.034284	1.031531	1.0288	1.026125	1.023514	1.020891	1.018226
1.039847	1.037082	1.034295	1.031509	1.028726	1.026034	1.023445	1.020849	1.018213
1.039778	1.037031	1.034278	1.031501	1.028715	1.026006	1.02338	1.020765	1.018152
1.039681	1.036953	1.034239	1.03149	1.028726	1.026003	1.023319	1.02067	1.018078
1.039641	1.036893	1.034165	1.031429	1.028696	1.025985	1.023293	1.020642	1.018066
1.039642	1.036845	1.034061	1.03133	1.028638	1.025961	1.023296	1.020666	1.0181
1.039652	1.036824	1.034004	1.031276	1.028611	1.025955	1.023307	1.020682	1.0181
1.039675	1.036836	1.034006	1.03128	1.028625	1.025973	1.023328	1.020687	1.018052
1.039699	1.036857	1.034022	1.031297	1.028645	1.025994	1.02335	1.020696	1.018016
1.039726	1.036879	1.03404	1.031316	1.02867	1.026022	1.023379	1.02072	1.018023
1.039746	1.0369	1.034061	1.031333	1.02868	1.026031	1.023392	1.020738	1.018041
1.039734	1.036909	1.034082	1.03133	1.028629	1.025966	1.023349	1.020718	1.01804
1.039706	1.036912	1.034103	1.031317	1.028549	1.025866	1.023279	1.020682	1.018035
1.039706	1.036926	1.034123	1.031319	1.028519	1.025828	1.023259	1.020681	1.018049
1.039729	1.036947	1.034141	1.031333	1.02853	1.025842	1.023283	1.020712	1.01808
1.039753	1.036969	1.034159	1.03135	1.028546	1.02586	1.023307	1.02074	1.018108
1.039775	1.03699	1.034179	1.031368	1.028563	1.025877	1.023326	1.020759	1.018129

1.039795	1.037009	1.034197	1.031386	1.028581	1.025895	1.023344	1.020777	1.018149
1.039811	1.037025	1.034212	1.031401	1.028596	1.02591	1.02336	1.020796	1.01817
1.039826	1.037039	1.034227	1.031416	1.028611	1.025926	1.023378	1.020816	1.018192
1.039837	1.037052	1.034242	1.031431	1.028625	1.025942	1.023396	1.020839	1.018218
1.039849	1.037066	1.034257	1.031448	1.028644	1.025962	1.023419	1.020865	1.018248
1.039892	1.037096	1.03428	1.031486	1.02871	1.026038	1.023482	1.020914	1.018286
1.039974	1.037146	1.034311	1.031547	1.028833	1.026177	1.023591	1.020989	1.018334
1.040035	1.037185	1.034338	1.031596	1.028927	1.026283	1.023674	1.021048	1.018375
1.040055	1.0372	1.034354	1.03162	1.028964	1.026323	1.023705	1.021071	1.018393
1.040064	1.037211	1.034368	1.031637	1.028982	1.026341	1.02372	1.021088	1.018422
1.040078	1.037223	1.034377	1.031646	1.028995	1.026357	1.023737	1.021119	1.018499
1.040094	1.037241	1.034396	1.031664	1.02901	1.026372	1.023753	1.021154	1.018593
1.04011	1.037288	1.03448	1.031749	1.029074	1.026418	1.023782	1.021181	1.018648
1.040129	1.03736	1.034616	1.031886	1.029175	1.026487	1.023821	1.021203	1.018677
1.040204	1.037459	1.034736	1.031992	1.029244	1.026543	1.023885	1.021272	1.018742
1.040355	1.037599	1.034842	1.03206	1.029269	1.026573	1.023976	1.021403	1.018859
1.040479	1.037709	1.03492	1.032119	1.029316	1.026632	1.024078	1.021527	1.018961
1.040518	1.037743	1.034951	1.032181	1.029428	1.026763	1.024196	1.021622	1.019014
1.040539	1.037757	1.034966	1.032235	1.029545	1.026898	1.024303	1.021698	1.01906
1.040601	1.037794	1.034985	1.032263	1.029601	1.026963	1.024355	1.021748	1.019135
1.040676	1.037841	1.035012	1.032287	1.029633	1.026996	1.024379	1.021781	1.019218
1.040721	1.037901	1.035098	1.032375	1.029711	1.027059	1.02442	1.021813	1.01927
1.04074	1.037974	1.035238	1.032522	1.029827	1.027144	1.024471	1.021841	1.019294
1.040806	1.038071	1.035368	1.032644	1.029917	1.027218	1.024545	1.021913	1.019352
1.040946	1.038209	1.035482	1.032726	1.029959	1.027265	1.024648	1.022047	1.019458
1.041087	1.038332	1.035572	1.032795	1.030015	1.027334	1.024761	1.022186	1.019576
1.04118	1.038389	1.0356	1.032847	1.030124	1.027465	1.024878	1.022291	1.019688
1.041268	1.038442	1.035631	1.032909	1.030253	1.027615	1.024999	1.022393	1.019802
1.041396	1.038579	1.035776	1.033048	1.030374	1.027731	1.025123	1.022519	1.019921
1.041544	1.038763	1.035986	1.03323	1.030497	1.027833	1.025249	1.022658	1.02004
1.041663	1.038884	1.036109	1.033356	1.030624	1.027961	1.025378	1.022777	1.020125
1.041753	1.03894	1.036142	1.033421	1.030756	1.028116	1.025509	1.022873	1.020171
1.041818	1.038996	1.036204	1.033508	1.030883	1.028257	1.02564	1.022986	1.020259
1.04183	1.039059	1.036333	1.033646	1.030999	1.028371	1.025772	1.023139	1.020435
1.041875	1.039146	1.036472	1.033788	1.031115	1.028482	1.025898	1.023283	1.020602
1.042046	1.039287	1.03658	1.033896	1.031242	1.02861	1.026009	1.023375	1.020673
1.042269	1.039461	1.03669	1.034	1.031371	1.02874	1.026114	1.023453	1.020719
1.042455	1.039643	1.036855	1.034143	1.031487	1.028845	1.026225	1.023573	1.020848
1.042623	1.039836	1.03706	1.034314	1.031595	1.028934	1.02634	1.02372	1.021033
1.042762	1.04	1.037244	1.034489	1.031742	1.029063	1.02646	1.023844	1.021187
1.042866	1.040128	1.037409	1.034674	1.031938	1.029239	1.026579	1.023933	1.021304
1.042976	1.040252	1.037553	1.03484	1.032126	1.029421	1.026722	1.024049	1.021424
1.043118	1.04038	1.037665	1.034968	1.032289	1.029603	1.02691	1.024226	1.021557
1.043261	1.040508	1.037773	1.035086	1.032433	1.02977	1.0271	1.024417	1.021702
1.043374	1.04063	1.03791	1.035217	1.032549	1.02989	1.027247	1.024583	1.021874
1.043482	1.040752	1.038058	1.035351	1.032649	1.029985	1.02737	1.024735	1.022056
1.043645	1.040896	1.038182	1.035467	1.032765	1.0301	1.027481	1.024853	1.022202
1.043855	1.041057	1.038285	1.035567	1.032892	1.030228	1.027577	1.024937	1.022317
1.04405	1.041231	1.038428	1.035712	1.033056	1.030391	1.027718	1.025063	1.022441
1.044217	1.041416	1.038627	1.035923	1.033277	1.030612	1.027929	1.025249	1.022576

1.044393	1.041615	1.03884	1.036136	1.033479	1.030808	1.028127	1.025437	1.022724
1.044616	1.041842	1.03906	1.036318	1.033603	1.030914	1.028258	1.025591	1.022893
1.044832	1.042063	1.039275	1.036491	1.033712	1.031005	1.028378	1.025744	1.023075
1.044961	1.042208	1.039449	1.036666	1.033874	1.031166	1.028553	1.025932	1.023274
1.045043	1.042314	1.0396	1.036842	1.034065	1.031366	1.028754	1.026134	1.023481
1.04519	1.042478	1.039789	1.037041	1.034266	1.031555	1.02891	1.026273	1.023637
1.0454	1.042703	1.040018	1.037268	1.034483	1.031735	1.029019	1.026345	1.023737
0.869871	0.867609	0.865352	1.037452	1.034671	1.031907	1.029149	1.026453	1.023869
0.870053	0.867748	0.86544	1.037552	1.034805	1.032063	1.029316	1.026633	1.024065
0.870233	0.867888	0.865537	1.037659	1.034945	1.032228	1.029501	1.026829	1.02426
0.870419	0.868066	0.865704	1.03784	1.035139	1.032427	1.029697	1.027011	1.024401
0.870597	0.868253	0.865899	1.038054	1.035356	1.032641	1.029902	1.027194	1.02453
0.870734	0.868414	0.866083	1.038272	1.03557	1.032849	1.030103	1.027382	1.024696
0.87084	0.86856	0.866267	1.038497	1.035786	1.033056	1.030301	1.027574	1.024885
0.870982	0.868723	0.866451	1.038719	1.036001	1.033262	1.030498	1.027766	1.025077
0.871163	0.868908	0.866638	1.038937	1.036213	1.033468	1.030694	1.027957	1.025273
0.871347	0.869093	0.866824	1.039154	1.036426	1.033675	1.030891	1.028149	1.025468
0.871522	0.869274	0.867009	1.039372	1.036641	1.033883	1.03109	1.028342	1.025661
0.871693	0.869451	0.867192	1.039588	1.036854	1.034091	1.031288	1.028533	1.025852
0.871859	0.869619	0.867365	1.039798	1.037063	1.034295	1.031483	1.02872	1.026036
0.872019	0.869783	0.867532	1.040002	1.03727	1.034498	1.031676	1.028905	1.026216
0.872175	0.869943	0.867698	1.040205	1.037474	1.034699	1.031868	1.029089	1.026395
0.872325	0.870099	0.867861	1.040406	1.037676	1.034898	1.032059	1.029273	1.026572
0.872503	0.87027	0.868028	1.040605	1.037877	1.035097	1.03225	1.029462	1.026777
0.872724	0.870464	0.868198	1.040802	1.038078	1.035296	1.032438	1.029659	1.027023
			0.866098	0.86383	1.035496	1.03263	1.029858	1.027264
			0.866256	0.863989	1.035693	1.032827	1.030057	1.027471
			0.866432	0.864161	1.035897	1.033031	1.030258	1.027666
			0.866676	0.86439	1.036128	1.033239	1.03045	1.027851
			0.866957	0.864648	1.036377	1.033458	1.030646	1.028035
			0.867168	0.864851	1.036614	1.033715	1.03091	1.028277
			0.867313	0.865003	1.036842	1.034013	1.031245	1.028579
			0.867475	0.86516	1.037059	1.034281	1.031539	1.028847
			0.867677	0.86533	1.037248	1.034486	1.031753	1.029044
			0.867883	0.865521	1.037464	1.034699	1.031964	1.029247
			0.868069	0.865753	1.037763	1.034975	1.032223	1.029517
			0.868257	0.866002	1.03809	1.035266	1.032489	1.0298
			0.868476	0.866235	0.863925	0.861531	1.032704	1.030019
			0.868716	0.866458	0.864125	0.861701	1.032888	1.0302
			0.868929	0.866648	0.864309	0.861897	1.033135	1.030432
			0.869112	0.866797	0.864468	0.862118	1.033456	1.030718
			0.607381	0.60576	0.864656	0.862348	1.03377	1.03101
			0.607503	0.605909	0.864906	0.862584	1.034045	1.031293
			0.60763	0.606066	0.865167	0.862825	1.03432	1.031576
			0.607781	0.606224	0.865392	0.863068	1.034629	1.03187
			0.607944	0.606384	0.865609	0.863312	1.034946	1.03216
			0.608103	0.606545	0.865848	0.863556	0.861238	0.858873
			0.608262	0.606708	0.866106	0.863801	0.861458	0.859046
			0.60842	0.606868	0.86634	0.864013	0.861651	0.859228
			0.60858	0.60703	0.866545	0.86418	0.861804	0.859417

0.608735	0.607178	0.866735	0.864356	0.861981	0.859625
0.608875	0.607289	0.866892	0.864567	0.862229	0.859867
0.609018	0.607406	0.867064	0.864796	0.862495	0.860119
0.609186	0.607593	0.867331	0.865036	0.862717	0.86035
0.609368	0.607816	0.86765	0.865289	0.862926	0.860575
0.609536	0.608001	0.867927	0.865562	0.863189	0.860821
0.609691	0.608151	0.868172	0.865859	0.863506	0.861086
0.609847	0.608303	0.868398	0.866129	0.863798	0.861362
0.610005	0.608461	0.868602	0.866354	0.864052	0.861658
0.610166	0.60862	0.868805	0.866568	0.864287	0.861939
		0.60722	0.605648	0.864499	0.862172
		0.607379	0.605808	0.864705	0.862387
		0.607532	0.605967	0.864936	0.862611
		0.607684	0.606127	0.865184	0.862841
		0.607869	0.606315	0.865462	0.863116
		0.608094	0.606536	0.865779	0.863447
		0.608303	0.606739	0.866062	0.863746
		0.608467	0.606898	0.866266	0.863968
		0.60863	0.607058	0.866475	0.864191
		0.608834	0.607265	0.866767	0.86449
		0.609048	0.607487	0.867087	0.86481
		0.609226	0.60767	0.867345	0.865061
		0.609375	0.607827	0.867559	0.86526
		0.609553	0.608009	0.867799	0.865495
		0.609768	0.608225	0.868075	0.865777
		0.609988	0.608443	0.606889	0.605307
		0.610207	0.608659	0.607103	0.605529
		0.610417	0.608864	0.607306	0.605741
				0.607473	0.605911
				0.607623	0.606062
				0.60781	0.606249
				0.608028	0.606468
				0.608246	0.606687
				0.60846	0.606902
				0.608673	0.607117
				0.608887	0.607331
				0.609101	0.607546
				0.609317	0.607763
				0.609533	0.607981
				0.609751	0.6082
				0.609968	0.608419
				0.610188	0.608639
				0.610406	0.608854
				0.610638	0.609082

						0.616668	0.615076
						0.616311	0.614717
						0.615955	0.614359
						0.615598	0.614003
						0.615241	0.613644
						0.614885	0.613283
						0.614529	0.612926
						0.614165	0.612571
						0.613814	0.612227
				0.616874	0.615182	0.613509	0.611909
				0.616538	0.614883	0.613233	0.611608
				0.61619	0.614561	0.858112	0.855878
				0.615831	0.61422	0.85763	0.85539
				0.615471	0.613873	0.857144	0.854917
				0.615105	0.613518	0.856654	0.854473
				0.614758	0.613169	0.856172	0.85403
				0.614458	0.612837	0.855705	0.853569
				0.614176	0.612518	0.855254	0.85311
				0.613877	0.612221	0.854837	0.85268
				0.61357	0.611943	0.854445	0.852271
				0.613276	0.611661	1.035623	1.032898
				0.612999	0.611379	1.035163	1.032453
		0.615964	0.614336	0.85765	0.855414	1.255563	1.252577
		0.615609	0.614009	0.857176	0.854934	1.255138	1.252154
		0.615256	0.613675	0.856695	0.854454	1.254717	1.251743
		0.614948	0.613344	0.85624	0.854023	1.254369	1.251426
		0.614659	0.613017	0.855799	0.853615	1.254056	1.251149
		0.61436	0.612714	0.855376	0.853199	1.25374	1.250826
		0.614055	0.612431	0.854969	0.852781	1.253427	1.250474
		0.613756	0.612139	0.854553	0.852364	1.253136	1.250151
		0.613467	0.611834	0.854123	0.85195	1.252877	1.249867
		0.613174	0.611532	0.853698	0.851536	1.252612	1.249584
		0.612868	0.611242	0.853288	0.851119	1.252323	1.249281
		0.612561	0.610954	0.852881	0.850703	1.252027	1.24898
		0.856882	0.854658	1.034571	1.03185	1.251726	1.248705
		0.856462	0.854231	1.034117	1.031414	1.251426	1.248445
		0.856034	0.853811	1.033688	1.031013	1.251172	1.248192
		0.855597	0.853398	1.033278	1.030648	1.250962	1.247949
0.61434	0.612675	0.855188	0.853009	1.032889	1.030267	1.250719	1.247681
0.614089	0.612399	0.854822	0.85266	1.032533	1.02986	1.250424	1.247378
0.613832	0.612131	0.854456	0.852301	1.032159	1.02944	1.25011	1.247061
0.61354	0.611868	0.854064	0.851893	1.255996	1.252876	1.249775	1.246727
0.613236	0.611601	0.853658	0.851466	1.255704	1.252564	1.249447	1.246401
0.857696	0.855451	0.853235	0.851046	1.255422	1.252307	1.249207	1.246154
0.857272	0.855013	0.8528	0.850629	1.255145	1.252083	1.249023	1.245957
0.856886	0.854617	0.852407	0.850249	1.254883	1.251828	1.248774	1.245707
0.856547	0.854268	0.852058	0.849908	1.254635	1.251537	1.248451	1.245392
0.856186	0.853912	1.034669	1.032014	1.254402	1.251274	1.248167	1.24512
0.855768	0.853527	1.034294	1.03167	1.254201	1.251076	1.247972	1.244943

	0.855351	0.853146	1.033916	1.031306	1.25398	1.250872	1.247783	1.244774
	0.85498	0.852794	1.033537	1.030885	1.253671	1.250586	1.247527	1.244545
	0.854631	0.852455	1.033162	1.030443	1.253334	1.250277	1.247255	1.2443
0.61205	0.854287	0.852119	1.032807	1.030056	1.253074	1.250036	1.247035	1.244096
0.611803	0.85395	0.851786	1.032469	1.029708	1.25287	1.249844	1.246855	1.243921
0.611549	0.853586	0.851413	1.032081	1.029329	1.25264	1.249649	1.246689	1.243762
0.611284	0.853182	0.850986	1.031622	1.028903	1.252372	1.24945	1.246546	1.243631
0.855015	1.264164	1.261121	1.258092	1.255075	1.252116	1.249237	1.246369	1.243466
0.854666	1.263935	1.260876	1.257849	1.254848	1.251891	1.248988	1.246102	1.243213
0.854329	1.263729	1.260658	1.257634	1.25465	1.251688	1.248744	1.245827	1.242949
0.85399	1.263507	1.260441	1.257431	1.254469	1.251515	1.248555	1.245625	1.242753
0.853653	1.263282	1.260232	1.257241	1.254303	1.251362	1.248398	1.245461	1.242592
0.853317	1.263062	1.260019	1.25704	1.254119	1.25119	1.248233	1.245302	1.242443
0.852982	1.262844	1.259802	1.256829	1.253919	1.251004	1.248066	1.245154	1.24231
0.852645	1.262643	1.259608	1.25664	1.253733	1.250824	1.247897	1.244994	1.242156
0.852306	1.262468	1.259449	1.256484	1.25357	1.250653	1.247721	1.244813	1.24197
0.851968	1.262292	1.259288	1.256329	1.253409	1.250488	1.247552	1.244638	1.241783
0.851632	1.262093	1.259099	1.256152	1.253243	1.250331	1.247405	1.244492	1.24162
0.851297	1.26189	1.258905	1.255969	1.253075	1.250175	1.24726	1.244351	1.24146
0.85096	1.26169	1.25872	1.255792	1.2529	1.250002	1.247089	1.244179	1.241282
0.850622	1.261496	1.258541	1.255619	1.252723	1.24982	1.246903	1.243989	1.241094
0.850304	1.261338	1.258377	1.255453	1.252561	1.249656	1.246731	1.243809	1.240906
0.850008	1.261222	1.258225	1.255291	1.25241	1.249508	1.246571	1.243633	1.24071
1.034065	1.261099	1.258096	1.25516	1.252283	1.249382	1.246437	1.24349	1.240555
1.033735	1.260952	1.258002	1.255085	1.252199	1.249293	1.246354	1.243412	1.240479
1.033396	1.260803	1.257908	1.255009	1.252109	1.249195	1.246261	1.243324	1.240396
1.033056	1.260652	1.257781	1.254884	1.251966	1.249038	1.246098	1.243158	1.240226
1.032728	1.260508	1.257646	1.254742	1.251802	1.248857	1.245907	1.24296	1.240023
1.032466	1.260404	1.257538	1.254621	1.251656	1.248692	1.245732	1.242779	1.239837
1.032255	1.26033	1.257447	1.25451	1.25152	1.248537	1.245565	1.242606	1.239661
1.031992	1.260203	1.257308	1.25437	1.251384	1.2484	1.245422	1.242457	1.239511
1.031663	1.260006	1.25711	1.254194	1.251251	1.248294	1.245314	1.242344	1.239399
1.031342	1.259817	1.256909	1.254005	1.251098	1.248162	1.245182	1.242209	1.23927
1.031065	1.259674	1.256722	1.2538	1.250898	1.247966	1.244984	1.242013	1.239085
1.030788	1.259537	1.256548	1.253605	1.250699	1.247766	1.244782	1.241813	1.238898
1.030464	1.259371	1.256398	1.253461	1.250556	1.247621	1.244633	1.241665	1.238762
1.030127	1.259199	1.256268	1.253348	1.250441	1.247504	1.244513	1.241545	1.23865
1.029856	1.027179	1.024524	1.021865	1.019206	1.247388	1.244407	1.241442	1.23853
1.029645	1.02696	1.024284	1.021629	1.018994	1.247278	1.244317	1.24136	1.238406
1.029415	1.026713	1.02401	1.021354	1.018738	1.247122	1.244179	1.241234	1.238269
1.029157	1.026421	1.023679	1.021013	1.018412	1.246892	1.243962	1.24104	1.23811
1.028876	1.026122	1.023366	1.020694	1.018095	1.246685	1.243762	1.240857	1.237968
1.028548	1.025825	1.023123	1.02046	1.017833	1.246569	1.24364	1.240737	1.237874
1.028219	1.025538	1.022902	1.020252	1.017594	1.246482	1.243546	1.24064	1.237797
1.027962	1.025293	1.022659	1.020013	1.017359	1.246365	1.243435	1.240538	1.237705
1.027748	1.025074	1.022409	1.019762	1.01713	1.246237	1.243322	1.240439	1.237608
1.027511	1.024839	1.022171	1.019524	1.016896	1.246127	1.24322	1.240345	1.237519
1.02725	1.02459	1.021944	1.019299	1.016657	1.246036	1.24313	1.240255	1.237439
1.027004	1.024348	1.021713	1.019069	1.016419	1.245936	1.243037	1.240169	1.237357
1.02679	1.024125	1.021469	1.018823	1.016185	1.24581	1.242936	1.240089	1.237269

1.026574	1.023901	1.021228	1.018579	1.015949	1.245692	1.242837	1.240006	1.237183
1.026324	1.023656	1.021004	1.018354	1.015707	1.245612	1.242748	1.239914	1.237106
1.026073	1.02341	1.020785	1.018132	1.015461	1.245543	1.242663	1.239818	1.237031
1.025881	1.023198	1.020547	1.017885	1.015218	1.245449	1.24257	1.239726	1.236947
1.025733	1.023009	1.020298	1.017621	1.014977	1.245337	1.242472	1.239638	1.236858
1.025562	1.022825	1.020087	1.017395	1.014742	1.245238	1.242384	1.239561	1.236779
1.025365	1.02265	1.019924	1.017212	1.014509	1.24515	1.242306	1.239495	1.236715
1.02515	1.022458	1.019752	1.017033	1.014297	1.245086	1.24224	1.239429	1.236647
1.024906	1.022231	1.019554	1.016852	1.014127	1.245067	1.242188	1.239344	1.23655
1.024658	1.021993	1.019337	1.016657	1.013958	1.245049	1.242146	1.239275	1.236472
1.024424	1.021752	1.019085	1.016417	1.013748	1.244981	1.242112	1.239271	1.236479
1.024204	1.021516	1.018822	1.016157	1.013517	1.244891	1.242081	1.239293	1.236522
1.024029	1.021332	1.018621	1.015942	1.013286	1.244818	1.242025	1.239254	1.23649
1.023897	1.021197	1.018474	1.015761	1.013053	1.244756	1.241946	1.239163	1.236397
1.023711	1.021014	1.018297	1.015576	1.012847	1.24472	1.241904	1.239113	1.23634
1.023442	1.02076	1.018079	1.015387	1.012683	1.244726	1.241923	1.239133	1.236351
1.023184	1.020506	1.017843	1.015178	1.01251	1.244706	1.241915	1.239124	1.23633
1.022997	1.020286	1.017586	1.014921	1.012288	1.244602	1.241809	1.239014	1.236209
1.022825	1.020078	1.017332	1.014663	1.012062	1.244481	1.241683	1.238883	1.236064
1.022613	1.019872	1.017129	1.014473	1.011893	1.244421	1.241624	1.238819	1.235974
1.022384	1.019673	1.016958	1.014321	1.011752	1.244389	1.241596	1.238786	1.235909
1.022188	1.019496	1.016797	1.014156	1.011565	1.008982	1.006406	1.238703	1.235826
1.022025	1.019342	1.016648	1.013981	1.011333	1.008722	1.006163	1.238569	1.235723
1.021863	1.019188	1.016499	1.013813	1.011125	1.008495	1.005949	1.238477	1.235648
1.021709	1.019039	1.016353	1.013664	1.010966	1.008332	1.005789	1.238463	1.235618
1.02153	1.018865	1.016191	1.013509	1.010818	1.008186	1.005643	1.23846	1.235599
1.021278	1.018625	1.015984	1.013327	1.010657	1.008036	1.005491	1.23843	1.235575
1.021007	1.018366	1.015765	1.013139	1.010496	1.00789	1.005341	1.238391	1.235549
1.020797	1.018167	1.015589	1.012978	1.010344	1.00774	1.005186	1.349997	1.346638
1.020627	1.018006	1.015441	1.012834	1.010197	1.007589	1.005027	1.349873	1.346513
1.020463	1.01785	1.015297	1.012693	1.010054	1.00744	1.00487	1.34975	1.346388
1.020304	1.0177	1.015157	1.012556	1.009914	1.007295	1.004714	1.349623	1.346261
1.020148	1.017548	1.015011	1.012412	1.00977	1.007148	1.004559	1.147494	1.144274
1.019994	1.017392	1.014852	1.012255	1.009617	1.006997	1.004407	1.147269	1.14405
1.01984	1.017235	1.014689	1.012093	1.00946	1.006843	1.004255	1.147049	1.143832
1.019684	1.017078	1.014531	1.011934	1.009301	1.006686	1.004102	1.146833	1.14362
1.019527	1.016922	1.014375	1.011776	1.009141	1.006527	1.003948	1.14662	1.143412
1.019363	1.01676	1.014214	1.011617	1.008984	1.006371	1.003794	1.146406	1.143201
1.019193	1.016592	1.014051	1.011458	1.008829	1.006218	1.003639	1.146192	1.142988
1.019072	1.016469	1.013916	1.011315	1.008679	1.006064	1.003486	1.145982	1.142779
1.019024	1.016412	1.013824	1.011196	1.008537	1.00591	1.003335	1.145778	1.142573
1.01896	1.01634	1.01372	1.011069	1.008392	1.005756	1.003184	1.145573	1.142367
1.018824	1.016201	1.013569	1.010911	1.008227	1.00559	1.003024	1.145361	1.142156
1.018673	1.016044	1.013406	1.010749	1.008073	1.005441	1.002877	1.145155	1.141947
1.018561	1.015912	1.013255	1.010611	1.007976	1.005365	1.002788	1.144979	1.141751
1.018464	1.015786	1.013107	1.010485	1.007912	1.005328	1.00273	1.144821	1.141568
1.01833	1.01564	1.012953	1.010344	1.007803	1.005235	1.002622	1.144659	1.141416
1.018162	1.015473	1.012789	1.010185	1.007652	1.005088	1.002469	1.144496	1.1413
1.017998	1.015323	1.012663	1.010066	1.007526	1.004954	1.002321	1.144316	1.14115
1.017834	1.015195	1.012594	1.010011	1.007451	1.004852	1.002186	1.144097	1.140934

1.0177	1.015092	1.012537	1.009958	1.007368	1.004744	1.002062	1.143901	1.140727
1.017635	1.015035	1.012467	1.009862	1.007229	1.004595	1.001953	1.143786	1.140592
1.017598	1.014991	1.012388	1.009749	1.007079	1.004443	1.001857	1.143695	1.140478
1.017535	1.014905	1.012267	1.009624	1.006974	1.004354	1.001779	1.143555	1.140324
1.017455	1.014792	1.012119	1.00949	1.006898	1.004306	1.001712	1.143385	1.140148
1.017342	1.014678	1.012015	1.009399	1.006827	1.004235	1.001613	1.143212	1.13999
1.017184	1.014559	1.011961	1.009361	1.006766	1.004144	1.001476	1.143029	1.13985
1.017057	1.014461	1.011903	1.009305	1.006678	1.004031	1.001346	1.142872	1.139717
1.017002	1.01441	1.011833	1.009204	1.00653	1.003873	1.001237	1.142769	1.139597
1.016959	1.014358	1.011745	1.009085	1.00638	1.003725	1.001141	1.142668	1.139471
1.016891	1.014261	1.011609	1.008949	1.006279	1.003645	1.001062	1.142511	1.139304
1.016808	1.014143	1.011456	1.008812	1.006202	1.003596	1.000995	1.142342	1.139132
1.016686	1.014029	1.011364	1.008731	1.006126	1.003524	1.000923	1.142243	1.139037
1.016531	1.013915	1.011316	1.008695	1.006055	1.003437	1.00085	1.142199	1.139005
1.016446	1.013842	1.01126	1.008638	1.005986	1.003362	1.000778	1.142106	1.138917
1.016466	1.013828	1.011188	1.008552	1.005921	1.003304	1.000708	1.141939	1.138744
1.016454	1.013794	1.011114	1.008469	1.00585	1.00324	1.000637	1.141793	1.138596
1.016317	1.013681	1.011042	1.008405	1.005767	1.003152	1.000565	1.141729	1.138544
1.016169	1.01356	1.010969	1.008341	1.005682	1.003061	1.000494	1.14167	1.138499
1.01613	1.013502	1.010883	1.00825	1.005606	1.002992	1.000422	1.141524	1.138353
1.016144	1.013477	1.010797	1.008152	1.005534	1.002933	1.00035	1.141339	1.138162
1.01613	1.013457	1.010758	1.008097	1.005465	1.002857	1.000279	1.141224	1.138047
1.016095	1.013447	1.010765	1.008085	1.005399	1.002766	1.000209	1.141176	1.138003
1.016037	1.013411	1.010753	1.008069	1.005355	1.002706	1.000147	0.997614	0.995085
1.015946	1.013335	1.010708	1.008045	1.005349	1.002695	1.000094	0.997532	0.995025
1.015856	1.013253	1.010644	1.008005	1.005338	1.002689	1.000056	0.997467	0.994966
1.015783	1.013171	1.01055	1.007924	1.00529	1.002666	1.000046	0.997458	0.994927
1.015729	1.013102	1.010457	1.007833	1.005225	1.002628	1.000041	0.997466	0.9949
1.015728	1.013093	1.010429	1.007785	1.005154	1.002554	0.999995	0.997446	0.994885
1.015762	1.013121	1.010443	1.007764	1.005077	1.002454	0.999923	0.997409	0.994879
1.015733	1.013094	1.010426	1.007742	1.005038	1.002404	0.999866	0.997355	0.994847
1.015631	1.013003	1.010377	1.007723	1.005044	1.002408	0.999823	0.997277	0.994787
1.015557	1.012925	1.010313	1.00768	1.005032	1.002404	0.999793	0.997223	0.994732
1.015552	1.012885	1.010224	1.00759	1.004979	1.002381	0.999789	0.99722	0.994693
1.015557	1.012859	1.010148	1.007503	1.004913	1.002339	0.999778	0.997223	0.994661
1.015524	1.012836	1.010124	1.007463	1.00484	1.002255	0.999718	0.997189	0.994635
1.015485	1.012823	1.010131	1.007449	1.004771	1.002161	0.999647	0.997148	0.994617
1.01549	1.012839	1.010151	1.007457	1.004752	1.002131	0.999632	0.997149	0.994625
1.015529	1.012878	1.010186	1.007487	1.004773	1.002151	0.999659	0.997181	0.994653
1.015512	1.012864	1.010185	1.007495	1.004789	1.002164	0.999655	0.997162	0.994636
1.015416	1.012776	1.010131	1.00747	1.004793	1.002166	0.999605	0.997071	0.994555
1.015341	1.012707	1.010091	1.007454	1.004801	1.002172	0.999568	0.996999	0.994492
1.015334	1.012704	1.0101	1.007469	1.004819	1.002187	0.99957	0.99699	0.994487
1.015351	1.012724	1.010124	1.007493	1.00484	1.002205	0.999585	0.997004	0.994501
1.015366	1.012743	1.010142	1.007511	1.004858	1.002222	0.9996	0.997017	0.994512
1.015386	1.012768	1.010161	1.007529	1.004876	1.00224	0.999619	0.997035	0.994527
1.01541	1.012795	1.010186	1.007553	1.0049	1.002264	0.999642	0.997056	0.994544
1.01544	1.012826	1.010219	1.007587	1.004934	1.002296	0.99967	0.997081	0.994564
1.015463	1.012834	1.010207	1.007572	1.00493	1.002302	0.999686	0.997102	0.994582
1.015475	1.012798	1.010111	1.007467	1.004858	1.002264	0.99968	0.997118	0.994599

1.015491	1.012782	1.010055	1.007404	1.004816	1.002244	0.99968	0.997134	0.994616
1.015518	1.012834	1.010135	1.007488	1.004883	1.002292	0.99971	0.99715	0.994634
1.015551	1.012908	1.01026	1.007619	1.004983	1.002361	0.999748	0.997168	0.994655
1.015584	1.012958	1.01033	1.007688	1.005033	1.002397	0.999775	0.997192	0.994684
1.015618	1.012997	1.010373	1.007726	1.005058	1.002415	0.999796	0.997219	0.994719
1.015648	1.013028	1.010407	1.007757	1.00508	1.002433	0.999816	0.997242	0.994749
1.015675	1.013048	1.01043	1.007778	1.005098	1.002449	0.999832	0.997261	0.994773
1.015701	1.013069	1.010451	1.007798	1.005115	1.002465	0.999849	0.99728	0.994797
1.015716	1.013084	1.010468	1.007815	1.005132	1.00248	0.99986	0.99729	0.994811
1.015756	1.013123	1.0105	1.007841	1.005151	1.002498	0.999886	0.997324	0.994849
1.015873	1.013233	1.01058	1.007895	1.005178	1.002525	0.999957	0.997433	0.994957
1.016018	1.013369	1.010677	1.007959	1.005208	1.002556	1.000044	0.997567	0.995088
1.0161	1.013447	1.010735	1.007999	1.00523	1.002578	1.00009	0.997635	0.995155
1.016139	1.013484	1.010765	1.008024	1.005249	1.002594	1.000109	0.997657	0.995177
1.016197	1.013528	1.010795	1.008058	1.005305	1.00266	1.000166	0.997703	0.995212
1.016283	1.013582	1.010819	1.0081	1.005407	1.00279	1.000274	0.997781	0.995263
1.016354	1.013639	1.010872	1.008171	1.005517	1.002914	1.000372	0.99785	0.995321
1.016381	1.013695	1.010982	1.008298	1.005633	1.003006	1.000422	0.997876	0.995382
1.016412	1.013763	1.011108	1.008433	1.005739	1.003084	1.00047	0.997913	0.995457
1.016512	1.013873	1.011217	1.008527	1.005803	1.00314	1.000555	0.998025	0.995574
1.016642	1.013999	1.011316	1.0086	1.005847	1.003189	1.000661	0.998174	0.99571
1.016715	1.014068	1.01137	1.008661	1.00593	1.003291	1.000779	0.998296	0.995803
1.016741	1.014084	1.011381	1.008706	1.006045	1.00344	1.000912	0.998397	0.995861
1.016787	1.01413	1.011435	1.008783	1.006161	1.003569	1.001015	0.998472	0.995922
1.016861	1.014219	1.011556	1.008912	1.00628	1.003667	1.00107	0.998503	0.995986
1.016954	1.014322	1.011682	1.009035	1.00638	1.003746	1.001127	0.998554	0.996068
1.017073	1.014438	1.011787	1.009117	1.006427	1.003787	1.001213	0.998677	0.996194
1.017199	1.014552	1.011877	1.009183	1.006465	1.003831	1.001313	0.998823	0.996327
1.017304	1.01463	1.011926	1.009238	1.006557	1.003946	1.001434	1.158937	1.156036
1.017394	1.014685	1.011951	1.009287	1.00668	1.004104	1.001569	1.159016	1.156062
1.01745	1.01474	1.01202	1.009376	1.006795	1.004234	1.001697	1.15911	1.156151
1.017462	1.014787	1.012134	1.009507	1.006905	1.004337	1.001818	1.159222	1.15631
1.017532	1.014873	1.01225	1.009631	1.007019	1.004449	1.001938	1.159324	1.15643
1.017719	1.015029	1.012357	1.00973	1.007144	1.004584	1.00206	1.159404	1.156465
1.017898	1.015181	1.012466	1.009831	1.007266	1.004715	1.00218	1.159481	1.156504
1.017957	1.01526	1.012579	1.009952	1.007374	1.004818	1.002293	1.159571	1.156626
1.017987	1.015323	1.012696	1.01008	1.007477	1.004913	1.002402	1.15966	1.156766
1.018121	1.01545	1.01281	1.010191	1.007593	1.005028	1.00251	1.159732	1.156828
1.018327	1.015622	1.012924	1.010291	1.007715	1.005156	1.002616	1.159791	1.156838
1.018509	1.015796	1.013073	1.010423	1.007834	1.005266	1.002721	1.159864	1.156906
1.018665	1.015975	1.013265	1.010591	1.007944	1.005348	1.002821	1.159956	1.157049
1.018801	1.016133	1.013446	1.010762	1.008076	1.005457	1.002931	1.160042	1.157178
1.018896	1.016243	1.013594	1.01093	1.008253	1.005623	1.003051	1.160101	1.157249
1.018997	1.016348	1.013726	1.011086	1.008435	1.005803	1.003187	1.160172	1.157316
1.019164	1.016491	1.01384	1.011203	1.008583	1.00597	1.003354	1.160312	1.157421
1.019361	1.016654	1.013948	1.011304	1.008713	1.006127	1.003535	1.160482	1.157545
1.019533	1.016825	1.014104	1.011444	1.008835	1.006245	1.003675	1.160611	1.157679
1.019687	1.017007	1.014303	1.011618	1.008945	1.006327	1.003781	1.160709	1.157825
1.019819	1.017159	1.014479	1.011789	1.009085	1.006446	1.003894	1.160791	1.15794
1.019911	1.017264	1.014624	1.011958	1.009268	1.006619	1.004018	1.160847	1.158004

1.020024	1.017377	1.014755	1.012109	1.009446	1.006797	1.004156	1.160928	1.158088
1.020194	1.01752	1.014861	1.012214	1.00958	1.006953	1.004316	1.161076	1.158244
1.020391	1.017684	1.014973	1.012319	1.009714	1.007112	1.004492	1.16125	1.158418
1.020596	1.017878	1.015148	1.012494	1.009905	1.00731	1.004688	1.161424	1.158552
1.020807	1.018092	1.015363	1.012711	1.010123	1.007524	1.004894	1.161599	1.158666
1.020988	1.01829	1.015575	1.012912	1.010292	1.007675	1.005047	1.161735	1.15879
1.021135	1.018474	1.015786	1.0131	1.01041	1.007756	1.005145	1.161831	1.15892
1.0213	1.018666	1.016	1.013296	1.010555	1.007874	1.005274	1.161952	1.159068
1.021511	1.018887	1.016224	1.013512	1.010751	1.008058	1.005459	1.162122	1.159242
1.021703	1.019085	1.016432	1.013723	1.01096	1.008264	1.00566	1.162303	1.159421
1.021812	1.019207	1.016589	1.013905	1.011162	1.008474	1.00586	1.162475	1.159592
1.021897	1.019307	1.016732	1.014082	1.011368	1.008691	1.006065	1.162649	1.159763
1.022047	1.01947	1.016924	1.01429	1.011583	1.008906	1.006269	1.16283	1.159939
1.022239	1.019676	1.017149	1.014519	1.011805	1.009121	1.006475	1.163016	1.160119
1.022435	1.019884	1.017371	1.014745	1.012025	1.009334	1.006683	1.163202	1.160298
1.022638	1.020095	1.01759	1.014965	1.012242	1.009547	1.006891	1.16339	1.160478
1.02284	1.020305	1.017807	1.015185	1.01246	1.009762	1.007102	1.163578	1.160659
1.023039	1.020513	1.018027	1.01541	1.012684	1.009982	1.007315	1.163768	1.160842
1.023233	1.020715	1.018239	1.015627	1.012902	1.010199	1.007528	1.16396	1.161028
1.023416	1.020901	1.018431	1.015824	1.013103	1.010403	1.007734	1.164155	1.161218
1.023593	1.021078	1.01861	1.016007	1.013294	1.0106	1.007937	1.164352	1.161409
1.023768	1.021252	1.018786	1.016189	1.013484	1.010798	1.008139	1.164547	1.161599
1.02394	1.021422	1.018958	1.016366	1.013673	1.010994	1.008341	1.164741	1.161787
1.02416	1.021638	1.01916	1.016562	1.013866	1.011189	1.008542	1.164935	1.161974
1.024452	1.02192	1.019407	1.016784	1.014068	1.011381	1.008741	1.165129	1.162159
1.024728	1.022187	1.019643	1.016999	1.014268	1.011575	1.008941	1.165322	1.162344
1.02494	1.022391	1.019832	1.017183	1.014452	1.011762	1.009134	1.165505	1.162527
1.025128	1.02257	1.020005	1.017361	1.014647	1.011967	1.00934	1.165698	1.162716
1.025311	1.02275	1.020185	1.017567	1.014902	1.012246	1.009605	1.165939	1.162929
1.025491	1.022926	1.020366	1.017786	1.015189	1.012565	1.009901	1.166208	1.163158
1.025705	1.02312	1.020546	1.017981	1.015425	1.012821	1.010145	1.166439	1.163393
1.025956	1.023332	1.020724	1.018151	1.015611	1.013017	1.010337	1.166629	1.163635
1.026188	1.02355	1.020939	1.018362	1.01582	1.013231	1.010561	1.166861	1.163898
1.026374	1.023771	1.02121	1.018644	1.01608	1.013487	1.010851	1.167176	1.164207
1.026578	1.024007	1.021489	1.018927	1.016334	1.013734	1.011129	1.167473	1.164491
1.026859	1.024283	1.021752	1.019166	1.016539	1.013924	1.011333	1.167672	1.164684
1.027158	1.024565	1.022	1.019387	1.016735	1.014107	1.01152	1.167839	1.164848
1.027387	1.024783	1.022204	1.0196	1.016979	1.014365	1.011764	1.16806	1.165077
1.027572	1.02496	1.022377	1.019807	1.017255	1.014672	1.012042	1.168309	1.165341
1.027786	1.025175	1.022601	1.02005	1.017527	1.014949	1.012288	1.168518	1.165555
1.028032	1.025431	1.022886	1.020338	1.017799	1.015197	1.012495	1.168674	1.165705
1.028296	1.025698	1.023166	1.020611	1.018048	1.015426	1.012715	1.168867	1.165891
1.028586	1.025978	1.023427	1.020845	1.018245	1.015621	1.012961	1.169141	1.166157
1.028876	1.026251	1.02367	1.021064	1.018445	1.015829	1.013222	1.169432	1.166443
1.029145	1.026484	1.023868	1.02127	1.018696	1.016107	1.013498	1.169691	1.166709
1.029398	1.026699	1.024051	1.021475	1.018971	1.016417	1.013783	1.169943	1.166973
0.856523	1.026915	1.024286	1.021726	1.019238	1.016693	1.014063	1.170211	1.167243
0.856663	1.027129	1.024563	1.022015	1.019501	1.016948	1.014342	1.170491	1.167518
0.856841	1.027371	1.024831	1.022291	1.019768	1.017214	1.014616	1.17076	1.167788
0.857071	1.027649	1.025084	1.022545	1.020044	1.017498	1.014886	1.171013	1.16805

0.857308	1.027934	1.025345	1.022804	1.020317	1.017777	1.015157	1.171271	1.168314
0.857534	1.02821	1.025631	1.023084	1.020578	1.018033	1.015435	1.17155	1.168587
0.857763	1.028489	1.02592	1.023365	1.020837	1.018287	1.015711	1.171831	1.168861
0.858006	1.028777	1.026187	1.023626	1.021104	1.018557	1.015977	1.172095	1.169127
0.858254	1.029069	1.026444	1.023878	1.021373	1.018832	1.016237	1.172351	1.16939
0.858482	0.856213	0.853999	1.024141	1.021632	1.019091	1.016502	1.172622	1.169661
0.858689	0.856417	0.854221	1.024414	1.02188	1.019333	1.016772	1.172906	1.169938
0.858938	0.856651	0.854443	1.024679	1.022134	1.019586	1.017038	1.173179	1.17021
0.85926	0.856932	0.854655	1.024918	1.02239	1.01985	1.017289	1.173424	1.170469
0.859575	0.857219	0.854886	1.02518	1.02267	1.020137	1.017562	1.173692	1.170739
0.859833	0.857491	0.855169	1.025515	1.02301	1.020478	1.017901	1.174036	1.171039
0.860065	0.857753	0.855463	1.02587	1.023362	1.020828	1.018256	1.174401	1.171355
0.860283	0.857981	0.855707	1.026178	1.023647	1.021107	1.018558	1.174721	1.171683
0.86049	0.858188	0.85592	1.026456	1.023889	1.021342	1.018828	1.175014	1.172021
0.860754	0.85843	0.856135	0.85392	0.851778	1.021595	1.019091	1.175286	1.172328
0.861084	0.858711	0.856347	0.85411	0.851983	1.021861	1.019339	1.175528	1.172595
0.861393	0.858991	0.856585	0.854332	0.852213	1.022155	1.019618	1.175805	1.172889
0.861638	0.859254	0.856862	0.854615	0.85249	1.022503	1.019959	1.176153	1.173249
0.861883	0.859524	0.857156	0.85491	0.852767	1.022841	1.0203	1.176505	1.173602
0.862187	0.859829	0.857458	0.855184	0.852992	1.023095	1.020574	1.176792	1.173879
0.862503	0.860141	0.857759	0.855451	0.853201	1.023323	1.020831	1.177061	1.17413
0.862746	0.86039	0.85802	0.855712	0.853455	0.85129	0.849251	1.177374	1.174435
0.862931	0.86059	0.858247	0.855969	0.853743	0.851592	0.849543	1.17772	1.174779
0.863162	0.860833	0.858509	0.856247	0.854037	0.851891	0.849831	1.178065	1.175127
0.863452	0.861134	0.858822	0.856561	0.854344	0.85219	0.85012	1.178416	1.175486
0.603716	0.861431	0.859113	0.856847	0.854626	0.852466	0.850384	1.178731	1.175813
0.603941	0.861697	0.859335	0.857056	0.854848	0.852689	0.850589	1.178963	1.176065
0.604156	0.861954	0.85955	0.85726	0.855064	0.852908	0.85079	1.179189	1.176311
0.604334	0.862207	0.859821	0.857538	0.855339	0.853176	0.85105	1.179495	1.176627
0.604493	0.862458	0.860121	0.857854	0.855643	0.853471	0.85134	1.179845	1.17698
0.604685	0.86273	0.860417	0.858157	0.855939	0.85376	0.851624	1.042099	1.039604
0.604905	0.863021	0.860714	0.858455	0.856232	0.854048	0.851906	1.042386	1.039885
0.605125	0.863312	0.86101	0.858751	0.856524	0.854335	0.852188	1.042671	1.040169
0.605342	0.863599	0.861304	0.859045	0.856814	0.854621	0.852469	1.042952	1.040454
0.605558	0.863884	0.861595	0.859338	0.857104	0.854907	0.85275	1.043234	1.040739
0.605775	0.864164	0.861882	0.859627	0.857392	0.855193	0.853033	1.043522	1.041026
0.605992	0.864442	0.862167	0.859915	0.85768	0.855479	0.853316	1.043804	1.041306
0.60621	0.864724	0.862455	0.860205	0.85797	0.855767	0.853596	1.04406	1.04156
0.60643	0.865009	0.862742	0.860495	0.858262	0.856056	0.853876	1.044298	1.041796
0.606651	0.865292	0.863027	0.860783	0.858553	0.856347	0.85416	1.04455	1.042041
0.606872	0.865573	0.863309	0.861069	0.858846	0.85664	0.854445	1.04481	1.04229
0.607093	0.865854	0.863593	0.861355	0.859135	0.856935	0.85475	0.852593	0.850482
0.607306	0.866127	0.863869	0.861632	0.859412	0.857227	0.855089	0.852965	0.85084
0.60753	0.866418	0.864167	0.861931	0.859706	0.857532	0.855429	0.853331	0.851192
	0.606226	0.604657	0.862295	0.860059	0.85787	0.855747	0.853631	0.851481
	0.606503	0.60494	0.862679	0.860432	0.858219	0.856053	0.853901	0.851743
	0.606743	0.605185	0.862998	0.860748	0.858527	0.856345	0.854178	0.852015
	0.606957	0.605401	0.863271	0.861026	0.858807	0.856625	0.854456	0.852289
	0.607173	0.605616	0.863554	0.861337	0.859133	0.856943	0.854766	0.8526
	0.607387	0.605827	0.863851	0.861696	0.859522	0.857315	0.855118	0.85296

0.60762	0.606057	0.864157	0.86204	0.859885	0.857666	0.855457	0.853303
0.607895	0.60633	0.864481	0.862341	0.860172	0.857952	0.855743	0.853584
0.608173	0.606608	0.864802	0.862625	0.860438	0.858229	0.856032	0.853866
0.608403	0.606843	0.865087	0.862897	0.860719	0.858554	0.85639	0.854217
0.608614	0.607063	0.86536	0.863166	0.861007	0.858897	0.856777	0.854596
0.60886	0.607315	0.605747	0.604162	0.861324	0.859218	0.857101	0.854915
0.609136	0.607595	0.606032	0.604452	0.861667	0.85952	0.857371	0.855181
		0.606312	0.604739	0.862026	0.859847	0.857671	0.855482
		0.606585	0.605021	0.862413	0.860212	0.858018	0.855837
		0.606843	0.605285	0.862779	0.860576	0.85838	0.856205
		0.607066	0.605505	0.863079	0.860921	0.858759	0.856576
		0.607284	0.605717	0.863362	0.861254	0.859132	0.856942
		0.607539	0.605976	0.863685	0.861565	0.859441	0.857276
		0.607817	0.606261	0.864031	0.861863	0.859712	0.857588
		0.60809	0.606542	0.604973	0.60337	0.860024	0.857902
		0.60836	0.606819	0.605255	0.603661	0.860376	0.858213
				0.605536	0.60395	0.86074	0.858546
				0.605814	0.604232	0.861118	0.858916
				0.606091	0.604513	0.861493	0.859293
				0.606369	0.604794	0.861847	0.859651
				0.606647	0.605075	0.862193	0.860007
				0.606918	0.605349	0.862545	0.860384
				0.607185	0.605619	0.862899	0.860775
				0.607455	0.605891	0.604323	0.602764
				0.607725	0.606164	0.604592	0.603009
				0.608012	0.606454	0.604881	0.60328
				0.608333	0.606784	0.605216	0.603619
				0.608653	0.607114	0.605554	0.603966
						0.605849	0.604265
						0.606122	0.604541
						0.606398	0.604821
						0.606673	0.605099
						0.606949	0.605389
						0.607224	0.605693
						0.607512	0.605999
						0.607829	0.606298
						0.608157	0.6066
						0.608479	0.606922
						0.608797	0.607254

							0.858161
							0.857679
							0.85714
							0.85653
				0.615601	0.613979	0.612363	1.035478
				0.615197	0.613584	0.611985	1.034843
				0.614789	0.613198	0.611625	1.03424
				0.614411	0.612839	0.611283	1.033665
				0.614049	0.612495	0.610952	1.033107
		0.616836	0.61526	0.859793	0.857683	0.855567	1.2453
		0.616471	0.614913	0.85931	0.857198	0.855066	1.244782
		0.616117	0.614567	0.858816	0.856686	0.854529	1.244241
		0.615777	0.614223	0.858312	0.856139	0.853941	1.243668
		0.615431	0.613864	0.857782	0.855577	0.853354	1.243103
		0.61507	0.613469	0.857203	0.854999	0.85279	1.242566
		0.614694	0.613061	0.85661	0.854422	0.852243	1.242053
		0.614291	0.612662	1.250498	1.247489	1.244511	1.241581
		0.613872	0.612268	1.249938	1.246985	1.244059	1.241138
		0.613475	0.611894	1.249431	1.246507	1.243602	1.240667
		0.613098	0.611541	1.248979	1.246057	1.243141	1.24017
0.616044	0.614353	0.858262	0.856111	1.248531	1.245614	1.242694	1.239696
0.615694	0.614002	0.857761	0.85561	1.248076	1.245176	1.24227	1.239269
0.615346	0.613653	0.857262	0.85511	1.247627	1.244739	1.241843	1.238842
0.615	0.613301	0.856758	0.854609	1.247193	1.244296	1.241389	1.238386
0.614647	0.612946	0.856251	0.854107	1.246763	1.24385	1.240924	1.237918
0.614255	0.612582	1.252181	1.249245	1.246317	1.243399	1.240462	1.237455
0.613835	0.61221	1.251664	1.248765	1.245862	1.242945	1.240001	1.236993
0.613462	0.611856	1.251215	1.248316	1.245412	1.242493	1.239548	1.236539
0.613143	0.611521	1.250848	1.247903	1.244966	1.242044	1.239103	1.236093
0.612829	0.611188	1.250466	1.247484	1.244522	1.241596	1.238661	1.235652
0.612509	0.610856	1.250032	1.247044	1.244077	1.241152	1.238219	1.235216

	0.612182	0.610522	1.249586	1.246598	1.243632	1.240709	1.23778	1.234783
0.613475	0.611839	0.610183	1.249148	1.246155	1.243187	1.240267	1.237344	1.234352
0.613115	0.611485	0.609841	1.248712	1.245713	1.242742	1.239827	1.236912	1.233924
0.612758	1.036712	1.033942	1.248276	1.245273	1.2423	1.239389	1.23648	1.233502
0.612402	1.036156	1.033392	1.247841	1.244835	1.241862	1.238953	1.236049	1.233085
0.612047	1.0356	1.032842	1.247412	1.2444	1.241424	1.238521	1.235627	1.232672
0.611692	1.035038	1.032283	1.246984	1.243957	1.240976	1.238092	1.235218	1.232263
0.611344	1.252559	1.249584	1.246585	1.243548	1.24056	1.237676	1.234806	1.231857
0.61101	1.25215	1.249225	1.246252	1.243221	1.24022	1.23729	1.234382	1.231458
0.61068	1.251758	1.248876	1.245929	1.242905	1.239892	1.236908	1.233954	1.231058
0.610345	1.251387	1.248498	1.245543	1.24251	1.239486	1.236485	1.233521	1.23064
0.610009	1.251029	1.248104	1.245123	1.242076	1.239046	1.236048	1.233091	1.230216
0.853651	1.250643	1.247684	1.244698	1.241675	1.238665	1.235673	1.232714	1.229827
0.853161	1.250227	1.247236	1.24426	1.241303	1.238342	1.235358	1.232389	1.229471
0.852705	1.249845	1.246829	1.243853	1.240924	1.237991	1.235029	1.232073	1.229141
0.852307	1.249524	1.24649	1.243489	1.240521	1.237577	1.234667	1.231769	1.228851
0.851907	1.249193	1.24616	1.243144	1.240136	1.237176	1.234301	1.231448	1.228549
0.851463	1.248798	1.245808	1.242813	1.239805	1.236836	1.23394	1.231069	1.228177
0.851006	1.248384	1.245444	1.242483	1.239494	1.236524	1.233589	1.230678	1.227791
0.850561	1.247998	1.245051	1.242103	1.239154	1.236213	1.233278	1.230359	1.227471
0.850125	1.247629	1.244638	1.241692	1.2388	1.235907	1.232993	1.230082	1.227189
1.030187	1.24728	1.24427	1.241323	1.238447	1.235569	1.232658	1.229746	1.226849
1.029761	1.246957	1.243953	1.240996	1.238093	1.23519	1.232263	1.229341	1.22644
1.249603	1.246622	1.243641	1.240685	1.237757	1.23483	1.23189	1.228962	1.226061
1.249191	1.246247	1.243321	1.24039	1.237452	1.234515	1.231572	1.228644	1.22575
1.248797	1.245888	1.243007	1.240097	1.237153	1.234211	1.231272	1.228348	1.22546
1.248514	1.245608	1.242711	1.239789	1.236838	1.233895	1.23097	1.228059	1.225168
1.248271	1.245355	1.242419	1.239475	1.23652	1.233581	1.230672	1.227778	1.224881
1.247941	1.245027	1.2421	1.239165	1.236217	1.233283	1.230376	1.227483	1.224593
1.247547	1.244644	1.241761	1.238856	1.235926	1.233	1.230082	1.227178	1.224304
1.247187	1.244275	1.241404	1.238517	1.235616	1.232712	1.2298	1.226897	1.224024
1.24687	1.243922	1.241013	1.238129	1.235275	1.232415	1.229535	1.226648	1.223756
1.246563	1.243593	1.240663	1.237782	1.234959	1.232128	1.229267	1.22639	1.223484
1.246246	1.243296	1.240407	1.237543	1.234712	1.231868	1.228988	1.226094	1.223193
1.245946	1.243022	1.240177	1.237327	1.234477	1.231609	1.228704	1.225793	1.222901
1.245703	1.242777	1.239907	1.237042	1.234186	1.231317	1.228419	1.225516	1.222622
1.24549	1.242548	1.239622	1.236728	1.23387	1.23101	1.228133	1.225249	1.22235
1.245235	1.242282	1.23934	1.236435	1.233574	1.230716	1.227846	1.224972	1.22208
1.244945	1.241985	1.239057	1.236159	1.233295	1.230433	1.227559	1.224686	1.221813
1.244649	1.241685	1.238771	1.235879	1.233013	1.230149	1.227274	1.224403	1.221543
1.244349	1.241388	1.238478	1.235588	1.232721	1.229858	1.226987	1.22412	1.221259
1.244044	1.241089	1.238185	1.235299	1.232434	1.229573	1.226708	1.223848	1.220991
1.243721	1.240778	1.237888	1.23501	1.232148	1.229293	1.226438	1.223593	1.220763
1.243405	1.240475	1.237603	1.234737	1.231881	1.229033	1.226185	1.223353	1.220552
1.243146	1.240226	1.237376	1.234527	1.231684	1.22884	1.225985	1.223141	1.220337
1.242925	1.240012	1.237187	1.234359	1.231533	1.228693	1.225819	1.222949	1.220119
1.242671	1.239764	1.236951	1.234135	1.231321	1.228485	1.225601	1.222715	1.219871
1.242378	1.239474	1.236657	1.233843	1.231039	1.228209	1.225322	1.222432	1.219585
1.242129	1.239233	1.236414	1.233598	1.230793	1.227962	1.22507	1.222178	1.219333
1.24197	1.239088	1.236278	1.233459	1.230635	1.227788	1.224886	1.22199	1.219155

1.241821	1.23895	1.236145	1.233319	1.230475	1.22761	1.224702	1.221804	1.218978
1.241614	1.238742	1.235921	1.233083	1.23023	1.227362	1.224458	1.221565	1.218731
1.24139	1.238514	1.235669	1.232817	1.229959	1.22709	1.224196	1.221311	1.218465
1.241197	1.238324	1.235476	1.232615	1.22974	1.226861	1.223973	1.221096	1.218247
1.241026	1.238162	1.235324	1.232458	1.229558	1.226663	1.223779	1.22091	1.218063
1.240866	1.238003	1.235167	1.232298	1.22939	1.226487	1.223596	1.220724	1.21788
1.240727	1.237855	1.235007	1.232137	1.229244	1.226346	1.223436	1.220543	1.217702
1.240561	1.237685	1.234826	1.231953	1.229066	1.226169	1.223243	1.220337	1.217503
1.24033	1.237461	1.2346	1.231717	1.228809	1.225898	1.222971	1.220071	1.217251
1.240092	1.23723	1.234367	1.231473	1.228542	1.225615	1.222693	1.219805	1.217
1.239906	1.237041	1.234169	1.231268	1.228334	1.225407	1.222491	1.21961	1.36878
1.239748	1.236876	1.233989	1.231084	1.228154	1.225235	1.222326	1.21945	1.368486
1.239605	1.236725	1.233819	1.230901	1.227965	1.225043	1.222138	1.219269	1.368173
1.239481	1.236591	1.233662	1.230723	1.227769	1.224836	1.221933	1.219071	1.367845
1.23933	1.236435	1.233494	1.230546	1.227583	1.224645	1.221741	1.218881	1.367523
1.239139	1.236246	1.233312	1.23037	1.227414	1.224479	1.221568	1.2187	1.367205
1.238942	1.236048	1.233121	1.230187	1.227242	1.224313	1.221399	1.218527	1.366897
1.238755	1.235848	1.232915	1.229983	1.227049	1.224131	1.221224	1.218361	1.366606
1.23857	1.235648	1.232707	1.229776	1.226853	1.223948	1.221054	1.218204	1.366325
1.238385	1.235462	1.232523	1.229595	1.226676	1.22378	1.220901	1.218065	1.366047
1.238201	1.235284	1.232352	1.229429	1.226511	1.22362	1.22076	1.21794	1.365772
1.238007	1.235094	1.232173	1.229256	1.226337	1.223449	1.220604	1.217797	1.36549
1.237795	1.234885	1.23198	1.22907	1.226149	1.223262	1.220428	1.217634	1.3652
1.237629	1.23472	1.231825	1.228922	1.226006	1.22312	1.220281	1.21748	1.364918
1.237553	1.234642	1.231743	1.228846	1.225949	1.223065	1.220183	1.217334	1.36464
1.237472	1.234559	1.231654	1.228763	1.225887	1.223007	1.220096	1.217211	1.364389
1.237302	1.23439	1.231489	1.228604	1.225736	1.222872	1.219992	1.217137	1.364212
1.237096	1.234187	1.231294	1.228416	1.225551	1.222707	1.219882	1.217082	1.364058
1.23691	1.234005	1.231119	1.228248	1.225388	1.222555	1.219759	1.216985	1.459046
1.236733	1.233832	1.230956	1.228091	1.225236	1.222409	1.219626	1.216859	1.45867
1.236586	1.233692	1.230825	1.227968	1.225116	1.222294	1.21952	1.216756	1.458322
1.236477	1.233593	1.230741	1.227891	1.225042	1.22222	1.219452	1.216687	1.458008
1.236355	1.23348	1.230637	1.227795	1.22495	1.222132	1.219365	1.216597	1.457703
1.236185	1.233314	1.23047	1.227634	1.224804	1.221995	1.219221	1.216451	1.457401
1.236013	1.233146	1.230299	1.227468	1.224655	1.221855	1.219073	1.2163	1.457106
1.235892	1.23303	1.230184	1.227358	1.224554	1.221761	1.218973	1.216197	1.456824
1.23579	1.232934	1.230092	1.22727	1.224472	1.221681	1.218893	1.216113	1.456545
1.235648	1.232793	1.229963	1.227147	1.224348	1.221558	1.218772	1.215995	1.456227
1.235474	1.232612	1.229803	1.226995	1.224189	1.221395	1.218617	1.215848	1.455874
1.235322	1.232465	1.229674	1.226872	1.224058	1.332893	1.329649	1.326418	1.455558
1.235201	1.232368	1.229591	1.226792	1.223967	1.332697	1.32945	1.326214	1.455293
1.235103	1.232291	1.229518	1.226721	1.223894	1.332517	1.329269	1.326032	1.455048
1.235039	1.232228	1.22944	1.226645	1.223842	1.332347	1.329112	1.325886	1.454821
1.234985	1.232171	1.229364	1.226571	1.223794	1.332186	1.328962	1.325745	1.454595
1.234903	1.232093	1.229286	1.226495	1.223724	1.33203	1.328795	1.32556	1.454333
1.234806	1.232003	1.229208	1.22642	1.223642	1.331878	1.328619	1.325348	1.454049
1.234722	1.231927	1.229139	1.226352	1.223568	1.331702	1.328426	1.325137	1.453765
1.234653	1.231864	1.229081	1.226294	1.223507	1.331503	1.328216	1.324924	1.453477
1.234574	1.231792	1.229015	1.337902	1.334585	1.331281	1.327997	1.324719	1.453205
1.234472	1.231696	1.228936	1.337669	1.334321	1.331008	1.327762	1.324528	1.452963

1.234377	1.231604	1.228853	1.337443	1.334079	1.330765	1.327543	1.324335	1.452718
1.234318	1.23154	1.22877	1.337294	1.333958	1.330647	1.327378	1.324123	1.45244
1.234269	1.231483	1.228685	1.337165	1.333869	1.330566	1.327232	1.32391	1.452155
1.234192	1.231403	1.228596	1.336969	1.333673	1.330373	1.327049	1.323734	1.451901
1.234097	1.231308	1.228504	1.33673	1.333406	1.330107	1.326842	1.323585	1.451666
1.234014	1.231226	1.228423	1.336547	1.333212	1.329907	1.326652	1.323406	1.451422
1.233949	1.23116	1.228356	1.336436	1.333108	1.329788	1.326478	1.323182	1.451159
1.233878	1.231089	1.228286	1.336306	1.332995	1.329675	1.326329	1.322998	1.450933
1.233778	1.23099	1.228195	1.336105	1.332837	1.329555	1.326228	1.322908	1.450789
1.233698	1.230909	1.228116	1.335905	1.332667	1.329418	1.326124	1.322832	1.450652
1.233706	1.230907	1.228094	1.335773	1.332489	1.329219	1.325957	1.322703	1.45045
1.233751	1.230938	1.228095	1.335673	1.332312	1.328997	1.325762	1.322547	1.450217
1.233721	1.230904	1.22805	1.335562	1.332184	1.328848	1.325589	1.322358	1.449972
1.233631	1.230817	1.227968	1.335448	1.332098	1.32876	1.325433	1.322134	1.449711
1.233567	1.230743	1.227882	1.335292	1.331969	1.328643	1.325293	1.321971	1.449505
1.233557	1.230697	1.227792	1.335072	1.331776	1.328487	1.325181	1.321898	1.449382
1.233516	1.230635	1.227706	1.334868	1.331579	1.328313	1.325054	1.321816	1.449249
1.233388	1.23053	1.227642	1.334736	1.331402	1.32811	1.324872	1.321662	1.449043
1.233235	1.230407	1.227574	1.334619	1.331238	1.327912	1.324687	1.321497	1.448826
1.233116	1.230281	1.227455	1.334458	1.331091	1.327779	1.324567	1.321387	1.448661
1.233014	1.230154	1.227312	1.334279	1.330961	1.327685	1.324482	1.321302	1.448521
1.232934	1.230076	1.227238	1.334138	1.330845	1.32758	1.324359	1.321159	1.448335
1.232876	1.230048	1.227232	1.334036	1.330745	1.327467	1.324198	1.320958	1.448101
1.232825	1.230015	1.227216	1.333935	1.330654	1.327372	1.324065	1.320792	1.447897
1.232774	1.229964	1.227177	1.333839	1.330585	1.327315	1.323986	1.320687	1.447742
1.232732	1.22991	1.227118	1.333714	1.33049	1.32724	1.323913	1.320608	1.447612
1.232709	1.229856	1.227012	1.333505	1.330307	1.327095	1.323813	1.320546	1.447509
1.232691	1.229803	1.226897	1.333275	1.330098	1.326925	1.323706	1.320495	1.447417
1.343272	1.339868	1.336445	1.333135	1.32995	1.570914	1.564914	1.559151	1.65656
1.343151	1.339763	1.336364	1.333055	1.329842	1.570568	1.564609	1.558886	1.656276
1.343031	1.339654	1.336271	1.332967	1.32975	1.570238	1.564322	1.558621	1.655965
1.342909	1.339537	1.33616	1.332871	1.329678	1.56993	1.564055	1.558354	1.655619
1.141049	1.339429	1.336058	1.332781	1.329608	1.569621	1.563791	1.558098	1.655296
1.140826	1.339335	1.335972	1.332699	1.329529	1.569301	1.563522	1.557862	1.655022
1.140609	1.339257	1.335895	1.332623	1.329451	1.568979	1.563252	1.55763	1.654767
1.140399	1.339203	1.335827	1.332549	1.32938	1.568663	1.562977	1.557384	1.654519
1.140193	1.339165	1.335769	1.332482	1.329313	1.568348	1.562698	1.557131	1.65428
1.139985	1.339119	1.335725	1.332434	1.329255	1.56803	1.56241	1.556878	1.654053
1.139775	1.339068	1.335695	1.332404	1.329204	1.56771	1.562114	1.556622	1.653837
1.139566	1.13634	1.13311	1.332368	1.32916	1.5674	1.561831	1.556369	1.551023
1.139357	1.136126	1.13289	1.332315	1.32912	1.567086	1.561556	1.556115	1.550778
1.13915	1.135926	1.132701	1.332288	1.329101	1.566839	1.561318	1.555875	1.550533
1.138947	1.13576	1.132589	1.332324	1.329127	1.566746	1.561146	1.555647	1.550284
1.138746	1.135598	1.132488	1.332372	1.329158	1.566674	1.560999	1.55545	1.550067
1.138536	1.135397	1.132311	1.129214	1.126104	1.566445	1.560836	1.555336	1.736772
1.13833	1.135182	1.132098	1.129011	1.125918	1.566136	1.560664	1.555266	1.736709
1.138188	1.135031	1.131926	1.128826	1.125728	1.565877	1.560454	1.555093	1.736504
1.138114	1.134945	1.131791	1.128654	1.125528	1.565654	1.560199	1.55482	1.736165
1.137996	1.134823	1.131643	1.12849	1.125362	1.565495	1.560023	1.554622	1.735865
1.137796	1.134636	1.13147	1.128343	1.125259	1.565448	1.559995	1.554576	1.735651

1.137591	1.134438	1.131287	1.128186	1.125144	1.565372	1.559939	1.554513	1.73545
1.13743	1.134257	1.131088	1.127983	1.124954	1.414956	1.409374	1.403815	1.735216
1.137281	1.134083	1.130895	1.127778	1.124745	1.414578	1.408994	1.403465	1.734992
1.137109	1.133922	1.130761	1.12765	1.124593	1.41433	1.408751	1.403241	1.734859
1.136926	1.133772	1.13067	1.127572	1.124476	1.414159	1.408588	1.403094	1.734784
1.136785	1.133647	1.130559	1.127457	1.124335	1.413905	1.408336	1.402851	1.734613
1.136694	1.133553	1.130429	1.127301	1.124164	1.413549	1.407975	1.402491	1.734327
1.13659	1.13344	1.130284	1.127143	1.124011	1.413246	1.407667	1.402178	1.734079
1.13645	1.133284	1.130113	1.126983	1.123895	1.413064	1.40748	1.401973	1.733923
1.136296	1.133119	1.129951	1.126838	1.123787	1.412912	1.407327	1.401807	1.733803
1.136118	1.132964	1.129839	1.126739	1.123667	1.412727	1.407152	1.401649	1.613972
1.135942	1.13282	1.129749	1.12666	1.123547	1.412539	1.406979	1.401502	1.61381
1.135849	1.132718	1.129632	1.126543	1.123448	1.412382	1.406834	1.401347	1.613562
1.135824	1.132653	1.129496	1.126397	1.123366	1.412251	1.406712	1.401187	1.613248
1.13574	1.132561	1.129389	1.126289	1.123272	1.412084	1.406511	1.40095	1.612929
1.135566	1.13243	1.129327	1.126236	1.123161	1.411865	1.406187	1.400587	1.612591
1.135419	1.13231	1.129251	1.126166	1.123053	1.411661	1.405925	1.400306	1.612319
1.135375	1.132237	1.129125	1.126032	1.122965	1.41152	1.405855	1.400256	1.612179
1.135337	1.132165	1.128992	1.125891	1.122874	1.411382	1.405818	1.400256	1.612096
1.135193	1.13204	1.128898	1.1258	1.122753	1.411181	1.405648	1.400133	1.612031
1.135	1.131891	1.12882	1.125731	1.122617	1.410949	1.405415	1.399951	1.611969
1.134886	1.131776	1.128703	1.125614	1.122503	1.410755	1.405221	1.399758	1.61175
1.134841	1.131688	1.128547	1.125451	1.122407	1.410594	1.405059	1.399547	1.611374
0.992558	0.989999	0.98742	1.125335	1.122305	1.410409	1.404874	1.399348	1.611105
0.992535	0.989997	0.98743	1.125296	1.122182	1.410162	1.404641	1.39917	1.611036
0.992496	0.989979	0.987437	1.125272	1.122083	1.409965	1.404431	1.398992	1.610957
0.992424	0.989914	0.987403	1.125243	1.122057	1.409926	1.404291	1.39878	1.610722
0.992349	0.989837	0.987352	1.125199	1.122049	1.409931	1.40419	1.398587	1.610456
0.992329	0.9898	0.987289	1.417578	1.412848	1.630114	1.623365	1.616721	1.75487
0.992343	0.989787	0.987218	1.417335	1.412742	1.630007	1.623367	1.616743	1.754758
0.992336	0.989777	0.987187	1.417173	1.412581	1.629798	1.623245	1.616688	1.754654
0.992312	0.989776	0.987202	1.417094	1.412346	1.629459	1.622986	1.616559	1.75457
0.992267	0.989749	0.987199	1.417045	1.412186	1.629222	1.622754	1.616383	1.754427
0.992184	0.989667	0.987147	1.417024	1.412171	1.629186	1.622588	1.616119	1.754154
0.992108	0.989591	0.987096	1.417012	1.412196	1.629215	1.622477	1.615889	1.753903
0.992084	0.989576	0.987095	1.417003	1.412199	1.629244	1.622454	1.615829	1.753824
0.992087	0.989587	0.987105	1.416977	1.412193	1.629276	1.622484	1.615853	1.753818
0.992094	0.989574	0.987059	1.416853	1.412135	1.629229	1.622486	1.615866	1.753753
0.992109	0.989547	0.986971	1.416648	1.412035	1.629122	1.622477	1.615884	1.753654
0.992096	0.989519	0.986916	1.416519	1.411971	1.407361	1.402632	1.397885	1.573214
0.992044	0.989487	0.986905	1.416494	1.411967	1.407371	1.402656	1.397915	1.573194
0.992005	0.989468	0.986905	1.416469	1.411918	1.407317	1.402634	1.397929	1.573174
0.992007	0.989474	0.986911	1.416395	1.411743	1.407108	1.402506	1.397903	1.573129
0.992022	0.989488	0.986922	1.416325	1.411563	1.406889	1.402367	1.397868	1.573082
0.992032	0.989497	0.986931	1.416303	1.411511	1.406825	1.402323	1.397852	1.573045
0.992042	0.989506	0.98694	1.416307	1.411526	1.406844	1.402329	1.397848	1.573014
0.992055	0.989517	0.98695	1.416308	1.411532	1.507186	1.502516	1.497883	1.572991
0.99207	0.98953	0.986963	1.416308	1.411538	1.507183	1.502519	1.497891	1.572976
0.992084	0.989541	0.986973	1.416305	1.411541	1.507176	1.502519	1.497897	1.572959
0.992092	0.989546	0.986977	1.416296	1.411537	1.507165	1.502514	1.497897	1.57294

0.992105	0.989557	0.986989	1.416296	1.411539	1.507155	1.502506	1.497893	1.572918
0.99213	0.989585	0.987017	1.416319	1.411555	1.507149	1.502497	1.497883	1.572889
0.992163	0.989622	0.987055	1.416354	1.411579	1.507145	1.502488	1.49787	1.572858
0.992201	0.989664	0.987097	1.41639	1.411606	1.507142	1.502481	1.497862	1.572828
0.992243	0.98971	0.987145	1.41643	1.411637	1.507142	1.502477	1.497856	1.572798
0.99228	0.98975	0.987186	1.319542	1.315049	1.507135	1.502467	1.497847	1.572772
0.99231	0.989784	0.98722	1.319601	1.315072	1.507111	1.502443	1.497831	1.572748
0.99234	0.989816	0.987253	1.319682	1.315162	1.507171	1.502478	1.49784	1.572734
0.992362	0.989843	0.987284	1.319805	1.315382	1.507395	1.502626	1.49789	1.572734
0.9924	0.989881	0.987321	1.319923	1.315595	1.507609	1.502774	1.497948	1.57274
0.992492	0.989952	0.987366	1.319978	1.315674	1.507662	1.502827	1.497993	1.572739
0.992601	0.990038	0.987425	1.320029	1.315707	1.50765	1.502839	1.498029	1.572744
0.992663	0.990113	0.987524	1.431645	1.42709	1.507675	1.502847	1.498044	1.657584
0.992691	0.990182	0.987655	1.431875	1.427221	1.507717	1.502846	1.498041	1.657648
0.992721	0.990234	0.987747	1.432022	1.427313	1.507761	1.502854	1.498036	1.657665
0.992748	0.99026	0.987787	1.432062	1.427355	1.507809	1.502867	1.498014	1.657608
0.992803	0.990312	0.987837	1.432097	1.427387	1.507849	1.502913	1.49806	1.657608
0.992911	0.990427	0.987933	1.432162	1.427399	1.507849	1.503011	1.498251	1.657754
0.993035	0.990551	0.988028	1.432231	1.427436	1.507881	1.503139	1.498467	1.657917
0.993144	0.990634	0.988073	1.432283	1.427568	1.508048	1.503297	1.498593	1.657967
0.993244	0.990697	0.988097	1.43234	1.427752	1.50828	1.503467	1.498675	1.65797
0.993303	0.990751	0.988165	1.432475	1.42791	1.508419	1.503559	1.498726	1.658005
0.993323	0.990794	0.988269	1.432683	1.428053	1.508483	1.503582	1.498741	1.658062
0.99338	0.990867	0.988373	1.288006	1.284502	1.37988	1.376038	1.372234	1.658162
0.993492	0.990989	0.98848	1.28802	1.284473	1.379856	1.37608	1.372339	1.658331
0.993611	0.991105	0.988568	1.288021	1.28446	1.37986	1.376136	1.372438	1.658472
0.993722	0.991185	0.988606	1.288012	1.284512	1.379946	1.376203	1.372472	1.658484
0.99382	0.991249	0.988635	1.288013	1.284587	1.380053	1.376279	1.372503	1.658495
1.153124	1.150187	1.147232	1.288074	1.284652	1.471378	1.467403	1.463425	1.658692
1.153112	1.150199	1.147311	1.288175	1.284716	1.471399	1.467481	1.463571	1.658992
1.153196	1.150283	1.1474	1.288253	1.284792	1.471465	1.467554	1.46365	1.659143
1.153384	1.150447	1.147502	1.28831	1.284881	1.471579	1.467625	1.463665	1.659144
1.153516	1.150567	1.147594	1.288375	1.284959	1.471657	1.467663	1.46366	1.65916
1.15352	1.150588	1.147666	1.288457	1.285016	1.471654	1.467629	1.463618	1.659219
1.153533	1.150617	1.14774	1.28854	1.285075	1.471664	1.467625	1.463616	1.659319
1.153676	1.150749	1.147838	1.288606	1.285161	1.471774	1.467752	1.463743	1.659479
1.153852	1.150908	1.147942	1.288669	1.285255	1.471913	1.467926	1.463927	1.659689
1.15391	1.150974	1.148026	1.288747	1.285321	1.471973	1.468027	1.464073	1.659965
1.15389	1.15098	1.148097	1.288836	1.285369	1.47198	1.468084	1.464199	1.660296
1.153956	1.151052	1.148181	1.288908	1.285434	1.383903	1.380251	1.376627	1.660522
1.154131	1.151205	1.148274	1.288955	1.285518	1.383999	1.380316	1.376662	1.660617
1.154294	1.151361	1.148392	1.289031	1.285604	1.384082	1.38038	1.376709	1.660723
1.1544	1.151497	1.14856	1.28917	1.285681	1.384112	1.380426	1.376781	1.660882
1.154486	1.151619	1.148729	1.289327	1.285772	1.38416	1.380495	1.37688	1.661085
1.154562	1.151702	1.148845	1.289473	1.285915	1.384307	1.380648	1.377035	1.66138
1.154636	1.15177	1.148935	1.28961	1.286082	1.384503	1.380837	1.377212	1.661714
1.154763	1.151882	1.149028	1.408738	1.404875	1.490672	1.486671	1.482678	1.66192
1.154936	1.152032	1.149119	1.408767	1.404975	1.490789	1.486731	1.482681	1.662019
1.15508	1.152175	1.14924	1.408861	1.405117	1.490941	1.486849	1.48276	1.662158
1.155175	1.152301	1.1494	1.40905	1.40533	1.49116	1.487069	1.482957	1.662354

1.155283	1.152439	1.149575	1.409246	1.405513	1.491335	1.48726	1.483152	1.593811
1.155452	1.15262	1.149767	1.409401	1.405585	1.491367	1.487328	1.483276	1.594074
1.155623	1.152797	1.149953	1.409547	1.405639	1.491379	1.48738	1.483393	1.594361
1.155721	1.152896	1.150078	1.409697	1.405775	1.491516	1.487539	1.483576	1.594669
1.15578	1.152953	1.150169	1.40985	1.40596	1.491725	1.487753	1.483787	1.594985
1.155892	1.15307	1.150306	1.410041	1.406161	1.491923	1.487908	1.483902	1.595187
1.156052	1.153248	1.150491	1.410276	1.406387	1.492118	1.488006	1.483915	1.595269
1.15622	1.153415	1.150643	1.410446	1.406571	1.492293	1.488125	1.483983	1.479966
1.156388	1.153551	1.150727	1.410491	1.406674	1.492428	1.488281	1.484153	1.480147
1.156559	1.15369	1.150819	1.410547	1.406785	1.492571	1.488457	1.484352	1.480354
1.15673	1.153852	1.150971	1.410717	1.406971	1.492762	1.488651	1.484547	1.480557
1.156901	1.154026	1.151149	1.410934	1.407189	1.492974	1.488858	1.484749	1.480755
1.157074	1.274452	1.271204	1.501209	1.497216	1.493183	1.489067	1.484948	1.480906
1.15725	1.274631	1.271384	1.501425	1.497431	1.493395	1.489282	1.485147	1.481024
1.157425	1.274809	1.271565	1.501642	1.497646	1.493606	1.489492	1.485347	1.481177
1.157598	1.274987	1.271746	1.501859	1.49786	1.493817	1.489697	1.485545	1.481371
1.157773	1.275166	1.271926	1.502075	1.498071	1.494025	1.489902	1.485749	1.481577
1.15795	1.275347	1.272106	1.502287	1.498275	1.494226	1.49011	1.485962	1.481782
1.158129	1.275527	1.272284	1.502496	1.498477	1.494426	1.490317	1.486179	1.482006
1.15831	1.275706	1.272456	1.5027	1.498676	1.494628	1.49052	1.486397	1.482276
1.158493	1.275884	1.272626	1.502902	1.498876	1.494829	1.490722	1.486615	1.482564
1.158675	1.276061	1.272795	1.503104	1.499074	1.495028	1.490921	1.486822	1.482804
1.158857	1.276238	1.272965	1.503303	1.499272	1.495224	1.491117	1.487019	1.483004
1.159039	1.276432	1.273178	1.392015	1.388375	1.384734	1.381057	1.377389	1.483203
1.159222	1.276655	1.273459	1.392387	1.388694	1.385004	1.381286	1.377588	1.483403
1.159405	1.276871	1.273722	1.392731	1.388996	1.385266	1.381513	1.377789	1.483604
1.159586	1.277059	1.273917	1.392972	1.389228	1.385486	1.381717	1.377979	1.483792
1.159769	1.277237	1.274088	1.393175	1.389433	1.385693	1.381928	1.378193	1.484007
1.159954	1.277409	1.274257	1.39338	1.389634	1.385908	1.382193	1.378502	1.484326
1.160148	1.277584	1.274426	1.393582	1.389829	1.386123	1.382484	1.378864	1.484702
1.16039	1.277826	1.274652	1.393826	1.390042	1.386328	1.382724	1.379141	1.48499
1.160681	1.278136	1.274938	1.394112	1.390269	1.386516	1.382911	1.379338	1.485191
1.160968	1.278426	1.275197	1.394389	1.390533	1.386767	1.383145	1.379562	1.485429
1.161252	1.278679	1.275397	1.39463	1.390846	1.38712	1.383468	1.379847	1.485749
1.161509	1.278916	1.275604	1.394896	1.391173	1.387479	1.383789	1.380126	1.486056
1.161696	1.279138	1.275865	1.39524	1.391502	1.387782	1.384043	1.380337	1.486269
1.161864	1.279359	1.276146	1.395606	1.391823	1.388058	1.38428	1.380546	1.486469
1.162104	1.279625	1.276423	1.395912	1.392084	1.3883	1.384554	1.380855	1.486779
1.162388	1.279919	1.276696	1.396181	1.392306	1.388516	1.38485	1.381232	1.487163
1.162614	1.280153	1.276922	1.39644	1.392577	1.388802	1.385163	1.381568	1.377924
1.162771	1.280313	1.277088	1.396682	1.392903	1.389173	1.385502	1.381866	1.378245
1.162953	1.280503	1.277286	1.396957	1.393233	1.389525	1.385812	1.38213	1.378527
1.163195	1.280761	1.27756	1.397302	1.393561	1.389825	1.38606	1.382333	1.378727
1.16346	1.281044	1.277853	1.397652	1.393869	1.390097	1.386301	1.382551	1.378937
1.163733	1.281332	1.27813	1.397936	1.394106	1.390319	1.386573	1.382877	1.37925
1.164015	1.281627	1.278407	1.398205	1.394328	1.390534	1.386867	1.38325	1.379609
1.164285	1.281912	1.278691	1.398551	1.394682	1.390888	1.387205	1.383571	1.379932
1.164546	1.282189	1.278981	1.398958	1.395142	1.391355	1.387582	1.383857	1.380235
1.164814	1.282472	1.279264	1.399307	1.395505	1.391719	1.387922	1.384174	1.380556
1.165091	1.282762	1.279537	1.399568	1.395716	1.391923	1.388206	1.384539	1.380907

1.165364	1.283049	1.279812	1.399856	1.395973	1.392174	1.388506	1.384889	1.381246
1.165625	1.283326	1.280098	1.400247	1.396393	1.392593	1.388855	1.385171	1.381542
1.165884	1.2836	1.280383	1.400652	1.396849	1.393055	1.389233	1.385462	1.381841
1.166155	1.283885	1.280655	1.400982	1.397227	1.393464	1.389645	1.385858	1.382197
1.166433	1.284173	1.28092	1.401277	1.397568	1.393846	1.390076	1.386311	1.382587
1.166702	1.284459	1.281198	1.401604	1.39789	1.394178	1.390449	1.38672	1.382986
1.166962	1.284742	1.281489	1.401959	1.398189	1.394458	1.390768	1.387094	1.383401
1.167229	1.285027	1.281772	1.278548	1.275359	1.272183	1.269022	1.265856	1.383774
1.167506	1.285319	1.28204	1.27881	1.275634	1.272467	1.269299	1.266127	1.384077
1.167782	1.285609	1.282312	1.279076	1.275909	1.272749	1.269576	1.2664	1.384379
1.168041	1.28588	1.282592	1.279354	1.276172	1.273004	1.269847	1.266687	1.384711
1.168308	1.286162	1.282891	1.279652	1.276447	1.273269	1.27013	1.26699	1.385069
1.168637	1.286522	1.283249	1.280006	1.276792	1.273609	1.270477	1.267344	1.385492
1.169006	1.286934	1.283649	1.280398	1.277183	1.274	1.270865	1.267731	1.385958
1.16934	1.287298	1.283992	1.280734	1.277525	1.274345	1.271202	1.26806	1.386347
1.169635	1.287606	1.28427	1.281002	1.27781	1.274639	1.271478	1.268321	1.386649
1.169949	1.287941	1.284582	1.281307	1.278123	1.274955	1.271783	1.268613	1.386971
1.170322	1.288347	1.284982	1.2817	1.278509	1.275334	1.272164	1.268987	1.387349
1.170678	1.288742	1.285381	1.282094	1.278887	1.275701	1.272537	1.26936	1.387744
1.170941	1.289042	1.285701	1.282412	1.279178	1.275975	1.272822	1.269664	1.38814
1.171171	1.289308	1.285995	1.282707	1.27944	1.276219	1.273077	1.269945	1.388544
1.171467	1.28964	1.286342	1.283053	1.279767	1.276533	1.273394	1.270273	1.38897
1.171811	1.29002	1.286728	1.283438	1.280141	1.276899	1.273754	1.270635	1.389414
1.172163	1.290407	1.287117	1.283825	1.280523	1.277268	1.274092	1.270946	1.267794
1.17253	1.290811	1.28752	1.284225	1.280923	1.277644	1.274397	1.271185	1.268035
1.172872	1.169936	1.166998	1.164056	1.161105	1.277996	1.274704	1.27145	1.268304
1.17315	1.170209	1.167248	1.164299	1.161361	1.278285	1.275019	1.271791	1.268659
1.173422	1.170476	1.16749	1.164535	1.161609	1.278569	1.275346	1.27216	1.269041
1.173754	1.170807	1.167812	1.164853	1.161928	1.278913	1.275698	1.272523	1.269407
1.174116	1.171171	1.168175	1.165213	1.162284	1.279288	1.276068	1.272889	1.269773
1.037108	1.171526	1.168531	1.165568	1.162635	1.279661	1.276435	1.273252	1.270136
1.037388	1.171877	1.168886	1.165923	1.162986	1.280037	1.276801	1.273611	1.270498
1.037673	1.17223	1.169242	1.166281	1.163343	1.28042	1.277174	1.273974	1.270859
1.037963	1.172587	1.169602	1.166642	1.163707	1.280814	1.277554	1.27434	1.27122
1.038251	1.172942	1.169958	1.167	1.164068	1.281207	1.277937	1.27471	1.271581
1.038536	1.173291	1.170305	1.167348	1.164419	1.281592	1.278316	1.275082	1.271945
1.038813	1.173634	1.170647	1.16769	1.164765	1.281972	1.278694	1.275455	1.272309
1.039067	1.173963	1.170982	1.168031	1.165112	1.282355	1.279074	1.275828	1.272671
1.039303	1.174283	1.171313	1.16837	1.165457	1.282737	1.279454	1.276201	1.273033
1.039545	1.174606	1.171646	1.16871	1.165799	1.283111	1.279826	1.27657	1.273393
1.039787	1.174928	1.171977	1.169044	1.166132	1.283472	1.280185	1.276926	1.273746
0.84839	0.84627	0.844135	1.169415	1.166498	1.16358	1.160644	1.157731	1.274139
0.848719	0.846601	0.844485	1.169858	1.16693	1.164005	1.161079	1.158174	1.274607
0.849047	0.846931	0.84483	1.170294	1.167354	1.164424	1.161511	1.158615	1.275073
0.849329	0.84721	0.84511	1.170651	1.167707	1.164775	1.161874	1.158987	1.275471
0.849593	0.847469	0.845363	1.170973	1.168028	1.165098	1.162208	1.159329	1.275839
0.849863	0.847734	0.845623	1.171299	1.168358	1.165432	1.162547	1.15967	1.276211
0.850133	0.848001	0.845886	1.171623	1.16869	1.165771	1.162886	1.160007	1.276581
0.850446	0.848308	0.846182	1.171983	1.169067	1.166158	1.163265	1.160378	1.276995
0.850818	0.848671	0.846525	1.172394	1.169504	1.166611	1.163701	1.160796	1.277472

0.851167	0.849013	0.846852	1.172785	1.169918	1.167039	1.164116	1.161196	1.277925
0.851443	0.849285	0.847121	1.173109	1.170257	1.167387	1.16446	1.161534	1.278298
0.851713	0.849553	0.847391	1.173434	1.170585	1.16772	1.164796	1.161873	1.278668
0.852045	0.849884	0.847732	1.035895	1.033369	1.168097	1.165191	1.162286	1.279117
0.8524	0.850241	0.848102	1.036239	1.0337	1.168485	1.165606	1.162729	1.2796
0.852712	0.85055	0.848413	1.036511	1.033968	1.168821	1.165958	1.163105	1.28004
0.852984	0.850819	0.848673	1.036721	1.034181	1.169107	1.166254	1.163423	1.280445
0.853294	0.851124	0.848966	1.036976	1.034436	1.169439	1.166586	1.163762	1.160962
0.853663	0.851486	0.84931	1.037301	1.034752	1.169838	1.166973	1.164133	1.161314
0.854037	0.851855	0.849669	0.847501	0.845354	1.032541	1.030018	1.027509	1.16168
0.854387	0.852206	0.85003	0.847862	0.8457	1.03286	1.030338	1.027833	1.162086
0.854734	0.852555	0.850393	0.848225	0.846047	1.033181	1.030659	1.028157	1.162503
0.855097	0.852922	0.850747	0.848572	0.846395	1.033505	1.030974	1.028466	1.162901
0.855467	0.853296	0.851094	0.848911	0.846744	1.033832	1.031287	1.028766	1.163289
0.855791	0.853633	0.851446	0.849268	0.847097	1.034162	1.031608	1.02908	1.026587
0.856064	0.853929	0.851803	0.849647	0.847454	1.034496	1.031938	1.029409	1.026908
0.856364	0.854233	0.852136	0.849995	0.847802	1.03483	1.03227	1.029736	1.02723
0.856716	0.854551	0.852411	0.850271	0.848131	1.035169	1.032607	1.030062	1.027555
0.857084	0.854891	0.85271	0.850566	0.848465	1.035501	1.032934	1.030378	1.027872
0.857451	0.855276	0.853118	0.850969	0.848832	1.035812	1.033233	1.030675	1.028168
0.857827	0.855683	0.853563	0.851407	0.849209	1.036112	1.033518	1.030957	1.028449
0.85823	0.856083	0.853941	0.851769	0.849563	1.036414	1.033802	1.031226	1.028716
0.858652	0.856479	0.854275	0.852082	0.849901	1.036714	1.034083	1.031484	1.028968
0.601203	0.856821	0.854605	0.852411	0.850237	1.037009	1.034366	1.031751	1.029226
0.601425	0.857089	0.854932	0.852761	0.850572	1.037295	1.034649	1.032029	1.029491
0.601676	0.857384	0.855259	0.853102	0.850905	0.848724	0.846577	0.844446	1.029758
0.602013	0.857762	0.855592	0.853417	0.851235	0.849063	0.846908	0.844767	1.030013
0.602362	0.858153	0.855926	0.853732	0.851572	0.849417	0.847253	0.845101	1.030293
0.602661	0.858492	0.856252	0.854064	0.851936	0.849807	0.847651	0.845501	1.030656
0.60294	0.858814	0.85658	0.85441	0.852314	0.850212	0.848071	0.84593	1.031057
0.603224	0.859171	0.856958	0.854792	0.852677	0.850565	0.848434	0.846306	1.031386
0.60351	0.859557	0.857381	0.855207	0.85303	0.850873	0.84875	0.84664	1.031658
0.603814	0.602237	0.600656	0.855585	0.853372	0.851194	0.849086	0.846998	1.031963
0.604148	0.602565	0.600955	0.855908	0.853695	0.851531	0.849456	0.8474	1.032326
0.604471	0.60288	0.601246	0.856234	0.854035	0.851889	0.849827	0.847785	1.032674
0.604751	0.603155	0.601526	0.856592	0.854423	0.852286	0.850191	0.848112	1.032954
0.605026	0.603428	0.601813	0.85697	0.854823	0.852688	0.850547	0.848422	1.033226
0.605345	0.60375	0.602142	0.85737	0.855192	0.853031	0.850878	0.848753	0.846682
0.605687	0.6041	0.602495	0.85779	0.855547	0.853344	0.851198	0.849098	0.847064
	0.604421	0.602833	0.601227	0.599595	0.853714	0.851565	0.849473	0.847457
	0.604714	0.603158	0.601567	0.599932	0.85414	0.851982	0.849882	0.847867
	0.605014	0.603468	0.601884	0.600248	0.854546	0.852394	0.850295	0.848269
	0.605328	0.603755	0.602156	0.60052	0.854903	0.852785	0.850708	0.848654
	0.605649	0.604047	0.602434	0.600799	0.855267	0.853179	0.851123	0.849043
	0.605976	0.604371	0.602758	0.601126	0.855678	0.853593	0.851537	0.849448
	0.606306	0.604707	0.603099	0.601473	0.856109	0.854015	0.85195	0.849861
	0.606631	0.605037	0.603433	0.601811	0.856534	0.854433	0.85236	0.850267
	0.606953	0.605364	0.603764	0.602148	0.856958	0.85485	0.852768	0.850671
			0.604096	0.602484	0.600896	0.599352	0.597827	0.851074
			0.604427	0.602821	0.601233	0.599682	0.598148	0.851477

0.604759	0.603157	0.60157	0.600012	0.59847	0.85188
0.605098	0.603496	0.601906	0.600342	0.598791	0.85228
0.605427	0.603831	0.602243	0.600674	0.599116	0.852684
0.60572	0.604151	0.602582	0.601014	0.599452	0.853098
0.606003	0.604465	0.602921	0.601359	0.599795	0.853519
0.60634	0.604803	0.603259	0.601702	0.600141	0.598575
0.606723	0.605158	0.603597	0.602045	0.600492	0.598923
0.607085	0.605505	0.603935	0.602387	0.600841	0.599269
0.607417	0.605839	0.604272	0.60273	0.601188	0.599613
0.607744	0.606171	0.60461	0.603073	0.601534	0.599957
0.608076	0.606505	0.604947	0.603415	0.60188	0.600302
0.60841	0.60684	0.605284	0.603756	0.602225	0.60065
		0.605614	0.604089	0.602565	0.601007
		0.605945	0.604419	0.602902	0.601369
		0.606305	0.604765	0.603237	0.601714
		0.606695	0.605125	0.603568	0.60204
		0.60709	0.6055	0.603923	0.602382
		0.60749	0.605901	0.604314	0.602751
		0.607877	0.606299	0.604712	0.603132
		0.608227	0.606672	0.605106	0.603529
		0.60856	0.60703	0.605492	0.603928
		0.608894	0.607368	0.605843	0.604298
		0.609229	0.607692	0.606169	0.604646
					0.604982
					0.6053
					0.605636
					0.606003
					0.606392
					0.606813
					0.607247
					0.60762
					0.607947

		0.85889
		0.858325
		0.85774
		0.857136
		1.034881
		1.034109
		1.033329
		1.03255
		1.031769
0.857547	0.855403	1.030978
0.856903	0.854748	1.030183
1.03486	1.032147	1.237348
1.03426	1.031569	1.23682
1.033625	1.030955	1.236254
1.032891	1.030232	1.235583

						1.03213	1.029474	1.23488
			0.859844	0.857668	0.855503	1.03138	1.028707	1.234175
			0.859323	0.857101	0.854889	1.03064	1.027943	1.233476
			1.038252	1.035473	1.032701	1.238938	1.235892	1.232873
			1.037508	1.034785	1.032067	1.238391	1.235378	1.232357
			1.03675	1.034066	1.031386	1.237776	1.234784	1.231777
			1.035965	1.033294	1.030632	1.237058	1.234071	1.231101
			1.035224	1.032546	1.029877	1.236336	1.233343	1.230404
			1.034607	1.031877	1.029155	1.235649	1.232624	1.229691
			1.034015	1.031231	1.028452	1.234989	1.231937	1.229006
	0.858869	0.856732	1.033313	1.030543	1.027783	1.234389	1.231369	1.228461
	0.858262	0.856106	1.032558	1.029838	1.027137	1.23383	1.230875	1.228001
	1.037303	1.034595	1.242277	1.239229	1.236208	1.233228	1.230293	1.227438
	1.036691	1.034013	1.241763	1.238673	1.235602	1.232583	1.229619	1.226766
	1.036046	1.033381	1.241195	1.238089	1.235	1.231974	1.229009	1.226166
	1.035331	1.032636	1.240505	1.237437	1.234401	1.231436	1.22853	1.225716
	1.034624	1.031903	1.239829	1.236797	1.233811	1.2309	1.228046	1.225258
	1.033979	1.031278	1.239277	1.236235	1.233235	1.230309	1.227442	1.224654
	1.033363	1.03069	1.238768	1.235704	1.23267	1.229711	1.226813	1.224016
0.856023	1.244235	1.241231	1.238206	1.23514	1.232112	1.229172	1.226297	1.223506
0.855542	1.243722	1.240666	1.237615	1.234562	1.231564	1.228677	1.225858	1.223083
0.854997	1.243149	1.240089	1.237052	1.234033	1.23107	1.228206	1.225398	1.222608
0.854371	1.242495	1.239494	1.236518	1.233568	1.230649	1.227769	1.224914	1.222072
1.032774	1.241883	1.23893	1.236	1.233093	1.230197	1.227301	1.224413	1.221535
1.032164	1.241377	1.238438	1.235507	1.23258	1.229664	1.226763	1.223876	1.221013
1.031589	1.240896	1.237954	1.235008	1.232049	1.22911	1.226212	1.223343	1.220502
1.031018	1.240392	1.237423	1.23446	1.231496	1.228563	1.2257	1.222867	1.220021
1.03045	1.239876	1.23687	1.233889	1.230933	1.228022	1.225203	1.222411	1.219548
1.242326	1.23933	1.236313	1.233338	1.230415	1.227533	1.224714	1.221909	1.219027
1.241766	1.238756	1.235747	1.232798	1.229933	1.227088	1.224233	1.22137	1.218464
1.241204	1.238199	1.235219	1.232294	1.229446	1.226603	1.22372	1.220821	1.217912
1.240634	1.237661	1.234742	1.231836	1.228944	1.226054	1.223153	1.220253	1.217372
1.240091	1.237151	1.234278	1.231386	1.228451	1.22552	1.222609	1.219715	1.216859
1.239605	1.23669	1.233823	1.230931	1.227994	1.225058	1.222143	1.219247	1.216392
1.239143	1.236244	1.233362	1.230462	1.227532	1.224606	1.2217	1.218809	1.215947
1.238682	1.235772	1.232857	1.229938	1.227007	1.224092	1.221223	1.21837	1.215502
1.238223	1.235284	1.232326	1.229381	1.226447	1.223549	1.220733	1.217937	1.215061
1.237722	1.234764	1.231793	1.22885	1.225937	1.223063	1.220269	1.217491	1.214612
1.237177	1.234207	1.231254	1.22834	1.225472	1.222633	1.219832	1.217035	1.214157
1.236669	1.233689	1.230747	1.227851	1.225014	1.222193	1.219374	1.216548	1.21367
1.236227	1.233239	1.230291	1.227391	1.224555	1.221728	1.218874	1.216009	1.213126
1.235796	1.232801	1.229845	1.226937	1.224098	1.221261	1.218377	1.215481	1.212596
1.23534	1.232344	1.229385	1.226477	1.22364	1.220805	1.217916	1.215015	1.212139
1.234877	1.231882	1.228922	1.226015	1.223183	1.220353	1.21747	1.214578	1.211716
1.234415	1.23142	1.228458	1.225552	1.222724	1.219901	1.217026	1.214143	1.211295
1.233953	1.230957	1.227992	1.225087	1.222264	1.219449	1.216586	1.213718	1.210883
1.233499	1.230501	1.227534	1.224629	1.221813	1.219007	1.216155	1.213298	1.210474
1.23305	1.23005	1.227081	1.224178	1.221369	1.218572	1.215728	1.21288	1.210064
1.232611	1.229611	1.226641	1.223741	1.22094	1.218153	1.215323	1.212489	1.209678
1.232182	1.229185	1.226215	1.223319	1.220528	1.217756	1.214952	1.212144	1.209338

1.231758	1.228769	1.225804	1.222916	1.220134	1.217375	1.214592	1.211805	1.209001
1.231332	1.228358	1.225417	1.222547	1.219773	1.217016	1.214228	1.211433	1.208624
1.230909	1.227953	1.225044	1.222194	1.219427	1.216668	1.213864	1.211046	1.20823
1.2305	1.227554	1.224653	1.221812	1.219054	1.216299	1.213488	1.21066	1.207837
1.230101	1.22716	1.224252	1.221409	1.218661	1.215914	1.213103	1.210271	1.207442
1.229697	1.226759	1.223847	1.221006	1.218268	1.215529	1.212719	1.209884	1.207051
1.229278	1.226338	1.223432	1.220599	1.217874	1.215146	1.212338	1.2095	1.206661
1.22888	1.225946	1.223045	1.220216	1.217493	1.214766	1.211959	1.209122	1.206278
1.228536	1.225628	1.222729	1.21989	1.21714	1.214389	1.211577	1.208741	1.205899
1.228198	1.225319	1.222424	1.219575	1.216794	1.214017	1.211205	1.208379	1.20554
1.227808	1.224944	1.222056	1.219208	1.216419	1.213643	1.210867	1.208083	1.205246
1.22739	1.224535	1.221659	1.218822	1.216036	1.213276	1.210551	1.207821	1.204986
1.226987	1.224145	1.221307	1.218497	1.21572	1.212962	1.210238	1.207502	1.204653
1.226593	1.223769	1.220998	1.218233	1.215472	1.212705	1.209931	1.207132	1.204253
1.226237	1.223419	1.220683	1.217939	1.215179	1.212399	1.209585	1.206742	1.203854
1.225941	1.223112	1.220359	1.217594	1.214807	1.212002	1.20917	1.20632	1.203462
1.225643	1.222802	1.220019	1.217229	1.214425	1.211608	1.208767	1.205918	1.203087
1.22529	1.222445	1.219637	1.216843	1.21407	1.211283	1.208446	1.20559	1.202746
1.224927	1.222079	1.219249	1.216455	1.213722	1.210972	1.208148	1.205293	1.202428
1.224607	1.221755	1.218916	1.216113	1.213363	1.210607	1.207806	1.204983	1.202134
1.22431	1.221455	1.218621	1.215803	1.213001	1.210212	1.20744	1.20467	1.201857
1.223966	1.22112	1.218309	1.215497	1.212671	1.20986	1.207092	1.204335	1.201536
1.223562	1.220744	1.217984	1.215203	1.212378	1.209555	1.206761	1.203973	1.20116
1.223189	1.220383	1.217643	1.21488	1.212076	1.209262	1.206448	1.203631	1.200808
1.222884	1.220057	1.217269	1.214497	1.211748	1.208985	1.20617	1.203335	1.200509
1.222597	1.219746	1.216906	1.214117	1.21141	1.208689	1.205884	1.203049	1.200228
1.222295	1.21944	1.216602	1.213803	1.21106	1.208319	1.205543	1.202754	1.199956
1.221994	1.219143	1.216325	1.213516	1.210708	1.207921	1.20518	1.202452	1.199686
1.221714	1.218859	1.216028	1.213206	1.210382	1.207585	1.204841	1.202119	1.347079
1.221449	1.218587	1.21572	1.212881	1.210079	1.207295	1.204518	1.20176	1.346532
1.221169	1.2183	1.215421	1.212577	1.209783	1.207006	1.20422	1.201449	1.346052
1.220864	1.21799	1.215132	1.212299	1.209494	1.20671	1.203948	1.201203	1.345663
1.220566	1.21769	1.21485	1.212029	1.209222	1.206439	1.203693	1.200963	1.345271
1.220291	1.217419	1.214572	1.211758	1.208983	1.206223	1.203458	1.2007	1.344826
1.220022	1.217157	1.214304	1.211497	1.208754	1.206015	1.203227	1.200437	1.344377
1.219738	1.216885	1.214059	1.211264	1.208511	1.205761	1.202974	1.200186	1.343965
1.219451	1.216612	1.213826	1.211045	1.208263	1.205486	1.202712	1.199943	1.343573
1.219188	1.216354	1.213574	1.210795	1.208009	1.205229	1.202459	1.199694	1.343182
1.218947	1.216111	1.213303	1.210514	1.207748	1.204986	1.20221	1.199438	1.342792
1.218694	1.215867	1.213061	1.210279	1.207528	1.204779	1.349873	1.346132	1.34242
1.21841	1.215614	1.212867	1.210123	1.207379	1.204629	1.349588	1.345817	1.342079
1.218148	1.215378	1.212676	1.209962	1.207223	1.204472	1.349282	1.345494	1.341726
1.217951	1.215186	1.212464	1.209739	1.207009	1.204265	1.348891	1.345121	1.341324
1.217774	1.215004	1.212243	1.209496	1.206773	1.204034	1.348468	1.344724	1.340904
1.217556	1.214782	1.212015	1.209263	1.206536	1.203792	1.348066	1.344315	1.435733
1.217314	1.214534	1.211781	1.209032	1.206294	1.203536	1.34767	1.343893	1.435231
1.217054	1.214275	1.211536	1.208793	1.206046	1.203283	1.347276	1.343499	1.434761
1.216767	1.213997	1.211274	1.208541	1.205787	1.203026	1.346881	1.343132	1.434329
1.216521	1.213757	1.211043	1.357709	1.353978	1.350256	1.346512	1.342772	1.645447
1.21636	1.213599	1.210875	1.357419	1.35369	1.349957	1.346182	1.342408	1.644625

1.216195	1.213435	1.210703	1.357123	1.353403	1.349667	1.345866	1.342058	1.643808
1.215938	1.213179	1.210455	1.356746	1.353054	1.349341	1.345554	1.341753	1.643123
1.215652	1.212892	1.210185	1.356343	1.352679	1.348996	1.345236	1.341457	1.642477
1.215426	1.212667	1.209969	1.355991	1.352311	1.348617	1.34486	1.341095	1.641724
1.215242	1.212486	1.209792	1.355675	1.351945	1.348208	1.344439	1.340683	1.640896
1.215066	1.212314	1.209624	1.355353	1.351604	1.347847	1.344061	1.340296	1.640126
1.214902	1.212158	1.209473	1.355026	1.351299	1.347558	1.343743	1.339943	1.63944
1.214718	1.21198	1.209292	1.354679	1.35097	1.347243	1.438081	1.433928	1.638732
1.214485	1.211744	1.209037	1.354285	1.350564	1.346836	1.437611	1.433482	1.63793
1.214248	1.211502	1.208772	1.353894	1.35016	1.346431	1.43715	1.433058	1.637142
1.364912	1.361087	1.357312	1.353563	1.34984	1.346126	1.436758	1.432687	1.636486
1.364622	1.360795	1.357013	1.353267	1.349566	1.345873	1.436406	1.432351	1.635905
1.364321	1.360502	1.356722	1.448232	1.444161	1.440114	1.646985	1.641113	1.720024
1.364013	1.36021	1.356445	1.447865	1.44379	1.439742	1.646351	1.640498	1.719391
1.363703	1.359911	1.356155	1.447487	1.443417	1.439376	1.645726	1.639893	1.718761
1.363385	1.359596	1.355845	1.44709	1.443036	1.439016	1.645114	1.639303	1.718137
1.363079	1.454945	1.450793	1.446695	1.442658	1.438657	1.644502	1.638711	1.717508
1.362805	1.454573	1.450417	1.446317	1.442283	1.438291	1.643881	1.638107	1.716866
1.362541	1.454207	1.450047	1.445946	1.441915	1.437928	1.64326	1.637497	1.716217
1.362255	1.453814	1.449662	1.445573	1.44156	1.437588	1.64265	1.636886	1.715566
1.361958	1.453406	1.44927	1.445201	1.441216	1.437264	1.642047	1.636271	1.714911
1.361669	1.453012	1.448891	1.444838	1.440869	1.436928	1.641433	1.635649	1.714247
1.361387	1.452633	1.448523	1.44448	1.440519	1.436582	1.640806	1.635013	1.71357
1.361102	1.452255	1.448169	1.444139	1.440175	1.436235	1.640189	1.634408	1.712917
1.360795	1.451863	1.447823	1.443816	1.439828	1.435874	1.63957	1.633842	1.712288
1.360521	1.451502	1.447503	1.443516	1.439508	1.435543	1.638995	1.633312	1.7117
1.360354	1.451233	1.447242	1.443262	1.439262	1.435301	1.63854	1.632851	1.711206
1.360222	1.450997	1.446999	1.443021	1.439041	1.435092	1.638123	1.632406	1.710739
1.454806	1.450686	1.446686	1.655175	1.649203	1.643394	1.721981	1.716054	1.925612
1.45444	1.450325	1.446323	1.654527	1.648578	1.64278	1.721319	1.715389	1.924829
1.454092	1.449977	1.445976	1.653896	1.647968	1.642179	1.720666	1.714733	1.924077
1.453761	1.449641	1.445645	1.653276	1.647368	1.641587	1.720014	1.714078	1.923357
1.453454	1.449333	1.445336	1.652702	1.646811	1.641036	1.719413	1.713472	1.97748
1.453184	1.449073	1.445065	1.652227	1.646351	1.64058	1.718923	1.71297	1.97686
1.452928	1.448827	1.444805	1.651753	1.645885	1.640116	1.718433	1.712476	1.976247
1.452665	1.448563	1.444523	1.65116	1.645275	1.639502	1.717801	1.711874	1.975549
1.452397	1.448293	1.444234	1.65052	1.64461	1.638831	1.717116	1.711234	1.974823
1.452105	1.448017	1.443962	1.649982	1.644083	1.638308	1.716552	1.710687	1.974184
1.451791	1.447735	1.443705	1.64953	1.643674	1.637912	1.716089	1.710217	1.97362
1.451489	1.44745	1.44344	1.649075	1.643247	1.637491	1.715618	1.709739	1.973035
1.451203	1.44716	1.443165	1.64862	1.642797	1.637034	1.715138	1.709253	1.972415
1.450937	1.446882	1.442886	1.648123	1.642297	1.636529	1.799392	1.792965	1.971778
1.450711	1.446638	1.442606	1.647522	1.641682	1.635917	1.798678	1.792262	1.971109
1.45049	1.4464	1.442326	1.646904	1.641053	1.635296	1.797943	1.791552	1.970447
1.450224	1.658275	1.652276	1.730662	1.724633	1.718684	1.797334	1.790975	1.969869
1.44993	1.657677	1.651753	1.730177	1.724168	1.718225	1.7968	1.790477	1.969345
1.449641	1.657143	1.651278	1.729699	1.723692	1.717746	1.796265	1.78995	2.01849
1.449352	1.656669	1.650853	1.729233	1.723209	1.717248	1.795733	1.789397	2.017914
1.44909	1.65622	1.650422	1.728759	1.722734	1.716774	1.795207	1.78886	2.017355
1.448876	1.655801	1.649965	1.728265	1.722278	1.716346	1.794684	1.788349	2.016825

1.448661	1.65538	1.649509	1.727767	1.721816	1.715913	1.794169	1.787852	2.016306
1.448398	1.654917	1.649065	1.812987	1.806488	1.800067	1.793667	1.787363	2.015791
1.448121	1.654444	1.648625	1.812403	1.805936	1.799551	1.79317	1.786881	2.015281
1.447868	1.653997	1.648183	1.811871	1.805395	1.799007	1.792653	1.7864	2.014772
1.44763	1.653568	1.647739	1.811374	1.804861	1.798443	1.792123	1.785919	2.014265
1.447396	1.653133	1.6473	1.810839	1.804329	1.797916	1.791613	1.785431	2.013752
1.447161	1.652679	1.646856	1.810244	1.803795	1.797436	1.791129	1.784931	2.013232
1.446953	1.652281	1.646473	1.809719	1.803283	1.79694	1.790635	1.78444	2.012717
1.446799	1.652	1.646222	1.80935	1.8028	1.796371	1.790096	1.783971	2.012217
1.446649	1.651726	1.645971	1.809005	1.802348	1.795833	1.789578	1.783507	2.011714
1.446455	1.651348	1.645579	1.808572	1.801959	1.795459	1.789153	1.783013	2.011168
1.446238	1.650919	1.64512	1.808093	1.801593	1.79516	1.788779	1.782526	2.01062
1.446018	1.820579	1.814037	1.807559	1.801131	1.794763	1.850769	1.84441	2.091421
1.445791	1.820048	1.813457	1.806967	1.80059	1.794293	1.850402	1.844133	2.091104
1.445599	1.819591	1.812978	1.806471	1.800077	1.79379	1.849972	1.843794	2.090762
1.445462	1.819251	1.812659	1.806122	1.799608	1.793232	1.849442	1.843357	2.090375
1.445312	1.818858	1.812302	1.805758	1.799159	1.792719	1.848944	1.842924	2.089993
1.445101	1.818272	1.811781	1.805296	1.798745	1.792343	1.84858	1.842568	2.089664
1.444883	1.817655	1.811208	1.804786	1.798325	1.791994	1.848234	1.842202	2.089314
1.44473	1.817191	1.810668	1.804218	1.797812	1.791534	1.847761	1.841703	2.088821
1.444609	1.816811	1.810143	1.803618	1.797254	1.791024	1.847227	1.841137	2.088248
1.444452	1.816395	1.809692	1.80315	1.7968	1.790586	1.846791	1.840701	2.087783
1.444258	1.815949	1.809315	1.802809	1.796445	1.790217	1.846449	1.840391	2.087415
1.444079	1.815536	1.808959	1.80248	1.796095	1.789848	1.904911	1.898823	2.123668
1.443927	1.815178	1.80862	1.802145	1.795738	1.789477	1.904546	1.89849	2.123354
1.44379	1.814836	1.808286	1.801804	1.795361	1.789076	1.904147	1.898127	2.12303
1.44367	1.814489	1.807944	1.801439	1.794924	1.788587	1.903646	1.897674	2.122633
1.443556	1.814142	1.807598	1.801066	1.794475	1.788081	1.903125	1.897202	2.122218
1.650825	1.813801	1.807257	1.800716	1.794095	1.787684	1.997619	1.991574	2.162833
1.650542	1.813457	1.806915	1.800377	1.793763	1.787362	1.997332	1.991298	2.162491
1.650194	1.813003	1.806485	1.799978	1.793402	1.787036	1.997043	1.991021	2.162158
1.649764	1.812409	1.805944	1.799503	1.793004	1.786704	1.996753	1.990744	2.161836
1.649372	1.811874	1.805448	1.799058	1.792627	1.786387	2.038525	2.032444	2.207744
1.649065	1.81148	1.80506	1.798689	1.792299	1.786093	2.038204	2.03212	2.207373
1.648801	1.811144	1.804712	1.798347	1.791985	1.785807	2.037888	2.031799	2.207001
1.64856	1.810811	1.804355	1.797987	1.791661	1.78552	2.037588	2.031501	2.206642
1.648349	1.810516	1.804025	1.797645	1.791343	1.785233	2.037294	2.031214	2.206289
1.648181	1.810333	1.803813	1.797403	1.79107	1.784941	2.036983	2.030918	2.205942
1.648054	1.810252	1.803707	1.797249	1.790836	1.784646	2.036658	2.030616	2.205602
1.545774	1.725978	1.719878	1.79704	1.790594	1.784376	2.036349	2.030313	2.205249
1.54553	1.725675	1.7196	1.796753	1.790331	1.784127	2.036046	2.029994	2.20487
1.545276	1.725346	1.719295	1.796449	1.790087	1.783927	2.035799	2.029724	2.204523
1.545031	1.725027	1.718979	1.796148	1.789899	1.783833	2.035674	2.029568	2.204253
1.544817	1.724724	1.718664	1.795842	1.78971	1.783743	2.035564	2.029435	2.204006
1.730626	1.724496	1.718393	1.795531	1.78944	1.783517	2.035332	2.029223	2.203725
1.730496	1.724308	1.718144	1.795219	1.789127	1.783215	2.035032	2.028968	2.20343
1.730275	1.846268	1.839898	1.954402	1.948026	1.941832	2.034729	2.028692	2.203134
1.729969	1.846036	1.839636	1.954102	1.947723	1.941532	2.034412	2.028389	2.202836
1.729666	1.845803	1.839422	1.953874	1.947502	1.941308	2.03415	2.028109	2.20253
1.729367	1.845553	1.839276	1.953752	1.947409	1.941212	2.033978	2.027856	2.202198

1.729114	1.845351	1.839167	1.953657	1.947322	1.94111	2.033816	2.027639	2.201906
1.72897	1.845276	1.83911	1.953557	1.947153	1.940887	2.033604	2.027478	2.201719
1.728873	1.845244	1.839056	1.95344	1.946957	1.940639	2.033393	2.027351	2.201575
1.728752	1.845137	1.838886	1.953232	1.94677	1.940484	2.033258	2.027243	2.201396
1.72862	1.844987	1.838639	1.952961	1.946587	1.940389	2.033173	2.02715	2.2012
1.728418	1.844807	1.838445	1.884898	1.878923	1.873103	2.033042	2.026987	2.200976
1.728129	1.844586	1.838306	1.884791	1.878833	1.873002	2.032862	2.02674	2.200714
1.727897	1.844417	1.838197	1.884688	1.87871	1.872849	2.032665	2.026519	2.20048
1.727795	1.844361	1.838146	1.88458	1.878512	1.87259	2.032443	2.026362	2.20031
1.727719	1.844314	1.838072	1.884438	1.878298	1.872337	2.032238	2.026231	2.200151
1.607697	1.84419	1.837867	1.939934	1.933773	1.927796	2.032114	2.026126	2.199964
1.607454	1.84403	1.837615	1.939628	1.933572	1.927696	2.032024	2.026025	2.199762
1.607129	1.951967	1.945412	1.991037	1.984903	1.97893	2.078131	2.071925	2.199522
1.606732	1.95174	1.945308	1.990982	1.984821	1.978794	2.077911	2.071628	2.19925
1.606428	1.951584	1.945205	1.990896	1.984736	1.978699	2.077784	2.071478	2.199087
1.606252	1.951529	1.945095	1.990759	1.98464	1.978659	2.077793	2.071544	2.199082
1.606075	1.864751	1.858118	1.990642	1.984553	1.97861	2.077769	2.071554	2.199041
1.60581	1.864493	1.857964	1.990591	1.984485	1.978506	2.077594	2.071322	2.198831
1.60557	1.864244	1.857818	1.990546	1.984423	1.978407	2.077414	2.071077	2.198617
1.605517	1.864161	1.85765	1.990427	1.98435	1.978386	2.077395	2.071067	2.198579
1.605545	1.864151	1.857473	1.990279	1.984274	1.978402	2.077453	2.071173	2.19863
1.605333	1.922474	1.915762	1.909317	1.903217	1.897261	2.077416	2.071173	2.198624
1.60489	1.922106	1.915577	1.90922	1.903051	1.897027	2.077297	2.071088	2.198576
1.604595	1.863516	1.857163	1.850954	1.844883	1.838954	2.021968	2.016137	2.115894
1.604564	1.863429	1.856946	1.85069	1.844714	1.838869	2.021914	2.016006	2.115686
1.604551	1.863382	1.856783	1.850475	1.844559	1.83877	2.021852	2.015889	2.115504
1.604432	1.863334	1.856765	1.850439	1.844421	1.838555	1.94613	1.940143	2.115461
1.604266	1.863264	1.856784	1.850462	1.844311	1.838335	1.945924	1.940049	2.115458
1.748498	1.863058	1.856679	1.976179	1.969645	1.96326	2.006714	2.000611	2.115377
1.748202	1.862767	1.8565	1.976087	1.969642	1.963287	2.006638	2.00039	2.115259
1.748036	1.862602	1.856337	1.975925	1.969559	1.963255	2.00657	2.000254	2.050548
1.748019	1.862574	1.856184	1.975673	1.969382	1.963159	2.006509	2.000208	2.050527
1.747962	1.862543	1.85607	1.975488	1.969249	1.963088	2.006473	2.000192	2.050504
1.747783	1.862459	1.856008	1.975436	1.969232	1.963099	2.006502	2.000214	2.050453
1.747611	1.862391	1.855984	1.975425	1.969211	1.963067	2.006491	2.000216	2.050392
1.747567	1.862409	1.856018	1.975408	1.969071	1.962842	2.006317	2.000134	1.993857
1.747557	1.862442	1.856067	1.975394	1.968897	1.962553	2.006077	1.999998	1.993801
1.747412	1.862341	1.856008	1.975341	1.968816	1.962433	2.00592	1.999806	1.99364
1.747167	1.862137	1.855868	1.975259	1.968802	1.962437	2.005818	1.999564	1.993396
1.568231	1.740735	1.734707	1.872942	1.866726	1.86057	2.005777	1.999437	1.993265
1.568194	1.740722	1.73471	1.872938	1.866731	1.860573	2.005815	1.999462	1.993279
1.568177	1.740722	1.734681	1.872869	1.866678	1.86054	2.005835	1.999489	1.993301
1.56815	1.740677	1.734526	1.872619	1.866485	1.860415	2.005778	1.999444	1.993282
1.568128	1.740636	1.734367	1.872361	1.86628	1.860279	2.005713	1.999394	1.99325
1.56811	1.740628	1.734327	1.872281	1.866207	1.860219	2.005702	1.999386	1.993195
1.568096	1.74064	1.73435	1.872295	1.866204	1.860197	2.005719	1.999399	1.993131
1.568081	1.810418	1.803972	1.872295	1.866195	1.860177	2.005735	1.999412	2.04959
1.568067	1.810419	1.803978	1.872292	1.866188	1.860165	2.005755	1.999427	2.049598
1.568051	1.810417	1.803981	1.872288	1.86618	1.860152	2.005773	1.99944	2.049606
1.568033	1.810413	1.803981	1.872283	1.866172	1.860138	2.005787	1.999451	2.049611

1.568013	1.810401	1.803972	1.872268	1.866154	1.860117	2.005796	1.99946	2.049616
1.567988	1.810374	1.803945	1.872233	1.866119	1.860081	2.005798	1.999464	2.049617
1.567961	1.810339	1.803908	1.872189	1.866075	1.860038	2.005799	1.999472	2.049623
1.567934	1.810305	1.803871	1.872144	1.866028	1.859993	2.005812	1.999509	2.049648
1.567907	1.810271	1.803834	1.872096	1.865979	1.859945	2.005833	1.999563	2.049689
1.56789	1.810247	1.803798	1.87205	1.865928	1.859895	2.00584	1.999598	2.049751
1.567883	1.810232	1.803755	1.871992	1.865867	1.859839	2.005831	1.999613	2.049842
1.567874	1.810233	1.803781	1.872029	1.86588	1.859828	2.005841	1.999624	2.049899
1.567861	1.810259	1.803939	1.872247	1.866034	1.8599	2.005886	1.999633	2.049892
1.567847	1.81028	1.804086	1.872451	1.866181	1.859974	2.005939	1.999643	2.049872
1.567814	1.810235	1.804071	1.872452	1.866189	1.85998	2.00598	1.999641	2.049849
1.567798	1.810195	1.804013	1.872383	1.866144	1.859957	2.006024	1.99966	2.04985
1.652475	1.810331	1.804104	1.872419	1.866133	1.859926	2.062808	2.056304	2.049988
1.652676	1.8106	1.804299	1.872523	1.866139	1.859887	2.062886	2.056511	2.050236
1.652771	1.810735	1.804396	1.872586	1.866201	1.85996	2.06303	2.056709	2.050432
1.652729	1.810699	1.804369	1.872601	1.866341	1.860188	2.063269	2.056904	2.050564
1.652701	1.810669	1.80433	1.872586	1.866428	1.860349	2.063446	2.057047	2.050675
1.652761	1.81072	1.804295	1.872505	1.866362	1.86031	2.063459	2.057092	2.05078
1.652828	1.810781	1.804289	1.872453	1.866291	1.860237	2.063441	2.0571	2.050857
1.652825	1.810786	1.80437	1.872569	1.866377	1.860281	2.063508	2.057107	2.050854
1.652803	1.810783	1.804504	1.872768	1.866531	1.860368	2.063612	2.057121	2.050824
1.652884	1.810891	1.804635	1.872881	1.866584	1.860377	2.063698	2.057229	2.05093
1.653058	1.811101	1.804773	1.872929	1.866555	1.86032	2.063772	2.057423	2.051155
1.653194	1.648149	1.643048	1.872945	1.866575	1.860364	2.017649	2.011433	2.051348
1.653274	1.648166	1.643011	1.872905	1.86667	1.86057	2.017902	2.011625	2.051465
1.65332	1.648169	1.643008	1.872916	1.866787	1.86077	2.018153	2.011827	2.051622
1.653293	1.648177	1.643125	1.873081	1.866907	1.860856	2.018324	2.012051	2.051915
1.653284	1.648214	1.643276	1.873286	1.867036	1.860924	2.018476	2.012274	2.052228
1.653479	1.648373	1.643363	1.873382	1.86716	1.861079	2.018666	2.012474	2.052416
1.653791	1.648606	1.643423	1.873429	1.867286	1.861278	2.018869	2.012652	2.052529
1.653934	1.648735	1.643534	1.873559	1.867425	1.861413	2.019011	2.012746	2.052626
1.653898	1.648755	1.643699	1.873778	1.867578	1.861479	2.019079	2.012738	2.05269
1.653931	1.648827	1.643836	1.873958	1.867728	1.861591	2.019233	2.012864	2.05285
1.654124	1.649018	1.643904	1.87404	1.867866	1.8618	2.019558	2.013259	2.053192
1.654321	1.649204	1.643983	1.874138	1.868012	1.862008	2.019875	2.013637	2.053526
1.65439	1.649275	1.644141	1.874351	1.868183	1.862127	2.020018	2.013731	2.05367
1.654445	1.649327	1.644325	1.874598	1.868365	1.862229	2.020124	2.013757	2.05377
1.654671	1.649506	1.64445	1.874744	1.868536	1.862431	2.02041	2.014035	2.054028
1.655021	1.649776	1.644539	1.874829	1.868702	1.8627	2.02082	2.014485	2.054395
1.65532	1.650063	1.644737	1.727863	1.722864	1.717932	1.925323	1.919273	2.054768
1.65557	1.650386	1.645073	1.728159	1.722998	1.717957	1.925453	1.919501	2.05516
1.655754	1.650633	1.64538	1.728468	1.723191	1.718057	1.92562	1.91969	2.055489
1.655803	1.650707	1.645597	1.728774	1.723509	1.718341	1.925884	1.91981	2.055674
1.655868	1.650769	1.645769	1.729032	1.72383	1.718667	1.926185	1.919958	2.055854
1.656131	1.650972	1.645881	1.729127	1.72402	1.718943	1.926508	1.92028	2.056173
1.656487	1.651245	1.645973	1.729152	1.724146	1.719187	1.926834	1.920679	2.05656
1.656775	1.740949	1.735289	1.810333	1.804958	1.79968	1.927052	1.920975	2.056943
1.657019	1.741266	1.735647	1.810662	1.805119	1.799719	1.927174	1.921192	2.057336
1.657186	1.741482	1.735938	1.810973	1.805346	1.79987	1.927362	1.921377	2.057659
1.657229	1.741545	1.736131	1.811262	1.805676	1.800191	1.927647	1.92151	2.057869

1.588776	1.741638	1.736293	1.811492	1.805975	1.800514	1.927948	1.921688	2.05811
1.589032	1.741867	1.736413	1.811577	1.806134	1.800748	1.92822	1.921982	2.058474
1.589347	1.742155	1.73655	1.811651	1.806274	1.800969	1.928504	1.922329	2.058878
1.589651	1.742468	1.736818	1.811904	1.806534	1.801242	1.928842	1.922683	2.059247
1.589958	1.742808	1.737168	1.812262	1.806862	1.801544	1.929217	1.923054	2.059608
1.590209	1.743107	1.737486	1.732043	1.726843	1.721739	1.716678	1.711656	1.917214
1.5904	1.743367	1.737778	1.732357	1.727171	1.722072	1.717004	1.711969	1.917592
1.476012	1.743643	1.738083	1.732665	1.727449	1.722322	1.717257	1.712237	1.917928
1.476201	1.743951	1.73841	1.732964	1.727631	1.722416	1.717377	1.712426	1.918182
1.476404	1.744269	1.738744	1.733267	1.727826	1.722526	1.717509	1.71262	1.918442
1.476617	1.683198	1.677914	1.733597	1.728119	1.722787	1.717764	1.71288	1.918775
1.476813	1.683507	1.678224	1.733926	1.728448	1.723111	1.718072	1.713169	1.919138
1.476902	1.683669	1.678436	1.734204	1.728747	1.72342	1.718375	1.71346	1.919494
1.476915	1.683724	1.678576	1.734445	1.729031	1.723731	1.718686	1.71376	1.919852
1.477012	1.683897	1.678795	1.734715	1.729326	1.72404	1.718994	1.714057	1.920209
1.477202	1.684202	1.679102	1.735018	1.729632	1.724348	1.719296	1.714352	1.920565
1.477411	1.684527	1.679423	1.735334	1.729945	1.724658	1.7196	1.71465	1.920925
1.477603	1.684814	1.67972	1.735654	1.730264	1.724974	1.719909	1.714953	1.921296
1.47783	1.685151	1.680051	1.735996	1.730593	1.725291	1.720219	1.715259	1.921669
1.478164	1.685646	1.680485	1.67533	1.670134	1.665036	1.720525	1.715565	1.922034
1.478542	1.686207	1.680965	1.675734	1.670482	1.665343	1.720831	1.715871	1.922397
1.478822	1.686629	1.681362	1.6761	1.670811	1.665642	1.721132	1.716173	1.922758
1.479027	1.686943	1.681696	1.676438	1.671125	1.665934	1.721428	1.716471	1.923117
1.479229	1.583545	1.579096	1.676778	1.671494	1.666318	1.721806	1.716818	1.791438
1.479436	1.583766	1.579312	1.677128	1.671945	1.666842	1.722304	1.717233	1.791812
1.479639	1.583983	1.579526	1.677468	1.672373	1.667332	1.72278	1.717642	1.792187
1.479829	1.584188	1.579737	1.677789	1.672711	1.667684	1.723151	1.718007	1.792538
1.480034	1.584403	1.579953	1.678101	1.67302	1.667992	1.723486	1.718353	1.792893
1.480309	1.584673	1.580182	1.6784	1.673334	1.668323	1.663339	1.658389	1.793273
1.480623	1.584976	1.580428	1.678705	1.673654	1.668661	1.663658	1.658692	1.793676
1.48088	1.585259	1.580727	1.679133	1.674058	1.669045	1.664052	1.659107	1.794149
1.481081	1.585519	1.581078	1.679686	1.674548	1.669479	1.664525	1.659639	1.7947
1.481342	1.58583	1.581439	1.680199	1.674999	1.669874	1.664943	1.660096	1.795173
1.481718	1.586235	1.581817	1.680637	1.675361	1.670179	1.665239	1.660407	1.795508
1.482079	1.586618	1.582164	1.681041	1.675732	1.670526	1.665567	1.660721	1.795831
1.482322	1.58688	1.582415	1.681393	1.676174	1.671036	1.666054	1.661146	1.741754
1.482531	1.587108	1.582644	1.681747	1.676648	1.6716	1.666585	1.661597	1.742158
1.482803	1.587412	1.582972	1.682224	1.677139	1.672084	1.667011	1.661957	1.742549
1.483113	1.587765	1.583365	1.682785	1.677645	1.67252	1.667373	1.662266	1.74294
1.374274	1.588146	1.58375	1.579349	1.574929	1.570515	1.667762	1.662681	1.657763
1.374644	1.588571	1.584138	1.579689	1.5752	1.570751	1.668174	1.663221	1.658294
1.374966	1.58894	1.584476	1.58	1.575488	1.571036	1.668622	1.663738	1.658804
1.375187	1.589186	1.584711	1.580256	1.575819	1.571428	1.669133	1.664164	1.659238
1.375398	1.589423	1.584951	1.580526	1.576169	1.571838	1.669652	1.664594	1.659675
1.375671	1.589741	1.585301	1.580895	1.576529	1.572192	1.670129	1.665114	1.660186
1.375977	1.590101	1.5857	1.581307	1.576901	1.572533	1.670593	1.665663	1.660721
1.376312	1.590489	1.586077	1.581679	1.577281	1.572918	1.671087	1.666138	1.661195
1.376676	1.590907	1.586443	1.582026	1.577668	1.573337	1.671603	1.666557	1.661623
1.377013	1.591301	1.586823	1.582398	1.578048	1.573723	1.672098	1.667024	1.66209
1.37731	1.591659	1.587223	1.582812	1.578419	1.574061	1.672565	1.667567	1.66262

1.377616	1.592023	1.587611	1.583206	1.578785	1.574405	1.673031	1.66808	1.663125
1.377959	1.592422	1.587964	1.583539	1.579146	1.574789	1.673512	1.66849	1.663548
1.378306	1.592819	1.588309	1.583861	1.579503	1.575174	1.673991	1.66889	1.663961
1.378609	1.593163	1.588672	1.584232	1.579857	1.575516	1.674449	1.669392	1.664445
1.378898	1.593487	1.589044	1.584623	1.580207	1.575837	1.674897	1.669938	1.664962
1.379266	1.593889	1.589414	1.584975	1.580556	1.576188	1.571896	1.567647	1.665418
1.379708	1.594363	1.58978	1.585285	1.580899	1.576563	1.572243	1.567956	1.665815
1.38012	1.498522	1.494258	1.585669	1.581306	1.576989	1.572651	1.568334	1.666272
1.380481	1.498948	1.494725	1.586176	1.58182	1.577502	1.573161	1.568828	1.666823
1.380816	1.499346	1.49518	1.586682	1.582319	1.577989	1.573654	1.569321	1.667399
1.381108	1.499686	1.495563	1.587115	1.582715	1.578354	1.574038	1.569742	1.667982
1.381402	1.500018	1.49592	1.58751	1.583068	1.578677	1.574381	1.570128	1.668552
1.381798	1.500433	1.496287	1.587859	1.583418	1.579032	1.574729	1.570471	1.669025
1.38226	1.500901	1.496659	1.588176	1.583762	1.579405	1.575075	1.570783	1.669437
1.382672	1.501349	1.497081	1.588594	1.584191	1.579844	1.575496	1.571181	1.669945
1.383034	1.501782	1.497562	1.589128	1.584723	1.580365	1.576006	1.571678	1.670564
1.383373	1.502178	1.49801	1.58963	1.585209	1.580832	1.576471	1.572148	1.671154
1.383671	1.502502	1.49837	1.590031	1.585572	1.581164	1.576823	1.572531	1.671652
1.383986	1.502843	1.498739	1.590435	1.585938	1.5815	1.577181	1.57292	1.672156
1.384382	1.503295	1.499198	1.590921	1.58641	1.581958	1.577641	1.573385	1.569094
1.38482	1.5038	1.4997	1.591443	1.586929	1.582472	1.578149	1.573886	1.569586
1.385257	1.504299	1.5002	1.591969	1.587449	1.582985	1.578659	1.574393	1.570087
1.385701	1.504803	1.500706	1.592502	1.587977	1.583506	1.579178	1.574909	1.570601
1.264643	1.382474	1.378907	1.375314	1.371663	1.36804	1.579631	1.575343	1.571036
1.264917	1.382814	1.379201	1.375593	1.371981	1.368388	1.57999	1.575659	1.571357
1.265212	1.383173	1.379527	1.375907	1.372325	1.368754	1.580381	1.576015	1.571714
1.265574	1.383595	1.37995	1.37633	1.372751	1.369179	1.580869	1.576493	1.572185
1.265956	1.38404	1.380404	1.376789	1.373204	1.369626	1.581393	1.577014	1.572696
1.266324	1.384479	1.380843	1.377229	1.373649	1.370073	1.581913	1.577526	1.573202
1.266689	1.384921	1.381281	1.377668	1.374095	1.370524	1.582437	1.578045	1.573716
1.267057	1.385364	1.38172	1.378105	1.374538	1.370971	1.582953	1.578556	1.574222
1.267426	1.38581	1.382159	1.378541	1.374977	1.371413	1.583462	1.57906	1.574723
1.267791	1.386252	1.382597	1.378978	1.375418	1.371856	1.583972	1.579568	1.575227
1.26815	1.386689	1.383036	1.37942	1.375863	1.372303	1.584489	1.580085	1.575737
1.268506	1.387122	1.38347	1.379856	1.376302	1.372744	1.584999	1.580596	1.576244
1.268862	1.387551	1.383896	1.38028	1.376727	1.37317	1.58549	1.581092	1.576743
1.269215	1.387976	1.384315	1.380697	1.377145	1.37359	1.585972	1.581582	1.577239
1.269565	1.388394	1.384727	1.381108	1.377564	1.374016	1.586461	1.582071	1.577733
1.269912	1.388808	1.385132	1.381513	1.377982	1.374445	1.586952	1.582559	1.578225
1.270263	1.38923	1.38554	1.381917	1.378395	1.374868	1.371248	1.367615	1.578718
1.270612	1.389656	1.385943	1.382309	1.378796	1.375281	1.371666	1.368036	1.579203
1.270994	1.267841	1.264688	1.382759	1.379238	1.37572	1.37212	1.368508	1.579748
1.271435	1.268282	1.26515	1.383318	1.379758	1.376212	1.372645	1.369078	1.580405
1.271873	1.268718	1.265608	1.383877	1.380277	1.376702	1.373165	1.369643	1.581056
1.272253	1.269091	1.265983	1.384339	1.380718	1.377129	1.373606	1.370104	1.581587
1.272609	1.269438	1.266323	1.384762	1.381138	1.377548	1.374027	1.37053	1.58208
1.272974	1.269795	1.26667	1.3852	1.381618	1.37806	1.374528	1.371006	1.582647
1.273343	1.270157	1.267016	1.385642	1.382139	1.378642	1.375084	1.371516	1.583264
1.273767	1.270574	1.267412	1.386105	1.382627	1.379151	1.375579	1.371987	1.583824
1.274269	1.27107	1.267874	1.386594	1.383075	1.379571	1.375991	1.372404	1.584302

1.274741	1.271537	1.268317	1.387072	1.383507	1.379973	1.376408	1.37285	1.584817
1.275116	1.271911	1.268689	1.3875	1.383907	1.380369	1.376866	1.373386	1.585447
1.27548	1.272277	1.26906	1.387948	1.384324	1.380776	1.377331	1.373923	1.586078
1.275912	1.272707	1.269504	1.388503	1.384829	1.381231	1.377774	1.374365	1.491788
1.276377	1.273172	1.269984	1.389114	1.385385	1.381722	1.378215	1.374766	1.492215
1.276843	1.273648	1.270452	1.389665	1.385931	1.382255	1.378707	1.375216	1.492723
1.277317	1.274138	1.270914	1.390169	1.386476	1.382832	1.379246	1.375704	1.493298
1.158173	1.274591	1.271371	1.268166	1.264984	1.261824	1.37978	1.376223	1.37271
1.15851	1.274986	1.271829	1.268655	1.265449	1.262265	1.380312	1.376798	1.373269
1.158855	1.27538	1.272269	1.269116	1.265887	1.262681	1.380811	1.377337	1.373805
1.159248	1.275803	1.272667	1.269493	1.266252	1.263039	1.381225	1.377754	1.37427
1.159659	1.276241	1.273057	1.269853	1.266607	1.263394	1.381629	1.378142	1.374707
1.160068	1.276697	1.273493	1.270279	1.267041	1.263831	1.38214	1.378602	1.375147
1.160478	1.277166	1.273957	1.270743	1.267522	1.264319	1.382714	1.379104	1.37559
1.024121	1.277623	1.274421	1.271213	1.267996	1.264793	1.383265	1.379634	1.376107
1.024434	1.278066	1.274888	1.271692	1.268466	1.265254	1.383793	1.380197	1.376707
1.02475	1.278515	1.275351	1.272163	1.268935	1.265718	1.384331	1.380744	1.377265
1.025076	1.278981	1.275808	1.272619	1.269404	1.266194	1.384897	1.381253	1.377726
1.025395	1.279441	1.276262	1.273071	1.269866	1.266661	1.385455	1.381761	1.378191
1.025695	1.279876	1.276715	1.273531	1.270312	1.267093	1.263877	1.260689	1.378731
1.025981	1.280297	1.277164	1.27399	1.270752	1.267515	1.264296	1.261116	1.379305
1.026251	1.16364	1.160825	1.274432	1.271197	1.267966	1.264766	1.261605	1.379892
1.026506	1.163971	1.161166	1.27486	1.271646	1.268437	1.265274	1.262141	1.380492
1.026759	1.164292	1.161499	1.275298	1.272122	1.268944	1.265785	1.26264	1.381049
1.027009	1.1646	1.161819	1.275744	1.272637	1.269501	1.266304	1.263094	1.381551
1.027266	1.024829	1.022447	1.276206	1.273137	1.270021	1.266798	1.263546	1.382065
1.027524	1.02511	1.022773	1.276703	1.2736	1.270452	1.267237	1.264001	1.382615
1.027806	1.025409	1.023106	1.277192	1.274044	1.270862	1.26767	1.264471	1.383193
1.028155	1.025739	1.023409	1.277617	1.274467	1.271298	1.268144	1.264988	1.261817
1.028536	1.026083	1.023698	1.278011	1.274882	1.271749	1.268638	1.265524	1.262367
1.028852	1.026381	1.023974	1.161254	1.158487	1.155725	1.269135	1.266004	1.262841
1.029114	1.026642	1.024237	1.161603	1.158898	1.156176	1.269637	1.266437	1.263249
1.029409	1.026935	1.024534	1.161979	1.159295	1.156586	1.270111	1.26688	1.263687
1.029759	1.027282	1.024885	1.16239	1.159665	1.156926	1.270537	1.267332	1.264168
1.030096	1.027619	1.025232	1.162807	1.160047	1.157281	1.270986	1.267812	1.264666
1.030361	1.027894	1.02554	1.163224	1.160476	1.157716	1.271515	1.268345	1.265176
1.030629	1.02817	1.025839	1.16363	1.160902	1.158157	1.27206	1.268896	1.265697
0.844645	1.028525	1.026149	1.163982	1.161248	1.158512	1.27255	1.269428	1.266239
0.845067	1.02893	1.026469	1.164309	1.161553	1.158821	1.27301	1.269946	1.266787
0.845483	1.029341	1.026841	1.164723	1.161938	1.159186	1.273464	1.270401	1.267258
0.845898	1.029767	1.027271	1.165227	1.162404	1.159604	1.273905	1.270787	1.267647
0.846284	0.844234	0.842131	1.025207	1.02279	1.02038	1.157209	1.154382	1.268071
0.846619	0.844555	0.842467	1.025539	1.023149	1.020754	1.157648	1.154793	1.268563
0.84696	0.844882	0.842809	1.025878	1.023515	1.021138	1.158104	1.155226	1.269078
0.847355	0.845276	0.84321	1.026272	1.023909	1.021526	1.158557	1.155659	1.269598
0.847771	0.845696	0.843635	1.026689	1.024317	1.02192	1.159016	1.156099	1.270127
0.848176	0.846105	0.844051	0.842004	0.839972	0.837912	1.159481	1.156538	1.270647
0.848577	0.84651	0.844466	0.842426	0.840398	0.838338	1.15995	1.156981	1.271163
0.848977	0.846913	0.844879	0.842847	0.840824	0.838764	1.160429	1.157437	1.271694
0.849375	0.847314	0.845291	0.843267	0.84125	0.839193	1.160919	1.157911	1.272241

0.849773	0.847714	0.845699	0.843683	0.841672	0.839618	1.161404	1.158381	1.272786
0.85017	0.848112	0.846103	0.844093	0.842088	0.840039	1.161878	1.158838	1.27332
0.850569	0.84851	0.846503	0.844495	0.842497	0.840453	1.16234	1.15929	1.273851
0.850977	0.848911	0.846897	0.844887	0.842894	0.840857	1.022532	1.019901	1.156749
0.851388	0.849313	0.847287	0.845272	0.843283	0.841254	1.022875	1.020257	1.157187
0.597013	0.849714	0.847676	0.845657	0.843672	0.841649	0.839521	0.837351	1.157626
0.597353	0.850113	0.848064	0.84604	0.844059	0.842044	0.839923	0.837761	1.158066
0.597692	0.850514	0.848454	0.846425	0.844447	0.842437	0.840325	0.83817	1.158508
0.598029	0.850916	0.848846	0.846812	0.844836	0.842831	0.840722	0.838571	1.158943
0.598369	0.851321	0.849241	0.8472	0.845224	0.843223	0.841128	0.838992	1.159412
0.598709	0.85172	0.849629	0.847583	0.845604	0.843613	0.841558	0.839467	1.159962
0.599058	0.852134	0.850031	0.847976	0.845987	0.844002	0.841987	0.839946	1.160519
0.599437	0.852609	0.850483	0.848404	0.846386	0.844382	0.84238	0.840363	1.02074
0.599831	0.85312	0.850969	0.848857	0.846798	0.844763	0.842755	0.840744	1.021064
0.600195	0.598671	0.597143	0.849335	0.847246	0.84518	0.84315	0.841128	0.839052
0.60053	0.599023	0.597523	0.849842	0.847732	0.845635	0.843565	0.841509	0.839456
0.600866	0.599367	0.597885	0.850314	0.84819	0.846071	0.843973	0.841893	0.839854
0.601207	0.599697	0.59822	0.85073	0.848594	0.846463	0.844357	0.842274	0.840236
0.601562	0.600036	0.598552	0.851134	0.848999	0.846871	0.844763	0.842676	0.84063
0.601951	0.600406	0.598892	0.851546	0.849445	0.847348	0.845234	0.843127	0.841057
0.602353	0.600792	0.599241	0.851966	0.849903	0.847842	0.845727	0.843602	0.841506
0.602743	0.601184	0.59962	0.598089	0.596607	0.595132	0.846179	0.844081	0.841982
0.603124	0.601582	0.60002	0.598475	0.596952	0.595449	0.846606	0.844565	0.84248
0.603479	0.601952	0.600407	0.598865	0.597321	0.595799	0.847039	0.84501	0.842936
0.6038	0.602292	0.600783	0.599262	0.59772	0.596187	0.847475	0.845405	0.843345
0.604125	0.602626	0.601141	0.59964	0.59811	0.596578	0.59505	0.593532	0.843762
0.604459	0.602945	0.601462	0.599974	0.598476	0.596969	0.595443	0.593913	0.844204
0.604819	0.603286	0.601792	0.600307	0.598833	0.597349	0.595833	0.594305	0.844664
0.605248	0.603705	0.602185	0.600676	0.599182	0.59769	0.59619	0.594688	0.845165
0.605703	0.604155	0.602604	0.601063	0.59953	0.598016	0.596533	0.595065	0.845677
0.60608	0.604536	0.602991	0.601449	0.599905	0.598378	0.596888	0.595416	0.59395
0.606396	0.604866	0.603358	0.601839	0.600302	0.598769	0.59725	0.595745	0.594264
	0.605214	0.603711	0.602203	0.600679	0.599156	0.597633	0.596118	0.594631
	0.605584	0.604042	0.602522	0.601026	0.599539	0.598047	0.596558	0.595082
	0.605969	0.604399	0.602868	0.601388	0.599921	0.598449	0.596979	0.59551
	0.606377	0.604826	0.603297	0.601796	0.600304	0.598809	0.597319	0.595841
	0.606791	0.605262	0.603736	0.602209	0.600686	0.599165	0.597652	0.596164
	0.607186	0.605633	0.604096	0.602576	0.601066	0.59956	0.598061	0.596579
	0.607575	0.605976	0.604421	0.602923	0.601446	0.599972	0.598505	0.597035
	0.607985	0.606378	0.604815	0.603309	0.601827	0.600353	0.598888	0.597419
	0.608416	0.606835	0.605274	0.603733	0.60221	0.600704	0.599214	0.597734
			0.605692	0.604135	0.602594	0.601077	0.599578	0.598094
			0.606025	0.604488	0.602975	0.601485	0.60001	0.598535
			0.606377	0.604857	0.603371	0.601907	0.600456	0.598986
			0.606818	0.605294	0.603803	0.602344	0.600898	0.599417
			0.607294	0.605754	0.604244	0.602779	0.601328	0.599833
			0.60776	0.606195	0.604655	0.603164	0.601693	0.600198
			0.608224	0.606625	0.605047	0.603516	0.602014	0.600529
			0.608632	0.607027	0.605441	0.6039	0.602391	0.600914
			0.608973	0.607391	0.605835	0.604317	0.602828	0.601359

0.604745	0.603269	0.6018
0.605188	0.60371	0.602226
0.60562	0.60413	0.602631
0.606	0.604483	0.602982
0.606359	0.604811	0.603316
0.606759	0.605206	0.603712
0.607188	0.605651	0.604156
		0.604595
		0.605024
		0.605454
		0.605885
		0.606317

								0.618493
								0.618022
								0.617556
								0.617101
								0.616615
								0.858364
								0.857553
								0.856822
								0.856137
						0.859748	0.857624	1.032525
						0.859085	0.856941	1.031707
						0.858412	0.856252	1.030884
						0.857727	0.855552	1.030057
						0.857034	0.854848	1.029225
						0.856349	0.854158	1.028396
						0.855649	0.853458	1.027552
			0.861271	0.859157	0.857028	1.03209	1.029384	1.381769
			0.860494	0.858341	0.856186	1.031061	1.028368	1.380661
			0.859738	0.857568	0.855402	1.030122	1.027439	1.379658
			0.859006	0.856848	0.854691	1.029296	1.026617	1.378785
			0.858292	0.856148	0.854001	1.232476	1.22954	1.37794
			0.857603	0.855463	0.85332	1.231739	1.228818	1.377095
			0.856925	0.854788	0.85265	1.231015	1.22811	1.376259
			0.85624	0.85411	0.851983	1.230301	1.227412	1.375429
			0.855555	0.853435	0.851321	1.229597	1.226723	1.374603
	0.859124	0.856997	1.032459	1.029815	1.02718	1.380672	1.377223	1.373777
	0.858455	0.856324	1.031658	1.029021	1.026396	1.379839	1.376399	1.372953
	0.857791	0.855659	1.236106	1.233222	1.230372	1.379005	1.37557	1.372122
	0.857132	0.855004	1.235447	1.232551	1.229684	1.378168	1.374736	1.371285
	0.856478	0.854354	1.234793	1.231883	1.228997	1.37733	1.373898	1.370443
	0.855822	0.853707	1.23412	1.231202	1.228304	1.376487	1.373054	1.369595
	0.855174	0.853064	1.23344	1.230519	1.227612	1.375644	1.372206	1.368745
0.856697	1.032283	1.029627	1.23275	1.229833	1.226934	1.374812	1.37136	1.367894
0.856101	1.031551	1.028866	1.232057	1.229148	1.226264	1.373986	1.370513	1.367043
0.855516	1.237428	1.234397	1.231405	1.228482	1.225586	1.373144	1.369658	1.366189
0.854946	1.236848	1.233812	1.230799	1.227832	1.224892	1.372276	1.368787	1.365326
1.032203	1.236238	1.233213	1.230199	1.22722	1.224261	1.371489	1.367979	1.364504
1.031448	1.235582	1.232589	1.229611	1.226677	1.22375	1.370854	1.367277	1.363743
1.030676	1.234903	1.231943	1.229002	1.226116	1.223227	1.370218	1.366595	1.363013
1.029904	1.386948	1.383441	1.379943	1.376482	1.373008	1.369463	1.365881	1.362312
1.029127	1.386098	1.38259	1.37909	1.375618	1.372146	1.368653	1.365146	1.361621
1.028346	1.385244	1.38174	1.378245	1.374767	1.371296	1.367826	1.364356	1.360875
1.027563	1.384384	1.380886	1.377396	1.373914	1.370441	1.366976	1.363521	1.360089
1.234358	1.383561	1.380046	1.376546	1.373059	1.369589	1.36613	1.362689	1.359289
1.233764	1.382776	1.37921	1.375677	1.372189	1.36873	1.365283	1.361857	1.358473
1.233165	1.382024	1.378435	1.374886	1.371387	1.36792	1.364468	1.36104	1.357659
1.232536	1.381331	1.377792	1.374261	1.370722	1.367198	1.363702	1.360238	1.356846

1.231887	1.380627	1.377152	1.373649	1.370083	1.366511	1.362973	1.359466	1.356056
1.231196	1.379814	1.376366	1.372895	1.36937	1.36583	1.362291	1.358763	1.355321
1.230493	1.378954	1.375513	1.372073	1.36862	1.365151	1.361636	1.358102	1.354625
1.229875	1.378167	1.3747	1.371255	1.367832	1.364406	1.360928	1.357427	1.353964
1.229332	1.377439	1.37391	1.370428	1.367004	1.363602	1.360176	1.356748	1.35334
1.22876	1.37674	1.373206	1.369716	1.366274	1.362859	1.359441	1.356031	1.352646
1.228144	1.376089	1.372638	1.369183	1.365698	1.362208	1.358722	1.355246	1.35183
1.227509	1.375406	1.372032	1.368617	1.365109	1.361571	1.358036	1.354508	1.351059
1.226831	1.374604	1.371253	1.367871	1.364413	1.360926	1.357432	1.353932	1.350468
1.22617	1.373794	1.370441	1.367078	1.363679	1.360261	1.356824	1.353371	1.349903
1.225642	1.373137	1.369749	1.366357	1.362937	1.359507	1.356069	1.352624	1.349177
1.225192	1.372563	1.369121	1.365669	1.362188	1.358705	1.355232	1.351773	1.34836
1.224646	1.371898	1.368455	1.364997	1.361509	1.358012	1.354507	1.351009	1.347588
1.224001	1.371138	1.367752	1.364344	1.360907	1.357437	1.353896	1.350325	1.34685
1.22342	1.370433	1.367069	1.363677	1.360254	1.356788	1.353235	1.349642	1.346152
1.222977	1.369852	1.366431	1.36298	1.359489	1.355979	1.352455	1.348935	1.345513
1.222523	1.369257	1.365786	1.362284	1.358733	1.355185	1.351691	1.348242	1.344885
1.221928	1.368529	1.365088	1.361612	1.358088	1.354558	1.351061	1.347595	1.344224
1.221298	1.367768	1.364376	1.360946	1.357472	1.353976	1.350468	1.346964	1.343561
1.220784	1.367119	1.363708	1.360259	1.356761	1.353252	1.349764	1.3463	1.342934
1.220346	1.366542	1.363068	1.359559	1.35599	1.352438	1.34899	1.345617	1.342333
1.219838	1.365891	1.362404	1.358885	1.355307	1.351754	1.348325	1.344983	1.341728
1.21924	1.365144	1.361703	1.358238	1.354728	1.351228	1.347792	1.344408	1.341115
1.218666	1.364441	1.361037	1.357614	1.354151	1.350686	1.347245	1.343833	1.340515
1.218169	1.363862	1.360469	1.357041	1.353551	1.350059	1.34662	1.343225	1.339934
1.217685	1.363289	1.359886	1.356442	1.352923	1.34941	1.345988	1.342631	1.339373
1.217169	1.362612	1.359158	1.355693	1.352211	1.348758	1.345396	1.342099	1.338851
1.216639	1.361894	1.358371	1.354888	1.351471	1.348108	1.344823	1.341592	1.338344
1.216091	1.361215	1.357691	1.354221	1.350844	1.347524	1.344261	1.341038	1.445144
1.215526	1.360565	1.357092	1.353665	1.350311	1.346998	1.34371	1.340446	1.44444
1.214993	1.359953	1.356517	1.353113	1.349757	1.346432	1.343129	1.339849	1.443757
1.214504	1.359393	1.355972	1.352562	1.349168	1.345804	1.342499	1.339238	1.443097
1.21403	1.358836	1.355414	1.351992	1.348569	1.345181	1.341889	1.338662	1.55651
1.213567	1.358257	1.354808	1.351372	1.347953	1.344585	1.341336	1.338165	1.555836
1.213106	1.357666	1.354187	1.350743	1.347347	1.344017	1.340815	1.337695	1.555178
1.212624	1.357042	1.353568	1.350145	1.346796	1.343513	1.340327	1.337202	1.554446
1.212134	1.356402	1.352953	1.349568	1.346282	1.343052	1.339861	1.336697	1.55367
1.211667	1.355814	1.352382	1.349021	1.345772	1.342567	1.339353	1.33614	1.552863
1.211225	1.35528	1.351858	1.348505	1.345269	1.342063	1.338805	1.335527	1.552014
1.210756	1.354723	1.351329	1.347995	1.344765	1.341554	1.466082	1.461914	1.551215
1.210229	1.354111	1.350777	1.347487	1.344265	1.341049	1.465403	1.461261	1.550511
1.209719	1.353513	1.350234	1.346979	1.343749	1.340516	1.464704	1.460594	1.549806
1.209281	1.35298	1.349723	1.346465	1.34319	1.339908	1.463945	1.459839	1.549022
1.208878	1.352477	1.349225	1.345952	1.34262	1.339282	1.463178	1.45907	1.548227
1.208472	1.351971	1.348723	1.345443	1.342088	1.338728	1.462494	1.4584	1.642296
1.208072	1.351468	1.348219	1.344939	1.341581	1.338223	1.461868	1.457797	1.641563
1.207673	1.35097	1.347728	1.344451	1.34109	1.337729	1.46124	1.457174	1.557515
1.207271	1.350475	1.34725	1.343983	1.34062	1.33725	1.460611	1.456531	1.556796
1.206886	1.349988	1.346769	1.472536	1.46833	1.46412	1.459978	1.455886	1.556076
1.206535	1.349514	1.346275	1.471884	1.467681	1.463474	1.459331	1.455239	1.555359

1.206185	1.34904	1.345777	1.471225	1.467025	1.462823	1.458685	1.454597	1.554648
1.205806	1.348551	1.345278	1.470573	1.466378	1.462181	1.458048	1.453969	1.740872
1.205413	1.348053	1.344776	1.469924	1.465734	1.461541	1.457418	1.45335	1.739834
1.205016	1.347552	1.344275	1.469279	1.465095	1.460906	1.456788	1.452726	1.738804
1.204614	1.347048	1.343774	1.46864	1.464459	1.460275	1.456159	1.452101	1.737779
1.204214	1.201401	1.198616	1.343576	1.339784	1.335991	1.45555	1.451498	1.736758
1.203815	1.201	1.198219	1.343012	1.339237	1.335465	1.454969	1.450923	1.735741
1.203427	1.20061	1.197834	1.342456	1.338701	1.334951	1.454393	1.450348	1.795942
1.20305	1.200239	1.197471	1.341919	1.338183	1.334447	1.453804	1.449745	1.794835
1.202692	1.199881	1.197114	1.341385	1.337669	1.333952	1.45323	1.449159	1.793751
1.202374	1.34823	1.344517	1.34083	1.337145	1.333471	1.452718	1.44866	1.792771
1.202077	1.347751	1.34398	1.340264	1.336615	1.332996	1.452239	1.44821	1.791852
1.201724	1.347245	1.343435	1.339696	1.336069	1.332487	1.741902	1.735912	2.042071
1.201315	1.346715	1.342877	1.339121	1.335504	1.331944	1.740879	1.734904	2.040957
1.200932	1.346208	1.342374	1.338614	1.334982	1.331412	1.739877	1.733953	2.039894
1.200596	1.345744	1.341958	1.338212	1.334522	1.330895	1.73892	1.733118	2.038929
1.200266	1.345269	1.341544	1.337825	1.334085	1.330404	1.737981	1.732262	2.037932
1.199907	1.34473	1.341072	1.337405	1.333666	1.329967	1.798065	1.792121	2.036792
1.199555	1.344182	1.340578	1.33696	1.333244	1.329548	1.797077	1.79104	2.035602
1.199266	1.343708	1.340081	1.336454	1.332761	1.329088	1.796037	1.78996	2.034449
1.199018	1.343279	1.339576	1.335901	1.332236	1.328606	1.794967	1.788877	2.033317
1.198711	1.34281	1.339055	1.335353	1.331726	1.328147	1.794001	1.787908	2.032265
1.198335	1.342292	1.338507	1.3348	1.331227	1.327716	1.793158	1.787065	2.0313
1.197983	1.34179	1.338004	1.430353	1.426439	1.422591	1.7792	1.773179	2.030364
1.197687	1.341315	1.337585	1.429819	1.425869	1.421992	1.778232	1.772342	2.029474
1.197413	1.340869	1.337188	1.429313	1.42532	1.421403	1.777247	1.771466	2.028552
1.197151	1.340477	1.336769	1.428789	1.424801	1.420884	1.776281	1.770456	2.027484
1.196899	1.34011	1.336348	1.428265	1.424299	1.420396	1.775331	1.769399	2.02636
1.343411	1.339707	1.335945	1.723618	1.717624	1.711709	1.907369	1.901143	2.0733
1.34293	1.339278	1.335557	1.722743	1.716762	1.710848	1.906446	1.900281	2.072337
1.342455	1.33882	1.335132	1.721872	1.715907	1.709999	1.90554	1.899403	2.071393
1.341984	1.338314	1.334646	1.721003	1.715065	1.709166	1.904655	1.898498	2.070466
1.341539	1.433652	1.429627	1.720141	1.714222	1.708335	1.903767	1.897599	2.069546
1.341151	1.433176	1.429145	1.71929	1.713376	1.707505	1.902862	1.896722	2.068627
1.34077	1.432716	1.428675	1.718442	1.712529	1.706674	1.901957	1.895847	2.067711
1.340324	1.432159	1.428155	1.717587	1.711686	1.705836	1.901066	1.894953	2.121196
1.339852	1.431564	1.427614	1.716728	1.710843	1.704994	1.900182	1.894054	2.120231
1.339443	1.431042	1.42711	1.715864	1.709985	1.704149	1.899294	1.8932	2.066783
1.339093	1.430588	1.426637	1.714997	1.709114	1.703301	1.898401	1.892389	2.065859
1.338735	1.640764	1.634958	1.714122	1.70825	1.702465	1.89752	1.891557	2.064927
1.338367	1.639941	1.634108	1.713236	1.707392	1.701643	1.896652	1.890681	2.063979
1.337973	1.639085	1.633249	1.712355	1.706547	1.700836	1.895799	1.889814	2.063038
1.337514	1.638209	1.632407	1.711491	1.705719	1.700042	1.894957	1.888979	2.0621
1.337042	1.637326	1.631574	1.710643	1.704911	1.699272	1.89414	1.888182	2.061195
1.431523	1.721577	1.715629	1.709813	1.704144	1.698568	1.893394	1.887489	2.060404
1.431015	1.720687	1.71477	1.709004	1.703409	1.697909	1.892696	1.886865	2.059688
1.430578	1.719919	1.714009	1.927014	1.920941	1.914961	2.071671	2.06522	2.058868
1.430222	1.719294	1.713362	1.92623	1.920135	1.914139	2.07069	2.064261	2.057935
1.639525	1.718611	1.712668	1.925431	1.919322	1.913319	2.069729	2.063312	2.057006
1.638706	1.717772	1.711844	1.924577	1.918484	1.912499	2.068819	2.062392	2.056095

1.637885	1.716933	1.711035	1.923751	1.91767	1.911699	2.067946	2.061518	2.055228
1.637189	1.716221	1.710375	1.923052	1.916952	1.910954	2.067139	2.060752	2.054474
1.636534	1.71555	1.709762	1.922393	1.916265	1.91023	2.066358	2.060029	2.053766
1.635808	1.714791	1.709032	1.921607	1.915482	1.909451	2.065495	2.059212	2.052964
1.635032	1.713971	1.708219	1.920728	1.914631	1.908634	2.064572	2.05833	2.052097
1.634317	1.713226	1.707479	1.919928	1.913854	1.907887	2.063733	2.057517	2.051298
1.633691	1.712585	1.706839	1.919235	1.913176	1.90723	2.063	2.056797	2.050587
1.633024	1.711909	1.706185	1.985118	1.978632	1.972265	2.062276	2.056076	2.049879
1.632217	1.711109	1.705465	1.984309	1.977874	1.971546	2.061539	2.055333	2.049156
1.631414	1.710302	1.704726	1.983464	1.977081	1.970795	2.060771	2.054558	2.048403
1.630765	1.709596	1.703981	1.982539	1.976183	1.969938	2.059897	2.053689	2.047554
1.6302	1.708947	1.70324	1.981579	1.975239	1.96903	2.058974	2.052779	2.046661
1.714135	1.70832	1.702567	2.077198	2.070743	2.064418	2.058163	2.051992	2.045887
1.713527	1.707721	1.701957	2.07643	2.069986	2.063674	2.057448	2.051312	2.045215
1.712912	1.707114	1.701351	2.075679	2.069252	2.062956	2.056749	2.050632	2.044542
1.712286	1.706493	1.70074	2.074938	2.068537	2.062263	2.05606	2.049937	2.043853
1.711654	1.790462	1.78418	2.074205	2.06783	2.061576	2.055374	2.049242	2.043162
1.711015	1.789754	1.783499	2.07348	2.06712	2.060877	2.054678	2.048543	2.042467
1.710373	1.789045	1.782818	2.072755	2.066408	2.060174	2.053979	2.047846	2.041772
1.709737	1.788339	1.782128	2.072019	2.065686	2.059465	2.053282	2.047158	2.15544
1.709102	1.787634	1.781431	2.071274	2.064957	2.058752	2.052585	2.046477	2.154733
1.708454	1.786917	1.780728	2.070528	2.064232	2.058047	2.051889	2.045783	2.154016
1.707792	1.786187	1.780018	2.06978	2.063513	2.057352	2.051195	2.045078	2.153288
1.707132	1.849528	1.843335	2.069037	2.062794	2.056651	2.050495	2.044369	2.15256
1.706463	1.848754	1.842603	2.068295	2.062071	2.05594	2.049783	2.043649	2.151827
1.705838	1.848018	1.841891	2.067566	2.061351	2.055227	2.049077	2.042948	2.151109
1.70534	1.847399	1.841224	2.066849	2.060624	2.054503	2.048382	2.042294	2.150423
1.704884	1.846824	1.840588	2.066158	2.059916	2.053796	2.047712	2.041678	2.149769
1.918982	2.016971	2.01049	2.065546	2.059313	2.053198	2.047132	2.041118	2.149189
1.918211	2.016235	2.00986	2.064996	2.058791	2.052685	2.046624	2.040607	2.148669
1.917483	2.015523	2.009204	2.064381	2.058188	2.052086	2.120125	2.113999	2.257687
1.916804	2.014831	2.008508	2.063677	2.057477	2.051377	2.119441	2.113339	2.256906
1.970932	2.014183	2.007852	2.063002	2.056784	2.050671	2.11875	2.112668	2.256114
1.970349	2.013621	2.007299	2.062417	2.056155	2.049995	2.118066	2.111995	2.255325
1.969766	2.013058	2.006754	2.06185	2.055557	2.049358	2.117412	2.111341	2.254558
1.969079	2.012381	2.006112	2.061239	2.054981	2.048807	2.116846	2.110763	2.253871
1.968352	2.011659	2.005435	2.060618	2.054427	2.048308	2.11633	2.110221	2.253222
1.967714	2.011026	2.004843	2.060064	2.053895	2.047785	2.115781	2.10963	2.252507
1.967151	2.010469	2.004326	2.059573	2.053389	2.047248	2.115211	2.108999	2.251736
1.966553	2.009861	2.003737	2.059001	2.05282	2.046678	2.114636	2.108405	2.251039
1.96589	2.00916	2.00302	2.058294	2.052149	2.046053	2.114053	2.107868	2.250454
1.965241	2.008492	2.002341	2.132006	2.125791	2.11964	2.185743	2.17941	2.249872
1.964646	2.007937	2.001802	2.131469	2.125256	2.119103	2.185154	2.178811	2.249241
1.964083	2.007429	2.001316	2.130973	2.124756	2.118592	2.184573	2.178193	2.24858
1.963553	2.006916	2.000815	2.130459	2.124243	2.118071	2.18396	2.177535	2.247857
1.96305	2.006408	2.000316	2.12994	2.123727	2.117544	2.183329	2.176851	2.2471
2.012108	2.005868	1.999786	2.129406	2.123176	2.116971	2.218547	2.211976	2.246429
2.011528	2.005292	1.999227	2.128858	2.122587	2.116343	2.217871	2.211374	2.245847
2.010978	2.004739	1.998656	2.128281	2.122003	2.115757	2.217237	2.210785	2.245266
2.010482	2.004224	1.998052	2.127635	2.121417	2.11525	2.216678	2.210201	2.244663

2.009995	2.003728	1.997489	2.12704	2.120871	2.114765	2.21613	2.209629	2.244068
2.009486	2.084036	2.077743	2.198947	2.192545	2.186196	2.215549	2.209073	2.2435
2.008973	2.08356	2.077365	2.198571	2.192087	2.18564	2.214962	2.208523	2.24294
2.008473	2.083067	2.07685	2.197993	2.191525	2.185098	2.214401	2.207955	2.242357
2.007981	2.082563	2.076247	2.197285	2.190896	2.184567	2.213856	2.207376	2.241759
2.007488	2.0821	2.075765	2.196734	2.190346	2.184021	2.213285	2.206802	2.241173
2.006996	2.081688	2.075437	2.196377	2.189884	2.183441	2.212671	2.206229	2.240599
2.006501	2.081258	2.07508	2.146469	2.140024	2.133616	2.209002	2.202564	2.240042
2.006007	2.080775	2.074622	2.14601	2.139627	2.13326	2.208561	2.202031	2.23949
2.005507	2.080277	2.074132	2.145509	2.139213	2.132918	2.20815	2.201537	2.238982
2.004981	2.079781	2.073637	2.144964	2.138704	2.132454	2.207688	2.201118	2.238589
2.004451	2.079286	2.073137	2.144395	2.138145	2.131929	2.207197	2.200731	2.238249
2.085066	2.156558	2.150186	2.143871	2.137602	2.131377	2.206643	2.200254	2.23781
2.084651	2.156029	2.149685	2.143384	2.137064	2.130792	2.206028	2.199703	2.237292
2.08427	2.15557	2.149239	2.142943	2.136601	2.130305	2.205488	2.199166	2.236774
2.083925	2.155201	2.148868	2.142572	2.136251	2.129963	2.205053	2.198644	2.236251
2.083603	2.154856	2.148494	2.188315	2.181886	2.175486	2.204633	2.198161	2.235769
2.083328	2.154526	2.148064	2.187782	2.181375	2.175023	2.204185	2.197745	2.235377
2.083033	2.154179	2.147623	2.187241	2.180848	2.17454	2.20374	2.197367	2.235022
2.082591	2.153744	2.147217	2.186826	2.18042	2.174104	2.203329	2.197017	2.234662
2.082061	2.153248	2.146824	2.186475	2.180041	2.173683	2.202931	2.196682	2.234303
2.081571	2.152725	2.146369	2.186044	2.179589	2.173204	2.202462	2.196259	2.233901
2.081105	2.152162	2.145845	2.185532	2.179063	2.172661	2.201918	2.195746	2.233452
2.117221	2.197879	2.191465	2.185065	2.178583	2.172169	2.201419	2.195268	2.233029
2.116913	2.197525	2.191094	2.184679	2.178183	2.171761	2.201007	2.19487	2.232666
2.116626	2.197211	2.19076	2.184322	2.177813	2.171381	2.200608	2.194464	2.232283
2.116302	2.196883	2.190433	2.183985	2.17746	2.171002	2.200171	2.193974	2.231797
2.115967	2.196551	2.190107	2.183652	2.177115	2.170633	2.199736	2.193471	2.23129
2.15646	2.196176	2.189731	2.183277	2.176757	2.170287	2.199363	2.193069	2.230887
2.156091	2.195771	2.189316	2.182869	2.176385	2.169955	2.199031	2.192737	2.230555
2.155748	2.195377	2.188865	2.182386	2.175925	2.169537	2.198644	2.192395	2.230235
2.155437	2.194995	2.18836	2.181799	2.175348	2.169013	2.198189	2.192042	2.229929
2.201227	2.194626	2.187906	2.181286	2.174834	2.16853	2.197754	2.191689	2.22962
2.20087	2.194276	2.18756	2.180936	2.174465	2.168145	2.197368	2.191333	2.229294
2.200507	2.19393	2.18724	2.18063	2.174141	2.167799	2.197007	2.190984	2.228965
2.200138	2.193561	2.18688	2.180279	2.173797	2.167463	2.196665	2.190652	2.266281
2.199772	2.193191	2.186516	2.179925	2.17346	2.167143	2.196339	2.19033	2.265947
2.199439	2.192866	2.186194	2.179604	2.173135	2.166816	2.196005	2.190008	2.265619
2.199133	2.192579	2.185907	2.179309	2.172821	2.166485	2.195666	2.189687	2.265297
2.198804	2.192281	2.185655	2.179086	2.172588	2.166228	2.195375	2.189375	2.223495
2.198436	2.19197	2.185461	2.178972	2.172474	2.166077	2.195148	2.189068	2.22314
2.198076	2.191643	2.185229	2.17881	2.172325	2.165912	2.194924	2.188781	2.222805
2.197745	2.191286	2.184886	2.178494	2.172047	2.165669	2.194676	2.18852	2.222506
2.197429	2.190929	2.184512	2.178128	2.171723	2.165394	2.19442	2.188285	2.222244
2.197122	2.190602	2.184171	2.177784	2.171395	2.165098	2.194165	2.188096	2.222059
2.19683	2.190303	2.183851	2.177452	2.171063	2.164786	2.193906	2.187929	2.221917
2.196588	2.225666	2.219113	2.21261	2.206123	2.199751	2.233748	2.227668	2.255346
2.1964	2.225524	2.218969	2.212442	2.205908	2.199484	2.233427	2.227328	2.255026
2.196158	2.189745	2.183281	2.176838	2.170388	2.164048	2.233165	2.227052	2.254758
2.195807	2.189391	2.182938	2.17653	2.170157	2.163893	2.233012	2.226902	2.254594

2.19548	2.189048	2.182594	2.176211	2.169909	2.163719	2.232841	2.226738	2.25443
2.195283	2.188827	2.18233	2.175918	2.169617	2.163439	2.232539	2.226458	2.254203
2.195127	2.188644	2.182101	2.17565	2.169327	2.16314	2.232206	2.226143	2.253953
2.194902	2.188403	2.181881	2.175449	2.169136	2.162951	2.231971	2.225874	2.253694
2.19464	2.188134	2.181676	2.175301	2.169013	2.162832	2.231797	2.225633	2.253431
2.194417	2.187928	2.181506	2.175154	2.168858	2.162662	2.231598	2.225434	2.253244
2.194236	2.187792	2.18138	2.175016	2.168668	2.162428	2.231366	2.225279	2.253142
2.194062	2.187644	2.18122	2.174841	2.168478	2.162235	2.231175	2.225147	2.2192
2.193895	2.187458	2.180981	2.174584	2.168289	2.162127	2.23106	2.225042	2.21909
2.193714	2.187251	2.180737	2.174339	2.16812	2.16204	2.230962	2.224938	2.218976
2.193473	2.187007	2.180547	2.1742	2.168003	2.161935	2.230834	2.224804	2.218841
2.193213	2.186756	2.180387	2.174109	2.167913	2.161827	2.230698	2.224657	2.218691
2.193015	2.186593	2.180255	2.216442	2.210115	2.203893	2.230558	2.224511	2.218526
2.192864	2.1865	2.180151	2.216314	2.209983	2.203756	2.230414	2.224363	2.218345
2.192729	2.186383	2.180039	2.216192	2.209869	2.203642	2.230269	2.224186	2.218148
2.192612	2.186222	2.179912	2.216078	2.209781	2.203563	2.230118	2.223953	2.217916
2.1925	2.186081	2.179792	2.215961	2.209682	2.203473	2.229986	2.223779	2.217745
2.192384	2.186004	2.17969	2.21583	2.209542	2.203339	2.229901	2.223767	2.217729
2.192271	2.185934	2.179593	2.2157	2.209402	2.203206	2.229824	2.223773	2.21773
2.192159	2.185795	2.179482	2.215598	2.209316	2.203124	2.229704	2.223614	2.21757
2.192058	2.185634	2.179369	2.215507	2.209253	2.203069	2.22957	2.223386	2.217342
2.192032	2.185583	2.179285	2.215389	2.209147	2.202984	2.229489	2.223313	2.217262
2.192087	2.185644	2.179231	2.215245	2.209001	2.202875	2.229459	2.223385	2.217316
2.109626	2.103356	2.097051	2.215121	2.208878	2.202773	2.229405	2.223401	2.21734
2.109342	2.103086	2.096913	2.215031	2.208797	2.202682	2.229306	2.223315	2.217307
2.109097	2.102854	2.096789	2.214944	2.208722	2.202603	2.229205	2.223206	2.217243
2.109087	2.102834	2.096701	2.214823	2.208621	2.202531	2.22911	2.22308	2.217123
2.109155	2.102892	2.096647	2.21472	2.208537	2.20248	2.229038	2.222975	2.217002
2.109137	2.150575	2.144159	2.214728	2.208548	2.202496	2.229058	2.223006	2.216994
2.109081	2.150544	2.144187	2.214814	2.208625	2.202557	2.229139	2.223124	2.217057
2.044306	2.150555	2.144245	2.214909	2.208671	2.202546	2.229124	2.223138	2.217071
2.044294	2.150621	2.144349	2.215025	2.208685	2.20245	2.228999	2.223042	2.217041
2.044227	2.150611	2.144377	2.21507	2.208691	2.202404	2.22891	2.22293	2.216964
2.044028	2.150438	2.144257	2.214992	2.208701	2.202479	2.228913	2.222802	2.216798
2.043841	2.150275	2.144113	2.214874	2.208685	2.202551	2.228934	2.222711	2.216659
1.987537	2.131172	2.124711	2.247585	2.241327	2.235145	2.228929	2.222736	2.216663
1.98755	2.131163	2.124566	2.247457	2.241198	2.235056	2.228919	2.222821	2.216739
1.987464	2.131083	2.124463	2.247423	2.241157	2.235022	2.228929	2.222892	2.216813
1.98731	2.130961	2.124401	2.247465	2.241182	2.235022	2.228954	2.222961	2.216897
1.987225	2.13089	2.12437	2.198012	2.191575	2.185249	2.228923	2.222967	2.216933
1.987228	2.130884	2.124377	2.198016	2.191493	2.185083	2.228808	2.222884	2.216901
1.98724	2.130886	2.124389	2.198029	2.191438	2.184963	2.228738	2.222832	2.216889
1.987253	2.130896	2.12441	2.198066	2.191458	2.184959	2.228788	2.22288	2.216948
1.987235	2.130871	2.124399	2.198081	2.191483	2.184986	2.228872	2.222954	2.217023
1.987101	2.081326	2.074987	2.197998	2.191435	2.18497	2.178724	2.172626	2.21708
1.986912	2.081086	2.074795	2.197865	2.191356	2.184941	2.178702	2.172591	2.21712
2.043119	2.080978	2.074702	2.197805	2.191324	2.184928	2.178657	2.172492	2.217085
2.043131	2.080982	2.074692	2.197805	2.19133	2.184928	2.178588	2.172327	2.216975
2.043147	2.080993	2.074693	2.19782	2.191346	2.184935	2.178549	2.172225	2.166089
2.043152	2.080996	2.074695	2.197846	2.191363	2.184942	2.178546	2.172213	2.166071

2.043161	2.081004	2.074705	2.154184	2.147873	2.141654	2.178577	2.172247	2.166099
2.043172	2.081016	2.074719	2.154235	2.14794	2.141731	2.178652	2.172312	2.166146
2.043187	2.081032	2.074737	2.154294	2.148018	2.141825	2.178754	2.172416	2.166234
2.0432	2.081032	2.074741	2.154335	2.148068	2.141892	2.178864	2.172579	2.166406
2.043219	2.081034	2.074745	2.154374	2.148105	2.141945	2.178982	2.172782	2.166635
2.043331	2.081157	2.074849	2.068644	2.062538	2.05654	2.179056	2.172899	2.166767
2.043551	2.081419	2.075066	2.068798	2.062623	2.056564	2.17908	2.172927	2.166799
2.043704	2.081609	2.075241	2.068937	2.062705	2.056589	2.179117	2.172949	2.166819
2.04372	2.081649	2.075325	2.069037	2.062764	2.056598	2.17917	2.172967	2.166834
2.043703	2.081647	2.075371	2.069115	2.062836	2.056651	2.179265	2.17302	2.166873
2.043689	2.081642	2.075386	2.069167	2.062963	2.056841	2.136333	2.130285	2.166989
2.043694	2.081645	2.075389	2.069201	2.0631	2.057073	2.13657	2.130474	2.167136
2.043827	2.081748	2.075439	2.069222	2.063149	2.05716	2.136686	2.13059	2.16724
2.044061	2.081928	2.075525	2.069234	2.063134	2.057145	2.136718	2.130657	2.167317
2.044219	2.082058	2.075645	2.069346	2.063232	2.057228	2.136816	2.130742	2.167414
2.044272	2.082112	2.075805	2.069585	2.063478	2.057445	2.136999	2.130844	2.16753
2.04435	2.082206	2.075981	2.069816	2.063697	2.057626	2.137177	2.130977	2.167682
2.044535	2.082422	2.076191	2.069996	2.063807	2.057679	2.137307	2.131162	2.167895
2.044707	2.082626	2.076368	2.070131	2.063885	2.057719	2.137438	2.131366	2.168125
2.044728	2.082656	2.076403	2.070184	2.063981	2.05786	2.137627	2.131568	2.168323
2.044686	2.082606	2.076362	2.070179	2.06407	2.058037	2.137833	2.131765	2.168507
2.044775	2.082658	2.076363	2.070161	2.064099	2.058123	2.052207	2.046351	2.125867
2.044976	2.082793	2.076391	2.070117	2.064069	2.058132	2.052223	2.046372	2.125992
2.04512	2.082896	2.076473	2.070185	2.064127	2.058187	2.052286	2.046452	2.126147
2.045142	2.082919	2.076625	2.070421	2.064315	2.058307	2.052415	2.046622	2.126349
2.04524	2.083016	2.076805	2.070654	2.064515	2.058461	2.052576	2.046808	2.126553
2.045587	2.083334	2.077001	2.070769	2.064656	2.05865	2.052774	2.046997	2.126744
2.045974	2.08369	2.077208	2.070874	2.064797	2.058854	2.052975	2.047167	2.126919
2.046123	2.083834	2.077399	2.071095	2.065006	2.059031	2.0531	2.047236	2.165941
2.04614	2.083863	2.077581	2.071379	2.065251	2.059195	2.053189	2.047254	2.166032
2.046259	2.084002	2.077753	2.071572	2.065441	2.059376	2.053373	2.047444	2.166251
2.046476	2.08425	2.07791	2.071668	2.065572	2.059575	2.053661	2.047825	2.166614
2.046693	2.084471	2.078091	2.071822	2.065738	2.059769	2.053895	2.048101	2.166895
2.046881	2.08461	2.078314	2.072101	2.065979	2.059944	2.053998	2.048136	2.166987
2.047082	2.084767	2.078542	2.072376	2.066226	2.060138	2.054126	2.048198	2.167103
2.047326	2.085032	2.078744	2.07254	2.066431	2.060398	2.054416	2.048503	2.167415
2.047583	2.085328	2.078941	2.072676	2.066622	2.060674	2.054759	2.048897	2.167803
2.047793	2.085519	2.079154	2.072905	2.066832	2.060871	2.055	2.049205	2.16814
2.047975	2.085642	2.07938	2.073195	2.067052	2.061013	2.055174	2.049464	2.168451
2.048275	2.085893	2.079613	2.073416	2.067257	2.06121	2.055372	2.049675	2.08789
2.048725	2.086299	2.079843	2.073534	2.067439	2.061479	2.055603	2.04982	2.088033
2.049137	2.086703	2.080129	2.073733	2.067651	2.06172	2.055817	2.04999	2.088194
2.049431	2.087067	2.080536	2.074125	2.067914	2.061855	2.055973	2.050233	2.08843
2.049676	2.087381	2.080942	2.074554	2.068214	2.062018	2.05615	2.05049	2.088674
2.04988	2.087559	2.081211	2.074897	2.068574	2.062365	2.056442	2.050705	2.08886
2.050073	2.087683	2.081416	2.075196	2.068959	2.062798	2.056795	2.050918	2.089037
2.050409	2.087973	2.08166	2.075426	2.069239	2.063132	2.101255	2.095206	2.089319
2.050869	2.088401	2.081929	2.075587	2.069429	2.063392	2.101565	2.095557	2.089692
2.051265	2.088798	2.082241	2.075841	2.069699	2.063702	2.101902	2.095927	2.090052
2.05155	2.089131	2.082604	2.076224	2.070094	2.064102	2.102293	2.096327	2.090389

2.051852	2.089481	2.083	2.076643	2.070492	2.06447	2.102642	2.096693	2.090713
2.052257	2.089912	2.083452	2.077077	2.07083	2.064712	2.10286	2.096957	2.091012
2.052669	2.090333	2.083896	2.077505	2.071156	2.064936	2.103057	2.0972	2.09131
2.052962	2.090606	2.084211	2.077852	2.071495	2.06526	2.103367	2.097522	2.091653
2.053197	2.090799	2.084455	2.078156	2.071843	2.065644	2.103739	2.097879	2.092012
1.910996	2.047184	2.04096	2.034783	2.028584	2.02249	2.104076	2.098154	2.092277
1.911367	2.047626	2.041394	2.035207	2.029008	2.022896	2.10438	2.098343	2.09244
1.911726	2.048062	2.041831	2.03564	2.029437	2.02331	2.104717	2.098601	2.092676
1.912039	2.048464	2.042268	2.036097	2.029888	2.023743	2.105118	2.098984	2.093045
1.912357	2.048858	2.042669	2.036501	2.030292	2.024146	2.105515	2.099391	2.093447
1.912709	2.049243	2.042963	2.036736	2.03055	2.024453	2.105849	2.099763	2.093835
1.913076	2.04963	2.04323	2.036928	2.030768	2.024732	2.106166	2.100131	2.094227
1.913434	2.097281	2.090723	2.084279	2.078011	2.071873	2.106519	2.100511	2.094617
1.91379	2.097716	2.091174	2.084731	2.078431	2.072259	2.106898	2.100898	2.095007
1.914146	2.098139	2.091607	2.085166	2.078852	2.072662	2.107291	2.101288	2.095394
1.914502	2.098546	2.092017	2.085581	2.079272	2.073084	2.107702	2.101682	2.095778
1.914865	2.098968	2.09244	2.086003	2.079696	2.073505	2.108111	2.102074	2.096159
1.915242	2.09942	2.092874	2.086418	2.080099	2.0739	2.108499	2.102456	2.096538
1.91562	2.099883	2.09334	2.086877	2.08054	2.074318	2.108902	2.102842	2.096916
1.915982	2.100349	2.093892	2.087476	2.081104	2.074821	2.109357	2.103245	2.09729
1.916339	2.100813	2.094476	2.08813	2.081722	2.075364	2.10984	2.103657	2.097666
1.916702	2.057799	2.051585	2.088642	2.082241	2.075871	2.110311	2.104068	2.098043
1.91707	2.058196	2.05197	2.089044	2.082685	2.076352	2.110775	2.104479	2.098422
1.786206	1.968309	1.962272	2.089447	2.083112	2.076806	2.111247	2.104957	2.09889
1.786537	1.968645	1.962609	2.089858	2.083519	2.077223	2.111735	2.105539	2.099496
1.786871	1.968991	1.962952	2.090282	2.083933	2.07764	2.112205	2.106083	2.100059
1.787188	1.969335	1.963291	2.090719	2.084361	2.078055	2.11261	2.106476	2.100452
1.787533	1.969722	1.963662	2.091183	2.084822	2.078505	2.113029	2.106854	2.100813
1.787986	1.970244	1.964136	2.091711	2.085376	2.079084	2.113594	2.107378	2.101288
1.788504	1.970849	1.964673	2.092275	2.08598	2.079732	2.114233	2.107972	2.101816
1.788996	1.971388	1.965145	2.092769	2.086477	2.080244	2.114744	2.108473	2.10231
1.78947	1.971872	1.965555	2.093189	2.086865	2.080622	2.115131	2.108888	2.102777
1.78989	1.972325	1.966003	2.093689	2.08734	2.081073	2.115563	2.109306	2.103219
1.790228	1.972743	1.966534	2.094338	2.087981	2.08167	2.116083	2.109725	2.103604
1.790551	1.973141	1.967041	2.094975	2.088614	2.082266	2.116627	2.110198	2.104042
1.736531	1.851753	1.846468	2.051996	2.045701	2.039423	2.117163	2.110802	2.104648
1.736886	1.852084	1.84678	2.052451	2.046146	2.039876	2.117712	2.111455	2.105313
1.737341	1.852554	1.847195	2.052982	2.046692	2.040446	2.118303	2.112042	2.105902
1.737871	1.853121	1.84768	2.053562	2.047303	2.041093	2.118924	2.112591	2.106447
1.652972	1.853627	1.848177	1.963265	1.957189	1.951148	2.119501	2.113098	2.106937
1.653409	1.854061	1.84869	1.963882	1.957762	1.951654	2.120029	2.113545	2.107351
1.653863	1.854521	1.849191	1.964466	1.958293	1.952122	2.120557	2.114053	2.107838
1.654359	1.855056	1.849672	1.964987	1.95874	1.952516	2.121084	2.114688	2.108483
1.654858	1.855586	1.850126	1.965469	1.959176	1.952933	2.12164	2.11535	2.109159
1.655327	1.80626	1.800842	1.845016	1.839582	1.834192	2.081531	2.075272	2.109761
1.655786	1.806669	1.801189	1.845379	1.840043	1.834748	2.082202	2.075852	2.110349
1.65627	1.807167	1.801699	1.845904	1.840543	1.835222	2.082826	2.076498	2.111005
1.656773	1.807735	1.80234	1.846564	1.84108	1.835637	2.083417	2.077196	2.111714
1.657259	1.808235	1.802854	1.847091	1.841576	1.836103	2.084036	2.077843	2.112368
1.657718	1.80863	1.803174	1.847415	1.84201	1.836649	2.084698	2.078413	2.112941

1.658181	1.809054	1.803548	1.79814	1.792932	1.787793	2.000074	1.993886	2.072839
1.658669	1.809596	1.804127	1.798714	1.793415	1.788185	2.000595	1.994471	2.073483
1.659152	1.810147	1.804746	1.799344	1.793935	1.78859	2.001129	1.995066	2.074137
1.659584	1.81059	1.805256	1.799907	1.794506	1.789145	2.001772	1.995613	2.074733
1.659992	1.810983	1.805713	1.800435	1.795105	1.789779	2.002479	1.996149	2.075314
1.660445	1.811439	1.806157	1.800886	1.79561	1.790337	1.834317	1.828813	2.076006
1.660941	1.811952	1.806579	1.801256	1.796029	1.790831	1.834907	1.829451	2.076809
1.66142	1.812435	1.807028	1.801682	1.796458	1.791279	1.835436	1.830045	2.07756
1.661851	1.812846	1.807506	1.802195	1.796899	1.791648	1.83586	1.830567	2.078222
1.662315	1.813301	1.808028	1.802751	1.797388	1.792061	1.836297	1.831057	2.078852
1.662887	1.8139	1.80864	1.803368	1.797994	1.79264	1.787405	1.782249	1.993807
1.66349	1.814547	1.80927	1.803984	1.798624	1.793267	1.78795	1.782681	1.99432
1.664018	1.815115	1.809771	1.804445	1.799115	1.793801	1.788488	1.783208	1.994993
1.664507	1.815643	1.810197	1.804809	1.799521	1.794279	1.789025	1.783798	1.99577
1.665061	1.816219	1.810727	1.805296	1.799973	1.794716	1.789501	1.784338	1.996464
1.665695	1.816859	1.81138	1.805917	1.800469	1.795095	1.789899	1.784818	1.997062
1.666265	1.817424	1.811985	1.806528	1.801003	1.79555	1.790368	1.785341	1.841117
1.66669	1.81783	1.81247	1.807083	1.801587	1.796149	1.790978	1.785954	1.841732
1.667114	1.81823	1.812944	1.807631	1.80219	1.796789	1.791624	1.786583	1.842366
1.564796	1.818753	1.813496	1.808214	1.802796	1.797412	1.792244	1.78719	1.842992
1.565276	1.81933	1.814067	1.808788	1.803392	1.798027	1.792849	1.787771	1.843599
1.565769	1.819906	1.814562	1.809242	1.803892	1.798582	1.79338	1.788249	1.844095
1.56628	1.82049	1.815001	1.809598	1.804314	1.799088	1.793852	1.78864	1.844501
1.566726	1.665024	1.659971	1.740448	1.735398	1.73041	1.794385	1.789127	1.845003
1.567082	1.665561	1.660548	1.741081	1.736038	1.731046	1.794998	1.789737	1.845629
1.567464	1.666117	1.661144	1.741732	1.736691	1.731693	1.795624	1.790367	1.846275
1.56793	1.666721	1.661743	1.742359	1.737327	1.732337	1.796247	1.790981	1.846906
1.568428	1.667343	1.66235	1.742986	1.737961	1.732979	1.796869	1.791596	1.847539
1.568927	1.564644	1.560343	1.743611	1.738588	1.733611	1.79748	1.792208	1.848171
1.569434	1.565142	1.560827	1.744235	1.739211	1.734237	1.798083	1.792818	1.848802
1.569935	1.565637	1.561315	1.744862	1.739836	1.734863	1.798686	1.793428	1.849433
1.570434	1.566132	1.561805	1.74549	1.740462	1.735488	1.799288	1.794038	1.850065
1.570931	1.566624	1.562291	1.746113	1.741083	1.73611	1.799888	1.794641	1.850689
1.571428	1.567111	1.56277	1.746728	1.741699	1.736732	1.800488	1.795235	1.851302
1.571926	1.567599	1.563248	1.74734	1.742311	1.737348	1.801082	1.795825	1.851912
1.572427	1.568094	1.563725	1.654565	1.650084	1.645648	1.801661	1.796407	1.852516
1.57293	1.568591	1.564205	1.655058	1.650572	1.646135	1.802239	1.796993	1.853124
1.573431	1.56909	1.56469	1.655563	1.651065	1.64662	1.802828	1.797589	1.853741
1.573932	1.569591	1.565182	1.656085	1.651569	1.647109	1.803429	1.7982	1.854372
1.574427	1.570104	1.565735	1.561414	1.557179	1.552997	1.735429	1.730419	1.79376
1.574908	1.570625	1.566356	1.562098	1.557834	1.553606	1.7362	1.731259	1.794569
1.575438	1.57117	1.566955	1.562729	1.558441	1.55418	1.736925	1.73203	1.795308
1.576063	1.571761	1.567506	1.563248	1.558944	1.554674	1.737552	1.732657	1.795904
1.576686	1.572345	1.568035	1.563735	1.559419	1.555146	1.738156	1.733249	1.79647
1.577209	1.572857	1.568533	1.564221	1.559892	1.555609	1.645986	1.641619	1.797067
1.577707	1.573357	1.56903	1.564712	1.560375	1.556082	1.646492	1.642121	1.797678
1.578306	1.573963	1.569618	1.565283	1.560947	1.556654	1.647084	1.642682	1.798336
1.578975	1.574648	1.570273	1.565915	1.561588	1.557304	1.647742	1.64329	1.799032
1.579568	1.575251	1.570859	1.566487	1.562165	1.557882	1.648332	1.643848	1.799684
1.580055	1.575742	1.571348	1.566973	1.562651	1.558365	1.648828	1.644328	1.800266

1.580563	1.576251	1.571869	1.5675	1.563167	1.558866	1.649357	1.644858	1.800907
1.581162	1.576848	1.572503	1.56815	1.563777	1.559432	1.649978	1.645509	1.80169
1.581758	1.577443	1.573138	1.568808	1.564408	1.560031	1.650638	1.646194	1.8025
1.487824	1.577939	1.573655	1.569357	1.565008	1.560675	1.651331	1.646849	1.803239
1.488259	1.578397	1.574122	1.569855	1.565593	1.561338	1.652037	1.647487	1.803938
1.488783	1.578951	1.574663	1.570392	1.566149	1.561911	1.557632	1.553353	1.643618
1.489384	1.579587	1.575266	1.570956	1.566676	1.562404	1.558102	1.553807	1.644106
1.369234	1.58021	1.57588	1.571553	1.567239	1.562935	1.558625	1.554333	1.644667
1.369741	1.580797	1.576503	1.572197	1.567856	1.563521	1.559229	1.554972	1.645341
1.37025	1.581398	1.577127	1.572837	1.568483	1.564135	1.559856	1.555621	1.646027
1.370792	1.582059	1.57775	1.573433	1.569103	1.564781	1.560488	1.556223	1.64667
1.371324	1.582717	1.578367	1.574025	1.569726	1.565436	1.561124	1.556819	1.647308
1.371763	1.488934	1.484999	1.574663	1.57036	1.566056	1.561745	1.557442	1.647968
1.372153	1.489378	1.485548	1.575326	1.571002	1.566659	1.562357	1.55808	1.648641
1.372663	1.489952	1.486131	1.575944	1.571629	1.567293	1.562988	1.558702	1.649298
1.373304	1.490667	1.486754	1.576515	1.572242	1.567962	1.56364	1.559306	1.649936
1.373876	1.491308	1.487352	1.5771	1.572849	1.568599	1.564274	1.559922	1.650595
1.374291	1.491776	1.487894	1.577724	1.573453	1.569176	1.564875	1.560568	1.651298
1.374716	1.492258	1.488439	1.578346	1.574053	1.569747	1.565468	1.561203	1.651994
1.37529	1.37185	1.368418	1.578929	1.574643	1.570355	1.566067	1.561784	1.652629
1.375912	1.372462	1.368945	1.579505	1.575232	1.570971	1.566665	1.56235	1.653253
1.376454	1.37298	1.369465	1.580127	1.575838	1.571564	1.567258	1.562951	1.558722
1.376945	1.373438	1.36998	1.580782	1.576455	1.572142	1.567847	1.563579	1.559393
1.377455	1.373928	1.370483	1.487439	1.483444	1.479452	1.568478	1.564256	1.560087
1.377991	1.374463	1.370971	1.487986	1.483997	1.48003	1.569161	1.565003	1.560818
1.378521	1.374988	1.371464	1.488544	1.484575	1.480639	1.569845	1.565708	1.561503
1.379018	1.375465	1.371956	1.489117	1.485203	1.481312	1.570517	1.566309	1.562082
1.379536	1.375962	1.372478	1.489724	1.485849	1.481987	1.57117	1.566882	1.562644
1.25864	1.376561	1.37308	1.369634	1.366217	1.362799	1.57176	1.567471	1.563262
1.259183	1.37721	1.373726	1.370256	1.366765	1.36328	1.572329	1.568073	1.563909
1.259655	1.377803	1.374345	1.370884	1.367365	1.363848	1.573003	1.568751	1.564577
1.260067	1.378351	1.374947	1.371524	1.368011	1.364488	1.573767	1.569498	1.565267
1.260518	1.37892	1.375513	1.372093	1.368594	1.365089	1.574505	1.570243	1.565984
1.261029	1.37953	1.376034	1.372557	1.369081	1.365625	1.575201	1.570993	1.566751
1.261539	1.380115	1.376546	1.373023	1.369573	1.366162	1.362783	1.35942	1.56747
1.262004	1.380614	1.377051	1.373544	1.370121	1.366728	1.363316	1.359898	1.568045
1.262471	1.38111	1.377574	1.374097	1.370697	1.36731	1.363848	1.360357	1.568591
1.263	1.381711	1.37817	1.374692	1.371302	1.36792	1.364437	1.360913	1.569245
1.263561	1.382373	1.378815	1.375322	1.371927	1.368543	1.365053	1.36152	1.569956
1.26406	1.382994	1.379448	1.375949	1.372513	1.369087	1.365593	1.362077	1.570629
1.264495	1.383579	1.380074	1.376575	1.373057	1.369546	1.366053	1.362582	1.571265
1.26495	1.261887	1.258893	1.377201	1.373627	1.370058	1.366563	1.363119	1.571929
1.265447	1.262391	1.259411	1.377823	1.374245	1.370668	1.367166	1.363715	1.572642
1.26596	1.262906	1.259931	1.378446	1.374874	1.3713	1.36779	1.364327	1.573369
1.266483	1.263432	1.260462	1.379072	1.375492	1.371908	1.368394	1.36493	1.361545
1.267012	1.263964	1.260997	1.3797	1.376111	1.372517	1.369	1.365536	1.362149
1.267531	1.264485	1.261529	1.380335	1.376744	1.373144	1.36962	1.366146	1.362748
1.268042	1.265001	1.26206	1.380974	1.377386	1.373785	1.370251	1.366761	1.363346
1.268563	1.265521	1.262588	1.381608	1.378025	1.374425	1.370885	1.367384	1.363956
1.269097	1.266047	1.263115	1.382237	1.37866	1.375065	1.371524	1.368017	1.364578

1.269624	1.266565	1.263634	1.382861	1.379291	1.375703	1.372163	1.368653	1.365204
1.270137	1.267068	1.264141	1.38348	1.37992	1.376341	1.372804	1.369292	1.365829
1.270643	1.267562	1.264635	1.384088	1.38054	1.376973	1.373441	1.36993	1.366457
1.153786	1.268046	1.265108	1.262164	1.259148	1.256114	1.37407	1.370566	1.367089
1.154198	1.268524	1.265568	1.262619	1.259619	1.256606	1.374691	1.371199	1.367722
1.154616	1.269012	1.266043	1.26309	1.2601	1.2571	1.375302	1.371816	1.36834
1.155041	1.269506	1.266529	1.263573	1.260587	1.257592	1.3759	1.372417	1.368945
1.155468	1.152578	1.149858	1.26406	1.261098	1.258129	1.255111	1.252067	1.369581
1.155887	1.152984	1.150249	1.264551	1.261646	1.258735	1.25573	1.252672	1.370264
1.156344	1.153423	1.150659	1.265044	1.262182	1.259319	1.256328	1.253266	1.370943
1.156889	1.153939	1.151115	1.26554	1.262677	1.259827	1.256844	1.253791	1.37157
1.157449	1.154472	1.151581	1.266035	1.263154	1.260305	1.257337	1.254309	1.37221
1.018083	1.154942	1.152014	1.149204	1.146585	1.14403	1.257865	1.254899	1.251861
1.018429	1.155384	1.152434	1.149603	1.146968	1.144413	1.258402	1.255522	1.252518
0.836942	1.155864	1.152893	1.150034	1.147362	1.144781	1.258899	1.256084	1.253124
0.837407	1.156382	1.15339	1.150493	1.147761	1.145131	1.259356	1.25659	1.253688
0.837844	1.156875	1.153886	1.150972	1.148194	1.145516	1.259821	1.257069	1.254202
0.838234	1.157327	1.15438	1.151483	1.148676	1.145951	1.260296	1.25751	1.254642
0.838618	1.157763	1.154856	1.151983	1.149166	1.146414	1.260802	1.257968	1.255084
0.839016	1.158182	1.15529	1.152437	1.149647	1.146912	1.261378	1.258496	1.255582
0.839429	1.158602	1.155709	1.152869	1.150117	1.147416	1.261978	1.259057	1.256111
0.839882	0.837819	0.835794	1.153294	1.15054	1.147844	1.262531	1.25962	1.256672
0.840368	0.838282	0.836219	1.153715	1.150933	1.148223	1.263055	1.26019	1.25726
0.84084	0.838761	0.836698	1.154232	1.151413	1.148662	1.146021	1.143452	1.257802
0.841301	0.839267	0.837247	1.154871	1.152	1.149169	1.146452	1.143813	1.258293
0.841745	0.839739	0.837749	1.008085	1.005565	1.003039	1.146904	1.144203	1.25879
0.84215	0.840125	0.838138	1.008569	1.006096	1.003614	1.147399	1.144664	1.259306
0.84257	0.84052	0.838519	1.009031	1.006603	1.004167	1.147885	1.145137	1.259844
0.843083	0.841023	0.838987	1.00954	1.007084	1.004636	1.148286	1.14557	1.260425
0.843627	0.841566	0.839486	1.010069	1.007561	1.00508	1.148666	1.145997	1.261029
0.59249	0.842022	0.839948	0.83789	0.83586	0.833851	1.003197	1.000774	1.261607
0.592801	0.842414	0.840384	0.838371	0.83639	0.834413	1.00383	1.001343	1.262167
0.593166	0.842871	0.840847	0.838843	0.836873	0.834903	1.004403	1.001902	1.262763
0.593615	0.843428	0.841354	0.8393	0.837283	0.835285	1.004885	1.002446	1.263411
0.594041	0.843959	0.841849	0.83976	0.837711	0.835694	1.005388	1.00299	1.264042
0.594372	0.844377	0.842295	0.840236	0.838218	0.83622	1.005988	1.003545	1.264606
0.594695	0.844786	0.842736	0.840711	0.83873	0.836755	1.006593	1.004099	1.265163
0.595109	0.593626	0.592125	0.841168	0.839161	0.837169	0.835165	0.833159	1.002159
0.595565	0.594066	0.592535	0.841617	0.839554	0.837529	0.835547	0.833598	1.002732
0.595948	0.594449	0.592917	0.842086	0.840012	0.837981	0.836013	0.834089	1.003323
0.596264	0.594778	0.593273	0.842572	0.840527	0.838518	0.836561	0.834635	1.003939
0.596623	0.595145	0.59366	0.592175	0.590693	0.589217	0.837062	0.835118	1.004491
0.597061	0.595585	0.594105	0.592617	0.591111	0.589605	0.837472	0.835485	1.004929
0.597505	0.596022	0.594538	0.59304	0.591514	0.589985	0.837886	0.835866	1.005389
0.597914	0.596408	0.594897	0.593386	0.591869	0.59036	0.83836	0.836357	1.005987
0.598309	0.59678	0.59524	0.593716	0.592219	0.590739	0.838859	0.83688	1.00662
0.598691	0.597174	0.595642	0.594124	0.592631	0.591151	0.589679	0.588217	0.835384
0.599063	0.597581	0.596082	0.594581	0.593082	0.591585	0.590088	0.588595	0.835789
0.599468	0.598004	0.596525	0.595031	0.593516	0.591996	0.59048	0.588971	0.836227
0.599911	0.59845	0.596979	0.595481	0.593936	0.592382	0.590851	0.589339	0.836697

0.600342	0.598873	0.597397	0.595893	0.594333	0.592767	0.591234	0.589726	0.588271
0.600743	0.599249	0.597745	0.596228	0.594689	0.593151	0.591638	0.590147	0.588698
0.60113	0.599617	0.59809	0.596567	0.595053	0.593548	0.592056	0.590578	0.589131
0.601499	0.600004	0.598495	0.596986	0.595481	0.593981	0.592488	0.591005	0.589553
0.601864	0.600401	0.598926	0.59744	0.595938	0.594432	0.592929	0.591432	0.58997
0.602266	0.600812	0.599353	0.59788	0.596383	0.594876	0.593368	0.59186	0.590381
0.602696	0.601238	0.599782	0.598314	0.596823	0.595319	0.593806	0.592288	0.590786
0.603124	0.601661	0.600209	0.598746	0.597261	0.595761	0.594238	0.592701	0.59118
0.603548	0.602084	0.600637	0.599179	0.597706	0.596211	0.594668	0.593099	0.591557
0.60397	0.602501	0.601051	0.599592	0.598121	0.596625	0.595072	0.593489	0.59194
0.604395	0.602913	0.601443	0.599964	0.598468	0.596957	0.595417	0.593864	0.592336
0.604814	0.603315	0.601823	0.600323	0.598804	0.59728	0.595761	0.594247	0.592744
	0.603699	0.602194	0.600692	0.599187	0.597682	0.596177	0.594673	0.593174
	0.604071	0.60256	0.601067	0.599595	0.598124	0.59663	0.595124	0.593618
	0.604452	0.602948	0.601467	0.600013	0.59856	0.597073	0.595569	0.594059
	0.604836	0.603355	0.601893	0.600444	0.598993	0.597512	0.596012	0.5945
			0.602317	0.600872	0.599425	0.597948	0.596453	0.594942
			0.602735	0.601294	0.599854	0.598383	0.596893	0.595385
			0.603153	0.601714	0.600279	0.598814	0.59733	0.595826
			0.603573	0.602133	0.600699	0.599238	0.597762	0.596264
			0.603996	0.602551	0.601117	0.59966	0.598191	0.5967
			0.604427	0.602975	0.601537	0.600085	0.598624	0.59714
			0.604865	0.603404	0.601959	0.60051	0.599058	0.59758
						0.600934	0.599491	0.598021
						0.601359	0.599925	0.598461
						0.601781	0.600354	0.598897
						0.602199	0.600776	0.599325
						0.602618	0.601198	0.599752
						0.603034	0.601614	0.60017
						0.603463	0.602039	0.600596
						0.603962	0.60253	0.601079
						0.604516	0.603072	0.601606
								0.602088
								0.602514
								0.602931
								0.603355
								0.603778
								0.604204
								0.604632

							0.861335
							0.860544
							1.226187
							1.225224
							1.224233
							1.223086
							1.221875
					0.860264	0.85808	1.220663
					0.859444	0.85724	1.219444
					1.22519	1.221757	1.218353
					1.22426	1.220823	1.217416
			0.618566	0.617079	1.223257	1.21982	1.216417
			0.617992	0.616492	1.222084	1.218649	1.215256
			0.617403	0.615886	1.220872	1.217456	1.214087
			0.6168	0.615282	1.219686	1.21634	1.375771
			0.6162	0.614688	1.218521	1.215256	1.37479
			0.857632	0.855419	1.217449	1.214173	1.373767
			0.856951	0.854726	1.216456	1.213098	1.372718
0.619266	0.617733	0.616203	1.222255	1.218809	1.378733	1.375188	1.371714
0.618667	0.617138	0.615609	1.221047	1.217611	1.377643	1.374175	1.370771
0.618098	0.616571	0.615042	1.219891	1.216464	1.376593	1.373181	1.369825
0.61762	0.616091	0.614558	1.218893	1.21547	1.375651	1.372215	1.368832

	0.61716	0.615632	0.614097	1.217953	1.214532	1.374752	1.371277	1.367853
	0.616653	0.615144	0.613622	1.21702	1.213596	1.373867	1.370396	1.366971
	0.616124	0.614641	0.613136	1.216089	1.212662	1.372986	1.369539	1.366131
0.617051	1.034984	1.032298	1.029584	1.37913	1.37551	1.371997	1.368566	1.365169
0.616542	1.034034	1.031311	1.028581	1.377986	1.374397	1.370902	1.367482	1.364092
0.616048	1.033134	1.030384	1.027645	1.376932	1.373374	1.369897	1.366489	1.363104
0.615587	1.032331	1.029574	1.026837	1.376048	1.372517	1.369054	1.365651	1.362266
0.615098	1.031497	1.028736	1.026006	1.375135	1.371632	1.368194	1.364815	1.361443
0.856159	1.219905	1.216463	1.213065	1.374029	1.370561	1.367184	1.363889	1.360576
0.855338	1.218712	1.21527	1.21188	1.372898	1.369462	1.366148	1.362938	1.359685
0.854598	1.217676	1.214246	1.210871	1.371951	1.368537	1.365226	1.362006	1.452514
0.853908	1.216736	1.213328	1.209973	1.371122	1.36772	1.364375	1.361084	1.451415
1.029769	1.380927	1.377304	1.373744	1.370289	1.366899	1.363539	1.360211	1.45041
1.028951	1.380025	1.376427	1.372893	1.369456	1.36608	1.362722	1.35939	1.449504
1.028132	1.379133	1.375557	1.372046	1.368626	1.365263	1.361909	1.358572	1.560957
1.027314	1.378268	1.374715	1.371226	1.367824	1.364474	1.361119	1.357771	1.559962
1.026483	1.37738	1.37384	1.370362	1.366973	1.363632	1.360276	1.356918	1.558905
1.025618	1.376409	1.372845	1.369351	1.36596	1.362625	1.359266	1.355901	1.557643
1.024737	1.375406	1.371811	1.368294	1.364895	1.361558	1.358196	1.354826	1.556312
1.378081	1.374447	1.37088	1.36738	1.36398	1.360633	1.357263	1.353885	1.555157
1.377068	1.373519	1.370023	1.36657	1.363173	1.359807	1.356426	1.35304	1.554129
1.376131	1.372637	1.369183	1.365757	1.36236	1.35898	1.355591	1.352196	1.553113
1.375286	1.371811	1.368361	1.364934	1.361534	1.358149	1.354753	1.351353	1.552108
1.374463	1.370999	1.367548	1.364115	1.360709	1.357318	1.353917	1.350513	1.551108
1.373638	1.370188	1.366741	1.363306	1.35989	1.356485	1.353079	1.349675	1.550103
1.372818	1.369377	1.365932	1.362494	1.359068	1.35565	1.352241	1.34884	1.549102
1.371993	1.368555	1.365106	1.361663	1.358232	1.354811	1.556432	1.552252	1.548105
1.371167	1.367726	1.364271	1.360822	1.357389	1.35397	1.555414	1.551245	1.547113
1.370337	1.366892	1.363437	1.359989	1.356559	1.353144	1.554408	1.550247	1.716703
1.369503	1.366054	1.362603	1.359162	1.35574	1.352332	1.553415	1.549258	1.715606
1.368663	1.365211	1.361765	1.358332	1.354918	1.351519	1.552427	1.548277	1.714515
1.367817	1.36436	1.360919	1.357494	1.35409	1.350702	1.551442	1.547303	1.71343
1.366966	1.363508	1.360073	1.356657	1.353265	1.349889	1.550465	1.546341	1.712352
1.366114	1.362655	1.359228	1.355824	1.352446	1.349086	1.549503	1.545397	1.711285
1.365261	1.361806	1.358387	1.354995	1.351631	1.348287	1.548547	1.544459	1.710223
1.364416	1.360967	1.357557	1.354173	1.350819	1.347485	1.547584	1.543513	1.709155
1.363576	1.360137	1.356734	1.353358	1.350009	1.346682	1.546617	1.542563	1.708083
1.362735	1.359309	1.355916	1.352548	1.349205	1.345882	1.545653	1.541613	1.707007
1.361892	1.358484	1.355103	1.351743	1.348407	1.345087	1.544692	1.540662	1.705927
1.361063	1.357656	1.354288	1.350942	1.347611	1.344295	1.631426	1.627139	1.704844
1.360242	1.356808	1.353461	1.350143	1.34682	1.343505	1.630385	1.626097	1.703757
1.359457	1.355991	1.35265	1.349346	1.346028	1.342716	1.629344	1.625053	1.702666
1.358747	1.355259	1.351876	1.348538	1.345223	1.341926	1.628303	1.624007	1.701574
1.358074	1.354575	1.351135	1.347745	1.344424	1.341138	1.627262	1.622959	1.700478
1.357381	1.447753	1.443967	1.440221	1.547026	1.542987	1.626205	1.621898	1.850218
1.356678	1.447039	1.443263	1.439502	1.546123	1.542032	1.625139	1.62083	1.848981
1.355931	1.446288	1.442546	1.438801	1.545308	1.541194	1.624186	1.619831	1.84781
1.35513	1.445496	1.44182	1.438134	1.544616	1.540512	1.623377	1.618912	1.846716
1.354323	1.44468	1.441054	1.437417	1.631356	1.626999	1.622546	1.618021	1.845657
1.353527	1.44384	1.440224	1.436598	1.630371	1.626036	1.621626	1.617159	1.84464

1.352738	1.442986	1.439366	1.43574	1.629326	1.625014	1.620665	1.616288	1.843621
1.351957	1.442116	1.438497	1.434879	1.628279	1.623979	1.619667	1.615345	1.915115
1.351196	1.441254	1.43763	1.434016	1.627221	1.622924	1.618638	1.61436	1.913904
1.35053	1.440511	1.436857	1.433213	1.62619	1.621877	1.617601	1.613356	1.912646
1.349952	1.439877	1.436169	1.432461	1.625179	1.620832	1.61655	1.612325	1.911337
1.349286	1.439176	1.435459	1.431735	1.62426	1.619899	1.615603	1.611362	1.910116
1.348472	1.438367	1.434711	1.431047	1.623481	1.619139	1.614809	1.61049	1.909017
1.347684	1.437558	1.433946	1.430331	1.622667	1.618357	1.781097	1.77609	1.90795
1.347036	1.436799	1.433143	1.429507	1.621677	1.617416	1.780041	1.775115	1.90689
1.346418	1.436065	1.432356	1.428687	1.620665	1.616442	1.778955	1.774128	1.905821
1.345724	1.533287	1.529299	1.525343	1.619814	1.615546	1.777917	1.773078	1.90466
1.344989	1.532552	1.528633	1.524715	1.619051	1.614694	1.776907	1.772	1.903454
1.344236	1.531714	1.527846	1.523971	1.618234	1.613879	1.912925	1.907608	2.028928
1.343453	1.530763	1.526932	1.523114	1.617371	1.613113	1.91189	1.906638	2.027875
1.342748	1.5299	1.526075	1.52227	1.616472	1.612274	1.910788	1.905576	2.026741
1.342181	1.529211	1.525333	1.521465	1.615507	1.611282	1.909552	1.904326	2.025439
1.341618	1.52853	1.52461	1.520687	1.614575	1.610316	1.908338	1.903087	2.02413
1.340943	1.527729	1.523845	1.519958	1.780716	1.7758	1.907285	1.902009	2.169537
1.340247	1.526905	1.523079	1.519249	1.779803	1.774927	1.906286	1.900996	2.168043
1.339659	1.52619	1.522346	1.518495	1.778802	1.773921	1.905196	1.899958	2.166621
1.339145	1.525549	1.521638	1.517716	1.777748	1.772833	1.904054	1.898916	2.165265
1.338569	1.52485	1.520927	1.516991	1.776787	1.771864	1.902997	1.897915	2.163929
1.337914	1.524074	1.520205	1.51633	1.775938	1.771041	1.902045	1.896971	2.162631
1.337288	1.627592	1.623118	1.618662	1.775083	1.770196	2.090159	2.083751	2.161266
1.336744	1.626813	1.622353	1.617901	1.77419	1.769259	2.088772	2.082315	2.15975
1.336217	1.626045	1.621577	1.617109	1.773266	1.768303	2.087369	2.08087	2.158227
1.33566	1.625203	1.620694	1.616198	1.772268	1.767363	2.086041	2.079569	2.156844
1.335089	1.624334	1.619771	1.615243	1.771244	1.766435	2.084749	2.078333	2.155523
1.441516	1.784992	1.780053	1.775161	1.906317	1.901148	2.12246	2.115906	2.154166
1.44082	1.784073	1.779195	1.77435	1.905396	1.900185	2.121159	2.114579	2.152785
1.440161	1.7832	1.778349	1.773518	1.904442	1.899211	2.119837	2.113246	2.151419
1.439548	1.782388	1.777515	1.772652	1.903434	1.898217	2.118479	2.111898	2.15007
1.552291	1.781576	1.776669	1.771766	2.091366	2.08483	2.117131	2.110574	2.148745
1.551612	1.780743	1.775793	1.770855	2.090053	2.083516	2.115816	2.109314	2.147473
1.550923	1.77988	1.774893	1.769929	2.088738	2.082217	2.114519	2.108063	2.146201
1.550158	1.778938	1.773947	1.768992	2.087433	2.080975	2.113233	2.106745	2.144835
1.549342	1.777945	1.772973	1.768053	2.086143	2.079771	2.111961	2.105398	2.143425
1.548511	1.776977	1.772051	1.767179	2.084918	2.078575	2.110715	2.104127	2.142118
1.547655	1.776027	1.771176	1.766371	2.083765	2.077393	2.109501	2.102933	2.140912
1.54685	1.775116	1.770313	1.765551	2.082573	2.07617	2.108243	2.101703	2.139686
1.546129	1.77426	1.769464	1.764706	2.081312	2.07488	2.106903	2.100387	2.138391
1.545427	1.773419	1.76861	1.763838	2.080023	2.073576	2.105567	2.099095	2.137125
1.544697	1.920899	1.914713	1.908595	2.117315	2.110757	2.104292	2.097914	2.135969
1.543971	1.919773	1.913524	1.907328	2.115952	2.109437	2.10305	2.09678	2.134854
1.637761	1.918679	1.912409	1.906185	2.114692	2.108192	2.101841	2.095624	2.133683
1.637036	1.917606	1.911346	1.905128	2.113507	2.107005	2.100663	2.094464	2.132484
1.55323	2.093559	2.086929	2.080352	2.112318	2.105818	2.143887	2.137589	2.17981
1.552529	2.092387	2.085765	2.079195	2.111124	2.104631	2.142688	2.136415	2.178595
1.551828	2.091218	2.084607	2.078046	2.109938	2.103452	2.141498	2.135252	2.209519
1.551128	2.090053	2.083458	2.076909	2.108761	2.102277	2.140312	2.134097	2.208293

1.550434	2.088894	2.082316	2.07578	2.107598	2.101121	2.139145	2.132961	2.207083
1.735092	2.126369	2.119672	2.113017	2.106458	2.10001	2.138027	2.131866	2.205907
1.734064	2.125181	2.118495	2.111857	2.105332	2.098922	2.136932	2.130793	2.204749
1.733054	2.124005	2.117326	2.110698	2.104193	2.097808	2.135811	2.129698	2.20357
1.73206	2.122838	2.116164	2.10954	2.103046	2.096677	2.13467	2.128588	2.202377
1.731053	2.121654	2.114986	2.108371	2.101893	2.095545	2.133531	2.127481	2.201184
1.730026	2.120444	2.113786	2.107188	2.100733	2.094413	2.132395	2.126376	2.199988
1.790045	2.119235	2.112586	2.106004	2.099574	2.093284	2.131264	2.125276	2.198799
1.788958	2.118037	2.111387	2.104811	2.098409	2.092155	2.130134	2.124179	2.197621
1.787882	2.116846	2.110202	2.103638	2.097262	2.09104	2.129015	2.123089	2.19645
1.786846	2.115664	2.109052	2.102527	2.096168	2.089958	2.176081	2.169854	2.195283
1.785836	2.114494	2.107934	2.101465	2.095119	2.088908	2.174933	2.168719	2.194122
2.035526	2.113354	2.106866	2.100469	2.138273	2.131923	2.173832	2.167596	2.192976
2.034393	2.112241	2.105852	2.099547	2.137348	2.130989	2.172781	2.166486	2.191846
2.033316	2.111161	2.104818	2.098561	2.136366	2.130028	2.171717	2.165367	2.227089
2.032333	2.110129	2.103742	2.097461	2.135278	2.129004	2.17061	2.164215	2.225878
2.031338	2.109095	2.102656	2.096344	2.134184	2.127991	2.169536	2.163104	2.224703
2.030244	2.108005	2.10155	2.095239	2.133133	2.127051	2.168577	2.162119	2.223636
2.029124	2.106914	2.100464	2.094167	2.132107	2.12612	2.167637	2.161167	2.222605
2.028064	2.105914	2.099505	2.093226	2.179305	2.172977	2.166559	2.160105	2.221498
2.027041	2.104972	2.098633	2.09238	2.178261	2.171831	2.165396	2.158981	2.220353
2.025999	2.103954	2.097691	2.091495	2.177245	2.170754	2.164315	2.157931	2.219292
2.024928	2.102843	2.096673	2.090578	2.176271	2.169759	2.16333	2.156971	2.218332
2.023921	2.101789	2.095658	2.089611	2.175245	2.168738	2.162318	2.15597	2.217333
2.023043	2.10086	2.094653	2.088546	2.174101	2.167635	2.16122	2.154858	2.216232
2.022174	2.099966	2.093681	2.087505	2.172969	2.166545	2.160134	2.153754	2.215123
2.021218	2.150672	2.144064	2.137551	2.171972	2.165553	2.159142	2.152759	2.184508
2.020219	2.149723	2.143151	2.136648	2.17104	2.16461	2.158198	2.151819	2.183499
2.067118	2.148653	2.142165	2.135734	2.170113	2.163668	2.157255	2.150879	2.182537
2.066043	2.147486	2.141125	2.134821	2.169201	2.162737	2.156322	2.14995	2.181618
2.065051	2.146395	2.14007	2.133815	2.168217	2.161794	2.193526	2.187083	2.180699
2.064171	2.145404	2.13899	2.132677	2.167136	2.160845	2.192613	2.186152	2.179775
2.063292	2.144432	2.13794	2.131574	2.166055	2.159853	2.191654	2.185211	2.178858
2.062351	2.143441	2.136957	2.130589	2.165004	2.158764	2.190591	2.184259	2.177974
2.061414	2.142466	2.136011	2.129652	2.163981	2.15767	2.189502	2.183282	2.17707
2.114593	2.14154	2.135086	2.128722	2.163009	2.156673	2.188457	2.18223	2.259644
2.113668	2.140644	2.134184	2.12781	2.162078	2.155737	2.187442	2.181148	2.258545
2.06037	2.139734	2.133282	2.126913	2.161163	2.154832	2.186504	2.180199	2.257586
2.059455	2.138816	2.132381	2.12603	2.160268	2.15396	2.185642	2.179373	2.256754
2.058541	2.137907	2.131496	2.125169	2.159405	2.153127	2.184801	2.178538	2.255896
2.057625	2.13701	2.130627	2.12433	2.158585	2.15235	2.183989	2.177674	2.254978
2.05672	2.136129	2.12978	2.123515	2.157773	2.151558	2.183151	2.176794	2.254055
2.055823	2.135271	2.128975	2.122745	2.156956	2.1507	2.182235	2.175887	2.25315
2.054953	2.134433	2.128182	2.121978	2.156123	2.149806	2.181285	2.174968	2.25225
2.054163	2.133625	2.127364	2.121144	2.155232	2.148891	2.236616	2.230256	2.288941
2.053426	2.132838	2.126528	2.120263	2.154301	2.147953	2.235692	2.229346	2.287993
2.05261	2.125947	2.119615	2.113329	2.191241	2.184801	2.234779	2.228466	2.28708
2.05171	2.125036	2.118688	2.112389	2.190277	2.183842	2.233875	2.227612	2.286197
2.050812	2.124129	2.117771	2.111467	2.189327	2.182906	2.260282	2.254026	2.285358
2.049925	2.12323	2.116868	2.110564	2.188384	2.181998	2.25941	2.25324	2.284596

2.049072	2.12236	2.116005	2.109707	2.18748	2.181118	2.25855	2.252451	2.283825
2.048307	2.121571	2.115242	2.10896	2.186671	2.180281	2.257685	2.251596	2.282954
2.047576	2.120811	2.114515	2.108255	2.1859	2.179463	2.25682	2.250713	2.282042
2.046761	2.11997	2.11369	2.107445	2.241533	2.23503	2.293439	2.28723	2.281155
2.045888	2.11907	2.112794	2.106561	2.240634	2.234142	2.292495	2.286312	2.280282
2.045089	2.118252	2.111979	2.105755	2.239815	2.233339	2.291639	2.285469	2.279452
2.044385	2.117534	2.111267	2.105052	2.239101	2.232646	2.290896	2.28472	2.278674
2.043698	2.116835	2.110563	2.104346	2.198787	2.192457	2.290164	2.283986	2.277918
2.043019	2.116149	2.109847	2.103608	2.198047	2.191745	2.289417	2.283256	2.277195
2.042314	2.115435	2.1091	2.102836	2.197273	2.191003	2.288643	2.282503	2.276456
2.041497	2.215796	2.209241	2.202761	2.264982	2.258593	2.28778	2.281649	2.275613
2.040627	2.214883	2.208312	2.201824	2.264009	2.257671	2.286876	2.280749	2.274724
2.039856	2.214056	2.207484	2.200999	2.263142	2.256833	2.286055	2.279947	2.273935
2.039168	2.213302	2.206741	2.200272	2.262367	2.256067	2.285307	2.279227	2.273233
2.038491	2.212564	2.206007	2.199544	2.261592	2.255306	2.284568	2.278518	2.300441
2.037818	2.21184	2.20527	2.1988	2.260804	2.254542	2.283831	2.277814	2.299778
2.037143	2.211119	2.204537	2.198059	2.260021	2.253783	2.2831	2.277113	2.299114
2.036456	2.210392	2.203803	2.197323	2.259243	2.253032	2.282372	2.276407	2.298444
2.035764	2.209662	2.203072	2.196596	2.258473	2.252287	2.281645	2.275699	2.297771
2.149141	2.27744	2.2707	2.264077	2.25772	2.251546	2.280918	2.274988	2.297099
2.148418	2.276634	2.269921	2.263325	2.256979	2.25081	2.280192	2.274276	2.296427
2.147699	2.275838	2.269148	2.262575	2.256242	2.250085	2.244095	2.238267	2.295752
2.146984	2.275054	2.268382	2.261827	2.255512	2.249369	2.243392	2.237572	2.295075
2.146264	2.274266	2.267612	2.261078	2.254785	2.248663	2.242694	2.236875	2.294394
2.145534	2.273469	2.266834	2.260324	2.254063	2.247971	2.242006	2.23617	2.293709
2.144806	2.272667	2.266054	2.259571	2.253341	2.247277	2.241316	2.235466	2.293026
2.144075	2.271842	2.265264	2.258822	2.252613	2.246563	2.240611	2.234766	2.292345
2.143366	2.27103	2.264483	2.258078	2.251885	2.245845	2.239905	2.234072	2.291671
2.142766	2.270329	2.263761	2.257344	2.251161	2.245141	2.239215	2.233393	2.291009
2.142252	2.269714	2.263085	2.25662	2.250443	2.24445	2.23854	2.23273	2.290361
2.250991	2.269042	2.262415	2.255956	2.249795	2.243822	2.268078	2.262194	2.314037
2.250204	2.268301	2.261754	2.255373	2.249241	2.243277	2.267565	2.261704	2.313523
2.249408	2.267547	2.261073	2.254764	2.248661	2.24271	2.267026	2.261176	2.312964
2.248619	2.266776	2.260337	2.254063	2.247988	2.242055	2.266393	2.260527	2.31227
2.247861	2.266034	2.259611	2.253356	2.247307	2.241399	2.265758	2.259868	2.311557
2.247206	2.265404	2.258977	2.252721	2.246705	2.240836	2.265225	2.259308	2.310933
2.2466	2.264827	2.258382	2.252115	2.246132	2.240305	2.264728	2.258787	2.310346
2.2459	2.264147	2.257715	2.25146	2.245492	2.239679	2.264135	2.258201	2.309729
2.245119	2.26338	2.256985	2.250765	2.244794	2.238971	2.263458	2.257559	2.309089
2.244418	2.262692	2.256325	2.250126	2.244147	2.238309	2.262829	2.256966	2.308489
2.243837	2.262124	2.255765	2.249571	2.243578	2.237724	2.262283	2.256456	2.307959
2.243272	2.261579	2.255224	2.249027	2.243015	2.23714	2.261726	2.255922	2.307398
2.242693	2.261035	2.254677	2.248463	2.24242	2.236508	2.261088	2.255276	2.306717
2.242088	2.260468	2.254114	2.247889	2.241822	2.235879	2.26045	2.254617	2.306021
2.241378	2.259788	2.253474	2.247286	2.241242	2.235313	2.259915	2.254067	2.305437
2.240607	2.259041	2.25279	2.246668	2.240676	2.234789	2.259444	2.253584	2.30492
2.239938	2.258396	2.25218	2.246096	2.27079	2.264863	2.258963	2.253104	2.304412
2.23937	2.25785	2.251643	2.245568	2.2703	2.264367	2.258475	2.252632	2.303916
2.238809	2.257313	2.251113	2.245045	2.269811	2.26388	2.315369	2.309388	2.303412
2.238237	2.256766	2.250574	2.244511	2.269312	2.263405	2.31486	2.308869	2.302891

2.237667	2.256221	2.250044	2.24399	2.26882	2.262929	2.314347	2.308345	2.302363
2.237101	2.255675	2.249531	2.243504	2.26835	2.262436	2.313815	2.307813	2.301832
2.236539	2.255133	2.249028	2.243032	2.267889	2.261942	2.31328	2.30728	2.301297
2.235985	2.254608	2.248519	2.242535	2.26742	2.261479	2.312778	2.30676	2.300755
2.235437	2.254095	2.248007	2.242024	2.266946	2.261039	2.312299	2.306252	2.30021
2.234883	2.253571	2.247504	2.241535	2.266478	2.260573	2.311805	2.30576	2.299719
2.234314	2.25303	2.247009	2.241076	2.266018	2.260077	2.311292	2.305291	2.299301
2.233771	2.252511	2.246518	2.240605	2.265552	2.25959	2.310783	2.304809	2.29886
2.233262	2.252025	2.246011	2.24008	2.265063	2.259137	2.310292	2.30429	2.29835
2.232797	2.251579	2.24554	2.239583	2.264589	2.258687	2.309798	2.303766	2.297827
2.232421	2.251218	2.245186	2.239219	2.264174	2.258197	2.309263	2.30326	2.297338
2.232085	2.250893	2.244886	2.238916	2.263794	2.257705	2.308723	2.302763	2.296858
2.231649	2.250473	2.244493	2.23854	2.263427	2.257316	2.308271	2.302265	2.296343
2.231131	2.249974	2.244026	2.238112	2.263077	2.257016	2.307896	2.301768	2.295797
2.23064	2.249504	2.243555	2.237647	2.296712	2.290612	2.307508	2.301329	2.322015
2.230185	2.249074	2.243074	2.237123	2.29621	2.29016	2.307101	2.300978	2.321654
2.229758	2.24867	2.242633	2.236645	2.295727	2.28969	2.30667	2.300619	2.321304
2.22938	2.248311	2.242293	2.236305	2.295325	2.28921	2.306176	2.300189	2.32091
2.229011	2.24795	2.24197	2.235999	2.294953	2.288748	2.305687	2.299751	2.320513
2.228585	2.247501	2.24155	2.235613	2.294575	2.288372	2.305302	2.299376	2.320152
2.228132	2.247008	2.241082	2.23519	2.294204	2.288053	2.304979	2.299031	2.319804
2.227719	2.246597	2.240676	2.234796	2.293849	2.287733	2.304629	2.298634	2.31939
2.227342	2.246263	2.240329	2.234431	2.293516	2.287422	2.30426	2.298184	2.318904
2.226982	2.245931	2.239964	2.234034	2.293136	2.287069	2.303878	2.297765	2.31847
2.226652	2.245592	2.239558	2.23357	2.292668	2.286638	2.303472	2.297399	2.318127
2.226302	2.24524	2.239153	2.233121	2.292213	2.28621	2.303074	2.297054	2.317805
2.225867	2.280783	2.274602	2.26847	2.291852	2.285857	2.302728	2.296736	2.317471
2.225411	2.280353	2.274213	2.268112	2.291523	2.285514	2.302382	2.296415	2.317129
2.22503	2.279976	2.273854	2.267756	2.291144	2.285082	2.301932	2.296005	2.316746
2.2247	2.279636	2.273518	2.267408	2.29074	2.284592	2.301413	2.295535	2.316334
2.224379	2.303729	2.297557	2.291387	2.313254	2.307013	2.300988	2.295135	2.315965
2.224067	2.303389	2.297215	2.291045	2.312939	2.306699	2.300671	2.294811	2.315642
2.223759	2.303056	2.29688	2.290709	2.312633	2.3064	2.300372	2.294508	2.315338
2.223452	2.302732	2.296555	2.290379	2.31232	2.306089	2.300068	2.294214	2.315049
2.223144	2.302409	2.296232	2.290054	2.312013	2.305785	2.299771	2.293927	2.314765
2.260344	2.302073	2.295906	2.289737	2.311719	2.305492	2.299478	2.293634	2.31447
2.260008	2.301733	2.295578	2.289423	2.311431	2.305205	2.29919	2.293342	2.314172
2.259683	2.301401	2.29525	2.289098	2.311128	2.304908	2.2989	2.293058	2.313886
2.259366	2.301076	2.29492	2.288765	2.310814	2.304605	2.298609	2.292782	2.313608
2.217682	2.300748	2.294602	2.288456	2.310525	2.304322	2.298328	2.292501	2.313323
2.217337	2.300415	2.294301	2.288182	2.310265	2.304052	2.298046	2.292202	2.313016
2.217	2.300082	2.293998	2.287909	2.310028	2.30383	2.297817	2.291949	2.312742
2.216668	2.299736	2.293672	2.28762	2.309833	2.303714	2.297712	2.291805	2.312548
2.216368	2.299409	2.293351	2.287326	2.309636	2.303604	2.297619	2.291681	2.312372
2.216167	2.299165	2.293073	2.287025	2.309365	2.303365	2.297396	2.291465	2.312147
2.216022	2.298969	2.292819	2.28672	2.309054	2.303054	2.297101	2.291201	2.311897
2.249369	2.327305	2.32105	2.31485	2.308762	2.302756	2.296812	2.290935	2.311655
2.249081	2.327029	2.320781	2.314581	2.30848	2.302459	2.296519	2.290661	2.311416
2.248814	2.326768	2.320546	2.314366	2.308257	2.302217	2.296267	2.290404	2.31117
2.248587	2.326528	2.320359	2.314229	2.308123	2.302063	2.296077	2.290163	2.310897

2.248382	2.326319	2.320197	2.314108	2.308	2.301919	2.295905	2.289954	2.310658
2.248209	2.326183	2.320066	2.31397	2.307838	2.30173	2.295723	2.289802	2.310515
2.248039	2.326061	2.319934	2.313818	2.307662	2.301535	2.29555	2.28968	2.310412
2.2478	2.32584	2.319721	2.313614	2.307476	2.30137	2.295405	2.289554	2.310278
2.247519	2.325556	2.319454	2.313374	2.307283	2.301225	2.295281	2.289429	2.31013
2.247318	2.325352	2.319259	2.313192	2.307124	2.30109	2.295143	2.289273	2.309956
2.247205	2.325234	2.319142	2.313075	2.307009	2.30097	2.294994	2.289077	2.309747
2.21335	2.234666	2.228823	2.223002	2.286201	2.280211	2.294831	2.288897	2.309567
2.213218	2.23451	2.228659	2.222828	2.286006	2.279985	2.29464	2.288759	2.309452
2.213085	2.23435	2.228484	2.222637	2.285797	2.279757	2.294454	2.288629	2.309343
2.212953	2.234188	2.228282	2.222404	2.285592	2.279585	2.294307	2.288492	2.309191
2.212812	2.234014	2.228063	2.222156	2.285392	2.27944	2.294178	2.28835	2.30902
2.212613	2.233782	2.227839	2.221948	2.285235	2.279303	2.294036	2.288183	2.308839
2.212368	2.233502	2.227609	2.22177	2.285113	2.279176	2.293888	2.287995	2.308649
2.212162	2.233292	2.227429	2.221614	2.284967	2.279003	2.293717	2.287833	2.3085
2.212005	2.233177	2.227317	2.221481	2.284773	2.278745	2.2935	2.287698	2.308406
2.211877	2.233067	2.227209	2.221363	2.284631	2.278569	2.29335	2.287591	2.308318
2.211786	2.232918	2.227079	2.221268	2.284623	2.278604	2.293356	2.287514	2.308198
2.211702	2.232769	2.226944	2.221172	2.284635	2.278679	2.293402	2.287464	2.308096
2.211576	2.232652	2.226815	2.221035	2.247244	2.241437	2.293377	2.287465	2.308086
2.211434	2.23255	2.226688	2.220877	2.247078	2.241281	2.293318	2.287486	2.308115
2.211329	2.232418	2.226556	2.220748	2.246963	2.241162	2.293219	2.287389	2.308016
2.211254	2.232252	2.226417	2.220644	2.24689	2.241071	2.293076	2.287175	2.307792
2.211231	2.232172	2.226312	2.220523	2.24679	2.240976	2.29298	2.287056	2.307666
2.211292	2.232233	2.226249	2.220349	2.246627	2.240871	2.292966	2.287106	2.307712
2.211328	2.232287	2.226215	2.220228	2.246499	2.240767	2.292946	2.287158	2.307772
2.211245	2.232241	2.226228	2.220269	2.246483	2.240648	2.292846	2.287111	2.333015
2.21112	2.232147	2.22624	2.220351	2.24651	2.240551	2.292739	2.287032	2.33297
2.211024	2.232002	2.226141	2.220306	2.246514	2.240563	2.25711	2.251441	2.332875
2.210943	2.231822	2.225973	2.220184	2.246507	2.240645	2.257158	2.251363	2.332747
2.210935	2.231767	2.225866	2.220047	2.246431	2.240637	2.257171	2.251349	2.332707
2.211017	2.231856	2.225821	2.21988	2.246268	2.240531	2.257152	2.251411	2.332763
2.211034	2.231898	2.2258	2.219792	2.246164	2.240429	2.257113	2.251449	2.332816
2.210908	2.231826	2.225813	2.219855	2.246179	2.240342	2.257034	2.251416	2.332835
2.210768	2.23172	2.225816	2.219936	2.246227	2.240292	2.256979	2.251386	2.332852
2.210699	2.231607	2.225748	2.219921	2.246248	2.240322	2.285373	2.279724	2.332896
2.21067	2.2315	2.225662	2.219876	2.246273	2.2404	2.285457	2.27977	2.332938
2.210694	2.231481	2.22565	2.219882	2.246333	2.240493	2.285523	2.279766	2.33291
2.210768	2.231534	2.225693	2.219924	2.246422	2.240605	2.285583	2.279728	2.332827
2.210832	2.23159	2.225712	2.219912	2.246439	2.240651	2.285622	2.279728	2.300539
2.210878	2.231643	2.22569	2.21982	2.246352	2.240604	2.285627	2.279774	2.300598
2.210935	2.231708	2.225698	2.219774	2.276109	2.270328	2.285657	2.279842	2.300669
2.211016	2.231789	2.225776	2.219847	2.276186	2.270402	2.285746	2.279931	2.300724
2.211102	2.231876	2.225879	2.219959	2.276305	2.270507	2.285857	2.280032	2.300785
2.211189	2.231971	2.225975	2.220051	2.276403	2.270602	2.28596	2.28013	2.300867
2.211266	2.232061	2.226068	2.220142	2.276501	2.270701	2.286066	2.280229	2.300962
2.21126	2.232081	2.226129	2.220236	2.304736	2.298849	2.312885	2.30696	2.30106
2.211174	2.232033	2.226158	2.220331	2.304852	2.298955	2.313001	2.307069	2.301163
2.160132	2.232019	2.2262	2.22042	2.304957	2.299055	2.31311	2.307172	2.301261
2.160111	2.232061	2.226262	2.2205	2.305048	2.299146	2.31321	2.307268	2.301352

2.160124	2.263843	2.257952	2.252106	2.305134	2.299234	2.313308	2.307364	2.301444
2.160143	2.263888	2.258008	2.252178	2.305221	2.299323	2.313407	2.307464	2.301543
2.160197	2.263956	2.258075	2.25225	2.305305	2.299411	2.313508	2.307566	2.301646
2.160341	2.218663	2.212602	2.206616	2.305386	2.299501	2.313612	2.30767	2.30175
2.160544	2.218795	2.212665	2.206621	2.305465	2.299591	2.313718	2.307778	2.301857
2.160667	2.191193	2.185071	2.179041	2.173175	2.167401	2.265855	2.260073	2.301978
2.160707	2.191226	2.185095	2.179059	2.173195	2.16743	2.265953	2.260189	2.302114
2.160741	2.191261	2.185133	2.179097	2.173228	2.167458	2.222661	2.216783	2.302257
2.160783	2.191313	2.185193	2.179155	2.173262	2.167464	2.222671	2.216809	2.302408
2.16083	2.191364	2.185271	2.179249	2.173338	2.167511	2.222711	2.216851	2.30256
2.160888	2.191403	2.185381	2.179427	2.173526	2.167683	2.222847	2.216945	2.302693
2.160954	2.191439	2.185499	2.179627	2.173748	2.167897	2.223023	2.217073	2.302836
2.161021	2.191486	2.185558	2.179705	2.173839	2.168003	2.223155	2.217238	2.263997
2.16109	2.191542	2.185576	2.179696	2.173838	2.168033	2.223259	2.217438	2.26425
2.161206	2.191661	2.185674	2.179771	2.1739	2.168089	2.223341	2.217563	2.26441
2.161373	2.191857	2.185876	2.179959	2.174043	2.168176	2.223386	2.217585	2.264446
2.161566	2.192061	2.186072	2.180135	2.17419	2.168288	2.223472	2.217641	2.264505
2.16179	2.192252	2.186214	2.18024	2.174306	2.168432	2.22365	2.217804	2.264659
2.16201	2.19243	2.186348	2.180343	2.17443	2.168595	2.223851	2.217987	2.264835
2.16217	2.192564	2.186495	2.180506	2.174603	2.168772	2.224014	2.218115	2.264978
2.162303	2.19268	2.18665	2.180699	2.174799	2.168953	2.224162	2.218225	2.265115
2.119851	2.192845	2.186831	2.180886	2.174964	2.169088	2.190552	2.184726	2.265294
2.120037	2.193054	2.187037	2.181073	2.175105	2.16918	2.190645	2.184868	2.265512
2.120234	2.193257	2.18722	2.181229	2.175238	2.169296	2.190767	2.185018	2.265711
2.120449	2.193452	2.187364	2.181333	2.175355	2.169444	2.190931	2.185183	2.265882
2.120645	2.19363	2.187511	2.18146	2.175501	2.169622	2.191106	2.185337	2.26604
2.120782	2.193763	2.187678	2.181662	2.175722	2.169845	2.191275	2.185434	2.266161
2.120907	2.193896	2.187861	2.181894	2.175968	2.170084	2.191455	2.185545	2.266303
2.159896	2.194096	2.188075	2.182114	2.2105	2.204516	2.224141	2.218156	2.266587
2.160084	2.194337	2.188311	2.182333	2.210706	2.204713	2.224387	2.218482	2.266955
2.160305	2.194569	2.188533	2.182543	2.210906	2.204903	2.224579	2.218692	2.267208
2.160565	2.194794	2.18874	2.182742	2.2111	2.205087	2.224717	2.218783	2.267341
2.160803	2.195024	2.188962	2.182953	2.211287	2.205242	2.22484	2.218895	2.237028
2.16099	2.195268	2.189213	2.183182	2.211444	2.205321	2.224926	2.21906	2.237249
2.161182	2.195508	2.189462	2.183419	2.211635	2.205457	2.225065	2.219252	2.237477
2.161406	2.155464	2.149605	2.143766	2.211935	2.205798	2.225363	2.219456	2.23766
2.161663	2.155646	2.149807	2.144016	2.212269	2.206212	2.225728	2.219692	2.237852
2.162007	2.155968	2.150055	2.14421	2.212504	2.206495	2.226037	2.220016	2.214128
2.162418	2.156391	2.15034	2.144368	2.212674	2.206693	2.226316	2.220408	2.214528
2.082073	2.156744	2.150659	2.14464	2.178565	2.172632	2.194032	2.188292	2.214863
2.082288	2.157017	2.151028	2.145059	2.178915	2.172841	2.1942	2.188497	2.215126
2.082477	2.157256	2.151353	2.14544	2.179266	2.173108	2.194417	2.188698	2.215352
2.082645	2.15745	2.151577	2.145708	2.179597	2.173496	2.194749	2.188907	2.215518
2.08282	2.157645	2.151774	2.145929	2.179899	2.173887	2.195103	2.189148	2.215704
2.083034	2.157896	2.151983	2.146109	2.140288	2.134506	2.195407	2.189485	2.216031
2.083282	2.158184	2.152208	2.14628	2.14046	2.134711	2.19568	2.189862	2.216421
2.083594	2.158521	2.152538	2.146589	2.140714	2.134908	2.195895	2.190135	2.216731
2.08397	2.158907	2.152964	2.147021	2.141034	2.135088	2.19605	2.190322	2.216981
2.084279	2.159217	2.153306	2.147384	2.14136	2.135357	2.196294	2.190535	2.217213
2.08448	2.1594	2.153512	2.147644	2.141695	2.135759	2.196666	2.190782	2.217412

2.084706	2.159623	2.153728	2.147883	2.14201	2.136156	2.159387	2.153551	2.217658
2.08504	2.159987	2.154014	2.148106	2.142257	2.136458	2.159715	2.153887	2.218016
2.085412	2.160408	2.154345	2.148354	2.142511	2.136753	2.16005	2.154262	2.218421
2.085786	2.16083	2.154739	2.148719	2.142878	2.137132	2.160437	2.154657	2.21881
2.086162	2.16126	2.155177	2.149159	2.143313	2.137558	2.160856	2.155073	2.219203
2.086461	2.080645	2.074791	2.069002	2.143724	2.137962	2.132216	2.126499	2.219616
2.086677	2.080913	2.075096	2.06934	2.144116	2.138352	2.132616	2.126917	2.220054
2.086943	2.08121	2.075424	2.069688	2.144488	2.138695	2.132961	2.127283	2.220453
2.0873	2.081564	2.075794	2.070054	2.144824	2.138953	2.133204	2.127556	2.220774
2.087685	2.081938	2.076177	2.07043	2.145174	2.139233	2.133467	2.127839	2.221101
2.088067	2.082317	2.076562	2.070816	2.145585	2.139628	2.13385	2.128212	2.186001
2.088456	2.082703	2.07695	2.071208	2.146022	2.140071	2.134283	2.128624	2.186407
2.088843	2.083088	2.077335	2.071593	2.146445	2.140494	2.134704	2.129038	2.159701
2.089231	2.083474	2.077718	2.071975	2.146863	2.140913	2.135124	2.129461	2.160128
2.089615	2.083856	2.078098	2.072354	2.147277	2.141328	2.13554	2.129875	2.160547
2.089995	2.084234	2.078475	2.072731	2.147686	2.141741	2.13595	2.130281	2.160956
2.090374	2.08461	2.078849	2.073103	2.148086	2.142142	2.136351	2.130678	2.161359
2.090752	2.084985	2.079216	2.073462	2.148467	2.142519	2.136729	2.131061	2.161752
2.091128	2.085358	2.079586	2.073827	2.148852	2.142903	2.137111	2.131443	2.162141
2.091498	2.085731	2.07997	2.074219	2.149269	2.143318	2.137512	2.13182	2.162515
2.091865	2.086102	2.080359	2.074623	2.149706	2.143761	2.137938	2.132212	2.162895
2.092231	2.086465	2.08073	2.075013	2.069268	2.063547	2.138451	2.132702	2.163366
2.092592	2.086821	2.081085	2.075391	2.069727	2.064099	2.139042	2.133279	2.163916
2.093037	2.087241	2.081473	2.075768	2.070142	2.064568	2.139546	2.133781	2.16442
2.093605	2.087753	2.081904	2.076128	2.070481	2.064909	2.139918	2.134174	2.164861
2.094141	2.088251	2.082359	2.076543	2.070869	2.06528	2.140308	2.134565	2.165286
2.094541	2.088681	2.082856	2.077092	2.071413	2.065789	2.140803	2.134997	2.165693
2.094907	2.089082	2.083346	2.077654	2.071977	2.066318	2.14132	2.135452	2.166117
2.095319	2.089461	2.083736	2.078065	2.072397	2.066747	2.141794	2.135956	2.166646
2.095756	2.089833	2.084079	2.078401	2.072744	2.067124	2.142244	2.136484	2.167228
2.096246	2.090312	2.08454	2.078839	2.073151	2.067503	2.142666	2.136927	2.167697
2.096792	2.0909	2.085115	2.079372	2.073608	2.067873	2.143049	2.13728	2.168052
2.097295	2.091437	2.085642	2.079875	2.07408	2.068312	2.06264	2.057049	2.168479
2.097707	2.091867	2.086063	2.0803	2.074558	2.068855	2.063206	2.057608	2.169038
2.098142	2.092304	2.086497	2.080746	2.075061	2.069421	2.063789	2.058173	2.169614
2.098688	2.092815	2.08702	2.081288	2.075613	2.069974	2.064307	2.058632	2.170124
2.099281	2.093365	2.087585	2.081869	2.076179	2.070514	2.064803	2.059074	2.170635
2.099877	2.093959	2.08817	2.082434	2.076697	2.070984	2.065285	2.059608	2.171228
2.100479	2.094588	2.088774	2.082994	2.077185	2.071409	2.065756	2.060201	2.171878
2.101002	2.095127	2.089295	2.083497	2.077688	2.071921	2.066291	2.060771	2.172503
2.101426	2.095558	2.089711	2.083923	2.0782	2.072534	2.066902	2.061313	2.1731
2.101894	2.096018	2.090177	2.08441	2.078742	2.073132	2.067499	2.061865	2.097574
2.102458	2.096548	2.090759	2.085036	2.079331	2.073665	2.068036	2.062444	2.098175
2.103068	2.097124	2.091374	2.085679	2.079931	2.074206	2.068578	2.063028	2.098776
2.103709	2.097772	2.091969	2.086232	2.080507	2.074821	2.069189	2.063604	2.099317
2.10437	2.098453	2.092566	2.08676	2.081077	2.075462	2.069823	2.064181	2.099847
2.104995	2.099058	2.093171	2.087364	2.081667	2.076037	2.070403	2.06478	2.100466
2.105594	2.099605	2.093781	2.088028	2.082273	2.076563	2.070942	2.065395	2.101157
2.106207	2.100196	2.094383	2.088638	2.082863	2.077128	2.071506	2.065975	2.101779
2.106846	2.100854	2.094971	2.089168	2.08343	2.077753	2.072108	2.066503	2.102302

2.066855	2.101489	2.095559	2.089717	2.084001	2.07836	2.072699	2.067043	2.102835
2.067427	2.10204	2.096154	2.090348	2.084595	2.0789	2.073246	2.067638	2.103441
2.067998	2.102584	2.096747	2.090984	2.085189	2.079433	2.07379	2.068238	2.104053
2.0686	2.103196	2.097319	2.091525	2.085749	2.080024	2.074372	2.068783	2.063205
2.06923	2.103851	2.097891	2.092033	2.0863	2.080645	2.074972	2.069307	2.063709
2.069932	2.104565	2.098572	2.092678	2.086917	2.08124	2.075554	2.069881	2.064273
2.070713	2.105346	2.099363	2.093454	2.087598	2.08181	2.076113	2.070499	2.064888
2.07142	2.106045	2.100086	2.09419	2.088286	2.082437	2.076739	2.071162	2.065551
2.071999	2.106605	2.100691	2.094852	2.088981	2.083156	2.077473	2.071902	2.066295
2.072568	2.107162	2.101273	2.095475	2.089653	2.083876	2.078203	2.072614	2.067007
1.987702	2.067089	2.061241	2.055502	2.090251	2.084527	2.078839	2.07319	2.067571
1.988265	2.067738	2.06183	2.056044	2.090838	2.08517	2.07946	2.073742	2.068105
1.98893	2.068463	2.062513	2.056696	2.091524	2.085886	2.080176	2.074439	2.068765
1.989664	2.069242	2.063259	2.057417	2.092269	2.086642	2.080946	2.075222	2.069503
1.990307	2.069932	2.063943	2.058095	2.092949	2.087303	2.081617	2.075923	2.070204
1.990838	2.070516	2.064551	2.058722	2.093551	2.087852	2.082176	2.076534	2.070867
1.835772	1.985379	1.979604	1.973966	2.094194	2.088452	2.082777	2.077169	2.07154
1.836367	1.986107	1.980302	1.974637	2.094936	2.089175	2.083491	2.077881	2.072254
1.836999	1.986879	1.981015	1.975295	2.095673	2.089909	2.084211	2.078581	2.072944
1.837637	1.987639	1.981652	1.975827	2.096298	2.090561	2.084847	2.079176	2.073529
1.838271	1.988391	1.982269	1.976325	2.096895	2.091194	2.085462	2.079744	2.074083
1.838812	1.989076	1.982931	1.976959	1.971353	1.965935	2.086153	2.080422	2.074755
1.839279	1.989706	1.983626	1.977695	1.972067	1.966602	2.086901	2.081183	2.075516
1.839811	1.990383	1.984336	1.978423	1.972774	1.967275	2.087659	2.08194	2.076269
1.840427	1.991118	1.98506	1.979135	1.973471	1.967954	2.088427	2.082686	2.077007
1.841061	1.835861	1.830621	1.825488	1.974167	1.968635	2.009008	2.003429	2.077749
1.841695	1.836486	1.831221	1.826063	1.974863	1.969312	2.009694	2.004105	2.078499
1.842331	1.837114	1.831825	1.826641	1.975561	1.969991	2.010384	2.004786	2.079254
1.84296	1.837735	1.832428	1.827226	1.976264	1.970674	2.011079	2.005476	2.080012
1.843586	1.838352	1.833033	1.827816	1.97697	1.97136	2.011779	2.006173	2.080775
1.844214	1.838974	1.83364	1.828409	1.977686	1.972059	2.012491	2.006878	2.081543
1.844846	1.839598	1.83425	1.829004	1.97841	1.97277	2.013213	2.007591	2.082316
1.845471	1.840219	1.834857	1.829597	1.824609	1.819758	1.967865	1.962331	2.002768
1.846088	1.840833	1.835459	1.830183	1.825179	1.820313	1.968549	1.963019	2.003475
1.846699	1.841442	1.83606	1.830772	1.825749	1.820865	1.969228	1.963702	2.004178
1.8473	1.842041	1.83666	1.831368	1.826326	1.821419	1.969902	1.964371	2.004867
1.847901	1.842638	1.837262	1.831971	1.826908	1.821975	1.970577	1.965039	2.005553
1.84851	1.843241	1.837866	1.83257	1.827491	1.822538	1.971256	1.965711	1.96026
1.849129	1.843853	1.838477	1.833178	1.828082	1.82311	1.971943	1.966395	1.960939
1.788668	1.844519	1.839169	1.833886	1.82875	1.823724	1.972697	1.967168	1.961712
1.789401	1.845249	1.839956	1.834712	1.829508	1.82439	1.97353	1.968046	1.962596
1.790087	1.845944	1.840691	1.835473	1.830212	1.82502	1.820112	1.815427	1.963414
1.790676	1.84656	1.841315	1.836097	1.830806	1.825575	1.820658	1.815982	1.964094
1.791247	1.847161	1.841914	1.836687	1.831374	1.826118	1.821185	1.8165	1.964739
1.791837	1.847767	1.842522	1.837291	1.831954	1.826667	1.821712	1.817012	1.965399
1.792451	1.848397	1.843145	1.837904	1.83255	1.827243	1.822261	1.817531	1.966069
1.793165	1.849152	1.843858	1.838577	1.833234	1.827947	1.822934	1.818142	1.966793
1.793959	1.850006	1.844642	1.839298	1.833988	1.828753	1.823707	1.818826	1.967564
1.794664	1.850757	1.84536	1.839986	1.83469	1.829479	1.824432	1.81953	1.93006
1.79525	1.851371	1.845984	1.840619	1.835313	1.830096	1.825092	1.820266	1.930932

1.795862	1.852002	1.846644	1.841304	1.835982	1.83074	1.825752	1.820969	1.931749
1.796563	1.852703	1.847402	1.84211	1.836762	1.831463	1.826421	1.82158	1.93242
1.797303	1.85345	1.848195	1.842942	1.837575	1.83223	1.827118	1.822185	1.933077
1.798085	1.854285	1.848988	1.843698	1.838359	1.833049	1.827893	1.822864	1.817981
1.798881	1.855156	1.849784	1.844431	1.83914	1.833899	1.828711	1.823584	1.818661
1.639316	1.794487	1.789353	1.784214	1.83995	1.834697	1.829486	1.824324	1.819373
1.639785	1.795065	1.790077	1.785056	1.84079	1.83545	1.830225	1.825088	1.820119
1.640311	1.795671	1.790749	1.785794	1.841575	1.836232	1.830998	1.825842	1.820847
1.640924	1.796315	1.791334	1.78637	1.842285	1.837082	1.831839	1.826576	1.821532
1.641568	1.797011	1.791949	1.786945	1.842963	1.837875	1.83264	1.827317	1.822243
1.642232	1.79781	1.792674	1.787594	1.843601	1.838501	1.833318	1.828095	1.823046
1.642903	1.798635	1.793441	1.788288	1.844248	1.839087	1.833956	1.828865	1.823857
1.643541	1.799364	1.794196	1.789058	1.845006	1.839787	1.834632	1.829542	1.824546
1.644161	1.800044	1.794952	1.789882	1.845835	1.840553	1.835333	1.830171	1.825172
1.6448	1.800782	1.795696	1.790634	1.846622	1.84134	1.836116	1.830945	1.825928
1.645457	1.801581	1.796426	1.791312	1.847364	1.842151	1.836987	1.831873	1.826825
1.646117	1.641687	1.637303	1.632959	1.848141	1.842984	1.83785	1.832746	1.827662
1.646775	1.642318	1.637932	1.633604	1.848985	1.843861	1.838686	1.833489	1.828364
1.647436	1.642962	1.638591	1.634283	1.849839	1.844712	1.83949	1.834212	1.829053
1.648091	1.643644	1.63932	1.635036	1.850678	1.845462	1.840224	1.834979	1.829815
1.648757	1.644352	1.640075	1.635808	1.851501	1.846172	1.840932	1.835754	1.830595
1.554561	1.645094	1.64078	1.63647	1.790935	1.785765	1.841693	1.836504	1.831329
1.555289	1.645865	1.641451	1.637057	1.791622	1.786536	1.842487	1.837235	1.832036
1.555975	1.646571	1.642109	1.637677	1.792335	1.78726	1.84324	1.837985	1.832798
1.556613	1.647192	1.642747	1.638331	1.793072	1.787912	1.843929	1.83875	1.833628
1.557236	1.647831	1.643417	1.63903	1.634605	1.630195	1.844677	1.839553	1.834467
1.557835	1.648523	1.644162	1.639814	1.635383	1.630947	1.84556	1.840431	1.835312
1.558445	1.649244	1.644919	1.640594	1.636167	1.631721	1.84647	1.841307	1.836137
1.559127	1.650009	1.645634	1.641263	1.636844	1.632421	1.847307	1.842076	1.836875
1.559839	1.650787	1.646326	1.641879	1.637477	1.633096	1.848124	1.842809	1.837584
1.560486	1.651466	1.64698	1.642525	1.638158	1.633824	1.848999	1.84365	1.838414
1.561078	1.652058	1.647594	1.643187	1.638874	1.634597	1.849924	1.844583	1.839347
1.561729	1.652731	1.648281	1.643894	1.639581	1.635299	1.771434	1.766636	1.840235
1.56248	1.653532	1.649074	1.644661	1.640263	1.635887	1.772031	1.767273	1.841043
1.563204	1.654326	1.649872	1.645439	1.640968	1.636508	1.772669	1.767938	1.841861
1.563812	1.655038	1.650629	1.646226	1.641751	1.637271	1.773461	1.768715	1.842729
1.564397	1.655731	1.651359	1.646987	1.642532	1.638064	1.774298	1.769532	1.84362
1.565057	1.656447	1.652029	1.647626	1.643191	1.638764	1.63439	1.630058	1.844515
1.565757	1.657176	1.652666	1.648194	1.643784	1.639421	1.635101	1.630822	1.845419
1.566451	1.562282	1.558101	1.553947	1.64446	1.640125	1.635833	1.631585	1.846333
1.567142	1.563005	1.558819	1.554653	1.645214	1.640872	1.636585	1.632348	1.847259
1.567843	1.563729	1.559544	1.555374	1.645986	1.641635	1.637346	1.633113	1.768848
1.568559	1.564448	1.560267	1.556099	1.646772	1.642415	1.638119	1.633878	1.769642
1.56928	1.565168	1.56099	1.556827	1.647563	1.6432	1.638897	1.634647	1.770441
1.358242	1.565893	1.561715	1.557552	1.648348	1.64398	1.639675	1.635424	1.771256
1.358843	1.566616	1.562436	1.558272	1.649131	1.644759	1.640454	1.636205	1.772078
1.359431	1.567313	1.563137	1.558982	1.649909	1.645538	1.641234	1.636987	1.772901
1.360009	1.567992	1.563825	1.559683	1.650682	1.646316	1.642015	1.63777	1.773724
1.360601	1.568683	1.564515	1.560376	1.651442	1.647075	1.642775	1.638529	1.707926
1.361207	1.56939	1.565208	1.561059	1.652185	1.647812	1.643508	1.639261	1.708711

1.361815	1.358421	1.354999	1.351605	1.652934	1.648556	1.644247	1.639999	1.709501
1.362416	1.359006	1.355579	1.352186	1.653699	1.64932	1.645009	1.640757	1.710306
1.36302	1.359594	1.356161	1.352766	1.654469	1.65009	1.645776	1.641521	1.711119
1.363637	1.360196	1.356752	1.35335	1.65524	1.650858	1.646543	1.642285	1.711937
1.36426	1.360807	1.357348	1.353935	1.656013	1.651628	1.647309	1.64305	1.712761
1.364876	1.361414	1.357936	1.354508	1.561133	1.557094	1.64807	1.643809	1.639487
1.365486	1.36202	1.358518	1.355069	1.561812	1.557777	1.648825	1.644565	1.640283
1.366113	1.362642	1.359146	1.355699	1.562556	1.558496	1.649598	1.645313	1.641044
1.366761	1.363286	1.359842	1.356432	1.563387	1.559256	1.650385	1.646041	1.641747
1.36741	1.363931	1.360525	1.357145	1.353749	1.350374	1.651218	1.646811	1.642478
1.368036	1.364555	1.361142	1.357765	1.35441	1.351081	1.652155	1.647686	1.6433
1.368679	1.365189	1.361744	1.358354	1.355049	1.351786	1.653102	1.648582	1.644145
1.24875	1.365863	1.362368	1.358941	1.355644	1.352405	1.653935	1.649393	1.644933
1.249396	1.36657	1.363009	1.359527	1.356212	1.352973	1.654705	1.650167	1.645704
1.250033	1.367332	1.363717	1.360175	1.35682	1.353555	1.655506	1.651021	1.646573
1.25067	1.368159	1.364495	1.360884	1.357463	1.354145	1.656333	1.651957	1.647546
1.251242	1.368909	1.365249	1.361617	1.358139	1.354758	1.470301	1.466693	1.648438
1.251711	1.369533	1.365963	1.362388	1.358864	1.355397	1.470975	1.467322	1.649181
1.252164	1.370122	1.366642	1.363135	1.35959	1.356064	1.471679	1.46796	1.649917
1.252646	1.249671	1.246644	1.243601	1.360287	1.356788	1.47248	1.468693	1.650726
1.253147	1.250161	1.247145	1.244123	1.360961	1.357522	1.473307	1.469466	1.651574
1.253694	1.250695	1.247665	1.244635	1.361579	1.358156	1.354765	1.351404	1.466489
1.254278	1.251262	1.248201	1.245139	1.362158	1.358728	1.355378	1.352096	1.467313
1.254852	1.251859	1.248808	1.245743	1.362841	1.359364	1.356005	1.352738	1.468067
1.255421	1.252499	1.249509	1.246472	1.363648	1.360071	1.356639	1.353313	1.468732
1.255964	1.253089	1.250149	1.247142	1.364425	1.360799	1.357322	1.35395	1.46945
1.25645	1.253565	1.250643	1.247668	1.365117	1.361557	1.358109	1.354738	1.470302
1.256947	1.25404	1.251116	1.248159	1.365773	1.362294	1.358884	1.355522	1.471152
1.257541	1.254628	1.25167	1.248682	1.245642	1.242591	1.35951	1.35615	1.471872
1.25817	1.255256	1.252254	1.249218	1.246153	1.243086	1.360087	1.356718	1.472537
1.258723	1.2558	1.252811	1.249796	1.246756	1.243708	1.360803	1.357372	1.473237
1.259218	1.25628	1.253352	1.250411	1.247434	1.244431	1.361627	1.358089	1.473958
1.259785	1.256836	1.253925	1.251007	1.248053	1.245069	1.36238	1.358808	1.474741
1.260461	1.257509	1.254547	1.251574	1.248583	1.245576	1.36302	1.359513	1.475597
1.261105	1.258147	1.255151	1.252143	1.249128	1.24611	1.243098	1.240095	1.356883
1.26162	1.258646	1.255683	1.25272	1.249743	1.246762	1.243783	1.240806	1.357713
1.262123	1.259135	1.256218	1.253312	1.250378	1.247433	1.244477	1.24151	1.358522
0.999689	1.259758	1.256859	1.253961	1.251005	1.248027	1.245045	1.242054	1.359174
1.000306	1.260446	1.257547	1.254629	1.251624	1.248582	1.245549	1.242518	1.359748
1.000893	1.261058	1.258122	1.255177	1.252168	1.249136	1.246112	1.243091	1.240121
1.001456	1.261601	1.25859	1.255603	1.252635	1.249681	1.246722	1.243763	1.240826
1.001994	1.138874	1.136023	1.1332	1.253174	1.250271	1.247346	1.244412	1.241482
1.002486	1.139546	1.136733	1.133937	1.25385	1.250948	1.247998	1.245016	1.242042
1.002999	1.14024	1.137455	1.134673	1.254513	1.251594	1.248618	1.2456	1.242597
1.003611	1.140978	1.138154	1.135327	1.255051	1.252098	1.24913	1.246155	1.24319
1.004238	1.141713	1.138838	1.135958	1.25556	1.252571	1.249623	1.246712	1.243803
0.833446	1.002325	0.999869	0.997413	1.133787	1.130987	1.25023	1.247337	1.244449
0.833813	1.002759	1.00035	0.997953	1.134498	1.131727	1.250911	1.24801	1.245122
0.834246	1.003283	1.000897	0.998527	1.135229	1.132474	1.25159	1.248678	1.245773
0.834749	1.003903	1.001515	0.999141	1.135983	1.133229	1.252272	1.249348	1.246408

0.586869	1.004534	1.002141	0.999758	1.136737	1.13398	1.131236	1.1285	1.247005
0.587292	1.005154	1.002762	1.000381	1.137504	1.134739	1.131954	1.12915	1.247536
0.587715	1.005772	1.003372	1.000983	1.138232	1.135455	1.132633	1.129776	1.248063
0.588131	0.834886	0.832927	0.830974	0.999099	0.996685	1.133224	1.130396	1.248637
0.588539	0.835391	0.833387	0.831388	0.999549	0.997097	1.133784	1.131018	1.249239
0.588927	0.587477	0.58603	0.584579	1.000066	0.997619	1.134446	1.131704	1.128984
0.589298	0.587827	0.586379	0.584939	1.000629	0.998221	1.135185	1.132441	1.129718
0.589668	0.588187	0.586745	0.58532	1.001215	0.998829	0.996453	0.994091	1.130453
0.590035	0.588555	0.587133	0.585727	1.001829	0.99944	0.997061	0.994694	1.131187
0.590417	0.588942	0.587533	0.586139	0.81834	0.816322	0.997669	0.995299	1.131922
0.590827	0.589359	0.587945	0.586546	0.818876	0.816861	0.998276	0.995901	1.132655
0.591251	0.589788	0.588362	0.586953	0.819411	0.8174	0.998883	0.996505	1.133389
0.591677	0.590208	0.588775	0.587359	0.58596	0.584567	0.999491	0.997109	0.994737
0.592108	0.590628	0.589186	0.587765	0.586368	0.58498	1.000101	0.997716	0.995339
0.59254	0.591051	0.5896	0.588173	0.586775	0.585389	0.816992	0.81497	0.995938
0.592974	0.591476	0.590016	0.588582	0.587181	0.585795	0.817524	0.815503	0.996536
0.593412	0.591908	0.590438	0.588996	0.58759	0.586201	0.818053	0.816036	0.814009
0.593856	0.592349	0.59087	0.589418	0.588003	0.586609	0.818579	0.816567	0.814545
0.594301	0.592792	0.591304	0.589842	0.588418	0.587018	0.819103	0.817098	0.815081
0.594744	0.593235	0.591738	0.590265	0.588834	0.587426	0.586027	0.584636	0.815618
0.595187	0.593678	0.592173	0.59069	0.58925	0.587836	0.586437	0.585053	0.816154
0.595632	0.594124	0.59261	0.591116	0.589666	0.588245	0.586845	0.585466	0.816686
0.596079	0.59457	0.593048	0.591543	0.590084	0.588655	0.587252	0.585875	0.817216
0.596525	0.595018	0.59349	0.591978	0.59051	0.589071	0.587664	0.586284	0.584888
0.596972	0.595467	0.593937	0.59242	0.590944	0.589495	0.58808	0.586694	0.585297
0.597414	0.595914	0.594385	0.592866	0.591381	0.589921	0.588497	0.587104	0.585706
0.597851	0.596357	0.594833	0.593314	0.591821	0.590349	0.588915	0.587514	0.586115
0.598286	0.596798	0.595281	0.593764	0.592262	0.590778	0.589334	0.587924	0.586523
0.598712	0.597232	0.595724	0.594211	0.592702	0.591205	0.589751	0.588332	0.586929
0.599142	0.597669	0.596168	0.594659	0.593143	0.591634	0.59017	0.588742	0.587336
0.599617	0.598135	0.596625	0.595107	0.593582	0.592063	0.59059	0.589152	0.587742
0.600126	0.598624	0.597092	0.595555	0.59402	0.592496	0.591013	0.589565	0.588148
0.600613	0.599116	0.597587	0.596051	0.594515	0.592987	0.591495	0.590035	0.588609
0.601075	0.599615	0.598123	0.596615	0.595088	0.593558	0.592059	0.590584	0.589144
0.601521	0.60009	0.59863	0.597148	0.595631	0.594102	0.592596	0.591106	0.589654
0.601944	0.600518	0.599068	0.597596	0.596087	0.594562	0.593051	0.591548	0.590084
0.602358	0.600927	0.599481	0.598015	0.596514	0.594995	0.59348	0.591965	0.590491
0.602775	0.601342	0.599903	0.598445	0.596952	0.595439	0.59392	0.592394	0.590909
0.603192	0.601755	0.600323	0.598874	0.597388	0.595881	0.59436	0.592827	0.591333
	0.602168	0.600742	0.599302	0.597822	0.596321	0.59481	0.593289	0.591803
	0.602578	0.601158	0.599727	0.598252	0.596757	0.595267	0.593778	0.592316
	0.603033	0.601614	0.600189	0.59872	0.597234	0.595754	0.594276	0.59282
	0.603557	0.602135	0.600711	0.599252	0.597777	0.59629	0.594791	0.593307
	0.604072	0.602643	0.601216	0.599767	0.598302	0.596809	0.595292	0.593781
	0.604517	0.603075	0.60164	0.600195	0.598739	0.597251	0.595737	0.594221
	0.604947	0.603493	0.60205	0.600608	0.599162	0.597683	0.596179	0.594666
	0.605421	0.603975	0.60254	0.601105	0.599666	0.598195	0.596699	0.595188
	0.605919	0.60449	0.603071	0.601645	0.600212	0.598748	0.597261	0.595754
				0.602117	0.600688	0.599232	0.597753	0.596252
				0.602518	0.601095	0.599644	0.598173	0.596679

0.602922	0.601499	0.600057	0.598603	0.597124
0.603337	0.601906	0.600481	0.599064	0.597619
0.603782	0.60234	0.600926	0.599537	0.598121
0.604303	0.602851	0.601428	0.600029	0.598603
0.604849	0.603387	0.601947	0.600528	0.59908
		0.602406	0.601	0.599565
		0.602826	0.601453	0.600055
		0.603293	0.601923	0.600531
		0.603819	0.602409	0.600989
				0.601463
				0.601989
				0.602508
				0.602945
				0.603351

								1.22163
								1.220412
								1.219184
								1.21792
								1.216664
						0.620235	0.618738	1.336862
						0.619638	0.618136	1.335541
						0.619037	0.617532	1.334218
						0.618435	0.616925	1.332896
				0.62072	0.619299	1.339303	1.335421	1.331587
				0.620126	0.618696	1.337982	1.33411	1.33029
				0.619541	0.618095	1.33666	1.332802	1.328999
				0.618977	0.617498	1.335334	1.331493	1.327712
				0.618409	0.616898	1.334012	1.330189	1.32643
				0.61781	0.616291	1.332701	1.32889	1.325148
				0.617195	0.61568	1.3314	1.327602	1.323877
	0.621015	0.61955	0.618079	1.222256	1.218852	1.330117	1.326347	1.322654
	0.620463	0.618972	0.617474	1.221055	1.217667	1.328851	1.32512	1.321473
	0.619884	0.618377	0.616867	1.219856	1.216484	1.327585	1.323886	1.574799
	0.619266	0.617758	0.616257	1.218657	1.2153	1.326313	1.32263	1.573444
	1.22765	1.22424	1.220836	1.217464	1.214121	1.325061	1.321407	1.57213
	1.226463	1.223052	1.219649	1.216274	1.212932	1.323839	1.320256	1.570918
	1.225273	1.221865	1.21847	1.215101	1.211772	1.322648	1.319133	1.569731
	1.224063	1.220657	1.217278	1.328789	1.325109	1.462439	1.458735	1.65962
	1.222854	1.219453	1.216099	1.3276	1.323991	1.461351	1.457643	1.658344
0.859193	1.221733	1.218366	1.215044	1.326464	1.322861	1.460235	1.45651	1.657028
0.858433	1.220699	1.217397	1.214117	1.325386	1.321728	1.459097	1.455336	1.655672
1.222911	1.219648	1.216375	1.213114	1.465666	1.461771	1.571734	1.567497	1.654323
1.221887	1.218566	1.215258	1.211975	1.464426	1.460551	1.570384	1.566153	1.652974
1.220835	1.217456	1.214112	1.210811	1.463175	1.459332	1.569051	1.564848	1.651676
1.219681	1.2163	1.212956	1.209657	1.461959	1.458147	1.56777	1.563642	1.732084
1.218482	1.215121	1.211798	1.208521	1.46078	1.457004	1.566533	1.562479	1.730852
1.217272	1.213934	1.210676	1.20747	1.459713	1.455977	1.56538	1.561299	1.729525
1.216053	1.212747	1.209582	1.20648	1.458727	1.455033	1.564287	1.560111	1.728141
1.214963	1.373746	1.37033	1.366986	1.457671	1.453986	1.563082	1.558851	1.726723
1.214028	1.372849	1.369402	1.366016	1.456543	1.452827	1.561752	1.557505	1.725255
1.21304	1.371907	1.368441	1.365023	1.455401	1.45166	1.560431	1.556199	1.723853
1.211898	1.370825	1.367374	1.363964	1.454233	1.45049	1.559146	1.554985	1.722589
1.210762	1.369759	1.36633	1.362935	1.453106	1.44936	1.557899	1.553796	1.721337
1.372302	1.463502	1.459695	1.455908	1.452129	1.448363	1.556745	1.552602	1.719998
1.371403	1.462578	1.458787	1.454996	1.451203	1.447412	1.555621	1.551398	1.71862
1.370399	1.461493	1.45771	1.45392	1.562876	1.558617	1.726397	1.721787	1.938983
1.36932	1.460291	1.456508	1.452724	1.561522	1.557259	1.724929	1.720337	1.937343
1.368324	1.459205	1.455425	1.45164	1.560293	1.556024	1.723588	1.719014	1.935809
1.367448	1.458293	1.454518	1.450724	1.559251	1.55497	1.722434	1.717877	1.934427
1.366543	1.45733	1.453558	1.449756	1.558153	1.553866	1.721226	1.716685	1.933002
1.365517	1.456174	1.452396	1.448596	1.556841	1.552557	1.719807	1.71528	1.9314

1.364489	1.455005	1.451219	1.447422	1.555517	1.551241	1.718379	1.713867	1.929793
1.363601	1.454022	1.450232	1.446433	1.554405	1.550138	1.717173	1.712676	1.928376
1.362774	1.45312	1.449328	1.445526	1.553386	1.549131	1.716065	1.711585	1.927044
1.361818	1.452074	1.448279	1.444475	1.552206	1.547962	1.714791	1.710327	1.925567
1.360741	1.450895	1.447098	1.443293	1.55088	1.546647	1.713365	1.708915	1.923956
1.359751	1.562418	1.558162	1.553902	1.721102	1.716543	1.712047	1.707612	1.922452
1.358905	1.561361	1.557105	1.552847	1.719936	1.715386	1.710903	1.706484	1.921122
1.358084	1.560341	1.556086	1.551831	1.718815	1.714276	1.709803	1.705397	1.919822
1.357248	1.559326	1.555076	1.550824	1.717715	1.71319	1.86116	1.856305	1.918508
1.356388	1.558279	1.554035	1.549786	1.716587	1.712079	1.85988	1.855023	1.917161
1.448808	1.557054	1.552808	1.548565	1.715269	1.710783	1.858451	1.853598	1.915664
1.447659	1.555716	1.551464	1.547229	1.713831	1.709369	1.856926	1.852081	1.914072
1.446622	1.55452	1.550266	1.546039	1.712543	1.708099	1.922728	1.917657	1.912622
1.445702	1.553471	1.549219	1.545	1.711408	1.706976	1.921402	1.916343	1.91132
1.556698	1.723865	1.719299	1.71477	1.710299	1.705877	1.920089	1.91504	1.910029
1.555704	1.722773	1.718211	1.713686	1.709222	1.704807	1.918779	1.91374	1.908738
1.554649	1.721622	1.717066	1.712547	1.708091	1.703686	1.917422	1.912394	1.9074
1.553385	1.720251	1.715709	1.711205	1.85877	1.853913	1.915906	1.910892	1.972678
1.55205	1.718806	1.714281	1.709798	1.857261	1.852419	1.914344	1.909347	1.971041
1.550901	1.717556	1.713044	1.708575	1.855926	1.851097	1.912957	1.907972	1.969578
1.549885	1.716445	1.711944	1.707484	1.854722	1.849905	1.911699	1.906725	1.968244
1.548886	1.715353	1.710863	1.706414	1.853531	1.848719	1.910448	1.905484	1.966917
1.547904	1.714279	1.709804	1.705364	1.852352	1.847535	1.909199	1.904247	1.965594
1.546928	1.713208	1.708747	1.704316	1.85118	1.846359	1.975002	1.969627	1.964273
1.545945	1.712126	1.707677	1.703256	1.850003	1.845185	1.973662	1.968294	1.96295
1.544964	1.711045	1.706606	1.702195	1.848831	1.844018	1.972331	1.966966	1.961626
1.543989	1.709969	1.705543	1.701142	1.847668	1.842868	1.971023	1.965654	2.026701
1.543019	1.708895	1.704482	1.700092	1.846511	1.841728	1.969727	1.964347	2.025329
1.712248	1.85981	1.854961	1.85014	1.911811	1.906816	1.968414	1.963008	2.02391
1.711159	1.858605	1.853767	1.848957	1.910574	1.905594	1.967089	1.961643	2.022448
1.710076	1.857406	1.852577	1.847773	1.909322	1.904338	1.965718	1.960249	2.020972
1.708999	1.856214	1.851394	1.846591	1.90805	1.903036	1.964282	1.958816	2.019481
1.707926	1.855021	1.850207	1.845403	1.973802	1.968317	1.962843	1.957378	2.017992
1.706856	1.85382	1.849007	1.844199	1.97242	1.966914	1.961418	1.955928	2.016499
1.705788	1.852619	1.847806	1.842995	1.971051	1.965542	1.960033	1.954516	2.015044
1.704719	1.851426	1.846617	1.841813	1.969735	1.964272	2.02505	2.01934	2.159669
1.70365	1.850239	1.845435	1.840646	1.968452	1.963068	2.023835	2.018133	2.15818
1.702575	1.849045	1.844248	1.839472	1.967141	1.961799	2.022546	2.016852	2.156609
1.701495	1.847841	1.843057	1.838292	1.965801	1.960463	2.021183	2.015498	2.154958
1.700414	1.84664	1.841865	1.837112	1.964467	1.959136	2.019829	2.01416	2.153314
1.699335	1.845443	1.840676	1.835932	1.963148	1.957837	2.018499	2.012852	2.151674
1.698254	1.844247	1.839488	1.834752	1.961833	1.956546	2.017187	2.011574	2.150088
1.697171	1.915554	1.910365	1.90523	2.026884	2.021416	2.162082	2.15534	2.148644
1.696085	1.914252	1.909085	1.903972	2.025536	2.020096	2.160498	2.153872	2.147253
1.845416	1.912948	1.907806	1.902716	2.024181	2.018758	2.158874	2.152313	2.14576
1.844194	1.911643	1.906529	1.901462	2.022823	2.017408	2.157222	2.150692	2.144198
1.842998	1.971065	1.965768	1.960525	2.021466	2.01606	2.155567	2.149057	2.142605
1.841822	1.969668	1.964393	1.959198	2.02011	2.014713	2.153906	2.147406	2.140976
1.840703	1.968354	1.963081	1.957903	2.018766	2.01337	2.152244	2.145748	2.139335
1.839678	1.967194	1.961871	1.956636	2.017418	2.012009	2.150559	2.144078	2.137686

1.838684	1.966096	1.960728	1.95543	2.016123	2.01069	2.148909	2.142421	2.136036
1.909831	2.031533	2.025982	2.020482	2.160626	2.153974	2.147366	2.140795	2.134368
1.908721	2.030421	2.024921	2.019442	2.15933	2.152597	2.145895	2.139209	2.13272
1.907523	2.029179	2.023719	2.018266	2.15789	2.151172	2.144464	2.137749	2.164386
1.906243	2.027823	2.022397	2.016983	2.15634	2.14972	2.143079	2.136405	2.162963
1.905015	2.026477	2.02106	2.015667	2.154733	2.148194	2.141632	2.135041	2.161583
1.903856	2.025139	2.019697	2.0143	2.153045	2.146563	2.140099	2.133649	2.160271
1.902747	2.171166	2.164496	2.157917	2.15139	2.144929	2.138533	2.1322	2.158886
1.901725	2.169779	2.163068	2.156425	2.149837	2.143317	2.13691	2.130611	2.157282
1.90071	2.16844	2.161701	2.155001	2.148351	2.141763	2.135321	2.129015	2.15563
1.899554	2.166895	2.160211	2.153567	2.146956	2.140395	2.133951	2.127616	2.2003
1.898324	2.165244	2.158661	2.152135	2.145621	2.13914	2.132714	2.126334	2.198809
2.023391	2.163703	2.157134	2.150635	2.144163	2.137734	2.131352	2.125009	2.197381
2.022306	2.162266	2.155617	2.149053	2.142571	2.136172	2.129865	2.123649	2.196031
2.02118	2.160837	2.154164	2.147575	2.141085	2.134692	2.1617	2.155164	2.194612
2.019966	2.159414	2.152829	2.146301	2.139812	2.13338	2.16028	2.1537	2.193038
2.018732	2.157952	2.151466	2.145014	2.138542	2.132085	2.158891	2.152274	2.1915
2.162899	2.156355	2.149912	2.143523	2.137104	2.130701	2.157526	2.150983	2.190178
2.161328	2.154742	2.148319	2.141981	2.135623	2.129295	2.156168	2.14973	2.188916
2.159923	2.153348	2.146938	2.140611	2.134258	2.127932	2.2008	2.194128	2.187475
2.158642	2.15211	2.145702	2.139351	2.132965	2.126595	2.199269	2.19256	2.185919
2.15733	2.150802	2.144368	2.137986	2.131601	2.125247	2.197813	2.191117	2.184491
2.15599	2.149412	2.14291	2.136483	2.13014	2.123881	2.196446	2.189831	2.183227
2.154613	2.148018	2.141488	2.135046	2.128739	2.122545	2.195073	2.188507	2.181921
2.153167	2.146649	2.140188	2.133799	2.127494	2.12127	2.193644	2.187039	2.18046
2.151713	2.145271	2.138884	2.132552	2.126255	2.120007	2.192228	2.185577	2.179006
2.15031	2.143847	2.137437	2.131102	2.173299	2.166837	2.190885	2.184259	2.177717
2.14893	2.142407	2.135942	2.129584	2.171786	2.165459	2.189581	2.183004	2.176494
2.147545	2.141011	2.134574	2.128257	2.170433	2.164151	2.188272	2.18169	2.211784
2.146157	2.139649	2.133307	2.127086	2.169211	2.162906	2.186966	2.180332	2.210325
2.144813	2.138344	2.132073	2.125917	2.198903	2.192282	2.18564	2.178983	2.208914
2.14353	2.13712	2.130888	2.124752	2.197623	2.19094	2.184278	2.177635	2.207567
2.142256	2.135885	2.129665	2.123532	2.196302	2.189599	2.182941	2.176326	2.242376
2.140974	2.134581	2.128321	2.122162	2.194875	2.188268	2.181676	2.175105	2.241123
2.139678	2.133255	2.126957	2.120781	2.193444	2.186953	2.180432	2.173898	2.239876
2.138319	2.180587	2.17405	2.167645	2.192158	2.185685	2.179165	2.172611	2.238528
2.136931	2.179202	2.172769	2.166453	2.190952	2.184444	2.177885	2.171279	2.237126
2.135643	2.177881	2.171502	2.165229	2.189695	2.183143	2.213068	2.206341	2.235766
2.13445	2.176622	2.170255	2.163982	2.188396	2.181787	2.211656	2.204963	2.234438
2.13327	2.175392	2.169011	2.162709	2.18707	2.180433	2.210276	2.203632	2.233155
2.13208	2.174193	2.167756	2.161379	2.185686	2.179074	2.208939	2.20236	2.231929
2.130908	2.173006	2.166525	2.160086	2.184351	2.177766	2.207654	2.201134	2.230732
2.129783	2.171834	2.165369	2.158936	2.183181	2.176584	2.206453	2.199962	2.229553
2.128663	2.170647	2.164219	2.157823	2.182067	2.175457	2.205299	2.198823	2.228391
2.127466	2.169346	2.162939	2.156579	2.217331	2.210683	2.204134	2.197678	2.227239
2.12622	2.167968	2.161574	2.155254	2.216036	2.209469	2.202969	2.196532	2.226095
2.173204	2.166688	2.160301	2.154001	2.214779	2.208262	2.201784	2.195341	2.22489
2.172017	2.165519	2.159132	2.152827	2.213561	2.207061	2.200572	2.194088	2.223602
2.202606	2.195788	2.189086	2.182484	2.212371	2.205882	2.19939	2.19289	2.250296
2.20141	2.194618	2.187935	2.181351	2.211222	2.204744	2.198276	2.191811	2.249191

2.200225	2.193455	2.186791	2.180224	2.210078	2.20361	2.197173	2.19076	2.248124
2.199056	2.19229	2.185628	2.179062	2.208897	2.202442	2.196034	2.189665	2.247014
2.197895	2.191127	2.184456	2.177886	2.207701	2.201261	2.194883	2.188559	2.245895
2.196724	2.226443	2.219691	2.213046	2.206515	2.200091	2.193739	2.187453	2.244772
2.195545	2.225246	2.218497	2.211855	2.205334	2.198927	2.192599	2.186344	2.243645
2.194365	2.22405	2.217306	2.210672	2.204173	2.197796	2.191501	2.185284	2.288657
2.19318	2.22285	2.216117	2.209498	2.203036	2.196707	2.190459	2.184291	2.287635
2.192006	2.221661	2.214939	2.208336	2.20191	2.195629	2.218947	2.212764	2.28661
2.190849	2.220495	2.213785	2.207195	2.200797	2.19455	2.217866	2.211707	2.285551
2.189702	2.219339	2.212637	2.206056	2.199683	2.19347	2.216781	2.210642	2.284483
2.188552	2.218171	2.211463	2.20488	2.198537	2.192372	2.264974	2.258786	2.283418
2.18741	2.217011	2.210289	2.203695	2.197381	2.191266	2.263881	2.257723	2.282356
2.186323	2.215947	2.209215	2.202599	2.196285	2.190182	2.262807	2.256688	2.315774
2.185294	2.214983	2.208243	2.20159	2.195248	2.189119	2.261752	2.255677	2.314737
2.22053	2.213952	2.207221	2.200561	2.194207	2.188065	2.260711	2.254699	2.313746
2.219347	2.212803	2.206108	2.199482	2.193146	2.187019	2.259692	2.253774	2.312832
2.218165	2.211632	2.204979	2.198401	2.192091	2.185983	2.258673	2.252837	2.311899
2.217048	2.210487	2.203862	2.197326	2.191034	2.184937	2.257622	2.251826	2.333901
2.215946	2.209347	2.202757	2.196277	2.190007	2.183918	2.256585	2.250797	2.332802
2.214798	2.208197	2.201699	2.195324	2.267889	2.261658	2.255633	2.24982	2.331744
2.213628	2.207048	2.200677	2.194439	2.26704	2.260799	2.254746	2.248876	2.330708
2.212555	2.20598	2.199656	2.19348	2.266124	2.25994	2.289979	2.28396	2.329697
2.211591	2.205	2.198631	2.192433	2.265134	2.25909	2.289138	2.283068	2.328717
2.210629	2.204055	2.197651	2.191424	2.264146	2.258192	2.288235	2.282142	2.327725
2.209651	2.203163	2.19676	2.190501	2.263166	2.257191	2.287193	2.281126	2.326686
2.208646	2.20224	2.195867	2.189608	2.262215	2.256196	2.286152	2.280125	2.325675
2.178204	2.255523	2.2493	2.243188	2.261342	2.255306	2.285231	2.279237	2.324773
2.177108	2.254382	2.248274	2.242298	2.260501	2.254467	2.284369	2.278404	2.323923
2.176128	2.253368	2.247282	2.241346	2.295627	2.289555	2.28353	2.277557	2.352841
2.175241	2.252448	2.246314	2.240344	2.294646	2.288703	2.282719	2.276707	2.351926
2.174356	2.251551	2.245389	2.239391	2.293668	2.287774	2.281824	2.275834	2.351019
2.173469	2.250679	2.244516	2.238497	2.292684	2.286736	2.280815	2.274936	2.350137
2.172587	2.249809	2.243658	2.237634	2.291739	2.28573	2.331382	2.32544	2.349206
2.171718	2.248922	2.242803	2.236806	2.29087	2.284823	2.330404	2.324428	2.348159
2.170839	2.248033	2.241954	2.235991	2.29003	2.283963	2.329463	2.32342	2.3471
2.253348	2.247155	2.241106	2.235158	2.289181	2.283127	2.328583	2.322499	2.346145
2.252369	2.24628	2.240259	2.234318	2.288333	2.282313	2.327741	2.321627	2.345247
2.251445	2.283018	2.276909	2.270874	2.287479	2.281463	2.356656	2.350469	2.344354
2.25057	2.282079	2.276001	2.270005	2.286619	2.28058	2.355725	2.349571	2.343474
2.249687	2.281148	2.275091	2.269122	2.285752	2.279711	2.354806	2.348665	2.342578
2.248784	2.28023	2.274177	2.268213	2.284872	2.278868	2.353911	2.347749	2.341654
2.247882	2.279317	2.27327	2.267313	2.283995	2.278019	2.353007	2.346829	2.340731
2.246989	2.278401	2.272379	2.266445	2.260542	2.254688	2.352065	2.345903	2.339822
2.246104	2.277493	2.271501	2.26559	2.25968	2.253803	2.35112	2.34498	2.338919
2.282722	2.276618	2.270639	2.264736	2.258832	2.252959	2.350218	2.344068	2.338009
2.281828	2.275764	2.269791	2.263885	2.257997	2.252146	2.349351	2.343171	2.337103
2.280941	2.274899	2.268937	2.263042	2.257187	2.251376	2.348548	2.342364	2.358014
2.280054	2.274013	2.268069	2.262204	2.256403	2.25066	2.347826	2.341665	2.357342
2.279222	2.273184	2.267249	2.261398	2.31346	2.307602	2.368917	2.36273	2.356623
2.278488	2.272462	2.266524	2.260654	2.312638	2.306725	2.368024	2.361872	2.355797

2.277754	2.271746	2.2658	2.259904	2.311805	2.305829	2.367112	2.360987	2.354942
2.276927	2.270947	2.264991	2.259075	2.310927	2.304948	2.366253	2.360119	2.35408
2.276065	2.27012	2.264155	2.258223	2.310042	2.304081	2.365422	2.359267	2.353226
2.275239	2.269342	2.263384	2.257447	2.333668	2.32768	2.364648	2.358501	2.352468
2.274436	2.268594	2.262657	2.256727	2.332952	2.326959	2.363922	2.357804	2.351784
2.273621	2.295329	2.289353	2.283383	2.332175	2.326167	2.3456	2.339633	2.35104
2.272783	2.294475	2.288519	2.282578	2.331309	2.325268	2.344686	2.338755	2.350208
2.271975	2.293655	2.287709	2.281787	2.330471	2.324416	2.343823	2.33792	2.349412
2.271245	2.292941	2.286987	2.281064	2.329734	2.32371	2.343116	2.337212	2.348719
2.270516	2.29224	2.286271	2.280338	2.329002	2.323018	2.342432	2.336531	2.348057
2.269686	2.29145	2.285471	2.279526	2.328161	2.322199	2.341639	2.335793	2.347383
2.268816	2.290628	2.284639	2.278681	2.327278	2.321329	2.340799	2.335029	2.346699
2.268038	2.289885	2.283894	2.277929	2.32649	2.320542	2.340005	2.33424	2.345939
2.26734	2.289212	2.283224	2.277261	2.325784	2.319825	2.339243	2.333421	2.345103
2.294486	2.343321	2.337203	2.331111	2.350506	2.344467	2.338521	2.332663	2.326897
2.293818	2.342613	2.336495	2.330407	2.349803	2.343784	2.337848	2.331992	2.326228
2.293151	2.341909	2.335791	2.329707	2.349103	2.343104	2.337185	2.331345	2.32559
2.292483	2.341207	2.335093	2.329015	2.348402	2.342412	2.336509	2.330694	2.324957
2.291815	2.340508	2.334397	2.328322	2.3477	2.341717	2.335832	2.330045	2.324327
2.29115	2.339811	2.333695	2.327616	2.346988	2.341023	2.335154	2.329384	2.323677
2.290487	2.339115	2.332989	2.326901	2.34627	2.340328	2.334475	2.328713	2.323014
2.289814	2.338408	2.332279	2.326189	2.345553	2.339627	2.333787	2.328038	2.322347
2.289132	2.337688	2.331564	2.325481	2.344836	2.338919	2.33309	2.327356	2.321677
2.288451	2.336973	2.33085	2.324771	2.344116	2.338209	2.332394	2.326676	2.32101
2.287774	2.336266	2.330138	2.324054	2.34339	2.337496	2.331698	2.326003	2.320349
2.287098	2.335559	2.329428	2.32334	2.342669	2.336789	2.331006	2.32533	2.319687
2.286414	2.334841	2.328714	2.322633	2.34196	2.336095	2.330323	2.324652	2.319014
2.285734	2.334124	2.328006	2.321938	2.341264	2.33541	2.329644	2.323973	2.318338
2.285068	2.333422	2.32732	2.321269	2.340585	2.334723	2.328957	2.323293	2.317663
2.284417	2.332734	2.326654	2.320624	2.339923	2.33404	2.328267	2.322613	2.31699
2.308004	2.33211	2.326048	2.320037	2.339327	2.33343	2.327642	2.321967	2.316331
2.307475	2.331572	2.325525	2.319531	2.33882	2.332918	2.327097	2.321358	2.315679
2.306903	2.356426	2.350302	2.344242	2.338271	2.332377	2.326545	2.320778	2.333751
2.306209	2.355721	2.349607	2.343563	2.33761	2.331739	2.32595	2.320244	2.333244
2.305492	2.354987	2.34888	2.34285	2.336917	2.331077	2.325338	2.319708	2.332744
2.304826	2.354278	2.348172	2.342157	2.336243	2.330425	2.324708	2.319097	2.332137
2.304186	2.353586	2.347478	2.341477	2.335581	2.329787	2.324076	2.318457	2.331486
2.303575	2.324322	2.318278	2.312342	2.334996	2.329234	2.323529	2.317889	2.330905
2.302992	2.323772	2.317733	2.311797	2.334484	2.328762	2.323058	2.317384	2.330381
2.302423	2.323209	2.317164	2.311219	2.33392	2.328222	2.322524	2.316836	2.329822
2.30188	2.322625	2.316548	2.310575	2.333262	2.327565	2.321878	2.316213	2.329197
2.30131	2.322026	2.315939	2.309958	2.332636	2.326937	2.321258	2.315611	2.32859
2.300654	2.32139	2.315357	2.309425	2.332126	2.32643	2.320749	2.315094	2.328053
2.299991	2.320759	2.314795	2.308929	2.331659	2.325967	2.320279	2.314606	2.327539
2.299423	2.320201	2.314267	2.308429	2.331173	2.325485	2.319794	2.314107	2.32701
2.298915	2.319691	2.313765	2.307936	2.330685	2.325001	2.319304	2.313603	2.326475
2.298414	2.319187	2.313269	2.307444	2.30173	2.2961	2.318789	2.313055	2.325902
2.297926	2.318695	2.31278	2.306954	2.301234	2.295592	2.31825	2.312461	2.325286
2.297429	2.318195	2.312283	2.306455	2.30072	2.295055	2.336367	2.330498	2.324713
2.296916	2.317681	2.311777	2.305947	2.300177	2.294466	2.335772	2.329963	2.324218

2.296394	2.317154	2.311251	2.305417	2.299627	2.293889	2.335197	2.329449	2.323741
2.295865	2.316611	2.310687	2.304838	2.299073	2.293382	2.334699	2.328951	2.323247
2.295326	2.316052	2.310107	2.304247	2.298526	2.292905	2.334233	2.328467	2.322753
2.294751	2.315452	2.309537	2.30372	2.298021	2.292413	2.333743	2.327978	2.322266
2.294151	2.314824	2.308977	2.303238	2.297548	2.291917	2.333242	2.327488	2.321787
2.293661	2.314328	2.308489	2.302757	2.297067	2.291432	2.305619	2.299928	2.32131
2.293308	2.313995	2.308084	2.302266	2.296567	2.290959	2.305142	2.299453	2.320836
2.292934	2.313651	2.307705	2.301833	2.296112	2.290494	2.304661	2.298976	2.320364
2.292475	2.313235	2.307351	2.301515	2.29574	2.290022	2.30415	2.298485	2.319896
2.291989	2.312787	2.306984	2.301209	2.295393	2.289581	2.303667	2.298005	2.319426
2.2915	2.312303	2.306546	2.300821	2.295033	2.289232	2.303289	2.297557	2.318934
2.291003	2.311794	2.306068	2.300387	2.294661	2.288925	2.302962	2.297136	2.318448
2.290505	2.311306	2.305587	2.299918	2.29423	2.288541	2.302591	2.296764	2.318064
2.290003	2.310831	2.305097	2.299411	2.293743	2.288097	2.302188	2.296442	2.317771
2.316142	2.310356	2.304624	2.298941	2.293273	2.287631	2.301744	2.296063	2.290433
2.315704	2.309866	2.304163	2.298518	2.29282	2.287122	2.301233	2.295599	2.290027
2.315299	2.30942	2.303741	2.298125	2.292406	2.286662	2.300759	2.295136	2.289597
2.314929	2.309067	2.303395	2.297782	2.292073	2.286338	2.300401	2.294703	2.289131
2.314585	2.308754	2.303072	2.297441	2.291752	2.286047	2.300078	2.294297	2.28868
2.314261	2.308443	2.302709	2.297022	2.291337	2.285666	2.299708	2.293942	2.288336
2.313949	2.308136	2.302328	2.296564	2.290876	2.285246	2.299321	2.293624	2.288057
2.313568	2.307778	2.301956	2.296172	2.290488	2.284874	2.298965	2.29331	2.287759
2.313115	2.307368	2.301588	2.295842	2.29017	2.284553	2.298643	2.293007	2.287448
2.312699	2.306979	2.301237	2.295521	2.289839	2.284192	2.298273	2.292661	2.28711
2.312363	2.306646	2.300922	2.29521	2.289475	2.283748	2.297807	2.292238	2.28673
2.312023	2.306293	2.300584	2.294883	2.289107	2.283319	2.297359	2.291818	2.286349
2.311615	2.305837	2.300155	2.2945	2.288749	2.282977	2.297019	2.291474	2.286011
2.311192	2.305362	2.299707	2.294104	2.288401	2.282675	2.296721	2.291149	2.285677
2.310822	2.305004	2.299357	2.293762	2.288076	2.282369	2.296393	2.290773	2.285277
2.31049	2.304725	2.299074	2.293458	2.287772	2.282068	2.296054	2.290368	2.284836
2.31017	2.32438	2.318666	2.312977	2.307232	2.301472	2.295741	2.290022	2.284472
2.309863	2.32408	2.318374	2.312691	2.306948	2.301186	2.295456	2.289742	2.284197
2.309565	2.323783	2.318083	2.312404	2.306664	2.300904	2.295181	2.289476	2.283938
2.30928	2.323493	2.317789	2.312109	2.306376	2.300628	2.294913	2.289213	2.283677
2.308999	2.323203	2.317497	2.311814	2.306089	2.300353	2.294646	2.288952	2.283418
2.308709	2.322912	2.317209	2.311531	2.305807	2.300071	2.294367	2.28868	2.283153
2.308417	2.322619	2.316924	2.311251	2.305526	2.299787	2.294085	2.288404	2.282885
2.30813	2.322326	2.316634	2.310965	2.305245	2.299511	2.293814	2.288139	2.282624
2.307847	2.322032	2.316339	2.310673	2.304962	2.299241	2.293552	2.287881	2.282368
2.307564	2.321744	2.316052	2.310387	2.304681	2.298966	2.293282	2.287617	2.282107
2.307278	2.321466	2.315777	2.31011	2.3044	2.298679	2.292997	2.287338	2.281837
2.307002	2.321185	2.315496	2.309831	2.304121	2.298401	2.292724	2.287074	2.281581
2.306748	2.320882	2.315192	2.30954	2.303846	2.298146	2.292482	2.286839	2.281344
2.306507	2.320586	2.314889	2.309249	2.303572	2.297896	2.292252	2.286629	2.281137
2.306267	2.320324	2.31461	2.308958	2.303285	2.297627	2.292021	2.286457	2.304559
2.306035	2.320091	2.314353	2.308677	2.303	2.297354	2.291792	2.286308	2.30446
2.305839	2.319915	2.31417	2.308472	2.302775	2.29711	2.291554	2.286101	2.304273
2.30568	2.319805	2.314066	2.308347	2.302611	2.296893	2.291304	2.285835	2.304002
2.305483	2.319635	2.313904	2.308179	2.302436	2.296707	2.291106	2.285623	2.303776
2.305204	2.319353	2.313632	2.307929	2.302239	2.296576	2.291004	2.285515	2.30364

2.304948	2.319081	2.313358	2.307668	2.302023	2.296423	2.29088	2.285392	2.303499
2.304795	2.318907	2.313146	2.307421	2.301763	2.296164	2.29063	2.285161	2.303292
2.304678	2.318764	2.31296	2.307191	2.301501	2.29588	2.290346	2.284895	2.303061
2.304517	2.318578	2.312778	2.307018	2.301323	2.29569	2.313805	2.308262	2.302831
2.304333	2.318372	2.312606	2.306887	2.301201	2.295557	2.313641	2.308063	2.302602
2.30416	2.318199	2.312454	2.306753	2.301059	2.295393	2.313465	2.307887	2.302423
2.303996	2.318062	2.312329	2.306621	2.300897	2.295192	2.313272	2.307737	2.3023
2.303847	2.317928	2.312188	2.306462	2.300724	2.295006	2.313102	2.3076	2.302183
2.30373	2.317794	2.312009	2.306247	2.300525	2.294854	2.312975	2.307483	2.302061
2.303605	2.317644	2.311822	2.306034	2.300336	2.294714	2.312854	2.307359	2.301927
2.303421	2.317439	2.311637	2.30588	2.324019	2.318319	2.312709	2.307187	2.301749
2.303217	2.317218	2.311461	2.305757	2.323899	2.318182	2.312551	2.306998	2.301558
2.303055	2.317067	2.311334	2.305641	2.323759	2.318	2.312364	2.306839	2.301429
2.302923	2.316971	2.311248	2.305538	2.323606	2.317782	2.312152	2.306702	2.301348
2.302808	2.316876	2.311157	2.305442	2.323499	2.317662	2.312036	2.306604	2.301265
2.302712	2.316777	2.311058	2.305356	2.323459	2.317687	2.312059	2.306562	2.301176
2.30262	2.316681	2.31096	2.305266	2.323397	2.317668	2.31204	2.306502	2.301089
2.30252	2.31659	2.310868	2.305159	2.323248	2.317469	2.311848	2.306374	2.301006
2.302427	2.316498	2.310777	2.305055	2.323099	2.317264	2.311649	2.30624	2.300919
2.302363	2.340421	2.334635	2.328861	2.323049	2.317248	2.311624	2.306163	2.300808
2.302313	2.340314	2.334532	2.328794	2.323048	2.317325	2.311686	2.306117	2.300691
2.302216	2.340213	2.334433	2.328704	2.322986	2.3173	2.311672	2.306092	2.300647
2.302074	2.340118	2.33434	2.328593	2.322871	2.317187	2.311596	2.306094	2.300676
2.301968	2.321444	2.315719	2.310024	2.304363	2.298745	2.311503	2.306028	2.30062
2.301916	2.321316	2.315602	2.309938	2.304283	2.298659	2.311385	2.305838	2.300403
2.301899	2.321232	2.315509	2.309852	2.304198	2.298573	2.311278	2.305679	2.300222
2.327124	2.321274	2.315474	2.309733	2.304074	2.298485	2.311222	2.305675	2.30023
2.327165	2.321353	2.315472	2.309632	2.303958	2.298399	2.311177	2.305714	2.300294
2.32712	2.321351	2.315489	2.30965	2.303936	2.298317	2.311085	2.305658	2.300269
2.327019	2.321302	2.315522	2.309744	2.303976	2.298243	2.31097	2.30555	2.300196
2.326943	2.321215	2.315482	2.309757	2.303989	2.298229	2.310931	2.305477	2.300118
2.326886	2.321076	2.315359	2.309684	2.303978	2.298286	2.310979	2.305435	2.300027
2.326871	2.321001	2.315268	2.309602	2.303945	2.298321	2.311021	2.305424	2.299984
2.326916	2.321044	2.315244	2.309512	2.303867	2.298296	2.311023	2.305451	2.300024
2.326985	2.321129	2.315261	2.309453	2.303805	2.298269	2.311025	2.305497	2.300089
2.327063	2.321224	2.315328	2.309483	2.303823	2.298289	2.311058	2.305559	2.300145
2.327138	2.321323	2.315422	2.30956	2.30388	2.298322	2.311093	2.30562	2.300196
2.327142	2.321361	2.315482	2.30963	2.303918	2.298308	2.311067	2.305618	2.300207
2.32709	2.32135	2.315522	2.309703	2.303948	2.298258	2.310995	2.305565	2.300185
2.294906	2.321356	2.31557	2.309784	2.304004	2.298258	2.292708	2.287346	2.300187
2.294942	2.321392	2.315638	2.309884	2.304096	2.298322	2.292754	2.287381	2.300227
2.294968	2.321399	2.315671	2.309953	2.304174	2.298396	2.29281	2.287403	2.300238
2.294941	2.321325	2.315618	2.309944	2.304204	2.298459	2.292851	2.287365	2.30016
2.294922	2.321256	2.315564	2.309928	2.30423	2.298527	2.292901	2.287335	2.300087
2.294977	2.321295	2.315603	2.309977	2.304294	2.298607	2.292977	2.287386	2.300124
2.295074	2.321394	2.315695	2.310062	2.304379	2.298696	2.293069	2.287482	2.300221
2.295169	2.32149	2.315788	2.310153	2.30447	2.29879	2.293161	2.287569	2.300305
2.295265	2.321591	2.31589	2.310256	2.304571	2.298888	2.293254	2.287653	2.300384
2.295358	2.321689	2.315988	2.310355	2.30467	2.298985	2.293348	2.287742	2.30047
2.295443	2.321777	2.316077	2.310446	2.304763	2.299078	2.293441	2.287836	2.300564

2.29553	2.289725	2.284105	2.27856	2.304856	2.299173	2.293537	2.287934	2.300664
2.295627	2.289819	2.284197	2.278649	2.304953	2.29927	2.293637	2.288037	2.300772
2.29573	2.28992	2.284295	2.278743	2.305053	2.299371	2.293739	2.288143	2.300882
2.295832	2.290022	2.284397	2.278846	2.30516	2.299474	2.293839	2.28824	2.300978
2.295938	2.290127	2.284504	2.278954	2.305272	2.299581	2.29394	2.288334	2.301071
2.296065	2.290253	2.284617	2.279053	2.305377	2.299693	2.294057	2.288454	2.283015
2.29621	2.290395	2.284728	2.279134	2.305472	2.29981	2.294189	2.288599	2.283157
2.296386	2.290586	2.284893	2.279265	2.305596	2.299935	2.29433	2.288772	2.283341
2.296619	2.290871	2.28516	2.279487	2.305776	2.30007	2.294479	2.288995	2.283602
2.29684	2.291137	2.285416	2.279711	2.305961	2.300212	2.29463	2.289208	2.283847
2.296966	2.291265	2.285557	2.279865	2.274167	2.268499	2.29476	2.289338	2.28398
2.297068	2.291346	2.285655	2.279989	2.274305	2.268644	2.294898	2.289448	2.284075
2.258087	2.291517	2.285797	2.280117	2.27446	2.268847	2.295119	2.289644	2.318751
2.258305	2.291746	2.285966	2.280243	2.274626	2.269095	2.295402	2.289902	2.318985
2.258459	2.252534	2.246623	2.240779	2.274777	2.269279	2.295609	2.290104	2.319197
2.258528	2.252631	2.246727	2.240878	2.274897	2.269369	2.295713	2.290231	2.319383
2.258606	2.252731	2.246849	2.241014	2.275061	2.269501	2.264034	2.258657	2.31956
2.258727	2.252842	2.247004	2.241218	2.275321	2.269749	2.264254	2.258829	2.319711
2.258871	2.252975	2.247178	2.24144	2.2756	2.270019	2.264497	2.259028	2.319887
2.259037	2.253163	2.247373	2.241634	2.235899	2.230196	2.264699	2.259254	2.320162
2.259223	2.253382	2.24758	2.241814	2.236056	2.230332	2.264892	2.259504	2.32049
2.259415	2.253578	2.247755	2.241969	2.236213	2.230504	2.265124	2.259753	2.320771
2.259618	2.253757	2.247904	2.242097	2.236365	2.230705	2.265388	2.260006	2.321017
2.259795	2.253918	2.248064	2.242266	2.236558	2.230926	2.225362	2.219866	2.321255
2.259933	2.254046	2.248239	2.242496	2.236807	2.231173	2.225579	2.220022	2.321468
2.260079	2.254198	2.248435	2.242738	2.237063	2.231424	2.225811	2.220219	2.291855
2.260254	2.254422	2.248667	2.242969	2.2373	2.231672	2.226087	2.220539	2.292256
2.260457	2.254672	2.248912	2.243198	2.237531	2.231912	2.226361	2.220877	2.292684
2.260711	2.254901	2.249136	2.243424	2.23775	2.232125	2.226571	2.221087	2.281772
2.261	2.255124	2.249353	2.243647	2.237961	2.232317	2.226739	2.221223	2.281934
2.261248	2.255366	2.249592	2.243876	2.238162	2.23248	2.226893	2.221394	2.282154
2.261456	2.255629	2.249854	2.244113	2.238348	2.232605	2.227023	2.221594	2.282434
2.231278	2.255878	2.250104	2.244349	2.238565	2.232798	2.227218	2.221814	2.282693
2.2315	2.2561	2.250326	2.244582	2.238841	2.23313	2.22754	2.222064	2.282902
2.231722	2.256319	2.250545	2.244812	2.239109	2.233445	2.227848	2.222309	2.283112
2.23192	2.256555	2.250778	2.24503	2.239294	2.23359	2.227996	2.222504	2.283363
2.232121	2.256794	2.251013	2.245249	2.239463	2.233698	2.22811	2.222688	2.283624
2.208389	2.257005	2.251232	2.245492	2.239727	2.23398	2.228378	2.222913	2.283835
2.208661	2.2572	2.251441	2.245754	2.240056	2.234381	2.228755	2.223169	2.284013
2.208964	2.257491	2.251697	2.245991	2.240326	2.234709	2.229094	2.223475	2.284293
2.209316	2.257903	2.252007	2.246186	2.240514	2.234949	2.229392	2.223852	2.284699
2.209624	2.227871	2.222019	2.216221	2.240744	2.235178	2.229655	2.224186	2.285087
2.209829	2.228154	2.222362	2.216601	2.241078	2.235399	2.229843	2.224411	2.2854
2.210017	2.228398	2.222686	2.216992	2.241437	2.235649	2.230045	2.224618	2.285679
2.210253	2.228596	2.22291	2.217264	2.283172	2.277	2.297617	2.291717	2.285911
2.210515	2.228781	2.223093	2.217484	2.283539	2.27745	2.298031	2.292004	2.28613
2.210825	2.229088	2.22336	2.217719	2.283852	2.277832	2.298419	2.292354	2.286456
2.211181	2.2295	2.223696	2.21796	2.284116	2.278163	2.298795	2.292762	2.286873
2.211494	2.229877	2.224039	2.218256	2.284443	2.278518	2.299178	2.293176	2.287288
2.211734	2.230189	2.224383	2.218621	2.284864	2.278922	2.299584	2.293604	2.287696

2.211994	2.230504	2.224739	2.21901	2.285292	2.279302	2.299954	2.294001	2.288088
2.212339	2.230864	2.225126	2.219415	2.285679	2.279596	2.300221	2.29432	2.288447
2.212703	2.23122	2.225505	2.219813	2.286059	2.279884	2.300479	2.294622	2.288795
2.213015	2.231501	2.225801	2.22014	2.286446	2.280267	2.300852	2.294989	2.289166
2.213308	2.231746	2.226053	2.220428	2.286839	2.280708	2.301293	2.295388	2.28954
2.213667	2.20784	2.202208	2.19666	2.258918	2.252894	2.301705	2.295733	2.28985
2.214094	2.208253	2.20261	2.197052	2.259356	2.25333	2.302093	2.296022	2.290088
2.214507	2.208672	2.203022	2.197451	2.259802	2.253772	2.302505	2.296365	2.290393
2.214882	2.209083	2.20343	2.197846	2.260254	2.254224	2.302958	2.296801	2.290814
2.215258	2.209494	2.203841	2.198245	2.260709	2.254678	2.30342	2.297261	2.291266
2.180265	2.209905	2.204251	2.19865	2.261163	2.255129	2.303874	2.29771	2.291709
2.180669	2.210315	2.204659	2.199055	2.261617	2.255579	2.304328	2.298161	2.292155
2.154013	2.210723	2.205061	2.199452	2.262066	2.256027	2.304781	2.298607	2.292593
2.154427	2.21113	2.205458	2.199843	2.262513	2.256473	2.305232	2.299048	2.293026
2.154837	2.211535	2.205857	2.200235	2.262956	2.256915	2.30568	2.29949	2.293463
2.155243	2.21194	2.206257	2.200631	2.263395	2.257351	2.306123	2.299933	2.293902
2.155643	2.176999	2.171406	2.165878	2.263832	2.257785	2.306564	2.300373	2.294339
2.156035	2.177386	2.171789	2.166255	2.264263	2.258214	2.307002	2.300802	2.294764
2.156421	2.17777	2.17217	2.166632	2.264695	2.258648	2.307446	2.301242	2.2952
2.156796	2.178148	2.172549	2.167015	2.265137	2.259099	2.307909	2.301708	2.295658
2.157175	2.178527	2.17293	2.167399	2.265582	2.259557	2.308387	2.302199	2.296146
2.157612	2.178933	2.173316	2.167774	2.210796	2.204965	2.28198	2.275937	2.296721
2.158099	2.179361	2.173706	2.168141	2.211181	2.205364	2.282474	2.276523	2.297376
2.158596	2.179846	2.174167	2.168577	2.211618	2.205788	2.282954	2.277048	2.297945
2.159111	2.180417	2.174735	2.169114	2.212127	2.206235	2.283402	2.277478	2.298385
2.159596	2.180951	2.17527	2.169631	2.212646	2.206723	2.254456	2.248649	2.298839
2.159993	2.18135	2.175674	2.170051	2.213156	2.207293	2.255056	2.249215	2.299391
2.160388	2.181728	2.176052	2.170447	2.213654	2.207866	2.25567	2.249798	2.299963
2.160904	2.190775	2.184992	2.179283	2.245314	2.239427	2.256144	2.250284	2.273667
2.161478	2.191314	2.185485	2.179725	2.245737	2.239815	2.256549	2.250721	2.274159
2.161952	2.191794	2.185981	2.180231	2.246268	2.240306	2.257028	2.251185	2.274643
2.162327	2.192219	2.186485	2.180806	2.246908	2.240894	2.257574	2.251668	2.275112
2.162745	2.192652	2.186965	2.181335	2.2475	2.241459	2.25813	2.252198	2.246365
2.163236	2.193088	2.187391	2.181768	2.247984	2.24196	2.258683	2.252796	2.24698
2.163763	2.193572	2.187854	2.182223	2.248484	2.242482	2.259256	2.253406	2.247602
2.164319	2.194166	2.188435	2.182781	2.249085	2.243093	2.259872	2.254	2.248164
2.164903	2.194809	2.189066	2.183384	2.249729	2.243735	2.2605	2.254582	2.248705
2.165505	2.195418	2.189673	2.183981	2.203532	2.19792	2.192349	2.186818	2.249233
2.166125	2.196015	2.190272	2.184583	2.20412	2.198474	2.192863	2.187284	2.249752
2.166735	2.196615	2.190864	2.185163	2.204673	2.198994	2.193369	2.187792	2.250335
2.167337	2.197222	2.191449	2.185723	2.205188	2.199469	2.193858	2.188347	2.250992
2.092013	2.197813	2.192012	2.186258	2.205701	2.199975	2.194379	2.188911	2.251639
2.09259	2.198383	2.192536	2.186744	2.206212	2.200544	2.194964	2.189472	2.252237
2.093146	2.198931	2.193053	2.187242	2.206742	2.201131	2.195563	2.190042	2.25284
2.093615	2.199416	2.193576	2.187819	2.207329	2.201704	2.196137	2.19063	2.2535
2.09407	2.199899	2.194119	2.188433	2.207943	2.202279	2.196707	2.191225	2.254178
2.094692	2.20056	2.194742	2.189018	2.208522	2.202867	2.197293	2.191798	2.2548
2.095448	2.201365	2.195437	2.189585	2.209076	2.203468	2.197892	2.192354	2.25538
2.096126	2.130503	2.124708	2.118985	2.209688	2.204055	2.198461	2.192912	2.255988
2.096697	2.131147	2.125407	2.119713	2.210377	2.204609	2.198974	2.193469	2.256642

2.097244	2.131744	2.126069	2.120423	2.211073	2.205213	2.199539	2.19404	2.257279
2.097789	2.132267	2.126638	2.121052	2.211761	2.205943	2.200233	2.194621	2.257839
2.098331	2.132773	2.127172	2.121636	2.212427	2.20669	2.200962	2.195233	2.258418
2.057621	2.13335	2.12772	2.122163	2.213023	2.207337	2.201638	2.195925	2.236291
2.058181	2.133957	2.128269	2.122652	2.213575	2.207927	2.202287	2.196658	2.237101
2.05873	2.134513	2.128817	2.123197	2.214145	2.208499	2.202884	2.197308	2.237842
2.059262	2.135016	2.12936	2.12379	2.214727	2.209044	2.203428	2.197882	2.238527
2.059877	2.135612	2.129948	2.124373	2.147447	2.141895	2.204	2.198458	2.239175
2.06063	2.136359	2.130595	2.124919	2.147983	2.142475	2.204615	2.199036	2.239762
2.06137	2.137115	2.131266	2.125502	2.14856	2.143096	2.205263	2.199649	2.240383
2.061985	2.137801	2.131957	2.12619	2.149252	2.143792	2.205967	2.200351	2.241137
2.062559	2.138449	2.132646	2.126911	2.149976	2.144499	2.206681	2.201076	2.24193
2.063159	2.139035	2.133257	2.127556	2.150604	2.145087	2.207304	2.20172	2.242643
2.06377	2.139577	2.133823	2.128162	2.15118	2.145605	2.207872	2.202316	2.243312
2.064432	2.140232	2.134486	2.128834	2.151828	2.146213	2.14072	2.135348	2.244014
2.06515	2.14101	2.135256	2.129579	2.152553	2.146915	2.141407	2.136026	2.244758
2.065871	2.14179	2.136027	2.130326	2.153287	2.147635	2.142099	2.136677	2.245457
2.066592	2.142548	2.136782	2.131073	2.154032	2.148369	2.142777	2.137252	2.246048
2.067287	2.143278	2.137504	2.131783	2.154745	2.149084	2.143449	2.137834	2.246661
2.067898	2.062311	2.056769	2.051284	2.086898	2.081465	2.144102	2.138495	2.133017
2.068484	2.062915	2.057345	2.051829	2.087455	2.082049	2.144753	2.139197	2.133744
2.06916	2.063592	2.058009	2.05248	2.088113	2.082708	2.145445	2.139908	2.134462
2.069905	2.064323	2.05874	2.05321	2.088844	2.083422	2.146169	2.140632	2.135185
2.070652	2.065064	2.05948	2.053947	2.089584	2.084143	2.146899	2.141363	2.135912
2.071397	2.065815	2.060228	2.05469	2.090327	2.08487	2.147635	2.142099	2.136644
2.072142	2.066563	2.060978	2.055437	2.091079	2.085605	2.148378	2.142837	2.137375
2.072882	2.067299	2.06172	2.056186	2.091839	2.086352	2.149127	2.143572	2.138098
2.073625	2.068035	2.062462	2.056935	2.092601	2.087104	2.149881	2.14431	2.138822
2.074379	2.068785	2.063207	2.057676	2.093354	2.087852	2.150636	2.145056	2.139556
2.07514	2.069541	2.063954	2.058413	2.094103	2.088597	2.151391	2.145805	2.140296
2.075899	2.070293	2.064701	2.059155	2.094855	2.089339	2.152142	2.14655	2.141034
2.076658	2.071041	2.065448	2.059901	2.095609	2.090078	2.152889	2.147291	2.14177
1.997337	2.071794	2.066195	2.060643	2.055154	2.049724	2.153632	2.148029	2.142504
1.998034	2.072555	2.066944	2.061378	2.055876	2.050435	2.154366	2.14876	2.143231
1.998732	2.073317	2.067694	2.062115	2.056603	2.051152	2.155109	2.149498	2.143965
1.999423	2.074073	2.068443	2.062856	2.057343	2.051893	2.15587	2.150242	2.144699
2.000115	2.074832	2.069198	2.063605	2.05809	2.052641	2.156643	2.151007	2.145461
1.954919	2.075606	2.069964	2.064357	2.058827	2.053365	2.128857	2.12332	2.146328
1.955591	2.076394	2.070743	2.065122	2.059567	2.05408	2.129646	2.124197	2.147262
1.956339	2.077232	2.071591	2.065975	2.060394	2.054866	2.130455	2.12502	2.148097
1.957176	2.07813	2.07252	2.066932	2.061322	2.055731	2.131289	2.125786	2.148825
1.95797	1.952583	1.94729	1.942019	2.062211	2.056576	2.093477	2.088013	2.149558
1.958664	1.953285	1.947982	1.942698	2.063001	2.057351	2.094252	2.088772	2.150313
1.959355	1.954006	1.948682	1.94337	2.063792	2.058147	2.095059	2.089561	2.151097
1.960125	1.95485	1.949499	1.944141	2.064667	2.059055	2.095979	2.090431	2.151953
1.960917	1.955726	1.950346	1.94494	2.065565	2.059996	2.096937	2.091329	2.152836
1.961649	1.956467	1.951082	1.94567	2.066388	2.060837	2.097789	2.092148	2.153648
1.962341	1.957111	1.95174	1.946354	2.067161	2.061608	2.098568	2.092913	2.154411
1.924842	1.957805	1.952464	1.947123	1.94179	1.936474	2.099411	2.093763	2.155267
1.925639	1.95857	1.953285	1.948013	1.942667	1.9373	2.100332	2.094721	2.156245

1.926401	1.959317	1.954066	1.948844	1.943514	1.938148	2.101279	2.09568	2.157211
1.927048	1.959985	1.954718	1.949503	1.944261	1.939019	2.102267	2.096607	2.158118
1.927699	1.960669	1.955368	1.950138	1.944979	1.939865	2.103231	2.097502	2.158989
1.813261	1.923201	1.917973	1.912812	1.945706	1.940589	1.981074	1.975649	2.123109
1.81398	1.924051	1.918798	1.913595	1.94644	1.941254	1.981736	1.976335	2.123866
1.814668	1.924848	1.919602	1.914406	1.947242	1.942001	1.982494	1.977129	2.124762
1.815336	1.925604	1.920398	1.915251	1.948105	1.942815	1.98333	1.978018	2.125781
1.816022	1.926389	1.921193	1.916068	1.910885	1.905697	1.938396	1.93318	2.055753
1.816734	1.927217	1.921986	1.916832	1.911672	1.90653	1.93927	1.934016	2.056608
1.817458	1.92805	1.922787	1.917605	1.91246	1.907353	1.94013	1.934852	2.057475
1.81819	1.928864	1.923601	1.91843	1.913267	1.908133	1.940932	1.935703	2.058401
1.818926	1.929682	1.924423	1.919264	1.914076	1.908905	1.941723	1.936551	2.059331
1.819644	1.930538	1.92525	1.920055	1.914867	1.909713	1.942558	1.937368	2.060184
1.820355	1.931417	1.92608	1.920823	1.915649	1.91054	1.943416	1.938165	2.060993
1.821084	1.816309	1.811561	1.806876	1.916438	1.911333	1.906205	1.901061	1.984595
1.821831	1.816948	1.812218	1.807575	1.917234	1.912091	1.906965	1.901862	1.985465
1.822572	1.817616	1.812885	1.808257	1.918026	1.912863	1.90775	1.902695	1.986374
1.8233	1.818354	1.813574	1.808894	1.918796	1.91365	1.908576	1.903584	1.987357
1.824019	1.819089	1.814267	1.809544	1.91959	1.914476	1.909431	1.90447	1.988317
1.824723	1.819753	1.81494	1.810245	1.92045	1.91539	1.910335	1.9053	1.938171
1.825423	1.820401	1.81562	1.810978	1.921343	1.916331	1.911246	1.906103	1.938936
1.826137	1.821108	1.816355	1.811739	1.922227	1.917175	1.912061	1.9069	1.93975
1.826863	1.821858	1.817133	1.812525	1.92311	1.917951	1.912807	1.907686	1.940591
1.827661	1.822672	1.817919	1.813268	1.808586	1.803924	1.913585	1.908463	1.903348
1.828553	1.823574	1.81872	1.813955	1.809261	1.804633	1.914391	1.909215	1.904077
1.829416	1.824427	1.819496	1.814653	1.809961	1.805373	1.915251	1.910033	1.904876
1.830187	1.825145	1.820209	1.815383	1.810721	1.806172	1.916218	1.910998	1.905832
1.830933	1.825843	1.820924	1.816135	1.811493	1.80696	1.917177	1.911972	1.906808
1.831685	1.826612	1.821711	1.816927	1.812243	1.807645	1.917987	1.9128	1.907668
1.832447	1.827419	1.822531	1.817733	1.812977	1.80828	1.918727	1.913569	1.908481
1.833298	1.828269	1.823322	1.818455	1.813665	1.808953	1.804322	1.799774	1.909341
1.83423	1.829161	1.82409	1.819104	1.814308	1.809651	1.805054	1.800529	1.910236
1.835137	1.830055	1.824901	1.819828	1.815022	1.810394	1.805815	1.801305	1.911151
1.836003	1.830951	1.825773	1.820661	1.815835	1.811195	1.806615	1.802115	1.912107
1.836863	1.831837	1.826654	1.821523	1.816678	1.812016	1.807415	1.802896	1.913012
1.837739	1.832719	1.827532	1.822391	1.81753	1.812842	1.808186	1.803578	1.913769
1.838604	1.833569	1.828389	1.823258	1.818387	1.813676	1.808958	1.804247	1.914503
1.839408	1.834314	1.829164	1.824081	1.819221	1.814503	1.809762	1.805008	1.915365
1.84018	1.834999	1.829892	1.824882	1.820045	1.815328	1.810585	1.805823	1.916302
1.841026	1.835797	1.830707	1.825727	1.820892	1.816166	1.811414	1.806643	1.801965
1.841944	1.836707	1.831608	1.826618	1.821765	1.817017	1.812251	1.807472	1.802785
1.764032	1.837616	1.832508	1.827506	1.822633	1.817861	1.813081	1.808298	1.803604
1.764811	1.838496	1.833385	1.82838	1.823488	1.818689	1.813898	1.809116	1.804417
1.765596	1.839381	1.834267	1.829259	1.824346	1.819518	1.814712	1.80993	1.805228
1.7664	1.840284	1.835168	1.830156	1.825216	1.820353	1.815526	1.810736	1.806029
1.767214	1.841197	1.836079	1.831062	1.826097	1.821198	1.816348	1.811545	1.80683
1.76803	1.842112	1.83699	1.831968	1.826994	1.82208	1.817211	1.812382	1.80765
1.768851	1.843032	1.837903	1.832878	1.827904	1.822992	1.818105	1.813241	1.808483
1.703296	1.764847	1.760155	1.755542	1.828813	1.823898	1.818992	1.814091	1.809307
1.704088	1.765654	1.760962	1.756347	1.829722	1.824797	1.819868	1.814932	1.810122

1.704881	1.766458	1.761764	1.757144	1.830626	1.825695	1.820745	1.815773	1.810937
1.705678	1.767253	1.762552	1.757925	1.831519	1.826587	1.821614	1.8166	1.811731
1.706481	1.768058	1.763345	1.758707	1.832414	1.827481	1.822497	1.817459	1.812566
1.70729	1.768884	1.764156	1.759502	1.833318	1.828386	1.82342	1.818423	1.813537
1.708114	1.769737	1.76499	1.760314	1.834232	1.829297	1.824363	1.819433	1.814569
1.635086	1.770675	1.765901	1.761181	1.835176	1.830215	1.825276	1.820366	1.815502
1.63598	1.77169	1.766883	1.762098	1.836149	1.83114	1.826168	1.82124	1.81636
1.636804	1.706349	1.701751	1.697153	1.758287	1.753685	1.827054	1.822112	1.817229
1.637524	1.707135	1.702531	1.697924	1.759067	1.754442	1.827921	1.822979	1.818119
1.638244	1.707908	1.703324	1.69874	1.759902	1.755252	1.828828	1.823868	1.819013
1.639016	1.70872	1.704212	1.699709	1.760884	1.756182	1.829805	1.824788	1.819894
1.639801	1.709538	1.705111	1.700701	1.761896	1.75716	1.830837	1.825748	1.820793
1.64056	1.710341	1.705936	1.701565	1.762815	1.758132	1.83195	1.826785	1.82175
1.641317	1.711145	1.706725	1.702361	1.76368	1.759099	1.833111	1.827876	1.822755
1.642156	1.712013	1.707552	1.703159	1.698794	1.694455	1.755345	1.750669	1.823819
1.643079	1.712942	1.708405	1.703944	1.699548	1.695208	1.756135	1.751579	1.824954
1.643971	1.63954	1.635189	1.63089	1.700388	1.696005	1.756928	1.752429	1.825995
1.644806	1.640458	1.63616	1.631878	1.701382	1.696883	1.757728	1.753172	1.826863
1.645616	1.641343	1.637098	1.632843	1.702381	1.697797	1.758571	1.753939	1.827729
1.646395	1.642109	1.637872	1.633639	1.703294	1.698761	1.69426	1.689781	1.828697
1.647175	1.64284	1.638588	1.634369	1.704166	1.699729	1.695251	1.690729	1.829718
1.462787	1.64365	1.639355	1.63511	1.704985	1.700583	1.696139	1.691654	1.830806
1.463573	1.64451	1.640154	1.635853	1.705766	1.701362	1.696962	1.69257	1.831944
1.464339	1.645411	1.641049	1.636731	1.70668	1.702226	1.697811	1.693435	1.689096
1.465082	1.646365	1.642069	1.637781	1.707756	1.703187	1.698681	1.694228	1.689842
1.465833	1.462236	1.458658	1.455047	1.634382	1.629976	1.699594	1.69509	1.690662
1.466605	1.462938	1.459319	1.455696	1.635158	1.630833	1.700585	1.696109	1.691668
1.467376	1.463641	1.459983	1.456348	1.635937	1.631692	1.70158	1.697135	1.692686
1.468134	1.464435	1.460809	1.457193	1.636873	1.632575	1.702496	1.698008	1.693547
1.468878	1.465255	1.461688	1.458103	1.637866	1.633468	1.703391	1.69884	1.694367
1.469581	1.46597	1.46242	1.458856	1.638748	1.634361	1.704376	1.699832	1.695352
1.470247	1.466603	1.46304	1.45949	1.639545	1.635256	1.705432	1.700951	1.696469
1.471001	1.467336	1.463764	1.460217	1.456602	1.452954	1.706422	1.701958	1.697472
1.471875	1.468217	1.464649	1.46109	1.457425	1.453706	1.70731	1.702795	1.698302
1.353576	1.46908	1.465498	1.461918	1.458218	1.454457	1.708217	1.703667	1.699172
1.354329	1.469838	1.466192	1.462574	1.458899	1.455202	1.709198	1.704684	1.700214
1.35506	1.470575	1.466864	1.463211	1.459577	1.455968	1.710215	1.705738	1.701285
1.355727	1.471353	1.467646	1.463999	1.460394	1.456821	1.453189	1.449504	1.70224
1.356365	1.472156	1.468484	1.464861	1.461276	1.457712	1.454049	1.450292	1.703145
1.237204	1.473008	1.469311	1.465653	1.462049	1.458473	1.454811	1.451067	1.704115
1.237913	1.473917	1.470135	1.466384	1.462718	1.459106	1.455472	1.451824	1.705141
1.238556	1.474751	1.470933	1.467145	1.463451	1.45982	1.456208	1.452621	1.540303
1.239073	1.475441	1.471673	1.467953	1.46429	1.460674	1.457074	1.453495	1.541288
1.2396	1.476168	1.472452	1.468795	1.465163	1.46156	1.457955	1.454352	1.542232
1.240226	1.2373	1.234433	1.231599	1.466049	1.462442	1.458798	1.45512	1.543051
1.240893	1.238001	1.235135	1.232287	1.466939	1.463311	1.459616	1.455856	1.543835
1.241566	1.23869	1.235828	1.232969	1.467791	1.464099	1.460374	1.456613	1.544702
1.242247	1.239377	1.236517	1.233646	1.468613	1.464832	1.46109	1.457381	1.545622
1.242875	1.239983	1.237105	1.234219	1.469374	1.465585	1.461859	1.458191	1.454563
1.243446	1.240497	1.237575	1.234669	1.470053	1.466354	1.462684	1.459046	1.455435

1.244002	1.241021	1.238082	1.235174	1.366927	1.363638	1.463536	1.459916	1.456312
1.244542	1.241584	1.238678	1.235805	1.367713	1.364415	1.464417	1.460797	1.45719
1.245096	1.242174	1.23931	1.236474	1.368538	1.365218	1.465312	1.461683	1.458067
1.245704	1.242809	1.23996	1.237133	1.369347	1.366013	1.466204	1.462569	1.458942
1.246343	1.243472	1.240628	1.237797	1.370158	1.366812	1.467098	1.463453	1.459812
1.126289	1.244128	1.241286	1.238456	1.235623	1.232791	1.467987	1.464336	1.460669
1.127018	1.244783	1.241941	1.239112	1.236284	1.233458	1.468873	1.465217	1.461515
1.127745	1.245437	1.242593	1.239764	1.23694	1.234118	1.469745	1.466046	1.462305
1.128471	1.246088	1.243242	1.240414	1.237593	1.234772	1.470594	1.466799	1.46301
1.129198	1.126496	1.123825	1.121166	1.238239	1.235419	1.367354	1.363858	1.46376
1.129926	1.127219	1.124544	1.121884	1.238871	1.236055	1.36814	1.364631	1.464629
1.130655	1.127942	1.125262	1.122598	1.239499	1.236687	1.368934	1.36543	1.46554
0.992374	1.128668	1.125982	1.123314	1.240133	1.237331	1.369726	1.366229	1.466445
0.99297	1.129395	1.126702	1.124028	1.240765	1.237972	1.37051	1.367025	1.46735
0.993565	1.130123	1.127425	1.124742	1.24138	1.23856	1.371229	1.36776	1.468214
0.99416	1.130851	1.12815	1.125455	1.241976	1.239094	1.371879	1.368433	1.469034
0.811968	1.131581	1.128875	1.126172	1.123439	1.120692	1.236801	1.233931	1.365786
0.81251	1.132315	1.129598	1.126892	1.124187	1.121483	1.237511	1.234635	1.36663
0.813051	1.13305	1.130324	1.127615	1.124939	1.122279	1.238226	1.235355	1.367508
0.813593	0.811562	0.809513	0.807474	1.125668	1.123016	1.238885	1.236064	1.368424
0.814134	0.812106	0.810059	0.808017	1.126391	1.123733	1.239518	1.236753	1.369337
0.814671	0.812647	0.810603	0.808561	1.127118	1.124451	1.121803	1.119178	1.370152
0.815206	0.813185	0.811146	0.809106	1.127846	1.125165	1.122507	1.119877	1.370901
0.583475	0.813722	0.811687	0.809649	0.80762	0.805601	1.123214	1.120578	1.371651
0.583891	0.814258	0.812226	0.810188	0.808154	0.806125	1.12392	1.121281	1.372398
0.584306	0.814794	0.812766	0.810729	0.808689	0.806651	0.976602	0.974234	1.119359
0.584721	0.81533	0.813307	0.811274	0.809229	0.807182	0.977269	0.974893	1.120064
0.585136	0.815864	0.813847	0.811817	0.80977	0.807717	0.977943	0.975557	1.12077
0.585549	0.584155	0.582733	0.581292	0.810311	0.808258	0.806238	0.804248	0.97386
0.585961	0.584574	0.583158	0.581723	0.810851	0.808803	0.806781	0.804781	0.974529
0.586368	0.584984	0.583574	0.582146	0.811385	0.809342	0.807317	0.805308	0.97519
0.586772	0.585389	0.583985	0.582564	0.811915	0.809877	0.807851	0.805832	0.975848
0.587225	0.58584	0.584442	0.58303	0.812511	0.81048	0.808451	0.80642	0.804418
0.587747	0.586357	0.584965	0.583563	0.813201	0.811176	0.809142	0.807096	0.80508
0.588244	0.586847	0.585461	0.58407	0.582646	0.581201	0.809802	0.807746	0.805719
0.588664	0.587263	0.585883	0.584501	0.583084	0.581645	0.81037	0.808312	0.80628
0.589062	0.587657	0.58628	0.584906	0.583496	0.582063	0.810907	0.808851	0.806816
0.589467	0.588054	0.58668	0.585311	0.583907	0.582482	0.811439	0.809387	0.807351
0.58988	0.588458	0.587083	0.585718	0.58432	0.582902	0.811972	0.809926	0.80789
0.590354	0.588933	0.587552	0.586181	0.584788	0.583382	0.581947	0.580484	0.808509
0.590886	0.589474	0.588082	0.586698	0.58531	0.583918	0.582494	0.58104	0.809204
0.591386	0.589968	0.588564	0.587171	0.585788	0.584408	0.582994	0.581549	0.809844
0.591835	0.590386	0.58897	0.587574	0.586195	0.584826	0.583422	0.581985	0.810399
0.592271	0.590793	0.589365	0.587966	0.58659	0.58523	0.583833	0.582405	0.580952
0.592697	0.591204	0.589765	0.588359	0.586985	0.585632	0.584242	0.582822	0.581376
0.593136	0.591635	0.590185	0.58877	0.587394	0.586044	0.58466	0.58325	0.581812
0.593654	0.592146	0.590685	0.589258	0.587874	0.58652	0.58514	0.583741	0.582313
0.59422	0.592707	0.591234	0.589794	0.588398	0.587033	0.585655	0.584269	0.582854
0.594721	0.593205	0.591722	0.590268	0.588862	0.587488	0.586111	0.584737	0.583334
0.595152	0.593636	0.592143	0.590677	0.58926	0.587879	0.586504	0.585141	0.583749

0.595613	0.594104	0.592602	0.591122	0.589694	0.588302	0.586927	0.585574	0.584191
0.596145	0.594658	0.593147	0.591647	0.590205	0.588802	0.587424	0.586077	0.584704
0.596676	0.595206	0.593688	0.592172	0.590716	0.5893	0.587916	0.586572	0.585205
0.597147	0.595667	0.594144	0.592623	0.591158	0.589728	0.588336	0.586986	0.585619
0.597601	0.596104	0.59458	0.59306	0.591586	0.590143	0.58874	0.587379	0.586009
0.5981	0.596615	0.595096	0.593576	0.592092	0.590634	0.589218	0.587846	0.586473
0.59863	0.597175	0.595667	0.594147	0.592653	0.591178	0.589748	0.588363	0.586987
0.599121	0.597678	0.596178	0.594661	0.593158	0.591666	0.590229	0.588842	0.587472
0.599558	0.598104	0.596608	0.595098	0.593585	0.592079	0.590641	0.589269	0.587921
0.600018	0.598558	0.597068	0.595565	0.594046	0.592528	0.591084	0.589711	0.588368
0.600552	0.5991	0.59762	0.596123	0.594601	0.593071	0.591604	0.590194	0.588817
0.601082	0.599643	0.598177	0.596691	0.595174	0.593641	0.592151	0.590698	0.589281
0.601521	0.600094	0.598653	0.597197	0.595706	0.594196	0.592706	0.591231	0.589791
0.601925	0.600507	0.599095	0.597675	0.59622	0.594743	0.593263	0.591776	0.590323
	0.600985	0.599575	0.59816	0.596708	0.595234	0.593756	0.592271	0.590819
	0.601514	0.600084	0.598645	0.597169	0.595672	0.594191	0.592721	0.591286
	0.602045	0.600604	0.599154	0.597667	0.59616	0.594672	0.593199	0.591761
	0.602574	0.601138	0.599696	0.598219	0.596721	0.595221	0.593715	0.592241
	0.603088	0.601664	0.600238	0.598776	0.59729	0.595783	0.594251	0.592746
	0.603565	0.602168	0.600769	0.599321	0.597843	0.59634	0.594807	0.593295
	0.604029	0.602654	0.601279	0.599847	0.598381	0.596891	0.595372	0.593864
				0.600309	0.598875	0.597414	0.595925	0.594429
				0.600732	0.59934	0.597917	0.596469	0.594994
				0.601199	0.599817	0.598411	0.596987	0.595529
				0.60171	0.600303	0.59889	0.597475	0.596034
						0.599383	0.597964	0.59653
						0.5999	0.598459	0.597014
						0.600425	0.598964	0.597508
						0.600949	0.599492	0.598042
						0.601474	0.600031	0.598593
								0.599135
								0.599673
								0.600205
								0.600728
								0.60125
								0.601772
								0.602297

						1.219912
						1.218555
						1.217224
						1.337778
						1.336415
						1.335038
						1.333656
				1.339628	1.335933	1.466919
				1.338187	1.334468	1.465222
				1.336737	1.333006	1.673199
				1.335253	1.331544	1.671588
				1.333792	1.330112	1.670012
		1.218103	1.21459	1.332426	1.328768	1.668517
		1.21683	1.213369	1.331108	1.327466	1.667059
		1.215592	1.212147	1.468078	1.464188	1.66552
		1.214386	1.210928	1.466754	1.462817	1.663915
1.220034	1.216581	1.334553	1.330786	1.580322	1.575905	1.662377
1.218722	1.215229	1.333086	1.329388	1.5788	1.574441	1.660934
1.21743	1.213897	1.470475	1.466539	1.668202	1.663823	1.659505
1.216205	1.212647	1.469077	1.46512	1.666579	1.662284	1.658044

		1.215	1.211433	1.467708	1.463728	1.664979	1.660763	1.656598
		1.213763	1.210226	1.466408	1.462439	1.663529	1.659361	1.655235
		1.212515	1.209035	1.465157	1.461217	1.662169	1.658027	1.653913
1.218176	1.214743	1.332455	1.328696	1.463904	1.459985	1.660776	1.656618	1.65249
1.216965	1.213541	1.331149	1.327403	1.462655	1.458748	1.659354	1.655138	1.650967
1.215727	1.212298	1.329802	1.326083	1.666728	1.662303	1.657957	1.653697	1.649491
1.214424	1.21097	1.328371	1.324711	1.665256	1.660911	1.656604	1.652332	1.648106
1.213137	1.209663	1.32696	1.323358	1.663806	1.659529	1.655256	1.650977	1.646737
1.333043	1.329287	1.325649	1.322081	1.662422	1.658163	1.653894	1.649604	1.882302
1.331734	1.327996	1.324381	1.320838	1.661068	1.656803	1.652525	1.648222	1.88065
1.330419	1.326694	1.462646	1.458953	1.659707	1.655435	1.651144	1.646821	1.878978
1.329106	1.325397	1.461427	1.457761	1.658348	1.654062	1.649752	1.645405	1.877292
1.32781	1.324117	1.460215	1.456565	1.656979	1.65268	1.648356	1.643994	1.944432
1.326531	1.322857	1.459012	1.455365	1.655599	1.651289	1.646957	1.642587	1.942686
1.325262	1.321607	1.662631	1.658469	1.654211	1.649895	1.645559	1.641189	1.940959
1.324	1.320366	1.661247	1.657072	1.65281	1.648495	1.644163	1.639801	1.93925
1.322745	1.319132	1.659868	1.655674	1.651409	1.647099	1.642776	1.638427	1.937562
1.321484	1.31789	1.658487	1.65428	1.650016	1.645718	1.877495	1.872496	1.935905
1.320236	1.316661	1.657122	1.652902	1.648637	1.644348	1.875897	1.870928	1.934267
1.319057	1.315514	1.65584	1.651593	1.647306	1.642993	1.874291	1.869351	1.932623
1.317938	1.314436	1.65463	1.650346	1.646017	1.641652	1.87268	1.867768	1.930977
1.5707	1.566624	1.653358	1.649057	1.644707	1.640314	1.871086	1.866189	1.929325
1.569352	1.56528	1.651998	1.647709	1.643367	1.638982	1.869513	1.864609	1.92766
1.568032	1.563948	1.65064	1.646352	1.877962	1.872921	1.936255	1.931108	1.926021
1.566806	1.562689	1.64933	1.645003	1.876356	1.87133	1.934598	1.929482	1.92443
1.565596	1.561438	1.648023	1.643653	1.874748	1.869747	1.932957	1.927884	1.922874
1.655342	1.651038	1.646678	1.642297	1.873153	1.868192	1.931352	1.92633	1.921358
1.654022	1.649681	1.645313	1.640941	1.871571	1.866662	1.929775	1.924809	1.919873
1.652684	1.648329	1.725268	1.720677	1.938386	1.933268	1.928233	1.923297	1.918374
1.651324	1.646976	1.723833	1.719262	1.936804	1.931735	1.926728	1.9218	1.916868
1.649977	1.64564	1.722432	1.717896	1.935246	1.930208	1.925213	1.920274	1.915323
1.648631	1.644308	1.721067	1.716605	1.933724	1.928693	1.923685	1.918708	1.913724
1.647343	1.643031	1.71974	1.715323	1.932191	1.927152	1.922127	1.917116	1.912109
1.72753	1.722989	1.940896	1.935725	1.930607	1.925538	1.920496	1.915487	1.910491
1.726335	1.7218	1.93941	1.934163	1.928998	1.923897	1.918846	1.91385	1.908878
1.724994	1.720445	1.93779	1.932537	1.927376	1.922288	1.917246	1.912253	1.973165
1.723559	1.718985	1.936081	1.930865	1.925743	1.920698	1.915682	1.91069	1.971527
1.722113	1.717529	1.934402	1.929225	1.924149	1.91916	1.914186	1.909218	1.96998
1.720637	1.716057	1.932736	1.927605	1.922592	1.917682	1.912773	1.907861	1.968549
1.719252	1.714693	1.931169	1.92607	1.921089	1.916212	1.911329	1.906443	1.96704
1.718052	1.71354	1.92979	1.92469	1.919679	1.914745	1.909814	1.904888	1.965358
1.716857	1.712388	1.928409	1.923311	1.918264	1.91326	1.908272	1.903299	1.963645
1.715507	1.711039	1.926834	1.921773	1.916728	1.911702	1.972757	1.967342	1.961983
1.714093	1.709611	1.925185	1.920178	1.915154	1.91013	1.971106	1.965698	1.960355
1.933771	1.928631	1.923614	1.918662	1.913681	1.908692	1.969599	1.964183	2.025133
1.93216	1.927059	1.922092	1.917199	1.912281	1.90736	1.968209	1.962777	2.02363
1.930652	1.925573	1.920613	1.915724	1.910828	1.90594	1.96672	1.961282	2.022061
1.929288	1.924207	1.919196	1.914237	1.909304	1.904399	1.965095	1.959658	2.020387
1.92788	1.922801	1.917747	1.91273	1.907769	1.902858	1.963472	1.95806	2.018761
1.926298	1.921233	1.91618	1.911161	1.906224	1.901353	1.961909	1.956573	2.017291

1.924707	1.91966	1.914622	1.909621	1.90471	1.899874	1.960364	1.9551	2.015834
1.923299	1.91826	1.913236	1.908244	1.969413	1.964102	1.95881	1.953537	2.014221
1.921971	1.916936	1.911932	1.906955	1.968013	1.962623	1.957267	1.951944	2.012546
1.920504	1.915486	1.910518	1.905582	1.96656	1.961173	2.022027	2.016502	2.120134
1.918909	1.913919	1.909008	1.904142	1.965069	1.959759	2.020619	2.015111	2.118469
1.917415	1.912436	1.907542	1.902697	1.963546	1.958272	2.019113	2.013627	2.116693
1.916085	1.91109	1.906148	1.901242	1.961967	1.956652	2.01743	2.011969	2.114693
1.914785	1.909776	1.904788	1.899822	1.960421	1.955061	2.015775	2.01034	2.112741
1.913478	1.908473	1.969803	1.964392	1.959001	1.95363	2.014301	2.008892	2.11104
1.91214	1.907147	1.968379	1.962984	1.957601	1.952234	2.012869	2.007481	2.109391
1.91065	1.905665	1.966807	1.961424	2.0223	2.016762	2.120367	2.113871	2.144471
1.909065	1.904088	1.965146	1.959777	2.020621	2.015107	2.118311	2.111855	2.142454
1.907625	1.902658	1.963636	1.958277	2.019091	2.013602	2.116482	2.110068	2.140654
1.906337	1.901383	1.962288	1.956937	2.017718	2.012256	2.114895	2.108525	2.139084
1.905057	1.900115	1.960935	1.955594	2.016347	2.010912	2.150313	2.143865	2.137537
1.903772	1.898839	1.959554	1.954226	2.014964	2.009557	2.148688	2.142294	2.136002
1.902441	1.897513	1.958123	1.952808	2.013526	2.008142	2.146988	2.140651	2.134396
1.967274	1.961898	1.956551	1.951244	2.011922	2.006553	2.145044	2.138766	2.132553
1.965647	1.96028	1.954941	1.949642	2.01027	2.004911	2.143028	2.136806	2.130631
1.964194	1.958837	2.019695	2.014214	2.154126	2.147635	2.141281	2.135088	2.128932
1.96287	1.957525	2.018338	2.012873	2.152501	2.146058	2.139734	2.133546	2.127394
1.961552	1.956218	2.016993	2.011544	2.150886	2.144484	2.138185	2.132004	2.15738
1.960236	1.954911	2.015655	2.010227	2.149278	2.142907	2.136629	2.130457	2.155777
1.958924	1.95361	2.014321	2.008911	2.14767	2.14133	2.135069	2.128897	2.154157
1.957615	1.952315	2.012987	2.007589	2.146048	2.139747	2.133503	2.127316	2.152506
1.956301	1.951011	2.011643	2.006254	2.14442	2.138156	2.13192	2.125705	2.15081
2.021178	2.015695	2.155715	2.149196	2.142816	2.136568	2.130315	2.124042	2.149012
2.019788	2.014294	2.154028	2.147552	2.141195	2.134954	2.128675	2.122338	2.14716
2.018347	2.01283	2.152218	2.145741	2.139394	2.133171	2.158781	2.15204	2.185258
2.016856	2.011307	2.150294	2.143771	2.137419	2.131225	2.156824	2.150204	2.183505
2.015361	2.009795	2.148429	2.141921	2.135587	2.129414	2.154984	2.148456	2.181815
2.013856	2.008293	2.146646	2.140257	2.133974	2.127808	2.153317	2.146831	2.180201
2.012378	2.006839	2.144929	2.138647	2.132413	2.126252	2.151699	2.145243	2.178612
2.010954	2.005482	2.143301	2.137042	2.130828	2.124681	2.15008	2.143655	2.17702
2.009573	2.00417	2.141724	2.13546	2.129256	2.123123	2.148476	2.142078	2.175434
2.153011	2.14651	2.140143	2.133895	2.1277	2.121571	2.146871	2.140498	2.173842
2.151476	2.144932	2.138571	2.132345	2.126157	2.120027	2.145272	2.138918	2.172248
2.149895	2.143341	2.136992	2.130785	2.156642	2.150124	2.183481	2.177041	2.250149
2.148272	2.14174	2.135408	2.129216	2.155046	2.148551	2.181886	2.175446	2.248457
2.146646	2.140129	2.133814	2.127641	2.153443	2.146976	2.180296	2.173862	2.246775
2.144998	2.138477	2.132191	2.126058	2.15183	2.145398	2.178711	2.172294	2.24511
2.143407	2.136876	2.130596	2.124475	2.150207	2.143814	2.177123	2.170728	2.243447
2.141971	2.135417	2.129071	2.122874	2.148543	2.142209	2.175527	2.169153	2.24178
2.140605	2.134034	2.127603	2.121301	2.146889	2.140602	2.173925	2.167571	2.240109
2.139185	2.132661	2.158637	2.151885	2.145356	2.139025	2.251756	2.245053	2.238423
2.137734	2.131309	2.157289	2.15052	2.143922	2.137473	2.250015	2.243325	2.236725
2.136218	2.129872	2.155881	2.149159	2.142544	2.136027	2.248378	2.241665	2.23506
2.134626	2.128338	2.15441	2.147814	2.141244	2.13471	2.246855	2.240067	2.233409
2.133012	2.126772	2.152887	2.146409	2.222806	2.215894	2.245397	2.238573	2.231885
2.131389	2.125186	2.151292	2.144888	2.221278	2.214479	2.244044	2.237273	2.230607

2.12976	2.123589	2.149667	2.143319	2.219692	2.213021	2.242667	2.235973	2.229355
2.128117	2.121991	2.148044	2.141743	2.218056	2.211426	2.241095	2.234453	2.227902
2.126488	2.120405	2.146421	2.140153	2.216378	2.209747	2.239415	2.232817	2.226345
2.157712	2.151203	2.190767	2.184243	2.251042	2.244353	2.237735	2.231168	2.224755
2.156231	2.149661	2.189111	2.182574	2.249314	2.242637	2.236037	2.229491	2.22312
2.154851	2.148261	2.187599	2.181017	2.247666	2.240971	2.23437	2.22784	2.221506
2.153625	2.147074	2.186296	2.179611	2.246109	2.239343	2.232711	2.226193	2.219896
2.152337	2.145848	2.184989	2.178247	2.244635	2.237827	2.231175	2.224662	2.218385
2.150818	2.144421	2.183569	2.176909	2.24331	2.236547	2.229909	2.223372	2.217063
2.149231	2.142919	2.182086	2.175544	2.241992	2.235307	2.228709	2.222167	2.215836
2.193631	2.18705	2.180499	2.173996	2.240438	2.233821	2.227308	2.220879	2.214639
2.19207	2.185431	2.17885	2.172344	2.238748	2.232195	2.225795	2.219548	2.213467
2.190643	2.18399	2.177374	2.170827	2.237148	2.230596	2.224247	2.218113	2.240684
2.189368	2.182743	2.176077	2.169439	2.235621	2.229	2.222637	2.216556	2.239167
2.188002	2.181407	2.211539	2.204734	2.23418	2.227537	2.221172	2.215109	2.237718
2.186434	2.179875	2.210053	2.203362	2.232876	2.226299	2.219954	2.213857	2.236394
2.184881	2.178337	2.208547	2.201969	2.231565	2.225079	2.218776	2.212662	2.235136
2.183515	2.176918	2.243242	2.236604	2.230092	2.223698	2.217481	2.211446	2.270302
2.182209	2.175545	2.24175	2.235071	2.228592	2.222292	2.216173	2.210245	2.269128
2.1808	2.174171	2.240359	2.233715	2.227289	2.221054	2.21498	2.209073	2.267914
2.179329	2.172805	2.239042	2.232482	2.226118	2.219927	2.213862	2.207925	2.266678
2.177922	2.17142	2.237629	2.231101	2.224783	2.218649	2.241009	2.235043	2.291589
2.176589	2.170003	2.236087	2.229524	2.223232	2.217176	2.239598	2.233754	2.290356
2.175254	2.168631	2.234622	2.228039	2.221774	2.215782	2.238243	2.232482	2.289104
2.173875	2.167339	2.233332	2.226778	2.220553	2.214604	2.237051	2.231264	2.287799
2.172517	2.166088	2.2321	2.225585	2.219392	2.213466	2.235882	2.230059	2.286502
2.171271	2.164881	2.230866	2.224355	2.218156	2.212214	2.234605	2.228836	2.285281
2.170061	2.163679	2.229628	2.223123	2.216907	2.210933	2.233299	2.227612	2.284092
2.205218	2.198725	2.228278	2.221866	2.243965	2.237955	2.232097	2.226412	2.282846
2.203686	2.197176	2.226827	2.220587	2.242765	2.236827	2.230982	2.225232	2.281554
2.20225	2.195747	2.22547	2.219351	2.241571	2.235673	2.229837	2.224058	2.314617
2.200949	2.194498	2.224258	2.218188	2.240391	2.234473	2.228637	2.222882	2.313392
2.235738	2.229256	2.22303	2.216984	2.239176	2.233269	2.227455	2.221736	2.3122
2.234447	2.227922	2.221675	2.21563	2.237851	2.232063	2.226333	2.22066	2.311059
2.233157	2.226589	2.22032	2.214276	2.236531	2.230875	2.225232	2.219596	2.309918
2.231841	2.225317	2.219102	2.213115	2.235379	2.229754	2.286159	2.280364	2.308689
2.230515	2.224089	2.217969	2.212069	2.234324	2.228672	2.285008	2.279161	2.307411
2.229241	2.222907	2.244947	2.23904	2.233233	2.227542	2.283813	2.277958	2.306168
2.228017	2.221778	2.243836	2.237946	2.232117	2.226368	2.282574	2.276749	2.304952
2.226815	2.220644	2.242698	2.236811	2.230971	2.225199	2.315525	2.309627	2.340888
2.225639	2.2195	2.241519	2.235607	2.229778	2.224041	2.314314	2.308477	2.339781
2.224463	2.218333	2.240316	2.234395	2.22859	2.222903	2.313132	2.307343	2.338687
2.223248	2.217095	2.239073	2.233203	2.227446	2.221806	2.311986	2.306214	2.337587
2.222036	2.215851	2.237833	2.232032	2.226326	2.220729	2.310854	2.305074	2.336469
2.220902	2.214734	2.236699	2.230914	2.225225	2.219647	2.347006	2.341124	2.335268
2.219821	2.213704	2.235642	2.229838	2.224143	2.218563	2.345834	2.339893	2.334006
2.218678	2.212622	2.234559	2.228769	2.286435	2.280632	2.344619	2.338667	2.332789
2.217453	2.211473	2.233443	2.227705	2.285272	2.279378	2.343341	2.337434	2.331614
2.244142	2.238162	2.232353	2.226661	2.324278	2.318259	2.342126	2.336255	2.33048
2.24306	2.237102	2.231319	2.225649	2.323211	2.317189	2.341035	2.335165	2.329396

2.24201	2.236065	2.230298	2.224642	2.32216	2.316154	2.339985	2.334113	2.328343
2.24093	2.235008	2.275083	2.269333	2.350961	2.344889	2.338922	2.333063	2.327304
2.239843	2.233948	2.274048	2.268309	2.349872	2.343815	2.337864	2.332024	2.326282
2.238751	2.232882	2.273008	2.267279	2.348778	2.342739	2.336808	2.330991	2.325268
2.237654	2.231811	2.271962	2.266241	2.347679	2.341661	2.335753	2.329965	2.324264
2.282624	2.276724	2.328551	2.322683	2.346584	2.340581	2.334691	2.328923	2.323239
2.281603	2.275699	2.327464	2.321601	2.345494	2.3395	2.333619	2.32786	2.322185
2.280581	2.274676	2.326381	2.320523	2.344405	2.338416	2.332541	2.32679	2.321123
2.279537	2.273645	2.325298	2.319449	2.343312	2.337321	2.33145	2.32571	2.320056
2.278488	2.272613	2.324217	2.318377	2.342221	2.336229	2.330363	2.324635	2.318992
2.277438	2.271577	2.323133	2.317309	2.341144	2.335166	2.32931	2.32359	2.317954
2.276389	2.270539	2.32205	2.316244	2.340075	2.334117	2.328276	2.322562	2.31693
2.30972	2.303778	2.320966	2.315177	2.338999	2.333053	2.327223	2.321519	2.315895
2.308668	2.302709	2.319881	2.314107	2.337916	2.331974	2.326151	2.320462	2.314851
2.307662	2.30168	2.318822	2.313047	2.336839	2.330909	2.325096	2.31941	2.313806
2.306733	2.300715	2.317802	2.312002	2.335769	2.329857	2.324049	2.318353	2.312748
2.305793	2.299752	2.316784	2.310958	2.334713	2.32884	2.323049	2.317338	2.311725
2.327753	2.32166	2.34548	2.339534	2.333676	2.327901	2.322158	2.316429	2.310788
2.326684	2.320617	2.34441	2.338455	2.332653	2.326983	2.321292	2.315553	2.309884
2.325692	2.319682	2.343441	2.337449	2.331643	2.325989	2.320313	2.314588	2.326205
2.324747	2.318815	2.342538	2.336493	2.330643	2.324948	2.319262	2.313574	2.325205
2.323769	2.317871	2.34157	2.335512	2.32964	2.323914	2.318239	2.312618	2.344001
2.322757	2.316842	2.340529	2.334497	2.328622	2.322876	2.317237	2.311729	2.343223
2.321732	2.315801	2.339502	2.33353	2.327667	2.321902	2.316276	2.310826	2.34239
2.320675	2.31475	2.338521	2.332669	2.326837	2.321038	2.315372	2.309866	2.34142
2.319647	2.313725	2.337551	2.331799	2.326006	2.320204	2.314505	2.308921	2.340439
2.318723	2.312775	2.336562	2.330796	2.325053	2.31935	2.334564	2.328884	2.339539
2.317856	2.311874	2.335579	2.329747	2.324052	2.318482	2.333783	2.328075	2.338678
2.346702	2.340632	2.334659	2.328788	2.323087	2.317535	2.33289	2.327231	2.3216
2.345817	2.339773	2.333792	2.327894	2.322142	2.316521	2.331909	2.326363	2.320821
2.344917	2.338878	2.33291	2.327022	2.321245	2.315572	2.330969	2.325462	2.319956
2.344006	2.337938	2.332014	2.326184	2.320414	2.314709	2.330079	2.32451	2.318967
2.343061	2.33698	2.331091	2.325309	2.31956	2.31386	2.329223	2.323592	2.318011
2.342045	2.335997	2.330103	2.324313	2.318609	2.312991	2.328415	2.322762	2.317166
2.341026	2.335024	2.329126	2.323327	2.317667	2.312131	2.327624	2.321965	2.316366
2.340088	2.334119	2.328274	2.322528	2.338058	2.332435	2.326809	2.321164	2.333361
2.339196	2.333253	2.327481	2.321815	2.337363	2.331652	2.325987	2.320369	2.332569
2.338305	2.332365	2.326603	2.320949	2.336537	2.330843	2.32519	2.319574	2.331763
2.33742	2.331461	2.325648	2.319945	2.335594	2.330017	2.32442	2.31878	2.330944
2.336529	2.330566	2.32472	2.318982	2.334664	2.329144	2.32359	2.317973	2.330144
2.335628	2.329685	2.32384	2.318092	2.333754	2.328186	2.32265	2.317143	2.329378
2.334726	2.328807	2.322978	2.317236	2.332893	2.327277	2.321748	2.316323	2.328603
2.333828	2.327925	2.322123	2.316413	2.332129	2.326539	2.321008	2.315542	2.327783
2.332936	2.327049	2.321283	2.315617	2.33141	2.325858	2.320316	2.314774	2.326953
2.332048	2.326192	2.341983	2.336306	2.330674	2.325098	2.319541	2.313994	2.326155
2.33117	2.325353	2.341218	2.335581	2.329934	2.324299	2.318722	2.313208	2.325373
2.352036	2.346173	2.340443	2.334802	2.329144	2.323495	2.335658	2.330088	2.324566
2.351343	2.345444	2.339658	2.333959	2.328298	2.322684	2.334838	2.329256	2.323729
2.350616	2.344697	2.338875	2.333138	2.32747	2.321875	2.33402	2.328439	2.322914
2.34981	2.343905	2.338093	2.33236	2.326677	2.321051	2.333183	2.327633	2.322133

2.348982	2.343105	2.337322	2.331611	2.325923	2.320269	2.332389	2.326866	2.321387
2.348149	2.342311	2.336575	2.330913	2.325254	2.319614	2.331728	2.326193	2.320706
2.347323	2.341525	2.335839	2.330232	2.324613	2.319005	2.331115	2.325556	2.320049
2.346572	2.340779	2.33509	2.329478	2.32387	2.318284	2.330389	2.324831	2.319328
2.34588	2.340065	2.334331	2.32867	2.323051	2.317482	2.329583	2.324044	2.318561
2.345135	2.339309	2.333557	2.327876	2.339986	2.334351	2.328775	2.323259	2.317802
2.344309	2.338486	2.332753	2.32709	2.339179	2.333527	2.327955	2.322466	2.317049
2.343524	2.337712	2.331999	2.326355	2.338428	2.332765	2.327194	2.32172	2.316326
2.342849	2.337056	2.331353	2.325717	2.337784	2.332126	2.326552	2.321067	2.315654
2.342209	2.336436	2.33074	2.325104	2.33717	2.331523	2.325947	2.320446	2.315008
2.341558	2.335796	2.3301	2.324458	2.336518	2.330884	2.325318	2.31982	2.314382
2.340898	2.335144	2.329442	2.32379	2.335843	2.330226	2.324674	2.319187	2.313763
2.340158	2.334418	2.328711	2.323046	2.335096	2.329501	2.323961	2.318474	2.313057
2.339339	2.333622	2.327905	2.322225	2.334278	2.328708	2.32318	2.317685	2.312268
2.321245	2.31563	2.327165	2.321481	2.333529	2.327969	2.322449	2.316957	2.311548
2.32058	2.314969	2.326526	2.320853	2.332887	2.327312	2.321793	2.316321	2.310932
2.319944	2.314334	2.343641	2.337921	2.332271	2.326684	2.321166	2.315714	2.310344
2.319315	2.313705	2.343013	2.337292	2.331647	2.32607	2.32056	2.315113	2.309748
2.318691	2.31308	2.342388	2.336664	2.331028	2.325468	2.319968	2.314522	2.30916
2.318045	2.312436	2.341755	2.336037	2.330408	2.324855	2.319364	2.313929	2.308577
2.317384	2.31178	2.341117	2.33541	2.329787	2.324239	2.318757	2.313337	2.307999
2.316723	2.311123	2.324064	2.318397	2.329158	2.32362	2.318151	2.312748	2.307424
2.31606	2.310464	2.323396	2.317727	2.328518	2.322998	2.317547	2.312162	2.306853
2.315396	2.309802	2.322727	2.317061	2.327884	2.32238	2.316944	2.311573	2.306277
2.314732	2.309133	2.32206	2.316408	2.327265	2.32177	2.316343	2.310979	2.305692
2.314066	2.308465	2.321392	2.315755	2.326646	2.32116	2.31574	2.310384	2.305106
2.313399	2.307803	2.320725	2.315091	2.309556	2.304106	2.315135	2.309791	2.304525
2.312731	2.307143	2.320057	2.314424	2.308899	2.303464	2.314528	2.309199	2.303946
2.312059	2.306475	2.319384	2.313756	2.308244	2.302829	2.313924	2.308604	2.303359
2.311386	2.305801	2.318706	2.313087	2.307591	2.302198	2.313322	2.308006	2.302768
2.310723	2.305142	2.318047	2.312443	2.306958	2.301575	2.296249	2.290973	2.302196
2.310064	2.304489	2.3174	2.311817	2.306339	2.300952	2.295633	2.290382	2.301638
2.328058	2.322416	2.316801	2.311238	2.305765	2.300374	2.295062	2.289833	2.301123
2.327548	2.321899	2.316295	2.310748	2.305282	2.299894	2.294588	2.289369	2.300693
2.32705	2.321394	2.315798	2.31026	2.304802	2.299418	2.294116	2.288904	2.30026
2.326451	2.3208	2.315204	2.309665	2.304212	2.298837	2.293543	2.288334	2.31468
2.325814	2.320176	2.314576	2.309028	2.303576	2.29821	2.292921	2.287713	2.314078
2.32525	2.319627	2.314026	2.308469	2.303002	2.297617	2.292317	2.287105	2.297713
2.324748	2.319145	2.313544	2.307975	2.302479	2.297049	2.291721	2.286498	2.297099
2.324191	2.318595	2.313006	2.307448	2.301954	2.296518	2.291188	2.285967	2.296562
2.323543	2.317933	2.312371	2.306862	2.301417	2.296035	2.290743	2.285542	2.296135
2.322912	2.317287	2.311751	2.306287	2.30089	2.295562	2.290307	2.285129	2.295721
2.322371	2.316748	2.311226	2.305781	2.300401	2.295088	2.28985	2.284689	2.295282
2.321851	2.316228	2.310712	2.305278	2.299905	2.294598	2.28937	2.284224	2.294817
2.32128	2.315624	2.310106	2.304686	2.299327	2.294037	2.288821	2.283682	2.29427
2.320685	2.314979	2.309455	2.304048	2.298705	2.293436	2.288232	2.283093	2.293674
2.320114	2.314414	2.308894	2.303495	2.298164	2.292913	2.287721	2.282586	2.29316
2.319562	2.31393	2.308426	2.303024	2.297703	2.292466	2.287286	2.282159	2.292729
2.319034	2.313447	2.30796	2.302565	2.297254	2.292026	2.286854	2.281734	2.292297
2.318537	2.312945	2.307477	2.302105	2.296804	2.291577	2.286407	2.281293	2.291847

2.318052	2.312449	2.306996	2.301647	2.296356	2.291133	2.285966	2.280856	2.291398
2.317566	2.311973	2.306525	2.301182	2.295903	2.290696	2.285539	2.280434	2.290956
2.317086	2.311507	2.306061	2.300718	2.29545	2.290263	2.285118	2.280017	2.290521
2.316607	2.311038	2.305603	2.300271	2.295011	2.289828	2.284689	2.279596	2.2901
2.316131	2.310568	2.30515	2.299836	2.294581	2.289392	2.284256	2.279174	2.28969
2.315662	2.310108	2.304702	2.2994	2.29415	2.288964	2.283831	2.278755	2.28927
2.3152	2.309657	2.304257	2.298959	2.293719	2.288544	2.283416	2.27834	2.288834
2.314742	2.309213	2.303819	2.298526	2.293291	2.288123	2.283001	2.277929	2.288413
2.314292	2.308778	2.303395	2.298107	2.292872	2.287698	2.282581	2.277525	2.288024
2.313839	2.308343	2.30297	2.297689	2.292454	2.287275	2.282162	2.277121	2.287638
2.313362	2.307891	2.302527	2.297253	2.292028	2.286859	2.281753	2.276711	2.287228
2.312883	2.307434	2.302079	2.296814	2.291601	2.286448	2.281348	2.276299	2.286808
2.312469	2.306996	2.301647	2.296398	2.291188	2.28603	2.280931	2.275889	2.286397
2.312112	2.306573	2.301226	2.295999	2.290784	2.285606	2.280502	2.275479	2.285992
2.284838	2.279356	2.300866	2.295649	2.290436	2.285253	2.280138	2.275094	2.285581
2.284501	2.279075	2.300599	2.295375	2.290173	2.285011	2.27987	2.274734	2.285146
2.284143	2.278779	2.30031	2.295073	2.289881	2.284742	2.27959	2.274398	2.284758
2.283719	2.278399	2.299916	2.294655	2.289469	2.284353	2.279236	2.274103	2.284483
2.283293	2.278004	2.299506	2.294227	2.289038	2.28393	2.278855	2.273811	2.284236
2.282955	2.27767	2.299185	2.293915	2.288702	2.283545	2.278459	2.273455	2.283909
2.282664	2.277365	2.298907	2.293665	2.288416	2.283178	2.278055	2.273068	2.283546
2.282333	2.277003	2.271779	2.266627	2.288095	2.282846	2.277722	2.272744	2.30405
2.281963	2.276582	2.271361	2.266237	2.287741	2.282549	2.277458	2.272482	2.303773
2.281603	2.276202	2.270982	2.265874	2.287409	2.282267	2.277196	2.272199	2.30346
2.281261	2.27589	2.270681	2.265574	2.287128	2.282014	2.276929	2.271865	2.303067
2.280931	2.2756	2.270387	2.265262	2.286825	2.281735	2.276646	2.271533	2.302694
2.280629	2.275317	2.270058	2.264868	2.286419	2.281362	2.276309	2.271239	2.30242
2.280325	2.27503	2.269718	2.264459	2.285993	2.280968	2.275962	2.270964	2.302187
2.279944	2.274676	2.269382	2.264139	2.285685	2.28067	2.297526	2.292478	2.301931
2.279512	2.274278	2.269048	2.263879	2.285455	2.280436	2.297286	2.292237	2.301669
2.279156	2.27394	2.268742	2.263608	2.258574	2.25363	2.297028	2.291996	2.301426
2.278886	2.273672	2.268466	2.263322	2.258287	2.253349	2.296755	2.291762	2.301213
2.278629	2.273414	2.268199	2.263041	2.281202	2.276179	2.296446	2.291487	2.300962
2.278366	2.273148	2.267932	2.262769	2.280897	2.275822	2.296064	2.291125	2.300627
2.278107	2.272885	2.267671	2.262505	2.280601	2.275476	2.29569	2.290765	2.300289
2.277848	2.272631	2.267417	2.262248	2.280336	2.275199	2.295409	2.290497	2.300027
2.27759	2.272381	2.267169	2.262001	2.280093	2.274964	2.295181	2.290273	2.2998
2.27733	2.272125	2.290285	2.285049	2.279871	2.27475	2.269752	2.264898	2.299547
2.277069	2.271865	2.290041	2.284831	2.279671	2.274562	2.269555	2.264665	2.299277
2.27681	2.27161	2.289798	2.284608	2.279475	2.2744	2.269401	2.264485	2.299063
2.27655	2.271363	2.289555	2.284377	2.279289	2.274282	2.269313	2.264384	2.298931
2.276298	2.271114	2.289309	2.284143	2.279091	2.274139	2.269196	2.264257	2.259336
2.276041	2.270843	2.289045	2.283899	2.278863	2.273926	2.268988	2.264043	2.259111
2.275812	2.270593	2.288795	2.283661	2.278627	2.273686	2.268745	2.263799	2.258867
2.299163	2.293856	2.288612	2.283449	2.278396	2.273442	2.268499	2.263566	2.258646
2.299072	2.29375	2.28847	2.283259	2.278173	2.273197	2.268251	2.263335	2.258433
2.298895	2.29358	2.288314	2.283115	2.278022	2.273024	2.268057	2.263121	2.258215
2.298636	2.293352	2.288151	2.283023	2.277946	2.272923	2.26792	2.262924	2.257992
2.298406	2.293131	2.287966	2.282881	2.277816	2.272782	2.267759	2.262728	2.287434
2.298233	2.292922	2.287739	2.282646	2.277584	2.272562	2.267548	2.262524	2.287254

2.298072	2.292738	2.287533	2.282422	2.277355	2.272342	2.267341	2.262338	2.287106
2.297916	2.292623	2.287421	2.282292	2.277205	2.272166	2.267164	2.262193	2.287024
2.297754	2.292519	2.287326	2.282184	2.277077	2.27201	2.267006	2.262071	2.286968
2.297535	2.292315	2.287133	2.282002	2.276913	2.271869	2.266882	2.261957	2.286879
2.297281	2.292049	2.286878	2.281774	2.276728	2.27174	2.266781	2.26185	2.286772
2.29709	2.291846	2.286665	2.281557	2.276524	2.271562	2.266623	2.261704	2.286647
2.296971	2.291714	2.286497	2.281345	2.27629	2.271322	2.266395	2.261509	2.286498
2.296847	2.291577	2.286344	2.281173	2.276106	2.271131	2.266208	2.261341	2.286369
2.296695	2.291404	2.286195	2.281064	2.276014	2.271043	2.266116	2.261234	2.286285
2.296541	2.291241	2.286066	2.280982	2.275952	2.270983	2.266047	2.261144	2.286209
2.296391	2.291125	2.285972	2.280905	2.275878	2.2709	2.296334	2.291207	2.28612
2.296251	2.291036	2.2859	2.280835	2.275803	2.270811	2.296274	2.291129	2.28602
2.29615	2.290957	2.285824	2.28075	2.275709	2.270706	2.296174	2.291007	2.285886
2.296085	2.290892	2.285747	2.280655	2.275601	2.270586	2.29604	2.290848	2.285722
2.296009	2.290819	2.285671	2.280569	2.275495	2.270452	2.295901	2.290715	2.307271
2.295918	2.290734	2.285594	2.280496	2.275389	2.270288	2.295742	2.290612	2.307208
2.295829	2.290649	2.285515	2.280422	2.275299	2.270165	2.295634	2.290537	2.307151
2.295752	2.290571	2.285434	2.280341	2.275241	2.27015	2.295649	2.290506	2.307075
2.295673	2.290489	2.28535	2.280257	2.275187	2.270146	2.295678	2.290483	2.307001
2.295565	2.290392	2.285261	2.280173	2.306136	2.30082	2.295579	2.290417	2.30695
2.295446	2.290287	2.285168	2.280088	2.306035	2.300668	2.295428	2.290334	2.30691
2.295371	2.290189	2.285073	2.28001	2.305997	2.300643	2.295403	2.290292	2.306846
2.295339	2.290096	2.284976	2.279937	2.30602	2.300735	2.295493	2.290285	2.306755
2.295261	2.289997	2.284881	2.279859	2.306013	2.300784	2.295558	2.290313	2.306735
2.295093	2.289887	2.284788	2.279773	2.30595	2.300755	2.295574	2.2904	2.306832
2.294946	2.289778	2.284693	2.279683	2.305881	2.300705	2.295565	2.290466	2.306924
2.294893	2.289666	2.284579	2.279589	2.305831	2.30065	2.295519	2.290449	2.306934
2.294882	2.289577	2.284477	2.279498	2.305785	2.300593	2.295461	2.290398	2.306906
2.294877	2.289577	2.284446	2.279425	2.305724	2.300557	2.295433	2.290354	2.306849
2.294883	2.289639	2.284465	2.279368	2.305662	2.300539	2.295426	2.290312	2.306772
2.294856	2.28966	2.316287	2.310912	2.305681	2.300572	2.317158	2.311948	2.30678
2.294788	2.289634	2.316348	2.311014	2.305793	2.300668	2.317249	2.312063	2.306886
2.294752	2.289621	2.316414	2.311123	2.305896	2.30073	2.317289	2.312126	2.306956
2.294786	2.289651	2.3165	2.311236	2.305966	2.300706	2.317215	2.312076	2.306943
2.294825	2.289663	2.316555	2.311315	2.306012	2.300679	2.317138	2.312001	2.306893
2.294812	2.289589	2.316503	2.311298	2.306023	2.300719	2.317153	2.311953	2.306807
2.294785	2.289491	2.316408	2.31123	2.306006	2.300775	2.317201	2.311917	2.306712
2.29479	2.28948	2.31638	2.31116	2.305947	2.300765	2.317207	2.311915	2.306701
2.294819	2.289536	2.316396	2.311081	2.305851	2.300706	2.317186	2.31194	2.306756
2.294862	2.289608	2.316459	2.311084	2.305839	2.300708	2.317202	2.311983	2.306809
2.294933	2.289705	2.316584	2.311192	2.305934	2.30079	2.317268	2.312046	2.306854
2.294969	2.289769	2.316692	2.311304	2.306037	2.300872	2.317331	2.31211	2.306906
2.294909	2.289744	2.316732	2.311376	2.306112	2.300929	2.31738	2.312178	2.306983
2.294847	2.289712	2.316765	2.311444	2.306177	2.300962	2.3174	2.312223	2.307049
2.294885	2.289756	2.316846	2.311528	2.306223	2.300937	2.317338	2.312189	2.30705
2.294977	2.289838	2.316949	2.311621	2.30626	2.300886	2.317237	2.312117	2.30702
2.295058	2.289916	2.31705	2.311716	2.328061	2.322555	2.317227	2.312115	2.307035
2.295136	2.289994	2.317152	2.311813	2.328147	2.322633	2.317295	2.312172	2.307088
2.29522	2.290077	2.317259	2.311915	2.328238	2.322718	2.317375	2.312245	2.307157
2.295312	2.290166	2.317377	2.312027	2.328336	2.322807	2.317459	2.312329	2.307244

2.295408	2.290258	2.317493	2.312138	2.328434	2.322899	2.317548	2.312419	2.307334
2.295512	2.290355	2.317601	2.312242	2.328525	2.322986	2.317632	2.312502	2.307417
2.295617	2.290454	2.317705	2.312343	2.328617	2.323078	2.317722	2.312588	2.307498
2.295711	2.290547	2.317808	2.312449	2.328719	2.323184	2.317823	2.312672	2.307569
2.295801	2.290636	2.317909	2.312557	2.328829	2.323305	2.31794	2.312761	2.30764
2.277793	2.272679	2.318011	2.312669	2.328964	2.323483	2.318128	2.312919	2.307766
2.277907	2.272768	2.31811	2.312785	2.329131	2.32373	2.318402	2.313157	2.307956
2.278063	2.272892	2.318228	2.312896	2.329268	2.323924	2.318619	2.313354	2.308125
2.278296	2.27308	2.318375	2.312991	2.329342	2.324014	2.318722	2.313467	2.308243
2.278524	2.273271	2.318532	2.313095	2.329414	2.324081	2.318794	2.313556	2.308348
2.278663	2.273412	2.318678	2.313224	2.329516	2.324165	2.318874	2.313645	2.308461
2.278771	2.273538	2.31883	2.313378	2.329646	2.324266	2.318961	2.313735	2.308574
2.313177	2.307679	2.340804	2.3353	2.329843	2.324437	2.319108	2.313857	2.308682
2.313365	2.307841	2.340997	2.335547	2.330097	2.324665	2.319302	2.314005	2.308788
2.313579	2.308061	2.341228	2.335792	2.330334	2.324878	2.319496	2.314188	2.308957
2.313832	2.308363	2.341514	2.336041	2.330551	2.325066	2.319682	2.314411	2.309206
2.314055	2.308621	2.341756	2.336255	2.330755	2.325268	2.319893	2.314648	2.309457
2.314189	2.30875	2.341885	2.336404	2.330944	2.325515	2.320163	2.314899	2.30968
2.314328	2.308866	2.341997	2.336535	2.331119	2.325753	2.320427	2.31514	2.309894
2.31458	2.309086	2.342179	2.336686	2.331262	2.325908	2.320596	2.315325	2.310093
2.314886	2.309352	2.342395	2.336852	2.3314	2.326031	2.320725	2.315485	2.310288
2.31514	2.30958	2.326799	2.32132	2.331623	2.326243	2.320934	2.315703	2.310513
2.315353	2.309777	2.32703	2.321608	2.331924	2.326531	2.32121	2.315967	2.310766
2.31559	2.310025	2.327297	2.321902	2.332229	2.326829	2.321494	2.316232	2.311009
2.315859	2.310349	2.327613	2.322198	2.332534	2.327136	2.321789	2.316496	2.31124
2.286368	2.280981	2.32793	2.322494	2.332831	2.32743	2.322067	2.316743	2.311458
2.286713	2.281268	2.328211	2.32278	2.333107	2.327686	2.322302	2.316949	2.311649
2.287071	2.281556	2.328491	2.323066	2.333373	2.327922	2.322515	2.317144	2.311843
2.276018	2.270362	2.328808	2.323358	2.333635	2.328135	2.32271	2.317363	2.312085
2.27626	2.270672	2.329147	2.323654	2.333897	2.328338	2.3229	2.3176	2.31236
2.276509	2.270942	2.296072	2.290498	2.334202	2.328637	2.323192	2.317884	2.312638
2.276765	2.271172	2.296332	2.290793	2.334556	2.329044	2.3236	2.318224	2.312922
2.277012	2.271408	2.265906	2.260472	2.319105	2.313652	2.323932	2.318525	2.313198
2.277247	2.27167	2.266148	2.260676	2.319319	2.313819	2.324094	2.318743	2.313459
2.277477	2.271921	2.266383	2.260879	2.319541	2.314002	2.324274	2.318965	2.313715
2.277704	2.272124	2.266601	2.261127	2.286159	2.280595	2.324624	2.319251	2.313954
2.277928	2.272313	2.266812	2.261384	2.286477	2.280966	2.325036	2.319572	2.314205
2.278144	2.272541	2.267033	2.261599	2.286728	2.281265	2.293194	2.287684	2.31455
2.278357	2.272794	2.26726	2.261787	2.286936	2.281519	2.293493	2.288049	2.314965
2.278605	2.273018	2.267471	2.261993	2.287155	2.28175	2.293753	2.288358	2.282979
2.27889	2.273192	2.267653	2.262223	2.287387	2.28195	2.293962	2.288599	2.283261
2.279203	2.27343	2.267878	2.262457	2.257052	2.251688	2.276787	2.271482	2.283514
2.279563	2.273805	2.268186	2.262673	2.257257	2.251938	2.277049	2.271698	2.283713
2.279921	2.27421	2.268529	2.262919	2.257492	2.252219	2.277341	2.27194	2.283929
2.280203	2.274548	2.268874	2.263254	2.257826	2.252552	2.277685	2.272283	2.284263
2.28046	2.274863	2.26923	2.263643	2.258212	2.25291	2.27805	2.27267	2.28465
2.280791	2.275207	2.269596	2.264027	2.258565	2.253194	2.278319	2.272966	2.284969
2.281183	2.275574	2.269974	2.264416	2.258895	2.253415	2.278507	2.273189	2.285239
2.281545	2.275889	2.270301	2.26477	2.259228	2.253697	2.2483	2.243058	2.268154
2.281854	2.276117	2.270546	2.265072	2.259567	2.254063	2.248645	2.243311	2.268355

2.282176	2.276379	2.270815	2.265376	2.25991	2.25445	2.249022	2.243605	2.268597
2.28255	2.276755	2.271179	2.265725	2.260271	2.254844	2.249421	2.243974	2.268945
2.282942	2.277173	2.271569	2.266071	2.26062	2.255226	2.24982	2.244369	2.269334
2.283337	2.277576	2.271916	2.266345	2.260889	2.255537	2.250161	2.24473	2.269699
2.283733	2.277977	2.272245	2.266585	2.261117	2.255808	2.25047	2.245079	2.27006
2.284068	2.278338	2.272593	2.266908	2.261438	2.256141	2.250817	2.245447	2.270442
2.284336	2.278658	2.272957	2.267314	2.261851	2.256535	2.251202	2.245835	2.270844
2.284656	2.279006	2.273335	2.26772	2.262257	2.256921	2.25158	2.24622	2.240893
2.285064	2.279404	2.273727	2.268108	2.262638	2.257295	2.251954	2.246608	2.24129
2.285499	2.27982	2.274128	2.268495	2.263006	2.257639	2.252294	2.246967	2.241666
2.285932	2.280242	2.274537	2.268884	2.263345	2.257907	2.252538	2.247246	2.241987
2.286372	2.280671	2.274953	2.269276	2.26368	2.25816	2.252761	2.247504	2.24229
2.286805	2.281097	2.275368	2.269677	2.264058	2.258506	2.253093	2.247845	2.242644
2.287233	2.28152	2.275784	2.270086	2.264467	2.258918	2.253503	2.248241	2.24303
2.287664	2.281946	2.276204	2.270504	2.264887	2.259346	2.253927	2.248648	2.243422
2.288097	2.282373	2.27663	2.27093	2.265317	2.259783	2.254359	2.249059	2.243815
2.288528	2.2828	2.277057	2.271362	2.265767	2.26026	2.254839	2.249511	2.244238
2.288951	2.283225	2.277487	2.271804	2.266252	2.260812	2.255409	2.250041	2.244717
2.28938	2.283651	2.277915	2.272243	2.266732	2.261355	2.255973	2.250573	2.245205
2.289816	2.284068	2.278328	2.272661	2.267158	2.261795	2.256423	2.251029	2.245657
2.290273	2.284494	2.278744	2.273074	2.267562	2.262185	2.256816	2.251446	2.246093
2.290818	2.284998	2.279218	2.273521	2.267984	2.262586	2.257211	2.251855	2.246525
2.291442	2.285568	2.279741	2.273996	2.268418	2.262986	2.257598	2.25225	2.246951
2.291998	2.286105	2.280274	2.274523	2.268922	2.263451	2.258037	2.252677	2.247379
2.292453	2.28659	2.280821	2.275133	2.269531	2.264011	2.258549	2.253139	2.2478
2.292911	2.287068	2.281343	2.275704	2.270103	2.264551	2.259059	2.253626	2.248262
2.293417	2.287534	2.281789	2.276142	2.270541	2.264996	2.259532	2.254155	2.248825
2.293947	2.288022	2.282237	2.276558	2.270949	2.265418	2.259989	2.254679	2.249393
2.267772	2.261947	2.282799	2.277073	2.271429	2.265869	2.260423	2.255113	2.249831
2.268337	2.262565	2.28342	2.277639	2.271947	2.266337	2.260849	2.255502	2.250203
2.268845	2.263095	2.283966	2.278176	2.272476	2.266857	2.261351	2.255976	2.250651
2.269293	2.263539	2.284441	2.278686	2.273018	2.26743	2.26193	2.25653	2.251169
2.240648	2.234998	2.284957	2.279235	2.273583	2.268002	2.262494	2.257067	2.251678
2.241232	2.235554	2.285557	2.279862	2.274196	2.268571	2.263022	2.257554	2.25215
2.241819	2.236108	2.286146	2.280466	2.274783	2.269119	2.263542	2.258054	2.252649
2.242339	2.23659	2.286639	2.280949	2.275265	2.269609	2.264053	2.258605	2.253226
2.242846	2.23707	2.287119	2.281405	2.275723	2.270092	2.264571	2.259179	2.253834
2.243401	2.237649	2.287706	2.281959	2.276274	2.270657	2.265146	2.259755	2.254409
2.243988	2.238295	2.288364	2.282579	2.276885	2.271279	2.26576	2.260337	2.254969
2.24459	2.238912	2.289014	2.283222	2.277517	2.271892	2.266358	2.260923	2.255543
2.245208	2.239494	2.289656	2.283898	2.278174	2.272495	2.266934	2.261513	2.256135
2.245832	2.240096	2.23447	2.228915	2.278823	2.273112	2.267532	2.262109	2.25672
2.246461	2.240749	2.235095	2.2295	2.279443	2.273759	2.268176	2.262715	2.257281
2.247091	2.2414	2.235719	2.230086	2.280065	2.274408	2.268823	2.263324	2.25785
2.247716	2.241996	2.23633	2.230723	2.280721	2.275033	2.269432	2.263933	2.258465
2.248339	2.242574	2.236936	2.231377	2.281386	2.27565	2.270029	2.264542	2.259096
2.248962	2.243197	2.237551	2.231981	2.282018	2.276284	2.270655	2.265148	2.259681
2.249583	2.243854	2.238171	2.232547	2.282625	2.276932	2.271305	2.265751	2.260231
2.250197	2.244474	2.238777	2.233136	2.227579	2.222101	2.271926	2.266356	2.260819
2.250803	2.245036	2.239362	2.233759	2.228188	2.222658	2.272505	2.266967	2.261468

2.251407	2.245613	2.23995	2.234369	2.228786	2.223222	2.273093	2.267572	2.262099
2.252002	2.246248	2.240555	2.234927	2.229346	2.223819	2.273717	2.268156	2.262662
2.252613	2.246894	2.241166	2.235487	2.22991	2.224424	2.274349	2.268745	2.263222
2.230467	2.224742	2.241765	2.236106	2.23052	2.225005	2.247606	2.242112	2.263844
2.23115	2.22531	2.242371	2.236766	2.231163	2.225588	2.248178	2.242737	2.264506
2.231859	2.225985	2.243064	2.237463	2.231851	2.226258	2.248852	2.24342	2.265197
2.232601	2.226763	2.243843	2.238206	2.232591	2.227015	2.249626	2.244163	2.265925
2.233306	2.227508	2.244576	2.238891	2.233271	2.227717	2.222231	2.216817	2.239402
2.233941	2.22818	2.245215	2.239465	2.23384	2.228322	2.222843	2.217395	2.239967
2.234592	2.228854	2.245866	2.240064	2.234421	2.228908	2.223426	2.21796	2.24053
2.235336	2.229587	2.24661	2.240793	2.235097	2.2295	2.22398	2.218539	2.241143
2.236096	2.230318	2.247369	2.241561	2.235809	2.230111	2.224548	2.219141	2.241789
2.236765	2.23095	2.248036	2.24226	2.236515	2.230809	2.225241	2.219832	2.242492
2.237381	2.231522	2.24865	2.242921	2.237224	2.23157	2.22602	2.220588	2.243241
2.238062	2.232185	2.249333	2.243615	2.237941	2.232319	2.226778	2.221328	2.24398
2.238819	2.232952	2.250095	2.24435	2.238671	2.233061	2.227522	2.22206	2.244718
2.23954	2.233694	2.250828	2.245051	2.239368	2.233771	2.22823	2.222747	2.245403
2.240163	2.234354	2.251476	2.245665	2.239983	2.234412	2.228869	2.223341	2.245976
2.2408	2.235021	2.252137	2.2463	2.240618	2.235065	2.229515	2.223947	2.246564
2.127715	2.122485	2.229924	2.224135	2.241372	2.235805	2.230243	2.224665	2.21917
2.128426	2.123176	2.230707	2.224938	2.242182	2.236588	2.231011	2.225437	2.219937
2.129134	2.123872	2.231491	2.225727	2.24298	2.23737	2.231782	2.226203	2.220694
2.12985	2.124579	2.232284	2.226515	2.243777	2.238157	2.23256	2.226972	2.221452
2.130567	2.125286	2.170476	2.164851	2.244572	2.238945	2.233339	2.227743	2.248031
2.131286	2.125992	2.17123	2.165596	2.245365	2.239732	2.234119	2.228515	2.248796
2.132005	2.126698	2.171984	2.166341	2.246156	2.240517	2.234898	2.229287	2.249563
2.132716	2.1274	2.172736	2.167088	2.246948	2.241301	2.235675	2.230059	2.250332
2.133429	2.128103	2.17349	2.167836	2.247739	2.242084	2.236451	2.23083	2.2511
2.13415	2.128812	2.174248	2.168587	2.248532	2.242868	2.237226	2.2316	2.251865
2.134877	2.129525	2.17501	2.169342	2.249326	2.243651	2.238001	2.232368	2.252629
2.135606	2.130246	2.175773	2.170097	2.164521	2.159038	2.238774	2.233135	2.253393
2.136339	2.130975	2.176537	2.170853	2.165264	2.159765	2.239544	2.233901	2.254158
2.137069	2.131699	2.177297	2.171606	2.166007	2.160497	2.240318	2.234669	2.254924
2.137788	2.132411	2.178048	2.172351	2.166747	2.16123	2.241095	2.235436	2.255686
2.138517	2.133134	2.178806	2.173103	2.167494	2.161975	2.241885	2.236218	2.256463
2.139257	2.133877	2.128527	2.123234	2.168239	2.162734	2.242691	2.237015	2.257254
2.140024	2.134645	2.129282	2.12397	2.169015	2.163517	2.243522	2.237842	2.258078
2.140875	2.135478	2.130121	2.124819	2.169905	2.164367	2.244417	2.238749	2.258991
2.141782	2.136352	2.131014	2.12574	2.170867	2.165261	2.245353	2.239708	2.259964
2.142606	2.137166	2.131832	2.126563	2.171728	2.16609	2.160551	2.155135	2.260855
2.143343	2.137917	2.13257	2.127282	2.172483	2.16685	2.161313	2.155896	2.261661
2.144079	2.13866	2.133305	2.128005	2.173239	2.167605	2.162066	2.156641	2.262461
2.144816	2.139387	2.134038	2.128749	2.174009	2.168349	2.162795	2.157363	2.263261
2.14559	2.140156	2.134808	2.129521	2.174812	2.169138	2.16357	2.158117	2.264085
2.146474	2.141063	2.135685	2.130353	2.175682	2.170057	2.164489	2.158971	2.264969
2.147394	2.142015	2.136602	2.13122	2.176595	2.171031	2.165466	2.159877	2.265892
2.148204	2.142835	2.137449	2.132098	2.177536	2.171941	2.16636	2.160781	2.210372
2.148936	2.143561	2.138248	2.132989	2.178503	2.172809	2.167201	2.161691	2.211348
2.149744	2.144332	2.139036	2.13381	2.179377	2.173629	2.168006	2.162535	2.212238
2.150645	2.145148	2.139801	2.134537	2.180126	2.174377	2.168755	2.163288	2.213012

2.151569	2.146019	2.14061	2.135289	2.12996	2.124644	2.169561	2.164072	2.189116
2.152517	2.146995	2.141522	2.136114	2.130779	2.125515	2.170503	2.164946	2.189962
2.153441	2.147964	2.142452	2.136982	2.131646	2.126421	2.171475	2.165859	2.190853
2.117651	2.112275	2.143318	2.137914	2.132574	2.127294	2.172369	2.166789	2.191811
2.118375	2.112998	2.144152	2.138874	2.133532	2.128159	2.17324	2.167735	2.192805
2.119221	2.113797	2.144965	2.139709	2.134387	2.12904	2.174189	2.168662	2.193725
2.120167	2.114652	2.145754	2.14043	2.135154	2.129936	2.175208	2.169574	2.194579
2.050311	2.044954	2.14661	2.141215	2.135939	2.130775	2.176141	2.170482	2.195481
2.051198	2.045858	2.147562	2.142093	2.136744	2.131507	2.176921	2.171384	2.196469
2.052086	2.046758	2.148532	2.143014	2.137604	2.132295	2.177744	2.172292	2.197438
2.052969	2.0476	2.149475	2.143966	2.138568	2.133272	2.178758	2.173213	2.198314
2.053846	2.048431	2.150423	2.144951	2.139578	2.134302	2.179817	2.17414	2.199172
2.054703	2.049304	2.151414	2.145979	2.14058	2.135229	2.180751	2.175072	2.200104
2.055547	2.050194	2.152425	2.147029	2.141577	2.136104	2.181624	2.176007	2.201073
1.979261	1.974018	2.086714	2.081501	2.142469	2.136989	2.182569	2.176929	2.201977
1.980074	1.974761	2.087425	2.082168	2.143244	2.137882	2.183591	2.177834	2.20281
1.980948	1.975584	2.088233	2.08293	2.144083	2.138782	2.133463	2.128098	2.203692
1.981934	1.976558	2.089237	2.083894	2.145058	2.13969	2.134338	2.12899	2.204657
1.982911	1.97754	2.090258	2.084872	2.146052	2.140608	2.135226	2.129911	2.205668
1.93296	1.927778	2.05017	2.044844	2.080384	2.075123	2.136164	2.130888	2.206741
1.933747	1.928593	2.05102	2.045653	2.081219	2.076002	2.137131	2.131887	2.207831
1.934548	1.929386	2.051865	2.046507	2.082111	2.076925	2.138119	2.132842	2.208816
1.935351	1.930155	2.052701	2.04739	2.083046	2.077882	2.139126	2.133766	2.209726
1.898214	1.893133	1.976476	1.971317	2.083977	2.078787	2.073591	2.068365	2.210674
1.89897	1.89393	1.977388	1.972254	2.084914	2.079627	2.07439	2.069193	2.211664
1.899789	1.894773	1.9783	1.973153	2.085813	2.080466	2.075209	2.070048	2.154995
1.900736	1.89569	1.979177	1.97394	2.086618	2.081307	2.076085	2.070962	2.156004
1.901708	1.896632	1.98007	1.974737	2.08744	2.082179	2.076989	2.071881	2.157002
1.902614	1.897571	1.981051	1.975682	2.088413	2.083137	2.077912	2.07274	2.157902
1.903487	1.898497	1.982062	1.976692	2.08946	2.084144	2.078857	2.073584	2.158774
1.904323	1.89931	1.894226	1.889154	2.052612	2.047369	2.079841	2.074525	2.159794
1.905118	1.900018	1.894946	1.889929	2.053552	2.048349	2.080866	2.075551	2.16094
1.905974	1.900821	1.895756	1.890765	2.054488	2.049265	2.081802	2.076504	2.162031
1.906934	1.901776	1.896704	1.891693	2.055413	2.050077	2.082593	2.077335	2.163025
1.907857	1.902719	1.897656	1.892637	1.976037	1.970787	2.083422	2.078193	2.110072
1.908637	1.903545	1.898537	1.893571	1.977028	1.97174	2.084404	2.079163	2.111049
1.909392	1.904352	1.899406	1.894504	1.978044	1.972739	2.08544	2.080175	2.112055
1.910257	1.905232	1.900308	1.895431	1.979036	1.973715	2.086456	2.081178	2.11306
1.911183	1.906152	1.901229	1.896355	1.98002	1.974687	2.087472	2.082185	2.114071
1.797401	1.792933	1.902156	1.897281	1.892288	1.887219	2.050554	2.045328	2.115072
1.798208	1.793726	1.903089	1.898209	1.893203	1.888115	2.051526	2.046288	2.11606
1.799013	1.794518	1.90402	1.899137	1.894123	1.889026	2.052519	2.047267	2.117063
1.799811	1.795299	1.904945	1.900064	1.895055	1.889964	2.05355	2.048279	2.118093
1.800609	1.796084	1.905869	1.900988	1.895985	1.890904	2.054585	2.049297	2.119128
1.801409	1.796881	1.906792	1.901904	1.896902	1.891828	2.055604	2.050302	2.120155
1.802212	1.797685	1.907717	1.90282	1.897817	1.892747	2.056617	2.051303	2.121179
1.803028	1.798497	1.794072	1.789711	1.898742	1.893671	1.981119	1.976019	2.0865
1.803853	1.799316	1.794879	1.790504	1.899675	1.894598	1.982072	1.97696	2.087494
1.80467	1.800129	1.795679	1.791289	1.900604	1.895526	1.983031	1.977907	2.088497
1.80548	1.800936	1.796473	1.792067	1.90153	1.896454	1.983995	1.978861	2.089509

1.806285	1.801733	1.797259	1.792842	1.78839	1.783926	1.892385	1.887453	2.090522
1.807063	1.802502	1.798025	1.793608	1.789151	1.784678	1.893294	1.888349	2.091519
1.807872	1.80329	1.798801	1.79438	1.789925	1.785459	1.894241	1.889282	2.092546
1.808796	1.804157	1.799625	1.795174	1.790741	1.786341	1.895318	1.89033	2.093656
1.809771	1.805063	1.800472	1.79598	1.791578	1.787264	1.896451	1.891439	2.094823
1.81067	1.805921	1.801299	1.796783	1.792388	1.788105	1.783791	1.779428	2.024343
1.811509	1.806745	1.80211	1.797585	1.793177	1.78888	1.784597	1.780327	2.025549
1.812394	1.80764	1.802988	1.798433	1.793992	1.78966	1.785379	1.781166	2.026663
1.813354	1.808648	1.803962	1.799336	1.794834	1.790442	1.786131	1.781926	2.027654
1.814294	1.809626	1.804931	1.800269	1.795716	1.791255	1.786899	1.782674	2.028619
1.815154	1.810489	1.80585	1.801254	1.796677	1.792127	1.787701	1.783422	2.029579
1.815996	1.811309	1.806735	1.802228	1.797648	1.793033	1.788543	1.784197	2.030557
1.816872	1.812128	1.807556	1.803082	1.79854	1.793969	1.789478	1.785069	1.839261
1.817773	1.812944	1.808337	1.803866	1.799384	1.794922	1.790467	1.786004	1.840178
1.818751	1.81383	1.809169	1.804667	1.800198	1.795787	1.791368	1.786927	1.841135
1.81981	1.814782	1.81004	1.805469	1.80097	1.796561	1.792184	1.787846	1.842146
1.82084	1.81578	1.810997	1.806378	1.801836	1.797388	1.793008	1.788714	1.784417
1.821809	1.816848	1.8121	1.807475	1.80287	1.798308	1.793843	1.789487	1.785176
1.822765	1.817896	1.813178	1.80855	1.803902	1.79926	1.794718	1.790282	1.785935
1.823703	1.818814	1.814071	1.809428	1.80481	1.800235	1.795702	1.7912	1.786788
1.824663	1.819718	1.814922	1.810247	1.805675	1.801207	1.796711	1.79216	1.787686
1.825769	1.820812	1.815941	1.811167	1.806553	1.802079	1.797593	1.793069	1.788618
1.826972	1.822027	1.817062	1.812147	1.807435	1.802888	1.798397	1.793952	1.789582
1.684804	1.680521	1.818084	1.813126	1.808383	1.803812	1.799308	1.794868	1.790503
1.68554	1.681274	1.818994	1.814108	1.809414	1.804876	1.800351	1.795821	1.791368
1.686326	1.682045	1.819903	1.815068	1.810405	1.805885	1.801349	1.796772	1.792269
1.687256	1.682891	1.820823	1.815957	1.811265	1.806725	1.802208	1.797701	1.793261
1.688211	1.683776	1.821789	1.816887	1.812159	1.807586	1.803078	1.798635	1.794253
1.689105	1.684711	1.764518	1.759796	1.813273	1.808668	1.804114	1.799602	1.795157
1.68999	1.685675	1.765684	1.760999	1.814466	1.809828	1.805204	1.800577	1.796028
1.690948	1.686603	1.682307	1.678071	1.815465	1.81079	1.806142	1.801508	1.79695
1.691963	1.687504	1.683137	1.678848	1.816316	1.811601	1.806962	1.802401	1.79791
1.692929	1.688428	1.684035	1.679723	1.759226	1.754631	1.807876	1.803319	1.798833
1.693813	1.689376	1.685024	1.68074	1.760379	1.755762	1.808927	1.804267	1.799693
1.694738	1.690357	1.686028	1.68175	1.761505	1.756858	1.75223	1.747601	1.800596
1.695799	1.691413	1.687034	1.682682	1.762478	1.757774	1.753137	1.748562	1.801603
1.696867	1.692457	1.688022	1.6836	1.763442	1.758686	1.754053	1.749554	1.802657
1.697776	1.69334	1.688924	1.684541	1.764537	1.759782	1.755149	1.750653	1.746234
1.698613	1.694147	1.689788	1.685499	1.765706	1.760981	1.756347	1.751811	1.747351
1.699576	1.695111	1.690773	1.686515	1.682262	1.678031	1.757522	1.752901	1.748362
1.700654	1.696216	1.691874	1.687592	1.683336	1.67911	1.758691	1.753931	1.749267
1.536378	1.532464	1.692945	1.688606	1.684321	1.680083	1.759765	1.754943	1.750222
1.537393	1.533482	1.693965	1.689509	1.685151	1.68087	1.76067	1.755917	1.751257
1.538348	1.534435	1.694938	1.690403	1.685994	1.681684	1.761616	1.756939	1.752344
1.539134	1.535213	1.695811	1.69133	1.68696	1.682679	1.762765	1.758094	1.753494
1.539875	1.535947	1.696673	1.692288	1.687982	1.683748	1.763994	1.759306	1.754678
1.540751	1.536839	1.697682	1.693325	1.689034	1.684806	1.680582	1.676351	1.75584
1.541712	1.537829	1.698792	1.694422	1.690118	1.685873	1.681636	1.6774	1.756993
1.450979	1.44742	1.534946	1.531095	1.691199	1.686931	1.682678	1.678438	1.758136
1.451852	1.448289	1.535927	1.532076	1.69228	1.687985	1.683714	1.679476	1.759282

1.452725	1.449154	1.536901	1.533047	1.693335	1.688994	1.684689	1.680434	1.676164
1.453595	1.450015	1.537871	1.534017	1.694359	1.689926	1.685549	1.681239	1.676943
1.454459	1.450865	1.538817	1.534948	1.695345	1.690838	1.686404	1.682051	1.677741
1.455324	1.451707	1.539712	1.535784	1.611116	1.606883	1.687348	1.68302	1.678729
1.456175	1.452527	1.540572	1.536573	1.611934	1.607772	1.688342	1.684066	1.679808
1.456971	1.453269	1.5414	1.537403	1.612826	1.608714	1.68938	1.685121	1.680869
1.457722	1.453947	1.542195	1.538257	1.613773	1.609702	1.690462	1.686197	1.681932
1.458473	1.454678	1.451011	1.447424	1.535316	1.531538	1.606665	1.602652	1.68299
1.459216	1.455474	1.451838	1.448274	1.536273	1.532498	1.607662	1.603639	1.684043
1.460003	1.456308	1.452691	1.449136	1.537233	1.533454	1.60865	1.604622	1.685098
1.460876	1.457184	1.45356	1.449993	1.538186	1.534398	1.609624	1.605605	1.686161
1.461784	1.458083	1.454439	1.450849	1.539133	1.535336	1.610589	1.606583	1.687225
1.462686	1.458976	1.455311	1.451694	1.54007	1.536264	1.611548	1.607552	1.688277
1.46359	1.459872	1.456182	1.452535	1.541	1.537186	1.612502	1.608515	1.689322
1.464473	1.460764	1.45706	1.453389	1.449797	1.446274	1.534343	1.530616	1.60549
1.465334	1.461652	1.457944	1.454253	1.450649	1.447114	1.53528	1.531547	1.606442
1.362442	1.35911	1.458831	1.455134	1.451517	1.447966	1.536222	1.532478	1.60739
1.363245	1.359881	1.459714	1.45603	1.452404	1.448828	1.537167	1.53341	1.608337
1.364082	1.360682	1.460619	1.456943	1.453304	1.449701	1.538118	1.534341	1.609278
1.364979	1.361545	1.461554	1.457855	1.454189	1.450557	1.539049	1.535252	1.531487
1.365894	1.362443	1.462531	1.458805	1.455111	1.45145	1.540013	1.536184	1.532397
1.366763	1.363363	1.359976	1.356596	1.456188	1.452497	1.541121	1.537215	1.533363
1.367603	1.364299	1.360977	1.357641	1.457366	1.453643	1.542326	1.538317	1.534374
1.368407	1.36516	1.361876	1.358564	1.458406	1.454667	1.450938	1.447203	1.535448
1.369174	1.365945	1.36267	1.359362	1.459305	1.455563	1.451858	1.448184	1.5366
1.116758	1.11415	1.363442	1.360141	1.356785	1.353382	1.452766	1.449131	1.537693
1.117464	1.11486	1.364212	1.360928	1.357585	1.354192	1.453676	1.450024	1.538669
1.118167	1.115563	1.364967	1.361703	1.358378	1.355002	1.454585	1.4509	1.539613
0.97152	0.96919	1.365705	1.362461	1.359159	1.355809	1.352453	1.349076	1.540569
0.972179	0.969842	1.366433	1.363211	1.359935	1.356615	1.353264	1.349861	1.541529
0.972832	0.97049	1.147787	1.144802	1.360713	1.357421	1.354076	1.350657	1.449867
0.973483	0.971136	1.148607	1.145616	1.361495	1.358226	1.354891	1.351463	1.450764
0.802456	0.800517	1.149508	1.146516	1.36235	1.3591	1.35578	1.352362	1.348971
0.803105	0.801159	1.15052	1.147534	1.36331	1.360077	1.356777	1.353393	1.350018
0.803732	0.801778	1.151493	1.148511	1.145545	1.142595	1.357712	1.35436	1.351005
0.804282	0.802319	1.152345	1.149363	1.146393	1.143436	1.358483	1.355155	1.351817
0.804809	0.802835	1.15316	1.150176	1.147201	1.144239	1.359207	1.355898	1.352576
0.805334	0.803349	1.020842	1.018155	1.148015	1.145052	1.359958	1.356668	1.353359
0.805863	0.803867	1.021558	1.018867	1.148836	1.145874	1.360727	1.357453	1.354156
0.806472	0.804463	0.802511	0.800596	1.149749	1.14679	1.143842	1.14091	1.355054
0.807156	0.805133	0.803165	0.801236	1.150751	1.147795	1.14485	1.14192	1.35605
0.80779	0.805756	0.803775	0.801832	1.018649	1.016001	1.145794	1.142864	1.356961
0.808343	0.806305	0.804312	0.802356	1.019363	1.016709	1.146621	1.143692	1.357723
0.579457	0.577978	0.804842	0.80288	1.020087	1.017433	1.147471	1.14454	1.141609
0.579884	0.578406	0.805387	0.803441	1.020893	1.018263	1.148446	1.145513	1.142582
0.580326	0.578853	0.805948	0.804019	1.021714	1.019108	1.149439	1.146505	1.143572
0.58084	0.57937	0.806569	0.804609	1.022474	1.019859	1.150314	1.147375	1.144442
0.581397	0.57993	0.807225	0.805207	1.0232	1.020557	1.151123	1.148181	1.145247
0.581891	0.580428	0.807824	0.805773	1.023938	1.021291	1.018658	1.01604	1.14614
0.582316	0.580859	0.808356	0.806297	1.024677	1.02206	1.019455	1.016868	1.147128

0.582769	0.581319	0.579825	0.578327	0.804909	0.803011	1.020246	1.017664	1.148066
0.583293	0.581855	0.580374	0.578881	0.805568	0.803618	1.021003	1.01838	1.148886
0.583805	0.58238	0.580918	0.57944	0.80624	0.804245	1.02178	1.01911	1.149715
0.584225	0.582814	0.581381	0.579935	0.806879	0.804895	1.022638	1.019944	1.017275
0.584621	0.583224	0.581819	0.580408	0.807503	0.805547	1.023517	1.020807	1.018127
0.585094	0.583708	0.582308	0.580898	0.579469	0.578025	1.024296	1.021568	1.018882
0.585621	0.584242	0.582831	0.581397	0.579938	0.578456	1.025007	1.022261	1.019576
0.586125	0.584763	0.583352	0.58191	0.580439	0.578942	1.025813	1.023055	1.020359
0.586607	0.585275	0.583879	0.582446	0.580985	0.579498	1.026744	1.023975	1.021251
0.587067	0.58575	0.584367	0.582946	0.581503	0.580036	0.578584	0.57714	1.022152
0.587487	0.586152	0.584768	0.583361	0.581943	0.580512	0.579083	0.577652	1.023046
0.587911	0.58655	0.585165	0.583772	0.582376	0.580976	0.579569	0.578147	1.02391
0.5884	0.587024	0.585646	0.584266	0.582873	0.581469	0.580048	0.578602	1.02467
0.588919	0.587535	0.586166	0.5848	0.5834	0.581974	0.580526	0.579041	1.025386
0.589417	0.588036	0.586675	0.585318	0.58392	0.582491	0.581037	0.579543	0.868447
0.589901	0.588535	0.587181	0.585828	0.584439	0.58302	0.581576	0.580097	0.869292
0.590369	0.589	0.587651	0.586309	0.584936	0.583539	0.582113	0.580648	0.579186
0.590806	0.589408	0.588065	0.586747	0.585406	0.58405	0.582648	0.581191	0.579727
0.591271	0.589842	0.588492	0.587184	0.585864	0.584543	0.583167	0.581725	0.580269
0.591805	0.590356	0.588974	0.587634	0.58631	0.585005	0.583653	0.582249	0.580818
0.592368	0.590901	0.589481	0.588098	0.586757	0.585455	0.584122	0.582758	0.581356
0.59292	0.591432	0.589992	0.588589	0.587231	0.585913	0.584579	0.583232	0.581852
0.593472	0.591963	0.590514	0.589101	0.587722	0.586376	0.585028	0.583684	0.582319
0.594015	0.592508	0.59105	0.589622	0.588227	0.586862	0.585502	0.584149	0.582786
0.594552	0.593068	0.591602	0.590152	0.588745	0.587374	0.586001	0.584625	0.583247
0.595074	0.593611	0.592143	0.590682	0.589267	0.587887	0.586504	0.585116	0.583729
0.595566	0.594116	0.59266	0.59121	0.589791	0.588396	0.587008	0.58563	0.584247
0.596065	0.594624	0.59318	0.59174	0.590316	0.588906	0.587514	0.586148	0.58477
0.596607	0.595172	0.593726	0.592278	0.590842	0.589415	0.588015	0.586652	0.585279
0.597164	0.595729	0.594275	0.592816	0.591367	0.589926	0.588519	0.587154	0.585784
0.597695	0.596248	0.59479	0.59333	0.591883	0.590446	0.589038	0.587665	0.58629
0.59821	0.596742	0.595282	0.593829	0.592394	0.590974	0.589568	0.58818	0.586794
0.598733	0.597258	0.595801	0.594355	0.592925	0.591508	0.590099	0.588698	0.587303
0.599265	0.5978	0.596353	0.594914	0.593479	0.592048	0.590627	0.589215	0.587815
0.599797	0.598345	0.596909	0.595478	0.594039	0.592597	0.591164	0.589742	0.588335
0.600327	0.598887	0.597462	0.596041	0.594604	0.593156	0.591716	0.590283	0.588865
0.600859	0.599428	0.598013	0.596602	0.595169	0.59372	0.592275	0.590832	0.589404
		0.598561	0.597157	0.59573	0.594285	0.59284	0.591389	0.589952
		0.599105	0.597707	0.596287	0.594851	0.593407	0.591952	0.590507
		0.599646	0.598253	0.596838	0.59541	0.593969	0.592509	0.591062
		0.600183	0.598793	0.597384	0.595963	0.594526	0.593062	0.591617
				0.597927	0.596513	0.595079	0.593614	0.592173
				0.598463	0.597056	0.595628	0.594168	0.592728
				0.599011	0.597601	0.596173	0.594719	0.593284
				0.59959	0.598153	0.596714	0.595265	0.593835
				0.600174	0.598706	0.597253	0.595812	0.594392
						0.597796	0.596378	0.594978
						0.598343	0.596953	0.595578
						0.598886	0.597504	0.596137
						0.599423	0.598028	0.596655

0.599991	0.59858	0.597194
0.600605	0.599183	0.597777
0.601228	0.599788	0.598359
		0.5989
		0.599434
		0.600012
		0.600619
		0.601204
		0.601759
		0.602334
		0.602953
		0.603586

						0.598374	0.596446	0.594554
						0.597748	0.595803	0.593887
						0.597075	0.595134	0.593214
						0.596341	0.594429	0.592529
				0.599366	0.597487	1.486536	1.482461	1.478385
				0.598709	0.596815	1.485021	1.48092	1.476818
				0.598062	0.596155	1.483493	1.479368	1.475246
				0.597353	0.595458	1.481837	1.4777	1.47357
				0.596622	0.594752	1.480126	1.475985	1.471857
				0.595929	0.594085	1.570554	1.566186	1.561848
				0.595267	0.593451	1.568861	1.564529	1.560235
		0.598399	0.596486	1.48362	1.479481	1.567198	1.562886	1.55862
		0.597732	0.595855	1.482043	1.477935	1.565576	1.561252	1.556984
		0.597069	0.595216	1.572567	1.568255	1.667768	1.663347	1.658982
		0.596417	0.594546	1.570928	1.566592	1.666156	1.66178	1.657459
		0.595765	0.593868	1.569296	1.564929	1.664553	1.660226	1.655954
		1.2149	1.211504	1.567637	1.563291	1.662975	1.658683	1.654442
		1.213594	1.210186	1.565968	1.561669	1.661417	1.65715	1.652931
		1.333869	1.330156	1.668548	1.664187	1.659878	1.655633	1.651429
		1.332413	1.328737	1.666989	1.66265	1.65836	1.654134	1.649937
1.216456	1.213031	1.569667	1.565392	1.665419	1.661114	1.656846	1.652627	1.648424
1.215118	1.211721	1.56802	1.563713	1.663822	1.659571	1.655332	1.6511	1.646868
1.213795	1.210415	1.566378	1.562048	1.662235	1.658029	1.653817	1.649576	1.645323
1.334037	1.330326	1.669308	1.664963	1.660689	1.656493	1.89793	1.892998	1.888065
1.332632	1.328882	1.667723	1.663417	1.65916	1.654959	1.896117	1.891183	1.886256
1.331255	1.327496	1.666187	1.661868	1.657611	1.653423	1.894279	1.889363	1.884456
1.329898	1.326154	1.664688	1.660322	1.656053	1.651888	1.892425	1.887541	1.882664
1.462638	1.458371	1.663186	1.658823	1.654551	1.650372	1.890589	1.885712	1.880848
1.461012	1.456836	1.661682	1.657382	1.653113	1.648873	1.888763	1.883867	1.879003
1.668801	1.664454	1.660171	1.655937	1.821832	1.817032	1.88699	1.88206	1.877171
1.667203	1.662875	1.658638	1.654464	1.820156	1.815429	1.885318	1.880321	1.875357
1.665637	1.661323	1.657115	1.65298	1.818462	1.813817	1.883652	1.878605	1.873577
1.66416	1.659858	1.655637	1.651477	1.891689	1.886814	1.944557	1.939361	1.934187
1.662726	1.658438	1.654187	1.649973	1.889871	1.884991	1.942689	1.937584	1.932475
1.661228	1.65699	1.652755	1.648539	1.888136	1.883206	1.9408	1.93574	1.930682
1.659682	1.655524	1.651343	1.647165	1.886473	1.881449	1.938888	1.933839	1.928825
1.658169	1.654044	1.649901	1.64576	1.947468	1.942221	1.937044	1.931974	1.92696
1.656698	1.652537	1.648411	1.644312	1.945633	1.940455	1.935297	1.930155	1.925073
1.655256	1.651062	1.81618	1.811562	1.943778	1.938676	1.933553	1.928368	1.923237
1.653839	1.649664	1.814556	1.809883	1.941884	1.936833	1.931759	1.926618	1.921512

1.652448	1.648299	1.812963	1.808225	1.940001	1.934984	1.929959	1.924892	1.919837
1.651098	1.646945	1.948587	1.943336	1.938206	1.933194	1.928193	1.923181	1.918173
1.649768	1.645597	1.946866	1.941607	1.936466	1.931435	1.92644	1.921479	1.916522
1.648346	1.644178	1.945098	1.939859	1.934712	1.929645	1.924642	1.919723	1.914816
1.646831	1.642687	1.943283	1.938095	1.932945	1.927822	1.922794	1.917912	1.913057
1.645355	1.641224	1.941494	1.936354	1.931213	1.926058	1.921014	1.916146	1.911319
1.643959	1.63982	1.939754	1.93465	1.929531	1.924387	1.919346	1.914465	1.909633
1.642576	1.638426	1.938023	1.932956	1.927865	1.92274	1.9177	1.912789	1.907933
1.877441	1.872643	1.936289	1.931271	1.926203	1.92108	1.916021	1.911052	1.906152
1.875768	1.870954	1.934543	1.929578	1.924537	1.91942	1.914342	1.909311	1.904359
1.874064	1.869223	1.932736	1.927816	1.922819	1.917748	1.912692	1.90764	1.902663
1.87234	1.867465	1.93089	1.926009	1.921066	1.916071	1.911063	1.906018	1.901031
1.939221	1.934096	1.92913	1.924271	1.919359	1.914406	1.909426	1.904387	1.899393
1.93751	1.93242	1.927467	1.922609	1.917701	1.912755	1.90778	1.902747	1.897745
1.935816	1.93076	1.92582	1.920964	1.916055	1.911105	1.906129	1.901105	1.896105
1.934127	1.92909	1.924167	1.919324	1.914415	1.909449	1.904468	1.899462	1.894482
1.932453	1.927429	1.922519	1.917687	1.912776	1.907793	1.902809	1.897819	1.892863
1.930804	1.925781	1.920871	1.916038	1.911125	1.90614	1.901158	1.896174	1.891232
1.929169	1.92414	1.919224	1.914383	1.909469	1.904492	1.899516	1.894536	1.889604
1.927528	1.922495	1.917573	1.912724	1.907816	1.902857	2.028676	2.023079	2.017549
1.925884	1.920847	1.915918	1.91106	1.906164	1.901235	2.02693	2.021353	2.015839
1.924247	1.919219	1.914281	1.909407	1.904517	1.899616	2.025185	2.019638	2.014145
1.922609	1.917607	1.912657	1.907753	1.902864	1.897995	2.023441	2.017934	2.012471
1.921003	1.916032	1.911077	1.90615	1.901254	1.896393	2.021699	2.016228	2.010798
1.919453	1.914517	1.909578	1.90465	1.899732	1.894824	2.019952	2.014504	2.009111
1.917933	1.913026	1.908103	1.903177	1.898234	1.89327	2.018216	2.012783	2.007425
1.916433	1.911526	1.906593	1.901647	2.027639	2.022038	2.016515	2.011088	2.00575
1.914947	1.910022	1.905064	1.900088	2.025918	2.020339	2.014834	2.00941	2.004085
1.91343	1.908478	1.9035	1.898509	2.024183	2.01862	2.013137	2.007738	2.002439
1.911889	1.906896	1.901901	1.896907	2.022431	2.016881	2.011422	2.006071	2.00081
1.910318	1.905302	1.965355	1.959956	2.020688	2.015167	2.009743	2.004439	1.99921
1.908709	1.90369	1.963644	1.958248	2.018948	2.013487	2.008115	2.002851	1.997651
1.907096	1.902089	1.96196	1.95657	2.01724	2.011837	2.006514	2.001283	1.996105
1.905501	1.900518	1.96033	1.954942	2.015583	2.010221	2.11317	2.106897	2.100774
1.903924	1.898976	1.958741	1.953363	2.013973	2.008637	2.111265	2.10506	2.098978
1.967833	1.962541	2.023411	2.017886	2.012451	2.0071	2.109396	2.103194	2.09711
1.966232	1.960986	2.02188	2.016417	2.01099	2.005596	2.107557	2.101314	2.095199
1.96468	1.959423	2.020278	2.014829	2.118648	2.112057	2.142779	2.136446	2.130247
1.963194	1.957853	2.018598	2.013108	2.116479	2.109949	2.140711	2.134472	2.128348
1.961655	1.956267	2.016916	2.011399	2.114345	2.107881	2.138683	2.132541	2.1265
1.959999	1.954647	2.01524	2.009721	2.112289	2.10589	2.136712	2.130673	2.124731
1.958326	1.953033	2.013597	2.008102	2.110341	2.104003	2.134827	2.128859	2.122983
1.9567	1.951461	2.012051	2.006634	2.108645	2.102347	2.133131	2.127134	2.121214
1.955103	1.949909	2.010543	2.005221	2.107043	2.100782	2.131511	2.125441	2.119431
2.019684	2.014295	2.11788	2.111482	2.142249	2.135924	2.129708	2.12363	2.117594
2.018131	2.012687	2.115843	2.109408	2.140165	2.133923	2.127772	2.121729	2.115711
2.016534	2.011059	2.113815	2.107382	2.13813	2.131951	2.168256	2.162127	2.156022
2.01486	2.009388	2.11177	2.105397	2.136136	2.13	2.166295	2.160244	2.154223
2.013249	2.007788	2.109849	2.10354	2.134266	2.128156	2.164421	2.158404	2.152423
2.011809	2.006366	2.1082	2.101945	2.132647	2.12654	2.162739	2.15665	2.150596

2.010386	2.004964	2.106589	2.100388	2.131067	2.124954	2.161079	2.154896	2.148751
2.008787	2.003386	2.104715	2.09858	2.129255	2.123142	2.187696	2.181094	2.174574
2.007117	2.001735	2.102736	2.096669	2.127348	2.121237	2.185663	2.179076	2.17259
2.113573	2.10717	2.13789	2.131744	2.125611	2.119505	2.183812	2.177237	2.170762
2.111951	2.105588	2.136278	2.13015	2.12403	2.117932	2.18213	2.17557	2.169089
2.110229	2.103913	2.134574	2.128453	2.193508	2.186898	2.219587	2.212932	2.206347
2.1083	2.102043	2.132682	2.126554	2.191454	2.184863	2.217527	2.210878	2.204306
2.106418	2.10022	2.130836	2.124698	2.189451	2.182874	2.215514	2.208885	2.202341
2.104777	2.09863	2.129221	2.123083	2.187713	2.181138	2.213756	2.207176	2.200675
2.103182	2.097083	2.127647	2.121512	2.186029	2.179455	2.212055	2.205542	2.199104
2.138192	2.132018	2.125847	2.119711	2.184111	2.177554	2.210156	2.203737	2.197401
2.136219	2.130083	2.123924	2.117784	2.182066	2.175536	2.208152	2.201841	2.195628
2.134447	2.12833	2.153305	2.146755	2.180211	2.173698	2.246991	2.240483	2.234096
2.132886	2.12677	2.151645	2.145112	2.178555	2.172046	2.245213	2.238639	2.232187
2.131343	2.125221	2.15001	2.143498	2.176929	2.170432	2.243483	2.236857	2.230353
2.129809	2.123677	2.1484	2.141911	2.175337	2.168864	2.241819	2.235191	2.228683
2.128202	2.122059	2.146721	2.140256	2.173679	2.167235	2.240106	2.233491	2.226995
2.126361	2.120208	2.184764	2.178244	2.210772	2.204263	2.238163	2.231571	2.225096
2.124443	2.118284	2.182728	2.176233	2.208752	2.202284	2.23615	2.229588	2.223144
2.122753	2.116595	2.18093	2.174449	2.206949	2.200511	2.279074	2.272061	2.265194
2.121227	2.115078	2.179306	2.172827	2.205306	2.198888	2.277259	2.270291	2.263469
2.150804	2.144294	2.177709	2.171239	2.244242	2.237592	2.275479	2.268548	2.26177
2.149225	2.142739	2.176144	2.169691	2.2426	2.235954	2.273738	2.266836	2.260097
2.14763	2.141169	2.174565	2.168131	2.240951	2.23431	2.271998	2.265127	2.258429
2.146011	2.139587	2.172975	2.166563	2.239299	2.232671	2.270266	2.263429	2.256773
2.144333	2.137935	2.171312	2.164924	2.237579	2.230971	2.268481	2.261688	2.255081
2.142497	2.136077	2.169424	2.16307	2.235645	2.229068	2.266513	2.259785	2.253243
2.140584	2.13412	2.16743	2.161112	2.233608	2.22707	2.264462	2.257812	2.251347
2.178607	2.172061	2.244979	2.23834	2.231772	2.225272	2.262594	2.256019	2.249629
2.176927	2.17043	2.243278	2.236646	2.230103	2.223641	2.260881	2.254381	2.248068
2.175299	2.168847	2.24162	2.234993	2.22847	2.22204	2.259205	2.252778	2.246539
2.173727	2.167311	2.239996	2.233369	2.226857	2.220445	2.257544	2.251193	2.245031
2.172172	2.165786	2.238386	2.231766	2.225273	2.21889	2.255932	2.249647	2.243551
2.170607	2.164247	2.236769	2.230173	2.22372	2.217395	2.254391	2.248153	2.2421
2.169041	2.162702	2.23515	2.228586	2.222184	2.215929	2.252888	2.246689	2.240671
2.167464	2.161143	2.233514	2.226984	2.220633	2.214449	2.31253	2.306259	2.300159
2.165882	2.159575	2.231866	2.225374	2.219078	2.212968	2.310978	2.304769	2.298714
2.243435	2.236788	2.230231	2.223776	2.217535	2.211501	2.309438	2.303285	2.297268
2.24176	2.235136	2.228607	2.222187	2.216002	2.210046	2.307911	2.301806	2.295821
2.240092	2.233491	2.226994	2.220616	2.214487	2.208603	2.331426	2.325318	2.31931
2.238436	2.23185	2.22539	2.219065	2.212994	2.207175	2.329892	2.32383	2.317853
2.236786	2.230218	2.223802	2.217535	2.211519	2.205757	2.32836	2.322337	2.316385
2.235143	2.228606	2.222238	2.216029	2.210064	2.204347	2.326826	2.320821	2.314882
2.233501	2.227002	2.220688	2.214543	2.208626	2.202944	2.325291	2.319295	2.313362
2.231845	2.225386	2.219133	2.213061	2.207195	2.201544	2.323759	2.317768	2.311839
2.230178	2.223762	2.217578	2.211588	2.205774	2.200149	2.322229	2.316238	2.310312
2.228524	2.22214	2.216016	2.210107	2.269349	2.263608	2.320707	2.314718	2.308794
2.226861	2.220493	2.214429	2.208608	2.267865	2.262175	2.319198	2.313211	2.307286
2.225324	2.21896	2.212923	2.207147	2.266391	2.26074	2.317681	2.311698	2.305777
2.22403	2.217631	2.211555	2.205736	2.264919	2.259293	2.316147	2.310169	2.304258

2.222793	2.21638	2.210262	2.204383	2.263484	2.257856	2.314609	2.308638	2.302743
2.221446	2.215128	2.209028	2.203117	2.288597	2.282848	2.313058	2.307103	2.301242
2.220043	2.213877	2.207831	2.201912	2.287273	2.281433	2.311517	2.30558	2.299757
2.218561	2.212514	2.20656	2.200713	2.286005	2.280107	2.329433	2.323456	2.317608
2.217001	2.211052	2.20523	2.199528	2.284797	2.278866	2.328061	2.322039	2.316157
2.215451	2.209585	2.268737	2.262985	2.283525	2.277619	2.326751	2.320731	2.314841
2.213903	2.208111	2.267267	2.26157	2.282162	2.276371	2.325541	2.319588	2.313727
2.212427	2.206676	2.265804	2.260135	2.280759	2.275069	2.324272	2.318397	2.312584
2.211073	2.205295	2.264352	2.258689	2.279303	2.273645	2.322819	2.317013	2.311271
2.209811	2.203993	2.26296	2.257277	2.27785	2.27219	2.321306	2.315558	2.309887
2.208667	2.202869	2.261758	2.25601	2.276501	2.270806	2.319835	2.314104	2.308464
2.2076	2.201857	2.260679	2.25484	2.275224	2.269474	2.318395	2.31265	2.307011
2.234844	2.229105	2.285733	2.279836	2.274021	2.268278	2.317127	2.311345	2.305667
2.233402	2.227749	2.284422	2.278638	2.272911	2.267239	2.316049	2.310195	2.304428
2.231986	2.226376	2.283054	2.27735	2.271695	2.266091	2.346046	2.340096	2.33421
2.23062	2.224978	2.281586	2.275894	2.270276	2.264733	2.344691	2.338838	2.333036
2.229326	2.223646	2.280166	2.274455	2.268845	2.263336	2.343276	2.337525	2.33182
2.264417	2.258634	2.278957	2.273179	2.267518	2.261971	2.341801	2.336082	2.330421
2.263309	2.257561	2.277828	2.271976	2.266252	2.260644	2.340333	2.334616	2.328975
2.262089	2.256336	2.276596	2.27078	2.318735	2.313001	2.339076	2.333364	2.327721
2.260801	2.25502	2.275307	2.269604	2.317593	2.311921	2.33797	2.33226	2.326592
2.285666	2.279837	2.274085	2.268415	2.316382	2.310727	2.33674	2.33103	2.325361
2.284513	2.278729	2.272953	2.267214	2.315083	2.309386	2.335351	2.32964	2.324003
2.283311	2.277555	2.306079	2.300212	2.313804	2.308093	2.334011	2.328288	2.322658
2.281966	2.27619	2.304724	2.298951	2.312601	2.306954	2.332833	2.327062	2.321371
2.280627	2.27483	2.303371	2.29769	2.311398	2.305814	2.331657	2.325855	2.320113
2.27945	2.27368	2.302165	2.296443	2.310113	2.304513	2.362742	2.356954	2.351209
2.278335	2.27261	2.301017	2.295206	2.308804	2.303156	2.361361	2.35565	2.349991
2.277098	2.271387	2.299769	2.293981	2.307582	2.301944	2.360111	2.354405	2.34875
2.275764	2.270039	2.298442	2.292767	2.306431	2.300848	2.358967	2.353206	2.347489
2.308717	2.302899	2.2972	2.291597	2.340166	2.334563	2.357797	2.352008	2.346261
2.307539	2.301768	2.296095	2.290502	2.339055	2.333406	2.356579	2.350802	2.345073
2.306379	2.300631	2.294962	2.28936	2.337901	2.33223	2.355366	2.349612	2.343908
2.305205	2.299405	2.293696	2.288063	2.336627	2.331015	2.354178	2.348455	2.342769
2.304019	2.298158	2.292406	2.286743	2.335338	2.329806	2.353006	2.347311	2.341636
2.302789	2.296938	2.328108	2.322419	2.33418	2.328689	2.351872	2.346164	2.340474
2.301535	2.295732	2.326938	2.321298	2.333087	2.327617	2.350753	2.345013	2.339296
2.300317	2.294543	2.325763	2.320122	2.331901	2.32642	2.349524	2.343806	2.338117
2.299131	2.293374	2.324585	2.318893	2.33062	2.325093	2.348178	2.342538	2.336933
2.335009	2.329184	2.323412	2.3177	2.329401	2.323855	2.346919	2.341328	2.335773
2.333866	2.327999	2.322236	2.316561	2.328287	2.322776	2.345815	2.340221	2.334656
2.332746	2.326844	2.321095	2.315458	2.32721	2.321737	2.344747	2.33913	2.333536
2.331669	2.325785	2.320038	2.314393	2.326142	2.320685	2.343665	2.338004	2.332377
2.330594	2.324753	2.319012	2.313354	2.32509	2.31963	2.342572	2.336866	2.331209
2.329428	2.323635	2.317935	2.312312	2.324036	2.318527	2.341409	2.335705	2.330066
2.328199	2.322465	2.31683	2.311274	2.322987	2.317394	2.3402	2.33453	2.328943
2.327024	2.321345	2.315757	2.31024	2.321953	2.316326	2.310777	2.305323	2.29997
2.325907	2.320281	2.314722	2.309221	2.320947	2.315341	2.309806	2.304352	2.298999
2.324818	2.319229	2.313673	2.308156	2.319884	2.314307	2.308798	2.303362	2.298025
2.323754	2.318176	2.312577	2.30699	2.318694	2.313146	2.307679	2.302302	2.29702

2.322712	2.317136	2.31149	2.305831	2.317507	2.311982	2.306555	2.301238	2.29601
2.321686	2.316116	2.310461	2.304784	2.316446	2.31092	2.305502	2.300213	2.295002
2.320674	2.315112	2.309464	2.303794	2.315447	2.309911	2.304488	2.29921	2.294001
2.319671	2.314115	2.308472	2.302804	2.314453	2.308918	2.303499	2.298234	2.293032
2.318678	2.313127	2.307489	2.301823	2.313472	2.307947	2.302539	2.297282	2.292091
2.317662	2.312118	2.306485	2.300826	2.312478	2.306967	2.301572	2.29633	2.291151
2.316615	2.31108	2.305456	2.299805	2.311465	2.305972	2.300597	2.295373	2.290211
2.315562	2.310034	2.304419	2.298778	2.310446	2.304974	2.317555	2.3123	2.307104
2.314505	2.308987	2.30338	2.297748	2.309423	2.303972	2.316558	2.31133	2.306157
2.313451	2.307942	2.302343	2.296719	2.3084	2.30297	2.31556	2.310359	2.30521
2.312419	2.306915	2.301321	2.295702	2.307387	2.301974	2.314564	2.309383	2.304251
2.311399	2.305899	2.300308	2.294692	2.306378	2.300981	2.313569	2.308403	2.303285
2.310371	2.304877	2.29929	2.29368	2.305368	2.299986	2.330788	2.325595	2.320448
2.309334	2.303847	2.298267	2.292662	2.304354	2.298988	2.329809	2.324632	2.319501
2.308303	2.30283	2.297265	2.291675	2.321293	2.315877	2.328847	2.323684	2.318566
2.307272	2.301834	2.296293	2.290722	2.320321	2.314899	2.327901	2.322753	2.317649
2.30626	2.300844	2.295336	2.289804	2.319393	2.313964	2.326989	2.321842	2.316739
2.305291	2.299857	2.294398	2.28895	2.336991	2.331506	2.326145	2.320963	2.31583
2.304338	2.298869	2.29346	2.288108	2.336194	2.330708	2.325327	2.320098	2.314927
2.320579	2.315042	2.329149	2.323779	2.335299	2.329835	2.32446	2.31921	2.31403
2.319557	2.313995	2.328139	2.322805	2.334344	2.328919	2.32357	2.318315	2.313143
2.338296	2.332659	2.327197	2.321856	2.333404	2.328043	2.322736	2.317473	2.312291
2.337515	2.331839	2.326326	2.32093	2.332477	2.327218	2.321973	2.316699	2.311486
2.336707	2.331034	2.342434	2.336926	2.331567	2.326366	2.32116	2.315885	2.310658
2.335817	2.330234	2.341622	2.336073	2.330683	2.325456	2.320239	2.314976	2.309766
2.334905	2.329408	2.340795	2.335227	2.329807	2.324532	2.319295	2.31406	2.308886
2.333986	2.328486	2.33987	2.334329	2.32891	2.323608	2.318377	2.31321	2.308101
2.33307	2.327526	2.338912	2.333423	2.32802	2.322696	2.317475	2.312383	2.307345
2.316007	2.310468	2.322729	2.317304	2.3272	2.32186	2.316625	2.311522	2.306488
2.315221	2.309656	2.321929	2.316542	2.326439	2.321088	2.31582	2.310638	2.305556
2.314362	2.308798	2.321074	2.315707	2.325622	2.32031	2.315052	2.309826	2.304705
2.313392	2.307863	2.320131	2.314764	2.324725	2.319528	2.314343	2.309116	2.303964
2.31246	2.306971	2.319227	2.313853	2.323837	2.31871	2.31358	2.308384	2.303238
2.311646	2.306192	2.318433	2.313048	2.322995	2.317808	2.312671	2.307566	2.302512
2.310873	2.305447	2.317673	2.312281	2.322177	2.3169	2.311736	2.306719	2.301763
2.327809	2.322322	2.316885	2.311504	2.321384	2.316077	2.310893	2.305868	2.300925
2.32701	2.321516	2.316092	2.310733	2.320611	2.315302	2.310099	2.305015	2.30004
2.326213	2.320729	2.315314	2.309964	2.31984	2.314532	2.309321	2.304213	2.29922
2.325416	2.319958	2.314549	2.309198	2.319072	2.313773	2.308567	2.30346	2.298456
2.324631	2.319182	2.313779	2.308429	2.318299	2.313006	2.307806	2.302711	2.297709
2.323867	2.318398	2.312998	2.307657	2.317517	2.312223	2.307029	2.301958	2.296977
2.323095	2.317613	2.312216	2.306883	2.316736	2.311444	2.306261	2.301213	2.296256
2.322288	2.31683	2.31144	2.30611	2.315962	2.310686	2.305517	2.300482	2.295537
2.321472	2.316044	2.310664	2.305337	2.315191	2.309936	2.304786	2.299762	2.294828
2.320674	2.315251	2.309876	2.304557	2.314415	2.309181	2.304053	2.299048	2.294137
2.319885	2.31445	2.30908	2.303774	2.313636	2.308427	2.303325	2.298345	2.293463
2.319081	2.313649	2.308287	2.302993	2.312865	2.307687	2.302617	2.297675	2.292824
2.318257	2.312845	2.307496	2.302213	2.312101	2.306958	2.30193	2.297043	2.29223
2.317451	2.312052	2.306715	2.301449	2.311354	2.306252	2.301259	2.296403	2.291605
2.316667	2.311262	2.305939	2.3007	2.31063	2.305582	2.300618	2.295743	2.29092

2.315917	2.310505	2.305194	2.299975	2.309922	2.304916	2.299968	2.295066	2.290217
2.315239	2.309833	2.304521	2.2993	2.309234	2.304222	2.299271	2.294366	2.289517
2.314591	2.309196	2.303879	2.298643	2.308552	2.303515	2.298551	2.293652	2.288816
2.313884	2.308502	2.30318	2.297929	2.307825	2.302788	2.351558	2.346447	2.341409
2.313137	2.30777	2.302442	2.297175	2.307064	2.302045	2.350836	2.345733	2.34071
2.312411	2.307074	2.301761	2.296498	2.306388	2.301377	2.350186	2.345095	2.340084
2.311712	2.306433	2.30116	2.295927	2.305824	2.300811	2.349634	2.34456	2.339558
2.311018	2.305771	2.300534	2.295337	2.305241	2.300225	2.349058	2.343999	2.339006
2.31031	2.305034	2.299814	2.294659	2.304566	2.299545	2.348376	2.34333	2.338349
2.309619	2.304304	2.299093	2.293974	2.303883	2.298864	2.347695	2.342655	2.337681
2.308994	2.30368	2.298473	2.293362	2.303276	2.298281	2.347133	2.342081	2.337097
2.308396	2.303103	2.297898	2.29278	2.302698	2.297739	2.346622	2.34155	2.336545
2.307713	2.302444	2.297251	2.292137	2.302056	2.297114	2.318043	2.313068	2.308156
2.306951	2.301714	2.296539	2.291437	2.301355	2.296416	2.317334	2.312364	2.307461
2.306251	2.301038	2.295881	2.290793	2.30071	2.295774	2.316685	2.311721	2.306823
2.305649	2.300447	2.295304	2.290232	2.30015	2.29522	2.31613	2.311172	2.306275
2.305069	2.299875	2.294747	2.289691	2.299609	2.294682	2.31559	2.310639	2.305745
2.304481	2.299296	2.294182	2.289142	2.299058	2.294128	2.315029	2.310086	2.305201
2.303899	2.298722	2.29362	2.288594	2.298505	2.293572	2.314467	2.309532	2.304657
2.303323	2.298152	2.293057	2.288038	2.297945	2.293014	2.313907	2.30898	2.304112
2.302752	2.297587	2.292495	2.287479	2.29738	2.292455	2.313348	2.308428	2.303567
2.302186	2.297027	2.291942	2.286932	2.296826	2.291901	2.31279	2.307874	2.303018
2.301625	2.296475	2.2914	2.286399	2.296285	2.291353	2.312231	2.307315	2.302463
2.301062	2.295921	2.290855	2.28586	2.295738	2.290803	2.311675	2.306763	2.301916
2.300491	2.295365	2.290302	2.285305	2.295177	2.290247	2.311122	2.30622	2.301382
2.299919	2.294805	2.289747	2.28475	2.294617	2.289694	2.310573	2.305681	2.300852
2.299347	2.294242	2.289194	2.284208	2.294073	2.289157	2.310035	2.305147	2.300322
2.298775	2.293677	2.28864	2.283668	2.293534	2.288624	2.309502	2.304616	2.299793
2.298201	2.293114	2.288085	2.283117	2.292981	2.288082	2.308964	2.304087	2.299271
2.297625	2.292554	2.287529	2.282559	2.292419	2.287532	2.308422	2.303558	2.298754
2.297058	2.29199	2.286969	2.282006	2.291865	2.286989	2.307883	2.303028	2.298232
2.29649	2.291409	2.286399	2.281457	2.291321	2.286451	2.307345	2.302494	2.297702
2.295969	2.290876	2.285863	2.280922	2.29078	2.285918	2.306813	2.301969	2.297185
2.295545	2.290446	2.285396	2.280402	2.290227	2.285371	2.306273	2.30145	2.296682
2.295123	2.290027	2.284948	2.279909	2.289703	2.284852	2.305761	2.300957	2.296207
2.309512	2.304387	2.299289	2.294233	2.28928	2.284426	2.333656	2.328774	2.323939
2.308926	2.30383	2.298795	2.293818	2.288903	2.284046	2.333289	2.32841	2.32358
2.292604	2.287565	2.298273	2.293325	2.288433	2.283595	2.332861	2.327978	2.323144
2.292013	2.286999	2.297724	2.292765	2.287886	2.283086	2.332383	2.327487	2.322641
2.291485	2.286479	2.297221	2.292254	2.287383	2.282609	2.331928	2.327027	2.322176
2.291044	2.286024	2.296787	2.291825	2.286959	2.282188	2.331517	2.326622	2.321779
2.290619	2.285582	2.296361	2.291394	2.286526	2.281754	2.331087	2.326202	2.321369
2.290185	2.285141	2.29591	2.290908	2.286021	2.281247	2.330583	2.325711	2.320887
2.289732	2.284689	2.295451	2.290417	2.285514	2.280739	2.330077	2.325218	2.3204
2.289202	2.28418	2.279175	2.274206	2.285092	2.280324	2.275616	2.270963	2.266352
2.288627	2.283637	2.278684	2.273778	2.284721	2.279965	2.275259	2.270597	2.265973
2.288122	2.283146	2.278218	2.273344	2.284333	2.279592	2.274891	2.270223	2.265587
2.287686	2.282706	2.277779	2.27291	2.283936	2.279214	2.274522	2.26985	2.265198
2.287251	2.282263	2.277327	2.272447	2.267647	2.262929	2.274101	2.269434	2.264783
2.286803	2.28181	2.27685	2.271935	2.26711	2.262377	2.273583	2.26894	2.264319

2.286348	2.281342	2.276363	2.271427	2.266585	2.261839	2.273078	2.268456	2.263863
2.285865	2.280822	2.275856	2.27096	2.266144	2.261409	2.293657	2.288932	2.284242
2.285385	2.280304	2.275361	2.270522	2.26574	2.261024	2.29329	2.288555	2.283856
2.284983	2.279918	2.274974	2.270123	2.265337	2.260622	2.29291	2.288176	2.283477
2.284636	2.279625	2.274664	2.269758	2.264942	2.260217	2.292528	2.287802	2.283109
2.28423	2.279231	2.274272	2.269362	2.264542	2.259816	2.296342	2.29153	2.286752
2.283744	2.278702	2.273769	2.268922	2.264138	2.259422	2.295938	2.291116	2.286329
2.283298	2.278236	2.273316	2.2685	2.263732	2.259021	2.295527	2.2907	2.285911
2.282961	2.277938	2.272991	2.26811	2.263312	2.2586	2.295098	2.290287	2.285511
2.282638	2.277667	2.272697	2.267753	2.262917	2.258187	2.294666	2.289871	2.28511
2.28225	2.277308	2.272371	2.267457	2.262605	2.25781	2.294235	2.289438	2.284689
2.281834	2.276911	2.27203	2.267188	2.262333	2.25746	2.293815	2.289007	2.28426
2.281425	2.276508	2.271655	2.26685	2.282539	2.2776	2.317408	2.31241	2.307483
2.281017	2.276094	2.271246	2.266455	2.282179	2.277313	2.317171	2.312091	2.307082
2.280604	2.275685	2.270838	2.266051	2.281796	2.27698	2.31689	2.311788	2.306747
2.280173	2.275279	2.270432	2.265638	2.281386	2.27658	2.316547	2.311511	2.306511
2.279775	2.274882	2.290669	2.285814	2.280981	2.276172	2.31618	2.311211	2.30626
2.279446	2.274486	2.290263	2.285431	2.280597	2.275764	2.315776	2.310838	2.305928
2.279147	2.274116	2.289883	2.285072	2.280236	2.27538	2.315384	2.310452	2.305565
2.278841	2.273821	2.289585	2.284763	2.300786	2.295842	2.3151	2.310118	2.305191
2.278541	2.273578	2.289341	2.284486	2.300503	2.295587	2.314878	2.309814	2.304812
2.299019	2.294023	2.289076	2.284176	2.324744	2.319671	2.31461	2.309531	2.304509
2.298766	2.293783	2.288797	2.28383	2.324372	2.319325	2.3143	2.309268	2.304284
2.298471	2.293509	2.28851	2.283512	2.324045	2.319009	2.314006	2.309016	2.304062
2.298096	2.293174	2.288208	2.283243	2.3238	2.318759	2.313756	2.308787	2.303838
2.297732	2.29284	2.287913	2.282991	2.323571	2.318505	2.313488	2.308535	2.303593
2.297457	2.292559	2.28765	2.282756	2.323329	2.318193	2.313137	2.308199	2.303287
2.297211	2.292286	2.287389	2.282518	2.32308	2.31787	2.31277	2.307844	2.302966
2.296922	2.29196	2.287061	2.282208	2.322799	2.317597	2.312503	2.307581	2.302707
2.296611	2.291601	2.28669	2.28185	2.322496	2.317358	2.312304	2.307379	2.302489
2.296342	2.2913	2.286381	2.281546	2.322228	2.317125	2.312092	2.30716	2.302259
2.296123	2.291066	2.28614	2.281301	2.321999	2.316899	2.311865	2.306926	2.302022
2.29589	2.290843	2.312219	2.307196	2.321779	2.316676	2.311633	2.306669	2.301747
2.295612	2.290619	2.312032	2.306997	2.321562	2.316454	2.311387	2.306363	2.301393
2.295333	2.290396	2.311849	2.3068	2.321349	2.316236	2.311146	2.306064	2.301045
2.295093	2.290174	2.31165	2.306596	2.321136	2.316028	2.310934	2.305832	2.300794
2.294867	2.289948	2.311443	2.306387	2.320923	2.315824	2.310735	2.305632	2.300589
2.294618	2.289706	2.311228	2.306172	2.3207	2.315603	2.310515	2.305412	2.300368
2.29435	2.289448	2.311005	2.30595	2.320467	2.315368	2.31028	2.30518	2.30014
2.294114	2.289197	2.310778	2.305727	2.32024	2.315145	2.310062	2.304967	2.29993
2.293928	2.288958	2.310544	2.305504	2.320019	2.314939	2.309866	2.304774	2.299732
2.254414	2.249531	2.297084	2.292109	2.3198	2.314735	2.309676	2.304603	2.299572
2.254175	2.249281	2.296863	2.291901	2.319585	2.314528	2.309493	2.304478	2.299493
2.253929	2.249035	2.29665	2.291695	2.319369	2.314316	2.309307	2.304353	2.299419
2.253714	2.248821	2.296442	2.291474	2.319129	2.314087	2.309093	2.304162	2.299248
2.253517	2.248632	2.29625	2.291256	2.318886	2.313853	2.308865	2.303935	2.299022
2.253332	2.248484	2.272667	2.267721	2.318713	2.313658	2.308653	2.303708	2.298788
2.253161	2.248381	2.272631	2.267669	2.318611	2.313501	2.30845	2.303474	2.298539
2.282436	2.277506	2.294085	2.289022	2.336023	2.330791	2.346804	2.341585	2.336429
2.282242	2.277303	2.293884	2.288845	2.335883	2.330717	2.346748	2.34151	2.336317

2.282072	2.277107	2.29368	2.288659	2.335727	2.330624	2.346675	2.341427	2.336207
2.281982	2.276997	2.293536	2.288493	2.335541	2.33044	2.346486	2.341248	2.336041
2.28192	2.276912	2.293413	2.288343	2.335356	2.330233	2.346261	2.341035	2.335852
2.281815	2.27679	2.293286	2.288231	2.314836	2.309758	2.346091	2.340846	2.33565
2.281688	2.27665	2.293162	2.288149	2.314767	2.309664	2.34595	2.340668	2.335438
2.281569	2.276538	2.293056	2.288057	2.314672	2.309544	2.345804	2.34052	2.335289
2.281457	2.276458	2.292972	2.287958	2.314551	2.309394	2.34565	2.340407	2.335214
2.281355	2.276376	2.292878	2.287843	2.295882	2.290825	2.329074	2.323946	2.318856
2.281271	2.276288	2.292758	2.2877	2.295735	2.290711	2.328965	2.323831	2.31873
2.281183	2.276188	2.292628	2.287559	2.295594	2.290604	2.32886	2.323713	2.318593
2.281069	2.276054	2.292494	2.287449	2.295488	2.290499	2.328735	2.323572	2.31844
2.280943	2.275908	2.29236	2.287355	2.295396	2.290391	2.328599	2.323423	2.318284
2.280817	2.275799	2.292255	2.287257	2.295287	2.290258	2.308324	2.303233	2.298188
2.280691	2.275717	2.292173	2.287159	2.295166	2.290104	2.308168	2.303105	2.298097
2.302178	2.297136	2.292081	2.287037	2.295027	2.289967	2.30804	2.303002	2.298018
2.302121	2.297064	2.291969	2.286871	2.294852	2.289845	2.307948	2.302931	2.297952
2.302067	2.297001	2.291883	2.286754	2.294727	2.289752	2.307869	2.302865	2.297887
2.301995	2.29695	2.291863	2.286769	2.294734	2.28971	2.307797	2.30279	2.297816
2.301925	2.296901	2.291849	2.286798	2.294756	2.289677	2.307729	2.302716	2.297745
2.301872	2.296837	2.291765	2.286691	2.294643	2.289591	2.307656	2.302649	2.297678
2.301829	2.296766	2.291652	2.286527	2.294474	2.289484	2.307583	2.302585	2.297614
2.301768	2.296714	2.291607	2.286487	2.294424	2.289419	2.30751	2.302516	2.297549
2.30169	2.296679	2.291624	2.286561	2.294483	2.28939	2.307433	2.302439	2.297484
2.301651	2.296636	2.291612	2.286599	2.294524	2.289397	2.30741	2.302391	2.297419
2.30167	2.296564	2.291537	2.286567	2.29453	2.289462	2.307472	2.302381	2.297338
2.301706	2.296526	2.291479	2.286519	2.294509	2.289508	2.307532	2.302392	2.297294
2.301749	2.296587	2.291495	2.286458	2.294428	2.289468	2.307535	2.30243	2.297348
2.301786	2.296674	2.291539	2.286409	2.294342	2.289399	2.307511	2.302468	2.297431
2.301762	2.296688	2.291572	2.286447	2.294356	2.28936	2.307458	2.302446	2.297445
2.301699	2.296659	2.2916	2.286541	2.294433	2.289337	2.307385	2.302385	2.297418
2.301675	2.296611	2.291575	2.286564	2.294465	2.28935	2.307375	2.302352	2.297371
2.301692	2.296534	2.291486	2.286512	2.294452	2.289408	2.307439	2.302345	2.297293
2.301717	2.296505	2.291434	2.286453	2.294412	2.289431	2.307481	2.302354	2.297261
2.301754	2.296577	2.29146	2.286394	2.294326	2.28938	2.307475	2.302392	2.297323
2.301772	2.296654	2.291505	2.286357	2.294252	2.289311	2.307441	2.302415	2.29739
2.301721	2.296654	2.291525	2.28638	2.294249	2.289249	2.307357	2.302366	2.297382
2.301646	2.296626	2.291542	2.286439	2.294288	2.2892	2.307261	2.302294	2.297349
2.30164	2.296632	2.291575	2.286506	2.294352	2.289222	2.307257	2.302292	2.29736
2.301684	2.296658	2.291621	2.286586	2.294443	2.289302	2.307322	2.302341	2.297401
2.301705	2.296644	2.291617	2.286618	2.294493	2.289366	2.307375	2.302363	2.297397
2.301688	2.296569	2.291545	2.286584	2.29449	2.289405	2.307405	2.302342	2.297327
2.301691	2.296524	2.291496	2.286557	2.294486	2.289446	2.307445	2.302344	2.297289
2.301751	2.296561	2.291521	2.286577	2.294518	2.289508	2.307518	2.302408	2.29734
2.301821	2.296625	2.291567	2.286597	2.294538	2.289554	2.307585	2.302483	2.297419
2.301855	2.296679	2.291589	2.286561	2.294488	2.28953	2.307592	2.302522	2.297483
2.301875	2.296735	2.291608	2.286505	2.294407	2.289472	2.307569	2.302545	2.297544
2.301918	2.296799	2.291656	2.286517	2.294407	2.289479	2.307587	2.30258	2.297588
2.301979	2.296871	2.291731	2.28659	2.294475	2.289542	2.307638	2.302622	2.297615
2.302052	2.296951	2.291814	2.286673	2.294555	2.289615	2.307701	2.302679	2.297662
2.302142	2.297044	2.291906	2.286763	2.294641	2.289697	2.307778	2.302755	2.297737

2.302233	2.297136	2.291997	2.286853	2.294728	2.289781	2.307859	2.302839	2.297825
2.302314	2.297213	2.292073	2.286928	2.294804	2.28986	2.307941	2.302927	2.297918
2.302391	2.297289	2.292148	2.287005	2.294881	2.289939	2.308024	2.303018	2.298019
2.302466	2.297371	2.292236	2.287096	2.294968	2.290013	2.308096	2.303105	2.298127
2.302541	2.297459	2.292334	2.287203	2.295066	2.29009	2.308165	2.303192	2.29824
2.302646	2.297554	2.292451	2.287361	2.282341	2.27739	2.308285	2.303297	2.298338
2.302784	2.297656	2.292593	2.287582	2.282585	2.277604	2.308461	2.303421	2.298417
2.302917	2.297764	2.292722	2.287759	2.282777	2.277783	2.308617	2.303542	2.298506
2.303028	2.297863	2.29281	2.287835	2.282857	2.277881	2.308724	2.303648	2.298603
2.303148	2.297991	2.29292	2.287915	2.282929	2.277967	2.308825	2.303766	2.298732
2.303313	2.298204	2.29313	2.28809	2.283075	2.278082	2.308946	2.303934	2.298942
2.30349	2.298441	2.293368	2.288294	2.283242	2.278205	2.309074	2.304115	2.299176
2.303606	2.298572	2.293503	2.288428	2.28337	2.278321	2.309189	2.304243	2.299316
2.303681	2.298628	2.293565	2.288514	2.283472	2.278434	2.309299	2.304333	2.299389
2.303826	2.298751	2.293679	2.288628	2.283598	2.278587	2.309458	2.304476	2.299514
2.304062	2.298959	2.293856	2.288771	2.28375	2.278789	2.309682	2.304689	2.299706
2.304297	2.299169	2.294045	2.288945	2.28393	2.279	2.309905	2.304899	2.299898
2.30449	2.299339	2.294229	2.289162	2.284157	2.279216	2.310104	2.305078	2.300062
2.30468	2.299516	2.294425	2.289395	2.284393	2.279425	2.310291	2.305251	2.300227
2.304895	2.299742	2.294651	2.289613	2.284594	2.279596	2.310456	2.305427	2.300418
2.305123	2.299992	2.294893	2.289824	2.284777	2.279751	2.310613	2.305607	2.300622
2.305355	2.300226	2.29511	2.290015	2.284959	2.279939	2.310809	2.305803	2.300818
2.305594	2.300449	2.295308	2.290185	2.285135	2.280156	2.311039	2.306017	2.301009
2.305816	2.300651	2.295499	2.290373	2.28533	2.280369	2.311254	2.306214	2.301187
2.306009	2.300818	2.295678	2.290588	2.285553	2.280578	2.311445	2.306384	2.30134
2.306204	2.301	2.295873	2.29081	2.285775	2.280775	2.311625	2.306555	2.301507
2.306414	2.301235	2.29611	2.291036	2.285981	2.280945	2.311789	2.306737	2.301713
2.306639	2.301491	2.29636	2.291258	2.286176	2.281111	2.311958	2.306933	2.30194
2.306892	2.301743	2.296583	2.291437	2.286347	2.281308	2.312176	2.307164	2.30218
2.307166	2.301999	2.296797	2.291597	2.286509	2.281527	2.312426	2.30742	2.302432
2.307442	2.302273	2.297065	2.291857	2.286762	2.281774	2.312672	2.307664	2.302676
2.307722	2.302569	2.297394	2.292228	2.287117	2.282055	2.312915	2.307898	2.302911
2.307993	2.302846	2.297684	2.292535	2.287416	2.282321	2.313165	2.30814	2.303153
2.308252	2.303083	2.297886	2.292694	2.287582	2.282547	2.313427	2.308402	2.303407
2.308507	2.303322	2.298096	2.292871	2.287766	2.282778	2.313688	2.308661	2.303661
2.308754	2.303598	2.298402	2.293205	2.288086	2.283037	2.31392	2.30889	2.303901
2.309009	2.303884	2.298736	2.293594	2.288459	2.283321	2.314161	2.30912	2.304139
2.309306	2.304138	2.299004	2.293909	2.288797	2.283665	2.278603	2.273637	2.268728
2.309638	2.304371	2.29923	2.294177	2.289117	2.284056	2.279008	2.273955	2.268958
2.277583	2.272234	2.299515	2.294441	2.289394	2.284384	2.279362	2.274289	2.269261
2.277924	2.272614	2.299872	2.29469	2.289609	2.284633	2.279658	2.27465	2.269658
2.278232	2.272968	2.300231	2.294982	2.289868	2.284886	2.279927	2.274976	2.270027
2.278464	2.273249	2.300582	2.295373	2.290235	2.285163	2.280152	2.275221	2.270312
2.278686	2.273497	2.300909	2.295767	2.29062	2.285463	2.280394	2.275458	2.27057
2.278958	2.273711	2.268516	2.263368	2.290949	2.285817	2.280746	2.275758	2.270825
2.279255	2.273914	2.268709	2.263598	2.29125	2.286196	2.281152	2.27609	2.271085
2.27957	2.274213	2.26898	2.263836	2.258737	2.253691	2.281509	2.276438	2.271418
2.279904	2.274596	2.269315	2.264075	2.258951	2.253949	2.281832	2.276804	2.271815
2.262916	2.257706	2.269648	2.264358	2.25922	2.254235	2.282167	2.277174	2.272207
2.26315	2.257997	2.269969	2.2647	2.259568	2.25457	2.28253	2.277557	2.272594

2.263406	2.258292	2.270295	2.265058	2.259924	2.254891	2.249887	2.244906	2.239931
2.263746	2.258629	2.270646	2.265419	2.260255	2.255147	2.250105	2.245153	2.240218
2.264106	2.258961	2.270986	2.265771	2.260578	2.2554	2.250321	2.245394	2.240499
2.264417	2.259217	2.271252	2.266067	2.260892	2.25572	2.250646	2.245725	2.240836
2.264709	2.259435	2.271479	2.266335	2.261202	2.25608	2.251036	2.246108	2.241203
2.265054	2.25974	2.271788	2.266659	2.261542	2.25644	2.251406	2.246471	2.241555
2.265451	2.260129	2.272176	2.267036	2.261911	2.256804	2.251763	2.246819	2.2419
2.235596	2.230364	2.255341	2.250256	2.262289	2.257169	2.252112	2.247139	2.2422
2.235987	2.230747	2.255732	2.250642	2.262672	2.257536	2.252445	2.247407	2.242417
2.236369	2.231129	2.256127	2.251032	2.263059	2.257907	2.252786	2.247688	2.242652
2.236721	2.231506	2.256522	2.251418	2.263445	2.258286	2.253155	2.248039	2.242988
2.237065	2.231881	2.256917	2.251804	2.263833	2.258669	2.253535	2.248419	2.243367
2.237434	2.232265	2.257313	2.252191	2.26422	2.259048	2.253907	2.248787	2.243731
2.237821	2.232653	2.257709	2.252579	2.264608	2.259426	2.254277	2.249152	2.244094
2.238209	2.23304	2.227939	2.222891	2.24783	2.242696	2.25466	2.249534	2.244473
2.238595	2.233423	2.228318	2.223266	2.248216	2.243083	2.25506	2.249932	2.244864
2.238997	2.233809	2.228695	2.22364	2.248601	2.24347	2.255465	2.250355	2.245296
2.239426	2.234193	2.229063	2.224009	2.248982	2.243855	2.255881	2.250827	2.245812
2.239866	2.234588	2.229438	2.22438	2.249361	2.244235	2.256291	2.251293	2.246321
2.2403	2.234999	2.229828	2.22475	2.249731	2.244601	2.25667	2.251684	2.246724
2.24074	2.235432	2.23024	2.225133	2.250105	2.244965	2.257037	2.25204	2.247075
2.241209	2.235933	2.230731	2.225588	2.250536	2.245365	2.257424	2.252409	2.247434
2.241706	2.236497	2.231293	2.226108	2.251016	2.245795	2.257824	2.252781	2.247791
2.242164	2.236987	2.231783	2.22658	2.221417	2.216284	2.241117	2.236108	2.231156
2.242556	2.237363	2.232165	2.226984	2.221856	2.216776	2.241647	2.236613	2.231621
2.242984	2.237762	2.232557	2.227387	2.222282	2.21724	2.242146	2.237096	2.232079
2.243528	2.238266	2.233022	2.227809	2.222673	2.217612	2.242528	2.237485	2.232485
2.244085	2.238788	2.233508	2.228252	2.223077	2.217977	2.242888	2.237852	2.232876
2.244527	2.239238	2.23398	2.228752	2.223573	2.218437	2.243324	2.238261	2.233269
2.24491	2.239652	2.234446	2.229282	2.224116	2.218947	2.243799	2.238694	2.233667
2.245338	2.24007	2.234877	2.229739	2.224584	2.219409	2.214309	2.209304	2.204374
2.245804	2.240485	2.235268	2.230123	2.224976	2.219827	2.214756	2.209788	2.204884
2.246294	2.240959	2.235723	2.230561	2.225415	2.220285	2.215232	2.210282	2.205385
2.246808	2.241521	2.236281	2.231089	2.225933	2.220812	2.215758	2.210788	2.205863
2.247345	2.242101	2.23686	2.231644	2.226475	2.22135	2.216283	2.211285	2.206331
2.247913	2.242651	2.237411	2.232203	2.227023	2.221869	2.216774	2.211746	2.206775
2.248498	2.243199	2.237958	2.232762	2.227569	2.222377	2.217251	2.212198	2.207221
2.249072	2.243769	2.238511	2.23329	2.228072	2.222854	2.217717	2.21268	2.207723
2.249643	2.244351	2.239065	2.233795	2.228546	2.223309	2.218176	2.213182	2.208264
2.250207	2.244902	2.239587	2.234282	2.229023	2.2238	2.218683	2.213702	2.208793
2.250765	2.245417	2.24007	2.234743	2.229498	2.224331	2.219244	2.214242	2.209309
2.25131	2.24592	2.240558	2.235232	2.230004	2.224872	2.242189	2.237101	2.232077
2.25182	2.246392	2.241049	2.235775	2.230562	2.225413	2.24273	2.237649	2.232629
2.252351	2.246899	2.241573	2.236338	2.231134	2.225962	2.243275	2.238198	2.233181
2.252984	2.247543	2.242188	2.236903	2.231682	2.226524	2.243843	2.238748	2.233714
2.253654	2.248238	2.242839	2.237474	2.232226	2.227093	2.244419	2.239298	2.23424
2.254221	2.248792	2.243394	2.238038	2.232789	2.227646	2.244969	2.239843	2.234781
2.25471	2.24924	2.243876	2.238597	2.233366	2.228187	2.2455	2.240386	2.235333
2.255282	2.249799	2.244437	2.239166	2.233937	2.228752	2.246063	2.240938	2.235874
2.255975	2.250517	2.245104	2.239741	2.234486	2.229342	2.246664	2.241492	2.236383

2.256653	2.251229	2.245784	2.240354	2.235081	2.229964	2.247294	2.242088	2.236944
2.257245	2.251867	2.246455	2.241051	2.235785	2.230652	2.247976	2.242777	2.237625
2.257818	2.252472	2.247105	2.241756	2.236501	2.23134	2.248655	2.243476	2.238329
2.258409	2.253031	2.247679	2.242366	2.237117	2.23193	2.249242	2.244075	2.238944
2.259013	2.253569	2.248213	2.242927	2.237678	2.232469	2.249782	2.244623	2.239515
2.259688	2.25422	2.248828	2.2435	2.238236	2.233038	2.25036	2.24519	2.240079
2.260436	2.25498	2.249517	2.244077	2.238781	2.233625	2.250968	2.245768	2.240627
2.23401	2.228642	2.250199	2.244704	2.26202	2.256798	2.280748	2.27544	2.27019
2.2346	2.229273	2.250865	2.24541	2.262744	2.257496	2.281452	2.276159	2.270921
2.235176	2.229874	2.251513	2.246116	2.263469	2.258185	2.282142	2.276869	2.271647
2.235762	2.230434	2.225113	2.219822	2.264116	2.258798	2.28275	2.277494	2.272286
2.236369	2.230996	2.225692	2.220445	2.264747	2.259398	2.283347	2.278102	2.272903
2.237051	2.231657	2.226353	2.221117	2.265429	2.260076	2.284038	2.278781	2.27357
2.237788	2.232385	2.227067	2.221816	2.266137	2.260801	2.284785	2.279499	2.274261
2.238519	2.233105	2.227752	2.222455	2.241187	2.235932	2.28547	2.280157	2.274895
2.239253	2.233823	2.228404	2.223019	2.241733	2.236508	2.28608	2.280742	2.275456
2.239941	2.234513	2.229062	2.223629	2.242336	2.237127	2.286727	2.281374	2.276071
2.240537	2.235149	2.229733	2.224334	2.243059	2.237836	2.287454	2.282101	2.276797
2.241141	2.235788	2.230416	2.225065	2.243815	2.238573	2.288206	2.282858	2.277554
2.213814	2.208531	2.231118	2.225785	2.244547	2.2393	2.264885	2.259604	2.254365
2.21455	2.209234	2.231832	2.226506	2.245278	2.24003	2.265633	2.260346	2.255102
2.215284	2.209946	2.232554	2.227228	2.246008	2.240758	2.266378	2.261088	2.255837
2.216026	2.21067	2.233284	2.227951	2.246736	2.241485	2.267123	2.261828	2.256572
2.242505	2.237051	2.258208	2.252795	2.247462	2.24221	2.267867	2.262569	2.257309
2.243251	2.237776	2.258939	2.253521	2.248184	2.242933	2.268609	2.26331	2.258047
2.243998	2.238503	2.259672	2.254249	2.24891	2.243657	2.269352	2.264051	2.258786
2.244748	2.239234	2.26041	2.254984	2.249641	2.244386	2.270098	2.264793	2.259525
2.245499	2.239967	2.26115	2.255721	2.250374	2.245117	2.270846	2.265537	2.260265
2.246248	2.240698	2.235261	2.229918	2.251107	2.24585	2.271599	2.266287	2.261009
2.246996	2.241431	2.235984	2.230635	2.25184	2.246586	2.272356	2.267039	2.261756
2.247752	2.242178	2.236721	2.231362	2.252577	2.247323	2.273112	2.267792	2.262504
2.248515	2.242938	2.23747	2.232097	2.253318	2.24806	2.273867	2.268545	2.263254
2.249278	2.243698	2.238219	2.232832	2.254058	2.248796	2.27462	2.269295	2.264001
2.250037	2.244453	2.238963	2.233561	2.254793	2.249527	2.275367	2.270035	2.264739
2.250808	2.245217	2.239716	2.2343	2.255537	2.250265	2.27612	2.270784	2.265487
2.25159	2.24599	2.240476	2.235045	2.256286	2.251008	2.27688	2.271544	2.266248
2.2524	2.246786	2.241258	2.235815	2.25706	2.251771	2.27766	2.27233	2.267037
2.253291	2.247647	2.24211	2.236665	2.231323	2.226085	2.278501	2.273189	2.267911
2.254234	2.248552	2.243008	2.237569	2.232213	2.226943	2.279383	2.274098	2.268841
2.255113	2.249413	2.243852	2.238396	2.233026	2.227745	2.280218	2.274941	2.269691
2.255923	2.25022	2.244633	2.239139	2.233757	2.228486	2.280999	2.275712	2.270452
2.256749	2.251068	2.245457	2.239916	2.234516	2.229257	2.281814	2.276509	2.271236
2.257619	2.252005	2.246378	2.240776	2.235352	2.2301	2.282711	2.27738	2.27209
2.258496	2.252935	2.247292	2.241638	2.236188	2.230935	2.283593	2.27824	2.272939
2.259349	2.253764	2.248098	2.242423	2.236951	2.231673	2.259328	2.254067	2.248859
2.260204	2.254561	2.248873	2.243198	2.237707	2.232391	2.260046	2.254798	2.249604
2.204804	2.199286	2.249747	2.244098	2.238594	2.233227	2.260872	2.255627	2.250437
2.205741	2.200172	2.250689	2.245089	2.239579	2.234152	2.261777	2.256529	2.251337
2.206608	2.201011	2.251574	2.24599	2.240489	2.235064	2.229752	2.224588	2.219482
2.207379	2.201777	2.252374	2.246768	2.241299	2.235966	2.230698	2.225483	2.220321

2.183546	2.178004	2.197062	2.191596	2.242115	2.236832	2.23159	2.226356	2.221172
2.184428	2.178932	2.197978	2.192445	2.242968	2.237616	2.232354	2.227188	2.222069
2.185347	2.179887	2.198929	2.19334	2.243853	2.2384	2.233095	2.227994	2.222943
2.186271	2.18077	2.199848	2.19429	2.244801	2.239273	2.233906	2.228763	2.223699
2.187205	2.181631	2.200763	2.195273	2.245786	2.240195	2.234757	2.229519	2.224396
2.188125	2.182554	2.201702	2.196202	2.190715	2.185231	2.235648	2.230343	2.225164
2.189032	2.183532	2.202662	2.197086	2.191584	2.186147	2.236583	2.231228	2.225986
2.189948	2.184466	2.203603	2.198003	2.19249	2.187053	2.237508	2.232126	2.22685
2.190882	2.185323	2.204513	2.198981	2.193459	2.187937	2.238404	2.233033	2.227766
2.19181	2.186193	2.205427	2.199945	2.194416	2.188832	2.183404	2.178204	2.173108
2.192717	2.187149	2.206374	2.200841	2.195308	2.189768	2.184354	2.179111	2.173972
2.193616	2.188114	2.207322	2.20172	2.196187	2.190713	2.185327	2.180028	2.174835
2.194517	2.188985	2.208219	2.20264	2.197113	2.191631	2.186236	2.180928	2.175733
2.195417	2.189806	2.209088	2.203579	2.198063	2.192536	2.187116	2.181826	2.176656
2.196309	2.190691	2.209996	2.204497	2.198985	2.193455	2.188039	2.182765	2.177605
2.197191	2.191644	2.210944	2.205392	2.199875	2.194386	2.189002	2.18375	2.178586
2.198089	2.192571	2.187021	2.181486	2.200795	2.195349	2.189988	2.184725	2.179532
2.198997	2.193419	2.187878	2.182392	2.201769	2.19637	2.191013	2.185677	2.180412
2.199948	2.194301	2.188768	2.183328	2.202759	2.197384	2.192016	2.186611	2.181282
2.201002	2.195328	2.189782	2.184332	2.203749	2.198329	2.192928	2.187503	2.182172
2.202092	2.196409	2.19084	2.185355	2.204733	2.199246	2.193806	2.188387	2.183081
2.203081	2.197412	2.191817	2.186288	2.205663	2.200172	2.194738	2.189342	2.184051
2.204004	2.198367	2.192743	2.187162	2.206554	2.2011	2.195703	2.190348	2.185071
2.204969	2.19936	2.193727	2.188122	2.20753	2.202085	2.19669	2.191328	2.186033
2.205988	2.200408	2.194792	2.189195	2.208615	2.203138	2.197699	2.192267	2.186914
2.149471	2.144036	2.195814	2.190237	2.209684	2.204198	2.198733	2.193249	2.187849
2.150392	2.144868	2.196717	2.19117	2.210682	2.205253	2.199823	2.194343	2.188924
2.1513	2.145689	2.197611	2.192095	2.211668	2.206285	2.200891	2.195433	2.190016
2.152183	2.146578	2.198595	2.193099	2.187654	2.182272	2.20182	2.196387	2.191003
2.153069	2.147509	2.199626	2.194143	2.188657	2.183172	2.202692	2.197286	2.191949
2.15407	2.148502	2.143095	2.137817	2.1897	2.184165	2.203679	2.198273	2.192937
2.155172	2.149553	2.144125	2.13884	2.190784	2.185238	2.204762	2.199331	2.193954
2.156254	2.150608	2.145134	2.139792	2.191788	2.186251	2.205811	2.200383	2.194995
2.157296	2.151661	2.146103	2.140626	2.192662	2.187164	2.2068	2.201418	2.196066
2.104911	2.099788	2.147089	2.141494	2.136038	2.130715	2.207797	2.202459	2.197142
2.105892	2.100769	2.148122	2.142487	2.137001	2.131655	2.208834	2.203509	2.198208
2.106887	2.101757	2.149178	2.14353	2.138021	2.132639	2.209896	2.20457	2.199275
2.107886	2.102748	2.15024	2.144571	2.139045	2.13365	2.185818	2.180546	2.175308
2.108889	2.103744	2.151311	2.145622	2.140085	2.134687	2.186911	2.181617	2.176357
2.109887	2.10474	2.152391	2.146678	2.141125	2.135719	2.130439	2.125323	2.120245
2.110879	2.105736	2.153478	2.147739	2.142166	2.136747	2.131452	2.126315	2.12122
2.111881	2.106737	2.154571	2.148808	2.143214	2.137778	2.132464	2.127303	2.122189
2.112901	2.10775	2.155673	2.14989	2.144274	2.138812	2.133472	2.128283	2.123145
2.113924	2.108763	2.156773	2.150972	2.145336	2.139849	2.134484	2.129265	2.124103
2.114941	2.10977	2.157865	2.152045	2.146392	2.140891	2.135507	2.13026	2.125072
2.115955	2.110774	2.15895	2.153112	2.147444	2.141933	2.136533	2.131261	2.126049
2.081326	2.076196	2.160025	2.154167	2.148484	2.142963	2.137552	2.132266	2.127039
2.082309	2.077168	2.161093	2.155213	2.149515	2.143984	2.138565	2.133275	2.12804
2.083302	2.078153	2.162179	2.156282	2.150568	2.145019	2.139589	2.134295	2.129062
2.084307	2.079152	2.163279	2.157365	2.151631	2.146059	2.140617	2.135323	2.130109

2.085312	2.080155	2.074908	2.069636	2.152777	2.147162	2.141685	2.136371	2.131154
2.086304	2.081161	2.075977	2.070791	2.15409	2.148388	2.142827	2.137451	2.132185
2.087312	2.082168	2.077041	2.071944	2.155406	2.149623	2.143981	2.138534	2.13321
2.088355	2.083158	2.078032	2.072967	2.156553	2.150743	2.145065	2.139575	2.134213
2.089433	2.084154	2.078997	2.07393	2.157625	2.151822	2.146138	2.140615	2.135218
2.019112	2.013965	2.080028	2.07493	2.125831	2.120124	2.147332	2.141763	2.136304
2.020287	2.015071	2.081111	2.07595	2.126961	2.121355	2.148628	2.143003	2.137458
2.021384	2.016125	2.082169	2.076957	2.128063	2.12251	2.14984	2.144224	2.138661
2.022366	2.01709	2.083176	2.07793	2.129117	2.123553	2.150932	2.145426	2.139945
2.023345	2.018086	2.084222	2.078943	2.130195	2.124597	2.119094	2.113715	2.108355
2.024376	2.019195	2.08538	2.080044	2.131319	2.125664	2.120126	2.114739	2.109402
2.02542	2.020321	2.086562	2.081189	2.132501	2.126793	2.121208	2.115768	2.110414
1.834863	1.830513	2.059302	2.054082	2.133821	2.128126	2.122512	2.11697	2.111517
1.835701	1.831301	2.060346	2.055232	2.135216	2.129573	2.123939	2.118268	2.112672
1.836612	1.832159	2.061404	2.056298	2.136426	2.130807	2.125182	2.119499	2.113872
1.837601	1.833084	2.062469	2.05727	2.137449	2.131835	2.126251	2.120676	2.115128
1.780051	1.775697	1.976145	1.971154	2.081496	2.076363	2.127344	2.121811	2.116306
1.780881	1.77662	1.977362	1.972402	2.082777	2.077533	2.128487	2.122875	2.11734
1.781696	1.777519	1.978542	1.973617	2.084043	2.078723	2.129678	2.12397	2.11838
1.782514	1.778314	1.979515	1.974601	2.085129	2.079876	2.130974	2.125198	2.119526
1.783353	1.779099	1.980441	1.975516	2.086153	2.081005	2.132286	2.126473	2.120727
1.784301	1.780046	1.775783	1.771543	2.058783	2.05369	2.133431	2.127676	2.121981
1.785324	1.781097	1.776784	1.772441	2.059856	2.054673	2.134472	2.128841	2.123282
1.786246	1.782017	1.777696	1.773342	2.061006	2.055799	2.135683	2.130068	2.124528
1.78704	1.782773	1.7785	1.774247	2.062252	2.057102	2.137103	2.131369	2.125705
1.787897	1.783603	1.779353	1.775153	2.063459	2.058332	2.138452	2.132651	2.126919
1.788924	1.784637	1.780341	1.77606	2.064558	2.059359	2.139587	2.133861	2.128211
1.789947	1.785666	1.781324	1.776963	2.065649	2.060378	2.140721	2.135086	2.129533
1.790801	1.78649	1.782167	1.777855	1.876901	1.872407	2.056401	2.051354	2.046341
1.791593	1.78724	1.782957	1.778738	1.877901	1.873437	2.057666	2.052611	2.047564
1.792501	1.788133	1.783835	1.779603	1.878812	1.874336	2.058743	2.053684	2.048639
1.793496	1.789133	1.784779	1.780452	1.879652	1.875133	2.059673	2.054609	2.049591
1.794422	1.790056	1.785686	1.781335	1.83539	1.830946	1.925301	1.920768	1.916285
1.795232	1.79085	1.786525	1.782257	1.836394	1.831982	1.926423	1.921878	1.917376
1.796097	1.791702	1.787411	1.783208	1.83742	1.833042	1.927559	1.922978	1.918434
1.797116	1.792722	1.788424	1.784209	1.838464	1.834105	1.92866	1.923983	1.919344
1.798192	1.793798	1.78947	1.785204	1.839482	1.835131	1.929718	1.924955	1.920231
1.741881	1.737583	1.790465	1.786107	1.840374	1.835995	1.87673	1.872183	1.867676
1.742984	1.73866	1.791431	1.78696	1.841199	1.836774	1.877537	1.873075	1.868661
1.743948	1.739593	1.792331	1.787852	1.842124	1.837703	1.878503	1.874074	1.869691
1.744773	1.74038	1.793158	1.788772	1.843139	1.838766	1.879617	1.875175	1.870768
1.745685	1.74127	1.736957	1.732746	1.844181	1.839832	1.88072	1.876268	1.871838
1.746742	1.742333	1.73802	1.7338	1.845257	1.840894	1.881805	1.877359	1.872914
1.74785	1.743442	1.739117	1.734872	1.846324	1.841919	1.882827	1.878377	1.873919
1.748979	1.744545	1.740205	1.735946	1.847357	1.842854	1.838396	1.834	1.829625
1.750127	1.745653	1.741286	1.737	1.848358	1.84375	1.839212	1.834764	1.830377
1.751264	1.746753	1.74232	1.737954	1.796847	1.792318	1.840123	1.835687	1.831317
1.752392	1.747841	1.743317	1.738837	1.797742	1.793254	1.841093	1.836708	1.832375
1.753472	1.748849	1.744273	1.739755	1.798696	1.794245	1.842104	1.837743	1.833423
1.754502	1.749763	1.745176	1.740705	1.799709	1.795292	1.84316	1.838796	1.834465

1.671804	1.667483	1.746114	1.741688	1.737365	1.733154	1.844231	1.839853	1.835505
1.672604	1.668322	1.747112	1.742708	1.738403	1.734208	1.845313	1.840911	1.836538
1.67344	1.66921	1.748158	1.743758	1.739458	1.735267	1.846393	1.841967	1.837572
1.674437	1.670206	1.749269	1.74484	1.740516	1.736306	1.795291	1.790948	1.786628
1.675515	1.671263	1.750423	1.745943	1.74158	1.737339	1.79635	1.792007	1.787693
1.676572	1.672305	1.668133	1.664043	1.742646	1.738386	1.797431	1.793084	1.788768
1.677626	1.673344	1.669142	1.665012	1.743716	1.739444	1.798527	1.794173	1.789852
1.678681	1.674391	1.670159	1.665988	1.744786	1.740497	1.799618	1.795261	1.790937
1.679737	1.675444	1.671184	1.666968	1.745853	1.741544	1.800702	1.796348	1.792026
1.680798	1.676507	1.672221	1.667965	1.746935	1.742595	1.801781	1.797424	1.7931
1.681873	1.677586	1.673281	1.668989	1.748039	1.743652	1.802851	1.798474	1.794141
1.682948	1.678666	1.674346	1.670025	1.749157	1.744717	1.80392	1.799521	1.795177
1.684007	1.679727	1.675399	1.671062	1.666864	1.662795	1.805002	1.800585	1.796234
1.685056	1.680776	1.676446	1.672103	1.667881	1.663766	1.806091	1.801659	1.797302
1.60149	1.597479	1.677493	1.673152	1.668907	1.664745	1.807185	1.802733	1.798365
1.602445	1.598439	1.67854	1.674209	1.669945	1.665732	1.808285	1.803808	1.799426
1.603392	1.599389	1.679576	1.675257	1.670977	1.66672	1.745636	1.741313	1.737098
1.604333	1.600327	1.680597	1.676289	1.671996	1.667701	1.746682	1.742331	1.738098
1.605267	1.60126	1.681616	1.677322	1.673021	1.668701	1.747758	1.743369	1.739107
1.527749	1.524022	1.682639	1.678363	1.674069	1.669744	1.748902	1.744455	1.740138
1.528655	1.524934	1.683664	1.679407	1.675124	1.670806	1.750078	1.745566	1.741184
1.529589	1.525854	1.60008	1.596101	1.676156	1.67184	1.667605	1.663467	1.65944
1.530553	1.526791	1.60103	1.597056	1.677187	1.672872	1.668623	1.664445	1.660376
1.531612	1.527841	1.524095	1.520376	1.678349	1.674044	1.669786	1.665568	1.661454
1.532788	1.529026	1.525281	1.521557	1.679667	1.675379	1.671116	1.666857	1.662695
1.533895	1.530138	1.526394	1.522667	1.518924	1.515166	1.672366	1.66808	1.66388
1.534861	1.531097	1.527352	1.523628	1.519889	1.516138	1.673445	1.669152	1.664933
1.535785	1.532009	1.528261	1.52454	1.520807	1.517062	1.67447	1.670178	1.665948
1.536717	1.53292	1.529165	1.525447	1.52172	1.517988	1.6755	1.671208	1.666969
1.537649	1.533827	1.530062	1.526345	1.522624	1.518905	1.676519	1.672231	1.667987
1.446258	1.442717	1.530986	1.527265	1.523544	1.519829	1.516104	1.512349	1.508618
1.447135	1.443572	1.531933	1.528204	1.524477	1.520759	1.517036	1.513293	1.509567
1.345653	1.342395	1.532997	1.529253	1.525516	1.521791	1.518066	1.514332	1.51061
1.346682	1.343393	1.53422	1.530455	1.526701	1.522965	1.519234	1.515504	1.511784
1.347658	1.344345	1.443186	1.439704	1.527832	1.524085	1.520351	1.516626	1.512911
1.348471	1.345148	1.444068	1.440559	1.528797	1.525047	1.521314	1.517596	1.513887
1.349237	1.345912	1.444908	1.441374	1.529716	1.525963	1.522234	1.518532	1.514839
1.350023	1.346694	1.343418	1.34018	1.530661	1.526894	1.523176	1.519524	1.515885
1.350825	1.347493	1.344207	1.340955	1.531629	1.527844	1.524136	1.520541	1.516968
1.351734	1.348408	1.345116	1.341849	1.440576	1.437077	1.525214	1.521595	1.518001
1.352748	1.349433	1.346139	1.34286	1.441694	1.438184	1.526405	1.522685	1.518995
1.353673	1.350368	1.347073	1.343785	1.340537	1.337329	1.527518	1.523731	1.519974
1.354443	1.351144	1.347847	1.344552	1.3413	1.338089	1.528486	1.524687	1.520916
1.138669	1.135726	1.348649	1.345351	1.342095	1.33888	1.529491	1.525692	1.521919
1.13965	1.136714	1.349623	1.346322	1.343059	1.339834	1.530673	1.526873	1.523097
1.140649	1.137721	1.350618	1.347318	1.344051	1.340813	1.531882	1.528082	1.524306
1.141521	1.138597	1.246192	1.243108	1.344889	1.341639	1.338407	1.335183	1.331994
1.142325	1.139401	1.24688	1.243819	1.345643	1.342384	1.339142	1.335906	1.332718
1.143215	1.140289	1.137346	1.134395	1.241565	1.238509	1.339994	1.336758	1.333566
1.144201	1.141267	1.138329	1.135386	1.242462	1.239412	1.340973	1.337748	1.334543

1.145134	1.142194	1.139259	1.136323	1.243293	1.240247	1.341884	1.338688	1.335497
1.145944	1.143002	1.140066	1.137133	1.243976	1.240931	1.342634	1.339509	1.336388
1.146766	1.143825	1.140888	1.137956	1.244672	1.241629	1.343393	1.340321	1.337256
1.014632	1.011999	1.141842	1.138907	1.135964	1.133009	1.239491	1.236585	1.23369
1.015492	1.012873	1.142849	1.139911	1.136969	1.134022	1.240384	1.237423	1.234468
1.01627	1.013681	1.143823	1.140897	1.137963	1.13502	1.241267	1.238286	1.23531
1.016995	1.014448	1.144782	1.141876	1.138955	1.136016	1.242148	1.239171	1.236198
1.017768	1.015214	1.012623	1.010018	1.139863	1.136925	1.134005	1.131111	1.128233
1.018597	1.015974	1.013337	1.0107	1.140656	1.137718	1.134818	1.131976	1.129152
1.01945	1.016769	1.014092	1.011426	1.141496	1.138556	1.135666	1.132849	1.130051
1.020337	1.017639	1.014949	1.012268	1.142459	1.139518	1.136612	1.133754	1.130915
1.021211	1.018518	1.015828	1.013144	1.143465	1.140521	1.137594	1.134685	1.131791
1.021996	1.019336	1.01667	1.014006	1.144475	1.141527	1.138588	1.135659	1.132744
1.022741	1.020126	1.017494	1.014859	1.145485	1.142535	1.139593	1.136661	1.133741
0.866106	0.863806	1.018274	1.015636	1.013001	1.010369	1.140568	1.137655	1.134743
0.866893	0.864522	1.019013	1.01634	1.013709	1.01112	1.141519	1.138646	1.135759
0.577738	0.576314	1.019813	1.017117	1.014477	1.011892	1.142446	1.139602	1.136742
0.578268	0.576826	1.020702	1.018002	1.015332	1.01269	1.143332	1.140509	1.137683
0.578803	0.577349	0.864529	0.862177	1.016212	1.01352	1.010887	1.008343	1.005822
0.579348	0.577879	0.865344	0.862982	1.017097	1.014404	1.011752	1.009152	1.006584
0.579891	0.578414	0.866165	0.863789	1.01799	1.01531	1.012646	1.009983	1.00736
0.580412	0.578956	0.866998	0.864601	0.862236	0.859903	1.013528	1.010848	1.008208
0.580918	0.579499	0.867836	0.865416	0.863036	0.860696	1.014409	1.011737	1.009102
0.581405	0.580009	0.868641	0.866223	0.863841	0.861491	1.015283	1.012621	1.009988
0.581869	0.580484	0.869415	0.867024	0.864649	0.862287	1.016149	1.0135	1.01087
0.582345	0.58096	0.579567	0.57817	0.86545	0.863085	0.860738	0.858421	0.85611
0.582846	0.581441	0.580041	0.578646	0.866233	0.863883	0.861541	0.859215	0.856893
0.583354	0.581932	0.580526	0.57913	0.867023	0.864684	0.862346	0.860009	0.857676
0.583866	0.582446	0.581046	0.579656	0.867842	0.865494	0.863146	0.8608	0.85846
0.584381	0.582973	0.581581	0.580199	0.868673	0.866307	0.863945	0.861589	0.859244
0.584892	0.583492	0.58211	0.580736	0.869494	0.867115	0.86474	0.862375	0.860023
0.5854	0.584009	0.582636	0.581271	0.870312	0.86792	0.865533	0.863158	0.860799
0.585911	0.584528	0.583161	0.581805	0.580432	0.579044	0.866336	0.86395	0.861582
0.586425	0.585047	0.583687	0.582337	0.580971	0.579591	0.867151	0.864754	0.862373
0.586944	0.585567	0.584209	0.582864	0.581505	0.580133	0.867968	0.865557	0.863163
0.587466	0.586085	0.584726	0.583384	0.58203	0.580666	0.868781	0.866355	0.863949
0.587995	0.586606	0.585244	0.583902	0.582551	0.581194	0.869593	0.867153	0.864737
0.588534	0.587134	0.585765	0.584417	0.583067	0.581717	0.580353	0.578962	0.577572
0.589081	0.587673	0.586294	0.584937	0.583584	0.582237	0.580876	0.579488	0.578107
0.589643	0.588243	0.586861	0.585494	0.584128	0.582764	0.581396	0.580012	0.578639
0.590221	0.588842	0.587461	0.586083	0.584696	0.583299	0.58191	0.580531	0.579168
0.590789	0.589422	0.588041	0.586653	0.58525	0.583828	0.582428	0.58106	0.579712
0.591344	0.589974	0.588588	0.587194	0.585782	0.584348	0.582948	0.581605	0.580282
0.591897	0.590523	0.589132	0.587734	0.586315	0.58487	0.58347	0.582142	0.580837
0.592448	0.591072	0.589682	0.588284	0.586857	0.585398	0.583986	0.582649	0.581342
0.593007	0.591631	0.590239	0.588838	0.587405	0.585937	0.584513	0.583159	0.581839
0.593601	0.592225	0.590821	0.589399	0.587963	0.586507	0.585087	0.583721	0.582384
0.594212	0.592838	0.591416	0.589966	0.588528	0.587097	0.585689	0.58431	0.582952
0.59478	0.593412	0.591987	0.590528	0.58909	0.587667	0.58626	0.584868	0.583491
0.595305	0.593949	0.592534	0.591084	0.589647	0.588219	0.586802	0.585394	0.584001

0.595836	0.594479	0.593077	0.591649	0.590221	0.588792	0.587364	0.585931	0.584515
0.596385	0.595001	0.593616	0.59223	0.590826	0.589406	0.587966	0.586488	0.58503
0.596937	0.595531	0.594162	0.592813	0.591429	0.590015	0.588567	0.587056	0.585568
0.597479	0.596086	0.594732	0.593399	0.592016	0.590587	0.589133	0.587637	0.58616
0.598025	0.596654	0.595307	0.593977	0.592589	0.591145	0.589692	0.588228	0.586779
0.5986	0.597223	0.595864	0.594518	0.593133	0.591711	0.590278	0.588835	0.587392
0.599194	0.597794	0.596407	0.595033	0.593656	0.592281	0.590882	0.589452	0.588004
0.599766	0.598349	0.596949	0.595566	0.5942	0.592856	0.591478	0.59005	0.588594
0.600308	0.598881	0.597487	0.596122	0.594771	0.593442	0.592069	0.590625	0.589156
0.60087	0.599429	0.598037	0.59668	0.595335	0.594009	0.592638	0.591195	0.589729
0.601472	0.60001	0.598605	0.597239	0.595882	0.594539	0.59317	0.591762	0.590331
0.60209	0.600607	0.599182	0.597796	0.596421	0.595061	0.593696	0.592328	0.590939
		0.599763	0.598338	0.596949	0.595599	0.594249	0.592903	0.591534
		0.600348	0.598878	0.597476	0.596143	0.594813	0.59348	0.592124
		0.600926	0.599442	0.598031	0.596696	0.59537	0.594051	0.592713
		0.601498	0.600023	0.598609	0.597256	0.595923	0.594619	0.593299
		0.602094	0.600618	0.599193	0.597819	0.596475	0.595179	0.593869
		0.602721	0.601224	0.599779	0.598382	0.597024	0.595727	0.594419
				0.600377	0.598958	0.597583	0.596277	0.594965
				0.600996	0.599557	0.598163	0.596834	0.595512
				0.601625	0.600166	0.598751	0.597394	0.59606
				0.602249	0.600768	0.599331	0.597953	0.596608
				0.602875	0.60137	0.599911	0.598515	0.597158
						0.600512	0.599097	0.597722
						0.601135	0.599698	0.5983
						0.601762	0.600305	0.598884
						0.602392	0.600917	0.599475
						0.603022	0.601533	0.600072

				0.596303	0.594453	0.592628
				0.595569	0.59372	0.591892
				0.594846	0.592994	0.591157
				0.594116	0.592267	0.59043
				0.593385	0.591542	0.589707
		0.596438	0.59453	1.482363	1.478347	1.474382
		0.595705	0.593803	1.480696	1.476692	1.472735
		0.59497	0.593073	1.479036	1.475045	1.471097
		0.594232	0.592339	1.477383	1.473407	1.46947
0.597427	0.595435	1.483847	1.479769	1.475734	1.471773	1.467847
0.596676	0.594681	1.482171	1.478105	1.474086	1.47014	1.466229

		0.595932	0.593933	1.480511	1.476452	1.472442	1.468509	1.464611
		0.595193	0.593201	1.478868	1.474811	1.665006	1.660797	1.65665
		0.594461	0.592485	1.477241	1.473186	1.663325	1.659116	1.654966
		0.593745	0.591808	1.475636	1.471613	1.661707	1.657489	1.653317
		0.593045	0.591175	1.474056	1.470101	1.660162	1.655924	1.65171
0.5961	0.594209	1.480409	1.476428	1.564379	1.560235	1.658567	1.654317	1.650078
0.595437	0.593524	1.478907	1.47488	1.562687	1.55853	1.656871	1.652616	1.648381
0.594776	0.592846	1.477394	1.473318	1.560981	1.556809	1.655161	1.650921	1.646712
0.594068	0.59215	1.47576	1.471666	1.661943	1.657693	1.900534	1.895636	1.890778
0.593351	0.591459	1.474089	1.469994	1.660207	1.655988	1.898528	1.893709	1.888934
1.480641	1.476582	1.564351	1.56004	1.658572	1.654357	1.896573	1.89177	1.887012
1.47916	1.475057	1.562725	1.558461	1.657008	1.652781	1.894662	1.889826	1.885037
1.47759	1.473457	1.664025	1.659669	1.746054	1.741504	1.892741	1.88789	1.883084
1.475904	1.471756	1.662243	1.657921	1.744185	1.73967	1.8908	1.885955	1.881152
1.566133	1.561749	1.660502	1.65621	1.742357	1.737891	1.888901	1.884062	1.879256
1.564467	1.560111	1.658878	1.654604	1.740638	1.736242	1.887097	1.882254	1.877422
1.562807	1.558492	1.65728	1.653022	1.738945	1.734614	1.885296	1.880452	1.875599
1.664363	1.659946	1.74621	1.741651	1.892925	1.888152	1.883385	1.878562	1.873725
1.662607	1.658247	1.7444	1.739882	1.891016	1.886219	1.881435	1.876639	1.871833
1.66095	1.656637	1.74268	1.73821	1.889164	1.884369	1.942472	1.937488	1.932509
1.659377	1.655102	1.741039	1.736624	1.887364	1.88259	1.94061	1.935604	1.930598
1.657819	1.65358	1.73941	1.735029	1.885577	1.880802	1.938732	1.933716	1.9287
1.656266	1.652066	1.737788	1.73341	1.883809	1.878999	1.93683	1.931823	1.926821
1.745083	1.740605	1.891612	1.886832	1.882025	1.877179	1.93491	1.929916	1.924934
1.743445	1.738975	1.889825	1.885026	1.8802	1.875334	1.932972	1.927992	1.923033
1.741823	1.737353	1.888042	1.883204	1.878349	1.873465	1.931012	1.926044	1.921108
1.740192	1.735744	1.886223	1.881357	1.939165	1.934069	1.929015	1.924037	1.919105
1.738559	1.734142	1.884381	1.879488	1.937182	1.932066	1.926995	1.922	1.917061
1.892315	1.887425	1.882516	1.877597	1.935195	1.930088	1.925031	1.920051	1.915131
1.89046	1.885557	1.880627	1.875681	1.933199	1.92813	1.923119	1.91819	1.913313
1.888599	1.883671	1.94144	1.936312	1.931235	1.926207	1.921239	1.916346	1.9115
1.886724	1.881739	1.939429	1.934351	1.929319	1.924333	1.919394	1.914508	1.909664
1.884872	1.87985	1.937467	1.932425	1.927427	1.922475	1.917562	1.912681	1.907839
1.883088	1.878101	1.935629	1.930545	1.925534	1.920604	1.915719	1.910865	1.906046
1.881332	1.876406	1.933849	1.92871	1.92367	1.918748	1.913881	1.909056	1.904265
1.94223	1.937147	1.932046	1.926935	1.921899	1.916946	1.912056	1.907238	1.902464
1.940352	1.935282	1.930235	1.925211	1.9202	1.915188	1.910244	1.905418	1.900653
1.938468	1.933419	1.928412	1.923442	1.918463	1.913457	1.908505	1.903654	1.898862
1.936578	1.93156	1.926575	1.921621	1.916684	1.911763	1.906858	1.901957	1.897088
1.934684	1.929692	1.924726	1.91979	1.914891	1.910039	1.905179	1.900263	1.895358
1.932773	1.927801	1.922862	1.917965	1.913092	1.90825	1.903411	1.89856	1.893711
1.930888	1.925915	1.920994	1.916131	1.911281	1.906438	1.901617	1.896841	1.892062
1.929089	1.924052	1.919117	1.914272	1.909445	1.90463	1.899836	1.895078	1.890319
1.927336	1.922218	1.917251	1.912409	1.907598	1.902819	1.898054	1.89329	1.888531
1.925569	1.920464	1.915482	1.910598	1.90577	1.901005	2.025505	2.020158	2.014832
1.923799	1.918786	1.913797	1.908831	1.903955	1.899189	2.023503	2.0182	2.012927
1.921988	1.917047	1.912079	1.907098	1.902203	1.897411	2.066064	2.060614	2.055187
1.92011	1.915215	1.91031	1.905412	1.900536	1.895675	2.064043	2.058607	2.053211
1.918265	1.913384	1.908532	1.903719	1.962499	1.957177	2.06208	2.056644	2.051262
1.916511	1.911585	1.906742	1.90198	1.960704	1.955422	2.060225	2.054767	2.049362

1.914806	1.90982	1.904972	1.90024	1.958906	1.953688	2.058415	2.052922	2.047476
1.913142	1.908136	1.903283	1.898554	1.95713	1.951964	2.056596	2.051054	2.045547
1.911509	1.906507	1.901646	1.896897	1.955361	1.950241	2.054774	2.049179	2.043607
1.909839	1.90486	1.96393	1.958713	2.019452	2.014158	2.052872	2.047298	2.041742
1.908132	1.903199	1.962155	1.956881	2.017536	2.01223	2.05089	2.045413	2.039953
1.90644	1.901553	1.960407	1.95509	2.059848	2.054353	2.152329	2.145846	2.13947
1.90479	1.899946	1.958714	1.953367	2.057953	2.052392	2.149868	2.143443	2.137159
1.903115	1.898313	1.957008	1.951664	2.0561	2.050484	2.147484	2.141094	2.13488
1.901349	1.896592	1.955235	1.949954	2.054296	2.04868	2.14528	2.138919	2.132746
1.899562	1.894841	1.953439	1.94825	2.052522	2.04693	2.143165	2.136821	2.130668
1.961894	1.956786	2.017558	2.012255	2.050742	2.045161	2.141029	2.134689	2.128537
1.960136	1.954994	2.01575	2.010521	2.048962	2.043391	2.138899	2.132552	2.126388
1.958372	1.953192	2.013907	2.008731	2.149929	2.143293	2.17966	2.173207	2.16692
1.956596	1.951368	2.012015	2.006868	2.147536	2.14105	2.177505	2.171128	2.164888
2.020873	2.015446	2.010177	2.005051	2.145217	2.138864	2.175387	2.169077	2.162872
2.019117	2.013699	2.008444	2.003332	2.143054	2.136782	2.173322	2.167047	2.16085
2.017378	2.011988	2.00675	2.00165	2.140951	2.134746	2.171284	2.165035	2.158837
2.015626	2.010272	2.048667	2.043378	2.138857	2.13272	2.169258	2.163032	2.156841
2.013877	2.008566	2.046901	2.041636	2.136786	2.130713	2.167244	2.161039	2.154855
2.012154	2.006865	2.045133	2.039892	2.134717	2.128707	2.210284	2.203981	2.197682
2.010453	2.005169	2.043365	2.038149	2.132655	2.126705	2.208249	2.201959	2.195661
2.008766	2.003481	2.142847	2.136687	2.173351	2.167285	2.206222	2.199941	2.193643
2.007092	2.001795	2.14078	2.134689	2.171342	2.165288	2.204205	2.197927	2.191627
2.005432	2.000138	2.138752	2.132717	2.169349	2.163305	2.265072	2.258325	2.251587
2.003785	1.998529	2.136747	2.130738	2.167342	2.161323	2.262947	2.256204	2.249462
2.002151	1.996947	2.134787	2.128796	2.165361	2.159353	2.26083	2.254089	2.247346
2.00053	1.995387	2.132917	2.12696	2.163462	2.157407	2.258688	2.251949	2.245224
1.998918	1.99384	2.131101	2.125183	2.161609	2.155475	2.256533	2.249798	2.243102
2.14134	2.135224	2.171845	2.165797	2.159694	2.153517	2.254381	2.24765	2.240973
2.139333	2.133241	2.169836	2.163816	2.157726	2.151538	2.252231	2.2455	2.238835
2.137383	2.131321	2.167868	2.16184	2.22299	2.216318	2.312837	2.305825	2.298916
2.135514	2.129512	2.165982	2.159882	2.220857	2.214191	2.310604	2.30363	2.296778
2.133664	2.127701	2.164088	2.157919	2.218727	2.21208	2.3084	2.301488	2.294712
2.131799	2.125818	2.162126	2.155931	2.216603	2.209994	2.30623	2.299426	2.292762
2.129925	2.123906	2.160141	2.153945	2.214498	2.207938	2.3041	2.297413	2.290866
2.128006	2.121979	2.158175	2.152	2.252948	2.246339	2.342767	2.335674	2.328736
2.126056	2.120038	2.156221	2.150087	2.250922	2.244365	2.340624	2.333571	2.326653
2.166574	2.160418	2.154292	2.148208	2.248944	2.242424	2.338482	2.331451	2.324545
2.164633	2.158469	2.152381	2.146365	2.247024	2.240526	2.336347	2.329314	2.322409
2.162769	2.156617	2.217539	2.211099	2.307464	2.300684	2.334195	2.327169	2.320277
2.161046	2.154953	2.215739	2.209205	2.30539	2.298589	2.33201	2.325008	2.318153
2.159323	2.153276	2.213916	2.207282	2.303286	2.296477	2.329823	2.322855	2.316049
2.157465	2.151386	2.211882	2.205237	2.301133	2.294369	2.327634	2.320704	2.313954
2.155546	2.149403	2.209758	2.20314	2.298964	2.292277	2.325471	2.318593	2.311907
2.153626	2.147447	2.207677	2.201089	2.296862	2.290275	2.323437	2.316655	2.310052
2.151695	2.145498	2.205617	2.199069	2.294813	2.288349	2.321511	2.314863	2.308359
2.216924	2.210286	2.244455	2.23769	2.292895	2.286479	2.319574	2.313005	2.30657
2.215098	2.208547	2.242693	2.235965	2.291158	2.284688	2.317618	2.311057	2.304652
2.213238	2.206757	2.24087	2.234186	2.329381	2.322426	2.340481	2.333889	2.327471
2.211248	2.204763	2.238794	2.232162	2.327275	2.320375	2.338449	2.331881	2.325496

2.209231	2.202741	2.2367	2.230131	2.325176	2.318353	2.33646	2.329923	2.32357
2.207262	2.20085	2.296715	2.290115	2.32332	2.316541	2.334657	2.328154	2.321828
2.205323	2.199023	2.294924	2.288441	2.321582	2.314835	2.332951	2.326488	2.320188
2.20347	2.197186	2.293047	2.286621	2.319704	2.312992	2.331113	2.324697	2.318436
2.201703	2.195349	2.291105	2.284676	2.317705	2.311027	2.329155	2.322795	2.316587
2.240364	2.233771	2.28915	2.282759	2.315761	2.309153	2.327316	2.321005	2.314839
2.238257	2.231723	2.287158	2.280891	2.313922	2.307449	2.325688	2.31942	2.313272
2.236242	2.229766	2.285237	2.279058	2.31208	2.305721	2.324026	2.317799	2.311675
2.23453	2.228053	2.323241	2.316619	2.334752	2.328385	2.346488	2.340265	2.334148
2.232933	2.22645	2.321435	2.314738	2.332801	2.32643	2.34452	2.338341	2.332279
2.231264	2.224845	2.319726	2.313018	2.331057	2.324699	2.342783	2.336638	2.330615
2.229564	2.223251	2.318087	2.311404	2.329443	2.323103	2.341181	2.335064	2.32907
2.227756	2.221496	2.316262	2.309622	2.327673	2.321369	2.33945	2.33337	2.32741
2.225819	2.219554	2.314227	2.307651	2.32573	2.319478	2.337572	2.331536	2.325615
2.223962	2.217695	2.336795	2.330245	2.348126	2.341889	2.335782	2.329796	2.323918
2.222296	2.216042	2.335004	2.328511	2.346428	2.340247	2.334196	2.328261	2.322429
2.220624	2.214393	2.333219	2.326784	2.344734	2.338609	2.332612	2.326736	2.320959
2.218755	2.212558	2.331263	2.324891	2.342865	2.336785	2.330847	2.325054	2.319371
2.216842	2.210689	2.329281	2.322973	2.340965	2.334926	2.32904	2.323326	2.317735
2.316163	2.309523	2.327507	2.32126	2.33927	2.333268	2.327404	2.321684	2.316082
2.314464	2.307877	2.325893	2.319704	2.337731	2.331762	2.325895	2.320102	2.31441
2.312794	2.306267	2.324315	2.318181	2.336223	2.330286	2.324428	2.318607	2.312873
2.311153	2.304696	2.322776	2.316693	2.334747	2.328843	2.323013	2.317221	2.311505
2.333896	2.327469	2.345331	2.339252	2.333272	2.327392	2.321586	2.315831	2.310145
2.33225	2.325884	2.343777	2.337753	2.331808	2.325936	2.32014	2.314415	2.30876
2.330567	2.32426	2.342178	2.336204	2.330288	2.324418	2.318628	2.312931	2.307309
2.328729	2.322483	2.340413	2.334468	2.328571	2.322707	2.316924	2.311246	2.305646
2.326829	2.320644	2.338576	2.332648	2.326763	2.320905	2.31513	2.309464	2.303881
2.325117	2.318987	2.336922	2.33101	2.325139	2.319295	2.313531	2.307872	2.302296
2.323567	2.317485	2.335424	2.329527	2.323672	2.317848	2.312098	2.306439	2.30086
2.346028	2.339949	2.333956	2.328064	2.322222	2.316422	2.310692	2.305041	2.299467
2.344504	2.338483	2.33251	2.326607	2.320772	2.315004	2.309305	2.303675	2.298114
2.343003	2.337029	2.331073	2.325159	2.31933	2.313588	2.307918	2.302313	2.296773
2.341519	2.335572	2.329626	2.323708	2.317882	2.312153	2.306502	2.300928	2.295419
2.340048	2.334116	2.328178	2.322263	2.316442	2.310719	2.305081	2.299537	2.294061
2.33858	2.332655	2.326732	2.320834	2.315024	2.309305	2.303674	2.298146	2.292687
2.337116	2.331192	2.325287	2.319416	2.313623	2.307907	2.302277	2.296754	2.291302
2.335647	2.329723	2.323832	2.317987	2.312215	2.306518	2.3009	2.295378	2.289917
2.334173	2.328246	2.322369	2.316547	2.310803	2.305142	2.299545	2.294021	2.288537
2.332703	2.326773	2.320907	2.315105	2.309385	2.303757	2.298176	2.292635	2.287116
2.331245	2.325309	2.31945	2.313661	2.307957	2.302354	2.296777	2.291189	2.285618
2.32978	2.323844	2.317993	2.312219	2.30653	2.300947	2.295374	2.289746	2.284133
2.328288	2.322366	2.316534	2.310781	2.305111	2.299545	2.325236	2.319485	2.313743
2.326781	2.320883	2.315074	2.309347	2.303698	2.298147	2.323807	2.318075	2.312348
2.325273	2.319399	2.313616	2.307918	2.302293	2.29676	2.322387	2.316664	2.310942
2.323761	2.317913	2.31216	2.306494	2.300895	2.295382	2.320974	2.315258	2.30954
2.322255	2.316432	2.310706	2.305072	2.2995	2.294008	2.319574	2.313898	2.308214
2.320756	2.314954	2.309255	2.303653	2.298111	2.292641	2.318193	2.312598	2.306991
2.319261	2.313485	2.307811	2.302236	2.327899	2.322341	2.3168	2.311271	2.305729
2.317766	2.312027	2.306379	2.300822	2.326453	2.320906	2.315379	2.309874	2.30436

2.316277	2.310578	2.304955	2.299413	2.32501	2.319468	2.313949	2.308452	2.302954
2.314801	2.309132	2.303535	2.298014	2.323578	2.318035	2.312519	2.30703	2.301556
2.313336	2.307694	2.302122	2.296624	2.322153	2.316605	2.311088	2.305602	2.30015
2.311883	2.306265	2.331872	2.326297	2.320736	2.315184	2.309665	2.304182	2.298745
2.310436	2.304843	2.330426	2.324877	2.319329	2.313772	2.308248	2.302767	2.297338
2.309109	2.303502	2.329025	2.323461	2.317907	2.312352	2.306835	2.30136	2.295939
2.307953	2.302264	2.327671	2.322031	2.316445	2.310913	2.305424	2.299959	2.294548
2.337974	2.332144	2.326372	2.320656	2.315028	2.3095	2.304023	2.298566	2.293166
2.336704	2.330945	2.325172	2.319395	2.313708	2.308121	2.302607	2.297151	2.291773
2.335382	2.329729	2.323986	2.318181	2.312451	2.306801	2.30124	2.295778	2.290413
2.333963	2.328365	2.322687	2.316956	2.311271	2.305631	2.331713	2.326176	2.320736
2.332479	2.326909	2.321319	2.315725	2.310138	2.304551	2.330667	2.325096	2.319599
2.331066	2.325483	2.319918	2.314383	2.30885	2.303312	2.329479	2.323941	2.318464
2.329714	2.324067	2.318465	2.312916	2.3074	2.301912	2.328157	2.32272	2.317348
2.328444	2.322761	2.317123	2.311538	2.337843	2.33229	2.326815	2.321444	2.316149
2.327317	2.32165	2.315983	2.310325	2.336584	2.330959	2.325437	2.320062	2.314791
2.326158	2.320522	2.314847	2.309149	2.33538	2.329696	2.324129	2.31873	2.313455
2.324788	2.319184	2.313568	2.307949	2.334241	2.3286	2.323046	2.317615	2.312288
2.323356	2.317777	2.312235	2.30673	2.33311	2.327548	2.322034	2.316577	2.311193
2.322076	2.316466	2.342949	2.33736	2.331811	2.326297	2.320836	2.31543	2.310091
2.320895	2.315213	2.34164	2.335973	2.330396	2.324914	2.319518	2.314216	2.308992
2.351906	2.346145	2.340421	2.334727	2.329132	2.323639	2.341103	2.335752	2.33049
2.350642	2.344987	2.339325	2.333665	2.328058	2.322494	2.339861	2.334454	2.32916
2.349353	2.343766	2.338155	2.332533	2.326937	2.321347	2.338661	2.333225	2.32791
2.348019	2.342391	2.336783	2.331203	2.325665	2.320158	2.337539	2.332155	2.326867
2.346705	2.341021	2.335403	2.329846	2.324362	2.318947	2.336408	2.33109	2.325845
2.345501	2.339834	2.3342	2.328602	2.323096	2.317681	2.335141	2.329861	2.324664
2.344346	2.33872	2.333071	2.32741	2.321855	2.316404	2.333833	2.328579	2.323432
2.34311	2.337493	2.331863	2.326229	2.320694	2.315256	2.332685	2.327457	2.322334
2.341812	2.336179	2.330596	2.325063	2.319607	2.314219	2.331672	2.326466	2.321346
2.340568	2.334934	2.32939	2.323929	2.318524	2.313165	2.330617	2.325412	2.320284
2.339402	2.333793	2.328277	2.322846	2.317454	2.312083	2.329491	2.324251	2.3191
2.338252	2.332655	2.327149	2.321726	2.316342	2.310981	2.328369	2.32311	2.317944
2.337097	2.331467	2.325932	2.32048	2.315112	2.309829	2.327274	2.322053	2.316904
2.335944	2.330278	2.32471	2.319228	2.31388	2.308685	2.326203	2.321028	2.315897
2.334785	2.32913	2.323581	2.318124	2.312808	2.307656	2.325184	2.320002	2.314852
2.333624	2.328008	2.322504	2.317103	2.311823	2.306685	2.324192	2.318977	2.313794
2.332478	2.326898	2.32142	2.316036	2.310762	2.305614	2.323098	2.317899	2.31274
2.331349	2.325805	2.320334	2.31493	2.30963	2.304441	2.321896	2.316764	2.311687
2.330217	2.324689	2.319228	2.313828	2.308526	2.303325	2.346238	2.341059	2.335946
2.329078	2.32353	2.318085	2.31273	2.30747	2.302309	2.345219	2.340046	2.334924
2.327937	2.322377	2.316957	2.311655	2.306443	2.301328	2.344234	2.339048	2.333897
2.326788	2.321261	2.315881	2.310627	2.322183	2.317046	2.357943	2.352678	2.347441
2.325646	2.320173	2.314837	2.309627	2.321195	2.31607	2.356931	2.351627	2.346352
2.324542	2.319118	2.330613	2.325383	2.320191	2.315039	2.380218	2.374833	2.369492
2.32347	2.318095	2.329605	2.324385	2.319178	2.31397	2.379097	2.373725	2.368421
2.29475	2.289633	2.328597	2.323354	2.318126	2.312899	2.378015	2.372671	2.367406
2.293799	2.2887	2.32759	2.322279	2.317023	2.311819	2.376972	2.371673	2.366451
2.310778	2.305638	2.326585	2.321232	2.31596	2.310773	2.375956	2.370698	2.365512
2.309782	2.304654	2.325596	2.320261	2.314995	2.309796	2.374972	2.36974	2.364577

2.308771	2.303642	2.324582	2.319282	2.314035	2.308832	2.374005	2.368791	2.363643
2.307722	2.302557	2.323471	2.318202	2.312991	2.307833	2.386752	2.381526	2.376358
2.306664	2.301448	2.322327	2.317086	2.311919	2.306826	2.385816	2.380595	2.375425
2.305666	2.300435	2.321283	2.316048	2.327522	2.322431	2.384885	2.379637	2.374436
2.304718	2.299497	2.32032	2.315069	2.326575	2.321505	2.383958	2.378654	2.373397
2.303771	2.29856	2.319361	2.314102	2.375189	2.36996	2.383036	2.377703	2.372416
2.302822	2.297617	2.3184	2.313143	2.374267	2.369046	2.382115	2.37679	2.371512
2.301878	2.296678	2.334249	2.328962	2.373358	2.368145	2.38121	2.375899	2.370635
2.300943	2.29575	2.333337	2.32806	2.372475	2.367273	2.380334	2.375031	2.369774
2.300008	2.294822	2.332427	2.327163	2.371597	2.366409	2.379468	2.374175	2.368928
2.3171	2.311875	2.331502	2.326249	2.370698	2.365526	2.378588	2.373312	2.368082
2.316166	2.31095	2.330568	2.325328	2.369789	2.364637	2.377704	2.372452	2.367242
2.315232	2.310026	2.329638	2.324416	2.368896	2.363762	2.376834	2.371603	2.366414
2.314297	2.309104	2.328711	2.323511	2.368016	2.362901	2.375977	2.370768	2.365599
2.313369	2.308182	2.303104	2.298114	2.367127	2.362031	2.375113	2.369924	2.364774
2.31245	2.307257	2.302185	2.29721	2.366218	2.361145	2.374233	2.369061	2.363926
2.31154	2.306346	2.301278	2.296312	2.365312	2.360261	2.373357	2.368206	2.36309
2.310632	2.305452	2.300387	2.295418	2.364405	2.359385	2.372497	2.367375	2.362282
2.309739	2.304588	2.299529	2.294551	2.363522	2.358524	2.371648	2.366562	2.361501
2.308889	2.303802	2.298766	2.293782	2.362727	2.357706	2.370813	2.365776	2.36077
2.308071	2.303072	2.298071	2.293084	2.361996	2.356919	2.369989	2.365011	2.360077
2.307243	2.302277	2.349721	2.344551	2.379424	2.374239	2.36916	2.364221	2.359336
2.306406	2.301411	2.348857	2.343721	2.378595	2.3734	2.368317	2.3634	2.358544
2.305554	2.300535	2.348001	2.342924	2.377812	2.372614	2.367522	2.362599	2.357743
2.304663	2.29965	2.347182	2.342214	2.377135	2.371928	2.366808	2.361833	2.356935
2.303793	2.298789	2.346381	2.341498	2.376445	2.371252	2.366119	2.36109	2.356139
2.30302	2.298005	2.345594	2.340685	2.375623	2.370509	2.365432	2.360384	2.355394
2.302284	2.297261	2.344824	2.339838	2.374746	2.369732	2.364736	2.359687	2.354668
2.301474	2.296508	2.344092	2.339071	2.373929	2.368934	2.363955	2.358914	2.353893
2.30061	2.295755	2.343393	2.338362	2.373151	2.368111	2.363105	2.358079	2.353084
2.299778	2.294963	2.34263	2.337601	2.351676	2.346739	2.362248	2.357262	2.352317
2.298985	2.294111	2.341771	2.336761	2.350821	2.345866	2.361377	2.356471	2.351616
2.298222	2.293292	2.340949	2.335957	2.350007	2.345043	2.36055	2.355689	2.350887
2.297509	2.292564	2.340238	2.335257	2.349297	2.344334	2.359813	2.354902	2.350062
2.296797	2.291866	2.339568	2.334592	2.348624	2.343664	2.359116	2.354136	2.349232
2.296018	2.291162	2.338904	2.333917	2.347927	2.34297	2.358422	2.353423	2.348494
2.295202	2.29045	2.338242	2.333242	2.347229	2.342273	2.357741	2.352746	2.347808
2.294389	2.28966	2.337495	2.332546	2.346548	2.341595	2.357058	2.35207	2.347132
2.293573	2.28879	2.336667	2.331833	2.345888	2.340936	2.356375	2.351398	2.34647
2.292795	2.287975	2.335868	2.331086	2.345168	2.34024	2.355691	2.350725	2.345803
2.292072	2.287248	2.335116	2.330273	2.344339	2.339478	2.355001	2.350047	2.345122
2.291367	2.286551	2.334401	2.329504	2.343547	2.338736	2.354315	2.349371	2.344444
2.290657	2.285852	2.333731	2.328872	2.342914	2.338085	2.353636	2.348698	2.343781
2.289963	2.285176	2.333097	2.328293	2.342333	2.337462	2.35296	2.348027	2.343123
2.289308	2.284558	2.332498	2.32768	2.34169	2.336798	2.352282	2.347354	2.342459
2.288683	2.28398	2.331926	2.327056	2.341015	2.336113	2.351604	2.34668	2.341794
2.288059	2.283359	2.331304	2.32641	2.340342	2.335443	2.330593	2.325808	2.321062
2.287441	2.282694	2.330626	2.325738	2.339669	2.334789	2.329952	2.325163	2.320409
2.286799	2.28202	2.329949	2.32507	2.338999	2.334132	2.329304	2.324517	2.319761
2.286115	2.281345	2.329288	2.324415	2.33833	2.33346	2.328634	2.323861	2.319121

2.285418	2.280668	2.32863	2.323768	2.337671	2.332797	2.327971	2.323212	2.318489
2.284727	2.279988	2.32797	2.323123	2.337022	2.332152	2.327332	2.322577	2.317865
2.284037	2.279311	2.327316	2.322492	2.336393	2.331532	2.326716	2.321962	2.317254
2.336442	2.331541	2.345551	2.340681	2.335825	2.330976	2.326164	2.321402	2.316682
2.33577	2.330904	2.34495	2.34013	2.335309	2.330475	2.325664	2.320888	2.316143
2.335153	2.330296	2.344343	2.339538	2.334729	2.329907	2.325102	2.320322	2.315565
2.334611	2.329727	2.343729	2.338891	2.334068	2.329256	2.324463	2.31969	2.314939
2.334047	2.32914	2.343102	2.338234	2.333396	2.32859	2.323807	2.319044	2.314304
2.333396	2.32849	2.342435	2.337564	2.332724	2.327915	2.323136	2.318386	2.313667
2.332739	2.327845	2.34178	2.336913	2.332075	2.327263	2.322486	2.317745	2.313043
2.332164	2.327285	2.34121	2.336339	2.331502	2.326699	2.321927	2.31718	2.312466
2.331622	2.326763	2.340681	2.335808	2.330975	2.326188	2.321421	2.31666	2.311919
2.303333	2.298576	2.340101	2.335256	2.330442	2.325665	2.320902	2.316133	2.31138
2.302647	2.297906	2.339479	2.33469	2.329912	2.325142	2.320379	2.31561	2.310855
2.302011	2.297273	2.338864	2.334094	2.329329	2.324566	2.319806	2.315035	2.310278
2.301453	2.296698	2.338256	2.333443	2.328657	2.323903	2.319151	2.314375	2.309611
2.300917	2.296148	2.337675	2.332818	2.328009	2.323259	2.318515	2.313738	2.308973
2.300381	2.295618	2.337147	2.332279	2.327463	2.322709	2.317967	2.313201	2.308447
2.29985	2.2951	2.336641	2.331775	2.326958	2.322198	2.317454	2.312701	2.307964
2.299312	2.294568	2.336119	2.331262	2.326448	2.321683	2.33789	2.333029	2.328188
2.29877	2.294029	2.335591	2.33075	2.325944	2.321176	2.337355	2.332493	2.327654
2.298227	2.293492	2.335063	2.33023	2.325428	2.320661	2.336817	2.331958	2.327123
2.297681	2.292957	2.334534	2.329699	2.324899	2.320137	2.336276	2.331425	2.326596
2.297141	2.292426	2.334009	2.329177	2.324379	2.319619	2.335739	2.330892	2.326067
2.296611	2.291898	2.333491	2.328672	2.323879	2.319114	2.335208	2.33036	2.325536
2.296085	2.291373	2.332974	2.328167	2.323379	2.318609	2.334678	2.329829	2.325005
2.295558	2.290851	2.332456	2.327648	2.32286	2.31809	2.334142	2.329296	2.324477
2.295035	2.290332	2.331939	2.327127	2.322336	2.317567	2.333604	2.328763	2.323948
2.294518	2.289819	2.331431	2.326622	2.321831	2.317055	2.33307	2.328227	2.323414
2.294006	2.28931	2.330929	2.326129	2.321338	2.31655	2.332537	2.32769	2.322875
2.313315	2.308535	2.330425	2.325627	2.320835	2.316044	2.350626	2.345654	2.340719
2.312781	2.308012	2.329918	2.325111	2.320318	2.315535	2.350138	2.345156	2.34021
2.312256	2.307493	2.329415	2.324605	2.31981	2.31503	2.363511	2.358456	2.353434
2.311736	2.306967	2.328907	2.32411	2.319315	2.314514	2.363004	2.357957	2.352944
2.311245	2.306467	2.328424	2.323641	2.318845	2.314024	2.362514	2.357472	2.352464
2.337724	2.33285	2.328024	2.323242	2.33934	2.33439	2.375782	2.370676	2.365608
2.337362	2.332492	2.327664	2.322874	2.338947	2.334003	2.375397	2.370254	2.365151
2.336922	2.332062	2.327229	2.322423	2.338473	2.333542	2.374971	2.369841	2.364748
2.336417	2.331573	2.326732	2.321901	2.337929	2.333019	2.374514	2.369439	2.364399
2.33595	2.33112	2.326274	2.321424	2.384284	2.379143	2.374043	2.368999	2.363992
2.335554	2.330737	2.32589	2.321027	2.383863	2.378677	2.373544	2.368493	2.363491
2.335139	2.330327	2.325481	2.320618	2.38345	2.378234	2.373075	2.36801	2.363004
2.334635	2.329811	2.324976	2.320137	2.383015	2.377828	2.372688	2.36762	2.362605
2.334119	2.329279	2.324454	2.319646	2.382585	2.377441	2.372328	2.367254	2.362223
2.282533	2.277888	2.324019	2.319222	2.382203	2.377056	2.371931	2.366831	2.361771
2.282142	2.277495	2.323629	2.318831	2.381843	2.376674	2.371519	2.366381	2.361286
2.281725	2.277056	2.359023	2.35407	2.381414	2.376251	2.3711	2.365959	2.360859
2.281283	2.27657	2.358548	2.353572	2.380908	2.375782	2.370671	2.365562	2.360488
2.280842	2.276111	2.358118	2.353126	2.380453	2.375351	2.370267	2.365184	2.360133
2.2804	2.2757	2.357766	2.352773	2.380093	2.374987	2.369903	2.36483	2.359792

2.279971	2.275308	2.357435	2.352446	2.37976	2.374643	2.369551	2.364483	2.359449
2.297912	2.293208	2.371361	2.366324	2.379416	2.374295	2.369198	2.364127	2.35909
2.297529	2.292837	2.371019	2.366	2.379074	2.37395	2.368846	2.363766	2.358718
2.297149	2.292457	2.370689	2.365667	2.378729	2.3736	2.368489	2.363392	2.358326
2.296776	2.292073	2.370367	2.365328	2.37838	2.373248	2.368127	2.363007	2.357914
2.318803	2.313981	2.370029	2.364987	2.378024	2.372885	2.367751	2.362604	2.357487
2.318451	2.31362	2.369671	2.364646	2.377662	2.372509	2.367355	2.362172	2.357027
2.318095	2.313259	2.369312	2.364295	2.377295	2.372139	2.366979	2.361787	2.356635
2.317734	2.312903	2.368958	2.363927	2.376925	2.371786	2.366651	2.361501	2.356381
2.31737	2.312545	2.368602	2.363556	2.376554	2.371435	2.36633	2.361233	2.356156
2.30338	2.298533	2.34439	2.339423	2.376184	2.371072	2.36598	2.360912	2.355867
2.302995	2.298145	2.344011	2.339062	2.375815	2.370702	2.365617	2.360567	2.355549
2.302599	2.297758	2.343644	2.338695	2.375446	2.370332	2.365251	2.360211	2.355209
2.302191	2.297372	2.343287	2.338322	2.375076	2.369961	2.36488	2.359843	2.354846
2.301834	2.296998	2.330723	2.325746	2.374719	2.369595	2.364507	2.359472	2.35448
2.301549	2.29663	2.330324	2.325378	2.374368	2.369222	2.364118	2.359089	2.354109
2.318232	2.313189	2.349872	2.344827	2.374048	2.368888	2.363771	2.358733	2.353747
2.317946	2.312934	2.349619	2.344561	2.373799	2.368647	2.363522	2.358432	2.35339
2.317645	2.312694	2.349385	2.344306	2.373557	2.368421	2.363296	2.358157	2.353062
2.317285	2.312373	2.349047	2.343953	2.373226	2.368106	2.363002	2.357892	2.352822
2.316894	2.312012	2.348658	2.343553	2.372851	2.367743	2.362671	2.357633	2.352633
2.316586	2.311706	2.348336	2.343231	2.361721	2.356561	2.362325	2.357322	2.352364
2.316361	2.311458	2.348082	2.342985	2.361463	2.356231	2.361956	2.356955	2.352015
2.316122	2.311178	2.347792	2.342714	2.361195	2.35593	2.36164	2.356635	2.351697
2.315844	2.310826	2.347422	2.342381	2.360899	2.355666	2.361413	2.356403	2.351452
2.315562	2.310491	2.347071	2.342055	2.360605	2.355415	2.361202	2.356175	2.351197
2.31529	2.310242	2.326141	2.321199	2.336754	2.331693	2.350022	2.344927	2.339878
2.315027	2.310028	2.325921	2.320948	2.336487	2.331432	2.349761	2.344613	2.33951
2.314785	2.309795	2.325646	2.320629	2.336146	2.331108	2.349464	2.344328	2.339233
2.31456	2.309556	2.325341	2.320267	2.335756	2.33074	2.349142	2.344062	2.33902
2.314336	2.309335	2.325081	2.319972	2.335443	2.330437	2.348866	2.343816	2.338801
2.314123	2.309143	2.324879	2.319755	2.335215	2.330205	2.348642	2.343594	2.338579
2.313865	2.308911	2.324654	2.319539	2.335002	2.329989	2.34843	2.343374	2.338348
2.313509	2.30859	2.324366	2.319298	2.334788	2.329779	2.348221	2.343151	2.338099
2.313155	2.308265	2.324075	2.319058	2.334576	2.329571	2.348014	2.342929	2.337851
2.312898	2.308015	2.32383	2.318834	2.334363	2.329354	2.347799	2.342709	2.337623
2.312689	2.307799	2.323607	2.318617	2.334147	2.329132	2.347581	2.342491	2.337406
2.312469	2.307577	2.323376	2.318387	2.333917	2.328903	2.347362	2.342273	2.337188
2.312247	2.307354	2.323142	2.318148	2.333674	2.328667	2.34714	2.342055	2.336971
2.312035	2.307135	2.322911	2.317916	2.333439	2.328434	2.346917	2.341832	2.336746
2.311822	2.306907	2.322678	2.317692	2.333217	2.328207	2.346693	2.3416	2.336508
2.311653	2.306716	2.322467	2.317476	2.312513	2.307586	2.322964	2.317928	2.312888
2.311581	2.306608	2.322308	2.317267	2.312277	2.307352	2.322737	2.317706	2.312674
2.311521	2.306514	2.322162	2.317066	2.312045	2.30712	2.322513	2.31749	2.312469
2.311362	2.306351	2.321978	2.316866	2.311832	2.306895	2.322284	2.31727	2.312267
2.311147	2.306149	2.321779	2.316675	2.311639	2.306683	2.32206	2.317052	2.312067
2.310923	2.305944	2.321605	2.316538	2.311512	2.306534	2.321886	2.316854	2.311853
2.310682	2.30573	2.321456	2.316458	2.311458	2.306451	2.321764	2.316676	2.311625
2.33133	2.326285	2.321284	2.316326	2.311342	2.306322	2.321615	2.316494	2.311409
2.331176	2.326086	2.321059	2.316091	2.311109	2.306106	2.321413	2.316293	2.311204

2.331038	2.325915	2.320861	2.31587	2.310881	2.305892	2.321213	2.31611	2.311032
2.330907	2.325822	2.320773	2.315761	2.310751	2.305738	2.321048	2.315973	2.310929
2.33077	2.325743	2.320709	2.315678	2.310647	2.305607	2.320904	2.31586	2.310855
2.330575	2.325563	2.320538	2.315514	2.310496	2.305477	2.320792	2.315756	2.310752
2.330342	2.32532	2.320302	2.315301	2.310316	2.30535	2.320702	2.31566	2.310633
2.330179	2.325139	2.320107	2.315096	2.310116	2.305172	2.320546	2.315512	2.310489
2.330097	2.325028	2.319957	2.314895	2.309883	2.304929	2.320307	2.315299	2.310312
2.330004	2.324911	2.319816	2.314726	2.309693	2.304726	2.299795	2.294892	2.290019
2.329866	2.324756	2.319668	2.314606	2.309587	2.304612	2.299677	2.294781	2.289917
2.329723	2.32461	2.319539	2.314509	2.309502	2.304512	2.299561	2.294665	2.289801
2.329599	2.324515	2.319453	2.314419	2.309389	2.30435	2.29936	2.294453	2.289593
2.329488	2.324446	2.319392	2.31434	2.309277	2.304182	2.299146	2.294219	2.289358
2.329399	2.324373	2.319327	2.314275	2.309211	2.304116	2.299071	2.294114	2.289222
2.329332	2.324301	2.319261	2.314223	2.309183	2.304132	2.299107	2.29411	2.289162
2.329269	2.324231	2.319187	2.314144	2.309108	2.304074	2.299059	2.294055	2.289093
2.329208	2.324165	2.319103	2.314033	2.308972	2.303912	2.29889	2.293924	2.289007
2.329148	2.324094	2.319009	2.313905	2.308821	2.303748	2.29873	2.293799	2.288923
2.329084	2.324008	2.318888	2.31374	2.308646	2.303612	2.298633	2.293717	2.288848
2.329021	2.323925	2.318777	2.313593	2.308496	2.303501	2.298557	2.293647	2.288777
2.309957	2.304938	2.318736	2.313566	2.308466	2.303447	2.298483	2.293572	2.288706
2.309903	2.304903	2.318729	2.313598	2.308498	2.303425	2.298414	2.293497	2.288634
2.309847	2.304843	2.318663	2.313527	2.308424	2.30335	2.298337	2.293418	2.288553
2.309789	2.30476	2.318543	2.31336	2.30825	2.303224	2.298253	2.293336	2.288462
2.309728	2.304694	2.318465	2.313268	2.308154	2.303137	2.298174	2.293254	2.288377
2.309652	2.304658	2.318464	2.313308	2.308191	2.303107	2.298088	2.293167	2.288302
2.309603	2.304626	2.318462	2.313354	2.308241	2.303107	2.298039	2.293102	2.28823
2.309618	2.304572	2.318398	2.313328	2.308249	2.303146	2.298082	2.293084	2.288141
2.309662	2.304529	2.31833	2.313279	2.308234	2.303189	2.29815	2.293089	2.288069
2.309701	2.304559	2.299481	2.294451	2.308168	2.303157	2.298151	2.293102	2.288079
2.309738	2.304637	2.299523	2.2944	2.30807	2.303078	2.298109	2.29312	2.288145
2.309727	2.304665	2.299553	2.294404	2.30805	2.30303	2.298051	2.293095	2.288159
2.309657	2.304643	2.299576	2.294474	2.308117	2.30301	2.297965	2.293018	2.288118
2.309615	2.304607	2.299567	2.294512	2.308166	2.303014	2.297927	2.292965	2.288066
2.309636	2.304548	2.299497	2.29448	2.308169	2.303058	2.297983	2.292963	2.287998
2.30967	2.304505	2.299431	2.294428	2.308145	2.303093	2.298049	2.292981	2.287953
2.309685	2.304518	2.299419	2.294371	2.289369	2.284424	2.298049	2.293002	2.287988
2.3097	2.304566	2.299442	2.294322	2.289292	2.284372	2.29803	2.293032	2.288064
2.309747	2.304641	2.299509	2.294353	2.289304	2.284388	2.298054	2.293078	2.288126
2.309818	2.304743	2.299616	2.294449	2.289386	2.284454	2.298109	2.293138	2.288186
2.292459	2.28753	2.299685	2.294524	2.28945	2.284481	2.298107	2.293144	2.288207
2.292398	2.287502	2.2997	2.294565	2.289481	2.284448	2.298023	2.293073	2.288171
2.292366	2.287494	2.299726	2.294618	2.289529	2.284446	2.297981	2.293038	2.288163
2.292417	2.28755	2.299799	2.294702	2.289616	2.284525	2.29805	2.293106	2.288229
2.292494	2.287622	2.299883	2.294796	2.289717	2.284631	2.298157	2.293207	2.288322
2.29256	2.287682	2.299961	2.294891	2.28982	2.284729	2.279711	2.274835	2.270022
2.292622	2.28773	2.300029	2.294979	2.289919	2.284825	2.279803	2.274926	2.270114
2.292643	2.28772	2.300021	2.294999	2.289964	2.284895	2.279889	2.275014	2.270198
2.292624	2.287649	2.29994	2.294952	2.289957	2.284938	2.279969	2.275099	2.270275
2.292641	2.287635	2.299921	2.294953	2.289984	2.284998	2.280054	2.275186	2.270358
2.292714	2.287705	2.3	2.295033	2.290066	2.285087	2.280148	2.275281	2.270452

2.292807	2.287803	2.282855	2.277952	2.290157	2.285176	2.280238	2.27537	2.270541
2.292905	2.287902	2.282943	2.27802	2.290225	2.285249	2.280315	2.275448	2.270619
2.293017	2.288023	2.283053	2.278104	2.2903	2.285319	2.280384	2.275518	2.27069
2.293159	2.288198	2.283227	2.278251	2.290423	2.285407	2.280448	2.275579	2.270756
2.29332	2.288408	2.283443	2.278439	2.290575	2.285505	2.280506	2.275632	2.270817
2.293437	2.288553	2.283592	2.278573	2.290694	2.285593	2.28057	2.275694	2.270885
2.293508	2.288632	2.283672	2.278654	2.29078	2.285668	2.280638	2.275763	2.270959
2.293583	2.288702	2.283741	2.278727	2.290863	2.285752	2.28072	2.275841	2.271034
2.293663	2.288763	2.283799	2.278794	2.290947	2.285842	2.280812	2.275917	2.271096
2.293775	2.288848	2.283874	2.278869	2.291039	2.285953	2.280933	2.276023	2.271182
2.293973	2.289013	2.284009	2.278975	2.273972	2.268995	2.281127	2.276208	2.271341
2.2942	2.289206	2.284169	2.279096	2.274088	2.269158	2.281343	2.27642	2.271529
2.29434	2.28934	2.284291	2.279202	2.274188	2.269262	2.281469	2.276554	2.271672
2.294419	2.289434	2.284391	2.279302	2.274277	2.269326	2.281529	2.276633	2.271786
2.294539	2.289554	2.284518	2.27944	2.274415	2.269444	2.281639	2.276738	2.271895
2.294708	2.289703	2.284675	2.279627	2.274614	2.269633	2.28181	2.276873	2.271992
2.294888	2.289874	2.284851	2.279823	2.274814	2.269821	2.264874	2.259983	2.255152
2.295066	2.290076	2.285057	2.280019	2.274996	2.26998	2.265021	2.260146	2.255335
2.295249	2.290285	2.285266	2.280209	2.27517	2.270138	2.265174	2.260323	2.255539
2.295442	2.290473	2.285445	2.280374	2.275332	2.270314	2.265362	2.260516	2.255732
2.295638	2.29065	2.285608	2.280526	2.275489	2.270497	2.265566	2.260717	2.255921
2.295821	2.290816	2.285772	2.280696	2.275665	2.27068	2.265751	2.260891	2.256084
2.295992	2.290971	2.285932	2.28088	2.275858	2.270864	2.265921	2.261043	2.256222
2.296163	2.291143	2.28611	2.281068	2.276046	2.271038	2.266084	2.261205	2.256389
2.296333	2.291339	2.286312	2.281261	2.276227	2.271198	2.266236	2.261382	2.256597
2.296517	2.291546	2.286517	2.281447	2.276399	2.271362	2.266403	2.261575	2.256821
2.29673	2.291756	2.286706	2.281599	2.276543	2.271534	2.266606	2.261797	2.257056
2.29696	2.291978	2.286905	2.281763	2.276701	2.271725	2.266829	2.262031	2.257295
2.297205	2.292226	2.28716	2.282027	2.276962	2.271968	2.267056	2.262256	2.257523
2.297463	2.292491	2.287448	2.282348	2.277284	2.272244	2.26729	2.262481	2.257746
2.297706	2.292734	2.28769	2.28259	2.277524	2.272481	2.267525	2.262713	2.257977
2.297934	2.292951	2.287882	2.282745	2.277677	2.272677	2.26776	2.262955	2.258216
2.298173	2.293189	2.288112	2.282964	2.277893	2.272907	2.283854	2.278995	2.274199
2.298436	2.293467	2.288418	2.283308	2.278236	2.273196	2.2841	2.279232	2.274439
2.298699	2.293747	2.288729	2.283662	2.278595	2.273507	2.284369	2.279483	2.27468
2.298945	2.294002	2.288999	2.28395	2.278901	2.273834	2.284703	2.279775	2.274924
2.299184	2.294246	2.28925	2.284211	2.279182	2.274158	2.28505	2.280079	2.275172
2.276561	2.271698	2.28949	2.284454	2.279428	2.274402	2.285294	2.28033	2.275429
2.27678	2.271933	2.289718	2.28468	2.279642	2.27459	2.285469	2.280547	2.275696
2.277053	2.272177	2.289953	2.284924	2.279897	2.274852	2.285743	2.280843	2.276012
2.277393	2.272421	2.290188	2.285188	2.280199	2.275208	2.286142	2.28124	2.276393
2.277736	2.272708	2.290462	2.285468	2.280499	2.275552	2.286519	2.281609	2.276743
2.278063	2.273075	2.290805	2.285757	2.280762	2.275827	2.286801	2.281879	2.276999
2.278377	2.273454	2.29117	2.286069	2.281044	2.276109	2.28708	2.282147	2.277255
2.27865	2.27377	2.291513	2.286429	2.297404	2.292405	2.308686	2.303672	2.298691
2.278912	2.274064	2.291853	2.286818	2.297821	2.292811	2.309088	2.304081	2.299097
2.279245	2.274401	2.292213	2.287202	2.298207	2.293158	2.309407	2.3044	2.299424
2.279639	2.274775	2.29259	2.287586	2.298573	2.293456	2.309657	2.304644	2.299689
2.28001	2.275103	2.292921	2.287932	2.298919	2.293774	2.309953	2.304917	2.29995
2.280341	2.275358	2.293176	2.288222	2.299235	2.29412	2.310315	2.305226	2.300197

2.280669	2.275634	2.293453	2.28852	2.299556	2.29448	2.310701	2.305569	2.300487
2.281004	2.275989	2.29382	2.288874	2.299915	2.294867	2.311114	2.305973	2.300873
2.281349	2.276373	2.29421	2.289233	2.300271	2.295246	2.311524	2.306391	2.301293
2.250091	2.245089	2.294557	2.289535	2.30056	2.295552	2.311859	2.306755	2.301681
2.250455	2.245442	2.294889	2.289811	2.300815	2.29582	2.312155	2.307091	2.302058
2.250814	2.245805	2.265442	2.260295	2.301145	2.296156	2.312506	2.307457	2.302434
2.251174	2.246182	2.26583	2.260675	2.301546	2.296556	2.312909	2.307851	2.302808
2.251487	2.246519	2.266194	2.261052	2.301958	2.296964	2.313317	2.308251	2.303195
2.251714	2.246783	2.266507	2.261408	2.30237	2.29737	2.313721	2.308658	2.303604
2.251954	2.247053	2.266822	2.261766	2.302783	2.297777	2.314129	2.309067	2.304016
2.252288	2.247389	2.284903	2.279803	2.297994	2.29287	2.314539	2.309474	2.304418
2.252662	2.247753	2.28528	2.280182	2.298382	2.293257	2.31495	2.309879	2.304818
2.253025	2.248115	2.285656	2.280558	2.298767	2.293639	2.288523	2.283409	2.278306
2.25339	2.248482	2.286035	2.280933	2.299148	2.294017	2.288898	2.283781	2.278675
2.253763	2.248845	2.286406	2.281301	2.299522	2.294388	2.289268	2.284152	2.279048
2.254137	2.249197	2.286762	2.281658	2.29989	2.294752	2.289631	2.284521	2.279425
2.254553	2.249585	2.287143	2.282023	2.300254	2.295112	2.289992	2.284892	2.279809
2.255056	2.250044	2.287576	2.282402	2.300611	2.295464	2.290351	2.285266	2.280208
2.255558	2.25051	2.288018	2.28279	2.300976	2.295819	2.29071	2.285637	2.280604
2.255967	2.250919	2.264694	2.25954	2.301357	2.29618	2.291059	2.285991	2.280969
2.256332	2.251303	2.265087	2.259936	2.301759	2.296558	2.291417	2.286344	2.281322
2.256696	2.251681	2.265495	2.260381	2.302233	2.297013	2.291847	2.286746	2.2817
2.257053	2.252052	2.265914	2.260871	2.302771	2.297536	2.292339	2.287192	2.282097
2.257447	2.252436	2.266316	2.261307	2.303244	2.298008	2.292804	2.287646	2.282538
2.257891	2.252829	2.266683	2.261659	2.303614	2.298402	2.29323	2.288116	2.283046
2.277352	2.272189	2.29186	2.286723	2.304002	2.298797	2.293644	2.288566	2.283531
2.277815	2.272693	2.292382	2.287219	2.304468	2.299221	2.294037	2.288948	2.283918
2.278269	2.273204	2.292922	2.287739	2.304961	2.299668	2.294444	2.289334	2.284297
2.278681	2.273631	2.293369	2.288195	2.305444	2.300155	2.294931	2.289817	2.284777
2.279073	2.274016	2.293773	2.288626	2.305932	2.300672	2.295468	2.290355	2.285307
2.264164	2.259164	2.294244	2.289115	2.30645	2.301198	2.295992	2.290857	2.285781
2.264686	2.259667	2.294781	2.289663	2.307002	2.301738	2.296508	2.291326	2.2862
2.265193	2.260148	2.29528	2.290164	2.307507	2.302245	2.29701	2.291808	2.286659
2.26566	2.26057	2.295696	2.290567	2.307919	2.302686	2.297484	2.292309	2.287183
2.266127	2.261011	2.296135	2.29099	2.308354	2.303145	2.297972	2.292828	2.28773
2.223999	2.219058	2.28236	2.277256	2.286389	2.281278	2.298511	2.293376	2.288287
2.22448	2.21959	2.282934	2.277817	2.286959	2.281846	2.299075	2.293939	2.288849
2.224992	2.220109	2.283492	2.278377	2.287528	2.282409	2.299643	2.294498	2.289397
2.22553	2.220629	2.284049	2.278946	2.288104	2.282975	2.300219	2.295057	2.289934
2.226065	2.221159	2.258733	2.253715	2.274412	2.269317	2.300782	2.295603	2.29046
2.226599	2.221705	2.259288	2.254253	2.274957	2.269861	2.301331	2.296135	2.290973
2.227134	2.222243	2.259839	2.254794	2.275506	2.270408	2.301878	2.296657	2.29147
2.227673	2.222763	2.260378	2.255345	2.276071	2.270959	2.302418	2.297149	2.29192
2.22821	2.223283	2.260916	2.255895	2.276636	2.271513	2.302972	2.297667	2.292406
2.228742	2.22382	2.261465	2.256431	2.277185	2.272075	2.303569	2.298284	2.293035
2.229272	2.224363	2.262015	2.256961	2.277728	2.272639	2.304186	2.298946	2.293731
2.229806	2.224888	2.262555	2.257502	2.25247	2.247464	2.291423	2.286243	2.281087
2.230341	2.2254	2.263087	2.258051	2.253021	2.247993	2.291976	2.28681	2.281679
2.230872	2.225923	2.263625	2.258591	2.253561	2.248528	2.292534	2.287372	2.282251
2.231386	2.226459	2.264171	2.259116	2.254082	2.249069	2.293101	2.287929	2.282801

2.231934	2.227002	2.250606	2.245589	2.25462	2.249616	2.293669	2.288487	2.28335
2.232552	2.227541	2.251143	2.246157	2.255191	2.250152	2.294218	2.289037	2.283906
2.233194	2.228103	2.251706	2.246755	2.255792	2.250715	2.294789	2.289602	2.284469
2.233822	2.228742	2.252366	2.247408	2.256443	2.251372	2.270976	2.265819	2.260712
2.234445	2.229424	2.253079	2.248092	2.257122	2.25208	2.271684	2.266458	2.261277
2.235037	2.230052	2.253721	2.2487	2.257723	2.252701	2.272335	2.267104	2.261914
2.235596	2.23063	2.254297	2.249236	2.258249	2.253243	2.272938	2.267763	2.262624
2.265058	2.259993	2.25491	2.249829	2.258828	2.253802	2.273526	2.268388	2.263289
2.265774	2.260684	2.255599	2.250528	2.259504	2.254394	2.274087	2.26895	2.263867
2.26648	2.261359	2.256272	2.251221	2.260185	2.255009	2.274678	2.26953	2.264449
2.267095	2.261942	2.256856	2.251831	2.260813	2.255653	2.27536	2.2702	2.265102
2.26769	2.262507	2.257423	2.25242	2.26143	2.256316	2.276075	2.270896	2.265766
2.26835	2.263163	2.25807	2.253058	2.262073	2.256985	2.276761	2.271538	2.266357
2.269046	2.263871	2.258765	2.253721	2.262727	2.257658	2.277432	2.272151	2.266907
2.269682	2.264518	2.259399	2.254327	2.263328	2.258278	2.278076	2.272786	2.267527
2.270246	2.265092	2.259962	2.254862	2.263861	2.258831	2.278687	2.273442	2.268223
2.270859	2.265705	2.260564	2.255447	2.250394	2.24542	2.279325	2.274117	2.268933
2.271569	2.266398	2.261251	2.256135	2.251077	2.24609	2.280014	2.274815	2.269644
2.272311	2.267118	2.261964	2.256852	2.25179	2.246786	2.280722	2.275525	2.270359
2.273043	2.267837	2.262674	2.257555	2.252486	2.247477	2.281435	2.276239	2.271072
2.273781	2.268564	2.26339	2.258259	2.253184	2.248174	2.282157	2.276957	2.271786
2.274517	2.26929	2.264106	2.258964	2.253882	2.24887	2.282877	2.277676	2.272502
2.275254	2.270017	2.264822	2.25967	2.254581	2.249568	2.283598	2.278396	2.273219
2.252094	2.246919	2.265547	2.260382	2.255284	2.250268	2.28432	2.279117	2.273936
2.25283	2.247652	2.266281	2.261103	2.255995	2.250971	2.285042	2.279838	2.274655
2.253567	2.248387	2.243232	2.238106	2.256707	2.251675	2.285765	2.280559	2.275374
2.254303	2.249119	2.243956	2.238819	2.25742	2.252379	2.286488	2.28128	2.276093
2.25504	2.249852	2.244682	2.239534	2.258134	2.253084	2.287212	2.282002	2.276814
2.255781	2.250589	2.245411	2.240251	2.258852	2.253793	2.28794	2.282728	2.277537
2.256524	2.251328	2.246141	2.240971	2.259571	2.254504	2.288669	2.283455	2.278261
2.257266	2.252063	2.246868	2.241688	2.236585	2.231572	2.289398	2.28418	2.278982
2.258008	2.252795	2.247591	2.242402	2.237291	2.232272	2.290126	2.284903	2.279699
2.258753	2.253538	2.248328	2.24313	2.23801	2.232982	2.255513	2.250384	2.245274
2.2595	2.254296	2.249082	2.243868	2.238735	2.233695	2.256239	2.25111	2.246001
2.260254	2.255059	2.24985	2.244639	2.2395	2.234443	2.257	2.251872	2.246767
2.261007	2.255808	2.250632	2.245481	2.240364	2.235277	2.257846	2.252722	2.247623
2.261782	2.256571	2.251425	2.246338	2.241245	2.236127	2.258708	2.25359	2.248499
2.262638	2.257395	2.252225	2.247118	2.24201	2.236879	2.259478	2.254366	2.249283
2.263547	2.25826	2.253032	2.247853	2.242704	2.237574	2.260193	2.255087	2.250012
2.264388	2.259084	2.253825	2.248602	2.243426	2.238292	2.260926	2.255821	2.250754
2.265155	2.25986	2.254595	2.249357	2.244169	2.239026	2.261667	2.25656	2.251502
2.265954	2.260682	2.255418	2.250164	2.244964	2.239819	2.262464	2.257348	2.252285
2.266839	2.261612	2.256346	2.251057	2.24584	2.240704	2.263355	2.258213	2.253116
2.267713	2.262522	2.257266	2.251967	2.246745	2.241611	2.264265	2.259103	2.253978
2.268478	2.263287	2.258074	2.252854	2.247663	2.242495	2.265146	2.259996	2.254885
2.269214	2.264001	2.258839	2.253723	2.248574	2.243361	2.266008	2.260881	2.2558
2.270045	2.264802	2.259633	2.254526	2.249377	2.244152	2.266809	2.261685	2.256614
2.270945	2.265662	2.260442	2.255277	2.250098	2.244884	2.267561	2.262426	2.257352
2.240732	2.235543	2.261299	2.256086	2.250885	2.245684	2.240542	2.235482	2.230498
2.241595	2.236443	2.262215	2.256966	2.251751	2.246568	2.241433	2.236347	2.231321

2.242468	2.237333	2.263128	2.257866	2.252645	2.247465	2.242327	2.237228	2.232177
2.243368	2.238184	2.264005	2.258779	2.253564	2.248349	2.243185	2.238101	2.23307
2.244253	2.23903	2.264882	2.259692	2.254485	2.249237	2.244048	2.238981	2.233972
2.218609	2.213539	2.265799	2.26058	2.255364	2.250134	2.244957	2.239872	2.234841
2.219367	2.214399	2.266724	2.261456	2.256226	2.251037	2.245886	2.24077	2.235699
2.220124	2.21517	2.267562	2.262335	2.257128	2.251938	2.246781	2.241661	2.236585
2.220872	2.215856	2.268321	2.263218	2.258066	2.252838	2.247647	2.242547	2.237497
2.221681	2.216613	2.269114	2.264061	2.258942	2.253721	2.248533	2.243437	2.238386
2.222576	2.217471	2.26995	2.264827	2.259705	2.254576	2.249456	2.244335	2.239239
2.223495	2.218371	2.270838	2.265648	2.26051	2.255441	2.250366	2.245223	2.240091
2.224406	2.219291	2.271811	2.266635	2.261479	2.256347	2.251223	2.246081	2.240963
2.22533	2.220237	2.272824	2.267671	2.262492	2.257267	2.25207	2.246933	2.241838
2.226286	2.221207	2.273826	2.268627	2.263413	2.258169	2.252958	2.247805	2.2427
2.227263	2.222193	2.274824	2.269543	2.264289	2.259065	2.253869	2.24869	2.243556
2.172578	2.167637	2.250819	2.245592	2.265194	2.259965	2.254765	2.249575	2.244434
2.173491	2.168463	2.251705	2.2465	2.266128	2.260867	2.255643	2.250457	2.245332
2.174388	2.169307	2.252607	2.247406	2.267054	2.261778	2.256547	2.251373	2.246265
2.175259	2.170189	2.25353	2.248293	2.26794	2.262687	2.257484	2.252345	2.247259
2.176137	2.171099	2.254489	2.249223	2.26887	2.263637	2.258456	2.253331	2.248247
2.177057	2.17206	2.203315	2.198205	2.269941	2.264689	2.259475	2.254295	2.249155
2.177996	2.173037	2.204353	2.19927	2.271064	2.265786	2.260526	2.255267	2.250051
2.178943	2.173948	2.205287	2.200225	2.272097	2.26684	2.261587	2.256308	2.251062
2.179897	2.174811	2.206148	2.201105	2.273072	2.267869	2.26266	2.2574	2.252154
2.180819	2.175688	2.207043	2.202022	2.274059	2.268863	2.26366	2.258412	2.253174
2.181691	2.176576	2.207977	2.20299	2.275068	2.269817	2.26457	2.25932	2.2541
2.182612	2.177497	2.208921	2.203932	2.198915	2.193854	2.265486	2.260245	2.255045
2.183645	2.178471	2.209856	2.204789	2.199743	2.194715	2.26644	2.261237	2.256059
2.184702	2.17947	2.210815	2.205659	2.20058	2.195589	2.267412	2.262248	2.257091
2.18571	2.180489	2.21185	2.206656	2.201546	2.196529	2.268403	2.263255	2.258119
2.186695	2.181511	2.212913	2.207709	2.202572	2.197506	2.269412	2.264263	2.25914
2.187658	2.182444	2.177297	2.172218	2.203542	2.198474	2.221115	2.216079	2.211068
2.188596	2.183297	2.178134	2.173089	2.20447	2.199441	2.222109	2.216994	2.211897
2.18959	2.184233	2.17905	2.174015	2.205434	2.200426	2.223117	2.217951	2.212798
2.190662	2.185286	2.180081	2.175018	2.206445	2.201435	2.224142	2.218967	2.213804
2.191748	2.186367	2.181143	2.176047	2.207479	2.202456	2.19744	2.192354	2.187283
2.192822	2.187444	2.182209	2.177089	2.20853	2.20349	2.198459	2.193361	2.188281
2.193896	2.188525	2.183282	2.178142	2.209592	2.204532	2.199482	2.194369	2.189279
2.194969	2.189598	2.184345	2.179188	2.21065	2.205568	2.200499	2.19537	2.190267
2.19604	2.190668	2.185406	2.180233	2.211707	2.206604	2.201515	2.196368	2.191251
2.197111	2.191738	2.186468	2.181282	2.212772	2.207652	2.202544	2.197378	2.192243
2.198182	2.192809	2.187532	2.182337	2.213844	2.208711	2.203588	2.1984	2.193244
2.199245	2.19387	2.188586	2.183382	2.214909	2.209764	2.204628	2.199424	2.194251
2.200294	2.194915	2.189624	2.184409	2.215956	2.210801	2.205656	2.200443	2.195263
2.201344	2.195959	2.190659	2.185435	2.217006	2.211846	2.206695	2.201476	2.196287
2.148048	2.142814	2.191703	2.186475	2.218076	2.212923	2.20777	2.202532	2.197319
2.149039	2.143796	2.192751	2.187519	2.219153	2.21401	2.208861	2.203612	2.19838
2.150041	2.144778	2.19379	2.188552	2.183407	2.17838	2.209946	2.204734	2.199534
2.151054	2.145767	2.194829	2.18958	2.184432	2.179408	2.211028	2.205889	2.200757
2.123816	2.118604	2.141654	2.13653	2.1855	2.180452	2.212096	2.207	2.201911
2.124924	2.119779	2.142846	2.137656	2.18662	2.181514	2.21315	2.208058	2.202985

2.126005	2.120907	2.143995	2.138759	2.187732	2.182571	2.214201	2.209107	2.204045
2.127015	2.121911	2.14502	2.139777	2.188789	2.183597	2.21524	2.210151	2.205115
2.128002	2.122876	2.146019	2.140798	2.189873	2.18465	2.2163	2.211203	2.206177
2.128981	2.123849	2.147067	2.141913	2.136779	2.131644	2.217413	2.212267	2.20721
2.129961	2.124819	2.148124	2.143058	2.137953	2.132774	2.218575	2.213358	2.208241
2.130987	2.125792	2.149132	2.144106	2.13904	2.133906	2.219828	2.214549	2.209352
2.132051	2.126768	2.150097	2.145076	2.140061	2.135049	2.221168	2.215832	2.210531
2.133189	2.127818	2.151115	2.146057	2.141052	2.136114	2.13116	2.126113	2.121068
2.134429	2.128957	2.152191	2.147044	2.141996	2.137068	2.132153	2.127192	2.122226
2.135657	2.130142	2.153331	2.148088	2.142979	2.138027	2.133123	2.128231	2.123339
2.136811	2.131392	2.154587	2.149241	2.144046	2.139026	2.134081	2.129187	2.124321
2.137931	2.132633	2.155859	2.150441	2.145174	2.140083	2.135081	2.13015	2.125276
2.139019	2.133721	2.157005	2.151638	2.146397	2.141301	2.136269	2.131265	2.126312
2.140091	2.134724	2.158082	2.152835	2.147672	2.142609	2.137561	2.132464	2.127396
2.108372	2.102977	2.130492	2.12531	2.148784	2.143737	2.1387	2.133603	2.128521
2.109587	2.104094	2.131543	2.12627	2.149728	2.144689	2.139693	2.13469	2.129697
2.110766	2.105253	2.132688	2.127362	2.1508	2.145722	2.140713	2.135754	2.130815
2.111875	2.106483	2.134004	2.1287	2.152117	2.146912	2.14179	2.136768	2.131807
2.112972	2.107684	2.135288	2.130015	2.15343	2.148128	2.142912	2.13783	2.132838
2.114062	2.108732	2.103458	2.098253	2.15457	2.149311	2.144128	2.139061	2.134063
2.115178	2.10976	2.104445	2.099237	2.155649	2.150472	2.145359	2.140331	2.135336
2.116436	2.110993	2.105623	2.100332	2.156741	2.151548	2.146434	2.141417	2.136439
2.117793	2.112359	2.106921	2.101492	2.157837	2.152568	2.147411	2.142389	2.137438
2.119035	2.113587	2.108127	2.10267	2.159031	2.153721	2.148533	2.143506	2.138558
2.120135	2.114644	2.109219	2.103864	2.160337	2.155029	2.14983	2.144799	2.13983
2.121297	2.115774	2.110376	2.105095	2.099847	2.09461	2.151084	2.146025	2.141023
2.122617	2.117097	2.111698	2.106408	2.10111	2.09577	2.152194	2.147056	2.142008
2.123964	2.118438	2.113018	2.107691	2.102337	2.096915	2.153308	2.148093	2.142999
2.125253	2.119647	2.114168	2.108793	2.103433	2.098063	2.154547	2.14932	2.144203
2.126514	2.120813	2.115262	2.109833	2.104487	2.099224	2.155848	2.150628	2.14549
2.127721	2.122046	2.116497	2.11105	2.105707	2.100481	2.15716	2.15188	2.146664
2.128879	2.123329	2.117838	2.112405	2.107058	2.101819	2.158491	2.153097	2.147756
2.130131	2.124652	2.119168	2.113695	2.108308	2.10303	2.159708	2.154273	2.148893
2.131531	2.126049	2.120492	2.114891	2.109402	2.104046	2.160751	2.155376	2.150082
2.132868	2.127361	2.121751	2.116078	2.110529	2.105119	2.099806	2.094578	2.089446
2.133986	2.128434	2.122852	2.11727	2.111781	2.106388	2.10108	2.095853	2.090715
2.135082	2.129485	2.123949	2.118491	2.113085	2.107731	2.102432	2.097175	2.09199
1.916637	1.912141	2.125243	2.119813	2.114428	2.109092	2.103777	2.098438	2.09315
1.91772	1.913248	2.126644	2.12119	2.11579	2.11046	2.105117	2.09967	2.094253
1.918812	1.91434	2.128005	2.122478	2.117022	2.111654	2.106286	2.100833	2.095414
1.919927	1.915434	2.129341	2.123687	2.118127	2.112674	2.107281	2.101921	2.096621
1.921	1.91646	2.130613	2.124899	2.11929	2.113794	2.108392	2.103093	2.097868
1.922021	1.917378	2.131779	2.12612	2.120555	2.115086	2.109699	2.1044	2.099163
1.922997	1.918287	2.13296	2.127371	2.12186	2.116426	2.111056	2.105742	2.100477
1.870244	1.865774	1.963371	1.958791	2.123191	2.117766	2.112396	2.107069	2.101788
1.871083	1.866709	1.964438	1.959885	2.124542	2.119118	2.113743	2.108404	2.103107
1.872062	1.867701	1.965516	1.960967	2.125877	2.120459	2.115085	2.109743	2.104439
1.873134	1.868738	1.966611	1.96205	2.127209	2.121795	2.116427	2.111088	2.105782
1.874201	1.869784	1.865444	1.861169	2.128554	2.123144	2.117779	2.112439	2.107122
1.875265	1.870839	1.866488	1.862199	2.129913	2.124507	2.119143	2.1138	2.108459

1.876326	1.871897	1.867536	1.863234	2.105215	2.099851	2.120499	2.115147	2.109786
1.877381	1.872953	1.868587	1.864275	2.106565	2.101184	2.121841	2.116481	2.111114
1.878437	1.874014	1.869641	1.865316	2.107913	2.10251	2.12317	2.117784	2.112399
1.834244	1.829912	1.8707	1.866352	1.914307	1.909941	2.124473	2.119011	2.113558
1.83529	1.830961	1.871761	1.867386	1.915358	1.910984	2.125766	2.12021	2.114674
1.836339	1.832006	1.872818	1.86842	1.916413	1.912028	2.127082	2.12149	2.115925
1.837393	1.833048	1.873871	1.869454	1.917469	1.913073	2.128414	2.122829	2.117277
1.786616	1.78231	1.874924	1.870496	1.918535	1.914127	2.103625	2.098089	2.092589
1.787692	1.783368	1.875977	1.871547	1.919614	1.915196	2.104962	2.099397	2.093876
1.788757	1.784423	1.780106	1.775806	1.868236	1.863939	1.911824	1.90733	1.902844
1.789801	1.785475	1.781162	1.776863	1.869292	1.864963	1.91287	1.908436	1.904015
1.790841	1.786528	1.782221	1.777924	1.870351	1.865983	1.913909	1.909537	1.905188
1.791899	1.787592	1.783286	1.778987	1.823243	1.818902	1.914931	1.910567	1.906245
1.792965	1.78866	1.784353	1.780052	1.824319	1.81994	1.915948	1.911562	1.90724
1.794025	1.789722	1.785413	1.781107	1.825392	1.820997	1.816676	1.812482	1.808366
1.795081	1.79078	1.786466	1.782152	1.826462	1.82207	1.817746	1.813532	1.809391
1.796131	1.79183	1.787514	1.783197	1.827534	1.823148	1.818821	1.814582	1.810411
1.797169	1.792871	1.788557	1.784243	1.82861	1.824232	1.819899	1.815629	1.81142
1.79821	1.793909	1.789597	1.78529	1.829689	1.825314	1.820975	1.816673	1.812429
1.79926	1.794947	1.790635	1.78634	1.782047	1.777746	1.822036	1.817708	1.813435
1.800318	1.79599	1.791677	1.787394	1.783104	1.778795	1.823099	1.818748	1.814452
1.801378	1.797039	1.792719	1.788436	1.784146	1.77984	1.824168	1.819805	1.815492
1.802453	1.798104	1.793774	1.789482	1.785191	1.780895	1.825254	1.820887	1.816564
1.80367	1.799301	1.794961	1.79067	1.786382	1.782093	1.826484	1.822117	1.817786
1.805053	1.800653	1.796304	1.792024	1.787744	1.783459	1.827886	1.823522	1.819188
1.806371	1.801931	1.797565	1.793289	1.789014	1.784733	1.780457	1.776173	1.771911
1.807542	1.803045	1.798649	1.794363	1.790085	1.785813	1.781543	1.777256	1.772984
1.808674	1.804118	1.799689	1.795387	1.791104	1.786837	1.782573	1.778286	1.77401
1.732817	1.728575	1.80076	1.796442	1.792149	1.787882	1.783619	1.779336	1.775061
1.73386	1.729582	1.801836	1.797499	1.793195	1.788922	1.78466	1.78039	1.776129
1.73491	1.730595	1.802915	1.798558	1.79424	1.789959	1.785709	1.781489	1.777286
1.73597	1.731616	1.804001	1.79962	1.795288	1.790996	1.786765	1.782623	1.778514
1.666976	1.662807	1.805234	1.800816	1.79646	1.79216	1.787932	1.783804	1.779713
1.668274	1.664087	1.806664	1.80219	1.7978	1.793498	1.789251	1.785053	1.780881
1.509185	1.505483	1.808055	1.803524	1.799098	1.794789	1.790521	1.786258	1.782012
1.510162	1.506455	1.809261	1.804684	1.800222	1.795894	1.791608	1.787323	1.783056
1.511124	1.507421	1.810468	1.805843	1.801344	1.796992	1.79269	1.788396	1.784125
1.51221	1.508533	1.73459	1.730286	1.802648	1.798276	1.793961	1.789661	1.785388
1.513342	1.509702	1.735943	1.731597	1.804029	1.799635	1.795309	1.791007	1.786737
1.514375	1.51074	1.591098	1.587167	1.805264	1.800837	1.796489	1.792184	1.78792
1.515323	1.511666	1.592062	1.588136	1.806373	1.801901	1.79752	1.793212	1.788956
1.516265	1.512589	1.593043	1.58915	1.737739	1.73344	1.79868	1.794361	1.790098
1.517186	1.513497	1.594048	1.590237	1.739078	1.734771	1.800068	1.795724	1.79143
1.518175	1.514475	1.59511	1.591346	1.587603	1.583874	1.801415	1.797045	1.792722
1.519343	1.515622	1.596292	1.592455	1.588647	1.584858	1.802523	1.798131	1.793805
1.520552	1.516823	1.597519	1.593585	1.589689	1.585822	1.803595	1.79918	1.794853
1.521612	1.517927	1.598709	1.594779	1.590866	1.586953	1.735103	1.730855	1.726711
1.522592	1.518982	1.599879	1.596007	1.592114	1.588175	1.736438	1.73215	1.727976
1.523651	1.520027	1.600986	1.597113	1.593216	1.589269	1.585381	1.581599	1.577903
1.524784	1.521052	1.602015	1.59808	1.594154	1.590221	1.586344	1.582537	1.578819

1.43498	1.431439	1.518375	1.514646	1.595183	1.591259	1.587387	1.583555	1.579808
1.43605	1.432537	1.519577	1.515822	1.596417	1.592482	1.58859	1.584726	1.580935
1.437095	1.433616	1.520764	1.516988	1.597648	1.593711	1.589812	1.585928	1.582104
1.438037	1.434541	1.521761	1.517965	1.598697	1.594811	1.590957	1.587099	1.583283
1.438953	1.435425	1.522709	1.518896	1.599702	1.595871	1.59207	1.588248	1.584455
1.232323	1.229334	1.432882	1.42932	1.600851	1.596986	1.593154	1.589311	1.585505
1.233212	1.230221	1.433977	1.430409	1.602103	1.598138	1.594214	1.590308	1.586465
1.234083	1.231109	1.343587	1.340211	1.603372	1.599342	1.595356	1.591411	1.587541
1.234942	1.232006	1.344662	1.341279	1.604666	1.600606	1.596592	1.59264	1.588753
1.127235	1.124419	1.345678	1.34231	1.442694	1.439039	1.597863	1.593906	1.589998
1.128067	1.125231	1.346561	1.343261	1.443772	1.440168	1.599173	1.595196	1.591244
1.128902	1.126039	1.347425	1.344188	1.444826	1.441282	1.600476	1.596489	1.592506
1.129837	1.126956	1.232183	1.229304	1.445858	1.442332	1.528512	1.52469	1.520857
1.130827	1.127931	1.233039	1.23011	1.446881	1.443351	1.529668	1.525896	1.522109
1.131822	1.128909	1.126034	1.123194	1.447956	1.444397	1.440872	1.437392	1.433927
1.132832	1.129896	1.127009	1.124159	1.449076	1.445461	1.441892	1.438404	1.43495
1.133825	1.13089	1.127998	1.125135	1.229755	1.226815	1.44296	1.439441	1.435968
1.134801	1.131898	1.129007	1.126121	1.230637	1.227694	1.444091	1.440509	1.43697
1.135754	1.132891	1.130007	1.127106	1.124239	1.121411	1.445241	1.441609	1.438011
1.136661	1.13384	1.130975	1.128078	1.125209	1.122375	1.446391	1.442756	1.439142
1.137565	1.134781	1.131936	1.129049	1.126184	1.123344	1.447545	1.443922	1.44031
1.138536	1.135763	1.132924	1.130042	1.127174	1.124319	1.346471	1.3432	1.339943
1.139546	1.136763	1.133922	1.131044	1.128171	1.125298	1.347478	1.344208	1.340956
1.007405	1.004852	1.134868	1.132011	1.129147	1.12627	1.256834	1.253636	1.250455
1.008241	1.00563	1.135758	1.132938	1.130101	1.127235	1.257926	1.254723	1.251532
1.009088	1.00644	1.136685	1.133892	1.131071	1.128206	1.259016	1.255806	1.252603
1.009956	1.007311	1.137681	1.134895	1.13207	1.129189	1.2601	1.256877	1.253665
1.010828	1.008195	1.138697	1.135911	1.133079	1.13018	1.261188	1.257952	1.254729
1.011693	1.009066	1.139706	1.136925	1.134092	1.131185	1.262292	1.25904	1.255803
1.012555	1.009933	1.140718	1.13794	1.135107	1.132197	1.263405	1.260136	1.256881
0.857686	0.855374	1.008231	1.005719	1.136116	1.133208	1.264521	1.261234	1.257958
0.858462	0.856152	1.009087	1.006579	1.137122	1.134219	1.26564	1.262333	1.259035
0.85924	0.856929	1.009941	1.007438	1.138127	1.13523	1.26676	1.263435	1.260116
0.860022	0.857705	1.010791	1.008295	1.139132	1.13624	1.267879	1.264537	1.261199
0.860808	0.85849	0.85624	0.85405	1.006621	1.004042	1.134328	1.13135	1.128381
0.861597	0.859286	0.857032	0.854829	1.007473	1.004903	1.135336	1.132356	1.129386
0.862395	0.860101	0.857843	0.855621	1.008335	1.005766	1.136344	1.133367	1.130404
0.863214	0.86096	0.858704	0.856456	1.009233	1.006645	1.13735	1.134378	1.131423
0.864045	0.861838	0.859585	0.857308	1.010146	1.007527	1.138354	1.135395	1.132454
0.864867	0.86266	0.860401	0.858115	0.855809	0.853474	1.005753	1.00312	1.000496
0.865681	0.863435	0.861165	0.858885	0.856583	0.854244	1.006614	1.00404	1.001476
0.578386	0.577069	0.861948	0.859662	0.857357	0.855021	1.007477	1.004937	1.002405
0.578971	0.577661	0.862763	0.860448	0.858131	0.855804	1.008337	1.005794	1.003259
0.579539	0.578238	0.86358	0.86124	0.858916	0.856608	0.854323	0.852078	0.849844
0.580053	0.578767	0.864381	0.862034	0.859721	0.857451	0.855191	0.852934	0.850675
0.580552	0.579275	0.865184	0.862842	0.860546	0.85831	0.856072	0.853801	0.851517
0.581076	0.579782	0.866004	0.863693	0.861411	0.859169	0.856918	0.854627	0.852325
0.581609	0.580282	0.866835	0.864568	0.8623	0.860028	0.857745	0.855432	0.853121
0.582125	0.580778	0.579476	0.578211	0.863134	0.860844	0.858551	0.856252	0.853959
0.582624	0.581268	0.579968	0.578714	0.863914	0.861617	0.859335	0.857085	0.854843

0.583129	0.581773	0.580475	0.579227	0.864719	0.862409	0.860127	0.857905	0.855692
0.58364	0.582296	0.581006	0.579765	0.865573	0.863234	0.860928	0.858692	0.856471
0.584174	0.582837	0.581545	0.580298	0.866434	0.864077	0.861754	0.859496	0.857259
0.584761	0.583407	0.582087	0.580803	0.867274	0.864937	0.862628	0.860363	0.858114
0.585369	0.583986	0.58263	0.581299	0.868118	0.86581	0.863525	0.861257	0.858998
0.585954	0.58453	0.583151	0.58181	0.869002	0.866694	0.864408	0.862144	0.859886
0.586527	0.585052	0.583659	0.58233	0.869915	0.867588	0.865286	0.863031	0.860781
0.5871	0.585605	0.584201	0.582869	0.581578	0.580342	0.866179	0.863913	0.861651
0.587671	0.58619	0.58478	0.583427	0.58212	0.580872	0.867092	0.864795	0.862493
0.588258	0.586791	0.585373	0.583995	0.582667	0.581402	0.867985	0.865658	0.863326
0.588875	0.587409	0.585976	0.584568	0.583217	0.581937	0.86884	0.866489	0.864145
0.589498	0.588035	0.586587	0.585148	0.58377	0.582468	0.869676	0.867313	0.864973
0.59011	0.588656	0.587198	0.585734	0.584324	0.582976	0.87049	0.86815	0.865843
0.590716	0.589275	0.587811	0.586324	0.58488	0.583478	0.871298	0.868995	0.866731
0.591322	0.589893	0.588421	0.586908	0.58544	0.584015	0.582654	0.581398	0.580176
0.591925	0.59051	0.589027	0.587486	0.586002	0.58458	0.583217	0.581939	0.580696
0.592501	0.591089	0.589611	0.588073	0.586588	0.58516	0.583785	0.582481	0.581216
0.593037	0.591613	0.590157	0.58867	0.587204	0.585757	0.584354	0.583021	0.581731
0.59358	0.592159	0.590726	0.589278	0.587826	0.586359	0.584931	0.58357	0.582256
0.594159	0.592785	0.591364	0.589902	0.588436	0.586957	0.585514	0.584133	0.582799
0.594744	0.593422	0.592013	0.590534	0.589053	0.587563	0.586107	0.584707	0.583354
0.5953	0.593999	0.592616	0.59117	0.589699	0.588192	0.586711	0.58529	0.583914
0.595843	0.594544	0.593194	0.591806	0.59036	0.588836	0.587329	0.585884	0.584484
0.596396	0.595093	0.593761	0.592408	0.590988	0.589482	0.58798	0.586515	0.585086
0.596957	0.595644	0.594314	0.592976	0.591586	0.590133	0.588665	0.587184	0.585721
0.597522	0.596195	0.594866	0.593541	0.592175	0.590763	0.589323	0.587836	0.586352
0.598092	0.596747	0.595418	0.594109	0.592759	0.591365	0.589937	0.588457	0.586972
0.598667	0.597302	0.595971	0.594671	0.593333	0.591951	0.590535	0.589068	0.58759
0.599247	0.597865	0.596524	0.595223	0.593891	0.592524	0.591124	0.589674	0.588205
0.599834	0.598436	0.597082	0.595771	0.59444	0.593089	0.591708	0.590275	0.58882
0.600436	0.599019	0.597649	0.596322	0.59499	0.593659	0.592297	0.59088	0.589433
0.601052	0.599615	0.598224	0.596876	0.595542	0.594231	0.592891	0.591486	0.590047
0.601697	0.600235	0.598823	0.597456	0.596115	0.594815	0.593489	0.5921	0.590675
0.602376	0.600883	0.59945	0.598065	0.596715	0.595414	0.594095	0.592725	0.591324
		0.600075	0.598673	0.59731	0.595999	0.59468	0.59333	0.591953
		0.600683	0.599261	0.59788	0.59655	0.595225	0.593889	0.592539
		0.601303	0.599859	0.598458	0.597105	0.595769	0.594446	0.593121
		0.601969	0.600496	0.599071	0.597699	0.596355	0.595041	0.593731
		0.602653	0.601149	0.599699	0.598309	0.596955	0.59565	0.59435
				0.600308	0.598897	0.597532	0.596234	0.59495
				0.600905	0.59947	0.598091	0.596801	0.595535
				0.601541	0.600083	0.598682	0.597375	0.596101
				0.602233	0.600749	0.599314	0.597957	0.596639
						0.599957	0.598554	0.597193
						0.600605	0.599182	0.597797
						0.601254	0.599816	0.598412
						0.601885	0.600423	0.598997
						0.602511	0.601022	0.599575

							0.592635	0.590838
							0.591906	0.590133
							0.591146	0.58938
							0.590325	0.588543
							0.589526	0.587729
				0.594409	0.592494	0.590625	1.224011	1.220701
				0.593556	0.591709	0.589903	1.222564	1.219317
				0.592798	0.590984	0.589199	1.221192	1.217953
				0.592105	0.590301	0.58851	1.219867	1.216606
		0.595059	0.593217	1.228235	1.225015	1.221785	1.469467	1.465485
		0.594311	0.592493	1.226894	1.223675	1.220439	1.467795	1.46379
		0.593578	0.591771	1.477986	1.474045	1.470097	1.661655	1.657172
		0.592864	0.591056	1.476332	1.472365	1.468383	1.659668	1.655145
		0.592153	0.59033	1.474665	1.470656	1.46663	1.657653	1.653116
0.595136	0.59329	1.228003	1.224733	1.47296	1.468874	1.464792	1.744908	1.740363
0.594414	0.592581	1.226657	1.22335	1.471239	1.467072	1.462938	1.74289	1.738431
0.593692	0.591864	1.47763	1.473622	1.665345	1.660669	1.656042	1.897523	1.892649
0.592972	0.59114	1.475948	1.471944	1.663448	1.658799	1.6542	1.895537	1.890674
0.592236	0.590381	1.474236	1.470207	1.75112	1.746471	1.74187	1.893526	1.888695
0.591478	0.589575	1.472483	1.468382	1.749064	1.744485	1.739971	1.891476	1.886711
1.478755	1.474797	1.470749	1.466602	1.74706	1.74254	1.738098	1.889442	1.884725
1.477068	1.473106	1.469065	1.464934	1.745184	1.740699	1.736288	1.887445	1.882732
1.47539	1.471428	1.467405	1.46331	1.743362	1.738899	1.734502	1.885464	1.880742
1.473725	1.469767	1.750747	1.746115	1.897965	1.893069	1.888241	1.883487	1.878767
1.472071	1.46812	1.748908	1.744305	1.895969	1.891084	1.886263	1.881516	1.876801
1.470423	1.466478	1.747066	1.742486	1.893963	1.889086	1.884272	1.879534	1.87483
1.468782	1.464842	1.745222	1.740661	1.89195	1.887078	1.882269	1.877544	1.872855
1.660907	1.656735	1.899916	1.8949	1.889943	1.885076	1.880273	1.875558	1.870884
1.659243	1.655085	1.897915	1.892903	1.887949	1.883084	1.878285	1.873579	1.868917
1.65758	1.653441	1.89592	1.890907	1.885954	1.881093	1.8763	1.934504	1.929683
1.655907	1.651796	1.893921	1.888904	1.88395	1.879096	1.874315	1.932446	1.927644

1.65423	1.650147	1.891922	1.886901	1.881946	1.877101	1.872333	1.930396	1.925614
1.899606	1.89482	1.889918	1.884895	1.879944	1.87511	1.870356	1.928354	1.923598
1.897575	1.892803	1.88791	1.882892	1.877948	1.873125	1.868387	1.926322	1.921593
1.895575	1.890787	1.885903	1.880919	1.939016	1.934004	1.929084	1.924304	1.919596
1.893605	1.888768	1.883891	1.878972	1.93702	1.932001	1.927068	1.922297	1.917604
1.891642	1.886769	1.881908	1.877063	1.935072	1.930052	1.925105	1.920326	1.915628
1.889669	1.884786	1.879965	1.875211	1.933201	1.928193	1.923231	1.91842	1.913676
1.887729	1.882843	1.878051	1.873363	1.931319	1.926326	1.921356	1.916511	1.911723
1.88588	1.880981	1.876178	1.871476	1.92934	1.924356	1.919395	1.914543	1.909744
1.884062	1.879154	1.874324	1.869572	1.927317	1.922345	1.917404	1.912555	1.907757
1.88216	1.87727	1.935194	1.930236	1.925294	1.920351	1.91544	1.910588	1.90578
1.880201	1.875351	1.9332	1.928214	1.923266	1.918362	1.913491	1.908633	1.90381
1.878278	1.873481	1.931266	1.92626	1.921307	1.916433	1.91159	1.959748	1.954693
1.876398	1.871676	1.929415	1.924395	1.919435	1.914583	1.909757	1.95782	1.952735
1.937304	1.932454	1.927523	1.922509	1.917552	1.912706	1.907885	1.955866	1.950777
1.935349	1.930462	1.925527	1.920545	1.915605	1.910738	1.9059	1.953821	1.948783
1.933394	1.928445	1.923504	1.918571	1.91366	1.90877	1.903914	1.95178	1.946806
1.931415	1.926426	1.921501	1.916645	1.91179	1.906902	1.902043	1.949845	1.944913
1.929431	1.92441	1.91951	1.914735	1.909942	1.905072	1.900221	1.947955	1.943053
1.92749	1.922465	1.91756	1.912779	1.907993	1.903158	1.89834	1.946006	1.941104
1.925587	1.920581	1.915646	1.910781	1.905956	1.901172	1.896413	1.944007	1.939077
1.923697	1.918708	1.913763	1.908858	1.956872	1.951928	1.947011	2.066315	2.060982
1.921826	1.916845	1.911917	1.90704	1.954971	1.950039	1.945136	2.064328	2.059025
1.919951	1.914981	1.910077	1.90524	1.953106	1.948173	1.94327	2.148559	2.142908
1.918067	1.913111	1.908233	1.903433	1.951261	1.946312	1.941392	2.146474	2.140857
1.916162	1.911229	1.906376	1.901606	1.949402	1.944438	1.939501	2.144375	2.138775
1.914189	1.909303	1.904471	1.899695	1.947422	1.942481	1.93756	2.142177	2.136568
1.912181	1.907354	1.90254	1.897737	1.945376	1.940478	1.935591	2.139925	2.134292
1.910273	1.905469	1.953338	1.948365	2.063514	2.058091	2.052687	2.137763	2.132146
1.908463	1.903645	1.951452	1.946503	2.061526	2.056072	2.050639	2.135688	2.130129
1.906661	1.901844	1.949584	1.944651	2.059532	2.054062	2.048617	2.17736	2.171669
1.904849	1.900064	1.94773	1.942797	2.057498	2.05205	2.046632	2.175259	2.169579
1.903045	1.89829	1.945882	1.940945	2.055471	2.050043	2.04465	2.173165	2.167499
1.901259	1.896501	2.064448	2.058951	2.053481	2.048036	2.042635	2.171064	2.165437
1.89948	1.894711	2.062421	2.056955	2.051501	2.046035	2.040619	2.168967	2.163382
1.897692	1.892934	2.060415	2.054942	2.140174	2.134546	2.128975	2.289553	2.283079
1.895898	1.891166	2.058421	2.052918	2.138084	2.132512	2.126991	2.287132	2.280665
2.067342	2.061888	2.056409	2.0509	2.13602	2.130477	2.124981	2.28472	2.278287
2.06531	2.059844	2.054371	2.04889	2.133987	2.128438	2.122938	2.282316	2.275951
2.063324	2.057824	2.052343	2.046882	2.261506	2.254933	2.248469	2.279925	2.273615
2.061423	2.055843	2.050323	2.044857	2.258993	2.252486	2.246087	2.277549	2.271255
2.059539	2.053896	2.04834	2.04286	2.256507	2.25006	2.243725	2.275187	2.268904
2.057609	2.052008	2.046456	2.040939	2.254085	2.247666	2.241371	2.307926	2.301605
2.055659	2.050147	2.044622	2.039069	2.251721	2.245307	2.239034	2.305569	2.299278
2.053688	2.048212	2.042727	2.037222	2.249459	2.243023	2.23672	2.303204	2.296927
2.051696	2.046218	2.040789	2.035404	2.247295	2.240807	2.234427	2.300832	2.294558
2.049719	2.044244	2.228677	2.222201	2.245077	2.238614	2.232229	2.298538	2.292222
2.047757	2.042289	2.226272	2.219851	2.242778	2.236455	2.230172	2.296352	2.289913
2.045825	2.040382	2.22393	2.217527	2.277711	2.271415	2.265134	2.337532	2.330368
2.043958	2.038573	2.221723	2.215271	2.27536	2.269091	2.262865	2.335142	2.328034

2.042101	2.03678	2.219547	2.213057	2.27304	2.266756	2.26055	2.332736	2.325743
2.142025	2.135761	2.246531	2.240086	2.270853	2.264514	2.258254	2.330237	2.32335
2.139621	2.133394	2.244179	2.237918	2.268743	2.262328	2.255972	2.327692	2.320909
2.137363	2.131129	2.241849	2.235682	2.302142	2.295662	2.289225	2.32525	2.318472
2.135248	2.128955	2.239527	2.233376	2.299829	2.293471	2.287127	2.322904	2.316027
2.220994	2.214464	2.237286	2.23111	2.297516	2.291255	2.284996	2.338736	2.331568
2.218731	2.212335	2.235156	2.228898	2.295196	2.28898	2.282787	2.336347	2.329262
2.216498	2.210248	2.233076	2.226744	2.292931	2.286734	2.280585	2.333985	2.327012
2.214371	2.208204	2.268078	2.261629	2.333955	2.327142	2.320441	2.364388	2.357312
2.212276	2.206168	2.266004	2.259576	2.331688	2.324858	2.318151	2.362023	2.355056
2.210094	2.204034	2.263848	2.257479	2.347892	2.340756	2.333767	2.359526	2.352659
2.207863	2.201836	2.261641	2.255363	2.345487	2.338296	2.331252	2.356941	2.350164
2.20572	2.199705	2.259486	2.253281	2.343144	2.335935	2.328855	2.354449	2.347675
2.203679	2.197654	2.2574	2.25125	2.340881	2.333689	2.326589	2.352048	2.345172
2.240509	2.234357	2.32082	2.313805	2.37144	2.364095	2.356839	2.349731	2.342786
2.238374	2.232141	2.318236	2.311305	2.368964	2.361741	2.354588	2.347528	2.340605
2.23625	2.229937	2.315671	2.308802	2.366474	2.359379	2.352348	2.345363	2.338492
2.234186	2.22785	2.313242	2.306388	2.364017	2.357013	2.350078	2.343158	2.336342
2.232152	2.225819	2.310893	2.304031	2.361591	2.354659	2.347807	2.340953	2.3342
2.279151	2.27244	2.34327	2.336214	2.359262	2.35238	2.345582	2.338784	2.332084
2.276987	2.270291	2.340942	2.333938	2.357018	2.350165	2.343396	2.336645	2.32999
2.274823	2.268138	2.338608	2.331658	2.354775	2.347961	2.341232	2.385161	2.378264
2.272656	2.265975	2.336256	2.329362	2.352524	2.34576	2.339088	2.382984	2.376186
2.348474	2.341056	2.333924	2.327084	2.350289	2.343579	2.336967	2.380827	2.374126
2.346077	2.33871	2.331626	2.324832	2.348076	2.34142	2.334867	2.378677	2.372059
2.343708	2.336394	2.329357	2.322605	2.345886	2.339287	2.332792	2.376547	2.370001
2.341373	2.334112	2.327123	2.32041	2.367484	2.360921	2.35446	2.396704	2.390181
2.339066	2.331861	2.324918	2.318243	2.365325	2.358824	2.352422	2.39461	2.38813
2.336755	2.329614	2.322721	2.316081	2.363172	2.356739	2.350405	2.392553	2.386119
2.334436	2.327368	2.320529	2.313921	2.361023	2.354666	2.34841	2.390534	2.38415
2.332185	2.325206	2.318426	2.311848	2.358943	2.352628	2.34642	2.388513	2.382185
2.330015	2.323155	2.316448	2.309898	2.356961	2.350634	2.344424	2.386474	2.380215
2.327913	2.321153	2.314507	2.307982	2.35501	2.348666	2.342449	2.384451	2.378256
2.325906	2.319181	2.337626	2.331091	2.376493	2.370119	2.363878	2.382461	2.376316
2.323938	2.317218	2.335654	2.329138	2.374516	2.368161	2.361945	2.380496	2.374396
2.321905	2.315195	2.333628	2.327146	2.372519	2.366202	2.360026	2.378559	2.372505
2.319834	2.31313	2.331564	2.325127	2.37051	2.364247	2.358125	2.37665	2.370643
2.317742	2.311054	2.357936	2.351482	2.368503	2.362303	2.356248	2.374789	2.368856
2.315619	2.308956	2.355832	2.349432	2.36649	2.360362	2.354388	2.372978	2.367155
2.313521	2.306908	2.353787	2.347439	2.364532	2.358481	2.352587	2.371199	2.365448
2.311463	2.304933	2.351824	2.345516	2.362637	2.356685	2.350877	2.36946	2.363707
2.309442	2.303017	2.349936	2.34368	2.360827	2.354949	2.349195	2.367726	2.361951
2.332337	2.326014	2.371437	2.365241	2.359185	2.35329	2.3475	2.365962	2.360171
2.33037	2.324186	2.36971	2.363621	2.357609	2.35166	2.345798	2.364185	2.358382
2.328529	2.322359	2.36788	2.361841	2.355864	2.34992	2.344055	2.362403	2.356609
2.326798	2.320541	2.365977	2.359945	2.353994	2.348096	2.342278	2.360616	2.354847
2.324997	2.31872	2.364095	2.358058	2.352118	2.346255	2.340486	2.358848	2.353148
2.3231	2.316888	2.362229	2.356163	2.350213	2.344371	2.338654	2.357093	2.351533
2.345726	2.339563	2.360427	2.354366	2.348424	2.342598	2.336915	2.355387	2.349915
2.343817	2.337785	2.358755	2.352786	2.3469	2.341083	2.335383	2.35378	2.348257

2.341948	2.336027	2.357102	2.351237	2.345418	2.339615	2.333896	2.352187	2.346577
2.340184	2.334254	2.355337	2.349511	2.343731	2.337968	2.332275	2.350498	2.344846
2.33848	2.332476	2.353513	2.347694	2.341942	2.336239	2.330601	2.348774	2.3431
2.363735	2.357637	2.351679	2.345865	2.34015	2.334541	2.328996	2.347154	2.341486
2.361861	2.355772	2.349821	2.344005	2.338337	2.332861	2.327452	2.345633	2.340001
2.360103	2.354037	2.3481	2.342285	2.33664	2.331226	2.325882	2.344045	2.338431
2.358543	2.352516	2.346599	2.340787	2.335124	2.329662	2.324269	2.342346	2.336714
2.356955	2.350972	2.345083	2.339286	2.333612	2.32809	2.322639	2.340627	2.334988
2.355155	2.349217	2.343369	2.337614	2.331962	2.326432	2.320976	2.338918	2.333331
2.353318	2.347423	2.341622	2.335919	2.330304	2.324777	2.319327	2.337224	2.331691
2.351671	2.345802	2.340039	2.334388	2.328804	2.323273	2.317806	2.335597	2.330038
2.350134	2.344278	2.338548	2.33295	2.327395	2.32186	2.316366	2.334015	2.32838
2.348481	2.342644	2.336939	2.331376	2.325845	2.320314	2.314818	2.36117	2.355434
2.346694	2.340877	2.335192	2.329645	2.324132	2.318616	2.313146	2.359421	2.353766
2.345012	2.33922	2.333551	2.328011	2.322505	2.317	2.311546	2.357716	2.352096
2.343543	2.337776	2.332116	2.326569	2.321056	2.315554	2.31009	2.356083	2.350388
2.342113	2.336386	2.330742	2.325184	2.31966	2.314158	2.30868	2.354503	2.348738
2.340614	2.334977	2.329375	2.323811	2.318277	2.312772	2.307294	2.353016	2.347275
2.339075	2.333538	2.327986	2.32242	2.316879	2.31137	2.305897	2.35155	2.345874
2.337426	2.331921	2.326389	2.320832	2.345379	2.339765	2.334194	2.349896	2.344276
2.335687	2.330162	2.324627	2.31909	2.343602	2.337992	2.332431	2.348102	2.342539
2.3341	2.328548	2.323007	2.317486	2.341965	2.336363	2.330815	2.346451	2.340927
2.332702	2.327116	2.321565	2.316056	2.340504	2.334916	2.32938	2.344978	2.339472
2.331327	2.325718	2.32016	2.314659	2.339072	2.333502	2.327986	2.343547	2.338054
2.329948	2.324342	2.318787	2.313283	2.337656	2.332113	2.326624	2.342154	2.336673
2.32851	2.32292	2.317373	2.311868	2.336197	2.330681	2.325221	2.340724	2.335255
2.326857	2.321291	2.31576	2.310265	2.334552	2.329054	2.323612	2.339092	2.333648
2.325099	2.319563	2.314055	2.308576	2.332824	2.327336	2.321908	2.337367	2.331953
2.323512	2.317995	2.342348	2.336782	2.331262	2.325786	2.320369	2.335804	2.330407
2.322066	2.316561	2.34089	2.335344	2.32984	2.324376	2.318969	2.334375	2.328984
2.32066	2.315159	2.339459	2.333928	2.32844	2.32299	2.317594	2.332974	2.32759
2.31929	2.313783	2.338044	2.332526	2.327053	2.32162	2.316241	2.331598	2.326222
2.317935	2.312424	2.336645	2.331134	2.325671	2.32025	2.314884	2.330218	2.324854
2.316582	2.311079	2.335259	2.329747	2.324284	2.318867	2.31351	2.328827	2.323487
2.315226	2.309735	2.333873	2.328356	2.322888	2.317464	2.312105	2.327396	2.32208
2.343607	2.338019	2.332469	2.326957	2.321476	2.316008	2.310604	2.305312	2.300104
2.342172	2.336589	2.331044	2.32554	2.320041	2.31451	2.30904	2.303706	2.298472
2.3407	2.335083	2.329514	2.323996	2.318495	2.312979	2.307525	2.302198	2.29697
2.339189	2.333491	2.327865	2.322308	2.31682	2.3114	2.306054	2.300786	2.295596
2.337658	2.331918	2.326267	2.320694	2.315226	2.309884	2.304622	2.299407	2.294256
2.336091	2.330389	2.324769	2.319226	2.313787	2.308471	2.303236	2.298055	2.292937
2.334547	2.328891	2.323315	2.317814	2.312406	2.307103	2.30188	2.296725	2.291636
2.333091	2.327441	2.321885	2.316422	2.311046	2.305758	2.300547	2.324566	2.319475
2.33168	2.32602	2.320478	2.315054	2.309709	2.304434	2.299233	2.323294	2.318214
2.330256	2.324589	2.319061	2.313672	2.308358	2.303105	2.297921	2.322017	2.316944
2.328834	2.323161	2.317645	2.312286	2.307002	2.301775	2.296613	2.320738	2.315666
2.327493	2.321798	2.316272	2.310913	2.334936	2.329724	2.324573	2.344827	2.339653
2.326259	2.320516	2.314949	2.309552	2.333577	2.32839	2.323272	2.343522	2.338364
2.325	2.319237	2.313645	2.308209	2.33223	2.32706	2.321968	2.342211	2.337068
2.323653	2.317925	2.312337	2.306876	2.330893	2.325733	2.320657	2.340885	2.335753

2.322285	2.316621	2.311058	2.305581	2.329594	2.324427	2.31935	2.339559	2.33444
2.320946	2.315375	2.339244	2.333747	2.328376	2.323167	2.318056	2.338243	2.333145
2.319609	2.314147	2.338097	2.332607	2.327215	2.321948	2.316782	2.336941	2.331865
2.318233	2.312833	2.336866	2.331448	2.326088	2.320788	2.315578	2.335692	2.330604
2.316823	2.311447	2.335567	2.33028	2.325	2.31969	2.314438	2.334492	2.329361
2.344922	2.339546	2.334254	2.329055	2.323844	2.318564	2.313321	2.333338	2.328176
2.343548	2.338195	2.332927	2.327754	2.322591	2.317404	2.312241	2.332251	2.327068
2.342183	2.336851	2.331601	2.326439	2.346764	2.341547	2.336352	2.331148	2.325981
2.34081	2.335513	2.330287	2.325138	2.345458	2.340262	2.335104	2.329979	2.324906
2.339463	2.334191	2.328981	2.323839	2.344144	2.338949	2.333815	2.328776	2.323815
2.338186	2.332886	2.327665	2.322528	2.342821	2.337651	2.33255	2.327554	2.322642
2.336967	2.331611	2.326361	2.321224	2.341502	2.336361	2.331292	2.326314	2.321415
2.335825	2.330466	2.325202	2.320037	2.340268	2.335115	2.330043	2.34874	2.343771
2.334778	2.329468	2.3242	2.318973	2.339119	2.333908	2.328794	2.347507	2.342553
2.333634	2.328379	2.323135	2.317899	2.338009	2.332781	2.327651	2.346374	2.341408
2.332304	2.327111	2.321942	2.316798	2.336964	2.331796	2.326694	2.345412	2.340377
2.330965	2.325811	2.320704	2.31565	2.335878	2.330783	2.325726	2.344457	2.339377
2.32973	2.324537	2.319419	2.314383	2.33462	2.329579	2.32458	2.343393	2.338371
2.328554	2.323298	2.318143	2.313091	2.333313	2.328308	2.323366	2.342278	2.337351
2.327462	2.322215	2.342439	2.337251	2.356201	2.351102	2.346073	2.34112	2.336229
2.326439	2.321248	2.341468	2.336232	2.355154	2.349991	2.344907	2.339926	2.335036
2.325289	2.320151	2.340388	2.33517	2.354123	2.348952	2.343855	2.338853	2.333943
2.323975	2.31889	2.339177	2.334063	2.353121	2.348015	2.342952	2.337931	2.33297
2.322729	2.317667	2.337971	2.332921	2.35206	2.347008	2.341985	2.336974	2.332002
2.321671	2.316564	2.336805	2.331706	2.35085	2.345818	2.340841	2.335902	2.331014
2.320644	2.315494	2.335671	2.330514	2.349654	2.34462	2.33967	2.334798	2.329999
2.31951	2.314409	2.334608	2.32948	2.348654	2.343584	2.338592	2.333703	2.328901
2.318344	2.313324	2.333576	2.328511	2.347734	2.342614	2.337555	2.332616	2.327774
2.342672	2.337557	2.356895	2.351775	2.346674	2.341567	2.336521	2.331595	2.326764
2.341628	2.336441	2.355763	2.350603	2.345504	2.340461	2.335494	2.330633	2.325852
2.340536	2.335333	2.354681	2.349515	2.344423	2.339408	2.334474	2.329639	2.32488
2.339364	2.334238	2.353681	2.348564	2.343488	2.338437	2.333455	2.328585	2.323803
2.338211	2.333139	2.352658	2.34758	2.342521	2.337457	2.332454	2.327563	2.322766
2.337136	2.332016	2.351538	2.346445	2.341404	2.336409	2.33148	2.326632	2.321865
2.336085	2.330898	2.350408	2.345291	2.340268	2.335359	2.330524	2.325729	2.320995
2.359815	2.354547	2.349363	2.344257	2.339256	2.334388	2.329589	2.338529	2.333756
2.358775	2.353529	2.348369	2.343289	2.338309	2.333458	2.328662	2.337605	2.332797
2.357757	2.352536	2.347386	2.342302	2.350836	2.345925	2.341075	2.349352	2.344522
2.356762	2.351574	2.346423	2.341301	2.349783	2.344828	2.339959	2.348318	2.343576
2.36943	2.364211	2.359018	2.353843	2.362001	2.357008	2.352115	2.347341	2.342651
2.368406	2.363164	2.357974	2.352827	2.361044	2.356091	2.35123	2.346459	2.341759
2.367375	2.362116	2.356938	2.351834	2.360122	2.355214	2.350381	2.345601	2.340871
2.366331	2.361091	2.355946	2.350893	2.359252	2.354364	2.349535	2.344733	2.339971
2.365285	2.360081	2.354978	2.349978	2.358404	2.353522	2.348685	2.343858	2.339064
2.364252	2.359095	2.367457	2.362465	2.357523	2.352624	2.347765	2.342931	2.338137
2.363229	2.35813	2.366547	2.361568	2.356621	2.351687	2.346797	2.341968	2.337198
2.362246	2.357177	2.365629	2.360644	2.355689	2.350748	2.345858	2.341053	2.336315
2.36131	2.356242	2.364706	2.359688	2.354721	2.349802	2.344948	2.340191	2.335497
2.373942	2.36883	2.363757	2.358722	2.353758	2.348873	2.344061	2.339344	2.334683
2.373	2.367834	2.362747	2.357734	2.352805	2.347968	2.343202	2.33851	2.333864

2.37206	2.36685	2.361756	2.356773	2.35188	2.347088	2.34236	2.337676	2.333028
2.371157	2.365967	2.360885	2.355905	2.35102	2.346249	2.341534	2.336831	2.33215
2.370259	2.36512	2.360064	2.355085	2.350198	2.345431	2.34071	2.335971	2.331247
2.369288	2.364193	2.359175	2.354231	2.349358	2.344567	2.339817	2.335064	2.330335
2.368255	2.363204	2.358236	2.353358	2.348509	2.343669	2.338866	2.334115	2.32941
2.36728	2.362259	2.357328	2.3525	2.347675	2.342809	2.337975	2.333222	2.328529
2.366383	2.36137	2.356458	2.351661	2.346858	2.341998	2.337164	2.332404	2.327701
2.365515	2.360509	2.355611	2.350837	2.346052	2.341203	2.336374	2.331609	2.326897
2.364667	2.359675	2.354793	2.350035	2.345262	2.34042	2.335593	2.330827	2.326112
2.363833	2.358855	2.353985	2.349238	2.344475	2.339638	2.334814	2.330049	2.325333
2.362997	2.358024	2.353161	2.348422	2.343665	2.338834	2.334015	2.329249	2.324532
2.362165	2.357192	2.352332	2.347597	2.342845	2.33802	2.333205	2.328441	2.323723
2.361348	2.356381	2.351525	2.346794	2.342045	2.337224	2.332412	2.327649	2.322931
2.360548	2.355591	2.350741	2.346011	2.341264	2.336445	2.331635	2.326872	2.322155
2.359737	2.35479	2.349947	2.34522	2.340473	2.335656	2.330848	2.326088	2.321374
2.358903	2.353969	2.349133	2.344409	2.339665	2.334848	2.330042	2.325287	2.320579
2.358078	2.35315	2.34832	2.3436	2.338858	2.334043	2.329238	2.324488	2.319786
2.357275	2.352341	2.347513	2.342802	2.338067	2.333252	2.328448	2.323703	2.319009
2.356498	2.35155	2.346718	2.342011	2.33728	2.332467	2.327665	2.322925	2.318234
2.355777	2.350813	2.345961	2.341228	2.336482	2.331674	2.326879	2.322134	2.317436
2.355098	2.350115	2.345233	2.340453	2.335679	2.330879	2.326097	2.321342	2.316628
2.354374	2.349391	2.344494	2.339679	2.334893	2.330128	2.32538	2.320622	2.31589
2.353604	2.348637	2.343739	2.338902	2.334124	2.329428	2.324745	2.319992	2.315234
2.352827	2.347898	2.343011	2.338156	2.333376	2.328715	2.324065	2.319321	2.314555
2.352054	2.347203	2.342343	2.33747	2.332662	2.327963	2.323285	2.318548	2.313803
2.351282	2.346498	2.341665	2.336784	2.331951	2.327202	2.322483	2.31776	2.313052
2.350524	2.345736	2.340908	2.336041	2.331212	2.326439	2.321707	2.317024	2.31237
2.349766	2.344942	2.340111	2.335276	2.330468	2.325683	2.320946	2.316304	2.311706
2.348974	2.344124	2.339309	2.334534	2.329764	2.324974	2.320221	2.335388	2.330633
2.348154	2.343283	2.338497	2.333806	2.329092	2.324305	2.319528	2.334612	2.329802
2.347394	2.342509	2.337727	2.333059	2.328369	2.323611	2.318851	2.333873	2.329025
2.346719	2.341821	2.337007	2.332277	2.327571	2.322889	2.318204	2.333194	2.328327
2.346023	2.341143	2.336311	2.33152	2.326788	2.322149	2.317512	2.332479	2.327613
2.345243	2.340453	2.335649	2.330828	2.326057	2.321373	2.316704	2.331653	2.326824
2.344446	2.339743	2.334978	2.330153	2.325355	2.320607	2.315886	2.330818	2.326029
2.343687	2.338969	2.334224	2.329461	2.324697	2.319926	2.315173	2.33008	2.325292
2.34295	2.338162	2.333427	2.328757	2.324057	2.319286	2.314517	2.329395	2.324588
2.342247	2.337418	2.332666	2.328001	2.323321	2.31859	2.313858	2.328717	2.323904
2.341578	2.336728	2.331932	2.327192	2.322492	2.317847	2.313207	2.328054	2.323245
2.340912	2.336055	2.331231	2.326435	2.341611	2.336858	2.332127	2.327352	2.322572
2.340242	2.3354	2.330575	2.325763	2.340879	2.336076	2.331315	2.326587	2.321885
2.339574	2.334744	2.329922	2.325108	2.340166	2.335311	2.330513	2.325815	2.321172
2.338907	2.334067	2.329242	2.324431	2.33945	2.334586	2.329777	2.32506	2.320401
2.338243	2.33339	2.328561	2.323757	2.338747	2.333887	2.329074	2.324319	2.319619
2.337586	2.332738	2.327911	2.323106	2.338071	2.333205	2.328383	2.323613	2.318895
2.336934	2.332102	2.32728	2.322469	2.337415	2.332542	2.32771	2.322935	2.31821
2.336278	2.331447	2.326628	2.32182	2.336746	2.331879	2.327053	2.322284	2.317562
2.335616	2.330769	2.325949	2.321155	2.336062	2.331217	2.326415	2.321662	2.316953
2.334965	2.33011	2.32529	2.3205	2.335385	2.33056	2.325781	2.321047	2.316355
2.334325	2.329484	2.32466	2.31985	2.334708	2.3299	2.325143	2.320434	2.315764

2.3337	2.328881	2.32406	2.319233	2.334067	2.329265	2.324518	2.319823	2.315167
2.333099	2.32831	2.343321	2.3384	2.333524	2.328703	2.323929	2.347684	2.342878
2.332513	2.327754	2.342775	2.337884	2.333015	2.328164	2.323351	2.347102	2.342251
2.33193	2.327165	2.342169	2.337277	2.332405	2.327549	2.322728	2.34649	2.341625
2.331351	2.326554	2.341518	2.336603	2.331721	2.326879	2.322071	2.345851	2.340995
2.33075	2.325937	2.340864	2.33594	2.331055	2.326223	2.321425	2.345224	2.340359
2.330121	2.325309	2.340202	2.335284	2.330406	2.325581	2.320786	2.344604	2.339702
2.349423	2.344487	2.339567	2.334661	2.329791	2.324968	2.320172	2.344022	2.339098
2.348805	2.343898	2.339001	2.334113	2.32925	2.324414	2.319608	2.34352	2.338615
2.348191	2.343312	2.338437	2.333567	2.328711	2.323862	2.319046	2.343019	2.338143
2.347558	2.342669	2.337795	2.332936	2.35723	2.352255	2.347308	2.342412	2.337548
2.346921	2.342006	2.33712	2.332266	2.356607	2.35162	2.346659	2.341761	2.336899
2.346331	2.341408	2.336514	2.33165	2.356024	2.351047	2.346096	2.341193	2.336324
2.345786	2.340872	2.33597	2.331081	2.355475	2.35053	2.345609	2.3407	2.335811
2.326283	2.321486	2.335381	2.330481	2.354895	2.34997	2.345068	2.340154	2.335254
2.325597	2.3208	2.334706	2.32982	2.354256	2.349329	2.344426	2.33951	2.334612
2.324939	2.32014	2.363663	2.358635	2.353646	2.348714	2.343806	2.33889	2.333995
2.324391	2.319584	2.363121	2.35811	2.353133	2.348198	2.343285	2.338368	2.333471
2.323885	2.319072	2.362613	2.357621	2.352657	2.347721	2.342804	2.337886	2.332988
2.323362	2.318554	2.362123	2.357129	2.352163	2.347229	2.342314	2.350072	2.345142
2.322836	2.318042	2.361652	2.356641	2.351666	2.346736	2.341829	2.349589	2.344664
2.32231	2.31752	2.361163	2.356151	2.351174	2.346244	2.341337	2.349098	2.344175
2.321781	2.316987	2.360656	2.355657	2.350688	2.345754	2.34084	2.348596	2.343672
2.339312	2.334413	2.360155	2.355163	2.36294	2.357979	2.35304	2.3481	2.343177
2.338826	2.333935	2.359669	2.354666	2.362438	2.357482	2.35255	2.347617	2.3427
2.338339	2.333456	2.359185	2.354171	2.361939	2.356986	2.35206	2.347134	2.342224
2.351505	2.346576	2.358695	2.353687	2.361456	2.356494	2.351561	2.346639	2.341739
2.351	2.346077	2.358201	2.353204	2.360978	2.356005	2.35106	2.346141	2.341249
2.350496	2.345574	2.370512	2.365488	2.36049	2.355518	2.350573	2.345651	2.340755
2.349992	2.345067	2.37	2.364983	2.359994	2.355034	2.350095	2.345166	2.340258
2.363077	2.358121	2.353186	2.348271	2.359493	2.354542	2.34961	2.344682	2.339771
2.362565	2.357617	2.352685	2.34777	2.358985	2.35404	2.349116	2.344197	2.339296
2.362059	2.357113	2.352183	2.347269	2.358479	2.353536	2.348618	2.343709	2.338816
2.361562	2.356604	2.351671	2.346763	2.357972	2.353031	2.348113	2.343208	2.338321
2.361074	2.356103	2.351167	2.346266	2.357473	2.35253	2.347611	2.342709	2.337827
2.360591	2.35562	2.350684	2.345784	2.356981	2.352034	2.347113	2.342217	2.337344
2.360124	2.355158	2.350224	2.345324	2.356508	2.351554	2.34663	2.341741	2.336878
2.359725	2.354757	2.349826	2.344935	2.356113	2.351146	2.34621	2.341328	2.336477
2.359389	2.354414	2.349487	2.344614	2.355794	2.350806	2.345849	2.340975	2.336139
2.358996	2.354024	2.349102	2.344234	2.355412	2.350425	2.345465	2.340585	2.335741
2.358508	2.353555	2.348637	2.343756	2.354928	2.349973	2.345039	2.340131	2.335243
2.358031	2.353097	2.348182	2.343288	2.354453	2.349531	2.344622	2.339689	2.33476
2.357639	2.352718	2.347807	2.342904	2.354057	2.349153	2.344258	2.339317	2.334368
2.357258	2.352345	2.347436	2.342532	2.353676	2.348778	2.343889	2.33895	2.333997
2.356792	2.351874	2.34697	2.342082	2.353238	2.348325	2.343424	2.338501	2.333573
2.356284	2.351354	2.346455	2.341593	2.352773	2.34783	2.342905	2.338007	2.333126
2.355855	2.350924	2.346027	2.341171	2.336325	2.331474	2.326642	2.337573	2.332716
2.355503	2.350583	2.345685	2.340814	2.335957	2.331106	2.326277	2.337202	2.332346
2.355162	2.350249	2.345347	2.340459	2.335587	2.330724	2.325885	2.336799	2.331937
2.354824	2.349909	2.345	2.340101	2.335207	2.330303	2.325425	2.336318	2.331442

2.354479	2.349557	2.344638	2.339727	2.334814	2.32988	2.324974	2.33585	2.330962
2.354111	2.349176	2.344241	2.339309	2.334388	2.329474	2.324589	2.335465	2.330575
2.353726	2.348776	2.343825	2.338875	2.333957	2.32908	2.324234	2.335116	2.33023
2.353317	2.348353	2.343404	2.338471	2.333571	2.328712	2.323881	2.334762	2.32988
2.352887	2.347912	2.342977	2.338087	2.333219	2.328367	2.323536	2.33441	2.329534
2.352459	2.347497	2.342583	2.337722	2.332873	2.328022	2.323187	2.334058	2.329183
2.352021	2.347112	2.34223	2.337382	2.332537	2.327678	2.322834	2.333705	2.328825
2.351638	2.346748	2.341878	2.337038	2.332195	2.327331	2.322482	2.333351	2.328468
2.351354	2.346398	2.341501	2.336671	2.33184	2.326981	2.322135	2.332995	2.328118
2.351103	2.34608	2.341147	2.336309	2.331478	2.326625	2.321785	2.332637	2.327764
2.350839	2.345834	2.34088	2.335977	2.331101	2.32625	2.32142	2.332273	2.327392
2.35057	2.345621	2.340654	2.335666	2.330728	2.32587	2.321048	2.331905	2.327013
2.350256	2.345338	2.340383	2.335389	2.330433	2.325536	2.320678	2.315857	2.311072
2.349902	2.344998	2.340078	2.335146	2.330206	2.325239	2.320308	2.315475	2.310705
2.349541	2.344644	2.339748	2.33486	2.329943	2.324956	2.319996	2.315141	2.310351
2.349172	2.344273	2.339385	2.334515	2.329626	2.324696	2.319771	2.314871	2.310005
2.348807	2.343909	2.339024	2.334157	2.329289	2.324415	2.319533	2.31461	2.309691
2.348441	2.343559	2.338674	2.333789	2.328919	2.324072	2.319224	2.314338	2.309449
2.348103	2.343236	2.338346	2.33344	2.328557	2.323715	2.318886	2.31405	2.309216
2.347866	2.342988	2.338093	2.333185	2.328287	2.323395	2.318526	2.313703	2.308905
2.347687	2.342783	2.337879	2.332983	2.328066	2.323095	2.318154	2.313323	2.308552
2.347418	2.342492	2.337584	2.332699	2.327787	2.322808	2.317854	2.313001	2.30821
2.347059	2.342115	2.337206	2.332337	2.327455	2.322538	2.317626	2.312734	2.307872
2.346735	2.341782	2.336863	2.33198	2.327107	2.322243	2.317377	2.312474	2.307578
2.346492	2.341541	2.336591	2.331638	2.32673	2.321896	2.317076	2.312211	2.307349
2.346238	2.3413	2.33633	2.331323	2.326385	2.321565	2.316777	2.311955	2.307138
2.345893	2.340982	2.336039	2.33106	2.326139	2.321316	2.316524	2.311719	2.30692
2.345533	2.340653	2.335749	2.330825	2.325934	2.321093	2.316277	2.31148	2.306694
2.345276	2.3404	2.335503	2.330589	2.325693	2.320813	2.315964	2.311177	2.306419
2.345089	2.340198	2.335287	2.330359	2.325435	2.320501	2.315609	2.310831	2.306109
2.333841	2.32892	2.33506	2.330134	2.325204	2.320242	2.315328	2.310554	2.305849
2.333607	2.32867	2.334827	2.329922	2.325007	2.320047	2.315131	2.310354	2.305644
2.333351	2.328384	2.334553	2.329672	2.324781	2.319843	2.314942	2.310166	2.30545
2.333053	2.328025	2.334193	2.329339	2.324486	2.319596	2.314738	2.309975	2.305257
2.332758	2.327674	2.333841	2.329008	2.324189	2.319349	2.314535	2.309787	2.305068
2.332518	2.327418	2.333593	2.328759	2.323947	2.319128	2.314335	2.309596	2.30488
2.332305	2.32721	2.333397	2.328551	2.323733	2.318921	2.314138	2.309405	2.304693
2.332086	2.326988	2.322007	2.317143	2.323521	2.318717	2.313943	2.309212	2.3045
2.331865	2.326761	2.321779	2.316919	2.323316	2.318519	2.313749	2.309018	2.304303
2.331639	2.326534	2.321554	2.316698	2.323114	2.31832	2.313552	2.308819	2.304101
2.331401	2.326301	2.321328	2.316482	2.322915	2.318121	2.31335	2.308615	2.303894
2.331174	2.326083	2.321114	2.31627	2.322716	2.317921	2.313148	2.30841	2.303685
2.330971	2.325885	2.320913	2.316053	2.322502	2.317713	2.312947	2.308209	2.30348
2.330783	2.325711	2.320737	2.315858	2.322303	2.317512	2.312747	2.308006	2.303274
2.330617	2.325588	2.320628	2.315735	2.310875	2.30604	2.301237	2.307795	2.303062
2.330463	2.325493	2.320555	2.315652	2.310766	2.30588	2.30103	2.307581	2.302847
2.330267	2.325331	2.320411	2.315511	2.310616	2.305709	2.300834	2.307376	2.30263
2.330029	2.325107	2.320201	2.315316	2.310432	2.305526	2.300646	2.307176	2.302406
2.329802	2.32488	2.31998	2.315111	2.310246	2.305366	2.300503	2.295683	2.290888
2.329588	2.324645	2.319744	2.314894	2.310063	2.305247	2.300436	2.295617	2.290796

2.32941	2.324443	2.319535	2.314691	2.309882	2.305117	2.30035	2.295536	2.290696
2.329313	2.32433	2.3194	2.314526	2.309696	2.304925	2.30016	2.295349	2.290521
2.329245	2.324249	2.319296	2.314386	2.309523	2.304723	2.299939	2.29513	2.290317
2.305774	2.300825	2.319165	2.314267	2.309407	2.304591	2.299786	2.294952	2.290117
2.305637	2.300671	2.319023	2.314167	2.30933	2.304503	2.299672	2.294798	2.289917
2.305498	2.300538	2.318898	2.314058	2.309223	2.304379	2.299527	2.294645	2.289759
2.305355	2.300427	2.318795	2.313939	2.309085	2.304213	2.299346	2.294492	2.28965
2.305222	2.300316	2.318684	2.313807	2.308936	2.304053	2.299185	2.294353	2.289541
2.305119	2.300204	2.318555	2.313651	2.30877	2.303912	2.299071	2.294241	2.289421
2.305	2.300078	2.318418	2.313496	2.308612	2.303781	2.298965	2.294129	2.289289
2.304806	2.29991	2.318271	2.31337	2.308497	2.303664	2.298839	2.293985	2.289127
2.304587	2.299727	2.318123	2.313256	2.308399	2.303551	2.298702	2.293828	2.288955
2.304444	2.299592	2.29475	2.289923	2.308274	2.303406	2.298542	2.293678	2.288828
2.304358	2.299494	2.294637	2.289795	2.30813	2.303236	2.29836	2.293532	2.288735
2.304279	2.299402	2.294526	2.289655	2.307975	2.30308	2.298215	2.293414	2.288652
2.304202	2.299314	2.29441	2.289488	2.307792	2.302935	2.298116	2.293334	2.288583
2.304131	2.299236	2.294315	2.289364	2.30766	2.302823	2.298029	2.293256	2.288507
2.304063	2.299174	2.294271	2.289352	2.307656	2.302781	2.297945	2.29316	2.28841
2.303996	2.299114	2.29423	2.289348	2.307661	2.302746	2.297863	2.293062	2.288308
2.303921	2.299028	2.294128	2.28922	2.284341	2.279493	2.274689	2.292973	2.288213
2.303841	2.29893	2.293998	2.289036	2.28414	2.279345	2.274598	2.292888	2.288122
2.303759	2.298847	2.293914	2.288951	2.284055	2.27926	2.274514	2.292802	2.288044
2.303674	2.298777	2.293873	2.28896	2.284079	2.279232	2.274435	2.292714	2.287978
2.283538	2.278733	2.293805	2.288909	2.284032	2.279166	2.274351	2.292624	2.287902
2.283457	2.278638	2.293684	2.28874	2.283848	2.279025	2.274258	2.29253	2.287805
2.283375	2.278546	2.293574	2.288596	2.283691	2.2789	2.274167	2.292434	2.287706
2.283276	2.278469	2.293523	2.288579	2.283682	2.278847	2.274071	2.292326	2.287611
2.283188	2.278401	2.293487	2.2886	2.283721	2.278831	2.273994	2.29223	2.287523
2.283167	2.278333	2.293412	2.288557	2.283707	2.278831	2.273995	2.269258	2.264581
2.283194	2.278272	2.293319	2.288482	2.283665	2.278848	2.274052	2.269275	2.264528
2.283207	2.278261	2.293289	2.288426	2.283606	2.278827	2.274067	2.287828	2.282987
2.283208	2.278309	2.29333	2.288386	2.283517	2.278758	2.274036	2.287832	2.283032
2.283187	2.278335	2.293363	2.288375	2.283478	2.278726	2.274026	2.28785	2.283088
2.283117	2.278301	2.293359	2.288403	2.283527	2.278775	2.274071	2.287908	2.283157
2.283066	2.278273	2.293361	2.288452	2.2836	2.278831	2.274105	2.287944	2.283202
2.283107	2.278314	2.29341	2.288516	2.283656	2.278831	2.274054	2.300018	2.295247
2.283187	2.278375	2.293469	2.288582	2.283704	2.278815	2.273978	2.299926	2.295166
2.283228	2.278374	2.293457	2.288588	2.283721	2.278825	2.273973	2.29989	2.295093
2.283246	2.278328	2.29339	2.288547	2.283712	2.278854	2.27402	2.299892	2.29502
2.283258	2.278322	2.273482	2.268739	2.283723	2.278896	2.27408	2.299925	2.295004
2.283261	2.278366	2.273541	2.268785	2.283768	2.27896	2.27416	2.3	2.29506
2.283284	2.278425	2.273603	2.268817	2.283788	2.278995	2.274214	2.300061	2.295124
2.283349	2.278488	2.273638	2.268794	2.283731	2.278952	2.274199	2.300072	2.295167
2.283434	2.27856	2.273678	2.268777	2.28368	2.278908	2.274178	2.300079	2.295212
2.28352	2.278646	2.273753	2.268834	2.283726	2.278951	2.274223	2.300133	2.295279
2.283612	2.278739	2.273849	2.268931	2.283825	2.27904	2.274302	2.300211	2.295357
2.283696	2.278824	2.273933	2.269015	2.314933	2.310016	2.305149	2.300282	2.295427
2.283772	2.278901	2.27401	2.269091	2.315009	2.31009	2.30522	2.300349	2.29549
2.283854	2.278984	2.274093	2.269173	2.315092	2.31017	2.305296	2.300422	2.295559
2.283948	2.279079	2.274188	2.269265	2.315185	2.31026	2.305382	2.300504	2.295638

2.265727	2.26093	2.274278	2.269353	2.315274	2.310346	2.305466	2.300586	2.295718
2.265804	2.261006	2.274351	2.269424	2.315348	2.310421	2.305542	2.300663	2.295794
2.265875	2.261074	2.274415	2.269488	2.315416	2.310491	2.305614	2.300736	2.295869
2.26594	2.261138	2.274475	2.269545	2.315476	2.310554	2.305679	2.300802	2.295936
2.266001	2.261198	2.274532	2.2696	2.315533	2.310612	2.30574	2.300864	2.295998
2.266072	2.261271	2.274605	2.269675	2.297521	2.292672	2.287871	2.300934	2.296074
2.266151	2.261358	2.274694	2.269768	2.297612	2.292747	2.287934	2.301007	2.296163
2.266231	2.261448	2.274797	2.269893	2.297747	2.292869	2.288039	2.301108	2.29626
2.2663	2.261538	2.274922	2.270077	2.297966	2.293076	2.288217	2.301255	2.29637
2.266385	2.261634	2.275043	2.27025	2.298171	2.293274	2.288394	2.301403	2.296482
2.266516	2.261733	2.256978	2.252253	2.298256	2.293368	2.288497	2.301505	2.296576
2.266671	2.261847	2.25706	2.252312	2.298304	2.293429	2.288575	2.301597	2.296676
2.266827	2.262017	2.291843	2.286942	2.298441	2.29355	2.288689	2.30173	2.296837
2.266988	2.262231	2.29208	2.287163	2.298639	2.293711	2.288826	2.301894	2.297048
2.267114	2.262384	2.292247	2.28733	2.28244	2.27758	2.272767	2.302087	2.297262
2.267191	2.262452	2.292322	2.287428	2.282571	2.277761	2.27299	2.302326	2.297486
2.267301	2.262545	2.292413	2.287526	2.282687	2.277912	2.273172	2.30252	2.297673
2.267494	2.262719	2.29257	2.287643	2.282774	2.277987	2.273248	2.302608	2.297786
2.267707	2.262912	2.292746	2.287779	2.282876	2.278066	2.273314	2.302677	2.297873
2.267889	2.263075	2.292908	2.287958	2.283063	2.278242	2.273471	2.302806	2.297967
2.26806	2.263232	2.293072	2.288164	2.283294	2.278465	2.273669	2.302964	2.298067
2.268226	2.263412	2.293265	2.288371	2.283502	2.278655	2.273839	2.285217	2.2804
2.268385	2.263613	2.293483	2.288581	2.283697	2.278821	2.273987	2.285387	2.280615
2.283444	2.278635	2.293698	2.288773	2.283872	2.278991	2.274162	2.285583	2.280842
2.283673	2.278857	2.293899	2.288924	2.284003	2.279157	2.27437	2.285812	2.28108
2.267758	2.263016	2.294118	2.289114	2.284181	2.279356	2.274595	2.269889	2.265227
2.268004	2.26326	2.294391	2.289421	2.284497	2.279637	2.274835	2.270111	2.265439
2.268252	2.263506	2.294673	2.289748	2.284839	2.279936	2.275082	2.270334	2.265647
2.289415	2.284589	2.294903	2.289964	2.285049	2.280162	2.275321	2.297507	2.29271
2.289639	2.284814	2.295105	2.290121	2.285193	2.28035	2.275557	2.297749	2.292928
2.289873	2.285047	2.295343	2.290359	2.285431	2.280589	2.275796	2.297987	2.293167
2.290118	2.285289	2.295618	2.290683	2.28577	2.28088	2.276038	2.298219	2.29343
2.290363	2.28553	2.28069	2.275844	2.286052	2.28114	2.276277	2.298452	2.293679
2.290605	2.285768	2.280904	2.276011	2.286201	2.281327	2.276506	2.298686	2.293899
2.290846	2.286008	2.281126	2.276195	2.286372	2.281527	2.276739	2.298919	2.294117
2.291081	2.286253	2.281393	2.276501	2.286695	2.281808	2.276975	2.299138	2.294347
2.291318	2.286501	2.281675	2.276847	2.287074	2.282128	2.277229	2.299364	2.294583
2.291572	2.286744	2.281926	2.277126	2.287387	2.282453	2.277552	2.299644	2.294817
2.291842	2.286987	2.28216	2.277365	2.287661	2.282789	2.277927	2.299966	2.295052
2.292163	2.287295	2.282448	2.277624	2.287918	2.283082	2.278255	2.300285	2.295343
2.292553	2.287689	2.282807	2.277903	2.288146	2.283321	2.278527	2.300606	2.295707
2.29291	2.288051	2.283151	2.278204	2.273321	2.268539	2.263805	2.300899	2.296046
2.293169	2.288319	2.283446	2.27855	2.27368	2.268838	2.264044	2.301128	2.296314
2.293419	2.288563	2.283718	2.278889	2.274046	2.269154	2.264301	2.301357	2.296559
2.29374	2.288823	2.283959	2.279155	2.302473	2.297503	2.29256	2.301651	2.296806
2.294092	2.289093	2.284191	2.279389	2.302732	2.297824	2.292924	2.301979	2.297057
2.29442	2.289417	2.284497	2.279658	2.302979	2.298105	2.293242	2.302305	2.29737
2.294732	2.289791	2.284867	2.279953	2.303208	2.298351	2.293525	2.302634	2.297737
2.295019	2.290126	2.285212	2.280272	2.303495	2.298641	2.293829	2.289006	2.2842
2.295259	2.290389	2.285509	2.280619	2.303864	2.299	2.294171	2.289356	2.284559

2.295536	2.290678	2.285824	2.280979	2.304241	2.299351	2.294493	2.289681	2.284893
2.295915	2.291055	2.286195	2.281342	2.30458	2.29964	2.29474	2.289929	2.285164
2.296331	2.291458	2.286584	2.281713	2.304919	2.299928	2.294985	2.290171	2.285427
2.296722	2.29184	2.286964	2.282101	2.305298	2.300286	2.295321	2.2905	2.285755
2.297105	2.29221	2.287337	2.282493	2.305695	2.300683	2.295713	2.290881	2.286122
2.297461	2.29253	2.287646	2.282817	2.306033	2.301047	2.296097	2.291264	2.286495
2.297787	2.292793	2.287885	2.283067	2.306309	2.301378	2.296477	2.291656	2.28688
2.29814	2.293102	2.288175	2.283361	2.306615	2.301722	2.296857	2.292043	2.287263
2.298543	2.293495	2.288559	2.283737	2.306977	2.302091	2.297233	2.292422	2.287642
2.298956	2.293906	2.288966	2.284135	2.307357	2.302469	2.29761	2.2928	2.288021
2.299354	2.294301	2.289359	2.284528	2.307742	2.302852	2.297993	2.29318	2.288397
2.29975	2.294694	2.289752	2.284923	2.308131	2.30324	2.298378	2.29356	2.288774
2.300145	2.295086	2.290141	2.285312	2.308512	2.303623	2.298762	2.293943	2.289153
2.300541	2.295478	2.29053	2.285696	2.308889	2.304003	2.299147	2.294327	2.289534
2.300943	2.295883	2.290931	2.286086	2.309266	2.304382	2.299528	2.294708	2.289914
2.301349	2.296296	2.291339	2.286475	2.309637	2.304754	2.299904	2.295086	2.290292
2.274726	2.26966	2.291786	2.286905	2.310043	2.305145	2.300283	2.295461	2.290669
2.275168	2.270152	2.292313	2.287418	2.310516	2.305569	2.300664	2.295829	2.29104
2.275605	2.270638	2.292837	2.287933	2.310997	2.306003	2.301053	2.296203	2.291414
2.275985	2.271038	2.266098	2.261167	2.311426	2.306419	2.301454	2.296589	2.291786
2.276339	2.271394	2.266462	2.261547	2.311834	2.306835	2.301872	2.296993	2.292172
2.276703	2.271754	2.266828	2.261929	2.312255	2.307286	2.302347	2.297466	2.292628
2.277072	2.272109	2.267187	2.262309	2.312683	2.307768	2.302874	2.298001	2.293147
2.307786	2.302716	2.297689	2.292706	2.287762	2.282862	2.277981	2.298477	2.293623
2.308295	2.303201	2.298141	2.293113	2.288134	2.283218	2.278335	2.298866	2.294037
2.308788	2.303686	2.298607	2.293546	2.288539	2.283601	2.278707	2.273841	2.269007
2.309189	2.30412	2.299064	2.294021	2.28902	2.284074	2.279161	2.274264	2.269395
2.309581	2.304553	2.29953	2.294515	2.289526	2.284572	2.279637	2.274705	2.269796
2.31006	2.305043	2.300024	2.295005	2.290007	2.285031	2.280079	2.275149	2.270248
2.31058	2.305558	2.300529	2.295493	2.290476	2.285476	2.280511	2.275601	2.270734
2.311033	2.306001	2.300971	2.295944	2.290935	2.285937	2.280974	2.276071	2.271212
2.311419	2.306371	2.301348	2.296355	2.291379	2.286412	2.281468	2.276563	2.271685
2.311858	2.306798	2.301777	2.296801	2.291841	2.28689	2.281955	2.27704	2.272144
2.312388	2.307325	2.302297	2.297307	2.292335	2.287373	2.282426	2.277491	2.272576
2.312942	2.307877	2.302836	2.297824	2.292829	2.287848	2.282886	2.277938	2.273017
2.313491	2.308418	2.303362	2.298326	2.293308	2.288299	2.28332	2.278388	2.273496
2.31404	2.308955	2.30388	2.298819	2.293776	2.288743	2.283754	2.278845	2.273993
2.314573	2.309468	2.304371	2.299283	2.294228	2.289206	2.284233	2.279337	2.274496
2.315092	2.309961	2.304841	2.299727	2.294669	2.289681	2.284744	2.279854	2.275008
2.315592	2.310437	2.305306	2.300196	2.295148	2.290178	2.285259	2.280375	2.275529
2.316069	2.310888	2.305761	2.300689	2.295668	2.290701	2.285779	2.280901	2.276061
2.316545	2.311359	2.306243	2.301199	2.296197	2.291229	2.286303	2.281426	2.276589
2.317009	2.311868	2.306773	2.301727	2.296723	2.291758	2.286833	2.28195	2.277105
2.317506	2.312399	2.307317	2.302265	2.297254	2.29229	2.287364	2.282474	2.27762
2.318098	2.31294	2.30784	2.302799	2.297797	2.292826	2.287892	2.282999	2.278142
2.318744	2.313506	2.30837	2.30334	2.298344	2.293363	2.288419	2.283523	2.278666
2.319382	2.314136	2.308975	2.3039	2.298873	2.293888	2.288946	2.284045	2.279183
2.320023	2.314822	2.309642	2.304477	2.299388	2.294403	2.289473	2.284566	2.279695
2.290433	2.285366	2.280287	2.275192	2.299969	2.294958	2.290008	2.285095	2.280224
2.291005	2.285953	2.280903	2.275855	2.30064	2.295562	2.290544	2.285623	2.280767

2.291567	2.286522	2.281497	2.276496	2.301303	2.296185	2.291122	2.286194	2.281342
2.292125	2.287074	2.282056	2.277075	2.301911	2.296829	2.291791	2.286856	2.281982
2.292684	2.287623	2.282605	2.277633	2.302494	2.297468	2.292474	2.28753	2.282626
2.279939	2.274918	2.283167	2.278191	2.303047	2.298043	2.293067	2.288111	2.283185
2.280498	2.275489	2.283742	2.278754	2.303586	2.298589	2.293619	2.28865	2.283705
2.281137	2.276118	2.284374	2.279386	2.304208	2.299207	2.294231	2.289255	2.284299
2.281856	2.276807	2.285066	2.280088	2.304912	2.299894	2.294899	2.28992	2.284962
2.28253	2.277456	2.285718	2.280747	2.305571	2.300538	2.295528	2.290552	2.285598
2.283114	2.278026	2.286294	2.281321	2.306146	2.301099	2.296079	2.291112	2.28618
2.283705	2.278608	2.286882	2.281903	2.306724	2.301671	2.296648	2.291688	2.28677
2.260041	2.255028	2.274256	2.269305	2.307369	2.302325	2.297313	2.292349	2.287422
2.260704	2.255701	2.274936	2.269966	2.308023	2.302995	2.297999	2.293027	2.288083
2.26128	2.256281	2.275526	2.270546	2.308604	2.303586	2.2986	2.293623	2.28867
2.261806	2.256807	2.276064	2.271081	2.309144	2.304133	2.299154	2.294173	2.289216
2.262415	2.257405	2.252445	2.247539	2.297506	2.292538	2.287603	2.294793	2.289839
2.263111	2.258079	2.25311	2.248207	2.298188	2.293216	2.288277	2.295488	2.290545
2.263823	2.258772	2.253794	2.24889	2.298884	2.293907	2.288966	2.296195	2.29126
2.264532	2.259474	2.254488	2.249576	2.299578	2.294602	2.289662	2.296902	2.291962
2.265245	2.260181	2.255188	2.250267	2.300276	2.2953	2.290362	2.297609	2.292665
2.265954	2.260884	2.255885	2.250957	2.27823	2.273322	2.268453	2.286117	2.281206
2.266664	2.261587	2.256582	2.251648	2.278918	2.274003	2.269127	2.286804	2.281894
2.267374	2.262291	2.257279	2.252336	2.279599	2.27468	2.269802	2.287492	2.282583
2.268086	2.262997	2.257976	2.253022	2.280277	2.275354	2.270475	2.28818	2.283272
2.268799	2.263702	2.258672	2.253707	2.280955	2.276029	2.271149	2.288866	2.283955
2.269511	2.264405	2.259366	2.254392	2.281633	2.276704	2.271821	2.289548	2.284633
2.270224	2.26511	2.260063	2.25508	2.282313	2.277379	2.272492	2.267623	2.262784
2.27094	2.265821	2.260766	2.255771	2.282996	2.278053	2.273158	2.268285	2.263442
2.271657	2.266533	2.261471	2.256465	2.283679	2.278727	2.273825	2.268946	2.2641
2.272376	2.267247	2.262176	2.257159	2.284361	2.279404	2.274496	2.269611	2.264759
2.273096	2.267961	2.262883	2.257855	2.285045	2.280082	2.275169	2.270278	2.26542
2.273813	2.268674	2.263588	2.258551	2.253587	2.248713	2.243893	2.270943	2.266081
2.274526	2.269384	2.264292	2.259246	2.254272	2.249387	2.244559	2.271604	2.266743
2.275255	2.270106	2.265009	2.259958	2.254976	2.250078	2.245238	2.272279	2.267421
2.275993	2.270835	2.265732	2.260681	2.255695	2.250779	2.245923	2.272962	2.268109
2.276769	2.271602	2.266495	2.261445	2.256453	2.25152	2.246648	2.273683	2.268832
2.277628	2.272453	2.267343	2.262294	2.257297	2.252347	2.247458	2.274485	2.269635
2.278507	2.273328	2.268214	2.263163	2.25816	2.253197	2.248292	2.275309	2.270454
2.258004	2.252884	2.269009	2.263944	2.25893	2.253964	2.249055	2.287361	2.282454
2.258744	2.253633	2.269765	2.264676	2.259647	2.254687	2.249781	2.288092	2.283155
2.259512	2.254433	2.249375	2.244332	2.260427	2.255466	2.250559	2.28888	2.283921
2.260306	2.255291	2.250254	2.245192	2.261276	2.256308	2.251395	2.289735	2.28476
2.261106	2.256116	2.251087	2.246015	2.262098	2.257117	2.252191	2.290552	2.28557
2.2619	2.25686	2.25181	2.246748	2.262846	2.257841	2.252892	2.291274	2.286305
2.26272	2.257618	2.252543	2.247493	2.263606	2.258578	2.253606	2.292008	2.287052
2.263613	2.258476	2.253384	2.248335	2.243327	2.238351	2.23343	2.292838	2.287882
2.264528	2.259376	2.254273	2.249218	2.244202	2.239216	2.234285	2.293714	2.28875
2.26537	2.260251	2.255156	2.250081	2.245053	2.240074	2.23515	2.263789	2.258886
2.266158	2.261114	2.256041	2.250934	2.245888	2.240932	2.236028	2.264678	2.259755
2.266954	2.261926	2.256855	2.251734	2.246681	2.241732	2.23684	2.245399	2.24054
2.267754	2.262666	2.257569	2.252458	2.247412	2.242452	2.237564	2.246208	2.241425

2.268587	2.263444	2.258323	2.253222	2.248181	2.243203	2.238305	2.247007	2.242269
2.26948	2.264318	2.259189	2.254091	2.249043	2.24404	2.23911	2.247795	2.243019
2.270394	2.265228	2.260096	2.254996	2.249936	2.24491	2.239947	2.248597	2.243762
2.271293	2.266135	2.260999	2.255884	2.250813	2.245783	2.240811	2.249456	2.244586
2.27219	2.267048	2.261911	2.256775	2.25169	2.246666	2.241698	2.25035	2.245457
2.273095	2.267951	2.262809	2.257668	2.252576	2.247546	2.242572	2.25124	2.246346
2.274006	2.268844	2.263697	2.258563	2.253472	2.248425	2.243437	2.25213	2.247258
2.274908	2.269739	2.264588	2.259456	2.254364	2.249311	2.24432	2.253042	2.248184
2.275796	2.270636	2.265486	2.260343	2.255249	2.250204	2.245224	2.253982	2.249133
2.276682	2.271531	2.266383	2.261237	2.256144	2.251113	2.246145	2.254927	2.25007
2.277567	2.272413	2.267271	2.26214	2.257062	2.252053	2.247095	2.255867	2.250967
2.278455	2.273299	2.268166	2.263056	2.257997	2.253003	2.248045	2.256798	2.251849
2.279352	2.274211	2.269095	2.264006	2.258951	2.25393	2.248941	2.257698	2.252738
2.280257	2.275142	2.270048	2.264979	2.259918	2.254845	2.249804	2.258579	2.253628
2.239406	2.234461	2.271	2.265926	2.260852	2.255754	2.250688	2.25947	2.254509
2.240326	2.235408	2.271955	2.266848	2.26175	2.256653	2.251588	2.260365	2.255376
2.241262	2.236339	2.272894	2.267768	2.262662	2.257578	2.252525	2.261307	2.256298
2.242223	2.237235	2.273803	2.268689	2.263604	2.258552	2.253527	2.262336	2.257324
2.243174	2.238125	2.274713	2.269618	2.264556	2.259527	2.25452	2.263356	2.258347
2.244059	2.23901	2.275643	2.270573	2.265519	2.260468	2.255435	2.264282	2.25927
2.244938	2.239907	2.276588	2.271536	2.266481	2.261391	2.256319	2.265175	2.260164
2.245926	2.240873	2.27755	2.272471	2.267396	2.262295	2.257221	2.266112	2.261119
2.246993	2.241893	2.27853	2.273389	2.268279	2.263183	2.258131	2.267075	2.262114
2.24801	2.242898	2.279536	2.274364	2.269229	2.264122	2.259064	2.268023	2.26306
2.248963	2.243889	2.280583	2.275415	2.270267	2.265123	2.26002	2.268947	2.263937
2.249918	2.24485	2.281591	2.27643	2.271281	2.266123	2.260997	2.269904	2.264856
2.250891	2.245746	2.282495	2.27734	2.272212	2.2671	2.262015	2.270948	2.26589
2.251879	2.246651	2.283403	2.278244	2.273132	2.268063	2.263019	2.271987	2.266932
2.252908	2.247668	2.284434	2.279237	2.274089	2.268986	2.263921	2.272909	2.267873
2.253948	2.248732	2.285522	2.280274	2.27507	2.269899	2.264784	2.273785	2.268775
2.254904	2.249721	2.272627	2.267425	2.276098	2.270895	2.265749	2.274755	2.269745
2.255785	2.250649	2.273632	2.268469	2.277171	2.271963	2.266799	2.275801	2.270768
2.256728	2.251618	2.274659	2.269521	2.278242	2.273026	2.267846	2.276844	2.271793
2.257764	2.252646	2.275719	2.270581	2.279303	2.274074	2.268879	2.277875	2.272815
2.209769	2.204778	2.276792	2.271648	2.280367	2.275124	2.269916	2.27891	2.27384
2.210782	2.205791	2.277875	2.272722	2.281438	2.276183	2.270961	2.279953	2.274869
2.211798	2.206809	2.278962	2.273801	2.282512	2.277245	2.27201	2.281001	2.275902
2.212802	2.207814	2.280042	2.274876	2.283585	2.278306	2.273058	2.282051	2.276943
2.2138	2.208814	2.281119	2.27595	2.284658	2.279367	2.274106	2.283105	2.277991
2.214804	2.209816	2.236036	2.230984	2.285734	2.280433	2.275162	2.284165	2.279044
2.215813	2.210821	2.237076	2.232024	2.286812	2.281506	2.276227	2.285232	2.280102
2.189226	2.184304	2.238113	2.23306	2.287886	2.282574	2.277288	2.286296	2.281157
2.190225	2.185289	2.239144	2.234087	2.288948	2.28363	2.278338	2.28735	2.282204
2.191235	2.186281	2.24018	2.23512	2.290017	2.284696	2.279398	2.288408	2.28325
2.192246	2.187273	2.24122	2.236171	2.24692	2.241704	2.236508	2.275242	2.270088
2.193283	2.188284	2.242273	2.237236	2.247996	2.24277	2.237559	2.276318	2.271135
2.194417	2.189368	2.211968	2.207072	2.233246	2.228101	2.222962	2.233573	2.228471
2.195624	2.190507	2.213067	2.208116	2.234349	2.229278	2.224202	2.234793	2.229621
2.196764	2.1916	2.214135	2.20914	2.235416	2.230392	2.225363	2.235973	2.230793
2.197822	2.192628	2.215152	2.21013	2.236435	2.231429	2.226435	2.237114	2.231999

2.198891	2.193705	2.188627	2.183648	2.237487	2.232472	2.227486	2.238218	2.233161
2.200018	2.194904	2.189828	2.184779	2.238597	2.233534	2.228517	2.239262	2.234229
2.201136	2.196102	2.191043	2.185948	2.239755	2.23463	2.229561	2.2403	2.235272
2.202178	2.197182	2.192165	2.187125	2.240991	2.235803	2.230665	2.241377	2.236333
2.20319	2.198206	2.19324	2.188299	2.242262	2.237021	2.231812	2.242484	2.237403
2.204255	2.199245	2.194283	2.189381	2.243439	2.238225	2.23302	2.243658	2.238514
2.20536	2.200286	2.195291	2.190384	2.244543	2.239423	2.23429	2.244893	2.239661
2.206524	2.201379	2.196333	2.191392	2.186501	2.181673	2.17684	2.246092	2.240836
2.207772	2.202533	2.197409	2.192396	2.187465	2.18264	2.177837	2.247246	2.242057
2.209002	2.20372	2.198542	2.193455	2.18847	2.183616	2.178815	2.248361	2.243244
2.210155	2.20496	2.199789	2.194627	2.189564	2.184638	2.179785	2.249397	2.244322
2.211276	2.20619	2.201048	2.195841	2.190716	2.185717	2.1808	2.250445	2.245385
2.161774	2.156837	2.202173	2.197024	2.191938	2.186942	2.182004	2.251655	2.246531
2.162807	2.157803	2.203226	2.198195	2.193202	2.18825	2.183314	2.252955	2.247728
2.163926	2.158856	2.204327	2.199313	2.194339	2.189403	2.184478	2.229347	2.224198
2.165132	2.159987	2.205466	2.200369	2.195345	2.190403	2.185505	2.230512	2.225456
2.125807	2.120775	2.156078	2.151107	2.196459	2.19148	2.186564	2.199931	2.195059
2.126848	2.121914	2.157315	2.152378	2.197783	2.192712	2.187687	2.200986	2.196047
2.12791	2.123049	2.158534	2.153625	2.19909	2.193952	2.188844	2.202086	2.197082
2.129085	2.124145	2.159615	2.154672	2.200195	2.195101	2.190045	2.20333	2.198343
2.13029	2.125233	2.160665	2.155672	2.201257	2.196234	2.191262	2.204616	2.199666
2.131404	2.126357	2.161834	2.156846	2.151905	2.147003	2.142149	2.205781	2.20082
2.132465	2.127504	2.163075	2.158124	2.15317	2.148171	2.143211	2.206873	2.201873
2.133582	2.128618	2.164225	2.159271	2.154309	2.149296	2.14432	2.189729	2.184769
2.134772	2.129691	2.165262	2.160255	2.155293	2.150364	2.145479	2.190972	2.186024
2.135919	2.130784	2.166356	2.161313	2.156353	2.151488	2.146667	2.141839	2.137032
2.136947	2.131912	2.167582	2.16256	2.157608	2.15275	2.147913	2.143018	2.138123
2.137992	2.133072	2.168844	2.163835	2.158875	2.153997	2.149123	2.144167	2.1392
2.139205	2.134304	2.129375	2.124419	2.159993	2.15506	2.150155	2.145221	2.140302
2.140476	2.135551	2.130566	2.125519	2.161058	2.156059	2.151126	2.146248	2.141424
2.141597	2.136635	2.131637	2.126604	2.162196	2.157197	2.152266	2.147411	2.142612
2.142604	2.137585	2.132604	2.12767	2.163388	2.158439	2.153537	2.148682	2.143857
2.143706	2.138659	2.13369	2.128809	2.164616	2.1597	2.154808	2.149924	2.145047
2.144932	2.1399	2.134939	2.130056	2.165905	2.160998	2.156094	2.151132	2.146158
2.146199	2.141177	2.136203	2.131283	2.167145	2.162222	2.157299	2.152292	2.147269
2.147511	2.142474	2.137444	2.132422	2.168243	2.163255	2.158304	2.153344	2.148405
2.148803	2.143734	2.138645	2.133533	2.169319	2.164265	2.159288	2.154396	2.149567
2.149934	2.144806	2.139707	2.134636	2.170494	2.165444	2.16047	2.155595	2.150787
2.150984	2.145784	2.1407	2.13574	2.171725	2.166716	2.161758	2.156876	2.152046
2.152167	2.146929	2.141845	2.136923	2.132042	2.127165	2.12233	2.15814	2.153292
2.153468	2.148226	2.143129	2.138183	2.133283	2.1284	2.12356	2.159398	2.154534
2.092661	2.087501	2.144426	2.139451	2.134526	2.129634	2.12478	2.160625	2.155716
2.093938	2.088753	2.145714	2.140713	2.135767	2.130872	2.125999	2.161812	2.156803
2.095229	2.090022	2.147016	2.141983	2.137006	2.132086	2.127181	2.162973	2.157887
2.096529	2.091313	2.148344	2.143276	2.138249	2.13325	2.128277	2.164096	2.159026
2.09784	2.092617	2.149677	2.144561	2.139473	2.134384	2.129344	2.165212	2.160194
2.099168	2.093935	2.119681	2.114507	2.140619	2.135505	2.130462	2.166382	2.161392
2.100505	2.095257	2.120958	2.115656	2.141711	2.136615	2.131612	2.16759	2.162621
2.101803	2.096492	2.122165	2.1168	2.142847	2.137771	2.132802	2.127893	2.123055
2.103054	2.097622	2.123283	2.117928	2.144021	2.138977	2.134036	2.129139	2.124303

2.104318	2.0988	2.093401	2.088101	2.113923	2.108944	2.104071	2.130396	2.12556
2.105635	2.100101	2.094676	2.089337	2.11517	2.110184	2.10531	2.131659	2.126822
2.106926	2.101412	2.095983	2.090615	2.116458	2.111448	2.106556	2.132921	2.128081
2.108093	2.102636	2.097245	2.091907	2.11779	2.112738	2.107799	2.102933	2.098138
2.109222	2.103835	2.098502	2.093216	2.11915	2.114045	2.109044	2.104153	2.099347
2.110473	2.105104	2.099787	2.094521	2.120488	2.115342	2.110296	2.105382	2.100562
2.111815	2.106424	2.101095	2.095825	2.121812	2.116634	2.111554	2.106617	2.101778
2.113142	2.107731	2.102392	2.097125	2.123138	2.117927	2.112812	2.107851	2.102992
2.114431	2.109005	2.103665	2.098416	2.124465	2.11922	2.114066	2.109079	2.1042
2.11578	2.110328	2.104975	2.099725	2.125797	2.120523	2.115336	2.11032	2.105416
2.117263	2.11175	2.106342	2.101036	2.127107	2.121815	2.116616	2.111575	2.106643
2.118772	2.113221	2.107767	2.102406	2.12847	2.123156	2.117935	2.112858	2.10789
2.094111	2.088701	2.109315	2.103959	2.098634	2.093306	2.08806	2.114187	2.109153
2.095465	2.090189	2.110919	2.105602	2.100263	2.094849	2.089496	2.115567	2.110453
2.017532	2.012605	2.112351	2.107058	2.101737	2.096341	2.090991	2.117069	2.111908
2.018696	2.013748	2.113626	2.108349	2.10308	2.0978	2.092556	2.118699	2.113519
1.933898	1.929113	2.088828	2.083618	2.104394	2.09919	2.094014	2.120213	2.115022
1.935063	1.930267	2.090099	2.084884	2.105686	2.100485	2.09531	2.121543	2.116342
1.936234	1.931426	2.091373	2.08615	2.106969	2.101754	2.096566	2.122832	2.117627
1.809281	1.805217	1.975936	1.97105	2.108256	2.103031	2.097836	2.124144	2.118942
1.810285	1.806215	1.977131	1.972238	2.109546	2.104308	2.099104	2.125456	2.12026
1.811308	1.807219	1.978321	1.973414	2.079744	2.074523	2.069335	2.126766	2.121568
1.812358	1.808241	1.979524	1.974599	2.081048	2.075822	2.070631	2.128089	2.122882
1.813555	1.809402	1.882043	1.877366	1.971054	1.9662	1.961393	2.066917	2.061784
1.814927	1.810728	1.883526	1.878835	1.972579	1.967706	1.962879	2.068542	2.063392
1.816207	1.811968	1.807822	1.803771	1.97401	1.969116	1.964267	2.070064	2.064903
1.817287	1.813022	1.808858	1.804798	1.975238	1.970315	1.965442	2.071365	2.066205
1.818322	1.814037	1.809857	1.805785	1.976412	1.971466	1.966574	2.072616	2.067457
1.819386	1.815079	1.810876	1.806778	1.929439	1.924636	1.919891	1.994846	1.989987
1.820477	1.816156	1.811932	1.807802	1.930605	1.925807	1.921069	1.996062	1.991184
1.821706	1.817406	1.813177	1.809014	1.931985	1.927176	1.922429	1.997454	1.992546
1.823045	1.818793	1.814573	1.810377	1.933535	1.928704	1.923936	1.998986	1.994039
1.824291	1.820048	1.815818	1.811594	1.807437	1.803376	1.79936	1.920523	1.91578
1.825423	1.821132	1.81687	1.812626	1.808458	1.804396	1.800379	1.921703	1.916932
1.777761	1.773517	1.817903	1.813642	1.809462	1.805391	1.801372	1.922879	1.918122
1.778795	1.774547	1.818947	1.814668	1.810469	1.806369	1.802343	1.924089	1.919445
1.779866	1.775623	1.820047	1.81575	1.811531	1.807399	1.803361	1.925343	1.9208
1.781127	1.776882	1.821336	1.817028	1.812792	1.808639	1.804574	1.926711	1.92211
1.782475	1.778229	1.822717	1.818401	1.81415	1.809981	1.805881	1.928137	1.923408
1.783659	1.779413	1.823932	1.819606	1.815341	1.811148	1.807025	1.802995	1.799027
1.784699	1.780456	1.825001	1.820667	1.816388	1.812164	1.808029	1.804046	1.800153
1.785834	1.781586	1.777344	1.773104	1.817539	1.81329	1.809134	1.805154	1.801273
1.787143	1.782877	1.77863	1.774403	1.818875	1.814605	1.810407	1.806346	1.80237
1.788415	1.784133	1.779882	1.775661	1.820176	1.815908	1.811692	1.807567	1.803505
1.78949	1.785204	1.780946	1.776714	1.821277	1.817072	1.812919	1.808813	1.804753
1.790536	1.786251	1.781988	1.77774	1.822348	1.818207	1.814121	1.810051	1.806011
1.791766	1.787485	1.783219	1.778966	1.774782	1.770688	1.766646	1.811179	1.807107
1.793098	1.788824	1.784561	1.780311	1.776094	1.771909	1.767766	1.812246	1.808115
1.725004	1.720966	1.785851	1.781617	1.777393	1.773168	1.768975	1.813439	1.809265
1.726063	1.722109	1.787098	1.782898	1.778696	1.774481	1.770286	1.814768	1.810561

1.727168	1.723238	1.788296	1.784104	1.779908	1.775699	1.771508	1.767357	1.763246
1.728358	1.72434	1.789405	1.78517	1.780947	1.776719	1.772533	1.768434	1.764398
1.729596	1.725473	1.790541	1.78626	1.782007	1.777761	1.773577	1.769515	1.765533
1.57956	1.575912	1.722573	1.718471	1.783259	1.779013	1.774818	1.770709	1.766664
1.580725	1.57705	1.723843	1.71974	1.784597	1.780355	1.776144	1.771957	1.767807
1.581779	1.578119	1.725082	1.721	1.716937	1.712879	1.708842	1.773226	1.769032
1.582753	1.579142	1.726305	1.722262	1.718215	1.714135	1.710069	1.774521	1.770322
1.583805	1.580177	1.666194	1.662288	1.719362	1.715306	1.711265	1.775801	1.771617
1.584948	1.581219	1.667247	1.66327	1.720338	1.716371	1.712432	1.777072	1.772924
1.586128	1.582307	1.578582	1.574949	1.721362	1.717437	1.713557	1.709642	1.70572
1.587332	1.583465	1.579701	1.576035	1.722529	1.718528	1.714598	1.710719	1.70687
1.588567	1.58467	1.580876	1.577183	1.72376	1.71966	1.715653	1.711782	1.707984
1.589843	1.585917	1.58209	1.578357	1.664061	1.660068	1.656163	1.712906	1.709047
1.591139	1.587196	1.58334	1.579561	1.66536	1.661352	1.657421	1.714065	1.71009
1.592395	1.588467	1.584594	1.580764	1.577033	1.573432	1.569907	1.65473	1.65086
1.593618	1.589736	1.585858	1.581971	1.578188	1.574552	1.571004	1.655956	1.652085
1.432519	1.429093	1.587082	1.583171	1.579357	1.575684	1.572104	1.657185	1.653312
1.433501	1.430083	1.588252	1.584364	1.58055	1.576832	1.573205	1.65841	1.654534
1.434509	1.431072	1.51666	1.512937	1.581743	1.577991	1.574324	1.570796	1.567359
1.435591	1.432089	1.51775	1.514059	1.582913	1.579157	1.575475	1.571922	1.568456
1.436711	1.433134	1.518855	1.51519	1.584093	1.580337	1.576643	1.573059	1.569555
1.43785	1.434247	1.43074	1.42733	1.512755	1.509083	1.505453	1.574184	1.570645
1.439009	1.435409	1.431898	1.428477	1.513995	1.510281	1.506606	1.575309	1.571732
1.44016	1.436566	1.433053	1.429624	1.426193	1.422725	1.419283	1.504173	1.500628
1.441306	1.437723	1.434211	1.430776	1.427336	1.423856	1.420394	1.505346	1.501751
1.362194	1.358653	1.43535	1.431911	1.428464	1.424979	1.421504	1.506526	1.502882
1.363397	1.359854	1.436454	1.433013	1.429565	1.426085	1.422605	1.507707	1.504016
1.364597	1.361052	1.437552	1.434112	1.430665	1.427191	1.423708	1.508895	1.50516
1.252601	1.249433	1.438662	1.435224	1.431779	1.428308	1.42482	1.421281	1.417747
1.253668	1.250493	1.439777	1.436342	1.432898	1.429429	1.425936	1.422375	1.41881
1.254733	1.251551	1.361182	1.357797	1.434013	1.430543	1.427047	1.423475	1.419897
1.255797	1.252608	1.362379	1.358995	1.435126	1.431654	1.428156	1.424583	1.421005
1.256868	1.253674	1.250569	1.247562	1.356799	1.35338	1.349942	1.425693	1.422116
1.257941	1.254743	1.251631	1.248613	1.357986	1.35457	1.351135	1.426807	1.423231
1.259038	1.255848	1.252731	1.249699	1.359205	1.355775	1.352328	1.427915	1.424344
1.260176	1.257019	1.253911	1.250861	1.360492	1.357012	1.353519	1.429004	1.425439
1.261324	1.258195	1.25509	1.252022	1.361783	1.358252	1.354716	1.430105	1.426553
1.262447	1.259298	1.256179	1.253099	1.249991	1.246825	1.243654	1.352431	1.348979
1.263571	1.26038	1.257233	1.254138	1.251031	1.247884	1.244744	1.353687	1.350278
1.264765	1.261538	1.258353	1.255211	1.252087	1.248974	1.245877	1.354983	1.351579
1.266019	1.262759	1.259524	1.256307	1.253151	1.25009	1.247052	1.356318	1.352887
1.26722	1.263943	1.260676	1.257407	1.254225	1.251187	1.248176	1.357595	1.354148
1.26834	1.26507	1.261795	1.258505	1.255303	1.252245	1.24922	1.358786	1.355341
1.269464	1.266194	1.262919	1.259628	1.256414	1.253329	1.250277	1.247196	1.244105
1.270637	1.267334	1.264055	1.260792	1.25759	1.254483	1.2514	1.248282	1.245159
1.27182	1.268489	1.265211	1.261981	1.258793	1.255659	1.252539	1.249385	1.24623
1.036995	1.034184	1.266415	1.263192	1.25999	1.256808	1.253643	1.250486	1.247347
1.037964	1.035193	1.267649	1.264417	1.261188	1.257948	1.254737	1.251595	1.248495
1.038973	1.036214	1.268858	1.265602	1.262353	1.2591	1.255881	1.252742	1.249647
1.040025	1.03725	1.270045	1.266749	1.263483	1.260262	1.257075	1.25393	1.25081

0.853417	0.851112	1.035471	1.032676	1.264641	1.261443	1.25827	1.255106	1.251951
0.854196	0.851898	1.036437	1.033668	1.265854	1.262656	1.259466	1.256256	1.253048
0.854985	0.852699	1.037411	1.034667	1.267069	1.263859	1.260645	1.2574	1.254157
0.85584	0.85356	1.038422	1.035675	1.162196	1.159192	1.156192	1.258539	1.255323
0.856723	0.854443	1.039448	1.036684	1.163305	1.16027	1.157253	1.259686	1.256517
0.857602	0.855306	1.040468	1.037671	1.034891	1.032131	1.029397	1.155412	1.152485
0.858484	0.856159	1.041487	1.038643	1.035841	1.033106	1.030405	1.156572	1.153644
0.859338	0.856997	1.042485	1.039626	1.036821	1.034102	1.031419	1.028747	1.026088
0.860162	0.857813	1.043454	1.040618	1.037831	1.035122	1.032441	1.029759	1.027085
0.860989	0.85865	1.044446	1.04163	1.038856	1.036142	1.033452	1.030761	1.02808
0.861829	0.859532	1.045486	1.042676	1.039896	1.037151	1.034434	1.031748	1.029082
0.862681	0.86042	1.046539	1.04373	1.040938	1.038161	1.035415	1.03273	1.030076
0.863555	0.861284	1.047585	1.044766	1.041963	1.039176	1.036419	1.033717	1.031048
0.86444	0.86214	1.04863	1.045799	1.042984	1.040195	1.037433	1.034705	1.032008
0.865306	0.863007	1.049669	1.046844	1.044027	1.041218	1.038435	1.035693	1.032986
0.866158	0.863881	1.050703	1.047899	1.045086	1.042246	1.039429	1.03668	1.033975
0.867012	0.864746	1.051744	1.048949	1.046137	1.043285	1.040451	1.037684	1.034961
0.867869	0.865598	1.052795	1.049991	1.047176	1.044339	1.041511	1.038709	1.035937
0.868723	0.866449	0.864171	0.861893	1.048217	1.045395	1.042573	1.039741	1.036925
0.869566	0.867306	0.865033	0.86275	1.049267	1.046446	1.043616	1.040769	1.037933
0.870411	0.868161	0.865892	0.863607	1.050324	1.0475	1.044662	1.041808	1.038959
0.871273	0.868995	0.866729	0.864482	1.051386	1.048564	1.045722	1.042866	1.040011
0.872151	0.869824	0.86756	0.865368	1.052454	1.049638	1.046797	1.043944	1.041086
0.583721	0.582408	0.868437	0.866262	1.053535	1.050741	1.047918	1.045068	1.042203
0.584332	0.582999	0.869361	0.867166	1.054633	1.051875	1.04909	1.046241	1.043363
0.584943	0.583585	0.87026	0.86804	1.055698	1.052968	1.050211	1.047374	1.044497
0.585544	0.584156	0.871113	0.868863	1.056711	1.053992	1.051252	1.04844	1.045587
0.586146	0.584728	0.87196	0.869688	1.057716	1.055	1.052269	1.049485	1.046663
0.586751	0.585309	0.872809	0.87054	1.058727	1.056007	1.053281	1.050521	1.04773
0.587358	0.585896	0.873678	0.871416	1.059753	1.057024	1.054299	1.051559	1.048796
0.587967	0.58649	0.58508	0.583734	0.870086	0.867836	0.865597	1.052649	1.049894
0.588578	0.587093	0.58568	0.584336	0.871021	0.868765	0.866525	1.053772	1.051011
0.589215	0.587732	0.586308	0.584938	0.871918	0.869658	0.867424	1.054845	1.052082
0.589882	0.588411	0.586966	0.585538	0.872766	0.870506	0.868288	1.05586	1.053098
0.590536	0.589087	0.587631	0.586158	0.584737	0.583393	0.582093	1.056897	1.054141
0.591158	0.589751	0.588305	0.586813	0.585367	0.583993	0.582667	1.057995	1.055254
0.59177	0.590398	0.588967	0.587469	0.586008	0.584605	0.583251	1.059096	1.056366
0.592384	0.591009	0.58958	0.58809	0.586625	0.585198	0.583817	1.060141	1.057407
0.592995	0.591603	0.590174	0.588702	0.587241	0.585792	0.584384	1.061176	1.05843
0.593604	0.592221	0.590804	0.589349	0.58789	0.586419	0.584981	0.583623	0.582322
0.594212	0.592856	0.59146	0.590024	0.588567	0.587072	0.585603	0.58422	0.582895
0.59479	0.59346	0.592091	0.590684	0.589241	0.587737	0.58625	0.584844	0.583491
0.595327	0.594024	0.592689	0.591327	0.589916	0.588424	0.586932	0.585503	0.584116
0.595875	0.59459	0.593279	0.591949	0.590564	0.589088	0.587601	0.586158	0.584745
0.596469	0.595182	0.593868	0.592533	0.591151	0.589693	0.588223	0.586782	0.585363
0.597073	0.595779	0.594456	0.593109	0.591726	0.590289	0.58884	0.587408	0.585988
0.597654	0.596365	0.59505	0.593717	0.59235	0.590933	0.5895	0.58807	0.586643
0.598227	0.596947	0.595648	0.594338	0.592994	0.591598	0.590179	0.588751	0.587316
0.598823	0.597533	0.596235	0.594938	0.59361	0.592235	0.590835	0.589418	0.587987
0.599438	0.598121	0.596812	0.59552	0.594203	0.592848	0.59147	0.590074	0.588658

0.600079	0.598733	0.597406	0.596105	0.594792	0.593456	0.592101	0.590726	0.589327
0.600752	0.599377	0.598024	0.596699	0.595378	0.594061	0.592731	0.591378	0.589997
0.601435	0.60003	0.598649	0.597296	0.595963	0.594659	0.593352	0.592019	0.590658
		0.599268	0.597889	0.596543	0.595247	0.593956	0.592642	0.591301
		0.599895	0.598489	0.597128	0.595832	0.594549	0.593252	0.591934
		0.600551	0.599117	0.597735	0.596425	0.595139	0.593856	0.592561
		0.601228	0.599767	0.598358	0.597023	0.595723	0.594451	0.593181
				0.598983	0.597623	0.596308	0.595043	0.593792
				0.599609	0.598224	0.596891	0.595629	0.594393
				0.600251	0.598838	0.597484	0.596216	0.594986
				0.600905	0.599463	0.598083	0.5968	0.595568
				0.601587	0.600116	0.598705	0.597397	0.596149
							0.59803	0.596741
							0.59868	0.597338
							0.599316	0.597937
							0.599945	0.598538

							0.889344	0.886414
							0.888027	0.885131
							1.070083	1.06698
							1.068507	1.065442
				0.592599	0.590685	0.588842	1.221558	1.218378
				0.591812	0.589936	0.588116	1.220243	1.217083
				0.591042	0.589201	0.587398	1.218939	1.215789
				1.227215	1.223939	1.220691	1.217486	1.214318
				1.225837	1.222523	1.219229	1.215989	1.212792
		0.592429	0.590508	1.224401	1.221111	1.217836	1.699455	1.694776
		0.591561	0.589707	1.222923	1.219703	1.216492	1.697407	1.692801
		1.227889	1.224684	1.221487	1.21828	1.215078	1.767645	1.762994
		1.226562	1.223342	1.220111	1.216835	1.213563	1.765401	1.760756
0.59289	0.591066	1.225201	1.221969	1.705158	1.70038	1.695618	1.827938	1.823141
0.592028	0.590225	1.223686	1.22047	1.702968	1.698256	1.693563	1.825777	1.820998
0.591182	0.589393	1.222169	1.218969	1.700785	1.696153	1.691546	1.823662	1.818896
1.227258	1.224031	1.220813	1.217604	1.835988	1.831153	1.826347	1.891935	1.887109
1.225956	1.222752	1.219535	1.216301	1.833847	1.829027	1.824242	1.88986	1.885014
1.22463	1.221425	1.704072	1.699321	1.831633	1.826809	1.822034	1.887719	1.882933
1.223294	1.220069	1.702005	1.697227	1.829357	1.824505	1.819729	1.885511	1.880867
1.221879	1.218634	1.837117	1.832096	1.897819	1.892896	1.888065	1.883389	1.878827
1.220334	1.217069	1.834741	1.829838	1.895685	1.890842	1.886069	1.881414	1.876841
1.21881	1.215531	1.832411	1.827613	1.893574	1.888803	1.884078	1.879423	1.874816
1.46821	1.464207	1.901117	1.896267	1.891471	1.886708	1.881985	1.877302	1.872652
1.466533	1.462552	1.899074	1.894202	1.889378	1.884603	1.879866	1.875152	1.870463
1.835829	1.830927	1.896999	1.892116	1.887282	1.882508	1.877779	1.873077	1.868405
1.833721	1.828815	1.89492	1.890027	1.885188	1.880422	1.87571	1.871047	1.866425
1.902701	1.897737	1.892817	1.887939	1.883119	1.878381	1.873695	1.93213	1.927321
1.900558	1.895591	1.890687	1.885847	1.881072	1.876387	1.871742	1.930061	1.925198
1.898405	1.893455	1.888581	1.883792	1.879061	1.874405	1.86977	1.981934	1.976822
1.896237	1.891333	1.886513	1.881796	1.87711	1.872437	1.86776	1.97971	1.974539
1.894085	1.889241	1.884477	1.879816	1.875157	1.870451	1.865721	1.977463	1.972254
1.891975	1.887206	1.882485	1.877826	1.936304	1.931395	1.926446	1.975176	1.969987
1.889902	1.885209	1.880518	1.875829	1.934184	1.929227	1.924239	1.97288	1.967747
1.887884	1.883225	1.878533	1.873793	1.932012	1.927036	1.922052	2.08994	2.084475
1.885916	1.881258	1.876539	1.871727	1.929795	1.924821	1.919879	2.087646	2.082272
1.883946	1.879275	1.93768	1.93266	1.927645	1.922674	1.917757	2.085385	2.080079
1.881976	1.877279	1.935595	1.930588	1.925585	1.920611	1.915694	2.08316	2.077893
1.879989	1.875249	1.933467	1.928481	2.04036	2.035012	2.029729	2.080959	2.075716
1.87796	1.873151	1.931243	1.926277	2.038059	2.032789	2.02759	2.07879	2.073563
1.875932	1.871058	1.929024	1.924075	2.035761	2.030578	2.025468	2.076622	2.071393
1.873947	1.869053	1.926923	1.92198	2.033549	2.028415	2.023351	2.074407	2.069138
1.871987	1.867099	1.924889	1.919946	2.031388	2.026284	2.021243	2.072174	2.066846
1.932921	1.927859	1.922862	1.917933	2.029257	2.024173	2.019149	2.069984	2.064629
1.930874	1.925827	1.92085	1.915947	2.027157	2.022085	2.017067	2.067831	2.062476
1.928832	1.923802	1.972441	1.967369	2.081434	2.07619	2.07099	2.065684	2.06033
1.926793	1.921781	1.970369	1.965322	2.079274	2.074042	2.068847	2.063544	2.058187
1.924763	1.919772	1.968307	1.963281	2.077121	2.071897	2.066704	2.15578	2.150165
1.922746	1.917781	1.966256	1.961246	2.074971	2.069749	2.064552	2.15353	2.147908

1.920741	1.915805	1.964214	1.959213	2.072824	2.067601	2.062398	2.151275	2.145646
1.918751	1.913842	2.025049	2.019825	2.070676	2.065454	2.06025	2.149027	2.143393
1.916773	1.91189	2.022963	2.017742	2.068528	2.063308	2.058105	2.146782	2.141147
1.914794	1.909929	2.020863	2.015649	2.124256	2.118873	2.113498	2.229075	2.223022
1.912815	1.907961	2.018748	2.013544	2.122039	2.11666	2.111289	2.226694	2.220655
1.964097	1.959031	2.072696	2.067351	2.119822	2.114445	2.109078	2.224318	2.218298
1.962034	1.956966	2.070532	2.065189	2.117607	2.112229	2.106862	2.221942	2.21595
1.95997	1.954905	2.068373	2.063031	2.115391	2.110014	2.104652	2.219576	2.213614
1.957898	1.952849	2.124102	2.118611	2.234788	2.228939	2.223128	2.299147	2.292434
1.955829	1.950809	2.121896	2.116398	2.232387	2.226554	2.220765	2.29648	2.289773
1.953785	1.948827	2.119775	2.114263	2.230056	2.224211	2.218414	2.293804	2.28713
1.951762	1.946892	2.117718	2.112189	2.227781	2.221901	2.216075	2.291131	2.284509
2.012433	2.007411	2.115588	2.110065	2.225488	2.219594	2.213758	2.288496	2.281956
2.010412	2.00534	2.113367	2.107871	2.22316	2.217277	2.211458	2.285898	2.279489
2.070168	2.064873	2.232713	2.226776	2.303183	2.296419	2.289798	2.283338	2.277008
2.06805	2.062732	2.230345	2.224515	2.300668	2.29395	2.287314	2.280834	2.27447
2.065952	2.060618	2.228007	2.222271	2.298157	2.291492	2.284853	2.27834	2.271913
2.11562	2.110126	2.225726	2.219991	2.29551	2.288888	2.282289	2.310976	2.304504
2.113579	2.108069	2.223481	2.217701	2.292803	2.286227	2.279697	2.308382	2.301909
2.191659	2.185803	2.22122	2.215418	2.290141	2.283648	2.277216	2.305912	2.29943
2.189338	2.183559	2.218949	2.213139	2.287508	2.281135	2.274832	2.303551	2.297054
2.18711	2.181373	2.21673	2.210927	2.284964	2.278638	2.272386	2.301116	2.294636
2.185019	2.179266	2.214581	2.208805	2.282531	2.276146	2.269828	2.298554	2.292137
2.224162	2.218275	2.29319	2.286627	2.280134	2.273693	2.267308	2.296023	2.289662
2.222033	2.216147	2.290742	2.284222	2.277763	2.271322	2.264931	2.293627	2.287286
2.219879	2.214003	2.288285	2.281801	2.275375	2.268959	2.262588	2.291258	2.284922
2.217593	2.211767	2.285727	2.279258	2.307971	2.301549	2.295163	2.331212	2.324261
2.215234	2.209477	2.283113	2.276648	2.305352	2.298978	2.292645	2.328502	2.321499
2.212987	2.20726	2.28058	2.274152	2.302868	2.296512	2.290192	2.325862	2.318871
2.210848	2.205109	2.278118	2.271764	2.300516	2.294145	2.287801	2.32329	2.316375
2.28868	2.282143	2.27571	2.269401	2.298165	2.291793	2.285443	2.320744	2.313908
2.286244	2.279767	2.273367	2.267051	2.295785	2.289438	2.283121	2.318216	2.311457
2.28383	2.277411	2.27104	2.26471	2.293404	2.28709	2.280813	2.315712	2.309009
2.281431	2.275033	2.30375	2.297374	2.333853	2.326914	2.320042	2.313253	2.306545
2.279046	2.272656	2.301363	2.29499	2.331229	2.324358	2.317566	2.310795	2.304072
2.276657	2.270296	2.298976	2.292553	2.328543	2.32169	2.314939	2.372523	2.365325
2.27427	2.267949	2.296589	2.290082	2.325815	2.318947	2.312209	2.36967	2.36266
2.307006	2.300648	2.337424	2.330277	2.323236	2.316351	2.309606	2.366913	2.360039
2.30467	2.298301	2.334933	2.327848	2.32084	2.313929	2.307145	2.364253	2.357463
2.302329	2.295958	2.332421	2.325386	2.352685	2.345543	2.338537	2.361678	2.354932
2.299964	2.29361	2.329823	2.322782	2.350007	2.342989	2.336118	2.359216	2.352453
2.297603	2.29127	2.327237	2.3202	2.347356	2.340464	2.33373	2.356791	2.350014
2.338719	2.331724	2.324758	2.317795	2.344918	2.338027	2.331288	2.369008	2.362116
2.336156	2.329221	2.322329	2.315473	2.342586	2.335651	2.328852	2.36646	2.359684
2.333611	2.326703	2.354151	2.347144	2.370498	2.363314	2.356263	2.409699	2.402876
2.33108	2.324177	2.351555	2.344642	2.367997	2.360944	2.353998	2.407304	2.400364
2.328535	2.321667	2.349002	2.342157	2.380525	2.373377	2.36633	2.404934	2.397989
2.325951	2.319174	2.346511	2.339698	2.377933	2.370854	2.363898	2.402597	2.395804
2.323438	2.316707	2.360488	2.353486	2.375351	2.368326	2.361443	2.400242	2.393622
2.321081	2.314259	2.357824	2.350921	2.372795	2.365806	2.358958	2.397814	2.391343

2.318787	2.311865	2.355203	2.348383	2.370251	2.363295	2.356483	2.395379	2.389037
2.350591	2.343498	2.352826	2.345946	2.413183	2.406259	2.399502	2.392999	2.386717
2.348213	2.341284	2.350598	2.343585	2.410581	2.403738	2.397111	2.390659	2.384398
2.3623	2.355281	2.348295	2.341291	2.408152	2.401339	2.394759	2.388376	2.382199
2.359682	2.352789	2.345934	2.339075	2.405896	2.399063	2.392444	2.386146	2.380116
2.357157	2.350307	2.37217	2.365161	2.403612	2.396817	2.390221	2.384007	2.378089
2.354771	2.347828	2.369579	2.36268	2.401232	2.394578	2.388119	2.381994	2.376133
2.352463	2.345424	2.367053	2.360242	2.398879	2.392379	2.386068	2.380013	2.374173
2.350243	2.343193	2.364733	2.357968	2.396635	2.390243	2.384034	2.377995	2.372118
2.348053	2.34104	2.362511	2.355771	2.394438	2.388138	2.382019	2.37598	2.370053
2.345754	2.338845	2.405472	2.39872	2.392212	2.386007	2.379991	2.374008	2.368106
2.343382	2.336633	2.403209	2.396466	2.38998	2.383865	2.377956	2.372063	2.366235
2.340973	2.334368	2.400909	2.394239	2.38781	2.381743	2.375878	2.370054	2.364302
2.3385	2.332027	2.398556	2.392031	2.385704	2.379633	2.37374	2.367964	2.3623
2.336142	2.329772	2.396278	2.389887	2.383655	2.377583	2.371664	2.365912	2.360298
2.334	2.32768	2.394134	2.387835	2.381673	2.375625	2.369706	2.363948	2.358323
2.331934	2.325652	2.392047	2.385831	2.379734	2.37371	2.367792	2.362009	2.356344
2.377401	2.371134	2.389991	2.383867	2.377833	2.371816	2.365875	2.360045	2.354318
2.375276	2.369077	2.387946	2.381915	2.375945	2.369937	2.363971	2.35809	2.352291
2.373133	2.366937	2.385784	2.379852	2.373971	2.368013	2.362075	2.356196	2.350381
2.370977	2.364731	2.383525	2.377699	2.371928	2.366054	2.360187	2.354355	2.348568
2.39395	2.387526	2.381392	2.375618	2.369908	2.3641	2.358296	2.352508	2.34675
2.391917	2.385539	2.379424	2.373632	2.367916	2.362148	2.356395	2.350645	2.344917
2.389909	2.383589	2.3775	2.371687	2.365958	2.360221	2.354512	2.348797	2.343099
2.387887	2.381631	2.375582	2.369778	2.364056	2.358342	2.352661	2.346976	2.341308
2.385871	2.379679	2.373674	2.367886	2.362178	2.356488	2.350832	2.345175	2.339535
2.383855	2.377713	2.371745	2.365977	2.360292	2.35464	2.349025	2.3434	2.337786
2.381846	2.375743	2.369809	2.364066	2.35841	2.352803	2.347236	2.341643	2.336053
2.379874	2.373799	2.367891	2.362173	2.356544	2.350974	2.345443	2.339875	2.334303
2.377936	2.371876	2.365988	2.360295	2.354693	2.34915	2.343646	2.338097	2.33254
2.376011	2.369978	2.364114	2.358444	2.352864	2.347345	2.341862	2.336327	2.330778
2.374091	2.368098	2.362264	2.356612	2.351048	2.345554	2.340094	2.334567	2.329016
2.372194	2.366262	2.360469	2.354833	2.349279	2.343792	2.338337	2.332811	2.32726
2.370334	2.364511	2.358786	2.353166	2.347604	2.342087	2.336593	2.331059	2.325518
2.368496	2.362787	2.357133	2.351533	2.345962	2.340399	2.334852	2.329306	2.323778
2.366648	2.36098	2.355364	2.349806	2.344256	2.338668	2.333086	2.327536	2.322021
2.364805	2.359129	2.35353	2.348024	2.342511	2.33691	2.331304	2.325755	2.320256
2.363052	2.357377	2.351776	2.346271	2.340756	2.335144	2.32953	2.32399	2.318512
2.361404	2.355736	2.350115	2.344551	2.338987	2.333368	2.327764	2.322238	2.316784
2.359734	2.354074	2.348435	2.342816	2.337211	2.331593	2.326013	2.32052	2.315112
2.358001	2.35235	2.346703	2.341048	2.335421	2.329817	2.324273	2.318849	2.31353
2.356241	2.350596	2.344946	2.339271	2.333639	2.32806	2.322561	2.317206	2.311972
2.354453	2.348806	2.343159	2.337489	2.331877	2.326347	2.320906	2.344468	2.339181
2.352653	2.346999	2.34136	2.335716	2.330141	2.324667	2.319281	2.342825	2.337542
2.350881	2.345224	2.339602	2.334006	2.328474	2.323016	2.317636	2.341139	2.335861
2.349131	2.343471	2.337875	2.332349	2.326867	2.321394	2.315981	2.339422	2.334144
2.34747	2.341821	2.336232	2.330713	2.354198	2.348618	2.343096	2.337712	2.332442
2.345934	2.340317	2.334712	2.329111	2.352476	2.346889	2.341382	2.336009	2.330752
2.344363	2.338763	2.333141	2.327474	2.350735	2.345153	2.339674	2.334323	2.329091
2.342673	2.337054	2.331418	2.325743	2.348958	2.343396	2.337948	2.332636	2.32745

2.340939	2.335289	2.329648	2.323995	2.347195	2.341672	2.33627	2.331004	2.325868
2.339182	2.333516	2.327892	2.322294	2.34552	2.340079	2.334756	2.32954	2.324433
2.337422	2.331745	2.326147	2.320624	2.343899	2.33856	2.33333	2.328166	2.323075
2.335807	2.330121	2.353345	2.34775	2.342281	2.336981	2.331792	2.326655	2.321583
2.334335	2.328641	2.351801	2.346173	2.340673	2.335355	2.330157	2.325027	2.319974
2.332768	2.327063	2.35017	2.344543	2.339044	2.333722	2.328521	2.323401	2.318364
2.331029	2.325299	2.348369	2.342802	2.33736	2.33206	2.326873	2.321768	2.316747
2.329296	2.323563	2.34661	2.341109	2.335728	2.330461	2.325295	2.320203	2.31519
2.356472	2.350686	2.345052	2.339594	2.334254	2.32903	2.323898	2.318814	2.31379
2.354849	2.349131	2.343552	2.338129	2.332822	2.327642	2.322546	2.317469	2.312427
2.353157	2.34748	2.341937	2.336542	2.33126	2.326107	2.321032	2.315958	2.310908
2.351432	2.345785	2.340269	2.334896	2.329633	2.324494	2.319429	2.314357	2.309303
2.349777	2.344194	2.338727	2.333382	2.328137	2.323008	2.317946	2.312876	2.307821
2.3482	2.342724	2.337331	2.33202	2.326792	2.321667	2.316601	2.311531	2.306479
2.346586	2.341186	2.335851	2.330577	2.325371	2.32025	2.31518	2.336133	2.330983
2.344856	2.339473	2.334167	2.328936	2.323766	2.318652	2.31358	2.334509	2.329369
2.343174	2.337787	2.332494	2.327301	2.322162	2.317055	2.311984	2.332888	2.327758
2.341717	2.336321	2.331028	2.325849	2.320724	2.315624	2.310557	2.331446	2.326315
2.340337	2.33493	2.32963	2.324451	2.31933	2.314237	2.309176	2.330051	2.324917
2.338773	2.33338	2.328088	2.322909	2.317786	2.312695	2.307637	2.328494	2.323365
2.33708	2.331722	2.326449	2.321266	2.316137	2.311043	2.305986	2.326822	2.321706
2.335503	2.330178	2.324923	2.319743	2.340776	2.335578	2.330419	2.325286	2.320191
2.334073	2.328778	2.323544	2.318374	2.339394	2.334194	2.329035	2.323922	2.318859
2.332672	2.327396	2.322177	2.317019	2.338033	2.332833	2.327675	2.322581	2.317548
2.331295	2.326017	2.320803	2.315659	2.336674	2.331479	2.326326	2.321246	2.316231
2.32988	2.324595	2.319383	2.314251	2.335262	2.330074	2.324929	2.319863	2.314867
2.328288	2.323009	2.3178	2.312667	2.333653	2.328472	2.323338	2.343244	2.338273
2.326614	2.321348	2.316143	2.311002	2.331953	2.326778	2.32166	2.341639	2.336693
2.325084	2.319836	2.314642	2.309502	2.330433	2.325271	2.320171	2.340212	2.33529
2.323676	2.318448	2.313271	2.30814	2.329064	2.323921	2.318843	2.338938	2.334038
2.322294	2.317084	2.338119	2.332893	2.352832	2.347705	2.342649	2.35878	2.353867
2.320936	2.315739	2.336762	2.331548	2.351544	2.346435	2.341396	2.357587	2.352684
2.319579	2.314393	2.335402	2.330199	2.350245	2.345158	2.34014	2.356393	2.351501
2.318229	2.313052	2.334045	2.328844	2.348922	2.343862	2.338876	2.355196	2.350324
2.316837	2.311671	2.332648	2.32745	2.347556	2.342524	2.337571	2.353961	2.349111
2.294963	2.289892	2.356366	2.351174	2.34606	2.34105	2.336123	2.352575	2.34775
2.293329	2.288277	2.354754	2.349582	2.344487	2.339495	2.334587	2.351098	2.3463
2.319217	2.314071	2.353284	2.348137	2.364612	2.359582	2.354636	2.349774	2.344989
2.317844	2.31271	2.351955	2.346843	2.363394	2.358388	2.353457	2.348602	2.343818
2.343529	2.338385	2.350648	2.345567	2.362192	2.357212	2.352304	2.347459	2.342679
2.342233	2.337085	2.349335	2.34428	2.360972	2.356028	2.351153	2.346328	2.341558
2.340951	2.335801	2.348038	2.343005	2.359761	2.354848	2.350005	2.345201	2.340445
2.339673	2.334533	2.346761	2.341744	2.358562	2.353663	2.348836	2.344053	2.339321
2.3384	2.333276	2.345499	2.340496	2.357375	2.352482	2.347663	2.342899	2.338193
2.337118	2.33202	2.344247	2.339269	2.356206	2.351324	2.346511	2.341755	2.337058
2.335831	2.330767	2.343004	2.338058	2.355054	2.350183	2.345374	2.340618	2.335917
2.334549	2.329512	2.363782	2.3588	2.353885	2.349032	2.344238	2.33949	2.334792
2.333277	2.328257	2.362557	2.35759	2.352694	2.347871	2.343107	2.338376	2.333688
2.331998	2.326998	2.361328	2.356378	2.351501	2.346702	2.341962	2.33725	2.332578
2.330704	2.325732	2.360094	2.355168	2.350312	2.345522	2.34079	2.336098	2.331451

2.329412	2.324468	2.35886	2.353959	2.349122	2.34434	2.339613	2.33494	2.330319
2.328138	2.323218	2.357628	2.352748	2.34793	2.343164	2.338453	2.333796	2.329191
2.326876	2.321977	2.356401	2.351538	2.346737	2.341991	2.337299	2.332656	2.328064
2.348928	2.343979	2.339107	2.334316	2.345549	2.340815	2.336138	2.331511	2.326935
2.347709	2.342787	2.337934	2.333147	2.344364	2.339637	2.33497	2.33036	2.325804
2.346539	2.341618	2.336769	2.331988	2.343191	2.338474	2.333818	2.341212	2.336655
2.345424	2.340461	2.335594	2.330825	2.342025	2.337325	2.332686	2.340114	2.335558
2.344355	2.339365	2.334482	2.329713	2.340896	2.336198	2.331562	2.339023	2.33447
2.343362	2.3384	2.333516	2.328709	2.339829	2.335093	2.330429	2.337925	2.333391
2.34237	2.337457	2.332584	2.327743	2.338802	2.334022	2.329321	2.336846	2.332327
2.341256	2.336379	2.331543	2.326743	2.337808	2.333018	2.328292	2.335823	2.331279
2.340067	2.335223	2.330439	2.325725	2.336837	2.332061	2.32732	2.334845	2.330256
2.33888	2.334066	2.329321	2.324656	2.335795	2.331066	2.326364	2.333933	2.329338
2.337684	2.332902	2.328184	2.323534	2.334683	2.330041	2.325435	2.333103	2.32854
2.336532	2.33175	2.327039	2.322401	2.345882	2.341286	2.336732	2.332189	2.327659
2.335443	2.330603	2.32587	2.321254	2.344767	2.340187	2.33565	2.33113	2.326629
2.334395	2.3295	2.324738	2.320131	2.343661	2.339078	2.334539	2.330028	2.325546
2.333405	2.328491	2.323704	2.319063	2.342579	2.337984	2.33344	2.328925	2.324442
2.332431	2.327526	2.322725	2.318041	2.341535	2.336918	2.332358	2.327825	2.323325
2.331361	2.326522	2.321766	2.3171	2.325659	2.321089	2.316564	2.326789	2.32225
2.330226	2.325499	2.320829	2.316222	2.324831	2.320229	2.315647	2.325802	2.321207
2.329145	2.324454	2.333075	2.328482	2.323905	2.319311	2.314726	2.324856	2.320244
2.32812	2.323372	2.331998	2.327408	2.322858	2.318323	2.313807	2.323966	2.319388
2.32713	2.322347	2.33098	2.326377	2.321831	2.317326	2.312851	2.323026	2.318479
2.326201	2.321457	2.33012	2.325475	2.320877	2.316323	2.311807	2.321955	2.317425
2.325253	2.320564	2.329267	2.324591	2.319947	2.31534	2.310776	2.320885	2.316356
2.324171	2.319516	2.328279	2.323642	2.319031	2.314443	2.309883	2.319952	2.315384
2.323037	2.318404	2.327231	2.322663	2.318116	2.313576	2.309046	2.319075	2.314456
2.335525	2.330847	2.326224	2.32166	2.317125	2.312608	2.308101	2.318124	2.313514
2.334636	2.329911	2.325241	2.320631	2.316068	2.311554	2.307072	2.317117	2.312569
2.333698	2.328972	2.324295	2.319668	2.315094	2.310584	2.306114	2.316156	2.311631
2.33267	2.328014	2.323391	2.318795	2.31424	2.309743	2.305272	2.31527	2.310703
2.331682	2.327089	2.322511	2.317936	2.325892	2.321344	2.316814	2.32608	2.321445
2.330835	2.326258	2.321675	2.31707	2.324973	2.32038	2.315814	2.32508	2.320494
2.33001	2.325428	2.320826	2.316187	2.324039	2.319401	2.314803	2.324075	2.319546
2.341714	2.337053	2.332397	2.327739	2.323103	2.318488	2.313907	2.323162	2.318617
2.340701	2.335991	2.331334	2.326733	2.322162	2.317606	2.313068	2.322296	2.317699
2.339751	2.335035	2.330376	2.325782	2.321218	2.316672	2.312143	2.307614	2.303094
2.33886	2.334179	2.32952	2.324882	2.320271	2.315685	2.311133	2.306635	2.302181
2.337968	2.333304	2.328644	2.323978	2.31934	2.314737	2.31018	2.325722	2.321191
2.337062	2.332381	2.327713	2.32305	2.318426	2.313855	2.309334	2.324862	2.320336
2.336153	2.331454	2.326784	2.322137	2.317535	2.312992	2.308496	2.32399	2.319453
2.335241	2.330545	2.325888	2.321273	2.316689	2.312127	2.307599	2.323012	2.318445
2.334328	2.329645	2.325008	2.320425	2.315855	2.311263	2.306686	2.32201	2.317412
2.333415	2.328758	2.324134	2.319547	2.31497	2.310374	2.305796	2.321087	2.316506
2.332504	2.327883	2.323268	2.318651	2.314052	2.309469	2.304919	2.320216	2.315686
2.331632	2.327006	2.322383	2.317752	2.333268	2.328576	2.323929	2.319358	2.314846
2.330804	2.326123	2.321471	2.316845	2.332346	2.327692	2.323082	2.318517	2.313989
2.329985	2.325267	2.320594	2.315971	2.33145	2.326821	2.322236	2.317665	2.313113
2.329178	2.324468	2.319795	2.315157	2.330594	2.325969	2.321386	2.316794	2.31221

2.328354	2.323665	2.319002	2.314359	2.329751	2.325115	2.320518	2.315905	2.311294
2.327469	2.322792	2.318148	2.31354	2.328898	2.324232	2.319596	2.31497	2.310363
2.326554	2.321889	2.317272	2.312716	2.328045	2.323341	2.318656	2.314024	2.309435
2.325654	2.321017	2.316428	2.3119	2.327202	2.322494	2.317799	2.313162	2.308572
2.324761	2.320166	2.315606	2.31109	2.326368	2.321683	2.317008	2.31237	2.307764
2.3239	2.319333	2.314783	2.310251	2.325485	2.320816	2.316164	2.311539	2.306943
2.323074	2.318519	2.313953	2.309363	2.324526	2.319863	2.315236	2.310649	2.306099
2.32227	2.317721	2.333101	2.328335	2.323604	2.318942	2.31433	2.309771	2.30526
2.321487	2.316946	2.332289	2.327523	2.322789	2.31812	2.313501	2.308944	2.304435
2.320712	2.316176	2.331492	2.326737	2.322008	2.317333	2.312701	2.308136	2.30362
2.319914	2.315383	2.330668	2.325922	2.321201	2.316529	2.311898	2.307335	2.302819
2.319106	2.314579	2.329835	2.325098	2.320387	2.315722	2.311099	2.30654	2.302027
2.318316	2.313792	2.329014	2.32428	2.31957	2.314908	2.310286	2.305729	2.301219
2.317542	2.313021	2.328206	2.323466	2.318751	2.314084	2.309459	2.304903	2.300398
2.316763	2.312244	2.327395	2.322654	2.317937	2.313266	2.308638	2.304084	2.299582
2.315973	2.311459	2.326579	2.321844	2.31713	2.312459	2.30783	2.303278	2.298781
2.315184	2.310672	2.325763	2.321031	2.316321	2.311651	2.307022	2.302472	2.297978
2.314407	2.309889	2.324946	2.32021	2.315497	2.310828	2.306204	2.301656	2.297164
2.313631	2.309107	2.324132	2.31939	2.314674	2.310007	2.305386	2.30084	2.296348
2.332677	2.328013	2.323324	2.318589	2.313876	2.309205	2.304577	2.300031	2.295543
2.331839	2.327193	2.322522	2.317803	2.313099	2.308419	2.303778	2.299231	2.294747
2.331053	2.326396	2.321729	2.317041	2.31236	2.307676	2.303021	2.298458	2.293956
2.330326	2.325624	2.320951	2.316311	2.311669	2.306988	2.302317	2.29772	2.29317
2.329586	2.324844	2.320161	2.315556	2.310945	2.30627	2.301592	2.311299	2.30666
2.328795	2.324031	2.31934	2.314746	2.31015	2.305485	2.300816	2.310556	2.305923
2.328013	2.323231	2.318527	2.313927	2.309332	2.304678	2.300024	2.309803	2.30518
2.327302	2.322495	2.317764	2.313132	2.308515	2.303866	2.299226	2.319117	2.314424
2.326612	2.321785	2.317023	2.312346	2.307698	2.303057	2.298434	2.318332	2.313625
2.325842	2.321028	2.316268	2.311567	2.321593	2.316896	2.312229	2.317642	2.31292
2.325011	2.32024	2.315503	2.310796	2.320855	2.316215	2.311609	2.317029	2.31229
2.324233	2.319489	2.314769	2.31006	2.320146	2.315523	2.310941	2.328009	2.323252
2.323533	2.318796	2.314079	2.309371	2.319466	2.314807	2.310199	2.327311	2.32264
2.32282	2.318085	2.313375	2.308688	2.318813	2.31412	2.309476	2.326605	2.321989
2.322043	2.317304	2.312618	2.308001	2.318215	2.313505	2.308815	2.32589	2.321236
2.321261	2.316517	2.311855	2.307303	2.317609	2.312899	2.308182	2.325194	2.320477
2.320527	2.315789	2.326169	2.321529	2.329221	2.324499	2.319776	2.324583	2.319832
2.319822	2.315095	2.325482	2.32079	2.328456	2.323811	2.319182	2.324012	2.319245
2.31914	2.31442	2.324829	2.320105	2.327739	2.323103	2.318497	2.323355	2.31862
2.318485	2.313766	2.324212	2.319467	2.32706	2.32237	2.317726	2.322619	2.317967
2.317828	2.313111	2.336005	2.331188	2.326409	2.321681	2.317001	2.321899	2.317282
2.317171	2.312453	2.335356	2.33056	2.325795	2.321059	2.316357	2.321207	2.316542
2.31649	2.311788	2.334715	2.329938	2.325184	2.320447	2.315733	2.320537	2.31582
2.31575	2.311115	2.334117	2.329323	2.324548	2.319812	2.315101	2.3199	2.315175
2.314989	2.310426	2.333516	2.328705	2.323911	2.319178	2.314475	2.319282	2.314561
2.342228	2.337545	2.33283	2.328061	2.3233	2.318569	2.313859	2.31866	2.31394
2.341527	2.336802	2.332092	2.3274	2.322703	2.317975	2.313249	2.318041	2.313322
2.340897	2.336151	2.331436	2.326759	2.322078	2.317357	2.312633	2.317422	2.312704
2.340344	2.335602	2.330869	2.32614	2.321422	2.31671	2.312011	2.316806	2.312085
2.339798	2.335049	2.330295	2.325525	2.320776	2.316067	2.311383	2.316178	2.311459
2.339249	2.334468	2.329692	2.324913	2.320157	2.315437	2.310743	2.315528	2.310815

2.33868	2.333867	2.329073	2.324295	2.31954	2.314811	2.310106	2.314881	2.310173
2.338056	2.33323	2.328428	2.323652	2.318903	2.314184	2.30949	2.326178	2.321435
2.337406	2.332574	2.327772	2.323001	2.318261	2.313559	2.308882	2.325567	2.320823
2.336771	2.331934	2.327131	2.322366	2.317632	2.312927	2.30825	2.324941	2.32022
2.336144	2.331304	2.326502	2.321745	2.317012	2.312292	2.3076	2.324306	2.319627
2.335511	2.330684	2.325898	2.321161	2.328977	2.324227	2.3195	2.323733	2.319057
2.33486	2.33007	2.325325	2.320631	2.328477	2.323758	2.319047	2.323247	2.318518
2.334255	2.329482	2.324755	2.320084	2.327946	2.323251	2.318556	2.322735	2.317967
2.333759	2.328944	2.324182	2.319481	2.327324	2.32263	2.317946	2.322138	2.317382
2.333272	2.328409	2.323603	2.318863	2.32668	2.321978	2.317297	2.32151	2.316783
2.332677	2.327808	2.335568	2.330802	2.326068	2.321347	2.31665	2.32087	2.316175
2.332038	2.327182	2.334954	2.330204	2.325474	2.32073	2.316007	2.320228	2.315564
2.331468	2.326628	2.334408	2.32966	2.32493	2.320182	2.315454	2.319662	2.314991
2.330959	2.326139	2.333927	2.329169	2.324433	2.3197	2.314984	2.319167	2.314453
2.343027	2.338202	2.333412	2.32866	2.323928	2.31921	2.314505	2.31867	2.313927
2.342398	2.337602	2.332841	2.32812	2.323414	2.318711	2.314013	2.318174	2.313423
2.341789	2.337009	2.332265	2.327563	2.322872	2.318178	2.313484	2.317643	2.312888
2.341256	2.336464	2.331703	2.326975	2.322264	2.317563	2.312868	2.317019	2.312254
2.340758	2.335943	2.331153	2.326388	2.321646	2.316934	2.312235	2.316377	2.311605
2.340266	2.335437	2.330635	2.32586	2.32111	2.316391	2.311688	2.315825	2.311058
2.339785	2.334946	2.33014	2.325369	2.320623	2.3159	2.311192	2.315327	2.310573
2.339296	2.334455	2.338762	2.333931	2.329125	2.324342	2.319578	2.314822	2.310077
2.338797	2.333964	2.338269	2.33343	2.328616	2.323829	2.319064	2.314311	2.309574
2.338304	2.333474	2.337775	2.332929	2.328111	2.323322	2.318557	2.332876	2.327921
2.337823	2.332981	2.337273	2.332428	2.327612	2.322827	2.318064	2.332359	2.327396
2.337345	2.332493	2.336776	2.33193	2.327115	2.322333	2.317573	2.331846	2.326875
2.336865	2.332018	2.336295	2.331438	2.326614	2.321832	2.317075	2.331333	2.326365
2.336384	2.331547	2.335821	2.33095	2.326114	2.32133	2.316576	2.33082	2.325859
2.335893	2.331063	2.335338	2.330467	2.325628	2.320837	2.316075	2.330301	2.325346
2.335395	2.330571	2.334851	2.32999	2.325154	2.320351	2.315574	2.329779	2.324829
2.334905	2.330079	2.334357	2.329504	2.324673	2.319866	2.315082	2.329263	2.324311
2.334426	2.329586	2.333855	2.329008	2.324184	2.319383	2.314602	2.328757	2.323791
2.333946	2.329097	2.333358	2.328512	2.323691	2.318895	2.314118	2.32825	2.323276
2.333457	2.328616	2.332875	2.328019	2.32319	2.318395	2.313622	2.327741	2.322771
2.33297	2.328137	2.332393	2.327527	2.322689	2.317892	2.313121	2.327227	2.322266
2.332486	2.327644	2.331896	2.327035	2.3222	2.317395	2.312614	2.326701	2.321746
2.332014	2.327155	2.331398	2.326547	2.321718	2.316901	2.312106	2.32617	2.321222
2.331607	2.326729	2.330951	2.326084	2.321241	2.316414	2.311611	2.325657	2.320712
2.331263	2.326362	2.330549	2.325644	2.320769	2.315931	2.311126	2.325158	2.320213
2.330861	2.325958	2.330138	2.325223	2.320339	2.315493	2.310683	2.324703	2.319764
2.330363	2.325495	2.329713	2.324835	2.319976	2.315125	2.310302	2.324312	2.319395
2.329874	2.325025	2.329271	2.324427	2.319593	2.314742	2.309912	2.32391	2.319005
2.329452	2.324561	2.328783	2.323945	2.319121	2.314279	2.309454	2.323432	2.318519
2.329052	2.324112	2.3283	2.323456	2.318634	2.313803	2.308991	2.322946	2.318014
2.328645	2.323715	2.327901	2.323049	2.318223	2.313403	2.308601	2.322533	2.31758
2.328239	2.323351	2.327552	2.322688	2.317851	2.31304	2.308249	2.322159	2.317185
2.327846	2.322973	2.327172	2.322288	2.317433	2.312616	2.307825	2.321727	2.316756
2.327471	2.322585	2.326762	2.321847	2.316965	2.312127	2.307324	2.321232	2.316291
2.327059	2.322174	2.326346	2.321423	2.316531	2.31168	2.30687	2.320781	2.315863
2.326574	2.321718	2.32592	2.321028	2.316158	2.311313	2.306501	2.32041	2.315503

2.326103	2.321277	2.325509	2.32065	2.315807	2.310971	2.30616	2.32006	2.315152
2.325721	2.320905	2.325143	2.320292	2.315456	2.310629	2.305822	2.319702	2.314778
2.32538	2.320564	2.324798	2.319948	2.315113	2.310293	2.305491	2.319342	2.31439
2.325031	2.320215	2.324447	2.319603	2.314771	2.309949	2.305138	2.318952	2.313961
2.324686	2.319867	2.324096	2.31926	2.314431	2.3096	2.304771	2.318541	2.313502
2.324336	2.319518	2.323748	2.318916	2.314087	2.309256	2.304423	2.318179	2.31313
2.323981	2.319171	2.323405	2.318568	2.313741	2.308918	2.304101	2.317882	2.312871
2.323626	2.318822	2.323059	2.31822	2.313393	2.308576	2.303769	2.317561	2.312572
2.323277	2.318472	2.322706	2.317872	2.313045	2.308225	2.30341	2.317167	2.312147
2.322924	2.318118	2.322349	2.317518	2.312692	2.307869	2.303045	2.316768	2.311715
2.322552	2.317752	2.321985	2.317148	2.312321	2.307506	2.302694	2.316426	2.311392
2.322172	2.317379	2.321614	2.316769	2.311941	2.307137	2.302345	2.316103	2.311107
2.321804	2.317006	2.321238	2.316396	2.311569	2.306764	2.30197	2.31572	2.310729
2.321444	2.316633	2.320856	2.316026	2.311204	2.306385	2.301571	2.315289	2.310276
2.32108	2.316265	2.320485	2.315655	2.310834	2.306015	2.301201	2.314911	2.309899
2.320697	2.315903	2.32013	2.315282	2.310454	2.305656	2.300876	2.314616	2.309643
2.320348	2.315551	2.319774	2.314916	2.310087	2.305303	2.300547	2.314312	2.309376
2.320078	2.315207	2.319391	2.314554	2.309743	2.304944	2.300169	2.313933	2.309024
2.31983	2.314883	2.319029	2.314216	2.309423	2.304605	2.299803	2.31355	2.308651
2.319548	2.314616	2.318765	2.313946	2.309148	2.304336	2.299537	2.327814	2.322807
2.319246	2.314382	2.318559	2.313718	2.3089	2.304108	2.299326	2.327547	2.322459
2.318914	2.314095	2.318291	2.31343	2.308597	2.303818	2.299053	2.327263	2.322167
2.318544	2.313752	2.317963	2.313084	2.308239	2.303466	2.298722	2.326966	2.321933
2.302793	2.298101	2.317658	2.312774	2.307922	2.303132	2.298379	2.326642	2.321666
2.302574	2.29787	2.317408	2.31254	2.307684	2.302833	2.298012	2.326266	2.32133
2.302367	2.297636	2.317148	2.312302	2.307451	2.302552	2.297676	2.325912	2.320998
2.302125	2.297335	2.316817	2.311994	2.307171	2.302292	2.297429	2.325653	2.320726
2.301871	2.297022	2.316475	2.31167	2.306879	2.302045	2.297222	2.32543	2.320469
2.301612	2.296771	2.316222	2.311409	2.306615	2.301803	2.296999	2.325172	2.320161
2.301347	2.296562	2.31603	2.311187	2.306367	2.301564	2.296771	2.324898	2.31982
2.301107	2.296349	2.315815	2.310929	2.306076	2.301281	2.296506	2.324623	2.319533
2.300897	2.296132	2.31557	2.310623	2.305726	2.300938	2.296193	2.324344	2.319305
2.300697	2.295924	2.30071	2.295826	2.305421	2.300632	2.295903	2.324082	2.319088
2.300506	2.295735	2.300517	2.295625	2.30521	2.300407	2.295665	2.323847	2.318871
2.300319	2.29555	2.300332	2.295443	2.305027	2.300208	2.29545	2.323626	2.318656
2.300131	2.295359	2.300137	2.295245	2.320226	2.31534	2.310515	2.323413	2.31844
2.299943	2.295165	2.299937	2.295044	2.320016	2.315134	2.310312	2.323206	2.318223
2.299749	2.294968	2.299737	2.294842	2.319805	2.314923	2.310101	2.322991	2.317999
2.29955	2.294768	2.299535	2.294639	2.319591	2.314708	2.309884	2.32277	2.317768
2.299345	2.294561	2.299325	2.294429	2.319372	2.314488	2.309664	2.322552	2.317549
2.299134	2.294345	2.299106	2.294209	2.319144	2.314262	2.309441	2.322339	2.317346
2.298921	2.294126	2.298884	2.293986	2.318913	2.314034	2.309216	2.322123	2.317142
2.298709	2.293904	2.298658	2.293758	2.318678	2.313801	2.308988	2.321902	2.316932
2.298496	2.293682	2.298433	2.293534	2.318448	2.313571	2.30876	2.321677	2.316717
2.29828	2.293463	2.298217	2.293325	2.304533	2.299753	2.295036	2.321445	2.316492
2.298064	2.293246	2.298008	2.29313	2.304337	2.299544	2.29481	2.321213	2.316263
2.297845	2.293035	2.297817	2.292974	2.304199	2.299398	2.294642	2.321018	2.316044
2.297621	2.292829	2.297645	2.292861	2.304122	2.299318	2.294535	2.320861	2.315834
2.297435	2.292637	2.297465	2.292714	2.303996	2.299194	2.2944	2.320699	2.315634
2.297311	2.292462	2.297259	2.292496	2.30377	2.298984	2.294208	2.320518	2.315436

2.297192	2.292307	2.297078	2.292297	2.303555	2.298776	2.294014	2.320344	2.315265
2.297046	2.292195	2.313113	2.308257	2.303432	2.298627	2.293842	2.328969	2.323888
2.296885	2.292088	2.313033	2.308175	2.303335	2.298498	2.293686	2.328829	2.323792
2.285314	2.280537	2.31286	2.308013	2.303186	2.298368	2.293572	2.328728	2.323696
2.285087	2.280298	2.312629	2.307803	2.303007	2.298237	2.293486	2.328652	2.323601
2.284921	2.280123	2.312448	2.307613	2.302815	2.298061	2.293331	2.328507	2.32346
2.284826	2.280018	2.312323	2.307438	2.302598	2.297825	2.293092	2.32828	2.323266
2.284723	2.279905	2.301972	2.297064	2.302424	2.297633	2.292887	2.328074	2.323071
2.28459	2.279752	2.301818	2.296941	2.302331	2.297536	2.292772	2.327927	2.32288
2.284446	2.2796	2.301674	2.296834	2.302258	2.297456	2.29267	2.327798	2.322711
2.284292	2.279477	2.301568	2.296734	2.291918	2.287092	2.282279	2.315523	2.31054
2.28414	2.279371	2.301482	2.29664	2.291805	2.28695	2.282114	2.315389	2.310454
2.284033	2.279285	2.301394	2.29652	2.302225	2.297329	2.292462	2.315295	2.310384
2.283963	2.279217	2.301307	2.296382	2.302052	2.297184	2.292365	2.315236	2.310332
2.301383	2.296571	2.301239	2.296301	2.301963	2.297099	2.292289	2.306051	2.301147
2.301318	2.29651	2.301204	2.296309	2.301997	2.2971	2.29224	2.305985	2.301074
2.301242	2.296436	2.301147	2.296285	2.301995	2.297079	2.292187	2.305922	2.301003
2.301141	2.296335	2.301042	2.296162	2.301863	2.296972	2.292112	2.305868	2.300943
2.301029	2.296214	2.300911	2.296007	2.3017	2.296845	2.29203	2.305813	2.300883
2.300909	2.296056	2.311397	2.306474	2.301586	2.296752	2.291953	2.305743	2.30081
2.300787	2.295886	2.311219	2.306339	2.301493	2.296671	2.291876	2.305665	2.300732
2.300712	2.295817	2.311149	2.306253	2.301395	2.296577	2.29179	2.305589	2.300657
2.300683	2.295846	2.311184	2.306213	2.301291	2.296468	2.291695	2.305515	2.300583
2.300631	2.29584	2.311194	2.306199	2.301247	2.296402	2.291611	2.30544	2.300516
2.300541	2.295767	2.311157	2.306223	2.301302	2.296399	2.291533	2.305354	2.300459
2.300443	2.295678	2.311101	2.306232	2.301349	2.29641	2.291489	2.305295	2.300405
2.311596	2.306788	2.311026	2.306178	2.301319	2.29642	2.291528	2.305306	2.300343
2.311505	2.3067	2.310943	2.306097	2.301256	2.29642	2.291585	2.305342	2.300295
2.311415	2.30662	2.310869	2.306014	2.301174	2.296363	2.291561	2.305352	2.300318
2.311333	2.306551	2.310804	2.305929	2.301076	2.296269	2.291485	2.305348	2.300387
2.311316	2.306533	2.310787	2.30591	2.30105	2.296218	2.291413	2.32705	2.322057
2.311372	2.306576	2.310827	2.30597	2.301108	2.296211	2.291336	2.326953	2.321999
2.311425	2.306597	2.310835	2.305993	2.301136	2.296213	2.291303	2.326892	2.321934
2.311459	2.306555	2.310759	2.305927	2.301097	2.296224	2.291356	2.326908	2.321862
2.311472	2.306512	2.310687	2.305848	2.301032	2.296217	2.291405	2.326937	2.321812
2.290414	2.28554	2.310699	2.3058	2.300941	2.296142	2.291366	2.326941	2.321839
2.290375	2.285572	2.310739	2.305768	2.300847	2.296042	2.291288	2.326929	2.321895
2.290307	2.285543	2.310731	2.305757	2.300824	2.29599	2.291209	2.326875	2.321889
2.290214	2.28547	2.310689	2.305767	2.300858	2.295971	2.291124	2.326786	2.321842
2.290182	2.285444	2.310679	2.305798	2.300912	2.295996	2.291109	2.308944	2.304196
2.290228	2.285486	2.310718	2.305864	2.300998	2.296079	2.291181	2.309004	2.30425
2.290281	2.285514	2.310732	2.305898	2.323893	2.318902	2.313926	2.309077	2.30431
2.290304	2.285476	2.310664	2.305846	2.323866	2.318916	2.31398	2.30914	2.304363
2.290332	2.285443	2.310598	2.305789	2.323829	2.318923	2.31403	2.309193	2.304396
2.290393	2.285484	2.290445	2.285674	2.323852	2.31896	2.314075	2.309209	2.304362
2.290473	2.285567	2.290529	2.285753	2.323906	2.319013	2.314118	2.309208	2.304297
2.290542	2.285636	2.290602	2.285825	2.323961	2.319069	2.314172	2.309242	2.304301
2.290604	2.285698	2.29067	2.285894	2.32402	2.319129	2.314234	2.309306	2.304367
2.290671	2.285765	2.290743	2.285968	2.324084	2.319194	2.3143	2.309374	2.304437
2.290748	2.285842	2.290825	2.28605	2.324153	2.319264	2.31437	2.309444	2.304506

2.290826	2.285919	2.314855	2.309997	2.305156	2.300297	2.29543	2.309514	2.304576
2.290903	2.285994	2.314928	2.310068	2.305227	2.300368	2.295501	2.309578	2.304638
2.290977	2.286067	2.314998	2.310137	2.305295	2.300436	2.29557	2.309642	2.304702
2.291043	2.286131	2.315061	2.310199	2.305358	2.300501	2.29564	2.309711	2.304774
2.291105	2.286192	2.315119	2.310257	2.305416	2.300566	2.295711	2.309785	2.304857
2.291184	2.286273	2.315196	2.310324	2.305479	2.300634	2.295793	2.309886	2.304989
2.29128	2.286372	2.315285	2.310396	2.305542	2.300705	2.295885	2.310018	2.305181
2.29139	2.286505	2.315421	2.310515	2.305643	2.300795	2.295973	2.310127	2.305334
2.291518	2.286697	2.315646	2.31072	2.305814	2.300916	2.296048	2.310185	2.305405
2.291645	2.286881	2.31586	2.310922	2.305987	2.301043	2.296126	2.310238	2.305457
2.291737	2.286976	2.315964	2.311036	2.306104	2.301146	2.296212	2.291381	2.286621
2.29183	2.287048	2.31603	2.311117	2.3062	2.301248	2.296314	2.291473	2.286697
2.316801	2.311917	2.316164	2.311232	2.306306	2.301377	2.29647	2.291631	2.286842
2.317024	2.312122	2.316344	2.311367	2.306419	2.301527	2.29667	2.291843	2.287042
2.31723	2.312304	2.316514	2.311527	2.306579	2.301705	2.296869	2.292038	2.287219
2.317419	2.312456	2.316668	2.311716	2.306798	2.301922	2.29707	2.292213	2.287362
2.317588	2.312615	2.31684	2.311916	2.307017	2.302129	2.297254	2.292381	2.287516
2.317736	2.31281	2.317064	2.312133	2.307216	2.3023	2.2974	2.292535	2.2877
2.317861	2.31299	2.317276	2.312336	2.307401	2.302462	2.297544	2.292695	2.287894
2.31794	2.313079	2.31739	2.312468	2.307552	2.302631	2.29773	2.292889	2.28809
2.318004	2.313134	2.317462	2.31257	2.307687	2.302805	2.297935	2.293098	2.288283
2.318163	2.313289	2.317625	2.312739	2.307864	2.302992	2.298128	2.293279	2.288443
2.318407	2.313532	2.317863	2.312966	2.308077	2.303192	2.298314	2.293439	2.288571
2.31865	2.313775	2.318103	2.31319	2.308283	2.303379	2.298482	2.293595	2.288721
2.318886	2.31401	2.318342	2.31341	2.308481	2.303543	2.298618	2.293741	2.2889
2.319115	2.314234	2.318566	2.313613	2.308663	2.303706	2.298771	2.293908	2.289097
2.319329	2.314436	2.318758	2.313776	2.308811	2.303876	2.298971	2.294121	2.28931
2.319535	2.314621	2.318931	2.313928	2.308958	2.304056	2.299193	2.294354	2.289536
2.305444	2.300551	2.319079	2.314097	2.309152	2.304265	2.299414	2.294583	2.28977
2.305634	2.300694	2.319217	2.31428	2.309376	2.304495	2.29964	2.294814	2.290013
2.30588	2.30094	2.319465	2.314515	2.309601	2.304721	2.299871	2.295045	2.290241
2.306186	2.301301	2.31983	2.314803	2.309824	2.304943	2.300107	2.295275	2.290453
2.306467	2.301624	2.320171	2.315108	2.310091	2.305192	2.300345	2.295504	2.290677
2.306694	2.301866	2.320454	2.315442	2.310449	2.305497	2.300582	2.29573	2.290924
2.30691	2.302086	2.320707	2.315744	2.310777	2.305785	2.300818	2.295959	2.291172
2.307141	2.302307	2.320918	2.315924	2.310942	2.305979	2.30105	2.296199	2.291405
2.307374	2.302526	2.321115	2.31607	2.311063	2.306147	2.30128	2.29644	2.291632
2.290013	2.285241	2.307776	2.302803	2.311331	2.306394	2.301503	2.29666	2.29186
2.290229	2.285486	2.308069	2.303147	2.311698	2.306697	2.301724	2.296867	2.292088
2.290498	2.285735	2.308331	2.303445	2.312024	2.30701	2.302012	2.297131	2.292329
2.290834	2.285984	2.308548	2.303687	2.312302	2.307342	2.302383	2.297463	2.292576
2.291164	2.286262	2.308805	2.303938	2.299089	2.294231	2.289374	2.297805	2.292864
2.291465	2.286599	2.30915	2.304216	2.299319	2.294483	2.289671	2.298131	2.293219
2.291749	2.286941	2.309516	2.304516	2.299568	2.294742	2.289963	2.298463	2.2936
2.291996	2.287227	2.309842	2.304852	2.29991	2.295082	2.290302	2.298821	2.293983
2.292233	2.287493	2.310156	2.305213	2.300301	2.29546	2.290654	2.299182	2.294362
2.292545	2.28781	2.310498	2.305583	2.30068	2.295799	2.290946	2.286156	2.281406
2.29292	2.288171	2.310865	2.305965	2.301056	2.296111	2.291186	2.28638	2.28165
2.293278	2.288492	2.311184	2.306301	2.301401	2.296432	2.291475	2.286644	2.281895
2.293605	2.288748	2.311423	2.306567	2.301701	2.296768	2.291833	2.286963	2.282137

2.293922	2.289019	2.294848	2.29007	2.302005	2.297114	2.292213	2.287313	2.282421
2.294224	2.28935	2.2952	2.290413	2.30235	2.297486	2.292614	2.287709	2.282792
2.294529	2.289705	2.295581	2.290767	2.302692	2.297849	2.293006	2.288112	2.283198
2.294868	2.290057	2.295932	2.291071	2.302966	2.298134	2.293321	2.293271	2.288385
2.29523	2.290414	2.296275	2.291354	2.303206	2.298378	2.293593	2.293615	2.288784
2.281728	2.276974	2.29664	2.291691	2.303524	2.298694	2.293919	2.293991	2.289186
2.282109	2.27735	2.297029	2.29208	2.303918	2.29908	2.294296	2.294396	2.28959
2.282489	2.277727	2.29742	2.292474	2.304317	2.299475	2.294685	2.294811	2.289996
2.282869	2.278106	2.297811	2.292863	2.304709	2.29987	2.295081	2.295234	2.290402
2.283247	2.278484	2.298203	2.293254	2.305103	2.300267	2.295478	2.295659	2.290815
2.283624	2.278862	2.298596	2.293646	2.305498	2.300659	2.295868	2.296082	2.291243
2.283999	2.279239	2.298988	2.294038	2.305893	2.301049	2.296255	2.296502	2.291674
2.284375	2.279611	2.286205	2.281312	2.311427	2.306558	2.30174	2.296913	2.292091
2.284751	2.27998	2.286582	2.281689	2.311843	2.306969	2.302146	2.297317	2.292499
2.285128	2.280353	2.286963	2.282067	2.312254	2.307377	2.302552	2.29772	2.292901
2.285505	2.280728	2.287345	2.282441	2.312658	2.307781	2.302957	2.298122	2.293297
2.285883	2.281107	2.276311	2.271475	2.273392	2.268691	2.264039	2.298524	2.293695
2.286257	2.281488	2.276697	2.271863	2.273808	2.269088	2.264418	2.298913	2.29409
2.286634	2.281873	2.277091	2.27227	2.274252	2.269514	2.264821	2.299319	2.294496
2.287006	2.282255	2.277497	2.272721	2.274766	2.270021	2.265304	2.299782	2.294922
2.287387	2.282642	2.277909	2.273189	2.275307	2.270562	2.265823	2.300276	2.295369
2.287823	2.283051	2.278308	2.2736	2.275765	2.271026	2.26629	2.289005	2.284153
2.288306	2.283479	2.278695	2.273963	2.276156	2.271429	2.266718	2.289484	2.284672
2.288775	2.283934	2.28612	2.281345	2.276596	2.27186	2.267146	2.289939	2.28515
2.289225	2.28443	2.28667	2.281884	2.277107	2.272328	2.267569	2.290352	2.285562
2.289657	2.284903	2.287197	2.282411	2.277622	2.272812	2.268019	2.290797	2.286001
2.29005	2.285291	2.287628	2.282869	2.278105	2.273309	2.268525	2.291326	2.286533
2.29045	2.285673	2.288048	2.28332	2.278589	2.273819	2.269055	2.291881	2.287086
2.290928	2.286146	2.288555	2.283836	2.279113	2.274347	2.269582	2.292405	2.287577
2.29145	2.286667	2.289102	2.284381	2.279654	2.274885	2.27011	2.292917	2.288044
2.266369	2.261548	2.289591	2.284859	2.280128	2.275372	2.270613	2.293438	2.288561
2.266813	2.261952	2.290023	2.285267	2.280532	2.275807	2.271092	2.293968	2.289126
2.267258	2.262384	2.265872	2.261096	2.280994	2.276281	2.271587	2.277716	2.272967
2.267699	2.262858	2.266399	2.261632	2.281555	2.276823	2.272106	2.278249	2.273513
2.268157	2.263351	2.266946	2.262189	2.282138	2.277385	2.272641	2.278785	2.274045
2.268653	2.263857	2.267483	2.262734	2.257987	2.253221	2.248458	2.279317	2.274534
2.269167	2.264373	2.268023	2.26328	2.258535	2.25377	2.248999	2.279861	2.275027
2.269678	2.264889	2.268569	2.263822	2.259077	2.254318	2.249556	2.280455	2.275622
2.270192	2.265408	2.269119	2.264363	2.259614	2.254866	2.250123	2.281083	2.276285
2.270712	2.265926	2.269664	2.264907	2.260158	2.255412	2.250671	2.258013	2.253204
2.27124	2.266442	2.270205	2.265458	2.260712	2.255955	2.251195	2.258518	2.253675
2.271765	2.26696	2.270749	2.266005	2.261262	2.2565	2.251729	2.259049	2.254187
2.272281	2.267481	2.271298	2.266548	2.261803	2.257048	2.252291	2.259651	2.254812
2.272795	2.268001	2.271846	2.267089	2.262341	2.257594	2.252852	2.260252	2.255437
2.273314	2.268517	2.272387	2.267631	2.262882	2.258131	2.253381	2.260778	2.255945
2.273835	2.269031	2.272925	2.268172	2.263423	2.258665	2.253901	2.261283	2.256419
2.274349	2.269545	2.273468	2.268713	2.263964	2.259211	2.254455	2.261863	2.25701
2.274859	2.270058	2.274015	2.269253	2.264505	2.259767	2.255036	2.2625	2.257691
2.275383	2.270573	2.274555	2.269791	2.265046	2.260315	2.255597	2.2631	2.258322
2.275916	2.27108	2.275075	2.270321	2.265586	2.26085	2.256125	2.263646	2.258882

2.276481	2.271627	2.275634	2.27087	2.266128	2.261389	2.256664	2.264197	2.259436
2.277116	2.272267	2.276281	2.27145	2.266657	2.261927	2.257227	2.264773	2.259993
2.277755	2.272919	2.276948	2.272053	2.267212	2.262491	2.257814	2.265373	2.260575
2.278312	2.273488	2.277565	2.27269	2.267863	2.263136	2.258449	2.266027	2.261241
2.278829	2.274013	2.278154	2.273345	2.268566	2.263825	2.25911	2.266712	2.261954
2.279401	2.274553	2.27872	2.273946	2.269195	2.264447	2.259717	2.267341	2.262601
2.28002	2.275097	2.279258	2.274494	2.269757	2.265006	2.260272	2.267917	2.263187
2.28064	2.275682	2.279855	2.275087	2.270351	2.265603	2.260873	2.268535	2.263804
2.281247	2.276322	2.280546	2.275757	2.271007	2.266273	2.261561	2.269232	2.264483
2.281866	2.276981	2.281254	2.276433	2.271656	2.266932	2.262237	2.269917	2.26515
2.282521	2.277648	2.281941	2.277065	2.27224	2.267508	2.262818	2.270515	2.265742
2.283179	2.278312	2.282621	2.277692	2.272822	2.268075	2.263382	2.271097	2.266325
2.283772	2.278923	2.28328	2.278359	2.27349	2.268733	2.264028	2.271757	2.266992
2.284328	2.279502	2.283925	2.279052	2.274217	2.269452	2.264727	2.272468	2.267714
2.284954	2.28013	2.284598	2.279752	2.274936	2.270167	2.265432	2.260723	2.25604
2.285655	2.280813	2.285299	2.280458	2.275648	2.270882	2.266146	2.261432	2.256741
2.286364	2.281506	2.28601	2.281172	2.276364	2.271598	2.26686	2.262141	2.257443
2.287063	2.282201	2.286731	2.281895	2.277088	2.272315	2.26757	2.262846	2.258144
2.287763	2.2829	2.287457	2.282621	2.277812	2.273033	2.268279	2.263551	2.258846
2.288464	2.283599	2.288182	2.283344	2.278532	2.273747	2.268988	2.264258	2.259554
2.289164	2.284298	2.288908	2.284066	2.27925	2.274459	2.269696	2.264965	2.260262
2.289863	2.284992	2.289628	2.284784	2.279966	2.275173	2.270407	2.265672	2.260965
2.290561	2.285684	2.290344	2.2855	2.28068	2.275887	2.271119	2.266378	2.261664
2.291253	2.286371	2.291056	2.286211	2.281392	2.276597	2.271826	2.267081	2.262361
2.291939	2.287053	2.291763	2.286919	2.2821	2.277303	2.272529	2.267781	2.263056
2.280424	2.275573	2.292467	2.287624	2.282804	2.278005	2.273228	2.268478	2.263752
2.281093	2.276238	2.293169	2.288322	2.2835	2.2787	2.273924	2.269174	2.26445
2.281761	2.276902	2.293869	2.289019	2.284194	2.279393	2.274617	2.269869	2.265147
2.28243	2.277566	2.294567	2.289714	2.284887	2.280087	2.275312	2.270562	2.265836
2.2831	2.278232	2.295266	2.29041	2.285582	2.280782	2.276007	2.271254	2.266523
2.283776	2.278911	2.284366	2.27954	2.28629	2.281486	2.276707	2.271952	2.267221
2.284458	2.279601	2.28508	2.280252	2.287007	2.282195	2.277409	2.272654	2.267927
2.285152	2.280297	2.285806	2.280994	2.287769	2.282952	2.278156	2.273401	2.268678
2.285852	2.280994	2.286544	2.281784	2.288607	2.283789	2.27898	2.274224	2.269505
2.286585	2.281714	2.287294	2.282566	2.289424	2.284605	2.279787	2.27503	2.270314
2.264791	2.259964	2.28806	2.283301	2.27855	2.273758	2.268975	2.275752	2.271036
2.265594	2.260741	2.288831	2.284018	2.279227	2.274431	2.269658	2.276444	2.271727
2.277567	2.27271	2.289579	2.284735	2.27992	2.275119	2.270349	2.265629	2.260952
2.278268	2.273432	2.290317	2.285454	2.280622	2.275816	2.271047	2.266325	2.261649
2.279032	2.27421	2.291112	2.286235	2.281389	2.276581	2.27181	2.267081	2.262394
2.279868	2.275054	2.291974	2.287082	2.282225	2.277418	2.272648	2.267903	2.26319
2.280675	2.275857	2.271053	2.266253	2.283072	2.278261	2.273481	2.268728	2.264003
2.281402	2.276564	2.271774	2.267044	2.283932	2.279108	2.274299	2.26955	2.264844
2.28214	2.277276	2.272492	2.267815	2.284762	2.279926	2.275094	2.27035	2.265662
2.282957	2.278072	2.273262	2.268548	2.285488	2.280653	2.275831	2.271082	2.266386
2.283811	2.278908	2.27406	2.269274	2.28618	2.281352	2.276551	2.271793	2.267077
2.284659	2.279754	2.274892	2.270071	2.265289	2.260544	2.255831	2.272574	2.267836
2.285509	2.280615	2.275754	2.270923	2.266128	2.261379	2.256662	2.273406	2.268641
2.286378	2.281478	2.27661	2.271773	2.266971	2.262213	2.257485	2.274238	2.269461
2.287281	2.282344	2.277454	2.27262	2.267818	2.263042	2.258295	2.275067	2.270299

2.288144	2.283201	2.278306	2.273468	2.268661	2.263872	2.259113	2.254392	2.249705
2.288913	2.284049	2.279189	2.274323	2.269488	2.264702	2.25995	2.255221	2.250519
2.289664	2.284878	2.280057	2.275172	2.270316	2.265534	2.260788	2.256051	2.251333
2.27167	2.266908	2.280837	2.275994	2.271168	2.266375	2.261608	2.256868	2.252154
2.272483	2.267657	2.281583	2.276804	2.27203	2.267221	2.262422	2.25768	2.25298
2.273361	2.268516	2.26372	2.258982	2.272874	2.268057	2.26325	2.258503	2.253799
2.2743	2.269475	2.264662	2.259855	2.273703	2.268885	2.26409	2.259334	2.254611
2.243384	2.238643	2.265571	2.260721	2.274543	2.269723	2.264935	2.260176	2.255447
2.244311	2.239523	2.266419	2.261576	2.275401	2.270575	2.265782	2.261028	2.256309
2.245222	2.240392	2.267264	2.262429	2.257624	2.252838	2.248085	2.261898	2.257193
2.246098	2.24126	2.268131	2.263279	2.258466	2.253691	2.248958	2.262794	2.258112
2.24696	2.242125	2.269003	2.264135	2.259315	2.254556	2.249846	2.263702	2.259035
2.247838	2.242992	2.269865	2.265008	2.260201	2.255457	2.250757	2.246082	2.241436
2.248722	2.243862	2.270724	2.265895	2.261114	2.256382	2.251682	2.246973	2.242271
2.249601	2.244746	2.271618	2.266805	2.262034	2.257294	2.252576	2.247846	2.243118
2.250471	2.245644	2.272547	2.267744	2.262966	2.258194	2.253438	2.248698	2.243973
2.251388	2.246567	2.241794	2.237075	2.263879	2.259084	2.254307	2.249566	2.244853
2.252393	2.247532	2.242724	2.237973	2.264752	2.259956	2.25519	2.250466	2.245777
2.253394	2.24849	2.243646	2.238866	2.265626	2.260834	2.256082	2.251371	2.246696
2.254305	2.249384	2.244531	2.239754	2.266526	2.261744	2.256996	2.252266	2.24756
2.255194	2.250266	2.245415	2.24065	2.267442	2.262665	2.257913	2.253154	2.248405
2.256159	2.251234	2.246377	2.241597	2.26837	2.263563	2.258783	2.254013	2.249263
2.257175	2.252263	2.247397	2.242583	2.269311	2.264448	2.25962	2.254849	2.25013
2.25813	2.253236	2.248379	2.243562	2.27028	2.265388	2.260529	2.255749	2.251033
2.259001	2.254136	2.249311	2.244534	2.271285	2.266397	2.261527	2.256725	2.25198
2.25991	2.255056	2.25025	2.245503	2.272283	2.267402	2.262529	2.257709	2.252933
2.260923	2.256038	2.251216	2.246474	2.273266	2.268391	2.263516	2.258682	2.25388
2.261951	2.257034	2.252189	2.247432	2.27422	2.269351	2.264483	2.259645	2.25483
2.262907	2.258005	2.25315	2.248346	2.243568	2.238798	2.234049	2.260575	2.255784
2.263838	2.258971	2.254111	2.249244	2.244415	2.239648	2.234924	2.261491	2.256743
2.264814	2.25996	2.255096	2.250198	2.245344	2.240572	2.235852	2.262434	2.257706
2.265825	2.260968	2.2561	2.251201	2.246342	2.241556	2.236823	2.263398	2.258673
2.26684	2.261978	2.257106	2.252203	2.247339	2.242546	2.237805	2.264374	2.259648
2.267855	2.262989	2.258112	2.253198	2.248327	2.243536	2.238797	2.265367	2.260634
2.268873	2.264001	2.259116	2.254196	2.24932	2.244528	2.239791	2.266361	2.261619
2.269889	2.265007	2.260117	2.255198	2.250322	2.245524	2.240778	2.267345	2.262598
2.270909	2.266014	2.261118	2.256202	2.251327	2.24652	2.241763	2.268326	2.263574
2.271939	2.267031	2.262127	2.257207	2.252329	2.247516	2.242752	2.238038	2.233366
2.272976	2.268056	2.263141	2.258214	2.253329	2.248511	2.243742	2.239019	2.234337
2.274019	2.269086	2.264159	2.259222	2.254329	2.249503	2.244728	2.24	2.235315
2.275067	2.27012	2.265181	2.26023	2.255325	2.250492	2.245711	2.240982	2.236297
2.276113	2.271157	2.266208	2.261248	2.256332	2.251485	2.246693	2.241962	2.237283
2.277154	2.272196	2.26724	2.26227	2.257341	2.252473	2.247662	2.242934	2.238271
2.278195	2.273237	2.268282	2.263316	2.258384	2.253494	2.24866	2.243923	2.239263
2.265053	2.260129	2.269348	2.264421	2.259512	2.254603	2.249732	2.244959	2.24026
2.266092	2.261174	2.270418	2.265541	2.260664	2.255742	2.250834	2.246014	2.241257
2.267183	2.262219	2.271449	2.266592	2.261733	2.256809	2.251889	2.247039	2.242241
2.268318	2.263268	2.272454	2.267596	2.262746	2.257832	2.252918	2.248049	2.24322
2.269489	2.26437	2.273513	2.268633	2.263781	2.258894	2.254011	2.249132	2.244267
2.270712	2.265541	2.274636	2.269712	2.264843	2.260008	2.255186	2.250303	2.245396

2.228084	2.222992	2.275751	2.27078	2.26589	2.261093	2.256323	2.251454	2.246538
2.229149	2.224034	2.276815	2.271802	2.266887	2.262104	2.257369	2.25255	2.247692
2.230194	2.225082	2.277895	2.272844	2.267898	2.263106	2.25838	2.253608	2.24882
2.231277	2.22622	2.279073	2.273983	2.268988	2.264146	2.259379	2.254609	2.249853
2.232372	2.227392	2.280289	2.27517	2.270126	2.265211	2.260375	2.255583	2.250836
2.23346	2.228486	2.239511	2.234507	2.271286	2.266331	2.261441	2.256612	2.251839
2.234543	2.229517	2.240547	2.23562	2.272475	2.267502	2.262566	2.257683	2.252849
2.235676	2.23059	2.241605	2.236702	2.273605	2.268644	2.263711	2.258815	2.253954
2.236884	2.231718	2.242685	2.237731	2.274647	2.269742	2.264889	2.260039	2.255202
2.238079	2.232874	2.243809	2.238793	2.233885	2.229115	2.224418	2.261215	2.256405
2.239211	2.234074	2.245026	2.239956	2.234979	2.230141	2.225386	2.262218	2.257426
2.240324	2.235271	2.246254	2.241148	2.236111	2.231188	2.226352	2.263204	2.258414
2.241435	2.236369	2.24736	2.242293	2.237287	2.232368	2.227517	2.264365	2.259523
2.242554	2.237422	2.248411	2.243421	2.238493	2.233619	2.228784	2.265617	2.260694
2.243775	2.238621	2.249594	2.24459	2.239648	2.234763	2.229918	2.225075	2.220249
2.245107	2.239967	2.250911	2.245798	2.240752	2.2358	2.23092	2.226111	2.221365
2.246337	2.241213	2.25215	2.24699	2.241897	2.23691	2.232008	2.227204	2.222482
2.247363	2.242261	2.253241	2.248158	2.243128	2.238173	2.233273	2.228415	2.223598
2.248417	2.243312	2.254317	2.2493	2.24433	2.23941	2.234519	2.229617	2.22472
2.249685	2.244497	2.255443	2.250382	2.245386	2.240463	2.235587	2.230713	2.225856
2.251018	2.245734	2.2566	2.251465	2.246418	2.241479	2.236614	2.231783	2.226995
2.227392	2.222197	2.257825	2.252684	2.247626	2.242675	2.237793	2.232936	2.228114
2.228523	2.223407	2.259106	2.253996	2.248948	2.243983	2.239065	2.234137	2.229221
2.229741	2.224667	2.260396	2.255264	2.250185	2.245187	2.240235	2.235282	2.230347
2.231066	2.225997	2.261707	2.256486	2.251323	2.246265	2.241279	2.236353	2.231488
2.232346	2.227268	2.246941	2.241685	2.252479	2.247385	2.24238	2.237477	2.232663
2.233501	2.228396	2.248083	2.242864	2.253688	2.248616	2.243627	2.23873	2.233909
2.234651	2.229523	2.249232	2.244065	2.254927	2.249875	2.24489	2.239983	2.235137
2.156066	2.151232	2.250527	2.245376	2.256218	2.251112	2.246061	2.241107	2.236228
2.157253	2.152451	2.251873	2.246723	2.257522	2.25234	2.247202	2.24219	2.237275
2.158444	2.153605	2.228226	2.22312	2.258713	2.25353	2.248396	2.243378	2.238453
2.159646	2.154716	2.229366	2.22422	2.259812	2.254686	2.249628	2.244645	2.239734
2.140145	2.135226	2.18567	2.18066	2.261021	2.255925	2.250902	2.245932	2.24102
2.141229	2.136335	2.186864	2.181901	2.262377	2.257274	2.25223	2.24724	2.242304
2.142338	2.137481	2.188101	2.183182	2.263762	2.25864	2.253564	2.248547	2.243586
2.143523	2.138691	2.189386	2.18449	2.265138	2.259998	2.254895	2.249859	2.244879
2.144742	2.139931	2.190694	2.185805	2.266508	2.261339	2.256201	2.251141	2.246138
2.145975	2.141171	2.19199	2.187089	2.201177	2.196154	2.191146	2.252308	2.247268
2.147223	2.142415	2.19328	2.188349	2.202394	2.197306	2.192228	2.253415	2.248332
2.148459	2.143648	2.194538	2.189558	2.203562	2.198454	2.193365	2.254624	2.249528
2.149693	2.144879	2.195769	2.190716	2.204676	2.199592	2.194548	2.255924	2.250843
2.150845	2.146011	2.161831	2.156881	2.18681	2.181813	2.176862	2.216916	2.211972
2.15186	2.146974	2.162804	2.15793	2.187982	2.183033	2.178117	2.218188	2.213245
2.15289	2.147964	2.163812	2.159009	2.18918	2.184275	2.17939	2.219473	2.214527
2.154039	2.149125	2.164991	2.160185	2.190405	2.185526	2.180671	2.220773	2.215816
2.155243	2.150359	2.166246	2.16141	2.191655	2.186791	2.181959	2.222079	2.217111
2.156462	2.151595	2.167497	2.162646	2.192925	2.188065	2.183241	2.197154	2.192261
2.157705	2.152848	2.168764	2.163901	2.194215	2.189347	2.184519	2.198418	2.193531
2.158967	2.154107	2.170031	2.165159	2.195509	2.190627	2.185788	2.199674	2.194797
2.16025	2.155374	2.171299	2.166419	2.196808	2.191904	2.187046	2.200922	2.196059

2.161539	2.156644	2.17257	2.167684	2.198113	2.193185	2.188308	2.20217	2.197314
2.162831	2.157917	2.173845	2.168952	2.199423	2.194477	2.189581	2.203422	2.198562
2.164122	2.15919	2.175122	2.170222	2.200734	2.195768	2.190854	2.204668	2.199803
2.1654	2.160461	2.176401	2.171491	2.166621	2.161782	2.156989	2.187237	2.182419
2.166671	2.16173	2.17768	2.172758	2.167872	2.163009	2.158191	2.18848	2.183661
2.126941	2.122113	2.17895	2.174013	2.169113	2.16424	2.159414	2.189743	2.184912
2.128168	2.12333	2.180213	2.175258	2.170345	2.165475	2.160654	2.191021	2.186172
2.098157	2.09336	2.181474	2.176509	2.171589	2.166719	2.161897	2.192301	2.187429
2.099348	2.094537	2.18273	2.177767	2.172847	2.167975	2.163146	2.193582	2.188684
2.100547	2.095726	2.163063	2.158158	2.174109	2.16923	2.16439	2.194861	2.189941
2.101761	2.096938	2.164334	2.159416	2.175369	2.170474	2.16562	2.19613	2.191198
2.10299	2.098165	2.165616	2.16068	2.176631	2.17172	2.166852	2.197404	2.192465
2.104218	2.099386	2.137704	2.132792	2.177873	2.172954	2.16808	2.163246	2.158454
2.105474	2.100629	2.13897	2.134032	2.179131	2.174206	2.169332	2.164493	2.159693
2.106899	2.102033	2.097189	2.09235	2.180573	2.175639	2.170756	2.16591	2.161103
2.108491	2.103598	2.098734	2.093885	2.182197	2.17725	2.172354	2.167497	2.162681
2.109975	2.105054	2.100171	2.095316	2.155883	2.150962	2.14609	2.168965	2.164141
2.111278	2.106332	2.101432	2.096574	2.157191	2.152247	2.147349	2.170236	2.165409
2.112545	2.107573	2.102654	2.097789	2.15845	2.153484	2.148563	2.171461	2.166628
2.113845	2.10884	2.103894	2.099009	2.159708	2.154726	2.149791	2.172699	2.167856
2.115146	2.110106	2.105131	2.100224	2.160957	2.155962	2.151016	2.173933	2.169077
2.116431	2.111353	2.106351	2.101434	2.096566	2.09172	2.086923	2.147352	2.142522
2.11772	2.1126	2.107573	2.102655	2.097784	2.092922	2.088106	2.148573	2.143725
2.119157	2.114008	2.10896	2.104034	2.099155	2.094282	2.089452	2.149967	2.1451
2.12077	2.115603	2.110538	2.105598	2.100708	2.095828	2.090991	2.151564	2.146678
2.093168	2.088007	2.112036	2.107072	2.102163	2.097279	2.092439	2.153067	2.148163
2.09451	2.089355	2.113352	2.10835	2.103409	2.098522	2.093686	2.154362	2.149443
2.095801	2.090651	2.114619	2.109578	2.104606	2.099715	2.094882	2.155602	2.150668
2.097092	2.09193	2.086828	2.081783	2.105821	2.100923	2.096086	2.156843	2.151888
2.098407	2.093228	2.088109	2.083048	2.107065	2.102159	2.097313	2.158107	2.153131
2.099909	2.094723	2.089591	2.084507	2.079502	2.074606	2.069776	2.093856	2.089034
2.10156	2.096373	2.091231	2.086121	2.081089	2.076173	2.071328	2.095384	2.090571
1.990525	1.985707	2.092729	2.087601	2.082551	2.077614	2.072751	2.067929	2.063156
1.991725	1.986894	2.094023	2.088889	2.08383	2.078871	2.073987	2.069161	2.064394
1.992984	1.988145	2.095395	2.090255	2.085185	2.080202	2.075293	2.070455	2.065683
1.994435	1.989607	2.097024	2.091875	2.086786	2.081775	2.076833	2.071968	2.067172
1.995906	1.99108	2.098663	2.093505	2.088397	2.083356	2.078379	2.073485	2.068662
1.951978	1.947245	2.100039	2.094873	2.089754	2.084681	2.079671	2.074758	2.069926
1.953207	1.948405	2.101306	2.096131	2.091005	2.085903	2.080861	2.075934	2.0711
1.919976	1.915234	2.02827	2.023376	2.092452	2.087334	2.082276	2.077333	2.072481
1.921356	1.916628	2.029786	2.024887	2.094067	2.088949	2.083889	2.078927	2.074045
1.922675	1.917948	1.988759	1.983962	2.095561	2.090438	2.085377	2.080398	2.075493
1.923873	1.919124	1.989997	1.985153	2.096831	2.091698	2.086636	2.081646	2.076729
1.874914	1.870266	1.950166	1.94536	2.060688	2.055645	2.050683	2.08294	2.078005
1.876347	1.871691	1.951629	1.946788	2.06226	2.057192	2.052205	2.08445	2.079466
1.877811	1.873146	1.953127	1.94827	2.06391	2.058821	2.053809	2.086042	2.081007
1.879051	1.874362	1.954416	1.949608	1.985972	1.981211	1.976498	2.087634	2.082585
1.88017	1.875449	1.955593	1.950872	1.987371	1.98264	1.97794	2.089229	2.08419
1.805161	1.801125	1.872082	1.867575	1.988725	1.983977	1.979261	2.053378	2.048443
1.806437	1.802386	1.873479	1.868869	1.990016	1.985201	1.98044	2.054621	2.049677

1.807709	1.803656	1.87491	1.870222	1.991395	1.98652	1.981713	2.055966	2.051004
1.808954	1.80493	1.876391	1.871676	1.992939	1.988019	1.983164	2.05751	2.052515
1.810161	1.80615	1.877818	1.873113	1.994505	1.989559	1.984667	2.059118	2.054088
1.811266	1.807209	1.879048	1.874424	1.996011	1.991072	1.986166	1.981348	1.976599
1.812346	1.808216	1.880206	1.875675	1.997474	1.992565	1.987667	1.982846	1.978083
1.813548	1.809362	1.805305	1.801409	1.872389	1.867837	1.863302	1.984324	1.979558
1.81484	1.810604	1.806479	1.802478	1.87353	1.869037	1.8646	1.985786	1.981029
1.767436	1.763263	1.807713	1.803639	1.874796	1.870285	1.865854	1.987179	1.982456
1.768753	1.764563	1.809016	1.804911	1.876213	1.871583	1.867041	1.988479	1.983834
1.770045	1.765861	1.810333	1.806201	1.877657	1.87293	1.868292	1.910819	1.906375
1.771294	1.767149	1.811654	1.807485	1.879096	1.874349	1.869683	1.912162	1.907603
1.772502	1.768408	1.812961	1.808763	1.880538	1.875792	1.871118	1.913547	1.908853
1.773632	1.769588	1.814202	1.810002	1.881954	1.877202	1.872519	1.914978	1.910236
1.774728	1.770731	1.815414	1.811229	1.88337	1.878608	1.873916	1.916445	1.911695
1.707345	1.703579	1.767945	1.76387	1.808359	1.804266	1.800213	1.870645	1.866003
1.708574	1.704808	1.769242	1.765174	1.809687	1.805558	1.801464	1.872063	1.867423
1.709783	1.705977	1.770465	1.766413	1.810969	1.806822	1.802702	1.87347	1.868831
1.710952	1.707044	1.771563	1.767544	1.812172	1.808035	1.803914	1.874856	1.870217
1.651889	1.64805	1.704229	1.700436	1.813393	1.809263	1.80514	1.876256	1.871605
1.653103	1.649251	1.705425	1.701642	1.814693	1.810547	1.806403	1.877687	1.873004
1.654327	1.650484	1.706667	1.702882	1.816018	1.811852	1.807684	1.879138	1.874411
1.655537	1.651689	1.707878	1.704093	1.700295	1.696385	1.692457	1.804844	1.800748
1.656742	1.652885	1.709079	1.705291	1.701494	1.697589	1.693662	1.806117	1.801981
1.569334	1.565936	1.650293	1.646627	1.702661	1.698766	1.694848	1.807376	1.803201
1.570433	1.567013	1.651457	1.64777	1.703794	1.699914	1.696014	1.80862	1.804405
1.499362	1.495954	1.652619	1.648914	1.704934	1.701066	1.697182	1.693245	1.689283
1.500459	1.49702	1.653777	1.650063	1.706091	1.702231	1.698359	1.694427	1.690464
1.501566	1.498093	1.654946	1.651221	1.707258	1.7034	1.699533	1.695608	1.691653
1.502683	1.499176	1.495792	1.492559	1.584084	1.580417	1.576759	1.696778	1.692834
1.50382	1.500286	1.496882	1.493638	1.58526	1.581575	1.577905	1.69794	1.694011
1.416466	1.413154	1.498067	1.494794	1.586488	1.582755	1.579048	1.639652	1.635805
1.417584	1.41429	1.499325	1.496008	1.587755	1.583953	1.580189	1.640775	1.636941
1.418691	1.415388	1.500517	1.497163	1.493823	1.490445	1.487076	1.577616	1.573985
1.419783	1.416438	1.501629	1.498246	1.494887	1.491495	1.488128	1.578788	1.575251
1.420876	1.417491	1.4142	1.411024	1.495952	1.492553	1.489192	1.579967	1.576486
1.421958	1.418547	1.415229	1.412019	1.497032	1.493626	1.490264	1.581128	1.577625
1.423067	1.419638	1.416292	1.41304	1.498128	1.494722	1.491366	1.582322	1.578762
1.42426	1.420826	1.417446	1.414117	1.410861	1.407701	1.404604	1.583621	1.579984
1.425497	1.422061	1.418646	1.415232	1.41191	1.408755	1.405682	1.584973	1.581245
1.34817	1.344765	1.419792	1.41635	1.413004	1.409832	1.406744	1.491594	1.488279
1.349437	1.345971	1.420899	1.417478	1.414141	1.410933	1.407798	1.49271	1.489367
1.350671	1.347167	1.343739	1.340401	1.415258	1.412015	1.408838	1.405697	1.402598
1.351859	1.348343	1.344905	1.341559	1.416332	1.413059	1.409859	1.406733	1.403672
1.353047	1.349541	1.346103	1.342745	1.417434	1.414124	1.410889	1.407765	1.404729
1.354251	1.35079	1.347374	1.344012	1.418618	1.415245	1.411938	1.408773	1.405716
1.355476	1.352062	1.348669	1.345299	1.419821	1.416388	1.41301	1.409795	1.406699
1.356753	1.353341	1.349936	1.346534	1.343147	1.339761	1.336415	1.410881	1.407751
1.358064	1.354627	1.35119	1.347745	1.344333	1.340968	1.337652	1.412014	1.408848
1.246539	1.243428	1.352419	1.348968	1.345558	1.342209	1.338908	1.335655	1.332449
1.247663	1.244497	1.353616	1.350197	1.346821	1.343483	1.340184	1.336922	1.333696

1.248778	1.245592	1.354857	1.351456	1.348097	1.344765	1.341464	1.338189	1.334941
1.249873	1.246726	1.356168	1.352756	1.349384	1.346051	1.342748	1.339459	1.336187
1.250989	1.247883	1.357499	1.35407	1.350677	1.347336	1.344024	1.340715	1.337417
1.252167	1.249063	1.24597	1.242883	1.351971	1.348613	1.345282	1.341942	1.338607
1.253374	1.250258	1.247148	1.24404	1.353265	1.349882	1.346523	1.343151	1.339785
1.254578	1.251448	1.248323	1.2452	1.242089	1.238996	1.235918	1.344347	1.340996
1.255785	1.252639	1.249497	1.246361	1.243228	1.240083	1.236958	1.345533	1.342228
1.151848	1.148897	1.250666	1.247496	1.244332	1.241167	1.238031	1.346752	1.343465
1.152985	1.150035	1.251826	1.248592	1.245386	1.24224	1.239139	1.348009	1.344709
1.154118	1.151171	1.252998	1.249732	1.246501	1.243357	1.240266	1.23722	1.234218
1.155247	1.152304	1.254195	1.250969	1.247754	1.244561	1.241404	1.23833	1.235317
1.15637	1.153434	1.255393	1.252209	1.249012	1.245777	1.242561	1.239454	1.236424
1.028385	1.025732	1.151604	1.148592	1.250136	1.246923	1.243733	1.240613	1.237548
1.029353	1.026736	1.152724	1.149668	1.251221	1.248056	1.244928	1.241801	1.238693
1.030326	1.027712	1.153846	1.150814	1.147797	1.144846	1.141918	1.243034	1.239898
1.031303	1.028664	1.154969	1.152016	1.149059	1.14611	1.143161	1.244309	1.241157
1.032273	1.029623	1.026992	1.024393	1.150271	1.147327	1.144367	1.245547	1.242381
1.033235	1.030594	1.027972	1.025374	1.151414	1.148479	1.145521	1.246733	1.243552
1.034203	1.031561	1.028938	1.026341	1.152536	1.149611	1.14666	1.143674	1.140666
1.035183	1.032507	1.029874	1.027299	1.153647	1.150736	1.147797	1.144818	1.141815
1.03618	1.033458	1.030808	1.028253	1.154749	1.151852	1.148927	1.145963	1.142971
1.037205	1.034441	1.031766	1.029212	1.026658	1.024044	1.021401	1.147104	1.144133
1.038257	1.03545	1.032746	1.030178	1.027621	1.025021	1.0224	1.148249	1.145303
1.039354	1.036517	1.033784	1.031183	1.028609	1.026026	1.023434	1.149429	1.1465
1.0405	1.037645	1.034881	1.032227	1.029621	1.027061	1.024508	1.150648	1.147727
1.041624	1.038749	1.035957	1.033261	1.030628	1.028076	1.025542	1.022923	1.020255
1.042709	1.039804	1.036986	1.03427	1.031619	1.029049	1.026503	1.023884	1.021224
1.043787	1.040861	1.038022	1.035287	1.032617	1.030027	1.027466	1.024849	1.022204
1.044867	1.041941	1.039089	1.036321	1.033626	1.031035	1.028483	1.025883	1.023262
1.045953	1.04304	1.040183	1.037383	1.03466	1.032062	1.029515	1.026933	1.024336
1.04707	1.044187	1.041335	1.038514	1.035757	1.03311	1.030517	1.027923	1.025338
1.048207	1.045365	1.042531	1.039697	1.036904	1.03418	1.031506	1.028882	1.026298
1.049293	1.046482	1.043669	1.040849	1.038052	1.035287	1.032561	1.029913	1.027318
1.05032	1.047527	1.044743	1.04197	1.039206	1.03644	1.033697	1.031027	1.028406
1.051374	1.048598	1.045832	1.043081	1.04033	1.037561	1.034807	1.032125	1.029488
1.0525	1.049739	1.046966	1.044176	1.041389	1.0386	1.035838	1.03316	1.030541
1.053626	1.050886	1.048108	1.045278	1.042454	1.039649	1.036879	1.034201	1.031587
1.054689	1.051992	1.049245	1.046426	1.043604	1.040797	1.038015	1.035299	1.032631
1.055729	1.053077	1.050368	1.047581	1.04478	1.041984	1.039194	1.036427	1.033681
1.056803	1.054136	1.051425	1.048654	1.045874	1.043111	1.040348	1.037564	1.034775
1.0579	1.05517	1.052427	1.049661	1.046908	1.044198	1.041488	1.038714	1.035907
1.059021	1.056247	1.053482	1.05072	1.047983	1.045303	1.042624	1.039854	1.037034
1.060168	1.057379	1.054607	1.051848	1.049114	1.046435	1.043755	1.04098	1.038153
1.061329	1.058525	1.055744	1.052985	1.050253	1.047569	1.044884	1.042105	1.039274
1.062496	1.059665	1.056869	1.05411	1.05138	1.048697	1.046011	1.043233	1.040403
1.06368	1.060818	1.058002	1.055239	1.052508	1.049823	1.047138	1.044362	1.041535
0.585242	0.583863	1.059159	1.056381	1.05364	1.050951	1.048265	1.04549	1.042665
0.585896	0.584489	1.060332	1.057531	1.054774	1.052079	1.049392	1.046617	1.043794
0.586554	0.585118	1.061506	1.058684	1.055912	1.053211	1.050524	1.047747	1.044923
0.587216	0.58575	1.062683	1.05984	1.057052	1.054346	1.051658	1.04888	1.046054

0.587881	0.586392	1.063873	1.061009	1.058203	1.055489	1.052797	1.050015	1.047185
0.588551	0.587046	1.065079	1.062193	1.059367	1.056642	1.053942	1.051153	1.048319
0.589217	0.587706	1.066295	1.063388	1.060543	1.057803	1.055092	1.052294	1.049454
0.589875	0.588373	1.067519	1.064596	1.061733	1.058976	1.056247	1.053435	1.050584
0.590526	0.589042	1.068748	1.065813	1.062934	1.060158	1.05741	1.054584	1.051722
0.591173	0.589707	0.588241	0.586765	1.06414	1.061349	1.058586	1.055755	1.052894
0.591814	0.590369	0.588909	0.587419	1.065352	1.062547	1.059772	1.056944	1.054094
0.592444	0.591021	0.589571	0.588079	1.066567	1.063751	1.060967	1.058151	1.055322
0.593063	0.591661	0.590226	0.588742	1.067782	1.064958	1.062172	1.059378	1.056586
0.593675	0.592306	0.590895	0.589425	1.069031	1.066188	1.063383	1.060595	1.05782
0.594285	0.592969	0.591596	0.590147	1.070347	1.067461	1.064606	1.061787	1.058994
0.594886	0.593621	0.592285	0.59086	1.071657	1.06873	1.065824	1.062972	1.060158
0.595473	0.594234	0.592921	0.591517	0.861446	0.859126	0.856804	1.064153	1.061344
0.596054	0.594825	0.593529	0.592149	0.862409	0.860097	0.857773	1.06534	1.06254
0.596637	0.59541	0.594133	0.592792	0.591407	0.589956	0.588486	1.066577	1.063739
0.597221	0.595987	0.594727	0.593439	0.592101	0.59068	0.589219	1.067853	1.064943
0.59784	0.596576	0.595314	0.594061	0.592763	0.591377	0.589942	0.858654	0.856313
0.5985	0.597176	0.595888	0.594651	0.593385	0.592048	0.59066	0.859711	0.857398
0.599177	0.5978	0.596481	0.595241	0.59399	0.59269	0.591347	0.860735	0.858468
0.59987	0.598461	0.597112	0.595839	0.594573	0.593289	0.591978	0.861699	0.859484
0.60057	0.599141	0.597763	0.59645	0.595157	0.593877	0.592588	0.862635	0.860472
		0.598434	0.597093	0.595778	0.594492	0.593209	0.863556	0.861428
		0.599118	0.597754	0.596419	0.59512	0.593831	0.864467	0.862362
		0.599776	0.598403	0.59706	0.595754	0.594461	0.593143	0.591808
		0.600412	0.599043	0.597703	0.596396	0.595099	0.593774	0.592431
				0.598341	0.59703	0.595734	0.594418	0.59309
				0.598962	0.59765	0.596367	0.595089	0.593813
				0.599602	0.598274	0.596991	0.595738	0.594507
				0.60028	0.5989	0.597576	0.596313	0.595094
				0.600985	0.599548	0.598172	0.596881	0.595653
				0.601721	0.60027	0.59887	0.597545	0.59628
				0.602482	0.601039	0.599631	0.598267	0.596942
							0.598937	0.597585
							0.59955	0.598207
							0.600196	0.598841
							0.600899	0.599486
							0.601629	0.600159
							0.602376	0.60089
							0.603135	0.601647
							0.603873	0.602366
							0.604599	0.603063

							0.589027	0.587142
							0.588134	0.586246
							0.883282	0.880386
							0.881936	0.879051
					0.589181	0.587289	1.219089	1.215882
					0.588294	0.586399	1.217562	1.214358
					0.883872	0.880974	1.365813	1.362149
					0.88268	0.879862	1.364311	1.360702
					0.881512	0.878769	1.362839	1.359286
		0.888822	0.885938	0.883101	1.218275	1.215195	1.696926	1.692439
		0.887725	0.884816	0.881941	1.216891	1.21375	1.694731	1.690229
		0.886479	0.883574	0.880698	1.365128	1.361496	1.692506	1.688014
		0.885095	0.88222	0.879376	1.363375	1.359768	1.690244	1.685781
0.889595	0.88665	1.221893	1.218689	1.21552	1.697106	1.692533	1.68803	1.683592
0.888253	0.885333	1.22036	1.217169	1.21401	1.694895	1.690349	1.685866	1.681444
0.886977	0.884087	1.218902	1.215724	1.212574	1.692764	1.688247	1.683784	1.679375
1.069599	1.066498	1.367706	1.364072	1.36047	1.690796	1.686314	1.754065	1.749535

1.06831	1.065237	1.366279	1.362666	1.359081	1.688869	1.684421	1.75213	1.747607
1.221269	1.218097	1.700415	1.695813	1.691258	1.759066	1.754533	1.814592	1.809928
1.219752	1.216589	1.698142	1.693572	1.689047	1.75682	1.752287	1.812259	1.80761
1.368505	1.364882	1.695892	1.691354	1.686861	1.819285	1.814611	1.879575	1.874868
1.366724	1.363124	1.693647	1.689143	1.684686	1.816968	1.812311	1.877289	1.872589
1.700642	1.696042	1.763848	1.759281	1.754752	1.814738	1.810096	2.112497	2.107129
1.698638	1.694063	1.761839	1.757278	1.752754	1.812674	1.808045	2.110212	2.104824
1.696642	1.692095	1.759841	1.755285	1.750763	1.81061	1.80599	2.107932	2.102514
1.766842	1.762242	1.822363	1.817675	1.81301	1.878211	1.873529	2.105443	2.100007
1.764575	1.760002	1.820046	1.815374	1.810721	1.87595	1.87127	2.102886	2.097431
1.827254	1.822551	1.88791	1.883218	1.878538	1.873855	1.869174	2.100467	2.094966
1.825195	1.820493	1.885889	1.881221	1.876562	1.871885	1.867197	2.098147	2.092578
1.823026	1.818334	1.883776	1.879123	1.874478	2.106735	2.101285	2.095765	2.090181
1.820698	1.816028	1.881523	1.876875	1.872237	2.104204	2.098772	2.093292	2.087769
1.888631	1.883966	1.879321	1.874678	1.870045	2.101714	2.096296	2.090846	2.085365
1.88654	1.88191	1.877292	1.872662	1.868033	2.099403	2.093979	2.088503	2.08298
1.884491	1.879881	1.875279	1.870659	1.866034	2.097094	2.09166	2.08616	2.080599
1.882372	1.877727	2.0525	2.047314	2.042116	2.09458	2.089146	2.083688	2.078207
1.880237	1.87553	2.049981	2.044779	2.039576	2.091979	2.086547	2.081161	2.075812
1.878179	1.873454	2.047614	2.042399	2.037186	2.089551	2.084116	2.118763	2.113352
1.876192	1.871487	2.045381	2.040157	2.034932	2.087281	2.081836	2.116406	2.110936
1.937542	1.932688	2.101251	2.095795	2.090355	2.084919	2.079498	2.114034	2.108571
1.935469	1.93056	2.098829	2.093282	2.087779	2.082371	2.077032	2.111605	2.106264
1.93334	1.928397	2.096404	2.090797	2.085255	2.079878	2.074613	2.109223	2.104002
2.046623	2.041302	2.094019	2.088458	2.082955	2.117564	2.112222	2.155264	2.14989
2.044166	2.038911	2.091656	2.086179	2.080746	2.115332	2.10999	2.15294	2.147599
2.041843	2.036595	2.089332	2.083913	2.078529	2.113076	2.107714	2.201953	2.196416
2.039598	2.034324	2.087032	2.081661	2.076318	2.110819	2.105425	2.199404	2.193825
2.037302	2.031992	2.084656	2.079305	2.073996	2.108494	2.103148	2.196957	2.191382
2.034956	2.029593	2.082189	2.076826	2.071542	2.106085	2.100878	2.194617	2.189093
2.090796	2.085243	2.119774	2.114311	2.108956	2.10375	2.098638	2.192293	2.186812
2.088386	2.082873	2.117405	2.112022	2.106741	2.101566	2.096462	2.189989	2.184536
2.085993	2.080545	2.115092	2.109793	2.104576	2.099402	2.094265	2.187641	2.182195
2.083645	2.078278	2.112838	2.107581	2.102377	2.196225	2.190669	2.224619	2.218877
2.081341	2.076058	2.110627	2.105386	2.100167	2.193808	2.188182	2.222001	2.216204
2.079116	2.073871	2.108419	2.103161	2.09791	2.191366	2.185732	2.21947	2.213674
2.076961	2.071718	2.10622	2.100915	2.095616	2.188899	2.183304	2.216999	2.211244
2.074791	2.069526	2.158692	2.153179	2.147688	2.226173	2.220348	2.214576	2.208858
2.072599	2.067277	2.15631	2.150807	2.145341	2.223726	2.217943	2.212208	2.206523
2.110206	2.104782	2.153975	2.148479	2.143029	2.221314	2.215567	2.209861	2.204196
2.108042	2.10264	2.151765	2.146246	2.140779	2.218946	2.213228	2.207533	2.201869
2.105862	2.100496	2.149566	2.144035	2.138553	2.216594	2.21089	2.205197	2.199525
2.158308	2.152773	2.231743	2.225842	2.219994	2.214223	2.208508	2.283306	2.276841
2.155905	2.150387	2.229246	2.223412	2.217618	2.211845	2.206101	2.280524	2.274045
2.153583	2.14807	2.226801	2.221003	2.215235	2.209448	2.203676	2.313067	2.306526
2.151327	2.145809	2.224397	2.218609	2.212844	2.207032	2.201229	2.31022	2.303745
2.149082	2.143559	2.222008	2.216232	2.210477	2.285301	2.278685	2.307457	2.301033
2.146843	2.141319	2.219634	2.213876	2.208141	2.282626	2.276041	2.304804	2.298407
2.229011	2.223102	2.299563	2.292999	2.286543	2.279984	2.273439	2.302196	2.295815
2.226611	2.220712	2.296826	2.290319	2.283901	2.27738	2.270864	2.299607	2.293232

2.224213	2.218326	2.294108	2.287658	2.28128	2.274797	2.26831	2.29704	2.290668
2.221828	2.215961	2.291406	2.285	2.278655	2.30744	2.300909	2.330134	2.323332
2.219453	2.213611	2.28872	2.282352	2.276034	2.304822	2.298322	2.327399	2.320664
2.29919	2.292551	2.286057	2.279724	2.273434	2.302216	2.295734	2.324663	2.317985
2.296501	2.289891	2.283421	2.277118	2.270853	2.299622	2.293143	2.321923	2.315295
2.293826	2.287239	2.316095	2.30977	2.303482	2.297041	2.290572	2.319218	2.312635
2.291148	2.284584	2.313435	2.307151	2.3009	2.294483	2.28803	2.316566	2.310023
2.288481	2.281936	2.310783	2.304534	2.298315	2.291925	2.285498	2.313933	2.307427
2.285818	2.279295	2.308137	2.301904	2.295703	2.289346	2.282958	2.350246	2.343161
2.28317	2.276664	2.305502	2.299276	2.293086	2.286763	2.280421	2.347398	2.340381
2.315817	2.309283	2.302883	2.296669	2.290497	2.319257	2.312567	2.344561	2.337608
2.313206	2.306676	2.30028	2.29408	2.287928	2.316573	2.309923	2.341735	2.334847
2.310679	2.304156	2.297756	2.291541	2.28538	2.353112	2.345929	2.364778	2.357937
2.308262	2.301751	2.295334	2.289064	2.282856	2.350239	2.343107	2.361994	2.355216
2.305807	2.29931	2.292886	2.286579	2.280343	2.34739	2.340323	2.389843	2.382952
2.30324	2.296754	2.290345	2.284047	2.277828	2.344557	2.337569	2.387036	2.380186
2.300644	2.294177	2.287794	2.28152	2.275333	2.341763	2.334874	2.384293	2.37747
2.298059	2.291635	2.327305	2.320382	2.313592	2.339042	2.332298	2.381701	2.374878
2.295489	2.28912	2.324591	2.317691	2.310943	2.336373	2.329786	2.37919	2.372358
2.293023	2.286688	2.354748	2.347509	2.340437	2.390075	2.383363	2.376588	2.369794
2.290651	2.284335	2.35207	2.344861	2.337808	2.387351	2.380648	2.373918	2.3672
2.33052	2.323608	2.349324	2.342176	2.335176	2.384631	2.377934	2.371268	2.364659
2.327854	2.320897	2.346458	2.339409	2.332513	2.38189	2.375206	2.368637	2.362194
2.325212	2.318228	2.37604	2.368866	2.361871	2.379225	2.372552	2.366053	2.359729
2.322641	2.315688	2.373299	2.366246	2.359365	2.376729	2.370044	2.363535	2.357193
2.320074	2.313183	2.37061	2.363671	2.356898	2.374288	2.367615	2.361081	2.354678
2.317393	2.310597	2.367855	2.360976	2.354275	2.371763	2.365219	2.394905	2.38831
2.31466	2.307982	2.365088	2.358236	2.351581	2.369202	2.362853	2.392552	2.385967
2.376728	2.3695	2.386601	2.37976	2.373105	2.366669	2.360386	2.390056	2.383534
2.37406	2.366947	2.38411	2.377297	2.370639	2.364153	2.35781	2.387411	2.381006
2.371428	2.364404	2.381618	2.374854	2.36822	2.397863	2.391295	2.384866	2.378577
2.368824	2.361848	2.379101	2.372427	2.365873	2.39546	2.388885	2.382513	2.376329
2.36622	2.359278	2.376558	2.369962	2.363486	2.393043	2.386497	2.38019	2.374101
2.363564	2.356653	2.387513	2.380756	2.37414	2.390456	2.384007	2.377781	2.371761
2.360905	2.354025	2.384819	2.378056	2.371456	2.387839	2.381517	2.375376	2.369413
2.382344	2.375491	2.405121	2.398375	2.391794	2.385391	2.379158	2.373091	2.367192
2.37978	2.372994	2.402609	2.395922	2.3894	2.383063	2.376899	2.370899	2.365063
2.390921	2.384013	2.400107	2.393499	2.387054	2.380784	2.374682	2.368748	2.362983
2.388328	2.381473	2.397609	2.391107	2.384767	2.378561	2.372507	2.366641	2.360957
2.40861	2.401788	2.395148	2.388731	2.382479	2.376351	2.37037	2.364569	2.358943
2.406042	2.399299	2.392728	2.386342	2.380135	2.374121	2.368277	2.36254	2.356921
2.403516	2.396853	2.390352	2.383997	2.377832	2.371924	2.366204	2.360524	2.354908
2.401078	2.394472	2.388035	2.381766	2.375678	2.369812	2.364117	2.358479	2.352915
2.398688	2.392133	2.385763	2.379602	2.373606	2.367751	2.362037	2.35643	2.350935
2.396233	2.389773	2.383497	2.37742	2.371497	2.36569	2.360007	2.354427	2.348953
2.393727	2.387399	2.381243	2.375234	2.369369	2.363636	2.358021	2.352463	2.346972
2.391376	2.3851	2.379005	2.373068	2.367271	2.361588	2.356012	2.350479	2.345001
2.389235	2.382882	2.376756	2.370909	2.365211	2.359546	2.35396	2.34846	2.343046
2.387129	2.380746	2.37461	2.368812	2.363164	2.357512	2.351926	2.346453	2.341086
2.384987	2.378742	2.372673	2.366819	2.361103	2.355475	2.349945	2.344486	2.339102

2.382831	2.376774	2.370804	2.364882	2.359078	2.353464	2.347984	2.342531	2.337122
2.380611	2.374703	2.368845	2.362972	2.357179	2.351534	2.345999	2.340547	2.335181
2.378365	2.372583	2.366843	2.361081	2.355355	2.349655	2.344017	2.338561	2.333266
2.376227	2.370481	2.364788	2.359105	2.353447	2.347766	2.342122	2.336661	2.331358
2.374191	2.368385	2.362667	2.357041	2.351464	2.345875	2.340318	2.334848	2.329455
2.372211	2.366382	2.360647	2.35502	2.349463	2.343944	2.338473	2.333027	2.327608
2.370309	2.364537	2.358804	2.353076	2.347428	2.341935	2.336547	2.331181	2.325851
2.368389	2.362681	2.356965	2.351161	2.345438	2.33995	2.334607	2.329316	2.324091
2.366341	2.360681	2.355017	2.349274	2.343591	2.338071	2.332672	2.3274	2.322254
2.364265	2.358629	2.353018	2.347384	2.341788	2.336237	2.330759	2.325484	2.320391
2.362302	2.356598	2.350965	2.345401	2.339889	2.334388	2.328944	2.323669	2.318545
2.36041	2.354577	2.348871	2.343354	2.337931	2.332537	2.3272	2.321925	2.31671
2.3585	2.352629	2.346896	2.341371	2.335965	2.330648	2.325411	2.320169	2.314934
2.35657	2.350767	2.345052	2.339448	2.333974	2.328706	2.323568	2.318399	2.313225
2.354631	2.348912	2.343237	2.337587	2.332067	2.326827	2.321751	2.316643	2.311536
2.352689	2.347049	2.341438	2.335815	2.330308	2.325073	2.319999	2.314923	2.309874
2.350727	2.345166	2.339629	2.334064	2.328595	2.323352	2.31825	2.313191	2.30819
2.348702	2.34321	2.337747	2.332261	2.326849	2.321588	2.316438	2.31138	2.306418
2.346668	2.341231	2.33584	2.330447	2.325104	2.319822	2.314611	2.309541	2.304599
2.344764	2.339355	2.334004	2.328679	2.32339	2.318104	2.312861	2.336905	2.331831
2.342967	2.33756	2.332225	2.326947	2.321702	2.316427	2.311171	2.335117	2.32995
2.341159	2.335733	2.3304	2.325168	2.319973	2.314729	2.309492	2.333372	2.328152
2.33932	2.333848	2.3285	2.323315	2.318185	2.313002	2.307822	2.331679	2.326461
2.337503	2.331997	2.326638	2.321485	2.316403	2.340419	2.335205	2.356245	2.35095
2.335729	2.330228	2.324873	2.319725	2.314656	2.338663	2.333493	2.354534	2.349253
2.33398	2.328497	2.323152	2.317999	2.312929	2.336918	2.331784	2.352825	2.347563
2.332249	2.326776	2.321439	2.316288	2.311225	2.335185	2.330075	2.35111	2.345867
2.330533	2.32507	2.319741	2.314597	2.309541	2.333466	2.32837	2.349396	2.344174
2.3288	2.323351	2.318037	2.31291	2.307872	2.331765	2.326686	2.347702	2.342502
2.327053	2.321622	2.31633	2.31123	2.306219	2.330083	2.325022	2.34603	2.340849
2.325303	2.319888	2.343682	2.33853	2.333463	2.3284	2.323362	2.344361	2.339201
2.323547	2.318143	2.341909	2.336782	2.331743	2.326709	2.321701	2.342695	2.337558
2.321798	2.316407	2.340145	2.33504	2.330028	2.325023	2.320044	2.341029	2.335914
2.320068	2.314696	2.338404	2.333318	2.328327	2.349497	2.344442	2.339359	2.334257
2.318346	2.312998	2.33668	2.331611	2.326637	2.347796	2.34276	2.337691	2.332599
2.316613	2.3113	2.334967	2.329917	2.324958	2.346106	2.34109	2.336035	2.33095
2.314877	2.309609	2.333271	2.328239	2.323295	2.344427	2.339429	2.334386	2.329308
2.342091	2.336803	2.331639	2.326625	2.321684	2.342783	2.337777	2.332735	2.327665
2.340372	2.335178	2.330081	2.325085	2.320138	2.341182	2.336137	2.331082	2.326021
2.338709	2.333579	2.328524	2.323534	2.318575	2.339566	2.334492	2.329429	2.324376
2.337136	2.33201	2.326949	2.321945	2.316969	2.337914	2.332836	2.327775	2.322728
2.335576	2.330441	2.325362	2.320339	2.315346	2.33625	2.331177	2.326123	2.321085
2.333969	2.328836	2.323752	2.318716	2.313711	2.334582	2.329513	2.324467	2.319443
2.332336	2.32721	2.322128	2.317083	2.312069	2.332912	2.327851	2.322821	2.317822
2.330666	2.325559	2.346611	2.341462	2.336349	2.331262	2.326213	2.321225	2.316291
2.328963	2.323885	2.344929	2.339798	2.334702	2.329627	2.324597	2.31967	2.314839
2.327277	2.322228	2.343261	2.338148	2.333071	2.328024	2.323028	2.348889	2.343948
2.325606	2.320582	2.341598	2.336498	2.331444	2.326451	2.321521	2.347374	2.342444
2.323974	2.318986	2.339997	2.334918	2.329884	2.324921	2.32002	2.345843	2.340911
2.322392	2.317476	2.33852	2.333486	2.32847	2.323471	2.318508	2.344251	2.339317

2.320861	2.316003	2.337071	2.332073	2.327066	2.322021	2.316986	2.342645	2.337707
2.319424	2.31453	2.335547	2.330529	2.32551	2.320466	2.31544	2.341063	2.33612
2.318037	2.313059	2.333985	2.328916	2.323872	2.318854	2.313878	2.339493	2.334551
2.316539	2.311529	2.332397	2.327296	2.322235	2.317238	2.312308	2.337932	2.333043
2.314943	2.30994	2.330778	2.325657	2.320584	2.315607	2.310721	2.336376	2.331595
2.339365	2.334289	2.329216	2.324108	2.319048	2.314095	2.309237	2.334885	2.330149
2.337761	2.332753	2.327748	2.322707	2.317701	2.312775	2.30792	2.333499	2.328702
2.336203	2.331235	2.326277	2.321297	2.316344	2.311443	2.306591	2.332102	2.32724
2.334756	2.329731	2.324739	2.319767	2.314834	2.309947	2.305108	2.352945	2.348071
2.33333	2.328223	2.323175	2.318203	2.313288	2.308419	2.303602	2.351467	2.3466
2.331772	2.326643	2.321599	2.316677	2.311823	2.307008	2.302241	2.350135	2.345268
2.330141	2.325024	2.320018	2.315171	2.310402	2.305663	2.300963	2.34889	2.344023
2.328639	2.323527	2.318531	2.3137	2.308952	2.357221	2.352383	2.377136	2.372202
2.327283	2.322169	2.317154	2.312273	2.307476	2.355733	2.350892	2.375705	2.370796
2.325859	2.320754	2.315733	2.310817	2.305986	2.354246	2.349416	2.374301	2.369428
2.324265	2.31919	2.314192	2.309278	2.304456	2.352765	2.347989	2.372975	2.36816
2.322676	2.317636	2.312669	2.307774	2.302974	2.35132	2.346584	2.371661	2.366893
2.321247	2.31624	2.335956	2.33109	2.3263	2.369145	2.364336	2.370305	2.36554
2.319863	2.314888	2.33467	2.329842	2.32507	2.36788	2.36301	2.368955	2.364174
2.318328	2.313378	2.333235	2.328467	2.323752	2.366598	2.361742	2.367708	2.362935
2.316686	2.311757	2.331694	2.327004	2.322376	2.365311	2.360522	2.366541	2.36179
2.339982	2.335071	2.350871	2.346158	2.341514	2.363966	2.359215	2.365271	2.36054
2.338709	2.333803	2.349626	2.344853	2.340148	2.362533	2.35778	2.363855	2.359142
2.337453	2.332558	2.348412	2.343592	2.338836	2.361159	2.3564	2.362488	2.357791
2.33618	2.331311	2.347222	2.342412	2.337661	2.359952	2.355189	2.361286	2.356599
2.33486	2.330021	2.345995	2.341214	2.336485	2.358758	2.353996	2.360103	2.355427
2.354243	2.349393	2.344595	2.33984	2.335136	2.357402	2.352656	2.35879	2.354135
2.352726	2.347894	2.343118	2.338391	2.333716	2.355985	2.351264	2.357432	2.352801
2.351381	2.346561	2.341799	2.337088	2.332434	2.354698	2.350007	2.356199	2.351581
2.350184	2.34537	2.340613	2.33591	2.331267	2.353523	2.348867	2.355074	2.350459
2.349007	2.344204	2.339455	2.334754	2.330116	2.352358	2.347729	2.353952	2.349346
2.347839	2.343057	2.338322	2.33362	2.32898	2.351206	2.346595	2.352839	2.348257
2.34667	2.341907	2.337182	2.332474	2.327825	2.350023	2.345413	2.351671	2.347107
2.345506	2.340748	2.336018	2.331288	2.326611	2.348748	2.344104	2.350351	2.345789
2.344307	2.339553	2.334817	2.330075	2.325377	2.347456	2.342777	2.349008	2.344441
2.342967	2.338227	2.333516	2.328816	2.324162	2.346245	2.341588	2.357664	2.353077
2.341541	2.336823	2.332153	2.327531	2.322959	2.345085	2.340483	2.35661	2.352016
2.340248	2.33555	2.330909	2.32633	2.321804	2.343941	2.339375	2.35555	2.350962
2.339087	2.334408	2.329784	2.325216	2.3207	2.342813	2.338265	2.354489	2.349924
2.337956	2.333291	2.32868	2.324123	2.319615	2.352222	2.347669	2.353431	2.348881
2.336842	2.33218	2.327577	2.323039	2.318551	2.351174	2.346618	2.352375	2.347819
2.335739	2.331081	2.326486	2.321965	2.317492	2.350131	2.345573	2.351326	2.346763
2.334634	2.329991	2.325407	2.320889	2.316419	2.349072	2.344527	2.350291	2.345737
2.333529	2.328907	2.324334	2.319816	2.315344	2.34801	2.343481	2.349262	2.344719
2.332409	2.327808	2.334835	2.330312	2.32583	2.346961	2.342434	2.348219	2.343679
2.331278	2.326701	2.333785	2.329281	2.324813	2.345921	2.341386	2.347166	2.342624
2.33016	2.325595	2.332724	2.328235	2.323778	2.355276	2.350684	2.346113	2.341562
2.329059	2.324491	2.331647	2.327167	2.322722	2.354216	2.349637	2.345064	2.340499
2.339694	2.335113	2.330582	2.326107	2.321669	2.353153	2.34858	2.343998	2.33941
2.338624	2.334063	2.329546	2.325075	2.320632	2.352086	2.347501	2.342891	2.338261

2.33755	2.333013	2.32851	2.324043	2.319597	2.351018	2.34642	2.341783	2.337115
2.336465	2.331936	2.327443	2.322987	2.318552	2.349957	2.34536	2.340716	2.336033
2.335373	2.330848	2.326361	2.32192	2.3175	2.348897	2.344309	2.339671	2.334993
2.334289	2.329772	2.325291	2.320852	2.316437	2.347822	2.343251	2.338642	2.334003
2.33321	2.328705	2.324229	2.319781	2.315361	2.346732	2.342189	2.337631	2.333066
2.332129	2.327636	2.323169	2.318718	2.314297	2.345647	2.34112	2.336595	2.332078
2.331041	2.326566	2.322113	2.31767	2.313252	2.344572	2.340042	2.335521	2.331014
2.329959	2.325496	2.321052	2.316617	2.312204	2.343496	2.338963	2.352879	2.348239
2.328888	2.324421	2.319975	2.315545	2.311139	2.342413	2.337887	2.351776	2.347151
2.327826	2.323349	2.318898	2.314471	2.310072	2.341328	2.336811	2.35067	2.346061
2.326764	2.322282	2.317831	2.31341	2.309017	2.340245	2.335725	2.349546	2.344941
2.325715	2.321227	2.316776	2.312358	2.307968	2.339162	2.334636	2.34841	2.343803
2.324778	2.320253	2.327151	2.322645	2.318173	2.338078	2.333557	2.347288	2.342678
2.323961	2.319362	2.326184	2.321618	2.317104	2.336989	2.332489	2.346181	2.341564
2.323086	2.318462	2.325248	2.320639	2.316085	2.335934	2.331431	2.345073	2.34045
2.322101	2.317544	2.324368	2.319738	2.315144	2.334915	2.330363	2.34394	2.339316
2.321062	2.316579	2.323457	2.318837	2.31423	2.333942	2.329347	2.342855	2.338217
2.319971	2.315515	2.322434	2.317881	2.313335	2.351321	2.34661	2.341907	2.337213
2.31885	2.314401	2.321355	2.316888	2.312429	2.350426	2.345732	2.341007	2.336253
2.317755	2.313303	2.320264	2.315828	2.311405	2.349385	2.34472	2.340016	2.335277
2.316677	2.312211	2.319156	2.314713	2.310294	2.348239	2.343616	2.338966	2.334295
2.315693	2.311202	2.318112	2.313646	2.309211	2.347099	2.342495	2.337877	2.33325
2.314831	2.310292	2.317141	2.312624	2.308148	2.345955	2.341345	2.336734	2.332129
2.31393	2.309377	2.335565	2.33091	2.326292	2.344871	2.340238	2.335616	2.331011
2.312914	2.308423	2.334626	2.329994	2.325379	2.343906	2.339214	2.334548	2.329905
2.311875	2.307447	2.333672	2.329075	2.324474	2.342965	2.338229	2.333524	2.328844
2.310875	2.306426	2.332599	2.32802	2.323449	2.341968	2.337271	2.332585	2.327907
2.309896	2.305394	2.331483	2.326914	2.322375	2.340945	2.336322	2.331681	2.327026
2.334421	2.329803	2.353872	2.349184	2.34453	2.349373	2.344725	2.340075	2.335427
2.333518	2.328941	2.352956	2.348221	2.343508	2.348288	2.343594	2.338945	2.33434
2.343394	2.338795	2.361553	2.356774	2.352014	2.347274	2.342564	2.33792	2.333336
2.342401	2.337774	2.360503	2.35577	2.351062	2.346366	2.341691	2.337059	2.332468
2.359642	2.354889	2.359477	2.354775	2.350104	2.345438	2.340788	2.336164	2.331563
2.358688	2.353961	2.358546	2.35381	2.349098	2.344401	2.339728	2.335098	2.330508
2.357744	2.353048	2.357627	2.35285	2.348088	2.343352	2.338646	2.334005	2.329423
2.356742	2.352039	2.35662	2.351864	2.347123	2.3424	2.3377	2.333042	2.328424
2.355715	2.350985	2.355571	2.350865	2.34618	2.341497	2.336821	2.332145	2.327471
2.354743	2.350009	2.354589	2.349884	2.345202	2.340524	2.335855	2.331188	2.326523
2.353825	2.349105	2.353671	2.348918	2.344186	2.33948	2.334803	2.330171	2.325581
2.352916	2.3482	2.352751	2.347968	2.343206	2.338489	2.333812	2.329206	2.324667
2.352022	2.34728	2.351817	2.347038	2.342289	2.337591	2.332937	2.328342	2.323804
2.351098	2.346347	2.350884	2.346119	2.341388	2.336707	2.332067	2.32747	2.322917
2.350072	2.345375	2.349956	2.345209	2.340482	2.335789	2.331126	2.326503	2.321918
2.349028	2.344398	2.349032	2.344302	2.339573	2.334862	2.330168	2.325513	2.320895
2.357491	2.352811	2.34812	2.343384	2.338649	2.333937	2.329244	2.344882	2.340035
2.356643	2.351934	2.347214	2.342459	2.337714	2.333012	2.328344	2.343968	2.339146
2.355769	2.351028	2.346285	2.341523	2.336784	2.332103	2.327463	2.343061	2.338249
2.35487	2.350089	2.345325	2.340572	2.335855	2.331207	2.326604	2.342166	2.337346
2.34264	2.337885	2.344377	2.339643	2.334952	2.330329	2.325748	2.34126	2.336421
2.341707	2.336958	2.343457	2.338758	2.334098	2.329484	2.324897	2.340339	2.335459

2.340767	2.33604	2.342554	2.337887	2.333251	2.328634	2.324027	2.339394	2.334479
2.339838	2.335139	2.341668	2.337012	2.332376	2.327736	2.323098	2.338401	2.333489
2.33892	2.334255	2.340795	2.336137	2.331485	2.326813	2.322138	2.337385	2.332496
2.33804	2.333394	2.339929	2.335243	2.33056	2.325875	2.321195	2.336407	2.331537
2.337194	2.332556	2.33907	2.334334	2.329606	2.32492	2.320262	2.335457	2.330608
2.336352	2.331713	2.338208	2.333448	2.328699	2.324007	2.319353	2.35465	2.349765
2.335513	2.330868	2.337346	2.332601	2.327861	2.323154	2.318473	2.353737	2.34886
2.334669	2.330004	2.325336	2.320655	2.315978	2.3223	2.317605	2.352834	2.347944
2.333805	2.329094	2.324399	2.31973	2.315076	2.321414	2.316742	2.351941	2.346995
2.332945	2.328185	2.323462	2.3188	2.314167	2.320527	2.315889	2.351064	2.346056
2.332121	2.327347	2.322611	2.317938	2.313303	2.360136	2.355204	2.35022	2.345194
2.331318	2.326547	2.321808	2.317116	2.312464	2.359285	2.35437	2.349396	2.344379
2.330497	2.325729	2.320989	2.316288	2.31163	2.35843	2.353525	2.348559	2.343547
2.329662	2.324897	2.320158	2.315458	2.310801	2.357572	2.352671	2.34771	2.342704
2.328833	2.324069	2.342535	2.337749	2.333012	2.356723	2.351825	2.346869	2.34187
2.328014	2.323252	2.341748	2.336963	2.332227	2.355886	2.350994	2.346045	2.341053
2.327194	2.322432	2.340958	2.336174	2.331441	2.355048	2.350162	2.34522	2.340235
2.326363	2.321603	2.340151	2.33537	2.330639	2.345011	2.340168	2.344379	2.3394
2.32553	2.320771	2.33934	2.334562	2.329834	2.344165	2.339329	2.343538	2.338562
2.315126	2.310432	2.317252	2.312588	2.307973	2.343335	2.338506	2.342711	2.337737
2.314356	2.30966	2.316479	2.31182	2.307209	2.342515	2.337695	2.341895	2.336921
2.325155	2.320415	2.315708	2.311047	2.306435	2.34169	2.336885	2.341086	2.336117
2.324365	2.319633	2.314931	2.31026	2.305641	2.340854	2.336078	2.340284	2.335322
2.323598	2.318891	2.314205	2.309523	2.304891	2.340044	2.335276	2.339489	2.334552
2.32288	2.318231	2.313579	2.308884	2.304223	2.339279	2.334482	2.338705	2.333831
2.322159	2.317569	2.312952	2.308254	2.30357	2.338527	2.333689	2.337916	2.3331
2.321373	2.316819	2.312231	2.307551	2.302873	2.337759	2.332886	2.328069	2.32331
2.320573	2.316035	2.311468	2.30682	2.302164	2.336993	2.332085	2.327252	2.322489
2.319846	2.315282	2.310712	2.306104	2.301486	2.336263	2.331318	2.32646	2.321678
2.319175	2.31455	2.309955	2.305397	2.300832	2.335562	2.330579	2.325688	2.32087
2.318537	2.313861	2.309234	2.304686	2.300137	2.334825	2.329829	2.324916	2.320068
2.31795	2.313236	2.308564	2.303973	2.299391	2.334036	2.329055	2.324136	2.319263
2.317317	2.312583	2.307883	2.303248	2.298634	2.333247	2.328305	2.32339	2.318497
2.316551	2.311834	2.307142	2.302487	2.297865	2.332481	2.32762	2.322724	2.317802
2.315772	2.311081	2.306408	2.30174	2.297113	2.331724	2.326936	2.322065	2.317133
2.315114	2.310429	2.305755	2.301075	2.296431	2.330964	2.326161	2.321318	2.316451
2.314517	2.309828	2.305148	2.300459	2.295796	2.330212	2.325346	2.32053	2.315764
2.313904	2.309208	2.304523	2.299844	2.295185	2.329525	2.324607	2.330088	2.325328
2.313288	2.308578	2.303889	2.299241	2.294606	2.328901	2.323934	2.32937	2.324554
2.312618	2.307903	2.303215	2.298578	2.293958	2.32823	2.323267	2.328681	2.323818
2.311858	2.307153	2.30247	2.297817	2.293193	2.327476	2.322605	2.328039	2.323143
2.311115	2.306421	2.301743	2.29707	2.29244	2.326716	2.321923	2.327384	2.322482
2.310467	2.305777	2.32076	2.315868	2.311014	2.336326	2.331498	2.326655	2.32181
2.309855	2.305166	2.320126	2.315233	2.310367	2.335621	2.330747	2.325921	2.321143
2.309231	2.304533	2.330556	2.325652	2.320781	2.334986	2.33012	2.325291	2.3205
2.308608	2.303894	2.329879	2.324995	2.320154	2.334398	2.329568	2.324723	2.319873
2.307988	2.303271	2.329224	2.324339	2.319499	2.333737	2.328916	2.324076	2.319228
2.307372	2.302665	2.328593	2.323682	2.318809	2.332991	2.328149	2.323339	2.318565
2.318613	2.313885	2.327959	2.32304	2.318153	2.332297	2.327439	2.322641	2.317903
2.317972	2.31325	2.32731	2.322418	2.317559	2.331714	2.32686	2.322037	2.317246

2.317334	2.31262	2.326668	2.321809	2.316982	2.331152	2.3263	2.321448	2.3166
2.316714	2.312013	2.326049	2.32121	2.316398	2.330548	2.325678	2.320822	2.315979
2.316108	2.311423	2.325447	2.32062	2.315811	2.329928	2.325035	2.320184	2.315368
2.315526	2.310861	2.32487	2.32003	2.315202	2.329294	2.324395	2.319542	2.31473
2.314965	2.310324	2.324316	2.31944	2.314573	2.328646	2.323752	2.318894	2.314072
2.314397	2.309759	2.323718	2.318826	2.313942	2.328	2.323103	2.318244	2.313422
2.31382	2.309158	2.323063	2.318181	2.313311	2.327357	2.322442	2.317586	2.312782
2.313239	2.308553	2.32241	2.317531	2.312671	2.326712	2.321796	2.330866	2.326017
2.312653	2.307951	2.321772	2.316871	2.311998	2.326048	2.321175	2.33024	2.325374
2.312067	2.307361	2.321156	2.316235	2.311348	2.325404	2.320576	2.329637	2.324756
2.311486	2.306806	2.3206	2.31569	2.310807	2.324847	2.320009	2.329068	2.324209
2.310909	2.306269	2.320072	2.315189	2.310319	2.324333	2.319461	2.328518	2.323698
2.310331	2.305686	2.319468	2.314606	2.309755	2.323757	2.318868	2.327919	2.32312
2.309752	2.305062	2.318794	2.313951	2.309127	2.323129	2.318236	2.327276	2.322484
2.309199	2.304483	2.318175	2.313334	2.30852	2.322522	2.31764	2.326671	2.321876
2.308694	2.303987	2.317654	2.312789	2.307956	2.321961	2.31711	2.326135	2.321322
2.308163	2.303471	2.317122	2.312236	2.307384	2.321389	2.316566	2.325584	2.320752
2.307532	2.302854	2.316504	2.311615	2.306758	2.320758	2.315948	2.324949	2.320103
2.306885	2.30222	2.31587	2.310988	2.306133	2.320126	2.315319	2.324302	2.319446
2.306337	2.301664	2.315298	2.310427	2.305584	2.319571	2.31476	2.32373	2.318872
2.305849	2.301157	2.314765	2.30991	2.305085	2.319064	2.314244	2.323203	2.31835
2.305356	2.300659	2.31425	2.309399	2.304579	2.318552	2.313731	2.322681	2.317832
2.304862	2.300175	2.313756	2.308897	2.304068	2.318035	2.313221	2.322167	2.317322
2.342147	2.337173	2.332222	2.327281	2.322373	2.331376	2.326506	2.321648	2.316807
2.34161	2.336646	2.331706	2.326776	2.321875	2.330863	2.325984	2.321124	2.316284
2.341074	2.336117	2.331185	2.326266	2.321372	2.330345	2.325456	2.320591	2.315752
2.34055	2.33559	2.330655	2.325736	2.320844	2.329807	2.324919	2.320054	2.315212
2.340032	2.335064	2.330122	2.325199	2.320304	2.329256	2.324367	2.319501	2.314657
2.339513	2.334544	2.329598	2.324671	2.319765	2.328682	2.323763	2.318888	2.314053
2.338995	2.334028	2.329081	2.324149	2.319227	2.328091	2.323119	2.31823	2.313411
2.338473	2.33351	2.328567	2.323637	2.318717	2.327566	2.322588	2.317694	2.312872
2.337944	2.332987	2.328055	2.32314	2.318243	2.327129	2.322203	2.317316	2.312465
2.337421	2.332466	2.327541	2.322639	2.317762	2.326678	2.321796	2.316918	2.312045
2.33691	2.331946	2.327018	2.322123	2.317256	2.326167	2.32129	2.316419	2.311556
2.336401	2.33143	2.326496	2.321605	2.316742	2.32564	2.320757	2.315892	2.311045
2.335884	2.330917	2.325987	2.321094	2.31623	2.325122	2.320244	2.315379	2.310527
2.335364	2.330409	2.325485	2.320589	2.31572	2.324606	2.319733	2.314864	2.310002
2.334853	2.329904	2.324984	2.320089	2.31522	2.324091	2.319213	2.314342	2.309481
2.334347	2.329401	2.324484	2.319596	2.314728	2.323577	2.318683	2.313812	2.308964
2.333894	2.328943	2.337888	2.332954	2.328039	2.323116	2.3182	2.313318	2.308464
2.333525	2.328554	2.337485	2.332573	2.327675	2.322736	2.31779	2.312873	2.307979
2.333137	2.328156	2.337076	2.332174	2.327283	2.322338	2.317376	2.312439	2.30752
2.332652	2.327691	2.336608	2.331685	2.326778	2.321847	2.316915	2.312004	2.30711
2.332146	2.327216	2.336134	2.331181	2.326246	2.321331	2.316436	2.311561	2.306705
2.331706	2.326803	2.335721	2.330748	2.325786	2.320852	2.315941	2.311066	2.306225
2.3313	2.32642	2.335339	2.330351	2.325363	2.320391	2.315439	2.310546	2.305706
2.330864	2.32598	2.334888	2.329909	2.324929	2.319954	2.314995	2.310099	2.305261
2.330396	2.325479	2.334364	2.32942	2.324485	2.319543	2.31461	2.309727	2.304887
2.329965	2.325019	2.333883	2.328961	2.324057	2.319137	2.314223	2.309354	2.304521
2.329598	2.324635	2.333479	2.328559	2.323659	2.318738	2.313824	2.30897	2.304165

2.32924	2.324274	2.333104	2.328173	2.323262	2.31833	2.313409	2.308561	2.303777
2.328862	2.323924	2.332754	2.327793	2.322844	2.317888	2.312949	2.308085	2.303288
2.328467	2.323566	2.3324	2.327407	2.322416	2.317437	2.31248	2.307593	2.302769
2.341856	2.3369	2.331944	2.326959	2.321979	2.317013	2.312067	2.30718	2.302344
2.341348	2.336369	2.331415	2.326468	2.321535	2.316607	2.311695	2.306821	2.301983
2.340939	2.335918	2.330946	2.326021	2.321122	2.316215	2.311319	2.306458	2.301627
2.340655	2.335563	2.330543	2.325627	2.320746	2.315837	2.310936	2.306086	2.301279
2.340339	2.335214	2.330169	2.32525	2.320372	2.315464	2.310566	2.305722	2.300927
2.33991	2.33484	2.329828	2.324889	2.319987	2.315094	2.310223	2.305378	2.30056
2.339475	2.334465	2.329489	2.324531	2.319606	2.314727	2.309879	2.305032	2.300189
2.339136	2.334096	2.329102	2.324154	2.319243	2.314358	2.309501	2.304655	2.299824
2.338832	2.33373	2.328699	2.323772	2.318886	2.313989	2.309108	2.304266	2.299459
2.338452	2.333365	2.32834	2.323403	2.318505	2.31361	2.308734	2.303894	2.299085
2.33801	2.333	2.328017	2.323044	2.318105	2.313221	2.308375	2.303534	2.298703
2.337642	2.332675	2.327717	2.322733	2.317772	2.312871	2.308008	2.303158	2.298324
2.337388	2.332414	2.327454	2.322496	2.317543	2.312574	2.307616	2.302744	2.297947
2.337123	2.332134	2.327169	2.322239	2.317302	2.312289	2.307265	2.310255	2.305429
2.336776	2.331779	2.326815	2.321903	2.316991	2.312013	2.30702	2.30998	2.305066
2.336402	2.331402	2.326442	2.321538	2.316645	2.311725	2.306794	2.309733	2.304725
2.336022	2.331046	2.326096	2.321166	2.316256	2.311365	2.306483	2.309443	2.304446
2.335642	2.330701	2.325765	2.320792	2.315842	2.310966	2.306124	2.30913	2.304203
2.335342	2.330403	2.325465	2.320485	2.315523	2.310621	2.305754	2.308774	2.303898
2.335128	2.330157	2.325201	2.320249	2.3153	2.310328	2.305364	2.308369	2.303534
2.334878	2.329877	2.324904	2.319975	2.315043	2.31004	2.305027	2.308012	2.303175
2.33455	2.329522	2.324537	2.319623	2.314718	2.309756	2.304782	2.307742	2.302823
2.33422	2.329178	2.324183	2.319264	2.314367	2.309455	2.304542	2.30749	2.3025
2.333943	2.328918	2.323915	2.318935	2.313988	2.317046	2.312155	2.307215	2.302239
2.333674	2.328679	2.323675	2.318624	2.313611	2.316681	2.311835	2.306938	2.302011
2.315211	2.31033	2.305437	2.300484	2.295566	2.316392	2.31155	2.306672	2.301776
2.31485	2.309993	2.305135	2.300225	2.295341	2.316156	2.311286	2.306411	2.301543
2.314559	2.309709	2.313494	2.308577	2.303675	2.315899	2.310988	2.306111	2.301269
2.314349	2.309482	2.313254	2.308344	2.30344	2.315615	2.310645	2.305758	2.300944
2.314149	2.309269	2.31303	2.308123	2.303215	2.315354	2.31034	2.305443	2.300644
2.313941	2.309063	2.312824	2.307918	2.30301	2.315137	2.310115	2.305207	2.300398
2.313731	2.308854	2.312616	2.307714	2.30281	2.314937	2.309916	2.305002	2.300177
2.313511	2.30863	2.312394	2.307506	2.302615	2.314739	2.309715	2.304798	2.299969
2.313283	2.30839	2.312153	2.307282	2.302411	2.31454	2.309518	2.304602	2.299772
2.313037	2.308104	2.311847	2.30699	2.302143	2.314297	2.309305	2.304402	2.299572
2.312773	2.307777	2.311484	2.306635	2.301819	2.314018	2.30908	2.3042	2.29937
2.321247	2.316177	2.311189	2.306334	2.301525	2.31375	2.308853	2.30399	2.299158
2.321047	2.315975	2.310977	2.306095	2.301266	2.313493	2.308622	2.303768	2.298933
2.320858	2.315808	2.310815	2.305909	2.301051	2.313262	2.308393	2.303538	2.298701
2.320678	2.315685	2.310723	2.305805	2.300918	2.313084	2.308171	2.303297	2.29846
2.320495	2.315562	2.310639	2.305716	2.300803	2.312919	2.307951	2.303052	2.298214
2.320283	2.315371	2.310461	2.305543	2.300626	2.312724	2.307725	2.302813	2.297974
2.320053	2.315138	2.31023	2.305325	2.300417	2.312514	2.307499	2.302578	2.297737
2.319822	2.314907	2.310004	2.305107	2.300207	2.312303	2.30728	2.321515	2.316592
2.319586	2.314673	2.309776	2.304886	2.299992	2.312088	2.307065	2.321279	2.31634
2.319364	2.314444	2.309547	2.304662	2.299779	2.311891	2.306891	2.321094	2.316139
2.31915	2.314209	2.309304	2.304426	2.299565	2.311728	2.306787	2.320999	2.316023

2.31897	2.314009	2.30909	2.304209	2.299358	2.311558	2.306668	2.320889	2.315902
2.318861	2.313889	2.308949	2.304035	2.299152	2.311339	2.306451	2.320673	2.31571
2.318782	2.313802	2.30884	2.303886	2.298964	2.311118	2.306212	2.320427	2.315492
2.318679	2.313678	2.308703	2.303751	2.298831	2.310977	2.306053	2.320238	2.315283
2.318564	2.313537	2.308554	2.303631	2.298741	2.310888	2.305945	2.320082	2.315079
2.318429	2.31341	2.308436	2.303526	2.298644	2.329994	2.324958	2.319933	2.314919
2.318274	2.313305	2.308359	2.303442	2.29854	2.329852	2.324802	2.319789	2.31481
2.3181	2.313165	2.308243	2.303329	2.298423	2.329716	2.324664	2.319663	2.314707
2.317887	2.312947	2.308036	2.303151	2.298278	2.329592	2.324564	2.31957	2.314604
2.317692	2.312742	2.307835	2.302977	2.298138	2.329476	2.324472	2.319478	2.314493
2.317585	2.312637	2.307731	2.302876	2.298039	2.329373	2.324365	2.31936	2.314355
2.317524	2.312582	2.307674	2.302808	2.297957	2.329273	2.324251	2.319228	2.314205
2.317461	2.312523	2.327803	2.322853	2.317916	2.329151	2.324113	2.319089	2.314076
2.317403	2.312469	2.327745	2.322773	2.317808	2.329011	2.323957	2.318942	2.313962
2.317342	2.312403	2.327663	2.322667	2.31768	2.328877	2.32383	2.318828	2.313866
2.317278	2.312324	2.327551	2.322526	2.317523	2.328746	2.323742	2.318755	2.313787
2.308568	2.303584	2.327424	2.322387	2.317385	2.328633	2.323662	2.318689	2.31372
2.308477	2.303446	2.327264	2.32226	2.317298	2.328553	2.323578	2.318615	2.313668
2.308389	2.303319	2.327122	2.322154	2.317229	2.328484	2.3235	2.318544	2.313616
2.308332	2.303287	2.327097	2.322102	2.31715	2.328406	2.323442	2.318486	2.313542
2.308291	2.303301	2.327128	2.32208	2.317072	2.328325	2.323392	2.318431	2.313455
2.317403	2.312418	2.319007	2.313957	2.308948	2.328249	2.32331	2.318346	2.31337
2.317287	2.312247	2.318825	2.313832	2.308881	2.328177	2.323199	2.318235	2.313285
2.317204	2.312147	2.318719	2.31374	2.308802	2.32809	2.323097	2.318132	2.313192
2.31718	2.312174	2.318748	2.313698	2.308689	2.32797	2.323014	2.318048	2.313084
2.317149	2.312198	2.318786	2.313683	2.308614	2.327876	2.322932	2.317962	2.312981
2.317051	2.312134	2.318766	2.313704	2.308653	2.320335	2.315299	2.317844	2.312899
2.316952	2.312047	2.318721	2.313728	2.308724	2.320347	2.315212	2.317737	2.312827
2.316949	2.311992	2.307073	2.302199	2.297324	2.320359	2.31522	2.317718	2.312751
2.317008	2.311956	2.306975	2.302111	2.297272	2.320373	2.315291	2.317758	2.312677
2.317031	2.311962	2.306956	2.302061	2.297202	2.320328	2.315296	2.310217	2.305098
2.317021	2.312019	2.307029	2.302048	2.297102	2.320211	2.315233	2.310206	2.305147
2.316979	2.312037	2.307073	2.30205	2.297052	2.320127	2.315151	2.310156	2.305156
2.31688	2.311972	2.30705	2.302076	2.297103	2.320112	2.31504	2.31003	2.305076
2.316796	2.311893	2.306998	2.302088	2.297161	2.320122	2.314957	2.309917	2.30498
2.316806	2.311841	2.306912	2.302032	2.297146	2.32013	2.314968	2.309898	2.304902
2.316857	2.311806	2.306822	2.301957	2.297108	2.320148	2.31503	2.309931	2.304843
2.316872	2.311813	2.306823	2.301957	2.297115	2.320178	2.315097	2.309991	2.304859
2.316867	2.311859	2.306896	2.302013	2.297152	2.320221	2.315176	2.31008	2.304938
2.299421	2.294624	2.306962	2.302043	2.297146	2.309536	2.304593	2.310132	2.305004
2.299472	2.294675	2.307014	2.302031	2.297077	2.309463	2.304552	2.310134	2.305049
2.299525	2.29473	2.307074	2.302041	2.297043	2.309422	2.30453	2.310143	2.3051
2.29958	2.294798	2.307164	2.302117	2.297102	2.30947	2.304572	2.310188	2.305167
2.299611	2.294845	2.307241	2.302201	2.297187	2.309544	2.304631	2.310242	2.305237
2.299559	2.294806	2.307244	2.302232	2.297242	2.309599	2.304677	2.310294	2.305307
2.299468	2.294726	2.30721	2.302242	2.297288	2.309647	2.304712	2.31034	2.305369
2.299459	2.29472	2.307232	2.302283	2.29734	2.309672	2.304698	2.310324	2.305374
2.299521	2.294775	2.3073	2.30235	2.297395	2.309674	2.304632	2.310245	2.305324
2.29959	2.294839	2.307376	2.302424	2.29746	2.309703	2.304618	2.310221	2.305311
2.299658	2.294906	2.307458	2.302504	2.297535	2.309772	2.304682	2.310279	2.305356

2.299727	2.294975	2.307541	2.302585	2.297617	2.309857	2.304771	2.299806	2.294943
2.299789	2.295036	2.307616	2.302666	2.297704	2.309945	2.304859	2.299885	2.295007
2.299852	2.295097	2.307689	2.302747	2.297794	2.310045	2.304968	2.299989	2.295092
2.299921	2.295155	2.307755	2.30282	2.297885	2.31017	2.305137	2.300166	2.295248
2.3	2.295218	2.30782	2.30289	2.297975	2.310311	2.305344	2.30039	2.295448
2.30014	2.29534	2.290555	2.285774	2.281015	2.293172	2.288322	2.300535	2.295588
2.300352	2.29553	2.290709	2.285871	2.28106	2.293219	2.28838	2.300601	2.295667
2.300521	2.29569	2.290846	2.285968	2.28112	2.293276	2.288436	2.30066	2.295739
2.300603	2.295783	2.290943	2.286055	2.281194	2.29335	2.288496	2.300721	2.295817
2.300661	2.295856	2.29103	2.286154	2.281299	2.293458	2.288584	2.3008	2.295899
2.300718	2.295924	2.291122	2.286293	2.281477	2.293649	2.288747	2.300931	2.295996
2.30078	2.295991	2.291212	2.286443	2.281678	2.293869	2.288939	2.301083	2.296101
2.300901	2.296086	2.291295	2.286537	2.281789	2.294001	2.289074	2.310176	2.305112
2.301066	2.296202	2.291372	2.286593	2.281837	2.294073	2.289171	2.31028	2.305209
2.30122	2.296343	2.291498	2.286706	2.281938	2.29418	2.289279	2.310396	2.305343
2.301355	2.29651	2.291687	2.286892	2.28211	2.294334	2.289396	2.310522	2.305521
2.282693	2.277913	2.291878	2.287074	2.282276	2.294496	2.28954	2.310672	2.305708
2.28289	2.278111	2.292052	2.287219	2.2824	2.294652	2.289728	2.310863	2.305895
2.283095	2.278303	2.292214	2.287358	2.282526	2.29482	2.289933	2.311065	2.306077
2.28328	2.278462	2.292348	2.287508	2.282696	2.29502	2.290137	2.311245	2.306235
2.283453	2.278605	2.29247	2.287664	2.282888	2.295237	2.290339	2.311417	2.306384
2.283606	2.278765	2.273955	2.269191	2.264447	2.278306	2.273552	2.311577	2.306551
2.283737	2.278939	2.274158	2.269394	2.264638	2.278459	2.273678	2.311726	2.306734
2.283894	2.279119	2.27435	2.269576	2.264804	2.278608	2.273829	2.313766	2.308802
2.284088	2.279309	2.274527	2.269722	2.26493	2.278748	2.274014	2.314017	2.309052
2.28429	2.279493	2.274692	2.269868	2.26507	2.260349	2.255672	2.332372	2.327336
2.284483	2.27964	2.274818	2.270023	2.265263	2.260557	2.255889	2.332619	2.327594
2.284686	2.279799	2.27496	2.2702	2.26548	2.260783	2.256113	2.33287	2.327855
2.284931	2.280059	2.275224	2.270443	2.265703	2.261006	2.256343	2.333128	2.328108
2.2852	2.280377	2.275558	2.270729	2.265935	2.261232	2.256579	2.33339	2.328356
2.285427	2.280605	2.275786	2.270959	2.266165	2.265147	2.260503	2.318894	2.313869
2.285608	2.280735	2.275901	2.271128	2.266393	2.265407	2.260754	2.319148	2.314138
2.28582	2.280927	2.276086	2.271331	2.266618	2.26566	2.261004	2.319402	2.314397
2.286096	2.281247	2.276414	2.271598	2.266821	2.265888	2.261251	2.319658	2.314637
2.286376	2.281576	2.276759	2.271891	2.267056	2.266137	2.2615	2.31991	2.314877
2.286616	2.281839	2.277046	2.272211	2.26739	2.266454	2.261746	2.30526	2.300389
2.286843	2.282075	2.277307	2.272537	2.267759	2.266801	2.262007	2.305488	2.300636
2.287075	2.282306	2.28142	2.276677	2.271932	2.26714	2.262343	2.305782	2.300887
2.287308	2.282529	2.281668	2.276937	2.272214	2.267476	2.262734	2.306125	2.301143
2.287538	2.282762	2.281933	2.277199	2.272481	2.267774	2.263072	2.306452	2.30144
2.287752	2.282996	2.282211	2.277456	2.27272	2.268023	2.263346	2.306763	2.30179
2.288018	2.283272	2.282522	2.277753	2.273002	2.268291	2.263608	2.307049	2.302118
2.288383	2.283627	2.2829	2.278139	2.273382	2.268616	2.263858	2.307285	2.302385
2.288776	2.283994	2.283277	2.278528	2.273773	2.268955	2.264128	2.307526	2.302637
2.289144	2.284297	2.283571	2.278827	2.274089	2.269288	2.264474	2.307835	2.302901
2.289498	2.284578	2.283835	2.279088	2.274369	2.269616	2.264857	2.308179	2.303174
2.29397	2.289047	2.284174	2.279388	2.27464	2.269907	2.265189	2.308509	2.303493
2.29428	2.289425	2.284574	2.279717	2.274895	2.270164	2.265482	2.308833	2.303853
2.294569	2.289765	2.284942	2.28006	2.275208	2.270476	2.265806	2.309168	2.304216
2.294817	2.290039	2.285252	2.280416	2.275601	2.270868	2.266181	2.309526	2.304585

2.295106	2.290341	2.285579	2.280786	2.276005	2.271261	2.266541	2.29357	2.288712
2.295497	2.290726	2.285961	2.281172	2.276386	2.271601	2.266832	2.293846	2.289003
2.29593	2.291146	2.286367	2.281571	2.276769	2.271942	2.267122	2.294117	2.28929
2.296355	2.291568	2.286783	2.281984	2.277178	2.272334	2.267494	2.294467	2.289644
2.296784	2.291996	2.287207	2.282409	2.277601	2.272758	2.267917	2.294863	2.29004
2.284413	2.279673	2.287618	2.282825	2.278023	2.273178	2.268332	2.29526	2.290438
2.284814	2.280072	2.28802	2.283239	2.278447	2.273599	2.268745	2.29566	2.290843
2.285204	2.280436	2.288373	2.283603	2.278829	2.273995	2.269155	2.296064	2.291247
2.285573	2.280738	2.288635	2.283876	2.279135	2.274348	2.269559	2.296473	2.291649
2.285953	2.28106	2.288919	2.284163	2.279445	2.2747	2.269964	2.296882	2.29205
2.286376	2.281469	2.289313	2.284538	2.279808	2.27508	2.270372	2.297288	2.292454
2.286818	2.281925	2.289769	2.284966	2.28021	2.27548	2.270781	2.29769	2.292857
2.287258	2.282406	2.27759	2.272826	2.268095	2.275919	2.27119	2.298076	2.293248
2.287697	2.282913	2.278137	2.273368	2.268609	2.276388	2.271598	2.29845	2.29363
2.288116	2.283368	2.278616	2.273849	2.269078	2.276829	2.272002	2.298827	2.29401
2.28851	2.283764	2.279016	2.274259	2.269494	2.277239	2.272396	2.299203	2.294387
2.288903	2.284152	2.279404	2.274656	2.269901	2.277647	2.272801	2.29959	2.294768
2.289301	2.284546	2.279798	2.275055	2.270306	2.278058	2.273214	2.299983	2.295143
2.289702	2.284941	2.280191	2.275451	2.27071	2.27848	2.273653	2.300405	2.295546
2.290105	2.285332	2.28058	2.275846	2.271122	2.278935	2.274157	2.300904	2.296028
2.290521	2.285734	2.280977	2.276246	2.27154	2.279403	2.274686	2.301436	2.296547
2.291003	2.286205	2.281434	2.27668	2.271957	2.267283	2.262639	2.301892	2.297009
2.291544	2.286737	2.281942	2.277143	2.272375	2.267674	2.263017	2.302287	2.29743
2.292038	2.287236	2.282437	2.277626	2.272842	2.268125	2.263453	2.302718	2.297857
2.292456	2.287675	2.282904	2.278128	2.273373	2.26866	2.263975	2.3032	2.298289
2.292897	2.288135	2.283387	2.278644	2.273912	2.2692	2.264498	2.30369	2.298742
2.293426	2.28867	2.283925	2.279177	2.274437	2.269703	2.264978	2.304168	2.299232
2.293969	2.289213	2.284464	2.279706	2.274953	2.2702	2.265457	2.304654	2.299741
2.282792	2.278052	2.284926	2.280182	2.275446	2.270706	2.265974	2.294314	2.289443
2.283231	2.278479	2.285354	2.280633	2.275927	2.271217	2.266513	2.29485	2.289968
2.283745	2.278991	2.285862	2.281145	2.276445	2.271742	2.267043	2.285664	2.280845
2.284331	2.279585	2.286446	2.281716	2.276999	2.272281	2.267567	2.286167	2.281319
2.284909	2.280172	2.275446	2.270726	2.266016	2.272811	2.268077	2.286663	2.281808
2.285473	2.280745	2.276021	2.271289	2.26656	2.273322	2.268558	2.287143	2.282321
2.28601	2.281285	2.276562	2.271826	2.267091	2.27383	2.26905	2.287638	2.282849
2.286474	2.281739	2.277017	2.272297	2.267581	2.27434	2.269582	2.288174	2.283395
2.286932	2.282178	2.277453	2.272753	2.268066	2.274858	2.270135	2.288731	2.283952
2.287519	2.28273	2.277982	2.273279	2.268599	2.263917	2.259245	2.289294	2.284513
2.288192	2.283357	2.27857	2.273852	2.269166	2.264485	2.259819	2.289864	2.285078
2.288781	2.283942	2.27915	2.274428	2.269738	2.265054	2.260387	2.290426	2.285639
2.28927	2.284478	2.279723	2.275008	2.270315	2.265625	2.260947	2.290979	2.286196
2.267466	2.26269	2.280293	2.275584	2.270892	2.266195	2.261509	2.28121	2.276488
2.268092	2.263266	2.280857	2.276148	2.271463	2.266766	2.262076	2.281769	2.277042
2.268718	2.263846	2.281428	2.276714	2.272031	2.267333	2.262644	2.282329	2.277596
2.269232	2.264395	2.259604	2.254874	2.250174	2.267886	2.263205	2.282881	2.278147
2.269716	2.264944	2.260191	2.255439	2.250709	2.268442	2.263767	2.283433	2.278702
2.270324	2.265567	2.260824	2.256071	2.251331	2.269057	2.264346	2.283996	2.279269
2.271027	2.266252	2.261497	2.25676	2.252024	2.269722	2.264939	2.284568	2.279848
2.253574	2.248855	2.262124	2.257398	2.252671	2.247886	2.243093	2.285195	2.280473
2.254139	2.249414	2.262686	2.257967	2.253257	2.248517	2.243777	2.285903	2.281166

2.254695	2.249972	2.263248	2.258525	2.253822	2.249122	2.244434	2.266336	2.261594
2.255251	2.250545	2.263829	2.259081	2.254357	2.249667	2.245002	2.266917	2.262178
2.255829	2.251139	2.264428	2.259656	2.254905	2.250208	2.245544	2.26747	2.262738
2.256496	2.251792	2.292274	2.287369	2.282483	2.277626	2.272799	2.268024	2.263297
2.25721	2.252478	2.292951	2.288049	2.283163	2.278262	2.273377	2.26858	2.263857
2.257857	2.253106	2.293582	2.288687	2.283809	2.278904	2.274008	2.269203	2.264474
2.258443	2.253681	2.294169	2.289286	2.284429	2.279553	2.27469	2.269891	2.265147
2.259059	2.254298	2.294799	2.289919	2.285068	2.280213	2.275372	2.270568	2.265795
2.259739	2.254998	2.295508	2.290617	2.285748	2.280892	2.276052	2.271219	2.266393
2.260406	2.255685	2.296204	2.291301	2.286414	2.281556	2.276716	2.271862	2.266999
2.260999	2.256286	2.29681	2.291905	2.287013	2.282162	2.277334	2.272496	2.267654
2.261582	2.256871	2.297397	2.292493	2.287604	2.28276	2.277944	2.273133	2.268331
2.262248	2.257529	2.281517	2.276682	2.271863	2.283423	2.278604	2.273801	2.269013
2.262969	2.258233	2.282204	2.277377	2.272568	2.284128	2.279298	2.27449	2.269704
2.263685	2.258942	2.282903	2.278079	2.273274	2.284836	2.28	2.27519	2.270403
2.2644	2.259658	2.283617	2.278791	2.273982	2.28555	2.280713	2.275901	2.271112
2.265116	2.260376	2.284333	2.2795	2.274685	2.286256	2.28142	2.276606	2.271816
2.265832	2.261095	2.285045	2.280202	2.275375	2.286951	2.282116	2.277305	2.272516
2.26655	2.261814	2.285757	2.280902	2.276063	2.287637	2.282796	2.277981	2.273193
2.267272	2.262535	2.28647	2.281605	2.276753	2.288302	2.283424	2.27859	2.273794
2.267996	2.263255	2.287182	2.282311	2.277447	2.288968	2.284048	2.279188	2.274383
2.268712	2.263968	2.287893	2.283024	2.278165	2.289699	2.284786	2.279926	2.275111
2.269422	2.264674	2.288603	2.283744	2.278904	2.290482	2.285616	2.280772	2.27595
2.270132	2.265382	2.289313	2.284458	2.279628	2.291237	2.2864	2.28157	2.276748
2.270842	2.26609	2.290024	2.285166	2.280335	2.291954	2.287124	2.282303	2.277493
2.271554	2.2668	2.290737	2.285875	2.281041	2.292665	2.287837	2.283022	2.278221
2.272271	2.267513	2.291455	2.286587	2.28175	2.29338	2.288555	2.28374	2.278938
2.272988	2.268226	2.292174	2.287302	2.28246	2.294095	2.28927	2.284452	2.279646
2.273695	2.268933	2.292889	2.288015	2.283171	2.278376	2.273613	2.28516	2.280352
2.2744	2.269638	2.293604	2.288731	2.283886	2.279085	2.274316	2.285865	2.281056
2.275117	2.270355	2.294328	2.289456	2.284611	2.279803	2.275024	2.286578	2.281775
2.275842	2.271079	2.295059	2.290189	2.285344	2.280525	2.275734	2.287294	2.282505
2.276613	2.271846	2.295832	2.290963	2.286117	2.281291	2.276491	2.288058	2.283277
2.277459	2.272683	2.296671	2.291799	2.286953	2.282125	2.277322	2.288897	2.284115
2.278288	2.273511	2.297507	2.29263	2.287782	2.282952	2.278148	2.28973	2.284941
2.279036	2.274269	2.298282	2.293398	2.28854	2.283712	2.278912	2.290492	2.285689
2.279757	2.275011	2.299048	2.294157	2.289288	2.284461	2.279666	2.291243	2.286423
2.280499	2.275799	2.29987	2.294979	2.290099	2.285271	2.280474	2.275695	2.270936
2.281252	2.27661	2.300721	2.295834	2.290946	2.286114	2.281312	2.276529	2.271768
2.282023	2.277384	2.301508	2.296629	2.291746	2.286909	2.282099	2.277314	2.272554
2.282811	2.278114	2.302224	2.297357	2.292493	2.287649	2.282826	2.27804	2.273288
2.27274	2.268034	2.302978	2.298117	2.293268	2.288422	2.283591	2.278802	2.274052
2.273603	2.268867	2.303808	2.298946	2.294102	2.289259	2.284431	2.279637	2.274874
2.274455	2.269713	2.304663	2.299799	2.294955	2.290117	2.285293	2.280494	2.275717
2.261728	2.257113	2.276699	2.271962	2.267231	2.262515	2.257814	2.281348	2.276567
2.26244	2.257893	2.277557	2.272816	2.268071	2.26336	2.258667	2.282207	2.277427
2.263184	2.258626	2.267465	2.262796	2.258121	2.264217	2.259517	2.283067	2.278287
2.263946	2.259323	2.268165	2.26357	2.258974	2.265084	2.260363	2.283929	2.279148
2.264738	2.260064	2.268901	2.264327	2.259764	2.265905	2.261207	2.284795	2.280009
2.265565	2.26086	2.269677	2.265055	2.260468	2.266668	2.262052	2.28567	2.280873

2.266397	2.261672	2.27048	2.26581	2.261192	2.256634	2.252106	2.28652	2.281729
2.26722	2.262495	2.271322	2.266627	2.26198	2.257394	2.252845	2.287307	2.282568
2.268045	2.263324	2.272177	2.267468	2.262799	2.258177	2.253595	2.288098	2.283408
2.268879	2.264145	2.273011	2.268305	2.263645	2.259036	2.254467	2.288981	2.284274
2.269718	2.264966	2.273841	2.269147	2.264507	2.259931	2.255392	2.289903	2.285151
2.249108	2.244426	2.27469	2.269984	2.265335	2.260754	2.256214	2.281164	2.27644
2.249917	2.245248	2.275555	2.270819	2.266131	2.261515	2.256946	2.281929	2.277249
2.250747	2.246077	2.276419	2.271668	2.266963	2.262327	2.25774	2.282736	2.278072
2.251602	2.246902	2.277266	2.272531	2.267848	2.263219	2.258632	2.283611	2.278916
2.252484	2.247762	2.27815	2.273424	2.26875	2.264124	2.259533	2.284495	2.27977
2.253418	2.248709	2.279133	2.274376	2.269667	2.265012	2.260396	2.285358	2.280632
2.254357	2.249668	2.280134	2.27534	2.270586	2.265896	2.261252	2.286223	2.281504
2.255218	2.250532	2.281047	2.27624	2.27147	2.26677	2.26212	2.287103	2.282394
2.256032	2.25134	2.281908	2.277101	2.272335	2.267639	2.262995	2.287995	2.283298
2.256867	2.252159	2.262164	2.257438	2.252754	2.268527	2.263886	2.288894	2.284189
2.257714	2.25298	2.262994	2.258287	2.253628	2.269432	2.264796	2.289802	2.285067
2.24015	2.235453	2.263885	2.259183	2.254529	2.249918	2.245342	2.290701	2.285944
2.241097	2.236422	2.264888	2.260161	2.255469	2.250816	2.246196	2.291572	2.286815
2.242034	2.237387	2.265892	2.261148	2.256424	2.251732	2.247071	2.292464	2.287704
2.242889	2.238252	2.249261	2.244591	2.239939	2.252698	2.248027	2.293425	2.28864
2.243715	2.239085	2.250145	2.245513	2.240898	2.253675	2.249008	2.294415	2.289603
2.244579	2.239963	2.251055	2.24641	2.241785	2.237169	2.232569	2.295378	2.290577
2.245468	2.24087	2.251978	2.247286	2.242617	2.238006	2.233434	2.296326	2.291566
2.246376	2.241787	2.252918	2.248203	2.24351	2.238891	2.234323	2.297281	2.292544
2.247309	2.242722	2.253886	2.249176	2.244483	2.23984	2.23524	2.298245	2.293515
2.248239	2.243632	2.239033	2.234412	2.229809	2.240782	2.236165	2.264709	2.260086
2.249144	2.244473	2.239838	2.235229	2.230653	2.241683	2.237088	2.26563	2.260958
2.250057	2.245321	2.240646	2.236045	2.231494	2.242586	2.238021	2.266559	2.261833
2.251013	2.246255	2.241562	2.236954	2.232401	2.243525	2.238973	2.267519	2.262772
2.251992	2.247234	2.242533	2.23791	2.233342	2.244485	2.239937	2.268496	2.263744
2.252962	2.248198	2.243489	2.238861	2.234288	2.245451	2.2409	2.269469	2.26471
2.253927	2.249155	2.244441	2.239815	2.235242	2.246425	2.241863	2.270441	2.265677
2.254898	2.250119	2.2454	2.240772	2.236197	2.247401	2.242831	2.271416	2.266647
2.255878	2.251093	2.246369	2.241735	2.237154	2.24838	2.243804	2.272396	2.267622
2.256857	2.252067	2.247337	2.242696	2.238108	2.249355	2.244775	2.273374	2.268594
2.25783	2.253035	2.248298	2.243649	2.239053	2.250323	2.245738	2.274343	2.269558
2.258801	2.253999	2.249256	2.244599	2.239996	2.251288	2.246698	2.27531	2.270519
2.259769	2.25496	2.25021	2.245548	2.24094	2.252255	2.247659	2.276277	2.271481
2.260737	2.255919	2.251162	2.246495	2.241883	2.253222	2.248621	2.277245	2.272445
2.261712	2.256886	2.252119	2.247442	2.242821	2.254189	2.249591	2.278225	2.273419
2.262692	2.257856	2.253077	2.248382	2.243749	2.255153	2.25057	2.279216	2.274399
2.23258	2.227849	2.254082	2.249367	2.244713	2.240155	2.235649	2.280225	2.275419
2.233596	2.228907	2.255169	2.250427	2.245735	2.241147	2.236619	2.281266	2.276507
2.234607	2.229959	2.256249	2.251489	2.246765	2.242144	2.237586	2.282304	2.277593
2.235587	2.230948	2.25725	2.252487	2.247755	2.24311	2.238527	2.283307	2.278622
2.236555	2.231913	2.25822	2.25347	2.248746	2.244083	2.239472	2.284304	2.279628
2.237522	2.232885	2.228267	2.223645	2.219043	2.245129	2.240482	2.285336	2.280631
2.238488	2.23386	2.229266	2.224699	2.220141	2.246228	2.241541	2.286393	2.281631
2.239505	2.234846	2.230243	2.225703	2.22118	2.247296	2.242621	2.287496	2.282679
2.240577	2.235839	2.231187	2.226647	2.222151	2.248336	2.243736	2.288658	2.283783

2.241682	2.236872	2.232165	2.2276	2.223102	2.249333	2.244798	2.289797	2.2849
2.242839	2.237975	2.233206	2.228571	2.224022	2.250258	2.245755	2.290881	2.286035
2.243989	2.239099	2.234284	2.229576	2.224961	2.251176	2.246679	2.291932	2.287151
2.245057	2.240213	2.23541	2.230659	2.225985	2.221444	2.216994	2.292942	2.288198
2.24608	2.241315	2.236555	2.231781	2.227057	2.222451	2.217931	2.293931	2.289207
2.247082	2.242345	2.237611	2.232858	2.228141	2.223504	2.218934	2.294958	2.290211
2.248058	2.24331	2.238594	2.233904	2.229243	2.224602	2.219995	2.296014	2.291205
2.249132	2.244348	2.239612	2.234933	2.23029	2.225661	2.221061	2.282429	2.277657
2.250356	2.245493	2.240681	2.235936	2.231256	2.226664	2.222138	2.283586	2.278833
2.251549	2.246639	2.241773	2.236964	2.232239	2.227662	2.223181	2.256488	2.251839
2.252601	2.24774	2.242898	2.238064	2.233295	2.228662	2.224122	2.257464	2.252828
2.253618	2.248822	2.24402	2.239184	2.234386	2.229688	2.225069	2.258429	2.253793
2.254691	2.249871	2.245066	2.240263	2.235501	2.230811	2.226182	2.259534	2.254854
2.255792	2.250905	2.246079	2.24133	2.236637	2.231989	2.227384	2.260713	2.255967
2.256937	2.252038	2.247201	2.242445	2.237745	2.233092	2.228483	2.261829	2.257073
2.25813	2.253273	2.248435	2.243608	2.238825	2.234122	2.229481	2.262887	2.258175
2.217756	2.21303	2.249614	2.244752	2.23993	2.235201	2.230546	2.263998	2.259308
2.218809	2.214045	2.250672	2.245853	2.241084	2.236386	2.231747	2.26522	2.260508
2.219875	2.215078	2.251743	2.246958	2.242229	2.237552	2.232923	2.266414	2.261675
2.221037	2.216253	2.252933	2.248099	2.243313	2.238592	2.233928	2.267448	2.262705
2.22222	2.217461	2.254151	2.249251	2.244385	2.239605	2.234895	2.268447	2.263703
2.223318	2.21855	2.213786	2.208996	2.204243	2.240732	2.236013	2.269589	2.264832
2.224365	2.219568	2.214807	2.210069	2.205376	2.241931	2.237226	2.270822	2.266043
2.225478	2.220677	2.21592	2.211204	2.206529	2.243107	2.238382	2.27199	2.267181
2.226667	2.221893	2.217145	2.21242	2.207715	2.244261	2.239468	2.273078	2.268227
2.227866	2.223092	2.218333	2.213588	2.208855	2.204114	2.199394	2.274203	2.269325
2.229075	2.224228	2.219409	2.214624	2.209877	2.205172	2.200512	2.275407	2.270534
2.230262	2.225352	2.220484	2.21567	2.210918	2.206255	2.201658	2.276643	2.271777
2.231353	2.226481	2.221647	2.216854	2.212112	2.207444	2.202832	2.277894	2.273011
2.232417	2.227623	2.222857	2.218104	2.21338	2.208686	2.204026	2.279155	2.274243
2.233601	2.228828	2.224082	2.219349	2.214633	2.20992	2.205224	2.243148	2.238306
2.23488	2.230087	2.225327	2.2206	2.215886	2.211156	2.206428	2.244343	2.239436
2.236157	2.231348	2.226565	2.221808	2.217061	2.212302	2.207549	2.245489	2.240569
2.237427	2.232615	2.227802	2.222959	2.218126	2.213317	2.20854	2.246557	2.241697
2.238685	2.233852	2.229003	2.224096	2.219203	2.214367	2.209584	2.247675	2.242865
2.239932	2.235023	2.230126	2.225223	2.220353	2.215544	2.210792	2.248923	2.24411
2.241148	2.236168	2.231236	2.22636	2.221537	2.216771	2.212058	2.250221	2.245389
2.24227	2.237314	2.232408	2.22755	2.222743	2.217995	2.213297	2.251506	2.246667
2.243346	2.238463	2.233614	2.228774	2.223973	2.219232	2.214538	2.252795	2.247951
2.244541	2.239669	2.234833	2.230005	2.225215	2.22048	2.215786	2.254084	2.249226
2.245844	2.240929	2.236066	2.231247	2.226472	2.22174	2.217043	2.255374	2.250494
2.247165	2.242215	2.237325	2.232503	2.22773	2.222994	2.218292	2.256657	2.251758
2.248494	2.243526	2.238617	2.233774	2.228983	2.224238	2.219531	2.257933	2.253014
2.249831	2.244848	2.239921	2.235055	2.230241	2.225481	2.220763	2.259204	2.254271
2.251168	2.246168	2.241223	2.236339	2.231506	2.226728	2.221992	2.260479	2.25555
2.252511	2.247495	2.242533	2.237631	2.232779	2.227976	2.223216	2.261744	2.256816
2.213478	2.208589	2.243861	2.23894	2.234064	2.229226	2.224427	2.262965	2.258002
2.214773	2.209878	2.245202	2.240262	2.235358	2.23048	2.22563	2.264157	2.259133
2.216066	2.211166	2.246539	2.241577	2.236646	2.231741	2.226862	2.265395	2.260328
2.217357	2.212453	2.247873	2.242885	2.237927	2.233009	2.228121	2.266678	2.261584

2.218639	2.213728	2.20884	2.203974	2.199139	2.234285	2.229395	2.267996	2.262898
2.219905	2.214981	2.210082	2.205209	2.200368	2.235574	2.230686	2.269367	2.2643
2.221164	2.216228	2.211321	2.206442	2.2016	2.236864	2.231976	2.270742	2.265714
2.196173	2.191312	2.21257	2.207685	2.202834	2.238145	2.233243	2.272073	2.267065
2.1974	2.192539	2.213822	2.208929	2.204068	2.239422	2.234501	2.273384	2.268387
2.198624	2.19376	2.215064	2.210162	2.205291	2.200455	2.195661	2.230914	2.226135
2.199845	2.194974	2.216299	2.211387	2.206506	2.201666	2.19687	2.232169	2.227369
2.201065	2.196191	2.217539	2.212619	2.207728	2.202881	2.198076	2.193331	2.188641
2.202282	2.197411	2.218786	2.213861	2.208961	2.2041	2.199278	2.194513	2.189804
2.203503	2.198633	2.220033	2.215101	2.210192	2.205319	2.200483	2.195704	2.190979
2.204729	2.199855	2.221276	2.21633	2.211408	2.206531	2.201696	2.196914	2.192183
2.205962	2.201082	2.222522	2.217561	2.212628	2.207748	2.202913	2.198129	2.193395
2.188827	2.183974	2.197418	2.192532	2.187674	2.208974	2.204133	2.199339	2.194591
2.190106	2.185234	2.198641	2.193754	2.188895	2.210212	2.205357	2.200548	2.195782
2.191559	2.186664	2.200012	2.195083	2.190177	2.211477	2.206583	2.20175	2.196977
2.193187	2.188263	2.201528	2.196514	2.191518	2.212763	2.207802	2.202939	2.198167
2.194699	2.189758	2.202969	2.197927	2.192899	2.214137	2.209141	2.20426	2.199485
2.196021	2.191086	2.204288	2.199311	2.194349	2.215658	2.210677	2.205798	2.201011
2.19729	2.192359	2.205557	2.200646	2.195753	2.217137	2.21218	2.207305	2.202502
2.16304	2.158251	2.206781	2.201883	2.197007	2.192117	2.187244	2.208603	2.203784
2.164245	2.159436	2.207985	2.203079	2.198202	2.193316	2.188448	2.209826	2.20499
2.165448	2.160636	2.209208	2.204294	2.199411	2.194529	2.189668	2.211068	2.206219
2.166663	2.161857	2.210454	2.205525	2.20063	2.195749	2.190894	2.212317	2.207457
2.168048	2.163238	2.193872	2.188965	2.184088	2.197111	2.192256	2.213703	2.208832
2.169632	2.164809	2.195495	2.190562	2.185663	2.198639	2.193778	2.215251	2.210368
2.143312	2.138516	2.197022	2.192068	2.187151	2.200084	2.195215	2.190373	2.185559
2.144576	2.139765	2.198331	2.193361	2.188429	2.201321	2.196436	2.191591	2.186784
2.145787	2.140962	2.1996	2.194631	2.189701	2.202567	2.197675	2.192829	2.188028
2.146996	2.142166	2.165189	2.160443	2.155744	2.204006	2.199143	2.194306	2.189494
2.148229	2.143397	2.166461	2.161779	2.15715	2.205509	2.200683	2.195853	2.191023
2.149667	2.14482	2.140052	2.135383	2.130767	2.206893	2.202047	2.197199	2.192353
2.151265	2.146394	2.141575	2.13681	2.132094	2.208196	2.203289	2.198403	2.193541
2.152727	2.147833	2.142978	2.138157	2.133386	2.209523	2.204597	2.199707	2.194853
2.153996	2.14908	2.14421	2.139382	2.134618	2.210874	2.205992	2.201145	2.196338
2.129962	2.125058	2.145503	2.140677	2.13592	2.131257	2.126668	2.202563	2.197773
2.131504	2.126588	2.147	2.142167	2.137386	2.132656	2.127979	2.203863	2.199023
2.133048	2.128123	2.148511	2.14368	2.138885	2.134089	2.12932	2.205179	2.20027
2.090773	2.085953	2.124575	2.119798	2.115055	2.135528	2.130773	2.206675	2.201721
2.091961	2.087143	2.125827	2.121113	2.116444	2.136963	2.132267	2.208241	2.203255
2.093343	2.08851	2.127222	2.122481	2.117788	2.138284	2.133587	2.184651	2.179687
2.094897	2.090033	2.128738	2.123886	2.119081	2.139495	2.134748	2.185799	2.180838
2.070641	2.065848	2.086686	2.08189	2.077135	2.115651	2.110878	2.187082	2.182124
2.071915	2.067212	2.088136	2.083373	2.078631	2.117196	2.112403	2.1886	2.183636
2.073207	2.06856	2.089544	2.084805	2.080075	2.11869	2.113886	2.10913	2.104418
2.074608	2.069887	2.090829	2.086044	2.081284	2.119927	2.115126	2.110413	2.105779
2.076072	2.071241	2.092111	2.087264	2.082462	2.121126	2.11633	2.111657	2.107098
2.07762	2.07274	2.093586	2.088705	2.083874	2.079105	2.074405	2.11303	2.108429
2.079223	2.074328	2.095177	2.09028	2.085426	2.080626	2.075882	2.11448	2.109765
2.080676	2.075803	2.096689	2.091812	2.086965	2.082141	2.077354	2.115958	2.111186
2.081966	2.077165	2.098132	2.093319	2.088512	2.083669	2.078833	2.11747	2.112692

2.083313	2.078522	2.099522	2.094735	2.089953	2.08512	2.080287	2.075558	2.070915
2.084772	2.079871	2.100808	2.095979	2.091196	2.086433	2.081706	2.077033	2.07241
2.08629	2.081276	2.102141	2.097252	2.092448	2.087736	2.083097	2.078474	2.073876
2.05067	2.04573	2.103673	2.098734	2.093877	2.089116	2.084435	2.079811	2.075249
2.052262	2.047327	2.105296	2.100311	2.09539	2.090539	2.085766	2.081113	2.076576
2.05386	2.048902	2.106884	2.101871	2.096918	2.092022	2.087198	2.082495	2.077904
2.055468	2.050473	2.108462	2.103438	2.098473	2.09356	2.088708	2.083931	2.079226
1.977743	1.973035	2.047098	2.042215	2.037392	2.0951	2.090229	2.085409	2.08064
1.979173	1.974498	2.04869	2.043769	2.038898	2.096637	2.09176	2.086935	2.082165
1.980546	1.975917	2.05025	2.045317	2.040425	2.098195	2.093304	2.088472	2.083699
1.981802	1.977251	2.051753	2.046867	2.042003	2.0998	2.094869	2.090005	2.085204
1.983059	1.978556	2.053213	2.048386	2.043568	2.1014	2.096435	2.091541	2.086709
1.984444	1.979856	1.975314	1.970794	1.966289	2.067822	2.06297	2.09308	2.088233
1.985901	1.981165	1.976537	1.972055	1.967616	2.069293	2.064474	2.094621	2.089765
1.908477	1.903864	1.9779	1.973424	1.969001	2.070806	2.065983	2.096147	2.091282
1.909941	1.905304	1.979384	1.974882	1.970433	2.072352	2.067495	2.097662	2.092787
1.911395	1.906736	1.98086	1.976337	1.971867	1.998989	1.994367	2.064199	2.0594
1.912829	1.908144	1.98231	1.977777	1.973301	2.000445	1.995823	2.065753	2.060915
1.914265	1.909559	1.983766	1.97922	1.974734	1.970185	1.965629	2.06731	2.062427
1.915715	1.910997	1.985241	1.980674	1.976168	1.971613	1.967053	2.068851	2.063919
1.917169	1.912443	1.986722	1.982128	1.977596	1.973033	1.968471	2.070388	2.065412
1.918626	1.913887	1.90927	1.904813	1.900427	1.974428	1.969871	1.99692	1.992214
1.920084	1.915331	1.910692	1.906188	1.90176	1.975807	1.97126	1.998334	1.993633
1.921526	1.916762	1.912109	1.907579	1.903128	1.977214	1.972662	1.999757	1.995063
1.922949	1.918177	1.913519	1.908986	1.904529	1.97865	1.974077	2.00119	1.996506
1.8015	1.797459	1.914943	1.910406	1.905941	1.936313	1.93181	2.00261	1.997934
1.802754	1.798678	1.916371	1.911826	1.907353	1.937724	1.933181	2.004002	1.999335
1.804032	1.799934	1.917849	1.913283	1.908787	1.939147	1.934559	2.005397	2.000735
1.741859	1.737984	1.91945	1.91483	1.910267	1.940598	1.935961	1.975008	1.970423
1.743103	1.739274	1.921093	1.916408	1.911765	1.942065	1.937382	1.976418	1.971829
1.691341	1.68761	1.852327	1.848221	1.844136	1.908546	1.903892	1.934237	1.929766
1.692487	1.688749	1.85362	1.849507	1.845417	1.909989	1.905324	1.935719	1.931298
1.634403	1.630713	1.795937	1.79196	1.788011	1.911459	1.906827	1.937257	1.93282
1.635722	1.631987	1.797281	1.793243	1.789256	1.912964	1.908426	1.938871	1.934334
1.572948	1.569363	1.695293	1.691335	1.68743	1.86065	1.856312	1.940437	1.93582
1.574085	1.570522	1.696554	1.692573	1.688642	1.862041	1.857698	1.94188	1.937253
1.575203	1.57166	1.69781	1.693827	1.689885	1.86346	1.859074	1.943305	1.93868
1.576375	1.5728	1.632942	1.629164	1.625421	1.739832	1.735994	1.909736	1.90512
1.57757	1.573948	1.634095	1.630393	1.626719	1.741223	1.737313	1.911242	1.90658
1.578772	1.575161	1.571592	1.568071	1.564559	1.690072	1.686185	1.858928	1.854418
1.579984	1.57643	1.572894	1.56935	1.565795	1.691345	1.687409	1.860369	1.855953
1.58126	1.577712	1.574169	1.570594	1.567005	1.692635	1.688687	1.768615	1.764275
1.582633	1.579016	1.575409	1.571785	1.568171	1.693947	1.690044	1.770185	1.765762
1.489612	1.486342	1.576626	1.572978	1.56936	1.637359	1.633625	1.771753	1.767231
1.490696	1.487413	1.577793	1.574191	1.570624	1.638723	1.63497	1.773265	1.768651
1.491764	1.488477	1.578973	1.575426	1.57191	1.640091	1.636306	1.774773	1.770087
1.404665	1.401631	1.580275	1.576713	1.573172	1.569565	1.565936	1.691441	1.687512
1.405745	1.402712	1.581649	1.578031	1.574422	1.570768	1.567109	1.692755	1.688883
1.40684	1.403794	1.488768	1.485502	1.482239	1.571983	1.56835	1.636482	1.632742
1.407956	1.404884	1.489923	1.486601	1.483304	1.573205	1.56966	1.637892	1.634102

1.331726	1.328548	1.402856	1.399746	1.396667	1.481205	1.478052	1.639284	1.635476
1.332933	1.329696	1.403851	1.400758	1.397711	1.482368	1.479196	1.640634	1.636873
1.33413	1.330848	1.404862	1.40179	1.398773	1.483545	1.480346	1.641983	1.638265
1.335304	1.332028	1.328807	1.325656	1.322548	1.396847	1.393867	1.64336	1.639623
1.336477	1.333227	1.330024	1.326872	1.323756	1.397922	1.39493	1.644742	1.640971
1.337697	1.334452	1.331248	1.328087	1.324958	1.321848	1.318764	1.560949	1.557574
1.338949	1.335697	1.332482	1.329305	1.326159	1.323034	1.319937	1.562205	1.558849
1.340192	1.336934	1.333708	1.330516	1.327354	1.32422	1.321117	1.318055	1.315033
1.341425	1.338159	1.334923	1.331716	1.328541	1.325405	1.322304	1.319233	1.316193
1.342661	1.339391	1.336147	1.332927	1.32974	1.326599	1.323495	1.320414	1.317357
1.343902	1.340635	1.337391	1.334161	1.330962	1.327809	1.324691	1.321595	1.318524
1.345148	1.341884	1.338641	1.335403	1.332192	1.329023	1.325888	1.322779	1.319696
1.346393	1.343131	1.339886	1.336638	1.333415	1.330232	1.327083	1.323962	1.32087
1.347651	1.344381	1.34113	1.337874	1.334641	1.331444	1.328279	1.325147	1.322048
1.236821	1.233801	1.342359	1.339105	1.335879	1.332665	1.329476	1.326335	1.323238
1.238036	1.234939	1.343577	1.340334	1.337129	1.333895	1.330674	1.327524	1.324438
1.239237	1.236107	1.344862	1.341617	1.338415	1.335169	1.331931	1.32877	1.325676
1.240415	1.237316	1.346247	1.342981	1.339754	1.336507	1.333273	1.330096	1.326968
1.241591	1.238514	1.235485	1.232513	1.229569	1.337828	1.334602	1.331408	1.328244
1.242765	1.239664	1.236614	1.233632	1.230682	1.339082	1.335854	1.332642	1.329446
1.243944	1.240803	1.237722	1.23473	1.231776	1.340322	1.337097	1.333871	1.330649
1.141155	1.138158	1.238846	1.235839	1.232883	1.229956	1.227056	1.33518	1.331946
1.142322	1.139291	1.239984	1.236959	1.233999	1.231101	1.228243	1.336527	1.333292
1.143524	1.140487	1.13752	1.134666	1.131882	1.232261	1.229395	1.337816	1.334579
1.144764	1.141749	1.138782	1.135895	1.133064	1.23344	1.230518	1.339051	1.335811
1.145959	1.142972	1.140013	1.137095	1.13422	1.131408	1.128642	1.228687	1.225748
1.147071	1.144122	1.141177	1.138227	1.135314	1.132484	1.129711	1.229821	1.22692
1.148189	1.145271	1.14234	1.13937	1.136431	1.133591	1.130815	1.230979	1.228097
1.149387	1.146475	1.143549	1.140583	1.137642	1.134793	1.131999	1.129229	1.126488
1.1506	1.147684	1.144759	1.141809	1.138881	1.136028	1.133217	1.1304	1.127585
1.02271	1.020036	1.145894	1.142965	1.14006	1.137217	1.134408	1.131577	1.128733
1.02367	1.020997	1.146982	1.144079	1.141204	1.138381	1.135586	1.132761	1.129916
1.024685	1.022011	1.148125	1.14524	1.142381	1.139549	1.136735	1.133905	1.131064
1.025759	1.023082	1.149324	1.146451	1.143594	1.14072	1.137848	1.134998	1.132168
1.026834	1.02416	1.021508	1.018896	1.016291	1.141901	1.138987	1.136123	1.133301
1.0279	1.025239	1.022591	1.01997	1.017353	1.1431	1.140181	1.137317	1.134497
1.028953	1.026302	1.02366	1.021035	1.018415	1.144305	1.14139	1.138527	1.135706
1.029967	1.02731	1.024674	1.022074	1.019478	1.016853	1.014222	1.139718	1.136894
1.030972	1.028301	1.025671	1.023107	1.020546	1.017921	1.015275	1.140907	1.138076
1.032042	1.02936	1.026729	1.024176	1.021625	1.018994	1.016337	1.142104	1.139268
1.033161	1.030472	1.027834	1.025272	1.022713	1.020073	1.017406	1.143307	1.140466
1.034279	1.031582	1.028937	1.026371	1.023806	1.021157	1.018479	1.015846	1.013245
1.03539	1.032686	1.030036	1.02747	1.024904	1.022246	1.019556	1.016915	1.01431
1.036505	1.033792	1.031134	1.028566	1.025998	1.023334	1.020636	1.017988	1.015377
1.037628	1.034899	1.032229	1.029653	1.02708	1.024415	1.021717	1.019066	1.016448
1.038754	1.036009	1.033325	1.030738	1.028158	1.025493	1.022799	1.020145	1.017521
1.039879	1.037123	1.034427	1.031828	1.029238	1.02657	1.023879	1.021224	1.018595
1.041005	1.038242	1.035535	1.032921	1.030319	1.027648	1.024957	1.022301	1.01967
1.042132	1.039367	1.036653	1.034026	1.031409	1.02873	1.026033	1.023375	1.020745
1.043262	1.040498	1.037781	1.03514	1.032509	1.029815	1.027106	1.024445	1.021821

1.044393	1.041632	1.038912	1.036259	1.033611	1.030902	1.028179	1.025513	1.022894
1.045527	1.042771	1.040049	1.037381	1.034715	1.031986	1.029244	1.026573	1.023961
1.04666	1.043907	1.041184	1.038505	1.035826	1.033083	1.030326	1.027647	1.025031
1.047784	1.045034	1.042312	1.039633	1.03695	1.034205	1.031444	1.028745	1.026096
1.048915	1.04616	1.043436	1.040759	1.038081	1.035343	1.032586	1.029869	1.02718
1.050074	1.047288	1.044547	1.041873	1.039209	1.036493	1.03376	1.031042	1.028332
1.051256	1.048423	1.045651	1.042982	1.040336	1.037653	1.034958	1.032252	1.029535
1.052478	1.049611	1.046809	1.044122	1.041469	1.038796	1.036123	1.033433	1.030729
1.053749	1.05086	1.048028	1.045298	1.042608	1.039922	1.037252	1.034584	1.031922
1.054989	1.052094	1.049242	1.046469	1.043739	1.041043	1.038378	1.035725	1.033087
1.05616	1.053278	1.050424	1.047608	1.044839	1.042148	1.039505	1.036854	1.034205
1.057322	1.054466	1.051619	1.048771	1.045968	1.043272	1.040636	1.037979	1.035313
1.058527	1.055705	1.052876	1.050016	1.047187	1.04445	1.041767	1.039093	1.036437
1.059743	1.056955	1.054149	1.051296	1.048454	1.045663	1.042912	1.040215	1.037571
1.060926	1.058144	1.055353	1.052534	1.049715	1.046908	1.04412	1.041397	1.038732
1.062086	1.059287	1.056506	1.053744	1.050981	1.048184	1.045381	1.042629	1.039916
1.063303	1.060469	1.057675	1.054936	1.052204	1.049421	1.046626	1.043872	1.041152
1.064588	1.061694	1.058854	1.0561	1.05337	1.05061	1.047852	1.045139	1.042464
1.065886	1.062953	1.060076	1.05729	1.05454	1.051789	1.049057	1.04637	1.043725
1.067186	1.064267	1.061376	1.058532	1.055723	1.052949	1.050209	1.047509	1.044849
1.068468	1.065585	1.062698	1.05981	1.056946	1.054139	1.051373	1.048642	1.045947
0.859252	0.857019	1.064001	1.061145	1.058292	1.055457	1.052641	1.049858	1.047105
0.860212	0.858013	1.065288	1.062501	1.059695	1.056838	1.05396	1.051118	1.048301
0.861157	0.858978	1.066498	1.063744	1.060965	1.058117	1.055238	1.052396	1.049579
0.86208	0.859909	1.067633	1.064882	1.06212	1.05931	1.056483	1.053697	1.050943
0.863065	0.860897	1.068818	1.066048	1.063283	1.060497	1.057709	1.054956	1.052231
0.864159	0.861984	1.070081	1.067259	1.064453	1.061666	1.058894	1.056136	1.05339
0.86522	0.863051	0.860839	0.858559	0.856295	1.062886	1.060121	1.057345	1.054565
0.86614	0.864017	0.861854	0.859628	0.857402	1.064246	1.061474	1.058674	1.05585
0.867022	0.864948	0.862845	0.860696	0.858532	1.065636	1.062852	1.060027	1.057164
0.595004	0.593724	0.863796	0.861677	0.859543	1.066893	1.064101	1.061278	1.058424
0.595632	0.594342	0.864722	0.86261	0.860489	1.068081	1.065284	1.062477	1.059661
0.596265	0.594984	0.865724	0.863623	0.861514	0.859364	0.857197	1.063775	1.060946
0.596906	0.595657	0.86681	0.864726	0.862626	0.860498	0.858347	1.065182	1.062283
0.59754	0.596303	0.595056	0.593782	0.592481	0.861574	0.859438	1.066541	1.063602
0.598147	0.59689	0.595638	0.594381	0.593104	0.862524	0.860393	1.06778	1.064867
0.598777	0.597489	0.596223	0.594978	0.593721	0.863475	0.86135	1.069032	1.066146
0.599486	0.598167	0.596884	0.595643	0.5944	0.86454	0.862427	0.860273	0.85808
0.600228	0.598875	0.59757	0.596325	0.595089	0.865631	0.863533	0.861377	0.85917
0.600924	0.599542	0.598213	0.596952	0.59571	0.594451	0.593174	0.862385	0.860197
0.601594	0.600185	0.59883	0.597541	0.596281	0.595027	0.593772	0.863326	0.861182
0.602324	0.600885	0.5995	0.598182	0.596901	0.595648	0.594408	0.864332	0.862216
0.603134	0.601661	0.600239	0.598889	0.597585	0.596328	0.595098	0.865426	0.863321
0.603919	0.602422	0.600972	0.599592	0.598262	0.596994	0.595762	0.5945	0.593219
0.604613	0.603115	0.601654	0.600247	0.598889	0.597598	0.59635	0.59509	0.59383
0.605301	0.603812	0.602349	0.600918	0.59953	0.59821	0.596937	0.595675	0.594432
		0.603125	0.601668	0.600245	0.598896	0.597597	0.596328	0.595094
		0.603945	0.602463	0.601006	0.599626	0.598299	0.597018	0.595787
		0.604754	0.603266	0.601792	0.600386	0.599027	0.597725	0.596479
		0.60556	0.604083	0.602608	0.60118	0.599786	0.598453	0.597177

0.601926	0.600501	0.59914	0.597835
0.602591	0.601136	0.599749	0.59842
0.603282	0.601807	0.600397	0.599042
0.604073	0.602592	0.601165	0.599779
0.604894	0.60342	0.601979	0.600562
		0.602777	0.60133
		0.603575	0.602102
		0.604353	0.602883
		0.605113	0.60368

							0.586492	0.584624
							0.585626	0.58376
							1.217774	1.21474
							1.216255	1.213243
					0.586575	0.58482	1.485215	1.481556
					0.585725	0.583961	1.483382	1.479706
					0.584858	0.583075	1.481546	1.477848
					1.216519	1.213393	1.695524	1.69114
					1.215005	1.211864	1.69327	1.688855
		0.883285	0.88041	0.877554	1.362583	1.358984	1.691042	1.686614
		0.882006	0.879122	0.876262	1.360859	1.357273	1.688832	1.684401
		1.220023	1.216807	1.213618	1.69561	1.691104	1.686639	1.68221
		1.218482	1.215288	1.212122	1.6934	1.688914	1.684463	1.68004
0.883888	0.881004	1.366638	1.362989	1.359383	1.6912	1.686732	1.915	1.909898
0.88261	0.879736	1.364929	1.361301	1.357714	1.689014	1.684554	1.912565	1.907491
0.881342	0.878477	1.363234	1.359624	1.356054	1.686835	1.682386	1.910147	1.905117
1.218828	1.215632	1.698183	1.693628	1.689119	1.684652	1.680231	1.966809	1.961643
1.217318	1.21413	1.695948	1.691407	1.686916	1.682476	1.678089	1.964354	1.959279
1.365388	1.361744	1.6937	1.689193	1.684738	1.913046	1.907968	1.961848	1.956817
1.363622	1.360006	1.691442	1.686986	1.682582	1.910589	1.905518	1.959304	1.954279
1.69833	1.693754	1.689247	1.684845	1.680499	1.967306	1.962056	2.057546	2.052373
1.696116	1.691576	1.687118	1.682784	1.678509	1.965042	1.959858	2.055333	2.050091
1.693987	1.689481	1.918789	1.913779	1.908828	1.962764	1.957629	2.104206	2.098826
1.692028	1.687543	1.916607	1.911562	1.906568	1.960384	1.955249	2.101738	2.096355
1.690087	1.685622	1.914433	1.909339	1.904285	1.957965	1.952815	2.099207	2.09384
1.687974	1.68353	1.971281	1.965989	1.960741	2.056251	2.05097	2.149411	2.143904
1.685766	1.681345	1.968754	1.963499	1.958287	2.053757	2.048477	2.146747	2.141247
1.755866	1.751332	2.067185	2.061831	2.056513	2.102227	2.096854	2.144052	2.138552
1.753586	1.749058	2.064558	2.059213	2.053905	2.099585	2.094215	2.141323	2.135815
1.971649	1.966354	2.113126	2.10772	2.102352	2.09698	2.091615	2.138631	2.133119
1.969103	1.963851	2.110551	2.10515	2.099775	2.094406	2.089048	2.135968	2.130456
1.966659	1.961444	2.108075	2.102675	2.097291	2.091923	2.086571	2.133399	2.127894
2.065456	2.060116	2.105801	2.100405	2.095018	2.14204	2.136532	2.168332	2.162777

2.063267	2.057929	2.103575	2.098182	2.092796	2.139734	2.134216	2.165997	2.16047
2.111913	2.106516	2.101136	2.095747	2.090367	2.137212	2.131694	2.163454	2.157947
2.109363	2.103955	2.098563	2.093179	2.087809	2.134556	2.129047	2.160782	2.155292
2.106811	2.101397	2.095997	2.090615	2.085251	2.169213	2.163651	2.158134	2.152659
2.104237	2.098824	2.093422	2.088034	2.082672	2.166521	2.160989	2.155493	2.15003
2.101748	2.096337	2.090936	2.085548	2.080191	2.16393	2.158425	2.204439	2.198848
2.099431	2.094026	2.088633	2.083249	2.0779	2.161538	2.156049	2.202023	2.196457
2.097106	2.091706	2.086324	2.080954	2.075624	2.159149	2.153674	2.199606	2.194064
2.094593	2.089199	2.173207	2.167634	2.16209	2.156587	2.151122	2.197006	2.191478
2.092008	2.086616	2.170503	2.164954	2.159437	2.153951	2.148497	2.194328	2.188811
2.089504	2.08408	2.167813	2.162276	2.156785	2.202796	2.197213	2.19167	2.186167
2.087054	2.081569	2.165122	2.159588	2.154122	2.200087	2.194536	2.189016	2.183528
2.084651	2.07917	2.162585	2.157064	2.151619	2.197535	2.192009	2.186511	2.181042
2.082312	2.076923	2.160261	2.154767	2.149335	2.195199	2.189688	2.18421	2.178766
2.169015	2.163422	2.157886	2.15242	2.147008	2.19282	2.187326	2.220466	2.214783
2.166454	2.160829	2.155283	2.149843	2.144468	2.190234	2.18476	2.217845	2.212171
2.16389	2.158233	2.152675	2.147259	2.141922	2.187641	2.182188	2.215216	2.209544
2.161414	2.155819	2.201755	2.196202	2.190722	2.224153	2.218469	2.212786	2.207105
2.158976	2.153486	2.199423	2.193892	2.188414	2.221773	2.216116	2.210439	2.204745
2.156459	2.151006	2.196902	2.191394	2.185932	2.219222	2.213573	2.207912	2.202239
2.153866	2.148393	2.194211	2.188724	2.183292	2.216514	2.210858	2.20522	2.199602
2.202842	2.197202	2.230987	2.225264	2.219599	2.213921	2.208254	2.202629	2.197047
2.200434	2.194834	2.22856	2.222865	2.21722	2.211541	2.205857	2.200224	2.194643
2.198071	2.1925	2.226156	2.220463	2.214813	2.209129	2.203439	2.197804	2.192227
2.195723	2.190122	2.223667	2.217905	2.212185	2.206492	2.20083	2.347563	2.340705
2.193362	2.187731	2.221169	2.215326	2.209527	2.203824	2.1982	2.344258	2.337452
2.230355	2.224539	2.218723	2.212882	2.20707	2.201371	2.19576	2.341227	2.334479
2.227715	2.222013	2.216308	2.21052	2.204737	2.199051	2.193438	2.338363	2.331675
2.22522	2.219556	2.21389	2.208145	2.202395	2.349207	2.342227	2.335394	2.32872
2.222882	2.217176	2.211479	2.205769	2.200062	2.346242	2.33919	2.332307	2.325595
2.220533	2.214768	2.209022	2.203324	2.197647	2.343216	2.336178	2.329302	2.322582
2.21815	2.2123	2.206477	2.200741	2.195064	2.340058	2.333205	2.326453	2.319793
2.215705	2.209804	2.203938	2.198178	2.192508	2.336949	2.330279	2.323651	2.317055
2.213102	2.207265	2.282016	2.275512	2.269108	2.33407	2.327439	2.320837	2.314256
2.210447	2.204727	2.279209	2.272829	2.266529	2.331298	2.324642	2.318028	2.311449
2.207951	2.202311	2.3489	2.34203	2.335231	2.328486	2.321802	2.31519	2.308644
2.205568	2.19999	2.346071	2.339227	2.332431	2.325663	2.318937	2.312332	2.305843
2.283887	2.277557	2.343195	2.33636	2.329564	2.322804	2.316091	2.374284	2.367282
2.281207	2.274875	2.340263	2.333412	2.32661	2.319891	2.313255	2.371305	2.364404
2.313933	2.307504	2.337313	2.330458	2.323671	2.317017	2.310482	2.368375	2.361566
2.31122	2.304771	2.334347	2.327511	2.320772	2.314215	2.307812	2.365498	2.358769
2.308489	2.302027	2.331379	2.32459	2.317927	2.311473	2.305191	2.362656	2.355979
2.305684	2.299238	2.328414	2.321732	2.315191	2.37347	2.366608	2.359828	2.353142
2.302848	2.29644	2.32547	2.318927	2.312531	2.370594	2.363784	2.357012	2.350282
2.33618	2.329331	2.322644	2.316195	2.309883	2.367714	2.360915	2.354151	2.347421
2.333267	2.326522	2.319925	2.313536	2.307255	2.364836	2.358008	2.351248	2.344554
2.330412	2.323765	2.382639	2.375681	2.368845	2.361998	2.35518	2.348433	2.341752
2.327622	2.321064	2.379766	2.372843	2.366024	2.359231	2.352483	2.345757	2.33904
2.324864	2.318379	2.376902	2.369996	2.363178	2.356434	2.349758	2.373314	2.366469
2.32211	2.315672	2.373977	2.367062	2.360227	2.353508	2.346884	2.370458	2.363693

2.319385	2.312982	2.371056	2.36413	2.357275	2.35057	2.343983	2.367583	2.360924
2.316725	2.310364	2.368212	2.361309	2.354471	2.34777	2.341182	2.364771	2.358187
2.314113	2.307796	2.36542	2.358562	2.351763	2.345059	2.338444	2.362001	2.355478
2.350354	2.343491	2.362655	2.355836	2.349064	2.372598	2.365872	2.359283	2.352827
2.347491	2.34071	2.359922	2.353136	2.346381	2.369881	2.363166	2.356617	2.350232
2.370583	2.363861	2.357195	2.350438	2.343701	2.367176	2.360484	2.378207	2.371884
2.367752	2.361083	2.354463	2.347731	2.341015	2.364474	2.357819	2.375599	2.369409
2.364948	2.358325	2.351745	2.345035	2.338341	2.361788	2.35518	2.37302	2.366957
2.36218	2.3556	2.379415	2.372589	2.365794	2.35912	2.352562	2.370443	2.364464
2.359438	2.352897	2.376692	2.369887	2.363109	2.356479	2.349982	2.367899	2.361978
2.387263	2.380601	2.373975	2.367202	2.360461	2.378034	2.371605	2.365443	2.359554
2.384452	2.377843	2.371267	2.364537	2.357844	2.375481	2.369162	2.363063	2.357182
2.381678	2.37511	2.368575	2.361889	2.35525	2.372941	2.36672	2.360715	2.354928
2.378945	2.372403	2.365897	2.359255	2.352671	2.370404	2.364267	2.358399	2.35282
2.37623	2.369715	2.38736	2.380746	2.374199	2.367921	2.361864	2.367851	2.362328
2.373507	2.367036	2.384713	2.378177	2.371718	2.365519	2.359537	2.365534	2.360071
2.370822	2.364385	2.382088	2.375638	2.369277	2.363161	2.357255	2.36325	2.357814
2.368227	2.361781	2.379472	2.373096	2.366833	2.372629	2.366678	2.361001	2.355626
2.365691	2.359213	2.376877	2.370578	2.364421	2.37025	2.364389	2.358789	2.353477
2.36315	2.356667	2.38645	2.380123	2.373971	2.367988	2.362183	2.356628	2.351345
2.360613	2.354143	2.383879	2.377711	2.371736	2.365836	2.360057	2.354516	2.349233
2.382123	2.37566	2.381393	2.375332	2.369476	2.363654	2.357926	2.352415	2.347134
2.379751	2.373321	2.379016	2.372987	2.367169	2.361417	2.355778	2.350318	2.34505
2.389528	2.383	2.37666	2.370656	2.364863	2.359181	2.353635	2.348231	2.342978
2.386919	2.380486	2.374242	2.368297	2.362557	2.356948	2.351486	2.346138	2.340909
2.384311	2.37799	2.371861	2.365977	2.360287	2.354749	2.349366	2.34407	2.338863
2.381899	2.375687	2.369656	2.363808	2.358138	2.352656	2.347346	2.342088	2.336881
2.379603	2.373499	2.367559	2.361735	2.356072	2.350636	2.34539	2.340157	2.33494
2.377242	2.371226	2.365368	2.35961	2.354	2.348599	2.343371	2.374497	2.369037
2.374815	2.368869	2.363085	2.357437	2.351926	2.346546	2.341288	2.372332	2.366917
2.372478	2.36658	2.360852	2.355299	2.349881	2.344529	2.339257	2.370223	2.364843
2.370299	2.364414	2.358705	2.353226	2.347889	2.342577	2.337322	2.368213	2.362849
2.368138	2.362277	2.356595	2.351168	2.345887	2.340622	2.335406	2.366236	2.360871
2.365873	2.360104	2.354494	2.349082	2.343808	2.338605	2.33348	2.364283	2.358874
2.363602	2.357947	2.352421	2.347009	2.341716	2.336569	2.33154	2.362322	2.356864
2.361447	2.35587	2.350403	2.345007	2.339709	2.334558	2.32952	2.360242	2.354807
2.359378	2.353857	2.348431	2.34306	2.337764	2.332564	2.327443	2.358077	2.352716
2.357351	2.351862	2.346461	2.341118	2.33584	2.330615	2.325443	2.355991	2.350686
2.355374	2.349886	2.344487	2.339181	2.333942	2.328719	2.323529	2.35399	2.348727
2.353399	2.347924	2.342536	2.337255	2.33204	2.326829	2.321645	2.352031	2.346784
2.351399	2.345978	2.340631	2.335343	2.330114	2.324928	2.319791	2.350127	2.344846
2.349405	2.344036	2.338728	2.333435	2.328192	2.323031	2.317938	2.348223	2.342908
2.347446	2.342083	2.336782	2.331519	2.326307	2.356875	2.351553	2.372547	2.367146
2.345503	2.340127	2.33482	2.329599	2.324434	2.354928	2.349556	2.370505	2.365135
2.34354	2.338181	2.332888	2.327678	2.32252	2.352934	2.347555	2.368484	2.36316
2.341563	2.336243	2.33098	2.325755	2.320573	2.350902	2.345547	2.366475	2.361215
2.339607	2.334311	2.32906	2.323823	2.318625	2.348888	2.343575	2.364502	2.35928
2.337685	2.332382	2.327116	2.321863	2.316659	2.346887	2.341645	2.362576	2.357351
2.335756	2.330459	2.325198	2.319953	2.314761	2.344941	2.339739	2.360662	2.355443
2.333785	2.328543	2.323342	2.318157	2.313011	2.343092	2.337838	2.358727	2.353564

2.331817	2.326636	2.321505	2.316394	2.311307	2.341289	2.335973	2.356819	2.351704
2.32991	2.324745	2.355347	2.35005	2.344783	2.36567	2.360283	2.381281	2.376024
2.328037	2.322866	2.353374	2.348123	2.342921	2.363844	2.358536	2.379487	2.374131
2.326135	2.320989	2.351445	2.346212	2.341031	2.361966	2.356724	2.37766	2.372301
2.3242	2.31911	2.349558	2.344314	2.339111	2.360036	2.354854	2.375805	2.370539
2.32234	2.317262	2.347651	2.342409	2.337209	2.38437	2.379131	2.373927	2.368757
2.320606	2.315449	2.345684	2.34048	2.335346	2.382432	2.377179	2.372012	2.366935
2.318885	2.31367	2.343761	2.338577	2.333482	2.380491	2.375235	2.3701	2.365094
2.317112	2.311948	2.36818	2.362843	2.357588	2.378507	2.373318	2.368224	2.363233
2.315314	2.31024	2.366425	2.361013	2.35566	2.376536	2.37142	2.366363	2.361367
2.313472	2.308457	2.39087	2.385395	2.379952	2.374679	2.369534	2.364483	2.359535
2.3116	2.306623	2.388953	2.383572	2.378208	2.372907	2.367667	2.362602	2.357729
2.309807	2.304813	2.386992	2.381685	2.376408	2.371133	2.365885	2.36083	2.355986
2.3081	2.30302	2.384965	2.379715	2.37454	2.369358	2.364202	2.359185	2.354319
2.335647	2.3304	2.38302	2.377802	2.372696	2.367593	2.362528	2.368777	2.363821
2.333966	2.328711	2.381235	2.376014	2.370914	2.365852	2.360848	2.367112	2.36208
2.33227	2.327041	2.379499	2.37426	2.369138	2.364102	2.359147	2.365429	2.360339
2.356719	2.351466	2.377742	2.372476	2.367313	2.362293	2.357388	2.363726	2.358659
2.354857	2.349694	2.375983	2.370692	2.365482	2.360466	2.355592	2.361998	2.357
2.352961	2.347872	2.374186	2.368929	2.363731	2.358693	2.353777	2.360215	2.355296
2.351027	2.346005	2.372358	2.367185	2.362045	2.356963	2.35194	2.35838	2.353557
2.34919	2.344211	2.370568	2.36546	2.36037	2.35527	2.350183	2.356627	2.351852
2.347481	2.342512	2.368826	2.363755	2.358705	2.353619	2.348532	2.354984	2.350197
2.345802	2.340838	2.367103	2.362061	2.357043	2.351977	2.346901	2.353357	2.348538
2.344121	2.339177	2.365411	2.360389	2.355389	2.35033	2.345252	2.351705	2.346832
2.342445	2.33751	2.363705	2.3587	2.353718	2.348674	2.343607	2.350064	2.345138
2.340748	2.335779	2.361898	2.356919	2.351982	2.35853	2.353462	2.348465	2.343525
2.339046	2.334019	2.360041	2.355089	2.35021	2.356841	2.351848	2.346894	2.341964
2.368641	2.363399	2.358273	2.353329	2.348474	2.355168	2.350238	2.34532	2.3404
2.366948	2.361711	2.356585	2.351629	2.346767	2.353507	2.348631	2.343747	2.338841
2.365263	2.360034	2.354913	2.349953	2.34509	2.35187	2.347034	2.342175	2.337285
2.363587	2.358365	2.353249	2.348301	2.343451	2.350266	2.345445	2.340602	2.335728
2.361909	2.356694	2.351588	2.346657	2.341826	2.348676	2.343867	2.339038	2.334181
2.360215	2.355013	2.349921	2.345008	2.340197	2.34709	2.342302	2.337494	2.332655
2.358516	2.353328	2.348252	2.34336	2.338571	2.34551	2.340747	2.335959	2.331138
2.356822	2.351638	2.358419	2.353509	2.348709	2.343931	2.339191	2.334424	2.32962
2.355131	2.349946	2.356756	2.351873	2.347106	2.342355	2.337638	2.332891	2.328104
2.35345	2.348283	2.355133	2.350268	2.345518	2.340781	2.336079	2.331349	2.326583
2.351779	2.346648	2.353549	2.34869	2.343939	2.339205	2.334508	2.329795	2.325057
2.350118	2.345047	2.352018	2.347166	2.342406	2.337666	2.332962	2.346745	2.342011
2.348468	2.343502	2.350578	2.345736	2.340955	2.336193	2.331459	2.345272	2.340543
2.346822	2.341955	2.349133	2.344306	2.339511	2.334729	2.329965	2.343808	2.339082
2.345154	2.340328	2.347573	2.342778	2.338007	2.333229	2.328455	2.34234	2.337622
2.343501	2.338684	2.345972	2.341212	2.336476	2.331711	2.326938	2.340873	2.336169
2.341965	2.337147	2.344449	2.33968	2.334937	2.348913	2.344131	2.339406	2.334731
2.340525	2.335694	2.342981	2.33817	2.333386	2.347398	2.342636	2.33794	2.333306
2.351235	2.34635	2.341497	2.336657	2.331849	2.345904	2.341162	2.35588	2.35133
2.349759	2.344859	2.339991	2.335141	2.330328	2.344429	2.339704	2.354513	2.350035
2.34825	2.343342	2.33847	2.333623	2.32882	2.362427	2.357747	2.353188	2.348761
2.346686	2.34178	2.336918	2.33209	2.327318	2.361063	2.356455	2.351933	2.347509

2.345106	2.340208	2.335364	2.330568	2.32584	2.359725	2.355188	2.350695	2.346254
2.34354	2.338649	2.372479	2.367705	2.363011	2.358413	2.3539	2.358204	2.353731
2.341992	2.337112	2.371038	2.366323	2.361692	2.357117	2.352601	2.356924	2.352425
2.361554	2.356698	2.369707	2.364998	2.360365	2.355787	2.351265	2.355606	2.351097
2.360229	2.355425	2.368485	2.363732	2.359034	2.354426	2.34989	2.354249	2.349741
2.358863	2.354089	2.367197	2.362428	2.357701	2.362001	2.35744	2.352908	2.348404
2.357426	2.352644	2.365797	2.361055	2.356358	2.360688	2.356112	2.351576	2.347079
2.355972	2.351177	2.364376	2.359677	2.355033	2.359412	2.354839	2.350307	2.345815
2.372517	2.367694	2.362954	2.358319	2.353753	2.35822	2.3537	2.349191	2.344689
2.371099	2.366278	2.361557	2.356981	2.352487	2.357046	2.352588	2.348107	2.343596
2.369815	2.365	2.360281	2.355699	2.351199	2.355785	2.351338	2.346866	2.342366
2.368619	2.363813	2.359086	2.354456	2.349898	2.354471	2.350002	2.34553	2.341055
2.367335	2.362541	2.357817	2.353171	2.348589	2.353167	2.348678	2.344216	2.339784
2.365951	2.361175	2.356466	2.351834	2.347262	2.351862	2.347354	2.342913	2.338548
2.364614	2.359861	2.355171	2.350556	2.345995	2.350622	2.346105	2.341671	2.33733
2.363393	2.358675	2.354009	2.349404	2.344846	2.349509	2.345	2.340543	2.336141
2.362167	2.357481	2.352839	2.348244	2.343689	2.348388	2.34389	2.33941	2.334945
2.360822	2.356152	2.351524	2.346941	2.342395	2.347123	2.342624	2.338146	2.333686
2.359451	2.354787	2.350171	2.345599	2.341063	2.345817	2.341313	2.336846	2.332417
2.358219	2.353564	2.348954	2.344387	2.339855	2.344639	2.340132	2.335666	2.331243
2.357089	2.352442	2.347836	2.34327	2.338736	2.343551	2.339041	2.334571	2.330142
2.355857	2.351228	2.346635	2.342072	2.337536	2.342385	2.337879	2.333409	2.328974
2.354483	2.349886	2.345318	2.340759	2.336221	2.341103	2.336611	2.332145	2.327704
2.353151	2.348578	2.34403	2.33948	2.334948	2.339869	2.335392	2.330934	2.326493
2.351967	2.347398	2.342858	2.338328	2.333818	2.338784	2.334316	2.329864	2.32543
2.350801	2.346228	2.34169	2.337178	2.332692	2.33771	2.333261	2.328827	2.324408
2.349519	2.344942	2.349934	2.345414	2.340932	2.336503	2.332119	2.327734	2.323353
2.348198	2.343615	2.348627	2.344098	2.33962	2.33524	2.330934	2.326612	2.322276
2.346988	2.342413	2.347457	2.342924	2.338439	2.334055	2.329746	2.34411	2.339678
2.345873	2.341318	2.346407	2.341873	2.337371	2.332933	2.328546	2.342808	2.338364
2.354445	2.349889	2.345353	2.340822	2.336313	2.331841	2.327403	2.34158	2.337118
2.353399	2.348836	2.344294	2.339774	2.335277	2.330805	2.32636	2.340482	2.335996
2.35229	2.347723	2.343179	2.338668	2.334183	2.32972	2.325279	2.339354	2.334849
2.351004	2.346453	2.341922	2.337413	2.332927	2.32847	2.32404	2.338069	2.333564
2.349684	2.345152	2.340638	2.336131	2.331644	2.327195	2.322776	2.33676	2.332262
2.348521	2.343993	2.339487	2.334993	2.33052	2.344632	2.340099	2.335585	2.331087
2.347453	2.342921	2.338418	2.333944	2.329492	2.343563	2.339018	2.334494	2.329991
2.346402	2.341868	2.337365	2.332899	2.328456	2.342491	2.337947	2.333421	2.328912
2.345376	2.340841	2.336333	2.331862	2.327418	2.341421	2.336894	2.332374	2.327857
2.344343	2.339813	2.335307	2.330834	2.326385	2.340352	2.335834	2.331319	2.326802
2.343285	2.338774	2.334283	2.329813	2.325362	2.339282	2.334757	2.330245	2.325744
2.342233	2.337737	2.33326	2.328792	2.324339	2.338209	2.333671	2.329159	2.324672
2.34121	2.336714	2.350844	2.346262	2.341692	2.337134	2.33259	2.328074	2.323589
2.340196	2.335693	2.349776	2.345188	2.340612	2.336045	2.33149	2.326965	2.322472
2.339158	2.334654	2.348696	2.3441	2.339507	2.334908	2.330311	2.325755	2.321239
2.338101	2.333601	2.347603	2.342995	2.338378	2.333731	2.329068	2.324464	2.319915
2.337024	2.3325	2.346433	2.341806	2.337174	2.332517	2.327847	2.323234	2.318677
2.335928	2.331343	2.345162	2.340507	2.33587	2.331248	2.326644	2.322076	2.31754
2.334808	2.330178	2.343908	2.339237	2.334604	2.330017	2.325472	2.320946	2.316437
2.333638	2.329017	2.342718	2.338058	2.33344	2.328871	2.324346	2.319841	2.315357

2.332477	2.327873	2.341557	2.336918	2.33232	2.327762	2.323243	2.318752	2.314289
2.349832	2.345099	2.34041	2.335792	2.33122	2.326671	2.322151	2.317663	2.313205
2.348746	2.34399	2.339289	2.334687	2.330139	2.325595	2.32107	2.316578	2.312118
2.347724	2.342947	2.338221	2.333601	2.32904	2.324496	2.319979	2.336788	2.332037
2.346769	2.341969	2.337204	2.332536	2.327928	2.323377	2.31888	2.335622	2.330886
2.345759	2.340957	2.336177	2.33147	2.326823	2.322266	2.317784	2.334454	2.329729
2.344662	2.339886	2.335121	2.330395	2.32572	2.32116	2.31669	2.333283	2.328563
2.343559	2.338822	2.334083	2.329347	2.32465	2.320079	2.315603	2.332111	2.327397
2.342498	2.337809	2.333103	2.32836	2.323637	2.319032	2.314514	2.330926	2.326234
2.341436	2.336796	2.332128	2.327394	2.32266	2.31802	2.313448	2.329756	2.325078
2.340332	2.335721	2.331089	2.326405	2.321713	2.317065	2.312452	2.328642	2.32393
2.339202	2.334607	2.330008	2.3254	2.320784	2.316148	2.311505	2.327572	2.322798
2.338078	2.333487	2.328906	2.324343	2.319779	2.31518	2.310561	2.326553	2.321736
2.336957	2.332358	2.327778	2.323231	2.318699	2.314163	2.309628	2.325596	2.320749
2.335838	2.331233	2.326655	2.322118	2.317608	2.334128	2.329382	2.324595	2.319759
2.334709	2.330115	2.325549	2.321021	2.316522	2.332978	2.328257	2.323517	2.318756
2.333602	2.329006	2.324442	2.319918	2.315427	2.331811	2.327103	2.322405	2.317722
2.332546	2.327902	2.323307	2.318788	2.314316	2.330635	2.325944	2.321271	2.316623
2.331526	2.32682	2.322182	2.317661	2.313205	2.329457	2.324778	2.320119	2.315484
2.330546	2.325811	2.321141	2.316596	2.312121	2.32829	2.323612	2.318958	2.314332
2.3296	2.324859	2.320166	2.31558	2.31106	2.327138	2.322449	2.317789	2.313163
2.328595	2.32389	2.319218	2.314625	2.310084	2.326078	2.321361	2.316677	2.31203
2.327522	2.322908	2.318312	2.313753	2.309218	2.325133	2.320359	2.315624	2.310928
2.326432	2.321884	2.317351	2.312829	2.308313	2.332784	2.327967	2.323183	2.318428
2.325311	2.320772	2.316263	2.311776	2.307304	2.331744	2.326981	2.32223	2.317489
2.324223	2.319669	2.315159	2.310694	2.306256	2.330662	2.325962	2.321256	2.316545
2.332655	2.328037	2.34376	2.338969	2.334224	2.329508	2.324822	2.320138	2.315457
2.33176	2.327102	2.342717	2.337872	2.333074	2.328329	2.323632	2.318956	2.314303
2.330784	2.326142	2.341702	2.336859	2.332053	2.327283	2.32255	2.317848	2.313179
2.329749	2.325167	2.34072	2.335925	2.331145	2.32635	2.321553	2.316794	2.312069
2.328766	2.324204	2.339714	2.334935	2.330165	2.325366	2.320554	2.315778	2.311033
2.327871	2.32326	2.338668	2.333855	2.329068	2.324296	2.319543	2.314814	2.310104
2.326949	2.322307	2.337633	2.332796	2.327998	2.323246	2.318535	2.313839	2.309154
2.325923	2.321338	2.336654	2.331845	2.327054	2.322284	2.317534	2.312804	2.308088
2.324869	2.320349	2.335666	2.330897	2.326124	2.321333	2.316533	2.31176	2.307008
2.344115	2.339331	2.33457	2.329817	2.325068	2.320299	2.315519	2.320714	2.315972
2.343054	2.338215	2.333419	2.328671	2.323951	2.319222	2.314497	2.319776	2.315088
2.342052	2.337198	2.332388	2.327643	2.322931	2.318204	2.313479	2.328097	2.3234
2.341112	2.336278	2.331475	2.326731	2.322008	2.317245	2.312465	2.327068	2.322346
2.340178	2.335347	2.330535	2.325774	2.321027	2.316243	2.311445	2.326063	2.321335
2.339253	2.334384	2.329527	2.324711	2.319921	2.315155	2.310413	2.32511	2.320416
2.338298	2.333406	2.328519	2.323658	2.31883	2.314077	2.309383	2.324152	2.31949
2.337246	2.332397	2.327547	2.322692	2.317856	2.332596	2.327848	2.323137	2.318469
2.336175	2.331385	2.326591	2.321758	2.316923	2.331669	2.326864	2.322119	2.317435
2.335215	2.330433	2.325642	2.320801	2.315956	2.33072	2.325915	2.321175	2.3165
2.33433	2.329521	2.3247	2.319834	2.314973	2.329765	2.324996	2.320284	2.315634
2.333424	2.32858	2.343445	2.338532	2.333638	2.328833	2.324094	2.3336	2.328916
2.332499	2.327608	2.342456	2.337561	2.332704	2.327927	2.323215	2.332734	2.328071
2.33155	2.326627	2.341478	2.336603	2.331779	2.32702	2.322318	2.331833	2.32717
2.330562	2.325634	2.340526	2.335675	2.330872	2.326106	2.321379	2.330852	2.326147

2.329566	2.324647	2.339592	2.334755	2.329958	2.325182	2.320433	2.329866	2.325123
2.348481	2.343564	2.338678	2.333819	2.328995	2.324224	2.319499	2.328937	2.324212
2.347551	2.342661	2.337784	2.33289	2.328027	2.323265	2.318579	2.328039	2.323357
2.346653	2.341785	2.336922	2.332029	2.327161	2.322396	2.317709	2.327163	2.322501
2.345784	2.340935	2.336091	2.331227	2.32638	2.321605	2.316885	2.326312	2.321655
2.344896	2.340041	2.335201	2.330364	2.325549	2.320782	2.316056	2.325459	2.320781
2.343982	2.339087	2.334223	2.32941	2.324637	2.319908	2.315217	2.324599	2.319859
2.34305	2.33813	2.333255	2.328469	2.323736	2.319042	2.314383	2.323751	2.318965
2.342077	2.337181	2.332341	2.327586	2.322884	2.318212	2.31357	2.322938	2.318159
2.341116	2.336254	2.331456	2.32673	2.322053	2.317391	2.312748	2.322116	2.317363
2.340246	2.335389	2.3306	2.325893	2.32123	2.316548	2.311865	2.321211	2.316482
2.339432	2.334566	2.32977	2.325074	2.320417	2.315697	2.310951	2.320263	2.315562
2.338604	2.333737	2.328942	2.324254	2.319602	2.328972	2.32414	2.319378	2.314684
2.337767	2.332907	2.328118	2.323434	2.318785	2.328145	2.323316	2.31855	2.313843
2.33694	2.332088	2.327305	2.322623	2.317975	2.327327	2.322503	2.317735	2.313019
2.336131	2.331288	2.32651	2.321829	2.31718	2.326522	2.321701	2.316934	2.312218
2.33532	2.330484	2.325712	2.32103	2.31638	2.325712	2.320895	2.316132	2.311417
2.334489	2.329658	2.324889	2.320209	2.315558	2.324882	2.320069	2.31531	2.310598
2.333655	2.328826	2.32406	2.31938	2.31473	2.324045	2.319238	2.314483	2.309775
2.332832	2.328006	2.323243	2.318563	2.313914	2.32322	2.318418	2.3228	2.31808
2.332016	2.327194	2.322432	2.317753	2.313104	2.322401	2.317606	2.321996	2.317281
2.331212	2.326384	2.321617	2.316938	2.312292	2.321582	2.316793	2.321189	2.316479
2.330415	2.325571	2.320793	2.316116	2.311477	2.320761	2.315978	2.320381	2.315676
2.329652	2.324789	2.319989	2.315304	2.310662	2.329118	2.324325	2.319577	2.314871
2.328956	2.324063	2.319226	2.31451	2.309844	2.32829	2.323514	2.318769	2.31405
2.328251	2.323336	2.318469	2.313722	2.309033	2.327476	2.322729	2.317991	2.313255
2.327466	2.322554	2.317686	2.312932	2.308241	2.326709	2.322021	2.317302	2.312544
2.326641	2.321745	2.316894	2.312143	2.307459	2.325961	2.321342	2.316649	2.311871
2.325815	2.320934	2.339418	2.334592	2.329834	2.325178	2.320597	2.315924	2.31115
2.324982	2.320116	2.33861	2.333782	2.329018	2.32437	2.319804	2.315148	2.310397
2.324166	2.319337	2.337854	2.333026	2.328252	2.323591	2.319011	2.314365	2.309652
2.323366	2.318608	2.337172	2.332346	2.327547	2.322846	2.318213	2.31357	2.308923
2.323978	2.319289	2.33647	2.331662	2.326866	2.322131	2.317443	2.312789	2.308181
2.323257	2.318536	2.335685	2.330941	2.326217	2.321476	2.316735	2.312043	2.307411
2.322566	2.317791	2.334889	2.330203	2.325555	2.320818	2.316037	2.311306	2.306628
2.321908	2.317098	2.33414	2.329444	2.324795	2.320061	2.315287	2.310546	2.305836
2.321267	2.316434	2.33342	2.328677	2.323984	2.319255	2.314515	2.30978	2.305042
2.320558	2.315755	2.324934	2.3202	2.315507	2.318502	2.313788	2.309057	2.304297
2.319784	2.315065	2.3243	2.319545	2.314809	2.317795	2.313104	2.308375	2.303597
2.319035	2.314356	2.323613	2.31885	2.314094	2.317068	2.312379	2.307659	2.302903
2.318326	2.313612	2.322832	2.318078	2.31334	2.316294	2.311577	2.306883	2.302218
2.317644	2.312886	2.322059	2.317318	2.312602	2.315538	2.310791	2.306113	2.301514
2.330989	2.326152	2.321356	2.316622	2.311919	2.307196	2.302473	2.305385	2.300754
2.330347	2.325498	2.320686	2.315956	2.311258	2.306534	2.301803	2.304679	2.299985
2.329688	2.324848	2.320041	2.315297	2.310583	2.305858	2.301136	2.303997	2.299273
2.329022	2.324206	2.319417	2.314649	2.309904	2.305181	2.300478	2.303334	2.298599
2.32837	2.323561	2.318777	2.314004	2.309251	2.304524	2.299819	2.302667	2.297931
2.327735	2.322915	2.318118	2.313361	2.308626	2.303889	2.299158	2.301995	2.297272
2.327083	2.32226	2.31746	2.312714	2.307988	2.303247	2.298505	2.293795	2.289114
2.326391	2.32159	2.316811	2.312056	2.30732	2.302589	2.297869	2.293163	2.288468

2.325705	2.320922	2.316161	2.311397	2.306649	2.301927	2.297227	2.292527	2.28782
2.325069	2.320273	2.315501	2.31074	2.305996	2.301267	2.296552	2.291852	2.287162
2.324452	2.319632	2.31484	2.310085	2.305351	2.300607	2.295866	2.291164	2.286499
2.323805	2.318992	2.314203	2.30944	2.304695	2.299947	2.295205	2.290502	2.285837
2.323139	2.318353	2.313583	2.308803	2.304033	2.299287	2.294562	2.28986	2.285175
2.322481	2.317706	2.312943	2.308164	2.303392	2.298646	2.293921	2.289218	2.284533
2.321834	2.317043	2.312272	2.307517	2.302773	2.298021	2.293271	2.288569	2.283909
2.321194	2.316394	2.311622	2.30689	2.302173	2.297422	2.29266	2.287959	2.283315
2.320548	2.315767	2.311017	2.306304	2.301607	2.296878	2.292136	2.287437	2.282777
2.319928	2.315165	2.310434	2.30573	2.301041	2.296332	2.291615	2.28692	2.28224
2.319389	2.314615	2.309866	2.305139	2.300427	2.295711	2.290999	2.286309	2.281636
2.318891	2.31409	2.309305	2.304539	2.299789	2.295054	2.290335	2.28565	2.280998
2.318319	2.313502	2.3087	2.303923	2.299165	2.294416	2.289683	2.284999	2.280364
2.317684	2.312858	2.308051	2.303291	2.29855	2.293792	2.289034	2.284346	2.279729
2.317072	2.312246	2.30744	2.302683	2.297946	2.293189	2.288432	2.283747	2.279137
2.316512	2.311695	2.306894	2.302117	2.297361	2.292623	2.287907	2.283236	2.27861
2.315934	2.311131	2.306337	2.301541	2.296765	2.292044	2.287367	2.282712	2.278083
2.315283	2.310496	2.305714	2.300908	2.296118	2.291401	2.286737	2.282108	2.27752
2.314625	2.309851	2.305081	2.300271	2.295475	2.290754	2.286091	2.281483	2.276937
2.314048	2.309272	2.304502	2.299703	2.294918	2.290192	2.285512	2.280887	2.276324
2.313524	2.308733	2.303957	2.29918	2.294418	2.289683	2.284974	2.28031	2.275692
2.313005	2.308203	2.303421	2.298656	2.293908	2.289172	2.284452	2.279769	2.275121
2.312494	2.307684	2.302896	2.298133	2.293391	2.288662	2.28395	2.279268	2.274614
2.311981	2.307169	2.302378	2.297614	2.292873	2.28815	2.283446	2.278766	2.27411
2.311463	2.306659	2.301874	2.297104	2.292355	2.287633	2.282934	2.278257	2.273601
2.310935	2.30614	2.301361	2.296589	2.291834	2.28711	2.282412	2.277736	2.273081
2.310394	2.305599	2.300823	2.296061	2.291315	2.286588	2.281878	2.284867	2.280184
2.309836	2.305038	2.300262	2.295514	2.290783	2.28605	2.281324	2.284304	2.279626
2.309235	2.304431	2.29965	2.294906	2.29018	2.285444	2.280709	2.283683	2.279006
2.308601	2.303788	2.298999	2.294251	2.289524	2.284784	2.280046	2.283017	2.278337
2.308063	2.303256	2.29847	2.293712	2.288969	2.284217	2.279467	2.282424	2.27773
2.307646	2.302867	2.298098	2.29332	2.288542	2.283762	2.278986	2.281913	2.277189
2.30722	2.302461	2.297705	2.292914	2.288115	2.283324	2.27854	2.28145	2.276704
2.306736	2.301971	2.297213	2.292428	2.287644	2.282881	2.278132	2.281055	2.276307
2.306229	2.301454	2.29669	2.291917	2.287155	2.282425	2.277717	2.28066	2.27592
2.305709	2.300932	2.296169	2.291401	2.286646	2.281921	2.277221	2.280174	2.275454
2.305177	2.300401	2.29564	2.290875	2.286121	2.281391	2.276681	2.279641	2.274949
2.304656	2.299875	2.29511	2.290346	2.285594	2.288579	2.283832	2.279118	2.274436
2.304143	2.299351	2.294576	2.289812	2.285064	2.28805	2.283308	2.278595	2.27391
2.303636	2.298837	2.294058	2.289294	2.28455	2.287535	2.282795	2.292421	2.287649
2.303128	2.298327	2.293552	2.288791	2.28405	2.287032	2.282287	2.291904	2.287134
2.302653	2.297849	2.293075	2.288318	2.283581	2.286559	2.28181	2.291419	2.286654
2.302253	2.297438	2.292655	2.287904	2.283176	2.286151	2.281396	2.291	2.28625
2.301867	2.297043	2.29225	2.287504	2.282784	2.285755	2.280998	2.290597	2.285862
2.3014	2.296587	2.299601	2.294815	2.290051	2.285293	2.280547	2.290137	2.285393
2.300891	2.2961	2.299125	2.294328	2.289548	2.284797	2.280071	2.28965	2.284882
2.300449	2.295665	2.29869	2.293886	2.289098	2.284355	2.279644	2.289214	2.284431
2.300071	2.295281	2.298295	2.293489	2.288704	2.283968	2.27927	2.288831	2.28404
2.299704	2.294902	2.297901	2.293085	2.288292	2.283555	2.278859	2.288415	2.283625
2.299356	2.294533	2.297505	2.292659	2.287836	2.283077	2.278367	2.287923	2.283153

2.298975	2.294141	2.297098	2.292233	2.287389	2.282612	2.277887	2.287442	2.282689
2.298488	2.293678	2.296658	2.291817	2.286993	2.282222	2.277494	2.287035	2.282281
2.29797	2.293195	2.296212	2.291411	2.286622	2.281867	2.277139	2.286662	2.281898
2.297544	2.292783	2.295813	2.291029	2.286257	2.281508	2.27678	2.286286	2.281513
2.29718	2.29242	2.295447	2.290668	2.2859	2.281154	2.276426	2.285914	2.281131
2.304662	2.29986	2.295077	2.290303	2.285541	2.280791	2.276052	2.285519	2.280719
2.304315	2.299497	2.2947	2.289932	2.28518	2.28042	2.27566	2.2851	2.280269
2.303962	2.299138	2.294335	2.289569	2.284816	2.28004	2.275253	2.284669	2.279826
2.30359	2.298788	2.293999	2.289224	2.28445	2.279641	2.274814	2.284212	2.279402
2.303216	2.298434	2.29366	2.288876	2.284083	2.279248	2.274391	2.283775	2.278995
2.302851	2.298061	2.293281	2.2885	2.283715	2.278897	2.274062	2.301612	2.296725
2.302488	2.297678	2.292884	2.288111	2.283346	2.278562	2.273769	2.301293	2.296354
2.302113	2.297303	2.292508	2.287733	2.282965	2.278177	2.27338	2.300887	2.295959
2.301727	2.296932	2.292147	2.287363	2.282575	2.277752	2.272911	2.30041	2.295546
2.301348	2.296556	2.291773	2.286989	2.282199	2.277369	2.272519	2.3	2.295148
2.300982	2.296171	2.291376	2.286608	2.281847	2.277053	2.272244	2.299687	2.294765
2.30061	2.295786	2.290985	2.286228	2.281488	2.276731	2.271966	2.299386	2.294415
2.300203	2.295399	2.29062	2.285851	2.281097	2.276356	2.271626	2.299069	2.294133
2.299812	2.295023	2.290262	2.285478	2.280706	2.275971	2.271263	2.298737	2.293861
2.299519	2.29468	2.28988	2.285102	2.280348	2.275608	2.270886	2.298352	2.293516
2.299284	2.294364	2.289495	2.284733	2.280015	2.275262	2.270502	2.29794	2.293131
2.299005	2.294073	2.28919	2.284428	2.279713	2.293888	2.289038	2.297595	2.292762
2.298689	2.293814	2.288969	2.284192	2.279446	2.29362	2.288799	2.297319	2.292404
2.298348	2.293524	2.288715	2.283922	2.279148	2.293315	2.288519	2.297026	2.292074
2.297959	2.293166	2.288387	2.283579	2.278781	2.292936	2.288163	2.296702	2.291797
2.297598	2.292821	2.288057	2.28324	2.278428	2.292561	2.287785	2.296352	2.291508
2.297341	2.292551	2.287777	2.282968	2.278161	2.292242	2.287397	2.295945	2.291139
2.297128	2.292307	2.287504	2.282711	2.27792	2.291945	2.287017	2.29553	2.29075
2.296894	2.292021	2.287177	2.28239	2.27762	2.291639	2.286707	2.295209	2.290428
2.296651	2.291712	2.286815	2.282029	2.277284	2.291333	2.286449	2.294952	2.290147
2.296387	2.291433	2.286521	2.28173	2.276987	2.291043	2.28619	2.294681	2.289837
2.296097	2.291189	2.286307	2.281503	2.276734	2.290774	2.285929	2.294393	2.289487
2.295821	2.290952	2.30512	2.300228	2.29536	2.290509	2.285673	2.294119	2.289166
2.295574	2.290716	2.304881	2.299984	2.295108	2.290262	2.285438	2.293878	2.28891
2.295343	2.290485	2.304637	2.299725	2.294835	2.289992	2.285183	2.293631	2.288668
2.295127	2.290261	2.318051	2.313007	2.307988	2.303067	2.298213	2.293325	2.288397
2.294923	2.290042	2.317787	2.312678	2.307597	2.302669	2.297846	2.292997	2.288119
2.294718	2.28983	2.317544	2.312402	2.307288	2.302351	2.297534	2.292701	2.287855
2.294514	2.289625	2.317318	2.312168	2.307046	2.302095	2.297263	2.292431	2.2876
2.313397	2.308433	2.317086	2.311933	2.306809	2.30185	2.297007	2.292172	2.28735
2.313159	2.308195	2.316842	2.311693	2.306572	2.301612	2.296765	2.291927	2.287101
2.312916	2.307955	2.316601	2.31146	2.306346	2.301385	2.296531	2.291687	2.286857
2.312668	2.307718	2.316369	2.311236	2.306125	2.301159	2.296293	2.291442	2.286612
2.312418	2.307481	2.316141	2.311025	2.305927	2.300956	2.296073	2.291211	2.286374
2.312171	2.307242	2.315916	2.310845	2.305792	2.300823	2.295916	2.291026	2.286153
2.311925	2.307001	2.315692	2.310682	2.30569	2.300729	2.295795	2.290868	2.285945
2.311674	2.306754	2.315452	2.310475	2.305517	2.300569	2.295633	2.290693	2.285738
2.311415	2.306497	2.315195	2.310228	2.305278	2.30035	2.295438	2.290503	2.285533
2.311198	2.306268	2.31495	2.309982	2.305035	2.300119	2.295227	2.290307	2.285351
2.311051	2.306075	2.314714	2.309733	2.304784	2.299874	2.294996	2.290109	2.285211

2.310911	2.305906	2.314514	2.309519	2.304564	2.299648	2.294768	2.289903	2.285057
2.310745	2.305773	2.314397	2.3094	2.304429	2.299481	2.294558	2.313483	2.308496
2.310565	2.305644	2.3143	2.309307	2.30432	2.299339	2.294368	2.313236	2.308237
2.310349	2.305438	2.314111	2.30913	2.304158	2.299189	2.29423	2.313064	2.308054
2.310111	2.305186	2.313865	2.308902	2.303962	2.299037	2.294127	2.312943	2.30792
2.309941	2.305005	2.313674	2.308702	2.30376	2.298847	2.293962	2.312763	2.307744
2.309848	2.304904	2.313543	2.30853	2.303543	2.29861	2.293721	2.312513	2.307521
2.309753	2.304798	2.31341	2.308373	2.303361	2.298412	2.293514	2.312284	2.3073
2.309635	2.304656	2.313249	2.30823	2.303242	2.298298	2.293391	2.312108	2.307086
2.309508	2.304511	2.313095	2.308102	2.303144	2.298204	2.293284	2.311947	2.306889
2.309363	2.304383	2.299429	2.29452	2.289634	2.284748	2.279869	2.311775	2.306723
2.309214	2.304263	2.299333	2.294421	2.289523	2.28462	2.279722	2.311608	2.30658
2.309094	2.304151	2.299224	2.2943	2.289387	2.284488	2.279605	2.311474	2.306457
2.308998	2.304049	2.299108	2.294161	2.289231	2.284352	2.279511	2.311367	2.306353
2.308904	2.303935	2.298977	2.294028	2.289103	2.284239	2.279421	2.311268	2.306258
2.308806	2.303795	2.298812	2.293892	2.289012	2.284159	2.279335	2.311177	2.306176
2.308729	2.303693	2.298695	2.293789	2.28893	2.309216	2.304253	2.311083	2.306084
2.308699	2.303691	2.298702	2.293757	2.288849	2.309111	2.304154	2.310977	2.30597
2.30867	2.303695	2.298719	2.293733	2.288773	2.309008	2.304054	2.310864	2.30584
2.308579	2.303585	2.2986	2.293637	2.288704	2.308916	2.303949	2.310738	2.30568
2.308459	2.303422	2.29842	2.293507	2.288638	2.308828	2.303843	2.310607	2.30551
2.308373	2.303336	2.298335	2.293421	2.288552	2.308726	2.303748	2.310513	2.305417
2.308319	2.303323	2.298339	2.293376	2.288448	2.30861	2.303665	2.310456	2.305396
2.308237	2.303256	2.298278	2.293299	2.288352	2.308498	2.30357	2.31038	2.305351
2.308101	2.303082	2.298091	2.293159	2.288272	2.308396	2.303456	2.31027	2.30526
2.307979	2.302935	2.297934	2.293036	2.28819	2.30829	2.303341	2.310154	2.305153
2.30793	2.302916	2.297924	2.292981	2.28808	2.308159	2.30323	2.310043	2.305034
2.307898	2.302933	2.29796	2.292959	2.287983	2.308035	2.303124	2.309931	2.304909
2.307802	2.302874	2.29794	2.292958	2.287986	2.307978	2.303017	2.309804	2.304795
2.307673	2.302774	2.297889	2.292973	2.288053	2.307967	2.302916	2.309672	2.304688
2.307628	2.302698	2.323053	2.318022	2.313	2.307942	2.302868	2.309599	2.304593
2.307678	2.302644	2.322909	2.317894	2.312917	2.307906	2.302881	2.309591	2.304508
2.300125	2.29503	2.322814	2.317791	2.312826	2.307851	2.302879	2.309587	2.304456
2.300091	2.295046	2.322815	2.317745	2.312722	2.307754	2.302828	2.309566	2.30445
2.300032	2.295058	2.322833	2.317721	2.312642	2.307673	2.302778	2.309549	2.304468
2.299936	2.295005	2.322801	2.317706	2.312636	2.307669	2.302772	2.309556	2.304504
2.299836	2.294934	2.322757	2.317703	2.312668	2.3077	2.302777	2.309564	2.304543
2.299826	2.294924	2.316289	2.311251	2.306231	2.307706	2.302742	2.309519	2.304524
2.299888	2.294959	2.316291	2.311265	2.306255	2.307699	2.302676	2.309433	2.304461
2.299945	2.294974	2.316259	2.311241	2.306247	2.307686	2.302643	2.309389	2.304424
2.299991	2.294954	2.316176	2.311162	2.306192	2.30766	2.302651	2.309402	2.304429
2.300047	2.294961	2.316124	2.31111	2.306154	2.301177	2.2962	2.309419	2.304422
2.300123	2.295028	2.316152	2.311128	2.306166	2.30119	2.29622	2.309417	2.30437
2.300205	2.295115	2.316208	2.311174	2.3062	2.301219	2.296247	2.309422	2.304324
2.300284	2.295191	2.316259	2.311222	2.306245	2.301262	2.296288	2.309453	2.304338
2.300355	2.295262	2.316313	2.311277	2.306299	2.301315	2.296338	2.309501	2.304387
2.319094	2.313941	2.316359	2.311322	2.30634	2.301358	2.296387	2.30359	2.298506
2.319073	2.313961	2.316398	2.31136	2.306369	2.301395	2.296441	2.303674	2.298591
2.319073	2.313994	2.316438	2.311385	2.30637	2.301396	2.296454	2.303729	2.298665
2.319109	2.314047	2.316478	2.311379	2.306312	2.301324	2.296393	2.30373	2.298718

2.319159	2.314109	2.316531	2.311404	2.306303	2.301749	2.296833	2.303754	2.29878
2.319213	2.314174	2.316614	2.311518	2.306443	2.301912	2.296966	2.303863	2.298872
2.319285	2.314246	2.316706	2.311657	2.306628	2.302122	2.297141	2.304001	2.298975
2.319403	2.314335	2.316774	2.311737	2.306727	2.302254	2.297269	2.304114	2.299066
2.319553	2.314435	2.31683	2.311785	2.306781	2.302346	2.297374	2.304216	2.299154
2.319668	2.314518	2.316881	2.31183	2.30683	2.302435	2.297473	2.292494	2.28749
2.319749	2.314581	2.316924	2.311869	2.306871	2.302516	2.297562	2.292589	2.28759
2.319826	2.314652	2.317616	2.312563	2.307571	2.302599	2.297655	2.292695	2.287717
2.319905	2.314733	2.317716	2.31265	2.307645	2.302678	2.297752	2.292823	2.287895
2.319987	2.314833	2.317846	2.312768	2.307747	2.302779	2.297859	2.292953	2.288067
2.310582	2.305558	2.318043	2.312965	2.307926	2.302932	2.297981	2.293061	2.288176
2.310678	2.305711	2.318263	2.313189	2.308136	2.303114	2.298121	2.293171	2.288268
2.318741	2.313716	2.318403	2.313343	2.308301	2.303288	2.2983	2.293344	2.288423
2.318822	2.313779	2.318487	2.313448	2.308439	2.303461	2.29851	2.293563	2.288621
2.318952	2.313897	2.31862	2.313577	2.308569	2.303602	2.298668	2.293728	2.288782
2.319143	2.314084	2.318815	2.313735	2.308685	2.303696	2.298754	2.293818	2.288894
2.320435	2.315382	2.31901	2.313904	2.308827	2.303818	2.298863	2.293925	2.289011
2.320628	2.315553	2.319175	2.314082	2.309023	2.304013	2.299046	2.294089	2.289145
2.320813	2.31572	2.319343	2.314271	2.309236	2.304227	2.299245	2.294268	2.289295
2.320986	2.315905	2.319543	2.314474	2.309437	2.304416	2.299414	2.294431	2.289465
2.321155	2.316101	2.31976	2.314686	2.309635	2.304596	2.299576	2.294595	2.289654
2.321353	2.31631	2.311285	2.30627	2.301274	2.304777	2.299763	2.294789	2.28986
2.321574	2.316532	2.311498	2.306456	2.301434	2.304956	2.299972	2.295012	2.290082
2.321801	2.316742	2.311691	2.306642	2.301619	2.305158	2.300194	2.295244	2.290315
2.322026	2.316922	2.311843	2.306819	2.301836	2.305391	2.300429	2.295486	2.290565
2.322266	2.317134	2.312036	2.307028	2.302071	2.305631	2.300668	2.295725	2.290807
2.322541	2.317439	2.312355	2.307318	2.302321	2.305864	2.300903	2.295956	2.291024
2.322822	2.31776	2.312697	2.307626	2.302579	2.306101	2.301139	2.296179	2.291226
2.323066	2.31799	2.312919	2.307862	2.30283	2.306352	2.30137	2.296386	2.291401
2.323291	2.318173	2.31308	2.30806	2.303079	2.306612	2.3016	2.296586	2.291565
2.323542	2.31842	2.313324	2.308304	2.303324	2.298364	2.293426	2.296833	2.291808
2.323819	2.318735	2.313657	2.308597	2.303565	2.298601	2.293688	2.297131	2.292138
2.324075	2.319007	2.313937	2.308858	2.303802	2.298839	2.29394	2.29741	2.292445
2.324284	2.319181	2.314094	2.309046	2.304034	2.299081	2.294174	2.297644	2.292697
2.324498	2.319367	2.314265	2.309243	2.304267	2.29932	2.294404	2.297871	2.292933
2.324764	2.319657	2.314565	2.309511	2.304494	2.299539	2.294636	2.298111	2.29316
2.325044	2.319982	2.31491	2.309812	2.304733	2.299764	2.294871	2.298354	2.293383
2.325283	2.32025	2.315209	2.310123	2.305044	2.300049	2.295112	2.29858	2.293619
2.325495	2.320483	2.315481	2.310446	2.305409	2.300379	2.295362	2.298796	2.293863
2.311138	2.306132	2.315728	2.310725	2.305727	2.300697	2.295656	2.290692	2.285812
2.311501	2.306412	2.315937	2.310946	2.30599	2.301003	2.296006	2.291037	2.28609
2.311855	2.306715	2.316187	2.311188	2.306243	2.30129	2.296345	2.291383	2.286395
2.312169	2.307058	2.316529	2.311484	2.306484	2.301536	2.29663	2.291699	2.286735
2.312465	2.307403	2.316891	2.311803	2.306745	2.301794	2.296917	2.292017	2.287093
2.31273	2.307699	2.31721	2.312135	2.307083	2.302134	2.297257	2.292365	2.287462
2.312988	2.307981	2.317517	2.31248	2.307463	2.302515	2.297617	2.29272	2.287831
2.313319	2.308317	2.303337	2.298344	2.293363	2.302867	2.297931	2.293022	2.288149
2.313711	2.308697	2.303705	2.298718	2.29374	2.303199	2.298207	2.293278	2.288421
2.314099	2.309056	2.304038	2.299056	2.294088	2.303525	2.298513	2.293576	2.288723
2.314478	2.309375	2.304312	2.299332	2.294389	2.303839	2.298862	2.293939	2.289076

2.299986	2.294999	2.304612	2.299634	2.294707	2.289766	2.284831	2.294308	2.289422
2.300299	2.295341	2.304996	2.30002	2.295086	2.290146	2.285211	2.294654	2.289721
2.300611	2.2957	2.305411	2.300434	2.295486	2.290539	2.285598	2.295003	2.290022
2.300968	2.296075	2.305813	2.300831	2.295874	2.290929	2.285996	2.295383	2.290383
2.301352	2.296463	2.306215	2.301221	2.296255	2.291316	2.286397	2.295779	2.29078
2.301742	2.296848	2.306607	2.301583	2.296588	2.291646	2.286743	2.281812	2.276847
2.302139	2.297232	2.306989	2.301914	2.296867	2.291915	2.28703	2.282132	2.27722
2.302536	2.29762	2.307383	2.302271	2.297187	2.292225	2.287349	2.282474	2.277603
2.302929	2.298014	2.307796	2.302675	2.297581	2.29261	2.287728	2.282857	2.278004
2.303323	2.29841	2.308214	2.303091	2.297993	2.293016	2.288125	2.283253	2.278406
2.303722	2.298804	2.308623	2.303495	2.298393	2.293414	2.288524	2.283651	2.278803
2.304121	2.299198	2.309032	2.303902	2.298797	2.293818	2.288929	2.284054	2.279197
2.304514	2.299593	2.309453	2.304322	2.299213	2.294226	2.289326	2.284444	2.279586
2.304903	2.29999	2.309882	2.304753	2.299642	2.294642	2.289722	2.28483	2.279971
2.30529	2.300388	2.310318	2.305211	2.300117	2.295109	2.290164	2.28525	2.280369
2.305675	2.300791	2.310764	2.305705	2.300654	2.295645	2.290666	2.285712	2.280782
2.306057	2.301186	2.296318	2.291436	2.286553	2.296149	2.291151	2.286169	2.281198
2.306424	2.301562	2.296704	2.291831	2.286955	2.296579	2.291588	2.2866	2.28161
2.306812	2.301947	2.297087	2.292211	2.287334	2.296984	2.292007	2.287026	2.282035
2.307266	2.302367	2.297479	2.292591	2.287712	2.297384	2.292418	2.287458	2.282502
2.307755	2.302811	2.297886	2.29298	2.288095	2.297784	2.292824	2.28789	2.282985
2.308222	2.30328	2.298351	2.293438	2.288542	2.298231	2.293246	2.288308	2.283421
2.308676	2.303773	2.29887	2.293957	2.289043	2.298717	2.29368	2.288713	2.28382
2.30911	2.304229	2.299345	2.294441	2.289528	2.29921	2.29415	2.289168	2.284266
2.309509	2.304625	2.299751	2.294866	2.289979	2.299706	2.294667	2.289691	2.28478
2.309934	2.305045	2.300175	2.295308	2.290444	2.300214	2.295196	2.290223	2.285294
2.310433	2.305547	2.30068	2.29582	2.290963	2.300757	2.295732	2.290737	2.285769
2.310956	2.306067	2.301196	2.296336	2.291478	2.301294	2.296261	2.291246	2.286242
2.311461	2.306541	2.301643	2.29677	2.291908	2.301757	2.296744	2.291742	2.286744
2.31196	2.306997	2.302061	2.297166	2.292298	2.302185	2.297205	2.292233	2.287265
2.303953	2.299042	2.302535	2.297637	2.292768	2.287915	2.28308	2.292751	2.287796
2.304425	2.299543	2.303061	2.298177	2.293316	2.288458	2.28361	2.293296	2.288341
2.304924	2.300065	2.303599	2.298724	2.293864	2.288999	2.284137	2.293834	2.288869
2.305464	2.300611	2.304145	2.299266	2.294399	2.289527	2.284655	2.294356	2.289364
2.306018	2.301167	2.304697	2.299811	2.294934	2.290049	2.285164	2.294871	2.289863
2.295932	2.291132	2.305252	2.300356	2.295462	2.290553	2.285638	2.295362	2.290387
2.296485	2.291692	2.30581	2.300903	2.295993	2.29106	2.286119	2.295864	2.290929
2.297048	2.29225	2.306366	2.301457	2.296546	2.309822	2.304796	2.313756	2.308712
2.297619	2.292809	2.30692	2.302015	2.297116	2.310418	2.305432	2.314418	2.309323
2.29818	2.293364	2.307471	2.302564	2.297666	2.310971	2.30599	2.315	2.309902
2.298732	2.293914	2.308016	2.303102	2.298192	2.31147	2.306449	2.315482	2.310442
2.299286	2.294465	2.298457	2.293581	2.288708	2.312001	2.306962	2.316018	2.311005
2.299852	2.295022	2.299011	2.294146	2.28929	2.312609	2.307602	2.316678	2.311607
2.300416	2.29558	2.299569	2.294715	2.289877	2.313229	2.308263	2.317366	2.312239
2.300962	2.296135	2.300131	2.295276	2.290439	2.313809	2.308868	2.31802	2.312914
2.301506	2.296692	2.300696	2.295834	2.290992	2.314375	2.309451	2.31866	2.313608
2.274551	2.269837	2.292452	2.287665	2.2829	2.314938	2.310022	2.305115	2.30022
2.275131	2.270409	2.293	2.288218	2.283464	2.315498	2.310581	2.305678	2.30079
2.275754	2.271028	2.293604	2.288805	2.284034	2.316061	2.311145	2.306243	2.301356
2.276437	2.271715	2.294289	2.289433	2.2846	2.316617	2.311708	2.306805	2.301908

2.277104	2.272385	2.285456	2.280596	2.275745	2.307826	2.302954	2.307399	2.302497
2.277713	2.272991	2.286073	2.281249	2.276436	2.308517	2.303624	2.308065	2.30317
2.2783	2.273573	2.28667	2.281896	2.277136	2.309221	2.304306	2.308744	2.303859
2.258576	2.25385	2.28725	2.282494	2.277754	2.309839	2.304918	2.309361	2.304484
2.259138	2.254411	2.287824	2.283069	2.278334	2.310417	2.305502	2.309953	2.305081
2.259753	2.255032	2.269421	2.264657	2.259913	2.31105	2.306142	2.310599	2.30572
2.260421	2.255712	2.270116	2.265335	2.26057	2.311732	2.306838	2.311298	2.306398
2.261054	2.256351	2.270768	2.265977	2.261199	2.287119	2.282229	2.30261	2.297737
2.261618	2.256909	2.271338	2.266546	2.261768	2.287688	2.282792	2.303178	2.298304
2.2622	2.257483	2.271919	2.267129	2.262354	2.288274	2.283368	2.30376	2.298891
2.262863	2.258138	2.272568	2.267782	2.263012	2.288931	2.284016	2.304419	2.29956
2.263561	2.258828	2.273249	2.268466	2.263701	2.28962	2.284696	2.305113	2.300264
2.26425	2.259515	2.273936	2.269152	2.264386	2.281053	2.276224	2.280501	2.275659
2.264942	2.260207	2.274636	2.269846	2.265075	2.281748	2.276924	2.281199	2.276345
2.26564	2.260902	2.275335	2.270539	2.265761	2.282434	2.27761	2.281884	2.27702
2.266345	2.261602	2.276036	2.27123	2.266443	2.283113	2.278285	2.282558	2.27769
2.267044	2.262293	2.276726	2.271915	2.26712	2.283777	2.27893	2.283196	2.278326
2.26774	2.262974	2.277404	2.272594	2.267796	2.284419	2.279514	2.283758	2.278896
2.268413	2.263636	2.278063	2.273255	2.268457	2.285055	2.280105	2.284334	2.279476
2.269011	2.264235	2.278667	2.273862	2.26907	2.285685	2.28076	2.285	2.280132
2.269597	2.264828	2.279268	2.274464	2.26968	2.28633	2.28146	2.285723	2.280838
2.270322	2.265555	2.280003	2.275191	2.2704	2.287069	2.282228	2.286504	2.281615
2.271155	2.266389	2.280847	2.276017	2.271206	2.287885	2.283056	2.287341	2.282456
2.271951	2.267182	2.281643	2.276805	2.271984	2.288667	2.283839	2.288128	2.283247
2.272699	2.267921	2.282376	2.277542	2.272727	2.289404	2.284564	2.28885	2.283977
2.27343	2.268644	2.283095	2.278265	2.273455	2.290127	2.285274	2.280448	2.275653
2.274145	2.269357	2.28381	2.278981	2.274171	2.29084	2.285979	2.281147	2.276347
2.274851	2.270064	2.284521	2.279691	2.274879	2.291548	2.286682	2.281844	2.277038
2.275556	2.270773	2.266005	2.261248	2.256513	2.292255	2.287389	2.282552	2.277747
2.276262	2.271484	2.266718	2.261956	2.257214	2.292963	2.2881	2.283268	2.278472
2.276984	2.272203	2.267434	2.262671	2.257927	2.293683	2.28882	2.284002	2.279236
2.277719	2.272929	2.268152	2.263389	2.258649	2.294411	2.289546	2.284751	2.28004
2.278493	2.273697	2.268916	2.264154	2.259417	2.295185	2.290312	2.285521	2.280827
2.27933	2.274533	2.269751	2.264992	2.260256	2.29603	2.291139	2.286321	2.281585
2.280154	2.27536	2.270581	2.265823	2.261085	2.296866	2.291962	2.287115	2.282331
2.280898	2.276116	2.271345	2.266582	2.261834	2.297623	2.292724	2.287868	2.283056
2.281627	2.27686	2.272101	2.267331	2.262572	2.298369	2.29348	2.288621	2.283788
2.282429	2.27767	2.272918	2.268149	2.263387	2.299187	2.294294	2.289428	2.284585
2.283272	2.278517	2.273769	2.269006	2.264249	2.300049	2.295145	2.290271	2.285426
2.284065	2.279305	2.274561	2.269825	2.265097	2.300912	2.295995	2.291115	2.286274
2.284803	2.280029	2.275288	2.270604	2.265938	2.301783	2.296849	2.291967	2.28714
2.285569	2.280782	2.276038	2.271376	2.266738	2.302604	2.297666	2.292784	2.287959
2.286387	2.281595	2.276838	2.272137	2.267459	2.303328	2.298411	2.293532	2.288688
2.287227	2.282432	2.277659	2.272913	2.268186	2.304053	2.299158	2.294282	2.289418
2.288079	2.283276	2.278495	2.273734	2.268994	2.304861	2.299967	2.29509	2.290225
2.288943	2.284131	2.279342	2.27458	2.269839	2.305708	2.300806	2.295926	2.291067
2.289805	2.284985	2.280188	2.275421	2.270677	2.306559	2.301655	2.296775	2.29192
2.290669	2.285841	2.281036	2.276262	2.271515	2.30742	2.302517	2.297639	2.292787
2.275234	2.270465	2.281885	2.277107	2.272357	2.30828	2.30338	2.298504	2.293652
2.276088	2.271314	2.282736	2.277954	2.273201	2.309136	2.304245	2.299371	2.294516

2.276945	2.272168	2.283591	2.278804	2.274047	2.309993	2.305106	2.300233	2.295376
2.277813	2.273031	2.284452	2.279658	2.274895	2.310857	2.305961	2.301084	2.296229
2.27868	2.273895	2.285314	2.280513	2.275742	2.311719	2.306813	2.301932	2.29708
2.279535	2.274746	2.286169	2.28136	2.276582	2.312569	2.30766	2.30278	2.297933
2.280382	2.275591	2.28702	2.282205	2.277419	2.313414	2.308505	2.303629	2.298788
2.281228	2.276436	2.287871	2.283051	2.278261	2.314272	2.309362	2.30449	2.299659
2.282071	2.27728	2.28872	2.283898	2.279105	2.315142	2.310227	2.305361	2.300544
2.282917	2.278131	2.289581	2.284753	2.279955	2.275216	2.270528	2.284891	2.280214
2.283763	2.278986	2.290449	2.285611	2.2808	2.276065	2.271391	2.285787	2.281131
2.275044	2.270315	2.291343	2.286499	2.28168	2.276945	2.272274	2.286692	2.282044
2.275915	2.271214	2.292289	2.287458	2.282641	2.277893	2.273197	2.287604	2.282937
2.276802	2.272124	2.293248	2.288433	2.283621	2.278857	2.27413	2.288516	2.283822
2.277697	2.273014	2.277876	2.27313	2.268387	2.27976	2.275018	2.289404	2.284705
2.278599	2.273892	2.27875	2.274	2.269257	2.280633	2.275886	2.290281	2.285589
2.279481	2.274764	2.270053	2.265351	2.26066	2.281561	2.276817	2.291221	2.28652
2.280343	2.275628	2.270931	2.266258	2.261602	2.282549	2.277816	2.292228	2.287498
2.28121	2.276502	2.271817	2.267159	2.262518	2.274607	2.269908	2.293207	2.288469
2.282088	2.277395	2.272719	2.268045	2.263384	2.275476	2.270783	2.294122	2.28942
2.282979	2.278292	2.273615	2.268923	2.264243	2.276338	2.271653	2.29503	2.290354
2.283887	2.279168	2.274471	2.269782	2.265116	2.277235	2.27257	2.295963	2.291255
2.284816	2.280051	2.275324	2.270643	2.266001	2.27815	2.273507	2.296909	2.292148
2.285787	2.280996	2.276245	2.271558	2.266916	2.279082	2.274452	2.289624	2.284867
2.286794	2.281989	2.277217	2.272514	2.267858	2.280032	2.275404	2.290597	2.285844
2.287784	2.282975	2.278193	2.273475	2.268801	2.280968	2.276318	2.291517	2.286773
2.28876	2.28396	2.279179	2.274445	2.269744	2.281881	2.277176	2.292365	2.287637
2.289703	2.284915	2.280143	2.275408	2.270697	2.282816	2.278066	2.293247	2.288528
2.290577	2.285806	2.28106	2.276353	2.271666	2.283798	2.279031	2.294215	2.289485
2.291451	2.286694	2.281974	2.277298	2.272642	2.284796	2.280022	2.295214	2.290469
2.277577	2.272868	2.28293	2.278266	2.273621	2.268932	2.264224	2.326509	2.321645
2.27856	2.273845	2.283908	2.279245	2.274603	2.269912	2.2652	2.327528	2.32266
2.259999	2.255345	2.270148	2.265517	2.260902	2.270887	2.266172	2.328543	2.323672
2.260964	2.256313	2.271136	2.266499	2.261876	2.271858	2.26714	2.329556	2.324681
2.261931	2.257282	2.272124	2.267481	2.26285	2.258179	2.253491	2.330569	2.325692
2.262903	2.258253	2.273112	2.268463	2.263826	2.25915	2.254458	2.331584	2.326707
2.263872	2.25922	2.254602	2.250014	2.245438	2.26012	2.255424	2.332597	2.327721
2.264831	2.260179	2.255561	2.25097	2.246391	2.261087	2.256387	2.333604	2.328728
2.265788	2.261135	2.256516	2.251926	2.247347	2.262058	2.257354	2.334615	2.329736
2.266748	2.262093	2.257474	2.252889	2.248317	2.263043	2.258334	2.323908	2.319047
2.267708	2.26305	2.258433	2.253857	2.249296	2.264041	2.259329	2.324938	2.320061
2.268674	2.264004	2.259382	2.254818	2.250276	2.265059	2.260367	2.290848	2.28612
2.269641	2.264948	2.260316	2.25577	2.251258	2.266102	2.261456	2.291973	2.287211
2.270658	2.265943	2.261291	2.256739	2.252232	2.26712	2.262512	2.293062	2.288279
2.271762	2.267017	2.262325	2.257729	2.253184	2.268088	2.263503	2.294079	2.289291
2.272869	2.268111	2.263389	2.258746	2.254152	2.269059	2.264483	2.295086	2.290311
2.273925	2.269198	2.264489	2.259815	2.255177	2.250601	2.246075	2.296123	2.291397
2.274954	2.270273	2.265594	2.260899	2.25622	2.251613	2.247059	2.297164	2.292498
2.275948	2.271286	2.266622	2.261918	2.257223	2.252595	2.24802	2.280544	2.275974
2.276914	2.272252	2.267594	2.262894	2.258201	2.253562	2.248968	2.281512	2.276948
2.277923	2.273241	2.268578	2.263897	2.259228	2.254588	2.249977	2.282527	2.277942
2.278976	2.274251	2.269572	2.264932	2.260319	2.255686	2.251056	2.283592	2.278947

2.280064	2.275295	2.270587	2.265963	2.261378	2.256763	2.252143	2.284695	2.280012
2.281208	2.276392	2.271631	2.266964	2.262355	2.257777	2.253234	2.28587	2.281204
2.28235	2.277505	2.272701	2.267979	2.263326	2.258773	2.2543	2.287022	2.282389
2.283429	2.278613	2.273818	2.269059	2.264355	2.259771	2.255278	2.288026	2.283423
2.284471	2.279712	2.274951	2.270169	2.265414	2.260773	2.256218	2.288964	2.284384
2.285473	2.280738	2.276002	2.271236	2.266489	2.261826	2.257228	2.289966	2.285369
2.286433	2.281698	2.276984	2.272273	2.267583	2.262922	2.258293	2.291011	2.286362
2.287483	2.282727	2.278003	2.273307	2.268637	2.263987	2.259361	2.292099	2.28743
2.288671	2.283857	2.279073	2.274325	2.269623	2.264995	2.260428	2.293246	2.288609
2.289839	2.284993	2.280172	2.275386	2.270654	2.266034	2.261501	2.294381	2.289768
2.290898	2.286101	2.281321	2.276554	2.271822	2.26717	2.262583	2.295448	2.290813
2.291928	2.287193	2.282468	2.277734	2.273013	2.268325	2.263669	2.296508	2.291845
2.292985	2.288239	2.283514	2.278792	2.274087	2.269408	2.264756	2.297634	2.292977
2.294058	2.289266	2.284514	2.279793	2.275109	2.270458	2.265845	2.298791	2.294155
2.295187	2.290385	2.285626	2.280907	2.276224	2.271563	2.266931	2.299898	2.295254
2.296371	2.291594	2.286849	2.282131	2.277433	2.272722	2.268017	2.300953	2.296274
2.270505	2.265807	2.288034	2.283303	2.278583	2.273843	2.269102	2.302043	2.297347
2.271702	2.266956	2.289139	2.284363	2.279613	2.274886	2.270187	2.303202	2.298529
2.272868	2.268085	2.290236	2.285427	2.280655	2.275939	2.271275	2.304355	2.299697
2.273941	2.269183	2.291369	2.286579	2.281814	2.277075	2.272367	2.267742	2.2632
2.27499	2.270269	2.292504	2.287747	2.283	2.278234	2.273468	2.268791	2.264201
2.27613	2.271379	2.266659	2.261965	2.257291	2.279332	2.274586	2.269915	2.265313
2.277325	2.272505	2.267725	2.262997	2.258313	2.280395	2.275721	2.271091	2.266501
2.27847	2.273633	2.268842	2.264106	2.259422	2.281526	2.276884	2.272272	2.267687
2.279548	2.274758	2.270014	2.265302	2.26063	2.282737	2.278084	2.273465	2.268879
2.280676	2.275916	2.271204	2.266509	2.261844	2.257203	2.252589	2.274619	2.270023
2.281908	2.277136	2.272412	2.267719	2.263052	2.258375	2.253704	2.275694	2.271061
2.283168	2.278364	2.273609	2.268905	2.264228	2.25952	2.254805	2.276765	2.272103
2.284405	2.279551	2.274748	2.270006	2.265303	2.260599	2.255911	2.277899	2.27325
2.285634	2.280721	2.275863	2.271073	2.266341	2.26166	2.257029	2.27907	2.274451
2.286831	2.281891	2.277014	2.272217	2.267486	2.262817	2.25821	2.25364	2.249106
2.288001	2.283059	2.278193	2.273417	2.268711	2.264052	2.259444	2.254872	2.250336
2.253852	2.24903	2.279418	2.274657	2.269963	2.265303	2.260685	2.256101	2.251552
2.255061	2.250266	2.280702	2.275945	2.271244	2.266572	2.261936	2.257329	2.252754
2.2563	2.251526	2.282003	2.277241	2.272526	2.267836	2.263176	2.258541	2.253933
2.257574	2.252801	2.283306	2.278532	2.273796	2.269083	2.264396	2.259728	2.255076
2.258867	2.254088	2.284612	2.279817	2.275057	2.270315	2.265596	2.260893	2.256202
2.241887	2.237173	2.25062	2.245913	2.241235	2.271524	2.266761	2.26203	2.257326
2.243157	2.238418	2.251877	2.247148	2.242446	2.272712	2.267902	2.26315	2.25845
2.244414	2.239651	2.25312	2.248354	2.243613	2.238864	2.234123	2.264298	2.259618
2.245659	2.240875	2.254352	2.24953	2.244736	2.239975	2.235252	2.265471	2.260826
2.246895	2.242075	2.255551	2.250693	2.245871	2.241114	2.236414	2.266677	2.262053
2.248116	2.243231	2.256693	2.251832	2.247027	2.242291	2.23762	2.267924	2.2633
2.249344	2.244403	2.257857	2.252995	2.248208	2.243493	2.238846	2.269186	2.264555
2.250614	2.24565	2.259113	2.25423	2.249428	2.244714	2.240081	2.235485	2.230928
2.25188	2.246909	2.260397	2.255494	2.250674	2.245956	2.241327	2.236728	2.232163
2.253048	2.248086	2.261627	2.25674	2.251934	2.247215	2.242574	2.237965	2.23339
2.254155	2.249214	2.262827	2.25798	2.253209	2.248489	2.243825	2.239199	2.234613
2.255327	2.250391	2.245538	2.240796	2.236129	2.249752	2.245072	2.240435	2.235841
2.256561	2.251611	2.24675	2.242009	2.237344	2.251	2.246317	2.241675	2.237076

2.257866	2.252901	2.248022	2.243258	2.238571	2.252252	2.247559	2.24291	2.238306
2.259278	2.254292	2.249375	2.24455	2.239799	2.253486	2.248783	2.244125	2.239517
2.260709	2.255718	2.250775	2.245895	2.24108	2.254768	2.250041	2.245363	2.24074
2.262088	2.257138	2.252224	2.247343	2.242507	2.256187	2.251393	2.246659	2.241987
2.26344	2.258549	2.253685	2.248828	2.243993	2.25767	2.252801	2.248003	2.243274
2.264752	2.259878	2.255033	2.250193	2.245371	2.259099	2.25423	2.249426	2.244683
2.266026	2.26114	2.256293	2.251466	2.246671	2.260495	2.255684	2.250919	2.246196
2.267296	2.262398	2.257547	2.252727	2.247947	2.24321	2.238516	2.252321	2.247611
2.268559	2.263654	2.258798	2.253977	2.249197	2.244458	2.23976	2.253606	2.248899
2.269832	2.264914	2.260048	2.255222	2.250439	2.245693	2.240986	2.254867	2.250157
2.271129	2.266187	2.261301	2.256469	2.251684	2.246934	2.242223	2.256136	2.251411
2.272432	2.267465	2.262557	2.257716	2.252927	2.248171	2.243452	2.257396	2.252657
2.237877	2.233017	2.263817	2.258964	2.254164	2.249396	2.244664	2.258644	2.253906
2.239111	2.234241	2.265083	2.260216	2.255401	2.250616	2.245869	2.259885	2.255157
2.240351	2.235464	2.266355	2.261473	2.256644	2.251846	2.247087	2.261138	2.25641
2.241587	2.236679	2.267627	2.262729	2.257887	2.253079	2.248312	2.262395	2.257661
2.242947	2.23802	2.233155	2.228368	2.22364	2.254418	2.24964	2.263749	2.259001
2.244506	2.239564	2.234682	2.229874	2.225124	2.255929	2.251131	2.265256	2.260486
2.246038	2.241084	2.236186	2.231356	2.226584	2.25742	2.252606	2.26675	2.261957
2.247387	2.242423	2.237511	2.232658	2.227859	2.258752	2.253928	2.249149	2.244416
2.248666	2.243692	2.238768	2.233893	2.229072	2.260035	2.255211	2.250424	2.245674
2.249944	2.244953	2.240015	2.235129	2.230308	2.225582	2.220936	2.251808	2.247072
2.251219	2.246205	2.241252	2.236365	2.231556	2.22687	2.222283	2.253252	2.248548
2.252636	2.247611	2.242646	2.237745	2.232923	2.228223	2.223623	2.254633	2.249923
2.254216	2.249193	2.24422	2.239294	2.234429	2.229656	2.224961	2.25595	2.251191
2.25573	2.250706	2.245725	2.240777	2.235875	2.231041	2.226267	2.221545	2.216877
2.257067	2.252035	2.247042	2.242079	2.237159	2.2323	2.227496	2.222741	2.218037
2.258418	2.253378	2.248373	2.243398	2.238465	2.233587	2.228761	2.223983	2.219248
2.259964	2.254914	2.249899	2.244915	2.239967	2.235062	2.2302	2.225396	2.220649
2.261564	2.256506	2.251481	2.246487	2.241527	2.236594	2.231695	2.22687	2.222118
2.262986	2.257922	2.25289	2.247883	2.242906	2.237955	2.233034	2.22819	2.223421
2.264281	2.259213	2.254176	2.249154	2.244158	2.239194	2.234264	2.229404	2.224611
2.190045	2.185287	2.255575	2.250543	2.245538	2.240565	2.235626	2.230749	2.225931
2.19155	2.186777	2.257137	2.252105	2.247101	2.242122	2.237172	2.232274	2.227425
2.192989	2.188199	2.258635	2.253601	2.248597	2.243616	2.238662	2.233758	2.228901
2.194201	2.18939	2.259924	2.25488	2.249872	2.244895	2.239954	2.235079	2.230269
2.195401	2.19057	2.261204	2.256148	2.251134	2.24616	2.24123	2.23638	2.231612
2.196821	2.19198	2.238915	2.23391	2.228948	2.247602	2.24266	2.237779	2.23296
2.198331	2.193485	2.240455	2.235438	2.230461	2.249135	2.24417	2.239234	2.234319
2.199667	2.194818	2.241868	2.236878	2.231931	2.250656	2.245697	2.240742	2.235785
2.200848	2.196002	2.243174	2.238248	2.233375	2.252181	2.247252	2.242313	2.237353
2.202156	2.197299	2.192524	2.187864	2.183265	2.229916	2.225061	2.243763	2.238807
2.203668	2.198773	2.193945	2.189204	2.184518	2.231193	2.2263	2.245012	2.240067
2.180231	2.175359	2.195411	2.190593	2.18582	2.232511	2.227577	2.246296	2.241353
2.181661	2.17683	2.196928	2.19209	2.187281	2.233993	2.229033	2.247762	2.242797
2.183051	2.17827	2.198428	2.1936	2.188784	2.235532	2.230559	2.249302	2.244311
2.162154	2.157373	2.174797	2.170056	2.165341	2.237042	2.232081	2.250856	2.24585
2.163423	2.158593	2.175989	2.171308	2.166679	2.238541	2.233612	2.252431	2.24742
2.164814	2.159937	2.155143	2.150457	2.145835	2.188227	2.183584	2.230176	2.225253
2.166328	2.161404	2.156554	2.151795	2.147109	2.1895	2.184906	2.231627	2.226747

2.130081	2.125301	2.158015	2.153187	2.148435	2.143796	2.13925	2.233038	2.228196
2.131588	2.126781	2.159529	2.154654	2.149847	2.145143	2.140526	2.234389	2.229574
2.133084	2.128266	2.161068	2.156158	2.151302	2.146527	2.141828	2.235744	2.230935
2.134523	2.129731	2.162616	2.157682	2.152785	2.147966	2.143217	2.195434	2.190628
2.135924	2.131177	2.164162	2.159219	2.154294	2.149444	2.144655	2.196877	2.192012
2.11679	2.112181	2.165652	2.160726	2.155806	2.150936	2.14611	2.198365	2.193448
2.118106	2.113525	2.167099	2.162213	2.157325	2.152448	2.147587	2.199896	2.194928
2.119535	2.114957	2.1686	2.163736	2.158866	2.153978	2.149085	2.144272	2.139534
2.121103	2.116505	2.170178	2.165314	2.160441	2.155533	2.150608	2.145762	2.140989
2.079037	2.074513	2.133637	2.128945	2.124256	2.157068	2.152127	2.147255	2.142446
2.080492	2.075882	2.134968	2.130286	2.125631	2.158543	2.153614	2.148733	2.143889
2.081948	2.077253	2.136298	2.131626	2.127007	2.160021	2.155111	2.150225	2.145346
2.083449	2.078721	2.137763	2.133094	2.128485	2.161573	2.156666	2.151766	2.146856
2.084972	2.080236	2.139293	2.134622	2.13001	2.163158	2.158248	2.153331	2.148393
2.086479	2.081734	2.140794	2.136119	2.131502	2.164708	2.159798	2.154869	2.149905
2.087976	2.083221	2.142278	2.137595	2.132971	2.166231	2.161323	2.156385	2.151399
2.089473	2.084707	2.101719	2.09714	2.092621	2.129734	2.125005	2.157906	2.152893
2.090977	2.086192	2.103146	2.098552	2.094024	2.131198	2.126487	2.159439	2.154386
2.057632	2.052926	2.104566	2.099955	2.095415	2.13265	2.127953	2.160962	2.155887
2.059088	2.054368	2.105966	2.101337	2.096784	2.134071	2.12938	2.162462	2.157405
2.060549	2.055813	2.107368	2.10272	2.098152	2.135486	2.130791	2.16395	2.158927
2.062047	2.057283	2.087332	2.082727	2.078199	2.136917	2.132203	2.127441	2.122619
2.063573	2.058778	2.088816	2.084201	2.079663	2.138362	2.133614	2.128845	2.124041
1.990455	1.985945	2.055651	2.051109	2.046637	2.13984	2.135053	2.130268	2.125474
1.99192	1.987457	2.057305	2.052725	2.048191	2.141355	2.136518	2.131707	2.126913
1.993365	1.98893	2.058909	2.054299	2.049718	2.067724	2.063062	2.133165	2.128392
1.994768	1.990325	2.060408	2.055784	2.051191	2.069134	2.064451	2.134651	2.129945
1.996166	1.991709	2.061893	2.057254	2.05265	2.070539	2.065844	2.136138	2.131489
1.997568	1.99311	1.988695	1.98429	1.979901	2.071934	2.067223	2.106939	2.102366
1.998991	1.994531	1.990106	1.985668	1.981244	2.073338	2.06862	2.108377	2.10378
2.000502	1.99602	1.991564	1.987081	1.982617	2.074792	2.070109	2.109923	2.10529
2.00208	1.997561	1.993058	1.98852	1.984014	2.076287	2.071667	2.111549	2.106866
2.003616	1.999061	1.994527	1.98998	1.985474	1.981103	1.976826	2.068581	2.063969
2.005107	2.000515	1.995973	1.991474	1.987019	1.982612	1.978248	2.07003	2.065376
1.931277	1.92682	1.997391	1.992929	1.988518	1.984083	1.979648	2.071482	2.066813
1.932685	1.928176	1.998742	1.994283	1.989891	1.985459	1.981025	2.072967	2.06834
1.934099	1.929552	2.000123	1.99565	1.991254	1.986822	1.982393	2.074444	2.069859
1.935545	1.931029	2.001658	1.997158	1.992717	1.988238	1.98376	2.00475	2.000391
1.937022	1.932561	2.003268	1.998727	1.994218	1.989678	1.985138	2.006033	2.001642
1.938572	1.934089	1.963732	1.959229	1.954753	1.991113	1.986592	2.007436	2.003005
1.940193	1.935622	1.965216	1.960652	1.956142	1.992547	1.988114	2.008939	2.004458
1.891016	1.886473	1.9667	1.962116	1.957605	1.994037	1.989636	1.985228	1.980811
1.892482	1.887895	1.968177	1.963634	1.959178	1.995603	1.991147	1.986726	1.98234
1.81002	1.805507	1.935493	1.93106	1.926715	1.956313	1.951895	1.988231	1.983857
1.811432	1.806995	1.937095	1.932611	1.928195	1.957826	1.953438	1.989769	1.985336
1.812874	1.808515	1.938727	1.934178	1.929675	1.959338	1.954984	1.991304	1.986797
1.767106	1.762756	1.846434	1.842008	1.837633	1.960894	1.956499	1.99279	1.988242
1.768717	1.764264	1.847905	1.843515	1.839196	1.962472	1.957993	1.994245	1.989677
1.770272	1.765781	1.849449	1.84504	1.840702	1.875726	1.871305	1.954967	1.950507
1.771769	1.767304	1.851064	1.846586	1.842156	1.877161	1.872707	1.956408	1.952005

1.721764	1.717348	1.76444	1.760033	1.755677	1.839173	1.834802	1.869792	1.865534
1.723489	1.71895	1.765898	1.761427	1.75704	1.840622	1.836301	1.871313	1.866989
1.72521	1.720567	1.767393	1.762872	1.758463	1.842116	1.837816	1.872842	1.868463
1.635875	1.632104	1.717624	1.713173	1.708813	1.843684	1.839286	1.874316	1.869986
1.637218	1.633483	1.719296	1.714853	1.710473	1.845302	1.840774	1.875801	1.871531
1.638594	1.634849	1.720893	1.716456	1.712087	1.802647	1.798134	1.877402	1.873059
1.63999	1.636206	1.722449	1.718017	1.713681	1.804304	1.799815	1.879089	1.874584
1.585878	1.58205	1.63381	1.630121	1.626472	1.758133	1.75378	1.880781	1.876187
1.587283	1.583502	1.635208	1.631482	1.627781	1.75966	1.755392	1.882492	1.877884
1.411451	1.40822	1.581199	1.577397	1.57361	1.713916	1.709748	1.752669	1.748326
1.412714	1.409487	1.582609	1.578824	1.575061	1.71549	1.711206	1.754132	1.749877
1.413982	1.410755	1.584019	1.580255	1.57652	1.717082	1.712673	1.755594	1.75141
1.415261	1.412023	1.585482	1.581705	1.577949	1.574205	1.570479	1.757169	1.752929
1.41655	1.413292	1.586965	1.583162	1.579374	1.575634	1.571932	1.758804	1.754451
1.417843	1.414564	1.411317	1.40811	1.404931	1.577108	1.573425	1.626597	1.622773
1.419141	1.415838	1.412577	1.409378	1.406218	1.578622	1.574958	1.628176	1.624336
1.420481	1.417164	1.413892	1.410687	1.407524	1.404379	1.401257	1.572756	1.569052
1.421882	1.418564	1.415286	1.412054	1.408857	1.405686	1.402542	1.574151	1.570467
1.423271	1.419953	1.41667	1.413412	1.410182	1.406986	1.403819	1.486221	1.482885
1.424594	1.421275	1.417988	1.414714	1.411468	1.408259	1.405081	1.487577	1.484214
1.425921	1.422594	1.419299	1.416015	1.412758	1.409539	1.406353	1.488954	1.485562
1.328777	1.325691	1.420668	1.417376	1.414113	1.410883	1.407682	1.404495	1.401319
1.330107	1.326984	1.422064	1.418765	1.415502	1.412259	1.409042	1.405851	1.402685
1.33138	1.328224	1.423412	1.420106	1.416841	1.413588	1.410356	1.40716	1.404003
1.332599	1.329413	1.424712	1.421401	1.418133	1.414869	1.411624	1.408425	1.405274
1.333857	1.33065	1.426065	1.422744	1.419466	1.416192	1.412937	1.409729	1.406572
1.335189	1.331976	1.42751	1.42417	1.42087	1.417583	1.414319	1.411095	1.407913
1.336515	1.333296	1.428954	1.4256	1.422286	1.418991	1.415721	1.412482	1.409273
1.226314	1.223371	1.430327	1.426987	1.423689	1.420399	1.417127	1.413877	1.410649
1.227415	1.224458	1.431687	1.42836	1.425082	1.421799	1.418528	1.415273	1.412033
1.125927	1.123154	1.33387	1.330768	1.327718	1.423139	1.419875	1.41663	1.413404
1.127103	1.124323	1.335205	1.33203	1.328903	1.424439	1.421187	1.417963	1.41477
1.128255	1.125488	1.122743	1.119995	1.11729	1.425792	1.422538	1.419319	1.416143
1.129379	1.126648	1.123919	1.121132	1.118373	1.427204	1.42393	1.420701	1.417525
1.130526	1.127818	1.125099	1.122282	1.11948	1.242614	1.239795	1.422091	1.418909
1.131721	1.12901	1.126278	1.123437	1.120605	1.243871	1.241023	1.423488	1.420299
1.132925	1.130203	1.127458	1.124601	1.121749	1.245144	1.242261	1.424886	1.42168
1.134108	1.131381	1.128631	1.125774	1.122916	1.120193	1.117563	1.426267	1.423014
1.135286	1.132554	1.129803	1.126952	1.124098	1.121352	1.118681	1.427652	1.424345
1.136472	1.133734	1.130976	1.128127	1.125272	1.122504	1.1198	1.243103	1.240229
1.137664	1.134917	1.132152	1.1293	1.12644	1.123655	1.120925	1.244419	1.241545
1.138859	1.136102	1.133328	1.130471	1.127607	1.124811	1.122068	1.245734	1.242851
1.140056	1.137288	1.134503	1.131638	1.128768	1.125974	1.123235	1.247048	1.244141
1.141258	1.138481	1.135686	1.132812	1.129933	1.127139	1.124406	1.248362	1.245429
1.142464	1.139684	1.136884	1.133997	1.131104	1.128305	1.12557	1.249671	1.246713
1.143674	1.140893	1.13809	1.135188	1.132279	1.129471	1.126732	1.250982	1.248003
1.016011	1.013492	1.139296	1.136378	1.133449	1.130631	1.127887	1.125185	1.122533
1.017084	1.014566	1.140506	1.137571	1.134622	1.131791	1.129039	1.126334	1.123685
1.01816	1.015641	1.141719	1.138781	1.135826	1.132978	1.130201	1.127483	1.124835
1.019239	1.016719	1.142936	1.140007	1.137057	1.134185	1.131368	1.128631	1.125984

1.020314	1.017791	1.015251	1.012638	1.010002	1.135442	1.132582	1.129814	1.127147
1.021385	1.018856	1.016325	1.013765	1.011185	1.136773	1.133863	1.131048	1.128329
1.022453	1.01992	1.017399	1.014886	1.012355	1.138098	1.135151	1.132292	1.129519
1.023504	1.020982	1.018477	1.015978	1.01346	1.010844	1.008166	1.133512	1.130698
1.024567	1.022051	1.019556	1.017056	1.014537	1.011934	1.009273	1.134745	1.131896
1.025693	1.023144	1.020623	1.01812	1.015613	1.013039	1.010422	1.13605	1.133169
1.026866	1.024257	1.021688	1.019175	1.016681	1.014149	1.011594	1.137402	1.134496
1.028056	1.025416	1.022815	1.020272	1.017758	1.015239	1.01272	1.010153	1.007529
1.029271	1.026631	1.024011	1.021412	1.018838	1.016304	1.013797	1.011253	1.00867
1.030454	1.027824	1.025199	1.02257	1.019957	1.017397	1.014876	1.012339	1.009786
1.031573	1.028966	1.026363	1.023749	1.021139	1.018544	1.015961	1.013401	1.010864
1.03268	1.03009	1.027509	1.024923	1.022331	1.019709	1.017071	1.014481	1.011942
1.033807	1.03121	1.028628	1.026063	1.023497	1.020884	1.018244	1.015633	1.013049
1.034943	1.032324	1.02973	1.027182	1.024643	1.022058	1.019443	1.01682	1.014179
1.036082	1.033438	1.030827	1.028271	1.025737	1.023178	1.020606	1.017999	1.015347
1.037227	1.034554	1.031918	1.029336	1.026787	1.024258	1.021745	1.019177	1.016549
1.038445	1.035743	1.033074	1.030452	1.027869	1.025339	1.022848	1.020307	1.017714
1.039766	1.03703	1.03431	1.031627	1.028983	1.026412	1.023896	1.021369	1.018832
1.04104	1.038293	1.035552	1.032835	1.030151	1.027539	1.024983	1.02245	1.019944
1.042175	1.039475	1.036779	1.034096	1.031428	1.028788	1.026171	1.023591	1.02105
1.043273	1.040624	1.037984	1.035346	1.032709	1.03005	1.027382	1.024757	1.022175
1.044399	1.041749	1.039117	1.036485	1.033856	1.031206	1.028547	1.025933	1.02337
1.045546	1.042868	1.040218	1.037572	1.03494	1.03231	1.029691	1.027117	1.024596
1.04681	1.044095	1.041405	1.038727	1.036068	1.033432	1.03082	1.028252	1.025737
1.048186	1.04542	1.042668	1.039935	1.037226	1.034559	1.031928	1.029339	1.026798
1.049486	1.046704	1.04393	1.041179	1.038452	1.035767	1.033118	1.030508	1.02794
1.05065	1.047912	1.045186	1.042477	1.039782	1.037102	1.034437	1.031809	1.029217
1.051818	1.049113	1.046427	1.043756	1.041094	1.038421	1.035746	1.0331	1.030485
1.053064	1.050324	1.047615	1.04494	1.042282	1.03961	1.036935	1.034277	1.031635
1.054338	1.051553	1.048808	1.046118	1.043456	1.040783	1.038111	1.035444	1.032777
1.055612	1.052847	1.050118	1.047431	1.044767	1.042092	1.039416	1.036737	1.034052
1.056891	1.054181	1.051493	1.048819	1.046153	1.043475	1.040791	1.038101	1.035404
1.058165	1.055446	1.052754	1.050086	1.047427	1.044752	1.042068	1.039373	1.036667
1.059432	1.056635	1.05389	1.05122	1.048581	1.045915	1.043237	1.040543	1.037832
1.060708	1.057863	1.055083	1.052409	1.049781	1.047125	1.044459	1.041771	1.039058
1.061999	1.059179	1.056415	1.053738	1.051103	1.048458	1.045813	1.043135	1.040423
1.0633	1.060497	1.057742	1.055056	1.052409	1.049773	1.047149	1.044484	1.041777
1.064607	1.061737	1.058928	1.056219	1.053567	1.050936	1.048328	1.045677	1.042983
1.065929	1.062971	1.060089	1.057349	1.054689	1.052058	1.049461	1.046824	1.044149
1.067282	1.064316	1.061417	1.058646	1.055953	1.053308	1.050711	1.048085	1.045434
1.068664	1.065746	1.062867	1.060064	1.05732	1.054651	1.052042	1.049424	1.046803
1.070076	1.067187	1.064318	1.061481	1.058688	1.055989	1.053363	1.050744	1.048142
1.071535	1.068653	1.065778	1.062902	1.060061	1.057328	1.054676	1.052048	1.049453
0.862274	0.860145	1.067188	1.064285	1.061408	1.058641	1.055955	1.053308	1.050712
0.863236	0.861113	1.068477	1.065569	1.06268	1.059879	1.057146	1.054471	1.051864
0.864194	0.862076	1.069769	1.066866	1.063974	1.06114	1.058355	1.055645	1.05302
0.865257	0.863145	1.071202	1.068304	1.065409	1.062543	1.059705	1.056957	1.054307
0.86637	0.864266	1.072704	1.069815	1.066919	1.064023	1.061135	1.058347	1.055664
0.867468	0.865372	0.863267	0.861149	0.859025	1.065489	1.062576	1.059754	1.057024
0.868562	0.866473	0.864378	0.862262	0.860138	1.066955	1.06404	1.061189	1.058399

0.596557	0.595315	0.865418	0.863304	0.861182	1.068348	1.065433	1.062557	1.059714
0.597128	0.595888	0.866345	0.864235	0.862117	1.06962	1.066705	1.063808	1.060916
0.597733	0.596483	0.867302	0.865195	0.863082	0.860969	0.858854	1.065107	1.062181
0.598443	0.597169	0.868381	0.866276	0.864171	0.862057	0.859938	1.066568	1.063633
0.599195	0.59789	0.869508	0.867403	0.865305	0.863189	0.861066	1.068092	1.065162
0.599935	0.598601	0.597301	0.596042	0.594795	0.864304	0.862181	1.069601	1.066674
0.600679	0.599316	0.597996	0.596726	0.595478	0.865418	0.863299	1.071112	1.068193
0.601451	0.600061	0.598713	0.597427	0.596168	0.866532	0.864415	1.072602	1.069693
0.602257	0.60084	0.599459	0.598146	0.596867	0.867649	0.865531	1.074075	1.071175
0.60302	0.601579	0.600172	0.59884	0.597548	0.868733	0.86663	0.864517	0.862391
0.603695	0.602232	0.60081	0.599476	0.598192	0.869762	0.867715	0.865633	0.863513
0.604379	0.602904	0.601471	0.600131	0.598845	0.870778	0.868766	0.866708	0.864603
0.605152	0.603684	0.602242	0.600867	0.599536	0.598249	0.597002	0.867667	0.865594
0.605958	0.604507	0.603062	0.601644	0.600252	0.598909	0.597608	0.868594	0.866555
		0.603862	0.602422	0.600997	0.599621	0.598287	0.86964	0.867608
		0.604656	0.603209	0.601767	0.600373	0.599018	0.870766	0.86872
		0.605444	0.603997	0.602549	0.601141	0.599765	0.87189	0.869812
		0.606224	0.604789	0.603346	0.601927	0.600528	0.873006	0.870874
					0.602717	0.601299	0.874134	0.871954
					0.603504	0.602072	0.875277	0.873072
					0.604289	0.602849	0.876432	0.874209
					0.60507	0.603634	0.602218	0.600816
					0.605847	0.604422	0.603004	0.601584
							0.603787	0.602357
							0.604569	0.603135

							0.584021	0.582251
							0.583063	0.581324
					0.585821	0.583975	1.061218	1.05824
					0.584966	0.583155	1.059801	1.056802
					0.584093	0.582325	1.481729	1.478022
					0.583181	0.581453	1.479786	1.476106
					0.582272	0.580574	1.477867	1.474197
			0.585039	0.583182	1.059764	1.056798	1.589158	1.585167
			0.584224	0.582358	1.058327	1.055342	1.587155	1.583148
			1.062893	1.05988	1.479825	1.476023	1.778426	1.773739
			1.061317	1.058391	1.47784	1.474131	1.776061	1.771336
0.586908	0.585143	0.583394	1.483297	1.479568	1.691356	1.687054	1.839335	1.834513
0.586085	0.584266	0.582469	1.481379	1.477629	1.689026	1.684785	1.836924	1.832134
1.065821	1.062743	1.059694	1.479499	1.475736	1.778113	1.773556	1.968288	1.963095
1.064375	1.061303	1.058262	1.477661	1.473917	1.77578	1.771207	1.96574	1.960584
1.062923	1.059867	1.056844	1.47584	1.472117	1.773475	1.768861	1.963174	1.958049
1.485441	1.481598	1.477765	1.689	1.684578	1.836654	1.831933	1.960511	1.955416
1.483506	1.479714	1.475917	1.686803	1.682352	1.834171	1.829414	1.957797	1.952725
1.697944	1.69353	1.6891	1.775845	1.771036	1.965624	1.960354	1.955168	1.950076
1.695705	1.691343	1.686962	1.773575	1.768772	1.963138	1.957839	1.952612	1.947456
1.693485	1.689164	1.684821	1.912177	1.90703	1.960657	1.955345	2.115233	2.109575
1.691295	1.686998	1.682679	1.909781	1.904631	1.958172	1.95287	2.112494	2.106874
1.689088	1.684809	1.680511	1.907362	1.902219	1.955682	1.950398	2.109768	2.104201
1.778298	1.773691	1.769074	1.9638	1.958463	1.953167	1.947898	2.185057	2.179441
1.775827	1.771252	1.766675	1.961218	1.955919	1.950653	1.945404	2.182284	2.1767
1.914733	1.909815	1.904913	1.958647	1.953401	2.112834	2.107168	2.179597	2.174037
1.912219	1.907308	1.902425	1.956083	1.950901	2.110108	2.104511	2.176974	2.17143
1.96886	1.963752	1.958682	1.95356	1.948429	2.185542	2.179922	2.174343	2.168813
1.966314	1.961221	1.956173	1.951082	1.945987	2.182836	2.177257	2.171705	2.166187
1.9638	1.958717	1.953682	2.10021	2.09497	2.180151	2.174598	2.169063	2.163551
1.961308	1.956223	1.951191	2.097652	2.092451	2.177497	2.171954	2.166422	2.160905
1.958863	1.953772	1.948734	2.095114	2.08993	2.174837	2.169297	2.163763	2.15824
1.956516	1.951416	1.94635	2.183116	2.177636	2.172123	2.166588	2.161059	2.155536
1.954209	1.949103	1.944004	2.180465	2.174927	2.16939	2.163859	2.158337	2.152817
2.103474	2.098212	2.092935	2.17781	2.172245	2.166699	2.161172	2.207179	2.201522
2.100859	2.095648	2.090418	2.175156	2.169586	2.164041	2.158515	2.204454	2.198816
2.151285	2.145904	2.140521	2.172492	2.166921	2.16138	2.15586	2.20174	2.196126
2.148742	2.143316	2.137903	2.16981	2.164247	2.158713	2.153202	2.199033	2.193448
2.146205	2.140731	2.13528	2.167131	2.161578	2.207578	2.201938	2.19634	2.190785
2.143628	2.138131	2.132656	2.164463	2.158925	2.204867	2.199245	2.193669	2.18814
2.141029	2.135519	2.130026	2.161797	2.156277	2.202166	2.196564	2.191008	2.185502
2.175819	2.170229	2.16466	2.159127	2.153622	2.199461	2.193878	2.188338	2.182844
2.173123	2.167538	2.161976	2.156454	2.150966	2.19676	2.191197	2.185672	2.180185
2.170392	2.164822	2.159277	2.205231	2.199631	2.194066	2.188537	2.183041	2.177571
2.167621	2.162076	2.156561	2.202472	2.1969	2.191373	2.185892	2.180438	2.174994
2.164892	2.15937	2.153882	2.199758	2.19422	2.188743	2.183327	2.215748	2.210087
2.162195	2.156688	2.151225	2.197076	2.191587	2.186188	2.180875	2.213281	2.207689
2.159606	2.154114	2.148672	2.194495	2.189044	2.183695	2.178446	2.210808	2.205263
2.15726	2.151783	2.146354	2.192132	2.186681	2.181304	2.176	2.208241	2.202675

2.154978	2.149518	2.1441	2.189826	2.184363	2.178932	2.173535	2.205629	2.200018
2.203925	2.198351	2.192816	2.187321	2.18186	2.21431	2.20863	2.202983	2.197363
2.201238	2.195687	2.190174	2.184692	2.179241	2.211609	2.205941	2.200306	2.194697
2.198562	2.193031	2.187535	2.182066	2.176625	2.20892	2.203262	2.318168	2.311222
2.195878	2.190361	2.18488	2.179424	2.173994	2.206223	2.200578	2.31479	2.307942
2.193299	2.187796	2.182327	2.214951	2.209278	2.203627	2.197998	2.311568	2.304806
2.190927	2.185436	2.179976	2.212522	2.206859	2.201224	2.195617	2.308626	2.301922
2.188554	2.183074	2.177624	2.210091	2.204439	2.198824	2.193243	2.305709	2.299054
2.185983	2.180518	2.175082	2.207471	2.201837	2.316184	2.309322	2.336023	2.329332
2.183328	2.17788	2.172461	2.204777	2.199162	2.312832	2.306053	2.332772	2.326123
2.219095	2.213406	2.207751	2.202122	2.196529	2.309544	2.302832	2.357833	2.351185
2.216404	2.210732	2.205095	2.199488	2.193918	2.306294	2.299635	2.354605	2.347962
2.21386	2.208202	2.202581	2.316836	2.309987	2.336694	2.329978	2.351555	2.3449
2.211519	2.205875	2.200267	2.313983	2.307195	2.333895	2.327197	2.34875	2.342045
2.209127	2.20349	2.197891	2.311047	2.304319	2.359264	2.35257	2.345889	2.339188
2.206521	2.200885	2.19529	2.307807	2.30114	2.356061	2.349408	2.342804	2.336232
2.203894	2.19825	2.19265	2.304514	2.297892	2.35278	2.346175	2.339664	2.333248
2.201435	2.195762	2.190131	2.334738	2.327996	2.349558	2.343007	2.336563	2.330232
2.199052	2.193344	2.187674	2.331623	2.324835	2.346383	2.339889	2.333502	2.327224
2.349539	2.342475	2.335478	2.328613	2.321867	2.343442	2.337001	2.330664	2.324435
2.346239	2.339303	2.332446	2.325713	2.319091	2.340731	2.33434	2.328046	2.321858
2.343054	2.336195	2.329422	2.350933	2.344361	2.337907	2.33157	2.360978	2.354302
2.34007	2.333195	2.326398	2.347866	2.341279	2.334852	2.32858	2.357813	2.351184
2.337075	2.330196	2.323391	2.344833	2.338249	2.331853	2.325638	2.354697	2.348114
2.333899	2.32711	2.320405	2.341933	2.335452	2.329126	2.322949	2.351814	2.345281
2.330719	2.324036	2.317449	2.339078	2.332716	2.326459	2.32031	2.34898	2.342492
2.327789	2.321119	2.314538	2.336157	2.329813	2.359118	2.352494	2.345947	2.339479
2.325011	2.318304	2.311669	2.333216	2.326833	2.355912	2.349328	2.342809	2.336345
2.322086	2.315434	2.308856	2.366553	2.359647	2.352917	2.346357	2.339856	2.333398
2.318994	2.312497	2.306096	2.363667	2.356834	2.350145	2.343594	2.337102	2.330655
2.344056	2.337656	2.331363	2.36081	2.354053	2.347393	2.340824	2.364332	2.35773
2.341298	2.334967	2.328733	2.357986	2.351301	2.344634	2.33799	2.36136	2.354708
2.33858	2.332278	2.326062	2.355111	2.348494	2.341833	2.335143	2.358402	2.35172
2.372356	2.36545	2.358676	2.352032	2.345483	2.338892	2.332277	2.355545	2.348919
2.369286	2.362334	2.355523	2.348899	2.342407	2.335907	2.329417	2.352746	2.346221
2.366275	2.359374	2.352607	2.346004	2.339526	2.36305	2.356478	2.349999	2.343564
2.363312	2.356532	2.349868	2.343282	2.336783	2.360264	2.353725	2.347302	2.340958
2.360424	2.353732	2.347131	2.340538	2.333995	2.357406	2.350879	2.368978	2.362746
2.357625	2.350984	2.3444	2.337763	2.331144	2.354438	2.347885	2.366036	2.360046
2.354849	2.348226	2.341635	2.365006	2.358194	2.375983	2.369437	2.363245	2.357465
2.352089	2.345427	2.338792	2.362106	2.355342	2.373211	2.366821	2.360769	2.355105
2.349316	2.342607	2.335932	2.359217	2.352539	2.370524	2.364327	2.35842	2.352835
2.346469	2.339757	2.333098	2.356393	2.349805	2.36789	2.36185	2.39445	2.388622
2.343587	2.336895	2.330286	2.35362	2.347137	2.365317	2.359418	2.392024	2.386242
2.370922	2.364136	2.35746	2.375293	2.368893	2.362798	2.356987	2.389568	2.38387
2.368063	2.361335	2.354729	2.372642	2.366359	2.360335	2.354559	2.387086	2.381505
2.365248	2.358575	2.352038	2.370012	2.363827	2.357891	2.352195	2.384691	2.379202
2.362481	2.355854	2.349381	2.367381	2.361263	2.355442	2.349904	2.382413	2.376976
2.384092	2.377493	2.371059	2.36481	2.358769	2.353062	2.347672	2.380183	2.374788
2.381371	2.374846	2.368492	2.362347	2.356418	2.350809	2.345507	2.377975	2.372631

2.378679	2.372234	2.365965	2.359939	2.354141	2.348625	2.343388	2.375796	2.370494
2.375992	2.369611	2.363422	2.357521	2.351866	2.3845	2.379025	2.373644	2.368359
2.373323	2.367	2.360887	2.355117	2.349614	2.382259	2.376858	2.371515	2.366226
2.37072	2.364475	2.358449	2.352791	2.347408	2.380031	2.374688	2.369366	2.364047
2.36818	2.36203	2.356103	2.35054	2.345246	2.377817	2.372516	2.367196	2.361815
2.365718	2.359668	2.353835	2.348347	2.343113	2.375621	2.370354	2.375135	2.369632
2.363367	2.357417	2.351663	2.34622	2.341007	2.373444	2.368205	2.372968	2.367484
2.361037	2.355195	2.349527	2.344123	2.338921	2.371282	2.366063	2.37081	2.365362
2.358644	2.352937	2.347391	2.379953	2.374481	2.379287	2.373995	2.368666	2.363251
2.356242	2.350671	2.345252	2.377777	2.372328	2.37711	2.371825	2.366514	2.361137
2.353863	2.348372	2.343037	2.385806	2.380317	2.374909	2.369592	2.36428	2.358947
2.351508	2.346054	2.340771	2.383489	2.378091	2.372694	2.367316	2.361986	2.356702
2.360928	2.355392	2.350021	2.381257	2.375911	2.370523	2.365116	2.359778	2.354515
2.358918	2.353397	2.347999	2.379124	2.373783	2.368403	2.363006	2.35767	2.352392
2.356878	2.351388	2.345988	2.377018	2.371677	2.366305	2.360925	2.355594	2.350301
2.354682	2.349268	2.343934	2.37492	2.369588	2.364224	2.358848	2.353521	2.348229
2.352467	2.347133	2.341875	2.372829	2.367508	2.362161	2.356803	2.351488	2.346199
2.387135	2.38158	2.376096	2.370723	2.365426	2.360124	2.35483	2.349549	2.34425
2.384946	2.379421	2.373961	2.368612	2.363341	2.358097	2.352888	2.347654	2.342345
2.382752	2.377265	2.371838	2.366502	2.361237	2.356021	2.350861	2.345664	2.340375
2.38056	2.375114	2.369723	2.36439	2.359115	2.353905	2.348763	2.343596	2.338354
2.37838	2.372969	2.367609	2.362272	2.356979	2.351776	2.346657	2.367625	2.362331
2.376211	2.370824	2.365482	2.360133	2.354816	2.349624	2.344545	2.365527	2.360296
2.374062	2.368709	2.363393	2.358032	2.352688	2.347497	2.342442	2.363429	2.358264
2.371938	2.36666	2.361403	2.356032	2.350646	2.345428	2.340353	2.361317	2.356215
2.369837	2.364625	2.359423	2.354052	2.348637	2.343393	2.338289	2.359224	2.354188
2.367762	2.362538	2.35733	2.351982	2.346602	2.367505	2.362296	2.357203	2.35225
2.365707	2.360423	2.355173	2.349864	2.344563	2.365474	2.360285	2.355235	2.35037
2.363635	2.358309	2.353032	2.34778	2.342572	2.363498	2.358323	2.353291	2.348453
2.361542	2.356187	2.350896	2.345723	2.340631	2.361585	2.356419	2.351378	2.346504
2.359489	2.354118	2.348819	2.369892	2.364772	2.359635	2.354494	2.349456	2.344549
2.357505	2.35213	2.346821	2.367849	2.362714	2.357597	2.352512	2.347503	2.342578
2.355527	2.350168	2.34487	2.365848	2.360679	2.355578	2.350549	2.345573	2.340635
2.353513	2.348212	2.342976	2.363932	2.35875	2.353668	2.348683	2.380365	2.375268
2.351486	2.346253	2.341093	2.362041	2.356857	2.351793	2.346841	2.378502	2.3734
2.375798	2.370487	2.365249	2.360044	2.354897	2.349851	2.344901	2.376537	2.371473
2.373722	2.368395	2.36314	2.35798	2.352898	2.34787	2.342899	2.374507	2.369513
2.371703	2.366382	2.361133	2.356006	2.350967	2.382713	2.377594	2.372556	2.367618
2.369753	2.364463	2.359241	2.354136	2.349115	2.380834	2.375715	2.370693	2.365796
2.367803	2.362557	2.357375	2.352283	2.347262	2.378961	2.373873	2.368876	2.363995
2.365829	2.360652	2.355537	2.350439	2.345388	2.37708	2.372085	2.367133	2.362217
2.363848	2.358727	2.35367	2.348569	2.343496	2.375185	2.37028	2.365371	2.360422
2.361852	2.356723	2.351665	2.372876	2.367741	2.373237	2.368345	2.363455	2.358538
2.359855	2.35469	2.349604	2.3708	2.365737	2.371275	2.36636	2.361477	2.356619
2.357913	2.352741	2.347652	2.379446	2.374397	2.369407	2.36448	2.359598	2.354766
2.356017	2.350861	2.345784	2.377583	2.372588	2.367617	2.362681	2.357796	2.352965
2.380468	2.37525	2.370109	2.375745	2.370778	2.365828	2.360905	2.356026	2.351191
2.378501	2.373343	2.368261	2.373932	2.368956	2.364029	2.359155	2.354301	2.349451
2.376557	2.371447	2.366411	2.372125	2.36715	2.362241	2.357398	2.352564	2.347716
2.374668	2.369557	2.364522	2.370301	2.365381	2.360479	2.355601	2.350765	2.345967

2.372791	2.367676	2.362638	2.368483	2.363619	2.35872	2.353802	2.348962	2.34422
2.370862	2.365799	2.360821	2.366696	2.361821	2.356933	2.352042	2.347222	2.342488
2.368919	2.363933	2.35904	2.364926	2.360007	2.355133	2.350303	2.345517	2.340768
2.378024	2.373003	2.368073	2.363131	2.358222	2.353366	2.34856	2.343796	2.339066
2.376327	2.371232	2.366225	2.361309	2.356466	2.351632	2.346814	2.342062	2.337383
2.374625	2.369503	2.364454	2.359534	2.354701	2.349878	2.345074	2.340344	2.3357
2.372905	2.367841	2.362815	2.35783	2.352898	2.348069	2.343339	2.338655	2.33401
2.371156	2.366164	2.361181	2.356153	2.35114	2.346301	2.341617	2.336964	2.33232
2.369339	2.3644	2.359474	2.354494	2.349516	2.34466	2.339908	2.335228	2.330622
2.367501	2.362601	2.357729	2.352831	2.347938	2.343071	2.338226	2.333502	2.328933
2.365718	2.360807	2.355932	2.35109	2.346276	2.341451	2.33662	2.331893	2.327294
2.363973	2.359017	2.354099	2.349297	2.344562	2.339817	2.335071	2.330366	2.325695
2.362264	2.357303	2.352377	2.34757	2.342837	2.33813	2.333455	2.328792	2.324118
2.360601	2.355684	2.350787	2.345917	2.341088	2.336373	2.331763	2.327168	2.322572
2.358907	2.354035	2.349179	2.344281	2.339397	2.334669	2.330078	2.325521	2.320996
2.357126	2.352298	2.347508	2.342664	2.337825	2.333079	2.328413	2.323829	2.319349
2.355353	2.350547	2.345803	2.34103	2.336265	2.331516	2.32678	2.322162	2.317699
2.353673	2.348822	2.344043	2.339309	2.33462	2.329923	2.325224	2.320611	2.316099
2.352039	2.347117	2.342269	2.337558	2.332936	2.328313	2.323703	2.319115	2.31453
2.350392	2.345472	2.340608	2.335877	2.331238	2.326647	2.322114	2.317576	2.312998
2.348741	2.343878	2.339039	2.33425	2.32952	2.324932	2.320475	2.316009	2.311503
2.34709	2.342284	2.337483	2.332673	2.327896	2.323304	2.318878	2.314454	2.310013
2.345445	2.340691	2.335946	2.331163	2.326397	2.321796	2.31734	2.31292	2.308534
2.343778	2.33907	2.334382	2.329643	2.32491	2.320302	2.315804	2.329068	2.324719
2.34205	2.33738	2.332748	2.328064	2.323381	2.318774	2.314229	2.327524	2.323202
2.340329	2.335685	2.331099	2.326472	2.32185	2.31725	2.312666	2.325987	2.32167
2.338708	2.334067	2.329497	2.324913	2.320343	2.315762	2.31117	2.324515	2.320154
2.337145	2.33249	2.327914	2.323364	2.318844	2.314292	2.309714	2.323087	2.318653
2.335578	2.330895	2.326299	2.321772	2.317299	2.330725	2.326183	2.321683	2.317215
2.334011	2.329284	2.324649	2.320137	2.315709	2.329236	2.324765	2.320306	2.315841
2.332456	2.327705	2.323048	2.318549	2.314152	2.327768	2.323354	2.318929	2.314469
2.330913	2.326169	2.321521	2.317033	2.312652	2.326334	2.321953	2.317547	2.313089
2.329383	2.324654	2.320019	2.315542	2.311171	2.324913	2.320553	2.31616	2.311707
2.32787	2.323146	2.318516	2.332221	2.32786	2.323495	2.319146	2.314763	2.310322
2.326367	2.32165	2.317026	2.33079	2.326442	2.322085	2.317739	2.313363	2.308935
2.34316	2.338444	2.333824	2.329375	2.325035	2.320684	2.316342	2.380329	2.375389
2.341704	2.337012	2.332412	2.327974	2.32364	2.319292	2.314951	2.378873	2.373914
2.340247	2.335576	2.330994	2.326568	2.322242	2.337064	2.33275	2.377413	2.372454
2.338787	2.334135	2.32957	2.325159	2.320844	2.335722	2.331405	2.375951	2.37101
2.337328	2.332695	2.328146	2.323748	2.319444	2.342732	2.3384	2.374477	2.369565
2.33587	2.331251	2.326716	2.322332	2.31804	2.341404	2.337076	2.372985	2.368115
2.334418	2.329816	2.325299	2.320924	2.316636	2.340072	2.33575	2.371485	2.366663
2.332974	2.328401	2.323909	2.338872	2.334584	2.338732	2.33442	2.369976	2.365206
2.331542	2.327007	2.322545	2.337535	2.333222	2.337397	2.333092	2.36847	2.363751
2.349495	2.345031	2.340627	2.344787	2.340447	2.336111	2.331784	2.367011	2.362317
2.348157	2.343751	2.339394	2.343595	2.339232	2.334865	2.330494	2.365589	2.3609
2.346858	2.342482	2.338143	2.342369	2.337985	2.333594	2.329196	2.364171	2.359495
2.34561	2.341231	2.336871	2.341102	2.336696	2.332291	2.327886	2.362756	2.358102
2.353038	2.348633	2.344234	2.339815	2.335392	2.33098	2.326576	2.361346	2.35672
2.351807	2.347374	2.342944	2.338506	2.334071	2.329658	2.325265	2.359934	2.355342

2.350557	2.346096	2.341639	2.337189	2.33275	2.328342	2.323963	2.358536	2.353983
2.349264	2.34479	2.340326	2.335879	2.33145	2.32705	2.322678	2.374383	2.369795
2.347941	2.343464	2.339006	2.334575	2.330168	2.325782	2.321417	2.373007	2.368465
2.346608	2.342135	2.337692	2.333295	2.328932	2.32458	2.320242	2.37171	2.367138
2.34526	2.340799	2.336379	2.332036	2.327742	2.323445	2.319154	2.370495	2.36582
2.343937	2.3395	2.335111	2.330803	2.326545	2.341277	2.336888	2.369209	2.364454
2.342634	2.338249	2.333908	2.329608	2.325338	2.339994	2.335579	2.367809	2.363019
2.341382	2.337029	2.332714	2.328407	2.324112	2.338688	2.334249	2.366378	2.361566
2.340236	2.335854	2.331507	2.327172	2.322853	2.337362	2.332924	2.364937	2.360101
2.339116	2.334686	2.330289	2.32592	2.321577	2.33603	2.331608	2.363507	2.35866
2.337887	2.333441	2.329025	2.324645	2.320297	2.334728	2.330342	2.362172	2.357356
2.336597	2.332153	2.327737	2.323359	2.319018	2.333448	2.32911	2.360895	2.356139
2.335364	2.330945	2.326545	2.341042	2.336602	2.332212	2.327871	2.359552	2.354813
2.334192	2.329828	2.325467	2.339925	2.33546	2.331029	2.326626	2.358138	2.353371
2.333002	2.32867	2.324334	2.338751	2.334273	2.329812	2.325364	2.376868	2.371772
2.331767	2.32742	2.323082	2.337449	2.332978	2.328514	2.324057	2.375374	2.370258
2.330518	2.326146	2.321798	2.336125	2.331674	2.327224	2.322775	2.373936	2.368813
2.329259	2.324867	2.320509	2.334834	2.330436	2.326025	2.321608	2.372633	2.367543
2.328007	2.3236	2.319233	2.333563	2.329223	2.324857	2.320478	2.371387	2.366342
2.32684	2.322438	2.318072	2.332347	2.327995	2.323626	2.31925	2.384936	2.379854
2.325739	2.321358	2.317001	2.331177	2.326761	2.322352	2.317953	2.383499	2.378406
2.343346	2.338868	2.334413	2.329968	2.325538	2.321121	2.316718	2.382144	2.377049
2.34202	2.337544	2.333104	2.328703	2.32433	2.319951	2.31557	2.38091	2.375827
2.340756	2.336278	2.331841	2.327463	2.323123	2.340529	2.335862	2.379619	2.374544
2.33965	2.335159	2.330698	2.326282	2.321896	2.339177	2.334494	2.378162	2.373082
2.338593	2.334089	2.329602	2.325137	2.32069	2.337836	2.333131	2.376686	2.371598
2.33752	2.333019	2.328523	2.324044	2.319579	2.336611	2.331897	2.375353	2.370261
2.336432	2.331941	2.327447	2.32297	2.318505	2.335435	2.330719	2.374073	2.36898
2.335226	2.330736	2.326241	2.321767	2.317306	2.334141	2.329423	2.372663	2.367574
2.333906	2.329408	2.32491	2.320443	2.315994	2.332741	2.328024	2.371135	2.366055
2.332654	2.328162	2.323675	2.319215	2.314772	2.331435	2.326722	2.369718	2.364637
2.331523	2.327057	2.322599	2.318141	2.313691	2.330273	2.325572	2.368471	2.363374
2.330374	2.32594	2.321519	2.338587	2.333851	2.329139	2.324447	2.367252	2.362147
2.329106	2.324715	2.320347	2.337376	2.332693	2.328005	2.323308	2.366019	2.360931
2.327821	2.323464	2.31914	2.336126	2.331504	2.32684	2.322138	2.364753	2.359695
2.326638	2.322259	2.317915	2.334799	2.33018	2.3735	2.368403	2.363342	2.358317
2.325519	2.321084	2.316679	2.333425	2.328775	2.371957	2.366876	2.361842	2.356856
2.324427	2.319967	2.315535	2.332172	2.327505	2.370563	2.365484	2.360466	2.355518
2.323368	2.318914	2.314488	2.331045	2.326373	2.36932	2.364224	2.359212	2.354305
2.322314	2.317867	2.313446	2.329924	2.325245	2.368093	2.362989	2.357983	2.353105
2.321266	2.316818	2.31239	2.328786	2.324096	2.366863	2.36177	2.356777	2.35191
2.320204	2.315756	2.311324	2.327635	2.322935	2.365623	2.360539	2.35555	2.35068
2.319124	2.314681	2.310251	2.326475	2.321765	2.364358	2.359245	2.354228	2.349331
2.318005	2.313569	2.309145	2.325282	2.320567	2.363068	2.357927	2.352881	2.347949
2.338131	2.333392	2.328675	2.323965	2.319264	2.361721	2.356636	2.351621	2.346685
2.336707	2.331961	2.327249	2.322552	2.317879	2.360328	2.355362	2.350424	2.345505
2.335389	2.330645	2.325941	2.321254	2.316595	2.359016	2.354117	2.349227	2.344328
2.334195	2.329467	2.324774	2.320091	2.315432	2.357796	2.352904	2.348027	2.343153
2.333038	2.328323	2.323638	2.31896	2.314303	2.356616	2.351717	2.346842	2.34198
2.331904	2.327189	2.322502	2.317833	2.313189	2.355474	2.35057	2.345685	2.340805

2.330778	2.326063	2.321376	2.316717	2.312087	2.354344	2.349431	2.344533	2.339634
2.329628	2.324925	2.32025	2.331826	2.327143	2.353184	2.348261	2.343361	2.338472
2.328469	2.323783	2.319125	2.330699	2.326019	2.352012	2.347078	2.34218	2.337315
2.32732	2.322644	2.317995	2.329565	2.324892	2.350843	2.345898	2.341001	2.336153
2.326181	2.321507	2.31686	2.328426	2.323763	2.349677	2.344722	2.339824	2.334989
2.325034	2.320366	2.315724	2.327282	2.322625	2.348497	2.343533	2.338637	2.33382
2.323873	2.319217	2.314585	2.326133	2.321479	2.347297	2.342322	2.337431	2.33264
2.322716	2.318069	2.313446	2.324981	2.320327	2.353048	2.34806	2.343161	2.338373
2.321571	2.316928	2.312306	2.323826	2.319173	2.351858	2.346893	2.342008	2.337221
2.320428	2.315788	2.311167	2.32267	2.318018	2.350673	2.34574	2.340872	2.336079
2.319263	2.314633	2.310027	2.321518	2.316865	2.349482	2.344575	2.339724	2.334936
2.318093	2.313473	2.308889	2.320367	2.315714	2.348291	2.343406	2.338572	2.333795
2.316992	2.312347	2.307748	2.327199	2.322532	2.347115	2.342249	2.337426	2.332649
2.315959	2.311253	2.306599	2.326013	2.321361	2.345951	2.341102	2.336287	2.331501
2.314956	2.310205	2.305504	2.324872	2.320213	2.344801	2.339955	2.335138	2.330345
2.313996	2.309227	2.304485	2.32379	2.319081	2.343648	2.338786	2.333963	2.329174
2.313017	2.308255	2.3035	2.322757	2.318006	2.342548	2.337655	2.33281	2.328008
2.311958	2.307254	2.30255	2.321808	2.317061	2.341581	2.336623	2.331715	2.326855
2.310854	2.306225	2.301595	2.320875	2.316157	2.340662	2.335637	2.330662	2.325727
2.318278	2.313654	2.309035	2.319812	2.31513	2.339655	2.334642	2.337429	2.332471
2.3171	2.312475	2.307863	2.318656	2.314018	2.33859	2.333643	2.336498	2.331523
2.315941	2.311304	2.306691	2.317485	2.312875	2.337457	2.332557	2.343035	2.338068
2.31479	2.310132	2.305509	2.316289	2.311691	2.336244	2.331368	2.341934	2.337083
2.313709	2.309024	2.30438	2.315131	2.310523	2.342925	2.338078	2.340804	2.336047
2.312762	2.308039	2.303346	2.314035	2.309381	2.341743	2.336903	2.339639	2.334917
2.311826	2.307082	2.302354	2.31299	2.308292	2.340609	2.335761	2.338486	2.333772
2.310773	2.306074	2.301386	2.321081	2.316395	2.347178	2.342239	2.337398	2.332672
2.309665	2.30504	2.300424	2.320195	2.315534	2.346256	2.341252	2.33636	2.331608
2.308541	2.303943	2.299362	2.319182	2.314546	2.345252	2.340283	2.33541	2.330658
2.307394	2.302787	2.298213	2.318063	2.313458	2.344188	2.339341	2.334545	2.329814
2.330947	2.32628	2.321654	2.325643	2.320989	2.34312	2.338335	2.333585	2.328876
2.330069	2.325417	2.320785	2.324724	2.320003	2.342034	2.337222	2.332471	2.327791
2.337888	2.333209	2.328531	2.32379	2.319033	2.340995	2.336158	2.331398	2.326726
2.33681	2.332122	2.327456	2.322764	2.318076	2.340081	2.33527	2.330504	2.325782
2.33571	2.331004	2.326344	2.32171	2.317106	2.339179	2.334406	2.329639	2.324864
2.334757	2.330015	2.325314	2.32066	2.316048	2.338126	2.333375	2.328634	2.323891
2.333878	2.329092	2.324326	2.319614	2.314948	2.336997	2.332259	2.327557	2.322889
2.332903	2.328121	2.323356	2.318637	2.31396	2.335983	2.331225	2.326516	2.321859
2.331836	2.327108	2.322407	2.317733	2.313085	2.335078	2.330263	2.3255	2.320793
2.330817	2.326115	2.321444	2.316795	2.312169	2.334144	2.329317	2.324538	2.319804
2.329898	2.325159	2.320445	2.315779	2.311152	2.333134	2.328378	2.323652	2.318949
2.328972	2.324206	2.319459	2.314779	2.310151	2.332139	2.327455	2.322782	2.318106
2.327949	2.323226	2.318526	2.313867	2.30925	2.33123	2.326564	2.321893	2.317197
2.32691	2.322251	2.317619	2.312989	2.308381	2.330344	2.325673	2.320987	2.316265
2.325974	2.321331	2.316712	2.312081	2.307463	2.329387	2.324696	2.320013	2.315328
2.325106	2.320452	2.315812	2.311159	2.306518	2.328387	2.323663	2.318992	2.314385
2.324245	2.319569	2.314904	2.310241	2.305596	2.327443	2.322711	2.31805	2.313485
2.323401	2.318689	2.313988	2.309326	2.304699	2.326565	2.321856	2.317206	2.312642
2.349747	2.34488	2.340018	2.335205	2.330426	2.325686	2.320985	2.316334	2.311756
2.348692	2.343876	2.339074	2.334293	2.329532	2.324784	2.320051	2.315369	2.310758

2.347641	2.342884	2.338144	2.333389	2.328634	2.323875	2.319114	2.314406	2.309766
2.346734	2.341993	2.337259	2.33249	2.327712	2.322955	2.318215	2.313522	2.308888
2.345892	2.341145	2.33639	2.331591	2.326782	2.322034	2.317336	2.312677	2.308064
2.345027	2.34025	2.335469	2.330669	2.325871	2.321142	2.31647	2.311829	2.307225
2.344153	2.339324	2.334506	2.329726	2.324976	2.320274	2.315619	2.310985	2.306382
2.34323	2.33838	2.333557	2.328798	2.324084	2.319406	2.314767	2.318148	2.313511
2.342229	2.337406	2.33262	2.327884	2.323191	2.318535	2.313918	2.317288	2.312599
2.341267	2.336468	2.331712	2.326997	2.322318	2.325698	2.321065	2.316398	2.311672
2.340428	2.335619	2.330854	2.32615	2.321485	2.324837	2.32014	2.31544	2.31073
2.339618	2.334798	2.330021	2.325322	2.320664	2.323981	2.319211	2.314479	2.309797
2.338741	2.333948	2.329191	2.324497	2.319834	2.323136	2.318349	2.313605	2.308917
2.337836	2.333087	2.328366	2.323673	2.318993	2.322291	2.317518	2.312776	2.308071
2.336946	2.332226	2.327523	2.330856	2.326127	2.321387	2.316636	2.311913	2.30722
2.336068	2.331362	2.32666	2.329952	2.325167	2.32042	2.315705	2.31102	2.306367
2.343299	2.338588	2.333873	2.329077	2.324255	2.319502	2.314804	2.310143	2.305524
2.342473	2.337767	2.333052	2.328254	2.323428	2.318666	2.313956	2.309294	2.304692
2.341652	2.336949	2.332235	2.32744	2.322615	2.317846	2.313122	2.308453	2.303865
2.340815	2.336119	2.331406	2.326608	2.321777	2.317006	2.312279	2.307613	2.303034
2.339975	2.335286	2.330575	2.325775	2.320938	2.316165	2.311439	2.306777	2.302205
2.339147	2.334461	2.329752	2.324951	2.320111	2.315334	2.310604	2.305943	2.301381
2.338327	2.333641	2.328934	2.324132	2.319291	2.314509	2.309772	2.305112	2.300562
2.337505	2.332818	2.32811	2.323306	2.318463	2.31368	2.308943	2.304286	2.299743
2.336682	2.331992	2.327282	2.322474	2.317627	2.312848	2.308118	2.303466	2.298925
2.335851	2.331156	2.326443	2.321631	2.316781	2.312006	2.307288	2.302644	2.298106
2.334997	2.330297	2.325584	2.320766	2.315913	2.311145	2.306444	2.301814	2.297283
2.334161	2.329448	2.324727	2.319904	2.31505	2.310288	2.3056	2.300981	2.29646
2.333395	2.328646	2.323895	2.319064	2.314211	2.309448	2.304756	2.30014	2.295631
2.332662	2.327866	2.323076	2.318239	2.313397	2.308631	2.303926	2.299306	2.294805
2.331904	2.327069	2.322251	2.317437	2.312641	2.307885	2.303165	2.298528	2.294009
2.331131	2.326261	2.321423	2.316657	2.311935	2.307201	2.30246	2.297796	2.293238
2.330381	2.325481	2.320616	2.315863	2.311172	2.306455	2.301725	2.316118	2.311422
2.329672	2.32474	2.319834	2.315047	2.31033	2.305626	2.300944	2.315332	2.310606
2.328931	2.323985	2.319055	2.314235	2.309485	2.304796	2.30017	2.314561	2.30981
2.328113	2.323176	2.318256	2.31342	2.308651	2.303988	2.29943	2.313833	2.309062
2.327271	2.322362	2.317474	2.312633	2.307846	2.303203	2.298694	2.313103	2.308321
2.326442	2.321596	2.31677	2.311934	2.307126	2.302462	2.29793	2.31231	2.30753
2.325619	2.320852	2.3161	2.311283	2.306459	2.301756	2.297157	2.311488	2.306722
2.324826	2.320071	2.315343	2.310571	2.305796	2.301087	2.296433	2.310721	2.305971
2.324058	2.319255	2.314505	2.309807	2.305147	2.30046	2.295757	2.310003	2.305274
2.323336	2.318485	2.313701	2.309035	2.304435	2.299764	2.295041	2.309255	2.304531
2.322687	2.31779	2.312948	2.308245	2.303619	2.298947	2.294245	2.308444	2.303701
2.322014	2.317103	2.312233	2.30749	2.30282	2.298142	2.293468	2.307652	2.302885
2.321227	2.31639	2.311584	2.306833	2.302122	2.297436	2.292776	2.306935	2.302142
2.320408	2.315662	2.310944	2.306203	2.301466	2.296773	2.29212	2.306249	2.301435
2.319641	2.314899	2.310198	2.3055	2.300815	2.31513	2.310345	2.305554	2.300745
2.318905	2.314114	2.309387	2.304754	2.300171	2.314486	2.309667	2.304861	2.300074
2.318202	2.313379	2.308629	2.30401	2.299461	2.313779	2.308978	2.304184	2.2994
2.317537	2.312695	2.307921	2.30326	2.29867	2.312999	2.308278	2.303526	2.298723
2.31687	2.312027	2.307247	2.302559	2.297938	2.31226	2.307579	2.302849	2.298042
2.316185	2.311371	2.306617	2.30195	2.297341	2.31162	2.306884	2.302132	2.297351

2.315504	2.310719	2.30599	2.301345	2.296746	2.310982	2.306192	2.301415	2.296659
2.314849	2.310057	2.305319	2.300654	2.296035	2.310262	2.305501	2.300738	2.29597
2.314201	2.309394	2.304639	2.299942	2.295293	2.309521	2.304813	2.300075	2.295282
2.281213	2.276637	2.272099	2.274938	2.270429	2.308855	2.304135	2.29939	2.2946
2.280547	2.275999	2.271494	2.274359	2.269869	2.308244	2.303463	2.298689	2.293922
2.279912	2.275385	2.270902	2.294768	2.290163	2.307598	2.302787	2.297998	2.293239
2.279311	2.274804	2.270327	2.294153	2.28951	2.306905	2.302108	2.297324	2.292552
2.278728	2.274223	2.26974	2.287045	2.282399	2.306209	2.30142	2.296639	2.291861
2.278167	2.273632	2.269121	2.286397	2.281738	2.305521	2.300706	2.295919	2.291163
2.277605	2.273037	2.268496	2.285742	2.281077	2.304836	2.299998	2.295204	2.290467
2.277005	2.272434	2.267885	2.285095	2.280416	2.30415	2.299327	2.294536	2.289779
2.276385	2.271827	2.267285	2.284458	2.279764	2.303473	2.298684	2.2939	2.289105
2.275771	2.271232	2.266705	2.283866	2.279171	2.302865	2.298083	2.2933	2.288498
2.275158	2.270647	2.266147	2.283318	2.278636	2.302322	2.297526	2.292739	2.287955
2.296633	2.292004	2.287388	2.282732	2.278061	2.296167	2.291393	2.292142	2.287377
2.296068	2.291397	2.286744	2.28208	2.277417	2.295518	2.290734	2.291477	2.286734
2.295512	2.290804	2.286114	2.281439	2.276776	2.294873	2.290086	2.290827	2.286097
2.294962	2.290253	2.285552	2.280865	2.276191	2.294283	2.28951	2.290255	2.285519
2.2944	2.2897	2.284998	2.280302	2.275613	2.293698	2.288947	2.289697	2.284946
2.293762	2.289067	2.28437	2.279671	2.274978	2.29305	2.288315	2.289068	2.284305
2.29308	2.288386	2.283701	2.279005	2.274313	2.29237	2.287648	2.288402	2.283625
2.292468	2.287768	2.283084	2.278393	2.273709	2.291756	2.287037	2.287788	2.283006
2.291927	2.287212	2.282518	2.277836	2.273169	2.29121	2.28648	2.287225	2.282448
2.291397	2.286668	2.281965	2.277289	2.272635	2.290672	2.285938	2.281208	2.276471
2.29087	2.286133	2.281423	2.276747	2.272099	2.290134	2.28541	2.280684	2.275946
2.290339	2.285596	2.280879	2.276204	2.271559	2.289591	2.284877	2.280157	2.275421
2.289795	2.28505	2.280331	2.275658	2.271017	2.289039	2.284327	2.303292	2.29844
2.289231	2.284486	2.279768	2.275098	2.270461	2.28847	2.283756	2.3027	2.297857
2.288604	2.283858	2.27914	2.274478	2.269851	2.287851	2.283123	2.302037	2.29721
2.287926	2.283179	2.278463	2.273811	2.269198	2.28719	2.282441	2.30132	2.296512
2.287303	2.282559	2.277849	2.273202	2.268594	2.286579	2.281821	2.30067	2.295868
2.286738	2.282006	2.27731	2.272662	2.268047	2.286025	2.281272	2.300098	2.295289
2.286229	2.281494	2.276798	2.272143	2.267521	2.285496	2.280752	2.299554	2.294731
2.285804	2.281028	2.276291	2.271623	2.267001	2.284981	2.280243	2.299014	2.294175
2.285398	2.280586	2.275814	2.271133	2.26651	2.284495	2.279763	2.298504	2.293649
2.284945	2.280156	2.2754	2.270716	2.26608	2.261466	2.256878	2.298066	2.293199
2.284467	2.279729	2.275017	2.270335	2.265682	2.261063	2.25648	2.297662	2.292788
2.283963	2.279253	2.274565	2.269888	2.265229	2.27854	2.273876	2.297182	2.292319
2.283431	2.278732	2.274057	2.269388	2.264733	2.278027	2.273346	2.296641	2.291805
2.282909	2.278211	2.273538	2.268873	2.264223	2.2775	2.272806	2.296089	2.291272
2.282392	2.277683	2.273	2.268337	2.263694	2.276956	2.272258	2.295526	2.290712
2.281912	2.27719	2.272491	2.26782	2.263172	2.284167	2.279401	2.294969	2.290158
2.281515	2.276783	2.272063	2.267357	2.262667	2.283615	2.278852	2.294426	2.289619
2.281132	2.276394	2.271658	2.266915	2.262183	2.283084	2.278324	2.293906	2.289105
2.280659	2.275931	2.271207	2.266475	2.261749	2.282625	2.277861	2.293443	2.288654
2.28014	2.275429	2.270734	2.266036	2.261347	2.282212	2.27744	2.293018	2.288244
2.279673	2.27495	2.270249	2.283532	2.278784	2.281741	2.276973	2.272235	2.267533
2.279261	2.274492	2.269748	2.283001	2.278256	2.281209	2.276458	2.271722	2.267
2.27884	2.274051	2.269286	2.282516	2.27777	2.280715	2.275971	2.271233	2.266495
2.278392	2.273631	2.268891	2.282114	2.277361	2.280291	2.275536	2.270791	2.266051

2.277951	2.273222	2.268509	2.28173	2.276974	2.279885	2.275114	2.270357	2.26561
2.277544	2.272821	2.268116	2.281337	2.276596	2.279487	2.274692	2.269909	2.265133
2.277152	2.272421	2.267713	2.28093	2.276209	2.27908	2.274264	2.269455	2.264647
2.29487	2.290027	2.28521	2.280455	2.275734	2.278594	2.273788	2.268986	2.264182
2.294449	2.289568	2.284712	2.27993	2.275193	2.278051	2.273277	2.268507	2.263732
2.294008	2.28912	2.28426	2.287165	2.282353	2.277566	2.272808	2.268055	2.263298
2.293536	2.288684	2.283862	2.286759	2.281939	2.277148	2.272387	2.267634	2.262882
2.293084	2.288264	2.283474	2.286367	2.281543	2.276747	2.271978	2.267222	2.262473
2.292678	2.287867	2.283082	2.285973	2.281155	2.276355	2.271573	2.266812	2.262069
2.292285	2.287478	2.282694	2.285582	2.280769	2.275965	2.271173	2.266406	2.261668
2.291879	2.287083	2.282311	2.285185	2.280363	2.275559	2.27077	2.266004	2.261261
2.291473	2.286686	2.28193	2.284784	2.279951	2.275146	2.270368	2.265604	2.260853
2.291086	2.286295	2.281536	2.284381	2.27955	2.274745	2.269964	2.281488	2.276665
2.290714	2.28591	2.281129	2.283975	2.279159	2.274353	2.269558	2.281064	2.276246
2.290325	2.285517	2.280732	2.283571	2.27876	2.273954	2.269155	2.280642	2.275825
2.289897	2.285107	2.280349	2.283171	2.278347	2.273542	2.268756	2.280223	2.275401
2.28951	2.28472	2.279968	2.282774	2.277943	2.273139	2.268357	2.279801	2.274975
2.28923	2.284382	2.27957	2.282367	2.277556	2.272746	2.26794	2.279358	2.274538
2.288979	2.28407	2.279191	2.281982	2.277192	2.272376	2.267543	2.278929	2.274109
2.288667	2.283772	2.2789	2.281681	2.276884	2.272066	2.267235	2.278579	2.273712
2.288322	2.283485	2.27866	2.28143	2.27661	2.271792	2.266979	2.278276	2.273339
2.295671	2.290811	2.285963	2.281117	2.276279	2.287712	2.282839	2.277942	2.272992
2.295255	2.290413	2.285593	2.280742	2.27589	2.287315	2.282458	2.277583	2.272676
2.294894	2.290058	2.285248	2.280397	2.275539	2.286938	2.282066	2.277197	2.272329
2.294637	2.289791	2.284965	2.28012	2.27527	2.286603	2.28166	2.276762	2.271924
2.294373	2.289513	2.284666	2.279828	2.27499	2.286268	2.28127	2.276343	2.271516
2.294015	2.289144	2.284282	2.279454	2.274635	2.285911	2.280932	2.276002	2.271135
2.293629	2.288748	2.283877	2.279052	2.274246	2.285539	2.280611	2.275691	2.270772
2.293296	2.288419	2.283544	2.27869	2.273848	2.285135	2.280246	2.275352	2.270435
2.292992	2.288128	2.283258	2.278351	2.273438	2.284708	2.279856	2.275001	2.270124
2.292648	2.287796	2.282939	2.278021	2.273089	2.284345	2.279512	2.274676	2.269823
2.292252	2.287408	2.282578	2.277697	2.272808	2.284056	2.279221	2.274383	2.269536
2.291894	2.287052	2.282239	2.293579	2.288656	2.283776	2.278935	2.274094	2.269252
2.291613	2.286762	2.281944	2.293277	2.288366	2.283491	2.278649	2.273812	2.26898
2.291361	2.286498	2.281668	2.292987	2.288079	2.2832	2.27835	2.273511	2.26869
2.291102	2.286239	2.281401	2.292701	2.287785	2.282884	2.277997	2.273142	2.268333
2.290849	2.285989	2.281144	2.29242	2.287491	2.28256	2.277625	2.272747	2.267948
2.290595	2.285736	2.280886	2.292142	2.287209	2.282264	2.277308	2.272418	2.267619
2.290343	2.285482	2.280628	2.291868	2.286936	2.281991	2.277034	2.272141	2.267334
2.290087	2.285226	2.280371	2.291595	2.286664	2.28172	2.276764	2.27187	2.267059
2.289828	2.284969	2.280117	2.291325	2.286392	2.281449	2.276495	2.271603	2.266796
2.305861	2.300923	2.295989	2.291055	2.286122	2.28118	2.276228	2.271338	2.266533
2.305591	2.300653	2.29572	2.290788	2.285856	2.280913	2.275959	2.271068	2.266264
2.305321	2.300382	2.295451	2.290521	2.285593	2.280651	2.275696	2.270803	2.265995
2.305048	2.300106	2.29518	2.290256	2.285335	2.280398	2.275444	2.270547	2.265727
2.304777	2.299829	2.294907	2.289991	2.285082	2.280153	2.275206	2.270306	2.265466
2.304517	2.29956	2.294638	2.289729	2.284836	2.279928	2.27501	2.270119	2.265262
2.304265	2.299296	2.294368	2.28947	2.284597	2.279725	2.274858	2.26999	2.265117
2.304054	2.299074	2.294134	2.289228	2.284355	2.279501	2.27467	2.26982	2.264942
2.303911	2.29892	2.293954	2.289014	2.284104	2.279239	2.274419	2.269581	2.264715

2.303757	2.298762	2.293777	2.288806	2.283857	2.278974	2.274153	2.26932	2.264468
2.303517	2.298538	2.293565	2.288584	2.283616	2.29977	2.294518	2.28927	2.284015
2.303258	2.298297	2.293343	2.288366	2.283391	2.299494	2.294217	2.288956	2.283703
2.303063	2.298091	2.293131	2.288167	2.283214	2.299284	2.29399	2.288709	2.283431
2.302909	2.297906	2.292925	2.287985	2.283074	2.299125	2.293816	2.288509	2.283186
2.302735	2.297732	2.292753	2.287828	2.282934	2.298948	2.293618	2.288293	2.282958
2.302543	2.297576	2.292626	2.287705	2.282801	2.298756	2.293392	2.288057	2.282747
2.302335	2.297392	2.292464	2.287547	2.282638	2.298545	2.293171	2.287838	2.282547
2.302095	2.297144	2.292218	2.287313	2.282424	2.298312	2.292975	2.28766	2.282365
2.301868	2.296902	2.291971	2.287069	2.282191	2.298059	2.292764	2.287472	2.282173
2.301706	2.296736	2.291794	2.286865	2.281954	2.29777	2.292486	2.28721	2.281931
2.301578	2.296605	2.291647	2.286679	2.281721	2.297475	2.292189	2.286923	2.281671
2.301454	2.296456	2.291472	2.286498	2.281541	2.297262	2.29197	2.286704	2.281459
2.301338	2.296299	2.291281	2.286324	2.281406	2.297116	2.291811	2.286535	2.281282
2.30124	2.29619	2.29116	2.286202	2.28129	2.296968	2.291656	2.286371	2.281106
2.301172	2.296151	2.291138	2.286155	2.281197	2.29681	2.2915	2.286211	2.280933
2.301088	2.296088	2.291092	2.286093	2.281104	2.296662	2.291349	2.286051	2.280753
2.300964	2.295951	2.290947	2.285963	2.281	2.296535	2.291203	2.285879	2.280542
2.300818	2.295787	2.290776	2.285817	2.280891	2.296412	2.291062	2.285715	2.280343
2.300641	2.295625	2.290636	2.285692	2.280779	2.296272	2.290924	2.285583	2.280226
2.300454	2.295464	2.290509	2.285575	2.280663	2.296124	2.290789	2.285468	2.280149
2.300355	2.295351	2.290384	2.285452	2.280548	2.295984	2.290647	2.284267	2.278972
2.300342	2.295284	2.29026	2.285322	2.280432	2.295853	2.290498	2.284132	2.278802
2.300309	2.295229	2.290174	2.285218	2.280316	2.295712	2.290357	2.284025	2.278687
2.30024	2.295194	2.290158	2.285164	2.280201	2.295553	2.290232	2.283969	2.278669
2.300148	2.29513	2.290111	2.285091	2.280083	2.295396	2.290104	2.283909	2.278653
2.300015	2.294973	2.289938	2.284932	2.279954	2.295255	2.289955	2.283801	2.278582
2.299872	2.294796	2.289738	2.284759	2.279827	2.295123	2.289806	2.283685	2.278493
2.299771	2.294705	2.289653	2.284662	2.279714	2.294103	2.288834	2.283602	2.2784
2.299696	2.294667	2.289637	2.284616	2.279615	2.294011	2.288771	2.283544	2.278307
2.299596	2.294594	2.289594	2.284577	2.279568	2.293978	2.288746	2.283519	2.278274
2.299464	2.294483	2.289525	2.28455	2.279583	2.294015	2.288769	2.283538	2.278312
2.299376	2.294391	2.289444	2.2845	2.279574	2.294029	2.288764	2.283526	2.27832
2.299363	2.294327	2.289334	2.284397	2.279501	2.293976	2.288683	2.283439	2.278254
2.299385	2.294296	2.289246	2.284306	2.279429	2.293923	2.28861	2.283357	2.278174
2.299433	2.294319	2.28924	2.283936	2.279087	2.293929	2.288621	2.28335	2.278109
2.299492	2.294371	2.289276	2.283992	2.279126	2.293951	2.288663	2.283374	2.278056
2.299497	2.294392	2.289301	2.284029	2.279126	2.293922	2.288657	2.283377	2.278045
2.299463	2.294395	2.289327	2.284057	2.279097	2.293858	2.288621	2.283368	2.27807
2.299443	2.294409	2.289366	2.284109	2.279109	2.293831	2.288603	2.283368	2.278103
2.299449	2.294445	2.289428	2.284197	2.279172	2.293852	2.288614	2.283385	2.278149
2.299211	2.294256	2.289291	2.284271	2.27924	2.293881	2.288615	2.28338	2.278171
2.299166	2.294228	2.289293	2.284302	2.279295	2.293898	2.288574	2.283314	2.278129
2.299123	2.294195	2.289286	2.284328	2.279354	2.293924	2.288542	2.283253	2.278085
2.299159	2.294232	2.289328	2.284384	2.279428	2.293979	2.288575	2.283275	2.278107
2.29924	2.294306	2.289396	2.284459	2.279512	2.294053	2.288648	2.283343	2.278167
2.299321	2.294387	2.289478	2.284543	2.279601	2.294127	2.288721	2.283413	2.278234
2.299407	2.294478	2.289575	2.28464	2.279695	2.294206	2.288799	2.283491	2.278314
2.299494	2.294572	2.289671	2.284737	2.27979	2.294285	2.288877	2.28357	2.278394
2.299584	2.294668	2.289764	2.284831	2.279884	2.294365	2.288956	2.283648	2.278472

2.293828	2.28891	2.283996	2.284922	2.279975	2.294442	2.289032	2.283723	2.278547
2.293909	2.288991	2.284079	2.285005	2.280058	2.294511	2.2891	2.283791	2.278615
2.293988	2.289067	2.28416	2.285085	2.280136	2.294576	2.289164	2.283854	2.278679
2.294065	2.289144	2.28424	2.285164	2.280213	2.294637	2.289225	2.283915	2.27874
2.294142	2.289222	2.28432	2.285242	2.280288	2.294698	2.289284	2.283974	2.278801
2.29423	2.289304	2.284397	2.279455	2.274499	2.294775	2.289362	2.28405	2.278868
2.294326	2.289385	2.284466	2.27953	2.274587	2.294869	2.289457	2.284138	2.278936
2.294459	2.289504	2.284568	2.279625	2.274681	2.294972	2.28958	2.284267	2.279045
2.294661	2.289691	2.284726	2.279753	2.274782	2.295084	2.289756	2.284471	2.279227
2.294858	2.289879	2.284891	2.279888	2.274887	2.295196	2.289924	2.284666	2.279407
2.294977	2.290006	2.285022	2.280007	2.274987	2.289926	2.284653	2.284764	2.279519
2.29507	2.290111	2.28514	2.280123	2.275095	2.290017	2.284724	2.284831	2.279607
2.295206	2.290232	2.285253	2.280254	2.275254	2.290178	2.284867	2.29909	2.293793
2.295371	2.290359	2.285359	2.280395	2.275453	2.290392	2.285063	2.299257	2.293928
2.295517	2.29048	2.285463	2.280516	2.275608	2.29056	2.285232	2.29447	2.289128
2.295637	2.290588	2.285556	2.280606	2.275701	2.290666	2.285365	2.294616	2.289295
2.295758	2.290711	2.285679	2.280725	2.275816	2.290786	2.285508	2.294768	2.289466
2.295879	2.290859	2.285859	2.280911	2.275999	2.29096	2.285695	2.294948	2.289643
2.296014	2.291026	2.28606	2.281115	2.276192	2.291137	2.285875	2.295116	2.28981
2.29619	2.291214	2.286253	2.281289	2.276337	2.291256	2.285987	2.295217	2.289927
2.296393	2.291417	2.286445	2.281454	2.276465	2.291356	2.286073	2.295291	2.290023
2.296598	2.291604	2.286613	2.281619	2.276634	2.291523	2.28623	2.295433	2.290168
2.296809	2.291779	2.286759	2.281782	2.276834	2.291746	2.286445	2.295634	2.290354
2.297039	2.291997	2.286966	2.281992	2.277054	2.291972	2.286666	2.295839	2.290541
2.297299	2.292284	2.287271	2.282276	2.277298	2.29219	2.286886	2.296039	2.290727
2.297546	2.292554	2.287559	2.28255	2.277544	2.306972	2.301594	2.296242	2.290908
2.297752	2.292748	2.287748	2.282757	2.277777	2.307208	2.301817	2.29644	2.291061
2.297936	2.292916	2.28791	2.282946	2.278007	2.307449	2.302045	2.296645	2.291223
2.298094	2.293088	2.288106	2.28316	2.278236	2.307677	2.302272	2.296874	2.291462
2.298238	2.293263	2.28832	2.283386	2.278465	2.3079	2.302499	2.297117	2.291746
2.29847	2.293487	2.288537	2.283607	2.278695	2.308127	2.302725	2.297341	2.291969
2.298795	2.29376	2.288756	2.283823	2.278927	2.308358	2.302947	2.297543	2.292125
2.299107	2.294045	2.289008	2.28406	2.279157	2.308581	2.303173	2.297765	2.292331
2.299385	2.294351	2.289328	2.284343	2.279384	2.308787	2.303407	2.298029	2.292635
2.29964	2.294632	2.289625	2.284611	2.279606	2.308992	2.303637	2.298292	2.292946
2.288211	2.283253	2.278302	2.284798	2.279815	2.290079	2.285108	2.280146	2.275194
2.288409	2.283416	2.278441	2.284963	2.280023	2.290289	2.285317	2.280363	2.275429
2.288654	2.283665	2.278691	2.285199	2.280247	2.290502	2.285541	2.280594	2.275659
2.288932	2.283974	2.279016	2.285486	2.280484	2.290721	2.285777	2.280836	2.275884
2.289193	2.28426	2.279327	2.28579	2.280772	2.290997	2.286057	2.281117	2.276162
2.289425	2.284512	2.279618	2.286113	2.281119	2.291344	2.286393	2.28145	2.276511
2.289699	2.284795	2.279921	2.301755	2.296705	2.291693	2.286716	2.28176	2.276837
2.290057	2.285144	2.28026	2.3021	2.297063	2.292027	2.28699	2.282003	2.277091
2.290443	2.285505	2.280591	2.30243	2.297404	2.292346	2.287261	2.282246	2.277331
2.290815	2.285827	2.280862	2.302691	2.29768	2.292638	2.287571	2.282552	2.277595
2.291183	2.286134	2.28111	2.302918	2.297918	2.292907	2.287894	2.282887	2.277873
2.283244	2.278258	2.273284	2.303184	2.298155	2.293151	2.288178	2.283192	2.278174
2.283539	2.278586	2.273629	2.303477	2.298384	2.293368	2.288427	2.283476	2.278498
2.283854	2.27893	2.273993	2.303808	2.298669	2.293644	2.288719	2.283788	2.278835
2.284204	2.279302	2.274386	2.304191	2.299034	2.294001	2.289074	2.284141	2.279187

2.284538	2.279653	2.274757	2.293734	2.28864	2.294375	2.289439	2.284499	2.279544
2.284809	2.279933	2.27506	2.29406	2.288998	2.294744	2.289801	2.284858	2.279912
2.285079	2.280205	2.275351	2.294376	2.289352	2.295109	2.29015	2.285201	2.280266
2.285426	2.28055	2.275698	2.287147	2.282186	2.295445	2.290443	2.285473	2.280549
2.28582	2.280938	2.276076	2.287512	2.282538	2.295767	2.290706	2.285706	2.28079
2.302789	2.29782	2.292865	2.287887	2.282904	2.296116	2.291021	2.286003	2.281089
2.303179	2.298217	2.293258	2.288275	2.283284	2.296492	2.291388	2.286362	2.28144
2.30357	2.298612	2.29365	2.288663	2.283666	2.296873	2.291764	2.286732	2.281804
2.303959	2.299003	2.294039	2.28905	2.284049	2.297255	2.292142	2.287107	2.282177
2.304345	2.299389	2.294423	2.289433	2.28443	2.297637	2.292521	2.287483	2.282552
2.30472	2.299763	2.294798	2.289809	2.284806	2.287291	2.282222	2.287847	2.282915
2.305089	2.300132	2.295167	2.290181	2.285182	2.287668	2.282592	2.288212	2.283277
2.291941	2.286995	2.282043	2.290554	2.285562	2.288054	2.282976	2.288589	2.283642
2.292323	2.287376	2.282426	2.290928	2.285947	2.288448	2.283373	2.288977	2.284012
2.292701	2.287753	2.282808	2.291301	2.286335	2.281356	2.276365	2.289419	2.284438
2.293074	2.288122	2.283186	2.291675	2.286728	2.281786	2.276848	2.289927	2.284933
2.293458	2.288498	2.283567	2.292048	2.287116	2.282202	2.277305	2.290408	2.285407
2.29385	2.288877	2.283938	2.292407	2.287488	2.282584	2.277699	2.290819	2.285824
2.294268	2.289285	2.284335	2.292784	2.287865	2.282961	2.278074	2.291204	2.286218
2.294752	2.289771	2.28481	2.279853	2.274911	2.283343	2.278446	2.280968	2.276056
2.295266	2.290294	2.285324	2.280335	2.275346	2.283737	2.278824	2.281344	2.276438
2.295716	2.290761	2.285805	2.280817	2.275823	2.284191	2.27927	2.300265	2.295254
2.296116	2.291188	2.286264	2.281306	2.276337	2.284698	2.279774	2.300753	2.295698
2.296558	2.291646	2.286743	2.281805	2.276854	2.271913	2.266978	2.301238	2.296165
2.297067	2.292157	2.287257	2.282324	2.277376	2.272422	2.267459	2.301708	2.296659
2.297567	2.292651	2.28775	2.282822	2.277884	2.272926	2.267948	2.295538	2.290569
2.298008	2.293075	2.28817	2.283261	2.278354	2.273418	2.268457	2.296052	2.291087
2.298447	2.293495	2.288585	2.283694	2.27882	2.273911	2.268975	2.296574	2.291605
2.298947	2.293991	2.289077	2.284195	2.279332	2.274432	2.2695	2.284901	2.279917
2.299481	2.294527	2.289609	2.284727	2.279864	2.274962	2.270027	2.285414	2.280399
2.300011	2.295036	2.290094	2.285199	2.280331	2.275433	2.270513	2.28591	2.280899
2.30054	2.295521	2.290533	2.285612	2.280732	2.275844	2.270957	2.286385	2.281414
2.301054	2.296015	2.291009	2.286074	2.281185	2.276303	2.271435	2.286888	2.281944
2.301534	2.296521	2.291544	2.286617	2.281725	2.276842	2.271972	2.287435	2.282496
2.302027	2.297045	2.292102	2.287185	2.282292	2.277406	2.272528	2.287995	2.283057
2.302572	2.297604	2.292669	2.287753	2.282857	2.277968	2.273089	2.288552	2.283622
2.303141	2.29818	2.293245	2.288327	2.283427	2.278536	2.273656	2.289114	2.284189
2.303708	2.298748	2.293816	2.288895	2.283992	2.299563	2.294616	2.289678	2.28475
2.30428	2.299315	2.294386	2.289462	2.284553	2.30012	2.29518	2.290244	2.285306
2.304857	2.299889	2.294957	2.29003	2.285118	2.300684	2.295743	2.290805	2.285865
2.305439	2.30047	2.295528	2.290599	2.285687	2.301258	2.296302	2.291359	2.286428
2.30602	2.30105	2.296101	2.29117	2.286256	2.301829	2.296864	2.291917	2.286991
2.306581	2.301613	2.296675	2.291741	2.286821	2.30239	2.297433	2.292487	2.287553
2.307168	2.302192	2.297255	2.292316	2.287391	2.302955	2.298003	2.293057	2.288114
2.307842	2.30282	2.297834	2.292885	2.287967	2.303529	2.298556	2.293601	2.288667
2.308553	2.303476	2.298428	2.293468	2.288558	2.304119	2.299117	2.294149	2.289224
2.309211	2.304142	2.299094	2.294127	2.289205	2.304766	2.299761	2.294776	2.289816
2.309832	2.304821	2.299823	2.294851	2.2899	2.305463	2.300473	2.295471	2.290438
2.310431	2.305455	2.300489	2.295516	2.290549	2.306114	2.301135	2.296132	2.29108
2.310993	2.306025	2.30107	2.296101	2.291136	2.306702	2.30173	2.296751	2.291751

2.31159	2.306607	2.30164	2.296673	2.291715	2.307283	2.302309	2.29735	2.292404
2.312276	2.307236	2.302214	2.297239	2.292294	2.307866	2.302874	2.297913	2.292993
2.312984	2.307888	2.302811	2.297825	2.29289	2.308466	2.303455	2.298488	2.29358
2.313647	2.308561	2.303488	2.298497	2.293551	2.309131	2.304123	2.299156	2.294243
2.314292	2.309251	2.304216	2.299219	2.294248	2.30983	2.304836	2.299872	2.294942
2.314959	2.309944	2.304929	2.29991	2.294899	2.310469	2.305484	2.300523	2.295582
2.315648	2.310646	2.305636	2.300577	2.295508	2.311051	2.306072	2.301113	2.296167
2.316305	2.311313	2.306312	2.301241	2.296152	2.311685	2.306704	2.301744	2.296797
2.3169	2.311917	2.306934	2.301899	2.296851	2.312402	2.307408	2.302441	2.2975
2.317517	2.312539	2.307569	2.302571	2.297569	2.313142	2.308135	2.303161	2.298222
2.303995	2.299139	2.294291	2.303279	2.298294	2.313881	2.308873	2.303892	2.29894
2.304709	2.299842	2.294983	2.304	2.299023	2.314621	2.309615	2.304629	2.299658
2.305405	2.300531	2.295667	2.304713	2.299745	2.315351	2.310342	2.305349	2.300371
2.306097	2.301214	2.296345	2.305418	2.300458	2.316069	2.311053	2.306053	2.301076
2.281307	2.276437	2.271586	2.306067	2.301114	2.316731	2.311705	2.306701	2.301729
2.281954	2.277033	2.272134	2.306645	2.3017	2.317322	2.312283	2.307278	2.302318
2.273447	2.268527	2.263629	2.31527	2.310254	2.317947	2.312901	2.327781	2.322652
2.274038	2.269156	2.264291	2.31597	2.31094	2.318641	2.313601	2.328476	2.323346
2.274639	2.269804	2.264983	2.316703	2.311655	2.319365	2.314333	2.329209	2.324071
2.275292	2.270478	2.265678	2.317431	2.312374	2.307321	2.302275	2.329947	2.324802
2.275988	2.271183	2.266393	2.318179	2.313115	2.308062	2.30302	2.330713	2.325558
2.276759	2.271954	2.267163	2.302908	2.297886	2.308865	2.303831	2.320259	2.315072
2.277598	2.272781	2.267979	2.303765	2.298738	2.309722	2.304699	2.321152	2.315941
2.27839	2.273561	2.268748	2.304571	2.299545	2.310532	2.305511	2.32198	2.316757
2.279122	2.274281	2.269456	2.305311	2.300294	2.311282	2.306253	2.322726	2.317505
2.279834	2.274985	2.270152	2.290967	2.286088	2.304893	2.299866	2.323452	2.318235
2.280528	2.27568	2.270847	2.291667	2.286782	2.305611	2.300582	2.324174	2.318955
2.281221	2.276374	2.271543	2.292367	2.287474	2.306327	2.301297	2.324895	2.319676
2.281932	2.277078	2.272239	2.293069	2.288175	2.307056	2.302024	2.325627	2.320413
2.28266	2.277797	2.272946	2.293782	2.28889	2.307799	2.302765	2.326374	2.321167
2.283445	2.278578	2.273719	2.29456	2.289666	2.299714	2.294714	2.320927	2.315739
2.284286	2.279422	2.274555	2.295401	2.290505	2.300576	2.295568	2.321794	2.316614
2.285086	2.280228	2.275363	2.321707	2.316572	2.325213	2.319942	2.32263	2.317446
2.285819	2.280968	2.276113	2.32246	2.317306	2.325972	2.320714	2.323406	2.318199
2.28654	2.281705	2.27687	2.323226	2.31805	2.32674	2.321497	2.324194	2.318964
2.287268	2.282474	2.277682	2.324068	2.318874	2.327581	2.322352	2.325055	2.319803
2.288008	2.283253	2.2785	2.324919	2.319711	2.328435	2.323215	2.325924	2.320657
2.288794	2.284019	2.27925	2.325682	2.320481	2.329224	2.323994	2.318776	2.313551
2.289616	2.284783	2.279962	2.326397	2.321215	2.329979	2.324727	2.319504	2.314304
2.290458	2.285593	2.280741	2.327177	2.322	2.330791	2.325527	2.320302	2.31512
2.291331	2.286456	2.28159	2.328022	2.322839	2.331662	2.326394	2.321172	2.316004
2.28315	2.278349	2.273555	2.303344	2.298399	2.293471	2.28856	2.322063	2.316898
2.283885	2.279144	2.274411	2.304225	2.299262	2.294331	2.289425	2.322972	2.31779
2.284617	2.279925	2.275244	2.305092	2.300129	2.295201	2.290295	2.323881	2.318684
2.285415	2.280693	2.275995	2.305896	2.300995	2.296085	2.291165	2.324763	2.319572
2.286245	2.281463	2.276719	2.306673	2.30185	2.296966	2.292031	2.325635	2.320459
2.287091	2.282284	2.277514	2.307466	2.30265	2.297783	2.292877	2.326523	2.321344
2.287956	2.283145	2.27836	2.308269	2.303406	2.29855	2.293706	2.327422	2.322227
2.28882	2.284005	2.279214	2.309118	2.30424	2.299386	2.294557	2.328307	2.32311
2.289682	2.284865	2.280078	2.310031	2.305183	2.300316	2.295436	2.32917	2.32399

2.290541	2.285722	2.280938	2.310925	2.306095	2.301219	2.296307	2.316014	2.310957
2.291393	2.286571	2.281778	2.311745	2.306891	2.302023	2.297148	2.316891	2.311814
2.292246	2.287425	2.282625	2.312567	2.307683	2.302821	2.297986	2.317772	2.312671
2.293106	2.288295	2.283504	2.313468	2.308583	2.303709	2.298849	2.318636	2.313526
2.293972	2.289178	2.284406	2.31441	2.309534	2.304636	2.299722	2.319496	2.314381
2.294856	2.290082	2.285325	2.315341	2.310445	2.305524	2.300583	2.320369	2.315242
2.295757	2.291007	2.286262	2.316268	2.311326	2.306381	2.301434	2.321253	2.316108
2.296669	2.291925	2.287179	2.309294	2.304392	2.307248	2.30229	2.322138	2.31699
2.297598	2.292834	2.288063	2.310176	2.305273	2.308119	2.303143	2.32301	2.317886
2.298519	2.293735	2.288944	2.311067	2.306177	2.309024	2.304037	2.323926	2.318812
2.299409	2.29462	2.289834	2.311994	2.307135	2.310008	2.305028	2.324947	2.319801
2.30029	2.295504	2.290731	2.312933	2.308105	2.31101	2.306042	2.325994	2.320801
2.301184	2.296412	2.291655	2.340856	2.335741	2.337428	2.332185	2.326955	2.321731
2.302086	2.297336	2.292594	2.341802	2.336648	2.338335	2.333103	2.327872	2.322623
2.303014	2.298254	2.293501	2.342723	2.337562	2.339266	2.334052	2.328832	2.323584
2.30397	2.299162	2.29437	2.343611	2.338478	2.340218	2.335033	2.329839	2.324616
2.304937	2.3001	2.295282	2.344545	2.339424	2.334311	2.329208	2.330815	2.32562
2.305922	2.301097	2.296279	2.345565	2.34042	2.33527	2.330116	2.331719	2.326551
2.306888	2.302081	2.297272	2.34659	2.341425	2.336242	2.33104	2.332637	2.327488
2.307779	2.302976	2.298184	2.347563	2.342423	2.337249	2.332044	2.333646	2.32849
2.308649	2.303844	2.299069	2.348512	2.343406	2.338256	2.333068	2.334684	2.329515
2.309593	2.304774	2.299986	2.322287	2.317455	2.312595	2.307712	2.335659	2.330511
2.310588	2.305745	2.300923	2.323189	2.318311	2.313444	2.308589	2.336594	2.331489
2.30397	2.299158	2.29436	2.324137	2.319229	2.314356	2.309514	2.337563	2.332488
2.304866	2.300094	2.295332	2.325138	2.320219	2.315341	2.310496	2.338571	2.333513
2.305782	2.301044	2.296313	2.32615	2.321226	2.316344	2.311494	2.33278	2.327791
2.30675	2.302021	2.297301	2.327161	2.322233	2.31735	2.312501	2.333793	2.328805
2.307739	2.303009	2.298291	2.328173	2.323244	2.31836	2.313512	2.334808	2.329818
2.337161	2.332327	2.327508	2.322618	2.317706	2.319363	2.314516	2.33582	2.330829
2.338179	2.333333	2.328504	2.323609	2.318697	2.320363	2.315516	2.336832	2.331839
2.339194	2.334339	2.3295	2.324601	2.319687	2.314824	2.310001	2.337857	2.332859
2.340207	2.335344	2.330497	2.325592	2.320675	2.315811	2.310992	2.338894	2.333887
2.320867	2.316113	2.311373	2.326594	2.32167	2.316804	2.311985	2.339935	2.334923
2.321881	2.317126	2.312383	2.327608	2.322675	2.317802	2.31298	2.340984	2.335969
2.322896	2.318142	2.3134	2.32863	2.323689	2.318809	2.313978	2.336418	2.331415
2.323905	2.319157	2.314418	2.329656	2.324706	2.319814	2.314967	2.337441	2.332443
2.324914	2.320173	2.315441	2.330693	2.325746	2.320843	2.315972	2.338473	2.333473
2.325921	2.321189	2.316473	2.331767	2.326852	2.321947	2.317045	2.312215	2.307482
2.326932	2.322206	2.317511	2.332861	2.327998	2.323098	2.318161	2.31329	2.308506
2.316303	2.311587	2.30691	2.333914	2.329084	2.32419	2.319241	2.314346	2.309523
2.317387	2.312627	2.307916	2.334928	2.330111	2.325227	2.320285	2.315384	2.310529
2.318461	2.313661	2.308918	2.315826	2.311141	2.32627	2.321356	2.316461	2.311583
2.319494	2.314668	2.309904	2.31682	2.31216	2.32734	2.322484	2.317614	2.312714
2.320552	2.315703	2.310916	2.317831	2.313179	2.3284	2.323599	2.318761	2.313861
2.3217	2.316823	2.311993	2.307268	2.302609	2.329416	2.324645	2.319843	2.314994
2.322873	2.31798	2.313112	2.308337	2.303623	2.330423	2.32566	2.320891	2.31611
2.323975	2.31912	2.314271	2.309463	2.304691	2.320637	2.315869	2.321905	2.317155
2.32503	2.320251	2.315464	2.310636	2.305801	2.321716	2.316887	2.32289	2.318147
2.310932	2.306255	2.301574	2.311763	2.306927	2.322836	2.317959	2.313143	2.308404
2.311923	2.30724	2.302578	2.312838	2.30808	2.324012	2.319095	2.314231	2.309432

2.312983	2.308271	2.303597	2.313897	2.309191	2.325154	2.320237	2.315357	2.310523
2.314193	2.309412	2.304664	2.314937	2.31021	2.326213	2.321377	2.316554	2.311742
2.315413	2.310576	2.305759	2.31599	2.31122	2.327254	2.322496	2.317733	2.312958
2.296426	2.29172	2.287017	2.317113	2.312294	2.307562	2.30291	2.318772	2.314019
2.297447	2.292801	2.288146	2.318267	2.313402	2.30862	2.30391	2.319757	2.315017
2.280794	2.276236	2.271678	2.319377	2.314533	2.309753	2.305027	2.320872	2.316108
2.281753	2.277191	2.272651	2.320454	2.315689	2.310952	2.306242	2.322091	2.317268
2.282801	2.278216	2.273663	2.306483	2.301817	2.312074	2.307379	2.323246	2.318412
2.28398	2.279343	2.274726	2.307518	2.302815	2.313072	2.308394	2.324307	2.319536
2.285141	2.280474	2.275814	2.308596	2.303856	2.314103	2.309421	2.32536	2.320633
2.286187	2.281554	2.276933	2.309779	2.305038	2.31527	2.31052	2.326425	2.321663
2.287219	2.282628	2.278058	2.310981	2.306249	2.316467	2.311649	2.327514	2.322702
2.288347	2.283736	2.279144	2.312091	2.307354	2.317588	2.312783	2.328672	2.323859
2.289521	2.284859	2.280212	2.313159	2.308414	2.318679	2.313933	2.329879	2.325087
2.290617	2.285958	2.281314	2.297577	2.292889	2.31985	2.31513	2.310429	2.305743
2.291635	2.287028	2.282447	2.298771	2.294096	2.321103	2.31638	2.311655	2.306912
2.292712	2.288141	2.283599	2.299959	2.295276	2.322309	2.317573	2.312831	2.308066
2.293907	2.289341	2.284789	2.301133	2.296399	2.323421	2.318654	2.313912	2.309189
2.295082	2.290513	2.285949	2.302285	2.297517	2.324539	2.319748	2.31501	2.310331
2.258711	2.254286	2.249874	2.282398	2.277787	2.293968	2.289314	2.316223	2.311551
2.259685	2.255265	2.250875	2.283461	2.278906	2.295152	2.290515	2.317465	2.312784
2.260787	2.256363	2.251975	2.284602	2.280064	2.296326	2.291664	2.31862	2.313911
2.261975	2.257539	2.253135	2.285792	2.281247	2.297496	2.292785	2.319723	2.314971
2.263151	2.258684	2.254246	2.286912	2.282354	2.298617	2.293912	2.304996	2.300287
2.264324	2.259798	2.255298	2.287943	2.283365	2.299676	2.295042	2.306194	2.301514
2.265451	2.260894	2.256365	2.289016	2.28443	2.300782	2.2962	2.307409	2.302755
2.266479	2.261952	2.257464	2.290179	2.285613	2.301986	2.297405	2.308639	2.304003
2.267517	2.263029	2.258592	2.291381	2.286838	2.303225	2.298627	2.309869	2.305234
2.268674	2.264196	2.259767	2.292606	2.288068	2.304468	2.299853	2.311085	2.306403
2.269892	2.265409	2.26097	2.293847	2.289307	2.305714	2.301075	2.312284	2.307537
2.271109	2.266612	2.262155	2.29507	2.290521	2.306923	2.30224	2.313432	2.308668
2.272336	2.267815	2.263331	2.296281	2.291717	2.308102	2.303357	2.314533	2.30979
2.273537	2.268978	2.264456	2.297421	2.292834	2.309223	2.304468	2.31566	2.310939
2.27471	2.270093	2.265512	2.29846	2.293839	2.310259	2.305566	2.316816	2.312119
2.249344	2.244757	2.24021	2.299521	2.294879	2.311332	2.306694	2.302075	2.297468
2.250453	2.245852	2.241302	2.300667	2.296036	2.312512	2.307882	2.303272	2.298675
2.251556	2.246959	2.242423	2.301855	2.297244	2.31374	2.309101	2.304487	2.299896
2.252681	2.248106	2.243594	2.278934	2.274392	2.314982	2.310334	2.305714	2.301119
2.253823	2.249283	2.244803	2.280188	2.275637	2.316244	2.311584	2.306952	2.302348
2.255011	2.250489	2.246022	2.281445	2.276883	2.317509	2.312838	2.308195	2.30358
2.256243	2.251726	2.247253	2.282708	2.278132	2.318779	2.314098	2.309444	2.304813
2.257483	2.252964	2.248483	2.283971	2.279383	2.297106	2.292474	2.310693	2.306049
2.258726	2.2542	2.24971	2.285232	2.280636	2.298372	2.293728	2.311939	2.307287
2.259971	2.255435	2.250936	2.286493	2.28189	2.299639	2.294984	2.313188	2.308528
2.261217	2.25667	2.25216	2.287754	2.283145	2.300913	2.29625	2.314447	2.309773
2.262462	2.257903	2.253383	2.289015	2.284399	2.302187	2.297516	2.315707	2.31102
2.228854	2.224359	2.219902	2.290273	2.285646	2.303449	2.298768	2.294112	2.28948
2.230067	2.225562	2.221096	2.291528	2.286888	2.304703	2.300011	2.295349	2.290717
2.231287	2.22677	2.222292	2.292783	2.28813	2.305958	2.301257	2.296589	2.291954
2.232514	2.227984	2.223492	2.294039	2.289372	2.307214	2.302508	2.297834	2.293191

2.233736	2.229195	2.22469	2.264251	2.259635	2.285972	2.281371	2.299078	2.294429
2.234942	2.230394	2.225882	2.265486	2.260854	2.287197	2.282588	2.300317	2.295668
2.236154	2.2316	2.227082	2.266729	2.262081	2.288431	2.283812	2.301559	2.296908
2.237369	2.232807	2.228288	2.267973	2.263308	2.258699	2.25414	2.302801	2.298142
2.238616	2.234046	2.229526	2.269249	2.264567	2.259942	2.255368	2.304057	2.299382
2.240012	2.235433	2.230902	2.270675	2.265975	2.261311	2.256679	2.305367	2.300653
2.241532	2.236943	2.232395	2.272227	2.267508	2.262786	2.258062	2.306723	2.301952
2.242951	2.238351	2.233788	2.273678	2.268954	2.264212	2.259455	2.279031	2.274297
2.244237	2.239626	2.235053	2.275	2.270288	2.265577	2.26087	2.280514	2.275787
2.245489	2.240865	2.236282	2.276283	2.271584	2.266902	2.26224	2.281948	2.277235
2.246728	2.242087	2.237491	2.277546	2.272851	2.268173	2.263514	2.283247	2.278543
2.247959	2.243302	2.238693	2.278796	2.274102	2.26942	2.264755	2.284504	2.279806
2.249206	2.244536	2.239912	2.280046	2.275343	2.270661	2.266003	2.285774	2.281074
2.250461	2.245784	2.241149	2.281309	2.276595	2.271913	2.267264	2.287057	2.282349
2.251723	2.247072	2.242463	2.282687	2.277983	2.273304	2.268651	2.288461	2.28375
2.25299	2.248397	2.243853	2.284179	2.279504	2.274832	2.270166	2.289985	2.285274
2.254327	2.249749	2.245221	2.240708	2.23622	2.27626	2.271584	2.266938	2.262327
2.255777	2.25114	2.246549	2.241986	2.237455	2.277521	2.272845	2.2682	2.263591
2.257211	2.252512	2.247856	2.243238	2.238661	2.278748	2.27407	2.269427	2.26482
2.258516	2.253795	2.249115	2.244468	2.239861	2.279974	2.275287	2.270641	2.266038
2.259806	2.255076	2.250385	2.245718	2.241089	2.281231	2.276532	2.27188	2.267279
2.261247	2.256504	2.251796	2.247114	2.24247	2.282654	2.277939	2.273275	2.268667
2.262774	2.25802	2.253294	2.248603	2.24395	2.284185	2.279451	2.274771	2.270149
2.264183	2.25942	2.254684	2.249987	2.24533	2.285608	2.28085	2.276152	2.271519
2.265469	2.260698	2.255962	2.251262	2.2466	2.286911	2.282122	2.277402	2.272763
2.266739	2.261961	2.257229	2.25254	2.247892	2.28826	2.283456	2.278724	2.274074
2.267989	2.263205	2.258486	2.253837	2.249244	2.289719	2.284942	2.280216	2.275547
2.269291	2.264499	2.259785	2.255164	2.250612	2.291186	2.28643	2.281707	2.277015
2.237013	2.232307	2.227672	2.256566	2.251977	2.247392	2.24282	2.283044	2.278328
2.238508	2.233774	2.2291	2.257999	2.253348	2.248712	2.244097	2.28434	2.279599
2.239846	2.235102	2.230423	2.259373	2.254725	2.250091	2.245479	2.285778	2.281011
2.241076	2.236335	2.231674	2.260707	2.256109	2.251514	2.246936	2.287318	2.282523
2.242424	2.237671	2.233002	2.228438	2.223952	2.252884	2.248311	2.288754	2.283933
2.243924	2.239136	2.234423	2.22979	2.225227	2.254171	2.249566	2.29004	2.285196
2.224108	2.219391	2.214735	2.231186	2.226554	2.221978	2.21746	2.291388	2.286528
2.225518	2.220828	2.216185	2.232676	2.228013	2.223409	2.218863	2.292906	2.288043
2.226896	2.222232	2.217606	2.234151	2.229476	2.22486	2.220299	2.294471	2.289617
2.2282	2.223506	2.218861	2.235455	2.230784	2.226191	2.221669	2.250642	2.246045
2.229476	2.224724	2.220043	2.236682	2.232028	2.227471	2.223009	2.252075	2.247511
2.230896	2.226099	2.221378	2.238042	2.233375	2.228803	2.224324	2.219868	2.215431
2.232447	2.227612	2.222845	2.239514	2.234809	2.230174	2.225611	2.22109	2.216613
2.233901	2.229059	2.224281	2.24099	2.236272	2.231597	2.226969	2.222395	2.217884
2.235186	2.230389	2.225661	2.242467	2.237776	2.233095	2.228431	2.223826	2.219293
2.236487	2.23172	2.227027	2.243923	2.239261	2.234588	2.22991	2.225288	2.220736
2.237906	2.233102	2.228378	2.245323	2.240685	2.236035	2.231385	2.226769	2.222191
2.239382	2.234519	2.229742	2.246709	2.242074	2.237443	2.23283	2.228229	2.223633
2.240896	2.235996	2.231173	2.226468	2.221851	2.238766	2.234145	2.229552	2.224983
2.242447	2.237522	2.232653	2.227862	2.223142	2.240034	2.235379	2.230789	2.226272
2.243994	2.239051	2.23415	2.229302	2.224514	2.241398	2.236703	2.232089	2.227568
2.245543	2.240591	2.235664	2.230789	2.225965	2.242856	2.238111	2.233445	2.228858

2.247051	2.242114	2.23719	2.232299	2.227446	2.24435	2.239563	2.234851	2.230207
2.2485	2.24362	2.238739	2.233838	2.228948	2.245875	2.24106	2.236321	2.231649
2.249916	2.245093	2.240264	2.235372	2.230466	2.247426	2.242582	2.237816	2.233121
2.233991	2.229192	2.224401	2.236872	2.232002	2.249004	2.244113	2.239303	2.234576
2.235383	2.2306	2.225848	2.238343	2.233535	2.250591	2.245655	2.240801	2.23604
2.23683	2.232012	2.227246	2.222475	2.217707	2.230192	2.225345	2.242322	2.237531
2.238319	2.233424	2.228602	2.223849	2.219139	2.231638	2.226828	2.243864	2.239046
2.191512	2.18669	2.181953	2.22529	2.22061	2.215863	2.211067	2.245416	2.24057
2.193018	2.188178	2.183429	2.226812	2.222127	2.217387	2.21261	2.24698	2.242105
2.194534	2.189678	2.18492	2.206341	2.201688	2.218921	2.214154	2.232317	2.227393
2.196042	2.191169	2.186402	2.207857	2.203211	2.220465	2.215694	2.233902	2.228962
2.197566	2.19267	2.187887	2.209372	2.204731	2.222007	2.21723	2.235484	2.230531
2.199134	2.194191	2.189367	2.210869	2.206225	2.223529	2.218763	2.237071	2.232101
2.200727	2.195726	2.190849	2.212358	2.207704	2.225036	2.220289	2.238655	2.23367
2.145024	2.140255	2.135597	2.187617	2.183004	2.204478	2.199776	2.240219	2.235234
2.146495	2.141707	2.137027	2.189096	2.184446	2.205936	2.201236	2.241763	2.236794
2.147962	2.143154	2.138456	2.190586	2.185905	2.207412	2.202706	2.219987	2.215125
2.149419	2.144588	2.139874	2.192085	2.187389	2.208914	2.204192	2.221491	2.216636
2.150896	2.146043	2.141309	2.193601	2.188883	2.210421	2.205676	2.20091	2.196104
2.152416	2.147538	2.142769	2.195128	2.190372	2.211913	2.207138	2.202366	2.197581
2.153957	2.149063	2.144264	2.196686	2.191883	2.213417	2.208605	2.20382	2.199056
2.155498	2.150628	2.145838	2.198322	2.193463	2.188635	2.183848	2.20529	2.200518
2.157043	2.152222	2.147462	2.200009	2.195086	2.190197	2.185344	2.206773	2.20198
2.158569	2.153769	2.149018	2.144297	2.139612	2.191742	2.186851	2.208295	2.203511
2.16007	2.155264	2.150504	2.145763	2.141056	2.193268	2.188366	2.209851	2.205113
2.123678	2.119038	2.114439	2.147256	2.142522	2.194826	2.189924	2.211449	2.206722
2.12527	2.12061	2.115971	2.14881	2.144041	2.196445	2.191553	2.213113	2.208333
2.126847	2.122172	2.117499	2.150361	2.145556	2.198062	2.193178	2.214767	2.209929
2.128303	2.12364	2.118969	2.151858	2.147009	2.199609	2.194722	2.18989	2.185112
2.12972	2.12507	2.120409	2.153342	2.148458	2.201149	2.196249	2.191402	2.186608
2.100675	2.096057	2.091442	2.11711	2.112406	2.145257	2.140609	2.192973	2.188135
2.102188	2.097489	2.092821	2.11856	2.113955	2.146877	2.142178	2.194581	2.189685
2.103678	2.098961	2.094287	2.089712	2.085201	2.14845	2.143709	2.196181	2.191265
2.105132	2.100475	2.095857	2.091259	2.086689	2.149965	2.145192	2.197767	2.192879
2.106629	2.10201	2.097422	2.092793	2.088163	2.113794	2.109152	2.19937	2.194501
2.108233	2.103581	2.098946	2.094277	2.089609	2.115245	2.110622	2.201006	2.196122
2.109828	2.105135	2.100448	2.095749	2.091065	2.11672	2.11212	2.202657	2.197743
2.066686	2.062107	2.057544	2.097231	2.092582	2.087987	2.08344	2.146645	2.14195
2.06803	2.063462	2.058927	2.09872	2.094129	2.089544	2.084968	2.148207	2.143462
2.069484	2.064914	2.060384	2.100242	2.095656	2.091054	2.086441	2.081872	2.077337
2.071025	2.066444	2.061903	2.101795	2.097171	2.092528	2.087869	2.083282	2.07877
2.001469	1.997088	1.992739	2.058853	2.054306	2.093997	2.089336	2.084749	2.080242
2.002949	1.998522	1.994116	2.0603	2.055701	2.095454	2.090858	2.0863	2.081764
2.004408	1.999934	1.995479	2.061746	2.057119	2.096939	2.09239	2.087854	2.083301
2.005794	2.001282	1.996807	2.063195	2.058603	2.098485	2.093909	2.089366	2.084847
2.007161	2.002623	1.998143	2.06466	2.060114	2.100056	2.095434	2.090875	2.086392
2.008555	2.004028	1.999559	2.066179	2.061614	2.057078	2.052575	2.092414	2.087911
2.009967	2.005469	2.001022	2.067733	2.063118	2.058568	2.054077	2.09397	2.089416
1.986661	1.98226	1.977904	1.997997	1.993589	2.060089	2.055575	2.095508	2.090935
1.98819	1.983729	1.979316	1.999397	1.995043	2.061644	2.057066	2.097026	2.092466

1.98971	1.985221	1.980779	2.000824	1.996477	2.063186	2.058576	2.098576	2.094007
1.991195	1.986745	1.982337	2.002298	1.997866	2.064693	2.060115	2.100181	2.09556
1.992702	1.988279	1.983892	2.003771	1.999264	2.066209	2.061656	2.101786	2.097118
1.916155	1.911771	1.907422	1.971819	1.967334	1.996292	1.991923	2.103339	2.098673
1.917757	1.913295	1.908868	1.97325	1.968809	1.99774	1.993306	2.104887	2.100235
1.868741	1.864395	1.86009	1.974724	1.970222	1.965766	1.961355	2.061628	2.057074
1.870171	1.865893	1.861658	1.976233	1.971603	1.967127	1.962785	2.063198	2.058596
1.871716	1.867461	1.863253	1.858918	1.854569	1.968626	1.964288	1.959945	1.955557
1.873392	1.869114	1.864886	1.860557	1.856221	1.970288	1.965883	1.961497	1.957109
1.875071	1.870746	1.866473	1.862158	1.857857	1.927019	1.922661	1.963006	1.958626
1.876724	1.872314	1.867954	1.863663	1.859422	1.928509	1.924052	1.964403	1.96005
1.878347	1.873865	1.869427	1.865147	1.860946	1.929962	1.925434	1.965798	1.96145
1.83842	1.83403	1.829685	1.866652	1.862394	1.93138	1.926886	1.922475	1.918169
1.839942	1.835589	1.83128	1.868174	1.863815	1.932787	1.928375	1.92397	1.919539
1.841572	1.837221	1.832914	1.828575	1.824253	1.861067	1.856876	1.925483	1.921004
1.843289	1.838915	1.834588	1.830236	1.825905	1.862695	1.858483	1.927021	1.922551
1.754989	1.750734	1.746537	1.831842	1.827516	1.823266	1.819088	1.928494	1.924043
1.756577	1.752257	1.747997	1.833365	1.829059	1.824783	1.82054	1.929863	1.925444
1.625022	1.621238	1.617496	1.834903	1.830616	1.826321	1.822028	1.887852	1.88356
1.626434	1.622664	1.618947	1.836507	1.832236	1.827944	1.823644	1.889387	1.885075
1.627861	1.624111	1.620423	1.83813	1.833858	1.829573	1.825284	1.890959	1.886622
1.547011	1.543571	1.540178	1.672111	1.668339	1.83111	1.826841	1.82263	1.818494
1.548548	1.545074	1.541634	1.673622	1.669789	1.832606	1.828363	1.824183	1.820081
1.471333	1.468021	1.464733	1.599304	1.59575	1.834186	1.82993	1.825733	1.821608
1.472693	1.469408	1.466142	1.600859	1.597291	1.835845	1.831533	1.827275	1.823069
1.403447	1.400355	1.397284	1.542646	1.539221	1.595261	1.591743	1.828875	1.824603
1.404759	1.401629	1.398526	1.544029	1.540635	1.596731	1.593247	1.830564	1.826255
1.406089	1.40292	1.399785	1.545427	1.542057	1.598208	1.594762	1.832269	1.827932
1.407446	1.404266	1.401121	1.468271	1.465046	1.599714	1.596278	1.679176	1.675027
1.408821	1.405637	1.402488	1.469684	1.466436	1.601234	1.597796	1.680862	1.676692
1.410198	1.407006	1.403843	1.471085	1.467824	1.530321	1.527035	1.595846	1.592323
1.411581	1.408378	1.405195	1.472482	1.469213	1.531758	1.528459	1.597355	1.593835
1.412962	1.409745	1.406544	1.403395	1.400286	1.467367	1.464191	1.526521	1.523187
1.41434	1.411108	1.407888	1.404727	1.401605	1.468704	1.465474	1.527834	1.524494
1.415717	1.412472	1.409239	1.406066	1.40293	1.47005	1.466779	1.529176	1.525825
1.417101	1.413852	1.410611	1.407423	1.404269	1.471418	1.46813	1.530577	1.527208
1.418468	1.415214	1.41197	1.408774	1.405609	1.472796	1.469501	1.532005	1.528615
1.41977	1.416505	1.413262	1.410079	1.406934	1.403796	1.400668	1.46761	1.464377
1.421062	1.417782	1.41454	1.411376	1.40826	1.405136	1.402012	1.468996	1.465747
1.422482	1.419186	1.415928	1.412749	1.409623	1.406504	1.403399	1.470439	1.467167
1.424004	1.420689	1.417402	1.414183	1.411015	1.407895	1.404823	1.471932	1.468632
1.239996	1.23715	1.234336	1.4156	1.412398	1.409268	1.406209	1.403136	1.400021
1.241259	1.238383	1.235543	1.416991	1.413765	1.410617	1.407543	1.40446	1.401341
1.242519	1.239612	1.236746	1.418381	1.415137	1.41197	1.408876	1.40578	1.40266
1.243777	1.240841	1.237952	1.419772	1.416523	1.413341	1.410226	1.407115	1.403989
1.245044	1.242082	1.23917	1.421171	1.417916	1.414722	1.411587	1.408463	1.405336
1.246337	1.243355	1.24042	1.237592	1.234835	1.416104	1.412963	1.409842	1.406731
1.247651	1.244654	1.241698	1.238838	1.236047	1.417495	1.414353	1.41124	1.408154
1.248984	1.245987	1.243022	1.24013	1.237298	1.234538	1.231849	1.412627	1.409548
1.250339	1.247357	1.244393	1.24147	1.238587	1.235786	1.23306	1.414006	1.410916

1.251682	1.248708	1.245747	1.242798	1.239876	1.237039	1.234278	1.415392	1.412285
1.253001	1.250024	1.247063	1.244096	1.241148	1.238284	1.235494	1.416778	1.413652
1.254322	1.25134	1.248382	1.245406	1.242445	1.239559	1.236739	1.418192	1.415034
1.255648	1.252674	1.24973	1.246763	1.243806	1.240901	1.238042	1.235239	1.232498
1.256993	1.254018	1.251083	1.248131	1.245189	1.242272	1.239376	1.236531	1.233738
1.25839	1.255374	1.252407	1.249459	1.246537	1.243623	1.240718	1.237855	1.23503
1.259828	1.256744	1.253719	1.250766	1.247866	1.244965	1.242072	1.239206	1.236363
1.132937	1.130114	1.127344	1.252105	1.249189	1.246285	1.243401	1.24054	1.237698
1.134258	1.131416	1.12861	1.253475	1.250498	1.247574	1.244704	1.241861	1.239041
1.007231	1.004664	1.002112	1.254873	1.251843	1.248891	1.246013	1.24317	1.240359
1.008328	1.005777	1.003237	1.25631	1.253243	1.25025	1.247325	1.244447	1.241614
1.00941	1.006872	1.004346	1.257759	1.254673	1.251647	1.248676	1.245756	1.24289
1.010486	1.007941	1.005418	1.259203	1.256132	1.253109	1.250131	1.247187	1.24427
1.011569	1.009005	1.006477	1.260643	1.257601	1.254595	1.251625	1.248664	1.245695
1.012718	1.010126	1.00757	1.153832	1.151012	1.255999	1.253024	1.250057	1.247088
1.013919	1.01129	1.008687	1.155106	1.152258	1.257344	1.254354	1.251394	1.248462
1.015108	1.012482	1.00987	1.15647	1.15359	1.258764	1.255738	1.252758	1.249829
1.016284	1.013717	1.011147	1.157959	1.155039	1.260284	1.257188	1.254149	1.251174
1.017433	1.014909	1.012376	1.009825	1.007264	1.153537	1.150628	1.255574	1.252565
1.018518	1.015989	1.013457	1.01091	1.008357	1.154868	1.15197	1.257058	1.25406
1.01961	1.01706	1.014517	1.011968	1.00942	1.156179	1.153303	1.258548	1.255567
1.02081	1.018243	1.015675	1.013098	1.010523	1.15747	1.154592	1.259956	1.256964
1.022058	1.019475	1.016875	1.014264	1.011649	1.158762	1.155872	1.261341	1.258323
1.023205	1.020633	1.018045	1.015446	1.012842	1.160165	1.157271	1.154409	1.15158
1.024264	1.021728	1.019192	1.016648	1.014102	1.161674	1.158781	1.155909	1.153059
1.025393	1.022867	1.02035	1.017831	1.015311	1.163108	1.160216	1.157337	1.154468
1.026642	1.024083	1.021532	1.018979	1.016429	1.164407	1.161517	1.158631	1.155742
1.027882	1.02529	1.022706	1.020128	1.017558	1.015006	1.012471	1.159952	1.157049
1.029015	1.02642	1.023848	1.021303	1.018777	1.016247	1.013716	1.161418	1.158512
1.030141	1.027544	1.024984	1.022478	1.020003	1.0175	1.014976	1.162907	1.160005
1.031389	1.028752	1.026151	1.023617	1.021125	1.01862	1.016111	1.16426	1.16136
1.03271	1.030009	1.027337	1.024739	1.022189	1.019667	1.017177	1.165539	1.162642
1.033957	1.031228	1.028527	1.025906	1.023341	1.020819	1.018343	1.166928	1.164032
1.035122	1.032401	1.029716	1.027119	1.024582	1.022078	1.019613	1.168431	1.165533
1.036347	1.033631	1.030951	1.028362	1.025833	1.023335	1.020875	1.169917	1.167012
1.037705	1.034974	1.032272	1.029653	1.027091	1.024577	1.022116	1.171354	1.168428
1.039056	1.036312	1.033588	1.030931	1.028325	1.025787	1.023318	1.172763	1.169825
1.04027	1.037529	1.034799	1.032113	1.029467	1.026901	1.024413	1.02191	1.019382
1.041451	1.038721	1.035991	1.03328	1.030595	1.027998	1.025482	1.022984	1.020506
1.042756	1.040036	1.037306	1.034581	1.031871	1.029245	1.026692	1.024179	1.021712
1.044149	1.04144	1.038713	1.035982	1.033258	1.030603	1.028006	1.025461	1.022979
1.045507	1.042814	1.040097	1.037368	1.034637	1.031956	1.029317	1.026739	1.024238
1.046832	1.044161	1.041465	1.038746	1.036016	1.033312	1.030629	1.028016	1.025492
1.0481	1.045451	1.042777	1.04007	1.037342	1.034624	1.031908	1.029265	1.026713
1.049254	1.046626	1.043976	1.041277	1.038551	1.035828	1.033102	1.030438	1.02785
1.050406	1.047794	1.045165	1.042478	1.039758	1.037037	1.03431	1.03163	1.029004
1.051682	1.049077	1.046464	1.043794	1.041092	1.038379	1.035651	1.032954	1.030289
1.05302	1.050415	1.047812	1.045162	1.042486	1.039786	1.037061	1.034351	1.03165
1.054347	1.051731	1.049131	1.046497	1.043846	1.041163	1.038449	1.035737	1.033017
1.055675	1.053038	1.050432	1.047812	1.045187	1.042525	1.03983	1.037125	1.034399

1.056944	1.054282	1.051665	1.049049	1.046442	1.043805	1.041145	1.038466	1.035757
1.058104	1.055416	1.052784	1.050162	1.047561	1.044959	1.042362	1.039742	1.037089
1.059334	1.056611	1.053949	1.051315	1.048714	1.046135	1.043581	1.041	1.038383
1.060761	1.057983	1.055269	1.052618	1.050017	1.047427	1.044854	1.04225	1.039598
1.062272	1.059434	1.056658	1.053985	1.051377	1.048762	1.046152	1.043509	1.040809
1.063768	1.06088	1.05805	1.055346	1.052721	1.050092	1.047473	1.044825	1.042128
1.065274	1.062343	1.059465	1.056725	1.054071	1.05143	1.048814	1.046179	1.043513
1.066772	1.063825	1.06092	1.058138	1.055436	1.052771	1.050152	1.047523	1.044881
1.068261	1.065327	1.062419	1.059588	1.056816	1.054115	1.051487	1.048862	1.046237
1.069746	1.066827	1.063918	1.061045	1.058212	1.055476	1.052828	1.050197	1.04758
1.071237	1.068326	1.065407	1.062502	1.059624	1.056851	1.054172	1.051522	1.048904
1.072698	1.069807	1.066891	1.06397	1.061061	1.058253	1.055533	1.052856	1.050228
1.074082	1.071246	1.068368	1.065459	1.062539	1.059699	1.056925	1.054208	1.051563
1.07543	1.072663	1.06984	1.066957	1.06404	1.061172	1.058339	1.055578	1.052908
0.865592	0.863588	0.861569	1.068451	1.06553	1.06264	1.059767	1.05697	1.054269
0.866698	0.864702	0.862691	1.069942	1.067016	1.064111	1.061212	1.058384	1.055645
0.867767	0.865765	0.863757	1.07139	1.068494	1.065596	1.062685	1.059832	1.057051
0.868786	0.866762	0.864751	1.072787	1.069964	1.067101	1.064192	1.06132	1.058492
0.86983	0.867784	0.86577	0.863759	0.861748	1.068594	1.065692	1.062809	1.059943
0.870929	0.868875	0.866859	0.864851	0.862848	1.070062	1.067169	1.06428	1.061394
0.872051	0.869989	0.867967	0.865954	0.863947	1.071514	1.068633	1.065748	1.062852
0.873177	0.871096	0.869059	0.867045	0.865043	1.072948	1.070088	1.067214	1.064323
0.874317	0.872211	0.870154	0.868135	0.866137	1.074367	1.071532	1.068677	1.065801
0.875479	0.873346	0.871263	0.869234	0.867236	1.075783	1.072969	1.070132	1.067266
0.876663	0.874498	0.872383	0.870342	0.868339	1.077193	1.0744	1.071579	1.068723
0.877878	0.8757	0.873563	0.87149	0.869453	0.867374	0.865264	1.073021	1.070173
0.879136	0.876973	0.874826	0.872695	0.870578	0.868469	0.866368	1.074459	1.071616
0.604218	0.602854	0.601488	0.873902	0.871715	0.869576	0.867478	1.075892	1.073059
0.605002	0.603637	0.602263	0.875079	0.872866	0.870707	0.868593	1.077311	1.074499
0.605766	0.604405	0.60303	0.876249	0.874026	0.871851	0.869714	1.078736	1.07595
			0.877429	0.875188	0.872995	0.870838	0.868711	0.866615
			0.878612	0.876354	0.874145	0.871972	0.869843	0.867768
			0.603941	0.602517	0.875321	0.87315	0.871027	0.868964
			0.604707	0.603287	0.876522	0.874375	0.872266	0.870208
					0.877722	0.875585	0.873479	0.871418
					0.878905	0.876749	0.874628	0.872558
					0.880104	0.877933	0.875796	0.873703
					0.605062	0.603709	0.877053	0.874907
					0.605896	0.604544	0.878334	0.876132
							0.879546	0.877343
							0.880714	0.878546
							0.605494	0.604154
							0.606257	0.604915

							0.582402	0.580653
							0.581424	0.57967
							0.58049	0.57872
							0.579625	0.577822
					0.582285	0.580528	1.592441	1.588292
					0.581411	0.579615	1.590292	1.586155
					0.580531	0.578695	1.588141	1.584024
					0.57961	0.577789	1.780526	1.775761
					0.578667	0.576892	1.777995	1.773251
			0.581339	0.579541	1.589864	1.585813	1.77546	1.77076
			0.580415	0.57866	1.58773	1.583652	1.772919	1.768287
0.584963	0.583133	0.581297	1.480651	1.476799	1.779823	1.77508	2.02088	2.015613
0.5841	0.582296	0.580474	1.47869	1.474899	1.777405	1.772621	2.018074	2.012832
0.58322	0.581444	0.579647	1.476722	1.472983	1.774993	1.770195	2.01532	2.010088
0.58231	0.580542	0.578762	1.587671	1.583594	1.838142	1.833308	2.012674	2.007418

0.581386	0.579617	0.577852	1.585548	1.581448	1.835622	1.830886	2.010078	2.004796
1.481951	1.478233	1.474542	1.776932	1.772232	2.017918	2.012676	2.070198	2.064707
1.479964	1.476203	1.472483	1.774378	1.769747	2.01515	2.009968	2.067421	2.06202
1.591292	1.587178	1.583126	1.837485	1.832804	2.012421	2.007262	2.064589	2.059248
1.589287	1.585159	1.58107	1.834966	1.830343	2.009732	2.004547	2.061679	2.056369
1.781166	1.776367	1.771601	1.965438	1.960417	2.069684	2.064233	2.111026	2.105635
1.778745	1.77399	1.769256	1.962902	1.957875	2.066934	2.061519	2.108261	2.102797
1.776328	1.77163	1.766947	1.960403	1.955344	2.064181	2.058818	2.105529	2.1
1.839593	1.834839	1.830099	1.957897	1.95278	2.113538	2.108143	2.102735	2.097246
1.837152	1.832449	1.827763	1.955393	1.950212	2.110614	2.10526	2.099911	2.094511
1.968168	1.963071	1.958007	2.066705	2.06116	2.107785	2.102395	2.137308	2.131909
1.965507	1.960429	1.955408	2.063944	2.058467	2.105037	2.099539	2.134336	2.128984
1.962908	1.957815	1.952785	2.113248	2.10776	2.102257	2.096724	2.16882	2.163465
1.960391	1.955233	1.950123	2.110274	2.104868	2.099436	2.093967	2.16608	2.160658
1.957892	1.952677	1.947491	2.107324	2.101963	2.174239	2.168794	2.16336	2.157903
1.955403	1.950157	1.944923	2.104444	2.099042	2.171319	2.165972	2.160628	2.155239
1.952898	1.947653	1.942407	2.101629	2.096161	2.168425	2.163148	2.157885	2.152591
2.115737	2.110109	2.104486	2.1767	2.17112	2.165665	2.160348	2.155078	2.149848
2.112828	2.107324	2.101823	2.174078	2.168497	2.162996	2.157573	2.152244	2.147061
2.109891	2.104485	2.099101	2.171404	2.165865	2.160364	2.154887	2.200897	2.195576
2.106913	2.101584	2.096323	2.168686	2.163236	2.157777	2.152292	2.1982	2.192851
2.181983	2.176602	2.171324	2.165973	2.160592	2.15517	2.149686	2.195498	2.190112
2.179294	2.173909	2.168619	2.163283	2.157934	2.152527	2.147043	2.192762	2.187323
2.176639	2.17123	2.165901	2.160568	2.155247	2.149863	2.1444	2.190047	2.184566
2.17391	2.168464	2.163089	2.157768	2.152496	2.198457	2.192908	2.187387	2.181902
2.171181	2.165694	2.160269	2.154957	2.149732	2.195688	2.190221	2.184755	2.179284
2.168527	2.163023	2.157574	2.203719	2.198359	2.192968	2.187548	2.182121	2.176674
2.165924	2.160419	2.154965	2.201021	2.195659	2.190282	2.184889	2.179492	2.174077
2.16331	2.157802	2.15234	2.198317	2.192957	2.187595	2.182235	2.213974	2.208331
2.160688	2.155174	2.149704	2.195614	2.190261	2.184918	2.179592	2.21128	2.20564
2.209651	2.203981	2.198358	2.192879	2.187512	2.182184	2.176909	2.208557	2.202946
2.206916	2.201236	2.195595	2.19007	2.184642	2.179321	2.174131	2.205766	2.200236
2.204167	2.198486	2.192838	2.187277	2.181796	2.176484	2.17137	2.202986	2.197538
2.201397	2.195742	2.190125	2.184586	2.179123	2.210969	2.205631	2.20029	2.19488
2.198624	2.193009	2.187441	2.181953	2.17654	2.208327	2.202982	2.197638	2.192244
2.1959	2.190316	2.184785	2.179333	2.173956	2.205678	2.200333	2.194992	2.18961
2.193217	2.18766	2.182156	2.176733	2.171387	2.20304	2.197696	2.192357	2.186982
2.190553	2.185024	2.179548	2.211452	2.205865	2.200391	2.19505	2.30942	2.302779
2.187907	2.182412	2.176966	2.208795	2.2032	2.197726	2.19239	2.30602	2.299441
2.185273	2.179807	2.174387	2.206138	2.200542	2.195068	2.189732	2.364671	2.358072
2.182653	2.177203	2.171794	2.203465	2.197885	2.192414	2.187063	2.361287	2.354775
2.180035	2.174601	2.169205	2.200804	2.195253	2.189793	2.184429	2.357975	2.351538
2.215015	2.209354	2.203743	2.198187	2.192685	2.305976	2.299424	2.354814	2.348396
2.212278	2.20666	2.201105	2.195606	2.190164	2.302848	2.296377	2.351755	2.345321
2.209609	2.204036	2.198532	2.19308	2.187681	2.361677	2.355142	2.348668	2.342232
2.206999	2.201476	2.196026	2.190612	2.185241	2.358624	2.352061	2.345565	2.339137
2.204481	2.198974	2.193531	2.306832	2.300267	2.355519	2.348957	2.342464	2.336052
2.202114	2.196562	2.191047	2.303653	2.297097	2.352317	2.345808	2.339364	2.332981
2.19971	2.19412	2.188547	2.300477	2.29393	2.349119	2.342665	2.336271	2.329919
2.197115	2.191544	2.185999	2.359103	2.352502	2.345985	2.33956	2.333191	2.32686

2.194444	2.188912	2.183427	2.355914	2.349358	2.342875	2.336469	2.330118	2.323808
2.311015	2.304244	2.297596	2.352713	2.346199	2.33975	2.33337	2.362564	2.355736
2.30768	2.300975	2.2944	2.349511	2.343037	2.336623	2.330269	2.359239	2.352452
2.337887	2.331167	2.324571	2.346348	2.339902	2.333512	2.327178	2.355921	2.349182
2.334652	2.327986	2.321427	2.343207	2.336773	2.330396	2.324074	2.352585	2.3459
2.359871	2.353231	2.346683	2.340199	2.333784	2.327428	2.321131	2.349428	2.342797
2.356987	2.350389	2.343881	2.337433	2.331052	2.324738	2.318492	2.346598	2.340016
2.354107	2.347547	2.341074	2.334664	2.328324	2.322055	2.315862	2.343802	2.337267
2.351011	2.344482	2.338037	2.331661	2.325359	2.354044	2.34737	2.340788	2.334301
2.347819	2.341318	2.334899	2.328558	2.322296	2.350818	2.344209	2.337685	2.331243
2.344626	2.338163	2.331788	2.325493	2.319278	2.347627	2.341071	2.334592	2.328177
2.341418	2.335	2.328682	2.322441	2.316279	2.34445	2.337938	2.331492	2.325089
2.338344	2.331935	2.325636	2.35451	2.347872	2.341314	2.33484	2.328425	2.322039
2.335434	2.328974	2.322636	2.351278	2.344707	2.3382	2.331759	2.325375	2.319006
2.332576	2.32611	2.319768	2.348197	2.341681	2.335214	2.328795	2.392544	2.385717
2.329751	2.323399	2.31715	2.345414	2.338927	2.33248	2.326068	2.389639	2.382858
2.326924	2.320709	2.314572	2.342691	2.336223	2.329787	2.323374	2.386776	2.380065
2.359584	2.352884	2.346272	2.339744	2.333296	2.326872	2.320454	2.38371	2.377124
2.356361	2.349737	2.3432	2.336712	2.33028	2.323866	2.31744	2.380562	2.374131
2.353368	2.346799	2.340309	2.333845	2.32742	2.391111	2.38419	2.377522	2.3712
2.35059	2.344054	2.337586	2.331131	2.324701	2.388189	2.381231	2.374565	2.36831
2.347712	2.341203	2.334753	2.328305	2.32188	2.385226	2.378354	2.371785	2.365633
2.344632	2.338145	2.331712	2.325276	2.318867	2.382172	2.37558	2.36926	2.363267
2.341597	2.335126	2.328704	2.32228	2.31589	2.379144	2.372823	2.36675	2.360934
2.338799	2.332334	2.325905	2.349128	2.342576	2.37617	2.369968	2.386971	2.381316
2.336042	2.329583	2.323151	2.346326	2.339785	2.373289	2.367165	2.384248	2.378773
2.333052	2.326615	2.320227	2.383674	2.376982	2.393561	2.387554	2.381835	2.376461
2.329934	2.323531	2.317218	2.380679	2.374281	2.391069	2.385228	2.379621	2.374291
2.356846	2.350333	2.343937	2.377809	2.371587	2.388504	2.382802	2.377293	2.371994
2.354066	2.347601	2.341264	2.375063	2.368891	2.385851	2.380266	2.374841	2.369557
2.351235	2.344846	2.338613	2.395258	2.389145	2.383328	2.377833	2.372469	2.367195
2.348222	2.341955	2.335901	2.392605	2.386699	2.381019	2.375589	2.370262	2.365005
2.345262	2.339131	2.333268	2.39004	2.384346	2.378792	2.373394	2.368078	2.362823
2.367048	2.361022	2.355282	2.387628	2.382077	2.376586	2.371161	2.365804	2.360511
2.36443	2.358536	2.352934	2.38529	2.379848	2.374393	2.368922	2.363511	2.358168
2.400358	2.394247	2.388429	2.382847	2.377491	2.372099	2.366669	2.361282	2.355939
2.397692	2.391636	2.385865	2.380339	2.375045	2.369732	2.364404	2.359098	2.353791
2.395088	2.389138	2.383449	2.377974	2.372704	2.367433	2.362166	2.356908	2.351618
2.392542	2.38677	2.381212	2.375786	2.3705	2.365227	2.359972	2.354718	2.349412
2.390068	2.384453	2.379011	2.373613	2.36829	2.362996	2.357737	2.379817	2.374443
2.387693	2.382186	2.376823	2.371412	2.366009	2.360665	2.35538	2.377442	2.372185
2.385358	2.37992	2.374605	2.36918	2.363717	2.358338	2.353033	2.375083	2.369955
2.382989	2.377555	2.372237	2.366848	2.361446	2.356114	2.350841	2.37289	2.367815
2.380623	2.375156	2.369803	2.364475	2.359193	2.353951	2.348738	2.370793	2.365732
2.378314	2.37286	2.367508	2.362221	2.357004	2.351821	2.346664	2.377676	2.372536
2.376055	2.370652	2.365328	2.360069	2.354875	2.349729	2.344628	2.375597	2.37042
2.37383	2.368476	2.363184	2.357947	2.352766	2.383904	2.378663	2.373467	2.368294
2.371639	2.36633	2.361075	2.355857	2.350681	2.381732	2.376451	2.37125	2.366141
2.369477	2.364201	2.358972	2.363733	2.358478	2.379545	2.374242	2.369049	2.364011
2.367354	2.362093	2.356863	2.361569	2.356284	2.377316	2.372061	2.366922	2.361934

2.365242	2.359981	2.354737	2.359385	2.354076	2.375084	2.369905	2.364835	2.359889
2.363105	2.357816	2.352542	2.35716	2.351889	2.372902	2.367793	2.362782	2.357877
2.360949	2.355617	2.350305	2.354911	2.349717	2.370756	2.365716	2.36076	2.355891
2.368746	2.363336	2.357966	2.352727	2.347593	2.368645	2.363658	2.358736	2.353872
2.366432	2.361074	2.355788	2.350604	2.345517	2.366573	2.361624	2.356716	2.351822
2.364184	2.358865	2.353639	2.374682	2.369557	2.364517	2.359572	2.354661	2.349758
2.36205	2.356737	2.35152	2.372569	2.367503	2.362471	2.357471	2.352532	2.347662
2.359957	2.354647	2.349429	2.37048	2.365467	2.360442	2.355394	2.35043	2.345592
2.357876	2.352579	2.347372	2.368416	2.363438	2.358439	2.353407	2.348451	2.343607
2.355798	2.350525	2.345337	2.36637	2.361417	2.35645	2.351462	2.34653	2.341667
2.353662	2.348441	2.343302	2.364331	2.359393	2.354451	2.349501	2.344596	2.339734
2.351484	2.346338	2.341272	2.362301	2.357368	2.352446	2.347535	2.34266	2.337814
2.375547	2.370365	2.365272	2.360252	2.355306	2.350394	2.345518	2.340681	2.335871
2.373387	2.368249	2.363203	2.358177	2.353191	2.348277	2.343435	2.338645	2.333901
2.371258	2.366152	2.361145	2.356121	2.351115	2.346201	2.341379	2.372736	2.367909
2.369158	2.364076	2.359099	2.354102	2.349123	2.344221	2.339395	2.370712	2.365925
2.367097	2.36203	2.357074	2.352107	2.347163	2.342277	2.337442	2.368714	2.363956
2.365103	2.360041	2.355093	2.350135	2.345202	2.340326	2.335502	2.366749	2.362007
2.363149	2.358086	2.35314	2.348183	2.343252	2.338385	2.33358	2.364808	2.360073
2.361156	2.356107	2.351173	2.346231	2.341315	2.372651	2.367714	2.362861	2.358135
2.359135	2.354111	2.349198	2.34428	2.339389	2.370701	2.365763	2.360912	2.356195
2.357129	2.352129	2.347233	2.342355	2.337516	2.368815	2.363867	2.359003	2.354275
2.355148	2.350165	2.345285	2.340472	2.335725	2.367029	2.362053	2.357151	2.352381
2.353173	2.348207	2.343336	2.374912	2.370104	2.365215	2.360225	2.355298	2.350492
2.351182	2.346232	2.341365	2.372923	2.368153	2.363293	2.358324	2.35341	2.348597
2.349215	2.344276	2.339406	2.370927	2.366168	2.361328	2.356392	2.351507	2.346703
2.347344	2.342407	2.337513	2.368974	2.3642	2.359363	2.354445	2.349586	2.344811
2.34553	2.34059	2.335662	2.367047	2.362237	2.357388	2.352486	2.347655	2.342924
2.380196	2.375126	2.370059	2.365125	2.360282	2.355427	2.350548	2.345745	2.341044
2.378184	2.373155	2.368141	2.363206	2.35833	2.353472	2.348623	2.343851	2.339167
2.376171	2.3712	2.366258	2.361333	2.356429	2.351576	2.346768	2.342029	2.337354
2.374161	2.369279	2.364444	2.359542	2.354614	2.349776	2.345031	2.340332	2.335647
2.372179	2.367366	2.362614	2.35774	2.352806	2.347988	2.343294	2.33863	2.333944
2.370263	2.365447	2.3607	2.355843	2.350936	2.346137	2.341452	2.336804	2.332159
2.368379	2.363529	2.35875	2.353913	2.349061	2.344285	2.339586	2.334945	2.330356
2.36648	2.36161	2.35681	2.352021	2.347259	2.342524	2.337807	2.333163	2.328617
2.364577	2.359694	2.354873	2.350151	2.345508	2.340825	2.336085	2.331428	2.32692
2.362726	2.357835	2.352996	2.348298	2.343702	2.339051	2.334326	2.329683	2.325194
2.360936	2.356041	2.351184	2.346456	2.341824	2.337181	2.332517	2.32792	2.323428
2.359146	2.354264	2.349411	2.344656	2.339978	2.335338	2.330735	2.326186	2.321692
2.357341	2.352502	2.347703	2.342938	2.338211	2.333572	2.329025	2.32452	2.320029
2.355521	2.350725	2.34598	2.341216	2.336461	2.331826	2.327321	2.322851	2.318371
2.353664	2.348873	2.344128	2.339387	2.334667	2.330052	2.32555	2.390003	2.384828
2.351793	2.34699	2.342225	2.337519	2.332869	2.328283	2.323763	2.387944	2.382821
2.349965	2.345167	2.340408	2.335736	2.331136	2.326579	2.322064	2.385993	2.380913
2.348171	2.343391	2.338657	2.334018	2.329457	2.324931	2.320436	2.384128	2.379086
2.3464	2.341645	2.336944	2.332329	2.327787	2.323284	2.318817	2.3823	2.377266
2.344653	2.339934	2.335276	2.33067	2.326117	2.321628	2.317205	2.380518	2.375449
2.342925	2.338237	2.333613	2.32902	2.324468	2.319992	2.315596	2.378718	2.373629
2.341223	2.336544	2.331924	2.327362	2.322859	2.318399	2.313984	2.37684	2.371794

2.339529	2.334858	2.330237	2.32571	2.321259	2.316816	2.312376	2.374952	2.369961
2.337809	2.333168	2.328581	2.324071	2.319627	2.315199	2.310785	2.373143	2.368162
2.336082	2.331482	2.326944	2.322441	2.31798	2.313567	2.309202	2.371369	2.366377
2.334398	2.329819	2.3253	2.320809	2.316355	2.379632	2.374539	2.38358	2.378619
2.332759	2.32818	2.323651	2.319176	2.314751	2.377806	2.372633	2.381687	2.376767
2.331105	2.326539	2.322015	2.317553	2.313143	2.375949	2.370732	2.397426	2.392488
2.329418	2.324888	2.320405	2.315949	2.311522	2.374025	2.368826	2.395588	2.39071
2.327734	2.323237	2.31879	2.314343	2.309909	2.372121	2.366974	2.393821	2.388984
2.326075	2.321585	2.317141	2.312718	2.308322	2.384627	2.379582	2.392189	2.387328
2.324427	2.319934	2.315484	2.311089	2.306743	2.382897	2.377961	2.390617	2.385724
2.322765	2.318283	2.313855	2.309476	2.305146	2.381097	2.376265	2.388996	2.384163
2.321102	2.316635	2.312245	2.307875	2.303541	2.379259	2.374529	2.387349	2.382639
2.319512	2.315032	2.310632	2.306269	2.301954	2.3775	2.372814	2.385672	2.381042
2.318009	2.313481	2.309009	2.304651	2.300385	2.375834	2.371121	2.383953	2.379365
2.316485	2.311942	2.307436	2.366178	2.36144	2.37416	2.369446	2.382258	2.377671
2.314897	2.310415	2.305957	2.364477	2.359719	2.372425	2.367789	2.380624	2.375977
2.313287	2.308878	2.30449	2.362825	2.358034	2.370716	2.366146	2.378993	2.37428
2.311656	2.307288	2.302963	2.36122	2.356463	2.369117	2.364508	2.377302	2.372581
2.310027	2.30568	2.30141	2.359641	2.354948	2.367576	2.362883	2.375597	2.37089
2.326005	2.321634	2.317343	2.358	2.353387	2.366012	2.361296	2.392838	2.388142
2.32459	2.320166	2.3158	2.356311	2.351796	2.364439	2.359743	2.391345	2.386632
2.323185	2.318735	2.314324	2.354693	2.350229	2.380349	2.375603	2.389835	2.385079
2.321795	2.317354	2.312936	2.353171	2.348704	2.378778	2.374037	2.388308	2.383465
2.365922	2.361051	2.356277	2.351656	2.347156	2.396272	2.39154	2.386754	2.381855
2.36432	2.359502	2.354757	2.350106	2.345536	2.394643	2.389926	2.385163	2.380287
2.362711	2.357949	2.353237	2.348556	2.343909	2.393002	2.388285	2.383542	2.378718
2.361046	2.356344	2.3517	2.347027	2.34235	2.391436	2.386644	2.381866	2.377086
2.35936	2.354716	2.350153	2.34551	2.34083	2.389916	2.385011	2.380169	2.375433
2.357782	2.35315	2.34861	2.366758	2.362095	2.388427	2.383473	2.378607	2.373899
2.356304	2.351645	2.347072	2.365304	2.360661	2.386974	2.382028	2.377171	2.372478
2.377684	2.373011	2.368414	2.363803	2.359196	2.385502	2.380584	2.375744	2.371047
2.376254	2.371502	2.366826	2.36222	2.357674	2.383988	2.379119	2.374302	2.36957
2.374821	2.370004	2.365262	2.36066	2.356164	2.382485	2.37767	2.372878	2.368114
2.373391	2.368561	2.363805	2.3592	2.354709	2.381022	2.376255	2.387243	2.382392
2.371954	2.367137	2.362395	2.357787	2.353285	2.379583	2.374859	2.38583	2.380984
2.370492	2.365692	2.360973	2.356379	2.351886	2.378167	2.373474	2.384415	2.379565
2.369013	2.364238	2.359556	2.354987	2.350513	2.376774	2.3721	2.383003	2.378143
2.383156	2.3783	2.373541	2.384656	2.380039	2.391139	2.386374	2.381576	2.376709
2.381709	2.376866	2.37212	2.38325	2.378664	2.389727	2.384946	2.380135	2.375262
2.380272	2.375448	2.370718	2.381856	2.377287	2.388306	2.383512	2.378688	2.37381
2.378844	2.374052	2.369346	2.380481	2.375903	2.386869	2.382069	2.377239	2.37235
2.377417	2.372658	2.367978	2.379103	2.374509	2.38542	2.380615	2.375783	2.370891
2.375981	2.371251	2.366588	2.393409	2.388708	2.383956	2.37915	2.374322	2.369443
2.374542	2.369837	2.365187	2.391952	2.387236	2.382476	2.377669	2.37285	2.367997
2.3731	2.368414	2.363777	2.390479	2.385743	2.380971	2.37616	2.389308	2.384248
2.371655	2.366983	2.362356	2.388991	2.38423	2.379443	2.374624	2.387685	2.382681
2.386735	2.382007	2.377324	2.387551	2.382758	2.377947	2.373108	2.386077	2.381114
2.385306	2.380622	2.375974	2.386183	2.381345	2.376495	2.371619	2.384486	2.379547
2.383882	2.379222	2.374586	2.384777	2.379908	2.393152	2.387984	2.382913	2.377993
2.382443	2.37776	2.373088	2.383263	2.378402	2.391554	2.386412	2.381364	2.376459

2.38102	2.376296	2.371569	2.381721	2.376868	2.389941	2.384842	2.37983	2.374945
2.379658	2.374904	2.370122	2.380223	2.375331	2.388308	2.383264	2.378304	2.37345
2.378326	2.373542	2.368703	2.378741	2.373788	2.386666	2.381688	2.376792	2.371977
2.391996	2.387125	2.382199	2.395415	2.390163	2.38507	2.380143	2.375302	2.37054
2.390555	2.385642	2.380699	2.393811	2.388576	2.383512	2.378627	2.373834	2.36914
2.389087	2.384134	2.379173	2.392206	2.387019	2.382001	2.37716	2.372408	2.367752
2.387585	2.382595	2.37761	2.39059	2.38549	2.380543	2.375762	2.371042	2.36638
2.38608	2.381062	2.376061	2.389001	2.383995	2.379117	2.374381	2.36968	2.364995
2.403109	2.397785	2.392513	2.387448	2.382541	2.377719	2.372998	2.36829	2.363571
2.401551	2.396225	2.390964	2.385949	2.381119	2.376334	2.371608	2.366881	2.362127
2.400167	2.39486	2.389604	2.384577	2.37972	2.374915	2.370176	2.365436	2.360667
2.398898	2.393624	2.388375	2.383296	2.378344	2.37348	2.368716	2.363965	2.359196
2.397482	2.392247	2.38703	2.381943	2.376955	2.372061	2.36727	2.362514	2.357774
2.395909	2.390722	2.385569	2.380518	2.375551	2.370654	2.36583	2.361072	2.356399
2.39434	2.389192	2.384095	2.37909	2.374163	2.369287	2.364458	2.359702	2.355056
2.392784	2.387662	2.382612	2.377666	2.372811	2.367993	2.363213	2.358468	2.353762
2.391304	2.386195	2.381171	2.376264	2.371459	2.366688	2.361959	2.357226	2.352459
2.390027	2.384913	2.379867	2.374927	2.370078	2.365287	2.360567	2.355843	2.35108
2.38883	2.3837	2.378614	2.373611	2.368681	2.363846	2.359117	2.354403	2.349674
2.387466	2.382338	2.377247	2.372225	2.367268	2.362417	2.357684	2.352985	2.348302
2.385988	2.380874	2.375801	2.370791	2.36584	2.360993	2.356257	2.351576	2.346952
2.384608	2.379497	2.374431	2.369436	2.364508	2.35967	2.354927	2.350245	2.345635
2.38337	2.378248	2.373171	2.368197	2.363308	2.358484	2.35373	2.349022	2.344365
2.382071	2.376942	2.371859	2.366908	2.362062	2.357258	2.352501	2.347774	2.343072
2.380584	2.375456	2.37038	2.36546	2.360658	2.355884	2.351141	2.346416	2.341695
2.37908	2.373958	2.368896	2.364005	2.359243	2.354497	2.349774	2.34506	2.340331
2.377735	2.372626	2.367583	2.36271	2.357969	2.353242	2.348535	2.343831	2.339105
2.376453	2.371364	2.366345	2.361485	2.356751	2.352035	2.347345	2.342653	2.337941
2.375047	2.369988	2.365003	2.360154	2.355417	2.350705	2.346022	2.341348	2.336671
2.37353	2.368513	2.363572	2.358732	2.35398	2.349262	2.344581	2.33993	2.33531
2.359621	2.354715	2.34989	2.345136	2.340453	2.347922	2.343232	2.338581	2.333978
2.358354	2.353485	2.348707	2.343975	2.339297	2.346741	2.342027	2.337341	2.332686
2.357129	2.352287	2.347544	2.342829	2.338157	2.345581	2.340847	2.33613	2.331428
2.355931	2.351101	2.346366	2.341655	2.336984	2.344395	2.339664	2.334947	2.330239
2.35472	2.349894	2.345156	2.340444	2.335772	2.343171	2.338449	2.333742	2.329047
2.353371	2.348554	2.343816	2.339104	2.334428	2.341807	2.337084	2.332384	2.327706
2.351942	2.347136	2.342398	2.337684	2.333003	2.340357	2.33563	2.330935	2.326282
2.350637	2.345839	2.3411	2.336385	2.331703	2.339041	2.334321	2.329635	2.324995
2.349461	2.344668	2.339922	2.335209	2.330529	2.337862	2.333161	2.328488	2.323847
2.348285	2.343497	2.33875	2.334038	2.329361	2.336688	2.332003	2.327342	2.322707
2.3471	2.342321	2.33758	2.332869	2.32819	2.33551	2.330834	2.326185	2.321567
2.345868	2.341094	2.33636	2.331652	2.326973	2.334285	2.329619	2.324981	2.320378
2.3445	2.339719	2.334987	2.330283	2.325613	2.33292	2.32827	2.323644	2.31904
2.343091	2.3383	2.333567	2.32887	2.324214	2.331516	2.326883	2.322266	2.317658
2.341818	2.337027	2.332298	2.327609	2.322964	2.330263	2.325637	2.321024	2.316416
2.34064	2.335858	2.331136	2.326456	2.321822	2.329119	2.324491	2.319877	2.315273
2.339474	2.3347	2.329982	2.32531	2.320684	2.327978	2.323354	2.318744	2.314141
2.338321	2.333554	2.328839	2.32417	2.319547	2.326838	2.322227	2.317624	2.313019
2.337162	2.332404	2.327696	2.323034	2.318418	2.325706	2.321104	2.316508	2.311903
2.335977	2.331232	2.326546	2.3219	2.317296	2.324583	2.31998	2.315384	2.310785

2.334795	2.330062	2.325397	2.320765	2.316175	2.32346	2.318856	2.314261	2.30967
2.333643	2.328915	2.324253	2.319626	2.315041	2.322324	2.317731	2.313145	2.308559
2.332507	2.32778	2.323113	2.318487	2.313904	2.321183	2.316606	2.312032	2.30745
2.331363	2.326645	2.321982	2.317362	2.312786	2.32006	2.315487	2.31729	2.312705
2.330215	2.325509	2.32086	2.316251	2.311686	2.318953	2.314373	2.316167	2.311587
2.335968	2.33126	2.326607	2.321993	2.317422	2.324228	2.319636	2.329046	2.32448
2.334807	2.330114	2.325472	2.320869	2.316304	2.323108	2.318524	2.327968	2.323404
2.333647	2.328965	2.324331	2.319736	2.315181	2.321982	2.317409	2.326888	2.32232
2.332492	2.327808	2.323173	2.318587	2.314048	2.320849	2.316286	2.325794	2.321218
2.331341	2.32665	2.322012	2.317434	2.31291	2.319709	2.315155	2.324693	2.320107
2.330199	2.325509	2.320869	2.316292	2.311771	2.332694	2.328153	2.32359	2.318999
2.329064	2.324376	2.319736	2.315157	2.310631	2.331573	2.32704	2.322484	2.31789
2.327918	2.323232	2.318595	2.329079	2.324559	2.320063	2.315601	2.321375	2.316781
2.326761	2.322079	2.317447	2.327972	2.323458	2.318965	2.314503	2.320265	2.315673
2.325601	2.320924	2.316302	2.326867	2.322357	2.317863	2.313395	2.319146	2.314562
2.324438	2.319773	2.315162	2.32577	2.321253	2.316752	2.312272	2.318011	2.313445
2.32327	2.318616	2.314021	2.324672	2.32015	2.315642	2.311151	2.316878	2.31233
2.337322	2.332675	2.328112	2.323564	2.319047	2.31454	2.310046	2.315764	2.311226
2.336174	2.33153	2.326991	2.322454	2.317945	2.313443	2.308947	2.314657	2.310126
2.335115	2.330444	2.325885	2.321343	2.316839	2.312338	2.307843	2.313543	2.309015
2.334127	2.329406	2.324796	2.320235	2.315732	2.311233	2.306737	2.312427	2.307898
2.333178	2.328448	2.323811	2.319222	2.314688	2.310164	2.305649	2.311319	2.306794
2.33229	2.327598	2.322961	2.318328	2.313715	2.30913	2.304573	2.310212	2.305699
2.331339	2.326686	2.322063	2.317416	2.312763	2.308142	2.303549	2.309154	2.304634
2.330247	2.325626	2.321037	2.31644	2.311843	2.307238	2.302615	2.308176	2.303599
2.329119	2.324521	2.319964	2.315423	2.3109	2.306329	2.301702	2.307233	2.302606
2.328015	2.323418	2.318869	2.314346	2.309849	2.305318	2.300749	2.306314	2.301701
2.326934	2.322322	2.317765	2.313238	2.308744	2.304253	2.29977	2.305397	2.300828
2.32597	2.321326	2.31673	2.312176	2.307664	2.303177	2.298724	2.304372	2.299828
2.325113	2.320417	2.315753	2.311148	2.306595	2.302082	2.297616	2.303254	2.298723
2.324184	2.319484	2.314805	2.310187	2.305618	2.30108	2.29658	2.302183	2.297632
2.323146	2.318513	2.313905	2.309329	2.304778	2.30022	2.295652	2.301177	2.296556
2.322106	2.317523	2.312971	2.30843	2.303902	2.299336	2.29473	2.300204	2.295534
2.321109	2.316494	2.311922	2.307379	2.302864	2.29833	2.293778	2.299284	2.294642
2.320131	2.315471	2.310864	2.306306	2.301793	2.29729	2.292803	2.298357	2.293764
2.319185	2.314541	2.309938	2.305361	2.300812	2.29628	2.29177	2.303785	2.299185
2.318253	2.313656	2.309079	2.304482	2.299877	2.295286	2.290707	2.302657	2.298062
2.317242	2.312674	2.308125	2.303542	2.298942	2.294344	2.289743	2.301651	2.297034
2.316147	2.311589	2.307076	2.302545	2.298011	2.293457	2.288882	2.300767	2.296095
2.315141	2.310587	2.306088	2.301576	2.297066	2.292529	2.287963	2.312399	2.307636
2.314298	2.309742	2.305224	2.300668	2.296095	2.291515	2.286923	2.311328	2.306613
2.313465	2.308897	2.30435	2.299752	2.295125	2.290509	2.285897	2.310276	2.305604
2.312529	2.307929	2.303358	2.29877	2.294178	2.289588	2.284998	2.31607	2.311367
2.31157	2.306937	2.302342	2.297776	2.293238	2.288689	2.28413	2.31518	2.310438
2.310665	2.306036	2.301432	2.296849	2.292287	2.295655	2.291082	2.305475	2.300804
2.309794	2.305194	2.300591	2.295967	2.291331	2.294653	2.290048	2.304422	2.299813
2.308926	2.304325	2.299705	2.295056	2.290386	2.306793	2.302113	2.303446	2.29888
2.308071	2.30343	2.298766	2.294108	2.289451	2.305863	2.301212	2.302563	2.298019
2.307168	2.302511	2.297834	2.301137	2.29649	2.304948	2.300309	2.30166	2.297121
2.30616	2.301543	2.296922	2.300255	2.295613	2.304048	2.299376	2.30069	2.296128

2.30516	2.300587	2.296024	2.299382	2.294739	2.303146	2.298439	2.299714	2.295127
2.312279	2.3077	2.303127	2.298497	2.293833	2.308181	2.303443	2.29877	2.294192
2.311464	2.306866	2.302262	2.297604	2.292912	2.30723	2.302504	2.297846	2.293286
2.310619	2.305989	2.301347	2.296676	2.291988	2.306293	2.301594	2.296952	2.29238
2.309759	2.305081	2.30039	2.295717	2.291058	2.305364	2.300711	2.296086	2.291477
2.308859	2.30416	2.299454	2.307913	2.303207	2.304463	2.29982	2.295194	2.290567
2.307906	2.303218	2.298544	2.307016	2.302347	2.303596	2.298912	2.294258	2.289641
2.306958	2.302291	2.297654	2.306139	2.3015	2.302744	2.298027	2.29335	2.288738
2.306043	2.301398	2.296785	2.305283	2.300667	2.301918	2.297207	2.292527	2.287893
2.305147	2.300525	2.295933	2.304431	2.299822	2.301076	2.296388	2.291716	2.287064
2.304268	2.299664	2.295087	2.303553	2.298902	2.300139	2.295475	2.290831	2.286205
2.303407	2.298816	2.29425	2.302665	2.297947	2.299155	2.294517	2.28991	2.285334
2.302566	2.297984	2.293423	2.30773	2.302962	2.298259	2.293622	2.289025	2.284473
2.301746	2.297171	2.292608	2.306906	2.302154	2.297448	2.292784	2.288169	2.283622
2.314131	2.309473	2.304821	2.306038	2.301312	2.296616	2.291947	2.287322	2.282759
2.313289	2.308585	2.303881	2.305091	2.300399	2.295736	2.291102	2.286485	2.281871
2.312449	2.307703	2.302952	2.304152	2.299487	2.294853	2.290257	2.285652	2.280993
2.311611	2.306858	2.302095	2.303281	2.298613	2.293986	2.28941	2.293452	2.288737
2.310777	2.306031	2.30127	2.302441	2.297759	2.29313	2.288565	2.292619	2.287914
2.315893	2.311126	2.306341	2.301614	2.296926	2.292296	2.287736	2.291796	2.287098
2.315066	2.310307	2.305527	2.300802	2.296113	2.291481	2.286917	2.29098	2.286292
2.314234	2.309481	2.304707	2.299982	2.29529	2.290657	2.286092	2.29715	2.292419
2.313397	2.30865	2.30388	2.299154	2.294457	2.289823	2.285262	2.296308	2.291589
2.31256	2.307816	2.303048	2.298321	2.293621	2.288987	2.284429	2.295462	2.290755
2.311723	2.306979	2.302208	2.297481	2.292783	2.288151	2.283594	2.294614	2.289918
2.310886	2.306142	2.301369	2.296641	2.291943	2.287312	2.282757	2.293763	2.289076
2.310047	2.305304	2.300528	2.295795	2.291088	2.286456	2.281911	2.28467	2.280035
2.309211	2.304473	2.299701	2.29496	2.290239	2.285604	2.281067	2.283819	2.279185
2.308389	2.30368	2.298942	2.294201	2.289458	2.284807	2.280258	2.28299	2.278358
2.307579	2.302917	2.298241	2.293505	2.288734	2.284056	2.279479	2.28218	2.277548
2.306758	2.302124	2.297489	2.292765	2.287987	2.283295	2.278695	2.281377	2.276765
2.305917	2.30129	2.296673	2.291965	2.287203	2.282515	2.277899	2.280579	2.276019
2.305093	2.300455	2.295838	2.291152	2.286429	2.289103	2.28442	2.279796	2.275268
2.304314	2.299639	2.294995	2.290344	2.285696	2.288386	2.283687	2.279041	2.274492
2.303546	2.298827	2.294145	2.289527	2.284957	2.287666	2.282957	2.278289	2.273702
2.302754	2.298005	2.293294	2.288686	2.284153	2.286871	2.282169	2.277498	2.27289
2.301957	2.297188	2.29245	2.287838	2.283314	2.286034	2.281353	2.276693	2.272068
2.30122	2.296436	2.291677	2.287043	2.282498	2.285215	2.280574	2.291662	2.286949
2.30054	2.29575	2.290969	2.286295	2.281697	2.284407	2.279827	2.29094	2.286208
2.299808	2.295042	2.290282	2.285586	2.280941	2.283626	2.279065	2.290176	2.285435
2.298984	2.294297	2.289627	2.284939	2.280249	2.282873	2.278267	2.289331	2.2846
2.298162	2.293534	2.288936	2.28427	2.27957	2.282148	2.27749	2.288501	2.283783
2.297393	2.29274	2.288131	2.283508	2.278886	2.281473	2.276784	2.287748	2.283037
2.296657	2.291949	2.287296	2.282713	2.278186	2.280801	2.276098	2.287028	2.282322
2.295964	2.291227	2.286539	2.281941	2.277412	2.280036	2.275375	2.28632	2.281611
2.295306	2.290552	2.28583	2.281181	2.276591	2.279211	2.274628	2.285622	2.280906
2.294631	2.289876	2.285146	2.287746	2.283053	2.294126	2.289494	2.284854	2.280167
2.293935	2.2892	2.284493	2.287062	2.282329	2.29334	2.288656	2.284006	2.279394
2.293248	2.288526	2.283833	2.286381	2.281626	2.292585	2.287847	2.283175	2.278603
2.292584	2.287857	2.283151	2.28569	2.280944	2.291877	2.287096	2.282384	2.277783

2.291916	2.287183	2.282465	2.284999	2.280264	2.291178	2.286367	2.281622	2.276977
2.291217	2.286495	2.281792	2.284308	2.279563	2.290453	2.285647	2.280899	2.276233
2.290508	2.2858	2.281118	2.283615	2.278853	2.289721	2.284936	2.2802	2.275523
2.289827	2.285113	2.280422	2.282904	2.278146	2.289002	2.28422	2.279486	2.274815
2.289167	2.284429	2.279708	2.282181	2.277438	2.288292	2.283501	2.278766	2.274114
2.288492	2.283746	2.279013	2.281472	2.276735	2.287582	2.282797	2.278067	2.273415
2.287796	2.283062	2.278344	2.280783	2.276035	2.286864	2.282106	2.277387	2.272709
2.287103	2.282379	2.277673	2.295772	2.290947	2.286169	2.281442	2.276738	2.272041
2.286425	2.281698	2.276986	2.295066	2.290265	2.285516	2.280824	2.276146	2.271459
2.285748	2.281018	2.276301	2.294369	2.2896	2.28488	2.280213	2.275555	2.270885
2.285046	2.280328	2.275635	2.293703	2.288959	2.284246	2.279565	2.274895	2.27023
2.284344	2.279643	2.274984	2.293054	2.28833	2.283611	2.278896	2.2742	2.269533
2.290974	2.286211	2.281488	2.292392	2.28766	2.282934	2.27821	2.294709	2.289637
2.290425	2.285632	2.280866	2.291719	2.286955	2.282218	2.277508	2.293952	2.288867
2.289843	2.285028	2.280228	2.291041	2.286257	2.28151	2.276803	2.293182	2.288098
2.289197	2.284373	2.279558	2.290348	2.285565	2.28081	2.276083	2.292375	2.287317
2.304299	2.299402	2.294511	2.289683	2.284901	2.280142	2.275403	2.291611	2.286573
2.303689	2.298804	2.293925	2.289095	2.284302	2.279544	2.274822	2.290967	2.285917
2.30308	2.298212	2.293357	2.288526	2.283719	2.278965	2.274269	2.290356	2.285291
2.302402	2.297552	2.29272	2.28789	2.283072	2.278319	2.273637	2.289667	2.284623
2.301688	2.296856	2.292046	2.28722	2.282394	2.277641	2.272965	2.288936	2.283935
2.301042	2.296216	2.291415	2.286597	2.281778	2.277026	2.272343	2.288235	2.28323
2.300464	2.295632	2.290825	2.28602	2.281227	2.276475	2.271769	2.287558	2.282502
2.299902	2.295064	2.290251	2.285456	2.280682	2.275934	2.271212	2.299879	2.294739
2.299348	2.294507	2.289692	2.284902	2.280136	2.275393	2.270674	2.299253	2.294106
2.298794	2.293952	2.289135	2.284347	2.279586	2.274849	2.270136	2.29863	2.293486
2.298234	2.29339	2.28857	2.283787	2.279034	2.2743	2.269586	2.297988	2.292849
2.297658	2.292814	2.28799	2.283211	2.278467	2.273735	2.269016	2.297324	2.292196
2.297022	2.292174	2.287343	2.282568	2.277835	2.293915	2.288778	2.29659	2.291479
2.296339	2.291486	2.286644	2.281874	2.277153	2.293167	2.288005	2.295799	2.290713
2.291074	2.286252	2.281443	2.28124	2.27652	2.292463	2.287292	2.295072	2.29
2.290491	2.285683	2.280889	2.28068	2.275944	2.291813	2.286653	2.294422	2.289349
2.289925	2.285128	2.280348	2.280134	2.275385	2.304136	2.298936	2.293786	2.288712
2.289356	2.284566	2.279799	2.279584	2.274831	2.303497	2.298292	2.293141	2.288072
2.288817	2.284024	2.279258	2.27904	2.274287	2.302873	2.297662	2.292508	2.287445
2.288347	2.283526	2.27873	2.273972	2.269246	2.302259	2.297051	2.291899	2.286834
2.287917	2.283057	2.278219	2.273444	2.268719	2.30166	2.296462	2.291316	2.286245
2.287458	2.282599	2.277759	2.272979	2.268246	2.301107	2.295926	2.29079	2.285709
2.286978	2.282156	2.277348	2.272572	2.267823	2.300597	2.295443	2.290322	2.285225
2.286468	2.281672	2.276888	2.272118	2.267362	2.296633	2.291514	2.289802	2.2847
2.285916	2.281128	2.276354	2.271591	2.266841	2.296037	2.290919	2.289206	2.284113
2.285364	2.280579	2.275808	2.30584	2.300607	2.295427	2.290306	2.288588	2.283505
2.284828	2.280043	2.275269	2.305229	2.299992	2.294809	2.28968	2.287957	2.28288
2.284316	2.279526	2.274745	2.304631	2.299392	2.294204	2.289066	2.287333	2.282256
2.283874	2.279076	2.274282	2.304088	2.298865	2.29368	2.288532	2.28678	2.281678
2.283474	2.278666	2.273858	2.30358	2.298381	2.293202	2.288044	2.286268	2.281132
2.283001	2.278192	2.273387	2.303022	2.297824	2.29265	2.2875	2.282373	2.27726
2.28245	2.277654	2.272866	2.302414	2.297194	2.292022	2.286901	2.28181	2.276742
2.281929	2.277143	2.272371	2.301831	2.296589	2.291412	2.286302	2.281229	2.276194
2.281478	2.2767	2.271938	2.301308	2.296046	2.290845	2.285704	2.280611	2.275576

2.281028	2.276251	2.271491	2.300777	2.295507	2.290292	2.285127	2.280017	2.274977
2.280523	2.275728	2.27096	2.300178	2.294925	2.289733	2.284601	2.279512	2.274467
2.28001	2.275191	2.270408	2.299564	2.294341	2.289186	2.284106	2.279049	2.273999
2.29422	2.289322	2.284457	2.299017	2.293819	2.288684	2.283619	2.278572	2.273526
2.29378	2.288886	2.284017	2.298519	2.293344	2.288218	2.283146	2.278094	2.273056
2.274808	2.270026	2.26527	2.298042	2.29287	2.287746	2.282675	2.277621	2.272576
2.274387	2.269608	2.264853	2.297591	2.292399	2.28727	2.28221	2.277157	2.272089
2.273969	2.269192	2.264436	2.297132	2.291928	2.297787	2.292677	2.293077	2.287895
2.27355	2.268774	2.264017	2.296646	2.291457	2.297312	2.292182	2.292553	2.287344
2.27313	2.268352	2.263593	2.296154	2.290979	2.296826	2.291671	2.292011	2.286787
2.272704	2.267921	2.263156	2.295667	2.290483	2.296314	2.291131	2.291453	2.286252
2.272276	2.267486	2.262713	2.295178	2.289972	2.295784	2.290577	2.29089	2.285729
2.271854	2.267058	2.262274	2.294672	2.28944	2.295241	2.290045	2.290374	2.285231
2.271438	2.266636	2.261838	2.294149	2.288884	2.294682	2.289531	2.289899	2.284755
2.271018	2.266217	2.261418	2.293679	2.288408	2.294199	2.289049	2.289421	2.284281
2.270594	2.265803	2.261021	2.293288	2.288047	2.293823	2.288598	2.28892	2.2838
2.270168	2.265386	2.260621	2.275768	2.270659	2.29346	2.288183	2.288462	2.283341
2.26974	2.264961	2.260199	2.275306	2.270231	2.293075	2.287828	2.288105	2.282921
2.269312	2.264531	2.259771	2.274828	2.269781	2.292672	2.287481	2.287773	2.282525
2.268877	2.264094	2.259345	2.274357	2.26931	2.292217	2.287062	2.287387	2.28215
2.268446	2.263658	2.258921	2.273887	2.268826	2.29173	2.286602	2.28697	2.281788
2.288899	2.283674	2.278524	2.273419	2.268364	2.291272	2.286139	2.286517	2.281377
2.288564	2.283285	2.278063	2.272949	2.26792	2.290839	2.285664	2.286022	2.280917
2.288205	2.282904	2.277645	2.272526	2.267509	2.290436	2.285238	2.285578	2.280475
2.287795	2.282532	2.277302	2.272185	2.267152	2.290085	2.2849	2.28523	2.280069
2.287361	2.282143	2.276956	2.271842	2.266785	2.289721	2.284555	2.284886	2.279678
2.286889	2.281694	2.276542	2.271424	2.266341	2.273121	2.268059	2.284488	2.279308
2.286415	2.281232	2.276109	2.270986	2.26588	2.272649	2.267589	2.284064	2.278938
2.286016	2.280837	2.275718	2.270595	2.265486	2.272237	2.267139	2.283603	2.278515
2.285672	2.280486	2.275351	2.270232	2.265136	2.271865	2.266699	2.283115	2.278059
2.285328	2.280119	2.274953	2.269833	2.264754	2.271482	2.2663	2.282712	2.277671
2.284984	2.279729	2.274512	2.269386	2.264331	2.271084	2.265949	2.282405	2.277364
2.284649	2.27936	2.274104	2.268973	2.263932	2.270706	2.265611	2.28211	2.277065
2.284332	2.279036	2.273766	2.268628	2.263585	2.270361	2.265278	2.281803	2.276759
2.284004	2.278716	2.273452	2.268311	2.26326	2.270032	2.264951	2.281493	2.276439
2.283621	2.278366	2.273132	2.268001	2.262945	2.269712	2.264627	2.266168	2.261125
2.283216	2.278006	2.272816	2.267701	2.262641	2.269401	2.264307	2.265826	2.26073
2.282849	2.277664	2.2725	2.267392	2.262329	2.269086	2.263991	2.265503	2.260382
2.28251	2.277334	2.272183	2.267079	2.262011	2.268769	2.26368	2.265196	2.260073
2.282185	2.277015	2.271871	2.266767	2.261698	2.268456	2.263373	2.264893	2.259769
2.281878	2.276708	2.271563	2.26646	2.26139	2.26815	2.263071	2.264594	2.259469
2.281573	2.276402	2.271256	2.266152	2.261083	2.267844	2.262768	2.264293	2.259169
2.281262	2.276093	2.270948	2.265844	2.260775	2.267536	2.262462	2.263988	2.258863
2.28095	2.275781	2.270637	2.265534	2.260466	2.267228	2.262155	2.263681	2.258554
2.280631	2.275462	2.270322	2.265223	2.260158	2.266921	2.261848	2.263372	2.258241
2.280313	2.275141	2.270005	2.264909	2.259849	2.266615	2.261542	2.263063	2.257926
2.280042	2.274847	2.269692	2.264592	2.259539	2.26631	2.261243	2.262766	2.257628
2.27982	2.274581	2.269383	2.264271	2.259226	2.266006	2.260951	2.262481	2.257344
2.279586	2.274317	2.269089	2.263963	2.258917	2.265702	2.260658	2.262202	2.257084
2.27933	2.274051	2.268806	2.263664	2.258603	2.265386	2.260357	2.261929	2.256862

2.279061	2.273792	2.26855	2.263399	2.25832	2.265096	2.260071	2.261659	2.256633
2.278775	2.27355	2.268353	2.263211	2.258116	2.264873	2.259815	2.261377	2.256348
2.278482	2.273309	2.268168	2.263043	2.257937	2.264676	2.259579	2.261107	2.256055
2.278194	2.27303	2.267908	2.262794	2.257699	2.264449	2.259362	2.258969	2.253927
2.277911	2.272728	2.2676	2.262497	2.257426	2.264205	2.25916	2.258831	2.253774
2.277673	2.272479	2.267343	2.262235	2.257161	2.262301	2.25729	2.258635	2.253576
2.277484	2.272292	2.267145	2.26201	2.2569	2.262049	2.257026	2.25837	2.253325
2.277299	2.272104	2.266945	2.261795	2.256664	2.26183	2.256796	2.258131	2.253089
2.277105	2.271894	2.266722	2.261579	2.256469	2.261677	2.256639	2.257958	2.252891
2.276904	2.271683	2.266503	2.261373	2.256287	2.26154	2.256498	2.257799	2.25271
2.276681	2.271476	2.266309	2.261185	2.256103	2.261385	2.256328	2.257618	2.252541
2.276451	2.271272	2.266126	2.261008	2.255917	2.261223	2.256151	2.257436	2.252385
2.276247	2.27108	2.265944	2.260819	2.255714	2.261047	2.255982	2.257278	2.25224
2.276065	2.270898	2.265762	2.260624	2.255496	2.260858	2.255817	2.257138	2.252105
2.275879	2.270703	2.265561	2.260422	2.255297	2.260699	2.255678	2.257011	2.251966
2.27569	2.27049	2.265329	2.260206	2.255121	2.260578	2.255573	2.256896	2.251813
2.274305	2.269121	2.263988	2.258914	2.253893	2.26047	2.255476	2.256793	2.251688
2.274107	2.268943	2.263844	2.258788	2.253778	2.260365	2.255375	2.256707	2.251631
2.273929	2.268786	2.263721	2.25868	2.253674	2.260265	2.255277	2.256625	2.251582
2.273854	2.268698	2.263614	2.258572	2.253576	2.260171	2.255183	2.256524	2.251467
2.273831	2.268646	2.263521	2.25847	2.253484	2.260079	2.255091	2.256414	2.251323
2.273723	2.268544	2.263423	2.258374	2.253388	2.259981	2.254992	2.269048	2.263895
2.273536	2.268393	2.263323	2.258285	2.253288	2.259877	2.254886	2.268942	2.263829
2.273417	2.268281	2.26322	2.258184	2.253185	2.259772	2.25478	2.268829	2.26373
2.273412	2.268227	2.263103	2.258056	2.253075	2.259668	2.254675	2.268699	2.263565
2.273418	2.268198	2.263021	2.257954	2.252973	2.259563	2.254572	2.268578	2.263418
2.273384	2.268193	2.263031	2.257929	2.25288	2.259442	2.254467	2.268485	2.263357
2.27333	2.268189	2.263067	2.257934	2.252807	2.259335	2.254369	2.268401	2.263321
2.273237	2.268126	2.263039	2.257922	2.252796	2.259306	2.254292	2.268287	2.263232
2.273119	2.268024	2.262973	2.257901	2.252827	2.25933	2.254233	2.268161	2.263116
2.273073	2.267958	2.262892	2.257839	2.252807	2.272774	2.26759	2.268102	2.26303
2.27311	2.267928	2.262784	2.257726	2.252734	2.272721	2.267595	2.268121	2.262977
2.273125	2.267908	2.26272	2.257645	2.252654	2.275558	2.270462	2.270611	2.265407
2.273088	2.267907	2.262744	2.257634	2.25257	2.275437	2.27036	2.270547	2.265386
2.273023	2.267889	2.26277	2.257637	2.252505	2.275331	2.270252	2.270464	2.265361
2.272918	2.267806	2.26273	2.257619	2.2525	2.27531	2.270183	2.27037	2.265296
2.272805	2.267699	2.262659	2.25759	2.25252	2.275325	2.270138	2.27028	2.265222
2.272774	2.267641	2.262579	2.257527	2.2525	2.275309	2.270122	2.270261	2.265205
2.272808	2.267618	2.262487	2.257436	2.252453	2.275276	2.270134	2.270299	2.265233
2.272857	2.267635	2.262462	2.257409	2.252448	2.275273	2.27016	2.270335	2.265242
2.272928	2.267705	2.26252	2.257462	2.252497	2.275311	2.270202	2.270365	2.265222
2.272979	2.267768	2.262589	2.271527	2.266482	2.275353	2.27025	2.265139	2.259993
2.27297	2.267789	2.262637	2.271573	2.266521	2.275397	2.27031	2.265204	2.260037
2.272954	2.267806	2.262684	2.271613	2.266546	2.275422	2.270352	2.265258	2.260087
2.272981	2.267845	2.262732	2.271627	2.266518	2.275377	2.270321	2.265246	2.260102
2.273033	2.267898	2.262781	2.271631	2.266466	2.275301	2.270253	2.265201	2.260102
2.273098	2.267963	2.262843	2.271664	2.266471	2.275292	2.270245	2.265204	2.260129
2.273178	2.268041	2.262918	2.271724	2.266527	2.275342	2.270289	2.265245	2.260179
2.273258	2.268119	2.262994	2.271788	2.266588	2.275401	2.270341	2.265295	2.260233
2.273337	2.268197	2.263071	2.271853	2.266652	2.275463	2.270402	2.265355	2.260291

2.287519	2.282313	2.27712	2.271915	2.266714	2.275523	2.270461	2.265412	2.260345
2.287576	2.282369	2.277174	2.271966	2.266761	2.275569	2.270507	2.265456	2.260385
2.28763	2.282422	2.277227	2.272018	2.266811	2.275616	2.27055	2.265496	2.260422
2.287685	2.282483	2.277295	2.272089	2.266884	2.275682	2.270598	2.265534	2.260464
2.287739	2.282549	2.277372	2.272176	2.266975	2.275763	2.270655	2.265574	2.26051
2.287792	2.282611	2.27745	2.272276	2.267102	2.265201	2.260127	2.255073	2.250029
2.287839	2.282666	2.277528	2.272395	2.267274	2.26539	2.260297	2.255214	2.250126
2.28792	2.282739	2.277601	2.272491	2.26741	2.265543	2.260443	2.255342	2.250222
2.288061	2.282838	2.277661	2.272544	2.267478	2.265623	2.260533	2.255436	2.250306
2.288208	2.282946	2.277727	2.272594	2.267528	2.265679	2.260604	2.255519	2.250396
2.288318	2.283045	2.277808	2.272658	2.267578	2.265725	2.260661	2.255596	2.250512
2.288416	2.283148	2.277908	2.272742	2.267637	2.265774	2.260715	2.25567	2.250633
2.288525	2.283273	2.278052	2.272885	2.267764	2.265886	2.260804	2.255746	2.250715
2.288642	2.283417	2.278235	2.273077	2.267947	2.266047	2.26092	2.255825	2.25077
2.288788	2.283579	2.278416	2.273256	2.268111	2.266198	2.261054	2.255943	2.250875
2.288971	2.283766	2.278597	2.273421	2.268252	2.266332	2.261206	2.256109	2.251041
2.289153	2.283944	2.278764	2.273577	2.268396	2.266476	2.261367	2.256281	2.25121
2.289323	2.284096	2.278898	2.273715	2.268552	2.266641	2.261538	2.256449	2.25136
2.289483	2.28424	2.279029	2.273854	2.268712	2.266811	2.261706	2.25661	2.251504
2.284666	2.279433	2.274231	2.274016	2.268879	2.266975	2.261851	2.256745	2.251645
2.284789	2.27958	2.274398	2.274188	2.269048	2.267134	2.261985	2.256867	2.251785
2.284938	2.279738	2.274563	2.269398	2.264247	2.267273	2.262123	2.257009	2.25194
2.28511	2.279907	2.27473	2.269549	2.264375	2.267394	2.262263	2.257167	2.252106
2.285281	2.280067	2.274882	2.269696	2.264519	2.26754	2.262428	2.257341	2.252271
2.285451	2.280214	2.27501	2.269833	2.264684	2.267722	2.262624	2.257533	2.252423
2.285612	2.28036	2.27515	2.269986	2.264866	2.26322	2.258133	2.272334	2.267149
2.285735	2.280501	2.275325	2.27018	2.265071	2.263431	2.258338	2.272552	2.267397
2.285871	2.280658	2.27552	2.270391	2.265289	2.26365	2.258547	2.272774	2.267659
2.286114	2.280887	2.27573	2.270598	2.265501	2.263865	2.25876	2.268874	2.263743
2.286419	2.281159	2.275954	2.270808	2.265714	2.264078	2.258976	2.269076	2.263905
2.286641	2.281381	2.276174	2.271026	2.265931	2.264292	2.259187	2.269287	2.264114
2.286772	2.281546	2.276391	2.271256	2.266152	2.264507	2.259392	2.269508	2.264374
2.286967	2.28175	2.27661	2.271478	2.26637	2.264722	2.259601	2.269723	2.264606
2.287287	2.282024	2.276821	2.271674	2.26658	2.264937	2.259816	2.269925	2.264769
2.287623	2.282323	2.277063	2.271894	2.266796	2.26515	2.260033	2.270134	2.264948
2.287911	2.282638	2.277392	2.272188	2.26702	2.265347	2.260244	2.270373	2.265217
2.288177	2.282956	2.277752	2.272514	2.267263	2.265553	2.260458	2.270622	2.265518
2.288412	2.283218	2.278046	2.272819	2.267567	2.281162	2.275977	2.270842	2.265763
2.288621	2.28344	2.278297	2.273115	2.267917	2.281514	2.27624	2.271044	2.265975
2.288899	2.283696	2.278536	2.273373	2.268216	2.281833	2.276534	2.279697	2.27457
2.289264	2.283993	2.278755	2.273579	2.268452	2.282115	2.276871	2.280049	2.274853
2.28961	2.284301	2.279013	2.273816	2.268688	2.282378	2.27719	2.280392	2.275154
2.289897	2.284621	2.279357	2.274131	2.268937	2.282607	2.277456	2.280686	2.275473
2.290159	2.284933	2.279717	2.274469	2.269209	2.282851	2.277722	2.280976	2.275805
2.290399	2.285197	2.280021	2.274789	2.269532	2.28318	2.278045	2.281298	2.276156
2.290634	2.285443	2.280306	2.275108	2.269888	2.283554	2.278392	2.28163	2.276511
2.290936	2.285746	2.280616	2.291492	2.286233	2.283916	2.278708	2.281916	2.276809
2.291297	2.286096	2.280946	2.291838	2.286605	2.284275	2.279001	2.282163	2.277058
2.29166	2.286433	2.28125	2.292147	2.286941	2.284613	2.279313	2.282453	2.277341
2.292018	2.286742	2.281506	2.292395	2.28722	2.284918	2.27965	2.282804	2.27768

2.292384	2.287066	2.281787	2.292666	2.287513	2.285234	2.280001	2.283168	2.27802
2.292773	2.287439	2.282137	2.293013	2.287861	2.285587	2.280367	2.283523	2.278324
2.293155	2.287822	2.282517	2.293394	2.288235	2.285959	2.280744	2.283884	2.278633
2.293477	2.288171	2.282891	2.293781	2.288619	2.286343	2.28113	2.284261	2.278988
2.293766	2.288504	2.28327	2.294181	2.289016	2.286739	2.281525	2.28465	2.279373
2.310371	2.305065	2.299788	2.294575	2.28941	2.287133	2.28192	2.285041	2.27976
2.310742	2.305441	2.300174	2.294966	2.289802	2.287528	2.282318	2.285436	2.280151
2.311124	2.305824	2.30056	2.295353	2.290191	2.287919	2.282713	2.285829	2.280541
2.311517	2.306212	2.300944	2.295737	2.290578	2.288308	2.283104	2.286218	2.280928
2.311912	2.306601	2.301328	2.296119	2.290961	2.288693	2.283491	2.286604	2.281313
2.3123	2.306988	2.301712	2.296501	2.291341	2.289072	2.283868	2.28698	2.281689
2.312686	2.307373	2.302096	2.296883	2.291719	2.289447	2.284243	2.287355	2.282066
2.313068	2.307753	2.302479	2.297264	2.292098	2.289827	2.284628	2.287744	2.282461
2.313448	2.30813	2.30286	2.297644	2.292477	2.29021	2.28502	2.288144	2.28287
2.296904	2.291906	2.286935	2.298021	2.292854	2.290592	2.285409	2.288546	2.283302
2.297377	2.292332	2.287312	2.29839	2.293228	2.290972	2.285795	2.288953	2.283765
2.297839	2.292756	2.287698	2.298771	2.293606	2.291351	2.286177	2.289351	2.284203
2.298261	2.293164	2.288084	2.299159	2.293978	2.291716	2.286546	2.289725	2.284586
2.298671	2.293576	2.288495	2.299578	2.294378	2.292102	2.286926	2.2901	2.284957
2.299078	2.294017	2.28897	2.300076	2.294857	2.292558	2.287344	2.290489	2.285334
2.299482	2.294469	2.289472	2.300611	2.295376	2.293052	2.28779	2.290896	2.285723
2.299885	2.294885	2.289913	2.301084	2.295859	2.293536	2.288266	2.291362	2.286181
2.300285	2.295274	2.290304	2.30151	2.296317	2.29402	2.288774	2.29188	2.286697
2.300734	2.295713	2.290739	2.301968	2.296798	2.294518	2.289286	2.292395	2.287197
2.301247	2.296223	2.291241	2.302478	2.297323	2.295045	2.289807	2.292905	2.28767
2.30177	2.296737	2.291737	2.302976	2.297822	2.295545	2.290308	2.307637	2.302279
2.302283	2.297217	2.292178	2.30341	2.29824	2.295964	2.290753	2.308091	2.302752
2.302787	2.297692	2.292618	2.303847	2.298653	2.296376	2.291193	2.308544	2.303231
2.303258	2.298172	2.293112	2.304366	2.299156	2.293967	2.288802	2.306936	2.301643
2.303712	2.298657	2.29364	2.304935	2.299712	2.29451	2.289332	2.307448	2.302145
2.282902	2.277934	2.273005	2.305496	2.300263	2.295049	2.289857	2.30795	2.302629
2.283448	2.278487	2.273557	2.306054	2.300815	2.295591	2.290382	2.308445	2.303095
2.277017	2.272122	2.267249	2.306607	2.301355	2.311699	2.30637	2.308924	2.303565
2.277571	2.272676	2.267798	2.307155	2.301878	2.312182	2.306827	2.309372	2.304042
2.278131	2.273231	2.268345	2.307696	2.302386	2.312652	2.307287	2.309833	2.304534
2.278699	2.273791	2.268889	2.306832	2.301444	2.321021	2.315661	2.310343	2.305057
2.279269	2.274354	2.269436	2.307375	2.301939	2.321478	2.31616	2.310876	2.305595
2.279828	2.274916	2.270005	2.307989	2.302559	2.322062	2.316707	2.311399	2.306124
2.28038	2.275479	2.270589	2.308651	2.303265	2.322737	2.317296	2.311925	2.306653
2.28094	2.27604	2.271156	2.325081	2.319604	2.314136	2.308668	2.312526	2.307219
2.281509	2.276599	2.271703	2.325653	2.320206	2.314777	2.309367	2.313218	2.307823
2.282074	2.277156	2.272252	2.326225	2.320792	2.315392	2.310035	2.313887	2.308447
2.282629	2.277712	2.272811	2.326812	2.321363	2.315966	2.310633	2.314495	2.3091
2.283184	2.278268	2.273371	2.327399	2.321929	2.316529	2.311207	2.315075	2.30974
2.283746	2.278828	2.273925	2.297866	2.29284	2.31711	2.311779	2.306463	2.301148
2.284312	2.27939	2.274481	2.29841	2.293394	2.317694	2.312344	2.307018	2.301712
2.284876	2.279955	2.275056	2.298971	2.29394	2.318259	2.31291	2.307586	2.302275
2.28544	2.280524	2.275646	2.299545	2.294483	2.318809	2.313475	2.30816	2.302831
2.286065	2.281151	2.276279	2.300168	2.295092	2.319429	2.31407	2.308736	2.3034
2.286777	2.281861	2.276973	2.300859	2.295795	2.320143	2.314694	2.3093	2.303976

2.30699	2.301932	2.296908	2.301533	2.296486	2.320866	2.315353	2.309912	2.304593
2.307595	2.30253	2.297495	2.302129	2.297104	2.321559	2.316059	2.310621	2.305286
2.308191	2.303121	2.298077	2.302719	2.297713	2.32225	2.316787	2.311365	2.306001
2.30885	2.303782	2.298744	2.303388	2.298377	2.32296	2.31752	2.312096	2.306678
2.309535	2.304475	2.29945	2.304091	2.299064	2.323674	2.318253	2.312824	2.307343
2.310163	2.30511	2.300095	2.304733	2.299693	2.31099	2.305665	2.313512	2.308025
2.310737	2.30569	2.300684	2.305316	2.300263	2.311592	2.306279	2.314161	2.308722
2.311364	2.306318	2.301312	2.305944	2.300888	2.312253	2.30694	2.314839	2.309435
2.31208	2.307026	2.30201	2.306648	2.301603	2.313012	2.307682	2.315566	2.310173
2.312812	2.307747	2.302718	2.307364	2.302331	2.313784	2.308433	2.316299	2.310911
2.313523	2.30845	2.303417	2.30807	2.303045	2.298021	2.292996	2.317022	2.311641
2.31423	2.309143	2.3041	2.308757	2.303738	2.298713	2.293678	2.317726	2.312353
2.314932	2.309805	2.304717	2.309366	2.304357	2.299332	2.294288	2.305158	2.299874
2.315628	2.310444	2.30529	2.309924	2.304924	2.299901	2.294849	2.305743	2.30047
2.316288	2.311095	2.305927	2.310558	2.305557	2.300532	2.295479	2.306406	2.301134
2.316905	2.311757	2.30663	2.311273	2.306258	2.301229	2.296183	2.307153	2.301869
2.317549	2.312435	2.307344	2.311998	2.306972	2.30194	2.296901	2.307915	2.302618
2.318243	2.313135	2.308051	2.312711	2.307685	2.302654	2.297617	2.308674	2.303366
2.318962	2.313853	2.30877	2.313434	2.308408	2.303379	2.298344	2.309446	2.304127
2.319688	2.314582	2.309503	2.314167	2.309134	2.304103	2.299075	2.310231	2.304896
2.320435	2.315333	2.31026	2.314922	2.309878	2.304844	2.299824	2.311039	2.305685
2.321246	2.316151	2.311088	2.315749	2.310694	2.305656	2.30064	2.31191	2.306537
2.32211	2.317024	2.311975	2.316637	2.31157	2.306526	2.301511	2.312835	2.307442
2.322929	2.317844	2.3128	2.317463	2.312391	2.307342	2.302322	2.297296	2.292242
2.32369	2.318597	2.313543	2.318208	2.313141	2.308089	2.303055	2.298019	2.292967
2.313058	2.307942	2.302864	2.318937	2.313871	2.308816	2.303774	2.298734	2.293685
2.313778	2.308663	2.303585	2.319666	2.31459	2.309534	2.304499	2.299464	2.294415
2.314498	2.309384	2.304306	2.320396	2.31531	2.310252	2.305226	2.3002	2.29516
2.315237	2.310118	2.305033	2.310404	2.305298	2.310989	2.305959	2.300944	2.295946
2.315995	2.310868	2.305771	2.311142	2.30604	2.311745	2.306705	2.301699	2.296757
2.316814	2.311674	2.306562	2.311938	2.306845	2.301769	2.296706	2.302494	2.297552
2.317695	2.312538	2.307406	2.312791	2.307713	2.302635	2.297552	2.30333	2.298336
2.31229	2.307141	2.302018	2.31361	2.308534	2.303452	2.298362	2.29329	2.28825
2.313028	2.307889	2.302782	2.314361	2.309265	2.304178	2.299099	2.294032	2.288972
2.313775	2.30865	2.303563	2.315132	2.310015	2.304922	2.299854	2.294792	2.289716
2.3146	2.309491	2.304425	2.315992	2.310856	2.305752	2.300682	2.295615	2.290527
2.315444	2.310347	2.305298	2.316872	2.311731	2.30662	2.301542	2.296464	2.29137
2.31624	2.311137	2.306085	2.311901	2.306825	2.307507	2.302407	2.29731	2.292218
2.317014	2.311893	2.306823	2.312687	2.307694	2.308407	2.303279	2.29816	2.293076
2.317846	2.312706	2.307614	2.313481	2.3085	2.303463	2.298366	2.299012	2.293924
2.318739	2.31358	2.308455	2.31428	2.309233	2.304196	2.299176	2.299874	2.294768
2.319635	2.314465	2.30932	2.315109	2.310001	2.304947	2.29996	2.300683	2.295571
2.320518	2.315353	2.310212	2.315986	2.310842	2.305744	2.3007	2.301385	2.296295
2.321404	2.316246	2.311112	2.316878	2.31171	2.30657	2.301457	2.302095	2.297026
2.314411	2.309278	2.304166	2.317763	2.312591	2.307437	2.302295	2.297189	2.292132
2.315309	2.310166	2.30504	2.31865	2.313486	2.308329	2.303174	2.298058	2.292992
2.316191	2.311054	2.305934	2.319552	2.314378	2.309212	2.304048	2.298923	2.293847
2.31706	2.31194	2.306842	2.320464	2.315272	2.310094	2.304927	2.299794	2.294707
2.317941	2.312826	2.307732	2.321361	2.316161	2.310978	2.305807	2.300672	2.295584
2.31884	2.313713	2.308601	2.322241	2.317046	2.311863	2.306686	2.301552	2.296477

2.319728	2.31459	2.309465	2.315725	2.310566	2.312748	2.307576	2.302451	2.297392
2.320584	2.315445	2.310325	2.316591	2.311431	2.313629	2.308478	2.303374	2.298342
2.321437	2.316296	2.311184	2.317457	2.312301	2.314519	2.309395	2.304315	2.299299
2.322312	2.31716	2.312041	2.31833	2.313194	2.315438	2.310344	2.305276	2.300238
2.323198	2.318032	2.3129	2.319211	2.314105	2.316378	2.311311	2.306245	2.301163
2.324082	2.318916	2.31379	2.320123	2.315043	2.31733	2.312251	2.307167	2.30206
2.324963	2.31981	2.314708	2.321065	2.316009	2.318295	2.31317	2.308048	2.302928
2.325873	2.320733	2.315646	2.322012	2.316952	2.311863	2.306741	2.308935	2.303801
2.32682	2.321697	2.316616	2.322968	2.317861	2.31275	2.307632	2.309829	2.304669
2.313756	2.308758	2.303793	2.323908	2.318761	2.313636	2.308536	2.310748	2.305576
2.314706	2.30967	2.304672	2.324801	2.319651	2.31454	2.309477	2.311727	2.306577
2.315655	2.310574	2.305539	2.325689	2.320552	2.315462	2.310429	2.312717	2.307599
2.316555	2.311465	2.306432	2.326627	2.321508	2.316416	2.311357	2.313637	2.308534
2.317432	2.312355	2.307345	2.327599	2.322503	2.317398	2.312278	2.314523	2.309427
2.31839	2.31331	2.308299	2.328596	2.323515	2.318407	2.313265	2.30815	2.303081
2.319435	2.314336	2.309297	2.329621	2.324551	2.319453	2.314324	2.309211	2.304124
2.320458	2.315358	2.310307	2.330648	2.325556	2.320454	2.315339	2.310234	2.305147
2.321424	2.316356	2.311326	2.331667	2.326498	2.32136	2.316253	2.311173	2.306126
2.322389	2.317352	2.312343	2.332689	2.327449	2.322276	2.31717	2.312104	2.307087
2.323386	2.318355	2.313352	2.322231	2.317018	2.311868	2.306785	2.313071	2.308024
2.324395	2.319353	2.314345	2.32326	2.318066	2.312912	2.307801	2.31406	2.308961
2.325395	2.32032	2.315271	2.310221	2.305179	2.313909	2.30881	2.315079	2.309965
2.326391	2.321263	2.316147	2.311091	2.306082	2.314879	2.309821	2.316126	2.311018
2.327405	2.322248	2.317092	2.31203	2.307034	2.315881	2.310848	2.317178	2.312073
2.328442	2.323284	2.318121	2.31305	2.308044	2.316921	2.311895	2.318237	2.313131
2.329477	2.324323	2.319159	2.314083	2.309067	2.317969	2.312947	2.319297	2.314187
2.330501	2.325348	2.320184	2.315107	2.310089	2.319022	2.314	2.320355	2.315239
2.331522	2.32637	2.321208	2.316131	2.311114	2.320076	2.315054	2.321412	2.316287
2.33254	2.32739	2.322229	2.317153	2.312135	2.321126	2.316104	2.322465	2.317331
2.333557	2.328408	2.323248	2.318172	2.313155	2.322175	2.317152	2.323515	2.318372
2.334579	2.329429	2.324269	2.319193	2.314175	2.323223	2.318197	2.324561	2.319409
2.335606	2.330451	2.325289	2.320212	2.315195	2.324269	2.31924	2.325602	2.32044
2.336644	2.33149	2.326329	2.321247	2.31622	2.325318	2.32029	2.326657	2.321489
2.337696	2.332546	2.327386	2.322291	2.317244	2.326364	2.321349	2.327728	2.322558
2.338756	2.333622	2.328478	2.323372	2.318296	2.313305	2.308414	2.328805	2.323646
2.339823	2.334739	2.329642	2.32453	2.319412	2.314386	2.309464	2.329883	2.324771
2.340889	2.335857	2.330815	2.325701	2.320544	2.315482	2.310519	2.330958	2.325898
2.329529	2.324574	2.319621	2.326795	2.32163	2.316549	2.311553	2.332009	2.326972
2.330546	2.325585	2.320643	2.327846	2.322697	2.31761	2.312583	2.333051	2.328018
2.331568	2.326606	2.321679	2.328929	2.323817	2.318734	2.313674	2.33415	2.329096
2.332589	2.32763	2.322726	2.330044	2.324995	2.319928	2.314831	2.335307	2.330206
2.333658	2.32868	2.323766	2.325865	2.320877	2.321077	2.31596	2.336447	2.331304
2.334794	2.329756	2.324784	2.326882	2.321919	2.322139	2.317023	2.337537	2.332362
2.335965	2.330868	2.325828	2.327907	2.322947	2.323183	2.318086	2.338645	2.333449
2.337177	2.332045	2.326944	2.328986	2.323993	2.318983	2.313952	2.339838	2.33463
2.338392	2.333247	2.328101	2.330099	2.325057	2.320045	2.315072	2.341051	2.335847
2.339508	2.334399	2.329277	2.324195	2.319142	2.321139	2.316152	2.342177	2.337016
2.340552	2.335516	2.330471	2.325396	2.320303	2.322258	2.317208	2.343248	2.338157
2.341588	2.336584	2.331582	2.326534	2.321456	2.323408	2.318317	2.339939	2.334869
2.342599	2.33758	2.332589	2.327598	2.32261	2.324603	2.319491	2.341121	2.335989

2.316469	2.311706	2.306981	2.328658	2.323723	2.325758	2.32066	2.3156	2.310612
2.317681	2.312865	2.30807	2.329723	2.32475	2.326817	2.321805	2.316813	2.311848
2.318902	2.314047	2.30919	2.330813	2.32578	2.327863	2.322931	2.318008	2.313075
2.309259	2.304464	2.299661	2.331983	2.326903	2.328961	2.324009	2.319076	2.314149
2.310292	2.305562	2.300824	2.333186	2.328071	2.330089	2.325074	2.320099	2.315165
2.311365	2.306635	2.301908	2.324788	2.31972	2.324191	2.319213	2.321255	2.316289
2.312463	2.307683	2.302925	2.325861	2.320877	2.325398	2.320461	2.322518	2.317498
2.313592	2.308795	2.304026	2.326995	2.322036	2.317097	2.312186	2.323709	2.318675
2.314773	2.310012	2.305256	2.328223	2.323193	2.318201	2.313251	2.324779	2.319798
2.315916	2.311191	2.306457	2.329437	2.324356	2.319327	2.314347	2.316852	2.311915
2.316931	2.312221	2.307519	2.330566	2.325535	2.320538	2.315577	2.31808	2.3131
2.31794	2.313237	2.308564	2.331686	2.326716	2.321761	2.316824	2.319326	2.314297
2.319096	2.314402	2.309736	2.332882	2.32788	2.322904	2.317957	2.313013	2.308057
2.320334	2.315643	2.310968	2.334116	2.329039	2.324013	2.319036	2.314098	2.309206
2.321518	2.316807	2.312114	2.335299	2.330224	2.325194	2.320207	2.31527	2.310408
2.322655	2.317897	2.313174	2.33643	2.331432	2.326445	2.321465	2.316528	2.311666
2.323807	2.319025	2.314286	2.337611	2.332667	2.327701	2.322707	2.317749	2.312868
2.324986	2.320226	2.3155	2.338888	2.333957	2.328968	2.323908	2.318888	2.313954
2.326182	2.321437	2.316715	2.340152	2.335213	2.330201	2.325099	2.320039	2.315064
2.327414	2.322632	2.317862	2.341302	2.33633	2.331325	2.326279	2.321266	2.316303
2.328641	2.323814	2.318991	2.342431	2.337417	2.332429	2.327469	2.322527	2.317596
2.309216	2.304515	2.299828	2.343664	2.338644	2.333663	2.328724	2.3238	2.318881
2.310264	2.305613	2.300997	2.344968	2.339964	2.334983	2.330027	2.325086	2.320166
2.311409	2.306783	2.302199	2.34628	2.341281	2.336285	2.33129	2.326312	2.321367
2.312665	2.308034	2.303437	2.347607	2.342603	2.337573	2.33251	2.327468	2.322461
2.313935	2.309295	2.304679	2.348915	2.343885	2.338822	2.333719	2.32864	2.323598
2.315213	2.310569	2.305936	2.350199	2.345092	2.339997	2.334909	2.329853	2.324832
2.316459	2.311803	2.30715	2.35144	2.346277	2.341167	2.336113	2.331095	2.326108
2.317595	2.312901	2.308223	2.333834	2.328809	2.342394	2.33737	2.332376	2.327407
2.318679	2.313939	2.309239	2.334935	2.329998	2.343651	2.338658	2.333686	2.328728
2.303932	2.29924	2.294594	2.336141	2.331233	2.344924	2.339955	2.335	2.330053
2.305085	2.30042	2.295797	2.337428	2.33251	2.346216	2.341264	2.336323	2.331385
2.306261	2.30162	2.297018	2.308205	2.30362	2.347521	2.342575	2.337639	2.332704
2.307459	2.302839	2.298253	2.30946	2.304868	2.348841	2.343887	2.338944	2.334006
2.308674	2.304068	2.299492	2.310717	2.30612	2.350166	2.345203	2.340252	2.335309
2.309905	2.305301	2.300723	2.311966	2.307367	2.351493	2.346529	2.341571	2.336621
2.311146	2.306538	2.301955	2.313213	2.308615	2.352819	2.347853	2.342891	2.337935
2.312384	2.307775	2.303192	2.298626	2.29408	2.335456	2.330579	2.344197	2.339241
2.313624	2.309013	2.304431	2.299861	2.295309	2.336757	2.33187	2.345496	2.340545
2.314862	2.310247	2.305662	2.30109	2.296537	2.338063	2.333168	2.346803	2.341849
2.3161	2.311476	2.306886	2.302314	2.297764	2.339374	2.334473	2.348117	2.343154
2.317343	2.31271	2.308114	2.30354	2.29899	2.340683	2.335778	2.349434	2.344466
2.318592	2.313952	2.30935	2.30477	2.300214	2.34199	2.337082	2.350752	2.34579
2.319843	2.315194	2.310583	2.305995	2.301434	2.343297	2.338386	2.35207	2.347112
2.305136	2.300546	2.295995	2.307207	2.302647	2.330196	2.325327	2.334793	2.329928
2.306367	2.301759	2.29719	2.308415	2.303857	2.331487	2.326613	2.336091	2.331222
2.307605	2.302982	2.298397	2.309631	2.305064	2.332775	2.3279	2.337393	2.33252
2.308846	2.304212	2.299613	2.310853	2.30627	2.334062	2.329187	2.338696	2.33382
2.310082	2.305441	2.300831	2.312081	2.307488	2.302927	2.298401	2.339998	2.335117
2.311313	2.306667	2.302053	2.313319	2.308719	2.30415	2.299613	2.341299	2.336411

2.28981	2.285217	2.280657	2.314558	2.30995	2.305372	2.300826	2.3282	2.323355
2.291048	2.286449	2.281881	2.315795	2.311175	2.306588	2.302038	2.329491	2.324638
2.292286	2.287682	2.283107	2.317036	2.312403	2.307808	2.303251	2.330782	2.325921
2.293514	2.288909	2.284334	2.302367	2.297804	2.309034	2.304462	2.332063	2.327197
2.294743	2.290138	2.285567	2.303601	2.29903	2.310268	2.305677	2.333349	2.328479
2.295987	2.291377	2.286809	2.282261	2.277739	2.311506	2.306905	2.334667	2.329783
2.297245	2.292625	2.28806	2.283503	2.278969	2.312749	2.308144	2.336012	2.331109
2.298605	2.293976	2.289408	2.284844	2.280301	2.314102	2.30949	2.337466	2.332544
2.300105	2.295471	2.290893	2.286324	2.281778	2.315609	2.310984	2.339076	2.334134
2.272573	2.267995	2.263469	2.287759	2.283211	2.2955	2.290921	2.340636	2.335675
2.273885	2.269293	2.264749	2.289046	2.284498	2.296803	2.29222	2.342033	2.337052
2.27515	2.270543	2.26598	2.290286	2.285737	2.298056	2.29347	2.343376	2.338378
2.276411	2.271791	2.267212	2.26268	2.258193	2.299309	2.294717	2.344718	2.339713
2.277675	2.273042	2.26845	2.263907	2.25941	2.300568	2.295968	2.346065	2.341058
2.27907	2.274425	2.269818	2.265265	2.260761	2.285056	2.280534	2.327805	2.322836
2.280593	2.275935	2.271313	2.266752	2.262242	2.286544	2.282017	2.329418	2.324442
2.282027	2.277363	2.272732	2.268162	2.263643	2.259154	2.254697	2.330939	2.325962
2.283319	2.278656	2.274025	2.269445	2.264911	2.260415	2.255961	2.332306	2.327337
2.284576	2.279915	2.275285	2.270696	2.266147	2.261644	2.25719	2.333645	2.328695
2.285819	2.281159	2.276525	2.271927	2.267364	2.262855	2.2584	2.310754	2.305902
2.287083	2.282423	2.277787	2.273181	2.268606	2.264089	2.259628	2.312106	2.307307
2.288488	2.283833	2.279203	2.274592	2.270007	2.265476	2.260997	2.296975	2.292215
2.289981	2.285333	2.280715	2.276104	2.271509	2.26696	2.262455	2.298514	2.293667
2.291369	2.286725	2.282113	2.277502	2.272901	2.268339	2.263811	2.299981	2.295068
2.292639	2.287995	2.283385	2.278774	2.274172	2.2696	2.265053	2.301362	2.296405
2.293967	2.289318	2.284706	2.280094	2.275492	2.270914	2.266355	2.302821	2.297824
2.295438	2.290778	2.286161	2.281545	2.276942	2.272362	2.267802	2.304443	2.299406
2.296903	2.292228	2.287602	2.28298	2.278376	2.273796	2.269239	2.306064	2.301003
2.273664	2.269071	2.264525	2.284261	2.279654	2.275075	2.270519	2.307528	2.302488
2.274914	2.2703	2.265738	2.285495	2.280887	2.276309	2.271754	2.308947	2.303942
2.276299	2.271667	2.267091	2.286867	2.28226	2.277677	2.273113	2.268605	2.264178
2.277779	2.27313	2.268542	2.288337	2.283733	2.279143	2.274563	2.270019	2.26552
2.279166	2.2745	2.269905	2.289738	2.28516	2.280587	2.27602	2.271468	2.266928
2.280411	2.275735	2.271142	2.291042	2.286528	2.282012	2.277499	2.272977	2.268431
2.281731	2.277037	2.272431	2.267939	2.263544	2.283391	2.278912	2.274418	2.269889
2.283236	2.278506	2.273852	2.269301	2.26484	2.284667	2.280171	2.275691	2.271221
2.284806	2.280042	2.275334	2.270709	2.26616	2.285948	2.281419	2.276939	2.272519
2.286377	2.2816	2.276862	2.272195	2.267591	2.287365	2.282806	2.278298	2.273845
2.287945	2.283166	2.27841	2.273718	2.26908	2.288855	2.284266	2.279715	2.275188
2.289368	2.284575	2.279809	2.275125	2.270509	2.290329	2.285736	2.281168	2.276607
2.290653	2.285839	2.281069	2.276429	2.271892	2.2918	2.287225	2.282663	2.278097
2.292033	2.2872	2.282417	2.277782	2.27326	2.268774	2.264329	2.284122	2.27957
2.293562	2.288709	2.283901	2.279204	2.274601	2.270084	2.265668	2.285536	2.281019
2.265441	2.260679	2.255952	2.28067	2.275975	2.271411	2.266992	2.28691	2.28243
2.266954	2.262187	2.257453	2.282185	2.277433	2.272807	2.268311	2.288211	2.283769
2.268465	2.263703	2.258971	2.283731	2.278936	2.274243	2.269648	2.289503	2.28508
2.269932	2.265196	2.260479	2.285281	2.280455	2.275718	2.27106	2.290884	2.28641
2.271364	2.26667	2.26198	2.286839	2.281996	2.277228	2.272529	2.292325	2.287751
2.223099	2.218677	2.214277	2.258736	2.254004	2.278758	2.274025	2.293802	2.289155
2.224351	2.219954	2.215604	2.260193	2.255497	2.280308	2.275552	2.29532	2.290622

2.225662	2.221269	2.216945	2.261655	2.256996	2.281867	2.277088	2.27237	2.267731
2.22708	2.222666	2.21833	2.263145	2.258521	2.283444	2.278629	2.273874	2.269206
2.228525	2.224071	2.2197	2.264606	2.260017	2.285004	2.280169	2.27539	2.270699
2.229942	2.225423	2.220991	2.265968	2.261423	2.256809	2.25211	2.276924	2.272207
2.231363	2.22677	2.222266	2.267303	2.262796	2.258224	2.253577	2.278469	2.27373
2.232818	2.228181	2.223636	2.268733	2.264228	2.25967	2.255058	2.28002	2.275259
2.234299	2.229639	2.225073	2.270231	2.265698	2.261137	2.256551	2.281577	2.276796
2.235789	2.231105	2.226516	2.271733	2.267179	2.262611	2.258036	2.253433	2.248751
2.23729	2.232577	2.227961	2.273239	2.268673	2.264096	2.259513	2.254903	2.250214
2.2388	2.234058	2.229413	2.274746	2.270162	2.265572	2.260983	2.256368	2.251678
2.240319	2.235546	2.230864	2.276242	2.271627	2.267023	2.262443	2.257836	2.253149
2.241844	2.237043	2.232328	2.27775	2.273098	2.268477	2.2639	2.259299	2.254618
2.227172	2.22234	2.217595	2.279294	2.274605	2.269956	2.265361	2.260753	2.256083
2.228724	2.223885	2.219133	2.280861	2.276135	2.271453	2.266826	2.262202	2.257545
2.230284	2.225433	2.220662	2.216008	2.211456	2.272969	2.268298	2.263648	2.259
2.231851	2.226983	2.222188	2.217521	2.212962	2.274503	2.269775	2.26509	2.260449
2.233419	2.228536	2.223719	2.219038	2.214467	2.261914	2.257148	2.26655	2.261903
2.234984	2.230085	2.225243	2.220544	2.215958	2.263468	2.258675	2.268024	2.26335
2.236555	2.231659	2.226814	2.222098	2.217483	2.265051	2.260229	2.255466	2.250768
2.238139	2.233296	2.228498	2.223765	2.21909	2.266697	2.261833	2.25705	2.252374
2.239721	2.234942	2.230204	2.225458	2.22072	2.268366	2.263458	2.258654	2.254002
2.241266	2.236513	2.231805	2.22706	2.222304	2.270015	2.265063	2.260223	2.255551
2.242796	2.238047	2.233351	2.228614	2.223865	2.271659	2.266668	2.261785	2.257067
2.223472	2.218747	2.214067	2.230187	2.225427	2.273312	2.268335	2.263445	2.258677
2.225155	2.220399	2.215667	2.231775	2.226985	2.274969	2.270064	2.265206	2.260382
2.226818	2.22203	2.217258	2.212486	2.207726	2.22385	2.219205	2.266953	2.262083
2.228433	2.223616	2.218833	2.214078	2.209354	2.22551	2.220838	2.268656	2.263754
2.23003	2.225185	2.220391	2.215654	2.210969	2.227156	2.222455	2.270344	2.265432
2.206658	2.201872	2.197134	2.217164	2.212494	2.207833	2.203181	2.272032	2.267151
2.208168	2.203357	2.198595	2.218665	2.213995	2.209342	2.204704	2.273703	2.268869
2.209698	2.204892	2.200144	2.220261	2.215565	2.210883	2.206208	2.222438	2.217848
2.211244	2.206464	2.201757	2.221923	2.217181	2.212442	2.207696	2.223902	2.21931
2.186429	2.181716	2.177074	2.223557	2.218778	2.214012	2.209247	2.204561	2.199997
2.188059	2.183311	2.178617	2.225154	2.220349	2.215588	2.21087	2.206209	2.201612
2.189687	2.184902	2.18016	2.226764	2.221945	2.217193	2.21251	2.207867	2.203245
2.191287	2.186471	2.181699	2.228396	2.223588	2.218838	2.214152	2.209503	2.20488
2.192877	2.188039	2.183252	2.230053	2.225257	2.220507	2.215805	2.211141	2.206514
2.19446	2.18962	2.184843	2.180141	2.175507	2.222185	2.217474	2.212793	2.208135
2.196041	2.191212	2.186459	2.181763	2.177127	2.223876	2.219152	2.214448	2.209745
2.197644	2.192822	2.188083	2.183389	2.178747	2.225581	2.220826	2.216086	2.211339
2.199263	2.194449	2.189718	2.185022	2.180372	2.2273	2.222498	2.217709	2.212916
2.175821	2.17105	2.166356	2.186643	2.181974	2.203966	2.199195	2.219318	2.214509
2.177514	2.172712	2.167977	2.188242	2.183541	2.205559	2.200758	2.220895	2.216119
2.118422	2.113854	2.109349	2.164885	2.160211	2.180399	2.175673	2.222514	2.217756
2.120047	2.115442	2.110889	2.166516	2.16183	2.182027	2.177303	2.224227	2.219425
2.121673	2.117034	2.112438	2.168161	2.163467	2.18368	2.178967	2.225981	2.221125
2.083441	2.078972	2.074544	2.169808	2.165101	2.185319	2.180611	2.202687	2.197895
2.084908	2.080462	2.076067	2.171462	2.166737	2.186956	2.182251	2.204407	2.199628
2.086413	2.081961	2.077562	2.112617	2.108118	2.16367	2.159009	2.2061	2.201326
2.087952	2.083468	2.079029	2.114169	2.109666	2.165291	2.160621	2.207767	2.202992

2.089485	2.084982	2.080524	2.115732	2.111214	2.166919	2.162236	2.185979	2.181242
2.090995	2.086509	2.082076	2.117316	2.112755	2.168556	2.163856	2.187658	2.182908
2.092514	2.088034	2.083614	2.118895	2.114302	2.170201	2.165483	2.189341	2.184578
2.094064	2.089539	2.085072	2.080635	2.076236	2.111353	2.10689	2.162427	2.157761
2.095627	2.091045	2.086517	2.082091	2.077749	2.112927	2.108426	2.164056	2.159374
2.097174	2.092581	2.088043	2.083594	2.07922	2.114457	2.109954	2.165681	2.160986
2.098717	2.094142	2.089627	2.085131	2.080669	2.115964	2.111481	2.167307	2.162604
2.100361	2.095775	2.091247	2.08672	2.082216	2.117571	2.113077	2.168994	2.164284
2.102128	2.097496	2.092917	2.088376	2.083881	2.119299	2.114757	2.170755	2.166041
2.006613	2.002181	1.997795	2.090001	2.085514	2.081072	2.076675	2.111884	2.107436
2.008132	2.003661	1.999223	2.091537	2.087041	2.08258	2.078154	2.113418	2.108946
2.009609	2.005113	2.000646	2.093069	2.088564	2.084089	2.07964	2.114963	2.110462
1.958484	1.954149	1.949869	2.067523	2.063037	2.085698	2.081234	2.116619	2.112097
1.959813	1.955513	1.951304	2.069156	2.064677	2.087339	2.082866	2.118315	2.113775
1.961288	1.956978	1.952764	2.000804	1.996483	2.061747	2.05728	2.052862	2.048501
1.962885	1.95853	1.954251	2.002286	1.997874	2.063211	2.058752	2.054337	2.049947
1.919657	1.915396	1.911192	1.951404	1.947077	1.99498	1.990734	2.055868	2.05146
1.921094	1.916846	1.912647	1.952907	1.948579	1.996543	1.992261	2.057492	2.053077
1.922547	1.918289	1.914074	1.909834	1.905596	1.998084	1.993756	2.05908	2.054665
1.924053	1.919718	1.915421	1.91116	1.906935	1.999505	1.995123	2.060534	2.056131
1.925582	1.92116	1.916771	1.912483	1.908271	2.00092	1.996484	2.061982	2.057588
1.927131	1.922673	1.918236	1.913926	1.909706	1.949953	1.94562	1.993613	1.989422
1.928687	1.924223	1.919766	1.915438	1.911198	1.951463	1.947107	1.995151	1.990921
1.848336	1.844058	1.839794	1.916937	1.912685	1.908421	1.904128	1.996702	1.992426
1.849698	1.845455	1.841253	1.91843	1.914173	1.909903	1.905606	1.998273	1.99394
1.820399	1.816299	1.812264	1.838591	1.83448	1.911396	1.907095	1.958555	1.954274
1.822011	1.817883	1.813828	1.840101	1.835996	1.912915	1.908603	1.960124	1.955825
1.823652	1.819493	1.815409	1.841616	1.837497	1.914407	1.910091	1.961682	1.957381
1.825271	1.821105	1.817011	1.843119	1.838928	1.880277	1.876054	1.907279	1.903132
1.826903	1.822733	1.81863	1.844629	1.840354	1.881669	1.877467	1.908714	1.904587
1.789373	1.785094	1.780885	1.846182	1.84188	1.837667	1.833543	1.91019	1.906065
1.791179	1.786863	1.782612	1.847766	1.843474	1.839247	1.835081	1.911695	1.907562
1.677633	1.673578	1.669571	1.84934	1.845051	1.840816	1.836628	1.877856	1.873776
1.679242	1.675204	1.671203	1.8509	1.84661	1.842373	1.838185	1.879412	1.87532
1.594641	1.591221	1.587829	1.77089	1.766567	1.843938	1.839751	1.835632	1.831612
1.596108	1.592693	1.589309	1.772552	1.768192	1.845496	1.841311	1.837192	1.833155
1.597593	1.59418	1.590802	1.774236	1.769853	1.847091	1.842897	1.838767	1.834707
1.52668	1.523431	1.52023	1.711854	1.707723	1.811911	1.807611	1.840378	1.836277
1.5281	1.524848	1.52165	1.713511	1.709434	1.813733	1.80937	1.842005	1.837857
1.529566	1.526286	1.523063	1.51985	1.516655	1.770799	1.766465	1.806748	1.80252
1.531072	1.527744	1.524472	1.521236	1.518036	1.772438	1.768104	1.808408	1.80417
1.466782	1.463587	1.460444	1.522627	1.519422	1.648993	1.644978	1.810052	1.805804
1.468169	1.464957	1.461793	1.524026	1.520816	1.650509	1.646468	1.811662	1.807406
1.469553	1.466327	1.463143	1.525431	1.522219	1.65204	1.647992	1.643987	1.640048
1.470928	1.467683	1.464483	1.526833	1.523634	1.653606	1.649597	1.645606	1.641629
1.472321	1.46906	1.465843	1.528254	1.525059	1.655174	1.651212	1.647244	1.64324
1.473788	1.470512	1.467269	1.52972	1.526482	1.523264	1.52007	1.648811	1.644841
1.475296	1.47201	1.46874	1.531221	1.527917	1.524667	1.521479	1.650344	1.646438
1.406486	1.403419	1.400366	1.467019	1.46383	1.526123	1.522908	1.651882	1.64799
1.407862	1.404827	1.401809	1.468545	1.465307	1.527628	1.524349	1.65342	1.649498

1.409227	1.406211	1.403218	1.470042	1.466796	1.529163	1.525834	1.567872	1.564498
1.410581	1.407562	1.404575	1.471495	1.4683	1.530745	1.52738	1.569282	1.565862
1.411938	1.408904	1.405914	1.472923	1.469782	1.532312	1.528938	1.570723	1.567262
1.413296	1.410239	1.407241	1.474319	1.471202	1.53382	1.530488	1.572212	1.568725
1.414678	1.411585	1.408567	1.4757	1.472591	1.535297	1.532022	1.57371	1.570211
1.232261	1.229566	1.226925	1.477082	1.47397	1.470859	1.467758	1.530229	1.526915
1.233563	1.230815	1.228118	1.47847	1.475339	1.472224	1.46913	1.531688	1.528438
1.234889	1.232116	1.229387	1.409649	1.406644	1.473624	1.470516	1.533135	1.529913
1.236249	1.233482	1.230743	1.411173	1.408087	1.475057	1.471908	1.534558	1.531326
1.237574	1.234813	1.232074	1.229348	1.22664	1.476535	1.473357	1.536043	1.532788
1.23882	1.236063	1.233337	1.230619	1.227919	1.478093	1.474912	1.537654	1.534352
1.240072	1.237308	1.234584	1.231875	1.22919	1.479644	1.476465	1.539265	1.53593
1.241399	1.238589	1.235827	1.233098	1.230407	1.315454	1.31263	1.474734	1.471574
1.242766	1.239899	1.237085	1.234326	1.231622	1.316731	1.313896	1.476138	1.473027
1.244154	1.241277	1.238447	1.235673	1.23295	1.23026	1.227602	1.408865	1.405871
1.245566	1.242712	1.239894	1.237113	1.234367	1.23163	1.228898	1.410281	1.407229
1.246939	1.244088	1.241269	1.238478	1.235713	1.232952	1.230189	1.315007	1.312162
1.248249	1.245375	1.242542	1.239737	1.236961	1.234208	1.231473	1.31642	1.313574
1.24961	1.246709	1.243854	1.241024	1.238226	1.235469	1.232753	1.230087	1.227484
1.251104	1.248179	1.245286	1.242408	1.239552	1.236753	1.234011	1.231323	1.228695
1.252613	1.249668	1.246739	1.243816	1.240905	1.238063	1.235289	1.232572	1.229913
1.253999	1.251047	1.248113	1.245185	1.24227	1.239427	1.236657	1.233936	1.231259
1.255339	1.252387	1.249462	1.246547	1.24365	1.240823	1.238068	1.235352	1.23266
1.256804	1.253835	1.250894	1.247958	1.245037	1.242184	1.239401	1.236663	1.233962
1.25838	1.255381	1.252402	1.249414	1.246429	1.243513	1.240665	1.237881	1.235174
1.259887	1.256868	1.253867	1.250854	1.247843	1.244893	1.241999	1.239181	1.236462
1.261265	1.25824	1.255244	1.252254	1.249278	1.246344	1.243446	1.240617	1.237879
1.154173	1.15135	1.148565	1.253681	1.250736	1.247813	1.244905	1.242056	1.239287
1.155628	1.152784	1.149973	1.255198	1.252238	1.24928	1.246317	1.24341	1.240583
1.157114	1.154248	1.151406	1.256729	1.253742	1.250744	1.247725	1.244761	1.241874
1.158465	1.155584	1.152722	1.149905	1.147125	1.25217	1.24916	1.246198	1.243296
1.159747	1.156854	1.15398	1.151161	1.148386	1.253577	1.250617	1.247692	1.244802
1.161135	1.15824	1.155361	1.152533	1.149746	1.255062	1.252122	1.249208	1.246314
1.162637	1.15975	1.156877	1.154034	1.151217	1.256637	1.253687	1.250761	1.247843
1.164108	1.161217	1.158337	1.155477	1.152638	1.149839	1.147085	1.252255	1.249319
1.165499	1.16257	1.159654	1.156774	1.153924	1.151114	1.148343	1.253622	1.250679
1.166883	1.16393	1.160986	1.158093	1.155238	1.152417	1.14963	1.255012	1.252063
1.168302	1.165379	1.16246	1.159574	1.156714	1.15388	1.151073	1.256555	1.253595
1.169742	1.16687	1.163995	1.161126	1.158261	1.155416	1.152589	1.258171	1.255197
1.171216	1.168364	1.165511	1.162648	1.159782	1.156929	1.15409	1.151276	1.148495
1.172719	1.169869	1.167022	1.164162	1.161295	1.158439	1.155593	1.152769	1.149974
1.174211	1.171359	1.168516	1.165658	1.162793	1.159936	1.157085	1.154254	1.151447
1.1757	1.172844	1.170003	1.167147	1.164286	1.16143	1.158579	1.155742	1.152922
1.177137	1.174274	1.171432	1.168577	1.165719	1.162865	1.160013	1.157173	1.154343
1.178458	1.175584	1.172738	1.169883	1.167029	1.164175	1.161319	1.158474	1.155638
1.179789	1.1769	1.174046	1.171189	1.168338	1.165485	1.162626	1.159778	1.156939
1.027676	1.025145	1.022659	1.172634	1.169787	1.166937	1.164082	1.161234	1.158396
1.029	1.026441	1.023935	1.174151	1.171308	1.168462	1.165612	1.162768	1.159931
1.03034	1.027749	1.025211	1.17566	1.172814	1.169968	1.16712	1.164277	1.161437
1.031705	1.029076	1.026496	1.177171	1.174318	1.171466	1.168619	1.165775	1.162929

1.033066	1.030413	1.0278	1.1787	1.175832	1.172972	1.170123	1.167276	1.164426
1.034432	1.031774	1.029135	1.180252	1.17736	1.174485	1.171632	1.168783	1.165929
1.035751	1.033098	1.030452	1.027869	1.025333	1.17601	1.173149	1.170294	1.167436
1.036948	1.034318	1.03171	1.029154	1.026638	1.177561	1.174681	1.171809	1.168944
1.03813	1.035521	1.032953	1.030426	1.027936	1.179111	1.176216	1.17333	1.170457
1.039447	1.036818	1.034225	1.03168	1.029175	1.18061	1.177736	1.174863	1.171984
1.040844	1.038172	1.035517	1.032924	1.030381	1.182079	1.179246	1.176402	1.17352
1.042226	1.039538	1.036854	1.034224	1.031638	1.183569	1.180747	1.177915	1.175042
1.043599	1.040924	1.038241	1.035581	1.032941	1.185075	1.182233	1.179396	1.176546
1.044955	1.042294	1.039619	1.036944	1.034272	1.186619	1.183759	1.180913	1.178074
1.046285	1.043636	1.040976	1.038307	1.035636	1.188217	1.185348	1.182492	1.179638
1.047608	1.044969	1.042325	1.039667	1.037004	1.189833	1.186956	1.184089	1.181217
1.048937	1.046303	1.043669	1.041015	1.038353	1.035768	1.033269	1.18568	1.182796
1.05027	1.047635	1.045008	1.042356	1.039695	1.037113	1.03462	1.187273	1.18438
1.051608	1.048967	1.046342	1.043692	1.041034	1.038455	1.035964	1.188866	1.185965
1.052952	1.0503	1.047673	1.045023	1.04237	1.039793	1.037303	1.190457	1.187551
1.054321	1.051649	1.049013	1.046363	1.043719	1.041143	1.038643	1.192047	1.189134
1.055715	1.053015	1.050361	1.047711	1.045079	1.042498	1.039974	1.193629	1.190712
1.057126	1.054393	1.051716	1.049072	1.046465	1.043888	1.041345	1.038812	1.036293
1.058551	1.055782	1.053077	1.050456	1.047905	1.045352	1.042798	1.04024	1.037673
1.059991	1.057187	1.054451	1.051847	1.049345	1.046814	1.044256	1.041675	1.039058
1.061455	1.058618	1.055843	1.053224	1.050723	1.048192	1.045634	1.043049	1.040418
1.062933	1.060068	1.057254	1.054604	1.052075	1.04953	1.046976	1.044395	1.041766
1.064401	1.06153	1.0587	1.056019	1.05345	1.050884	1.048329	1.045749	1.043112
1.065865	1.063004	1.060177	1.057464	1.054839	1.052243	1.049687	1.047105	1.044455
1.067327	1.064497	1.061694	1.058962	1.056287	1.053652	1.051065	1.048462	1.045806
1.068789	1.066016	1.063272	1.060537	1.057819	1.055128	1.052468	1.049818	1.047162
1.070252	1.067525	1.064824	1.062091	1.059344	1.056609	1.05388	1.051187	1.048541
1.071713	1.068986	1.066281	1.063552	1.060812	1.058062	1.05529	1.052575	1.049963
1.073185	1.070445	1.067719	1.06499	1.062262	1.059506	1.056707	1.053982	1.051402
1.074698	1.071963	1.069229	1.066479	1.063717	1.060941	1.058136	1.055405	1.052815
1.076238	1.073513	1.070772	1.067987	1.06517	1.06237	1.059578	1.056849	1.054223
1.07778	1.075031	1.072262	1.069453	1.066615	1.063817	1.061055	1.05834	1.055691
1.079328	1.076522	1.073699	1.070874	1.068048	1.065278	1.062566	1.059882	1.057222
0.869345	0.867212	0.865061	1.072327	1.06952	1.066771	1.064087	1.061413	1.058732
0.870479	0.868344	0.866196	1.07383	1.071052	1.068313	1.065617	1.06292	1.060197
0.87161	0.86948	0.867348	1.075361	1.072605	1.069864	1.06714	1.064414	1.061661
0.872769	0.870637	0.86852	1.07692	1.074164	1.071402	1.068631	1.065877	1.063144
0.873947	0.871815	0.869714	1.078495	1.075724	1.072933	1.070114	1.067338	1.064641
0.875161	0.873038	0.870944	0.868863	0.866794	1.074445	1.071625	1.068852	1.066159
0.876403	0.874296	0.872202	0.870084	0.867955	1.075943	1.073156	1.070403	1.067694
0.877613	0.875509	0.873412	0.871281	0.869132	1.07749	1.074714	1.071956	1.069213
0.878779	0.876666	0.874566	0.87245	0.870328	1.079098	1.076305	1.073515	1.070709
0.879974	0.877843	0.875727	0.873615	0.871513	0.869437	0.867395	1.075065	1.072218
0.881241	0.879076	0.876912	0.874776	0.87266	0.87058	0.868545	1.076586	1.07375
0.882511	0.880315	0.878113	0.875953	0.873824	0.871735	0.869695	1.07812	1.075305
0.606755	0.605371	0.603989	0.877179	0.875064	0.87297	0.870898	0.868831	0.86676
0.607514	0.606158	0.604811	0.878436	0.876343	0.874242	0.872129	0.870037	0.867989
			0.604283	0.60293	0.87549	0.873371	0.871268	0.8692
			0.605104	0.603754	0.876724	0.874627	0.872524	0.8704

0.605924	0.604576	0.877964	0.87586	0.873744	0.871591
0.606744	0.605399	0.879221	0.877063	0.874911	0.872766
		0.604834	0.603426	0.876089	0.87396
		0.605605	0.604222	0.877306	0.875203
		0.606377	0.605028	0.878544	0.87646
		0.607183	0.605845	0.604507	0.603161
		0.608008	0.606668	0.605326	0.603977
				0.606143	0.604795
				0.60696	0.605615
				0.607775	0.606434
				0.608587	0.60725
				0.6094	0.608065

								0.577429
								0.576505
								0.575561
					0.579922	0.578175	0.576423	1.222878
					0.578996	0.577214	0.575448	1.220967
					0.578125	0.576307	0.574515	1.594437
					0.57729	0.575438	0.573611	1.592164
			0.579939	0.578184	1.225916	1.222485	1.219076	1.769262
			0.578976	0.577207	1.224013	1.220642	1.217291	1.766743
	0.581617	0.579816	1.05509	1.052158	1.778053	1.77336	1.768733	1.95853
	0.580717	0.578922	1.053555	1.050635	1.77549	1.770818	1.76621	1.955755
	0.579825	0.57803	1.052043	1.049135	1.772948	1.768308	1.763725	1.953012
	0.578918	0.577095	1.779902	1.775126	1.965523	1.960423	1.955379	1.950378
	0.578	0.576141	1.777371	1.7726	1.962797	1.957744	1.952752	1.947799
0.578891	1.05332	1.050417	1.774765	1.770036	1.960026	1.954997	1.950039	2.062298
0.577905	1.051702	1.04883	1.772091	1.767438	1.957214	1.95219	1.947255	2.059372
0.576945	1.778917	1.77417	1.964603	1.959506	2.009002	2.003814	1.998721	2.056455
0.576021	1.776389	1.771673	1.961905	1.956814	2.006242	2.001093	1.99602	2.053539
1.584162	1.773884	1.769204	2.013933	2.008696	2.066541	2.061186	2.05589	2.103012
1.582048	1.771366	1.766712	2.011145	2.005962	2.063683	2.058391	2.053136	2.100157
1.579946	1.768865	1.76424	2.008385	2.003259	2.060852	2.055619	2.050405	2.097341
1.77104	1.961324	1.956242	2.068683	2.063356	2.058072	2.052835	2.047616	2.094487
1.768554	1.958658	1.953667	2.065871	2.060594	2.055327	2.05005	2.0448	2.091621
1.766105	2.010646	2.005494	2.063052	2.057795	2.104834	2.099445	2.094084	2.129229
1.763696	2.007934	2.00279	2.060228	2.05496	2.101926	2.09659	2.091275	2.126371
2.010404	2.068036	2.062712	2.057413	2.052146	2.099047	2.093743	2.088455	2.123511
2.007667	2.065198	2.05989	2.054614	2.049374	2.096223	2.090905	2.085606	2.120639
2.004946	2.062367	2.057074	2.05182	2.046605	2.0934	2.088068	2.08276	2.117773
2.002244	2.059515	2.054265	2.101161	2.095846	2.130924	2.125556	2.120219	2.114925

1.999576	2.056675	2.05146	2.098305	2.092971	2.127999	2.122666	2.117366	2.11209
2.059259	2.106098	2.100737	2.135806	2.130431	2.125087	2.119774	2.114497	2.197466
2.056624	2.103327	2.09783	2.132812	2.127488	2.122185	2.116881	2.111621	2.194579
2.053902	2.100542	2.095009	2.129921	2.124586	2.156295	2.150989	2.145729	2.191715
2.051078	2.097743	2.092292	2.127149	2.121721	2.15342	2.148167	2.142966	2.188878
2.100285	2.135257	2.129839	2.161526	2.156018	2.202088	2.196701	2.191369	2.186046
2.097381	2.132339	2.127012	2.158782	2.153344	2.199334	2.193878	2.188491	2.183193
2.094516	2.129438	2.124166	2.156021	2.150683	2.196613	2.191097	2.185654	2.180369
2.091782	2.163856	2.158507	2.153198	2.147935	2.193853	2.188377	2.182949	2.177629
2.089109	2.161121	2.155677	2.150357	2.145153	2.191075	2.185689	2.180312	2.174942
2.126519	2.158382	2.152938	2.19896	2.193593	2.188254	2.18294	2.177627	2.172273
2.123675	2.155645	2.150278	2.196197	2.190752	2.18539	2.180144	2.174911	2.169627
2.158175	2.152878	2.147592	2.193452	2.187988	2.182616	2.177374	2.17216	2.239683
2.155305	2.150053	2.144867	2.190736	2.185336	2.179969	2.17464	2.169354	2.236765
2.152505	2.198594	2.193308	2.187991	2.182661	2.177314	2.171923	2.166578	2.233863
2.149875	2.195831	2.190475	2.185157	2.179883	2.174583	2.169229	2.163898	2.231021
2.147291	2.193109	2.187657	2.182315	2.177072	2.171818	2.166533	2.161244	2.228214
2.144619	2.190382	2.184921	2.179555	2.174271	2.242026	2.23651	2.231002	2.263692
2.141909	2.187664	2.182239	2.176847	2.171478	2.239085	2.233603	2.228158	2.260876
2.190304	2.184954	2.179578	2.174187	2.168776	2.236239	2.230742	2.225306	2.258013
2.187565	2.182257	2.176947	2.171581	2.166161	2.233482	2.227919	2.222439	2.255102
2.184799	2.179522	2.174274	2.205451	2.199818	2.230769	2.225159	2.219638	2.358216
2.181969	2.176718	2.171532	2.202675	2.197145	2.228116	2.222491	2.216932	2.354734
2.179173	2.173925	2.168769	2.199868	2.194444	2.225446	2.219836	2.214269	2.351346
2.176482	2.171191	2.165986	2.233564	2.228139	2.368464	2.361558	2.354753	2.348102
2.17385	2.1685	2.163218	2.230664	2.225276	2.364856	2.358134	2.351494	2.344944
2.171248	2.202415	2.196849	2.227882	2.222498	2.361359	2.354782	2.348272	2.341793
2.168677	2.199765	2.194201	2.225183	2.219773	2.357935	2.35148	2.345086	2.338665
2.202699	2.197098	2.191537	2.367827	2.361069	2.354497	2.34815	2.341866	2.335525
2.200003	2.194408	2.188852	2.364337	2.357569	2.351028	2.344778	2.338603	2.332369
2.197326	2.191746	2.186197	2.360916	2.354163	2.347656	2.341469	2.335369	2.329227
2.194686	2.189136	2.183596	2.357607	2.350913	2.34445	2.33828	2.332206	2.326132
2.192061	2.186544	2.181014	2.354349	2.347727	2.341304	2.33512	2.329039	2.323014
2.189438	2.364698	2.357811	2.351099	2.34454	2.338136	2.331901	2.32578	2.31978
2.186822	2.36133	2.354521	2.347875	2.341376	2.334982	2.32868	2.322502	2.316511
2.184205	2.358018	2.35128	2.344689	2.338233	2.33186	2.325547	2.319361	2.347636
2.181588	2.354753	2.348085	2.34154	2.335111	2.328766	2.322487	2.316328	2.344365
2.296186	2.351496	2.344895	2.338402	2.33201	2.325698	2.319454	2.313313	2.341092
2.292917	2.348236	2.341702	2.33527	2.328932	2.32266	2.316447	2.310307	2.337791
2.351536	2.345016	2.338541	2.332161	2.325871	2.354482	2.347748	2.341105	2.334549
2.348327	2.341863	2.335428	2.329085	2.322827	2.351214	2.344529	2.337932	2.331421
2.345165	2.33875	2.332349	2.326036	2.319802	2.34796	2.341328	2.334788	2.328341
2.342035	2.335655	2.329297	2.358136	2.35139	2.344728	2.338141	2.331649	2.325244
2.338939	2.332583	2.326268	2.354891	2.348157	2.341517	2.334973	2.328526	2.322156
2.335849	2.329515	2.323234	2.35164	2.344943	2.338343	2.331843	2.325439	2.360685
2.332769	2.326454	2.320195	2.348386	2.34175	2.335205	2.328752	2.322389	2.35752
2.329704	2.358588	2.351825	2.345153	2.338571	2.332075	2.325662	2.319339	2.354373
2.32666	2.355319	2.348583	2.341946	2.335403	2.328943	2.322554	2.316269	2.351222
2.323623	2.352064	2.345362	2.338761	2.332257	2.325834	2.319476	2.31324	2.348142
2.320584	2.348807	2.342154	2.335598	2.329137	2.36469	2.358014	2.3515	2.345181

2.317552	2.345563	2.338965	2.332459	2.326044	2.361504	2.354891	2.348471	2.342316
2.348988	2.342343	2.335792	2.329336	2.322974	2.358363	2.351863	2.34557	2.36688
2.345748	2.339145	2.332639	2.326236	2.319934	2.35527	2.348918	2.342779	2.36417
2.342532	2.335983	2.329535	2.365255	2.358677	2.379863	2.373509	2.367368	2.361476
2.339318	2.332838	2.326466	2.362136	2.355678	2.376928	2.370663	2.364605	2.358798
2.336273	2.329847	2.32353	2.359119	2.352737	2.374016	2.367844	2.361878	2.37937
2.333533	2.327131	2.320825	2.356261	2.349856	2.371091	2.365026	2.359181	2.376743
2.33082	2.324435	2.318133	2.353425	2.347006	2.368201	2.362244	2.356521	2.374153
2.327894	2.321533	2.31525	2.378027	2.371561	2.365379	2.35952	2.353893	2.371588
2.324875	2.318541	2.312289	2.374972	2.368657	2.362606	2.356845	2.351308	2.369059
2.321837	2.385026	2.378345	2.371932	2.365766	2.382996	2.377334	2.37187	2.366585
2.318769	2.381796	2.375193	2.368899	2.362889	2.380244	2.374736	2.369394	2.364161
2.315748	2.378647	2.37216	2.365995	2.36013	2.377585	2.372197	2.366947	2.361755
2.312755	2.375568	2.369242	2.363218	2.357484	2.375009	2.369699	2.364509	2.359346
2.379043	2.372642	2.36648	2.383671	2.378034	2.37258	2.367327	2.362174	2.357032
2.376255	2.369971	2.363954	2.38126	2.375762	2.370404	2.365186	2.360046	2.354919
2.373557	2.367393	2.361515	2.378927	2.373556	2.36828	2.363083	2.357945	2.352829
2.370765	2.364736	2.358986	2.376472	2.371187	2.365968	2.360789	2.355659	2.350557
2.367952	2.362066	2.356442	2.373978	2.36875	2.363566	2.3584	2.353278	2.348192
2.36516	2.382406	2.376892	2.371527	2.366331	2.361168	2.356014	2.350902	2.373108
2.362376	2.379738	2.374391	2.369106	2.363917	2.358758	2.353614	2.348512	2.370678
2.359809	2.37726	2.372029	2.366801	2.361614	2.356457	2.35132	2.346229	2.368376
2.357558	2.375062	2.369879	2.364677	2.359491	2.354335	2.349199	2.344121	2.36628
2.355353	2.372887	2.36772	2.36253	2.357346	2.352194	2.347059	2.341994	2.364164
2.375869	2.370561	2.365382	2.360197	2.355024	2.377215	2.371995	2.366857	2.361844
2.373488	2.368217	2.363012	2.357826	2.352664	2.37482	2.36961	2.364485	2.359498
2.371247	2.365996	2.360778	2.355591	2.350434	2.372574	2.367416	2.362333	2.357343
2.369087	2.36384	2.358617	2.353428	2.348272	2.37041	2.365335	2.360313	2.355304
2.366795	2.361559	2.356341	2.351162	2.346018	2.368147	2.363132	2.358173	2.35322
2.364354	2.359132	2.353934	2.348775	2.343651	2.365764	2.360793	2.355906	2.351094
2.361989	2.356783	2.351606	2.373727	2.368557	2.36349	2.358536	2.353684	2.348959
2.359805	2.354613	2.349455	2.371585	2.366478	2.361435	2.356446	2.351551	2.346795
2.357623	2.352444	2.347309	2.369451	2.36441	2.359401	2.354399	2.349473	2.344674
2.355282	2.35011	2.345004	2.367142	2.362157	2.357219	2.352314	2.347469	2.342701
2.352905	2.347741	2.342672	2.364802	2.359866	2.355007	2.35023	2.345498	2.340778
2.350677	2.372767	2.367668	2.362668	2.357771	2.352936	2.348168	2.343439	2.347429
2.348557	2.370674	2.365641	2.360686	2.355817	2.350969	2.346124	2.34132	2.345255
2.346397	2.368505	2.363505	2.358589	2.353766	2.357721	2.352815	2.347952	2.343172
2.344172	2.366219	2.36121	2.356326	2.351575	2.355571	2.350759	2.345971	2.341204
2.369139	2.364002	2.358997	2.362955	2.358191	2.353452	2.348727	2.344006	2.339245
2.367013	2.361951	2.356993	2.360932	2.356161	2.351431	2.346748	2.342053	2.337263
2.364927	2.359955	2.355056	2.358973	2.354172	2.349425	2.344754	2.340069	2.335265
2.362845	2.366772	2.361871	2.356992	2.352139	2.347349	2.342643	2.337955	2.333219
2.360773	2.364716	2.359878	2.355	2.350093	2.345251	2.340483	2.335783	2.331155
2.367493	2.362577	2.35775	2.352898	2.348035	2.343216	2.338436	2.333739	2.329178
2.365322	2.360357	2.355492	2.350691	2.345962	2.341238	2.336485	2.331804	2.327279
2.363194	2.358218	2.353346	2.348579	2.343921	2.339248	2.334513	2.32984	2.335012
2.361121	2.3562	2.351369	2.34661	2.341929	2.337237	2.332494	2.327811	2.332973
2.359075	2.354215	2.349428	2.344667	2.339939	2.345063	2.340284	2.335566	2.356717
2.357044	2.35223	2.347479	2.342706	2.337921	2.343043	2.338302	2.33363	2.354748

2.355032	2.350248	2.345518	2.340731	2.335897	2.341029	2.336338	2.331723	2.352824
2.353042	2.348245	2.343483	2.338692	2.333876	2.33905	2.334404	2.329832	2.424925
2.351066	2.346227	2.341402	2.336614	2.331859	2.337098	2.332496	2.327958	2.422623
2.34904	2.344187	2.339348	2.344595	2.339848	2.360908	2.356255	2.351647	2.420292
2.34696	2.342119	2.337315	2.342618	2.337927	2.358972	2.354344	2.349735	2.417934
2.344892	2.340077	2.335316	2.366448	2.361725	2.357043	2.352406	2.34778	2.434356
2.342849	2.338077	2.333357	2.364477	2.359794	2.355113	2.350411	2.345738	2.431948
2.340829	2.336102	2.331426	2.362528	2.357872	2.353185	2.348423	2.34371	2.429572
2.338842	2.344307	2.339626	2.360605	2.355953	2.351267	2.3465	2.341785	2.42728
2.336883	2.342383	2.337751	2.358691	2.354025	2.349338	2.344594	2.339902	2.42504
2.334947	2.366145	2.361452	2.356742	2.352029	2.420565	2.41497	2.409472	2.422815
2.333034	2.364202	2.359516	2.354771	2.349983	2.418095	2.412608	2.407229	2.420614
2.331121	2.362264	2.357577	2.352817	2.347997	2.415736	2.410315	2.40501	2.418432
2.329209	2.360336	2.355646	2.350894	2.346091	2.41351	2.4081	2.402815	2.416269
2.363131	2.358389	2.353676	2.348935	2.344169	2.411303	2.405915	2.400653	2.414125
2.361177	2.356397	2.35162	2.346884	2.342184	2.409078	2.403752	2.398536	2.412008
2.359228	2.354406	2.349566	2.344836	2.3402	2.406862	2.401608	2.396442	2.409898
2.357291	2.352462	2.347607	2.342885	2.338274	2.423341	2.418093	2.412913	2.407766
2.355363	2.350544	2.345701	2.340988	2.336383	2.421133	2.415945	2.410809	2.405633
2.353435	2.348632	2.343807	2.339111	2.334522	2.418964	2.413817	2.408715	2.403549
2.35151	2.346728	2.34193	2.337258	2.332692	2.416833	2.411706	2.40663	2.401508
2.349587	2.344831	2.340073	2.4067	2.401348	2.41472	2.409618	2.40457	2.399483
2.347664	2.342933	2.338228	2.404571	2.399242	2.41262	2.407553	2.402537	2.397472
2.345754	2.341062	2.336425	2.4025	2.397177	2.410551	2.405512	2.400522	2.395474
2.343865	2.339243	2.334704	2.400552	2.395191	2.40853	2.403499	2.398522	2.393487
2.341992	2.337445	2.333002	2.398629	2.393232	2.406534	2.401501	2.396531	2.391512
2.340135	2.33563	2.331231	2.415214	2.409795	2.404535	2.399498	2.394529	2.389536
2.338291	2.333808	2.329422	2.413112	2.40776	2.402539	2.397496	2.392524	2.38757
2.336442	2.331985	2.327622	2.411009	2.405715	2.400534	2.395499	2.390546	2.385661
2.334583	2.330156	2.325828	2.408902	2.403658	2.39852	2.393507	2.388594	2.383813
2.332773	2.328354	2.324036	2.406793	2.401604	2.396511	2.391523	2.386644	2.381938
2.331038	2.326591	2.322245	2.40467	2.399538	2.394492	2.389532	2.384679	2.380003
2.329321	2.324845	2.320465	2.40258	2.397514	2.392519	2.387581	2.382741	2.378069
2.327572	2.39227	2.387126	2.400568	2.3956	2.390663	2.385728	2.380873	2.376177
2.325827	2.390243	2.385123	2.398601	2.393729	2.388849	2.383914	2.379036	2.374302
2.324133	2.388278	2.383165	2.396644	2.391803	2.386951	2.382041	2.377173	2.372414
2.322474	2.386362	2.381248	2.394702	2.389847	2.385	2.380133	2.375299	2.370523
2.320767	2.402916	2.397764	2.392757	2.387886	2.383054	2.378255	2.373474	2.368683
2.318994	2.400887	2.395786	2.390796	2.385911	2.381107	2.376413	2.371715	2.366908
2.317253	2.398901	2.393858	2.388888	2.383991	2.379199	2.37456	2.369908	2.383049
2.315591	2.397015	2.39204	2.387089	2.382169	2.377346	2.372662	2.367981	2.381203
2.313944	2.39514	2.390221	2.385298	2.380383	2.375535	2.370778	2.366043	2.37936
2.379734	2.393184	2.388277	2.38341	2.378593	2.391932	2.387124	2.382348	2.377623
2.377779	2.391206	2.386286	2.381482	2.376798	2.390268	2.38542	2.380608	2.375929
2.37591	2.389315	2.384376	2.379578	2.374918	2.388476	2.383633	2.378828	2.374169
2.374114	2.387495	2.382529	2.377688	2.372966	2.386577	2.38178	2.377019	2.372356
2.372299	2.385678	2.380726	2.394289	2.38952	2.384765	2.380007	2.375283	2.370611
2.370445	2.383855	2.378982	2.39264	2.387836	2.38307	2.378346	2.373647	2.368956
2.368609	2.39514	2.390338	2.390994	2.386186	2.38142	2.37671	2.372021	2.367325
2.366826	2.393397	2.388591	2.389309	2.384561	2.379823	2.375082	2.370369	2.365703

2.365054	2.391656	2.386827	2.387601	2.382924	2.378218	2.373441	2.368699	2.364069
2.363257	2.389887	2.385046	2.385847	2.381177	2.37648	2.371716	2.366984	2.374605
2.361446	2.388106	2.383259	2.384068	2.379371	2.374671	2.369946	2.365244	2.372901
2.373723	2.386348	2.381532	2.382368	2.377647	2.372942	2.368246	2.363567	2.387634
2.371939	2.384611	2.379863	2.380742	2.375996	2.371282	2.36661	2.361951	2.386014
2.387657	2.382897	2.378199	2.379124	2.374372	2.381992	2.377363	2.372755	2.384398
2.385939	2.381209	2.376526	2.377498	2.372779	2.380451	2.375778	2.37114	2.382778
2.384248	2.379539	2.374867	2.37588	2.371182	2.378904	2.37419	2.369528	2.381158
2.382558	2.377872	2.373242	2.374285	2.369548	2.377299	2.372594	2.367945	2.37953
2.380905	2.376227	2.371624	2.372689	2.367898	2.375678	2.370997	2.366375	2.377904
2.379371	2.374649	2.369964	2.383552	2.37881	2.3741	2.369419	2.364801	2.376296
2.377923	2.373119	2.368279	2.381906	2.37722	2.372544	2.367852	2.363228	2.374703
2.376387	2.371561	2.366654	2.380316	2.375604	2.387105	2.382365	2.377694	2.373133
2.37476	2.36998	2.365106	2.378794	2.373952	2.385368	2.380721	2.376143	2.371593
2.373077	2.386902	2.382111	2.377265	2.372351	2.383694	2.379097	2.374569	2.370036
2.371318	2.385236	2.380509	2.375718	2.37086	2.38215	2.377498	2.372929	2.368431
2.36955	2.383544	2.378862	2.37414	2.369379	2.38064	2.37593	2.371308	2.366832
2.367844	2.381879	2.377161	2.372476	2.367819	2.379103	2.374423	2.369803	2.365284
2.36617	2.380232	2.375445	2.370782	2.366226	2.377552	2.372941	2.368349	2.363765
2.38344	2.378651	2.373843	2.385189	2.380544	2.375945	2.371395	2.366851	2.362265
2.381911	2.377128	2.372334	2.383592	2.378895	2.374288	2.3698	2.365323	2.360785
2.38032	2.375557	2.370806	2.382017	2.37731	2.372701	2.368227	2.363768	2.377015
2.378636	2.37391	2.36925	2.380474	2.375813	2.37121	2.366678	2.36217	2.375323
2.376981	2.37228	2.367682	2.378915	2.374311	2.369726	2.365152	2.3606	2.373643
2.375429	2.370708	2.366079	2.377292	2.372758	2.368222	2.363669	2.359111	2.372013
2.373906	2.369168	2.364496	2.375673	2.371191	2.3667	2.362187	2.357641	2.370408
2.372339	2.367648	2.363013	2.374132	2.369624	2.365126	2.360641	2.356121	2.368823
2.370766	2.366154	2.361595	2.372643	2.368063	2.363529	2.359065	2.354581	2.367255
2.369276	2.380426	2.375804	2.371184	2.366571	2.379775	2.375119	2.37046	2.365697
2.36787	2.378985	2.374377	2.369762	2.365145	2.37825	2.373575	2.368904	2.364152
2.366432	2.377514	2.372928	2.368326	2.363716	2.376726	2.372008	2.367304	2.362562
2.364919	2.375978	2.371438	2.366863	2.362268	2.375185	2.370386	2.365617	2.360895
2.363426	2.374452	2.369945	2.365394	2.36082	2.373653	2.368783	2.363954	2.359235
2.377607	2.372993	2.368478	2.363936	2.359385	2.37215	2.367256	2.362393	2.357629
2.37619	2.371551	2.367006	2.362464	2.357941	2.370649	2.36576	2.360881	2.35606
2.374751	2.370069	2.365473	2.378525	2.373846	2.369101	2.364242	2.359379	2.354541
2.373304	2.368565	2.363901	2.376859	2.372222	2.367522	2.362717	2.357895	2.353065
2.371857	2.367093	2.362401	2.375274	2.370648	2.365973	2.36121	2.356422	2.351598
2.37041	2.365656	2.360977	2.373774	2.369127	2.36445	2.359722	2.354964	2.350144
2.368958	2.381906	2.377051	2.37229	2.367629	2.362949	2.358239	2.353498	2.348682
2.367494	2.380367	2.375545	2.370803	2.366153	2.361478	2.356761	2.352014	2.347202
2.366036	2.37884	2.374044	2.369322	2.364686	2.360014	2.355283	2.350525	2.345715
2.364604	2.377342	2.372552	2.367839	2.363213	2.358542	2.353799	2.349031	2.344222
2.363183	2.375859	2.371067	2.366357	2.361737	2.357066	2.352312	2.347536	2.342728
2.379252	2.374374	2.369587	2.364876	2.36025	2.355576	2.350824	2.346054	2.341254
2.377743	2.37289	2.36811	2.363397	2.358754	2.354076	2.349336	2.344583	2.339796
2.376222	2.371391	2.366621	2.361906	2.357252	2.35257	2.347841	2.343105	2.338344
2.374685	2.369872	2.365113	2.360401	2.35574	2.351058	2.346336	2.341616	2.336897
2.373154	2.368356	2.363609	2.358899	2.354231	2.349549	2.344833	2.340131	2.335454
2.371634	2.366847	2.362109	2.357397	2.352718	2.348035	2.343331	2.338651	2.334011

2.370132	2.36536	2.360633	2.355921	2.351228	2.34654	2.341845	2.337181	2.332571
2.368663	2.363923	2.359226	2.35452	2.349814	2.345112	2.340403	2.335734	2.331139
2.367225	2.362523	2.35786	2.353163	2.348441	2.343722	2.338989	2.334304	2.329713
2.365831	2.361143	2.356476	2.351768	2.347026	2.342294	2.33756	2.332878	2.328295
2.364483	2.359789	2.355081	2.350344	2.345577	2.340837	2.336119	2.331458	2.326884
2.363117	2.358407	2.353663	2.348899	2.344111	2.339369	2.334676	2.330041	2.325477
2.361722	2.356988	2.352215	2.347428	2.34262	2.337879	2.333225	2.328627	2.324069
2.360301	2.355543	2.350751	2.345954	2.341144	2.336415	2.331792	2.327223	2.322677
2.35884	2.354064	2.349267	2.344484	2.339709	2.335003	2.330379	2.325816	2.32131
2.357361	2.352574	2.347781	2.343029	2.338309	2.333636	2.329005	2.32444	2.319971
2.355892	2.351105	2.346325	2.341612	2.336958	2.332336	2.327736	2.32318	2.318689
2.354431	2.349657	2.344896	2.34023	2.335649	2.331087	2.326536	2.321995	2.317445
2.353048	2.348304	2.343569	2.33892	2.334347	2.32979	2.325243	2.320697	2.316122
2.351753	2.347062	2.342363	2.337697	2.33306	2.328445	2.323852	2.319278	2.314714
2.350441	2.345779	2.341102	2.336422	2.331737	2.327079	2.322452	2.31786	2.313308
2.349077	2.344396	2.339721	2.335038	2.330349	2.325687	2.321054	2.31646	2.311909
2.347701	2.342991	2.338308	2.333625	2.328944	2.324294	2.319675	2.315096	2.310555
2.346322	2.341596	2.336893	2.332207	2.327537	2.322915	2.318349	2.313822	2.309323
2.344948	2.340218	2.335494	2.330806	2.326148	2.321559	2.317054	2.312588	2.308139
2.343627	2.338927	2.334223	2.329542	2.324879	2.320284	2.315776	2.311308	2.306862
2.342346	2.337706	2.333053	2.328386	2.323704	2.319076	2.314515	2.309998	2.305523
2.341048	2.336429	2.331796	2.327136	2.322447	2.317803	2.313215	2.308671	2.304177
2.339728	2.335078	2.330424	2.325756	2.321074	2.316436	2.311852	2.307312	2.302812
2.33839	2.33372	2.329061	2.324401	2.31974	2.315119	2.310544	2.306009	2.301505
2.337002	2.332364	2.327763	2.323153	2.31854	2.31395	2.309383	2.304845	2.300331
2.335636	2.331031	2.326479	2.321917	2.317352	2.312792	2.308233	2.303692	2.299172
2.334402	2.329765	2.325173	2.320598	2.316046	2.311495	2.306938	2.302397	2.297883
2.333233	2.328541	2.323869	2.319255	2.314695	2.310142	2.305582	2.301045	2.296542
2.331996	2.327314	2.322639	2.318019	2.313447	2.308887	2.30433	2.299798	2.295301
2.330706	2.326093	2.321482	2.316882	2.31229	2.307719	2.303168	2.298643	2.294148
2.329397	2.324818	2.320245	2.315658	2.311058	2.306482	2.301932	2.297409	2.292919
2.328057	2.323452	2.318867	2.314281	2.309694	2.305123	2.300563	2.296034	2.291556
2.326751	2.322118	2.317517	2.312927	2.308353	2.303788	2.299222	2.29469	2.29022
2.325557	2.320922	2.31632	2.311729	2.30715	2.302586	2.298029	2.293505	2.289032
2.324376	2.319747	2.315148	2.310554	2.305968	2.301404	2.296862	2.292349	2.287871
2.323053	2.318429	2.313829	2.309236	2.304652	2.300093	2.295558	2.291052	2.294503
2.321653	2.317036	2.312434	2.307845	2.303267	2.298714	2.294183	2.289683	2.293126
2.320378	2.315766	2.311167	2.306578	2.301998	2.297446	2.292924	2.288433	2.291869
2.319229	2.314623	2.310031	2.305439	2.300846	2.296292	2.291782	2.287305	2.290734
2.318091	2.313488	2.308897	2.304302	2.299704	2.29515	2.290649	2.286181	2.303145
2.316964	2.312358	2.307756	2.303162	2.298573	2.294024	2.289522	2.285055	2.30204
2.315786	2.311176	2.306564	2.301971	2.297393	2.292852	2.28835	2.283884	2.30089
2.314446	2.309834	2.30522	2.300631	2.29606	2.299442	2.294917	2.290426	2.299602
2.313055	2.308442	2.303831	2.299246	2.294681	2.298054	2.29354	2.28906	2.298274
2.311812	2.307198	2.302587	2.298006	2.29345	2.31049	2.30599	2.301523	2.297085
2.310673	2.306059	2.301446	2.296869	2.292322	2.30939	2.304897	2.300435	2.295994
2.309542	2.304931	2.300323	2.295752	2.291213	2.308303	2.303812	2.29935	2.294911
2.308418	2.303815	2.299219	2.294657	2.290125	2.307233	2.302734	2.298266	2.293834
2.307302	2.302706	2.298121	2.308097	2.303574	2.30616	2.301657	2.297186	2.292757
2.306191	2.3016	2.297018	2.307021	2.302498	2.305074	2.30058	2.296114	2.291679

2.305084	2.300495	2.295913	2.305938	2.301413	2.303977	2.299493	2.295034	2.290591
2.303975	2.299388	2.294804	2.304857	2.300327	2.308187	2.303699	2.299234	2.294788
2.302867	2.298277	2.293689	2.303767	2.299227	2.307061	2.302569	2.298104	2.293666
2.308116	2.317479	2.312881	2.30829	2.303701	2.305849	2.301337	2.296862	2.304817
2.307	2.316388	2.311772	2.307136	2.302478	2.304558	2.300007	2.295512	2.303458
2.319901	2.315275	2.310619	2.305949	2.301259	2.303305	2.298749	2.294256	2.302189
2.31881	2.314126	2.309397	2.304708	2.300043	2.302105	2.297595	2.293139	2.301054
2.317711	2.312982	2.308197	2.303496	2.298859	2.300937	2.296473	2.292054	2.299955
2.316599	2.311866	2.307079	2.302376	2.297733	2.299802	2.295355	2.290955	2.298856
2.315481	2.310759	2.30599	2.30129	2.296639	2.298693	2.294253	2.289864	2.297769
2.314366	2.309643	2.304877	2.300186	2.29555	2.310083	2.305584	2.301133	2.296685
2.313253	2.308526	2.303759	2.299081	2.294472	2.308995	2.304501	2.300052	2.295605
2.312145	2.307422	2.302661	2.297992	2.293396	2.307899	2.303406	2.298958	2.294515
2.311041	2.306327	2.301577	2.296913	2.292318	2.306794	2.302299	2.297851	2.293415
2.309945	2.305262	2.300548	2.295895	2.29129	2.305723	2.301205	2.296742	2.292312
2.308862	2.304249	2.299616	2.29498	2.290335	2.304698	2.300123	2.295618	2.291195
2.30778	2.303231	2.298674	2.294064	2.289402	2.303708	2.299078	2.294527	2.290098
2.306693	2.302163	2.297631	2.299625	2.295002	2.302774	2.298112	2.293515	2.289041
2.305601	2.301072	2.296545	2.298574	2.294043	2.301851	2.29718	2.292551	2.28802
2.304496	2.299974	2.295459	2.297499	2.293006	2.300844	2.296217	2.29161	2.287055
2.30338	2.298867	2.294364	2.296399	2.291907	2.299775	2.295234	2.290694	2.286135
2.302282	2.297769	2.293263	2.30776	2.303202	2.298675	2.294195	2.289712	2.292656
2.301205	2.296681	2.292153	2.306614	2.302052	2.297539	2.293091	2.288655	2.291661
2.300135	2.295595	2.291049	2.305472	2.300904	2.296399	2.291977	2.287579	2.290631
2.299048	2.294489	2.289941	2.304333	2.29976	2.295263	2.290868	2.286498	2.28955
2.298002	2.29342	2.288865	2.30322	2.298639	2.294143	2.289762	2.285408	2.288445
2.297091	2.298978	2.294346	2.302169	2.297563	2.293049	2.288656	2.284298	2.287324
2.296237	2.298086	2.293397	2.301164	2.296531	2.291991	2.287569	2.283191	2.286199
2.295259	2.297115	2.292441	2.300205	2.295585	2.291039	2.286582	2.282164	2.291401
2.294184	2.296085	2.291488	2.299295	2.294723	2.290185	2.285684	2.281206	2.290364
2.29309	2.295008	2.290462	2.298303	2.293779	2.29592	2.291409	2.286905	2.289384
2.291958	2.293851	2.289322	2.297191	2.292719	2.294913	2.290458	2.286	2.288493
2.290894	2.305216	2.30061	2.296086	2.291643	2.293874	2.289464	2.285047	2.287558
2.290009	2.304271	2.29961	2.295047	2.290575	2.292793	2.288377	2.283967	2.286481
2.289158	2.303369	2.298655	2.294048	2.289535	2.291725	2.287283	2.28286	2.285364
2.294585	2.302383	2.297705	2.299794	2.295279	2.290784	2.286306	2.281843	2.284297
2.293499	2.301357	2.296761	2.2989	2.294411	2.289914	2.285394	2.280877	2.283258
2.292458	2.307005	2.302435	2.297914	2.293442	2.296002	2.291433	2.286863	2.282286
2.291452	2.305949	2.301358	2.296832	2.292373	2.294954	2.290444	2.285929	2.281391
2.302898	2.304945	2.300349	2.295816	2.291346	2.293918	2.289433	2.284946	2.280438
2.301937	2.304032	2.299475	2.294932	2.290408	2.292912	2.288378	2.283861	2.279364
2.300981	2.303117	2.298589	2.29404	2.289477	2.291923	2.287347	2.282804	2.278314
2.306705	2.302114	2.297571	2.29303	2.288496	2.29096	2.286413	2.281892	2.277414
2.305725	2.301095	2.296524	2.291992	2.287504	2.290005	2.285505	2.28102	2.276551
2.296163	2.300152	2.295581	2.291043	2.28654	2.289016	2.284514	2.280028	2.275563
2.295244	2.299259	2.294706	2.29015	2.285595	2.288008	2.283472	2.278966	2.2745
2.294352	2.29837	2.293811	2.289241	2.284661	2.287042	2.282499	2.27799	2.273524
2.293501	2.297491	2.292895	2.288312	2.28374	2.286125	2.281607	2.277118	2.272655
2.292604	2.296579	2.291971	2.287391	2.282833	2.285219	2.280711	2.276226	2.271756
2.291601	2.295593	2.291036	2.286489	2.281951	2.284319	2.279781	2.27526	2.270759

2.290593	2.294604	2.290104	2.28559	2.281069	2.283416	2.278845	2.27429	2.269761
2.28967	2.293687	2.289206	2.284694	2.280161	2.282491	2.277917	2.273363	2.284067
2.28878	2.292794	2.288309	2.283788	2.279239	2.281558	2.276996	2.272458	2.283164
2.287852	2.291837	2.28733	2.289752	2.285189	2.280638	2.276097	2.271571	2.290008
2.286898	2.290829	2.286282	2.288716	2.284224	2.279728	2.275219	2.270702	2.289068
2.285966	2.289875	2.285316	2.287755	2.283301	2.278829	2.274329	2.269808	2.288115
2.285061	2.288992	2.28446	2.286898	2.282437	2.27795	2.273423	2.26888	2.28714
2.284175	2.288128	2.283622	2.286048	2.281564	2.292369	2.287748	2.283122	2.286178
2.283308	2.287267	2.282772	2.285166	2.280644	2.291406	2.286792	2.282184	2.28525
2.282455	2.286412	2.281913	2.28427	2.279712	2.290436	2.28584	2.28126	2.28434
2.281611	2.285552	2.281016	2.283345	2.278786	2.289499	2.284923	2.280364	2.283442
2.280776	2.284692	2.280101	2.282408	2.277866	2.288583	2.284031	2.279488	2.282554
2.279938	2.283853	2.279245	2.281537	2.276997	2.287699	2.283158	2.278617	2.281642
2.279101	2.283036	2.278448	2.280732	2.276179	2.286846	2.282306	2.277754	2.280707
2.278227	2.289092	2.28448	2.279902	2.275355	2.285999	2.281453	2.276886	2.299437
2.277287	2.288149	2.283569	2.279023	2.274514	2.285158	2.280601	2.27602	2.298472
2.276363	2.287216	2.28266	2.278142	2.273668	2.284307	2.279728	2.275126	2.297487
2.284045	2.286351	2.281793	2.292527	2.287993	2.283424	2.278796	2.274158	2.296422
2.283228	2.285518	2.280948	2.291653	2.28712	2.282533	2.277853	2.273177	2.295341
2.282419	2.284697	2.280124	2.290806	2.286271	2.289285	2.284531	2.279786	2.294341
2.281622	2.283892	2.279319	2.28998	2.285439	2.288431	2.283675	2.278927	2.293394
2.28771	2.283082	2.278508	2.289147	2.284601	2.287573	2.282818	2.27807	2.292453
2.286892	2.282267	2.277692	2.288311	2.28376	2.286714	2.281961	2.277217	2.291521
2.286069	2.281447	2.276871	2.287468	2.282914	2.305871	2.30073	2.295626	2.302228
2.285241	2.280621	2.276043	2.286619	2.282061	2.304921	2.299785	2.294683	2.301269
2.284407	2.279788	2.275207	2.285762	2.281201	2.303965	2.298834	2.293734	2.300305
2.275419	2.278938	2.274356	2.284891	2.280328	2.302995	2.297869	2.292771	2.299326
2.274568	2.278083	2.273499	2.284014	2.27945	2.302023	2.296902	2.291805	2.298345
2.273741	2.292421	2.287757	2.283147	2.27859	2.301079	2.295964	2.290867	2.297382
2.272934	2.291578	2.286898	2.282286	2.277744	2.300158	2.29505	2.289952	2.296434
2.272171	2.290787	2.286084	2.281461	2.276912	2.299243	2.294167	2.289091	2.295537
2.271473	2.290068	2.285333	2.280681	2.2761	2.298336	2.29333	2.28831	2.294715
2.270752	2.28933	2.284577	2.300829	2.295856	2.309044	2.304032	2.298998	2.293871
2.269958	2.288517	2.283775	2.299937	2.294935	2.308083	2.303088	2.298072	2.292949
2.269137	2.287682	2.282969	2.299056	2.294025	2.307125	2.302129	2.297116	2.292002
2.268311	2.286863	2.282202	2.29823	2.293171	2.30621	2.301198	2.296175	2.300488
2.267479	2.286048	2.281448	2.297429	2.292346	2.305318	2.300277	2.295233	2.299542
2.282267	2.285238	2.280654	2.296573	2.291484	2.304406	2.299338	2.294284	2.298618
2.28149	2.284435	2.279822	2.295665	2.290589	2.303475	2.298381	2.293323	2.297715
2.280703	2.283624	2.278986	2.294761	2.289723	2.302592	2.297477	2.292404	2.296818
2.279883	2.282791	2.278137	2.293863	2.288915	2.301795	2.296652	2.291539	2.295916
2.279086	2.281984	2.277316	2.292979	2.288094	2.300985	2.295839	2.290708	2.295042
2.278351	2.273684	2.269046	2.292115	2.287203	2.300087	2.295007	2.289926	2.294234
2.277642	2.27297	2.268319	2.291276	2.286301	2.299164	2.294162	2.289147	2.293445
2.27692	2.272255	2.267614	2.290489	2.285468	2.298278	2.293276	2.288276	2.292607
2.276196	2.271547	2.26693	2.28974	2.284679	2.297415	2.292364	2.287347	2.291739
2.275484	2.270844	2.266227	2.288965	2.2839	2.296587	2.291499	2.286459	2.290868
2.27479	2.270153	2.265504	2.288164	2.283132	2.2958	2.290684	2.285612	2.289989
2.274051	2.290505	2.28546	2.305215	2.300112	2.304509	2.299341	2.294209	2.289137
2.273222	2.289651	2.284694	2.304445	2.299308	2.303688	2.298535	2.293415	2.288335

2.272391	2.288783	2.283909	2.303662	2.298502	2.302871	2.297734	2.292627	2.287547
2.271625	2.287925	2.28304	2.302798	2.29769	2.302084	2.296936	2.291817	2.286749
2.270894	2.28708	2.28215	2.301912	2.296878	2.301306	2.296141	2.291003	2.285953
2.27019	2.286305	2.281376	2.301116	2.296077	2.300501	2.29534	2.290208	2.285164
2.269515	2.285585	2.280688	2.300387	2.295286	2.299677	2.294536	2.289427	2.284379
2.268815	2.284814	2.279926	2.299601	2.294488	2.29887	2.293734	2.288634	2.283592
2.268074	2.283966	2.279055	2.298735	2.293681	2.298085	2.292937	2.287824	2.2828
2.267377	2.283162	2.278223	2.292058	2.287066	2.297296	2.292142	2.287023	2.282012
2.266792	2.282483	2.27751	2.29129	2.286268	2.296486	2.291348	2.286246	2.281232
2.266222	2.281825	2.276819	2.290536	2.285472	2.295674	2.290558	2.285478	2.280461
2.26557	2.28109	2.276058	2.289742	2.284675	2.294876	2.289773	2.284708	2.279703
2.264877	2.280319	2.275271	2.288934	2.283881	2.294084	2.288994	2.283943	2.278958
2.284603	2.292508	2.287419	2.288151	2.283086	2.293286	2.288218	2.283194	2.278238
2.283814	2.291728	2.286667	2.287387	2.282288	2.292481	2.287444	2.28246	2.277544
2.283045	2.290971	2.285939	2.296519	2.291361	2.291734	2.286711	2.281744	2.276841
2.2823	2.290245	2.285248	2.295843	2.290698	2.291078	2.286043	2.281057	2.276128
2.281581	2.28953	2.28455	2.295154	2.290028	2.290417	2.285367	2.280359	2.275402
2.280904	2.288812	2.283803	2.294389	2.289285	2.289684	2.284625	2.279607	2.274649
2.280248	2.288105	2.283054	2.293613	2.288528	2.288937	2.283874	2.278852	2.273899
2.279597	2.287436	2.282371	2.292912	2.287825	2.288238	2.283185	2.278171	2.273212
2.27895	2.28679	2.281727	2.292253	2.287151	2.287565	2.282532	2.277533	2.272562
2.278246	2.286085	2.281036	2.291551	2.286437	2.286851	2.281834	2.276846	2.271865
2.277475	2.285316	2.280293	2.290801	2.285676	2.28609	2.281085	2.276105	2.271114
2.289632	2.284588	2.279582	2.290083	2.284958	2.279896	2.274923	2.269974	2.270399
2.288992	2.283941	2.278931	2.28943	2.284318	2.279262	2.274281	2.269323	2.269753
2.288372	2.283314	2.278294	2.28879	2.283695	2.278646	2.273654	2.268688	2.269124
2.287739	2.282676	2.277649	2.288142	2.28306	2.278018	2.273021	2.26805	2.268494
2.287095	2.282029	2.276993	2.287483	2.282413	2.277377	2.272377	2.267403	2.267854
2.286397	2.281327	2.276279	2.28676	2.281707	2.276678	2.27167	2.266689	2.277071
2.285657	2.280583	2.275519	2.285989	2.280955	2.275935	2.270914	2.265924	2.276315
2.284957	2.279885	2.274816	2.285276	2.280249	2.275231	2.270207	2.265214	2.27561
2.284306	2.279241	2.274184	2.284633	2.279596	2.274574	2.269557	2.264571	2.274967
2.283669	2.278612	2.273565	2.284006	2.27896	2.273935	2.268923	2.263943	2.274339
2.283036	2.277983	2.272939	2.283377	2.278334	2.27331	2.268299	2.26332	2.273718
2.282415	2.277366	2.272325	2.282757	2.277716	2.272693	2.26768	2.262701	2.273102
2.281804	2.276761	2.271726	2.282146	2.277092	2.272062	2.26705	2.262074	2.27248
2.281209	2.276172	2.271147	2.281551	2.276477	2.271435	2.266422	2.261447	2.271857
2.280662	2.275632	2.27062	2.281015	2.275924	2.270862	2.265825	2.260833	2.266336
2.28016	2.275139	2.270145	2.280536	2.275428	2.270338	2.265258	2.260229	2.265722
2.279627	2.274609	2.269622	2.280011	2.2749	2.269803	2.264711	2.259672	2.265158
2.279048	2.274026	2.269031	2.279418	2.274323	2.269251	2.264199	2.25919	2.264673
2.278449	2.28388	2.278823	2.278805	2.273726	2.268678	2.263665	2.258688	2.26417
2.277831	2.283261	2.278203	2.278184	2.273106	2.268066	2.263066	2.258102	2.263589
2.277209	2.282638	2.27758	2.277557	2.27248	2.267441	2.262445	2.257486	2.26298
2.27661	2.282024	2.276964	2.276937	2.271866	2.266831	2.261836	2.256879	2.262381
2.276028	2.281422	2.276356	2.276325	2.271263	2.266234	2.261236	2.256277	2.261787
2.272175	2.280875	2.275799	2.275764	2.270714	2.265691	2.260688	2.255726	2.261243
2.271695	2.280392	2.275298	2.275258	2.270225	2.26521	2.260199	2.25523	2.260753
2.271176	2.279873	2.274775	2.27473	2.269703	2.264692	2.259684	2.254716	2.26024
2.270569	2.279274	2.274197	2.274147	2.269108	2.264096	2.259107	2.254156	2.259668

2.269971	2.278682	2.273625	2.27357	2.268514	2.2635	2.258535	2.253602	2.259102
2.269451	2.275184	2.270141	2.273044	2.267977	2.262965	2.258022	2.253106	2.258597
2.268968	2.274693	2.269641	2.272536	2.267459	2.262446	2.257521	2.252621	2.258107
2.268491	2.274203	2.269136	2.272008	2.26691	2.261885	2.256962	2.252067	2.257559
2.268025	2.27372	2.268632	2.27147	2.266343	2.261298	2.256364	2.251467	2.256974
2.267535	2.273211	2.268104	2.268364	2.263251	2.260757	2.255818	2.250922	2.256439
2.26702	2.272674	2.267546	2.26781	2.262738	2.260269	2.255334	2.250437	2.255963
2.282713	2.277537	2.272376	2.272208	2.267146	2.270356	2.265376	2.260431	2.257098
2.282147	2.27699	2.271867	2.27172	2.266665	2.269883	2.264921	2.259987	2.256653
2.281588	2.276458	2.271375	2.271251	2.266196	2.269419	2.264471	2.259549	2.256212
2.281081	2.275964	2.270891	2.27078	2.26574	2.26687	2.26193	2.257014	2.255763
2.280601	2.275495	2.270417	2.270316	2.265293	2.266426	2.261475	2.256552	2.255311
2.280119	2.275025	2.269957	2.269863	2.264841	2.265972	2.261022	2.256098	2.254858
2.27964	2.274559	2.269509	2.26942	2.264386	2.26551	2.260569	2.255651	2.254403
2.279172	2.274098	2.269055	2.26897	2.263937	2.265057	2.260115	2.255196	2.252029
2.27872	2.273643	2.268587	2.268509	2.263495	2.264617	2.25966	2.254727	2.251563
2.278267	2.273185	2.268123	2.26805	2.263051	2.264173	2.259204	2.254259	2.251096
2.277785	2.272703	2.267668	2.267603	2.262595	2.263708	2.258746	2.253803	2.25063
2.277319	2.272232	2.26722	2.26716	2.262141	2.263247	2.258289	2.253349	2.250164
2.276942	2.271827	2.266775	2.266707	2.261705	2.262814	2.257833	2.252873	2.249689
2.276617	2.271464	2.26634	2.266257	2.261286	2.262403	2.257386	2.252394	2.249213
2.276245	2.271096	2.265959	2.265876	2.26091	2.262023	2.256998	2.251989	2.248779
2.275833	2.270731	2.265637	2.265568	2.260583	2.261679	2.256673	2.251664	2.248388
2.275405	2.270339	2.26529	2.265232	2.260224	2.263125	2.258116	2.253095	2.248002
2.274947	2.269893	2.264886	2.264831	2.259796	2.262682	2.25768	2.252675	2.247619
2.274513	2.269465	2.264484	2.264432	2.259378	2.26225	2.257236	2.252236	2.247225
2.274166	2.269122	2.264134	2.264086	2.259039	2.261896	2.256823	2.251779	2.24679
2.273847	2.268802	2.263791	2.263747	2.258718	2.261564	2.256425	2.251329	2.246352
2.273465	2.268417	2.263384	2.263342	2.25833	2.261185	2.256051	2.25096	2.242868
2.273047	2.267992	2.262935	2.262893	2.257898	2.260774	2.255697	2.25065	2.242582
2.272675	2.267615	2.262543	2.262486	2.257469	2.260341	2.255305	2.250293	2.242252
2.272357	2.267294	2.262215	2.262122	2.257036	2.259875	2.254861	2.249877	2.241865
2.272045	2.266983	2.261906	2.261789	2.256647	2.259457	2.254455	2.249487	2.241506
2.271739	2.266686	2.261618	2.261499	2.256338	2.259133	2.254125	2.249156	2.241208
2.271412	2.266368	2.261318	2.261211	2.256053	2.258844	2.253827	2.248849	2.240934
2.256111	2.265966	2.260945	2.26087	2.255745	2.258555	2.253539	2.248555	2.240669
2.255671	2.265532	2.260544	2.26051	2.255435	2.258273	2.253261	2.24827	2.240412
2.255302	2.271634	2.266618	2.265201	2.260112	2.255063	2.250067	2.245096	2.240145
2.254988	2.271329	2.266306	2.26492	2.259834	2.254784	2.249779	2.244806	2.239873
2.254682	2.271035	2.266006	2.26465	2.25956	2.254497	2.249465	2.244473	2.239554
2.254382	2.270749	2.26572	2.264392	2.25929	2.2542	2.249109	2.244076	2.239166
2.254081	2.267784	2.262781	2.264135	2.259024	2.253911	2.248774	2.243705	2.249493
2.253774	2.267518	2.262516	2.263871	2.258758	2.25364	2.248492	2.243414	2.249186
2.253464	2.267248	2.262247	2.263605	2.258496	2.253382	2.248238	2.243162	2.248915
2.253146	2.266973	2.261974	2.26334	2.25824	2.253135	2.248	2.242926	2.248654
2.252826	2.266696	2.2617	2.263075	2.25799	2.252898	2.24778	2.242714	2.248416
2.252524	2.252392	2.247434	2.262808	2.257737	2.252669	2.24759	2.24255	2.248232
2.252238	2.252125	2.247148	2.262535	2.257482	2.252448	2.247432	2.242439	2.248107
2.251991	2.251899	2.246901	2.262291	2.257234	2.262985	2.257962	2.252953	2.247943
2.25181	2.251744	2.246719	2.262091	2.256993	2.262713	2.257703	2.252713	2.247715

2.251614	2.251578	2.246536	2.261895	2.25676	2.262445	2.257435	2.252453	2.247466
2.251333	2.251334	2.246303	2.261676	2.256531	2.262195	2.257173	2.252185	2.247203
2.251029	2.251065	2.246051	2.261451	2.256315	2.261968	2.256928	2.251926	2.246943
2.24891	2.253332	2.248285	2.248241	2.24321	2.261803	2.256755	2.251735	2.24673
2.24873	2.253117	2.248035	2.248009	2.243041	2.261683	2.256632	2.251592	2.246552
2.248525	2.252893	2.247792	2.247775	2.242839	2.249336	2.244357	2.239377	2.246387
2.24829	2.252657	2.247551	2.247533	2.242595	2.249088	2.244136	2.239186	2.246237
2.248062	2.252437	2.247341	2.258838	2.253835	2.248865	2.243934	2.239006	2.246094
2.247846	2.25224	2.247179	2.258682	2.253678	2.248709	2.243784	2.23886	2.245969
2.247651	2.252063	2.247038	2.258543	2.253532	2.248556	2.243627	2.238701	2.24583
2.247496	2.251915	2.2469	2.258388	2.253347	2.248347	2.2434	2.238468	2.245628
2.247364	2.251785	2.246766	2.258228	2.253151	2.248122	2.243153	2.238213	2.245405
2.247227	2.25164	2.246606	2.253402	2.248348	2.243341	2.238392	2.233472	2.245244
2.247091	2.251484	2.246425	2.253212	2.248186	2.243203	2.238272	2.233359	2.245129
2.246938	2.263228	2.258109	2.253051	2.248048	2.243081	2.238154	2.233237	2.245013
2.246751	2.263041	2.25796	2.252926	2.247942	2.242978	2.238037	2.233104	2.244894
2.246607	2.262891	2.257832	2.252812	2.247835	2.24287	2.237913	2.232962	2.232573
2.24657	2.262831	2.257739	2.252699	2.247711	2.242736	2.237772	2.232812	2.232402
2.246548	2.262784	2.257655	2.25259	2.247587	2.242598	2.237622	2.23265	2.232222
2.246423	2.262653	2.257542	2.252483	2.247476	2.242471	2.237458	2.232456	2.232035
2.246253	2.262485	2.257416	2.252377	2.247373	2.242351	2.23729	2.232253	2.231851
2.258762	2.262377	2.257307	2.252266	2.24726	2.242236	2.237173	2.232134	2.242953
2.258729	2.262324	2.257212	2.25215	2.247139	2.242128	2.237107	2.232098	2.242898
2.258641	2.262228	2.2571	2.252032	2.247018	2.242012	2.237006	2.232007	2.242803
2.258446	2.262046	2.256958	2.251908	2.246898	2.24188	2.236833	2.231806	2.242629
2.258278	2.261885	2.256821	2.251785	2.246781	2.241755	2.236679	2.231633	2.242474
2.258239	2.261819	2.256706	2.251654	2.246662	2.241657	2.236618	2.23159	2.238322
2.25824	2.261791	2.256616	2.25154	2.246551	2.241567	2.236582	2.231589	2.238284
2.258185	2.261754	2.256592	2.251497	2.24646	2.241455	2.236494	2.23153	2.238251
2.2581	2.261716	2.256614	2.251505	2.246387	2.241335	2.236379	2.231442	2.238224
2.257999	2.261637	2.256578	2.251481	2.246349	2.241278	2.236294	2.231341	2.238153
2.257872	2.261506	2.256483	2.25143	2.246357	2.241296	2.236239	2.23122	2.238029
2.260233	2.261408	2.256383	2.251355	2.246333	2.253153	2.24802	2.242925	2.244734
2.260242	2.261376	2.256281	2.251235	2.246238	2.253095	2.248007	2.242934	2.244696
2.260264	2.261367	2.256205	2.251131	2.246134	2.253017	2.24799	2.242956	2.24468
2.260234	2.261356	2.256198	2.251101	2.246058	2.25292	2.247924	2.242926	2.244661
2.260191	2.261353	2.25623	2.251115	2.246004	2.252823	2.247844	2.24288	2.244649
2.260181	2.261368	2.256275	2.251154	2.246003	2.252797	2.247816	2.242861	2.244653
2.260194	2.261398	2.256336	2.251219	2.246049	2.252827	2.247826	2.24286	2.244669
2.260176	2.261388	2.256352	2.251248	2.246082	2.252855	2.24782	2.242826	2.244643
2.26011	2.261322	2.256306	2.251227	2.246098	2.252876	2.247784	2.242742	2.244557
2.254878	2.256592	2.251617	2.251207	2.246117	2.252906	2.247771	2.242689	2.244499
2.254897	2.256594	2.251609	2.251216	2.246157	2.252963	2.247813	2.242715	2.244519
2.254937	2.256611	2.251604	2.251216	2.246184	2.25301	2.247865	2.242766	2.244565
2.254967	2.256615	2.251562	2.251159	2.246144	2.252997	2.247881	2.242805	2.244601
2.255	2.25662	2.251508	2.251078	2.246073	2.252953	2.247882	2.242841	2.244634
2.255047	2.256652	2.251509	2.263021	2.257984	2.259785	2.254712	2.249661	2.244639
2.255106	2.25671	2.251559	2.263067	2.25802	2.259813	2.254743	2.249683	2.244614
2.255164	2.256768	2.251613	2.26312	2.258067	2.259853	2.254783	2.249715	2.244616
2.25522	2.256823	2.251667	2.263177	2.258123	2.259905	2.254833	2.249761	2.244655

2.255271	2.256872	2.251717	2.263232	2.258178	2.259958	2.254886	2.249815	2.244713
2.255308	2.256906	2.25175	2.26327	2.258219	2.260002	2.254938	2.249875	2.24478
2.255343	2.25694	2.251785	2.263309	2.258258	2.260045	2.254992	2.249941	2.244864
2.255392	2.256996	2.251846	2.263366	2.258303	2.260086	2.255046	2.250018	2.244978
2.25545	2.257068	2.251927	2.263441	2.258357	2.260131	2.255103	2.250101	2.245113
2.244997	2.25715	2.252042	2.263569	2.258466	2.260222	2.255177	2.250169	2.245206
2.245063	2.257247	2.252198	2.26376	2.258637	2.260365	2.255268	2.250218	2.245254
2.245134	2.257336	2.252324	2.263913	2.258783	2.260494	2.255361	2.25028	2.245307
2.245206	2.2574	2.252379	2.26398	2.258869	2.260587	2.255447	2.250355	2.245366
2.245297	2.25748	2.25244	2.264039	2.258941	2.260668	2.255543	2.250457	2.245455
2.245445	2.270633	2.265526	2.271011	2.265862	2.260746	2.255667	2.250623	2.245623
2.245609	2.270813	2.265686	2.271139	2.265943	2.260821	2.255798	2.250808	2.245819
2.245703	2.27092	2.265797	2.271239	2.266025	2.260897	2.255888	2.250915	2.245937
2.245747	2.270976	2.265874	2.271321	2.266109	2.260976	2.25595	2.250967	2.246001
2.245841	2.271071	2.265968	2.271418	2.266225	2.261099	2.256064	2.251069	2.246095
2.246	2.271215	2.266078	2.271529	2.266377	2.261277	2.256242	2.251234	2.246225
2.24616	2.271367	2.266211	2.271666	2.266545	2.261461	2.25642	2.251396	2.246364
2.246292	2.271513	2.266377	2.271845	2.26673	2.261639	2.256571	2.251526	2.246503
2.246425	2.271668	2.266558	2.272037	2.266913	2.261808	2.256717	2.251658	2.246652
2.246577	2.265729	2.260671	2.266908	2.261799	2.261951	2.256867	2.25182	2.246823
2.246741	2.265914	2.260857	2.267079	2.261938	2.262082	2.257021	2.251998	2.247007
2.246906	2.266083	2.261014	2.267229	2.26209	2.26224	2.257192	2.252176	2.247175
2.247074	2.26624	2.261145	2.267359	2.262252	2.26242	2.257378	2.252357	2.247329
2.247226	2.266391	2.261296	2.267519	2.262437	2.262614	2.257556	2.252518	2.24748
2.247342	2.266524	2.261472	2.267722	2.262653	2.262822	2.257712	2.252635	2.247617
2.261997	2.274336	2.269282	2.267938	2.262877	2.26304	2.257896	2.252793	2.247788
2.262269	2.2746	2.26951	2.268153	2.263097	2.263271	2.258157	2.253073	2.248047
2.262563	2.274884	2.26975	2.268373	2.263318	2.263506	2.258433	2.253376	2.248324
2.258636	2.275077	2.269963	2.268598	2.263542	2.263723	2.258631	2.25356	2.248518
2.258766	2.275224	2.270163	2.268826	2.263768	2.263934	2.25879	2.253686	2.248672
2.258975	2.275435	2.270378	2.269048	2.263996	2.264166	2.259024	2.253918	2.248896
2.259268	2.275714	2.270609	2.269264	2.264226	2.264423	2.259337	2.254261	2.24919
2.259514	2.272438	2.267314	2.269482	2.264454	2.264672	2.259629	2.254582	2.249493
2.259648	2.272586	2.267509	2.269705	2.264679	2.264905	2.259873	2.254846	2.249802
2.259803	2.27275	2.267709	2.269928	2.264902	2.265132	2.260103	2.255089	2.250096
2.260092	2.263331	2.258289	2.262897	2.257923	2.265354	2.260327	2.255319	2.25034
2.260429	2.263636	2.258527	2.26311	2.258134	2.265575	2.260548	2.255542	2.250561
2.26071	2.263931	2.258831	2.263401	2.258381	2.2658	2.26078	2.255782	2.250792
2.260958	2.264225	2.259186	2.263752	2.258658	2.266031	2.261021	2.256035	2.251026
2.269507	2.26448	2.259486	2.264068	2.258963	2.263402	2.258389	2.253401	2.251312
2.269715	2.264684	2.259721	2.264346	2.259305	2.263781	2.258751	2.253742	2.251667
2.269965	2.264915	2.259951	2.264606	2.259624	2.264138	2.259091	2.254062	2.251995
2.270292	2.265205	2.260186	2.264833	2.259878	2.264416	2.259357	2.254314	2.252245
2.270652	2.265529	2.260444	2.265073	2.260128	2.264684	2.259618	2.254567	2.252483
2.271027	2.265888	2.260768	2.265385	2.260436	2.264999	2.259943	2.25489	2.261297
2.271407	2.266266	2.261131	2.265736	2.260768	2.265327	2.26029	2.255241	2.261584
2.271728	2.266603	2.261478	2.266074	2.261066	2.265606	2.260581	2.255544	2.261875
2.271996	2.266905	2.261818	2.266405	2.261338	2.265842	2.260823	2.255809	2.262172
2.272284	2.267218	2.262165	2.266753	2.261647	2.260643	2.255661	2.250693	2.262488
2.272609	2.267559	2.262534	2.267131	2.262011	2.260997	2.256012	2.25104	2.262832

2.27292	2.267878	2.262875	2.267489	2.262375	2.273517	2.268453	2.263404	2.263179
2.273176	2.268127	2.263142	2.267781	2.262708	2.273872	2.2688	2.263743	2.263526
2.273433	2.268372	2.263396	2.268061	2.263037	2.274226	2.269137	2.264062	2.263855
2.273765	2.268693	2.263713	2.268388	2.263381	2.274566	2.269428	2.264314	2.264113
2.274144	2.269061	2.264067	2.26874	2.263735	2.274899	2.269696	2.264529	2.264329
2.274524	2.269433	2.26443	2.269103	2.264097	2.275248	2.270008	2.264812	2.264611
2.27491	2.269813	2.264806	2.269478	2.264467	2.275611	2.270363	2.265159	2.264954
2.275295	2.270194	2.265181	2.269854	2.264842	2.275984	2.270737	2.265531	2.26532
2.275679	2.270572	2.265552	2.270229	2.265222	2.276365	2.271123	2.265917	2.265696
2.276062	2.270949	2.265923	2.270605	2.265605	2.276754	2.271532	2.266336	2.266107
2.276438	2.271322	2.266293	2.284151	2.279057	2.277154	2.271983	2.266825	2.266591
2.276816	2.271698	2.266665	2.284516	2.279428	2.277555	2.272443	2.267331	2.267097
2.277213	2.272084	2.267035	2.284874	2.27979	2.277933	2.272845	2.267754	2.267528
2.277626	2.272481	2.267407	2.285228	2.280145	2.278295	2.273207	2.268119	2.267908
2.278081	2.272929	2.267832	2.276638	2.271572	2.275429	2.270367	2.265312	2.268283
2.278594	2.273445	2.268322	2.277074	2.271949	2.275776	2.270713	2.265668	2.26865
2.27907	2.28794	2.282703	2.277504	2.272335	2.276138	2.271071	2.266031	2.269024
2.279463	2.288335	2.283104	2.277901	2.27272	2.276515	2.271437	2.266393	2.269404
2.279833	2.288707	2.283494	2.278301	2.273125	2.276922	2.271834	2.266782	2.269811
2.28021	2.28909	2.283898	2.278728	2.273577	2.27739	2.2723	2.267245	2.270285
2.280597	2.289482	2.284315	2.279174	2.274058	2.277894	2.272803	2.267742	2.270789
2.281049	2.289932	2.284781	2.27965	2.274543	2.278383	2.273272	2.268189	2.271227
2.281558	2.290435	2.285291	2.280156	2.275036	2.278865	2.273717	2.268598	2.271614
2.282039	2.290897	2.285749	2.280613	2.275496	2.279329	2.274171	2.269036	2.272044
2.282472	2.291294	2.286126	2.280995	2.275904	2.279767	2.274635	2.269517	2.272535
2.296955	2.291708	2.28652	2.281391	2.276322	2.280212	2.275106	2.270006	2.273029
2.297438	2.292187	2.286986	2.281857	2.276797	2.280698	2.275599	2.270498	2.273501
2.297937	2.292684	2.28747	2.282334	2.277273	2.281177	2.276083	2.270981	2.273969
2.296364	2.293158	2.287916	2.282757	2.277676	2.272617	2.267581	2.262539	2.269493
2.296853	2.293624	2.288349	2.283159	2.278047	2.27298	2.267968	2.262961	2.269956
2.297322	2.29409	2.288818	2.28362	2.27849	2.273416	2.268412	2.263418	2.270445
2.297768	2.294554	2.28932	2.284136	2.279001	2.273919	2.268906	2.263909	2.270959
2.298239	2.295041	2.289836	2.284662	2.279522	2.274435	2.269414	2.264411	2.271479
2.29875	2.295563	2.290362	2.285189	2.280046	2.274955	2.269931	2.264926	2.272006
2.299276	2.296098	2.290895	2.28572	2.280573	2.275478	2.270452	2.265445	2.272536
2.299806	2.296635	2.291435	2.286256	2.281098	2.275997	2.27097	2.265965	2.269831
2.300343	2.297176	2.291977	2.286793	2.281624	2.276517	2.27149	2.266485	2.270354
2.300885	2.297717	2.292514	2.287326	2.282152	2.27704	2.27201	2.267002	2.270875
2.30143	2.298257	2.293047	2.287855	2.282682	2.277567	2.272531	2.267518	2.271394
2.301964	2.298782	2.293577	2.288387	2.283212	2.278095	2.273053	2.268033	2.271911
2.302475	2.299283	2.294102	2.28892	2.283745	2.278624	2.273575	2.268549	2.272426
2.303041	2.299826	2.294634	2.289448	2.284277	2.279156	2.274099	2.269064	2.272943
2.303719	2.300449	2.295166	2.289951	2.2848	2.279689	2.274621	2.269575	2.273457
2.304404	2.301092	2.295727	2.290479	2.285335	2.280227	2.27515	2.270095	2.273981
2.295834	2.29967	2.294338	2.291099	2.285899	2.280758	2.275686	2.270639	2.265584
2.296421	2.300283	2.295009	2.291761	2.286488	2.281303	2.276239	2.271203	2.266134
2.296986	2.300849	2.29561	2.292379	2.287112	2.281926	2.276853	2.271807	2.266738
2.297525	2.301376	2.296158	2.292968	2.28777	2.282617	2.277518	2.272447	2.26739
2.298093	2.292836	2.28762	2.293535	2.288387	2.283259	2.278143	2.273054	2.268006
2.298694	2.293419	2.288172	2.294067	2.288945	2.283831	2.278707	2.273609	2.268566

2.299323	2.294034	2.288764	2.285854	2.280727	2.28441	2.279286	2.274188	2.269144
2.299998	2.29471	2.289448	2.286534	2.281389	2.285043	2.279934	2.274847	2.269791
2.30068	2.295395	2.29015	2.287229	2.282058	2.285677	2.280587	2.275514	2.270444
2.301301	2.296009	2.290777	2.287849	2.282662	2.286253	2.281169	2.2761	2.271021
2.3019	2.296595	2.291375	2.28844	2.283241	2.286805	2.28172	2.276651	2.271566
2.302572	2.297261	2.292036	2.289093	2.283891	2.278731	2.273625	2.268537	2.272173
2.30331	2.298	2.292754	2.289804	2.284605	2.279442	2.27432	2.26922	2.272836
2.304057	2.298747	2.293485	2.290525	2.285324	2.280154	2.275015	2.269902	2.264813
2.304807	2.299497	2.294229	2.291256	2.286039	2.280856	2.275705	2.270586	2.265502
2.305553	2.30024	2.294964	2.291981	2.286752	2.281553	2.27638	2.271244	2.266162
2.306291	2.300969	2.295679	2.292689	2.287464	2.282246	2.277014	2.271829	2.266741
2.307009	2.301678	2.29637	2.293377	2.288162	2.282928	2.277638	2.272402	2.267306
2.29462	2.302328	2.297003	2.294007	2.288801	2.283577	2.278297	2.273065	2.267961
2.29523	2.302938	2.297596	2.294599	2.289402	2.284203	2.278977	2.273786	2.268674
2.295895	2.303604	2.298257	2.295257	2.290057	2.284868	2.279673	2.274507	2.269388
2.296618	2.304328	2.29899	2.295985	2.290769	2.285573	2.280387	2.275228	2.270105
2.297354	2.305065	2.299733	2.296722	2.291492	2.286287	2.281103	2.275946	2.270819
2.298092	2.305802	2.300468	2.297452	2.292215	2.287004	2.281814	2.276653	2.271522
2.29884	2.306548	2.301213	2.29819	2.292946	2.287729	2.282534	2.277367	2.272231
2.299591	2.299429	2.294128	2.298936	2.29368	2.288456	2.283265	2.2781	2.272954
2.300361	2.300201	2.294904	2.299701	2.29443	2.289199	2.284015	2.278855	2.273697
2.301193	2.301035	2.295745	2.300527	2.295237	2.289998	2.284822	2.279667	2.2745
2.302077	2.301922	2.296639	2.301405	2.296094	2.290844	2.285676	2.280529	2.275354
2.287199	2.297613	2.292368	2.292184	2.286903	2.291637	2.286471	2.281327	2.276151
2.287926	2.298365	2.293108	2.292921	2.287635	2.292359	2.287189	2.282043	2.276873
2.288647	2.299115	2.293841	2.293651	2.288362	2.293077	2.287899	2.282748	2.277586
2.289373	2.299875	2.294584	2.294391	2.2891	2.293804	2.288613	2.283456	2.278303
2.290123	2.300661	2.295351	2.295154	2.289862	2.294555	2.289349	2.284183	2.279041
2.290948	2.301527	2.296188	2.295985	2.290699	2.287935	2.282743	2.277597	2.27985
2.291812	2.302434	2.297064	2.296854	2.291575	2.288807	2.283597	2.278439	2.280698
2.292608	2.303274	2.297895	2.297684	2.292389	2.289609	2.284397	2.279238	2.281488
2.293346	2.304056	2.298688	2.298478	2.293147	2.290347	2.285149	2.279999	2.282228
2.283221	2.304844	2.299508	2.299307	2.293945	2.291128	2.285941	2.280801	2.275676
2.283931	2.305648	2.300381	2.300201	2.294812	2.291983	2.286808	2.281677	2.276547
2.284666	2.306462	2.301236	2.301073	2.295672	2.292837	2.287661	2.282533	2.277411
2.28546	2.297278	2.291991	2.301843	2.296465	2.293634	2.288428	2.283286	2.278215
2.286286	2.298102	2.292747	2.302597	2.297254	2.294428	2.289183	2.284019	2.278999
2.287135	2.298983	2.29359	2.303445	2.298117	2.292834	2.287582	2.282409	2.279787
2.288003	2.299903	2.294489	2.304351	2.299029	2.29374	2.288469	2.283265	2.280578
2.288845	2.283773	2.278713	2.290044	2.284743	2.294644	2.289364	2.284142	2.281416
2.289663	2.284611	2.279587	2.290974	2.285648	2.295546	2.290268	2.285046	2.282304
2.290458	2.285419	2.280417	2.291866	2.286544	2.286873	2.281582	2.276346	2.283201
2.291212	2.286166	2.281148	2.29267	2.287419	2.287798	2.28249	2.277231	2.284096
2.291977	2.286929	2.2819	2.293492	2.288297	2.288721	2.2834	2.278122	2.284996
2.287105	2.287789	2.282796	2.294438	2.289206	2.289612	2.284305	2.279038	2.276694
2.287968	2.288686	2.283739	2.295424	2.290125	2.290495	2.285209	2.279962	2.277594
2.288815	2.283837	2.278898	2.29631	2.291022	2.291405	2.286117	2.280865	2.278491
2.289657	2.284642	2.279663	2.297129	2.291905	2.292334	2.287028	2.281755	2.279387
2.290532	2.285506	2.280514	2.298026	2.292807	2.287582	2.282324	2.277098	2.280289
2.291442	2.286444	2.281478	2.299024	2.293737	2.288473	2.283226	2.278013	2.281198

2.292375	2.287392	2.282436	2.294847	2.289508	2.289369	2.28413	2.278927	2.282109
2.29334	2.288338	2.283344	2.295783	2.290435	2.290285	2.285036	2.279828	2.283016
2.294301	2.289271	2.284234	2.296704	2.291358	2.291204	2.285946	2.280734	2.283933
2.29521	2.290164	2.285117	2.280094	2.275089	2.286973	2.281725	2.276525	2.282064
2.296088	2.291032	2.285992	2.280968	2.275959	2.287893	2.28266	2.277477	2.283037
2.296961	2.291899	2.286861	2.281842	2.276839	2.288835	2.283621	2.27845	2.278437
2.297826	2.292761	2.287725	2.282714	2.277728	2.289798	2.284606	2.279445	2.279397
2.29869	2.293627	2.288602	2.283606	2.27864	2.290779	2.285586	2.280417	2.27523
2.299538	2.294486	2.289487	2.28452	2.279588	2.291788	2.286557	2.281354	2.276162
2.300434	2.295388	2.290406	2.285453	2.280535	2.292788	2.287522	2.28229	2.2771
2.301444	2.29639	2.291384	2.286401	2.281446	2.293732	2.28846	2.283233	2.278061
2.302486	2.297423	2.292389	2.287369	2.282363	2.294683	2.289411	2.284194	2.279034
2.303445	2.298401	2.293385	2.288369	2.283355	2.295733	2.290442	2.285202	2.280009
2.304364	2.299354	2.294377	2.289387	2.28439	2.296843	2.291526	2.286245	2.280992
2.298049	2.300314	2.29533	2.290344	2.28536	2.297901	2.29259	2.287303	2.28202
2.299059	2.301278	2.296236	2.291231	2.286262	2.298909	2.293637	2.288379	2.283097
2.300072	2.302265	2.297181	2.292158	2.28719	2.299926	2.294678	2.28944	2.284166
2.301089	2.303299	2.298209	2.293171	2.288176	2.300965	2.295714	2.290481	2.285221
2.302082	2.304315	2.299233	2.294189	2.289176	2.302016	2.29674	2.291494	2.286247
2.302997	2.305238	2.300175	2.295151	2.29016	2.285193	2.280252	2.275322	2.287197
2.303893	2.306136	2.301096	2.296101	2.291149	2.286182	2.281176	2.276191	2.288126
2.304878	2.307121	2.302084	2.297097	2.29216	2.287191	2.282157	2.277146	2.289142
2.305926	2.308167	2.30312	2.298127	2.29319	2.288219	2.283181	2.278165	2.29022
2.306978	2.301925	2.296906	2.299171	2.29423	2.289255	2.284212	2.27919	2.291306
2.308033	2.302982	2.297966	2.300233	2.295285	2.290302	2.285252	2.280222	2.292402
2.309086	2.304037	2.299023	2.301293	2.296338	2.291347	2.286288	2.281252	2.293494
2.310131	2.305082	2.30007	2.302344	2.297383	2.292386	2.287316	2.28227	2.294572
2.311172	2.306121	2.301112	2.303388	2.298423	2.293418	2.288338	2.283282	2.295644
2.312208	2.307155	2.302145	2.304423	2.299451	2.294438	2.289347	2.284282	2.296706
2.313239	2.308183	2.303173	2.305452	2.300474	2.295453	2.290352	2.285278	2.297766
2.314267	2.309207	2.304197	2.306482	2.301504	2.296482	2.291376	2.286295	2.298846
2.315289	2.310227	2.305217	2.307511	2.302541	2.297521	2.292415	2.287327	2.299941
2.316332	2.311261	2.306243	2.308542	2.303581	2.298574	2.293487	2.28841	2.283372
2.317392	2.312304	2.307268	2.309572	2.304626	2.299647	2.294612	2.289569	2.284515
2.318488	2.313385	2.308323	2.303334	2.298418	2.30071	2.29572	2.290711	2.285649
2.319659	2.314544	2.309442	2.304416	2.299458	2.301737	2.296765	2.291774	2.286715
2.32084	2.315719	2.310579	2.305509	2.300498	2.302752	2.297785	2.292808	2.287764
2.321942	2.316821	2.311667	2.306571	2.301521	2.303761	2.298804	2.293852	2.288848
2.322998	2.317884	2.312735	2.307625	2.302542	2.304772	2.299822	2.294892	2.289944
2.324067	2.318975	2.313861	2.308752	2.303641	2.305847	2.300861	2.295913	2.29099
2.325148	2.320095	2.315049	2.309955	2.304818	2.306985	2.301919	2.296909	2.291983
2.326216	2.321188	2.316195	2.311129	2.306002	2.308157	2.303027	2.297958	2.293005
2.327248	2.322228	2.317264	2.312247	2.307192	2.309382	2.304214	2.299087	2.294067
2.32831	2.32328	2.318317	2.313336	2.30835	2.310584	2.30541	2.300254	2.295175
2.32946	2.324385	2.319371	2.314375	2.309405	2.304394	2.299319	2.294242	2.296366
2.330657	2.325531	2.32045	2.315417	2.310432	2.305446	2.300453	2.295446	2.297586
2.331869	2.326755	2.321666	2.316603	2.311565	2.306545	2.301546	2.29655	2.29873
2.333099	2.328039	2.322981	2.317891	2.31277	2.307675	2.302609	2.297584	2.299825
2.329845	2.329223	2.324186	2.319097	2.31396	2.308839	2.30373	2.298674	2.300924
2.330908	2.330279	2.325256	2.320207	2.31514	2.310052	2.304923	2.299826	2.302018

2.30567	2.326962	2.321937	2.321309	2.316283	2.311223	2.306106	2.301002	2.295943
2.3069	2.328191	2.323086	2.322415	2.317349	2.312294	2.307249	2.302216	2.297185
2.308132	2.329432	2.324258	2.32354	2.318417	2.313355	2.308376	2.303414	2.298423
2.309223	2.330583	2.32544	2.324727	2.319571	2.31448	2.309477	2.304502	2.299524
2.310255	2.331697	2.326626	2.325941	2.320767	2.315641	2.310576	2.305555	2.30058
2.311349	2.332823	2.327757	2.322654	2.31752	2.32429	2.319185	2.314122	2.309113
2.312487	2.333953	2.32884	2.323751	2.318689	2.325528	2.320463	2.315423	2.310416
2.313647	2.335136	2.330005	2.324919	2.319876	2.322809	2.317761	2.312734	2.311635
2.314834	2.336398	2.331299	2.326195	2.321088	2.323964	2.318875	2.313819	2.312716
2.307001	2.337639	2.332564	2.32745	2.322299	2.325138	2.320018	2.31494	2.313834
2.308139	2.338783	2.333692	2.328593	2.323488	2.32637	2.321269	2.316201	2.315105
2.309277	2.33992	2.334804	2.329726	2.324685	2.327624	2.322547	2.31749	2.316403
2.303118	2.341152	2.336034	2.330965	2.325943	2.328884	2.323778	2.318687	2.313645
2.304337	2.342437	2.337325	2.332256	2.327228	2.330145	2.324985	2.319844	2.314763
2.305566	2.308002	2.303038	2.333472	2.328424	2.331337	2.326183	2.32105	2.315966
2.306812	2.309212	2.30419	2.334615	2.329528	2.332453	2.327365	2.322301	2.317251
2.307993	2.31039	2.305356	2.327834	2.322735	2.333634	2.32859	2.323568	2.318543
2.309053	2.311505	2.306548	2.329101	2.324018	2.33493	2.329887	2.324864	2.31985
2.310142	2.312649	2.307763	2.330392	2.325328	2.336244	2.33118	2.326134	2.321116
2.311384	2.306522	2.301702	2.331696	2.32664	2.337535	2.332414	2.327313	2.32226
2.312688	2.307812	2.302963	2.332992	2.327935	2.338801	2.333622	2.328465	2.323371
2.313971	2.30906	2.304158	2.334199	2.329115	2.332663	2.327505	2.322378	2.324582
2.315245	2.310283	2.305309	2.335344	2.330216	2.333771	2.328677	2.323619	2.325859
2.316425	2.311452	2.306474	2.336553	2.331413	2.334983	2.329927	2.324905	2.319892
2.317485	2.312549	2.307646	2.337828	2.332716	2.336308	2.331263	2.326244	2.321227
2.318605	2.3137	2.308854	2.304006	2.299164	2.337661	2.332616	2.327588	2.32256
2.319858	2.314961	2.310118	2.305281	2.300457	2.339029	2.333972	2.328927	2.323886
2.321161	2.316265	2.31141	2.306574	2.301762	2.340402	2.335326	2.330256	2.325192
2.32247	2.317575	2.312714	2.307878	2.303068	2.341765	2.336668	2.331568	2.326455
2.323796	2.318899	2.31403	2.309187	2.304371	2.343116	2.337993	2.332859	2.327689
2.325124	2.320219	2.315336	2.310478	2.305647	2.344429	2.339269	2.334101	2.328913
2.326456	2.321539	2.316635	2.311755	2.306902	2.345709	2.3405	2.3353	2.330123
2.327773	2.322842	2.317915	2.313007	2.308116	2.346953	2.341723	2.336519	2.331366
2.32907	2.324127	2.319181	2.314232	2.309278	2.34815	2.342933	2.337759	2.332648
2.330363	2.325394	2.320412	2.315426	2.310433	2.316581	2.311615	2.306697	2.333958
2.331655	2.326624	2.321566	2.316558	2.31159	2.317783	2.312852	2.307966	2.335297
2.332949	2.327855	2.32272	2.317697	2.312766	2.319013	2.314118	2.30926	2.336653
2.334253	2.329142	2.323982	2.318944	2.314003	2.32027	2.315397	2.310559	2.338011
2.335563	2.33046	2.325306	2.320254	2.315282	2.32155	2.316691	2.311868	2.339371
2.336867	2.331769	2.326621	2.32156	2.316566	2.322839	2.317982	2.313164	2.34072
2.338165	2.333072	2.327932	2.322868	2.317862	2.324137	2.319273	2.31445	2.342057
2.339475	2.334386	2.329254	2.324183	2.319158	2.325434	2.320564	2.315738	2.337702
2.340804	2.335715	2.330577	2.32549	2.320436	2.326712	2.321847	2.317029	2.339029
2.342134	2.337059	2.331936	2.32684	2.321758	2.328027	2.323153	2.318328	2.340364
2.325056	2.338433	2.33338	2.328297	2.323181	2.329415	2.324487	2.319619	2.341691
2.326361	2.339812	2.334841	2.329786	2.324651	2.330856	2.325867	2.320947	2.343054
2.327662	2.341147	2.336208	2.331196	2.326117	2.332366	2.327366	2.322427	2.317569
2.328959	2.342453	2.337513	2.332557	2.327585	2.333926	2.328947	2.324009	2.319143
2.330254	2.343759	2.338821	2.333892	2.328975	2.335372	2.330403	2.325466	2.320599
2.331547	2.345065	2.340127	2.335199	2.330279	2.336692	2.331724	2.326791	2.321929

2.318537	2.327984	2.323142	2.336501	2.331574	2.337997	2.333024	2.328091	2.323229
2.319815	2.329275	2.324435	2.337808	2.332884	2.339317	2.33433	2.329384	2.324515
2.321094	2.330567	2.325728	2.339114	2.334189	2.34063	2.335632	2.330673	2.325793
2.322371	2.331857	2.327016	2.340412	2.335478	2.34193	2.336933	2.331973	2.32708
2.323653	2.333151	2.328306	2.34171	2.336764	2.343227	2.338237	2.33328	2.328372
2.324947	2.334453	2.329604	2.343018	2.338067	2.344541	2.339551	2.334591	2.329674
2.326253	2.335764	2.330911	2.344337	2.339385	2.345869	2.340873	2.335908	2.330984
2.327668	2.322826	2.318014	2.327456	2.322595	2.347301	2.342305	2.337336	2.332404
2.329239	2.324378	2.31955	2.329001	2.324139	2.348885	2.343893	2.338926	2.333981
2.330759	2.325881	2.321037	2.330495	2.325628	2.350412	2.345423	2.340457	2.335503
2.332113	2.327221	2.322367	2.331829	2.326949	2.351762	2.346773	2.341808	2.336856
2.333419	2.328513	2.323649	2.333113	2.328218	2.353056	2.348064	2.343098	2.338151
2.334743	2.329823	2.324942	2.334407	2.329504	2.337227	2.332312	2.327429	2.339444
2.336084	2.33115	2.32625	2.335715	2.330805	2.338535	2.333604	2.328708	2.34074
2.317899	2.332613	2.327698	2.337164	2.33224	2.339977	2.335028	2.330119	2.342169
2.319497	2.334211	2.329285	2.338753	2.333808	2.341551	2.336582	2.331659	2.343726
2.32102	2.335736	2.330802	2.325876	2.320962	2.343056	2.338066	2.333126	2.328264
2.322403	2.337123	2.332177	2.32724	2.322313	2.344423	2.339408	2.334448	2.32958
2.323785	2.338523	2.333577	2.328634	2.323695	2.345824	2.340787	2.335807	2.330928
2.301103	2.320381	2.315513	2.330192	2.325236	2.347388	2.342341	2.337347	2.332444
2.302574	2.321909	2.31708	2.331781	2.326811	2.348989	2.343936	2.338929	2.333996
2.287528	2.299218	2.294442	2.313634	2.308704	2.350456	2.345393	2.340372	2.335412
2.288898	2.300566	2.295772	2.314984	2.310062	2.351851	2.346778	2.341741	2.336756
2.290243	2.301923	2.29715	2.316403	2.31151	2.339921	2.334885	2.329877	2.338213
2.29155	2.303279	2.298576	2.317895	2.313064	2.341532	2.336492	2.331466	2.339796
2.292935	2.304683	2.299998	2.295271	2.290526	2.309715	2.304788	2.299871	2.328683
2.294461	2.306159	2.301388	2.296617	2.291861	2.31107	2.306141	2.301224	2.330053
2.296017	2.307667	2.302807	2.297982	2.293204	2.312429	2.307506	2.302599	2.331447
2.297523	2.309198	2.304331	2.299497	2.294704	2.28989	2.285036	2.280189	2.314379
2.299012	2.310732	2.305884	2.301052	2.296245	2.291434	2.286613	2.281791	2.316008
2.259787	2.312181	2.307298	2.302445	2.297629	2.292839	2.288086	2.283327	2.302461
2.261054	2.313566	2.308612	2.303723	2.2989	2.294146	2.289488	2.284824	2.304019
2.262412	2.298442	2.293492	2.30512	2.300284	2.295533	2.290899	2.286264	2.281536
2.263892	2.300079	2.295057	2.306665	2.301804	2.297014	2.292314	2.287621	2.282862
2.265357	2.301724	2.296653	2.308254	2.303368	2.298539	2.293774	2.289024	2.284236
2.266758	2.303341	2.298269	2.309884	2.304975	2.300119	2.295324	2.290549	2.285754
2.26812	2.30492	2.299859	2.311498	2.306573	2.301703	2.296896	2.29211	2.287318
2.26942	2.306381	2.301318	2.31298	2.308074	2.303223	2.298433	2.293663	2.288882
2.270692	2.30779	2.302718	2.314401	2.309521	2.3047	2.299942	2.295199	2.29044
2.272072	2.267572	2.263107	2.299264	2.294399	2.306111	2.301336	2.296598	2.291882
2.273547	2.269019	2.264514	2.300803	2.295814	2.307452	2.302617	2.297866	2.293216
2.275023	2.270485	2.265955	2.302394	2.29731	2.308893	2.304002	2.299221	2.294582
2.2765	2.27197	2.267435	2.304048	2.298917	2.310474	2.305539	2.30071	2.295994
2.27795	2.27344	2.268916	2.30572	2.300566	2.312112	2.307133	2.302249	2.297451
2.279348	2.274877	2.270384	2.307396	2.302242	2.313789	2.308755	2.303815	2.298976
2.280707	2.276278	2.271829	2.309061	2.30393	2.315492	2.310405	2.30541	2.30054
2.282008	2.277608	2.273219	2.310671	2.305586	2.317184	2.312061	2.307019	2.302109
2.28327	2.278886	2.274566	2.312238	2.307218	2.31887	2.313726	2.308644	2.303693
2.284607	2.280201	2.275887	2.271523	2.26713	2.303803	2.298739	2.293718	2.305297
2.286017	2.281546	2.277171	2.272808	2.268472	2.305374	2.300342	2.295334	2.306922

2.263181	2.282934	2.278507	2.274138	2.269838	2.306955	2.301954	2.296961	2.308556
2.264628	2.284382	2.279929	2.27555	2.271256	2.308562	2.303586	2.298604	2.310195
2.266096	2.285856	2.281382	2.276992	2.272693	2.310179	2.305223	2.300251	2.311839
2.267571	2.287334	2.282839	2.278437	2.274135	2.311796	2.306857	2.301893	2.313482
2.269056	2.288822	2.284307	2.279893	2.275586	2.313419	2.308492	2.303532	2.315126
2.270552	2.290315	2.285775	2.281347	2.277039	2.272706	2.268334	2.263923	2.316772
2.272058	2.291814	2.287244	2.282803	2.278495	2.274161	2.269782	2.265365	2.31842
2.244102	2.293322	2.288725	2.284265	2.279946	2.275607	2.271229	2.266814	2.320072
2.245556	2.294839	2.290217	2.285733	2.28139	2.277041	2.272675	2.26827	2.32173
2.247016	2.296366	2.29172	2.287211	2.282839	2.278476	2.274117	2.269723	2.306631
2.248483	2.297902	2.293235	2.288701	2.284297	2.279914	2.275553	2.271164	2.308262
2.249956	2.299449	2.29476	2.290199	2.285763	2.281358	2.276989	2.272601	2.309889
2.25143	2.246843	2.242321	2.291691	2.287231	2.282808	2.278425	2.274034	2.311503
2.252906	2.248311	2.243769	2.293197	2.288713	2.284269	2.279867	2.275466	2.313112
2.254384	2.249814	2.245296	2.294793	2.290261	2.285774	2.281336	2.276911	2.272459
2.255862	2.251349	2.246895	2.296471	2.291871	2.287321	2.282829	2.278366	2.27391
2.257327	2.252849	2.248442	2.270372	2.265849	2.288874	2.284333	2.279836	2.27539
2.258756	2.254288	2.249902	2.271879	2.267353	2.290432	2.285841	2.281313	2.276902
2.246151	2.255753	2.251366	2.273379	2.268849	2.29199	2.287377	2.282835	2.278443
2.247778	2.257317	2.252888	2.274903	2.270348	2.293546	2.288972	2.284456	2.280031
2.249428	2.258898	2.254417	2.276429	2.271843	2.295096	2.290576	2.286093	2.281623
2.250964	2.246405	2.241887	2.277937	2.273323	2.268803	2.264411	2.260056	2.283147
2.252443	2.24788	2.243373	2.279437	2.2748	2.270258	2.265847	2.261479	2.284631
2.253997	2.249401	2.244875	2.240351	2.235837	2.2718	2.267353	2.262947	2.286139
2.255622	2.250962	2.246384	2.241872	2.23743	2.273434	2.268929	2.264456	2.287666
2.257265	2.252558	2.247941	2.243427	2.23902	2.262042	2.25747	2.252928	2.248443
2.25891	2.254191	2.249569	2.245035	2.240594	2.263619	2.259024	2.254471	2.250002
2.260582	2.255853	2.251217	2.246649	2.242155	2.265179	2.26058	2.256035	2.251584
2.262318	2.257553	2.252851	2.248228	2.243683	2.266726	2.262166	2.257653	2.253192
2.264066	2.259261	2.254484	2.249804	2.245211	2.268279	2.263765	2.259289	2.254806
2.213293	2.260903	2.256112	2.251418	2.246811	2.269898	2.265371	2.260879	2.256369
2.214772	2.2625	2.257739	2.253062	2.248462	2.271566	2.266983	2.262437	2.257899
2.195494	2.264147	2.259411	2.254733	2.250111	2.24554	2.241022	2.236547	2.259445
2.197069	2.265852	2.261138	2.256438	2.251758	2.247132	2.242565	2.23806	2.261008
2.198669	2.194165	2.189713	2.258128	2.253392	2.248722	2.244124	2.239603	2.262595
2.200295	2.195763	2.191271	2.259773	2.254992	2.2503	2.245715	2.241207	2.264218
2.20192	2.197354	2.192816	2.261408	2.256602	2.2519	2.247327	2.24283	2.265854
2.203506	2.198909	2.194343	2.244602	2.239853	2.253568	2.248963	2.244434	2.267478
2.205071	2.200445	2.195863	2.246237	2.24153	2.255275	2.250618	2.246039	2.269102
2.206627	2.201987	2.197407	2.247904	2.243209	2.256972	2.252289	2.247678	2.243183
2.20817	2.203531	2.19897	2.249599	2.244894	2.258666	2.253974	2.249344	2.244803
2.209756	2.20511	2.200545	2.196037	2.191591	2.241906	2.237239	2.232626	2.246446
2.211397	2.206732	2.202126	2.197605	2.193169	2.243613	2.238927	2.23429	2.248118
2.21305	2.208368	2.203731	2.199192	2.194749	2.24531	2.240616	2.235966	2.231395
2.214679	2.209997	2.205375	2.200819	2.196329	2.246968	2.242294	2.237658	2.233054
2.216327	2.211642	2.207036	2.202459	2.197916	2.248627	2.243972	2.239351	2.234717
2.193146	2.213326	2.208673	2.204067	2.199511	2.250323	2.245648	2.24101	2.236367
2.194876	2.215041	2.210308	2.20567	2.201121	2.252051	2.247335	2.242661	2.238021
2.196577	2.191853	2.187167	2.207357	2.202804	2.198289	2.193806	2.189355	2.239768
2.198252	2.193551	2.188897	2.209131	2.204567	2.200042	2.195554	2.191095	2.24161

2.176547	2.195231	2.190593	2.210866	2.206287	2.20175	2.197257	2.192793	2.243413
2.178199	2.196876	2.192225	2.212524	2.207928	2.203379	2.198881	2.194412	2.245142
2.179853	2.198517	2.193844	2.214162	2.209547	2.204984	2.200476	2.196001	2.246841
2.153134	2.176821	2.172169	2.190841	2.186272	2.206573	2.202044	2.197555	2.248505
2.15473	2.178471	2.173797	2.192449	2.187852	2.20817	2.203615	2.199107	2.250167
2.15633	2.180125	2.175441	2.170825	2.166275	2.209851	2.205273	2.200742	2.196282
2.157939	2.181785	2.177102	2.172495	2.167963	2.211592	2.206991	2.202434	2.197952
2.159612	2.183507	2.178807	2.174184	2.169633	2.165113	2.160612	2.156153	2.199571
2.161365	2.185306	2.18057	2.175897	2.171283	2.166712	2.162181	2.157696	2.201123
2.103026	2.1584	2.153744	2.177597	2.172932	2.168323	2.163776	2.159276	2.180884
2.104515	2.159997	2.155341	2.179253	2.174574	2.169963	2.165429	2.160941	2.182585
2.106006	2.161601	2.156947	2.180924	2.176239	2.171626	2.167101	2.162618	2.184297
2.107622	2.163313	2.158644	2.182671	2.177963	2.173321	2.168757	2.164238	2.185924
2.109284	2.165068	2.160379	2.184457	2.179724	2.175044	2.170421	2.165847	2.187529
2.044186	2.106403	2.101998	2.186224	2.181497	2.176804	2.172144	2.167529	2.162971
2.045607	2.107913	2.103529	2.187983	2.183285	2.178601	2.173917	2.169272	2.164687
2.047104	2.084302	2.079932	2.160782	2.156192	2.180347	2.175648	2.170989	2.166399
2.048714	2.08595	2.081536	2.162453	2.157819	2.182013	2.17731	2.172659	2.168094
2.050307	2.045999	2.041734	2.078742	2.074351	2.183708	2.179002	2.174357	2.169808
2.051803	2.047533	2.04331	2.0804	2.075991	2.185491	2.180779	2.176129	2.171564
2.053288	2.049063	2.044894	2.082082	2.077663	2.187311	2.18259	2.177925	2.173327
1.985322	2.050626	2.046479	2.042258	2.037978	2.168359	2.163612	2.158906	2.154237
1.986789	2.052207	2.048065	2.04384	2.039552	2.170204	2.165431	2.160688	2.155958
1.988253	1.984202	1.980229	2.045416	2.041128	2.07821	2.073821	2.069443	2.157724
1.989713	1.985652	1.981691	2.04699	2.042709	2.079867	2.075465	2.071075	2.15953
1.950093	1.987103	1.9831	2.0485	2.044237	2.039992	2.035775	2.031567	2.161346
1.951605	1.988544	1.984413	2.049898	2.045671	2.041467	2.037289	2.03311	2.163169
1.953137	1.990014	1.985761	2.051326	2.047117	2.042941	2.0388	2.03465	2.164991
1.899031	1.95042	1.946203	1.983075	1.979015	2.044449	2.04032	2.036187	2.073187
1.900495	1.951958	1.947719	1.984583	1.980461	2.045981	2.041852	2.037725	2.074802
1.901972	1.95349	1.949218	1.986088	1.981939	1.977865	1.97389	1.96992	2.03504
1.903461	1.955029	1.950722	1.987605	1.983446	1.979356	1.975352	1.971355	2.036574
1.86973	1.956587	1.952252	1.948054	1.943977	1.980849	1.976829	1.972821	2.038161
1.871256	1.958162	1.953802	1.949572	1.945455	1.982338	1.978319	1.974322	2.03981
1.827621	1.868648	1.864501	1.951157	1.946985	1.942908	1.938954	1.935028	1.9718
1.829157	1.870232	1.866144	1.952864	1.948604	1.944453	1.940445	1.936484	1.973278
1.830697	1.871817	1.867788	1.954576	1.950242	1.946019	1.941949	1.937942	1.97475
1.832234	1.873351	1.86933	1.924057	1.919769	1.947574	1.943455	1.939408	1.935427
1.833774	1.874866	1.87083	1.925631	1.921358	1.949144	1.944979	1.940886	1.936883
1.798363	1.831304	1.827337	1.927244	1.923008	1.91881	1.914644	1.910549	1.938362
1.800001	1.832854	1.82887	1.928881	1.924708	1.920519	1.91628	1.912104	1.939861
1.801625	1.797511	1.793456	1.871359	1.867357	1.922165	1.917888	1.913664	1.941369
1.803224	1.799095	1.79502	1.872846	1.868843	1.923725	1.919448	1.915213	1.942876
1.63617	1.80069	1.796594	1.792547	1.788555	1.925285	1.921034	1.916812	1.91266
1.6377	1.802301	1.798189	1.794129	1.790129	1.926889	1.922703	1.918519	1.914313
1.639267	1.803946	1.799807	1.795725	1.791705	1.928493	1.924374	1.920236	1.915995
1.640889	1.689601	1.685681	1.797322	1.793262	1.836636	1.832625	1.828609	1.917652
1.642539	1.691266	1.687275	1.798937	1.794819	1.838152	1.834136	1.830144	1.919294
1.644111	1.640195	1.636276	1.800643	1.796455	1.792363	1.78839	1.784464	1.889652
1.645612	1.641748	1.637904	1.802441	1.798165	1.793993	1.78996	1.785993	1.891235

1.561169	1.643276	1.639475	1.688275	1.684316	1.795682	1.791581	1.787554	1.830726
1.562493	1.644764	1.640954	1.689829	1.685951	1.797467	1.793273	1.789146	1.832266
1.563849	1.646267	1.642422	1.691353	1.687549	1.799224	1.794982	1.790794	1.833872
1.565267	1.647806	1.64389	1.640065	1.636333	1.685235	1.681391	1.677582	1.788539
1.566721	1.649392	1.645401	1.641525	1.637763	1.686723	1.682974	1.679249	1.790347
1.523598	1.564745	1.561321	1.643159	1.639304	1.688237	1.684485	1.68077	1.739581
1.525185	1.566274	1.562873	1.644908	1.640917	1.689768	1.685954	1.682205	1.741045
1.526694	1.567714	1.564328	1.560934	1.55753	1.638619	1.634817	1.631084	1.680001
1.528112	1.569053	1.565681	1.562319	1.558967	1.640312	1.63649	1.632711	1.681599
1.529558	1.570421	1.567039	1.563689	1.560371	1.641952	1.638113	1.634304	1.683196
1.531069	1.571849	1.568409	1.565025	1.561694	1.643449	1.639605	1.635804	1.684778
1.532607	1.573317	1.569825	1.566403	1.563047	1.644965	1.641099	1.637292	1.686338
1.468429	1.530884	1.527608	1.56793	1.564554	1.561203	1.557864	1.554572	1.635015
1.469939	1.532491	1.529243	1.569513	1.566119	1.562726	1.559317	1.555947	1.636462
1.402915	1.533974	1.53075	1.570978	1.567578	1.564178	1.560764	1.557384	1.554059
1.404237	1.535367	1.532165	1.572363	1.568966	1.565585	1.562215	1.558875	1.555567
1.309374	1.402674	1.39975	1.573832	1.57043	1.567045	1.563676	1.560333	1.557017
1.310777	1.404193	1.401267	1.575422	1.572001	1.56858	1.565148	1.561743	1.558381
1.224922	1.309395	1.306669	1.518878	1.515543	1.570094	1.566624	1.563179	1.559786
1.226112	1.310657	1.307923	1.520249	1.516928	1.571525	1.568094	1.564683	1.561303
1.227304	1.31192	1.309173	1.521616	1.51831	1.572957	1.569575	1.566208	1.562846
1.228627	1.313303	1.310539	1.404001	1.401076	1.516517	1.513228	1.509949	1.564344
1.230009	1.314741	1.311955	1.405524	1.402591	1.518054	1.514738	1.511427	1.565822
1.231302	1.228684	1.226106	1.310525	1.307796	1.5195	1.51618	1.512868	1.50955
1.232517	1.22988	1.227276	1.311779	1.309038	1.520864	1.517559	1.514271	1.510986
1.233797	1.231142	1.228516	1.225944	1.223423	1.440521	1.437397	1.434295	1.512453
1.235192	1.232519	1.229876	1.227288	1.224751	1.441973	1.438841	1.435729	1.513979
1.23657	1.233879	1.231227	1.228624	1.226069	1.310382	1.307676	1.304985	1.434001
1.237818	1.235115	1.232469	1.229857	1.227282	1.311661	1.308939	1.306236	1.435281
1.239061	1.236346	1.233706	1.231086	1.228491	1.31293	1.310188	1.30747	1.436552
1.240462	1.237728	1.235068	1.23243	1.22982	1.227251	1.224728	1.222237	1.437956
1.241964	1.239206	1.236509	1.233847	1.231224	1.228642	1.226103	1.223598	1.439444
1.243461	1.240674	1.237943	1.235259	1.232625	1.23003	1.227474	1.224952	1.44092
1.244963	1.242144	1.23938	1.236677	1.234035	1.231428	1.228851	1.22631	1.442394
1.246417	1.243568	1.24077	1.238043	1.235387	1.232765	1.23017	1.227612	1.37202
1.247766	1.244891	1.242059	1.239303	1.236619	1.233976	1.231368	1.228804	1.373293
1.249141	1.246243	1.243378	1.24059	1.237874	1.235207	1.232587	1.230016	1.374588
1.25066	1.247738	1.244846	1.242025	1.239271	1.236577	1.233946	1.231365	1.228824
1.252247	1.249306	1.246389	1.243535	1.24074	1.238015	1.235373	1.232776	1.230206
1.145739	1.250877	1.247944	1.245062	1.242225	1.239464	1.236792	1.23417	1.23158
1.147203	1.252465	1.249524	1.246616	1.243737	1.240934	1.238219	1.235564	1.23296
1.148661	1.254046	1.251097	1.24817	1.245263	1.242425	1.239667	1.236974	1.234352
1.150122	1.255631	1.252672	1.249732	1.24681	1.243945	1.241138	1.2384	1.235754
1.151531	1.148748	1.145989	1.251256	1.248335	1.245458	1.242618	1.239845	1.23717
1.152823	1.150039	1.147285	1.252693	1.249807	1.246949	1.244109	1.24132	1.238606
1.154121	1.151337	1.148583	1.254123	1.251268	1.24843	1.245597	1.2428	1.24005
1.155573	1.152772	1.149995	1.147261	1.144568	1.249893	1.247055	1.244254	1.241492
1.157102	1.154279	1.151469	1.148695	1.145954	1.251351	1.248502	1.245696	1.242933
1.158603	1.155772	1.152949	1.150152	1.147377	1.252854	1.249977	1.247146	1.244362
1.16009	1.157261	1.154442	1.151634	1.148836	1.254394	1.251473	1.2486	1.245779

1.161582	1.158753	1.155934	1.153119	1.150308	1.147522	1.144758	1.142037	1.247228
1.163082	1.160246	1.15742	1.154602	1.151792	1.148996	1.146206	1.143453	1.248714
1.164584	1.161742	1.158908	1.156088	1.153279	1.150477	1.147671	1.144893	1.14215
1.166087	1.163239	1.1604	1.157575	1.154762	1.151954	1.149143	1.146358	1.143606
1.167594	1.16474	1.161896	1.159064	1.156245	1.153434	1.150623	1.147838	1.145085
1.16911	1.166248	1.163396	1.160557	1.157732	1.154915	1.152103	1.149313	1.146552
1.170633	1.167761	1.164899	1.162052	1.159221	1.156399	1.153584	1.150789	1.148019
1.172159	1.169281	1.166406	1.163551	1.160713	1.157886	1.155067	1.152268	1.149493
1.173688	1.170807	1.167916	1.165052	1.162208	1.159376	1.156552	1.153749	1.150972
1.175227	1.172341	1.169439	1.166563	1.163709	1.160869	1.158039	1.155233	1.152458
1.176779	1.173886	1.170977	1.168089	1.165217	1.162363	1.159527	1.156719	1.153949
1.178339	1.175437	1.172524	1.169624	1.166731	1.163864	1.161024	1.158216	1.155455
1.179907	1.176997	1.174077	1.171163	1.168252	1.165375	1.162535	1.159736	1.156994
1.181481	1.178562	1.175634	1.172707	1.16978	1.166895	1.164059	1.161269	1.158544
1.183056	1.180126	1.177184	1.174249	1.171318	1.16843	1.165588	1.162792	1.160059
1.184634	1.181688	1.17873	1.175791	1.172867	1.169978	1.167125	1.164313	1.161554
1.186211	1.183254	1.180283	1.177342	1.174424	1.17154	1.168691	1.165874	1.163096
1.187787	1.184823	1.181845	1.178904	1.175992	1.173119	1.170292	1.167489	1.1647
1.033763	1.186396	1.183414	1.180474	1.177567	1.1747	1.171885	1.169085	1.166282
1.035097	1.187969	1.184986	1.182045	1.17914	1.176269	1.173438	1.170621	1.167798
1.036434	1.189551	1.18657	1.183631	1.180731	1.177853	1.175001	1.172158	1.169311
1.037778	1.191154	1.18818	1.185252	1.182371	1.179498	1.17663	1.173762	1.170875
1.039126	1.19277	1.189805	1.186893	1.184034	1.181167	1.178286	1.175392	1.172463
1.040466	1.194382	1.191423	1.188513	1.185651	1.182778	1.179888	1.176984	1.174041
1.041799	1.19599	1.193038	1.190117	1.187231	1.184342	1.181447	1.178545	1.175613
1.043153	1.197636	1.194686	1.191755	1.188847	1.185938	1.183027	1.180115	1.177191
1.044526	1.199327	1.196379	1.193442	1.190519	1.187588	1.184639	1.181696	1.178771
1.045931	1.043356	1.040811	1.19513	1.192185	1.189231	1.186256	1.183296	1.180373
1.047392	1.044818	1.042254	1.196796	1.193805	1.190827	1.187863	1.184921	1.182015
1.048862	1.046283	1.043693	1.198456	1.19542	1.192423	1.18948	1.186565	1.183676
1.050265	1.04768	1.045086	1.20011	1.197067	1.194069	1.191132	1.188223	1.185332
1.051637	1.049043	1.046453	1.201762	1.198736	1.195747	1.192809	1.189893	1.186989
1.053073	1.050456	1.047848	1.203439	1.200426	1.19744	1.194492	1.191561	1.18864
1.054574	1.051917	1.049264	1.205141	1.202138	1.199151	1.196181	1.193227	1.190284
1.056056	1.053376	1.050704	1.048073	1.045481	1.200863	1.197874	1.194898	1.191939
1.057485	1.054813	1.05217	1.049554	1.046966	1.202577	1.199567	1.196572	1.193609
1.058929	1.056269	1.053654	1.051044	1.048445	1.204287	1.201264	1.198258	1.195291
1.060439	1.057782	1.055155	1.05252	1.04988	1.205983	1.202968	1.199966	1.196988
1.061976	1.059314	1.05666	1.053991	1.051306	1.207678	1.204677	1.201685	1.198693
1.063494	1.060812	1.058131	1.055451	1.052771	1.050123	1.04752	1.044929	1.200392
1.065002	1.06229	1.059579	1.056904	1.054261	1.051632	1.049021	1.046405	1.202089
1.066484	1.06376	1.061051	1.058385	1.055759	1.053132	1.050499	1.047858	1.203795
1.067927	1.065216	1.062551	1.059901	1.057271	1.054625	1.05195	1.049279	1.205508
1.069403	1.066696	1.064052	1.061404	1.05876	1.056099	1.053404	1.050725	1.048089
1.070939	1.068217	1.065548	1.062873	1.060199	1.057532	1.054868	1.052221	1.049603
1.072506	1.069758	1.067042	1.064333	1.061634	1.058968	1.056344	1.053735	1.051131
0.864692	1.071304	1.068524	1.065791	1.063102	1.060449	1.057842	1.055247	1.052644
0.865941	1.072858	1.070008	1.067256	1.06459	1.061956	1.059355	1.056762	1.054153
0.867131	1.074399	1.071536	1.068776	1.066107	1.063469	1.060862	1.058265	1.055658
0.868275	1.07593	1.073101	1.070343	1.067651	1.064992	1.062367	1.05976	1.057161

0.869443	0.867342	0.86527	1.07193	1.069203	1.066516	1.063876	1.06126	1.058662
0.870641	0.868566	0.866522	1.073537	1.070756	1.068034	1.065386	1.062769	1.060161
0.871863	0.869803	0.867768	1.075154	1.072331	1.069575	1.066907	1.064275	1.061656
0.873122	0.871045	0.868977	1.076773	1.073941	1.071159	1.068437	1.065761	1.063141
0.874387	0.872287	0.870181	1.0784	1.07557	1.072767	1.069984	1.067257	1.064629
0.601822	0.873511	0.871415	0.869338	0.867279	1.074384	1.071566	1.068801	1.066134
0.602633	0.874728	0.872662	0.870579	0.868482	1.076014	1.073175	1.070378	1.067655
0.603448	0.875953	0.873879	0.871793	0.869697	1.077647	1.074795	1.071974	1.06921
0.604269	0.877187	0.875067	0.872979	0.870921	1.079285	1.076428	1.073594	1.070799
0.605089	0.603738	0.602388	0.874186	0.87214	0.870078	0.867981	0.865895	1.072406
0.605909	0.604559	0.603208	0.875432	0.873347	0.871269	0.869197	0.86713	1.074025
0.606726	0.605381	0.604033	0.876674	0.874556	0.872464	0.870414	0.868365	1.07567
	0.606194	0.604853	0.877872	0.875768	0.873682	0.871621	0.869569	1.077358
	0.607012	0.605679	0.879069	0.876992	0.874917	0.872835	0.870773	1.079067
			0.605189	0.60383	0.876193	0.874099	0.872026	0.870013
			0.606067	0.604717	0.877496	0.875395	0.873311	0.871281
			0.606913	0.605569	0.878737	0.876643	0.874565	0.872521
			0.607724	0.606385	0.879901	0.877836	0.875787	0.873729
					0.605854	0.604504	0.603163	0.874937
					0.606662	0.605317	0.603982	0.876153
					0.607481	0.606141	0.604813	0.877378
								0.87862
								0.879875
								0.606032
								0.606838

								0.576449
								0.575536
								0.574618
								0.573714
								0.572816
					0.576831	0.575187	0.573549	1.484387
					0.575922	0.574281	0.572645	1.482107
					0.575017	0.573367	0.571728	1.479843
					0.57412	0.572438	0.570784	1.477599
			0.576794	0.574994	1.487388	1.483292	1.479251	1.584414
			0.575895	0.574086	1.485108	1.481038	1.477013	1.582044
			0.574966	0.573163	1.482815	1.478784	1.474789	1.579685
	0.577475	0.575787	1.488485	1.484514	1.59013	1.585828	1.581547	1.965067
	0.576543	0.574866	1.486171	1.482233	1.587745	1.583481	1.57923	1.962188
	0.575625	0.573934	1.483887	1.479975	1.901699	1.896787	1.891882	2.061335
	0.574722	0.572988	1.481636	1.477741	1.898986	1.894147	1.889326	2.058466
0.575595	1.487519	1.483407	1.768612	1.764066	2.021233	2.016076	2.010952	2.108467
0.574653	1.485242	1.481162	1.766024	1.761464	2.018166	2.013078	2.008034	2.105423
0.573716	1.48297	1.478942	1.763475	1.758911	2.015165	2.010116	2.005124	2.102387
1.219493	1.770157	1.765587	1.95477	1.949698	2.062309	2.057098	2.051964	2.099411
1.217665	1.767589	1.763119	1.952128	1.947117	2.059466	2.054215	2.049049	2.096462
1.590136	1.902549	1.897736	2.017191	2.011995	2.109424	2.104033	2.098711	2.093517
1.58786	1.899935	1.895071	2.014237	2.009171	2.106556	2.101161	2.095799	2.090579
1.764654	1.953881	1.948845	2.061289	2.056154	2.103621	2.098236	2.092866	2.087629
1.762225	1.951245	1.946205	2.058363	2.053229	2.100602	2.095241	2.089895	2.084651
1.953585	2.016116	2.010844	2.10812	2.102842	2.097578	2.092267	2.086973	2.122309
1.950801	2.013199	2.007995	2.10512	2.099813	2.094558	2.089353	2.084167	2.119468
1.94804	2.010301	2.005172	2.102156	2.096822	2.091569	2.086456	2.081363	2.116638
1.945426	2.057359	2.052116	2.099275	2.093932	2.088665	2.083534	2.078431	2.113682
1.942888	2.054564	2.049355	2.096443	2.091105	2.085819	2.080619	2.075453	2.110684
2.057025	2.104231	2.098898	2.093583	2.088286	2.123545	2.118259	2.113011	2.163013
2.054234	2.101391	2.096013	2.090707	2.085485	2.12075	2.115453	2.110188	2.16013
2.051381	2.098478	2.093095	2.128352	2.123102	2.117872	2.112617	2.10739	2.157268
2.048424	2.095448	2.090121	2.125344	2.120072	2.114861	2.109732	2.104633	2.154443
2.097729	2.092443	2.087169	2.122362	2.117069	2.167198	2.162001	2.156836	2.184182
2.094827	2.089537	2.084283	2.119449	2.114167	2.164198	2.158988	2.153812	2.181198
2.091977	2.08668	2.081432	2.116576	2.111319	2.161247	2.155991	2.150771	2.178193
2.089145	2.124282	2.118984	2.113728	2.108514	2.191106	2.185814	2.180553	2.175305
2.086335	2.121442	2.116142	2.110908	2.105747	2.188297	2.183007	2.17774	2.172489
2.123906	2.118602	2.113326	2.196095	2.190766	2.185468	2.180191	2.174936	2.169693
2.121038	2.115763	2.110539	2.193248	2.187915	2.182621	2.177372	2.172149	2.166925
2.118181	2.168211	2.162865	2.190396	2.185076	2.179797	2.174569	2.169366	2.236322
2.115339	2.165287	2.159945	2.187522	2.182253	2.17701	2.171786	2.166582	2.233426
2.112505	2.162374	2.157038	2.184666	2.179444	2.174235	2.169014	2.163805	2.230533
2.109674	2.192452	2.187128	2.181859	2.176651	2.210402	2.205113	2.199834	2.266098

2.106853	2.189606	2.184324	2.179077	2.173872	2.207574	2.202302	2.197038	2.263113
2.192088	2.186779	2.18153	2.176302	2.1711	2.23831	2.232809	2.227317	2.260117
2.189245	2.18397	2.178748	2.173536	2.168336	2.235394	2.229902	2.224415	2.257115
2.186407	2.181152	2.175946	2.209664	2.204397	2.271011	2.265416	2.259822	2.254188
2.183575	2.178322	2.173115	2.206797	2.201556	2.268054	2.26248	2.256914	2.251354
2.180756	2.17551	2.170304	2.237573	2.232147	2.265099	2.259545	2.254007	2.320448
2.177955	2.172732	2.167535	2.234696	2.229261	2.262136	2.256598	2.251077	2.317043
2.175167	2.169963	2.164779	2.231832	2.226384	2.259179	2.253661	2.248162	2.313671
2.172377	2.239826	2.23438	2.267334	2.261767	2.329061	2.322796	2.316613	2.344107
2.169608	2.236917	2.23147	2.264361	2.258806	2.325669	2.319465	2.313353	2.340807
2.166929	2.234092	2.228591	2.261388	2.255854	2.356717	2.350174	2.343752	2.337487
2.164331	2.231341	2.225739	2.258414	2.252911	2.353119	2.346679	2.340366	2.334167
2.234158	2.266928	2.261164	2.328027	2.321755	2.3496	2.343243	2.337019	2.33087
2.231287	2.26405	2.258374	2.324845	2.318544	2.346155	2.339865	2.333714	2.327601
2.228401	2.261153	2.255581	2.356056	2.349346	2.342814	2.336555	2.330431	2.324335
2.225502	2.258208	2.252732	2.352632	2.346081	2.339641	2.333337	2.327139	2.321038
2.222635	2.255282	2.249878	2.349209	2.342845	2.33652	2.33017	2.323889	2.31777
2.258019	2.358803	2.352206	2.345771	2.339524	2.333301	2.32702	2.320775	2.314629
2.255305	2.355436	2.348779	2.342345	2.336169	2.330038	2.323884	2.31774	2.311565
2.252527	2.352072	2.345479	2.339075	2.332881	2.326755	2.320673	2.314607	2.342015
2.249692	2.348722	2.342316	2.335963	2.329652	2.323442	2.317383	2.311384	2.338695
2.35172	2.345351	2.33911	2.332828	2.326479	2.320229	2.314158	2.30817	2.33533
2.348218	2.341914	2.335795	2.32962	2.323374	2.317169	2.311032	2.304963	2.331871
2.344811	2.338545	2.332506	2.326424	2.320299	2.314158	2.307961	2.301809	2.32846
2.341608	2.335369	2.329341	2.3233	2.317254	2.345058	2.338315	2.331632	2.366742
2.33851	2.332279	2.326219	2.320187	2.314201	2.341823	2.335144	2.328498	2.363406
2.335392	2.329148	2.323035	2.351379	2.344933	2.338475	2.331912	2.325376	2.360147
2.332285	2.326009	2.319821	2.347889	2.341447	2.335048	2.328646	2.32228	2.356962
2.329199	2.322923	2.316692	2.344484	2.338006	2.331626	2.32534	2.319117	2.353756
2.326136	2.319896	2.313655	2.341165	2.334601	2.328187	2.321977	2.315877	2.35053
2.323085	2.35135	2.344582	2.337903	2.331294	2.366488	2.359966	2.353607	2.347286
2.320062	2.348124	2.341359	2.3347	2.328132	2.363172	2.356588	2.350191	2.343978
2.317008	2.344881	2.338152	2.33154	2.325039	2.359955	2.353307	2.346858	2.340736
2.313831	2.341552	2.334935	2.328407	2.32196	2.356769	2.350159	2.343755	2.337712
2.310608	2.338194	2.331729	2.325308	2.318915	2.353634	2.347109	2.34079	2.334815
2.341285	2.334926	2.328593	2.36382	2.35711	2.350527	2.344076	2.337834	2.331919
2.338013	2.331727	2.325515	2.360666	2.353993	2.347453	2.341074	2.334908	2.329047
2.334723	2.328489	2.322375	2.357471	2.350883	2.344432	2.33816	2.3321	2.353309
2.331381	2.325172	2.319133	2.354207	2.347769	2.341471	2.335353	2.329437	2.350707
2.328121	2.363562	2.357263	2.350979	2.34469	2.338547	2.332583	2.326813	2.371605
2.325034	2.36034	2.354051	2.347825	2.341648	2.335631	2.329797	2.32416	2.368992
2.322014	2.357199	2.35091	2.344738	2.338671	2.332777	2.327066	2.321552	2.366415
2.318961	2.354054	2.347821	2.341744	2.335822	2.357147	2.351428	2.345892	2.391604
2.315911	2.350933	2.344785	2.338826	2.333064	2.354479	2.348876	2.343434	2.389119
2.35413	2.347836	2.341772	2.363159	2.357405	2.375188	2.369673	2.364295	2.386655
2.350994	2.34476	2.338781	2.360209	2.354591	2.372476	2.367095	2.361834	2.384216
2.347902	2.369113	2.363117	2.380758	2.375232	2.369845	2.364568	2.359393	2.381783
2.344838	2.366104	2.360265	2.378012	2.372609	2.367321	2.362106	2.356972	2.379349
2.341875	2.363209	2.357517	2.375356	2.370056	2.364847	2.359675	2.354569	2.37693
2.339081	2.360489	2.354895	2.372781	2.36755	2.390029	2.384805	2.379639	2.374538

2.336401	2.357883	2.352367	2.370275	2.36509	2.387558	2.382366	2.37723	2.372162
2.360988	2.378614	2.373141	2.367814	2.362653	2.385097	2.379934	2.374827	2.369789
2.358393	2.376078	2.370681	2.365399	2.360243	2.38265	2.377511	2.372433	2.367424
2.355801	2.373534	2.368211	2.362976	2.357837	2.380217	2.375108	2.370061	2.365081
2.353206	2.370981	2.365728	2.360545	2.355434	2.377798	2.372724	2.367711	2.36276
2.373828	2.36846	2.363265	2.358127	2.353045	2.375393	2.370349	2.365368	2.360447
2.371293	2.365988	2.360834	2.355729	2.350672	2.373	2.367978	2.36302	2.358128
2.368793	2.363545	2.358419	2.353341	2.348311	2.370621	2.365623	2.360689	2.355831
2.366316	2.361115	2.356007	2.378365	2.37328	2.368256	2.363292	2.358394	2.353574
2.363865	2.358705	2.353611	2.375947	2.370894	2.365905	2.360981	2.356125	2.351351
2.361437	2.356315	2.351246	2.373558	2.36852	2.363555	2.358667	2.353861	2.34916
2.359034	2.353945	2.34891	2.371196	2.36616	2.36121	2.356357	2.351608	2.347003
2.356649	2.351608	2.346636	2.368929	2.363933	2.359019	2.354199	2.34948	2.34491
2.354264	2.349301	2.34444	2.366792	2.36189	2.357042	2.352248	2.347524	2.342907
2.351967	2.374362	2.369481	2.364628	2.359805	2.355015	2.350252	2.345531	2.340873
2.349853	2.372174	2.367227	2.362352	2.357555	2.352801	2.348078	2.343393	2.338747
2.34776	2.369997	2.364963	2.360047	2.355257	2.350528	2.345844	2.341201	2.33658
2.345501	2.367705	2.362677	2.357768	2.352987	2.348272	2.343612	2.338994	2.334391
2.343157	2.365353	2.360377	2.3555	2.350726	2.346017	2.341373	2.336771	2.332183
2.368004	2.362994	2.358069	2.353231	2.348484	2.343798	2.339169	2.334579	2.355247
2.365585	2.360612	2.355743	2.350955	2.346257	2.341606	2.336993	2.332407	2.353033
2.36331	2.358375	2.353551	2.348804	2.344143	2.339519	2.33492	2.330339	2.350928
2.361267	2.356368	2.35157	2.346847	2.342207	2.3376	2.333011	2.328439	2.348999
2.359205	2.354345	2.349578	2.344878	2.340252	2.335655	2.331073	2.326505	2.347041
2.356934	2.352128	2.347422	2.342759	2.338145	2.333551	2.328962	2.32439	2.432603
2.354622	2.349864	2.345215	2.340586	2.335981	2.331386	2.326786	2.322207	2.429853
2.352447	2.347682	2.343032	2.338406	2.333809	2.329219	2.324611	2.320031	2.427138
2.350358	2.345553	2.340865	2.336221	2.33163	2.327049	2.322435	2.317859	2.42445
2.348318	2.343526	2.338825	2.334175	2.329582	2.439052	2.433224	2.427486	2.421951
2.346344	2.341622	2.336935	2.332288	2.327684	2.43661	2.430821	2.425137	2.419663
2.344308	2.339639	2.334976	2.339054	2.334428	2.434106	2.428379	2.422763	2.423507
2.342124	2.337469	2.332846	2.336922	2.332293	2.431416	2.425781	2.420261	2.421066
2.339963	2.335311	2.330718	2.334795	2.330173	2.428761	2.423219	2.417789	2.418637
2.337994	2.342031	2.337416	2.332817	2.328223	2.432624	2.427125	2.421718	2.416319
2.336088	2.340105	2.335472	2.330881	2.326328	2.430291	2.424848	2.419474	2.414045
2.342711	2.338033	2.333397	2.432711	2.42707	2.427852	2.422447	2.417107	2.432817
2.34054	2.335863	2.331233	2.430032	2.424478	2.425348	2.419968	2.414658	2.430413
2.338454	2.333789	2.32918	2.433892	2.428381	2.422954	2.4176	2.412312	2.428064
2.336479	2.331841	2.327277	2.431562	2.426099	2.42071	2.415386	2.41011	2.425791
2.334505	2.329884	2.325355	2.429209	2.423785	2.43955	2.434176	2.428842	2.423504
2.332478	2.32784	2.323313	2.426709	2.421312	2.437053	2.431689	2.426384	2.421141
2.33046	2.325814	2.321285	2.424226	2.418849	2.434561	2.429213	2.423942	2.418795
2.328506	2.333474	2.328926	2.443054	2.437624	2.432266	2.42696	2.421724	2.416567
2.326591	2.331642	2.327127	2.440809	2.435391	2.430055	2.424807	2.419611	2.414403
2.324696	2.428455	2.422894	2.438453	2.433021	2.427694	2.422503	2.417374	2.412209
2.322827	2.426151	2.420541	2.436006	2.430533	2.425204	2.420071	2.415035	2.409995
2.330528	2.444836	2.439169	2.433609	2.428146	2.422839	2.417749	2.412772	2.407828
2.328466	2.442285	2.436746	2.431291	2.42591	2.420663	2.415596	2.410637	2.405743
2.3521	2.439772	2.434346	2.428994	2.42371	2.418528	2.413469	2.408501	2.403625
2.350153	2.437407	2.432	2.426702	2.421519	2.416398	2.411311	2.406289	2.401378

2.348261	2.435103	2.429682	2.424411	2.419312	2.414242	2.409132	2.404064	2.399108
2.419477	2.432804	2.427366	2.422086	2.416982	2.411929	2.406882	2.40188	2.396942
2.417205	2.430517	2.425058	2.419745	2.414582	2.409526	2.404592	2.399721	2.394839
2.414854	2.428166	2.422753	2.417468	2.412309	2.407274	2.402402	2.397597	2.392752
2.412412	2.425741	2.420446	2.415253	2.410161	2.405171	2.400311	2.395508	2.390686
2.428759	2.423384	2.41818	2.413064	2.40804	2.403095	2.398241	2.393429	2.388606
2.426465	2.421146	2.415968	2.410892	2.405928	2.401026	2.396187	2.391369	2.386503
2.424212	2.418951	2.413795	2.408752	2.403834	2.39896	2.394119	2.389283	2.384382
2.421962	2.416757	2.411669	2.406669	2.401771	2.396892	2.391997	2.38711	2.382222
2.419737	2.414583	2.409568	2.404606	2.399709	2.394807	2.389847	2.38491	2.38006
2.41755	2.412431	2.407447	2.402489	2.397563	2.392643	2.387689	2.382775	2.391396
2.415397	2.410301	2.405313	2.400334	2.395356	2.390419	2.385521	2.380688	2.38935
2.413265	2.408171	2.403151	2.398151	2.393163	2.388242	2.383401	2.378637	2.391693
2.411157	2.406038	2.400942	2.395921	2.390976	2.386109	2.381326	2.37662	2.389733
2.409052	2.403924	2.398785	2.393761	2.388854	2.384036	2.379308	2.374653	2.387795
2.406949	2.401849	2.396746	2.391744	2.386844	2.382043	2.377371	2.37276	2.385874
2.404835	2.399773	2.394731	2.389769	2.38488	2.380095	2.37547	2.370891	2.383971
2.402671	2.397632	2.392649	2.387741	2.382904	2.383152	2.378547	2.373976	2.382094
2.400494	2.395474	2.39055	2.385706	2.380942	2.38127	2.376675	2.372106	2.38022
2.398415	2.393414	2.388519	2.383715	2.37901	2.379396	2.374766	2.370158	2.378257
2.396419	2.391437	2.386545	2.381762	2.377107	2.377532	2.372825	2.368136	2.376214
2.394429	2.389468	2.384586	2.379823	2.375203	2.375667	2.370917	2.366184	2.374237
2.392435	2.387496	2.382638	2.377896	2.373293	2.373798	2.36906	2.364334	2.372359
2.390452	2.385535	2.380704	2.37598	2.371389	2.371931	2.36721	2.362499	2.370501
2.388482	2.383587	2.378783	2.374079	2.369499	2.370071	2.365346	2.360635	2.368628
2.386527	2.381653	2.376871	2.372183	2.367612	2.368214	2.363487	2.358776	2.366765
2.384586	2.379728	2.374952	2.375527	2.370956	2.379019	2.374269	2.369534	2.376206
2.382666	2.377822	2.373038	2.373642	2.369087	2.377142	2.372417	2.367705	2.374435
2.380831	2.376003	2.371196	2.371805	2.367234	2.386598	2.381913	2.377246	2.372674
2.379092	2.374281	2.369428	2.370017	2.365397	2.384761	2.380109	2.375486	2.370925
2.377295	2.372507	2.367639	2.368228	2.363571	2.382937	2.378315	2.373729	2.369176
2.375391	2.370639	2.365809	2.366437	2.361759	2.381131	2.376525	2.37196	2.367414
2.37346	2.368735	2.363947	2.364625	2.359943	2.379336	2.374758	2.37022	2.36568
2.371542	2.366812	2.362034	2.374878	2.370242	2.377557	2.373048	2.368569	2.381021
2.369623	2.364876	2.360099	2.373028	2.368437	2.375781	2.37135	2.366937	2.379344
2.367702	2.380641	2.375872	2.371201	2.366628	2.373968	2.369579	2.365203	2.377569
2.365781	2.378789	2.374047	2.369391	2.364818	2.372133	2.367758	2.363397	2.375726
2.363907	2.376994	2.372298	2.367662	2.363078	2.387406	2.382924	2.378465	2.373881
2.362091	2.375271	2.370649	2.366037	2.361425	2.385634	2.38111	2.376616	2.37203
2.378214	2.373538	2.368967	2.364379	2.359761	2.383869	2.379303	2.37477	2.370192
2.376404	2.371743	2.36718	2.362614	2.358038	2.382087	2.377488	2.372923	2.368382
2.374616	2.369966	2.36539	2.360839	2.356317	2.380316	2.375689	2.371091	2.366577
2.37294	2.368295	2.363689	2.376761	2.372195	2.378595	2.373941	2.369304	2.37515
2.371308	2.366665	2.362024	2.375045	2.370502	2.376883	2.372209	2.367537	2.373386
2.369569	2.364939	2.360308	2.373282	2.368727	2.385381	2.380781	2.376182	2.389385
2.367743	2.363139	2.358553	2.371484	2.366882	2.383556	2.378981	2.374412	2.387491
2.365979	2.37899	2.374357	2.380848	2.376289	2.381792	2.377233	2.372685	2.385659
2.364296	2.377263	2.372648	2.379213	2.374638	2.380108	2.375555	2.371018	2.38391
2.362654	2.386703	2.382151	2.377589	2.373014	2.39657	2.391735	2.386939	2.382171
2.361073	2.385116	2.380528	2.375964	2.371428	2.394856	2.389976	2.385138	2.380407

2.359486	2.383521	2.378897	2.374329	2.369832	2.393139	2.388217	2.383337	2.378638
2.37007	2.381818	2.37721	2.372645	2.368135	2.391293	2.386403	2.381555	2.37686
2.368342	2.380059	2.375495	2.370937	2.366384	2.389381	2.384564	2.37979	2.375081
2.382965	2.378371	2.373836	2.369286	2.364714	2.387563	2.382795	2.378069	2.373362
2.381333	2.376746	2.372228	2.367688	2.363116	2.38583	2.381089	2.376393	2.371701
2.379729	2.375145	2.370628	2.384552	2.379712	2.38414	2.379412	2.374727	2.370049
2.378155	2.37357	2.369023	2.382829	2.378054	2.382503	2.377767	2.373067	2.368395
2.37658	2.372	2.367434	2.381125	2.376407	2.380868	2.376127	2.371413	2.366745
2.374966	2.370411	2.365877	2.379471	2.374748	2.37919	2.374466	2.369769	2.3651
2.373345	2.368821	2.364334	2.377836	2.373091	2.3775	2.372797	2.368122	2.36345
2.371758	2.36725	2.362776	2.376176	2.371445	2.375837	2.371124	2.366436	2.361756
2.370194	2.365693	2.361212	2.374503	2.369807	2.374188	2.369446	2.364722	2.360033
2.368634	2.364137	2.359656	2.372851	2.368157	2.363463	2.35874	2.354039	2.358371
2.367079	2.362585	2.358111	2.371226	2.366493	2.361777	2.357078	2.352419	2.356782
2.365525	2.37899	2.3743	2.369583	2.36483	2.360108	2.355431	2.350804	2.35518
2.363975	2.377336	2.372617	2.3679	2.363182	2.358486	2.353813	2.34918	2.353521
2.362414	2.375674	2.370927	2.36621	2.361524	2.356857	2.352197	2.347567	2.351874
2.360811	2.373968	2.369253	2.364542	2.359828	2.355158	2.35055	2.345985	2.350308
2.3592	2.372254	2.367583	2.362878	2.358118	2.353434	2.348894	2.344413	2.348773
2.357676	2.370615	2.365902	2.36118	2.356435	2.351774	2.347256	2.342804	2.347184
2.356222	2.369038	2.364211	2.359456	2.354772	2.350161	2.345631	2.341165	2.345555
2.372273	2.367451	2.362576	2.357804	2.353137	2.357413	2.352856	2.348364	2.343937
2.370632	2.365853	2.361023	2.356251	2.351529	2.355751	2.351216	2.346758	2.342336
2.368964	2.364224	2.359452	2.354699	2.349951	2.354133	2.349601	2.345152	2.340737
2.367258	2.362521	2.357804	2.353107	2.34842	2.352608	2.348024	2.343516	2.339116
2.365565	2.360817	2.356139	2.351498	2.346897	2.351109	2.346475	2.341906	2.337523
2.36397	2.359201	2.354494	2.358692	2.354117	2.349557	2.344952	2.340404	2.336031
2.362434	2.35764	2.352865	2.357028	2.352491	2.347985	2.343453	2.338968	2.334602
2.360899	2.356101	2.351305	2.355444	2.35092	2.346441	2.341964	2.337525	2.333144
2.359375	2.354593	2.349812	2.353936	2.349402	2.344926	2.340491	2.336085	2.331667
2.357815	2.353063	2.348315	2.352424	2.347859	2.34337	2.33897	2.334601	2.330181
2.356201	2.3515	2.346803	2.350892	2.346259	2.341729	2.337363	2.333053	2.328693
2.354572	2.349914	2.345274	2.349356	2.34468	2.340121	2.335767	2.331488	2.327188
2.352929	2.34829	2.343709	2.347814	2.343176	2.33863	2.334237	2.329919	2.32564
2.3513	2.346666	2.342135	2.346274	2.34171	2.337198	2.33275	2.328367	2.324091
2.349755	2.34512	2.340606	2.344761	2.340261	2.335788	2.331319	2.326903	2.322616
2.348271	2.343628	2.339103	2.343263	2.33883	2.334403	2.329935	2.325502	2.321191
2.346794	2.342127	2.337567	2.341717	2.337342	2.332966	2.328524	2.324098	2.319755
2.345327	2.340614	2.335986	2.340107	2.335785	2.331468	2.327083	2.32269	2.318306
2.34386	2.339125	2.334462	2.33856	2.334267	2.329985	2.325644	2.321281	2.316867
2.342389	2.337677	2.333045	2.337134	2.33283	2.328542	2.324216	2.319868	2.315446
2.340916	2.336239	2.331658	2.335746	2.331421	2.327116	2.322794	2.318452	2.31403
2.339432	2.334786	2.330251	2.334341	2.330013	2.325702	2.321379	2.317035	2.312611
2.337949	2.333332	2.328842	2.332937	2.328611	2.324297	2.319968	2.315616	2.311192
2.336487	2.331893	2.327432	2.331524	2.327203	2.322889	2.318553	2.314194	2.309771
2.335043	2.330464	2.32602	2.330104	2.325792	2.321479	2.317136	2.31277	2.30835
2.333618	2.329055	2.324618	2.328688	2.324377	2.320063	2.315716	2.311346	2.30693
2.332218	2.327671	2.32323	2.327277	2.322959	2.318641	2.314291	2.309922	2.305512
2.330821	2.326288	2.321839	2.325861	2.321536	2.317214	2.312866	2.308501	2.304097
2.329415	2.324891	2.320432	2.324431	2.320104	2.315783	2.311442	2.307085	2.302685

2.328007	2.323491	2.319024	2.323	2.31867	2.31435	2.310017	2.305671	2.301276
2.326596	2.322088	2.317622	2.321577	2.317233	2.312906	2.308583	2.304252	2.29987
2.325183	2.320686	2.31623	2.320166	2.315802	2.311463	2.307148	2.302832	2.298466
2.323778	2.319306	2.314883	2.318812	2.314434	2.310078	2.305747	2.301418	2.297055
2.32238	2.317948	2.313583	2.317518	2.31313	2.308749	2.304377	2.30001	2.295636
2.320981	2.316572	2.31224	2.316175	2.311788	2.307398	2.302998	2.298603	2.294226
2.319567	2.315156	2.310822	2.314747	2.310383	2.306007	2.301601	2.297199	2.292829
2.31818	2.313762	2.309412	2.313319	2.308966	2.304602	2.300199	2.295799	2.29144
2.316857	2.312445	2.308074	2.311946	2.307565	2.303184	2.298789	2.294405	2.290057
2.31556	2.311149	2.306754	2.310584	2.306163	2.30176	2.297379	2.29302	2.288688
2.31424	2.318069	2.313625	2.309187	2.304752	2.300346	2.29598	2.291647	2.287351
2.312911	2.316684	2.31222	2.307772	2.303336	2.298939	2.294592	2.290288	2.286041
2.311557	2.315296	2.310822	2.306372	2.301941	2.29756	2.293243	2.288972	2.284751
2.310175	2.313902	2.309424	2.304979	2.30056	2.296203	2.291932	2.287704	2.283487
2.308796	2.312526	2.308066	2.303641	2.299247	2.29491	2.290656	2.286435	2.282205
2.307408	2.311164	2.306772	2.302403	2.298056	2.293734	2.289439	2.28516	2.28089
2.306067	2.309841	2.305498	2.30117	2.296861	2.292547	2.288206	2.283867	2.279558
2.304863	2.308609	2.304221	2.299864	2.295544	2.291223	2.286879	2.282535	2.278209
2.303708	2.307409	2.302942	2.298529	2.294176	2.28984	2.285507	2.281181	2.276853
2.302436	2.306113	2.301631	2.297199	2.292821	2.288469	2.284138	2.279824	2.27552
2.301081	2.304748	2.300295	2.295869	2.291469	2.2871	2.282765	2.278462	2.274203
2.299726	2.303394	2.298983	2.294584	2.290194	2.285828	2.281488	2.27718	2.280232
2.298362	2.302048	2.297701	2.293361	2.289027	2.284694	2.280346	2.276007	2.279014
2.29705	2.300736	2.296421	2.292114	2.287819	2.283508	2.279158	2.2748	2.290842
2.295859	2.299497	2.295138	2.2908	2.28649	2.282173	2.277827	2.273474	2.289497
2.294682	2.298264	2.293844	2.289461	2.285121	2.280791	2.276458	2.272126	2.28815
2.293396	2.296949	2.292508	2.288107	2.283751	2.286722	2.282428	2.278156	2.292389
2.292068	2.295605	2.291161	2.286754	2.282385	2.285353	2.281109	2.276899	2.291195
2.290834	2.294358	2.289915	2.285505	2.281125	2.290039	2.285801	2.281595	2.301392
2.289682	2.293199	2.288764	2.284354	2.279967	2.288888	2.284598	2.280336	2.300058
2.288459	2.291966	2.287536	2.290449	2.286043	2.287679	2.283353	2.279056	2.298726
2.287111	2.290604	2.286171	2.289073	2.284679	2.286355	2.282023	2.277726	2.297389
2.285787	2.289266	2.284835	2.29377	2.289409	2.297768	2.293374	2.289019	2.303433
2.284595	2.28807	2.283655	2.292632	2.288276	2.296618	2.292222	2.287854	2.302181
2.283426	2.286899	2.282501	2.291528	2.287184	2.295513	2.291123	2.286745	2.300971
2.290031	2.29903	2.294639	2.290291	2.285993	2.29435	2.289991	2.285629	2.299802
2.288666	2.297698	2.293305	2.288985	2.28475	2.293157	2.288842	2.284513	2.298659
2.287416	2.296479	2.292088	2.287769	2.283534	2.291933	2.287623	2.283299	2.297405
2.286284	2.295374	2.290989	2.286642	2.282341	2.290669	2.286323	2.281976	2.296029
2.298694	2.294281	2.289899	2.298251	2.29384	2.289445	2.285069	2.280703	2.298714
2.297603	2.293198	2.288822	2.297159	2.29273	2.288318	2.283931	2.279556	2.297524
2.296466	2.292067	2.287694	2.29602	2.291585	2.287166	2.282773	2.278395	2.296322
2.295185	2.290786	2.286407	2.294717	2.29028	2.30447	2.299955	2.295461	2.294974
2.293861	2.289458	2.28507	2.293364	2.288928	2.303072	2.298563	2.294076	2.293594
2.292671	2.288267	2.283877	2.292151	2.287716	2.301819	2.297317	2.292838	2.292359
2.291576	2.287173	2.282785	2.291037	2.286601	2.300669	2.296177	2.291708	2.291229
2.290494	2.298843	2.294381	2.289934	2.285503	2.303544	2.299055	2.294588	2.290121
2.289426	2.297759	2.29329	2.288846	2.284425	2.302436	2.297958	2.2935	2.28904
2.288353	2.29667	2.292195	2.292617	2.288198	2.301323	2.296851	2.292398	2.287945
2.287265	2.295563	2.291088	2.291512	2.287111	2.300211	2.295738	2.291281	2.286831

2.286167	2.294443	2.289961	2.29038	2.285986	2.299051	2.294576	2.290114	2.285664
2.290354	2.298177	2.293649	2.28917	2.284741	2.297738	2.293264	2.28881	2.284356
2.289236	2.297019	2.292447	2.287921	2.28344	2.296356	2.291885	2.287441	2.282982
2.300329	2.295797	2.291241	2.29428	2.289746	2.295115	2.290643	2.286198	2.281736
2.298995	2.294512	2.290026	2.293108	2.288618	2.29399	2.289513	2.285055	2.280594
2.29774	2.293292	2.288857	2.291966	2.287509	2.292877	2.288398	2.283931	2.279471
2.296598	2.292162	2.287749	2.290858	2.286413	2.291767	2.28729	2.282822	2.278363
2.295495	2.291065	2.286662	2.299641	2.295148	2.290664	2.286186	2.281718	2.277258
2.294408	2.289983	2.285581	2.298527	2.294041	2.289561	2.285081	2.280608	2.276144
2.293335	2.288915	2.28451	2.297418	2.292937	2.288459	2.283975	2.279496	2.275026
2.292257	2.287841	2.28344	2.296314	2.291835	2.296149	2.29161	2.287077	2.294078
2.291177	2.286767	2.282375	2.295214	2.290735	2.295031	2.290481	2.285938	2.292923
2.290093	2.29321	2.288772	2.294107	2.289623	2.2939	2.289348	2.284802	2.291774
2.289004	2.292114	2.287684	2.292996	2.288502	2.292759	2.28821	2.283671	2.290632
2.287915	2.291015	2.286588	2.291877	2.287374	2.291613	2.287068	2.282537	2.28949
2.286824	2.289913	2.285482	2.290747	2.28624	2.290464	2.285924	2.281399	2.288347
2.285735	2.288807	2.284373	2.289613	2.285103	2.289313	2.284776	2.280257	2.2872
2.284637	2.287682	2.283261	2.288484	2.283971	2.288163	2.283624	2.279106	2.291007
2.283555	2.286563	2.28215	2.287355	2.282838	2.287013	2.28247	2.277951	2.289851
2.282543	2.285494	2.281048	2.286213	2.281695	2.291192	2.286646	2.28213	2.288696
2.281587	2.284466	2.279956	2.285065	2.280546	2.290037	2.285493	2.280981	2.287543
2.288059	2.283482	2.27892	2.289643	2.2851	2.288894	2.284348	2.279842	2.299853
2.287133	2.282558	2.27795	2.288603	2.284005	2.287749	2.283202	2.278704	2.298683
2.286161	2.291247	2.286566	2.287602	2.282975	2.297831	2.293206	2.288634	2.297537
2.285101	2.290215	2.285593	2.286671	2.282069	2.296898	2.292226	2.287593	2.296422
2.284005	2.289145	2.284593	2.285722	2.281159	2.295976	2.291269	2.286584	2.295342
2.2829	2.293729	2.289189	2.284648	2.280107	2.294927	2.290256	2.285596	2.294323
2.281784	2.29259	2.288042	2.283506	2.278981	2.293813	2.28921	2.284614	2.293335
2.286948	2.291446	2.286895	2.282362	2.277846	2.306179	2.301562	2.296952	2.292299
2.285847	2.290292	2.285737	2.281204	2.276691	2.304986	2.300391	2.295814	2.291227
2.284832	2.289227	2.28465	2.280099	2.275572	2.30381	2.299223	2.294665	2.308501
2.283946	2.288292	2.283658	2.279058	2.274488	2.302644	2.298048	2.293494	2.30723
2.283029	2.287348	2.282685	2.278059	2.273464	2.301546	2.296927	2.292358	2.305976
2.281984	2.286326	2.281717	2.277129	2.272561	2.300596	2.295928	2.291297	2.304775
2.280893	2.285265	2.280728	2.276195	2.271669	2.299673	2.294964	2.290274	2.303616
2.2798	2.284151	2.279638	2.289621	2.285058	2.298623	2.293943	2.289276	2.302544
2.278706	2.283009	2.278494	2.288462	2.28392	2.297501	2.292888	2.288292	2.30152
2.277719	2.281975	2.277429	2.287353	2.282796	2.315121	2.310172	2.305265	2.310951
2.276842	2.281051	2.276439	2.286281	2.281673	2.313833	2.308879	2.303981	2.309689
2.275916	2.2801	2.275462	2.296188	2.291486	2.31264	2.307665	2.302748	2.308446
2.274875	2.279079	2.274492	2.295226	2.290557	2.311616	2.30659	2.301607	2.307239
2.27385	2.278068	2.273526	2.294263	2.289625	2.310592	2.305532	2.300501	2.306072
2.27295	2.277131	2.272556	2.293234	2.288577	2.309435	2.304403	2.29941	2.304985
2.272079	2.276209	2.271584	2.292189	2.287501	2.308249	2.303263	2.298331	2.303928
2.271098	2.28965	2.284972	2.310528	2.30548	2.317785	2.312744	2.307749	2.308497
2.270049	2.288593	2.283969	2.309463	2.304458	2.31674	2.311672	2.306635	2.30734
2.269075	2.287582	2.282963	2.30836	2.303364	2.315627	2.310565	2.305529	2.306224
2.268196	2.286627	2.281951	2.307206	2.30218	2.314428	2.309412	2.304434	2.305159
2.267283	2.285655	2.280955	2.306092	2.301046	2.313271	2.308278	2.303332	2.304072
2.266266	2.284622	2.27998	2.305055	2.300026	2.312213	2.307192	2.302209	2.302921

2.265254	2.2836	2.279026	2.304047	2.299034	2.311179	2.30612	2.301093	2.301774
2.279503	2.282669	2.278115	2.303047	2.298016	2.310117	2.305045	2.30001	2.30069
2.278617	2.281773	2.277215	2.302044	2.296985	2.309047	2.303974	2.298948	2.299638
2.285393	2.300669	2.295738	2.300948	2.295913	2.307973	2.30292	2.297908	2.298592
2.284402	2.299543	2.294571	2.299775	2.294804	2.306894	2.30188	2.296889	2.297554
2.283426	2.298466	2.293479	2.309769	2.304777	2.305837	2.300834	2.295846	2.296504
2.282465	2.297456	2.292489	2.308758	2.303768	2.304814	2.299782	2.294768	2.295431
2.281528	2.296467	2.29152	2.307762	2.302756	2.303778	2.298721	2.293692	2.29437
2.280629	2.295495	2.290555	2.306755	2.301707	2.302696	2.297639	2.292628	2.293338
2.279745	2.294524	2.28958	2.305733	2.300643	2.301606	2.296562	2.291581	2.292325
2.278855	2.293528	2.288548	2.30466	2.299572	2.30054	2.295525	2.290575	2.291334
2.277958	2.292512	2.287482	2.303558	2.298497	2.29949	2.294513	2.289595	2.290356
2.277015	2.29146	2.286428	2.302483	2.297451	2.298465	2.29351	2.288605	2.289351
2.276021	2.290369	2.285378	2.30143	2.296431	2.297466	2.292519	2.287609	2.288323
2.294343	2.289323	2.284359	2.300402	2.295428	2.296474	2.291518	2.286592	2.287281
2.293376	2.288352	2.283383	2.299402	2.294445	2.295488	2.290497	2.285533	2.286214
2.292409	2.287391	2.28242	2.298416	2.293467	2.294503	2.289484	2.284493	2.285173
2.291382	2.298039	2.293045	2.297437	2.292489	2.293525	2.288515	2.283535	2.284211
2.290345	2.297024	2.292062	2.296462	2.291503	2.292534	2.287551	2.282599	2.283279
2.289357	2.305459	2.300464	2.29547	2.29047	2.291478	2.286522	2.28161	2.282311
2.288402	2.304479	2.299487	2.294469	2.289408	2.290379	2.285454	2.280591	2.281327
2.287458	2.303511	2.298522	2.293492	2.288399	2.294956	2.29003	2.285177	2.280359
2.28653	2.302559	2.297572	2.292543	2.28745	2.293992	2.289071	2.28422	2.279407
2.29719	2.301606	2.296621	2.291597	2.286511	2.293047	2.288127	2.283274	2.278461
2.296234	2.300646	2.295665	2.290648	2.285577	2.292112	2.287193	2.282336	2.27752
2.295273	2.299681	2.294704	2.289696	2.284641	2.291181	2.286262	2.281402	2.27658
2.294299	2.298703	2.293731	2.288732	2.283691	2.290235	2.28532	2.280461	2.279361
2.29332	2.29772	2.292752	2.287762	2.282735	2.289285	2.284376	2.279519	2.278419
2.29235	2.296743	2.291781	2.2868	2.281787	2.288341	2.283436	2.278581	2.27748
2.291386	2.29577	2.290814	2.285843	2.280844	2.287402	2.282501	2.277646	2.276542
2.290465	2.29483	2.289864	2.284895	2.279913	2.280821	2.275934	2.271092	2.270245
2.289602	2.293935	2.288935	2.283955	2.278991	2.27991	2.27501	2.270152	2.269306
2.288729	2.293036	2.288009	2.283028	2.278095	2.279033	2.274118	2.26924	2.268385
2.287808	2.29211	2.287082	2.282126	2.277252	2.278224	2.273294	2.268385	2.2675
2.286873	2.291178	2.286156	2.281229	2.276416	2.277421	2.272482	2.267546	2.266627
2.295323	2.290241	2.28522	2.280307	2.275527	2.280555	2.2756	2.270636	2.26573
2.294389	2.289309	2.284288	2.27938	2.274612	2.279646	2.274709	2.269754	2.264831
2.293511	2.288445	2.283418	2.278501	2.273715	2.278737	2.273833	2.26891	2.263979
2.292689	2.287648	2.282608	2.277664	2.272828	2.277823	2.27297	2.268105	2.263175
2.291832	2.286819	2.281801	2.276849	2.271968	2.276927	2.272093	2.267257	2.262332
2.290905	2.285931	2.280996	2.276071	2.271149	2.276058	2.271197	2.266343	2.261423
2.28999	2.28504	2.280171	2.275268	2.270322	2.275193	2.270295	2.265414	2.260508
2.289136	2.284163	2.279283	2.274377	2.269437	2.274305	2.269382	2.264485	2.259618
2.288312	2.283304	2.278385	2.273467	2.268548	2.273426	2.268482	2.263571	2.258742
2.287515	2.282509	2.277563	2.272634	2.267725	2.272604	2.267646	2.262718	2.257877
2.28674	2.281754	2.276787	2.27185	2.266946	2.271821	2.266857	2.261913	2.257025
2.285905	2.280946	2.276001	2.276903	2.27197	2.271023	2.266066	2.261124	2.256208
2.285002	2.280079	2.27521	2.276137	2.271178	2.270212	2.265277	2.260358	2.255432
2.284125	2.279215	2.274386	2.279345	2.274355	2.269384	2.264464	2.259561	2.254642
2.283303	2.278363	2.273497	2.278438	2.273472	2.268519	2.263592	2.258687	2.253805

2.282505	2.277531	2.272614	2.277534	2.272593	2.267663	2.262736	2.257833	2.252985
2.281722	2.276742	2.271805	2.276714	2.271777	2.266863	2.261973	2.257104	2.252252
2.280952	2.275976	2.271031	2.275932	2.27099	2.266087	2.261242	2.256413	2.251544
2.280168	2.281043	2.276083	2.275151	2.270201	2.265289	2.260436	2.255605	2.250755
2.279376	2.280258	2.275311	2.274378	2.269414	2.264479	2.259577	2.254712	2.249905
2.278593	2.279477	2.274534	2.273597	2.268627	2.263678	2.258745	2.253856	2.250273
2.277824	2.278705	2.273748	2.272804	2.267837	2.262888	2.257947	2.253046	2.249438
2.277052	2.277932	2.272966	2.272016	2.267052	2.262105	2.257167	2.252265	2.248641
2.276265	2.277146	2.272189	2.271241	2.266271	2.261325	2.256401	2.251516	2.247911
2.275485	2.276367	2.271418	2.270473	2.2655	2.260558	2.255653	2.250791	2.247208
2.274735	2.275611	2.270648	2.2697	2.264741	2.259817	2.254932	2.250087	2.246504
2.274007	2.274877	2.26989	2.268936	2.263998	2.259095	2.25423	2.249396	2.2458
2.273314	2.274189	2.269199	2.26824	2.263298	2.258388	2.253515	2.24867	2.245063
2.272658	2.27355	2.268573	2.267609	2.26264	2.257698	2.25279	2.247915	2.244295
2.271968	2.276904	2.271916	2.266932	2.261945	2.258254	2.253304	2.24839	2.243529
2.271227	2.276148	2.271164	2.26618	2.26119	2.257499	2.252546	2.247629	2.242769
2.270475	2.275379	2.270393	2.26541	2.260427	2.256742	2.251789	2.246872	2.242015
2.269722	2.274616	2.269615	2.264633	2.259669	2.255996	2.251035	2.24611	2.241259
2.268977	2.273865	2.268849	2.263868	2.258925	2.255264	2.2503	2.245369	2.240525
2.268281	2.273168	2.268161	2.263183	2.258235	2.254574	2.249627	2.244714	2.239868
2.267614	2.272504	2.267521	2.262547	2.257578	2.253911	2.248996	2.244114	2.239264
2.266902	2.271793	2.266828	2.261859	2.256876	2.253207	2.248313	2.243455	2.238618
2.266138	2.271028	2.266076	2.261112	2.256124	2.252456	2.247573	2.242735	2.237933
2.265416	2.270306	2.26536	2.260398	2.25541	2.251745	2.246866	2.242034	2.237244
2.264775	2.269663	2.264712	2.259751	2.254771	2.251107	2.24622	2.241372	2.236553
2.264155	2.269041	2.264081	2.259121	2.254152	2.250494	2.245597	2.240731	2.235879
2.263531	2.268413	2.263445	2.258487	2.253535	2.24989	2.244991	2.240116	2.235253
2.262897	2.267775	2.262797	2.257843	2.252911	2.249282	2.244387	2.239513	2.234649
2.272073	2.267075	2.262086	2.257133	2.252216	2.248605	2.243736	2.238886	2.234033
2.27133	2.266329	2.261327	2.256373	2.251468	2.247875	2.243049	2.238242	2.233409
2.270631	2.265631	2.260626	2.256986	2.252058	2.247175	2.242356	2.237558	2.232734
2.269986	2.264991	2.259996	2.256356	2.251414	2.246509	2.24165	2.236816	2.231988
2.269357	2.264368	2.259382	2.255741	2.25079	2.245866	2.240973	2.236106	2.231271
2.268739	2.263752	2.258768	2.255129	2.250179	2.245253	2.240351	2.235474	2.230633
2.268128	2.263143	2.258159	2.254522	2.249575	2.244652	2.239749	2.234869	2.230022
2.267511	2.262527	2.257543	2.253908	2.248966	2.244045	2.23914	2.234258	2.229408
2.266894	2.261912	2.256928	2.253295	2.248356	2.243436	2.238532	2.23365	2.228799
2.261403	2.261305	2.25633	2.252697	2.247747	2.242819	2.237913	2.233031	2.228182
2.26079	2.260703	2.255744	2.25211	2.247137	2.242191	2.237282	2.232403	2.227558
2.260222	2.260145	2.255201	2.251567	2.246577	2.241612	2.236687	2.231795	2.226945
2.259722	2.259657	2.254727	2.251094	2.246092	2.241105	2.236139	2.231207	2.226338
2.25921	2.259155	2.254233	2.2506	2.245594	2.240596	2.235606	2.230648	2.225766
2.258633	2.258582	2.253657	2.250027	2.245031	2.240048	2.235084	2.23015	2.22527
2.258036	2.257985	2.253049	2.249421	2.244437	2.239477	2.234554	2.229658	2.224787
2.257446	2.258755	2.25378	2.248805	2.243826	2.238875	2.233967	2.229086	2.224222
2.256863	2.258165	2.253168	2.248181	2.2432	2.238249	2.233342	2.228465	2.223611
2.25633	2.257629	2.252616	2.247611	2.242608	2.237642	2.232728	2.22785	2.223004
2.255853	2.257153	2.252127	2.247095	2.242049	2.237046	2.232116	2.227233	2.222395
2.255341	2.256645	2.251621	2.246581	2.241514	2.236492	2.231549	2.22666	2.221827
2.254751	2.256065	2.251068	2.246053	2.241008	2.236001	2.231056	2.226161	2.221333

2.254164	2.255488	2.250521	2.245533	2.240516	2.235525	2.230575	2.225671	2.220842
2.253644	2.25498	2.250028	2.245048	2.240032	2.235035	2.230063	2.225133	2.220287
2.253144	2.254491	2.249544	2.244566	2.239547	2.23454	2.229545	2.22459	2.219723
2.252604	2.249233	2.244302	2.244017	2.239017	2.234026	2.229043	2.224095	2.219228
2.25204	2.24866	2.243706	2.243423	2.238454	2.233499	2.228552	2.223635	2.218782
2.251519	2.248136	2.243168	2.242888	2.237932	2.232993	2.228066	2.223169	2.218329
2.251048	2.247667	2.242701	2.242424	2.237457	2.232513	2.22759	2.2227	2.217874
2.252164	2.24721	2.242246	2.241968	2.236987	2.232031	2.227101	2.222208	2.213651
2.251713	2.246756	2.241788	2.241503	2.23651	2.231537	2.226583	2.221666	2.213119
2.251268	2.246306	2.241333	2.241037	2.236027	2.231034	2.226061	2.221124	2.212589
2.250825	2.24586	2.240877	2.240565	2.235516	2.230503	2.225545	2.220633	2.212134
2.250382	2.245416	2.240422	2.240093	2.235002	2.229969	2.225042	2.22017	2.211717
2.249929	2.244961	2.239967	2.239642	2.234557	2.229527	2.224595	2.219718	2.211297
2.249466	2.244499	2.239512	2.239211	2.234172	2.229164	2.224199	2.219278	2.210878
2.247094	2.244035	2.239051	2.23876	2.233737	2.225161	2.220207	2.215295	2.220457
2.246631	2.243568	2.238579	2.238276	2.233216	2.224656	2.21974	2.214873	2.220031
2.246164	2.243101	2.238111	2.237804	2.232724	2.224188	2.219295	2.214453	2.219602
2.245688	2.242631	2.237657	2.237378	2.23234	2.223849	2.218915	2.214026	2.219154
2.24521	2.242161	2.237207	2.236962	2.231983	2.223546	2.218567	2.21362	2.218718
2.24474	2.239792	2.234848	2.231806	2.226878	2.223195	2.218237	2.213296	2.218349
2.244275	2.239328	2.234381	2.231348	2.22644	2.22282	2.21792	2.21302	2.218022
2.243808	2.238852	2.233915	2.230892	2.225994	2.232477	2.227566	2.222649	2.217678
2.243334	2.238362	2.233448	2.230437	2.225537	2.232051	2.227148	2.222249	2.217324
2.242908	2.234696	2.229805	2.224925	2.220057	2.231637	2.226732	2.221836	2.216944
2.242556	2.234352	2.2294	2.224498	2.219655	2.231239	2.226314	2.2214	2.216516
2.242213	2.234028	2.229026	2.224106	2.219281	2.230863	2.225924	2.220992	2.2161
2.241823	2.233695	2.228709	2.223794	2.218964	2.230534	2.225605	2.220676	2.215747
2.241425	2.233367	2.228423	2.223523	2.218672	2.23022	2.22531	2.220393	2.215422
2.237973	2.233058	2.228143	2.233891	2.228941	2.228489	2.223587	2.218687	2.221185
2.237665	2.23276	2.227871	2.233597	2.228585	2.228096	2.223198	2.218321	2.220848
2.237322	2.232428	2.227562	2.233279	2.228252	2.227755	2.222852	2.217978	2.220525
2.236932	2.23205	2.227208	2.232935	2.227944	2.227473	2.222556	2.217664	2.220222
2.236572	2.231696	2.226864	2.232596	2.227643	2.222701	2.217782	2.212884	2.218717
2.236278	2.231399	2.22656	2.232285	2.227357	2.222417	2.217446	2.212494	2.218323
2.236007	2.231116	2.226255	2.231964	2.227056	2.222119	2.217107	2.21211	2.217932
2.235733	2.241479	2.236516	2.231583	2.226691	2.221773	2.216774	2.211789	2.226521
2.235463	2.24116	2.236142	2.231185	2.226302	2.221407	2.216449	2.211499	2.226232
2.235204	2.240875	2.235829	2.230857	2.225974	2.221085	2.216144	2.211205	2.225918
2.234953	2.240618	2.235565	2.230586	2.225694	2.220797	2.215858	2.210913	2.225587
2.234661	2.240332	2.235296	2.230322	2.225424	2.22708	2.222121	2.217153	2.22528
2.234306	2.24	2.235014	2.230066	2.225165	2.226811	2.221848	2.216877	2.225006
2.244605	2.239675	2.234732	2.229807	2.224905	2.226543	2.221578	2.216605	2.224741
2.2443	2.23938	2.234455	2.229539	2.224637	2.226269	2.221306	2.216336	2.224477
2.244018	2.239098	2.234179	2.229266	2.224365	2.225994	2.221035	2.216068	2.224216
2.243741	2.238817	2.233902	2.228992	2.224092	2.235149	2.230166	2.225174	2.22016
2.243479	2.238544	2.233626	2.228716	2.223818	2.234872	2.229894	2.224907	2.219896
2.243269	2.238308	2.23336	2.228436	2.223541	2.234597	2.229626	2.224647	2.219643
2.243113	2.238108	2.233102	2.22815	2.22326	2.234321	2.229362	2.224394	2.219399
2.242934	2.237901	2.232855	2.227881	2.222988	2.234048	2.229098	2.224146	2.219174
2.242713	2.237675	2.232615	2.227626	2.222716	2.233766	2.228828	2.223901	2.218977

2.242477	2.237449	2.232397	2.227405	2.222479	2.233513	2.228578	2.223665	2.218778
2.24223	2.237235	2.232229	2.227258	2.222323	2.233336	2.228373	2.223432	2.218538
2.24198	2.237022	2.232072	2.227128	2.22219	2.233186	2.228192	2.223217	2.2183
2.241747	2.236787	2.231849	2.242941	2.237964	2.232992	2.228018	2.223058	2.218135
2.241528	2.236541	2.231585	2.242671	2.237712	2.232772	2.227851	2.222938	2.218016
2.24135	2.23635	2.23138	2.24245	2.237484	2.232546	2.227646	2.222755	2.217837
2.241218	2.236221	2.231241	2.242286	2.237278	2.232305	2.227392	2.222498	2.217588
2.241089	2.236091	2.231098	2.242122	2.237091	2.232098	2.227171	2.222267	2.217353
2.240955	2.235939	2.230922	2.241936	2.236928	2.231953	2.227022	2.222105	2.217164
2.24081	2.235781	2.230746	2.241757	2.236779	2.231824	2.226887	2.221954	2.216989
2.24063	2.235616	2.230594	2.241611	2.23664	2.231682	2.226724	2.22177	2.216811
2.240437	2.235449	2.230452	2.241481	2.236505	2.231535	2.226552	2.221581	2.216641
2.240279	2.235299	2.23031	2.241338	2.236345	2.231363	2.226385	2.221423	2.216493
2.240149	2.235161	2.230171	2.241186	2.236163	2.23117	2.226219	2.22129	2.216365
2.240014	2.245084	2.240044	2.241018	2.235991	2.231001	2.226067	2.221154	2.216223
2.239875	2.244921	2.239857	2.240824	2.235828	2.230862	2.225935	2.221016	2.216062
2.227607	2.250781	2.245685	2.24065	2.235684	2.230736	2.225801	2.220865	2.215897
2.227413	2.250597	2.245533	2.240521	2.23557	2.23062	2.225644	2.220671	2.215715
2.227217	2.250422	2.245399	2.240412	2.23547	2.230513	2.2255	2.220493	2.215552
2.227051	2.250288	2.245283	2.240307	2.235369	2.230419	2.225424	2.220432	2.215479
2.226905	2.250181	2.24518	2.240208	2.23527	2.230332	2.225383	2.220427	2.215452
2.237964	2.239555	2.234626	2.240107	2.235169	2.230231	2.22528	2.220324	2.215351
2.237862	2.239434	2.234503	2.240007	2.235068	2.230116	2.22512	2.220129	2.215183
2.237751	2.239315	2.234384	2.239905	2.234968	2.230014	2.225007	2.220005	2.215066
2.237623	2.239199	2.234272	2.239802	2.234872	2.229936	2.224976	2.22001	2.215038
2.2375	2.239082	2.234158	2.239699	2.234774	2.229856	2.224943	2.220015	2.215019
2.233314	2.238948	2.234034	2.239601	2.234674	2.229757	2.224855	2.219943	2.214977
2.233214	2.238826	2.233912	2.239502	2.23457	2.229651	2.224746	2.219841	2.214916
2.233189	2.238777	2.233811	2.239385	2.234459	2.229547	2.224641	2.219733	2.214803
2.233214	2.238777	2.233728	2.239262	2.234345	2.229443	2.224533	2.219614	2.214656
2.233183	2.238751	2.233687	2.228699	2.223797	2.229331	2.22442	2.219509	2.214556
2.23309	2.238699	2.233696	2.228709	2.223728	2.229203	2.224297	2.219418	2.214511
2.23977	2.238631	2.233685	2.22871	2.223688	2.229128	2.224207	2.219331	2.214457
2.23967	2.238529	2.233618	2.228673	2.223683	2.229139	2.224162	2.21923	2.214363
2.239597	2.238446	2.233553	2.228637	2.223698	2.229186	2.224155	2.219161	2.214288
2.23959	2.238445	2.233544	2.228639	2.223734	2.218808	2.21382	2.208855	2.214288
2.239621	2.23848	2.233553	2.228646	2.223769	2.218863	2.213873	2.208898	2.214325
2.239651	2.238494	2.233516	2.228588	2.223722	2.218838	2.213882	2.208933	2.214347
2.239684	2.238496	2.233447	2.228481	2.223612	2.218748	2.213859	2.208967	2.214365
2.239677	2.238484	2.233412	2.228422	2.223524	2.218654	2.213807	2.208961	2.214374
2.23961	2.23845	2.233417	2.228421	2.223458	2.218545	2.21371	2.208897	2.214367
2.239565	2.234598	2.229622	2.228446	2.223431	2.218481	2.213648	2.208855	2.214375
2.239587	2.234629	2.229666	2.228494	2.223466	2.218504	2.213668	2.208874	2.214414
2.239627	2.234673	2.229719	2.228555	2.223527	2.218563	2.21372	2.208919	2.214468
2.239656	2.23471	2.229774	2.228619	2.223586	2.218616	2.213772	2.208969	2.214531
2.239675	2.234738	2.229823	2.228682	2.223648	2.218676	2.213832	2.209029	2.214603
2.239647	2.234708	2.22981	2.228693	2.223687	2.218735	2.213893	2.209087	2.214673
2.239571	2.234619	2.229738	2.228656	2.223706	2.218795	2.213957	2.209144	2.214743
2.239538	2.234578	2.229704	2.228646	2.223736	2.218854	2.214019	2.209201	2.214813
2.23957	2.234605	2.22973	2.22868	2.223785	2.218913	2.214079	2.209259	2.214884

2.23963	2.234662	2.229778	2.224901	2.220028	2.21897	2.214136	2.209316	2.204494
2.239702	2.234726	2.229825	2.224941	2.220074	2.219025	2.214191	2.209369	2.204547
2.2398	2.234817	2.229895	2.224998	2.220127	2.219079	2.214244	2.209422	2.2046
2.239952	2.234974	2.230035	2.225113	2.220205	2.219134	2.214295	2.209476	2.204653
2.24014	2.235176	2.230222	2.225266	2.220299	2.219188	2.214344	2.209531	2.204707
2.240266	2.235312	2.230352	2.225378	2.220375	2.219241	2.214393	2.209585	2.204763
2.24033	2.235383	2.230427	2.225449	2.220432	2.21929	2.214442	2.209637	2.204819
2.240385	2.235441	2.230492	2.225516	2.220496	2.215538	2.2107	2.205908	2.204875
2.240428	2.235485	2.230547	2.225581	2.220569	2.215612	2.210757	2.205948	2.20492
2.240497	2.235549	2.230615	2.225658	2.220664	2.215714	2.210843	2.206015	2.204986
2.240652	2.235682	2.23072	2.225761	2.220799	2.215872	2.211002	2.206163	2.205111
2.240839	2.235846	2.23085	2.225887	2.220959	2.216059	2.211193	2.206343	2.205263
2.240967	2.235987	2.231004	2.226053	2.221135	2.216234	2.211345	2.206472	2.205396
2.241056	2.236114	2.23118	2.22625	2.221325	2.216404	2.211473	2.206568	2.205517
2.241146	2.236217	2.231306	2.226388	2.221464	2.216536	2.211583	2.206659	2.205622
2.241234	2.236282	2.231361	2.226445	2.221536	2.21662	2.211667	2.206739	2.205698
2.241346	2.236376	2.231442	2.226521	2.221616	2.216706	2.211764	2.206845	2.205803
2.241503	2.236538	2.231602	2.226666	2.221728	2.216803	2.211885	2.206997	2.205969
2.241679	2.23672	2.231778	2.226827	2.221859	2.216922	2.212031	2.207176	2.206157
2.241857	2.236886	2.231921	2.226963	2.222009	2.217087	2.212218	2.207375	2.20253
2.242037	2.237048	2.232054	2.227096	2.222177	2.217286	2.212434	2.20759	2.202709
2.242189	2.237197	2.232208	2.227264	2.22237	2.217498	2.21265	2.207806	2.213718
2.242315	2.237333	2.23238	2.227464	2.222588	2.217726	2.212872	2.208024	2.213961
2.242463	2.237497	2.232573	2.227674	2.222803	2.217942	2.21308	2.208227	2.214166
2.242641	2.237699	2.232792	2.227895	2.223009	2.218134	2.213269	2.208412	2.214317
2.242839	2.237913	2.233016	2.228118	2.223218	2.229236	2.224302	2.219374	2.214453
2.243063	2.238126	2.233229	2.228333	2.223437	2.229429	2.224453	2.219488	2.214575
2.243297	2.238342	2.233443	2.228546	2.223658	2.229626	2.224612	2.219612	2.214711
2.24351	2.238561	2.233662	2.228765	2.223872	2.229828	2.22483	2.219844	2.214933
2.243712	2.238782	2.233885	2.228985	2.224082	2.230033	2.225082	2.220137	2.215204
2.243926	2.238993	2.234098	2.240124	2.235176	2.230231	2.225282	2.220339	2.215405
2.244146	2.239189	2.234299	2.240323	2.235377	2.23042	2.225424	2.220442	2.215529
2.244413	2.23943	2.234517	2.240521	2.235579	2.230621	2.225607	2.220609	2.215701
2.244763	2.239747	2.234752	2.240705	2.235774	2.230842	2.225874	2.22091	2.215968
2.245106	2.240073	2.235015	2.24092	2.235983	2.231064	2.226144	2.22122	2.216255
2.245369	2.240362	2.235335	2.24122	2.236219	2.231257	2.226351	2.221459	2.216528
2.245596	2.240631	2.23567	2.241553	2.236477	2.231457	2.226544	2.221671	2.216791
2.245817	2.251844	2.246853	2.241831	2.236765	2.231739	2.226786	2.221875	2.217008
2.246029	2.252038	2.24706	2.242074	2.237079	2.232082	2.227061	2.222071	2.217191
2.246307	2.252284	2.247276	2.242293	2.237342	2.232374	2.227341	2.222327	2.217422
2.246671	2.252593	2.247498	2.242476	2.237541	2.232605	2.22763	2.222658	2.217712
2.247007	2.252896	2.247751	2.242698	2.23775	2.232823	2.227901	2.222976	2.218009
2.247263	2.253172	2.248068	2.243009	2.237995	2.233026	2.228124	2.223237	2.218297
2.247494	2.253428	2.248389	2.243338	2.238262	2.233247	2.228351	2.223489	2.218589
2.256202	2.253646	2.248646	2.24362	2.238553	2.233543	2.22864	2.223774	2.218896
2.25642	2.253851	2.248878	2.243885	2.238865	2.23388	2.228957	2.224066	2.219202
2.2567	2.254127	2.249152	2.244171	2.239183	2.234209	2.229248	2.224316	2.219457
2.257034	2.254462	2.24946	2.244474	2.23951	2.234538	2.22952	2.224531	2.219664
2.257373	2.252301	2.247262	2.244743	2.239798	2.234836	2.2298	2.224788	2.219914
2.257711	2.2526	2.247498	2.244953	2.240022	2.235086	2.230085	2.225105	2.220229

2.258055	2.252911	2.247758	2.245191	2.240267	2.23535	2.230386	2.225437	2.220548
2.258415	2.253263	2.248089	2.24551	2.240583	2.235667	2.230713	2.225764	2.220836
2.258763	2.253619	2.248445	2.245865	2.240931	2.236008	2.231055	2.226098	2.221127
2.259044	2.253926	2.248786	2.24374	2.238804	2.236357	2.231403	2.226442	2.221453
2.259286	2.254209	2.249124	2.244107	2.239168	2.236715	2.231759	2.226795	2.221802
2.259579	2.254524	2.249471	2.24447	2.23953	2.237073	2.232115	2.227148	2.22215
2.259918	2.254868	2.249827	2.244831	2.239888	2.23743	2.232471	2.227503	2.222499
2.260272	2.255221	2.250185	2.245191	2.240246	2.235299	2.230332	2.225354	2.222848
2.260626	2.255571	2.250542	2.24555	2.240603	2.235653	2.230684	2.225703	2.223192
2.261012	2.255942	2.250904	2.245906	2.240957	2.236006	2.231036	2.226055	2.223543
2.261466	2.256359	2.251275	2.246253	2.241303	2.236357	2.231392	2.226417	2.223911
2.261944	2.256794	2.251654	2.246601	2.241649	2.236708	2.231749	2.226783	2.22429
2.262371	2.257207	2.252043	2.24697	2.242001	2.237052	2.232098	2.227147	2.22218
2.262766	2.257607	2.252442	2.247357	2.24236	2.237394	2.232443	2.227511	2.222588
2.263153	2.258016	2.252877	2.247796	2.242778	2.237791	2.232825	2.227886	2.222977
2.263531	2.258435	2.253358	2.248303	2.243272	2.238257	2.233249	2.228269	2.223336
2.263917	2.258849	2.253812	2.248775	2.243737	2.238708	2.233683	2.228682	2.223729
2.264311	2.259241	2.25419	2.24915	2.244122	2.239112	2.234124	2.229156	2.224206
2.264731	2.259648	2.254569	2.249519	2.244495	2.239502	2.234555	2.229625	2.224681
2.265215	2.260127	2.255033	2.24996	2.244903	2.239886	2.234932	2.230005	2.225066
2.265724	2.260632	2.255527	2.250428	2.245327	2.240272	2.235293	2.230352	2.225416
2.266152	2.261058	2.255957	2.250863	2.245768	2.240713	2.235722	2.230764	2.225811
2.266514	2.261419	2.256337	2.251273	2.246226	2.241202	2.236207	2.231228	2.226241
2.266921	2.261813	2.256724	2.251667	2.246643	2.241638	2.236651	2.231673	2.226677
2.267394	2.262253	2.257118	2.252031	2.246993	2.241991	2.23703	2.232086	2.227115
2.26787	2.262704	2.257539	2.252429	2.247376	2.242371	2.237429	2.232512	2.227565
2.268316	2.263152	2.258006	2.252907	2.247854	2.242846	2.237899	2.232975	2.228033
2.268765	2.263612	2.2585	2.253418	2.248366	2.243353	2.23839	2.233451	2.228504
2.264334	2.264108	2.259009	2.25393	2.248868	2.243842	2.23887	2.233919	2.228957
2.264834	2.264629	2.259533	2.254448	2.24937	2.244328	2.239344	2.234383	2.229401
2.265345	2.265152	2.260049	2.254954	2.249859	2.2448	2.239798	2.234822	2.229841
2.265871	2.265679	2.260559	2.255447	2.250337	2.245261	2.24023	2.235235	2.230275
2.266399	2.266208	2.261074	2.255942	2.250805	2.245707	2.240665	2.235671	2.230732
2.266927	2.266737	2.261596	2.256438	2.251245	2.246116	2.241098	2.236142	2.231226
2.267456	2.267268	2.262122	2.256944	2.251714	2.246564	2.241562	2.236633	2.231731
2.264782	2.267798	2.262648	2.257479	2.252275	2.247136	2.242106	2.237143	2.232233
2.265307	2.268328	2.263175	2.258022	2.252862	2.247742	2.242674	2.237663	2.23274
2.265825	2.268857	2.263702	2.258542	2.253365	2.248235	2.243173	2.238171	2.233248
2.266339	2.269383	2.26423	2.259048	2.253819	2.248662	2.243632	2.238674	2.233761
2.266853	2.26991	2.264763	2.259584	2.254353	2.249193	2.244156	2.239191	2.23428
2.267367	2.270437	2.265302	2.260153	2.254982	2.249846	2.244751	2.239719	2.234801
2.267882	2.270964	2.265838	2.260715	2.255594	2.250483	2.245361	2.240291	2.235366
2.268394	2.271488	2.266363	2.26125	2.256146	2.251061	2.245983	2.24095	2.23602
2.268912	2.272018	2.266896	2.261786	2.256684	2.251616	2.246591	2.241606	2.236671
2.26054	2.267593	2.26252	2.262339	2.257221	2.252145	2.247132	2.242163	2.23723
2.261072	2.268139	2.263081	2.262909	2.257767	2.25267	2.247649	2.242676	2.237747
2.261679	2.268755	2.263693	2.263523	2.258386	2.253279	2.248216	2.243203	2.23827
2.26235	2.269434	2.264347	2.264176	2.259065	2.253956	2.248822	2.243735	2.238793
2.262979	2.270068	2.264959	2.264785	2.259688	2.254585	2.249432	2.244322	2.239372
2.263545	2.270633	2.265502	2.265325	2.260233	2.255151	2.25005	2.244988	2.240033

2.26412	2.271213	2.266069	2.265887	2.260778	2.255701	2.250651	2.24564	2.240679
2.26475	2.27186	2.266723	2.266525	2.261351	2.256231	2.251198	2.246213	2.241248
2.265382	2.272512	2.26739	2.267181	2.261946	2.256782	2.251753	2.246782	2.241813
2.265949	2.273095	2.267987	2.2678	2.262581	2.257428	2.2524	2.247427	2.242455
2.266489	2.273649	2.268556	2.268409	2.263247	2.258133	2.253101	2.248112	2.243138
2.267098	2.274271	2.269185	2.269059	2.263924	2.258825	2.253785	2.248783	2.243809
2.267768	2.274957	2.26987	2.269748	2.264613	2.259511	2.25446	2.249447	2.244477
2.259747	2.267189	2.262137	2.265461	2.260371	2.260191	2.255113	2.250073	2.2451
2.260444	2.267867	2.262805	2.266133	2.261054	2.26087	2.255733	2.250632	2.245644
2.261112	2.268516	2.263442	2.266776	2.261709	2.261528	2.256352	2.251209	2.246207
2.261701	2.269086	2.264	2.267344	2.262291	2.262137	2.256987	2.251866	2.246863
2.262272	2.269638	2.264543	2.267897	2.262856	2.262736	2.257636	2.252559	2.247558
2.262915	2.262179	2.257074	2.268532	2.263494	2.263389	2.25831	2.253251	2.248249
2.263605	2.262872	2.257774	2.26922	2.264179	2.264078	2.259005	2.253952	2.248945
2.264302	2.263572	2.258476	2.262204	2.257149	2.264768	2.259697	2.254646	2.249637
2.26501	2.264282	2.259182	2.262915	2.257858	2.265459	2.260386	2.255332	2.250321
2.265718	2.264991	2.259885	2.263622	2.258564	2.266149	2.26108	2.256029	2.251015
2.266418	2.26569	2.260575	2.264316	2.259262	2.266836	2.261779	2.256737	2.251716
2.267122	2.266394	2.261272	2.265017	2.259963	2.267525	2.26249	2.257469	2.252445
2.267832	2.267108	2.261988	2.265738	2.260669	2.260898	2.255904	2.250929	2.253247
2.268559	2.26784	2.262728	2.266481	2.26139	2.261624	2.256683	2.251764	2.254071
2.26935	2.26425	2.259191	2.25845	2.253378	2.257131	2.252219	2.247336	2.247517
2.270193	2.265093	2.26004	2.259303	2.254202	2.25793	2.252962	2.248028	2.248209
2.270987	2.265885	2.260829	2.260094	2.254977	2.258694	2.253688	2.248722	2.248908
2.271715	2.266608	2.26154	2.260807	2.255687	2.259408	2.254393	2.249419	2.249616
2.272438	2.267326	2.262243	2.26151	2.256392	2.260123	2.255099	2.25012	2.250338
2.273168	2.268051	2.26295	2.262218	2.257105	2.260845	2.255804	2.250818	2.25108
2.273921	2.268798	2.26368	2.262946	2.257841	2.261589	2.25653	2.251533	2.251833
2.274746	2.269619	2.264484	2.263749	2.25865	2.262404	2.257324	2.252301	2.254439
2.275609	2.270478	2.26533	2.264592	2.259497	2.263254	2.258152	2.2531	2.255199
2.276397	2.271269	2.266125	2.265389	2.260282	2.264035	2.258925	2.253861	2.255938
2.277116	2.271996	2.266877	2.266146	2.261012	2.264752	2.259646	2.254589	2.256656
2.270577	2.272772	2.267668	2.266943	2.261792	2.265525	2.260415	2.255356	2.25257
2.271436	2.273624	2.268521	2.267805	2.262648	2.266383	2.261255	2.256178	2.253384
2.272311	2.274494	2.269383	2.268676	2.263523	2.267266	2.262122	2.257025	2.254225
2.27318	2.275371	2.270238	2.269535	2.264403	2.268166	2.263008	2.257892	2.25508
2.274032	2.276233	2.27108	2.270378	2.265271	2.269056	2.263897	2.258773	2.25595
2.274804	2.277007	2.271863	2.266751	2.26167	2.260963	2.255892	2.250853	2.256835
2.275526	2.277726	2.272611	2.267504	2.262394	2.261696	2.256703	2.251742	2.257729
2.276324	2.271239	2.266173	2.268307	2.263182	2.262482	2.257499	2.252556	2.258564
2.277197	2.272104	2.267031	2.269161	2.264037	2.263319	2.258274	2.253283	2.259334
2.278086	2.272984	2.267904	2.27003	2.264909	2.266885	2.261778	2.256727	2.260114
2.278979	2.273871	2.268782	2.270908	2.26579	2.267766	2.262636	2.257555	2.260912
2.279875	2.274762	2.269667	2.271795	2.266681	2.268662	2.263522	2.258421	2.261746
2.271549	2.275647	2.270557	2.272688	2.267577	2.269563	2.264425	2.259325	2.262655
2.272424	2.276532	2.271451	2.273583	2.268475	2.270467	2.265344	2.26026	2.263607
2.273314	2.268182	2.263092	2.26725	2.262172	2.271366	2.266274	2.261214	2.26456
2.274217	2.269071	2.263952	2.268113	2.263038	2.27226	2.267212	2.262188	2.265521
2.275115	2.269962	2.264834	2.259731	2.254648	2.273132	2.268094	2.26308	2.266417
2.276003	2.270852	2.265738	2.260627	2.255508	2.273966	2.268896	2.263863	2.267226

2.276903	2.271759	2.266665	2.261552	2.25641	2.255893	2.250843	2.245841	2.259653
2.27782	2.272697	2.26763	2.262535	2.257402	2.256876	2.251794	2.246752	2.260549
2.278754	2.273646	2.268596	2.263516	2.258399	2.257872	2.252764	2.247689	2.261465
2.276913	2.274594	2.269514	2.264419	2.259302	2.258776	2.253663	2.248583	2.255144
2.277889	2.275542	2.270409	2.265283	2.26016	2.259636	2.254532	2.249464	2.256037
2.273322	2.276474	2.271319	2.266172	2.26103	2.260497	2.255403	2.250352	2.250313
2.274241	2.277391	2.272238	2.267078	2.261903	2.261351	2.256271	2.251243	2.251236
2.270056	2.278324	2.27318	2.272637	2.267432	2.262276	2.257195	2.252168	2.252167
2.270998	2.279281	2.274159	2.273638	2.268457	2.263307	2.258203	2.253138	2.253102
2.271951	2.275652	2.270535	2.274627	2.269474	2.264337	2.259216	2.254123	2.254059
2.27293	2.276624	2.271481	2.275554	2.270413	2.265293	2.260195	2.255122	2.255071
2.273913	2.277592	2.272415	2.276464	2.271333	2.266231	2.26116	2.256113	2.256087
2.274855	2.278525	2.273347	2.277396	2.27229	2.267198	2.262104	2.257031	2.257013
2.275778	2.279441	2.274278	2.278341	2.273266	2.26818	2.263035	2.257909	2.25789
2.27677	2.28042	2.275249	2.275172	2.270112	2.269169	2.264007	2.25886	2.258837
2.277831	2.281462	2.276257	2.276166	2.27111	2.270171	2.265026	2.259891	2.259857
2.278899	2.276938	2.271749	2.277166	2.272089	2.267014	2.261893	2.256782	2.255834
2.279968	2.277998	2.272787	2.278163	2.273023	2.267909	2.262804	2.257726	2.256799
2.281016	2.279047	2.27383	2.279176	2.273973	2.268819	2.263724	2.258673	2.257768
2.281993	2.280048	2.274866	2.280221	2.274995	2.26982	2.264721	2.259672	2.258768
2.282949	2.281035	2.275903	2.281284	2.27605	2.270863	2.265753	2.260695	2.259784
2.283966	2.28206	2.276952	2.282342	2.277097	2.2719	2.266786	2.261726	2.256727
2.285024	2.283112	2.27801	2.2834	2.278146	2.272941	2.267826	2.262766	2.257764
2.286094	2.289675	2.284556	2.284462	2.279198	2.273984	2.268867	2.263806	2.2588
2.287179	2.290746	2.285625	2.28553	2.280254	2.275031	2.26991	2.264847	2.259836
2.288259	2.291812	2.28669	2.286594	2.281307	2.276077	2.270953	2.265888	2.260876
2.289325	2.292864	2.287742	2.287646	2.282349	2.277112	2.27199	2.26693	2.261925
2.290384	2.293912	2.288791	2.2887	2.283398	2.278157	2.273032	2.267973	2.262975
2.291436	2.294959	2.289849	2.289768	2.284471	2.279227	2.274082	2.269006	2.264012
2.292486	2.296006	2.29091	2.290845	2.285563	2.280321	2.275148	2.270042	2.265046
2.293545	2.297058	2.291973	2.291935	2.286694	2.281471	2.276273	2.27113	2.266107
2.29461	2.298113	2.293038	2.293037	2.287862	2.282676	2.277453	2.272262	2.26719
2.278385	2.296396	2.291318	2.289006	2.28391	2.283831	2.278598	2.27338	2.268268
2.279489	2.297517	2.292384	2.290048	2.284969	2.284911	2.279685	2.274464	2.269323
2.280593	2.298645	2.29345	2.291084	2.286016	2.285978	2.280776	2.275572	2.270413
2.281652	2.29975	2.294503	2.292117	2.287067	2.287065	2.281915	2.276755	2.271591
2.282712	2.300872	2.295575	2.293167	2.288122	2.288147	2.283054	2.277947	2.272794
2.283835	2.302075	2.296726	2.294274	2.289188	2.289202	2.284134	2.279068	2.273955
2.28499	2.303325	2.29793	2.295426	2.29027	2.290244	2.28518	2.280145	2.275089
2.286077	2.304527	2.299141	2.296619	2.291413	2.291346	2.286251	2.281203	2.276172
2.2871	2.305687	2.300369	2.297862	2.29262	2.292503	2.287338	2.282232	2.277198
2.288115	2.306814	2.301551	2.299068	2.293826	2.288616	2.283434	2.278306	2.278249
2.289117	2.307883	2.302652	2.300206	2.295024	2.289845	2.284638	2.279461	2.279343
2.290158	2.308975	2.303749	2.301324	2.296194	2.291051	2.285846	2.280647	2.280475
2.291304	2.293046	2.288134	2.302418	2.297283	2.292157	2.287003	2.281851	2.281674
2.292502	2.294192	2.28921	2.303529	2.298359	2.293231	2.28814	2.283054	2.282897
2.293689	2.295395	2.290408	2.302042	2.296821	2.294375	2.289263	2.284177	2.284051
2.294876	2.296645	2.291695	2.303403	2.298095	2.295564	2.290377	2.285244	2.28516
2.295995	2.297792	2.292872	2.304668	2.29934	2.296778	2.291547	2.286377	2.281293
2.297023	2.298808	2.293911	2.305816	2.300553	2.298026	2.292788	2.287585	2.282447

2.290931	2.299878	2.294977	2.290089	2.285219	2.299249	2.294019	2.28881	2.283641
2.292176	2.301095	2.296134	2.291199	2.286293	2.300391	2.295198	2.290038	2.284903
2.293433	2.302334	2.297317	2.292336	2.28739	2.301553	2.296388	2.291272	2.286171
2.294559	2.303487	2.29849	2.293529	2.288603	2.302853	2.297656	2.292499	2.287372
2.295643	2.304615	2.299667	2.294748	2.289865	2.304209	2.298956	2.293726	2.288551
2.304142	2.305812	2.300849	2.295921	2.291034	2.305456	2.300199	2.294966	2.289792
2.305429	2.307064	2.302037	2.297058	2.292131	2.306617	2.301397	2.296218	2.291083
2.30664	2.301626	2.296605	2.298219	2.293283	2.291463	2.286592	2.281751	2.292335
2.307728	2.30274	2.297763	2.299421	2.294527	2.292716	2.287802	2.282901	2.293516
2.308849	2.303875	2.298923	2.30061	2.295745	2.293943	2.289003	2.284067	2.294734
2.31011	2.305098	2.300087	2.301739	2.296848	2.295043	2.290149	2.285267	2.296056
2.31139	2.306337	2.301263	2.302873	2.297949	2.296138	2.291301	2.28649	2.297415
2.308625	2.307507	2.302468	2.304101	2.299179	2.294307	2.289496	2.284711	2.298765
2.309724	2.308653	2.303698	2.305386	2.300483	2.295616	2.29079	2.285985	2.30011
2.310928	2.305957	2.301046	2.306674	2.301781	2.296906	2.292046	2.287203	2.285394
2.312229	2.307262	2.302342	2.307977	2.303083	2.29819	2.293273	2.288365	2.286504
2.313534	2.308554	2.303601	2.302607	2.297722	2.299402	2.294468	2.289543	2.28766
2.314844	2.309823	2.304796	2.303753	2.298817	2.300484	2.295603	2.290748	2.288899
2.316106	2.311055	2.305981	2.304908	2.299931	2.301592	2.296765	2.291978	2.290165
2.317233	2.312202	2.307179	2.302194	2.297243	2.302847	2.298032	2.293252	2.291446
2.318324	2.313333	2.308393	2.303465	2.29854	2.304169	2.299344	2.294546	2.292728
2.319542	2.314575	2.309671	2.304766	2.299854	2.30184	2.297004	2.292186	2.293969
2.320844	2.315893	2.310995	2.306095	2.301191	2.303173	2.2983	2.293435	2.295179
2.314906	2.317204	2.312297	2.307394	2.302496	2.297603	2.292714	2.28783	2.292948
2.316231	2.318512	2.313582	2.308668	2.303776	2.298873	2.29393	2.288992	2.294101
2.317548	2.312567	2.307615	2.309888	2.304988	2.300076	2.295111	2.290158	2.295277
2.318853	2.313822	2.308797	2.311015	2.306072	2.30115	2.296228	2.291335	2.296493
2.32013	2.31505	2.309963	2.312134	2.307158	2.302232	2.297363	2.292536	2.297736
2.321342	2.31624	2.311151	2.313325	2.308369	2.303463	2.298605	2.293788	2.295807
2.322523	2.317415	2.312355	2.314556	2.309645	2.304769	2.299906	2.295076	2.2971
2.323738	2.31864	2.313603	2.308638	2.303756	2.306073	2.301213	2.296379	2.291593
2.324978	2.319903	2.31489	2.309943	2.305071	2.307389	2.302535	2.297703	2.292905
2.326257	2.321204	2.316205	2.311264	2.306389	2.301538	2.296693	2.291869	2.294215
2.327585	2.32255	2.317551	2.312602	2.307708	2.302845	2.298005	2.293188	2.295524
2.328928	2.323907	2.318905	2.313946	2.309031	2.304152	2.299315	2.294503	2.289696
2.330275	2.325258	2.320256	2.31529	2.310356	2.305464	2.300622	2.295809	2.291001
2.331628	2.326611	2.321608	2.316634	2.311684	2.306778	2.30193	2.297114	2.292304
2.332984	2.327965	2.322956	2.317974	2.313013	2.308097	2.303245	2.298426	2.293612
2.334344	2.329319	2.324302	2.319311	2.314342	2.30942	2.304563	2.29974	2.294922
2.335694	2.330669	2.32565	2.320653	2.315675	2.310744	2.305881	2.301054	2.296232
2.337036	2.332015	2.326998	2.321999	2.31701	2.312069	2.307199	2.302367	2.297544
2.332731	2.333357	2.328343	2.323342	2.318347	2.313398	2.308516	2.303673	2.298849
2.334053	2.334692	2.329682	2.324683	2.319688	2.314732	2.309831	2.304968	2.300143
2.335384	2.336031	2.331022	2.326022	2.321029	2.316067	2.311145	2.306262	2.301434
2.336713	2.337365	2.332349	2.327347	2.322357	2.317393	2.312456	2.307556	2.302721
2.338078	2.338729	2.333697	2.328686	2.323693	2.318724	2.313774	2.308861	2.304016
2.312752	2.340215	2.335141	2.330092	2.325067	2.320074	2.315109	2.310187	2.305335
2.314304	2.341787	2.336654	2.331551	2.326476	2.321446	2.316467	2.311541	2.306681
2.315754	2.337626	2.332532	2.333057	2.327975	2.322939	2.317955	2.313021	2.30815
2.3171	2.339055	2.334032	2.334628	2.329591	2.324581	2.319601	2.314658	2.30977

2.31841	2.340438	2.335472	2.336126	2.331126	2.32614	2.321166	2.316217	2.311312
2.319693	2.341767	2.336813	2.337488	2.332498	2.327521	2.322554	2.317607	2.312688
2.320961	2.34307	2.338116	2.3388	2.333814	2.32884	2.323879	2.318933	2.314001
2.322231	2.344371	2.339417	2.34011	2.335124	2.330151	2.325186	2.320235	2.315293
2.323505	2.345671	2.340712	2.341413	2.336424	2.331446	2.326475	2.321517	2.316568
2.324794	2.335152	2.330233	2.337067	2.332113	2.332764	2.32779	2.322829	2.317878
2.326098	2.336488	2.33156	2.338393	2.333436	2.334099	2.329125	2.324165	2.319215
2.327507	2.337927	2.332993	2.339827	2.334864	2.335536	2.33056	2.325601	2.320653
2.329067	2.339514	2.334578	2.341415	2.336439	2.337116	2.332137	2.327179	2.322233
2.330579	2.325716	2.320903	2.342953	2.337973	2.33866	2.333676	2.328715	2.323771
2.331933	2.327064	2.32224	2.344323	2.339356	2.34006	2.335062	2.330085	2.325142
2.333233	2.328359	2.323525	2.345645	2.340704	2.341436	2.336428	2.331439	2.326498
2.334531	2.329659	2.324826	2.347	2.342104	2.337203	2.332261	2.327322	2.328003
2.335833	2.330965	2.326138	2.348372	2.343527	2.338675	2.333777	2.328871	2.32957
2.337265	2.332396	2.327565	2.338042	2.333224	2.340105	2.335208	2.330305	2.331016
2.338825	2.33395	2.329106	2.339574	2.334674	2.341499	2.336564	2.331638	2.332354
2.323452	2.335426	2.330573	2.34105	2.336093	2.342878	2.337911	2.332969	2.333704
2.324769	2.336754	2.331899	2.342413	2.337445	2.344217	2.339232	2.334283	2.33507
2.32611	2.338105	2.333251	2.343809	2.338844	2.345616	2.340621	2.335669	2.336487
2.327601	2.339608	2.334757	2.345357	2.340403	2.347186	2.342195	2.337229	2.332325
2.329122	2.341137	2.33629	2.346935	2.341996	2.348799	2.343823	2.338853	2.333883
2.33051	2.325685	2.320927	2.34837	2.343436	2.338524	2.333634	2.328748	2.33544
2.331828	2.326988	2.322226	2.349726	2.344794	2.339905	2.335079	2.330263	2.337004
2.333261	2.328411	2.323648	2.35121	2.346274	2.341382	2.336561	2.331751	2.338498
2.334821	2.329965	2.325206	2.352832	2.347881	2.342959	2.338075	2.333198	2.339906
2.323774	2.331489	2.326743	2.338743	2.333949	2.344553	2.339627	2.334702	2.341384
2.32515	2.332919	2.328216	2.340265	2.335509	2.346175	2.341257	2.336332	2.34302
2.326548	2.334352	2.329665	2.341747	2.337021	2.34775	2.34285	2.33794	2.344649
2.309496	2.335848	2.331082	2.326366	2.321706	2.349157	2.344263	2.339373	2.334494
2.311096	2.337384	2.332501	2.327708	2.323014	2.350509	2.345616	2.340749	2.335922
2.297606	2.338979	2.334034	2.329194	2.32447	2.352011	2.347113	2.342245	2.337408
2.2992	2.340627	2.335654	2.330787	2.326036	2.35362	2.34871	2.343824	2.33893
2.276779	2.324716	2.319798	2.332362	2.3276	2.355237	2.350323	2.345426	2.340504
2.278099	2.326145	2.321304	2.333925	2.329177	2.35688	2.351966	2.347065	2.342146
2.279464	2.306784	2.302029	2.31803	2.313317	2.325968	2.321306	2.316659	2.343766
2.280973	2.308275	2.30345	2.319416	2.314652	2.327295	2.322684	2.318108	2.34533
2.282532	2.309805	2.304888	2.320804	2.315976	2.328598	2.324026	2.319513	2.346849
2.284099	2.296081	2.291173	2.322332	2.317447	2.330024	2.32541	2.320876	2.348294
2.285671	2.297691	2.29278	2.323938	2.319002	2.33152	2.32682	2.322218	2.349694
2.287171	2.29926	2.294371	2.30475	2.299831	2.315726	2.310999	2.306369	2.335342
2.288607	2.300798	2.295956	2.306373	2.301454	2.317338	2.312556	2.307857	2.336795
2.290009	2.28536	2.280672	2.30797	2.303064	2.298207	2.293431	2.288721	2.321805
2.291355	2.286727	2.282122	2.309526	2.30465	2.299799	2.294996	2.290248	2.323361
2.292738	2.288116	2.283574	2.311077	2.306233	2.301394	2.29657	2.291789	2.307732
2.294233	2.289608	2.285082	2.312657	2.307836	2.303002	2.298148	2.293329	2.309265
2.295775	2.291131	2.286592	2.314222	2.309425	2.304602	2.299728	2.29488	2.310812
2.297304	2.292615	2.288029	2.300333	2.295626	2.306152	2.301297	2.296455	2.291711
2.298839	2.294085	2.289426	2.301742	2.297079	2.307672	2.30286	2.298048	2.29329
2.300403	2.295603	2.290891	2.303221	2.298584	2.309224	2.304436	2.299638	2.294862
2.301999	2.297169	2.292425	2.30477	2.30014	2.310806	2.306025	2.301224	2.296424

2.303599	2.298743	2.293975	2.306337	2.301709	2.312395	2.307617	2.302815	2.297997
2.305198	2.300316	2.295531	2.307916	2.303286	2.31399	2.309216	2.304415	2.299587
2.306799	2.301893	2.297093	2.309499	2.304865	2.315585	2.310814	2.306015	2.301181
2.308403	2.303468	2.298645	2.311069	2.306436	2.301792	2.297077	2.292335	2.302774
2.310014	2.305051	2.300201	2.31264	2.308004	2.303362	2.298649	2.293909	2.304367
2.311646	2.306662	2.301786	2.314231	2.309575	2.304921	2.300211	2.295478	2.305957
2.313296	2.308297	2.303395	2.315838	2.311148	2.306472	2.301765	2.297043	2.307546
2.314946	2.30993	2.305	2.300208	2.295575	2.308036	2.303323	2.298602	2.309129
2.316596	2.311558	2.306596	2.301781	2.297136	2.309614	2.304883	2.300151	2.310705
2.301574	2.313197	2.308205	2.303366	2.298703	2.311195	2.306448	2.301707	2.29696
2.303221	2.314853	2.309823	2.304951	2.300259	2.312764	2.308017	2.30328	2.298527
2.30487	2.316524	2.311478	2.30658	2.301847	2.314352	2.309596	2.304859	2.300107
2.306499	2.318219	2.313234	2.308338	2.303535	2.315997	2.311195	2.306434	2.301714
2.308122	2.319926	2.315039	2.310157	2.305273	2.317676	2.312809	2.308013	2.303336
2.26802	2.321607	2.316751	2.31187	2.306953	2.319341	2.314433	2.309605	2.30493
2.26948	2.323265	2.318387	2.313496	2.308586	2.320998	2.316066	2.311208	2.3065
2.270984	2.324976	2.320069	2.315158	2.310234	2.322666	2.317734	2.312872	2.308133
2.27255	2.32677	2.321834	2.316882	2.311898	2.324334	2.319439	2.314618	2.309857
2.274117	2.328551	2.323584	2.318604	2.313593	2.32605	2.321177	2.316374	2.311585
2.275655	2.33026	2.325267	2.320302	2.315359	2.327886	2.322971	2.318105	2.313265
2.277178	2.331944	2.326923	2.32198	2.317126	2.329736	2.324766	2.319823	2.314938
2.278686	2.274243	2.269819	2.323609	2.318779	2.331448	2.326472	2.32152	2.316641
2.280181	2.275721	2.271262	2.325214	2.320368	2.333081	2.328124	2.323198	2.318355
2.281675	2.277214	2.272764	2.32691	2.32203	2.31718	2.312309	2.307477	2.320019
2.283162	2.278719	2.274328	2.3287	2.323764	2.318855	2.313935	2.309064	2.321625
2.24401	2.280256	2.275893	2.330482	2.325492	2.320537	2.3156	2.310719	2.323295
2.245593	2.281842	2.277452	2.332228	2.327188	2.322214	2.317323	2.31249	2.32508
2.247191	2.283438	2.279003	2.333971	2.328896	2.323913	2.319065	2.314277	2.32689
2.24877	2.273326	2.268826	2.276084	2.271676	2.325655	2.320798	2.316004	2.328674
2.250339	2.274924	2.27038	2.277602	2.273228	2.327424	2.322537	2.317718	2.330453
2.251879	2.247429	2.243016	2.267531	2.263142	2.3292	2.324297	2.319466	2.332229
2.253397	2.24897	2.244607	2.269203	2.264775	2.33099	2.326074	2.321234	2.334001
2.254949	2.250535	2.246192	2.270857	2.266422	2.323392	2.31841	2.313496	2.335752
2.25654	2.252126	2.247769	2.272488	2.268089	2.32535	2.32029	2.315276	2.337466
2.258142	2.253726	2.249355	2.274125	2.269741	2.26535	2.260921	2.256487	2.312065
2.259748	2.255328	2.250961	2.275784	2.271356	2.26694	2.262533	2.258132	2.313942
2.261362	2.256935	2.252568	2.27744	2.272967	2.268527	2.264141	2.259773	2.315834
2.262992	2.258548	2.254151	2.24978	2.245436	2.270173	2.265755	2.26135	2.317629
2.264628	2.260163	2.255725	2.251343	2.247029	2.271841	2.267373	2.262907	2.3194
2.238765	2.26176	2.25732	2.252933	2.248607	2.273468	2.269002	2.264539	2.321286
2.240335	2.263343	2.258933	2.254543	2.250174	2.275062	2.270641	2.266233	2.323271
2.24194	2.264955	2.260539	2.256137	2.251746	2.24739	2.243086	2.238802	2.325211
2.243594	2.266607	2.262129	2.257701	2.253327	2.24898	2.244651	2.240344	2.327085
2.226885	2.268267	2.263737	2.259281	2.25491	2.250563	2.246211	2.241884	2.266686
2.228499	2.269913	2.265385	2.260909	2.256489	2.252106	2.247759	2.243442	2.268293
2.230119	2.271569	2.267049	2.262553	2.258077	2.253655	2.249311	2.245005	2.269901
2.23176	2.245649	2.241175	2.264167	2.25969	2.25526	2.250887	2.246552	2.242234
2.233426	2.247314	2.242788	2.265779	2.261327	2.256907	2.252487	2.248102	2.243782
2.235166	2.230602	2.226082	2.267491	2.26303	2.258597	2.254153	2.249743	2.245411
2.236986	2.2324	2.227858	2.269304	2.264803	2.26034	2.255891	2.25148	2.247125

2.238768	2.23416	2.229593	2.253793	2.249282	2.244823	2.240405	2.236031	2.248805
2.240479	2.235846	2.231247	2.255459	2.2509	2.246411	2.242004	2.237643	2.250421
2.242168	2.237514	2.232884	2.257109	2.252509	2.247991	2.243581	2.239222	2.252007
2.243846	2.239177	2.234517	2.258754	2.254141	2.249602	2.245144	2.240743	2.236364
2.245525	2.240848	2.236165	2.260416	2.255797	2.251237	2.246719	2.242263	2.237883
2.191859	2.242581	2.237898	2.262178	2.257525	2.252932	2.248391	2.243914	2.239514
2.193511	2.244355	2.239693	2.264011	2.259307	2.254674	2.250133	2.245657	2.241226
2.195104	2.24606	2.24142	2.236793	2.232168	2.256378	2.251834	2.24736	2.242923
2.196617	2.247676	2.243063	2.238442	2.233798	2.258032	2.253484	2.249017	2.244605
2.176388	2.193729	2.189341	2.240111	2.235457	2.259723	2.25516	2.250681	2.246273
2.178074	2.195401	2.190976	2.241845	2.237193	2.261494	2.256891	2.252362	2.247906
2.179777	2.197084	2.192614	2.243578	2.238937	2.26328	2.258643	2.254065	2.249554
2.181389	2.176891	2.172431	2.245243	2.240623	2.265028	2.260393	2.255806	2.251265
2.182978	2.178455	2.173969	2.246893	2.242296	2.266767	2.262153	2.257575	2.253015
2.158461	2.180097	2.175586	2.171164	2.16684	2.268536	2.263938	2.259372	2.254794
2.160151	2.1818	2.177266	2.172825	2.168483	2.27033	2.265747	2.261198	2.256603
2.161858	2.183532	2.178978	2.174513	2.170139	2.254165	2.249595	2.245067	2.258342
2.163582	2.185297	2.180729	2.176232	2.171806	2.255942	2.251293	2.246701	2.259975
2.165312	2.187063	2.182481	2.177955	2.173483	2.169067	2.164708	2.160403	2.243782
2.167049	2.188818	2.184215	2.179667	2.175173	2.170733	2.166352	2.162027	2.245547
2.168775	2.190554	2.185936	2.181374	2.17687	2.17242	2.168023	2.163684	2.247349
2.14961	2.165873	2.161339	2.183065	2.178568	2.174114	2.169689	2.165319	2.249138
2.151269	2.167498	2.162974	2.184749	2.180272	2.175821	2.171362	2.166954	2.250929
2.152996	2.148373	2.143836	2.166878	2.162411	2.17754	2.173047	2.168601	2.197767
2.154788	2.150142	2.145579	2.168638	2.164164	2.179273	2.174745	2.17026	2.199442
2.156593	2.151929	2.14734	2.14283	2.13841	2.161444	2.156895	2.152387	2.182962
2.158407	2.153729	2.149119	2.144588	2.140149	2.1632	2.158638	2.154116	2.184715
2.160224	2.155535	2.150909	2.146361	2.141902	2.164968	2.160396	2.155861	2.186483
2.068808	2.157339	2.152709	2.148149	2.143672	2.16675	2.162155	2.157593	2.153131
2.07042	2.159143	2.154513	2.149946	2.145456	2.168549	2.163936	2.15935	2.154852
2.030802	2.160933	2.156303	2.151737	2.147249	2.142766	2.138248	2.133747	2.156684
2.032321	2.162717	2.158086	2.153526	2.149051	2.144589	2.140111	2.13564	2.158596
2.033911	2.070943	2.066644	2.155326	2.150845	2.146386	2.141932	2.137477	2.13298
2.035584	2.072663	2.068311	2.157137	2.152632	2.148159	2.143709	2.139258	2.134748
1.967778	2.033008	2.028759	2.158944	2.154407	2.149915	2.145466	2.141022	2.136513
1.969268	2.034601	2.030316	2.160726	2.156151	2.151638	2.147195	2.142771	2.13829
1.970745	2.036192	2.031891	2.162534	2.157917	2.153376	2.148929	2.144515	2.140065
1.931482	1.968166	1.96409	2.164422	2.159761	2.155179	2.150696	2.146262	2.141829
1.932933	1.969668	1.965648	2.166354	2.161654	2.157026	2.152492	2.148018	2.14359
1.934389	1.930428	1.926492	1.963159	1.959162	2.158923	2.15434	2.149817	2.14536
1.93585	1.931872	1.927938	1.964649	1.96071	2.160867	2.156236	2.151651	2.147137
1.937318	1.933322	1.929388	1.96613	1.962212	2.108633	2.104107	2.09961	2.14897
1.938784	1.934775	1.930844	1.967598	1.963653	2.110432	2.105942	2.101471	2.150875
1.908575	1.936252	1.932309	1.928387	1.924483	2.002339	1.998365	1.994406	2.098829
1.91014	1.937754	1.933775	1.929832	1.925923	2.003894	1.999901	1.995935	2.100641
1.911754	1.939302	1.935268	1.931293	1.927377	2.005458	2.001422	1.997429	2.102433
1.913447	1.909321	1.905254	1.93279	1.928838	1.924943	1.921093	1.917298	2.10417
1.915182	1.911036	1.906872	1.934325	1.930323	1.926388	1.922504	1.918682	2.105875
1.885547	1.912684	1.908515	1.935949	1.93193	1.927963	1.924021	1.920138	1.96011
1.887143	1.914278	1.910191	1.937667	1.93366	1.929666	1.925641	1.921658	1.961646

1.826767	1.88465	1.880587	1.907811	1.903856	1.931302	1.927251	1.923229	1.963271
1.828279	1.886219	1.882156	1.909326	1.905368	1.932825	1.92884	1.924882	1.96504
1.829853	1.887839	1.883746	1.910844	1.906865	1.934316	1.930391	1.926497	1.966779
1.784563	1.889618	1.885445	1.881327	1.877265	1.904407	1.900513	1.896663	1.924054
1.786348	1.891441	1.887188	1.882999	1.878872	1.905909	1.901975	1.898099	1.925468
1.735676	1.85007	1.845905	1.884759	1.88062	1.87654	1.87254	1.868582	1.895797
1.737166	1.851758	1.847691	1.886576	1.882458	1.878375	1.874348	1.87034	1.897421
1.676301	1.734833	1.731027	1.888247	1.884143	1.880063	1.876018	1.871986	1.899001
1.677813	1.736305	1.732442	1.889744	1.88565	1.881582	1.87753	1.873504	1.900531
1.679365	1.737849	1.73394	1.89134	1.887248	1.883184	1.879121	1.875092	1.871126
1.680996	1.73955	1.73563	1.893165	1.889066	1.884989	1.880908	1.876845	1.872805
1.682605	1.741237	1.737321	1.894998	1.890886	1.88679	1.882694	1.878602	1.874487
1.631304	1.680323	1.676621	1.805761	1.801766	1.888403	1.884302	1.880213	1.876117
1.632687	1.681727	1.678044	1.807322	1.803309	1.889965	1.885863	1.881789	1.877738
1.550793	1.630538	1.62691	1.809055	1.805025	1.846703	1.842667	1.838655	1.879427
1.552303	1.632227	1.628576	1.810912	1.806867	1.848509	1.844462	1.840421	1.881167
1.55374	1.633822	1.630149	1.679257	1.675574	1.804573	1.800576	1.796577	1.837977
1.555067	1.635277	1.631585	1.680715	1.677029	1.806157	1.802145	1.798146	1.839575
1.556444	1.553187	1.550004	1.629401	1.625764	1.807795	1.803768	1.799765	1.795797
1.557962	1.554695	1.551489	1.631046	1.627394	1.809613	1.805569	1.801551	1.797577
1.559509	1.556228	1.552995	1.632707	1.629037	1.81144	1.807381	1.803347	1.799352
1.560977	1.557656	1.554376	1.551148	1.547977	1.62687	1.623227	1.61962	1.800965
1.562415	1.559046	1.55571	1.552443	1.54925	1.628286	1.624639	1.621029	1.802514
1.506244	1.560504	1.557151	1.553864	1.550647	1.629838	1.626179	1.622559	1.747966
1.507714	1.562021	1.558684	1.555394	1.552154	1.631509	1.62783	1.624195	1.74974
1.509197	1.505956	1.502732	1.556926	1.553665	1.550443	1.54727	1.544133	1.622204
1.510703	1.507457	1.504235	1.558458	1.555181	1.551943	1.548752	1.545599	1.62387
1.430882	1.508929	1.505704	1.559951	1.556661	1.553408	1.550198	1.547027	1.543899
1.432175	1.510317	1.507076	1.561334	1.558036	1.554769	1.551536	1.548342	1.545199
1.433464	1.511697	1.508437	1.562704	1.559397	1.556118	1.552862	1.549643	1.546482
1.434858	1.431755	1.428657	1.506669	1.503451	1.557579	1.554306	1.551069	1.547889
1.43632	1.433203	1.4301	1.508208	1.504972	1.559113	1.555825	1.552574	1.549375
1.437777	1.43465	1.431544	1.428452	1.425374	1.503276	1.500075	1.496905	1.550871
1.439238	1.436104	1.432996	1.429897	1.426803	1.504804	1.501594	1.498414	1.552382
1.369029	1.437522	1.434422	1.431326	1.428232	1.42516	1.422114	1.419094	1.496751
1.370299	1.438867	1.4358	1.432734	1.429671	1.426613	1.423552	1.420507	1.498268
1.371589	1.440217	1.437167	1.434121	1.431081	1.428034	1.424959	1.421894	1.499764
1.226326	1.441626	1.438538	1.435461	1.432398	1.429336	1.426256	1.423196	1.420196
1.22767	1.443072	1.439924	1.436797	1.433691	1.430604	1.42752	1.424471	1.421492
1.229027	1.372857	1.369858	1.438229	1.435098	1.431989	1.42889	1.425825	1.422822
1.230403	1.374306	1.371312	1.439734	1.436588	1.433458	1.430335	1.427236	1.424176
1.231784	1.293003	1.290174	1.44122	1.43806	1.434915	1.43178	1.428664	1.425572
1.233171	1.294349	1.291492	1.442674	1.439503	1.436354	1.433219	1.430107	1.427018
1.234561	1.231984	1.229444	1.372556	1.369542	1.43781	1.434674	1.431563	1.428474
1.235951	1.233352	1.230807	1.374001	1.370982	1.439301	1.436154	1.433032	1.429926
1.23735	1.234729	1.232177	1.37546	1.372435	1.440806	1.437644	1.434507	1.431382
1.238777	1.236135	1.233557	1.294058	1.29119	1.442305	1.439127	1.435978	1.432841
1.240216	1.237554	1.234943	1.29543	1.292546	1.443809	1.440614	1.437451	1.434306
1.241628	1.23895	1.236326	1.296816	1.293917	1.370356	1.367295	1.364272	1.435786
1.243015	1.240326	1.237706	1.298214	1.295301	1.37182	1.368739	1.365695	1.437279

1.244428	1.241717	1.239085	1.299611	1.296688	1.293791	1.290913	1.288059	1.438774
1.245868	1.243118	1.240452	1.300995	1.29807	1.29517	1.29228	1.289409	1.440267
1.13946	1.244561	1.241857	1.239219	1.236656	1.296556	1.293652	1.290768	1.366924
1.140899	1.246082	1.243335	1.240655	1.238046	1.297944	1.295029	1.292141	1.368365
1.14237	1.247644	1.244858	1.24213	1.239461	1.299348	1.296418	1.293524	1.369814
1.143827	1.24921	1.246406	1.243644	1.240919	1.300789	1.297826	1.294909	1.292006
1.145283	1.250788	1.247981	1.245191	1.242409	1.302258	1.299253	1.2963	1.293376
1.146749	1.144061	1.14142	1.246739	1.243909	1.30374	1.300692	1.297702	1.29476
1.148224	1.145527	1.142871	1.248289	1.245416	1.305234	1.302141	1.299113	1.296156
1.149712	1.147004	1.14433	1.249852	1.246953	1.244105	1.241333	1.238636	1.297575
1.151208	1.148489	1.145793	1.251428	1.248527	1.245662	1.242835	1.240081	1.299026
1.152724	1.149997	1.147282	1.253027	1.250121	1.247237	1.24436	1.24155	1.300495
1.154282	1.151559	1.148835	1.146108	1.143372	1.248802	1.245881	1.243026	1.301961
1.15585	1.153134	1.150408	1.147656	1.144868	1.250373	1.247417	1.244527	1.303451
1.157359	1.154641	1.151917	1.149156	1.146348	1.251959	1.248992	1.246089	1.305026
1.158827	1.156102	1.153382	1.150625	1.14782	1.253559	1.2506	1.2477	1.306668
1.160343	1.157598	1.154861	1.152097	1.149294	1.146515	1.14377	1.141075	1.308293
1.161922	1.159141	1.156359	1.15357	1.150769	1.147994	1.145251	1.142548	1.309884
1.163481	1.160669	1.157851	1.155048	1.152259	1.149494	1.146758	1.14405	1.24966
1.164978	1.162151	1.159323	1.156529	1.153769	1.15103	1.148307	1.145602	1.251338
1.166468	1.163631	1.160804	1.158023	1.155294	1.152581	1.14987	1.147168	1.253035
1.167992	1.165141	1.162317	1.159546	1.156833	1.154133	1.151429	1.148725	1.146004
1.169536	1.166672	1.163855	1.16109	1.158383	1.155685	1.152982	1.150274	1.147533
1.171101	1.168234	1.165422	1.162652	1.159932	1.15722	1.154501	1.151779	1.149034
1.172687	1.169827	1.167016	1.164234	1.161484	1.15874	1.155989	1.153244	1.15051
1.174276	1.171415	1.168595	1.165794	1.163014	1.160245	1.157478	1.154727	1.152007
1.175863	1.172987	1.170139	1.167312	1.164506	1.161724	1.158967	1.156234	1.153534
1.177472	1.174584	1.171711	1.16886	1.166027	1.16323	1.16048	1.157762	1.155074
1.17913	1.176241	1.173356	1.170484	1.167618	1.164795	1.162035	1.159314	1.15662
1.180803	1.177917	1.175026	1.172138	1.169245	1.166398	1.163622	1.160887	1.158176
1.182455	1.179565	1.17667	1.173782	1.170894	1.168041	1.165243	1.162479	1.159741
1.184097	1.181201	1.178305	1.175426	1.172562	1.169717	1.16689	1.164087	1.161317
1.185733	1.182834	1.179945	1.177074	1.174221	1.171376	1.168527	1.165696	1.162904
1.187358	1.184461	1.18159	1.178727	1.175874	1.173021	1.170155	1.167306	1.164501
1.189001	1.186101	1.183234	1.180371	1.177512	1.174655	1.171786	1.168934	1.166128
1.19067	1.187761	1.184878	1.182001	1.179128	1.176268	1.173417	1.170588	1.167796
1.192349	1.189425	1.186519	1.183626	1.180744	1.177885	1.175058	1.172254	1.169474
1.194028	1.191083	1.188153	1.185249	1.182369	1.179522	1.176717	1.173933	1.171148
1.19571	1.192742	1.189789	1.186878	1.184007	1.181172	1.178385	1.175613	1.172816
1.197385	1.194404	1.191447	1.188537	1.185677	1.182847	1.180049	1.177261	1.174456
1.199058	1.196072	1.193122	1.19022	1.187372	1.18454	1.18171	1.178889	1.176074
1.20076	1.197775	1.194829	1.191923	1.189061	1.186213	1.183365	1.180525	1.177696
1.202493	1.199517	1.196572	1.193647	1.190742	1.187859	1.185009	1.182171	1.179318
1.045482	1.201275	1.19832	1.195374	1.192435	1.189526	1.186669	1.183828	1.180962
1.047002	1.20305	1.200054	1.197087	1.194147	1.191236	1.18836	1.185503	1.182646
1.04853	1.204828	1.2018	1.198814	1.195875	1.192959	1.19006	1.187183	1.184333
1.050039	1.206591	1.203583	1.200594	1.197622	1.194673	1.19175	1.188852	1.185977
1.051541	1.20835	1.205383	1.202396	1.199382	1.196391	1.193445	1.190524	1.187614
1.053051	1.210115	1.207154	1.204168	1.201149	1.198147	1.195178	1.192236	1.189321
1.054567	1.211884	1.208903	1.205916	1.202922	1.199934	1.196944	1.193981	1.191086

1.056073	1.053466	1.050848	1.207678	1.204692	1.201707	1.198708	1.195734	1.192838
1.057564	1.054964	1.052362	1.209461	1.206454	1.203454	1.200464	1.197496	1.194568
1.059055	1.05646	1.053872	1.211242	1.208222	1.205213	1.202226	1.199256	1.196298
1.060554	1.057962	1.055372	1.213005	1.210008	1.207007	1.203996	1.200996	1.198032
1.06205	1.059457	1.056866	1.214767	1.211796	1.208808	1.205772	1.202742	1.199773
1.063523	1.060926	1.058351	1.055765	1.053171	1.21058	1.207552	1.204524	1.201531
1.064988	1.062382	1.05983	1.057257	1.054663	1.212339	1.209337	1.206329	1.203303
1.066498	1.063868	1.061306	1.058733	1.056148	1.214117	1.211127	1.208128	1.205088
1.068049	1.06538	1.062778	1.060192	1.057624	1.215912	1.212922	1.209919	1.206884
1.069625	1.066916	1.064268	1.061662	1.059105	1.056553	1.053998	1.051425	1.208721
1.071217	1.068467	1.065768	1.063137	1.060583	1.058049	1.05553	1.052998	1.210627
1.072849	1.070065	1.067316	1.064654	1.062087	1.059556	1.057059	1.054557	1.212516
1.074556	1.071756	1.068965	1.066262	1.063656	1.061093	1.058578	1.056065	1.214318
1.076292	1.073482	1.070655	1.067908	1.065248	1.062638	1.060087	1.057549	1.216085
0.868017	1.075153	1.072293	1.069508	1.066796	1.064146	1.061578	1.059034	1.217852
0.869265	1.076792	1.073907	1.071085	1.068322	1.065633	1.063056	1.060515	1.219616
0.870485	1.078443	1.075545	1.072694	1.069883	1.067158	1.064566	1.062021	1.059441
0.871669	1.080104	1.077203	1.074331	1.071474	1.068711	1.066101	1.063553	1.060989
0.872877	0.870825	0.868779	1.076016	1.073137	1.070343	1.067688	1.065099	1.062529
0.874138	0.872112	0.870081	1.077787	1.074921	1.072101	1.069343	1.066644	1.064023
0.875407	0.873401	0.871376	1.079558	1.076723	1.07389	1.071036	1.068228	1.06555
0.876651	0.874633	0.872592	1.081242	1.078449	1.075635	1.072759	1.069912	1.067195
0.877883	0.875841	0.873778	1.08289	1.080141	1.077359	1.074497	1.071645	1.068898
0.604686	0.877051	0.875013	0.873003	0.87103	1.079039	1.076185	1.073334	1.070568
0.605502	0.878259	0.876283	0.874294	0.872295	1.080679	1.077835	1.074995	1.072219
	0.879514	0.87755	0.875555	0.873529	1.082315	1.079506	1.076698	1.073905
	0.880824	0.878806	0.876771	0.874714	1.083943	1.081209	1.07846	1.075637
	0.606751	0.605397	0.878007	0.875929	0.873893	0.871937	0.869989	1.077372
	0.607623	0.606269	0.879299	0.87722	0.875184	0.873223	0.871272	1.079104
	0.608487	0.607142	0.880608	0.878536	0.876502	0.874517	0.872544	1.080815
			0.881888	0.879832	0.877803	0.875793	0.87379	1.082454
			0.883166	0.881128	0.879105	0.877065	0.875024	1.084055
			0.608302	0.607034	0.880423	0.878361	0.876298	0.874227
			0.609171	0.607903	0.881757	0.879679	0.877608	0.875531
					0.883107	0.881009	0.878923	0.876837
					0.884481	0.882355	0.880242	0.878144
					0.885852	0.883693	0.881552	0.879445
								0.880722
								0.882003
								0.88334
								0.884718

					0.572903
					0.572082
					0.571242
					0.570386
			0.572886	0.571202	1.481334
			0.572034	0.570351	1.478989
			0.571168	0.569479	1.476626
0.575319	0.573674	0.572003	0.570281	0.568544	1.589154

			0.574425	0.572805	0.571146	0.56938	0.567587	1.586412
	0.576794	0.575162	0.573527	0.571904	0.570244	1.477627	1.473557	1.847849
	0.575887	0.574261	0.572623	0.570976	0.569304	1.475278	1.471203	1.844854
	0.574985	0.573358	1.484918	1.480962	1.47698	1.852201	1.846982	1.900601
	0.574095	0.572455	1.482585	1.478605	1.474603	1.84906	1.843784	1.897463
0.574829	0.573193	0.571542	1.480262	1.476262	1.472247	1.904827	1.899504	2.109402
0.573905	0.572265	0.570618	1.477951	1.473947	1.469942	1.901875	1.896631	2.106331
0.572977	0.571331	0.569681	1.475645	1.471642	1.467652	1.898951	1.893822	2.103329
0.572072	1.481157	1.477259	1.852465	1.847295	1.84216	1.955608	1.950589	2.100268
0.571175	1.478882	1.474959	1.849456	1.84423	1.83907	1.952591	1.947693	2.097198
1.480522	1.585628	1.581409	1.965157	1.959884	1.954692	2.104479	2.099258	2.094139
1.478257	1.583242	1.579028	1.96223	1.95704	1.95192	2.10144	2.096197	2.091087
1.476011	1.909783	1.904687	2.06152	2.056164	2.050866	2.098488	2.093217	2.088093
1.473793	1.906831	1.901801	2.058546	2.053313	2.04811	2.095655	2.090348	2.085181
1.580299	1.963689	1.958721	2.10862	2.103382	2.098137	2.092809	2.087481	2.082276
1.577925	1.960775	1.955865	2.105546	2.100355	2.09515	2.089854	2.084547	2.079337
1.575558	1.957882	1.953021	2.102471	2.097304	2.092123	2.086862	2.081588	2.076386
1.959974	2.056767	2.051693	2.099411	2.094276	2.089122	2.083879	2.078616	2.169647
1.957111	2.053748	2.048709	2.096361	2.091257	2.086128	2.080892	2.075632	2.166517
2.055982	2.1036	2.098439	2.093316	2.088234	2.083128	2.07791	2.072666	2.163409
2.053104	2.10061	2.095413	2.090272	2.085203	2.08012	2.074929	2.069714	2.16032
2.102983	2.097635	2.092407	2.087254	2.082199	2.077139	2.168032	2.162598	2.157252
2.099981	2.09465	2.089412	2.08426	2.079232	2.074197	2.164935	2.159508	2.154184
2.097012	2.091707	2.086459	2.081305	2.07629	2.071274	2.161878	2.156479	2.15118
2.094143	2.088874	2.083599	2.119046	2.113946	2.108872	2.1589	2.153596	2.148323
2.091309	2.086078	2.080777	2.116149	2.111005	2.105921	2.155955	2.150771	2.145525
2.088398	2.083186	2.077895	2.168661	2.163341	2.158097	2.152951	2.147842	2.14263
2.085436	2.080232	2.074977	2.165608	2.160275	2.155025	2.149911	2.144849	2.139675
2.082466	2.117846	2.112611	2.162639	2.157297	2.152034	2.146925	2.141881	2.178707
2.079475	2.114868	2.109766	2.15978	2.15443	2.149139	2.144004	2.138943	2.175774
2.117056	2.167311	2.162123	2.15689	2.151548	2.146247	2.141085	2.136	2.242916
2.114182	2.164319	2.159127	2.1539	2.148585	2.143304	2.138125	2.133017	2.239842
2.111331	2.16134	2.156116	2.150888	2.145621	2.140382	2.135195	2.130063	2.236781
2.108403	2.158319	2.15309	2.147892	2.142725	2.137568	2.203554	2.198275	2.26433
2.105461	2.155299	2.15007	2.144912	2.139858	2.134799	2.200767	2.19548	2.261355
2.157675	2.15239	2.147155	2.174404	2.169308	2.164221	2.238677	2.233327	2.258389
2.154843	2.149578	2.144334	2.171577	2.166399	2.161265	2.23567	2.230442	2.255442
2.152005	2.179281	2.174015	2.207722	2.202416	2.197172	2.263241	2.257883	2.252452
2.149159	2.176471	2.171237	2.204915	2.199588	2.194313	2.2602	2.2548	2.249388
2.178875	2.173621	2.168414	2.273902	2.268283	2.262708	2.257203	2.251756	2.246363
2.17592	2.17068	2.16547	2.270768	2.265265	2.259789	2.254294	2.248827	2.243458
2.172953	2.167726	2.162507	2.26762	2.262233	2.256863	2.251396	2.245931	2.240589
2.170076	2.236977	2.23146	2.264571	2.259178	2.253812	2.248382	2.242965	2.342327
2.167257	2.23402	2.228543	2.261584	2.256117	2.250698	2.245313	2.239975	2.338691
2.164468	2.269724	2.264155	2.258628	2.253136	2.247704	2.347917	2.341492	2.335173
2.161713	2.266788	2.261223	2.255705	2.250233	2.244824	2.344368	2.338013	2.33177
2.230847	2.263852	2.258298	2.2528	2.247361	2.241984	2.340903	2.334615	2.328428
2.22796	2.260907	2.255373	2.249905	2.244515	2.239184	2.337539	2.331311	2.325153
2.225068	2.257958	2.252457	2.247027	2.241681	2.236389	2.334204	2.328037	2.321901
2.260513	2.255006	2.249572	2.349865	2.343395	2.337043	2.330849	2.324755	2.318652

2.257518	2.252044	2.246683	2.346342	2.339918	2.333612	2.327483	2.321454	2.315387
2.254498	2.321267	2.315166	2.342717	2.336386	2.330159	2.324057	2.318037	2.31201
2.251465	2.317708	2.311639	2.339028	2.332822	2.326693	2.320591	2.31454	2.308555
2.24856	2.348257	2.341807	2.335505	2.329372	2.323308	2.317229	2.311187	2.338267
2.245814	2.344962	2.338486	2.332169	2.326047	2.320008	2.313982	2.307999	2.334808
2.314257	2.341624	2.335178	2.328883	2.322776	2.316757	2.310774	2.304836	2.372869
2.310843	2.338063	2.331785	2.325613	2.319557	2.313563	2.307592	2.301657	2.369278
2.307474	2.334549	2.328423	2.322365	2.316357	2.310386	2.304423	2.298485	2.365725
2.337644	2.331334	2.325174	2.319099	2.313115	2.307174	2.333934	2.327559	2.362186
2.334539	2.328268	2.322	2.315854	2.309873	2.303955	2.330515	2.324165	2.358676
2.331333	2.32513	2.318891	2.312742	2.30671	2.30075	2.368417	2.361731	2.355212
2.328064	2.321954	2.315851	2.309749	2.303616	2.297557	2.364792	2.358245	2.351791
2.324801	2.318753	2.312737	2.339911	2.333232	2.326665	2.361299	2.354812	2.348407
2.32155	2.315519	2.309516	2.336532	2.329998	2.323539	2.357974	2.351422	2.345045
2.318297	2.312279	2.306285	2.374956	2.368146	2.3614	2.354709	2.348126	2.341775
2.315023	2.309036	2.303084	2.371329	2.364651	2.358047	2.351475	2.344988	2.338657
2.311765	2.305802	2.29989	2.367712	2.361146	2.354677	2.348253	2.341906	2.335619
2.308561	2.335628	2.329247	2.364199	2.357692	2.351293	2.344972	2.338746	2.332593
2.305403	2.33216	2.325873	2.360752	2.35427	2.347906	2.341667	2.335559	2.329594
2.335325	2.370482	2.363834	2.357306	2.350877	2.344579	2.338408	2.33239	2.357369
2.332149	2.367157	2.360417	2.353859	2.34751	2.341309	2.335188	2.329226	2.354276
2.32892	2.363822	2.357072	2.350522	2.344214	2.338075	2.332042	2.326191	2.351311
2.325571	2.360437	2.353821	2.347342	2.340991	2.334844	2.328969	2.323332	2.348533
2.32223	2.35705	2.350595	2.344218	2.337857	2.331725	2.325997	2.32055	2.345825
2.360121	2.353645	2.347325	2.341078	2.334832	2.328802	2.32314	2.317755	2.343096
2.356719	2.350276	2.344076	2.337973	2.331906	2.326025	2.320408	2.315038	2.34043
2.353464	2.347066	2.340941	2.334959	2.329088	2.323387	2.317868	2.312554	2.385277
2.350327	2.343978	2.337893	2.332018	2.326366	2.320876	2.315488	2.310252	2.38281
2.347217	2.340933	2.334879	2.329091	2.323613	2.318304	2.343547	2.338352	2.380396
2.344139	2.337934	2.331891	2.326159	2.320812	2.315661	2.341044	2.336033	2.378051
2.341062	2.334972	2.329	2.323347	2.318089	2.313044	2.385797	2.380743	2.375681
2.337941	2.33203	2.326244	2.320714	2.315481	2.31046	2.383148	2.378218	2.373251
2.334871	2.329145	2.323572	2.31818	2.312967	2.307948	2.380523	2.375668	2.370784
2.33195	2.326363	2.320967	2.393681	2.388266	2.383013	2.377979	2.373092	2.368257
2.329119	2.323649	2.318419	2.391122	2.385799	2.380589	2.375497	2.370525	2.365711
2.326286	2.347803	2.342598	2.388566	2.383339	2.378187	2.373095	2.368094	2.363263
2.323466	2.345023	2.339991	2.38602	2.380899	2.37582	2.370769	2.365776	2.360893
2.347674	2.393569	2.388514	2.383498	2.378468	2.373461	2.368454	2.363478	2.38428
2.345167	2.391034	2.38599	2.381006	2.376051	2.371116	2.366148	2.361198	2.381988
2.366133	2.388548	2.383484	2.378509	2.37362	2.368755	2.363835	2.358925	2.37971
2.363639	2.386048	2.380945	2.375968	2.371142	2.366351	2.361493	2.356638	2.377414
2.361173	2.383572	2.378431	2.373447	2.36867	2.363943	2.359152	2.354362	2.375132
2.386334	2.381128	2.375995	2.371025	2.366274	2.361583	2.356856	2.352134	2.372903
2.383884	2.378713	2.373614	2.368668	2.363919	2.359244	2.354576	2.349928	2.3707
2.381458	2.376323	2.371254	2.366317	2.361537	2.356848	2.35223	2.347657	2.368445
2.379058	2.373959	2.368921	2.363978	2.359131	2.354393	2.349816	2.345323	2.366139
2.37666	2.371596	2.366591	2.361658	2.356778	2.35202	2.372971	2.368437	2.363864
2.374252	2.369221	2.364257	2.359359	2.354505	2.34977	2.370693	2.366176	2.361638
2.371859	2.36686	2.361932	2.357072	2.35226	2.347559	2.368455	2.363948	2.359436
2.369499	2.364529	2.359625	2.354792	2.350016	2.345349	2.366227	2.361741	2.357257

2.367158	2.362214	2.35733	2.352523	2.347784	2.343153	2.364017	2.359553	2.355093
2.364816	2.359901	2.355045	2.350268	2.345563	2.340964	2.36181	2.357367	2.352923
2.36248	2.357596	2.352772	2.348026	2.343353	2.338782	2.35961	2.355187	2.350753
2.360165	2.355313	2.350522	2.371301	2.366553	2.361906	2.357415	2.35301	2.348584
2.357872	2.353051	2.348298	2.369065	2.36433	2.359695	2.355223	2.350837	2.346416
2.355588	2.350801	2.346083	2.366838	2.362124	2.357503	2.353042	2.348662	2.42679
2.353303	2.348549	2.343867	2.364611	2.359927	2.355327	2.350861	2.34647	2.423972
2.351042	2.346322	2.34167	2.362403	2.357761	2.353188	2.348713	2.344301	2.421181
2.348824	2.34413	2.339492	2.360215	2.355646	2.351131	2.346649	2.342205	2.418471
2.346647	2.341981	2.337354	2.358065	2.35357	2.349112	2.344621	2.340144	2.415806
2.344542	2.339936	2.335348	2.356039	2.351528	2.347047	2.42437	2.418655	2.413129
2.342502	2.337981	2.333454	2.354117	2.34952	2.344951	2.421613	2.415906	2.410453
2.340444	2.36128	2.356696	2.352093	2.347446	2.342832	2.418891	2.413237	2.407853
2.338367	2.359131	2.354541	2.349937	2.34529	2.340685	2.416217	2.410666	2.405341
2.336268	2.356957	2.352349	2.430813	2.424922	2.419132	2.413549	2.408124	2.408845
2.334139	2.354778	2.350145	2.427977	2.422064	2.416298	2.410844	2.405583	2.406402
2.331987	2.352585	2.347927	2.425132	2.41923	2.413512	2.408181	2.403067	2.403979
2.32981	2.350374	2.34571	2.422322	2.416536	2.410933	2.411589	2.406541	2.417207
2.327616	2.348153	2.343496	2.419538	2.41393	2.40848	2.40916	2.404091	2.414749
2.350589	2.428352	2.42248	2.422621	2.417189	2.411902	2.406734	2.401681	2.417582
2.348368	2.425528	2.419687	2.419901	2.414587	2.409412	2.404314	2.399305	2.415185
2.346259	2.428637	2.422909	2.417398	2.412163	2.407058	2.407875	2.402871	2.433163
2.34433	2.426248	2.420646	2.415221	2.410009	2.404916	2.405767	2.400791	2.431018
2.342381	2.423858	2.418384	2.413046	2.407851	2.402761	2.403638	2.398679	2.428842
2.426837	2.421281	2.415917	2.416647	2.411464	2.406366	2.41636	2.411354	2.426484
2.424145	2.418662	2.41339	2.41421	2.409075	2.404007	2.413969	2.408957	2.424055
2.421529	2.416114	2.410887	2.411755	2.406653	2.40161	2.431725	2.426662	2.421617
2.418968	2.413618	2.408401	2.409289	2.40421	2.399189	2.429247	2.424198	2.419173
2.416565	2.417363	2.412138	2.428133	2.422999	2.417925	2.426935	2.421903	2.4169
2.414343	2.415197	2.41001	2.425985	2.420872	2.415816	2.424815	2.419797	2.414819
2.418198	2.412962	2.407807	2.42376	2.418671	2.413637	2.422624	2.417628	2.412682
2.415779	2.410565	2.405432	2.421357	2.416296	2.411291	2.420269	2.4153	2.410402
2.413345	2.408139	2.403018	2.418914	2.41388	2.408906	2.417877	2.412942	2.408091
2.410972	2.426777	2.421584	2.430499	2.425438	2.420443	2.415489	2.410594	2.405778
2.408634	2.424361	2.419168	2.428061	2.423017	2.418048	2.413132	2.40828	2.403492
2.427369	2.42207	2.416908	2.4258	2.420785	2.415846	2.410966	2.406149	2.401385
2.425093	2.419881	2.414771	2.42368	2.418703	2.413797	2.408951	2.404164	2.399418
2.422803	2.417634	2.412553	2.421461	2.416518	2.411644	2.406825	2.402061	2.397332
2.420487	2.415298	2.410214	2.419095	2.414183	2.409342	2.404541	2.39979	2.395078
2.418198	2.413013	2.407939	2.4168	2.411916	2.407101	2.402314	2.397573	2.392876
2.415963	2.410865	2.405844	2.414713	2.409852	2.405051	2.400274	2.395539	2.390855
2.413743	2.408734	2.403771	2.412649	2.407805	2.403013	2.398244	2.393516	2.388845
2.411485	2.406476	2.401541	2.410406	2.405572	2.400789	2.39603	2.391314	2.386664
2.409226	2.404181	2.399259	2.408099	2.403269	2.398492	2.393744	2.389043	2.384417
2.407064	2.415814	2.410848	2.405951	2.401125	2.396352	2.391614	2.386926	2.382319
2.404992	2.413776	2.40883	2.403943	2.399119	2.394348	2.389619	2.384943	2.380349
2.402932	2.411725	2.406769	2.401869	2.397031	2.392253	2.38754	2.382889	2.382166
2.400902	2.409658	2.404637	2.399677	2.394792	2.389991	2.385304	2.3807	2.38003
2.398808	2.407529	2.402478	2.397491	2.392581	2.38777	2.383111	2.378549	2.377933
2.396536	2.405255	2.400288	2.39537	2.390498	2.385718	2.381089	2.376554	2.375996

2.394227	2.40295	2.398097	2.393277	2.38847	2.383736	2.379131	2.374605	2.374095
2.392061	2.40078	2.39597	2.3912	2.386462	2.381775	2.381172	2.376599	2.372075
2.389978	2.398689	2.393881	2.389135	2.384471	2.379832	2.37922	2.374574	2.369996
2.38791	2.396599	2.391768	2.387018	2.382379	2.37777	2.377208	2.372579	2.368002
2.385869	2.394518	2.389629	2.384837	2.380172	2.375574	2.375126	2.37061	2.366091
2.383788	2.392409	2.38751	2.386853	2.382182	2.377598	2.373105	2.368667	2.364195
2.381629	2.390241	2.385415	2.384852	2.380227	2.375677	2.3712	2.366768	2.362319
2.379469	2.388088	2.383352	2.382894	2.378322	2.373802	2.369316	2.364859	2.36041
2.377357	2.386004	2.381338	2.380964	2.376433	2.371927	2.3674	2.36288	2.358386
2.375281	2.383966	2.379361	2.379059	2.374555	2.370048	2.36547	2.360876	2.356327
2.386644	2.386275	2.381704	2.377156	2.37263	2.368095	2.363506	2.358908	2.364924
2.384667	2.38437	2.379819	2.375259	2.370671	2.366087	2.361516	2.356963	2.363088
2.387056	2.382453	2.37789	2.373316	2.368708	2.364119	2.359576	2.355066	2.37127
2.385137	2.380525	2.375907	2.37131	2.366736	2.362193	2.357694	2.353227	2.369576
2.383201	2.378577	2.373927	2.369327	2.364793	2.360296	2.355832	2.351393	2.384161
2.381214	2.376578	2.371964	2.367401	2.362901	2.358433	2.353988	2.349557	2.38235
2.379239	2.374594	2.370026	2.365504	2.361036	2.356588	2.352143	2.347698	2.380514
2.377359	2.372713	2.368142	2.363627	2.359183	2.354745	2.361233	2.356754	2.378632
2.375518	2.370879	2.366292	2.361769	2.357344	2.352908	2.359382	2.354832	2.376733
2.373593	2.368985	2.364428	2.370937	2.36654	2.362121	2.383969	2.3794	2.374917
2.371597	2.367045	2.362556	2.369155	2.36476	2.360339	2.382218	2.377661	2.373171
2.369648	2.376206	2.371782	2.36737	2.362974	2.35855	2.380471	2.375931	2.37144
2.367765	2.374379	2.369977	2.36558	2.361184	2.356761	2.378735	2.374216	2.369728
2.365906	2.372573	2.368183	2.380977	2.376473	2.371957	2.376966	2.372474	2.367999
2.364059	2.370787	2.366402	2.379116	2.374562	2.370009	2.375111	2.370658	2.366222
2.362226	2.369017	2.364634	2.377265	2.372655	2.368059	2.373243	2.368822	2.36443
2.371697	2.367253	2.362884	2.375462	2.370836	2.366221	2.371462	2.36703	2.380235
2.369929	2.365496	2.361147	2.373689	2.369071	2.364451	2.36973	2.36526	2.378352
2.368168	2.380822	2.376395	2.371899	2.367283	2.362658	2.367987	2.363495	2.376481
2.366415	2.379021	2.374592	2.370094	2.365477	2.360848	2.366236	2.361736	2.374619
2.364664	2.37722	2.372785	2.378138	2.373582	2.369017	2.382092	2.377366	2.372777
2.362902	2.37541	2.370968	2.376385	2.371811	2.367237	2.380218	2.375531	2.370961
2.361161	2.373613	2.369153	2.374629	2.370038	2.365457	2.37835	2.373703	2.369153
2.376414	2.371859	2.367355	2.372876	2.368268	2.36368	2.376479	2.371858	2.367334
2.374707	2.370113	2.365557	2.371124	2.366512	2.361929	2.374635	2.370029	2.365524
2.372921	2.368313	2.363735	2.3872	2.382378	2.377599	2.372906	2.368285	2.363766
2.37108	2.366473	2.361894	2.385331	2.380631	2.375952	2.371264	2.366605	2.362047
2.369243	2.364632	2.360036	2.383427	2.378812	2.374211	2.369542	2.364876	2.360306
2.367406	2.362782	2.358149	2.381473	2.376905	2.372358	2.367727	2.363092	2.358543
2.365597	2.389152	2.384296	2.379563	2.375015	2.370497	2.365899	2.3613	2.356774
2.363854	2.38737	2.382497	2.377746	2.37318	2.368653	2.364069	2.359495	2.354986
2.362101	2.385586	2.38072	2.375962	2.371366	2.366818	2.362255	2.357717	2.353227
2.370705	2.383703	2.378891	2.374163	2.369551	2.365001	2.360498	2.356035	2.351572
2.368881	2.381779	2.37705	2.372375	2.367754	2.363205	2.358772	2.354396	2.349961
2.384603	2.379894	2.375259	2.370643	2.366016	2.361456	2.357038	2.352682	2.348263
2.382697	2.378038	2.373511	2.368961	2.364334	2.359752	2.355302	2.350912	2.346498
2.380869	2.376232	2.371744	2.367223	2.362612	2.358034	2.353565	2.349151	2.344746
2.379142	2.374487	2.369941	2.365397	2.360823	2.356283	2.351817	2.347398	2.343005
2.377441	2.372772	2.368164	2.363594	2.359058	2.354561	2.350103	2.345684	2.341307
2.375748	2.371097	2.366461	2.361885	2.357385	2.352923	2.348468	2.344047	2.339695

2.374043	2.369417	2.36477	2.360198	2.355728	2.351296	2.346852	2.342437	2.33811
2.372255	2.367634	2.363006	2.358452	2.353997	2.349582	2.345169	2.340788	2.336475
2.370426	2.365803	2.361214	2.356688	2.352236	2.347831	2.343461	2.339128	2.334824
2.368688	2.364074	2.359517	2.355014	2.350566	2.346169	2.341826	2.337525	2.333229
2.367037	2.362443	2.357913	2.353428	2.348983	2.344589	2.340262	2.335977	2.331687
2.365398	2.36082	2.356311	2.351843	2.347409	2.343022	2.3387	2.334418	2.330136
2.363761	2.359196	2.354697	2.350243	2.345832	2.341462	2.337132	2.332837	2.328569
2.362121	2.357568	2.353082	2.348642	2.344251	2.339892	2.33555	2.331237	2.326976
2.36047	2.35593	2.351477	2.347055	2.342658	2.338286	2.333929	2.3296	2.325323
2.358807	2.354279	2.34986	2.345456	2.341049	2.336662	2.332295	2.327953	2.323655
2.357108	2.352582	2.348171	2.343781	2.339398	2.335033	2.330683	2.326351	2.322052
2.355387	2.350856	2.346433	2.342054	2.337716	2.3334	2.329084	2.32478	2.320491
2.353752	2.349221	2.344774	2.34039	2.336079	2.331793	2.327498	2.323208	2.318926
2.352222	2.347696	2.343205	2.338793	2.33449	2.330217	2.325924	2.321634	2.317357
2.350656	2.346141	2.341636	2.337216	2.332915	2.328645	2.324355	2.320067	2.315786
2.348975	2.344479	2.340033	2.335649	2.331345	2.327067	2.322789	2.318514	2.314217
2.347305	2.342827	2.338438	2.33409	2.329781	2.325494	2.321224	2.316956	2.312642
2.345765	2.34129	2.336877	2.332516	2.328217	2.323938	2.319655	2.31537	2.325731
2.344281	2.339805	2.335342	2.33095	2.326659	2.322387	2.318083	2.313771	2.324095
2.342738	2.338293	2.333851	2.329454	2.325121	2.320807	2.316491	2.312179	2.322472
2.341156	2.336769	2.332399	2.328016	2.3236	2.319206	2.314884	2.310592	2.320859
2.339565	2.335217	2.330901	2.326541	2.322104	2.317679	2.313326	2.309006	2.319241
2.337961	2.333627	2.329338	2.325018	2.32065	2.316258	2.311832	2.307408	2.317602
2.336369	2.332043	2.327764	2.323472	2.319168	2.314819	2.310351	2.30586	2.316008
2.334796	2.330481	2.326182	2.321887	2.317611	2.313296	2.308872	2.304416	2.314506
2.33325	2.328944	2.324621	2.320312	2.316034	2.311738	2.307381	2.302997	2.313042
2.331763	2.327464	2.323151	2.318828	2.314489	2.310151	2.305833	2.301516	2.311575
2.33031	2.326015	2.321731	2.317393	2.312962	2.308552	2.304256	2.300004	2.310107
2.328809	2.324508	2.320247	2.31592	2.311475	2.307048	2.302734	2.298471	2.308579
2.327263	2.322943	2.318706	2.314416	2.310032	2.305638	2.30126	2.29691	2.306989
2.325757	2.321419	2.317163	2.31288	2.308549	2.304196	2.314244	2.309781	2.305446
2.324324	2.319965	2.315614	2.311285	2.306992	2.302696	2.312775	2.308353	2.303986
2.322887	2.318521	2.314093	2.30972	2.305439	2.301176	2.311292	2.306927	2.302539
2.321388	2.31705	2.312634	2.308253	2.303927	2.299634	2.309749	2.305436	2.31131
2.319875	2.315576	2.311209	2.306835	2.302448	2.2981	2.30819	2.303917	2.309836
2.318402	2.314123	2.309796	2.305438	2.301026	2.296646	2.306672	2.302379	2.308318
2.31695	2.312679	2.308394	2.30406	2.299648	2.295248	2.305182	2.300826	2.306771
2.315464	2.311186	2.306931	2.302627	2.298242	2.293853	2.303728	2.299333	2.324093
2.313935	2.309633	2.305398	2.301129	2.296804	2.292464	2.302316	2.297907	2.322617
2.312437	2.308112	2.303885	2.299643	2.29537	2.291074	2.300906	2.296488	2.321141
2.310998	2.306665	2.302435	2.298203	2.29396	2.289689	2.299492	2.295059	2.319645
2.309579	2.305235	2.300987	2.296752	2.292532	2.288284	2.298067	2.293632	2.318158
2.308154	2.303782	2.299484	2.295227	2.291029	2.286816	2.296612	2.29221	2.316703
2.306733	2.30233	2.297972	2.293685	2.289503	2.285324	2.295144	2.290791	2.315263
2.305316	2.300908	2.296533	2.292236	2.288052	2.28388	2.293697	2.28937	2.313815
2.303904	2.299509	2.295152	2.29086	2.286659	2.282471	2.292265	2.287949	2.312364
2.302493	2.298108	2.293764	2.303663	2.299393	2.295134	2.290846	2.286537	2.310919
2.301083	2.296704	2.292364	2.30224	2.297978	2.293727	2.289443	2.285136	2.309484
2.299675	2.295301	2.290965	2.300819	2.296567	2.292323	2.307559	2.303123	2.319106
2.298266	2.293898	2.289571	2.299404	2.295159	2.290918	2.306107	2.30166	2.317635

2.296861	2.292499	2.28818	2.297991	2.29375	2.28951	2.304652	2.300195	2.31616
2.295468	2.291112	2.286793	2.296576	2.292337	2.288099	2.3032	2.298741	2.314693
2.294081	2.289731	2.285409	2.29516	2.290921	2.286684	2.301747	2.297292	2.313228
2.29268	2.288337	2.284019	2.293743	2.289501	2.285261	2.311846	2.307337	2.329144
2.291266	2.286931	2.282624	2.292324	2.288076	2.283828	2.310359	2.305854	2.327659
2.289865	2.285538	2.28124	2.290914	2.286655	2.282399	2.308877	2.30438	2.32618
2.288482	2.284161	2.279864	2.289503	2.285231	2.280967	2.3074	2.302919	2.324714
2.287109	2.2828	2.278514	2.288126	2.283839	2.279561	2.305937	2.301461	2.323246
2.285737	2.281455	2.277211	2.286821	2.28252	2.278216	2.310049	2.305563	2.321768
2.284384	2.280131	2.275931	2.285542	2.281224	2.276889	2.308629	2.30411	2.320287
2.283088	2.278853	2.27465	2.296815	2.292414	2.287996	2.307193	2.302662	2.318815
2.281833	2.277606	2.27337	2.295462	2.291028	2.286588	2.305746	2.301219	2.317348
2.280562	2.290221	2.285875	2.317938	2.313381	2.308829	2.304294	2.299781	2.319314
2.279279	2.288882	2.284501	2.31648	2.311907	2.307351	2.302833	2.298348	2.317897
2.27797	2.287517	2.283111	2.315018	2.310441	2.30589	2.313947	2.309415	2.316495
2.276623	2.286119	2.281702	2.313556	2.308986	2.304447	2.31249	2.30797	2.315102
2.275262	2.284712	2.280293	2.312106	2.307559	2.303043	2.311077	2.30657	2.313744
2.273892	2.302096	2.297633	2.310676	2.306184	2.301723	2.313547	2.309092	2.312475
2.272528	2.300689	2.296234	2.309275	2.304843	2.30044	2.312311	2.307885	2.311232
2.271226	2.299377	2.294943	2.312069	2.307657	2.303271	2.310988	2.306556	2.309887
2.269973	2.298142	2.293739	2.310842	2.306382	2.301943	2.309604	2.30514	2.308468
2.276036	2.301637	2.297253	2.309541	2.305052	2.30058	2.311562	2.307076	2.310125
2.274754	2.300308	2.295906	2.308147	2.30365	2.299174	2.310126	2.305639	2.308698
2.286462	2.306358	2.301883	2.306739	2.302242	2.297771	2.30871	2.304237	2.307317
2.285101	2.30496	2.30048	2.30532	2.30083	2.296377	2.307333	2.302909	2.306036
2.283758	2.303593	2.29911	2.303935	2.299453	2.295017	2.305988	2.301607	2.304775
2.288037	2.302367	2.297881	2.306394	2.301903	2.297455	2.304696	2.300297	2.303456
2.286893	2.301208	2.296718	2.305206	2.300707	2.296241	2.303431	2.298984	2.302108
2.297025	2.309261	2.304724	2.303908	2.299403	2.294927	2.302089	2.297626	2.300739
2.295671	2.307866	2.303333	2.302514	2.298005	2.293527	2.300676	2.296223	2.299344
2.294344	2.306516	2.302001	2.312932	2.308361	2.303823	2.306933	2.302461	2.298023
2.293065	2.30525	2.300776	2.311718	2.307149	2.302607	2.305723	2.301259	2.296822
2.299085	2.307676	2.303228	2.310479	2.305913	2.301372	2.304495	2.300039	2.295605
2.297783	2.306333	2.301869	2.309095	2.304537	2.300008	2.303142	2.298694	2.294264
2.2965	2.304994	2.300498	2.307692	2.303144	2.29863	2.301775	2.297334	2.292908
2.295306	2.303765	2.299257	2.306434	2.301896	2.297393	2.300539	2.296101	2.305257
2.294162	2.302598	2.298086	2.305254	2.300727	2.296236	2.299376	2.294937	2.304077
2.29291	2.301329	2.296813	2.303969	2.299454	2.294973	2.29811	2.29367	2.302793
2.291541	2.299948	2.295429	2.302567	2.298065	2.293596	2.296732	2.29229	2.301395
2.294225	2.298623	2.294109	2.301236	2.296746	2.292287	2.295422	2.290978	2.300065
2.293037	2.29743	2.292938	2.300065	2.295579	2.29112	2.294251	2.289802	2.298868
2.291839	2.296228	2.291759	2.29889	2.294417	2.289969	2.293103	2.288658	2.297707
2.290502	2.29489	2.290431	2.297569	2.293148	2.288752	2.291918	2.287504	2.296549
2.289134	2.29352	2.289067	2.296207	2.291842	2.287505	2.290709	2.286331	2.295376
2.287902	2.303918	2.299397	2.29494	2.290566	2.286222	2.28942	2.285045	2.294073
2.28677	2.302746	2.29822	2.293738	2.289308	2.284908	2.288069	2.283673	2.292671
2.285663	2.301604	2.297075	2.292575	2.288105	2.283667	2.300287	2.295824	2.291365
2.284589	2.300502	2.295971	2.291462	2.286971	2.282512	2.299104	2.294638	2.290177
2.283499	2.299385	2.294853	2.290338	2.285836	2.281368	2.297934	2.293465	2.289003
2.28239	2.298247	2.293713	2.289197	2.284698	2.280232	2.296767	2.292294	2.287832

2.281225	2.297052	2.292516	2.288004	2.283515	2.279058	2.295565	2.291087	2.286627
2.279909	2.295704	2.291171	2.286666	2.282192	2.277747	2.29423	2.289751	2.303461
2.278525	2.294283	2.289755	2.285261	2.280804	2.276375	2.292837	2.28836	2.301954
2.277276	2.293001	2.288475	2.283986	2.279541	2.275123	2.291562	2.287086	2.304409
2.276139	2.29183	2.287303	2.282816	2.278379	2.273968	2.290383	2.28591	2.303136
2.27502	2.290681	2.286155	2.28167	2.277239	2.272833	2.293491	2.289018	2.30188
2.273913	2.289551	2.285025	2.280543	2.276117	2.271715	2.29235	2.287874	2.300635
2.272808	2.288423	2.283903	2.293319	2.288838	2.284378	2.309016	2.30419	2.309495
2.27169	2.287283	2.282776	2.292182	2.287704	2.283247	2.30778	2.30296	2.308249
2.270568	2.286141	2.281649	2.291046	2.286572	2.282115	2.306546	2.301733	2.307008
2.289517	2.285	2.280525	2.301294	2.296788	2.292299	2.305306	2.3005	2.305762
2.288366	2.283859	2.279402	2.300157	2.295648	2.291155	2.304062	2.299263	2.304513
2.287221	2.287676	2.283232	2.299015	2.294505	2.290011	2.302818	2.298027	2.303262
2.286082	2.286544	2.282107	2.297868	2.293359	2.288866	2.301574	2.29679	2.30201
2.284944	2.298896	2.294403	2.314834	2.309972	2.305135	2.30033	2.295551	2.300754
2.283808	2.297747	2.293254	2.313575	2.308719	2.303886	2.299085	2.294306	2.299491
2.282671	2.296597	2.292105	2.312314	2.307464	2.302637	2.297839	2.293062	2.29823
2.286496	2.295447	2.290957	2.311054	2.306208	2.301385	2.306617	2.301792	2.296979
2.285357	2.294296	2.289806	2.309791	2.304949	2.300132	2.305353	2.300537	2.295732
2.284217	2.293142	2.288651	2.308523	2.303687	2.298878	2.304088	2.299281	2.294488
2.283076	2.291986	2.287492	2.307251	2.302423	2.297623	2.302822	2.298024	2.293246
2.295335	2.309399	2.304545	2.305981	2.301161	2.29637	2.301556	2.296765	2.292002
2.294168	2.308128	2.303277	2.304712	2.299902	2.295119	2.30029	2.295503	2.290756
2.293008	2.306857	2.302012	2.313522	2.308666	2.303836	2.299024	2.29424	2.289506
2.291849	2.305577	2.300748	2.312249	2.3074	2.302574	2.297761	2.292976	2.288248
2.290719	2.304318	2.299496	2.31098	2.306134	2.301312	2.296499	2.291715	2.286992
2.289658	2.303115	2.298258	2.309697	2.304854	2.30004	2.295236	2.29046	2.285745
2.288642	2.301956	2.297042	2.308421	2.303574	2.298769	2.293975	2.289212	2.284504
2.287632	2.30085	2.295899	2.307216	2.302338	2.297511	2.292716	2.287963	2.283258
2.286637	2.299798	2.29482	2.306072	2.301138	2.296264	2.29146	2.286713	2.28201
2.30361	2.309262	2.304254	2.304955	2.300002	2.295105	2.290287	2.285527	2.280809
2.302361	2.308057	2.303129	2.303884	2.298964	2.294076	2.289232	2.284427	2.279664
2.301105	2.306828	2.301968	2.302772	2.297891	2.293024	2.288174	2.283346	2.278556
2.299866	2.305565	2.300721	2.301542	2.296692	2.291861	2.287055	2.28227	2.27751
2.298668	2.304325	2.299469	2.300286	2.295455	2.290657	2.285902	2.281176	2.276462
2.297604	2.303221	2.298324	2.299106	2.29425	2.28944	2.284695	2.279991	2.275301
2.296619	2.302195	2.297242	2.297966	2.293063	2.288217	2.283459	2.278757	2.274079
2.306029	2.306771	2.301795	2.296855	2.291954	2.287104	2.282326	2.2776	2.272902
2.304807	2.305588	2.30067	2.295781	2.290929	2.286103	2.281297	2.276515	2.271763
2.303576	2.30438	2.299505	2.294657	2.289847	2.285048	2.28024	2.27544	2.274357
2.302334	2.303126	2.298261	2.293432	2.288646	2.28388	2.279122	2.274375	2.273319
2.301137	2.301915	2.297053	2.292232	2.287454	2.282706	2.277987	2.273285	2.272256
2.300072	2.300856	2.296	2.291171	2.286364	2.281584	2.276837	2.272111	2.271071
2.299048	2.299842	2.294991	2.290148	2.285307	2.280485	2.275691	2.270923	2.269856
2.303585	2.298705	2.293861	2.289031	2.28421	2.279404	2.274615	2.269843	2.268754
2.302387	2.297493	2.292658	2.287856	2.283089	2.278336	2.273585	2.268834	2.267723
2.301261	2.296356	2.291506	2.286702	2.281949	2.277217	2.276186	2.271433	2.266644
2.300223	2.295307	2.290411	2.285565	2.280779	2.276027	2.274994	2.270264	2.265506
2.299152	2.294236	2.289325	2.284461	2.279653	2.274886	2.273849	2.269132	2.264391
2.297985	2.293089	2.288232	2.283407	2.278615	2.273854	2.272821	2.268103	2.263345

2.296821	2.291949	2.287156	2.282377	2.2776	2.272843	2.271804	2.267074	2.262295
2.295747	2.290894	2.286133	2.281365	2.276568	2.271781	2.270703	2.265942	2.261164
2.294713	2.289876	2.285128	2.280356	2.275529	2.270704	2.269574	2.264774	2.260006
2.293658	2.28881	2.284048	2.279272	2.274456	2.26964	2.268497	2.263683	2.258907
2.29259	2.287709	2.282909	2.278125	2.273355	2.268585	2.26746	2.262651	2.257855
2.291534	2.286644	2.281829	2.280762	2.275994	2.271227	2.266424	2.26161	2.256801
2.290489	2.285622	2.280831	2.279772	2.274998	2.270215	2.26539	2.260554	2.255738
2.289465	2.284625	2.279853	2.278794	2.273999	2.269192	2.264347	2.259496	2.254684
2.288479	2.283658	2.278885	2.277807	2.272973	2.268132	2.263279	2.258435	2.253646
2.287506	2.282698	2.277909	2.276804	2.27193	2.267059	2.262208	2.257382	2.252619
2.286526	2.281706	2.276883	2.275747	2.270861	2.265988	2.261155	2.256355	2.251602
2.28554	2.280694	2.275826	2.274659	2.269778	2.264922	2.260116	2.255344	2.250593
2.284516	2.279655	2.274774	2.273602	2.268739	2.2639	2.259092	2.254311	2.249562
2.283454	2.278587	2.273723	2.272569	2.267741	2.262922	2.258082	2.253256	2.248506
2.282398	2.277536	2.272696	2.271563	2.266759	2.261953	2.257095	2.252244	2.247494
2.281347	2.276509	2.271704	2.270592	2.265798	2.260997	2.256149	2.251306	2.246556
2.280324	2.275508	2.270731	2.269632	2.26483	2.260023	2.25519	2.250368	2.245626
2.27936	2.278259	2.273474	2.26867	2.263823	2.258983	2.254164	2.249374	2.244662
2.278424	2.277318	2.272532	2.267708	2.262809	2.25793	2.253124	2.24837	2.243691
2.277476	2.276373	2.271579	2.266746	2.261836	2.25695	2.252149	2.247406	2.243593
2.276526	2.275428	2.270619	2.265781	2.260886	2.256015	2.251214	2.246466	2.242649
2.275576	2.274466	2.269621	2.264767	2.259888	2.255038	2.250256	2.245522	2.241695
2.274626	2.273483	2.268578	2.263693	2.258832	2.254014	2.249272	2.244573	2.240724
2.273677	2.272507	2.267556	2.262649	2.2578	2.253007	2.248301	2.243635	2.239771
2.272729	2.271545	2.26658	2.261666	2.25682	2.252038	2.247356	2.242716	2.238851
2.271784	2.270593	2.265625	2.260711	2.255867	2.25109	2.246426	2.241804	2.237941
2.274545	2.269651	2.264684	2.259773	2.254932	2.250162	2.246385	2.241754	2.23703
2.273607	2.268717	2.263755	2.258849	2.254014	2.249248	2.245473	2.240843	2.236119
2.272668	2.26778	2.26282	2.257919	2.253093	2.248334	2.244561	2.239931	2.235205
2.271728	2.26684	2.261882	2.256986	2.252172	2.247423	2.243651	2.239018	2.234288
2.265455	2.265912	2.26096	2.256069	2.251258	2.246511	2.242739	2.238104	2.233369
2.26453	2.264996	2.260053	2.255164	2.250346	2.245592	2.24182	2.237186	2.232446
2.26361	2.264088	2.259169	2.254292	2.249466	2.2447	2.240918	2.236275	2.231527
2.2627	2.263194	2.258332	2.253488	2.248655	2.243867	2.240057	2.235378	2.230616
2.261791	2.262297	2.257495	2.252687	2.247851	2.243043	2.239202	2.234485	2.229715
2.260863	2.261368	2.256594	2.251806	2.246977	2.242169	2.238318	2.233589	2.228836
2.259932	2.260426	2.25566	2.250883	2.246066	2.24127	2.237419	2.232692	2.227969
2.259052	2.259527	2.254749	2.250906	2.246084	2.241279	2.236517	2.231781	2.227073
2.258222	2.258667	2.253855	2.249993	2.245179	2.240383	2.235607	2.230854	2.226147
2.257373	2.2578	2.252962	2.249093	2.244305	2.239532	2.234748	2.229975	2.225254
2.25648	2.256908	2.252056	2.248201	2.243477	2.238756	2.23397	2.229169	2.224407
2.255599	2.256037	2.251178	2.247334	2.242652	2.237968	2.233191	2.228381	2.223583
2.254775	2.255233	2.250362	2.246503	2.241793	2.237096	2.232355	2.227592	2.22279
2.253961	2.254444	2.249575	2.245699	2.240938	2.236205	2.231499	2.226792	2.221995
2.253089	2.248294	2.2435	2.244927	2.240137	2.235369	2.230643	2.225921	2.22112
2.252184	2.24741	2.242706	2.244178	2.239372	2.234567	2.229785	2.225005	2.220196
2.251328	2.24655	2.241873	2.243366	2.238579	2.233776	2.22896	2.224137	2.219324
2.250523	2.245712	2.240993	2.242484	2.237757	2.233006	2.228177	2.223323	2.218509
2.249732	2.24607	2.241288	2.24162	2.23692	2.232196	2.227361	2.222491	2.217677
2.248948	2.245299	2.2405	2.240799	2.236058	2.231302	2.226467	2.22161	2.216791

2.248175	2.244542	2.239733	2.24	2.235209	2.230412	2.225578	2.220736	2.215913
2.247417	2.243776	2.238968	2.239225	2.234417	2.229603	2.224761	2.219915	2.215089
2.246667	2.243012	2.238209	2.238466	2.233653	2.228832	2.22398	2.219123	2.214294
2.245905	2.242262	2.237454	2.237704	2.232886	2.228059	2.223203	2.218342	2.21351
2.245136	2.241521	2.236702	2.236943	2.232122	2.227292	2.222435	2.217573	2.212742
2.24552	2.240737	2.235934	2.231128	2.226325	2.221511	2.221666	2.216805	2.211975
2.244655	2.239888	2.235143	2.230373	2.225564	2.220738	2.220895	2.216036	2.211207
2.243835	2.239074	2.234365	2.229615	2.2248	2.219965	2.220125	2.215269	2.210443
2.243126	2.23836	2.23362	2.228849	2.224031	2.219197	2.219359	2.214507	2.209685
2.242447	2.237669	2.232883	2.228081	2.22326	2.21843	2.218593	2.213746	2.20893
2.24173	2.236938	2.232133	2.227319	2.222491	2.21766	2.217826	2.212983	2.208171
2.240999	2.23619	2.231378	2.226558	2.221724	2.21689	2.21706	2.21222	2.207414
2.240247	2.23543	2.230615	2.225793	2.220957	2.216122	2.216296	2.211464	2.206666
2.239475	2.234656	2.229844	2.225024	2.220187	2.215355	2.215534	2.210712	2.205927
2.238708	2.233893	2.229088	2.224271	2.219429	2.214594	2.209777	2.204982	2.205206
2.23795	2.233141	2.22835	2.223535	2.218677	2.21383	2.209026	2.204255	2.204508
2.237202	2.232404	2.227629	2.222823	2.217956	2.213103	2.208305	2.203546	2.203816
2.23646	2.231684	2.226937	2.222153	2.217307	2.212463	2.207652	2.202871	2.203126
2.235739	2.23098	2.226254	2.221493	2.216674	2.211845	2.207017	2.202203	2.202433
2.235071	2.230296	2.225549	2.22078	2.215976	2.211159	2.206324	2.201497	2.201719
2.234442	2.229631	2.224836	2.220039	2.215242	2.210437	2.205601	2.200772	2.200994
2.233802	2.228983	2.224164	2.219352	2.21455	2.209746	2.20492	2.200101	2.20032
2.233157	2.228358	2.223542	2.218724	2.213904	2.20909	2.204283	2.19949	2.199701
2.232486	2.227697	2.222887	2.218068	2.213235	2.208413	2.203622	2.19885	2.199057
2.23176	2.22696	2.222158	2.217344	2.212506	2.207682	2.202899	2.198138	2.198346
2.231046	2.226231	2.221436	2.216628	2.21179	2.206966	2.202188	2.197433	2.197643
2.230407	2.225584	2.220787	2.21598	2.211148	2.20633	2.201552	2.196795	2.192038
2.2298	2.224973	2.220168	2.215361	2.210539	2.20573	2.200949	2.196185	2.191431
2.229185	2.224356	2.219543	2.214733	2.209924	2.205124	2.200338	2.195566	2.190814
2.228568	2.223738	2.218915	2.214104	2.209308	2.204518	2.199726	2.194944	2.190194
2.227902	2.223071	2.21824	2.213427	2.20864	2.203859	2.199064	2.194276	2.189528
2.227168	2.222338	2.2175	2.212684	2.207904	2.203129	2.198334	2.193543	2.188797
2.226458	2.22163	2.216789	2.211973	2.207195	2.202423	2.197629	2.19284	2.188094
2.225815	2.220989	2.216156	2.211343	2.206561	2.201785	2.196996	2.192212	2.187463
2.225197	2.220372	2.215549	2.210741	2.205953	2.201173	2.196388	2.191611	2.186858
2.224581	2.219757	2.214937	2.210131	2.205343	2.200563	2.19578	2.191005	2.182107
2.223972	2.219148	2.214328	2.209523	2.204739	2.199961	2.195178	2.190403	2.181529
2.223357	2.218534	2.213714	2.20891	2.204127	2.199352	2.19457	2.189795	2.180946
2.222738	2.217916	2.213095	2.208291	2.203511	2.198737	2.193957	2.189182	2.180359
2.222126	2.217307	2.212491	2.207688	2.202901	2.198123	2.193341	2.188569	2.179773
2.221515	2.216699	2.211896	2.207096	2.202292	2.197498	2.192717	2.18795	2.179184
2.220936	2.216123	2.211333	2.206535	2.201715	2.196904	2.188087	2.18333	2.178605
2.220432	2.215619	2.210837	2.20604	2.201213	2.196385	2.187564	2.182774	2.178033
2.219941	2.215129	2.210351	2.205554	2.200725	2.195887	2.187064	2.182242	2.177486
2.219381	2.214569	2.209788	2.204991	2.200168	2.19534	2.186562	2.181758	2.201233
2.218781	2.213969	2.209179	2.204384	2.199573	2.194768	2.186062	2.181304	2.200785
2.218187	2.213376	2.208577	2.199832	2.195055	2.190286	2.185531	2.180791	2.200268
2.217592	2.212783	2.207972	2.199252	2.194487	2.189729	2.184967	2.180214	2.199679
2.217037	2.212228	2.207408	2.198712	2.193957	2.189206	2.184435	2.17967	2.199115
2.216551	2.211743	2.206914	2.198242	2.193494	2.188747	2.18397	2.179193	2.198604

2.216062	2.211254	2.206424	2.197778	2.19303	2.188281	2.183504	2.178723	2.198105
2.215495	2.206833	2.202044	2.197266	2.1925	2.187741	2.207284	2.202439	2.197598
2.214915	2.206282	2.201515	2.196744	2.191956	2.187181	2.20672	2.201907	2.197094
2.214415	2.205806	2.201039	2.196264	2.191464	2.186682	2.206213	2.201415	2.196613
2.213972	2.205383	2.2006	2.195812	2.191008	2.186224	2.205743	2.200947	2.196147
2.213526	2.204959	2.200159	2.205273	2.200408	2.195567	2.205229	2.200427	2.195632
2.213084	2.20454	2.19972	2.204798	2.199899	2.195026	2.204653	2.199837	2.195052
2.208885	2.204085	2.199258	2.204315	2.199399	2.194509	2.204112	2.199284	2.194506
2.208341	2.203546	2.198743	2.20381	2.198916	2.194043	2.203649	2.198822	2.194041
2.207795	2.203006	2.198229	2.203311	2.198447	2.193599	2.203215	2.198391	2.193606
2.207337	2.212496	2.207674	2.217403	2.212509	2.207629	2.202778	2.197953	2.193168
2.206924	2.21207	2.207233	2.216954	2.212068	2.207197	2.202346	2.19752	2.192736
2.206508	2.21164	2.206797	2.216508	2.211626	2.206759	2.201911	2.197086	2.1923
2.206096	2.211213	2.206368	2.21607	2.211186	2.206319	2.201474	2.196651	2.191861
2.215626	2.210784	2.205938	2.215634	2.21075	2.205883	2.201041	2.196221	2.191428
2.215196	2.210352	2.205505	2.2152	2.210317	2.205452	2.200613	2.195795	2.191003
2.214763	2.209919	2.205072	2.214765	2.209885	2.205023	2.200187	2.195371	2.190582
2.214322	2.209482	2.204639	2.214329	2.209451	2.204592	2.19976	2.19495	2.190163
2.213884	2.209043	2.204204	2.213892	2.209017	2.204162	2.199334	2.194528	2.189746
2.213458	2.208595	2.203763	2.213454	2.208584	2.203732	2.198907	2.194104	2.189324
2.213046	2.208148	2.203321	2.213018	2.208153	2.203306	2.198482	2.193679	2.1889
2.212688	2.213846	2.208961	2.212586	2.207732	2.202896	2.198067	2.193255	2.188475
2.212389	2.213534	2.208577	2.212153	2.207316	2.2025	2.197659	2.192829	2.188045
2.212053	2.22173	2.216717	2.211776	2.206944	2.202135	2.197293	2.192455	2.187655
2.211648	2.221359	2.216403	2.211492	2.206647	2.201823	2.196997	2.192173	2.187331
2.211232	2.22097	2.21608	2.211206	2.206347	2.201504	2.196692	2.191884	2.187013
2.210827	2.220549	2.215693	2.210838	2.20597	2.201117	2.196307	2.191507	2.18666
2.210437	2.220131	2.215292	2.210449	2.205575	2.200716	2.195898	2.191097	2.18629
2.216177	2.219793	2.21495	2.210106	2.205244	2.200387	2.195533	2.190691	2.185893
2.215886	2.219513	2.214645	2.209791	2.204953	2.200104	2.195195	2.190286	2.185475
2.215598	2.219221	2.214313	2.209438	2.204617	2.199783	2.194863	2.189935	2.185119
2.215318	2.218921	2.213947	2.209036	2.204224	2.199416	2.194535	2.189647	2.184835
2.213832	2.218605	2.213598	2.208667	2.203855	2.199061	2.194217	2.189368	2.184552
2.213458	2.218258	2.213286	2.208372	2.20355	2.198746	2.193922	2.189094	2.184248
2.213078	2.21791	2.212989	2.208099	2.203254	2.198427	2.193616	2.188805	2.183936
2.221637	2.217594	2.212704	2.207817	2.202924	2.198054	2.193247	2.188458	2.183605
2.221326	2.217284	2.212421	2.207531	2.202584	2.197668	2.192858	2.188089	2.183269
2.220971	2.216074	2.211227	2.207204	2.202262	2.197348	2.192539	2.187771	2.182965
2.220583	2.215667	2.210834	2.206841	2.201953	2.197084	2.192276	2.187493	2.182687
2.220242	2.215313	2.210487	2.206514	2.201664	2.196825	2.192019	2.187225	2.182418
2.219964	2.215032	2.210204	2.206235	2.201396	2.196568	2.191763	2.186967	2.182158
2.219702	2.21477	2.209939	2.205971	2.201137	2.196314	2.191509	2.186712	2.181902
2.219439	2.214509	2.209678	2.205713	2.200882	2.19606	2.191254	2.186455	2.181645
2.219181	2.214253	2.209424	2.205462	2.20063	2.195806	2.190999	2.186199	2.181389
2.215148	2.213995	2.209168	2.204345	2.199508	2.194679	2.190742	2.185943	2.181136
2.214887	2.21374	2.208913	2.204088	2.199248	2.194417	2.190484	2.185687	2.180884
2.214639	2.213495	2.208659	2.203827	2.198985	2.194153	2.190225	2.185429	2.18063
2.214403	2.213257	2.208401	2.203558	2.198717	2.193889	2.189965	2.18517	2.180372
2.214202	2.213062	2.208188	2.203326	2.198467	2.193626	2.188811	2.184012	2.180116
2.214061	2.212942	2.20805	2.203156	2.198246	2.193361	2.18854	2.183748	2.179863

2.213908	2.212808	2.207904	2.202985	2.198029	2.193106	2.188275	2.183488	2.17961
2.213673	2.208798	2.20392	2.202771	2.197801	2.192861	2.188017	2.183221	2.179349
2.213419	2.208542	2.203674	2.202539	2.197575	2.192637	2.187781	2.182971	2.1791
2.213235	2.208335	2.203438	2.202304	2.197376	2.192472	2.187616	2.182792	2.184247
2.213092	2.208155	2.203204	2.202063	2.197195	2.192348	2.187502	2.182665	2.184082
2.212905	2.207951	2.20297	2.201826	2.196984	2.192164	2.187327	2.182492	2.183904
2.21267	2.207719	2.202732	2.201585	2.196732	2.191907	2.187082	2.182268	2.183715
2.212438	2.207493	2.202518	2.201381	2.196515	2.191679	2.186851	2.182044	2.18351
2.212216	2.207278	2.202345	2.201237	2.196372	2.191523	2.186665	2.181822	2.183271
2.212015	2.207084	2.20219	2.20111	2.196243	2.19138	2.186494	2.181618	2.183046
2.211856	2.206936	2.202048	2.200971	2.196081	2.191201	2.186323	2.181463	2.182889
2.211722	2.206812	2.201913	2.200826	2.195911	2.191016	2.186161	2.181335	2.182764
2.211584	2.206669	2.201749	2.196844	2.191951	2.187085	2.186018	2.181204	2.182619
2.211447	2.206513	2.201563	2.19665	2.191786	2.186949	2.185893	2.181076	2.182463
2.211288	2.206346	2.201395	2.196488	2.191647	2.186826	2.185764	2.180927	2.182297
2.211098	2.206161	2.201246	2.196367	2.191541	2.186719	2.185624	2.18074	2.182109
2.210921	2.205995	2.201114	2.19626	2.191444	2.18662	2.185508	2.180597	2.181963
2.210775	2.205872	2.201004	2.19616	2.191349	2.186534	2.185454	2.180572	2.181919
2.210651	2.205768	2.200905	2.196064	2.191253	2.186445	2.185403	2.180559	2.18189
2.210553	2.205661	2.200801	2.195961	2.191144	2.186329	2.185284	2.180434	2.18177
2.210474	2.205559	2.2007	2.195855	2.191024	2.186193	2.185127	2.180253	2.181607
2.210378	2.205463	2.200603	2.195746	2.190885	2.186029	2.184967	2.180105	2.181474
2.210267	2.205375	2.20051	2.195632	2.190721	2.185831	2.184796	2.179979	2.181366
2.210165	2.205281	2.200419	2.195538	2.190614	2.185713	2.184683	2.179871	2.181258
2.210071	2.205168	2.200326	2.195475	2.190601	2.185727	2.184657	2.179775	2.181136
2.21	2.205077	2.200239	2.19541	2.190588	2.185747	2.184655	2.179717	2.181045
2.209989	2.205056	2.200169	2.195316	2.190511	2.1857	2.180825	2.175938	2.181032
2.20998	2.205044	2.200103	2.195217	2.190416	2.185627	2.180809	2.17598	2.181047
2.209865	2.204937	2.200017	2.195141	2.190329	2.185535	2.180741	2.175943	2.181026
2.209684	2.204775	2.199922	2.195082	2.190248	2.185429	2.180638	2.175857	2.180985
2.209594	2.204704	2.199882	2.19506	2.190224	2.185395	2.180576	2.175769	2.180914
2.209602	2.204735	2.199906	2.195083	2.19027	2.185442	2.180558	2.17567	2.180803
2.209598	2.204748	2.199907	2.19508	2.190281	2.185458	2.180544	2.175614	2.180725
2.20954	2.204698	2.199843	2.195009	2.190214	2.185406	2.180533	2.175642	2.18072
2.209486	2.204648	2.199786	2.194943	2.190135	2.185328	2.180504	2.175669	2.180724
2.209483	2.204651	2.199804	2.194951	2.190085	2.18523	2.180417	2.175615	2.180695
2.209497	2.204667	2.199847	2.194986	2.190049	2.185133	2.18031	2.175527	2.180651
2.209471	2.20463	2.19983	2.19498	2.19004	2.185111	2.180258	2.175445	2.177103
2.209418	2.204553	2.199769	2.194946	2.190053	2.185149	2.180246	2.175362	2.177007
2.209403	2.204529	2.199743	2.194933	2.190079	2.1852	2.180267	2.175336	2.176964
2.209438	2.204573	2.199767	2.194953	2.190127	2.185271	2.180331	2.175381	2.176986
2.209484	2.204621	2.199782	2.194953	2.190147	2.185314	2.180384	2.175436	2.177016
2.209531	2.204644	2.199749	2.19489	2.190095	2.185286	2.180391	2.175475	2.177026
2.209585	2.204669	2.19972	2.194831	2.19004	2.18525	2.180395	2.175517	2.177042
2.209647	2.20472	2.199751	2.194849	2.190057	2.185273	2.180434	2.175573	2.177084
2.209715	2.204786	2.199816	2.194911	2.190115	2.185327	2.180493	2.175637	2.177141
2.209783	2.204852	2.19988	2.194974	2.190174	2.185386	2.180555	2.175705	2.185857
2.209852	2.20492	2.199948	2.195041	2.190242	2.185454	2.180625	2.175776	2.185908
2.209921	2.204989	2.200015	2.195109	2.190309	2.185521	2.180693	2.175846	2.185955
2.209991	2.205058	2.200084	2.195176	2.190376	2.185588	2.180759	2.175912	2.186

2.199671	2.205125	2.200149	2.19524	2.19044	2.185651	2.190275	2.185389	2.18604
2.199724	2.205186	2.200208	2.195298	2.190497	2.185708	2.190312	2.185425	2.186071
2.199775	2.205243	2.200264	2.195354	2.190552	2.185763	2.190346	2.185458	2.186099
2.199828	2.2053	2.200319	2.195407	2.190606	2.185818	2.190382	2.185492	2.186127
2.199881	2.205357	2.200374	2.19546	2.19066	2.185873	2.190417	2.185527	2.186155
2.199938	2.205423	2.200442	2.195527	2.190719	2.185925	2.190451	2.185566	2.180638
2.200001	2.205497	2.200521	2.195604	2.190777	2.185969	2.190481	2.185609	2.18069
2.200064	2.195221	2.190346	2.205143	2.200265	2.195404	2.190532	2.185656	2.180752
2.200125	2.195315	2.190494	2.205321	2.200426	2.195533	2.190619	2.185707	2.180828
2.200196	2.195411	2.190635	2.205489	2.200583	2.195664	2.190712	2.185764	2.180906
2.200286	2.195485	2.190711	2.205559	2.200659	2.195743	2.190781	2.185821	2.180961
2.200394	2.195564	2.190771	2.205598	2.200708	2.195804	2.190849	2.185892	2.181023
2.200537	2.195707	2.190905	2.205701	2.200792	2.195879	2.190945	2.186018	2.181153
2.200711	2.1959	2.191092	2.205849	2.200899	2.195965	2.191065	2.18619	2.181337
2.200841	2.206397	2.201545	2.196677	2.191779	2.186898	2.191213	2.18636	2.181507
2.200911	2.20646	2.201632	2.196791	2.191923	2.187065	2.191396	2.186531	2.181658
2.201006	2.206544	2.201737	2.196919	2.192078	2.187238	2.191572	2.186689	2.181803
2.201172	2.206696	2.201902	2.197091	2.19225	2.187403	2.191717	2.186811	2.181934
2.201352	2.206855	2.202065	2.197255	2.192413	2.18756	2.191856	2.186934	2.18207
2.197699	2.206947	2.202146	2.197339	2.192516	2.187684	2.192001	2.187094	2.182238
2.19782	2.207023	2.202201	2.197392	2.192594	2.187795	2.19215	2.187272	2.182421
2.208774	2.20721	2.202351	2.197521	2.192726	2.187936	2.192313	2.187442	2.182584
2.209061	2.207497	2.202586	2.197716	2.192903	2.188103	2.192488	2.18761	2.182732
2.209288	2.207729	2.202799	2.197909	2.193081	2.188268	2.183444	2.178622	2.182874
2.209418	2.207867	2.202966	2.198093	2.193259	2.188431	2.183582	2.178737	2.183005
2.209542	2.208003	2.203136	2.198279	2.19343	2.188582	2.183717	2.178861	2.183152
2.209697	2.208182	2.20333	2.198465	2.193569	2.188686	2.183838	2.179019	2.183341
2.209867	2.208376	2.203533	2.198657	2.19372	2.188807	2.183979	2.179198	2.183547
2.210067	2.208573	2.203735	2.198871	2.193958	2.189059	2.184208	2.179394	2.183738
2.210287	2.208775	2.203939	2.1991	2.19425	2.189391	2.184496	2.179608	2.183929
2.210485	2.205619	2.200803	2.199316	2.194496	2.189659	2.184761	2.179857	2.184164
2.21066	2.205818	2.201006	2.199522	2.1947	2.189869	2.185012	2.18015	2.184452
2.210848	2.206014	2.201204	2.199723	2.194896	2.190067	2.185227	2.180385	2.175549
2.211048	2.206189	2.201387	2.199916	2.195092	2.190255	2.18538	2.1805	2.175672
2.21128	2.206391	2.201581	2.200109	2.195286	2.190445	2.18554	2.18063	2.17581
2.211585	2.206679	2.201803	2.200293	2.195472	2.190649	2.185778	2.1809	2.176066
2.211913	2.206999	2.202048	2.200491	2.195665	2.19086	2.18604	2.181216	2.176366
2.212168	2.207274	2.202334	2.197428	2.192575	2.187751	2.186254	2.181463	2.176632
2.212372	2.207522	2.202653	2.197765	2.192838	2.187943	2.186439	2.181668	2.176882
2.21258	2.207743	2.20292	2.198057	2.193116	2.188198	2.186671	2.181878	2.17711
2.212788	2.207926	2.203125	2.198297	2.193415	2.188532	2.186957	2.182089	2.177305
2.213033	2.208144	2.203334	2.198521	2.193695	2.188852	2.187255	2.182338	2.177539
2.213343	2.208434	2.203565	2.198724	2.193923	2.189115	2.187548	2.182654	2.177855
2.213677	2.208756	2.20382	2.198941	2.194151	2.18937	2.187846	2.182994	2.178192
2.214016	2.209092	2.204125	2.199224	2.19443	2.189654	2.184844	2.18003	2.178502
2.214353	2.209437	2.204459	2.199545	2.194732	2.189945	2.185151	2.180354	2.1788
2.214632	2.209737	2.204777	2.19986	2.195011	2.190193	2.185406	2.180628	2.179079
2.214861	2.209997	2.205084	2.200177	2.195273	2.190403	2.185613	2.180855	2.17934
2.215114	2.210271	2.2054	2.200506	2.195567	2.190663	2.185869	2.181121	2.179628
2.215411	2.210576	2.205737	2.200859	2.195909	2.190991	2.186191	2.181439	2.17995

2.215697	2.210861	2.206047	2.201189	2.19625	2.191336	2.186534	2.181775	2.176988
2.21593	2.211078	2.206281	2.201452	2.196556	2.191678	2.186884	2.182121	2.177332
2.216163	2.21129	2.206503	2.201702	2.196857	2.192025	2.187241	2.182474	2.177682
2.216465	2.211581	2.206794	2.202002	2.197182	2.19237	2.18759	2.18282	2.178027
2.216808	2.211918	2.207125	2.202332	2.19752	2.192716	2.187936	2.183163	2.178369
2.21715	2.212256	2.20746	2.202668	2.19786	2.193061	2.188281	2.183505	2.178711
2.217492	2.212596	2.207803	2.203012	2.198205	2.193405	2.188623	2.183845	2.179051
2.217835	2.212938	2.208146	2.203357	2.19855	2.193749	2.188966	2.184187	2.179393
2.218175	2.213277	2.208488	2.2037	2.198893	2.194093	2.189311	2.184533	2.17974
2.218527	2.213626	2.208833	2.204043	2.199237	2.194438	2.189657	2.184881	2.180088
2.218897	2.213989	2.209177	2.204376	2.199573	2.19478	2.190002	2.185229	2.18044
2.219289	2.214374	2.209537	2.20472	2.199916	2.195126	2.190349	2.185578	2.180792
2.217214	2.214819	2.209967	2.20513	2.200301	2.195488	2.190698	2.18592	2.181135
2.217681	2.215308	2.210449	2.205589	2.200718	2.195865	2.191052	2.186261	2.181475
2.218094	2.213223	2.208366	2.206018	2.201143	2.196283	2.19146	2.186658	2.181861
2.21844	2.213571	2.208732	2.206413	2.201581	2.196756	2.191938	2.187125	2.182301
2.218816	2.21394	2.209103	2.206798	2.201997	2.197198	2.192387	2.187571	2.183767
2.219275	2.214373	2.209498	2.207165	2.202353	2.197554	2.192754	2.187958	2.184183
2.219739	2.214816	2.209908	2.207549	2.202718	2.197909	2.193113	2.188331	2.184586
2.220134	2.21523	2.210351	2.205492	2.200654	2.19583	2.193521	2.188714	2.184954
2.2205	2.21563	2.210804	2.205976	2.201137	2.196295	2.193954	2.189105	2.185312
2.22088	2.216012	2.211204	2.206392	2.201566	2.196733	2.191878	2.187025	2.185729
2.221268	2.216375	2.211559	2.206754	2.201955	2.197151	2.192321	2.187487	2.186194
2.221687	2.216783	2.211959	2.207155	2.202374	2.19759	2.192775	2.187951	2.184415
2.222148	2.217252	2.212423	2.207619	2.202843	2.198064	2.19325	2.188421	2.184863
2.222619	2.217727	2.212886	2.208072	2.203293	2.198512	2.1937	2.188874	2.185307
2.223093	2.218186	2.21331	2.208467	2.203667	2.198878	2.194082	2.189284	2.185738
2.223562	2.218639	2.213731	2.208861	2.204037	2.199239	2.194459	2.18969	2.186174
2.224001	2.219076	2.214178	2.209314	2.204491	2.199691	2.19491	2.190142	2.186639
2.224424	2.219507	2.214643	2.209805	2.204993	2.200196	2.195405	2.190623	2.187124
2.224875	2.219973	2.21513	2.210306	2.205496	2.200697	2.195902	2.191116	2.187617
2.225351	2.220473	2.215641	2.210821	2.206004	2.201198	2.196406	2.191624	2.188119
2.225842	2.220981	2.216154	2.211333	2.206513	2.201703	2.198218	2.19342	2.19517
2.226351	2.221496	2.216663	2.211839	2.207021	2.202212	2.198723	2.193915	2.195663
2.226865	2.222012	2.217173	2.212346	2.207529	2.20272	2.199226	2.19441	2.196157
2.227373	2.222522	2.217685	2.212856	2.208035	2.203222	2.199726	2.194909	2.196658
2.22788	2.223029	2.218195	2.213366	2.20854	2.203722	2.200223	2.195408	2.19716
2.228386	2.223534	2.218698	2.213869	2.209042	2.204224	2.200722	2.195903	2.197657
2.228894	2.224038	2.219197	2.214366	2.209543	2.204726	2.201221	2.196395	2.198151
2.229418	2.224557	2.219703	2.214863	2.210042	2.205228	2.201721	2.196893	2.198649
2.229957	2.22509	2.220209	2.215354	2.210535	2.205726	2.202222	2.197396	2.199149
2.230527	2.225654	2.220758	2.215887	2.211051	2.206232	2.202728	2.19791	2.199658
2.231153	2.226273	2.221388	2.216503	2.211606	2.206734	2.203232	2.198429	2.20017
2.231776	2.22689	2.222019	2.217125	2.212181	2.207267	2.203764	2.19897	2.200706
2.232331	2.227442	2.222569	2.217685	2.212772	2.207884	2.204376	2.199568	2.201306
2.232856	2.227967	2.223083	2.218217	2.213371	2.208537	2.205022	2.200189	2.201931
2.233393	2.228504	2.223608	2.220113	2.21528	2.210454	2.205603	2.200752	2.202494
2.233936	2.229047	2.224135	2.220633	2.21581	2.210993	2.206135	2.201273	2.203009
2.234515	2.229624	2.224712	2.221208	2.216382	2.211562	2.206703	2.20184	2.20356
2.235144	2.230251	2.225362	2.221862	2.217016	2.212178	2.207327	2.202473	2.204161

2.235761	2.230865	2.225995	2.2225	2.217634	2.212779	2.207938	2.203096	2.204767
2.236313	2.231414	2.226552	2.223058	2.218182	2.213321	2.20849	2.203666	2.205357
2.236869	2.231967	2.227107	2.223611	2.218729	2.213864	2.209038	2.204228	2.20595
2.237507	2.232601	2.227737	2.224238	2.219349	2.214477	2.209644	2.20483	2.206566
2.23819	2.23328	2.228408	2.224903	2.220008	2.215129	2.210279	2.205448	2.207189
2.238866	2.233951	2.229067	2.225554	2.220661	2.215775	2.210892	2.206025	2.201199
2.239542	2.234623	2.229721	2.226199	2.221313	2.216421	2.211489	2.206561	2.201715
2.240173	2.235249	2.230333	2.226806	2.221927	2.217038	2.212088	2.207137	2.20228
2.240725	2.235799	2.230874	2.227347	2.222476	2.217604	2.212689	2.207774	2.202927
2.24129	2.236362	2.231433	2.227905	2.223038	2.218182	2.213304	2.208432	2.203595
2.241931	2.236999	2.232072	2.228543	2.223673	2.218815	2.213949	2.209091	2.204258
2.242606	2.237668	2.232748	2.229217	2.224338	2.219474	2.21461	2.209757	2.204925
2.243285	2.239743	2.234803	2.229889	2.225006	2.220138	2.215274	2.210422	2.20559
2.243974	2.240427	2.235484	2.230568	2.225682	2.220812	2.215943	2.211087	2.206254
2.24466	2.241109	2.236162	2.231242	2.226354	2.221482	2.216609	2.211749	2.206915
2.245341	2.241786	2.236834	2.23191	2.227021	2.222147	2.21727	2.212405	2.207571
2.246029	2.24247	2.237519	2.232593	2.227698	2.222818	2.217939	2.213074	2.208239
2.246717	2.243156	2.238214	2.233289	2.228378	2.223485	2.218612	2.213757	2.208917
2.247432	2.243869	2.238938	2.234015	2.229086	2.224178	2.219311	2.214466	2.20962
2.248222	2.244658	2.239733	2.234808	2.229868	2.224949	2.22008	2.215236	2.210386
2.249039	2.245474	2.240551	2.235625	2.230675	2.225747	2.220873	2.216026	2.211173
2.242487	2.246215	2.241289	2.236359	2.231406	2.226473	2.221593	2.21674	2.211885
2.243194	2.246901	2.241968	2.237033	2.23208	2.227146	2.222259	2.217397	2.212542
2.243908	2.247591	2.242648	2.237707	2.232759	2.227829	2.222931	2.218056	2.213201
2.244626	2.248282	2.243324	2.238377	2.23344	2.228517	2.223605	2.218712	2.213858
2.245371	2.242199	2.237215	2.239083	2.234155	2.229238	2.224312	2.219401	2.215007
2.246175	2.243024	2.238028	2.239866	2.234943	2.230028	2.225091	2.220165	2.215773
2.246982	2.243854	2.238854	2.240664	2.235741	2.230823	2.225879	2.220945	2.216553
2.249551	2.244605	2.239616	2.241407	2.23647	2.23154	2.226598	2.22167	2.217273
2.250268	2.245315	2.24035	2.242128	2.237171	2.232223	2.22729	2.222376	2.217971
2.250978	2.246032	2.241111	2.23617	2.231189	2.226216	2.228036	2.223135	2.218731
2.251673	2.246751	2.241896	2.236995	2.232008	2.227026	2.22883	2.223942	2.219548
2.247608	2.2475	2.242662	2.237774	2.232791	2.227812	2.229593	2.224709	2.220333
2.248419	2.248287	2.243388	2.238468	2.233504	2.228547	2.230294	2.2254	2.221062
2.249257	2.2491	2.244139	2.239186	2.234237	2.229299	2.231012	2.226108	2.221792
2.250102	2.249938	2.244962	2.239999	2.235049	2.23011	2.225172	2.220256	2.222056
2.250959	2.250794	2.245818	2.240853	2.235895	2.23095	2.226014	2.221095	2.222827
2.251821	2.25165	2.246669	2.241702	2.236745	2.231799	2.226862	2.221939	2.223629
2.252686	2.252509	2.247525	2.242558	2.237603	2.23266	2.227721	2.222794	2.224454
2.253539	2.24855	2.243595	2.243413	2.238458	2.233516	2.228577	2.22365	2.21874
2.254383	2.249421	2.244453	2.244267	2.23931	2.234367	2.229432	2.224508	2.219595
2.2552	2.250258	2.245298	2.245123	2.240164	2.235219	2.230286	2.225365	2.220449
2.255956	2.251022	2.246116	2.245981	2.241023	2.236073	2.231139	2.226217	2.221301
2.25674	2.251803	2.24694	2.246838	2.241881	2.236925	2.23199	2.227066	2.222152
2.257644	2.252693	2.247804	2.247691	2.242729	2.237772	2.232838	2.227916	2.222999
2.258602	2.253627	2.248684	2.248542	2.243574	2.238618	2.233684	2.228763	2.223845
2.259525	2.254522	2.249551	2.249395	2.244422	2.239465	2.234528	2.229606	2.224688
2.260431	2.255396	2.250411	2.250249	2.245272	2.240313	2.23537	2.230444	2.225529
2.261315	2.256269	2.251274	2.251112	2.246136	2.241177	2.236226	2.23129	2.226378
2.262168	2.25714	2.252141	2.251984	2.247013	2.242055	2.237089	2.232137	2.227229

2.254664	2.258015	2.253016	2.248047	2.243119	2.238201	2.237989	2.233026	2.228117
2.25554	2.258892	2.253897	2.248943	2.244045	2.239156	2.23896	2.234002	2.229079
2.256428	2.259781	2.254793	2.249854	2.244981	2.240116	2.239939	2.234988	2.230051
2.250099	2.260693	2.25572	2.250785	2.245899	2.241019	2.240848	2.235901	2.230956
2.251007	2.261622	2.256665	2.251728	2.246807	2.241895	2.241727	2.236783	2.231837
2.24533	2.25587	2.250895	2.25264	2.247704	2.242783	2.242634	2.237709	2.232773
2.24627	2.25681	2.251795	2.253527	2.248588	2.243678	2.243563	2.238671	2.23376
2.247194	2.257731	2.252692	2.247702	2.242774	2.237881	2.244489	2.239605	2.234707
2.248083	2.258611	2.253577	2.248599	2.243695	2.238815	2.245412	2.240498	2.235589
2.249001	2.243996	2.239042	2.249513	2.244615	2.239734	2.240032	2.23509	2.23647
2.250005	2.244983	2.240001	2.250449	2.245508	2.240594	2.240897	2.235971	2.237381
2.251028	2.245995	2.240986	2.251408	2.24641	2.241454	2.241767	2.236865	2.238308
2.251974	2.24696	2.241972	2.252406	2.24738	2.242395	2.242706	2.237805	2.239262
2.252879	2.247902	2.242963	2.253431	2.248393	2.243388	2.243691	2.238776	2.240237
2.253828	2.248863	2.243948	2.24828	2.243276	2.238297	2.239439	2.23454	2.234864
2.254819	2.249842	2.244929	2.249278	2.244283	2.239307	2.240435	2.235513	2.235799
2.250815	2.25082	2.245883	2.250231	2.245245	2.24028	2.241414	2.236491	2.236756
2.251801	2.25179	2.246784	2.251103	2.246125	2.241184	2.242355	2.237472	2.237749
2.252796	2.25277	2.247698	2.251988	2.24701	2.242089	2.243297	2.238456	2.238753
2.253807	2.253781	2.248685	2.243655	2.23871	2.233831	2.238213	2.233421	2.239752
2.254828	2.254808	2.249708	2.244666	2.239699	2.234802	2.239197	2.234414	2.240752
2.251768	2.255833	2.250726	2.245676	2.240696	2.235788	2.240192	2.23541	2.241751
2.2528	2.256859	2.251748	2.246693	2.241706	2.236791	2.241198	2.236409	2.24275
2.253832	2.254227	2.24911	2.247711	2.24272	2.2378	2.242208	2.23741	2.238033
2.254861	2.255259	2.250137	2.248726	2.243737	2.238816	2.243224	2.238413	2.239034
2.2559	2.256303	2.251181	2.249758	2.244763	2.239835	2.244243	2.239422	2.240044
2.256958	2.25737	2.252246	2.250806	2.245791	2.240845	2.245258	2.240437	2.241067
2.25802	2.258449	2.253335	2.251884	2.246845	2.241874	2.246285	2.24146	2.242101
2.259073	2.259538	2.254467	2.249405	2.24434	2.239334	2.24735	2.242495	2.237625
2.260122	2.260629	2.255616	2.250581	2.245498	2.240459	2.248435	2.243537	2.238682
2.261162	2.256213	2.251271	2.251684	2.246602	2.24155	2.236592	2.231698	2.239704
2.262194	2.257225	2.252293	2.252727	2.247662	2.242616	2.237628	2.232696	2.240696
2.263225	2.258238	2.253313	2.253773	2.24874	2.243714	2.238705	2.233736	2.241719
2.264239	2.259242	2.254333	2.254832	2.249856	2.24487	2.239846	2.234839	2.242789
2.265295	2.260279	2.255362	2.255881	2.250952	2.246004	2.240976	2.235945	2.240578
2.266439	2.261377	2.256397	2.256893	2.251984	2.247063	2.242045	2.237013	2.24163
2.267624	2.262516	2.25746	2.257915	2.253003	2.248094	2.243097	2.238085	2.242694
2.268821	2.263705	2.258599	2.259012	2.254058	2.249129	2.244172	2.239216	2.243834
2.270027	2.264929	2.259792	2.260162	2.255138	2.250167	2.245258	2.240376	2.245017
2.271157	2.266092	2.260979	2.26135	2.256276	2.25126	2.246346	2.241478	2.236556
2.272212	2.2672	2.26217	2.262588	2.257478	2.252407	2.247435	2.242518	2.237621
2.273256	2.26827	2.263303	2.263771	2.258663	2.253575	2.248559	2.243594	2.238697
2.274283	2.26928	2.264339	2.264858	2.259819	2.25478	2.249747	2.244736	2.239791
2.275343	2.270307	2.265365	2.265916	2.260941	2.255956	2.25093	2.245899	2.240911
2.276496	2.271415	2.266425	2.261466	2.256536	2.251616	2.252051	2.247074	2.24209
2.277694	2.272572	2.26752	2.262517	2.257563	2.252647	2.253149	2.248248	2.243287
2.278887	2.27378	2.268724	2.263703	2.258713	2.253764	2.254257	2.249358	2.244419
2.280084	2.275033	2.27001	2.264987	2.259949	2.25494	2.255371	2.250426	2.245511
2.276275	2.276202	2.271194	2.266181	2.261144	2.256129	2.256547	2.251582	2.246668
2.277363	2.277261	2.272251	2.267264	2.262293	2.257341	2.257804	2.252849	2.247909

2.278511	2.278378	2.273346	2.268361	2.263427	2.258515	2.259015	2.254075	2.26191
2.279794	2.279642	2.274547	2.269505	2.264528	2.25959	2.260095	2.255172	2.26305
2.281091	2.280928	2.27578	2.270685	2.265656	2.26068	2.261182	2.256271	2.264188
2.282274	2.282124	2.276999	2.271921	2.266894	2.261914	2.262417	2.257494	2.265384
2.283421	2.283295	2.278229	2.273195	2.268188	2.263213	2.263716	2.258767	2.266603
2.284666	2.284555	2.279506	2.274475	2.269452	2.264454	2.259494	2.254557	2.267789
2.28599	2.285889	2.280827	2.275769	2.270701	2.265656	2.260651	2.255682	2.268951
2.287246	2.287148	2.282079	2.277011	2.271931	2.266873	2.261851	2.256868	2.270163
2.288388	2.288285	2.283221	2.278173	2.273132	2.268112	2.263114	2.258146	2.271451
2.289576	2.284485	2.279461	2.279363	2.274353	2.269364	2.264381	2.259417	2.272724
2.290905	2.28581	2.280767	2.280656	2.275645	2.270647	2.265632	2.260626	2.273914
2.292283	2.287182	2.282108	2.281969	2.276939	2.271919	2.266869	2.261825	2.275097
2.293642	2.288517	2.28339	2.283209	2.278137	2.273094	2.268062	2.263052	2.271895
2.29499	2.289834	2.284645	2.284416	2.27929	2.274222	2.269235	2.264297	2.273181
2.280566	2.291078	2.285896	2.285677	2.280537	2.27546	2.270487	2.265569	2.280508
2.281633	2.292241	2.287137	2.286986	2.281872	2.276804	2.271815	2.266873	2.281816
2.282771	2.293473	2.288412	2.288301	2.283209	2.278145	2.273129	2.268145	2.283079
2.284035	2.294825	2.289746	2.289624	2.284547	2.279483	2.274417	2.269365	2.284264
2.285328	2.296195	2.291077	2.290924	2.285844	2.280772	2.27567	2.270573	2.285452
2.28661	2.297533	2.292358	2.292155	2.287025	2.281924	2.276839	2.271781	2.286692
2.287882	2.298853	2.29362	2.293367	2.288177	2.283044	2.277994	2.273	2.28796
2.289094	2.300128	2.294884	2.294633	2.289439	2.284305	2.279258	2.274271	2.289254
2.290262	2.30137	2.296148	2.295936	2.290774	2.285661	2.280598	2.275585	2.290573
2.288023	2.30264	2.297451	2.297274	2.292134	2.287033	2.281957	2.27692	2.29191
2.289189	2.303937	2.298794	2.298651	2.293525	2.288426	2.283338	2.278281	2.293266
2.290389	2.288967	2.284185	2.29507	2.28999	2.284925	2.284724	2.279652	2.294632
2.291643	2.290251	2.28548	2.296459	2.291376	2.286306	2.28611	2.281031	2.296006
2.292921	2.291557	2.286786	2.297854	2.292765	2.287689	2.287497	2.282414	2.297382
2.291026	2.289429	2.284671	2.299252	2.294158	2.289074	2.288883	2.28379	2.298749
2.292326	2.290728	2.285971	2.300651	2.295553	2.290463	2.29027	2.285166	2.300115
2.286842	2.29202	2.287265	2.306517	2.301383	2.296253	2.311971	2.306729	2.301489
2.288135	2.293307	2.288555	2.307913	2.302786	2.297658	2.313383	2.308126	2.30287
2.289431	2.294596	2.28984	2.309301	2.304181	2.299058	2.312753	2.307489	2.30426
2.290731	2.295888	2.291119	2.310678	2.305568	2.300455	2.314165	2.308893	2.305663
2.284904	2.297181	2.292398	2.312053	2.306945	2.301836	2.311286	2.306047	2.307063
2.286208	2.298472	2.293681	2.313428	2.308304	2.303186	2.312649	2.307421	2.308456
2.287511	2.299764	2.294966	2.314804	2.309657	2.304525	2.314	2.308783	2.309841
2.288815	2.287735	2.282968	2.299508	2.294689	2.289874	2.315352	2.310134	2.309148
2.29012	2.289032	2.28426	2.300792	2.295963	2.291139	2.316702	2.311475	2.310497
2.291428	2.290333	2.28555	2.302072	2.297234	2.292403	2.318055	2.312818	2.311847
2.292739	2.291636	2.286838	2.303347	2.298503	2.293668	2.319411	2.314161	2.313196
2.294047	2.292938	2.288131	2.298557	2.293706	2.288863	2.320766	2.315506	2.314544
2.295348	2.294239	2.289431	2.29986	2.29499	2.29013	2.322118	2.31685	2.315887
2.296645	2.295537	2.290732	2.301166	2.29628	2.291404	2.323476	2.3182	2.317235
2.29793	2.296821	2.292023	2.296007	2.29114	2.286279	2.304122	2.299159	2.314313
2.299219	2.298108	2.293318	2.297311	2.292442	2.287575	2.305421	2.300449	2.315673
2.300533	2.299416	2.294626	2.298624	2.293759	2.288893	2.306741	2.301756	2.312107
2.301873	2.300747	2.295952	2.299953	2.295098	2.290238	2.308088	2.303085	2.31353
2.303327	2.302192	2.297394	2.301402	2.296552	2.291694	2.309547	2.304533	2.315069
2.304925	2.303779	2.298981	2.303	2.298148	2.293288	2.311145	2.306126	2.31675

2.306445	2.305287	2.300488	2.304516	2.299664	2.294803	2.306945	2.301946	2.30265
2.307798	2.306626	2.301824	2.305857	2.301004	2.296144	2.308314	2.303316	2.304025
2.309092	2.307908	2.303102	2.307142	2.302292	2.297436	2.309637	2.304642	2.305358
2.310372	2.305519	2.300732	2.308425	2.303588	2.298743	2.310962	2.305964	2.306683
2.31164	2.306784	2.301997	2.309701	2.304883	2.300056	2.312293	2.307297	2.308019
2.312947	2.308088	2.303294	2.311013	2.306229	2.301442	2.313725	2.30876	2.309496
2.314286	2.309422	2.304616	2.312354	2.307616	2.302886	2.315239	2.310328	2.311086
2.315726	2.310854	2.306031	2.313767	2.309028	2.304303	2.316687	2.311799	2.312566
2.317307	2.312421	2.307573	2.315282	2.310476	2.305688	2.318049	2.313142	2.313902
2.318847	2.313951	2.30908	2.31347	2.30862	2.303789	2.319394	2.314462	2.309513
2.320227	2.315326	2.310442	2.314825	2.30996	2.305113	2.320717	2.315766	2.310812
2.321589	2.316688	2.311796	2.316181	2.311314	2.306464	2.322071	2.317105	2.312146
2.323089	2.337502	2.332468	2.335883	2.330875	2.325889	2.323566	2.318598	2.313636
2.324643	2.339063	2.334042	2.337473	2.332452	2.327454	2.325133	2.32017	2.315206
2.326087	2.340527	2.335507	2.338945	2.333917	2.328913	2.326587	2.321622	2.316657
2.327431	2.341902	2.336873	2.340309	2.335282	2.330278	2.327939	2.322968	2.318001
2.328804	2.343317	2.338296	2.341743	2.336715	2.331707	2.329359	2.324383	2.319418
2.330228	2.344805	2.339829	2.34331	2.338276	2.333258	2.330906	2.325931	2.32097
2.331671	2.346292	2.341347	2.344857	2.339818	2.334791	2.332434	2.327459	2.322503
2.327468	2.347714	2.342757	2.346265	2.341223	2.336193	2.333827	2.328848	2.323893
2.328926	2.349143	2.344155	2.34765	2.342605	2.337578	2.335203	2.33022	2.325266
2.330472	2.350701	2.345675	2.34916	2.34412	2.339101	2.334106	2.329135	2.326782
2.332074	2.352338	2.347265	2.35074	2.345708	2.340705	2.335716	2.330748	2.328386
2.333571	2.353865	2.34876	2.352228	2.347198	2.342207	2.337244	2.332305	2.329959
2.334946	2.355267	2.350142	2.353608	2.348573	2.343597	2.338683	2.33381	2.33151
2.3364	2.356743	2.351597	2.355057	2.350013	2.345036	2.340139	2.335291	2.33301
2.338028	2.358375	2.353193	2.356637	2.351575	2.346574	2.341628	2.336725	2.33441
2.33967	2.360039	2.354835	2.358271	2.353191	2.348162	2.343159	2.338193	2.335832
2.329617	2.356677	2.351534	2.359951	2.354857	2.349805	2.344779	2.339786	2.337393
2.331115	2.358266	2.353171	2.361636	2.356535	2.351468	2.346438	2.341439	2.339022
2.332589	2.359767	2.354681	2.363178	2.358094	2.353047	2.348045	2.343073	2.340657
2.334037	2.361199	2.356092	2.364611	2.359562	2.354563	2.349618	2.344701	2.342307
2.335572	2.362731	2.357598	2.366116	2.361049	2.356044	2.351112	2.34622	2.341301
2.337218	2.364389	2.359223	2.367704	2.36255	2.357469	2.352494	2.34759	2.34271
2.338862	2.35548	2.35033	2.364676	2.359481	2.354356	2.353935	2.348997	2.344127
2.340475	2.357156	2.352007	2.366352	2.361138	2.355983	2.355523	2.350528	2.345605
2.342052	2.358813	2.353684	2.368048	2.362836	2.357665	2.357172	2.352114	2.347122
2.343557	2.360408	2.355324	2.360751	2.355589	2.350455	2.350326	2.345277	2.348697
2.345009	2.361954	2.356933	2.362416	2.35727	2.352132	2.351994	2.346913	2.350316
2.330711	2.363435	2.358485	2.364029	2.358922	2.353803	2.353663	2.34855	2.351941
2.332071	2.36485	2.359981	2.365597	2.360551	2.355472	2.355336	2.350192	2.353578
2.317044	2.366312	2.36146	2.356568	2.351612	2.346614	2.351996	2.346878	2.355228
2.318571	2.367842	2.362908	2.357994	2.353105	2.348186	2.353614	2.348529	2.356896
2.303078	2.354878	2.349934	2.359449	2.354608	2.349752	2.355228	2.350181	2.358565
2.304589	2.356468	2.351501	2.36102	2.35618	2.351335	2.356846	2.351826	2.360227
2.306116	2.358084	2.353113	2.362639	2.357783	2.352929	2.358467	2.353469	2.361886
2.287033	2.344492	2.339535	2.349818	2.34503	2.340247	2.360102	2.355116	2.363549
2.288575	2.346112	2.341145	2.351428	2.346645	2.341868	2.361746	2.356763	2.365213
2.290111	2.33044	2.325487	2.337878	2.333117	2.328363	2.35289	2.347937	2.362176
2.291639	2.332055	2.327104	2.339496	2.334725	2.329964	2.354503	2.34955	2.363807

2.293185	2.333686	2.328728	2.323854	2.319086	2.314328	2.326741	2.321881	2.365441
2.294758	2.335336	2.330356	2.325468	2.320699	2.315943	2.328355	2.323493	2.367082
2.296343	2.337	2.33199	2.327084	2.322314	2.317559	2.329971	2.325105	2.368723
2.29793	2.338673	2.333634	2.328706	2.323927	2.319168	2.31434	2.309484	2.336863
2.299522	2.340356	2.335285	2.330334	2.325543	2.320777	2.315944	2.311087	2.338481
2.301114	2.296349	2.291651	2.305478	2.3009	2.296344	2.317552	2.312695	2.30781
2.302706	2.297929	2.293199	2.307056	2.302467	2.297908	2.319163	2.314307	2.309424
2.304303	2.299537	2.294819	2.308726	2.304101	2.299506	2.320791	2.31593	2.311044
2.305905	2.301184	2.296535	2.310519	2.305823	2.301149	2.322439	2.317566	2.31267
2.292226	2.302817	2.29822	2.312275	2.30752	2.302783	2.324088	2.319202	2.314309
2.293786	2.304392	2.299802	2.313908	2.309137	2.304385	2.325735	2.320836	2.315977
2.295367	2.30598	2.301374	2.315519	2.310736	2.305976	2.327376	2.322467	2.317643
2.297034	2.307673	2.303031	2.317186	2.312355	2.307558	2.328992	2.324082	2.319264
2.298738	2.309408	2.304718	2.318873	2.313983	2.309139	2.330604	2.325698	2.320872
2.300352	2.311043	2.30634	2.320536	2.315619	2.310756	2.332268	2.327373	2.322536
2.30189	2.312594	2.307909	2.322179	2.317264	2.312404	2.333978	2.329101	2.324247
2.303479	2.298829	2.294193	2.323824	2.318931	2.314091	2.335715	2.330828	2.325952
2.30515	2.300453	2.295769	2.325463	2.320626	2.315831	2.337485	2.332549	2.32764
2.306827	2.30209	2.297374	2.327136	2.322337	2.317571	2.33925	2.334264	2.329329
2.308464	2.303722	2.299035	2.328888	2.324075	2.319282	2.340976	2.335953	2.331017
2.310108	2.305368	2.300719	2.33066	2.325812	2.320977	2.342695	2.33765	2.332718
2.311819	2.307067	2.302385	2.313055	2.308359	2.303685	2.344442	2.339414	2.334469
2.313567	2.308796	2.304045	2.314679	2.309957	2.30528	2.346205	2.341217	2.33625
2.315248	2.31047	2.30569	2.316319	2.311592	2.306918	2.347967	2.342984	2.337994
2.316859	2.312086	2.307313	2.31797	2.313264	2.308599	2.349732	2.344718	2.3397
2.318514	2.313738	2.308975	2.304258	2.299598	2.294971	2.351486	2.346444	2.341416
2.320244	2.315452	2.310703	2.30598	2.301277	2.296603	2.353218	2.348159	2.343144
2.322011	2.317198	2.312448	2.307705	2.302949	2.298227	2.354957	2.349892	2.344893
2.323813	2.318984	2.314186	2.309393	2.304584	2.299829	2.35672	2.35168	2.346681
2.325628	2.320781	2.315918	2.31107	2.306217	2.301439	2.358503	2.353492	2.348486
2.327391	2.322519	2.31762	2.312751	2.307908	2.303136	2.360316	2.35527	2.35026
2.329117	2.324216	2.319298	2.314436	2.30964	2.304897	2.362157	2.357032	2.352017
2.33082	2.325902	2.320998	2.316151	2.311373	2.306637	2.332048	2.327116	2.353819
2.332479	2.327563	2.322718	2.317902	2.313105	2.308349	2.333842	2.32893	2.355677
2.307103	2.320666	2.315807	2.319657	2.314848	2.31008	2.335645	2.330736	2.357531
2.308984	2.322546	2.317612	2.321393	2.316606	2.311855	2.337455	2.332494	2.359352
2.31088	2.324444	2.319437	2.323146	2.318374	2.313636	2.339269	2.334257	2.36118
2.312638	2.326242	2.321261	2.316346	2.311509	2.30671	2.341076	2.336074	2.349757
2.314355	2.328015	2.323102	2.31821	2.313317	2.308466	2.342889	2.337916	2.351632
2.316206	2.3299	2.325002	2.320111	2.315194	2.310312	2.337231	2.3322	2.334727
2.318168	2.331878	2.326958	2.32205	2.317133	2.312237	2.339166	2.334043	2.336532
2.32011	2.315128	2.310255	2.323947	2.319036	2.31414	2.341118	2.335943	2.330886
2.322021	2.317022	2.312084	2.32578	2.320886	2.316016	2.343098	2.337925	2.332865
2.262337	2.318895	2.31391	2.327615	2.322721	2.317863	2.345054	2.339902	2.33484
2.263917	2.32073	2.315757	2.329489	2.324549	2.319655	2.346943	2.341834	2.336766
2.265489	2.32256	2.317605	2.331365	2.326374	2.321439	2.348812	2.343741	2.338667
2.237942	2.324426	2.319423	2.333191	2.328203	2.32327	2.350702	2.345603	2.340533
2.239507	2.326319	2.321237	2.335003	2.330043	2.325132	2.352604	2.347443	2.342381
2.241129	2.304128	2.299077	2.318135	2.313258	2.308428	2.354558	2.34937	2.344299
2.242814	2.306093	2.301064	2.320143	2.315219	2.310345	2.356565	2.351381	2.346286

2.244463	2.240149	2.235862	2.322123	2.317151	2.312232	2.358561	2.353383	2.348257
2.246044	2.241715	2.237432	2.32403	2.319009	2.314046	2.360515	2.355344	2.350178
2.247609	2.243274	2.238999	2.325938	2.320883	2.315889	2.362499	2.357323	2.352123
2.232024	2.227751	2.223546	2.303696	2.29871	2.293779	2.331694	2.326778	2.354139
2.233571	2.229322	2.22514	2.305611	2.300649	2.295732	2.333716	2.328773	2.356196
2.235183	2.230907	2.226688	2.244566	2.240279	2.236025	2.32391	2.318998	2.358279
2.236846	2.232507	2.22821	2.246071	2.241795	2.237572	2.325852	2.320972	2.360384
2.23853	2.234159	2.229809	2.225533	2.221342	2.217212	2.282325	2.277608	2.351932
2.240245	2.235876	2.231502	2.22719	2.222955	2.218774	2.284109	2.27931	2.353836
2.241923	2.237554	2.233173	2.228844	2.224581	2.220365	2.238091	2.233862	2.355785
2.243513	2.239131	2.234765	2.230453	2.226205	2.221996	2.239742	2.23549	2.35784
2.245105	2.240706	2.236359	2.232067	2.227841	2.223645	2.241419	2.237151	2.359935
2.246776	2.242361	2.238019	2.233731	2.229508	2.225309	2.243103	2.238813	2.341522
2.248492	2.244058	2.239709	2.235414	2.23118	2.226968	2.244778	2.240472	2.343601
2.250235	2.24577	2.241393	2.237062	2.232775	2.228522	2.224286	2.220084	2.32604
2.252009	2.247502	2.243076	2.238683	2.234306	2.229987	2.225748	2.221577	2.327971
2.253739	2.249218	2.244773	2.240347	2.235914	2.231546	2.227292	2.223124	2.329925
2.2554	2.250904	2.246485	2.24207	2.237624	2.233232	2.228946	2.224742	2.331901
2.239262	2.252608	2.24821	2.243804	2.239352	2.234944	2.230626	2.226384	2.244227
2.241016	2.254357	2.249947	2.245527	2.241063	2.23664	2.232301	2.228035	2.245887
2.242801	2.256129	2.251694	2.247255	2.24278	2.238345	2.233989	2.229705	2.247563
2.244588	2.257909	2.253453	2.248997	2.244515	2.240072	2.235701	2.231398	2.24926
2.246383	2.259699	2.255221	2.250749	2.24626	2.24181	2.237428	2.233109	2.250973
2.193434	2.243651	2.239199	2.252498	2.247991	2.243524	2.239134	2.234808	2.230567
2.195082	2.2454	2.24095	2.254242	2.249704	2.245211	2.240815	2.236493	2.232254
2.178574	2.247173	2.242727	2.238268	2.233756	2.229283	2.242559	2.238219	2.23396
2.180286	2.248984	2.244543	2.240099	2.235618	2.23116	2.244407	2.240008	2.235694
2.18201	2.250802	2.246361	2.241929	2.23748	2.233039	2.24626	2.241803	2.237436
2.148755	2.179341	2.175109	2.227814	2.223365	2.21892	2.230379	2.225933	2.239178
2.15043	2.181038	2.176782	2.229593	2.225145	2.220702	2.232144	2.227685	2.240917
2.152208	2.182813	2.178498	2.231389	2.226933	2.222485	2.217985	2.213494	2.225009
2.15406	2.184645	2.180243	2.233193	2.22872	2.224263	2.219734	2.215212	2.226703
2.128481	2.186461	2.18198	2.235001	2.230514	2.226058	2.221535	2.21702	2.212588
2.130222	2.188257	2.183704	2.236806	2.232313	2.227874	2.223399	2.218933	2.214483
2.131984	2.154912	2.150435	2.180992	2.176701	2.172478	2.225244	2.220826	2.216375
2.13379	2.156742	2.1522	2.182744	2.178393	2.174134	2.227038	2.222662	2.218245
2.135604	2.158595	2.15401	2.184554	2.180123	2.175802	2.228822	2.224466	2.220081
2.137405	2.132945	2.128448	2.186472	2.181935	2.177508	2.2306	2.226217	2.221834
2.139196	2.134769	2.130312	2.188446	2.183801	2.179256	2.232396	2.227952	2.223552
2.140953	2.136535	2.132113	2.15514	2.150623	2.146177	2.176689	2.172388	2.225327
2.14268	2.138255	2.133868	2.156955	2.152475	2.148038	2.178554	2.174146	2.227144
2.144463	2.140013	2.135622	2.131239	2.126842	2.122482	2.180416	2.175954	2.229049
2.146314	2.141813	2.13737	2.132959	2.128572	2.12423	2.182275	2.177832	2.231076
2.094335	2.143654	2.139154	2.134706	2.13031	2.125968	2.149056	2.14476	2.175365
2.096179	2.145559	2.141005	2.136503	2.132057	2.127673	2.150765	2.146454	2.177108
2.098023	2.147478	2.142893	2.138349	2.13385	2.129414	2.152496	2.148149	2.178832
2.099796	2.095384	2.090937	2.140291	2.135783	2.131314	2.126887	2.122526	2.180601
2.101529	2.097171	2.092809	2.142283	2.137791	2.133302	2.128804	2.124343	2.182407
1.956243	2.098901	2.094584	2.090242	2.085861	2.081472	2.1307	2.126211	2.184326
1.957714	2.10057	2.096266	2.091949	2.087603	2.083252	2.132589	2.128136	2.186359

1.959284	2.030086	2.02597	2.093656	2.08932	2.084987	2.080654	2.076332	2.125596
1.961015	2.031859	2.027651	2.095362	2.090992	2.086643	2.082312	2.078002	2.127328
1.962732	2.033641	2.029359	2.097111	2.092707	2.088339	2.084004	2.079698	2.129086
1.920176	1.960294	1.956295	2.0676	2.063247	2.058926	2.085848	2.081532	2.077217
1.9216	1.96186	1.957915	2.069469	2.065118	2.060792	2.087729	2.083397	2.079067
1.891914	1.963385	1.959414	2.030298	2.026127	2.021981	2.058239	2.053959	2.080817
1.893435	1.964885	1.960844	2.031854	2.027706	2.023588	2.059921	2.055641	2.082514
1.895001	1.891068	1.887193	1.929757	1.925916	1.922123	2.021165	2.017052	2.053146
1.896619	1.892734	1.888875	1.931528	1.927648	1.923817	2.022974	2.018869	2.055036
1.867206	1.894353	1.890502	1.933251	1.929334	1.925467	1.999993	1.995942	2.056879
1.86879	1.895838	1.89197	1.934818	1.930877	1.926986	2.001647	1.997593	2.058594
1.870379	1.897332	1.893449	1.936401	1.932442	1.928532	2.003316	1.999252	2.060323
1.872036	1.868042	1.864128	1.891259	1.887458	1.883693	1.926379	1.922543	1.996967
1.873718	1.869762	1.865869	1.892959	1.889157	1.885382	1.928139	1.924272	1.998794
1.875363	1.871378	1.867469	1.863594	1.85975	1.855931	1.929767	1.92587	1.922024
1.876984	1.872923	1.868969	1.86507	1.861228	1.857414	1.931302	1.927378	1.92351
1.833843	1.874565	1.870577	1.866658	1.862815	1.859001	1.904395	1.90041	1.925108
1.83552	1.876329	1.872319	1.868383	1.864539	1.860724	1.906218	1.902222	1.926846
1.79186	1.833251	1.829298	1.870143	1.866289	1.862466	1.85863	1.854805	1.900096
1.793637	1.835	1.831045	1.871938	1.86806	1.864216	1.860374	1.856549	1.901943
1.79539	1.836718	1.832763	1.873704	1.869796	1.865925	1.862077	1.858252	1.903758
1.796985	1.83828	1.83432	1.830406	1.826543	1.822721	1.863615	1.859789	1.905413
1.798513	1.839773	1.835805	1.831876	1.827985	1.824139	1.865076	1.861248	1.906994
1.744135	1.796222	1.792303	1.833497	1.829584	1.825716	1.821912	1.818148	1.859016
1.74589	1.798012	1.794083	1.835246	1.831317	1.827432	1.823604	1.819816	1.860737
1.618592	1.743864	1.740113	1.791964	1.788085	1.784246	1.825306	1.821494	1.817713
1.620252	1.745668	1.741884	1.793755	1.789866	1.786016	1.827028	1.823186	1.819393
1.540809	1.618279	1.614727	1.739835	1.736134	1.732468	1.828695	1.824829	1.821021
1.542099	1.619722	1.616136	1.741363	1.73765	1.733976	1.830204	1.826326	1.822502
1.543367	1.621142	1.617528	1.742868	1.739139	1.735453	1.831689	1.827802	1.823962
1.544757	1.622708	1.619078	1.744552	1.740793	1.737081	1.73341	1.729777	1.825579
1.546221	1.624364	1.620728	1.746346	1.742549	1.738802	1.735117	1.731476	1.827293
1.547691	1.544555	1.541466	1.618781	1.61521	1.611699	1.736824	1.733175	1.729544
1.549168	1.54601	1.542908	1.620424	1.616808	1.613256	1.738535	1.734877	1.731241
1.493603	1.547486	1.544367	1.541277	1.538213	1.535173	1.692586	1.688985	1.732949
1.495108	1.548997	1.545851	1.542741	1.539667	1.536619	1.694328	1.690674	1.734664
1.496605	1.550507	1.54734	1.544212	1.541128	1.538073	1.696089	1.692381	1.736393
1.41724	1.494947	1.491867	1.545696	1.542602	1.539533	1.616297	1.612748	1.738138
1.418563	1.496387	1.493312	1.547172	1.54407	1.540991	1.61795	1.614369	1.7399
1.419865	1.416928	1.414013	1.491637	1.488628	1.485643	1.539368	1.536345	1.693906
1.421151	1.418174	1.415241	1.49296	1.489972	1.487017	1.540795	1.537804	1.695691
1.422509	1.419501	1.416544	1.494342	1.491339	1.48838	1.485461	1.482565	1.536226
1.423954	1.420928	1.417936	1.495793	1.492727	1.489719	1.486798	1.483923	1.537625
1.425409	1.422367	1.419346	1.41637	1.413434	1.410567	1.488172	1.4853	1.482408
1.426841	1.423784	1.420752	1.417765	1.414818	1.411936	1.489625	1.486726	1.483827
1.428277	1.425206	1.422167	1.419175	1.416227	1.413337	1.491109	1.488176	1.485262
1.429726	1.426644	1.423593	1.420589	1.417632	1.41473	1.4119	1.409121	1.486709
1.431186	1.428094	1.425028	1.42201	1.419042	1.416128	1.413282	1.410486	1.48817
1.432658	1.429553	1.426471	1.423438	1.420456	1.41753	1.414674	1.411867	1.409075
1.434141	1.431023	1.427924	1.424874	1.421876	1.418936	1.416075	1.413265	1.410455

1.435623	1.432491	1.429378	1.426315	1.423302	1.420349	1.417482	1.414665	1.411839
1.437097	1.433952	1.430831	1.427758	1.424733	1.421768	1.418892	1.416067	1.413228
1.36389	1.435423	1.432292	1.42921	1.426173	1.423197	1.42031	1.417473	1.414624
1.365315	1.436915	1.433771	1.430674	1.427622	1.424632	1.421729	1.418879	1.416025
1.36675	1.438417	1.435258	1.432148	1.429088	1.426087	1.423167	1.420299	1.417438
1.289129	1.439917	1.436746	1.433634	1.430589	1.427597	1.424662	1.421767	1.41888
1.29049	1.441418	1.438238	1.435131	1.432115	1.429143	1.426192	1.423264	1.42034
1.291867	1.367986	1.364952	1.436628	1.433616	1.430643	1.427679	1.424731	1.421782
1.293259	1.369426	1.366381	1.438123	1.435088	1.432095	1.42912	1.426166	1.423205
1.294671	1.291796	1.288954	1.439664	1.436603	1.433582	1.430585	1.427611	1.424633
1.296108	1.293242	1.290433	1.441284	1.438199	1.435141	1.432098	1.429077	1.426073
1.297556	1.294692	1.291913	1.442905	1.439796	1.436702	1.433614	1.430545	1.427515
1.298987	1.296108	1.293332	1.36941	1.366424	1.363447	1.435096	1.431994	1.428945
1.300437	1.297531	1.294741	1.370862	1.367858	1.364863	1.436571	1.433446	1.430384
1.30198	1.29904	1.296212	1.293391	1.290556	1.287731	1.43807	1.434937	1.431877
1.303595	1.300613	1.297726	1.294855	1.29198	1.289131	1.439587	1.43646	1.433412
1.305185	1.302157	1.299214	1.296301	1.293407	1.29055	1.3662	1.363229	1.434955
1.306732	1.303657	1.300662	1.297721	1.294834	1.291992	1.367696	1.364714	1.436503
1.246831	1.305209	1.302151	1.299171	1.296282	1.293445	1.290637	1.287862	1.438066
1.248473	1.30686	1.303712	1.300667	1.297752	1.294904	1.292098	1.289329	1.439655
1.250137	1.308549	1.305322	1.302213	1.299254	1.296377	1.29356	1.290788	1.441247
1.143285	1.310229	1.306983	1.303838	1.300814	1.297872	1.295011	1.292207	1.367744
1.144785	1.311915	1.308682	1.305514	1.302411	1.299385	1.296461	1.293608	1.369225
1.146287	1.214363	1.211269	1.307164	1.304	1.300909	1.297927	1.295026	1.292183
1.147787	1.215927	1.212808	1.308797	1.305586	1.302444	1.299404	1.296454	1.293609
1.149308	1.146608	1.143906	1.310472	1.307222	1.304035	1.300943	1.297943	1.29508
1.150857	1.148164	1.14546	1.312205	1.308921	1.305694	1.302553	1.299503	1.2966
1.152407	1.149721	1.147018	1.214562	1.211428	1.208343	1.304207	1.301113	1.298159
1.153947	1.151257	1.148551	1.216169	1.213019	1.209915	1.305892	1.302767	1.299755
1.155487	1.152787	1.15008	1.217777	1.214623	1.211512	1.307612	1.304462	1.301389
1.157028	1.154319	1.151615	1.148931	1.146267	1.143629	1.309372	1.306194	1.303067
1.158574	1.155854	1.153156	1.150485	1.147849	1.145225	1.311163	1.307957	1.30478
1.160144	1.15742	1.154732	1.152066	1.149424	1.146789	1.213427	1.210349	1.306482
1.161734	1.159014	1.156344	1.153674	1.150996	1.148323	1.215047	1.211949	1.308175
1.163358	1.160634	1.157958	1.155272	1.152562	1.149859	1.147179	1.144516	1.309878
1.165033	1.162289	1.159566	1.156845	1.15412	1.151405	1.148705	1.146023	1.311591
1.166713	1.163946	1.161176	1.158419	1.15568	1.152955	1.150236	1.147537	1.313324
1.168368	1.165582	1.162791	1.160012	1.157247	1.1545	1.15177	1.149066	1.215325
1.170011	1.167209	1.164407	1.161614	1.158823	1.156055	1.153318	1.150614	1.216989
1.171643	1.168831	1.166017	1.163213	1.160416	1.157643	1.15491	1.15221	1.218689
1.173264	1.170447	1.167622	1.164814	1.162023	1.159259	1.156538	1.153843	1.220421
1.174875	1.172054	1.169232	1.166431	1.163653	1.160898	1.15817	1.155461	1.152752
1.176467	1.173643	1.170844	1.168065	1.165311	1.162565	1.159808	1.157058	1.154343
1.178094	1.175268	1.17248	1.16971	1.166964	1.164218	1.161443	1.15867	1.155948
1.179798	1.176971	1.174164	1.171371	1.168594	1.165827	1.163065	1.160314	1.157579
1.181504	1.178676	1.175852	1.173037	1.170229	1.167441	1.164693	1.161965	1.159219
1.183126	1.180297	1.177491	1.17469	1.171887	1.169098	1.166339	1.163595	1.160842
1.184728	1.181897	1.179121	1.176346	1.173565	1.170786	1.168004	1.165228	1.162471
1.186438	1.183607	1.180827	1.178055	1.175288	1.172518	1.169724	1.166928	1.164165
1.188239	1.185407	1.182596	1.179808	1.177053	1.174293	1.171493	1.168685	1.165915

1.189992	1.187155	1.18433	1.18153	1.178769	1.176007	1.173211	1.170408	1.16763
1.191674	1.188822	1.18601	1.183211	1.180418	1.177634	1.174859	1.172086	1.169295
1.193363	1.190492	1.187683	1.184878	1.182062	1.179261	1.176498	1.173743	1.170945
1.195098	1.192206	1.189358	1.186531	1.183724	1.180931	1.178149	1.175373	1.172584
1.196842	1.193931	1.191038	1.188188	1.185395	1.182614	1.179807	1.177001	1.174225
1.198563	1.195633	1.192739	1.189884	1.187079	1.184287	1.181472	1.178662	1.175892
1.200278	1.197331	1.194455	1.191604	1.188777	1.185963	1.18315	1.18035	1.17758
1.202039	1.199076	1.196191	1.193335	1.190503	1.187689	1.184882	1.182087	1.179307
1.203841	1.200865	1.197947	1.195076	1.192259	1.189464	1.18667	1.18388	1.181075
1.205688	1.202696	1.199737	1.196836	1.194012	1.191214	1.188419	1.185626	1.182805
1.207608	1.204594	1.201578	1.198624	1.195752	1.192915	1.190093	1.187285	1.18446
1.209506	1.206475	1.203419	1.200419	1.197497	1.194617	1.191769	1.188947	1.186124
1.211297	1.208264	1.205214	1.202207	1.199253	1.196344	1.193493	1.190679	1.187867
1.213048	1.21002	1.206997	1.203999	1.201021	1.198091	1.195239	1.192433	1.189631
1.214821	1.211803	1.208793	1.2058	1.202817	1.199873	1.196996	1.194162	1.191348
1.216605	1.213599	1.210599	1.207612	1.204635	1.201684	1.198766	1.19588	1.193039
1.056855	1.215439	1.212439	1.209444	1.206452	1.203479	1.200528	1.19761	1.194753
1.058432	1.21733	1.21432	1.211302	1.208264	1.205248	1.202275	1.199344	1.196487
1.059984	1.2192	1.216191	1.213164	1.210102	1.20706	1.204071	1.201126	1.198257
1.061464	1.22101	1.218035	1.215036	1.211994	1.20896	1.205959	1.20299	1.200085
1.062961	1.222817	1.219866	1.216893	1.21388	1.210866	1.207862	1.204874	1.201929
1.064562	1.061976	1.059444	1.218691	1.215693	1.212698	1.209706	1.206722	1.203748
1.066216	1.063568	1.060961	1.220473	1.217484	1.214507	1.211534	1.208562	1.205565
1.067853	1.065173	1.062531	1.222334	1.219344	1.216368	1.213396	1.210423	1.207415
1.069485	1.066794	1.064145	1.224256	1.221256	1.21827	1.215285	1.212301	1.20929
1.07113	1.068409	1.065736	1.063104	1.060522	1.057958	1.217154	1.214149	1.211133
1.072784	1.070002	1.067279	1.064631	1.06209	1.059558	1.218989	1.215951	1.212922
1.074468	1.071641	1.068869	1.0662	1.06368	1.061163	1.220845	1.217782	1.214743
1.07622	1.07338	1.070567	1.067859	1.065308	1.062768	1.222751	1.219683	1.216645
1.077973	1.075141	1.072306	1.069565	1.066966	1.064387	1.224682	1.221615	1.218578
1.079638	1.076833	1.074031	1.071296	1.068659	1.066039	1.226625	1.223544	1.220502
1.081261	1.078498	1.075761	1.073053	1.070382	1.067718	1.228585	1.225484	1.22243
0.872163	1.080204	1.077499	1.074803	1.072114	1.069422	1.066705	1.063992	1.22437
0.873462	1.081946	1.079246	1.076549	1.073858	1.071154	1.068404	1.065652	1.22632
0.874766	1.083681	1.080987	1.078291	1.075594	1.072878	1.070103	1.067323	1.228281
0.876066	1.085398	1.082717	1.080027	1.077324	1.074597	1.071807	1.06901	1.230259
0.877371	1.087117	1.084448	1.081757	1.079035	1.076288	1.073491	1.070689	1.232236
0.878676	0.87666	0.874679	1.083479	1.080701	1.077908	1.07511	1.072327	1.069616
0.879987	0.877981	0.875994	1.085199	1.082363	1.07952	1.076725	1.073964	1.071267
0.881315	0.879301	0.87731	1.086929	1.084083	1.081231	1.078424	1.075653	1.072955
0.882656	0.880624	0.87863	1.088664	1.085844	1.083008	1.080182	1.07738	1.074672
	0.881967	0.879957	0.877931	0.875877	0.873813	1.081987	1.079157	1.076424
	0.883331	0.88129	0.879255	0.877232	0.875198	1.083851	1.080995	1.078218
			0.880591	0.878585	0.876569	1.085684	1.082815	1.080003
			0.881946	0.879932	0.877905	1.087441	1.084578	1.081747
			0.883305	0.881276	0.879235	1.089191	1.086351	1.08351
			0.884658	0.882619	0.880575	0.878511	0.876434	1.085361
			0.886009	0.883962	0.881919	0.879885	0.877845	1.087244
						0.881245	0.879215	0.87713
						0.882595	0.880557	0.878469

0.879817
0.881181
0.607374
0.608227
0.60907

								0.568632
								0.56771
								0.566777
								1.593048
								1.590446
						1.05692	1.053756	1.587793
						1.055293	1.052134	1.585106
			1.063156	1.059952	1.056748	1.59104	1.586675	1.844936
			1.061248	1.058041	1.054861	1.588093	1.583727	1.841598
			1.059371	1.056164	1.053007	1.585205	1.580854	1.838341
	0.571345	0.569665	1.595706	1.591281	1.586887	1.84539	1.840318	1.893766
	0.570463	0.568819	1.593026	1.588632	1.584264	1.842438	1.837412	1.890923
0.571245	1.060666	1.057491	1.590347	1.585982	1.581655	1.839493	1.834543	2.005598
0.570403	1.059002	1.055796	1.587681	1.583349	1.579074	1.83658	1.831728	2.002665
0.569558	1.593792	1.589395	1.848673	1.843585	1.838568	1.892195	1.88739	2.097194
0.56871	1.591151	1.586766	1.845682	1.840622	1.835647	1.889351	1.884621	2.094209
1.477351	1.85291	1.847765	1.901292	1.896234	1.891267	2.101221	2.096165	2.091135
1.474974	1.849848	1.844712	1.898262	1.893218	1.888267	2.097956	2.09291	2.087884
1.472574	1.846745	1.841631	1.895222	1.890219	1.885305	2.094727	2.089682	2.084653
1.584632	1.902252	1.897153	2.009421	2.004292	1.999243	2.091679	2.086646	2.081615

1.581833	1.899047	1.894015	2.006152	2.001173	1.996275	2.088721	2.083697	2.078661
1.842499	1.896064	1.891101	2.100749	2.09572	2.090763	2.085752	2.080729	2.116511
1.839599	1.89325	1.888361	2.097777	2.092761	2.087804	2.082793	2.077763	2.113483
1.895427	2.105151	2.099934	2.094818	2.089816	2.084854	2.079831	2.074784	2.166085
1.892448	2.10203	2.096917	2.091872	2.086887	2.081916	2.076872	2.071793	2.162931
2.104118	2.098953	2.093922	2.088929	2.083948	2.078958	2.114656	2.109489	2.159775
2.101084	2.095953	2.090961	2.085985	2.08098	2.075957	2.111597	2.106429	2.156623
2.098103	2.092989	2.08801	2.083031	2.077995	2.072938	2.108523	2.103361	2.153467
2.095112	2.090031	2.085041	2.120856	2.115739	2.1106	2.160886	2.155566	2.19256
2.092127	2.08708	2.082064	2.117802	2.112671	2.107524	2.157695	2.152373	2.189351
2.089121	2.084098	2.07907	2.17045	2.165153	2.159844	2.154519	2.1492	2.186164
2.086096	2.081087	2.076059	2.167282	2.161984	2.156677	2.151353	2.14604	2.182991
2.083093	2.118835	2.113745	2.16413	2.158846	2.153556	2.190467	2.185103	2.224022
2.080124	2.115791	2.110705	2.160999	2.155742	2.150489	2.187396	2.182073	2.220934
2.077164	2.168347	2.163107	2.157885	2.152667	2.14746	2.184365	2.179085	2.217884
2.074199	2.165212	2.159976	2.154778	2.149616	2.144472	2.181365	2.176114	2.214848
2.071239	2.16209	2.156866	2.151697	2.146595	2.141514	2.17839	2.173161	2.211827
2.164278	2.158995	2.153805	2.19086	2.185726	2.180609	2.219416	2.214065	2.208799
2.161171	2.155923	2.150783	2.18783	2.182725	2.17763	2.216375	2.211038	2.205772
2.158084	2.152868	2.147774	2.184809	2.179731	2.174656	2.213333	2.208002	2.228578
2.155016	2.149831	2.14478	2.181801	2.176748	2.171689	2.210289	2.204957	2.225573
2.151972	2.188941	2.183851	2.222848	2.217693	2.212523	2.233189	2.227853	2.252377
2.148939	2.185888	2.180829	2.219769	2.214627	2.209465	2.230181	2.224845	2.249288
2.145955	2.182872	2.177825	2.216694	2.211562	2.206412	2.227181	2.221853	2.246224
2.143077	2.179927	2.174849	2.213615	2.208495	2.20337	2.224198	2.218896	2.243201
2.140246	2.177018	2.17189	2.210542	2.205434	2.20034	2.221233	2.215972	2.24022
2.137356	2.217959	2.212679	2.233619	2.228527	2.223461	2.248067	2.242697	2.237325
2.134434	2.214926	2.209632	2.230592	2.22552	2.220494	2.245095	2.239829	2.2345
2.173456	2.238188	2.232859	2.257777	2.252497	2.24729	2.242084	2.236887	2.302686
2.170626	2.235348	2.229998	2.254793	2.249472	2.244231	2.239026	2.233853	2.299247
2.237726	2.262865	2.257307	2.251814	2.246455	2.241175	2.307528	2.30169	2.328573
2.234702	2.259783	2.254272	2.248802	2.243414	2.238102	2.304003	2.298248	2.324998
2.231658	2.256678	2.251234	2.245809	2.240404	2.235068	2.300532	2.294849	2.321465
2.258966	2.253606	2.248249	2.242893	2.237498	2.232152	2.330005	2.324003	2.317999
2.255915	2.250553	2.245287	2.240011	2.234644	2.229303	2.326552	2.320524	2.314575
2.252912	2.247543	2.242307	2.342038	2.335611	2.329259	2.323077	2.317028	2.311113
2.249956	2.244573	2.239314	2.338347	2.332013	2.325756	2.319592	2.313524	2.307622
2.246988	2.31362	2.307573	2.334694	2.328477	2.32234	2.316214	2.310142	2.304238
2.243985	2.310146	2.30411	2.331068	2.325019	2.319051	2.31299	2.306922	2.300989
2.241024	2.34012	2.333749	2.327547	2.321608	2.315751	2.309763	2.30374	2.371029
2.238168	2.33667	2.330374	2.32421	2.31823	2.312338	2.306437	2.300557	2.367506
2.235337	2.333285	2.327084	2.320961	2.314896	2.30892	2.303093	2.297363	2.364014
2.336012	2.329812	2.323744	2.317709	2.311629	2.305613	2.332251	2.326091	2.360369
2.332423	2.326312	2.320393	2.314465	2.308405	2.302371	2.328703	2.322508	2.356647
2.328967	2.322902	2.317012	2.311114	2.305094	2.299092	2.36604	2.359464	2.352983
2.325639	2.319578	2.313595	2.307643	2.30169	2.295776	2.362341	2.355757	2.349365
2.322344	2.316287	2.310249	2.337172	2.330798	2.324489	2.358723	2.352157	2.377098
2.31907	2.313025	2.307012	2.333681	2.327353	2.321098	2.355217	2.348702	2.373571
2.315812	2.309785	2.303819	2.330258	2.323958	2.317738	2.351753	2.345313	2.37012
2.312569	2.339669	2.33325	2.367969	2.361295	2.354723	2.348285	2.341962	2.366762

2.30932	2.336174	2.329839	2.364444	2.357766	2.351189	2.344829	2.338626	2.36345
2.305999	2.332634	2.326316	2.360776	2.354175	2.347677	2.34136	2.335191	2.360058
2.302629	2.329063	2.322719	2.357019	2.350549	2.344189	2.33789	2.331695	2.356626
2.331905	2.366675	2.359974	2.353405	2.347028	2.340767	2.365532	2.359279	2.353342
2.328458	2.363074	2.356445	2.349945	2.343618	2.337412	2.362204	2.356051	2.350209
2.366147	2.359519	2.352977	2.346558	2.340275	2.334118	2.358959	2.352937	2.347202
2.362565	2.35599	2.349559	2.343237	2.336992	2.330881	2.355794	2.34994	2.344339
2.359039	2.352522	2.346189	2.339957	2.333762	2.327713	2.352706	2.347026	2.341569
2.355605	2.349159	2.342853	2.367688	2.361484	2.355449	2.349677	2.344136	2.338829
2.35223	2.345863	2.33956	2.364372	2.358277	2.352375	2.346726	2.341312	2.336153
2.348841	2.342548	2.336315	2.36116	2.355186	2.349418	2.343909	2.338637	2.333586
2.345447	2.339227	2.333119	2.358041	2.3522	2.346571	2.341218	2.336099	2.331122
2.342112	2.366955	2.36086	2.35496	2.349267	2.343789	2.338577	2.333587	2.376059
2.338836	2.363711	2.357691	2.351895	2.346369	2.341068	2.335981	2.331088	2.373529
2.335627	2.360527	2.354604	2.348927	2.343558	2.338412	2.333433	2.32862	2.396823
2.332472	2.357367	2.35162	2.346114	2.34087	2.335827	2.330935	2.326191	2.394302
2.32941	2.354307	2.348735	2.343395	2.338259	2.333301	2.328478	2.323785	2.391797
2.326529	2.351476	2.345964	2.340693	2.33567	2.330822	2.373204	2.368374	2.389294
2.323775	2.348806	2.3433	2.338048	2.33313	2.328388	2.370702	2.365874	2.386801
2.351662	2.346124	2.340744	2.383015	2.377961	2.373069	2.368208	2.363424	2.384348
2.348769	2.343441	2.338304	2.380611	2.375515	2.370542	2.36572	2.361022	2.381936
2.345963	2.340803	2.335858	2.378185	2.373096	2.368097	2.388999	2.384245	2.379516
2.343278	2.338215	2.333371	2.375689	2.370703	2.365773	2.386617	2.381788	2.377059
2.340664	2.33568	2.330896	2.373177	2.368303	2.363465	2.384265	2.379375	2.374638
2.338101	2.380442	2.375508	2.370649	2.365856	2.361103	2.381923	2.377072	2.372316
2.335593	2.377918	2.372981	2.36813	2.363397	2.358719	2.379579	2.374805	2.370038
2.380292	2.375363	2.370483	2.391452	2.386647	2.381896	2.377168	2.372465	2.367741
2.377741	2.372804	2.368015	2.389012	2.384186	2.379407	2.374714	2.370082	2.365439
2.375295	2.37036	2.365618	2.386616	2.3818	2.377022	2.372329	2.367698	2.363098
2.372968	2.368045	2.363305	2.384279	2.379508	2.374762	2.370018	2.3653	2.360703
2.370643	2.391538	2.386692	2.38191	2.377183	2.372481	2.367723	2.362966	2.358368
2.368291	2.38917	2.384264	2.379448	2.374762	2.370128	2.365441	2.360755	2.356155
2.365915	2.386792	2.381845	2.377002	2.372339	2.367751	2.363148	2.35856	2.353974
2.363489	2.384401	2.379506	2.374683	2.369979	2.365352	2.360792	2.35629	2.351768
2.361035	2.381992	2.377195	2.372421	2.367656	2.362957	2.35842	2.35399	2.349551
2.358576	2.37952	2.374808	2.370106	2.365365	2.360673	2.356131	2.35169	2.390144
2.356108	2.376994	2.372365	2.367756	2.363108	2.358481	2.353907	2.349385	2.387683
2.379353	2.374585	2.370003	2.365453	2.360877	2.356308	2.351727	2.347162	2.385295
2.377085	2.372333	2.367753	2.363217	2.358684	2.35416	2.349603	2.345045	2.382997
2.374839	2.370098	2.365501	2.360963	2.356468	2.351991	2.371179	2.366441	2.436906
2.372556	2.367807	2.363173	2.358624	2.35418	2.349773	2.368919	2.364243	2.434214
2.370287	2.36553	2.360857	2.35629	2.351878	2.347525	2.36663	2.362028	2.431537
2.368079	2.363327	2.358643	2.354057	2.349609	2.345233	2.383255	2.378667	2.428825
2.365906	2.361171	2.356493	2.351889	2.347366	2.342923	2.380817	2.376291	2.4261
2.363708	2.359012	2.354354	2.373573	2.368808	2.364124	2.433997	2.42868	2.423394
2.361489	2.356855	2.352232	2.371377	2.366615	2.361926	2.431258	2.425931	2.420705
2.359286	2.354704	2.350111	2.428808	2.423052	2.417398	2.428535	2.423201	2.418026
2.357106	2.352559	2.347986	2.426098	2.420347	2.414692	2.425798	2.420472	2.415339
2.354941	2.350419	2.345861	2.423384	2.41765	2.412014	2.423104	2.417799	2.412703
2.352794	2.372062	2.3673	2.42066	2.414967	2.409379	2.420483	2.415227	2.41017

2.350653	2.369839	2.365075	2.417933	2.4123	2.406787	2.417918	2.41272	2.407693
2.348495	2.426665	2.420886	2.431746	2.426164	2.420711	2.415409	2.410246	2.405218
2.346328	2.423885	2.418107	2.428976	2.423515	2.41819	2.412951	2.407811	2.40276
2.344159	2.421113	2.415375	2.426271	2.420904	2.415674	2.410496	2.405395	2.400346
2.341988	2.418347	2.412688	2.423631	2.418329	2.41316	2.408045	2.403002	2.397982
2.421171	2.415594	2.410039	2.421041	2.415794	2.41067	2.405604	2.400606	2.421788
2.418354	2.412839	2.407424	2.418507	2.413307	2.408205	2.403168	2.398196	2.419357
2.41558	2.410133	2.40485	2.416005	2.410849	2.405767	2.400747	2.395785	2.416922
2.412919	2.423942	2.418682	2.41351	2.408409	2.403367	2.424615	2.419539	2.419257
2.410327	2.421375	2.416161	2.411035	2.405988	2.400989	2.42219	2.417097	2.416823
2.407752	2.418843	2.413682	2.408597	2.40358	2.398598	2.424476	2.419414	2.414397
2.405204	2.416352	2.411245	2.406193	2.401185	2.396201	2.422055	2.416999	2.41198
2.402692	2.419362	2.414335	2.434831	2.429749	2.42469	2.419627	2.414581	2.409588
2.400213	2.416895	2.411927	2.432412	2.427323	2.422249	2.417188	2.412157	2.407223
2.403774	2.41962	2.414657	2.429989	2.424896	2.419812	2.414758	2.409748	2.404886
2.401383	2.417228	2.412262	2.427548	2.42246	2.417383	2.412333	2.407336	2.40256
2.399014	2.414854	2.409869	2.425106	2.420025	2.414964	2.40994	2.404983	2.400295
2.412247	2.43277	2.427713	2.422661	2.417581	2.412545	2.407616	2.402791	2.398179
2.409833	2.430339	2.42528	2.420231	2.415154	2.410151	2.405351	2.400701	2.396154
2.412644	2.427911	2.422907	2.41791	2.412866	2.407899	2.403165	2.39859	2.394081
2.410216	2.425484	2.420591	2.415695	2.410712	2.405784	2.401058	2.396471	2.391975
2.42811	2.423148	2.418294	2.413447	2.408527	2.403654	2.398938	2.394337	2.38985
2.425958	2.420958	2.416023	2.411135	2.406278	2.401485	2.396787	2.392177	2.387699
2.423781	2.418751	2.41374	2.408816	2.404015	2.399298	2.394626	2.390022	2.38555
2.421432	2.416411	2.411404	2.406499	2.401745	2.397082	2.392458	2.387894	2.383424
2.419019	2.414024	2.409059	2.404196	2.399482	2.394859	2.390287	2.385775	2.381305
2.416601	2.411644	2.406741	2.401932	2.397246	2.392644	2.388107	2.383631	2.379162
2.41418	2.409273	2.404453	2.399709	2.395042	2.390448	2.385934	2.381482	2.377016
2.411935	2.407069	2.402308	2.397615	2.39298	2.388405	2.383903	2.379456	2.374985
2.409885	2.405053	2.400329	2.395674	2.391082	2.386541	2.382039	2.377572	2.373092
2.40779	2.402991	2.398293	2.393663	2.389105	2.384592	2.380091	2.375608	2.371121
2.405569	2.400808	2.396118	2.391496	2.386955	2.382458	2.377964	2.373482	2.368991
2.403314	2.398587	2.393906	2.389289	2.384759	2.380271	2.375783	2.371307	2.366817
2.401034	2.396336	2.39168	2.387081	2.382555	2.378068	2.373585	2.369116	2.368065
2.398768	2.394093	2.38947	2.384893	2.380368	2.375876	2.371398	2.366937	2.365925
2.396675	2.392019	2.387418	2.382858	2.378338	2.373849	2.372869	2.368398	2.373379
2.394718	2.390073	2.385488	2.380942	2.376431	2.371947	2.370989	2.366522	2.371591
2.392645	2.388012	2.38344	2.378905	2.374405	2.369932	2.369002	2.364545	2.369698
2.390408	2.385792	2.381231	2.37671	2.372228	2.367774	2.366879	2.362439	2.367675
2.388224	2.383614	2.379047	2.378144	2.373643	2.369176	2.364731	2.360313	2.365631
2.386217	2.38159	2.376966	2.376031	2.371486	2.36699	2.362555	2.358171	2.363572
2.384224	2.379585	2.374915	2.373958	2.369372	2.364849	2.360424	2.356071	2.361548
2.382071	2.381195	2.376559	2.371955	2.367397	2.362894	2.358481	2.354136	2.359673
2.379857	2.379063	2.374516	2.369993	2.365493	2.361029	2.356631	2.352281	2.357869
2.37778	2.377027	2.372487	2.367972	2.363483	2.35903	2.364542	2.360234	2.373257
2.375825	2.375077	2.370471	2.365899	2.361384	2.356919	2.362487	2.358187	2.371149
2.377642	2.373078	2.368458	2.363873	2.359354	2.354889	2.360516	2.356222	2.380778
2.375513	2.370987	2.366442	2.361924	2.357444	2.353003	2.358693	2.354399	2.3789
2.373426	2.368929	2.36444	2.369981	2.365563	2.36118	2.374108	2.369721	2.377007
2.371516	2.36701	2.362469	2.368018	2.363575	2.35918	2.372046	2.367674	2.374977

2.369637	2.365113	2.360506	2.366055	2.36158	2.357169	2.369969	2.365617	2.37294
2.367596	2.363079	2.358511	2.364156	2.359701	2.355305	2.36806	2.363724	2.379627
2.365471	2.360973	2.356504	2.362302	2.357896	2.353533	2.366252	2.36193	2.377893
2.363467	2.369222	2.364828	2.377688	2.373229	2.368798	2.364417	2.360076	2.376073
2.361581	2.367388	2.362997	2.375814	2.371385	2.366973	2.362563	2.358165	2.374164
2.359712	2.365568	2.361156	2.373899	2.369484	2.365088	2.381088	2.376668	2.389468
2.357864	2.363763	2.359285	2.371902	2.367472	2.363086	2.379179	2.374825	2.387548
2.355969	2.361922	2.357403	2.369914	2.365473	2.361096	2.377283	2.372999	2.385641
2.353914	2.359955	2.355501	2.377275	2.372877	2.368535	2.375484	2.371216	2.383731
2.35181	2.357955	2.353606	2.375455	2.371094	2.366765	2.373712	2.369435	2.381806
2.360472	2.382382	2.378007	2.37365	2.36928	2.364928	2.371851	2.36755	2.379802
2.358678	2.3806	2.376232	2.371863	2.367452	2.363052	2.369931	2.365593	2.377739
2.366905	2.378826	2.374444	2.370057	2.365632	2.361218	2.38492	2.380323	2.375786
2.365229	2.377064	2.37264	2.368227	2.363817	2.359434	2.383059	2.378486	2.373964
2.379719	2.375276	2.370824	2.366401	2.362017	2.357671	2.381217	2.37667	2.372163
2.377879	2.373426	2.368985	2.364584	2.360235	2.355927	2.379375	2.374855	2.370366
2.376019	2.371565	2.36715	2.362782	2.358469	2.354195	2.377532	2.373022	2.368545
2.37416	2.369732	2.365349	2.389327	2.384764	2.38022	2.37566	2.371106	2.366613
2.372304	2.367916	2.363571	2.387468	2.382923	2.378382	2.373779	2.369159	2.364639
2.370497	2.366127	2.361811	2.385616	2.381083	2.376547	2.371938	2.367306	2.362786
2.368733	2.364363	2.360072	2.383774	2.379247	2.374718	2.370133	2.365532	2.361034
2.366987	2.380281	2.375755	2.381936	2.377393	2.372853	2.368293	2.363737	2.359274
2.365262	2.378439	2.373921	2.380103	2.375509	2.370925	2.366389	2.361896	2.357485
2.363538	2.376605	2.372092	2.378274	2.373645	2.369031	2.364519	2.360079	2.355712
2.361805	2.374779	2.370276	2.376467	2.37185	2.367251	2.362755	2.358331	2.353979
2.360067	2.372953	2.368449	2.374645	2.370061	2.365498	2.36102	2.356606	2.352262
2.375662	2.371105	2.366561	2.372738	2.368185	2.36367	2.359233	2.354858	2.350535
2.373811	2.369245	2.36464	2.370784	2.366265	2.361807	2.357424	2.353103	2.348803
2.371965	2.367405	2.362777	2.368912	2.364415	2.359991	2.35564	2.351349	2.347051
2.370125	2.365582	2.360969	2.367117	2.362632	2.358216	2.353877	2.349594	2.345276
2.368297	2.363771	2.359177	2.365343	2.360869	2.356462	2.352133	2.347857	2.343527
2.366488	2.361972	2.357392	2.36358	2.359121	2.354728	2.350409	2.346142	2.341821
2.364689	2.360185	2.355623	2.361834	2.357389	2.353009	2.348699	2.344441	2.340133
2.362896	2.358417	2.353877	2.360112	2.355677	2.351301	2.347001	2.342753	2.338455
2.361111	2.356656	2.352142	2.358402	2.353973	2.3496	2.34531	2.341073	2.336784
2.359336	2.35488	2.350386	2.35667	2.352251	2.347886	2.343608	2.339385	2.335105
2.357568	2.353094	2.348617	2.354926	2.350518	2.346164	2.3419	2.337692	2.333423
2.355808	2.35133	2.34687	2.353203	2.348801	2.344452	2.340198	2.336002	2.331743
2.354056	2.34959	2.345142	2.351495	2.347094	2.342744	2.338497	2.334312	2.330063
2.352303	2.347859	2.343442	2.349827	2.34543	2.341077	2.336828	2.332641	2.328395
2.35053	2.346126	2.341785	2.348233	2.343847	2.339486	2.335218	2.331003	2.326748
2.348782	2.344412	2.340136	2.346641	2.342268	2.337903	2.333615	2.329371	2.325111
2.347121	2.342745	2.338461	2.344969	2.340608	2.336256	2.331969	2.327721	2.3486
2.345499	2.3411	2.336775	2.343262	2.338913	2.334583	2.330305	2.326065	2.346927
2.343815	2.339419	2.335083	2.341571	2.337236	2.332923	2.328646	2.324402	2.345224
2.342082	2.337709	2.333385	2.339889	2.33557	2.331269	2.326988	2.322732	2.343498
2.340359	2.336008	2.331694	2.33822	2.333934	2.329662	2.339413	2.335082	2.341805
2.338642	2.334308	2.330001	2.336556	2.332336	2.328126	2.337853	2.333491	2.340161
2.336971	2.332656	2.328355	2.334931	2.330751	2.326577	2.336278	2.331893	2.347046
2.3354	2.331105	2.326802	2.333371	2.329161	2.324959	2.334621	2.330237	2.345326

2.333852	2.329578	2.325283	2.331846	2.327586	2.323331	2.332953	2.32858	2.343611
2.33222	2.338852	2.334601	2.344412	2.340064	2.335709	2.331351	2.326991	2.341955
2.330552	2.337213	2.333047	2.34289	2.338524	2.334134	2.329778	2.325434	2.340331
2.328948	2.335616	2.331461	2.341284	2.336912	2.332513	2.33913	2.334718	2.338657
2.327404	2.334056	2.329841	2.339591	2.335227	2.330846	2.337434	2.33301	2.336933
2.325863	2.346583	2.342259	2.337925	2.333568	2.3292	2.335759	2.33132	2.335232
2.324327	2.345034	2.340685	2.33633	2.331974	2.32761	2.334134	2.329675	2.333581
2.322757	2.343449	2.339084	2.334716	2.330358	2.325993	2.332491	2.328022	2.331922
2.321089	2.341744	2.337378	2.333008	2.32864	2.32427	2.339122	2.334615	2.330205
2.319393	2.340004	2.335641	2.331273	2.326893	2.322516	2.337349	2.332874	2.328482
2.317778	2.338347	2.333975	2.329602	2.325214	2.320838	2.335652	2.331212	2.326835
2.316216	2.336742	2.332355	2.327969	2.323578	2.319208	2.334006	2.329607	2.325241
2.314652	2.335142	2.330739	2.326345	2.321957	2.317599	2.332373	2.328003	2.323651
2.313088	2.33355	2.329131	2.324728	2.320353	2.316013	2.330752	2.326399	2.322066
2.31151	2.331943	2.327522	2.323123	2.318761	2.314437	2.329142	2.324803	2.320485
2.309906	2.330302	2.325909	2.321536	2.317183	2.312865	2.327543	2.323217	2.318908
2.308298	2.328658	2.324294	2.31995	2.315611	2.311305	2.325954	2.321637	2.317334
2.321318	2.32704	2.322673	2.337546	2.33312	2.328729	2.324377	2.32006	2.315759
2.319702	2.325434	2.321054	2.335892	2.331502	2.327149	2.322808	2.318485	2.314184
2.318083	2.323826	2.319461	2.334269	2.329896	2.325558	2.321226	2.316909	2.325727
2.316462	2.322221	2.317891	2.332671	2.328299	2.323959	2.319636	2.315332	2.324127
2.314855	2.320635	2.316324	2.331072	2.326706	2.322368	2.318051	2.313754	2.322531
2.313263	2.319073	2.314758	2.329465	2.325112	2.320788	2.316473	2.312173	2.32094
2.311687	2.317515	2.313204	2.327873	2.323529	2.319212	2.314898	2.310596	2.31935
2.310115	2.315932	2.311665	2.326316	2.321967	2.317637	2.313325	2.309029	2.317755
2.308576	2.314375	2.310141	2.324765	2.320408	2.316066	2.311756	2.307465	2.316163
2.307126	2.312921	2.308631	2.323173	2.318824	2.3145	2.310192	2.3059	2.314584
2.305726	2.311526	2.307138	2.321571	2.317233	2.312938	2.308632	2.304334	2.313012
2.304261	2.329032	2.324515	2.320051	2.315686	2.311368	2.307058	2.302768	2.311432
2.302737	2.327532	2.323065	2.318613	2.314181	2.309787	2.305468	2.301201	2.309846
2.301205	2.325991	2.321572	2.317145	2.312685	2.30825	2.303914	2.299642	2.318636
2.29966	2.324383	2.320006	2.315624	2.311198	2.306779	2.302411	2.298082	2.317031
2.298139	2.322791	2.318433	2.314081	2.3097	2.305318	2.300931	2.296555	2.315463
2.306899	2.321281	2.316878	2.312505	2.308162	2.303829	2.299463	2.295097	2.313974
2.305474	2.319805	2.315342	2.310932	2.306619	2.302337	2.298008	2.293672	2.312522
2.304002	2.318319	2.313853	2.309441	2.305136	2.300866	2.319886	2.315451	2.315986
2.302504	2.316833	2.3124	2.308007	2.30369	2.299405	2.318409	2.313999	2.314504
2.319729	2.315348	2.310943	2.306562	2.302218	2.297902	2.316885	2.312503	2.313001
2.318245	2.313869	2.309488	2.305109	2.300715	2.296349	2.315307	2.31096	2.311478
2.316747	2.312368	2.308008	2.314645	2.310176	2.305731	2.31865	2.314299	2.309928
2.315211	2.31082	2.306485	2.313131	2.308675	2.304231	2.317104	2.312702	2.30833
2.313682	2.309276	2.304957	2.311614	2.307185	2.30276	2.315584	2.311125	2.306745
2.3122	2.307782	2.303465	2.310122	2.305727	2.30133	2.314117	2.30963	2.30524
2.310742	2.30631	2.301983	2.30863	2.304273	2.299915	2.312674	2.308175	2.303775
2.309279	2.31585	2.311447	2.324411	2.320026	2.315639	2.311183	2.306701	2.302299
2.307817	2.314345	2.309886	2.322806	2.318433	2.314076	2.309657	2.305217	2.300824
2.306365	2.312861	2.308369	2.321254	2.316889	2.312553	2.308162	2.303753	2.299367
2.304927	2.311404	2.306905	2.319767	2.315407	2.311082	2.306706	2.302314	2.297934
2.3145	2.327312	2.32277	2.3183	2.313944	2.309625	2.305261	2.300885	2.296511
2.313035	2.325844	2.321309	2.316845	2.31249	2.308173	2.303822	2.299462	2.295095

2.311568	2.324375	2.319853	2.315399	2.311045	2.30673	2.30239	2.298046	2.293686
2.3101	2.322902	2.318391	2.313948	2.309604	2.305297	2.300969	2.296635	2.295584
2.308631	2.321427	2.316928	2.312498	2.308167	2.303873	2.299554	2.295228	2.294205
2.324518	2.319959	2.315469	2.311049	2.306726	2.302441	2.30146	2.297158	2.292826
2.323045	2.318498	2.314015	2.309601	2.305282	2.301003	2.300063	2.295779	2.29145
2.321577	2.317038	2.312561	2.308152	2.30384	2.299567	2.298661	2.294386	2.290058
2.320115	2.31558	2.311106	2.306703	2.302401	2.298136	2.297251	2.292969	2.288641
2.318655	2.314124	2.309652	2.305252	2.300959	2.296703	2.29584	2.291551	2.287221
2.317195	2.312672	2.308195	2.307199	2.30293	2.2987	2.294431	2.290145	2.285815
2.315735	2.311222	2.30674	2.30576	2.301501	2.297282	2.293021	2.288741	2.284412
2.314272	2.313183	2.308721	2.304335	2.300074	2.295856	2.291599	2.287327	2.283005
2.31281	2.311748	2.307301	2.302923	2.298655	2.294426	2.290171	2.285906	2.281595
2.314808	2.310347	2.305928	2.301568	2.297291	2.293045	2.288774	2.284492	2.28018
2.31341	2.308979	2.304607	2.300274	2.295991	2.29172	2.287412	2.283085	2.278758
2.312039	2.307638	2.303298	2.29898	2.294678	2.290376	2.286038	2.281682	2.291169
2.310701	2.306334	2.302005	2.297678	2.293334	2.288986	2.284632	2.280279	2.289759
2.30938	2.305028	2.300696	2.296352	2.291969	2.287584	2.283225	2.278886	2.288368
2.308068	2.303684	2.299328	2.294966	2.290573	2.286186	2.281832	2.277506	2.286996
2.306756	2.30232	2.29793	2.293551	2.289163	2.284791	2.280452	2.276144	2.285645
2.305395	2.300944	2.296538	2.295214	2.290826	2.286465	2.28215	2.277875	2.287029
2.303996	2.299555	2.295146	2.293821	2.289439	2.285092	2.280819	2.276597	2.285751
2.305666	2.30124	2.296851	2.292484	2.288124	2.283796	2.292988	2.288723	2.303027
2.304248	2.299855	2.295531	2.291223	2.28691	2.282607	2.291759	2.287437	2.301558
2.302885	2.298518	2.294229	2.289952	2.285667	2.281377	2.290488	2.286118	2.310784
2.301646	2.297283	2.292951	2.288634	2.28433	2.280028	2.289113	2.284735	2.30925
2.300421	2.296055	2.291669	2.287299	2.282963	2.278641	2.287702	2.28333	2.307712
2.299097	2.294724	2.29033	2.29947	2.295061	2.290669	2.286289	2.281926	2.306189
2.297723	2.293343	2.288966	2.298099	2.293674	2.289265	2.284885	2.280533	2.304685
2.296342	2.291971	2.287631	2.296782	2.29238	2.287985	2.302001	2.297279	2.303245
2.294944	2.290599	2.28632	2.295515	2.291172	2.286821	2.300714	2.295966	2.301871
2.293621	2.302841	2.298529	2.294227	2.289915	2.285587	2.310041	2.305229	2.300459
2.292418	2.3016	2.297246	2.292906	2.288574	2.284235	2.308559	2.303749	2.298974
2.291198	2.300344	2.295946	2.29157	2.287222	2.282877	2.307081	2.302287	2.297516
2.289857	2.298981	2.294577	2.290205	2.285881	2.281569	2.305673	2.300921	2.296169
2.288502	2.297609	2.293208	2.288849	2.284552	2.280276	2.30428	2.299571	2.29484
2.300795	2.296362	2.291954	2.287584	2.283273	2.278981	2.302845	2.29813	2.293399
2.29962	2.295183	2.290764	2.286374	2.282024	2.277689	2.30139	2.296642	2.291899
2.298341	2.293905	2.289484	2.3142	2.309451	2.304722	2.299977	2.295231	2.290494
2.296949	2.29252	2.288104	2.312696	2.30799	2.303312	2.298611	2.293905	2.289191
2.295622	2.291197	2.286784	2.311245	2.306554	2.301894	2.297215	2.292532	2.291195
2.294426	2.289998	2.28558	2.309894	2.305159	2.300452	2.295747	2.291051	2.289726
2.293263	2.288831	2.284408	2.308572	2.303789	2.29903	2.294293	2.289578	2.288263
2.292103	2.287668	2.283242	2.307261	2.302464	2.29769	2.296301	2.291585	2.28691
2.290927	2.286489	2.282062	2.305938	2.301143	2.296369	2.294979	2.290257	2.28559
2.289622	2.303447	2.298665	2.304505	2.299712	2.294942	2.293563	2.288846	2.284186
2.28822	2.301929	2.297153	2.302988	2.298202	2.293441	2.292083	2.28738	2.282729
2.286915	2.311195	2.306369	2.304926	2.300156	2.295412	2.290696	2.28601	2.281367
2.285726	2.309877	2.305058	2.303629	2.298863	2.294124	2.289423	2.284756	2.28012
2.284554	2.311924	2.307123	2.302346	2.297585	2.292853	2.288167	2.283517	2.278888
2.283388	2.31063	2.305834	2.301063	2.296311	2.291591	2.286918	2.282282	2.277659

2.282187	2.309302	2.304511	2.299747	2.295006	2.290297	2.285635	2.281011	2.276393
2.298668	2.30785	2.303064	2.298305	2.293573	2.288873	2.284221	2.279604	2.274992
2.297163	2.306345	2.301562	2.29681	2.292085	2.287394	2.282749	2.278138	2.273529
2.29963	2.304956	2.300179	2.295433	2.290717	2.286034	2.281394	2.276786	2.27218
2.298362	2.303662	2.298891	2.294152	2.289445	2.28477	2.280133	2.275527	2.270922
2.297111	2.302391	2.297627	2.292896	2.2882	2.283535	2.278901	2.274294	2.269689
2.295871	2.301139	2.296383	2.291663	2.286981	2.282328	2.277697	2.273087	2.26848
2.304679	2.299897	2.295148	2.290436	2.285768	2.281128	2.276498	2.271883	2.26727
2.303436	2.298657	2.293911	2.289204	2.28455	2.279921	2.275291	2.270667	2.266044
2.302197	2.29742	2.292675	2.287972	2.283326	2.278705	2.274072	2.269438	2.264801
2.300955	2.296182	2.29144	2.286738	2.282089	2.277463	2.272826	2.268186	2.267225
2.299712	2.294943	2.290205	2.285501	2.280839	2.276196	2.271552	2.266907	2.265912
2.298464	2.293696	2.288959	2.284245	2.279554	2.274878	2.27021	2.265547	2.264539
2.297212	2.292442	2.287705	2.282973	2.278234	2.273503	2.268792	2.264097	2.263093
2.295955	2.291178	2.286426	2.281674	2.276903	2.27214	2.267409	2.262704	2.261708
2.294688	2.289893	2.285104	2.280326	2.275554	2.270802	2.26609	2.261412	2.260428
2.293425	2.288614	2.283791	2.27899	2.274224	2.269491	2.264805	2.260158	2.259185
2.292177	2.287363	2.282526	2.277716	2.27295	2.268224	2.26355	2.258921	2.25795
2.290939	2.286128	2.281288	2.276475	2.271709	2.266984	2.262318	2.257699	2.256727
2.289708	2.284905	2.280066	2.275251	2.270476	2.265744	2.264749	2.260118	2.255497
2.288484	2.283694	2.278863	2.274049	2.26926	2.264512	2.263506	2.258879	2.254263
2.287261	2.282495	2.277697	2.272901	2.268101	2.263332	2.262299	2.257649	2.253027
2.286041	2.281315	2.27658	2.271822	2.267013	2.262211	2.261125	2.25642	2.251783
2.284813	2.280121	2.275439	2.270721	2.265924	2.261115	2.259991	2.255236	2.250577
2.283568	2.278897	2.274243	2.26956	2.264818	2.260054	2.258919	2.254128	2.249431
2.28232	2.277662	2.273023	2.268369	2.263687	2.258979	2.257852	2.253048	2.248322
2.281079	2.276427	2.271792	2.270839	2.266163	2.261469	2.256731	2.251969	2.247251
2.279841	2.275189	2.27055	2.269591	2.264927	2.260258	2.255577	2.250889	2.246199
2.278598	2.273948	2.269309	2.268347	2.263691	2.259039	2.254392	2.249748	2.245095
2.277352	2.272704	2.268066	2.267101	2.26245	2.257807	2.253176	2.248556	2.24395
2.276134	2.271474	2.266831	2.265857	2.261208	2.256573	2.251953	2.247351	2.243595
2.27494	2.270247	2.265588	2.264602	2.259959	2.255334	2.250725	2.246134	2.242392
2.273801	2.272771	2.268066	2.263388	2.258735	2.254106	2.249498	2.244914	2.241178
2.272773	2.271729	2.26698	2.262254	2.25755	2.25288	2.24826	2.243683	2.239952
2.27176	2.270715	2.265931	2.261161	2.256407	2.251691	2.247053	2.242474	2.238737
2.270628	2.269601	2.264842	2.260088	2.255342	2.250622	2.245963	2.241349	2.237575
2.269429	2.268432	2.263731	2.259028	2.254322	2.249624	2.244944	2.24028	2.23645
2.268237	2.267234	2.262555	2.257879	2.253205	2.248534	2.243868	2.239208	2.235358
2.26704	2.265996	2.261307	2.25664	2.25199	2.247358	2.242744	2.238141	2.234307
2.269577	2.26483	2.260118	2.255438	2.250791	2.246173	2.241591	2.23703	2.233216
2.268556	2.263797	2.259035	2.254305	2.249617	2.24497	2.240384	2.235838	2.232054
2.267518	2.262758	2.257966	2.253202	2.248484	2.243811	2.239209	2.234657	2.230887
2.266345	2.26161	2.256858	2.252131	2.247444	2.242786	2.238974	2.23436	2.229749
2.265133	2.260426	2.255734	2.251064	2.246426	2.241798	2.237965	2.233296	2.228632
2.264001	2.259276	2.254576	2.249911	2.245295	2.240695	2.236878	2.232222	2.227562
2.262918	2.258144	2.253396	2.248702	2.244093	2.239519	2.235744	2.231146	2.226526
2.261829	2.257045	2.252288	2.247586	2.242964	2.238382	2.234608	2.230022	2.225413
2.260732	2.255987	2.251269	2.246579	2.241921	2.237285	2.233465	2.228833	2.224204
2.259641	2.254918	2.250225	2.246388	2.241701	2.237022	2.232352	2.227691	2.223041
2.258564	2.253814	2.249098	2.245251	2.240589	2.235945	2.231301	2.226659	2.221996

2.257483	2.25271	2.247972	2.244117	2.239481	2.234873	2.230258	2.225639	2.220967
2.25638	2.251634	2.246927	2.243086	2.238433	2.233798	2.229165	2.224533	2.219868
2.255267	2.250566	2.24591	2.242091	2.237409	2.232728	2.228061	2.223402	2.218751
2.254165	2.249469	2.244826	2.241019	2.236349	2.231677	2.227008	2.222344	2.217705
2.253071	2.248347	2.243691	2.239886	2.235262	2.230643	2.225998	2.221343	2.216715
2.251995	2.247261	2.242607	2.238811	2.234219	2.229632	2.224991	2.220326	2.215687
2.250938	2.24622	2.241593	2.23782	2.233245	2.228662	2.223994	2.219283	2.214601
2.249903	2.246048	2.241423	2.236824	2.232248	2.227655	2.22297	2.218234	2.213523
2.248901	2.245052	2.240404	2.235774	2.231161	2.226542	2.221871	2.217171	2.21248
2.247908	2.244063	2.239383	2.234717	2.230065	2.225419	2.220769	2.216117	2.211453
2.246893	2.243045	2.238358	2.233688	2.22904	2.2244	2.219755	2.215108	2.210442
2.245867	2.242013	2.237335	2.232679	2.228057	2.22344	2.218791	2.214124	2.209439
2.244842	2.240999	2.236343	2.231706	2.227091	2.222472	2.217805	2.213111	2.208414
2.243814	2.239998	2.235383	2.23077	2.226147	2.221505	2.216805	2.212073	2.207365
2.24282	2.239019	2.234428	2.229824	2.225178	2.220504	2.21578	2.21103	2.206327
2.241876	2.23807	2.233479	2.228862	2.224171	2.219452	2.214717	2.209976	2.205302
2.240946	2.237129	2.232519	2.227875	2.223144	2.218391	2.213656	2.208938	2.204298
2.240011	2.236185	2.231522	2.226833	2.222086	2.217333	2.212623	2.207944	2.203328
2.239075	2.235241	2.230515	2.225779	2.221027	2.216289	2.211611	2.206975	2.202376
2.238975	2.234297	2.229549	2.224793	2.220035	2.2153	2.210636	2.206019	2.201414
2.238028	2.23335	2.228605	2.223851	2.21909	2.214352	2.209691	2.205078	2.20045
2.237045	2.232355	2.227615	2.222871	2.218127	2.213404	2.208749	2.204135	2.199495
2.236017	2.2313	2.226569	2.221849	2.217144	2.212459	2.207814	2.203193	2.198557
2.235017	2.230278	2.22555	2.220845	2.21617	2.211513	2.206865	2.202226	2.19759
2.23408	2.229327	2.22459	2.21988	2.215213	2.21056	2.205884	2.201204	2.196557
2.233163	2.228401	2.223652	2.218932	2.214266	2.209611	2.204907	2.200188	2.19553
2.232248	2.227479	2.22272	2.217993	2.213328	2.208673	2.203963	2.199236	2.194574
2.231337	2.226565	2.221797	2.217063	2.212396	2.207743	2.203037	2.198316	2.193655
2.230419	2.225642	2.220869	2.216131	2.211465	2.206815	2.202114	2.197399	2.19274
2.229497	2.224715	2.219939	2.2152	2.210536	2.20589	2.201196	2.19649	2.191834
2.22857	2.223785	2.219007	2.21427	2.20961	2.204968	2.200281	2.195581	2.190928
2.227636	2.222846	2.218073	2.213341	2.208685	2.204049	2.199366	2.194669	2.190018
2.226712	2.221919	2.217144	2.212415	2.207766	2.203138	2.19846	2.193767	2.189116
2.225802	2.221005	2.216216	2.211482	2.206847	2.202238	2.197569	2.192878	2.18822
2.224913	2.220115	2.21531	2.210565	2.205941	2.201346	2.19669	2.19201	2.187347
2.224071	2.219286	2.214466	2.209704	2.205063	2.200461	2.19583	2.191192	2.186536
2.223253	2.21849	2.213658	2.208876	2.204204	2.199582	2.194982	2.190399	2.185758
2.222387	2.217641	2.212812	2.208025	2.20333	2.198687	2.194102	2.18955	2.184916
2.221478	2.216747	2.211935	2.207154	2.202441	2.197778	2.193193	2.188652	2.184019
2.220571	2.215848	2.211069	2.20631	2.201586	2.196903	2.192306	2.187755	2.183117
2.219671	2.214944	2.210228	2.205515	2.200789	2.196084	2.191456	2.186867	2.182211
2.21879	2.214051	2.209381	2.204706	2.199984	2.195267	2.190609	2.185979	2.181312
2.217957	2.213193	2.208512	2.203833	2.199119	2.194408	2.189733	2.185079	2.180423
2.217136	2.212345	2.207632	2.202935	2.198236	2.193547	2.188864	2.184192	2.17955
2.216266	2.211469	2.206736	2.202037	2.197379	2.192734	2.188056	2.183368	2.17871
2.215368	2.210581	2.205831	2.201137	2.196535	2.191949	2.187288	2.182589	2.177897
2.214517	2.209739	2.204981	2.200283	2.19569	2.191117	2.186477	2.181801	2.177099
2.213721	2.208951	2.20419	2.199476	2.194838	2.19023	2.185622	2.18101	2.176325
2.212896	2.208138	2.203399	2.19869	2.194016	2.189371	2.184769	2.180186	2.175511
2.211999	2.207265	2.202598	2.197937	2.193251	2.18857	2.183927	2.179297	2.174609

2.211107	2.20639	2.201782	2.197165	2.192485	2.187789	2.183103	2.178416	2.173713
2.21028	2.205554	2.200925	2.196306	2.191669	2.187018	2.182328	2.177614	2.181834
2.209487	2.204739	2.200057	2.195414	2.19083	2.186244	2.181569	2.176845	2.181059
2.208703	2.203946	2.199239	2.194577	2.189985	2.185402	2.180752	2.176064	2.186004
2.207934	2.203172	2.198458	2.193778	2.189133	2.184508	2.179892	2.175278	2.185251
2.207168	2.202404	2.197686	2.192994	2.188317	2.183664	2.179053	2.174463	2.184453
2.206403	2.201639	2.196922	2.192226	2.187546	2.182881	2.178235	2.173601	2.183589
2.205642	2.200881	2.196164	2.191468	2.186786	2.182117	2.177446	2.172781	2.182764
2.204889	2.20013	2.195412	2.190714	2.186029	2.181361	2.176709	2.172071	2.182052
2.204139	2.199382	2.194663	2.189964	2.185277	2.18061	2.175982	2.171376	2.181354
2.203386	2.198632	2.193912	2.18921	2.184521	2.17985	2.175209	2.170587	2.180558
2.202635	2.197884	2.193162	2.188458	2.183767	2.17909	2.174421	2.169762	2.179725
2.201898	2.19715	2.192422	2.187713	2.183025	2.178351	2.182668	2.177981	2.178969
2.201173	2.196428	2.191689	2.186974	2.182292	2.17763	2.181971	2.177304	2.17828
2.200474	2.195741	2.191002	2.186283	2.181598	2.176932	2.186964	2.18228	2.177572
2.199815	2.195109	2.190388	2.185671	2.180965	2.176268	2.186269	2.181555	2.176828
2.199149	2.194463	2.189756	2.194117	2.189367	2.18462	2.185569	2.180826	2.176083
2.198444	2.19375	2.189042	2.193406	2.188655	2.183906	2.184846	2.180093	2.175341
2.197723	2.193012	2.188296	2.192662	2.187918	2.183177	2.184119	2.179369	2.174615
2.197	2.19228	2.187558	2.191918	2.187183	2.182457	2.183412	2.178686	2.169314
2.196275	2.191552	2.186822	2.191172	2.186447	2.18174	2.182716	2.178028	2.168706
2.19559	2.190864	2.186138	2.196211	2.191457	2.186721	2.182018	2.177335	2.168041
2.194952	2.190222	2.185514	2.195591	2.190818	2.186053	2.18132	2.176606	2.167317
2.194293	2.189559	2.184864	2.19494	2.190152	2.185366	2.176028	2.17131	2.175148
2.193576	2.188839	2.184145	2.194211	2.189418	2.184624	2.175307	2.170589	2.174408
2.19287	2.188131	2.183434	2.193489	2.188694	2.1839	2.174608	2.169894	2.173698
2.187286	2.187495	2.182793	2.188257	2.183489	2.178723	2.173989	2.169272	2.173065
2.186687	2.186891	2.182181	2.187656	2.182898	2.178143	2.173408	2.168687	2.172471
2.18608	2.186282	2.181562	2.187049	2.1823	2.177557	2.181381	2.176619	2.171866
2.18547	2.185674	2.180942	2.18644	2.181703	2.176973	2.180784	2.176011	2.17126
2.184811	2.180696	2.175973	2.185785	2.181058	2.176339	2.180136	2.175358	2.170608
2.184084	2.179999	2.17527	2.185067	2.180348	2.175638	2.179422	2.174642	2.169893
2.18338	2.193916	2.189137	2.192996	2.188245	2.183504	2.178733	2.173954	2.169205
2.18274	2.193292	2.188521	2.192376	2.187623	2.18288	2.178113	2.17334	2.168591
2.182125	2.192694	2.187933	2.191784	2.18703	2.182284	2.17752	2.172753	2.168005
2.177387	2.192102	2.187347	2.191192	2.18644	2.181694	2.176932	2.172166	2.167419
2.176811	2.19152	2.18677	2.190608	2.185859	2.181116	2.176354	2.171587	2.16684
2.17623	2.195689	2.190925	2.19002	2.185275	2.180535	2.175774	2.171007	2.166261
2.175647	2.195093	2.190332	2.189431	2.18469	2.179954	2.175194	2.170426	2.165681
2.175065	2.194498	2.189741	2.188842	2.184105	2.179372	2.174613	2.169845	2.1651
2.174482	2.193903	2.189147	2.188249	2.183517	2.178788	2.174028	2.169257	2.164513
2.173906	2.193315	2.188563	2.187669	2.182938	2.178209	2.173451	2.168681	2.163936
2.173333	2.192727	2.187989	2.187102	2.182365	2.177629	2.172877	2.168119	2.16337
2.172781	2.192161	2.187438	2.18656	2.181814	2.177069	2.172325	2.167582	2.162829
2.196429	2.191666	2.186952	2.182239	2.177502	2.172766	2.17184	2.167105	2.162351
2.195978	2.191215	2.186506	2.181796	2.177058	2.17232	2.171396	2.166665	2.161913
2.195468	2.19071	2.186001	2.181291	2.176554	2.171816	2.170891	2.166161	2.161414
2.1949	2.190151	2.185438	2.180725	2.175992	2.171257	2.170329	2.165595	2.160854
2.194337	2.189587	2.184869	2.180154	2.175426	2.170697	2.169764	2.165024	2.160288
2.193789	2.189017	2.184292	2.179577	2.17486	2.170142	2.169202	2.164446	2.159713

2.19326	2.188467	2.183736	2.179021	2.174315	2.169607	2.168659	2.163891	2.15916
2.192764	2.187977	2.183245	2.17853	2.17383	2.169127	2.168177	2.163404	2.158677
2.192287	2.187514	2.18278	2.178061	2.173361	2.16866	2.167712	2.162941	2.158217
2.191815	2.187037	2.18228	2.17754	2.172825	2.168117	2.167178	2.162421	2.157701
2.19135	2.186555	2.181761	2.176986	2.172245	2.167522	2.166598	2.161866	2.157151
2.190848	2.186058	2.181257	2.176475	2.171725	2.166995	2.162279	2.157577	2.156638
2.190298	2.185539	2.180769	2.176011	2.171274	2.166549	2.161826	2.157111	2.156169
2.189773	2.185037	2.180298	2.175563	2.170836	2.166113	2.161381	2.156651	2.155701
2.189302	2.184571	2.17985	2.175128	2.170398	2.165667	2.160928	2.156189	2.155223
2.188855	2.184122	2.179411	2.174695	2.169958	2.165215	2.160467	2.155718	2.154739
2.188417	2.183683	2.178971	2.174251	2.169505	2.164749	2.159982	2.155212	2.154233
2.187987	2.183251	2.17853	2.173798	2.169041	2.164271	2.159483	2.154692	2.153722
2.187547	2.182804	2.178074	2.173331	2.168555	2.163769	2.158983	2.154205	2.153253
2.187099	2.182346	2.177606	2.17285	2.168047	2.163244	2.15848	2.153746	2.152819
2.18666	2.181892	2.177126	2.17235	2.167542	2.162745	2.158004	2.153302	2.152394
2.186234	2.181441	2.176616	2.171807	2.167029	2.162279	2.157562	2.152876	2.151979
2.185814	2.181008	2.176149	2.171321	2.16656	2.161836	2.157133	2.152457	2.147807
2.185398	2.180609	2.175791	2.17098	2.166179	2.161406	2.156698	2.152037	2.147392
2.184984	2.180221	2.175462	2.170681	2.165837	2.161008	2.156283	2.151622	2.146977
2.184565	2.179813	2.175075	2.170314	2.165498	2.160684	2.155929	2.151216	2.146548
2.184143	2.179395	2.174658	2.169916	2.165165	2.160406	2.155616	2.150822	2.146119
2.183721	2.178976	2.174239	2.169509	2.164793	2.160067	2.155284	2.15048	2.145755
2.183296	2.178554	2.173817	2.169091	2.164381	2.159672	2.15494	2.150196	2.145458
2.182883	2.178129	2.173392	2.168669	2.163963	2.159265	2.15457	2.149874	2.150854
2.182479	2.177685	2.172956	2.168246	2.16354	2.158846	2.154162	2.149485	2.150497
2.182102	2.177272	2.172536	2.167825	2.163121	2.15843	2.153749	2.149077	2.150119
2.181783	2.176947	2.172154	2.167401	2.162703	2.158029	2.153343	2.14866	2.149707
2.181482	2.176657	2.171799	2.166996	2.162304	2.157646	2.152952	2.148251	2.149289
2.181126	2.176325	2.171471	2.166666	2.16197	2.157308	2.152617	2.147923	2.148936
2.180736	2.175971	2.171168	2.166395	2.161686	2.157003	2.152325	2.147652	2.148627
2.180384	2.175637	2.170873	2.16612	2.161382	2.156663	2.157766	2.153059	2.148303
2.180079	2.17533	2.170594	2.165847	2.161054	2.156277	2.157369	2.152688	2.147959
2.179759	2.175	2.170285	2.165547	2.160727	2.155919	2.157	2.152329	2.147625
2.179393	2.174607	2.169908	2.165196	2.160405	2.155623	2.156695	2.152015	2.147318
2.17903	2.17422	2.169528	2.164836	2.16009	2.155347	2.156405	2.151701	2.147003
2.178714	2.17391	2.169208	2.164516	2.159796	2.155074	2.156095	2.15134	2.146627
2.178425	2.173636	2.168912	2.164204	2.159504	2.154798	2.155779	2.150966	2.146232
2.178142	2.173348	2.168583	2.163846	2.159155	2.154468	2.155451	2.150639	2.145908
2.177871	2.173055	2.16823	2.16345	2.158757	2.15409	2.155112	2.15035	2.14564
2.177604	2.172774	2.167913	2.168977	2.164235	2.159531	2.154801	2.150073	2.145378
2.177342	2.17251	2.167645	2.168694	2.163952	2.159251	2.154529	2.14981	2.145119
2.177084	2.172252	2.167389	2.16843	2.163688	2.158988	2.154268	2.149553	2.144863
2.176828	2.171996	2.167134	2.168169	2.163428	2.158729	2.154011	2.149297	2.144607
2.176574	2.171743	2.166882	2.167915	2.163175	2.158476	2.153758	2.149044	2.144353
2.176325	2.171496	2.166632	2.167657	2.162918	2.15822	2.153503	2.14879	2.1441
2.176079	2.171251	2.166384	2.167401	2.162661	2.157964	2.153247	2.148536	2.143847
2.175828	2.171002	2.166133	2.167144	2.162404	2.157708	2.152995	2.148287	2.1436
2.175573	2.170748	2.16588	2.166886	2.162148	2.157454	2.152745	2.148043	2.143358
2.175321	2.1705	2.165638	2.166641	2.161898	2.1572	2.152494	2.147796	2.143114
2.175075	2.170261	2.165405	2.166402	2.161646	2.156935	2.152228	2.147539	2.142863

2.17483	2.170029	2.165193	2.166194	2.161427	2.156699	2.151985	2.147293	2.142617
2.174581	2.169808	2.165025	2.166056	2.161288	2.156541	2.1518	2.147072	2.142377
2.174338	2.169593	2.164867	2.165935	2.16117	2.156409	2.151639	2.146873	2.142156
2.179408	2.175252	2.17049	2.165736	2.160978	2.156223	2.151467	2.146716	2.142002
2.1792	2.175021	2.170245	2.165489	2.16074	2.156005	2.15129	2.14659	2.141893
2.17902	2.17484	2.170053	2.165286	2.160526	2.155787	2.151087	2.146412	2.141726
2.178878	2.174721	2.169923	2.165133	2.160337	2.155562	2.150847	2.146172	2.141492
2.17871	2.173909	2.169107	2.164976	2.160162	2.155367	2.150641	2.145959	2.141275
2.178475	2.173684	2.168899	2.164792	2.160001	2.155225	2.150503	2.145814	2.141112
2.178243	2.173457	2.168692	2.164613	2.159852	2.1551	2.150381	2.145679	2.140958
2.178083	2.173299	2.168541	2.164473	2.159719	2.154971	2.150235	2.145509	2.140784
2.177951	2.173163	2.168406	2.164341	2.159589	2.154838	2.150083	2.145331	2.14061
2.177781	2.172977	2.168211	2.163459	2.158715	2.153975	2.149924	2.145178	2.140466
2.177586	2.172753	2.167968	2.163213	2.158488	2.153777	2.14976	2.145044	2.140347
2.177412	2.172573	2.167781	2.163024	2.158306	2.153608	2.149611	2.144914	2.14022
2.177263	2.172448	2.167666	2.162911	2.158185	2.153476	2.149484	2.144792	2.140085
2.17714	2.17234	2.167565	2.16281	2.158074	2.153352	2.148642	2.14394	2.139937
2.177058	2.172236	2.167458	2.1627	2.157959	2.153228	2.148493	2.143761	2.139761
2.176986	2.172139	2.167354	2.162592	2.157841	2.153097	2.148339	2.14358	2.139589
2.176884	2.172044	2.167258	2.162483	2.1577	2.152927	2.14817	2.143432	2.139462
2.176767	2.171952	2.167166	2.162377	2.157553	2.152743	2.148003	2.143307	2.139363
2.176662	2.171861	2.167074	2.162282	2.157458	2.152649	2.147906	2.143207	2.138532
2.176565	2.17177	2.166981	2.162198	2.157416	2.152643	2.14788	2.143132	2.138434
2.176465	2.171674	2.166885	2.162105	2.157337	2.152577	2.147807	2.143044	2.138337
2.176354	2.171569	2.166782	2.161993	2.157187	2.152393	2.147644	2.142928	2.138242
2.176252	2.171463	2.166679	2.161885	2.157052	2.152235	2.1475	2.142816	2.138145
2.176169	2.171346	2.166571	2.161798	2.156999	2.152207	2.147449	2.142717	2.138028
2.176107	2.171244	2.16647	2.161716	2.156968	2.152219	2.147433	2.142638	2.137921
2.176095	2.171217	2.166396	2.161614	2.156887	2.152172	2.147405	2.142621	2.146092
2.176118	2.171242	2.166345	2.161506	2.156782	2.152094	2.147374	2.142645	2.14609
2.176089	2.17123	2.166319	2.161461	2.156711	2.152006	2.147306	2.142616	2.146057
2.176004	2.171182	2.166327	2.161489	2.156677	2.151899	2.147193	2.142531	2.145995
2.175919	2.171111	2.166304	2.161493	2.156652	2.151834	2.155323	2.150622	2.145909
2.175837	2.171	2.166217	2.161439	2.156638	2.151847	2.155282	2.15051	2.145778
2.175776	2.170905	2.166123	2.161365	2.156617	2.151869	2.155262	2.150422	2.145662
2.175771	2.170893	2.166062	2.161274	2.15655	2.151839	2.155236	2.150402	2.145623
2.175789	2.170918	2.166021	2.161186	2.156474	2.151795	2.155217	2.150422	2.145626
2.172299	2.17091	2.166009	2.169408	2.164666	2.159964	2.155216	2.150449	2.145649
2.172235	2.170882	2.166024	2.169436	2.164682	2.159963	2.155229	2.150488	2.145695
2.172203	2.170874	2.166049	2.169462	2.164679	2.159926	2.155197	2.15048	2.145703
2.172216	2.170895	2.166092	2.169489	2.164647	2.159834	2.1551	2.150409	2.145663
2.172217	2.179166	2.174332	2.169494	2.164618	2.159768	2.155026	2.150347	2.145625
2.17217	2.179077	2.174261	2.169447	2.164596	2.159767	2.155026	2.150336	2.145624
2.172127	2.17899	2.174187	2.169398	2.164586	2.159791	2.155043	2.150327	2.145614
2.172144	2.175886	2.1711	2.169399	2.164601	2.159811	2.15503	2.150263	2.145536
2.172197	2.175918	2.171128	2.16943	2.164634	2.159837	2.15501	2.150179	2.14543
2.18086	2.175957	2.171166	2.169471	2.164675	2.159872	2.15502	2.150154	2.145393
2.180911	2.176008	2.171216	2.169526	2.164726	2.159917	2.155056	2.150182	2.145414
2.180959	2.176056	2.171264	2.16958	2.164776	2.159963	2.155098	2.150219	2.145447
2.181004	2.1761	2.171308	2.169629	2.164826	2.160012	2.155145	2.150263	2.14549

2.181044	2.176141	2.171348	2.169675	2.164872	2.160058	2.155192	2.150309	2.145536
2.181076	2.176174	2.171381	2.169715	2.164911	2.160097	2.15523	2.150348	2.145576
2.181104	2.176203	2.171411	2.169751	2.164948	2.160135	2.15527	2.150391	2.145621
2.181132	2.176231	2.171441	2.169789	2.164989	2.16018	2.15532	2.150446	2.145674
2.18116	2.17626	2.17147	2.169826	2.165031	2.160229	2.155379	2.150514	2.145738
2.175679	2.170806	2.166033	2.166725	2.161947	2.15717	2.155457	2.150626	2.145858
2.17574	2.170864	2.166071	2.166745	2.161973	2.157215	2.155557	2.150791	2.146044
2.17583	2.170963	2.166153	2.166804	2.162024	2.157269	2.155646	2.150923	2.146192
2.175975	2.171138	2.166317	2.166932	2.162114	2.157324	2.155697	2.150979	2.146255
2.176112	2.171304	2.166478	2.167066	2.162222	2.157405	2.155768	2.151039	2.146313
2.176174	2.171374	2.166558	2.167154	2.162342	2.157552	2.155921	2.151176	2.145995
2.176217	2.171414	2.166609	2.167225	2.162464	2.15772	2.1561	2.151335	2.146117
2.176335	2.171518	2.166695	2.167294	2.162542	2.157814	2.156203	2.151439	2.146207
2.176506	2.171665	2.166803	2.167359	2.162591	2.157855	2.156255	2.151505	2.146274
2.176659	2.171804	2.166933	2.167472	2.16269	2.157941	2.156339	2.151584	2.146356
2.176783	2.171927	2.167087	2.167643	2.162854	2.158087	2.156464	2.151674	2.146451
2.176918	2.172066	2.167251	2.167818	2.163019	2.158232	2.156597	2.151789	2.146571
2.177079	2.172242	2.167426	2.167982	2.163155	2.158345	2.156725	2.151945	2.146736
2.177249	2.172425	2.167598	2.168137	2.163288	2.158462	2.156864	2.152116	2.146911
2.17742	2.172589	2.167739	2.162906	2.158099	2.15332	2.153853	2.149124	2.147063
2.177589	2.172742	2.167866	2.163024	2.158244	2.153495	2.154028	2.149285	2.147205
2.177734	2.172878	2.168008	2.163173	2.158403	2.153661	2.154181	2.149418	2.147333
2.177854	2.172997	2.16816	2.16335	2.158578	2.153822	2.154317	2.149523	2.147446
2.177995	2.173147	2.168333	2.163534	2.158745	2.153968	2.154451	2.149655	2.14759
2.178165	2.173339	2.168531	2.163723	2.158892	2.154076	2.154572	2.149823	2.147779
2.178349	2.173544	2.168739	2.163922	2.159065	2.154225	2.154729	2.150009	2.147979
2.178546	2.173748	2.168948	2.164142	2.159313	2.154495	2.154974	2.150216	2.148173
2.178751	2.173955	2.169159	2.164366	2.159574	2.154786	2.155237	2.150431	2.14837
2.178953	2.174162	2.169365	2.164566	2.159756	2.154952	2.150167	2.145403	2.145616
2.179155	2.174368	2.169569	2.164754	2.159896	2.155053	2.150292	2.145585	2.14581
2.179365	2.174566	2.16977	2.16496	2.160103	2.155258	2.150492	2.145776	2.145993
2.179584	2.174754	2.16997	2.165185	2.160378	2.15557	2.150765	2.145972	2.146159
2.170717	2.174948	2.170168	2.165401	2.160632	2.155858	2.15104	2.14621	2.146368
2.170883	2.175152	2.170363	2.165591	2.160838	2.156087	2.151311	2.146525	2.146651
2.171056	2.175359	2.170564	2.165787	2.161035	2.156296	2.151563	2.146832	2.146944
2.171264	2.17555	2.170771	2.166005	2.161237	2.156482	2.151961	2.147245	2.147203
2.171499	2.175754	2.170995	2.166242	2.161451	2.156671	2.152145	2.147445	2.147444
2.171779	2.176043	2.171279	2.16652	2.161729	2.156944	2.152383	2.147645	2.142933
2.172095	2.176398	2.171609	2.16683	2.162056	2.15728	2.152657	2.147841	2.143104
2.172369	2.176702	2.171891	2.167308	2.162528	2.157749	2.152925	2.14809	2.143332
2.172587	2.176941	2.172108	2.167507	2.162733	2.157972	2.153191	2.148404	2.14363
2.172825	2.177193	2.172352	2.167735	2.162948	2.158184	2.153439	2.148705	2.143929
2.173124	2.1775	2.172673	2.168041	2.163199	2.158382	2.153645	2.148951	2.144203
2.173428	2.177801	2.172998	2.16836	2.163468	2.158601	2.153863	2.149195	2.144477
2.173681	2.168906	2.164192	2.168632	2.163753	2.158895	2.154157	2.149486	2.14478
2.173916	2.169114	2.164409	2.168887	2.164052	2.159232	2.154489	2.149793	2.145088
2.17419	2.169384	2.164677	2.16917	2.164363	2.159563	2.154796	2.150054	2.145339
2.174495	2.169707	2.164986	2.169477	2.164686	2.159895	2.155086	2.150277	2.145541
2.174811	2.170026	2.165275	2.169746	2.164968	2.160193	2.155372	2.15054	2.145798
2.175136	2.17033	2.165524	2.169954	2.165184	2.160436	2.155649	2.150857	2.146131

2.17219	2.170644	2.165792	2.161752	2.157042	2.152372	2.155941	2.151192	2.146483
2.17253	2.17098	2.166116	2.162061	2.157345	2.152675	2.156264	2.151533	2.146831
2.172876	2.171326	2.166463	2.162401	2.157679	2.153003	2.156603	2.151881	2.147184
2.173219	2.17244	2.167562	2.162737	2.158012	2.153335	2.156949	2.152232	2.147536
2.173561	2.172781	2.167904	2.16308	2.158354	2.153676	2.157302	2.152585	2.147888
2.173903	2.173123	2.168246	2.163422	2.158696	2.154017	2.157654	2.152938	2.14824
2.174246	2.173466	2.168589	2.163765	2.159038	2.154359	2.158006	2.15329	2.148591
2.17459	2.170808	2.16595	2.164109	2.159382	2.154703	2.158359	2.15364	2.154452
2.174935	2.171149	2.16629	2.164453	2.159728	2.155051	2.158712	2.15399	2.154797
2.175284	2.171494	2.166638	2.164805	2.160081	2.155402	2.159066	2.15434	2.155142
2.175639	2.171847	2.166998	2.165169	2.160434	2.155744	2.159412	2.154686	2.155486
2.175996	2.172208	2.167372	2.165552	2.160805	2.156099	2.159767	2.155039	2.155834
2.176349	2.17258	2.167783	2.165993	2.161244	2.156522	2.151817	2.147134	2.15619
2.176699	2.172961	2.168221	2.166477	2.161732	2.156994	2.152256	2.147527	2.156555
2.177074	2.173339	2.168624	2.163914	2.159193	2.154472	2.158671	2.153912	2.156974
2.177475	2.173707	2.168978	2.164266	2.159557	2.154858	2.159088	2.154368	2.157462
2.178911	2.174107	2.169362	2.164641	2.159931	2.155239	2.159489	2.154797	2.157476
2.179366	2.174579	2.169827	2.165086	2.160343	2.155617	2.159849	2.155153	2.157846
2.179812	2.175047	2.170292	2.165537	2.160766	2.15601	2.160221	2.155511	2.158208
2.180182	2.175424	2.170682	2.165943	2.161195	2.156458	2.160672	2.15595	2.15863
2.180523	2.175764	2.171038	2.166326	2.161623	2.156927	2.161152	2.156422	2.159079
2.180929	2.176157	2.171414	2.172943	2.168226	2.163523	2.158821	2.154122	2.151941
2.181384	2.176588	2.171803	2.173296	2.168562	2.163857	2.159175	2.15451	2.152363
2.179594	2.177017	2.172215	2.173696	2.168958	2.164255	2.159579	2.154927	2.1528
2.180015	2.177433	2.172653	2.174154	2.169433	2.16474	2.160056	2.155385	2.153258
2.180444	2.177867	2.173113	2.174636	2.169928	2.165238	2.160542	2.155849	2.153715
2.180894	2.178335	2.173601	2.175136	2.170422	2.16572	2.161015	2.156312	2.154164
2.18136	2.178823	2.1741	2.175641	2.170915	2.166196	2.16148	2.156767	2.154606
2.181847	2.177088	2.172365	2.176124	2.17139	2.16666	2.161926	2.157191	2.152421
2.18235	2.177592	2.172852	2.176592	2.171851	2.167114	2.162357	2.157597	2.152836
2.182843	2.178079	2.173325	2.175033	2.170278	2.16553	2.162782	2.158026	2.153282
2.183329	2.17855	2.173784	2.175482	2.1707	2.165932	2.163197	2.158474	2.153759
2.190342	2.185523	2.180711	2.175911	2.171121	2.166353	2.161724	2.157025	2.154244
2.190839	2.185998	2.181127	2.176294	2.171528	2.166802	2.1622	2.157516	2.154737
2.19134	2.186486	2.181577	2.17672	2.171971	2.167272	2.162687	2.15801	2.155231
2.191842	2.187002	2.182127	2.177283	2.172499	2.167756	2.163161	2.158489	2.153827
2.192346	2.187531	2.182713	2.177897	2.17307	2.168267	2.163649	2.158969	2.154308
2.192852	2.18805	2.183252	2.178454	2.173643	2.168846	2.164199	2.159469	2.154786
2.193359	2.188565	2.183766	2.178981	2.174226	2.169474	2.164794	2.159987	2.155266
2.193858	2.189068	2.184274	2.179501	2.174774	2.170048	2.165371	2.160547	2.155803
2.194348	2.189558	2.184776	2.180014	2.175285	2.170563	2.165933	2.161162	2.156407
2.194844	2.19005	2.185274	2.180518	2.175785	2.171066	2.166474	2.161748	2.156997
2.19534	2.190536	2.18576	2.181006	2.176278	2.171563	2.166981	2.162267	2.157543
2.195865	2.191049	2.186259	2.181497	2.17677	2.172061	2.16748	2.162767	2.158072
2.196473	2.19164	2.186799	2.181997	2.177266	2.172563	2.167983	2.163268	2.158581
2.197114	2.192265	2.187365	2.182516	2.177776	2.173076	2.168498	2.163777	2.159089
2.197682	2.19284	2.187951	2.183269	2.178512	2.173794	2.16907	2.164359	2.15967
2.198195	2.19338	2.188557	2.183925	2.179154	2.174409	2.169693	2.165001	2.160309
2.198719	2.193908	2.18913	2.184534	2.179754	2.174992	2.170279	2.165597	2.160903
2.199254	2.194412	2.189647	2.18507	2.180289	2.175522	2.170806	2.166121	2.161424

2.199818	2.194956	2.190195	2.185626	2.180846	2.176079	2.171359	2.166668	2.161967
2.200438	2.195589	2.190825	2.186255	2.181474	2.176707	2.171985	2.167292	2.162585
2.201079	2.196254	2.191481	2.186904	2.182124	2.17736	2.172636	2.167937	2.163225
2.201721	2.196905	2.19212	2.187537	2.182764	2.178007	2.173277	2.168567	2.163851
2.202361	2.197548	2.192749	2.18816	2.183397	2.178649	2.173912	2.169186	2.164467
2.196408	2.191625	2.186847	2.188732	2.183972	2.179228	2.174485	2.16975	2.165031
2.196929	2.192148	2.187368	2.189252	2.184489	2.179742	2.174995	2.170256	2.165541
2.197494	2.192712	2.187932	2.189817	2.18505	2.180299	2.175549	2.170808	2.166096
2.19813	2.193343	2.188566	2.190456	2.185683	2.180925	2.176174	2.171436	2.166724
2.198787	2.193996	2.189221	2.191115	2.186337	2.181574	2.176823	2.172086	2.167372
2.199446	2.194652	2.189876	2.191772	2.186994	2.18223	2.177477	2.172738	2.168022
2.200112	2.195316	2.190538	2.192435	2.187658	2.182895	2.17814	2.173397	2.168677
2.200777	2.19598	2.191199	2.193097	2.188319	2.183555	2.178797	2.17405	2.169326
2.201443	2.196645	2.19186	2.193759	2.188979	2.184213	2.179453	2.174702	2.169974
2.202105	2.197307	2.19252	2.194419	2.189639	2.184872	2.180108	2.175353	2.170622
2.202764	2.197966	2.193175	2.195075	2.190295	2.185529	2.180761	2.176001	2.171267
2.203428	2.198629	2.19384	2.195744	2.190961	2.186192	2.181423	2.176663	2.171927
2.204092	2.19929	2.194512	2.196424	2.191632	2.186852	2.182088	2.177337	2.1726
2.204781	2.199975	2.195208	2.19713	2.192328	2.187538	2.182778	2.178038	2.173299
2.205539	2.200731	2.195968	2.191615	2.186835	2.182065	2.183536	2.178798	2.174058
2.206323	2.201512	2.196748	2.192399	2.187617	2.182846	2.184318	2.179578	2.174838
2.207036	2.202224	2.197455	2.193108	2.188327	2.183558	2.18503	2.180286	2.175545
2.207698	2.202886	2.198109	2.19376	2.188984	2.18422	2.185689	2.18094	2.176198
2.208366	2.203554	2.198764	2.19441	2.189642	2.184887	2.186352	2.181595	2.176854
2.209037	2.204224	2.199416	2.195054	2.190296	2.185554	2.187014	2.182244	2.177504
2.210188	2.205367	2.200535	2.195734	2.190986	2.186257	2.187715	2.182939	2.178201
2.210962	2.206141	2.2013	2.196494	2.191755	2.187037	2.188502	2.183731	2.17899
2.211743	2.206921	2.202078	2.197272	2.192536	2.187826	2.189304	2.184551	2.17981
2.212447	2.207622	2.202792	2.197995	2.193249	2.188533	2.190041	2.185338	2.180613
2.213121	2.208292	2.203484	2.1987	2.193936	2.189206	2.19075	2.186107	2.181404
2.21387	2.209037	2.204234	2.199452	2.194678	2.189937	2.191475	2.186825	2.182121
2.214686	2.209848	2.205036	2.200244	2.195469	2.190716	2.19221	2.18749	2.182763
2.215493	2.210662	2.205838	2.201036	2.196264	2.191509	2.192973	2.188206	2.183461
2.216285	2.211483	2.206644	2.201829	2.197069	2.192326	2.193785	2.189006	2.184246
2.217053	2.212268	2.207422	2.202602	2.197847	2.193115	2.194584	2.189818	2.185054
2.217277	2.212482	2.20766	2.203318	2.198552	2.193813	2.189105	2.184418	2.185868
2.217977	2.21315	2.208348	2.204024	2.199241	2.194493	2.189824	2.185199	2.186677
2.218753	2.213911	2.209105	2.204785	2.200001	2.19525	2.190573	2.18594	2.187426
2.219577	2.214728	2.209906	2.205581	2.200804	2.196055	2.191342	2.186658	2.188142
2.213847	2.215543	2.210715	2.20639	2.201615	2.196868	2.19215	2.187457	2.199583
2.214692	2.216357	2.211534	2.207214	2.202437	2.197694	2.193004	2.188348	2.20047
2.215541	2.210669	2.205833	2.208036	2.203258	2.198515	2.193837	2.189198	2.201322
2.216395	2.21152	2.206675	2.208847	2.204066	2.199318	2.194615	2.189943	2.202082
2.217248	2.21237	2.207518	2.20966	2.20488	2.200129	2.195401	2.190695	2.202848
2.218091	2.213212	2.208362	2.209996	2.205232	2.200497	2.196247	2.191546	2.203698
2.218932	2.214054	2.20921	2.210827	2.206073	2.201349	2.19712	2.192435	2.204579
2.219778	2.214905	2.21007	2.205272	2.20052	2.195795	2.197993	2.19329	2.205429
2.220629	2.215766	2.210939	2.206153	2.201426	2.196717	2.198868	2.194126	2.206261
2.22149	2.216639	2.211827	2.207051	2.202327	2.197615	2.193523	2.188746	2.201578
2.222358	2.217527	2.21274	2.207973	2.203226	2.198483	2.194381	2.189601	2.202406

2.223249	2.218424	2.213646	2.208881	2.204112	2.199347	2.194584	2.189821	2.203262
2.224184	2.219334	2.214533	2.209751	2.204974	2.200211	2.19547	2.19074	2.204181
2.225124	2.220243	2.215412	2.21061	2.205833	2.201082	2.196363	2.191664	2.205106
2.226015	2.221121	2.216277	2.211472	2.206712	2.20198	2.210043	2.205224	2.200582
2.226891	2.221993	2.217142	2.212339	2.207602	2.20289	2.210948	2.206106	2.201455
2.227835	2.222934	2.21807	2.213251	2.208498	2.203771	2.21183	2.206982	2.202338
2.22884	2.223939	2.219054	2.214204	2.2094	2.204627	2.212689	2.207846	2.203225
2.229801	2.224908	2.220025	2.215166	2.210334	2.205531	2.213592	2.208744	2.204127
2.230687	2.225814	2.220973	2.216146	2.211321	2.206512	2.214562	2.209687	2.20504
2.231579	2.226723	2.221908	2.217102	2.212289	2.207485	2.215536	2.210648	2.205978
2.232522	2.227669	2.222818	2.217986	2.213175	2.208387	2.216477	2.211623	2.206962
2.233484	2.228629	2.223729	2.218859	2.214048	2.209276	2.217412	2.212603	2.20796
2.234452	2.2296	2.224693	2.219814	2.214991	2.21021	2.218359	2.213568	2.208937
2.235423	2.230576	2.225686	2.220813	2.215974	2.211172	2.219313	2.214521	2.2099
2.230018	2.231497	2.226622	2.221765	2.216935	2.212134	2.220261	2.215438	2.210811
2.230891	2.232357	2.227497	2.222666	2.217874	2.213098	2.221202	2.216314	2.211663
2.2318	2.226898	2.222052	2.223592	2.218829	2.214074	2.222164	2.217225	2.212554
2.232776	2.227863	2.223014	2.224565	2.219813	2.215067	2.223159	2.218199	2.213517
2.233773	2.228853	2.223996	2.225555	2.220808	2.216066	2.224166	2.219196	2.214507
2.234766	2.229841	2.224982	2.234611	2.229768	2.224931	2.225173	2.220196	2.215503
2.235763	2.230836	2.225974	2.235616	2.230773	2.225935	2.226182	2.221202	2.216508
2.236761	2.231832	2.226966	2.236619	2.231773	2.226933	2.221991	2.217002	2.217507
2.23776	2.232829	2.227958	2.237621	2.232771	2.227928	2.222981	2.217989	2.218502
2.233074	2.233825	2.22895	2.238622	2.23377	2.228925	2.223979	2.218987	2.214045
2.234072	2.234818	2.22994	2.239622	2.234771	2.229926	2.224982	2.219992	2.215044
2.235082	2.235822	2.230934	2.240621	2.23577	2.23093	2.225999	2.221025	2.216075
2.236107	2.236835	2.231926	2.241608	2.236762	2.231938	2.227043	2.222113	2.217172
2.237153	2.237874	2.23294	2.24261	2.23776	2.232944	2.228083	2.2232	2.218273
2.232756	2.233337	2.228401	2.243664	2.23878	2.233938	2.229084	2.224227	2.219313
2.23387	2.234476	2.229517	2.244747	2.239812	2.234925	2.230064	2.225223	2.220324
2.234926	2.235554	2.230593	2.245816	2.240838	2.23591	2.231045	2.226221	2.221348
2.235934	2.236582	2.231636	2.246874	2.241857	2.236889	2.232021	2.227217	2.222381
2.236947	2.237611	2.232703	2.243135	2.238117	2.233137	2.233036	2.228215	2.223391
2.237976	2.238651	2.233812	2.244299	2.239273	2.234259	2.234095	2.229205	2.224361
2.235711	2.255237	2.250353	2.24544	2.24043	2.235408	2.235194	2.230234	2.225355
2.236719	2.256235	2.251379	2.246506	2.241558	2.236586	2.236354	2.231343	2.22641
2.237742	2.25724	2.252383	2.247534	2.242656	2.237753	2.237526	2.232488	2.227504
2.238847	2.255922	2.251006	2.24853	2.243678	2.23882	2.233888	2.228917	2.228646
2.240007	2.257048	2.252048	2.249504	2.244652	2.239824	2.234959	2.230072	2.229821
2.231607	2.258209	2.253155	2.248168	2.24328	2.238435	2.23599	2.231147	2.230927
2.232751	2.259412	2.254332	2.249301	2.24435	2.239446	2.23697	2.232134	2.231963
2.233861	2.260567	2.255496	2.250457	2.245474	2.240532	2.23802	2.233167	2.228316
2.234901	2.261628	2.256626	2.251649	2.246692	2.241752	2.239198	2.234287	2.229397
2.235958	2.262692	2.257743	2.252817	2.24789	2.242964	2.240382	2.235429	2.230505
2.237105	2.258841	2.25388	2.253879	2.248953	2.244044	2.239125	2.234207	2.23167
2.23829	2.260002	2.254967	2.254921	2.249981	2.245083	2.240202	2.235343	2.232842
2.23946	2.252611	2.247604	2.256094	2.251119	2.246185	2.241282	2.23641	2.231555
2.240627	2.253842	2.248857	2.257356	2.25233	2.247327	2.242361	2.23743	2.232553
2.241801	2.255034	2.250047	2.258554	2.253509	2.248483	2.243493	2.238538	2.23364
2.242984	2.256178	2.251158	2.259668	2.254647	2.249656	2.244691	2.239753	2.234837

2.256875	2.257337	2.252299	2.260818	2.255806	2.25083	2.245877	2.240948	2.236023
2.258051	2.258544	2.253532	2.262069	2.257018	2.251991	2.247007	2.242058	2.23714
2.259224	2.259748	2.254764	2.263324	2.258237	2.253164	2.248147	2.243176	2.238268
2.260387	2.260896	2.255904	2.250917	2.24592	2.24094	2.249362	2.244386	2.239482
2.261551	2.262026	2.257012	2.252029	2.247081	2.242158	2.250614	2.245639	2.240731
2.262737	2.263215	2.258196	2.253217	2.248291	2.243388	2.251855	2.246861	2.241928
2.263942	2.26445	2.259441	2.254466	2.249543	2.244633	2.253092	2.248061	2.243085
2.265177	2.265696	2.260676	2.255685	2.250745	2.245819	2.25429	2.24926	2.244271
2.26646	2.266961	2.261893	2.256854	2.251865	2.246909	2.255424	2.250456	2.245497
2.267714	2.268198	2.263105	2.258039	2.253014	2.248035	2.243129	2.238275	2.24673
2.268868	2.269352	2.264303	2.259274	2.254256	2.249272	2.244364	2.239505	2.247963
2.270015	2.270503	2.265512	2.260533	2.255534	2.250554	2.245631	2.240743	2.249193
2.266835	2.271757	2.266774	2.261804	2.256819	2.251844	2.246891	2.241956	2.250401
2.268134	2.273069	2.268067	2.263083	2.258108	2.253138	2.248148	2.243155	2.251599
2.275437	2.274376	2.269332	2.264311	2.259318	2.254337	2.24934	2.244348	2.252819
2.276761	2.275687	2.270575	2.265489	2.260445	2.255436	2.250463	2.245528	2.254058
2.278019	2.276938	2.271798	2.266687	2.261621	2.256601	2.251648	2.246751	2.247069
2.27916	2.278079	2.272985	2.267919	2.26288	2.25788	2.252935	2.248037	2.248365
2.280312	2.279238	2.274193	2.269177	2.264174	2.259198	2.254257	2.249348	2.249674
2.281565	2.280508	2.275471	2.270466	2.265485	2.260529	2.255591	2.250674	2.250988
2.282866	2.281831	2.276788	2.271779	2.266813	2.261871	2.256933	2.252005	2.252299
2.284186	2.283171	2.278125	2.273112	2.268144	2.263198	2.25825	2.253308	2.24837
2.28553	2.284534	2.279486	2.274464	2.26948	2.264514	2.25955	2.25459	2.249618
2.286872	2.281845	2.276827	2.275808	2.270804	2.265812	2.260821	2.255832	2.250837
2.288212	2.283178	2.278165	2.277149	2.27212	2.267094	2.262062	2.25703	2.252019
2.289553	2.284503	2.279484	2.278464	2.273405	2.268344	2.263282	2.258226	2.253212
2.290896	2.285818	2.280781	2.279744	2.274642	2.269544	2.264472	2.259424	2.25443
2.292237	2.287125	2.282055	2.280994	2.275856	2.270732	2.265665	2.260641	2.255673
2.293571	2.288417	2.283289	2.282191	2.277049	2.271938	2.266894	2.261902	2.256957
2.294905	2.289705	2.28451	2.283371	2.278238	2.273159	2.268147	2.263193	2.258271
2.296254	2.291025	2.285792	2.28463	2.279491	2.27442	2.269429	2.264504	2.259596
2.297616	2.29237	2.287122	2.285951	2.280797	2.275716	2.270737	2.265836	2.260935
2.298993	2.293736	2.288479	2.287302	2.282129	2.277032	2.272052	2.267159	2.262262
2.30039	2.295126	2.289862	2.288677	2.28348	2.278359	2.273366	2.268467	2.263568
2.30179	2.296529	2.291273	2.290094	2.284879	2.279733	2.274714	2.269788	2.264877
2.303192	2.29795	2.292732	2.291585	2.28636	2.281183	2.27611	2.271117	2.266172
2.304588	2.29937	2.294192	2.293085	2.287865	2.282673	2.277552	2.272491	2.267507
2.303907	2.300754	2.295582	2.294504	2.289335	2.284191	2.279078	2.273999	2.268982
2.305264	2.302117	2.296933	2.295877	2.290784	2.285721	2.280649	2.275582	2.270539
2.306622	2.301419	2.296231	2.293157	2.288124	2.28313	2.282107	2.277064	2.272027
2.307979	2.302778	2.297582	2.294505	2.289458	2.284457	2.283463	2.278459	2.273463
2.309329	2.304129	2.298935	2.295859	2.290793	2.285778	2.284798	2.279818	2.274848
2.310668	2.305471	2.300291	2.297225	2.292142	2.287105	2.286122	2.281138	2.276168
2.312007	2.30681	2.301644	2.298588	2.293488	2.28843	2.287438	2.282445	2.277469
2.309108	2.308146	2.302979	2.297845	2.292742	2.287678	2.288751	2.28375	2.278769
2.310455	2.30949	2.304317	2.299179	2.294068	2.288996	2.290072	2.285064	2.280077
2.306864	2.310859	2.305686	2.300545	2.295426	2.290344	2.289682	2.284674	2.281418
2.30827	2.312254	2.307083	2.301943	2.296815	2.291724	2.291063	2.286051	2.282791
2.309787	2.309051	2.303878	2.303448	2.298315	2.293217	2.288183	2.283197	2.284273
2.311438	2.31069	2.305524	2.305086	2.299951	2.294847	2.289802	2.2848	2.285887

2.297665	2.312278	2.307109	2.306654	2.30152	2.296414	2.291357	2.286339	2.285676
2.299043	2.313742	2.308553	2.308068	2.302936	2.297834	2.292765	2.28773	2.287068
2.300384	2.315174	2.309958	2.30944	2.30431	2.299214	2.294135	2.289083	2.288422
2.301714	2.316606	2.311365	2.306174	2.301041	2.295945	2.295512	2.290448	2.289786
2.303054	2.318053	2.312786	2.307576	2.302444	2.297352	2.296903	2.291831	2.291169
2.304526	2.315116	2.309858	2.309109	2.30397	2.298875	2.298411	2.293338	2.288313
2.306104	2.316788	2.311512	2.31075	2.305598	2.300495	2.300016	2.294948	2.289917
2.307578	2.302622	2.297696	2.312313	2.307155	2.302046	2.296949	2.291877	2.291453
2.308918	2.303967	2.299048	2.313773	2.308613	2.303502	2.298403	2.293327	2.292886
2.304556	2.305289	2.300376	2.315215	2.310063	2.30496	2.29987	2.294802	2.289753
2.305859	2.306597	2.301685	2.316642	2.311522	2.30646	2.301411	2.296383	2.291348
2.307196	2.307937	2.303024	2.318101	2.313001	2.307971	2.302958	2.297968	2.292947
2.308683	2.309424	2.304508	2.319689	2.314546	2.309471	2.304438	2.299442	2.294415
2.310247	2.310987	2.306066	2.321346	2.316129	2.310972	2.305892	2.300868	2.295825
2.311697	2.312437	2.307513	2.307544	2.30266	2.297813	2.307414	2.302378	2.297328
2.313044	2.313786	2.30886	2.308919	2.304081	2.299284	2.308997	2.303963	2.298917
2.314466	2.309542	2.304638	2.310358	2.305529	2.300741	2.310545	2.305494	2.300441
2.316027	2.311103	2.306196	2.311902	2.307013	2.30216	2.312016	2.306917	2.301843
2.317567	2.312651	2.307753	2.313457	2.308521	2.30361	2.313519	2.308369	2.303272
2.318965	2.314073	2.309226	2.310026	2.305156	2.300295	2.31515	2.309957	2.304843
2.320341	2.315473	2.310674	2.31152	2.306688	2.30185	2.316805	2.311575	2.306449
2.321847	2.316961	2.312133	2.312957	2.30811	2.303263	2.303246	2.298338	2.307956
2.323434	2.318505	2.3136	2.31436	2.309469	2.304594	2.304579	2.299677	2.309413
2.325021	2.320083	2.315142	2.310217	2.305316	2.300436	2.306025	2.301117	2.310945
2.326619	2.321709	2.316772	2.311839	2.306915	2.302007	2.307583	2.302659	2.312549
2.328147	2.323261	2.318349	2.313432	2.308511	2.303598	2.309177	2.30424	2.314206
2.329533	2.324667	2.319815	2.314959	2.310089	2.305215	2.31082	2.305878	2.315939
2.330928	2.326066	2.321256	2.316447	2.311622	2.306787	2.312428	2.307495	2.317675
2.332459	2.327563	2.322712	2.317873	2.313031	2.308197	2.313886	2.309011	2.304134
2.334057	2.329112	2.324188	2.319286	2.3144	2.309548	2.315281	2.310471	2.305653
2.33568	2.330713	2.325751	2.320812	2.315891	2.311009	2.311402	2.306602	2.307111
2.337334	2.332362	2.327387	2.322424	2.317471	2.312547	2.31288	2.308016	2.308516
2.336372	2.333963	2.329005	2.324044	2.319073	2.314118	2.314413	2.309498	2.309969
2.337862	2.335511	2.330608	2.32568	2.320706	2.31573	2.316012	2.311063	2.311479
2.339323	2.337012	2.332164	2.327278	2.322321	2.317346	2.312396	2.307465	2.307737
2.340753	2.338435	2.333637	2.328805	2.323899	2.318962	2.314012	2.309058	2.309313
2.342198	2.339852	2.33508	2.330292	2.325444	2.32056	2.315625	2.31066	2.310913
2.343733	2.341347	2.336537	2.331738	2.326929	2.322099	2.317199	2.312259	2.312527
2.345325	2.342888	2.338005	2.333166	2.328386	2.323609	2.318754	2.31386	2.314155
2.346931	2.341973	2.337063	2.334683	2.329914	2.325161	2.320332	2.315464	2.315775
2.348559	2.343591	2.338671	2.33628	2.331504	2.326748	2.321928	2.317073	2.31739
2.350191	2.34521	2.340285	2.337885	2.333106	2.328349	2.323531	2.318681	2.319003
2.351825	2.346824	2.341895	2.339492	2.334719	2.329967	2.325146	2.320287	2.320609
2.353461	2.34844	2.343507	2.341098	2.336331	2.331584	2.32676	2.321893	2.316973
2.355095	2.350057	2.345115	2.342699	2.337932	2.333189	2.328367	2.323501	2.318578
2.356732	2.351676	2.346724	2.344297	2.339529	2.334786	2.329967	2.325105	2.320184
2.358374	2.353303	2.348339	2.343458	2.338692	2.333955	2.331555	2.3267	2.321783
2.360022	2.354935	2.349959	2.345066	2.340285	2.335536	2.333134	2.328288	2.323378
2.357012	2.356578	2.351583	2.346672	2.341878	2.337121	2.334714	2.329875	2.324971
2.358657	2.358232	2.353211	2.348277	2.343472	2.338709	2.336295	2.331461	2.326562

2.360299	2.359882	2.354838	2.349884	2.34507	2.340302	2.335492	2.330664	2.328152
2.361936	2.361521	2.356458	2.35149	2.346671	2.341902	2.337087	2.332252	2.329738
2.363573	2.363164	2.358087	2.353106	2.348279	2.343505	2.338683	2.333845	2.331329
2.331887	2.364821	2.359731	2.354734	2.349886	2.345095	2.340273	2.335445	2.332931
2.333519	2.366488	2.361397	2.356389	2.351512	2.346693	2.34187	2.337054	2.334542
2.302904	2.363439	2.358414	2.35813	2.35322	2.348354	2.343505	2.338675	2.333822
2.304519	2.365116	2.360169	2.359939	2.35499	2.350063	2.345171	2.340307	2.33547
2.306141	2.312587	2.307809	2.361678	2.356707	2.351745	2.346824	2.341937	2.337103
2.307764	2.314222	2.309434	2.363326	2.358353	2.35339	2.348459	2.343557	2.338712
2.309421	2.315897	2.311088	2.338355	2.333405	2.328471	2.35011	2.345205	2.34035
2.311155	2.31767	2.312829	2.340078	2.335094	2.330134	2.351789	2.346903	2.342046
2.312886	2.319442	2.314573	2.341812	2.336801	2.331821	2.353489	2.34862	2.343759
2.314523	2.321107	2.316242	2.311374	2.306495	2.301639	2.355227	2.350337	2.345456
2.316118	2.322721	2.317873	2.313034	2.308208	2.303396	2.356979	2.352051	2.347147
2.317756	2.32436	2.3195	2.31466	2.309856	2.305066	2.332097	2.327187	2.348822
2.319428	2.326016	2.321118	2.316253	2.311445	2.306661	2.33372	2.328823	2.350484
2.321099	2.327683	2.322772	2.317895	2.313074	2.30828	2.310137	2.305312	2.325612
2.322761	2.329361	2.324474	2.319609	2.314767	2.309939	2.311779	2.306924	2.32721
2.324439	2.319591	2.314792	2.321332	2.316466	2.311606	2.313441	2.308569	2.328845
2.326151	2.321309	2.316494	2.323039	2.318142	2.313261	2.31513	2.310292	2.330572
2.327876	2.323035	2.318194	2.324736	2.319818	2.314927	2.316842	2.312047	2.332339
2.329593	2.324723	2.319854	2.326405	2.321507	2.316642	2.311838	2.307078	2.308999
2.331308	2.32639	2.321493	2.328061	2.323205	2.318389	2.313584	2.3088	2.310743
2.333008	2.32807	2.323177	2.329775	2.324926	2.320115	2.315316	2.310537	2.312493
2.334692	2.329757	2.324905	2.331551	2.326668	2.321819	2.317033	2.31229	2.31425
2.336407	2.331478	2.32664	2.333319	2.328423	2.323557	2.318769	2.314029	2.315994
2.338174	2.333249	2.32837	2.335051	2.330191	2.32536	2.320541	2.31574	2.317714
2.339956	2.335034	2.330117	2.336799	2.331964	2.327158	2.322308	2.317453	2.319436
2.341729	2.336804	2.331904	2.338609	2.333733	2.328885	2.324034	2.319195	2.314402
2.343504	2.338575	2.333704	2.340437	2.335503	2.330594	2.325749	2.320946	2.316144
2.345294	2.340364	2.335473	2.342211	2.337276	2.332366	2.327504	2.322678	2.317866
2.347093	2.342165	2.337222	2.343951	2.339051	2.334178	2.329286	2.324399	2.319575
2.348882	2.34394	2.338983	2.345721	2.340818	2.335942	2.331042	2.326146	2.321314
2.350653	2.345678	2.340758	2.347532	2.342573	2.337642	2.332766	2.327928	2.323091
2.352444	2.34744	2.342533	2.337641	2.332713	2.327815	2.334501	2.329693	2.324849
2.35428	2.349262	2.344304	2.339378	2.33447	2.329595	2.336271	2.331408	2.326552
2.356121	2.351088	2.346075	2.341115	2.336231	2.331383	2.338048	2.333125	2.328258
2.344666	2.352853	2.347849	2.342892	2.337986	2.333115	2.339806	2.3349	2.330029
2.346444	2.354607	2.349629	2.344688	2.339742	2.334831	2.341562	2.336699	2.331827
2.329722	2.356453	2.351434	2.346461	2.341507	2.336594	2.343332	2.338451	2.333578
2.33157	2.358378	2.353259	2.348215	2.343279	2.338395	2.345114	2.340166	2.335292
2.32592	2.340087	2.334915	2.35006	2.345122	2.340243	2.346956	2.341966	2.337083
2.327891	2.342118	2.336927	2.352031	2.347063	2.342156	2.348883	2.343888	2.338983
2.329846	2.344133	2.33894	2.347536	2.342494	2.337509	2.339115	2.3342	2.340892
2.331735	2.346075	2.340893	2.349499	2.344406	2.339371	2.340945	2.336058	2.342757
2.333607	2.348005	2.342842	2.351465	2.346321	2.341233	2.342775	2.337915	2.34462
2.335499	2.349991	2.344846	2.339721	2.334582	2.329492	2.338194	2.333283	2.340397
2.337402	2.352004	2.346876	2.341754	2.336601	2.331486	2.340169	2.335223	2.342346
2.339323	2.353998	2.348863	2.343734	2.33858	2.333458	2.342119	2.337124	2.344256
2.341267	2.355978	2.35081	2.345662	2.340523	2.335412	2.344045	2.338983	2.346124

2.343185	2.338154	2.333167	2.347593	2.34247	2.337373	2.345998	2.340896	2.348047
2.345039	2.339981	2.335012	2.349533	2.344431	2.339352	2.348005	2.342903	2.350064
2.346929	2.341845	2.336884	2.351488	2.34639	2.341318	2.350004	2.344919	2.352097
2.348936	2.343827	2.338823	2.353461	2.348324	2.343224	2.338167	2.333151	2.341877
2.351002	2.34587	2.340805	2.355461	2.350267	2.345127	2.340073	2.335088	2.343856
2.353079	2.347927	2.342823	2.357519	2.352293	2.347127	2.342055	2.337059	2.345847
2.355173	2.350004	2.344876	2.359623	2.35438	2.349196	2.34409	2.339055	2.347846
2.34674	2.341585	2.336468	2.361725	2.356468	2.351265	2.346134	2.341069	2.349843
2.348678	2.343556	2.338471	2.363831	2.358565	2.35334	2.348193	2.343106	2.351837
2.350637	2.345522	2.340444	2.356129	2.350864	2.345638	2.350231	2.345134	2.340045
2.352648	2.347487	2.342354	2.358081	2.352816	2.347605	2.35222	2.347135	2.342033
2.35469	2.349472	2.344276	2.360041	2.354773	2.349576	2.35421	2.349136	2.344029
2.33632	2.351516	2.346287	2.341122	2.336042	2.331029	2.346474	2.341398	2.34604
2.338378	2.353596	2.348346	2.343159	2.338052	2.333012	2.348522	2.343425	2.348064
2.320923	2.35568	2.350414	2.345207	2.340081	2.335022	2.350592	2.345468	2.340319
2.322905	2.357774	2.352497	2.347274	2.342136	2.33706	2.352687	2.347526	2.342352
2.324891	2.359861	2.354568	2.349327	2.344178	2.339089	2.354779	2.349589	2.344389
2.32688	2.361936	2.356619	2.351356	2.346198	2.341102	2.356863	2.35165	2.346422
2.240102	2.34339	2.338101	2.353397	2.348229	2.343125	2.358961	2.353735	2.348481
2.241743	2.345447	2.34014	2.355464	2.350285	2.345171	2.36109	2.355862	2.350584
2.243395	2.347515	2.342194	2.357549	2.352355	2.347229	2.363234	2.35801	2.352718
2.245059	2.34959	2.344258	2.359645	2.354427	2.349283	2.344218	2.33921	2.354887
2.246736	2.35168	2.346343	2.361763	2.356514	2.351344	2.346279	2.341287	2.357082
2.226404	2.333991	2.328765	2.343241	2.338005	2.332839	2.348367	2.343363	2.35925
2.228091	2.336148	2.330963	2.345503	2.340209	2.334968	2.350483	2.34544	2.361395
2.229778	2.338302	2.333121	2.347704	2.342373	2.337085	2.352588	2.347496	2.363517
2.231457	2.340436	2.335196	2.349789	2.344452	2.339159	2.354658	2.349511	2.365597
2.233144	2.342586	2.337274	2.351872	2.346543	2.341263	2.356757	2.351549	2.367691
2.234851	2.344776	2.339404	2.354019	2.348718	2.343465	2.3382	2.332965	2.348507
2.236566	2.346986	2.341558	2.356193	2.350918	2.345692	2.340387	2.335077	2.350598
2.220674	2.233983	2.229793	2.338375	2.333167	2.328011	2.342507	2.33717	2.352698
2.222365	2.235668	2.231465	2.340529	2.335263	2.330051	2.344592	2.339264	2.354818
2.208224	2.219827	2.215622	2.342703	2.337378	2.332122	2.346725	2.341431	2.336204
2.210051	2.221588	2.217328	2.344891	2.339512	2.334224	2.348914	2.34369	2.338491
2.211908	2.207519	2.203211	2.280645	2.275974	2.271378	2.351088	2.3459	2.340718
2.21381	2.209409	2.205035	2.282567	2.277833	2.273188	2.353207	2.347992	2.342808
2.215693	2.211298	2.206881	2.284532	2.279728	2.27502	2.355342	2.35007	2.344866
2.217476	2.213107	2.20872	2.257292	2.252419	2.247627	2.357529	2.352181	2.346931
2.219213	2.214879	2.210552	2.259386	2.25445	2.249567	2.359776	2.354338	2.349025
2.220975	2.216642	2.212335	2.208024	2.203671	2.199342	2.246839	2.242154	2.351275
2.222749	2.218392	2.214078	2.209789	2.20551	2.201255	2.248967	2.244222	2.353657
2.224622	2.220233	2.215881	2.211572	2.207316	2.20309	2.251021	2.246256	2.33427
2.226633	2.222197	2.217756	2.213368	2.20906	2.204804	2.252961	2.24824	2.336675
2.171138	2.224152	2.219661	2.215223	2.210866	2.20657	2.25493	2.250224	2.27313
2.17288	2.226043	2.221612	2.217211	2.212838	2.208499	2.256992	2.252209	2.275116
2.17459	2.227899	2.223547	2.2192	2.214825	2.210454	2.25909	2.25422	2.27712
2.176285	2.229684	2.225348	2.221024	2.216662	2.212304	2.20796	2.203635	2.251514
2.177984	2.231443	2.227085	2.222762	2.21842	2.214097	2.209802	2.205535	2.253677
2.179826	2.175468	2.17127	2.224543	2.220178	2.215851	2.211562	2.207312	2.255695
2.181807	2.177367	2.173051	2.22635	2.221923	2.217555	2.213236	2.208968	2.257579

2.121222	2.179244	2.174847	2.228217	2.223754	2.219367	2.215025	2.21074	2.259567
2.122945	2.181054	2.176648	2.230169	2.225727	2.221358	2.217015	2.212716	2.261735
2.124699	2.182894	2.178469	2.232129	2.227696	2.223337	2.218993	2.214686	2.263912
2.072911	2.13755	2.133116	2.175902	2.171655	2.1675	2.220795	2.216496	2.265986
2.074744	2.139443	2.13491	2.177677	2.173364	2.169162	2.222572	2.218284	2.268052
2.07649	2.1127	2.108236	2.132322	2.128064	2.123909	2.224516	2.220223	2.215928
2.078191	2.114491	2.110079	2.134233	2.129946	2.125746	2.226557	2.222249	2.217938
2.048866	2.075674	2.071396	2.136077	2.131746	2.127503	2.123383	2.119354	2.21984
2.05072	2.077501	2.073149	2.137842	2.133451	2.129161	2.125012	2.120969	2.221614
2.052544	2.048215	2.043876	2.111081	2.106713	2.10245	2.126719	2.122649	2.118595
2.054281	2.049974	2.045664	2.112966	2.108587	2.104298	2.128568	2.12445	2.120374
2.056035	2.051758	2.047483	2.114893	2.110513	2.106206	2.130479	2.126309	2.122203
1.992901	2.053641	2.049366	2.087873	2.08354	2.079271	2.103934	2.099789	2.124082
1.994736	2.05555	2.051263	2.08982	2.08549	2.08122	2.105868	2.101682	2.125973
1.918223	1.992376	1.988281	2.048792	2.044595	2.040448	2.078795	2.074616	2.127732
1.91969	1.993998	1.989914	2.050478	2.046259	2.042083	2.080475	2.076272	2.129393
1.921265	1.995701	1.991627	2.029682	2.025465	2.021281	2.08225	2.078026	2.073853
1.922978	1.997503	1.993437	2.031581	2.027348	2.023144	2.084141	2.079899	2.075695
1.89616	1.92087	1.917073	1.991213	1.987129	1.983067	2.020889	2.016777	2.077573
1.897978	1.922609	1.918798	1.993078	1.989	1.984939	2.022822	2.01867	2.079489
1.899766	1.92432	1.920493	1.994912	1.990845	1.986789	2.024732	2.02054	2.081386
1.901409	1.897459	1.893569	1.918233	1.914459	1.910713	1.984416	1.980357	2.062548
1.902986	1.899024	1.895108	1.919697	1.915916	1.912164	1.986024	1.981951	2.064268
1.855188	1.900723	1.896779	1.892881	1.889044	1.88524	1.987729	1.983648	2.021542
1.856909	1.902537	1.898562	1.894636	1.890783	1.886967	1.989512	1.985431	2.023408
1.813965	1.90437	1.900375	1.896426	1.892546	1.888706	1.884872	1.881062	1.983215
1.815642	1.906232	1.902228	1.898256	1.89433	1.890444	1.886602	1.882802	1.985048
1.817264	1.813524	1.809804	1.900081	1.896119	1.892195	1.888346	1.884554	1.880785
1.81873	1.815009	1.811353	1.901875	1.897919	1.893983	1.890125	1.886321	1.882539
1.820169	1.816463	1.812865	1.903634	1.899693	1.895756	1.891894	1.88808	1.884293
1.82176	1.818022	1.814382	1.810744	1.807057	1.803381	1.893562	1.889784	1.886024
1.823444	1.819645	1.815901	1.812181	1.808458	1.804778	1.895168	1.89145	1.887734
1.725938	1.821311	1.817509	1.813738	1.809984	1.806284	1.855383	1.851701	1.88938
1.727636	1.823025	1.819209	1.815417	1.811637	1.807897	1.857028	1.853248	1.89095
1.729339	1.824748	1.820919	1.81711	1.81331	1.809541	1.805814	1.802138	1.892583
1.731042	1.826466	1.822614	1.818787	1.814981	1.811207	1.807468	1.803773	1.894329
1.732754	1.828194	1.824321	1.820478	1.816665	1.812886	1.809146	1.805446	1.896123
1.73448	1.730844	1.727231	1.822199	1.818365	1.814565	1.810815	1.807108	1.803428
1.73622	1.732568	1.728951	1.823938	1.820077	1.816249	1.812488	1.808777	1.805087
1.690258	1.734299	1.73067	1.727053	1.723425	1.719813	1.814164	1.810445	1.806748
1.691983	1.736037	1.732392	1.728764	1.725128	1.721511	1.815843	1.812115	1.808414
1.533204	1.690045	1.686457	1.730482	1.726839	1.723216	1.817538	1.81379	1.81008
1.534633	1.69177	1.688153	1.732207	1.728556	1.724928	1.819248	1.815469	1.811743
1.479515	1.693512	1.689862	1.73394	1.73028	1.726648	1.723035	1.719447	1.813415
1.48094	1.695275	1.691583	1.73568	1.732014	1.72838	1.724754	1.72115	1.815097
1.482373	1.697048	1.693316	1.737432	1.733756	1.730117	1.726479	1.722861	1.816794
1.483811	1.480886	1.477924	1.691349	1.687727	1.684149	1.728202	1.724579	1.768107
1.485255	1.482317	1.479346	1.693087	1.689435	1.685829	1.729927	1.726305	1.769795
1.406306	1.483756	1.480779	1.623243	1.619996	1.61678	1.683934	1.680385	1.724446
1.407656	1.485203	1.482223	1.624838	1.621575	1.618342	1.685624	1.68206	1.7262

1.409018	1.486661	1.483672	1.576174	1.572971	1.569797	1.687331	1.68375	1.72795
1.410392	1.488129	1.485128	1.57775	1.574536	1.571352	1.689051	1.685448	1.72968
1.411778	1.408938	1.4061	1.483592	1.480616	1.47767	1.619827	1.616634	1.683663
1.41318	1.41033	1.407468	1.485052	1.482067	1.479117	1.621434	1.618262	1.685467
1.414592	1.411732	1.408847	1.486522	1.483529	1.480579	1.623058	1.619909	1.687296
1.416007	1.413126	1.410229	1.488006	1.485006	1.482049	1.574557	1.57148	1.618381
1.417424	1.414519	1.411619	1.489502	1.486499	1.483534	1.576154	1.573058	1.619955
1.418837	1.415918	1.413022	1.410163	1.407349	1.404573	1.48214	1.479249	1.621573
1.420245	1.417323	1.414438	1.411593	1.408798	1.406032	1.483708	1.480791	1.623234
1.42166	1.418738	1.415869	1.413038	1.410255	1.407493	1.485272	1.482327	1.574859
1.423089	1.42017	1.417322	1.414508	1.411723	1.40895	1.48682	1.483842	1.576479
1.424522	1.421604	1.418772	1.415967	1.413178	1.410393	1.488359	1.485357	1.57811
1.425942	1.423018	1.420182	1.417376	1.414587	1.411802	1.408987	1.406161	1.483892
1.427375	1.424441	1.421589	1.418771	1.415978	1.413196	1.410392	1.407584	1.485436
1.428878	1.425936	1.423054	1.420207	1.417391	1.414596	1.411804	1.40902	1.406242
1.430434	1.427485	1.424561	1.421672	1.418819	1.416	1.41322	1.410468	1.407698
1.431974	1.42901	1.426057	1.42314	1.420263	1.417427	1.414644	1.4119	1.409129
1.433493	1.430506	1.427538	1.424609	1.421724	1.418879	1.416078	1.413312	1.41053
1.435019	1.432008	1.429031	1.426094	1.423198	1.420338	1.417513	1.414718	1.411925
1.436565	1.43353	1.430556	1.427614	1.424692	1.421799	1.418941	1.416115	1.413314
1.438112	1.435052	1.432079	1.429132	1.426186	1.423265	1.420376	1.41752	1.41471
1.364721	1.43654	1.433549	1.430593	1.427649	1.42473	1.42183	1.418955	1.416129
1.366156	1.438026	1.435007	1.432038	1.429105	1.426201	1.423297	1.420409	1.417563
1.2894	1.439595	1.436547	1.433551	1.430609	1.427697	1.42478	1.421873	1.419002
1.29086	1.441239	1.438154	1.435121	1.432153	1.429218	1.426276	1.423346	1.420448
1.292336	1.367774	1.364806	1.436702	1.433703	1.430738	1.427773	1.424824	1.421905
1.293823	1.3693	1.366324	1.438293	1.435257	1.432253	1.429265	1.4263	1.423371
1.295328	1.292536	1.289789	1.439891	1.436817	1.433773	1.430765	1.427791	1.511893
1.296845	1.294004	1.291242	1.441496	1.438381	1.435299	1.432276	1.4293	1.513604
1.29839	1.29549	1.292703	1.443098	1.43995	1.436837	1.433803	1.430828	1.515334
1.299991	1.297023	1.294174	1.369416	1.366405	1.363439	1.435364	1.43238	1.517082
1.301636	1.298593	1.29566	1.370908	1.367901	1.36495	1.43695	1.433951	1.518846
1.303291	1.300193	1.29719	1.294279	1.29148	1.288749	1.438541	1.435524	1.520611
1.30496	1.301823	1.298761	1.295802	1.292985	1.290242	1.44014	1.437101	1.522379
1.306647	1.303482	1.300374	1.297371	1.294513	1.291735	1.36658	1.363664	1.524158
1.308353	1.30517	1.302036	1.298991	1.296057	1.293208	1.368101	1.365191	1.525954
1.310081	1.306885	1.303731	1.300647	1.297636	1.294712	1.369635	1.366724	1.527753
1.21223	1.308629	1.305453	1.302335	1.299271	1.296288	1.293438	1.290687	1.529536
1.213894	1.310394	1.307198	1.304051	1.300945	1.29791	1.294991	1.292166	1.531323
1.21556	1.212471	1.209421	1.305782	1.302635	1.29955	1.296577	1.293696	1.465985
1.217236	1.214115	1.211067	1.30753	1.304347	1.301218	1.298198	1.295267	1.467762
1.150051	1.215803	1.212729	1.309286	1.306086	1.302932	1.299864	1.296871	1.401264
1.151655	1.217539	1.214405	1.311047	1.307853	1.304703	1.301582	1.298508	1.403075
1.153263	1.219283	1.216106	1.212985	1.209953	1.206967	1.303315	1.300185	1.404934
1.15486	1.221019	1.217839	1.214701	1.211619	1.208581	1.305043	1.301911	1.406853
1.156466	1.22276	1.219587	1.21644	1.213303	1.210209	1.306792	1.303668	1.408824
1.158088	1.224509	1.221332	1.218174	1.215015	1.211896	1.208867	1.205903	1.410851
1.15973	1.226271	1.223086	1.219919	1.216755	1.213626	1.210571	1.207573	1.412923
1.161426	1.228082	1.224879	1.221699	1.218538	1.215408	1.212321	1.209276	1.415005
1.163172	1.229936	1.226707	1.22351	1.220363	1.217241	1.214117	1.211009	1.417101

1.164868	1.162113	1.15936	1.225295	1.222143	1.219017	1.215875	1.212742	1.332105
1.166491	1.163718	1.160978	1.227039	1.223858	1.220706	1.217576	1.21447	1.334106
1.168119	1.165335	1.1626	1.228795	1.225583	1.222403	1.219281	1.216202	1.336132
1.169788	1.167008	1.164246	1.230588	1.227344	1.224138	1.221012	1.217938	1.338206
1.17147	1.168701	1.165912	1.232408	1.229138	1.225909	1.222767	1.219683	1.340296
1.173151	1.170392	1.167611	1.23427	1.230996	1.227752	1.224574	1.221443	1.342356
1.174836	1.172087	1.169332	1.23616	1.232893	1.22964	1.226416	1.223217	1.344415
1.176539	1.173779	1.171027	1.168279	1.165533	1.162793	1.228238	1.225022	1.346554
1.178262	1.175469	1.172698	1.169936	1.167178	1.164433	1.230043	1.226858	1.348776
1.179966	1.177152	1.174366	1.171595	1.16883	1.166083	1.231865	1.228689	1.350965
1.181627	1.178816	1.176028	1.173256	1.170501	1.167759	1.233712	1.230499	1.353077
1.183307	1.180503	1.17771	1.174934	1.172179	1.169434	1.235569	1.232319	1.35521
1.185064	1.182258	1.179448	1.176647	1.173858	1.171081	1.168319	1.165575	1.357428
1.186841	1.184034	1.181208	1.178381	1.17555	1.172735	1.169965	1.167229	1.359694
1.188557	1.185756	1.182944	1.180125	1.177288	1.174463	1.171683	1.168936	1.361994
1.190238	1.187446	1.184667	1.181878	1.179059	1.176241	1.173451	1.170682	1.364329
1.191949	1.189157	1.186379	1.183595	1.180788	1.17798	1.17519	1.172413	1.308717
1.193693	1.190889	1.188074	1.185265	1.182464	1.179672	1.176892	1.174121	1.310845
1.195453	1.192634	1.189794	1.186968	1.184172	1.181389	1.178617	1.175851	1.313015
1.197234	1.194394	1.191565	1.18875	1.185952	1.183166	1.180391	1.177622	1.315268
1.199021	1.196155	1.193335	1.190533	1.18774	1.184956	1.182178	1.179402	1.317544
1.200786	1.197884	1.195047	1.192245	1.189481	1.18673	1.183958	1.181174	1.319809
1.202554	1.199614	1.196746	1.193936	1.191208	1.188499	1.185733	1.182937	1.322069
1.204384	1.20142	1.198523	1.195691	1.192957	1.190245	1.187468	1.184659	1.324289
1.206265	1.203289	1.200362	1.197495	1.194722	1.191973	1.18917	1.186345	1.326476
1.208111	1.205135	1.202205	1.199325	1.196517	1.193732	1.19091	1.188075	1.185257
1.2099	1.206939	1.204044	1.201181	1.198348	1.195529	1.192696	1.189862	1.187048
1.211723	1.208771	1.2059	1.203047	1.200192	1.197346	1.1945	1.191662	1.188847
1.21363	1.210673	1.207781	1.204909	1.20204	1.199179	1.196315	1.193457	1.190635
1.215568	1.2126	1.20968	1.206781	1.203897	1.201024	1.198138	1.195257	1.192425
1.217492	1.214519	1.211589	1.208675	1.205768	1.20287	1.199961	1.197059	1.194219
1.219417	1.216441	1.21351	1.210586	1.207652	1.204722	1.201789	1.198867	1.196017
1.221349	1.218367	1.215432	1.212499	1.209544	1.206589	1.203636	1.200696	1.197831
1.223288	1.220297	1.217358	1.214417	1.211445	1.208471	1.205501	1.202546	1.19966
1.225237	1.222237	1.219291	1.216341	1.213358	1.210368	1.20738	1.204403	1.201495
1.2272	1.224187	1.221232	1.218273	1.215284	1.212285	1.209271	1.20626	1.203329
1.229168	1.226146	1.223181	1.220214	1.21722	1.214213	1.211178	1.208138	1.205183
1.066963	1.22811	1.22514	1.222165	1.219163	1.216147	1.213099	1.210042	1.207063
1.068625	1.230084	1.227108	1.224125	1.221117	1.218093	1.215041	1.211979	1.208976
1.070319	1.232078	1.229083	1.226088	1.223084	1.220069	1.217028	1.213977	1.210956
1.072038	1.234087	1.231066	1.228056	1.225062	1.222067	1.21905	1.216021	1.212988
1.073763	1.071121	1.068498	1.230031	1.22704	1.224054	1.22105	1.218037	1.215004
1.075497	1.072816	1.070178	1.232014	1.229017	1.22603	1.22303	1.220025	1.217007
1.077228	1.074513	1.071866	1.234003	1.230998	1.228003	1.225004	1.222005	1.218999
1.078933	1.076202	1.073564	1.236003	1.232985	1.229975	1.226974	1.223979	1.220977
1.080666	1.077916	1.075275	1.238002	1.234971	1.231948	1.228944	1.225951	1.222949
1.082491	1.079703	1.077008	1.074351	1.07172	1.069115	1.230921	1.227923	1.224918
1.084353	1.081523	1.078755	1.076043	1.073403	1.070801	1.2329	1.229894	1.226885
0.875004	1.083305	1.080491	1.07774	1.075082	1.072471	1.234864	1.231857	1.228851
0.876342	1.085063	1.082221	1.079442	1.07676	1.074132	1.236816	1.233814	1.230817

0.877704	1.086881	1.084041	1.081248	1.078525	1.075849	1.07321	1.070609	1.23279
0.879102	1.088784	1.085988	1.083197	1.080401	1.077633	1.074937	1.072295	1.234757
0.606093	0.878431	0.87636	1.085112	1.08227	1.079442	1.076704	1.074029	1.23678
0.606959	0.879796	0.877725	1.086898	1.084078	1.081272	1.07854	1.075861	1.238933
0.60781	0.881142	0.879066	1.088649	1.085866	1.0831	1.08038	1.077699	1.241089
	0.882489	0.880426	0.878352	0.87626	0.874178	1.082138	1.079431	1.076792
	0.88383	0.881788	0.879725	0.877625	0.875523	1.083859	1.081113	1.078438
			0.881081	0.87899	0.876888	0.874792	0.872708	1.08012
			0.88242	0.880354	0.87827	0.876163	0.874048	1.081826
			0.608259	0.606974	0.605676	0.877544	0.875413	1.083579
			0.609159	0.607856	0.606544	0.878922	0.876794	1.085373
						0.606126	0.604818	0.876108
						0.6071	0.605801	0.877675
						0.608083	0.606795	0.879267
								0.606398
								0.607251
								0.608102
								0.608944

								0.873999
								0.872222
						0.875672	0.873095	1.587498
						0.87395	0.871389	1.584797
						0.872206	0.869658	1.582089
				0.875542	0.873018	1.587687	1.583492	1.840225
				0.873882	0.871305	1.58492	1.58068	1.836954
				1.228797	1.225126	1.843781	1.838791	1.957491
				1.226405	1.222799	1.840536	1.835649	1.954393
	1.23522	1.231478	1.22777	1.58508	1.580799	1.837386	1.832546	2.004029
	1.233106	1.229366	1.225655	1.582473	1.578164	1.834344	1.829472	2.000749
0.567018	1.59279	1.588493	1.584201	1.841398	1.836334	1.954961	1.949944	1.997568
0.566123	1.589971	1.58572	1.581483	1.838355	1.833366	1.952	1.947038	1.994609
0.565208	1.58714	1.582924	1.578738	1.835292	1.830398	1.949055	1.944166	1.991691
1.588705	1.846941	1.841969	1.837051	1.890357	1.885497	1.998449	1.993506	1.988606
1.586069	1.843907	1.838864	1.8339	1.887245	1.882457	1.995226	1.990336	1.985461
1.583412	1.840901	1.835872	1.830934	2.00207	1.997104	1.992187	1.987325	2.079374
1.580748	1.837935	1.832994	1.828143	1.999145	1.994215	1.989318	1.984461	2.076441
1.839866	1.893208	1.888315	1.883508	1.99613	1.991233	2.083376	2.078391	2.132409
1.836596	1.890018	1.885207	1.880477	1.992927	1.988069	2.080161	2.075153	2.129028
1.833416	1.88692	1.882186	1.87752	1.989781	1.984944	2.076977	2.071949	2.125685
1.888883	2.001502	1.996592	1.991725	2.083956	2.078979	2.073968	2.068933	2.160241
1.886121	1.998593	1.993733	1.988895	2.081044	2.076039	2.071016	2.065978	2.157141
2.00062	2.09303	2.088052	2.083086	2.078086	2.073073	2.164554	2.159275	2.154012
1.99774	2.09011	2.085107	2.080112	2.075104	2.070088	2.161412	2.156116	2.150871
2.092176	2.08715	2.082093	2.077034	2.168642	2.16337	2.158088	2.152802	2.189922
2.089214	2.084162	2.079008	2.07384	2.165188	2.159843	2.154548	2.1493	2.186431
2.086151	2.081086	2.075888	2.070671	2.161803	2.156423	2.193491	2.188239	2.227529
2.082897	2.077853	2.072716	2.067563	2.158566	2.153217	2.190296	2.185115	2.22438
2.079653	2.074629	2.069561	2.064487	2.155389	2.150099	2.187205	2.182099	2.221335
2.076595	2.168097	2.162797	2.157507	2.152238	2.147018	2.184148	2.17911	2.218307

2.073617	2.164941	2.159624	2.15434	2.149126	2.143985	2.18114	2.176167	2.215312
2.111382	2.161774	2.156462	2.151203	2.188302	2.183174	2.222388	2.217341	2.212319
2.108328	2.158612	2.153319	2.148097	2.185217	2.180157	2.21933	2.214336	2.209328
2.16076	2.197811	2.192501	2.187264	2.182138	2.177102	2.21619	2.211199	2.206182
2.157613	2.194653	2.189396	2.184198	2.179064	2.173993	2.212935	2.207881	2.202827
2.154472	2.191502	2.186289	2.181126	2.220106	2.214885	2.209725	2.204622	2.199536
2.151338	2.188352	2.183151	2.178009	2.216941	2.211777	2.206643	2.201543	2.196447
2.148209	2.185207	2.180021	2.174903	2.213798	2.208701	2.203604	2.198518	2.19342
2.187273	2.182059	2.176928	2.171863	2.210708	2.205639	2.200553	2.195463	2.244839
2.184092	2.178923	2.173865	2.168866	2.207653	2.202592	2.197506	2.192408	2.241726
2.18096	2.219892	2.214763	2.209682	2.204606	2.199543	2.219814	2.214681	2.238635
2.177872	2.21679	2.211705	2.206647	2.201572	2.196495	2.216786	2.211665	2.235566
2.218831	2.213705	2.208647	2.203606	2.224002	2.218879	2.243022	2.237737	2.232508
2.215749	2.210629	2.205583	2.20055	2.220976	2.215859	2.239931	2.234661	2.229456
2.212695	2.23318	2.228108	2.223052	2.247409	2.242122	2.236858	2.231614	2.297635
2.209664	2.230179	2.225098	2.220034	2.244308	2.239031	2.233804	2.228617	2.294209
2.206645	2.227191	2.222097	2.217023	2.241219	2.235957	2.230771	2.225646	2.29083
2.203608	2.224186	2.219085	2.214013	2.238146	2.232928	2.299001	2.293237	2.287478
2.200565	2.221176	2.216071	2.211006	2.23509	2.229932	2.29558	2.289858	2.284151
2.223342	2.24775	2.242457	2.237225	2.232059	2.226941	2.292154	2.28645	2.312571
2.220331	2.244654	2.23939	2.234191	2.229051	2.223952	2.288718	2.283004	2.308925
2.246937	2.24159	2.236363	2.2312	2.29682	2.29109	2.317451	2.311366	2.305354
2.243881	2.238573	2.233391	2.228271	2.293499	2.287822	2.314013	2.307918	2.301903
2.240848	2.235575	2.230436	2.225354	2.290209	2.284582	2.310611	2.304514	2.298504
2.237829	2.304001	2.298207	2.292514	2.286892	2.281309	2.307184	2.301106	2.326761
2.234842	2.300548	2.2948	2.289159	2.283579	2.278034	2.303761	2.297715	2.323167
2.231938	2.297207	2.291474	2.28585	2.312134	2.3063	2.300358	2.294347	2.391395
2.2291	2.293956	2.288212	2.282579	2.308694	2.302881	2.29697	2.291001	2.387573
2.296622	2.323127	2.317054	2.3111	2.305258	2.29948	2.3658	2.358965	2.383806
2.293276	2.319649	2.31358	2.307628	2.301825	2.296098	2.362007	2.35524	2.380083
2.322336	2.316185	2.310126	2.304181	2.298415	2.292739	2.389855	2.38311	2.37646
2.318827	2.312723	2.306683	2.300757	2.295031	2.289409	2.386118	2.379548	2.373002
2.315364	2.309311	2.303293	2.297385	2.291683	2.286097	2.382416	2.376022	2.369583
2.312016	2.306042	2.300037	2.294121	2.360009	2.353589	2.378711	2.372427	2.366077
2.308725	2.30284	2.296852	2.290922	2.356323	2.349912	2.37502	2.36881	2.362539
2.305344	2.372949	2.366054	2.359247	2.352673	2.346267	2.371348	2.365201	2.359007
2.301892	2.369086	2.362315	2.355611	2.349063	2.342654	2.367693	2.361594	2.355475
2.298501	2.365259	2.358626	2.352048	2.376909	2.370398	2.364121	2.358046	2.352013
2.295181	2.361466	2.355002	2.348588	2.373438	2.366931	2.360652	2.354563	2.348632
2.364307	2.357751	2.351411	2.345126	2.369971	2.363496	2.357229	2.351138	2.345311
2.360743	2.354162	2.347841	2.341593	2.366423	2.360021	2.353796	2.347728	2.341998
2.357243	2.350646	2.344301	2.338056	2.362882	2.356591	2.35044	2.344427	2.338798
2.353685	2.378498	2.372027	2.365683	2.359436	2.353309	2.347291	2.341397	2.335885
2.350113	2.374943	2.368473	2.362156	2.356058	2.350131	2.344291	2.338565	2.333182
2.346608	2.37147	2.365013	2.35872	2.352731	2.346952	2.341281	2.335746	2.358883
2.34317	2.368087	2.361653	2.355377	2.349453	2.343771	2.338264	2.332946	2.356192
2.370885	2.364659	2.358294	2.35209	2.34625	2.340673	2.335339	2.330247	2.3536
2.367309	2.361104	2.354872	2.348827	2.343103	2.33765	2.33252	2.327691	2.351158
2.363799	2.357616	2.351545	2.345683	2.340093	2.334769	2.32981	2.325181	2.348732
2.360451	2.354338	2.348439	2.342761	2.337319	2.332119	2.355584	2.350893	2.368835

2.357201	2.351191	2.345466	2.339968	2.334686	2.329618	2.353068	2.348323	2.366257
2.353982	2.348134	2.342541	2.337178	2.33207	2.327167	2.350633	2.345864	2.385716
2.35081	2.345173	2.339683	2.334421	2.329495	2.324778	2.348274	2.343493	2.383197
2.347708	2.342238	2.33689	2.331754	2.326934	2.322306	2.390439	2.385548	2.380738
2.344663	2.339316	2.33416	2.329181	2.324378	2.319724	2.387794	2.383074	2.378356
2.341742	2.336506	2.331516	2.326672	2.394853	2.389935	2.385185	2.380581	2.375938
2.338986	2.333854	2.328981	2.324232	2.39232	2.38741	2.382655	2.378033	2.373406
2.336336	2.331306	2.326514	2.32183	2.389835	2.384951	2.380179	2.375507	2.370881
2.333762	2.328847	2.324092	2.319429	2.387344	2.382524	2.37777	2.373083	2.368468
2.331259	2.326457	2.32171	2.317045	2.38487	2.38013	2.375408	2.37072	2.366118
2.32877	2.39705	2.392088	2.387204	2.382427	2.377724	2.373037	2.368382	2.363801
2.326301	2.394432	2.389562	2.384767	2.380012	2.375312	2.370667	2.366075	2.361524
2.371069	2.391904	2.387086	2.382339	2.377614	2.372937	2.368318	2.363755	2.359227
2.368641	2.3895	2.38467	2.379911	2.375234	2.370617	2.366007	2.361418	2.356907
2.391942	2.387101	2.382275	2.377514	2.372867	2.368284	2.363675	2.359063	2.398291
2.389438	2.384633	2.379884	2.375183	2.370523	2.365898	2.361276	2.356668	2.395723
2.386941	2.382166	2.377503	2.372865	2.368179	2.363496	2.358864	2.354276	2.393156
2.384502	2.379773	2.375126	2.37049	2.365789	2.361089	2.356481	2.351945	2.390685
2.382098	2.377418	2.372752	2.368088	2.363381	2.358689	2.354122	2.349655	2.388272
2.379678	2.375022	2.370369	2.36572	2.361045	2.356389	2.395286	2.39053	2.385854
2.377243	2.372588	2.367977	2.363385	2.358779	2.354188	2.392932	2.38812	2.38343
2.374824	2.370182	2.365597	2.361042	2.3565	2.351975	2.390587	2.385774	2.381082
2.372432	2.367831	2.363234	2.358675	2.35418	2.349726	2.388243	2.383528	2.378856
2.370049	2.365475	2.360876	2.356324	2.351867	2.347467	2.385888	2.381275	2.376633
2.367648	2.363046	2.358508	2.354025	2.349588	2.345197	2.383497	2.378927	2.430059
2.365269	2.360624	2.356153	2.351744	2.347322	2.342928	2.381095	2.376544	2.427294
2.362993	2.358349	2.353851	2.349426	2.387912	2.383306	2.435129	2.429782	2.424468
2.360789	2.356178	2.351594	2.347092	2.385465	2.380935	2.43235	2.426939	2.4216
2.358535	2.353979	2.349394	2.344879	2.383096	2.378558	2.429521	2.424147	2.418849
2.356223	2.351754	2.347269	2.342808	2.380814	2.376165	2.426622	2.421413	2.416233
2.35392	2.392584	2.387925	2.38327	2.434692	2.429122	2.423805	2.418693	2.4136
2.351633	2.390115	2.385515	2.380934	2.431998	2.426473	2.421137	2.415952	2.410867
2.349383	2.387681	2.383101	2.378566	2.42929	2.423868	2.41853	2.413264	2.408167
2.34723	2.385394	2.38075	2.376167	2.426475	2.421199	2.415944	2.410722	2.405624
2.345117	2.383172	2.378434	2.373766	2.423626	2.418507	2.413371	2.408251	2.403162
2.385525	2.437262	2.431637	2.426124	2.420877	2.41578	2.410715	2.405703	2.400679
2.383118	2.434479	2.42896	2.423508	2.418206	2.413022	2.407992	2.403103	2.398194
2.380723	2.431635	2.426236	2.420886	2.415594	2.410385	2.405362	2.400495	2.395647
2.378333	2.428695	2.423441	2.418254	2.413062	2.407914	2.402847	2.397856	2.392998
2.431264	2.425823	2.420661	2.415592	2.410507	2.405447	2.400373	2.395301	2.390428
2.428561	2.423103	2.41791	2.412841	2.40784	2.402899	2.397915	2.392917	2.38805
2.425926	2.420474	2.415218	2.410111	2.405176	2.400347	2.395478	2.390601	2.385752
2.423311	2.417919	2.412664	2.407553	2.402641	2.397852	2.39305	2.388264	2.405079
2.420727	2.41543	2.410205	2.4051	2.400182	2.395386	2.390629	2.385925	2.402762
2.418153	2.412949	2.407755	2.402656	2.397721	2.392903	2.409818	2.405122	2.400389
2.415594	2.410484	2.405325	2.40023	2.395264	2.390404	2.407282	2.402636	2.397968
2.413021	2.407998	2.402895	2.39783	2.392855	2.387967	2.404788	2.400136	2.399811
2.410411	2.40547	2.400455	2.39546	2.390512	2.38562	2.402347	2.397604	2.397279
2.407827	2.402951	2.398025	2.393106	2.388191	2.383303	2.399948	2.395124	2.394795
2.405323	2.400489	2.395634	2.390775	2.407421	2.402476	2.40196	2.397106	2.39751

2.402848	2.398035	2.393242	2.38844	2.405066	2.400128	2.399643	2.394826	2.395243
2.400331	2.417144	2.412345	2.407551	2.407098	2.402172	2.402326	2.397587	2.392995
2.397799	2.414531	2.40978	2.405055	2.404671	2.399811	2.400026	2.395357	2.39078
2.395347	2.416545	2.411785	2.40707	2.407198	2.402434	2.397755	2.393141	2.388591
2.392984	2.414137	2.409361	2.404648	2.404826	2.400139	2.395512	2.390936	2.386429
2.416701	2.416569	2.41181	2.407129	2.402479	2.397876	2.393302	2.388764	2.384297
2.414283	2.414162	2.409394	2.404727	2.400153	2.395648	2.391137	2.386636	2.382197
2.411865	2.411762	2.407005	2.402364	2.397861	2.393443	2.388993	2.384535	2.38012
2.414252	2.409379	2.404667	2.400077	2.395617	2.391243	2.386849	2.382458	2.391735
2.411837	2.407004	2.402363	2.397839	2.393412	2.38906	2.384716	2.380391	2.389652
2.409413	2.404613	2.400056	2.395616	2.391238	2.386918	2.382598	2.378287	2.387515
2.406982	2.402211	2.397753	2.393414	2.389096	2.384812	2.380493	2.376155	2.385336
2.404631	2.399906	2.395503	2.391219	2.386955	2.382716	2.378397	2.374021	2.383148
2.402369	2.39771	2.393301	2.389012	2.384807	2.380636	2.376316	2.371889	2.380949
2.400159	2.395581	2.391173	2.386872	2.382689	2.378541	2.387857	2.383303	2.386789
2.397992	2.393528	2.389163	2.384864	2.38063	2.376409	2.385668	2.381117	2.384663
2.395866	2.391502	2.387185	2.382888	2.378583	2.374262	2.383459	2.378921	2.382539
2.393777	2.389433	2.385142	2.380851	2.376499	2.372114	2.381252	2.376711	2.380412
2.391717	2.387348	2.383068	2.378779	2.374392	2.36996	2.379042	2.374492	2.378286
2.38965	2.385273	2.380973	2.376658	2.372248	2.367794	2.385137	2.380614	2.378307
2.38758	2.383207	2.378854	2.374488	2.370067	2.365619	2.382998	2.378479	2.37622
2.385485	2.381117	2.376722	2.372313	2.377108	2.372694	2.383057	2.378562	2.374167
2.383359	2.378998	2.37458	2.370143	2.374977	2.370547	2.380942	2.376512	2.372157
2.381215	2.37685	2.372418	2.367965	2.372845	2.368405	2.378839	2.374477	2.370165
2.379053	2.37466	2.37022	2.365766	2.383372	2.378874	2.376753	2.372444	2.368175
2.376882	2.372454	2.368008	2.363561	2.381224	2.376755	2.374692	2.370425	2.366197
2.374709	2.379708	2.375281	2.370868	2.381418	2.376994	2.372666	2.368419	2.364229
2.372542	2.377593	2.373148	2.368734	2.379341	2.374981	2.370679	2.366436	2.36228
2.3705	2.378362	2.373907	2.369494	2.377402	2.373086	2.368808	2.364575	2.360422
2.368603	2.376547	2.372102	2.367706	2.375623	2.371331	2.367075	2.36286	2.358675
2.366629	2.374658	2.370236	2.365865	2.373787	2.36951	2.365268	2.361064	2.373562
2.364488	2.372609	2.368228	2.363897	2.371822	2.367549	2.363309	2.359105	2.37152
2.362311	2.370528	2.366184	2.361887	2.369813	2.365541	2.3613	2.357088	2.369427
2.363562	2.368469	2.364137	2.359852	2.367783	2.363527	2.359284	2.355057	2.385605
2.361454	2.366444	2.362105	2.357814	2.365744	2.361501	2.357254	2.353011	2.383388
2.368972	2.376898	2.372463	2.36807	2.363754	2.359485	2.371775	2.367427	2.381189
2.367235	2.375157	2.370658	2.366186	2.361803	2.357477	2.369674	2.365336	2.379004
2.365378	2.373309	2.368813	2.364333	2.376538	2.372084	2.385954	2.381444	2.376938
2.363371	2.37133	2.366927	2.36253	2.374704	2.370255	2.384042	2.37953	2.375029
2.361334	2.369315	2.364991	2.360671	2.37282	2.368393	2.382098	2.377583	2.373082
2.359277	2.367261	2.362956	2.358662	2.370775	2.366386	2.380003	2.37548	2.370973
2.357253	2.365234	2.360932	2.35665	2.368726	2.364381	2.377906	2.37338	2.368863
2.355381	2.379937	2.375546	2.371181	2.384984	2.380484	2.375971	2.371457	2.374695
2.353583	2.378078	2.373697	2.369347	2.383078	2.378591	2.374097	2.369602	2.372872
2.368879	2.376099	2.37173	2.367393	2.381027	2.376545	2.379875	2.375414	2.370917
2.366782	2.374018	2.369662	2.365337	2.378852	2.374367	2.377744	2.373293	2.368851
2.37639	2.372027	2.367686	2.363368	2.38461	2.380116	2.375642	2.371187	2.366776
2.374534	2.370188	2.365864	2.361547	2.382674	2.378109	2.373589	2.369103	2.364678
2.37266	2.394331	2.38984	2.385328	2.380736	2.376123	2.371574	2.367073	2.362644
2.370638	2.392261	2.387778	2.383279	2.378715	2.374139	2.369626	2.365163	2.360778

2.368608	2.390177	2.385696	2.381209	2.376685	2.372162	2.367698	2.363281	2.358951
2.375332	2.388263	2.383774	2.379273	2.374728	2.370182	2.365703	2.361278	2.356973
2.373621	2.386441	2.381938	2.377408	2.372812	2.368206	2.363681	2.359222	2.354925
2.371794	2.384491	2.379979	2.375446	2.37086	2.366272	2.361765	2.357325	2.353046
2.369844	2.382407	2.377889	2.373381	2.36887	2.364381	2.359958	2.355594	2.351343
2.384908	2.380371	2.375856	2.371371	2.366918	2.362504	2.358149	2.353848	2.349614
2.382986	2.378453	2.373956	2.36949	2.365049	2.360646	2.356313	2.35204	2.347798
2.381072	2.376536	2.372048	2.367595	2.363165	2.358776	2.354462	2.350213	2.345967
2.379106	2.374527	2.370008	2.365548	2.361162	2.356839	2.352575	2.348367	2.344147
2.377127	2.372501	2.367943	2.363473	2.35914	2.354897	2.350686	2.346517	2.342329
2.375159	2.370563	2.366015	2.361559	2.357268	2.353072	2.348882	2.344716	2.355185
2.373201	2.36869	2.364187	2.359764	2.355506	2.351333	2.347141	2.342949	2.353346
2.371308	2.366853	2.362395	2.358002	2.353743	2.349553	2.345337	2.341114	2.362656
2.369493	2.365056	2.360646	2.35628	2.351979	2.347717	2.343449	2.339184	2.360664
2.367694	2.363268	2.358892	2.354544	2.35021	2.345896	2.341596	2.337312	2.358737
2.36591	2.36149	2.357113	2.352763	2.348428	2.344115	2.339832	2.335579	2.356958
2.364099	2.359688	2.355313	2.350967	2.346646	2.342353	2.338101	2.333886	2.355218
2.362174	2.357788	2.353463	2.349168	2.344877	2.340605	2.35089	2.346622	2.353439
2.360206	2.355851	2.351595	2.347368	2.343116	2.338869	2.349128	2.344855	2.351645
2.358363	2.354026	2.349797	2.345597	2.341364	2.337128	2.358498	2.354149	2.372339
2.356626	2.352294	2.348059	2.343851	2.33962	2.335386	2.356677	2.352297	2.37043
2.354893	2.350574	2.346329	2.342106	2.337864	2.333621	2.354842	2.350459	2.36855
2.353152	2.348858	2.344607	2.340364	2.336092	2.331821	2.352982	2.348635	2.366708
2.351421	2.347145	2.342876	2.338609	2.334315	2.33003	2.351141	2.346838	2.364895
2.349706	2.345426	2.341115	2.336812	2.332528	2.328272	2.349348	2.345086	2.363121
2.347994	2.343703	2.339351	2.33502	2.330754	2.326537	2.347582	2.34336	2.361364
2.346269	2.341979	2.337629	2.333301	2.354437	2.350099	2.354623	2.350349	2.359593
2.344532	2.340247	2.335927	2.331625	2.352695	2.34836	2.352864	2.348608	2.357816
2.342754	2.338461	2.334166	2.329896	2.350927	2.346617	2.364686	2.360379	2.356048
2.340927	2.336612	2.332341	2.328112	2.349135	2.344871	2.362908	2.358611	2.354293
2.339148	2.334822	2.330566	2.326364	2.347367	2.343139	2.361121	2.356813	2.352497
2.337451	2.33313	2.328874	2.324674	2.345635	2.341422	2.359312	2.354955	2.350623
2.335781	2.33147	2.327212	2.323009	2.343924	2.33972	2.357517	2.353114	2.348766
2.33411	2.355157	2.35078	2.346463	2.350912	2.346633	2.355773	2.351357	2.347003
2.332446	2.353442	2.349075	2.344767	2.349192	2.344926	2.354048	2.349635	2.345279
2.330774	2.351719	2.34736	2.34306	2.347457	2.343199	2.352302	2.347891	2.343539
2.329099	2.349994	2.34564	2.341346	2.345712	2.34146	2.350543	2.346139	2.341794
2.327427	2.348271	2.343925	2.339637	2.34397	2.339724	2.348789	2.344394	2.340057
2.325755	2.346548	2.342211	2.337932	2.342233	2.337992	2.34704	2.342655	2.338327
2.324097	2.344839	2.340506	2.336228	2.353945	2.349644	2.345296	2.340924	2.336606
2.322458	2.343142	2.338803	2.33452	2.352183	2.347895	2.343562	2.339207	2.334899
2.32084	2.341473	2.337121	2.332826	2.350429	2.346149	2.341829	2.337491	2.333195
2.34423	2.348447	2.344003	2.339608	2.3487	2.3444	2.340082	2.335765	2.331482
2.34262	2.34683	2.342367	2.337941	2.346985	2.342653	2.338333	2.334035	2.329767
2.340951	2.345143	2.340678	2.336245	2.34526	2.340921	2.336604	2.332316	2.328053
2.339235	2.343401	2.338947	2.334525	2.34353	2.339202	2.334891	2.330604	2.326339
2.337533	2.341665	2.337223	2.332816	2.34181	2.337497	2.333203	2.328936	2.324678
2.335858	2.339941	2.335504	2.331111	2.340096	2.335802	2.331548	2.327336	2.323099
2.342628	2.338222	2.333809	2.329443	2.338415	2.334128	2.329897	2.325719	2.321494
2.340896	2.336508	2.332165	2.327859	2.336807	2.332494	2.328239	2.324035	2.319797

2.339171	2.334801	2.330531	2.326285	2.335209	2.330866	2.326576	2.322328	2.318071
2.337489	2.333108	2.328854	2.324626	2.333534	2.329194	2.324893	2.320623	2.31636
2.335833	2.331427	2.327155	2.322921	2.331818	2.327502	2.323203	2.31892	2.314657
2.334159	2.343096	2.338737	2.334425	2.330127	2.325849	2.32156	2.317268	2.313006
2.332467	2.341399	2.337026	2.33271	2.328457	2.324236	2.319967	2.315669	2.311409
2.330805	2.339737	2.335355	2.33103	2.326792	2.322594	2.318336	2.314042	2.320125
2.329202	2.338136	2.333745	2.329398	2.325125	2.32089	2.316627	2.312353	2.318425
2.327584	2.336522	2.332142	2.32779	2.32348	2.319199	2.314929	2.310675	2.316737
2.325885	2.334829	2.330511	2.326209	2.321893	2.317586	2.313315	2.309073	2.315122
2.324167	2.333112	2.328866	2.324624	2.320315	2.315993	2.311722	2.307487	2.313524
2.322515	2.331435	2.327189	2.32295	2.318649	2.314337	2.320413	2.316123	2.311851
2.320908	2.329783	2.325489	2.321214	2.316926	2.312644	2.318704	2.314404	2.310132
2.31932	2.328163	2.323839	2.319541	2.31526	2.310996	2.317044	2.312745	2.308478
2.317755	2.326579	2.322244	2.317935	2.313656	2.3094	2.31544	2.311154	2.306894
2.316191	2.324998	2.320659	2.316344	2.312062	2.307806	2.31384	2.309568	2.305318
2.31462	2.323412	2.319077	2.314762	2.310474	2.30621	2.312239	2.307983	2.303743
2.313047	2.321823	2.317492	2.313176	2.308875	2.304597	2.310616	2.306375	2.302145
2.311476	2.320228	2.315891	2.311563	2.317565	2.313206	2.308917	2.304684	2.305414
2.309904	2.318627	2.314279	2.309933	2.315889	2.311494	2.307192	2.302965	2.303689
2.321374	2.317017	2.312645	2.308281	2.314225	2.309845	2.305552	2.301331	2.302046
2.319769	2.315398	2.31099	2.306608	2.312567	2.308245	2.303978	2.299763	2.300466
2.318175	2.313801	2.309386	2.305	2.310954	2.306654	2.307343	2.303106	2.298899
2.316595	2.312239	2.307855	2.303489	2.309401	2.305071	2.30574	2.301528	2.297346
2.31501	2.310666	2.306305	2.301956	2.30784	2.303494	2.30415	2.299957	2.295795
2.313401	2.309043	2.304665	2.300312	2.306216	2.30192	2.302582	2.298388	2.294235
2.311794	2.307422	2.303025	2.29867	2.304598	2.300354	2.301025	2.296827	2.29268
2.310225	2.316201	2.31177	2.307375	2.307976	2.303688	2.299457	2.29528	2.291147
2.308675	2.314658	2.310274	2.30591	2.306465	2.302127	2.297893	2.293745	2.289626
2.307102	2.313087	2.308743	2.304415	2.304966	2.300624	2.296383	2.292224	2.288098
2.30551	2.31149	2.307184	2.302899	2.303486	2.299182	2.294928	2.290715	2.286561
2.314254	2.314857	2.310549	2.30626	2.30198	2.297715	2.293465	2.289232	2.288102
2.312682	2.313294	2.308974	2.30468	2.300426	2.2962	2.291987	2.28779	2.286668
2.311141	2.311756	2.307424	2.303121	2.298877	2.294673	2.290493	2.286341	2.285239
2.30966	2.310269	2.305943	2.301639	2.29737	2.293137	2.288967	2.28485	2.283791
2.3082	2.308792	2.304478	2.300179	2.295879	2.291607	2.287434	2.283341	2.282328
2.311603	2.307254	2.302952	2.298672	2.294389	2.290131	2.289034	2.28493	2.294533
2.310061	2.30568	2.301388	2.297136	2.292902	2.28869	2.28759	2.283419	2.292988
2.308543	2.304147	2.29984	2.295586	2.291386	2.287218	2.286143	2.281954	2.291497
2.307052	2.30266	2.298305	2.294013	2.289835	2.285715	2.284698	2.280545	2.290069
2.305543	2.30117	2.296794	2.29248	2.288304	2.284197	2.283225	2.27911	2.28863
2.303988	2.299657	2.295317	2.291019	2.286806	2.282647	2.281693	2.277613	2.287162
2.302438	2.298147	2.293859	2.28959	2.285339	2.281119	2.280166	2.276102	2.285675
2.300939	2.296666	2.292419	2.288181	2.287088	2.282861	2.278702	2.274598	2.284149
2.299461	2.295195	2.290987	2.286784	2.285718	2.281475	2.277274	2.273104	2.282613
2.297968	2.2937	2.289517	2.285341	2.284318	2.280086	2.289596	2.285338	2.300427
2.296467	2.292187	2.288018	2.283865	2.282901	2.278702	2.288206	2.283934	2.298875
2.294995	2.293911	2.289768	2.28565	2.281493	2.277321	2.29908	2.294728	2.308488
2.293556	2.292497	2.288357	2.284246	2.280107	2.275952	2.297669	2.293291	2.306892
2.292132	2.291092	2.286937	2.282815	2.278688	2.274552	2.296239	2.291855	2.305309
2.290716	2.289686	2.285478	2.281315	2.277191	2.273079	2.294766	2.290418	2.303757

2.289307	2.288286	2.284022	2.279811	2.275684	2.271594	2.293284	2.288981	2.302213
2.291224	2.286899	2.282611	2.278378	2.28799	2.283858	2.309947	2.305311	2.300655
2.289846	2.285519	2.281225	2.276985	2.286576	2.282433	2.308374	2.303747	2.299094
2.288463	2.284129	2.27983	2.275585	2.297405	2.29319	2.306813	2.302194	2.297545
2.287075	2.282733	2.278429	2.274183	2.295971	2.291761	2.305264	2.300651	2.296007
2.285677	2.281332	2.277029	2.272785	2.294538	2.29033	2.303712	2.299107	2.294468
2.284264	2.279924	2.27563	2.271392	2.293107	2.288895	2.302155	2.297557	2.292927
2.282848	2.278514	2.274229	2.269999	2.291677	2.287461	2.300599	2.296006	2.291384
2.281438	2.29092	2.286589	2.282314	2.308162	2.303622	2.299047	2.294454	2.289835
2.28003	2.289496	2.285171	2.280903	2.306604	2.302072	2.297499	2.292904	2.288286
2.27863	2.288085	2.283758	2.27949	2.305044	2.30052	2.295953	2.291365	2.286751
2.277236	2.286682	2.28235	2.278077	2.303482	2.298968	2.29441	2.289832	2.285224
2.275836	2.285274	2.280945	2.276672	2.301927	2.297411	2.292856	2.288283	2.283682
2.274425	2.283858	2.27954	2.275273	2.300374	2.295845	2.291286	2.286714	2.282124
2.286799	2.312814	2.308106	2.303446	2.29886	2.294312	2.289739	2.285156	2.280567
2.285411	2.311305	2.306635	2.302003	2.297411	2.29284	2.288238	2.283617	2.279014
2.284047	2.309822	2.305186	2.300575	2.295971	2.291372	2.286739	2.282083	2.277465
2.282719	2.308385	2.303756	2.299135	2.296363	2.291724	2.28707	2.282405	2.277789
2.281416	2.306966	2.302329	2.297683	2.294872	2.290189	2.285519	2.280861	2.276257
2.282802	2.307368	2.302712	2.298042	2.293342	2.288636	2.283967	2.279325	2.274739
2.281503	2.305889	2.301195	2.296494	2.291778	2.287067	2.28241	2.277795	2.273234
2.298386	2.304379	2.29966	2.294942	2.290222	2.285518	2.280877	2.276288	2.271752
2.29688	2.302828	2.298101	2.293383	2.288672	2.283984	2.279362	2.274795	2.270288
2.306026	2.301283	2.296552	2.291841	2.287151	2.282493	2.277897	2.273357	2.26887
2.304494	2.299752	2.295019	2.29032	2.285672	2.28107	2.276523	2.272025	2.267545
2.302974	2.298248	2.293522	2.288837	2.28423	2.279677	2.275169	2.270704	2.266219
2.301496	2.29681	2.292115	2.28745	2.282845	2.278283	2.273763	2.269281	2.264781
2.300047	2.295409	2.290754	2.286111	2.28149	2.276891	2.272333	2.267809	2.263284
2.298605	2.293968	2.289317	2.284678	2.28006	2.275461	2.270889	2.266342	2.265146
2.297175	2.292495	2.287816	2.283166	2.278566	2.273995	2.269429	2.264872	2.263678
2.295724	2.291013	2.286318	2.281666	2.277092	2.272559	2.271363	2.266825	2.262284
2.294226	2.28951	2.284815	2.280178	2.275646	2.271168	2.270028	2.265534	2.26101
2.292763	2.288047	2.28336	2.278737	2.27758	2.27314	2.268682	2.26422	2.263446
2.291413	2.286696	2.282022	2.277403	2.276229	2.271755	2.267274	2.262797	2.262009
2.290084	2.28537	2.280712	2.276095	2.274889	2.27036	2.265839	2.26133	2.260525
2.288649	2.283943	2.279299	2.27469	2.273479	2.268927	2.264386	2.259857	2.25905
2.287158	2.282464	2.277835	2.273236	2.272035	2.267475	2.262921	2.258377	2.257575
2.285761	2.284443	2.279834	2.275252	2.270672	2.266103	2.261538	2.256981	2.256167
2.284467	2.28316	2.27856	2.273983	2.269399	2.264822	2.260247	2.255678	2.254834
2.286493	2.281834	2.27724	2.272665	2.268079	2.263497	2.262629	2.258033	2.253473
2.285046	2.280402	2.275811	2.271235	2.26665	2.262068	2.261189	2.256592	2.252031
2.283606	2.278979	2.274388	2.269809	2.265225	2.260645	2.259759	2.255166	2.250613
2.282276	2.277662	2.27307	2.268488	2.263904	2.259325	2.258439	2.253856	2.249316
2.280976	2.276375	2.271781	2.267195	2.262609	2.258032	2.257149	2.25258	2.248058
2.279583	2.274989	2.270396	2.265808	2.261221	2.256644	2.25576	2.251204	2.246709
2.27813	2.273539	2.268947	2.26436	2.259771	2.255193	2.254305	2.249762	2.2453
2.276769	2.272177	2.267586	2.263	2.262106	2.257508	2.252942	2.248405	2.243955
2.275518	2.270923	2.266333	2.261748	2.26085	2.256253	2.251688	2.247152	2.242684
2.274281	2.269684	2.265094	2.26051	2.259609	2.255016	2.250454	2.24592	2.24225
2.273052	2.268453	2.26386	2.259276	2.258374	2.253789	2.249236	2.24471	2.241033

2.271787	2.267187	2.262592	2.258007	2.257105	2.252529	2.247985	2.24347	2.239791
2.270386	2.265787	2.261191	2.256607	2.255702	2.251135	2.246601	2.242096	2.238412
2.268925	2.264327	2.259731	2.25515	2.254241	2.249682	2.245158	2.240664	2.236976
2.267578	2.266688	2.262074	2.257477	2.252901	2.248349	2.243833	2.239349	2.235658
2.266322	2.26543	2.260823	2.256232	2.251661	2.247113	2.242605	2.238131	2.234437
2.265088	2.264191	2.25959	2.255006	2.25044	2.245898	2.242196	2.237714	2.233232
2.263877	2.262974	2.258378	2.2538	2.249242	2.244706	2.241005	2.236527	2.232046
2.26266	2.261746	2.257153	2.252582	2.248031	2.243505	2.239803	2.235327	2.230845
2.261421	2.260493	2.255904	2.25134	2.246801	2.242286	2.238584	2.234107	2.229623
2.260157	2.259209	2.254616	2.250054	2.245526	2.241025	2.237323	2.232845	2.228357
2.262521	2.257853	2.253227	2.248645	2.244124	2.239641	2.235939	2.231461	2.226971
2.261163	2.25645	2.251777	2.247163	2.242645	2.238183	2.234481	2.230004	2.225512
2.259797	2.255088	2.250413	2.245798	2.242081	2.237611	2.23313	2.228652	2.224158
2.258414	2.253762	2.24913	2.244546	2.240835	2.236365	2.231883	2.227401	2.222902
2.257076	2.252469	2.247877	2.243323	2.239617	2.235145	2.230662	2.226176	2.221673
2.255807	2.251216	2.24665	2.242118	2.238415	2.233944	2.22946	2.224971	2.220462
2.254566	2.249983	2.245438	2.240925	2.237222	2.232751	2.228264	2.22377	2.219257
2.253334	2.248758	2.244229	2.239728	2.236021	2.231545	2.227054	2.222553	2.218036
2.252113	2.247542	2.243025	2.238534	2.23482	2.230339	2.22584	2.221331	2.21681
2.250887	2.247133	2.24261	2.238112	2.233622	2.229138	2.224633	2.220113	2.215585
2.249659	2.245909	2.241399	2.236911	2.232426	2.227941	2.223429	2.218897	2.214361
2.248436	2.244693	2.24019	2.235707	2.231223	2.226736	2.222218	2.217676	2.213134
2.247215	2.243486	2.238984	2.234499	2.230012	2.225523	2.220999	2.21645	2.211904
2.246011	2.242282	2.237778	2.23329	2.2288	2.224305	2.219776	2.215221	2.210672
2.244822	2.241063	2.236564	2.232084	2.227587	2.223082	2.218545	2.213984	2.209434
2.243663	2.239867	2.23536	2.230877	2.22637	2.221855	2.217311	2.212746	2.208197
2.242564	2.238733	2.234179	2.229657	2.225137	2.220623	2.216078	2.211512	2.206965
2.241502	2.23764	2.233022	2.228441	2.223904	2.219389	2.214842	2.210278	2.205738
2.240432	2.236574	2.23192	2.227293	2.222712	2.218157	2.213597	2.209039	2.204508
2.239361	2.235538	2.23087	2.226203	2.221554	2.216925	2.212341	2.207795	2.203278
2.239042	2.234451	2.2298	2.225127	2.220436	2.215751	2.211135	2.206576	2.202056
2.237859	2.2333	2.228706	2.224075	2.219374	2.21465	2.209989	2.205374	2.200825
2.236654	2.232119	2.227576	2.222988	2.218299	2.213568	2.208884	2.204231	2.199658
2.235439	2.230917	2.226388	2.221823	2.217178	2.212499	2.207847	2.203213	2.198631
2.234221	2.229698	2.225167	2.220615	2.216022	2.21141	2.20681	2.202215	2.197638
2.233015	2.228465	2.223927	2.219383	2.214811	2.210231	2.205663	2.201104	2.196553
2.231824	2.227228	2.222674	2.218127	2.21356	2.208992	2.20445	2.199926	2.195416
2.230694	2.226059	2.221466	2.216891	2.212313	2.207748	2.203221	2.198726	2.194246
2.229627	2.224957	2.220299	2.215664	2.21106	2.206489	2.201969	2.197494	2.193037
2.228548	2.223871	2.219182	2.214511	2.209881	2.20529	2.20076	2.196285	2.191832
2.227443	2.222811	2.218148	2.213483	2.208823	2.204184	2.199613	2.195099	2.190623
2.226318	2.221724	2.217095	2.212448	2.207776	2.203107	2.198509	2.193964	2.189464
2.225146	2.220536	2.215916	2.211293	2.20666	2.202036	2.197467	2.19294	2.188433
2.223971	2.219327	2.214705	2.210102	2.205521	2.200963	2.196441	2.191949	2.187444
2.222895	2.218239	2.213592	2.20897	2.204384	2.199831	2.19532	2.190847	2.18636
2.221885	2.217228	2.212542	2.207872	2.203247	2.198661	2.19414	2.189676	2.185216
2.220786	2.216139	2.211455	2.206785	2.20216	2.19757	2.19304	2.188562	2.192698
2.21958	2.214957	2.21033	2.205717	2.201137	2.196578	2.192036	2.187511	2.191597
2.218404	2.213787	2.209192	2.20462	2.200079	2.195554	2.191013	2.186463	2.190521
2.217309	2.212651	2.208028	2.203443	2.19892	2.194429	2.189918	2.185398	2.189488

2.216242	2.211548	2.20689	2.202286	2.197772	2.193308	2.188824	2.184337	2.188459
2.215169	2.210496	2.205849	2.201253	2.196751	2.192294	2.187804	2.183297	2.187395
2.214108	2.20948	2.204859	2.200278	2.195781	2.191317	2.186805	2.182263	2.186318
2.21309	2.208479	2.203855	2.199262	2.194742	2.190253	2.185734	2.181199	2.18526
2.21211	2.207495	2.202848	2.198221	2.193654	2.189121	2.184605	2.18011	2.184214
2.21107	2.206447	2.201801	2.197174	2.192593	2.188044	2.183525	2.179034	2.183147
2.20994	2.205305	2.2007	2.196122	2.191578	2.187053	2.182517	2.177977	2.182046
2.208826	2.204182	2.199606	2.195063	2.190547	2.186042	2.181496	2.176922	2.180961
2.207789	2.20314	2.19855	2.19399	2.189456	2.184936	2.180401	2.175856	2.179921
2.206776	2.202126	2.197511	2.192923	2.188364	2.183829	2.179308	2.174802	2.178901
2.205754	2.201097	2.196477	2.191889	2.187342	2.182822	2.178305	2.173796	2.177893
2.204734	2.200068	2.195454	2.190881	2.186363	2.181872	2.177353	2.172817	2.176894
2.203707	2.19905	2.194459	2.189909	2.185404	2.180917	2.185068	2.180477	2.175866
2.202672	2.198039	2.193492	2.188975	2.18447	2.179963	2.184088	2.179448	2.174813
2.201662	2.197058	2.192537	2.188035	2.183515	2.17898	2.183081	2.178412	2.173767
2.200691	2.19612	2.191602	2.187085	2.18253	2.177951	2.182031	2.177363	2.17273
2.199732	2.195183	2.190654	2.186113	2.181525	2.176912	2.180984	2.176331	2.171714
2.198777	2.194226	2.189667	2.185095	2.180491	2.175874	2.179966	2.175347	2.17644
2.19782	2.193251	2.188658	2.184057	2.17945	2.174844	2.178968	2.174386	2.175479
2.196826	2.192235	2.187631	2.18303	2.178438	2.173854	2.177998	2.173431	2.174525
2.195811	2.191189	2.186591	2.18201	2.177446	2.172894	2.177052	2.172482	2.173578
2.194835	2.190201	2.185604	2.18103	2.176481	2.171944	2.176095	2.171498	2.172581
2.193907	2.189278	2.184677	2.180096	2.175547	2.171006	2.175124	2.170467	2.171522
2.192955	2.188339	2.183745	2.179167	2.183378	2.178804	2.174158	2.169463	2.170498
2.191939	2.187346	2.182786	2.178234	2.182446	2.177859	2.173213	2.168523	2.169554
2.190926	2.186354	2.181827	2.177298	2.181503	2.176893	2.172252	2.167584	2.168619
2.189972	2.185403	2.180884	2.176354	2.180525	2.175871	2.176926	2.172258	2.167632
2.189049	2.184474	2.179951	2.175406	2.179534	2.174829	2.175872	2.171227	2.166631
2.188131	2.183553	2.179024	2.174469	2.178578	2.173849	2.174883	2.170249	2.16567
2.187225	2.182641	2.178104	2.173541	2.177647	2.172916	2.173945	2.169313	2.164739
2.186318	2.181731	2.177185	2.172614	2.17672	2.171989	2.173014	2.168387	2.163816
2.185409	2.18082	2.176266	2.17169	2.175796	2.171068	2.172091	2.167469	2.162903
2.184502	2.179907	2.175347	2.170765	2.174872	2.170147	2.171169	2.166554	2.161991
2.183589	2.178983	2.174419	2.169837	2.173944	2.169219	2.17024	2.165633	2.161077
2.182694	2.17807	2.173498	2.168912	2.173019	2.168297	2.169316	2.164715	2.160163
2.181862	2.177211	2.172607	2.167996	2.172104	2.167391	2.168403	2.163797	2.159244
2.181061	2.176379	2.171732	2.167087	2.171199	2.166503	2.167507	2.162887	2.158328
2.180206	2.175508	2.170839	2.166184	2.176004	2.171325	2.166668	2.162033	2.157458
2.179303	2.174602	2.16993	2.165286	2.17515	2.170529	2.165887	2.161235	2.156632
2.178399	2.182562	2.177858	2.173193	2.174275	2.1697	2.165073	2.160416	2.155797
2.177496	2.181656	2.176937	2.172266	2.17336	2.168814	2.164203	2.159554	2.154933
2.17661	2.180777	2.176043	2.171359	2.172451	2.167916	2.163323	2.158697	2.154085
2.175769	2.179967	2.175218	2.170511	2.17158	2.167027	2.162457	2.157884	2.153296
2.174941	2.179172	2.174413	2.169683	2.170718	2.166136	2.161587	2.157074	2.152516
2.174081	2.184014	2.179243	2.174495	2.169833	2.165227	2.160689	2.156212	2.151674
2.173206	2.183097	2.178341	2.173613	2.168938	2.164312	2.159776	2.155316	2.150793
2.172366	2.182229	2.177489	2.172778	2.168098	2.163461	2.158909	2.15443	2.144981
2.171568	2.181417	2.176692	2.171996	2.167321	2.162678	2.158089	2.153548	2.144109
2.170747	2.18059	2.175871	2.171186	2.166533	2.161907	2.157297	2.152706	2.143269
2.169866	2.179715	2.174986	2.170305	2.165707	2.161148	2.156549	2.151929	2.142476

2.168992	2.178849	2.17411	2.169429	2.164874	2.16037	2.155792	2.15117	2.141711
2.177088	2.178036	2.173302	2.16862	2.164043	2.159518	2.154964	2.150398	2.140981
2.176312	2.177252	2.172528	2.167844	2.163219	2.158641	2.154108	2.149617	2.140259
2.181229	2.176479	2.171761	2.167077	2.16243	2.157824	2.148443	2.14397	2.139465
2.180473	2.17572	2.171006	2.166323	2.161671	2.157051	2.147652	2.143122	2.138615
2.179698	2.17496	2.170254	2.165571	2.160917	2.156288	2.146884	2.142316	2.137796
2.178894	2.174201	2.169504	2.164821	2.160167	2.155533	2.146142	2.141561	2.137015
2.178106	2.17344	2.168751	2.16407	2.159417	2.154782	2.145408	2.140821	2.144685
2.177362	2.172675	2.16799	2.163317	2.158666	2.154032	2.144679	2.140088	2.143928
2.176624	2.171912	2.167228	2.162562	2.157914	2.153281	2.14395	2.13936	2.143182
2.17585	2.171154	2.166474	2.161808	2.152428	2.147814	2.143214	2.138626	2.142438
2.175064	2.170399	2.165723	2.161053	2.151692	2.147073	2.142474	2.137891	2.141697
2.174308	2.169644	2.164967	2.160296	2.150954	2.146334	2.150172	2.145559	2.14095
2.173579	2.168888	2.164206	2.159537	2.150216	2.145596	2.149423	2.144804	2.140198
2.172843	2.163425	2.158761	2.154113	2.157963	2.15331	2.148674	2.144056	2.139457
2.17209	2.162688	2.158022	2.153374	2.157211	2.152554	2.147924	2.143317	2.138727
2.171339	2.161957	2.157291	2.152643	2.156467	2.151806	2.147181	2.142586	2.13801
2.170587	2.161225	2.156566	2.151924	2.15573	2.151063	2.146443	2.141861	2.137306
2.169855	2.160515	2.155861	2.151223	2.155013	2.150343	2.145726	2.141153	2.136616
2.164583	2.168414	2.163714	2.159031	2.154353	2.149694	2.145076	2.140494	2.135949
2.163991	2.167816	2.163095	2.158396	2.153731	2.149092	2.14447	2.139866	2.135297
2.163333	2.167148	2.162422	2.15772	2.153065	2.148438	2.143815	2.139203	2.134622
2.162606	2.166409	2.161694	2.157004	2.152353	2.147727	2.143108	2.138499	2.133918
2.170395	2.165665	2.160962	2.156286	2.151649	2.147038	2.142429	2.137829	2.13325
2.169648	2.164918	2.160222	2.155565	2.150966	2.146399	2.141817	2.137231	2.132654
2.168934	2.164203	2.159511	2.154865	2.150294	2.145761	2.141202	2.13663	2.132055
2.168303	2.163572	2.158874	2.154219	2.14963	2.145079	2.140517	2.135954	2.13138
2.167716	2.162984	2.158276	2.153602	2.14898	2.144392	2.139817	2.135258	2.130687
2.167122	2.162393	2.157676	2.152991	2.14836	2.143766	2.139187	2.134628	2.130056
2.166528	2.161806	2.15708	2.152388	2.147766	2.143185	2.138608	2.134043	2.129469
2.165884	2.161167	2.156438	2.151745	2.147131	2.142561	2.137997	2.133444	2.12888
2.165172	2.160459	2.155735	2.151048	2.146439	2.141879	2.137342	2.132833	2.128295
2.164485	2.159774	2.155058	2.150379	2.145772	2.141214	2.13669	2.1322	2.127676
2.163866	2.159154	2.154448	2.149777	2.145166	2.140596	2.136048	2.131523	2.12698
2.163273	2.158561	2.153866	2.149201	2.144586	2.140001	2.135421	2.130851	2.126283
2.162686	2.157976	2.153286	2.148625	2.14401	2.139421	2.13483	2.130242	2.125662
2.16211	2.157402	2.152715	2.148057	2.143443	2.138855	2.134261	2.129667	2.125083
2.161532	2.156827	2.152146	2.147492	2.142879	2.138288	2.133691	2.129093	2.124506
2.160954	2.156253	2.151579	2.146931	2.142317	2.137723	2.133123	2.128525	2.123937
2.160375	2.155677	2.151009	2.146367	2.141752	2.137156	2.132556	2.127958	2.12337
2.159791	2.155097	2.150434	2.145796	2.141183	2.136588	2.131989	2.127393	2.122806
2.15921	2.154515	2.149857	2.145224	2.140613	2.136018	2.131421	2.126826	2.12224
2.158625	2.153921	2.149271	2.144647	2.140039	2.135445	2.130848	2.126252	2.121666
2.158061	2.153343	2.148694	2.144075	2.139468	2.134874	2.130277	2.125681	2.121095
2.157576	2.152841	2.148164	2.143519	2.138903	2.134309	2.129715	2.125125	2.120541
2.157139	2.152389	2.147666	2.142978	2.138349	2.133755	2.129165	2.124586	2.120002
2.156648	2.151902	2.147176	2.142486	2.137852	2.133256	2.128668	2.124095	2.124648
2.156104	2.151384	2.146701	2.142047	2.137421	2.132819	2.128231	2.123659	2.124202
2.155555	2.150856	2.146203	2.141573	2.136953	2.132348	2.127759	2.123185	2.12372
2.154999	2.150312	2.145656	2.141022	2.136405	2.131805	2.127217	2.12264	2.123169

2.154463	2.149783	2.145119	2.140474	2.135853	2.131251	2.126658	2.122076	2.1226
2.15399	2.149316	2.14465	2.139995	2.135346	2.130711	2.1261	2.121511	2.122025
2.153536	2.148864	2.1442	2.139537	2.134857	2.130182	2.12555	2.120953	2.121458
2.153016	2.148346	2.143695	2.139044	2.134366	2.129689	2.130225	2.12558	2.120952
2.152454	2.147784	2.143154	2.13853	2.133879	2.129227	2.129764	2.125118	2.120491
2.151936	2.147268	2.142651	2.138042	2.133407	2.128769	2.129296	2.124633	2.119997
2.151474	2.146813	2.142198	2.13759	2.132959	2.128321	2.12882	2.124117	2.119456
2.151004	2.14634	2.141722	2.137112	2.132486	2.127853	2.128336	2.123614	2.118941
2.150496	2.145809	2.141177	2.136563	2.131948	2.127336	2.127834	2.123138	2.118486
2.149988	2.145281	2.140636	2.136018	2.131413	2.12682	2.127338	2.122682	2.118055
2.149503	2.144812	2.14017	2.135553	2.136164	2.131518	2.126879	2.122249	2.117636
2.149034	2.144375	2.139744	2.135129	2.135724	2.131072	2.126443	2.121833	2.117226
2.148591	2.143948	2.139318	2.134702	2.135289	2.13064	2.126015	2.121409	2.125486
2.148171	2.143534	2.138899	2.134276	2.134862	2.130224	2.125597	2.120982	2.125044
2.147753	2.143118	2.138479	2.133854	2.134435	2.129802	2.125175	2.120558	2.124599
2.147333	2.142695	2.138058	2.133433	2.134006	2.129366	2.124744	2.120136	2.124149
2.143182	2.147589	2.142906	2.138236	2.133578	2.128935	2.124317	2.119718	2.123713
2.142768	2.147171	2.142481	2.137805	2.133152	2.128517	2.123903	2.119308	2.12331
2.142354	2.146756	2.142057	2.137375	2.132728	2.128104	2.123495	2.118901	2.122919
2.141929	2.14633	2.141636	2.136957	2.132311	2.127688	2.123083	2.118495	2.122518
2.141503	2.145901	2.141216	2.136546	2.131899	2.127273	2.12267	2.118089	2.122112
2.141101	2.145472	2.140789	2.136127	2.131484	2.126861	2.122259	2.117677	2.121694
2.14072	2.145038	2.140353	2.135701	2.131066	2.126451	2.121848	2.117259	2.121262
2.146041	2.141281	2.136599	2.131956	2.130655	2.126051	2.121447	2.116848	2.120834
2.145729	2.140978	2.136239	2.131542	2.130244	2.125655	2.121048	2.116436	2.120405
2.145406	2.140676	2.1359	2.131163	2.129866	2.125286	2.120676	2.116056	2.120003
2.145017	2.140315	2.135576	2.130867	2.129569	2.124978	2.12037	2.115758	2.11911
2.144605	2.139929	2.135253	2.130598	2.129299	2.124689	2.120084	2.115485	2.118796
2.144215	2.139527	2.134884	2.13026	2.125641	2.121032	2.119735	2.115141	2.118444
2.143839	2.139112	2.134483	2.12988	2.125259	2.120645	2.119347	2.11475	2.118069
2.143495	2.138757	2.134131	2.129534	2.124917	2.120302	2.11899	2.114368	2.117682
2.143188	2.13847	2.133838	2.129231	2.124622	2.120013	2.118668	2.11399	2.117272
2.142891	2.138191	2.133538	2.128909	2.124306	2.119708	2.118348	2.113638	2.116897
2.14261	2.137908	2.133203	2.128528	2.123926	2.119351	2.118014	2.113328	2.11659
2.142315	2.137612	2.132865	2.128152	2.123549	2.11899	2.117686	2.113037	2.116301
2.141956	2.13727	2.132538	2.127837	2.123235	2.118675	2.117386	2.112757	2.115998
2.141573	2.136911	2.132221	2.127554	2.122949	2.118372	2.117092	2.112479	2.115689
2.141252	2.136598	2.131927	2.127269	2.122637	2.118025	2.113431	2.108854	2.115357
2.140981	2.136322	2.131654	2.126985	2.122309	2.117642	2.113035	2.108473	2.115005
2.140716	2.136054	2.131387	2.126712	2.122005	2.117303	2.112686	2.108134	2.114682
2.140455	2.135792	2.131126	2.126451	2.121735	2.117023	2.112402	2.10785	2.114399
2.140198	2.135533	2.130869	2.126193	2.121475	2.116759	2.112136	2.107584	2.111438
2.13994	2.135275	2.13061	2.125935	2.121215	2.116497	2.111876	2.107326	2.111168
2.139685	2.135018	2.130355	2.12568	2.120961	2.116242	2.111622	2.107075	2.110903
2.139431	2.134766	2.130104	2.125431	2.12071	2.115989	2.111368	2.106821	2.11064
2.13918	2.134517	2.129858	2.125186	2.120463	2.115739	2.111116	2.106568	2.110379
2.138933	2.13427	2.129612	2.124942	2.120218	2.115492	2.110866	2.106316	2.110118
2.138691	2.134026	2.129368	2.124697	2.119973	2.115247	2.110619	2.106065	2.109855
2.138447	2.133783	2.129127	2.124457	2.119736	2.115013	2.110381	2.105817	2.109593
2.138201	2.133542	2.128889	2.124223	2.119508	2.114788	2.110146	2.105562	2.109324

2.137959	2.133304	2.128654	2.123994	2.119293	2.114588	2.10994	2.105337	2.109077
2.137714	2.133061	2.128418	2.123773	2.119109	2.114444	2.109807	2.10519	2.108888
2.137485	2.13283	2.128186	2.123553	2.11893	2.114313	2.109691	2.105065	2.108717
2.137321	2.132648	2.127974	2.123319	2.118708	2.114116	2.109503	2.104877	2.108504
2.137201	2.132501	2.127775	2.123076	2.118458	2.113874	2.109267	2.104649	2.108267
2.137034	2.132324	2.127572	2.122848	2.118218	2.113629	2.109104	2.104481	2.108039
2.136813	2.132111	2.127361	2.122632	2.117983	2.113373	2.108841	2.104232	2.107821
2.136597	2.131901	2.127165	2.122446	2.117785	2.113155	2.108609	2.103996	2.107598
2.1364	2.131695	2.126993	2.122306	2.117651	2.113014	2.108432	2.103778	2.10735
2.136219	2.131507	2.126837	2.122182	2.117532	2.112885	2.108272	2.103581	2.10712
2.13606	2.131362	2.126704	2.122053	2.117385	2.112714	2.108095	2.103417	2.113499
2.135919	2.131242	2.126583	2.121921	2.117228	2.112531	2.10792	2.103276	2.113361
2.135788	2.131113	2.126437	2.121758	2.117065	2.112375	2.10777	2.103141	2.113206
2.135669	2.130982	2.126271	2.121569	2.116894	2.112237	2.107642	2.103016	2.113044
2.13553	2.130831	2.12611	2.121403	2.116741	2.112104	2.114813	2.110123	2.105425
2.135363	2.130651	2.125949	2.121264	2.116613	2.111978	2.114644	2.109921	2.105221
2.1352	2.130486	2.125807	2.121143	2.116489	2.11184	2.114468	2.109729	2.105034
2.13505	2.130359	2.1257	2.121042	2.116352	2.111656	2.114268	2.109563	2.104894
2.134914	2.130251	2.125609	2.120951	2.116226	2.111487	2.114087	2.109418	2.104771
2.134806	2.130157	2.125516	2.12086	2.123999	2.119224	2.114001	2.10931	2.104649
2.134718	2.130073	2.125425	2.12077	2.123937	2.119207	2.113964	2.109224	2.104532
2.133885	2.129979	2.125331	2.120676	2.123827	2.119097	2.113852	2.109113	2.104418
2.133784	2.129878	2.125235	2.120576	2.123672	2.118903	2.11367	2.108979	2.104308
2.133684	2.137697	2.133015	2.128311	2.123543	2.11876	2.11353	2.108857	2.104195
2.133589	2.137583	2.132899	2.128202	2.123466	2.118718	2.113471	2.108759	2.104078
2.133492	2.137469	2.132783	2.128091	2.12338	2.118659	2.113398	2.108654	2.10396
2.133384	2.136898	2.132208	2.127504	2.123233	2.118485	2.11323	2.108518	2.103844
2.133278	2.136782	2.132092	2.12738	2.123079	2.118295	2.113049	2.108378	2.103728
2.141363	2.136653	2.131971	2.127276	2.12252	2.117749	2.112965	2.108266	2.103602
2.14128	2.136521	2.131848	2.127183	2.122476	2.11775	2.112941	2.108175	2.103475
2.141231	2.136454	2.131757	2.127079	2.122396	2.117707	2.112905	2.108128	2.10341
2.141228	2.136463	2.131697	2.126955	2.122276	2.117619	2.112859	2.108133	2.103413
2.141193	2.13645	2.131652	2.126874	2.121819	2.117157	2.112482	2.107803	2.102592
2.141091	2.136382	2.131628	2.126874	2.12177	2.117052	2.112377	2.107735	2.102558
2.140979	2.136294	2.131597	2.126883	2.12174	2.11697	2.112288	2.10767	2.102526
2.140892	2.136186	2.131518	2.12684	2.121712	2.116954	2.112277	2.107657	2.102526
2.140824	2.136078	2.131426	2.126781	2.121693	2.116974	2.1123	2.107658	2.102528
2.140811	2.136042	2.131391	2.126756	2.121688	2.11699	2.112299	2.10761	2.102209
2.140844	2.136064	2.131398	2.126755	2.121697	2.117009	2.112283	2.107527	2.102091
2.140865	2.136081	2.131385	2.126715	2.121662	2.116992	2.112257	2.107476	2.102019
2.140867	2.136089	2.131339	2.12662	2.121569	2.116925	2.112217	2.107467	2.102002
2.140871	2.136104	2.13131	2.126548	2.121492	2.116864	2.112184	2.107472	2.102009
2.140897	2.136141	2.131326	2.126542	2.121473	2.116845	2.112181	2.107498	2.102046
2.14091	2.136168	2.131352	2.126559	2.121469	2.116825	2.112169	2.107513	2.102079
2.140853	2.136135	2.131347	2.126568	2.121441	2.116752	2.112092	2.107459	2.102054
2.140763	2.136073	2.131333	2.126582	2.121408	2.11666	2.111988	2.107374	2.102001
2.140731	2.135661	2.130934	2.126189	2.121407	2.116627	2.111948	2.107343	2.101498
2.140748	2.135676	2.130957	2.126218	2.121433	2.116647	2.111964	2.107358	2.101512
2.140779	2.135703	2.130988	2.126253	2.121466	2.116678	2.111994	2.107388	2.101539
2.14082	2.135742	2.131027	2.126292	2.121506	2.116718	2.112035	2.10743	2.101583

2.140867	2.135785	2.131069	2.126333	2.121548	2.116761	2.11208	2.107476	2.10163
2.14091	2.135826	2.131109	2.126373	2.121587	2.116799	2.112118	2.107516	2.101674
2.140955	2.135867	2.131151	2.126415	2.121628	2.116841	2.112159	2.107557	2.101718
2.140998	2.135902	2.13119	2.12646	2.121679	2.116894	2.112208	2.107595	2.101756
2.141046	2.135936	2.13123	2.12651	2.121737	2.116959	2.112266	2.107636	2.101793
2.14115	2.136017	2.131294	2.126566	2.121816	2.117071	2.11238	2.107731	2.101869
2.141318	2.13615	2.131382	2.126629	2.121921	2.117236	2.112557	2.107887	2.10199
2.141458	2.136265	2.131467	2.126696	2.122014	2.117369	2.112699	2.108016	2.102097
2.141533	2.136339	2.131534	2.126755	2.122069	2.117423	2.112757	2.108082	2.102168
2.141595	2.136404	2.131611	2.126839	2.122143	2.117482	2.112813	2.108143	2.102239
2.141231	2.136476	2.131727	2.126996	2.1223	2.117621	2.112212	2.107512	2.102329
2.141311	2.136549	2.131854	2.127176	2.122482	2.117785	2.112351	2.107621	2.102437
2.141384	2.136617	2.131939	2.127278	2.12259	2.117896	2.112473	2.10776	2.102589
2.141454	2.136684	2.131994	2.127326	2.122647	2.117971	2.112586	2.107925	2.102775
2.141556	2.136796	2.132096	2.127415	2.122726	2.118045	2.112674	2.108039	2.102902
2.141697	2.136964	2.132258	2.127557	2.122833	2.118112	2.112722	2.108083	2.102949
2.141851	2.137134	2.132417	2.127695	2.12295	2.118206	2.112801	2.108151	2.103011
2.142017	2.137292	2.132545	2.127801	2.12307	2.118353	2.112954	2.108292	2.103134
2.14218	2.137438	2.132667	2.127911	2.123203	2.118519	2.113127	2.10845	2.103272
2.142307	2.137553	2.132796	2.128057	2.123359	2.118681	2.113277	2.108575	2.103392
2.142418	2.137657	2.132932	2.128225	2.123531	2.118843	2.113419	2.108691	2.103513
2.142553	2.137803	2.133099	2.128403	2.12369	2.118975	2.113547	2.108829	2.103667
2.142706	2.137986	2.133293	2.128593	2.12384	2.119077	2.113657	2.108985	2.103851
2.142879	2.138178	2.133492	2.12879	2.123318	2.118536	2.113368	2.108692	2.104038
2.143075	2.13838	2.133694	2.128997	2.12356	2.118809	2.113626	2.108904	2.104224
2.143277	2.138583	2.133894	2.129201	2.123789	2.119062	2.113869	2.109114	2.104414
2.143474	2.13878	2.13409	2.129389	2.12395	2.119198	2.114017	2.109297	2.104614
2.14367	2.138975	2.134282	2.129572	2.1241	2.119316	2.11415	2.109477	2.104816
2.140932	2.139168	2.134473	2.129764	2.124308	2.119538	2.114369	2.109679	2.105011
2.14112	2.139359	2.134662	2.129961	2.124545	2.119815	2.114631	2.109892	2.105202
2.141301	2.139547	2.13485	2.130149	2.124736	2.120008	2.114825	2.110085	2.105396
2.141473	2.13973	2.135035	2.130328	2.124877	2.120111	2.114944	2.110255	2.105593
2.141663	2.139915	2.135223	2.130518	2.125053	2.120273	2.11511	2.110437	2.105784
2.141874	2.140093	2.135409	2.130728	2.125301	2.120553	2.115367	2.110634	2.105951
2.142111	2.1403	2.13561	2.13094	2.125552	2.120844	2.115643	2.110859	2.106144
2.142395	2.140591	2.135846	2.131129	2.125751	2.121075	2.115903	2.111142	2.111009
2.142693	2.140913	2.136105	2.131324	2.12594	2.121284	2.116152	2.111445	2.111314
2.13825	2.138195	2.133408	2.128636	2.126174	2.121483	2.116357	2.111689	2.111577
2.138442	2.138427	2.133691	2.128948	2.126442	2.121672	2.11653	2.111894	2.111813
2.138648	2.138065	2.133358	2.128638	2.12671	2.121916	2.121366	2.116698	2.112033
2.138864	2.138237	2.133545	2.128859	2.126981	2.122228	2.121656	2.116917	2.112224
2.139111	2.138445	2.133746	2.129069	2.127238	2.122532	2.121951	2.117167	2.112455
2.139404	2.138726	2.133977	2.12926	2.127451	2.122782	2.122227	2.11747	2.11277
2.139719	2.139043	2.134239	2.129475	2.127678	2.123038	2.122512	2.117798	2.113111
2.140049	2.134946	2.130149	2.125391	2.132746	2.128094	2.122832	2.118128	2.113429
2.140381	2.135299	2.130508	2.125749	2.133097	2.12844	2.123172	2.118466	2.113745
2.140655	2.1356	2.130833	2.126093	2.133443	2.128777	2.12351	2.118813	2.114085
2.140876	2.135854	2.131135	2.126433	2.133789	2.129112	2.12385	2.119168	2.114447
2.141141	2.136138	2.131448	2.126765	2.127402	2.122734	2.124142	2.119477	2.114772
2.141468	2.136462	2.131777	2.127087	2.127675	2.122951	2.124357	2.119714	2.115041

2.141813	2.142145	2.137444	2.132728	2.12796	2.123192	2.124593	2.119967	2.115319
2.14216	2.142489	2.137786	2.133067	2.12829	2.123513	2.124912	2.120287	2.115642
2.142512	2.142839	2.138133	2.133411	2.128638	2.123865	2.125265	2.120634	2.115984
2.142862	2.142989	2.138266	2.133527	2.128739	2.123951	2.119266	2.114658	2.116333
2.143213	2.143338	2.138613	2.133874	2.129089	2.124305	2.119621	2.115011	2.116691
2.143563	2.143686	2.13896	2.13422	2.129437	2.124655	2.11997	2.115358	2.117044
2.143912	2.144033	2.139306	2.134567	2.129784	2.125003	2.120316	2.1157	2.117393
2.149752	2.144379	2.139653	2.134914	2.13013	2.125348	2.120659	2.116042	2.114281
2.150097	2.144725	2.14	2.13526	2.130473	2.125687	2.120998	2.116383	2.114632
2.150442	2.145072	2.140348	2.135609	2.130821	2.126033	2.121344	2.116728	2.114984
2.150786	2.145419	2.140699	2.135964	2.131179	2.126393	2.121697	2.117071	2.115326
2.151133	2.145768	2.141052	2.136323	2.131549	2.126771	2.122071	2.117429	2.115677
2.151479	2.146317	2.14162	2.13692	2.132196	2.127474	2.122512	2.117855	2.116088
2.151829	2.146663	2.14197	2.137289	2.132615	2.127949	2.123003	2.118331	2.116542
2.152235	2.147058	2.142349	2.137662	2.133008	2.12837	2.123433	2.11876	2.114074
2.152713	2.147512	2.14276	2.138034	2.133363	2.128722	2.123791	2.119135	2.114478
2.152716	2.147956	2.143184	2.138437	2.133752	2.1291	2.124163	2.11951	2.114869
2.153102	2.148364	2.143627	2.138907	2.13422	2.129552	2.124586	2.119895	2.115231
2.153474	2.148757	2.144061	2.139374	2.134693	2.130014	2.125022	2.120296	2.115605
2.153866	2.149132	2.144437	2.13976	2.135093	2.13043	2.125452	2.120734	2.116045
2.15427	2.149504	2.144795	2.140117	2.135468	2.130834	2.125885	2.121194	2.116517
2.147195	2.149935	2.145218	2.140537	2.135896	2.131276	2.126336	2.121652	2.116971
2.147645	2.150416	2.145695	2.141005	2.136366	2.131748	2.126804	2.122112	2.117416
2.148098	2.150885	2.146148	2.141441	2.136793	2.132171	2.127227	2.122543	2.11785
2.148558	2.151335	2.146563	2.141822	2.137151	2.132516	2.127582	2.122929	2.118265
2.149007	2.151773	2.146982	2.142223	2.137534	2.132888	2.127961	2.123332	2.118695
2.149428	2.152188	2.147418	2.142682	2.137998	2.133349	2.128419	2.123791	2.11916
2.149844	2.152607	2.14787	2.143167	2.138494	2.133846	2.12891	2.124273	2.119641
2.147692	2.145575	2.140916	2.136287	2.138989	2.134341	2.129404	2.124762	2.120128
2.148143	2.146059	2.141414	2.136791	2.139492	2.134845	2.129908	2.125259	2.120621
2.148615	2.146552	2.141908	2.137282	2.139991	2.135344	2.130404	2.12575	2.126651
2.149109	2.147056	2.142401	2.137764	2.140489	2.135839	2.130892	2.126235	2.127124
2.149602	2.147555	2.142895	2.138253	2.140987	2.136336	2.131385	2.126723	2.127594
2.150088	2.148042	2.14339	2.138753	2.141488	2.136837	2.131884	2.127213	2.128052
2.150575	2.14853	2.143885	2.139254	2.14199	2.13734	2.132386	2.127711	2.128528
2.14918	2.146428	2.141798	2.137183	2.142496	2.137842	2.132891	2.128228	2.129053
2.149671	2.146926	2.142292	2.137673	2.143002	2.138343	2.133397	2.128751	2.129596
2.150152	2.147411	2.142781	2.138165	2.143501	2.138842	2.133899	2.129257	2.13011
2.150626	2.14789	2.143268	2.13866	2.143997	2.139339	2.134399	2.129755	2.130608
2.151129	2.146486	2.141873	2.137275	2.134558	2.129963	2.135192	2.13057	2.131107
2.151662	2.146974	2.142367	2.13778	2.135056	2.130449	2.135677	2.131065	2.131607
2.152217	2.147505	2.142898	2.138316	2.133702	2.12909	2.1362	2.131575	2.132108
2.152807	2.148119	2.143504	2.138909	2.134305	2.129703	2.136792	2.1321	2.132595
2.153393	2.148738	2.144111	2.139502	2.134908	2.130317	2.137398	2.132649	2.133111
2.153921	2.149276	2.144644	2.140028	2.135435	2.130852	2.140584	2.13583	2.135704
2.154432	2.149788	2.145153	2.140533	2.135934	2.131351	2.141136	2.136441	2.136352
2.155002	2.150352	2.14572	2.141098	2.136472	2.131858	2.141654	2.136995	2.136
2.155619	2.150955	2.146332	2.141709	2.137036	2.132364	2.14214	2.137508	2.136533
2.156197	2.151525	2.146908	2.142289	2.137605	2.132918	2.142687	2.138064	2.135339
2.156715	2.152043	2.147428	2.142821	2.138175	2.133533	2.143313	2.138681	2.135956

2.157256	2.152582	2.147963	2.143363	2.138756	2.134159	2.143952	2.139306	2.136579
2.157866	2.153185	2.148555	2.143951	2.139363	2.134791	2.14459	2.13993	2.137205
2.158501	2.153809	2.149159	2.144541	2.139961	2.135406	2.14521	2.140536	2.137813
2.159131	2.154426	2.149733	2.145076	2.140492	2.135945	2.138472	2.133849	2.138816
2.159757	2.155041	2.150295	2.145589	2.14099	2.136444	2.138975	2.134354	2.139323
2.160331	2.15562	2.150873	2.146163	2.141557	2.137003	2.139531	2.134915	2.139893
2.160852	2.156164	2.151463	2.146791	2.142185	2.137616	2.140135	2.135526	2.140517
2.161412	2.156739	2.152072	2.147427	2.142822	2.138242	2.140753	2.136147	2.141151
2.162037	2.157365	2.152704	2.148066	2.14346	2.138877	2.141383	2.136775	2.14179
2.162681	2.158006	2.153345	2.148707	2.1441	2.139517	2.14202	2.137406	2.142431
2.163327	2.15865	2.153988	2.149349	2.151938	2.147289	2.145472	2.140827	2.143067
2.163978	2.159297	2.154632	2.14999	2.152579	2.147929	2.146113	2.141461	2.143703
2.164624	2.15994	2.155271	2.150626	2.153216	2.148565	2.14675	2.142094	2.137462
2.165267	2.160579	2.155908	2.15126	2.153851	2.149199	2.147386	2.142726	2.138091
2.165913	2.161222	2.156545	2.151895	2.154487	2.149838	2.148031	2.143374	2.138735
2.166558	2.161865	2.157183	2.152528	2.155123	2.150482	2.148685	2.144033	2.139391
2.167214	2.162517	2.15783	2.153173	2.155768	2.151131	2.149349	2.144717	2.140081
2.167874	2.163168	2.158481	2.153826	2.156414	2.151776	2.150026	2.145445	2.140836
2.168561	2.163847	2.15916	2.154505	2.157083	2.15244	2.150713	2.146172	2.141585
2.169317	2.1646	2.159908	2.155246	2.15781	2.153153	2.151414	2.146854	2.142257
2.170099	2.16538	2.160682	2.15601	2.15856	2.153889	2.152122	2.147514	2.142894
2.170809	2.166091	2.161392	2.156718	2.162178	2.15748	2.152811	2.14817	2.143534
2.171465	2.166751	2.162054	2.157384	2.162859	2.158168	2.153486	2.148819	2.144172
2.172129	2.174805	2.170043	2.165311	2.163551	2.158881	2.154198	2.149514	2.144859
2.172794	2.17548	2.170709	2.165978	2.164256	2.159632	2.154964	2.150274	2.145615
2.173498	2.176186	2.17141	2.166678	2.164971	2.160364	2.155706	2.151019	2.146359
2.174276	2.176953	2.172181	2.167441	2.165695	2.161037	2.156368	2.1517	2.147042
2.175079	2.177742	2.172976	2.168229	2.166433	2.161707	2.157022	2.152374	2.147721
2.175868	2.181533	2.176747	2.171975	2.167198	2.162439	2.157746	2.153106	2.148457
2.176647	2.182312	2.177532	2.172762	2.167974	2.1632	2.1585	2.153859	2.149216
2.177368	2.183044	2.178256	2.17348	2.168691	2.163918	2.159211	2.154558	2.149924
2.178033	2.18373	2.178922	2.174129	2.169352	2.164598	2.159884	2.155207	2.150586
2.178737	2.184444	2.179628	2.174828	2.170056	2.165308	2.160587	2.155895	2.151274
2.179502	2.185202	2.180397	2.175607	2.170824	2.166059	2.161333	2.156642	2.152003
2.180291	2.185981	2.181186	2.176404	2.171612	2.166833	2.162103	2.157413	2.152752
2.181097	2.18678	2.181979	2.177193	2.172405	2.167631	2.162896	2.158195	2.153519
2.181909	2.187591	2.18278	2.177987	2.173206	2.168442	2.163703	2.158988	2.1543
2.182688	2.188396	2.183584	2.178787	2.174005	2.169241	2.164501	2.159782	2.155089
2.183451	2.1892	2.184393	2.179591	2.174805	2.170035	2.165295	2.160581	2.155886
2.1948	2.190009	2.185197	2.180391	2.175604	2.170835	2.166092	2.161374	2.156677
2.195647	2.190823	2.185997	2.181184	2.176402	2.171641	2.166893	2.162161	2.15746
2.196478	2.191637	2.186801	2.181983	2.177201	2.172444	2.167693	2.162953	2.158252
2.197269	2.192449	2.187614	2.182791	2.178002	2.173236	2.168486	2.163753	2.159059
2.198062	2.193261	2.188426	2.183599	2.178803	2.174031	2.169283	2.164561	2.159876
2.198893	2.194075	2.189229	2.184396	2.179602	2.174837	2.170094	2.165376	2.160702
2.199736	2.194889	2.190029	2.185191	2.180404	2.175652	2.170916	2.166201	2.161538
2.200559	2.195695	2.190833	2.185998	2.181218	2.176476	2.171753	2.167054	2.162395
2.201371	2.196497	2.19164	2.186815	2.182043	2.17731	2.172605	2.167929	2.16327
2.196698	2.197308	2.192457	2.187643	2.182883	2.178162	2.173466	2.168796	2.164129
2.197512	2.198123	2.193281	2.188482	2.183741	2.179039	2.17434	2.169651	2.164967

2.198365	2.198979	2.194136	2.189337	2.184604	2.17991	2.175205	2.1705	2.165805
2.199299	2.199916	2.195049	2.190223	2.18547	2.180759	2.176042	2.171331	2.166644
2.200244	2.200865	2.195971	2.191112	2.186334	2.181602	2.176878	2.17217	2.167496
2.195713	2.201742	2.196838	2.191968	2.18718	2.182443	2.177732	2.173051	2.168381
2.196588	2.20259	2.197686	2.192817	2.188026	2.183289	2.178599	2.173956	2.169288
2.197488	2.203479	2.198588	2.193726	2.188926	2.184176	2.179492	2.174866	2.170202
2.198407	2.204403	2.199538	2.19469	2.189876	2.185102	2.18041	2.175787	2.171127
2.199313	2.200228	2.195368	2.19052	2.190808	2.186029	2.181324	2.176679	2.17201
2.200189	2.201089	2.196239	2.191412	2.191705	2.186956	2.182229	2.177525	2.172825
2.201088	2.201969	2.197121	2.192308	2.192601	2.187879	2.183135	2.178382	2.173654
2.202051	2.202918	2.198061	2.193245	2.193516	2.188801	2.184057	2.179299	2.174561
2.203039	2.203896	2.199026	2.194196	2.194432	2.189708	2.184968	2.180223	2.175486
2.204021	2.199129	2.194274	2.189452	2.190349	2.185583	2.185819	2.181082	2.176361
2.204999	2.200112	2.195248	2.190405	2.191258	2.186441	2.186635	2.181906	2.177212
2.205924	2.201051	2.196204	2.191367	2.192202	2.187354	2.187509	2.182779	2.178098
2.206789	2.201942	2.197139	2.192339	2.193181	2.188323	2.188442	2.183701	2.179017
2.207684	2.202855	2.198085	2.193315	2.194165	2.189301	2.184495	2.179733	2.179949
2.208638	2.203808	2.19905	2.194292	2.195147	2.190282	2.185473	2.180707	2.180892
2.209616	2.20478	2.200025	2.195271	2.19613	2.191265	2.186455	2.181686	2.18184
2.210608	2.205767	2.201009	2.196252	2.197111	2.192244	2.187432	2.182661	2.177952
2.21161	2.206765	2.201999	2.197236	2.198094	2.193225	2.188411	2.183638	2.178927
2.212608	2.207762	2.202994	2.198229	2.193397	2.188554	2.189404	2.184617	2.1799
2.213603	2.208759	2.203992	2.199229	2.194404	2.189566	2.190408	2.1856	2.180874
2.209142	2.209753	2.204989	2.200235	2.195432	2.190615	2.191458	2.186631	2.181889
2.210124	2.210738	2.205983	2.20125	2.196487	2.191715	2.192567	2.187723	2.182954
2.211137	2.206781	2.202016	2.197282	2.197526	2.192791	2.193657	2.188806	2.184018
2.212212	2.207829	2.203028	2.198268	2.198525	2.193809	2.194688	2.189847	2.185049
2.213299	2.20889	2.204045	2.19925	2.199511	2.194807	2.195705	2.190886	2.186087
2.214342	2.209925	2.205046	2.200223	2.195491	2.190802	2.19108	2.186322	2.187176
2.215371	2.210957	2.206051	2.201204	2.196472	2.191797	2.192116	2.187407	2.188283
2.216434	2.211522	2.206603	2.201733	2.197486	2.192783	2.188088	2.183409	2.189336
2.217524	2.212646	2.207727	2.202833	2.198529	2.193761	2.18905	2.184391	2.190343
2.218575	2.213732	2.20884	2.203952	2.199612	2.19479	2.190051	2.18538	2.186705
2.21957	2.214768	2.20994	2.205101	2.200752	2.195887	2.1911	2.186375	2.187696
2.220558	2.215774	2.211004	2.206216	2.201881	2.197003	2.192182	2.187401	2.182674
2.221549	2.216738	2.211994	2.207251	2.202968	2.198135	2.19331	2.188488	2.183726
2.222562	2.217702	2.21296	2.208243	2.204021	2.199254	2.194444	2.189601	2.184806
2.223651	2.218741	2.213954	2.209212	2.205015	2.200292	2.195518	2.190709	2.185918
2.224792	2.219831	2.214967	2.210165	2.205969	2.201278	2.196554	2.191815	2.187051
2.225919	2.220953	2.216041	2.211191	2.206972	2.202271	2.197563	2.192857	2.18812
2.227039	2.222116	2.217182	2.212287	2.208015	2.203261	2.198532	2.193831	2.189124
2.223473	2.223247	2.218315	2.213408	2.208573	2.203784	2.199569	2.194851	2.190149
2.224527	2.224305	2.219415	2.214551	2.209742	2.204964	2.200728	2.195958	2.191213
2.225605	2.225381	2.220524	2.215699	2.210913	2.206147	2.201897	2.197085	2.192303
2.226788	2.226562	2.221681	2.216825	2.212014	2.207229	2.202985	2.198197	2.193434
2.227994	2.227768	2.222848	2.217947	2.2131	2.20829	2.204059	2.199311	2.19458
2.226719	2.224234	2.219363	2.214505	2.214248	2.209434	2.205197	2.200436	2.195693
2.227729	2.225266	2.220458	2.215661	2.215433	2.210632	2.206377	2.201568	2.196787
2.2288	2.223983	2.219187	2.214406	2.211999	2.207244	2.207516	2.202697	2.197907
2.229942	2.225077	2.220243	2.21544	2.213028	2.208295	2.2086	2.20382	2.19906

2.2311	2.226207	2.221346	2.216525	2.214104	2.209384	2.209147	2.204385	2.200203
2.232254	2.22739	2.222542	2.217728	2.215303	2.210579	2.210314	2.205515	2.201305
2.233426	2.228597	2.223765	2.218957	2.216519	2.21178	2.211483	2.206651	2.202414
2.234649	2.229818	2.224966	2.220132	2.215341	2.210572	2.208116	2.203341	2.203575
2.235889	2.231039	2.226158	2.22129	2.21646	2.21166	2.209212	2.204494	2.204763
2.237055	2.23219	2.22732	2.222465	2.217629	2.212819	2.20807	2.203371	2.200963
2.238159	2.233278	2.228454	2.223652	2.218836	2.214035	2.209291	2.204592	2.20216
2.239314	2.234424	2.229626	2.22485	2.22004	2.215234	2.210479	2.205763	2.203314
2.240544	2.235654	2.230857	2.226074	2.221241	2.216403	2.21161	2.206851	2.204392
2.241781	2.236884	2.23207	2.227269	2.222423	2.217574	2.212759	2.207973	2.203234
2.242993	2.238067	2.233204	2.228371	2.22355	2.218749	2.213963	2.209194	2.204468
2.244201	2.239246	2.234334	2.229473	2.224685	2.219944	2.215198	2.210459	2.205747
2.245407	2.240454	2.235544	2.230689	2.225916	2.221193	2.216463	2.211738	2.207026
2.246615	2.241681	2.236803	2.231974	2.227206	2.222477	2.21775	2.213031	2.208311
2.247858	2.242945	2.238086	2.233269	2.228494	2.223751	2.219019	2.2143	2.209567
2.249134	2.244247	2.239399	2.234581	2.229789	2.225021	2.220276	2.215552	2.210799
2.242208	2.245554	2.240703	2.235871	2.231052	2.226251	2.221482	2.21674	2.211975
2.243512	2.246867	2.241995	2.237132	2.232269	2.227418	2.222604	2.217821	2.213053
2.244815	2.248166	2.243264	2.238366	2.233465	2.228575	2.223728	2.218916	2.214154
2.246102	2.249434	2.244493	2.239561	2.234645	2.229752	2.224911	2.220117	2.215371
2.247373	2.25068	2.245699	2.240739	2.235821	2.230943	2.226128	2.22137	2.216642
2.243436	2.251893	2.246895	2.241931	2.237026	2.232171	2.227377	2.22264	2.217923
2.244635	2.253081	2.248083	2.243132	2.238253	2.233431	2.228657	2.223933	2.219221
2.245832	2.254287	2.249303	2.244373	2.239512	2.234706	2.229944	2.225224	2.220514
2.247023	2.25551	2.250557	2.245655	2.240805	2.236001	2.231237	2.226514	2.221802
2.248236	2.248478	2.243591	2.238749	2.242111	2.237303	2.232535	2.227806	2.223091
2.249485	2.249758	2.244889	2.240057	2.243427	2.238607	2.233833	2.2291	2.224382
2.250759	2.25106	2.246202	2.241373	2.244749	2.239917	2.235135	2.230394	2.225672
2.25206	2.252378	2.247521	2.242689	2.246071	2.241233	2.23644	2.231688	2.22696
2.253383	2.25371	2.248847	2.244007	2.247396	2.242553	2.237749	2.232982	2.228246
2.25471	2.255041	2.250171	2.245323	2.24872	2.243872	2.239058	2.234275	2.229531
2.256043	2.256374	2.251494	2.246637	2.250043	2.245192	2.240366	2.235567	2.230815
2.257368	2.257702	2.252814	2.247949	2.24311	2.238295	2.241679	2.236866	2.232106
2.258681	2.259023	2.254128	2.249256	2.244418	2.239606	2.242998	2.238174	2.233404
2.259991	2.260341	2.255439	2.250562	2.245724	2.240914	2.244315	2.239486	2.234706
2.261283	2.261639	2.256738	2.251862	2.247021	2.242206	2.245619	2.240791	2.236006
2.262603	2.262955	2.258051	2.253173	2.248326	2.243506	2.246931	2.242108	2.237319
2.264026	2.2591	2.254197	2.249328	2.249653	2.24483	2.248267	2.243446	2.238651
2.265514	2.260528	2.255582	2.250683	2.251008	2.246184	2.249632	2.244812	2.240013
2.266992	2.261997	2.257047	2.252144	2.252473	2.247644	2.242835	2.23805	2.241478
2.268473	2.263517	2.258599	2.253717	2.254053	2.249216	2.244402	2.239612	2.243051
2.269891	2.264964	2.260071	2.255208	2.25555	2.250709	2.245892	2.241098	2.244547
2.271214	2.266295	2.261416	2.256564	2.256914	2.252074	2.247255	2.242457	2.245916
2.272512	2.267592	2.262719	2.257872	2.25823	2.253392	2.248573	2.243771	2.24724
2.27381	2.268887	2.264007	2.259156	2.25952	2.254686	2.249866	2.245061	2.240276
2.275112	2.270182	2.26529	2.260429	2.260796	2.255967	2.251147	2.24634	2.241552
2.276445	2.271506	2.266604	2.261735	2.256902	2.252098	2.252455	2.247648	2.242858
2.277808	2.272859	2.267947	2.263069	2.25823	2.253422	2.253787	2.248981	2.244189
2.279281	2.274322	2.269399	2.264511	2.259665	2.254851	2.255219	2.250411	2.245615
2.280888	2.275921	2.270985	2.266085	2.26123	2.256409	2.256773	2.25196	2.24716

2.280678	2.277457	2.272509	2.267597	2.262733	2.257906	2.258267	2.253451	2.248651
2.28207	2.278851	2.273889	2.268963	2.264092	2.25926	2.259623	2.25481	2.250017
2.283425	2.280207	2.275232	2.270294	2.265414	2.260577	2.260944	2.256135	2.251354
2.284787	2.279806	2.274828	2.269886	2.266749	2.261902	2.257088	2.25231	2.252706
2.286165	2.281179	2.276196	2.271247	2.268103	2.263243	2.258425	2.253648	2.254071
2.283335	2.282671	2.277686	2.272732	2.269582	2.264706	2.259877	2.255094	2.255511
2.284923	2.284261	2.279276	2.274321	2.271164	2.266268	2.261424	2.256627	2.257013
2.286446	2.285785	2.280799	2.275839	2.270901	2.265995	2.262904	2.258096	2.258464
2.287871	2.28721	2.282218	2.277252	2.272302	2.267381	2.264284	2.259466	2.259834
2.284721	2.288665	2.283668	2.278695	2.273734	2.268801	2.263929	2.25911	2.261244
2.286306	2.290238	2.285233	2.280251	2.27528	2.270338	2.265457	2.26063	2.262773
2.287896	2.291816	2.286804	2.281814	2.276835	2.271885	2.266997	2.262162	2.264316
2.28936	2.288902	2.283924	2.278969	2.278276	2.27332	2.268424	2.263579	2.264183
2.290767	2.290296	2.285316	2.280359	2.27967	2.274712	2.269807	2.264952	2.265577
2.292271	2.287243	2.282254	2.277292	2.281172	2.276219	2.271306	2.266432	2.261644
2.293861	2.288835	2.283846	2.278886	2.282774	2.277832	2.272909	2.268009	2.263175
2.295389	2.290365	2.285373	2.280416	2.280199	2.275279	2.274492	2.269587	2.264727
2.296795	2.291775	2.286775	2.281823	2.28166	2.276802	2.276043	2.271169	2.26632
2.29823	2.293213	2.288206	2.283254	2.283123	2.278301	2.277556	2.272707	2.267868
2.299807	2.294791	2.289773	2.2848	2.284635	2.279764	2.278982	2.27412	2.269271
2.301423	2.296411	2.291391	2.286399	2.286181	2.281237	2.280401	2.275513	2.270645
2.302958	2.297976	2.292993	2.288025	2.283067	2.278127	2.277886	2.272972	2.272152
2.304449	2.299512	2.294589	2.289669	2.284727	2.279784	2.279536	2.274596	2.273742
2.305952	2.301003	2.296093	2.291196	2.286276	2.281357	2.281136	2.276208	2.271297
2.307455	2.302433	2.297488	2.292586	2.287703	2.282843	2.282693	2.27782	2.272943
2.309026	2.303935	2.298949	2.294027	2.289149	2.284312	2.284199	2.27936	2.274511
2.310705	2.305563	2.300539	2.295577	2.290645	2.285756	2.285614	2.280758	2.275929
2.312415	2.307242	2.302181	2.297175	2.292186	2.287236	2.287051	2.282164	2.277337
2.299256	2.308945	2.303851	2.298807	2.293799	2.288832	2.283903	2.279014	2.278841
2.300831	2.31066	2.305542	2.300467	2.295456	2.290489	2.285539	2.280612	2.280395
2.302339	2.312314	2.307205	2.30213	2.297116	2.292139	2.287175	2.282229	2.281994
2.303791	2.31392	2.308848	2.303799	2.298784	2.293795	2.288824	2.283874	2.283636
2.305236	2.31548	2.310435	2.305415	2.300419	2.295443	2.290477	2.285522	2.285282
2.306666	2.316975	2.311948	2.306968	2.302018	2.297089	2.292137	2.287172	2.28693
2.302871	2.318512	2.313472	2.308503	2.303588	2.298707	2.293776	2.288816	2.283879
2.304417	2.320145	2.315033	2.310012	2.305108	2.300266	2.295369	2.290444	2.285523
2.306001	2.321826	2.316633	2.311547	2.306637	2.301818	2.296952	2.292069	2.287168
2.307598	2.307966	2.303121	2.298356	2.308253	2.303416	2.298559	2.293706	2.288817
2.309211	2.309566	2.304717	2.299945	2.309922	2.305042	2.300179	2.295347	2.290467
2.310814	2.305912	2.30108	2.296323	2.311604	2.306691	2.301816	2.296986	2.292106
2.31241	2.307496	2.302668	2.297915	2.313306	2.308365	2.30347	2.298623	2.293735
2.314004	2.309078	2.30425	2.299497	2.315004	2.310046	2.305131	2.300262	2.295361
2.315593	2.310654	2.305821	2.301066	2.316696	2.311735	2.306801	2.301903	2.296985
2.31199	2.312237	2.307393	2.302631	2.297969	2.293371	2.308471	2.303543	2.298606
2.313591	2.313838	2.308973	2.304192	2.299528	2.294932	2.310132	2.305176	2.300221
2.315198	2.315447	2.310557	2.305754	2.301084	2.29649	2.311792	2.306809	2.301837
2.3168	2.317055	2.312146	2.307324	2.302646	2.298047	2.313458	2.30845	2.303463
2.3184	2.318664	2.31374	2.308901	2.304213	2.299605	2.315129	2.310098	2.305096
2.319999	2.320273	2.315337	2.310484	2.305782	2.301161	2.301664	2.297004	2.306745
2.321597	2.32188	2.316935	2.312068	2.307349	2.302712	2.30321	2.298556	2.308411

2.323195	2.323496	2.318566	2.313705	2.308966	2.304298	2.299635	2.294988	2.310084
2.32479	2.325129	2.320258	2.315438	2.310671	2.305946	2.301241	2.296561	2.311761
2.32639	2.326765	2.32195	2.317167	2.312374	2.307597	2.302852	2.298137	2.313447
2.327998	2.323131	2.318371	2.313638	2.313995	2.309202	2.304441	2.299707	2.300125
2.32962	2.324753	2.319976	2.315225	2.315585	2.310791	2.306022	2.301277	2.301709
2.328948	2.32644	2.321642	2.316861	2.317206	2.312388	2.307602	2.302845	2.303276
2.330661	2.328176	2.32335	2.318526	2.318844	2.313989	2.309181	2.304415	2.304834
2.332326	2.32985	2.325013	2.320171	2.32048	2.315602	2.310789	2.306029	2.301339
2.333927	2.331445	2.326622	2.321791	2.322111	2.317223	2.312425	2.307697	2.303017
2.335546	2.330742	2.325926	2.321109	2.323753	2.318875	2.314089	2.309378	2.304699
2.337222	2.332397	2.327558	2.322733	2.325411	2.320578	2.315797	2.311062	2.30636
2.338912	2.334065	2.329206	2.324373	2.327083	2.322293	2.317513	2.312747	2.308019
2.340589	2.335733	2.330882	2.32606	2.328772	2.323982	2.319194	2.314416	2.309681
2.342264	2.337407	2.332578	2.327773	2.330472	2.325659	2.32086	2.316078	2.311346
2.34395	2.339101	2.334278	2.329472	2.326921	2.322134	2.322525	2.317753	2.313025
2.345646	2.340816	2.335985	2.331162	2.328582	2.323772	2.324185	2.319438	2.314715
2.320825	2.34249	2.337662	2.332837	2.328007	2.323193	2.325853	2.321116	2.316391
2.322419	2.344098	2.33929	2.334487	2.329674	2.324873	2.327533	2.322786	2.318048
2.324038	2.345724	2.340932	2.336149	2.33135	2.326558	2.329212	2.324447	2.319696
2.325734	2.347414	2.342629	2.337852	2.333049	2.328247	2.330882	2.326088	2.321333
2.327469	2.349137	2.344347	2.339567	2.33475	2.329931	2.332547	2.327727	2.322972
2.304217	2.324393	2.319612	2.314843	2.33642	2.331581	2.334199	2.329386	2.32464
2.305984	2.326163	2.321336	2.316518	2.338071	2.333213	2.335846	2.33106	2.326327
2.307725	2.314103	2.309286	2.304488	2.31334	2.308542	2.335599	2.330824	2.328022
2.309438	2.315784	2.310925	2.306105	2.314999	2.310254	2.337334	2.332539	2.329724
2.311166	2.306342	2.301509	2.296721	2.316693	2.311966	2.326953	2.322169	2.331441
2.312927	2.30813	2.3033	2.298497	2.318445	2.31366	2.32866	2.323916	2.333181
2.314682	2.309908	2.305083	2.300269	2.320198	2.315359	2.330373	2.325662	2.334917
2.309655	2.311598	2.306758	2.30194	2.308223	2.303468	2.318957	2.314237	2.322647
2.311349	2.313265	2.308408	2.303589	2.309906	2.305191	2.320701	2.315938	2.324334
2.313071	2.315004	2.310147	2.305324	2.300574	2.295876	2.302193	2.297551	2.31294
2.314813	2.316789	2.311946	2.307116	2.302311	2.297546	2.303867	2.299266	2.314695
2.316542	2.318539	2.313724	2.308914	2.30409	2.299296	2.305618	2.301026	2.316481
2.318254	2.320244	2.315479	2.310726	2.30593	2.301148	2.307467	2.302845	2.318309
2.319972	2.315161	2.310453	2.305767	2.307731	2.302978	2.298299	2.293678	2.307067
2.321704	2.316897	2.312139	2.307417	2.309436	2.30474	2.300058	2.295395	2.3088
2.323444	2.318642	2.313828	2.309062	2.311122	2.30647	2.301788	2.297095	2.310517
2.325192	2.320373	2.315558	2.310787	2.312838	2.308163	2.30348	2.298801	2.312241
2.326948	2.322108	2.317317	2.312559	2.314576	2.309845	2.30516	2.300517	2.313973
2.328714	2.32389	2.319119	2.314371	2.316383	2.311625	2.306936	2.302302	2.297674
2.330489	2.325713	2.320962	2.316225	2.318253	2.313489	2.308792	2.30415	2.299516
2.3323	2.327537	2.322782	2.318038	2.313287	2.308553	2.310606	2.305947	2.301306
2.334164	2.329364	2.324566	2.319788	2.315032	2.310302	2.312347	2.30766	2.303013
2.336023	2.331178	2.326346	2.321545	2.316787	2.312064	2.314107	2.309395	2.30474
2.337829	2.332948	2.328125	2.323343	2.318599	2.313887	2.315945	2.311221	2.306555
2.339637	2.334727	2.329919	2.325159	2.320425	2.315718	2.317794	2.313066	2.308389
2.335361	2.336587	2.331762	2.326983	2.322221	2.317487	2.319571	2.314848	2.310165
2.337315	2.338497	2.333639	2.328819	2.324009	2.319229	2.321317	2.316603	2.311915
2.339243	2.334281	2.329383	2.324517	2.325859	2.321049	2.316306	2.31162	2.313703
2.341145	2.336213	2.331332	2.326473	2.327773	2.322947	2.318175	2.313448	2.315523

2.343079	2.338149	2.333254	2.328382	2.323511	2.318659	2.31385	2.309079	2.31737
2.345061	2.340086	2.33512	2.330195	2.325333	2.320518	2.315737	2.310991	2.319266
2.347065	2.342045	2.337009	2.332029	2.327164	2.322371	2.317606	2.312877	2.321136
2.336926	2.344053	2.338986	2.333967	2.329065	2.324233	2.319429	2.314663	2.317108
2.338927	2.346087	2.341007	2.335959	2.331006	2.326111	2.321249	2.316426	2.318871
2.340933	2.348125	2.343042	2.337977	2.332987	2.328045	2.323144	2.318286	2.313515
2.342941	2.350167	2.345091	2.340023	2.335005	2.330024	2.325093	2.320213	2.315413
2.3449	2.352132	2.347073	2.342023	2.337002	2.332009	2.32706	2.322153	2.317328
2.346799	2.354005	2.348976	2.343971	2.338978	2.334004	2.329048	2.324112	2.319265
2.334964	2.343675	2.338735	2.333835	2.340948	2.335998	2.331034	2.326067	2.321198
2.336915	2.345611	2.340656	2.335752	2.342907	2.337979	2.333005	2.328003	2.323109
2.338891	2.34758	2.3426	2.337681	2.344874	2.339964	2.334978	2.329944	2.325027
2.340887	2.349577	2.344583	2.339654	2.346873	2.341967	2.336969	2.331912	2.326973
2.342901	2.351598	2.346591	2.341651	2.34889	2.343979	2.338969	2.333895	2.328938
2.335149	2.353621	2.3486	2.343645	2.350902	2.345985	2.340969	2.335888	2.330916
2.337165	2.35565	2.350612	2.345643	2.352914	2.347989	2.34297	2.337892	2.332907
2.339177	2.348438	2.343392	2.338419	2.35492	2.349989	2.344969	2.339894	2.334896
2.341175	2.350427	2.345367	2.340385	2.356916	2.351983	2.34696	2.34188	2.336868
2.343198	2.338016	2.332969	2.328007	2.337457	2.332599	2.348972	2.343908	2.338889
2.345254	2.340034	2.33496	2.329981	2.339443	2.334592	2.351038	2.346034	2.341027
2.347356	2.342102	2.336989	2.331979	2.341444	2.336595	2.353114	2.348175	2.343186
2.349549	2.344284	2.339105	2.334027	2.343467	2.338592	2.346406	2.341494	2.345252
2.3518	2.346539	2.34128	2.33611	2.345508	2.340589	2.34841	2.343498	2.347271
2.354001	2.348742	2.343431	2.338197	2.347555	2.342588	2.350414	2.345501	2.340548
2.356163	2.350903	2.345562	2.340286	2.349606	2.344585	2.352414	2.347499	2.342577
2.358298	2.353047	2.347712	2.342425	2.33727	2.332214	2.341644	2.336776	2.344575
2.360396	2.355176	2.349902	2.344645	2.339425	2.334265	2.343637	2.338713	2.346512
2.362491	2.357294	2.35208	2.346863	2.341608	2.336376	2.345694	2.340695	2.348472
2.343481	2.3594	2.354209	2.349025	2.343809	2.338603	2.347888	2.342778	2.3505
2.345522	2.361501	2.356316	2.351159	2.346008	2.340866	2.350138	2.344926	2.352588
2.347562	2.363593	2.358405	2.353262	2.348135	2.343024	2.352333	2.347128	2.354786
2.349615	2.365688	2.360485	2.355341	2.350215	2.345116	2.3545	2.349375	2.357067
2.331038	2.367921	2.362661	2.357467	2.352312	2.347201	2.356626	2.351549	2.346441
2.33332	2.370314	2.364943	2.359639	2.35442	2.349271	2.358703	2.353643	2.348573
2.335554	2.372674	2.367235	2.361854	2.356574	2.351373	2.346258	2.341222	2.350668
2.337667	2.37494	2.369523	2.364141	2.358804	2.353522	2.348342	2.343252	2.352688
2.33974	2.377166	2.371785	2.366423	2.361054	2.355717	2.350483	2.345337	2.354749
2.341791	2.336681	2.331585	2.326515	2.363284	2.357996	2.352773	2.347608	2.356995
2.343844	2.338709	2.333598	2.32854	2.365506	2.360306	2.35512	2.349957	2.359322
2.346004	2.340801	2.335653	2.330578	2.367688	2.362493	2.357319	2.352173	2.361569
2.348252	2.342944	2.337739	2.332622	2.369838	2.364587	2.359406	2.354295	2.363764
2.328765	2.345196	2.339955	2.334803	2.352676	2.347427	2.36159	2.356454	2.351354
2.331193	2.3476	2.34235	2.33717	2.355109	2.349787	2.363905	2.35866	2.353486
2.268381	2.328208	2.322925	2.317701	2.334342	2.329211	2.366229	2.360891	2.355653
2.270322	2.330443	2.32515	2.319924	2.336526	2.331385	2.368519	2.363163	2.357913
2.272271	2.332685	2.327373	2.322136	2.338691	2.333538	2.370798	2.365437	2.360184
2.246759	2.335138	2.32977	2.324465	2.331299	2.326051	2.344598	2.339212	2.362349
2.248908	2.337695	2.332251	2.326852	2.333562	2.328207	2.346801	2.341348	2.364482
2.250933	2.293596	2.288749	2.28394	2.323757	2.318363	2.349156	2.343668	2.358813
2.252846	2.295601	2.290748	2.285938	2.326086	2.320734	2.351656	2.346163	2.361295

2.254842	2.297681	2.292815	2.287991	2.328442	2.323082	2.317783	2.312543	2.343199
2.256983	2.299896	2.295	2.290132	2.330827	2.325365	2.320013	2.314753	2.345487
2.259124	2.302104	2.297183	2.292281	2.33323	2.327689	2.32229	2.317007	2.347821
2.261158	2.256377	2.25165	2.246949	2.289463	2.284583	2.324719	2.319409	2.339088
2.263184	2.258377	2.253651	2.248962	2.29159	2.286741	2.327219	2.321881	2.341664
2.211637	2.2605	2.255758	2.251059	2.293789	2.288947	2.284154	2.279407	2.344241
2.213631	2.262698	2.257929	2.253209	2.296034	2.29119	2.286382	2.28161	2.346826
2.215526	2.264833	2.260031	2.25528	2.298186	2.293333	2.288505	2.283705	2.323858
2.217308	2.266896	2.262052	2.25726	2.300229	2.295363	2.290509	2.285677	2.326109
2.11457	2.269005	2.264126	2.259296	2.254552	2.249862	2.292559	2.287702	2.282905
2.116349	2.271202	2.266305	2.261453	2.25668	2.251958	2.294731	2.289861	2.28505
2.118168	2.273439	2.268513	2.26363	2.258814	2.25405	2.296899	2.292021	2.287195
2.120017	2.179202	2.174839	2.170513	2.26086	2.256038	2.29894	2.29406	2.289223
2.121865	2.181158	2.176715	2.172309	2.262888	2.258001	2.300952	2.296071	2.291225
2.123582	2.119433	2.115257	2.11111	2.228205	2.223287	2.303081	2.298193	2.293336
2.125198	2.121043	2.116931	2.112848	2.230348	2.225385	2.305301	2.3004	2.29553
2.06973	2.122753	2.11867	2.114621	2.176002	2.171225	2.275851	2.27093	2.297775
2.071528	2.124583	2.120486	2.116434	2.178096	2.173372	2.278209	2.273233	2.300076
2.073373	2.069262	2.065264	2.061325	2.180219	2.175535	2.227276	2.222444	2.288715
2.075278	2.071146	2.067106	2.063131	2.182394	2.177708	2.229562	2.224698	2.291035
2.077169	2.073019	2.068934	2.064917	2.184554	2.179843	2.231805	2.226924	2.293333
2.058336	2.074741	2.070624	2.066576	2.092522	2.088547	2.177099	2.172419	2.295515
2.060035	2.076398	2.072255	2.06818	2.094129	2.09013	2.178991	2.174334	2.297633
2.01743	2.057652	2.053516	2.049445	2.065888	2.061932	2.157155	2.152516	2.252225
2.019275	2.059537	2.055386	2.051296	2.067685	2.06368	2.159185	2.154516	2.254271
1.979228	2.061462	2.057296	2.053186	2.049115	2.045097	2.134941	2.130274	2.176547
1.981045	2.063424	2.059243	2.055116	2.051019	2.046972	2.137154	2.132404	2.178655
1.87704	1.978923	1.975032	1.971186	2.052947	2.048876	2.04487	2.040924	2.155837
1.878783	1.980763	1.97685	1.972981	2.054883	2.050793	2.046748	2.04275	2.157971
1.880535	1.982615	1.978679	1.974784	2.056831	2.052725	2.048644	2.044594	2.160143
1.882285	1.960051	1.956044	1.952073	2.019732	2.01577	2.050555	2.046474	2.146263
1.884029	1.961964	1.957931	1.953931	2.021607	2.017629	2.052481	2.048382	2.148569
1.885721	1.882043	1.878317	1.874609	1.951864	1.947959	2.054411	2.050292	2.077129
1.887357	1.883736	1.880044	1.876352	1.953746	1.949809	2.056344	2.052202	2.079016
1.888988	1.885383	1.881732	1.878072	1.874406	1.870742	1.996801	1.992757	2.080924
1.890608	1.886943	1.883342	1.879745	1.876092	1.872426	1.998723	1.994667	2.082862
1.892269	1.888531	1.884963	1.881417	1.87778	1.87412	2.000656	1.996585	2.084811
1.799769	1.890253	1.886667	1.883115	1.879486	1.875836	1.95327	1.949324	2.02895
1.801417	1.892047	1.888417	1.884831	1.881203	1.877566	1.955135	1.951162	2.03086
1.803071	1.852318	1.848707	1.845145	1.882933	1.879296	1.917711	1.913925	2.032778
1.80474	1.854103	1.850452	1.846851	1.884675	1.881027	1.919447	1.915637	2.034704
1.806407	1.802804	1.799291	1.795815	1.844971	1.84138	1.92119	1.917364	1.913606
1.80807	1.804462	1.800935	1.797442	1.846689	1.843068	1.922942	1.919108	1.915331
1.809736	1.806122	1.802587	1.799081	1.84842	1.844766	1.885954	1.882177	1.91706
1.811405	1.807782	1.804247	1.800735	1.850167	1.846472	1.887656	1.883864	1.918784
1.813082	1.809446	1.805912	1.802396	1.851924	1.848192	1.889369	1.885559	1.920516
1.764492	1.811104	1.807564	1.804046	1.800472	1.796883	1.891106	1.88727	1.88349
1.76616	1.812774	1.809224	1.805698	1.802117	1.798518	1.892861	1.888995	1.885198
1.720902	1.764344	1.760887	1.75745	1.803789	1.800168	1.894627	1.890735	1.886936
1.722721	1.766158	1.762662	1.75917	1.805488	1.801832	1.896405	1.89249	1.8887

1.724503	1.767921	1.764396	1.760868	1.807178	1.803496	1.848175	1.844408	1.890481
1.726217	1.769598	1.766067	1.762532	1.808851	1.805156	1.84986	1.846096	1.892266
1.680234	1.72447	1.720994	1.717513	1.760619	1.757047	1.851565	1.847823	1.894095
1.681998	1.726239	1.722722	1.719207	1.76226	1.758683	1.853293	1.849617	1.896014
1.683784	1.728023	1.724456	1.720904	1.763911	1.760341	1.855051	1.851437	1.897959
1.61527	1.681998	1.678475	1.67498	1.76561	1.762075	1.81053	1.807036	1.84956
1.616827	1.683701	1.680152	1.676647	1.767337	1.763855	1.812347	1.808796	1.851299
1.618404	1.68541	1.68183	1.678308	1.77816	1.774699	1.814095	1.810517	1.806916
1.619997	1.68712	1.683503	1.679957	1.779864	1.776408	1.815773	1.812197	1.808592
1.571645	1.618357	1.615153	1.612012	1.737572	1.734156	1.817462	1.813876	1.810273
1.573249	1.619966	1.616769	1.613632	1.73938	1.73591	1.819181	1.815552	1.811957
1.57488	1.621612	1.61842	1.61528	1.741199	1.737669	1.820909	1.817253	1.813666
1.48092	1.623308	1.6201	1.61693	1.678395	1.67521	1.781498	1.777944	1.815443
1.482487	1.625034	1.621799	1.61859	1.680008	1.676774	1.783186	1.779686	1.817244
1.40347	1.576787	1.573597	1.570427	1.681655	1.678396	1.739333	1.735874	1.77788
1.404914	1.578475	1.575259	1.572066	1.683329	1.680061	1.741143	1.737627	1.779556
1.406338	1.403549	1.400758	1.397986	1.639631	1.636412	1.678515	1.675345	1.781236
1.407738	1.404951	1.402167	1.399399	1.641325	1.638081	1.680166	1.676932	1.782911
1.409137	1.406355	1.403576	1.400811	1.643013	1.639747	1.681825	1.678545	1.675346
1.410542	1.407771	1.404991	1.40222	1.644679	1.641397	1.683488	1.680203	1.676987
1.411948	1.409183	1.4064	1.403622	1.64634	1.643051	1.685166	1.681885	1.678653
1.413352	1.410576	1.407788	1.405009	1.486811	1.483947	1.641482	1.638259	1.680318
1.414756	1.41196	1.409167	1.406389	1.48825	1.485414	1.643179	1.639941	1.681997
1.416165	1.413352	1.41056	1.407789	1.489751	1.486889	1.579434	1.57634	1.638409
1.417578	1.414748	1.411964	1.409207	1.491308	1.488374	1.581051	1.577977	1.640148
1.419013	1.416169	1.413385	1.41063	1.49287	1.48988	1.48697	1.484129	1.576515
1.420473	1.417622	1.414825	1.412058	1.494427	1.49141	1.488465	1.485583	1.578088
1.508652	1.505439	1.50225	1.499101	1.496004	1.492957	1.489967	1.487033	1.579649
1.510335	1.507105	1.503915	1.500759	1.497624	1.494522	1.491473	1.488472	1.581198
1.512033	1.508784	1.505596	1.502432	1.499255	1.496096	1.492988	1.489925	1.582763
1.513782	1.510516	1.507285	1.504073	1.500851	1.49765	1.494512	1.49143	1.488448
1.515563	1.51228	1.508985	1.505704	1.502436	1.499202	1.496051	1.492975	1.489979
1.517315	1.514015	1.510698	1.507392	1.504095	1.50083	1.49765	1.494544	1.491514
1.51904	1.515722	1.512425	1.509137	1.505829	1.502535	1.499307	1.496135	1.49305
1.520784	1.517446	1.51415	1.510864	1.507553	1.504251	1.500995	1.497781	1.494656
1.522568	1.519204	1.515864	1.512545	1.509245	1.505968	1.502723	1.499508	1.496373
1.524352	1.520965	1.517586	1.514237	1.510941	1.507683	1.504448	1.501241	1.498099
1.526103	1.5227	1.519326	1.515981	1.512661	1.509372	1.506124	1.502915	1.499757
1.527857	1.524442	1.521082	1.517745	1.514394	1.511061	1.507791	1.504574	1.501395
1.462621	1.526249	1.522853	1.519482	1.516122	1.512787	1.509498	1.50625	1.503047
1.464426	1.528105	1.524639	1.521206	1.517851	1.51454	1.511231	1.507937	1.504706
1.398032	1.462857	1.459467	1.456116	1.519612	1.516281	1.512955	1.509643	1.506393
1.399773	1.464575	1.461225	1.457903	1.521407	1.518003	1.514659	1.511366	1.508104
1.401562	1.466337	1.462997	1.459686	1.523214	1.519761	1.516405	1.513125	1.509851
1.403417	1.468168	1.464767	1.461413	1.52501	1.521577	1.518225	1.514941	1.511657
1.40533	1.470059	1.46658	1.463169	1.526841	1.523434	1.520078	1.516769	1.513467
1.407321	1.403862	1.400486	1.397183	1.461669	1.458367	1.521939	1.518558	1.515212
1.409364	1.405866	1.40243	1.399071	1.463564	1.460231	1.523812	1.52034	1.516944
1.411394	1.407846	1.404354	1.400942	1.465424	1.462045	1.525669	1.522176	1.51876
1.413423	1.409814	1.406269	1.402807	1.467262	1.463822	1.527514	1.524057	1.520647

1.328511	1.411819	1.408215	1.404694	1.401285	1.397966	1.462241	1.458919	1.522534
1.330498	1.41386	1.410185	1.406594	1.403145	1.399801	1.464098	1.460769	1.52442
1.332516	1.328915	1.325309	1.321786	1.40507	1.401692	1.465985	1.462617	1.526299
1.334587	1.330973	1.327344	1.323783	1.407103	1.403655	1.467891	1.464429	1.528146
1.336667	1.333044	1.329409	1.325827	1.409188	1.405669	1.469833	1.466263	1.530012
1.338679	1.335031	1.331401	1.327822	1.41127	1.407718	1.404196	1.400715	1.464682
1.340669	1.336987	1.333368	1.329808	1.413364	1.409797	1.406232	1.402684	1.466627
1.342769	1.339056	1.335421	1.33184	1.328294	1.324791	1.408223	1.404641	1.401199
1.344976	1.341234	1.337555	1.333914	1.330282	1.326687	1.410168	1.406585	1.403125
1.347161	1.343403	1.339696	1.336015	1.332322	1.328662	1.325102	1.32163	1.405121
1.349282	1.34553	1.341835	1.338157	1.334449	1.330763	1.327175	1.323669	1.40722
1.351422	1.347671	1.34397	1.340285	1.336575	1.332883	1.329277	1.325741	1.409344
1.353631	1.349848	1.346076	1.34233	1.338617	1.334943	1.331335	1.327785	1.411431
1.355879	1.352054	1.348193	1.344373	1.340657	1.337007	1.333402	1.329844	1.413532
1.358157	1.354306	1.350408	1.346553	1.342814	1.339146	1.335528	1.331961	1.328363
1.360463	1.356593	1.35269	1.348827	1.345051	1.34134	1.337698	1.33412	1.330505
1.305193	1.358815	1.354918	1.351065	1.347282	1.343555	1.339884	1.336267	1.33262
1.307227	1.360953	1.357076	1.353261	1.349505	1.345795	1.34209	1.3384	1.334706
1.309327	1.36317	1.359289	1.355485	1.351742	1.348039	1.344295	1.340528	1.336789
1.311555	1.365542	1.361605	1.357752	1.353991	1.350275	1.34648	1.342636	1.338857
1.313823	1.367963	1.363969	1.360059	1.356264	1.35252	1.348677	1.344772	1.34096
1.316082	1.370366	1.366354	1.362418	1.358577	1.354783	1.350912	1.346995	1.343163
1.318342	1.372773	1.368763	1.364815	1.360922	1.357064	1.353172	1.349265	1.34542
1.320556	1.316895	1.313329	1.309808	1.363284	1.359372	1.355448	1.351521	1.347659
1.322733	1.319078	1.315542	1.312042	1.365666	1.361707	1.357741	1.353774	1.349892
1.18245	1.179678	1.176955	1.174247	1.368058	1.364069	1.360064	1.356051	1.352144
1.184248	1.181477	1.178743	1.176022	1.370458	1.366464	1.362422	1.358351	1.354412
1.186054	1.183283	1.18054	1.177805	1.315213	1.311664	1.364798	1.360685	1.356715
1.187847	1.185079	1.182337	1.179598	1.317434	1.313882	1.367173	1.363054	1.359058
1.18964	1.186875	1.184138	1.181398	1.319681	1.316119	1.369576	1.365468	1.36145
1.191436	1.188675	1.185938	1.183201	1.322016	1.318411	1.372047	1.367961	1.363927
1.193234	1.190475	1.187739	1.185007	1.324409	1.320743	1.374566	1.370504	1.366457
1.195036	1.192271	1.189539	1.186814	1.326804	1.32309	1.319451	1.315874	1.368927
1.19684	1.194062	1.191336	1.188621	1.329207	1.325454	1.321769	1.318138	1.371341
1.198652	1.195861	1.193136	1.190424	1.187688	1.184941	1.324118	1.320431	1.373813
1.200471	1.197669	1.194937	1.192222	1.189489	1.186749	1.326507	1.322763	1.376368
1.202305	1.199487	1.196741	1.194019	1.191289	1.188559	1.328925	1.325133	1.378963
1.204151	1.201306	1.198544	1.195815	1.19309	1.190372	1.331379	1.327559	1.323818
1.206024	1.203146	1.200359	1.197615	1.194892	1.192183	1.333849	1.330016	1.326241
1.20796	1.205036	1.202205	1.199427	1.196691	1.193984	1.336287	1.332468	1.328677
1.209945	1.206969	1.204079	1.201252	1.198492	1.195778	1.338709	1.334925	1.331128
1.211948	1.208943	1.206002	1.203122	1.200325	1.197584	1.194855	1.192145	1.333577
1.213974	1.210965	1.207976	1.205039	1.202188	1.199398	1.196652	1.193951	1.336022
1.215986	1.212978	1.209961	1.206985	1.204089	1.201253	1.198483	1.195772	1.338471
1.217968	1.214962	1.211951	1.20897	1.206045	1.203169	1.200358	1.197605	1.340875
1.219939	1.216937	1.213936	1.21096	1.208017	1.205112	1.202269	1.199481	1.343382
1.221906	1.218902	1.215899	1.21292	1.209974	1.207069	1.204235	1.201464	1.198715
1.223871	1.220863	1.217853	1.21487	1.211928	1.209032	1.206221	1.203483	1.200745
1.225846	1.222839	1.219822	1.216827	1.213871	1.210958	1.208129	1.205373	1.202627
1.227828	1.224828	1.221802	1.218789	1.215805	1.212856	1.209981	1.207169	1.204401

1.229808	1.226811	1.223783	1.22076	1.217754	1.214775	1.211857	1.208994	1.206198
1.231772	1.228776	1.225754	1.222732	1.219714	1.216712	1.21376	1.210853	1.20802
1.23379	1.23079	1.227767	1.22474	1.221705	1.21868	1.215696	1.212747	1.209876
1.235938	1.232922	1.22987	1.226808	1.223737	1.220673	1.217651	1.214665	1.211751
1.238088	1.23506	1.231987	1.228901	1.225808	1.222719	1.219669	1.216653	1.213697
1.074224	1.237083	1.234047	1.231002	1.22795	1.224895	1.22185	1.218814	1.215824
1.075831	1.23904	1.236069	1.233099	1.23012	1.227131	1.22411	1.221065	1.218049
1.077466	1.24097	1.238027	1.235095	1.232152	1.229198	1.2262	1.223166	1.220142
1.079115	1.242864	1.23992	1.236998	1.234061	1.231117	1.228141	1.22514	1.222124
1.080816	1.078133	1.075558	1.073026	1.235967	1.233036	1.230082	1.22711	1.224108
1.082566	1.079834	1.077196	1.074618	1.237863	1.234959	1.232027	1.229078	1.226093
0.873972	1.081627	1.078922	1.076288	1.073756	1.071282	1.234016	1.231087	1.228123
0.875522	1.0836	1.080836	1.078142	1.075566	1.073061	1.236126	1.233218	1.230279
0.877109	1.085628	1.082814	1.080064	1.077429	1.07487	1.238255	1.235364	1.232448
0.60508	0.876436	0.874302	0.872211	1.079198	1.076571	1.240239	1.237351	1.234448
0.605946	0.877842	0.875702	0.873583	1.08093	1.078223	1.242147	1.23925	1.23635
0.60681	0.605512	0.604207	0.60291	1.082783	1.08003	1.077397	1.074862	1.238354
0.607663	0.606386	0.605117	0.603852	1.084766	1.082003	1.079312	1.076679	1.240463
	0.607288	0.606042	0.604793	0.875959	0.873909	1.081208	1.078495	1.075891
	0.608254	0.606997	0.605731	0.877462	0.875351	1.083026	1.080264	1.077603
	0.609202	0.607934	0.606649	0.878936	0.876777	1.084851	1.082062	1.079354
				0.606209	0.604897	0.876083	0.873983	1.081216
				0.60707	0.605774	0.877536	0.875445	1.083144
						0.605443	0.604183	1.085094
						0.60642	0.605137	1.087073
						0.607346	0.606069	0.604778
						0.608207	0.606974	0.605722
								0.606651
								0.607548
								0.608433

								1.585681
								1.582777
						1.588474	1.584169	1.579957
						1.585614	1.58137	1.577213
				0.873035	0.870439	1.582835	1.578641	1.95714
				0.871357	0.868804	1.580183	1.576022	1.954049
				0.869674	0.867145	1.577516	1.57337	1.950927
	0.875523	0.873018	0.870506	1.583005	1.578871	1.834195	1.829278	1.947602
	0.873818	0.871321	0.868806	1.580242	1.576067	1.830948	1.825981	1.944238
	1.589978	1.585782	1.581615	1.837551	1.832631	1.951067	1.94598	1.993968
	1.587273	1.58302	1.578792	1.834294	1.829365	1.947756	1.942769	1.990737
0.87136	1.584497	1.580225	1.57599	1.954587	1.949552	1.997531	1.992523	1.98762
0.869529	1.581634	1.577385	1.5732	1.951336	1.946437	1.994413	1.989502	1.984641
1.583148	1.839549	1.834535	1.829596	2.001092	1.996174	1.99129	1.986451	1.981622
1.580492	1.836544	1.831533	1.826585	1.997907	1.992975	1.988096	1.983281	1.978476
1.577831	1.833532	1.828542	1.823602	1.994749	1.989793	1.984915	1.980123	1.975344
1.835237	1.953846	1.948774	1.943765	1.991512	1.986606	1.981789	1.977071	2.023369
1.831928	1.950463	1.945476	1.940573	1.988289	1.983454	1.978711	1.974071	2.020449
1.952395	2.000128	1.995101	1.990154	1.985276	1.98047	1.975733	1.971076	2.121569
1.949425	1.997165	1.992201	1.987289	1.982432	1.977624	1.972842	1.968093	2.118299
1.99905	1.994107	1.989192	1.984311	1.979469	1.974661	2.125224	2.120158	2.195204
1.995802	1.990896	1.986014	1.981162	1.976332	1.971529	2.121955	2.116944	2.191961
1.992646	1.987766	1.982905	1.978069	2.128843	2.123742	2.198892	2.193798	2.188698
1.989724	1.984869	1.980018	1.975178	2.125747	2.120615	2.195642	2.190479	2.185345
1.986836	1.981996	1.977144	1.97229	2.122652	2.117487	2.192396	2.187169	2.182007
1.983758	2.075703	2.070718	2.065727	2.15702	2.151796	2.189039	2.183888	2.223296
1.980604	2.072475	2.067474	2.06247	2.153599	2.148386	2.185643	2.180634	2.220082
2.074385	2.166051	2.160804	2.155563	2.19276	2.187495	2.182385	2.177426	2.216831
2.071439	2.162948	2.157682	2.152428	2.189592	2.184357	2.179247	2.174258	2.213549
2.127218	2.159721	2.154451	2.149209	2.186379	2.181225	2.220544	2.215437	2.210398
2.123813	2.156267	2.15101	2.145817	2.183055	2.178072	2.21743	2.212437	2.207464
2.120455	2.152864	2.147635	2.142499	2.179788	2.174941	2.214311	2.209413	2.204492
2.15494	2.192065	2.186812	2.181655	2.221025	2.216048	2.211105	2.206214	2.201286
2.151845	2.188946	2.183749	2.178642	2.217914	2.212889	2.207908	2.202985	2.198026
2.148752	2.185857	2.180722	2.175675	2.214884	2.209871	2.20488	2.199921	2.194928
2.14567	2.182801	2.177735	2.172755	2.211914	2.20694	2.201946	2.196943	2.191907
2.18473	2.223999	2.218871	2.213813	2.208804	2.203837	2.198828	2.193785	2.188713
2.181325	2.220577	2.215529	2.210534	2.205537	2.200546	2.195509	2.190428	2.185326
2.22237	2.217289	2.212298	2.207341	2.202343	2.197324	2.192258	2.187143	2.212786
2.219285	2.21425	2.20928	2.204327	2.199316	2.19427	2.189174	2.184023	2.20958
2.216291	2.211287	2.206314	2.201342	2.196308	2.191235	2.186116	2.180943	2.206437
2.213301	2.208312	2.203315	2.198304	2.193244	2.188148	2.213894	2.208558	2.225867

2.210331	2.205339	2.200296	2.195227	2.190132	2.185016	2.210685	2.20539	2.222789
2.207334	2.202309	2.197191	2.192038	2.217954	2.21262	2.230011	2.224772	2.290598
2.204316	2.199233	2.194016	2.188756	2.214529	2.209153	2.226608	2.221473	2.286886
2.201148	2.196042	2.190795	2.185514	2.233986	2.228612	2.294737	2.288993	2.283368
2.197781	2.192693	2.187508	2.182311	2.230824	2.225557	2.291316	2.285641	2.280077
2.194474	2.243571	2.238249	2.232951	2.227727	2.222568	2.287973	2.282373	2.314861
2.191355	2.240398	2.235101	2.229849	2.224688	2.219605	2.284648	2.279151	2.311582
2.188303	2.237302	2.232028	2.226815	2.221705	2.216683	2.281365	2.275954	2.308312
2.239512	2.234228	2.229003	2.223845	2.289268	2.283651	2.278138	2.272742	2.334029
2.236431	2.231192	2.226025	2.220926	2.285983	2.280426	2.27493	2.269504	2.330478
2.233379	2.299848	2.29403	2.288298	2.282658	2.277099	2.338648	2.332676	2.355864
2.230354	2.296404	2.29065	2.284965	2.279306	2.273686	2.334847	2.328825	2.351749
2.227338	2.292972	2.287262	2.281606	2.275937	2.270282	2.331128	2.325117	2.385368
2.224328	2.289548	2.283857	2.278206	2.272539	2.266885	2.327513	2.321608	2.381436
2.291854	2.28613	2.280451	2.274806	2.33601	2.329927	2.390919	2.384206	2.377622
2.288422	2.282693	2.277027	2.271412	2.332398	2.326434	2.38702	2.380403	2.373908
2.285026	2.279284	2.27363	2.268046	2.328844	2.323008	2.383197	2.376675	2.370268
2.281713	2.307482	2.301505	2.295622	2.354531	2.348273	2.379407	2.372967	2.366633
2.278453	2.30409	2.298132	2.292257	2.350841	2.344649	2.375663	2.369296	2.363025
2.306569	2.332555	2.326104	2.319769	2.384858	2.378362	2.371964	2.365671	2.359461
2.302958	2.328754	2.322407	2.316163	2.381066	2.374635	2.368308	2.362093	2.355941
2.29941	2.397212	2.390449	2.383812	2.377319	2.370956	2.364696	2.358544	2.352449
2.295965	2.393209	2.386544	2.380019	2.373615	2.367331	2.36113	2.355015	2.348974
2.292573	2.389269	2.382693	2.376273	2.369953	2.363742	2.357597	2.351519	2.345537
2.320329	2.385403	2.378908	2.372578	2.366326	2.360174	2.354084	2.348055	2.342136
2.316766	2.381609	2.375186	2.368933	2.362739	2.356635	2.350601	2.344633	2.338789
2.384551	2.377902	2.371541	2.365344	2.359191	2.353124	2.347152	2.341269	2.363866
2.380834	2.374275	2.367967	2.361806	2.355684	2.349645	2.343741	2.337964	2.360665
2.377146	2.370658	2.364397	2.358281	2.352209	2.346228	2.340427	2.334795	2.357623
2.373474	2.367036	2.360822	2.354759	2.348758	2.342868	2.337208	2.33177	2.354755
2.369889	2.363484	2.357302	2.351286	2.345374	2.339605	2.362438	2.357129	2.351977
2.366449	2.360049	2.353866	2.347872	2.342068	2.336462	2.359426	2.354277	2.349266
2.363055	2.356665	2.350485	2.344518	2.338836	2.333412	2.356509	2.351514	2.346627
2.359609	2.353273	2.347132	2.341222	2.33567	2.330422	2.353652	2.348822	2.367003
2.356154	2.349902	2.343829	2.338002	2.332591	2.327518	2.350874	2.346203	2.364449
2.352715	2.346569	2.340616	2.334922	2.357985	2.353012	2.348219	2.343639	2.361908
2.349284	2.343268	2.337484	2.331974	2.355179	2.350374	2.345681	2.341134	2.359388
2.345985	2.340144	2.334528	2.32918	2.35248	2.347782	2.366015	2.361453	2.356886
2.342855	2.337252	2.331796	2.326567	2.349902	2.34523	2.363453	2.358934	2.354406
2.339777	2.362814	2.357459	2.352279	2.370226	2.365512	2.360905	2.356427	2.373701
2.336651	2.35986	2.354715	2.349714	2.367671	2.362944	2.358354	2.353917	2.371171
2.333604	2.356951	2.352013	2.347194	2.365159	2.360413	2.355833	2.351427	2.368657
2.330805	2.35423	2.349438	2.34475	2.362723	2.357973	2.375185	2.370673	2.366184
2.328182	2.351637	2.346949	2.342358	2.360337	2.355597	2.372737	2.368207	2.36374
2.353868	2.371809	2.367152	2.362582	2.379774	2.374959	2.370285	2.365741	2.382421
2.351238	2.369178	2.364565	2.360038	2.377226	2.372475	2.367828	2.363272	2.379911
2.348697	2.38869	2.383984	2.379366	2.374697	2.370033	2.365426	2.360865	2.377457
2.346278	2.386137	2.381411	2.376788	2.372203	2.367667	2.363119	2.358554	2.375092
2.3439	2.383651	2.378897	2.374254	2.36973	2.365298	2.360807	2.356257	2.372752
2.364092	2.381261	2.376494	2.371808	2.367279	2.362859	2.379416	2.374861	2.39317

2.361668	2.378901	2.374134	2.369407	2.364849	2.360403	2.376933	2.372455	2.390698
2.381059	2.376413	2.37168	2.366982	2.362433	2.357988	2.374513	2.370127	2.388318
2.378498	2.373841	2.369171	2.364552	2.360035	2.355604	2.372136	2.367855	2.386005
2.376015	2.371354	2.366745	2.362197	2.378655	2.374163	2.39253	2.388092	2.383614
2.373624	2.368962	2.364417	2.359935	2.376364	2.371867	2.390111	2.385622	2.381128
2.371223	2.36658	2.36208	2.357647	2.374064	2.369599	2.387733	2.383189	2.378672
2.368754	2.364168	2.359682	2.355269	2.371718	2.36737	2.385435	2.380847	2.376305
2.3663	2.361775	2.357295	2.35289	2.36936	2.365118	2.383116	2.378506	2.373951
2.363932	2.359444	2.354978	2.350577	2.3896	2.385158	2.380648	2.376076	2.371548
2.361612	2.35715	2.352701	2.348304	2.387135	2.382626	2.378117	2.373613	2.369131
2.359301	2.398648	2.39396	2.38932	2.384729	2.380184	2.375675	2.371203	2.421888
2.357006	2.396197	2.391555	2.386963	2.382376	2.377812	2.373299	2.368833	2.41909
2.354728	2.393778	2.38916	2.38459	2.380018	2.375466	2.426718	2.421482	2.416307
2.352484	2.391419	2.386788	2.382198	2.37766	2.373164	2.42398	2.418692	2.413519
2.393633	2.38901	2.384375	2.379777	2.375266	2.370815	2.42122	2.415943	2.410802
2.391065	2.386453	2.381851	2.37729	2.372783	2.368327	2.418344	2.413264	2.408241
2.388487	2.383879	2.379319	2.374803	2.370296	2.365824	2.415453	2.410591	2.405697
2.386033	2.381442	2.376905	2.372411	2.422891	2.417657	2.412632	2.407824	2.402974
2.383651	2.379082	2.374559	2.370074	2.420113	2.414896	2.409862	2.405014	2.400162
2.381266	2.433129	2.427802	2.422562	2.41736	2.412219	2.407199	2.4023	2.397433
2.37888	2.430336	2.425002	2.419743	2.414635	2.409635	2.404646	2.399673	2.394774
2.376528	2.427529	2.422225	2.416997	2.411953	2.40703	2.40207	2.397078	2.392179
2.374216	2.424657	2.41946	2.414364	2.409332	2.404367	2.399427	2.394511	2.389671
2.371932	2.421847	2.416735	2.411743	2.406716	2.401715	2.396797	2.391959	2.387183
2.424583	2.419229	2.414083	2.40905	2.404054	2.39912	2.394235	2.389398	2.384652
2.421948	2.416668	2.411464	2.406354	2.401391	2.39654	2.391694	2.386852	2.382133
2.419178	2.413955	2.40881	2.403746	2.398783	2.393912	2.389101	2.384351	2.39584
2.416326	2.411157	2.40614	2.401196	2.396215	2.39126	2.386485	2.381887	2.39341
2.413619	2.40849	2.403506	2.398593	2.393643	2.388719	2.383988	2.379443	2.411805
2.411076	2.405969	2.400904	2.395916	2.391058	2.386308	2.381627	2.377018	2.409257
2.408524	2.403462	2.398357	2.393325	2.388528	2.383889	2.401012	2.396328	2.40676
2.405903	2.400945	2.3959	2.390899	2.38608	2.381389	2.398497	2.393927	2.404368
2.403276	2.398407	2.393454	2.388526	2.383687	2.378929	2.396012	2.391549	2.401993
2.400664	2.395773	2.390929	2.386135	2.38136	2.37663	2.408732	2.404111	2.399545
2.39808	2.393124	2.388386	2.383742	2.379065	2.374405	2.406408	2.401701	2.397094
2.395611	2.39066	2.385924	2.381293	2.39833	2.393711	2.404072	2.399384	2.394774
2.393242	2.388346	2.383525	2.378798	2.395835	2.391339	2.40174	2.397148	2.392562
2.390814	2.407735	2.402844	2.398029	2.393424	2.388962	2.39936	2.394849	2.390306
2.388299	2.405306	2.400518	2.395766	2.391123	2.386567	2.396902	2.39246	2.387993
2.385795	2.402839	2.398158	2.393495	2.388845	2.384227	2.398049	2.393598	2.389151
2.383325	2.400278	2.395679	2.391134	2.386568	2.382016	2.395779	2.391235	2.386752
2.380907	2.397736	2.39319	2.388743	2.384278	2.379827	2.393544	2.388898	2.384366
2.400166	2.395373	2.390799	2.386335	2.400344	2.395822	2.396054	2.391459	2.386959
2.397901	2.393118	2.388469	2.383925	2.397889	2.393431	2.393726	2.389227	2.384763
2.395596	2.395066	2.390361	2.385744	2.400252	2.395811	2.391379	2.386968	2.38256
2.393263	2.392805	2.388141	2.383537	2.397947	2.393429	2.389005	2.384684	2.380356
2.39516	2.395655	2.391069	2.386519	2.395693	2.391136	2.386719	2.382447	2.385347
2.392752	2.393359	2.388833	2.384324	2.393492	2.388968	2.384565	2.380287	2.383268
2.390365	2.391067	2.38661	2.382156	2.391328	2.386841	2.382441	2.378129	2.381171
2.393133	2.402667	2.398183	2.393701	2.389207	2.384717	2.380288	2.375915	2.378997

2.390853	2.400347	2.395949	2.39156	2.387098	2.382603	2.378141	2.3737	2.376811
2.388565	2.397992	2.393657	2.38935	2.384942	2.380485	2.383455	2.379025	2.374705
2.386286	2.395625	2.391332	2.387094	2.382752	2.378361	2.381429	2.376995	2.372652
2.384094	2.393373	2.389058	2.384809	2.380504	2.37618	2.379362	2.374959	2.370627
2.381996	2.391248	2.386835	2.382479	2.378184	2.373932	2.377251	2.372918	2.368634
2.379915	2.389141	2.384653	2.380213	2.383557	2.379363	2.375136	2.370882	2.368402
2.377834	2.387028	2.38253	2.378068	2.381446	2.377224	2.373027	2.368863	2.366465
2.375758	2.384912	2.380417	2.375949	2.379356	2.375091	2.370914	2.366833	2.364517
2.387257	2.382779	2.378271	2.37379	2.377234	2.372936	2.36877	2.364743	2.362506
2.385144	2.380633	2.37611	2.371616	2.375109	2.370789	2.366631	2.362641	2.360473
2.382997	2.386329	2.381833	2.377371	2.373003	2.368718	2.366457	2.362474	2.35849
2.380821	2.38422	2.379702	2.37523	2.370912	2.366711	2.364511	2.360531	2.356543
2.378626	2.382094	2.377592	2.373145	2.370797	2.366637	2.362568	2.358606	2.373149
2.376405	2.379948	2.375508	2.371133	2.368832	2.364673	2.360627	2.356709	2.371232
2.382269	2.379801	2.37542	2.371113	2.366878	2.362721	2.358688	2.354792	2.3878
2.380123	2.377683	2.373358	2.369117	2.364922	2.360788	2.356752	2.352828	2.385666
2.377996	2.375591	2.371312	2.367125	2.362966	2.358856	2.354812	2.350842	2.383516
2.375915	2.373575	2.369315	2.365141	2.361	2.356908	2.352854	2.348851	2.396539
2.373864	2.371605	2.367349	2.363164	2.35903	2.354946	2.350882	2.346849	2.394316
2.373932	2.369624	2.365387	2.361205	2.357062	2.35296	2.384034	2.3797	2.392097
2.371899	2.367638	2.363431	2.359262	2.355096	2.350948	2.381837	2.377501	2.389881
2.369882	2.365651	2.361466	2.357307	2.353124	2.348943	2.379646	2.375297	2.38766
2.367883	2.363657	2.359484	2.355332	2.351141	2.346942	2.377453	2.373073	2.385418
2.365904	2.361682	2.357509	2.353354	2.349165	2.34497	2.375298	2.370886	2.383211
2.363947	2.35974	2.355539	2.351356	2.382078	2.377658	2.390021	2.385534	2.381087
2.362014	2.35783	2.3536	2.349384	2.379988	2.375604	2.38796	2.383459	2.378998
2.360113	2.35598	2.351771	2.347554	2.377963	2.373527	2.385844	2.381344	2.376887
2.358239	2.354172	2.350011	2.345811	2.37598	2.371438	2.383699	2.37921	2.374771
2.356374	2.387405	2.382947	2.378459	2.373904	2.369317	2.381549	2.377076	2.372656
2.35453	2.385302	2.380803	2.376288	2.371732	2.367161	2.379391	2.374938	2.37054
2.369237	2.383161	2.378629	2.374095	2.369548	2.365005	2.377239	2.372808	2.400596
2.367178	2.380975	2.376434	2.371896	2.367363	2.362847	2.375089	2.370681	2.398471
2.365085	2.378769	2.37423	2.369701	2.365193	2.360713	2.372964	2.368576	2.396366
2.381078	2.376559	2.372035	2.367529	2.363061	2.35863	2.377867	2.373485	2.394273
2.378861	2.374349	2.369848	2.365377	2.36096	2.356588	2.375872	2.371494	2.392227
2.376664	2.372163	2.36768	2.363241	2.366453	2.362177	2.373962	2.369648	2.390352
2.374486	2.369998	2.365531	2.361124	2.364448	2.36027	2.372126	2.36792	2.388617
2.372429	2.375552	2.371118	2.366747	2.362502	2.358351	2.384394	2.380198	2.386779
2.370534	2.373708	2.369293	2.364927	2.360637	2.356417	2.382387	2.378231	2.384818
2.368593	2.371812	2.367414	2.363058	2.358742	2.354478	2.391212	2.387015	2.382807
2.366475	2.369734	2.365357	2.361033	2.356755	2.352532	2.389176	2.384962	2.380748
2.364346	2.367636	2.363267	2.358967	2.354743	2.350589	2.387145	2.382905	2.378677
2.370148	2.365656	2.361244	2.356918	2.368749	2.364593	2.385124	2.380875	2.385296
2.368281	2.363738	2.359265	2.354897	2.366728	2.362639	2.383124	2.378873	2.383248
2.366363	2.361854	2.357407	2.353058	2.364881	2.3608	2.381219	2.376973	2.38131
2.36441	2.360013	2.355667	2.351391	2.363197	2.359068	2.379407	2.375171	2.379479
2.362406	2.358092	2.353842	2.349647	2.386064	2.381791	2.386205	2.381883	2.377556
2.360308	2.356023	2.351863	2.347761	2.384109	2.379823	2.384175	2.379819	2.375472
2.358279	2.354019	2.349921	2.345882	2.382161	2.377871	2.382168	2.377781	2.373413
2.356469	2.35225	2.348153	2.344107	2.380311	2.376034	2.380295	2.375898	2.371509

2.354716	2.35054	2.346422	2.342339	2.378464	2.374201	2.378432	2.374036	2.369637
2.352807	2.348682	2.344563	2.34046	2.376487	2.372215	2.376408	2.372029	2.367661
2.350825	2.346752	2.342637	2.338521	2.374438	2.370143	2.374293	2.369943	2.365629
2.348963	2.371024	2.366784	2.36254	2.381039	2.376645	2.372276	2.367926	2.363628
2.347228	2.369193	2.364952	2.360711	2.379151	2.374747	2.370357	2.365974	2.36165
2.345455	2.367315	2.363067	2.358829	2.377213	2.372814	2.368424	2.364039	2.359714
2.343586	2.365332	2.361066	2.356823	2.375153	2.370777	2.36643	2.362105	2.357839
2.341713	2.363356	2.359075	2.354831	2.373111	2.368766	2.364462	2.360196	2.35598
2.339903	2.37577	2.371429	2.367128	2.371217	2.366899	2.362605	2.358333	2.3541
2.338111	2.373912	2.369582	2.36529	2.369368	2.365079	2.360787	2.356493	2.352227
2.350881	2.371981	2.367637	2.36334	2.367401	2.36315	2.358895	2.35464	2.350398
2.348991	2.370003	2.365628	2.361319	2.365359	2.361153	2.356958	2.352784	2.348604
2.358234	2.376506	2.372063	2.367692	2.36343	2.359253	2.355093	2.350961	2.346808
2.356305	2.374583	2.370196	2.365873	2.361639	2.357474	2.35332	2.349188	2.345019
2.354432	2.372707	2.36837	2.364086	2.359863	2.35569	2.351524	2.347375	2.343187
2.352674	2.37091	2.366595	2.36232	2.358071	2.353848	2.349641	2.345455	2.341257
2.35094	2.369121	2.364814	2.360541	2.356266	2.352001	2.347759	2.343539	2.339333
2.349145	2.367253	2.362939	2.358668	2.354414	2.350181	2.345957	2.341742	2.337538
2.347325	2.365352	2.361027	2.356759	2.352545	2.348377	2.344195	2.340005	2.335805
2.36788	2.363483	2.359178	2.354935	2.350745	2.346599	2.342432	2.338253	2.334051
2.365997	2.361637	2.357381	2.353182	2.349002	2.344846	2.340676	2.336497	2.332289
2.364156	2.359832	2.355605	2.351423	2.347233	2.343049	2.33886	2.334666	2.330456
2.362369	2.358082	2.353854	2.34965	2.345422	2.341186	2.336951	2.332716	2.328507
2.360603	2.356341	2.352099	2.347863	2.343598	2.339321	2.335057	2.330799	2.337015
2.358853	2.354596	2.350324	2.346049	2.341766	2.337481	2.333229	2.329003	2.33523
2.357101	2.352835	2.348532	2.344225	2.339936	2.335664	2.33144	2.327264	2.3335
2.355298	2.351007	2.346702	2.342406	2.338137	2.333894	2.329696	2.325543	2.331767
2.353467	2.349141	2.344851	2.340591	2.336361	2.33216	2.327988	2.323846	2.330045
2.35168	2.34734	2.343055	2.338808	2.334603	2.330433	2.326277	2.322138	2.32832
2.349939	2.345608	2.341314	2.33706	2.332865	2.328717	2.324567	2.320424	2.326596
2.348163	2.343851	2.339567	2.335318	2.331132	2.326991	2.322839	2.318681	2.324838
2.346314	2.342035	2.337791	2.333578	2.329402	2.325254	2.321085	2.316898	2.323026
2.344476	2.340227	2.336022	2.331843	2.327667	2.323498	2.31931	2.315101	2.321207
2.342715	2.338471	2.33428	2.330109	2.325907	2.321695	2.327863	2.323617	2.31941
2.340988	2.33674	2.332553	2.328379	2.324144	2.319884	2.326026	2.321802	2.317629
2.339251	2.335007	2.330828	2.326658	2.322412	2.318135	2.324253	2.320038	2.315881
2.337514	2.333278	2.329107	2.324944	2.320704	2.31643	2.322527	2.318313	2.31416
2.335784	2.331555	2.327389	2.323232	2.318997	2.314728	2.320809	2.316601	2.312452
2.33406	2.329837	2.325675	2.321521	2.317291	2.313029	2.319101	2.314902	2.310757
2.332345	2.328126	2.323967	2.319816	2.315592	2.311337	2.317401	2.31321	2.30907
2.330642	2.326425	2.322269	2.318121	2.313903	2.309655	2.315708	2.311524	2.30739
2.328942	2.324728	2.320573	2.316429	2.312217	2.307977	2.314021	2.309842	2.305714
2.327236	2.323026	2.318873	2.31473	2.320841	2.316557	2.312333	2.308161	2.30404
2.325527	2.321322	2.317171	2.31303	2.319134	2.314862	2.310647	2.306483	2.302369
2.323813	2.31961	2.315467	2.311338	2.317435	2.313171	2.308964	2.304807	2.300698
2.322093	2.31789	2.313763	2.309651	2.315742	2.311485	2.307284	2.303132	2.299029
2.320421	2.316206	2.312074	2.307961	2.314041	2.309792	2.305598	2.301451	2.297353
2.31882	2.314575	2.310408	2.306265	2.312327	2.308088	2.303901	2.299761	2.295668
2.317202	2.312935	2.308739	2.30457	2.310616	2.306389	2.302209	2.298071	2.298971
2.31551	2.311247	2.307047	2.302875	2.30891	2.304695	2.300518	2.296375	2.297258

2.313796	2.309548	2.305353	2.301185	2.307217	2.30302	2.298847	2.294696	2.295559
2.312098	2.318174	2.313937	2.309733	2.305555	2.301402	2.297244	2.29308	2.293911
2.310411	2.316483	2.312248	2.30805	2.303914	2.299818	2.295681	2.291503	2.292296
2.308775	2.31484	2.310602	2.306401	2.302272	2.29819	2.299026	2.294813	2.290664
2.307194	2.31325	2.309004	2.304786	2.300633	2.296524	2.297338	2.293145	2.289013
2.315866	2.311622	2.307377	2.303155	2.298985	2.294852	2.295657	2.291506	2.287404
2.314162	2.309919	2.305689	2.301484	2.297313	2.293173	2.293994	2.289922	2.285868
2.312468	2.308224	2.304012	2.299826	2.295655	2.29151	2.292342	2.288336	2.284323
2.310852	2.306609	2.302406	2.298227	2.298986	2.294799	2.290706	2.28671	2.282705
2.309255	2.305014	2.300815	2.29664	2.297383	2.293185	2.28908	2.285069	2.281059
2.307592	2.30336	2.299161	2.294984	2.295725	2.291545	2.287447	2.283429	2.279428
2.305891	2.301671	2.297467	2.293286	2.294035	2.289895	2.285814	2.281792	2.277802
2.304246	2.304991	2.300759	2.296554	2.292402	2.288294	2.284225	2.280198	2.276205
2.302665	2.303402	2.299169	2.294966	2.290831	2.286748	2.282687	2.278652	2.274637
2.30109	2.301818	2.297592	2.2934	2.289279	2.28521	2.281154	2.277117	2.273085
2.299516	2.30023	2.296028	2.291861	2.287748	2.283677	2.279624	2.275595	2.27156
2.297922	2.298625	2.294451	2.290313	2.286206	2.282131	2.278079	2.274055	2.270019
2.301172	2.296966	2.292812	2.288693	2.284599	2.280531	2.276477	2.272443	2.268398
2.299471	2.295289	2.291152	2.287048	2.282968	2.278911	2.274854	2.270801	2.266745
2.297843	2.293677	2.289557	2.285466	2.281393	2.277337	2.273277	2.269213	2.278846
2.296273	2.292117	2.288011	2.283933	2.279861	2.275799	2.271733	2.267664	2.277302
2.294721	2.290578	2.286483	2.282411	2.278338	2.27427	2.28397	2.279864	2.278409
2.293191	2.289065	2.284974	2.280901	2.276825	2.272752	2.282454	2.278348	2.276909
2.291664	2.287555	2.283467	2.27939	2.275309	2.27123	2.283579	2.27949	2.294401
2.29013	2.28604	2.28195	2.277865	2.273778	2.269693	2.282068	2.277975	2.292739
2.288597	2.284521	2.280429	2.276336	2.272244	2.268153	2.280553	2.276457	2.291073
2.287068	2.282994	2.278905	2.274813	2.287265	2.283144	2.279038	2.274946	2.289432
2.285541	2.281462	2.277377	2.273289	2.285767	2.281631	2.277522	2.273438	2.287801
2.284011	2.282971	2.278893	2.274811	2.28425	2.280114	2.276005	2.271921	2.286149
2.282478	2.281458	2.277367	2.273273	2.282716	2.278593	2.274488	2.270397	2.284479
2.284015	2.293589	2.28945	2.285312	2.281187	2.277073	2.291694	2.28724	2.282823
2.282521	2.292042	2.287911	2.283796	2.279673	2.275553	2.29003	2.285599	2.281195
2.281046	2.29052	2.286386	2.282278	2.278154	2.274032	2.288369	2.283958	2.279566
2.279613	2.289076	2.284891	2.28073	2.276604	2.272507	2.286721	2.282306	2.277915
2.278189	2.287658	2.283418	2.279192	2.275058	2.270982	2.285074	2.28065	2.276257
2.290381	2.286205	2.281971	2.277738	2.292295	2.287836	2.283401	2.278992	2.274611
2.288867	2.284731	2.280543	2.27634	2.290728	2.286178	2.281714	2.277333	2.272972
2.287358	2.28322	2.279063	2.274894	2.289151	2.284575	2.280092	2.275698	2.271334
2.285851	2.281658	2.277521	2.273397	2.287567	2.283035	2.278543	2.274085	2.26969
2.284368	2.299374	2.29489	2.29044	2.285964	2.281482	2.276996	2.272498	2.268086
2.282934	2.29782	2.293285	2.28878	2.284316	2.279883	2.275426	2.270941	2.266545
2.281497	2.296286	2.291702	2.287139	2.282681	2.278286	2.273862	2.269408	2.265032
2.280003	2.294702	2.290129	2.285571	2.281122	2.276739	2.272334	2.267906	2.263533
2.278492	2.293103	2.288567	2.284039	2.279596	2.275203	2.270808	2.266413	2.26204
2.295978	2.302619	2.298049	2.293482	2.288958	2.284465	2.280013	2.275601	2.280892
2.294423	2.301046	2.296491	2.291927	2.287356	2.282795	2.278336	2.273978	2.279324
2.303953	2.299429	2.294892	2.290347	2.285763	2.28117	2.276702	2.272355	2.277719
2.302302	2.297749	2.293237	2.288736	2.284178	2.279604	2.275121	2.270725	2.276059
2.30067	2.296086	2.291592	2.287125	2.282602	2.278062	2.273574	2.26913	2.274428
2.299089	2.294483	2.28999	2.285536	2.281048	2.276554	2.272075	2.267604	2.272872

2.297527	2.292903	2.288397	2.283942	2.279488	2.275045	2.270584	2.266104	2.271348
2.295961	2.29132	2.28678	2.282301	2.277869	2.273475	2.269046	2.264585	2.269831
2.294399	2.289745	2.285161	2.280641	2.276225	2.271878	2.267489	2.263065	2.268325
2.29285	2.28819	2.283585	2.279048	2.274643	2.270326	2.26596	2.261558	2.266823
2.291315	2.286656	2.282049	2.277512	2.273116	2.268811	2.264455	2.260063	2.265325
2.289781	2.285127	2.280527	2.275995	2.271603	2.267301	2.262949	2.258561	2.267547
2.288249	2.283605	2.279017	2.274496	2.270102	2.265792	2.261436	2.257047	2.266023
2.286715	2.282082	2.277511	2.273003	2.268607	2.264286	2.259922	2.255528	2.264492
2.285172	2.28055	2.275998	2.271508	2.267115	2.262787	2.258413	2.254004	2.262954
2.283629	2.279016	2.274483	2.27001	2.26562	2.261287	2.256903	2.252478	2.261413
2.282096	2.277486	2.272962	2.268498	2.264111	2.259776	2.255389	2.250957	2.259882
2.280569	2.275959	2.271437	2.266976	2.262591	2.258258	2.253871	2.249437	2.258357
2.279038	2.274436	2.269917	2.265456	2.261069	2.256733	2.256509	2.252055	2.25683
2.277502	2.272918	2.268402	2.263938	2.259545	2.2552	2.254964	2.250513	2.255301
2.275964	2.271397	2.266885	2.26242	2.259883	2.25553	2.255097	2.250653	2.258409
2.274423	2.269868	2.265359	2.260896	2.258359	2.254005	2.253562	2.249118	2.256889
2.272886	2.268344	2.26384	2.259377	2.256839	2.252481	2.252028	2.247586	2.255373
2.273226	2.268698	2.264196	2.25973	2.25532	2.250949	2.25361	2.249192	2.253866
2.271714	2.267202	2.262705	2.258238	2.253815	2.249426	2.252082	2.247675	2.252363
2.270215	2.265722	2.261249	2.256798	2.25632	2.251888	2.250595	2.246172	2.250856
2.268729	2.264259	2.259823	2.255403	2.254913	2.250454	2.249141	2.24468	2.249347
2.267273	2.262825	2.258407	2.253996	2.256619	2.252144	2.247671	2.243191	2.247852
2.26585	2.261427	2.257007	2.252578	2.255174	2.250666	2.246178	2.241701	2.246368
2.264448	2.263348	2.258918	2.254474	2.253701	2.249174	2.253842	2.249346	2.244908
2.263086	2.261948	2.257466	2.252977	2.252179	2.247657	2.252343	2.24788	2.243477
2.261713	2.26053	2.255998	2.251468	2.250652	2.246145	2.250856	2.246437	2.242072
2.260258	2.262816	2.258251	2.253692	2.249161	2.24466	2.249394	2.245031	2.240704
2.258757	2.261317	2.256765	2.252223	2.247699	2.243206	2.247963	2.243655	2.239359
2.260618	2.25983	2.255308	2.250804	2.246312	2.241845	2.246611	2.2423	2.237989
2.259137	2.258346	2.253875	2.249435	2.244999	2.240578	2.245339	2.240969	2.236601
2.25773	2.256925	2.25247	2.248054	2.243646	2.239253	2.244006	2.239593	2.235187
2.256441	2.255603	2.251108	2.246652	2.242229	2.237835	2.242576	2.238152	2.233735
2.258851	2.254272	2.249737	2.245238	2.240796	2.236397	2.241128	2.236702	2.232287
2.257429	2.252858	2.248307	2.243789	2.239344	2.234951	2.239678	2.235258	2.230856
2.255972	2.251424	2.246877	2.242357	2.237915	2.233526	2.238252	2.23384	2.229453
2.254539	2.250038	2.245532	2.241046	2.237449	2.233038	2.236939	2.232524	2.228125
2.253115	2.248674	2.244237	2.23981	2.236222	2.231801	2.235697	2.231273	2.226847
2.251714	2.24729	2.242888	2.238497	2.234911	2.230483	2.234375	2.229941	2.225496
2.250339	2.245885	2.241485	2.237106	2.23351	2.229081	2.232968	2.228521	2.224064
2.248947	2.244475	2.240086	2.235726	2.232136	2.22772	2.231608	2.227146	2.222678
2.247501	2.24304	2.238692	2.234384	2.230829	2.226456	2.230363	2.225879	2.221395
2.246091	2.241646	2.237327	2.233052	2.229522	2.225182	2.229103	2.224601	2.220103
2.244813	2.241192	2.236829	2.232502	2.228156	2.223801	2.227712	2.223202	2.218699
2.243586	2.239966	2.235559	2.231172	2.226782	2.222388	2.22628	2.221768	2.217265
2.242284	2.238692	2.234292	2.229898	2.225486	2.221061	2.224942	2.220431	2.21593
2.240937	2.237391	2.233034	2.228666	2.224246	2.219791	2.223667	2.219159	2.214662
2.239608	2.236067	2.231718	2.227352	2.222919	2.218445	2.222315	2.217811	2.21332
2.238297	2.23471	2.230325	2.225929	2.221479	2.216996	2.220861	2.216361	2.211876
2.237807	2.23338	2.228959	2.224533	2.220068	2.21558	2.219444	2.214946	2.210469
2.236578	2.232134	2.227692	2.223245	2.218769	2.214277	2.218145	2.213647	2.209177

2.235331	2.230877	2.226419	2.221955	2.217472	2.212978	2.216852	2.212353	2.207891
2.233949	2.229489	2.225019	2.220543	2.216056	2.211563	2.21544	2.210944	2.206489
2.232512	2.228048	2.22357	2.219087	2.214599	2.210109	2.21399	2.209498	2.205049
2.23119	2.22672	2.222235	2.217746	2.213257	2.208769	2.212657	2.208173	2.203728
2.229962	2.225484	2.220993	2.2165	2.21201	2.207526	2.211424	2.20695	2.202507
2.228752	2.224267	2.219771	2.215273	2.210784	2.206303	2.210213	2.205749	2.20131
2.22756	2.22307	2.218568	2.214067	2.20958	2.205105	2.209026	2.204574	2.200139
2.226356	2.221861	2.217355	2.212851	2.208365	2.203894	2.207827	2.203386	2.198957
2.225128	2.220629	2.216121	2.211617	2.20713	2.202659	2.206601	2.20217	2.197748
2.223857	2.219355	2.214845	2.210341	2.205854	2.201383	2.205332	2.200909	2.196495
2.222467	2.21796	2.213449	2.208946	2.204462	2.199996	2.203946	2.199528	2.195117
2.221002	2.216491	2.21198	2.207479	2.203	2.198541	2.20249	2.198073	2.193663
2.219642	2.215126	2.210614	2.206113	2.20164	2.197191	2.201142	2.196727	2.19232
2.218381	2.213861	2.209346	2.204845	2.200379	2.195941	2.199898	2.195487	2.191082
2.217145	2.212621	2.208103	2.203602	2.199144	2.194717	2.19868	2.194273	2.189868
2.215928	2.211399	2.206877	2.202377	2.197927	2.193513	2.197481	2.193076	2.18867
2.214718	2.210185	2.205662	2.201163	2.196721	2.192318	2.196292	2.191889	2.187482
2.213497	2.208965	2.204444	2.199949	2.195512	2.191118	2.195098	2.1907	2.186294
2.212273	2.207745	2.203229	2.198739	2.194308	2.189919	2.193904	2.189512	2.185108
2.211045	2.206519	2.202012	2.197533	2.201519	2.197111	2.19271	2.188321	2.183917
2.209816	2.20529	2.200795	2.196331	2.200322	2.195917	2.191517	2.187128	2.182723
2.208585	2.204062	2.199579	2.195129	2.199126	2.194722	2.190322	2.18593	2.181522
2.207354	2.202835	2.198365	2.193928	2.19793	2.193528	2.189125	2.184728	2.180316
2.206123	2.201609	2.197151	2.192727	2.196736	2.192336	2.18793	2.183524	2.179107
2.204889	2.200382	2.195938	2.191528	2.195547	2.191149	2.18674	2.182324	2.177899
2.203657	2.199159	2.194727	2.190329	2.194358	2.189962	2.185549	2.181123	2.17669
2.202434	2.197945	2.19352	2.189128	2.193164	2.188768	2.184352	2.179917	2.175478
2.201218	2.196737	2.192316	2.187926	2.191967	2.187571	2.18315	2.178708	2.174264
2.200002	2.195533	2.191114	2.186723	2.190765	2.186365	2.181941	2.177495	2.173049
2.198789	2.194332	2.189914	2.185519	2.189557	2.185151	2.180725	2.176279	2.171834
2.197577	2.193129	2.188713	2.184317	2.188352	2.183938	2.179507	2.175061	2.170619
2.196351	2.191913	2.187506	2.183117	2.187151	2.182726	2.17829	2.173844	2.169404
2.195175	2.199242	2.194803	2.190381	2.185948	2.181513	2.177072	2.172626	2.168188
2.194108	2.19813	2.193642	2.189178	2.184728	2.180291	2.175851	2.171406	2.166973
2.193078	2.197053	2.19251	2.187994	2.18352	2.179078	2.174633	2.170187	2.165757
2.192012	2.195995	2.19143	2.186879	2.182367	2.177885	2.173416	2.16896	2.164529
2.190927	2.194951	2.190384	2.18581	2.181256	2.176718	2.172213	2.167739	2.163302
2.18979	2.19385	2.189306	2.184748	2.180193	2.175641	2.171113	2.166605	2.162139
2.188602	2.192693	2.188204	2.183702	2.179188	2.174662	2.170122	2.165562	2.161038
2.187406	2.191515	2.187062	2.1826	2.178119	2.173619	2.169085	2.164511	2.165629
2.186191	2.190305	2.185857	2.181406	2.176941	2.172465	2.167966	2.163443	2.164578
2.185015	2.189117	2.184654	2.180194	2.175733	2.17127	2.166806	2.162341	2.163506
2.183949	2.188006	2.183495	2.178992	2.174511	2.170046	2.165597	2.161165	2.162361
2.182925	2.186935	2.182369	2.177814	2.173302	2.168822	2.164372	2.159957	2.16118
2.181857	2.18588	2.181315	2.176752	2.17221	2.167689	2.16321	2.158773	2.159983
2.180764	2.184841	2.180316	2.175771	2.171202	2.166621	2.16209	2.157603	2.158773
2.188223	2.183738	2.17923	2.174702	2.170137	2.165552	2.166682	2.162154	2.157661
2.18707	2.182551	2.178038	2.173528	2.169008	2.164487	2.165654	2.161161	2.156672
2.185961	2.181415	2.176885	2.172371	2.167874	2.163393	2.164584	2.160121	2.155643
2.184949	2.180404	2.175838	2.171275	2.166742	2.162232	2.163405	2.158951	2.154499

2.183943	2.179401	2.174804	2.170198	2.165629	2.161086	2.162233	2.157774	2.153338
2.18285	2.178292	2.173708	2.169125	2.164577	2.160054	2.161188	2.156694	2.152227
2.181727	2.177149	2.172593	2.168058	2.163552	2.159066	2.160194	2.155653	2.15114
2.180668	2.176087	2.171525	2.166984	2.168147	2.163639	2.159118	2.154582	2.150074
2.179657	2.175087	2.170493	2.165905	2.16703	2.162494	2.157981	2.15349	2.14903
2.178597	2.17403	2.169429	2.164832	2.165946	2.161405	2.156896	2.152419	2.147972
2.17746	2.172882	2.168315	2.163766	2.16491	2.160405	2.155894	2.151376	2.146891
2.176348	2.17176	2.167216	2.162701	2.163864	2.159382	2.154872	2.150333	2.145823
2.175319	2.170731	2.166167	2.161627	2.162761	2.158258	2.153758	2.149263	2.144789
2.174316	2.169727	2.165137	2.160561	2.161661	2.157132	2.152646	2.148205	2.143776
2.173293	2.174396	2.169779	2.16518	2.160629	2.156114	2.151641	2.147213	2.142785
2.172265	2.173349	2.168739	2.164159	2.159638	2.15516	2.150699	2.146258	2.141808
2.171226	2.172303	2.167713	2.163161	2.158663	2.154206	2.149742	2.145277	2.140804
2.170174	2.171257	2.166702	2.162189	2.157712	2.153263	2.148784	2.144277	2.139774
2.169141	2.170231	2.16569	2.16119	2.156712	2.152253	2.147766	2.143251	2.138745
2.168136	2.169237	2.164677	2.160141	2.155624	2.151126	2.146644	2.142181	2.13772
2.167138	2.168241	2.163658	2.159089	2.15454	2.150014	2.145542	2.141126	2.136706
2.171814	2.167206	2.162615	2.158049	2.153515	2.149013	2.144554	2.140142	2.135717
2.170813	2.166172	2.161579	2.157026	2.152521	2.148057	2.143609	2.139181	2.134735
2.169856	2.165209	2.160609	2.156051	2.151554	2.147102	2.142645	2.138189	2.133721
2.168927	2.164292	2.159685	2.155114	2.150611	2.146155	2.141676	2.137179	2.132687
2.167949	2.163334	2.158743	2.154179	2.149662	2.145176	2.140676	2.136162	2.131666
2.16691	2.162326	2.157779	2.153249	2.148704	2.144158	2.139634	2.135128	2.130653
2.165897	2.161334	2.156819	2.152315	2.147753	2.143168	2.138629	2.13413	2.129672
2.164946	2.160379	2.155873	2.151379	2.146824	2.142244	2.137704	2.1332	2.128739
2.164009	2.159437	2.154923	2.150423	2.145879	2.141317	2.136788	2.132286	2.127825
2.163054	2.158495	2.153947	2.149408	2.144862	2.140321	2.135818	2.131351	2.126911
2.162099	2.157559	2.152966	2.148373	2.143822	2.139303	2.134832	2.130412	2.125999
2.161162	2.156633	2.152021	2.147402	2.142849	2.138339	2.133882	2.129484	2.119993
2.16024	2.155718	2.151104	2.146482	2.141926	2.137414	2.132957	2.128563	2.119074
2.159323	2.154805	2.150192	2.145571	2.141015	2.136502	2.132044	2.127649	2.118166
2.158411	2.153894	2.149286	2.144668	2.140115	2.135604	2.131145	2.126745	2.117273
2.1575	2.152985	2.148381	2.143769	2.139219	2.134708	2.125194	2.12081	2.116383
2.156588	2.152076	2.147477	2.142869	2.13832	2.133808	2.124309	2.119921	2.115492
2.155677	2.151167	2.146573	2.141969	2.13742	2.132906	2.123423	2.119033	2.114604
2.154762	2.150257	2.145667	2.141065	2.136517	2.132003	2.122537	2.118147	2.113717
2.153849	2.149349	2.14476	2.140161	2.135614	2.1311	2.121651	2.117263	2.112833
2.15296	2.148447	2.143862	2.13927	2.129714	2.125209	2.120762	2.11638	2.111956
2.152093	2.147551	2.14297	2.138389	2.128842	2.124319	2.119869	2.115497	2.111084
2.151225	2.146663	2.142101	2.137544	2.128011	2.123469	2.119007	2.114627	2.110218
2.150341	2.145778	2.141264	2.136759	2.127248	2.122683	2.118194	2.113777	2.109362
2.149479	2.144914	2.140433	2.135962	2.126476	2.121897	2.117384	2.112928	2.108503
2.148679	2.139133	2.134649	2.130178	2.125636	2.121064	2.11654	2.112056	2.107619
2.147895	2.138346	2.133823	2.129314	2.124774	2.120223	2.115698	2.111192	2.106741
2.147058	2.137523	2.132976	2.128447	2.12393	2.119424	2.114912	2.110391	2.105919
2.146192	2.136681	2.132117	2.127575	2.123094	2.118648	2.11416	2.10963	2.105136
2.140434	2.135876	2.131302	2.126744	2.122268	2.117838	2.113358	2.108832	2.112594
2.139624	2.135115	2.130538	2.125959	2.121456	2.116993	2.112504	2.107992	2.111749
2.13881	2.134324	2.129749	2.125161	2.120634	2.116139	2.11995	2.11544	2.110931
2.137973	2.133458	2.12889	2.124318	2.11978	2.115267	2.119088	2.114642	2.110176

2.137155	2.132601	2.12804	2.123485	2.118942	2.114417	2.118245	2.113846	2.109414
2.136402	2.131826	2.127261	2.122707	2.126499	2.12194	2.117444	2.113015	2.108579
2.135677	2.131092	2.12652	2.12196	2.125744	2.121193	2.116672	2.112179	2.107722
2.13492	2.130363	2.125792	2.121223	2.124997	2.120446	2.115913	2.111394	2.106917
2.134143	2.129646	2.125079	2.120499	2.124263	2.119707	2.115168	2.110648	2.10615
2.133343	2.137258	2.132689	2.128102	2.123534	2.118973	2.114431	2.109907	2.105394
2.132509	2.136391	2.13188	2.127367	2.122818	2.11825	2.113702	2.10917	2.104649
2.140094	2.135541	2.131062	2.126596	2.122071	2.117517	2.112974	2.108437	2.10391
2.139325	2.134754	2.130237	2.125744	2.121254	2.116767	2.112254	2.107714	2.10318
2.138577	2.133991	2.129422	2.124886	2.120422	2.115999	2.111518	2.106985	2.102451
2.137827	2.133232	2.128651	2.124101	2.119622	2.115186	2.110718	2.106224	2.101718
2.137083	2.132485	2.127909	2.123358	2.118843	2.114359	2.109893	2.105449	2.100982
2.136342	2.13175	2.127181	2.122634	2.118117	2.113626	2.109158	2.104718	2.100253
2.135604	2.131027	2.126467	2.121932	2.11744	2.112977	2.108504	2.104025	2.09953
2.134875	2.130314	2.125772	2.121254	2.116773	2.112317	2.107831	2.103319	2.098802
2.134153	2.129606	2.125098	2.120609	2.11612	2.111632	2.107122	2.102591	2.098064
2.133449	2.128917	2.124432	2.119959	2.115453	2.11093	2.1064	2.101858	2.097328
2.13278	2.128269	2.123772	2.119275	2.114747	2.110202	2.105662	2.101125	2.096598
2.132124	2.127629	2.123107	2.118573	2.114023	2.109464	2.104922	2.100394	2.095873
2.131451	2.126947	2.122405	2.117851	2.113296	2.108738	2.104198	2.099674	2.095783
2.13077	2.126239	2.121679	2.117115	2.112563	2.108018	2.103486	2.098966	2.095068
2.130079	2.125533	2.12096	2.116388	2.111839	2.107305	2.102788	2.098288	2.102722
2.129372	2.124822	2.120242	2.115665	2.111119	2.106595	2.102103	2.097645	2.102096
2.128697	2.124142	2.119562	2.114986	2.110438	2.105914	2.10143	2.096991	2.10144
2.128086	2.123521	2.118951	2.114385	2.109828	2.105281	2.100776	2.096313	2.100721
2.127473	2.122898	2.118338	2.113783	2.109216	2.104649	2.100119	2.095626	2.099987
2.126792	2.122212	2.117654	2.113102	2.108533	2.103961	2.100036	2.095526	2.098518
2.126096	2.121514	2.116954	2.112401	2.107834	2.103264	2.099335	2.094822	2.097793
2.125465	2.120883	2.11632	2.111764	2.107199	2.102634	2.098701	2.094188	2.100736
2.124882	2.120301	2.115734	2.111173	2.10661	2.102053	2.098118	2.093608	2.100137
2.124299	2.119719	2.115145	2.110577	2.10602	2.101474	2.102034	2.097476	2.09953
2.123723	2.119144	2.11456	2.109983	2.105434	2.100905	2.101452	2.096881	2.098919
2.123111	2.118533	2.113943	2.109359	2.104817	2.1003	2.100835	2.096254	2.098274
2.122416	2.117843	2.113252	2.108667	2.104127	2.099613	2.100137	2.095557	2.097559
2.121717	2.117147	2.112558	2.107977	2.103437	2.098922	2.099433	2.094856	2.09684
2.121094	2.116524	2.111939	2.107362	2.107949	2.103376	2.106479	2.101791	2.096191
2.12051	2.115939	2.111359	2.106787	2.107353	2.102781	2.105872	2.101181	2.09558
2.119931	2.115359	2.110783	2.106215	2.106763	2.102192	2.105271	2.100578	2.094977
2.119362	2.11479	2.110216	2.10565	2.106185	2.101613	2.10468	2.099988	2.094387
2.118795	2.119348	2.114721	2.110103	2.105607	2.101035	2.10409	2.0994	2.093799
2.11823	2.118772	2.114146	2.109527	2.105027	2.100456	2.1035	2.098811	2.09321
2.117665	2.118196	2.113569	2.108951	2.104448	2.099877	2.10291	2.098224	2.092622
2.117093	2.117615	2.112988	2.10837	2.103865	2.099298	2.102322	2.097638	2.092035
2.116523	2.117036	2.11241	2.107793	2.103286	2.09872	2.101735	2.097052	2.091449
2.11596	2.116458	2.111838	2.107229	2.102714	2.098139	2.10114	2.096461	2.09086
2.115408	2.115887	2.111278	2.106679	2.102153	2.097561	2.100545	2.095869	2.090269
2.120004	2.115373	2.110771	2.10618	2.101645	2.097042	2.099998	2.095304	2.08969
2.119555	2.114924	2.110325	2.105737	2.101197	2.096586	2.099501	2.094763	2.089117
2.119072	2.11444	2.109845	2.105259	2.10846	2.103736	2.098062	2.093302	2.088584
2.11852	2.113889	2.109294	2.104713	2.107909	2.103195	2.097542	2.092818	2.088123

2.117953	2.113324	2.108729	2.104148	2.10734	2.102639	2.097011	2.092325	2.087656
2.117391	2.112769	2.108168	2.103579	2.106761	2.102071	2.09645	2.091773	2.08711
2.116837	2.112225	2.107617	2.103019	2.106187	2.101506	2.095886	2.091206	2.086541
2.116341	2.111736	2.107126	2.102524	2.105682	2.101005	2.095384	2.090699	2.086022
2.115887	2.111287	2.106679	2.102079	2.105226	2.100549	2.094925	2.090231	2.085535
2.115393	2.110796	2.106194	2.101599	2.10473	2.10005	2.094427	2.089737	2.085041
2.114843	2.110246	2.105655	2.101072	2.104178	2.099491	2.093874	2.089206	2.084535
2.114326	2.109731	2.105146	2.10057	2.103657	2.098967	2.093352	2.088698	2.084044
2.113883	2.109295	2.104709	2.100129	2.103204	2.098522	2.092904	2.08824	2.083586
2.113466	2.108883	2.104292	2.099706	2.102774	2.098102	2.092479	2.087799	2.083135
2.113043	2.108455	2.103865	2.099282	2.102343	2.097675	2.092048	2.087358	2.082676
2.112622	2.108025	2.103435	2.098854	2.101908	2.097241	2.091611	2.086913	2.082213
2.120759	2.115524	2.110803	2.106092	2.101407	2.096739	2.091113	2.086424	2.08173
2.120316	2.115065	2.110304	2.105552	2.100848	2.096176	2.090559	2.085897	2.081231
2.119855	2.114583	2.109808	2.105045	2.100337	2.095665	2.090052	2.085403	2.080756
2.119357	2.114056	2.109308	2.104584	2.099894	2.095229	2.089614	2.084957	2.080311
2.118895	2.113575	2.108844	2.104145	2.099466	2.094807	2.089191	2.084528	2.079882
2.118523	2.113211	2.108451	2.103715	2.099025	2.094369	2.08876	2.08411	2.079471
2.11818	2.112886	2.108093	2.103311	2.098601	2.093939	2.088333	2.083697	2.079064
2.117802	2.112524	2.107744	2.102965	2.09823	2.093527	2.087901	2.083266	2.078638
2.117407	2.112147	2.107406	2.102655	2.097894	2.093132	2.087473	2.082829	2.078201
2.116998	2.111751	2.107034	2.102305	2.097546	2.092776	2.087097	2.082421	2.077774
2.116573	2.111333	2.106629	2.101917	2.097194	2.092463	2.086774	2.08204	2.077352
2.116146	2.110908	2.106212	2.101515	2.09682	2.092123	2.086439	2.081682	2.076968
2.115713	2.110472	2.105783	2.101098	2.096417	2.091738	2.08608	2.081357	2.076648
2.115294	2.110037	2.10535	2.100672	2.096001	2.091336	2.085706	2.081025	2.076331
2.114346	2.109606	2.104911	2.10024	2.094625	2.089969	2.085311	2.080651	2.075976
2.113977	2.10919	2.10448	2.099807	2.094196	2.089551	2.084904	2.080257	2.075603
2.113628	2.108834	2.104093	2.099388	2.093772	2.089136	2.084495	2.07985	2.075207
2.113296	2.108522	2.10374	2.098982	2.093352	2.088726	2.084085	2.079434	2.074792
2.112943	2.108194	2.103404	2.09863	2.092994	2.088368	2.083725	2.079071	2.074424
2.112561	2.107845	2.103086	2.098339	2.092709	2.088075	2.083428	2.078774	2.07411
2.112202	2.107508	2.102779	2.098056	2.092422	2.087767	2.083113	2.078463	2.073794
2.111897	2.107205	2.102491	2.097775	2.092108	2.087402	2.082732	2.0781	2.073453
2.111597	2.106895	2.102191	2.097482	2.091788	2.087038	2.082356	2.077739	2.073115
2.111254	2.106525	2.10183	2.09714	2.09146	2.086723	2.082047	2.07743	2.07281
2.110897	2.106134	2.101446	2.096781	2.091133	2.086433	2.081768	2.077139	2.072513
2.110565	2.105806	2.101127	2.096472	2.090837	2.086146	2.081471	2.076811	2.072168
2.110253	2.105525	2.100857	2.096204	2.090565	2.085865	2.081162	2.076454	2.071784
2.109958	2.105251	2.100588	2.095932	2.090295	2.085593	2.080874	2.076132	2.071444
2.109684	2.104984	2.100318	2.095661	2.090032	2.085342	2.080619	2.075858	2.071159
2.106741	2.10471	2.100031	2.09536	2.089737	2.085063	2.080349	2.075591	2.075843
2.106462	2.104415	2.099696	2.09499	2.089365	2.084712	2.080028	2.075311	2.075577
2.106185	2.104119	2.099358	2.094612	2.088982	2.084351	2.0797	2.075033	2.075319
2.105922	2.103851	2.099066	2.094298	2.088667	2.084049	2.079415	2.074769	2.075055
2.105667	2.1036	2.098803	2.094023	2.088392	2.083781	2.079153	2.074515	2.07479
2.105409	2.103347	2.09855	2.093769	2.088134	2.083518	2.078892	2.07426	2.074524
2.105148	2.103094	2.098308	2.093535	2.087892	2.08326	2.078629	2.074006	2.074257
2.104891	2.100178	2.095432	2.090694	2.087692	2.08304	2.084298	2.079612	2.073987
2.104637	2.09995	2.095251	2.090556	2.087559	2.082884	2.084094	2.079364	2.07371

2.104391	2.099717	2.095059	2.090404	2.087418	2.082724	2.083893	2.079122	2.073439
2.104158	2.099457	2.094812	2.09018	2.093156	2.08842	2.083663	2.078878	2.07318
2.103933	2.099191	2.094544	2.089924	2.092902	2.088176	2.083425	2.078645	2.072942
2.103701	2.098943	2.094286	2.089664	2.09038	2.08568	2.0832	2.078457	2.072769
2.103468	2.098709	2.094035	2.089397	2.090104	2.085419	2.082979	2.078298	2.072641
2.103266	2.10399	2.099264	2.094566	2.089864	2.085171	2.081796	2.077134	2.072455
2.103103	2.103859	2.099126	2.094399	2.089665	2.084934	2.081541	2.076877	2.0722
2.102917	2.103693	2.098958	2.094219	2.089472	2.084724	2.08132	2.076646	2.071963
2.102668	2.103437	2.098717	2.093999	2.089282	2.084567	2.081173	2.076481	2.071777
2.102425	2.103175	2.098464	2.093765	2.089081	2.084407	2.081026	2.076321	2.071602
2.108755	2.102973	2.098236	2.093515	2.088831	2.084171	2.078634	2.073947	2.071409
2.108601	2.102795	2.098021	2.093265	2.08857	2.083915	2.078389	2.073725	2.071215
2.108423	2.102599	2.097826	2.093077	2.088384	2.08373	2.078206	2.073551	2.07105
2.108231	2.102393	2.097649	2.092938	2.088256	2.083601	2.078073	2.073413	2.070908
2.100711	2.095197	2.090576	2.085988	2.088136	2.083476	2.077939	2.07327	2.070756
2.100542	2.095064	2.090456	2.085872	2.088022	2.083354	2.077808	2.073119	2.070592
2.10039	2.094943	2.090343	2.085757	2.087903	2.083223	2.077663	2.072955	2.068238
2.100265	2.094826	2.090227	2.085637	2.087773	2.083076	2.077493	2.072761	2.068037
2.100149	2.09471	2.090107	2.085508	2.08763	2.082913	2.077311	2.072563	2.067838
2.100024	2.094583	2.089971	2.08536	2.086609	2.08186	2.077121	2.072388	2.067678
2.099897	2.09445	2.089823	2.085195	2.086422	2.081659	2.076928	2.072228	2.067541
2.099777	2.094317	2.089661	2.085005	2.086231	2.081481	2.076767	2.072089	2.067419
2.099663	2.094181	2.08948	2.084785	2.08603	2.081321	2.076634	2.071973	2.067314
2.09955	2.094063	2.089348	2.084641	2.085887	2.081186	2.076507	2.071855	2.067203
2.099438	2.09397	2.089294	2.084617	2.085827	2.081072	2.076371	2.071728	2.067081
2.099326	2.093878	2.089246	2.084605	2.085792	2.080992	2.076266	2.071613	2.06696
2.099214	2.093772	2.089158	2.08454	2.085756	2.080976	2.076233	2.071522	2.066834
2.099101	2.09366	2.089057	2.084456	2.085717	2.080979	2.076226	2.071452	2.066725
2.098979	2.093546	2.088947	2.084355	2.085638	2.080926	2.076188	2.071422	2.066686
2.098853	2.093428	2.088829	2.08424	2.08553	2.080837	2.076133	2.071419	2.066695
2.098757	2.093309	2.088707	2.084123	2.085417	2.080735	2.076053	2.071373	2.066666
2.09869	2.093182	2.088575	2.084001	2.085295	2.080615	2.075943	2.071282	2.066602
2.097826	2.092498	2.087858	2.08326	2.085179	2.080503	2.075836	2.071181	2.066518
2.097816	2.09249	2.087803	2.083151	2.085061	2.080398	2.075731	2.071063	2.066401
2.097824	2.092515	2.087782	2.083075	2.084972	2.080319	2.07565	2.070967	2.066299
2.097854	2.092561	2.087809	2.083073	2.084959	2.080304	2.075633	2.070952	2.06627
2.097883	2.09261	2.087858	2.083113	2.084985	2.080316	2.075641	2.07097	2.066274
2.097577	2.092588	2.087864	2.083139	2.084989	2.08028	2.075594	2.070939	2.066252
2.097467	2.092509	2.08784	2.083162	2.084982	2.080211	2.075508	2.070875	2.066215
2.097374	2.092417	2.087786	2.083151	2.08497	2.080177	2.075468	2.070842	2.066196
2.097298	2.092301	2.087688	2.08309	2.084947	2.080187	2.075489	2.070853	2.066207
2.097257	2.092029	2.08742	2.082843	2.078427	2.073788	2.075515	2.070854	2.066196
2.097278	2.092039	2.087431	2.082859	2.078453	2.073819	2.075519	2.070806	2.06612
2.097318	2.092082	2.087471	2.082893	2.078491	2.073857	2.075528	2.070762	2.066046
2.097325	2.092112	2.087502	2.082919	2.078526	2.073903	2.075568	2.070779	2.066049
2.09732	2.092144	2.087531	2.082936	2.078554	2.073947	2.07562	2.070829	2.066096
2.096826	2.092164	2.087524	2.082899	2.078298	2.073708	2.075633	2.070866	2.066147
2.096845	2.092173	2.087483	2.082808	2.078204	2.07364	2.07561	2.070893	2.066207
2.096875	2.092194	2.087469	2.08276	2.078152	2.073604	2.075607	2.070926	2.066265
2.096916	2.09223	2.087496	2.082777	2.078166	2.073619	2.075636	2.070971	2.066319

2.096963	2.092276	2.08754	2.08282	2.078205	2.073653	2.075671	2.071014	2.066371
2.097011	2.092327	2.087594	2.082873	2.078248	2.073683	2.075702	2.071052	2.066418
2.097061	2.092385	2.087663	2.082949	2.078316	2.073732	2.075744	2.071093	2.066461
2.097107	2.092448	2.087761	2.083078	2.078443	2.073838	2.075825	2.071144	2.066497
2.097152	2.092515	2.087878	2.083242	2.07861	2.073981	2.075933	2.071203	2.066528
2.097209	2.092566	2.087954	2.083348	2.07872	2.074081	2.06943	2.064763	2.066555
2.097277	2.092597	2.087986	2.083394	2.078773	2.074138	2.069488	2.064816	2.066602
2.097348	2.09264	2.088022	2.083432	2.078815	2.074188	2.069549	2.064896	2.066691
2.097411	2.092693	2.088062	2.083461	2.078846	2.074233	2.069627	2.065029	2.066857
2.097492	2.092776	2.088129	2.083512	2.078891	2.074282	2.069702	2.065155	2.067018
2.097615	2.092922	2.088268	2.08363	2.078983	2.074341	2.06975	2.065212	2.067089
2.097763	2.093098	2.088437	2.083776	2.079096	2.074414	2.0698	2.065252	2.067131
2.097924	2.093258	2.088571	2.083882	2.079201	2.07453	2.069915	2.065357	2.067225
2.098099	2.093409	2.088682	2.083962	2.079302	2.074681	2.070082	2.065509	2.067355
2.098222	2.093523	2.088775	2.084038	2.079389	2.074795	2.070209	2.065639	2.067486
2.098281	2.093591	2.088843	2.084103	2.079451	2.074854	2.07028	2.065734	2.067614
2.098345	2.093661	2.088929	2.084204	2.079553	2.074952	2.070383	2.065854	2.067758
2.098439	2.093747	2.089051	2.084372	2.079737	2.075137	2.070566	2.066027	2.067931
2.098552	2.093854	2.089199	2.084568	2.079951	2.075352	2.070773	2.066217	2.068111
2.098688	2.094011	2.089378	2.084767	2.080156	2.075555	2.070968	2.066397	2.066319
2.098845	2.0942	2.089579	2.084972	2.080362	2.075756	2.071158	2.066571	2.066473
2.09902	2.094393	2.089778	2.085171	2.080557	2.075942	2.071329	2.066719	2.066605
2.099216	2.094593	2.089977	2.085364	2.080742	2.076114	2.071482	2.066843	2.066714
2.099409	2.094787	2.090167	2.085545	2.08091	2.076267	2.076142	2.071481	2.066852
2.099595	2.094973	2.090346	2.085713	2.081057	2.07639	2.076264	2.071636	2.067031
2.099783	2.095157	2.090515	2.085866	2.085736	2.081044	2.076401	2.071806	2.067224
2.099977	2.095335	2.090658	2.08598	2.085856	2.081197	2.076578	2.072	2.067427
2.100174	2.095517	2.090811	2.086108	2.085992	2.081369	2.076772	2.072202	2.067634
2.100373	2.095724	2.091035	2.086344	2.086212	2.081561	2.076953	2.072391	2.07257
2.100573	2.095947	2.091302	2.086647	2.086487	2.081773	2.077137	2.072575	2.072755
2.10077	2.100721	2.096089	2.091447	2.086752	2.082033	2.077378	2.072784	2.072938
2.100963	2.100913	2.096289	2.091664	2.087013	2.082346	2.077682	2.073015	2.073113
2.101151	2.101099	2.096481	2.091869	2.087252	2.08263	2.077972	2.073272	2.07333
2.101325	2.10128	2.096667	2.092063	2.087461	2.082859	2.07823	2.073572	2.073624
2.101512	2.101461	2.096849	2.092251	2.087656	2.083066	2.078467	2.073862	2.073919
2.106304	2.101637	2.097024	2.092436	2.087846	2.08326	2.078673	2.074089	2.074164
2.106547	2.101828	2.097205	2.092622	2.088031	2.083446	2.078864	2.074287	2.074383
2.106818	2.102091	2.097433	2.092813	2.088216	2.083642	2.083829	2.079205	2.075766
2.107112	2.102409	2.097698	2.09301	2.088403	2.083846	2.084027	2.079388	2.07595
2.107374	2.102699	2.097973	2.093262	2.08865	2.084097	2.084267	2.079626	2.076186
2.10759	2.102951	2.09826	2.093577	2.088969	2.084405	2.084562	2.079934	2.076486
2.107832	2.103214	2.098558	2.093907	2.089293	2.084704	2.084843	2.08023	2.076781
2.108139	2.103516	2.098882	2.094246	2.089599	2.084952	2.085067	2.080468	2.07704
2.108456	2.103815	2.099196	2.094574	2.089899	2.085204	2.085295	2.080705	2.077298
2.10873	2.104059	2.099448	2.094846	2.090186	2.085504	2.085589	2.080989	2.077589
2.108993	2.104286	2.099677	2.095096	2.09047	2.08583	2.085915	2.081292	2.07789
2.109307	2.104579	2.099969	2.095395	2.090782	2.086158	2.086226	2.081566	2.07815
2.109664	2.104929	2.100313	2.095733	2.091118	2.086492	2.086529	2.081817	2.078373
2.110006	2.10528	2.100658	2.096068	2.091455	2.086833	2.08685	2.082103	2.07864
2.110321	2.105629	2.101004	2.096403	2.091799	2.087193	2.087203	2.08244	2.078967

2.110639	2.105972	2.101334	2.096712	2.096865	2.092233	2.088819	2.08404	2.079314
2.110972	2.106299	2.101618	2.096954	2.097098	2.092487	2.089105	2.084366	2.079666
2.111312	2.106625	2.101898	2.097189	2.09732	2.092726	2.089379	2.084691	2.080024
2.111661	2.106968	2.102224	2.097497	2.097611	2.093019	2.08969	2.085029	2.08038
2.112017	2.107324	2.102579	2.097849	2.097942	2.093343	2.090021	2.085374	2.080736
2.112373	2.107681	2.10294	2.098211	2.098284	2.093673	2.090355	2.085719	2.081088
2.112727	2.108041	2.103308	2.098583	2.098635	2.094007	2.090691	2.086063	2.081437
2.109636	2.108408	2.103696	2.09899	2.099026	2.094383	2.091057	2.086417	2.081783
2.109994	2.108787	2.10412	2.099457	2.099486	2.094824	2.091474	2.086785	2.08212
2.11035	2.109162	2.104535	2.099912	2.099939	2.095265	2.091893	2.087157	2.082462
2.110693	2.109513	2.104899	2.10029	2.100322	2.095655	2.09228	2.087523	2.082811
2.11104	2.109857	2.105242	2.100635	2.100675	2.096021	2.092655	2.087895	2.083178
2.111418	2.10677	2.102155	2.097558	2.101017	2.096375	2.093034	2.088306	2.083607
2.111822	2.107132	2.102498	2.097895	2.101347	2.096716	2.093412	2.088744	2.084083
2.109365	2.107547	2.102903	2.098288	2.101716	2.097072	2.093781	2.089145	2.084506
2.109816	2.108032	2.103386	2.098751	2.102129	2.097441	2.094132	2.089495	2.084863
2.11024	2.113742	2.109053	2.104367	2.101291	2.096578	2.094516	2.089872	2.085238
2.110597	2.1141	2.109422	2.104753	2.101708	2.097026	2.094974	2.090319	2.085665
2.110957	2.114452	2.109779	2.105124	2.102113	2.09747	2.095436	2.090774	2.086102
2.11139	2.114868	2.110167	2.105485	2.102475	2.097843	2.095826	2.091177	2.086518
2.111856	2.11531	2.11057	2.105848	2.102826	2.098194	2.096193	2.091566	2.086931
2.112292	2.115729	2.110987	2.106266	2.103249	2.098618	2.093998	2.089396	2.08737
2.112708	2.116134	2.111418	2.106731	2.103731	2.099101	2.094478	2.089864	2.087832
2.113138	2.116565	2.111872	2.107211	2.104222	2.09959	2.094959	2.090333	2.088291
2.113584	2.117029	2.112354	2.107706	2.104724	2.100082	2.095441	2.090805	2.08875
2.114044	2.114896	2.110248	2.10562	2.105222	2.100567	2.095913	2.091261	2.086596
2.114522	2.115375	2.110735	2.106107	2.105708	2.101038	2.096362	2.091682	2.087006
2.115011	2.115861	2.11122	2.106586	2.106183	2.101494	2.096799	2.092094	2.087414
2.115502	2.116347	2.111696	2.107047	2.106636	2.101924	2.097223	2.092528	2.087862
2.115996	2.116832	2.112164	2.107492	2.107072	2.102338	2.09764	2.092974	2.088334
2.121974	2.119181	2.11447	2.109757	2.107501	2.102774	2.098088	2.093441	2.088815
2.122433	2.11963	2.114891	2.110158	2.107922	2.103229	2.098564	2.093928	2.089308
2.12288	2.120064	2.115316	2.110582	2.108366	2.1037	2.099051	2.094422	2.089805
2.123296	2.12046	2.115736	2.111039	2.108844	2.104188	2.099546	2.094921	2.090305
2.123745	2.120899	2.116191	2.111518	2.10934	2.10469	2.100051	2.095425	2.090806
2.124296	2.121463	2.116723	2.112012	2.107354	2.102736	2.100561	2.095919	2.091291
2.124882	2.122075	2.117296	2.112534	2.107869	2.103262	2.101087	2.096428	2.091788
2.125421	2.12264	2.117878	2.113127	2.108457	2.103836	2.101662	2.097009	2.092359
2.125932	2.12318	2.118472	2.113771	2.109099	2.104446	2.102273	2.097641	2.092983
2.126436	2.1237	2.119024	2.114353	2.109681	2.105014	2.102844	2.098219	2.093564
2.126929	2.124195	2.119527	2.114866	2.1102	2.105535	2.103371	2.098741	2.0941
2.12743	2.124701	2.120031	2.115369	2.110707	2.106051	2.10141	2.096784	2.094618
2.127938	2.125228	2.120541	2.11586	2.111204	2.106564	2.10192	2.097272	2.095102
2.128471	2.125777	2.121075	2.116377	2.111723	2.107094	2.102446	2.097781	2.095605
2.131034	2.126368	2.121675	2.116984	2.112323	2.107679	2.103029	2.098373	2.096192
2.13166	2.126978	2.122301	2.11763	2.112957	2.108286	2.103633	2.099	2.096818
2.131294	2.127539	2.122872	2.118211	2.11353	2.108842	2.104188	2.099569	2.097402
2.131822	2.128063	2.123402	2.118746	2.114058	2.10936	2.104706	2.100098	2.097957
2.130642	2.128633	2.123972	2.119315	2.114624	2.10992	2.105262	2.100649	2.096034
2.131266	2.129263	2.124596	2.119931	2.115239	2.110537	2.10587	2.101236	2.096615

2.131897	2.128965	2.12429	2.119617	2.115863	2.111165	2.106484	2.101816	2.09718
2.132531	2.129607	2.124924	2.120241	2.116492	2.111801	2.107094	2.102366	2.097692
2.133148	2.130232	2.125541	2.120848	2.117105	2.112424	2.107696	2.102917	2.098209
2.134176	2.130787	2.126087	2.121385	2.116711	2.112046	2.10827	2.103505	2.098802
2.134687	2.131303	2.126595	2.121888	2.117221	2.112573	2.10883	2.104115	2.09944
2.135253	2.130114	2.12542	2.120727	2.11779	2.113145	2.109418	2.104733	2.100076
2.135868	2.13072	2.126035	2.121352	2.118413	2.113756	2.110032	2.105362	2.100715
2.136495	2.131343	2.126663	2.121987	2.119047	2.11438	2.109717	2.105056	2.101354
2.137133	2.131982	2.127305	2.12263	2.119689	2.115016	2.110351	2.105692	2.101993
2.137775	2.132625	2.127949	2.123274	2.120334	2.115658	2.11099	2.106329	2.10263
2.138411	2.133263	2.128588	2.123914	2.120976	2.116298	2.111628	2.106964	2.102323
2.139046	2.133898	2.129225	2.124553	2.121618	2.11694	2.112268	2.107599	2.102955
2.132859	2.13453	2.129857	2.125186	2.120519	2.115854	2.112907	2.108234	2.103587
2.133485	2.135159	2.130487	2.125818	2.121152	2.116491	2.113545	2.10887	2.10422
2.134118	2.135791	2.131123	2.12646	2.121795	2.117131	2.114189	2.109518	2.104867
2.134755	2.136419	2.13176	2.12711	2.122443	2.11777	2.114832	2.110173	2.105525
2.135432	2.137588	2.132949	2.128322	2.123126	2.118447	2.11379	2.109154	2.10622
2.136183	2.138333	2.133698	2.129077	2.123878	2.119196	2.114541	2.109912	2.106982
2.136933	2.139081	2.134446	2.129825	2.124625	2.119944	2.115291	2.110665	2.107738
2.137609	2.132975	2.128384	2.123807	2.125294	2.120622	2.115971	2.111341	2.108415
2.138253	2.133624	2.129023	2.124435	2.125929	2.121269	2.11662	2.111982	2.109055
2.138901	2.134278	2.129667	2.125067	2.126568	2.121918	2.117269	2.112622	2.107982
2.139548	2.134932	2.13031	2.125699	2.127205	2.122563	2.117913	2.113255	2.108612
2.14024	2.135626	2.130998	2.126381	2.127892	2.12326	2.118611	2.113947	2.109301
2.140994	2.136379	2.13175	2.127132	2.128647	2.124027	2.119383	2.114716	2.110065
2.14173	2.13711	2.132485	2.127872	2.129389	2.124774	2.120139	2.115487	2.110842
2.142394	2.137761	2.133148	2.12855	2.130066	2.125437	2.120826	2.116236	2.111627
2.143055	2.138412	2.133809	2.129226	2.130739	2.126093	2.121502	2.116968	2.112392
2.143791	2.139145	2.134538	2.129948	2.131467	2.126814	2.122209	2.117655	2.113068
2.144564	2.139924	2.135304	2.130699	2.132225	2.12757	2.122934	2.11832	2.1137
2.14531	2.1407	2.136079	2.131464	2.126869	2.122289	2.123686	2.119041	2.114402
2.146037	2.14148	2.136869	2.132247	2.127646	2.123056	2.124465	2.119811	2.115164
2.146746	2.142212	2.137624	2.133023	2.128426	2.123829	2.125244	2.120588	2.115938
2.147422	2.142864	2.13833	2.133795	2.129216	2.124612	2.126019	2.121367	2.116718
2.148115	2.143526	2.139023	2.134536	2.129979	2.125387	2.126797	2.122148	2.1175
2.148864	2.144254	2.139715	2.135203	2.130675	2.126142	2.127591	2.122932	2.118276
2.14964	2.145012	2.140415	2.135854	2.13135	2.12688	2.128376	2.123712	2.119052
2.15042	2.145777	2.141156	2.13657	2.132052	2.127576	2.129092	2.124465	2.11983
2.151209	2.146556	2.141928	2.137329	2.132772	2.128248	2.129767	2.125202	2.120608
2.152	2.147346	2.142716	2.138112	2.133542	2.129002	2.131106	2.126561	2.121379
2.152792	2.148147	2.143521	2.138921	2.134366	2.129844	2.131944	2.127355	2.122142
2.153593	2.148959	2.144343	2.139752	2.135205	2.130685	2.132775	2.128147	2.122908
2.154405	2.149781	2.145187	2.140615	2.136056	2.131509	2.133575	2.128928	2.123682
2.155231	2.150616	2.146038	2.141476	2.1369	2.132319	2.134364	2.129708	2.124462
2.156079	2.151476	2.146888	2.142305	2.137707	2.133102	2.128499	2.123898	2.125246
2.156938	2.152344	2.147732	2.143117	2.138495	2.133871	2.129267	2.124681	2.126038
2.157787	2.153179	2.148546	2.14391	2.139278	2.134652	2.130052	2.125474	2.127454
2.15863	2.153991	2.149336	2.144685	2.140055	2.135441	2.130849	2.126278	2.128281
2.159465	2.154803	2.150132	2.145471	2.140843	2.136239	2.131658	2.1271	2.129123
2.160287	2.15561	2.150928	2.146262	2.141636	2.137041	2.132477	2.127945	2.129985

2.161122	2.156444	2.151762	2.147096	2.14247	2.137876	2.133315	2.128793	2.130836
2.161987	2.157335	2.152672	2.148017	2.143383	2.138767	2.134181	2.129625	2.131646
2.162862	2.158233	2.153592	2.148951	2.144312	2.139679	2.135066	2.130472	2.132464
2.163724	2.159077	2.154438	2.149809	2.14519	2.14058	2.135974	2.131373	2.133358
2.164586	2.159901	2.155256	2.150637	2.146045	2.141475	2.136892	2.132299	2.134287
2.165484	2.160784	2.156129	2.151502	2.146906	2.142333	2.137752	2.133165	2.128582
2.166417	2.161717	2.157048	2.152399	2.147768	2.143156	2.138559	2.133979	2.12942
2.167306	2.162613	2.157948	2.153298	2.148651	2.144015	2.139407	2.134826	2.130278
2.168122	2.163446	2.158817	2.154204	2.149573	2.144939	2.140324	2.135726	2.13117
2.16895	2.164282	2.159675	2.155088	2.150472	2.145848	2.141236	2.136633	2.132069
2.16984	2.165153	2.16052	2.15591	2.151294	2.146684	2.142093	2.137522	2.132965
2.170754	2.166046	2.161371	2.156724	2.152102	2.147506	2.142942	2.138413	2.133865
2.171657	2.166966	2.162275	2.157605	2.152977	2.148383	2.143828	2.13932	2.134779
2.172562	2.167908	2.163214	2.158528	2.153891	2.149291	2.144738	2.140239	2.135702
2.173479	2.168848	2.16415	2.159452	2.154809	2.150206	2.145653	2.141159	2.136626
2.17441	2.169786	2.165086	2.160381	2.155735	2.151128	2.146575	2.142082	2.137549
2.175346	2.170725	2.16602	2.161309	2.15666	2.152052	2.147498	2.143005	2.138473
2.176288	2.171664	2.166951	2.162232	2.157582	2.152976	2.148422	2.143929	2.139396
2.177233	2.172604	2.167882	2.163156	2.158503	2.153899	2.149347	2.144855	2.140323
2.173325	2.173545	2.168813	2.164077	2.159421	2.154818	2.15027	2.145785	2.141259
2.1743	2.17449	2.169749	2.165004	2.160343	2.155738	2.151193	2.146718	2.142202
2.175277	2.170628	2.165874	2.161111	2.161282	2.156661	2.152116	2.147653	2.143157
2.176257	2.171616	2.16687	2.162112	2.162237	2.157589	2.153039	2.14859	2.144121
2.177256	2.172612	2.16789	2.163156	2.163243	2.158569	2.153999	2.149536	2.14507
2.17828	2.173619	2.16894	2.164257	2.164315	2.159612	2.155006	2.150493	2.145999
2.179301	2.174618	2.169975	2.165335	2.160632	2.155903	2.15601	2.151448	2.146925
2.180296	2.17559	2.170964	2.16635	2.161654	2.156923	2.156981	2.15238	2.147841
2.181303	2.176573	2.171948	2.167344	2.162661	2.157944	2.157963	2.153335	2.148778
2.182371	2.177616	2.172966	2.168347	2.163686	2.159011	2.154346	2.149686	2.149784
2.183466	2.178688	2.173997	2.169347	2.164712	2.160093	2.155455	2.150794	2.150835
2.18452	2.179731	2.175008	2.17033	2.165709	2.161129	2.156522	2.151892	2.147268
2.18554	2.180751	2.176001	2.171296	2.166679	2.162125	2.157558	2.152987	2.148396
2.181927	2.181806	2.177044	2.17232	2.167692	2.16313	2.158576	2.154039	2.149474
2.182979	2.182917	2.178163	2.173424	2.168756	2.164137	2.159557	2.15502	2.150479
2.178016	2.179346	2.17461	2.169877	2.169855	2.165193	2.160583	2.156027	2.151488
2.179035	2.180362	2.175674	2.171001	2.171019	2.166369	2.16174	2.15713	2.152544
2.180059	2.181365	2.17672	2.172108	2.172177	2.16756	2.162922	2.15826	2.15362
2.181138	2.182416	2.177744	2.173115	2.168508	2.16392	2.164005	2.159355	2.154713
2.182249	2.183497	2.178755	2.174064	2.169453	2.164898	2.16503	2.160428	2.155815
2.183339	2.184594	2.179816	2.175079	2.170445	2.165879	2.161302	2.156721	2.156862
2.18441	2.185713	2.180927	2.176157	2.171474	2.166849	2.162246	2.157669	2.157848
2.185469	2.180771	2.176014	2.171262	2.172549	2.167888	2.163262	2.158671	2.154112
2.186497	2.18179	2.177073	2.172373	2.173692	2.169046	2.164406	2.15977	2.155167
2.187551	2.18283	2.178146	2.17349	2.174838	2.170207	2.165557	2.160888	2.156248
2.188697	2.18398	2.179279	2.174598	2.175926	2.171269	2.166618	2.161973	2.157355
2.189872	2.185162	2.18043	2.175705	2.177003	2.17231	2.167661	2.163055	2.158475
2.190974	2.18626	2.181535	2.176819	2.178126	2.173433	2.168778	2.164159	2.159566
2.192033	2.187306	2.182612	2.17794	2.179279	2.174608	2.169942	2.165278	2.160639
2.193144	2.188413	2.183721	2.179053	2.174392	2.169743	2.171061	2.166393	2.161745
2.194324	2.189596	2.184868	2.180153	2.17546	2.170788	2.172119	2.167498	2.162887

2.19548	2.190756	2.186011	2.181271	2.176557	2.171868	2.173211	2.168626	2.164042
2.196563	2.191836	2.187121	2.182419	2.177719	2.173032	2.174388	2.169809	2.165218
2.197649	2.192919	2.188241	2.183584	2.1789	2.174215	2.175574	2.170986	2.166378
2.198813	2.194087	2.189423	2.184774	2.180076	2.175362	2.176699	2.172073	2.167453
2.200014	2.195291	2.190627	2.185968	2.181247	2.176499	2.177805	2.173131	2.168499
2.196216	2.196453	2.191764	2.187087	2.182372	2.177645	2.172955	2.168295	2.169642
2.197381	2.197583	2.192849	2.18814	2.183457	2.178797	2.174146	2.169504	2.170863
2.198536	2.198736	2.193987	2.189268	2.184607	2.179986	2.175356	2.17072	2.172079
2.199667	2.199919	2.195199	2.1905	2.185848	2.181227	2.176592	2.171945	2.173286
2.198552	2.196164	2.19149	2.186829	2.187102	2.182472	2.177822	2.173154	2.168487
2.199796	2.197404	2.192732	2.188071	2.18835	2.183715	2.17904	2.174326	2.169626
2.201067	2.198654	2.193971	2.189299	2.189575	2.184928	2.180229	2.175478	2.170757
2.20233	2.197632	2.192912	2.188199	2.18655	2.181906	2.181334	2.176608	2.171913
2.203591	2.198859	2.194097	2.189338	2.187631	2.182952	2.182394	2.177729	2.173088
2.204816	2.200055	2.195273	2.190499	2.18577	2.181076	2.179451	2.174861	2.174302
2.206008	2.201218	2.196436	2.191675	2.186967	2.182302	2.180689	2.176107	2.175555
2.207176	2.202388	2.19762	2.192882	2.188197	2.183554	2.181948	2.177368	2.176819
2.208302	2.203566	2.198836	2.194127	2.189462	2.184829	2.183221	2.178641	2.178089
2.209452	2.204765	2.200072	2.195393	2.190742	2.186114	2.184501	2.179918	2.179361
2.210679	2.206005	2.201331	2.196669	2.192024	2.187395	2.183687	2.179097	2.176608
2.211947	2.20727	2.202606	2.197954	2.19331	2.188677	2.184969	2.180378	2.177879
2.213225	2.208547	2.203886	2.199239	2.194596	2.189962	2.185347	2.180753	2.177069
2.21452	2.209836	2.205174	2.200526	2.195883	2.191249	2.186633	2.182034	2.17835
2.215811	2.211125	2.20646	2.20181	2.197167	2.192534	2.187916	2.183313	2.179629
2.217099	2.212412	2.207745	2.203092	2.198448	2.193816	2.189196	2.18459	2.180905
2.218388	2.213701	2.20903	2.204373	2.199728	2.195096	2.190474	2.185863	2.181263
2.219679	2.21499	2.210315	2.205654	2.201007	2.196373	2.191747	2.187131	2.182529
2.220969	2.21628	2.211601	2.206935	2.202285	2.197648	2.19302	2.188398	2.183793
2.222258	2.217569	2.212886	2.208215	2.203563	2.198926	2.194295	2.189669	2.18506
2.223546	2.218858	2.214171	2.209495	2.204841	2.200204	2.195571	2.19094	2.186327
2.224833	2.220148	2.215458	2.210779	2.206121	2.201479	2.196842	2.19221	2.187595
2.22612	2.221438	2.216747	2.212065	2.207401	2.202752	2.198111	2.19348	2.188862
2.227407	2.222724	2.218033	2.213351	2.208683	2.204027	2.199383	2.19475	2.19013
2.228694	2.224003	2.219315	2.214637	2.209967	2.205307	2.200659	2.196023	2.191397
2.229983	2.225283	2.220596	2.215922	2.211251	2.206588	2.201937	2.197298	2.192667
2.231267	2.226556	2.221869	2.217199	2.212527	2.207861	2.203208	2.198568	2.193934
2.232564	2.227839	2.223153	2.218485	2.213813	2.209145	2.204491	2.19985	2.195213
2.233881	2.229145	2.224458	2.219792	2.215119	2.21045	2.205793	2.20115	2.196509
2.235229	2.230482	2.225792	2.221128	2.216454	2.211784	2.207125	2.202477	2.197833
2.236683	2.231927	2.227232	2.222564	2.217891	2.213222	2.208562	2.203909	2.19926
2.238249	2.233485	2.228782	2.224108	2.219435	2.214771	2.210109	2.205451	2.200797
2.239741	2.234971	2.230257	2.225574	2.2209	2.216241	2.211579	2.206917	2.202259
2.241111	2.236338	2.23161	2.226913	2.222238	2.217584	2.212923	2.208259	2.203598
2.242437	2.237662	2.23292	2.228207	2.22353	2.218878	2.214219	2.209553	2.20489
2.235507	2.238959	2.234205	2.22948	2.224796	2.220143	2.215483	2.210817	2.206156
2.236784	2.240244	2.235479	2.230742	2.226051	2.221393	2.21673	2.212065	2.207408
2.238086	2.241551	2.236778	2.232032	2.227332	2.222667	2.218001	2.213336	2.208682
2.23941	2.24288	2.2381	2.233346	2.228637	2.223962	2.219292	2.214628	2.209978
2.240832	2.236066	2.231316	2.22659	2.230045	2.225359	2.220684	2.216022	2.211372
2.242371	2.2376	2.232845	2.228114	2.231574	2.226876	2.222195	2.217536	2.212884

2.243865	2.239095	2.234337	2.2296	2.233065	2.228354	2.223668	2.219009	2.214354
2.245247	2.240485	2.235721	2.230972	2.234437	2.229716	2.225023	2.22036	2.215703
2.246606	2.241862	2.237101	2.232348	2.235817	2.231084	2.226383	2.221715	2.217054
2.247998	2.243293	2.238564	2.233834	2.229113	2.2244	2.22786	2.223186	2.218521
2.249401	2.244737	2.240051	2.235355	2.230637	2.225909	2.229376	2.224694	2.220022
2.250823	2.246149	2.241471	2.236787	2.232065	2.227324	2.230794	2.226102	2.221424
2.252262	2.247536	2.242843	2.238154	2.233422	2.228672	2.23214	2.227438	2.222753
2.253671	2.248916	2.244222	2.239543	2.234821	2.230083	2.225376	2.220697	2.224155
2.255029	2.250275	2.245605	2.240962	2.236282	2.231589	2.226897	2.222202	2.225662
2.256438	2.251686	2.247021	2.242388	2.237732	2.233068	2.228384	2.223676	2.22714
2.257976	2.253214	2.248497	2.243805	2.239119	2.234442	2.229747	2.225033	2.228502
2.259537	2.254772	2.25	2.245242	2.240515	2.235809	2.231098	2.226382	2.22986
2.259445	2.256276	2.251513	2.246753	2.242011	2.237281	2.232559	2.227844	2.223156
2.260879	2.257753	2.25303	2.248298	2.243551	2.238797	2.234067	2.229355	2.224675
2.256959	2.259201	2.254474	2.249741	2.244986	2.240222	2.235488	2.23078	2.226112
2.258417	2.260614	2.255847	2.251089	2.246323	2.24156	2.236829	2.232126	2.227475
2.259918	2.260499	2.255713	2.250945	2.247743	2.242978	2.238246	2.233541	2.228891
2.261496	2.262066	2.257276	2.252506	2.249302	2.244525	2.23978	2.235064	2.230387
2.263037	2.263605	2.258826	2.254067	2.250874	2.246096	2.241347	2.236623	2.231921
2.264426	2.265008	2.260263	2.25555	2.250816	2.246082	2.242933	2.238214	2.233505
2.265787	2.266384	2.26167	2.257	2.252309	2.247617	2.244502	2.239799	2.235096
2.267277	2.267864	2.263122	2.258427	2.253725	2.249035	2.24436	2.239702	2.236613
2.268848	2.269412	2.264604	2.259839	2.255093	2.250378	2.245716	2.241109	2.238079
2.2664	2.271002	2.266151	2.261338	2.256557	2.251813	2.247135	2.242523	2.237936
2.268054	2.272645	2.267774	2.26293	2.258118	2.253339	2.248612	2.243934	2.239318
2.269648	2.268818	2.263984	2.259167	2.259707	2.254901	2.250133	2.245397	2.240741
2.271129	2.270348	2.26556	2.260775	2.261327	2.256502	2.251709	2.246939	2.242239
2.272582	2.271839	2.267102	2.26236	2.262936	2.258112	2.253307	2.248515	2.243774
2.274049	2.269294	2.264579	2.259874	2.26449	2.259705	2.254911	2.250104	2.245336
2.275531	2.270731	2.266025	2.261356	2.266009	2.261283	2.256515	2.251704	2.246923
2.277091	2.272254	2.267513	2.262823	2.263757	2.25907	2.258077	2.253288	2.248513
2.278714	2.273843	2.269028	2.264271	2.265217	2.260571	2.259607	2.254861	2.25011
2.280347	2.275452	2.270593	2.265794	2.261113	2.25651	2.261155	2.256442	2.251706
2.281987	2.277084	2.272216	2.267406	2.262708	2.258084	2.262726	2.25803	2.253303
2.278962	2.278722	2.273852	2.269038	2.264324	2.259679	2.260603	2.255909	2.254897
2.280599	2.280357	2.275485	2.270669	2.265947	2.261292	2.262216	2.257516	2.256486
2.282237	2.281994	2.27712	2.272303	2.267576	2.262916	2.263838	2.259125	2.258072
2.283878	2.283632	2.278752	2.273929	2.269199	2.264535	2.265458	2.260735	2.255984
2.28552	2.28527	2.280383	2.275553	2.27082	2.266155	2.26708	2.262345	2.257585
2.287156	2.28691	2.282015	2.277177	2.27244	2.267771	2.263077	2.258369	2.259182
2.288786	2.28855	2.283648	2.278802	2.274057	2.269382	2.264681	2.259963	2.260775
2.290411	2.285489	2.28061	2.275785	2.27567	2.270989	2.266281	2.261556	2.262368
2.292032	2.287106	2.28222	2.277387	2.277275	2.27259	2.267877	2.263147	2.26396
2.293648	2.288719	2.283826	2.278986	2.278879	2.27419	2.269474	2.264739	2.259981
2.29526	2.290327	2.285428	2.280582	2.280482	2.275793	2.271074	2.266335	2.261574
2.296871	2.291936	2.287032	2.282183	2.28209	2.277398	2.272675	2.267932	2.263168
2.298492	2.293555	2.288653	2.283803	2.283708	2.278998	2.274266	2.26952	2.264757
2.30012	2.295183	2.290287	2.285441	2.285338	2.280601	2.275855	2.271106	2.266345
2.301756	2.296818	2.291949	2.287129	2.282347	2.2776	2.277484	2.272716	2.267944
2.303399	2.298459	2.293643	2.288876	2.284088	2.279302	2.279156	2.274349	2.269551

2.305053	2.300108	2.29532	2.290585	2.285795	2.280988	2.280826	2.275989	2.271176
2.306711	2.301751	2.296951	2.292209	2.287425	2.282633	2.282478	2.277627	2.272815
2.308393	2.303426	2.2986	2.293834	2.289051	2.284272	2.284133	2.279282	2.274481
2.295458	2.305183	2.300332	2.295528	2.290719	2.28592	2.285794	2.280973	2.276192
2.297092	2.306969	2.302091	2.297244	2.292403	2.287579	2.287468	2.282686	2.277926
2.298671	2.308674	2.303782	2.298924	2.29409	2.289284	2.284502	2.279745	2.279642
2.300214	2.310325	2.30543	2.30058	2.295781	2.291022	2.286252	2.281472	2.281349
2.296726	2.297211	2.292632	2.288077	2.29744	2.292701	2.287938	2.283156	2.283036
2.298391	2.29885	2.294218	2.28961	2.299051	2.294299	2.289542	2.284786	2.284703
2.300056	2.300491	2.295822	2.291177	2.300701	2.295923	2.291158	2.286414	2.281692
2.301692	2.302106	2.297447	2.292815	2.30244	2.297622	2.292824	2.288049	2.283306
2.30333	2.303728	2.299089	2.294478	2.304206	2.299345	2.294511	2.289703	2.284937
2.304994	2.300339	2.295717	2.291118	2.305955	2.301051	2.296202	2.291406	2.286645
2.306671	2.302022	2.297391	2.292776	2.307703	2.302762	2.297909	2.293143	2.2884
2.308345	2.303688	2.299041	2.29441	2.294788	2.29017	2.299656	2.294894	2.290154
2.310019	2.305341	2.300669	2.296021	2.296408	2.291826	2.301441	2.296661	2.291914
2.311678	2.306985	2.302308	2.297661	2.293066	2.288513	2.303228	2.298435	2.293679
2.313313	2.308612	2.303961	2.299345	2.294752	2.290187	2.305011	2.300218	2.295455
2.314953	2.310255	2.305626	2.301034	2.296443	2.291869	2.306802	2.301998	2.297225
2.316617	2.31194	2.307308	2.302708	2.298125	2.293564	2.30861	2.303766	2.29897
2.318292	2.313641	2.308999	2.304383	2.299806	2.295259	2.31042	2.305535	2.300715
2.31997	2.315328	2.310691	2.306076	2.301492	2.296934	2.292386	2.287849	2.302494
2.321654	2.31701	2.312385	2.307778	2.30318	2.298595	2.294045	2.289529	2.304293
2.32335	2.318704	2.314073	2.309459	2.304853	2.300261	2.295708	2.291193	2.30607
2.325057	2.32041	2.315754	2.311116	2.30651	2.301931	2.297375	2.292842	2.307824
2.326766	2.322109	2.31744	2.312789	2.308177	2.303596	2.299035	2.294493	2.305095
2.328474	2.323793	2.319135	2.314498	2.309866	2.30525	2.300679	2.296153	2.306892
2.330181	2.325478	2.320829	2.316201	2.311552	2.306906	2.302326	2.297811	2.308683
2.317928	2.327175	2.322507	2.317859	2.31321	2.308574	2.303988	2.299452	2.294941
2.319635	2.328878	2.324184	2.319509	2.314866	2.310249	2.305659	2.301093	2.296568
2.308245	2.318991	2.314304	2.309641	2.316556	2.311929	2.307328	2.302752	2.298219
2.309964	2.320681	2.31601	2.311367	2.318275	2.313615	2.309001	2.304428	2.299888
2.311735	2.320116	2.315449	2.310812	2.308468	2.303819	2.310734	2.306152	2.3016
2.313575	2.32197	2.317281	2.312619	2.310266	2.305627	2.312545	2.307936	2.303368
2.302399	2.310695	2.306006	2.30134	2.312044	2.307416	2.302806	2.298212	2.305123
2.304156	2.312469	2.307757	2.303063	2.313766	2.309148	2.304535	2.299926	2.306831
2.305888	2.314213	2.309486	2.304773	2.31547	2.310854	2.306239	2.301627	2.308534
2.307588	2.302943	2.298276	2.293624	2.301837	2.297218	2.307912	2.303328	2.298779
2.30928	2.30461	2.299969	2.295348	2.303549	2.298891	2.309577	2.305035	2.300519
2.293043	2.306377	2.301742	2.297127	2.292495	2.28787	2.298645	2.294117	2.302256
2.294883	2.308229	2.303583	2.298956	2.294327	2.289709	2.300467	2.295893	2.303992
2.29668	2.310043	2.305385	2.300746	2.296123	2.291518	2.299714	2.295129	2.305764
2.298409	2.311791	2.307122	2.30247	2.297859	2.293279	2.301505	2.296945	2.307597
2.300151	2.303127	2.298476	2.293843	2.299608	2.29504	2.290504	2.286002	2.296725
2.301956	2.304934	2.300293	2.295669	2.301413	2.296815	2.292246	2.287704	2.298408
2.303769	2.306741	2.302117	2.297512	2.303232	2.298603	2.293995	2.289403	2.300079
2.305522	2.308495	2.303885	2.299302	2.305034	2.300425	2.295816	2.291204	2.286643
2.307248	2.310224	2.305624	2.301064	2.306826	2.302262	2.297673	2.293057	2.288475
2.309021	2.304392	2.299837	2.295326	2.298283	2.293754	2.299444	2.294845	2.29027
2.310838	2.306201	2.301623	2.297081	2.300009	2.295443	2.301128	2.296568	2.292029

2.31268	2.308033	2.303436	2.29887	2.301778	2.29718	2.302858	2.29832	2.293804
2.31456	2.309896	2.305287	2.30071	2.30362	2.299004	2.30467	2.300122	2.295607
2.316422	2.311749	2.30714	2.302562	2.305481	2.300851	2.306507	2.301945	2.297424
2.312382	2.313553	2.308964	2.304401	2.307336	2.302693	2.298112	2.293589	2.299257
2.314173	2.315342	2.310778	2.306235	2.309192	2.304538	2.299952	2.295428	2.301096
2.308839	2.31136	2.306767	2.302195	2.311039	2.306377	2.301784	2.297253	2.302919
2.310698	2.313208	2.308613	2.304048	2.312878	2.308213	2.303613	2.299071	2.304732
2.312588	2.315092	2.310485	2.305911	2.309239	2.304534	2.299893	2.295311	2.296393
2.314515	2.317019	2.312386	2.307788	2.311128	2.30642	2.301775	2.297186	2.298221
2.316438	2.311746	2.307131	2.302549	2.305007	2.300352	2.303649	2.299056	2.294515
2.318337	2.313632	2.308997	2.304397	2.306855	2.302188	2.305505	2.300913	2.29637
2.320239	2.315521	2.31087	2.306253	2.308708	2.304027	2.30736	2.302769	2.298228
2.322168	2.317432	2.312766	2.308134	2.310584	2.305883	2.30923	2.304638	2.3001
2.324111	2.319355	2.314677	2.310032	2.312475	2.307751	2.311109	2.306513	2.301977
2.326061	2.321283	2.316597	2.311946	2.307231	2.302506	2.31299	2.308383	2.303844
2.32802	2.323216	2.318527	2.313875	2.309137	2.304378	2.314875	2.310249	2.305702
2.329977	2.325147	2.320452	2.315802	2.311069	2.306316	2.316828	2.31216	2.307579
2.331917	2.327064	2.322363	2.317723	2.313036	2.308344	2.318876	2.314123	2.30947
2.333904	2.32902	2.324296	2.319647	2.314996	2.310361	2.312826	2.308085	2.31141
2.335997	2.331056	2.326274	2.321577	2.316923	2.312313	2.314815	2.310102	2.313449
2.338117	2.333122	2.32827	2.323511	2.318838	2.314238	2.316782	2.312119	2.315504
2.340176	2.335156	2.330252	2.325439	2.320748	2.31615	2.311542	2.306935	2.317469
2.34221	2.337188	2.332239	2.327373	2.322657	2.318048	2.313435	2.30883	2.319387
2.335539	2.339287	2.334293	2.329362	2.324597	2.319946	2.315315	2.310716	2.321312
2.337652	2.341456	2.336414	2.331403	2.326562	2.321837	2.317175	2.312587	2.323241
2.339701	2.343556	2.338522	2.333494	2.328597	2.323793	2.319086	2.314483	2.32517
2.341644	2.345547	2.34061	2.335667	2.33074	2.325837	2.321056	2.316394	2.327075
2.343577	2.347506	2.34266	2.337811	2.332882	2.327926	2.323093	2.318375	2.329043
2.345538	2.340646	2.335831	2.33104	2.334959	2.330065	2.325254	2.32052	2.315822
2.347537	2.342586	2.337759	2.332992	2.336995	2.332204	2.327442	2.322709	2.317976
2.349647	2.344614	2.33973	2.334931	2.330156	2.32542	2.329479	2.324754	2.320024
2.351843	2.346712	2.341734	2.336857	2.332059	2.327336	2.331419	2.326709	2.322008
2.341277	2.348883	2.343845	2.338901	2.334058	2.329301	2.324563	2.319851	2.323957
2.343478	2.35115	2.346085	2.341082	2.336162	2.331313	2.32651	2.321753	2.325852
2.345626	2.353358	2.348283	2.343247	2.338276	2.333364	2.328517	2.323733	2.32957
2.347654	2.355424	2.350363	2.345341	2.340382	2.335479	2.330642	2.325872	2.33169
2.349694	2.35748	2.352426	2.34742	2.342475	2.337588	2.332767	2.328018	2.333824
2.351866	2.359615	2.35452	2.349484	2.344506	2.339591	2.334763	2.330027	2.335852
2.354105	2.361801	2.35665	2.351566	2.346539	2.341577	2.336726	2.331987	2.337833
2.356357	2.351191	2.346099	2.341064	2.34874	2.343743	2.338832	2.334006	2.339794
2.358631	2.353523	2.34844	2.34339	2.351079	2.346051	2.341049	2.336067	2.341732
2.346292	2.355761	2.350689	2.345637	2.353337	2.348282	2.343227	2.338161	2.34376
2.348392	2.357864	2.3528	2.347763	2.35547	2.350394	2.345338	2.340293	2.345908
2.350526	2.360001	2.354945	2.349921	2.357642	2.352549	2.358033	2.352991	2.348061
2.35278	2.362274	2.357225	2.352207	2.359952	2.354844	2.360276	2.355152	2.350146
2.35505	2.364564	2.359519	2.354502	2.362277	2.357162	2.362547	2.35734	2.352254
2.357213	2.366747	2.361692	2.356669	2.364479	2.359381	2.364771	2.35958	2.354482
2.35934	2.368888	2.363815	2.358781	2.366631	2.361561	2.366985	2.36186	2.356784
2.353605	2.356492	2.351442	2.346442	2.367064	2.362083	2.369265	2.364172	2.359089
2.356001	2.358809	2.353742	2.348744	2.369383	2.364399	2.371612	2.366525	2.36141

2.337872	2.3531	2.347988	2.342959	2.371628	2.366631	2.373868	2.368786	2.363662
2.340091	2.355309	2.350162	2.345107	2.37374	2.368712	2.375967	2.370893	2.365798
2.342369	2.357576	2.352374	2.347271	2.375863	2.370809	2.378084	2.373014	2.367947
2.33359	2.360021	2.354731	2.349539	2.371087	2.366008	2.38035	2.375276	2.370214
2.33614	2.362525	2.357131	2.351836	2.373366	2.368286	2.382648	2.377565	2.372499
2.338629	2.346907	2.341448	2.336101	2.360983	2.355929	2.365395	2.360371	2.374662
2.341092	2.349234	2.343678	2.338253	2.363116	2.358034	2.367497	2.36246	2.376757
2.318394	2.351644	2.346046	2.340573	2.355786	2.350636	2.369717	2.364665	2.372421
2.320707	2.354143	2.34856	2.34307	2.35826	2.353019	2.372052	2.366988	2.374778
2.27817	2.356663	2.351103	2.345612	2.340237	2.334965	2.365412	2.36031	2.377178
2.280298	2.359209	2.353675	2.348201	2.34279	2.337452	2.367907	2.362767	2.379621
2.282417	2.361725	2.356207	2.35075	2.345325	2.339956	2.370426	2.365237	2.382067
2.284423	2.364106	2.358574	2.353117	2.347732	2.342421	2.372934	2.367681	2.362498
2.286402	2.366452	2.360891	2.355419	2.350087	2.344862	2.375422	2.370099	2.364863
2.288498	2.327313	2.322298	2.31737	2.352487	2.347232	2.341954	2.336654	2.367128
2.290684	2.329627	2.324568	2.319578	2.354915	2.349545	2.344183	2.338826	2.369314
2.292921	2.304577	2.299789	2.295057	2.357422	2.351959	2.346531	2.341133	2.371638
2.295218	2.306862	2.302049	2.297282	2.360025	2.354494	2.349021	2.343604	2.37413
2.283851	2.309159	2.304325	2.299526	2.321558	2.316554	2.351566	2.346132	2.35722
2.28614	2.311453	2.306589	2.301759	2.323901	2.318862	2.354144	2.348694	2.359776
2.288413	2.313736	2.30885	2.303999	2.326262	2.3212	2.356754	2.351285	2.362362
2.29062	2.285759	2.280905	2.276077	2.328665	2.323566	2.359381	2.353876	2.348437
2.29278	2.287955	2.283129	2.278315	2.331078	2.325948	2.362022	2.356476	2.351006
2.247477	2.29003	2.285208	2.280413	2.305878	2.301085	2.323244	2.318255	2.353593
2.249411	2.291983	2.287147	2.28238	2.307936	2.303236	2.325585	2.320594	2.3562
2.171819	2.294064	2.289179	2.284387	2.27968	2.275047	2.327869	2.322902	2.358802
2.173905	2.296334	2.291346	2.286448	2.281684	2.277028	2.330056	2.325155	2.361391
2.151126	2.250943	2.246024	2.241181	2.283752	2.279049	2.303964	2.299126	2.322564
2.153237	2.253164	2.24821	2.243328	2.285962	2.281204	2.306263	2.30142	2.324882
2.155375	2.255409	2.250442	2.245542	2.288245	2.283424	2.308618	2.303747	2.327219
2.141434	2.189585	2.184742	2.179973	2.242944	2.238222	2.280929	2.276372	2.329595
2.143652	2.191765	2.18691	2.182137	2.245143	2.240368	2.283149	2.27858	2.332
2.073051	2.169308	2.16448	2.159744	2.215577	2.210778	2.285401	2.280794	2.305951
2.0749	2.171509	2.166655	2.161898	2.217827	2.213004	2.287686	2.283016	2.308316
2.07678	2.145464	2.14063	2.135899	2.15932	2.154652	2.210534	2.205954	2.310686
2.078704	2.147769	2.142868	2.13807	2.161464	2.156785	2.212749	2.208096	2.313046
2.080646	2.150105	2.145135	2.140264	2.163626	2.158934	2.214979	2.210263	2.31544
2.0249	2.078457	2.07435	2.070301	2.165815	2.161097	2.217226	2.212479	2.207794
2.026813	2.080392	2.076248	2.072158	2.168031	2.163275	2.219487	2.214726	2.210008
2.028725	2.024697	2.020653	2.01665	2.170277	2.165455	2.160728	2.156101	2.212237
2.030636	2.0266	2.022563	2.01856	2.172553	2.167643	2.162872	2.158245	2.214485
1.909924	2.028498	2.024466	2.020471	2.054628	2.050586	2.165109	2.160441	2.216752
1.911614	2.030387	2.026359	2.022381	2.056657	2.052627	2.16746	2.162698	2.219037
1.913312	1.961629	1.957833	1.954108	2.020378	2.016519	2.16982	2.164971	2.160187
1.915015	1.963412	1.959591	1.955844	2.022273	2.018433	2.17215	2.167245	2.162404
1.916733	1.965219	1.961379	1.957612	2.024181	2.020345	2.17448	2.169525	2.164634
1.879765	1.91474	1.911095	1.907515	1.955799	1.952166	2.098873	2.093949	2.166857
1.881472	1.916508	1.912896	1.909341	1.957695	1.953992	2.101226	2.096243	2.169091
1.883238	1.879607	1.876046	1.872526	1.90755	1.903969	2.042453	2.037607	2.156259
1.885062	1.88146	1.87785	1.874259	1.909284	1.905665	2.044788	2.039875	2.15866

1.886861	1.883263	1.879622	1.875987	1.872376	1.868784	1.95561	1.951824	2.037383
1.888609	1.884982	1.881343	1.87771	1.874062	1.87042	1.957405	1.953692	2.039803
1.890382	1.886708	1.883061	1.879427	1.87575	1.872069	1.868512	1.865077	2.042217
1.892234	1.888491	1.884801	1.881131	1.877429	1.87373	1.870152	1.866694	2.044529
1.894111	1.890296	1.886551	1.882838	1.879122	1.875426	1.871823	1.86831	2.046832
1.845821	1.892102	1.888311	1.884565	1.880867	1.877215	1.873598	1.870015	2.018342
1.847532	1.893913	1.890084	1.886312	1.882653	1.879069	1.875443	1.871775	2.020741
1.803278	1.845491	1.841811	1.838198	1.884472	1.880892	1.87725	1.873546	1.86991
1.804939	1.847181	1.843542	1.839966	1.886331	1.882689	1.879023	1.875329	1.871696
1.806637	1.80305	1.799565	1.796126	1.888176	1.884478	1.880796	1.877129	1.873505
1.808397	1.804861	1.801346	1.797852	1.889988	1.886247	1.882567	1.878949	1.875341
1.810167	1.806666	1.803115	1.799565	1.8918	1.888036	1.884358	1.880773	1.877169
1.811914	1.808381	1.804806	1.80124	1.797734	1.794273	1.886207	1.882586	1.878949
1.81365	1.810061	1.80647	1.802908	1.799431	1.796011	1.888076	1.884391	1.880708
1.774318	1.811751	1.80817	1.804627	1.80115	1.797721	1.839632	1.836057	1.882466
1.775969	1.813444	1.809894	1.80638	1.802886	1.799413	1.841314	1.837704	1.88422
1.777654	1.815186	1.81163	1.808105	1.804586	1.801083	1.797601	1.794136	1.886029
1.779378	1.816992	1.813385	1.809791	1.806238	1.80272	1.799254	1.79584	1.887905
1.672246	1.777596	1.774014	1.77044	1.807912	1.804387	1.800931	1.797548	1.794163
1.673856	1.779287	1.77569	1.772112	1.809636	1.80612	1.802655	1.799243	1.795851
1.675474	1.780966	1.777369	1.773805	1.811383	1.807869	1.804389	1.800944	1.797541
1.67708	1.782638	1.779065	1.775532	1.772007	1.76851	1.806091	1.802664	1.799268
1.678697	1.784322	1.780778	1.777279	1.773727	1.770173	1.807791	1.804395	1.801008
1.635161	1.677137	1.673975	1.670866	1.775454	1.771899	1.76844	1.765074	1.802708
1.636912	1.678907	1.675679	1.672497	1.777187	1.77368	1.770198	1.766742	1.804374
1.573416	1.635402	1.632209	1.629058	1.671017	1.667954	1.771947	1.768437	1.806084
1.574963	1.63702	1.633838	1.630701	1.672669	1.669578	1.773676	1.770169	1.807859
1.57651	1.573417	1.570386	1.567401	1.629257	1.6262	1.71667	1.713525	1.768437
1.578078	1.575002	1.571959	1.568955	1.630887	1.627823	1.71825	1.715088	1.770125
1.579664	1.576604	1.573545	1.570519	1.632524	1.629454	1.719832	1.716654	1.771824
1.485579	1.578206	1.575144	1.572114	1.569117	1.566152	1.673006	1.669892	1.77358
1.487071	1.579814	1.576754	1.573728	1.570705	1.5677	1.674645	1.671543	1.775374
1.488566	1.581426	1.578352	1.575321	1.572289	1.569276	1.566324	1.563434	1.670138
1.490058	1.583034	1.579934	1.576887	1.573866	1.57088	1.567941	1.565054	1.671873
1.491627	1.488671	1.485789	1.482966	1.575487	1.572511	1.569568	1.566664	1.563762
1.493322	1.490333	1.487402	1.48453	1.577184	1.574184	1.571204	1.568249	1.56531
1.495025	1.492004	1.489025	1.486101	1.578882	1.57585	1.572832	1.56983	1.566857
1.496648	1.493589	1.490575	1.487618	1.580521	1.577462	1.574426	1.57142	1.568437
1.49825	1.495155	1.492112	1.489127	1.582148	1.579058	1.57601	1.57301	1.570025
1.499887	1.496773	1.493699	1.490679	1.487738	1.484863	1.577605	1.574582	1.571585
1.501546	1.498424	1.49532	1.492262	1.489278	1.486357	1.57921	1.576146	1.573131
1.503208	1.500065	1.496947	1.493875	1.490866	1.487915	1.58089	1.577786	1.574744
1.504867	1.501685	1.498573	1.495518	1.49251	1.489549	1.58266	1.579518	1.576436
1.506571	1.503353	1.500236	1.497181	1.494159	1.491179	1.584416	1.581237	1.578116
1.508358	1.505114	1.501964	1.498868	1.495799	1.492765	1.586101	1.582882	1.579727
1.510159	1.506897	1.503703	1.500556	1.497439	1.494358	1.587797	1.584549	1.581364
1.511903	1.50863	1.50539	1.502195	1.499064	1.495988	1.492948	1.489945	1.583135
1.513635	1.510356	1.50707	1.50383	1.500697	1.497644	1.494621	1.491633	1.584965
1.515434	1.512136	1.508827	1.505562	1.502405	1.499326	1.496277	1.493262	1.586713
1.517288	1.51396	1.510647	1.507375	1.504176	1.501034	1.497925	1.494848	1.588401

1.519124	1.515755	1.512437	1.509165	1.505946	1.502773	1.499623	1.496494	1.49343
1.520925	1.517492	1.514168	1.510912	1.50771	1.504556	1.501395	1.498225	1.495125
1.522751	1.519277	1.515938	1.512679	1.509474	1.506321	1.50315	1.499958	1.496836
1.524629	1.521174	1.5178	1.514486	1.511223	1.508009	1.504815	1.501641	1.498527
1.526542	1.523117	1.519706	1.516331	1.512996	1.509704	1.506483	1.503332	1.500231
1.461319	1.525049	1.521632	1.518244	1.514866	1.511515	1.508259	1.505095	1.501977
1.463216	1.526988	1.523576	1.520198	1.516792	1.513393	1.510098	1.506902	1.503752
1.397919	1.528929	1.525485	1.522079	1.518652	1.515238	1.511923	1.508707	1.505526
1.399807	1.530868	1.527356	1.523888	1.520449	1.517051	1.513738	1.510511	1.507299
1.401756	1.465516	1.462044	1.458623	1.522303	1.518911	1.515591	1.512346	1.509103
1.403801	1.467536	1.464004	1.460525	1.524252	1.520843	1.517497	1.514219	1.510947
1.405852	1.469549	1.465968	1.462447	1.526222	1.522798	1.519426	1.516116	1.512814
1.407828	1.404294	1.40084	1.397476	1.528164	1.524747	1.521367	1.51803	1.514699
1.409811	1.406182	1.402701	1.399336	1.530115	1.526707	1.523319	1.519961	1.5166
1.324705	1.408232	1.4047	1.40129	1.464744	1.461418	1.525267	1.521895	1.518512
1.326822	1.410397	1.406799	1.403316	1.466701	1.463325	1.527217	1.523836	1.520434
1.328917	1.325284	1.321759	1.318341	1.402001	1.398729	1.529211	1.525793	1.522359
1.33099	1.327347	1.32381	1.320372	1.404082	1.400745	1.53125	1.527764	1.524282
1.333068	1.329422	1.325881	1.322428	1.406189	1.40279	1.465876	1.462428	1.526232
1.335142	1.331505	1.327962	1.324496	1.40831	1.404853	1.467878	1.464388	1.528217
1.337246	1.333611	1.330067	1.326589	1.410459	1.406949	1.469912	1.466378	1.53023
1.339419	1.335759	1.332205	1.328717	1.325247	1.321812	1.405589	1.402121	1.464822
1.341635	1.337939	1.334368	1.330866	1.327372	1.323907	1.407727	1.404193	1.46681
1.343861	1.340145	1.336538	1.332999	1.329487	1.326012	1.322543	1.319078	1.468868
1.346101	1.342378	1.338718	1.335122	1.331599	1.328133	1.324653	1.321161	1.470988
1.348357	1.344628	1.340931	1.337292	1.333742	1.330257	1.326761	1.323258	1.407071
1.350628	1.3469	1.343186	1.33952	1.335921	1.332379	1.32886	1.325368	1.40928
1.352915	1.349172	1.345449	1.341767	1.338124	1.334523	1.330976	1.327488	1.324006
1.355203	1.351419	1.347698	1.344022	1.340349	1.3367	1.33312	1.329609	1.326106
1.357525	1.353686	1.349958	1.346285	1.34259	1.338906	1.33529	1.331744	1.32821
1.359938	1.356035	1.352257	1.348544	1.344832	1.341144	1.3375	1.333899	1.330331
1.362408	1.358436	1.354583	1.350806	1.347081	1.343408	1.33974	1.336076	1.33247
1.364835	1.360816	1.35691	1.353086	1.349341	1.345664	1.341979	1.338286	1.334651
1.367222	1.363177	1.359237	1.35538	1.35161	1.347914	1.344219	1.34053	1.336874
1.369679	1.365614	1.361633	1.35773	1.353917	1.350181	1.346466	1.34278	1.339109
1.372242	1.368166	1.364127	1.360149	1.356264	1.352457	1.348708	1.345022	1.341345
1.374829	1.370736	1.366651	1.362615	1.358662	1.354783	1.350991	1.347292	1.343603
1.320173	1.373253	1.369168	1.365132	1.361137	1.357193	1.353346	1.349598	1.345876
1.322528	1.375773	1.371695	1.367675	1.363653	1.359659	1.355761	1.351958	1.3482
1.324904	1.378347	1.374229	1.370183	1.366155	1.36217	1.358268	1.354448	1.350672
1.327298	1.380963	1.376776	1.372676	1.368658	1.364721	1.360843	1.35703	1.353246
1.329726	1.325928	1.32222	1.318582	1.371145	1.367207	1.363323	1.3595	1.355705
1.332189	1.328379	1.324576	1.320835	1.373602	1.369616	1.365694	1.361847	1.358038
1.33466	1.330849	1.326982	1.323161	1.319514	1.315984	1.3681	1.364218	1.360384
1.337092	1.33331	1.329445	1.325604	1.321886	1.318256	1.370576	1.366652	1.362782
1.3396	1.335825	1.331964	1.328109	1.324316	1.320576	1.373103	1.369122	1.365204
1.195997	1.338477	1.334557	1.330645	1.326775	1.322951	1.375668	1.371604	1.367624
1.19801	1.34119	1.337198	1.333226	1.329292	1.325404	1.378306	1.374143	1.370089
1.199896	1.343826	1.339838	1.335879	1.331947	1.328052	1.324239	1.320501	1.372738
1.201688	1.346411	1.342483	1.338595	1.334721	1.330867	1.32702	1.32317	1.37554

1.20348	1.200785	1.198099	1.195409	1.337357	1.333534	1.329672	1.325764	1.321967
1.205274	1.202562	1.199879	1.197203	1.339809	1.336007	1.332157	1.328257	1.324423
1.207088	1.204348	1.201657	1.198986	1.342268	1.338462	1.334617	1.330739	1.326886
1.208911	1.206134	1.203436	1.200771	1.344774	1.340916	1.337058	1.333203	1.329351
1.210801	1.207979	1.205261	1.20259	1.347361	1.343438	1.339556	1.33572	1.331875
1.212877	1.210003	1.207236	1.204527	1.350164	1.346193	1.342282	1.338437	1.334584
1.215055	1.212126	1.209293	1.206526	1.353082	1.349071	1.345127	1.341251	1.337384
1.217116	1.214145	1.211257	1.208438	1.205703	1.203033	1.347806	1.34388	1.339986
1.21908	1.216079	1.213144	1.210275	1.207506	1.204813	1.35034	1.34634	1.342406
1.221061	1.218043	1.215073	1.212164	1.209364	1.206646	1.353005	1.348967	1.345003
1.223059	1.220042	1.217051	1.214115	1.211294	1.208559	1.355891	1.351878	1.347899
1.225112	1.222105	1.219101	1.216139	1.213285	1.210512	1.207809	1.205183	1.350773
1.227302	1.224311	1.221293	1.218293	1.215368	1.212502	1.209719	1.207024	1.353387
1.229509	1.22654	1.223516	1.220485	1.217486	1.214518	1.211643	1.208857	1.355969
1.231533	1.228586	1.225582	1.222558	1.21954	1.216533	1.21362	1.210796	1.358788
1.233451	1.230528	1.227559	1.22457	1.221566	1.21856	1.215643	1.212806	1.361755
1.235466	1.232565	1.229626	1.226666	1.223677	1.220674	1.217737	1.214857	1.212037
1.237587	1.23471	1.231797	1.228862	1.225889	1.222891	1.219915	1.216955	1.214045
1.073422	1.236781	1.233885	1.230974	1.228024	1.225046	1.22206	1.21906	1.216095
1.07506	1.238683	1.235791	1.232908	1.229996	1.227067	1.224123	1.22116	1.218213
1.076731	1.240607	1.237708	1.23484	1.23196	1.229078	1.22618	1.223266	1.220347
1.078558	1.242728	1.239806	1.236924	1.23405	1.231187	1.228299	1.22539	1.222454
1.080459	1.244924	1.241963	1.239048	1.236166	1.233311	1.230424	1.227512	1.224556
1.082327	1.079622	1.077009	1.074463	1.238147	1.235311	1.232453	1.229584	1.226664
1.084188	1.081369	1.078665	1.076051	1.240032	1.237221	1.234411	1.231617	1.228775
0.603466	1.083277	1.080498	1.077807	1.075248	1.072794	1.236424	1.233649	1.230839
0.604446	1.085398	1.082557	1.079774	1.077146	1.07463	1.238503	1.235676	1.232836
0.605403	0.604138	0.60284	0.601547	1.079057	1.076482	1.240606	1.237735	1.234868
0.606288	0.605011	0.603703	0.602402	1.08089	1.078281	1.242725	1.239866	1.237009
0.607159	0.605873	0.604563	0.603263	1.082755	1.080108	1.244856	1.242002	1.239151
	0.606808	0.605511	0.604213	0.890487	0.888293	1.079381	1.07682	1.241168
	0.607766	0.606492	0.605202	0.89215	0.889858	1.081252	1.078553	1.243142
				0.604857	0.603569	1.083245	1.08046	1.077797
				0.605787	0.604537	1.085355	1.082525	1.079779
				0.606695	0.605475	0.604223	0.602938	1.081791
				0.607573	0.606369	0.60514	0.603888	1.083847
						0.606038	0.60481	0.891242
						0.606907	0.605664	0.892706
						0.607773	0.606507	0.894149
								0.606167
								0.607114

		0.871557
		0.869947
0.873109	0.870726	1.582589
0.871378	0.868947	1.579732
0.869622	0.867136	1.576872
1.582026	1.578038	1.888907

						1.579156	1.575199	1.885679
				1.584613	1.580423	1.576336	1.572381	1.948577
				1.58175	1.577624	1.573563	1.569586	1.945207
		0.870874	0.868308	1.578954	1.574887	1.951746	1.946806	1.995369
		0.869121	0.866536	1.576236	1.572236	1.948596	1.943616	1.992049
		1.581678	1.57758	1.889068	1.884294	1.998892	1.993799	1.988762
		1.578912	1.574907	1.885974	1.881148	1.995506	1.990451	1.985481
		1.576159	1.572233	1.882873	1.877989	1.992114	1.987116	1.98223
1.581475	1.577352	1.573371	1.569451	1.945537	1.940549	1.988807	1.983856	1.979017
1.57864	1.574573	1.570575	1.566619	1.942196	1.937321	1.985555	1.980655	1.975847
1.575876	1.571852	1.948705	1.943808	1.992111	1.987208	1.982349	1.977528	2.023483
1.573183	1.56919	1.945536	1.940635	1.98886	1.983989	1.979191	1.974477	2.020544
1.952184	1.947276	1.995522	1.990549	1.985656	1.980833	2.026823	2.022197	2.017566
1.949017	1.944045	1.992216	1.987311	1.982492	1.977756	2.023831	2.019191	2.014527
1.945845	1.940839	1.988951	1.984121	1.979369	1.9747	2.020837	2.016159	2.011449
1.942598	1.937684	1.985769	1.981016	2.027108	2.02247	2.017792	2.013066	2.192454
1.939357	1.934578	1.98264	1.977953	2.024104	2.019433	2.014716	2.00994	2.188995
1.989075	1.984278	1.979553	1.97488	2.021057	2.016345	2.195929	2.190728	2.185551
1.985942	1.981217	1.976504	1.971809	2.017985	2.01322	2.192473	2.187268	2.182118
1.982858	1.978145	2.024341	2.019643	2.119026	2.113951	2.189022	2.18385	2.223351
1.979837	1.975065	2.021262	2.016533	2.11568	2.110578	2.185561	2.18047	2.219961
1.976789	1.97198	2.122486	2.117467	2.192568	2.187337	2.182196	2.177159	2.216613
1.973657	1.968868	2.11923	2.114264	2.189316	2.184127	2.179005	2.173949	2.213303
1.97055	1.965791	2.115998	2.111067	2.186072	2.180938	2.175841	2.170771	2.210024
2.018618	2.013864	2.192945	2.187831	2.182721	2.177618	2.216964	2.211807	2.206752
2.015729	2.010971	2.189685	2.18447	2.179337	2.17427	2.213579	2.208497	2.203519
2.116524	2.111455	2.186314	2.181093	2.220484	2.215387	2.210344	2.205348	2.200423
2.113185	2.108079	2.18286	2.177703	2.217106	2.212153	2.207228	2.202328	2.19744
2.189957	2.184737	2.179564	2.174464	2.213836	2.208949	2.204079	2.19923	2.194358
2.186768	2.181598	2.176478	2.171421	2.210704	2.20578	2.200883	2.196023	2.191139
2.183561	2.178448	2.217761	2.212617	2.20757	2.202611	2.197692	2.192826	2.187939
2.180228	2.175157	2.214429	2.209348	2.204339	2.1994	2.194511	2.18969	2.184843
2.176919	2.171898	2.211126	2.206103	2.201133	2.196218	2.191356	2.186569	2.181752
2.218163	2.213084	2.208052	2.203065	2.198106	2.193178	2.188284	2.183438	2.178567
2.215061	2.210071	2.20509	2.200122	2.195158	2.190199	2.185245	2.1803	2.17534
2.211869	2.206928	2.201968	2.197005	2.192032	2.187051	2.182081	2.177124	2.202443
2.208598	2.20367	2.198706	2.193733	2.188747	2.18375	2.178802	2.173911	2.199215
2.205449	2.200516	2.19554	2.190549	2.185543	2.180525	2.205994	2.200967	2.312712
2.202512	2.197554	2.19256	2.187545	2.182507	2.17745	2.202789	2.197735	2.308864
2.199528	2.194538	2.189519	2.184478	2.179418	2.17434	2.199579	2.194527	2.305074
2.196299	2.191279	2.186225	2.181153	2.206559	2.201342	2.272173	2.26652	2.301383
2.193007	2.187958	2.18287	2.177773	2.203117	2.19795	2.268312	2.262834	2.297758
2.189877	2.184802	2.210321	2.20506	2.199889	2.194797	2.305243	2.299655	2.309519
2.186822	2.181725	2.207146	2.201903	2.196793	2.191797	2.301758	2.296151	2.306001
2.183598	2.178491	2.203856	2.198673	2.286724	2.281148	2.31373	2.30815	2.302608
2.180181	2.175074	2.200428	2.195354	2.28305	2.277641	2.310259	2.304819	2.299367
2.207478	2.20224	2.290684	2.284986	2.317664	2.312203	2.30678	2.30141	2.324435
2.204317	2.199139	2.287223	2.28159	2.314189	2.30872	2.303264	2.297822	2.320627
2.20124	2.196134	2.283879	2.278309	2.310812	2.305301	2.299786	2.294256	2.316843
2.220714	2.215655	2.280587	2.275109	2.307531	2.301995	2.296459	2.290916	2.378018

2.217708	2.212723	2.277324	2.271938	2.304273	2.298711	2.293165	2.287635	2.37409
2.284915	2.279349	2.311877	2.306348	2.30078	2.295199	2.317806	2.312038	2.370017
2.281305	2.275816	2.308229	2.302692	2.297117	2.291522	2.313973	2.308225	2.365856
2.277868	2.272438	2.304736	2.299189	2.321985	2.316146	2.374772	2.368287	2.36191
2.27464	2.269252	2.301434	2.295875	2.318566	2.312775	2.370925	2.364516	2.358219
2.309279	2.303731	2.326803	2.320965	2.38025	2.373644	2.367171	2.360839	2.354615
2.305995	2.300429	2.323352	2.317529	2.376412	2.369911	2.363526	2.357263	2.351098
2.302709	2.297116	2.319891	2.314072	2.37257	2.36617	2.35987	2.353672	2.347565
2.328145	2.322275	2.344597	2.338288	2.368614	2.362241	2.355984	2.349845	2.343808
2.324628	2.318779	2.340837	2.334496	2.364625	2.358246	2.352013	2.345931	2.33997
2.349572	2.343313	2.373865	2.367247	2.360785	2.354457	2.34828	2.342254	2.364584
2.34548	2.339292	2.369807	2.363394	2.357072	2.350842	2.344747	2.338778	2.361182
2.378755	2.372268	2.365906	2.35965	2.353443	2.347299	2.341289	2.335399	2.357891
2.374898	2.368487	2.362207	2.356035	2.349899	2.343815	2.337887	2.332098	2.354696
2.371167	2.36483	2.358612	2.352496	2.346424	2.34041	2.362813	2.3571	2.351629
2.367541	2.361279	2.355102	2.349018	2.343012	2.337091	2.359599	2.354041	2.348737
2.363986	2.357794	2.351649	2.345589	2.339658	2.333848	2.356473	2.351079	2.345941
2.360414	2.354279	2.34818	2.342172	2.364626	2.358914	2.353422	2.34815	2.343119
2.356853	2.350766	2.344718	2.338776	2.361336	2.355792	2.35044	2.345272	2.340318
2.353326	2.347275	2.341268	2.335382	2.358023	2.352624	2.347428	2.34244	2.360814
2.34983	2.343798	2.337818	2.331971	2.354664	2.349384	2.344367	2.339648	2.3582
2.346381	2.3404	2.362794	2.356983	2.351521	2.346368	2.341503	2.336976	2.355643
2.342982	2.337109	2.35964	2.354012	2.348711	2.343704	2.338944	2.33447	2.353138
2.339631	2.333878	2.356567	2.351149	2.346021	2.341163	2.336494	2.332037	2.350673
2.336306	2.33066	2.353487	2.348269	2.34331	2.338602	2.357085	2.352622	2.348237
2.333043	2.327511	2.35047	2.345448	2.340651	2.336081	2.35461	2.350187	2.345827
2.358184	2.352726	2.347611	2.342755	2.361127	2.356599	2.352143	2.347757	2.365044
2.355142	2.349848	2.344888	2.340174	2.358589	2.354107	2.349689	2.345338	2.362602
2.352269	2.34713	2.342282	2.337653	2.356086	2.35165	2.368959	2.364549	2.360173
2.349587	2.344598	2.339812	2.335199	2.353623	2.349233	2.366528	2.362129	2.357761
2.346971	2.342106	2.360312	2.35567	2.351178	2.346829	2.364101	2.359712	2.399812
2.344372	2.339597	2.357809	2.3532	2.348742	2.344425	2.361663	2.357281	2.397198
2.341818	2.337106	2.35532	2.350746	2.346321	2.34203	2.35923	2.35486	2.3946
2.362204	2.357478	2.352846	2.34831	2.365527	2.361164	2.356813	2.352476	2.392065
2.359688	2.354984	2.350389	2.34589	2.363076	2.358731	2.354409	2.350115	2.389568
2.357194	2.352524	2.369644	2.36508	2.360639	2.356315	2.396138	2.391609	2.387068
2.354723	2.350097	2.367184	2.362636	2.358216	2.353913	2.393583	2.389081	2.384569
2.352264	2.347676	2.364727	2.360197	2.376976	2.372638	2.391045	2.386571	2.382088
2.349815	2.345255	2.362266	2.357754	2.374509	2.370204	2.388519	2.384074	2.379623
2.36903	2.364391	2.359826	2.355338	2.394945	2.390469	2.386021	2.381606	2.377186
2.366538	2.361936	2.357411	2.352961	2.392425	2.387983	2.383569	2.379187	2.374794
2.364073	2.35952	2.355035	2.350622	2.389941	2.385524	2.381137	2.376789	2.372421
2.361681	2.357205	2.373796	2.369315	2.3875	2.383072	2.378692	2.374376	2.370039
2.359338	2.354955	2.371517	2.367025	2.385095	2.380633	2.376246	2.371959	2.367655
2.378031	2.37366	2.391929	2.387254	2.382687	2.378206	2.373816	2.369544	2.365269
2.375565	2.371245	2.389452	2.384827	2.380278	2.375789	2.371399	2.367129	2.362881
2.373129	2.36884	2.38699	2.38243	2.377905	2.373409	2.369011	2.364729	2.415226
2.370746	2.366456	2.384563	2.380093	2.375597	2.371087	2.366667	2.362349	2.412349
2.368379	2.364079	2.382135	2.377747	2.373282	2.368766	2.36433	2.359978	2.409492
2.388579	2.384069	2.379677	2.375339	2.3709	2.366394	2.416757	2.411629	2.406633

2.386106	2.38159	2.377215	2.372903	2.368491	2.364015	2.413911	2.408791	2.40381
2.383719	2.37918	2.374788	2.370466	2.366092	2.361694	2.411201	2.406094	2.417841
2.381396	2.376823	2.372385	2.368022	2.363696	2.359412	2.408593	2.403504	2.415166
2.379024	2.374454	2.369982	2.365581	2.415823	2.410883	2.405908	2.400862	2.412471
2.376589	2.372066	2.367571	2.363128	2.412903	2.408034	2.403127	2.398151	2.409743
2.374177	2.3697	2.420319	2.41507	2.410049	2.405219	2.417078	2.412061	2.407067
2.371833	2.367387	2.41755	2.412306	2.407293	2.402471	2.414309	2.409405	2.404505
2.369491	2.365067	2.414815	2.409619	2.404617	2.399776	2.411569	2.406759	2.401945
2.367079	2.362667	2.412075	2.407033	2.402062	2.397164	2.408854	2.404051	2.422709
2.364648	2.360242	2.409344	2.404495	2.399566	2.394599	2.406162	2.401326	2.419944
2.416635	2.411499	2.40661	2.401836	2.413545	2.40845	2.403479	2.398647	2.417247
2.413881	2.408774	2.403874	2.399081	2.410748	2.405722	2.400806	2.396005	2.414599
2.411169	2.406117	2.401205	2.396378	2.408011	2.403066	2.39821	2.393437	2.412014
2.408498	2.403551	2.398622	2.393745	2.405352	2.400507	2.395714	2.390957	2.409499
2.40586	2.400981	2.396053	2.391157	2.402735	2.397968	2.393234	2.388515	2.427003
2.403273	2.398355	2.393467	2.388635	2.400173	2.39542	2.390733	2.38611	2.424582
2.400683	2.395708	2.390873	2.386127	2.397625	2.39287	2.388222	2.383698	2.422162
2.397987	2.39304	2.404607	2.399784	2.395006	2.390284	2.406634	2.402092	2.419601
2.395239	2.390364	2.401901	2.397101	2.392362	2.387689	2.404026	2.399478	2.41697
2.392566	2.387758	2.39927	2.394504	2.389814	2.3852	2.401527	2.39698	2.414436
2.389958	2.385218	2.396707	2.391985	2.387355	2.382813	2.399134	2.39459	2.411989
2.387405	2.382716	2.394187	2.389518	2.405833	2.401281	2.396748	2.392224	2.409584
2.384914	2.380246	2.391713	2.387115	2.403414	2.398858	2.394344	2.389873	2.407229
2.382457	2.377819	2.38927	2.384727	2.401006	2.396452	2.391957	2.38753	2.404883
2.380011	2.37545	2.407722	2.403122	2.398572	2.394075	2.411483	2.406974	2.402504
2.377587	2.373112	2.405279	2.400664	2.396145	2.39171	2.409105	2.404589	2.400126
2.3912	2.386626	2.402848	2.398276	2.393771	2.389328	2.406698	2.40222	2.397796
2.388769	2.384189	2.400431	2.395937	2.391435	2.38694	2.404277	2.399865	2.3955
2.407146	2.402545	2.39803	2.393567	2.38908	2.384588	2.401909	2.397528	2.393194
2.404684	2.400164	2.395648	2.391159	2.386703	2.382279	2.399602	2.39521	2.390873
2.402242	2.397773	2.393261	2.388763	2.384335	2.379963	2.397289	2.392906	2.391282
2.399829	2.395334	2.390851	2.386397	2.381985	2.377613	2.394937	2.390617	2.389046
2.397428	2.392905	2.388448	2.384038	2.379645	2.375276	2.392597	2.388335	2.386814
2.39502	2.390536	2.386082	2.381667	2.377307	2.372999	2.393093	2.388803	2.390732
2.39262	2.388194	2.383733	2.3793	2.374979	2.370752	2.39085	2.38653	2.388503
2.390265	2.385812	2.381372	2.376979	2.376046	2.371823	2.394817	2.39055	2.386316
2.387949	2.383402	2.379002	2.374696	2.373793	2.369548	2.392581	2.388362	2.384162
2.385669	2.381091	2.380117	2.375809	2.371529	2.367292	2.394375	2.390217	2.386074
2.383441	2.378917	2.377902	2.373514	2.369235	2.365056	2.392195	2.388029	2.383887
2.384668	2.380193	2.380679	2.376268	2.378935	2.374842	2.390012	2.385849	2.381715
2.382346	2.377964	2.378507	2.374139	2.376838	2.372674	2.387785	2.383674	2.379587
2.38001	2.375705	2.376326	2.372036	2.374777	2.370535	2.385584	2.381516	2.377468
2.3826	2.378309	2.381091	2.376909	2.392005	2.387714	2.383503	2.379389	2.375309
2.380328	2.375969	2.378854	2.374753	2.389887	2.385687	2.381492	2.377291	2.373144
2.37812	2.373746	2.376688	2.372588	2.387724	2.383599	2.379445	2.375241	2.372106
2.375977	2.37163	2.374581	2.370405	2.385513	2.381458	2.377372	2.373243	2.370154
2.381067	2.376795	2.372532	2.368307	2.38337	2.379328	2.375276	2.371213	2.387144
2.379034	2.374806	2.370563	2.366339	2.381327	2.377208	2.373133	2.369117	2.384954
2.376976	2.372796	2.368595	2.3644	2.379325	2.375131	2.371025	2.367034	2.382774
2.374835	2.370709	2.366575	2.362449	2.377361	2.373148	2.370146	2.366139	2.380647

2.372668	2.368587	2.364526	2.360482	2.375404	2.371202	2.368208	2.364167	2.378521
2.370536	2.366449	2.362429	2.358447	2.374567	2.370388	2.384921	2.38059	2.389581
2.368428	2.3643	2.360297	2.356361	2.37252	2.368409	2.382797	2.378381	2.387271
2.366386	2.362235	2.378514	2.374495	2.370444	2.366381	2.380649	2.376204	2.399693
2.364415	2.36026	2.376474	2.37239	2.368328	2.364295	2.378472	2.374062	2.397441
2.364198	2.360031	2.37447	2.37033	2.384825	2.38059	2.391624	2.387164	2.395209
2.362274	2.358104	2.372504	2.368349	2.382683	2.378443	2.389418	2.384975	2.393002
2.360349	2.356187	2.370556	2.366388	2.380558	2.376279	2.387183	2.382762	2.390787
2.358402	2.354281	2.368611	2.364402	2.378381	2.374016	2.384845	2.380469	2.402984
2.356443	2.352376	2.366664	2.362408	2.37619	2.37172	2.382467	2.37814	2.400663
2.354489	2.35044	2.38303	2.378533	2.389204	2.384616	2.380153	2.375834	2.398319
2.352535	2.348477	2.380902	2.376395	2.386989	2.382373	2.377886	2.373541	2.395955
2.369057	2.364914	2.393979	2.389379	2.384779	2.380173	2.402492	2.398027	2.393668
2.367094	2.362899	2.391756	2.387148	2.382574	2.378023	2.400304	2.395836	2.391475
2.383393	2.378939	2.389531	2.384912	2.38037	2.375884	2.39814	2.393676	2.389313
2.381258	2.376796	2.387306	2.382665	2.378152	2.373736	2.395974	2.391523	2.387156
2.37911	2.374647	2.385088	2.380436	2.375952	2.371597	2.393818	2.389387	2.385023
2.392038	2.387496	2.382887	2.378274	2.38906	2.384672	2.391665	2.387266	2.382925
2.389799	2.385258	2.3807	2.376155	2.38693	2.382537	2.38952	2.385162	2.380855
2.387572	2.383038	2.378521	2.374031	2.384801	2.380421	2.387399	2.383075	2.378795
2.385356	2.380831	2.37635	2.371907	2.382677	2.378325	2.385302	2.381005	2.376746
2.383156	2.378661	2.37421	2.3698	2.391918	2.387542	2.383216	2.378942	2.380228
2.380974	2.376541	2.372115	2.367716	2.38979	2.38544	2.381137	2.376885	2.378228
2.378816	2.374436	2.370033	2.365648	2.387678	2.383352	2.37907	2.374834	2.376231
2.376695	2.372327	2.383039	2.378629	2.385582	2.381283	2.382631	2.378422	2.374237
2.374594	2.370224	2.380922	2.376548	2.383502	2.379227	2.38063	2.376426	2.372245
2.372487	2.36813	2.390144	2.385758	2.387106	2.382855	2.37863	2.37443	2.370251
2.370382	2.366044	2.388031	2.383687	2.385098	2.380856	2.376635	2.372437	2.368258
2.368282	2.363968	2.391653	2.387368	2.383103	2.378864	2.374645	2.370444	2.37479
2.366187	2.361899	2.389602	2.385366	2.381122	2.376883	2.372659	2.368449	2.372758
2.396176	2.391822	2.387563	2.383361	2.379135	2.374904	2.37927	2.37499	2.384343
2.394094	2.389779	2.385535	2.381338	2.37713	2.372923	2.377268	2.37299	2.382372
2.392036	2.38776	2.383526	2.379327	2.375132	2.370947	2.375277	2.371014	2.380431
2.389987	2.385753	2.381531	2.377334	2.373145	2.368967	2.37329	2.369076	2.378545
2.387979	2.383779	2.37957	2.375375	2.371186	2.367007	2.371321	2.367161	2.376685
2.386114	2.381917	2.377717	2.373528	2.377881	2.373626	2.369415	2.365256	2.374793
2.384367	2.380145	2.37595	2.371771	2.376083	2.371801	2.367558	2.363362	2.372879
2.382523	2.378282	2.382702	2.378444	2.374165	2.369876	2.36562	2.361401	2.370918
2.38056	2.376308	2.380666	2.376382	2.372102	2.367829	2.363582	2.359361	2.368897
2.378555	2.3743	2.378599	2.37429	2.370009	2.365751	2.361518	2.357306	2.366871
2.376515	2.372276	2.376524	2.372194	2.367908	2.363657	2.359438	2.355253	2.364859
2.374461	2.370238	2.374443	2.370103	2.365815	2.361569	2.357364	2.353207	2.362856
2.380993	2.376691	2.372364	2.36804	2.36376	2.359517	2.355319	2.351176	2.360856
2.378925	2.37461	2.370303	2.366011	2.361746	2.357507	2.353314	2.349177	2.358879
2.376973	2.37265	2.368359	2.364092	2.35985	2.355635	2.351453	2.347312	2.357026
2.375133	2.370805	2.366526	2.362281	2.358072	2.353897	2.349732	2.345576	2.355292
2.373204	2.368877	2.364609	2.360385	2.356202	2.35206	2.347911	2.343749	2.353474
2.371122	2.366806	2.362555	2.35835	2.354187	2.350065	2.34593	2.34178	2.351525
2.369059	2.364746	2.360501	2.356306	2.352153	2.348041	2.343917	2.339778	2.349544
2.367121	2.362774	2.358505	2.354293	2.350132	2.346021	2.341898	2.337761	2.347542

2.365223	2.360846	2.356555	2.352324	2.34815	2.344033	2.339906	2.335765	2.345558
2.363295	2.358965	2.354705	2.350495	2.346325	2.342194	2.338056	2.333904	2.343708
2.361355	2.357117	2.352921	2.348756	2.344597	2.340449	2.336294	2.332124	2.341938
2.359394	2.355201	2.35104	2.346903	2.342754	2.3386	2.334436	2.330253	2.34961
2.357403	2.353205	2.349054	2.344932	2.340791	2.336642	2.332477	2.328283	2.347642
2.355471	2.351277	2.347132	2.343016	2.338879	2.33473	2.330567	2.326378	2.345747
2.353647	2.349492	2.345356	2.341234	2.337091	2.332934	2.328776	2.324613	2.344
2.351829	2.3477	2.343567	2.339436	2.335286	2.331122	2.326972	2.32284	2.342254
2.349918	2.345762	2.34162	2.337488	2.333335	2.329169	2.335357	2.331213	2.340384
2.347993	2.34379	2.339633	2.3355	2.331346	2.327182	2.333375	2.329258	2.338483
2.346165	2.341956	2.33779	2.333647	2.329495	2.325341	2.331529	2.327426	2.336676
2.344408	2.340217	2.336045	2.331886	2.327738	2.323601	2.329779	2.325678	2.334932
2.342617	2.338418	2.334224	2.330041	2.336238	2.332084	2.32796	2.323873	2.333144
2.340788	2.336544	2.332304	2.32808	2.334265	2.330139	2.326045	2.321989	2.331295
2.338934	2.334669	2.330406	2.326166	2.332344	2.328243	2.337568	2.333519	2.329478
2.337032	2.332807	2.328582	2.324379	2.330567	2.326485	2.335834	2.331796	2.327752
2.335142	2.330963	2.32678	2.322618	2.328811	2.324739	2.334104	2.330073	2.326026
2.333343	2.329157	2.335317	2.331103	2.32696	2.322876	2.332243	2.328223	2.324197
2.33159	2.327375	2.333493	2.329247	2.325078	2.320975	2.330336	2.326327	2.322328
2.329816	2.325581	2.331681	2.327437	2.323275	2.319181	2.32855	2.32454	2.320547
2.32803	2.323778	2.32988	2.325665	2.321535	2.317477	2.326865	2.322845	2.31884
2.326209	2.32198	2.3281	2.323924	2.319819	2.315781	2.325174	2.321129	2.317101
2.324329	2.320181	2.326347	2.322221	2.318137	2.314092	2.323471	2.31938	2.315308
2.332843	2.328712	2.324603	2.320519	2.316444	2.31238	2.321742	2.317617	2.313516
2.331076	2.326959	2.322873	2.318806	2.314717	2.310615	2.319955	2.315832	2.311744
2.329341	2.325213	2.321129	2.317065	2.31296	2.308826	2.318153	2.314051	2.309992
2.327578	2.323416	2.319309	2.315232	2.311135	2.30703	2.316389	2.312316	2.312851
2.325809	2.321595	2.317448	2.313344	2.309271	2.305231	2.314648	2.310612	2.311153
2.324068	2.319834	2.315665	2.311543	2.307483	2.303481	2.312934	2.308922	2.309455
2.322358	2.318136	2.31396	2.309826	2.30577	2.301783	2.311253	2.307251	2.307762
2.320616	2.316413	2.312248	2.308122	2.304071	2.300085	2.314107	2.310072	2.306031
2.318812	2.314631	2.310503	2.306419	2.302379	2.298382	2.31237	2.308296	2.304234
2.31701	2.312856	2.308768	2.304723	2.300688	2.29667	2.310617	2.306509	2.302434
2.315254	2.311138	2.30707	2.303034	2.298984	2.294929	2.308833	2.304727	2.303819
2.31352	2.309443	2.305388	2.301347	2.297276	2.293184	2.307045	2.30296	2.302075
2.311796	2.307741	2.303695	2.299655	2.300666	2.296525	2.305322	2.301256	2.300381
2.310085	2.30604	2.301996	2.297957	2.298955	2.294827	2.303645	2.2996	2.298731
2.308382	2.304338	2.30028	2.296225	2.297202	2.293093	2.301938	2.297932	2.297079
2.306687	2.302633	2.298541	2.294445	2.295392	2.291309	2.300191	2.296246	2.295426
2.305002	2.300942	2.296823	2.292697	2.29362	2.289561	2.298473	2.294573	2.293768
2.303328	2.299272	2.295151	2.291019	2.291931	2.287892	2.296815	2.292922	2.292095
2.30166	2.297611	2.293493	2.289364	2.290269	2.286252	2.295179	2.291283	2.290422
2.299993	2.295948	2.296834	2.292667	2.288599	2.284607	2.293541	2.289645	2.288763
2.298327	2.294287	2.295155	2.290988	2.286935	2.282969	2.29191	2.288011	2.28711
2.296662	2.292627	2.293487	2.289333	2.285291	2.281335	2.290277	2.286379	2.285462
2.294998	2.290969	2.29183	2.287699	2.283665	2.279707	2.288643	2.28475	2.283821
2.293326	2.289302	2.290164	2.286058	2.29495	2.290955	2.287001	2.283108	2.282166
2.291642	2.287621	2.288485	2.284405	2.293301	2.289305	2.285349	2.28145	2.280494
2.294914	2.290875	2.28681	2.282752	2.291649	2.287654	2.283694	2.279789	2.278818
2.293217	2.289195	2.285143	2.281097	2.289993	2.286002	2.282043	2.278131	2.277144

2.291532	2.287528	2.283486	2.279448	2.28834	2.284351	2.280391	2.276474	2.275471
2.289882	2.285879	2.281845	2.277815	2.286697	2.2827	2.278735	2.274819	2.273805
2.288254	2.284244	2.280217	2.276199	2.285068	2.281054	2.277081	2.273167	2.272143
2.286618	2.28261	2.278609	2.274623	2.283488	2.279451	2.275456	2.271521	2.2888
2.284971	2.280976	2.277023	2.273092	2.281962	2.277896	2.273864	2.269883	2.286961
2.283362	2.279366	2.275434	2.271526	2.280394	2.276309	2.272254	2.268239	2.286918
2.281819	2.277799	2.27384	2.269901	2.278752	2.274668	2.270609	2.266584	2.285123
2.280272	2.27623	2.272236	2.268257	2.277089	2.273011	2.268957	2.264929	2.283343
2.278661	2.274619	2.270605	2.266599	2.275417	2.271345	2.287959	2.283601	2.292772
2.277031	2.273003	2.268973	2.264944	2.273752	2.269688	2.286182	2.281834	2.29104
2.275436	2.271438	2.267402	2.263353	2.272154	2.268101	2.296048	2.29168	2.289343
2.27386	2.269905	2.26587	2.261805	2.270603	2.266562	2.294423	2.290044	2.287675
2.272268	2.268317	2.277183	2.273075	2.30151	2.297121	2.292744	2.288369	2.285985
2.270653	2.266662	2.27552	2.271426	2.299759	2.295369	2.290999	2.286645	2.284262
2.269056	2.265028	2.28606	2.281918	2.308006	2.303558	2.300697	2.296303	2.291939
2.267508	2.263459	2.284466	2.280338	2.306341	2.301887	2.299023	2.294657	2.290308
2.265953	2.261891	2.282874	2.278754	2.304673	2.300218	2.297352	2.293008	2.288675
2.264329	2.260266	2.311829	2.307375	2.302926	2.298487	2.29562	2.291275	2.286954
2.262682	2.258624	2.310051	2.305591	2.301153	2.296737	2.293871	2.289514	2.2852
2.27476	2.27068	2.308341	2.303876	2.299447	2.29505	2.29218	2.287812	2.283491
2.273222	2.26915	2.306685	2.302215	2.297793	2.29341	2.290536	2.286155	2.281814
2.274346	2.270295	2.295096	2.290684	2.296147	2.291767	2.288886	2.28451	2.280166
2.272833	2.268774	2.293444	2.289066	2.294509	2.290114	2.287226	2.28288	2.278555
2.289951	2.285525	2.291802	2.287448	2.283089	2.278733	2.285571	2.28125	2.276942
2.28831	2.283905	2.290185	2.285821	2.281465	2.277121	2.283936	2.279619	2.275316
2.286671	2.28229	2.28857	2.28419	2.279837	2.275509	2.282299	2.277975	2.27367
2.28503	2.280648	2.28693	2.282556	2.278205	2.273877	2.28063	2.276288	2.271969
2.283387	2.278994	2.285278	2.280919	2.276569	2.272232	2.278942	2.274578	2.270238
2.281744	2.277358	2.283644	2.279283	2.274927	2.27058	2.268057	2.263743	2.269666
2.2801	2.275739	2.282026	2.277647	2.273276	2.268915	2.26639	2.262115	2.268025
2.278459	2.274112	2.280405	2.276024	2.271658	2.267304	2.264776	2.260511	2.269866
2.276822	2.27247	2.278773	2.274423	2.270094	2.265786	2.263255	2.258945	2.268222
2.275186	2.270829	2.277147	2.272826	2.268529	2.264254	2.261717	2.25737	2.266578
2.273546	2.269204	2.275538	2.271225	2.266916	2.262615	2.260064	2.255745	2.264928
2.271905	2.267581	2.273934	2.269627	2.265299	2.260957	2.258387	2.254106	2.263272
2.270257	2.265936	2.272315	2.268046	2.263737	2.2594	2.262123	2.25776	2.26159
2.268605	2.26428	2.27069	2.266476	2.262208	2.257906	2.260586	2.256139	2.259893
2.266979	2.262668	2.269084	2.264879	2.260632	2.25636	2.259016	2.254543	2.258252
2.265374	2.261101	2.267494	2.263244	2.258999	2.254761	2.257416	2.252981	2.256674
2.263803	2.259561	2.265942	2.261658	2.257396	2.253151	2.255786	2.25139	2.255084
2.262286	2.258063	2.26446	2.260174	2.255861	2.251527	2.254096	2.249739	2.253462
2.260779	2.256557	2.26297	2.258693	2.254337	2.249918	2.252418	2.248078	2.251821
2.259236	2.254974	2.270912	2.266598	2.265957	2.261474	2.258991	2.25455	2.250144
2.257685	2.253366	2.269282	2.264985	2.264358	2.259915	2.257413	2.252907	2.248458
2.276459	2.272048	2.267689	2.263352	2.263687	2.259261	2.2558	2.251295	2.246844
2.274935	2.270538	2.26612	2.261698	2.261993	2.257592	2.254163	2.24971	2.245286
2.273364	2.26899	2.264549	2.260085	2.260338	2.255926	2.252504	2.2481	2.243713
2.271731	2.267392	2.262977	2.258529	2.258736	2.254257	2.25081	2.246454	2.24212
2.270099	2.265774	2.265117	2.260677	2.257141	2.252615	2.249145	2.244815	2.240516
2.268492	2.264129	2.263469	2.259058	2.255528	2.251018	2.247542	2.243191	2.238886

2.266905	2.262489	2.261819	2.257436	2.253924	2.249451	2.245977	2.241594	2.237268
2.265355	2.260908	2.260226	2.255856	2.252358	2.24792	2.244454	2.240057	2.23572
2.26383	2.25936	2.25866	2.254291	2.250809	2.246412	2.242959	2.23856	2.234215
2.262305	2.257807	2.257072	2.252683	2.249211	2.244859	2.241432	2.237051	2.23528
2.260783	2.256252	2.255463	2.251032	2.247566	2.243265	2.239877	2.235537	2.233787
2.262969	2.258396	2.254795	2.250322	2.245962	2.2417	2.240906	2.236611	2.2323
2.261443	2.256866	2.253249	2.24877	2.244418	2.240175	2.239402	2.235124	2.23082
2.259916	2.255344	2.251723	2.247247	2.242904	2.238673	2.237912	2.233643	2.229341
2.258384	2.253825	2.25021	2.245749	2.244135	2.239926	2.236441	2.232169	2.227857
2.256852	2.252308	2.248706	2.244266	2.242674	2.238469	2.234975	2.230694	2.226372
2.255325	2.250789	2.247195	2.242773	2.241197	2.236992	2.233491	2.229214	2.225819
2.253801	2.24927	2.245683	2.241277	2.239713	2.235502	2.231994	2.227725	2.224335
2.252282	2.24776	2.247948	2.243564	2.239256	2.23502	2.231375	2.227113	2.222808
2.250768	2.246259	2.24646	2.242077	2.237744	2.233459	2.229777	2.225515	2.221226
2.253893	2.249399	2.244978	2.240601	2.236258	2.23195	2.228245	2.223975	2.219689
2.252387	2.247911	2.243504	2.239144	2.234824	2.230545	2.226844	2.222553	2.218244
2.250885	2.246425	2.242031	2.237686	2.233393	2.229151	2.225458	2.221149	2.216813
2.249389	2.244937	2.240548	2.236207	2.231924	2.227696	2.224004	2.21969	2.215346
2.247897	2.243452	2.239066	2.234723	2.230442	2.226217	2.222522	2.218209	2.213867
2.246406	2.241979	2.237596	2.233252	2.228966	2.224732	2.221029	2.21672	2.212383
2.244918	2.240516	2.236139	2.231792	2.227493	2.223238	2.219521	2.215222	2.210894
2.243443	2.239067	2.234708	2.230373	2.226067	2.221788	2.218047	2.213734	2.2094
2.241979	2.237628	2.233306	2.229007	2.224702	2.220396	2.216617	2.212262	2.207901
2.240543	2.236217	2.231923	2.227644	2.223332	2.218996	2.215179	2.210788	2.206404
2.23915	2.234853	2.230565	2.226277	2.221939	2.217562	2.21371	2.209304	2.204914
2.237779	2.233501	2.229204	2.224891	2.220523	2.216108	2.212228	2.207818	2.203431
2.236419	2.232132	2.227805	2.223452	2.219058	2.214629	2.210739	2.206336	2.201959
2.235063	2.230748	2.226378	2.221977	2.217563	2.213134	2.209246	2.204859	2.2005
2.233667	2.229322	2.224921	2.220493	2.216073	2.211655	2.207774	2.2034	2.199057
2.232235	2.227854	2.223432	2.218994	2.214582	2.210187	2.206318	2.201955	2.197629
2.230791	2.226389	2.221956	2.217518	2.213122	2.208759	2.204913	2.200574	2.196262
2.229332	2.224925	2.22049	2.216059	2.211695	2.207384	2.203584	2.199292	2.194997
2.227887	2.223489	2.219065	2.214648	2.210307	2.206026	2.20225	2.197988	2.193699
2.226483	2.222115	2.217726	2.213341	2.208994	2.204678	2.200866	2.196579	2.19227
2.225102	2.220761	2.216409	2.212053	2.207692	2.203325	2.199459	2.19513	2.190791
2.223748	2.21938	2.215016	2.210656	2.20629	2.20192	2.198035	2.193679	2.189319
2.22241	2.217984	2.21358	2.209195	2.20483	2.200484	2.1966	2.192225	2.18785
2.221026	2.216567	2.212139	2.207738	2.203386	2.199077	2.195215	2.190855	2.186486
2.219587	2.215121	2.210681	2.206275	2.201951	2.197698	2.193886	2.189578	2.18524
2.218197	2.21373	2.209286	2.204877	2.200564	2.196332	2.192541	2.188263	2.183944
2.216913	2.212448	2.208007	2.203599	2.199262	2.194988	2.19116	2.186866	2.182535
2.215622	2.21116	2.206726	2.202321	2.197958	2.193634	2.189763	2.185452	2.181112
2.214215	2.209754	2.205329	2.200933	2.196564	2.192221	2.188343	2.184053	2.179741
2.212778	2.208317	2.203902	2.199518	2.19515	2.1908	2.186925	2.182661	2.178384
2.211442	2.206983	2.202574	2.198199	2.193831	2.189477	2.185586	2.1813	2.177008
2.210175	2.205717	2.201314	2.196945	2.19258	2.188225	2.184305	2.179961	2.175619
2.208839	2.204387	2.199987	2.19562	2.191255	2.1869	2.182957	2.178576	2.174206
2.207408	2.202967	2.198567	2.194195	2.189829	2.185472	2.181517	2.177121	2.172752
2.206017	2.201589	2.197194	2.192821	2.188453	2.184092	2.180126	2.175723	2.171354
2.204745	2.200337	2.195952	2.191583	2.18721	2.182837	2.178857	2.17445	2.170068

2.203477	2.19909	2.194722	2.190367	2.185996	2.181616	2.177627	2.173219	2.168822
2.202086	2.197717	2.193385	2.189072	2.184733	2.180377	2.176396	2.171984	2.16758
2.200652	2.196298	2.192006	2.187743	2.183444	2.179122	2.175155	2.170741	2.166333
2.19933	2.194973	2.190675	2.186406	2.1821	2.177772	2.173797	2.169383	2.164976
2.198102	2.193725	2.189381	2.185054	2.180702	2.176335	2.172332	2.167918	2.163517
2.196899	2.192505	2.188127	2.183757	2.17937	2.174973	2.170949	2.166537	2.162143
2.195723	2.191319	2.186926	2.182539	2.178137	2.173725	2.169692	2.165283	2.160895
2.194538	2.190127	2.185725	2.181327	2.176916	2.172495	2.168457	2.164054	2.159671
2.193333	2.188922	2.184512	2.180102	2.175686	2.171265	2.167226	2.16283	2.158454
2.192085	2.187676	2.18326	2.178842	2.174424	2.170006	2.165969	2.161582	2.157212
2.190711	2.186304	2.181884	2.177459	2.173039	2.168621	2.164583	2.160205	2.155842
2.18926	2.184855	2.180432	2.176003	2.171581	2.167163	2.163123	2.158753	2.154398
2.187918	2.183512	2.179087	2.174656	2.170234	2.165817	2.161775	2.157412	2.15853
2.186679	2.182272	2.177846	2.173414	2.168992	2.164577	2.160535	2.156177	2.157291
2.185464	2.181054	2.176627	2.172194	2.167774	2.163363	2.159321	2.154965	2.156075
2.184262	2.179848	2.175419	2.170986	2.16657	2.162165	2.158124	2.153769	2.154873
2.183069	2.17865	2.174219	2.169786	2.165374	2.160977	2.156936	2.15258	2.153676
2.181874	2.177448	2.173016	2.168585	2.164178	2.159791	2.155748	2.151389	2.152473
2.18068	2.176247	2.171813	2.167383	2.162981	2.158602	2.154557	2.150192	2.151263
2.179485	2.175047	2.170607	2.166173	2.16177	2.157393	2.158812	2.154424	2.150033
2.178289	2.173847	2.1694	2.164958	2.160549	2.156167	2.157572	2.153187	2.148785
2.177088	2.172644	2.168195	2.163745	2.159316	2.154905	2.156275	2.151875	2.147462
2.175884	2.171441	2.166991	2.162534	2.158068	2.153594	2.154902	2.150462	2.146038
2.174676	2.170234	2.165786	2.161326	2.156833	2.152312	2.153568	2.149096	2.144664
2.173464	2.169021	2.164578	2.160124	2.155627	2.151094	2.152329	2.147846	2.143409
2.17225	2.167806	2.16337	2.158925	2.154432	2.1499	2.151124	2.146637	2.142199
2.171035	2.166592	2.162162	2.157726	2.158868	2.154314	2.149915	2.145429	2.140992
2.169819	2.165378	2.160955	2.156528	2.157668	2.153116	2.148716	2.144233	2.139797
2.168605	2.164166	2.159751	2.155335	2.156475	2.151927	2.147521	2.143031	2.13859
2.167391	2.162956	2.158552	2.154147	2.155288	2.150746	2.146331	2.141825	2.137371
2.166179	2.161751	2.157355	2.152962	2.154116	2.149601	2.145193	2.140676	2.136205
2.16497	2.16055	2.156161	2.151782	2.152967	2.148512	2.144131	2.139605	2.135106
2.163762	2.159351	2.160603	2.156209	2.15181	2.147408	2.143057	2.138542	2.134032
2.162555	2.158152	2.159401	2.155011	2.150626	2.146247	2.141925	2.137462	2.132983
2.161347	2.156953	2.158196	2.153808	2.149429	2.14506	2.140764	2.136361	2.131926
2.160131	2.155749	2.15699	2.152604	2.148229	2.143865	2.139579	2.135203	2.130797
2.158913	2.154543	2.155783	2.1514	2.147025	2.142659	2.138373	2.134005	2.129622
2.157724	2.153337	2.154567	2.150192	2.14582	2.141454	2.137166	2.132802	2.128433
2.15656	2.152125	2.153341	2.14898	2.144612	2.140247	2.135956	2.13159	2.127225
2.161078	2.156573	2.152153	2.147784	2.14341	2.139041	2.134744	2.130376	2.126013
2.160016	2.155481	2.151016	2.146595	2.142195	2.137819	2.133516	2.129153	2.124797
2.158957	2.154416	2.149921	2.145457	2.14103	2.136638	2.132318	2.127948	2.123587
2.157862	2.153365	2.148896	2.144441	2.139996	2.13556	2.13119	2.126773	2.122378
2.156741	2.152306	2.147883	2.14346	2.139006	2.134526	2.130104	2.125637	2.121201
2.155563	2.151155	2.146759	2.142362	2.137929	2.133468	2.129053	2.12459	2.120146
2.154343	2.14994	2.145558	2.141188	2.136799	2.132397	2.128031	2.123611	2.119181
2.153209	2.148787	2.144389	2.140012	2.135637	2.131267	2.126928	2.122539	2.11813
2.152179	2.147701	2.14325	2.138823	2.134427	2.130059	2.125729	2.121361	2.116984
2.151135	2.146631	2.142153	2.137697	2.133279	2.128896	2.124552	2.120182	2.115817
2.150037	2.14558	2.141136	2.136699	2.132266	2.127837	2.123439	2.119019	2.114618

2.148922	2.144516	2.140115	2.135712	2.131273	2.126804	2.122358	2.117891	2.113452
2.147791	2.143377	2.138989	2.13461	2.130191	2.125741	2.121308	2.116858	2.11242
2.146661	2.142217	2.137831	2.133474	2.129083	2.124673	2.120277	2.115869	2.111446
2.145602	2.141166	2.136787	2.132435	2.128043	2.123626	2.119218	2.114804	2.110378
2.144605	2.140204	2.135834	2.131473	2.127055	2.122595	2.118141	2.113683	2.109239
2.143562	2.139172	2.134805	2.130441	2.126016	2.121545	2.117076	2.112608	2.108159
2.142446	2.138031	2.133658	2.129301	2.124899	2.120465	2.116033	2.111605	2.107173
2.141353	2.136919	2.13254	2.128187	2.1238	2.119391	2.114981	2.110579	2.106158
2.140338	2.135914	2.131541	2.127187	2.122781	2.118339	2.113894	2.109463	2.105031
2.139348	2.134936	2.130565	2.1262	2.121771	2.117293	2.112808	2.10834	2.103893
2.138343	2.13391	2.129509	2.125116	2.120688	2.116234	2.111763	2.107301	2.097641
2.137334	2.132864	2.128418	2.123988	2.119574	2.115175	2.110744	2.106308	2.096666
2.136311	2.131827	2.127369	2.122933	2.118532	2.114162	2.109742	2.105303	2.095667
2.135274	2.130794	2.126356	2.121947	2.11756	2.113197	2.108764	2.104294	2.094648
2.134252	2.129782	2.125351	2.120946	2.116556	2.11218	2.102545	2.098091	2.093633
2.13326	2.128804	2.124354	2.119911	2.115482	2.111064	2.101438	2.097032	2.092622
2.132269	2.127821	2.123352	2.118874	2.114414	2.109966	2.100354	2.095992	2.091624
2.131264	2.1268	2.122321	2.117841	2.113393	2.108969	2.099384	2.095027	2.090656
2.130253	2.125766	2.121288	2.116821	2.1124	2.108016	2.09846	2.094088	2.089695
2.129228	2.124743	2.120284	2.115847	2.10631	2.101952	2.097517	2.093117	2.0887
2.128196	2.123726	2.119304	2.114908	2.1054	2.101031	2.096569	2.092131	2.087683
2.127193	2.122746	2.118345	2.113965	2.104469	2.100077	2.095589	2.091133	2.087686
2.126219	2.121807	2.117412	2.113023	2.103519	2.099086	2.094573	2.090118	2.086682
2.125269	2.120878	2.111371	2.106976	2.102546	2.098088	2.093564	2.089125	2.085707
2.124346	2.119955	2.110425	2.105977	2.101528	2.097074	2.092568	2.088168	2.084779
2.123434	2.119033	2.10948	2.104986	2.100523	2.096079	2.091595	2.087234	2.083867
2.122517	2.118107	2.108565	2.104065	2.099598	2.095151	2.090669	2.086323	2.08295
2.121594	2.11717	2.107654	2.103169	2.098706	2.094258	2.089772	2.085428	2.082034
2.115576	2.111143	2.106684	2.102216	2.097773	2.093351	2.088875	2.084529	2.089619
2.114605	2.110128	2.105667	2.10122	2.096813	2.092438	2.087983	2.083628	2.088682
2.113664	2.109159	2.104697	2.100262	2.095875	2.09153	2.088069	2.083672	2.087702
2.112762	2.10825	2.103785	2.099351	2.094967	2.090629	2.087139	2.082692	2.086658
2.111871	2.107356	2.10289	2.098453	2.09407	2.089733	2.086216	2.081728	2.092754
2.11098	2.106465	2.101998	2.097561	2.093178	2.088841	2.085313	2.080814	2.091795
2.110093	2.10558	2.101112	2.096675	2.092291	2.087954	2.084418	2.079919	2.090864
2.109205	2.104691	2.100225	2.09579	2.091407	2.08707	2.09207	2.087451	2.089936
2.108319	2.103805	2.099341	2.094908	2.090527	2.08619	2.091156	2.086539	2.089017
2.107447	2.102935	2.098466	2.094026	2.089643	2.085308	2.097365	2.092724	2.088094
2.106586	2.10208	2.097599	2.093141	2.088753	2.084424	2.096441	2.091798	2.087169
2.105746	2.101261	2.096775	2.0923	2.096087	2.091716	2.095523	2.090883	2.086256
2.104941	2.100505	2.096023	2.09153	2.095279	2.090862	2.09461	2.089976	2.085353
2.104125	2.099733	2.095258	2.090754	2.094468	2.090009	2.093709	2.089092	2.084483
2.103259	2.098888	2.102638	2.098104	2.093611	2.089143	2.092833	2.088261	2.083683
2.102381	2.098014	2.101758	2.09723	2.092739	2.088273	2.091963	2.087443	2.082902
2.101535	2.097149	2.100882	2.096362	2.1002	2.095609	2.091059	2.08657	2.082053
2.100708	2.096284	2.100005	2.095498	2.099295	2.094691	2.09014	2.085664	2.081162
2.108104	2.103627	2.099153	2.094682	2.098454	2.093838	2.089274	2.084782	2.080276
2.107237	2.102754	2.098328	2.093923	2.097685	2.093054	2.088463	2.083921	2.07939
2.10641	2.101919	2.097514	2.093138	2.096894	2.092272	2.087669	2.083083	2.078524
2.105655	2.101147	2.096703	2.092286	2.096043	2.091479	2.086897	2.082287	2.077697

2.10491	2.100398	2.09591	2.09144	2.095184	2.090667	2.086106	2.081484	2.076872
2.104124	2.099657	2.095162	2.090662	2.094344	2.089802	2.08523	2.080614	2.076007
2.103321	2.098911	2.094427	2.089915	2.093517	2.088922	2.084326	2.079726	2.075135
2.102506	2.098097	2.093641	2.089163	2.092731	2.088111	2.083502	2.078906	2.074318
2.101678	2.097228	2.092816	2.088413	2.091978	2.087352	2.082737	2.078135	2.073542
2.100884	2.096405	2.091998	2.087618	2.091181	2.086578	2.081974	2.077368	2.072771
2.100133	2.095639	2.091187	2.08676	2.090326	2.085786	2.081219	2.076613	2.072011
2.099393	2.094889	2.099324	2.09474	2.089473	2.08497	2.080426	2.075825	2.071227
2.098656	2.094143	2.098536	2.093938	2.088641	2.084103	2.079546	2.074961	2.07038
2.097924	2.093406	2.097767	2.093158	2.087826	2.083239	2.078661	2.074092	2.069532
2.097197	2.092677	2.097005	2.092385	2.087041	2.082443	2.077862	2.073298	2.068751
2.096472	2.091954	2.096253	2.091623	2.086278	2.081687	2.07711	2.072543	2.068005
2.095743	2.091224	2.095499	2.09087	2.085524	2.080931	2.076353	2.071784	2.067246
2.09501	2.090489	2.094744	2.090129	2.084781	2.080181	2.075597	2.071028	2.066478
2.094278	2.089759	2.093993	2.089384	2.084036	2.079435	2.074848	2.070275	2.065714
2.093548	2.089038	2.093247	2.088629	2.083284	2.078692	2.074106	2.069524	2.064953
2.092818	2.088316	2.092501	2.08788	2.082539	2.077956	2.073372	2.068782	2.064202
2.092085	2.087581	2.09175	2.087143	2.081805	2.077222	2.072637	2.068041	2.06346
2.091354	2.086847	2.091003	2.086415	2.081083	2.076505	2.07192	2.067323	2.062743
2.09125	2.086732	2.089527	2.084949	2.080384	2.075833	2.071268	2.066679	2.065457
2.090534	2.086017	2.088799	2.084238	2.079703	2.075193	2.070656	2.066082	2.064849
2.098078	2.093452	2.08812	2.083556	2.079024	2.074523	2.0734	2.068791	2.064177
2.097468	2.092844	2.087497	2.082908	2.078351	2.073822	2.072675	2.068057	2.06344
2.09682	2.092199	2.086845	2.082243	2.081127	2.076543	2.071942	2.067317	2.0627
2.096094	2.091473	2.086131	2.081542	2.080415	2.075811	2.071199	2.066574	2.061959
2.095352	2.090732	2.085402	2.080833	2.079703	2.075092	2.070477	2.065855	2.061243
2.093879	2.089258	2.088177	2.083589	2.079003	2.074425	2.069831	2.065215	2.060604
2.093157	2.08854	2.087455	2.082872	2.078313	2.073778	2.069211	2.064602	2.059992
2.09606	2.091406	2.086792	2.082205	2.07764	2.073097	2.068528	2.063924	2.059318
2.095459	2.090798	2.086176	2.081577	2.076982	2.072395	2.067803	2.063202	2.058604
2.094855	2.090195	2.085566	2.080954	2.076341	2.071729	2.067124	2.062524	2.05793
2.094256	2.089605	2.084969	2.080345	2.075725	2.071113	2.066506	2.061904	2.057312
2.093621	2.088979	2.084338	2.079705	2.075085	2.070475	2.06587	2.061266	2.056675
2.092908	2.088269	2.083628	2.078993	2.074375	2.06977	2.065168	2.060562	2.055973
2.092191	2.087553	2.082913	2.07828	2.073666	2.069067	2.064467	2.059861	2.055274
2.091546	2.086911	2.082273	2.077641	2.073028	2.068431	2.063833	2.059227	2.054639
2.090938	2.086307	2.081671	2.077041	2.072429	2.067833	2.063235	2.058631	2.054041
2.090338	2.085711	2.081076	2.076445	2.071834	2.067239	2.062643	2.058041	2.053451
2.089752	2.085126	2.08049	2.075858	2.071248	2.066656	2.062062	2.057462	2.052873
2.089165	2.08454	2.079904	2.075271	2.070662	2.066072	2.061481	2.056883	2.052296
2.088575	2.08395	2.079315	2.074683	2.070075	2.065486	2.060897	2.056302	2.051719
2.087986	2.08336	2.078725	2.074095	2.069489	2.064901	2.060313	2.055722	2.051141
2.087399	2.082772	2.078137	2.073506	2.068901	2.064317	2.059732	2.055142	2.050563
2.086812	2.082185	2.077549	2.072917	2.068314	2.063734	2.059151	2.054563	2.049984
2.086223	2.081596	2.07696	2.072329	2.067728	2.063151	2.058571	2.053981	2.049401
2.085634	2.081008	2.076373	2.071744	2.067145	2.062571	2.057991	2.0534	2.048819
2.08505	2.080424	2.075794	2.071173	2.066575	2.061997	2.057416	2.052829	2.04825
2.084466	2.07984	2.07522	2.070613	2.066015	2.061425	2.056842	2.052264	2.047691
2.083928	2.079301	2.074688	2.07009	2.065491	2.060894	2.056309	2.051739	2.046438
2.08347	2.07884	2.074229	2.069632	2.065033	2.060436	2.055851	2.051285	2.045981

2.083007	2.078375	2.073763	2.069166	2.064567	2.059971	2.055388	2.050825	2.045517
2.082463	2.077833	2.073224	2.06863	2.064031	2.059433	2.054851	2.050291	2.044984
2.081893	2.077261	2.072655	2.068063	2.063465	2.058866	2.054285	2.049727	2.044422
2.08135	2.076698	2.072083	2.067489	2.062896	2.058307	2.053729	2.049165	2.043855
2.080825	2.076137	2.071506	2.066908	2.062321	2.057748	2.053176	2.048602	2.04328
2.080323	2.075626	2.070994	2.066397	2.06181	2.057239	2.052665	2.048085	2.042757
2.079844	2.075173	2.070556	2.065968	2.061376	2.056787	2.052204	2.047626	2.042298
2.07938	2.074732	2.07013	2.065548	2.06095	2.056344	2.051749	2.047163	2.041826
2.078936	2.074304	2.069707	2.065124	2.060521	2.055904	2.051287	2.046668	2.0413
2.078489	2.073862	2.069264	2.064679	2.060073	2.055452	2.050821	2.046171	2.040777
2.078004	2.073353	2.068743	2.064156	2.059557	2.054953	2.049584	2.044934	2.040304
2.077502	2.072815	2.068191	2.063601	2.059014	2.054434	2.049091	2.044468	2.03986
2.077018	2.072329	2.067707	2.06312	2.058536	2.05396	2.048626	2.044026	2.039435
2.076547	2.071883	2.067276	2.062698	2.058111	2.053522	2.048185	2.043606	2.039031
2.076098	2.071456	2.066856	2.062278	2.056945	2.052345	2.047758	2.043187	2.038615
2.075675	2.071052	2.066446	2.061854	2.056523	2.051935	2.047348	2.042764	2.038179
2.075259	2.070647	2.066037	2.061436	2.056105	2.051525	2.046937	2.042341	2.037742
2.074842	2.070225	2.065622	2.06103	2.055692	2.051102	2.046508	2.041915	2.03731
2.074425	2.0698	2.065205	2.060626	2.05528	2.050677	2.046078	2.041491	2.036883
2.074006	2.069386	2.064789	2.060204	2.054857	2.050259	2.045664	2.041075	2.036467
2.073585	2.068978	2.064372	2.059773	2.054429	2.049846	2.045257	2.040663	2.036059
2.07316	2.068559	2.063954	2.059354	2.054008	2.049426	2.044837	2.040243	2.035639
2.072728	2.06813	2.063534	2.058947	2.053593	2.048999	2.044405	2.039813	2.035207
2.072323	2.067708	2.063113	2.058533	2.053174	2.048575	2.04398	2.039392	2.040371
2.071955	2.06729	2.062679	2.058099	2.052744	2.048154	2.043567	2.038985	2.039966
2.071604	2.0669	2.062267	2.057674	2.052318	2.047739	2.04316	2.038582	2.039564
2.071272	2.066575	2.061911	2.057272	2.051897	2.047325	2.042751	2.03817	2.039142
2.070941	2.066272	2.061581	2.056893	2.051495	2.046928	2.042355	2.03777	2.038726
2.070568	2.065922	2.060483	2.055789	2.051164	2.046591	2.047617	2.042994	2.038372
2.070166	2.065542	2.060145	2.055498	2.050885	2.0463	2.047307	2.042692	2.038062
2.069784	2.065154	2.059778	2.055165	2.05056	2.045965	2.046953	2.042347	2.03772
2.069421	2.064751	2.059372	2.054779	2.050177	2.045574	2.046545	2.041951	2.037343
2.069079	2.064384	2.058999	2.054415	2.049814	2.045208	2.046158	2.041557	2.036953
2.068762	2.064087	2.058703	2.054113	2.049512	2.044907	2.045824	2.041172	2.036537
2.06846	2.063813	2.058422	2.053815	2.049211	2.044612	2.045503	2.040804	2.036138
2.068173	2.063535	2.058111	2.053459	2.054423	2.049803	2.045158	2.040471	2.035813
2.067889	2.063254	2.05779	2.053088	2.054024	2.049429	2.044813	2.040162	2.035528
2.067555	2.062934	2.063099	2.058352	2.053696	2.04911	2.044503	2.039865	2.035232
2.067178	2.06258	2.062772	2.058066	2.053426	2.048836	2.044223	2.039582	2.034933
2.066843	2.062262	2.062467	2.057789	2.053156	2.048558	2.043941	2.039303	2.034648
2.066564	2.061992	2.062189	2.05752	2.052886	2.048278	2.043661	2.039038	2.03439
2.071202	2.066581	2.061918	2.057248	2.052602	2.047974	2.043351	2.038742	2.034109
2.070941	2.066315	2.061646	2.056964	2.052287	2.047612	2.04297	2.038372	2.033763
2.070687	2.066055	2.061378	2.056679	2.051969	2.047245	2.04258	2.037991	2.033406
2.070425	2.065791	2.061113	2.056411	2.051685	2.046939	2.042262	2.037675	2.0331
2.070161	2.065526	2.06085	2.05615	2.051421	2.046666	2.041984	2.037395	2.032821
2.069895	2.065259	2.060583	2.055884	2.051152	2.046392	2.041708	2.03712	2.032548
2.069627	2.064989	2.060312	2.055611	2.050879	2.04612	2.041437	2.036852	2.032283
2.069356	2.064719	2.060042	2.055342	2.050613	2.045859	2.041178	2.036589	2.032017
2.069079	2.064451	2.059775	2.055077	2.050352	2.045606	2.040923	2.036322	2.031739

2.068806	2.064182	2.05951	2.054816	2.050105	2.045377	2.040699	2.036082	2.031484
2.068532	2.063902	2.059238	2.054565	2.049887	2.045204	2.040544	2.035914	2.03129
2.068275	2.063631	2.058972	2.054318	2.049675	2.045043	2.040407	2.035765	2.031116
2.068085	2.063415	2.058733	2.054058	2.049423	2.04482	2.040199	2.035556	2.030898
2.067943	2.063239	2.058513	2.053792	2.049145	2.044555	2.039945	2.035309	2.030656
2.067749	2.063029	2.058281	2.053536	2.048879	2.04429	2.039684	2.035055	2.030415
2.067498	2.062782	2.058033	2.053285	2.048619	2.044017	2.039409	2.034792	2.030176
2.067258	2.062543	2.057804	2.053068	2.048398	2.04378	2.039165	2.034546	2.02994
2.067049	2.062321	2.057607	2.052907	2.048244	2.043613	2.038977	2.034327	2.029698
2.066856	2.062121	2.057431	2.052765	2.048108	2.043461	2.038805	2.034129	2.029478
2.066679	2.061959	2.05728	2.05262	2.047951	2.04328	2.038615	2.033953	2.029313
2.066516	2.061819	2.057142	2.052472	2.047787	2.043092	2.038427	2.033796	2.029179
2.066359	2.061666	2.05698	2.052296	2.04761	2.042924	2.038268	2.033648	2.029034
2.06621	2.061507	2.056799	2.052096	2.047421	2.042771	2.038133	2.03351	2.028886
2.066045	2.061332	2.056619	2.051915	2.047251	2.04262	2.037989	2.033356	2.02872
2.06586	2.061134	2.056436	2.051759	2.047106	2.042476	2.037836	2.033178	2.028528
2.063504	2.058785	2.056265	2.051609	2.04696	2.042321	2.037671	2.033001	2.028349
2.063321	2.058623	2.056115	2.051463	2.046801	2.042135	2.037479	2.032832	2.028203
2.063149	2.058475	2.055972	2.051313	2.046636	2.041947	2.037292	2.032677	2.028076
2.063001	2.058333	2.055814	2.051128	2.046451	2.041782	2.037147	2.032553	2.027969
2.062873	2.058201	2.055655	2.050932	2.046262	2.041633	2.037028	2.032451	2.027876
2.062757	2.058086	2.055542	2.050815	2.046145	2.041517	2.036914	2.032341	2.027769
2.062655	2.05799	2.055473	2.050776	2.046098	2.041435	2.036808	2.032227	2.027651
2.062545	2.057882	2.053209	2.048532	2.046011	2.041335	2.036699	2.032114	2.027536
2.06242	2.057749	2.053052	2.04835	2.045842	2.041197	2.036582	2.032004	2.02743
2.062298	2.057623	2.05291	2.048191	2.045692	2.041067	2.036466	2.031893	2.027322
2.062181	2.057527	2.052838	2.048141	2.04563	2.040968	2.036345	2.031772	2.027204
2.062075	2.057441	2.052787	2.048126	2.045602	2.040892	2.036238	2.031657	2.027084
2.061997	2.057333	2.052693	2.048063	2.04556	2.040858	2.036192	2.03157	2.026966
2.061943	2.057218	2.052578	2.047975	2.045512	2.040854	2.036188	2.031506	2.026855
2.061904	2.057161	2.052502	2.047883	2.043261	2.038645	2.036156	2.031462	2.026794
2.061888	2.057172	2.052468	2.047779	2.043143	2.038551	2.036096	2.031444	2.026792
2.061847	2.057161	2.052436	2.047707	2.043053	2.038457	2.036018	2.031403	2.030702
2.061754	2.057098	2.052405	2.047702	2.043025	2.038366	2.035908	2.031315	2.030641
2.061655	2.057013	2.052358	2.047695	2.043005	2.038293	2.03581	2.031221	2.030561
2.061582	2.056905	2.052261	2.047628	2.042958	2.038263	2.035773	2.031144	2.030448
2.061526	2.056795	2.052147	2.047535	2.042899	2.038252	2.035766	2.031081	2.030329
2.061497	2.056753	2.05208	2.047444	2.042819	2.038209	2.033569	2.02888	2.030269
2.061493	2.056764	2.052049	2.047351	2.04272	2.038144	2.033533	2.028874	2.030256
2.061503	2.056792	2.052051	2.047312	2.042672	2.038108	2.033515	2.028884	2.03027
2.061538	2.056846	2.052098	2.047341	2.042689	2.038116	2.033527	2.028922	2.030323
2.061546	2.056876	2.052135	2.047378	2.04271	2.03811	2.033516	2.028933	2.030353
2.061484	2.05684	2.052127	2.047393	2.042706	2.038053	2.033435	2.028868	2.030315
2.061419	2.056797	2.052116	2.047413	2.042708	2.037999	2.033355	2.028798	2.030267
2.061424	2.056809	2.052142	2.047453	2.042741	2.038008	2.033352	2.028797	2.028334
2.061467	2.056851	2.052189	2.047507	2.042794	2.038054	2.033392	2.028832	2.028355
2.061518	2.056899	2.052239	2.04756	2.042845	2.0381	2.037601	2.032989	2.028384
2.061581	2.056956	2.052295	2.047614	2.042897	2.038152	2.037646	2.033031	2.028425
2.061641	2.057012	2.05235	2.047667	2.042949	2.038203	2.037689	2.033073	2.028466
2.061696	2.057066	2.052403	2.047719	2.043	2.038251	2.037729	2.033112	2.028504

2.061748	2.057118	2.052455	2.04777	2.04305	2.0383	2.03777	2.033153	2.029251
2.061797	2.057167	2.052505	2.04782	2.0431	2.03835	2.037814	2.033197	2.0293
2.061842	2.057214	2.052552	2.047868	2.043149	2.0384	2.037858	2.033242	2.029349
2.06188	2.057258	2.052597	2.047913	2.043193	2.038444	2.037897	2.033284	2.029396
2.061912	2.057299	2.052639	2.047955	2.043236	2.038487	2.037935	2.033323	2.029442
2.061933	2.05732	2.05268	2.048	2.043285	2.038541	2.037983	2.033366	2.033032
2.061969	2.057349	2.052715	2.048045	2.043339	2.038603	2.038037	2.033406	2.033063
2.062047	2.057413	2.052774	2.048102	2.043409	2.038697	2.038132	2.033486	2.033129
2.062201	2.057542	2.052874	2.048176	2.043504	2.03885	2.038299	2.033637	2.033262
2.062353	2.057673	2.052979	2.048256	2.043601	2.038998	2.038462	2.033789	2.033399
2.062428	2.05775	2.053032	2.048308	2.047841	2.043198	2.042871	2.038185	2.033486
2.062478	2.05781	2.053099	2.04838	2.047907	2.043255	2.042926	2.038249	2.033558
2.062561	2.057889	2.053197	2.048504	2.051715	2.047043	2.04303	2.038339	2.033644
2.062665	2.057982	2.053319	2.04867	2.051877	2.047192	2.043166	2.038446	2.033739
2.062794	2.058112	2.053468	2.048839	2.052331	2.047635	2.043302	2.038576	2.033869
2.06295	2.05829	2.053649	2.049015	2.052486	2.047767	2.043434	2.03873	2.034042
2.063115	2.058469	2.057942	2.053286	2.052634	2.047905	2.04358	2.038894	2.034219
2.063287	2.058638	2.05809	2.053412	2.052762	2.048056	2.043751	2.03907	2.034391
2.063456	2.058795	2.058229	2.053534	2.05289	2.048213	2.043927	2.039243	2.034553
2.061724	2.057128	2.058362	2.053685	2.053042	2.048372	2.044461	2.039762	2.034685
2.061847	2.057231	2.058493	2.053851	2.053206	2.048532	2.044614	2.03989	2.034804
2.061978	2.057367	2.062723	2.058046	2.054161	2.049459	2.044751	2.04003	2.034953
2.062112	2.057529	2.062886	2.058206	2.054312	2.049584	2.044872	2.040177	2.035127
2.06227	2.057703	2.063045	2.05835	2.054452	2.04972	2.045016	2.040345	2.035688
2.062459	2.057889	2.063191	2.058458	2.054565	2.049867	2.045191	2.040541	2.035898
2.062659	2.058082	2.063358	2.058598	2.054711	2.050037	2.04538	2.040744	2.03611
2.062859	2.058283	2.06358	2.058839	2.054951	2.050252	2.045581	2.040944	2.036311
2.063061	2.058487	2.063814	2.0591	2.055211	2.050479	2.045787	2.041145	2.03651
2.067947	2.063318	2.064856	2.060115	2.055387	2.050668	2.045985	2.041346	2.036712
2.068134	2.063499	2.065013	2.060241	2.055521	2.050839	2.04618	2.041547	2.036916
2.06832	2.063693	2.065217	2.060446	2.055722	2.051034	2.046372	2.041742	2.037115
2.068502	2.063901	2.06547	2.060732	2.055992	2.05125	2.046558	2.041929	2.037307
2.068706	2.064104	2.060613	2.05599	2.056264	2.051498	2.046785	2.042138	2.037503
2.06894	2.064284	2.060802	2.056211	2.056529	2.051801	2.047086	2.04238	2.037698
2.069191	2.064488	2.061001	2.056421	2.056781	2.052099	2.047392	2.042642	2.03792
2.069463	2.064771	2.061254	2.056624	2.056988	2.052338	2.047656	2.042928	2.038209
2.069737	2.065082	2.06153	2.056834	2.057185	2.052552	2.047899	2.04322	2.038525
2.071134	2.06649	2.061802	2.057105	2.057438	2.052772	2.048112	2.043463	2.038794
2.071333	2.066715	2.062074	2.057422	2.057731	2.052992	2.0483	2.04367	2.039033
2.071551	2.066924	2.062309	2.057692	2.058008	2.05325	2.048541	2.043898	2.039261
2.071788	2.067106	2.062493	2.057907	2.05827	2.053554	2.048847	2.044146	2.039467
2.072043	2.067323	2.062697	2.058115	2.058516	2.053846	2.049151	2.044415	2.039709
2.072322	2.067609	2.062949	2.058321	2.058726	2.054089	2.049422	2.044711	2.040021
2.072618	2.067929	2.063233	2.058549	2.058949	2.054336	2.049698	2.045024	2.040357
2.072939	2.068272	2.063561	2.058849	2.059244	2.054637	2.050008	2.045347	2.04068
2.073267	2.068626	2.063914	2.059191	2.059581	2.054968	2.050335	2.04568	2.041004
2.07354	2.068921	2.064226	2.059519	2.054904	2.050358	2.050664	2.046018	2.041343
2.073765	2.069164	2.064506	2.059841	2.055239	2.050685	2.050998	2.046363	2.041695
2.074029	2.069436	2.064802	2.060165	2.055559	2.050979	2.051286	2.046663	2.042009
2.074347	2.069748	2.065121	2.060488	2.055854	2.051219	2.051504	2.04689	2.042262

2.074686	2.070078	2.065451	2.060818	2.056157	2.051474	2.051739	2.047132	2.042526
2.075037	2.070423	2.065795	2.061158	2.056486	2.051785	2.052044	2.047438	2.04284
2.075398	2.070778	2.066145	2.061504	2.056826	2.052119	2.052378	2.047772	2.043175
2.075756	2.071132	2.066495	2.061846	2.057164	2.052454	2.052717	2.04811	2.043515
2.076114	2.071488	2.066845	2.062189	2.057504	2.052796	2.053067	2.048459	2.043863
2.076466	2.071839	2.067194	2.062536	2.057853	2.05315	2.053424	2.048808	2.044204
2.076815	2.072187	2.067543	2.062888	2.05821	2.053513	2.053787	2.049152	2.044535
2.077159	2.072533	2.067893	2.063245	2.058581	2.053905	2.054189	2.049539	2.044905
2.077494	2.072877	2.068245	2.06361	2.058978	2.054348	2.054661	2.05	2.04534
2.077833	2.073221	2.068596	2.063974	2.05937	2.054782	2.055124	2.050457	2.045777
2.078174	2.07356	2.068936	2.064321	2.059728	2.055156	2.050577	2.045993	2.04617
2.078533	2.073911	2.069283	2.064666	2.060074	2.055504	2.05093	2.046357	2.046546
2.078959	2.074324	2.069674	2.065031	2.060425	2.055849	2.051276	2.04671	2.04692
2.079435	2.074782	2.070098	2.065412	2.06078	2.056191	2.051614	2.047051	2.047291
2.079863	2.075209	2.070523	2.065831	2.06119	2.056588	2.051997	2.047418	2.047662
2.080234	2.075601	2.070953	2.066301	2.061669	2.057052	2.052435	2.047814	2.048026
2.080608	2.075984	2.071363	2.066746	2.062126	2.057507	2.052877	2.048231	2.048425
2.081002	2.07635	2.071728	2.067121	2.062517	2.057916	2.053305	2.048675	2.048886
2.081409	2.07673	2.072099	2.067495	2.062903	2.058325	2.053737	2.049132	2.049364
2.081835	2.077164	2.072534	2.067929	2.063342	2.058775	2.054193	2.049593	2.049827
2.082276	2.077626	2.072998	2.068386	2.063799	2.059234	2.054653	2.050053	2.050283
2.082727	2.078084	2.073442	2.068808	2.064208	2.059636	2.055056	2.050469	2.050716
2.08319	2.078542	2.073872	2.069202	2.064578	2.059989	2.055411	2.050847	2.051129
2.083638	2.078979	2.0743	2.069622	2.064989	2.060391	2.055812	2.051258	2.046697
2.084066	2.079385	2.074722	2.070075	2.065455	2.06086	2.056277	2.051711	2.047142
2.081902	2.077221	2.07516	2.070545	2.065939	2.061347	2.05676	2.052182	2.047599
2.08233	2.077673	2.075629	2.071029	2.066429	2.061835	2.057245	2.052664	2.04807
2.082768	2.078144	2.076115	2.071523	2.066926	2.062329	2.057736	2.053155	2.048553
2.083236	2.078629	2.076606	2.072013	2.067418	2.062824	2.058233	2.053648	2.049045
2.083723	2.079127	2.077105	2.072503	2.067909	2.063322	2.058733	2.054143	2.049544
2.084213	2.07962	2.0776	2.072997	2.068402	2.063815	2.059225	2.054633	2.050034
2.084705	2.08011	2.078091	2.073494	2.068898	2.064304	2.05971	2.05512	2.050512
2.085199	2.080601	2.076008	2.07142	2.069393	2.064795	2.060199	2.055611	2.051002
2.085696	2.081095	2.076503	2.071917	2.069889	2.06529	2.060695	2.05611	2.051511
2.086193	2.081589	2.076998	2.072414	2.070383	2.065784	2.061192	2.056607	2.052021
2.086681	2.082081	2.077489	2.072903	2.070874	2.066273	2.061681	2.057099	2.05252
2.087175	2.082574	2.077981	2.073393	2.071366	2.066762	2.062169	2.057587	2.053013
2.087706	2.08307	2.078474	2.073896	2.069303	2.064702	2.062655	2.058073	2.053501
2.088267	2.083572	2.078972	2.07441	2.069813	2.065196	2.063143	2.058557	2.053984
2.088839	2.084135	2.079529	2.074964	2.070365	2.065748	2.063684	2.05907	2.054476
2.089431	2.084773	2.080159	2.075566	2.070969	2.066373	2.064288	2.059612	2.05497
2.089994	2.085375	2.080759	2.076147	2.071552	2.066968	2.06234	2.05764	2.055507
2.090493	2.085892	2.081283	2.076676	2.072081	2.067494	2.062881	2.058227	2.056123
2.090988	2.086385	2.081785	2.077189	2.072593	2.067998	2.063403	2.058808	2.056743
2.091526	2.086879	2.082277	2.077698	2.073101	2.068495	2.063905	2.059334	2.054768
2.092088	2.087381	2.082774	2.078211	2.073613	2.068999	2.064407	2.059846	2.055289
2.092669	2.087958	2.083349	2.078782	2.074185	2.069574	2.064984	2.060423	2.055866
2.093272	2.0886	2.083987	2.079402	2.074807	2.070209	2.065623	2.061051	2.056485
2.0914	2.086774	2.084624	2.080023	2.075429	2.070844	2.066262	2.061679	2.057105
2.092008	2.087408	2.085261	2.080648	2.076056	2.071483	2.066904	2.062313	2.057731

2.092591	2.08801	2.085867	2.081245	2.076656	2.07209	2.067513	2.062917	2.05833
2.093108	2.08854	2.0864	2.081776	2.077186	2.072623	2.068046	2.063448	2.058861
2.093621	2.089058	2.086923	2.082301	2.077712	2.073147	2.06857	2.063971	2.059388
2.094206	2.089635	2.08751	2.082897	2.078309	2.073738	2.069159	2.064565	2.059984
2.094831	2.090243	2.088132	2.083533	2.078945	2.074367	2.069785	2.065197	2.06062
2.095461	2.090865	2.086274	2.081693	2.079581	2.075	2.070419	2.065835	2.06126
2.096101	2.091501	2.086909	2.082326	2.08022	2.075642	2.071064	2.066482	2.061908
2.09674	2.09214	2.087546	2.082961	2.080861	2.076286	2.07171	2.06713	2.062555
2.097379	2.092779	2.088184	2.083598	2.081503	2.076929	2.072354	2.067777	2.063203
2.098017	2.093418	2.088823	2.084236	2.082146	2.077571	2.072996	2.068421	2.063848
2.097712	2.093115	2.089461	2.084874	2.082788	2.078209	2.073632	2.069058	2.064486
2.098346	2.093751	2.090098	2.085511	2.083428	2.078844	2.074265	2.069691	2.065119
2.098977	2.094381	2.089785	2.085195	2.081564	2.076994	2.074896	2.07032	2.065747
2.099606	2.095008	2.090411	2.085822	2.08219	2.07762	2.075527	2.070947	2.066372
2.100244	2.095638	2.091043	2.086459	2.082827	2.078252	2.076163	2.071584	2.06701
2.100887	2.096266	2.091675	2.087102	2.083468	2.078883	2.076798	2.072225	2.067658
2.101567	2.096934	2.092346	2.08778	2.083195	2.078601	2.074975	2.070421	2.068341
2.10232	2.097677	2.093088	2.088522	2.083934	2.079333	2.075707	2.071162	2.069091
2.103072	2.098423	2.093829	2.089261	2.084671	2.080069	2.076445	2.071903	2.069837
2.103751	2.099104	2.094507	2.089933	2.085345	2.080752	2.076176	2.071621	2.070505
2.104399	2.099759	2.095156	2.090575	2.085994	2.081418	2.076844	2.072268	2.071146
2.103349	2.09873	2.095807	2.091218	2.086656	2.082119	2.077561	2.072972	2.071019
2.10399	2.099382	2.096454	2.091859	2.087326	2.082844	2.078314	2.073717	2.071764
2.104683	2.100078	2.097149	2.092548	2.088017	2.083543	2.079019	2.074425	2.07248
2.10544	2.100829	2.097903	2.093296	2.088732	2.084202	2.079653	2.075077	2.073151
2.106208	2.101586	2.09698	2.092389	2.089468	2.084889	2.080316	2.075752	2.073843
2.106976	2.102333	2.097738	2.093164	2.090245	2.085648	2.081064	2.076496	2.074591
2.107727	2.103066	2.09848	2.093924	2.091016	2.086425	2.08184	2.077261	2.075356
2.10841	2.103758	2.099163	2.094598	2.091717	2.087176	2.082614	2.078022	2.073435
2.109061	2.10443	2.099817	2.095229	2.092381	2.08791	2.083383	2.078778	2.07418
2.109768	2.105145	2.100523	2.095922	2.0914	2.086942	2.084076	2.079475	2.074881
2.110525	2.105896	2.101273	2.096666	2.092106	2.087585	2.084694	2.080115	2.075541
2.111293	2.106659	2.102035	2.097425	2.092838	2.088271	2.085362	2.080794	2.076231
2.11207	2.107432	2.102808	2.098199	2.093604	2.089025	2.086109	2.081534	2.076966
2.11285	2.10821	2.103587	2.098977	2.09438	2.089797	2.086879	2.082297	2.077725
2.113629	2.108989	2.104363	2.099749	2.095151	2.090569	2.085994	2.081427	2.078506
2.114407	2.10977	2.10514	2.100521	2.095923	2.091345	2.086775	2.082213	2.079302
2.115186	2.110544	2.105914	2.101294	2.096695	2.092115	2.087549	2.082993	2.078458
2.115965	2.111315	2.106685	2.102067	2.097467	2.092885	2.08832	2.083772	2.079253
2.116737	2.112088	2.10746	2.102843	2.098247	2.093669	2.089106	2.084552	2.080031
2.117503	2.112863	2.108236	2.103621	2.099032	2.094467	2.089902	2.085329	2.080783
2.11827	2.113639	2.109018	2.10441	2.099835	2.095287	2.090728	2.086148	2.081587
2.11904	2.114412	2.109802	2.105212	2.100661	2.096144	2.091609	2.087049	2.082491
2.119819	2.115195	2.1106	2.106031	2.1015	2.097002	2.092485	2.087944	2.083393
2.120612	2.116002	2.111428	2.10688	2.102345	2.097827	2.093297	2.088754	2.084202
2.121419	2.116826	2.112272	2.10774	2.103193	2.098638	2.094083	2.089526	2.084972
2.122863	2.118294	2.113117	2.108576	2.104017	2.099447	2.094878	2.090308	2.085748
2.123715	2.11916	2.113965	2.109395	2.104824	2.100252	2.095676	2.091091	2.086524
2.12456	2.120003	2.114797	2.110213	2.105642	2.101082	2.096514	2.09193	2.087365
2.125398	2.120815	2.115605	2.111026	2.106474	2.101946	2.097405	2.092846	2.088291

2.126227	2.121625	2.116419	2.11185	2.107312	2.102801	2.098279	2.093741	2.0892
2.127031	2.122434	2.117248	2.1127	2.108159	2.103627	2.099095	2.094561	2.090035
2.127846	2.123255	2.118086	2.113551	2.109	2.104442	2.099898	2.095369	2.090858
2.128724	2.124115	2.118922	2.114365	2.109806	2.105251	2.100718	2.096214	2.091717
2.129637	2.125002	2.119769	2.115171	2.110602	2.106061	2.101549	2.097078	2.092594
2.124002	2.119432	2.120665	2.11604	2.111459	2.106916	2.102409	2.097953	2.093479
2.124892	2.120366	2.121607	2.116965	2.11237	2.10781	2.103296	2.098844	2.094375
2.125784	2.121286	2.12316	2.118523	2.113287	2.108712	2.104181	2.099709	2.095229
2.12668	2.122192	2.124085	2.119456	2.114215	2.109621	2.105057	2.10053	2.096012
2.127567	2.12307	2.124975	2.120359	2.115124	2.110528	2.105943	2.101368	2.096814
2.128422	2.123886	2.125785	2.121186	2.115981	2.111423	2.106854	2.102265	2.097695
2.129276	2.124691	2.12658	2.121997	2.116825	2.112318	2.107777	2.103187	2.09861
2.13017	2.125563	2.121001	2.116473	2.117708	2.113226	2.108699	2.104106	2.099524
2.131089	2.126476	2.121907	2.117373	2.118612	2.114144	2.109624	2.10503	2.100447
2.132009	2.127393	2.122821	2.118284	2.119528	2.115064	2.110546	2.105952	2.101368
2.132934	2.128318	2.123747	2.119209	2.120455	2.115987	2.111466	2.106871	2.102287
2.133859	2.129244	2.124673	2.120135	2.122022	2.117559	2.112392	2.1078	2.103216
2.134779	2.130163	2.125593	2.121058	2.122952	2.11849	2.113326	2.108737	2.104151
2.135708	2.131091	2.12652	2.121983	2.123884	2.119424	2.114268	2.109692	2.105112
2.136649	2.132035	2.127454	2.122904	2.12481	2.120359	2.115224	2.110689	2.106135
2.137606	2.132998	2.128405	2.123837	2.125743	2.121296	2.116183	2.111697	2.107177
2.138594	2.134012	2.129415	2.124828	2.126715	2.122238	2.117119	2.112657	2.108158
2.139607	2.135065	2.130471	2.125865	2.127716	2.123185	2.118039	2.113582	2.109096
2.140587	2.136075	2.131489	2.126876	2.122317	2.117797	2.119609	2.11517	2.110037
2.141527	2.137034	2.132459	2.127852	2.123275	2.118718	2.12052	2.116092	2.110984
2.14246	2.137985	2.133431	2.128844	2.124263	2.119683	2.12147	2.117029	2.111923
2.143389	2.138942	2.13443	2.129888	2.12532	2.120729	2.122484	2.117981	2.112834
2.144327	2.139894	2.135422	2.130928	2.126386	2.121802	2.123535	2.118963	2.113764
2.145288	2.140824	2.136366	2.13191	2.127406	2.122865	2.124618	2.120007	2.114764
2.146273	2.141753	2.137291	2.13286	2.128399	2.123919	2.125717	2.121088	2.11581
2.142645	2.138055	2.138256	2.133802	2.129353	2.124913	2.126752	2.122147	2.117542
2.143764	2.139143	2.139252	2.134732	2.130267	2.125851	2.127732	2.123193	2.118631
2.14486	2.140241	2.135631	2.131043	2.131227	2.126801	2.128697	2.124195	2.119668
2.145925	2.141357	2.13675	2.132138	2.132248	2.12776	2.129629	2.125129	2.120624
2.146972	2.142443	2.137854	2.133246	2.128673	2.124124	2.130609	2.12609	2.121587
2.147988	2.143442	2.138893	2.134347	2.129805	2.125267	2.131716	2.127151	2.122602
2.149004	2.144425	2.139912	2.135434	2.130934	2.126425	2.13285	2.128241	2.123641
2.150076	2.145474	2.140944	2.136454	2.131957	2.127462	2.122949	2.118417	2.124703
2.151181	2.146561	2.141985	2.137439	2.132923	2.128435	2.123948	2.119467	2.12578
2.152256	2.147656	2.143078	2.138518	2.133986	2.129479	2.124983	2.120503	2.122654
2.153304	2.148768	2.144231	2.139694	2.135147	2.130591	2.126048	2.12152	2.123653
2.149593	2.145088	2.145336	2.140826	2.136277	2.131699	2.127134	2.122582	2.124709
2.150604	2.14607	2.146337	2.141853	2.137331	2.132785	2.12825	2.123729	2.125881
2.151643	2.147077	2.147358	2.142892	2.138393	2.133874	2.129364	2.124872	2.127045
2.152767	2.148213	2.143718	2.139247	2.139528	2.134984	2.130451	2.125931	2.128097
2.153918	2.149385	2.144891	2.140409	2.14068	2.136099	2.131529	2.12697	2.129123
2.154994	2.15045	2.145953	2.141473	2.136938	2.132364	2.132626	2.128074	2.123542
2.156024	2.151448	2.146945	2.142476	2.137966	2.133432	2.133733	2.12922	2.124702
2.157113	2.152522	2.148014	2.143546	2.139039	2.134508	2.129987	2.125479	2.125803
2.158272	2.153688	2.14918	2.144701	2.140166	2.135593	2.131037	2.126499	2.126824

2.159434	2.154848	2.150327	2.145828	2.141276	2.136686	2.132115	2.12756	2.123041
2.160583	2.155962	2.151397	2.146861	2.142319	2.13778	2.133246	2.128712	2.124206
2.161721	2.157074	2.152472	2.147902	2.143376	2.138892	2.134401	2.129894	2.125401
2.162826	2.158212	2.153618	2.149048	2.144527	2.140051	2.135569	2.131079	2.126586
2.163921	2.159364	2.1548	2.150246	2.145725	2.141231	2.136741	2.132258	2.12776
2.165078	2.160537	2.155986	2.15144	2.146904	2.142377	2.13786	2.133357	2.128847
2.166287	2.161731	2.157182	2.152639	2.148075	2.143498	2.138934	2.134384	2.129857
2.167476	2.162891	2.158326	2.15377	2.149195	2.144607	2.140033	2.135469	2.130941
2.168636	2.164002	2.15939	2.154798	2.150234	2.145695	2.141162	2.13663	2.132125
2.163818	2.159168	2.160471	2.155859	2.151308	2.146813	2.142319	2.137821	2.13334
2.164946	2.160297	2.161618	2.157029	2.15249	2.147998	2.143513	2.139035	2.134567
2.166082	2.161449	2.162803	2.158249	2.153719	2.149216	2.14473	2.140262	2.135801
2.167259	2.162646	2.164024	2.159484	2.154955	2.150443	2.145952	2.141487	2.137023
2.168468	2.163878	2.165277	2.16074	2.156207	2.151684	2.147182	2.14271	2.138236
2.1697	2.165122	2.166532	2.161993	2.157453	2.152918	2.148403	2.143918	2.139426
2.170959	2.166381	2.167794	2.163247	2.158696	2.154148	2.149617	2.145111	2.140594
2.172222	2.167642	2.163089	2.158551	2.159932	2.155367	2.150813	2.146279	2.141735
2.173488	2.168901	2.164338	2.159788	2.161158	2.156572	2.15199	2.147413	2.142839
2.174756	2.170161	2.165587	2.161022	2.162377	2.15776	2.153147	2.148533	2.143937
2.172024	2.167443	2.166837	2.162252	2.163578	2.158912	2.154266	2.149638	2.145045
2.173294	2.168709	2.16809	2.163484	2.16478	2.16006	2.155383	2.15075	2.146167
2.172482	2.167891	2.165391	2.160792	2.160053	2.155329	2.156573	2.151923	2.147336
2.173763	2.169172	2.166658	2.162051	2.161304	2.156574	2.157813	2.153141	2.148541
2.175043	2.170453	2.165849	2.161227	2.158645	2.153939	2.159054	2.154368	2.149756
2.176322	2.171732	2.167124	2.162496	2.159896	2.155182	2.160295	2.1556	2.150981
2.17668	2.172091	2.1684	2.163768	2.161157	2.156442	2.15176	2.147105	2.152224
2.17795	2.173364	2.169677	2.165046	2.162428	2.157718	2.153034	2.148369	2.153469
2.179216	2.174634	2.170952	2.166327	2.163712	2.159019	2.154344	2.149679	2.154763
2.180479	2.175898	2.172224	2.167613	2.162979	2.158324	2.155736	2.1511	2.156187
2.18174	2.177158	2.173494	2.1689	2.164305	2.159707	2.157161	2.152567	2.157667
2.183001	2.178415	2.173826	2.16924	2.165596	2.161025	2.156452	2.151878	2.155525
2.184261	2.179669	2.175081	2.170499	2.166861	2.162292	2.157729	2.153172	2.156842
2.185521	2.180923	2.176335	2.171756	2.168121	2.163553	2.158995	2.154448	2.151951
2.18678	2.182175	2.177587	2.173011	2.169378	2.16481	2.160252	2.155704	2.1532
2.188041	2.183429	2.17884	2.174266	2.16969	2.165117	2.161505	2.156956	2.152418
2.189301	2.184681	2.180091	2.175517	2.170941	2.166367	2.162756	2.158207	2.153669
2.190572	2.185946	2.181352	2.176777	2.172199	2.167625	2.164015	2.159467	2.154928
2.191865	2.187235	2.182635	2.178052	2.173473	2.168899	2.164334	2.159779	2.156202
2.193188	2.188555	2.183946	2.179353	2.17477	2.170197	2.165633	2.161077	2.157499
2.194613	2.189977	2.185359	2.180756	2.176168	2.171596	2.167031	2.162474	2.158895
2.196147	2.191506	2.186879	2.182266	2.177673	2.173099	2.168534	2.163974	2.160397
2.197605	2.192959	2.188323	2.183701	2.179104	2.174529	2.169961	2.165401	2.16085
2.198938	2.194286	2.189641	2.185008	2.180405	2.175829	2.171261	2.166699	2.162147
2.200229	2.195574	2.190925	2.186288	2.181683	2.177104	2.172534	2.167971	2.163419
2.2015	2.196851	2.19221	2.18758	2.182973	2.178386	2.173811	2.169249	2.164699
2.202759	2.19812	2.193496	2.188883	2.18428	2.179687	2.17511	2.17055	2.166002
2.204037	2.199411	2.194816	2.190241	2.185666	2.181096	2.176532	2.171973	2.167423
2.205334	2.20072	2.196163	2.191637	2.187106	2.182575	2.178034	2.173476	2.168922
2.206726	2.202109	2.19755	2.193023	2.188493	2.183967	2.179428	2.174872	2.17032
2.208231	2.203594	2.198986	2.194399	2.189824	2.185263	2.180706	2.176152	2.171611

2.209695	2.205043	2.200398	2.195763	2.191148	2.186551	2.181976	2.177422	2.172892
2.211044	2.206389	2.201737	2.19709	2.192456	2.187833	2.183242	2.178683	2.174164
2.212396	2.207742	2.20309	2.198442	2.193797	2.189157	2.184555	2.179992	2.17548
2.213858	2.209201	2.204546	2.199895	2.195251	2.190612	2.186011	2.181448	2.176928
2.215354	2.210694	2.206036	2.201386	2.196748	2.192122	2.187528	2.182971	2.178438
2.216755	2.212095	2.207436	2.202784	2.198149	2.193528	2.188947	2.184412	2.179897
2.218086	2.213429	2.208769	2.204117	2.199481	2.194861	2.190297	2.1858	2.181325
2.219486	2.214829	2.21017	2.205519	2.200887	2.196274	2.191713	2.187214	2.18274
2.220986	2.216326	2.211669	2.207025	2.202402	2.197798	2.193218	2.188661	2.184129
2.222457	2.217793	2.213138	2.208499	2.20389	2.199306	2.19472	2.190125	2.185552
2.223811	2.219143	2.214486	2.209853	2.20527	2.20073	2.196183	2.191623	2.187068
2.225165	2.220494	2.215835	2.211204	2.206641	2.202135	2.197624	2.193107	2.188581
2.218504	2.213872	2.21732	2.212677	2.208091	2.203552	2.199019	2.194493	2.189962
2.220037	2.215414	2.218875	2.214217	2.209588	2.204982	2.200398	2.195836	2.191285
2.221498	2.2169	2.220398	2.215758	2.21112	2.206486	2.201875	2.19729	2.19272
2.222897	2.218343	2.221901	2.21731	2.212696	2.208063	2.203446	2.198844	2.194255
2.224315	2.219767	2.215225	2.210689	2.214195	2.209582	2.204977	2.200383	2.195796
2.225755	2.221155	2.216581	2.212032	2.215566	2.211002	2.206446	2.201901	2.197352
2.227236	2.222581	2.217969	2.213394	2.216943	2.212407	2.207882	2.203373	2.198856
2.228796	2.224113	2.219473	2.214868	2.218396	2.213821	2.209268	2.204737	2.20022
2.230386	2.225692	2.221031	2.216394	2.219892	2.215258	2.210655	2.206081	2.201549
2.231941	2.227272	2.222603	2.217942	2.213302	2.20868	2.212147	2.20754	2.202981
2.233475	2.228857	2.224194	2.219515	2.214868	2.210243	2.213709	2.209074	2.204478
2.233379	2.22881	2.225743	2.22108	2.216437	2.211804	2.215273	2.210623	2.206005
2.234788	2.230267	2.227253	2.222643	2.218016	2.213369	2.216843	2.212192	2.207566
2.236203	2.231692	2.228719	2.22416	2.219558	2.214921	2.210296	2.205682	2.209136
2.23763	2.233068	2.230116	2.225601	2.221045	2.21646	2.211862	2.207247	2.210707
2.239089	2.234464	2.231511	2.227017	2.2225	2.217973	2.213412	2.208805	2.212279
2.240615	2.235951	2.231371	2.226848	2.223917	2.219433	2.21491	2.210337	2.213849
2.242183	2.237488	2.232847	2.228258	2.225325	2.220873	2.216386	2.211859	2.215417
2.243757	2.239037	2.234367	2.229751	2.22523	2.220792	2.217896	2.213393	2.208862
2.245343	2.240605	2.23593	2.23131	2.226777	2.222323	2.219428	2.214934	2.210411
2.24693	2.242177	2.237493	2.232865	2.22832	2.223851	2.219375	2.214896	2.21196
2.248517	2.24375	2.239053	2.234411	2.229856	2.225378	2.220903	2.216435	2.213514
2.250104	2.245326	2.240616	2.235961	2.231389	2.226893	2.22241	2.217948	2.215041
2.25169	2.246906	2.242185	2.237512	2.232907	2.228364	2.223855	2.219391	2.2165
2.253274	2.248487	2.243757	2.239068	2.234436	2.229856	2.225323	2.220848	2.217964
2.251181	2.246391	2.245331	2.240638	2.236017	2.231461	2.226929	2.222426	2.217922
2.252777	2.247984	2.246906	2.242215	2.237625	2.233123	2.228606	2.224067	2.219519
2.25437	2.249574	2.248475	2.24378	2.239201	2.234722	2.230213	2.225663	2.221099
2.255961	2.251162	2.250039	2.245337	2.240754	2.236272	2.231766	2.227229	2.22267
2.257549	2.252746	2.247985	2.243275	2.242304	2.237815	2.233309	2.22878	2.224229
2.259133	2.254322	2.249559	2.244846	2.243853	2.239352	2.23484	2.230314	2.225768
2.255179	2.250392	2.251141	2.24643	2.245411	2.240893	2.236372	2.231847	2.227307
2.256769	2.251982	2.252739	2.248027	2.246981	2.242439	2.237907	2.233388	2.228857
2.258364	2.253579	2.254352	2.249647	2.248572	2.244002	2.239455	2.234937	2.230412
2.259955	2.255179	2.25599	2.251323	2.24668	2.242066	2.241042	2.236499	2.231968
2.261545	2.256783	2.257647	2.253034	2.24839	2.243736	2.242656	2.238072	2.233525
2.263143	2.258389	2.25376	2.24919	2.250039	2.245372	2.240731	2.236111	2.235077
2.264748	2.259992	2.255349	2.250763	2.251621	2.246973	2.242334	2.23769	2.236619

2.266382	2.261635	2.256974	2.252363	2.253221	2.248583	2.243945	2.239294	2.238196
2.268058	2.26334	2.258664	2.254022	2.254863	2.250208	2.245569	2.240937	2.23983
2.269755	2.26506	2.260369	2.255694	2.25652	2.251852	2.247211	2.242597	2.24148
2.271468	2.266766	2.262053	2.257352	2.252689	2.24806	2.248888	2.244268	2.239674
2.27319	2.268465	2.263732	2.259013	2.254354	2.249744	2.250582	2.245945	2.241331
2.274887	2.270146	2.265413	2.260699	2.256039	2.251424	2.25226	2.247611	2.242985
2.276568	2.271813	2.267094	2.262405	2.257742	2.253104	2.253927	2.249269	2.244637
2.278257	2.273504	2.268792	2.264109	2.259435	2.254773	2.255589	2.250933	2.246302
2.279959	2.275231	2.270516	2.265814	2.261116	2.256424	2.257241	2.252603	2.247984
2.276998	2.272316	2.272215	2.267505	2.262795	2.258086	2.253428	2.24883	2.249661
2.278602	2.273921	2.273848	2.269163	2.26447	2.259773	2.255115	2.250503	2.251323
2.280215	2.275529	2.275479	2.270822	2.266149	2.261469	2.25681	2.252175	2.25298
2.281914	2.277218	2.277172	2.272517	2.267846	2.263167	2.258498	2.253835	2.254631
2.283663	2.278953	2.2789	2.274233	2.269552	2.264865	2.260181	2.255492	2.256284
2.285422	2.280712	2.276029	2.271362	2.271252	2.266542	2.261846	2.257159	2.257966
2.287198	2.282497	2.277793	2.273091	2.272947	2.268199	2.263493	2.258834	2.259674
2.288967	2.284266	2.27955	2.274831	2.274679	2.269914	2.2652	2.260543	2.255923
2.290725	2.28601	2.281296	2.276587	2.276466	2.271721	2.267001	2.262299	2.257643
2.29248	2.287755	2.283042	2.278342	2.273641	2.268943	2.268784	2.264052	2.259371
2.294236	2.289523	2.2848	2.280079	2.275352	2.27062	2.270465	2.265766	2.261107
2.295992	2.29129	2.286558	2.281817	2.277059	2.272288	2.272136	2.267478	2.262848
2.297745	2.293023	2.288298	2.283575	2.278826	2.274058	2.273907	2.269222	2.264572
2.299497	2.294737	2.290028	2.285344	2.280626	2.275885	2.275738	2.270991	2.266297
2.301256	2.296482	2.291772	2.287096	2.282396	2.277685	2.277562	2.272802	2.268094
2.303022	2.298257	2.293529	2.28883	2.28414	2.279461	2.27939	2.274663	2.269972
2.300299	2.295541	2.295278	2.290565	2.285877	2.281215	2.276558	2.2719	2.271813
2.302064	2.297276	2.297006	2.292304	2.287612	2.282936	2.278278	2.273639	2.27358
2.303828	2.299016	2.298738	2.29404	2.289342	2.284653	2.279991	2.27536	2.275325
2.290451	2.285982	2.300492	2.295766	2.291065	2.286392	2.281735	2.277093	2.277063
2.292099	2.28765	2.302255	2.297492	2.292788	2.288136	2.283485	2.278827	2.278797
2.293739	2.289284	2.299566	2.294812	2.294527	2.28986	2.290634	2.285934	2.285442
2.295377	2.290897	2.301321	2.296587	2.296279	2.291574	2.292362	2.287692	2.287241
2.297075	2.292585	2.303135	2.298402	2.298077	2.293352	2.294151	2.289484	2.284837
2.298849	2.294365	2.305025	2.300269	2.299935	2.295211	2.296016	2.291319	2.286644
2.300607	2.296124	2.291652	2.287201	2.297369	2.29266	2.29786	2.293133	2.288431
2.302302	2.297803	2.293315	2.28885	2.299137	2.294428	2.29963	2.294883	2.290166
2.303992	2.299478	2.294978	2.290501	2.300908	2.296193	2.301397	2.296638	2.291914
2.29427	2.289782	2.296716	2.292235	2.302757	2.298018	2.303228	2.298475	2.293752
2.296024	2.291543	2.298481	2.294002	2.304637	2.299863	2.305082	2.300344	2.295629
2.297745	2.29325	2.288766	2.284295	2.291235	2.286775	2.306887	2.30215	2.297443
2.29944	2.294914	2.290419	2.285948	2.29288	2.288404	2.308651	2.303907	2.299207
2.301189	2.296641	2.292131	2.287646	2.283166	2.278694	2.306392	2.301639	2.300999
2.303025	2.298468	2.293929	2.28941	2.28492	2.280458	2.308307	2.303512	2.302825
2.292187	2.287657	2.295725	2.291187	2.286694	2.28224	2.284813	2.280317	2.30468
2.293879	2.289372	2.297476	2.292969	2.288487	2.284029	2.286621	2.282106	2.306571
2.295554	2.291066	2.299211	2.294741	2.290272	2.285809	2.288426	2.283906	2.308486
2.282151	2.2777	2.288316	2.283864	2.291987	2.287533	2.290195	2.285696	2.301001
2.283941	2.279442	2.290026	2.285543	2.293665	2.289226	2.291943	2.287483	2.302953
2.285726	2.281213	2.27673	2.272281	2.295393	2.290948	2.293701	2.289267	2.291837
2.28751	2.283012	2.278527	2.274065	2.297165	2.292695	2.295466	2.291047	2.293622

2.289309	2.284827	2.280339	2.275865	2.283945	2.279491	2.297262	2.292845	2.295417
2.291128	2.286659	2.282166	2.277679	2.285764	2.281309	2.299103	2.294674	2.297231
2.292956	2.288498	2.283997	2.279497	2.287588	2.283135	2.300954	2.296509	2.299047
2.294793	2.290335	2.285823	2.281305	2.276856	2.272461	2.293449	2.289069	2.294044
2.296633	2.292171	2.287645	2.28311	2.278656	2.274263	2.295267	2.290877	2.295828
2.298457	2.293995	2.289459	2.284911	2.28045	2.276056	2.293215	2.288855	2.295848
2.300271	2.29581	2.291266	2.286709	2.282242	2.277843	2.295005	2.290646	2.297645
2.291961	2.287527	2.29308	2.288517	2.284043	2.279636	2.283771	2.279441	2.299455
2.293783	2.289344	2.2949	2.290338	2.285856	2.281435	2.285563	2.281245	2.30128
2.290045	2.285576	2.296719	2.292159	2.287668	2.28323	2.287351	2.283043	2.291713
2.291899	2.287426	2.298522	2.293959	2.289457	2.285001	2.289116	2.284827	2.293534
2.293756	2.289284	2.300342	2.295786	2.291275	2.286797	2.290899	2.286614	2.295349
2.295632	2.291171	2.286659	2.282132	2.293194	2.28868	2.292744	2.288424	2.284121
2.297514	2.293071	2.288607	2.284132	2.295157	2.290605	2.294622	2.290245	2.285902
2.299386	2.294955	2.290513	2.286061	2.281541	2.276967	2.296476	2.292046	2.287668
2.30125	2.296827	2.292388	2.287937	2.283421	2.278855	2.298314	2.293834	2.289418
2.303111	2.298681	2.294242	2.289799	2.285306	2.280773	2.285534	2.281019	2.291217
2.304959	2.300505	2.296068	2.291649	2.287204	2.282742	2.287524	2.28296	2.293069
2.306844	2.302349	2.297901	2.293493	2.289085	2.284684	2.289497	2.284922	2.280391
2.308812	2.304243	2.29976	2.295337	2.290934	2.286557	2.291413	2.28691	2.282411
2.310801	2.306156	2.30163	2.297181	2.292768	2.288397	2.293292	2.288881	2.284436
2.312737	2.308055	2.303494	2.299013	2.303886	2.299503	2.295123	2.290755	2.286353
2.31465	2.309958	2.305368	2.300852	2.305697	2.301299	2.296922	2.292571	2.288205
2.316594	2.311917	2.307328	2.302799	2.307609	2.303167	2.298759	2.294394	2.290036
2.318569	2.313936	2.309375	2.304857	2.309623	2.305098	2.300627	2.296216	2.291838
2.32052	2.315913	2.320661	2.31615	2.311611	2.307056	2.302548	2.298088	2.293675
2.322414	2.317809	2.322557	2.318056	2.313553	2.309057	2.304557	2.300038	2.295562
2.324346	2.319714	2.324436	2.319927	2.315455	2.311022	2.306545	2.302	2.297487
2.311144	2.30652	2.326325	2.321764	2.31728	2.312869	2.308431	2.30395	2.299484
2.313215	2.308498	2.328236	2.323602	2.319082	2.314669	2.310268	2.305878	2.301489
2.31527	2.310538	2.33024	2.325517	2.320939	2.316489	2.312079	2.307716	2.303359
2.317318	2.312626	2.332311	2.327491	2.322838	2.318321	2.313871	2.309496	2.305145
2.319301	2.314641	2.334367	2.329533	2.324843	2.320268	2.315779	2.31139	2.307029
2.321201	2.316572	2.336411	2.331659	2.326975	2.32235	2.317823	2.313419	2.309029
2.324902	2.320273	2.332084	2.327429	2.329066	2.324409	2.31985	2.315415	2.310993
2.326947	2.322243	2.334053	2.329384	2.331025	2.326367	2.321787	2.317297	2.312845
2.329021	2.32425	2.336054	2.331353	2.332996	2.328345	2.323746	2.319196	2.314708
2.331085	2.326344	2.330221	2.325511	2.335115	2.330453	2.325828	2.321231	2.316694
2.333139	2.328464	2.332387	2.327664	2.337287	2.332611	2.327958	2.323321	2.31873
2.335105	2.330452	2.325797	2.32116	2.339363	2.334676	2.330003	2.325335	2.320713
2.336999	2.332337	2.327701	2.3231	2.34137	2.336676	2.331989	2.327298	2.322662
2.338998	2.334325	2.329698	2.325116	2.329077	2.324426	2.334058	2.329352	2.324692
2.341142	2.33646	2.331829	2.327245	2.331229	2.326588	2.336247	2.331536	2.326835
2.343274	2.338571	2.333929	2.32934	2.333349	2.328717	2.330751	2.326046	2.328963
2.345296	2.340547	2.335894	2.331312	2.335355	2.330728	2.332772	2.328067	2.331001
2.347329	2.342523	2.337849	2.333269	2.337344	2.332719	2.334774	2.330073	2.333036
2.349499	2.344633	2.339929	2.33533	2.33942	2.334795	2.336866	2.332177	2.32749
2.351748	2.346816	2.342068	2.337435	2.341529	2.336902	2.338991	2.334319	2.329646
2.353983	2.348966	2.344148	2.339453	2.343544	2.338912	2.334283	2.329655	2.331692
2.356214	2.351092	2.346173	2.341387	2.345467	2.340831	2.336202	2.33158	2.333633

2.358437	2.353271	2.348279	2.343412	2.338688	2.3341	2.338195	2.333575	2.335643
2.360642	2.355525	2.3505	2.345567	2.340807	2.336198	2.340305	2.335686	2.337769
2.362865	2.357806	2.352767	2.347788	2.34299	2.338345	2.342453	2.337827	2.33992
2.365154	2.360113	2.355078	2.350088	2.34525	2.340538	2.34463	2.339984	2.335359
2.367443	2.362408	2.357385	2.3524	2.347519	2.342727	2.346795	2.34212	2.337484
2.36961	2.364578	2.359555	2.354561	2.349633	2.344764	2.348813	2.344114	2.339466
2.371705	2.366671	2.361643	2.356634	2.351657	2.346712	2.350745	2.346019	2.341357
2.367347	2.362293	2.363839	2.358813	2.353803	2.348812	2.343953	2.339246	2.343361
2.369698	2.364634	2.366148	2.361099	2.356072	2.351067	2.346172	2.341395	2.345475
2.372082	2.367004	2.361917	2.356841	2.358393	2.353381	2.348453	2.343607	2.347654
2.37449	2.369388	2.3643	2.359233	2.360766	2.35575	2.35079	2.345875	2.349892
2.376902	2.371778	2.366691	2.361636	2.363154	2.358138	2.353155	2.348186	2.352183
2.357389	2.35233	2.369084	2.364027	2.358984	2.353959	2.355533	2.350546	2.354538
2.359744	2.354684	2.371451	2.366388	2.361347	2.356332	2.357896	2.352918	2.356922
2.361983	2.356906	2.365172	2.360085	2.363562	2.358549	2.353568	2.348619	2.350281
2.364129	2.359024	2.3673	2.362204	2.365656	2.360639	2.355689	2.350819	2.352522
2.366402	2.361253	2.369541	2.364434	2.359356	2.354316	2.357865	2.353001	2.348158
2.368825	2.363612	2.371915	2.366796	2.361697	2.35663	2.360107	2.355153	2.350262
2.351899	2.346675	2.3609	2.355844	2.364086	2.358988	2.353924	2.348882	2.352405
2.354409	2.349137	2.363363	2.358273	2.366511	2.361371	2.356269	2.351193	2.354653
2.356962	2.35165	2.365869	2.360732	2.368963	2.363785	2.35865	2.353554	2.356953
2.343057	2.337757	2.368386	2.363189	2.37142	2.366222	2.361065	2.355942	2.350885
2.345614	2.340296	2.370922	2.365657	2.373889	2.368681	2.363508	2.358359	2.353277
2.348182	2.342833	2.353956	2.348677	2.37637	2.371142	2.365949	2.360782	2.355682
2.350759	2.345371	2.356465	2.351131	2.378862	2.373605	2.368392	2.363216	2.358105
2.353339	2.347916	2.358994	2.35362	2.38139	2.37609	2.370848	2.365662	2.360537
2.355917	2.350468	2.36155	2.356154	2.383963	2.378597	2.373314	2.368118	2.36298
2.317652	2.312748	2.364112	2.3587	2.353358	2.348084	2.375806	2.370591	2.365432
2.319949	2.315023	2.36666	2.361235	2.355872	2.350569	2.37834	2.373086	2.367893
2.32226	2.31731	2.369218	2.363776	2.358395	2.353076	2.380901	2.375598	2.370364
2.324616	2.319641	2.355258	2.349803	2.360921	2.355591	2.383476	2.378122	2.372844
2.327003	2.322005	2.357875	2.352351	2.363455	2.358117	2.386066	2.380659	2.375333
2.300934	2.295915	2.319363	2.314366	2.366011	2.360652	2.370143	2.364731	2.37784
2.303295	2.298275	2.321724	2.316692	2.368588	2.363197	2.372709	2.367249	2.380362
2.305653	2.300628	2.29556	2.290502	2.371191	2.36575	2.360352	2.35498	2.382919
2.307996	2.302965	2.297887	2.292817	2.373827	2.36831	2.362865	2.357483	2.385525
2.310355	2.305302	2.300215	2.29514	2.376482	2.370883	2.365391	2.360002	2.388148
2.203179	2.198601	2.302537	2.297464	2.321023	2.316021	2.367921	2.362512	2.372102
2.205318	2.200674	2.304867	2.299796	2.323353	2.318315	2.370484	2.36505	2.374682
2.207498	2.202806	2.233255	2.228288	2.297069	2.292018	2.373157	2.367701	2.377363
2.209715	2.204984	2.2356	2.230596	2.299421	2.294377	2.375924	2.370445	2.380124
2.211948	2.207183	2.202482	2.19784	2.227962	2.223074	2.311874	2.306936	2.382875
2.214196	2.209405	2.204665	2.199992	2.230333	2.225509	2.314416	2.309393	2.385609
2.15547	2.150822	2.206863	2.202162	2.232711	2.227918	2.247117	2.242143	2.373161
2.157638	2.152943	2.209063	2.20433	2.235061	2.230237	2.249457	2.244492	2.375901
2.15982	2.155081	2.211289	2.206538	2.237446	2.232555	2.25178	2.246836	2.378666
2.161999	2.157233	2.213572	2.20885	2.204195	2.199611	2.230008	2.225115	2.244269
2.164194	2.159408	2.215897	2.211222	2.206526	2.201834	2.232373	2.227451	2.246635
2.151272	2.146408	2.157007	2.152476	2.208836	2.204095	2.234804	2.229867	2.249073
2.153645	2.148748	2.159259	2.154682	2.211136	2.206399	2.237299	2.232356	2.251578

2.032677	2.0281	2.146327	2.141635	2.190995	2.186298	2.239763	2.234799	2.254032
2.035009	2.030326	2.148656	2.14388	2.193217	2.188484	2.24216	2.237157	2.256403
2.037361	2.032595	2.061879	2.057347	2.182014	2.177215	2.186018	2.18143	2.234564
2.039698	2.034932	2.064073	2.059389	2.18432	2.179543	2.188295	2.183652	2.236957
2.042039	2.037293	2.0663	2.061475	2.186674	2.181909	2.190601	2.185905	2.239388
2.013531	2.008787	2.034767	2.029983	2.09446	2.089901	2.179475	2.174732	2.241877
2.015839	2.011046	2.037098	2.032375	2.096798	2.092058	2.181775	2.17705	2.244417
1.866367	1.862897	2.008599	2.003898	2.063681	2.058936	2.184164	2.179417	2.174735
1.868143	1.864649	2.010956	2.006215	2.065965	2.061178	2.186638	2.181832	2.177102
1.869929	1.866401	2.013347	2.008556	2.003795	1.999085	2.058829	2.054346	2.179485
1.871719	1.868131	2.015772	2.010924	2.006144	2.001446	2.061157	2.056513	2.181862
1.873503	1.869863	2.01824	2.013326	2.008519	2.003826	2.063513	2.058738	2.184299
1.875256	1.871597	1.868033	1.864537	2.010905	2.006167	2.036755	2.032016	2.138068
1.877003	1.873345	1.869792	1.866308	2.013318	2.008522	2.039078	2.034392	2.140627
1.878782	1.875143	1.871575	1.868064	2.015781	2.010976	2.006242	2.001577	2.032082
1.880582	1.876981	1.873382	1.869818	2.018292	2.013505	2.008734	2.00395	2.034343
1.882394	1.878792	1.875189	1.871617	1.868133	1.864724	2.01118	2.006358	2.036697
1.884214	1.880563	1.876993	1.873473	1.869957	1.866457	2.01355	2.008805	2.039171
1.790743	1.787355	1.878815	1.875313	1.871773	1.868214	1.864731	1.861341	2.041602
1.792467	1.789105	1.880672	1.8771	1.873553	1.870031	1.866538	1.86307	2.043874
1.794194	1.790858	1.882531	1.878883	1.875329	1.871851	1.868352	1.864808	2.046177
1.795904	1.792549	1.789169	1.785796	1.877129	1.87361	1.870096	1.866572	2.013772
1.797606	1.794214	1.790838	1.787479	1.878935	1.875346	1.871813	1.868348	2.016462
1.799304	1.79591	1.792517	1.789134	1.839217	1.835734	1.873572	1.870101	1.866645
1.800997	1.79763	1.794201	1.790766	1.840949	1.837494	1.875364	1.871835	1.868338
1.802699	1.799329	1.795891	1.792444	1.789065	1.785737	1.877138	1.873584	1.870067
1.80441	1.800991	1.797585	1.794194	1.790793	1.787388	1.878872	1.875352	1.871854
1.765003	1.761615	1.799284	1.795933	1.792516	1.789055	1.785683	1.782424	1.873631
1.766678	1.763277	1.800983	1.797605	1.794189	1.790751	1.787369	1.784051	1.875346
1.768369	1.764957	1.80269	1.79927	1.795858	1.792458	1.789071	1.785685	1.877063
1.770085	1.766632	1.763247	1.759904	1.797558	1.794136	1.790731	1.787332	1.837607
1.771827	1.768317	1.764926	1.76159	1.799284	1.795812	1.792384	1.788999	1.839413
1.667021	1.663923	1.766672	1.763316	1.801065	1.797586	1.79415	1.790754	1.787419
1.668761	1.66566	1.768491	1.765088	1.802907	1.799465	1.796032	1.792604	1.789212
1.560849	1.557948	1.712187	1.709063	1.763449	1.76012	1.797869	1.794414	1.79098
1.562385	1.559478	1.713756	1.710617	1.765127	1.761802	1.799616	1.796141	1.792691
1.563917	1.561	1.71532	1.712171	1.766802	1.763461	1.801332	1.797855	1.794405
1.565474	1.562535	1.559621	1.556732	1.710634	1.707544	1.761732	1.758366	1.796159
1.567039	1.564079	1.561163	1.558275	1.712216	1.709086	1.763372	1.760036	1.797936
1.568603	1.565651	1.56274	1.559857	1.612309	1.609361	1.707607	1.704557	1.758468
1.570171	1.567245	1.564346	1.561469	1.613862	1.610913	1.709277	1.706212	1.760229
1.571777	1.56885	1.56594	1.56305	1.56016	1.557273	1.609515	1.606655	1.704791
1.573433	1.570469	1.56752	1.564596	1.561687	1.558797	1.610992	1.608155	1.706441
1.575073	1.572072	1.569095	1.566149	1.563231	1.560341	1.612479	1.609642	1.606802
1.576648	1.573625	1.57065	1.567718	1.564809	1.561925	1.613988	1.611094	1.608213
1.578248	1.575198	1.572224	1.569308	1.566409	1.563535	1.615518	1.612561	1.609633
1.579968	1.576865	1.573865	1.570935	1.568037	1.565177	1.562293	1.559368	1.611151
1.581745	1.578584	1.575546	1.572589	1.569684	1.566833	1.563947	1.561003	1.612712
1.583475	1.580294	1.577234	1.574252	1.571316	1.568431	1.565524	1.562577	1.614225
1.585172	1.582006	1.578933	1.575925	1.572939	1.569982	1.567039	1.564103	1.615705

1.490441	1.487515	1.580662	1.577621	1.574593	1.571585	1.568617	1.565686	1.5628
1.492117	1.489169	1.582428	1.579347	1.57629	1.573265	1.570284	1.56735	1.564456
1.493809	1.490836	1.584203	1.581074	1.577988	1.574945	1.571954	1.569021	1.566121
1.495492	1.492497	1.585968	1.582778	1.57965	1.576578	1.573578	1.570662	1.567774
1.497186	1.494168	1.587745	1.584497	1.581325	1.578221	1.575208	1.572305	1.569426
1.498907	1.495861	1.492802	1.489764	1.583079	1.579935	1.576892	1.573972	1.571073
1.500645	1.497567	1.494492	1.491443	1.584881	1.581697	1.578615	1.575657	1.572719
1.502368	1.499244	1.496149	1.493094	1.490097	1.487157	1.580382	1.577373	1.574386
1.504074	1.500888	1.497773	1.494718	1.491739	1.488831	1.582198	1.57912	1.576071
1.505827	1.502593	1.499447	1.496373	1.493394	1.490503	1.58401	1.580886	1.577791
1.507651	1.504391	1.501194	1.498064	1.495057	1.492158	1.585803	1.582669	1.579554
1.509499	1.506216	1.502977	1.499799	1.496756	1.493829	1.587604	1.584469	1.581341
1.511346	1.50803	1.504786	1.501608	1.498526	1.495533	1.492575	1.489661	1.583141
1.513203	1.509853	1.506615	1.503451	1.500332	1.497263	1.494259	1.49134	1.584955
1.515074	1.511686	1.508433	1.505263	1.502125	1.499029	1.495994	1.493036	1.490075
1.516957	1.513529	1.51025	1.507061	1.503917	1.500826	1.497768	1.494749	1.491743
1.518855	1.515402	1.512091	1.508872	1.505716	1.502627	1.49955	1.496478	1.49343
1.520768	1.517305	1.513956	1.510692	1.507519	1.504434	1.501339	1.49822	1.495128
1.522697	1.519219	1.515836	1.512536	1.509341	1.506243	1.503132	1.499991	1.496875
1.524644	1.521133	1.517729	1.514412	1.511187	1.508049	1.504924	1.501807	1.498706
1.526609	1.523057	1.519634	1.516304	1.513046	1.509862	1.506721	1.503629	1.500549
1.461247	1.457753	1.521539	1.518195	1.514907	1.511685	1.508519	1.505418	1.502332
1.463181	1.459642	1.523459	1.520094	1.516777	1.513522	1.510326	1.507199	1.504094
1.465184	1.461591	1.525421	1.522009	1.51866	1.515385	1.512165	1.509003	1.505875
1.467248	1.463594	1.527421	1.523939	1.520555	1.517272	1.514031	1.510827	1.507666
1.403441	1.399884	1.462118	1.458668	1.522498	1.519182	1.515907	1.512666	1.509476
1.405658	1.402082	1.464224	1.460671	1.524497	1.521114	1.51779	1.51452	1.511306
1.320509	1.317045	1.46635	1.462703	1.459213	1.45586	1.519689	1.516394	1.513157
1.322591	1.319097	1.468448	1.464724	1.461164	1.457743	1.521581	1.518275	1.515026
1.324677	1.32116	1.470572	1.466796	1.463177	1.459689	1.523533	1.520199	1.516923
1.326798	1.323289	1.319773	1.316293	1.465361	1.461819	1.525665	1.522223	1.518853
1.328943	1.325453	1.321961	1.318499	1.467648	1.464058	1.527909	1.52432	1.520826
1.331094	1.327579	1.324084	1.320624	1.46985	1.466216	1.46263	1.45908	1.522906
1.333251	1.329672	1.32615	1.322678	1.471984	1.468307	1.464709	1.461192	1.525097
1.335435	1.331803	1.328255	1.324764	1.321295	1.317856	1.466799	1.46328	1.459836
1.337645	1.333986	1.330417	1.326903	1.323402	1.319925	1.4689	1.465312	1.461804
1.33989	1.336213	1.332612	1.32906	1.325528	1.322022	1.471024	1.467366	1.46379
1.342162	1.338475	1.33482	1.331201	1.327643	1.324138	1.320671	1.317229	1.46587
1.344486	1.340793	1.337075	1.333377	1.329786	1.326282	1.322803	1.319336	1.467996
1.346947	1.343228	1.339449	1.335675	1.332013	1.328441	1.324914	1.321426	1.47013
1.349501	1.345746	1.341912	1.338065	1.334312	1.330631	1.327034	1.323529	1.472289
1.351944	1.348175	1.344339	1.340491	1.336705	1.332969	1.329324	1.325784	1.322297
1.354266	1.350508	1.346735	1.342966	1.339216	1.335481	1.331813	1.328215	1.324671
1.356593	1.352835	1.349103	1.34539	1.341666	1.337937	1.334247	1.330593	1.326994
1.358954	1.355169	1.351423	1.347706	1.343975	1.340239	1.336522	1.332815	1.329164
1.361337	1.357518	1.353745	1.350005	1.346262	1.342522	1.338785	1.335036	1.331339
1.363727	1.359889	1.35609	1.35233	1.348583	1.344853	1.341114	1.33735	1.333623
1.36616	1.362302	1.358474	1.35469	1.350939	1.347222	1.343488	1.339726	1.33598
1.368754	1.364855	1.360998	1.357193	1.353428	1.349703	1.345965	1.342207	1.338453
1.371482	1.367527	1.363637	1.359814	1.356026	1.352278	1.34853	1.34478	1.341028

1.318332	1.314783	1.36617	1.362317	1.358504	1.354738	1.350981	1.347235	1.343485
1.320674	1.317014	1.368543	1.364648	1.360806	1.357027	1.353263	1.349516	1.345765
1.323044	1.319288	1.370988	1.367036	1.363165	1.359383	1.355617	1.351866	1.348114
1.325468	1.321659	1.373595	1.369564	1.365679	1.361934	1.358189	1.354437	1.350685
1.327976	1.324135	1.376318	1.372194	1.368276	1.364542	1.360801	1.35704	1.353282
1.330688	1.326828	1.323037	1.319329	1.370939	1.36709	1.36327	1.359476	1.355697
1.333506	1.329642	1.325775	1.32195	1.37366	1.369631	1.36569	1.36185	1.358042
1.336127	1.332274	1.328372	1.324485	1.376374	1.372227	1.368212	1.364354	1.360541
1.33857	1.334747	1.330848	1.326947	1.379081	1.374865	1.370817	1.366969	1.363176
1.341147	1.337315	1.333427	1.32954	1.3257	1.321903	1.373533	1.369625	1.365786
1.343957	1.340051	1.33619	1.332358	1.328502	1.324636	1.376397	1.372309	1.36831
1.346751	1.342776	1.338924	1.335126	1.331263	1.327362	1.379286	1.37505	1.370914
1.349343	1.345349	1.341454	1.337613	1.333754	1.329894	1.382105	1.377875	1.373728
1.351923	1.347925	1.343968	1.340057	1.336202	1.332402	1.384937	1.380751	1.376621
1.354733	1.350726	1.346729	1.34277	1.338894	1.335089	1.331312	1.327558	1.379428
1.357683	1.353664	1.349635	1.345638	1.341722	1.337874	1.334036	1.330204	1.382211
1.209291	1.206582	1.352385	1.348357	1.3444	1.340506	1.336649	1.332837	1.329021
1.211192	1.208394	1.354956	1.350906	1.346905	1.34296	1.339131	1.335454	1.331762
1.213168	1.210299	1.357665	1.353589	1.349546	1.345555	1.341717	1.338079	1.334431
1.215278	1.212385	1.36064	1.356544	1.352463	1.348428	1.344498	1.340691	1.336919
1.217411	1.214499	1.363679	1.359526	1.35541	1.351358	1.347345	1.343359	1.339445
1.219472	1.216506	1.213598	1.210744	1.358163	1.354194	1.35021	1.346183	1.342217
1.221525	1.218507	1.215561	1.212675	1.360899	1.357015	1.353082	1.349063	1.345081
1.223663	1.220658	1.217689	1.21476	1.363878	1.359852	1.355831	1.35179	1.347802
1.225864	1.222919	1.219944	1.216962	1.367046	1.362705	1.358494	1.354417	1.350436
1.227976	1.225073	1.222112	1.219125	1.216171	1.213239	1.361377	1.357229	1.353203
1.229969	1.227084	1.224168	1.221237	1.218312	1.21539	1.364534	1.360269	1.356129
1.231996	1.229119	1.226234	1.223341	1.220435	1.21752	1.214632	1.211767	1.359096
1.234142	1.231262	1.228356	1.225433	1.222498	1.219551	1.216645	1.213777	1.362047
1.236295	1.233419	1.230494	1.22754	1.224575	1.221596	1.218672	1.215804	1.365054
1.238343	1.235515	1.232632	1.229717	1.226763	1.223774	1.220824	1.217914	1.368166
1.240349	1.237582	1.234767	1.231923	1.229003	1.226019	1.223046	1.220076	1.371374
1.075305	1.072887	1.236841	1.234052	1.231175	1.228228	1.225263	1.222267	1.21931
1.077143	1.074601	1.238859	1.236116	1.233293	1.230416	1.227487	1.224489	1.221506
1.079064	1.076427	1.240926	1.238176	1.235379	1.232557	1.229669	1.226697	1.223716
1.081104	1.078414	1.243059	1.240224	1.237413	1.234635	1.231798	1.228891	1.225949
0.889045	0.88686	1.077739	1.075124	1.239469	1.236702	1.233896	1.231049	1.228153
0.890547	0.888403	1.079656	1.077042	1.241597	1.238777	1.235955	1.233138	1.230284
0.892022	0.889924	1.081582	1.079002	1.243758	1.240864	1.238006	1.235201	1.23238
0.604937	0.603724	0.889328	0.887246	1.078302	1.075678	1.240103	1.237284	1.234466
0.605855	0.604612	0.890818	0.888774	1.080195	1.077586	1.242233	1.239378	1.236541
		0.604315	0.603128	0.888227	0.886096	1.076934	1.074315	1.238648
		0.605259	0.604072	0.8898	0.887687	1.078909	1.076222	1.240786
				0.891337	0.889242	1.08086	1.078153	1.242952
				0.892804	0.89074	1.082761	1.080114	1.245153
				0.894285	0.892226	1.084649	1.082066	1.247368
				0.606486	0.605265	0.891615	0.889552	1.081374
				0.607422	0.606169	0.893081	0.891042	1.083266
						0.89463	0.892593	0.890552
						0.896243	0.894187	0.892136

0.893724
0.895313
0.896914
0.89853
0.900158

						0.870372	0.867968	0.865527
						0.868548	0.866041	0.863524
				0.871594	0.869185	1.579706	1.575549	1.571436
				0.869839	0.867385	1.576797	1.572649	1.568542
				0.868071	0.865578	1.573916	1.569787	1.565695
				1.072897	1.06989	1.884022	1.879173	1.87436
				1.070671	1.067686	1.880795	1.875956	1.871166
		0.867678	0.865132	1.648688	1.644649	1.997777	1.992717	1.987715
		0.865875	0.8633	1.645852	1.641838	1.99431	1.989308	1.984371
0.869086	0.866601	1.575739	1.571608	1.880638	1.875886	1.990875	1.985929	1.981054
0.867372	0.86477	1.572906	1.568786	1.877408	1.872689	1.987468	1.982578	1.977763
1.578415	1.574238	1.883923	1.879005	1.993947	1.988991	1.984102	1.979269	1.974512
1.575586	1.571431	1.880721	1.875803	1.990506	1.985602	1.980773	1.976006	1.97131
1.57276	1.56864	1.877542	1.872656	1.987141	1.982286	1.977506	1.972791	1.968141
1.884074	1.879233	1.993972	1.988908	1.983959	1.97912	1.97435	1.969641	1.964997

1.880858	1.876047	1.990641	1.985737	1.980869	1.976039	1.971262	1.966538	1.961878
1.943631	1.938711	1.987299	1.982492	1.977696	1.972914	2.018689	2.013982	2.009322
1.940294	1.935425	1.983952	1.979201	1.974471	1.969769	2.015632	2.01093	2.006253
1.99038	1.985454	1.980656	1.975954	2.021824	2.017203	2.160464	2.155453	2.150456
1.987066	1.982162	1.977403	1.972753	2.018703	2.014106	2.157162	2.15222	2.147274
1.983817	1.978959	2.024814	2.020217	2.015613	2.011007	2.153834	2.148946	2.144048
1.980653	1.975909	2.021835	2.017215	2.012571	2.007904	2.150453	2.145568	2.140695
1.977526	1.972897	2.018889	2.014229	2.009534	2.004796	2.147058	2.14216	2.137304
1.974361	1.969774	2.01582	2.011154	2.153789	2.148696	2.143677	2.138778	2.133938
1.971197	1.966607	2.012692	2.008031	2.150426	2.145332	2.140314	2.135418	2.130592
2.018851	2.01425	2.157111	2.152101	2.183809	2.17867	2.218184	2.213104	2.208094
2.015886	2.011225	2.153798	2.14874	2.180399	2.175361	2.214856	2.209798	2.204794
2.012876	2.008161	2.187215	2.182049	2.221616	2.216541	2.211505	2.206489	2.201515
2.009793	2.005033	2.183768	2.178592	2.218078	2.213057	2.208086	2.203144	2.198248
2.006679	2.001894	2.180341	2.175192	2.214613	2.209629	2.204705	2.199823	2.19499
2.187258	2.182075	2.176966	2.171918	2.211309	2.206347	2.201432	2.196546	2.191709
2.183805	2.178656	2.173636	2.168711	2.208083	2.203136	2.198218	2.193313	2.188453
2.180412	2.175334	2.214779	2.209794	2.204839	2.199921	2.19503	2.19016	2.185317
2.177065	2.172084	2.211492	2.206528	2.201604	2.196723	2.191874	2.187067	2.182263
2.218238	2.213201	2.208214	2.203282	2.198394	2.193555	2.188735	2.183944	2.179135
2.214892	2.20989	2.204943	2.200052	2.19521	2.190424	2.185623	2.180796	2.175937
2.211583	2.206616	2.201711	2.196864	2.192051	2.187283	2.182476	2.177602	2.172693
2.208304	2.203383	2.198532	2.19374	2.188935	2.184129	2.179274	2.174339	2.169389
2.205063	2.200192	2.195383	2.190627	2.185812	2.180945	2.176034	2.171051	2.166079
2.201865	2.197061	2.192266	2.187489	2.182634	2.177705	2.220969	2.215752	2.210582
2.198709	2.193961	2.189158	2.184328	2.17942	2.174428	2.217488	2.212286	2.207164
2.195607	2.190823	2.18598	2.1811	2.176151	2.171122	2.214014	2.208894	2.203869
2.192553	2.187661	2.182748	2.177815	2.172834	2.16779	2.210544	2.205564	2.200678
2.189421	2.184461	2.179517	2.174574	2.217559	2.212336	2.305672	2.299952	2.294371
2.186176	2.1812	2.176279	2.171388	2.214293	2.209183	2.302083	2.296373	2.290773
2.18297	2.177994	2.173087	2.168223	2.310025	2.304224	2.298483	2.292788	2.287181
2.179905	2.174943	2.170003	2.16508	2.30616	2.300393	2.2947	2.289065	2.283525
2.176843	2.171896	2.166923	2.161939	2.302307	2.296571	2.290932	2.285375	2.279917
2.173619	2.16865	2.239032	2.233724	2.298456	2.292872	2.287365	2.281912	2.276517
2.170329	2.16533	2.235511	2.230282	2.294646	2.289255	2.283906	2.278563	2.273218
2.197302	2.192191	2.302459	2.296661	2.291078	2.285723	2.295327	2.289983	2.284627
2.194192	2.189198	2.29905	2.293335	2.287744	2.282281	2.291831	2.286417	2.281019
2.306936	2.301219	2.295535	2.289896	2.299403	2.293835	2.288325	2.282889	2.27749
2.303101	2.297418	2.29179	2.28622	2.295791	2.290251	2.284786	2.279424	2.2741
2.299342	2.293701	2.288119	2.282599	2.29223	2.286725	2.281299	2.275989	2.270718
2.295795	2.290277	2.29998	2.294442	2.288921	2.283412	2.368734	2.362367	2.35611
2.292324	2.286929	2.296708	2.291177	2.285657	2.280138	2.364818	2.358447	2.352191
2.304083	2.298674	2.293187	2.287664	2.373784	2.367248	2.360812	2.354473	2.348261
2.300507	2.295042	2.289536	2.284018	2.369611	2.363143	2.356772	2.35049	2.344349
2.297083	2.291577	2.314007	2.30826	2.365605	2.359214	2.352916	2.346697	2.340617
2.293838	2.288299	2.310599	2.304885	2.36178	2.355477	2.349262	2.343115	2.337084
2.318638	2.312835	2.370736	2.364264	2.357918	2.351695	2.345557	2.339483	2.333521
2.314854	2.309088	2.366575	2.360184	2.353907	2.347742	2.341671	2.335664	2.329803
2.31109	2.305366	2.362473	2.35617	2.349964	2.343856	2.337853	2.331917	2.326165
2.371474	2.365004	2.358664	2.352432	2.346294	2.340248	2.33431	2.328437	2.322768

2.367618	2.361238	2.35497	2.348806	2.34274	2.336768	2.33091	2.325122	2.31955
2.363615	2.357315	2.351116	2.345022	2.339056	2.333213	2.355553	2.349914	2.344483
2.359524	2.3533	2.347168	2.341144	2.335299	2.329628	2.352164	2.346806	2.341641
2.355651	2.349499	2.343424	2.337454	2.359794	2.354209	2.348838	2.343699	2.338749
2.352037	2.345955	2.339923	2.333981	2.356356	2.350829	2.34555	2.340553	2.335757
2.3485	2.34248	2.336504	2.330616	2.35305	2.347605	2.342428	2.337566	2.332906
2.345021	2.339042	2.33314	2.327349	2.349894	2.344592	2.339564	2.334867	2.330347
2.341527	2.335597	2.329798	2.324147	2.346831	2.341698	2.336827	2.332275	2.327868
2.337832	2.33199	2.35447	2.348982	2.343766	2.338822	2.334094	2.329615	2.325254
2.334077	2.328353	2.351003	2.345761	2.340761	2.336016	2.33142	2.326972	2.322616
2.358772	2.35315	2.347837	2.342787	2.337957	2.333362	2.352174	2.347717	2.343331
2.355529	2.35009	2.344938	2.340039	2.335338	2.330855	2.349703	2.345288	2.340928
2.3524	2.34714	2.342151	2.337405	2.356119	2.351655	2.347248	2.34287	2.338539
2.349361	2.344274	2.339464	2.334894	2.353655	2.349201	2.344806	2.340465	2.33617
2.346456	2.341535	2.336873	2.332435	2.351211	2.346748	2.342348	2.338022	2.333748
2.343748	2.338993	2.334418	2.33001	2.348744	2.344258	2.339828	2.335459	2.331159
2.341123	2.336512	2.331991	2.327577	2.346253	2.341755	2.337301	2.332888	2.328557
2.338391	2.333852	2.352538	2.348109	2.343675	2.339239	2.357458	2.353008	2.348635
2.335626	2.33111	2.349781	2.345403	2.341047	2.33672	2.354977	2.350612	2.346304
2.356143	2.351615	2.347179	2.34284	2.338532	2.334262	2.372833	2.368444	2.364097
2.353614	2.349119	2.344724	2.340416	2.336127	2.331861	2.370352	2.366013	2.361719
2.351138	2.346687	2.342328	2.338041	2.376679	2.372276	2.390717	2.386198	2.381734
2.348704	2.344319	2.339988	2.335708	2.374265	2.369893	2.388236	2.383713	2.379249
2.346298	2.341972	2.337667	2.333394	2.371863	2.367517	2.38576	2.381225	2.376752
2.343898	2.339605	2.356796	2.352449	2.392301	2.387761	2.383235	2.378697	2.374215
2.341511	2.337236	2.354392	2.350061	2.38975	2.38521	2.380684	2.376151	2.371667
2.360662	2.356314	2.373304	2.368927	2.387184	2.382623	2.378094	2.373593	2.369148
2.358228	2.353882	2.370834	2.366473	2.384603	2.380006	2.375474	2.371027	2.366652
2.355811	2.351471	2.391159	2.386584	2.382007	2.37742	2.4013	2.396733	2.392248
2.353419	2.349091	2.388577	2.383956	2.379388	2.374862	2.398696	2.394199	2.389784
2.395204	2.390607	2.385976	2.38134	2.3768	2.372348	2.396146	2.391713	2.387355
2.39255	2.387918	2.38331	2.378734	2.374262	2.369892	2.393658	2.389271	2.384947
2.389914	2.385249	2.380674	2.376173	2.371773	2.367478	2.391211	2.386861	2.382561
2.387388	2.38273	2.37818	2.373714	2.369354	2.365104	2.388788	2.38447	2.380193
2.384924	2.380294	2.375768	2.371321	2.366985	2.362763	2.386386	2.382095	2.377837
2.382454	2.377854	2.373356	2.368937	2.392643	2.388282	2.436108	2.431087	2.426128
2.379988	2.375421	2.370956	2.366569	2.390204	2.385849	2.433181	2.428207	2.4233
2.377542	2.373011	2.368573	2.364208	2.387772	2.383431	2.43029	2.425359	2.420497
2.37512	2.370628	2.36621	2.361852	2.385345	2.381032	2.427444	2.42255	2.417725
2.372718	2.368258	2.363853	2.3595	2.434561	2.429551	2.440392	2.435419	2.430507
2.37034	2.365889	2.361492	2.357143	2.431652	2.426701	2.437521	2.432581	2.427678
2.367977	2.36353	2.359138	2.354796	2.428768	2.423873	2.43467	2.429762	2.424867
2.365611	2.361177	2.411116	2.406027	2.425919	2.421065	2.431833	2.42696	2.422098
2.36325	2.358837	2.408291	2.403256	2.423103	2.41828	2.42901	2.424176	2.419361
2.360915	2.356554	2.430502	2.42534	2.435994	2.43106	2.426199	2.421403	2.416637
2.358608	2.354335	2.427813	2.422654	2.433228	2.428266	2.4234	2.418638	2.413923
2.410266	2.405336	2.425105	2.419965	2.430471	2.425489	2.420627	2.415913	2.411257
2.40745	2.402594	2.42234	2.417252	2.427709	2.422722	2.41788	2.413235	2.408655
2.404638	2.399841	2.419566	2.414533	2.424957	2.419984	2.415174	2.410593	2.406084
2.401833	2.397096	2.432333	2.427205	2.422192	2.417277	2.412519	2.407976	2.403518

2.399051	2.39436	2.429506	2.424419	2.419461	2.414616	2.409916	2.405397	2.400976
2.412951	2.408147	2.426747	2.421765	2.416874	2.412059	2.407382	2.402878	2.398478
2.41023	2.405393	2.42404	2.41921	2.414395	2.409587	2.40491	2.400412	2.396018
2.407501	2.402635	2.421311	2.416606	2.41187	2.407098	2.422531	2.417987	2.413541
2.404747	2.399854	2.418543	2.41394	2.409286	2.404584	2.420016	2.415477	2.411027
2.40207	2.397163	2.415842	2.411296	2.406712	2.4021	2.417547	2.413009	2.408545
2.399535	2.394632	2.413262	2.408698	2.404166	2.39968	2.415165	2.410618	2.40612
2.397029	2.392156	2.410732	2.406131	2.401637	2.397268	2.412786	2.408236	2.40371
2.417791	2.412921	2.408195	2.403585	2.419086	2.414725	2.41032	2.405785	2.401264
2.415124	2.410367	2.405681	2.401072	2.416538	2.412197	2.407815	2.403312	2.398819
2.412525	2.407877	2.423218	2.418564	2.414052	2.409693	2.405322	2.400886	2.396461
2.409982	2.405441	2.420792	2.416151	2.411623	2.407206	2.402833	2.398497	2.394177
2.407451	2.40297	2.41834	2.413758	2.409235	2.404766	2.400373	2.39609	2.391832
2.40492	2.400435	2.415845	2.411397	2.406914	2.402389	2.397949	2.393647	2.389393
2.422351	2.417794	2.413355	2.409002	2.404553	2.400006	2.395544	2.391223	2.386968
2.41993	2.415346	2.410883	2.406503	2.402063	2.397559	2.393135	2.388827	2.384597
2.417534	2.412943	2.408436	2.403991	2.399554	2.395124	2.390762	2.386476	2.382272
2.415066	2.410564	2.406061	2.401576	2.397157	2.392801	2.388494	2.384223	2.380021
2.412563	2.408196	2.403722	2.39921	2.394817	2.390539	2.386288	2.382034	2.377827
2.410056	2.405722	2.401275	2.396789	2.392431	2.388196	2.383996	2.379811	2.375656
2.407539	2.403139	2.398718	2.394311	2.389998	2.385774	2.381621	2.377563	2.373519
2.405085	2.400638	2.396246	2.391909	2.387632	2.383411	2.37928	2.375288	2.371304
2.402728	2.398274	2.393914	2.389626	2.38537	2.381141	2.376992	2.372963	2.368952
2.400401	2.395964	2.391633	2.387379	2.383138	2.378912	2.374744	2.370661	2.366604
2.398068	2.393681	2.389368	2.385118	2.380897	2.376708	2.378873	2.374792	2.370737
2.395747	2.391422	2.387125	2.382868	2.378666	2.374522	2.376778	2.372676	2.368595
2.393449	2.389152	2.384879	2.38064	2.382824	2.378731	2.395762	2.391641	2.387542
2.39117	2.386882	2.382636	2.378429	2.380691	2.376582	2.393643	2.389561	2.385495
2.388888	2.384621	2.383014	2.378802	2.380646	2.376508	2.391542	2.387467	2.38341
2.386599	2.38237	2.380787	2.376575	2.378493	2.37438	2.389465	2.385364	2.381287
2.387008	2.382778	2.384663	2.380486	2.376345	2.372244	2.399534	2.395368	2.391238
2.384763	2.380514	2.382484	2.378346	2.374216	2.370097	2.397385	2.393184	2.389026
2.382524	2.37826	2.380308	2.376206	2.372079	2.367933	2.395217	2.391	2.386831
2.386507	2.382304	2.378156	2.374043	2.374119	2.369971	2.393019	2.388851	2.384733
2.384318	2.380162	2.376013	2.371873	2.371927	2.367751	2.390822	2.386728	2.382683
2.382141	2.377994	2.378025	2.373926	2.388478	2.384312	2.418455	2.414062	2.409733
2.379973	2.375807	2.375861	2.371804	2.386431	2.382309	2.416256	2.411833	2.407473
2.381924	2.377794	2.373717	2.369676	2.384354	2.380252	2.41344	2.409022	2.404664
2.379773	2.37568	2.3716	2.367531	2.382226	2.378106	2.411133	2.406781	2.402481
2.377631	2.373566	2.369487	2.365404	2.39805	2.393684	2.408876	2.404561	2.400293
2.375501	2.371428	2.367369	2.363319	2.395915	2.391603	2.406752	2.402378	2.398057
2.373376	2.369293	2.365255	2.361245	2.393808	2.389555	2.404664	2.400225	2.395843
2.371236	2.367184	2.364127	2.360109	2.403907	2.399606	2.428416	2.423899	2.419429
2.369097	2.365092	2.362053	2.358017	2.40167	2.3974	2.426172	2.42173	2.417311
2.368033	2.364002	2.378472	2.37423	2.399456	2.395172	2.423873	2.419502	2.415149
2.366015	2.361902	2.376241	2.37205	2.39726	2.392915	2.421513	2.417217	2.412951
2.382715	2.378322	2.387308	2.383051	2.428171	2.423658	2.419237	2.414965	2.410735
2.380582	2.376231	2.385172	2.380897	2.425977	2.421508	2.417098	2.412764	2.408481
2.378463	2.37416	2.383046	2.378731	2.423767	2.41936	2.414975	2.410592	2.406261
2.376369	2.372084	2.395559	2.391099	2.421458	2.417123	2.412802	2.408459	2.404148

2.374262	2.369988	2.393322	2.388801	2.419139	2.414863	2.410607	2.406334	2.402073
2.385257	2.380941	2.405655	2.401105	2.416934	2.412629	2.408372	2.404152	2.39995
2.382953	2.378675	2.403385	2.398924	2.414804	2.410414	2.406113	2.401937	2.397802
2.395276	2.390916	2.401069	2.396696	2.412661	2.408255	2.403943	2.399768	2.395647
2.392964	2.388547	2.398695	2.394417	2.410511	2.406165	2.401871	2.397646	2.393476
2.390696	2.38624	2.396358	2.392124	2.408326	2.404049	2.399792	2.395547	2.391352
2.388499	2.384042	2.394087	2.389804	2.406068	2.401863	2.397673	2.393482	2.389314
2.38632	2.381887	2.391862	2.387514	2.403822	2.399669	2.395537	2.391413	2.38729
2.398528	2.3941	2.389694	2.38532	2.40166	2.397492	2.393367	2.389293	2.385221
2.39629	2.391938	2.38756	2.383184	2.399553	2.395337	2.391196	2.38716	2.383141
2.394009	2.389726	2.385402	2.381069	2.397497	2.393258	2.389108	2.385087	2.381093
2.391692	2.387477	2.383231	2.37898	2.39549	2.391246	2.387086	2.383059	2.379066
2.389434	2.385265	2.38108	2.376901	2.393488	2.389243	2.392968	2.388843	2.38476
2.387251	2.383101	2.37896	2.374838	2.391495	2.387251	2.390908	2.386719	2.382585
2.385084	2.380936	2.391376	2.387316	2.389481	2.385258	2.392981	2.388755	2.384588
2.382903	2.378733	2.389218	2.385198	2.387419	2.383254	2.391015	2.386775	2.382576
2.380745	2.376547	2.387069	2.383078	2.385351	2.381249	2.38906	2.384821	2.380601
2.378646	2.374441	2.385002	2.381024	2.387835	2.38378	2.387092	2.382861	2.378641
2.376589	2.372388	2.382985	2.379008	2.385875	2.38183	2.38512	2.380905	2.376697
2.37454	2.370344	2.385825	2.38186	2.391393	2.387294	2.383156	2.378951	2.374749
2.372505	2.368316	2.383867	2.379896	2.389412	2.385326	2.381199	2.376999	2.372798
2.376015	2.371847	2.381911	2.377934	2.387433	2.383358	2.388408	2.384182	2.379953
2.374026	2.369866	2.379955	2.375972	2.385453	2.381388	2.386451	2.382242	2.378029
2.37204	2.367886	2.378002	2.374013	2.383478	2.379417	2.384489	2.380301	2.376106
2.370054	2.365908	2.383822	2.379769	2.38151	2.377444	2.382518	2.378351	2.374178
2.368069	2.363928	2.38184	2.377788	2.379546	2.375473	2.380544	2.376397	2.372245
2.366078	2.36194	2.379846	2.375801	2.37758	2.373506	2.378576	2.374439	2.370299
2.364084	2.359947	2.377846	2.37381	2.375614	2.371543	2.376612	2.372477	2.368343
2.370564	2.366378	2.375853	2.37182	2.373649	2.369589	2.374658	2.370523	2.366389
2.368542	2.364368	2.373861	2.369824	2.371681	2.367642	2.372717	2.368578	2.364439
2.380149	2.376001	2.371912	2.367869	2.369743	2.365704	2.370771	2.366628	2.362485
2.378219	2.374113	2.370038	2.365991	2.367863	2.363781	2.368811	2.364666	2.360527
2.376311	2.372237	2.368171	2.364116	2.365985	2.361861	2.366853	2.362704	2.358567
2.374425	2.370335	2.366257	2.36219	2.373251	2.36908	2.364912	2.360753	2.356608
2.372547	2.368422	2.364322	2.360237	2.371302	2.367139	2.362975	2.358804	2.35465
2.370639	2.366495	2.362372	2.358265	2.369331	2.365177	2.36102	2.356848	2.352693
2.368709	2.364553	2.360407	2.356273	2.36734	2.363197	2.359051	2.354886	2.350739
2.366748	2.362597	2.358441	2.35429	2.365357	2.36121	2.357066	2.352914	2.348783
2.364752	2.360626	2.356477	2.35232	2.363384	2.35921	2.355058	2.350927	2.346824
2.362745	2.358637	2.354498	2.350346	2.361415	2.357223	2.356543	2.352427	2.348346
2.36073	2.356616	2.352488	2.348355	2.359446	2.355251	2.354566	2.350454	2.346387
2.35872	2.354594	2.350475	2.346363	2.357487	2.353308	2.352632	2.348531	2.344478
2.35672	2.352593	2.348475	2.344368	2.355539	2.351424	2.350802	2.346742	2.34272
2.354746	2.350621	2.346498	2.342388	2.353611	2.349582	2.349032	2.345026	2.341041
2.35289	2.348761	2.354136	2.349978	2.345897	2.341893	2.34722	2.343233	2.339261
2.351148	2.347012	2.352364	2.348196	2.344095	2.34006	2.34538	2.341381	2.337401
2.349319	2.345173	2.350512	2.346346	2.342237	2.338185	2.343507	2.339505	2.335525
2.347355	2.343192	2.348532	2.344386	2.340291	2.336245	2.341588	2.337602	2.333636
2.345365	2.341193	2.346537	2.342414	2.338336	2.334302	2.33967	2.3357	2.331748
2.343371	2.33921	2.344557	2.34045	2.336386	2.332366	2.337757	2.333794	2.32985

2.341404	2.337265	2.342618	2.338525	2.334476	2.330471	2.335884	2.331926	2.327986
2.339574	2.335464	2.340829	2.336757	2.336388	2.332385	2.334161	2.330201	2.326258
2.337828	2.333751	2.339133	2.335086	2.334728	2.330732	2.332531	2.328565	2.324615
2.345472	2.341376	2.337317	2.333294	2.332946	2.32896	2.330783	2.326819	2.322867
2.343505	2.339415	2.335376	2.331382	2.331045	2.327071	2.328919	2.324963	2.321017
2.341599	2.337497	2.333456	2.329466	2.329128	2.325166	2.327037	2.323089	2.319147
2.339817	2.335666	2.331582	2.327551	2.32719	2.323236	2.325129	2.321187	2.317252
2.338053	2.33387	2.329753	2.325688	2.325303	2.321355	2.323265	2.319327	2.315397
2.336232	2.332098	2.328015	2.323972	2.323587	2.319632	2.321552	2.317611	2.313681
2.3344	2.330339	2.326309	2.322306	2.321928	2.317963	2.319891	2.315945	2.312012
2.3326	2.328546	2.324518	2.320514	2.320125	2.316162	2.318107	2.314161	2.310226
2.330824	2.326729	2.322669	2.31864	2.318229	2.314277	2.316246	2.312302	2.308366
2.329032	2.324929	2.320866	2.316836	2.316417	2.312471	2.312968	2.309007	2.305054
2.327217	2.32315	2.319122	2.315125	2.314715	2.310775	2.31126	2.307303	2.303357
2.32542	2.321374	2.317365	2.313385	2.312973	2.309035	2.309507	2.305556	2.301618
2.32367	2.319591	2.315552	2.311543	2.311098	2.307158	2.307613	2.303669	2.299742
2.321923	2.317813	2.313743	2.309703	2.309223	2.305282	2.305719	2.301784	2.297869
2.320124	2.316046	2.315521	2.311478	2.311876	2.307916	2.303977	2.300056	2.296152
2.318308	2.314291	2.313795	2.309782	2.310182	2.306226	2.302295	2.298391	2.294497
2.316553	2.312561	2.312057	2.308032	2.308401	2.304432	2.300496	2.296607	2.292728
2.31485	2.310859	2.310311	2.306242	2.306553	2.30255	2.298599	2.294722	2.290865
2.3131	2.309102	2.312898	2.3088	2.304752	2.300745	2.296795	2.292923	2.289071
2.311272	2.307257	2.31105	2.307018	2.30302	2.299054	2.295125	2.291251	2.287389
2.30946	2.305439	2.309231	2.30526	2.301311	2.297387	2.293479	2.289591	2.285704
2.307712	2.30372	2.307505	2.303552	2.299626	2.295732	2.291827	2.287896	2.283957
2.305997	2.302038	2.305809	2.301852	2.297933	2.294056	2.290148	2.286178	2.282195
2.30885	2.304876	2.304081	2.3001	2.296161	2.292266	2.288355	2.284404	2.280444
2.307156	2.303172	2.30234	2.29832	2.294344	2.290411	2.286494	2.282597	2.2787
2.305443	2.301438	2.300586	2.296566	2.292587	2.288646	2.284732	2.280854	2.27698
2.303713	2.299673	2.298817	2.294834	2.290884	2.286967	2.283065	2.279176	2.275288
2.301966	2.297916	2.297068	2.293124	2.289198	2.285298	2.281395	2.277479	2.273562
2.300188	2.296173	2.29535	2.29144	2.287532	2.283633	2.27971	2.275743	2.271772
2.29842	2.294452	2.293652	2.289765	2.285862	2.28195	2.278002	2.273992	2.269979
2.299831	2.295891	2.29198	2.288092	2.28417	2.280216	2.276239	2.272224	2.268217
2.298129	2.294227	2.29032	2.286415	2.282461	2.278458	2.274455	2.270455	2.266476
2.296457	2.292568	2.28864	2.284694	2.280716	2.276702	2.291348	2.286979	2.282667
2.294814	2.290917	2.286948	2.282943	2.278943	2.274944	2.289448	2.285118	2.28084
2.293167	2.289262	2.28527	2.28123	2.277218	2.273224	2.287576	2.283279	2.279026
2.291521	2.287608	2.283613	2.279569	2.27555	2.271549	2.285737	2.281465	2.277227
2.289852	2.285918	2.281921	2.277885	2.292613	2.28823	2.294729	2.290475	2.286245
2.288134	2.284153	2.280153	2.27614	2.29072	2.286373	2.292941	2.288719	2.284512
2.286413	2.282385	2.278381	2.274391	2.288829	2.28452	2.291156	2.286954	2.282762
2.284731	2.280679	2.276669	2.272684	2.286968	2.282684	2.289368	2.285146	2.280936
2.283067	2.279004	2.274988	2.270998	2.285125	2.280862	2.287585	2.283323	2.27908
2.28141	2.277336	2.273313	2.26932	2.283294	2.27906	2.285827	2.281548	2.277287
2.279762	2.275678	2.271648	2.267651	2.281476	2.277278	2.284094	2.279813	2.275543
2.278104	2.274017	2.288291	2.283937	2.290745	2.286568	2.282364	2.278082	2.273806
2.276435	2.272353	2.286472	2.282127	2.288985	2.284829	2.280638	2.276356	2.272075
2.274763	2.270687	2.284658	2.280329	2.287235	2.283091	2.282098	2.27782	2.273538
2.273089	2.269016	2.282844	2.278543	2.285493	2.281351	2.280361	2.276083	2.271802

2.271415	2.267343	2.281034	2.276765	2.283757	2.279611	2.278621	2.274345	2.270064
2.269747	2.265674	2.279231	2.274988	2.283567	2.279439	2.276888	2.272612	2.268332
2.268081	2.264007	2.277434	2.273213	2.281849	2.27772	2.275157	2.270881	2.266602
2.284363	2.279939	2.288491	2.284292	2.280122	2.275994	2.273422	2.269146	2.264866
2.282542	2.278148	2.286752	2.282553	2.278386	2.274262	2.271683	2.267406	2.263125
2.282537	2.278188	2.285022	2.280817	2.278256	2.274123	2.269944	2.265667	2.261387
2.280767	2.276443	2.283297	2.279076	2.276502	2.272377	2.268204	2.263931	2.259653
2.27902	2.274726	2.281599	2.277355	2.274761	2.270635	2.266464	2.262195	2.257922
2.288493	2.28424	2.279962	2.275685	2.273058	2.268906	2.264719	2.260451	2.256182
2.286818	2.28262	2.278343	2.274033	2.27137	2.267183	2.262976	2.258714	2.254456
2.285129	2.280939	2.276663	2.27235	2.269667	2.265456	2.261246	2.257023	2.2528
2.283431	2.279216	2.274944	2.270651	2.267959	2.263729	2.259526	2.255363	2.251193
2.281718	2.277484	2.282483	2.278179	2.275067	2.270765	2.266498	2.262294	2.258085
2.27998	2.275735	2.28076	2.276523	2.273422	2.269094	2.264796	2.260559	2.256324
2.287597	2.28329	2.27905	2.274852	2.271752	2.26741	2.26662	2.262327	2.258042
2.285952	2.28162	2.277358	2.273136	2.270016	2.26568	2.264874	2.260562	2.256261
2.28432	2.279977	2.275684	2.271421	2.268276	2.263953	2.263137	2.258811	2.254495
2.282646	2.278348	2.275213	2.270899	2.266586	2.262275	2.261452	2.257112	2.252785
2.280955	2.276723	2.273581	2.269235	2.264913	2.260613	2.259784	2.255434	2.251099
2.279257	2.275038	2.271886	2.267534	2.263206	2.258901	2.258057	2.253709	2.249378
2.277551	2.273305	2.270142	2.265803	2.261474	2.257151	2.256284	2.251948	2.247633
2.275878	2.271608	2.268436	2.26411	2.263258	2.258901	2.254558	2.250233	2.24593
2.274251	2.269958	2.26678	2.262468	2.261607	2.257242	2.252897	2.248579	2.244287
2.272631	2.268326	2.265137	2.260827	2.259955	2.255591	2.251249	2.246935	2.242649
2.271018	2.266722	2.263508	2.259173	2.258285	2.253939	2.249609	2.245291	2.241002
2.269388	2.265105	2.261864	2.257501	2.256596	2.252269	2.247952	2.243632	2.239342
2.267684	2.263402	2.263604	2.259213	2.254854	2.250524	2.246213	2.241913	2.237641
2.26594	2.261651	2.261828	2.257445	2.253085	2.248741	2.244431	2.240163	2.235919
2.265336	2.261014	2.2601	2.255724	2.251365	2.247018	2.242712	2.238466	2.234241
2.263683	2.259339	2.258413	2.254045	2.249691	2.245347	2.241047	2.236815	2.232601
2.265491	2.261114	2.256743	2.252377	2.248031	2.2437	2.23941	2.235183	2.230972
2.263842	2.259468	2.255091	2.250719	2.246383	2.242081	2.237809	2.233574	2.229351
2.262193	2.257821	2.253441	2.249066	2.244742	2.240467	2.236211	2.231968	2.227735
2.260535	2.256151	2.251774	2.24741	2.243097	2.238836	2.23459	2.230354	2.226124
2.258871	2.254474	2.250104	2.245757	2.241456	2.237202	2.232963	2.228736	2.224511
2.257194	2.252811	2.248451	2.244113	2.239821	2.235576	2.231341	2.227109	2.222877
2.255508	2.251156	2.246808	2.242477	2.238192	2.233955	2.22972	2.225474	2.221229
2.253846	2.249482	2.245145	2.240839	2.236567	2.232332	2.228094	2.223843	2.219592
2.252208	2.24778	2.243455	2.239201	2.234949	2.230705	2.226462	2.222215	2.217969
2.250592	2.246128	2.241802	2.237564	2.233319	2.229074	2.224829	2.220585	2.21634
2.249009	2.244571	2.240216	2.235919	2.231655	2.22743	2.223201	2.21895	2.214699
2.247423	2.243031	2.238656	2.234301	2.230012	2.225793	2.221568	2.217311	2.213055
2.245786	2.241451	2.237105	2.232763	2.228452	2.224173	2.219908	2.215654	2.21141
2.244132	2.239853	2.235556	2.231257	2.226931	2.222572	2.218252	2.213999	2.209767
2.242511	2.23823	2.233957	2.229696	2.225383	2.221014	2.219241	2.214966	2.210719
2.240914	2.236585	2.232319	2.228096	2.22382	2.219494	2.217741	2.21341	2.209108
2.239337	2.234991	2.230716	2.226487	2.224787	2.220516	2.216219	2.21187	2.207547
2.237788	2.233467	2.229158	2.22486	2.223158	2.218923	2.214664	2.210347	2.206049
2.236229	2.231944	2.227614	2.223265	2.22156	2.217345	2.213114	2.208832	2.204562
2.234631	2.230388	2.226077	2.221736	2.220045	2.215822	2.211591	2.20733	2.203072

2.233036	2.228829	2.224549	2.220234	2.218556	2.214307	2.210061	2.205817	2.201569
2.231494	2.227298	2.225626	2.221339	2.217049	2.21275	2.208476	2.204249	2.200025
2.229979	2.225775	2.224118	2.219845	2.215534	2.211174	2.206865	2.202649	2.198453
2.231026	2.226797	2.222552	2.218297	2.213988	2.209617	2.205295	2.201062	2.196858
2.229478	2.225184	2.22093	2.216696	2.212412	2.208074	2.203759	2.199482	2.195236
2.22795	2.223607	2.219341	2.215117	2.210853	2.20655	2.202247	2.197937	2.193655
2.226454	2.222089	2.217812	2.213583	2.20933	2.205059	2.200769	2.196443	2.192132
2.224965	2.220584	2.216288	2.212041	2.207794	2.203553	2.199283	2.194959	2.190634
2.223468	2.219068	2.214737	2.210449	2.206197	2.201987	2.198693	2.194402	2.190098
2.221972	2.217555	2.213188	2.208854	2.204592	2.200411	2.197148	2.192899	2.188626
2.221412	2.216984	2.212603	2.208255	2.203995	2.199831	2.195647	2.19142	2.18716
2.219922	2.215486	2.211104	2.206758	2.202504	2.198347	2.194171	2.189955	2.185699
2.218411	2.213992	2.209614	2.205266	2.201012	2.196856	2.192686	2.188482	2.184236
2.216875	2.212503	2.208137	2.203782	2.199522	2.195364	2.191196	2.187009	2.182774
2.215361	2.211017	2.206658	2.202298	2.198027	2.193847	2.189669	2.185492	2.181272
2.213895	2.209532	2.20517	2.200811	2.196514	2.192278	2.188066	2.183892	2.179693
2.212437	2.208049	2.203682	2.19933	2.195011	2.190723	2.186476	2.182297	2.178111
2.210961	2.206567	2.202205	2.197864	2.193543	2.189242	2.184978	2.180779	2.176579
2.20948	2.205085	2.200733	2.196406	2.192096	2.187803	2.183535	2.179307	2.17508
2.207997	2.203604	2.199259	2.194945	2.190657	2.186396	2.182142	2.177893	2.173637
2.206511	2.202123	2.197786	2.193483	2.189226	2.185021	2.180796	2.176532	2.172242
2.205023	2.200644	2.196311	2.192012	2.187771	2.183597	2.179388	2.175118	2.170811
2.203531	2.199169	2.194835	2.190528	2.186284	2.182111	2.177905	2.17364	2.169337
2.202042	2.197696	2.193364	2.189052	2.1848	2.180614	2.176402	2.172142	2.167846
2.200558	2.196223	2.191897	2.187589	2.183325	2.179112	2.174883	2.170626	2.166341
2.199083	2.194758	2.190444	2.186145	2.181871	2.177625	2.173372	2.16911	2.164828
2.197622	2.193311	2.189018	2.184744	2.180471	2.176203	2.171923	2.167625	2.163318
2.196176	2.19188	2.187614	2.183369	2.179101	2.174813	2.170503	2.166155	2.16181
2.194756	2.190482	2.186226	2.181983	2.177702	2.173384	2.169046	2.164676	2.160319
2.19336	2.189115	2.184857	2.180593	2.176282	2.171923	2.16756	2.16319	2.158844
2.191998	2.187745	2.183465	2.179166	2.174827	2.170444	2.166071	2.161714	2.15739
2.190693	2.186377	2.182041	2.177688	2.173321	2.168937	2.164577	2.160247	2.155955
2.189354	2.184982	2.180598	2.176202	2.171817	2.167439	2.163098	2.158802	2.154548
2.187909	2.183524	2.179128	2.174722	2.170335	2.165958	2.161637	2.157392	2.153184
2.186423	2.182042	2.177653	2.173259	2.168882	2.164514	2.160211	2.156009	2.151837
2.184945	2.18057	2.176207	2.171853	2.167509	2.163171	2.158881	2.154666	2.150469
2.183471	2.179105	2.174781	2.170486	2.16619	2.161896	2.157613	2.153348	2.14909
2.182102	2.177728	2.173405	2.169115	2.164828	2.160547	2.156264	2.151977	2.14769
2.18085	2.176449	2.172086	2.167746	2.163425	2.159125	2.154833	2.150547	2.146264
2.179548	2.175127	2.170734	2.166354	2.162007	2.157693	2.153399	2.149129	2.144864
2.178128	2.173698	2.169294	2.164906	2.160556	2.156243	2.151965	2.147735	2.143517
2.176706	2.17228	2.167877	2.163489	2.159141	2.154831	2.150566	2.146362	2.142175
2.175372	2.170981	2.166591	2.162202	2.157853	2.15354	2.154718	2.150459	2.146218
2.174061	2.169715	2.165344	2.160958	2.156607	2.152291	2.153443	2.149133	2.144838
2.17269	2.168355	2.16399	2.159606	2.155256	2.150938	2.152073	2.147742	2.143426
2.171278	2.166932	2.162563	2.158183	2.153835	2.149517	2.150641	2.146309	2.14199
2.169864	2.165529	2.161181	2.156828	2.152492	2.148173	2.149287	2.144956	2.140636
2.16844	2.164151	2.159857	2.155563	2.151255	2.146933	2.148039	2.143711	2.139392
2.167055	2.162787	2.158523	2.154267	2.155439	2.151092	2.146752	2.142429	2.138111
2.165734	2.161428	2.15714	2.152868	2.154023	2.149679	2.145343	2.141023	2.136708

2.164448	2.160094	2.155769	2.151467	2.152598	2.148258	2.143926	2.13961	2.135297
2.163196	2.158829	2.154491	2.150174	2.151292	2.146953	2.142623	2.138307	2.133995
2.161953	2.157591	2.153248	2.148922	2.150031	2.145692	2.141362	2.137044	2.132732
2.160601	2.156244	2.1519	2.147571	2.148671	2.144332	2.140003	2.135684	2.131373
2.159151	2.154802	2.150463	2.146135	2.147226	2.142888	2.138559	2.134241	2.12993
2.157783	2.153441	2.154555	2.150205	2.145863	2.141527	2.1372	2.132882	2.128571
2.156539	2.1522	2.153308	2.14896	2.144619	2.140288	2.135964	2.131647	2.127335
2.155318	2.150982	2.152085	2.147737	2.143399	2.139071	2.134749	2.130434	2.126122
2.154103	2.149767	2.150867	2.146519	2.142182	2.137855	2.133534	2.12922	2.124908
2.152862	2.148527	2.149622	2.145276	2.140939	2.136611	2.13229	2.127977	2.123665
2.151494	2.147158	2.148246	2.143902	2.139566	2.135238	2.130917	2.126604	2.122293
2.150051	2.145715	2.146794	2.142452	2.138117	2.133789	2.129468	2.125156	2.120845
2.154161	2.149802	2.145453	2.141113	2.136779	2.132451	2.12813	2.123817	2.119505
2.152924	2.148565	2.144218	2.139879	2.135546	2.131219	2.126897	2.122582	2.11827
2.15171	2.147353	2.143007	2.13867	2.134338	2.130012	2.125689	2.12137	2.117052
2.150511	2.146158	2.141815	2.137482	2.133152	2.128827	2.124502	2.120174	2.115845
2.149314	2.144962	2.14062	2.136288	2.13196	2.127637	2.123309	2.118972	2.114632
2.148105	2.143746	2.139404	2.135073	2.130746	2.126423	2.122093	2.117754	2.113403
2.146881	2.142509	2.138159	2.133825	2.129495	2.125172	2.12084	2.116503	2.112143
2.145623	2.141216	2.136834	2.132471	2.128125	2.123801	2.119472	2.115138	2.110784
2.144336	2.139878	2.135447	2.131035	2.126666	2.12234	2.118017	2.113687	2.10935
2.143009	2.13855	2.13411	2.129686	2.12531	2.120982	2.11666	2.112333	2.108006
2.141627	2.137226	2.132827	2.128435	2.124073	2.119741	2.115415	2.111089	2.106765
2.140288	2.135937	2.131575	2.127213	2.122865	2.11853	2.114199	2.109873	2.105551
2.139042	2.134706	2.130357	2.126008	2.121665	2.117328	2.112996	2.10867	2.104347
2.137833	2.133501	2.129156	2.124814	2.120473	2.116135	2.111801	2.107475	2.103152
2.136628	2.132297	2.127954	2.123613	2.119273	2.114934	2.1106	2.106275	2.101952
2.135433	2.1311	2.126755	2.122411	2.118069	2.113731	2.109398	2.105073	2.100751
2.134226	2.129896	2.125551	2.121205	2.116864	2.112525	2.108192	2.103868	2.099547
2.133011	2.128688	2.124344	2.119997	2.115655	2.111317	2.106985	2.102662	2.098341
2.131824	2.127482	2.123132	2.118788	2.114447	2.110109	2.105777	2.101454	2.097133
2.130668	2.126268	2.121905	2.117574	2.113238	2.108902	2.10457	2.100245	2.095923
2.129546	2.125091	2.120704	2.116367	2.112029	2.107693	2.103362	2.099036	2.094713
2.12848	2.123986	2.119553	2.115163	2.110803	2.106476	2.102154	2.097828	2.093504
2.127428	2.122915	2.118437	2.113983	2.109592	2.105265	2.100946	2.096619	2.092295
2.126336	2.121851	2.117362	2.112873	2.108443	2.104074	2.099727	2.095401	2.091084
2.12522	2.120794	2.116317	2.111809	2.107339	2.102902	2.09851	2.094182	2.089873
2.124064	2.119675	2.115227	2.110741	2.106262	2.101784	2.097353	2.092993	2.088661
2.122866	2.118498	2.114099	2.109677	2.10522	2.100723	2.096255	2.09183	2.087441
2.121659	2.117304	2.112942	2.108568	2.104141	2.099655	2.095177	2.090709	2.086275
2.120446	2.116098	2.111749	2.107395	2.103004	2.098577	2.09413	2.089653	2.085188
2.119234	2.114887	2.110542	2.106197	2.101838	2.097468	2.093062	2.088599	2.084129
2.118013	2.113665	2.109324	2.104989	2.100646	2.096299	2.091926	2.087512	2.083086
2.116816	2.11246	2.108112	2.103776	2.099438	2.095097	2.090751	2.086397	2.082034
2.115733	2.111338	2.106956	2.102586	2.09823	2.093886	2.08424	2.079932	2.075618
2.114732	2.110281	2.105843	2.101417	2.097025	2.092667	2.08303	2.078726	2.074423
2.113684	2.109225	2.104775	2.100331	2.095917	2.09153	2.081881	2.077554	2.073236
2.112588	2.108181	2.10377	2.099354	2.094928	2.09049	2.080798	2.076412	2.072045
2.111454	2.107089	2.102713	2.098326	2.093904	2.089445	2.079739	2.07532	2.07092
2.11025	2.105895	2.101535	2.097173	2.092784	2.088368	2.078711	2.074322	2.069931

2.109063	2.104699	2.100339	2.095987	2.091629	2.087265	2.077669	2.073318	2.068946
2.108	2.103593	2.099197	2.094814	2.085199	2.080869	2.077568	2.073208	2.068833
2.106991	2.10253	2.098085	2.093654	2.08403	2.079702	2.076397	2.072035	2.067669
2.105924	2.101465	2.097022	2.092592	2.082983	2.078655	2.075333	2.070947	2.066558
2.104816	2.100406	2.096005	2.091614	2.082037	2.077706	2.074354	2.069923	2.065483
2.103744	2.099346	2.089728	2.085364	2.081011	2.076676	2.073311	2.068871	2.064421
2.10273	2.098286	2.08864	2.084235	2.079862	2.075523	2.072173	2.067781	2.06338
2.101697	2.097224	2.087565	2.083132	2.079766	2.075398	2.079386	2.074918	2.07045
2.100593	2.096155	2.086541	2.082135	2.078749	2.074335	2.078244	2.073751	2.069271
2.099481	2.095086	2.085523	2.08115	2.077752	2.073298	2.077127	2.072598	2.068095
2.093237	2.088844	2.084458	2.080077	2.07668	2.072246	2.076063	2.071542	2.067044
2.09223	2.087793	2.083373	2.078966	2.075578	2.071199	2.075038	2.070544	2.066061
2.091213	2.086759	2.082333	2.077927	2.07455	2.070198	2.074024	2.069526	2.06503
2.090188	2.08574	2.081334	2.076958	2.073592	2.069246	2.073031	2.068497	2.063958
2.089181	2.084744	2.081357	2.076975	2.080952	2.076483	2.071988	2.067445	2.062895
2.088203	2.083783	2.080374	2.075956	2.07986	2.075357	2.070855	2.066351	2.061846
2.087228	2.082818	2.079385	2.074932	2.07877	2.074241	2.069737	2.065273	2.060812
2.086247	2.08182	2.078369	2.073909	2.077723	2.07321	2.068721	2.06427	2.059815
2.08526	2.080809	2.077346	2.072892	2.076695	2.072207	2.067732	2.06328	2.058815
2.084251	2.079796	2.076339	2.071904	2.075679	2.071173	2.066679	2.062203	2.057724
2.083229	2.078781	2.075342	2.070937	2.074674	2.070125	2.065589	2.061074	2.056577
2.083233	2.07879	2.082719	2.078194	2.073648	2.069079	2.064531	2.06001	2.055516
2.082267	2.077858	2.081733	2.077157	2.072591	2.068031	2.063502	2.059015	2.054549
2.081322	2.076935	2.080765	2.076152	2.071573	2.067021	2.06952	2.065032	2.060559
2.080406	2.076022	2.07983	2.075215	2.070632	2.066074	2.068561	2.064071	2.059591
2.07949	2.075095	2.078884	2.074281	2.069703	2.065146	2.067618	2.063113	2.058612
2.078538	2.074105	2.077867	2.073285	2.075774	2.071213	2.066663	2.062122	2.057584
2.077575	2.073096	2.076827	2.072272	2.074777	2.070253	2.065711	2.061126	2.056544
2.085029	2.080421	2.075854	2.07131	2.073819	2.069318	2.064783	2.060175	2.055569
2.084097	2.079497	2.07493	2.070385	2.072891	2.068403	2.063874	2.059259	2.054644
2.083124	2.078539	2.081048	2.076492	2.071967	2.067482	2.062955	2.058343	2.053731
2.082088	2.077531	2.080065	2.075554	2.071049	2.066557	2.062029	2.057433	2.052836
2.088164	2.083602	2.079091	2.074616	2.070119	2.065608	2.061071	2.056493	2.051915
2.087208	2.082652	2.078146	2.073674	2.069159	2.064603	2.060043	2.055478	2.050925
2.086279	2.081723	2.077215	2.072736	2.068196	2.063596	2.05901	2.054455	2.049926
2.085353	2.0808	2.076293	2.071813	2.067265	2.062644	2.058049	2.053499	2.048981
2.084436	2.079885	2.075379	2.070901	2.06635	2.061725	2.057127	2.052579	2.048065
2.083516	2.078967	2.074463	2.069986	2.065435	2.060808	2.056209	2.051664	2.047154
2.082592	2.078047	2.073545	2.069069	2.064521	2.059896	2.0553	2.050757	2.04625
2.081677	2.077128	2.072628	2.068156	2.063613	2.058995	2.054402	2.049857	2.045347
2.080762	2.076202	2.071705	2.067244	2.06271	2.058101	2.053511	2.048956	2.044436
2.079881	2.075304	2.070802	2.066343	2.061819	2.057231	2.052651	2.048085	2.043551
2.079074	2.074475	2.06995	2.065466	2.060952	2.05641	2.051858	2.047285	2.04273
2.078289	2.07367	2.069114	2.064597	2.06009	2.0556	2.051081	2.046503	2.041926
2.077446	2.072821	2.068247	2.063703	2.059197	2.054737	2.050237	2.045657	2.041072
2.076569	2.071952	2.067367	2.0628	2.058289	2.05384	2.04935	2.044771	2.040189
2.075709	2.071121	2.066532	2.061945	2.057418	2.052956	2.048459	2.043885	2.03931
2.074866	2.070333	2.065752	2.061145	2.056588	2.052083	2.047561	2.042994	2.038429
2.07402	2.069519	2.064943	2.060328	2.055751	2.05121	2.046671	2.042126	2.037585
2.073169	2.068648	2.064068	2.05946	2.054879	2.050321	2.04579	2.041299	2.036809

2.072311	2.067761	2.063184	2.058598	2.054023	2.049456	2.044933	2.040484	2.036035
2.071431	2.066871	2.062326	2.057792	2.053238	2.048662	2.047408	2.042906	2.038416
2.070552	2.065988	2.06148	2.057005	2.052479	2.047902	2.046618	2.042077	2.03756
2.06973	2.065161	2.06065	2.056175	2.051664	2.04712	2.045838	2.041274	2.03673
2.068953	2.06438	2.059836	2.055316	2.050811	2.046328	2.045071	2.040491	2.035918
2.068186	2.063612	2.059048	2.054496	2.049982	2.045513	2.044257	2.039671	2.035087
2.067434	2.062866	2.058294	2.053725	2.049181	2.044663	2.043376	2.038798	2.034225
2.066659	2.062098	2.057531	2.052963	2.051692	2.047104	2.041877	2.037294	2.032722
2.065816	2.061261	2.056723	2.052194	2.050929	2.046337	2.041098	2.036508	2.03193
2.064976	2.060434	2.055924	2.051434	2.050179	2.045592	2.040346	2.03575	2.031165
2.064228	2.059717	2.055209	2.050703	2.049431	2.044838	2.039588	2.034999	2.030419
2.063522	2.05905	2.054531	2.049985	2.048687	2.044086	2.038835	2.034259	2.029689
2.062761	2.058288	2.053776	2.049242	2.047943	2.043344	2.03809	2.033513	2.028944
2.061952	2.057443	2.052955	2.048478	2.047199	2.042611	2.037352	2.032765	2.028186
2.061158	2.056623	2.055444	2.050955	2.046426	2.04186	2.036609	2.03202	2.027437
2.060388	2.055842	2.05463	2.050119	2.045599	2.041076	2.035857	2.031282	2.026704
2.059637	2.055088	2.053847	2.049316	2.0448	2.040305	2.035107	2.030542	2.025967
2.058903	2.054365	2.053128	2.048609	2.044093	2.039584	2.034367	2.029793	2.025214
2.058195	2.05367	2.052441	2.047939	2.043417	2.038878	2.033629	2.029041	2.024457
2.060866	2.056294	2.051759	2.047247	2.04203	2.037457	2.032878	2.028293	2.023714
2.060242	2.055646	2.051085	2.046546	2.041303	2.036709	2.032122	2.027548	2.02298
2.059566	2.054963	2.050384	2.045821	2.040562	2.035967	2.031381	2.026813	2.02225
2.058839	2.054244	2.049654	2.045068	2.039807	2.035228	2.030655	2.026086	2.021522
2.058107	2.053521	2.048924	2.044325	2.039062	2.034499	2.029941	2.025384	2.020828
2.057369	2.052787	2.048191	2.043592	2.038327	2.033776	2.029238	2.024719	2.020199
2.056655	2.052074	2.047482	2.042889	2.037623	2.033074	2.028549	2.024059	2.019568
2.056013	2.051429	2.046843	2.04226	2.036987	2.032421	2.027881	2.023381	2.01888
2.055397	2.050808	2.046229	2.041658	2.036377	2.031788	2.027221	2.022693	2.018168
2.054722	2.050132	2.045557	2.040992	2.035706	2.031109	2.026531	2.021986	2.017448
2.054009	2.04942	2.044846	2.040282	2.034995	2.0304	2.02582	2.021265	2.016718
2.05334	2.048756	2.044185	2.039622	2.034332	2.029739	2.025159	2.0206	2.016049
2.05273	2.048157	2.043588	2.039024	2.033728	2.029136	2.024558	2.020001	2.015452
2.052103	2.047541	2.042983	2.038428	2.033132	2.028544	2.023968	2.019412	2.014864
2.051411	2.046865	2.042337	2.03782	2.032543	2.027963	2.023389	2.018827	2.014273
2.050717	2.046184	2.04168	2.037195	2.031935	2.027364	2.022793	2.018224	2.013665
2.05008	2.045541	2.040307	2.035794	2.031255	2.026692	2.022125	2.017555	2.012993
2.049474	2.044919	2.039649	2.035098	2.030544	2.025986	2.021425	2.016856	2.012294
2.048882	2.044322	2.039031	2.03446	2.029896	2.02534	2.020782	2.016215	2.011655
2.048306	2.043748	2.03845	2.033871	2.029303	2.024744	2.020186	2.015622	2.011065
2.047732	2.043176	2.037872	2.033289	2.028719	2.02416	2.019603	2.015041	2.010486
2.047157	2.042602	2.037294	2.032711	2.028142	2.023586	2.019031	2.014471	2.009918
2.046581	2.042027	2.036717	2.032136	2.027568	2.023015	2.018462	2.013904	2.009353
2.046003	2.04145	2.036138	2.031557	2.026992	2.02244	2.01789	2.013333	2.008783
2.045425	2.040871	2.035557	2.030979	2.026415	2.021865	2.017316	2.01276	2.008212
2.044844	2.040292	2.034977	2.030401	2.025838	2.021288	2.016738	2.012184	2.007637
2.044263	2.039713	2.034397	2.029824	2.025261	2.020709	2.016159	2.011605	2.00706
2.04369	2.039136	2.033816	2.029243	2.024681	2.020129	2.01558	2.011027	2.006483
2.043122	2.038557	2.03323	2.028656	2.024095	2.019547	2.015	2.010447	2.005904
2.04185	2.037261	2.032673	2.028087	2.02352	2.018971	2.014426	2.009878	2.00534
2.041385	2.03678	2.032163	2.027539	2.022949	2.018391	2.013847	2.009313	2.004788

2.040917	2.036303	2.031665	2.027015	2.022408	2.017842	2.013298	2.008778	2.004264
2.04039	2.035785	2.031163	2.026532	2.021936	2.017372	2.012828	2.00831	2.003796
2.039837	2.035248	2.030656	2.026064	2.021489	2.01693	2.012387	2.007866	2.003347
2.039277	2.0347	2.030123	2.025547	2.020983	2.016431	2.01189	2.007361	2.002835
2.038709	2.034142	2.02957	2.024998	2.02044	2.015897	2.011358	2.006816	2.002282
2.038191	2.033631	2.029062	2.024491	2.019937	2.015401	2.010862	2.006314	2.001771
2.037737	2.033182	2.028612	2.024038	2.019485	2.014953	2.010416	2.005865	2.001311
2.037264	2.03271	2.028142	2.023569	2.019017	2.014486	2.00995	2.005404	2.000848
2.036723	2.032162	2.0276	2.023042	2.018495	2.013958	2.009425	2.004895	2.000359
2.036186	2.031619	2.027062	2.022517	2.017973	2.013431	2.008899	2.004387	1.999873
2.03572	2.031158	2.0266	2.022051	2.017505	2.012962	2.013901	2.009353	2.004802
2.035292	2.030738	2.026175	2.021613	2.017062	2.012519	2.01344	2.008896	2.004349
2.034869	2.030311	2.025742	2.021171	2.016605	2.012043	2.012934	2.008386	2.003841
2.034458	2.029884	2.025308	2.020729	2.016142	2.011542	2.012391	2.007831	2.003286
2.034033	2.029448	2.024859	2.020268	2.021158	2.016506	2.011889	2.007325	2.00278
2.033593	2.029002	2.024394	2.019777	2.020655	2.016036	2.011443	2.006885	2.002341
2.033144	2.028541	2.02392	2.019293	2.020168	2.015584	2.011016	2.006463	2.001917
2.032677	2.028041	2.023427	2.018829	2.019719	2.015155	2.010598	2.00604	2.001488
2.032217	2.02755	2.022947	2.018384	2.019288	2.014736	2.010184	2.005622	2.001065
2.031812	2.027154	2.028058	2.023444	2.01886	2.014308	2.009759	2.005204	2.000654
2.031437	2.026811	2.027692	2.023043	2.018437	2.013876	2.009327	2.004787	2.00025
2.03102	2.026399	2.027268	2.022617	2.018012	2.013452	2.008905	2.004367	1.999832
2.030562	2.025916	2.026783	2.022167	2.017583	2.013034	2.00849	2.003943	1.999401
2.035678	2.030983	2.026342	2.021737	2.017162	2.012622	2.008081	2.003527	1.998978
2.035304	2.030635	2.025978	2.021332	2.016741	2.012206	2.00767	2.003113	1.998561
2.034937	2.0303	2.025634	2.020957	2.016352	2.011818	2.007283	2.002727	1.998173
2.034536	2.029927	2.02529	2.020643	2.016046	2.011501	2.006958	2.00241	1.997854
2.034129	2.02954	2.024942	2.020343	2.015765	2.011205	2.006652	2.002114	1.997559
2.033751	2.029137	2.024546	2.019971	2.015405	2.010848	2.006297	2.001762	1.99721
2.033391	2.028723	2.024117	2.01955	2.014994	2.010451	2.005911	2.001373	1.996826
2.033038	2.028353	2.023728	2.019143	2.014582	2.010049	2.005517	2.000977	1.996434
2.032694	2.028035	2.023385	2.018744	2.01416	2.009634	2.005108	2.000567	1.996026
2.032337	2.02771	2.023051	2.018379	2.013779	2.009253	2.004729	2.000188	1.995647
2.031942	2.027352	2.022718	2.018066	2.013474	2.008944	2.004414	1.999877	1.995329
2.031556	2.026996	2.022392	2.017773	2.01319	2.008644	2.004104	1.99957	1.995021
2.031234	2.026676	2.022085	2.017482	2.012887	2.008298	2.00373	1.999203	1.994669
2.03094	2.026366	2.021781	2.01719	2.012578	2.007938	2.003339	1.998819	1.994307
2.030615	2.026011	2.021427	2.016856	2.012251	2.007608	2.003009	1.998496	1.993993
2.030268	2.025617	2.021031	2.016485	2.011909	2.007301	2.002728	1.998223	1.99372
2.029953	2.025269	2.02068	2.016148	2.011591	2.00701	2.002454	1.997948	1.993439
2.029682	2.024982	2.020388	2.015857	2.011306	2.006739	2.002188	1.99767	1.993152
2.029408	2.024711	2.020116	2.015583	2.011032	2.006469	2.001915	1.997374	1.992838
2.029099	2.02444	2.019857	2.01532	2.010764	2.006191	2.001616	1.997033	1.992463
2.028787	2.024173	2.019606	2.015065	2.0105	2.005913	2.001314	1.996688	1.992083
2.0285	2.023906	2.019345	2.014803	2.010235	2.005643	2.001034	1.996388	1.991768
2.028227	2.023639	2.019081	2.014539	2.00997	2.005376	2.000765	1.996115	1.991491
2.027957	2.023373	2.018815	2.014272	2.009703	2.005111	2.000499	1.995844	1.991215
2.027692	2.023107	2.018548	2.014003	2.009434	2.004846	2.000235	1.995576	1.990945
2.027425	2.02284	2.018281	2.013735	2.009167	2.004579	1.999969	1.99531	1.990678
2.02715	2.022571	2.018013	2.013468	2.0089	2.00431	1.999699	1.995042	1.990413

2.026886	2.022302	2.017744	2.013202	2.008634	2.004042	1.999432	1.994781	1.990155
2.026654	2.022033	2.017466	2.012931	2.008368	2.003779	1.999172	1.994526	1.989902
2.026436	2.021771	2.017191	2.012662	2.008103	2.00352	1.998919	1.994284	1.989667
2.026204	2.021521	2.016929	2.012392	2.007834	2.003261	2.002547	1.997894	1.993254
2.025966	2.021285	2.016681	2.012126	2.007566	2.003004	2.002306	1.997703	1.993103
2.02575	2.021092	2.016486	2.011914	2.007341	2.002767	2.00206	1.99748	1.992903
2.025562	2.020952	2.016356	2.011767	2.007166	2.002548	2.001803	1.997216	1.992644
2.025359	2.020785	2.016197	2.011601	2.006988	2.002352	2.00158	1.996982	1.992404
2.025114	2.020546	2.015966	2.011383	2.006793	2.002196	2.001428	1.996815	1.992212
2.02488	2.020305	2.015727	2.011153	2.006588	2.002033	2.001277	1.996655	1.992032
2.024712	2.020126	2.015527	2.010927	2.010255	2.005656	2.001057	1.99645	1.991842
2.024573	2.01997	2.015344	2.010709	2.010008	2.005405	2.000813	1.996233	1.991653
2.024414	2.019792	2.015161	2.010529	2.009816	2.005209	2.00062	1.996052	1.991485
2.024241	2.0196	2.014982	2.010382	2.009667	2.005056	2.000464	1.995899	1.991334
2.02407	2.019427	2.01482	2.010237	2.00951	2.004883	2.000279	1.995709	1.991145
2.023897	2.019279	2.01468	2.010095	2.009341	2.00468	2.000048	1.995464	1.990896
2.023739	2.019144	2.014544	2.009943	2.009164	2.004484	1.999836	1.995242	1.990672
2.023609	2.01902	2.014397	2.009757	2.008965	2.004304	1.999674	1.995086	1.990519
2.023494	2.018906	2.01426	2.009584	2.008781	2.004146	1.999536	1.994958	1.990393
2.02339	2.018801	2.018066	2.013357	2.008681	2.004033	1.999789	1.995195	1.990616
2.023293	2.018701	2.017979	2.0133	2.008625	2.003949	1.999685	1.995081	1.990493
2.023186	2.018593	2.017862	2.013181	2.008505	2.003831	1.999572	1.994966	1.990371
2.02307	2.018476	2.017716	2.013005	2.008329	2.003684	1.99945	1.994851	1.99025
2.022957	2.018364	2.017589	2.012867	2.008191	2.003557	1.999336	1.994742	1.990141
2.022851	2.018261	2.017497	2.012801	2.008126	2.003467	1.999236	1.99464	1.990048
2.022743	2.018155	2.017398	2.012723	2.008049	2.003373	1.999135	1.99454	1.989958
2.022624	2.018032	2.017252	2.01256	2.007889	2.003236	1.999023	1.994437	1.989865
2.022502	2.017907	2.017102	2.012386	2.007718	2.003094	1.998908	1.994332	1.989767
2.022386	2.017804	2.017004	2.012302	2.007632	2.002988	1.998793	1.994217	1.989655
2.022275	2.017715	2.016939	2.012276	2.007601	2.00291	1.998683	1.994097	1.989532
2.022194	2.017619	2.016852	2.012221	2.007559	2.002861	1.998626	1.994012	1.989423
2.022144	2.017512	2.016735	2.012137	2.007509	2.002851	1.998629	1.993964	1.989322
2.025987	2.021281	2.016644	2.012049	2.007892	2.003264	1.998616	1.993928	1.989256
2.025957	2.02127	2.016602	2.011949	2.007782	2.003179	1.998561	1.993903	1.989247
2.025916	2.021261	2.016572	2.011866	2.007683	2.003088	1.99849	1.993875	1.989249
2.025823	2.021199	2.01653	2.011839	2.007651	2.003017	1.998402	1.993814	1.98922
2.02571	2.021114	2.016482	2.011835	2.00765	2.002961	1.998315	1.993748	1.989184
2.025629	2.021017	2.016404	2.011795	2.007631	2.002939	1.998291	1.993728	1.989173
2.025569	2.020908	2.016303	2.011732	2.007605	2.002947	1.998318	1.993744	1.989179
2.025551	2.020854	2.016246	2.011694	2.007591	2.002961	1.998342	1.993742	1.989153
2.025589	2.020866	2.016247	2.01169	2.0076	2.002989	1.998365	1.993715	1.989079
2.02562	2.02089	2.016254	2.011674	2.007587	2.002993	1.998372	1.993691	1.989027
2.02561	2.020899	2.016239	2.01161	2.007513	2.002937	1.998338	1.99368	1.989035
2.025597	2.020914	2.016229	2.011549	2.007439	2.002877	1.998301	1.993679	1.989062
2.02369	2.019029	2.016786	2.012066	2.007426	2.002869	1.998299	1.99369	1.989076
2.023718	2.019065	2.016825	2.012096	2.00745	2.002891	1.998319	1.993712	1.989091
2.023751	2.019103	2.01687	2.012136	2.007487	2.002927	2.001755	1.997146	1.992518
2.023793	2.019145	2.016922	2.012189	2.007541	2.00298	2.001803	1.997195	1.992565
2.023833	2.019186	2.016972	2.01224	2.010998	2.006429	2.001849	1.997241	1.99261
2.02387	2.019223	2.017017	2.012286	2.01104	2.006471	2.001891	1.997283	1.992652

2.024606	2.019946	2.015244	2.010522	2.011082	2.006513	2.001933	1.997325	1.992694
2.024653	2.019994	2.015291	2.010568	2.011128	2.006558	2.001978	1.997369	1.992738
2.024703	2.020042	2.015338	2.010615	2.011174	2.006604	2.002023	1.997415	1.992784
2.02475	2.020089	2.018923	2.014191	2.011216	2.006647	2.002068	1.997462	1.992834
2.024796	2.020134	2.018964	2.014231	2.011258	2.006689	2.002112	1.997509	1.992886
2.028382	2.02372	2.019011	2.01428	2.011308	2.006731	2.002152	1.997555	1.992944
2.028417	2.023763	2.019061	2.014336	2.011362	2.006768	2.002183	1.997596	1.993007
2.028474	2.023817	2.019127	2.014425	2.009773	2.005171	2.002252	1.99766	1.993076
2.028572	2.023887	2.019221	2.01457	2.009938	2.005326	2.002388	1.997761	1.993145
2.028675	2.023963	2.019316	2.014712	2.0101	2.005484	2.002532	1.997877	1.993235
[truncated: 917,907 more chars]
