# Supplementary material for: Artefact-free topography based scleral-asymmetry
Source: PLoS One. 2019 Jul 26;14(7):e0219789. doi: 10.1371/journal.pone.0219789 (PMC6660081; doi:10.1371/journal.pone.0219789)
Supplement: S3 Table — (ZIP) [file Eye std OS.pdf › Eye std OS.pdf]

0.559745	0.557481
0.55958	0.557296
0.559411	0.557125
0.559245	0.556965
0.559078	0.556804
0.558912	0.556643
0.558745	0.556482
0.558564	0.556315
0.558407	0.556158
0.558327	0.556027
0.55828	0.555911
0.558168	0.555783
0.558006	0.555649
0.557837	0.555495
0.557662	0.555315
0.557487	0.555136
0.557312	0.554958
0.557149	0.554804
0.759917	0.756569
0.759969	0.756636
0.759939	0.75663
0.75983	0.756555
0.759748	0.756475
0.759723	0.756396

		0.759699	0.756329
		0.5562	0.553845
		0.556021	0.55368
		0.555885	0.553592
		0.555774	0.553554
		0.555682	0.553456
		0.555615	0.553282
		0.555531	0.553148
		0.555399	0.553111
		0.555274	0.553077
0.535234	0.533572	0.924923	0.921415
0.535101	0.533496	0.924868	0.921297
0.534969	0.533353	0.924742	0.921132
0.534841	0.533161	0.924563	0.920927
0.534709	0.533012	0.924439	0.920778
0.534573	0.532929	0.924397	0.920706
0.534432	0.532823	0.924336	0.920639
0.534283	0.532634	0.924202	0.920554
0.534131	0.532434	0.924053	0.920456
0.533979	0.532307	0.923941	0.920329
0.533824	0.53221	0.923843	0.920184
0.533663	0.532056	0.755944	0.753096
0.533494	0.531848	0.755845	0.752975
0.533354	0.531678	0.755741	0.752862
0.533264	0.531571	0.755617	0.752764
0.533166	0.531476	0.755518	0.752675
0.533016	0.531376	0.755487	0.752592
0.532849	0.531266	0.75548	0.752522
0.532694	0.531103	0.755438	0.752482
0.532545	0.530906	0.755376	0.752463
0.532427	0.530762	0.755291	0.752415
0.532345	0.530677	0.755173	0.752338
0.53224	0.530586	0.755082	0.752258
0.532078	0.530464	0.755038	0.752174
0.531918	0.530346	0.755021	0.752106
0.531816	0.530261	0.755033	0.752081
0.531734	0.530183	0.755059	0.752081
0.531622	0.530054	0.755052	0.752085
0.531492	0.529886	0.755021	0.752098
0.531383	0.529757	0.754984	0.752105
0.531304	0.529679	0.754935	0.752102
0.531225	0.529601	0.7549	0.752103
0.531133	0.5295	0.754901	0.75212
0.531038	0.529394	0.754905	0.752131
0.530942	0.529292	0.754877	0.752109
0.530848	0.529193	0.754836	0.752069
0.53076	0.529103	0.754818	0.752034
0.53068	0.529023	0.754819	0.752001
0.530599	0.528942	0.754827	0.751989
0.530517	0.528859	0.754843	0.75201

0.530433	0.528774	0.754858	0.752034
0.530344	0.528683	0.754864	0.752045
0.530254	0.528592	0.754867	0.752053
0.53017	0.528513	0.754866	0.752052
0.530088	0.52844	0.754863	0.752048
0.529992	0.52835	0.754876	0.752065
0.529879	0.528241	0.754907	0.752105
0.529773	0.528136	0.754937	0.752157
0.529681	0.528044	0.754965	0.752229
0.529596	0.527956	0.754984	0.752288
0.529513	0.527862	0.754974	0.752294
0.529438	0.527774	0.754957	0.752277
0.52938	0.527731	0.754973	0.752285
0.529337	0.527721	0.755012	0.752308
0.52927	0.527674	0.75506	0.75234
0.52917	0.527577	0.755121	0.752388
0.529086	0.527483	0.755163	0.752424
0.529047	0.527414	0.755158	0.752427
0.529013	0.527348	0.755152	0.752433
0.528945	0.527265	0.75519	0.752477
0.528864	0.527181	0.755254	0.752545
0.528805	0.52714	0.755336	0.752633
0.528763	0.527139	0.755437	0.752743
		0.554405	0.552202
		0.554606	0.552398
		0.554804	0.552577
		0.554969	0.552706
		0.555126	0.552836
		0.555293	0.553052
		0.555467	0.553317
		0.555669	0.553524
		0.555905	0.553672
		0.556108	0.553828
		0.556241	0.554007
		0.556384	0.554194
		0.556611	0.554382
		0.556861	0.554575
		0.55704	0.554787
		0.557172	0.555011
		0.55735	0.555188
		0.557589	0.555305
		0.557805	0.555443
		0.557952	0.55565
		0.558096	0.55586
		0.558292	0.555998
		0.558518	0.556116
0.540497	0.538208	0.765757	0.762374
0.54051	0.538214	0.765989	0.762632
0.540551	0.538239	0.766187	0.76288
0.540628	0.538291	0.76633	0.763116

0.540722	0.538365	0.766493	0.763331
0.540842	0.538469	0.766726	0.763506
0.540961	0.538585	0.766984	0.763673
0.541031	0.538682	0.767237	0.76387
0.541072	0.53877	0.767492	0.764086
0.541125	0.538856	0.76774	0.764327
0.541186	0.538938	0.767985	0.764596
0.541266	0.539026	0.53675	0.534436
0.541378	0.539132	0.536856	0.534551
0.541487	0.539221	0.53694	0.534654
0.541561	0.539252	0.536966	0.534726
0.541624	0.539261	0.536969	0.534784
0.541705	0.539294	0.536992	0.53485
0.541799	0.539341	0.537028	0.53492
0.541906	0.539413	0.537089	0.535002
0.542025	0.539513	0.537181	0.535097
0.542153	0.539621	0.537279	0.535197
0.542284	0.539729	0.537375	0.535299
0.542425	0.539844	0.537479	0.535405
0.542575	0.539966	0.537586	0.535514
0.542739	0.540104	0.537708	0.535632
0.542934	0.540296	0.537885	0.535776
0.543159	0.540536	0.538111	0.535945
0.543375	0.540764	0.538325	0.536109
0.543578	0.540975	0.538523	0.536264

0.530433	0.528774	0.754858	0.752034
0.530344	0.528683	0.754864	0.752045
0.530254	0.528592	0.754867	0.752053
0.53017	0.528513	0.754866	0.752052
0.530088	0.52844	0.754863	0.752048
0.529992	0.52835	0.754876	0.752065
0.529879	0.528241	0.754907	0.752105
0.529773	0.528136	0.754937	0.752157
0.529681	0.528044	0.754965	0.752229
0.529596	0.527956	0.754984	0.752288
0.529513	0.527862	0.754974	0.752294
0.529438	0.527774	0.754957	0.752277
0.52938	0.527731	0.754973	0.752285
0.529337	0.527721	0.755012	0.752308
0.52927	0.527674	0.75506	0.75234
0.52917	0.527577	0.755121	0.752388
0.529086	0.527483	0.755163	0.752424
0.529047	0.527414	0.755158	0.752427
0.529013	0.527348	0.755152	0.752433
0.528945	0.527265	0.75519	0.752477
0.528864	0.527181	0.755254	0.752545
0.528805	0.52714	0.755336	0.752633
0.528763	0.527139	0.755437	0.752743
		0.554405	0.552202
		0.554606	0.552398
		0.554804	0.552577
		0.554969	0.552706
		0.555126	0.552836
		0.555293	0.553052
		0.555467	0.553317
		0.555669	0.553524
		0.555905	0.553672
		0.556108	0.553828
		0.556241	0.554007
		0.556384	0.554194
		0.556611	0.554382
		0.556861	0.554575
		0.55704	0.554787
		0.557172	0.555011
		0.55735	0.555188
		0.557589	0.555305
		0.557805	0.555443
		0.557952	0.55565
		0.558096	0.55586
		0.558292	0.555998
		0.558518	0.556116
0.540497	0.538208	0.765757	0.762374
0.54051	0.538214	0.765989	0.762632
0.540551	0.538239	0.766187	0.76288
0.540628	0.538291	0.76633	0.763116

0.540722	0.538365	0.766493	0.763331
0.540842	0.538469	0.766726	0.763506
0.540961	0.538585	0.766984	0.763673
0.541031	0.538682	0.767237	0.76387
0.541072	0.53877	0.767492	0.764086
0.541125	0.538856	0.76774	0.764327
0.541186	0.538938	0.767985	0.764596
0.541266	0.539026	0.53675	0.534436
0.541378	0.539132	0.536856	0.534551
0.541487	0.539221	0.53694	0.534654
0.541561	0.539252	0.536966	0.534726
0.541624	0.539261	0.536969	0.534784
0.541705	0.539294	0.536992	0.53485
0.541799	0.539341	0.537028	0.53492
0.541906	0.539413	0.537089	0.535002
0.542025	0.539513	0.537181	0.535097
0.542153	0.539621	0.537279	0.535197
0.542284	0.539729	0.537375	0.535299
0.542425	0.539844	0.537479	0.535405
0.542575	0.539966	0.537586	0.535514
0.542739	0.540104	0.537708	0.535632
0.542934	0.540296	0.537885	0.535776
0.543159	0.540536	0.538111	0.535945
0.543375	0.540764	0.538325	0.536109
0.543578	0.540975	0.538523	0.536264

				0.507569	0.504883
				0.507349	0.504675
				0.507144	0.504493
				0.506921	0.504282
				0.506701	0.50407
				0.904242	0.900593
				0.903821	0.900165
				0.903404	0.899745
				0.902988	0.899334
				0.902575	0.898926
				0.902153	0.89851
				0.901746	0.898103
				0.901404	0.897728
				0.901105	0.897378
				0.900774	0.897049
				0.900413	0.896748
		0.907077	0.903563	1.011301	1.007491
		0.90666	0.903155	1.010865	1.007047
		0.906249	0.902746	1.010421	1.0066
		0.905918	0.902366	1.295559	1.291094
		0.905614	0.902003	1.294979	1.290554
		0.90527	0.901671	1.294467	1.290037
		0.904898	0.901364	1.29401	1.289537
		0.90451	0.901032	1.293541	1.289019
		0.904092	0.900671	1.293063	1.28848
		0.903712	0.900302	1.292555	1.28794
		0.903418	0.899939	1.291999	1.287412
		0.903139	0.899564	1.291419	1.286883
		0.902806	0.899142	1.290809	1.286334
		0.902447	0.898708	1.290196	1.28578
		0.902076	0.898374	1.289703	1.285269
		0.90169	0.898129	1.289318	1.284794
		0.901368	0.897842	1.288878	1.28431
		0.901158	0.897488	1.288343	1.283806
1.121933	1.118125	1.296976	1.292376	1.445452	1.440457
1.121633	1.117736	1.296471	1.291881	1.444899	1.439909
1.12135	1.117322	1.295939	1.291383	1.444349	1.439361
1.120962	1.116929	1.295455	1.2909	1.443782	1.43879
1.120522	1.116546	1.294992	1.290422	1.443206	1.438209
1.120177	1.116184	1.294503	1.289905	1.442629	1.437665
1.11993	1.115841	1.293981	1.289336	1.442045	1.437154
1.119648	1.115535	1.293537	1.28886	1.441527	1.436675
1.11928	1.115294	1.293239	1.288542	1.441098	1.436222

			1.11892	1.115037	1.29294	1.288258	1.440729	1.43581
			1.118643	1.114679	1.292511	1.28794	1.440438	1.435469
			1.118388	1.114292	1.292045	1.287605	1.440168	1.435162
			1.118061	1.113993	1.291653	1.287208	1.439767	1.43482
			1.117685	1.113751	1.291308	1.286764	1.439274	1.434458
			1.117353	1.113445	1.290909	1.286351	1.438825	1.434064
			1.117067	1.113053	1.290439	1.28598	1.438434	1.433633
			1.116816	1.112709	1.289992	1.285589	1.438016	1.433192
			1.116628	1.112481	1.289595	1.285131	1.437519	1.432745
			1.11643	1.112286	1.28924	1.284689	1.437024	1.432305
0.50853	0.507019	0.505473	1.203254	1.198964	1.36378	1.358901	1.436598	1.431891
0.508288	0.506793	0.50526	1.20286	1.198692	1.36353	1.358608	1.436216	1.4315
0.50806	0.506551	0.505015	1.202497	1.19833	1.36316	1.358287	1.435887	1.431145
0.507844	0.506291	0.504737	1.202154	1.197888	1.362693	1.35795	1.435613	1.430825
0.507625	0.506055	0.504491	1.201841	1.197495	1.362257	1.357593	1.43529	1.430491
0.507405	0.505859	0.504296	1.20157	1.197172	1.361866	1.357209	1.434899	1.430131
0.507175	0.50565	0.504091	1.201289	1.196876	1.361509	1.356826	1.434493	1.429775
0.506917	0.505394	0.503839	1.200965	1.196597	1.361198	1.356456	1.43409	1.429442
0.506658	0.505127	0.503575	1.200624	1.196329	1.360915	1.356103	1.433691	1.42911
0.506434	0.50489	0.503332	1.200284	1.196058	1.360642	1.355791	1.433316	1.428746
0.506231	0.504666	0.503095	1.199941	1.195782	1.360377	1.355508	1.432961	1.428369
			1.011322	1.007562	1.289377	1.284614	1.43264	1.42804
			1.011064	1.007248	1.288994	1.284314	1.432354	1.427758
			1.010841	1.00698	1.288647	1.284032	1.432076	1.427477
			1.010669	1.006797	1.288362	1.283777	1.431799	1.427187
			1.010511	1.006636	1.288102	1.283537	1.431529	1.426897
0.92853	0.925268	0.921984	1.129373	1.12519	1.287856	1.28331	1.497019	1.491964
0.928165	0.924949	0.921716	1.129187	1.12501	1.287622	1.28309	1.496743	1.49166
0.927854	0.92466	0.921448	1.128988	1.124817	1.287379	1.282863	1.496468	1.491383
0.927578	0.92439	0.921179	1.128779	1.124617	1.287132	1.282633	1.496197	1.491129
0.927308	0.924123	0.920913	1.128572	1.124419	1.2869	1.282419	1.495937	1.490879
0.927045	0.92386	0.92065	1.128367	1.124224	1.286682	1.282224	1.495689	1.490633
0.926783	0.923596	0.920385	1.128162	1.124029	1.286468	1.282032	1.495447	1.490394
0.92652	0.923331	0.920117	1.127955	1.123833	1.286255	1.281837	1.495213	1.490165
0.926257	0.923062	0.919845	1.127751	1.123636	1.286042	1.281641	1.49498	1.489941
0.925982	0.92278	0.919564	1.127556	1.123441	1.285826	1.281439	1.494743	1.489712
0.925713	0.922498	0.91928	1.127366	1.123248	1.285611	1.281234	1.494506	1.489486
0.925507	0.922255	0.919003	1.034403	1.030438	1.130391	1.126359	1.356151	1.351499
0.925355	0.922045	0.918729	1.034203	1.030265	1.130257	1.126226	1.355964	1.351358
0.925169	0.921839	0.9185	1.034037	1.030095	1.227847	1.223266	1.355778	1.351211
0.924936	0.92164	0.918336	1.033919	1.029919	1.227672	1.223108	1.355586	1.351053
0.924683	0.92142	0.918152	1.033805	1.029775	1.227533	1.222981	1.355416	1.350897
0.924402	0.921145	0.917895	1.033681	1.029707	1.227474	1.222926	1.355294	1.350745
0.924127	0.92086	0.917612	1.033545	1.029647	1.227433	1.222887	1.355186	1.350604
1.045409	1.041346	1.037312	1.033369	1.0295	1.227334	1.222799	1.355066	1.3505
1.045311	1.041208	1.037136	1.033179	1.029313	1.227209	1.222686	1.354937	1.350414
1.045178	1.041093	1.037032	1.033073	1.029196	1.227103	1.22256	1.354761	1.350272
1.045016	1.041005	1.037005	1.033055	1.029149	1.227012	1.222417	1.354536	1.350075
1.044828	1.040871	1.036918	1.1451	1.140604	1.226924	1.222311	1.35436	1.349909
1.044595	1.040652	1.03672	1.144943	1.14046	1.226845	1.222264	1.354267	1.349799

1.04439	1.040446	1.036518	1.144769	1.140295	1.22675	1.222228	1.354196	1.349704
0.922297	0.919034	0.915771	1.144593	1.140088	1.2266	1.222171	1.35413	1.349608
0.922139	0.918874	0.915603	1.144418	1.139888	1.22645	1.22211	1.354059	1.349511
0.921979	0.91867	0.915363	1.144265	1.139802	1.226404	1.222045	1.353931	1.349392
0.921819	0.918441	0.915082	1.144131	1.139796	1.226427	1.221979	1.353766	1.349259
0.921605	0.918234	0.914893	1.144052	1.139756	1.226408	1.221925	1.289185	1.28483
0.921318	0.91805	0.914816	1.144041	1.139677	1.226338	1.221881	1.289111	1.284756
0.921094	0.917881	0.914699	1.144008	1.139603	1.226285	1.221852	1.289042	1.284672
0.921016	0.917742	0.914477	1.143916	1.13955	1.226276	1.22185	1.288974	1.284565
0.920939	0.917594	0.914241	1.143814	1.139502	1.226272	1.221843	1.288896	1.284452
1.052861	1.048953	1.045058	1.041234	1.037468	1.297961	1.293324	1.440694	1.435857
1.052677	1.048764	1.044879	1.041092	1.037381	1.297873	1.293207	1.440514	1.435699
1.052496	1.0486	1.044739	1.040951	1.037225	1.297767	1.293134	1.440423	1.435595
1.052312	1.048453	1.044631	1.040812	1.037017	1.297644	1.293094	1.440403	1.435535
1.05215	1.048285	1.044467	1.040649	1.036857	1.467171	1.461865	1.581161	1.575605
1.052009	1.048078	1.044215	1.040448	1.036766	1.467085	1.46174	1.580972	1.575421
1.051892	1.047907	1.044	1.040267	1.036667	1.40278	1.397736	1.580803	1.575257
1.051824	1.047828	1.043896	1.040138	1.036513	1.402585	1.397561	1.580708	1.575154
1.051753	1.047767	1.043827	1.040034	1.036359	1.402386	1.397384	1.580624	1.575066
1.051601	1.047648	1.043735	1.039948	1.036263	1.402214	1.397201	1.580486	1.574953
1.05141	1.047502	1.043637	1.039874	1.036193	1.402054	1.397013	1.580326	1.574831
0.750295	0.747562	0.744891	0.905729	0.902335	1.401881	1.396832	1.580202	1.574713
0.750179	0.747447	0.744784	0.905601	0.902173	1.401696	1.396658	1.580111	1.574599
0.750073	0.747345	0.744689	0.905501	0.902043	1.401512	1.396476	1.579991	1.574473
0.749984	0.74727	0.74462	0.905451	0.901962	1.401319	1.396277	1.579818	1.574328
0.749895	0.747193	0.744552	0.90539	0.901899	1.401158	1.396095	1.579655	1.574189
0.749783	0.747089	0.744472	0.90527	0.901848	1.401071	1.395956	1.525667	1.520463
0.74968	0.746985	0.744388	0.905134	0.901803	1.401018	1.395845	1.525512	1.520309
0.749641	0.74692	0.744298	1.028918	1.025233	1.466797	1.461388	1.568991	1.563362
0.749646	0.746883	0.744206	1.028838	1.025154	1.466722	1.461314	1.568926	1.563239
0.749621	0.746851	0.744154	1.028762	1.025043	1.466606	1.461246	1.568895	1.563165
0.749568	0.746826	0.744145	1.028688	1.024887	1.46644	1.461188	1.568912	1.563147
0.749498	0.74679	0.74414	1.028638	1.024774	1.466299	1.461108	1.620669	1.614593
0.749401	0.746733	0.744134	1.028642	1.024745	1.466213	1.460987	1.620501	1.614489
0.749311	0.746671	0.744115	1.028623	1.024732	1.466146	1.460859	1.620305	1.614369
0.74926	0.74661	0.744055	1.028492	1.024682	1.393306	1.388111	1.620128	1.614245
0.749236	0.746555	0.743976	1.028319	1.024619	1.393238	1.388007	1.619966	1.614118
0.749241	0.746525	0.743909	1.028208	1.024551	1.393181	1.387935	1.61987	1.614005
0.749274	0.746518	0.743848	1.028139	1.024476	1.393134	1.38789	1.61983	1.613902
0.749303	0.746527	0.743821	1.028094	1.02442	1.206364	1.202047	1.4886	1.483181
0.749327	0.746554	0.743835	1.028078	1.024391	1.206347	1.20206	1.488611	1.483162
0.749349	0.746582	0.743856	1.028058	1.024344	1.20631	1.202062	1.488625	1.483161
0.749377	0.746608	0.743874	1.02801	1.024244	1.20623	1.202045	1.488651	1.48319
0.749394	0.746626	0.743891	1.027962	1.024141	1.206139	1.202015	1.48866	1.48322
0.90388	0.900762	0.897652	1.239345	1.234938	1.430401	1.425286	1.420137	1.414847
0.90391	0.900811	0.897713	1.239351	1.234909	1.430283	1.425178	1.420058	1.414822
0.903938	0.900854	0.897776	1.239371	1.234913	1.430228	1.425119	1.53662	1.531214
0.903961	0.900891	0.89784	1.239406	1.234949	1.430234	1.425102	1.536571	1.531191
0.904	0.900935	0.897898	1.239433	1.234977	1.430239	1.425095	1.536539	1.53118
0.904066	0.900992	0.897946	1.239448	1.234991	1.430234	1.425099	1.536529	1.531185

0.904132	0.901047	0.89799	1.239459	1.235	1.430225	1.4251	1.536519	1.531187
0.904184	0.90109	0.898026	1.239461	1.234994	1.430192	1.425082	1.536493	1.531168
0.90423	0.901131	0.898061	1.239467	1.234997	1.430169	1.425064	1.536464	1.531141
0.904272	0.901172	0.898105	1.239505	1.235065	1.430211	1.425069	1.536436	1.531112
0.904311	0.901215	0.898152	1.239562	1.235173	1.430297	1.425093	1.536414	1.531083
0.904372	0.901262	0.898189	1.239597	1.235247	1.430383	1.42515	1.536442	1.531085
0.904453	0.901312	0.898214	1.239609	1.23529	1.430476	1.425247	1.536527	1.531121
0.904545	0.901373	0.898244	1.233497	1.22931	1.375478	1.370619	1.536595	1.531165
0.904653	0.90145	0.898283	1.233474	1.229295	1.37546	1.37063	1.536625	1.53122
0.904753	0.901528	0.89833	1.233463	1.229273	1.375424	1.370621	1.536632	1.531268
0.904819	0.901592	0.898388	1.23349	1.229259	1.375371	1.370573	1.536603	1.531287
0.90487	0.901653	0.898455	1.23354	1.229255	1.375319	1.37052	1.536567	1.531295
1.014877	1.01138	1.007908	1.304241	1.299761	1.375326	1.370545	1.536596	1.531319
1.014913	1.011439	1.007986	1.304301	1.299811	1.375381	1.37063	1.536674	1.531355
1.01497	1.011502	1.008057	1.304359	1.299872	1.375459	1.370712	1.536753	1.531416
1.015058	1.011574	1.008123	1.304417	1.29995	1.375572	1.370792	1.536833	1.531507
1.125863	1.121847	1.117874	1.304464	1.300019	1.375665	1.37086	1.536917	1.531609
1.125848	1.121839	1.117884	1.304494	1.300073	1.37572	1.370911	1.537015	1.531724
1.125824	1.121825	1.117893	1.304513	1.300105	1.375741	1.370942	1.537099	1.53183
1.125841	1.121842	1.117913	1.304523	1.300088	1.375703	1.370927	1.537121	1.531885
1.125883	1.121877	1.117941	1.304529	1.300046	1.375635	1.370891	1.537114	1.531913
1.125949	1.121936	1.117985	1.304559	1.300049	1.375624	1.37091	1.53715	1.531945
1.126041	1.12202	1.118046	1.304609	1.30009	1.375659	1.370975	1.537219	1.531977
0.905709	0.902328	0.898973	1.141127	1.137081	1.295727	1.291416	1.537297	1.532055
0.905737	0.902338	0.898974	1.141174	1.137158	1.295804	1.291486	1.537377	1.532194
0.905774	0.902365	0.898996	1.14123	1.137232	1.295884	1.291568	1.537484	1.532351
0.905844	0.90244	0.899071	1.141295	1.137271	1.295948	1.291661	1.537647	1.532514
0.905928	0.902535	0.899167	1.141373	1.13731	1.296013	1.291761	1.537825	1.532674
0.906024	0.902626	0.899249	1.141473	1.137398	1.2961	1.29186	1.537951	1.532795
0.906126	0.902717	0.899328	1.141589	1.137515	1.2962	1.29196	1.538052	1.532893
0.906187	0.902782	0.899402	1.141689	1.137611	1.430311	1.42579	1.634025	1.628697
0.906203	0.902819	0.899474	1.141774	1.137687	1.430335	1.425833	1.634147	1.628814
0.906239	0.902863	0.899534	1.14185	1.137767	1.430361	1.42587	1.634246	1.628925
0.906311	0.902916	0.899572	1.141912	1.137857	1.430388	1.425893	1.634294	1.629018
0.90641	0.902988	0.899614	1.141979	1.13795	1.430425	1.425923	1.634348	1.629108
0.906548	0.90309	0.899673	1.142058	1.138035	1.430486	1.425987	1.634456	1.629194
0.906705	0.903215	0.899757	1.142158	1.138127	1.430558	1.426062	1.634578	1.629283
0.757798	0.754649	0.751529	1.142325	1.138245	1.37317	1.368826	1.580925	1.575927
0.758005	0.754889	0.751779	1.142546	1.138386	1.373213	1.368849	1.580996	1.576073
0.758159	0.755068	0.751983	1.142729	1.13856	1.373332	1.368916	1.581084	1.576185
0.758245	0.755175	0.752137	1.142868	1.138784	1.37355	1.369035	1.581181	1.576247
0.75835	0.755281	0.752262	0.89704	0.893713	1.108354	1.104509	1.47271	1.468065
0.758525	0.755414	0.752345	0.897109	0.89379	1.10838	1.104597	1.472951	1.468231
0.758703	0.755548	0.752427	0.897168	0.893847	1.108384	1.104679	1.473197	1.46842
0.758811	0.755663	0.752565	0.897233	0.893917	1.108391	1.1047	1.473325	1.468598
0.7589	0.755776	0.752725	0.897301	0.893993	1.108399	1.104691	1.473395	1.468766
0.75908	0.755921	0.752843	0.749784	0.74676	1.187933	1.183627	1.420734	1.416289
0.759337	0.756094	0.752927	0.749899	0.746975	1.188051	1.183683	1.420804	1.416339
0.759605	0.756307	0.753069	0.750037	0.747147	1.188166	1.183782	1.420946	1.416436
0.759895	0.756581	0.753292	0.750203	0.747249	1.188278	1.183946	1.421192	1.416599

0.760144	0.756837	0.753532	0.750389	0.747358	1.188383	1.184101	1.421437	1.416789
0.760292	0.757016	0.753755	0.750592	0.747505	1.188466	1.184198	1.421623	1.417006
0.760412	0.757178	0.753984	0.750811	0.747676	1.188559	1.184284	1.42179	1.417233
0.76058	0.757382	0.754236	0.751054	0.747877	1.188719	1.184409	1.421961	1.41744
0.760775	0.757606	0.754497	0.751311	0.748102	1.188921	1.184554	1.422128	1.417634
0.761003	0.757818	0.754696	0.751515	0.748323	1.189128	1.18469	1.422289	1.417804
0.761266	0.758018	0.754828	0.751666	0.74854	1.189345	1.184818	1.422437	1.417946
0.532097	0.529739	0.527367	0.751834	0.748759	0.87631	0.872335	1.101236	1.097497
0.532212	0.529821	0.527414	0.752038	0.748983	0.876578	0.872601	1.101504	1.097719
0.532328	0.529916	0.527483	0.752257	0.749199	0.876842	0.872877	1.101788	1.097947
0.532441	0.530031	0.527585	0.752484	0.749386	0.877111	0.873153	1.102066	1.098179
0.532553	0.530156	0.527706	0.752723	0.74957	0.877388	0.873433	1.102349	1.098421
0.532662	0.530274	0.527823	0.752982	0.749797	0.877695	0.873722	1.102639	1.098687
0.532767	0.530391	0.527941	0.75326	0.750054	0.878024	0.874018	1.102936	1.098973
0.721108	0.717866	0.714486	0.886487	0.882404	0.878354	0.874309	1.103226	1.099255
0.721325	0.718098	0.714731	0.88685	0.882758	0.878683	0.874591	1.103506	1.099533
0.721545	0.718332	0.714978	0.887215	0.883116	0.879018	0.874883	1.103799	1.099817
0.721759	0.718563	0.715223	0.887583	0.883475	0.879358	0.875192	1.104116	1.100107
0.721976	0.718794	0.715468	0.887952	0.883839	0.879708	0.875514	1.104446	1.100406
0.722201	0.719027	0.715713	0.888318	0.884215	0.880077	0.875856	1.104783	1.100713
0.722431	0.719261	0.715957	0.88868	0.884596	0.880456	0.876212	1.105132	1.101035
0.722656	0.719489	0.716197	0.889033	0.884967	0.880827	0.876566	0.872533	0.868863
0.72288	0.719713	0.716434	0.889378	0.885329	0.881194	0.876922	0.872879	0.869206
0.723128	0.719948	0.716672	0.889723	0.885691	0.88156	0.877276	0.873226	0.869563
0.72341	0.720196	0.716907	0.890065	0.886052	0.881924	0.877624	0.87357	0.869939
0.723689	0.720455	0.717164	0.71382	0.710419	0.88229	0.877982	0.873927	0.870314
0.723942	0.720728	0.717462	0.714112	0.710686	0.882655	0.878356	0.874303	0.870671
0.724185	0.72101	0.717783	0.714425	0.710965	0.883028	0.878747	0.874696	0.871029
0.724421	0.7213	0.718121	0.71477	0.711287	0.883425	0.879159	0.875106	0.871403
0.724655	0.721596	0.718468	0.715129	0.711632	0.88384	0.879591	0.875537	0.871791
0.724929	0.721874	0.718745	0.715407	0.711913	1.102124	1.09774	1.093456	1.089337
0.725242	0.722134	0.71895	0.715603	0.712132	1.102407	1.09808	1.093813	1.08964
0.725571	0.722418	0.719184	0.715832	0.712384	1.102683	1.09838	1.094121	1.089926
0.725927	0.722745	0.719471	0.716119	0.712692	1.102943	1.09861	1.094343	1.090179
0.726268	0.723069	0.719776	0.716431	0.713027	1.103219	1.098839	1.094556	1.09043
0.726535	0.723341	0.720077	0.71676	0.713386	1.10355	1.099111	1.094799	1.090698
0.726799	0.723613	0.720394	0.717106	0.713758	1.103896	1.099404	1.095062	1.09097
0.727179	0.723977	0.720756	0.717462	0.71411	1.1042	1.099713	1.095362	1.091232
0.727639	0.724402	0.721151	0.717828	0.714451	1.104481	1.100038	1.095695	1.091488
0.728072	0.724802	0.721517	0.718177	0.714793	1.104788	1.100373	1.096032	1.091773
0.728482	0.725178	0.721852	0.718504	0.715133	1.105122	1.100717	1.096375	1.092088
0.537189	0.534826	0.532469	0.530138	0.527822	0.97988	0.97601	1.096725	1.092418
0.537359	0.535026	0.532722	0.530399	0.52807	0.980153	0.976289	1.097083	1.092767
0.537538	0.535236	0.532987	0.53067	0.528321	0.980438	0.976574	1.097448	1.093114
			0.530923	0.528587	0.980719	0.976839	1.097831	1.093437
			0.531171	0.528858	0.980998	0.977093	1.098213	1.093745
			0.531428	0.529118	0.981268	0.977324	1.098531	1.094027
			0.53169	0.529369	0.981529	0.977535	1.098796	1.094285
			0.53193	0.529592	0.981771	0.977753	1.099095	1.094571
			0.532134	0.529772	0.981988	0.977979	1.099442	1.094888

0.532355	0.529976	0.982201	0.978212	1.0998	1.095229
0.532625	0.53024	0.982406	0.978453	1.100168	1.095599
0.532913	0.530536	0.982627	0.978701	1.100533	1.095982
0.533193	0.530859	0.982916	0.978954	1.100856	1.096354
0.533473	0.531198	0.983237	0.979214	1.101165	1.096725
0.533745	0.531485	0.717695	0.714221	0.887959	0.884089
0.534011	0.531721	0.718101	0.714721	0.88859	0.884637
0.534272	0.531965	0.718499	0.715177	0.889189	0.885205
0.534526	0.532232	0.718894	0.715561	0.889735	0.885811
0.534779	0.532499	0.719288	0.715935	0.890246	0.886392
0.535028	0.532748	0.719681	0.716327	0.712996	0.70969
0.535288	0.532999	0.720078	0.71673	0.713402	0.710081
0.535597	0.533276	0.720481	0.717156	0.713842	0.710505
0.535942	0.533572	0.720893	0.717601	0.714307	0.710949
0.536254	0.533882	0.721357	0.718059	0.714763	0.711413
0.536531	0.534206	0.72188	0.718533	0.715212	0.711902
0.536814	0.534537	0.722392	0.718996	0.715648	0.712373
0.537111	0.534881	0.722867	0.719424	0.716051	0.7128
0.537421	0.535215	0.723336	0.719861	0.71647	0.713228
0.537746	0.535506	0.723818	0.720352	0.716962	0.713697
0.538085	0.535781	0.724303	0.720864	0.717486	0.714181
0.538437	0.536087	0.724764	0.721346	0.717975	0.71464
0.538803	0.536416	0.725209	0.721808	0.718443	0.715084
0.539177	0.536769	0.72571	0.722316	0.718951	0.715575
0.539565	0.537154	0.726286	0.722886	0.719514	0.716128
0.539933	0.537536	0.726865	0.723449	0.720065	0.716681
0.540253	0.537896	0.727424	0.723976	0.720573	0.717212
0.540569	0.538254	0.72797	0.724484	0.721061	0.717727
		0.536277	0.533861	0.721523	0.718201
		0.536648	0.534196	0.721974	0.718656
		0.537023	0.534553	0.722486	0.719163
		0.5374	0.534931	0.723054	0.719717
		0.537779	0.53531	0.723623	0.720272
		0.538159	0.535687	0.724185	0.720822
		0.538541	0.536066	0.724749	0.721375
		0.538928	0.536449	0.725315	0.721934
		0.539314	0.536834	0.725883	0.722497
		0.539692	0.537216	0.726457	0.723064
		0.540065	0.537597	0.727034	0.723634
				0.727608	0.724204
				0.728175	0.724773
				0.728766	0.725359
				0.729395	0.72597
				0.730044	0.726597
				0.537793	0.535446
				0.538217	0.535859
				0.538603	0.536246
				0.538956	0.53661

0.904132	0.901047	0.89799	1.239459	1.235	1.430225	1.4251	1.536519	1.531187
0.904184	0.90109	0.898026	1.239461	1.234994	1.430192	1.425082	1.536493	1.531168
0.90423	0.901131	0.898061	1.239467	1.234997	1.430169	1.425064	1.536464	1.531141
0.904272	0.901172	0.898105	1.239505	1.235065	1.430211	1.425069	1.536436	1.531112
0.904311	0.901215	0.898152	1.239562	1.235173	1.430297	1.425093	1.536414	1.531083
0.904372	0.901262	0.898189	1.239597	1.235247	1.430383	1.42515	1.536442	1.531085
0.904453	0.901312	0.898214	1.239609	1.23529	1.430476	1.425247	1.536527	1.531121
0.904545	0.901373	0.898244	1.233497	1.22931	1.375478	1.370619	1.536595	1.531165
0.904653	0.90145	0.898283	1.233474	1.229295	1.37546	1.37063	1.536625	1.53122
0.904753	0.901528	0.89833	1.233463	1.229273	1.375424	1.370621	1.536632	1.531268
0.904819	0.901592	0.898388	1.23349	1.229259	1.375371	1.370573	1.536603	1.531287
0.90487	0.901653	0.898455	1.23354	1.229255	1.375319	1.37052	1.536567	1.531295
1.014877	1.01138	1.007908	1.304241	1.299761	1.375326	1.370545	1.536596	1.531319
1.014913	1.011439	1.007986	1.304301	1.299811	1.375381	1.37063	1.536674	1.531355
1.01497	1.011502	1.008057	1.304359	1.299872	1.375459	1.370712	1.536753	1.531416
1.015058	1.011574	1.008123	1.304417	1.29995	1.375572	1.370792	1.536833	1.531507
1.125863	1.121847	1.117874	1.304464	1.300019	1.375665	1.37086	1.536917	1.531609
1.125848	1.121839	1.117884	1.304494	1.300073	1.37572	1.370911	1.537015	1.531724
1.125824	1.121825	1.117893	1.304513	1.300105	1.375741	1.370942	1.537099	1.53183
1.125841	1.121842	1.117913	1.304523	1.300088	1.375703	1.370927	1.537121	1.531885
1.125883	1.121877	1.117941	1.304529	1.300046	1.375635	1.370891	1.537114	1.531913
1.125949	1.121936	1.117985	1.304559	1.300049	1.375624	1.37091	1.53715	1.531945
1.126041	1.12202	1.118046	1.304609	1.30009	1.375659	1.370975	1.537219	1.531977
0.905709	0.902328	0.898973	1.141127	1.137081	1.295727	1.291416	1.537297	1.532055
0.905737	0.902338	0.898974	1.141174	1.137158	1.295804	1.291486	1.537377	1.532194
0.905774	0.902365	0.898996	1.14123	1.137232	1.295884	1.291568	1.537484	1.532351
0.905844	0.90244	0.899071	1.141295	1.137271	1.295948	1.291661	1.537647	1.532514
0.905928	0.902535	0.899167	1.141373	1.13731	1.296013	1.291761	1.537825	1.532674
0.906024	0.902626	0.899249	1.141473	1.137398	1.2961	1.29186	1.537951	1.532795
0.906126	0.902717	0.899328	1.141589	1.137515	1.2962	1.29196	1.538052	1.532893
0.906187	0.902782	0.899402	1.141689	1.137611	1.430311	1.42579	1.634025	1.628697
0.906203	0.902819	0.899474	1.141774	1.137687	1.430335	1.425833	1.634147	1.628814
0.906239	0.902863	0.899534	1.14185	1.137767	1.430361	1.42587	1.634246	1.628925
0.906311	0.902916	0.899572	1.141912	1.137857	1.430388	1.425893	1.634294	1.629018
0.90641	0.902988	0.899614	1.141979	1.13795	1.430425	1.425923	1.634348	1.629108
0.906548	0.90309	0.899673	1.142058	1.138035	1.430486	1.425987	1.634456	1.629194
0.906705	0.903215	0.899757	1.142158	1.138127	1.430558	1.426062	1.634578	1.629283
0.757798	0.754649	0.751529	1.142325	1.138245	1.37317	1.368826	1.580925	1.575927
0.758005	0.754889	0.751779	1.142546	1.138386	1.373213	1.368849	1.580996	1.576073
0.758159	0.755068	0.751983	1.142729	1.13856	1.373332	1.368916	1.581084	1.576185
0.758245	0.755175	0.752137	1.142868	1.138784	1.37355	1.369035	1.581181	1.576247
0.75835	0.755281	0.752262	0.89704	0.893713	1.108354	1.104509	1.47271	1.468065
0.758525	0.755414	0.752345	0.897109	0.89379	1.10838	1.104597	1.472951	1.468231
0.758703	0.755548	0.752427	0.897168	0.893847	1.108384	1.104679	1.473197	1.46842
0.758811	0.755663	0.752565	0.897233	0.893917	1.108391	1.1047	1.473325	1.468598
0.7589	0.755776	0.752725	0.897301	0.893993	1.108399	1.104691	1.473395	1.468766
0.75908	0.755921	0.752843	0.749784	0.74676	1.187933	1.183627	1.420734	1.416289
0.759337	0.756094	0.752927	0.749899	0.746975	1.188051	1.183683	1.420804	1.416339
0.759605	0.756307	0.753069	0.750037	0.747147	1.188166	1.183782	1.420946	1.416436
0.759895	0.756581	0.753292	0.750203	0.747249	1.188278	1.183946	1.421192	1.416599

0.760144	0.756837	0.753532	0.750389	0.747358	1.188383	1.184101	1.421437	1.416789
0.760292	0.757016	0.753755	0.750592	0.747505	1.188466	1.184198	1.421623	1.417006
0.760412	0.757178	0.753984	0.750811	0.747676	1.188559	1.184284	1.42179	1.417233
0.76058	0.757382	0.754236	0.751054	0.747877	1.188719	1.184409	1.421961	1.41744
0.760775	0.757606	0.754497	0.751311	0.748102	1.188921	1.184554	1.422128	1.417634
0.761003	0.757818	0.754696	0.751515	0.748323	1.189128	1.18469	1.422289	1.417804
0.761266	0.758018	0.754828	0.751666	0.74854	1.189345	1.184818	1.422437	1.417946
0.532097	0.529739	0.527367	0.751834	0.748759	0.87631	0.872335	1.101236	1.097497
0.532212	0.529821	0.527414	0.752038	0.748983	0.876578	0.872601	1.101504	1.097719
0.532328	0.529916	0.527483	0.752257	0.749199	0.876842	0.872877	1.101788	1.097947
0.532441	0.530031	0.527585	0.752484	0.749386	0.877111	0.873153	1.102066	1.098179
0.532553	0.530156	0.527706	0.752723	0.74957	0.877388	0.873433	1.102349	1.098421
0.532662	0.530274	0.527823	0.752982	0.749797	0.877695	0.873722	1.102639	1.098687
0.532767	0.530391	0.527941	0.75326	0.750054	0.878024	0.874018	1.102936	1.098973
0.721108	0.717866	0.714486	0.886487	0.882404	0.878354	0.874309	1.103226	1.099255
0.721325	0.718098	0.714731	0.88685	0.882758	0.878683	0.874591	1.103506	1.099533
0.721545	0.718332	0.714978	0.887215	0.883116	0.879018	0.874883	1.103799	1.099817
0.721759	0.718563	0.715223	0.887583	0.883475	0.879358	0.875192	1.104116	1.100107
0.721976	0.718794	0.715468	0.887952	0.883839	0.879708	0.875514	1.104446	1.100406
0.722201	0.719027	0.715713	0.888318	0.884215	0.880077	0.875856	1.104783	1.100713
0.722431	0.719261	0.715957	0.88868	0.884596	0.880456	0.876212	1.105132	1.101035
0.722656	0.719489	0.716197	0.889033	0.884967	0.880827	0.876566	0.872533	0.868863
0.72288	0.719713	0.716434	0.889378	0.885329	0.881194	0.876922	0.872879	0.869206
0.723128	0.719948	0.716672	0.889723	0.885691	0.88156	0.877276	0.873226	0.869563
0.72341	0.720196	0.716907	0.890065	0.886052	0.881924	0.877624	0.87357	0.869939
0.723689	0.720455	0.717164	0.71382	0.710419	0.88229	0.877982	0.873927	0.870314
0.723942	0.720728	0.717462	0.714112	0.710686	0.882655	0.878356	0.874303	0.870671
0.724185	0.72101	0.717783	0.714425	0.710965	0.883028	0.878747	0.874696	0.871029
0.724421	0.7213	0.718121	0.71477	0.711287	0.883425	0.879159	0.875106	0.871403
0.724655	0.721596	0.718468	0.715129	0.711632	0.88384	0.879591	0.875537	0.871791
0.724929	0.721874	0.718745	0.715407	0.711913	1.102124	1.09774	1.093456	1.089337
0.725242	0.722134	0.71895	0.715603	0.712132	1.102407	1.09808	1.093813	1.08964
0.725571	0.722418	0.719184	0.715832	0.712384	1.102683	1.09838	1.094121	1.089926
0.725927	0.722745	0.719471	0.716119	0.712692	1.102943	1.09861	1.094343	1.090179
0.726268	0.723069	0.719776	0.716431	0.713027	1.103219	1.098839	1.094556	1.09043
0.726535	0.723341	0.720077	0.71676	0.713386	1.10355	1.099111	1.094799	1.090698
0.726799	0.723613	0.720394	0.717106	0.713758	1.103896	1.099404	1.095062	1.09097
0.727179	0.723977	0.720756	0.717462	0.71411	1.1042	1.099713	1.095362	1.091232
0.727639	0.724402	0.721151	0.717828	0.714451	1.104481	1.100038	1.095695	1.091488
0.728072	0.724802	0.721517	0.718177	0.714793	1.104788	1.100373	1.096032	1.091773
0.728482	0.725178	0.721852	0.718504	0.715133	1.105122	1.100717	1.096375	1.092088
0.537189	0.534826	0.532469	0.530138	0.527822	0.97988	0.97601	1.096725	1.092418
0.537359	0.535026	0.532722	0.530399	0.52807	0.980153	0.976289	1.097083	1.092767
0.537538	0.535236	0.532987	0.53067	0.528321	0.980438	0.976574	1.097448	1.093114
			0.530923	0.528587	0.980719	0.976839	1.097831	1.093437
			0.531171	0.528858	0.980998	0.977093	1.098213	1.093745
			0.531428	0.529118	0.981268	0.977324	1.098531	1.094027
			0.53169	0.529369	0.981529	0.977535	1.098796	1.094285
			0.53193	0.529592	0.981771	0.977753	1.099095	1.094571
			0.532134	0.529772	0.981988	0.977979	1.099442	1.094888

0.532355	0.529976	0.982201	0.978212	1.0998	1.095229
0.532625	0.53024	0.982406	0.978453	1.100168	1.095599
0.532913	0.530536	0.982627	0.978701	1.100533	1.095982
0.533193	0.530859	0.982916	0.978954	1.100856	1.096354
0.533473	0.531198	0.983237	0.979214	1.101165	1.096725
0.533745	0.531485	0.717695	0.714221	0.887959	0.884089
0.534011	0.531721	0.718101	0.714721	0.88859	0.884637
0.534272	0.531965	0.718499	0.715177	0.889189	0.885205
0.534526	0.532232	0.718894	0.715561	0.889735	0.885811
0.534779	0.532499	0.719288	0.715935	0.890246	0.886392
0.535028	0.532748	0.719681	0.716327	0.712996	0.70969
0.535288	0.532999	0.720078	0.71673	0.713402	0.710081
0.535597	0.533276	0.720481	0.717156	0.713842	0.710505
0.535942	0.533572	0.720893	0.717601	0.714307	0.710949
0.536254	0.533882	0.721357	0.718059	0.714763	0.711413
0.536531	0.534206	0.72188	0.718533	0.715212	0.711902
0.536814	0.534537	0.722392	0.718996	0.715648	0.712373
0.537111	0.534881	0.722867	0.719424	0.716051	0.7128
0.537421	0.535215	0.723336	0.719861	0.71647	0.713228
0.537746	0.535506	0.723818	0.720352	0.716962	0.713697
0.538085	0.535781	0.724303	0.720864	0.717486	0.714181
0.538437	0.536087	0.724764	0.721346	0.717975	0.71464
0.538803	0.536416	0.725209	0.721808	0.718443	0.715084
0.539177	0.536769	0.72571	0.722316	0.718951	0.715575
0.539565	0.537154	0.726286	0.722886	0.719514	0.716128
0.539933	0.537536	0.726865	0.723449	0.720065	0.716681
0.540253	0.537896	0.727424	0.723976	0.720573	0.717212
0.540569	0.538254	0.72797	0.724484	0.721061	0.717727
		0.536277	0.533861	0.721523	0.718201
		0.536648	0.534196	0.721974	0.718656
		0.537023	0.534553	0.722486	0.719163
		0.5374	0.534931	0.723054	0.719717
		0.537779	0.53531	0.723623	0.720272
		0.538159	0.535687	0.724185	0.720822
		0.538541	0.536066	0.724749	0.721375
		0.538928	0.536449	0.725315	0.721934
		0.539314	0.536834	0.725883	0.722497
		0.539692	0.537216	0.726457	0.723064
		0.540065	0.537597	0.727034	0.723634
				0.727608	0.724204
				0.728175	0.724773
				0.728766	0.725359
				0.729395	0.72597
				0.730044	0.726597
				0.537793	0.535446
				0.538217	0.535859
				0.538603	0.536246
				0.538956	0.53661

						0.735919	0.73227
						0.735275	0.731681
						0.734625	0.731065
						0.733938	0.730385
						0.733322	0.729748
						0.732902	0.72925
						0.732537	0.728807
						1.108732	1.104539
						1.107958	1.103885
						1.107242	1.103227
						1.10659	1.102565
				0.737113	0.733422	1.212866	1.20859
				0.73644	0.732825	1.21214	1.207891
				0.735809	0.732234	1.211387	1.207176
				0.73535	0.731687	1.210553	1.206403
				0.734954	0.731164	1.209702	1.205613
				1.110769	1.106499	1.208995	1.204898
				1.109977	1.105852	1.208403	1.204241
				1.109272	1.1052	1.207732	1.20356
				1.108699	1.104537	1.20694	1.202836
				1.108086	1.103878	1.206186	1.202137
				1.10732	1.103225	1.205563	1.201508
				1.106547	1.102586	1.204966	1.200892
		0.500777	0.497947	1.105925	1.101985	1.204299	1.200222
		0.500354	0.49766	1.105376	1.101403	1.203601	1.19953
		0.499976	0.497318	1.104758	1.100779	1.202909	1.198876
		0.499637	0.496926	1.104081	1.100116	1.202216	1.198256
		0.874247	0.870572	1.103414	1.099484	1.201562	1.197621
		0.873817	0.870043	1.102761	1.098904	1.200976	1.196957
		0.87334	0.869515	1.102136	1.09833	1.200373	1.196285
		1.109692	1.105571	1.316219	1.311374	1.43488	1.429734
		1.108951	1.104961	1.315468	1.310597	1.434044	1.428936
0.503104	0.500379	1.108305	1.104336	1.314649	1.309783	1.433209	1.428145
0.502737	0.500096	1.10772	1.103708	1.313794	1.30895	1.432379	1.427362
0.502441	0.499854	1.107167	1.103136	1.313011	1.308172	1.543775	1.538458
0.502225	0.499665	1.106659	1.10263	1.312308	1.307451	1.542972	1.537646
0.501992	0.499432	1.106114	1.102095	1.311596	1.306741	1.542195	1.536856
0.501698	0.499096	1.105486	1.101485	1.310847	1.306036	1.541455	1.536101

	0.50141	0.498767	1.10486	1.100877	1.310092	1.305325	1.540711	1.535352
	0.501181	0.498536	1.104316	1.100333	1.309336	1.304578	1.539912	1.534573
	0.500981	0.498352	1.1038	1.099815	1.308587	1.303824	1.539101	1.533795
	0.897639	0.89389	1.209245	1.205059	1.375695	1.370781	1.603219	1.597761
	0.897161	0.89334	1.208585	1.204456	1.374983	1.370068	1.602482	1.597042
	0.896647	0.892814	1.20794	1.20382	1.374205	1.369316	1.601719	1.596307
	0.896081	0.892317	1.207311	1.203139	1.373338	1.36851	1.600927	1.595554
0.502259	1.109016	1.104963	1.350644	1.345805	1.49064	1.48542	1.60012	1.59478
0.502059	1.108481	1.104434	1.349928	1.345078	1.489809	1.484599	1.599285	1.59396
0.501897	1.107966	1.103915	1.349231	1.344381	1.489014	1.483805	1.598461	1.593144
0.501701	1.107454	1.103407	1.348547	1.343706	1.488247	1.483059	1.597707	1.592398
0.5015	1.106949	1.102909	1.347876	1.343053	1.487509	1.482348	1.596995	1.59169
0.896962	1.212314	1.20803	1.347212	1.342408	1.55655	1.551226	1.596282	1.590988
0.896525	1.211748	1.207465	1.346555	1.341773	1.555815	1.550503	1.595575	1.590294
0.896099	1.288659	1.284166	1.345916	1.341152	1.555093	1.54979	1.594878	1.589603
0.89569	1.288072	1.283587	1.3453	1.34055	1.554389	1.54909	1.594192	1.588913
0.895283	1.28749	1.283012	1.344688	1.339953	1.553689	1.548396	1.593513	1.588228
0.89487	1.286905	1.28243	1.344071	1.339354	1.552989	1.547705	1.59284	1.587553
0.894456	1.286319	1.281846	1.343452	1.338756	1.55229	1.547016	1.592169	1.586882
0.894036	1.285719	1.281254	1.342828	1.338155	1.551587	1.546318	1.591489	1.586204
0.893618	1.285116	1.28066	1.342204	1.337554	1.550882	1.545618	1.590807	1.585527
0.893281	1.284587	1.280118	1.40897	1.404151	1.55019	1.54495	1.640677	1.635055
0.893039	1.28414	1.279634	1.408428	1.403571	1.549507	1.544311	1.640043	1.634419
1.003627	1.283666	1.279134	1.407889	1.403024	1.548859	1.543697	1.63943	1.633806
1.003175	1.283125	1.278595	1.407353	1.402535	1.548275	1.543123	1.638847	1.633229
1.002726	1.282574	1.278042	1.4068	1.40204	1.547685	1.542536	1.638251	1.632646
1.28661	1.282042	1.277466	1.482823	1.477897	1.597681	1.59245	1.690742	1.685046
1.28611	1.281517	1.276893	1.482153	1.477264	1.596984	1.591776	1.69004	1.684378
1.285588	1.281006	1.276398	1.481579	1.476688	1.596334	1.591135	1.689358	1.683727
1.28505	1.280505	1.275961	1.481073	1.476151	1.595716	1.590513	1.688687	1.683087
1.284487	1.353939	1.349184	1.480535	1.47561	1.645984	1.640447	1.688041	1.682457
1.283884	1.35333	1.348632	1.479956	1.475068	1.645426	1.63986	1.687422	1.681842
1.283315	1.35275	1.348093	1.479376	1.474511	1.644855	1.639278	1.686818	1.681223
1.282822	1.352245	1.347597	1.478801	1.47391	1.644224	1.638683	1.686226	1.680588
1.282354	1.351763	1.347111	1.478239	1.473327	1.643602	1.638096	1.685638	1.679952
1.281874	1.351267	1.346603	1.47771	1.472848	1.643061	1.637539	1.685039	1.679326
1.281389	1.350768	1.346094	1.477202	1.472421	1.642561	1.636997	1.684438	1.678708
1.280864	1.350253	1.34562	1.476702	1.471949	1.642043	1.636453	1.683849	1.678107
1.2803	1.349722	1.345179	1.476212	1.471446	1.641518	1.635908	1.683272	1.677521
1.27978	1.349219	1.344738	1.475701	1.470903	1.640944	1.635336	1.682688	1.676937
1.279323	1.34876	1.344296	1.475159	1.470292	1.64029	1.634719	1.682094	1.676351
1.435535	1.430744	1.426052	1.474618	1.469712	1.639661	1.634114	1.681501	1.675772
1.434996	1.430205	1.425511	1.474089	1.469242	1.639126	1.633557	1.68091	1.675201
1.434451	1.429663	1.424978	1.473579	1.468804	1.638625	1.633022	1.680328	1.674642
1.433884	1.429143	1.424521	1.473118	1.468316	1.711954	1.706197	1.843985	1.837895
1.43331	1.428641	1.42411	1.47269	1.467806	1.711441	1.70568	1.843408	1.837312
1.432805	1.428166	1.423661	1.472226	1.467332	1.710935	1.70517	1.842828	1.836731
1.432371	1.42772	1.42317	1.471723	1.466893	1.710435	1.704669	1.842246	1.836153
1.431933	1.427283	1.422717	1.47125	1.466443	1.787328	1.781403	1.841672	1.83558
1.431465	1.426851	1.42234	1.470837	1.46596	1.786802	1.780872	1.841113	1.835015

1.431014	1.426426	1.421962	1.470424	1.465486	1.78628	1.780344	1.840557	1.834455
1.43061	1.425992	1.421516	1.469958	1.465064	1.785765	1.779825	1.839996	1.833899
1.430244	1.425575	1.421056	1.469477	1.464653	1.785252	1.779307	1.839435	1.833345
1.429947	1.425244	1.420654	1.469013	1.464183	1.784723	1.778775	1.838874	1.832786
1.429704	1.424976	1.420294	1.468563	1.463676	1.784184	1.778233	1.838313	1.832223
1.42936	1.424652	1.419969	1.528177	1.523038	1.727681	1.721869	1.877591	1.871241
1.428894	1.424264	1.419696	1.52788	1.522652	1.727138	1.72132	1.876966	1.870626
1.428439	1.423864	1.419374	1.527553	1.522294	1.726649	1.720808	1.876369	1.87004
1.428049	1.423462	1.418942	1.52714	1.521982	1.726269	1.720361	1.875814	1.869495
1.427671	1.42306	1.41849	1.526697	1.521652	1.725889	1.719933	1.875298	1.868986
1.427276	1.422662	1.418083	1.588013	1.582859	1.798826	1.792676	1.874844	1.86853
1.426877	1.422273	1.417705	1.587537	1.582346	1.798231	1.792187	1.874423	1.868103
1.426494	1.421915	1.417389	1.587158	1.581906	1.797672	1.791681	1.873948	1.867637
1.426123	1.421586	1.417132	1.586863	1.581521	1.797134	1.791157	1.87343	1.86714
1.42577	1.486214	1.481531	1.586514	1.581162	1.855093	1.848821	1.872914	1.866661
1.425435	1.485829	1.48111	1.586082	1.580842	1.854668	1.84834	1.872407	1.866216
1.425123	1.485467	1.480693	1.585641	1.580505	1.854226	1.847852	1.871897	1.865748
1.424857	1.485158	1.480317	1.585208	1.580109	1.853704	1.84732	1.871369	1.865205
1.42459	1.484865	1.479972	1.584795	1.579697	1.853158	1.846787	1.870856	1.864656
1.424247	1.484544	1.479677	1.584447	1.5793	1.852627	1.846309	1.870417	1.864183
1.423863	1.484214	1.47942	1.584144	1.578913	1.852101	1.845859	1.870019	1.863755
1.423531	1.483901	1.479169	1.58385	1.578553	1.807976	1.80205	1.82874	1.822739
1.423248	1.483606	1.478926	1.583566	1.578218	1.807477	1.801516	1.828219	1.822287
1.422962	1.483316	1.478684	1.58329	1.577908	1.807021	1.801026	1.903071	1.896901
1.422659	1.483034	1.478451	1.583031	1.577634	1.806627	1.800607	1.902592	1.896453
1.422354	1.482741	1.478194	1.582752	1.577361	1.806252	1.800214	1.90214	1.896028
1.487063	1.548489	1.543799	1.582408	1.577059	1.805866	1.79983	1.901719	1.895626
1.486734	1.548127	1.543433	1.58204	1.576748	1.805482	1.799456	1.901316	1.895239
1.486454	1.547819	1.54311	1.581702	1.576444	1.880537	1.874265	1.938544	1.932236
1.486209	1.54755	1.542815	1.581383	1.576143	1.880134	1.873868	1.938113	1.931819
1.485964	1.547289	1.542542	1.58109	1.575861	1.879745	1.873479	1.937688	1.931402
1.485719	1.547038	1.542295	1.58083	1.5756	1.87937	1.873099	1.937267	1.930985
1.48548	1.546789	1.542047	1.580572	1.575347	1.949771	1.943266	1.936856	1.930574
1.485251	1.546535	1.541785	1.580307	1.575101	1.949381	1.942875	1.936459	1.930173
1.485028	1.546283	1.541522	1.580043	1.574855	1.948998	1.942491	1.936072	1.92978
1.484803	1.546035	1.541269	1.579782	1.574599	1.948614	1.94211	1.935691	1.929398
1.484585	1.545793	1.541021	1.579525	1.574339	1.948231	1.941733	1.935316	1.929024
1.346957	1.47775	1.473138	1.579263	1.574089	1.947855	1.941359	1.934941	1.928648
1.346849	1.477567	1.472907	1.578998	1.573846	1.947484	1.940987	1.934567	1.928272
1.346733	1.477403	1.472719	1.587234	1.582037	1.925883	1.919426	1.934199	1.927896
1.346608	1.477268	1.472592	1.58705	1.581795	1.925504	1.919055	1.933838	1.927521
1.346468	1.477121	1.472461	1.58687	1.581573	1.925144	1.918699	1.933484	1.927151
1.346291	1.476928	1.472294	1.586676	1.581388	1.924819	1.918362	1.93313	1.92678
1.34612	1.476728	1.472111	1.586469	1.581211	1.924517	1.918047	1.932796	1.926427
1.346021	1.533391	1.528499	1.767606	1.76151	1.924232	1.917774	1.932521	1.926133
1.345961	1.53324	1.528284	1.767279	1.761241	1.923961	1.917527	1.93228	1.925875
1.345851	1.533082	1.528102	1.76699	1.760981	1.923666	1.917247	1.932003	1.925594
1.345697	1.532919	1.527952	1.766732	1.760725	1.923348	1.916936	1.931694	1.925296
1.345549	1.585364	1.580258	1.766484	1.760486	1.923047	1.916632	1.931387	1.924992
1.345417	1.585179	1.580074	1.766238	1.760268	1.922773	1.91634	1.931084	1.924677

1.345287	1.584999	1.579897	1.766011	1.760064	1.922513	1.916061	1.930795	1.924376
1.345149	1.536699	1.531721	1.765824	1.759882	1.922264	1.915806	1.930529	1.924109
1.345015	1.536549	1.53159	1.765654	1.759706	1.922021	1.915564	1.930282	1.923864
1.344908	1.536421	1.531473	1.765467	1.759501	1.921764	1.915326	1.930052	1.923635
1.34482	1.536311	1.531366	1.765272	1.759278	1.9215	1.915094	1.929838	1.92342
1.280549	1.60963	1.604182	1.675604	1.669807	1.881716	1.875566	1.892232	1.886052
1.280468	1.609433	1.603928	1.675348	1.669624	1.881511	1.875359	1.892019	1.885816
1.280365	1.609218	1.60369	1.675118	1.669452	1.881299	1.87514	1.918764	1.912413
1.280223	1.608972	1.603493	1.674947	1.669299	1.881065	1.874889	1.91848	1.912145
1.280081	1.608736	1.603315	1.674785	1.669133	1.880823	1.874643	1.918216	1.911895
1.431104	1.608564	1.60316	1.633721	1.628098	1.880567	1.87444	1.918042	1.911712
1.430974	1.608418	1.603011	1.633522	1.627859	1.880315	1.874259	1.917907	1.91156
1.430847	1.644713	1.638985	1.790181	1.784177	1.908681	1.902497	1.917722	1.911373
1.430726	1.644476	1.638718	1.789903	1.784011	1.908534	1.902318	1.917502	1.911163
1.570094	1.644288	1.638524	1.789676	1.783833	1.908362	1.902141	1.917317	1.910979
1.569931	1.644166	1.63843	1.789519	1.783635	1.908155	1.90197	1.917183	1.910835
1.569783	1.644043	1.638328	1.823863	1.817788	1.882136	1.876017	1.917029	1.910676
1.569661	1.643873	1.638152	1.823621	1.817599	1.881956	1.875812	1.916806	1.910464
1.569556	1.643712	1.637973	1.823374	1.81741	1.881774	1.875599	1.916571	1.910245
1.569467	1.64362	1.637854	1.823162	1.817182	1.881555	1.875398	1.91639	1.91006
1.569387	1.643568	1.637775	1.822977	1.816942	1.88132	1.8752	1.916231	1.909892
1.569282	1.643502	1.637748	1.822863	1.816774	1.881128	1.874997	1.916035	1.90972
1.569152	1.643428	1.637779	1.822821	1.816674	1.880974	1.874787	1.915803	1.909545
1.569028	1.650469	1.644731	1.822728	1.816583	1.85023	1.844136	1.86846	1.86242
1.568914	1.650322	1.644573	1.822545	1.816506	1.850191	1.844033	1.868305	1.862232
1.568804	1.650175	1.644405	1.822347	1.816399	1.850129	1.843945	1.868185	1.862072
1.515349	1.650048	1.644269	1.822156	1.816204	1.849957	1.843846	1.868127	1.861983
1.515204	1.649934	1.64416	1.821983	1.815987	1.849745	1.843733	1.868076	1.861916
1.55785	1.649843	1.644123	1.821887	1.815839	1.849557	1.843554	1.867925	1.861803
1.557681	1.649768	1.644134	1.82184	1.815733	1.84938	1.843334	1.867711	1.861663
1.557558	1.649679	1.644069	1.821723	1.815637	1.849269	1.843175	1.867531	1.861503
1.557481	1.649571	1.643919	1.821531	1.815562	1.849238	1.84308	1.867381	1.861316
1.608612	1.649492	1.643781	1.82134	1.815447	1.849162	1.84299	1.867268	1.861164
1.608562	1.649465	1.643675	1.821157	1.815247	1.848982	1.84289	1.867212	1.861076
1.608517	1.649457	1.643605	1.82101	1.815051	1.848787	1.842784	1.867162	1.861011
1.608455	1.602749	1.597127	1.792332	1.786352	1.808972	1.803085	1.867074	1.860946
1.608379	1.60271	1.597114	1.792311	1.786273	1.808859	1.802967	1.866969	1.860886
1.608262	1.60264	1.597109	1.792321	1.786248	1.808799	1.802868	1.86686	1.86082
1.60811	1.602548	1.597119	1.792363	1.786274	1.808789	1.802784	1.866746	1.86075
1.477895	1.602473	1.597099	1.792361	1.786292	1.808796	1.802733	1.836228	1.830321
1.477838	1.602418	1.597042	1.7923	1.786296	1.808823	1.80272	1.836155	1.830227
1.477813	1.602373	1.59698	1.792243	1.786302	1.808855	1.802722	1.836107	1.830154
1.47783	1.602327	1.596916	1.792213	1.786313	1.808882	1.802731	1.83609	1.83012
1.477864	1.602298	1.596869	1.792202	1.78633	1.80891	1.802748	1.836092	1.830109
1.409644	1.602316	1.596879	1.756199	1.750461	1.780398	1.774266	1.796662	1.79075
1.409671	1.60236	1.596918	1.756191	1.750448	1.780428	1.774294	1.796689	1.790777
1.525862	1.767711	1.761906	1.756132	1.750412	1.780461	1.774327	1.796719	1.790806
1.525859	1.767643	1.761793	1.756026	1.750355	1.780499	1.774366	1.796754	1.79084
1.525864	1.767598	1.761721	1.755948	1.750287	1.780494	1.77438	1.796785	1.790876
1.525884	1.767591	1.761713	1.755913	1.750197	1.780417	1.774354	1.79681	1.790914

1.525897	1.723654	1.717877	1.755896	1.750128	1.744315	1.738413	1.768365	1.76252
1.525889	1.723636	1.717877	1.755896	1.750112	1.744288	1.738383	1.768381	1.762538
1.52587	1.723611	1.717877	1.755905	1.75012	1.744288	1.738364	1.768395	1.762551
1.52585	1.723578	1.717865	1.755907	1.750129	1.744291	1.738342	1.768409	1.762565
1.525826	1.723538	1.717845	1.755905	1.750141	1.7443	1.73832	1.768423	1.762578
1.525811	1.723494	1.717813	1.755883	1.750124	1.744282	1.738287	1.768431	1.76259
1.525799	1.723444	1.717766	1.755839	1.750079	1.744235	1.738237	1.76843	1.762596
1.525817	1.723418	1.717727	1.755795	1.75003	1.74419	1.738208	1.76846	1.762625
1.525885	1.72343	1.717699	1.75575	1.749983	1.744157	1.738217	1.768544	1.762686
1.525964	1.723461	1.717693	1.755723	1.749941	1.744124	1.738232	1.768643	1.76277
1.52603	1.723509	1.717733	1.711946	1.706167	1.744076	1.738227	1.73243	1.726719
1.526088	1.723564	1.717793	1.711979	1.70614	1.744031	1.738217	1.732463	1.726781
1.526108	1.723585	1.717821	1.712	1.706144	1.744029	1.738219	1.732475	1.726809
1.526098	1.723583	1.717828	1.712018	1.706173	1.744062	1.73823	1.73247	1.726814
1.526138	1.723608	1.717854	1.712047	1.706207	1.744097	1.738257	1.732493	1.726845
1.526235	1.723662	1.717903	1.712091	1.706246	1.744134	1.738303	1.732548	1.72691
1.52635	1.723735	1.717955	1.712132	1.706285	1.700483	1.694724	1.732614	1.726978
1.52648	1.723832	1.717998	1.712156	1.706315	1.700525	1.694787	1.732695	1.727044
1.526605	1.723932	1.718047	1.712185	1.706349	1.700568	1.694845	1.732767	1.727103
1.526692	1.724008	1.718117	1.712249	1.706404	1.700612	1.694868	1.732785	1.727137
1.526753	1.724072	1.7182	1.712331	1.70647	1.700655	1.694874	1.732779	1.727163
1.526785	1.724126	1.718284	1.712413	1.706519	1.700683	1.694897	1.732819	1.727232
1.526784	1.724168	1.718372	1.712496	1.706554	1.700697	1.694933	1.732897	1.727339
1.526859	1.724244	1.718454	1.712566	1.706594	1.700726	1.694981	1.689348	1.683873
1.527048	1.724371	1.718527	1.712615	1.706642	1.700779	1.695046	1.689421	1.683929
1.527243	1.724505	1.718603	1.712668	1.706697	1.700836	1.695104	1.689477	1.683974
1.527391	1.724621	1.718686	1.712736	1.706767	1.700895	1.695128	1.689479	1.683993
1.527521	1.724734	1.718776	1.712812	1.706836	1.700949	1.69515	1.689483	1.684022
1.527634	1.724844	1.718878	1.712889	1.706869	1.70097	1.695207	1.689577	1.684127
1.527734	1.724951	1.718989	1.712968	1.706885	1.700976	1.695288	1.689725	1.68428
1.623357	1.725055	1.719075	1.713046	1.706961	1.701057	1.695381	1.689831	1.684392
1.623458	1.725153	1.719132	1.713119	1.707099	1.701216	1.695488	1.689893	1.684462
1.623575	1.72527	1.71923	1.713223	1.707233	1.701357	1.695601	1.689981	1.684555
1.623719	1.725423	1.719406	1.713383	1.707343	1.701446	1.695716	1.690123	1.684698
1.62385	1.725576	1.719582	1.713546	1.707459	1.701542	1.695834	1.690264	1.684839
1.623912	1.725692	1.719691	1.713666	1.707605	1.701693	1.695952	1.690355	1.684935
1.623964	1.725804	1.719784	1.713774	1.707761	1.701859	1.696073	1.690436	1.68502
1.570908	1.725966	1.719925	1.713904	1.707884	1.701982	1.696202	1.69057	1.685148
1.571122	1.726165	1.7201	1.714049	1.707988	1.702076	1.696338	1.69074	1.685307
1.571273	1.726361	1.720322	1.714257	1.708149	1.702207	1.696463	1.690869	1.685447
1.571347	1.726557	1.720606	1.714548	1.708379	1.702375	1.696569	1.690942	1.685563
1.463472	1.520123	1.515003	1.714814	1.708622	1.702581	1.696711	1.691041	1.685683
1.463553	1.520199	1.515088	1.715003	1.708879	1.702856	1.696926	1.691194	1.685809
1.463667	1.520288	1.515143	1.715166	1.709118	1.703126	1.697159	1.691381	1.685955
1.463878	1.520479	1.515261	1.715319	1.709278	1.703302	1.697371	1.691612	1.686154
1.464131	1.520717	1.515414	1.715473	1.709402	1.703434	1.69757	1.691862	1.68638
1.411854	1.45944	1.454469	1.715695	1.709584	1.703593	1.697733	1.692036	1.686568
1.411917	1.4596	1.454746	1.715979	1.709811	1.703768	1.697861	1.692145	1.686725
1.411988	1.459721	1.45495	1.716227	1.710052	1.703986	1.698028	1.69228	1.686891
1.412063	1.459781	1.455049	1.716419	1.71031	1.704262	1.698247	1.692449	1.687066

1.412189	1.45987	1.455136	1.7166	1.710562	1.704542	1.698488	1.692647	1.687253
1.412422	1.460047	1.455235	1.716776	1.710792	1.704803	1.698745	1.692886	1.68745
1.412695	1.460267	1.455359	1.716961	1.711011	1.705048	1.69901	1.693151	1.687663
1.412941	1.460503	1.455564	1.610404	1.604882	1.70525	1.699263	1.693437	1.687909
1.413177	1.460756	1.455823	1.610662	1.605064	1.705426	1.699508	1.693736	1.688174
1.413375	1.460977	1.456073	1.610923	1.605271	1.705638	1.699736	1.693978	1.688405
1.413536	1.46117	1.456316	1.61119	1.6055	1.705883	1.699944	1.694162	1.688601
1.093844	1.409106	1.404455	1.45159	1.446449	1.706149	1.70017	1.694361	1.688814
1.094035	1.409272	1.40471	1.451871	1.446719	1.706432	1.70042	1.694589	1.689056
1.094222	1.409427	1.404942	1.452136	1.446995	1.706736	1.700697	1.694844	1.689317
1.094421	1.409567	1.405113	1.452337	1.447244	1.707065	1.701013	1.695141	1.6896
1.094633	1.409709	1.405262	1.452515	1.447484	1.70741	1.701354	1.695464	1.6899
1.094883	1.409903	1.405455	1.452718	1.44773	1.601616	1.596005	1.695791	1.690207
1.095166	1.410133	1.405675	1.452937	1.447978	1.601919	1.596311	1.696125	1.690523
1.095451	1.410371	1.405908	1.453171	1.448233	1.602224	1.596618	1.696464	1.690842
1.09574	1.410617	1.406157	1.453423	1.448497	1.60253	1.596928	1.696808	1.691164
1.096026	1.410862	1.406407	1.453677	1.448763	1.602839	1.59724	1.697154	1.691492
1.0963	1.411103	1.406654	1.45393	1.449031	1.603154	1.597555	1.697501	1.691824
1.096577	1.411343	1.406899	1.454181	1.449297	1.603469	1.597874	1.697854	1.692163
1.096863	1.343601	1.339364	1.454421	1.449554	1.551602	1.546362	1.592696	1.587411
1.097161	1.343818	1.339581	1.454657	1.449806	1.551884	1.546665	1.593029	1.587723
0.865423	1.344062	1.339805	1.454895	1.450063	1.55217	1.546964	1.593355	1.588033
0.865759	1.344329	1.340034	1.455132	1.450323	1.552457	1.54726	1.593675	1.588342
0.866122	1.344628	1.340297	1.455397	1.450586	1.44575	1.440866	1.593997	1.588649
0.866524	1.344975	1.34061	1.455696	1.450845	1.445994	1.441132	1.594318	1.588954
0.866911	1.258877	1.25467	1.456001	1.451125	1.446262	1.44141	1.594646	1.589262
0.867249	1.259124	1.254929	1.456303	1.451444	1.446581	1.441705	1.594972	1.589564
0.867572	1.259348	1.255171	1.456599	1.451779	1.446928	1.442023	1.595319	1.589887
0.867905	1.259567	1.255385	1.394215	1.389646	1.447296	1.442386	1.544022	1.53888
0.868244	1.259785	1.255585	1.39445	1.389965	1.447676	1.442779	1.544451	1.539294
1.085336	1.162497	1.158559	1.394704	1.390246	1.448001	1.443146	1.544866	1.539705
1.085577	1.162725	1.158793	1.394975	1.390489	1.448271	1.443488	1.545273	1.540115
1.085839	1.162979	1.159051	1.395269	1.390754	1.448562	1.443837	1.545677	1.540526
1.086128	1.163267	1.159342	1.395596	1.391058	1.448892	1.444206	1.54609	1.540945
1.086423	1.163559	1.159628	1.395928	1.391377	1.449236	1.444564	1.546476	1.541342
1.08672	1.163838	1.159875	1.396246	1.391702	1.44959	1.444878	1.546793	1.54168
1.087007	1.164105	1.160108	1.396562	1.392035	1.44995	1.445178	1.547087	1.541997
1.087233	1.083337	1.079537	1.253306	1.249177	1.387831	1.383252	1.440723	1.435971
1.087417	1.083538	1.079793	1.253601	1.249496	1.388169	1.38358	1.441068	1.436318
1.087651	1.083779	1.080067	1.253903	1.249801	1.388499	1.383911	1.441425	1.436651
1.087939	1.084066	1.080363	1.254215	1.250089	1.388821	1.384244	1.441795	1.436963
1.088245	1.084356	1.080642	1.157552	1.153647	1.324452	1.320048	1.442168	1.437305
1.088575	1.084643	1.08088	1.157789	1.153915	1.324796	1.320418	1.442538	1.4377
1.088896	1.084922	1.081116	1.158028	1.154189	1.325152	1.320793	1.442915	1.438114
1.08916	1.085176	1.081383	1.158305	1.154481	1.325521	1.32116	1.443315	1.438527
1.089398	1.085418	1.081663	1.158601	1.154783	1.325898	1.321528	1.443726	1.438945
1.089654	1.085676	1.081936	1.078243	1.074629	1.247735	1.243493	1.317547	1.313137
1.089922	1.085943	1.082205	1.078518	1.074916	1.248051	1.24384	1.317973	1.313551
1.0902	1.086215	1.082476	1.078798	1.075214	1.248385	1.244181	1.318372	1.313955
1.090482	1.086483	1.082741	1.07908	1.075531	1.248748	1.244514	1.31873	1.314345

1.090796	1.086782	1.083031	1.079372	1.075837	1.249092	1.244842	1.319101	1.314739
1.091161	1.087136	1.083367	1.079683	1.076115	1.249383	1.245169	1.319524	1.315151
1.091556	1.087519	1.083722	1.080001	1.076383	1.24966	1.245485	1.319947	1.31556
1.091974	1.087919	1.084076	1.080316	1.076663	1.249958	1.245774	1.241572	1.237324
1.092408	1.088333	1.084432	1.080631	1.076945	1.250263	1.246047	1.24183	1.237622
0.880307	1.088707	1.084781	1.080947	1.077218	1.250546	1.246327	1.242116	1.237925
0.880771	1.08904	1.085123	1.081263	1.07748	1.250806	1.246611	1.242427	1.238233
0.881298	1.08942	1.085466	1.081579	1.077775	1.073982	1.070187	1.242713	1.238526
0.881938	1.089881	1.085799	1.08188	1.07812	1.074326	1.070452	1.242942	1.238786
0.882571	1.090346	1.086167	1.082218	1.078479	1.074677	1.070741	1.243213	1.239067
0.706419	1.090776	1.086627	1.082653	1.078839	1.074998	1.071088	1.243623	1.239415
0.706805	1.091181	1.08711	1.083117	1.0792	1.07531	1.071447	1.24407	1.239787
0.707211	1.09153	1.08751	1.083512	1.079543	1.075618	1.071744	1.244414	1.24014
0.707626	1.091839	1.087858	1.083866	1.079877	1.075924	1.072005	1.244696	1.240484
0.708091	0.881376	0.877741	1.084217	1.080245	1.076275	1.07228	1.244955	1.240792
0.708615	0.881934	0.878212	1.084557	1.080649	1.076678	1.072565	1.245176	1.241055
0.709121	0.882508	0.878731	1.084936	1.081054	1.077072	1.072886	1.24547	1.241354
0.709582	0.883098	0.879336	1.085387	1.081453	1.07744	1.073265	1.245896	1.241728
0.71003	0.883683	0.879965	1.085855	1.081846	1.077798	1.073662	1.246358	1.242123
0.710481	0.884238	0.880583	1.086303	1.082222	1.078143	1.074046	1.246786	1.2425
0.710928	0.884775	0.881195	1.08674	1.082595	1.078489	1.074433	1.247209	1.242881
0.711362	0.708165	0.705027	1.087146	1.08299	1.07888	1.074838	1.150935	1.146798
0.711784	0.708598	0.705488	1.087525	1.083402	1.079309	1.075258	1.151344	1.147207
0.712257	0.709064	0.705954	1.087872	1.083798	1.079729	1.075664	1.151743	1.147613
0.712795	0.709573	0.706431	1.088183	1.084176	1.080142	1.076059	1.152136	1.14802
0.713344	0.710087	0.706899	1.08848	1.084537	1.080536	1.076431	1.072378	1.068458
0.713892	0.710592	0.70734	1.088758	1.084869	1.080893	1.076762	1.072686	1.068787
0.714429	0.711097	0.70779	1.089049	1.085197	1.081239	1.077091	1.073001	1.069115
0.714922	0.711599	0.708297	1.089406	1.085548	1.081597	1.077472	1.073396	1.069493
0.715393	0.712102	0.708837	1.089806	1.085915	1.081959	1.077879	1.073835	1.069899
0.715901	0.71262	0.709368	0.963688	0.960195	1.082336	1.078287	1.074264	1.070311
0.716441	0.713152	0.709895	0.964015	0.960486	1.082728	1.078698	1.074691	1.070731
0.716982	0.713685	0.710422	0.964345	0.960777	1.083116	1.079102	1.075111	1.07115
0.71752	0.714215	0.710947	0.964679	0.961067	1.083496	1.079498	1.075522	1.071567
0.718063	0.71475	0.711474	0.965015	0.961362	1.083881	1.079891	1.075927	1.071982
0.718614	0.715292	0.712006	0.965352	0.961668	1.084275	1.080283	1.076324	1.072394
0.719171	0.715838	0.71254	0.965686	0.96198	1.084677	1.080678	1.076718	1.072805
0.719728	0.716386	0.713077	0.966006	0.962294	1.085088	1.081081	1.077117	1.073214
0.720289	0.716936	0.713616	0.966315	0.962611	1.085506	1.081493	1.077523	1.073625
0.720853	0.717491	0.714157	0.966617	0.962922	0.9593	0.955739	1.077971	1.074065
0.72142	0.718047	0.714698	0.966908	0.963228	0.959625	0.956091	1.078462	1.074535
0.721997	0.71862	0.715267	0.967233	0.963542	0.959943	0.956444	1.078968	1.075023
0.722585	0.719216	0.715879	0.96761	0.963861	0.960243	0.956795	1.079502	1.075539
0.723189	0.719831	0.716514	0.968015	0.964199	0.960552	0.957135	1.080014	1.076043
0.533109	0.720478	0.717177	0.713852	0.710512	0.960891	0.957445	1.080444	1.076492
0.533512	0.721134	0.717845	0.714509	0.711136	0.96125	0.957743	1.080835	1.076914
0.533904	0.721741	0.718457	0.71512	0.711743	0.961627	0.958051	1.08123	1.077339
0.534287	0.722313	0.719026	0.715699	0.712342	0.962022	0.958366	1.081623	1.077762
	0.722901	0.719598	0.716281	0.712956	0.962433	0.958706	0.955111	0.951782
	0.723507	0.72017	0.716866	0.713592	0.962874	0.959078	0.95542	0.952096

0.724134	0.720769	0.717464	0.714215	0.963279	0.959447	0.955752	0.952413
0.724803	0.721417	0.718087	0.714804	0.963606	0.959793	0.956104	0.952719
0.72547	0.72208	0.71872	0.715384	0.963908	0.96014	0.956478	0.953038
0.726076	0.72272	0.719349	0.715965	0.96421	0.960506	0.956891	0.953409
0.726659	0.723356	0.719986	0.716556	0.96452	0.960883	0.957321	0.953803
0.727305	0.724025	0.720661	0.717225	0.964912	0.961258	0.957678	0.954146
0.728004	0.724724	0.721374	0.717966	0.965379	0.961629	0.957971	0.954446
0.536497	0.534295	0.72206	0.718689	0.965854	0.962044	0.958329	0.954791
0.536932	0.534679	0.722705	0.719385	0.966341	0.962525	0.958788	0.955203
0.537359	0.535077	0.723339	0.72005	0.716723	0.713316	0.959259	0.955628
0.537779	0.535536	0.723964	0.720646	0.717326	0.713987	0.959707	0.956051
0.538203	0.536017	0.724596	0.721235	0.717912	0.714636	0.960134	0.956461
		0.725273	0.721914	0.71858	0.715272	0.960489	0.956812
		0.72598	0.722648	0.719298	0.715901	0.960804	0.957127
		0.726684	0.723368	0.720012	0.71658	0.961211	0.957486
		0.727388	0.724076	0.720726	0.717312	0.961712	0.957884
		0.536186	0.53392	0.721442	0.718041	0.962209	0.958314
		0.536662	0.53443	0.722162	0.718754	0.962678	0.958784
		0.537134	0.534931	0.722878	0.719465	0.96314	0.959272
		0.53761	0.535403	0.723583	0.720184	0.963607	0.959767
		0.538084	0.535859	0.724277	0.720896	0.964065	0.960262
		0.538542	0.536308	0.72494	0.72157	0.964481	0.960716
		0.538987	0.536751	0.725581	0.722216	0.964865	0.96114
		0.539458	0.537233	0.726254	0.72288	0.719522	0.716221
		0.539959	0.537765	0.726964	0.723563	0.720178	0.71686
		0.540476	0.538285	0.727682	0.724268	0.720868	0.717532
		0.541016	0.538764	0.728404	0.725016	0.721636	0.718291
		0.541557	0.539234	0.729118	0.725756	0.7224	0.719052
				0.537385	0.535045	0.723068	0.719726
				0.537867	0.535487	0.72369	0.720359
				0.538346	0.535942	0.724333	0.721016
				0.538824	0.536406	0.724986	0.721688
				0.539322	0.5369	0.725669	0.722377
				0.539839	0.537427	0.726384	0.723087
				0.540385	0.537981	0.727133	0.723811
				0.540982	0.538578	0.72794	0.724545
				0.541597	0.539181	0.728762	0.725288
				0.542181	0.539725	0.72954	0.726046
				0.542752	0.540235	0.730293	0.726811
				0.543331	0.540763	0.538238	0.535813
				0.54392	0.541305	0.53874	0.536311
						0.539273	0.536836
						0.53985	0.5374
						0.54044	0.53798
						0.541024	0.538561
						0.54161	0.539147
						0.542196	0.539732
						0.542784	0.540319
						0.543373	0.540908
						0.543964	0.541503

1.525897	1.723654	1.717877	1.755896	1.750128	1.744315	1.738413	1.768365	1.76252
1.525889	1.723636	1.717877	1.755896	1.750112	1.744288	1.738383	1.768381	1.762538
1.52587	1.723611	1.717877	1.755905	1.75012	1.744288	1.738364	1.768395	1.762551
1.52585	1.723578	1.717865	1.755907	1.750129	1.744291	1.738342	1.768409	1.762565
1.525826	1.723538	1.717845	1.755905	1.750141	1.7443	1.73832	1.768423	1.762578
1.525811	1.723494	1.717813	1.755883	1.750124	1.744282	1.738287	1.768431	1.76259
1.525799	1.723444	1.717766	1.755839	1.750079	1.744235	1.738237	1.76843	1.762596
1.525817	1.723418	1.717727	1.755795	1.75003	1.74419	1.738208	1.76846	1.762625
1.525885	1.72343	1.717699	1.75575	1.749983	1.744157	1.738217	1.768544	1.762686
1.525964	1.723461	1.717693	1.755723	1.749941	1.744124	1.738232	1.768643	1.76277
1.52603	1.723509	1.717733	1.711946	1.706167	1.744076	1.738227	1.73243	1.726719
1.526088	1.723564	1.717793	1.711979	1.70614	1.744031	1.738217	1.732463	1.726781
1.526108	1.723585	1.717821	1.712	1.706144	1.744029	1.738219	1.732475	1.726809
1.526098	1.723583	1.717828	1.712018	1.706173	1.744062	1.73823	1.73247	1.726814
1.526138	1.723608	1.717854	1.712047	1.706207	1.744097	1.738257	1.732493	1.726845
1.526235	1.723662	1.717903	1.712091	1.706246	1.744134	1.738303	1.732548	1.72691
1.52635	1.723735	1.717955	1.712132	1.706285	1.700483	1.694724	1.732614	1.726978
1.52648	1.723832	1.717998	1.712156	1.706315	1.700525	1.694787	1.732695	1.727044
1.526605	1.723932	1.718047	1.712185	1.706349	1.700568	1.694845	1.732767	1.727103
1.526692	1.724008	1.718117	1.712249	1.706404	1.700612	1.694868	1.732785	1.727137
1.526753	1.724072	1.7182	1.712331	1.70647	1.700655	1.694874	1.732779	1.727163
1.526785	1.724126	1.718284	1.712413	1.706519	1.700683	1.694897	1.732819	1.727232
1.526784	1.724168	1.718372	1.712496	1.706554	1.700697	1.694933	1.732897	1.727339
1.526859	1.724244	1.718454	1.712566	1.706594	1.700726	1.694981	1.689348	1.683873
1.527048	1.724371	1.718527	1.712615	1.706642	1.700779	1.695046	1.689421	1.683929
1.527243	1.724505	1.718603	1.712668	1.706697	1.700836	1.695104	1.689477	1.683974
1.527391	1.724621	1.718686	1.712736	1.706767	1.700895	1.695128	1.689479	1.683993
1.527521	1.724734	1.718776	1.712812	1.706836	1.700949	1.69515	1.689483	1.684022
1.527634	1.724844	1.718878	1.712889	1.706869	1.70097	1.695207	1.689577	1.684127
1.527734	1.724951	1.718989	1.712968	1.706885	1.700976	1.695288	1.689725	1.68428
1.623357	1.725055	1.719075	1.713046	1.706961	1.701057	1.695381	1.689831	1.684392
1.623458	1.725153	1.719132	1.713119	1.707099	1.701216	1.695488	1.689893	1.684462
1.623575	1.72527	1.71923	1.713223	1.707233	1.701357	1.695601	1.689981	1.684555
1.623719	1.725423	1.719406	1.713383	1.707343	1.701446	1.695716	1.690123	1.684698
1.62385	1.725576	1.719582	1.713546	1.707459	1.701542	1.695834	1.690264	1.684839
1.623912	1.725692	1.719691	1.713666	1.707605	1.701693	1.695952	1.690355	1.684935
1.623964	1.725804	1.719784	1.713774	1.707761	1.701859	1.696073	1.690436	1.68502
1.570908	1.725966	1.719925	1.713904	1.707884	1.701982	1.696202	1.69057	1.685148
1.571122	1.726165	1.7201	1.714049	1.707988	1.702076	1.696338	1.69074	1.685307
1.571273	1.726361	1.720322	1.714257	1.708149	1.702207	1.696463	1.690869	1.685447
1.571347	1.726557	1.720606	1.714548	1.708379	1.702375	1.696569	1.690942	1.685563
1.463472	1.520123	1.515003	1.714814	1.708622	1.702581	1.696711	1.691041	1.685683
1.463553	1.520199	1.515088	1.715003	1.708879	1.702856	1.696926	1.691194	1.685809
1.463667	1.520288	1.515143	1.715166	1.709118	1.703126	1.697159	1.691381	1.685955
1.463878	1.520479	1.515261	1.715319	1.709278	1.703302	1.697371	1.691612	1.686154
1.464131	1.520717	1.515414	1.715473	1.709402	1.703434	1.69757	1.691862	1.68638
1.411854	1.45944	1.454469	1.715695	1.709584	1.703593	1.697733	1.692036	1.686568
1.411917	1.4596	1.454746	1.715979	1.709811	1.703768	1.697861	1.692145	1.686725
1.411988	1.459721	1.45495	1.716227	1.710052	1.703986	1.698028	1.69228	1.686891
1.412063	1.459781	1.455049	1.716419	1.71031	1.704262	1.698247	1.692449	1.687066

1.412189	1.45987	1.455136	1.7166	1.710562	1.704542	1.698488	1.692647	1.687253
1.412422	1.460047	1.455235	1.716776	1.710792	1.704803	1.698745	1.692886	1.68745
1.412695	1.460267	1.455359	1.716961	1.711011	1.705048	1.69901	1.693151	1.687663
1.412941	1.460503	1.455564	1.610404	1.604882	1.70525	1.699263	1.693437	1.687909
1.413177	1.460756	1.455823	1.610662	1.605064	1.705426	1.699508	1.693736	1.688174
1.413375	1.460977	1.456073	1.610923	1.605271	1.705638	1.699736	1.693978	1.688405
1.413536	1.46117	1.456316	1.61119	1.6055	1.705883	1.699944	1.694162	1.688601
1.093844	1.409106	1.404455	1.45159	1.446449	1.706149	1.70017	1.694361	1.688814
1.094035	1.409272	1.40471	1.451871	1.446719	1.706432	1.70042	1.694589	1.689056
1.094222	1.409427	1.404942	1.452136	1.446995	1.706736	1.700697	1.694844	1.689317
1.094421	1.409567	1.405113	1.452337	1.447244	1.707065	1.701013	1.695141	1.6896
1.094633	1.409709	1.405262	1.452515	1.447484	1.70741	1.701354	1.695464	1.6899
1.094883	1.409903	1.405455	1.452718	1.44773	1.601616	1.596005	1.695791	1.690207
1.095166	1.410133	1.405675	1.452937	1.447978	1.601919	1.596311	1.696125	1.690523
1.095451	1.410371	1.405908	1.453171	1.448233	1.602224	1.596618	1.696464	1.690842
1.09574	1.410617	1.406157	1.453423	1.448497	1.60253	1.596928	1.696808	1.691164
1.096026	1.410862	1.406407	1.453677	1.448763	1.602839	1.59724	1.697154	1.691492
1.0963	1.411103	1.406654	1.45393	1.449031	1.603154	1.597555	1.697501	1.691824
1.096577	1.411343	1.406899	1.454181	1.449297	1.603469	1.597874	1.697854	1.692163
1.096863	1.343601	1.339364	1.454421	1.449554	1.551602	1.546362	1.592696	1.587411
1.097161	1.343818	1.339581	1.454657	1.449806	1.551884	1.546665	1.593029	1.587723
0.865423	1.344062	1.339805	1.454895	1.450063	1.55217	1.546964	1.593355	1.588033
0.865759	1.344329	1.340034	1.455132	1.450323	1.552457	1.54726	1.593675	1.588342
0.866122	1.344628	1.340297	1.455397	1.450586	1.44575	1.440866	1.593997	1.588649
0.866524	1.344975	1.34061	1.455696	1.450845	1.445994	1.441132	1.594318	1.588954
0.866911	1.258877	1.25467	1.456001	1.451125	1.446262	1.44141	1.594646	1.589262
0.867249	1.259124	1.254929	1.456303	1.451444	1.446581	1.441705	1.594972	1.589564
0.867572	1.259348	1.255171	1.456599	1.451779	1.446928	1.442023	1.595319	1.589887
0.867905	1.259567	1.255385	1.394215	1.389646	1.447296	1.442386	1.544022	1.53888
0.868244	1.259785	1.255585	1.39445	1.389965	1.447676	1.442779	1.544451	1.539294
1.085336	1.162497	1.158559	1.394704	1.390246	1.448001	1.443146	1.544866	1.539705
1.085577	1.162725	1.158793	1.394975	1.390489	1.448271	1.443488	1.545273	1.540115
1.085839	1.162979	1.159051	1.395269	1.390754	1.448562	1.443837	1.545677	1.540526
1.086128	1.163267	1.159342	1.395596	1.391058	1.448892	1.444206	1.54609	1.540945
1.086423	1.163559	1.159628	1.395928	1.391377	1.449236	1.444564	1.546476	1.541342
1.08672	1.163838	1.159875	1.396246	1.391702	1.44959	1.444878	1.546793	1.54168
1.087007	1.164105	1.160108	1.396562	1.392035	1.44995	1.445178	1.547087	1.541997
1.087233	1.083337	1.079537	1.253306	1.249177	1.387831	1.383252	1.440723	1.435971
1.087417	1.083538	1.079793	1.253601	1.249496	1.388169	1.38358	1.441068	1.436318
1.087651	1.083779	1.080067	1.253903	1.249801	1.388499	1.383911	1.441425	1.436651
1.087939	1.084066	1.080363	1.254215	1.250089	1.388821	1.384244	1.441795	1.436963
1.088245	1.084356	1.080642	1.157552	1.153647	1.324452	1.320048	1.442168	1.437305
1.088575	1.084643	1.08088	1.157789	1.153915	1.324796	1.320418	1.442538	1.4377
1.088896	1.084922	1.081116	1.158028	1.154189	1.325152	1.320793	1.442915	1.438114
1.08916	1.085176	1.081383	1.158305	1.154481	1.325521	1.32116	1.443315	1.438527
1.089398	1.085418	1.081663	1.158601	1.154783	1.325898	1.321528	1.443726	1.438945
1.089654	1.085676	1.081936	1.078243	1.074629	1.247735	1.243493	1.317547	1.313137
1.089922	1.085943	1.082205	1.078518	1.074916	1.248051	1.24384	1.317973	1.313551
1.0902	1.086215	1.082476	1.078798	1.075214	1.248385	1.244181	1.318372	1.313955
1.090482	1.086483	1.082741	1.07908	1.075531	1.248748	1.244514	1.31873	1.314345

1.090796	1.086782	1.083031	1.079372	1.075837	1.249092	1.244842	1.319101	1.314739
1.091161	1.087136	1.083367	1.079683	1.076115	1.249383	1.245169	1.319524	1.315151
1.091556	1.087519	1.083722	1.080001	1.076383	1.24966	1.245485	1.319947	1.31556
1.091974	1.087919	1.084076	1.080316	1.076663	1.249958	1.245774	1.241572	1.237324
1.092408	1.088333	1.084432	1.080631	1.076945	1.250263	1.246047	1.24183	1.237622
0.880307	1.088707	1.084781	1.080947	1.077218	1.250546	1.246327	1.242116	1.237925
0.880771	1.08904	1.085123	1.081263	1.07748	1.250806	1.246611	1.242427	1.238233
0.881298	1.08942	1.085466	1.081579	1.077775	1.073982	1.070187	1.242713	1.238526
0.881938	1.089881	1.085799	1.08188	1.07812	1.074326	1.070452	1.242942	1.238786
0.882571	1.090346	1.086167	1.082218	1.078479	1.074677	1.070741	1.243213	1.239067
0.706419	1.090776	1.086627	1.082653	1.078839	1.074998	1.071088	1.243623	1.239415
0.706805	1.091181	1.08711	1.083117	1.0792	1.07531	1.071447	1.24407	1.239787
0.707211	1.09153	1.08751	1.083512	1.079543	1.075618	1.071744	1.244414	1.24014
0.707626	1.091839	1.087858	1.083866	1.079877	1.075924	1.072005	1.244696	1.240484
0.708091	0.881376	0.877741	1.084217	1.080245	1.076275	1.07228	1.244955	1.240792
0.708615	0.881934	0.878212	1.084557	1.080649	1.076678	1.072565	1.245176	1.241055
0.709121	0.882508	0.878731	1.084936	1.081054	1.077072	1.072886	1.24547	1.241354
0.709582	0.883098	0.879336	1.085387	1.081453	1.07744	1.073265	1.245896	1.241728
0.71003	0.883683	0.879965	1.085855	1.081846	1.077798	1.073662	1.246358	1.242123
0.710481	0.884238	0.880583	1.086303	1.082222	1.078143	1.074046	1.246786	1.2425
0.710928	0.884775	0.881195	1.08674	1.082595	1.078489	1.074433	1.247209	1.242881
0.711362	0.708165	0.705027	1.087146	1.08299	1.07888	1.074838	1.150935	1.146798
0.711784	0.708598	0.705488	1.087525	1.083402	1.079309	1.075258	1.151344	1.147207
0.712257	0.709064	0.705954	1.087872	1.083798	1.079729	1.075664	1.151743	1.147613
0.712795	0.709573	0.706431	1.088183	1.084176	1.080142	1.076059	1.152136	1.14802
0.713344	0.710087	0.706899	1.08848	1.084537	1.080536	1.076431	1.072378	1.068458
0.713892	0.710592	0.70734	1.088758	1.084869	1.080893	1.076762	1.072686	1.068787
0.714429	0.711097	0.70779	1.089049	1.085197	1.081239	1.077091	1.073001	1.069115
0.714922	0.711599	0.708297	1.089406	1.085548	1.081597	1.077472	1.073396	1.069493
0.715393	0.712102	0.708837	1.089806	1.085915	1.081959	1.077879	1.073835	1.069899
0.715901	0.71262	0.709368	0.963688	0.960195	1.082336	1.078287	1.074264	1.070311
0.716441	0.713152	0.709895	0.964015	0.960486	1.082728	1.078698	1.074691	1.070731
0.716982	0.713685	0.710422	0.964345	0.960777	1.083116	1.079102	1.075111	1.07115
0.71752	0.714215	0.710947	0.964679	0.961067	1.083496	1.079498	1.075522	1.071567
0.718063	0.71475	0.711474	0.965015	0.961362	1.083881	1.079891	1.075927	1.071982
0.718614	0.715292	0.712006	0.965352	0.961668	1.084275	1.080283	1.076324	1.072394
0.719171	0.715838	0.71254	0.965686	0.96198	1.084677	1.080678	1.076718	1.072805
0.719728	0.716386	0.713077	0.966006	0.962294	1.085088	1.081081	1.077117	1.073214
0.720289	0.716936	0.713616	0.966315	0.962611	1.085506	1.081493	1.077523	1.073625
0.720853	0.717491	0.714157	0.966617	0.962922	0.9593	0.955739	1.077971	1.074065
0.72142	0.718047	0.714698	0.966908	0.963228	0.959625	0.956091	1.078462	1.074535
0.721997	0.71862	0.715267	0.967233	0.963542	0.959943	0.956444	1.078968	1.075023
0.722585	0.719216	0.715879	0.96761	0.963861	0.960243	0.956795	1.079502	1.075539
0.723189	0.719831	0.716514	0.968015	0.964199	0.960552	0.957135	1.080014	1.076043
0.533109	0.720478	0.717177	0.713852	0.710512	0.960891	0.957445	1.080444	1.076492
0.533512	0.721134	0.717845	0.714509	0.711136	0.96125	0.957743	1.080835	1.076914
0.533904	0.721741	0.718457	0.71512	0.711743	0.961627	0.958051	1.08123	1.077339
0.534287	0.722313	0.719026	0.715699	0.712342	0.962022	0.958366	1.081623	1.077762
	0.722901	0.719598	0.716281	0.712956	0.962433	0.958706	0.955111	0.951782
	0.723507	0.72017	0.716866	0.713592	0.962874	0.959078	0.95542	0.952096

0.724134	0.720769	0.717464	0.714215	0.963279	0.959447	0.955752	0.952413
0.724803	0.721417	0.718087	0.714804	0.963606	0.959793	0.956104	0.952719
0.72547	0.72208	0.71872	0.715384	0.963908	0.96014	0.956478	0.953038
0.726076	0.72272	0.719349	0.715965	0.96421	0.960506	0.956891	0.953409
0.726659	0.723356	0.719986	0.716556	0.96452	0.960883	0.957321	0.953803
0.727305	0.724025	0.720661	0.717225	0.964912	0.961258	0.957678	0.954146
0.728004	0.724724	0.721374	0.717966	0.965379	0.961629	0.957971	0.954446
0.536497	0.534295	0.72206	0.718689	0.965854	0.962044	0.958329	0.954791
0.536932	0.534679	0.722705	0.719385	0.966341	0.962525	0.958788	0.955203
0.537359	0.535077	0.723339	0.72005	0.716723	0.713316	0.959259	0.955628
0.537779	0.535536	0.723964	0.720646	0.717326	0.713987	0.959707	0.956051
0.538203	0.536017	0.724596	0.721235	0.717912	0.714636	0.960134	0.956461
		0.725273	0.721914	0.71858	0.715272	0.960489	0.956812
		0.72598	0.722648	0.719298	0.715901	0.960804	0.957127
		0.726684	0.723368	0.720012	0.71658	0.961211	0.957486
		0.727388	0.724076	0.720726	0.717312	0.961712	0.957884
		0.536186	0.53392	0.721442	0.718041	0.962209	0.958314
		0.536662	0.53443	0.722162	0.718754	0.962678	0.958784
		0.537134	0.534931	0.722878	0.719465	0.96314	0.959272
		0.53761	0.535403	0.723583	0.720184	0.963607	0.959767
		0.538084	0.535859	0.724277	0.720896	0.964065	0.960262
		0.538542	0.536308	0.72494	0.72157	0.964481	0.960716
		0.538987	0.536751	0.725581	0.722216	0.964865	0.96114
		0.539458	0.537233	0.726254	0.72288	0.719522	0.716221
		0.539959	0.537765	0.726964	0.723563	0.720178	0.71686
		0.540476	0.538285	0.727682	0.724268	0.720868	0.717532
		0.541016	0.538764	0.728404	0.725016	0.721636	0.718291
		0.541557	0.539234	0.729118	0.725756	0.7224	0.719052
				0.537385	0.535045	0.723068	0.719726
				0.537867	0.535487	0.72369	0.720359
				0.538346	0.535942	0.724333	0.721016
				0.538824	0.536406	0.724986	0.721688
				0.539322	0.5369	0.725669	0.722377
				0.539839	0.537427	0.726384	0.723087
				0.540385	0.537981	0.727133	0.723811
				0.540982	0.538578	0.72794	0.724545
				0.541597	0.539181	0.728762	0.725288
				0.542181	0.539725	0.72954	0.726046
				0.542752	0.540235	0.730293	0.726811
				0.543331	0.540763	0.538238	0.535813
				0.54392	0.541305	0.53874	0.536311
						0.539273	0.536836
						0.53985	0.5374
						0.54044	0.53798
						0.541024	0.538561
						0.54161	0.539147
						0.542196	0.539732
						0.542784	0.540319
						0.543373	0.540908
						0.543964	0.541503

					0.551104
					0.550487
					0.549942
					0.549483
					0.778167
					0.77716
					0.776186
					0.775204
					0.774205
		0.553509	0.551202	0.548848	1.214615
		0.553048	0.550635	0.548182	1.213327
		0.552528	0.55007	0.547579	1.212055
		0.551917	0.549505	0.54707	1.210809
		0.551322	0.548954	0.546569	1.209577
		0.550807	0.548435	0.546038	1.208361
		0.550314	0.547926	0.545506	1.207161
		0.878782	0.875901	0.872971	1.397788
		0.878132	0.875259	0.872338	1.396412
		0.877495	0.874623	0.871708	1.45587
		0.876867	0.873992	0.871082	1.4546
0.552419	0.550081	1.106453	1.102662	1.098867	1.45336
0.551949	0.549591	1.105634	1.101832	1.098038	1.452155
0.551477	0.549101	1.104814	1.101002	1.097208	1.450963
0.551009	0.548615	1.103989	1.100169	1.09638	1.560067
0.550531	0.548125	1.103162	1.099334	1.095551	1.558805
0.879044	0.876138	1.318708	1.313923	1.309194	1.557541
0.878395	0.875504	1.317633	1.312847	1.308114	1.556278
0.877758	0.874869	1.441724	1.436506	1.431355	1.555036
0.877134	0.874232	1.440635	1.435432	1.430308	1.553812
0.87651	0.873589	1.439548	1.434364	1.42927	1.552625
0.875886	0.87294	1.438455	1.433298	1.428235	1.551493

			0.875258	0.872287	1.437361	1.432236	1.427204	1.550385
			0.874617	0.871632	1.436267	1.431174	1.426176	1.549279
			0.873968	0.870975	1.435174	1.430116	1.425151	1.548178
	0.55038	0.547836	1.207678	1.203577	1.434082	1.42906	1.424129	1.571997
	0.549881	0.547316	1.206761	1.202707	1.432993	1.428008	1.423108	1.570915
	0.549392	0.546851	1.205895	1.201864	1.431924	1.426971	1.422104	1.569848
	0.548923	0.546472	1.205092	1.201044	1.430871	1.42595	1.421123	1.568801
	0.989245	0.985543	1.377202	1.372008	1.494852	1.489687	1.484637	1.567757
	0.988483	0.984849	1.376207	1.371052	1.493845	1.48866	1.483596	1.566716
	0.987734	0.984153	1.375217	1.370127	1.492876	1.487655	1.482555	1.565665
	0.987034	0.983472	1.3742	1.369189	1.491914	1.486657	1.481511	1.564566
	0.986371	0.982801	1.373169	1.368249	1.490968	1.485677	1.480478	1.563445
0.728643	1.208423	1.204292	1.372123	1.367293	1.518336	1.512977	1.507665	1.56239
0.728137	1.207663	1.203486	1.371055	1.36632	1.517455	1.512145	1.506844	1.561393
0.727586	1.206903	1.20275	1.370071	1.365362	1.516538	1.511275	1.506001	1.606077
0.726959	1.206147	1.202133	1.369219	1.364416	1.515547	1.510331	1.505117	1.605114
0.726329	1.205377	1.201505	1.368386	1.363506	1.514584	1.509388	1.504207	1.604136
0.725719	1.204551	1.200762	1.367512	1.362679	1.513734	1.508487	1.503258	1.603088
0.725152	1.203734	1.199984	1.366613	1.361872	1.512926	1.507605	1.502299	1.602021
1.10043	1.203054	1.199244	1.428949	1.424075	1.512089	1.506736	1.501388	1.601009
1.099936	1.202469	1.198526	1.427974	1.423154	1.511237	1.505878	1.500508	1.600028
1.099355	1.201837	1.197869	1.427088	1.422255	1.510326	1.504974	1.499607	1.781872
1.098677	1.201155	1.197287	1.426305	1.421375	1.509341	1.504013	1.498681	1.7807
1.204422	1.371945	1.367001	1.425518	1.420528	1.508402	1.503082	1.49777	1.779573
1.203727	1.371016	1.366109	1.424691	1.419734	1.507565	1.502215	1.496882	1.778529
1.203042	1.370101	1.365221	1.423867	1.418948	1.506752	1.501373	1.496021	1.777522
1.202364	1.369276	1.364416	1.455033	1.449996	1.616949	1.611381	1.605839	1.889235
1.201684	1.368473	1.363633	1.454269	1.449182	1.616054	1.610493	1.604958	1.888189
1.200956	1.431345	1.42643	1.453468	1.448396	1.615157	1.609597	1.604066	1.887146
1.200189	1.430351	1.425517	1.452638	1.447631	1.61426	1.608698	1.603168	1.886109
1.199481	1.429421	1.424612	1.523091	1.517965	1.784174	1.778223	1.772282	1.885061
1.198851	1.428575	1.423714	1.522147	1.517062	1.783212	1.777263	1.771308	1.884
1.198221	1.427751	1.422847	1.521243	1.516185	1.782244	1.776294	1.770328	1.882935
1.197555	1.426928	1.422031	1.520443	1.515391	1.781263	1.775302	1.769332	1.881868
1.196882	1.426121	1.421243	1.519676	1.51461	1.780274	1.774303	1.768334	1.8808
1.19621	1.425344	1.42048	1.518886	1.513773	1.779277	1.773308	1.767346	1.879735
1.195544	1.424586	1.41973	1.518087	1.51291	1.778275	1.772313	1.766363	1.878674
1.194932	1.423812	1.418931	1.613391	1.607944	1.817507	1.811392	1.805303	1.874963
1.194377	1.423028	1.418086	1.612492	1.607056	1.816475	1.810379	1.804317	1.873945
1.193747	1.422218	1.417256	1.611595	1.606157	1.815455	1.809373	1.803334	1.872941
1.192985	1.421363	1.416449	1.610696	1.605233	1.814454	1.808373	1.802343	1.871945
1.192235	1.420525	1.415657	1.609817	1.604329	1.813461	1.807387	1.801371	1.870974
1.424697	1.647012	1.641373	1.784268	1.778277	1.888904	1.882481	1.876186	1.870053
1.423943	1.646108	1.640503	1.783382	1.777353	1.887849	1.881479	1.87525	1.869147
1.423197	1.645245	1.639632	1.782498	1.776481	1.886901	1.880538	1.87431	1.868207
1.422462	1.644414	1.638765	1.781622	1.775649	1.886027	1.879639	1.873373	1.867247
1.533248	1.694867	1.689007	1.821108	1.814998	1.885122	1.878714	1.872419	1.866295
1.532436	1.693954	1.688082	1.820167	1.814091	1.884185	1.877757	1.871439	1.865345
1.531635	1.693061	1.687189	1.853424	1.84716	1.883246	1.876823	1.870508	1.864435
1.530854	1.692209	1.686357	1.852542	1.846247	1.882314	1.875939	1.869671	1.863597

1.530085	1.691364	1.685538	1.851672	1.845335	1.881373	1.875056	1.868848	1.862766
1.529323	1.690489	1.684691	1.850766	1.844394	1.880392	1.874124	1.867965	1.861874
1.528575	1.689616	1.683845	1.849856	1.843459	1.879414	1.873187	1.867071	1.860972
1.592373	1.58701	1.581676	1.774261	1.768154	1.878546	1.872337	1.866235	1.860138
1.59167	1.586305	1.580959	1.77346	1.767398	1.877766	1.871552	1.865442	1.859357
1.590953	1.585584	1.580218	1.772643	1.766627	1.876974	1.870756	1.864637	1.914719
1.590223	1.584844	1.579445	1.77181	1.765843	1.87617	1.869948	1.863823	1.913876
1.589469	1.584085	1.578662	1.770961	1.765031	1.875335	1.869108	1.862975	1.913001
1.588669	1.583289	1.577861	1.770074	1.764162	1.874435	1.868197	1.862051	1.912056
1.587869	1.582496	1.57707	1.769196	1.763291	1.873528	1.867284	1.861131	1.91111
1.587128	1.763806	1.757746	1.768386	1.762478	1.872675	1.866449	1.860311	1.935268
1.586421	1.762994	1.756954	1.767617	1.761699	1.871852	1.865656	1.859547	1.934435
1.585725	1.762204	1.756167	1.766849	1.760933	1.871043	1.864867	1.858776	1.933611
1.585043	1.761437	1.755391	1.766088	1.760181	1.87025	1.864086	1.858006	1.932803
1.584357	1.760666	1.754617	1.765333	1.759434	1.925714	1.919373	1.913127	1.931997
1.583659	1.759882	1.75384	1.764583	1.75869	1.924882	1.91855	1.912316	1.931191
1.582966	1.759103	1.753071	1.763841	1.757955	1.924059	1.917736	1.911513	1.930389
1.582286	1.758342	1.752312	1.763107	1.75723	1.923245	1.916933	1.910721	1.929591
1.581612	1.757591	1.751564	1.762383	1.756516	1.922442	1.91614	1.909939	1.928798
1.580935	1.756844	1.750825	1.761672	1.755816	1.921649	1.915359	1.909169	1.928013
1.580259	1.756104	1.750098	1.760977	1.75513	1.920867	1.914589	1.90841	1.927233
1.629439	1.75539	1.74941	1.760319	1.754463	1.888693	1.882527	1.87646	1.926449
1.628797	1.754701	1.748764	1.759698	1.753812	1.887932	1.881764	1.8757	1.925657
1.628182	1.754037	1.748132	1.759094	1.753196	1.887214	1.881039	1.87497	1.96407
1.627608	1.753413	1.747522	1.75852	1.752645	1.886573	1.880375	1.874281	1.963308
1.627035	1.752785	1.746892	1.757924	1.75208	1.885929	1.879725	1.873622	1.962583
1.67934	1.805753	1.799692	1.89789	1.891502	1.885213	1.879061	1.873005	1.961926
1.678704	1.805059	1.798962	1.897086	1.890717	1.884468	1.878388	1.8724	1.961294
1.678085	1.804389	1.798256	1.896307	1.889957	1.910656	1.904367	1.898172	2.015476
1.677476	1.803735	1.797561	1.89554	1.88921	1.909901	1.903619	1.897432	2.014668
1.676867	1.803093	1.796889	1.894809	1.888501	1.909183	1.90291	1.896728	2.013878
1.676263	1.802473	1.796248	1.894124	1.887844	1.908517	1.90225	1.896069	2.013108
1.675632	1.801826	1.795608	1.893436	1.887175	1.949967	1.943533	1.937185	2.012357
1.674944	1.801113	1.794952	1.89272	1.886454	1.949206	1.94282	1.93652	2.011631
1.67425	1.800395	1.794302	1.892008	1.885735	1.94845	1.94211	1.935858	2.010915
1.673602	1.799731	1.793681	1.891339	1.885068	1.947758	1.94144	1.935206	1.986211
1.672979	1.799092	1.793072	1.890687	1.884426	1.947099	1.940788	1.934553	1.98549
1.672371	1.798443	1.792423	1.889992	1.883764	1.946419	1.940096	1.933848	1.984749
1.671779	1.797789	1.791739	1.889258	1.883085	1.945722	1.939371	1.933094	1.98399
1.671194	1.861156	1.854928	1.93357	1.927221	2.003187	1.996462	1.989808	1.983256
1.670612	1.860535	1.854346	1.932973	1.92663	2.002486	1.995767	1.98912	1.982558
1.670046	1.859939	1.853791	1.932401	1.926061	2.001813	1.995098	1.988451	1.981872
1.669509	1.859366	1.853245	1.931826	1.925501	2.001193	1.994463	1.987793	1.981193
1.668986	1.858805	1.852707	1.931257	1.924948	2.000589	1.993838	1.987139	1.980516
1.831812	1.89828	1.891974	1.930686	1.924389	1.999952	1.993181	1.986461	1.979832
1.831229	1.897687	1.891397	1.930114	1.923826	1.999296	1.992505	1.985769	1.979144
1.830654	1.897097	1.890825	1.929534	1.923241	1.998623	1.991823	1.985083	1.978458
1.830086	1.896511	1.890261	1.928946	1.922632	1.997931	1.991134	1.9844	1.977772
1.829521	1.941225	1.934765	1.989457	1.982793	1.997238	1.990441	1.983714	1.9771
1.828957	1.94062	1.934191	1.98884	1.982135	1.996541	1.989735	1.983016	1.976444

1.828398	1.940018	1.933618	1.988226	1.981491	1.995863	1.98905	1.982337	1.975804
1.827854	1.939427	1.933036	1.987607	1.980883	1.995236	1.988426	1.981716	1.975186
1.827314	1.938839	1.932453	1.986988	1.980289	1.994631	1.987827	1.981118	1.97458
1.826761	1.938243	1.931878	1.986375	1.979669	1.993994	1.987191	1.980487	1.973962
1.826201	1.937641	1.931309	1.985768	1.979033	1.993339	1.986535	1.979837	1.973338
1.864971	1.909746	1.903566	1.961242	1.95462	1.992728	1.985918	1.979218	1.972738
1.864376	1.909162	1.902992	1.960641	1.954065	1.992171	1.985345	1.978627	1.97216
1.863813	1.908604	1.902435	1.96005	1.953505	1.991614	1.984793	1.978076	1.971616
1.863299	1.908082	1.901901	1.959466	1.952919	1.991041	1.984269	1.977597	1.971139
1.862811	1.90759	1.901403	1.958909	1.952338	1.990461	1.983737	1.977113	1.97066
1.862339	1.994286	1.987603	1.980867	1.97406	1.989862	1.983141	1.976534	1.970104
1.861882	1.993787	1.98712	1.98037	1.973516	1.98926	1.982518	1.97591	1.96951
1.86141	1.993262	1.986583	1.979827	1.972973	1.988708	1.98194	1.975311	1.968929
1.860929	1.992717	1.986003	1.979247	1.972436	1.9882	1.981395	1.974727	1.968352
1.860487	1.992185	1.985428	1.978659	1.971868	1.987659	1.98086	1.974194	1.967824
1.860114	1.991676	1.98486	1.978055	1.97125	1.987066	1.980335	1.973733	1.967363
1.859698	1.982561	1.975722	1.977477	1.97065	1.986476	1.979813	1.973277	1.966909
1.859149	1.981984	1.975182	1.976959	1.970105	1.985904	1.97928	1.97279	1.966431
1.858567	1.981395	1.97465	1.976464	1.969592	1.985362	1.97876	1.972298	1.965951
1.858047	1.980806	1.974097	1.975951	1.969107	1.984891	1.978285	1.971817	1.965482
1.857565	1.980222	1.973538	1.975436	1.96865	1.984472	1.977842	1.971342	1.965018
1.816792	1.979695	1.973024	1.974958	1.968214	1.961549	1.954981	1.948541	1.964544
1.816427	1.979223	1.972555	1.974518	1.9678	1.961146	1.954553	1.94808	1.964055
1.89079	1.978763	1.972086	1.974072	1.967384	1.960746	1.954137	1.947642	1.983786
1.890378	1.978309	1.971609	1.973616	1.966968	1.960357	1.953742	1.947232	1.983343
1.88998	1.977857	1.971127	1.973145	1.96653	1.959946	1.953344	1.946838	1.982922
1.889594	1.977394	1.970624	1.963937	1.957337	1.959474	1.952919	1.946458	1.982526
1.889214	1.976932	1.97012	1.963413	1.956812	1.958981	1.952488	1.946086	1.98214
1.925964	1.976487	1.96965	1.96293	1.956328	1.98001	1.973449	1.966977	1.98173
1.925559	1.976055	1.969205	1.962478	1.955872	1.979554	1.973019	1.966568	1.981304
1.925151	1.975628	1.968775	1.962048	1.955444	1.979125	1.972599	1.966152	1.980864
1.92474	1.975206	1.968363	1.961644	1.955051	1.978726	1.972192	1.965728	1.980406
1.924332	1.974785	1.96795	1.983564	1.976898	1.978331	1.971791	1.965313	1.979963
1.923926	1.974359	1.96753	1.983131	1.976474	1.97793	1.971396	1.964917	1.979542
1.923528	1.973938	1.967112	1.982698	1.976051	1.97753	1.971005	1.96453	1.979145
1.923144	1.973528	1.9667	1.982269	1.975628	1.96906	1.962551	1.956096	1.978786
1.922769	1.973124	1.966293	1.981842	1.975206	1.968647	1.962149	1.955713	1.978453
1.92239	1.972718	1.965885	1.981413	1.974783	1.968234	1.96175	1.955335	1.978127
1.92201	1.97231	1.965477	1.980983	1.974359	1.967821	1.961354	1.954963	1.977812
1.921628	1.940101	1.933458	1.98059	1.97396	1.96742	1.96096	1.954583	1.956399
1.92124	1.939721	1.933121	1.980242	1.973588	1.967028	1.960558	1.954181	1.956031
1.920857	1.939351	1.932796	1.979914	1.973246	1.96667	1.960192	1.953811	1.955679
1.920476	1.938991	1.932474	1.979604	1.97295	1.96638	1.959901	1.95351	1.955371
1.920113	1.938644	1.932157	1.979295	1.972664	1.966108	1.959634	1.953237	1.955085
1.919796	1.962064	1.955483	1.978951	1.972333	1.965791	1.959336	1.952953	1.946638
1.919511	1.961738	1.955141	1.97859	1.971982	1.965457	1.959027	1.952668	1.946368
1.919224	1.961422	1.954824	1.978266	1.971664	1.965145	1.958717	1.952359	1.946065
1.918939	1.961121	1.954533	1.977978	1.971379	1.964853	1.958403	1.952025	1.945727
1.918642	1.960805	1.954228	1.977678	1.971091	1.964571	1.95811	1.951718	1.945417
1.918318	1.960459	1.953893	1.977352	1.970797	1.964301	1.957846	1.951453	1.94515

1.918008	1.960122	1.953562	1.977025	1.970498	1.964027	1.957591	1.95121	1.944903
1.917742	1.959815	1.953253	1.976708	1.970184	1.963727	1.957333	1.950989	1.944677
1.917499	1.959527	1.952958	1.976402	1.969871	1.963424	1.957076	1.950776	1.94446
1.917265	1.959261	1.952679	1.97612	1.969592	1.963153	1.956821	1.950535	1.94422
1.917041	1.959013	1.952414	1.975858	1.969338	1.962904	1.956566	1.950274	1.943966
1.879905	1.958755	1.952158	1.945681	1.939331	1.962664	1.956308	1.950001	1.943703
1.879646	1.958484	1.95191	1.945451	1.939115	1.962435	1.956043	1.949706	1.943425
1.906096	1.958223	1.951671	1.945224	1.938891	1.962205	1.9558	1.949449	1.943174
1.905853	1.957984	1.951443	1.944993	1.938637	1.961958	1.955587	1.949267	1.942974
1.905624	1.957751	1.951213	1.944762	1.938396	1.961733	1.955398	1.949104	1.942785
1.905413	1.957509	1.950952	1.944517	1.938212	1.961582	1.955239	1.948922	1.94258
1.905216	1.957272	1.95069	1.944279	1.938058	1.961463	1.955094	1.948735	1.942369
1.905025	1.957078	1.950508	1.944107	1.937898	1.961299	1.954914	1.948535	1.942156
1.904842	1.956921	1.950392	1.943991	1.937737	1.961102	1.954706	1.948322	1.941939
1.904664	1.956752	1.950237	1.943839	1.937575	1.960927	1.954515	1.948115	1.941719
1.904494	1.956561	1.950022	1.943631	1.93741	1.960783	1.954344	1.947908	1.941484
1.90432	1.956375	1.949824	1.943439	1.937248	1.960637	1.954189	1.947734	1.94129
1.904135	1.95621	1.949693	1.943313	1.937089	1.960472	1.954054	1.94763	1.941184
1.903946	1.956044	1.949566	1.943191	1.93693	1.960307	1.953926	1.947542	1.941104
1.903752	1.955843	1.949363	1.943	1.93677	1.930561	1.924345	1.918121	1.940985
1.903562	1.95563	1.949129	1.94278	1.936608	1.93044	1.924216	1.917977	1.94085
1.903418	1.955478	1.948966	1.942611	1.936436	1.93027	1.924058	1.917831	1.94071
1.903322	1.955385	1.948867	1.942484	1.936251	1.930054	1.923871	1.917685	1.940565
1.856444	1.955278	1.948784	1.942391	1.936109	1.929877	1.923696	1.917524	1.94042
1.856246	1.955143	1.948719	1.942348	1.936032	1.929759	1.923534	1.917334	1.940273
1.856057	1.955003	1.948638	1.942292	1.935969	1.929673	1.923402	1.917166	1.940135
1.855915	1.954857	1.948495	1.942172	1.935897	1.929628	1.923331	1.917059	1.940005
1.855801	1.954721	1.948337	1.94203	1.935817	1.929589	1.923284	1.916986	1.939893
1.855726	1.933164	1.926769	1.941907	1.9357	1.929486	1.923208	1.916931	1.91068
1.855678	1.933118	1.92669	1.941794	1.935558	1.929342	1.923117	1.916894	1.910641
1.855557	1.933024	1.926613	1.94171	1.935447	1.929216	1.923004	1.916803	1.910567
1.855353	1.932877	1.926541	1.941659	1.935366	1.929102	1.922865	1.916653	1.910455
1.855169	1.932739	1.926466	1.94161	1.935305	1.92902	1.92275	1.916518	1.910348
1.855029	1.932622	1.92638	1.941554	1.935265	1.928981	1.922677	1.916413	1.910256
1.854924	1.932531	1.926297	1.941494	1.935238	1.928967	1.922635	1.916339	1.91018
1.854874	1.93249	1.926223	1.920001	1.913839	1.928978	1.922636	1.916316	1.910131
1.854859	1.93248	1.92616	1.919932	1.913819	1.928998	1.92266	1.916325	1.910102
1.854847	1.932479	1.92612	1.919877	1.913776	1.928974	1.922655	1.916332	1.910086
1.854844	1.932492	1.926101	1.919834	1.913715	1.928916	1.922632	1.916341	1.910084
1.824486	1.871359	1.865336	1.895697	1.889743	1.907505	1.901301	1.89509	1.910095
1.824388	1.871317	1.865343	1.895707	1.889695	1.907431	1.901269	1.895108	1.910126
1.824302	1.871288	1.865366	1.895732	1.889662	1.907363	1.901225	1.895101	1.91014
1.824259	1.871295	1.86541	1.895767	1.889645	1.907306	1.901157	1.895037	1.91011
1.824239	1.871321	1.865464	1.895811	1.889644	1.907266	1.901093	1.894965	1.910073
1.784953	1.871363	1.865519	1.89586	1.889663	1.907252	1.901066	1.894934	1.888818
1.784979	1.871419	1.865576	1.895915	1.8897	1.907262	1.901064	1.894929	1.888829
1.785008	1.871477	1.865637	1.895977	1.889749	1.907294	1.901087	1.894949	1.888858
1.785042	1.871539	1.865702	1.896046	1.889814	1.907351	1.901138	1.894996	1.888907
1.785077	1.871599	1.865764	1.896112	1.889875	1.907405	1.901188	1.895044	1.888957
1.785116	1.871658	1.865824	1.896174	1.889931	1.907454	1.901234	1.895089	1.889004

1.756767	1.836324	1.830569	1.896231	1.889982	1.907498	1.901276	1.895131	1.889051
1.75679	1.836374	1.830618	1.896279	1.890024	1.907533	1.90131	1.895167	1.889092
1.756809	1.83642	1.830664	1.896323	1.890062	1.907565	1.901342	1.895201	1.889134
1.75683	1.836468	1.830711	1.896368	1.890099	1.907595	1.901374	1.895239	1.889181
1.756852	1.836516	1.830759	1.896415	1.890138	1.907627	1.901409	1.89528	1.889231
1.756872	1.80572	1.799984	1.896468	1.890207	1.884021	1.877974	1.872018	1.889284
1.756888	1.805755	1.80002	1.896529	1.890308	1.884143	1.878071	1.872083	1.889336
1.756912	1.805788	1.800052	1.89659	1.890411	1.884272	1.878191	1.872182	1.889415
1.756935	1.805808	1.800066	1.89664	1.890509	1.884407	1.878344	1.872336	1.889537
1.756987	1.805857	1.800108	1.896709	1.89061	1.884537	1.878493	1.872492	1.889668
1.721086	1.779217	1.773495	1.863756	1.857705	1.884641	1.878599	1.872606	1.889787
1.721168	1.779389	1.773653	1.863905	1.857829	1.884737	1.878688	1.872699	1.8899
1.721215	1.779508	1.773778	1.864051	1.857973	1.884868	1.878786	1.872773	1.889999
1.721239	1.779596	1.773884	1.864198	1.858133	1.885022	1.878888	1.87283	1.890086
1.721282	1.779692	1.773976	1.864318	1.858256	1.88514	1.878985	1.87291	1.890181
1.721351	1.779801	1.77405	1.864401	1.858329	1.885212	1.879074	1.873017	1.890287
1.721415	1.745686	1.740058	1.841271	1.835222	1.852357	1.846309	1.840343	1.890399
1.721459	1.745742	1.740122	1.841402	1.835343	1.852476	1.846425	1.840459	1.890516
1.721502	1.745807	1.740205	1.841554	1.835482	1.85261	1.846552	1.840582	1.890646
1.721563	1.716057	1.710614	1.801377	1.795525	1.852746	1.846689	1.840727	1.8908
1.721638	1.716157	1.710727	1.801559	1.795675	1.852891	1.846837	1.840886	1.89097
1.721738	1.716262	1.710818	1.80172	1.795829	1.853048	1.846995	1.841046	1.891129
1.721866	1.716374	1.71089	1.801862	1.795987	1.853218	1.847164	1.841209	1.891281
1.67848	1.716465	1.71097	1.73511	1.729412	1.830231	1.824191	1.818245	1.860265
1.678518	1.716522	1.71106	1.735232	1.729543	1.830412	1.82434	1.818365	1.860385
1.678555	1.716584	1.711157	1.735362	1.72967	1.830591	1.824502	1.818513	1.860533
1.678607	1.716675	1.71127	1.735496	1.729765	1.830748	1.824695	1.818742	1.860762
1.678674	1.716779	1.711384	1.73563	1.729861	1.830916	1.824905	1.818993	1.861012
1.67878	1.673429	1.668094	1.706003	1.700462	1.758823	1.753078	1.747407	1.839446
1.678912	1.673534	1.668164	1.706088	1.700606	1.759037	1.75325	1.747529	1.839629
1.679028	1.673652	1.668284	1.706219	1.700747	1.759215	1.753395	1.747641	1.839815
1.679127	1.673785	1.668456	1.706401	1.700883	1.759355	1.753506	1.747737	1.840002
1.679237	1.673916	1.668611	1.706569	1.701032	1.759524	1.753651	1.747861	1.840186
1.679372	1.674039	1.668719	1.706699	1.701203	1.759753	1.753855	1.748024	1.840352
1.679506	1.674163	1.668832	1.706834	1.701373	1.759982	1.754075	1.748218	1.840549
1.679619	1.674299	1.668997	1.663634	1.658209	1.725562	1.719807	1.714115	1.812188
1.679726	1.674437	1.669173	1.663812	1.658342	1.725705	1.720005	1.714373	1.812457
1.679847	1.674559	1.669299	1.663945	1.658485	1.7259	1.720196	1.714564	1.8127
1.679979	1.674669	1.66939	1.664047	1.658636	1.726127	1.720381	1.714709	1.81293
1.680131	1.674816	1.669521	1.664175	1.658775	1.653342	1.647819	1.642369	1.743737
1.680312	1.675008	1.669695	1.664325	1.658891	1.653444	1.647949	1.642531	1.743936
1.680477	1.675197	1.669897	1.664517	1.659043	1.653572	1.648095	1.642698	1.744143
1.680598	1.675365	1.670147	1.664788	1.659267	1.653753	1.648266	1.642866	1.744364
1.680718	1.675525	1.67039	1.665062	1.659513	1.653967	1.648456	1.643036	1.744596
1.680873	1.675673	1.670554	1.665252	1.659745	1.654215	1.648661	1.643198	1.669749
1.68105	1.675821	1.670683	1.665404	1.659975	1.654487	1.648883	1.643363	1.669978
1.68125	1.676009	1.670846	1.665576	1.6602	1.654744	1.649123	1.643573	1.670211
1.681472	1.676232	1.671033	1.66576	1.660419	1.654991	1.64938	1.643822	1.670447
1.681685	1.676457	1.671251	1.665976	1.660638	1.655219	1.649631	1.644086	1.670711
1.681889	1.676686	1.671507	1.66623	1.660852	1.655415	1.649874	1.644372	1.671015

1.682078	1.676905	1.671773	1.666503	1.661081	1.65562	1.650114	1.644649	1.639318
1.682239	1.677099	1.672044	1.666799	1.661344	1.655858	1.650357	1.644904	1.63958
1.682397	1.677283	1.672305	1.667091	1.661617	1.656109	1.650597	1.645139	1.639832
1.682586	1.677472	1.672527	1.667335	1.661872	1.656362	1.65082	1.645337	1.640054
1.682793	1.677661	1.67272	1.667544	1.662117	1.656621	1.651036	1.645512	1.640255
1.683005	1.67785	1.672886	1.667724	1.662358	1.656896	1.651278	1.645713	1.640455
1.683219	1.678036	1.673019	1.667869	1.662593	1.657189	1.651547	1.645937	1.640652
1.683453	1.678246	1.673179	1.66803	1.662816	1.657457	1.651808	1.646177	1.640869
1.683716	1.678491	1.673383	1.668219	1.663019	1.657677	1.652048	1.646431	1.641116
1.683988	1.678747	1.673607	1.668426	1.663224	1.657892	1.652294	1.646704	1.641376
1.684256	1.678998	1.673841	1.66865	1.663445	1.658123	1.652564	1.647003	1.641644
1.684529	1.679254	1.674088	1.668891	1.663682	1.658368	1.652849	1.647318	1.641919
1.684813	1.679524	1.674347	1.66915	1.663951	1.658649	1.653149	1.647626	1.642195
1.685106	1.679803	1.674619	1.669426	1.664246	1.658959	1.653464	1.647934	1.642471
1.685402	1.680086	1.674895	1.669705	1.664539	1.659264	1.653778	1.648247	1.642758
1.685699	1.68037	1.675175	1.669986	1.664828	1.659561	1.654091	1.648566	1.643052
1.686003	1.68066	1.675456	1.670267	1.665117	1.659861	1.654404	1.648887	1.643364
1.686316	1.680956	1.675735	1.670543	1.665407	1.660166	1.654718	1.649211	1.643704
1.686635	1.681255	1.676015	1.67082	1.665698	1.660471	1.655033	1.649537	1.644054
1.582274	1.68155	1.67629	1.67109	1.665983	1.660772	1.655347	1.649864	1.644401
1.582554	1.68185	1.676572	1.671368	1.66627	1.661073	1.655662	1.650195	1.644749
1.582842	1.682178	1.676901	1.671692	1.666582	1.661381	1.655981	1.650529	1.645095
1.583138	1.682531	1.67727	1.672056	1.666916	1.661695	1.656304	1.650867	1.64544
1.583429	1.57844	1.573638	1.672425	1.667271	1.662039	1.656645	1.651207	1.645778
1.583713	1.578733	1.57396	1.672799	1.667657	1.662424	1.657006	1.651542	1.646103
1.583998	1.579019	1.574261	1.673161	1.668035	1.662809	1.657381	1.651901	1.646448
1.584277	1.579283	1.574517	1.673499	1.668387	1.663179	1.657777	1.652313	1.646838
1.584576	1.579555	1.574762	1.673821	1.66872	1.663533	1.658174	1.652742	1.647249
1.533808	1.579879	1.575032	1.570257	1.565583	1.663847	1.658536	1.653151	1.647664
1.534187	1.580237	1.57532	1.570504	1.565817	1.664139	1.658881	1.653551	1.648087
1.534573	1.580602	1.575641	1.570802	1.566115	1.664483	1.659244	1.653941	1.648508
1.534967	1.580977	1.575996	1.571151	1.566475	1.664876	1.659625	1.654317	1.648927
1.535369	1.581365	1.576367	1.571514	1.566841	1.66527	1.66001	1.654704	1.649342
1.535782	1.581775	1.576759	1.57189	1.567203	1.665651	1.660394	1.6551	1.649751
1.536185	1.582185	1.57715	1.572267	1.567566	1.666044	1.660797	1.655516	1.650165
1.536553	1.582582	1.577524	1.572627	1.567923	1.563202	1.558403	1.553539	1.650598
1.536908	1.582972	1.57789	1.572983	1.568281	1.563571	1.558802	1.553957	1.65104
1.43126	1.583351	1.578255	1.573343	1.568647	1.563944	1.559182	1.554345	1.651479
1.43161	1.583721	1.578618	1.573706	1.569018	1.564321	1.559548	1.554713	1.651919
1.431916	1.58407	1.578984	1.57408	1.569389	1.564686	1.559902	1.555064	1.550132
1.432164	1.584384	1.579351	1.574466	1.56976	1.565039	1.560238	1.555394	1.550491
1.432468	1.53361	1.528753	1.574855	1.570128	1.565392	1.560586	1.555744	1.550848
1.432886	1.534045	1.529154	1.575231	1.570483	1.565741	1.560959	1.556136	1.551206
1.433335	1.53451	1.52958	1.575629	1.570853	1.566101	1.56134	1.556534	1.551573
1.433756	1.534965	1.530033	1.576093	1.571274	1.566486	1.561707	1.5569	1.551966
1.434175	1.535422	1.530501	1.576587	1.571721	1.566891	1.56208	1.557262	1.552377
1.308736	1.429961	1.425399	1.57704	1.57216	1.567321	1.562503	1.557684	1.552808
1.309128	1.430388	1.425794	1.577463	1.572597	1.567773	1.562968	1.558154	1.553259
1.309536	1.430812	1.426206	1.577913	1.573034	1.568203	1.5634	1.558591	1.5537
1.30997	1.431228	1.426642	1.578401	1.573464	1.568589	1.563774	1.558974	1.554125

1.310394	1.431648	1.427078	1.578888	1.57392	1.569016	1.564184	1.559379	1.554555
1.310789	1.43208	1.427499	1.579357	1.574436	1.569555	1.564691	1.559841	1.554987
1.311177	1.432511	1.427916	1.579821	1.574956	1.57011	1.565225	1.560338	1.555443
1.233089	1.307291	1.303042	1.580294	1.575411	1.570567	1.565731	1.560886	1.555969
1.233437	1.307704	1.303421	1.580768	1.575833	1.570975	1.566221	1.561458	1.556533
1.233761	1.308097	1.303816	1.581222	1.576274	1.571413	1.566674	1.561933	1.557037
1.234061	1.308471	1.304228	1.581656	1.57673	1.571875	1.567088	1.562315	1.557491
1.234366	1.308848	1.304623	1.367597	1.363284	1.572321	1.567502	1.562709	1.557922
1.234683	1.309236	1.304984	1.367998	1.363698	1.572732	1.567916	1.563128	1.55832
1.234994	1.309622	1.305363	1.368423	1.364122	1.573157	1.56835	1.563571	1.558734
1.235269	1.30999	1.305805	1.368914	1.364561	1.573623	1.568827	1.56406	1.559205
1.235551	1.310369	1.306271	1.369428	1.365019	1.574123	1.569329	1.564566	1.559702
1.235916	1.231889	1.228003	1.302572	1.298363	1.529497	1.524792	1.520124	1.5602
1.236338	1.232312	1.228383	1.303014	1.29887	1.53011	1.525333	1.520596	1.560704
1.236718	1.232702	1.228747	1.303444	1.299357	1.530695	1.525869	1.521079	1.561182
1.237058	1.233059	1.22909	1.303859	1.299823	1.531257	1.5264	1.52157	1.561626
1.237386	1.233405	1.229452	1.304299	1.300279	1.531793	1.526932	1.522092	1.562101
1.237703	1.233744	1.229861	1.304792	1.300732	1.532295	1.527476	1.522674	1.562643
1.238018	1.234074	1.230265	1.305283	1.301168	1.532779	1.528006	1.523252	1.563195
1.23833	1.234386	1.230614	1.226754	1.222799	1.421884	1.41743	1.412983	1.51892
1.238654	1.234698	1.230942	1.227079	1.223101	1.422291	1.417851	1.413426	1.519443
1.142759	1.235065	1.231292	1.227423	1.22345	1.422757	1.418299	1.413861	1.519934
1.143168	1.235476	1.231657	1.227776	1.223833	1.423268	1.418764	1.414284	1.520399
1.143581	1.23589	1.232019	1.228127	1.22422	1.299125	1.294939	1.290766	1.410273
1.144002	1.236311	1.232384	1.22848	1.224619	1.299609	1.295366	1.291131	1.410679
1.064639	1.236709	1.232727	1.228812	1.224989	1.300073	1.295802	1.291533	1.411112
1.064993	1.237053	1.233018	1.229088	1.225294	1.300487	1.296252	1.292009	1.411601
1.065335	1.237387	1.233313	1.22937	1.225589	1.300888	1.296709	1.292512	1.412115
1.065687	1.237753	1.233682	1.229733	1.225935	1.222092	1.218116	1.21411	1.412637
1.066045	1.238138	1.234093	1.230142	1.226307	1.222446	1.218499	1.214527	1.413168
1.066427	1.14225	1.138415	1.230541	1.226689	1.222821	1.218886	1.214932	1.41369
1.066833	1.142645	1.138792	1.230935	1.227082	1.223215	1.219278	1.215325	1.414205
1.067244	1.143045	1.13917	1.231328	1.227477	1.223612	1.219672	1.215719	1.414718
1.067658	1.143449	1.139546	1.231718	1.227871	1.224011	1.22007	1.216116	1.41523
1.068078	1.143859	1.139924	1.232107	1.228262	1.224406	1.220465	1.216512	1.41574
1.068504	1.144279	1.140302	1.232492	1.228642	1.224786	1.220851	1.216905	1.350119
1.068932	1.144707	1.14069	1.232883	1.229021	1.225162	1.221233	1.217297	1.350577
1.069352	1.065482	1.061631	1.233299	1.229414	1.225541	1.221613	1.217686	1.351034
1.069768	1.065897	1.062038	1.233735	1.229818	1.225922	1.221994	1.218074	1.351494
1.070197	1.066331	1.062486	1.234205	1.23024	1.226311	1.222381	1.218472	1.35196
1.070638	1.066786	1.062976	1.23471	1.23068	1.226703	1.222773	1.218877	1.35243
1.071096	1.067254	1.063478	1.139078	1.135189	1.227131	1.223198	1.21931	1.215427
1.071577	1.067741	1.063992	1.139612	1.135677	1.227617	1.223684	1.2198	1.215898
1.072063	1.068231	1.064496	1.140134	1.136167	1.228113	1.224171	1.220282	1.216365
1.072535	1.068706	1.064961	1.140602	1.13662	1.228576	1.224606	1.220695	1.216784
1.073004	1.069177	1.065407	1.141045	1.137062	1.229031	1.225023	1.221079	1.217181
1.07348	1.069654	1.065859	1.062062	1.058256	1.2295	1.225455	1.221478	1.217588
1.073959	1.070135	1.066312	1.062508	1.05872	1.22998	1.225897	1.221885	1.217999
0.948573	1.070626	1.066781	1.062973	1.059206	1.230493	1.226369	1.222318	1.218437
0.948914	1.071132	1.067268	1.063461	1.059722	1.231052	1.226879	1.22278	1.218906

0.949227	1.071617	1.067761	1.063959	1.060224	1.231589	1.227392	1.223269	1.219384
0.94948	1.072057	1.068261	1.06447	1.060688	1.232056	1.227888	1.223791	1.219858
0.949729	1.072486	1.06876	1.064981	1.061141	1.232516	1.228392	1.224334	1.220347
0.950035	1.072933	1.069252	1.065483	1.061611	1.057772	1.054004	1.050292	1.220876
0.950369	1.073388	1.069732	1.06597	1.062084	1.058242	1.054503	1.050816	1.221424
0.950698	0.94741	0.944244	1.066398	1.062538	1.058715	1.054969	1.051271	1.221922
0.951024	0.947699	0.944461	1.066768	1.062976	1.059192	1.055405	1.051662	1.222376
0.951366	0.948013	0.944736	1.067183	1.063436	1.059679	1.05587	1.052099	1.22284
0.951728	0.948364	0.9451	1.067668	1.063928	1.060178	1.056379	1.052603	1.223316
0.952098	0.948722	0.945473	1.068174	1.064432	1.060682	1.0569	1.053128	1.223822
0.952474	0.949074	0.945811	1.068693	1.064942	1.06119	1.057424	1.053663	1.224388
0.95285	0.949426	0.946145	1.069213	1.065457	1.061703	1.057951	1.054202	1.224978
0.953208	0.949783	0.946498	1.069704	1.065968	1.06223	1.058475	1.054719	1.050945
0.953553	0.950139	0.946858	1.070177	1.066478	1.062764	1.058997	1.055224	1.051456
0.953868	0.950449	0.947189	0.943925	0.940661	1.06328	1.05952	1.05575	1.051984
0.954142	0.950702	0.947486	0.944257	0.941023	1.063778	1.060045	1.056299	1.052529
0.954488	0.951014	0.947796	0.944574	0.941359	1.064271	1.06056	1.056835	1.053067
0.95495	0.951423	0.948121	0.944857	0.941642	1.064761	1.061062	1.057345	1.053591
0.955455	0.951877	0.94848	0.945161	0.941935	1.065246	1.061556	1.057845	1.054104
0.955972	0.952366	0.948908	0.945547	0.942297	1.065725	1.062043	1.058341	1.054604
0.956497	0.952875	0.949368	0.945967	0.942686	1.066202	1.062524	1.058833	1.055094
0.95699	0.953353	0.949795	0.946354	0.943044	0.93977	0.936499	0.93325	1.055581
0.95746	0.95381	0.950201	0.946722	0.943385	0.9401	0.936852	0.933623	1.056065
0.712963	0.954214	0.950575	0.947077	0.943731	0.940444	0.937206	0.933984	1.056556
0.713588	0.95455	0.950909	0.947415	0.944081	0.940801	0.937556	0.934327	1.057055
0.714241	0.954904	0.951246	0.947752	0.944439	0.941173	0.93792	0.93468	1.05756
0.714978	0.955312	0.951594	0.948085	0.944811	0.941572	0.938306	0.935049	1.058067
0.715724	0.955744	0.951964	0.948433	0.945184	0.941968	0.9387	0.935441	1.058591
0.716407	0.956182	0.952375	0.948818	0.945539	0.942317	0.939095	0.935884	1.05916
0.717066	0.956635	0.952819	0.949232	0.94589	0.942645	0.93949	0.936349	1.05975
0.717749	0.957114	0.95329	0.949673	0.946273	0.942993	0.939862	0.936754	0.933516
0.718449	0.957617	0.953789	0.950143	0.946682	0.943357	0.940214	0.93711	0.93388
0.719151	0.715923	0.712718	0.950604	0.947096	0.943734	0.940573	0.937465	0.93425
0.719854	0.716616	0.713397	0.951043	0.947512	0.944127	0.940943	0.937824	0.934639
0.720547	0.717296	0.714076	0.951495	0.947938	0.944528	0.941314	0.938179	0.935017
0.721205	0.717945	0.714759	0.951972	0.94838	0.944932	0.941681	0.938519	0.935364
0.721869	0.718603	0.715459	0.952472	0.948836	0.945343	0.942052	0.938862	0.935706
0.722607	0.719343	0.716211	0.953019	0.949309	0.945752	0.942432	0.93923	0.936081
0.723389	0.72013	0.716994	0.953595	0.949799	0.946171	0.942828	0.939619	0.936475
0.533474	0.720873	0.717739	0.954111	0.95031	0.946667	0.943295	0.940053	0.936877
0.533976	0.721572	0.718446	0.954572	0.950848	0.947244	0.943837	0.940532	0.937286
0.534491	0.722283	0.719141	0.955009	0.951355	0.947796	0.944377	0.941032	0.937716
0.535025	0.723014	0.719814	0.95541	0.951801	0.948283	0.944896	0.941557	0.938172
0.535572	0.723767	0.720506	0.955831	0.952257	0.948776	0.945419	0.942088	0.938647
0.536134	0.724556	0.721258	0.718073	0.715012	0.949322	0.945958	0.942609	0.939139
0.536708	0.725366	0.722038	0.718842	0.715791	0.949887	0.946506	0.943131	0.939646
0.537286	0.726175	0.722812	0.719598	0.716551	0.950437	0.947056	0.943672	0.940162
0.537868	0.726988	0.723586	0.720351	0.717301	0.950979	0.947611	0.944227	0.940689
0.538454	0.536019	0.533615	0.7211	0.718049	0.951509	0.948165	0.944795	0.941236
0.53905	0.536585	0.53413	0.721846	0.718793	0.952029	0.948722	0.945381	0.941808

0.53712	0.534634	0.72259	0.719535	0.952535	0.949265	0.945954	0.942377
0.537583	0.535111	0.723339	0.720277	0.95303	0.949791	0.946509	0.94294
0.538055	0.535596	0.724078	0.721012	0.953511	0.950295	0.947038	0.943485
0.538622	0.536121	0.724778	0.721726	0.953969	0.950766	0.947523	0.943988
0.539242	0.536675	0.725473	0.722436	0.954414	0.951212	0.947975	0.944461
0.539883	0.537257	0.726264	0.723184	0.7202	0.717298	0.714382	0.944909
0.540549	0.537868	0.727135	0.723965	0.720918	0.718004	0.715097	0.94533
		0.536041	0.533908	0.721643	0.718716	0.715814	0.945747
		0.536619	0.534434	0.722379	0.719437	0.716532	0.946159
		0.537199	0.534959	0.723115	0.720158	0.717253	0.714199
		0.537762	0.535474	0.723849	0.720881	0.717982	0.714961
		0.53834	0.535993	0.724579	0.721599	0.718709	0.715729
		0.538981	0.536527	0.725285	0.722289	0.719413	0.716485
		0.539664	0.537079	0.725988	0.722968	0.720105	0.717231
		0.540352	0.537699	0.726793	0.723704	0.720796	0.717936
		0.541053	0.538388	0.727694	0.724492	0.72148	0.718595
				0.536587	0.534385	0.532324	0.719298
				0.537268	0.535015	0.532888	0.720073
				0.537932	0.535637	0.533455	0.720873
				0.53854	0.536213	0.533998	0.721687
				0.539121	0.536761	0.53452	0.722499
				0.539695	0.537278	0.534995	0.723249
				0.54026	0.53777	0.535434	0.723955
				0.540885	0.538311	0.535901	0.533837
				0.541575	0.538901	0.536385	0.534276
				0.542272	0.539534	0.536941	0.534782
				0.54297	0.540233	0.537617	0.535404
				0.543649	0.540938	0.538324	0.536057
							0.536671
							0.537274
							0.537874
							0.538469
							0.539086
							0.539727
							0.540386
							0.541059
							0.541748
							0.542457
							0.543182
							0.543904
							0.544624

1.756767	1.836324	1.830569	1.896231	1.889982	1.907498	1.901276	1.895131	1.889051
1.75679	1.836374	1.830618	1.896279	1.890024	1.907533	1.90131	1.895167	1.889092
1.756809	1.83642	1.830664	1.896323	1.890062	1.907565	1.901342	1.895201	1.889134
1.75683	1.836468	1.830711	1.896368	1.890099	1.907595	1.901374	1.895239	1.889181
1.756852	1.836516	1.830759	1.896415	1.890138	1.907627	1.901409	1.89528	1.889231
1.756872	1.80572	1.799984	1.896468	1.890207	1.884021	1.877974	1.872018	1.889284
1.756888	1.805755	1.80002	1.896529	1.890308	1.884143	1.878071	1.872083	1.889336
1.756912	1.805788	1.800052	1.89659	1.890411	1.884272	1.878191	1.872182	1.889415
1.756935	1.805808	1.800066	1.89664	1.890509	1.884407	1.878344	1.872336	1.889537
1.756987	1.805857	1.800108	1.896709	1.89061	1.884537	1.878493	1.872492	1.889668
1.721086	1.779217	1.773495	1.863756	1.857705	1.884641	1.878599	1.872606	1.889787
1.721168	1.779389	1.773653	1.863905	1.857829	1.884737	1.878688	1.872699	1.8899
1.721215	1.779508	1.773778	1.864051	1.857973	1.884868	1.878786	1.872773	1.889999
1.721239	1.779596	1.773884	1.864198	1.858133	1.885022	1.878888	1.87283	1.890086
1.721282	1.779692	1.773976	1.864318	1.858256	1.88514	1.878985	1.87291	1.890181
1.721351	1.779801	1.77405	1.864401	1.858329	1.885212	1.879074	1.873017	1.890287
1.721415	1.745686	1.740058	1.841271	1.835222	1.852357	1.846309	1.840343	1.890399
1.721459	1.745742	1.740122	1.841402	1.835343	1.852476	1.846425	1.840459	1.890516
1.721502	1.745807	1.740205	1.841554	1.835482	1.85261	1.846552	1.840582	1.890646
1.721563	1.716057	1.710614	1.801377	1.795525	1.852746	1.846689	1.840727	1.8908
1.721638	1.716157	1.710727	1.801559	1.795675	1.852891	1.846837	1.840886	1.89097
1.721738	1.716262	1.710818	1.80172	1.795829	1.853048	1.846995	1.841046	1.891129
1.721866	1.716374	1.71089	1.801862	1.795987	1.853218	1.847164	1.841209	1.891281
1.67848	1.716465	1.71097	1.73511	1.729412	1.830231	1.824191	1.818245	1.860265
1.678518	1.716522	1.71106	1.735232	1.729543	1.830412	1.82434	1.818365	1.860385
1.678555	1.716584	1.711157	1.735362	1.72967	1.830591	1.824502	1.818513	1.860533
1.678607	1.716675	1.71127	1.735496	1.729765	1.830748	1.824695	1.818742	1.860762
1.678674	1.716779	1.711384	1.73563	1.729861	1.830916	1.824905	1.818993	1.861012
1.67878	1.673429	1.668094	1.706003	1.700462	1.758823	1.753078	1.747407	1.839446
1.678912	1.673534	1.668164	1.706088	1.700606	1.759037	1.75325	1.747529	1.839629
1.679028	1.673652	1.668284	1.706219	1.700747	1.759215	1.753395	1.747641	1.839815
1.679127	1.673785	1.668456	1.706401	1.700883	1.759355	1.753506	1.747737	1.840002
1.679237	1.673916	1.668611	1.706569	1.701032	1.759524	1.753651	1.747861	1.840186
1.679372	1.674039	1.668719	1.706699	1.701203	1.759753	1.753855	1.748024	1.840352
1.679506	1.674163	1.668832	1.706834	1.701373	1.759982	1.754075	1.748218	1.840549
1.679619	1.674299	1.668997	1.663634	1.658209	1.725562	1.719807	1.714115	1.812188
1.679726	1.674437	1.669173	1.663812	1.658342	1.725705	1.720005	1.714373	1.812457
1.679847	1.674559	1.669299	1.663945	1.658485	1.7259	1.720196	1.714564	1.8127
1.679979	1.674669	1.66939	1.664047	1.658636	1.726127	1.720381	1.714709	1.81293
1.680131	1.674816	1.669521	1.664175	1.658775	1.653342	1.647819	1.642369	1.743737
1.680312	1.675008	1.669695	1.664325	1.658891	1.653444	1.647949	1.642531	1.743936
1.680477	1.675197	1.669897	1.664517	1.659043	1.653572	1.648095	1.642698	1.744143
1.680598	1.675365	1.670147	1.664788	1.659267	1.653753	1.648266	1.642866	1.744364
1.680718	1.675525	1.67039	1.665062	1.659513	1.653967	1.648456	1.643036	1.744596
1.680873	1.675673	1.670554	1.665252	1.659745	1.654215	1.648661	1.643198	1.669749
1.68105	1.675821	1.670683	1.665404	1.659975	1.654487	1.648883	1.643363	1.669978
1.68125	1.676009	1.670846	1.665576	1.6602	1.654744	1.649123	1.643573	1.670211
1.681472	1.676232	1.671033	1.66576	1.660419	1.654991	1.64938	1.643822	1.670447
1.681685	1.676457	1.671251	1.665976	1.660638	1.655219	1.649631	1.644086	1.670711
1.681889	1.676686	1.671507	1.66623	1.660852	1.655415	1.649874	1.644372	1.671015

1.682078	1.676905	1.671773	1.666503	1.661081	1.65562	1.650114	1.644649	1.639318
1.682239	1.677099	1.672044	1.666799	1.661344	1.655858	1.650357	1.644904	1.63958
1.682397	1.677283	1.672305	1.667091	1.661617	1.656109	1.650597	1.645139	1.639832
1.682586	1.677472	1.672527	1.667335	1.661872	1.656362	1.65082	1.645337	1.640054
1.682793	1.677661	1.67272	1.667544	1.662117	1.656621	1.651036	1.645512	1.640255
1.683005	1.67785	1.672886	1.667724	1.662358	1.656896	1.651278	1.645713	1.640455
1.683219	1.678036	1.673019	1.667869	1.662593	1.657189	1.651547	1.645937	1.640652
1.683453	1.678246	1.673179	1.66803	1.662816	1.657457	1.651808	1.646177	1.640869
1.683716	1.678491	1.673383	1.668219	1.663019	1.657677	1.652048	1.646431	1.641116
1.683988	1.678747	1.673607	1.668426	1.663224	1.657892	1.652294	1.646704	1.641376
1.684256	1.678998	1.673841	1.66865	1.663445	1.658123	1.652564	1.647003	1.641644
1.684529	1.679254	1.674088	1.668891	1.663682	1.658368	1.652849	1.647318	1.641919
1.684813	1.679524	1.674347	1.66915	1.663951	1.658649	1.653149	1.647626	1.642195
1.685106	1.679803	1.674619	1.669426	1.664246	1.658959	1.653464	1.647934	1.642471
1.685402	1.680086	1.674895	1.669705	1.664539	1.659264	1.653778	1.648247	1.642758
1.685699	1.68037	1.675175	1.669986	1.664828	1.659561	1.654091	1.648566	1.643052
1.686003	1.68066	1.675456	1.670267	1.665117	1.659861	1.654404	1.648887	1.643364
1.686316	1.680956	1.675735	1.670543	1.665407	1.660166	1.654718	1.649211	1.643704
1.686635	1.681255	1.676015	1.67082	1.665698	1.660471	1.655033	1.649537	1.644054
1.582274	1.68155	1.67629	1.67109	1.665983	1.660772	1.655347	1.649864	1.644401
1.582554	1.68185	1.676572	1.671368	1.66627	1.661073	1.655662	1.650195	1.644749
1.582842	1.682178	1.676901	1.671692	1.666582	1.661381	1.655981	1.650529	1.645095
1.583138	1.682531	1.67727	1.672056	1.666916	1.661695	1.656304	1.650867	1.64544
1.583429	1.57844	1.573638	1.672425	1.667271	1.662039	1.656645	1.651207	1.645778
1.583713	1.578733	1.57396	1.672799	1.667657	1.662424	1.657006	1.651542	1.646103
1.583998	1.579019	1.574261	1.673161	1.668035	1.662809	1.657381	1.651901	1.646448
1.584277	1.579283	1.574517	1.673499	1.668387	1.663179	1.657777	1.652313	1.646838
1.584576	1.579555	1.574762	1.673821	1.66872	1.663533	1.658174	1.652742	1.647249
1.533808	1.579879	1.575032	1.570257	1.565583	1.663847	1.658536	1.653151	1.647664
1.534187	1.580237	1.57532	1.570504	1.565817	1.664139	1.658881	1.653551	1.648087
1.534573	1.580602	1.575641	1.570802	1.566115	1.664483	1.659244	1.653941	1.648508
1.534967	1.580977	1.575996	1.571151	1.566475	1.664876	1.659625	1.654317	1.648927
1.535369	1.581365	1.576367	1.571514	1.566841	1.66527	1.66001	1.654704	1.649342
1.535782	1.581775	1.576759	1.57189	1.567203	1.665651	1.660394	1.6551	1.649751
1.536185	1.582185	1.57715	1.572267	1.567566	1.666044	1.660797	1.655516	1.650165
1.536553	1.582582	1.577524	1.572627	1.567923	1.563202	1.558403	1.553539	1.650598
1.536908	1.582972	1.57789	1.572983	1.568281	1.563571	1.558802	1.553957	1.65104
1.43126	1.583351	1.578255	1.573343	1.568647	1.563944	1.559182	1.554345	1.651479
1.43161	1.583721	1.578618	1.573706	1.569018	1.564321	1.559548	1.554713	1.651919
1.431916	1.58407	1.578984	1.57408	1.569389	1.564686	1.559902	1.555064	1.550132
1.432164	1.584384	1.579351	1.574466	1.56976	1.565039	1.560238	1.555394	1.550491
1.432468	1.53361	1.528753	1.574855	1.570128	1.565392	1.560586	1.555744	1.550848
1.432886	1.534045	1.529154	1.575231	1.570483	1.565741	1.560959	1.556136	1.551206
1.433335	1.53451	1.52958	1.575629	1.570853	1.566101	1.56134	1.556534	1.551573
1.433756	1.534965	1.530033	1.576093	1.571274	1.566486	1.561707	1.5569	1.551966
1.434175	1.535422	1.530501	1.576587	1.571721	1.566891	1.56208	1.557262	1.552377
1.308736	1.429961	1.425399	1.57704	1.57216	1.567321	1.562503	1.557684	1.552808
1.309128	1.430388	1.425794	1.577463	1.572597	1.567773	1.562968	1.558154	1.553259
1.309536	1.430812	1.426206	1.577913	1.573034	1.568203	1.5634	1.558591	1.5537
1.30997	1.431228	1.426642	1.578401	1.573464	1.568589	1.563774	1.558974	1.554125

1.310394	1.431648	1.427078	1.578888	1.57392	1.569016	1.564184	1.559379	1.554555
1.310789	1.43208	1.427499	1.579357	1.574436	1.569555	1.564691	1.559841	1.554987
1.311177	1.432511	1.427916	1.579821	1.574956	1.57011	1.565225	1.560338	1.555443
1.233089	1.307291	1.303042	1.580294	1.575411	1.570567	1.565731	1.560886	1.555969
1.233437	1.307704	1.303421	1.580768	1.575833	1.570975	1.566221	1.561458	1.556533
1.233761	1.308097	1.303816	1.581222	1.576274	1.571413	1.566674	1.561933	1.557037
1.234061	1.308471	1.304228	1.581656	1.57673	1.571875	1.567088	1.562315	1.557491
1.234366	1.308848	1.304623	1.367597	1.363284	1.572321	1.567502	1.562709	1.557922
1.234683	1.309236	1.304984	1.367998	1.363698	1.572732	1.567916	1.563128	1.55832
1.234994	1.309622	1.305363	1.368423	1.364122	1.573157	1.56835	1.563571	1.558734
1.235269	1.30999	1.305805	1.368914	1.364561	1.573623	1.568827	1.56406	1.559205
1.235551	1.310369	1.306271	1.369428	1.365019	1.574123	1.569329	1.564566	1.559702
1.235916	1.231889	1.228003	1.302572	1.298363	1.529497	1.524792	1.520124	1.5602
1.236338	1.232312	1.228383	1.303014	1.29887	1.53011	1.525333	1.520596	1.560704
1.236718	1.232702	1.228747	1.303444	1.299357	1.530695	1.525869	1.521079	1.561182
1.237058	1.233059	1.22909	1.303859	1.299823	1.531257	1.5264	1.52157	1.561626
1.237386	1.233405	1.229452	1.304299	1.300279	1.531793	1.526932	1.522092	1.562101
1.237703	1.233744	1.229861	1.304792	1.300732	1.532295	1.527476	1.522674	1.562643
1.238018	1.234074	1.230265	1.305283	1.301168	1.532779	1.528006	1.523252	1.563195
1.23833	1.234386	1.230614	1.226754	1.222799	1.421884	1.41743	1.412983	1.51892
1.238654	1.234698	1.230942	1.227079	1.223101	1.422291	1.417851	1.413426	1.519443
1.142759	1.235065	1.231292	1.227423	1.22345	1.422757	1.418299	1.413861	1.519934
1.143168	1.235476	1.231657	1.227776	1.223833	1.423268	1.418764	1.414284	1.520399
1.143581	1.23589	1.232019	1.228127	1.22422	1.299125	1.294939	1.290766	1.410273
1.144002	1.236311	1.232384	1.22848	1.224619	1.299609	1.295366	1.291131	1.410679
1.064639	1.236709	1.232727	1.228812	1.224989	1.300073	1.295802	1.291533	1.411112
1.064993	1.237053	1.233018	1.229088	1.225294	1.300487	1.296252	1.292009	1.411601
1.065335	1.237387	1.233313	1.22937	1.225589	1.300888	1.296709	1.292512	1.412115
1.065687	1.237753	1.233682	1.229733	1.225935	1.222092	1.218116	1.21411	1.412637
1.066045	1.238138	1.234093	1.230142	1.226307	1.222446	1.218499	1.214527	1.413168
1.066427	1.14225	1.138415	1.230541	1.226689	1.222821	1.218886	1.214932	1.41369
1.066833	1.142645	1.138792	1.230935	1.227082	1.223215	1.219278	1.215325	1.414205
1.067244	1.143045	1.13917	1.231328	1.227477	1.223612	1.219672	1.215719	1.414718
1.067658	1.143449	1.139546	1.231718	1.227871	1.224011	1.22007	1.216116	1.41523
1.068078	1.143859	1.139924	1.232107	1.228262	1.224406	1.220465	1.216512	1.41574
1.068504	1.144279	1.140302	1.232492	1.228642	1.224786	1.220851	1.216905	1.350119
1.068932	1.144707	1.14069	1.232883	1.229021	1.225162	1.221233	1.217297	1.350577
1.069352	1.065482	1.061631	1.233299	1.229414	1.225541	1.221613	1.217686	1.351034
1.069768	1.065897	1.062038	1.233735	1.229818	1.225922	1.221994	1.218074	1.351494
1.070197	1.066331	1.062486	1.234205	1.23024	1.226311	1.222381	1.218472	1.35196
1.070638	1.066786	1.062976	1.23471	1.23068	1.226703	1.222773	1.218877	1.35243
1.071096	1.067254	1.063478	1.139078	1.135189	1.227131	1.223198	1.21931	1.215427
1.071577	1.067741	1.063992	1.139612	1.135677	1.227617	1.223684	1.2198	1.215898
1.072063	1.068231	1.064496	1.140134	1.136167	1.228113	1.224171	1.220282	1.216365
1.072535	1.068706	1.064961	1.140602	1.13662	1.228576	1.224606	1.220695	1.216784
1.073004	1.069177	1.065407	1.141045	1.137062	1.229031	1.225023	1.221079	1.217181
1.07348	1.069654	1.065859	1.062062	1.058256	1.2295	1.225455	1.221478	1.217588
1.073959	1.070135	1.066312	1.062508	1.05872	1.22998	1.225897	1.221885	1.217999
0.948573	1.070626	1.066781	1.062973	1.059206	1.230493	1.226369	1.222318	1.218437
0.948914	1.071132	1.067268	1.063461	1.059722	1.231052	1.226879	1.22278	1.218906

0.949227	1.071617	1.067761	1.063959	1.060224	1.231589	1.227392	1.223269	1.219384
0.94948	1.072057	1.068261	1.06447	1.060688	1.232056	1.227888	1.223791	1.219858
0.949729	1.072486	1.06876	1.064981	1.061141	1.232516	1.228392	1.224334	1.220347
0.950035	1.072933	1.069252	1.065483	1.061611	1.057772	1.054004	1.050292	1.220876
0.950369	1.073388	1.069732	1.06597	1.062084	1.058242	1.054503	1.050816	1.221424
0.950698	0.94741	0.944244	1.066398	1.062538	1.058715	1.054969	1.051271	1.221922
0.951024	0.947699	0.944461	1.066768	1.062976	1.059192	1.055405	1.051662	1.222376
0.951366	0.948013	0.944736	1.067183	1.063436	1.059679	1.05587	1.052099	1.22284
0.951728	0.948364	0.9451	1.067668	1.063928	1.060178	1.056379	1.052603	1.223316
0.952098	0.948722	0.945473	1.068174	1.064432	1.060682	1.0569	1.053128	1.223822
0.952474	0.949074	0.945811	1.068693	1.064942	1.06119	1.057424	1.053663	1.224388
0.95285	0.949426	0.946145	1.069213	1.065457	1.061703	1.057951	1.054202	1.224978
0.953208	0.949783	0.946498	1.069704	1.065968	1.06223	1.058475	1.054719	1.050945
0.953553	0.950139	0.946858	1.070177	1.066478	1.062764	1.058997	1.055224	1.051456
0.953868	0.950449	0.947189	0.943925	0.940661	1.06328	1.05952	1.05575	1.051984
0.954142	0.950702	0.947486	0.944257	0.941023	1.063778	1.060045	1.056299	1.052529
0.954488	0.951014	0.947796	0.944574	0.941359	1.064271	1.06056	1.056835	1.053067
0.95495	0.951423	0.948121	0.944857	0.941642	1.064761	1.061062	1.057345	1.053591
0.955455	0.951877	0.94848	0.945161	0.941935	1.065246	1.061556	1.057845	1.054104
0.955972	0.952366	0.948908	0.945547	0.942297	1.065725	1.062043	1.058341	1.054604
0.956497	0.952875	0.949368	0.945967	0.942686	1.066202	1.062524	1.058833	1.055094
0.95699	0.953353	0.949795	0.946354	0.943044	0.93977	0.936499	0.93325	1.055581
0.95746	0.95381	0.950201	0.946722	0.943385	0.9401	0.936852	0.933623	1.056065
0.712963	0.954214	0.950575	0.947077	0.943731	0.940444	0.937206	0.933984	1.056556
0.713588	0.95455	0.950909	0.947415	0.944081	0.940801	0.937556	0.934327	1.057055
0.714241	0.954904	0.951246	0.947752	0.944439	0.941173	0.93792	0.93468	1.05756
0.714978	0.955312	0.951594	0.948085	0.944811	0.941572	0.938306	0.935049	1.058067
0.715724	0.955744	0.951964	0.948433	0.945184	0.941968	0.9387	0.935441	1.058591
0.716407	0.956182	0.952375	0.948818	0.945539	0.942317	0.939095	0.935884	1.05916
0.717066	0.956635	0.952819	0.949232	0.94589	0.942645	0.93949	0.936349	1.05975
0.717749	0.957114	0.95329	0.949673	0.946273	0.942993	0.939862	0.936754	0.933516
0.718449	0.957617	0.953789	0.950143	0.946682	0.943357	0.940214	0.93711	0.93388
0.719151	0.715923	0.712718	0.950604	0.947096	0.943734	0.940573	0.937465	0.93425
0.719854	0.716616	0.713397	0.951043	0.947512	0.944127	0.940943	0.937824	0.934639
0.720547	0.717296	0.714076	0.951495	0.947938	0.944528	0.941314	0.938179	0.935017
0.721205	0.717945	0.714759	0.951972	0.94838	0.944932	0.941681	0.938519	0.935364
0.721869	0.718603	0.715459	0.952472	0.948836	0.945343	0.942052	0.938862	0.935706
0.722607	0.719343	0.716211	0.953019	0.949309	0.945752	0.942432	0.93923	0.936081
0.723389	0.72013	0.716994	0.953595	0.949799	0.946171	0.942828	0.939619	0.936475
0.533474	0.720873	0.717739	0.954111	0.95031	0.946667	0.943295	0.940053	0.936877
0.533976	0.721572	0.718446	0.954572	0.950848	0.947244	0.943837	0.940532	0.937286
0.534491	0.722283	0.719141	0.955009	0.951355	0.947796	0.944377	0.941032	0.937716
0.535025	0.723014	0.719814	0.95541	0.951801	0.948283	0.944896	0.941557	0.938172
0.535572	0.723767	0.720506	0.955831	0.952257	0.948776	0.945419	0.942088	0.938647
0.536134	0.724556	0.721258	0.718073	0.715012	0.949322	0.945958	0.942609	0.939139
0.536708	0.725366	0.722038	0.718842	0.715791	0.949887	0.946506	0.943131	0.939646
0.537286	0.726175	0.722812	0.719598	0.716551	0.950437	0.947056	0.943672	0.940162
0.537868	0.726988	0.723586	0.720351	0.717301	0.950979	0.947611	0.944227	0.940689
0.538454	0.536019	0.533615	0.7211	0.718049	0.951509	0.948165	0.944795	0.941236
0.53905	0.536585	0.53413	0.721846	0.718793	0.952029	0.948722	0.945381	0.941808

0.53712	0.534634	0.72259	0.719535	0.952535	0.949265	0.945954	0.942377
0.537583	0.535111	0.723339	0.720277	0.95303	0.949791	0.946509	0.94294
0.538055	0.535596	0.724078	0.721012	0.953511	0.950295	0.947038	0.943485
0.538622	0.536121	0.724778	0.721726	0.953969	0.950766	0.947523	0.943988
0.539242	0.536675	0.725473	0.722436	0.954414	0.951212	0.947975	0.944461
0.539883	0.537257	0.726264	0.723184	0.7202	0.717298	0.714382	0.944909
0.540549	0.537868	0.727135	0.723965	0.720918	0.718004	0.715097	0.94533
		0.536041	0.533908	0.721643	0.718716	0.715814	0.945747
		0.536619	0.534434	0.722379	0.719437	0.716532	0.946159
		0.537199	0.534959	0.723115	0.720158	0.717253	0.714199
		0.537762	0.535474	0.723849	0.720881	0.717982	0.714961
		0.53834	0.535993	0.724579	0.721599	0.718709	0.715729
		0.538981	0.536527	0.725285	0.722289	0.719413	0.716485
		0.539664	0.537079	0.725988	0.722968	0.720105	0.717231
		0.540352	0.537699	0.726793	0.723704	0.720796	0.717936
		0.541053	0.538388	0.727694	0.724492	0.72148	0.718595
				0.536587	0.534385	0.532324	0.719298
				0.537268	0.535015	0.532888	0.720073
				0.537932	0.535637	0.533455	0.720873
				0.53854	0.536213	0.533998	0.721687
				0.539121	0.536761	0.53452	0.722499
				0.539695	0.537278	0.534995	0.723249
				0.54026	0.53777	0.535434	0.723955
				0.540885	0.538311	0.535901	0.533837
				0.541575	0.538901	0.536385	0.534276
				0.542272	0.539534	0.536941	0.534782
				0.54297	0.540233	0.537617	0.535404
				0.543649	0.540938	0.538324	0.536057
							0.536671
							0.537274
							0.537874
							0.538469
							0.539086
							0.539727
							0.540386
							0.541059
							0.541748
							0.542457
							0.543182
							0.543904
							0.544624

		0.737508
		0.73634
		0.735063
		0.73366
		0.900631
		0.899424
		0.898281
		0.897124
		0.895994
0.735925	0.731604	1.254553
0.734631	0.73062	1.253215
0.901994	0.897512	1.439164
0.901005	0.896618	1.43783
0.900028	0.895678	1.436436
0.898991	0.894609	1.434868

						0.897972	0.893517	1.433241
						0.897038	0.89249	1.431604
						0.896147	0.891489	1.429968
			0.55051	0.548155	0.54575	1.225016	1.219958	1.488001
			0.549863	0.5475	0.545094	1.223895	1.218827	1.486604
			0.77657	0.771851	0.767311	1.222673	1.217609	1.48513
			0.775513	0.770806	0.766262	1.221302	1.216258	1.483529
			0.905988	0.900829	0.895813	1.396061	1.390428	1.603029
			0.905007	0.899795	0.894723	1.394597	1.388955	1.601343
			0.904057	0.898783	0.893654	1.393181	1.387537	1.599713
			0.903046	0.897722	0.892561	1.391787	1.386206	1.598273
			0.901999	0.896631	0.891454	1.39042	1.38494	1.596956
	0.550202	0.547827	1.306912	1.301408	1.296053	1.457925	1.452248	1.66011
	0.549538	0.547169	1.305665	1.300151	1.294799	1.456439	1.450876	1.658661
	0.548934	0.546564	1.30439	1.298872	1.293533	1.45498	1.449533	1.657256
	0.548455	0.546079	1.303069	1.297545	1.29223	1.453567	1.448243	1.655946
	0.547989	0.545588	1.301749	1.296234	1.290956	1.452174	1.446957	1.654628
	0.547472	0.544994	1.300462	1.294987	1.28976	1.450775	1.445624	1.653209
	0.54693	0.544371	1.299197	1.293784	1.28862	1.449402	1.44429	1.65177
0.548787	1.104817	1.100912	1.465729	1.459477	1.453524	1.448124	1.443016	1.650437
0.548151	1.103908	1.100063	1.464217	1.45812	1.452303	1.44692	1.441787	1.649177
0.547579	1.102999	1.099188	1.462748	1.456784	1.451078	1.526279	1.520964	1.683176
0.547079	1.102084	1.098285	1.461331	1.455475	1.449851	1.52512	1.519781	1.681815
0.773127	1.303845	1.298399	1.550138	1.543928	1.537928	1.65702	1.651123	1.680471
0.772143	1.302645	1.297146	1.548609	1.542478	1.536564	1.655717	1.649868	1.679173
0.771175	1.30146	1.295907	1.547075	1.541024	1.535202	1.654417	1.648624	1.677891
0.770134	1.300234	1.294693	1.594501	1.588467	1.582644	1.714005	1.708	1.746674
0.769051	1.298993	1.293504	1.592975	1.587077	1.581371	1.712735	1.706768	1.745383
1.209132	1.465051	1.458732	1.591577	1.585762	1.580108	1.711455	1.70548	1.744045
1.207875	1.463558	1.457404	1.59029	1.584514	1.578858	1.710171	1.704145	1.742664
1.206629	1.517203	1.51111	1.651127	1.645081	1.639166	1.753891	1.747652	1.934684
1.205393	1.515841	1.509893	1.649894	1.643813	1.637853	1.752531	1.746308	1.933047
1.204183	1.514495	1.508676	1.648668	1.642566	1.636572	1.751208	1.744991	1.931441
1.203024	1.513145	1.507433	1.647423	1.641365	1.635387	1.749971	1.74371	1.929836
1.201879	1.511796	1.506174	1.646159	1.640166	1.634224	1.748768	1.742458	1.928234
1.391852	1.510425	1.504885	1.644839	1.638904	1.633001	1.747554	1.741242	1.926607
1.390471	1.509048	1.50358	1.643486	1.63761	1.631752	1.746338	1.740048	1.924969
1.449816	1.553884	1.548217	1.642187	1.636361	1.630531	1.855481	1.848968	1.923352
1.448558	1.552564	1.546963	1.640936	1.635151	1.629337	1.854168	1.847614	1.921759
1.447357	1.551318	1.545759	1.639717	1.633956	1.628141	1.852832	1.846214	1.999568
1.446228	1.550173	1.544621	1.638536	1.632776	1.626935	1.851452	1.844732	1.997738
1.445125	1.549063	1.54351	1.637372	1.631598	1.625727	1.850087	1.843284	1.995954
1.553832	1.628074	1.622309	1.686524	1.680632	1.674655	1.890795	1.88375	1.994373
1.552668	1.626961	1.621214	1.68536	1.679437	1.673435	1.889468	1.882445	1.992887
1.551505	1.625848	1.620114	1.684191	1.678239	1.672214	1.888137	1.881132	1.991402
1.550345	1.624737	1.619013	1.68302	1.67704	1.670992	1.886809	1.879827	1.989936
1.549199	1.623632	1.617911	1.805471	1.79924	1.792934	1.885485	1.878529	1.988478
1.548066	1.622535	1.616809	1.804238	1.797977	1.791649	1.884164	1.877236	1.987024
1.546942	1.621431	1.6157	1.803001	1.796715	1.790373	1.96755	1.960457	1.985583
1.545824	1.620315	1.614583	1.801761	1.795457	1.789107	1.966203	1.959138	1.98416

1.544712	1.619195	1.613458	1.800516	1.794203	1.787855	1.964865	1.957829	1.982746
1.543609	1.618079	1.612319	1.799258	1.792956	1.786626	1.963534	1.956518	1.981324
1.542511	1.616962	1.611171	1.797994	1.791714	1.785413	1.962213	1.955216	1.979913
1.566262	1.615837	1.61002	1.796729	1.790471	1.784203	1.960914	1.953957	1.978565
1.565167	1.614703	1.608865	1.795463	1.789229	1.782998	1.959634	1.952733	1.977271
1.564097	1.613592	1.607725	1.89402	1.887484	1.880956	1.916055	1.909408	2.046132
1.563055	1.612505	1.606597	1.892664	1.886147	1.879655	1.914808	1.90821	2.044729
1.56203	1.611446	1.605506	1.891346	1.884835	1.878367	1.913579	1.907038	2.04334
1.561038	1.610442	1.604484	1.890096	1.883561	1.877096	1.912376	1.905906	2.041966
1.560037	1.609439	1.603484	1.888878	1.88231	1.875841	1.911198	1.904808	2.040629
1.558958	1.77711	1.770796	1.914395	1.907768	1.901258	1.936877	1.930527	2.057489
1.557833	1.775905	1.76965	1.913167	1.90653	1.900029	1.935746	1.929495	2.056276
1.556728	1.774705	1.768539	1.912014	1.905362	1.898846	1.934603	1.92839	2.054968
1.555634	1.773502	1.767461	1.910941	1.904266	1.897711	1.93345	1.92722	2.053572
1.600193	1.89596	1.889549	1.937727	1.931068	1.92451	1.985046	1.97859	2.063203
1.599197	1.894746	1.888317	1.936491	1.929892	1.923374	1.983796	1.977293	2.061829
1.598215	1.893547	1.887072	1.935236	1.928716	1.922259	1.982577	1.976022	2.060466
1.597203	1.892332	1.885814	1.933974	1.927564	1.921197	1.981447	1.974829	2.02713
1.596191	1.891131	1.884572	1.932724	1.926415	1.920136	1.980347	1.973683	2.025819
1.595213	1.890004	1.883425	1.931544	1.925233	1.918967	1.979195	1.972592	2.024607
1.594254	1.888927	1.882346	1.930419	1.924034	1.91773	1.978013	1.971542	2.023461
1.775697	1.914549	1.907967	1.929341	1.922912	1.916567	1.98627	1.97965	1.986577
1.774477	1.913305	1.906864	1.928326	1.92188	1.915483	1.985004	1.978338	1.985279
1.773323	1.912088	1.905729	1.927253	1.920833	1.914429	1.98378	1.97706	2.043845
1.772305	1.910931	1.904519	1.926052	1.919744	1.913428	1.982647	1.975863	2.042636
1.771334	1.909819	1.903328	1.924841	1.918626	1.912394	1.981517	1.974703	2.041464
1.882678	1.936607	1.930105	1.92371	1.917456	1.911212	1.98032	1.973585	2.040328
1.881676	1.935603	1.929072	1.922624	1.916269	1.90997	1.97909	1.972476	2.039201
1.880641	1.934557	1.928041	1.921579	1.915163	1.908811	1.977837	1.971255	2.110764
1.879584	1.933487	1.927023	1.920576	1.91412	1.907712	1.976562	1.969947	2.109417
1.878536	1.932429	1.925984	1.944211	1.937522	1.930888	1.975367	1.968722	2.108101
1.877505	1.931388	1.924916	1.94313	1.936473	1.929863	1.974277	1.967603	2.106817
1.876462	1.930338	1.923853	1.942052	1.935419	1.92883	2.035301	2.028593	2.105577
1.875384	1.929257	1.922819	1.940992	1.934341	1.927745	2.034173	2.02751	2.104415
1.874303	1.928174	1.921786	1.93993	1.933258	1.926652	2.033049	2.026439	2.103271
1.873253	1.927119	1.920722	1.938839	1.932181	1.925591	2.031939	2.025337	2.102062
1.87222	1.92608	1.919649	1.937738	1.931108	1.924544	2.030841	2.024229	2.100833
1.86878	1.89517	1.889091	1.936675	1.930038	1.923478	2.029751	2.023177	2.09968
1.867772	1.894176	1.888144	1.935645	1.928969	1.922391	2.028669	2.022177	2.098597
1.866795	1.893213	1.887216	1.982016	1.975303	1.968716	2.075249	2.068622	2.097509
1.865857	1.892292	1.886317	1.98106	1.974372	1.9678	2.074248	2.067603	2.096408
1.86494	1.891381	1.88541	1.980089	1.97343	1.966876	2.07324	2.066569	2.095289
1.864044	1.890465	1.884464	1.979041	1.972399	1.965865	2.072174	2.065488	2.094129
1.863151	1.889541	1.8835	1.977964	1.971336	1.964822	2.071082	2.06439	2.092955
1.862207	1.968992	1.962477	2.042495	2.035704	2.029016	2.079417	2.072648	2.107263
1.861232	1.967925	1.961428	2.041398	2.034599	2.027909	2.078287	2.071544	2.10614
1.860292	1.966874	1.960366	2.040283	2.033515	2.026858	2.077195	2.070466	2.105059
1.859388	1.965833	1.959279	2.039137	2.032445	2.025861	2.076142	2.069416	2.104027
1.858503	2.009593	2.00296	2.085808	2.079067	2.072393	2.075107	2.068361	2.102976
1.857644	2.008619	2.002023	2.084824	2.078102	2.071428	2.074085	2.067283	2.101872

1.856778	2.007652	2.001104	2.083866	2.077157	2.070475	2.073075	2.066216	2.100774
1.855854	1.981814	1.975362	2.062845	2.056203	2.049573	2.072082	2.065198	2.099737
1.854922	1.980786	1.974386	2.061848	2.055222	2.048596	2.07109	2.064197	2.098723
1.854085	1.979853	1.973473	2.060855	2.054211	2.047568	2.070044	2.063158	2.097668
1.853321	1.978993	1.972608	2.059864	2.053174	2.046496	2.068956	2.06209	2.096586
1.908524	1.978131	1.971734	2.05035	2.043643	2.036907	2.067893	2.061029	2.095523
1.907709	1.977269	1.970852	2.049378	2.042661	2.035915	2.066867	2.059978	2.094487
1.906865	1.976379	1.969939	2.048377	2.041658	2.03491	2.065833	2.058923	2.093443
1.905961	1.975424	1.968956	2.047312	2.040594	2.033851	2.064758	2.057845	2.092357
1.905051	1.974465	1.967977	2.046248	2.039531	2.032795	2.063692	2.056785	2.091291
1.928971	2.047637	2.040961	2.045258	2.038531	2.031792	2.04359	2.036743	2.090316
1.928137	2.046702	2.040026	2.044311	2.037568	2.030824	2.042636	2.035815	2.089401
1.927323	2.045791	2.039109	2.043378	2.036622	2.029876	2.041707	2.034913	2.088506
1.926531	2.044908	2.038213	2.042462	2.035696	2.028952	2.040804	2.034041	2.087637
1.925741	2.055096	2.048318	2.041548	2.034775	2.028034	2.039906	2.033171	2.086767
1.924949	2.054203	2.047411	2.040632	2.033854	2.027119	2.039009	2.032298	2.085891
1.924161	2.053311	2.046507	2.039721	2.032942	2.026212	2.038118	2.031429	2.085016
1.923376	2.052422	2.04561	2.038819	2.03204	2.025318	2.037235	2.030565	2.084143
1.922595	2.051532	2.044715	2.037922	2.031145	2.024431	2.036357	2.029704	2.08327
1.921815	2.050638	2.04382	2.037029	2.030255	2.023549	2.035484	2.028847	2.082398
1.921038	2.04974	2.042926	2.036139	2.02937	2.022671	2.034614	2.027991	2.081525
1.920257	2.048834	2.042022	2.035241	2.028478	2.02179	2.033739	2.02713	2.080645
1.919469	2.047918	2.041107	2.034331	2.027577	2.020901	2.032855	2.026257	2.079754
1.957738	2.047014	2.04021	2.033442	2.026696	2.020032	2.013488	2.007046	2.061923
1.956963	2.046118	2.039329	2.032576	2.025837	2.019183	2.012665	2.006248	2.061084
1.956224	2.045255	2.038487	2.031749	2.025015	2.01837	2.011879	2.005489	2.060286
1.955555	2.04447	2.037727	2.031005	2.024271	2.017629	2.011162	2.004798	2.059573
1.954912	2.043717	2.036998	2.030292	2.023558	2.016918	2.010473	2.004131	2.05889
2.008806	2.042907	2.036197	2.048484	2.041727	2.035058	2.028579	2.022195	2.05812
2.008004	2.04206	2.035346	2.04765	2.040913	2.034264	2.027794	2.021415	2.057289
2.007214	2.041218	2.034496	2.046818	2.040105	2.033478	2.027011	2.02063	2.056463
2.006437	2.040377	2.033643	2.045985	2.039299	2.032697	2.026228	2.019836	2.055638
2.005678	2.039563	2.032814	2.045176	2.038524	2.031949	2.025473	2.019065	2.054843
2.004946	2.038795	2.032031	2.044414	2.037807	2.031267	2.024774	2.018338	2.054101
2.004228	2.038045	2.031268	2.04367	2.037103	2.030594	2.02409	2.01763	2.053379
1.979599	2.037287	2.030503	2.042914	2.036358	2.029861	2.023369	2.01692	2.036432
1.978895	2.036533	2.029748	2.042166	2.035605	2.029109	2.022641	2.016219	2.035704
1.97819	2.035796	2.02903	2.041474	2.034911	2.028415	2.021961	2.015552	2.035012
1.977486	2.035076	2.028347	2.040832	2.034269	2.027771	2.021322	2.014916	2.034352
1.976779	1.994101	1.987551	2.002237	1.995822	1.989452	2.020686	2.014271	2.033679
1.976066	1.993403	1.986871	2.001585	1.995202	1.98885	2.020057	2.01361	2.032983
1.975357	1.992701	1.986186	2.000923	1.994561	1.988223	2.019406	2.012938	2.032288
1.974657	1.99199	1.985489	2.000239	1.993868	1.987526	2.018694	2.01224	2.031605
1.973964	1.991284	1.984794	1.999559	1.993171	1.986821	2.017972	2.011544	2.030934
1.973281	1.966812	1.960427	1.998921	1.992536	1.986188	2.017305	2.010888	2.030287
1.972606	1.966149	1.959775	1.998312	1.991941	1.985602	2.016673	2.01026	2.029659
1.971927	1.965492	1.959154	1.997728	1.991369	1.985033	2.016062	2.009638	2.061258
1.971241	1.964838	1.958566	1.997172	1.990823	1.984483	2.015476	2.009024	2.060623
1.970588	1.964214	1.957979	1.996614	1.990273	1.983932	2.014889	2.008423	2.060003
1.969991	1.963635	1.957385	1.996048	1.989707	1.983371	2.014296	2.007841	2.059408

1.969403	1.963063	1.956796	1.995481	1.989141	1.98281	2.013703	2.00726	2.058814
1.968791	1.962465	1.95622	1.972456	1.966175	1.959894	2.013116	2.006662	2.058202
1.968175	1.96186	1.955648	1.971908	1.965623	1.959332	2.012531	2.006061	2.057587
1.967573	1.961268	1.955059	1.97134	1.965061	1.958778	2.011946	2.005486	2.056995
1.966982	1.960684	1.954459	1.970759	1.964494	1.958227	2.011362	2.004929	2.056418
1.96641	1.960121	1.953889	1.970202	1.963935	1.95767	1.973673	1.967359	2.046875
1.965856	1.959579	1.953348	1.969666	1.963382	1.957104	1.973117	1.966811	2.046273
1.965324	1.959058	1.95284	1.969166	1.962855	1.956553	1.972564	1.966264	2.045674
1.964829	1.958571	1.952384	1.968727	1.962368	1.956021	1.97201	1.965715	2.045085
1.964336	1.958088	1.951931	1.968298	1.961901	1.955517	1.971474	1.965173	2.044494
1.963807	1.957576	1.951425	1.967822	1.961447	1.95507	1.967744	1.961377	2.043881
1.963262	1.957049	1.950893	1.967324	1.960999	1.954651	1.967274	1.960846	2.043268
1.962716	1.95652	1.950365	2.007294	2.000812	1.994321	2.004879	1.998331	2.042724
1.962168	1.955986	1.949835	2.006755	2.000288	1.993816	2.004397	1.997893	2.042239
1.961644	1.955472	1.949336	2.023411	2.016903	2.010391	2.003899	1.997441	2.04177
1.961154	1.954987	1.948884	2.022942	2.016383	2.009838	2.003373	1.996966	2.041316
1.960673	1.954506	1.948422	2.022466	2.015873	2.009307	2.00286	1.996487	2.023388
1.960188	1.95401	1.947911	2.021946	2.015376	2.008836	2.00239	1.996016	2.022931
1.959711	1.953519	1.947387	2.021406	2.014882	2.008383	2.00193	1.995539	2.02246
1.959258	1.973176	1.966959	2.02085	2.014363	2.007897	2.001437	1.995029	2.021933
1.958819	1.972705	1.966433	2.020287	2.013837	2.007399	2.000932	1.994506	2.021375
1.958359	1.972225	1.965936	2.019787	2.013363	2.006947	2.000481	1.994047	2.020865
1.957874	1.971734	1.965465	2.019348	2.012943	2.00654	2.000085	1.993649	2.020397
1.977527	1.971263	1.965017	1.999267	1.992888	1.986519	1.980125	1.973752	1.986928
1.977076	1.970818	1.964594	1.998843	1.992441	1.98606	1.979703	1.973376	1.986575
1.976645	1.970391	1.964184	1.998423	1.991987	1.985587	1.979264	1.972987	1.986223
1.976243	1.969988	1.963782	1.997993	1.991512	1.985083	1.978778	1.972539	1.985826
1.975848	1.96959	1.963381	1.997561	1.991036	1.98458	1.978285	1.972076	1.985417
1.975416	1.969156	1.962962	1.99714	1.990606	1.984144	1.977854	1.971657	1.985029
1.974957	1.968696	1.962528	1.996725	1.990208	1.983756	1.977467	1.971266	1.984657
1.974482	1.968215	1.962064	1.996282	1.989796	1.983371	1.977092	1.970893	1.984303
1.973986	1.967704	1.961562	1.995803	1.989368	1.982988	1.976731	1.970542	1.98397
1.973509	1.967214	1.961076	1.995335	1.988946	1.982609	1.976372	1.970191	1.983636
1.973058	1.966753	1.960619	1.994893	1.98854	1.982233	1.976008	1.96983	1.983295
1.972648	1.966335	1.960197	1.994474	1.988144	1.98186	1.975644	1.969468	1.982952
1.97231	1.965992	1.959827	1.994083	1.987761	1.981488	1.975278	1.969109	1.982607
1.972019	1.965703	1.959501	1.993721	1.987392	1.981116	1.974912	1.96875	1.982262
1.971742	1.965438	1.959218	1.993402	1.987042	1.980744	1.974541	1.968391	1.981925
1.971483	1.965204	1.95898	1.993125	1.986713	1.98037	1.974165	1.968031	1.981593
1.950176	1.964933	1.958713	1.952472	1.946175	1.939928	1.933805	1.927755	1.961537
1.949839	1.964615	1.958407	1.952176	1.945887	1.939638	1.93349	1.927412	1.961189
1.949498	1.96428	1.95808	1.951862	1.945599	1.939366	1.933202	1.927099	1.960861
1.949158	1.963926	1.957726	1.951526	1.945307	1.939112	1.932959	1.92685	1.960575
1.948832	1.963576	1.957364	1.951175	1.945003	1.938853	1.932719	1.926615	1.960302
1.940385	1.963275	1.957022	1.950815	1.944661	1.938537	1.932424	1.926337	1.960018
1.940121	1.963002	1.956691	1.95045	1.944298	1.938188	1.932095	1.926034	1.959728
1.939823	1.962711	1.95638	1.950122	1.943958	1.937844	1.931757	1.925712	1.95943
1.939492	1.962403	1.956087	1.949828	1.943636	1.937497	1.931403	1.925364	1.959122
1.939183	1.962115	1.955814	1.949557	1.943345	1.937188	1.931091	1.925056	1.958831
1.93891	1.961855	1.955563	1.949311	1.943095	1.936934	1.93084	1.924809	1.958559

1.938653	1.961609	1.955326	1.949079	1.942864	1.936703	1.930613	1.924584	1.958309
1.938402	1.953822	1.947546	1.948855	1.942643	1.936484	1.930396	1.924366	1.95809
1.938159	1.95358	1.947305	1.948637	1.942431	1.936277	1.930189	1.924157	1.957888
1.937915	1.953333	1.947063	1.948415	1.942216	1.936067	1.92998	1.92395	1.957675
1.937671	1.953086	1.946822	1.948193	1.942	1.935857	1.929773	1.923745	1.95746
1.93743	1.952843	1.946577	1.947963	1.941778	1.935643	1.929568	1.923546	1.957257
1.937191	1.952606	1.946328	1.947724	1.941547	1.935424	1.929364	1.923356	1.957071
1.936952	1.952366	1.946079	1.947488	1.94132	1.935208	1.929157	1.923157	1.956877
1.936713	1.952119	1.945828	1.947259	1.9411	1.934995	1.928944	1.92294	1.956666
1.936476	1.95187	1.945579	1.947034	1.940884	1.934785	1.928731	1.922722	1.956451
1.936244	1.951623	1.945335	1.939115	1.932972	1.926882	1.928519	1.922512	1.956234
1.936016	1.951376	1.945093	1.938878	1.932739	1.926654	1.928314	1.922311	1.95602
1.935793	1.951136	1.944846	1.93864	1.932531	1.926472	1.928145	1.922127	1.955823
1.935574	1.9509	1.944597	1.938401	1.932345	1.926327	1.928006	1.92196	1.955639
1.935344	1.950672	1.944383	1.938206	1.932177	1.926179	1.927868	1.921808	1.955481
1.93509	1.950449	1.944215	1.938069	1.932032	1.926022	1.927729	1.921674	1.955353
1.934877	1.950253	1.944057	1.937933	1.931892	1.925874	1.919826	1.91379	1.955228
1.934754	1.950104	1.943892	1.937766	1.931745	1.925744	1.919692	1.913642	1.955077
1.934662	1.94998	1.943737	1.937601	1.9316	1.92562	1.919568	1.91351	1.95494
1.934551	1.949871	1.943616	1.937471	1.931466	1.925488	1.919458	1.913425	1.954865
1.934436	1.949774	1.943513	1.937359	1.931338	1.925357	1.919359	1.913367	1.954825
1.934301	1.949642	1.943377	1.937223	1.93121	1.925242	1.919262	1.913288	1.954772
1.934145	1.949476	1.943208	1.937065	1.931085	1.925144	1.91917	1.913192	1.95471
1.934014	1.927709	1.921484	1.936927	1.930957	1.925029	1.919061	1.913088	1.907087
1.933926	1.92764	1.921403	1.936812	1.930819	1.924878	1.918925	1.912976	1.906979
1.933837	1.927574	1.921342	1.936729	1.930704	1.924738	1.918791	1.912859	1.906867
1.93371	1.927473	1.921294	1.936702	1.93064	1.924634	1.918665	1.912723	1.906749
1.933578	1.927364	1.921247	1.936693	1.930603	1.924558	1.918558	1.912594	1.906639
1.904467	1.927258	1.921157	1.936631	1.930564	1.924529	1.918506	1.912512	1.906564
1.9044	1.927156	1.92104	1.936539	1.930529	1.924537	1.918496	1.912466	1.906518
1.904339	1.927086	1.92095	1.936451	1.930484	1.924531	1.918493	1.912453	1.906499
1.904286	1.927052	1.920886	1.936362	1.930427	1.924512	1.918501	1.912479	1.906509
1.904231	1.927025	1.920845	1.936305	1.930377	1.924478	1.918498	1.912506	1.906524
1.904174	1.927001	1.920838	1.9363	1.93034	1.924422	1.918481	1.912537	1.906547
1.904112	1.926972	1.920841	1.936314	1.930312	1.924361	1.918445	1.91254	1.90655
1.904037	1.898024	1.892094	1.936336	1.930296	1.924305	1.918373	1.912465	1.906495
1.903961	1.897954	1.892078	1.936359	1.930288	1.924257	1.918286	1.912351	1.906409
1.903908	1.897897	1.892043	1.936353	1.930282	1.924237	1.918229	1.912256	1.906325
1.903875	1.897848	1.89199	1.936323	1.930278	1.924243	1.918193	1.912169	1.906238
1.903866	1.89782	1.891941	1.914758	1.908853	1.902951	1.918194	1.912135	1.906201
1.903887	1.897816	1.891893	1.914709	1.908854	1.902997	1.918243	1.912166	1.906226
1.903911	1.897823	1.891858	1.914665	1.908845	1.903024	1.918286	1.912211	1.906264
1.90392	1.897836	1.891844	1.914629	1.908808	1.902997	1.918295	1.912251	1.906301
1.903932	1.897859	1.891845	1.914607	1.908772	1.902961	1.9183	1.912293	1.906339
1.882736	1.897892	1.891859	1.885948	1.880203	1.874495	1.918316	1.91233	1.906362
1.882771	1.897934	1.891888	1.885965	1.880209	1.874495	1.918342	1.912367	1.906381
1.882815	1.897984	1.891935	1.886006	1.880243	1.874523	1.91839	1.91242	1.906423
1.882869	1.898044	1.892	1.886072	1.880304	1.874579	1.918461	1.912489	1.906485
1.882923	1.876897	1.870879	1.886138	1.880366	1.874636	1.918529	1.912556	1.90655
1.882976	1.876954	1.870939	1.8862	1.880425	1.874691	1.918592	1.912618	1.906616

1.883029	1.877011	1.871001	1.886265	1.880486	1.874749	1.868996	1.863248	1.90668
1.883079	1.877069	1.871066	1.886333	1.880552	1.874808	1.869047	1.863291	1.906731
1.883131	1.87713	1.871135	1.886405	1.880621	1.874871	1.8691	1.863332	1.906786
1.883189	1.877195	1.871204	1.865315	1.859566	1.853853	1.869157	1.863381	1.906868
1.88325	1.877264	1.871279	1.865392	1.85964	1.853924	1.869217	1.863435	1.906969
1.883309	1.877339	1.87138	1.865503	1.859737	1.854002	1.869284	1.863496	1.90707
1.883364	1.87742	1.871511	1.865653	1.859857	1.854087	1.869356	1.863563	1.907174
1.883433	1.877507	1.871645	1.865809	1.859996	1.854201	1.848398	1.842627	1.907267
1.883517	1.877597	1.871776	1.865969	1.860167	1.854364	1.848513	1.842686	1.907337
1.883621	1.877703	1.871911	1.866125	1.860337	1.854529	1.848638	1.84276	1.907415
1.883755	1.877844	1.872052	1.866262	1.860467	1.854652	1.848757	1.842873	1.858127
1.883905	1.878003	1.872199	1.866397	1.860588	1.854765	1.848881	1.843009	1.858246
1.884042	1.878161	1.872363	1.866562	1.860748	1.854918	1.849028	1.843149	1.837393
1.884172	1.87832	1.872542	1.866751	1.860937	1.8551	1.849194	1.843297	1.837534
1.884289	1.878454	1.872694	1.866915	1.861108	1.855272	1.849363	1.84346	1.837695
1.884385	1.878556	1.872811	1.867049	1.861258	1.855437	1.84954	1.843646	1.837887
1.88449	1.878665	1.872933	1.867182	1.861397	1.855583	1.849692	1.843805	1.838051
1.884613	1.878795	1.873075	1.867324	1.861523	1.855692	1.849792	1.843901	1.838151
1.884751	1.87894	1.873225	1.867469	1.861649	1.855797	1.849883	1.843983	1.838233
1.884918	1.879108	1.873383	1.867617	1.861788	1.855927	1.850004	1.844094	1.838332
1.885101	1.879288	1.873547	1.86777	1.861938	1.856074	1.850141	1.844218	1.838438
1.885258	1.879448	1.873714	1.867942	1.862114	1.856247	1.850295	1.844347	1.838551
1.885394	1.879591	1.873884	1.868132	1.862315	1.856446	1.850465	1.844481	1.838671
1.854433	1.879744	1.874061	1.868324	1.862507	1.856633	1.850649	1.844659	1.838843
1.854578	1.87991	1.874251	1.868519	1.862678	1.85679	1.850842	1.8449	1.839088
1.854744	1.880085	1.874436	1.86871	1.862862	1.856976	1.851057	1.845148	1.839338
1.854953	1.880265	1.874592	1.868873	1.863083	1.857243	1.851314	1.845376	1.839557
1.855176	1.880456	1.874758	1.869047	1.863321	1.857531	1.851578	1.845594	1.839765
1.833625	1.849651	1.84404	1.869299	1.863565	1.857762	1.851791	1.845791	1.839964
1.833821	1.849882	1.84433	1.869598	1.863817	1.857965	1.851977	1.845974	1.840155
1.834026	1.850106	1.844573	1.869849	1.864067	1.858206	1.852201	1.846177	1.840343
1.834244	1.850327	1.844768	1.870052	1.864316	1.858489	1.852464	1.846399	1.840524
1.834434	1.850523	1.844971	1.839374	1.833727	1.827978	1.852717	1.84663	1.840734
1.834561	1.850669	1.845199	1.839643	1.83397	1.828192	1.852941	1.846869	1.840994
1.834715	1.850834	1.845428	1.839905	1.83421	1.828408	1.853169	1.847116	1.841261
1.806413	1.829332	1.823897	1.840105	1.834442	1.828671	1.85343	1.847366	1.841495
1.806682	1.829615	1.824114	1.840292	1.834672	1.828946	1.853703	1.847628	1.841731
1.806945	1.829925	1.824387	1.840537	1.834901	1.829174	1.823247	1.817267	1.842032
1.807213	1.830262	1.824702	1.840823	1.835129	1.829371	1.823491	1.817583	1.84238
1.738381	1.801867	1.796419	1.819403	1.813702	1.807946	1.823751	1.817871	1.842697
1.738546	1.802085	1.796685	1.819737	1.814023	1.808245	1.824023	1.818119	1.842973
1.738721	1.802303	1.796933	1.820048	1.814343	1.808564	1.824323	1.818391	1.812642
1.738927	1.802526	1.797141	1.820299	1.814645	1.808909	1.824673	1.818717	1.812936
1.739155	1.802761	1.797348	1.82054	1.814935	1.809245	1.825026	1.819063	1.813248
1.664569	1.803027	1.797596	1.792129	1.786616	1.781015	1.80367	1.797781	1.813599
1.664849	1.803307	1.797862	1.79238	1.786842	1.781233	1.803952	1.798124	1.813967
1.665065	1.803554	1.798125	1.792648	1.787096	1.781474	1.804232	1.79842	1.814282
1.665228	1.803774	1.798389	1.792936	1.787372	1.781732	1.804508	1.798676	1.814551
1.665424	1.770115	1.764783	1.793192	1.78764	1.782005	1.776225	1.770406	1.793234
1.665664	1.770366	1.764998	1.7934	1.787893	1.782299	1.776517	1.770683	1.793545

1.634107	1.770632	1.76522	1.79361	1.788144	1.782592	1.776819	1.770981	1.793874
1.634371	1.770917	1.765462	1.793839	1.788392	1.782867	1.777119	1.7713	1.794227
1.634649	1.771217	1.765721	1.794086	1.788648	1.783143	1.777427	1.771637	1.794597
1.634913	1.771533	1.76601	1.76054	1.75515	1.749698	1.777757	1.771993	1.766248
1.635167	1.77186	1.766321	1.760842	1.755454	1.750012	1.778101	1.772363	1.76661
1.635385	1.69871	1.693529	1.761151	1.755751	1.750305	1.744702	1.739028	1.766965
1.635563	1.698947	1.693801	1.761469	1.756043	1.750578	1.744999	1.73936	1.767317
1.635758	1.6992	1.694084	1.761784	1.756332	1.750849	1.745285	1.739673	1.73399
1.635987	1.699489	1.69439	1.762091	1.756617	1.751116	1.745555	1.739954	1.734292
1.636218	1.699773	1.69469	1.762396	1.756906	1.751394	1.745833	1.74024	1.734598
1.636429	1.670177	1.665345	1.76271	1.757212	1.751693	1.746138	1.740557	1.734926
1.636637	1.670387	1.665574	1.763019	1.757524	1.752008	1.746463	1.740893	1.735269
1.636859	1.670603	1.665782	1.763298	1.757827	1.752336	1.746807	1.741247	1.735626
1.637092	1.670823	1.665973	1.763554	1.758126	1.752677	1.74717	1.741619	1.735997
1.637338	1.671057	1.66618	1.76383	1.758438	1.753026	1.747535	1.741993	1.73637
1.637592	1.6713	1.666401	1.764123	1.758763	1.753382	1.747905	1.742368	1.736744
1.637884	1.671584	1.666655	1.738752	1.733604	1.728398	1.748277	1.742749	1.737124
1.638239	1.671933	1.666958	1.739064	1.733923	1.728732	1.748652	1.743138	1.737512
1.638615	1.672309	1.667289	1.7394	1.734253	1.729065	1.749026	1.743528	1.737901
1.638981	1.633826	1.628913	1.662728	1.657976	1.653213	1.749392	1.743907	1.738286
1.639346	1.634195	1.629275	1.663076	1.658291	1.653504	1.749759	1.744285	1.73867
1.639701	1.634549	1.629618	1.663414	1.658622	1.653833	1.75016	1.744683	1.739064
1.640047	1.634891	1.629949	1.663747	1.658969	1.654194	1.75059	1.745098	1.739465
1.640383	1.635219	1.630262	1.66406	1.659301	1.654546	1.725555	1.720121	1.739884
1.640705	1.635528	1.63055	1.664349	1.659612	1.654885	1.725971	1.720549	1.740327
1.641035	1.635838	1.630835	1.664631	1.659912	1.655211	1.726374	1.720968	1.740769
1.641382	1.63615	1.631115	1.664903	1.660194	1.655514	1.726746	1.721353	1.741194
1.641749	1.636482	1.631414	1.665187	1.660477	1.655808	1.727106	1.721733	1.741624
1.642149	1.636864	1.631774	1.626861	1.622178	1.617525	1.699025	1.694	1.758627
1.642577	1.637285	1.632176	1.627232	1.622493	1.617806	1.699336	1.69435	1.759073
1.643026	1.637728	1.63258	1.627599	1.622825	1.618118	1.699668	1.694706	1.759517
1.643502	1.638198	1.63299	1.627964	1.623172	1.618457	1.700019	1.695069	1.759963
1.643957	1.638652	1.633408	1.628353	1.62355	1.618827	1.700382	1.695424	1.760385
1.644379	1.63908	1.633834	1.628777	1.623975	1.619242	1.70076	1.695764	1.760771
1.644787	1.639494	1.634264	1.629214	1.624407	1.619657	1.701138	1.696105	1.761152
1.645174	1.63988	1.634692	1.629656	1.624817	1.620033	1.7015	1.696453	1.734042
1.645564	1.640269	1.635122	1.630098	1.625222	1.6204	1.701862	1.696812	1.734437
1.64602	1.640724	1.63556	1.630519	1.625624	1.620789	1.702245	1.697202	1.734863
1.646522	1.641225	1.636006	1.630926	1.626023	1.621192	1.702642	1.697615	1.735316
1.545146	1.641706	1.636451	1.631346	1.626438	1.621606	1.667316	1.662306	1.692846
1.54555	1.642171	1.636897	1.631779	1.626873	1.622037	1.667709	1.662677	1.693256
1.545919	1.642606	1.637335	1.632216	1.6273	1.622454	1.668107	1.663074	1.693687
1.546223	1.642985	1.637759	1.632656	1.627703	1.622831	1.668508	1.663524	1.694151
1.546542	1.643371	1.638181	1.633092	1.628109	1.623219	1.668922	1.663985	1.694624
1.546975	1.542061	1.537203	1.633506	1.628543	1.623672	1.669362	1.664421	1.695083
1.547458	1.542544	1.537622	1.633918	1.628991	1.624152	1.669817	1.664855	1.695541
1.547903	1.542989	1.538054	1.634377	1.629444	1.624605	1.670272	1.665322	1.696015
1.548326	1.543411	1.538501	1.634873	1.629903	1.625039	1.670729	1.665816	1.696502
1.548771	1.543858	1.538947	1.534158	1.529516	1.524979	1.671181	1.666276	1.696967
1.549252	1.544343	1.53939	1.534578	1.529951	1.525422	1.671627	1.666686	1.697402

1.549719	1.544812	1.539834	1.535007	1.530385	1.525855	1.67207	1.667102	1.697835
1.550131	1.545229	1.54028	1.53546	1.530809	1.526251	1.672508	1.667554	1.698278
1.550544	1.54564	1.540726	1.535916	1.53123	1.526642	1.672944	1.66801	1.698721
1.551018	1.546086	1.54116	1.536342	1.531649	1.527054	1.580447	1.575784	1.699154
1.551529	1.546556	1.541595	1.536757	1.532072	1.527481	1.580843	1.576158	1.699582
1.552063	1.547083	1.542079	1.537214	1.532533	1.527942	1.581265	1.576546	1.664257
1.55263	1.54767	1.54261	1.537707	1.53303	1.528437	1.58171	1.576944	1.664676
1.553125	1.548197	1.543143	1.538236	1.533539	1.528925	1.582179	1.577397	1.572628
1.553501	1.548622	1.543685	1.538827	1.534068	1.529396	1.582683	1.577943	1.573144
1.553877	1.549033	1.544197	1.539386	1.53458	1.529863	1.583177	1.578468	1.573653
1.554319	1.549462	1.544627	1.539824	1.535038	1.530331	1.583619	1.57888	1.574085
1.554798	1.549913	1.545038	1.540224	1.535479	1.530801	1.584043	1.579253	1.57449
1.555336	1.550444	1.545519	1.54068	1.535952	1.531285	1.584486	1.579654	1.574903
1.555916	1.551027	1.546044	1.541168	1.536443	1.531778	1.584938	1.580071	1.575319
1.556409	1.551531	1.546563	1.541687	1.536943	1.53226	1.585415	1.580547	1.57578
1.556801	1.551949	1.547078	1.542247	1.537456	1.53273	1.585922	1.581092	1.576295
1.557213	1.552373	1.547579	1.542784	1.537954	1.533193	1.586414	1.581615	1.576797
1.557678	1.552816	1.548047	1.543262	1.538414	1.533637	1.586864	1.582076	1.577256
1.558175	1.553291	1.548525	1.54374	1.538883	1.534098	1.587321	1.582532	1.577716
1.514036	1.553838	1.549063	1.54427	1.539409	1.534619	1.587829	1.583026	1.578213
1.514609	1.554423	1.549623	1.544814	1.539957	1.535169	1.58836	1.583529	1.578722
1.515162	1.554984	1.550141	1.545312	1.540479	1.535706	1.588875	1.584006	1.579209
1.515704	1.555533	1.550631	1.545776	1.540982	1.536238	1.589382	1.584464	1.57968
1.405903	1.511487	1.50668	1.546247	1.541466	1.536731	1.532011	1.527346	1.522829
1.406317	1.511971	1.507156	1.546721	1.541921	1.53717	1.53245	1.52779	1.523262
1.406745	1.512464	1.507656	1.547226	1.542404	1.537632	1.532913	1.52826	1.523722
1.40721	1.512986	1.508206	1.547792	1.542951	1.538158	1.533435	1.528782	1.524234
1.407697	1.513524	1.508775	1.548377	1.543523	1.538713	1.533983	1.529323	1.524767
1.40821	1.403864	1.399592	1.504606	1.4999	1.495235	1.534535	1.529858	1.525295
1.408743	1.404385	1.400086	1.505162	1.500469	1.495814	1.535096	1.530395	1.525826
1.40927	1.404906	1.400591	1.50573	1.501041	1.496386	1.535652	1.530934	1.526354
1.409795	1.40543	1.401106	1.506309	1.501615	1.496952	1.536204	1.531473	1.526879
1.410313	1.405948	1.401619	1.506888	1.502189	1.497515	1.492855	1.488244	1.527404
1.410823	1.406456	1.402125	1.507465	1.50276	1.498078	1.493404	1.488781	1.527934
1.41133	1.406961	1.40263	1.50804	1.50333	1.498636	1.493946	1.489306	1.528455
1.345843	1.407465	1.403131	1.456959	1.452382	1.447821	1.494471	1.489806	1.528956
1.346301	1.407969	1.403631	1.457501	1.452917	1.448341	1.494993	1.490299	1.52945
1.346763	1.408478	1.404129	1.458038	1.453458	1.448876	1.495529	1.4908	1.486306
1.347229	1.40899	1.404629	1.458577	1.454005	1.449423	1.496076	1.491312	1.486803
1.347706	1.409523	1.405165	1.459174	1.45458	1.449975	1.496655	1.491892	1.48736
1.348191	1.410078	1.405736	1.459833	1.455183	1.450528	1.497266	1.492544	1.487981
1.211562	1.344502	1.340352	1.402015	1.397691	1.393396	1.497901	1.493205	1.488615
1.212013	1.345042	1.340928	1.402652	1.398299	1.393976	1.498561	1.493865	1.489259
1.212466	1.345577	1.34149	1.403276	1.398912	1.394574	1.499234	1.494516	1.489896
1.212903	1.346076	1.341983	1.403827	1.399491	1.395169	1.448409	1.443779	1.4905
1.213335	1.346559	1.342444	1.404345	1.400059	1.395771	1.449051	1.444351	1.491094
1.213768	1.347046	1.34292	1.338804	1.334709	1.330588	1.449704	1.44496	1.491711
1.214201	1.347535	1.343402	1.339298	1.33525	1.331164	1.450366	1.445596	1.492346
1.214662	1.348054	1.343915	1.339811	1.335771	1.331693	1.451013	1.446255	1.493005
1.215162	1.348614	1.344469	1.340347	1.336267	1.332163	1.451644	1.446945	1.493697

1.215637	1.349151	1.345012	1.34088	1.336758	1.332627	1.452266	1.447626	1.442975
1.216035	1.349617	1.345515	1.341396	1.337244	1.333091	1.452872	1.448278	1.44362
1.216434	1.350081	1.346015	1.341913	1.337742	1.333572	1.453486	1.448914	1.444256
1.216911	1.350611	1.346551	1.342453	1.338284	1.334108	1.396082	1.391847	1.444866
1.217431	1.351175	1.347103	1.343004	1.33885	1.334678	1.396681	1.392403	1.445471
1.217949	1.35173	1.347642	1.343541	1.339413	1.335256	1.397309	1.392999	1.446136
1.218474	1.352283	1.348171	1.344067	1.339976	1.335849	1.397965	1.39363	1.446856
1.218951	1.2151	1.211295	1.344597	1.340514	1.336395	1.332179	1.327934	1.447575
1.219346	1.215498	1.211771	1.345142	1.34102	1.336874	1.332698	1.328509	1.448301
1.219768	1.215907	1.212229	1.345664	1.34151	1.337342	1.333191	1.329044	1.448986
1.220293	1.216379	1.212634	1.346122	1.341979	1.337821	1.333646	1.329475	1.449569
1.22086	1.216885	1.213039	1.34657	1.34245	1.338312	1.334098	1.329885	1.45013
1.047188	1.217423	1.21352	1.347082	1.342967	1.338834	1.334613	1.330388	1.392909
1.047711	1.217987	1.214051	1.347635	1.343515	1.339381	1.335167	1.330947	1.393521
1.048235	1.218537	1.214579	1.348195	1.344067	1.339931	1.335726	1.331515	1.394153
1.048762	1.219074	1.215109	1.348765	1.344628	1.340487	1.336292	1.332096	1.394809
1.04929	1.219622	1.215635	1.349322	1.345183	1.341045	1.336858	1.332671	1.395451
1.049825	1.220195	1.216155	1.349854	1.345729	1.341607	1.337421	1.333228	1.396056
1.050351	1.220762	1.21667	1.350382	1.346268	1.342161	1.337984	1.333794	1.396674
1.050848	1.221294	1.217185	1.350925	1.346797	1.342689	1.338542	1.33439	1.330243
1.051332	1.221811	1.217695	1.351468	1.347322	1.343212	1.339104	1.335001	1.330869
1.051816	1.222334	1.218185	1.351986	1.347857	1.343771	1.339692	1.335616	1.331486
1.052298	1.22286	1.21866	1.352487	1.348397	1.344356	1.3403	1.336237	1.332101
1.052779	1.223379	1.219158	1.353018	1.348948	1.34493	1.340892	1.336842	1.332706
1.053251	1.223884	1.219679	1.353582	1.349511	1.345494	1.341467	1.337429	1.333299
1.053755	1.049954	1.046144	1.216157	1.212248	1.208453	1.342028	1.338012	1.333891
1.054323	1.050525	1.046668	1.216699	1.212737	1.208909	1.34257	1.338598	1.334485
1.054906	1.051109	1.047203	1.217251	1.213234	1.20937	1.343106	1.339177	1.33508
1.055462	1.051652	1.047731	1.217813	1.213762	1.209868	1.343647	1.339734	1.335679
1.056005	1.052178	1.048262	1.218388	1.21431	1.210386	1.344189	1.340278	1.336276
0.930216	1.052708	1.048813	1.218985	1.214864	1.210893	1.34473	1.34082	1.336851
0.930593	1.053238	1.049383	1.219603	1.215425	1.211394	1.345269	1.341356	1.337408
0.930992	1.053801	1.049976	1.220239	1.215996	1.211899	1.345816	1.3419	1.337966
0.93143	1.054413	1.050603	1.220896	1.216569	1.212396	1.346362	1.342448	1.338526
0.931855	1.055014	1.051215	1.221554	1.217171	1.212939	1.346941	1.343016	1.339092
0.932225	1.05557	1.05178	1.22221	1.217833	1.213585	1.347585	1.343619	1.339669
0.932583	1.056105	1.052319	1.222849	1.218507	1.214266	1.348254	1.344238	1.340255
0.932975	0.929768	0.926476	1.127776	1.123655	1.119628	1.348899	1.344856	1.34086
0.933381	0.930157	0.926822	1.128304	1.124231	1.12024	1.349536	1.345478	1.341477
0.933753	0.930518	0.927188	1.128886	1.124842	1.120861	1.350188	1.34609	1.342068
0.934092	0.930851	0.927576	1.129525	1.125488	1.12149	1.350852	1.346691	1.34263
0.934438	0.931179	0.927941	1.130134	1.126125	1.122133	1.213341	1.209304	1.343231
0.934792	0.93149	0.928258	1.130683	1.126741	1.122798	1.214028	1.209953	1.343894
0.935179	0.931831	0.928586	1.131217	1.127348	1.123463	1.214728	1.210626	1.344584
0.935635	0.932255	0.928976	1.131761	1.127946	1.124111	1.215415	1.211299	1.34529
0.936128	0.932715	0.929388	1.132305	1.128536	1.124749	1.216098	1.211977	1.346008
0.936607	0.933149	0.929766	1.02005	1.016803	1.013566	1.121484	1.117566	1.346717
0.937086	0.933574	0.930126	1.020441	1.017223	1.014024	1.122118	1.118202	1.347422
0.93759	0.934026	0.930513	1.020842	1.017627	1.014446	1.122729	1.11883	1.348128
0.938122	0.934505	0.930923	1.021248	1.018007	1.014823	1.123313	1.119453	1.348838

0.938666	0.935011	0.931376	1.021695	1.018414	1.015209	1.123892	1.120059	1.349534
0.939225	0.935555	0.931895	1.022208	1.018871	1.015621	1.124473	1.120636	1.350204
0.939777	0.936107	0.932438	1.02275	1.019354	1.01605	1.125057	1.121206	1.350868
0.9403	0.93664	0.932975	1.023297	1.019851	1.016501	1.125648	1.121794	1.273553
0.940801	0.937161	0.933512	1.023853	1.020363	1.016968	1.126244	1.122389	1.274201
0.941277	0.93766	0.934031	1.024399	1.02087	1.017435	1.014079	1.010793	1.27485
0.941726	0.938136	0.934537	1.024938	1.021375	1.017903	1.014522	1.011218	1.275501
0.942172	0.938607	0.935036	1.025473	1.021881	1.01838	1.014986	1.011674	1.120367
0.942613	0.939074	0.935528	1.026004	1.022388	1.018864	1.015473	1.012168	1.120999
0.711086	0.939541	0.936009	0.932521	0.929063	0.925715	1.015979	1.012684	1.121662
0.711891	0.940007	0.93646	0.932968	0.929526	0.926195	1.016515	1.013234	1.12238
0.712715	0.940485	0.936918	0.93342	0.929995	0.926681	1.017051	1.013782	1.123108
0.713543	0.941007	0.93745	0.933944	0.930487	0.927141	1.017538	1.014268	1.123785
0.714369	0.941552	0.938023	0.934511	0.930996	0.927592	1.017997	1.014716	1.124432
0.715124	0.712156	0.709058	0.935069	0.931526	0.928082	1.018483	1.015167	1.011863
0.715806	0.71289	0.709877	0.935627	0.932079	0.928609	1.01899	1.015616	1.012316
0.716496	0.713606	0.71066	0.936143	0.932612	0.929139	1.019507	1.016071	1.012762
0.717201	0.7143	0.711397	0.936596	0.933114	0.929667	1.020028	1.016523	1.01319
0.717933	0.715009	0.712123	0.93703	0.933601	0.930192	1.020566	1.017007	1.013638
0.718723	0.715774	0.71286	0.937448	0.934076	0.930722	1.021149	1.017561	1.014138
0.719536	0.716566	0.71361	0.937879	0.934544	0.931237	1.021738	1.018144	1.014667
0.720316	0.71736	0.714407	0.938407	0.935021	0.931677	0.928319	0.924992	1.015211
0.721076	0.718158	0.715239	0.939006	0.935504	0.932068	0.92873	0.925461	1.015768
0.531981	0.718899	0.716031	0.713046	0.709888	0.706702	0.929185	0.9259	1.016268
0.532411	0.719573	0.716782	0.713857	0.710738	0.707558	0.929686	0.926298	1.016697
0.53288	0.720252	0.717502	0.714625	0.711565	0.70842	0.930205	0.926737	1.017154
0.533431	0.720947	0.718167	0.715308	0.712341	0.709279	0.930751	0.927266	1.017677
0.534	0.721679	0.718847	0.715989	0.713099	0.710115	0.931281	0.927808	1.018223
0.534539	0.72251	0.719632	0.716747	0.71385	0.710878	0.931724	0.92829	1.018768
0.535064	0.723378	0.720461	0.717539	0.7146	0.711612	0.932139	0.928756	1.019318
0.53555	0.724131	0.721225	0.718317	0.71539	0.712415	0.932652	0.929258	0.925861
0.536003	0.724794	0.721943	0.719091	0.716214	0.71327	0.933242	0.929788	0.92636
0.536519	0.534324	0.532476	0.719833	0.716998	0.714096	0.711024	0.707868	0.926909
0.537115	0.534887	0.533012	0.720526	0.717727	0.714879	0.711866	0.708766	0.927528
0.537738	0.535471	0.533554	0.721251	0.718465	0.715655	0.712692	0.709647	0.928137
0.538358	0.536043	0.534077	0.722065	0.719257	0.716436	0.713486	0.710462	0.928676
0.539005	0.536639	0.534611	0.722898	0.720055	0.717212	0.714271	0.711268	0.929215
0.539716	0.537308	0.535195	0.723668	0.720806	0.717973	0.715076	0.712131	0.929831
0.540467	0.538021	0.535809	0.724416	0.721538	0.718726	0.715888	0.713017	0.930485
0.541192	0.538704	0.536407	0.725242	0.722324	0.719497	0.716677	0.713843	0.710845
0.541896	0.539362	0.536988	0.726143	0.72316	0.720281	0.717442	0.714609	0.711626
	0.540041	0.537601	0.727022	0.723996	0.721088	0.718244	0.71542	0.712448
	0.540748	0.538251	0.72783	0.724808	0.721923	0.719106	0.716311	0.713352
	0.541486	0.538948	0.728697	0.725657	0.722773	0.719972	0.717202	0.714253
	0.542291	0.539732	0.537365	0.535256	0.53324	0.720811	0.718048	0.715104
	0.543105	0.540541	0.538125	0.535895	0.533776	0.721639	0.718867	0.715928
	0.543823	0.541269	0.538833	0.536536	0.534355	0.722452	0.719644	0.716722
	0.544478	0.541947	0.539513	0.537179	0.534963	0.723257	0.720391	0.717495
	0.545161	0.542647	0.540213	0.53785	0.535602	0.724118	0.721218	0.718326
	0.545868	0.543367	0.540932	0.538551	0.536282	0.725043	0.722135	0.719223

0.541668	0.539249	0.536942	0.725965	0.723043	0.720105
0.542438	0.539932	0.537544	0.726863	0.723913	0.720947
0.543195	0.540616	0.538158	0.72776	0.724766	0.721773
0.543887	0.541307	0.538837	0.728655	0.725582	0.72257
0.544548	0.542007	0.539556	0.729566	0.726401	0.723373
0.54523	0.542733	0.540308	0.538049	0.535883	0.724283
0.545924	0.543482	0.541094	0.538806	0.536589	0.72528
			0.539559	0.537299	0.726279
			0.540303	0.538012	0.727276
			0.54105	0.538737	0.728277
			0.541811	0.539488	0.729278
			0.542563	0.540233	0.730282
					0.538589
					0.539238
					0.539911
					0.540601

1.883029	1.877011	1.871001	1.886265	1.880486	1.874749	1.868996	1.863248	1.90668
1.883079	1.877069	1.871066	1.886333	1.880552	1.874808	1.869047	1.863291	1.906731
1.883131	1.87713	1.871135	1.886405	1.880621	1.874871	1.8691	1.863332	1.906786
1.883189	1.877195	1.871204	1.865315	1.859566	1.853853	1.869157	1.863381	1.906868
1.88325	1.877264	1.871279	1.865392	1.85964	1.853924	1.869217	1.863435	1.906969
1.883309	1.877339	1.87138	1.865503	1.859737	1.854002	1.869284	1.863496	1.90707
1.883364	1.87742	1.871511	1.865653	1.859857	1.854087	1.869356	1.863563	1.907174
1.883433	1.877507	1.871645	1.865809	1.859996	1.854201	1.848398	1.842627	1.907267
1.883517	1.877597	1.871776	1.865969	1.860167	1.854364	1.848513	1.842686	1.907337
1.883621	1.877703	1.871911	1.866125	1.860337	1.854529	1.848638	1.84276	1.907415
1.883755	1.877844	1.872052	1.866262	1.860467	1.854652	1.848757	1.842873	1.858127
1.883905	1.878003	1.872199	1.866397	1.860588	1.854765	1.848881	1.843009	1.858246
1.884042	1.878161	1.872363	1.866562	1.860748	1.854918	1.849028	1.843149	1.837393
1.884172	1.87832	1.872542	1.866751	1.860937	1.8551	1.849194	1.843297	1.837534
1.884289	1.878454	1.872694	1.866915	1.861108	1.855272	1.849363	1.84346	1.837695
1.884385	1.878556	1.872811	1.867049	1.861258	1.855437	1.84954	1.843646	1.837887
1.88449	1.878665	1.872933	1.867182	1.861397	1.855583	1.849692	1.843805	1.838051
1.884613	1.878795	1.873075	1.867324	1.861523	1.855692	1.849792	1.843901	1.838151
1.884751	1.87894	1.873225	1.867469	1.861649	1.855797	1.849883	1.843983	1.838233
1.884918	1.879108	1.873383	1.867617	1.861788	1.855927	1.850004	1.844094	1.838332
1.885101	1.879288	1.873547	1.86777	1.861938	1.856074	1.850141	1.844218	1.838438
1.885258	1.879448	1.873714	1.867942	1.862114	1.856247	1.850295	1.844347	1.838551
1.885394	1.879591	1.873884	1.868132	1.862315	1.856446	1.850465	1.844481	1.838671
1.854433	1.879744	1.874061	1.868324	1.862507	1.856633	1.850649	1.844659	1.838843
1.854578	1.87991	1.874251	1.868519	1.862678	1.85679	1.850842	1.8449	1.839088
1.854744	1.880085	1.874436	1.86871	1.862862	1.856976	1.851057	1.845148	1.839338
1.854953	1.880265	1.874592	1.868873	1.863083	1.857243	1.851314	1.845376	1.839557
1.855176	1.880456	1.874758	1.869047	1.863321	1.857531	1.851578	1.845594	1.839765
1.833625	1.849651	1.84404	1.869299	1.863565	1.857762	1.851791	1.845791	1.839964
1.833821	1.849882	1.84433	1.869598	1.863817	1.857965	1.851977	1.845974	1.840155
1.834026	1.850106	1.844573	1.869849	1.864067	1.858206	1.852201	1.846177	1.840343
1.834244	1.850327	1.844768	1.870052	1.864316	1.858489	1.852464	1.846399	1.840524
1.834434	1.850523	1.844971	1.839374	1.833727	1.827978	1.852717	1.84663	1.840734
1.834561	1.850669	1.845199	1.839643	1.83397	1.828192	1.852941	1.846869	1.840994
1.834715	1.850834	1.845428	1.839905	1.83421	1.828408	1.853169	1.847116	1.841261
1.806413	1.829332	1.823897	1.840105	1.834442	1.828671	1.85343	1.847366	1.841495
1.806682	1.829615	1.824114	1.840292	1.834672	1.828946	1.853703	1.847628	1.841731
1.806945	1.829925	1.824387	1.840537	1.834901	1.829174	1.823247	1.817267	1.842032
1.807213	1.830262	1.824702	1.840823	1.835129	1.829371	1.823491	1.817583	1.84238
1.738381	1.801867	1.796419	1.819403	1.813702	1.807946	1.823751	1.817871	1.842697
1.738546	1.802085	1.796685	1.819737	1.814023	1.808245	1.824023	1.818119	1.842973
1.738721	1.802303	1.796933	1.820048	1.814343	1.808564	1.824323	1.818391	1.812642
1.738927	1.802526	1.797141	1.820299	1.814645	1.808909	1.824673	1.818717	1.812936
1.739155	1.802761	1.797348	1.82054	1.814935	1.809245	1.825026	1.819063	1.813248
1.664569	1.803027	1.797596	1.792129	1.786616	1.781015	1.80367	1.797781	1.813599
1.664849	1.803307	1.797862	1.79238	1.786842	1.781233	1.803952	1.798124	1.813967
1.665065	1.803554	1.798125	1.792648	1.787096	1.781474	1.804232	1.79842	1.814282
1.665228	1.803774	1.798389	1.792936	1.787372	1.781732	1.804508	1.798676	1.814551
1.665424	1.770115	1.764783	1.793192	1.78764	1.782005	1.776225	1.770406	1.793234
1.665664	1.770366	1.764998	1.7934	1.787893	1.782299	1.776517	1.770683	1.793545

1.634107	1.770632	1.76522	1.79361	1.788144	1.782592	1.776819	1.770981	1.793874
1.634371	1.770917	1.765462	1.793839	1.788392	1.782867	1.777119	1.7713	1.794227
1.634649	1.771217	1.765721	1.794086	1.788648	1.783143	1.777427	1.771637	1.794597
1.634913	1.771533	1.76601	1.76054	1.75515	1.749698	1.777757	1.771993	1.766248
1.635167	1.77186	1.766321	1.760842	1.755454	1.750012	1.778101	1.772363	1.76661
1.635385	1.69871	1.693529	1.761151	1.755751	1.750305	1.744702	1.739028	1.766965
1.635563	1.698947	1.693801	1.761469	1.756043	1.750578	1.744999	1.73936	1.767317
1.635758	1.6992	1.694084	1.761784	1.756332	1.750849	1.745285	1.739673	1.73399
1.635987	1.699489	1.69439	1.762091	1.756617	1.751116	1.745555	1.739954	1.734292
1.636218	1.699773	1.69469	1.762396	1.756906	1.751394	1.745833	1.74024	1.734598
1.636429	1.670177	1.665345	1.76271	1.757212	1.751693	1.746138	1.740557	1.734926
1.636637	1.670387	1.665574	1.763019	1.757524	1.752008	1.746463	1.740893	1.735269
1.636859	1.670603	1.665782	1.763298	1.757827	1.752336	1.746807	1.741247	1.735626
1.637092	1.670823	1.665973	1.763554	1.758126	1.752677	1.74717	1.741619	1.735997
1.637338	1.671057	1.66618	1.76383	1.758438	1.753026	1.747535	1.741993	1.73637
1.637592	1.6713	1.666401	1.764123	1.758763	1.753382	1.747905	1.742368	1.736744
1.637884	1.671584	1.666655	1.738752	1.733604	1.728398	1.748277	1.742749	1.737124
1.638239	1.671933	1.666958	1.739064	1.733923	1.728732	1.748652	1.743138	1.737512
1.638615	1.672309	1.667289	1.7394	1.734253	1.729065	1.749026	1.743528	1.737901
1.638981	1.633826	1.628913	1.662728	1.657976	1.653213	1.749392	1.743907	1.738286
1.639346	1.634195	1.629275	1.663076	1.658291	1.653504	1.749759	1.744285	1.73867
1.639701	1.634549	1.629618	1.663414	1.658622	1.653833	1.75016	1.744683	1.739064
1.640047	1.634891	1.629949	1.663747	1.658969	1.654194	1.75059	1.745098	1.739465
1.640383	1.635219	1.630262	1.66406	1.659301	1.654546	1.725555	1.720121	1.739884
1.640705	1.635528	1.63055	1.664349	1.659612	1.654885	1.725971	1.720549	1.740327
1.641035	1.635838	1.630835	1.664631	1.659912	1.655211	1.726374	1.720968	1.740769
1.641382	1.63615	1.631115	1.664903	1.660194	1.655514	1.726746	1.721353	1.741194
1.641749	1.636482	1.631414	1.665187	1.660477	1.655808	1.727106	1.721733	1.741624
1.642149	1.636864	1.631774	1.626861	1.622178	1.617525	1.699025	1.694	1.758627
1.642577	1.637285	1.632176	1.627232	1.622493	1.617806	1.699336	1.69435	1.759073
1.643026	1.637728	1.63258	1.627599	1.622825	1.618118	1.699668	1.694706	1.759517
1.643502	1.638198	1.63299	1.627964	1.623172	1.618457	1.700019	1.695069	1.759963
1.643957	1.638652	1.633408	1.628353	1.62355	1.618827	1.700382	1.695424	1.760385
1.644379	1.63908	1.633834	1.628777	1.623975	1.619242	1.70076	1.695764	1.760771
1.644787	1.639494	1.634264	1.629214	1.624407	1.619657	1.701138	1.696105	1.761152
1.645174	1.63988	1.634692	1.629656	1.624817	1.620033	1.7015	1.696453	1.734042
1.645564	1.640269	1.635122	1.630098	1.625222	1.6204	1.701862	1.696812	1.734437
1.64602	1.640724	1.63556	1.630519	1.625624	1.620789	1.702245	1.697202	1.734863
1.646522	1.641225	1.636006	1.630926	1.626023	1.621192	1.702642	1.697615	1.735316
1.545146	1.641706	1.636451	1.631346	1.626438	1.621606	1.667316	1.662306	1.692846
1.54555	1.642171	1.636897	1.631779	1.626873	1.622037	1.667709	1.662677	1.693256
1.545919	1.642606	1.637335	1.632216	1.6273	1.622454	1.668107	1.663074	1.693687
1.546223	1.642985	1.637759	1.632656	1.627703	1.622831	1.668508	1.663524	1.694151
1.546542	1.643371	1.638181	1.633092	1.628109	1.623219	1.668922	1.663985	1.694624
1.546975	1.542061	1.537203	1.633506	1.628543	1.623672	1.669362	1.664421	1.695083
1.547458	1.542544	1.537622	1.633918	1.628991	1.624152	1.669817	1.664855	1.695541
1.547903	1.542989	1.538054	1.634377	1.629444	1.624605	1.670272	1.665322	1.696015
1.548326	1.543411	1.538501	1.634873	1.629903	1.625039	1.670729	1.665816	1.696502
1.548771	1.543858	1.538947	1.534158	1.529516	1.524979	1.671181	1.666276	1.696967
1.549252	1.544343	1.53939	1.534578	1.529951	1.525422	1.671627	1.666686	1.697402

1.549719	1.544812	1.539834	1.535007	1.530385	1.525855	1.67207	1.667102	1.697835
1.550131	1.545229	1.54028	1.53546	1.530809	1.526251	1.672508	1.667554	1.698278
1.550544	1.54564	1.540726	1.535916	1.53123	1.526642	1.672944	1.66801	1.698721
1.551018	1.546086	1.54116	1.536342	1.531649	1.527054	1.580447	1.575784	1.699154
1.551529	1.546556	1.541595	1.536757	1.532072	1.527481	1.580843	1.576158	1.699582
1.552063	1.547083	1.542079	1.537214	1.532533	1.527942	1.581265	1.576546	1.664257
1.55263	1.54767	1.54261	1.537707	1.53303	1.528437	1.58171	1.576944	1.664676
1.553125	1.548197	1.543143	1.538236	1.533539	1.528925	1.582179	1.577397	1.572628
1.553501	1.548622	1.543685	1.538827	1.534068	1.529396	1.582683	1.577943	1.573144
1.553877	1.549033	1.544197	1.539386	1.53458	1.529863	1.583177	1.578468	1.573653
1.554319	1.549462	1.544627	1.539824	1.535038	1.530331	1.583619	1.57888	1.574085
1.554798	1.549913	1.545038	1.540224	1.535479	1.530801	1.584043	1.579253	1.57449
1.555336	1.550444	1.545519	1.54068	1.535952	1.531285	1.584486	1.579654	1.574903
1.555916	1.551027	1.546044	1.541168	1.536443	1.531778	1.584938	1.580071	1.575319
1.556409	1.551531	1.546563	1.541687	1.536943	1.53226	1.585415	1.580547	1.57578
1.556801	1.551949	1.547078	1.542247	1.537456	1.53273	1.585922	1.581092	1.576295
1.557213	1.552373	1.547579	1.542784	1.537954	1.533193	1.586414	1.581615	1.576797
1.557678	1.552816	1.548047	1.543262	1.538414	1.533637	1.586864	1.582076	1.577256
1.558175	1.553291	1.548525	1.54374	1.538883	1.534098	1.587321	1.582532	1.577716
1.514036	1.553838	1.549063	1.54427	1.539409	1.534619	1.587829	1.583026	1.578213
1.514609	1.554423	1.549623	1.544814	1.539957	1.535169	1.58836	1.583529	1.578722
1.515162	1.554984	1.550141	1.545312	1.540479	1.535706	1.588875	1.584006	1.579209
1.515704	1.555533	1.550631	1.545776	1.540982	1.536238	1.589382	1.584464	1.57968
1.405903	1.511487	1.50668	1.546247	1.541466	1.536731	1.532011	1.527346	1.522829
1.406317	1.511971	1.507156	1.546721	1.541921	1.53717	1.53245	1.52779	1.523262
1.406745	1.512464	1.507656	1.547226	1.542404	1.537632	1.532913	1.52826	1.523722
1.40721	1.512986	1.508206	1.547792	1.542951	1.538158	1.533435	1.528782	1.524234
1.407697	1.513524	1.508775	1.548377	1.543523	1.538713	1.533983	1.529323	1.524767
1.40821	1.403864	1.399592	1.504606	1.4999	1.495235	1.534535	1.529858	1.525295
1.408743	1.404385	1.400086	1.505162	1.500469	1.495814	1.535096	1.530395	1.525826
1.40927	1.404906	1.400591	1.50573	1.501041	1.496386	1.535652	1.530934	1.526354
1.409795	1.40543	1.401106	1.506309	1.501615	1.496952	1.536204	1.531473	1.526879
1.410313	1.405948	1.401619	1.506888	1.502189	1.497515	1.492855	1.488244	1.527404
1.410823	1.406456	1.402125	1.507465	1.50276	1.498078	1.493404	1.488781	1.527934
1.41133	1.406961	1.40263	1.50804	1.50333	1.498636	1.493946	1.489306	1.528455
1.345843	1.407465	1.403131	1.456959	1.452382	1.447821	1.494471	1.489806	1.528956
1.346301	1.407969	1.403631	1.457501	1.452917	1.448341	1.494993	1.490299	1.52945
1.346763	1.408478	1.404129	1.458038	1.453458	1.448876	1.495529	1.4908	1.486306
1.347229	1.40899	1.404629	1.458577	1.454005	1.449423	1.496076	1.491312	1.486803
1.347706	1.409523	1.405165	1.459174	1.45458	1.449975	1.496655	1.491892	1.48736
1.348191	1.410078	1.405736	1.459833	1.455183	1.450528	1.497266	1.492544	1.487981
1.211562	1.344502	1.340352	1.402015	1.397691	1.393396	1.497901	1.493205	1.488615
1.212013	1.345042	1.340928	1.402652	1.398299	1.393976	1.498561	1.493865	1.489259
1.212466	1.345577	1.34149	1.403276	1.398912	1.394574	1.499234	1.494516	1.489896
1.212903	1.346076	1.341983	1.403827	1.399491	1.395169	1.448409	1.443779	1.4905
1.213335	1.346559	1.342444	1.404345	1.400059	1.395771	1.449051	1.444351	1.491094
1.213768	1.347046	1.34292	1.338804	1.334709	1.330588	1.449704	1.44496	1.491711
1.214201	1.347535	1.343402	1.339298	1.33525	1.331164	1.450366	1.445596	1.492346
1.214662	1.348054	1.343915	1.339811	1.335771	1.331693	1.451013	1.446255	1.493005
1.215162	1.348614	1.344469	1.340347	1.336267	1.332163	1.451644	1.446945	1.493697

1.215637	1.349151	1.345012	1.34088	1.336758	1.332627	1.452266	1.447626	1.442975
1.216035	1.349617	1.345515	1.341396	1.337244	1.333091	1.452872	1.448278	1.44362
1.216434	1.350081	1.346015	1.341913	1.337742	1.333572	1.453486	1.448914	1.444256
1.216911	1.350611	1.346551	1.342453	1.338284	1.334108	1.396082	1.391847	1.444866
1.217431	1.351175	1.347103	1.343004	1.33885	1.334678	1.396681	1.392403	1.445471
1.217949	1.35173	1.347642	1.343541	1.339413	1.335256	1.397309	1.392999	1.446136
1.218474	1.352283	1.348171	1.344067	1.339976	1.335849	1.397965	1.39363	1.446856
1.218951	1.2151	1.211295	1.344597	1.340514	1.336395	1.332179	1.327934	1.447575
1.219346	1.215498	1.211771	1.345142	1.34102	1.336874	1.332698	1.328509	1.448301
1.219768	1.215907	1.212229	1.345664	1.34151	1.337342	1.333191	1.329044	1.448986
1.220293	1.216379	1.212634	1.346122	1.341979	1.337821	1.333646	1.329475	1.449569
1.22086	1.216885	1.213039	1.34657	1.34245	1.338312	1.334098	1.329885	1.45013
1.047188	1.217423	1.21352	1.347082	1.342967	1.338834	1.334613	1.330388	1.392909
1.047711	1.217987	1.214051	1.347635	1.343515	1.339381	1.335167	1.330947	1.393521
1.048235	1.218537	1.214579	1.348195	1.344067	1.339931	1.335726	1.331515	1.394153
1.048762	1.219074	1.215109	1.348765	1.344628	1.340487	1.336292	1.332096	1.394809
1.04929	1.219622	1.215635	1.349322	1.345183	1.341045	1.336858	1.332671	1.395451
1.049825	1.220195	1.216155	1.349854	1.345729	1.341607	1.337421	1.333228	1.396056
1.050351	1.220762	1.21667	1.350382	1.346268	1.342161	1.337984	1.333794	1.396674
1.050848	1.221294	1.217185	1.350925	1.346797	1.342689	1.338542	1.33439	1.330243
1.051332	1.221811	1.217695	1.351468	1.347322	1.343212	1.339104	1.335001	1.330869
1.051816	1.222334	1.218185	1.351986	1.347857	1.343771	1.339692	1.335616	1.331486
1.052298	1.22286	1.21866	1.352487	1.348397	1.344356	1.3403	1.336237	1.332101
1.052779	1.223379	1.219158	1.353018	1.348948	1.34493	1.340892	1.336842	1.332706
1.053251	1.223884	1.219679	1.353582	1.349511	1.345494	1.341467	1.337429	1.333299
1.053755	1.049954	1.046144	1.216157	1.212248	1.208453	1.342028	1.338012	1.333891
1.054323	1.050525	1.046668	1.216699	1.212737	1.208909	1.34257	1.338598	1.334485
1.054906	1.051109	1.047203	1.217251	1.213234	1.20937	1.343106	1.339177	1.33508
1.055462	1.051652	1.047731	1.217813	1.213762	1.209868	1.343647	1.339734	1.335679
1.056005	1.052178	1.048262	1.218388	1.21431	1.210386	1.344189	1.340278	1.336276
0.930216	1.052708	1.048813	1.218985	1.214864	1.210893	1.34473	1.34082	1.336851
0.930593	1.053238	1.049383	1.219603	1.215425	1.211394	1.345269	1.341356	1.337408
0.930992	1.053801	1.049976	1.220239	1.215996	1.211899	1.345816	1.3419	1.337966
0.93143	1.054413	1.050603	1.220896	1.216569	1.212396	1.346362	1.342448	1.338526
0.931855	1.055014	1.051215	1.221554	1.217171	1.212939	1.346941	1.343016	1.339092
0.932225	1.05557	1.05178	1.22221	1.217833	1.213585	1.347585	1.343619	1.339669
0.932583	1.056105	1.052319	1.222849	1.218507	1.214266	1.348254	1.344238	1.340255
0.932975	0.929768	0.926476	1.127776	1.123655	1.119628	1.348899	1.344856	1.34086
0.933381	0.930157	0.926822	1.128304	1.124231	1.12024	1.349536	1.345478	1.341477
0.933753	0.930518	0.927188	1.128886	1.124842	1.120861	1.350188	1.34609	1.342068
0.934092	0.930851	0.927576	1.129525	1.125488	1.12149	1.350852	1.346691	1.34263
0.934438	0.931179	0.927941	1.130134	1.126125	1.122133	1.213341	1.209304	1.343231
0.934792	0.93149	0.928258	1.130683	1.126741	1.122798	1.214028	1.209953	1.343894
0.935179	0.931831	0.928586	1.131217	1.127348	1.123463	1.214728	1.210626	1.344584
0.935635	0.932255	0.928976	1.131761	1.127946	1.124111	1.215415	1.211299	1.34529
0.936128	0.932715	0.929388	1.132305	1.128536	1.124749	1.216098	1.211977	1.346008
0.936607	0.933149	0.929766	1.02005	1.016803	1.013566	1.121484	1.117566	1.346717
0.937086	0.933574	0.930126	1.020441	1.017223	1.014024	1.122118	1.118202	1.347422
0.93759	0.934026	0.930513	1.020842	1.017627	1.014446	1.122729	1.11883	1.348128
0.938122	0.934505	0.930923	1.021248	1.018007	1.014823	1.123313	1.119453	1.348838

0.938666	0.935011	0.931376	1.021695	1.018414	1.015209	1.123892	1.120059	1.349534
0.939225	0.935555	0.931895	1.022208	1.018871	1.015621	1.124473	1.120636	1.350204
0.939777	0.936107	0.932438	1.02275	1.019354	1.01605	1.125057	1.121206	1.350868
0.9403	0.93664	0.932975	1.023297	1.019851	1.016501	1.125648	1.121794	1.273553
0.940801	0.937161	0.933512	1.023853	1.020363	1.016968	1.126244	1.122389	1.274201
0.941277	0.93766	0.934031	1.024399	1.02087	1.017435	1.014079	1.010793	1.27485
0.941726	0.938136	0.934537	1.024938	1.021375	1.017903	1.014522	1.011218	1.275501
0.942172	0.938607	0.935036	1.025473	1.021881	1.01838	1.014986	1.011674	1.120367
0.942613	0.939074	0.935528	1.026004	1.022388	1.018864	1.015473	1.012168	1.120999
0.711086	0.939541	0.936009	0.932521	0.929063	0.925715	1.015979	1.012684	1.121662
0.711891	0.940007	0.93646	0.932968	0.929526	0.926195	1.016515	1.013234	1.12238
0.712715	0.940485	0.936918	0.93342	0.929995	0.926681	1.017051	1.013782	1.123108
0.713543	0.941007	0.93745	0.933944	0.930487	0.927141	1.017538	1.014268	1.123785
0.714369	0.941552	0.938023	0.934511	0.930996	0.927592	1.017997	1.014716	1.124432
0.715124	0.712156	0.709058	0.935069	0.931526	0.928082	1.018483	1.015167	1.011863
0.715806	0.71289	0.709877	0.935627	0.932079	0.928609	1.01899	1.015616	1.012316
0.716496	0.713606	0.71066	0.936143	0.932612	0.929139	1.019507	1.016071	1.012762
0.717201	0.7143	0.711397	0.936596	0.933114	0.929667	1.020028	1.016523	1.01319
0.717933	0.715009	0.712123	0.93703	0.933601	0.930192	1.020566	1.017007	1.013638
0.718723	0.715774	0.71286	0.937448	0.934076	0.930722	1.021149	1.017561	1.014138
0.719536	0.716566	0.71361	0.937879	0.934544	0.931237	1.021738	1.018144	1.014667
0.720316	0.71736	0.714407	0.938407	0.935021	0.931677	0.928319	0.924992	1.015211
0.721076	0.718158	0.715239	0.939006	0.935504	0.932068	0.92873	0.925461	1.015768
0.531981	0.718899	0.716031	0.713046	0.709888	0.706702	0.929185	0.9259	1.016268
0.532411	0.719573	0.716782	0.713857	0.710738	0.707558	0.929686	0.926298	1.016697
0.53288	0.720252	0.717502	0.714625	0.711565	0.70842	0.930205	0.926737	1.017154
0.533431	0.720947	0.718167	0.715308	0.712341	0.709279	0.930751	0.927266	1.017677
0.534	0.721679	0.718847	0.715989	0.713099	0.710115	0.931281	0.927808	1.018223
0.534539	0.72251	0.719632	0.716747	0.71385	0.710878	0.931724	0.92829	1.018768
0.535064	0.723378	0.720461	0.717539	0.7146	0.711612	0.932139	0.928756	1.019318
0.53555	0.724131	0.721225	0.718317	0.71539	0.712415	0.932652	0.929258	0.925861
0.536003	0.724794	0.721943	0.719091	0.716214	0.71327	0.933242	0.929788	0.92636
0.536519	0.534324	0.532476	0.719833	0.716998	0.714096	0.711024	0.707868	0.926909
0.537115	0.534887	0.533012	0.720526	0.717727	0.714879	0.711866	0.708766	0.927528
0.537738	0.535471	0.533554	0.721251	0.718465	0.715655	0.712692	0.709647	0.928137
0.538358	0.536043	0.534077	0.722065	0.719257	0.716436	0.713486	0.710462	0.928676
0.539005	0.536639	0.534611	0.722898	0.720055	0.717212	0.714271	0.711268	0.929215
0.539716	0.537308	0.535195	0.723668	0.720806	0.717973	0.715076	0.712131	0.929831
0.540467	0.538021	0.535809	0.724416	0.721538	0.718726	0.715888	0.713017	0.930485
0.541192	0.538704	0.536407	0.725242	0.722324	0.719497	0.716677	0.713843	0.710845
0.541896	0.539362	0.536988	0.726143	0.72316	0.720281	0.717442	0.714609	0.711626
	0.540041	0.537601	0.727022	0.723996	0.721088	0.718244	0.71542	0.712448
	0.540748	0.538251	0.72783	0.724808	0.721923	0.719106	0.716311	0.713352
	0.541486	0.538948	0.728697	0.725657	0.722773	0.719972	0.717202	0.714253
	0.542291	0.539732	0.537365	0.535256	0.53324	0.720811	0.718048	0.715104
	0.543105	0.540541	0.538125	0.535895	0.533776	0.721639	0.718867	0.715928
	0.543823	0.541269	0.538833	0.536536	0.534355	0.722452	0.719644	0.716722
	0.544478	0.541947	0.539513	0.537179	0.534963	0.723257	0.720391	0.717495
	0.545161	0.542647	0.540213	0.53785	0.535602	0.724118	0.721218	0.718326
	0.545868	0.543367	0.540932	0.538551	0.536282	0.725043	0.722135	0.719223

0.541668	0.539249	0.536942	0.725965	0.723043	0.720105
0.542438	0.539932	0.537544	0.726863	0.723913	0.720947
0.543195	0.540616	0.538158	0.72776	0.724766	0.721773
0.543887	0.541307	0.538837	0.728655	0.725582	0.72257
0.544548	0.542007	0.539556	0.729566	0.726401	0.723373
0.54523	0.542733	0.540308	0.538049	0.535883	0.724283
0.545924	0.543482	0.541094	0.538806	0.536589	0.72528
			0.539559	0.537299	0.726279
			0.540303	0.538012	0.727276
			0.54105	0.538737	0.728277
			0.541811	0.539488	0.729278
			0.542563	0.540233	0.730282
					0.538589
					0.539238
					0.539911
					0.540601

								0.561381
								0.559714
								0.952738
								0.951057
								0.949385
								0.94771
								0.94603
								0.944279
								0.942575
						0.557651	0.552718	0.941125
						0.55629	0.551653	0.939857
						0.949204	0.943764	1.251818
						0.947621	0.942408	1.250448
						0.946104	0.941086	1.249076
						0.944673	0.939811	1.247708
						0.943301	0.938546	1.246302
						0.942011	0.937285	1.244814
						0.940742	0.935998	1.243267
			0.739592	0.734499	0.729844	1.252399	1.247078	1.53625
			0.737684	0.732864	0.728517	1.250705	1.24536	1.534236
			0.735951	0.731388	0.727304	1.249147	1.243778	1.532331
			0.734403	0.73009	0.726227	1.247763	1.242365	1.530557
			0.948418	0.943393	0.938726	1.338169	1.332612	1.586045
			0.947085	0.94213	0.93749	1.336694	1.331147	1.584278
			0.945855	0.940926	0.936267	1.335215	1.32969	1.582545
			0.944604	0.93971	0.935039	1.333726	1.328217	1.580831
			0.943368	0.938503	0.93381	1.332229	1.326736	1.579137
	0.736545	0.732027	1.255516	1.250064	1.244701	1.532743	1.526558	1.577437
	0.734994	0.73079	1.254093	1.24864	1.243245	1.531	1.524842	1.575733
	0.733602	0.729628	1.344282	1.338717	1.333168	1.529267	1.523124	1.734475
	0.732415	0.728562	1.342754	1.337207	1.331666	1.527544	1.5214	1.732704
	0.731327	0.727541	1.341216	1.335691	1.330169	1.525833	1.519692	1.764986
	0.730299	0.726546	1.339675	1.334173	1.328674	1.524141	1.518016	1.763259
	0.729333	0.72558	1.338134	1.33265	1.327179	1.52246	1.516359	1.761553
0.732719	1.256108	1.250688	1.336583	1.331108	1.325668	1.520775	1.514715	1.759873
0.731736	1.254872	1.249324	1.335039	1.32956	1.324154	1.519093	1.513083	1.758217
0.730753	1.344984	1.339246	1.499881	1.493742	1.487709	1.68122	1.67504	1.756562
0.729771	1.343694	1.337962	1.498336	1.492138	1.486051	1.679581	1.673419	1.754904
0.896337	1.34234	1.336659	1.496826	1.490605	1.484489	1.678028	1.671861	1.753296
0.895178	1.340885	1.335321	1.495362	1.489191	1.483093	1.676624	1.670401	1.751754
0.894019	1.339387	1.333933	1.493857	1.487749	1.481689	1.675229	1.668975	1.750259
0.892849	1.433939	1.428024	1.57941	1.573209	1.567053	1.720898	1.714617	1.863709
0.891681	1.432213	1.426318	1.57769	1.571531	1.565434	1.719335	1.713172	1.862195
1.249259	1.430482	1.424614	1.575978	1.569857	1.563814	1.717734	1.711639	1.889667
1.247835	1.428749	1.422906	1.574257	1.568173	1.562179	1.716097	1.710038	1.888032
1.433093	1.551641	1.545557	1.685911	1.679628	1.673449	1.714461	1.708442	1.886365
1.431672	1.550079	1.543929	1.684245	1.677959	1.671793	1.712818	1.706845	1.884659
1.43025	1.548566	1.542385	1.682675	1.676379	1.67021	1.711228	1.705279	1.915363
1.428778	1.547116	1.540984	1.681287	1.674961	1.668747	1.709717	1.703749	1.913635

1.427265	1.545648	1.539592	1.679924	1.673591	1.667351	1.708273	1.702271	1.911944
1.425648	1.544025	1.538041	1.678412	1.672171	1.66601	1.706924	1.700895	1.943229
1.423992	1.542338	1.536425	1.67684	1.670734	1.664701	1.705628	1.699579	1.94167
1.481965	1.66107	1.654933	1.675309	1.669263	1.663295	1.704237	1.698227	1.958658
1.480559	1.659588	1.653438	1.673804	1.667756	1.661805	1.702768	1.696853	1.957144
1.479141	1.65815	1.652025	1.67241	1.666346	1.660379	1.895602	1.889091	2.009352
1.477696	1.656773	1.650736	1.671185	1.665083	1.659048	1.893984	1.887409	2.007366
1.597012	1.681997	1.675962	1.669924	1.663811	1.65774	1.91355	1.906755	2.005431
1.595415	1.680484	1.674481	1.668485	1.662446	1.656443	1.912078	1.905272	2.003732
1.593842	1.678966	1.672969	1.667001	1.661047	1.655132	1.910607	1.903806	2.002071
1.592453	1.724805	1.7187	1.752769	1.746608	1.740461	1.936489	1.929621	2.074577
1.591176	1.723521	1.71737	1.75136	1.745135	1.738927	1.934679	1.927778	2.072558
1.654246	1.722175	1.716024	1.778555	1.772173	1.765784	1.933014	1.926076	2.070589
1.65282	1.720767	1.714668	1.777147	1.770793	1.764418	1.931495	1.924504	2.068653
1.651442	1.719402	1.713314	1.950839	1.943906	1.936953	1.98613	1.978322	2.101806
1.650165	1.718127	1.711968	1.949214	1.942215	1.935236	1.984286	1.976702	2.100162
1.648877	1.716841	1.710616	1.947588	1.940531	1.933536	1.982474	1.975117	2.098562
1.647478	1.746238	1.7399	1.946014	1.938975	1.932005	1.980814	1.973531	2.134056
1.646053	1.744784	1.738475	1.944451	1.937451	1.930518	1.979198	1.971956	2.132332
1.644729	1.743424	1.737079	1.942839	1.935816	1.928888	1.977452	1.970379	2.143851
1.643473	1.742124	1.735703	1.941196	1.934113	1.927169	1.975625	1.968806	2.142235
1.677297	1.926271	1.919213	1.99669	1.988734	1.981033	2.036818	2.029811	2.14057
1.675856	1.924634	1.917676	1.99504	1.987154	1.979495	2.0352	2.028189	2.138835
1.674444	1.923038	1.91616	1.993405	1.985595	1.977991	2.092823	2.085627	2.16211
1.673117	1.921565	1.914706	1.991771	1.984006	1.97645	2.091203	2.084087	2.160509
1.671807	1.920097	1.913226	1.990087	1.982398	1.974929	2.089623	2.082591	2.158949
1.740299	1.918513	1.911595	2.01823	2.010651	2.003314	2.088103	2.081116	2.157369
1.738915	1.916891	1.90991	2.016256	2.00892	2.001843	2.086617	2.07965	2.155782
1.737559	1.915369	1.908379	2.034154	2.026917	2.019914	2.106597	2.099561	2.196739
1.736222	1.913926	1.906971	2.032558	2.025403	2.01847	2.10503	2.097931	2.194987
1.926936	1.97239	1.964594	2.062461	2.05507	2.047921	2.103439	2.09634	2.205104
1.925345	1.9708	1.963016	2.06077	2.053397	2.046281	2.101804	2.0948	2.203481
1.923761	2.021454	2.013609	2.100373	2.092938	2.085771	2.127308	2.120167	2.201883
1.922173	2.019849	2.012005	2.098687	2.091327	2.084242	2.12578	2.118676	2.200305
1.920558	2.018216	2.010408	2.09704	2.089771	2.082774	2.124297	2.117195	2.198715
1.918836	2.016469	2.008809	2.095467	2.088276	2.081326	2.138102	2.130857	2.197057
1.917056	2.014663	2.007221	2.093953	2.086835	2.079906	2.136562	2.129268	2.195346
1.915393	2.013002	2.005711	2.092468	2.085409	2.078492	2.134989	2.127587	2.193564
1.913844	2.011483	2.00428	2.091017	2.084005	2.077089	2.133379	2.125803	2.191699
1.991642	2.00995	2.002852	2.106976	2.099917	2.092904	2.131788	2.1241	2.230898
1.989922	2.00835	2.001403	2.105513	2.098465	2.091436	2.130251	2.122548	2.229226
1.98825	2.00679	1.999981	2.104054	2.096985	2.089916	2.128678	2.121007	2.227585
1.986736	2.005341	1.998616	2.102593	2.095433	2.08827	2.126973	2.119379	2.225926
1.985306	2.003957	1.997288	2.101138	2.093866	2.086593	2.125237	2.117735	2.224276
1.983907	2.067674	2.060719	2.127102	2.119494	2.111874	2.161613	2.154032	2.222649
1.982547	2.066296	2.059348	2.12563	2.118009	2.110372	2.160051	2.152442	2.221043
1.981194	2.064923	2.057982	2.124158	2.116521	2.108866	2.158494	2.150871	2.21946
1.979849	2.063559	2.056624	2.122689	2.115033	2.107359	2.156946	2.149323	2.2179
1.978509	2.081143	2.074133	2.121208	2.113537	2.105851	2.192043	2.184342	2.216352
1.977173	2.079738	2.072717	2.11971	2.112029	2.104337	2.190455	2.182767	2.214813

1.975839	2.078325	2.071291	2.1182	2.110514	2.102822	2.188867	2.181194	2.213282
1.974503	2.076908	2.069856	2.116682	2.108994	2.101305	2.187281	2.179622	2.211754
1.97317	2.075483	2.068413	2.115155	2.107468	2.099788	2.185698	2.178053	2.210233
1.97185	2.074049	2.066961	2.113623	2.10594	2.098271	2.184123	2.176495	2.208725
1.970543	2.072605	2.065501	2.112086	2.104409	2.096756	2.182555	2.174947	2.207227
2.039174	2.081414	2.074296	2.136484	2.128761	2.121017	2.169765	2.162181	2.205733
2.037764	2.079957	2.072836	2.134962	2.12724	2.119499	2.16822	2.160649	2.204237
2.036357	2.078502	2.071382	2.173996	2.166219	2.158423	2.166704	2.159146	2.202764
2.03495	2.077043	2.069936	2.172448	2.16469	2.156919	2.165215	2.157678	2.201322
2.033563	2.075596	2.068497	2.170906	2.163186	2.155459	2.163775	2.156255	2.199913
2.050187	2.074182	2.067063	2.169371	2.161737	2.154105	2.173848	2.166307	2.19855
2.048867	2.072789	2.065636	2.167842	2.160321	2.152811	2.172591	2.165024	2.197228
2.047511	2.071388	2.064209	2.166306	2.158848	2.151417	2.171262	2.163729	2.195929
2.046119	2.069973	2.062778	2.16476	2.157319	2.149931	2.169867	2.162425	2.194656
2.055801	2.112466	2.105161	2.163276	2.155822	2.148445	2.168479	2.161151	2.193414
2.054548	2.11122	2.103865	2.161868	2.154364	2.146969	2.167115	2.159938	2.192228
2.053283	2.109971	2.102607	2.160521	2.152953	2.145515	2.165746	2.158696	2.191014
2.02012	2.151743	2.144414	2.159273	2.151632	2.144106	2.16435	2.157354	2.1897
2.018764	2.15035	2.143139	2.158043	2.150339	2.142723	2.162949	2.155967	2.188344
2.017486	2.148964	2.141779	2.156667	2.148964	2.141344	2.161565	2.154579	2.186993
2.016256	2.147577	2.140358	2.155194	2.147542	2.139977	2.160198	2.153186	2.185641
1.97977	2.129116	2.122155	2.165972	2.158393	2.150905	2.158916	2.151874	2.184327
1.978536	2.127797	2.120785	2.164496	2.157058	2.149716	2.157735	2.150645	2.183049
2.037084	2.126522	2.119468	2.163066	2.15572	2.148484	2.156539	2.149458	2.142537
2.035919	2.125314	2.118237	2.161703	2.154353	2.147145	2.155293	2.148336	2.141437
2.034783	2.124138	2.117048	2.160398	2.153012	2.145795	2.154023	2.147191	2.14033
2.033658	2.097354	2.090363	2.159175	2.151752	2.144497	2.152715	2.145907	2.161851
2.032541	2.096217	2.089229	2.157995	2.150538	2.143233	2.151402	2.144569	2.160555
2.103736	2.119472	2.112227	2.156741	2.149315	2.142039	2.15018	2.143305	2.159273
2.102507	2.118308	2.111025	2.155435	2.148094	2.140907	2.14903	2.142092	2.157992
2.101247	2.117096	2.109823	2.154185	2.146885	2.13974	2.147876	2.140939	2.156794
2.099938	2.115812	2.108617	2.15301	2.145688	2.138514	2.146707	2.139866	2.155709
2.098666	2.12852	2.12137	2.151837	2.144508	2.137322	2.145554	2.138777	2.154614
2.097497	2.127379	2.120208	2.150623	2.143354	2.136226	2.144433	2.137604	2.153432
2.096345	2.126258	2.119055	2.149419	2.142214	2.135149	2.143323	2.136431	2.15225
2.095116	2.125071	2.117908	2.110811	2.103821	2.096941	2.164956	2.158015	2.151157
2.093858	2.123859	2.116767	2.10972	2.102722	2.095831	2.163814	2.156964	2.150105
2.092671	2.122701	2.115617	2.10859	2.101624	2.094766	2.162702	2.15586	2.148999
2.091548	2.121591	2.11446	2.107423	2.100526	2.093743	2.161619	2.15471	2.147843
2.090416	2.098686	2.091577	2.090677	2.083886	2.07722	2.160514	2.153584	2.146712
2.089262	2.097522	2.090437	2.089584	2.082789	2.076121	2.159364	2.152504	2.145627
2.088098	2.096354	2.089295	2.088491	2.081706	2.075046	2.158231	2.151421	2.144541
2.086919	2.095187	2.088123	2.08736	2.080645	2.07405	2.157149	2.150284	2.143405
2.085736	2.094023	2.086955	2.086236	2.079607	2.073091	2.156103	2.149158	2.142282
2.100003	2.092865	2.085834	2.085182	2.078606	2.072138	2.122996	2.116279	2.141279
2.098848	2.091719	2.08475	2.084178	2.077635	2.071194	2.122073	2.115392	2.140355
2.097766	2.090652	2.083716	2.111546	2.1049	2.098348	2.121081	2.114445	2.139372
2.096769	2.089674	2.08274	2.110561	2.103856	2.097253	2.120011	2.113438	2.138334
2.095763	2.088689	2.08175	2.109556	2.102823	2.096197	2.118953	2.112398	2.137258
2.094715	2.087663	2.080704	2.108498	2.101809	2.09522	2.117927	2.1113	2.13611

2.093667	2.086639	2.079672	2.107462	2.100821	2.094271	2.116927	2.110227	2.134991
2.092653	2.08565	2.078715	2.106526	2.099886	2.093332	2.11598	2.109279	2.114464
2.091645	2.084668	2.077787	2.105635	2.098976	2.092396	2.115053	2.108382	2.113544
2.090583	2.083632	2.076817	2.104706	2.098024	2.09141	2.114074	2.10743	2.100762
2.089482	2.082554	2.07582	2.103756	2.097044	2.090387	2.113059	2.106442	2.099769
2.088435	2.08154	2.074864	2.102836	2.096108	2.089424	2.112093	2.105486	2.098814
2.087458	2.080608	2.073962	2.101958	2.095229	2.088535	2.111189	2.104568	2.097907
2.086477	2.079673	2.073055	2.095623	2.088905	2.082218	2.096991	2.090404	2.097007
2.085447	2.078692	2.072119	2.094709	2.088007	2.081325	2.096102	2.089502	2.096116
2.084429	2.077712	2.071164	2.093764	2.087085	2.080417	2.095197	2.088577	2.095202
2.083487	2.076769	2.070176	2.092725	2.086082	2.079455	2.094225	2.08756	2.094194
2.082593	2.075851	2.069174	2.091642	2.08504	2.078464	2.093221	2.0865	2.093141
2.081716	2.104221	2.097394	2.090655	2.084065	2.077509	2.092266	2.085525	2.092178
2.080861	2.10335	2.096505	2.089748	2.083146	2.076583	2.091351	2.084621	2.091289
2.080007	2.102485	2.095634	2.088867	2.082249	2.075674	2.090458	2.083744	2.090426
2.079148	2.101622	2.094779	2.088011	2.081376	2.074785	2.089585	2.082891	2.089587
2.078289	2.100761	2.093927	2.087161	2.080511	2.073908	2.088728	2.082056	2.088766
2.077432	2.099901	2.093066	2.086297	2.079642	2.073037	2.087881	2.081233	2.087959
2.076574	2.09904	2.092202	2.085431	2.078777	2.072178	2.087048	2.080425	2.087166
2.075709	2.098172	2.091336	2.08457	2.077925	2.071339	2.081159	2.074569	2.086389
2.074839	2.097299	2.090468	2.083711	2.077084	2.070517	2.080352	2.073778	2.085625
2.073965	2.096426	2.089606	2.082862	2.076247	2.069694	2.079545	2.072991	2.084864
2.073083	2.095552	2.088751	2.082024	2.075417	2.068872	2.07874	2.072209	2.084109
2.055289	2.094684	2.087898	2.081184	2.074585	2.06805	2.077934	2.071423	2.070193
2.054439	2.093818	2.087037	2.080331	2.073744	2.067222	2.077119	2.070622	2.069417
2.053617	2.092972	2.086192	2.079493	2.072916	2.066408	2.076317	2.069833	2.068649
2.052854	2.092168	2.085384	2.078689	2.072119	2.065621	2.07554	2.069071	2.067903
2.052115	2.091386	2.084607	2.07792	2.071351	2.064858	2.074789	2.06834	2.067184
2.051321	2.090598	2.083873	2.077224	2.070645	2.064138	2.074092	2.067683	2.0613
2.050491	2.089809	2.083177	2.076589	2.069991	2.063454	2.073438	2.067087	2.060696
2.049678	2.089016	2.082432	2.075878	2.06928	2.062739	2.072742	2.066421	2.060027
2.048876	2.088208	2.081619	2.07507	2.068496	2.06198	2.071993	2.065671	2.059278
2.048115	2.087441	2.080831	2.07427	2.067718	2.061226	2.071239	2.064909	2.058517
2.047429	2.086762	2.080112	2.073518	2.066966	2.060481	2.070485	2.064148	2.057755
2.046754	2.086093	2.079409	2.072788	2.06624	2.059764	2.069753	2.063403	2.057008
2.029897	2.070017	2.063364	2.072075	2.065566	2.059123	2.069094	2.062709	2.068276
2.029142	2.06924	2.062626	2.071382	2.064927	2.058522	2.068473	2.062045	2.067585
2.02842	2.068506	2.061939	2.070728	2.064287	2.057883	2.067819	2.061368	2.066888
2.027726	2.067804	2.061297	2.070114	2.063653	2.057217	2.067139	2.060683	2.066189
2.027035	2.052628	2.046245	2.054178	2.047724	2.041293	2.050099	2.043693	2.049896
2.026338	2.051906	2.045504	2.053422	2.046987	2.040589	2.049437	2.04308	2.04929
2.02566	2.051212	2.044785	2.052682	2.046273	2.039911	2.06133	2.054968	2.048685
2.025016	2.050585	2.044143	2.052019	2.045639	2.03931	2.060682	2.054296	2.048052
2.024389	2.049985	2.043535	2.051395	2.045038	2.038728	2.060035	2.05361	2.047402
2.023761	2.049369	2.042944	2.036573	2.030259	2.023964	2.044818	2.038393	2.046724
2.023137	2.048752	2.042367	2.036012	2.029656	2.023308	2.044112	2.037682	2.04603
2.054651	2.048144	2.041754	2.049261	2.042836	2.036417	2.043426	2.037016	2.045386
2.054057	2.047549	2.041105	2.048546	2.042116	2.03571	2.042757	2.036395	2.044794
2.053454	2.046939	2.040453	2.047843	2.041419	2.035033	2.042111	2.035779	2.044207
2.052829	2.046291	2.03979	2.04716	2.040763	2.034406	2.041493	2.03515	2.043613

2.052212	2.045661	2.039159	2.046511	2.040135	2.033799	2.040893	2.034536	2.043031
2.051636	2.058898	2.052391	2.045937	2.039547	2.033199	2.040306	2.033966	2.028044
2.051073	2.058339	2.051845	2.045391	2.038979	2.032609	2.039729	2.033411	2.027514
2.05048	2.057722	2.051227	2.044777	2.03838	2.032025	2.025625	2.019298	2.02696
2.049867	2.057068	2.050564	2.044122	2.037764	2.031448	2.025043	2.018696	2.026393
2.040507	2.034184	2.027912	2.043539	2.03718	2.030863	2.024458	2.018118	2.02585
2.039936	2.033645	2.027408	2.043044	2.036631	2.030257	2.023861	2.017562	2.02533
2.039358	2.033095	2.026896	2.042551	2.036109	2.029701	2.023308	2.017032	2.02483
2.03876	2.032508	2.026348	2.042038	2.035633	2.029247	2.022834	2.01652	2.024335
2.038157	2.031913	2.025782	2.041503	2.035153	2.02881	2.02239	2.016047	2.02388
2.037558	2.031322	2.025191	2.040912	2.034605	2.028316	2.021947	2.015653	2.023526
2.036964	2.030734	2.024589	2.040296	2.034024	2.027793	2.021503	2.015301	2.023224
2.036388	2.030148	2.024019	2.039734	2.033459	2.027236	2.021009	2.014895	2.009354
2.035828	2.029563	2.023473	2.039215	2.032902	2.026645	2.02047	2.014449	2.008959
2.035322	2.029044	2.022968	2.038717	2.032385	2.026113	2.019969	2.014002	2.008558
2.034886	2.028611	2.022517	2.03825	2.031925	2.025663	2.019518	2.013549	2.008141
2.017044	2.028197	2.02207	2.037772	2.031465	2.025229	2.019091	2.013127	2.007752
2.01663	2.027783	2.021597	2.037245	2.030968	2.024784	2.018688	2.012767	2.007431
2.016202	2.027359	2.021121	2.036718	2.03047	2.024338	2.018297	2.012434	2.007133
2.015708	2.026891	2.020661	2.036254	2.03001	2.023891	2.017897	2.012096	2.006818
2.015172	2.026391	2.020208	2.035826	2.029569	2.023438	2.01749	2.011759	2.006497
2.014634	2.025857	2.019724	2.035374	2.029094	2.022942	2.017045	2.011396	2.006177
2.014086	2.02528	2.019207	2.034896	2.028581	2.022396	2.016556	2.011008	2.005856
1.980657	2.007393	2.001392	2.012696	2.006495	2.000429	2.016103	2.010626	2.005531
1.980281	2.006978	2.00097	2.012275	2.006077	2.000021	2.015712	2.010261	2.005205
1.979929	2.006596	2.000576	2.011875	2.005683	1.999638	2.015335	2.009889	2.004865
1.979563	2.006225	2.000207	2.011506	2.005309	1.999258	2.014942	2.009485	2.004489
1.979199	2.005862	1.999846	2.011143	2.004942	1.998886	2.014552	2.009079	2.004109
1.978833	2.005482	1.999453	1.993436	1.987284	1.981282	2.014189	2.008728	2.003786
1.978468	2.00509	1.999035	1.993007	1.986876	1.980905	2.013845	2.008416	2.003504
1.978112	2.004693	1.998589	1.992536	1.986427	1.9805	2.013488	2.008105	2.003228
1.977764	2.004287	1.998105	1.992008	1.985929	1.980059	2.013117	2.007794	2.002957
1.977422	2.003893	1.997647	1.991513	1.985451	1.979624	2.012746	2.007485	2.002689
1.977086	2.003522	1.997246	1.991089	1.98502	1.979204	2.012376	2.007178	2.002423
1.976751	2.003159	1.996867	1.990697	1.984616	1.978802	2.012014	2.006872	2.002154
1.976406	2.002788	1.996486	1.990312	1.984235	1.978434	1.995374	1.990278	2.001873
1.976061	2.00242	1.996118	1.989948	1.98388	1.978094	1.995047	1.989971	2.001588
1.97573	2.002073	1.995779	1.989617	1.983555	1.977778	1.99474	1.989683	2.001318
1.975412	2.001745	1.995467	1.989317	1.98326	1.977488	1.994452	1.989413	2.001059
1.955414	2.001428	1.995184	1.98906	1.983007	1.977232	1.972003	1.967213	1.984529
1.95509	2.001126	1.994947	1.988867	1.982814	1.977023	1.971781	1.966977	1.984269
1.954769	2.000814	1.994688	1.988648	1.982603	1.97681	1.971565	1.966755	1.984022
1.954446	2.000465	1.994357	1.988343	1.982331	1.976569	1.971344	1.966545	1.983791
1.954132	2.000115	1.994006	1.988007	1.982031	1.976312	1.971122	1.966351	1.983584
1.953845	1.999801	1.993674	1.987672	1.981718	1.976036	1.970899	1.966187	1.98343
1.953577	1.999513	1.993354	1.987334	1.981395	1.975747	1.970675	1.966039	1.983306
1.953317	1.999261	1.993073	1.987036	1.981107	1.975482	1.970446	1.965854	1.983125
1.953066	1.999049	1.992836	1.986776	1.980851	1.975237	1.970213	1.965632	1.982889
1.952805	1.998806	1.992574	1.986505	1.980602	1.975014	1.970001	1.965425	1.982666
1.952517	1.998503	1.992262	1.986205	1.980355	1.974824	1.969826	1.965247	1.982467

1.952242	1.998199	1.991954	1.985911	1.98011	1.97463	1.969649	1.96507	1.982278
1.952005	1.997929	1.991678	1.985639	1.979855	1.974397	1.969433	1.964871	1.982096
1.951788	1.997676	1.991423	1.985386	1.979606	1.974157	1.969209	1.96467	1.98192
1.951576	1.997437	1.991199	1.985175	1.979399	1.973952	1.969014	1.964487	1.981744
1.951373	1.997212	1.990999	1.984995	1.97922	1.973771	1.968839	1.964319	1.981572
1.951177	1.945117	1.939039	1.984792	1.979025	1.973588	1.968675	1.964177	1.981442
1.95099	1.944923	1.938829	1.984564	1.978811	1.9734	1.968522	1.964064	1.981357
1.950792	1.94472	1.938617	1.984338	1.978608	1.973231	1.968386	1.96396	1.959735
1.950569	1.944496	1.938406	1.984118	1.978428	1.973092	1.968269	1.963855	1.959643
1.950343	1.944273	1.938197	1.983911	1.978261	1.972966	1.968163	1.963759	1.959555
1.950132	1.944065	1.937989	1.983722	1.978103	1.972838	1.968063	1.963682	1.959478
1.949929	1.943868	1.93779	1.98355	1.977956	1.972715	1.967967	1.963608	1.959399
1.949732	1.943681	1.937624	1.983398	1.977825	1.972601	1.967856	1.963492	1.959278
1.949542	1.943505	1.937487	1.983263	1.977709	1.972495	1.967735	1.963343	1.959121
1.949378	1.943346	1.937346	1.983129	1.977589	1.972385	1.967616	1.963208	1.958985
1.949248	1.943209	1.937199	1.982992	1.977461	1.972263	1.967496	1.963086	1.958872
1.949118	1.943076	1.937058	1.982859	1.977341	1.972155	1.967395	1.96299	1.958784
1.948965	1.942929	1.93693	1.982731	1.977235	1.972075	1.967333	1.96294	1.958733
1.948822	1.942792	1.936811	1.982611	1.97714	1.972007	1.967283	1.962905	1.958696
1.948728	1.942686	1.9367	1.98251	1.977052	1.971932	1.967216	1.962841	1.958637
1.948664	1.942603	1.936599	1.982422	1.976972	1.97186	1.967146	1.962772	1.958576
1.948603	1.942531	1.936514	1.982329	1.976893	1.9718	1.967105	1.962747	1.958554
1.948548	1.942476	1.936447	1.982231	1.976815	1.971753	1.96709	1.962765	1.958569
1.901074	1.942401	1.936364	1.930647	1.925354	1.92044	1.967067	1.962775	1.958588
1.900955	1.942287	1.936249	1.930541	1.925272	1.920387	1.967025	1.962772	1.958608
1.900845	1.942188	1.936147	1.930444	1.925199	1.920342	1.966985	1.962758	1.958617
1.900764	1.942138	1.936082	1.93037	1.925141	1.920305	1.966953	1.962721	1.958592
1.900701	1.942118	1.936047	1.930325	1.925105	1.920282	1.966934	1.962686	1.958562
1.900659	1.935572	1.929508	1.930343	1.925117	1.920288	1.966949	1.9627	1.958565
1.900635	1.935589	1.929551	1.93041	1.925167	1.920316	1.966989	1.962747	1.958589
1.900609	1.935594	1.92959	1.930481	1.925221	1.920346	1.96701	1.962781	1.980937
1.900581	1.93559	1.929632	1.930566	1.925285	1.920378	1.967014	1.962806	1.980957
1.900555	1.935568	1.929635	1.930602	1.92532	1.920405	1.967015	1.962808	1.98095
1.900533	1.935511	1.92957	1.930552	1.925306	1.920425	1.967014	1.962767	1.980888
1.900512	1.935457	1.929491	1.930477	1.925273	1.920438	1.967017	1.962731	1.980828
1.900493	1.894574	1.888675	1.923776	1.918599	1.913801	1.948828	1.944615	1.980816
1.900473	1.894575	1.888642	1.923709	1.918555	1.913792	1.948834	1.94464	1.980832
1.900435	1.894563	1.888634	1.923704	1.91855	1.913791	1.948847	1.94468	1.980874
1.900383	1.894544	1.888651	1.923749	1.918575	1.913796	1.948865	1.944734	1.980942
1.900363	1.894545	1.888681	1.923798	1.9186	1.913797	1.948866	1.944768	1.980993
1.900386	1.894575	1.88873	1.923847	1.91862	1.913787	1.948837	1.944772	1.981018
1.900411	1.894601	1.888777	1.923905	1.918656	1.913802	1.94883	1.944788	1.981049
1.900422	1.894612	1.888821	1.92398	1.918728	1.913865	1.948874	1.944838	1.981104
1.900426	1.894613	1.888852	1.924049	1.918807	1.913946	1.948937	1.944895	1.981162
1.900414	1.894589	1.888843	1.883431	1.878438	1.873846	1.943429	1.939365	1.964011
1.900397	1.894556	1.888814	1.88342	1.878468	1.873912	1.943481	1.939396	1.964034
1.900417	1.894565	1.888824	1.883439	1.878512	1.873977	1.967401	1.963196	1.964024
1.90047	1.894611	1.888866	1.883482	1.878567	1.874041	1.9674	1.963151	1.963981
1.900532	1.894671	1.888925	1.883541	1.878631	1.874109	1.935832	1.931687	1.963962
1.900604	1.894747	1.889002	1.883617	1.878707	1.87418	1.935882	1.931719	1.963973

1.900675	1.894824	1.889083	1.883701	1.878789	1.874259	1.935947	1.931777	1.959033
1.900726	1.89488	1.889153	1.883781	1.878874	1.874345	1.936033	1.931866	1.959094
1.900783	1.894944	1.889228	1.883867	1.878964	1.87444	1.936137	1.931983	1.959186
1.900888	1.895056	1.88933	1.883962	1.879062	1.874545	1.936265	1.932135	1.959324
1.901025	1.895203	1.889454	1.884067	1.879167	1.874659	1.936411	1.932314	1.959496
1.901165	1.89536	1.889603	1.884203	1.879287	1.874765	1.898897	1.895053	1.959625
1.901314	1.895535	1.889784	1.884374	1.879422	1.874864	1.898971	1.895137	1.959711
1.901443	1.895687	1.889948	1.884537	1.879561	1.874977	1.899055	1.895219	1.959787
1.901534	1.895798	1.890078	1.88468	1.879706	1.875116	1.899155	1.895298	1.959854
1.901617	1.895892	1.89019	1.884808	1.879848	1.875263	1.899267	1.895387	1.959926
1.852497	1.895971	1.890277	1.88491	1.879973	1.875409	1.8994	1.895507	1.960021
1.852579	1.896049	1.890359	1.885005	1.880094	1.875556	1.89954	1.89564	1.960125
1.831737	1.896172	1.890487	1.913594	1.90856	1.903896	1.926591	1.922483	1.98264
1.831876	1.89633	1.89065	1.913736	1.908684	1.904001	1.926677	1.922567	1.982721
1.832038	1.877963	1.872298	1.913898	1.908834	1.904137	1.926788	1.922669	1.982824
1.832232	1.878168	1.872502	1.914083	1.909014	1.904308	1.926928	1.922792	1.982951
1.832401	1.856194	1.85061	1.914269	1.909198	1.904486	1.927081	1.92293	1.951169
1.832513	1.856341	1.850781	1.914449	1.90938	1.904665	1.927249	1.923094	1.951325
1.832602	1.856467	1.850936	1.914616	1.909552	1.904838	1.927418	1.923263	1.951486
1.832689	1.827193	1.821811	1.897438	1.892415	1.887744	1.927572	1.923412	1.95162
1.832772	1.827269	1.821898	1.897539	1.892534	1.88788	1.927714	1.923551	1.951743
1.832877	1.827373	1.822014	1.897658	1.892652	1.887997	1.927834	1.923683	1.91959
1.832998	1.8275	1.822154	1.897791	1.892764	1.888093	1.92793	1.923806	1.919733
1.833161	1.827656	1.822302	1.877287	1.872303	1.86767	1.911985	1.907908	1.919883
1.833381	1.827845	1.822448	1.877433	1.872479	1.867877	1.912177	1.908087	1.920048
1.833612	1.828054	1.822627	1.877614	1.872678	1.868094	1.912377	1.908265	1.920207
1.833834	1.828285	1.822882	1.877865	1.872903	1.868287	1.912558	1.908418	1.920347
1.834053	1.828524	1.823156	1.878137	1.873141	1.868482	1.912737	1.908565	1.920482
1.834267	1.828746	1.823373	1.850828	1.845957	1.841425	1.893673	1.889529	1.904619
1.834476	1.828954	1.823561	1.851004	1.846154	1.841641	1.893864	1.889688	1.904767
1.834653	1.829136	1.823767	1.851218	1.846367	1.841844	1.894064	1.889873	1.904947
1.83479	1.829283	1.82399	1.851472	1.846596	1.842033	1.894274	1.890088	1.905163
1.834978	1.829474	1.824218	1.851707	1.846814	1.842228	1.894478	1.890289	1.905363
1.835263	1.829751	1.824445	1.851896	1.847012	1.842439	1.894667	1.890451	1.905517
1.835552	1.830031	1.824678	1.852089	1.84721	1.842647	1.894859	1.890624	1.905683
1.835764	1.830244	1.824921	1.852332	1.847428	1.842837	1.838655	1.834802	1.857997
1.835965	1.830445	1.825169	1.852589	1.847652	1.843023	1.838846	1.835017	1.858241
1.836249	1.830709	1.825407	1.852798	1.847863	1.843239	1.839057	1.83522	1.858469
1.836593	1.831019	1.825641	1.852977	1.848065	1.843475	1.839281	1.835416	1.858689
1.836932	1.831347	1.825919	1.8532	1.848274	1.843679	1.83948	1.835612	1.831907
1.837268	1.831703	1.826251	1.853471	1.848486	1.843841	1.839645	1.835809	1.832112
1.807028	1.83203	1.82658	1.853766	1.848727	1.844029	1.839821	1.835996	1.832302
1.807282	1.832287	1.826887	1.854095	1.849025	1.844277	1.840026	1.83616	1.832461
1.80754	1.832536	1.827181	1.854418	1.849331	1.844549	1.84025	1.836331	1.832621
1.807866	1.832831	1.827454	1.854671	1.849596	1.844822	1.840499	1.836541	1.832813
1.808224	1.83315	1.827719	1.854894	1.849844	1.8451	1.840765	1.836776	1.833027
1.808558	1.837398	1.831861	1.855148	1.850097	1.845359	1.841022	1.837027	1.833265
1.808878	1.837732	1.832171	1.855424	1.850353	1.845602	1.841273	1.837295	1.83353
1.787609	1.838061	1.832496	1.827181	1.82214	1.817426	1.841524	1.83756	1.83379
1.787928	1.838381	1.832842	1.82753	1.822445	1.817682	1.841777	1.837821	1.834048

1.788245	1.838691	1.833183	1.827882	1.822756	1.817944	1.842027	1.838069	1.834291
1.788555	1.838981	1.833507	1.828222	1.823067	1.818215	1.842268	1.83829	1.834507
1.78887	1.839269	1.83382	1.828551	1.823376	1.818492	1.84251	1.838501	1.834712
1.760519	1.819544	1.814108	1.828845	1.823669	1.818778	1.814405	1.810432	1.834923
1.760847	1.819859	1.814396	1.82912	1.823954	1.819073	1.814668	1.810648	1.835136
1.761183	1.8202	1.814717	1.809437	1.8043	1.799448	1.814936	1.810875	1.835348
1.761529	1.820566	1.81507	1.809775	1.804623	1.799751	1.815211	1.811112	1.835558
1.728263	1.794038	1.788572	1.8101	1.804936	1.800052	1.795595	1.791504	1.807571
1.728592	1.794374	1.788868	1.8104	1.805235	1.800354	1.795879	1.791761	1.807815
1.728926	1.794711	1.789163	1.810694	1.805522	1.80064	1.796154	1.792022	1.808069
1.729266	1.795043	1.789455	1.810988	1.805783	1.800878	1.796394	1.792278	1.808333
1.729612	1.795376	1.789752	1.811287	1.806041	1.801105	1.796626	1.792537	1.808605
1.729965	1.764273	1.758667	1.784626	1.779399	1.774485	1.796895	1.792806	1.789042
1.730323	1.764611	1.758984	1.784935	1.779686	1.774751	1.79719	1.793085	1.789311
1.730687	1.764957	1.759311	1.753881	1.748675	1.743789	1.770567	1.766487	1.789592
1.731054	1.765309	1.759646	1.754202	1.748993	1.7441	1.770864	1.766746	1.789887
1.731426	1.765669	1.760003	1.754554	1.74934	1.744435	1.771179	1.767019	1.790182
1.731801	1.76604	1.760393	1.754952	1.749725	1.744795	1.771512	1.767308	1.790472
1.732181	1.766417	1.760796	1.75537	1.750131	1.745175	1.77186	1.767604	1.79076
1.732566	1.766802	1.761202	1.755788	1.750548	1.745574	1.740982	1.736736	1.763958
1.732955	1.767193	1.761611	1.756208	1.750972	1.745984	1.741339	1.737017	1.764199
1.733344	1.767576	1.762005	1.756609	1.751371	1.746371	1.741688	1.737312	1.733351
1.733734	1.767955	1.762389	1.756994	1.751753	1.74674	1.742031	1.73762	1.733617
1.734135	1.768337	1.762766	1.757367	1.752123	1.747103	1.742372	1.73793	1.733891
1.734547	1.768718	1.763131	1.757721	1.752476	1.747453	1.742707	1.738241	1.734173
1.734974	1.769118	1.76351	1.758086	1.752841	1.747818	1.743053	1.738557	1.734457
1.735418	1.769551	1.763917	1.758474	1.753237	1.748218	1.743421	1.738874	1.734735
1.735878	1.770006	1.764347	1.758883	1.753651	1.748633	1.7438	1.739199	1.735018
1.752761	1.770485	1.76481	1.759329	1.754084	1.749049	1.744185	1.739545	1.735324
1.753236	1.770985	1.765301	1.759802	1.754533	1.749468	1.744575	1.739902	1.735643
1.753711	1.771488	1.765798	1.760281	1.754976	1.749871	1.744947	1.740248	1.735956
1.754192	1.772	1.766307	1.760769	1.755416	1.750257	1.745302	1.740582	1.736263
1.75464	1.772475	1.766781	1.761229	1.755841	1.750644	1.745661	1.740917	1.736571
1.755036	1.772892	1.767201	1.761646	1.756245	1.751028	1.746023	1.741253	1.736878
1.755425	1.7733	1.767604	1.762046	1.756646	1.751425	1.746397	1.741594	1.737191
1.728499	1.750161	1.744511	1.762427	1.757054	1.751856	1.746798	1.741941	1.737511
1.728908	1.750575	1.744901	1.762815	1.757471	1.7523	1.747212	1.742296	1.737841
1.729335	1.751005	1.745338	1.763271	1.75792	1.752735	1.74762	1.742671	1.73819
1.729779	1.751452	1.745812	1.763776	1.758392	1.753165	1.748027	1.743061	1.738555
1.68758	1.751944	1.746307	1.740788	1.735421	1.730199	1.748428	1.743451	1.738914
1.68805	1.752491	1.746832	1.741276	1.73586	1.730588	1.748821	1.743839	1.739267
1.688525	1.753027	1.747336	1.741744	1.736294	1.730991	1.749225	1.744229	1.739626
1.688993	1.753524	1.74779	1.742167	1.736722	1.731423	1.749647	1.744607	1.739983
1.689466	1.754018	1.748244	1.742593	1.737151	1.731858	1.75008	1.745009	1.740368
1.68995	1.727307	1.721665	1.743066	1.737589	1.732267	1.727064	1.722043	1.740826
1.690438	1.727827	1.722171	1.743561	1.738037	1.732677	1.727495	1.722521	1.741321
1.690915	1.728342	1.722672	1.744054	1.738515	1.733141	1.727958	1.722984	1.741787
1.691384	1.728852	1.723173	1.744549	1.739018	1.733652	1.728448	1.723441	1.742234
1.691855	1.729369	1.723687	1.745066	1.739529	1.734153	1.728937	1.723912	1.742693
1.692331	1.729895	1.724218	1.745609	1.74004	1.734631	1.729416	1.724403	1.74317

1.692794	1.687575	1.682141	1.719173	1.713723	1.708426	1.729911	1.724894	1.720136
1.693224	1.688023	1.682646	1.719754	1.714293	1.708966	1.730431	1.725364	1.720569
1.693646	1.688462	1.683148	1.720346	1.714882	1.709532	1.730973	1.725844	1.721007
1.694088	1.688923	1.68364	1.720919	1.715472	1.710122	1.731544	1.726372	1.721483
1.694537	1.68939	1.684123	1.721481	1.716062	1.710726	1.732135	1.72693	1.721986
1.659222	1.689835	1.684589	1.722018	1.716618	1.711293	1.732713	1.727498	1.722514
1.659628	1.690256	1.685036	1.722527	1.71714	1.711826	1.733282	1.728082	1.72307
1.56788	1.690703	1.685509	1.72306	1.717675	1.712358	1.733838	1.728639	1.7236
1.56832	1.691187	1.686022	1.723631	1.718229	1.712886	1.73437	1.729148	1.724082
1.568784	1.691694	1.686549	1.724213	1.7188	1.713434	1.734913	1.729661	1.724565
1.569269	1.692228	1.68708	1.681914	1.676698	1.671507	1.70878	1.703623	1.725073
1.56976	1.692776	1.687617	1.682448	1.677262	1.672095	1.709373	1.70417	1.725598
1.570219	1.606805	1.601984	1.682987	1.677793	1.672617	1.709931	1.704721	1.726145
1.570657	1.607265	1.602454	1.68353	1.678304	1.673097	1.710469	1.705276	1.726711
1.571098	1.607718	1.602939	1.598116	1.593196	1.588292	1.711012	1.705832	1.727275
1.57154	1.60816	1.603443	1.598656	1.5937	1.588747	1.711554	1.706386	1.727833
1.571987	1.608609	1.603935	1.599178	1.59421	1.589235	1.669517	1.664507	1.701861
1.572437	1.609065	1.604403	1.599664	1.594723	1.58977	1.670091	1.665073	1.702463
1.572905	1.609537	1.604862	1.600126	1.595226	1.590314	1.670676	1.665639	1.703066
1.573418	1.610042	1.605314	1.600551	1.595697	1.590837	1.586025	1.581265	1.703641
1.573953	1.610566	1.605764	1.60096	1.596151	1.591344	1.586522	1.581723	1.704203
1.57448	1.611087	1.606236	1.601398	1.596598	1.59181	1.586985	1.582175	1.704756
1.575002	1.61161	1.606725	1.601856	1.597039	1.592242	1.58742	1.58262	1.7053
1.518421	1.570715	1.565816	1.60235	1.597521	1.592712	1.587894	1.583102	1.626811
1.518838	1.571147	1.566311	1.602889	1.598055	1.59323	1.588414	1.58363	1.627372
1.519276	1.571593	1.566813	1.603434	1.598602	1.593769	1.588953	1.584168	1.579495
1.519775	1.572085	1.567323	1.603966	1.599151	1.594329	1.589506	1.584704	1.580021
1.520299	1.5726	1.567843	1.6045	1.599703	1.594895	1.590064	1.585242	1.580548
1.520827	1.573133	1.56838	1.605044	1.600249	1.595442	1.590607	1.585778	1.581073
1.521362	1.573681	1.568929	1.605594	1.600794	1.595981	1.591144	1.586312	1.581596
1.521883	1.574211	1.569471	1.606147	1.601347	1.596529	1.591683	1.586838	1.58211
1.522392	1.574727	1.570008	1.606702	1.601905	1.597083	1.592222	1.587355	1.582615
1.522907	1.575248	1.570543	1.607253	1.602464	1.597645	1.59277	1.587883	1.58313
1.523433	1.575775	1.571073	1.607793	1.603021	1.598216	1.593333	1.588425	1.583658
1.523958	1.576308	1.571608	1.608338	1.60358	1.598787	1.593899	1.588977	1.584195
1.524474	1.576846	1.572153	1.608891	1.604135	1.599341	1.594454	1.58953	1.584734
1.524985	1.577386	1.57271	1.609461	1.6047	1.599901	1.595018	1.590101	1.58529
1.481987	1.577921	1.573292	1.610074	1.605306	1.600493	1.595621	1.590719	1.585894
1.482483	1.578453	1.573891	1.610719	1.605943	1.601112	1.596252	1.591372	1.586535
1.483005	1.578988	1.574481	1.5699	1.565115	1.560282	1.59691	1.592037	1.587186
1.483552	1.57952	1.575062	1.570532	1.56578	1.560969	1.597602	1.592719	1.587855
1.484127	1.54039	1.536037	1.571134	1.566417	1.561634	1.598269	1.593373	1.588493
1.484742	1.540997	1.536608	1.571688	1.566998	1.562247	1.598882	1.593968	1.589069
1.485369	1.541621	1.537179	1.572231	1.56757	1.562856	1.599492	1.594562	1.589644
1.48599	1.542249	1.537758	1.572783	1.568161	1.563493	1.600136	1.595196	1.590261
1.486609	1.54288	1.538341	1.573344	1.568765	1.564144	1.600796	1.595851	1.590902
1.487212	1.543491	1.538932	1.534418	1.529971	1.525475	1.601473	1.596523	1.591561
1.487802	1.544086	1.53953	1.535033	1.530625	1.526166	1.602164	1.597212	1.592237
1.488393	1.544663	1.540121	1.535643	1.531251	1.526808	1.566331	1.561473	1.592927
1.488982	1.545213	1.540701	1.536249	1.531841	1.527388	1.566974	1.562187	1.593632

1.438328	1.485065	1.480855	1.536852	1.532432	1.527972	1.567613	1.562888	1.594337
1.438931	1.485674	1.481454	1.537463	1.533047	1.528599	1.568267	1.563574	1.595044
1.439551	1.486306	1.482056	1.538058	1.533653	1.529226	1.568908	1.564232	1.595732
1.440178	1.486953	1.482631	1.538592	1.53421	1.52982	1.569505	1.564828	1.560029
1.440819	1.487618	1.483204	1.539104	1.534744	1.530393	1.570074	1.565387	1.560623
1.441491	1.437014	1.432709	1.539704	1.535309	1.530936	1.526355	1.52167	1.561227
1.442193	1.437694	1.433366	1.540379	1.535896	1.531446	1.526887	1.522269	1.561833
1.442908	1.438394	1.434041	1.541066	1.536516	1.532006	1.527454	1.522875	1.562457
1.443645	1.439122	1.434732	1.541757	1.537175	1.53264	1.528072	1.523487	1.563107
1.444364	1.439845	1.435427	1.431113	1.426943	1.422828	1.528708	1.52411	1.563763
1.44503	1.440549	1.436116	1.431776	1.427579	1.423436	1.529347	1.524749	1.56442
1.445676	1.441236	1.436798	1.432443	1.428223	1.424057	1.529996	1.525395	1.565077
1.38882	1.441893	1.437463	1.433107	1.42887	1.424682	1.530655	1.526038	1.56573
1.389379	1.442529	1.438115	1.43377	1.42952	1.425315	1.531323	1.526682	1.56638
1.389992	1.4432	1.438777	1.434423	1.430168	1.425956	1.532002	1.527344	1.567042
1.390663	1.443909	1.439448	1.435067	1.430813	1.426609	1.532692	1.528025	1.567711
1.391308	1.4446	1.440109	1.435703	1.431446	1.427243	1.486787	1.482235	1.568401
1.391887	1.445248	1.440746	1.43633	1.432058	1.427848	1.487428	1.482913	1.56912
1.392474	1.445899	1.441386	1.43696	1.432667	1.428443	1.488061	1.48359	1.569846
1.326103	1.388954	1.384827	1.437604	1.433282	1.429037	1.488686	1.484234	1.570557
1.326719	1.389605	1.385422	1.438254	1.433899	1.429629	1.489309	1.484872	1.571271
1.327321	1.390243	1.386031	1.438899	1.434514	1.430229	1.48995	1.485551	1.57202
1.327917	1.390877	1.386651	1.439541	1.43513	1.430834	1.490603	1.486257	1.572795
1.328511	1.324341	1.320188	1.440184	1.435755	1.431451	1.42733	1.423331	1.573551
1.329103	1.324909	1.320699	1.440824	1.436389	1.432086	1.427966	1.423974	1.574286
1.329694	1.325483	1.321234	1.441493	1.437035	1.432712	1.428587	1.424601	1.531669
1.330276	1.326058	1.321812	1.442209	1.43769	1.433306	1.429171	1.425205	1.532342
1.330874	1.326661	1.322426	1.442959	1.438366	1.433907	1.429756	1.425807	1.533007
1.331532	1.327344	1.323097	1.318894	1.314744	1.310709	1.43037	1.426425	1.53368
1.332218	1.328067	1.3238	1.319557	1.315346	1.31125	1.431009	1.427057	1.534359
1.332854	1.328735	1.32447	1.320214	1.315973	1.31184	1.431707	1.427718	1.535068
1.333451	1.329363	1.325118	1.320871	1.316626	1.312477	1.43246	1.428408	1.535802
1.334039	1.329973	1.325743	1.321504	1.317261	1.313106	1.371421	1.367434	1.536538
1.334621	1.330567	1.32634	1.322104	1.317868	1.313715	1.372077	1.368034	1.537275
1.335195	1.331148	1.326928	1.322699	1.318478	1.314333	1.310385	1.306581	1.490018
1.335755	1.331709	1.327508	1.3233	1.319104	1.314972	1.310996	1.307142	1.49067
1.336319	1.332271	1.328092	1.32391	1.319741	1.315623	1.311618	1.307711	1.491327
1.336919	1.332876	1.328711	1.324544	1.320393	1.316284	1.312262	1.308322	1.435596
1.337539	1.333503	1.329352	1.325197	1.321055	1.31695	1.312918	1.308957	1.436285
1.338121	1.334096	1.329983	1.325853	1.321705	1.317583	1.313536	1.309559	1.36777
1.338663	1.334654	1.330606	1.326516	1.322342	1.318179	1.314112	1.310125	1.368388
1.339234	1.335229	1.331227	1.327169	1.322984	1.318797	1.314714	1.310713	1.369036
1.339849	1.335826	1.331841	1.327802	1.323634	1.319456	1.315364	1.311341	1.369732
1.340495	1.33645	1.332468	1.328439	1.324287	1.320124	1.316025	1.311986	1.370447
1.341179	1.337118	1.333127	1.329093	1.324941	1.320779	1.316679	1.312638	1.30877
1.341884	1.33781	1.333803	1.329757	1.325599	1.321434	1.317337	1.313301	1.309425
1.342579	1.338494	1.334481	1.33043	1.326271	1.322107	1.318008	1.313969	1.310084
1.343271	1.339178	1.335163	1.331113	1.326954	1.32279	1.318688	1.314643	1.310748
1.343961	1.33986	1.335846	1.331796	1.327634	1.323465	1.319358	1.315306	1.311404
1.344651	1.340542	1.336531	1.332481	1.32831	1.32413	1.320016	1.315959	1.312052

1.345332	1.341216	1.337208	1.33316	1.328982	1.324793	1.320671	1.316608	1.312695
1.345998	1.34188	1.337876	1.33383	1.329652	1.32546	1.321331	1.317257	1.313331
1.346657	1.342538	1.338535	1.33449	1.330313	1.326121	1.321986	1.317901	1.313963
1.269526	1.343188	1.339178	1.335129	1.33095	1.326756	1.32262	1.318534	1.314589
1.270157	1.343835	1.339814	1.335758	1.331578	1.327385	1.323251	1.319165	1.315213
1.27079	1.344489	1.340462	1.336406	1.332241	1.328061	1.32392	1.319817	1.315847
1.271425	1.345149	1.341117	1.337068	1.332931	1.328776	1.324622	1.320486	1.316489
1.116554	1.345814	1.341767	1.337721	1.333624	1.329509	1.325356	1.3212	1.317174
1.117137	1.346477	1.342404	1.33836	1.334321	1.330271	1.326137	1.321974	1.317918
1.117748	1.347168	1.343057	1.339001	1.335005	1.331008	1.326899	1.322744	1.318664
1.118411	1.347914	1.343743	1.339649	1.335658	1.331681	1.327598	1.32347	1.319381
1.119099	1.348683	1.344446	1.340304	1.336299	1.332328	1.328272	1.324182	1.320097
1.119788	1.271515	1.267396	1.340959	1.336941	1.332978	1.328952	1.324907	1.320842
1.120482	1.272253	1.268075	1.341623	1.337586	1.333623	1.329627	1.325631	1.321597
1.00859	1.273004	1.268795	1.342341	1.338261	1.334266	1.330275	1.326305	1.322305
1.009089	1.273769	1.269555	1.34311	1.338964	1.334903	1.330892	1.32693	1.322967
1.009562	1.274512	1.270312	1.343898	1.339702	1.335583	1.331541	1.327566	1.323615
1.009993	1.275219	1.271067	1.34472	1.340496	1.33633	1.33224	1.328216	1.32424
1.010416	1.275913	1.271798	1.345513	1.34128	1.337088	1.332963	1.328896	1.324887
1.01084	1.276595	1.272464	1.268372	1.264324	1.260317	1.33371	1.329639	1.325604
1.011278	1.277285	1.273116	1.269007	1.264979	1.261003	1.334462	1.330408	1.326352
1.011779	1.008437	1.0052	1.269699	1.265647	1.261659	1.335169	1.331149	1.3271
1.012326	1.008963	1.005691	1.270428	1.266323	1.262295	1.335846	1.331875	1.327849
1.012836	1.009478	1.006204	1.271202	1.267061	1.262994	1.33655	1.332569	1.328552
1.013307	1.009988	1.006755	1.272036	1.267876	1.263769	1.337284	1.333224	1.329197
1.013768	1.01046	1.007247	1.272829	1.268665	1.264534	1.338027	1.333899	1.329858
1.014215	1.010855	1.007611	1.273528	1.269383	1.265266	1.338784	1.334627	1.330564
1.014676	1.011254	1.007968	1.274212	1.270084	1.265981	1.339531	1.335362	1.331283
1.01519	1.011739	1.008422	1.274939	1.27079	1.266666	1.262597	1.258581	1.331994
1.015733	1.012265	1.00892	1.275688	1.271505	1.267346	1.263263	1.259247	1.332711
0.922481	1.012775	1.009404	1.006106	1.002895	0.999712	1.264004	1.259968	1.33345
0.922962	1.013279	1.009882	1.006578	1.003405	1.000262	1.264808	1.260735	1.334209
0.923496	1.01384	1.010403	1.007072	1.003901	1.000771	1.265602	1.261507	1.334986
0.924104	1.014481	1.010982	1.007593	1.004374	1.001213	1.266377	1.262281	1.335786
0.924704	1.015112	1.011555	1.008114	1.004857	1.001675	1.267161	1.263059	1.25907
0.925238	1.015671	1.012074	1.00861	1.005369	1.002211	1.267968	1.263834	1.259812
0.925773	1.016232	1.0126	1.009116	1.005891	1.002756	1.268784	1.26462	1.26056
0.926391	1.016884	1.013198	1.009664	1.006399	1.003241	1.00007	0.996914	1.261355
0.927048	1.017582	1.013841	1.010246	1.006909	1.003699	1.000553	0.997461	1.262177
0.707727	0.924176	0.920642	1.010871	1.007473	1.004205	1.00106	0.997999	1.262991
0.708535	0.924733	0.921241	1.011542	1.008089	1.004755	1.00159	0.99853	1.2638
0.709375	0.925303	0.921844	1.012213	1.008711	1.005315	1.002111	0.999025	1.264591
0.710282	0.925901	0.922458	1.01289	1.009341	1.005881	1.002609	0.999458	1.265349
0.711185	0.9265	0.923056	1.013537	1.009954	1.006451	1.003119	0.999908	1.266122
0.712043	0.92708	0.923594	1.014089	1.010508	1.007013	1.003668	1.000438	1.266953
0.712887	0.927664	0.924134	1.014634	1.011061	1.007582	1.004238	1.001001	1.267807
0.713743	0.928289	0.924782	1.015319	1.01171	1.008186	1.004811	1.00155	0.998359
0.714603	0.928942	0.925501	1.016097	1.012423	1.008815	1.005392	1.002098	0.998868
0.715467	0.712529	0.709507	0.922739	0.919185	0.915687	1.005994	1.002665	0.999412
0.716336	0.713396	0.710408	0.923386	0.919817	0.916291	1.006613	1.00325	0.999991

0.717187	0.714238	0.711265	0.923992	0.920433	0.91691	1.007259	1.003865	1.000598
0.718006	0.715039	0.712047	0.924523	0.921012	0.91754	1.007939	1.004523	1.00124
0.71882	0.715841	0.712833	0.925063	0.921607	0.918189	1.008639	1.005198	1.001891
0.719642	0.716687	0.713705	0.710713	0.707698	0.704705	1.009352	1.005882	1.00253
0.720479	0.71756	0.714615	0.711656	0.708667	0.705683	1.010072	1.006566	1.003158
0.721376	0.718455	0.715522	0.712579	0.709615	0.706647	0.916844	0.913443	1.003756
0.722324	0.719373	0.716431	0.713492	0.710552	0.707603	0.91747	0.913984	1.004327
0.723279	0.720294	0.717326	0.714376	0.711456	0.708528	0.705522	0.702491	1.004914
0.724243	0.72122	0.718204	0.715228	0.712326	0.709424	0.706419	0.703379	1.005521
0.725199	0.722134	0.719072	0.716066	0.713163	0.710273	0.707284	0.704263	1.00614
0.726134	0.723017	0.719922	0.716887	0.713944	0.711034	0.708084	0.705134	1.00677
0.727065	0.723897	0.720776	0.717715	0.714727	0.711788	0.708882	0.706011	1.007418
0.536269	0.7248	0.721662	0.718589	0.715583	0.712637	0.709754	0.706921	0.831847
0.536922	0.72572	0.722573	0.719496	0.716483	0.713537	0.710664	0.70785	0.832774
0.537613	0.726671	0.723521	0.720437	0.717399	0.714429	0.71156	0.708763	0.70595
0.538339	0.727649	0.724508	0.721415	0.718332	0.715318	0.712446	0.709665	0.706861
	0.53683	0.534554	0.722401	0.71928	0.716228	0.713343	0.710564	0.707764
	0.537595	0.5353	0.723387	0.720237	0.71716	0.714252	0.711456	0.708657
	0.53836	0.536049	0.724387	0.721216	0.718116	0.715178	0.712352	0.709546
	0.539113	0.536799	0.725409	0.722224	0.719106	0.71613	0.713256	0.710434
	0.539858	0.537547	0.726444	0.723251	0.720117	0.717095	0.714165	0.71132
	0.540587	0.538289	0.727483	0.72428	0.721126	0.718057	0.715067	0.712198
	0.541303	0.539024	0.728528	0.725315	0.722139	0.71902	0.715968	0.713074
			0.53747	0.535135	0.532809	0.719997	0.716895	0.713972
			0.538215	0.535891	0.533564	0.720988	0.717845	0.714893
			0.538953	0.536638	0.534316	0.72199	0.718817	0.715835
			0.539666	0.537357	0.535049	0.722996	0.719807	0.716797
			0.540399	0.538097	0.535804	0.724029	0.720831	0.717786
						0.725107	0.721899	0.718805
						0.726223	0.723009	0.719859
						0.7274	0.724183	0.720985
						0.728629	0.725411	0.722174
								0.723301
								0.724353
								0.725391
								0.726439
								0.72748
								0.728502
								0.729518

1.900675	1.894824	1.889083	1.883701	1.878789	1.874259	1.935947	1.931777	1.959033
1.900726	1.89488	1.889153	1.883781	1.878874	1.874345	1.936033	1.931866	1.959094
1.900783	1.894944	1.889228	1.883867	1.878964	1.87444	1.936137	1.931983	1.959186
1.900888	1.895056	1.88933	1.883962	1.879062	1.874545	1.936265	1.932135	1.959324
1.901025	1.895203	1.889454	1.884067	1.879167	1.874659	1.936411	1.932314	1.959496
1.901165	1.89536	1.889603	1.884203	1.879287	1.874765	1.898897	1.895053	1.959625
1.901314	1.895535	1.889784	1.884374	1.879422	1.874864	1.898971	1.895137	1.959711
1.901443	1.895687	1.889948	1.884537	1.879561	1.874977	1.899055	1.895219	1.959787
1.901534	1.895798	1.890078	1.88468	1.879706	1.875116	1.899155	1.895298	1.959854
1.901617	1.895892	1.89019	1.884808	1.879848	1.875263	1.899267	1.895387	1.959926
1.852497	1.895971	1.890277	1.88491	1.879973	1.875409	1.8994	1.895507	1.960021
1.852579	1.896049	1.890359	1.885005	1.880094	1.875556	1.89954	1.89564	1.960125
1.831737	1.896172	1.890487	1.913594	1.90856	1.903896	1.926591	1.922483	1.98264
1.831876	1.89633	1.89065	1.913736	1.908684	1.904001	1.926677	1.922567	1.982721
1.832038	1.877963	1.872298	1.913898	1.908834	1.904137	1.926788	1.922669	1.982824
1.832232	1.878168	1.872502	1.914083	1.909014	1.904308	1.926928	1.922792	1.982951
1.832401	1.856194	1.85061	1.914269	1.909198	1.904486	1.927081	1.92293	1.951169
1.832513	1.856341	1.850781	1.914449	1.90938	1.904665	1.927249	1.923094	1.951325
1.832602	1.856467	1.850936	1.914616	1.909552	1.904838	1.927418	1.923263	1.951486
1.832689	1.827193	1.821811	1.897438	1.892415	1.887744	1.927572	1.923412	1.95162
1.832772	1.827269	1.821898	1.897539	1.892534	1.88788	1.927714	1.923551	1.951743
1.832877	1.827373	1.822014	1.897658	1.892652	1.887997	1.927834	1.923683	1.91959
1.832998	1.8275	1.822154	1.897791	1.892764	1.888093	1.92793	1.923806	1.919733
1.833161	1.827656	1.822302	1.877287	1.872303	1.86767	1.911985	1.907908	1.919883
1.833381	1.827845	1.822448	1.877433	1.872479	1.867877	1.912177	1.908087	1.920048
1.833612	1.828054	1.822627	1.877614	1.872678	1.868094	1.912377	1.908265	1.920207
1.833834	1.828285	1.822882	1.877865	1.872903	1.868287	1.912558	1.908418	1.920347
1.834053	1.828524	1.823156	1.878137	1.873141	1.868482	1.912737	1.908565	1.920482
1.834267	1.828746	1.823373	1.850828	1.845957	1.841425	1.893673	1.889529	1.904619
1.834476	1.828954	1.823561	1.851004	1.846154	1.841641	1.893864	1.889688	1.904767
1.834653	1.829136	1.823767	1.851218	1.846367	1.841844	1.894064	1.889873	1.904947
1.83479	1.829283	1.82399	1.851472	1.846596	1.842033	1.894274	1.890088	1.905163
1.834978	1.829474	1.824218	1.851707	1.846814	1.842228	1.894478	1.890289	1.905363
1.835263	1.829751	1.824445	1.851896	1.847012	1.842439	1.894667	1.890451	1.905517
1.835552	1.830031	1.824678	1.852089	1.84721	1.842647	1.894859	1.890624	1.905683
1.835764	1.830244	1.824921	1.852332	1.847428	1.842837	1.838655	1.834802	1.857997
1.835965	1.830445	1.825169	1.852589	1.847652	1.843023	1.838846	1.835017	1.858241
1.836249	1.830709	1.825407	1.852798	1.847863	1.843239	1.839057	1.83522	1.858469
1.836593	1.831019	1.825641	1.852977	1.848065	1.843475	1.839281	1.835416	1.858689
1.836932	1.831347	1.825919	1.8532	1.848274	1.843679	1.83948	1.835612	1.831907
1.837268	1.831703	1.826251	1.853471	1.848486	1.843841	1.839645	1.835809	1.832112
1.807028	1.83203	1.82658	1.853766	1.848727	1.844029	1.839821	1.835996	1.832302
1.807282	1.832287	1.826887	1.854095	1.849025	1.844277	1.840026	1.83616	1.832461
1.80754	1.832536	1.827181	1.854418	1.849331	1.844549	1.84025	1.836331	1.832621
1.807866	1.832831	1.827454	1.854671	1.849596	1.844822	1.840499	1.836541	1.832813
1.808224	1.83315	1.827719	1.854894	1.849844	1.8451	1.840765	1.836776	1.833027
1.808558	1.837398	1.831861	1.855148	1.850097	1.845359	1.841022	1.837027	1.833265
1.808878	1.837732	1.832171	1.855424	1.850353	1.845602	1.841273	1.837295	1.83353
1.787609	1.838061	1.832496	1.827181	1.82214	1.817426	1.841524	1.83756	1.83379
1.787928	1.838381	1.832842	1.82753	1.822445	1.817682	1.841777	1.837821	1.834048

1.788245	1.838691	1.833183	1.827882	1.822756	1.817944	1.842027	1.838069	1.834291
1.788555	1.838981	1.833507	1.828222	1.823067	1.818215	1.842268	1.83829	1.834507
1.78887	1.839269	1.83382	1.828551	1.823376	1.818492	1.84251	1.838501	1.834712
1.760519	1.819544	1.814108	1.828845	1.823669	1.818778	1.814405	1.810432	1.834923
1.760847	1.819859	1.814396	1.82912	1.823954	1.819073	1.814668	1.810648	1.835136
1.761183	1.8202	1.814717	1.809437	1.8043	1.799448	1.814936	1.810875	1.835348
1.761529	1.820566	1.81507	1.809775	1.804623	1.799751	1.815211	1.811112	1.835558
1.728263	1.794038	1.788572	1.8101	1.804936	1.800052	1.795595	1.791504	1.807571
1.728592	1.794374	1.788868	1.8104	1.805235	1.800354	1.795879	1.791761	1.807815
1.728926	1.794711	1.789163	1.810694	1.805522	1.80064	1.796154	1.792022	1.808069
1.729266	1.795043	1.789455	1.810988	1.805783	1.800878	1.796394	1.792278	1.808333
1.729612	1.795376	1.789752	1.811287	1.806041	1.801105	1.796626	1.792537	1.808605
1.729965	1.764273	1.758667	1.784626	1.779399	1.774485	1.796895	1.792806	1.789042
1.730323	1.764611	1.758984	1.784935	1.779686	1.774751	1.79719	1.793085	1.789311
1.730687	1.764957	1.759311	1.753881	1.748675	1.743789	1.770567	1.766487	1.789592
1.731054	1.765309	1.759646	1.754202	1.748993	1.7441	1.770864	1.766746	1.789887
1.731426	1.765669	1.760003	1.754554	1.74934	1.744435	1.771179	1.767019	1.790182
1.731801	1.76604	1.760393	1.754952	1.749725	1.744795	1.771512	1.767308	1.790472
1.732181	1.766417	1.760796	1.75537	1.750131	1.745175	1.77186	1.767604	1.79076
1.732566	1.766802	1.761202	1.755788	1.750548	1.745574	1.740982	1.736736	1.763958
1.732955	1.767193	1.761611	1.756208	1.750972	1.745984	1.741339	1.737017	1.764199
1.733344	1.767576	1.762005	1.756609	1.751371	1.746371	1.741688	1.737312	1.733351
1.733734	1.767955	1.762389	1.756994	1.751753	1.74674	1.742031	1.73762	1.733617
1.734135	1.768337	1.762766	1.757367	1.752123	1.747103	1.742372	1.73793	1.733891
1.734547	1.768718	1.763131	1.757721	1.752476	1.747453	1.742707	1.738241	1.734173
1.734974	1.769118	1.76351	1.758086	1.752841	1.747818	1.743053	1.738557	1.734457
1.735418	1.769551	1.763917	1.758474	1.753237	1.748218	1.743421	1.738874	1.734735
1.735878	1.770006	1.764347	1.758883	1.753651	1.748633	1.7438	1.739199	1.735018
1.752761	1.770485	1.76481	1.759329	1.754084	1.749049	1.744185	1.739545	1.735324
1.753236	1.770985	1.765301	1.759802	1.754533	1.749468	1.744575	1.739902	1.735643
1.753711	1.771488	1.765798	1.760281	1.754976	1.749871	1.744947	1.740248	1.735956
1.754192	1.772	1.766307	1.760769	1.755416	1.750257	1.745302	1.740582	1.736263
1.75464	1.772475	1.766781	1.761229	1.755841	1.750644	1.745661	1.740917	1.736571
1.755036	1.772892	1.767201	1.761646	1.756245	1.751028	1.746023	1.741253	1.736878
1.755425	1.7733	1.767604	1.762046	1.756646	1.751425	1.746397	1.741594	1.737191
1.728499	1.750161	1.744511	1.762427	1.757054	1.751856	1.746798	1.741941	1.737511
1.728908	1.750575	1.744901	1.762815	1.757471	1.7523	1.747212	1.742296	1.737841
1.729335	1.751005	1.745338	1.763271	1.75792	1.752735	1.74762	1.742671	1.73819
1.729779	1.751452	1.745812	1.763776	1.758392	1.753165	1.748027	1.743061	1.738555
1.68758	1.751944	1.746307	1.740788	1.735421	1.730199	1.748428	1.743451	1.738914
1.68805	1.752491	1.746832	1.741276	1.73586	1.730588	1.748821	1.743839	1.739267
1.688525	1.753027	1.747336	1.741744	1.736294	1.730991	1.749225	1.744229	1.739626
1.688993	1.753524	1.74779	1.742167	1.736722	1.731423	1.749647	1.744607	1.739983
1.689466	1.754018	1.748244	1.742593	1.737151	1.731858	1.75008	1.745009	1.740368
1.68995	1.727307	1.721665	1.743066	1.737589	1.732267	1.727064	1.722043	1.740826
1.690438	1.727827	1.722171	1.743561	1.738037	1.732677	1.727495	1.722521	1.741321
1.690915	1.728342	1.722672	1.744054	1.738515	1.733141	1.727958	1.722984	1.741787
1.691384	1.728852	1.723173	1.744549	1.739018	1.733652	1.728448	1.723441	1.742234
1.691855	1.729369	1.723687	1.745066	1.739529	1.734153	1.728937	1.723912	1.742693
1.692331	1.729895	1.724218	1.745609	1.74004	1.734631	1.729416	1.724403	1.74317

1.692794	1.687575	1.682141	1.719173	1.713723	1.708426	1.729911	1.724894	1.720136
1.693224	1.688023	1.682646	1.719754	1.714293	1.708966	1.730431	1.725364	1.720569
1.693646	1.688462	1.683148	1.720346	1.714882	1.709532	1.730973	1.725844	1.721007
1.694088	1.688923	1.68364	1.720919	1.715472	1.710122	1.731544	1.726372	1.721483
1.694537	1.68939	1.684123	1.721481	1.716062	1.710726	1.732135	1.72693	1.721986
1.659222	1.689835	1.684589	1.722018	1.716618	1.711293	1.732713	1.727498	1.722514
1.659628	1.690256	1.685036	1.722527	1.71714	1.711826	1.733282	1.728082	1.72307
1.56788	1.690703	1.685509	1.72306	1.717675	1.712358	1.733838	1.728639	1.7236
1.56832	1.691187	1.686022	1.723631	1.718229	1.712886	1.73437	1.729148	1.724082
1.568784	1.691694	1.686549	1.724213	1.7188	1.713434	1.734913	1.729661	1.724565
1.569269	1.692228	1.68708	1.681914	1.676698	1.671507	1.70878	1.703623	1.725073
1.56976	1.692776	1.687617	1.682448	1.677262	1.672095	1.709373	1.70417	1.725598
1.570219	1.606805	1.601984	1.682987	1.677793	1.672617	1.709931	1.704721	1.726145
1.570657	1.607265	1.602454	1.68353	1.678304	1.673097	1.710469	1.705276	1.726711
1.571098	1.607718	1.602939	1.598116	1.593196	1.588292	1.711012	1.705832	1.727275
1.57154	1.60816	1.603443	1.598656	1.5937	1.588747	1.711554	1.706386	1.727833
1.571987	1.608609	1.603935	1.599178	1.59421	1.589235	1.669517	1.664507	1.701861
1.572437	1.609065	1.604403	1.599664	1.594723	1.58977	1.670091	1.665073	1.702463
1.572905	1.609537	1.604862	1.600126	1.595226	1.590314	1.670676	1.665639	1.703066
1.573418	1.610042	1.605314	1.600551	1.595697	1.590837	1.586025	1.581265	1.703641
1.573953	1.610566	1.605764	1.60096	1.596151	1.591344	1.586522	1.581723	1.704203
1.57448	1.611087	1.606236	1.601398	1.596598	1.59181	1.586985	1.582175	1.704756
1.575002	1.61161	1.606725	1.601856	1.597039	1.592242	1.58742	1.58262	1.7053
1.518421	1.570715	1.565816	1.60235	1.597521	1.592712	1.587894	1.583102	1.626811
1.518838	1.571147	1.566311	1.602889	1.598055	1.59323	1.588414	1.58363	1.627372
1.519276	1.571593	1.566813	1.603434	1.598602	1.593769	1.588953	1.584168	1.579495
1.519775	1.572085	1.567323	1.603966	1.599151	1.594329	1.589506	1.584704	1.580021
1.520299	1.5726	1.567843	1.6045	1.599703	1.594895	1.590064	1.585242	1.580548
1.520827	1.573133	1.56838	1.605044	1.600249	1.595442	1.590607	1.585778	1.581073
1.521362	1.573681	1.568929	1.605594	1.600794	1.595981	1.591144	1.586312	1.581596
1.521883	1.574211	1.569471	1.606147	1.601347	1.596529	1.591683	1.586838	1.58211
1.522392	1.574727	1.570008	1.606702	1.601905	1.597083	1.592222	1.587355	1.582615
1.522907	1.575248	1.570543	1.607253	1.602464	1.597645	1.59277	1.587883	1.58313
1.523433	1.575775	1.571073	1.607793	1.603021	1.598216	1.593333	1.588425	1.583658
1.523958	1.576308	1.571608	1.608338	1.60358	1.598787	1.593899	1.588977	1.584195
1.524474	1.576846	1.572153	1.608891	1.604135	1.599341	1.594454	1.58953	1.584734
1.524985	1.577386	1.57271	1.609461	1.6047	1.599901	1.595018	1.590101	1.58529
1.481987	1.577921	1.573292	1.610074	1.605306	1.600493	1.595621	1.590719	1.585894
1.482483	1.578453	1.573891	1.610719	1.605943	1.601112	1.596252	1.591372	1.586535
1.483005	1.578988	1.574481	1.5699	1.565115	1.560282	1.59691	1.592037	1.587186
1.483552	1.57952	1.575062	1.570532	1.56578	1.560969	1.597602	1.592719	1.587855
1.484127	1.54039	1.536037	1.571134	1.566417	1.561634	1.598269	1.593373	1.588493
1.484742	1.540997	1.536608	1.571688	1.566998	1.562247	1.598882	1.593968	1.589069
1.485369	1.541621	1.537179	1.572231	1.56757	1.562856	1.599492	1.594562	1.589644
1.48599	1.542249	1.537758	1.572783	1.568161	1.563493	1.600136	1.595196	1.590261
1.486609	1.54288	1.538341	1.573344	1.568765	1.564144	1.600796	1.595851	1.590902
1.487212	1.543491	1.538932	1.534418	1.529971	1.525475	1.601473	1.596523	1.591561
1.487802	1.544086	1.53953	1.535033	1.530625	1.526166	1.602164	1.597212	1.592237
1.488393	1.544663	1.540121	1.535643	1.531251	1.526808	1.566331	1.561473	1.592927
1.488982	1.545213	1.540701	1.536249	1.531841	1.527388	1.566974	1.562187	1.593632

1.438328	1.485065	1.480855	1.536852	1.532432	1.527972	1.567613	1.562888	1.594337
1.438931	1.485674	1.481454	1.537463	1.533047	1.528599	1.568267	1.563574	1.595044
1.439551	1.486306	1.482056	1.538058	1.533653	1.529226	1.568908	1.564232	1.595732
1.440178	1.486953	1.482631	1.538592	1.53421	1.52982	1.569505	1.564828	1.560029
1.440819	1.487618	1.483204	1.539104	1.534744	1.530393	1.570074	1.565387	1.560623
1.441491	1.437014	1.432709	1.539704	1.535309	1.530936	1.526355	1.52167	1.561227
1.442193	1.437694	1.433366	1.540379	1.535896	1.531446	1.526887	1.522269	1.561833
1.442908	1.438394	1.434041	1.541066	1.536516	1.532006	1.527454	1.522875	1.562457
1.443645	1.439122	1.434732	1.541757	1.537175	1.53264	1.528072	1.523487	1.563107
1.444364	1.439845	1.435427	1.431113	1.426943	1.422828	1.528708	1.52411	1.563763
1.44503	1.440549	1.436116	1.431776	1.427579	1.423436	1.529347	1.524749	1.56442
1.445676	1.441236	1.436798	1.432443	1.428223	1.424057	1.529996	1.525395	1.565077
1.38882	1.441893	1.437463	1.433107	1.42887	1.424682	1.530655	1.526038	1.56573
1.389379	1.442529	1.438115	1.43377	1.42952	1.425315	1.531323	1.526682	1.56638
1.389992	1.4432	1.438777	1.434423	1.430168	1.425956	1.532002	1.527344	1.567042
1.390663	1.443909	1.439448	1.435067	1.430813	1.426609	1.532692	1.528025	1.567711
1.391308	1.4446	1.440109	1.435703	1.431446	1.427243	1.486787	1.482235	1.568401
1.391887	1.445248	1.440746	1.43633	1.432058	1.427848	1.487428	1.482913	1.56912
1.392474	1.445899	1.441386	1.43696	1.432667	1.428443	1.488061	1.48359	1.569846
1.326103	1.388954	1.384827	1.437604	1.433282	1.429037	1.488686	1.484234	1.570557
1.326719	1.389605	1.385422	1.438254	1.433899	1.429629	1.489309	1.484872	1.571271
1.327321	1.390243	1.386031	1.438899	1.434514	1.430229	1.48995	1.485551	1.57202
1.327917	1.390877	1.386651	1.439541	1.43513	1.430834	1.490603	1.486257	1.572795
1.328511	1.324341	1.320188	1.440184	1.435755	1.431451	1.42733	1.423331	1.573551
1.329103	1.324909	1.320699	1.440824	1.436389	1.432086	1.427966	1.423974	1.574286
1.329694	1.325483	1.321234	1.441493	1.437035	1.432712	1.428587	1.424601	1.531669
1.330276	1.326058	1.321812	1.442209	1.43769	1.433306	1.429171	1.425205	1.532342
1.330874	1.326661	1.322426	1.442959	1.438366	1.433907	1.429756	1.425807	1.533007
1.331532	1.327344	1.323097	1.318894	1.314744	1.310709	1.43037	1.426425	1.53368
1.332218	1.328067	1.3238	1.319557	1.315346	1.31125	1.431009	1.427057	1.534359
1.332854	1.328735	1.32447	1.320214	1.315973	1.31184	1.431707	1.427718	1.535068
1.333451	1.329363	1.325118	1.320871	1.316626	1.312477	1.43246	1.428408	1.535802
1.334039	1.329973	1.325743	1.321504	1.317261	1.313106	1.371421	1.367434	1.536538
1.334621	1.330567	1.32634	1.322104	1.317868	1.313715	1.372077	1.368034	1.537275
1.335195	1.331148	1.326928	1.322699	1.318478	1.314333	1.310385	1.306581	1.490018
1.335755	1.331709	1.327508	1.3233	1.319104	1.314972	1.310996	1.307142	1.49067
1.336319	1.332271	1.328092	1.32391	1.319741	1.315623	1.311618	1.307711	1.491327
1.336919	1.332876	1.328711	1.324544	1.320393	1.316284	1.312262	1.308322	1.435596
1.337539	1.333503	1.329352	1.325197	1.321055	1.31695	1.312918	1.308957	1.436285
1.338121	1.334096	1.329983	1.325853	1.321705	1.317583	1.313536	1.309559	1.36777
1.338663	1.334654	1.330606	1.326516	1.322342	1.318179	1.314112	1.310125	1.368388
1.339234	1.335229	1.331227	1.327169	1.322984	1.318797	1.314714	1.310713	1.369036
1.339849	1.335826	1.331841	1.327802	1.323634	1.319456	1.315364	1.311341	1.369732
1.340495	1.33645	1.332468	1.328439	1.324287	1.320124	1.316025	1.311986	1.370447
1.341179	1.337118	1.333127	1.329093	1.324941	1.320779	1.316679	1.312638	1.30877
1.341884	1.33781	1.333803	1.329757	1.325599	1.321434	1.317337	1.313301	1.309425
1.342579	1.338494	1.334481	1.33043	1.326271	1.322107	1.318008	1.313969	1.310084
1.343271	1.339178	1.335163	1.331113	1.326954	1.32279	1.318688	1.314643	1.310748
1.343961	1.33986	1.335846	1.331796	1.327634	1.323465	1.319358	1.315306	1.311404
1.344651	1.340542	1.336531	1.332481	1.32831	1.32413	1.320016	1.315959	1.312052

1.345332	1.341216	1.337208	1.33316	1.328982	1.324793	1.320671	1.316608	1.312695
1.345998	1.34188	1.337876	1.33383	1.329652	1.32546	1.321331	1.317257	1.313331
1.346657	1.342538	1.338535	1.33449	1.330313	1.326121	1.321986	1.317901	1.313963
1.269526	1.343188	1.339178	1.335129	1.33095	1.326756	1.32262	1.318534	1.314589
1.270157	1.343835	1.339814	1.335758	1.331578	1.327385	1.323251	1.319165	1.315213
1.27079	1.344489	1.340462	1.336406	1.332241	1.328061	1.32392	1.319817	1.315847
1.271425	1.345149	1.341117	1.337068	1.332931	1.328776	1.324622	1.320486	1.316489
1.116554	1.345814	1.341767	1.337721	1.333624	1.329509	1.325356	1.3212	1.317174
1.117137	1.346477	1.342404	1.33836	1.334321	1.330271	1.326137	1.321974	1.317918
1.117748	1.347168	1.343057	1.339001	1.335005	1.331008	1.326899	1.322744	1.318664
1.118411	1.347914	1.343743	1.339649	1.335658	1.331681	1.327598	1.32347	1.319381
1.119099	1.348683	1.344446	1.340304	1.336299	1.332328	1.328272	1.324182	1.320097
1.119788	1.271515	1.267396	1.340959	1.336941	1.332978	1.328952	1.324907	1.320842
1.120482	1.272253	1.268075	1.341623	1.337586	1.333623	1.329627	1.325631	1.321597
1.00859	1.273004	1.268795	1.342341	1.338261	1.334266	1.330275	1.326305	1.322305
1.009089	1.273769	1.269555	1.34311	1.338964	1.334903	1.330892	1.32693	1.322967
1.009562	1.274512	1.270312	1.343898	1.339702	1.335583	1.331541	1.327566	1.323615
1.009993	1.275219	1.271067	1.34472	1.340496	1.33633	1.33224	1.328216	1.32424
1.010416	1.275913	1.271798	1.345513	1.34128	1.337088	1.332963	1.328896	1.324887
1.01084	1.276595	1.272464	1.268372	1.264324	1.260317	1.33371	1.329639	1.325604
1.011278	1.277285	1.273116	1.269007	1.264979	1.261003	1.334462	1.330408	1.326352
1.011779	1.008437	1.0052	1.269699	1.265647	1.261659	1.335169	1.331149	1.3271
1.012326	1.008963	1.005691	1.270428	1.266323	1.262295	1.335846	1.331875	1.327849
1.012836	1.009478	1.006204	1.271202	1.267061	1.262994	1.33655	1.332569	1.328552
1.013307	1.009988	1.006755	1.272036	1.267876	1.263769	1.337284	1.333224	1.329197
1.013768	1.01046	1.007247	1.272829	1.268665	1.264534	1.338027	1.333899	1.329858
1.014215	1.010855	1.007611	1.273528	1.269383	1.265266	1.338784	1.334627	1.330564
1.014676	1.011254	1.007968	1.274212	1.270084	1.265981	1.339531	1.335362	1.331283
1.01519	1.011739	1.008422	1.274939	1.27079	1.266666	1.262597	1.258581	1.331994
1.015733	1.012265	1.00892	1.275688	1.271505	1.267346	1.263263	1.259247	1.332711
0.922481	1.012775	1.009404	1.006106	1.002895	0.999712	1.264004	1.259968	1.33345
0.922962	1.013279	1.009882	1.006578	1.003405	1.000262	1.264808	1.260735	1.334209
0.923496	1.01384	1.010403	1.007072	1.003901	1.000771	1.265602	1.261507	1.334986
0.924104	1.014481	1.010982	1.007593	1.004374	1.001213	1.266377	1.262281	1.335786
0.924704	1.015112	1.011555	1.008114	1.004857	1.001675	1.267161	1.263059	1.25907
0.925238	1.015671	1.012074	1.00861	1.005369	1.002211	1.267968	1.263834	1.259812
0.925773	1.016232	1.0126	1.009116	1.005891	1.002756	1.268784	1.26462	1.26056
0.926391	1.016884	1.013198	1.009664	1.006399	1.003241	1.00007	0.996914	1.261355
0.927048	1.017582	1.013841	1.010246	1.006909	1.003699	1.000553	0.997461	1.262177
0.707727	0.924176	0.920642	1.010871	1.007473	1.004205	1.00106	0.997999	1.262991
0.708535	0.924733	0.921241	1.011542	1.008089	1.004755	1.00159	0.99853	1.2638
0.709375	0.925303	0.921844	1.012213	1.008711	1.005315	1.002111	0.999025	1.264591
0.710282	0.925901	0.922458	1.01289	1.009341	1.005881	1.002609	0.999458	1.265349
0.711185	0.9265	0.923056	1.013537	1.009954	1.006451	1.003119	0.999908	1.266122
0.712043	0.92708	0.923594	1.014089	1.010508	1.007013	1.003668	1.000438	1.266953
0.712887	0.927664	0.924134	1.014634	1.011061	1.007582	1.004238	1.001001	1.267807
0.713743	0.928289	0.924782	1.015319	1.01171	1.008186	1.004811	1.00155	0.998359
0.714603	0.928942	0.925501	1.016097	1.012423	1.008815	1.005392	1.002098	0.998868
0.715467	0.712529	0.709507	0.922739	0.919185	0.915687	1.005994	1.002665	0.999412
0.716336	0.713396	0.710408	0.923386	0.919817	0.916291	1.006613	1.00325	0.999991

0.717187	0.714238	0.711265	0.923992	0.920433	0.91691	1.007259	1.003865	1.000598
0.718006	0.715039	0.712047	0.924523	0.921012	0.91754	1.007939	1.004523	1.00124
0.71882	0.715841	0.712833	0.925063	0.921607	0.918189	1.008639	1.005198	1.001891
0.719642	0.716687	0.713705	0.710713	0.707698	0.704705	1.009352	1.005882	1.00253
0.720479	0.71756	0.714615	0.711656	0.708667	0.705683	1.010072	1.006566	1.003158
0.721376	0.718455	0.715522	0.712579	0.709615	0.706647	0.916844	0.913443	1.003756
0.722324	0.719373	0.716431	0.713492	0.710552	0.707603	0.91747	0.913984	1.004327
0.723279	0.720294	0.717326	0.714376	0.711456	0.708528	0.705522	0.702491	1.004914
0.724243	0.72122	0.718204	0.715228	0.712326	0.709424	0.706419	0.703379	1.005521
0.725199	0.722134	0.719072	0.716066	0.713163	0.710273	0.707284	0.704263	1.00614
0.726134	0.723017	0.719922	0.716887	0.713944	0.711034	0.708084	0.705134	1.00677
0.727065	0.723897	0.720776	0.717715	0.714727	0.711788	0.708882	0.706011	1.007418
0.536269	0.7248	0.721662	0.718589	0.715583	0.712637	0.709754	0.706921	0.831847
0.536922	0.72572	0.722573	0.719496	0.716483	0.713537	0.710664	0.70785	0.832774
0.537613	0.726671	0.723521	0.720437	0.717399	0.714429	0.71156	0.708763	0.70595
0.538339	0.727649	0.724508	0.721415	0.718332	0.715318	0.712446	0.709665	0.706861
	0.53683	0.534554	0.722401	0.71928	0.716228	0.713343	0.710564	0.707764
	0.537595	0.5353	0.723387	0.720237	0.71716	0.714252	0.711456	0.708657
	0.53836	0.536049	0.724387	0.721216	0.718116	0.715178	0.712352	0.709546
	0.539113	0.536799	0.725409	0.722224	0.719106	0.71613	0.713256	0.710434
	0.539858	0.537547	0.726444	0.723251	0.720117	0.717095	0.714165	0.71132
	0.540587	0.538289	0.727483	0.72428	0.721126	0.718057	0.715067	0.712198
	0.541303	0.539024	0.728528	0.725315	0.722139	0.71902	0.715968	0.713074
			0.53747	0.535135	0.532809	0.719997	0.716895	0.713972
			0.538215	0.535891	0.533564	0.720988	0.717845	0.714893
			0.538953	0.536638	0.534316	0.72199	0.718817	0.715835
			0.539666	0.537357	0.535049	0.722996	0.719807	0.716797
			0.540399	0.538097	0.535804	0.724029	0.720831	0.717786
						0.725107	0.721899	0.718805
						0.726223	0.723009	0.719859
						0.7274	0.724183	0.720985
						0.728629	0.725411	0.722174
								0.723301
								0.724353
								0.725391
								0.726439
								0.72748
								0.728502
								0.729518

							0.560889
							0.559205
							0.557516
							0.555814
							0.554113
					0.562183	0.557221	0.945256
					0.560436	0.555482	0.943377
					0.558851	0.55392	1.046321
					0.557552	0.552645	1.044815
					0.556221	0.551371	1.043289
					0.554633	0.549902	1.041564
					0.552949	0.548367	1.039777
			0.561098	0.556002	0.944152	0.938676	1.038038
			0.559326	0.554238	0.942246	0.936867	1.036332
			0.557787	0.552743	1.045194	1.039973	1.382255
			0.556521	0.551559	1.04379	1.038582	1.380647
			0.948858	0.943145	1.042308	1.037122	1.378994
			0.94699	0.941374	1.040607	1.035464	1.377197
			0.945071	0.93956	1.038879	1.033796	1.375394
			0.943169	0.93779	1.037242	1.032258	1.585907
			0.941312	0.936062	1.035655	1.030774	1.583924
0.56194	0.556621	0.551654	0.93971	0.934518	1.034155	1.029275	1.63676
0.560555	0.555387	0.550558	0.938321	0.933127	1.032734	1.027774	1.634678
0.953637	0.947804	0.942178	1.041384	1.036301	1.53348	1.527246	1.684859
0.951617	0.945925	0.940429	1.03971	1.034656	1.531394	1.525357	1.682914
0.949671	0.944127	0.938767	1.038122	1.033091	1.588842	1.58272	1.680971
0.947945	0.942558	0.937344	1.036763	1.031745	1.586925	1.580802	1.678965

	0.946332	0.941088	0.936002	1.035479	1.03047	1.585068	1.578915	1.676971
0.556031	0.944813	0.939664	0.934644	1.034162	1.029163	1.58321	1.577094	1.692354
0.554767	0.943363	0.938273	0.933279	1.032832	1.027844	1.581359	1.575306	1.690524
0.947158	1.046238	1.041265	1.03638	1.592227	1.585688	1.683763	1.677487	1.688584
0.945507	1.044642	1.039666	1.034785	1.590104	1.58366	1.681693	1.675431	1.686539
0.943918	1.043132	1.038167	1.033294	1.588062	1.581713	1.697055	1.690801	1.809632
0.942434	1.041772	1.036845	1.03199	1.586169	1.579912	1.69524	1.689042	1.807845
0.940931	1.040392	1.035504	1.030668	1.584256	1.57808	1.693399	1.687269	1.806061
0.939281	1.247142	1.241698	1.236318	1.58217	1.576058	1.691381	1.685348	1.804177
0.937639	1.245434	1.240027	1.234679	1.580085	1.57402	1.689334	1.683382	1.802243
0.936229	1.24392	1.23855	1.233234	1.578244	1.572202	1.687434	1.681444	1.953304
0.934978	1.242534	1.237202	1.231919	1.576566	1.570528	1.68563	1.679527	1.951113
1.246458	1.593783	1.587353	1.581035	1.678931	1.672666	1.683888	1.677752	1.94908
1.245077	1.591989	1.585606	1.579338	1.677189	1.670948	1.68222	1.676134	1.947215
1.243677	1.590168	1.583827	1.577606	1.692885	1.686658	1.88096	1.874441	2.007956
1.242252	1.5883	1.581999	1.57583	1.691069	1.684878	1.8791	1.872613	2.005975
1.240791	1.586401	1.580126	1.573996	1.68919	1.683039	1.877177	1.870731	2.003958
1.239271	1.584448	1.578158	1.572029	1.687171	1.681058	1.875096	1.868707	2.00183
1.237716	1.582484	1.57617	1.570031	1.685119	1.679045	1.872967	1.866639	1.99966
1.529853	1.58062	1.574341	1.56823	1.683286	1.677247	1.896355	1.889998	1.997571
1.52797	1.578832	1.572633	1.566579	1.681621	1.675613	1.89453	1.88819	1.995539
1.526148	1.6987	1.69232	1.686075	1.840643	1.834274	1.919449	1.913095	1.993574
1.524397	1.696882	1.69057	1.684391	1.838898	1.832557	1.917712	1.911355	1.99169
1.579751	1.69509	1.688837	1.682719	1.864484	1.858104	1.915991	1.909623	2.063937
1.578019	1.693327	1.687118	1.681047	1.862734	1.856387	1.914265	1.907882	2.061789
1.576314	1.691589	1.685417	1.679387	1.860988	1.854676	1.912544	1.906143	2.05964
1.574629	1.728772	1.72263	1.716627	1.919213	1.912442	1.998167	1.991105	2.057487
1.572962	1.727075	1.720963	1.71499	1.917362	1.910631	1.996288	1.989207	2.055335
1.571302	1.759302	1.753162	1.747148	1.915537	1.90882	1.994405	1.987318	2.102083
1.56965	1.757644	1.75156	1.745584	1.913736	1.907011	1.992519	1.985439	2.099851
1.728215	1.871446	1.865022	1.858674	1.937447	1.930706	1.99063	1.983568	2.097679
1.72651	1.869688	1.863299	1.85697	1.935657	1.92891	1.988738	1.981706	2.095582
1.758775	1.867926	1.861574	1.855266	1.933868	1.927114	2.060394	2.052586	2.155181
1.757077	1.866149	1.859841	1.853564	1.932073	1.925314	2.05825	2.050533	2.153079
1.75539	1.864373	1.858106	1.85186	1.930276	1.923516	2.056108	2.048487	2.15099
1.753735	1.862619	1.856375	1.850146	1.928474	1.921724	2.053969	2.046444	2.169446
1.752103	1.860881	1.854646	1.848424	1.926669	1.919939	2.051839	2.044408	2.167337
1.750479	1.859155	1.852918	1.846696	1.924874	1.918172	2.073561	2.066166	2.165239
1.74886	1.857439	1.85119	1.844961	1.923091	1.916426	2.071525	2.064173	2.163154
1.747266	1.855731	1.849463	1.843227	2.061094	2.053262	2.134428	2.12683	2.179442
1.74569	1.85401	1.847727	1.84149	2.058963	2.05123	2.132351	2.124807	2.1774
1.744162	1.852335	1.846017	1.839764	2.05684	2.049211	2.130293	2.122802	2.17537
1.857204	1.85076	1.844359	1.838039	2.078524	2.070972	2.128222	2.120794	2.173342
1.855715	1.849248	1.842752	1.836348	2.076398	2.068946	2.12616	2.118793	2.171317
1.883202	1.942529	1.935799	1.929145	2.101885	2.094411	2.184426	2.176863	2.169279
1.881693	1.941069	1.934315	1.927608	2.099881	2.092431	2.182373	2.174823	2.167235
1.880098	1.970501	1.96371	1.956947	2.098031	2.090601	2.180455	2.172871	2.193504
1.878399	1.968782	1.962133	1.9555	2.096382	2.088961	2.178708	2.17102	2.191515
1.908976	2.106013	2.098181	2.090506	2.152619	2.145026	2.176954	2.169208	2.189592
1.907221	2.103833	2.096127	2.088586	2.150698	2.143185	2.17514	2.167449	2.187792

1.905488	2.101646	2.094039	2.086611	2.148713	2.141288	2.173279	2.165661	2.185991
1.936623	2.09948	2.091953	2.084622	2.146704	2.139347	2.199497	2.191866	2.204339
1.934982	2.097357	2.089887	2.082631	2.144678	2.137378	2.197462	2.189809	2.202336
1.951788	2.095421	2.087972	2.080742	2.142727	2.135455	2.195456	2.187755	2.210729
1.950333	2.093658	2.086191	2.078941	2.140835	2.133566	2.193463	2.185688	2.208616
2.001769	2.124949	2.117403	2.110099	2.139058	2.131791	2.20088	2.193082	2.206567
1.999886	2.123081	2.115675	2.108486	2.137453	2.130191	2.199157	2.191228	2.204594
1.998037	2.121223	2.113946	2.106862	2.167877	2.160573	2.208277	2.200278	2.202692
1.996345	2.119384	2.112155	2.105109	2.166015	2.158693	2.20638	2.198452	2.20091
1.994701	2.117583	2.110384	2.103357	2.164115	2.156747	2.204432	2.19661	2.199149
2.066979	2.143012	2.135692	2.128487	2.196969	2.189209	2.202481	2.194662	2.197233
2.065202	2.141344	2.134132	2.126947	2.195226	2.187328	2.200529	2.192658	2.19524
2.063386	2.1728	2.165639	2.158448	2.203708	2.19578	2.209057	2.201147	2.213075
2.061517	2.170863	2.163762	2.156627	2.201894	2.194064	2.207348	2.199429	2.211343
2.094605	2.180487	2.17332	2.166152	2.20004	2.192267	2.20554	2.197612	2.209541
2.093037	2.178837	2.171591	2.164328	2.198112	2.190302	2.203528	2.195588	2.207587
2.091502	2.177208	2.169886	2.162534	2.196211	2.188352	2.201524	2.193568	2.205633
2.127001	2.175466	2.168165	2.160821	2.205227	2.197347	2.219266	2.211308	2.203756
2.1253	2.173705	2.166444	2.159129	2.203489	2.195602	2.217478	2.209499	2.20195
2.13679	2.172038	2.164713	2.157331	2.201623	2.193734	2.215657	2.207787	2.200305
2.135251	2.170433	2.162976	2.155464	2.199676	2.191789	2.213827	2.206165	2.198794
2.133642	2.168769	2.161263	2.153694	2.20813	2.200217	2.212047	2.204555	2.197272
2.131941	2.16703	2.159574	2.152039	2.206413	2.19845	2.21033	2.202966	2.195744
2.155025	2.165318	2.157906	2.150397	2.224118	2.216157	2.208594	2.201337	2.194174
2.153426	2.1637	2.156293	2.148766	2.222339	2.214318	2.206782	2.19961	2.192522
2.151836	2.162065	2.154642	2.147089	2.220546	2.2125	2.204991	2.197884	2.190859
2.150152	2.173227	2.165706	2.158113	2.218719	2.210763	2.203318	2.196262	2.189259
2.14843	2.171384	2.163798	2.156196	2.21689	2.209084	2.201716	2.19469	2.187686
2.189033	2.213992	2.206147	2.198332	2.215167	2.207465	2.200134	2.193103	2.186102
2.187322	2.212303	2.204453	2.196645	2.213534	2.205905	2.19858	2.191509	2.184513
2.197407	2.210638	2.202776	2.194966	2.211896	2.204325	2.197023	2.189953	2.182965
2.195777	2.208999	2.201119	2.1933	2.210249	2.202706	2.195452	2.188442	2.181474
2.19416	2.227313	2.219427	2.211644	2.208584	2.201103	2.193905	2.186951	2.179989
2.192555	2.225641	2.217656	2.209821	2.206869	2.19954	2.192412	2.185479	2.178491
2.190923	2.223942	2.215862	2.207987	2.205161	2.198002	2.19094	2.184004	2.17698
2.189172	2.222145	2.214076	2.206248	2.203531	2.196489	2.18945	2.182475	2.175422
2.187346	2.220293	2.212301	2.204577	2.201957	2.194998	2.187958	2.18092	2.173843
2.185548	2.218529	2.210628	2.202999	2.19591	2.189082	2.18212	2.175116	2.172344
2.183766	2.216843	2.209056	2.201519	2.194457	2.187625	2.180678	2.173692	2.170925
2.222977	2.215185	2.207504	2.200049	2.192987	2.186126	2.179189	2.172236	2.169493
2.221304	2.213547	2.205965	2.198583	2.191481	2.184547	2.177615	2.170716	2.168026
2.219663	2.211931	2.204422	2.197093	2.189961	2.182974	2.176048	2.169192	2.166551
2.218037	2.210331	2.202833	2.195518	2.188417	2.181466	2.174553	2.167698	2.183329
2.216432	2.208752	2.201244	2.193928	2.18688	2.179992	2.173094	2.166221	2.181839
2.214842	2.207197	2.199724	2.192438	2.185415	2.178545	2.171652	2.164773	2.180358
2.213268	2.205667	2.198255	2.191018	2.184005	2.177125	2.170232	2.16335	2.178884
2.211717	2.204157	2.196799	2.189606	2.182605	2.175722	2.168824	2.161933	2.17376
2.210189	2.202665	2.195355	2.188203	2.181218	2.174337	2.167431	2.160521	2.17232
2.208688	2.201206	2.193933	2.186807	2.179833	2.172953	2.166038	2.159112	2.17089
2.207221	2.19979	2.192534	2.185414	2.178443	2.17156	2.164639	2.157703	2.169465

2.205772	2.198396	2.191152	2.18403	2.177058	2.170168	2.163242	2.1563	2.168052
2.204327	2.197007	2.189782	2.182663	2.175686	2.168786	2.180766	2.173774	2.166657
2.202889	2.195628	2.188422	2.181307	2.174322	2.167408	2.179372	2.172383	2.165274
2.20146	2.194253	2.187062	2.179947	2.172953	2.166025	2.177972	2.170988	2.163897
2.20004	2.192883	2.185703	2.178585	2.17158	2.164638	2.17657	2.169591	2.162527
2.198616	2.191507	2.18434	2.177221	2.170209	2.163255	2.175171	2.168197	2.16116
2.197185	2.190121	2.18297	2.175855	2.168839	2.161877	2.173777	2.166806	2.159795
2.195761	2.188733	2.181597	2.174488	2.186394	2.17938	2.172386	2.16542	2.158436
2.194349	2.187341	2.180214	2.173109	2.185001	2.177987	2.171	2.164043	2.157088
2.192953	2.185957	2.178842	2.171745	2.183621	2.176604	2.16962	2.162674	2.155746
2.191586	2.184597	2.177508	2.170432	2.18228	2.175245	2.168254	2.161308	2.154407
2.190242	2.183254	2.176206	2.169161	2.180974	2.173907	2.1669	2.159948	2.153076
2.188913	2.181928	2.174944	2.167948	2.179719	2.172595	2.165564	2.158607	2.151774
2.187599	2.180617	2.173726	2.166802	2.178521	2.171312	2.164244	2.157283	2.150503
2.186311	2.179316	2.172478	2.165599	2.17729	2.170039	2.162952	2.155983	2.14924
2.185063	2.178023	2.171178	2.164304	2.175996	2.16877	2.161686	2.154707	2.147975
2.183814	2.17674	2.169864	2.162979	2.174695	2.167527	2.160458	2.153466	2.146731
2.18253	2.194403	2.187428	2.180467	2.173417	2.166352	2.159305	2.152283	2.145526
2.181242	2.193125	2.186103	2.179117	2.172146	2.165186	2.158167	2.151121	2.14434
2.180003	2.191931	2.184873	2.177843	2.170868	2.163919	2.156922	2.149907	2.143134
2.178793	2.190794	2.18371	2.176625	2.169589	2.162587	2.155608	2.148665	2.141923
2.177529	2.189567	2.18252	2.175445	2.168351	2.161278	2.154325	2.147472	2.140771
2.1762	2.188235	2.181305	2.174319	2.167162	2.159991	2.153072	2.146338	2.13969
2.135777	2.161188	2.154461	2.147671	2.165944	2.158721	2.151828	2.145172	2.138574
2.134605	2.159927	2.153166	2.14636	2.164647	2.157457	2.150576	2.143922	2.137369
2.133462	2.158708	2.151884	2.145038	2.163359	2.156232	2.149357	2.142677	2.136155
2.154987	2.157577	2.150693	2.143795	2.162156	2.15509	2.148219	2.141507	2.134976
2.153829	2.156477	2.149542	2.142594	2.160998	2.153993	2.147127	2.140384	2.133829
2.15259	2.155267	2.148364	2.141441	2.159861	2.152868	2.146023	2.139307	2.132776
2.151283	2.153971	2.14717	2.140334	2.158748	2.151729	2.144918	2.138276	2.131804
2.150037	2.1527	2.145929	2.139135	2.157586	2.150608	2.143833	2.137224	2.130806
2.148864	2.15145	2.144617	2.137809	2.156362	2.149525	2.142784	2.136142	2.129767
2.147725	2.150252	2.143342	2.136495	2.15512	2.148395	2.141696	2.135057	2.128738
2.146617	2.149152	2.142167	2.135245	2.153856	2.147147	2.140514	2.133982	2.127755
2.14552	2.148075	2.141032	2.134042	2.152612	2.145882	2.139308	2.132895	2.126768
2.144395	2.146928	2.139901	2.13292	2.151457	2.144691	2.138122	2.131747	2.125706
2.143258	2.145753	2.138782	2.131849	2.15036	2.143552	2.136962	2.130581	2.124615
2.142138	2.144618	2.137651	2.130733	2.149279	2.142507	2.135929	2.129547	2.123625
2.141031	2.143521	2.136511	2.129574	2.148216	2.141557	2.13502	2.128634	2.122726
2.139907	2.142399	2.135396	2.128484	2.147206	2.140624	2.134116	2.127725	2.121835
2.138747	2.141221	2.13431	2.127497	2.146276	2.139713	2.133203	2.126792	2.12093
2.137606	2.140076	2.133258	2.126535	2.145344	2.13878	2.132279	2.125882	2.120061
2.136519	2.129695	2.122934	2.116254	2.118948	2.112437	2.131321	2.125024	2.11927
2.13547	2.128701	2.121965	2.11529	2.117937	2.111395	2.130353	2.124188	2.118511
2.134468	2.127693	2.120938	2.114253	2.116906	2.110394	2.12941	2.123325	2.11772
2.1335	2.126682	2.119876	2.113168	2.115857	2.109412	2.12848	2.122452	2.116919
2.132465	2.125614	2.118812	2.112119	2.11481	2.108387	2.127529	2.121609	2.116157
2.131354	2.124478	2.117742	2.111109	2.113764	2.107311	2.126549	2.120795	2.115435
2.130257	2.12338	2.116678	2.11008	2.112725	2.106277	2.125611	2.119999	2.114727
2.129192	2.122356	2.115613	2.108987	2.11168	2.10532	2.12475	2.119229	2.114037

2.128159	2.121368	2.114582	2.107926	2.110669	2.104393	2.123904	2.118456	2.11334
2.107715	2.120413	2.113637	2.106993	2.109734	2.103468	2.123024	2.117638	2.112596
2.106772	2.119469	2.112733	2.106123	2.108844	2.102555	2.122138	2.116811	2.111841
2.094072	2.118452	2.111783	2.105234	2.107943	2.101636	2.112639	2.107384	2.111136
2.093041	2.117382	2.110805	2.104342	2.107045	2.100719	2.11178	2.10663	2.110469
2.09208	2.116401	2.109877	2.103468	2.106193	2.09989	2.111011	2.105934	2.109828
2.091207	2.115532	2.10901	2.102617	2.105397	2.09917	2.110352	2.105307	2.109217
2.090348	2.095262	2.088794	2.082469	2.095429	2.089319	2.109719	2.104698	2.108622
2.089488	2.094398	2.087934	2.08163	2.094664	2.088644	2.109095	2.104099	2.108053
2.088611	2.093522	2.087056	2.080767	2.09388	2.087959	2.108462	2.103485	2.107476
2.087664	2.092588	2.0861	2.07981	2.093009	2.087205	2.107764	2.102793	2.106825
2.086683	2.091628	2.08511	2.07881	2.092096	2.086417	2.107033	2.102066	2.106145
2.085764	2.0794	2.07299	2.066811	2.07202	2.066478	2.106343	2.101414	2.096764
2.084896	2.078552	2.072157	2.066001	2.071231	2.065743	2.105683	2.100826	2.096214
2.084051	2.077724	2.07134	2.065207	2.070462	2.065034	2.105043	2.100247	2.104568
2.083228	2.076915	2.070535	2.064421	2.069712	2.064359	2.10443	2.099672	2.103998
2.082423	2.076123	2.069749	2.063653	2.068974	2.06369	2.103819	2.099099	2.103427
2.081633	2.075349	2.068983	2.062905	2.06824	2.063009	2.103192	2.098515	2.102849
2.080856	2.074587	2.068234	2.062174	2.067519	2.062332	2.102565	2.097932	2.10227
2.08009	2.073835	2.067497	2.06146	2.056005	2.050965	2.094012	2.089427	2.101697
2.079334	2.073091	2.066774	2.060763	2.055344	2.050345	2.093383	2.088834	2.101128
2.078573	2.072344	2.066059	2.06009	2.054715	2.049762	2.066395	2.061943	2.100556
2.077809	2.071592	2.065355	2.059442	2.054121	2.049216	2.065815	2.061358	2.099977
2.063935	2.070838	2.064656	2.058804	2.053537	2.048679	2.065244	2.060789	2.099411
2.063153	2.070078	2.063961	2.058177	2.052959	2.048142	2.06468	2.060242	2.098867
2.062377	2.069321	2.063267	2.05755	2.052384	2.047608	2.064121	2.059701	2.098326
2.061617	2.068571	2.062567	2.056908	2.062917	2.058174	2.053649	2.049374	2.081066
2.060875	2.067832	2.06187	2.056265	2.062305	2.057614	2.053115	2.048844	2.080512
2.054932	2.067121	2.061192	2.055636	2.061699	2.057055	2.052581	2.048321	2.079979
2.054262	2.066433	2.060532	2.055019	2.0611	2.056496	2.05205	2.047805	2.079464
2.053556	2.07681	2.070958	2.065494	2.060544	2.055963	2.051546	2.047335	2.06118
2.052808	2.076082	2.070339	2.064974	2.060045	2.055459	2.051079	2.046926	2.060771
2.052055	2.07535	2.069703	2.064427	2.059533	2.054962	2.050613	2.046498	2.060337
2.051293	2.074599	2.069007	2.063795	2.058966	2.054456	2.050129	2.046005	2.059824
2.050554	2.07387	2.068316	2.063153	2.0584	2.053964	2.049654	2.045504	2.059297
2.061841	2.073228	2.067697	2.062569	2.057885	2.05352	2.049221	2.045042	2.0588
2.061192	2.072634	2.067119	2.062015	2.057394	2.053097	2.048808	2.044598	2.058319
2.06052	2.067501	2.061996	2.056901	2.056888	2.052625	2.048354	2.044147	2.05786
2.059833	2.066841	2.061394	2.056352	2.05637	2.052113	2.047865	2.043693	2.057422
2.043585	2.066232	2.060846	2.055856	2.055894	2.051627	2.0474	2.043268	2.057005
2.043007	2.065689	2.060371	2.055433	2.055477	2.051182	2.046974	2.042888	2.056624
2.042451	2.052255	2.047042	2.0422	2.055068	2.05075	2.046553	2.042499	2.056227
2.041915	2.05179	2.046593	2.041752	2.054635	2.050312	2.046113	2.042061	2.055768
2.041373	2.051327	2.04614	2.041296	2.054205	2.049891	2.045685	2.041615	2.055296
2.040761	2.050778	2.045638	2.040835	2.053798	2.049517	2.045308	2.041207	2.054861
2.04011	2.050181	2.04511	2.040375	2.053409	2.049176	2.044965	2.040824	2.054451
2.039504	2.049624	2.044627	2.039958	2.048326	2.0441	2.062424	2.058221	2.054084
2.038941	2.049102	2.044188	2.039585	2.047955	2.043707	2.062042	2.057889	2.053765
2.038403	2.048612	2.043758	2.039202	2.047584	2.043333	2.061674	2.057544	2.053428
2.037893	2.048168	2.043327	2.038781	2.047207	2.043	2.061333	2.057165	2.053045

2.037392	2.04773	2.042905	2.038371	2.046837	2.042666	2.060991	2.056786	2.052661
2.022495	2.047262	2.042497	2.03801	2.046481	2.042296	2.060619	2.056435	2.05231
2.021992	2.046788	2.042096	2.037666	2.046132	2.041918	2.060241	2.05609	2.051966
2.0215	2.046342	2.041689	2.037289	2.045775	2.041571	2.055579	2.051407	2.051591
2.021017	2.045915	2.041278	2.036891	2.045413	2.041245	2.05523	2.051023	2.051196
2.02053	2.045469	2.040871	2.036515	2.045056	2.040898	2.054865	2.050655	2.050838
2.02003	2.044992	2.040467	2.036168	2.044706	2.040526	2.054481	2.050307	2.05053
2.019562	2.030958	2.026512	2.022275	2.031587	2.027459	2.054102	2.049949	2.050206
2.019142	2.030583	2.026131	2.021885	2.031219	2.027119	2.053738	2.049563	2.049834
2.018761	2.030243	2.025775	2.021511	2.03086	2.026782	2.053375	2.049174	2.049456
2.018438	2.029935	2.025476	2.02121	2.030534	2.026427	2.052992	2.048809	2.064796
2.018147	2.029645	2.025208	2.020947	2.03023	2.026072	2.052606	2.048453	2.064449
2.004342	2.029313	2.024906	2.020666	2.016431	2.012274	2.052262	2.048089	2.060108
2.003964	2.028948	2.02458	2.020375	2.016161	2.012012	2.051956	2.047718	2.059694
2.003602	2.028606	2.024251	2.020057	2.035419	2.031218	2.051651	2.047384	2.059326
2.003259	2.028298	2.023914	2.019694	2.035058	2.030901	2.051351	2.047101	2.059021
2.002934	2.028005	2.023598	2.019352	2.0347	2.030565	2.051035	2.046819	2.058733
2.002647	2.027734	2.023329	2.019072	2.034363	2.030188	2.050668	2.046508	2.058444
2.002365	2.027461	2.023073	2.018818	2.034047	2.029817	2.050303	2.046196	2.058161
2.002035	2.014443	2.010164	2.006011	2.033772	2.029524	2.054809	2.050638	2.057886
2.001683	2.014085	2.009861	2.00576	2.033526	2.02928	2.054554	2.050367	2.057613
2.00137	2.013779	2.009575	2.005494	2.033269	2.029037	2.054287	2.050062	2.057298
2.001093	2.013522	2.009306	2.005216	2.033006	2.0288	2.054013	2.049719	2.056939
2.00082	2.013269	2.00903	2.004919	2.03272	2.028547	2.041928	2.037666	2.045216
2.000547	2.013016	2.008733	2.004586	2.032392	2.028268	2.041662	2.037407	2.044962
2.000266	2.012761	2.008445	2.004267	2.032071	2.027984	2.041395	2.037161	2.044721
1.999957	2.012497	2.008187	2.004006	2.031802	2.027718	2.041129	2.036908	2.044459
1.999642	2.012232	2.007945	2.003771	2.031548	2.027445	2.040852	2.036649	2.044193
1.999363	2.01198	2.007706	2.003531	2.019433	2.015346	2.040521	2.036365	2.04393
1.99911	2.011737	2.007473	2.003291	2.019128	2.01499	2.040152	2.036062	2.043669
1.998858	2.011499	2.007247	2.003065	2.018854	2.014685	2.039834	2.035776	2.064467
1.998609	2.011267	2.007029	2.002855	2.018617	2.014438	2.039573	2.035508	2.064184
1.998363	2.011036	2.00681	2.002642	2.018385	2.014205	2.039329	2.035256	2.063914
1.998121	2.010807	2.006586	2.00242	2.01815	2.013976	2.0391	2.035027	2.063669
1.997876	2.010576	2.00636	2.002197	2.017915	2.013749	2.038872	2.034801	2.063427
1.99762	2.010337	2.006132	2.001976	2.017676	2.01351	2.038625	2.034556	2.063165
1.997357	2.010092	2.005901	2.001753	2.017433	2.013265	2.038371	2.034302	2.062895
1.997097	2.009841	2.005659	2.001518	2.017184	2.01302	2.027046	2.023026	2.062627
1.996839	2.009582	2.005405	2.001271	2.016928	2.012774	2.026792	2.022775	2.06236
1.980336	1.992444	1.988337	1.984277	2.016681	2.012538	2.026548	2.022528	2.062096
1.980076	1.992193	1.988096	1.984048	2.016442	2.01231	2.026308	2.022283	2.061836
1.979821	1.991943	1.987857	1.983824	2.01622	2.012112	2.0261	2.022057	2.05057
1.979569	1.991687	1.987614	1.983608	2.016033	2.011979	2.025963	2.021869	2.050322
1.979333	1.991441	1.987376	1.983393	2.015848	2.011851	2.025833	2.021687	2.05008
1.979147	1.996694	1.99256	1.988533	2.015619	2.011649	2.02563	2.021477	2.049844
1.978989	1.996488	1.992328	1.988282	2.015367	2.011406	2.025387	2.02125	2.049613
1.978795	1.996266	1.992099	1.988049	2.033333	2.029262	2.025145	2.021011	2.039111
1.978566	1.996031	1.991874	1.98783	2.033096	2.02902	2.024898	2.020758	2.038861
1.978352	1.995812	1.991675	1.987643	2.032891	2.028797	2.024664	2.020519	2.038619
1.978159	1.995613	1.99151	1.987503	2.032735	2.028599	2.024446	2.020293	2.038379

1.977988	1.995439	1.991362	1.98737	2.032586	2.028414	2.024248	2.020096	2.03817
1.977852	1.995309	1.991224	1.987217	2.018695	2.014557	2.02408	2.019958	2.038033
1.977728	1.995192	1.991088	1.98706	2.018519	2.014391	2.023934	2.019842	2.037918
1.977568	1.995022	1.990924	1.986902	2.018367	2.014259	2.023806	2.019695	2.037742
1.977388	1.994822	1.990743	1.986744	2.018231	2.014153	2.023692	2.019532	2.03753
1.977248	1.994661	1.990601	1.986618	2.0181	2.014013	2.023538	2.019352	2.037333
1.97715	1.994542	1.990503	1.986531	2.017976	2.013835	2.023337	2.01915	2.037146
1.955748	1.994438	1.990402	1.986424	2.002162	1.998047	2.009497	2.005365	2.036986
1.955668	1.994355	1.990285	1.986272	2.001981	1.997862	2.009333	2.005237	2.036879
1.955587	1.994265	1.990157	1.986109	2.001798	1.997688	2.009179	2.00511	2.036764
1.955489	1.994135	1.990011	1.985955	2.001643	1.997554	2.009037	2.004943	2.03657
1.955378	1.993985	1.989858	1.985807	2.001503	1.997436	2.008897	2.004757	2.036344
1.955249	2.013895	2.009654	2.005489	2.027658	2.022848	2.032719	2.028022	2.023391
1.955102	2.013734	2.009532	2.00539	2.027516	2.02265	2.032502	2.027817	2.023213
1.954978	2.013603	2.009414	2.005273	2.027356	2.022456	2.032308	2.027649	2.041189
1.954884	2.013512	2.009296	2.005123	2.027162	2.022253	2.032134	2.027527	2.041065
1.954805	2.013426	2.009193	2.005	2.027018	2.02211	2.032004	2.027417	2.040941
1.954741	2.013327	2.009118	2.00495	2.026992	2.02209	2.031946	2.027297	2.040792
1.954687	2.013235	2.009052	2.004914	2.026993	2.022106	2.03192	2.027195	2.040652
1.954633	2.013172	2.008973	2.004825	2.026921	2.022072	2.017296	2.012595	2.040566
1.954585	2.013126	2.008892	2.004713	2.026817	2.022015	2.017264	2.012574	2.040507
1.954553	2.013065	2.008824	2.004641	2.02673	2.021925	2.01717	2.012477	2.040394
1.954535	2.012987	2.008771	2.004604	2.026656	2.021799	2.017015	2.012306	2.04023
1.954538	1.993753	1.989666	1.985618	2.026576	2.021701	2.016908	2.012198	2.040111
1.954569	1.993762	1.989621	1.985528	2.026478	2.021652	2.016889	2.012197	2.040067
1.954598	1.99378	1.989595	1.985462	2.026397	2.021602	2.016866	2.0122	2.040037
1.954602	1.993789	1.989611	1.985469	2.026359	2.021504	2.016769	2.012148	2.040002
1.954595	1.993785	1.989635	1.9855	2.026345	2.021411	2.016662	2.012075	2.039957
1.954576	1.99373	1.989607	1.985496	2.02635	2.021399	2.01662	2.011989	2.026075
1.954548	1.993642	1.989546	1.985472	2.026369	2.02144	2.016618	2.011895	2.025941
1.976782	1.993607	1.989498	1.98542	2.026335	2.021441	2.016612	2.011851	2.034396
1.976816	1.993628	1.989458	1.985332	2.02624	2.021403	2.016606	2.011862	2.034349
1.976832	1.993645	1.989438	1.985277	2.026163	2.021345	2.016577	2.011871	2.034324
1.976808	1.993648	1.989455	1.98529	2.026127	2.021249	2.016496	2.011859	2.034324
1.976772	1.993632	1.989469	1.985317	2.026116	2.021171	2.016424	2.011843	2.034328
1.976731	1.993565	1.98944	1.985324	2.008436	2.003564	2.016417	2.011834	2.034315
1.976695	1.993484	1.989399	1.985327	2.008474	2.003605	2.016441	2.011821	2.034289
1.976713	1.993473	1.989397	1.985337	2.008508	2.003647	2.016453	2.011772	2.034221
1.976776	1.993515	1.989423	1.985353	2.008542	2.003695	2.016459	2.011696	2.034117
1.976831	1.993553	1.989431	1.985337	2.008541	2.003725	2.016476	2.011667	2.034062
1.976874	1.993582	1.989409	1.985275	2.008489	2.003728	2.016503	2.011696	2.034069
1.976924	1.99362	1.989407	1.98524	2.008463	2.003741	2.016539	2.011741	2.034085
1.976991	1.993679	1.989453	1.985273	2.008505	2.003785	2.016587	2.011793	2.034084
1.97706	1.993743	1.989517	1.985334	2.008574	2.003842	2.016642	2.011852	2.03409
1.959978	1.993801	1.989577	1.985392	2.008642	2.003904	1.999148	1.994399	2.034119
1.960016	1.993854	1.989637	1.985456	2.008716	2.003972	1.999212	1.994458	2.034164
1.960029	1.99389	1.98969	1.985519	2.008793	2.004044	2.009066	2.004258	2.034211
1.960015	1.99391	1.989735	1.985583	2.008873	2.00412	2.009132	2.004319	2.034263
1.960014	1.993932	1.989778	1.985641	2.008948	2.004191	2.009194	2.004376	2.03431
1.960027	1.993955	1.989818	1.985694	2.009015	2.004254	2.009249	2.004424	2.03435

1.95507	1.977751	1.97367	1.969603	2.009075	2.004313	2.009301	2.004473	2.034393
1.955113	1.977782	1.973699	1.969632	2.009128	2.004368	2.009352	2.004527	2.034443
1.955179	1.977827	1.973731	1.969657	2.009175	2.004419	2.009403	2.004583	2.034497
1.955284	1.977902	1.97378	1.969688	2.009225	2.004471	2.009453	2.00464	2.034552
1.955419	1.977999	1.97384	1.96972	2.009274	2.004523	2.009502	2.004697	2.034608
1.955527	1.973637	1.969437	1.965282	1.994221	1.989502	2.009548	2.004751	2.018641
1.95561	1.973701	1.969477	1.965301	1.994252	1.989535	2.009591	2.004803	2.018695
1.955682	1.973758	1.969522	1.965333	1.994298	1.989576	2.009637	2.004857	2.018765
1.955742	1.973805	1.969568	1.965376	1.994362	1.989623	2.009685	2.004914	2.01886
1.955803	1.973854	1.969627	1.965444	1.994451	1.989685	2.009745	2.004982	2.018962
1.955875	1.973915	1.969722	1.965564	1.990761	1.985928	1.99533	1.990599	2.019049
1.955954	1.973984	1.96983	1.965702	1.990913	1.986028	1.995414	1.990693	2.019145
1.978304	1.974053	1.969906	1.965785	1.991006	1.986108	1.995513	1.990834	2.019299
1.978383	1.974125	1.969962	1.96583	1.991058	1.986176	1.995626	1.991013	2.019499
1.978498	1.974237	1.970048	1.96589	1.991126	1.986254	1.995726	1.991148	2.019644
1.978658	1.974402	1.970169	1.965967	1.991208	1.986337	1.995804	1.991224	2.019721
1.94709	1.974562	1.970296	1.966062	1.991323	1.986457	1.992376	1.98775	2.006064
1.947226	1.97469	1.970415	1.966179	1.991493	1.986647	1.992534	1.987852	2.006141
1.947362	1.974813	1.970542	1.966317	1.991684	1.986848	1.992702	1.987965	2.006235
1.947488	1.974947	1.970696	1.966484	1.991868	1.986997	1.992831	1.988084	2.006368
1.947612	1.97509	1.970865	1.966668	1.992049	1.987123	1.992946	1.988214	2.006528
1.915592	1.975255	1.971025	1.966819	1.992201	1.987259	1.993089	1.988382	1.983759
1.915749	1.975442	1.97118	1.966942	1.992327	1.987402	1.993254	1.988587	1.983961
1.915903	1.975606	1.971324	1.967069	1.99248	1.987573	1.99343	1.988768	1.984132
1.916053	1.975737	1.97145	1.967201	1.992669	1.987786	1.993619	1.988912	1.98425
1.916199	1.944249	1.940199	1.936181	1.992873	1.988	1.993806	1.989054	1.984371
1.916339	1.944376	1.940344	1.936342	1.993084	1.988183	1.993969	1.9892	1.984515
1.916478	1.944509	1.940499	1.936514	1.993302	1.988369	1.994139	1.989363	1.984681
1.900662	1.944664	1.940661	1.936684	1.993525	1.988602	1.994368	1.989586	1.984892
1.900822	1.944834	1.940832	1.936856	1.993751	1.98886	1.99463	1.989846	1.985134
1.901	1.930682	1.926725	1.922792	1.993966	1.989061	1.994828	1.990053	1.985343
1.901198	1.930852	1.926905	1.922979	1.99417	1.989203	1.99496	1.990206	1.985519
1.901388	1.931023	1.927083	1.923157	1.994363	1.989349	1.995101	1.990373	1.985707
1.901556	1.931199	1.927253	1.923315	1.994527	1.989498	1.99526	1.990571	1.985922
1.901731	1.931376	1.927424	1.923478	1.994717	1.989686	1.995451	1.990784	1.986142
1.854287	1.931547	1.9276	1.923667	1.953038	1.948178	1.995703	1.991	1.986345
1.854521	1.931727	1.927784	1.923866	1.95333	1.948492	1.995992	1.991234	1.986556
1.854763	1.931959	1.92799	1.924053	1.953575	1.94876	1.996286	1.991519	1.986823
1.855016	1.932231	1.928215	1.924236	1.953791	1.949002	1.99659	1.991845	1.987135
1.828395	1.898992	1.895074	1.891183	1.920498	1.916563	1.956961	1.952769	1.987438
1.828589	1.899223	1.895336	1.891461	1.920737	1.916758	1.957166	1.953004	1.987735
1.828778	1.899438	1.895581	1.891726	1.920981	1.916972	1.957383	1.953231	1.988008
1.828965	1.899629	1.89579	1.891955	1.921229	1.917229	1.95763	1.953443	1.988229
1.829153	1.89982	1.895989	1.892168	1.921469	1.917491	1.957888	1.953663	1.988452
1.829344	1.844629	1.841095	1.837565	1.935352	1.931299	1.927227	1.923142	1.97761
1.829542	1.844811	1.841302	1.837792	1.935547	1.931519	1.927467	1.923398	1.977924
1.829762	1.845015	1.841519	1.838023	1.935762	1.931725	1.927679	1.923629	1.978226
1.830003	1.845238	1.841746	1.838259	1.935992	1.931915	1.927867	1.92384	1.978522
1.830245	1.845462	1.841963	1.838474	1.936219	1.932123	1.928067	1.924039	1.978782
1.83049	1.845687	1.842165	1.83866	1.936447	1.932358	1.928283	1.92422	1.978983

1.83073	1.84591	1.842366	1.838843	1.898674	1.894819	1.928508	1.924412	1.950599
1.830956	1.846125	1.842567	1.839032	1.898845	1.895017	1.928737	1.924635	1.950861
1.831178	1.846342	1.842775	1.83923	1.899018	1.895216	1.928974	1.924876	1.951146
1.831409	1.846581	1.843003	1.839448	1.899225	1.895437	1.929227	1.925134	1.951436
1.831644	1.84683	1.843244	1.839683	1.899454	1.89567	1.92949	1.925406	1.951735
1.831861	1.828403	1.824916	1.821456	1.8843	1.880589	1.908985	1.905016	1.95204
1.832059	1.828606	1.82515	1.821713	1.884547	1.880817	1.909227	1.905266	1.952353
1.804145	1.828821	1.825386	1.821967	1.884781	1.881025	1.909443	1.905496	1.952657
1.80438	1.829058	1.825628	1.82221	1.884988	1.881195	1.909613	1.905691	1.952942
1.804628	1.829307	1.825876	1.822456	1.885201	1.881375	1.90979	1.905889	1.953227
1.804887	1.829569	1.826137	1.822714	1.885442	1.881599	1.893041	1.889196	1.934199
1.805155	1.829839	1.826404	1.822977	1.885697	1.881845	1.893276	1.889434	1.93449
1.785629	1.833524	1.829957	1.826396	1.848995	1.845292	1.893521	1.889682	1.93479
1.785895	1.833792	1.830219	1.826653	1.849254	1.845551	1.893777	1.889939	1.935097
1.786162	1.815675	1.812134	1.808604	1.823279	1.819657	1.894028	1.890194	1.935402
1.786431	1.815925	1.812374	1.80884	1.823519	1.819897	1.894275	1.890445	1.935703
1.786696	1.783297	1.779959	1.776645	1.823751	1.820132	1.879293	1.875584	1.920344
1.786952	1.78353	1.780179	1.77686	1.823972	1.820353	1.87952	1.875819	1.920641
1.787204	1.783757	1.780393	1.777069	1.824193	1.820579	1.879751	1.876056	1.920939
1.760418	1.78398	1.780605	1.77728	1.806138	1.802583	1.856219	1.85259	1.921238
1.760625	1.784201	1.780815	1.777491	1.806366	1.802834	1.856483	1.852846	1.921542
1.729806	1.784431	1.781032	1.777708	1.806605	1.803098	1.856769	1.853121	1.888163
1.730027	1.784667	1.781256	1.777931	1.806852	1.803373	1.857076	1.853414	1.888462
1.730258	1.726783	1.723449	1.720206	1.8071	1.803637	1.841094	1.837461	1.866082
1.730502	1.727001	1.723657	1.720408	1.807344	1.803882	1.841344	1.837718	1.866366
1.730751	1.727224	1.723868	1.720611	1.775366	1.772114	1.841594	1.837972	1.85061
1.730997	1.727444	1.724071	1.720804	1.775624	1.772379	1.841855	1.838228	1.850877
1.731247	1.727669	1.724274	1.720996	1.775882	1.772646	1.842118	1.838488	1.851148
1.731511	1.727899	1.72448	1.721186	1.717964	1.714813	1.842377	1.838767	1.835211
1.731785	1.728135	1.724689	1.721378	1.718153	1.715006	1.842636	1.839056	1.835513
1.73206	1.72838	1.724915	1.721591	1.71836	1.71521	1.793551	1.789999	1.835788
1.732337	1.728634	1.725156	1.721823	1.718581	1.715422	1.793754	1.790194	1.836034
1.732615	1.728892	1.725412	1.722078	1.718825	1.715654	1.747873	1.744488	1.836293
1.732888	1.72915	1.725684	1.722364	1.719102	1.715912	1.748142	1.744731	1.836572
1.733174	1.729424	1.72597	1.722659	1.719386	1.716178	1.748421	1.744991	1.836864
1.733491	1.729735	1.726269	1.722945	1.719659	1.71644	1.74869	1.745263	1.787617
1.733822	1.730061	1.726575	1.723232	1.719934	1.716708	1.74896	1.745546	1.7879
1.734152	1.730374	1.726873	1.72352	1.720218	1.716991	1.713789	1.710622	1.788192
1.734482	1.73068	1.727168	1.723809	1.72051	1.717285	1.714069	1.710876	1.788495
1.734795	1.730965	1.727457	1.724107	1.720801	1.717567	1.714346	1.711151	1.788801
1.735082	1.73122	1.727739	1.724415	1.721092	1.717831	1.714617	1.711451	1.789112
1.735394	1.731507	1.728035	1.724723	1.721389	1.718115	1.714904	1.711752	1.789426
1.735749	1.731852	1.728353	1.72502	1.721696	1.71844	1.715223	1.71205	1.789747
1.736131	1.73222	1.728686	1.725327	1.722011	1.718772	1.715546	1.712348	1.790065
1.736535	1.732584	1.729037	1.725668	1.722337	1.719083	1.715849	1.712647	1.790364
1.736958	1.732956	1.729399	1.726024	1.722669	1.719388	1.716146	1.712947	1.790658
1.737387	1.73335	1.729758	1.726356	1.722992	1.71971	1.71646	1.713252	1.790984
1.737825	1.733762	1.730116	1.726672	1.723308	1.720044	1.716788	1.713562	1.791334
1.738251	1.734155	1.730469	1.726994	1.723623	1.720362	1.717105	1.713878	1.79168
1.738663	1.734519	1.730816	1.727329	1.723935	1.720655	1.717406	1.714205	1.792017

1.715623	1.734887	1.731159	1.727655	1.724245	1.720955	1.71771	1.714523	1.792351
1.71603	1.735279	1.731498	1.727954	1.724551	1.721282	1.718029	1.714817	1.792681
1.716443	1.735677	1.731839	1.728252	1.724856	1.72161	1.718347	1.715106	1.793007
1.716857	1.736061	1.732191	1.728579	1.725162	1.721903	1.71864	1.715411	1.793324
1.717278	1.736442	1.732552	1.728923	1.725472	1.722182	1.718924	1.715725	1.793637
1.717734	1.713371	1.709517	1.705949	1.725801	1.722499	1.719238	1.716044	1.793973
1.718225	1.713798	1.709886	1.706273	1.72615	1.722851	1.719579	1.716368	1.794329
1.718702	1.714221	1.710252	1.706595	1.726495	1.723206	1.719929	1.716703	1.794698
1.719154	1.714634	1.710611	1.706912	1.726831	1.723561	1.720285	1.717053	1.795087
1.719603	1.715046	1.710971	1.707231	1.727168	1.723916	1.720644	1.71741	1.795471
1.720057	1.715449	1.711328	1.707555	1.704132	1.700957	1.721003	1.71777	1.795827
1.720522	1.715861	1.711695	1.70789	1.704457	1.701283	1.721361	1.718132	1.796176
1.721025	1.716317	1.7121	1.708252	1.704799	1.701617	1.721716	1.71849	1.796546
1.721556	1.716805	1.712532	1.708635	1.705154	1.701956	1.722067	1.718845	1.79693
1.722097	1.717308	1.712977	1.709024	1.70551	1.70229	1.722413	1.719198	1.797309
1.722645	1.717825	1.713433	1.70942	1.705865	1.702617	1.722753	1.719549	1.797685
1.69682	1.71837	1.713908	1.709825	1.706228	1.702952	1.69972	1.69659	1.765943
1.69742	1.718956	1.714406	1.710238	1.706602	1.703305	1.700047	1.696893	1.766276
1.69803	1.719553	1.714918	1.710666	1.706984	1.703658	1.700372	1.697195	1.7666
1.698625	1.72014	1.715442	1.711118	1.707373	1.703993	1.700682	1.697504	1.766914
1.699209	1.720721	1.715972	1.711585	1.707771	1.704324	1.70099	1.697819	1.767227
1.699762	1.72127	1.716486	1.712054	1.708181	1.704676	1.70132	1.698153	1.746889
1.700289	1.721794	1.716989	1.712527	1.708603	1.705044	1.701667	1.698502	1.747236
1.62208	1.722341	1.717507	1.713011	1.709037	1.705427	1.702027	1.698857	1.747591
1.622622	1.722913	1.718043	1.713505	1.709484	1.705829	1.702401	1.699218	1.747953
1.574918	1.697132	1.692388	1.687986	1.709934	1.706231	1.702777	1.699585	1.748321
1.57544	1.697725	1.692925	1.688461	1.710378	1.706623	1.703148	1.699959	1.748694
1.575964	1.698326	1.693471	1.688946	1.710829	1.707019	1.703523	1.700338	1.749072
1.576475	1.620213	1.615612	1.611325	1.711294	1.707428	1.703905	1.700715	1.749453
1.576979	1.620737	1.616109	1.611786	1.711773	1.707848	1.704295	1.701095	1.749838
1.577482	1.62127	1.61662	1.612265	1.638357	1.634624	1.704706	1.701492	1.750231
1.577985	1.621811	1.617144	1.612763	1.638836	1.635051	1.705137	1.701904	1.750633
1.578494	1.573971	1.569501	1.565301	1.609309	1.6057	1.705577	1.702323	1.751042
1.579012	1.574481	1.570009	1.565795	1.609791	1.606115	1.706025	1.702749	1.75146
1.579532	1.57499	1.570522	1.566296	1.610286	1.606544	1.706486	1.703179	1.751878
1.580043	1.575484	1.571022	1.566794	1.610788	1.606992	1.633322	1.630172	1.752285
1.580565	1.575984	1.571529	1.567299	1.611302	1.607453	1.633764	1.630561	1.752694
1.581128	1.57652	1.572067	1.567829	1.61183	1.607923	1.634219	1.630975	1.701144
1.581724	1.577083	1.572627	1.568378	1.612372	1.608404	1.634686	1.63141	1.701591
1.582342	1.577676	1.573209	1.568941	1.612934	1.608904	1.635174	1.631856	1.628684
1.582992	1.578305	1.57382	1.569525	1.613525	1.609429	1.635687	1.632318	1.629122
1.583611	1.578902	1.574398	1.570075	1.565875	1.561921	1.606301	1.603116	1.629538
1.584163	1.579432	1.574909	1.570563	1.56633	1.562337	1.606706	1.603464	1.629908
1.584714	1.579958	1.57541	1.571038	1.56678	1.562756	1.607114	1.603812	1.630278
1.585308	1.580523	1.575935	1.571526	1.567251	1.563212	1.607561	1.604197	1.630679
1.585926	1.581108	1.576474	1.572025	1.567739	1.563692	1.608035	1.604608	1.6311
1.586561	1.581715	1.577046	1.572564	1.56826	1.564197	1.608546	1.605063	1.631551
1.587215	1.582344	1.577647	1.573137	1.568811	1.564725	1.609093	1.605558	1.63203
1.587866	1.582961	1.578247	1.573718	1.569361	1.565238	1.561484	1.55807	1.6028
1.588504	1.583553	1.578839	1.574304	1.569903	1.565723	1.561925	1.558472	1.603212

1.589177	1.584197	1.579466	1.574909	1.57047	1.566241	1.562388	1.558873	1.603612
1.589926	1.584949	1.580159	1.575537	1.571076	1.566828	1.562901	1.559277	1.603987
1.590683	1.585727	1.580877	1.576189	1.571709	1.567442	1.563437	1.55969	1.604366
1.5551	1.586461	1.581601	1.576883	1.572368	1.568053	1.563973	1.560131	1.604781
1.555771	1.587168	1.582325	1.5776	1.573047	1.568671	1.564518	1.560589	1.605217
1.556397	1.587801	1.582987	1.57828	1.573715	1.569304	1.565079	1.56105	1.605649
1.556973	1.58836	1.583594	1.57893	1.574378	1.569952	1.565657	1.561512	1.606079
1.557598	1.552805	1.548101	1.5435	1.575001	1.570573	1.566231	1.562005	1.558276
1.558298	1.553513	1.548741	1.544076	1.575564	1.571157	1.566803	1.562542	1.558747
1.55901	1.554238	1.549405	1.544677	1.576136	1.571725	1.567358	1.56308	1.559233
1.55971	1.554959	1.550119	1.545361	1.576757	1.572263	1.567861	1.563579	1.559704
1.560403	1.555677	1.550852	1.546081	1.577407	1.572813	1.568372	1.564088	1.560189
1.561075	1.556369	1.551569	1.546807	1.542148	1.537597	1.568981	1.564664	1.560711
1.561732	1.557044	1.552279	1.547542	1.542876	1.538299	1.569658	1.565289	1.561266
1.562395	1.557716	1.552972	1.548254	1.543599	1.539022	1.53458	1.530271	1.561875
1.56306	1.558381	1.553643	1.54894	1.544317	1.539774	1.535329	1.53099	1.562541
1.563746	1.559072	1.554347	1.549657	1.545048	1.540513	1.536062	1.531703	1.563207
1.564463	1.559796	1.555103	1.550433	1.5458	1.541224	1.536757	1.532394	1.563861
1.565199	1.560546	1.555879	1.55122	1.546554	1.541931	1.537443	1.533073	1.564503
1.565954	1.561326	1.556658	1.551986	1.5473	1.54265	1.538137	1.533741	1.529497
1.566722	1.562123	1.557443	1.552747	1.548042	1.543369	1.538828	1.5344	1.530103
1.567491	1.562903	1.558217	1.553506	1.548771	1.544062	1.539496	1.535049	1.530723
1.568265	1.563672	1.558986	1.554263	1.549491	1.544736	1.540147	1.53569	1.531355
1.569036	1.564455	1.55978	1.555053	1.550239	1.545431	1.54082	1.53636	1.532018
1.569807	1.565255	1.560606	1.555882	1.551018	1.546143	1.541511	1.53706	1.53272
1.527344	1.566054	1.561423	1.556702	1.551813	1.5469	1.542243	1.537775	1.533422
1.528075	1.566852	1.562217	1.55749	1.552622	1.547728	1.543037	1.538498	1.534102
1.528796	1.567635	1.562998	1.55827	1.553439	1.54858	1.543856	1.539233	1.534787
1.529492	1.568378	1.563765	1.55906	1.554256	1.549414	1.544663	1.539981	1.535495
1.530174	1.569098	1.564522	1.559853	1.555071	1.55024	1.545469	1.540742	1.536222
1.530866	1.569823	1.565276	1.560637	1.555865	1.551034	1.546257	1.541517	1.536981
1.531565	1.57055	1.566027	1.561413	1.556638	1.551798	1.547029	1.542309	1.537772
1.532272	1.527959	1.52357	1.519082	1.557429	1.55258	1.547807	1.543082	1.538539
1.532993	1.528682	1.524325	1.519866	1.558249	1.55339	1.548588	1.54382	1.539262
1.485862	1.529388	1.52506	1.520631	1.559063	1.55421	1.549388	1.544581	1.540002
1.486494	1.530041	1.525742	1.521355	1.559856	1.555048	1.550232	1.545399	1.540791
1.487119	1.530682	1.526402	1.522054	1.560629	1.55588	1.551084	1.546245	1.541609
1.431444	1.531355	1.527067	1.522726	1.518198	1.513572	1.551901	1.547097	1.542454
1.432082	1.53205	1.527733	1.523382	1.518874	1.514288	1.552698	1.547959	1.543322
1.36389	1.532773	1.528439	1.524082	1.519584	1.51502	1.553499	1.548812	1.544186
1.364521	1.533524	1.529186	1.524826	1.520324	1.515763	1.5543	1.549657	1.545048
1.36517	1.481818	1.477559	1.47329	1.521076	1.516523	1.511984	1.507446	1.545908
1.365839	1.482557	1.478268	1.47398	1.52184	1.517311	1.512793	1.508275	1.546765
1.366521	1.483311	1.478988	1.474677	1.522608	1.518103	1.513604	1.509103	1.54762
1.30507	1.431967	1.427728	1.423466	1.523375	1.518885	1.514405	1.509926	1.548475
1.305709	1.432677	1.428419	1.424149	1.52414	1.519664	1.515201	1.510743	1.549327
1.306353	1.433388	1.429104	1.424818	1.472387	1.468015	1.515992	1.511553	1.550171
1.307002	1.434099	1.429784	1.42548	1.47307	1.468711	1.516778	1.512354	1.551008
1.307649	1.434814	1.430473	1.426151	1.473759	1.469406	1.465098	1.46083	1.508659
1.308294	1.435533	1.431172	1.426834	1.474452	1.470096	1.465791	1.461534	1.509446

1.30893	1.436253	1.43187	1.427517	1.475156	1.470795	1.466493	1.462245	1.510238
1.309553	1.436971	1.432566	1.428198	1.475875	1.471513	1.467215	1.462977	1.511042
1.310169	1.437692	1.433263	1.428879	1.476602	1.472237	1.467942	1.463712	1.51185
1.310782	1.438418	1.43396	1.429555	1.477329	1.472957	1.468661	1.464438	1.460133
1.311392	1.439147	1.434661	1.430234	1.478063	1.473679	1.469379	1.46516	1.460876
1.312006	1.308169	1.304316	1.300461	1.426581	1.422282	1.470096	1.46587	1.461604
1.312623	1.308773	1.304929	1.301084	1.427293	1.422968	1.47081	1.46657	1.46232
1.313268	1.309399	1.305566	1.301738	1.428044	1.423708	1.471566	1.467295	1.46304
1.313952	1.310053	1.30624	1.302438	1.428851	1.424522	1.472375	1.468051	1.463762
1.314643	1.310713	1.306909	1.303126	1.429652	1.425343	1.473201	1.46883	1.464504
1.315318	1.311357	1.307539	1.303762	1.430414	1.426157	1.474036	1.469639	1.465285
1.316006	1.312018	1.308172	1.304384	1.431156	1.426947	1.474864	1.470459	1.466085
1.316749	1.312742	1.308849	1.305021	1.431872	1.427663	1.475642	1.471266	1.466893
1.317525	1.313508	1.309556	1.305669	1.432574	1.428332	1.476387	1.472064	1.467708
1.318276	1.314271	1.310286	1.306355	1.433325	1.429011	1.477135	1.47284	1.468502
1.319009	1.315041	1.311046	1.30708	1.434122	1.429693	1.477879	1.473595	1.469278
1.319698	1.315759	1.311775	1.307803	1.434943	1.430432	1.478658	1.474343	1.470026
1.320318	1.316395	1.31246	1.308526	1.435804	1.431258	1.479483	1.475075	1.470723
1.320939	1.317015	1.313117	1.309222	1.43665	1.432103	1.480334	1.475835	1.471441
1.321605	1.317647	1.313736	1.309847	1.43743	1.432933	1.481215	1.476678	1.472244
1.322298	1.318293	1.314349	1.310449	1.438183	1.433748	1.482102	1.477551	1.473084
1.323026	1.318997	1.315025	1.311102	1.438936	1.434497	1.430119	1.425797	1.473914
1.323782	1.319745	1.315747	1.311789	1.439689	1.435201	1.430831	1.426558	1.474744
1.324511	1.320491	1.316494	1.312524	1.308578	1.304672	1.431595	1.427315	1.475565
1.325211	1.321238	1.317276	1.313321	1.309352	1.305401	1.432417	1.428063	1.47637
1.325905	1.321964	1.318028	1.314087	1.310109	1.306134	1.43325	1.42884	1.477201
1.326594	1.322644	1.318709	1.314775	1.310813	1.306857	1.434094	1.429677	1.478085
1.327292	1.323327	1.319385	1.315452	1.311506	1.30757	1.434924	1.430514	1.478973
1.328023	1.324067	1.320118	1.316172	1.312208	1.308252	1.435695	1.431279	1.479812
1.32877	1.32483	1.320874	1.31691	1.312917	1.308928	1.436447	1.432025	1.480639
1.329504	1.325562	1.321608	1.317648	1.313657	1.309668	1.437271	1.432842	1.481513
1.330229	1.326271	1.322329	1.318389	1.314426	1.31046	1.438151	1.433715	1.482423
1.330981	1.327009	1.323067	1.319134	1.315183	1.31123	1.439012	1.434592	1.483361
1.331773	1.327789	1.32383	1.319881	1.315921	1.311965	1.439845	1.435471	1.484336
1.25519	1.328554	1.324589	1.320636	1.316669	1.312709	1.308779	1.304876	1.485297
1.255891	1.329262	1.325328	1.321409	1.317448	1.313486	1.309556	1.305651	1.486217
1.256591	1.329961	1.326063	1.322182	1.318233	1.31427	1.310333	1.306414	1.487112
1.25733	1.330696	1.326809	1.322944	1.319003	1.315042	1.311086	1.307132	1.487976
1.258094	1.331451	1.327556	1.323692	1.31976	1.315806	1.311826	1.307826	1.488822
1.258867	1.254886	1.251113	1.24739	1.320468	1.31654	1.312566	1.30856	1.436002
1.259654	1.255635	1.251794	1.248022	1.321124	1.317242	1.313302	1.309328	1.436854
1.260443	1.256403	1.252507	1.248691	1.321816	1.317965	1.314051	1.310103	1.43771
1.261231	1.257199	1.253267	1.249419	1.322571	1.318729	1.314824	1.310883	1.438563
1.262028	1.258001	1.254039	1.250161	1.323331	1.319484	1.315586	1.311661	1.439426
1.262846	1.258797	1.254804	1.250892	1.24711	1.243399	1.316298	1.312423	1.308512
1.26367	1.25959	1.255567	1.251627	1.247812	1.244076	1.317006	1.313185	1.309313
0.995251	1.260362	1.256318	1.252364	1.248547	1.244818	1.31778	1.313974	1.310117
0.995716	1.261124	1.257064	1.253106	1.249309	1.245606	1.318598	1.314782	1.310923
0.996247	1.261948	1.257873	1.253905	1.250107	1.246407	1.319419	1.31559	1.311722
0.996847	1.262837	1.258751	1.254768	1.250949	1.247226	1.320249	1.316405	1.312516

0.997464	1.107646	1.103907	1.100275	1.251778	1.248023	1.244283	1.240573	1.313295
0.998096	1.108483	1.104678	1.100979	1.252578	1.248786	1.245023	1.241301	1.31405
0.998718	1.109311	1.105438	1.101668	1.253353	1.249516	1.245734	1.242015	1.314802
0.999294	0.996057	0.992788	0.989541	1.254078	1.250182	1.246397	1.24272	1.31557
0.999845	0.996555	0.993277	0.990025	1.254786	1.250826	1.247047	1.243425	1.31635
1.000386	0.99706	0.993773	0.990521	1.099971	1.096401	1.247768	1.244174	1.317156
1.000917	0.997567	0.994271	0.991028	1.100664	1.097053	1.248546	1.244959	1.317986
1.001472	0.998103	0.994809	0.991577	1.10139	1.097747	1.249359	1.245759	1.318821
1.002049	0.998668	0.995392	0.992176	1.102148	1.098487	1.250212	1.246576	1.31966
1.002657	0.999271	0.996002	0.992792	1.102933	1.099244	1.095783	1.092512	1.243765
1.003306	0.999923	0.996642	0.993412	1.103744	1.100003	1.096505	1.093206	1.24456
1.003979	1.000602	0.997299	0.994041	1.104573	1.100774	1.097231	1.093893	1.245347
0.828817	1.001279	0.997956	0.994675	0.991415	0.9882	1.097963	1.094562	1.246121
0.82976	1.001964	0.99862	0.995315	0.99204	0.988809	1.098701	1.095223	1.246887
0.703129	1.002654	0.999292	0.995965	0.992669	0.989416	1.099458	1.0959	1.092517
0.704046	1.003349	0.999972	0.996625	0.993302	0.99002	1.100231	1.096591	1.093161
0.704959	0.829539	0.826489	0.823429	0.993949	0.990635	1.10102	1.097302	1.093822
0.70587	0.830465	0.827433	0.824384	0.994616	0.991264	1.101825	1.098034	1.094503
0.706775	0.831387	0.828377	0.825343	0.995288	0.991905	1.102648	1.098787	1.095201
0.707674	0.832305	0.829321	0.826306	0.995955	0.992564	0.989207	0.985886	1.095909
0.708568	0.833218	0.83026	0.827268	0.996624	0.993232	0.989851	0.986489	1.096638
0.709453	0.834126	0.83119	0.828219	0.825149	0.822039	0.990497	0.987116	1.097415
0.710333	0.83503	0.832112	0.829161	0.826104	0.823001	0.991151	0.987768	1.09823
0.711224	0.835933	0.833024	0.830089	0.82705	0.823962	0.991829	0.988436	1.099067
0.712124	0.836835	0.833928	0.831003	0.827987	0.824925	0.992532	0.989121	1.099928
0.713034	0.710271	0.707539	0.704802	0.828919	0.825883	0.822799	0.819675	1.1008
0.713954	0.711164	0.708427	0.705697	0.829847	0.826839	0.823765	0.82064	1.10168
0.714884	0.712056	0.709313	0.706588	0.830771	0.82779	0.824729	0.821606	1.102572
0.715812	0.712927	0.710179	0.707466	0.831684	0.82873	0.825684	0.822567	0.988469
0.716762	0.713808	0.711049	0.708344	0.832595	0.829669	0.826639	0.823532	0.989171
0.717798	0.714774	0.711976	0.709252	0.833519	0.83062	0.827611	0.824522	0.821418
0.718906	0.715809	0.712949	0.710184	0.834454	0.831582	0.828596	0.825534	0.822444
0.719999	0.716864	0.713959	0.711159	0.83544	0.832582	0.829619	0.82659	0.823517
0.721074	0.717945	0.715019	0.712197	0.836501	0.833643	0.830701	0.827711	0.824661
0.722136	0.71901	0.716052	0.713198	0.837523	0.834657	0.831733	0.828781	0.825754
0.723181	0.720031	0.717013	0.714103	0.838444	0.835563	0.832649	0.829729	0.826722
0.724217	0.721037	0.71795	0.714977	0.839329	0.836429	0.833524	0.830636	0.827653
0.725243	0.722041	0.718892	0.715859	0.840217	0.837298	0.834409	0.831565	0.828614
0.726269	0.72305	0.719839	0.716747	0.841104	0.838163	0.835291	0.832496	0.829582
	0.724102	0.720842	0.717698	0.714799	0.712052	0.836185	0.833429	0.830556
	0.725194	0.721895	0.718703	0.715752	0.712959	0.837087	0.834364	0.831539
	0.726329	0.723011	0.719787	0.716785	0.713936	0.838043	0.835306	0.832492
	0.727531	0.724219	0.720982	0.717932	0.715016	0.83908	0.836258	0.8334
	0.728713	0.725416	0.722174	0.719076	0.716089	0.840114	0.837208	0.834304
	0.729804	0.726522	0.723272	0.720123	0.717068	0.841079	0.838134	0.835216
	0.730868	0.72761	0.724358	0.72116	0.718037	0.842044	0.83907	0.836144
	0.731975	0.728764	0.725533	0.722293	0.719102	0.843094	0.84007	0.837122
	0.733099	0.729949	0.726753	0.723479	0.720222	0.844197	0.841112	0.838133
				0.724584	0.721295	0.718233	0.715342	0.839123
				0.725605	0.722318	0.71924	0.716321	0.840089

0.726642	0.723364	0.720269	0.717314	0.841076
0.72771	0.724443	0.721325	0.718321	0.842096
0.728814	0.725562	0.722419	0.719358	0.843135
0.505293	0.502842	0.723589	0.720452	0.844179
0.50625	0.503833	0.724789	0.721577	0.845244
		0.725919	0.72269	0.719596
		0.727003	0.7238	0.720709
		0.503776	0.501302	0.721831
		0.504661	0.502166	0.722959
				0.724109
				0.725302
				0.726488
				0.503302
				0.504045

1.95507	1.977751	1.97367	1.969603	2.009075	2.004313	2.009301	2.004473	2.034393
1.955113	1.977782	1.973699	1.969632	2.009128	2.004368	2.009352	2.004527	2.034443
1.955179	1.977827	1.973731	1.969657	2.009175	2.004419	2.009403	2.004583	2.034497
1.955284	1.977902	1.97378	1.969688	2.009225	2.004471	2.009453	2.00464	2.034552
1.955419	1.977999	1.97384	1.96972	2.009274	2.004523	2.009502	2.004697	2.034608
1.955527	1.973637	1.969437	1.965282	1.994221	1.989502	2.009548	2.004751	2.018641
1.95561	1.973701	1.969477	1.965301	1.994252	1.989535	2.009591	2.004803	2.018695
1.955682	1.973758	1.969522	1.965333	1.994298	1.989576	2.009637	2.004857	2.018765
1.955742	1.973805	1.969568	1.965376	1.994362	1.989623	2.009685	2.004914	2.01886
1.955803	1.973854	1.969627	1.965444	1.994451	1.989685	2.009745	2.004982	2.018962
1.955875	1.973915	1.969722	1.965564	1.990761	1.985928	1.99533	1.990599	2.019049
1.955954	1.973984	1.96983	1.965702	1.990913	1.986028	1.995414	1.990693	2.019145
1.978304	1.974053	1.969906	1.965785	1.991006	1.986108	1.995513	1.990834	2.019299
1.978383	1.974125	1.969962	1.96583	1.991058	1.986176	1.995626	1.991013	2.019499
1.978498	1.974237	1.970048	1.96589	1.991126	1.986254	1.995726	1.991148	2.019644
1.978658	1.974402	1.970169	1.965967	1.991208	1.986337	1.995804	1.991224	2.019721
1.94709	1.974562	1.970296	1.966062	1.991323	1.986457	1.992376	1.98775	2.006064
1.947226	1.97469	1.970415	1.966179	1.991493	1.986647	1.992534	1.987852	2.006141
1.947362	1.974813	1.970542	1.966317	1.991684	1.986848	1.992702	1.987965	2.006235
1.947488	1.974947	1.970696	1.966484	1.991868	1.986997	1.992831	1.988084	2.006368
1.947612	1.97509	1.970865	1.966668	1.992049	1.987123	1.992946	1.988214	2.006528
1.915592	1.975255	1.971025	1.966819	1.992201	1.987259	1.993089	1.988382	1.983759
1.915749	1.975442	1.97118	1.966942	1.992327	1.987402	1.993254	1.988587	1.983961
1.915903	1.975606	1.971324	1.967069	1.99248	1.987573	1.99343	1.988768	1.984132
1.916053	1.975737	1.97145	1.967201	1.992669	1.987786	1.993619	1.988912	1.98425
1.916199	1.944249	1.940199	1.936181	1.992873	1.988	1.993806	1.989054	1.984371
1.916339	1.944376	1.940344	1.936342	1.993084	1.988183	1.993969	1.9892	1.984515
1.916478	1.944509	1.940499	1.936514	1.993302	1.988369	1.994139	1.989363	1.984681
1.900662	1.944664	1.940661	1.936684	1.993525	1.988602	1.994368	1.989586	1.984892
1.900822	1.944834	1.940832	1.936856	1.993751	1.98886	1.99463	1.989846	1.985134
1.901	1.930682	1.926725	1.922792	1.993966	1.989061	1.994828	1.990053	1.985343
1.901198	1.930852	1.926905	1.922979	1.99417	1.989203	1.99496	1.990206	1.985519
1.901388	1.931023	1.927083	1.923157	1.994363	1.989349	1.995101	1.990373	1.985707
1.901556	1.931199	1.927253	1.923315	1.994527	1.989498	1.99526	1.990571	1.985922
1.901731	1.931376	1.927424	1.923478	1.994717	1.989686	1.995451	1.990784	1.986142
1.854287	1.931547	1.9276	1.923667	1.953038	1.948178	1.995703	1.991	1.986345
1.854521	1.931727	1.927784	1.923866	1.95333	1.948492	1.995992	1.991234	1.986556
1.854763	1.931959	1.92799	1.924053	1.953575	1.94876	1.996286	1.991519	1.986823
1.855016	1.932231	1.928215	1.924236	1.953791	1.949002	1.99659	1.991845	1.987135
1.828395	1.898992	1.895074	1.891183	1.920498	1.916563	1.956961	1.952769	1.987438
1.828589	1.899223	1.895336	1.891461	1.920737	1.916758	1.957166	1.953004	1.987735
1.828778	1.899438	1.895581	1.891726	1.920981	1.916972	1.957383	1.953231	1.988008
1.828965	1.899629	1.89579	1.891955	1.921229	1.917229	1.95763	1.953443	1.988229
1.829153	1.89982	1.895989	1.892168	1.921469	1.917491	1.957888	1.953663	1.988452
1.829344	1.844629	1.841095	1.837565	1.935352	1.931299	1.927227	1.923142	1.97761
1.829542	1.844811	1.841302	1.837792	1.935547	1.931519	1.927467	1.923398	1.977924
1.829762	1.845015	1.841519	1.838023	1.935762	1.931725	1.927679	1.923629	1.978226
1.830003	1.845238	1.841746	1.838259	1.935992	1.931915	1.927867	1.92384	1.978522
1.830245	1.845462	1.841963	1.838474	1.936219	1.932123	1.928067	1.924039	1.978782
1.83049	1.845687	1.842165	1.83866	1.936447	1.932358	1.928283	1.92422	1.978983

1.83073	1.84591	1.842366	1.838843	1.898674	1.894819	1.928508	1.924412	1.950599
1.830956	1.846125	1.842567	1.839032	1.898845	1.895017	1.928737	1.924635	1.950861
1.831178	1.846342	1.842775	1.83923	1.899018	1.895216	1.928974	1.924876	1.951146
1.831409	1.846581	1.843003	1.839448	1.899225	1.895437	1.929227	1.925134	1.951436
1.831644	1.84683	1.843244	1.839683	1.899454	1.89567	1.92949	1.925406	1.951735
1.831861	1.828403	1.824916	1.821456	1.8843	1.880589	1.908985	1.905016	1.95204
1.832059	1.828606	1.82515	1.821713	1.884547	1.880817	1.909227	1.905266	1.952353
1.804145	1.828821	1.825386	1.821967	1.884781	1.881025	1.909443	1.905496	1.952657
1.80438	1.829058	1.825628	1.82221	1.884988	1.881195	1.909613	1.905691	1.952942
1.804628	1.829307	1.825876	1.822456	1.885201	1.881375	1.90979	1.905889	1.953227
1.804887	1.829569	1.826137	1.822714	1.885442	1.881599	1.893041	1.889196	1.934199
1.805155	1.829839	1.826404	1.822977	1.885697	1.881845	1.893276	1.889434	1.93449
1.785629	1.833524	1.829957	1.826396	1.848995	1.845292	1.893521	1.889682	1.93479
1.785895	1.833792	1.830219	1.826653	1.849254	1.845551	1.893777	1.889939	1.935097
1.786162	1.815675	1.812134	1.808604	1.823279	1.819657	1.894028	1.890194	1.935402
1.786431	1.815925	1.812374	1.80884	1.823519	1.819897	1.894275	1.890445	1.935703
1.786696	1.783297	1.779959	1.776645	1.823751	1.820132	1.879293	1.875584	1.920344
1.786952	1.78353	1.780179	1.77686	1.823972	1.820353	1.87952	1.875819	1.920641
1.787204	1.783757	1.780393	1.777069	1.824193	1.820579	1.879751	1.876056	1.920939
1.760418	1.78398	1.780605	1.77728	1.806138	1.802583	1.856219	1.85259	1.921238
1.760625	1.784201	1.780815	1.777491	1.806366	1.802834	1.856483	1.852846	1.921542
1.729806	1.784431	1.781032	1.777708	1.806605	1.803098	1.856769	1.853121	1.888163
1.730027	1.784667	1.781256	1.777931	1.806852	1.803373	1.857076	1.853414	1.888462
1.730258	1.726783	1.723449	1.720206	1.8071	1.803637	1.841094	1.837461	1.866082
1.730502	1.727001	1.723657	1.720408	1.807344	1.803882	1.841344	1.837718	1.866366
1.730751	1.727224	1.723868	1.720611	1.775366	1.772114	1.841594	1.837972	1.85061
1.730997	1.727444	1.724071	1.720804	1.775624	1.772379	1.841855	1.838228	1.850877
1.731247	1.727669	1.724274	1.720996	1.775882	1.772646	1.842118	1.838488	1.851148
1.731511	1.727899	1.72448	1.721186	1.717964	1.714813	1.842377	1.838767	1.835211
1.731785	1.728135	1.724689	1.721378	1.718153	1.715006	1.842636	1.839056	1.835513
1.73206	1.72838	1.724915	1.721591	1.71836	1.71521	1.793551	1.789999	1.835788
1.732337	1.728634	1.725156	1.721823	1.718581	1.715422	1.793754	1.790194	1.836034
1.732615	1.728892	1.725412	1.722078	1.718825	1.715654	1.747873	1.744488	1.836293
1.732888	1.72915	1.725684	1.722364	1.719102	1.715912	1.748142	1.744731	1.836572
1.733174	1.729424	1.72597	1.722659	1.719386	1.716178	1.748421	1.744991	1.836864
1.733491	1.729735	1.726269	1.722945	1.719659	1.71644	1.74869	1.745263	1.787617
1.733822	1.730061	1.726575	1.723232	1.719934	1.716708	1.74896	1.745546	1.7879
1.734152	1.730374	1.726873	1.72352	1.720218	1.716991	1.713789	1.710622	1.788192
1.734482	1.73068	1.727168	1.723809	1.72051	1.717285	1.714069	1.710876	1.788495
1.734795	1.730965	1.727457	1.724107	1.720801	1.717567	1.714346	1.711151	1.788801
1.735082	1.73122	1.727739	1.724415	1.721092	1.717831	1.714617	1.711451	1.789112
1.735394	1.731507	1.728035	1.724723	1.721389	1.718115	1.714904	1.711752	1.789426
1.735749	1.731852	1.728353	1.72502	1.721696	1.71844	1.715223	1.71205	1.789747
1.736131	1.73222	1.728686	1.725327	1.722011	1.718772	1.715546	1.712348	1.790065
1.736535	1.732584	1.729037	1.725668	1.722337	1.719083	1.715849	1.712647	1.790364
1.736958	1.732956	1.729399	1.726024	1.722669	1.719388	1.716146	1.712947	1.790658
1.737387	1.73335	1.729758	1.726356	1.722992	1.71971	1.71646	1.713252	1.790984
1.737825	1.733762	1.730116	1.726672	1.723308	1.720044	1.716788	1.713562	1.791334
1.738251	1.734155	1.730469	1.726994	1.723623	1.720362	1.717105	1.713878	1.79168
1.738663	1.734519	1.730816	1.727329	1.723935	1.720655	1.717406	1.714205	1.792017

1.715623	1.734887	1.731159	1.727655	1.724245	1.720955	1.71771	1.714523	1.792351
1.71603	1.735279	1.731498	1.727954	1.724551	1.721282	1.718029	1.714817	1.792681
1.716443	1.735677	1.731839	1.728252	1.724856	1.72161	1.718347	1.715106	1.793007
1.716857	1.736061	1.732191	1.728579	1.725162	1.721903	1.71864	1.715411	1.793324
1.717278	1.736442	1.732552	1.728923	1.725472	1.722182	1.718924	1.715725	1.793637
1.717734	1.713371	1.709517	1.705949	1.725801	1.722499	1.719238	1.716044	1.793973
1.718225	1.713798	1.709886	1.706273	1.72615	1.722851	1.719579	1.716368	1.794329
1.718702	1.714221	1.710252	1.706595	1.726495	1.723206	1.719929	1.716703	1.794698
1.719154	1.714634	1.710611	1.706912	1.726831	1.723561	1.720285	1.717053	1.795087
1.719603	1.715046	1.710971	1.707231	1.727168	1.723916	1.720644	1.71741	1.795471
1.720057	1.715449	1.711328	1.707555	1.704132	1.700957	1.721003	1.71777	1.795827
1.720522	1.715861	1.711695	1.70789	1.704457	1.701283	1.721361	1.718132	1.796176
1.721025	1.716317	1.7121	1.708252	1.704799	1.701617	1.721716	1.71849	1.796546
1.721556	1.716805	1.712532	1.708635	1.705154	1.701956	1.722067	1.718845	1.79693
1.722097	1.717308	1.712977	1.709024	1.70551	1.70229	1.722413	1.719198	1.797309
1.722645	1.717825	1.713433	1.70942	1.705865	1.702617	1.722753	1.719549	1.797685
1.69682	1.71837	1.713908	1.709825	1.706228	1.702952	1.69972	1.69659	1.765943
1.69742	1.718956	1.714406	1.710238	1.706602	1.703305	1.700047	1.696893	1.766276
1.69803	1.719553	1.714918	1.710666	1.706984	1.703658	1.700372	1.697195	1.7666
1.698625	1.72014	1.715442	1.711118	1.707373	1.703993	1.700682	1.697504	1.766914
1.699209	1.720721	1.715972	1.711585	1.707771	1.704324	1.70099	1.697819	1.767227
1.699762	1.72127	1.716486	1.712054	1.708181	1.704676	1.70132	1.698153	1.746889
1.700289	1.721794	1.716989	1.712527	1.708603	1.705044	1.701667	1.698502	1.747236
1.62208	1.722341	1.717507	1.713011	1.709037	1.705427	1.702027	1.698857	1.747591
1.622622	1.722913	1.718043	1.713505	1.709484	1.705829	1.702401	1.699218	1.747953
1.574918	1.697132	1.692388	1.687986	1.709934	1.706231	1.702777	1.699585	1.748321
1.57544	1.697725	1.692925	1.688461	1.710378	1.706623	1.703148	1.699959	1.748694
1.575964	1.698326	1.693471	1.688946	1.710829	1.707019	1.703523	1.700338	1.749072
1.576475	1.620213	1.615612	1.611325	1.711294	1.707428	1.703905	1.700715	1.749453
1.576979	1.620737	1.616109	1.611786	1.711773	1.707848	1.704295	1.701095	1.749838
1.577482	1.62127	1.61662	1.612265	1.638357	1.634624	1.704706	1.701492	1.750231
1.577985	1.621811	1.617144	1.612763	1.638836	1.635051	1.705137	1.701904	1.750633
1.578494	1.573971	1.569501	1.565301	1.609309	1.6057	1.705577	1.702323	1.751042
1.579012	1.574481	1.570009	1.565795	1.609791	1.606115	1.706025	1.702749	1.75146
1.579532	1.57499	1.570522	1.566296	1.610286	1.606544	1.706486	1.703179	1.751878
1.580043	1.575484	1.571022	1.566794	1.610788	1.606992	1.633322	1.630172	1.752285
1.580565	1.575984	1.571529	1.567299	1.611302	1.607453	1.633764	1.630561	1.752694
1.581128	1.57652	1.572067	1.567829	1.61183	1.607923	1.634219	1.630975	1.701144
1.581724	1.577083	1.572627	1.568378	1.612372	1.608404	1.634686	1.63141	1.701591
1.582342	1.577676	1.573209	1.568941	1.612934	1.608904	1.635174	1.631856	1.628684
1.582992	1.578305	1.57382	1.569525	1.613525	1.609429	1.635687	1.632318	1.629122
1.583611	1.578902	1.574398	1.570075	1.565875	1.561921	1.606301	1.603116	1.629538
1.584163	1.579432	1.574909	1.570563	1.56633	1.562337	1.606706	1.603464	1.629908
1.584714	1.579958	1.57541	1.571038	1.56678	1.562756	1.607114	1.603812	1.630278
1.585308	1.580523	1.575935	1.571526	1.567251	1.563212	1.607561	1.604197	1.630679
1.585926	1.581108	1.576474	1.572025	1.567739	1.563692	1.608035	1.604608	1.6311
1.586561	1.581715	1.577046	1.572564	1.56826	1.564197	1.608546	1.605063	1.631551
1.587215	1.582344	1.577647	1.573137	1.568811	1.564725	1.609093	1.605558	1.63203
1.587866	1.582961	1.578247	1.573718	1.569361	1.565238	1.561484	1.55807	1.6028
1.588504	1.583553	1.578839	1.574304	1.569903	1.565723	1.561925	1.558472	1.603212

1.589177	1.584197	1.579466	1.574909	1.57047	1.566241	1.562388	1.558873	1.603612
1.589926	1.584949	1.580159	1.575537	1.571076	1.566828	1.562901	1.559277	1.603987
1.590683	1.585727	1.580877	1.576189	1.571709	1.567442	1.563437	1.55969	1.604366
1.5551	1.586461	1.581601	1.576883	1.572368	1.568053	1.563973	1.560131	1.604781
1.555771	1.587168	1.582325	1.5776	1.573047	1.568671	1.564518	1.560589	1.605217
1.556397	1.587801	1.582987	1.57828	1.573715	1.569304	1.565079	1.56105	1.605649
1.556973	1.58836	1.583594	1.57893	1.574378	1.569952	1.565657	1.561512	1.606079
1.557598	1.552805	1.548101	1.5435	1.575001	1.570573	1.566231	1.562005	1.558276
1.558298	1.553513	1.548741	1.544076	1.575564	1.571157	1.566803	1.562542	1.558747
1.55901	1.554238	1.549405	1.544677	1.576136	1.571725	1.567358	1.56308	1.559233
1.55971	1.554959	1.550119	1.545361	1.576757	1.572263	1.567861	1.563579	1.559704
1.560403	1.555677	1.550852	1.546081	1.577407	1.572813	1.568372	1.564088	1.560189
1.561075	1.556369	1.551569	1.546807	1.542148	1.537597	1.568981	1.564664	1.560711
1.561732	1.557044	1.552279	1.547542	1.542876	1.538299	1.569658	1.565289	1.561266
1.562395	1.557716	1.552972	1.548254	1.543599	1.539022	1.53458	1.530271	1.561875
1.56306	1.558381	1.553643	1.54894	1.544317	1.539774	1.535329	1.53099	1.562541
1.563746	1.559072	1.554347	1.549657	1.545048	1.540513	1.536062	1.531703	1.563207
1.564463	1.559796	1.555103	1.550433	1.5458	1.541224	1.536757	1.532394	1.563861
1.565199	1.560546	1.555879	1.55122	1.546554	1.541931	1.537443	1.533073	1.564503
1.565954	1.561326	1.556658	1.551986	1.5473	1.54265	1.538137	1.533741	1.529497
1.566722	1.562123	1.557443	1.552747	1.548042	1.543369	1.538828	1.5344	1.530103
1.567491	1.562903	1.558217	1.553506	1.548771	1.544062	1.539496	1.535049	1.530723
1.568265	1.563672	1.558986	1.554263	1.549491	1.544736	1.540147	1.53569	1.531355
1.569036	1.564455	1.55978	1.555053	1.550239	1.545431	1.54082	1.53636	1.532018
1.569807	1.565255	1.560606	1.555882	1.551018	1.546143	1.541511	1.53706	1.53272
1.527344	1.566054	1.561423	1.556702	1.551813	1.5469	1.542243	1.537775	1.533422
1.528075	1.566852	1.562217	1.55749	1.552622	1.547728	1.543037	1.538498	1.534102
1.528796	1.567635	1.562998	1.55827	1.553439	1.54858	1.543856	1.539233	1.534787
1.529492	1.568378	1.563765	1.55906	1.554256	1.549414	1.544663	1.539981	1.535495
1.530174	1.569098	1.564522	1.559853	1.555071	1.55024	1.545469	1.540742	1.536222
1.530866	1.569823	1.565276	1.560637	1.555865	1.551034	1.546257	1.541517	1.536981
1.531565	1.57055	1.566027	1.561413	1.556638	1.551798	1.547029	1.542309	1.537772
1.532272	1.527959	1.52357	1.519082	1.557429	1.55258	1.547807	1.543082	1.538539
1.532993	1.528682	1.524325	1.519866	1.558249	1.55339	1.548588	1.54382	1.539262
1.485862	1.529388	1.52506	1.520631	1.559063	1.55421	1.549388	1.544581	1.540002
1.486494	1.530041	1.525742	1.521355	1.559856	1.555048	1.550232	1.545399	1.540791
1.487119	1.530682	1.526402	1.522054	1.560629	1.55588	1.551084	1.546245	1.541609
1.431444	1.531355	1.527067	1.522726	1.518198	1.513572	1.551901	1.547097	1.542454
1.432082	1.53205	1.527733	1.523382	1.518874	1.514288	1.552698	1.547959	1.543322
1.36389	1.532773	1.528439	1.524082	1.519584	1.51502	1.553499	1.548812	1.544186
1.364521	1.533524	1.529186	1.524826	1.520324	1.515763	1.5543	1.549657	1.545048
1.36517	1.481818	1.477559	1.47329	1.521076	1.516523	1.511984	1.507446	1.545908
1.365839	1.482557	1.478268	1.47398	1.52184	1.517311	1.512793	1.508275	1.546765
1.366521	1.483311	1.478988	1.474677	1.522608	1.518103	1.513604	1.509103	1.54762
1.30507	1.431967	1.427728	1.423466	1.523375	1.518885	1.514405	1.509926	1.548475
1.305709	1.432677	1.428419	1.424149	1.52414	1.519664	1.515201	1.510743	1.549327
1.306353	1.433388	1.429104	1.424818	1.472387	1.468015	1.515992	1.511553	1.550171
1.307002	1.434099	1.429784	1.42548	1.47307	1.468711	1.516778	1.512354	1.551008
1.307649	1.434814	1.430473	1.426151	1.473759	1.469406	1.465098	1.46083	1.508659
1.308294	1.435533	1.431172	1.426834	1.474452	1.470096	1.465791	1.461534	1.509446

1.30893	1.436253	1.43187	1.427517	1.475156	1.470795	1.466493	1.462245	1.510238
1.309553	1.436971	1.432566	1.428198	1.475875	1.471513	1.467215	1.462977	1.511042
1.310169	1.437692	1.433263	1.428879	1.476602	1.472237	1.467942	1.463712	1.51185
1.310782	1.438418	1.43396	1.429555	1.477329	1.472957	1.468661	1.464438	1.460133
1.311392	1.439147	1.434661	1.430234	1.478063	1.473679	1.469379	1.46516	1.460876
1.312006	1.308169	1.304316	1.300461	1.426581	1.422282	1.470096	1.46587	1.461604
1.312623	1.308773	1.304929	1.301084	1.427293	1.422968	1.47081	1.46657	1.46232
1.313268	1.309399	1.305566	1.301738	1.428044	1.423708	1.471566	1.467295	1.46304
1.313952	1.310053	1.30624	1.302438	1.428851	1.424522	1.472375	1.468051	1.463762
1.314643	1.310713	1.306909	1.303126	1.429652	1.425343	1.473201	1.46883	1.464504
1.315318	1.311357	1.307539	1.303762	1.430414	1.426157	1.474036	1.469639	1.465285
1.316006	1.312018	1.308172	1.304384	1.431156	1.426947	1.474864	1.470459	1.466085
1.316749	1.312742	1.308849	1.305021	1.431872	1.427663	1.475642	1.471266	1.466893
1.317525	1.313508	1.309556	1.305669	1.432574	1.428332	1.476387	1.472064	1.467708
1.318276	1.314271	1.310286	1.306355	1.433325	1.429011	1.477135	1.47284	1.468502
1.319009	1.315041	1.311046	1.30708	1.434122	1.429693	1.477879	1.473595	1.469278
1.319698	1.315759	1.311775	1.307803	1.434943	1.430432	1.478658	1.474343	1.470026
1.320318	1.316395	1.31246	1.308526	1.435804	1.431258	1.479483	1.475075	1.470723
1.320939	1.317015	1.313117	1.309222	1.43665	1.432103	1.480334	1.475835	1.471441
1.321605	1.317647	1.313736	1.309847	1.43743	1.432933	1.481215	1.476678	1.472244
1.322298	1.318293	1.314349	1.310449	1.438183	1.433748	1.482102	1.477551	1.473084
1.323026	1.318997	1.315025	1.311102	1.438936	1.434497	1.430119	1.425797	1.473914
1.323782	1.319745	1.315747	1.311789	1.439689	1.435201	1.430831	1.426558	1.474744
1.324511	1.320491	1.316494	1.312524	1.308578	1.304672	1.431595	1.427315	1.475565
1.325211	1.321238	1.317276	1.313321	1.309352	1.305401	1.432417	1.428063	1.47637
1.325905	1.321964	1.318028	1.314087	1.310109	1.306134	1.43325	1.42884	1.477201
1.326594	1.322644	1.318709	1.314775	1.310813	1.306857	1.434094	1.429677	1.478085
1.327292	1.323327	1.319385	1.315452	1.311506	1.30757	1.434924	1.430514	1.478973
1.328023	1.324067	1.320118	1.316172	1.312208	1.308252	1.435695	1.431279	1.479812
1.32877	1.32483	1.320874	1.31691	1.312917	1.308928	1.436447	1.432025	1.480639
1.329504	1.325562	1.321608	1.317648	1.313657	1.309668	1.437271	1.432842	1.481513
1.330229	1.326271	1.322329	1.318389	1.314426	1.31046	1.438151	1.433715	1.482423
1.330981	1.327009	1.323067	1.319134	1.315183	1.31123	1.439012	1.434592	1.483361
1.331773	1.327789	1.32383	1.319881	1.315921	1.311965	1.439845	1.435471	1.484336
1.25519	1.328554	1.324589	1.320636	1.316669	1.312709	1.308779	1.304876	1.485297
1.255891	1.329262	1.325328	1.321409	1.317448	1.313486	1.309556	1.305651	1.486217
1.256591	1.329961	1.326063	1.322182	1.318233	1.31427	1.310333	1.306414	1.487112
1.25733	1.330696	1.326809	1.322944	1.319003	1.315042	1.311086	1.307132	1.487976
1.258094	1.331451	1.327556	1.323692	1.31976	1.315806	1.311826	1.307826	1.488822
1.258867	1.254886	1.251113	1.24739	1.320468	1.31654	1.312566	1.30856	1.436002
1.259654	1.255635	1.251794	1.248022	1.321124	1.317242	1.313302	1.309328	1.436854
1.260443	1.256403	1.252507	1.248691	1.321816	1.317965	1.314051	1.310103	1.43771
1.261231	1.257199	1.253267	1.249419	1.322571	1.318729	1.314824	1.310883	1.438563
1.262028	1.258001	1.254039	1.250161	1.323331	1.319484	1.315586	1.311661	1.439426
1.262846	1.258797	1.254804	1.250892	1.24711	1.243399	1.316298	1.312423	1.308512
1.26367	1.25959	1.255567	1.251627	1.247812	1.244076	1.317006	1.313185	1.309313
0.995251	1.260362	1.256318	1.252364	1.248547	1.244818	1.31778	1.313974	1.310117
0.995716	1.261124	1.257064	1.253106	1.249309	1.245606	1.318598	1.314782	1.310923
0.996247	1.261948	1.257873	1.253905	1.250107	1.246407	1.319419	1.31559	1.311722
0.996847	1.262837	1.258751	1.254768	1.250949	1.247226	1.320249	1.316405	1.312516

0.997464	1.107646	1.103907	1.100275	1.251778	1.248023	1.244283	1.240573	1.313295
0.998096	1.108483	1.104678	1.100979	1.252578	1.248786	1.245023	1.241301	1.31405
0.998718	1.109311	1.105438	1.101668	1.253353	1.249516	1.245734	1.242015	1.314802
0.999294	0.996057	0.992788	0.989541	1.254078	1.250182	1.246397	1.24272	1.31557
0.999845	0.996555	0.993277	0.990025	1.254786	1.250826	1.247047	1.243425	1.31635
1.000386	0.99706	0.993773	0.990521	1.099971	1.096401	1.247768	1.244174	1.317156
1.000917	0.997567	0.994271	0.991028	1.100664	1.097053	1.248546	1.244959	1.317986
1.001472	0.998103	0.994809	0.991577	1.10139	1.097747	1.249359	1.245759	1.318821
1.002049	0.998668	0.995392	0.992176	1.102148	1.098487	1.250212	1.246576	1.31966
1.002657	0.999271	0.996002	0.992792	1.102933	1.099244	1.095783	1.092512	1.243765
1.003306	0.999923	0.996642	0.993412	1.103744	1.100003	1.096505	1.093206	1.24456
1.003979	1.000602	0.997299	0.994041	1.104573	1.100774	1.097231	1.093893	1.245347
0.828817	1.001279	0.997956	0.994675	0.991415	0.9882	1.097963	1.094562	1.246121
0.82976	1.001964	0.99862	0.995315	0.99204	0.988809	1.098701	1.095223	1.246887
0.703129	1.002654	0.999292	0.995965	0.992669	0.989416	1.099458	1.0959	1.092517
0.704046	1.003349	0.999972	0.996625	0.993302	0.99002	1.100231	1.096591	1.093161
0.704959	0.829539	0.826489	0.823429	0.993949	0.990635	1.10102	1.097302	1.093822
0.70587	0.830465	0.827433	0.824384	0.994616	0.991264	1.101825	1.098034	1.094503
0.706775	0.831387	0.828377	0.825343	0.995288	0.991905	1.102648	1.098787	1.095201
0.707674	0.832305	0.829321	0.826306	0.995955	0.992564	0.989207	0.985886	1.095909
0.708568	0.833218	0.83026	0.827268	0.996624	0.993232	0.989851	0.986489	1.096638
0.709453	0.834126	0.83119	0.828219	0.825149	0.822039	0.990497	0.987116	1.097415
0.710333	0.83503	0.832112	0.829161	0.826104	0.823001	0.991151	0.987768	1.09823
0.711224	0.835933	0.833024	0.830089	0.82705	0.823962	0.991829	0.988436	1.099067
0.712124	0.836835	0.833928	0.831003	0.827987	0.824925	0.992532	0.989121	1.099928
0.713034	0.710271	0.707539	0.704802	0.828919	0.825883	0.822799	0.819675	1.1008
0.713954	0.711164	0.708427	0.705697	0.829847	0.826839	0.823765	0.82064	1.10168
0.714884	0.712056	0.709313	0.706588	0.830771	0.82779	0.824729	0.821606	1.102572
0.715812	0.712927	0.710179	0.707466	0.831684	0.82873	0.825684	0.822567	0.988469
0.716762	0.713808	0.711049	0.708344	0.832595	0.829669	0.826639	0.823532	0.989171
0.717798	0.714774	0.711976	0.709252	0.833519	0.83062	0.827611	0.824522	0.821418
0.718906	0.715809	0.712949	0.710184	0.834454	0.831582	0.828596	0.825534	0.822444
0.719999	0.716864	0.713959	0.711159	0.83544	0.832582	0.829619	0.82659	0.823517
0.721074	0.717945	0.715019	0.712197	0.836501	0.833643	0.830701	0.827711	0.824661
0.722136	0.71901	0.716052	0.713198	0.837523	0.834657	0.831733	0.828781	0.825754
0.723181	0.720031	0.717013	0.714103	0.838444	0.835563	0.832649	0.829729	0.826722
0.724217	0.721037	0.71795	0.714977	0.839329	0.836429	0.833524	0.830636	0.827653
0.725243	0.722041	0.718892	0.715859	0.840217	0.837298	0.834409	0.831565	0.828614
0.726269	0.72305	0.719839	0.716747	0.841104	0.838163	0.835291	0.832496	0.829582
	0.724102	0.720842	0.717698	0.714799	0.712052	0.836185	0.833429	0.830556
	0.725194	0.721895	0.718703	0.715752	0.712959	0.837087	0.834364	0.831539
	0.726329	0.723011	0.719787	0.716785	0.713936	0.838043	0.835306	0.832492
	0.727531	0.724219	0.720982	0.717932	0.715016	0.83908	0.836258	0.8334
	0.728713	0.725416	0.722174	0.719076	0.716089	0.840114	0.837208	0.834304
	0.729804	0.726522	0.723272	0.720123	0.717068	0.841079	0.838134	0.835216
	0.730868	0.72761	0.724358	0.72116	0.718037	0.842044	0.83907	0.836144
	0.731975	0.728764	0.725533	0.722293	0.719102	0.843094	0.84007	0.837122
	0.733099	0.729949	0.726753	0.723479	0.720222	0.844197	0.841112	0.838133
				0.724584	0.721295	0.718233	0.715342	0.839123
				0.725605	0.722318	0.71924	0.716321	0.840089

0.726642	0.723364	0.720269	0.717314	0.841076
0.72771	0.724443	0.721325	0.718321	0.842096
0.728814	0.725562	0.722419	0.719358	0.843135
0.505293	0.502842	0.723589	0.720452	0.844179
0.50625	0.503833	0.724789	0.721577	0.845244
		0.725919	0.72269	0.719596
		0.727003	0.7238	0.720709
		0.503776	0.501302	0.721831
		0.504661	0.502166	0.722959
				0.724109
				0.725302
				0.726488
				0.503302
				0.504045

0.562728
0.561177
0.55962
0.55808

0.562728
0.561177
0.55962
0.55808

								0.55649
								0.554711
								0.552825
						0.560255	0.555605	0.999945
						0.558665	0.553987	0.998285
						0.557065	0.552375	0.996638
						0.555456	0.550758	0.994985
						0.55384	0.549146	0.993345
						0.729597	0.72475	1.202173
						0.727931	0.723118	1.200294
						0.940163	0.934673	1.289271
						0.938295	0.932841	1.287353
				0.73283	0.727896	1.205777	1.200205	1.285461
				0.731146	0.726271	1.203911	1.198359	1.283598
				0.943847	0.93829	1.292854	1.287229	1.57603
				0.941907	0.936417	1.290932	1.285323	1.573952
				0.939991	0.934554	1.289013	1.283415	1.57188
				0.93812	0.932716	1.287095	1.281484	1.569788
				0.936279	0.930896	1.285188	1.279567	1.567708
	0.951305	0.945583	0.939931	1.203725	1.198231	1.283323	1.277763	1.565723
	0.949346	0.943661	0.938078	1.201861	1.196396	1.281496	1.276057	1.563817
	0.947373	0.941736	0.936225	1.290641	1.285085	1.573329	1.567497	1.835399
	0.945367	0.939795	0.934365	1.288724	1.283168	1.571268	1.565552	1.833102
0.555945	1.048094	1.042661	1.03739	1.286824	1.281285	1.569239	1.56365	1.830854
0.554305	1.046245	1.040844	1.035598	1.284933	1.279438	1.567244	1.561809	1.828653
0.552685	1.044445	1.039068	1.033837	1.283085	1.277666	1.565308	1.560002	1.826474
0.551042	1.042653	1.037305	1.032108	1.443389	1.437852	1.688958	1.683116	1.824271
0.549408	1.040896	1.035578	1.030422	1.441555	1.43617	1.686989	1.681093	1.822058
0.93978	1.203756	1.19827	1.19291	1.439794	1.434485	1.684941	1.679016	1.819845
0.938096	1.202164	1.19673	1.191395	1.438117	1.43281	1.682825	1.676886	1.817629
1.041202	1.453169	1.447444	1.441798	1.692989	1.686785	1.828228	1.821768	1.815429
1.039607	1.451266	1.445498	1.439843	1.690745	1.684632	1.825972	1.819554	1.813235
1.037984	1.449346	1.44356	1.437919	1.688513	1.682486	1.82373	1.817373	1.811088
1.036256	1.447415	1.441708	1.436156	1.686376	1.680363	1.821499	1.81525	1.937508
1.034496	1.445481	1.439897	1.434469	1.684303	1.678286	1.819307	1.813165	1.935341
1.032774	1.443556	1.438098	1.432808	1.728848	1.722633	1.817236	1.811101	1.96506
1.031083	1.441647	1.436322	1.43118	1.726888	1.720733	1.815253	1.80906	1.962768
1.376471	1.639684	1.633653	1.627806	1.825898	1.819513	1.824956	1.818722	1.96047
1.374887	1.637732	1.631664	1.625774	1.823677	1.817359	1.822849	1.816641	1.958159
1.373289	1.686464	1.680141	1.673979	1.821476	1.815201	1.969477	1.962708	2.048285
1.371592	1.6844	1.6781	1.671944	1.819306	1.813036	1.967153	1.960438	2.045837
1.369911	1.682366	1.676099	1.669957	1.817187	1.810913	1.964858	1.958178	2.04338
1.579786	1.733291	1.726961	1.720721	1.827008	1.820715	1.962667	1.95593	2.040825
1.577928	1.731359	1.725052	1.718818	1.82506	1.818789	1.960521	1.953688	2.038232
1.630621	1.729325	1.723042	1.716823	1.887171	1.880769	1.998063	1.990835	2.110233
1.628571	1.727216	1.720955	1.714762	1.885086	1.878672	1.995766	1.988531	2.107534
1.678637	1.838249	1.831729	1.825309	1.883035	1.876607	1.993509	1.986278	2.104915
1.676755	1.836199	1.829727	1.823343	1.881032	1.874588	1.991305	1.984089	2.102391
1.674856	1.898362	1.891894	1.885469	2.003518	1.9963	2.041446	2.033635	2.118879
1.672849	1.896281	1.889831	1.883413	2.001257	1.994038	2.038963	2.031181	2.116343

1.670841	1.894209	1.887771	1.881354	1.998999	1.99178	2.036482	2.028742	2.113842
1.686183	1.892196	1.88576	1.879336	1.99679	1.989576	2.089728	2.081607	2.151976
1.684355	1.890211	1.883771	1.877336	1.994606	1.987399	2.087236	2.079239	2.149589
1.682423	1.888169	1.881735	1.875299	1.992384	1.985179	2.104631	2.096703	2.147121
1.680393	1.886072	1.879655	1.873226	1.990126	1.982917	2.101988	2.09418	2.144582
1.803343	1.993092	1.98602	1.978987	2.03789	2.030077	2.099478	2.091765	2.142143
1.801562	1.991003	1.983934	1.976906	2.035584	2.027792	2.097156	2.089505	2.139852
1.7998	1.988958	1.981889	1.97486	2.033327	2.025578	2.094909	2.087304	2.137603
1.797975	1.986955	1.979891	1.972864	2.093131	2.084941	2.135055	2.127201	2.153421
1.796108	1.984946	1.977887	1.970861	2.090734	2.082675	2.132804	2.124968	2.151181
1.946766	2.017655	2.010454	2.003287	2.103878	2.095949	2.130393	2.122585	2.148805
1.944603	2.015386	2.008177	2.001005	2.101234	2.093454	2.127891	2.120115	2.146347
1.942559	2.013204	2.005994	1.99883	2.113506	2.105736	2.159451	2.151713	2.16307
1.940643	2.011108	2.003904	1.996762	2.111161	2.103472	2.157189	2.149502	2.160895
2.000895	2.040274	2.03239	2.024635	2.108889	2.101254	2.154949	2.147294	2.158713
1.998912	2.038043	2.030219	2.022533	2.106709	2.099098	2.152729	2.14507	2.156504
1.996898	2.035766	2.028019	2.020418	2.104518	2.096922	2.150477	2.142806	2.154244
1.99476	2.11116	2.10291	2.094888	2.148575	2.140831	2.167118	2.159478	2.159326
1.99258	2.108443	2.100315	2.092428	2.146115	2.138401	2.164698	2.157052	2.156871
1.990491	2.105908	2.097879	2.0901	2.1438	2.136124	2.162433	2.154782	2.165298
1.988466	2.103511	2.095563	2.087869	2.141593	2.133961	2.160285	2.152627	2.163124
1.986519	2.101233	2.093349	2.085715	2.139444	2.131843	2.165655	2.157973	2.188493
1.984665	2.099093	2.091253	2.083649	2.137357	2.129776	2.163579	2.155905	2.186405
2.05608	2.158742	2.150723	2.142978	2.155115	2.147576	2.172205	2.164541	2.184319
2.054004	2.156593	2.148633	2.140915	2.153067	2.14552	2.170117	2.162451	2.182211
2.051932	2.15446	2.146564	2.138872	2.151034	2.143472	2.168027	2.160353	2.180095
2.049856	2.152321	2.144496	2.13684	2.157014	2.14941	2.193336	2.185645	2.200141
2.047782	2.150184	2.142434	2.13482	2.154993	2.147368	2.191231	2.183513	2.197986
2.094208	2.148049	2.140368	2.132796	2.177104	2.169465	2.189119	2.181351	2.203786
2.092049	2.145915	2.1383	2.130771	2.175067	2.167416	2.187002	2.179162	2.201551
2.089936	2.164395	2.156818	2.149315	2.173004	2.165324	2.184832	2.176906	2.199255
2.087878	2.162308	2.154752	2.147254	2.170907	2.163181	2.182591	2.174546	2.19686
2.147346	2.178636	2.171117	2.163631	2.168788	2.161	2.189248	2.181169	2.194512
2.145301	2.176606	2.169087	2.161568	2.166633	2.158743	2.186935	2.178906	2.192292
2.143272	2.174593	2.167065	2.159504	2.164482	2.156502	2.18466	2.17672	2.190165
2.161773	2.172577	2.16503	2.157439	2.18961	2.181609	2.203759	2.195792	2.197866
2.159726	2.170562	2.16299	2.155372	2.187563	2.179667	2.201837	2.193872	2.195925
2.157681	2.168545	2.160949	2.153302	2.19438	2.186572	2.199819	2.191911	2.193991
2.155638	2.166527	2.15891	2.151229	2.192258	2.184436	2.197706	2.189913	2.192069
2.171979	2.164486	2.156851	2.149147	2.190129	2.182295	2.205269	2.197577	2.190157
2.169923	2.162411	2.154767	2.147055	2.187995	2.180154	2.203138	2.195565	2.188233
2.167863	2.160322	2.152664	2.144951	2.185866	2.178043	2.20105	2.193604	2.186363
2.165805	2.206883	2.199209	2.19151	2.183755	2.176006	2.199068	2.191777	2.184632
2.163745	2.204762	2.197058	2.189349	2.181654	2.174011	2.197141	2.190007	2.182957
2.16168	2.202641	2.194907	2.187188	2.190117	2.182568	2.195199	2.188169	2.181203
2.15961	2.200521	2.192755	2.185024	2.188002	2.180566	2.193255	2.186289	2.179397
2.185783	2.198397	2.190598	2.182862	2.205809	2.198447	2.191308	2.184399	2.177563
2.183697	2.196254	2.18842	2.180688	2.203668	2.196417	2.189356	2.182499	2.175695
2.181675	2.204682	2.19681	2.189075	2.201574	2.19441	2.187411	2.180597	2.173818
2.179763	2.202662	2.194751	2.187007	2.199522	2.192411	2.185459	2.178691	2.171938

2.177892	2.200718	2.192794	2.185053	2.197568	2.190476	2.183545	2.1768	2.170055
2.19638	2.198895	2.191033	2.183334	2.195821	2.188688	2.18172	2.174937	2.168163
2.1945	2.197127	2.189375	2.181748	2.1942	2.187003	2.179969	2.173112	2.166281
2.202921	2.195184	2.187547	2.180035	2.192535	2.185377	2.178324	2.171386	2.164485
2.200793	2.193093	2.185585	2.178231	2.19085	2.183821	2.176785	2.169755	2.162766
2.198707	2.191021	2.183608	2.17638	2.189094	2.182171	2.175179	2.168134	2.161117
2.196666	2.188958	2.181597	2.174453	2.187227	2.180377	2.173471	2.16653	2.159577
2.194722	2.206787	2.199447	2.192335	2.185384	2.178577	2.171735	2.164879	2.157981
2.192969	2.205035	2.197696	2.190578	2.183628	2.176824	2.169973	2.163097	2.156194
2.191272	2.203361	2.196026	2.188895	2.181935	2.175117	2.168228	2.161289	2.154353
2.189437	2.201596	2.19434	2.187273	2.180347	2.173534	2.16661	2.159592	2.152596
2.18754	2.199799	2.192662	2.185702	2.178827	2.172024	2.165063	2.157956	2.150883
2.205451	2.198051	2.190975	2.184064	2.177199	2.170384	2.163417	2.156306	2.14923
2.203736	2.196347	2.189272	2.182353	2.175461	2.168612	2.161672	2.154648	2.147647
2.202008	2.194679	2.187628	2.180709	2.173777	2.16687	2.181449	2.174389	2.167354
2.200241	2.193069	2.186092	2.179194	2.172208	2.165198	2.179707	2.172619	2.16559
2.198465	2.191446	2.184547	2.177677	2.170652	2.163556	2.178001	2.170877	2.163846
2.196667	2.189726	2.182882	2.176054	2.169045	2.161948	2.176386	2.169266	2.162242
2.194891	2.187989	2.181187	2.174401	2.167423	2.160352	2.1748	2.167692	2.16068
2.193264	2.186371	2.179592	2.172817	2.165819	2.15871	2.17312	2.166001	2.159013
2.19175	2.184837	2.17806	2.171275	2.164223	2.157038	2.17138	2.164236	2.157281
2.190219	2.183279	2.176473	2.169655	2.16258	2.155378	2.169699	2.162562	2.17205
2.188673	2.181689	2.174813	2.167936	2.160879	2.153737	2.16809	2.161	2.170501
2.187111	2.180109	2.173176	2.166245	2.159191	2.152088	2.166467	2.159434	2.16896
2.185528	2.178564	2.171616	2.164648	2.157552	2.150407	2.164779	2.157806	2.167383
2.183932	2.177008	2.17005	2.163051	2.155921	2.14874	2.163106	2.156196	2.165827
2.182322	2.175377	2.16838	2.161354	2.154251	2.14713	2.158394	2.151597	2.144972
2.18071	2.173721	2.166678	2.159628	2.152579	2.145556	2.156886	2.150129	2.143534
2.179143	2.17216	2.165093	2.15802	2.150984	2.143995	2.15535	2.148621	2.142046
2.177615	2.170677	2.163604	2.15651	2.149457	2.142452	2.153798	2.147084	2.140519
2.176088	2.169166	2.1621	2.155005	2.166172	2.1591	2.15224	2.145574	2.139042
2.174562	2.167618	2.160582	2.153514	2.164632	2.157496	2.150656	2.144093	2.137634
2.17302	2.166034	2.159011	2.15197	2.163072	2.155926	2.149124	2.142643	2.151927
2.171437	2.164381	2.157325	2.150288	2.161458	2.154439	2.147707	2.141249	2.150527
2.169845	2.162721	2.155622	2.148585	2.159847	2.152989	2.146329	2.139863	2.149124
2.168272	2.1794	2.172272	2.165223	2.158319	2.151543	2.144908	2.138413	2.147674
2.166714	2.177866	2.170748	2.16371	2.15684	2.150104	2.14347	2.136932	2.146204
2.165208	2.176354	2.169234	2.162198	2.155344	2.148633	2.142018	2.13549	2.144801
2.163756	2.174867	2.167737	2.160696	2.153837	2.147127	2.140546	2.134082	2.143463
2.162318	2.173402	2.166233	2.159161	2.152314	2.145645	2.139113	2.132707	2.142148
2.160896	2.171967	2.164704	2.157545	2.150745	2.144193	2.137734	2.131364	2.140848
2.159465	2.170532	2.163202	2.155982	2.149231	2.142785	2.136393	2.13006	2.13958
2.176232	2.169086	2.161785	2.154598	2.147872	2.141449	2.135093	2.128804	2.138352
2.174737	2.167613	2.160393	2.153288	2.146586	2.140158	2.133826	2.127588	2.137163
2.173187	2.166043	2.158918	2.151932	2.145279	2.138874	2.132586	2.126408	2.136029
2.171595	2.164404	2.157393	2.150558	2.143974	2.137605	2.131371	2.125262	2.134942
2.166441	2.162857	2.155927	2.149198	2.142669	2.136336	2.130151	2.124101	2.13384
2.164981	2.16141	2.154523	2.14785	2.141362	2.135066	2.128923	2.122916	2.132712
2.163546	2.159998	2.153152	2.146528	2.140074	2.133808	2.127713	2.121771	2.131633
2.162125	2.158607	2.151813	2.145245	2.138818	2.132572	2.126539	2.120699	2.130639

2.160723	2.15724	2.150493	2.143977	2.137572	2.13134	2.125373	2.11965	2.129676
2.159337	2.155883	2.149175	2.142698	2.13631	2.130089	2.124188	2.118585	2.128701
2.15797	2.154547	2.147868	2.141423	2.135048	2.128839	2.123005	2.117523	2.127731
2.156628	2.153248	2.146591	2.140166	2.133811	2.127626	2.121857	2.116478	2.126764
2.15531	2.151985	2.145341	2.138925	2.132594	2.126445	2.120738	2.115448	2.125799
2.153998	2.147044	2.140458	2.134097	2.131412	2.125333	2.119696	2.114483	2.124884
2.15269	2.145786	2.139211	2.132868	2.130275	2.124307	2.118752	2.113597	2.124029
2.151389	2.144534	2.137969	2.131647	2.129151	2.1233	2.117831	2.112734	2.123197
2.150091	2.143281	2.136731	2.130433	2.128028	2.122287	2.116903	2.111874	2.122377
2.148802	2.142037	2.1355	2.129221	2.126909	2.121275	2.115978	2.11102	2.121566
2.147525	2.140809	2.13427	2.127997	2.125775	2.120257	2.115048	2.110157	2.120745
2.146265	2.139603	2.133052	2.126772	2.12464	2.119238	2.114117	2.109294	2.119925
2.145065	2.138474	2.131902	2.125595	2.123535	2.118236	2.113201	2.108454	2.119128
2.143928	2.137427	2.130819	2.124463	2.122458	2.11725	2.112298	2.107634	2.118352
2.142758	2.13633	2.129722	2.123359	2.117733	2.112628	2.126625	2.122033	2.117611
2.14153	2.135145	2.128586	2.122277	2.116706	2.111662	2.125741	2.121279	2.11693
2.140291	2.133936	2.127452	2.121229	2.115717	2.110723	2.124874	2.12052	2.116234
2.139067	2.132732	2.126338	2.120221	2.11477	2.109817	2.124013	2.119707	2.115461
2.137845	2.131521	2.125238	2.119251	2.113867	2.108949	2.123175	2.118885	2.114663
2.136607	2.130303	2.124165	2.118341	2.11303	2.10814	2.122391	2.118105	2.113901
2.135363	2.129084	2.123114	2.117475	2.112246	2.107382	2.121651	2.117353	2.113159
2.134182	2.127914	2.12206	2.116561	2.111432	2.106645	2.117637	2.113325	2.112418
2.13307	2.126794	2.120998	2.115589	2.110585	2.105939	2.116968	2.112608	2.111677
2.131967	2.125717	2.119974	2.114637	2.109733	2.105196	2.11626	2.111882	2.110942
2.130863	2.124705	2.11902	2.113728	2.108868	2.104372	2.115461	2.11112	2.110206
2.129749	2.12369	2.11808	2.112848	2.108023	2.103546	2.114657	2.110371	2.109493
2.128578	2.122572	2.117097	2.112008	2.107241	2.102779	2.113913	2.109684	2.108837
2.127407	2.121433	2.116112	2.111192	2.106491	2.102044	2.113202	2.109028	2.108209
2.126369	2.120431	2.115187	2.110355	2.105725	2.101341	2.112522	2.108358	2.107552
2.12544	2.119536	2.11431	2.109504	2.104952	2.100668	2.111873	2.107683	2.106876
2.124526	2.118689	2.113484	2.108691	2.104185	2.099959	2.111183	2.107008	2.102868
2.123626	2.1179	2.112718	2.107921	2.103417	2.099195	2.110437	2.106337	2.102235
2.122727	2.117108	2.111969	2.107187	2.118133	2.113864	2.109692	2.105648	2.101575
2.121816	2.116269	2.111216	2.106505	2.11745	2.113154	2.108967	2.104914	2.100841
2.120903	2.115431	2.110474	2.105842	2.116792	2.112476	2.108265	2.104179	2.100092
2.119991	2.114647	2.10975	2.105154	2.116126	2.111837	2.107619	2.103491	2.099386
2.119083	2.113888	2.109038	2.104459	2.115462	2.111224	2.107008	2.102832	2.098704
2.118195	2.113097	2.108314	2.103787	2.114804	2.110577	2.106368	2.10219	2.098061
2.117325	2.112275	2.107581	2.103137	2.114154	2.109902	2.105703	2.10157	2.097458
2.116482	2.111485	2.106854	2.102465	2.11349	2.10924	2.105051	2.100933	2.096826
2.115667	2.110746	2.106133	2.101752	2.112805	2.108606	2.104423	2.100265	2.096142
2.114876	2.110018	2.105422	2.101049	2.11212	2.107963	2.103784	2.099595	2.095458
2.114108	2.109273	2.104723	2.10039	2.11145	2.107276	2.103101	2.098936	2.094811
2.113362	2.108541	2.104038	2.099747	2.110788	2.106585	2.102413	2.098281	2.094171
2.112637	2.107862	2.103372	2.099085	2.11013	2.105951	2.101779	2.097624	2.101703
2.11193	2.10722	2.102724	2.098418	2.109479	2.105354	2.101181	2.09697	2.101008
2.111218	2.106547	2.10208	2.097796	2.108865	2.104762	2.100592	2.096363	2.100373
2.110496	2.105833	2.101437	2.097224	2.108293	2.104181	2.100017	2.095809	2.099803
2.10981	2.105169	2.100823	2.096654	2.107707	2.103573	2.107586	2.10338	2.12741
2.109191	2.104595	2.100259	2.096079	2.107087	2.102913	2.106906	2.10274	2.126725

2.108581	2.104041	2.0997	2.095491	2.106456	2.102253	2.106224	2.102085	2.126012
2.107934	2.103456	2.099101	2.09486	2.105818	2.101641	2.105588	2.101414	2.125243
2.107276	2.102861	2.09849	2.09422	2.105184	2.101051	2.104972	2.100742	2.12446
2.106634	2.102265	2.09791	2.093643	2.112708	2.108557	2.104361	2.100124	2.123773
2.106003	2.101665	2.097352	2.093116	2.112136	2.107953	2.103756	2.099547	2.123158
2.105365	2.101036	2.096768	2.092572	2.111539	2.107317	2.103122	2.098954	2.122535
2.10471	2.100361	2.096146	2.092005	2.110908	2.106632	2.102442	2.098337	2.121896
2.104084	2.09972	2.095537	2.091432	2.110279	2.10597	2.101789	2.097733	2.121265
2.103522	2.099159	2.094973	2.090865	2.109673	2.105374	2.101206	2.097163	2.120656
2.102974	2.098624	2.094416	2.090283	2.109059	2.104793	2.100642	2.096601	2.120049
2.102384	2.106598	2.102326	2.098123	2.1084	2.104178	2.100055	2.09603	2.119436
2.101781	2.106017	2.101702	2.097446	2.107723	2.103553	2.099461	2.095452	2.118812
2.092437	2.105444	2.10111	2.096829	2.107102	2.102958	2.098871	2.094846	2.11813
2.091886	2.104878	2.100546	2.096266	2.106529	2.102389	2.098282	2.094212	2.117391
2.10022	2.118909	2.114524	2.110189	2.10597	2.101833	2.097715	2.093618	2.116715
2.099659	2.11835	2.11397	2.109639	2.105425	2.101294	2.09718	2.093084	2.11613
2.0991	2.117794	2.113417	2.109089	2.104882	2.10076	2.112874	2.108393	2.115574
2.09854	2.11724	2.112859	2.108524	2.104328	2.100225	2.112315	2.107845	2.115023
2.097979	2.116684	2.112296	2.107955	2.103771	2.099689	2.111757	2.107302	2.114478
2.097412	2.116117	2.111727	2.107386	2.103212	2.099146	2.111188	2.106744	2.11392
2.096842	2.115542	2.111154	2.106816	2.102651	2.098599	2.110613	2.106178	2.113354
2.096274	2.107015	2.102626	2.09829	2.102094	2.098056	2.110043	2.105618	2.112791
2.09571	2.106446	2.102052	2.097711	2.10154	2.097516	2.109477	2.105063	2.112229
2.095151	2.105876	2.101479	2.097136	2.10099	2.096984	2.108918	2.104512	2.11167
2.0946	2.105302	2.100903	2.096562	2.100447	2.096463	2.10837	2.103964	2.111111
2.094049	2.104729	2.10033	2.095996	2.09991	2.095946	2.107824	2.103419	2.110555
2.076838	2.104155	2.099764	2.09544	2.099381	2.095427	2.107272	2.102874	2.110001
2.076283	2.103584	2.099206	2.094898	2.098862	2.094913	2.10672	2.102329	2.109447
2.075746	2.103029	2.098668	2.094379	2.098363	2.094412	2.106175	2.101782	2.108887
2.075224	2.102487	2.098149	2.093883	2.097884	2.093923	2.105635	2.101235	2.108324
2.057057	2.086078	2.081833	2.077662	2.089351	2.085377	2.105113	2.100715	2.107793
2.056604	2.085573	2.081377	2.077261	2.088946	2.084927	2.104612	2.100225	2.107293
2.056139	2.085064	2.080905	2.076832	2.105407	2.10095	2.115648	2.111183	2.113312
2.055632	2.084534	2.080387	2.076328	2.104868	2.100414	2.115146	2.110761	2.112913
2.055117	2.084003	2.079858	2.075801	2.104308	2.099875	2.114648	2.110339	2.112515
2.054613	2.076022	2.071964	2.067997	2.103765	2.099355	2.114139	2.10985	2.112029
2.054122	2.075535	2.071472	2.067505	2.103231	2.09885	2.113633	2.10933	2.1115
2.053678	2.058306	2.054301	2.050392	2.087722	2.083414	2.105989	2.101663	2.110992
2.053277	2.057915	2.053893	2.049965	2.087251	2.08299	2.105572	2.101193	2.110499
2.052876	2.057508	2.053477	2.049539	2.080178	2.076009	2.10511	2.100713	2.110011
2.052474	2.057073	2.053041	2.049104	2.079696	2.0755	2.104574	2.100207	2.109521
2.052058	2.047967	2.043996	2.040122	2.079234	2.074999	2.089669	2.085383	2.109057
2.051604	2.047529	2.043597	2.039758	2.078818	2.074539	2.089178	2.084952	2.108655
2.051149	2.0471	2.043211	2.039408	2.078419	2.074103	2.088712	2.084535	2.108262
2.050745	2.046722	2.042834	2.039028	2.091056	2.086633	2.082326	2.078136	2.1078
2.050373	2.046376	2.042467	2.038637	2.090623	2.086249	2.081931	2.077674	2.107299
2.05001	2.046016	2.042111	2.038283	2.075732	2.071414	2.081472	2.077201	2.085995
2.049657	2.045642	2.041769	2.037971	2.075327	2.070917	2.080947	2.076713	2.085522
2.049308	2.066	2.061637	2.057403	2.074886	2.07042	2.080442	2.076249	2.084891
2.048968	2.065656	2.061243	2.056943	2.074381	2.069927	2.079981	2.075828	2.084466

2.048619	2.065294	2.060829	2.056467	2.073875	2.069454	2.079544	2.075418	2.084044
2.048245	2.064847	2.060371	2.056006	2.073412	2.069028	2.079135	2.074994	2.083598
2.047858	2.064366	2.059899	2.055557	2.072972	2.068622	2.078742	2.074574	2.083149
2.047461	2.078647	2.074082	2.069644	2.065255	2.06094	2.06392	2.059725	2.082732
2.047057	2.07818	2.073661	2.069264	2.064861	2.060506	2.063488	2.059338	2.082338
2.046709	2.077787	2.073264	2.068858	2.064456	2.060105	2.063086	2.058938	2.081918
2.046428	2.077485	2.072891	2.068412	2.064031	2.059746	2.062722	2.058521	2.081462
2.046139	2.077183	2.072546	2.068014	2.063637	2.05938	2.062345	2.058113	2.067853
2.045811	2.076857	2.07225	2.067722	2.0633	2.058971	2.061919	2.057726	2.06745
2.045467	2.076505	2.071949	2.067448	2.062985	2.058578	2.061504	2.057346	2.067049
2.060682	2.076092	2.071578	2.067121	2.062676	2.05827	2.067524	2.063258	2.066596
2.060302	2.075649	2.07117	2.066763	2.062365	2.058001	2.067211	2.062857	2.06613
2.055961	2.075248	2.070764	2.066355	2.061993	2.057679	2.060045	2.0557	2.065742
2.055588	2.074881	2.070354	2.065898	2.061565	2.057318	2.05972	2.055403	2.06542
2.055222	2.074492	2.069955	2.065488	2.069516	2.065252	2.067272	2.062955	2.078307
2.05486	2.074056	2.069562	2.065144	2.069128	2.064808	2.066823	2.062556	2.07792
2.054524	2.073658	2.069197	2.064819	2.068763	2.064398	2.066392	2.062146	2.077512
2.054238	2.073353	2.068889	2.064506	2.068437	2.064078	2.066028	2.061725	2.07705
2.053977	2.073088	2.068598	2.064186	2.068116	2.063789	2.065696	2.061318	2.076591
2.053722	2.069259	2.064685	2.060194	2.074088	2.069754	2.065385	2.060983	2.076219
2.053474	2.068992	2.064341	2.059776	2.07368	2.069424	2.065092	2.060694	2.0759
2.053191	2.068708	2.06403	2.059432	2.073325	2.069098	2.064785	2.060402	2.069627
2.052874	2.068409	2.063752	2.05916	2.073022	2.068777	2.064469	2.060114	2.069308
2.041192	2.068102	2.063471	2.058894	2.072715	2.068432	2.064124	2.059807	2.068989
2.040926	2.067794	2.063185	2.058626	2.072386	2.068038	2.063728	2.059465	2.068671
2.040653	2.067463	2.062883	2.058354	2.072065	2.067659	2.063343	2.059121	2.068349
2.040335	2.06707	2.062537	2.058068	2.071769	2.067345	2.063021	2.058801	2.068022
2.040009	2.066666	2.062181	2.057778	2.071486	2.067062	2.062728	2.058482	2.067681
2.039729	2.05568	2.051224	2.046857	2.068085	2.063662	2.062427	2.058128	2.067291
2.03948	2.055414	2.050953	2.04658	2.067794	2.063383	2.062127	2.057751	2.066867
2.059762	2.055155	2.050694	2.046318	2.067513	2.063106	2.07547	2.070923	2.066505
2.059497	2.054905	2.05045	2.046076	2.067245	2.062831	2.075183	2.070634	2.066212
2.059236	2.054653	2.050205	2.045834	2.066977	2.062558	2.074904	2.070357	2.065933
2.058983	2.054395	2.049952	2.045589	2.066708	2.062289	2.07463	2.070079	2.065653
2.05873	2.054134	2.049697	2.045342	2.066438	2.062019	2.074355	2.069804	2.065377
2.058469	2.053874	2.049441	2.04509	2.056398	2.051984	2.071249	2.066696	2.0651
2.058202	2.053611	2.049183	2.044836	2.056117	2.0517	2.07095	2.066406	2.064823
2.057931	2.053339	2.04892	2.044582	2.055841	2.051424	2.070659	2.066118	2.064542
2.057655	2.05306	2.048652	2.044328	2.055569	2.051156	2.070373	2.065832	2.064259
2.057385	2.052787	2.048387	2.044074	2.055294	2.050884	2.060873	2.056341	2.063979
2.057122	2.052523	2.048127	2.043819	2.055016	2.050606	2.060578	2.056055	2.063702
2.0459	2.052257	2.047866	2.043565	2.054739	2.050331	2.060289	2.055778	2.060572
2.045627	2.051978	2.047595	2.043311	2.054468	2.050067	2.060013	2.055513	2.060308
2.045361	2.051705	2.047329	2.043059	2.054198	2.049805	2.059746	2.055268	2.060069
2.045116	2.051459	2.04708	2.042809	2.053921	2.049532	2.059493	2.055072	2.050701
2.04489	2.051237	2.046847	2.042564	2.053643	2.049255	2.05925	2.054905	2.050564
2.034467	2.058423	2.053951	2.049573	2.067955	2.063426	2.059004	2.05469	2.050363
2.034294	2.058303	2.053801	2.049374	2.067702	2.063177	2.058752	2.054431	2.050102
2.034095	2.058137	2.053617	2.049165	2.067467	2.062962	2.058534	2.054189	2.049845
2.033844	2.05789	2.053371	2.048931	2.067257	2.062805	2.058378	2.053986	2.049605

2.0336	2.057632	2.053119	2.048699	2.067043	2.062634	2.058208	2.053776	2.049368
2.033404	2.057398	2.052885	2.048477	2.066791	2.062388	2.057965	2.053529	2.049131
2.033226	2.057177	2.052667	2.048273	2.066535	2.062116	2.057693	2.053269	2.048894
2.033029	2.036654	2.032261	2.027988	2.066328	2.06187	2.057431	2.053013	2.048647
2.032826	2.036464	2.03211	2.027867	2.066152	2.061635	2.057172	2.05276	2.048397
2.03266	2.036314	2.031964	2.027713	2.065949	2.061405	2.056939	2.052548	2.048189
2.032538	2.036214	2.031827	2.027525	2.065713	2.061175	2.056731	2.052384	2.048034
2.032406	2.036083	2.031667	2.02733	2.0493	2.044837	2.049078	2.044778	2.047861
2.032248	2.035878	2.031456	2.027126	2.049115	2.044682	2.04893	2.044592	2.047636
2.032076	2.035663	2.031247	2.026938	2.04894	2.044526	2.048773	2.044395	2.047403
2.031887	2.035481	2.031091	2.026805	2.048772	2.044323	2.048558	2.04418	2.047185
2.031694	2.035316	2.030962	2.026695	2.048607	2.044099	2.048319	2.043961	2.04698
2.018812	2.035183	2.030829	2.026548	2.038137	2.033668	2.039459	2.035137	2.046812
2.018684	2.035078	2.030697	2.026377	2.037921	2.033454	2.039267	2.034985	2.046677
2.03648	2.051417	2.046816	2.042279	2.037758	2.033283	2.039104	2.034833	2.046526
2.036342	2.051271	2.046701	2.042194	2.037669	2.033171	2.038978	2.034676	2.046348
2.036211	2.051134	2.046582	2.042092	2.037563	2.03305	2.03885	2.034531	2.046185
2.036089	2.051022	2.046439	2.041913	2.037375	2.032868	2.038689	2.034404	2.046067
2.035973	2.050913	2.046295	2.04173	2.037186	2.032692	2.038537	2.034286	2.045955
2.035863	2.039222	2.034641	2.030123	2.037091	2.032597	2.038439	2.034163	2.03752
2.035757	2.039088	2.034536	2.030055	2.037042	2.032548	2.038374	2.034045	2.037355
2.03564	2.038963	2.034412	2.029937	2.036947	2.032482	2.038315	2.033962	2.037231
2.035512	2.038846	2.034271	2.029775	2.036812	2.032406	2.038269	2.033914	2.037148
2.035398	2.038725	2.034138	2.029632	2.03668	2.032297	2.038176	2.033823	2.037032
2.035299	2.038587	2.034015	2.029529	2.036561	2.032138	2.038005	2.03365	2.036851
2.035222	2.038471	2.033902	2.029424	2.036439	2.031983	2.037844	2.033493	2.036685
2.035187	2.038409	2.033797	2.02928	2.036301	2.031879	2.037764	2.033423	2.036591
2.035163	2.038371	2.033708	2.02914	2.036167	2.031788	2.037705	2.033379	2.036524
2.021351	2.038329	2.033657	2.029071	2.024594	2.020214	2.037596	2.033303	2.036452
2.021259	2.038285	2.033634	2.029048	2.02453	2.020087	2.037461	2.03321	2.036379
2.029653	2.038195	2.033568	2.029002	2.024473	2.020001	2.037367	2.033111	2.066435
2.029546	2.03805	2.033454	2.028937	2.024427	2.01996	2.037314	2.032997	2.066251
2.029472	2.037939	2.033348	2.02885	2.024364	2.019923	2.026364	2.022028	2.066109
2.029454	2.037895	2.033256	2.028721	2.024261	2.01988	2.026348	2.022006	2.066053
2.029455	2.037884	2.03319	2.028608	2.024167	2.019838	2.026337	2.022002	2.066018
2.029458	2.037895	2.033181	2.028576	2.02413	2.019806	2.026315	2.02199	2.06596
2.02946	2.037923	2.033205	2.028585	2.024121	2.019774	2.026286	2.021978	2.0659
2.029436	2.037938	2.033228	2.028599	2.024104	2.019716	2.026227	2.021951	2.056551
2.029391	2.037947	2.033255	2.028624	2.024087	2.019638	2.026145	2.021913	2.056516
2.02936	2.024722	2.020094	2.015522	2.024091	2.019603	2.01527	2.011099	2.056472
2.029353	2.024708	2.020099	2.015546	2.02412	2.019622	2.015275	2.011082	2.056425
2.029334	2.024672	2.020086	2.015563	2.024151	2.019651	2.015284	2.011052	2.056348
2.029277	2.024585	2.020036	2.015564	2.024173	2.01967	2.015272	2.010978	2.056206
2.029226	2.024501	2.019987	2.015563	2.024194	2.019693	2.015266	2.01091	2.056065
2.029228	2.024487	2.019981	2.015572	2.024215	2.019725	2.015289	2.010903	2.05601
2.029263	2.024513	2.02	2.015588	2.024238	2.019762	2.015328	2.010931	2.056003
2.029304	2.024549	2.020033	2.015621	2.024274	2.019804	2.015366	2.010959	2.055998
2.029354	2.024598	2.020082	2.015668	2.024323	2.019848	2.015405	2.01099	2.055999
2.0294	2.024644	2.020127	2.015712	2.024369	2.019894	2.015447	2.011026	2.056004
2.029437	2.024679	2.020162	2.015748	2.024411	2.019939	2.015492	2.011068	2.056012

2.029477	2.024717	2.020198	2.015783	2.011338	2.006911	2.015535	2.011108	2.046823
2.029527	2.024764	2.02024	2.01582	2.011372	2.006942	2.015569	2.011142	2.04682
2.029581	2.024816	2.020286	2.015859	2.011406	2.006971	2.015599	2.011173	2.046816
2.029634	2.024868	2.020336	2.015905	2.011446	2.007005	2.015629	2.0112	2.04681
2.02969	2.024923	2.020389	2.015955	2.01149	2.00704	2.01566	2.011226	2.046804
2.013799	2.024993	2.020451	2.016005	2.011532	2.007077	2.015697	2.011269	2.039958
2.013871	2.025075	2.020518	2.016053	2.011572	2.007115	2.015741	2.011326	2.039995
2.013973	2.025196	2.020616	2.016119	2.011623	2.00716	2.015797	2.011411	2.040073
2.014124	2.025389	2.02077	2.016217	2.011692	2.007216	2.015873	2.011544	2.040224
2.014275	2.025575	2.02092	2.016317	2.011768	2.00728	2.015955	2.011674	2.040368
2.014373	2.025679	2.02101	2.016391	2.011835	2.007346	2.002986	1.998761	2.040431
2.014457	2.02575	2.021077	2.016456	2.011905	2.007424	2.00306	1.998822	2.040462
2.014578	2.025842	2.021154	2.01653	2.011999	2.007548	2.003182	1.99891	2.040512
2.014726	2.025942	2.021234	2.016609	2.01211	2.007708	2.00334	1.999015	2.040571
2.014841	2.026032	2.021313	2.01669	2.012206	2.007827	2.003455	1.999102	2.040636
2.014915	2.026104	2.021386	2.016763	2.012276	2.007892	2.003519	1.999167	2.040709
2.001282	2.010274	2.005645	2.001107	2.012369	2.00796	2.003577	1.99923	2.02916
2.001371	2.010387	2.005789	2.001272	2.012518	2.008051	2.003641	1.99929	2.029191
2.001481	2.010521	2.005948	2.001445	2.012675	2.008152	2.003717	1.999367	2.029235
2.001632	2.010689	2.006095	2.001565	2.012785	2.008256	2.003828	1.999502	2.029353
2.001808	2.010873	2.006237	2.001661	2.012879	2.008368	2.003963	1.999668	2.029506
1.979221	1.992911	1.988483	1.984104	2.013	2.00851	2.004108	1.999804	2.029607
1.97937	1.993033	1.988592	1.984215	2.013144	2.00868	2.004267	1.999915	2.029666
1.979507	1.993158	1.98872	1.984354	2.013302	2.008842	2.004415	2.00003	2.029742
1.97962	1.993286	1.988874	1.984534	2.013475	2.008984	2.00454	2.000147	2.029837
1.979747	1.975225	1.970845	1.966536	1.997882	1.993435	2.004686	2.000288	2.029961
1.979911	1.975401	1.971006	1.966681	1.998058	1.993629	2.004899	2.000493	2.030142
1.980094	1.975591	1.971175	1.966828	1.998234	1.993826	2.005118	2.000708	2.030336
1.980285	1.975768	1.971351	1.967003	1.9984	1.993964	2.005258	2.000858	2.030477
1.980487	1.97594	1.971533	1.967194	1.998559	1.994067	2.005351	2.000971	2.03059
1.98069	1.976134	1.971717	1.967363	1.998707	1.994181	2.005466	2.001109	2.030729
1.980895	1.976349	1.971902	1.967508	1.998839	1.994302	2.005599	2.001268	2.030896
1.981101	1.976561	1.972097	1.967679	1.981997	1.977471	2.005758	2.001432	2.03105
1.981304	1.976756	1.972306	1.967901	1.982219	1.977662	2.005957	2.001599	2.031175
1.981512	1.976958	1.972519	1.968125	1.982443	1.977858	2.006159	2.00177	2.031304
1.981733	1.977187	1.972729	1.968309	1.982607	1.978011	2.006326	2.001946	2.031468
1.981963	1.977426	1.97294	1.968484	1.982761	1.978161	2.00649	2.002126	2.031642
1.982201	1.977641	1.97315	1.968695	1.96421	1.959743	1.988084	1.983765	2.031788
1.982448	1.977842	1.97336	1.96893	1.964458	1.959999	1.988316	1.983945	2.031918
1.982725	1.978094	1.973594	1.969152	1.964687	1.960242	1.988561	1.984158	2.032097
1.983042	1.978406	1.973851	1.969352	1.964888	1.960473	1.988824	1.984413	2.032334
1.983344	1.978717	1.974125	1.969582	1.965104	1.96069	1.974845	1.970469	2.030867
1.983605	1.979011	1.974429	1.969877	1.965352	1.960873	1.97502	1.97067	2.0311
1.983853	1.979287	1.974728	1.970184	1.965616	1.961067	1.975195	1.97086	2.03132
1.972986	1.979515	1.97498	1.970465	1.965894	1.961328	1.97542	1.971045	2.031486
1.973238	1.979725	1.975209	1.970729	1.966179	1.961623	1.975674	1.971234	2.031632
1.973525	1.968889	1.964361	1.959876	1.966426	1.961903	1.975949	1.971474	2.031848
1.973844	1.969205	1.964617	1.960074	1.966641	1.962174	1.976247	1.971759	2.032123
1.97414	1.969518	1.964898	1.960318	1.96689	1.96245	1.97654	1.972046	2.005192
1.9744	1.969826	1.965214	1.960628	1.96719	1.962738	1.976829	1.972333	2.005453

1.94618	1.970124	1.96553	1.96095	1.956428	1.951956	1.977102	1.972615	2.005723
1.946436	1.970408	1.965832	1.961263	1.956708	1.952184	1.977332	1.972879	2.006009
1.946693	1.970673	1.96612	1.961573	1.956988	1.952412	1.977562	1.973141	2.006299
1.946933	1.970896	1.966373	1.961868	1.957289	1.952703	1.948299	1.944066	2.006583
1.947167	1.971097	1.966607	1.962154	1.957603	1.953033	1.948622	1.944355	2.006861
1.947436	1.971358	1.966885	1.962459	1.957925	1.953366	1.948936	1.94462	1.998123
1.947739	1.971678	1.967204	1.962781	1.958256	1.953704	1.949244	1.944858	1.998318
1.948047	1.972011	1.967536	1.963112	1.958593	1.954048	1.949565	1.945126	1.998554
1.948354	1.972352	1.967878	1.963452	1.958938	1.954399	1.949906	1.945442	1.99886
1.948663	1.972697	1.968225	1.963795	1.959285	1.954751	1.950253	1.945776	1.999192
1.929727	1.944506	1.940174	1.935883	1.95963	1.9551	1.9506	1.946113	1.999529
1.930024	1.944826	1.940496	1.936205	1.959975	1.955449	1.950948	1.946457	1.999876
1.930327	1.945146	1.940815	1.936522	1.960318	1.955797	1.951296	1.946799	2.00022
1.930634	1.945466	1.941131	1.936835	1.96066	1.956144	1.951642	1.947139	2.000563
1.930936	1.945782	1.941444	1.937147	1.960996	1.956484	1.951981	1.947471	1.983852
1.931235	1.946092	1.941754	1.937456	1.961325	1.956817	1.952311	1.947795	1.984171
1.915914	1.927152	1.922889	1.91867	1.933411	1.929043	1.952634	1.948111	1.984482
1.916216	1.927457	1.92319	1.918965	1.933715	1.929342	1.952947	1.948414	1.98478
1.916518	1.927763	1.923491	1.91926	1.934018	1.929639	1.953259	1.948719	1.985083
1.916813	1.928059	1.923787	1.919557	1.934324	1.929943	1.95358	1.949043	1.944665
1.917111	1.928356	1.924085	1.919855	1.934633	1.930248	1.953909	1.949383	1.945008
1.884331	1.928685	1.924402	1.92016	1.915873	1.911577	1.926188	1.921838	1.945403
1.884631	1.92904	1.924736	1.920471	1.916169	1.911867	1.926513	1.92222	1.945846
1.862332	1.929423	1.925096	1.920801	1.916486	1.91218	1.926849	1.922585	1.946249
1.862642	1.929847	1.925493	1.921159	1.91683	1.91252	1.927195	1.92292	1.946595
1.847063	1.914651	1.910311	1.905985	1.917184	1.912883	1.908629	1.904414	1.91906
1.847341	1.915004	1.910652	1.906317	1.917545	1.913285	1.90903	1.90478	1.919408
1.847616	1.915342	1.910983	1.906648	1.917908	1.913689	1.909435	1.905146	1.919756
1.831719	1.894593	1.890277	1.885989	1.918258	1.914044	1.909782	1.905471	1.920086
1.832015	1.894941	1.890629	1.886347	1.918604	1.914373	1.910097	1.905775	1.920405
1.83229	1.881188	1.876914	1.87267	1.898939	1.894716	1.91042	1.906092	1.901902
1.832545	1.881516	1.877286	1.873081	1.899338	1.895061	1.910746	1.906417	1.902222
1.832805	1.881845	1.877642	1.873461	1.883285	1.879037	1.911093	1.906774	1.902575
1.833072	1.882173	1.877964	1.873784	1.883618	1.879389	1.911467	1.907178	1.902981
1.833353	1.882521	1.878298	1.874111	1.88396	1.879756	1.911857	1.907587	1.903387
1.784144	1.88291	1.878675	1.874482	1.870295	1.866124	1.892293	1.888041	1.903751
1.784423	1.883322	1.879077	1.874876	1.870695	1.866535	1.892702	1.888414	1.904102
1.784711	1.822341	1.818252	1.814211	1.871098	1.866918	1.893074	1.888774	1.904457
1.785008	1.822697	1.818627	1.814606	1.871507	1.867283	1.893424	1.889125	1.904816
1.785313	1.823071	1.818987	1.814953	1.827868	1.823643	1.880531	1.876264	1.885302
1.785631	1.823472	1.819333	1.815244	1.828167	1.823951	1.880895	1.876664	1.885718
1.785937	1.823841	1.81966	1.815537	1.828474	1.824275	1.864167	1.860018	1.886133
1.78622	1.824144	1.819954	1.815845	1.828805	1.824625	1.864571	1.86042	1.886535
1.786497	1.824436	1.82025	1.816167	1.829149	1.824985	1.864985	1.860828	1.886941
1.786772	1.824755	1.820577	1.816513	1.829505	1.825339	1.821198	1.817082	1.87413
1.787054	1.825097	1.820928	1.816879	1.829873	1.825696	1.821559	1.81746	1.874567
1.787376	1.825502	1.821313	1.81725	1.830253	1.826073	1.821932	1.81783	1.832824
1.787732	1.825961	1.821732	1.81763	1.830644	1.826465	1.822316	1.818197	1.833179
1.78809	1.82642	1.822172	1.818051	1.831057	1.826858	1.822695	1.818568	1.83355
1.78845	1.82688	1.822644	1.818524	1.831497	1.827245	1.823062	1.818945	1.833943

1.7888	1.827318	1.823102	1.818996	1.83195	1.827654	1.823449	1.81933	1.834334
1.789125	1.827706	1.823516	1.819446	1.83242	1.82811	1.82388	1.819722	1.834702
1.789442	1.828083	1.823913	1.819877	1.832886	1.828579	1.82433	1.820129	1.83508
1.789763	1.786243	1.782786	1.779347	1.816134	1.811983	1.824781	1.820571	1.816425
1.790085	1.786571	1.783104	1.779657	1.816518	1.8124	1.825234	1.821034	1.816879
1.790419	1.786901	1.783428	1.779976	1.816905	1.812789	1.825652	1.82147	1.817322
1.790762	1.787233	1.783757	1.780301	1.817293	1.813149	1.826034	1.821883	1.817759
1.791127	1.787588	1.7841	1.780632	1.817688	1.813538	1.826436	1.822288	1.818176
1.791522	1.78798	1.784463	1.780965	1.818085	1.813975	1.82687	1.82268	1.818555
1.791916	1.788374	1.784836	1.781315	1.818504	1.814428	1.82732	1.82309	1.818948
1.792284	1.788753	1.785218	1.781697	1.81897	1.814889	1.827791	1.82355	1.8194
1.792641	1.789122	1.785602	1.782094	1.819454	1.815351	1.828267	1.824029	1.819875
1.792996	1.789472	1.78597	1.78248	1.819919	1.81578	1.828708	1.82449	1.820338
1.793349	1.789808	1.786327	1.782863	1.820376	1.81619	1.829129	1.824943	1.820798
1.793711	1.790161	1.786694	1.783246	1.820843	1.816633	1.812485	1.808402	1.82124
1.794084	1.790532	1.787073	1.783632	1.821326	1.817112	1.812947	1.808831	1.821665
1.762544	1.790904	1.787445	1.784004	1.821793	1.817589	1.813405	1.809243	1.822068
1.762896	1.791278	1.78781	1.784359	1.822239	1.818057	1.813849	1.80962	1.822431
1.763239	1.791642	1.78816	1.784693	1.822663	1.818513	1.814291	1.810003	1.822804
1.763556	1.791974	1.788474	1.784992	1.781519	1.77805	1.814734	1.810431	1.823235
1.763864	1.792294	1.788775	1.785276	1.781817	1.778378	1.815179	1.810884	1.823699
1.743588	1.792634	1.789105	1.785599	1.782147	1.778722	1.815634	1.811348	1.824181
1.743917	1.792988	1.789456	1.78595	1.782499	1.779079	1.816096	1.811823	1.824682
1.74426	1.793359	1.789821	1.786313	1.782862	1.779445	1.816563	1.8123	1.808146
1.744618	1.793751	1.790203	1.786687	1.783235	1.779821	1.817038	1.812777	1.808623
1.744979	1.741676	1.738396	1.735154	1.783614	1.7802	1.817514	1.813255	1.809101
1.745336	1.742022	1.738741	1.735498	1.783997	1.780578	1.817988	1.813733	1.80958
1.745698	1.742371	1.739088	1.735845	1.784383	1.780957	1.818463	1.814212	1.810059
1.74607	1.742734	1.73944	1.736187	1.765666	1.762307	1.818943	1.814692	1.810536
1.746451	1.743107	1.739796	1.736528	1.766019	1.762661	1.819426	1.815174	1.811014
1.746836	1.743484	1.740161	1.73688	1.766377	1.763015	1.77864	1.775147	1.8115
1.747228	1.743866	1.740532	1.737241	1.76674	1.763368	1.779019	1.775536	1.811996
1.747628	1.744257	1.740911	1.737608	1.734365	1.73117	1.760335	1.756937	1.812489
1.748039	1.74466	1.741297	1.737977	1.734726	1.731525	1.760696	1.757293	1.81297
1.748453	1.745068	1.74169	1.738353	1.735091	1.731883	1.761063	1.757659	1.813471
1.748862	1.745479	1.742088	1.738735	1.735457	1.732235	1.761433	1.758047	1.814014
1.749275	1.745894	1.742493	1.739126	1.735831	1.732593	1.761812	1.758451	1.814584
1.697919	1.746311	1.742904	1.739532	1.736226	1.732974	1.76221	1.758873	1.797717
1.698345	1.746733	1.743325	1.739953	1.736638	1.733373	1.762623	1.759309	1.798318
1.625675	1.747183	1.743771	1.740394	1.737065	1.733783	1.763039	1.759732	1.756399
1.626124	1.747664	1.744244	1.740858	1.73751	1.734204	1.76346	1.760141	1.756801
1.62656	1.748146	1.744718	1.741321	1.737956	1.734631	1.731395	1.728249	1.757194
1.626964	1.748625	1.745188	1.741774	1.738397	1.73506	1.731803	1.728629	1.757575
1.627361	1.749101	1.745655	1.742226	1.738841	1.735497	1.732222	1.729016	1.757962
1.627763	1.683912	1.680683	1.677467	1.739297	1.735957	1.73267	1.729435	1.758376
1.628167	1.684341	1.68111	1.677892	1.739754	1.736422	1.733126	1.729865	1.758801
1.628584	1.684754	1.681519	1.6783	1.675134	1.672009	1.668927	1.665886	1.759217
1.62901	1.685154	1.681915	1.678697	1.675511	1.672358	1.669265	1.666229	1.759626
1.599964	1.685535	1.6823	1.679096	1.6759	1.672729	1.669625	1.666586	1.760047
1.600315	1.685896	1.682678	1.679502	1.676309	1.67313	1.670015	1.666961	1.760483

1.600657	1.686245	1.683038	1.679887	1.676705	1.67353	1.670408	1.667335	1.760912
1.600986	1.686574	1.683365	1.68023	1.677072	1.673926	1.670805	1.667706	1.761324
1.601318	1.686908	1.683692	1.680565	1.677427	1.674309	1.67119	1.66807	1.761727
1.601679	1.687285	1.684056	1.680926	1.67778	1.674654	1.67153	1.668405	1.665339
1.602054	1.687685	1.684441	1.681301	1.678134	1.674983	1.67185	1.668729	1.665666
1.602421	1.688072	1.684824	1.681691	1.678516	1.675352	1.67221	1.669086	1.666017
1.602779	1.68845	1.685209	1.682099	1.678925	1.675755	1.672605	1.669471	1.66639
1.555084	1.600098	1.597355	1.594767	1.679326	1.676161	1.67301	1.669871	1.66678
1.555446	1.60044	1.597676	1.595091	1.679713	1.676568	1.673428	1.670289	1.667192
1.555834	1.600809	1.598013	1.595419	1.6801	1.676973	1.673842	1.670707	1.667607
1.556244	1.601223	1.598385	1.595762	1.680488	1.677364	1.674236	1.671108	1.66801
1.556676	1.601661	1.598773	1.596112	1.680879	1.677753	1.674627	1.671506	1.668412
1.557118	1.602092	1.599156	1.596465	1.681278	1.678159	1.675035	1.671912	1.668814
1.557579	1.602529	1.599543	1.596821	1.681682	1.678575	1.675452	1.672322	1.669219
1.558093	1.554717	1.551886	1.549352	1.656298	1.653401	1.675858	1.672739	1.66964
1.558665	1.55519	1.552259	1.549641	1.656647	1.653735	1.67625	1.673163	1.670079
1.559237	1.555663	1.552636	1.549941	1.657005	1.654087	1.676661	1.673594	1.670521
1.559793	1.556122	1.553012	1.550253	1.657375	1.654473	1.677102	1.674035	1.670966
1.560342	1.556581	1.553395	1.550577	1.657761	1.654873	1.677551	1.674478	1.671408
1.525349	1.557034	1.553783	1.550915	1.658179	1.655283	1.652385	1.649513	1.671846
1.525875	1.557501	1.554187	1.551266	1.658612	1.655697	1.652782	1.649899	1.672278
1.526458	1.52259	1.519299	1.516405	1.659032	1.656088	1.653157	1.65027	1.672691
1.527087	1.523182	1.519778	1.516766	1.659446	1.656465	1.653515	1.65063	1.673088
1.527747	1.523809	1.520307	1.517186	1.616753	1.613969	1.653897	1.650993	1.648104
1.528447	1.524478	1.520893	1.517675	1.617194	1.614364	1.654303	1.651354	1.648446
1.529146	1.525151	1.52149	1.518182	1.586812	1.584017	1.654726	1.65174	1.648816
1.529822	1.525807	1.522082	1.518697	1.587286	1.584424	1.655172	1.652176	1.649247
1.530501	1.526464	1.522677	1.519215	1.587764	1.584824	1.655616	1.652616	1.649688
1.531198	1.52713	1.523266	1.519713	1.588215	1.585193	1.627648	1.624722	1.650091
1.53191	1.527806	1.523857	1.520207	1.588655	1.585547	1.628023	1.625103	1.650476
1.532654	1.528509	1.524475	1.520726	1.5891	1.585916	1.582966	1.580279	1.650857
1.533427	1.529237	1.525117	1.521265	1.589545	1.586294	1.583304	1.580605	1.651232
1.534191	1.529973	1.525783	1.521842	1.590035	1.586704	1.583667	1.580952	1.623404
1.534936	1.530719	1.52648	1.522463	1.590576	1.58715	1.584058	1.581327	1.623838
1.535684	1.53146	1.527186	1.523113	1.591151	1.587629	1.584475	1.58171	1.624269
1.536437	1.53218	1.527889	1.523788	1.591757	1.588148	1.584919	1.582086	1.624662
1.5372	1.532896	1.528596	1.524481	1.592388	1.588696	1.585387	1.582473	1.625055
1.537983	1.533623	1.529306	1.52517	1.593027	1.589258	1.585875	1.582886	1.625481
1.538785	1.534362	1.530022	1.525864	1.593679	1.589837	1.586383	1.583321	1.62593
1.53961	1.535142	1.530775	1.526584	1.594352	1.590437	1.586909	1.583771	1.580827
1.54046	1.535967	1.531566	1.527329	1.595047	1.591059	1.587457	1.584239	1.581238
1.541306	1.53679	1.532363	1.528088	1.595747	1.591678	1.587999	1.584703	1.581649
1.54214	1.537598	1.53316	1.528858	1.596442	1.592275	1.588516	1.585154	1.582053
1.542974	1.538409	1.533963	1.52964	1.597153	1.592893	1.589052	1.585613	1.582461
1.543814	1.539231	1.534775	1.530432	1.597894	1.593556	1.589628	1.586087	1.582864
1.544656	1.540059	1.535592	1.531231	1.598658	1.594259	1.590245	1.58659	1.583284
1.545495	1.540888	1.536408	1.532029	1.599445	1.595019	1.590937	1.587173	1.583779
1.546332	1.541719	1.537224	1.532829	1.600256	1.595829	1.591693	1.58782	1.584332
1.504065	1.542547	1.538039	1.533628	1.601068	1.59664	1.592451	1.588476	1.584897
1.50486	1.54337	1.538852	1.534428	1.601884	1.597452	1.593214	1.589144	1.585474

1.505659	1.544197	1.539666	1.535225	1.602694	1.598259	1.593975	1.589818	1.58606
1.506465	1.545022	1.540473	1.536011	1.603486	1.599053	1.594726	1.590485	1.586643
1.507283	1.545866	1.541296	1.536809	1.604284	1.599846	1.595481	1.591169	1.587248
1.455727	1.503603	1.499097	1.494667	1.605106	1.600653	1.596254	1.591892	1.587906
1.456516	1.504489	1.499961	1.495492	1.605946	1.601468	1.597039	1.592643	1.588599
1.457295	1.452947	1.448505	1.444093	1.606797	1.60229	1.597829	1.593401	1.589304
1.458069	1.453762	1.449321	1.44489	1.607661	1.603118	1.598625	1.594171	1.590025
1.458815	1.454535	1.450115	1.445693	1.57073	1.566245	1.599426	1.59495	1.590764
1.459522	1.455249	1.450879	1.4465	1.571568	1.567036	1.600221	1.595734	1.59152
1.460236	1.45596	1.451637	1.447308	1.526379	1.521871	1.601055	1.596549	1.592299
1.460981	1.456692	1.452407	1.448124	1.527251	1.522713	1.601964	1.597414	1.5931
1.461743	1.457433	1.453172	1.448926	1.528133	1.523583	1.602907	1.598313	1.59393
1.462517	1.458179	1.45391	1.44967	1.528956	1.524438	1.560635	1.556097	1.594808
1.463304	1.458936	1.454637	1.450381	1.529745	1.525285	1.561536	1.55701	1.595721
1.464103	1.459732	1.455416	1.451141	1.530532	1.526066	1.521631	1.517224	1.553429
1.464918	1.460571	1.456247	1.451945	1.53131	1.52678	1.522341	1.517992	1.554253
1.465703	1.461386	1.45707	1.45276	1.44844	1.444134	1.523069	1.518736	1.555039
1.466436	1.462162	1.45788	1.453593	1.449273	1.444946	1.523833	1.519441	1.555757
1.467168	1.462919	1.458665	1.454404	1.450097	1.44577	1.524625	1.520165	1.556489
1.467927	1.463649	1.459391	1.455142	1.450861	1.446569	1.525472	1.520962	1.516548
1.468706	1.46438	1.460106	1.45586	1.451605	1.447354	1.526334	1.521789	1.517344
1.469522	1.465182	1.460895	1.456637	1.45237	1.448108	1.527139	1.522597	1.518154
1.470369	1.466034	1.461735	1.457452	1.453146	1.448839	1.527908	1.523397	1.518979
1.471199	1.466871	1.46257	1.45828	1.453957	1.449627	1.528713	1.524198	1.519773
1.472009	1.467691	1.463402	1.459124	1.454809	1.450481	1.529558	1.524994	1.520527
1.472831	1.468513	1.464227	1.459956	1.45565	1.45133	1.447002	1.442658	1.521305
1.473687	1.469342	1.465039	1.460756	1.456455	1.452146	1.447816	1.443457	1.52213
1.474543	1.470169	1.465848	1.461557	1.45726	1.452963	1.448636	1.444273	1.522978
1.475364	1.47098	1.466669	1.462392	1.4581	1.453806	1.449479	1.445115	1.523834
1.476175	1.471785	1.467492	1.463237	1.458951	1.454656	1.450332	1.445973	1.524705
1.47701	1.472593	1.468299	1.464053	1.459774	1.455486	1.451172	1.446826	1.5256
1.477865	1.473403	1.469094	1.464846	1.460574	1.4563	1.452002	1.447678	1.52652
1.478762	1.474259	1.469923	1.465656	1.461376	1.457104	1.452814	1.448506	1.52741
1.479714	1.475177	1.470799	1.466491	1.462184	1.457896	1.453602	1.449302	1.528259
1.480664	1.476102	1.471678	1.467322	1.462988	1.458685	1.454388	1.450096	1.529109
1.481586	1.477008	1.472535	1.468127	1.463774	1.459471	1.455179	1.450899	1.529984
1.482496	1.477915	1.473396	1.468936	1.464565	1.460263	1.455976	1.451709	1.530862
1.483398	1.478836	1.474284	1.469779	1.465388	1.461076	1.456788	1.452529	1.531722
1.484295	1.479765	1.475188	1.470646	1.466232	1.461903	1.45761	1.453358	1.532576
1.431693	1.480684	1.476096	1.471532	1.467087	1.462725	1.458422	1.454183	1.533458
1.432543	1.481596	1.477008	1.472437	1.467954	1.463539	1.459222	1.455005	1.534367
1.433381	1.482493	1.477915	1.473351	1.468847	1.464398	1.460062	1.455838	1.53527
1.434189	1.483358	1.478801	1.474265	1.46977	1.46532	1.46096	1.456687	1.536169
1.435016	1.484248	1.479714	1.475203	1.470718	1.466264	1.461871	1.457534	1.537042
1.304549	1.485218	1.480701	1.476196	1.471699	1.467216	1.462772	1.458358	1.537841
1.305373	1.486218	1.481713	1.477206	1.472689	1.46817	1.463672	1.459185	1.538634
1.306192	1.487196	1.482695	1.478184	1.473643	1.46909	1.464565	1.460056	1.539527
1.307009	1.488162	1.483657	1.479137	1.474573	1.469989	1.465456	1.460963	1.54049
1.307803	1.489085	1.484563	1.48003	1.475465	1.470888	1.466372	1.461903	1.5415
1.308571	1.489957	1.4854	1.480848	1.476308	1.471785	1.46731	1.462877	1.54256

1.30934	1.436768	1.432334	1.427935	1.47719	1.472709	1.468271	1.463874	1.54364
1.310115	1.437649	1.433209	1.428808	1.478143	1.473671	1.469255	1.464894	1.544737
1.310902	1.438562	1.434127	1.42973	1.479137	1.474663	1.470257	1.46592	1.54583
1.311709	1.439514	1.435087	1.430691	1.480166	1.475687	1.471278	1.466938	1.546899
1.312532	1.440497	1.43608	1.431686	1.481223	1.476731	1.472306	1.467947	1.547946
1.313372	1.30955	1.305652	1.301739	1.482272	1.477757	1.473309	1.468924	1.46456
1.314228	1.310428	1.306546	1.302637	1.483318	1.478771	1.474292	1.469873	1.465483
1.315083	1.311302	1.307433	1.303528	1.484364	1.479793	1.475277	1.470811	1.466389
1.315937	1.312171	1.308313	1.304412	1.485409	1.480821	1.476264	1.47173	1.467269
1.240207	1.313033	1.309189	1.305297	1.432235	1.427753	1.477266	1.472672	1.468175
1.241009	1.313884	1.310063	1.306191	1.433247	1.428749	1.478301	1.473671	1.469147
1.241802	1.314725	1.310931	1.307085	1.434266	1.429749	1.47934	1.474685	1.470138
1.242582	1.315558	1.311792	1.307971	1.435275	1.430721	1.480349	1.475675	1.471112
1.243352	1.316381	1.312644	1.308851	1.436281	1.431685	1.481345	1.476654	1.472076
1.089349	1.317191	1.313483	1.30972	1.437293	1.432673	1.482353	1.477611	1.472995
1.089984	1.317988	1.314309	1.31058	1.438309	1.43368	1.483368	1.478539	1.473867
1.090625	1.242115	1.238617	1.235075	1.37057	1.366308	1.430041	1.425241	1.474829
1.091278	1.242873	1.239395	1.235894	1.371531	1.367335	1.431171	1.426332	1.475914
1.091935	1.243629	1.240162	1.236699	1.372472	1.368345	1.432306	1.427466	1.477057
1.092589	1.089382	1.086277	1.083158	1.373371	1.369288	1.433382	1.428609	1.478259
1.09325	1.090003	1.086907	1.083822	1.374244	1.370202	1.434433	1.429747	1.479474
1.093933	1.09062	1.087516	1.084454	1.3751	1.371114	1.435465	1.430809	1.480597
1.094635	1.091235	1.088108	1.08506	1.375936	1.372016	1.436477	1.431822	1.481664
1.095369	1.091886	1.088723	1.08567	1.168636	1.165164	1.437484	1.432854	1.482755
1.096139	1.092576	1.089361	1.086283	1.169346	1.165945	1.438486	1.433906	1.483871
1.096918	1.093271	1.09	1.086894	1.170043	1.166703	1.370653	1.366474	1.430328
1.097691	1.093951	1.090629	1.087502	1.17073	1.167439	1.371545	1.367437	1.431413
1.098487	1.094655	1.091269	1.088104	1.171396	1.168149	1.372418	1.368387	1.432494
0.98494	1.095401	1.091919	1.088676	1.172015	1.168813	1.165388	1.16178	1.433557
0.985606	1.096196	1.092598	1.089252	1.172622	1.169459	1.166103	1.162606	1.434611
0.818269	1.097082	1.093363	1.089898	1.173297	1.170146	1.166838	1.163433	1.435672
0.8193	1.098051	1.094203	1.090601	1.174025	1.170862	1.167585	1.164258	1.436736
0.820379	0.961072	0.957415	0.953967	1.174801	1.1716	1.168333	1.165067	1.437812
0.821529	0.962399	0.958651	0.955095	1.175634	1.172369	1.169093	1.165871	1.43892
0.822632	0.963706	0.959885	0.956229	1.176479	1.173121	1.169812	1.166616	1.163197
0.823614	0.964946	0.961063	0.957314	1.177303	1.173814	1.170447	1.167254	1.163882
0.824561	0.966158	0.962237	0.958411	1.178155	1.174512	1.171068	1.167865	1.164533
0.82554	0.822425	0.819239	0.816106	1.061372	1.057765	1.171737	1.168528	1.16523
0.826531	0.823431	0.820258	0.817119	1.062567	1.058794	1.172442	1.169217	1.165944
0.827553	0.824488	0.821332	0.818189	1.063815	1.059908	1.173231	1.169948	1.166668
0.828607	0.825596	0.822465	0.819319	1.06512	1.061107	1.174101	1.170721	1.167405
0.829604	0.826632	0.823524	0.820383	0.960852	0.957148	1.0586	1.055306	1.168109
0.830514	0.827555	0.824468	0.821339	0.962043	0.95825	1.059657	1.056256	1.168755
0.831415	0.828467	0.825402	0.822289	0.963228	0.959366	1.060745	1.057229	1.16942
0.832342	0.829415	0.826374	0.82328	0.964415	0.960512	1.061902	1.058257	1.170141
0.833286	0.830382	0.827367	0.824295	0.965623	0.961698	1.063108	1.059334	1.17091
0.834274	0.83139	0.828405	0.825361	0.822236	0.81907	1.064367	1.060483	1.056868
0.835289	0.832423	0.829469	0.826459	0.823358	0.820204	1.065672	1.061692	1.057988
0.836264	0.833399	0.830469	0.827491	0.824413	0.821275	1.066974	1.062914	1.059129
0.837193	0.83431	0.831399	0.82845	0.825397	0.82228	1.068275	1.064152	1.060289

0.838149	0.835253	0.832358	0.829436	0.826409	0.823315	1.069579	1.065412	1.061484
0.839163	0.836272	0.833395	0.830499	0.827499	0.824432	1.070891	1.066704	1.062722
0.840181	0.837283	0.834418	0.831544	0.828568	0.825528	1.072195	1.068005	1.063982
0.841152	0.838207	0.83534	0.832482	0.829526	0.826512	0.950394	0.946885	1.06524
0.842126	0.839117	0.836234	0.833385	0.830443	0.827452	0.951415	0.947929	1.066517
0.716646	0.840134	0.837221	0.834357	0.831421	0.828453	0.952468	0.949044	1.067855
0.717734	0.84123	0.838273	0.835374	0.832436	0.829488	0.953538	0.950206	1.06924
0.718807	0.842287	0.839284	0.83635	0.833404	0.83047	0.827536	0.824605	1.070577
0.719853	0.843289	0.840235	0.837265	0.834304	0.831375	0.828469	0.825593	1.07186
0.720928	0.844328	0.841229	0.838227	0.835254	0.832327	0.829433	0.826579	1.073114
0.722067	0.845456	0.842324	0.839301	0.836322	0.833397	0.830483	0.827587	1.074327
0.723214	0.846601	0.843443	0.840401	0.837416	0.834493	0.831555	0.828609	1.075541
0.500701	0.847684	0.844506	0.841447	0.838461	0.835544	0.832609	0.829666	0.953142
0.501432	0.848758	0.845559	0.84248	0.839485	0.83657	0.833649	0.830733	0.95426
	0.723342	0.720305	0.717379	0.840501	0.837552	0.834626	0.831735	0.955315
	0.724613	0.721487	0.718448	0.841505	0.838488	0.835543	0.832678	0.956316
	0.501722	0.499251	0.496862	0.842578	0.839502	0.836525	0.833656	0.957364
	0.502689	0.500151	0.497672	0.843739	0.840617	0.837593	0.834675	0.958472
	0.503633	0.501062	0.498524	0.844936	0.841777	0.83871	0.835739	0.959625
	0.504538	0.502001	0.499464	0.846165	0.842985	0.839888	0.836877	0.960832
	0.505407	0.502925	0.500413	0.847392	0.8442	0.841083	0.838044	0.962068
				0.498848	0.496546	0.84222	0.839191	0.963309
				0.499599	0.497285	0.843323	0.840327	0.964549
				0.500439	0.498054	0.84441	0.841417	0.965695
				0.501363	0.498843	0.845472	0.842452	0.966741
				0.502321	0.499703	0.674519	0.672115	0.840526
				0.50332	0.500668	0.675452	0.672951	0.841604
				0.504311	0.501663	0.676423	0.673827	0.84272
				0.505244	0.502638	0.677402	0.674755	0.84391
				0.506152	0.503616	0.678394	0.675713	0.845138
						0.502099	0.499547	0.674056
						0.503073	0.500441	0.674944
						0.504052	0.501384	0.675879
						0.505045	0.502387	0.676871
						0.506007	0.503387	0.677867
						0.506914	0.50436	0.678852
						0.507799	0.505324	0.679834
								0.503806
								0.504788
								0.505749
								0.506693
								0.507601
								0.508461

2.029477	2.024717	2.020198	2.015783	2.011338	2.006911	2.015535	2.011108	2.046823
2.029527	2.024764	2.02024	2.01582	2.011372	2.006942	2.015569	2.011142	2.04682
2.029581	2.024816	2.020286	2.015859	2.011406	2.006971	2.015599	2.011173	2.046816
2.029634	2.024868	2.020336	2.015905	2.011446	2.007005	2.015629	2.0112	2.04681
2.02969	2.024923	2.020389	2.015955	2.01149	2.00704	2.01566	2.011226	2.046804
2.013799	2.024993	2.020451	2.016005	2.011532	2.007077	2.015697	2.011269	2.039958
2.013871	2.025075	2.020518	2.016053	2.011572	2.007115	2.015741	2.011326	2.039995
2.013973	2.025196	2.020616	2.016119	2.011623	2.00716	2.015797	2.011411	2.040073
2.014124	2.025389	2.02077	2.016217	2.011692	2.007216	2.015873	2.011544	2.040224
2.014275	2.025575	2.02092	2.016317	2.011768	2.00728	2.015955	2.011674	2.040368
2.014373	2.025679	2.02101	2.016391	2.011835	2.007346	2.002986	1.998761	2.040431
2.014457	2.02575	2.021077	2.016456	2.011905	2.007424	2.00306	1.998822	2.040462
2.014578	2.025842	2.021154	2.01653	2.011999	2.007548	2.003182	1.99891	2.040512
2.014726	2.025942	2.021234	2.016609	2.01211	2.007708	2.00334	1.999015	2.040571
2.014841	2.026032	2.021313	2.01669	2.012206	2.007827	2.003455	1.999102	2.040636
2.014915	2.026104	2.021386	2.016763	2.012276	2.007892	2.003519	1.999167	2.040709
2.001282	2.010274	2.005645	2.001107	2.012369	2.00796	2.003577	1.99923	2.02916
2.001371	2.010387	2.005789	2.001272	2.012518	2.008051	2.003641	1.99929	2.029191
2.001481	2.010521	2.005948	2.001445	2.012675	2.008152	2.003717	1.999367	2.029235
2.001632	2.010689	2.006095	2.001565	2.012785	2.008256	2.003828	1.999502	2.029353
2.001808	2.010873	2.006237	2.001661	2.012879	2.008368	2.003963	1.999668	2.029506
1.979221	1.992911	1.988483	1.984104	2.013	2.00851	2.004108	1.999804	2.029607
1.97937	1.993033	1.988592	1.984215	2.013144	2.00868	2.004267	1.999915	2.029666
1.979507	1.993158	1.98872	1.984354	2.013302	2.008842	2.004415	2.00003	2.029742
1.97962	1.993286	1.988874	1.984534	2.013475	2.008984	2.00454	2.000147	2.029837
1.979747	1.975225	1.970845	1.966536	1.997882	1.993435	2.004686	2.000288	2.029961
1.979911	1.975401	1.971006	1.966681	1.998058	1.993629	2.004899	2.000493	2.030142
1.980094	1.975591	1.971175	1.966828	1.998234	1.993826	2.005118	2.000708	2.030336
1.980285	1.975768	1.971351	1.967003	1.9984	1.993964	2.005258	2.000858	2.030477
1.980487	1.97594	1.971533	1.967194	1.998559	1.994067	2.005351	2.000971	2.03059
1.98069	1.976134	1.971717	1.967363	1.998707	1.994181	2.005466	2.001109	2.030729
1.980895	1.976349	1.971902	1.967508	1.998839	1.994302	2.005599	2.001268	2.030896
1.981101	1.976561	1.972097	1.967679	1.981997	1.977471	2.005758	2.001432	2.03105
1.981304	1.976756	1.972306	1.967901	1.982219	1.977662	2.005957	2.001599	2.031175
1.981512	1.976958	1.972519	1.968125	1.982443	1.977858	2.006159	2.00177	2.031304
1.981733	1.977187	1.972729	1.968309	1.982607	1.978011	2.006326	2.001946	2.031468
1.981963	1.977426	1.97294	1.968484	1.982761	1.978161	2.00649	2.002126	2.031642
1.982201	1.977641	1.97315	1.968695	1.96421	1.959743	1.988084	1.983765	2.031788
1.982448	1.977842	1.97336	1.96893	1.964458	1.959999	1.988316	1.983945	2.031918
1.982725	1.978094	1.973594	1.969152	1.964687	1.960242	1.988561	1.984158	2.032097
1.983042	1.978406	1.973851	1.969352	1.964888	1.960473	1.988824	1.984413	2.032334
1.983344	1.978717	1.974125	1.969582	1.965104	1.96069	1.974845	1.970469	2.030867
1.983605	1.979011	1.974429	1.969877	1.965352	1.960873	1.97502	1.97067	2.0311
1.983853	1.979287	1.974728	1.970184	1.965616	1.961067	1.975195	1.97086	2.03132
1.972986	1.979515	1.97498	1.970465	1.965894	1.961328	1.97542	1.971045	2.031486
1.973238	1.979725	1.975209	1.970729	1.966179	1.961623	1.975674	1.971234	2.031632
1.973525	1.968889	1.964361	1.959876	1.966426	1.961903	1.975949	1.971474	2.031848
1.973844	1.969205	1.964617	1.960074	1.966641	1.962174	1.976247	1.971759	2.032123
1.97414	1.969518	1.964898	1.960318	1.96689	1.96245	1.97654	1.972046	2.005192
1.9744	1.969826	1.965214	1.960628	1.96719	1.962738	1.976829	1.972333	2.005453

1.94618	1.970124	1.96553	1.96095	1.956428	1.951956	1.977102	1.972615	2.005723
1.946436	1.970408	1.965832	1.961263	1.956708	1.952184	1.977332	1.972879	2.006009
1.946693	1.970673	1.96612	1.961573	1.956988	1.952412	1.977562	1.973141	2.006299
1.946933	1.970896	1.966373	1.961868	1.957289	1.952703	1.948299	1.944066	2.006583
1.947167	1.971097	1.966607	1.962154	1.957603	1.953033	1.948622	1.944355	2.006861
1.947436	1.971358	1.966885	1.962459	1.957925	1.953366	1.948936	1.94462	1.998123
1.947739	1.971678	1.967204	1.962781	1.958256	1.953704	1.949244	1.944858	1.998318
1.948047	1.972011	1.967536	1.963112	1.958593	1.954048	1.949565	1.945126	1.998554
1.948354	1.972352	1.967878	1.963452	1.958938	1.954399	1.949906	1.945442	1.99886
1.948663	1.972697	1.968225	1.963795	1.959285	1.954751	1.950253	1.945776	1.999192
1.929727	1.944506	1.940174	1.935883	1.95963	1.9551	1.9506	1.946113	1.999529
1.930024	1.944826	1.940496	1.936205	1.959975	1.955449	1.950948	1.946457	1.999876
1.930327	1.945146	1.940815	1.936522	1.960318	1.955797	1.951296	1.946799	2.00022
1.930634	1.945466	1.941131	1.936835	1.96066	1.956144	1.951642	1.947139	2.000563
1.930936	1.945782	1.941444	1.937147	1.960996	1.956484	1.951981	1.947471	1.983852
1.931235	1.946092	1.941754	1.937456	1.961325	1.956817	1.952311	1.947795	1.984171
1.915914	1.927152	1.922889	1.91867	1.933411	1.929043	1.952634	1.948111	1.984482
1.916216	1.927457	1.92319	1.918965	1.933715	1.929342	1.952947	1.948414	1.98478
1.916518	1.927763	1.923491	1.91926	1.934018	1.929639	1.953259	1.948719	1.985083
1.916813	1.928059	1.923787	1.919557	1.934324	1.929943	1.95358	1.949043	1.944665
1.917111	1.928356	1.924085	1.919855	1.934633	1.930248	1.953909	1.949383	1.945008
1.884331	1.928685	1.924402	1.92016	1.915873	1.911577	1.926188	1.921838	1.945403
1.884631	1.92904	1.924736	1.920471	1.916169	1.911867	1.926513	1.92222	1.945846
1.862332	1.929423	1.925096	1.920801	1.916486	1.91218	1.926849	1.922585	1.946249
1.862642	1.929847	1.925493	1.921159	1.91683	1.91252	1.927195	1.92292	1.946595
1.847063	1.914651	1.910311	1.905985	1.917184	1.912883	1.908629	1.904414	1.91906
1.847341	1.915004	1.910652	1.906317	1.917545	1.913285	1.90903	1.90478	1.919408
1.847616	1.915342	1.910983	1.906648	1.917908	1.913689	1.909435	1.905146	1.919756
1.831719	1.894593	1.890277	1.885989	1.918258	1.914044	1.909782	1.905471	1.920086
1.832015	1.894941	1.890629	1.886347	1.918604	1.914373	1.910097	1.905775	1.920405
1.83229	1.881188	1.876914	1.87267	1.898939	1.894716	1.91042	1.906092	1.901902
1.832545	1.881516	1.877286	1.873081	1.899338	1.895061	1.910746	1.906417	1.902222
1.832805	1.881845	1.877642	1.873461	1.883285	1.879037	1.911093	1.906774	1.902575
1.833072	1.882173	1.877964	1.873784	1.883618	1.879389	1.911467	1.907178	1.902981
1.833353	1.882521	1.878298	1.874111	1.88396	1.879756	1.911857	1.907587	1.903387
1.784144	1.88291	1.878675	1.874482	1.870295	1.866124	1.892293	1.888041	1.903751
1.784423	1.883322	1.879077	1.874876	1.870695	1.866535	1.892702	1.888414	1.904102
1.784711	1.822341	1.818252	1.814211	1.871098	1.866918	1.893074	1.888774	1.904457
1.785008	1.822697	1.818627	1.814606	1.871507	1.867283	1.893424	1.889125	1.904816
1.785313	1.823071	1.818987	1.814953	1.827868	1.823643	1.880531	1.876264	1.885302
1.785631	1.823472	1.819333	1.815244	1.828167	1.823951	1.880895	1.876664	1.885718
1.785937	1.823841	1.81966	1.815537	1.828474	1.824275	1.864167	1.860018	1.886133
1.78622	1.824144	1.819954	1.815845	1.828805	1.824625	1.864571	1.86042	1.886535
1.786497	1.824436	1.82025	1.816167	1.829149	1.824985	1.864985	1.860828	1.886941
1.786772	1.824755	1.820577	1.816513	1.829505	1.825339	1.821198	1.817082	1.87413
1.787054	1.825097	1.820928	1.816879	1.829873	1.825696	1.821559	1.81746	1.874567
1.787376	1.825502	1.821313	1.81725	1.830253	1.826073	1.821932	1.81783	1.832824
1.787732	1.825961	1.821732	1.81763	1.830644	1.826465	1.822316	1.818197	1.833179
1.78809	1.82642	1.822172	1.818051	1.831057	1.826858	1.822695	1.818568	1.83355
1.78845	1.82688	1.822644	1.818524	1.831497	1.827245	1.823062	1.818945	1.833943

1.7888	1.827318	1.823102	1.818996	1.83195	1.827654	1.823449	1.81933	1.834334
1.789125	1.827706	1.823516	1.819446	1.83242	1.82811	1.82388	1.819722	1.834702
1.789442	1.828083	1.823913	1.819877	1.832886	1.828579	1.82433	1.820129	1.83508
1.789763	1.786243	1.782786	1.779347	1.816134	1.811983	1.824781	1.820571	1.816425
1.790085	1.786571	1.783104	1.779657	1.816518	1.8124	1.825234	1.821034	1.816879
1.790419	1.786901	1.783428	1.779976	1.816905	1.812789	1.825652	1.82147	1.817322
1.790762	1.787233	1.783757	1.780301	1.817293	1.813149	1.826034	1.821883	1.817759
1.791127	1.787588	1.7841	1.780632	1.817688	1.813538	1.826436	1.822288	1.818176
1.791522	1.78798	1.784463	1.780965	1.818085	1.813975	1.82687	1.82268	1.818555
1.791916	1.788374	1.784836	1.781315	1.818504	1.814428	1.82732	1.82309	1.818948
1.792284	1.788753	1.785218	1.781697	1.81897	1.814889	1.827791	1.82355	1.8194
1.792641	1.789122	1.785602	1.782094	1.819454	1.815351	1.828267	1.824029	1.819875
1.792996	1.789472	1.78597	1.78248	1.819919	1.81578	1.828708	1.82449	1.820338
1.793349	1.789808	1.786327	1.782863	1.820376	1.81619	1.829129	1.824943	1.820798
1.793711	1.790161	1.786694	1.783246	1.820843	1.816633	1.812485	1.808402	1.82124
1.794084	1.790532	1.787073	1.783632	1.821326	1.817112	1.812947	1.808831	1.821665
1.762544	1.790904	1.787445	1.784004	1.821793	1.817589	1.813405	1.809243	1.822068
1.762896	1.791278	1.78781	1.784359	1.822239	1.818057	1.813849	1.80962	1.822431
1.763239	1.791642	1.78816	1.784693	1.822663	1.818513	1.814291	1.810003	1.822804
1.763556	1.791974	1.788474	1.784992	1.781519	1.77805	1.814734	1.810431	1.823235
1.763864	1.792294	1.788775	1.785276	1.781817	1.778378	1.815179	1.810884	1.823699
1.743588	1.792634	1.789105	1.785599	1.782147	1.778722	1.815634	1.811348	1.824181
1.743917	1.792988	1.789456	1.78595	1.782499	1.779079	1.816096	1.811823	1.824682
1.74426	1.793359	1.789821	1.786313	1.782862	1.779445	1.816563	1.8123	1.808146
1.744618	1.793751	1.790203	1.786687	1.783235	1.779821	1.817038	1.812777	1.808623
1.744979	1.741676	1.738396	1.735154	1.783614	1.7802	1.817514	1.813255	1.809101
1.745336	1.742022	1.738741	1.735498	1.783997	1.780578	1.817988	1.813733	1.80958
1.745698	1.742371	1.739088	1.735845	1.784383	1.780957	1.818463	1.814212	1.810059
1.74607	1.742734	1.73944	1.736187	1.765666	1.762307	1.818943	1.814692	1.810536
1.746451	1.743107	1.739796	1.736528	1.766019	1.762661	1.819426	1.815174	1.811014
1.746836	1.743484	1.740161	1.73688	1.766377	1.763015	1.77864	1.775147	1.8115
1.747228	1.743866	1.740532	1.737241	1.76674	1.763368	1.779019	1.775536	1.811996
1.747628	1.744257	1.740911	1.737608	1.734365	1.73117	1.760335	1.756937	1.812489
1.748039	1.74466	1.741297	1.737977	1.734726	1.731525	1.760696	1.757293	1.81297
1.748453	1.745068	1.74169	1.738353	1.735091	1.731883	1.761063	1.757659	1.813471
1.748862	1.745479	1.742088	1.738735	1.735457	1.732235	1.761433	1.758047	1.814014
1.749275	1.745894	1.742493	1.739126	1.735831	1.732593	1.761812	1.758451	1.814584
1.697919	1.746311	1.742904	1.739532	1.736226	1.732974	1.76221	1.758873	1.797717
1.698345	1.746733	1.743325	1.739953	1.736638	1.733373	1.762623	1.759309	1.798318
1.625675	1.747183	1.743771	1.740394	1.737065	1.733783	1.763039	1.759732	1.756399
1.626124	1.747664	1.744244	1.740858	1.73751	1.734204	1.76346	1.760141	1.756801
1.62656	1.748146	1.744718	1.741321	1.737956	1.734631	1.731395	1.728249	1.757194
1.626964	1.748625	1.745188	1.741774	1.738397	1.73506	1.731803	1.728629	1.757575
1.627361	1.749101	1.745655	1.742226	1.738841	1.735497	1.732222	1.729016	1.757962
1.627763	1.683912	1.680683	1.677467	1.739297	1.735957	1.73267	1.729435	1.758376
1.628167	1.684341	1.68111	1.677892	1.739754	1.736422	1.733126	1.729865	1.758801
1.628584	1.684754	1.681519	1.6783	1.675134	1.672009	1.668927	1.665886	1.759217
1.62901	1.685154	1.681915	1.678697	1.675511	1.672358	1.669265	1.666229	1.759626
1.599964	1.685535	1.6823	1.679096	1.6759	1.672729	1.669625	1.666586	1.760047
1.600315	1.685896	1.682678	1.679502	1.676309	1.67313	1.670015	1.666961	1.760483

1.600657	1.686245	1.683038	1.679887	1.676705	1.67353	1.670408	1.667335	1.760912
1.600986	1.686574	1.683365	1.68023	1.677072	1.673926	1.670805	1.667706	1.761324
1.601318	1.686908	1.683692	1.680565	1.677427	1.674309	1.67119	1.66807	1.761727
1.601679	1.687285	1.684056	1.680926	1.67778	1.674654	1.67153	1.668405	1.665339
1.602054	1.687685	1.684441	1.681301	1.678134	1.674983	1.67185	1.668729	1.665666
1.602421	1.688072	1.684824	1.681691	1.678516	1.675352	1.67221	1.669086	1.666017
1.602779	1.68845	1.685209	1.682099	1.678925	1.675755	1.672605	1.669471	1.66639
1.555084	1.600098	1.597355	1.594767	1.679326	1.676161	1.67301	1.669871	1.66678
1.555446	1.60044	1.597676	1.595091	1.679713	1.676568	1.673428	1.670289	1.667192
1.555834	1.600809	1.598013	1.595419	1.6801	1.676973	1.673842	1.670707	1.667607
1.556244	1.601223	1.598385	1.595762	1.680488	1.677364	1.674236	1.671108	1.66801
1.556676	1.601661	1.598773	1.596112	1.680879	1.677753	1.674627	1.671506	1.668412
1.557118	1.602092	1.599156	1.596465	1.681278	1.678159	1.675035	1.671912	1.668814
1.557579	1.602529	1.599543	1.596821	1.681682	1.678575	1.675452	1.672322	1.669219
1.558093	1.554717	1.551886	1.549352	1.656298	1.653401	1.675858	1.672739	1.66964
1.558665	1.55519	1.552259	1.549641	1.656647	1.653735	1.67625	1.673163	1.670079
1.559237	1.555663	1.552636	1.549941	1.657005	1.654087	1.676661	1.673594	1.670521
1.559793	1.556122	1.553012	1.550253	1.657375	1.654473	1.677102	1.674035	1.670966
1.560342	1.556581	1.553395	1.550577	1.657761	1.654873	1.677551	1.674478	1.671408
1.525349	1.557034	1.553783	1.550915	1.658179	1.655283	1.652385	1.649513	1.671846
1.525875	1.557501	1.554187	1.551266	1.658612	1.655697	1.652782	1.649899	1.672278
1.526458	1.52259	1.519299	1.516405	1.659032	1.656088	1.653157	1.65027	1.672691
1.527087	1.523182	1.519778	1.516766	1.659446	1.656465	1.653515	1.65063	1.673088
1.527747	1.523809	1.520307	1.517186	1.616753	1.613969	1.653897	1.650993	1.648104
1.528447	1.524478	1.520893	1.517675	1.617194	1.614364	1.654303	1.651354	1.648446
1.529146	1.525151	1.52149	1.518182	1.586812	1.584017	1.654726	1.65174	1.648816
1.529822	1.525807	1.522082	1.518697	1.587286	1.584424	1.655172	1.652176	1.649247
1.530501	1.526464	1.522677	1.519215	1.587764	1.584824	1.655616	1.652616	1.649688
1.531198	1.52713	1.523266	1.519713	1.588215	1.585193	1.627648	1.624722	1.650091
1.53191	1.527806	1.523857	1.520207	1.588655	1.585547	1.628023	1.625103	1.650476
1.532654	1.528509	1.524475	1.520726	1.5891	1.585916	1.582966	1.580279	1.650857
1.533427	1.529237	1.525117	1.521265	1.589545	1.586294	1.583304	1.580605	1.651232
1.534191	1.529973	1.525783	1.521842	1.590035	1.586704	1.583667	1.580952	1.623404
1.534936	1.530719	1.52648	1.522463	1.590576	1.58715	1.584058	1.581327	1.623838
1.535684	1.53146	1.527186	1.523113	1.591151	1.587629	1.584475	1.58171	1.624269
1.536437	1.53218	1.527889	1.523788	1.591757	1.588148	1.584919	1.582086	1.624662
1.5372	1.532896	1.528596	1.524481	1.592388	1.588696	1.585387	1.582473	1.625055
1.537983	1.533623	1.529306	1.52517	1.593027	1.589258	1.585875	1.582886	1.625481
1.538785	1.534362	1.530022	1.525864	1.593679	1.589837	1.586383	1.583321	1.62593
1.53961	1.535142	1.530775	1.526584	1.594352	1.590437	1.586909	1.583771	1.580827
1.54046	1.535967	1.531566	1.527329	1.595047	1.591059	1.587457	1.584239	1.581238
1.541306	1.53679	1.532363	1.528088	1.595747	1.591678	1.587999	1.584703	1.581649
1.54214	1.537598	1.53316	1.528858	1.596442	1.592275	1.588516	1.585154	1.582053
1.542974	1.538409	1.533963	1.52964	1.597153	1.592893	1.589052	1.585613	1.582461
1.543814	1.539231	1.534775	1.530432	1.597894	1.593556	1.589628	1.586087	1.582864
1.544656	1.540059	1.535592	1.531231	1.598658	1.594259	1.590245	1.58659	1.583284
1.545495	1.540888	1.536408	1.532029	1.599445	1.595019	1.590937	1.587173	1.583779
1.546332	1.541719	1.537224	1.532829	1.600256	1.595829	1.591693	1.58782	1.584332
1.504065	1.542547	1.538039	1.533628	1.601068	1.59664	1.592451	1.588476	1.584897
1.50486	1.54337	1.538852	1.534428	1.601884	1.597452	1.593214	1.589144	1.585474

1.505659	1.544197	1.539666	1.535225	1.602694	1.598259	1.593975	1.589818	1.58606
1.506465	1.545022	1.540473	1.536011	1.603486	1.599053	1.594726	1.590485	1.586643
1.507283	1.545866	1.541296	1.536809	1.604284	1.599846	1.595481	1.591169	1.587248
1.455727	1.503603	1.499097	1.494667	1.605106	1.600653	1.596254	1.591892	1.587906
1.456516	1.504489	1.499961	1.495492	1.605946	1.601468	1.597039	1.592643	1.588599
1.457295	1.452947	1.448505	1.444093	1.606797	1.60229	1.597829	1.593401	1.589304
1.458069	1.453762	1.449321	1.44489	1.607661	1.603118	1.598625	1.594171	1.590025
1.458815	1.454535	1.450115	1.445693	1.57073	1.566245	1.599426	1.59495	1.590764
1.459522	1.455249	1.450879	1.4465	1.571568	1.567036	1.600221	1.595734	1.59152
1.460236	1.45596	1.451637	1.447308	1.526379	1.521871	1.601055	1.596549	1.592299
1.460981	1.456692	1.452407	1.448124	1.527251	1.522713	1.601964	1.597414	1.5931
1.461743	1.457433	1.453172	1.448926	1.528133	1.523583	1.602907	1.598313	1.59393
1.462517	1.458179	1.45391	1.44967	1.528956	1.524438	1.560635	1.556097	1.594808
1.463304	1.458936	1.454637	1.450381	1.529745	1.525285	1.561536	1.55701	1.595721
1.464103	1.459732	1.455416	1.451141	1.530532	1.526066	1.521631	1.517224	1.553429
1.464918	1.460571	1.456247	1.451945	1.53131	1.52678	1.522341	1.517992	1.554253
1.465703	1.461386	1.45707	1.45276	1.44844	1.444134	1.523069	1.518736	1.555039
1.466436	1.462162	1.45788	1.453593	1.449273	1.444946	1.523833	1.519441	1.555757
1.467168	1.462919	1.458665	1.454404	1.450097	1.44577	1.524625	1.520165	1.556489
1.467927	1.463649	1.459391	1.455142	1.450861	1.446569	1.525472	1.520962	1.516548
1.468706	1.46438	1.460106	1.45586	1.451605	1.447354	1.526334	1.521789	1.517344
1.469522	1.465182	1.460895	1.456637	1.45237	1.448108	1.527139	1.522597	1.518154
1.470369	1.466034	1.461735	1.457452	1.453146	1.448839	1.527908	1.523397	1.518979
1.471199	1.466871	1.46257	1.45828	1.453957	1.449627	1.528713	1.524198	1.519773
1.472009	1.467691	1.463402	1.459124	1.454809	1.450481	1.529558	1.524994	1.520527
1.472831	1.468513	1.464227	1.459956	1.45565	1.45133	1.447002	1.442658	1.521305
1.473687	1.469342	1.465039	1.460756	1.456455	1.452146	1.447816	1.443457	1.52213
1.474543	1.470169	1.465848	1.461557	1.45726	1.452963	1.448636	1.444273	1.522978
1.475364	1.47098	1.466669	1.462392	1.4581	1.453806	1.449479	1.445115	1.523834
1.476175	1.471785	1.467492	1.463237	1.458951	1.454656	1.450332	1.445973	1.524705
1.47701	1.472593	1.468299	1.464053	1.459774	1.455486	1.451172	1.446826	1.5256
1.477865	1.473403	1.469094	1.464846	1.460574	1.4563	1.452002	1.447678	1.52652
1.478762	1.474259	1.469923	1.465656	1.461376	1.457104	1.452814	1.448506	1.52741
1.479714	1.475177	1.470799	1.466491	1.462184	1.457896	1.453602	1.449302	1.528259
1.480664	1.476102	1.471678	1.467322	1.462988	1.458685	1.454388	1.450096	1.529109
1.481586	1.477008	1.472535	1.468127	1.463774	1.459471	1.455179	1.450899	1.529984
1.482496	1.477915	1.473396	1.468936	1.464565	1.460263	1.455976	1.451709	1.530862
1.483398	1.478836	1.474284	1.469779	1.465388	1.461076	1.456788	1.452529	1.531722
1.484295	1.479765	1.475188	1.470646	1.466232	1.461903	1.45761	1.453358	1.532576
1.431693	1.480684	1.476096	1.471532	1.467087	1.462725	1.458422	1.454183	1.533458
1.432543	1.481596	1.477008	1.472437	1.467954	1.463539	1.459222	1.455005	1.534367
1.433381	1.482493	1.477915	1.473351	1.468847	1.464398	1.460062	1.455838	1.53527
1.434189	1.483358	1.478801	1.474265	1.46977	1.46532	1.46096	1.456687	1.536169
1.435016	1.484248	1.479714	1.475203	1.470718	1.466264	1.461871	1.457534	1.537042
1.304549	1.485218	1.480701	1.476196	1.471699	1.467216	1.462772	1.458358	1.537841
1.305373	1.486218	1.481713	1.477206	1.472689	1.46817	1.463672	1.459185	1.538634
1.306192	1.487196	1.482695	1.478184	1.473643	1.46909	1.464565	1.460056	1.539527
1.307009	1.488162	1.483657	1.479137	1.474573	1.469989	1.465456	1.460963	1.54049
1.307803	1.489085	1.484563	1.48003	1.475465	1.470888	1.466372	1.461903	1.5415
1.308571	1.489957	1.4854	1.480848	1.476308	1.471785	1.46731	1.462877	1.54256

1.30934	1.436768	1.432334	1.427935	1.47719	1.472709	1.468271	1.463874	1.54364
1.310115	1.437649	1.433209	1.428808	1.478143	1.473671	1.469255	1.464894	1.544737
1.310902	1.438562	1.434127	1.42973	1.479137	1.474663	1.470257	1.46592	1.54583
1.311709	1.439514	1.435087	1.430691	1.480166	1.475687	1.471278	1.466938	1.546899
1.312532	1.440497	1.43608	1.431686	1.481223	1.476731	1.472306	1.467947	1.547946
1.313372	1.30955	1.305652	1.301739	1.482272	1.477757	1.473309	1.468924	1.46456
1.314228	1.310428	1.306546	1.302637	1.483318	1.478771	1.474292	1.469873	1.465483
1.315083	1.311302	1.307433	1.303528	1.484364	1.479793	1.475277	1.470811	1.466389
1.315937	1.312171	1.308313	1.304412	1.485409	1.480821	1.476264	1.47173	1.467269
1.240207	1.313033	1.309189	1.305297	1.432235	1.427753	1.477266	1.472672	1.468175
1.241009	1.313884	1.310063	1.306191	1.433247	1.428749	1.478301	1.473671	1.469147
1.241802	1.314725	1.310931	1.307085	1.434266	1.429749	1.47934	1.474685	1.470138
1.242582	1.315558	1.311792	1.307971	1.435275	1.430721	1.480349	1.475675	1.471112
1.243352	1.316381	1.312644	1.308851	1.436281	1.431685	1.481345	1.476654	1.472076
1.089349	1.317191	1.313483	1.30972	1.437293	1.432673	1.482353	1.477611	1.472995
1.089984	1.317988	1.314309	1.31058	1.438309	1.43368	1.483368	1.478539	1.473867
1.090625	1.242115	1.238617	1.235075	1.37057	1.366308	1.430041	1.425241	1.474829
1.091278	1.242873	1.239395	1.235894	1.371531	1.367335	1.431171	1.426332	1.475914
1.091935	1.243629	1.240162	1.236699	1.372472	1.368345	1.432306	1.427466	1.477057
1.092589	1.089382	1.086277	1.083158	1.373371	1.369288	1.433382	1.428609	1.478259
1.09325	1.090003	1.086907	1.083822	1.374244	1.370202	1.434433	1.429747	1.479474
1.093933	1.09062	1.087516	1.084454	1.3751	1.371114	1.435465	1.430809	1.480597
1.094635	1.091235	1.088108	1.08506	1.375936	1.372016	1.436477	1.431822	1.481664
1.095369	1.091886	1.088723	1.08567	1.168636	1.165164	1.437484	1.432854	1.482755
1.096139	1.092576	1.089361	1.086283	1.169346	1.165945	1.438486	1.433906	1.483871
1.096918	1.093271	1.09	1.086894	1.170043	1.166703	1.370653	1.366474	1.430328
1.097691	1.093951	1.090629	1.087502	1.17073	1.167439	1.371545	1.367437	1.431413
1.098487	1.094655	1.091269	1.088104	1.171396	1.168149	1.372418	1.368387	1.432494
0.98494	1.095401	1.091919	1.088676	1.172015	1.168813	1.165388	1.16178	1.433557
0.985606	1.096196	1.092598	1.089252	1.172622	1.169459	1.166103	1.162606	1.434611
0.818269	1.097082	1.093363	1.089898	1.173297	1.170146	1.166838	1.163433	1.435672
0.8193	1.098051	1.094203	1.090601	1.174025	1.170862	1.167585	1.164258	1.436736
0.820379	0.961072	0.957415	0.953967	1.174801	1.1716	1.168333	1.165067	1.437812
0.821529	0.962399	0.958651	0.955095	1.175634	1.172369	1.169093	1.165871	1.43892
0.822632	0.963706	0.959885	0.956229	1.176479	1.173121	1.169812	1.166616	1.163197
0.823614	0.964946	0.961063	0.957314	1.177303	1.173814	1.170447	1.167254	1.163882
0.824561	0.966158	0.962237	0.958411	1.178155	1.174512	1.171068	1.167865	1.164533
0.82554	0.822425	0.819239	0.816106	1.061372	1.057765	1.171737	1.168528	1.16523
0.826531	0.823431	0.820258	0.817119	1.062567	1.058794	1.172442	1.169217	1.165944
0.827553	0.824488	0.821332	0.818189	1.063815	1.059908	1.173231	1.169948	1.166668
0.828607	0.825596	0.822465	0.819319	1.06512	1.061107	1.174101	1.170721	1.167405
0.829604	0.826632	0.823524	0.820383	0.960852	0.957148	1.0586	1.055306	1.168109
0.830514	0.827555	0.824468	0.821339	0.962043	0.95825	1.059657	1.056256	1.168755
0.831415	0.828467	0.825402	0.822289	0.963228	0.959366	1.060745	1.057229	1.16942
0.832342	0.829415	0.826374	0.82328	0.964415	0.960512	1.061902	1.058257	1.170141
0.833286	0.830382	0.827367	0.824295	0.965623	0.961698	1.063108	1.059334	1.17091
0.834274	0.83139	0.828405	0.825361	0.822236	0.81907	1.064367	1.060483	1.056868
0.835289	0.832423	0.829469	0.826459	0.823358	0.820204	1.065672	1.061692	1.057988
0.836264	0.833399	0.830469	0.827491	0.824413	0.821275	1.066974	1.062914	1.059129
0.837193	0.83431	0.831399	0.82845	0.825397	0.82228	1.068275	1.064152	1.060289

0.838149	0.835253	0.832358	0.829436	0.826409	0.823315	1.069579	1.065412	1.061484
0.839163	0.836272	0.833395	0.830499	0.827499	0.824432	1.070891	1.066704	1.062722
0.840181	0.837283	0.834418	0.831544	0.828568	0.825528	1.072195	1.068005	1.063982
0.841152	0.838207	0.83534	0.832482	0.829526	0.826512	0.950394	0.946885	1.06524
0.842126	0.839117	0.836234	0.833385	0.830443	0.827452	0.951415	0.947929	1.066517
0.716646	0.840134	0.837221	0.834357	0.831421	0.828453	0.952468	0.949044	1.067855
0.717734	0.84123	0.838273	0.835374	0.832436	0.829488	0.953538	0.950206	1.06924
0.718807	0.842287	0.839284	0.83635	0.833404	0.83047	0.827536	0.824605	1.070577
0.719853	0.843289	0.840235	0.837265	0.834304	0.831375	0.828469	0.825593	1.07186
0.720928	0.844328	0.841229	0.838227	0.835254	0.832327	0.829433	0.826579	1.073114
0.722067	0.845456	0.842324	0.839301	0.836322	0.833397	0.830483	0.827587	1.074327
0.723214	0.846601	0.843443	0.840401	0.837416	0.834493	0.831555	0.828609	1.075541
0.500701	0.847684	0.844506	0.841447	0.838461	0.835544	0.832609	0.829666	0.953142
0.501432	0.848758	0.845559	0.84248	0.839485	0.83657	0.833649	0.830733	0.95426
	0.723342	0.720305	0.717379	0.840501	0.837552	0.834626	0.831735	0.955315
	0.724613	0.721487	0.718448	0.841505	0.838488	0.835543	0.832678	0.956316
	0.501722	0.499251	0.496862	0.842578	0.839502	0.836525	0.833656	0.957364
	0.502689	0.500151	0.497672	0.843739	0.840617	0.837593	0.834675	0.958472
	0.503633	0.501062	0.498524	0.844936	0.841777	0.83871	0.835739	0.959625
	0.504538	0.502001	0.499464	0.846165	0.842985	0.839888	0.836877	0.960832
	0.505407	0.502925	0.500413	0.847392	0.8442	0.841083	0.838044	0.962068
				0.498848	0.496546	0.84222	0.839191	0.963309
				0.499599	0.497285	0.843323	0.840327	0.964549
				0.500439	0.498054	0.84441	0.841417	0.965695
				0.501363	0.498843	0.845472	0.842452	0.966741
				0.502321	0.499703	0.674519	0.672115	0.840526
				0.50332	0.500668	0.675452	0.672951	0.841604
				0.504311	0.501663	0.676423	0.673827	0.84272
				0.505244	0.502638	0.677402	0.674755	0.84391
				0.506152	0.503616	0.678394	0.675713	0.845138
						0.502099	0.499547	0.674056
						0.503073	0.500441	0.674944
						0.504052	0.501384	0.675879
						0.505045	0.502387	0.676871
						0.506007	0.503387	0.677867
						0.506914	0.50436	0.678852
						0.507799	0.505324	0.679834
								0.503806
								0.504788
								0.505749
								0.506693
								0.507601
								0.508461

								0.557367
								0.55542
								0.553598
								0.551905
						0.559033	0.554587	1.068696
						0.557259	0.552741	1.066805
						0.555537	0.550933	1.064955
						0.553907	0.549266	1.063215
						0.552291	0.54765	1.061517
						0.550503	0.545969	1.059761
						0.548599	0.544247	1.05797
				0.555862	0.551266	1.065378	1.06074	1.172775
				0.553998	0.54956	1.063546	1.05887	1.1708
				0.55216	0.547815	1.061704	1.057026	1.347048
				0.550373	0.545946	1.059817	1.055227	1.345118
				0.548637	0.54409	1.057954	1.05345	1.343212
		0.55612	0.551563	0.995849	0.991179	1.172701	1.16805	1.48572
		0.554302	0.549912	0.994195	0.989537	1.170922	1.166216	1.483559
		0.552594	0.548284	0.992509	0.98789	1.262368	1.257532	1.797317
		0.550969	0.54668	0.990801	0.986249	1.260601	1.255755	1.794687
0.558129	0.553665	0.998054	0.993553	1.058746	1.054047	1.368254	1.362906	1.792173
0.556577	0.552017	0.996304	0.991715	1.056834	1.052216	1.366208	1.361041	1.789806
0.555015	0.550383	0.994593	0.98993	1.264513	1.259708	1.742579	1.73625	1.926873
0.553452	0.548774	0.99296	0.98827	1.26263	1.257759	1.739972	1.733852	1.92439

0.551857	0.547175	0.99136	0.98667	1.260819	1.255882	1.73741	1.731471	1.921916
0.55016	0.545571	0.989752	0.985084	1.368616	1.363161	1.735032	1.729152	1.919455
0.548404	0.543967	0.988144	0.98352	1.366779	1.361445	1.732785	1.726884	1.917006
0.995495	0.991089	1.195821	1.190428	1.364934	1.359724	1.730602	1.724672	1.914576
0.993808	0.989389	1.193917	1.18858	1.363088	1.358012	1.728492	1.722519	1.912159
0.992113	0.987669	1.371782	1.366397	1.73893	1.732567	1.902893	1.896479	1.990304
0.990387	0.985905	1.369677	1.36443	1.73659	1.730372	1.900596	1.894216	1.987824
0.988689	0.984184	1.367625	1.362522	1.734275	1.728189	1.898318	1.891978	1.985371
1.196629	1.191288	1.365732	1.36075	1.732012	1.726031	1.896062	1.889752	2.004671
1.194775	1.189483	1.363916	1.359048	1.729797	1.723898	1.893824	1.887536	2.002212
1.283664	1.278297	1.567094	1.561813	1.798584	1.792557	1.991478	1.984719	1.999736
1.281738	1.276396	1.565063	1.559878	1.796411	1.79041	1.98905	1.982273	1.99725
1.279885	1.274598	1.563076	1.557953	1.847811	1.841561	2.008477	2.001637	2.010093
1.278111	1.27291	1.561137	1.556041	1.845478	1.83928	2.00604	1.999181	2.007508
1.570154	1.564496	1.776006	1.770066	1.958297	1.951937	2.003602	1.99672	2.004923
1.568153	1.562561	1.773809	1.767915	1.955928	1.949579	2.00117	1.994258	2.002334
1.56617	1.560652	1.771649	1.765783	1.95357	1.947219	1.998727	1.991785	1.99975
1.564188	1.558761	1.825271	1.819132	2.009685	2.003044	2.011832	2.004489	2.107744
1.562228	1.556902	1.823013	1.816864	2.007243	2.000577	2.009248	2.001904	2.104842
1.560361	1.555126	1.820829	1.814673	2.004832	1.998113	2.006651	1.999305	2.101934
1.558575	1.553425	1.818713	1.81255	2.002446	1.995649	2.004041	1.996695	2.099022
1.828996	1.822779	1.816572	1.81043	2.000047	1.993175	2.026032	2.018659	2.096154
1.826729	1.820534	1.814397	1.808323	1.997637	1.990692	2.023417	2.016063	2.093325
1.824494	1.8183	1.940861	1.934304	2.010933	2.003541	2.106667	2.098504	2.103772
1.822311	1.816103	1.938487	1.931869	2.008331	2.00094	2.103765	2.095707	2.101098
1.820149	1.813925	1.936104	1.929407	2.005705	1.998343	2.100902	2.092976	2.098501
1.817978	1.811764	1.933721	1.926961	2.027754	2.020387	2.098089	2.090339	2.153017
1.815806	1.809615	1.931334	1.924521	2.025163	2.017834	2.09533	2.087774	2.150486
1.813619	1.807443	1.981505	1.97456	2.071102	2.063447	2.10587	2.098427	2.147868
1.811416	1.805251	1.979059	1.972123	2.068448	2.060924	2.103327	2.09589	2.145176
1.809219	1.803057	2.004273	1.99731	2.065855	2.058419	2.141788	2.13397	2.142485
1.807011	1.800842	2.001863	1.994953	2.063354	2.055929	2.139079	2.13129	2.139809
1.804857	1.798678	1.999492	1.992608	2.060849	2.053433	2.136364	2.128604	2.137132
1.930736	1.923952	2.062382	2.054758	2.108266	2.100567	2.133632	2.125891	2.134433
1.928592	1.921794	2.059889	2.05216	2.105513	2.097918	2.130912	2.123186	2.131747
1.958244	1.951382	2.05732	2.049582	2.143947	2.136045	2.144499	2.136733	2.129118
1.955915	1.949041	2.054705	2.047017	2.141176	2.133369	2.141867	2.134118	2.126541
1.953578	1.946689	2.052096	2.044474	2.130939	2.12322	2.132342	2.124609	2.141469
1.951219	1.944309	2.049477	2.041937	2.12835	2.120769	2.129979	2.122304	2.139164
2.040448	2.032721	2.099093	2.091294	2.142715	2.135147	2.127569	2.11995	2.136821
2.038054	2.03036	2.096643	2.088945	2.140254	2.13262	2.125026	2.11746	2.134366
2.035661	2.02802	2.094229	2.08663	2.137815	2.130091	2.122467	2.114941	2.131881
2.033146	2.025565	2.134122	2.126273	2.135321	2.127578	2.137937	2.1304	2.147922
2.030582	2.023069	2.131462	2.123741	2.132824	2.125091	2.135446	2.127909	2.145428
2.102111	2.09423	2.145807	2.138133	2.130407	2.1227	2.133068	2.125538	2.143043
2.099476	2.091679	2.143252	2.135701	2.128054	2.120391	2.130785	2.123266	2.140746
2.096942	2.089232	2.140742	2.133231	2.14365	2.136033	2.147595	2.140055	2.132572
2.094536	2.086919	2.138289	2.130707	2.141148	2.133618	2.145207	2.137651	2.130162
2.111056	2.103434	2.135834	2.128186	2.138648	2.131203	2.142821	2.135261	2.180084
2.108591	2.101021	2.13332	2.125656	2.136157	2.128799	2.140472	2.132933	2.17776

2.106148	2.098621	2.130838	2.123182	2.133714	2.126409	2.138121	2.13061	2.17545
2.144013	2.136241	2.146528	2.138886	2.150503	2.143136	2.135723	2.128216	2.173044
2.141671	2.13393	2.14428	2.136691	2.148267	2.140787	2.133313	2.125802	2.170608
2.139249	2.131543	2.141965	2.134447	2.146023	2.138498	2.173984	2.166435	2.218699
2.136756	2.129087	2.139594	2.132172	2.143786	2.136271	2.171747	2.16423	2.216507
2.134361	2.126719	2.137255	2.129861	2.141478	2.133968	2.169437	2.161978	2.214259
2.132116	2.124486	2.134965	2.127488	2.13905	2.131529	2.166993	2.159626	2.2119
2.129903	2.122276	2.132698	2.125137	2.136644	2.129108	2.164565	2.157295	2.209563
2.145721	2.138083	2.149553	2.141957	2.141845	2.134288	2.162255	2.155069	2.227424
2.143478	2.135836	2.147314	2.139723	2.139597	2.132025	2.159982	2.152874	2.225224
2.141113	2.133474	2.144938	2.137344	2.17262	2.164996	2.191774	2.184623	2.230921
2.138671	2.131041	2.14248	2.134884	2.170144	2.162582	2.189391	2.182279	2.22853
2.155415	2.147786	2.14761	2.139999	2.167811	2.160327	2.209278	2.202161	2.226276
2.153236	2.145596	2.145404	2.137796	2.165632	2.158243	2.207187	2.200087	2.224169
2.151056	2.143412	2.153937	2.146314	2.188769	2.181456	2.221963	2.214822	2.22208
2.14886	2.141228	2.151733	2.144104	2.186579	2.179358	2.219867	2.212744	2.220011
2.146609	2.138989	2.149482	2.141862	2.184353	2.177217	2.217717	2.21061	2.217888
2.151652	2.144009	2.174707	2.167139	2.181993	2.174926	2.215389	2.208294	2.21558
2.149176	2.141529	2.172238	2.164735	2.179608	2.1726	2.213007	2.20592	2.21321
2.157588	2.149936	2.192134	2.184638	2.208264	2.201125	2.219133	2.212037	2.210996
2.1554	2.147759	2.189983	2.182571	2.206195	2.199077	2.21705	2.209941	2.208895
2.180776	2.173163	2.187869	2.18054	2.204165	2.197072	2.215023	2.207923	2.206882
2.178665	2.171049	2.185792	2.178547	2.202177	2.195112	2.213062	2.205998	2.204981
2.176566	2.168955	2.191736	2.184531	2.200202	2.193164	2.211107	2.204072	2.203072
2.17446	2.166868	2.189681	2.182547	2.198231	2.191216	2.209138	2.20211	2.201116
2.172356	2.164793	2.18763	2.180561	2.196252	2.189257	2.207149	2.200114	2.199112
2.192389	2.184802	2.185584	2.178568	2.203053	2.196066	2.205123	2.198048	2.197003
2.190266	2.182724	2.18353	2.176559	2.201006	2.194036	2.203059	2.195935	2.194836
2.196086	2.18856	2.181396	2.174458	2.198844	2.191894	2.200916	2.193818	2.192737
2.193847	2.186334	2.179186	2.172269	2.196569	2.18964	2.198691	2.191689	2.190691
2.191596	2.184147	2.208496	2.201403	2.194394	2.187487	2.196555	2.189618	2.188682
2.189319	2.182011	2.206384	2.199339	2.192364	2.185477	2.194545	2.187623	2.186714
2.187094	2.179926	2.204326	2.197336	2.190397	2.183526	2.192587	2.185665	2.18477
2.18497	2.177901	2.20231	2.195378	2.188472	2.181614	2.190662	2.183737	2.182847
2.182925	2.175933	2.200336	2.193457	2.186579	2.179727	2.18876	2.181826	2.180937
2.190646	2.183671	2.198394	2.19154	2.184674	2.177822	2.186838	2.179902	2.179019
2.188744	2.181802	2.196484	2.189635	2.182768	2.17591	2.184911	2.177974	2.1771
2.18685	2.179934	2.194578	2.187732	2.18086	2.173992	2.182982	2.176056	2.175199
2.184969	2.178071	2.192677	2.185831	2.178952	2.172068	2.181052	2.174149	2.173317
2.183095	2.176211	2.190777	2.18393	2.177042	2.170142	2.179126	2.172253	2.17145
2.181207	2.174335	2.188868	2.182025	2.175131	2.168218	2.177209	2.170373	2.169605
2.179364	2.172495	2.186982	2.180125	2.173217	2.166291	2.175291	2.168496	2.167765
2.177638	2.170735	2.185141	2.178226	2.171288	2.164351	2.17336	2.166609	2.165916
2.175969	2.169029	2.183342	2.176347	2.169366	2.162412	2.171428	2.164719	2.164067
2.174282	2.167368	2.18162	2.17454	2.167498	2.160497	2.169499	2.162826	2.16221
2.172589	2.165747	2.179962	2.172791	2.165677	2.158605	2.167576	2.160932	2.16035
2.170817	2.164021	2.178251	2.171076	2.163938	2.156823	2.165763	2.159122	2.15855
2.168953	2.162174	2.176486	2.169416	2.16231	2.155175	2.164074	2.157401	2.156805
2.167066	2.16029	2.174663	2.167684	2.176476	2.169353	2.162438	2.155752	2.155148
2.165178	2.158392	2.17276	2.165805	2.17468	2.167696	2.160873	2.154218	2.153646

2.16328	2.156478	2.170821	2.163869	2.172828	2.166002	2.159289	2.152678	2.152161
2.161368	2.154553	2.168878	2.16193	2.170934	2.164197	2.157556	2.15098	2.150539
2.159458	2.152629	2.166933	2.159981	2.169009	2.162329	2.15574	2.149196	2.148846
2.157617	2.150754	2.165026	2.158065	2.167105	2.160469	2.153921	2.147399	2.147123
2.155834	2.14892	2.163149	2.156174	2.165212	2.158606	2.152085	2.145576	2.145361
2.154147	2.147197	2.161397	2.154407	2.16343	2.15683	2.150326	2.143836	2.155189
2.152607	2.145639	2.159821	2.1528	2.16178	2.155155	2.148652	2.142184	2.153564
2.151019	2.144052	2.158252	2.151246	2.160207	2.153565	2.162336	2.155871	2.152061
2.14924	2.142311	2.156604	2.149722	2.158733	2.152107	2.160903	2.154501	2.15074
2.147401	2.140516	2.154916	2.148186	2.157266	2.150672	2.159519	2.153216	2.149531
2.145607	2.138719	2.153155	2.14651	2.155634	2.149082	2.158019	2.151904	2.148378
2.143838	2.136922	2.151353	2.144757	2.153906	2.147404	2.156458	2.150588	2.147275
2.142183	2.135267	2.149688	2.1431	2.167526	2.160992	2.154876	2.149193	2.14605
2.14065	2.133762	2.148159	2.141532	2.165885	2.159325	2.153254	2.147706	2.1447
2.160323	2.15339	2.162381	2.155702	2.164333	2.157782	2.15176	2.146308	2.143393
2.158604	2.151738	2.160801	2.154216	2.16292	2.156433	2.150463	2.14505	2.142155
2.156898	2.150093	2.159217	2.152725	2.16152	2.155135	2.149239	2.143867	2.14099
2.155308	2.14852	2.172932	2.166408	2.157338	2.15101	2.145151	2.139791	2.139947
2.153761	2.146984	2.171333	2.164799	2.155815	2.149662	2.143954	2.138719	2.138967
2.152172	2.145474	2.169819	2.163298	2.154396	2.148393	2.142801	2.137644	2.137945
2.150562	2.143987	2.168366	2.16187	2.153049	2.147181	2.141685	2.136576	2.136906
2.165345	2.15878	2.166846	2.160374	2.151633	2.145901	2.14052	2.135504	2.135901
2.163772	2.157185	2.165229	2.158779	2.15012	2.144528	2.139289	2.134425	2.134939
2.162232	2.155647	2.163684	2.157264	2.148681	2.143214	2.138098	2.133356	2.133963
2.160738	2.154228	2.162291	2.155912	2.14739	2.142027	2.136988	2.132291	2.13293
2.15927	2.152847	2.160952	2.15463	2.146169	2.140893	2.135917	2.131253	2.131915
2.138537	2.132239	2.159608	2.153365	2.144969	2.139762	2.134859	2.130276	2.131
2.137111	2.130843	2.15827	2.152131	2.143809	2.13866	2.133829	2.129341	2.130135
2.135617	2.129375	2.156888	2.150914	2.142702	2.137632	2.132861	2.128413	2.12923
2.134064	2.127844	2.155468	2.149716	2.141649	2.136674	2.131952	2.127498	2.128296
2.132573	2.126376	2.154096	2.148531	2.140585	2.135695	2.131031	2.126603	2.127408
2.131168	2.124991	2.152791	2.147363	2.139501	2.134677	2.13008	2.125728	2.126583
2.145394	2.139164	2.151499	2.146189	2.138416	2.133674	2.129153	2.124873	2.125771
2.143992	2.13778	2.15017	2.144963	2.137312	2.132712	2.128286	2.124049	2.124959
2.142613	2.136444	2.148883	2.143767	2.136236	2.131778	2.12744	2.12323	2.124138
2.141277	2.135235	2.14775	2.14272	2.135265	2.130875	2.126576	2.122368	2.123271
2.139979	2.13412	2.146724	2.141774	2.134368	2.129998	2.125706	2.121484	2.12238
2.138727	2.133023	2.145722	2.140854	2.133491	2.129124	2.124837	2.120625	2.121527
2.137523	2.131953	2.144753	2.13997	2.132643	2.128252	2.123966	2.119786	2.120709
2.136336	2.13089	2.125902	2.12126	2.131779	2.127396	2.123125	2.118975	2.119911
2.135163	2.129837	2.124906	2.120297	2.130877	2.126557	2.122329	2.118203	2.119139
2.133999	2.128774	2.123902	2.119337	2.129984	2.125731	2.121547	2.117438	2.118372
2.132822	2.12766	2.12288	2.118414	2.129127	2.124916	2.12076	2.116665	2.117605
2.131665	2.126551	2.121869	2.117514	2.12829	2.124108	2.119971	2.11588	2.116824
2.130598	2.12555	2.120933	2.116639	2.127456	2.123299	2.119167	2.115052	2.115982
2.129599	2.124627	2.120056	2.11579	2.12663	2.12249	2.11835	2.114193	2.115096
2.128601	2.123723	2.119206	2.114968	2.125815	2.121658	2.117508	2.113344	2.120868
2.127599	2.122838	2.118384	2.114177	2.125007	2.120793	2.116628	2.112503	2.120054
2.126632	2.121972	2.117579	2.113402	2.106862	2.102674	2.115784	2.111689	2.119257
2.12572	2.121128	2.116786	2.112643	2.106113	2.101907	2.115008	2.110915	2.118468

2.124831	2.120298	2.116002	2.11189	2.105375	2.101163	2.11426	2.110163	2.117693
2.123941	2.119474	2.11522	2.111131	2.104631	2.10042	2.113517	2.109427	2.123791
2.123057	2.118657	2.11444	2.110368	2.103887	2.099682	2.112783	2.108705	2.123038
2.12217	2.117833	2.113651	2.109598	2.103133	2.098938	2.112046	2.107979	2.122283
2.121281	2.117004	2.112857	2.10882	2.102373	2.098189	2.111307	2.107254	2.121531
2.120422	2.116189	2.112068	2.108046	2.101611	2.097433	2.12215	2.118017	2.120764
2.119598	2.115388	2.111286	2.107278	2.100848	2.096669	2.12137	2.117241	2.119982
2.118792	2.114597	2.110509	2.106508	2.100082	2.095901	2.120583	2.116457	2.11919
2.117999	2.113817	2.109734	2.105731	2.099307	2.095126	2.119789	2.115663	2.118385
2.117213	2.113042	2.108961	2.104953	2.098533	2.094354	2.118996	2.114868	2.117578
2.11642	2.112263	2.10819	2.104184	2.097774	2.093601	2.118217	2.114086	2.116783
2.115625	2.111483	2.10742	2.103419	2.097023	2.092858	2.117445	2.113312	2.115994
2.114848	2.110712	2.106651	2.102649	2.09856	2.094423	2.119529	2.115416	2.115205
2.114086	2.109948	2.105883	2.101874	2.097787	2.093656	2.118735	2.114628	2.114416
2.113348	2.109194	2.10512	2.1011	2.097016	2.092899	2.117951	2.113845	2.126506
2.112646	2.108453	2.10436	2.100327	2.096248	2.092149	2.117172	2.113061	2.125635
2.111942	2.107722	2.103609	2.099559	2.09549	2.091423	2.116423	2.112299	2.124778
2.111198	2.106994	2.102867	2.098798	2.094757	2.090749	2.122614	2.118418	2.123948
2.110449	2.106281	2.10214	2.098046	2.094032	2.090087	2.121946	2.117712	2.123136
2.10974	2.105609	2.101446	2.097307	2.093288	2.089364	2.121195	2.116964	2.122328
2.109055	2.104967	2.100774	2.09658	2.092535	2.088603	2.12039	2.116192	2.12153
2.108339	2.104273	2.100076	2.095866	2.091803	2.087851	2.132989	2.128403	2.120789
2.107587	2.103523	2.099347	2.095163	2.09109	2.087102	2.132129	2.127644	2.120114
2.106835	2.102765	2.098624	2.094485	2.090412	2.086393	2.13131	2.126891	2.128791
2.10608	2.102004	2.09792	2.093855	2.089796	2.08575	2.130545	2.126123	2.128023
2.105345	2.101255	2.097218	2.093216	2.089181	2.085131	2.129822	2.125379	2.127259
2.104658	2.100534	2.096493	2.092504	2.088507	2.084519	2.12916	2.1247	2.12654
2.104001	2.09984	2.095769	2.091763	2.087808	2.083906	2.128521	2.124056	2.12585
2.103355	2.099195	2.095098	2.091062	2.11465	2.110251	2.119679	2.115299	2.1252
2.102721	2.098593	2.09447	2.090387	2.113847	2.109434	2.118866	2.114607	2.124586
2.098759	2.094674	2.093833	2.089747	2.1131	2.108655	2.118065	2.113874	2.1239
2.098094	2.09398	2.093183	2.089154	2.112432	2.107923	2.117271	2.113081	2.123112
2.097409	2.093276	2.09252	2.08854	2.118849	2.11429	2.116515	2.112308	2.122331
2.096685	2.092564	2.091824	2.087851	2.118084	2.113591	2.115832	2.111597	2.121603
2.095958	2.091862	2.091135	2.087161	2.117321	2.112904	2.115169	2.110912	2.120901
2.095276	2.091212	2.090518	2.086563	2.123274	2.118816	2.129676	2.12523	2.134125
2.094621	2.090592	2.089937	2.086005	2.122609	2.118106	2.128923	2.124534	2.133458
2.093976	2.089945	2.121817	2.117397	2.121882	2.117409	2.12821	2.123816	2.132738
2.093343	2.089278	2.120996	2.116559	2.1211	2.116727	2.127535	2.12308	2.131969
2.092703	2.088623	2.120199	2.115732	2.120311	2.116011	2.126824	2.122352	2.117944
2.09205	2.087994	2.119444	2.114921	2.119502	2.115232	2.126046	2.121642	2.117278
2.091392	2.087359	2.118703	2.114153	2.118737	2.114479	2.125279	2.120928	2.116602
2.090729	2.086689	2.117963	2.11347	2.118085	2.113825	2.12458	2.120192	2.115857
2.090069	2.086018	2.117238	2.112824	2.117474	2.113203	2.123901	2.119455	2.1151
2.097586	2.093525	2.116557	2.112161	2.114613	2.110309	2.123191	2.118755	2.114413
2.096936	2.092921	2.115904	2.11149	2.113918	2.109593	2.122466	2.118078	2.113767
2.096314	2.092309	2.115219	2.11078	2.113198	2.108891	2.121759	2.117371	2.113065
2.095725	2.091694	2.114497	2.11002	2.112446	2.108203	2.121076	2.11663	2.112299
2.122825	2.118315	2.113779	2.109297	2.111723	2.107508	2.118411	2.113921	2.111579
2.122103	2.117563	2.113068	2.108644	2.111052	2.106781	2.117641	2.113224	2.11095

2.121372	2.116819	2.112376	2.108014	2.110406	2.106078	2.116898	2.112555	2.110349
2.120645	2.116141	2.111728	2.107389	2.109786	2.105465	2.116263	2.111925	2.120595
2.119929	2.115494	2.1111	2.106765	2.109175	2.104888	2.115674	2.111314	2.119966
2.119264	2.114847	2.110444	2.106096	2.10851	2.104263	2.11504	2.110667	2.119307
2.118642	2.114204	2.10977	2.105393	2.107806	2.103604	2.114378	2.109995	2.118628
2.117993	2.113523	2.109094	2.104732	2.107135	2.102933	2.12502	2.120527	2.119963
2.1173	2.112784	2.108406	2.104118	2.106502	2.102235	2.124271	2.11986	2.119343
2.116635	2.112089	2.107758	2.103534	2.105899	2.10158	2.123569	2.119224	2.118738
2.116035	2.111499	2.107189	2.102986	2.105342	2.101014	2.122968	2.118637	2.118137
2.115456	2.110944	2.106645	2.102449	2.104799	2.100481	2.122406	2.118071	2.117544
2.114867	2.110375	2.106083	2.101889	2.104237	2.099934	2.12184	2.117507	2.116966
2.114273	2.109803	2.105513	2.101314	2.103661	2.099384	2.121275	2.116941	2.116387
2.11364	2.109213	2.104914	2.100691	2.103039	2.098804	2.12067	2.116315	2.115739
2.112968	2.108603	2.104284	2.100019	2.102369	2.098194	2.120027	2.115632	2.115025
2.11234	2.108019	2.103688	2.099396	2.101742	2.097604	2.119404	2.11499	2.11436
2.111775	2.107471	2.103142	2.098847	2.101179	2.097039	2.118812	2.114414	2.113775
2.11123	2.106937	2.109198	2.104868	2.112535	2.108229	2.126165	2.121651	2.117217
2.110693	2.10641	2.108662	2.104334	2.111983	2.107681	2.125591	2.121074	2.116635
2.110162	2.105888	2.108132	2.103805	2.111437	2.107145	2.12503	2.120505	2.11606
2.109622	2.105363	2.107596	2.103268	2.110883	2.106602	2.124463	2.119935	2.115484
2.109078	2.104837	2.107058	2.102728	2.110324	2.106059	2.123895	2.119366	2.114909
2.10853	2.104302	2.106514	2.102184	2.109766	2.105519	2.123329	2.118796	2.114336
2.107977	2.103757	2.105961	2.101637	2.109207	2.104982	2.122764	2.118225	2.113763
2.107428	2.103217	2.105414	2.101096	2.108652	2.104445	2.122195	2.117654	2.113193
2.106884	2.102688	2.104879	2.100565	2.108104	2.103907	2.121619	2.117085	2.112631
2.106342	2.10216	2.104345	2.100036	2.107556	2.10337	2.121043	2.116516	2.112068
2.1058	2.101628	2.103805	2.0995	2.107005	2.102835	2.120473	2.115943	2.111497
2.105257	2.101095	2.103264	2.098963	2.106452	2.1023	2.119903	2.115369	2.110923
2.104709	2.100561	2.102725	2.098429	2.10999	2.105797	2.101585	2.097326	2.093139
2.104159	2.100026	2.102187	2.097899	2.109428	2.10524	2.101031	2.096775	2.09259
2.10363	2.099502	2.101661	2.097387	2.10888	2.104692	2.100486	2.096237	2.092057
2.103123	2.09899	2.101146	2.096888	2.108343	2.104153	2.099949	2.095709	2.091534
2.109086	2.104903	2.100655	2.096429	2.107843	2.103635	2.099429	2.095209	2.091046
2.108634	2.104406	2.100204	2.096047	2.107414	2.103155	2.098939	2.094764	2.090629
2.10819	2.103923	2.099764	2.095669	2.106988	2.102678	2.09845	2.094319	2.09021
2.107711	2.10345	2.099301	2.095213	2.106487	2.10216	2.097927	2.093807	2.112277
2.107221	2.10299	2.098832	2.09472	2.105949	2.101622	2.097387	2.093261	2.111695
2.106758	2.102566	2.102812	2.098618	2.105419	2.101093	2.096855	2.092721	2.111121
2.106321	2.102176	2.102382	2.09814	2.104892	2.100566	2.096325	2.09218	2.110547
2.10586	2.101739	2.101924	2.097663	2.104392	2.100082	2.095843	2.091682	2.110008
2.105361	2.101233	2.101421	2.097178	2.103926	2.09966	2.095434	2.091242	2.109514
2.104867	2.100716	2.100919	2.096713	2.103474	2.099235	2.095019	2.090816	2.109049
2.104409	2.100212	2.100445	2.096302	2.103038	2.098767	2.094551	2.090393	2.10863
2.103958	2.099715	2.099976	2.095894	2.102596	2.098281	2.094062	2.08996	2.108209
2.103483	2.099232	2.099486	2.095415	2.102091	2.097777	2.093563	2.08947	2.107698
2.102997	2.098763	2.098989	2.094895	2.101549	2.097263	2.093057	2.088946	2.107134
2.081768	2.077602	2.09853	2.09441	2.10105	2.096791	2.092593	2.088463	2.106611
2.081338	2.077199	2.098112	2.093957	2.100591	2.096362	2.092172	2.088021	2.106121
2.080666	2.076478	2.108721	2.104413	2.100152	2.095934	2.091749	2.087592	2.105659
2.080228	2.076029	2.108285	2.104004	2.099739	2.095495	2.091306	2.087179	2.105238

2.079796	2.075589	2.107855	2.103597	2.099328	2.095062	2.090869	2.086768	2.104814
2.079375	2.07519	2.103166	2.098894	2.098897	2.094647	2.090458	2.086343	2.104339
2.078959	2.074802	2.102752	2.098461	2.09846	2.094237	2.090053	2.085915	2.103855
2.078539	2.074378	2.089527	2.085268	2.098035	2.093803	2.089621	2.085498	2.103426
2.078116	2.073931	2.089091	2.084854	2.097616	2.093354	2.089171	2.085087	2.103036
2.077693	2.073507	2.081593	2.07738	2.092942	2.088679	2.088736	2.084662	2.102597
2.07727	2.073113	2.081152	2.07692	2.0925	2.088262	2.088321	2.08422	2.102094
2.063656	2.059495	2.080709	2.076467	2.079297	2.075098	2.083663	2.07954	2.101617
2.063228	2.059046	2.080261	2.076039	2.078875	2.074662	2.083229	2.07913	2.101217
2.0628	2.058595	2.079812	2.075612	2.078454	2.074228	2.082797	2.078722	2.100823
2.062366	2.058182	2.079367	2.075158	2.078016	2.073808	2.082387	2.0783	2.096785
2.061933	2.057782	2.078926	2.074699	2.077576	2.073397	2.081986	2.077873	2.096309
2.061522	2.057357	2.066072	2.06183	2.077167	2.072982	2.068843	2.064754	2.095864
2.061132	2.05691	2.065631	2.061433	2.076786	2.072565	2.068411	2.064335	2.095443
2.073857	2.069475	2.065231	2.061054	2.069333	2.065111	2.068014	2.063925	2.095006
2.073497	2.069132	2.064888	2.060703	2.068992	2.064776	2.067667	2.063525	2.094534
2.073129	2.068793	2.064541	2.060335	2.056113	2.05189	2.091227	2.086795	2.082425
2.072717	2.068426	2.064149	2.059901	2.055688	2.051508	2.090871	2.086431	2.082039
2.072304	2.068055	2.063753	2.059462	2.055254	2.051111	2.090504	2.086079	2.081683
2.071942	2.067701	2.063395	2.059094	2.054865	2.050692	2.090078	2.085699	2.081337
2.071607	2.067352	2.063057	2.058767	2.054504	2.050265	2.08962	2.085307	2.081002
2.065329	2.061077	2.062692	2.058432	2.054165	2.049899	2.071989	2.067703	2.080654
2.064954	2.060659	2.0623	2.058094	2.053851	2.049594	2.071652	2.067349	2.080296
2.064602	2.06028	2.061933	2.057755	2.053532	2.049289	2.071312	2.066975	2.07991
2.064292	2.059969	2.061611	2.057425	2.053205	2.048974	2.070951	2.066556	2.079474
2.063989	2.059672	2.061291	2.057076	2.052859	2.04865	2.070593	2.06615	2.079052
2.063677	2.059362	2.060937	2.056671	2.052463	2.048306	2.070251	2.065798	2.061422
2.063355	2.05905	2.060577	2.056256	2.052058	2.047957	2.069915	2.06547	2.061091
2.063001	2.058731	2.06025	2.055912	2.051718	2.047636	2.069582	2.065138	2.060748
2.062623	2.058407	2.059945	2.055617	2.051424	2.047332	2.069251	2.064809	2.060405
2.062282	2.058092	2.053803	2.049513	2.051126	2.047024	2.068917	2.06448	2.060073
2.061982	2.057787	2.053502	2.049218	2.050824	2.046713	2.068585	2.064158	2.059757
2.061694	2.057492	2.053207	2.048924	2.050513	2.046376	2.068217	2.063813	2.059432
2.061414	2.057209	2.052922	2.048631	2.050179	2.045985	2.067775	2.063418	2.059085
2.061138	2.056931	2.05264	2.048339	2.049848	2.045597	2.067337	2.063024	2.058735
2.060858	2.056647	2.052354	2.04805	2.049541	2.04527	2.066976	2.062674	2.0584
2.060578	2.056363	2.052069	2.047763	2.04925	2.044978	2.066659	2.06235	2.058073
2.060296	2.056079	2.051784	2.04748	2.043214	2.038978	2.066356	2.06204	2.057757
2.060011	2.055794	2.051501	2.047199	2.042942	2.038718	2.066072	2.061744	2.057453
2.059729	2.05551	2.051218	2.046922	2.042685	2.038493	2.065831	2.06148	2.057166
2.059447	2.055226	2.050935	2.046648	2.042449	2.038317	2.065655	2.06126	2.056902
2.05631	2.052084	2.050653	2.046373	2.042205	2.038122	2.065456	2.061029	2.056637
2.056037	2.051799	2.050368	2.046088	2.041929	2.037864	2.06518	2.060754	2.056359
2.05578	2.051522	2.050089	2.045806	2.041647	2.037584	2.064874	2.060462	2.056076
2.046388	2.042102	2.049826	2.045538	2.041374	2.037309	2.064571	2.060169	2.055793
2.046205	2.041864	2.049577	2.045286	2.041112	2.037035	2.064264	2.059873	2.055508
2.045982	2.041622	2.046476	2.042205	2.040899	2.036791	2.059115	2.054751	2.055231
2.045723	2.041377	2.046274	2.042048	2.040737	2.036578	2.05884	2.054468	2.054959
2.045466	2.04113	2.036894	2.032711	2.067414	2.062972	2.058567	2.054192	2.054692
2.045214	2.040872	2.036633	2.03245	2.06713	2.06269	2.058288	2.053916	2.054421

2.044977	2.040636	2.036382	2.032181	2.066835	2.06241	2.058022	2.053664	2.054176
2.044778	2.040463	2.036183	2.031941	2.058717	2.054305	2.057776	2.053466	2.054
2.044588	2.040304	2.035997	2.031707	2.058449	2.054052	2.057549	2.053289	2.053844
2.044355	2.04008	2.035766	2.031467	2.055728	2.051361	2.049527	2.045267	2.048828
2.044094	2.039814	2.035507	2.031224	2.055493	2.051175	2.049355	2.045048	2.048574
2.043859	2.039553	2.035247	2.030976	2.055233	2.050942	2.049137	2.044814	2.04052
2.043651	2.039293	2.034979	2.030717	2.054939	2.050652	2.048865	2.044559	2.040289
2.043443	2.039053	2.034741	2.030493	2.054682	2.050384	2.046097	2.041814	2.040082
2.043219	2.038841	2.034554	2.030333	2.0545	2.05016	2.045866	2.041625	2.039924
2.043003	2.038651	2.034389	2.030189	2.054337	2.049962	2.045659	2.041444	2.03976
2.042814	2.038489	2.034228	2.030021	2.054151	2.049802	2.045498	2.041246	2.039536
2.042643	2.038343	2.034075	2.029852	2.053963	2.049658	2.045352	2.041038	2.039286
2.042479	2.038181	2.033923	2.029708	2.053789	2.049466	2.045141	2.040805	2.036503
2.042325	2.038009	2.033773	2.029585	2.053627	2.049238	2.04488	2.040551	2.036269
2.042175	2.037858	2.033625	2.029437	2.053435	2.049007	2.044637	2.04033	2.03607
2.042028	2.037735	2.033479	2.029252	2.053195	2.048765	2.044413	2.040153	2.035919
2.041884	2.037606	2.033335	2.029085	2.052988	2.048559	2.044218	2.039983	2.035764
2.041743	2.037447	2.033196	2.028975	2.052875	2.048437	2.044076	2.039804	2.035566
2.041605	2.037287	2.033057	2.02887	2.052772	2.048327	2.043945	2.03963	2.035369
2.033188	2.028885	2.032913	2.028707	2.052577	2.048135	2.043763	2.039468	2.035217
2.033046	2.028762	2.032766	2.028519	2.052343	2.04791	2.043561	2.039314	2.035086
2.032908	2.028616	2.03262	2.028377	2.052189	2.047761	2.043411	2.039156	2.034921
2.032776	2.028449	2.032476	2.028277	2.052109	2.047682	2.043309	2.038992	2.034723
2.032638	2.028294	2.024056	2.019878	2.051997	2.047593	2.043218	2.038865	2.03457
2.032487	2.028164	2.02391	2.019707	2.051823	2.047485	2.043148	2.038801	2.034489
2.032341	2.028033	2.023762	2.019538	2.051644	2.047353	2.043052	2.038732	2.034422
2.032211	2.027872	2.023607	2.0194	2.051488	2.047169	2.042876	2.038613	2.034347
2.032098	2.027717	2.023458	2.019272	2.051338	2.046973	2.042678	2.038472	2.034256
2.032019	2.027622	2.023338	2.019126	2.04389	2.039545	2.042536	2.038314	2.034096
2.031966	2.027569	2.023242	2.018976	2.043724	2.039432	2.042429	2.038144	2.033889
2.061741	2.05709	2.052507	2.047995	2.043613	2.039343	2.042343	2.038027	2.033748
2.061626	2.05704	2.052471	2.047953	2.043566	2.039288	2.042284	2.037971	2.033677
2.061527	2.056984	2.05243	2.047913	2.043511	2.039205	2.03491	2.030628	2.033607
2.061471	2.056925	2.052379	2.047864	2.043428	2.039059	2.034749	2.030511	2.033532
2.061408	2.056839	2.052301	2.047801	2.043338	2.038913	2.034585	2.030378	2.033433
2.06129	2.056678	2.052163	2.04771	2.043256	2.038819	2.034468	2.030222	2.033263
2.061156	2.05649	2.051997	2.047595	2.043169	2.038745	2.034376	2.030065	2.033072
2.051793	2.047108	2.05186	2.047444	2.043036	2.038656	2.034304	2.029971	2.025699
2.051754	2.047046	2.051742	2.047261	2.042868	2.03856	2.034251	2.029927	2.025653
2.051716	2.047002	2.051666	2.047143	2.042755	2.038487	2.034203	2.029885	2.025603
2.051683	2.046981	2.051642	2.047107	2.042714	2.038445	2.034158	2.029842	2.025546
2.05163	2.046953	2.042351	2.037825	2.042685	2.038404	2.034112	2.0298	2.025497
2.05153	2.046899	2.042315	2.037795	2.042654	2.038364	2.03407	2.029766	2.025466
2.05143	2.046842	2.042277	2.037765	2.042617	2.038307	2.034008	2.029721	2.025434
2.051381	2.046799	2.042237	2.037721	2.042544	2.038183	2.033874	2.029629	2.025379
2.05136	2.046764	2.042196	2.037672	2.042455	2.03803	2.033706	2.029514	2.025311
2.051345	2.04674	2.042169	2.037641	2.033155	2.028714	2.033608	2.029437	2.025256
2.051339	2.046727	2.042155	2.037628	2.033135	2.028684	2.03357	2.029392	2.02521
2.051336	2.046718	2.042144	2.037615	2.03312	2.028662	2.024328	2.020151	2.025173
2.051337	2.046711	2.042134	2.037602	2.033104	2.028644	2.024308	2.020127	2.025147

2.042173	2.037572	2.042127	2.037592	2.033091	2.02863	2.024291	2.020107	2.025125
2.042171	2.037569	2.042126	2.037586	2.033079	2.02861	2.024268	2.020083	2.025103
2.042171	2.03757	2.042129	2.037583	2.033071	2.028597	2.02425	2.020064	2.025085
2.042167	2.037568	2.042131	2.037584	2.033072	2.0286	2.024252	2.020058	2.015869
2.042163	2.037565	2.042133	2.037588	2.033083	2.028619	2.024271	2.020065	2.015864
2.035335	2.030758	2.042141	2.037602	2.033117	2.028684	2.02434	2.020103	2.015874
2.035365	2.03078	2.042154	2.037627	2.033179	2.028804	2.024468	2.020178	2.0159
2.035422	2.030814	2.042169	2.037645	2.033222	2.028888	2.024561	2.020235	2.015926
2.035525	2.030866	2.042187	2.037647	2.033222	2.028901	2.024582	2.020259	2.015949
2.035628	2.030922	2.04221	2.037652	2.033219	2.028896	2.02458	2.020269	2.015969
2.035685	2.030969	2.033077	2.028547	2.033226	2.028894	2.024576	2.020269	2.015984
2.035727	2.031019	2.033112	2.028578	2.03325	2.028897	2.02457	2.020265	2.015998
2.035784	2.03109	2.033189	2.028674	2.033335	2.028938	2.024586	2.020277	2.016015
2.035851	2.031178	2.033301	2.028822	2.033468	2.02901	2.02462	2.020303	2.016034
2.035944	2.031298	2.033416	2.028935	2.03357	2.029079	2.024674	2.020367	2.016101
2.036076	2.031467	2.033543	2.029006	2.033631	2.02914	2.02475	2.020481	2.016231
2.024609	2.020068	2.033634	2.029058	2.024555	2.020118	2.024843	2.020591	2.016343
2.024639	2.020099	2.033656	2.029087	2.024616	2.020227	2.024968	2.020678	2.016397
2.024659	2.020104	2.033654	2.029105	2.024668	2.020327	2.025082	2.020751	2.016438
2.024732	2.020135	2.033656	2.02911	2.024681	2.020353	2.025123	2.020797	2.016494
2.024828	2.020176	2.033664	2.029116	2.024681	2.020346	2.02513	2.020832	2.01656
2.024907	2.02024	2.015689	2.01122	2.024749	2.020389	2.025177	2.020907	2.016657
2.02497	2.020324	2.015805	2.011369	2.024877	2.020472	2.025253	2.021017	2.016783
2.025063	2.020438	2.015934	2.011508	2.025	2.020581	2.02536	2.021122	2.01688
2.025194	2.020592	2.016074	2.011621	2.025108	2.02073	2.025514	2.021219	2.016931
2.025339	2.020749	2.016217	2.011742	2.014219	2.009902	2.01653	2.012214	2.016978
2.025489	2.020879	2.016354	2.011894	2.014337	2.009973	2.016576	2.012255	2.017035
2.025644	2.021008	2.016492	2.01205	2.014451	2.010024	2.0166	2.012295	2.017106
2.025801	2.02117	2.016638	2.012169	2.014529	2.010081	2.016659	2.012382	2.017218
2.025958	2.02135	2.016789	2.01227	2.014591	2.010142	2.016739	2.012497	2.017357
2.026105	2.021501	2.016938	2.012414	2.014712	2.010258	2.016854	2.012608	2.017474
2.026241	2.02162	2.017085	2.012604	2.014899	2.010437	2.017011	2.012718	2.01757
2.026383	2.021757	2.017233	2.012768	2.008312	2.003876	2.006211	2.00194	2.008575
2.026541	2.021935	2.017383	2.012874	2.0084	2.003965	2.006293	2.002054	2.008711
2.026698	2.02211	2.017535	2.012989	2.008502	2.004069	2.006388	2.002173	2.008847
2.026841	2.022243	2.017688	2.013173	2.008698	2.004265	2.00655	2.002295	2.008948
2.02698	2.022366	2.017843	2.013379	2.008926	2.004498	2.006745	2.00243	2.009051
2.027132	2.022525	2.018001	2.013536	2.009104	2.00471	2.006951	2.002615	2.009218
2.027294	2.02271	2.018162	2.013663	2.00925	2.004912	2.007168	2.002838	2.009433
2.027464	2.022874	2.018316	2.01381	2.009401	2.005078	2.007333	2.003028	2.009642
2.027641	2.023007	2.018459	2.01398	2.009559	2.005196	2.00743	2.00318	2.009852
2.026121	2.021444	2.018622	2.014153	2.00972	2.005334	2.001034	1.996835	1.999111
2.026355	2.021666	2.018817	2.014307	2.009875	2.00552	2.001219	1.996976	1.999207
2.026596	2.021914	2.019039	2.014482	2.010051	2.005731	2.001428	1.99713	1.999304
2.02681	2.02217	2.019298	2.014723	2.010291	2.005975	2.001666	1.997348	1.999481
2.027016	2.022436	2.019583	2.015004	2.010564	2.006235	2.001915	1.997595	1.999696
2.027254	2.022696	2.019858	2.015278	2.010813	2.006442	2.002106	1.997803	1.999897
2.027519	2.022953	2.020129	2.01555	2.011046	2.006606	2.002246	1.997982	2.00009
2.000648	1.99615	2.018594	2.014036	2.0113	2.006815	2.00243	1.998162	2.000258
2.000851	1.996315	2.018799	2.014301	2.011584	2.007089	2.002673	1.99834	2.000381

2.001074	1.996506	2.019022	2.014567	2.011872	2.007382	2.002943	1.998548	2.000533
2.001352	1.996773	2.019301	2.014843	2.012159	2.007681	2.00324	1.998821	2.000766
2.001647	1.997066	2.019594	2.015109	2.012434	2.007982	2.003549	1.999119	2.00103
2.001926	1.997335	2.007759	2.003249	2.012662	2.008256	2.003843	1.999404	1.995039
2.002198	1.997597	2.007985	2.003426	2.012865	2.008516	2.004132	1.999688	1.995299
1.99348	1.988898	2.008246	2.003659	2.013119	2.0088	2.00443	1.999984	1.995581
1.993714	1.989172	2.00854	2.003948	2.013423	2.009106	2.004738	2.000292	1.995886
1.993973	1.989455	1.993867	1.989344	1.999808	1.995493	2.00504	2.000596	1.996189
1.994269	1.989747	1.994156	1.989637	2.000106	1.995784	2.005342	2.000896	1.996488
1.994584	1.990048	1.994451	1.989929	2.000394	1.996052	2.005618	2.001182	1.996782
1.994913	1.990364	1.98579	1.981254	2.000644	1.996254	2.005824	2.001426	1.997062
1.995251	1.990687	1.986093	1.981529	2.000886	1.996442	2.006013	2.001662	1.997339
1.995583	1.991008	1.986401	1.981823	1.992789	1.988331	2.006282	2.001943	1.997633
1.995913	1.991327	1.986713	1.982132	1.993104	1.988646	2.006605	2.002256	1.997938
1.97938	1.974963	1.987026	1.982443	1.977955	1.973548	1.992929	1.988572	1.998256
1.979696	1.975277	1.987339	1.982753	1.97827	1.973875	1.993263	1.988898	1.998586
1.980006	1.975587	1.987653	1.983071	1.978603	1.974231	1.98527	1.980891	1.998925
1.980302	1.975886	1.987967	1.983401	1.978968	1.974646	1.985701	1.981272	1.999271
1.980601	1.976187	1.988284	1.983732	1.979332	1.975055	1.986128	1.981653	1.999621
1.940522	1.936445	1.950674	1.946438	1.979655	1.975391	1.986473	1.981993	1.977539
1.940854	1.936764	1.950996	1.946754	1.979961	1.97569	1.986778	1.982312	1.977869
1.941223	1.937105	1.951336	1.947093	1.980283	1.976002	1.98709	1.982631	1.978201
1.941626	1.937463	1.951692	1.947453	1.980618	1.976317	1.987401	1.982946	1.978534
1.942002	1.937815	1.933672	1.929584	1.980993	1.976657	1.972296	1.967889	1.978872
1.942339	1.938156	1.934053	1.930014	1.981428	1.977032	1.972635	1.968224	1.979209
1.91497	1.910966	1.934429	1.930426	1.981846	1.977404	1.972984	1.968581	1.979574
1.915301	1.911289	1.934776	1.930779	1.982198	1.977752	1.973346	1.968982	1.980005
1.915641	1.911622	1.935122	1.931114	1.982526	1.978093	1.973709	1.969387	1.980441
1.915989	1.911987	1.935502	1.931465	1.982862	1.978427	1.974046	1.969735	1.980799
1.916344	1.912368	1.935899	1.93182	1.9832	1.97876	1.974374	1.970053	1.981115
1.897912	1.894001	1.936261	1.932171	1.983564	1.979138	1.974747	1.970398	1.981437
1.898211	1.894286	1.93659	1.932518	1.983952	1.97956	1.975167	1.970766	1.981761
1.898532	1.894582	1.936934	1.932886	1.984349	1.979963	1.975561	1.971133	1.982108
1.898892	1.894901	1.937309	1.933297	1.984764	1.980328	1.975904	1.971489	1.982484
1.899255	1.895227	1.937683	1.933696	1.985163	1.980682	1.976246	1.971858	1.982886
1.899597	1.89555	1.920902	1.916964	1.985506	1.981029	1.97661	1.972262	1.967925
1.899937	1.895882	1.921234	1.917276	1.985834	1.98138	1.976991	1.972682	1.968371
1.900296	1.896243	1.921609	1.91763	1.971693	1.967306	1.977403	1.973101	1.968795
1.900669	1.896626	1.922015	1.918012	1.972087	1.967723	1.97784	1.973522	1.969207
1.881199	1.877196	1.922426	1.918416	1.972501	1.968135	1.978262	1.973947	1.969633
1.881599	1.877579	1.922841	1.918847	1.97294	1.96854	1.978665	1.974378	1.970076
1.882	1.877965	1.923261	1.919275	1.973379	1.968956	1.964613	1.960376	1.970503
1.882396	1.878358	1.923685	1.919687	1.973807	1.969394	1.965044	1.960776	1.970889
1.882796	1.878752	1.924111	1.9201	1.974238	1.969834	1.965477	1.96118	1.971282
1.869989	1.86595	1.906448	1.902481	1.974684	1.970255	1.965891	1.961611	1.971736
1.870399	1.866335	1.906869	1.902913	1.975137	1.970673	1.966302	1.962051	1.972212
1.828676	1.824638	1.895551	1.891613	1.960256	1.955803	1.966737	1.962473	1.958196
1.829048	1.825022	1.895987	1.892022	1.960706	1.956267	1.967192	1.96288	1.958566
1.829421	1.825396	1.842765	1.839012	1.951696	1.947271	1.952339	1.948031	1.958979
1.829795	1.825751	1.843158	1.839405	1.952155	1.9477	1.952768	1.948483	1.959455

1.830173	1.826115	1.822267	1.818538	1.936518	1.932047	1.953202	1.948931	1.95992
1.830555	1.826509	1.822633	1.818867	1.936949	1.932496	1.953651	1.949352	1.960333
1.830945	1.826908	1.823004	1.819201	1.937383	1.93295	1.954103	1.949775	1.960743
1.812335	1.808343	1.823375	1.819574	1.861067	1.857126	1.929007	1.924712	1.945934
1.812747	1.808718	1.823754	1.819973	1.861491	1.857526	1.929433	1.92517	1.946412
1.813178	1.809134	1.824167	1.820381	1.841719	1.837757	1.929917	1.925667	1.921428
1.813631	1.809591	1.824615	1.820801	1.842166	1.8382	1.930461	1.926205	1.921958
1.814075	1.810051	1.825058	1.821203	1.842593	1.838624	1.90262	1.898477	1.922444
1.814498	1.810509	1.825488	1.821572	1.842985	1.839014	1.903071	1.898887	1.922854
1.814926	1.810963	1.825918	1.821948	1.843372	1.839393	1.903503	1.89929	1.923265
1.815378	1.811411	1.826356	1.82236	1.843767	1.839757	1.885773	1.881596	1.895555
1.815834	1.811847	1.826791	1.822788	1.844174	1.840127	1.886138	1.881993	1.896005
1.816264	1.812248	1.827202	1.823217	1.844624	1.840549	1.886567	1.882425	1.878304
1.816679	1.812626	1.827596	1.823649	1.845104	1.84101	1.88704	1.882882	1.878749
1.817104	1.813038	1.809079	1.805204	1.845576	1.841481	1.887516	1.883336	1.879184
1.817541	1.813485	1.809514	1.805622	1.846043	1.841965	1.887998	1.883791	1.879613
1.817967	1.813932	1.809946	1.80603	1.846492	1.842431	1.888461	1.884216	1.880008
1.818364	1.814363	1.81036	1.806412	1.846906	1.842857	1.888883	1.884577	1.880334
1.818768	1.814798	1.810781	1.806806	1.84733	1.843285	1.889309	1.884945	1.880669
1.819207	1.815247	1.811229	1.80725	1.822254	1.818531	1.839733	1.835722	1.881085
1.819669	1.815707	1.811695	1.807722	1.822726	1.818979	1.840208	1.836176	1.881543
1.820147	1.816182	1.812173	1.808202	1.823203	1.819439	1.840692	1.836641	1.882004
1.820643	1.816672	1.812663	1.808694	1.823691	1.81991	1.841188	1.83712	1.882475
1.804159	1.800234	1.813151	1.809184	1.80534	1.801596	1.841686	1.837601	1.882952
1.804631	1.800702	1.813636	1.809674	1.805825	1.802068	1.842185	1.838085	1.883435
1.805102	1.801166	1.814117	1.810158	1.806305	1.802539	1.842687	1.838571	1.88392
1.805571	1.801627	1.814591	1.810632	1.806777	1.80301	1.843192	1.839059	1.8844
1.80604	1.802086	1.815061	1.811102	1.807246	1.803479	1.843696	1.839548	1.884882
1.806506	1.802543	1.815535	1.811577	1.807718	1.803945	1.844196	1.840035	1.885373
1.806973	1.803	1.816008	1.812052	1.808189	1.804408	1.844692	1.840522	1.885869
1.807444	1.803457	1.816475	1.812517	1.80865	1.804865	1.827595	1.823455	1.836916
1.807921	1.803914	1.816937	1.812972	1.809102	1.805318	1.828086	1.823947	1.837408
1.808399	1.804375	1.817403	1.81343	1.809555	1.805768	1.828572	1.824435	1.837897
1.808873	1.804841	1.81787	1.813887	1.810003	1.806209	1.829048	1.824911	1.838376
1.809362	1.805316	1.81835	1.814361	1.810468	1.806662	1.829532	1.825397	1.838867
1.809876	1.805801	1.818844	1.814861	1.810961	1.807139	1.803313	1.799471	1.82183
1.810412	1.806304	1.819359	1.815387	1.811477	1.807633	1.803796	1.799959	1.822358
1.793563	1.789471	1.819917	1.815956	1.812027	1.808146	1.804296	1.80048	1.822929
1.794136	1.790014	1.820505	1.816556	1.812603	1.808673	1.804809	1.801024	1.823529
1.753	1.749616	1.821066	1.817117	1.813144	1.809181	1.805302	1.801529	1.824082
1.753397	1.75001	1.821598	1.817638	1.81365	1.809667	1.805775	1.801993	1.824582
1.75379	1.750404	1.805601	1.80165	1.814147	1.810157	1.806256	1.802458	1.82509
1.75418	1.7508	1.806106	1.802113	1.814628	1.81065	1.806749	1.802933	1.825621
1.754577	1.751208	1.806632	1.802593	1.815127	1.811164	1.807262	1.803424	1.82616
1.755003	1.751648	1.807212	1.803135	1.799139	1.795219	1.807822	1.803949	1.826698
1.755439	1.752099	1.807815	1.803706	1.799703	1.79579	1.808399	1.804493	1.827241
1.755853	1.752518	1.790271	1.786252	1.80026	1.796328	1.792426	1.788544	1.8278
1.756254	1.752914	1.790793	1.786808	1.800815	1.796843	1.792925	1.789062	1.828371
1.756662	1.753313	1.791304	1.787325	1.801336	1.797338	1.793408	1.789552	1.81355
1.757081	1.753714	1.791797	1.787792	1.80181	1.797802	1.793864	1.790001	1.814026

1.7575	1.754121	1.750776	1.747453	1.802297	1.798291	1.794348	1.790467	1.814515
1.757917	1.754545	1.751187	1.747848	1.802824	1.798842	1.794895	1.790973	1.81504
1.758328	1.754966	1.751602	1.748254	1.803374	1.799413	1.795461	1.791502	1.815588
1.662354	1.659406	1.752013	1.748678	1.803952	1.79997	1.796007	1.792051	1.816165
1.662675	1.659726	1.752421	1.749111	1.804546	1.800525	1.796549	1.792614	1.816761
1.663016	1.660058	1.692746	1.689657	1.787014	1.783061	1.7971	1.79316	1.817341
1.663374	1.660402	1.693087	1.689993	1.787523	1.783602	1.797658	1.793693	1.817907
1.663749	1.660761	1.693445	1.690334	1.732538	1.728799	1.798208	1.794236	1.818481
1.664147	1.661139	1.69382	1.690674	1.732989	1.72925	1.798743	1.794794	1.819065
1.664549	1.661526	1.694213	1.691045	1.733485	1.729732	1.74654	1.742753	1.81965
1.664945	1.661916	1.694627	1.691482	1.734086	1.730303	1.747093	1.743271	1.820228
1.66534	1.66231	1.695053	1.691943	1.73472	1.7309	1.747661	1.743793	1.820808
1.665739	1.662706	1.695477	1.692378	1.689276	1.686185	1.727628	1.723834	1.821407
1.666141	1.663105	1.695903	1.692799	1.689692	1.686592	1.728146	1.724379	1.822018
1.666554	1.663508	1.660531	1.657598	1.690088	1.686988	1.72867	1.724902	1.773086
1.666979	1.663913	1.66092	1.657974	1.69046	1.687371	1.729201	1.725399	1.773639
1.667416	1.664339	1.661331	1.658367	1.690852	1.687762	1.729734	1.72592	1.742663
1.667873	1.664802	1.66178	1.658793	1.691286	1.688166	1.730257	1.72648	1.743253
1.668329	1.665265	1.662229	1.659221	1.691723	1.688582	1.730803	1.727059	1.743861
1.668765	1.665697	1.662646	1.659622	1.692132	1.689011	1.731408	1.727659	1.744476
1.66919	1.666115	1.663053	1.66002	1.692541	1.68945	1.732036	1.728263	1.745088
1.669601	1.666527	1.663475	1.660452	1.692995	1.689902	1.686812	1.683724	1.725036
1.669999	1.666935	1.663906	1.660909	1.693482	1.690364	1.687248	1.684125	1.725526
1.645209	1.642336	1.664342	1.661359	1.658391	1.65544	1.687672	1.684544	1.72607
1.645572	1.642726	1.664783	1.661801	1.658821	1.655849	1.688083	1.684985	1.726681
1.645958	1.643131	1.665227	1.662239	1.659247	1.656257	1.6885	1.685424	1.727286
1.646391	1.643563	1.665676	1.662666	1.659661	1.656666	1.688929	1.685857	1.727857
1.646831	1.643996	1.666127	1.663097	1.660082	1.657084	1.689369	1.686285	1.728411
1.647239	1.644411	1.641551	1.63869	1.660539	1.657533	1.689829	1.686706	1.683607
1.647629	1.64481	1.641965	1.639119	1.661011	1.657998	1.690301	1.687126	1.683991
1.647997	1.645171	1.642335	1.639507	1.661452	1.658443	1.655456	1.652487	1.684419
1.648341	1.645494	1.642666	1.639859	1.661868	1.658875	1.655896	1.652924	1.684884
1.620568	1.617781	1.643025	1.640227	1.637416	1.634602	1.656316	1.653346	1.685337
1.620984	1.618184	1.643427	1.640621	1.637791	1.634951	1.656704	1.653739	1.685761
1.6214	1.618591	1.643833	1.641025	1.638181	1.635317	1.657108	1.654148	1.6862
1.621783	1.618979	1.64423	1.641439	1.6386	1.635732	1.657562	1.654601	1.686679
1.622163	1.619363	1.644624	1.641857	1.639029	1.636165	1.658036	1.655071	1.687175
1.622562	1.619752	1.617009	1.614312	1.639444	1.636589	1.633759	1.63095	1.652612
1.622974	1.620145	1.6174	1.614713	1.639852	1.63701	1.634186	1.631376	1.653079
1.578068	1.575434	1.617809	1.615123	1.640262	1.637422	1.634599	1.631789	1.653537
1.578457	1.575812	1.618235	1.615542	1.640671	1.637823	1.634996	1.632189	1.653985
1.578849	1.576193	1.618661	1.61596	1.613183	1.610367	1.635404	1.632599	1.629829
1.579236	1.576573	1.619084	1.616376	1.6136	1.61079	1.635832	1.633026	1.630255
1.579625	1.576953	1.619508	1.616793	1.614016	1.611214	1.63626	1.633456	1.630684
1.580005	1.577324	1.619931	1.617203	1.614423	1.611623	1.608848	1.606094	1.631112
1.580395	1.5777	1.620362	1.61762	1.614832	1.612032	1.60926	1.606516	1.631542
1.580839	1.578112	1.575454	1.572885	1.615269	1.612464	1.60969	1.606945	1.63197
1.581325	1.578549	1.575867	1.573288	1.615727	1.612914	1.610133	1.607381	1.632398
1.581817	1.578985	1.576276	1.573688	1.616186	1.613367	1.610581	1.607824	1.605109
1.582313	1.579417	1.576681	1.574086	1.616645	1.613824	1.611035	1.608274	1.605557

1.582814	1.579851	1.577084	1.57448	1.617104	1.614284	1.611494	1.608731	1.606011
1.583311	1.580279	1.577478	1.574862	1.617556	1.61474	1.611952	1.609187	1.606466
1.583828	1.58072	1.577881	1.575249	1.618019	1.61521	1.612425	1.609658	1.606932
1.58439	1.581197	1.578312	1.575657	1.618512	1.615719	1.612937	1.610159	1.60742
1.584985	1.5817	1.578763	1.576081	1.619025	1.616246	1.613468	1.610679	1.607925
1.5856	1.582228	1.579237	1.576521	1.57386	1.571298	1.613977	1.611202	1.608451
1.586236	1.582781	1.579734	1.576975	1.574282	1.571699	1.61447	1.611731	1.609
1.586903	1.583372	1.580265	1.577455	1.574724	1.572114	1.61496	1.612236	1.609515
1.587605	1.584012	1.58084	1.577966	1.575187	1.572544	1.615444	1.612707	1.609981
1.588318	1.584655	1.58142	1.578489	1.575667	1.572991	1.61594	1.61318	1.610444
1.589024	1.585272	1.581989	1.579028	1.57617	1.57346	1.616458	1.613668	1.610917
1.589743	1.585888	1.582552	1.579561	1.576674	1.573939	1.616996	1.614174	1.611405
1.590497	1.586515	1.583092	1.580039	1.577134	1.57442	1.571763	1.569159	1.611928
1.591278	1.587157	1.583625	1.580489	1.577572	1.574903	1.572267	1.569655	1.612478
1.548961	1.544784	1.584227	1.580992	1.578034	1.57537	1.572741	1.570138	1.613041
1.549753	1.545512	1.584895	1.58154	1.578514	1.575819	1.573186	1.570613	1.613622
1.550531	1.546258	1.542456	1.539018	1.579038	1.576297	1.573642	1.571075	1.56855
1.551275	1.547014	1.543153	1.539628	1.579626	1.576822	1.574117	1.571515	1.568974
1.552036	1.547791	1.543871	1.540257	1.580227	1.57736	1.574605	1.571963	1.569402
1.512196	1.50804	1.54459	1.540871	1.537603	1.534741	1.575108	1.572446	1.569873
1.512987	1.508806	1.545323	1.541491	1.538147	1.53523	1.575616	1.572943	1.570362
1.513809	1.509611	1.546089	1.542141	1.538704	1.535708	1.532899	1.530289	1.570851
1.514655	1.510444	1.546879	1.542813	1.539273	1.536179	1.533317	1.530711	1.571343
1.515446	1.511213	1.506988	1.502936	1.539845	1.536664	1.53375	1.531133	1.528643
1.516171	1.511906	1.507635	1.503528	1.540414	1.53716	1.534195	1.531553	1.529049
1.516917	1.512619	1.508313	1.504165	1.54103	1.537689	1.534662	1.531989	1.529466
1.517713	1.513382	1.50905	1.504868	1.54171	1.538256	1.535154	1.532451	1.52991
1.518533	1.514172	1.509824	1.50562	1.542437	1.538864	1.535675	1.532924	1.530351
1.519368	1.514986	1.510638	1.506427	1.502557	1.498988	1.536251	1.533405	1.530761
1.52022	1.515819	1.511477	1.507267	1.503338	1.499662	1.536851	1.533888	1.531155
1.521083	1.516647	1.512295	1.508071	1.504064	1.500264	1.537392	1.534344	1.531548
1.52196	1.517474	1.5131	1.508849	1.504751	1.500811	1.537889	1.534778	1.531938
1.522841	1.518337	1.513944	1.509657	1.505475	1.501412	1.497819	1.494747	1.532326
1.523729	1.519249	1.514838	1.510506	1.506247	1.502076	1.498368	1.495162	1.532699
1.524617	1.520162	1.515737	1.511365	1.507042	1.502786	1.498973	1.495631	1.492655
1.525508	1.521059	1.516621	1.512219	1.507851	1.503535	1.499638	1.49618	1.493105
1.526392	1.521944	1.517499	1.513081	1.508688	1.504335	1.500367	1.496791	1.493611
1.527245	1.522795	1.518362	1.51396	1.509571	1.505212	1.501183	1.497475	1.494167
1.528084	1.52363	1.519221	1.514853	1.510489	1.506147	1.502064	1.498211	1.494761
1.528971	1.524523	1.520123	1.515764	1.511408	1.507072	1.502922	1.49891	1.495307
1.529904	1.52547	1.521067	1.516694	1.512334	1.507996	1.503768	1.499577	1.495805
1.530826	1.526406	1.521994	1.517602	1.51323	1.50888	1.504589	1.500269	1.496366
1.531738	1.527327	1.522897	1.518474	1.514072	1.509691	1.505354	1.500979	1.497
1.532613	1.528212	1.523776	1.51934	1.514922	1.510519	1.506158	1.501759	1.497726
1.533399	1.529008	1.524604	1.520211	1.515821	1.511436	1.507078	1.502675	1.498592
1.53417	1.529787	1.525424	1.521092	1.516743	1.51239	1.508049	1.503662	1.499539
1.535046	1.530659	1.526313	1.522008	1.517679	1.513339	1.509012	1.504649	1.500498
1.535996	1.531594	1.52725	1.522953	1.518628	1.514293	1.509977	1.505648	1.501476
1.536998	1.532581	1.528226	1.523917	1.519581	1.515235	1.510914	1.506598	1.502394
1.538059	1.533629	1.529248	1.524906	1.520541	1.516168	1.511825	1.5075	1.503249

1.53913	1.534679	1.530267	1.525886	1.521484	1.517074	1.512702	1.508364	1.504076
1.540204	1.535717	1.531272	1.526853	1.522403	1.517937	1.513524	1.50917	1.50486
1.541269	1.536744	1.532259	1.527796	1.523301	1.518788	1.514345	1.509979	1.50566
1.542312	1.537749	1.533208	1.528685	1.524164	1.519649	1.51521	1.510853	1.506542
1.543333	1.538734	1.534134	1.529552	1.525017	1.520522	1.516101	1.511762	1.507467
1.460211	1.455881	1.535062	1.530465	1.525927	1.521441	1.517024	1.512683	1.50839
1.461115	1.456775	1.535986	1.531412	1.526884	1.5224	1.517978	1.513622	1.50932
1.462014	1.457676	1.536942	1.532393	1.527872	1.523385	1.518954	1.514578	1.510262
1.4629	1.458584	1.537937	1.533414	1.528899	1.524401	1.519954	1.515557	1.511222
1.463809	1.459509	1.538948	1.534448	1.529937	1.525427	1.520962	1.516537	1.512179
1.464768	1.460465	1.539975	1.53549	1.530978	1.526454	1.521965	1.517503	1.513118
1.465741	1.461428	1.541006	1.536535	1.532021	1.527484	1.522972	1.518473	1.514059
1.466696	1.462374	1.542023	1.537576	1.533064	1.528515	1.523985	1.519459	1.51502
1.467639	1.463308	1.54303	1.53861	1.534106	1.529548	1.525005	1.52046	1.515997
1.468546	1.464216	1.544008	1.539617	1.535132	1.530588	1.526043	1.521483	1.516994
1.469414	1.465098	1.544959	1.540599	1.536145	1.531634	1.527101	1.522529	1.518011
1.470335	1.465995	1.545909	1.54157	1.537146	1.532674	1.528157	1.523575	1.519029
1.471325	1.466902	1.546851	1.542521	1.538125	1.533701	1.529203	1.524609	1.520037
1.472371	1.467858	1.547827	1.543495	1.53912	1.534741	1.530263	1.525659	1.521061
1.473501	1.468907	1.548873	1.544522	1.540156	1.535809	1.531343	1.526729	1.522102
1.474663	1.47	1.549961	1.545581	1.541218	1.536902	1.53245	1.52783	1.523176
1.475771	1.471081	1.46673	1.462575	1.542313	1.538047	1.53362	1.528999	1.524328
1.47685	1.472162	1.467775	1.463579	1.543429	1.539222	1.53483	1.530217	1.525539
1.477931	1.473219	1.468779	1.46453	1.544453	1.540275	1.535928	1.531382	1.526753
1.479012	1.474245	1.469739	1.465421	1.545377	1.541201	1.536912	1.532501	1.527983
1.42553	1.420806	1.470713	1.466318	1.546294	1.542107	1.537863	1.533568	1.529151
1.4266	1.421832	1.471718	1.467238	1.547227	1.54302	1.538796	1.534574	1.530222
1.42768	1.422877	1.472739	1.468168	1.548163	1.543927	1.53971	1.535541	1.531242
1.428754	1.423924	1.473758	1.469087	1.549088	1.54482	1.540605	1.536479	1.532227
1.429835	1.424994	1.474804	1.470032	1.550033	1.545722	1.541495	1.537395	1.533183
1.43095	1.426127	1.475946	1.471087	1.551065	1.546681	1.542419	1.538324	1.53414
1.432093	1.427315	1.477169	1.472234	1.552172	1.547691	1.54337	1.539259	1.535094
1.433253	1.428534	1.423651	1.418753	1.553383	1.548788	1.544396	1.540261	1.536106
1.43445	1.429807	1.424956	1.420051	1.554733	1.549999	1.54552	1.541354	1.537205
1.159455	1.155536	1.426211	1.421317	1.556056	1.551194	1.546626	1.542415	1.538257
1.160253	1.156437	1.427336	1.422464	1.557263	1.552297	1.547644	1.543366	1.539179
1.161013	1.157304	1.428414	1.423575	1.558447	1.553395	1.548662	1.544303	1.540073
1.161799	1.158177	1.429495	1.424696	1.474811	1.469947	1.549748	1.5453	1.541012
1.162587	1.159043	1.430571	1.425821	1.476002	1.471099	1.550886	1.546342	1.541983
1.163374	1.159908	1.319599	1.315041	1.477258	1.472323	1.552119	1.54748	1.543045
1.164163	1.160776	1.3207	1.316209	1.478576	1.473618	1.553446	1.548713	1.544193
1.164893	1.161568	1.321727	1.317335	1.424733	1.419845	1.554763	1.54994	1.545335
1.165534	1.162257	1.322653	1.318403	1.425912	1.421051	1.556047	1.551137	1.546442
1.166172	1.162921	1.15947	1.155865	1.315016	1.310346	1.557349	1.552363	1.547585
1.166842	1.16359	1.160218	1.156731	1.31609	1.311501	1.558704	1.553661	1.548812
1.167544	1.164268	1.160946	1.157549	1.317128	1.312646	1.560068	1.554988	1.55008
1.053619	1.050476	1.161679	1.158321	1.318105	1.313746	1.561379	1.55629	1.55134
1.05467	1.051475	1.162412	1.159055	1.319035	1.314811	1.562658	1.557585	1.55261
1.055727	1.05247	1.163154	1.159806	1.319956	1.315838	1.419628	1.414778	1.553926
1.056792	1.053457	1.1639	1.160567	1.320861	1.316825	1.420792	1.41602	1.555295

1.057885	1.054467	1.051212	1.048046	1.157924	1.154396	1.421913	1.417214	1.556636
1.059012	1.055501	1.052244	1.049113	1.158798	1.155254	1.422961	1.418323	1.557916
1.060169	1.056568	1.053295	1.05018	1.159664	1.156105	1.424006	1.419418	1.559185
1.061351	1.05767	1.054343	1.051192	1.160471	1.156947	1.425116	1.42056	1.415973
1.062569	1.058815	1.05541	1.052191	1.161259	1.157783	1.426239	1.421704	1.417147
1.063856	1.060026	1.056532	1.053225	1.162075	1.158607	1.318616	1.314408	1.418251
1.065203	1.061297	1.057701	1.054285	1.162912	1.159423	1.319532	1.315355	1.419304
1.06653	1.06258	1.058895	1.055376	1.163788	1.160292	1.320519	1.316362	1.42041
1.067838	1.063879	1.060118	1.056501	1.164708	1.161229	1.3216	1.317446	1.421581
1.069121	1.065166	1.061345	1.057647	1.054189	1.050935	1.158598	1.154956	1.422757
1.070357	1.066412	1.062555	1.058801	1.055293	1.051989	1.15944	1.155812	1.423926
1.071593	1.067662	1.063782	1.059984	1.056419	1.053046	1.160275	1.156656	1.425074
0.94982	0.946534	1.065065	1.061222	1.057592	1.054136	1.161158	1.157525	1.42615
0.950958	0.947687	1.066381	1.062502	1.058805	1.055256	1.162073	1.158416	1.427202
0.952029	0.948778	1.067705	1.063816	1.06007	1.05644	1.052909	1.049492	1.428359
0.953043	0.949819	1.069041	1.065167	1.061388	1.057686	1.054106	1.050672	1.429606
0.954068	0.950834	1.070332	1.066475	1.062686	1.05895	1.055339	1.051878	1.157751
0.9551	0.951804	1.071559	1.06772	1.063952	1.060241	1.056624	1.053117	1.158783
0.956175	0.952812	0.949578	0.946427	1.065197	1.061507	1.057887	1.054347	1.050845
0.957331	0.953918	0.950645	0.947462	1.066397	1.062693	1.059072	1.055545	1.052054
0.958532	0.955078	0.951761	0.948534	1.067603	1.063863	1.060229	1.056718	1.053246
0.959754	0.956266	0.952921	0.949661	1.068895	1.065077	1.061387	1.057844	1.054366
0.960992	0.957486	0.954121	0.950832	1.070245	1.066322	1.06255	1.058942	1.055442
0.962183	0.958708	0.955324	0.951994	0.94868	0.945392	1.063808	1.060112	1.056552
0.963325	0.959936	0.956535	0.953151	0.949825	0.946548	1.065163	1.061348	1.057685
0.837599	0.834691	0.957727	0.954308	0.950975	0.947706	1.066536	1.062634	1.058883
0.83868	0.835787	0.958891	0.955461	0.952124	0.948855	1.067916	1.063979	1.060169
0.839776	0.836876	0.96004	0.956623	0.953285	0.950007	1.069304	1.065352	1.061495
0.840914	0.837976	0.961177	0.957808	0.954474	0.951171	0.947871	0.944575	1.06284
0.84207	0.839073	0.962302	0.958998	0.955671	0.952337	0.94902	0.945723	1.064205
0.671611	0.66926	0.963414	0.960156	0.956838	0.953493	0.950168	0.946868	1.065573
0.672413	0.669992	0.964516	0.961292	0.957985	0.954641	0.951315	0.948011	1.066947
0.67329	0.670814	0.96567	0.962447	0.959136	0.955787	0.952457	0.949148	1.068318
0.674256	0.671744	0.966884	0.963625	0.960291	0.956931	0.953592	0.950278	1.069685
0.675229	0.672681	0.968104	0.964807	0.96145	0.95808	0.954733	0.951411	0.948099
0.67618	0.673588	0.969309	0.965987	0.962615	0.959238	0.955882	0.95255	0.949229
0.677127	0.674491	0.970522	0.96717	0.963782	0.960402	0.957038	0.953692	0.950361
0.501194	0.498683	0.672894	0.670509	0.964945	0.96157	0.958202	0.954835	0.951498
0.502121	0.499536	0.673766	0.671354	0.96611	0.962742	0.95937	0.95598	0.952639
0.503078	0.500462	0.674681	0.672236	0.967293	0.963914	0.960531	0.957124	0.953782
0.504062	0.501453	0.675635	0.673151	0.96849	0.965086	0.961685	0.958265	0.954927
0.505028	0.502443	0.676587	0.674058	0.671638	0.669316	0.962835	0.959407	0.956072
0.505964	0.503414	0.677519	0.674935	0.67248	0.670133	0.963974	0.960541	0.957206
		0.678496	0.675859	0.67336	0.670972	0.965126	0.9617	0.958371
		0.67958	0.676889	0.674326	0.671861	0.966307	0.962911	0.9596
		0.680685	0.677954	0.675327	0.672773	0.967501	0.964142	0.960852
		0.505102	0.50249	0.67631	0.673686	0.671191	0.668842	0.96209
		0.506046	0.50348	0.677291	0.674605	0.672079	0.669735	0.96332
				0.678266	0.675516	0.672945	0.670581	0.964491
				0.679232	0.676412	0.673784	0.671372	0.965597

0.50389	0.501163	0.674711	0.672249	0.827343
0.504929	0.502225	0.675777	0.673271	0.828322
0.505972	0.503325	0.676876	0.674314	0.829337
0.507001	0.504425	0.677952	0.675298	0.672744
0.508005	0.505516	0.67902	0.676273	0.673641
		0.504047	0.501429	0.674613
		0.505048	0.502437	0.675638
		0.506016	0.503432	0.676642
		0.506944	0.504406	0.67762
				0.502944
				0.504008
				0.505087
				0.506172
				0.507244

2.042173	2.037572	2.042127	2.037592	2.033091	2.02863	2.024291	2.020107	2.025125
2.042171	2.037569	2.042126	2.037586	2.033079	2.02861	2.024268	2.020083	2.025103
2.042171	2.03757	2.042129	2.037583	2.033071	2.028597	2.02425	2.020064	2.025085
2.042167	2.037568	2.042131	2.037584	2.033072	2.0286	2.024252	2.020058	2.015869
2.042163	2.037565	2.042133	2.037588	2.033083	2.028619	2.024271	2.020065	2.015864
2.035335	2.030758	2.042141	2.037602	2.033117	2.028684	2.02434	2.020103	2.015874
2.035365	2.03078	2.042154	2.037627	2.033179	2.028804	2.024468	2.020178	2.0159
2.035422	2.030814	2.042169	2.037645	2.033222	2.028888	2.024561	2.020235	2.015926
2.035525	2.030866	2.042187	2.037647	2.033222	2.028901	2.024582	2.020259	2.015949
2.035628	2.030922	2.04221	2.037652	2.033219	2.028896	2.02458	2.020269	2.015969
2.035685	2.030969	2.033077	2.028547	2.033226	2.028894	2.024576	2.020269	2.015984
2.035727	2.031019	2.033112	2.028578	2.03325	2.028897	2.02457	2.020265	2.015998
2.035784	2.03109	2.033189	2.028674	2.033335	2.028938	2.024586	2.020277	2.016015
2.035851	2.031178	2.033301	2.028822	2.033468	2.02901	2.02462	2.020303	2.016034
2.035944	2.031298	2.033416	2.028935	2.03357	2.029079	2.024674	2.020367	2.016101
2.036076	2.031467	2.033543	2.029006	2.033631	2.02914	2.02475	2.020481	2.016231
2.024609	2.020068	2.033634	2.029058	2.024555	2.020118	2.024843	2.020591	2.016343
2.024639	2.020099	2.033656	2.029087	2.024616	2.020227	2.024968	2.020678	2.016397
2.024659	2.020104	2.033654	2.029105	2.024668	2.020327	2.025082	2.020751	2.016438
2.024732	2.020135	2.033656	2.02911	2.024681	2.020353	2.025123	2.020797	2.016494
2.024828	2.020176	2.033664	2.029116	2.024681	2.020346	2.02513	2.020832	2.01656
2.024907	2.02024	2.015689	2.01122	2.024749	2.020389	2.025177	2.020907	2.016657
2.02497	2.020324	2.015805	2.011369	2.024877	2.020472	2.025253	2.021017	2.016783
2.025063	2.020438	2.015934	2.011508	2.025	2.020581	2.02536	2.021122	2.01688
2.025194	2.020592	2.016074	2.011621	2.025108	2.02073	2.025514	2.021219	2.016931
2.025339	2.020749	2.016217	2.011742	2.014219	2.009902	2.01653	2.012214	2.016978
2.025489	2.020879	2.016354	2.011894	2.014337	2.009973	2.016576	2.012255	2.017035
2.025644	2.021008	2.016492	2.01205	2.014451	2.010024	2.0166	2.012295	2.017106
2.025801	2.02117	2.016638	2.012169	2.014529	2.010081	2.016659	2.012382	2.017218
2.025958	2.02135	2.016789	2.01227	2.014591	2.010142	2.016739	2.012497	2.017357
2.026105	2.021501	2.016938	2.012414	2.014712	2.010258	2.016854	2.012608	2.017474
2.026241	2.02162	2.017085	2.012604	2.014899	2.010437	2.017011	2.012718	2.01757
2.026383	2.021757	2.017233	2.012768	2.008312	2.003876	2.006211	2.00194	2.008575
2.026541	2.021935	2.017383	2.012874	2.0084	2.003965	2.006293	2.002054	2.008711
2.026698	2.02211	2.017535	2.012989	2.008502	2.004069	2.006388	2.002173	2.008847
2.026841	2.022243	2.017688	2.013173	2.008698	2.004265	2.00655	2.002295	2.008948
2.02698	2.022366	2.017843	2.013379	2.008926	2.004498	2.006745	2.00243	2.009051
2.027132	2.022525	2.018001	2.013536	2.009104	2.00471	2.006951	2.002615	2.009218
2.027294	2.02271	2.018162	2.013663	2.00925	2.004912	2.007168	2.002838	2.009433
2.027464	2.022874	2.018316	2.01381	2.009401	2.005078	2.007333	2.003028	2.009642
2.027641	2.023007	2.018459	2.01398	2.009559	2.005196	2.00743	2.00318	2.009852
2.026121	2.021444	2.018622	2.014153	2.00972	2.005334	2.001034	1.996835	1.999111
2.026355	2.021666	2.018817	2.014307	2.009875	2.00552	2.001219	1.996976	1.999207
2.026596	2.021914	2.019039	2.014482	2.010051	2.005731	2.001428	1.99713	1.999304
2.02681	2.02217	2.019298	2.014723	2.010291	2.005975	2.001666	1.997348	1.999481
2.027016	2.022436	2.019583	2.015004	2.010564	2.006235	2.001915	1.997595	1.999696
2.027254	2.022696	2.019858	2.015278	2.010813	2.006442	2.002106	1.997803	1.999897
2.027519	2.022953	2.020129	2.01555	2.011046	2.006606	2.002246	1.997982	2.00009
2.000648	1.99615	2.018594	2.014036	2.0113	2.006815	2.00243	1.998162	2.000258
2.000851	1.996315	2.018799	2.014301	2.011584	2.007089	2.002673	1.99834	2.000381

2.001074	1.996506	2.019022	2.014567	2.011872	2.007382	2.002943	1.998548	2.000533
2.001352	1.996773	2.019301	2.014843	2.012159	2.007681	2.00324	1.998821	2.000766
2.001647	1.997066	2.019594	2.015109	2.012434	2.007982	2.003549	1.999119	2.00103
2.001926	1.997335	2.007759	2.003249	2.012662	2.008256	2.003843	1.999404	1.995039
2.002198	1.997597	2.007985	2.003426	2.012865	2.008516	2.004132	1.999688	1.995299
1.99348	1.988898	2.008246	2.003659	2.013119	2.0088	2.00443	1.999984	1.995581
1.993714	1.989172	2.00854	2.003948	2.013423	2.009106	2.004738	2.000292	1.995886
1.993973	1.989455	1.993867	1.989344	1.999808	1.995493	2.00504	2.000596	1.996189
1.994269	1.989747	1.994156	1.989637	2.000106	1.995784	2.005342	2.000896	1.996488
1.994584	1.990048	1.994451	1.989929	2.000394	1.996052	2.005618	2.001182	1.996782
1.994913	1.990364	1.98579	1.981254	2.000644	1.996254	2.005824	2.001426	1.997062
1.995251	1.990687	1.986093	1.981529	2.000886	1.996442	2.006013	2.001662	1.997339
1.995583	1.991008	1.986401	1.981823	1.992789	1.988331	2.006282	2.001943	1.997633
1.995913	1.991327	1.986713	1.982132	1.993104	1.988646	2.006605	2.002256	1.997938
1.97938	1.974963	1.987026	1.982443	1.977955	1.973548	1.992929	1.988572	1.998256
1.979696	1.975277	1.987339	1.982753	1.97827	1.973875	1.993263	1.988898	1.998586
1.980006	1.975587	1.987653	1.983071	1.978603	1.974231	1.98527	1.980891	1.998925
1.980302	1.975886	1.987967	1.983401	1.978968	1.974646	1.985701	1.981272	1.999271
1.980601	1.976187	1.988284	1.983732	1.979332	1.975055	1.986128	1.981653	1.999621
1.940522	1.936445	1.950674	1.946438	1.979655	1.975391	1.986473	1.981993	1.977539
1.940854	1.936764	1.950996	1.946754	1.979961	1.97569	1.986778	1.982312	1.977869
1.941223	1.937105	1.951336	1.947093	1.980283	1.976002	1.98709	1.982631	1.978201
1.941626	1.937463	1.951692	1.947453	1.980618	1.976317	1.987401	1.982946	1.978534
1.942002	1.937815	1.933672	1.929584	1.980993	1.976657	1.972296	1.967889	1.978872
1.942339	1.938156	1.934053	1.930014	1.981428	1.977032	1.972635	1.968224	1.979209
1.91497	1.910966	1.934429	1.930426	1.981846	1.977404	1.972984	1.968581	1.979574
1.915301	1.911289	1.934776	1.930779	1.982198	1.977752	1.973346	1.968982	1.980005
1.915641	1.911622	1.935122	1.931114	1.982526	1.978093	1.973709	1.969387	1.980441
1.915989	1.911987	1.935502	1.931465	1.982862	1.978427	1.974046	1.969735	1.980799
1.916344	1.912368	1.935899	1.93182	1.9832	1.97876	1.974374	1.970053	1.981115
1.897912	1.894001	1.936261	1.932171	1.983564	1.979138	1.974747	1.970398	1.981437
1.898211	1.894286	1.93659	1.932518	1.983952	1.97956	1.975167	1.970766	1.981761
1.898532	1.894582	1.936934	1.932886	1.984349	1.979963	1.975561	1.971133	1.982108
1.898892	1.894901	1.937309	1.933297	1.984764	1.980328	1.975904	1.971489	1.982484
1.899255	1.895227	1.937683	1.933696	1.985163	1.980682	1.976246	1.971858	1.982886
1.899597	1.89555	1.920902	1.916964	1.985506	1.981029	1.97661	1.972262	1.967925
1.899937	1.895882	1.921234	1.917276	1.985834	1.98138	1.976991	1.972682	1.968371
1.900296	1.896243	1.921609	1.91763	1.971693	1.967306	1.977403	1.973101	1.968795
1.900669	1.896626	1.922015	1.918012	1.972087	1.967723	1.97784	1.973522	1.969207
1.881199	1.877196	1.922426	1.918416	1.972501	1.968135	1.978262	1.973947	1.969633
1.881599	1.877579	1.922841	1.918847	1.97294	1.96854	1.978665	1.974378	1.970076
1.882	1.877965	1.923261	1.919275	1.973379	1.968956	1.964613	1.960376	1.970503
1.882396	1.878358	1.923685	1.919687	1.973807	1.969394	1.965044	1.960776	1.970889
1.882796	1.878752	1.924111	1.9201	1.974238	1.969834	1.965477	1.96118	1.971282
1.869989	1.86595	1.906448	1.902481	1.974684	1.970255	1.965891	1.961611	1.971736
1.870399	1.866335	1.906869	1.902913	1.975137	1.970673	1.966302	1.962051	1.972212
1.828676	1.824638	1.895551	1.891613	1.960256	1.955803	1.966737	1.962473	1.958196
1.829048	1.825022	1.895987	1.892022	1.960706	1.956267	1.967192	1.96288	1.958566
1.829421	1.825396	1.842765	1.839012	1.951696	1.947271	1.952339	1.948031	1.958979
1.829795	1.825751	1.843158	1.839405	1.952155	1.9477	1.952768	1.948483	1.959455

1.830173	1.826115	1.822267	1.818538	1.936518	1.932047	1.953202	1.948931	1.95992
1.830555	1.826509	1.822633	1.818867	1.936949	1.932496	1.953651	1.949352	1.960333
1.830945	1.826908	1.823004	1.819201	1.937383	1.93295	1.954103	1.949775	1.960743
1.812335	1.808343	1.823375	1.819574	1.861067	1.857126	1.929007	1.924712	1.945934
1.812747	1.808718	1.823754	1.819973	1.861491	1.857526	1.929433	1.92517	1.946412
1.813178	1.809134	1.824167	1.820381	1.841719	1.837757	1.929917	1.925667	1.921428
1.813631	1.809591	1.824615	1.820801	1.842166	1.8382	1.930461	1.926205	1.921958
1.814075	1.810051	1.825058	1.821203	1.842593	1.838624	1.90262	1.898477	1.922444
1.814498	1.810509	1.825488	1.821572	1.842985	1.839014	1.903071	1.898887	1.922854
1.814926	1.810963	1.825918	1.821948	1.843372	1.839393	1.903503	1.89929	1.923265
1.815378	1.811411	1.826356	1.82236	1.843767	1.839757	1.885773	1.881596	1.895555
1.815834	1.811847	1.826791	1.822788	1.844174	1.840127	1.886138	1.881993	1.896005
1.816264	1.812248	1.827202	1.823217	1.844624	1.840549	1.886567	1.882425	1.878304
1.816679	1.812626	1.827596	1.823649	1.845104	1.84101	1.88704	1.882882	1.878749
1.817104	1.813038	1.809079	1.805204	1.845576	1.841481	1.887516	1.883336	1.879184
1.817541	1.813485	1.809514	1.805622	1.846043	1.841965	1.887998	1.883791	1.879613
1.817967	1.813932	1.809946	1.80603	1.846492	1.842431	1.888461	1.884216	1.880008
1.818364	1.814363	1.81036	1.806412	1.846906	1.842857	1.888883	1.884577	1.880334
1.818768	1.814798	1.810781	1.806806	1.84733	1.843285	1.889309	1.884945	1.880669
1.819207	1.815247	1.811229	1.80725	1.822254	1.818531	1.839733	1.835722	1.881085
1.819669	1.815707	1.811695	1.807722	1.822726	1.818979	1.840208	1.836176	1.881543
1.820147	1.816182	1.812173	1.808202	1.823203	1.819439	1.840692	1.836641	1.882004
1.820643	1.816672	1.812663	1.808694	1.823691	1.81991	1.841188	1.83712	1.882475
1.804159	1.800234	1.813151	1.809184	1.80534	1.801596	1.841686	1.837601	1.882952
1.804631	1.800702	1.813636	1.809674	1.805825	1.802068	1.842185	1.838085	1.883435
1.805102	1.801166	1.814117	1.810158	1.806305	1.802539	1.842687	1.838571	1.88392
1.805571	1.801627	1.814591	1.810632	1.806777	1.80301	1.843192	1.839059	1.8844
1.80604	1.802086	1.815061	1.811102	1.807246	1.803479	1.843696	1.839548	1.884882
1.806506	1.802543	1.815535	1.811577	1.807718	1.803945	1.844196	1.840035	1.885373
1.806973	1.803	1.816008	1.812052	1.808189	1.804408	1.844692	1.840522	1.885869
1.807444	1.803457	1.816475	1.812517	1.80865	1.804865	1.827595	1.823455	1.836916
1.807921	1.803914	1.816937	1.812972	1.809102	1.805318	1.828086	1.823947	1.837408
1.808399	1.804375	1.817403	1.81343	1.809555	1.805768	1.828572	1.824435	1.837897
1.808873	1.804841	1.81787	1.813887	1.810003	1.806209	1.829048	1.824911	1.838376
1.809362	1.805316	1.81835	1.814361	1.810468	1.806662	1.829532	1.825397	1.838867
1.809876	1.805801	1.818844	1.814861	1.810961	1.807139	1.803313	1.799471	1.82183
1.810412	1.806304	1.819359	1.815387	1.811477	1.807633	1.803796	1.799959	1.822358
1.793563	1.789471	1.819917	1.815956	1.812027	1.808146	1.804296	1.80048	1.822929
1.794136	1.790014	1.820505	1.816556	1.812603	1.808673	1.804809	1.801024	1.823529
1.753	1.749616	1.821066	1.817117	1.813144	1.809181	1.805302	1.801529	1.824082
1.753397	1.75001	1.821598	1.817638	1.81365	1.809667	1.805775	1.801993	1.824582
1.75379	1.750404	1.805601	1.80165	1.814147	1.810157	1.806256	1.802458	1.82509
1.75418	1.7508	1.806106	1.802113	1.814628	1.81065	1.806749	1.802933	1.825621
1.754577	1.751208	1.806632	1.802593	1.815127	1.811164	1.807262	1.803424	1.82616
1.755003	1.751648	1.807212	1.803135	1.799139	1.795219	1.807822	1.803949	1.826698
1.755439	1.752099	1.807815	1.803706	1.799703	1.79579	1.808399	1.804493	1.827241
1.755853	1.752518	1.790271	1.786252	1.80026	1.796328	1.792426	1.788544	1.8278
1.756254	1.752914	1.790793	1.786808	1.800815	1.796843	1.792925	1.789062	1.828371
1.756662	1.753313	1.791304	1.787325	1.801336	1.797338	1.793408	1.789552	1.81355
1.757081	1.753714	1.791797	1.787792	1.80181	1.797802	1.793864	1.790001	1.814026

1.7575	1.754121	1.750776	1.747453	1.802297	1.798291	1.794348	1.790467	1.814515
1.757917	1.754545	1.751187	1.747848	1.802824	1.798842	1.794895	1.790973	1.81504
1.758328	1.754966	1.751602	1.748254	1.803374	1.799413	1.795461	1.791502	1.815588
1.662354	1.659406	1.752013	1.748678	1.803952	1.79997	1.796007	1.792051	1.816165
1.662675	1.659726	1.752421	1.749111	1.804546	1.800525	1.796549	1.792614	1.816761
1.663016	1.660058	1.692746	1.689657	1.787014	1.783061	1.7971	1.79316	1.817341
1.663374	1.660402	1.693087	1.689993	1.787523	1.783602	1.797658	1.793693	1.817907
1.663749	1.660761	1.693445	1.690334	1.732538	1.728799	1.798208	1.794236	1.818481
1.664147	1.661139	1.69382	1.690674	1.732989	1.72925	1.798743	1.794794	1.819065
1.664549	1.661526	1.694213	1.691045	1.733485	1.729732	1.74654	1.742753	1.81965
1.664945	1.661916	1.694627	1.691482	1.734086	1.730303	1.747093	1.743271	1.820228
1.66534	1.66231	1.695053	1.691943	1.73472	1.7309	1.747661	1.743793	1.820808
1.665739	1.662706	1.695477	1.692378	1.689276	1.686185	1.727628	1.723834	1.821407
1.666141	1.663105	1.695903	1.692799	1.689692	1.686592	1.728146	1.724379	1.822018
1.666554	1.663508	1.660531	1.657598	1.690088	1.686988	1.72867	1.724902	1.773086
1.666979	1.663913	1.66092	1.657974	1.69046	1.687371	1.729201	1.725399	1.773639
1.667416	1.664339	1.661331	1.658367	1.690852	1.687762	1.729734	1.72592	1.742663
1.667873	1.664802	1.66178	1.658793	1.691286	1.688166	1.730257	1.72648	1.743253
1.668329	1.665265	1.662229	1.659221	1.691723	1.688582	1.730803	1.727059	1.743861
1.668765	1.665697	1.662646	1.659622	1.692132	1.689011	1.731408	1.727659	1.744476
1.66919	1.666115	1.663053	1.66002	1.692541	1.68945	1.732036	1.728263	1.745088
1.669601	1.666527	1.663475	1.660452	1.692995	1.689902	1.686812	1.683724	1.725036
1.669999	1.666935	1.663906	1.660909	1.693482	1.690364	1.687248	1.684125	1.725526
1.645209	1.642336	1.664342	1.661359	1.658391	1.65544	1.687672	1.684544	1.72607
1.645572	1.642726	1.664783	1.661801	1.658821	1.655849	1.688083	1.684985	1.726681
1.645958	1.643131	1.665227	1.662239	1.659247	1.656257	1.6885	1.685424	1.727286
1.646391	1.643563	1.665676	1.662666	1.659661	1.656666	1.688929	1.685857	1.727857
1.646831	1.643996	1.666127	1.663097	1.660082	1.657084	1.689369	1.686285	1.728411
1.647239	1.644411	1.641551	1.63869	1.660539	1.657533	1.689829	1.686706	1.683607
1.647629	1.64481	1.641965	1.639119	1.661011	1.657998	1.690301	1.687126	1.683991
1.647997	1.645171	1.642335	1.639507	1.661452	1.658443	1.655456	1.652487	1.684419
1.648341	1.645494	1.642666	1.639859	1.661868	1.658875	1.655896	1.652924	1.684884
1.620568	1.617781	1.643025	1.640227	1.637416	1.634602	1.656316	1.653346	1.685337
1.620984	1.618184	1.643427	1.640621	1.637791	1.634951	1.656704	1.653739	1.685761
1.6214	1.618591	1.643833	1.641025	1.638181	1.635317	1.657108	1.654148	1.6862
1.621783	1.618979	1.64423	1.641439	1.6386	1.635732	1.657562	1.654601	1.686679
1.622163	1.619363	1.644624	1.641857	1.639029	1.636165	1.658036	1.655071	1.687175
1.622562	1.619752	1.617009	1.614312	1.639444	1.636589	1.633759	1.63095	1.652612
1.622974	1.620145	1.6174	1.614713	1.639852	1.63701	1.634186	1.631376	1.653079
1.578068	1.575434	1.617809	1.615123	1.640262	1.637422	1.634599	1.631789	1.653537
1.578457	1.575812	1.618235	1.615542	1.640671	1.637823	1.634996	1.632189	1.653985
1.578849	1.576193	1.618661	1.61596	1.613183	1.610367	1.635404	1.632599	1.629829
1.579236	1.576573	1.619084	1.616376	1.6136	1.61079	1.635832	1.633026	1.630255
1.579625	1.576953	1.619508	1.616793	1.614016	1.611214	1.63626	1.633456	1.630684
1.580005	1.577324	1.619931	1.617203	1.614423	1.611623	1.608848	1.606094	1.631112
1.580395	1.5777	1.620362	1.61762	1.614832	1.612032	1.60926	1.606516	1.631542
1.580839	1.578112	1.575454	1.572885	1.615269	1.612464	1.60969	1.606945	1.63197
1.581325	1.578549	1.575867	1.573288	1.615727	1.612914	1.610133	1.607381	1.632398
1.581817	1.578985	1.576276	1.573688	1.616186	1.613367	1.610581	1.607824	1.605109
1.582313	1.579417	1.576681	1.574086	1.616645	1.613824	1.611035	1.608274	1.605557

1.582814	1.579851	1.577084	1.57448	1.617104	1.614284	1.611494	1.608731	1.606011
1.583311	1.580279	1.577478	1.574862	1.617556	1.61474	1.611952	1.609187	1.606466
1.583828	1.58072	1.577881	1.575249	1.618019	1.61521	1.612425	1.609658	1.606932
1.58439	1.581197	1.578312	1.575657	1.618512	1.615719	1.612937	1.610159	1.60742
1.584985	1.5817	1.578763	1.576081	1.619025	1.616246	1.613468	1.610679	1.607925
1.5856	1.582228	1.579237	1.576521	1.57386	1.571298	1.613977	1.611202	1.608451
1.586236	1.582781	1.579734	1.576975	1.574282	1.571699	1.61447	1.611731	1.609
1.586903	1.583372	1.580265	1.577455	1.574724	1.572114	1.61496	1.612236	1.609515
1.587605	1.584012	1.58084	1.577966	1.575187	1.572544	1.615444	1.612707	1.609981
1.588318	1.584655	1.58142	1.578489	1.575667	1.572991	1.61594	1.61318	1.610444
1.589024	1.585272	1.581989	1.579028	1.57617	1.57346	1.616458	1.613668	1.610917
1.589743	1.585888	1.582552	1.579561	1.576674	1.573939	1.616996	1.614174	1.611405
1.590497	1.586515	1.583092	1.580039	1.577134	1.57442	1.571763	1.569159	1.611928
1.591278	1.587157	1.583625	1.580489	1.577572	1.574903	1.572267	1.569655	1.612478
1.548961	1.544784	1.584227	1.580992	1.578034	1.57537	1.572741	1.570138	1.613041
1.549753	1.545512	1.584895	1.58154	1.578514	1.575819	1.573186	1.570613	1.613622
1.550531	1.546258	1.542456	1.539018	1.579038	1.576297	1.573642	1.571075	1.56855
1.551275	1.547014	1.543153	1.539628	1.579626	1.576822	1.574117	1.571515	1.568974
1.552036	1.547791	1.543871	1.540257	1.580227	1.57736	1.574605	1.571963	1.569402
1.512196	1.50804	1.54459	1.540871	1.537603	1.534741	1.575108	1.572446	1.569873
1.512987	1.508806	1.545323	1.541491	1.538147	1.53523	1.575616	1.572943	1.570362
1.513809	1.509611	1.546089	1.542141	1.538704	1.535708	1.532899	1.530289	1.570851
1.514655	1.510444	1.546879	1.542813	1.539273	1.536179	1.533317	1.530711	1.571343
1.515446	1.511213	1.506988	1.502936	1.539845	1.536664	1.53375	1.531133	1.528643
1.516171	1.511906	1.507635	1.503528	1.540414	1.53716	1.534195	1.531553	1.529049
1.516917	1.512619	1.508313	1.504165	1.54103	1.537689	1.534662	1.531989	1.529466
1.517713	1.513382	1.50905	1.504868	1.54171	1.538256	1.535154	1.532451	1.52991
1.518533	1.514172	1.509824	1.50562	1.542437	1.538864	1.535675	1.532924	1.530351
1.519368	1.514986	1.510638	1.506427	1.502557	1.498988	1.536251	1.533405	1.530761
1.52022	1.515819	1.511477	1.507267	1.503338	1.499662	1.536851	1.533888	1.531155
1.521083	1.516647	1.512295	1.508071	1.504064	1.500264	1.537392	1.534344	1.531548
1.52196	1.517474	1.5131	1.508849	1.504751	1.500811	1.537889	1.534778	1.531938
1.522841	1.518337	1.513944	1.509657	1.505475	1.501412	1.497819	1.494747	1.532326
1.523729	1.519249	1.514838	1.510506	1.506247	1.502076	1.498368	1.495162	1.532699
1.524617	1.520162	1.515737	1.511365	1.507042	1.502786	1.498973	1.495631	1.492655
1.525508	1.521059	1.516621	1.512219	1.507851	1.503535	1.499638	1.49618	1.493105
1.526392	1.521944	1.517499	1.513081	1.508688	1.504335	1.500367	1.496791	1.493611
1.527245	1.522795	1.518362	1.51396	1.509571	1.505212	1.501183	1.497475	1.494167
1.528084	1.52363	1.519221	1.514853	1.510489	1.506147	1.502064	1.498211	1.494761
1.528971	1.524523	1.520123	1.515764	1.511408	1.507072	1.502922	1.49891	1.495307
1.529904	1.52547	1.521067	1.516694	1.512334	1.507996	1.503768	1.499577	1.495805
1.530826	1.526406	1.521994	1.517602	1.51323	1.50888	1.504589	1.500269	1.496366
1.531738	1.527327	1.522897	1.518474	1.514072	1.509691	1.505354	1.500979	1.497
1.532613	1.528212	1.523776	1.51934	1.514922	1.510519	1.506158	1.501759	1.497726
1.533399	1.529008	1.524604	1.520211	1.515821	1.511436	1.507078	1.502675	1.498592
1.53417	1.529787	1.525424	1.521092	1.516743	1.51239	1.508049	1.503662	1.499539
1.535046	1.530659	1.526313	1.522008	1.517679	1.513339	1.509012	1.504649	1.500498
1.535996	1.531594	1.52725	1.522953	1.518628	1.514293	1.509977	1.505648	1.501476
1.536998	1.532581	1.528226	1.523917	1.519581	1.515235	1.510914	1.506598	1.502394
1.538059	1.533629	1.529248	1.524906	1.520541	1.516168	1.511825	1.5075	1.503249

1.53913	1.534679	1.530267	1.525886	1.521484	1.517074	1.512702	1.508364	1.504076
1.540204	1.535717	1.531272	1.526853	1.522403	1.517937	1.513524	1.50917	1.50486
1.541269	1.536744	1.532259	1.527796	1.523301	1.518788	1.514345	1.509979	1.50566
1.542312	1.537749	1.533208	1.528685	1.524164	1.519649	1.51521	1.510853	1.506542
1.543333	1.538734	1.534134	1.529552	1.525017	1.520522	1.516101	1.511762	1.507467
1.460211	1.455881	1.535062	1.530465	1.525927	1.521441	1.517024	1.512683	1.50839
1.461115	1.456775	1.535986	1.531412	1.526884	1.5224	1.517978	1.513622	1.50932
1.462014	1.457676	1.536942	1.532393	1.527872	1.523385	1.518954	1.514578	1.510262
1.4629	1.458584	1.537937	1.533414	1.528899	1.524401	1.519954	1.515557	1.511222
1.463809	1.459509	1.538948	1.534448	1.529937	1.525427	1.520962	1.516537	1.512179
1.464768	1.460465	1.539975	1.53549	1.530978	1.526454	1.521965	1.517503	1.513118
1.465741	1.461428	1.541006	1.536535	1.532021	1.527484	1.522972	1.518473	1.514059
1.466696	1.462374	1.542023	1.537576	1.533064	1.528515	1.523985	1.519459	1.51502
1.467639	1.463308	1.54303	1.53861	1.534106	1.529548	1.525005	1.52046	1.515997
1.468546	1.464216	1.544008	1.539617	1.535132	1.530588	1.526043	1.521483	1.516994
1.469414	1.465098	1.544959	1.540599	1.536145	1.531634	1.527101	1.522529	1.518011
1.470335	1.465995	1.545909	1.54157	1.537146	1.532674	1.528157	1.523575	1.519029
1.471325	1.466902	1.546851	1.542521	1.538125	1.533701	1.529203	1.524609	1.520037
1.472371	1.467858	1.547827	1.543495	1.53912	1.534741	1.530263	1.525659	1.521061
1.473501	1.468907	1.548873	1.544522	1.540156	1.535809	1.531343	1.526729	1.522102
1.474663	1.47	1.549961	1.545581	1.541218	1.536902	1.53245	1.52783	1.523176
1.475771	1.471081	1.46673	1.462575	1.542313	1.538047	1.53362	1.528999	1.524328
1.47685	1.472162	1.467775	1.463579	1.543429	1.539222	1.53483	1.530217	1.525539
1.477931	1.473219	1.468779	1.46453	1.544453	1.540275	1.535928	1.531382	1.526753
1.479012	1.474245	1.469739	1.465421	1.545377	1.541201	1.536912	1.532501	1.527983
1.42553	1.420806	1.470713	1.466318	1.546294	1.542107	1.537863	1.533568	1.529151
1.4266	1.421832	1.471718	1.467238	1.547227	1.54302	1.538796	1.534574	1.530222
1.42768	1.422877	1.472739	1.468168	1.548163	1.543927	1.53971	1.535541	1.531242
1.428754	1.423924	1.473758	1.469087	1.549088	1.54482	1.540605	1.536479	1.532227
1.429835	1.424994	1.474804	1.470032	1.550033	1.545722	1.541495	1.537395	1.533183
1.43095	1.426127	1.475946	1.471087	1.551065	1.546681	1.542419	1.538324	1.53414
1.432093	1.427315	1.477169	1.472234	1.552172	1.547691	1.54337	1.539259	1.535094
1.433253	1.428534	1.423651	1.418753	1.553383	1.548788	1.544396	1.540261	1.536106
1.43445	1.429807	1.424956	1.420051	1.554733	1.549999	1.54552	1.541354	1.537205
1.159455	1.155536	1.426211	1.421317	1.556056	1.551194	1.546626	1.542415	1.538257
1.160253	1.156437	1.427336	1.422464	1.557263	1.552297	1.547644	1.543366	1.539179
1.161013	1.157304	1.428414	1.423575	1.558447	1.553395	1.548662	1.544303	1.540073
1.161799	1.158177	1.429495	1.424696	1.474811	1.469947	1.549748	1.5453	1.541012
1.162587	1.159043	1.430571	1.425821	1.476002	1.471099	1.550886	1.546342	1.541983
1.163374	1.159908	1.319599	1.315041	1.477258	1.472323	1.552119	1.54748	1.543045
1.164163	1.160776	1.3207	1.316209	1.478576	1.473618	1.553446	1.548713	1.544193
1.164893	1.161568	1.321727	1.317335	1.424733	1.419845	1.554763	1.54994	1.545335
1.165534	1.162257	1.322653	1.318403	1.425912	1.421051	1.556047	1.551137	1.546442
1.166172	1.162921	1.15947	1.155865	1.315016	1.310346	1.557349	1.552363	1.547585
1.166842	1.16359	1.160218	1.156731	1.31609	1.311501	1.558704	1.553661	1.548812
1.167544	1.164268	1.160946	1.157549	1.317128	1.312646	1.560068	1.554988	1.55008
1.053619	1.050476	1.161679	1.158321	1.318105	1.313746	1.561379	1.55629	1.55134
1.05467	1.051475	1.162412	1.159055	1.319035	1.314811	1.562658	1.557585	1.55261
1.055727	1.05247	1.163154	1.159806	1.319956	1.315838	1.419628	1.414778	1.553926
1.056792	1.053457	1.1639	1.160567	1.320861	1.316825	1.420792	1.41602	1.555295

1.057885	1.054467	1.051212	1.048046	1.157924	1.154396	1.421913	1.417214	1.556636
1.059012	1.055501	1.052244	1.049113	1.158798	1.155254	1.422961	1.418323	1.557916
1.060169	1.056568	1.053295	1.05018	1.159664	1.156105	1.424006	1.419418	1.559185
1.061351	1.05767	1.054343	1.051192	1.160471	1.156947	1.425116	1.42056	1.415973
1.062569	1.058815	1.05541	1.052191	1.161259	1.157783	1.426239	1.421704	1.417147
1.063856	1.060026	1.056532	1.053225	1.162075	1.158607	1.318616	1.314408	1.418251
1.065203	1.061297	1.057701	1.054285	1.162912	1.159423	1.319532	1.315355	1.419304
1.06653	1.06258	1.058895	1.055376	1.163788	1.160292	1.320519	1.316362	1.42041
1.067838	1.063879	1.060118	1.056501	1.164708	1.161229	1.3216	1.317446	1.421581
1.069121	1.065166	1.061345	1.057647	1.054189	1.050935	1.158598	1.154956	1.422757
1.070357	1.066412	1.062555	1.058801	1.055293	1.051989	1.15944	1.155812	1.423926
1.071593	1.067662	1.063782	1.059984	1.056419	1.053046	1.160275	1.156656	1.425074
0.94982	0.946534	1.065065	1.061222	1.057592	1.054136	1.161158	1.157525	1.42615
0.950958	0.947687	1.066381	1.062502	1.058805	1.055256	1.162073	1.158416	1.427202
0.952029	0.948778	1.067705	1.063816	1.06007	1.05644	1.052909	1.049492	1.428359
0.953043	0.949819	1.069041	1.065167	1.061388	1.057686	1.054106	1.050672	1.429606
0.954068	0.950834	1.070332	1.066475	1.062686	1.05895	1.055339	1.051878	1.157751
0.9551	0.951804	1.071559	1.06772	1.063952	1.060241	1.056624	1.053117	1.158783
0.956175	0.952812	0.949578	0.946427	1.065197	1.061507	1.057887	1.054347	1.050845
0.957331	0.953918	0.950645	0.947462	1.066397	1.062693	1.059072	1.055545	1.052054
0.958532	0.955078	0.951761	0.948534	1.067603	1.063863	1.060229	1.056718	1.053246
0.959754	0.956266	0.952921	0.949661	1.068895	1.065077	1.061387	1.057844	1.054366
0.960992	0.957486	0.954121	0.950832	1.070245	1.066322	1.06255	1.058942	1.055442
0.962183	0.958708	0.955324	0.951994	0.94868	0.945392	1.063808	1.060112	1.056552
0.963325	0.959936	0.956535	0.953151	0.949825	0.946548	1.065163	1.061348	1.057685
0.837599	0.834691	0.957727	0.954308	0.950975	0.947706	1.066536	1.062634	1.058883
0.83868	0.835787	0.958891	0.955461	0.952124	0.948855	1.067916	1.063979	1.060169
0.839776	0.836876	0.96004	0.956623	0.953285	0.950007	1.069304	1.065352	1.061495
0.840914	0.837976	0.961177	0.957808	0.954474	0.951171	0.947871	0.944575	1.06284
0.84207	0.839073	0.962302	0.958998	0.955671	0.952337	0.94902	0.945723	1.064205
0.671611	0.66926	0.963414	0.960156	0.956838	0.953493	0.950168	0.946868	1.065573
0.672413	0.669992	0.964516	0.961292	0.957985	0.954641	0.951315	0.948011	1.066947
0.67329	0.670814	0.96567	0.962447	0.959136	0.955787	0.952457	0.949148	1.068318
0.674256	0.671744	0.966884	0.963625	0.960291	0.956931	0.953592	0.950278	1.069685
0.675229	0.672681	0.968104	0.964807	0.96145	0.95808	0.954733	0.951411	0.948099
0.67618	0.673588	0.969309	0.965987	0.962615	0.959238	0.955882	0.95255	0.949229
0.677127	0.674491	0.970522	0.96717	0.963782	0.960402	0.957038	0.953692	0.950361
0.501194	0.498683	0.672894	0.670509	0.964945	0.96157	0.958202	0.954835	0.951498
0.502121	0.499536	0.673766	0.671354	0.96611	0.962742	0.95937	0.95598	0.952639
0.503078	0.500462	0.674681	0.672236	0.967293	0.963914	0.960531	0.957124	0.953782
0.504062	0.501453	0.675635	0.673151	0.96849	0.965086	0.961685	0.958265	0.954927
0.505028	0.502443	0.676587	0.674058	0.671638	0.669316	0.962835	0.959407	0.956072
0.505964	0.503414	0.677519	0.674935	0.67248	0.670133	0.963974	0.960541	0.957206
		0.678496	0.675859	0.67336	0.670972	0.965126	0.9617	0.958371
		0.67958	0.676889	0.674326	0.671861	0.966307	0.962911	0.9596
		0.680685	0.677954	0.675327	0.672773	0.967501	0.964142	0.960852
		0.505102	0.50249	0.67631	0.673686	0.671191	0.668842	0.96209
		0.506046	0.50348	0.677291	0.674605	0.672079	0.669735	0.96332
				0.678266	0.675516	0.672945	0.670581	0.964491
				0.679232	0.676412	0.673784	0.671372	0.965597

0.50389	0.501163	0.674711	0.672249	0.827343
0.504929	0.502225	0.675777	0.673271	0.828322
0.505972	0.503325	0.676876	0.674314	0.829337
0.507001	0.504425	0.677952	0.675298	0.672744
0.508005	0.505516	0.67902	0.676273	0.673641
		0.504047	0.501429	0.674613
		0.505048	0.502437	0.675638
		0.506016	0.503432	0.676642
		0.506944	0.504406	0.67762
				0.502944
				0.504008
				0.505087
				0.506172
				0.507244

						0.555403	0.550677	0.546072
						0.55346	0.548953	0.544521
						0.55146	0.547132	0.542861
						0.549361	0.545177	0.541067
				0.556845	0.551987	1.071415	1.06696	1.062583
				0.554958	0.550269	1.069617	1.065076	1.06061
				0.553028	0.548577	1.067854	1.063218	1.058655
				0.551028	0.54674	1.244343	1.239506	1.234749
				0.549013	0.544847	1.242228	1.237446	1.232756
				0.547195	0.54302	1.24017	1.235433	1.230806
				0.545552	0.541242	1.238152	1.233459	1.228897
		0.552688	0.548257	1.245717	1.240918	1.379875	1.374738	1.369746
		0.550937	0.546452	1.243722	1.239002	1.377805	1.372688	1.367716
		0.549184	0.544678	1.24174	1.237076	1.508171	1.50277	1.497494
		0.547353	0.543011	1.23981	1.235093	1.505926	1.500597	1.495391
		0.545527	0.541359	1.237887	1.233104	1.503682	1.498436	1.493309
0.55274	0.548163	1.062145	1.057616	1.379535	1.374337	1.562824	1.557538	1.552362
0.550954	0.546557	1.060343	1.055787	1.377374	1.372215	1.56052	1.555351	1.550279
0.549179	0.544833	1.058521	1.05398	1.375265	1.370163	1.776555	1.770622	1.76482
0.547417	0.54299	1.056682	1.052196	1.373213	1.368186	1.774085	1.768186	1.762404
1.064007	1.059391	1.237512	1.232836	1.44379	1.438714	1.828277	1.822081	1.815976
1.062152	1.057578	1.235548	1.230791	1.441565	1.436648	1.825748	1.819591	1.813497
1.06032	1.055771	1.233599	1.228793	1.439378	1.434599	1.823226	1.817107	1.81103
1.05853	1.053932	1.374854	1.369713	1.776153	1.770252	1.920689	1.914419	1.908169
1.056768	1.052105	1.372731	1.367726	1.773645	1.767801	1.918029	1.911791	1.905578
1.055022	1.050372	1.443333	1.438228	1.827812	1.821658	1.977437	1.97098	1.964521
1.053291	1.04871	1.441342	1.436237	1.825233	1.819135	1.974812	1.968346	1.961877
1.16798	1.163285	1.525891	1.520359	1.822754	1.816653	1.972167	1.965696	1.959226
1.166063	1.161471	1.523705	1.518352	1.820397	1.814209	1.969485	1.963019	1.956568
1.342104	1.337319	1.830495	1.824263	1.917886	1.911475	1.966877	1.960379	1.953907
1.340236	1.335501	1.827875	1.821745	1.915379	1.909077	1.964423	1.957807	1.951221
1.338418	1.333755	1.825298	1.819221	1.912852	1.906678	1.961989	1.95527	1.948577
1.480445	1.475269	1.822866	1.816743	1.972359	1.96596	2.014303	2.007068	1.999857
1.478437	1.473414	1.820513	1.814302	1.969714	1.963337	2.011529	2.004388	1.99724
1.791018	1.784903	1.91791	1.911556	1.96718	1.960728	2.008658	2.001586	1.994524
1.78846	1.782452	1.915427	1.909208	1.96474	1.958123	2.005695	1.998681	1.991739
1.785971	1.779998	1.912913	1.906796	2.002077	1.995156	2.007266	2.000252	1.993363
1.783563	1.777522	1.910343	1.904288	1.999464	1.992586	2.004598	1.997449	1.990469
1.920365	1.914004	1.946628	1.940351	1.996805	1.99	2.001959	1.994728	1.987684
1.917977	1.91165	1.944126	1.937723	1.994045	1.987329	1.999299	1.99214	1.985143

1.915628	1.909371	1.941684	1.935118	1.99128	1.984637	1.996635	1.989616	1.982719
1.913275	1.907104	2.002322	1.995412	2.007752	2.000824	2.066647	2.058829	2.051112
1.910927	1.904855	1.999873	1.992899	2.005105	1.998109	2.063578	2.055941	2.048409
1.908554	1.902544	1.997386	1.990391	2.002488	1.995426	2.107788	2.100006	2.092373
1.906155	1.900175	1.994868	1.987897	1.999907	1.992768	2.104559	2.096964	2.089529
1.983861	1.977433	1.992338	1.985394	1.997353	1.99019	2.116288	2.108855	2.101574
1.981349	1.974883	1.9898	1.982874	1.994826	1.987715	2.113412	2.105986	2.098721
1.978847	1.972328	1.987252	1.980353	1.992318	1.98529	2.110607	2.103137	2.095841
1.998071	1.991482	2.003935	1.996833	2.094238	2.086404	2.10781	2.10025	2.092892
1.995554	1.98891	2.001313	1.994275	2.091448	2.083707	2.105044	2.097378	2.089941
1.992985	1.986257	1.998628	1.991688	2.117982	2.110077	2.132119	2.124318	2.116733
1.99038	1.983547	1.995902	1.989085	2.115091	2.107291	2.129371	2.121567	2.113966
2.002787	1.995567	2.0981	2.090595	2.112228	2.104515	2.137584	2.129828	2.122247
2.000185	1.992962	2.095198	2.087778	2.109396	2.10175	2.134863	2.127174	2.11962
1.997597	1.990388	2.092355	2.085005	2.136563	2.128843	2.1572	2.149482	2.141871
1.99501	1.987824	2.089565	2.082278	2.133769	2.126078	2.154443	2.146793	2.139237
1.992444	1.985296	2.086819	2.079587	2.130996	2.123324	2.151688	2.144105	2.13661
2.099571	2.091601	2.084103	2.076928	2.139185	2.131535	2.148925	2.141411	2.133984
2.096739	2.088852	2.081418	2.074295	2.136447	2.1288	2.146176	2.138725	2.131362
2.093908	2.086112	2.137755	2.130316	2.13373	2.126133	2.172843	2.165356	2.15793
2.091082	2.083384	2.134959	2.127557	2.131033	2.123524	2.170222	2.16272	2.155279
2.088309	2.080709	2.14314	2.135755	2.128336	2.120905	2.167594	2.160087	2.152644
2.085585	2.078084	2.140406	2.133014	2.125628	2.118268	2.16495	2.157457	2.15003
2.096157	2.088762	2.137743	2.130319	2.122946	2.115638	2.162306	2.154829	2.147426
2.093629	2.086354	2.135207	2.1277	2.120304	2.113023	2.159664	2.152201	2.144827
2.091162	2.083986	2.132709	2.125126	2.117697	2.110422	2.157022	2.149577	2.142243
2.145311	2.137746	2.130144	2.122579	2.1346	2.127262	2.169282	2.161878	2.154617
2.142752	2.135118	2.127548	2.120052	2.132064	2.124671	2.166644	2.159273	2.15208
2.14013	2.132474	2.124932	2.117482	2.129492	2.122072	2.220262	2.212839	2.205598
2.137449	2.12981	2.122293	2.114873	2.126886	2.119463	2.217603	2.21026	2.203091
2.134768	2.127145	2.119651	2.112259	2.173669	2.166189	2.214984	2.207744	2.200659
2.132089	2.124463	2.116989	2.109634	2.171029	2.163592	2.212399	2.205305	2.198334
2.129433	2.121827	2.114373	2.10704	2.168427	2.161058	2.209883	2.202902	2.196013
2.126814	2.119285	2.129181	2.121819	2.165883	2.158641	2.207493	2.200526	2.193626
2.124233	2.116809	2.126717	2.119338	2.163398	2.156284	2.205153	2.198158	2.191209
2.121711	2.114397	2.142869	2.13546	2.161019	2.153929	2.209579	2.202574	2.195615
2.119241	2.11204	2.140559	2.133177	2.15872	2.151584	2.207152	2.200127	2.193156
2.134157	2.126949	2.138151	2.130791	2.212271	2.205019	2.208608	2.201559	2.194563
2.131772	2.124469	2.135632	2.128286	2.209726	2.202515	2.206141	2.199092	2.1921
2.129375	2.122005	2.133139	2.125811	2.21794	2.210781	2.211132	2.204068	2.197075
2.126936	2.119577	2.1307	2.12339	2.215444	2.208305	2.208681	2.201692	2.194782
2.124493	2.117174	2.128312	2.121043	2.213023	2.2059	2.206289	2.19936	2.192516
2.140558	2.133268	2.126024	2.118853	2.210795	2.203689	2.204053	2.197082	2.190201
2.138119	2.13091	2.123776	2.116729	2.208644	2.201553	2.201878	2.194831	2.187878
2.13571	2.128493	2.213601	2.206466	2.206326	2.199247	2.199559	2.192524	2.185578
2.133329	2.126032	2.211113	2.204012	2.203885	2.196817	2.197135	2.190176	2.183296
2.125115	2.117786	2.208662	2.20158	2.201453	2.194385	2.194708	2.187841	2.181049
2.122739	2.115453	2.20624	2.19916	2.199016	2.191932	2.192261	2.185515	2.178848
2.17265	2.165337	2.210863	2.20376	2.196665	2.189574	2.216257	2.209561	2.202932
2.170393	2.163153	2.208668	2.201579	2.194497	2.187417	2.214052	2.2073	2.200627

2.168159	2.161001	2.206504	2.19943	2.192366	2.185311	2.211893	2.205057	2.198313
2.16582	2.158744	2.204209	2.197144	2.19873	2.191726	2.209631	2.202769	2.196013
2.163448	2.156452	2.201863	2.194805	2.196414	2.189447	2.207337	2.200474	2.193731
2.211458	2.204314	2.199618	2.192562	2.212867	2.205909	2.205125	2.198275	2.191541
2.209286	2.202159	2.197466	2.190406	2.210694	2.203753	2.20299	2.196166	2.189442
2.207059	2.199947	2.220966	2.213898	2.208475	2.201552	2.200814	2.194018	2.187308
2.20472	2.197624	2.218627	2.211572	2.206162	2.199258	2.198553	2.191777	2.185084
2.202405	2.195324	2.216313	2.209276	2.203879	2.196993	2.196326	2.189581	2.182917
2.220271	2.213189	2.214129	2.207109	2.201728	2.194856	2.194234	2.18754	2.180925
2.218086	2.211016	2.211969	2.20497	2.199607	2.192746	2.192174	2.185547	2.178995
2.223778	2.216707	2.209684	2.202717	2.197372	2.190521	2.190002	2.183467	2.177005
2.221392	2.214328	2.207332	2.200404	2.195081	2.188237	2.187775	2.181342	2.174986
2.219147	2.212091	2.205118	2.19822	2.192918	2.186092	2.179428	2.172991	2.16664
2.217049	2.210002	2.203047	2.196171	2.190891	2.184093	2.177422	2.170922	2.164507
2.214977	2.207944	2.201002	2.194141	2.187322	2.180544	2.175446	2.168897	2.162431
2.212931	2.205918	2.198985	2.192128	2.185328	2.178584	2.173507	2.166959	2.160487
2.210838	2.203849	2.196927	2.190073	2.183292	2.176583	2.171529	2.16499	2.158529
2.208565	2.20161	2.194707	2.187864	2.181105	2.174428	2.169392	2.162852	2.156413
2.206233	2.199316	2.192437	2.185609	2.178872	2.172223	2.167204	2.16066	2.154253
2.20405	2.197167	2.190311	2.183502	2.176785	2.170158	2.165152	2.1586	2.152226
2.201975	2.195125	2.188293	2.181503	2.174805	2.168195	2.163199	2.156636	2.150298
2.199977	2.193142	2.18633	2.179562	2.172881	2.166283	2.159745	2.153165	2.146856
2.198076	2.191236	2.184437	2.177692	2.171026	2.164437	2.157904	2.151302	2.145021
2.196163	2.189316	2.182531	2.17581	2.169161	2.162582	2.156066	2.149474	2.143245
2.194205	2.187359	2.180591	2.173896	2.16727	2.160709	2.154228	2.147689	2.141542
2.192193	2.185345	2.178593	2.171925	2.165322	2.158777	2.152348	2.14591	2.139886
2.190054	2.183186	2.176442	2.169802	2.163217	2.156679	2.150323	2.144069	2.138242
2.187849	2.180956	2.174218	2.167605	2.161036	2.154499	2.14823	2.142215	2.136633
2.185753	2.178862	2.172139	2.165548	2.158992	2.152452	2.146263	2.140468	2.135117
2.183748	2.176881	2.170181	2.163607	2.157061	2.150512	2.144398	2.138816	2.13369
2.181785	2.174955	2.168281	2.161724	2.155195	2.148657	2.139531	2.134059	2.129058
2.179869	2.173094	2.166448	2.159907	2.1534	2.146883	2.137826	2.132522	2.127696
2.177971	2.171249	2.164628	2.158104	2.151644	2.145198	2.136233	2.131074	2.126393
2.176076	2.169388	2.162785	2.156279	2.149908	2.143624	2.134795	2.129751	2.125163
2.174187	2.167529	2.160938	2.154451	2.148191	2.142116	2.133448	2.128505	2.123988
2.172294	2.165668	2.159091	2.152619	2.146471	2.140605	2.132105	2.127283	2.122853
2.170401	2.163808	2.157243	2.150788	2.144755	2.13911	2.130786	2.126094	2.121756
2.168526	2.161964	2.155417	2.148986	2.143065	2.137622	2.129463	2.124898	2.120654
2.166668	2.160133	2.153608	2.147207	2.141393	2.136137	2.128133	2.123697	2.119548
2.164823	2.158315	2.151834	2.145494	2.136767	2.131595	2.126832	2.122506	2.118439
2.162994	2.156515	2.150103	2.143863	2.135244	2.13021	2.125564	2.121323	2.117319
2.161169	2.154718	2.148401	2.142293	2.13379	2.128879	2.124333	2.120161	2.116207
2.159332	2.152914	2.14672	2.140789	2.132415	2.127615	2.123153	2.119036	2.115119
2.157492	2.151109	2.145058	2.139331	2.131095	2.126397	2.12201	2.117936	2.114047
2.155645	2.149299	2.143391	2.13787	2.129776	2.125188	2.120882	2.116851	2.112988
2.153794	2.147486	2.141726	2.136415	2.128468	2.123997	2.119773	2.115784	2.111943
2.151998	2.145726	2.137162	2.13196	2.127166	2.122808	2.132548	2.128485	2.124545
2.150243	2.143999	2.135546	2.130517	2.125863	2.12162	2.13141	2.127384	2.123464
2.148608	2.142412	2.134046	2.129143	2.138632	2.134376	2.130272	2.126283	2.122387
2.147169	2.14104	2.132718	2.127863	2.137379	2.133178	2.129123	2.125177	2.121314

2.145793	2.139773	2.131494	2.126662	2.136178	2.132009	2.127988	2.124077	2.120245
2.144362	2.13854	2.144476	2.139595	2.135059	2.1309	2.154897	2.15069	2.146572
2.142909	2.137337	2.14337	2.138524	2.134005	2.129839	2.153773	2.149567	2.145454
2.141386	2.136036	2.142198	2.137465	2.133	2.128824	2.152686	2.148475	2.144362
2.139795	2.134649	2.140979	2.13643	2.132049	2.127857	2.151636	2.147413	2.143291
2.149621	2.144495	2.139745	2.135347	2.131057	2.126898	2.150643	2.146422	2.142293
2.148112	2.143118	2.138481	2.134191	2.130006	2.125952	2.149739	2.145541	2.141403
2.1467	2.141804	2.137249	2.133034	2.128933	2.124976	2.148797	2.144629	2.140496
2.145434	2.140587	2.136073	2.131893	2.127833	2.123922	2.147723	2.143593	2.139498
2.144272	2.139455	2.13496	2.130787	2.126741	2.122848	2.146602	2.142506	2.138456
2.143212	2.13845	2.133967	2.129775	2.125718	2.121818	2.1455	2.14141	2.137376
2.142229	2.137534	2.133061	2.128832	2.124746	2.120819	2.144408	2.140304	2.136269
2.141127	2.136524	2.132107	2.127902	2.123825	2.11989	2.143405	2.139276	2.135222
2.139908	2.135428	2.131115	2.126996	2.122966	2.119041	2.1425	2.138328	2.13423
2.138699	2.134314	2.130082	2.126031	2.122045	2.118138	2.141557	2.137383	2.13328
2.13751	2.133173	2.128979	2.124961	2.121007	2.117131	2.140536	2.136434	2.132396
2.13638	2.132073	2.127896	2.123888	2.119953	2.116101	2.139487	2.135463	2.131497
2.135383	2.131097	2.126916	2.122882	2.118934	2.115079	2.13841	2.134418	2.13049
2.134449	2.130176	2.125981	2.121912	2.117941	2.114072	2.137338	2.133363	2.129457
2.133462	2.12921	2.125034	2.120979	2.144619	2.140445	2.142622	2.138633	2.134712
2.13245	2.128224	2.124089	2.120079	2.143712	2.139549	2.141725	2.137756	2.133841
2.131484	2.12728	2.123162	2.119162	2.142757	2.138608	2.140785	2.136826	2.13292
2.130576	2.126388	2.122256	2.118219	2.141734	2.137606	2.139787	2.135828	2.131937
2.12965	2.125485	2.121353	2.117299	2.14075	2.136642	2.138824	2.134853	2.130963
2.128656	2.124529	2.120441	2.116431	2.139863	2.135775	2.137946	2.133939	2.130014
2.127676	2.123587	2.119541	2.115575	2.138987	2.134918	2.13708	2.133045	2.129093
2.1268	2.122729	2.11868	2.114694	2.13803	2.133979	2.136154	2.132152	2.12823
2.12597	2.121909	2.117839	2.113812	2.137052	2.133017	2.135211	2.131264	2.127396
2.125081	2.121032	2.116982	2.112976	2.136166	2.13214	2.134327	2.130379	2.126513
2.124147	2.120111	2.116113	2.112181	2.135356	2.131332	2.133491	2.129497	2.125591
2.123263	2.119232	2.115271	2.11139	2.14072	2.136656	2.142184	2.138043	2.134009
2.12245	2.118418	2.114471	2.110608	2.139872	2.135774	2.141257	2.137165	2.133183
2.121644	2.117602	2.11366	2.109803	2.139005	2.134896	2.140351	2.136312	2.132378
2.120815	2.116747	2.112795	2.108939	2.138095	2.134038	2.139509	2.135503	2.13158
2.119979	2.115889	2.111925	2.108066	2.137181	2.133191	2.138686	2.134702	2.130778
2.119132	2.115063	2.111107	2.10725	2.145955	2.141877	2.13785	2.13386	2.129931
2.118279	2.114254	2.110316	2.106463	2.145105	2.141031	2.137002	2.132994	2.129058
2.117445	2.113437	2.1095	2.105641	2.144186	2.14011	2.146924	2.142633	2.13847
2.116629	2.112611	2.10866	2.104782	2.143195	2.139109	2.145833	2.141667	2.137619
2.115822	2.111787	2.125568	2.121514	2.142265	2.138172	2.144816	2.140737	2.136766
2.115021	2.110958	2.124703	2.120695	2.141438	2.137345	2.143916	2.139865	2.135909
2.114234	2.110153	2.123864	2.119901	2.14064	2.136553	2.143062	2.139006	2.135037
2.113478	2.109407	2.123082	2.119125	2.139838	2.135774	2.142239	2.138134	2.134122
2.112726	2.108683	2.122328	2.118364	2.13904	2.134998	2.141425	2.137261	2.1332
2.111921	2.107924	2.121557	2.117606	2.138234	2.134169	2.140538	2.136382	2.132333
2.111082	2.107146	2.120779	2.116852	2.137424	2.133304	2.139606	2.135501	2.131505
2.116801	2.112807	2.115677	2.111773	2.13662	2.132483	2.138741	2.134662	2.13069
2.116008	2.112027	2.114898	2.111002	2.135834	2.131716	2.137953	2.133871	2.129892
2.115228	2.111257	2.114111	2.1102	2.145735	2.141381	2.149831	2.14557	2.141407
2.114458	2.110498	2.113303	2.109332	2.144721	2.140469	2.14896	2.144722	2.140567

2.113699	2.109748	2.112504	2.108474	2.143721	2.139558	2.14808	2.143869	2.139725
2.119769	2.11579	2.111746	2.107709	2.142848	2.138703	2.147203	2.143014	2.138879
2.119016	2.11504	2.11101	2.106992	2.142042	2.137879	2.14633	2.14216	2.138033
2.118264	2.114292	2.110278	2.106281	2.118107	2.114248	2.150377	2.146184	2.142031
2.117515	2.113548	2.109553	2.105582	2.11737	2.113503	2.149533	2.145342	2.141196
2.116751	2.11279	2.108815	2.10487	2.116621	2.112748	2.148684	2.144502	2.140366
2.115972	2.112016	2.10806	2.104141	2.115854	2.11198	2.147825	2.14366	2.13954
2.115182	2.111232	2.1195	2.115264	2.131115	2.12703	2.14697	2.142824	2.13872
2.11438	2.110438	2.118655	2.114466	2.130285	2.126206	2.146126	2.142	2.137911
2.113576	2.109642	2.117811	2.113668	2.129455	2.125383	2.145286	2.141181	2.137107
2.112782	2.108855	2.11698	2.112882	2.128636	2.124566	2.144447	2.14036	2.1363
2.111995	2.108075	2.11616	2.112105	2.127823	2.123753	2.143608	2.13954	2.135493
2.111217	2.107312	2.115359	2.111328	2.127004	2.122937	2.14277	2.138716	2.13468
2.110448	2.106565	2.114575	2.110552	2.126178	2.122117	2.14193	2.137888	2.133861
2.122159	2.117929	2.119962	2.11591	2.125373	2.121313	2.141106	2.137069	2.133046
2.121335	2.117158	2.119199	2.115161	2.124591	2.120524	2.1403	2.136263	2.132237
2.120518	2.116391	2.118448	2.114439	2.12384	2.119758	2.139507	2.135461	2.131428
2.119707	2.11562	2.117714	2.113767	2.144722	2.140362	2.136123	2.132071	2.128041
2.118905	2.114849	2.116981	2.113103	2.14404	2.139634	2.135352	2.131276	2.127234
2.118112	2.114075	2.129862	2.125825	2.143274	2.138877	2.134593	2.130491	2.126436
2.117331	2.113301	2.129033	2.124996	2.142452	2.138106	2.133848	2.129718	2.125645
2.116604	2.112572	2.128235	2.12418	2.141645	2.137363	2.133136	2.128968	2.12486
2.115939	2.111889	2.127464	2.123372	2.140852	2.136658	2.13247	2.128249	2.124079
2.124629	2.120574	2.12673	2.122583	2.140049	2.135917	2.13176	2.127511	2.123306
2.123932	2.119941	2.126058	2.121826	2.139224	2.135105	2.130964	2.126728	2.122534
2.123227	2.119294	2.12538	2.121081	2.138401	2.134272	2.13014	2.125942	2.121781
2.122499	2.11856	2.124618	2.120324	2.137592	2.133444	2.129322	2.12519	2.121084
2.121764	2.117784	2.12382	2.119573	2.136801	2.132623	2.128504	2.124445	2.120403
2.121064	2.117026	2.123049	2.118863	2.136066	2.131848	2.127707	2.123662	2.119644
2.120392	2.116275	2.122293	2.118187	2.13538	2.131112	2.126928	2.122848	2.118824
2.119677	2.115522	2.121525	2.117463	2.13465	2.130394	2.129436	2.125269	2.121165
2.118901	2.114759	2.120734	2.116669	2.133862	2.129706	2.128781	2.124564	2.120395
2.11814	2.114022	2.123427	2.119372	2.133066	2.128984	2.12809	2.123859	2.119657
2.117433	2.113341	2.122716	2.118637	2.132265	2.128176	2.127289	2.12313	2.118986
2.116745	2.11267	2.122022	2.117929	2.131483	2.127355	2.126457	2.122381	2.118311
2.129743	2.125447	2.121293	2.117253	2.130779	2.126599	2.136503	2.132279	2.128074
2.129023	2.12467	2.120555	2.116598	2.130117	2.125879	2.135657	2.131415	2.127219
2.1283	2.123948	2.119848	2.115915	2.129412	2.125181	2.134876	2.1306	2.12638
2.127573	2.123276	2.119171	2.115205	2.12867	2.124507	2.134159	2.129828	2.125549
2.113596	2.109344	2.118499	2.114525	2.146098	2.14175	2.133422	2.129073	2.12477
2.112917	2.108643	2.117827	2.113894	2.145362	2.140971	2.132636	2.128335	2.124069
2.112243	2.107962	2.117171	2.113265	2.144629	2.140205	2.131867	2.127606	2.123374
2.111557	2.107341	2.11655	2.112606	2.143874	2.139481	2.131164	2.126877	2.122625
2.110871	2.106735	2.115939	2.111945	2.143121	2.138772	2.130479	2.126156	2.121879
2.110197	2.106075	2.115298	2.111315	2.142401	2.138038	2.12975	2.125453	2.121206
2.109532	2.105386	2.11464	2.110703	2.141701	2.137293	2.128999	2.124766	2.120582
2.108852	2.104731	2.126981	2.122864	2.140978	2.136581	2.132264	2.128061	2.123899
2.108156	2.104114	2.126301	2.122146	2.140224	2.135901	2.131633	2.127428	2.123247
2.10748	2.103484	2.113572	2.109421	2.139504	2.135235	2.130995	2.126773	2.122567
2.106832	2.102808	2.112881	2.108781	2.138852	2.134587	2.130334	2.126073	2.121837

2.106208	2.102151	2.11221	2.108157	2.138209	2.133928	2.129653	2.125359	2.121101
2.116318	2.11213	2.11541	2.111288	2.13754	2.133223	2.128928	2.124646	2.120402
2.115719	2.111562	2.1148	2.11065	2.136856	2.132499	2.128187	2.12393	2.11971
2.115095	2.11097	2.11416	2.109966	2.136134	2.131794	2.127489	2.123213	2.11898
2.114456	2.110367	2.113501	2.109249	2.135381	2.131101	2.126822	2.122495	2.118222
2.115748	2.111609	2.112859	2.108582	2.13469	2.130439	2.126172	2.121818	2.117524
2.115134	2.110985	2.112245	2.107979	2.134081	2.129821	2.125548	2.121196	2.116903
2.114516	2.110344	2.132073	2.127605	2.133473	2.129187	2.124902	2.12057	2.116294
2.113873	2.109659	2.131375	2.126963	2.132831	2.128491	2.124185	2.119903	2.115671
2.113235	2.108979	2.130677	2.126318	2.132186	2.127796	2.123472	2.119239	2.115052
2.112639	2.108365	2.130038	2.125692	2.131566	2.127169	2.122845	2.118625	2.114448
2.112061	2.107785	2.129425	2.125067	2.13095	2.126575	2.122262	2.118032	2.113847
2.111435	2.10718	2.12769	2.123281	2.120023	2.115683	2.121646	2.117397	2.113196
2.110764	2.106556	2.12701	2.122542	2.119295	2.115011	2.121004	2.116723	2.112496
2.110123	2.105941	2.126353	2.121852	2.118618	2.114369	2.120383	2.116085	2.111844
2.109531	2.105339	2.125727	2.121236	2.11802	2.113772	2.119794	2.115501	2.111262
2.112889	2.108618	2.125114	2.120641	2.116266	2.111993	2.119215	2.114934	2.110701
2.112303	2.108029	2.124512	2.120046	2.115674	2.111397	2.118638	2.114367	2.110144
2.111722	2.107447	2.123917	2.119457	2.115087	2.110807	2.118065	2.113806	2.109593
2.111139	2.106857	2.123313	2.118861	2.114496	2.110219	2.117495	2.113242	2.109034
2.110556	2.106268	2.122707	2.118264	2.113905	2.109634	2.11693	2.112679	2.108471
2.109982	2.105696	2.122118	2.117678	2.113322	2.109053	2.116366	2.112119	2.107914
2.109419	2.105144	2.121546	2.117106	2.112749	2.108477	2.115805	2.111564	2.107364
2.108859	2.104595	2.120974	2.116532	2.112175	2.107903	2.10365	2.099389	2.095171
2.108304	2.104046	2.120395	2.115952	2.111596	2.107329	2.103078	2.098817	2.094597
2.10775	2.1035	2.119817	2.115372	2.111018	2.106755	2.102508	2.098244	2.094023
2.107193	2.102958	2.119245	2.114798	2.110444	2.106184	2.101938	2.097675	2.093453
2.106636	2.102417	2.118675	2.114225	2.109871	2.105613	2.101369	2.097106	2.092885
2.089098	2.085107	2.118097	2.113645	2.109293	2.105041	2.100801	2.096539	2.092318
2.088554	2.084565	2.117515	2.11306	2.108712	2.104469	2.100236	2.095972	2.091752
2.088019	2.084028	2.116935	2.112476	2.10813	2.103895	2.099667	2.095405	2.091188
2.087492	2.083493	2.116358	2.111892	2.107546	2.103316	2.099094	2.094835	2.090622
2.08699	2.082971	2.11579	2.11132	2.106972	2.102745	2.098527	2.094274	2.090068
2.08654	2.082474	2.115232	2.110755	2.106405	2.102181	2.097968	2.09372	2.08952
2.08609	2.081982	2.114691	2.110217	2.105865	2.101635	2.097422	2.093188	2.089
2.10791	2.103559	2.114182	2.109749	2.105397	2.101129	2.096902	2.092708	2.088557
2.107344	2.103019	2.113689	2.109312	2.104964	2.100643	2.096397	2.092255	2.088149
2.10678	2.102471	2.113155	2.108802	2.104461	2.100129	2.095881	2.091758	2.087669
2.106213	2.101914	2.112585	2.108229	2.103898	2.09959	2.095356	2.091224	2.08713
2.105691	2.101408	2.100531	2.096266	2.103347	2.099078	2.094863	2.090708	2.086594
2.105231	2.100984	2.100074	2.095749	2.102817	2.09861	2.094424	2.090225	2.086073
2.104781	2.100549	2.099609	2.09524	2.102294	2.098131	2.093965	2.089732	2.085551
2.104327	2.100047	2.099087	2.094722	2.101764	2.0976	2.093432	2.089195	2.085013
2.103861	2.099524	2.098556	2.094216	2.101249	2.097061	2.092882	2.088657	2.084488
2.103348	2.099013	2.094719	2.090468	2.100791	2.096568	2.092371	2.088173	2.084026
2.102808	2.098507	2.094258	2.090053	2.10037	2.096102	2.091885	2.087722	2.083604
2.102308	2.098037	2.093803	2.089606	2.099915	2.095651	2.091435	2.087267	2.08314
2.101846	2.097608	2.093361	2.089131	2.09943	2.095223	2.091028	2.086814	2.082636
2.101388	2.097153	2.092892	2.08864	2.087719	2.083638	2.090577	2.086337	2.082133
2.10093	2.096647	2.092372	2.088124	2.087191	2.083107	2.090042	2.085815	2.081632

2.100464	2.096133	2.091854	2.087622	2.086678	2.082575	2.089499	2.0853	2.081152
2.09997	2.095628	2.091372	2.08718	2.086231	2.08209	2.088991	2.084833	2.080725
2.099476	2.095139	2.090912	2.086762	2.085811	2.081628	2.088506	2.084389	2.080318
2.099049	2.094709	2.090477	2.08632	2.085363	2.081187	2.088068	2.083946	2.079873
2.098671	2.094325	2.090064	2.085866	2.0849	2.080765	2.087669	2.08351	2.079403
2.098235	2.09389	2.089626	2.085423	2.084452	2.080324	2.076231	2.072158	2.068139
2.097723	2.093384	2.089153	2.084997	2.084026	2.079856	2.075747	2.071715	2.067732
2.097245	2.092913	2.088698	2.084562	2.083587	2.079392	2.075275	2.071269	2.067308
2.09686	2.092527	2.08828	2.084094	2.083107	2.078944	2.074844	2.070817	2.066839
2.096489	2.092163	2.087883	2.083641	2.082635	2.0785	2.074414	2.070364	2.066368
2.092474	2.088177	2.08751	2.083259	2.079074	2.074951	2.073938	2.069911	2.065937
2.092033	2.087785	2.087146	2.082911	2.0787	2.074506	2.073452	2.069459	2.065522
2.091589	2.087347	2.086731	2.082531	2.078325	2.074107	2.073028	2.069021	2.065075
2.091139	2.086868	2.086272	2.082128	2.077955	2.073751	2.072658	2.068596	2.064603
2.090688	2.086408	2.08222	2.078092	2.077544	2.073368	2.072284	2.068196	2.064175
2.090234	2.085981	2.081776	2.077615	2.077069	2.072949	2.071907	2.067834	2.063809
2.078145	2.073923	2.081336	2.077152	2.073013	2.068919	2.073665	2.069596	2.065559
2.077722	2.073466	2.080891	2.076738	2.072597	2.068467	2.073189	2.069169	2.065182
2.077323	2.073022	2.080463	2.07635	2.072204	2.068023	2.072706	2.068728	2.064791
2.076967	2.072646	2.080091	2.075984	2.071836	2.067647	2.069113	2.065117	2.061178
2.076641	2.072314	2.079755	2.075634	2.071486	2.067312	2.068764	2.064705	2.060719
2.076304	2.071977	2.067766	2.063631	2.071098	2.06696	2.068425	2.064339	2.060329
2.075958	2.071641	2.067393	2.0632	2.07067	2.066595	2.0681	2.06402	2.060004
2.075595	2.071305	2.067037	2.062804	2.070275	2.06624	2.067769	2.063701	2.059686
2.075203	2.07097	2.066714	2.06247	2.06994	2.065905	2.067433	2.063377	2.059363
2.074815	2.070631	2.066391	2.06215	2.069617	2.065563	2.067076	2.063042	2.059041
2.057198	2.05303	2.066053	2.061822	2.057672	2.053599	2.063616	2.059657	2.055731
2.056849	2.052663	2.065701	2.061491	2.057329	2.053208	2.063197	2.059281	2.055403
2.05647	2.052251	2.065315	2.061145	2.056997	2.052865	2.062846	2.058949	2.055089
2.056074	2.051807	2.064902	2.060788	2.056673	2.052555	2.062542	2.058646	2.054784
2.055713	2.051418	2.047253	2.043179	2.056352	2.052247	2.050976	2.047097	2.043251
2.055392	2.051089	2.04692	2.042845	2.056035	2.051942	2.05067	2.046782	2.042928
2.055074	2.050774	2.046602	2.042521	2.055723	2.051638	2.050358	2.046445	2.042573
2.054749	2.05046	2.046293	2.042208	2.055417	2.051331	2.050027	2.046063	2.042155
2.054424	2.050151	2.045989	2.041899	2.055117	2.051029	2.0497	2.045685	2.041742
2.054102	2.04984	2.045681	2.041589	2.044354	2.040231	2.049398	2.045362	2.041402
2.053783	2.049531	2.045373	2.041281	2.044054	2.039936	2.04911	2.045069	2.041102
2.053472	2.049227	2.045071	2.040976	2.036878	2.03278	2.048823	2.044776	2.040804
2.05317	2.048929	2.044772	2.040676	2.036576	2.032477	2.048537	2.044488	2.040514
2.052875	2.048632	2.044473	2.040374	2.036276	2.032181	2.038101	2.034006	2.029984
2.052592	2.048338	2.044171	2.040072	2.035977	2.031891	2.037819	2.033732	2.029715
2.052309	2.048043	2.04387	2.039769	2.035679	2.031604	2.037544	2.03348	2.029478
2.052021	2.047744	2.043565	2.039464	2.035381	2.031319	2.037285	2.033271	2.029304
2.051735	2.04745	2.043266	2.039161	2.035083	2.031033	2.037025	2.033065	2.029135
2.051455	2.04717	2.042979	2.038862	2.034781	2.030736	2.036738	2.032803	2.028893
2.051183	2.046906	2.042706	2.03857	2.03448	2.030434	2.036438	2.03251	2.02861
2.05094	2.046693	2.04248	2.038308	2.0342	2.030154	2.029523	2.025609	2.021727
2.050726	2.046533	2.042303	2.038075	2.033941	2.029894	2.029252	2.025322	2.02143
2.050494	2.046334	2.042095	2.037837	2.033696	2.029663	2.029019	2.025064	2.021149
2.050222	2.046063	2.04183	2.037584	2.033469	2.02948	2.028847	2.024854	2.020896

2.04996	2.045783	2.041556	2.037328	2.033239	2.029287	2.028663	2.024638	2.020648
2.049749	2.045524	2.041285	2.037064	2.032979	2.029031	2.028403	2.024376	2.02039
2.049553	2.045275	2.041024	2.036809	2.032719	2.028756	2.028116	2.024096	2.020129
2.044545	2.04028	2.040803	2.036612	2.032515	2.028515	2.027843	2.023821	2.01986
2.044299	2.040056	2.040613	2.036454	2.032348	2.028293	2.027578	2.023549	2.019588
2.036273	2.032065	2.040424	2.036262	2.032144	2.028068	2.027335	2.023319	2.019368
2.036084	2.031909	2.040243	2.036033	2.031898	2.027834	2.027116	2.023139	2.019211
2.035895	2.031728	2.040041	2.035801	2.031663	2.027622	2.026918	2.022958	2.019036
2.035703	2.031492	2.039798	2.035571	2.031456	2.027452	2.026759	2.022766	2.01881
2.035499	2.031244	2.039553	2.035353	2.031262	2.027284	2.026592	2.022564	2.018576
2.035263	2.031009	2.031862	2.027708	2.031085	2.027081	2.026367	2.022336	2.018356
2.035016	2.030784	2.031667	2.027542	2.030911	2.026863	2.026117	2.022101	2.018147
2.03225	2.028038	2.026435	2.022322	2.023247	2.019202	2.025888	2.0219	2.017973
2.032053	2.027873	2.026255	2.022093	2.022999	2.018968	2.025673	2.021727	2.017829
2.031864	2.027688	2.026057	2.021869	2.022773	2.018763	2.018331	2.014414	2.01054
2.031685	2.027467	2.025828	2.021645	2.022569	2.018595	2.018178	2.014239	2.010347
2.031511	2.02727	2.025629	2.021452	2.022388	2.018433	2.018021	2.014069	2.010167
2.031344	2.027142	2.025514	2.021329	2.022245	2.018261	2.017832	2.013911	2.010034
2.031179	2.02702	2.025407	2.021215	2.022109	2.018091	2.017643	2.013754	2.009904
2.031009	2.026827	2.022627	2.01844	2.016953	2.012973	2.017488	2.013585	2.009724
2.030836	2.026602	2.022395	2.018225	2.016767	2.012827	2.017348	2.013409	2.009522
2.03067	2.026438	2.022237	2.018073	2.016619	2.012671	2.017179	2.01324	2.009353
2.03051	2.026331	2.022146	2.017978	2.016505	2.012506	2.016982	2.013076	2.009216
2.030362	2.026198	2.022029	2.017879	2.016406	2.012377	2.012132	2.008253	2.004418
2.030223	2.026006	2.021859	2.01777	2.016334	2.012309	2.012041	2.008108	2.004232
2.030105	2.025835	2.021695	2.017646	2.016242	2.012238	2.011963	2.007981	2.004066
2.030031	2.025742	2.02157	2.017481	2.016084	2.012126	2.011877	2.007896	2.003966
2.029964	2.025677	2.021465	2.017313	2.01591	2.011995	2.011778	2.007819	2.003891
2.029839	2.025592	2.02138	2.017205	2.013144	2.009196	2.011619	2.007695	2.0038
2.02968	2.025495	2.021307	2.017136	2.013046	2.00903	2.011419	2.007542	2.0037
2.029533	2.025355	2.021189	2.017047	2.012956	2.00891	2.011275	2.007394	2.003559
2.029398	2.025161	2.021017	2.01694	2.012881	2.008842	2.01119	2.007248	2.003368
2.029284	2.025002	2.020859	2.01681	2.012775	2.008758	2.011107	2.007124	2.003208
2.029211	2.024913	2.020733	2.016636	2.012602	2.008635	2.011021	2.007042	2.003112
2.029136	2.024846	2.020631	2.016476	2.01243	2.008497	2.010916	2.006962	2.003037
2.029011	2.024774	2.020563	2.016385	2.012313	2.008345	2.008269	2.004362	2.000488
2.028872	2.024705	2.020515	2.016332	2.012228	2.008194	2.008087	2.004234	2.000418
2.021525	2.017391	2.02046	2.016283	2.012169	2.008105	2.007979	2.004141	2.000344
2.021466	2.017321	2.020401	2.016245	2.012136	2.008066	2.007926	2.004074	2.000269
2.021386	2.017212	2.020307	2.016187	2.012095	2.008023	2.007868	2.003985	2.000158
2.021271	2.017046	2.020162	2.016104	2.012041	2.007974	2.0078	2.003862	1.999996
2.021177	2.016909	2.012801	2.008798	2.011982	2.007925	2.00774	2.003761	1.999863
2.021134	2.016851	2.012736	2.008729	2.011922	2.007881	2.007701	2.003715	1.999808
2.021106	2.016818	2.012684	2.008651	2.011845	2.007828	2.007663	2.003685	1.999779
2.021059	2.016765	2.0126	2.008524	2.011717	2.007741	2.007605	2.003638	1.999736
2.021007	2.01671	2.012508	2.008377	2.011565	2.007638	2.007538	2.003587	1.999691
2.020965	2.016672	2.012451	2.00829	2.00426	2.000364	2.007482	2.00354	1.999643
2.020929	2.016648	2.012427	2.008258	2.004221	2.000319	2.007433	2.003495	1.999591
2.020899	2.016626	2.012405	2.008231	2.004189	2.000282	2.00048	1.996554	1.992654
2.020875	2.016604	2.012382	2.008206	2.004162	2.000251	2.000444	1.996519	1.992617

2.020853	2.016582	2.012359	2.008183	2.004137	2.000224	1.996333	1.992439	1.988564
2.020829	2.016557	2.012332	2.008153	2.004107	2.000196	1.996307	1.992416	1.988543
2.020809	2.016534	2.012308	2.008128	2.004082	2.000171	1.996284	1.992395	1.988528
2.011599	2.007334	2.012294	2.008117	2.004069	2.000151	1.996261	1.992374	1.988515
2.01159	2.007324	2.01229	2.00812	2.004069	2.000138	1.996241	1.992353	1.988505
2.01159	2.007324	2.003136	1.999012	2.004113	2.000152	1.996231	1.992333	1.988485
2.011598	2.007333	2.003177	1.999102	2.004207	2.000198	1.996235	1.992313	1.988451
2.011612	2.007346	2.00321	1.999164	1.99513	1.991111	1.996239	1.992301	1.988429
2.011628	2.007354	2.003215	1.999169	1.995134	1.991114	1.996241	1.992298	1.988418
2.011655	2.007383	2.003232	1.999167	1.995125	1.991108	1.996244	1.992309	1.988431
2.011712	2.007476	2.003307	1.999194	1.995126	1.991101	1.996247	1.99235	1.9885
2.011779	2.007589	2.003399	1.999228	1.995128	1.991093	1.99625	1.9924	1.988587
2.01181	2.007634	2.003433	1.999239	1.995126	1.991085	1.987138	1.983321	1.979535
2.011817	2.00763	2.003426	1.999234	1.995121	1.991079	1.987129	1.983303	1.97951
2.011859	2.007644	2.003434	1.999246	1.995142	1.991115	1.987166	1.983317	1.979502
2.011945	2.007678	2.003455	1.999276	1.995193	1.991204	1.987264	1.983373	1.979514
2.012026	2.007723	2.003495	1.999329	1.995258	1.991285	1.987344	1.98342	1.97953
2.012073	2.007774	2.003564	1.999422	1.995344	1.99133	1.987361	1.983428	1.979542
2.012122	2.007842	2.003649	1.999521	1.995426	1.991364	1.987365	1.983437	1.979566
2.012208	2.007952	2.003746	1.999584	1.995467	1.991394	1.987394	1.983486	1.97963
2.012312	2.008082	2.003849	1.99963	1.995491	1.991423	1.987436	1.983554	1.979713
2.012406	2.008164	2.003914	1.999678	1.995536	1.991483	1.987503	1.983611	1.979755
2.012493	2.008205	2.003947	1.999722	1.995598	1.991572	1.987595	1.983659	1.979762
2.012566	2.008254	2.003999	1.99979	1.995672	1.991644	1.987655	1.983691	1.979773
2.012612	2.008313	2.004079	1.999896	1.995767	1.991688	1.987665	1.983697	1.979787
2.012667	2.008389	2.004168	1.999995	1.995848	1.991725	1.987676	1.983714	1.979821
2.012754	2.008501	2.004265	2.000053	1.995885	1.991754	1.987709	1.983772	1.979899
2.012855	2.008623	2.004361	2.000099	1.995911	1.991788	1.987758	1.983846	1.97999
2.012959	2.00871	2.004431	2.00015	1.995962	1.991858	1.987838	1.983915	1.980051
2.01307	2.008784	2.00449	2.000206	1.996029	1.991951	1.98794	1.983987	1.980099
2.013192	2.00891	2.004612	2.000319	1.996133	1.992047	1.988028	1.984071	1.980181
2.013327	2.009094	2.004803	2.000496	1.996281	1.992145	1.988101	1.984167	1.980298
2.004385	2.000212	2.004955	2.000642	1.996412	1.992253	1.988196	1.984271	1.980409
2.004485	2.000271	2.005012	2.000708	1.996498	1.992374	1.988331	1.984378	1.980494
2.00459	2.000339	2.005082	2.00079	1.996598	1.992502	1.98847	1.984492	1.980588
2.004714	2.000495	2.005255	2.000963	1.996757	1.992628	1.988577	1.984609	1.980717
2.004849	2.000678	2.005462	2.001174	1.996946	1.992765	1.988685	1.984733	1.980859
2.004993	2.000808	2.005614	2.001362	1.997144	1.992952	1.988855	1.984876	1.98098
2.005147	2.000904	2.005733	2.001538	1.997354	1.993179	1.989071	1.985036	1.98109
2.005329	2.001053	2.00588	2.00169	1.997522	1.993375	1.989277	1.985217	1.981242
2.005551	2.001266	2.006059	2.00181	1.997636	1.993538	1.989476	1.985427	1.981445
1.994877	1.990647	1.997204	1.992948	1.997769	1.993689	1.989647	1.985619	1.981647
1.995012	1.990833	1.997414	1.99315	1.997955	1.993824	1.989761	1.985767	1.981833
1.995136	1.991	1.997615	1.993363	1.998159	1.99397	1.989876	1.985905	1.982004
1.99528	1.991119	1.997755	1.993545	1.998363	1.994166	1.990056	1.986052	1.982132
1.995434	1.991217	1.997868	1.99371	1.998566	1.994388	1.990271	1.98621	1.982246
1.995615	1.991372	1.998014	1.99385	1.998728	1.994591	1.990493	1.986411	1.982425
1.99582	1.991573	1.998184	1.993966	1.998854	1.99478	1.990727	1.98665	1.982658
1.99601	1.991783	1.987554	1.983345	1.990003	1.986002	1.990957	1.986889	1.982893
1.996173	1.992001	1.987786	1.983572	1.990232	1.986227	1.991188	1.987127	1.983126

1.996351	1.992223	1.988023	1.983812	1.990469	1.986438	1.991396	1.987349	1.983354
1.99658	1.992451	1.988253	1.984043	1.990682	1.986599	1.991542	1.987529	1.98357
1.996819	1.992668	1.988471	1.984272	1.990898	1.986759	1.991686	1.987705	1.983785
1.990808	1.986642	1.988644	1.984488	1.980358	1.976248	1.982924	1.978997	1.97513
1.99101	1.986794	1.988794	1.984699	1.980603	1.976503	1.983184	1.979245	1.975362
1.991258	1.98701	1.988998	1.984932	1.980851	1.976756	1.983439	1.979486	1.975588
1.99155	1.987289	1.989256	1.985187	1.981104	1.977009	1.983693	1.979725	1.975812
1.991847	1.987577	1.989523	1.985451	1.981363	1.977264	1.973224	1.969261	1.965358
1.992141	1.987866	1.989794	1.985724	1.98163	1.977519	1.973451	1.969424	1.965472
1.992434	1.988158	1.990068	1.985998	1.981899	1.977777	1.973683	1.969599	1.965601
1.992722	1.988446	1.990339	1.986267	1.982165	1.978042	1.973939	1.969833	1.965814
1.99301	1.988734	1.990612	1.986535	1.982432	1.97831	1.974205	1.970093	1.966064
1.993307	1.989032	1.990891	1.986812	1.982706	1.978581	1.97447	1.970349	1.966309
1.993612	1.989338	1.991176	1.987095	1.982985	1.978854	1.974737	1.970607	1.966559
1.993924	1.989646	1.991458	1.987372	1.983259	1.979126	1.975006	1.970877	1.966825
1.994245	1.989956	1.991738	1.987642	1.983525	1.979395	1.975279	1.971156	1.967105
1.994568	1.990268	1.992016	1.987911	1.983791	1.979663	1.975556	1.971455	1.967417
1.994891	1.990575	1.992288	1.988179	1.984058	1.979931	1.975842	1.971795	1.967795
1.995219	1.990888	1.992564	1.988449	1.984325	1.980197	1.976125	1.97213	1.968167
1.97314	1.968813	1.987008	1.982906	1.984588	1.980457	1.976388	1.972407	1.968455
1.973472	1.96914	1.987331	1.983209	1.984855	1.980719	1.976645	1.972658	1.968703
1.973831	1.969519	1.980145	1.975994	1.985145	1.980999	1.976917	1.972923	1.968963
1.974213	1.969944	1.980567	1.976359	1.985455	1.981295	1.977202	1.973196	1.969228
1.974573	1.970322	1.966068	1.961837	1.985773	1.981624	1.977531	1.9735	1.969506
1.974895	1.97063	1.966373	1.962147	1.986101	1.982002	1.977924	1.973848	1.969802
1.975232	1.970938	1.966676	1.962457	1.97922	1.975172	1.978296	1.974185	1.970101
1.975628	1.971285	1.967	1.962771	1.979516	1.975464	1.978589	1.974474	1.970393
1.976029	1.971638	1.967333	1.963098	1.979819	1.975742	1.978858	1.974749	1.970683
1.976375	1.971972	1.967683	1.963474	1.980182	1.976064	1.971965	1.967856	1.9638
1.97669	1.972295	1.96804	1.963875	1.980573	1.976406	1.972268	1.968148	1.964086
1.977005	1.972611	1.968363	1.964211	1.96006	1.955914	1.972576	1.968473	1.964423
1.977314	1.972914	1.96865	1.964481	1.960332	1.956208	1.972887	1.968831	1.964812
1.97766	1.973261	1.968982	1.96479	1.960641	1.95654	1.97323	1.969192	1.965178
1.978059	1.973679	1.969385	1.965163	1.961012	1.956937	1.973627	1.969552	1.965498
1.978481	1.974112	1.969808	1.965565	1.961407	1.957339	1.974018	1.969904	1.965813
1.963556	1.959215	1.970227	1.965996	1.961823	1.957711	1.962471	1.958374	1.954315
1.96399	1.959631	1.970648	1.966435	1.962242	1.958071	1.962778	1.958683	1.95464
1.964424	1.960069	1.971072	1.966828	1.962609	1.958418	1.963099	1.959028	1.955008
1.964858	1.96052	1.971497	1.967189	1.962943	1.958755	1.96343	1.959399	1.955409
1.96528	1.960931	1.971888	1.967551	1.963298	1.959125	1.955025	1.951012	1.947042
1.965687	1.961296	1.97224	1.96791	1.963674	1.959533	1.955446	1.951407	1.947415
1.966087	1.961665	1.972607	1.968291	1.964069	1.959942	1.955859	1.951807	1.947803
1.966467	1.962049	1.973006	1.968712	1.964489	1.960337	1.956244	1.952214	1.948227
1.966862	1.962455	1.973426	1.969146	1.964916	1.960737	1.956632	1.952621	1.948651
1.967321	1.962911	1.973872	1.969567	1.96533	1.96116	1.957055	1.953026	1.94904
1.967803	1.963389	1.974333	1.969988	1.965741	1.961591	1.957491	1.95343	1.949417
1.953827	1.949454	1.959467	1.955166	1.966164	1.962003	1.957896	1.953836	1.949823
1.954195	1.949834	1.959874	1.955595	1.966596	1.9624	1.958277	1.954244	1.950251
1.954611	1.950257	1.960307	1.956024	1.967024	1.96282	1.958691	1.954657	1.95066
1.955096	1.95074	1.960775	1.956447	1.967437	1.963265	1.959144	1.95507	1.951035

1.955578	1.951231	1.946885	1.942556	1.967879	1.963731	1.959615	1.955507	1.951439
1.956023	1.951715	1.947384	1.943057	1.968393	1.964237	1.960113	1.955998	1.951914
1.956459	1.952189	1.94788	1.943567	1.968916	1.96474	1.960603	1.956495	1.95241
1.941667	1.937414	1.948319	1.944017	1.969377	1.965174	1.961026	1.956944	1.952884
1.942115	1.937826	1.948728	1.944435	1.969806	1.96557	1.961415	1.957367	1.953346
1.917138	1.912847	1.933981	1.929696	1.940621	1.936459	1.961842	1.957791	1.953771
1.917654	1.913341	1.934444	1.930112	1.941026	1.936888	1.962302	1.958209	1.954157
1.918146	1.913838	1.909493	1.905145	1.92633	1.922234	1.962781	1.958662	1.954588
1.918599	1.914343	1.91	1.905629	1.926819	1.922725	1.963293	1.959172	1.955093
1.919039	1.914822	1.910492	1.90612	1.927314	1.923209	1.963789	1.959676	1.955603
1.891425	1.887299	1.910917	1.906589	1.902371	1.898255	1.936231	1.932203	1.928214
1.891835	1.887668	1.911328	1.907054	1.902838	1.898673	1.936636	1.93263	1.92866
1.874136	1.869973	1.911795	1.907537	1.903319	1.899131	1.920436	1.91646	1.912528
1.874576	1.870411	1.912297	1.908029	1.903804	1.899612	1.920918	1.916929	1.912987
1.875003	1.870832	1.884848	1.880671	1.904246	1.900067	1.895974	1.891988	1.888054
1.875421	1.871236	1.885254	1.881044	1.904634	1.90049	1.896406	1.892375	1.888405
1.875818	1.871641	1.867433	1.863226	1.905057	1.900936	1.896857	1.892792	1.888793
1.876178	1.872051	1.867862	1.86366	1.905552	1.901427	1.897341	1.893267	1.889259
1.876544	1.872465	1.868301	1.864113	1.906071	1.901935	1.897839	1.893761	1.88975
1.876956	1.872883	1.86873	1.864558	1.86047	1.85646	1.898343	1.894255	1.890235
1.877394	1.873306	1.86916	1.865006	1.860924	1.856909	1.898853	1.894754	1.890725
1.87784	1.873742	1.869602	1.865463	1.861383	1.857359	1.899359	1.895252	1.891216
1.878296	1.87419	1.870054	1.865927	1.861847	1.857811	1.899863	1.89575	1.891709
1.878754	1.874634	1.870499	1.866382	1.862303	1.858259	1.854251	1.85026	1.846341
1.879214	1.875072	1.870933	1.866823	1.862747	1.858704	1.854695	1.850705	1.846786
1.879674	1.875512	1.87137	1.867266	1.863192	1.85915	1.855143	1.851154	1.847234
1.880135	1.875958	1.871814	1.867718	1.863647	1.859603	1.855594	1.851607	1.847684
1.880599	1.876407	1.872261	1.868169	1.864101	1.860059	1.856049	1.852061	1.848134
1.881067	1.876854	1.8727	1.868609	1.864545	1.860513	1.856509	1.852515	1.84858
1.88154	1.877304	1.873139	1.869044	1.864986	1.860969	1.856971	1.852969	1.849023
1.832948	1.829065	1.873598	1.869497	1.865438	1.861426	1.857429	1.853419	1.849463
1.833426	1.829528	1.874076	1.869966	1.865901	1.861885	1.857883	1.853865	1.849899
1.833906	1.829996	1.874554	1.870434	1.866363	1.862348	1.858341	1.854313	1.850333
1.834382	1.830467	1.875027	1.870893	1.866819	1.862812	1.858806	1.85476	1.850761
1.834868	1.830945	1.875509	1.871361	1.867283	1.863281	1.859273	1.855215	1.851201
1.817847	1.813939	1.876002	1.871841	1.867754	1.863749	1.859738	1.855676	1.851659
1.818356	1.814426	1.876509	1.872341	1.868245	1.864229	1.860212	1.856153	1.852139
1.818901	1.81494	1.877046	1.872892	1.868793	1.864756	1.860728	1.856679	1.852668
1.81947	1.815474	1.877603	1.873478	1.869378	1.865314	1.861271	1.857239	1.853232
1.820013	1.816002	1.81215	1.808411	1.869915	1.865841	1.861792	1.857765	1.853757
1.820524	1.816523	1.812657	1.808892	1.870402	1.866333	1.862286	1.858254	1.854241
1.821047	1.817058	1.813175	1.809377	1.870897	1.866849	1.862811	1.858761	1.854732
1.821598	1.817622	1.813716	1.809877	1.871422	1.86742	1.863401	1.859313	1.855247
1.822148	1.818181	1.814253	1.81038	1.871947	1.867983	1.86398	1.85986	1.855765
1.822675	1.8187	1.814762	1.810877	1.872443	1.868476	1.864474	1.860356	1.856266
1.823195	1.819203	1.81526	1.811377	1.872941	1.868949	1.864939	1.86084	1.856772
1.823731	1.819718	1.815776	1.811905	1.873508	1.869482	1.865455	1.861379	1.857328
1.824276	1.82024	1.816303	1.812454	1.874127	1.870057	1.866006	1.861957	1.857923
1.809491	1.8055	1.816825	1.812987	1.874716	1.870631	1.866573	1.862536	1.858509
1.809969	1.80598	1.817337	1.813501	1.875266	1.871206	1.867162	1.863117	1.859082

1.810462	1.806476	1.802606	1.798831	1.79514	1.791541	1.867736	1.863692	1.859657
1.810987	1.807002	1.803115	1.799316	1.795615	1.792021	1.868278	1.864262	1.860246
1.811533	1.807546	1.803639	1.799812	1.796096	1.792497	1.868803	1.864818	1.860828
1.812101	1.808103	1.804187	1.800348	1.796611	1.792976	1.856926	1.852991	1.849055
1.812686	1.808672	1.804749	1.800908	1.797145	1.793462	1.857397	1.853473	1.849564
1.813255	1.809228	1.8053	1.801455	1.797682	1.79398	1.857939	1.853987	1.850057
1.813813	1.809776	1.805843	1.801995	1.798223	1.79453	1.858546	1.854525	1.850531
1.814373	1.810322	1.806381	1.802529	1.798757	1.795069	1.859133	1.855081	1.851054
1.81494	1.810866	1.806913	1.803054	1.799278	1.79559	1.859683	1.855661	1.851654
1.815506	1.81141	1.807446	1.803584	1.799804	1.796111	1.844183	1.840232	1.836294
1.816062	1.811947	1.807983	1.804129	1.800348	1.796645	1.844753	1.840777	1.836824
1.816623	1.812492	1.808526	1.804679	1.800896	1.797181	1.845328	1.841323	1.837356
1.81721	1.813065	1.809084	1.805222	1.801426	1.797701	1.794033	1.790429	1.786857
1.817814	1.813659	1.809655	1.805762	1.801948	1.798215	1.794555	1.790986	1.787444
1.76901	1.76498	1.810238	1.806317	1.802491	1.798761	1.795106	1.791546	1.788009
1.76958	1.765566	1.810836	1.806885	1.803052	1.799339	1.79569	1.792111	1.788554
1.738854	1.735107	1.762225	1.758383	1.803633	1.799924	1.796271	1.792669	1.789093
1.739405	1.735615	1.762791	1.758971	1.804245	1.800517	1.796842	1.793215	1.789622
1.739977	1.736146	1.763378	1.759567	1.804857	1.8011	1.797402	1.793759	1.790157
1.740595	1.73676	1.764016	1.760159	1.756393	1.752717	1.797932	1.794301	1.790719
1.741227	1.737409	1.764677	1.760749	1.756931	1.753217	1.798458	1.794849	1.791291
1.721289	1.717588	1.765306	1.761323	1.757479	1.753763	1.799033	1.795419	1.791856
1.721822	1.718167	1.765917	1.761888	1.758033	1.754343	1.799645	1.796007	1.792417
1.722384	1.718746	1.766544	1.762487	1.75862	1.754934	1.751322	1.747776	1.744276
1.722987	1.719328	1.767194	1.763125	1.759243	1.755537	1.751898	1.748308	1.744776
1.723572	1.719887	1.767833	1.763777	1.759886	1.756151	1.752481	1.748852	1.745293
1.724111	1.720399	1.768441	1.764438	1.760556	1.756789	1.753085	1.74943	1.745847
1.724636	1.720907	1.769044	1.765098	1.761224	1.757419	1.753682	1.75001	1.746414
1.680518	1.677456	1.769658	1.765733	1.761848	1.757997	1.75423	1.750562	1.746975
1.680908	1.677867	1.770281	1.766359	1.762452	1.758552	1.754759	1.751105	1.747535
1.68134	1.67831	1.770931	1.767007	1.763082	1.759148	1.755328	1.751666	1.748086
1.68181	1.678783	1.771608	1.767675	1.763734	1.759776	1.75593	1.75224	1.748629
1.682268	1.679243	1.720157	1.716517	1.764355	1.760396	1.756541	1.752833	1.749198
1.682701	1.67968	1.72071	1.717023	1.764928	1.760995	1.757157	1.753445	1.749799
1.683144	1.680119	1.721271	1.717542	1.765512	1.761605	1.757784	1.75407	1.750414
1.683612	1.680575	1.721862	1.718116	1.76614	1.76224	1.758422	1.754703	1.751037
1.684093	1.68104	1.722469	1.718716	1.766789	1.762893	1.759074	1.755348	1.75167
1.649742	1.646899	1.678434	1.675368	1.767457	1.763572	1.759755	1.756014	1.752316
1.6502	1.64735	1.678906	1.675838	1.768141	1.764269	1.760454	1.756696	1.752973
1.650653	1.647796	1.679371	1.676297	1.768812	1.76495	1.761136	1.757366	1.753625
1.651099	1.648237	1.679828	1.676746	1.769471	1.765613	1.761801	1.758025	1.754275
1.627116	1.624427	1.680297	1.677204	1.770135	1.766278	1.762463	1.758679	1.754917
1.627539	1.624846	1.680784	1.677678	1.770808	1.766945	1.763122	1.759324	1.755546
1.627967	1.625271	1.681278	1.678159	1.771485	1.767614	1.763781	1.759969	1.756177
1.628397	1.6257	1.622972	1.620243	1.710951	1.707735	1.764443	1.760621	1.75682
1.62883	1.626132	1.623399	1.62066	1.711474	1.708242	1.765104	1.761276	1.75747
1.62926	1.626562	1.623823	1.621074	1.71199	1.708741	1.765756	1.761923	1.758115
1.629689	1.626993	1.624248	1.621488	1.712503	1.709236	1.766402	1.762566	1.75876
1.602459	1.599831	1.624691	1.621928	1.655125	1.652275	1.767075	1.763238	1.759437
1.602908	1.600283	1.625154	1.622398	1.655612	1.652745	1.76778	1.763944	1.760153

1.60336	1.600735	1.598085	1.595434	1.656127	1.653235	1.750703	1.746958	1.743264
1.603809	1.601183	1.598554	1.595933	1.656685	1.653752	1.751395	1.747622	1.743913
1.604265	1.601633	1.599022	1.596428	1.65724	1.654272	1.752094	1.748285	1.744554
1.604736	1.602089	1.599474	1.596883	1.621641	1.618918	1.688696	1.685564	1.682491
1.60522	1.602551	1.59992	1.59732	1.622083	1.619373	1.68922	1.686054	1.682951
1.605723	1.603027	1.60038	1.597771	1.595163	1.592558	1.689791	1.686589	1.683451
1.606247	1.603514	1.600849	1.598232	1.595631	1.593051	1.690403	1.687163	1.683985
1.60675	1.604001	1.601324	1.598698	1.596099	1.593531	1.667529	1.664322	1.661162
1.607224	1.604484	1.601803	1.599165	1.596559	1.59399	1.66812	1.66493	1.661774
1.607698	1.604973	1.602292	1.599646	1.597034	1.594459	1.63349	1.630527	1.627591
1.60818	1.605472	1.602795	1.600149	1.597535	1.594953	1.63406	1.631108	1.628178
1.608674	1.60598	1.60331	1.600668	1.598053	1.595468	1.634642	1.631696	1.62877
1.609191	1.606498	1.603834	1.601202	1.598594	1.596014	1.63526	1.632305	1.629369
1.609727	1.607026	1.604366	1.601747	1.59915	1.596579	1.635901	1.632929	1.629972
1.610278	1.607563	1.6049	1.602282	1.599683	1.597108	1.636519	1.633541	1.630573
1.610847	1.608112	1.605435	1.602805	1.600192	1.5976	1.637118	1.634144	1.631172
1.566033	1.563543	1.605978	1.603341	1.600716	1.598107	1.637717	1.634747	1.631774
1.566469	1.564002	1.606537	1.603903	1.601272	1.598646	1.638319	1.635351	1.632384
1.566906	1.564461	1.607091	1.604458	1.601823	1.599189	1.638923	1.635954	1.632991
1.567374	1.564928	1.607621	1.604976	1.602341	1.59972	1.639529	1.636552	1.633583
1.567855	1.565401	1.608147	1.605484	1.60285	1.600247	1.640136	1.637154	1.634178
1.568344	1.565888	1.563431	1.56099	1.603374	1.600768	1.640744	1.637778	1.634813
1.568839	1.566385	1.563927	1.561479	1.603908	1.601286	1.641353	1.638417	1.635476
1.526218	1.523851	1.564407	1.561962	1.604458	1.601827	1.641968	1.639041	1.636109
1.526615	1.524248	1.564869	1.562437	1.605028	1.602396	1.642589	1.639646	1.636705
1.527019	1.524648	1.522292	1.519964	1.605588	1.602952	1.643206	1.640258	1.637317
1.527449	1.525068	1.522706	1.520374	1.606123	1.603479	1.643815	1.640895	1.637977
1.52787	1.52548	1.523109	1.520772	1.606647	1.603996	1.644415	1.641525	1.63863
1.528252	1.525854	1.523479	1.521145	1.518805	1.516463	1.645007	1.642119	1.639223
1.528613	1.526208	1.523828	1.521499	1.519165	1.516831	1.645586	1.64269	1.639785
1.528976	1.526557	1.524167	1.521835	1.519504	1.517178	1.646129	1.64324	1.640347
1.529336	1.5269	1.524495	1.522154	1.519822	1.517507	1.646641	1.643768	1.640903
1.529695	1.527249	1.524839	1.522499	1.520173	1.517866	1.565029	1.56242	1.559819
1.530042	1.527601	1.5252	1.522881	1.520566	1.518265	1.565516	1.562908	1.560309
1.490091	1.487778	1.525578	1.523272	1.520965	1.518667	1.565996	1.563381	1.560779
1.490488	1.488145	1.525985	1.523672	1.521361	1.519063	1.566438	1.563797	1.561185
1.490925	1.488534	1.526398	1.524068	1.521746	1.519447	1.566864	1.5642	1.561581
1.491394	1.488934	1.526794	1.524431	1.522091	1.519786	1.567269	1.564616	1.56202
1.491885	1.48934	1.527177	1.524776	1.522413	1.520103	1.567668	1.565043	1.562482
1.49232	1.489692	1.487218	1.484936	1.522769	1.520465	1.568139	1.565513	1.562953
1.492698	1.489988	1.487498	1.485247	1.523154	1.520868	1.56868	1.566026	1.563436
1.493139	1.490334	1.487814	1.485579	1.52355	1.521272	1.569229	1.566532	1.5639
1.493658	1.490742	1.488176	1.485933	1.523957	1.521677	1.56979	1.567031	1.564338
1.494262	1.491216	1.488584	1.486312	1.524367	1.522069	1.570325	1.567507	1.564759
1.494987	1.491783	1.48906	1.48673	1.484441	1.482229	1.570777	1.567925	1.565154
1.495788	1.492411	1.489575	1.487161	1.484796	1.482516	1.57121	1.568332	1.565549
1.496613	1.493068	1.490093	1.487554	1.485116	1.482816	1.571716	1.568802	1.56599
1.497466	1.493755	1.49062	1.487925	1.485414	1.483125	1.572268	1.569318	1.566467
1.498258	1.494392	1.491126	1.488315	1.485745	1.483454	1.481236	1.479053	1.476904
1.498979	1.494963	1.491598	1.488719	1.486107	1.483803	1.481578	1.479391	1.477239

1.499714	1.495588	1.492126	1.489156	1.486491	1.484169	1.481934	1.479738	1.477582
1.500471	1.496299	1.492729	1.489619	1.486881	1.484549	1.482308	1.480099	1.47793
1.501275	1.497084	1.493408	1.490145	1.487317	1.484954	1.482692	1.480464	1.478282
1.502173	1.497973	1.494201	1.49079	1.487848	1.485398	1.483076	1.480823	1.478632
1.503128	1.498932	1.49507	1.491508	1.488438	1.48587	1.483467	1.481184	1.478984
1.504079	1.499888	1.495934	1.492222	1.489028	1.486354	1.483876	1.481564	1.479352
1.505037	1.500853	1.496808	1.492946	1.489629	1.486851	1.484301	1.481959	1.479733
1.505995	1.501812	1.497689	1.493703	1.490268	1.48737	1.484735	1.48236	1.480119
1.506956	1.502761	1.498576	1.494493	1.490942	1.487903	1.485168	1.482762	1.48051
1.507909	1.503706	1.499477	1.495324	1.491668	1.488483	1.485639	1.483185	1.480907
1.508849	1.504648	1.5004	1.496209	1.492463	1.489131	1.486164	1.483634	1.481307
1.509785	1.505583	1.501331	1.497126	1.493301	1.489823	1.48673	1.484111	1.481719
1.510725	1.506506	1.502255	1.498055	1.494164	1.490548	1.487332	1.484621	1.482161
1.511668	1.50742	1.503178	1.498998	1.495052	1.491304	1.487967	1.485159	1.482624
1.51262	1.50833	1.504093	1.499936	1.495937	1.492063	1.488606	1.4857	1.483086
1.513581	1.509237	1.505002	1.500871	1.496823	1.492825	1.489251	1.486244	1.483549
1.514546	1.510148	1.505907	1.501789	1.497703	1.493621	1.48995	1.486828	1.484031
1.515509	1.511065	1.506805	1.502682	1.498574	1.494458	1.490714	1.487452	1.484526
1.516485	1.51199	1.507704	1.503567	1.499445	1.495322	1.491524	1.488126	1.485065
1.517465	1.512906	1.508587	1.504437	1.500314	1.496206	1.492375	1.488862	1.485676
1.518481	1.513855	1.509495	1.505317	1.501192	1.497115	1.493265	1.489645	1.486335
1.519597	1.514923	1.510499	1.506251	1.502102	1.498052	1.494179	1.490447	1.487012
1.520791	1.516081	1.511578	1.507229	1.503041	1.499017	1.495122	1.49128	1.48772
1.522018	1.517291	1.512723	1.508279	1.504046	1.500025	1.496103	1.492182	1.488524
1.523293	1.518569	1.51395	1.509416	1.50513	1.501089	1.497136	1.493166	1.489436
1.524508	1.519794	1.515132	1.51052	1.506178	1.502102	1.498114	1.494115	1.490336
1.525617	1.520916	1.516221	1.511543	1.507144	1.503014	1.498989	1.494988	1.491188
1.526677	1.521999	1.517281	1.512551	1.508097	1.503902	1.499834	1.495837	1.492026
1.527709	1.523063	1.518338	1.513569	1.509063	1.5048	1.500682	1.496679	1.49285
1.528716	1.524116	1.519395	1.514604	1.510051	1.50571	1.501536	1.497524	1.493674
1.529734	1.525197	1.520502	1.51571	1.511112	1.50668	1.502438	1.498416	1.494549
1.530756	1.526298	1.521647	1.516875	1.512237	1.5077	1.503381	1.499347	1.495465
1.531829	1.527443	1.522836	1.518085	1.513415	1.50879	1.504397	1.500327	1.496411
1.53298	1.528659	1.524091	1.519363	1.514669	1.50997	1.505505	1.50137	1.497397
1.534076	1.529816	1.525291	1.520592	1.515886	1.511133	1.506606	1.502401	1.498371
1.535036	1.53084	1.526361	1.521701	1.516996	1.512207	1.507635	1.503372	1.499293
1.535956	1.531814	1.527385	1.522775	1.518084	1.513278	1.50867	1.504347	1.500215
1.536901	1.532796	1.528422	1.523879	1.51922	1.51441	1.509773	1.505381	1.501185
1.53786	1.533776	1.529459	1.524997	1.520381	1.515583	1.510923	1.506454	1.502183
1.538886	1.534801	1.530533	1.526154	1.521588	1.516817	1.512134	1.507565	1.503188
1.539978	1.535868	1.531643	1.52735	1.522843	1.518114	1.513408	1.508715	1.504199
1.541055	1.536908	1.532711	1.528489	1.52404	1.519365	1.51466	1.509887	1.505268
1.542088	1.537892	1.533707	1.529539	1.525149	1.520545	1.515873	1.511077	1.506401
1.543155	1.5389	1.534713	1.530581	1.526243	1.521713	1.517086	1.512298	1.507594
1.544309	1.539991	1.535785	1.531667	1.527368	1.522909	1.518337	1.513584	1.508882
1.545499	1.541109	1.536871	1.532751	1.528482	1.524089	1.519576	1.514872	1.510193
1.54667	1.542185	1.537907	1.53378	1.529532	1.525189	1.520722	1.516059	1.511402
1.547843	1.543249	1.538922	1.53478	1.530541	1.526236	1.521809	1.517185	1.512556
1.549093	1.544412	1.540024	1.535838	1.531592	1.527316	1.522929	1.518364	1.513779
1.550424	1.545678	1.541219	1.536957	1.532685	1.52843	1.524087	1.519602	1.515076

1.551739	1.54694	1.542411	1.538068	1.533759	1.529508	1.525199	1.520795	1.516339
1.552998	1.548154	1.543554	1.539125	1.534766	1.530495	1.52621	1.521898	1.517537
1.554264	1.549398	1.544739	1.54023	1.53582	1.531521	1.527243	1.522993	1.518701
1.411333	1.406733	1.546053	1.541479	1.537027	1.532704	1.528407	1.524132	1.519826
1.412567	1.408013	1.547424	1.5428	1.53831	1.533962	1.529628	1.52529	1.520936
1.413734	1.409235	1.548755	1.544102	1.539582	1.5352	1.530835	1.526468	1.522096
1.414854	1.410421	1.550066	1.545403	1.54086	1.536436	1.53204	1.527662	1.523291
1.415978	1.411562	1.407124	1.402693	1.542103	1.53763	1.533201	1.528815	1.524452
1.417102	1.41264	1.408191	1.403768	1.543295	1.53876	1.534294	1.529911	1.525567
1.418247	1.413754	1.409297	1.404874	1.544523	1.539938	1.535435	1.53104	1.526696
1.419444	1.414974	1.410511	1.406062	1.545838	1.541223	1.536682	1.53224	1.527848
1.420633	1.416202	1.411741	1.407273	1.547175	1.542544	1.537974	1.533482	1.529035
1.421738	1.417348	1.412916	1.408472	1.548492	1.543862	1.539282	1.534769	1.530293
1.422807	1.418451	1.414058	1.409657	1.549794	1.545177	1.540602	1.536087	1.531595
1.423921	1.419528	1.415133	1.41075	1.406384	1.402033	1.541872	1.53737	1.532882
1.42507	1.420574	1.41614	1.411758	1.407409	1.403096	1.543099	1.538628	1.534164
1.154036	1.150336	1.417229	1.412836	1.408479	1.404161	1.544317	1.539859	1.53541
1.155058	1.151352	1.41843	1.414014	1.409611	1.405224	1.545512	1.541046	1.536608
1.047365	1.043912	1.419672	1.415229	1.410785	1.406336	1.401977	1.397753	1.393572
1.048576	1.04512	1.420933	1.416475	1.412014	1.407552	1.403157	1.39885	1.394604
1.04978	1.046335	1.422209	1.417732	1.413265	1.408808	1.404387	1.399987	1.395662
1.050944	1.047547	1.285128	1.280811	1.41448	1.410036	1.405613	1.401186	1.396827
1.052078	1.048752	1.286539	1.282168	1.415681	1.411255	1.406847	1.40243	1.398064
1.05319	1.04989	1.287908	1.283518	1.41689	1.412468	1.408062	1.403653	1.39928
1.054273	1.050958	1.289237	1.284862	1.418108	1.413671	1.409255	1.404857	1.40048
1.055395	1.05203	1.048676	1.045355	1.281914	1.277668	1.410462	1.406065	1.401679
1.056573	1.053112	1.049759	1.046497	1.283277	1.279033	1.411696	1.407282	1.402876
1.057794	1.05423	1.050865	1.04764	1.284648	1.280399	1.412931	1.408499	1.404075
1.059064	1.055422	1.052029	1.048803	1.28602	1.281746	1.348027	1.343732	1.339459
1.06037	1.056663	1.05323	1.049981	1.287403	1.283097	1.349361	1.345063	1.340792
1.06169	1.057927	1.054458	1.051182	1.288821	1.284484	1.350726	1.346416	1.34214
1.063026	1.05922	1.055713	1.052403	1.290268	1.2859	1.352116	1.347787	1.343499
1.064369	1.06052	1.056971	1.05362	1.050244	1.046865	1.283007	1.278736	1.274511
1.065715	1.061827	1.058232	1.05483	1.051436	1.048069	1.284478	1.280183	1.275928
0.944774	0.941485	1.059507	1.056044	1.052625	1.049269	1.285952	1.281627	1.277341
0.945896	0.9426	1.06079	1.057251	1.053801	1.050459	1.287415	1.283052	1.278732
0.947028	0.943735	1.062105	1.058482	1.054992	1.05165	1.288874	1.284472	1.280122
0.948198	0.944936	1.063482	1.059771	1.056225	1.052856	1.153206	1.149241	1.145318
0.949388	0.946172	1.064903	1.061104	1.057492	1.054072	1.154524	1.150547	1.146627
0.950572	0.947397	1.066345	1.062476	1.058795	1.055297	1.15584	1.151878	1.147983
0.951754	0.948617	1.067813	1.063888	1.060133	1.056532	1.157154	1.153232	1.14938
0.952917	0.949802	1.069255	1.065307	1.061492	1.057784	1.054224	1.050861	1.04757
0.954052	0.95094	1.070666	1.066738	1.062875	1.059045	1.055416	1.052091	1.048845
0.9552	0.95207	0.94884	0.945559	1.064263	1.060346	1.056659	1.053332	1.050094
0.956386	0.953206	0.949966	0.946698	1.065644	1.061715	1.057991	1.054584	1.051279
0.95759	0.954352	0.951096	0.947835	1.067026	1.063122	1.059375	1.055857	1.052451
0.958805	0.955533	0.952252	0.948974	1.068411	1.064548	1.06079	1.057171	1.053664
0.960025	0.956734	0.953426	0.950119	1.069802	1.065992	1.062232	1.058514	1.054903
0.961187	0.957902	0.954601	0.951302	1.071221	1.067414	1.06363	1.059843	1.056162
0.962289	0.959036	0.955775	0.952526	1.07267	1.068817	1.064984	1.061156	1.057439

0.824663	0.822067	0.956966	0.953747	0.950505	0.947245	1.066358	1.062505	1.058761
0.825582	0.822948	0.958183	0.954958	0.951708	0.948436	1.067762	1.063902	1.060137
0.826528	0.823841	0.959401	0.95616	0.952905	0.949637	1.069197	1.065335	1.061547
0.670338	0.668033	0.9606	0.957342	0.954089	0.950848	1.070686	1.066818	1.062989
0.671222	0.668922	0.961797	0.958521	0.955275	0.952066	1.072194	1.068321	1.064447
0.67216	0.669824	0.823813	0.821141	0.956491	0.953282	0.950051	0.946776	0.943499
0.673138	0.670742	0.824727	0.822043	0.95773	0.954498	0.951249	0.947965	0.944685
0.674104	0.671656	0.825666	0.822954	0.820246	0.817557	0.95244	0.949164	0.945893
0.67505	0.672563	0.826631	0.823874	0.821145	0.81846	0.953621	0.950373	0.947129
0.500564	0.498294	0.827609	0.824799	0.822045	0.819362	0.954819	0.951587	0.948359
0.501561	0.499192	0.828585	0.825717	0.822937	0.820262	0.956051	0.952803	0.949564
0.50259	0.500133	0.829588	0.826657	0.823844	0.821167	0.957292	0.954027	0.950778
0.503665	0.501159	0.673946	0.671489	0.824793	0.822084	0.819384	0.816658	0.813941
0.504756	0.502226	0.67495	0.672409	0.825767	0.823013	0.820297	0.817603	0.814917
		0.500834	0.498534	0.826738	0.823964	0.821236	0.81854	0.815853
		0.501787	0.499464	0.827711	0.824936	0.8222	0.819471	0.816755
		0.50278	0.500414	0.70143	0.698906	0.823143	0.820402	0.817681
		0.50383	0.501382	0.702478	0.699773	0.824047	0.821331	0.818644
		0.504876	0.502375	0.500068	0.497929	0.824984	0.822269	0.819596
		0.50589	0.503407	0.501077	0.498871	0.826002	0.823221	0.820507
		0.506884	0.50445	0.502112	0.499842	0.827046	0.824183	0.821413
				0.50312	0.500782	0.700787	0.69814	0.695621
				0.504122	0.501717	0.701673	0.699004	0.696472
				0.505143	0.502698	0.702673	0.699919	0.697311
				0.506186	0.503723	0.703784	0.700883	0.698134
				0.507181	0.504739	0.704872	0.701879	0.699032
				0.508101	0.505732	0.705896	0.702913	0.70005
				0.509005	0.506717	0.706934	0.703962	0.701081
				0.509938	0.507702	0.505415	0.503053	0.500746
				0.510864	0.508675	0.506405	0.504021	0.501676
						0.50738	0.505003	0.502642
						0.508343	0.505995	0.503639
						0.509314	0.507013	0.504678
						0.510301	0.508066	0.505771
						0.511252	0.509083	0.506833
						0.512146	0.510034	0.507828
						0.513009	0.510953	0.5088

2.020853	2.016582	2.012359	2.008183	2.004137	2.000224	1.996333	1.992439	1.988564
2.020829	2.016557	2.012332	2.008153	2.004107	2.000196	1.996307	1.992416	1.988543
2.020809	2.016534	2.012308	2.008128	2.004082	2.000171	1.996284	1.992395	1.988528
2.011599	2.007334	2.012294	2.008117	2.004069	2.000151	1.996261	1.992374	1.988515
2.01159	2.007324	2.01229	2.00812	2.004069	2.000138	1.996241	1.992353	1.988505
2.01159	2.007324	2.003136	1.999012	2.004113	2.000152	1.996231	1.992333	1.988485
2.011598	2.007333	2.003177	1.999102	2.004207	2.000198	1.996235	1.992313	1.988451
2.011612	2.007346	2.00321	1.999164	1.99513	1.991111	1.996239	1.992301	1.988429
2.011628	2.007354	2.003215	1.999169	1.995134	1.991114	1.996241	1.992298	1.988418
2.011655	2.007383	2.003232	1.999167	1.995125	1.991108	1.996244	1.992309	1.988431
2.011712	2.007476	2.003307	1.999194	1.995126	1.991101	1.996247	1.99235	1.9885
2.011779	2.007589	2.003399	1.999228	1.995128	1.991093	1.99625	1.9924	1.988587
2.01181	2.007634	2.003433	1.999239	1.995126	1.991085	1.987138	1.983321	1.979535
2.011817	2.00763	2.003426	1.999234	1.995121	1.991079	1.987129	1.983303	1.97951
2.011859	2.007644	2.003434	1.999246	1.995142	1.991115	1.987166	1.983317	1.979502
2.011945	2.007678	2.003455	1.999276	1.995193	1.991204	1.987264	1.983373	1.979514
2.012026	2.007723	2.003495	1.999329	1.995258	1.991285	1.987344	1.98342	1.97953
2.012073	2.007774	2.003564	1.999422	1.995344	1.99133	1.987361	1.983428	1.979542
2.012122	2.007842	2.003649	1.999521	1.995426	1.991364	1.987365	1.983437	1.979566
2.012208	2.007952	2.003746	1.999584	1.995467	1.991394	1.987394	1.983486	1.97963
2.012312	2.008082	2.003849	1.99963	1.995491	1.991423	1.987436	1.983554	1.979713
2.012406	2.008164	2.003914	1.999678	1.995536	1.991483	1.987503	1.983611	1.979755
2.012493	2.008205	2.003947	1.999722	1.995598	1.991572	1.987595	1.983659	1.979762
2.012566	2.008254	2.003999	1.99979	1.995672	1.991644	1.987655	1.983691	1.979773
2.012612	2.008313	2.004079	1.999896	1.995767	1.991688	1.987665	1.983697	1.979787
2.012667	2.008389	2.004168	1.999995	1.995848	1.991725	1.987676	1.983714	1.979821
2.012754	2.008501	2.004265	2.000053	1.995885	1.991754	1.987709	1.983772	1.979899
2.012855	2.008623	2.004361	2.000099	1.995911	1.991788	1.987758	1.983846	1.97999
2.012959	2.00871	2.004431	2.00015	1.995962	1.991858	1.987838	1.983915	1.980051
2.01307	2.008784	2.00449	2.000206	1.996029	1.991951	1.98794	1.983987	1.980099
2.013192	2.00891	2.004612	2.000319	1.996133	1.992047	1.988028	1.984071	1.980181
2.013327	2.009094	2.004803	2.000496	1.996281	1.992145	1.988101	1.984167	1.980298
2.004385	2.000212	2.004955	2.000642	1.996412	1.992253	1.988196	1.984271	1.980409
2.004485	2.000271	2.005012	2.000708	1.996498	1.992374	1.988331	1.984378	1.980494
2.00459	2.000339	2.005082	2.00079	1.996598	1.992502	1.98847	1.984492	1.980588
2.004714	2.000495	2.005255	2.000963	1.996757	1.992628	1.988577	1.984609	1.980717
2.004849	2.000678	2.005462	2.001174	1.996946	1.992765	1.988685	1.984733	1.980859
2.004993	2.000808	2.005614	2.001362	1.997144	1.992952	1.988855	1.984876	1.98098
2.005147	2.000904	2.005733	2.001538	1.997354	1.993179	1.989071	1.985036	1.98109
2.005329	2.001053	2.00588	2.00169	1.997522	1.993375	1.989277	1.985217	1.981242
2.005551	2.001266	2.006059	2.00181	1.997636	1.993538	1.989476	1.985427	1.981445
1.994877	1.990647	1.997204	1.992948	1.997769	1.993689	1.989647	1.985619	1.981647
1.995012	1.990833	1.997414	1.99315	1.997955	1.993824	1.989761	1.985767	1.981833
1.995136	1.991	1.997615	1.993363	1.998159	1.99397	1.989876	1.985905	1.982004
1.99528	1.991119	1.997755	1.993545	1.998363	1.994166	1.990056	1.986052	1.982132
1.995434	1.991217	1.997868	1.99371	1.998566	1.994388	1.990271	1.98621	1.982246
1.995615	1.991372	1.998014	1.99385	1.998728	1.994591	1.990493	1.986411	1.982425
1.99582	1.991573	1.998184	1.993966	1.998854	1.99478	1.990727	1.98665	1.982658
1.99601	1.991783	1.987554	1.983345	1.990003	1.986002	1.990957	1.986889	1.982893
1.996173	1.992001	1.987786	1.983572	1.990232	1.986227	1.991188	1.987127	1.983126

1.996351	1.992223	1.988023	1.983812	1.990469	1.986438	1.991396	1.987349	1.983354
1.99658	1.992451	1.988253	1.984043	1.990682	1.986599	1.991542	1.987529	1.98357
1.996819	1.992668	1.988471	1.984272	1.990898	1.986759	1.991686	1.987705	1.983785
1.990808	1.986642	1.988644	1.984488	1.980358	1.976248	1.982924	1.978997	1.97513
1.99101	1.986794	1.988794	1.984699	1.980603	1.976503	1.983184	1.979245	1.975362
1.991258	1.98701	1.988998	1.984932	1.980851	1.976756	1.983439	1.979486	1.975588
1.99155	1.987289	1.989256	1.985187	1.981104	1.977009	1.983693	1.979725	1.975812
1.991847	1.987577	1.989523	1.985451	1.981363	1.977264	1.973224	1.969261	1.965358
1.992141	1.987866	1.989794	1.985724	1.98163	1.977519	1.973451	1.969424	1.965472
1.992434	1.988158	1.990068	1.985998	1.981899	1.977777	1.973683	1.969599	1.965601
1.992722	1.988446	1.990339	1.986267	1.982165	1.978042	1.973939	1.969833	1.965814
1.99301	1.988734	1.990612	1.986535	1.982432	1.97831	1.974205	1.970093	1.966064
1.993307	1.989032	1.990891	1.986812	1.982706	1.978581	1.97447	1.970349	1.966309
1.993612	1.989338	1.991176	1.987095	1.982985	1.978854	1.974737	1.970607	1.966559
1.993924	1.989646	1.991458	1.987372	1.983259	1.979126	1.975006	1.970877	1.966825
1.994245	1.989956	1.991738	1.987642	1.983525	1.979395	1.975279	1.971156	1.967105
1.994568	1.990268	1.992016	1.987911	1.983791	1.979663	1.975556	1.971455	1.967417
1.994891	1.990575	1.992288	1.988179	1.984058	1.979931	1.975842	1.971795	1.967795
1.995219	1.990888	1.992564	1.988449	1.984325	1.980197	1.976125	1.97213	1.968167
1.97314	1.968813	1.987008	1.982906	1.984588	1.980457	1.976388	1.972407	1.968455
1.973472	1.96914	1.987331	1.983209	1.984855	1.980719	1.976645	1.972658	1.968703
1.973831	1.969519	1.980145	1.975994	1.985145	1.980999	1.976917	1.972923	1.968963
1.974213	1.969944	1.980567	1.976359	1.985455	1.981295	1.977202	1.973196	1.969228
1.974573	1.970322	1.966068	1.961837	1.985773	1.981624	1.977531	1.9735	1.969506
1.974895	1.97063	1.966373	1.962147	1.986101	1.982002	1.977924	1.973848	1.969802
1.975232	1.970938	1.966676	1.962457	1.97922	1.975172	1.978296	1.974185	1.970101
1.975628	1.971285	1.967	1.962771	1.979516	1.975464	1.978589	1.974474	1.970393
1.976029	1.971638	1.967333	1.963098	1.979819	1.975742	1.978858	1.974749	1.970683
1.976375	1.971972	1.967683	1.963474	1.980182	1.976064	1.971965	1.967856	1.9638
1.97669	1.972295	1.96804	1.963875	1.980573	1.976406	1.972268	1.968148	1.964086
1.977005	1.972611	1.968363	1.964211	1.96006	1.955914	1.972576	1.968473	1.964423
1.977314	1.972914	1.96865	1.964481	1.960332	1.956208	1.972887	1.968831	1.964812
1.97766	1.973261	1.968982	1.96479	1.960641	1.95654	1.97323	1.969192	1.965178
1.978059	1.973679	1.969385	1.965163	1.961012	1.956937	1.973627	1.969552	1.965498
1.978481	1.974112	1.969808	1.965565	1.961407	1.957339	1.974018	1.969904	1.965813
1.963556	1.959215	1.970227	1.965996	1.961823	1.957711	1.962471	1.958374	1.954315
1.96399	1.959631	1.970648	1.966435	1.962242	1.958071	1.962778	1.958683	1.95464
1.964424	1.960069	1.971072	1.966828	1.962609	1.958418	1.963099	1.959028	1.955008
1.964858	1.96052	1.971497	1.967189	1.962943	1.958755	1.96343	1.959399	1.955409
1.96528	1.960931	1.971888	1.967551	1.963298	1.959125	1.955025	1.951012	1.947042
1.965687	1.961296	1.97224	1.96791	1.963674	1.959533	1.955446	1.951407	1.947415
1.966087	1.961665	1.972607	1.968291	1.964069	1.959942	1.955859	1.951807	1.947803
1.966467	1.962049	1.973006	1.968712	1.964489	1.960337	1.956244	1.952214	1.948227
1.966862	1.962455	1.973426	1.969146	1.964916	1.960737	1.956632	1.952621	1.948651
1.967321	1.962911	1.973872	1.969567	1.96533	1.96116	1.957055	1.953026	1.94904
1.967803	1.963389	1.974333	1.969988	1.965741	1.961591	1.957491	1.95343	1.949417
1.953827	1.949454	1.959467	1.955166	1.966164	1.962003	1.957896	1.953836	1.949823
1.954195	1.949834	1.959874	1.955595	1.966596	1.9624	1.958277	1.954244	1.950251
1.954611	1.950257	1.960307	1.956024	1.967024	1.96282	1.958691	1.954657	1.95066
1.955096	1.95074	1.960775	1.956447	1.967437	1.963265	1.959144	1.95507	1.951035

1.955578	1.951231	1.946885	1.942556	1.967879	1.963731	1.959615	1.955507	1.951439
1.956023	1.951715	1.947384	1.943057	1.968393	1.964237	1.960113	1.955998	1.951914
1.956459	1.952189	1.94788	1.943567	1.968916	1.96474	1.960603	1.956495	1.95241
1.941667	1.937414	1.948319	1.944017	1.969377	1.965174	1.961026	1.956944	1.952884
1.942115	1.937826	1.948728	1.944435	1.969806	1.96557	1.961415	1.957367	1.953346
1.917138	1.912847	1.933981	1.929696	1.940621	1.936459	1.961842	1.957791	1.953771
1.917654	1.913341	1.934444	1.930112	1.941026	1.936888	1.962302	1.958209	1.954157
1.918146	1.913838	1.909493	1.905145	1.92633	1.922234	1.962781	1.958662	1.954588
1.918599	1.914343	1.91	1.905629	1.926819	1.922725	1.963293	1.959172	1.955093
1.919039	1.914822	1.910492	1.90612	1.927314	1.923209	1.963789	1.959676	1.955603
1.891425	1.887299	1.910917	1.906589	1.902371	1.898255	1.936231	1.932203	1.928214
1.891835	1.887668	1.911328	1.907054	1.902838	1.898673	1.936636	1.93263	1.92866
1.874136	1.869973	1.911795	1.907537	1.903319	1.899131	1.920436	1.91646	1.912528
1.874576	1.870411	1.912297	1.908029	1.903804	1.899612	1.920918	1.916929	1.912987
1.875003	1.870832	1.884848	1.880671	1.904246	1.900067	1.895974	1.891988	1.888054
1.875421	1.871236	1.885254	1.881044	1.904634	1.90049	1.896406	1.892375	1.888405
1.875818	1.871641	1.867433	1.863226	1.905057	1.900936	1.896857	1.892792	1.888793
1.876178	1.872051	1.867862	1.86366	1.905552	1.901427	1.897341	1.893267	1.889259
1.876544	1.872465	1.868301	1.864113	1.906071	1.901935	1.897839	1.893761	1.88975
1.876956	1.872883	1.86873	1.864558	1.86047	1.85646	1.898343	1.894255	1.890235
1.877394	1.873306	1.86916	1.865006	1.860924	1.856909	1.898853	1.894754	1.890725
1.87784	1.873742	1.869602	1.865463	1.861383	1.857359	1.899359	1.895252	1.891216
1.878296	1.87419	1.870054	1.865927	1.861847	1.857811	1.899863	1.89575	1.891709
1.878754	1.874634	1.870499	1.866382	1.862303	1.858259	1.854251	1.85026	1.846341
1.879214	1.875072	1.870933	1.866823	1.862747	1.858704	1.854695	1.850705	1.846786
1.879674	1.875512	1.87137	1.867266	1.863192	1.85915	1.855143	1.851154	1.847234
1.880135	1.875958	1.871814	1.867718	1.863647	1.859603	1.855594	1.851607	1.847684
1.880599	1.876407	1.872261	1.868169	1.864101	1.860059	1.856049	1.852061	1.848134
1.881067	1.876854	1.8727	1.868609	1.864545	1.860513	1.856509	1.852515	1.84858
1.88154	1.877304	1.873139	1.869044	1.864986	1.860969	1.856971	1.852969	1.849023
1.832948	1.829065	1.873598	1.869497	1.865438	1.861426	1.857429	1.853419	1.849463
1.833426	1.829528	1.874076	1.869966	1.865901	1.861885	1.857883	1.853865	1.849899
1.833906	1.829996	1.874554	1.870434	1.866363	1.862348	1.858341	1.854313	1.850333
1.834382	1.830467	1.875027	1.870893	1.866819	1.862812	1.858806	1.85476	1.850761
1.834868	1.830945	1.875509	1.871361	1.867283	1.863281	1.859273	1.855215	1.851201
1.817847	1.813939	1.876002	1.871841	1.867754	1.863749	1.859738	1.855676	1.851659
1.818356	1.814426	1.876509	1.872341	1.868245	1.864229	1.860212	1.856153	1.852139
1.818901	1.81494	1.877046	1.872892	1.868793	1.864756	1.860728	1.856679	1.852668
1.81947	1.815474	1.877603	1.873478	1.869378	1.865314	1.861271	1.857239	1.853232
1.820013	1.816002	1.81215	1.808411	1.869915	1.865841	1.861792	1.857765	1.853757
1.820524	1.816523	1.812657	1.808892	1.870402	1.866333	1.862286	1.858254	1.854241
1.821047	1.817058	1.813175	1.809377	1.870897	1.866849	1.862811	1.858761	1.854732
1.821598	1.817622	1.813716	1.809877	1.871422	1.86742	1.863401	1.859313	1.855247
1.822148	1.818181	1.814253	1.81038	1.871947	1.867983	1.86398	1.85986	1.855765
1.822675	1.8187	1.814762	1.810877	1.872443	1.868476	1.864474	1.860356	1.856266
1.823195	1.819203	1.81526	1.811377	1.872941	1.868949	1.864939	1.86084	1.856772
1.823731	1.819718	1.815776	1.811905	1.873508	1.869482	1.865455	1.861379	1.857328
1.824276	1.82024	1.816303	1.812454	1.874127	1.870057	1.866006	1.861957	1.857923
1.809491	1.8055	1.816825	1.812987	1.874716	1.870631	1.866573	1.862536	1.858509
1.809969	1.80598	1.817337	1.813501	1.875266	1.871206	1.867162	1.863117	1.859082

1.810462	1.806476	1.802606	1.798831	1.79514	1.791541	1.867736	1.863692	1.859657
1.810987	1.807002	1.803115	1.799316	1.795615	1.792021	1.868278	1.864262	1.860246
1.811533	1.807546	1.803639	1.799812	1.796096	1.792497	1.868803	1.864818	1.860828
1.812101	1.808103	1.804187	1.800348	1.796611	1.792976	1.856926	1.852991	1.849055
1.812686	1.808672	1.804749	1.800908	1.797145	1.793462	1.857397	1.853473	1.849564
1.813255	1.809228	1.8053	1.801455	1.797682	1.79398	1.857939	1.853987	1.850057
1.813813	1.809776	1.805843	1.801995	1.798223	1.79453	1.858546	1.854525	1.850531
1.814373	1.810322	1.806381	1.802529	1.798757	1.795069	1.859133	1.855081	1.851054
1.81494	1.810866	1.806913	1.803054	1.799278	1.79559	1.859683	1.855661	1.851654
1.815506	1.81141	1.807446	1.803584	1.799804	1.796111	1.844183	1.840232	1.836294
1.816062	1.811947	1.807983	1.804129	1.800348	1.796645	1.844753	1.840777	1.836824
1.816623	1.812492	1.808526	1.804679	1.800896	1.797181	1.845328	1.841323	1.837356
1.81721	1.813065	1.809084	1.805222	1.801426	1.797701	1.794033	1.790429	1.786857
1.817814	1.813659	1.809655	1.805762	1.801948	1.798215	1.794555	1.790986	1.787444
1.76901	1.76498	1.810238	1.806317	1.802491	1.798761	1.795106	1.791546	1.788009
1.76958	1.765566	1.810836	1.806885	1.803052	1.799339	1.79569	1.792111	1.788554
1.738854	1.735107	1.762225	1.758383	1.803633	1.799924	1.796271	1.792669	1.789093
1.739405	1.735615	1.762791	1.758971	1.804245	1.800517	1.796842	1.793215	1.789622
1.739977	1.736146	1.763378	1.759567	1.804857	1.8011	1.797402	1.793759	1.790157
1.740595	1.73676	1.764016	1.760159	1.756393	1.752717	1.797932	1.794301	1.790719
1.741227	1.737409	1.764677	1.760749	1.756931	1.753217	1.798458	1.794849	1.791291
1.721289	1.717588	1.765306	1.761323	1.757479	1.753763	1.799033	1.795419	1.791856
1.721822	1.718167	1.765917	1.761888	1.758033	1.754343	1.799645	1.796007	1.792417
1.722384	1.718746	1.766544	1.762487	1.75862	1.754934	1.751322	1.747776	1.744276
1.722987	1.719328	1.767194	1.763125	1.759243	1.755537	1.751898	1.748308	1.744776
1.723572	1.719887	1.767833	1.763777	1.759886	1.756151	1.752481	1.748852	1.745293
1.724111	1.720399	1.768441	1.764438	1.760556	1.756789	1.753085	1.74943	1.745847
1.724636	1.720907	1.769044	1.765098	1.761224	1.757419	1.753682	1.75001	1.746414
1.680518	1.677456	1.769658	1.765733	1.761848	1.757997	1.75423	1.750562	1.746975
1.680908	1.677867	1.770281	1.766359	1.762452	1.758552	1.754759	1.751105	1.747535
1.68134	1.67831	1.770931	1.767007	1.763082	1.759148	1.755328	1.751666	1.748086
1.68181	1.678783	1.771608	1.767675	1.763734	1.759776	1.75593	1.75224	1.748629
1.682268	1.679243	1.720157	1.716517	1.764355	1.760396	1.756541	1.752833	1.749198
1.682701	1.67968	1.72071	1.717023	1.764928	1.760995	1.757157	1.753445	1.749799
1.683144	1.680119	1.721271	1.717542	1.765512	1.761605	1.757784	1.75407	1.750414
1.683612	1.680575	1.721862	1.718116	1.76614	1.76224	1.758422	1.754703	1.751037
1.684093	1.68104	1.722469	1.718716	1.766789	1.762893	1.759074	1.755348	1.75167
1.649742	1.646899	1.678434	1.675368	1.767457	1.763572	1.759755	1.756014	1.752316
1.6502	1.64735	1.678906	1.675838	1.768141	1.764269	1.760454	1.756696	1.752973
1.650653	1.647796	1.679371	1.676297	1.768812	1.76495	1.761136	1.757366	1.753625
1.651099	1.648237	1.679828	1.676746	1.769471	1.765613	1.761801	1.758025	1.754275
1.627116	1.624427	1.680297	1.677204	1.770135	1.766278	1.762463	1.758679	1.754917
1.627539	1.624846	1.680784	1.677678	1.770808	1.766945	1.763122	1.759324	1.755546
1.627967	1.625271	1.681278	1.678159	1.771485	1.767614	1.763781	1.759969	1.756177
1.628397	1.6257	1.622972	1.620243	1.710951	1.707735	1.764443	1.760621	1.75682
1.62883	1.626132	1.623399	1.62066	1.711474	1.708242	1.765104	1.761276	1.75747
1.62926	1.626562	1.623823	1.621074	1.71199	1.708741	1.765756	1.761923	1.758115
1.629689	1.626993	1.624248	1.621488	1.712503	1.709236	1.766402	1.762566	1.75876
1.602459	1.599831	1.624691	1.621928	1.655125	1.652275	1.767075	1.763238	1.759437
1.602908	1.600283	1.625154	1.622398	1.655612	1.652745	1.76778	1.763944	1.760153

1.60336	1.600735	1.598085	1.595434	1.656127	1.653235	1.750703	1.746958	1.743264
1.603809	1.601183	1.598554	1.595933	1.656685	1.653752	1.751395	1.747622	1.743913
1.604265	1.601633	1.599022	1.596428	1.65724	1.654272	1.752094	1.748285	1.744554
1.604736	1.602089	1.599474	1.596883	1.621641	1.618918	1.688696	1.685564	1.682491
1.60522	1.602551	1.59992	1.59732	1.622083	1.619373	1.68922	1.686054	1.682951
1.605723	1.603027	1.60038	1.597771	1.595163	1.592558	1.689791	1.686589	1.683451
1.606247	1.603514	1.600849	1.598232	1.595631	1.593051	1.690403	1.687163	1.683985
1.60675	1.604001	1.601324	1.598698	1.596099	1.593531	1.667529	1.664322	1.661162
1.607224	1.604484	1.601803	1.599165	1.596559	1.59399	1.66812	1.66493	1.661774
1.607698	1.604973	1.602292	1.599646	1.597034	1.594459	1.63349	1.630527	1.627591
1.60818	1.605472	1.602795	1.600149	1.597535	1.594953	1.63406	1.631108	1.628178
1.608674	1.60598	1.60331	1.600668	1.598053	1.595468	1.634642	1.631696	1.62877
1.609191	1.606498	1.603834	1.601202	1.598594	1.596014	1.63526	1.632305	1.629369
1.609727	1.607026	1.604366	1.601747	1.59915	1.596579	1.635901	1.632929	1.629972
1.610278	1.607563	1.6049	1.602282	1.599683	1.597108	1.636519	1.633541	1.630573
1.610847	1.608112	1.605435	1.602805	1.600192	1.5976	1.637118	1.634144	1.631172
1.566033	1.563543	1.605978	1.603341	1.600716	1.598107	1.637717	1.634747	1.631774
1.566469	1.564002	1.606537	1.603903	1.601272	1.598646	1.638319	1.635351	1.632384
1.566906	1.564461	1.607091	1.604458	1.601823	1.599189	1.638923	1.635954	1.632991
1.567374	1.564928	1.607621	1.604976	1.602341	1.59972	1.639529	1.636552	1.633583
1.567855	1.565401	1.608147	1.605484	1.60285	1.600247	1.640136	1.637154	1.634178
1.568344	1.565888	1.563431	1.56099	1.603374	1.600768	1.640744	1.637778	1.634813
1.568839	1.566385	1.563927	1.561479	1.603908	1.601286	1.641353	1.638417	1.635476
1.526218	1.523851	1.564407	1.561962	1.604458	1.601827	1.641968	1.639041	1.636109
1.526615	1.524248	1.564869	1.562437	1.605028	1.602396	1.642589	1.639646	1.636705
1.527019	1.524648	1.522292	1.519964	1.605588	1.602952	1.643206	1.640258	1.637317
1.527449	1.525068	1.522706	1.520374	1.606123	1.603479	1.643815	1.640895	1.637977
1.52787	1.52548	1.523109	1.520772	1.606647	1.603996	1.644415	1.641525	1.63863
1.528252	1.525854	1.523479	1.521145	1.518805	1.516463	1.645007	1.642119	1.639223
1.528613	1.526208	1.523828	1.521499	1.519165	1.516831	1.645586	1.64269	1.639785
1.528976	1.526557	1.524167	1.521835	1.519504	1.517178	1.646129	1.64324	1.640347
1.529336	1.5269	1.524495	1.522154	1.519822	1.517507	1.646641	1.643768	1.640903
1.529695	1.527249	1.524839	1.522499	1.520173	1.517866	1.565029	1.56242	1.559819
1.530042	1.527601	1.5252	1.522881	1.520566	1.518265	1.565516	1.562908	1.560309
1.490091	1.487778	1.525578	1.523272	1.520965	1.518667	1.565996	1.563381	1.560779
1.490488	1.488145	1.525985	1.523672	1.521361	1.519063	1.566438	1.563797	1.561185
1.490925	1.488534	1.526398	1.524068	1.521746	1.519447	1.566864	1.5642	1.561581
1.491394	1.488934	1.526794	1.524431	1.522091	1.519786	1.567269	1.564616	1.56202
1.491885	1.48934	1.527177	1.524776	1.522413	1.520103	1.567668	1.565043	1.562482
1.49232	1.489692	1.487218	1.484936	1.522769	1.520465	1.568139	1.565513	1.562953
1.492698	1.489988	1.487498	1.485247	1.523154	1.520868	1.56868	1.566026	1.563436
1.493139	1.490334	1.487814	1.485579	1.52355	1.521272	1.569229	1.566532	1.5639
1.493658	1.490742	1.488176	1.485933	1.523957	1.521677	1.56979	1.567031	1.564338
1.494262	1.491216	1.488584	1.486312	1.524367	1.522069	1.570325	1.567507	1.564759
1.494987	1.491783	1.48906	1.48673	1.484441	1.482229	1.570777	1.567925	1.565154
1.495788	1.492411	1.489575	1.487161	1.484796	1.482516	1.57121	1.568332	1.565549
1.496613	1.493068	1.490093	1.487554	1.485116	1.482816	1.571716	1.568802	1.56599
1.497466	1.493755	1.49062	1.487925	1.485414	1.483125	1.572268	1.569318	1.566467
1.498258	1.494392	1.491126	1.488315	1.485745	1.483454	1.481236	1.479053	1.476904
1.498979	1.494963	1.491598	1.488719	1.486107	1.483803	1.481578	1.479391	1.477239

1.499714	1.495588	1.492126	1.489156	1.486491	1.484169	1.481934	1.479738	1.477582
1.500471	1.496299	1.492729	1.489619	1.486881	1.484549	1.482308	1.480099	1.47793
1.501275	1.497084	1.493408	1.490145	1.487317	1.484954	1.482692	1.480464	1.478282
1.502173	1.497973	1.494201	1.49079	1.487848	1.485398	1.483076	1.480823	1.478632
1.503128	1.498932	1.49507	1.491508	1.488438	1.48587	1.483467	1.481184	1.478984
1.504079	1.499888	1.495934	1.492222	1.489028	1.486354	1.483876	1.481564	1.479352
1.505037	1.500853	1.496808	1.492946	1.489629	1.486851	1.484301	1.481959	1.479733
1.505995	1.501812	1.497689	1.493703	1.490268	1.48737	1.484735	1.48236	1.480119
1.506956	1.502761	1.498576	1.494493	1.490942	1.487903	1.485168	1.482762	1.48051
1.507909	1.503706	1.499477	1.495324	1.491668	1.488483	1.485639	1.483185	1.480907
1.508849	1.504648	1.5004	1.496209	1.492463	1.489131	1.486164	1.483634	1.481307
1.509785	1.505583	1.501331	1.497126	1.493301	1.489823	1.48673	1.484111	1.481719
1.510725	1.506506	1.502255	1.498055	1.494164	1.490548	1.487332	1.484621	1.482161
1.511668	1.50742	1.503178	1.498998	1.495052	1.491304	1.487967	1.485159	1.482624
1.51262	1.50833	1.504093	1.499936	1.495937	1.492063	1.488606	1.4857	1.483086
1.513581	1.509237	1.505002	1.500871	1.496823	1.492825	1.489251	1.486244	1.483549
1.514546	1.510148	1.505907	1.501789	1.497703	1.493621	1.48995	1.486828	1.484031
1.515509	1.511065	1.506805	1.502682	1.498574	1.494458	1.490714	1.487452	1.484526
1.516485	1.51199	1.507704	1.503567	1.499445	1.495322	1.491524	1.488126	1.485065
1.517465	1.512906	1.508587	1.504437	1.500314	1.496206	1.492375	1.488862	1.485676
1.518481	1.513855	1.509495	1.505317	1.501192	1.497115	1.493265	1.489645	1.486335
1.519597	1.514923	1.510499	1.506251	1.502102	1.498052	1.494179	1.490447	1.487012
1.520791	1.516081	1.511578	1.507229	1.503041	1.499017	1.495122	1.49128	1.48772
1.522018	1.517291	1.512723	1.508279	1.504046	1.500025	1.496103	1.492182	1.488524
1.523293	1.518569	1.51395	1.509416	1.50513	1.501089	1.497136	1.493166	1.489436
1.524508	1.519794	1.515132	1.51052	1.506178	1.502102	1.498114	1.494115	1.490336
1.525617	1.520916	1.516221	1.511543	1.507144	1.503014	1.498989	1.494988	1.491188
1.526677	1.521999	1.517281	1.512551	1.508097	1.503902	1.499834	1.495837	1.492026
1.527709	1.523063	1.518338	1.513569	1.509063	1.5048	1.500682	1.496679	1.49285
1.528716	1.524116	1.519395	1.514604	1.510051	1.50571	1.501536	1.497524	1.493674
1.529734	1.525197	1.520502	1.51571	1.511112	1.50668	1.502438	1.498416	1.494549
1.530756	1.526298	1.521647	1.516875	1.512237	1.5077	1.503381	1.499347	1.495465
1.531829	1.527443	1.522836	1.518085	1.513415	1.50879	1.504397	1.500327	1.496411
1.53298	1.528659	1.524091	1.519363	1.514669	1.50997	1.505505	1.50137	1.497397
1.534076	1.529816	1.525291	1.520592	1.515886	1.511133	1.506606	1.502401	1.498371
1.535036	1.53084	1.526361	1.521701	1.516996	1.512207	1.507635	1.503372	1.499293
1.535956	1.531814	1.527385	1.522775	1.518084	1.513278	1.50867	1.504347	1.500215
1.536901	1.532796	1.528422	1.523879	1.51922	1.51441	1.509773	1.505381	1.501185
1.53786	1.533776	1.529459	1.524997	1.520381	1.515583	1.510923	1.506454	1.502183
1.538886	1.534801	1.530533	1.526154	1.521588	1.516817	1.512134	1.507565	1.503188
1.539978	1.535868	1.531643	1.52735	1.522843	1.518114	1.513408	1.508715	1.504199
1.541055	1.536908	1.532711	1.528489	1.52404	1.519365	1.51466	1.509887	1.505268
1.542088	1.537892	1.533707	1.529539	1.525149	1.520545	1.515873	1.511077	1.506401
1.543155	1.5389	1.534713	1.530581	1.526243	1.521713	1.517086	1.512298	1.507594
1.544309	1.539991	1.535785	1.531667	1.527368	1.522909	1.518337	1.513584	1.508882
1.545499	1.541109	1.536871	1.532751	1.528482	1.524089	1.519576	1.514872	1.510193
1.54667	1.542185	1.537907	1.53378	1.529532	1.525189	1.520722	1.516059	1.511402
1.547843	1.543249	1.538922	1.53478	1.530541	1.526236	1.521809	1.517185	1.512556
1.549093	1.544412	1.540024	1.535838	1.531592	1.527316	1.522929	1.518364	1.513779
1.550424	1.545678	1.541219	1.536957	1.532685	1.52843	1.524087	1.519602	1.515076

1.551739	1.54694	1.542411	1.538068	1.533759	1.529508	1.525199	1.520795	1.516339
1.552998	1.548154	1.543554	1.539125	1.534766	1.530495	1.52621	1.521898	1.517537
1.554264	1.549398	1.544739	1.54023	1.53582	1.531521	1.527243	1.522993	1.518701
1.411333	1.406733	1.546053	1.541479	1.537027	1.532704	1.528407	1.524132	1.519826
1.412567	1.408013	1.547424	1.5428	1.53831	1.533962	1.529628	1.52529	1.520936
1.413734	1.409235	1.548755	1.544102	1.539582	1.5352	1.530835	1.526468	1.522096
1.414854	1.410421	1.550066	1.545403	1.54086	1.536436	1.53204	1.527662	1.523291
1.415978	1.411562	1.407124	1.402693	1.542103	1.53763	1.533201	1.528815	1.524452
1.417102	1.41264	1.408191	1.403768	1.543295	1.53876	1.534294	1.529911	1.525567
1.418247	1.413754	1.409297	1.404874	1.544523	1.539938	1.535435	1.53104	1.526696
1.419444	1.414974	1.410511	1.406062	1.545838	1.541223	1.536682	1.53224	1.527848
1.420633	1.416202	1.411741	1.407273	1.547175	1.542544	1.537974	1.533482	1.529035
1.421738	1.417348	1.412916	1.408472	1.548492	1.543862	1.539282	1.534769	1.530293
1.422807	1.418451	1.414058	1.409657	1.549794	1.545177	1.540602	1.536087	1.531595
1.423921	1.419528	1.415133	1.41075	1.406384	1.402033	1.541872	1.53737	1.532882
1.42507	1.420574	1.41614	1.411758	1.407409	1.403096	1.543099	1.538628	1.534164
1.154036	1.150336	1.417229	1.412836	1.408479	1.404161	1.544317	1.539859	1.53541
1.155058	1.151352	1.41843	1.414014	1.409611	1.405224	1.545512	1.541046	1.536608
1.047365	1.043912	1.419672	1.415229	1.410785	1.406336	1.401977	1.397753	1.393572
1.048576	1.04512	1.420933	1.416475	1.412014	1.407552	1.403157	1.39885	1.394604
1.04978	1.046335	1.422209	1.417732	1.413265	1.408808	1.404387	1.399987	1.395662
1.050944	1.047547	1.285128	1.280811	1.41448	1.410036	1.405613	1.401186	1.396827
1.052078	1.048752	1.286539	1.282168	1.415681	1.411255	1.406847	1.40243	1.398064
1.05319	1.04989	1.287908	1.283518	1.41689	1.412468	1.408062	1.403653	1.39928
1.054273	1.050958	1.289237	1.284862	1.418108	1.413671	1.409255	1.404857	1.40048
1.055395	1.05203	1.048676	1.045355	1.281914	1.277668	1.410462	1.406065	1.401679
1.056573	1.053112	1.049759	1.046497	1.283277	1.279033	1.411696	1.407282	1.402876
1.057794	1.05423	1.050865	1.04764	1.284648	1.280399	1.412931	1.408499	1.404075
1.059064	1.055422	1.052029	1.048803	1.28602	1.281746	1.348027	1.343732	1.339459
1.06037	1.056663	1.05323	1.049981	1.287403	1.283097	1.349361	1.345063	1.340792
1.06169	1.057927	1.054458	1.051182	1.288821	1.284484	1.350726	1.346416	1.34214
1.063026	1.05922	1.055713	1.052403	1.290268	1.2859	1.352116	1.347787	1.343499
1.064369	1.06052	1.056971	1.05362	1.050244	1.046865	1.283007	1.278736	1.274511
1.065715	1.061827	1.058232	1.05483	1.051436	1.048069	1.284478	1.280183	1.275928
0.944774	0.941485	1.059507	1.056044	1.052625	1.049269	1.285952	1.281627	1.277341
0.945896	0.9426	1.06079	1.057251	1.053801	1.050459	1.287415	1.283052	1.278732
0.947028	0.943735	1.062105	1.058482	1.054992	1.05165	1.288874	1.284472	1.280122
0.948198	0.944936	1.063482	1.059771	1.056225	1.052856	1.153206	1.149241	1.145318
0.949388	0.946172	1.064903	1.061104	1.057492	1.054072	1.154524	1.150547	1.146627
0.950572	0.947397	1.066345	1.062476	1.058795	1.055297	1.15584	1.151878	1.147983
0.951754	0.948617	1.067813	1.063888	1.060133	1.056532	1.157154	1.153232	1.14938
0.952917	0.949802	1.069255	1.065307	1.061492	1.057784	1.054224	1.050861	1.04757
0.954052	0.95094	1.070666	1.066738	1.062875	1.059045	1.055416	1.052091	1.048845
0.9552	0.95207	0.94884	0.945559	1.064263	1.060346	1.056659	1.053332	1.050094
0.956386	0.953206	0.949966	0.946698	1.065644	1.061715	1.057991	1.054584	1.051279
0.95759	0.954352	0.951096	0.947835	1.067026	1.063122	1.059375	1.055857	1.052451
0.958805	0.955533	0.952252	0.948974	1.068411	1.064548	1.06079	1.057171	1.053664
0.960025	0.956734	0.953426	0.950119	1.069802	1.065992	1.062232	1.058514	1.054903
0.961187	0.957902	0.954601	0.951302	1.071221	1.067414	1.06363	1.059843	1.056162
0.962289	0.959036	0.955775	0.952526	1.07267	1.068817	1.064984	1.061156	1.057439

0.824663	0.822067	0.956966	0.953747	0.950505	0.947245	1.066358	1.062505	1.058761
0.825582	0.822948	0.958183	0.954958	0.951708	0.948436	1.067762	1.063902	1.060137
0.826528	0.823841	0.959401	0.95616	0.952905	0.949637	1.069197	1.065335	1.061547
0.670338	0.668033	0.9606	0.957342	0.954089	0.950848	1.070686	1.066818	1.062989
0.671222	0.668922	0.961797	0.958521	0.955275	0.952066	1.072194	1.068321	1.064447
0.67216	0.669824	0.823813	0.821141	0.956491	0.953282	0.950051	0.946776	0.943499
0.673138	0.670742	0.824727	0.822043	0.95773	0.954498	0.951249	0.947965	0.944685
0.674104	0.671656	0.825666	0.822954	0.820246	0.817557	0.95244	0.949164	0.945893
0.67505	0.672563	0.826631	0.823874	0.821145	0.81846	0.953621	0.950373	0.947129
0.500564	0.498294	0.827609	0.824799	0.822045	0.819362	0.954819	0.951587	0.948359
0.501561	0.499192	0.828585	0.825717	0.822937	0.820262	0.956051	0.952803	0.949564
0.50259	0.500133	0.829588	0.826657	0.823844	0.821167	0.957292	0.954027	0.950778
0.503665	0.501159	0.673946	0.671489	0.824793	0.822084	0.819384	0.816658	0.813941
0.504756	0.502226	0.67495	0.672409	0.825767	0.823013	0.820297	0.817603	0.814917
		0.500834	0.498534	0.826738	0.823964	0.821236	0.81854	0.815853
		0.501787	0.499464	0.827711	0.824936	0.8222	0.819471	0.816755
		0.50278	0.500414	0.70143	0.698906	0.823143	0.820402	0.817681
		0.50383	0.501382	0.702478	0.699773	0.824047	0.821331	0.818644
		0.504876	0.502375	0.500068	0.497929	0.824984	0.822269	0.819596
		0.50589	0.503407	0.501077	0.498871	0.826002	0.823221	0.820507
		0.506884	0.50445	0.502112	0.499842	0.827046	0.824183	0.821413
				0.50312	0.500782	0.700787	0.69814	0.695621
				0.504122	0.501717	0.701673	0.699004	0.696472
				0.505143	0.502698	0.702673	0.699919	0.697311
				0.506186	0.503723	0.703784	0.700883	0.698134
				0.507181	0.504739	0.704872	0.701879	0.699032
				0.508101	0.505732	0.705896	0.702913	0.70005
				0.509005	0.506717	0.706934	0.703962	0.701081
				0.509938	0.507702	0.505415	0.503053	0.500746
				0.510864	0.508675	0.506405	0.504021	0.501676
						0.50738	0.505003	0.502642
						0.508343	0.505995	0.503639
						0.509314	0.507013	0.504678
						0.510301	0.508066	0.505771
						0.511252	0.509083	0.506833
						0.512146	0.510034	0.507828
						0.513009	0.510953	0.5088

				0.720279	0.716058	0.711979
				0.71834	0.714168	0.710142
				0.716406	0.712289	0.708327
				0.714471	0.710409	0.70651
				0.712546	0.708539	0.704699
		0.719587	0.715047	1.198472	1.193408	1.188484
		0.717655	0.71318	1.196192	1.191166	1.186272
		0.71572	0.711315	1.245995	1.240901	1.23594
		0.71377	0.709441	1.243656	1.238595	1.233685
0.720374	0.716101	1.199717	1.194485	1.310054	1.30499	1.300089
0.718404	0.71419	1.197415	1.192224	1.307751	1.30268	1.297786

		0.716427	0.712299	1.195136	1.189991	1.305481	1.300406	1.29552
		0.714452	0.710411	1.244911	1.239688	1.516935	1.511465	1.50617
		0.712482	0.708532	1.2426	1.237427	1.514619	1.509213	1.503963
		0.710549	0.706669	1.322224	1.317025	1.571931	1.566464	1.561132
		0.708643	0.704819	1.319921	1.314849	1.569626	1.564239	1.558958
0.715374	0.710946	1.245897	1.240852	1.379334	1.374335	1.567317	1.562014	1.556796
0.713562	0.709332	1.24381	1.23871	1.377085	1.372145	1.564983	1.559772	1.554639
0.711755	0.707725	1.241725	1.23658	1.374865	1.369978	1.562659	1.557532	1.55248
0.709841	0.705946	1.321294	1.316041	1.570992	1.565613	1.683379	1.677804	1.672348
0.707923	0.704109	1.318979	1.313798	1.568685	1.563326	1.680886	1.675378	1.669992
1.065699	1.061255	1.378426	1.373308	1.566403	1.561071	1.726336	1.720766	1.715321
1.063874	1.0593	1.376251	1.371249	1.564146	1.558845	1.72386	1.718314	1.712886
1.240331	1.235503	1.506111	1.500808	1.685024	1.679249	1.834527	1.828711	1.823003
1.23825	1.233478	1.503894	1.498586	1.68255	1.676856	1.831989	1.826147	1.820401
1.379806	1.374708	1.501675	1.496378	1.76991	1.764053	1.905935	1.899715	1.893596
1.377602	1.372574	1.49943	1.494189	1.767363	1.761566	1.903311	1.897084	1.890944
1.375436	1.370472	1.497184	1.492012	1.764842	1.759091	1.90069	1.894471	1.888323
1.505424	1.500132	1.684267	1.678535	1.833586	1.827717	1.966948	1.960456	1.954038
1.503167	1.497867	1.681755	1.67612	1.831037	1.825171	1.964108	1.957679	1.951318
1.500941	1.495711	1.769005	1.763165	1.905053	1.898859	1.977298	1.970616	1.963983
1.498745	1.493655	1.766563	1.760709	1.902444	1.896295	1.974528	1.967872	1.961256
1.557885	1.552725	1.820314	1.814137	1.968943	1.96249	1.97179	1.965139	1.958524
1.555699	1.550545	1.817776	1.811651	1.966294	1.959866	1.969094	1.962415	1.955775
1.771009	1.765148	1.915321	1.909023	1.963666	1.957242	2.005124	1.998253	1.991433
1.768577	1.762757	1.91271	1.906468	1.961066	1.954604	2.002249	1.995413	1.988645
1.766179	1.760395	1.910113	1.903913	1.958459	1.95195	1.99934	1.992554	1.985854
1.819958	1.813841	1.968758	1.962265	1.971603	1.964753	2.106386	2.098662	2.091043
1.817443	1.811368	1.966109	1.959558	1.968748	1.961916	2.103036	2.095284	2.087644
1.914861	1.90859	1.963419	1.956839	2.004503	1.99751	2.099753	2.091994	2.084352
1.912218	1.905933	1.960685	1.954103	2.001576	1.994613	2.096538	2.088788	2.081156
1.971119	1.964534	1.973991	1.96711	2.076889	2.069105	2.093329	2.085624	2.078038
1.968426	1.961796	1.971155	1.964305	2.07369	2.065892	2.090094	2.082494	2.075023
1.965763	1.959103	1.968356	1.961514	2.070492	2.062704	2.086907	2.079401	2.072028
1.963168	1.956529	2.004092	1.997022	2.067212	2.059556	2.113708	2.105963	2.098366
1.9606	1.954005	2.001335	1.99416	2.063952	2.056443	2.110617	2.102847	2.0952
1.974159	1.967267	2.076832	2.068736	2.092859	2.08523	2.107413	2.099706	2.092138
1.971416	1.964574	2.073796	2.065822	2.089938	2.082165	2.104151	2.096559	2.089151
2.007084	2.000109	2.070677	2.062838	2.116941	2.108907	2.118867	2.111302	2.10392
2.004214	1.997204	2.067452	2.059774	2.113909	2.106008	2.116027	2.10834	2.100822
2.001336	1.994318	2.064252	2.056691	2.110831	2.103077	2.151002	2.143224	2.135567
1.998424	1.991484	2.06113	2.053585	2.107645	2.100028	2.148094	2.140441	2.132867
1.995548	1.988676	2.058028	2.050497	2.104461	2.096947	2.145142	2.137659	2.130222
2.07032	2.062493	2.086814	2.079308	2.129641	2.122151	2.142077	2.134729	2.127444
2.06729	2.059334	2.08365	2.076303	2.126638	2.119082	2.138968	2.131737	2.124607
2.064283	2.056372	2.110401	2.10282	2.151017	2.14343	2.182418	2.175131	2.167953
2.061308	2.053586	2.107486	2.099719	2.147876	2.140436	2.179496	2.172187	2.164977
2.058267	2.050734	2.132762	2.124992	2.144871	2.13749	2.186862	2.179533	2.172296
2.055112	2.04778	2.129855	2.122179	2.142049	2.13459	2.183855	2.176626	2.169514
2.09894	2.091522	2.154471	2.14683	2.158373	2.150856	2.188894	2.181778	2.174801
2.095893	2.088417	2.151405	2.143913	2.155573	2.1481	2.18613	2.179024	2.172063

2.092964	2.085361	2.148346	2.140999	2.152784	2.14539	2.183438	2.176311	2.169327
2.133076	2.12536	2.145466	2.138182	2.150025	2.142695	2.180763	2.173587	2.166549
2.13032	2.122603	2.142694	2.135416	2.147276	2.140008	2.178108	2.170871	2.163763
2.155215	2.147431	2.159006	2.1517	2.189826	2.182582	2.197755	2.190587	2.183528
2.152422	2.144659	2.156209	2.148805	2.186873	2.179709	2.195	2.187964	2.181003
2.149614	2.141906	2.162936	2.155491	2.183994	2.176845	2.216589	2.209511	2.202518
2.146792	2.139178	2.160249	2.152795	2.181228	2.173983	2.213591	2.206658	2.19985
2.143945	2.136454	2.157581	2.150141	2.178524	2.171192	2.210668	2.20384	2.197178
2.15117	2.143864	2.154922	2.147501	2.19823	2.190934	2.207967	2.201163	2.194531
2.148267	2.14113	2.152259	2.144873	2.195632	2.188385	2.205381	2.19855	2.191883
2.163851	2.156784	2.160433	2.153059	2.193036	2.185853	2.202815	2.195924	2.189186
2.16106	2.153993	2.157672	2.150388	2.190448	2.183348	2.200286	2.193305	2.186458
2.158289	2.151169	2.202029	2.194907	2.212007	2.204838	2.204472	2.19749	2.190613
2.155534	2.148291	2.199158	2.19216	2.209276	2.202214	2.201946	2.194971	2.18807
2.163697	2.156315	2.196361	2.189459	2.206583	2.199609	2.207202	2.200198	2.193238
2.161027	2.153625	2.1937	2.186841	2.203954	2.197042	2.204676	2.197674	2.190692
2.158386	2.150991	2.191095	2.184267	2.201365	2.194494	2.202152	2.195154	2.188162
2.155748	2.148361	2.212613	2.205697	2.198812	2.191957	2.199624	2.19264	2.185659
2.153121	2.145753	2.209978	2.20313	2.196284	2.18943	2.197094	2.190129	2.183173
2.150496	2.143161	2.207355	2.200558	2.193744	2.186895	2.19456	2.187617	2.180689
2.147874	2.140586	2.204748	2.197987	2.191196	2.184353	2.192023	2.185104	2.178208
2.216547	2.20919	2.20217	2.195438	2.195473	2.188654	2.189495	2.182602	2.175742
2.213912	2.206607	2.199623	2.192916	2.192972	2.186143	2.186972	2.18011	2.173291
2.2113	2.204059	2.197106	2.190407	2.198205	2.191333	2.184473	2.177638	2.170855
2.208705	2.201537	2.194608	2.187896	2.19569	2.188844	2.182011	2.175197	2.168437
2.206141	2.199061	2.192154	2.185411	2.19319	2.186373	2.179571	2.172776	2.166037
2.203633	2.196682	2.196617	2.189833	2.190732	2.18392	2.18063	2.173865	2.167179
2.201164	2.194357	2.194323	2.187464	2.188302	2.181483	2.178191	2.171464	2.164826
2.198686	2.191948	2.191941	2.185057	2.189396	2.182538	2.175759	2.169071	2.16248
2.196199	2.189461	2.189476	2.182618	2.186946	2.180097	2.173335	2.166685	2.16014
2.200591	2.193835	2.198279	2.191367	2.184501	2.17767	2.204648	2.197635	2.190748
2.198261	2.191432	2.19582	2.188909	2.182058	2.175256	2.202185	2.195225	2.1884
2.195926	2.189027	2.193361	2.186455	2.179626	2.172865	2.199761	2.192868	2.186114
2.197413	2.190441	2.190884	2.183995	2.177201	2.170495	2.197381	2.190595	2.183936
2.194964	2.187974	2.188406	2.181544	2.174798	2.168162	2.195049	2.18837	2.181796
2.192496	2.1855	2.185942	2.179125	2.172444	2.165909	2.20011	2.193428	2.186826
2.19001	2.183023	2.183494	2.176736	2.170136	2.163719	2.197902	2.191195	2.184535
2.187554	2.180594	2.181117	2.174437	2.176302	2.169879	2.195642	2.188917	2.182227
2.18512	2.178206	2.178809	2.17224	2.174134	2.167684	2.193329	2.186592	2.179901
2.190119	2.183246	2.176565	2.170053	2.204677	2.19781	2.206938	2.200189	2.193515
2.187907	2.181103	2.174424	2.167858	2.202319	2.195453	2.204576	2.197804	2.191137
2.185712	2.178966	2.172278	2.165647	2.199942	2.19309	2.20222	2.195424	2.188769
2.183403	2.176681	2.178364	2.171686	2.213494	2.20665	2.199878	2.193058	2.186421
2.181057	2.174339	2.176029	2.169381	2.211111	2.204307	2.197569	2.190736	2.184127
2.187124	2.180399	2.191709	2.185034	2.208767	2.202042	2.19538	2.188578	2.182021
2.184835	2.178096	2.189375	2.182725	2.206456	2.199845	2.193291	2.186562	2.180075
2.200611	2.193843	2.187152	2.180534	2.204238	2.197649	2.191147	2.184529	2.178176
2.198442	2.191724	2.18508	2.1785	2.202143	2.195455	2.188925	2.18246	2.176318
2.196252	2.18959	2.182993	2.176452	2.200033	2.193248	2.186699	2.180408	2.174499
2.193968	2.187354	2.180795	2.174288	2.197809	2.190983	2.184462	2.178361	2.172696

2.19167	2.185109	2.17859	2.172119	2.195593	2.188752	2.182281	2.176375	2.170952
2.189422	2.182942	2.207032	2.200265	2.193518	2.186683	2.180269	2.17454	2.169322
2.187206	2.180811	2.204937	2.198214	2.191512	2.184714	2.178377	2.172815	2.167775
2.185028	2.178649	2.202749	2.196056	2.189423	2.182741	2.172493	2.167032	2.162085
2.182892	2.176462	2.200478	2.193803	2.187262	2.180771	2.170706	2.165384	2.160551
2.180735	2.174273	2.198221	2.191575	2.185147	2.178877	2.169015	2.163834	2.159109
2.178515	2.172057	2.195974	2.189392	2.183108	2.177096	2.167457	2.162425	2.157803
2.176364	2.169918	2.193789	2.18724	2.181082	2.175322	2.165906	2.161024	2.156509
2.174395	2.167956	2.191714	2.185088	2.174996	2.169407	2.164246	2.159523	2.155133
2.172498	2.16607	2.189716	2.183008	2.172948	2.167553	2.162591	2.15802	2.153753
2.170554	2.164159	2.187792	2.181154	2.171188	2.165925	2.161092	2.15663	2.152454
2.168588	2.162238	2.185925	2.179461	2.16963	2.164455	2.159698	2.155315	2.151208
2.160231	2.153893	2.183943	2.177693	2.16803	2.16297	2.158302	2.15399	2.149933
2.158052	2.151707	2.181827	2.175843	2.166385	2.161467	2.156901	2.152648	2.148621
2.155937	2.14959	2.175886	2.170098	2.164793	2.160019	2.155558	2.151364	2.147366
2.153967	2.147611	2.173975	2.168408	2.163291	2.158667	2.154319	2.150185	2.146215
2.152019	2.145695	2.172128	2.16676	2.161813	2.157325	2.153083	2.149014	2.145081
2.149988	2.143788	2.170292	2.165093	2.160284	2.155897	2.151742	2.147761	2.143902
2.147962	2.141942	2.168522	2.163469	2.158775	2.154465	2.150381	2.146484	2.1427
2.176958	2.170805	2.166936	2.162014	2.157419	2.153172	2.149127	2.145241	2.141455
2.175143	2.169213	2.165508	2.1607	2.156187	2.151989	2.147955	2.144021	2.140174
2.17337	2.167661	2.164123	2.159432	2.154999	2.150843	2.146821	2.142861	2.138976
2.171634	2.166148	2.162785	2.158217	2.153866	2.149744	2.145735	2.141775	2.137882
2.165403	2.160019	2.161456	2.157	2.152723	2.148633	2.144639	2.140686	2.136792
2.163767	2.158542	2.160122	2.155759	2.151547	2.147491	2.143516	2.139572	2.135682
2.162165	2.157077	2.158779	2.154493	2.150336	2.146309	2.142353	2.138423	2.13454
2.160561	2.155586	2.150941	2.14666	2.149037	2.145033	2.147667	2.143703	2.139786
2.158982	2.154104	2.149529	2.145285	2.1477	2.143712	2.146341	2.142394	2.138492
2.157514	2.152744	2.14824	2.144029	2.145783	2.141792	2.144607	2.140665	2.136771
2.156149	2.151496	2.147062	2.142879	2.14466	2.140667	2.143468	2.139542	2.135666
2.154817	2.150275	2.145908	2.141752	2.14356	2.139572	2.152802	2.148786	2.144821
2.153512	2.149075	2.144772	2.140644	2.142482	2.138507	2.151702	2.147693	2.143734
2.152239	2.147886	2.143638	2.139538	2.141409	2.137446	2.150609	2.146607	2.142657
2.150993	2.146689	2.142483	2.138414	2.14032	2.136367	2.149497	2.145513	2.141583
2.14978	2.145507	2.141331	2.137291	2.139229	2.135285	2.148383	2.144419	2.140511
2.148614	2.144369	2.140214	2.136184	2.143855	2.139789	2.147277	2.143325	2.139432
2.147485	2.143263	2.13912	2.135088	2.142735	2.138678	2.146177	2.142232	2.138347
2.146349	2.14215	2.138023	2.133993	2.148022	2.143932	2.145087	2.141144	2.137257
2.145209	2.141034	2.136924	2.132901	2.146897	2.142826	2.144009	2.140063	2.136165
2.144064	2.139915	2.147591	2.143466	2.14577	2.141718	2.142928	2.13897	2.135055
2.142907	2.138787	2.146454	2.142337	2.144635	2.140603	2.141836	2.137846	2.133904
2.141757	2.137666	2.145322	2.141212	2.143506	2.139492	2.140749	2.136733	2.132774
2.140633	2.136568	2.144208	2.140101	2.142388	2.138389	2.139675	2.135666	2.13172
2.139523	2.135482	2.143103	2.139002	2.141282	2.137295	2.138613	2.134627	2.130709
2.150259	2.146121	2.142003	2.13792	2.140201	2.136223	2.132272	2.128283	2.124368
2.149136	2.145004	2.140906	2.136854	2.139142	2.135168	2.131227	2.127263	2.123378
2.148014	2.143891	2.139813	2.135787	2.138085	2.134124	2.130205	2.126291	2.122455
2.146893	2.142782	2.138722	2.134718	2.13703	2.133093	2.129213	2.125378	2.121615
2.145776	2.141676	2.143963	2.139941	2.135975	2.132063	2.137685	2.133817	2.130021
2.144667	2.140572	2.142859	2.138859	2.134918	2.131028	2.136651	2.132841	2.1291

2.143562	2.139474	2.141761	2.13778	2.133859	2.129991	2.135613	2.13185	2.128158
2.142459	2.138392	2.140677	2.136705	2.132798	2.128951	2.145377	2.141336	2.137439
2.141361	2.13732	2.139603	2.135635	2.131739	2.127911	2.144196	2.140228	2.136416
2.14028	2.136254	2.138536	2.134578	2.140235	2.136302	2.143033	2.13913	2.135389
2.13921	2.135192	2.137476	2.133533	2.139174	2.13525	2.141884	2.13804	2.134358
2.138201	2.134169	2.136439	2.132501	2.138124	2.134222	2.140792	2.136985	2.133331
2.137273	2.133189	2.135424	2.131478	2.13708	2.133219	2.139771	2.135975	2.132314
2.142649	2.138499	2.144098	2.140017	2.136061	2.132237	2.138777	2.134981	2.131298
2.141693	2.1376	2.143178	2.139049	2.135073	2.131267	2.137776	2.133974	2.130269
2.14071	2.136697	2.142273	2.138111	2.13412	2.130331	2.136805	2.13299	2.129255
2.139649	2.135685	2.141283	2.137183	2.143853	2.139831	2.135928	2.132083	2.128294
2.13854	2.134606	2.140244	2.136264	2.142924	2.138978	2.135107	2.131226	2.127372
2.137477	2.133555	2.139196	2.135279	2.1419	2.138024	2.134204	2.13034	2.126485
2.136453	2.132522	2.138127	2.134223	2.140779	2.136968	2.133219	2.129432	2.125644
2.135492	2.131559	2.137128	2.133222	2.139712	2.135935	2.132224	2.128485	2.12474
2.134635	2.130717	2.136255	2.132319	2.138739	2.134942	2.131214	2.127467	2.123721
2.133766	2.129869	2.13539	2.131448	2.137819	2.133997	2.130239	2.126462	2.122692
2.132774	2.128904	2.145186	2.140991	2.136969	2.133157	2.141653	2.137634	2.133618
2.131746	2.1279	2.144097	2.140065	2.13614	2.132347	2.140791	2.136715	2.132631
2.140308	2.136323	2.143006	2.139072	2.135206	2.131423	2.139826	2.135766	2.131697
2.139379	2.135345	2.141914	2.138033	2.134203	2.130423	2.138794	2.134802	2.130804
2.13844	2.134418	2.140919	2.13708	2.133266	2.129468	2.137778	2.133809	2.12984
2.13749	2.133556	2.140044	2.136236	2.132416	2.128566	2.136773	2.132772	2.128783
2.136535	2.132677	2.139152	2.135361	2.131539	2.127667	2.135804	2.13178	2.127769
2.135558	2.131727	2.138174	2.134386	2.130586	2.126766	2.134912	2.1309	2.126878
2.134604	2.130788	2.1372	2.133397	2.129613	2.125849	2.134011	2.130018	2.125991
2.144381	2.140287	2.136309	2.132456	2.140884	2.136943	2.138162	2.134135	2.130081
2.143488	2.139445	2.135456	2.131541	2.139873	2.135901	2.137084	2.133056	2.129019
2.142512	2.138532	2.134573	2.130664	2.138952	2.134957	2.136099	2.132067	2.128037
2.141472	2.137564	2.133669	2.129824	2.13811	2.134095	2.135187	2.131151	2.127116
2.140506	2.136641	2.132788	2.128985	2.13725	2.133205	2.134242	2.130191	2.12615
2.139645	2.135788	2.131947	2.128153	2.136368	2.132268	2.133235	2.129154	2.125107
2.1388	2.134928	2.143457	2.139487	2.140564	2.1364	2.13225	2.128144	2.124087
2.137933	2.134007	2.142465	2.138485	2.139556	2.135444	2.131334	2.127227	2.123152
2.137064	2.133084	2.141466	2.137468	2.138537	2.134497	2.130441	2.126335	2.12224
2.136189	2.132222	2.140565	2.136551	2.137596	2.13358	2.129539	2.125406	2.121282
2.135313	2.131396	2.139718	2.135684	2.136691	2.132676	2.128628	2.124461	2.120309
2.134463	2.130561	2.138839	2.134766	2.135725	2.131699	2.131132	2.126921	2.122734
2.133641	2.129724	2.137932	2.133799	2.134696	2.130645	2.130063	2.125939	2.121848
2.132795	2.128853	2.137006	2.132855	2.133718	2.129649	2.12905	2.124987	2.120963
2.131907	2.127922	2.136043	2.131951	2.132825	2.128753	2.128131	2.124087	2.120075
2.131008	2.127	2.135101	2.131071	2.13196	2.127888	2.127239	2.123191	2.11917
2.142464	2.138308	2.139199	2.135157	2.134578	2.130456	2.126351	2.122267	2.118211
2.14153	2.137442	2.138342	2.134308	2.133708	2.129581	2.125459	2.121332	2.11724
2.140646	2.136583	2.137478	2.133453	2.13282	2.128653	2.124504	2.12039	2.116326
2.139801	2.135739	2.136616	2.132595	2.13192	2.12769	2.123508	2.119444	2.115449
2.138967	2.134912	2.135755	2.131699	2.130982	2.12675	2.12258	2.118547	2.114587
2.138149	2.134109	2.134892	2.130755	2.129994	2.125834	2.121727	2.117705	2.113739
2.142235	2.138175	2.134034	2.129839	2.129045	2.124943	2.120887	2.116874	2.112901
2.141384	2.137331	2.133191	2.128994	2.12818	2.124087	2.120041	2.116044	2.112077

2.14052	2.136461	2.132332	2.128158	2.127338	2.123244	2.119198	2.115213	2.111252
2.139619	2.13552	2.131402	2.127279	2.126474	2.122397	2.126628	2.122521	2.118441
2.138707	2.134554	2.13045	2.126388	2.12561	2.121552	2.125763	2.121651	2.117566
2.137845	2.133676	2.132895	2.128817	2.124757	2.120716	2.12489	2.120744	2.116628
2.137024	2.132869	2.132066	2.127977	2.123914	2.119886	2.12401	2.119804	2.115635
2.136205	2.13206	2.131238	2.127142	2.123077	2.119056	2.123137	2.118895	2.114694
2.135386	2.131244	2.130406	2.126313	2.122248	2.118225	2.122273	2.118025	2.113821
2.134571	2.130429	2.129575	2.125485	2.121421	2.117395	2.121414	2.117169	2.112971
2.133763	2.129616	2.128742	2.124657	2.120595	2.116568	2.12056	2.116321	2.11213
2.132957	2.128802	2.127907	2.123825	2.119767	2.115741	2.119709	2.115482	2.111304
2.132147	2.127981	2.127064	2.122983	2.127164	2.123012	2.121057	2.116802	2.112597
2.131334	2.127156	2.126216	2.122134	2.126289	2.122149	2.120193	2.115958	2.111772
2.13052	2.126336	2.125376	2.121296	2.125425	2.121296	2.119338	2.115125	2.110958
2.129704	2.12552	2.124542	2.120467	2.124571	2.120453	2.118494	2.114301	2.110154
2.128888	2.124701	2.123707	2.119638	2.123717	2.11961	2.117647	2.113475	2.109347
2.128069	2.123873	2.122862	2.118804	2.122861	2.118762	2.116794	2.112639	2.108526
2.127255	2.123056	2.122027	2.117974	2.122006	2.117917	2.115945	2.111809	2.107714
2.126445	2.122255	2.121204	2.117147	2.123317	2.119201	2.115105	2.110992	2.106916
2.125648	2.12148	2.120407	2.116335	2.122452	2.11835	2.114275	2.11019	2.10614
2.127981	2.123816	2.11968	2.115566	2.121612	2.117514	2.113458	2.109423	2.105419
2.127227	2.123141	2.119003	2.11483	2.120793	2.11669	2.112653	2.108682	2.104737
2.126444	2.122402	2.126396	2.122081	2.119983	2.115877	2.111847	2.107912	2.104001
2.125627	2.121594	2.125571	2.12128	2.119178	2.11507	2.111039	2.10711	2.103208
2.124813	2.120778	2.124751	2.120508	2.118412	2.114293	2.110249	2.106312	2.102408
2.124006	2.119965	2.123961	2.119801	2.117719	2.113564	2.109482	2.105523	2.101612
2.12322	2.119162	2.123172	2.119083	2.117019	2.112847	2.108738	2.104748	2.100816
2.120135	2.116037	2.12446	2.120343	2.116234	2.112121	2.108042	2.104012	2.100025
2.119428	2.115284	2.12362	2.119489	2.115406	2.111381	2.107359	2.103288	2.099236
2.118687	2.114571	2.122839	2.118663	2.11457	2.110586	2.106594	2.102512	2.098439
2.117923	2.113896	2.122103	2.117854	2.113722	2.109742	2.105764	2.101696	2.097637
2.127769	2.123604	2.121317	2.117035	2.112884	2.108902	2.104934	2.1009	2.096875
2.126919	2.122743	2.120458	2.116193	2.112051	2.10806	2.104103	2.100133	2.096174
2.126096	2.121894	2.119615	2.115385	2.111257	2.107248	2.103287	2.09936	2.09545
2.125343	2.121097	2.118829	2.114652	2.110537	2.106487	2.102489	2.098551	2.094648
2.124605	2.120327	2.118072	2.113942	2.10984	2.105755	2.10172	2.097751	2.093834
2.12383	2.119591	2.115409	2.111274	2.109106	2.105041	2.101014	2.097014	2.093063
2.123035	2.118871	2.114702	2.110535	2.108362	2.104345	2.100346	2.096315	2.092319
2.122212	2.118083	2.113944	2.109807	2.107651	2.103638	2.09964	2.095615	2.091618
2.121358	2.11723	2.113141	2.109093	2.106974	2.102925	2.098902	2.094916	2.090963
2.12055	2.116399	2.112335	2.10835	2.106265	2.102211	2.098185	2.094221	2.09029
2.119809	2.115594	2.111516	2.107552	2.105498	2.101497	2.097507	2.09353	2.089579
2.119092	2.114837	2.110744	2.106785	2.104745	2.100785	2.096824	2.092839	2.088875
2.118373	2.114163	2.110086	2.10612	2.10215	2.098166	2.0961	2.092142	2.088212
2.117674	2.113532	2.109473	2.10549	2.101493	2.097464	2.09537	2.091445	2.087558
2.117025	2.112901	2.108832	2.104815	2.1008	2.096773	2.094683	2.090757	2.086872
2.116413	2.112275	2.108178	2.104119	2.10009	2.096091	2.094019	2.090074	2.086167
2.115769	2.111612	2.107504	2.103436	2.099402	2.0954	2.093328	2.08939	2.085493
2.115093	2.110906	2.106804	2.102769	2.09874	2.094702	2.092605	2.088705	2.084852
2.114406	2.11021	2.10611	2.102088	2.098061	2.094008	2.091904	2.088027	2.084201
2.113706	2.109548	2.105437	2.101371	2.097335	2.093323	2.09125	2.087359	2.083514

2.113002	2.108879	2.104762	2.100661	2.09662	2.092646	2.090603	2.0867	2.08284
2.116216	2.112076	2.104057	2.099991	2.095965	2.091974	2.088035	2.08416	2.080337
2.115506	2.111339	2.10335	2.099346	2.095341	2.091315	2.087357	2.083523	2.079753
2.114794	2.11066	2.102701	2.098719	2.094728	2.090703	2.09252	2.088652	2.084842
2.114083	2.110026	2.102099	2.098111	2.094127	2.090129	2.091953	2.088078	2.084252
2.11341	2.109389	2.101472	2.097465	2.093484	2.089522	2.091363	2.087478	2.083632
2.112783	2.108752	2.100812	2.096766	2.092784	2.088876	2.090749	2.086844	2.082969
2.112159	2.108101	2.104028	2.09996	2.092097	2.088218	2.091317	2.087396	2.083503
2.111514	2.107414	2.103341	2.099303	2.091452	2.087545	2.090624	2.086736	2.082886
2.110874	2.106734	2.102669	2.09867	2.090832	2.086885	2.089936	2.086085	2.082283
2.110267	2.106119	2.102058	2.098073	2.09024	2.086288	2.089324	2.085479	2.081682
2.109674	2.10554	2.101485	2.097499	2.089669	2.085728	2.088753	2.0849	2.081085
2.109044	2.104944	2.100904	2.096921	2.092965	2.08903	2.088181	2.084321	2.080495
2.108381	2.104337	2.100322	2.096344	2.092393	2.088465	2.087614	2.083748	2.079913
2.107748	2.103733	2.099722	2.095732	2.091781	2.087872	2.087026	2.083144	2.07929
2.107159	2.10313	2.099093	2.095064	2.091106	2.087233	2.086408	2.082492	2.078599
2.106588	2.10254	2.098476	2.094413	2.090446	2.086602	2.08978	2.085822	2.081879
2.106031	2.101978	2.097904	2.093828	2.089855	2.086013	2.089187	2.085223	2.081275
2.105481	2.101425	2.09735	2.093272	2.089293	2.085443	2.088605	2.084646	2.080704
2.104921	2.100863	2.096788	2.092714	2.090161	2.086292	2.088031	2.084073	2.080136
2.104357	2.100297	2.096227	2.092162	2.089608	2.085733	2.087463	2.083509	2.079576
2.103797	2.099733	2.095665	2.091606	2.089051	2.085172	2.086898	2.082946	2.079019
2.103243	2.099171	2.095102	2.091045	2.08849	2.08461	2.086333	2.082386	2.078464
2.102686	2.098611	2.094542	2.09049	2.087934	2.084053	2.085773	2.08183	2.077913
2.102123	2.098051	2.093989	2.089945	2.087387	2.083502	2.085217	2.081279	2.077368
2.101559	2.097493	2.093437	2.089402	2.086844	2.082955	2.084665	2.080731	2.076827
2.100997	2.096932	2.092882	2.088856	2.086299	2.082411	2.084119	2.080191	2.076292
2.100436	2.096373	2.092329	2.088312	2.085757	2.08187	2.083578	2.079654	2.075761
2.088188	2.084115	2.091789	2.087777	2.085219	2.08133	2.083036	2.079118	2.075231
2.087632	2.083572	2.09126	2.087249	2.084685	2.080791	2.082495	2.078583	2.074704
2.087076	2.083027	2.090728	2.086719	2.08415	2.080254	2.081958	2.078051	2.074176
2.086519	2.082479	2.090192	2.086182	2.08361	2.079719	2.081425	2.07752	2.073647
2.085972	2.081938	2.077904	2.073882	2.083081	2.079192	2.080898	2.076993	2.073119
2.085431	2.081406	2.077377	2.073359	2.082558	2.078667	2.080371	2.076464	2.072589
2.084913	2.080886	2.076864	2.072859	2.082061	2.078161	2.079854	2.075944	2.072067
2.084451	2.080391	2.084725	2.080701	2.087274	2.083303	2.07936	2.07544	2.071561
2.084017	2.079911	2.08425	2.080279	2.086873	2.082861	2.07888	2.074952	2.071072
2.083535	2.079419	2.083757	2.079803	2.086407	2.082383	2.078397	2.074488	2.07063
2.083012	2.078919	2.08325	2.079281	2.085887	2.081875	2.077913	2.074051	2.070234
2.082502	2.078444	2.082765	2.078765	2.07477	2.070763	2.077419	2.073586	2.069796
2.082016	2.078013	2.08232	2.078264	2.074237	2.070234	2.076904	2.073077	2.069294
2.081522	2.077562	2.081859	2.077762	2.073716	2.069724	2.076411	2.072574	2.068783
2.080992	2.077044	2.081345	2.077249	2.073219	2.069266	2.07598	2.072115	2.068289
2.080461	2.076505	2.080813	2.076741	2.072739	2.068824	2.075561	2.071662	2.067797
2.079978	2.07599	2.080304	2.076274	2.07229	2.068362	2.075091	2.071174	2.067295
2.079523	2.075486	2.079803	2.075827	2.071858	2.067889	2.074591	2.070669	2.066792
2.079038	2.074979	2.07929	2.075335	2.071373	2.067394	2.063438	2.059508	2.055622
2.078523	2.074467	2.078762	2.074794	2.070833	2.066873	2.062942	2.059049	2.055194
2.078028	2.073988	2.078268	2.074275	2.070311	2.066374	2.06247	2.058597	2.054758
2.077566	2.073566	2.077832	2.073796	2.06982	2.065916	2.062037	2.058151	2.054295

2.07712	2.07315	2.077407	2.073341	2.069356	2.065473	2.061608	2.057709	2.053838
2.076687	2.072696	2.076956	2.072917	2.068939	2.065034	2.061154	2.05727	2.053417
2.076261	2.072236	2.076503	2.072508	2.068541	2.064601	2.060696	2.056832	2.053002
2.075826	2.071815	2.076078	2.072077	2.068108	2.064172	2.060267	2.056389	2.052542
2.075385	2.071419	2.075672	2.071633	2.067653	2.063747	2.059856	2.055942	2.05205
2.074951	2.070991	2.07524	2.071199	2.067218	2.063315	2.05942	2.055483	2.051569
2.074526	2.070522	2.074774	2.070779	2.06681	2.062873	2.058948	2.055002	2.051092
2.074094	2.070065	2.074318	2.070351	2.066393	2.062441	2.058503	2.05456	2.05066
2.073646	2.069652	2.073897	2.069903	2.065945	2.06203	2.058127	2.054201	2.050304
2.073196	2.069239	2.073475	2.069454	2.065498	2.061623	2.057758	2.053859	2.049975
2.072759	2.068781	2.073018	2.069026	2.065083	2.061198	2.057335	2.053477	2.049639
2.072327	2.068306	2.072549	2.068606	2.064681	2.060767	2.056893	2.05308	2.049299
2.071882	2.067866	2.072107	2.06817	2.064253	2.060347	2.056483	2.05268	2.04891
2.071426	2.067454	2.071687	2.067721	2.063802	2.059936	2.056099	2.052273	2.048479
2.060228	2.056343	2.071258	2.067295	2.063382	2.059527	2.055696	2.051866	2.048068
2.059823	2.05589	2.070806	2.066898	2.063004	2.059113	2.055258	2.051454	2.047688
2.061517	2.057517	2.070383	2.066524	2.062645	2.058724	2.054846	2.051055	2.047307
2.061152	2.057149	2.070027	2.066184	2.062312	2.05839	2.0545	2.050676	2.046897
2.060789	2.056804	2.069694	2.065848	2.061979	2.058069	2.054179	2.050316	2.046496
2.060378	2.056436	2.069336	2.065461	2.061589	2.057714	2.05385	2.049984	2.046154
2.059943	2.056063	2.068969	2.06505	2.06117	2.057339	2.053518	2.04967	2.045849
2.056413	2.052568	2.058605	2.054749	2.060788	2.056952	2.053136	2.049317	2.045525
2.056083	2.052229	2.05826	2.054409	2.06044	2.056549	2.052702	2.048927	2.045186
2.055747	2.051871	2.055033	2.051222	2.060106	2.056175	2.052304	2.048553	2.044844
2.05539	2.051471	2.054644	2.050881	2.059789	2.055852	2.05197	2.048217	2.044507
2.05504	2.051084	2.054264	2.05054	2.059471	2.055538	2.051651	2.04788	2.04415
2.054718	2.050755	2.046949	2.043272	2.050088	2.046222	2.05132	2.047509	2.043741
2.054409	2.050452	2.046637	2.042935	2.049746	2.045897	2.050989	2.047132	2.043319
2.054103	2.050146	2.046306	2.04256	2.049362	2.045546	2.048168	2.044305	2.040479
2.053801	2.049842	2.045967	2.042161	2.048948	2.045178	2.047844	2.04399	2.040163
2.053494	2.049542	2.04565	2.041806	2.038039	2.03437	2.047525	2.043679	2.039853
2.053188	2.04925	2.045357	2.041502	2.037727	2.034054	2.047212	2.043371	2.03955
2.052853	2.048945	2.045062	2.041205	2.037426	2.033747	2.046906	2.043068	2.039246
2.052463	2.048611	2.044754	2.040903	2.037127	2.033448	2.04661	2.042768	2.038936
2.052075	2.048275	2.044444	2.040603	2.036833	2.033154	2.046318	2.042472	2.03863
2.040826	2.037061	2.044136	2.040303	2.036536	2.032858	2.046026	2.04218	2.038334
2.040518	2.036747	2.043831	2.040004	2.036241	2.032563	2.045737	2.041892	2.038047
2.036974	2.033222	2.043539	2.039713	2.035951	2.032273	2.035383	2.031607	2.027828
2.036686	2.032939	2.043259	2.039429	2.035665	2.031986	2.035096	2.031324	2.02755
2.036412	2.032661	2.032092	2.028285	2.035384	2.031707	2.034817	2.031049	2.027279
2.036147	2.032386	2.031815	2.028007	2.03511	2.031437	2.034549	2.030783	2.027015
2.035907	2.032124	2.031545	2.027732	2.034838	2.031169	2.027503	2.023788	2.020073
2.035715	2.031885	2.031284	2.02746	2.034563	2.030898	2.027238	2.023529	2.019821
2.035528	2.031652	2.031029	2.027195	2.034295	2.030631	2.026975	2.023272	2.019572
2.024959	2.021033	2.030778	2.026941	2.023189	2.019543	2.026715	2.023018	2.019331
2.024688	2.020775	2.030532	2.026699	2.022946	2.01929	2.02646	2.022767	2.019094
2.02441	2.020509	2.027069	2.023284	2.022746	2.019068	2.02622	2.02252	2.018849
2.024122	2.020233	2.026836	2.023111	2.022593	2.018877	2.025994	2.022276	2.018596
2.023846	2.019967	2.016164	2.012427	2.022407	2.018669	2.01495	2.011249	2.007592
2.023588	2.01971	2.015908	2.012176	2.022163	2.018427	2.01471	2.011013	2.007358

2.023344	2.019474	2.015667	2.011921	2.021908	2.018182	2.014477	2.010793	2.007148
2.023123	2.019289	2.015476	2.011693	2.021665	2.017945	2.014257	2.010609	2.006993
2.022909	2.019114	2.015294	2.011469	2.021424	2.017717	2.014052	2.010441	2.006854
2.022658	2.018879	2.01506	2.011226	2.007461	2.003776	2.01388	2.010266	2.006668
2.022387	2.018605	2.014793	2.010972	2.007232	2.003593	2.013729	2.010089	2.006453
2.022156	2.018351	2.014532	2.010718	2.006992	2.003379	2.013537	2.00989	2.006243
2.021971	2.018116	2.014276	2.010459	2.006734	2.003127	2.013298	2.009663	2.006034
2.015135	2.011269	2.014042	2.010232	2.006508	2.002891	2.002898	1.99925	1.995616
2.014903	2.011042	2.013839	2.01006	2.006343	2.002694	2.002681	1.999063	1.995464
2.014673	2.010834	2.013656	2.009905	2.006189	2.002505	2.00247	1.998876	1.995305
2.014476	2.010665	2.013494	2.009729	2.005999	2.002298	2.002249	1.998653	1.995085
2.014298	2.010515	2.013346	2.009554	2.005804	2.002095	2.002036	1.998423	1.994842
2.014128	2.010342	2.013186	2.009406	2.005662	2.001951	1.99828	1.994662	1.991075
2.013969	2.010153	2.013018	2.009282	2.005562	2.001855	1.998173	1.994517	1.990891
2.013806	2.009984	2.012857	2.009127	2.005414	2.001716	1.998037	1.994373	1.990735
2.013637	2.009845	2.012708	2.008931	2.005199	2.001515	1.99786	1.994228	1.990617
2.006356	2.002596	2.01255	2.008731	2.004983	2.001317	1.997686	1.99408	1.990488
2.006207	2.002415	2.01236	2.008532	2.004789	2.00115	1.997539	1.993923	1.990317
2.006058	2.002231	2.012173	2.008345	2.00461	2.000991	1.997391	1.993761	1.990139
2.00589	2.002083	1.998275	1.994472	2.004462	2.000825	1.997214	1.993594	1.989985
2.005714	2.001951	1.998159	1.994354	2.004332	2.000657	1.997023	1.993423	1.989841
2.005544	2.00178	1.997988	1.994186	1.997425	1.993766	1.996858	1.993256	1.98967
2.00538	2.001572	1.997766	1.993971	1.997233	1.993607	1.996717	1.993091	1.989476
2.005216	2.001393	1.997585	1.993796	1.997068	1.993447	1.989845	1.986219	1.982603
2.005049	2.001268	1.997477	1.99369	1.996945	1.993281	1.98965	1.98604	1.982447
2.004892	2.00114	1.997366	1.99359	1.996836	1.993133	1.989473	1.985871	1.982292
2.004757	2.000964	1.997197	1.993463	1.996735	1.993031	1.989357	1.985721	1.982111
2.004638	2.000786	1.997018	1.993324	1.996631	1.992942	1.989266	1.985588	1.981933
1.999919	1.996072	1.996889	1.99317	1.98949	1.985855	1.989165	1.98548	1.981806
1.999845	1.996006	1.996791	1.993007	1.989304	1.985707	1.989061	1.985389	1.981714
1.999728	1.995918	1.996696	1.992882	1.98916	1.985554	1.988916	1.985264	1.981608
1.999565	1.99581	1.996608	1.992801	1.989059	1.985392	1.98872	1.985099	1.98149
1.999409	1.995675	1.99649	1.992706	1.988959	1.985251	1.988553	1.98494	1.981354
1.999269	1.995487	1.996309	1.992574	1.988856	1.985149	1.988442	1.984796	1.981181
1.999152	1.995312	1.996132	1.99243	1.988739	1.985054	1.988353	1.98467	1.981019
1.999079	1.99522	1.991438	1.987724	1.98858	1.984935	1.981298	1.977625	1.973977
1.999031	1.995168	1.991351	1.987572	1.988412	1.984813	1.981218	1.977554	1.973901
1.998974	1.995119	1.991288	1.987477	1.988303	1.984714	1.981129	1.977468	1.973814
1.998915	1.995078	1.991249	1.987429	1.988241	1.984631	1.981032	1.977375	1.973723
1.996351	1.992569	1.991203	1.98738	1.983645	1.980021	1.980906	1.977263	1.97363
1.996215	1.992484	1.991146	1.987328	1.983573	1.979888	1.980735	1.977121	1.973533
1.996101	1.992407	1.991091	1.98728	1.983509	1.979774	1.980591	1.976995	1.973441
1.996046	1.992355	1.991046	1.987238	1.983461	1.979709	1.98051	1.976911	1.973357
1.99601	1.992308	1.991	1.987196	1.983419	1.979661	1.980455	1.976844	1.973279
1.995963	1.992255	1.988496	1.984711	1.983372	1.979609	1.975906	1.972306	1.968752
1.995913	1.992193	1.988433	1.984654	1.983322	1.979559	1.975854	1.972247	1.968688
1.995838	1.992086	1.988328	1.984575	1.983264	1.979507	1.975802	1.972192	1.968628
1.995739	1.991933	1.988179	1.984474	1.983199	1.979452	1.975752	1.972141	1.968574
1.995663	1.99182	1.988065	1.984387	1.983136	1.979397	1.975701	1.972089	1.968518
1.995621	1.991764	1.988002	1.984321	1.983074	1.979341	1.975649	1.972034	1.968459

1.991877	1.988038	1.987962	1.984268	1.980566	1.97684	1.975598	1.97198	1.968402
1.991859	1.988019	1.987932	1.984227	1.980519	1.97679	1.975549	1.97193	1.96835
1.991854	1.988023	1.987925	1.984202	1.980481	1.976747	1.975502	1.971882	1.968302
1.984692	1.9809	1.987969	1.984207	1.980458	1.976712	1.97546	1.971836	1.968256
1.984725	1.98098	1.988047	1.984233	1.980447	1.976684	1.975422	1.971794	1.968212
1.984725	1.981006	1.981177	1.977343	1.980427	1.97665	1.972932	1.969303	1.965723
1.984689	1.980976	1.981146	1.977315	1.980398	1.97661	1.972883	1.969252	1.965672
1.984658	1.980941	1.981107	1.97728	1.973477	1.969691	1.972842	1.969207	1.965624
1.984637	1.980909	1.981067	1.977237	1.973434	1.969653	1.972807	1.969165	1.965576
1.984636	1.980887	1.98103	1.977196	1.973397	1.969633	1.972798	1.969145	1.96554
1.984681	1.980889	1.977081	1.973269	1.973382	1.969661	1.972855	1.969179	1.965536
1.984742	1.9809	1.97707	1.973251	1.973377	1.969706	1.972935	1.969231	1.965547
1.984754	1.980894	1.977054	1.973231	1.973357	1.969704	1.966059	1.962354	1.958666
1.984732	1.980875	1.977035	1.973211	1.97333	1.969671	1.966025	1.962325	1.95865
1.975663	1.971832	1.977041	1.973232	1.973349	1.969668	1.966004	1.962302	1.958632
1.975665	1.971842	1.977075	1.973305	1.973428	1.969705	1.965999	1.962281	1.958604
1.975681	1.97187	1.977121	1.97337	1.969636	1.965914	1.966005	1.962283	1.958607
1.975716	1.971933	1.977184	1.9734	1.969647	1.965925	1.96602	1.962326	1.958675
1.975761	1.971997	1.977241	1.973418	1.969648	1.965939	1.96605	1.962384	1.958754
1.97581	1.97202	1.968218	1.964414	1.969667	1.965984	1.966113	1.962437	1.958787
1.975861	1.972024	1.968211	1.964421	1.969695	1.966045	1.966192	1.962488	1.958801
1.975889	1.97204	1.968233	1.964461	1.969748	1.966094	1.966233	1.962515	1.958819
1.975895	1.972064	1.968277	1.964532	1.969825	1.966134	1.96624	1.96252	1.958835
1.975916	1.972107	1.968328	1.964584	1.960867	1.957176	1.966253	1.962546	1.958883
1.975955	1.972174	1.968387	1.964606	1.96087	1.957182	1.966271	1.962603	1.958978
1.976008	1.972243	1.968442	1.964625	1.960874	1.957198	1.962537	1.958929	1.955358
1.976076	1.972289	1.968473	1.964642	1.960894	1.957242	1.962609	1.958995	1.955416
1.976153	1.97234	1.96851	1.964674	1.960931	1.957302	1.962692	1.959066	1.955473
1.976226	1.972432	1.968606	1.964764	1.961013	1.95737	1.959438	1.955803	1.952204
1.9763	1.972548	1.968737	1.96489	1.961124	1.957449	1.959504	1.95589	1.952318
1.976382	1.972632	1.968822	1.964975	1.961208	1.957531	1.959591	1.955979	1.952409
1.976473	1.972682	1.968857	1.965014	1.961261	1.957615	1.959701	1.956069	1.952476
1.976572	1.972765	1.968936	1.965099	1.961352	1.957715	1.95981	1.956168	1.952563
1.976682	1.972913	1.969102	1.965267	1.961506	1.957831	1.959906	1.956276	1.952689
1.976797	1.973066	1.969276	1.965452	1.961681	1.957966	1.960016	1.956394	1.952819
1.976917	1.973174	1.969401	1.965617	1.961863	1.958138	1.960177	1.956522	1.952919
1.97704	1.973272	1.969513	1.965771	1.962041	1.958317	1.960353	1.956658	1.953017
1.977161	1.973401	1.96964	1.965891	1.962158	1.958436	1.960479	1.95679	1.953152
1.977281	1.973549	1.969776	1.965985	1.962231	1.958512	1.960574	1.956921	1.953313
1.977422	1.973686	1.969905	1.966106	1.96235	1.958636	1.960712	1.957079	1.953488
1.977586	1.973801	1.970022	1.96626	1.962527	1.958817	1.960902	1.957271	1.953681
1.977763	1.973943	1.970159	1.966416	1.962698	1.959	1.961097	1.957458	1.953856
1.977959	1.974136	1.970331	1.966552	1.962826	1.959161	1.961283	1.957616	1.953981
1.978153	1.974346	1.970525	1.966703	1.962963	1.959327	1.961472	1.957777	1.954109
1.978311	1.974548	1.970739	1.966906	1.96316	1.959524	1.961674	1.957975	1.954296
1.978455	1.974752	1.970967	1.967139	1.963387	1.959733	1.961877	1.958186	1.954509
1.978642	1.974957	1.971185	1.967364	1.963603	1.959914	1.962038	1.958356	1.954694
1.978865	1.975166	1.971396	1.967589	1.963814	1.960071	1.962162	1.958492	1.954857
1.979074	1.975343	1.971575	1.967793	1.96402	1.960244	1.962312	1.958631	1.954995
1.979258	1.975471	1.971704	1.96797	1.964221	1.960442	1.962499	1.95877	1.955093

1.979449	1.975614	1.971846	1.968144	1.964415	1.960643	1.9627	1.958931	1.955215
1.979664	1.975815	1.97204	1.968334	1.964606	1.960844	1.962914	1.959136	1.955409
1.979889	1.976038	1.972249	1.968519	1.964784	1.961038	1.963131	1.959358	1.95563
1.980117	1.976253	1.972437	1.968666	1.96492	1.961205	1.963331	1.959567	1.95584
1.980347	1.97647	1.972618	1.968794	1.965034	1.961356	1.963522	1.959772	1.95605
1.980577	1.976694	1.972822	1.968968	1.965198	1.961535	1.963723	1.959982	1.95626
1.980811	1.976927	1.97305	1.969187	1.96541	1.961741	1.963931	1.960195	1.956472
1.972125	1.968301	1.973273	1.969412	1.965633	1.961956	1.964146	1.960412	1.956688
1.972259	1.968478	1.973483	1.969637	1.965863	1.962179	1.96437	1.960635	1.95691
1.972402	1.968656	1.973693	1.969863	1.966096	1.962407	1.964599	1.960863	1.957137
1.972604	1.96885	1.973902	1.970085	1.985579	1.981732	1.98325	1.979337	1.975428
1.972835	1.969056	1.974116	1.970308	1.985812	1.981968	1.983494	1.979578	1.975665
1.962449	1.958694	1.974346	1.970541	1.986047	1.9822	1.98373	1.979813	1.975898
1.96269	1.958927	1.97459	1.97078	1.986285	1.982431	1.983962	1.980043	1.976128
1.962944	1.959165	1.965962	1.962201	1.986525	1.982667	1.9842	1.980279	1.976361
1.963207	1.959404	1.966196	1.962433	1.986767	1.98291	1.984447	1.980519	1.976596
1.963499	1.959662	1.955868	1.952125	1.98701	1.983151	1.984693	1.980763	1.976839
1.963846	1.959951	1.956122	1.952357	1.987248	1.983386	1.984936	1.981011	1.977099
1.964192	1.960244	1.956384	1.952599	1.987494	1.983625	1.985179	1.981258	1.977359
1.964474	1.960514	1.956646	1.952856	1.987756	1.983872	1.98542	1.981497	1.977601
1.964726	1.960772	1.956911	1.953129	1.988038	1.984132	1.985666	1.981736	1.977839
1.964983	1.96103	1.957186	1.953431	1.978927	1.975011	1.985941	1.981997	1.978091
1.965239	1.961286	1.957468	1.953762	1.979299	1.975343	1.986243	1.982276	1.978356
1.965506	1.96155	1.957745	1.954065	1.970942	1.967006	1.98654	1.982578	1.978666
1.965783	1.961819	1.958008	1.954326	1.971202	1.967258	1.986829	1.982914	1.979043
1.966073	1.962111	1.958289	1.95459	1.971457	1.967512	1.978057	1.974203	1.970389
1.966387	1.962449	1.958618	1.954882	1.971728	1.967777	1.978336	1.974479	1.970666
1.966705	1.962795	1.958955	1.955182	1.972009	1.96806	1.978634	1.974756	1.970925
1.966996	1.963095	1.959253	1.955473	1.972309	1.968391	1.978998	1.975092	1.971227
1.967276	1.963371	1.959533	1.955762	1.972625	1.968753	1.979401	1.975459	1.971549
1.967595	1.963664	1.959828	1.95608	1.972969	1.969096	1.97143	1.967504	1.96361
1.967952	1.96397	1.960136	1.956429	1.973345	1.969423	1.971726	1.967786	1.963888
1.961136	1.957131	1.960443	1.956753	1.973685	1.969735	1.972028	1.968094	1.964204
1.961441	1.957429	1.960734	1.957028	1.97396	1.970021	1.972341	1.968443	1.964582
1.961754	1.957751	1.961048	1.957313	1.974239	1.970323	1.972678	1.968808	1.964968
1.962104	1.958133	1.961421	1.957644	1.974564	1.970681	1.973067	1.969182	1.96532
1.962474	1.958538	1.961818	1.958	1.974915	1.971064	1.973476	1.969564	1.96567
1.962857	1.958911	1.955039	1.951238	1.975298	1.971435	1.973849	1.969945	1.966056
1.963253	1.959267	1.955396	1.951623	1.975708	1.971805	1.974204	1.970326	1.966466
1.951789	1.947821	1.955776	1.951995	1.976089	1.972181	1.974583	1.9707	1.966829
1.952162	1.948225	1.956183	1.952353	1.976436	1.972566	1.974995	1.971067	1.967141
1.95254	1.948616	1.956579	1.952717	1.970259	1.966421	1.975385	1.97142	1.967451
1.952929	1.948972	1.956944	1.953099	1.970654	1.966775	1.975719	1.971736	1.967763
1.953317	1.949327	1.957306	1.953476	1.971044	1.967128	1.976056	1.972067	1.9681
1.953692	1.949714	1.957692	1.953825	1.971386	1.967496	1.970272	1.966304	1.962346
1.954062	1.950111	1.958082	1.954159	1.971709	1.967865	1.970691	1.966742	1.962784
1.948611	1.944696	1.94653	1.942618	1.972056	1.968204	1.971043	1.967125	1.9632
1.949012	1.945056	1.946851	1.942955	1.972418	1.968516	1.971342	1.967467	1.963601
1.949409	1.945434	1.947188	1.943281	1.972756	1.968841	1.964979	1.961166	1.957368
1.949794	1.945838	1.947549	1.943588	1.973055	1.969183	1.965347	1.961499	1.957663

1.94743	1.943463	1.947913	1.943916	1.962242	1.95841	1.965704	1.961829	1.957965
1.947864	1.943849	1.948261	1.944286	1.962623	1.95874	1.966007	1.962148	1.958312
1.948317	1.944249	1.948625	1.944683	1.963037	1.959086	1.966314	1.962473	1.958668
1.948788	1.944707	1.949052	1.945111	1.963477	1.95949	1.966693	1.962825	1.959001
1.949271	1.945201	1.949516	1.945557	1.963932	1.959926	1.967115	1.963198	1.959331
1.949721	1.945678	1.944473	1.940509	1.964329	1.960328	1.956382	1.952467	1.948604
1.950138	1.946142	1.944921	1.940898	1.96467	1.960701	1.956785	1.95288	1.949019
1.950585	1.946614	1.945382	1.941319	1.965051	1.961071	1.957156	1.953269	1.949429
1.951085	1.947107	1.945869	1.941797	1.965495	1.961431	1.957465	1.953611	1.949821
1.95158	1.947581	1.946344	1.942282	1.96596	1.961816	1.957797	1.953966	1.950213
1.952027	1.947995	1.946768	1.942743	1.96643	1.962262	1.95822	1.95438	1.950622
1.952462	1.948396	1.947183	1.9432	1.966907	1.962735	1.958678	1.954811	1.951027
1.952928	1.948852	1.944827	1.940843	1.962719	1.958546	1.959112	1.955205	1.951387
1.953411	1.949343	1.945319	1.941331	1.963222	1.959027	1.959538	1.955582	1.95172
1.926017	1.92204	1.945788	1.941776	1.963672	1.959484	1.959971	1.955991	1.952103
1.926413	1.922482	1.946231	1.94217	1.964055	1.959909	1.960411	1.956434	1.952538
1.894882	1.891052	1.933131	1.929056	1.938588	1.934668	1.93372	1.929977	1.926302
1.89535	1.891519	1.933627	1.929555	1.939082	1.935143	1.934181	1.930438	1.926768
1.895837	1.892	1.934139	1.930077	1.939602	1.935637	1.934654	1.930908	1.927243
1.886349	1.882531	1.905849	1.901837	1.926589	1.92263	1.935144	1.931394	1.927725
1.886838	1.883022	1.906357	1.902344	1.927108	1.923141	1.935649	1.931891	1.928214
1.887324	1.883506	1.889584	1.885671	1.927629	1.923662	1.93327	1.929479	1.925769
1.88781	1.883986	1.890069	1.886161	1.928154	1.924194	1.933801	1.929988	1.926251
1.888304	1.884473	1.880601	1.876714	1.899908	1.896011	1.920834	1.917025	1.91329
1.888806	1.884969	1.881096	1.877209	1.900419	1.896518	1.92135	1.917526	1.913774
1.889311	1.885465	1.881587	1.8777	1.891645	1.887755	1.893182	1.889407	1.885704
1.889817	1.885956	1.882071	1.87818	1.892144	1.888259	1.893687	1.889891	1.886162
1.890321	1.886444	1.88255	1.878656	1.892641	1.888762	1.894191	1.890372	1.886618
1.844758	1.841001	1.883027	1.879132	1.875287	1.871498	1.894688	1.890851	1.887076
1.845192	1.841428	1.883501	1.879605	1.875757	1.871965	1.895181	1.891327	1.887534
1.84562	1.841845	1.883963	1.880065	1.876217	1.872428	1.886396	1.88254	1.878742
1.846042	1.842252	1.884413	1.880512	1.876666	1.872886	1.886881	1.883005	1.879184
1.846463	1.842659	1.838875	1.835119	1.87713	1.873353	1.86959	1.865791	1.862035
1.846876	1.843062	1.839282	1.835541	1.87761	1.873828	1.870057	1.866246	1.862478
1.847302	1.843479	1.839705	1.835986	1.878114	1.874322	1.87054	1.866726	1.862954
1.847755	1.843929	1.840168	1.836473	1.87867	1.874857	1.87106	1.86726	1.863503
1.848227	1.844391	1.84064	1.83697	1.879235	1.875399	1.871587	1.867807	1.864071
1.848721	1.844838	1.841075	1.837416	1.833746	1.830051	1.872076	1.868303	1.864575
1.849233	1.845282	1.84149	1.837831	1.834163	1.830467	1.872542	1.86877	1.865044
1.849734	1.84575	1.841935	1.838258	1.834581	1.83089	1.873019	1.869251	1.865529
1.850223	1.846243	1.842407	1.838691	1.834995	1.831313	1.873504	1.869743	1.86603
1.850725	1.84676	1.842908	1.839152	1.835438	1.831767	1.874023	1.870258	1.866538
1.851252	1.847313	1.843448	1.839653	1.835925	1.832276	1.874604	1.870808	1.867047
1.851782	1.847868	1.843996	1.840173	1.836432	1.832793	1.875187	1.871367	1.867575
1.852294	1.848391	1.844527	1.840707	1.836955	1.833282	1.829635	1.825979	1.822351
1.852804	1.8489	1.845045	1.841243	1.837481	1.833758	1.830081	1.82646	1.822876
1.853338	1.849399	1.845532	1.841731	1.837961	1.834214	1.830522	1.826914	1.823347
1.853891	1.849889	1.845992	1.842183	1.838406	1.834653	1.830957	1.827345	1.823779
1.854458	1.850425	1.846499	1.84266	1.838868	1.835118	1.831424	1.827798	1.824218
1.855043	1.851022	1.847066	1.843164	1.839344	1.835615	1.831932	1.828278	1.824662

1.855627	1.851613	1.847635	1.84369	1.839847	1.836124	1.832444	1.828772	1.825132
1.856203	1.852164	1.848182	1.844246	1.840395	1.83664	1.832938	1.829277	1.825651
1.856773	1.85271	1.848728	1.844811	1.840956	1.837165	1.833438	1.829791	1.826183
1.857335	1.853278	1.849293	1.845361	1.841496	1.8377	1.833965	1.830305	1.826685
1.857885	1.853852	1.849863	1.845907	1.842031	1.838242	1.834508	1.830822	1.827178
1.846072	1.84208	1.850408	1.84646	1.842583	1.838783	1.835037	1.831343	1.827692
1.846538	1.842565	1.850931	1.847018	1.84315	1.839324	1.835556	1.831868	1.828225
1.847043	1.843061	1.839135	1.83526	1.843708	1.839867	1.836084	1.832393	1.828747
1.847603	1.843563	1.839625	1.835767	1.844247	1.840413	1.836629	1.832915	1.829248
1.84818	1.844105	1.840153	1.836296	1.844795	1.840961	1.837172	1.83344	1.829755
1.848749	1.844727	1.840777	1.836886	1.845374	1.841512	1.837704	1.83397	1.830287
1.849331	1.84538	1.841431	1.837493	1.84596	1.842064	1.838234	1.834502	1.830825
1.849953	1.846009	1.84204	1.838059	1.834124	1.830232	1.838776	1.835028	1.831341
1.850599	1.846627	1.842626	1.838605	1.834649	1.830763	1.839326	1.835553	1.831848
1.83528	1.831343	1.843202	1.839164	1.835193	1.831291	1.839864	1.836092	1.832394
1.835856	1.831906	1.843764	1.839737	1.835756	1.831812	1.840385	1.836641	1.832979
1.836429	1.83248	1.844336	1.840302	1.836311	1.832353	1.828494	1.824791	1.821172
1.837001	1.833078	1.844924	1.840838	1.836835	1.832918	1.829092	1.825386	1.821749
1.837585	1.833688	1.845531	1.841399	1.837386	1.83351	1.829714	1.825989	1.82232
1.787174	1.78364	1.830329	1.826259	1.838023	1.834146	1.830336	1.826569	1.822857
1.787751	1.784217	1.830977	1.826927	1.838701	1.834805	1.830965	1.827147	1.823389
1.788304	1.78476	1.831575	1.827569	1.839368	1.835457	1.831593	1.82775	1.82397
1.788839	1.785277	1.832139	1.828196	1.840033	1.836105	1.83222	1.828371	1.824586
1.78938	1.785818	1.832728	1.828816	1.84067	1.836716	1.832809	1.828964	1.825189
1.789926	1.786387	1.833351	1.829431	1.841276	1.837281	1.833349	1.829523	1.825774
1.741838	1.738436	1.783385	1.779786	1.82608	1.822119	1.833899	1.830086	1.826355
1.742382	1.738975	1.783935	1.780318	1.826611	1.822674	1.834482	1.83067	1.826928
1.742934	1.739511	1.784486	1.780855	1.827149	1.823239	1.835079	1.831261	1.827502
1.743485	1.740051	1.73656	1.733036	1.827754	1.823841	1.835681	1.831858	1.828091
1.744035	1.740584	1.737102	1.733604	1.828392	1.824463	1.836291	1.832457	1.828682
1.744558	1.741078	1.737603	1.734142	1.779023	1.775427	1.821184	1.817357	1.813585
1.745056	1.741535	1.738069	1.734654	1.779605	1.776007	1.82178	1.817919	1.814117
1.74559	1.742036	1.738573	1.73519	1.731773	1.728304	1.8224	1.818508	1.814675
1.746172	1.742599	1.739132	1.735758	1.732352	1.728895	1.82305	1.819133	1.815268
1.746772	1.743179	1.739706	1.736334	1.732934	1.729492	1.726049	1.722586	1.719167
1.747374	1.743759	1.740277	1.736908	1.733516	1.730088	1.726652	1.723175	1.719736
1.747985	1.744346	1.740853	1.737482	1.734094	1.730682	1.727253	1.723764	1.720307
1.748608	1.744943	1.741432	1.738049	1.73466	1.731262	1.727841	1.72434	1.720863
1.749239	1.745549	1.742015	1.738613	1.735218	1.731834	1.728421	1.724907	1.721411
1.749871	1.746157	1.742603	1.739183	1.735783	1.732414	1.72901	1.725483	1.721968
1.750503	1.746769	1.743197	1.73976	1.736357	1.733004	1.729609	1.726068	1.722536
1.75113	1.747377	1.743787	1.740333	1.736923	1.73358	1.730192	1.726644	1.7231
1.751743	1.747973	1.744365	1.740891	1.737472	1.734132	1.730747	1.727198	1.723651
1.75236	1.748577	1.744951	1.741456	1.738025	1.734682	1.7313	1.727758	1.724217
1.752996	1.749205	1.745562	1.742043	1.738597	1.735248	1.731868	1.728345	1.724821
1.753643	1.749848	1.746189	1.742644	1.739179	1.735821	1.732443	1.728946	1.725449
1.754291	1.750498	1.746825	1.743251	1.739767	1.736397	1.733022	1.72956	1.726096
1.754945	1.751159	1.747472	1.743868	1.740363	1.736978	1.733606	1.730186	1.726762
1.755635	1.751861	1.748157	1.744515	1.740979	1.737573	1.734193	1.730798	1.7274
1.756372	1.752617	1.748893	1.745198	1.74162	1.738182	1.734783	1.731393	1.728006

1.757091	1.753349	1.74961	1.745881	1.742269	1.738797	1.735375	1.731986	1.728607
1.757765	1.754022	1.75028	1.746547	1.742918	1.739409	1.735962	1.732579	1.729218
1.758422	1.754674	1.750937	1.747217	1.74358	1.740035	1.736559	1.733174	1.729822
1.759069	1.755323	1.751599	1.747904	1.744268	1.740686	1.737175	1.733765	1.730398
1.759714	1.755967	1.752262	1.748602	1.744971	1.741359	1.737811	1.734364	1.730969
1.742737	1.739068	1.752928	1.749298	1.745679	1.742059	1.738494	1.735017	1.731593
1.743411	1.739708	1.753597	1.749993	1.746394	1.742786	1.739219	1.735718	1.73226
1.744129	1.740389	1.736774	1.733271	1.747084	1.743484	1.739921	1.736411	1.732939
1.744903	1.741118	1.737459	1.73391	1.747732	1.744137	1.740585	1.73709	1.73363
1.659297	1.656238	1.738179	1.734576	1.748398	1.744801	1.741255	1.737769	1.734317
1.659912	1.656851	1.738958	1.735298	1.749111	1.745502	1.741948	1.738447	1.734987
1.660534	1.65748	1.739756	1.73605	1.749856	1.746231	1.742662	1.739135	1.735654
1.661143	1.658078	1.740522	1.736815	1.733186	1.729644	1.743421	1.739855	1.736335
1.661745	1.658662	1.741271	1.737593	1.733977	1.730432	1.744208	1.740594	1.737024
1.662352	1.659239	1.721745	1.718075	1.734723	1.731171	1.727635	1.724067	1.720531
1.662964	1.659805	1.722461	1.718786	1.735422	1.731862	1.72832	1.724754	1.721219
1.663591	1.660412	1.723233	1.719549	1.715919	1.712345	1.729016	1.725451	1.72192
1.664246	1.66109	1.724102	1.720404	1.716741	1.713104	1.72973	1.726158	1.722635
1.664898	1.66177	1.724969	1.721256	1.717563	1.713877	1.730464	1.726884	1.723363
1.63064	1.627723	1.659318	1.656281	1.71834	1.714658	1.711031	1.707477	1.703982
1.631234	1.628315	1.659922	1.65687	1.719095	1.715438	1.711824	1.708257	1.704734
1.631863	1.628928	1.660547	1.657461	1.719833	1.716179	1.71257	1.709002	1.705473
1.632516	1.629555	1.661185	1.658051	1.720559	1.716897	1.713288	1.709726	1.706206
1.63315	1.630185	1.661835	1.658679	1.721354	1.717677	1.714055	1.710487	1.706958
1.63376	1.630818	1.6625	1.659354	1.722232	1.718531	1.714881	1.711292	1.707736
1.634381	1.63145	1.628513	1.625573	1.656919	1.653882	1.715677	1.712072	1.708498
1.635038	1.632089	1.629129	1.626158	1.657511	1.654431	1.716391	1.712781	1.709217
1.635683	1.632711	1.629727	1.62673	1.65809	1.654974	1.71711	1.713495	1.70994
1.636263	1.633274	1.630286	1.627294	1.658678	1.655572	1.717918	1.714292	1.71072
1.636813	1.633816	1.630833	1.627857	1.659274	1.656192	1.718763	1.71512	1.711517
1.637395	1.634418	1.631443	1.628464	1.62553	1.622638	1.677221	1.674086	1.670972
1.637996	1.635067	1.632103	1.629107	1.626144	1.623209	1.677836	1.674675	1.671543
1.6386	1.635696	1.632733	1.62972	1.626731	1.623758	1.678426	1.675262	1.672138
1.639211	1.636298	1.63332	1.630287	1.627273	1.624268	1.678973	1.675842	1.672763
1.60266	1.59981	1.633906	1.630864	1.627838	1.624812	1.621876	1.619095	1.616398
1.603153	1.600335	1.634505	1.63148	1.628465	1.625443	1.6225	1.619697	1.616974
1.603642	1.600864	1.635115	1.632116	1.629116	1.626101	1.62315	1.620311	1.617545
1.559448	1.556882	1.598581	1.595657	1.629772	1.626737	1.623756	1.620873	1.618065
1.559935	1.557389	1.599186	1.596278	1.630432	1.627365	1.624343	1.621412	1.618558
1.560409	1.557869	1.599747	1.596838	1.593883	1.59086	1.624925	1.621965	1.619084
1.560877	1.558326	1.600268	1.597342	1.594383	1.591376	1.625498	1.622529	1.619635
1.561317	1.558753	1.556106	1.553395	1.594912	1.591925	1.588964	1.586039	1.583187
1.561719	1.559139	1.556512	1.553855	1.595492	1.592528	1.58957	1.586612	1.583719
1.562112	1.559524	1.556923	1.554322	1.596084	1.593143	1.590189	1.587208	1.584282
1.562508	1.559936	1.557363	1.554802	1.596677	1.593752	1.590808	1.587834	1.584904
1.562911	1.560361	1.557814	1.555283	1.597268	1.594359	1.591428	1.588474	1.585553
1.563343	1.560797	1.558257	1.555741	1.553148	1.550462	1.592033	1.5891	1.586188
1.5638	1.561244	1.558699	1.556182	1.553603	1.550945	1.592629	1.589719	1.586817
1.564298	1.561715	1.559154	1.556631	1.554062	1.551437	1.548782	1.546088	1.543388
1.564841	1.562212	1.559624	1.557087	1.554525	1.551941	1.549314	1.546612	1.543888

1.565392	1.562715	1.560101	1.557551	1.554996	1.552443	1.54984	1.547131	1.544388
1.565937	1.563216	1.560581	1.55803	1.555481	1.552946	1.550356	1.54765	1.544899
1.566486	1.563722	1.561068	1.558512	1.555965	1.553439	1.550856	1.548154	1.5454
1.530052	1.527372	1.56156	1.558987	1.556431	1.553907	1.551326	1.548621	1.545865
1.530556	1.527844	1.562059	1.559464	1.556892	1.554362	1.551778	1.54907	1.546317
1.531071	1.528326	1.562569	1.559952	1.557364	1.554819	1.552231	1.549534	1.546798
1.531596	1.528818	1.563089	1.560451	1.557843	1.555277	1.552681	1.550008	1.5473
1.532131	1.529332	1.52667	1.524126	1.558328	1.555744	1.553144	1.550489	1.547805
1.532676	1.529871	1.527179	1.524587	1.558816	1.556222	1.553621	1.550977	1.548309
1.533222	1.530414	1.527695	1.525058	1.559315	1.556708	1.554105	1.551475	1.548826
1.533757	1.530948	1.52821	1.525543	1.559832	1.557206	1.554599	1.551994	1.549375
1.534297	1.531486	1.52873	1.526033	1.560355	1.557708	1.555096	1.552514	1.549924
1.534858	1.532042	1.529256	1.526514	1.523867	1.521332	1.555586	1.553013	1.55044
1.535432	1.532607	1.529787	1.526996	1.524316	1.521766	1.556075	1.553499	1.550935
1.535999	1.533159	1.530318	1.527503	1.5248	1.522226	1.556574	1.553983	1.551413
1.53656	1.533702	1.53085	1.528035	1.525317	1.522708	1.55708	1.554462	1.551874
1.537125	1.534251	1.531389	1.528572	1.525838	1.523194	1.520609	1.518085	1.515597
1.537684	1.534809	1.531937	1.529111	1.526358	1.523679	1.521065	1.518523	1.516025
1.538272	1.53538	1.532491	1.529654	1.52688	1.524169	1.521526	1.518966	1.516458
1.538912	1.535966	1.533044	1.530194	1.527401	1.524661	1.521991	1.519407	1.516882
1.53959	1.53657	1.533606	1.530741	1.52793	1.525163	1.522468	1.51986	1.517317
1.540282	1.53719	1.534184	1.531304	1.528474	1.525683	1.522966	1.520344	1.517791
1.541001	1.537832	1.534781	1.531885	1.529037	1.526224	1.523489	1.520859	1.518298
1.541825	1.538552	1.535445	1.532533	1.52967	1.526841	1.524089	1.52144	1.518858
1.542765	1.53936	1.536188	1.533262	1.530387	1.527549	1.524783	1.5221	1.519483
1.543695	1.540159	1.536914	1.533957	1.531061	1.528212	1.525433	1.522723	1.520076
1.544572	1.540918	1.537592	1.534578	1.531645	1.528781	1.52599	1.523263	1.520598
1.54544	1.541673	1.53826	1.535178	1.532199	1.529318	1.526517	1.523776	1.521099
1.546306	1.542427	1.538929	1.535777	1.53275	1.529847	1.527033	1.524278	1.521591
1.547181	1.543197	1.53961	1.536378	1.533298	1.530373	1.527546	1.524781	1.52209
1.548109	1.544025	1.54034	1.537006	1.533865	1.530931	1.528108	1.525349	1.522666
1.549082	1.544906	1.541114	1.537659	1.53445	1.531514	1.528707	1.525967	1.523304
1.492488	1.488705	1.542003	1.538423	1.535129	1.532158	1.529332	1.526579	1.52391
1.493452	1.48965	1.543035	1.539325	1.535925	1.532877	1.529989	1.527184	1.524475
1.494413	1.490606	1.54408	1.540246	1.536737	1.5336	1.530639	1.527781	1.525033
1.495331	1.491527	1.545071	1.541124	1.537512	1.534283	1.531249	1.528352	1.525582
1.496251	1.492454	1.546076	1.542025	1.538313	1.534982	1.531869	1.528935	1.526148
1.497215	1.493424	1.547163	1.543013	1.539198	1.535755	1.532554	1.529584	1.526783
1.498199	1.494408	1.548284	1.544048	1.540134	1.536573	1.533277	1.53027	1.527454
1.499149	1.495322	1.549319	1.545039	1.541054	1.537381	1.533987	1.530926	1.528078
1.500067	1.49617	1.550275	1.545994	1.541963	1.538182	1.534686	1.531558	1.528661
1.501039	1.497052	1.551273	1.547009	1.542945	1.539064	1.535467	1.532257	1.529292
1.502067	1.497963	1.552324	1.548104	1.544028	1.540064	1.536369	1.533061	1.530006
1.503172	1.498956	1.553433	1.549236	1.545133	1.541077	1.537277	1.533858	1.530702
1.504406	1.500105	1.554628	1.550385	1.546203	1.542023	1.538096	1.534552	1.531291
1.505685	1.501318	1.555874	1.551565	1.54729	1.542985	1.538929	1.535253	1.531879
1.506878	1.502474	1.55713	1.552792	1.548462	1.544069	1.539909	1.536104	1.532606
1.508033	1.503611	1.558401	1.554052	1.549686	1.545232	1.540987	1.537055	1.533425
1.50925	1.504792	1.559654	1.555252	1.55084	1.546354	1.542053	1.53801	1.534256
1.510531	1.506015	1.560881	1.556385	1.551915	1.547427	1.543104	1.538966	1.535101

1.511801	1.507256	1.562159	1.557572	1.553051	1.548572	1.544229	1.53999	1.535997
1.51305	1.508527	1.563538	1.558869	1.554304	1.549843	1.545476	1.541109	1.536947
1.514266	1.509778	1.564914	1.560178	1.55558	1.551139	1.546753	1.542276	1.537962
1.515409	1.510947	1.566185	1.561417	1.556807	1.552391	1.548004	1.54347	1.539065
1.516524	1.512089	1.567433	1.562648	1.558034	1.553632	1.549246	1.544688	1.540229
1.517698	1.513288	1.568768	1.563957	1.559311	1.55487	1.55045	1.54589	1.541413
1.51891	1.514526	1.570168	1.565325	1.560629	1.556105	1.551626	1.547085	1.542625
1.520091	1.515735	1.571595	1.566721	1.561983	1.5574	1.552875	1.548338	1.543871
1.521235	1.516917	1.573061	1.568159	1.563389	1.558772	1.554212	1.549658	1.545151
1.522368	1.518064	1.574489	1.569558	1.564765	1.560133	1.555557	1.550996	1.546461
1.523478	1.51914	1.575825	1.570865	1.566062	1.561444	1.556885	1.552346	1.547815
1.524608	1.52022	1.577141	1.572151	1.567338	1.562737	1.558199	1.553686	1.549169
1.525822	1.521386	1.578492	1.573463	1.568627	1.564019	1.559482	1.554976	1.550471
1.527087	1.522602	1.579868	1.574797	1.569927	1.565293	1.560742	1.556234	1.551742
1.528359	1.523847	1.581314	1.576203	1.571283	1.566583	1.561985	1.557472	1.552998
1.529644	1.525124	1.582828	1.577676	1.57269	1.567884	1.563208	1.558688	1.554237
1.530906	1.526398	1.521961	1.51758	1.574129	1.569227	1.564472	1.559922	1.555467
1.532134	1.527665	1.523233	1.518832	1.575604	1.570619	1.565785	1.561172	1.556673
1.533356	1.528926	1.524501	1.520083	1.577091	1.572042	1.567138	1.562463	1.557912
1.534569	1.530175	1.525756	1.521326	1.578574	1.573486	1.568538	1.563818	1.559222
1.535795	1.531422	1.527006	1.522571	1.580064	1.574953	1.569974	1.565214	1.560572
1.537083	1.532681	1.528258	1.523833	1.58157	1.576446	1.571441	1.566634	1.561943
1.538412	1.533947	1.529508	1.525103	1.583086	1.577959	1.572933	1.568078	1.563335
1.394962	1.390694	1.530746	1.526351	1.584574	1.579449	1.574407	1.569501	1.564705
1.396107	1.391769	1.531965	1.527574	1.586032	1.580914	1.57586	1.570902	1.566049
1.397269	1.392886	1.533223	1.528825	1.587508	1.582393	1.577328	1.572328	1.567429
1.398458	1.394068	1.53454	1.530121	1.589011	1.58389	1.578812	1.573787	1.568858
1.399658	1.395275	1.535884	1.531443	1.59054	1.585413	1.580325	1.575279	1.570324
1.400865	1.396486	1.53724	1.532792	1.528358	1.523927	1.581888	1.576821	1.571833
1.40208	1.397708	1.538608	1.534157	1.529721	1.525293	1.583481	1.578392	1.573368
1.403299	1.39893	1.394663	1.390484	1.531076	1.526656	1.585072	1.579955	1.574892
1.404525	1.400155	1.395883	1.391693	1.53243	1.528023	1.586667	1.58152	1.576413
1.340614	1.336395	1.397106	1.392912	1.533792	1.52939	1.524977	1.520489	1.516028
1.342003	1.33776	1.398329	1.394139	1.535161	1.530756	1.526332	1.521814	1.517321
1.343397	1.339144	1.33501	1.330981	1.487227	1.482853	1.527683	1.523144	1.518633
1.344789	1.340547	1.336406	1.332358	1.488555	1.484158	1.529026	1.524481	1.519971
1.346194	1.341979	1.337832	1.333753	1.4899	1.48547	1.530372	1.525828	1.521329
1.277346	1.273276	1.339322	1.335197	1.438458	1.434094	1.531732	1.527193	1.522708
1.278806	1.274802	1.340842	1.336665	1.439975	1.435568	1.533098	1.528573	1.524107
1.280275	1.276273	1.342303	1.338109	1.333898	1.329654	1.484947	1.480538	1.4762
1.281753	1.277702	1.343716	1.339537	1.335321	1.331036	1.486232	1.481874	1.477595
1.146988	1.143219	1.275017	1.270895	1.336724	1.332426	1.435532	1.431268	1.427063
1.1484	1.14459	1.27643	1.272279	1.338096	1.333819	1.43701	1.432726	1.428496
1.149786	1.145958	1.277856	1.273678	1.339486	1.335239	1.331045	1.326939	1.322879
1.151107	1.147313	1.279312	1.275118	1.340923	1.336712	1.332531	1.328371	1.324245
1.152406	1.148654	1.280778	1.276583	1.342389	1.338204	1.334028	1.329817	1.32563
1.153723	1.149966	1.28223	1.278077	1.273931	1.269792	1.335476	1.331246	1.327046
1.155052	1.151263	1.283676	1.279586	1.275452	1.271251	1.336896	1.33267	1.328487
1.052707	1.049392	1.285113	1.28103	1.276891	1.272668	1.33831	1.334108	1.32995
1.053964	1.050633	1.286537	1.282407	1.278249	1.274039	1.339713	1.33556	1.33144

1.055253	1.051888	1.287992	1.283816	1.279638	1.27544	1.271286	1.267227	1.26319
1.056581	1.053157	1.289501	1.285288	1.281088	1.276892	1.272732	1.268634	1.264557
1.057927	1.054426	1.291009	1.286772	1.28256	1.278369	1.274202	1.270059	1.265935
1.059272	1.055659	1.155613	1.151863	1.28404	1.279867	1.275705	1.271535	1.267381
1.060632	1.056906	1.156979	1.153253	1.285535	1.28138	1.277226	1.273043	1.26887
1.062028	1.058262	1.158429	1.154662	1.287048	1.282886	1.278734	1.274573	1.270416
1.063455	1.0597	1.159942	1.156088	1.288576	1.284393	1.280238	1.276125	1.272009
1.064908	1.061133	1.057536	1.054094	1.290091	1.285917	1.281776	1.277672	1.273556
1.06639	1.062555	1.058902	1.055397	1.291586	1.287467	1.283364	1.279222	1.275052
0.945094	0.941834	1.060298	1.056726	1.293104	1.288998	1.284905	1.280734	1.276531
0.946332	0.943113	1.061746	1.058096	1.294677	1.290497	1.286351	1.282176	1.277991
0.947577	0.944394	1.063213	1.059479	1.296247	1.291977	1.287772	1.283605	1.279456
0.948834	0.945633	1.064681	1.060844	1.297741	1.293428	1.289211	1.28507	1.280964
0.950092	0.946848	1.066149	1.062215	1.299221	1.294877	1.290659	1.286551	1.28249
0.813138	0.810411	1.067588	1.06364	1.215697	1.211706	1.292138	1.288031	1.283994
0.814035	0.811311	1.069002	1.065111	1.217264	1.213163	1.293643	1.289512	1.285481
0.814949	0.812216	0.947108	0.943846	1.062767	1.058977	1.295182	1.291027	1.286998
0.815889	0.813117	0.9483	0.945034	1.064194	1.060362	1.296748	1.292581	1.288558
0.816835	0.814053	0.811288	0.808532	1.065637	1.061784	1.213573	1.209765	1.206119
0.817785	0.815071	0.812307	0.809507	1.067093	1.063235	1.215156	1.211233	1.20747
0.818733	0.816096	0.813343	0.810509	1.068587	1.064722	1.216778	1.212731	1.208837
0.81966	0.817031	0.81431	0.811527	0.955806	0.952317	1.218367	1.214255	1.210302
0.820576	0.817922	0.815241	0.812552	0.957208	0.953632	1.219962	1.215814	1.211833
0.821504	0.818846	0.816177	0.813516	0.958526	0.95497	1.22167	1.217456	1.213394
0.822439	0.819801	0.817116	0.81442	0.959771	0.956338	1.223493	1.219182	1.214988
0.696508	0.694151	0.818039	0.815332	0.812646	0.809997	0.954264	0.950776	0.947324
0.697418	0.69493	0.818932	0.816268	0.8136	0.810929	0.955565	0.952097	0.948628
0.698346	0.695731	0.819833	0.817201	0.814544	0.81185	0.95683	0.953402	0.949939
0.699256	0.696602	0.820765	0.81811	0.815452	0.812787	0.958068	0.954678	0.951229
0.700186	0.69751	0.821718	0.819017	0.81635	0.813728	0.959285	0.955936	0.952507
0.701186	0.698431	0.695805	0.693308	0.817267	0.814673	0.960507	0.95718	0.953755
0.702242	0.699372	0.696687	0.694165	0.818199	0.815622	0.961725	0.958412	0.954986
0.703328	0.700356	0.69761	0.695054	0.819164	0.816581	0.813983	0.81128	0.808549
0.704452	0.701383	0.698575	0.695976	0.820165	0.817549	0.814945	0.812292	0.809619
0.50451	0.502172	0.699583	0.69693	0.694387	0.691968	0.815904	0.813287	0.810663
0.505517	0.503168	0.700642	0.697923	0.69532	0.692848	0.816848	0.814241	0.811642
0.506518	0.504181	0.701733	0.698935	0.696267	0.693743	0.817804	0.815196	0.812612
0.507548	0.505243	0.702849	0.699947	0.697208	0.694657	0.818808	0.816198	0.81362
0.508577	0.506319	0.703977	0.700971	0.698162	0.695586	0.819833	0.817213	0.814635
0.509535	0.507322	0.705069	0.702022	0.699163	0.696524	0.694	0.691585	0.689232
0.510437	0.508268	0.706132	0.703097	0.700205	0.697471	0.694867	0.692423	0.690069
		0.507032	0.504766	0.701285	0.698475	0.695795	0.693302	0.690916
		0.508066	0.505824	0.702417	0.699553	0.696802	0.694228	0.691766
		0.509062	0.50685	0.703531	0.700628	0.697824	0.695182	0.692655
		0.509959	0.507777	0.704577	0.701658	0.698831	0.696171	0.693624
		0.510841	0.508688	0.705611	0.702684	0.699844	0.697171	0.694607
				0.507543	0.505437	0.700867	0.698133	0.695509
				0.508538	0.506393	0.701904	0.69909	0.696387
				0.509527	0.50737	0.702991	0.700136	0.697378
				0.510511	0.508367	0.704129	0.701265	0.698475

1.991877	1.988038	1.987962	1.984268	1.980566	1.97684	1.975598	1.97198	1.968402
1.991859	1.988019	1.987932	1.984227	1.980519	1.97679	1.975549	1.97193	1.96835
1.991854	1.988023	1.987925	1.984202	1.980481	1.976747	1.975502	1.971882	1.968302
1.984692	1.9809	1.987969	1.984207	1.980458	1.976712	1.97546	1.971836	1.968256
1.984725	1.98098	1.988047	1.984233	1.980447	1.976684	1.975422	1.971794	1.968212
1.984725	1.981006	1.981177	1.977343	1.980427	1.97665	1.972932	1.969303	1.965723
1.984689	1.980976	1.981146	1.977315	1.980398	1.97661	1.972883	1.969252	1.965672
1.984658	1.980941	1.981107	1.97728	1.973477	1.969691	1.972842	1.969207	1.965624
1.984637	1.980909	1.981067	1.977237	1.973434	1.969653	1.972807	1.969165	1.965576
1.984636	1.980887	1.98103	1.977196	1.973397	1.969633	1.972798	1.969145	1.96554
1.984681	1.980889	1.977081	1.973269	1.973382	1.969661	1.972855	1.969179	1.965536
1.984742	1.9809	1.97707	1.973251	1.973377	1.969706	1.972935	1.969231	1.965547
1.984754	1.980894	1.977054	1.973231	1.973357	1.969704	1.966059	1.962354	1.958666
1.984732	1.980875	1.977035	1.973211	1.97333	1.969671	1.966025	1.962325	1.95865
1.975663	1.971832	1.977041	1.973232	1.973349	1.969668	1.966004	1.962302	1.958632
1.975665	1.971842	1.977075	1.973305	1.973428	1.969705	1.965999	1.962281	1.958604
1.975681	1.97187	1.977121	1.97337	1.969636	1.965914	1.966005	1.962283	1.958607
1.975716	1.971933	1.977184	1.9734	1.969647	1.965925	1.96602	1.962326	1.958675
1.975761	1.971997	1.977241	1.973418	1.969648	1.965939	1.96605	1.962384	1.958754
1.97581	1.97202	1.968218	1.964414	1.969667	1.965984	1.966113	1.962437	1.958787
1.975861	1.972024	1.968211	1.964421	1.969695	1.966045	1.966192	1.962488	1.958801
1.975889	1.97204	1.968233	1.964461	1.969748	1.966094	1.966233	1.962515	1.958819
1.975895	1.972064	1.968277	1.964532	1.969825	1.966134	1.96624	1.96252	1.958835
1.975916	1.972107	1.968328	1.964584	1.960867	1.957176	1.966253	1.962546	1.958883
1.975955	1.972174	1.968387	1.964606	1.96087	1.957182	1.966271	1.962603	1.958978
1.976008	1.972243	1.968442	1.964625	1.960874	1.957198	1.962537	1.958929	1.955358
1.976076	1.972289	1.968473	1.964642	1.960894	1.957242	1.962609	1.958995	1.955416
1.976153	1.97234	1.96851	1.964674	1.960931	1.957302	1.962692	1.959066	1.955473
1.976226	1.972432	1.968606	1.964764	1.961013	1.95737	1.959438	1.955803	1.952204
1.9763	1.972548	1.968737	1.96489	1.961124	1.957449	1.959504	1.95589	1.952318
1.976382	1.972632	1.968822	1.964975	1.961208	1.957531	1.959591	1.955979	1.952409
1.976473	1.972682	1.968857	1.965014	1.961261	1.957615	1.959701	1.956069	1.952476
1.976572	1.972765	1.968936	1.965099	1.961352	1.957715	1.95981	1.956168	1.952563
1.976682	1.972913	1.969102	1.965267	1.961506	1.957831	1.959906	1.956276	1.952689
1.976797	1.973066	1.969276	1.965452	1.961681	1.957966	1.960016	1.956394	1.952819
1.976917	1.973174	1.969401	1.965617	1.961863	1.958138	1.960177	1.956522	1.952919
1.97704	1.973272	1.969513	1.965771	1.962041	1.958317	1.960353	1.956658	1.953017
1.977161	1.973401	1.96964	1.965891	1.962158	1.958436	1.960479	1.95679	1.953152
1.977281	1.973549	1.969776	1.965985	1.962231	1.958512	1.960574	1.956921	1.953313
1.977422	1.973686	1.969905	1.966106	1.96235	1.958636	1.960712	1.957079	1.953488
1.977586	1.973801	1.970022	1.96626	1.962527	1.958817	1.960902	1.957271	1.953681
1.977763	1.973943	1.970159	1.966416	1.962698	1.959	1.961097	1.957458	1.953856
1.977959	1.974136	1.970331	1.966552	1.962826	1.959161	1.961283	1.957616	1.953981
1.978153	1.974346	1.970525	1.966703	1.962963	1.959327	1.961472	1.957777	1.954109
1.978311	1.974548	1.970739	1.966906	1.96316	1.959524	1.961674	1.957975	1.954296
1.978455	1.974752	1.970967	1.967139	1.963387	1.959733	1.961877	1.958186	1.954509
1.978642	1.974957	1.971185	1.967364	1.963603	1.959914	1.962038	1.958356	1.954694
1.978865	1.975166	1.971396	1.967589	1.963814	1.960071	1.962162	1.958492	1.954857
1.979074	1.975343	1.971575	1.967793	1.96402	1.960244	1.962312	1.958631	1.954995
1.979258	1.975471	1.971704	1.96797	1.964221	1.960442	1.962499	1.95877	1.955093

1.979449	1.975614	1.971846	1.968144	1.964415	1.960643	1.9627	1.958931	1.955215
1.979664	1.975815	1.97204	1.968334	1.964606	1.960844	1.962914	1.959136	1.955409
1.979889	1.976038	1.972249	1.968519	1.964784	1.961038	1.963131	1.959358	1.95563
1.980117	1.976253	1.972437	1.968666	1.96492	1.961205	1.963331	1.959567	1.95584
1.980347	1.97647	1.972618	1.968794	1.965034	1.961356	1.963522	1.959772	1.95605
1.980577	1.976694	1.972822	1.968968	1.965198	1.961535	1.963723	1.959982	1.95626
1.980811	1.976927	1.97305	1.969187	1.96541	1.961741	1.963931	1.960195	1.956472
1.972125	1.968301	1.973273	1.969412	1.965633	1.961956	1.964146	1.960412	1.956688
1.972259	1.968478	1.973483	1.969637	1.965863	1.962179	1.96437	1.960635	1.95691
1.972402	1.968656	1.973693	1.969863	1.966096	1.962407	1.964599	1.960863	1.957137
1.972604	1.96885	1.973902	1.970085	1.985579	1.981732	1.98325	1.979337	1.975428
1.972835	1.969056	1.974116	1.970308	1.985812	1.981968	1.983494	1.979578	1.975665
1.962449	1.958694	1.974346	1.970541	1.986047	1.9822	1.98373	1.979813	1.975898
1.96269	1.958927	1.97459	1.97078	1.986285	1.982431	1.983962	1.980043	1.976128
1.962944	1.959165	1.965962	1.962201	1.986525	1.982667	1.9842	1.980279	1.976361
1.963207	1.959404	1.966196	1.962433	1.986767	1.98291	1.984447	1.980519	1.976596
1.963499	1.959662	1.955868	1.952125	1.98701	1.983151	1.984693	1.980763	1.976839
1.963846	1.959951	1.956122	1.952357	1.987248	1.983386	1.984936	1.981011	1.977099
1.964192	1.960244	1.956384	1.952599	1.987494	1.983625	1.985179	1.981258	1.977359
1.964474	1.960514	1.956646	1.952856	1.987756	1.983872	1.98542	1.981497	1.977601
1.964726	1.960772	1.956911	1.953129	1.988038	1.984132	1.985666	1.981736	1.977839
1.964983	1.96103	1.957186	1.953431	1.978927	1.975011	1.985941	1.981997	1.978091
1.965239	1.961286	1.957468	1.953762	1.979299	1.975343	1.986243	1.982276	1.978356
1.965506	1.96155	1.957745	1.954065	1.970942	1.967006	1.98654	1.982578	1.978666
1.965783	1.961819	1.958008	1.954326	1.971202	1.967258	1.986829	1.982914	1.979043
1.966073	1.962111	1.958289	1.95459	1.971457	1.967512	1.978057	1.974203	1.970389
1.966387	1.962449	1.958618	1.954882	1.971728	1.967777	1.978336	1.974479	1.970666
1.966705	1.962795	1.958955	1.955182	1.972009	1.96806	1.978634	1.974756	1.970925
1.966996	1.963095	1.959253	1.955473	1.972309	1.968391	1.978998	1.975092	1.971227
1.967276	1.963371	1.959533	1.955762	1.972625	1.968753	1.979401	1.975459	1.971549
1.967595	1.963664	1.959828	1.95608	1.972969	1.969096	1.97143	1.967504	1.96361
1.967952	1.96397	1.960136	1.956429	1.973345	1.969423	1.971726	1.967786	1.963888
1.961136	1.957131	1.960443	1.956753	1.973685	1.969735	1.972028	1.968094	1.964204
1.961441	1.957429	1.960734	1.957028	1.97396	1.970021	1.972341	1.968443	1.964582
1.961754	1.957751	1.961048	1.957313	1.974239	1.970323	1.972678	1.968808	1.964968
1.962104	1.958133	1.961421	1.957644	1.974564	1.970681	1.973067	1.969182	1.96532
1.962474	1.958538	1.961818	1.958	1.974915	1.971064	1.973476	1.969564	1.96567
1.962857	1.958911	1.955039	1.951238	1.975298	1.971435	1.973849	1.969945	1.966056
1.963253	1.959267	1.955396	1.951623	1.975708	1.971805	1.974204	1.970326	1.966466
1.951789	1.947821	1.955776	1.951995	1.976089	1.972181	1.974583	1.9707	1.966829
1.952162	1.948225	1.956183	1.952353	1.976436	1.972566	1.974995	1.971067	1.967141
1.95254	1.948616	1.956579	1.952717	1.970259	1.966421	1.975385	1.97142	1.967451
1.952929	1.948972	1.956944	1.953099	1.970654	1.966775	1.975719	1.971736	1.967763
1.953317	1.949327	1.957306	1.953476	1.971044	1.967128	1.976056	1.972067	1.9681
1.953692	1.949714	1.957692	1.953825	1.971386	1.967496	1.970272	1.966304	1.962346
1.954062	1.950111	1.958082	1.954159	1.971709	1.967865	1.970691	1.966742	1.962784
1.948611	1.944696	1.94653	1.942618	1.972056	1.968204	1.971043	1.967125	1.9632
1.949012	1.945056	1.946851	1.942955	1.972418	1.968516	1.971342	1.967467	1.963601
1.949409	1.945434	1.947188	1.943281	1.972756	1.968841	1.964979	1.961166	1.957368
1.949794	1.945838	1.947549	1.943588	1.973055	1.969183	1.965347	1.961499	1.957663

1.94743	1.943463	1.947913	1.943916	1.962242	1.95841	1.965704	1.961829	1.957965
1.947864	1.943849	1.948261	1.944286	1.962623	1.95874	1.966007	1.962148	1.958312
1.948317	1.944249	1.948625	1.944683	1.963037	1.959086	1.966314	1.962473	1.958668
1.948788	1.944707	1.949052	1.945111	1.963477	1.95949	1.966693	1.962825	1.959001
1.949271	1.945201	1.949516	1.945557	1.963932	1.959926	1.967115	1.963198	1.959331
1.949721	1.945678	1.944473	1.940509	1.964329	1.960328	1.956382	1.952467	1.948604
1.950138	1.946142	1.944921	1.940898	1.96467	1.960701	1.956785	1.95288	1.949019
1.950585	1.946614	1.945382	1.941319	1.965051	1.961071	1.957156	1.953269	1.949429
1.951085	1.947107	1.945869	1.941797	1.965495	1.961431	1.957465	1.953611	1.949821
1.95158	1.947581	1.946344	1.942282	1.96596	1.961816	1.957797	1.953966	1.950213
1.952027	1.947995	1.946768	1.942743	1.96643	1.962262	1.95822	1.95438	1.950622
1.952462	1.948396	1.947183	1.9432	1.966907	1.962735	1.958678	1.954811	1.951027
1.952928	1.948852	1.944827	1.940843	1.962719	1.958546	1.959112	1.955205	1.951387
1.953411	1.949343	1.945319	1.941331	1.963222	1.959027	1.959538	1.955582	1.95172
1.926017	1.92204	1.945788	1.941776	1.963672	1.959484	1.959971	1.955991	1.952103
1.926413	1.922482	1.946231	1.94217	1.964055	1.959909	1.960411	1.956434	1.952538
1.894882	1.891052	1.933131	1.929056	1.938588	1.934668	1.93372	1.929977	1.926302
1.89535	1.891519	1.933627	1.929555	1.939082	1.935143	1.934181	1.930438	1.926768
1.895837	1.892	1.934139	1.930077	1.939602	1.935637	1.934654	1.930908	1.927243
1.886349	1.882531	1.905849	1.901837	1.926589	1.92263	1.935144	1.931394	1.927725
1.886838	1.883022	1.906357	1.902344	1.927108	1.923141	1.935649	1.931891	1.928214
1.887324	1.883506	1.889584	1.885671	1.927629	1.923662	1.93327	1.929479	1.925769
1.88781	1.883986	1.890069	1.886161	1.928154	1.924194	1.933801	1.929988	1.926251
1.888304	1.884473	1.880601	1.876714	1.899908	1.896011	1.920834	1.917025	1.91329
1.888806	1.884969	1.881096	1.877209	1.900419	1.896518	1.92135	1.917526	1.913774
1.889311	1.885465	1.881587	1.8777	1.891645	1.887755	1.893182	1.889407	1.885704
1.889817	1.885956	1.882071	1.87818	1.892144	1.888259	1.893687	1.889891	1.886162
1.890321	1.886444	1.88255	1.878656	1.892641	1.888762	1.894191	1.890372	1.886618
1.844758	1.841001	1.883027	1.879132	1.875287	1.871498	1.894688	1.890851	1.887076
1.845192	1.841428	1.883501	1.879605	1.875757	1.871965	1.895181	1.891327	1.887534
1.84562	1.841845	1.883963	1.880065	1.876217	1.872428	1.886396	1.88254	1.878742
1.846042	1.842252	1.884413	1.880512	1.876666	1.872886	1.886881	1.883005	1.879184
1.846463	1.842659	1.838875	1.835119	1.87713	1.873353	1.86959	1.865791	1.862035
1.846876	1.843062	1.839282	1.835541	1.87761	1.873828	1.870057	1.866246	1.862478
1.847302	1.843479	1.839705	1.835986	1.878114	1.874322	1.87054	1.866726	1.862954
1.847755	1.843929	1.840168	1.836473	1.87867	1.874857	1.87106	1.86726	1.863503
1.848227	1.844391	1.84064	1.83697	1.879235	1.875399	1.871587	1.867807	1.864071
1.848721	1.844838	1.841075	1.837416	1.833746	1.830051	1.872076	1.868303	1.864575
1.849233	1.845282	1.84149	1.837831	1.834163	1.830467	1.872542	1.86877	1.865044
1.849734	1.84575	1.841935	1.838258	1.834581	1.83089	1.873019	1.869251	1.865529
1.850223	1.846243	1.842407	1.838691	1.834995	1.831313	1.873504	1.869743	1.86603
1.850725	1.84676	1.842908	1.839152	1.835438	1.831767	1.874023	1.870258	1.866538
1.851252	1.847313	1.843448	1.839653	1.835925	1.832276	1.874604	1.870808	1.867047
1.851782	1.847868	1.843996	1.840173	1.836432	1.832793	1.875187	1.871367	1.867575
1.852294	1.848391	1.844527	1.840707	1.836955	1.833282	1.829635	1.825979	1.822351
1.852804	1.8489	1.845045	1.841243	1.837481	1.833758	1.830081	1.82646	1.822876
1.853338	1.849399	1.845532	1.841731	1.837961	1.834214	1.830522	1.826914	1.823347
1.853891	1.849889	1.845992	1.842183	1.838406	1.834653	1.830957	1.827345	1.823779
1.854458	1.850425	1.846499	1.84266	1.838868	1.835118	1.831424	1.827798	1.824218
1.855043	1.851022	1.847066	1.843164	1.839344	1.835615	1.831932	1.828278	1.824662

1.855627	1.851613	1.847635	1.84369	1.839847	1.836124	1.832444	1.828772	1.825132
1.856203	1.852164	1.848182	1.844246	1.840395	1.83664	1.832938	1.829277	1.825651
1.856773	1.85271	1.848728	1.844811	1.840956	1.837165	1.833438	1.829791	1.826183
1.857335	1.853278	1.849293	1.845361	1.841496	1.8377	1.833965	1.830305	1.826685
1.857885	1.853852	1.849863	1.845907	1.842031	1.838242	1.834508	1.830822	1.827178
1.846072	1.84208	1.850408	1.84646	1.842583	1.838783	1.835037	1.831343	1.827692
1.846538	1.842565	1.850931	1.847018	1.84315	1.839324	1.835556	1.831868	1.828225
1.847043	1.843061	1.839135	1.83526	1.843708	1.839867	1.836084	1.832393	1.828747
1.847603	1.843563	1.839625	1.835767	1.844247	1.840413	1.836629	1.832915	1.829248
1.84818	1.844105	1.840153	1.836296	1.844795	1.840961	1.837172	1.83344	1.829755
1.848749	1.844727	1.840777	1.836886	1.845374	1.841512	1.837704	1.83397	1.830287
1.849331	1.84538	1.841431	1.837493	1.84596	1.842064	1.838234	1.834502	1.830825
1.849953	1.846009	1.84204	1.838059	1.834124	1.830232	1.838776	1.835028	1.831341
1.850599	1.846627	1.842626	1.838605	1.834649	1.830763	1.839326	1.835553	1.831848
1.83528	1.831343	1.843202	1.839164	1.835193	1.831291	1.839864	1.836092	1.832394
1.835856	1.831906	1.843764	1.839737	1.835756	1.831812	1.840385	1.836641	1.832979
1.836429	1.83248	1.844336	1.840302	1.836311	1.832353	1.828494	1.824791	1.821172
1.837001	1.833078	1.844924	1.840838	1.836835	1.832918	1.829092	1.825386	1.821749
1.837585	1.833688	1.845531	1.841399	1.837386	1.83351	1.829714	1.825989	1.82232
1.787174	1.78364	1.830329	1.826259	1.838023	1.834146	1.830336	1.826569	1.822857
1.787751	1.784217	1.830977	1.826927	1.838701	1.834805	1.830965	1.827147	1.823389
1.788304	1.78476	1.831575	1.827569	1.839368	1.835457	1.831593	1.82775	1.82397
1.788839	1.785277	1.832139	1.828196	1.840033	1.836105	1.83222	1.828371	1.824586
1.78938	1.785818	1.832728	1.828816	1.84067	1.836716	1.832809	1.828964	1.825189
1.789926	1.786387	1.833351	1.829431	1.841276	1.837281	1.833349	1.829523	1.825774
1.741838	1.738436	1.783385	1.779786	1.82608	1.822119	1.833899	1.830086	1.826355
1.742382	1.738975	1.783935	1.780318	1.826611	1.822674	1.834482	1.83067	1.826928
1.742934	1.739511	1.784486	1.780855	1.827149	1.823239	1.835079	1.831261	1.827502
1.743485	1.740051	1.73656	1.733036	1.827754	1.823841	1.835681	1.831858	1.828091
1.744035	1.740584	1.737102	1.733604	1.828392	1.824463	1.836291	1.832457	1.828682
1.744558	1.741078	1.737603	1.734142	1.779023	1.775427	1.821184	1.817357	1.813585
1.745056	1.741535	1.738069	1.734654	1.779605	1.776007	1.82178	1.817919	1.814117
1.74559	1.742036	1.738573	1.73519	1.731773	1.728304	1.8224	1.818508	1.814675
1.746172	1.742599	1.739132	1.735758	1.732352	1.728895	1.82305	1.819133	1.815268
1.746772	1.743179	1.739706	1.736334	1.732934	1.729492	1.726049	1.722586	1.719167
1.747374	1.743759	1.740277	1.736908	1.733516	1.730088	1.726652	1.723175	1.719736
1.747985	1.744346	1.740853	1.737482	1.734094	1.730682	1.727253	1.723764	1.720307
1.748608	1.744943	1.741432	1.738049	1.73466	1.731262	1.727841	1.72434	1.720863
1.749239	1.745549	1.742015	1.738613	1.735218	1.731834	1.728421	1.724907	1.721411
1.749871	1.746157	1.742603	1.739183	1.735783	1.732414	1.72901	1.725483	1.721968
1.750503	1.746769	1.743197	1.73976	1.736357	1.733004	1.729609	1.726068	1.722536
1.75113	1.747377	1.743787	1.740333	1.736923	1.73358	1.730192	1.726644	1.7231
1.751743	1.747973	1.744365	1.740891	1.737472	1.734132	1.730747	1.727198	1.723651
1.75236	1.748577	1.744951	1.741456	1.738025	1.734682	1.7313	1.727758	1.724217
1.752996	1.749205	1.745562	1.742043	1.738597	1.735248	1.731868	1.728345	1.724821
1.753643	1.749848	1.746189	1.742644	1.739179	1.735821	1.732443	1.728946	1.725449
1.754291	1.750498	1.746825	1.743251	1.739767	1.736397	1.733022	1.72956	1.726096
1.754945	1.751159	1.747472	1.743868	1.740363	1.736978	1.733606	1.730186	1.726762
1.755635	1.751861	1.748157	1.744515	1.740979	1.737573	1.734193	1.730798	1.7274
1.756372	1.752617	1.748893	1.745198	1.74162	1.738182	1.734783	1.731393	1.728006

1.757091	1.753349	1.74961	1.745881	1.742269	1.738797	1.735375	1.731986	1.728607
1.757765	1.754022	1.75028	1.746547	1.742918	1.739409	1.735962	1.732579	1.729218
1.758422	1.754674	1.750937	1.747217	1.74358	1.740035	1.736559	1.733174	1.729822
1.759069	1.755323	1.751599	1.747904	1.744268	1.740686	1.737175	1.733765	1.730398
1.759714	1.755967	1.752262	1.748602	1.744971	1.741359	1.737811	1.734364	1.730969
1.742737	1.739068	1.752928	1.749298	1.745679	1.742059	1.738494	1.735017	1.731593
1.743411	1.739708	1.753597	1.749993	1.746394	1.742786	1.739219	1.735718	1.73226
1.744129	1.740389	1.736774	1.733271	1.747084	1.743484	1.739921	1.736411	1.732939
1.744903	1.741118	1.737459	1.73391	1.747732	1.744137	1.740585	1.73709	1.73363
1.659297	1.656238	1.738179	1.734576	1.748398	1.744801	1.741255	1.737769	1.734317
1.659912	1.656851	1.738958	1.735298	1.749111	1.745502	1.741948	1.738447	1.734987
1.660534	1.65748	1.739756	1.73605	1.749856	1.746231	1.742662	1.739135	1.735654
1.661143	1.658078	1.740522	1.736815	1.733186	1.729644	1.743421	1.739855	1.736335
1.661745	1.658662	1.741271	1.737593	1.733977	1.730432	1.744208	1.740594	1.737024
1.662352	1.659239	1.721745	1.718075	1.734723	1.731171	1.727635	1.724067	1.720531
1.662964	1.659805	1.722461	1.718786	1.735422	1.731862	1.72832	1.724754	1.721219
1.663591	1.660412	1.723233	1.719549	1.715919	1.712345	1.729016	1.725451	1.72192
1.664246	1.66109	1.724102	1.720404	1.716741	1.713104	1.72973	1.726158	1.722635
1.664898	1.66177	1.724969	1.721256	1.717563	1.713877	1.730464	1.726884	1.723363
1.63064	1.627723	1.659318	1.656281	1.71834	1.714658	1.711031	1.707477	1.703982
1.631234	1.628315	1.659922	1.65687	1.719095	1.715438	1.711824	1.708257	1.704734
1.631863	1.628928	1.660547	1.657461	1.719833	1.716179	1.71257	1.709002	1.705473
1.632516	1.629555	1.661185	1.658051	1.720559	1.716897	1.713288	1.709726	1.706206
1.63315	1.630185	1.661835	1.658679	1.721354	1.717677	1.714055	1.710487	1.706958
1.63376	1.630818	1.6625	1.659354	1.722232	1.718531	1.714881	1.711292	1.707736
1.634381	1.63145	1.628513	1.625573	1.656919	1.653882	1.715677	1.712072	1.708498
1.635038	1.632089	1.629129	1.626158	1.657511	1.654431	1.716391	1.712781	1.709217
1.635683	1.632711	1.629727	1.62673	1.65809	1.654974	1.71711	1.713495	1.70994
1.636263	1.633274	1.630286	1.627294	1.658678	1.655572	1.717918	1.714292	1.71072
1.636813	1.633816	1.630833	1.627857	1.659274	1.656192	1.718763	1.71512	1.711517
1.637395	1.634418	1.631443	1.628464	1.62553	1.622638	1.677221	1.674086	1.670972
1.637996	1.635067	1.632103	1.629107	1.626144	1.623209	1.677836	1.674675	1.671543
1.6386	1.635696	1.632733	1.62972	1.626731	1.623758	1.678426	1.675262	1.672138
1.639211	1.636298	1.63332	1.630287	1.627273	1.624268	1.678973	1.675842	1.672763
1.60266	1.59981	1.633906	1.630864	1.627838	1.624812	1.621876	1.619095	1.616398
1.603153	1.600335	1.634505	1.63148	1.628465	1.625443	1.6225	1.619697	1.616974
1.603642	1.600864	1.635115	1.632116	1.629116	1.626101	1.62315	1.620311	1.617545
1.559448	1.556882	1.598581	1.595657	1.629772	1.626737	1.623756	1.620873	1.618065
1.559935	1.557389	1.599186	1.596278	1.630432	1.627365	1.624343	1.621412	1.618558
1.560409	1.557869	1.599747	1.596838	1.593883	1.59086	1.624925	1.621965	1.619084
1.560877	1.558326	1.600268	1.597342	1.594383	1.591376	1.625498	1.622529	1.619635
1.561317	1.558753	1.556106	1.553395	1.594912	1.591925	1.588964	1.586039	1.583187
1.561719	1.559139	1.556512	1.553855	1.595492	1.592528	1.58957	1.586612	1.583719
1.562112	1.559524	1.556923	1.554322	1.596084	1.593143	1.590189	1.587208	1.584282
1.562508	1.559936	1.557363	1.554802	1.596677	1.593752	1.590808	1.587834	1.584904
1.562911	1.560361	1.557814	1.555283	1.597268	1.594359	1.591428	1.588474	1.585553
1.563343	1.560797	1.558257	1.555741	1.553148	1.550462	1.592033	1.5891	1.586188
1.5638	1.561244	1.558699	1.556182	1.553603	1.550945	1.592629	1.589719	1.586817
1.564298	1.561715	1.559154	1.556631	1.554062	1.551437	1.548782	1.546088	1.543388
1.564841	1.562212	1.559624	1.557087	1.554525	1.551941	1.549314	1.546612	1.543888

1.565392	1.562715	1.560101	1.557551	1.554996	1.552443	1.54984	1.547131	1.544388
1.565937	1.563216	1.560581	1.55803	1.555481	1.552946	1.550356	1.54765	1.544899
1.566486	1.563722	1.561068	1.558512	1.555965	1.553439	1.550856	1.548154	1.5454
1.530052	1.527372	1.56156	1.558987	1.556431	1.553907	1.551326	1.548621	1.545865
1.530556	1.527844	1.562059	1.559464	1.556892	1.554362	1.551778	1.54907	1.546317
1.531071	1.528326	1.562569	1.559952	1.557364	1.554819	1.552231	1.549534	1.546798
1.531596	1.528818	1.563089	1.560451	1.557843	1.555277	1.552681	1.550008	1.5473
1.532131	1.529332	1.52667	1.524126	1.558328	1.555744	1.553144	1.550489	1.547805
1.532676	1.529871	1.527179	1.524587	1.558816	1.556222	1.553621	1.550977	1.548309
1.533222	1.530414	1.527695	1.525058	1.559315	1.556708	1.554105	1.551475	1.548826
1.533757	1.530948	1.52821	1.525543	1.559832	1.557206	1.554599	1.551994	1.549375
1.534297	1.531486	1.52873	1.526033	1.560355	1.557708	1.555096	1.552514	1.549924
1.534858	1.532042	1.529256	1.526514	1.523867	1.521332	1.555586	1.553013	1.55044
1.535432	1.532607	1.529787	1.526996	1.524316	1.521766	1.556075	1.553499	1.550935
1.535999	1.533159	1.530318	1.527503	1.5248	1.522226	1.556574	1.553983	1.551413
1.53656	1.533702	1.53085	1.528035	1.525317	1.522708	1.55708	1.554462	1.551874
1.537125	1.534251	1.531389	1.528572	1.525838	1.523194	1.520609	1.518085	1.515597
1.537684	1.534809	1.531937	1.529111	1.526358	1.523679	1.521065	1.518523	1.516025
1.538272	1.53538	1.532491	1.529654	1.52688	1.524169	1.521526	1.518966	1.516458
1.538912	1.535966	1.533044	1.530194	1.527401	1.524661	1.521991	1.519407	1.516882
1.53959	1.53657	1.533606	1.530741	1.52793	1.525163	1.522468	1.51986	1.517317
1.540282	1.53719	1.534184	1.531304	1.528474	1.525683	1.522966	1.520344	1.517791
1.541001	1.537832	1.534781	1.531885	1.529037	1.526224	1.523489	1.520859	1.518298
1.541825	1.538552	1.535445	1.532533	1.52967	1.526841	1.524089	1.52144	1.518858
1.542765	1.53936	1.536188	1.533262	1.530387	1.527549	1.524783	1.5221	1.519483
1.543695	1.540159	1.536914	1.533957	1.531061	1.528212	1.525433	1.522723	1.520076
1.544572	1.540918	1.537592	1.534578	1.531645	1.528781	1.52599	1.523263	1.520598
1.54544	1.541673	1.53826	1.535178	1.532199	1.529318	1.526517	1.523776	1.521099
1.546306	1.542427	1.538929	1.535777	1.53275	1.529847	1.527033	1.524278	1.521591
1.547181	1.543197	1.53961	1.536378	1.533298	1.530373	1.527546	1.524781	1.52209
1.548109	1.544025	1.54034	1.537006	1.533865	1.530931	1.528108	1.525349	1.522666
1.549082	1.544906	1.541114	1.537659	1.53445	1.531514	1.528707	1.525967	1.523304
1.492488	1.488705	1.542003	1.538423	1.535129	1.532158	1.529332	1.526579	1.52391
1.493452	1.48965	1.543035	1.539325	1.535925	1.532877	1.529989	1.527184	1.524475
1.494413	1.490606	1.54408	1.540246	1.536737	1.5336	1.530639	1.527781	1.525033
1.495331	1.491527	1.545071	1.541124	1.537512	1.534283	1.531249	1.528352	1.525582
1.496251	1.492454	1.546076	1.542025	1.538313	1.534982	1.531869	1.528935	1.526148
1.497215	1.493424	1.547163	1.543013	1.539198	1.535755	1.532554	1.529584	1.526783
1.498199	1.494408	1.548284	1.544048	1.540134	1.536573	1.533277	1.53027	1.527454
1.499149	1.495322	1.549319	1.545039	1.541054	1.537381	1.533987	1.530926	1.528078
1.500067	1.49617	1.550275	1.545994	1.541963	1.538182	1.534686	1.531558	1.528661
1.501039	1.497052	1.551273	1.547009	1.542945	1.539064	1.535467	1.532257	1.529292
1.502067	1.497963	1.552324	1.548104	1.544028	1.540064	1.536369	1.533061	1.530006
1.503172	1.498956	1.553433	1.549236	1.545133	1.541077	1.537277	1.533858	1.530702
1.504406	1.500105	1.554628	1.550385	1.546203	1.542023	1.538096	1.534552	1.531291
1.505685	1.501318	1.555874	1.551565	1.54729	1.542985	1.538929	1.535253	1.531879
1.506878	1.502474	1.55713	1.552792	1.548462	1.544069	1.539909	1.536104	1.532606
1.508033	1.503611	1.558401	1.554052	1.549686	1.545232	1.540987	1.537055	1.533425
1.50925	1.504792	1.559654	1.555252	1.55084	1.546354	1.542053	1.53801	1.534256
1.510531	1.506015	1.560881	1.556385	1.551915	1.547427	1.543104	1.538966	1.535101

1.511801	1.507256	1.562159	1.557572	1.553051	1.548572	1.544229	1.53999	1.535997
1.51305	1.508527	1.563538	1.558869	1.554304	1.549843	1.545476	1.541109	1.536947
1.514266	1.509778	1.564914	1.560178	1.55558	1.551139	1.546753	1.542276	1.537962
1.515409	1.510947	1.566185	1.561417	1.556807	1.552391	1.548004	1.54347	1.539065
1.516524	1.512089	1.567433	1.562648	1.558034	1.553632	1.549246	1.544688	1.540229
1.517698	1.513288	1.568768	1.563957	1.559311	1.55487	1.55045	1.54589	1.541413
1.51891	1.514526	1.570168	1.565325	1.560629	1.556105	1.551626	1.547085	1.542625
1.520091	1.515735	1.571595	1.566721	1.561983	1.5574	1.552875	1.548338	1.543871
1.521235	1.516917	1.573061	1.568159	1.563389	1.558772	1.554212	1.549658	1.545151
1.522368	1.518064	1.574489	1.569558	1.564765	1.560133	1.555557	1.550996	1.546461
1.523478	1.51914	1.575825	1.570865	1.566062	1.561444	1.556885	1.552346	1.547815
1.524608	1.52022	1.577141	1.572151	1.567338	1.562737	1.558199	1.553686	1.549169
1.525822	1.521386	1.578492	1.573463	1.568627	1.564019	1.559482	1.554976	1.550471
1.527087	1.522602	1.579868	1.574797	1.569927	1.565293	1.560742	1.556234	1.551742
1.528359	1.523847	1.581314	1.576203	1.571283	1.566583	1.561985	1.557472	1.552998
1.529644	1.525124	1.582828	1.577676	1.57269	1.567884	1.563208	1.558688	1.554237
1.530906	1.526398	1.521961	1.51758	1.574129	1.569227	1.564472	1.559922	1.555467
1.532134	1.527665	1.523233	1.518832	1.575604	1.570619	1.565785	1.561172	1.556673
1.533356	1.528926	1.524501	1.520083	1.577091	1.572042	1.567138	1.562463	1.557912
1.534569	1.530175	1.525756	1.521326	1.578574	1.573486	1.568538	1.563818	1.559222
1.535795	1.531422	1.527006	1.522571	1.580064	1.574953	1.569974	1.565214	1.560572
1.537083	1.532681	1.528258	1.523833	1.58157	1.576446	1.571441	1.566634	1.561943
1.538412	1.533947	1.529508	1.525103	1.583086	1.577959	1.572933	1.568078	1.563335
1.394962	1.390694	1.530746	1.526351	1.584574	1.579449	1.574407	1.569501	1.564705
1.396107	1.391769	1.531965	1.527574	1.586032	1.580914	1.57586	1.570902	1.566049
1.397269	1.392886	1.533223	1.528825	1.587508	1.582393	1.577328	1.572328	1.567429
1.398458	1.394068	1.53454	1.530121	1.589011	1.58389	1.578812	1.573787	1.568858
1.399658	1.395275	1.535884	1.531443	1.59054	1.585413	1.580325	1.575279	1.570324
1.400865	1.396486	1.53724	1.532792	1.528358	1.523927	1.581888	1.576821	1.571833
1.40208	1.397708	1.538608	1.534157	1.529721	1.525293	1.583481	1.578392	1.573368
1.403299	1.39893	1.394663	1.390484	1.531076	1.526656	1.585072	1.579955	1.574892
1.404525	1.400155	1.395883	1.391693	1.53243	1.528023	1.586667	1.58152	1.576413
1.340614	1.336395	1.397106	1.392912	1.533792	1.52939	1.524977	1.520489	1.516028
1.342003	1.33776	1.398329	1.394139	1.535161	1.530756	1.526332	1.521814	1.517321
1.343397	1.339144	1.33501	1.330981	1.487227	1.482853	1.527683	1.523144	1.518633
1.344789	1.340547	1.336406	1.332358	1.488555	1.484158	1.529026	1.524481	1.519971
1.346194	1.341979	1.337832	1.333753	1.4899	1.48547	1.530372	1.525828	1.521329
1.277346	1.273276	1.339322	1.335197	1.438458	1.434094	1.531732	1.527193	1.522708
1.278806	1.274802	1.340842	1.336665	1.439975	1.435568	1.533098	1.528573	1.524107
1.280275	1.276273	1.342303	1.338109	1.333898	1.329654	1.484947	1.480538	1.4762
1.281753	1.277702	1.343716	1.339537	1.335321	1.331036	1.486232	1.481874	1.477595
1.146988	1.143219	1.275017	1.270895	1.336724	1.332426	1.435532	1.431268	1.427063
1.1484	1.14459	1.27643	1.272279	1.338096	1.333819	1.43701	1.432726	1.428496
1.149786	1.145958	1.277856	1.273678	1.339486	1.335239	1.331045	1.326939	1.322879
1.151107	1.147313	1.279312	1.275118	1.340923	1.336712	1.332531	1.328371	1.324245
1.152406	1.148654	1.280778	1.276583	1.342389	1.338204	1.334028	1.329817	1.32563
1.153723	1.149966	1.28223	1.278077	1.273931	1.269792	1.335476	1.331246	1.327046
1.155052	1.151263	1.283676	1.279586	1.275452	1.271251	1.336896	1.33267	1.328487
1.052707	1.049392	1.285113	1.28103	1.276891	1.272668	1.33831	1.334108	1.32995
1.053964	1.050633	1.286537	1.282407	1.278249	1.274039	1.339713	1.33556	1.33144

1.055253	1.051888	1.287992	1.283816	1.279638	1.27544	1.271286	1.267227	1.26319
1.056581	1.053157	1.289501	1.285288	1.281088	1.276892	1.272732	1.268634	1.264557
1.057927	1.054426	1.291009	1.286772	1.28256	1.278369	1.274202	1.270059	1.265935
1.059272	1.055659	1.155613	1.151863	1.28404	1.279867	1.275705	1.271535	1.267381
1.060632	1.056906	1.156979	1.153253	1.285535	1.28138	1.277226	1.273043	1.26887
1.062028	1.058262	1.158429	1.154662	1.287048	1.282886	1.278734	1.274573	1.270416
1.063455	1.0597	1.159942	1.156088	1.288576	1.284393	1.280238	1.276125	1.272009
1.064908	1.061133	1.057536	1.054094	1.290091	1.285917	1.281776	1.277672	1.273556
1.06639	1.062555	1.058902	1.055397	1.291586	1.287467	1.283364	1.279222	1.275052
0.945094	0.941834	1.060298	1.056726	1.293104	1.288998	1.284905	1.280734	1.276531
0.946332	0.943113	1.061746	1.058096	1.294677	1.290497	1.286351	1.282176	1.277991
0.947577	0.944394	1.063213	1.059479	1.296247	1.291977	1.287772	1.283605	1.279456
0.948834	0.945633	1.064681	1.060844	1.297741	1.293428	1.289211	1.28507	1.280964
0.950092	0.946848	1.066149	1.062215	1.299221	1.294877	1.290659	1.286551	1.28249
0.813138	0.810411	1.067588	1.06364	1.215697	1.211706	1.292138	1.288031	1.283994
0.814035	0.811311	1.069002	1.065111	1.217264	1.213163	1.293643	1.289512	1.285481
0.814949	0.812216	0.947108	0.943846	1.062767	1.058977	1.295182	1.291027	1.286998
0.815889	0.813117	0.9483	0.945034	1.064194	1.060362	1.296748	1.292581	1.288558
0.816835	0.814053	0.811288	0.808532	1.065637	1.061784	1.213573	1.209765	1.206119
0.817785	0.815071	0.812307	0.809507	1.067093	1.063235	1.215156	1.211233	1.20747
0.818733	0.816096	0.813343	0.810509	1.068587	1.064722	1.216778	1.212731	1.208837
0.81966	0.817031	0.81431	0.811527	0.955806	0.952317	1.218367	1.214255	1.210302
0.820576	0.817922	0.815241	0.812552	0.957208	0.953632	1.219962	1.215814	1.211833
0.821504	0.818846	0.816177	0.813516	0.958526	0.95497	1.22167	1.217456	1.213394
0.822439	0.819801	0.817116	0.81442	0.959771	0.956338	1.223493	1.219182	1.214988
0.696508	0.694151	0.818039	0.815332	0.812646	0.809997	0.954264	0.950776	0.947324
0.697418	0.69493	0.818932	0.816268	0.8136	0.810929	0.955565	0.952097	0.948628
0.698346	0.695731	0.819833	0.817201	0.814544	0.81185	0.95683	0.953402	0.949939
0.699256	0.696602	0.820765	0.81811	0.815452	0.812787	0.958068	0.954678	0.951229
0.700186	0.69751	0.821718	0.819017	0.81635	0.813728	0.959285	0.955936	0.952507
0.701186	0.698431	0.695805	0.693308	0.817267	0.814673	0.960507	0.95718	0.953755
0.702242	0.699372	0.696687	0.694165	0.818199	0.815622	0.961725	0.958412	0.954986
0.703328	0.700356	0.69761	0.695054	0.819164	0.816581	0.813983	0.81128	0.808549
0.704452	0.701383	0.698575	0.695976	0.820165	0.817549	0.814945	0.812292	0.809619
0.50451	0.502172	0.699583	0.69693	0.694387	0.691968	0.815904	0.813287	0.810663
0.505517	0.503168	0.700642	0.697923	0.69532	0.692848	0.816848	0.814241	0.811642
0.506518	0.504181	0.701733	0.698935	0.696267	0.693743	0.817804	0.815196	0.812612
0.507548	0.505243	0.702849	0.699947	0.697208	0.694657	0.818808	0.816198	0.81362
0.508577	0.506319	0.703977	0.700971	0.698162	0.695586	0.819833	0.817213	0.814635
0.509535	0.507322	0.705069	0.702022	0.699163	0.696524	0.694	0.691585	0.689232
0.510437	0.508268	0.706132	0.703097	0.700205	0.697471	0.694867	0.692423	0.690069
		0.507032	0.504766	0.701285	0.698475	0.695795	0.693302	0.690916
		0.508066	0.505824	0.702417	0.699553	0.696802	0.694228	0.691766
		0.509062	0.50685	0.703531	0.700628	0.697824	0.695182	0.692655
		0.509959	0.507777	0.704577	0.701658	0.698831	0.696171	0.693624
		0.510841	0.508688	0.705611	0.702684	0.699844	0.697171	0.694607
				0.507543	0.505437	0.700867	0.698133	0.695509
				0.508538	0.506393	0.701904	0.69909	0.696387
				0.509527	0.50737	0.702991	0.700136	0.697378
				0.510511	0.508367	0.704129	0.701265	0.698475

							0.473263	0.472067
							0.472591	0.471375
							0.71431	0.710266
							0.712293	0.708149
							0.710265	0.706064
				0.473383	0.472182	0.470957	0.849746	0.845755
				0.472599	0.471371	0.47014	0.847776	0.843923
				0.716284	0.712231	0.708278	0.845916	0.842128
				0.714361	0.710366	0.70645	0.844133	0.84037
		0.472981	0.471761	0.853871	0.849979	0.846159	1.25984	1.254965
		0.472297	0.471082	0.852035	0.848171	0.844368	1.257552	1.252674
		0.716809	0.712748	1.270076	1.265112	1.260205	1.301727	1.29674
		0.714897	0.710885	1.267761	1.262809	1.257913	1.299401	1.294453
		0.713002	0.709037	1.265455	1.260494	1.255596	1.297081	1.292222
0.471974	0.47077	0.852492	0.848632	1.263149	1.258113	1.253177	1.365713	1.360917
0.471296	0.470071	0.850658	0.846826	1.26085	1.255732	1.250759	1.363353	1.358797
0.715489	0.711412	1.268442	1.263523	1.305091	1.299853	1.294791	1.65903	1.653929
0.713596	0.70958	1.266144	1.261228	1.302781	1.297663	1.292724	1.656684	1.651592
0.711716	0.707751	1.263834	1.258883	1.451088	1.445793	1.440692	1.654331	1.649264
0.709844	0.70592	1.261501	1.256456	1.448502	1.443398	1.438499	1.651962	1.646947
1.269115	1.264202	1.30571	1.300465	1.567772	1.562654	1.557752	1.649598	1.644622
1.266828	1.26195	1.303431	1.298219	1.565373	1.560337	1.555501	1.647245	1.642276
1.264555	1.259713	1.301192	1.296064	1.563072	1.558086	1.553277	1.644895	1.639918
1.262279	1.25747	1.449912	1.44455	1.657867	1.652645	1.647558	1.749493	1.744189
1.260012	1.255233	1.447576	1.44236	1.655516	1.65031	1.645216	1.746996	1.741683
1.304219	1.29934	1.566735	1.561476	1.65316	1.647965	1.642862	1.801928	1.796359
1.301947	1.297079	1.56434	1.559185	1.650805	1.645614	1.6405	1.799328	1.793745
1.512857	1.50761	1.562011	1.556918	1.75591	1.750344	1.744874	1.863008	1.857259
1.510502	1.50538	1.559768	1.554684	1.753357	1.747822	1.742375	1.860339	1.854586
1.567704	1.562631	1.680167	1.674601	1.793156	1.787524	1.781972	1.907814	1.901601
1.565286	1.560339	1.67773	1.672188	1.790576	1.784956	1.779416	1.904956	1.898758

1.562899	1.558056	1.675308	1.669789	1.788011	1.782396	1.776861	1.902097	1.895924
1.560583	1.555781	1.754686	1.748942	1.850344	1.844553	1.838836	1.975416	1.968921
1.558322	1.553514	1.752115	1.746423	1.847727	1.841926	1.836197	1.972399	1.965917
1.678589	1.67331	1.791791	1.786078	1.912506	1.906086	1.899733	2.041605	2.034578
1.676205	1.670899	1.789226	1.783589	1.909729	1.903286	1.896907	2.038354	2.031356
1.721743	1.716277	1.851688	1.8459	2.010983	2.004119	1.997332	2.069702	2.06233
1.719354	1.713837	1.849095	1.843377	2.008129	2.001232	1.9944	2.066366	2.0591
1.716975	1.71142	1.846523	1.840857	2.00527	1.998348	1.991481	2.063064	2.05591
1.827428	1.821563	1.959938	1.953784	2.002327	1.995395	1.988527	2.087594	2.080036
1.824917	1.819055	1.957181	1.951012	1.999344	1.992424	1.98558	2.084111	2.076611
1.850661	1.844759	2.009764	2.003073	2.071694	2.063863	2.056108	2.111772	2.104205
1.848113	1.842264	2.006847	2.000084	2.068258	2.060519	2.052859	2.10836	2.100751
1.961646	1.955315	2.003969	1.997134	2.097671	2.089683	2.081819	2.105045	2.097386
1.958847	1.952642	2.001145	1.994227	2.094133	2.086342	2.078674	2.101851	2.09412
1.97165	1.965232	2.073916	2.065983	2.121775	2.114045	2.106436	2.098637	2.090897
1.96875	1.962346	2.070577	2.062719	2.118364	2.110637	2.103037	2.095315	2.087677
1.965863	1.95943	2.067196	2.059474	2.115029	2.107256	2.099618	2.092006	2.084527
1.962981	1.956484	2.097228	2.089596	2.111795	2.103969	2.096286	2.120704	2.113384
1.960119	1.953535	2.093787	2.086357	2.108611	2.100732	2.093004	2.117568	2.110456
2.072364	2.064622	2.090423	2.083061	2.105289	2.097448	2.089768	2.114418	2.107414
2.069201	2.061419	2.087123	2.079716	2.101851	2.094128	2.086575	2.111264	2.104282
2.099408	2.091574	2.113767	2.106089	2.130409	2.122808	2.115354	2.128775	2.121775
2.096184	2.088493	2.110638	2.102864	2.127154	2.119699	2.112392	2.12583	2.118792
2.092973	2.085387	2.107497	2.099692	2.123988	2.116638	2.109438	2.155564	2.148363
2.089803	2.082161	2.104214	2.096563	2.120974	2.113618	2.10641	2.152572	2.145484
2.086626	2.078902	2.100895	2.093435	2.117986	2.110608	2.103371	2.14957	2.142606
2.083351	2.075675	2.097608	2.09019	2.12622	2.11883	2.111561	2.157117	2.15015
2.080024	2.072471	2.094347	2.086893	2.122914	2.115658	2.108511	2.154029	2.147017
2.106433	2.098755	2.123111	2.115688	2.162527	2.155343	2.148242	2.176265	2.169252
2.103212	2.095649	2.120097	2.112702	2.15952	2.152388	2.145329	2.173329	2.166301
2.100054	2.092551	2.128552	2.121142	2.156572	2.149455	2.142408	2.170427	2.16344
2.096966	2.08942	2.125444	2.118182	2.153696	2.146531	2.139442	2.167546	2.160704
2.093907	2.086344	2.122379	2.115236	2.15082	2.14362	2.136506	2.164696	2.157989
2.108603	2.101159	2.162152	2.154985	2.174018	2.166911	2.159907	2.161882	2.155203
2.105534	2.098265	2.159255	2.152025	2.171028	2.164011	2.157096	2.159095	2.15241
2.140279	2.132968	2.182438	2.175224	2.176944	2.169939	2.163058	2.156317	2.149682
2.137307	2.130001	2.179422	2.172306	2.174087	2.167094	2.160224	2.153554	2.146998
2.164901	2.15761	2.176456	2.169374	2.171189	2.164266	2.15746	2.197709	2.191045
2.161839	2.154689	2.173547	2.166417	2.168232	2.161457	2.154782	2.194922	2.188185
2.184983	2.177803	2.170644	2.163504	2.165342	2.15867	2.152087	2.198129	2.191348
2.182157	2.174864	2.167699	2.160662	2.162581	2.155908	2.149317	2.19531	2.188585
2.179392	2.171979	2.164804	2.157867	2.159866	2.153172	2.146554	2.192496	2.185829
2.176621	2.169239	2.162068	2.155103	2.209888	2.203135	2.196447	2.197451	2.190707
2.173849	2.166572	2.159421	2.152384	2.207093	2.200393	2.193749	2.19469	2.187877
2.170992	2.163827	2.187282	2.180343	2.204427	2.197684	2.190993	2.191916	2.185124
2.168062	2.161027	2.184631	2.177806	2.201877	2.19501	2.188194	2.189134	2.182436
2.173247	2.166315	2.212878	2.206094	2.199285	2.192367	2.185486	2.18641	2.179738
2.170488	2.16351	2.210034	2.203358	2.196638	2.189774	2.182909	2.183759	2.177
2.190171	2.183207	2.207256	2.20062	2.201698	2.194859	2.188003	2.214586	2.207331
2.18752	2.180648	2.204648	2.197897	2.198897	2.192124	2.18536	2.211923	2.204711

2.18488	2.178131	2.202093	2.195202	2.196101	2.18937	2.182688	2.209266	2.202144
2.206384	2.19961	2.199479	2.192597	2.193462	2.18667	2.179951	2.206499	2.199506
2.203532	2.196856	2.196836	2.190041	2.190911	2.184005	2.177187	2.203679	2.196834
2.200792	2.194097	2.201875	2.195106	2.188299	2.181389	2.174558	2.200945	2.194122
2.198156	2.191322	2.199061	2.192361	2.185638	2.178827	2.172063	2.198285	2.191357
2.202252	2.19538	2.196325	2.189625	2.182941	2.176237	2.169565	2.195687	2.188709
2.199692	2.192834	2.193719	2.1869	2.180174	2.173577	2.167037	2.193165	2.186241
2.197129	2.190316	2.191159	2.184227	2.177454	2.170936	2.164508	2.19067	2.183838
2.2023	2.195485	2.188614	2.181661	2.208678	2.201758	2.194963	2.188183	2.181467
2.199677	2.192916	2.186082	2.179153	2.206078	2.199146	2.192363	2.185685	2.179103
2.196997	2.190305	2.183526	2.176644	2.203473	2.196494	2.189684	2.183092	2.176638
2.194274	2.187663	2.180955	2.174146	2.200885	2.193829	2.186958	2.180434	2.174102
2.191616	2.185033	2.178377	2.171651	2.19834	2.191277	2.18439	2.180736	2.174444
2.189032	2.18242	2.175795	2.169163	2.195846	2.188853	2.181997	2.17827	2.171861
2.186455	2.179795	2.207261	2.200276	2.196108	2.189256	2.182495	2.175855	2.169341
2.183853	2.177123	2.204482	2.197617	2.193585	2.186845	2.180163	2.173502	2.166938
2.181277	2.174484	2.201734	2.194976	2.191069	2.184439	2.177838	2.171168	2.164575
2.178769	2.171965	2.19913	2.192424	2.188591	2.182031	2.175485	2.1688	2.162187
2.176303	2.169518	2.196613	2.189933	2.186146	2.179629	2.173123	2.16643	2.159817
2.173846	2.167092	2.196868	2.190286	2.183763	2.177264	2.170775	2.171539	2.164935
2.171405	2.164697	2.194449	2.187925	2.181438	2.17493	2.168436	2.16922	2.16268
2.17249	2.165862	2.192033	2.185561	2.179104	2.172573	2.166081	2.166949	2.16056
2.17007	2.163488	2.189612	2.183186	2.176753	2.170183	2.163701	2.164734	2.158603
2.200804	2.193876	2.187205	2.180813	2.181808	2.175195	2.16872	2.162549	2.156699
2.198349	2.19146	2.184817	2.178444	2.17943	2.172784	2.166334	2.160363	2.154792
2.195917	2.189065	2.182444	2.176075	2.177052	2.170401	2.164001	2.158229	2.152924
2.193496	2.186684	2.18008	2.173703	2.174684	2.168093	2.161795	2.152175	2.147018
2.191092	2.184319	2.177728	2.17133	2.172324	2.165846	2.159696	2.150224	2.145242
2.188701	2.18197	2.182736	2.176286	2.169968	2.163668	2.157725	2.164328	2.159495
2.186321	2.179633	2.180379	2.173876	2.167612	2.16156	2.155888	2.162622	2.157906
2.191336	2.184687	2.178097	2.171537	2.181443	2.175632	2.170217	2.160956	2.156365
2.189052	2.1825	2.175926	2.169288	2.179199	2.17359	2.168411	2.159313	2.154872
2.186808	2.180318	2.173769	2.167109	2.177053	2.171627	2.166655	2.157706	2.153404
2.184613	2.178081	2.18762	2.18107	2.170851	2.165518	2.160642	2.156123	2.151936
2.182426	2.175812	2.185382	2.179006	2.168949	2.16375	2.158993	2.154581	2.150488
2.196157	2.18951	2.183132	2.176973	2.167107	2.162058	2.157425	2.159869	2.155804
2.193813	2.187136	2.180871	2.174977	2.16533	2.160442	2.15593	2.158439	2.154417
2.191469	2.184783	2.178637	2.173001	2.163573	2.158851	2.154469	2.157055	2.153088
2.189119	2.18243	2.176409	2.171033	2.161828	2.157282	2.153042	2.155723	2.151828
2.186812	2.180205	2.174332	2.169177	2.160159	2.155777	2.151665	2.15441	2.150553
2.184563	2.178165	2.172469	2.167467	2.15858	2.154352	2.150357	2.153115	2.149233
2.18237	2.176245	2.170749	2.16588	2.157096	2.152983	2.149068	2.151816	2.147898
2.180232	2.174431	2.164965	2.160197	2.155758	2.151669	2.147748	2.150479	2.146554
2.178163	2.172703	2.163467	2.158857	2.1545	2.150389	2.146419	2.149127	2.145205
2.176213	2.170974	2.161901	2.157431	2.159919	2.155785	2.151767	2.147785	2.143868
2.174373	2.169258	2.160296	2.155954	2.158517	2.15442	2.150418	2.146449	2.142541
2.172601	2.167612	2.158749	2.154499	2.157115	2.153049	2.14907	2.145134	2.141263
2.170897	2.166038	2.157258	2.153057	2.155698	2.151659	2.147712	2.143842	2.140048
2.165098	2.160327	2.155843	2.151685	2.154345	2.150328	2.146405	2.142576	2.138831
2.163491	2.15891	2.15455	2.150451	2.153132	2.14913	2.145204	2.141349	2.137569

2.161933	2.15753	2.153288	2.149246	2.151946	2.147959	2.144028	2.140137	2.13631
2.160426	2.156119	2.151949	2.14795	2.150666	2.146701	2.142785	2.138907	2.13509
2.158964	2.154704	2.150576	2.146613	2.14934	2.145404	2.141518	2.137678	2.1339
2.157507	2.153301	2.149214	2.145276	2.148017	2.144127	2.140289	2.136495	2.132756
2.156055	2.151904	2.147855	2.143931	2.146687	2.142864	2.139096	2.135356	2.131662
2.154691	2.150588	2.153248	2.14929	2.145413	2.141616	2.137881	2.13418	2.130528
2.153457	2.149393	2.152064	2.148111	2.144224	2.140389	2.136623	2.132937	2.129321
2.152233	2.148205	2.150882	2.146935	2.143038	2.139167	2.135372	2.131701	2.128118
2.150932	2.146939	2.149621	2.145682	2.141792	2.13793	2.13415	2.130511	2.126968
2.149624	2.145663	2.148349	2.144419	2.140543	2.136706	2.132955	2.129347	2.125837
2.148383	2.144454	2.147145	2.143222	2.139359	2.135548	2.13182	2.128214	2.124701
2.147183	2.143282	2.145978	2.142063	2.138213	2.134432	2.130725	2.127105	2.123565
2.145941	2.142057	2.14475	2.140843	2.137005	2.133244	2.129559	2.135445	2.131857
2.144648	2.140767	2.143448	2.139549	2.135722	2.131971	2.12831	2.134236	2.130725
2.143409	2.13953	2.1422	2.138315	2.1345	2.130756	2.127108	2.133047	2.129575
2.142273	2.138394	2.14106	2.137196	2.133395	2.129656	2.126002	2.131891	2.128375
2.141158	2.137283	2.139947	2.136106	2.132321	2.128587	2.124926	2.130764	2.127202
2.146599	2.14269	2.138827	2.135004	2.131235	2.127512	2.123861	2.129694	2.12614
2.145424	2.141537	2.137693	2.133888	2.130134	2.126424	2.12279	2.128638	2.125118
2.144173	2.1403	2.136471	2.13268	2.128941	2.125246	2.121631	2.136173	2.132597
2.142859	2.138997	2.135178	2.131399	2.127676	2.123995	2.120404	2.134929	2.131402
2.141635	2.13778	2.133968	2.130196	2.136036	2.132259	2.128582	2.133756	2.130275
2.140526	2.136673	2.132861	2.12909	2.134915	2.131141	2.127476	2.132668	2.129232
2.139425	2.135576	2.131768	2.127998	2.142706	2.138863	2.135134	2.131605	2.128202
2.138307	2.134468	2.13067	2.126909	2.141599	2.137783	2.134081	2.130566	2.127172
2.13716	2.133334	2.129547	2.125796	2.140467	2.13669	2.133023	2.129518	2.126125
2.135921	2.132106	2.138001	2.134146	2.139221	2.135495	2.131876	2.128388	2.124997
2.13464	2.130836	2.136707	2.132859	2.137927	2.134259	2.130695	2.127226	2.123838
2.143286	2.139384	2.13552	2.131684	2.136751	2.133129	2.129609	2.135588	2.132041
2.142168	2.138272	2.134421	2.130605	2.135674	2.132089	2.128604	2.134561	2.131018
2.140886	2.136991	2.133355	2.129565	2.13464	2.13109	2.127634	2.13356	2.130018
2.139809	2.135929	2.132318	2.128564	2.133649	2.130134	2.126704	2.132592	2.129044
2.138745	2.134881	2.131094	2.127375	2.132683	2.129192	2.125777	2.131618	2.12806
2.137687	2.133839	2.130084	2.126416	2.131735	2.12825	2.124834	2.130618	2.127054
2.136632	2.132799	2.129078	2.125464	2.130792	2.1273	2.12387	2.129586	2.126011
2.135568	2.131753	2.137061	2.133411	2.139645	2.135942	2.132301	2.128472	2.124868
2.134496	2.1307	2.136013	2.13241	2.13861	2.134874	2.131178	2.127302	2.123661
2.133405	2.129606	2.134907	2.131348	2.137525	2.1338	2.130097	2.138042	2.134195
2.13229	2.128465	2.133732	2.130213	2.136379	2.132716	2.129057	2.136952	2.1331
2.131181	2.127368	2.142523	2.138877	2.135274	2.131656	2.128031	2.135875	2.132022
2.130075	2.126347	2.141483	2.137847	2.134248	2.130638	2.127015	2.1348	2.130948
2.128999	2.125363	2.140496	2.136858	2.133249	2.129632	2.126001	2.133733	2.129885
2.127985	2.124403	2.139502	2.135869	2.132252	2.128622	2.124978	2.132673	2.128834
2.127009	2.123467	2.138523	2.134892	2.131266	2.127614	2.12395	2.131617	2.127789
2.145046	2.141264	2.137546	2.133918	2.142125	2.138257	2.134399	2.130554	2.126739
2.143975	2.140258	2.136573	2.132948	2.141083	2.1372	2.133329	2.129487	2.125685
2.142984	2.139281	2.1356	2.131967	2.140026	2.136132	2.132256	2.12842	2.124632
2.142092	2.138337	2.134623	2.130971	2.13895	2.135053	2.131181	2.127354	2.123581
2.141198	2.1374	2.133652	2.129971	2.137869	2.133969	2.130104	2.126286	2.122528
2.140261	2.136452	2.132681	2.128959	2.136778	2.132878	2.129019	2.125211	2.121467

2.139315	2.135516	2.131729	2.127959	2.135698	2.131793	2.127939	2.12414	2.120413
2.138379	2.134625	2.142661	2.138635	2.134653	2.130728	2.126869	2.123086	2.119385
2.137439	2.133748	2.141741	2.137665	2.133639	2.129685	2.125813	2.122046	2.118375
2.131662	2.127972	2.140758	2.136704	2.13268	2.128694	2.12479	2.126028	2.122336
2.130647	2.126954	2.139722	2.135756	2.131777	2.127751	2.123797	2.125012	2.121327
2.129628	2.125929	2.138664	2.134752	2.130812	2.126794	2.122841	2.124039	2.120353
2.128608	2.124906	2.137584	2.133669	2.129755	2.125821	2.121943	2.123128	2.11942
2.127582	2.123872	2.136493	2.132569	2.128676	2.124819	2.121015	2.122209	2.11851
2.126539	2.122814	2.135395	2.131478	2.1276	2.123758	2.119987	2.121239	2.117634
2.125503	2.121759	2.1343	2.13039	2.126522	2.12268	2.118932	2.12025	2.116753
2.136544	2.132594	2.133221	2.129306	2.125447	2.121634	2.117922	2.119255	2.115794
2.135525	2.131546	2.132162	2.128233	2.12438	2.120608	2.116936	2.118254	2.114785
2.134584	2.130589	2.126598	2.122602	2.128285	2.124501	2.12082	2.120551	2.117032
2.133735	2.129734	2.125708	2.121642	2.127276	2.123504	2.119839	2.119532	2.11599
2.132812	2.128814	2.124781	2.120694	2.126313	2.122561	2.118911	2.118558	2.114977
2.131751	2.127768	2.123777	2.119762	2.125429	2.121717	2.118077	2.117658	2.114005
2.130668	2.126696	2.122746	2.118806	2.124525	2.120851	2.117227	2.11676	2.113057
2.12962	2.125638	2.126844	2.122877	2.123495	2.119853	2.116265	2.115817	2.112146
2.128598	2.124601	2.125775	2.121795	2.122427	2.118808	2.115255	2.114846	2.111238
2.127667	2.123665	2.124808	2.120809	2.124696	2.121022	2.117429	2.113829	2.110237
2.126804	2.122805	2.123915	2.11989	2.123729	2.120023	2.1164	2.112775	2.109171
2.131	2.126963	2.122954	2.11896	2.1185	2.11476	2.111092	2.111781	2.108146
2.129912	2.125874	2.121914	2.118022	2.117616	2.113893	2.110211	2.110865	2.107165
2.128855	2.124795	2.120864	2.117056	2.11669	2.112981	2.109292	2.11772	2.113906
2.127894	2.123764	2.119809	2.116025	2.115658	2.11195	2.108268	2.116723	2.11296
2.126949	2.122763	2.118783	2.115004	2.11462	2.110901	2.107221	2.115707	2.112008
2.125957	2.121807	2.117852	2.114086	2.113674	2.109917	2.1062	2.114659	2.110974
2.124951	2.12088	2.116967	2.113211	2.112764	2.10896	2.105194	2.113603	2.109911
2.123982	2.119931	2.119371	2.115576	2.119977	2.116083	2.112233	2.112627	2.108931
2.123043	2.118967	2.118369	2.114563	2.118949	2.115103	2.111299	2.111718	2.108018
2.122085	2.118025	2.117417	2.113606	2.117963	2.11413	2.110338	2.106588	2.102864
2.121094	2.117121	2.116542	2.112737	2.117044	2.113161	2.109324	2.105564	2.101852
2.123502	2.119533	2.115645	2.111845	2.116113	2.112202	2.108338	2.104571	2.100865
2.12253	2.118544	2.11465	2.110851	2.115114	2.111253	2.107434	2.103674	2.099961
2.12157	2.117554	2.113644	2.109842	2.114108	2.110314	2.106555	2.102802	2.099072
2.120603	2.116622	2.120908	2.117006	2.113167	2.109399	2.105657	2.104066	2.100255
2.119639	2.115726	2.120013	2.116104	2.112257	2.108494	2.104748	2.10311	2.099259
2.118728	2.11484	2.119092	2.115156	2.111291	2.107529	2.103792	2.102148	2.098307
2.11787	2.113969	2.118151	2.114162	2.110265	2.106501	2.102783	2.101177	2.097397
2.116992	2.113065	2.117195	2.113193	2.111552	2.107747	2.103995	2.100234	2.096492
2.116075	2.112098	2.11621	2.112263	2.110645	2.106852	2.103102	2.09934	2.095591
2.115144	2.111135	2.115243	2.111357	2.109765	2.105981	2.102226	2.098451	2.094682
2.114194	2.11022	2.114336	2.110478	2.108889	2.105105	2.10134	2.097539	2.093741
2.113246	2.10933	2.113459	2.109617	2.108019	2.104227	2.100445	2.096619	2.092795
2.120485	2.116498	2.114849	2.110981	2.107139	2.103315	2.099506	2.101181	2.097338
2.119607	2.115627	2.113972	2.110107	2.106252	2.10238	2.098536	2.100235	2.096442
2.11873	2.114753	2.113079	2.1092	2.105335	2.101455	2.09761	2.099326	2.095562
2.117854	2.113877	2.11217	2.108255	2.104379	2.100536	2.096734	2.098458	2.094702
2.116975	2.112981	2.111243	2.107317	2.103446	2.099637	2.09587	2.097599	2.093844
2.116085	2.112037	2.11028	2.106397	2.102562	2.09877	2.095013	2.096745	2.092994

2.115198	2.111105	2.109332	2.105497	2.101699	2.097915	2.094157	2.095882	2.092125
2.116567	2.112439	2.108462	2.104637	2.100844	2.097061	2.093294	2.094983	2.091193
2.115695	2.111598	2.107632	2.103801	2.099998	2.096205	2.092422	2.094064	2.090234
2.114768	2.110725	2.106787	2.102957	2.099143	2.095318	2.091506	2.093136	2.089311
2.113793	2.109832	2.105934	2.102112	2.098282	2.094407	2.090553	2.092198	2.088415
2.112858	2.108952	2.105084	2.101271	2.102981	2.099036	2.095117	2.091296	2.087539
2.111973	2.108088	2.104236	2.100434	2.102143	2.09819	2.094262	2.090439	2.086685
2.11111	2.107237	2.103394	2.099601	2.101309	2.097354	2.093423	2.089597	2.085841
2.110262	2.106399	2.102564	2.098773	2.100476	2.096521	2.092589	2.088758	2.085
2.109428	2.105571	2.101738	2.097946	2.099643	2.095689	2.091758	2.087923	2.084161
2.1086	2.104744	2.100909	2.097113	2.098802	2.094849	2.090918	2.087079	2.083314
2.107781	2.10392	2.100081	2.096278	2.097959	2.094005	2.090075	2.086232	2.082464
2.106971	2.103106	2.099264	2.095458	2.097131	2.093176	2.089244	2.085398	2.081629
2.106172	2.102302	2.098458	2.094653	2.096318	2.092359	2.088424	2.084575	2.080806
2.105368	2.101495	2.097649	2.093844	2.095503	2.091541	2.087605	2.083761	2.08
2.104553	2.100678	2.096832	2.093028	2.094683	2.090719	2.086784	2.082957	2.079217
2.103744	2.099865	2.096016	2.092213	2.093862	2.089896	2.085966	2.082157	2.078442
2.102948	2.099057	2.095204	2.0914	2.093043	2.089074	2.085146	2.081358	2.07767
2.102168	2.098257	2.094394	2.090588	2.092226	2.088255	2.08433	2.080562	2.0769
2.101434	2.097476	2.093588	2.089776	2.091407	2.087439	2.083523	2.080917	2.077258
2.100732	2.096709	2.092786	2.088964	2.090589	2.086629	2.082725	2.080123	2.076477
2.09999	2.095939	2.097533	2.093661	2.089786	2.085853	2.081975	2.079369	2.075716
2.099205	2.095161	2.096747	2.092866	2.089001	2.085119	2.081285	2.078664	2.074978
2.098422	2.094406	2.095989	2.092084	2.088214	2.084367	2.080568	2.077941	2.074241
2.097662	2.093704	2.09528	2.09132	2.087414	2.083567	2.079781	2.077167	2.073489
2.096896	2.092994	2.094568	2.090561	2.086619	2.082766	2.078988	2.076395	2.072753
2.0961	2.092218	2.093798	2.089784	2.085841	2.081992	2.078228	2.075667	2.072069
2.095291	2.091411	2.093001	2.089008	2.085081	2.08124	2.077487	2.074959	2.071406
2.094485	2.090604	2.092213	2.088264	2.085493	2.081635	2.077862	2.07424	2.070702
2.093681	2.089794	2.091428	2.087546	2.084815	2.080947	2.077152	2.073517	2.069968
2.092919	2.089013	2.090651	2.086806	2.084104	2.080261	2.076478	2.072826	2.06925
2.092214	2.088266	2.089878	2.086034	2.083349	2.07958	2.075858	2.072184	2.068555
2.0915	2.087542	2.089135	2.085274	2.08259	2.078882	2.075213	2.071523	2.067856
2.090745	2.086854	2.08845	2.084546	2.081829	2.078137	2.074488	2.070802	2.067131
2.089977	2.086164	2.087774	2.083837	2.081088	2.077392	2.073748	2.070072	2.066414
2.089205	2.085414	2.087049	2.083151	2.080416	2.076706	2.073046	2.069383	2.065747
2.088437	2.084634	2.086301	2.082476	2.079782	2.076051	2.072365	2.068718	2.065108
2.093254	2.089381	2.085551	2.081764	2.079096	2.075385	2.071711	2.068066	2.064451
2.092569	2.088646	2.084795	2.081015	2.078367	2.074714	2.071087	2.06743	2.06378
2.091885	2.087944	2.085171	2.081369	2.077644	2.074023	2.070427	2.066774	2.063119
2.091195	2.087291	2.084516	2.08068	2.076929	2.073298	2.069706	2.066089	2.062481
2.09051	2.08664	2.083868	2.080012	2.076239	2.072581	2.068976	2.065387	2.061823
2.089838	2.085954	2.083183	2.079362	2.075599	2.071902	2.068256	2.064661	2.061108
2.08917	2.085263	2.082495	2.078722	2.074977	2.07124	2.067548	2.063932	2.060372
2.088492	2.084602	2.081839	2.078067	2.074321	2.070584	2.066885	2.063244	2.059654
2.087808	2.083958	2.081202	2.077406	2.073648	2.069934	2.066253	2.062583	2.058948
2.087136	2.083292	2.080539	2.076748	2.072993	2.069278	2.065594	2.061924	2.058289
2.086474	2.082599	2.079844	2.076094	2.072359	2.068613	2.064904	2.061266	2.057682
2.085816	2.081931	2.079174	2.075441	2.071713	2.067954	2.064237	2.060627	2.057081
2.08515	2.081307	2.078551	2.074779	2.071033	2.0673	2.063616	2.06002	2.05648

2.084502	2.080705	2.077951	2.074136	2.070369	2.066662	2.063007	2.059416	2.055869
2.084969	2.081181	2.077375	2.07355	2.069773	2.066061	2.062399	2.058789	2.055221
2.084391	2.080607	2.076806	2.072987	2.069206	2.065474	2.061787	2.05815	2.054556
2.083786	2.079984	2.076183	2.072381	2.068604	2.064848	2.061136	2.057495	2.053905
2.083163	2.079324	2.075518	2.071745	2.067979	2.064194	2.060455	2.056827	2.053262
2.082528	2.078683	2.074872	2.071096	2.070753	2.066933	2.063161	2.059502	2.055908
2.081872	2.078065	2.07425	2.070425	2.070057	2.066272	2.062535	2.058856	2.055226
2.081233	2.077457	2.07364	2.069776	2.069386	2.065633	2.061925	2.058232	2.054574
2.080635	2.07687	2.073054	2.069181	2.068783	2.065038	2.061338	2.057648	2.05399
2.080045	2.076275	2.072461	2.068597	2.0682	2.064452	2.06075	2.057071	2.053426
2.077875	2.074074	2.071809	2.06798	2.0676	2.063836	2.060119	2.056459	2.052842
2.077243	2.073398	2.07113	2.067351	2.066995	2.063209	2.059471	2.055834	2.052253
2.076635	2.072771	2.0705	2.066739	2.066395	2.062608	2.05887	2.055243	2.051676
2.07605	2.072186	2.06991	2.066143	2.0658	2.062031	2.058309	2.054683	2.05111
2.075433	2.071587	2.06931	2.065522	2.065174	2.061434	2.057741	2.054107	2.050517
2.074759	2.070957	2.068683	2.064856	2.064494	2.060802	2.057152	2.053501	2.049873
2.0741	2.070337	2.068066	2.064208	2.063835	2.060185	2.056572	2.052906	2.049247
2.073503	2.069749	2.067481	2.06362	2.063248	2.059615	2.056017	2.052349	2.048683
2.072934	2.069179	2.066913	2.063063	2.062695	2.059069	2.055476	2.051812	2.048147
2.07237	2.068619	2.064828	2.060993	2.062156	2.058532	2.054941	2.051281	2.04762
2.071816	2.068069	2.064288	2.060466	2.061632	2.058007	2.054414	2.050758	2.047105
2.075194	2.071433	2.063745	2.059936	2.061108	2.057483	2.05389	2.050238	2.04659
2.07464	2.070879	2.063199	2.059404	2.060582	2.056961	2.053369	2.04972	2.046075
2.074091	2.070332	2.066551	2.062743	2.060059	2.056439	2.052849	2.049203	2.045562
2.07355	2.069793	2.066017	2.062219	2.059538	2.055919	2.05233	2.048689	2.045054
2.073013	2.069259	2.065488	2.061698	2.059018	2.055401	2.051811	2.048175	2.044546
2.07248	2.068727	2.064961	2.061178	2.057152	2.053533	2.049941	2.047658	2.044033
2.07195	2.068197	2.064435	2.060661	2.056638	2.053016	2.049423	2.047141	2.043519
2.071423	2.06767	2.063911	2.060142	2.060022	2.056375	2.052756	2.046625	2.043008
2.070898	2.067145	2.063387	2.059622	2.059502	2.055854	2.052234	2.046111	2.042499
2.070372	2.06662	2.062864	2.059103	2.055399	2.051783	2.048197	2.040836	2.037272
2.069845	2.066095	2.062342	2.058584	2.054882	2.051262	2.047673	2.04032	2.036764
2.069318	2.06557	2.061819	2.058065	2.054364	2.05074	2.047149	2.04355	2.039975
2.068789	2.065042	2.061293	2.05754	2.053839	2.050215	2.046624	2.043029	2.039459
2.068266	2.064519	2.060771	2.05702	2.05332	2.049694	2.046102	2.042512	2.03895
2.067757	2.06401	2.060262	2.056513	2.052812	2.049178	2.045583	2.042002	2.038454
2.067264	2.063513	2.059765	2.05602	2.052319	2.048674	2.045071	2.041502	2.037974
2.066814	2.06304	2.059291	2.055568	2.051876	2.048209	2.044587	2.041037	2.037538
2.066406	2.062591	2.05884	2.055156	2.051481	2.047781	2.04413	2.040605	2.037146
2.065964	2.06213	2.058379	2.054715	2.05105	2.047336	2.043673	2.040162	2.036724
2.065471	2.061646	2.057898	2.054228	2.050563	2.046859	2.043207	2.039696	2.036255
2.064974	2.061173	2.057428	2.05374	2.050069	2.046389	2.042759	2.039241	2.035784
2.064498	2.06074	2.057001	2.053278	2.049595	2.045954	2.042358	2.038824	2.035332
2.064023	2.06031	2.056576	2.052819	2.049124	2.045523	2.041963	2.038413	2.034885
2.063524	2.059825	2.056096	2.052334	2.048641	2.045058	2.041516	2.037966	2.034433
2.063015	2.05931	2.055585	2.051837	2.048156	2.044577	2.041039	2.0375	2.033984
2.062547	2.058826	2.055104	2.05138	2.047712	2.044121	2.040575	2.037054	2.033565
2.06212	2.05837	2.054651	2.050964	2.047313	2.043693	2.040121	2.036627	2.03318
2.061695	2.057937	2.054219	2.050542	2.046892	2.043253	2.039667	2.03619	2.032769
2.061262	2.057538	2.05382	2.050106	2.046429	2.042785	2.039201	2.035723	2.032305

2.060831	2.057134	2.053411	2.049658	2.045957	2.042324	2.038753	2.035268	2.031838
2.049634	2.045902	2.052948	2.049186	2.045492	2.041895	2.038356	2.034858	2.0314
2.049199	2.045425	2.052464	2.048709	2.045036	2.041484	2.03798	2.034469	2.030982
2.048727	2.044943	2.051998	2.048269	2.044618	2.041076	2.03758	2.046579	2.042973
2.048227	2.044457	2.051542	2.047856	2.04423	2.040676	2.037168	2.046179	2.042616
2.047751	2.044005	2.051112	2.047442	2.043828	2.040281	2.036779	2.045786	2.042231
2.0473	2.043592	2.050712	2.047024	2.043407	2.039894	2.036421	2.045403	2.041809
2.046881	2.043187	2.0395	2.035819	2.043	2.039506	2.036048	2.04501	2.041393
2.046502	2.04277	2.039078	2.035427	2.042624	2.039111	2.035634	2.044596	2.041004
2.04615	2.042373	2.038671	2.035045	2.042253	2.038715	2.035215	2.044178	2.040613
2.045823	2.042035	2.038315	2.034662	2.041853	2.038319	2.034825	2.04377	2.040187
2.045508	2.041727	2.037989	2.034286	2.041449	2.037926	2.034445	2.043366	2.03975
2.045145	2.041395	2.037664	2.033945	2.039842	2.036271	2.032751	2.042956	2.039344
2.044742	2.041047	2.037346	2.033635	2.039521	2.0359	2.032334	2.042539	2.038963
2.044341	2.040667	2.036988	2.033305	2.039196	2.035549	2.031954	2.042146	2.038572
2.043948	2.04024	2.036571	2.032945	2.038869	2.035231	2.031626	2.041779	2.038155
2.043556	2.039824	2.036161	2.03257	2.02899	2.025401	2.021828	2.040477	2.036801
2.043156	2.039455	2.035793	2.032173	2.028583	2.025026	2.021487	2.040147	2.036469
2.042768	2.039107	2.035444	2.031779	2.028167	2.024635	2.021131	2.039814	2.036155
2.042431	2.03878	2.035116	2.031438	2.045541	2.041821	2.038144	2.042524	2.038873
2.042125	2.038466	2.034799	2.031126	2.045209	2.041428	2.037703	2.042108	2.038512
2.041791	2.038103	2.034434	2.030786	2.044875	2.041068	2.037315	2.041717	2.038128
2.041433	2.037692	2.03402	2.030422	2.044545	2.040742	2.036977	2.041346	2.037715
2.041079	2.037303	2.033623	2.030042	2.044177	2.040398	2.036646	2.032945	2.029301
2.04074	2.03696	2.033259	2.029634	2.043741	2.040013	2.036319	2.032633	2.028987
2.040389	2.036625	2.032911	2.029241	2.043316	2.039633	2.03599	2.032327	2.028688
2.039993	2.036273	2.032576	2.028899	2.042964	2.039293	2.035662	2.032011	2.02838
2.039589	2.035921	2.032253	2.028583	2.042643	2.038962	2.035322	2.031684	2.02807
2.039234	2.035586	2.031932	2.02827	2.042316	2.038598	2.034929	2.031315	2.027743
2.038912	2.035265	2.031614	2.027964	2.041992	2.038214	2.0345	2.030916	2.027404
2.036136	2.032491	2.031294	2.027656	2.041677	2.037864	2.034123	2.030557	2.027082
2.035829	2.032175	2.030974	2.027351	2.041372	2.037552	2.033804	2.030243	2.026777
2.03551	2.031834	2.028193	2.024593	2.041062	2.037247	2.0335	2.029943	2.026479
2.035164	2.031439	2.027796	2.024241	2.040736	2.036939	2.033201	2.02965	2.026186
2.034821	2.031048	2.027402	2.023889	2.040409	2.036633	2.032908	2.029362	2.025898
2.034511	2.03072	2.027071	2.023571	2.038287	2.034514	2.030794	2.029069	2.025607
2.034222	2.030427	2.026775	2.023272	2.037982	2.034214	2.0305	2.028776	2.025319
2.033943	2.030148	2.026493	2.022987	2.037688	2.033922	2.030212	2.026685	2.023242
2.033677	2.029886	2.02623	2.022717	2.037404	2.033636	2.029927	2.026405	2.022968
2.033416	2.029629	2.025972	2.022452	2.037127	2.033358	2.02965	2.02613	2.022696
2.03316	2.029374	2.025716	2.022192	2.036854	2.033087	2.029381	2.02586	2.022424
2.023015	2.0193	2.025472	2.021939	2.036587	2.032821	2.029116	2.025593	2.022155
2.022768	2.019063	2.025239	2.021694	2.036322	2.032555	2.028851	2.025328	2.021888
2.022532	2.018845	2.025026	2.021461	2.036064	2.032293	2.028588	2.025064	2.021624
2.02232	2.01867	2.02486	2.021256	2.035817	2.032035	2.028325	2.024799	2.021361
2.02212	2.01852	2.024722	2.021068	2.035579	2.031782	2.028066	2.024537	2.0211
2.021893	2.018322	2.014728	2.011112	2.035338	2.031536	2.027819	2.024284	2.02084
2.02164	2.018081	2.014492	2.010873	2.035096	2.031298	2.027581	2.024036	2.02058
2.021394	2.017836	2.029627	2.025864	2.034853	2.031076	2.027376	2.023818	2.020337
2.021155	2.017588	2.029366	2.025599	2.034605	2.030883	2.027225	2.023647	2.02012

2.020938	2.01735	2.029108	2.025337	2.034361	2.030685	2.027065	2.023471	2.019906
2.010388	2.006784	2.028862	2.025084	2.034113	2.030452	2.026846	2.02325	2.019679
2.010226	2.006574	2.028628	2.024848	2.03388	2.030215	2.026606	2.023012	2.01945
2.010029	2.006365	2.028416	2.02466	2.024968	2.021331	2.017748	2.022781	2.019217
2.009813	2.00616	2.028224	2.024508	2.024829	2.021155	2.01753	2.022552	2.018985
2.009613	2.00598	2.018465	2.014775	2.024654	2.02096	2.017319	2.022353	2.018798
2.009431	2.005831	2.018318	2.014586	2.024439	2.020744	2.017114	2.022186	2.018665
2.009256	2.005668	2.018149	2.014385	2.024224	2.020542	2.016931	2.022029	2.018519
2.009095	2.005476	2.017931	2.014163	2.024014	2.020368	2.016787	2.021882	2.018339
2.008924	2.005272	2.017705	2.013947	2.023819	2.020203	2.016643	2.021729	2.018148
2.008712	2.005063	2.017498	2.01377	2.014405	2.010806	2.00726	2.021538	2.017948
2.008485	2.004854	2.017303	2.013611	2.014258	2.010632	2.007055	2.021333	2.01775
1.998581	1.994945	2.017132	2.013431	2.014068	2.010432	2.00685	2.012503	2.009
1.998399	1.99479	2.016983	2.013237	2.013847	2.010211	2.006642	2.012329	2.008861
1.998236	1.994629	2.016814	2.013038	2.013633	2.010009	2.00646	2.00299	1.999572
1.998097	1.994453	2.016612	2.012824	2.013424	2.009832	2.006313	2.002836	1.999401
1.997956	1.994287	2.007795	2.003966	2.008947	2.005414	2.001951	1.998507	1.995097
1.997799	1.994152	2.007669	2.003836	2.008813	2.005261	2.001784	1.99835	1.994958
1.997636	1.994016	2.00755	2.003714	2.008686	2.005111	2.001615	1.998192	1.994819
1.997477	1.993844	2.007362	2.003523	2.008506	2.004945	2.001461	1.998031	1.994641
1.997316	1.993654	2.007143	2.003302	2.008302	2.004773	2.001312	1.997867	1.994446
1.997146	1.993486	2.006971	2.003129	1.999398	1.995832	1.992335	1.997699	1.994281
1.996968	1.99334	2.006844	2.003002	1.999258	1.995659	1.992138	1.997526	1.994142
1.985704	1.982135	2.006684	2.002842	1.999095	1.995486	1.991958	1.997359	1.993989
1.985535	1.981935	2.00646	2.002617	1.998882	1.99531	1.991812	1.997205	1.993809
1.985368	1.981744	2.006252	2.00241	1.998686	1.995141	1.991663	1.997048	1.993631
1.97851	1.974924	2.006122	2.002286	1.998556	1.994981	1.991478	1.988001	1.984561
1.97834	1.97479	2.006013	2.00219	1.998452	1.994834	1.991292	1.987821	1.984403
1.978194	1.974624	2.000416	1.996633	1.998346	1.994719	1.991159	1.987668	1.98423
1.978067	1.974444	2.000224	1.996491	1.998241	1.994626	1.991062	1.987535	1.984051
1.994052	1.990243	2.000069	1.996336	1.992674	1.989096	1.985564	1.987431	1.983921
1.993953	1.990144	1.999948	1.996161	1.99248	1.988956	1.985482	1.987359	1.983843
1.993836	1.99005	1.99985	1.996021	1.992323	1.988834	1.985398	1.981892	1.978387
1.993676	1.989952	1.999785	1.995947	1.99224	1.988749	1.985314	1.981814	1.978313
1.99352	1.989852	1.999724	1.99589	1.992177	1.988666	1.985212	1.981719	1.978229
1.993411	1.989756	1.996624	1.992784	1.9921	1.988548	1.985061	1.981582	1.978123
1.993319	1.989651	1.99653	1.992708	1.992023	1.988422	1.984891	1.981424	1.977999
1.9932	1.989499	1.996388	1.992607	1.991944	1.988325	1.984769	1.981284	1.977849
1.993065	1.989308	1.996208	1.992487	1.991865	1.98825	1.98468	1.981156	1.977674
1.992951	1.989164	1.988775	1.985137	1.991784	1.98818	1.984608	1.981057	1.977541
1.992866	1.989078	1.988693	1.985054	1.991708	1.988119	1.984557	1.980995	1.977459
1.992785	1.989002	1.988615	1.984953	1.991603	1.988039	1.9845	1.980937	1.977388
1.992697	1.988918	1.988514	1.984801	1.991435	1.98791	1.984417	1.980866	1.977312
1.992611	1.988837	1.988416	1.98465	1.991265	1.987778	1.984329	1.980795	1.977243
1.986076	1.982333	1.988341	1.984559	1.988119	1.984643	1.98121	1.980729	1.977179
1.986001	1.982261	1.988282	1.984502	1.988057	1.984579	1.981145	1.980666	1.977122
1.985932	1.982195	1.988226	1.984449	1.988005	1.984526	1.981092	1.980613	1.977071
1.985869	1.982134	1.988176	1.984405	1.987967	1.984488	1.981053	1.98057	1.977027
1.985805	1.982071	1.981952	1.978209	1.987927	1.984448	1.981011	1.980525	1.976979
1.985735	1.982004	1.981892	1.978153	1.987882	1.984403	1.980966	1.980475	1.976926

1.981658	1.97795	1.981832	1.978097	1.980628	1.977199	1.973812	1.977394	1.973848
1.981588	1.977881	1.981772	1.97804	1.980577	1.977146	1.973754	1.977334	1.973782
1.981519	1.977814	1.981713	1.977983	1.980526	1.977091	1.973695	1.977271	1.973713
1.981457	1.977753	1.981657	1.977928	1.974322	1.970912	1.967543	1.977212	1.97365
1.981398	1.977696	1.981605	1.977875	1.974267	1.970855	1.967482	1.977155	1.973589
1.981334	1.977635	1.981553	1.977829	1.974221	1.970802	1.96742	1.977095	1.973525
1.981265	1.977572	1.981502	1.977786	1.97418	1.97075	1.967356	1.97703	1.973456
1.981197	1.977509	1.98146	1.97777	1.974173	1.970722	1.967302	1.971194	1.967645
1.981127	1.977446	1.981432	1.977802	1.97423	1.970736	1.967264	1.971135	1.967579
1.981067	1.977391	1.981409	1.977833	1.974282	1.97075	1.967231	1.971082	1.967517
1.978974	1.975292	1.977625	1.974074	1.974266	1.970724	1.967192	1.963651	1.960128
1.978952	1.975269	1.977599	1.974036	1.974221	1.970682	1.967153	1.963607	1.960076
1.978943	1.975288	1.977624	1.974031	1.974193	1.970648	1.967122	1.963594	1.960084
1.978947	1.975343	1.977688	1.974047	1.974173	1.970618	1.967099	1.963606	1.960139
1.97256	1.968981	1.977706	1.97404	1.974152	1.97061	1.967108	1.963627	1.960165
1.972505	1.968927	1.977662	1.974001	1.974131	1.970633	1.967163	1.963661	1.960158
1.972482	1.968886	1.977622	1.973968	1.97039	1.966938	1.963507	1.963678	1.960143
1.972534	1.96889	1.977607	1.973947	1.970367	1.966913	1.963483	1.963653	1.960122
1.9726	1.968909	1.97761	1.973949	1.970364	1.966893	1.963448	1.96362	1.960101
1.972616	1.968919	1.971755	1.968127	1.970429	1.966925	1.963443	1.963603	1.960083
1.972614	1.968933	1.971797	1.96821	1.970528	1.966981	1.963453	1.963597	1.960075
1.972634	1.968982	1.969771	1.966173	1.970575	1.967008	1.963464	1.959944	1.956457
1.972672	1.969062	1.969855	1.96621	1.970579	1.967009	1.963478	1.959993	1.956544
1.972733	1.969134	1.969922	1.966245	1.964717	1.961169	1.957668	1.960051	1.956617
1.972827	1.969186	1.969956	1.966269	1.964746	1.961227	1.95775	1.960121	1.956657
1.97292	1.96925	1.970011	1.966322	1.964802	1.961298	1.957832	1.960194	1.956708
1.972986	1.969354	1.970137	1.966448	1.964916	1.961379	1.957887	1.960263	1.956804
1.97305	1.969465	1.970275	1.966588	1.965043	1.961467	1.957941	1.960335	1.956908
1.977642	1.974003	1.97034	1.966652	1.965112	1.961549	1.958032	1.954563	1.951125
1.977752	1.974057	1.970367	1.966677	1.96515	1.961629	1.958143	1.954649	1.951165
1.970088	1.966407	1.970453	1.966762	1.965233	1.961708	1.958219	1.954723	1.951238
1.970159	1.966527	1.9706	1.96691	1.965363	1.961786	1.958256	1.954783	1.951346
1.966949	1.963358	1.970716	1.967023	1.963371	1.959772	1.956228	1.954851	1.951437
1.96706	1.963428	1.970758	1.967057	1.963412	1.959848	1.956333	1.954937	1.951487
1.967172	1.963503	1.970808	1.967102	1.963466	1.959932	1.956439	1.955024	1.951541
1.967264	1.963616	1.963207	1.959525	1.963592	1.960025	1.956506	1.955097	1.951636
1.967356	1.963744	1.963358	1.959689	1.96375	1.960133	1.95657	1.955172	1.951743
1.967476	1.963848	1.963465	1.959826	1.956207	1.952599	1.949034	1.953197	1.949754
1.96762	1.963938	1.963546	1.95995	1.956365	1.952767	1.949197	1.953319	1.949821
1.967785	1.964073	1.963663	1.960064	1.956483	1.952906	1.94935	1.953459	1.94993
1.967982	1.964266	1.963824	1.960159	1.956548	1.953009	1.949498	1.953626	1.950093
1.968159	1.964451	1.960756	1.957069	1.956638	1.953111	1.949621	1.946115	1.942618
1.968279	1.964603	1.960923	1.957238	1.956792	1.953217	1.949691	1.946208	1.942755
1.968394	1.964739	1.961078	1.957414	1.956964	1.953336	1.949764	1.946293	1.942877
1.968547	1.964852	1.961188	1.957558	1.957129	1.95349	1.949897	1.946403	1.942965
1.968719	1.964957	1.961278	1.957685	1.957288	1.953661	1.950064	1.946529	1.943043
1.968897	1.96511	1.961412	1.957805	1.957405	1.953805	1.950232	1.946687	1.943178
1.969083	1.965304	1.961582	1.957914	1.957485	1.953929	1.950407	1.946875	1.943363
1.969237	1.965483	1.961759	1.958058	1.954434	1.950921	1.947445	1.947059	1.943551
1.96934	1.965636	1.961939	1.958247	1.954624	1.951102	1.947618	1.94724	1.943739

1.969465	1.965801	1.962129	1.958451	1.954827	1.951279	1.947772	1.947401	1.943918
1.969662	1.966003	1.962335	1.958661	1.955024	1.951425	1.947875	1.947517	1.944074
1.969885	1.966217	1.962545	1.958876	1.955225	1.951572	1.947977	1.94763	1.944227
1.970096	1.96642	1.962748	1.959087	1.955434	1.951762	1.948149	1.947798	1.944399
1.970304	1.966614	1.962942	1.959293	1.955649	1.951978	1.948362	1.947998	1.944585
1.970498	1.966774	1.963095	1.95947	1.955846	1.952183	1.94857	1.945096	1.941691
1.970676	1.966893	1.963203	1.959615	1.956025	1.952379	1.948777	1.945308	1.941902
1.970868	1.967042	1.963344	1.959781	1.956212	1.952579	1.948983	1.945518	1.942112
1.971086	1.967246	1.963542	1.959982	1.956416	1.952783	1.949188	1.945723	1.942319
1.971312	1.967466	1.963758	1.960195	1.956627	1.952992	1.949396	1.94593	1.942525
1.97154	1.967683	1.96397	1.96041	1.956845	1.95321	1.949613	1.946142	1.94273
1.971769	1.967903	1.964187	1.960629	1.957065	1.953431	1.949833	1.946354	1.942933
1.971996	1.968123	1.964402	1.96084	1.957274	1.95364	1.950041	1.946559	1.943133
1.972223	1.968344	1.964617	1.961047	1.957475	1.95384	1.95024	1.946758	1.943331
1.972454	1.968575	1.964842	1.961263	1.957683	1.95404	1.950435	1.94695	1.943522
1.972688	1.968809	1.965073	1.961487	1.957896	1.95424	1.950622	1.947133	1.943706
1.972941	1.969075	1.965336	1.961729	1.958119	1.954446	1.950816	1.947319	1.943889
1.973231	1.969404	1.965661	1.962008	1.958358	1.954665	1.951022	1.947511	1.944071
1.973522	1.969735	1.965989	1.962289	1.9586	1.954891	1.951235	1.94771	1.944258
1.973776	1.970004	1.966257	1.962538	1.958833	1.955114	1.951451	1.947917	1.944457
1.974014	1.970245	1.966497	1.962773	1.959063	1.955343	1.951678	1.948136	1.944666
1.974261	1.970488	1.966736	1.963007	1.9593	1.955602	1.951951	1.948393	1.944894
1.974511	1.970728	1.96697	1.963238	1.959542	1.955886	1.952267	1.948686	1.94514
1.974805	1.970993	1.967216	1.963476	1.959784	1.95615	1.952548	1.948949	1.945375
1.97516	1.971288	1.967475	1.963718	1.960015	1.956379	1.952778	1.949171	1.945592
1.975504	1.971591	1.967754	1.963987	1.960274	1.956621	1.953005	1.94939	1.94581
1.97579	1.971886	1.96806	1.96431	1.960595	1.956904	1.953248	1.949617	1.946032
1.976066	1.972191	1.968386	1.964652	1.960931	1.957199	1.9535	1.949857	1.946269
1.967378	1.963554	1.968735	1.964975	1.961229	1.957477	1.953765	1.950128	1.946554
1.967708	1.963918	1.969096	1.96529	1.96151	1.957751	1.954045	1.950424	1.946869
1.968011	1.964228	1.969406	1.965582	1.961797	1.958054	1.954362	1.950732	1.947156
1.968284	1.964484	1.969663	1.965851	1.962086	1.958386	1.95472	1.951055	1.947421
1.968595	1.96477	1.960968	1.957189	1.962396	1.958711	1.95505	1.951358	1.947685
1.968969	1.965106	1.961293	1.95753	1.962742	1.959025	1.95533	1.951625	1.94795
1.969355	1.965466	1.96164	1.957878	1.963082	1.959328	1.9556	1.951892	1.948227
1.96147	1.957638	1.962016	1.95821	1.954432	1.950679	1.946963	1.952177	1.948535
1.961828	1.958022	1.962396	1.958531	1.954712	1.95095	1.947239	1.952477	1.948854
1.9622	1.958367	1.962724	1.958828	1.954996	1.951255	1.947566	1.952799	1.949157
1.962579	1.958683	1.963018	1.959107	1.955281	1.951584	1.94793	1.95314	1.949453
1.962918	1.958991	1.9551	1.951236	1.955604	1.951917	1.94827	1.944573	1.940897
1.96321	1.959283	1.955408	1.951578	1.955971	1.952254	1.948581	1.944906	1.941266
1.963513	1.959599	1.955735	1.95192	1.95633	1.952596	1.948909	1.945246	1.941624
1.963843	1.959962	1.956097	1.952245	1.95665	1.952943	1.949278	1.945598	1.941945
1.964195	1.960334	1.956463	1.952574	1.956977	1.953292	1.949649	1.945951	1.942267
1.964575	1.960679	1.956799	1.952927	1.94914	1.945475	1.941854	1.946293	1.942625
1.964977	1.961024	1.957131	1.953293	1.949519	1.945818	1.942164	1.946633	1.942996
1.959248	1.955287	1.957511	1.953655	1.949867	1.94616	1.942503	1.946983	1.943344
1.959678	1.955727	1.957931	1.954017	1.950194	1.946503	1.942865	1.94734	1.943676
1.953517	1.949639	1.958357	1.954412	1.950564	1.946863	1.943218	1.939562	1.935937
1.953843	1.950031	1.958796	1.954858	1.950994	1.94724	1.943552	1.939911	1.936319

1.954163	1.950395	1.953064	1.949161	1.951427	1.947634	1.943909	1.940282	1.936719
1.954498	1.950702	1.953391	1.94954	1.951845	1.948053	1.944322	1.940699	1.93714
1.954836	1.951002	1.953705	1.949899	1.952248	1.948474	1.944751	1.941126	1.937558
1.955173	1.951358	1.954072	1.950253	1.946475	1.942739	1.939051	1.941516	1.937923
1.955517	1.951746	1.954469	1.950605	1.946805	1.943101	1.939442	1.941892	1.938264
1.955905	1.952155	1.954888	1.951006	1.947195	1.943501	1.93985	1.936195	1.932562
1.956337	1.952591	1.955335	1.951454	1.94764	1.943937	1.940277	1.936621	1.932986
1.956754	1.952991	1.955746	1.951885	1.948077	1.944349	1.940666	1.937019	1.933401
1.957139	1.953326	1.95609	1.95228	1.948493	1.944716	1.940988	1.937364	1.933793
1.957526	1.953673	1.956446	1.952677	1.948905	1.945084	1.941318	1.937712	1.934182
1.957944	1.95409	1.956875	1.953106	1.949328	1.945489	1.941707	1.938104	1.934585
1.958363	1.954528	1.957323	1.953533	1.94974	1.945899	1.942117	1.938507	1.934978
1.947573	1.943787	1.957728	1.953904	1.950093	1.946277	1.942515	1.938881	1.935316
1.947911	1.944155	1.958111	1.95424	1.950411	1.946636	1.94291	1.93924	1.935618
1.948293	1.944549	1.958525	1.954634	1.950797	1.947041	1.943329	1.939637	1.935983
1.948722	1.944971	1.95897	1.95509	1.951256	1.947493	1.943772	1.940074	1.936414
1.949166	1.945408	1.948692	1.944841	1.951725	1.947956	1.944226	1.940521	1.936854
1.94962	1.945864	1.949157	1.945296	1.952186	1.94842	1.944691	1.940974	1.937289
1.950082	1.946326	1.949627	1.945755	1.95265	1.948888	1.945159	1.941429	1.937726
1.950548	1.946779	1.950088	1.946217	1.953121	1.949354	1.945619	1.941876	1.93816
1.951017	1.947233	1.950548	1.94668	1.953594	1.94982	1.946075	1.942322	1.938595
1.947152	1.943394	1.951016	1.947142	1.943311	1.939547	1.935813	1.942772	1.939038
1.947639	1.943883	1.951491	1.947602	1.94376	1.939992	1.936256	1.943226	1.939488
1.948126	1.944368	1.951956	1.948056	1.944207	1.940439	1.936702	1.932951	1.929223
1.948616	1.944846	1.952412	1.948503	1.94465	1.940887	1.937152	1.933386	1.929638
1.934075	1.930301	1.952864	1.94895	1.945094	1.941332	1.937595	1.933813	1.930046
1.934551	1.930771	1.953313	1.949401	1.945541	1.941769	1.93802	1.934224	1.930443
1.935027	1.931241	1.953763	1.94985	1.945987	1.942204	1.938442	1.934633	1.93084
1.922566	1.918799	1.942876	1.938971	1.946422	1.94264	1.938878	1.935059	1.931252
1.923046	1.919275	1.94333	1.939408	1.946852	1.943077	1.939321	1.935492	1.93167
1.909704	1.905962	1.924737	1.920832	1.947288	1.943512	1.939754	1.935918	1.932087
1.910156	1.906411	1.925204	1.921298	1.947729	1.943945	1.940179	1.936337	1.932501
1.902167	1.898434	1.91246	1.908589	1.925221	1.92146	1.917718	1.93677	1.932936
1.902637	1.898904	1.912939	1.909062	1.925664	1.921897	1.918153	1.937217	1.933391
1.903142	1.899394	1.913436	1.909563	1.926142	1.922361	1.918607	1.937687	1.933876
1.903725	1.899918	1.913964	1.910124	1.923991	1.920164	1.91637	1.915318	1.911566
1.90433	1.900455	1.914504	1.910702	1.924567	1.920702	1.916871	1.915837	1.912113
1.888299	1.884443	1.907025	1.903245	1.912295	1.908442	1.904623	1.916312	1.912592
1.888792	1.884935	1.907533	1.903739	1.912756	1.908899	1.905074	1.916765	1.913033
1.889314	1.885466	1.892204	1.888417	1.905597	1.901766	1.897965	1.917267	1.913507
1.889867	1.886044	1.892782	1.888946	1.906081	1.902297	1.898539	1.917827	1.914017
1.890409	1.886598	1.893335	1.889464	1.901483	1.897745	1.894023	1.89526	1.891451
1.890915	1.887094	1.893829	1.889951	1.901976	1.898227	1.894496	1.895686	1.89188
1.891437	1.887586	1.894319	1.890449	1.90249	1.898714	1.894961	1.896113	1.892329
1.892029	1.888112	1.894839	1.891	1.887199	1.883432	1.879693	1.896603	1.892866
1.892646	1.888654	1.895375	1.891573	1.887787	1.883983	1.880212	1.897124	1.893438
1.893215	1.889209	1.885327	1.88157	1.888338	1.88454	1.880775	1.892838	1.889133
1.89375	1.889778	1.885894	1.882096	1.888862	1.885105	1.881376	1.89343	1.889676
1.869371	1.865512	1.886421	1.882597	1.889365	1.88563	1.881925	1.878208	1.874501
1.869863	1.865983	1.886894	1.883059	1.889833	1.886099	1.882408	1.878735	1.875079

1.852543	1.848675	1.887386	1.883552	1.890337	1.886588	1.882891	1.879247	1.875632
1.85309	1.849163	1.887922	1.884121	1.890926	1.887132	1.883389	1.879732	1.876123
1.853657	1.849681	1.888491	1.884713	1.891531	1.887696	1.883908	1.880223	1.876598
1.854208	1.850253	1.86431	1.860595	1.892085	1.888262	1.884481	1.88076	1.877088
1.854757	1.850855	1.864928	1.86114	1.892622	1.888832	1.885084	1.881324	1.877591
1.855315	1.851408	1.865504	1.861689	1.869721	1.866032	1.862387	1.881893	1.878162
1.855882	1.851919	1.866044	1.862241	1.870291	1.866573	1.862908	1.88247	1.878797
1.856444	1.85246	1.848594	1.844841	1.870853	1.867126	1.86346	1.859858	1.856303
1.856995	1.853056	1.84918	1.845362	1.871392	1.86769	1.864055	1.860453	1.856883
1.857548	1.853642	1.849761	1.845893	1.871953	1.868276	1.864664	1.861048	1.85745
1.858108	1.854168	1.850281	1.846439	1.872569	1.868901	1.865284	1.861634	1.85799
1.858673	1.854691	1.850804	1.847003	1.873209	1.869536	1.8659	1.86221	1.858522
1.859237	1.855288	1.851414	1.847609	1.856474	1.852802	1.849166	1.862763	1.85907
1.859805	1.85593	1.852077	1.848242	1.857077	1.853346	1.849662	1.863301	1.859627
1.828781	1.825238	1.852704	1.848835	1.857652	1.853901	1.850202	1.863867	1.860177
1.829368	1.825809	1.853299	1.849385	1.85819	1.854465	1.850787	1.864457	1.860716
1.829951	1.826368	1.853883	1.849957	1.858769	1.855054	1.85138	1.847642	1.843912
1.83052	1.826907	1.854451	1.850575	1.859423	1.855688	1.851986	1.848236	1.844496
1.831084	1.827451	1.855029	1.851203	1.860083	1.856315	1.852576	1.848824	1.845093
1.831636	1.82802	1.855648	1.851827	1.860705	1.856885	1.853103	1.849371	1.84568
1.832192	1.828604	1.856287	1.852447	1.861307	1.857434	1.853613	1.849909	1.846266
1.820319	1.81676	1.856922	1.853045	1.86189	1.858008	1.854188	1.85049	1.846855
1.820928	1.817361	1.857559	1.853626	1.862454	1.858598	1.854806	1.851101	1.847444
1.821526	1.817946	1.846774	1.842821	1.850328	1.846532	1.842801	1.851679	1.847998
1.822111	1.818517	1.847379	1.843418	1.850929	1.847112	1.843355	1.852215	1.848504
1.822683	1.819068	1.847963	1.844015	1.851545	1.847707	1.843923	1.852766	1.849029
1.823232	1.819583	1.848501	1.844605	1.852183	1.848338	1.844536	1.853372	1.84961
1.823779	1.820099	1.849041	1.845196	1.852821	1.848964	1.84514	1.853975	1.850202
1.824353	1.820655	1.84963	1.845791	1.841953	1.838066	1.834218	1.854515	1.850767
1.824937	1.821234	1.850248	1.846394	1.842527	1.838583	1.834692	1.855033	1.851327
1.8255	1.821814	1.818182	1.814614	1.843145	1.839171	1.835253	1.844764	1.841075
1.826044	1.822398	1.818787	1.815224	1.843799	1.839817	1.835887	1.845388	1.841674
1.826607	1.822982	1.819384	1.815828	1.844448	1.840461	1.83652	1.832731	1.829044
1.827192	1.823561	1.819965	1.81642	1.845083	1.841091	1.837138	1.833322	1.829605
1.812149	1.808532	1.820552	1.817019	1.845726	1.84173	1.837765	1.833924	1.830176
1.812766	1.809135	1.821161	1.817636	1.84639	1.84239	1.838415	1.834547	1.830769
1.81339	1.809744	1.821773	1.818257	1.847058	1.843055	1.839072	1.835182	1.831376
1.771204	1.767635	1.822366	1.818859	1.847703	1.843701	1.839717	1.835817	1.831996
1.771766	1.768186	1.822952	1.819453	1.848339	1.844341	1.840359	1.836458	1.83263
1.772352	1.768766	1.782834	1.779407	1.848997	1.845004	1.841026	1.837124	1.833291
1.772961	1.769378	1.783446	1.780003	1.849681	1.845692	1.841719	1.837819	1.833983
1.719745	1.716515	1.76647	1.763043	1.812522	1.8086	1.804706	1.838504	1.834667
1.720289	1.717049	1.767048	1.763607	1.813144	1.809222	1.80533	1.83916	1.835327
1.720847	1.717591	1.767632	1.764176	1.81377	1.809851	1.805964	1.839823	1.835992
1.721443	1.718163	1.768241	1.764766	1.814419	1.810505	1.806625	1.802826	1.799086
1.72206	1.718749	1.768862	1.765366	1.815082	1.811181	1.807314	1.803513	1.799764
1.722691	1.719333	1.71608	1.712934	1.762496	1.759058	1.755639	1.804252	1.800466
1.723334	1.719918	1.716632	1.713475	1.763111	1.759721	1.756344	1.805032	1.801189
1.723957	1.720504	1.717192	1.714013	1.763705	1.760343	1.756995	1.805776	1.801933
1.724556	1.721088	1.717753	1.714542	1.764271	1.760912	1.757583	1.806484	1.80271

1.72516	1.721691	1.718342	1.715101	1.71196	1.708946	1.705971	1.791101	1.787432
1.725786	1.722331	1.718978	1.715715	1.712548	1.7095	1.7065	1.791751	1.788092
1.726407	1.722974	1.719625	1.716348	1.713157	1.71007	1.707036	1.792391	1.788731
1.726994	1.723587	1.72025	1.716974	1.713771	1.710653	1.707588	1.793034	1.789383
1.727569	1.724188	1.720866	1.717601	1.714393	1.711247	1.708155	1.793684	1.790039
1.728182	1.724802	1.721481	1.718216	1.715005	1.711849	1.708746	1.757831	1.754498
1.728826	1.725426	1.722092	1.718822	1.715611	1.712461	1.70936	1.758502	1.755123
1.729488	1.726069	1.722715	1.719425	1.716199	1.713043	1.709939	1.70688	1.703857
1.730171	1.726731	1.723349	1.720017	1.716758	1.713579	1.710465	1.707421	1.704425
1.730859	1.727411	1.724005	1.720631	1.717336	1.714134	1.711007	1.70797	1.704989
1.731542	1.728119	1.724704	1.721288	1.717959	1.714742	1.711604	1.708543	1.705541
1.732222	1.728829	1.725414	1.721969	1.718613	1.715384	1.71223	1.709134	1.706094
1.732889	1.7295	1.726093	1.722663	1.719309	1.716062	1.712881	1.709745	1.706663
1.733551	1.73015	1.726756	1.723369	1.720036	1.716769	1.713553	1.710373	1.707244
1.734227	1.73081	1.727419	1.724059	1.720737	1.717453	1.714211	1.711007	1.707855
1.734915	1.731474	1.728078	1.724732	1.721415	1.718116	1.714858	1.71165	1.708499
1.735628	1.73217	1.728768	1.725427	1.722107	1.718786	1.715505	1.712285	1.709124
1.736376	1.73292	1.729515	1.726166	1.722826	1.719468	1.716147	1.712896	1.709707
1.73713	1.733667	1.730255	1.726899	1.723545	1.72016	1.716808	1.713526	1.710308
1.720621	1.717187	1.730935	1.727591	1.724248	1.720872	1.717521	1.714222	1.710974
1.721332	1.717843	1.731599	1.728268	1.724943	1.721587	1.718247	1.71494	1.711668
1.701968	1.698504	1.715151	1.711891	1.725634	1.72227	1.718924	1.715613	1.712338
1.7027	1.699228	1.715859	1.712578	1.726319	1.722932	1.71957	1.716262	1.713
1.703429	1.699924	1.716532	1.713228	1.726982	1.723595	1.720239	1.716934	1.713675
1.704153	1.70058	1.717157	1.71383	1.727614	1.724259	1.720935	1.717636	1.71437
1.704884	1.701274	1.697819	1.69452	1.711167	1.707903	1.704668	1.718324	1.715046
1.705626	1.702045	1.698585	1.695246	1.711865	1.708582	1.705329	1.718983	1.715681
1.706379	1.702839	1.699375	1.695985	1.712566	1.709252	1.705973	1.71963	1.716311
1.707158	1.703623	1.700128	1.696669	1.693276	1.689966	1.686702	1.720265	1.716966
1.707944	1.704403	1.70087	1.697338	1.693889	1.690557	1.68729	1.7209	1.717635
1.708676	1.705142	1.701598	1.69804	1.69457	1.691227	1.687955	1.684726	1.681545
1.709365	1.705845	1.702314	1.698767	1.695304	1.691962	1.688682	1.685431	1.682221
1.710104	1.7066	1.703076	1.69953	1.696059	1.692698	1.689394	1.686112	1.682869
1.710914	1.707427	1.703904	1.700341	1.696842	1.693438	1.690086	1.686758	1.683475
1.670371	1.667356	1.704723	1.701138	1.697607	1.694159	1.690764	1.687399	1.684091
1.671039	1.668016	1.705499	1.701874	1.698306	1.694825	1.691406	1.688037	1.684735
1.671689	1.668654	1.706252	1.7026	1.699006	1.695499	1.692062	1.688693	1.685402
1.672261	1.669231	1.706971	1.703359	1.699786	1.696263	1.6928	1.689412	1.686102
1.672791	1.669773	1.707673	1.704139	1.700614	1.697082	1.693591	1.690173	1.68683
1.673372	1.670365	1.708427	1.704947	1.701453	1.69791	1.694395	1.690953	1.687581
1.673998	1.671001	1.709229	1.705784	1.702308	1.698754	1.695218	1.691758	1.688361
1.617448	1.614807	1.668671	1.665715	1.703144	1.699577	1.696018	1.692529	1.689098
1.618015	1.615374	1.66934	1.666387	1.703956	1.70037	1.696781	1.693244	1.689759
1.618607	1.615951	1.670004	1.667045	1.704745	1.701142	1.69753	1.693958	1.690432
1.619245	1.616544	1.670658	1.667682	1.705506	1.701887	1.698267	1.694699	1.691179
1.619905	1.617143	1.671302	1.668302	1.706243	1.702619	1.699005	1.695457	1.691959
1.583373	1.58063	1.671931	1.668899	1.665847	1.66275	1.659645	1.69624	1.692735
1.583971	1.58117	1.672547	1.669476	1.666416	1.663361	1.660302	1.697045	1.693518
1.584544	1.581693	1.639981	1.636948	1.667005	1.663986	1.660965	1.697861	1.694326
1.585087	1.582196	1.640529	1.637501	1.667613	1.664626	1.661638	1.698692	1.695163

1.585638	1.582707	1.579916	1.577264	1.635097	1.63218	1.62926	1.659275	1.656217
1.586209	1.583234	1.580421	1.577769	1.635703	1.632794	1.629886	1.659956	1.656917
1.586783	1.583778	1.580948	1.578287	1.636318	1.633409	1.630504	1.660627	1.657607
1.543099	1.54033	1.581518	1.578826	1.63693	1.634016	1.631113	1.628172	1.625214
1.543574	1.540838	1.582112	1.579378	1.637545	1.63462	1.631715	1.628783	1.625839
1.544065	1.541336	1.582689	1.579927	1.638162	1.635227	1.632321	1.629399	1.626473
1.544571	1.54183	1.583257	1.580475	1.638781	1.635835	1.632928	1.630019	1.627113
1.545083	1.54234	1.539668	1.537058	1.578301	1.575691	1.573128	1.630627	1.627733
1.5456	1.542869	1.54018	1.537521	1.578812	1.576187	1.573618	1.631217	1.628327
1.546131	1.543408	1.540705	1.538009	1.579353	1.576705	1.574122	1.631811	1.628919
1.546686	1.543956	1.541243	1.538535	1.579943	1.577263	1.574649	1.632416	1.629512
1.547245	1.544512	1.541798	1.539088	1.580567	1.577849	1.575195	1.633035	1.630117
1.547787	1.545086	1.542385	1.539673	1.537027	1.53447	1.531976	1.600266	1.597534
1.548316	1.545664	1.542988	1.540278	1.537617	1.535018	1.532484	1.600892	1.598159
1.548813	1.546196	1.543543	1.540848	1.538187	1.535569	1.533013	1.601552	1.598812
1.54928	1.546686	1.544057	1.541388	1.538744	1.536127	1.533565	1.602249	1.599498
1.54975	1.547172	1.54456	1.541913	1.53928	1.536658	1.534086	1.531611	1.529201
1.550231	1.547659	1.545056	1.542422	1.539791	1.537151	1.534558	1.532073	1.529658
1.550714	1.548147	1.545549	1.542922	1.540291	1.537636	1.535025	1.532528	1.530103
1.551193	1.548636	1.546041	1.543413	1.540779	1.538123	1.535507	1.532991	1.530542
1.551682	1.549131	1.546537	1.543904	1.541268	1.538617	1.535999	1.533462	1.530982
1.515294	1.512844	1.547042	1.544416	1.541785	1.539134	1.53651	1.53395	1.531442
1.51578	1.513303	1.547557	1.544946	1.542323	1.539671	1.537038	1.534456	1.531919
1.516319	1.513821	1.511319	1.50882	1.5429	1.540246	1.537606	1.535006	1.532447
1.516921	1.514409	1.5119	1.509402	1.543525	1.540869	1.538222	1.53561	1.533035
1.517492	1.514965	1.512447	1.509946	1.544111	1.541455	1.538806	1.536185	1.533597
1.517994	1.515445	1.512915	1.510408	1.544616	1.541965	1.539321	1.536694	1.534096
1.518481	1.515916	1.513373	1.510858	1.545109	1.542462	1.539823	1.537192	1.534585
1.518972	1.516411	1.513863	1.511333	1.508802	1.506259	1.503722	1.537704	1.535089
1.519476	1.516926	1.514376	1.51183	1.509286	1.506738	1.504198	1.538231	1.535608
1.520052	1.517498	1.514944	1.512394	1.509845	1.507292	1.504744	1.502196	1.499668
1.520687	1.518118	1.515559	1.513013	1.510464	1.507904	1.505346	1.502787	1.500244
1.52128	1.518698	1.516129	1.513579	1.511024	1.508455	1.505888	1.503324	1.500777
1.52182	1.519225	1.516642	1.514077	1.511508	1.508924	1.506347	1.503788	1.501251
1.522353	1.519743	1.517145	1.514566	1.511988	1.509397	1.50682	1.504267	1.501741
1.522879	1.520252	1.517641	1.515053	1.512476	1.509902	1.507344	1.504803	1.502286
1.523424	1.520779	1.518157	1.515563	1.512989	1.510431	1.50789	1.505357	1.502841
1.524041	1.521384	1.518753	1.516155	1.513574	1.511005	1.50845	1.5059	1.503365
1.524696	1.522026	1.519388	1.516788	1.514198	1.511607	1.509024	1.506444	1.503881
1.525295	1.522612	1.519966	1.517368	1.514778	1.512186	1.509599	1.507006	1.504426
1.525847	1.523148	1.520497	1.517903	1.515323	1.512749	1.510177	1.507583	1.504995
1.526437	1.523718	1.521057	1.518466	1.515891	1.513324	1.510758	1.508164	1.505571
1.527097	1.52435	1.521672	1.51908	1.516501	1.513921	1.511342	1.508743	1.506146
1.527738	1.524967	1.522276	1.519683	1.517104	1.514519	1.511934	1.509333	1.506734
1.528281	1.525506	1.522816	1.520232	1.517667	1.515107	1.512545	1.509952	1.507352
1.528819	1.52604	1.52335	1.520771	1.518222	1.515692	1.513158	1.510576	1.507973
1.529476	1.526662	1.523947	1.521357	1.518803	1.516276	1.513745	1.511161	1.508554
1.53021	1.52734	1.524585	1.521971	1.519401	1.516859	1.514318	1.511727	1.509113
1.530955	1.52802	1.52522	1.522584	1.520002	1.51746	1.514921	1.512329	1.509711
1.531713	1.528706	1.52586	1.523203	1.520614	1.518084	1.51556	1.51297	1.510351

1.532494	1.529378	1.526466	1.523787	1.521193	1.518673	1.516166	1.51359	1.510984
1.533279	1.529998	1.526995	1.524298	1.521706	1.519194	1.516707	1.514167	1.511602
1.534126	1.530666	1.527559	1.524832	1.522229	1.519714	1.517239	1.51473	1.512205
1.535095	1.531475	1.52826	1.525476	1.522833	1.52029	1.517801	1.515293	1.51278
1.536144	1.532367	1.529036	1.526173	1.523471	1.520886	1.518372	1.515855	1.513344
1.537225	1.533294	1.529837	1.52687	1.524093	1.521471	1.518943	1.516434	1.513942
1.538347	1.534264	1.530671	1.527575	1.524709	1.522051	1.519516	1.517024	1.514562
1.539489	1.535244	1.531504	1.528265	1.5253	1.522611	1.520074	1.517589	1.515144
1.540643	1.536216	1.532312	1.528915	1.525848	1.523143	1.520616	1.518124	1.515681
1.541854	1.537279	1.533218	1.529645	1.526452	1.5237	1.521159	1.51865	1.516204
1.543156	1.538485	1.534278	1.530498	1.52714	1.524296	1.5217	1.519165	1.516719
1.544494	1.539774	1.535441	1.531451	1.527911	1.524944	1.522263	1.519684	1.517231
1.54582	1.541122	1.536704	1.532518	1.528794	1.525678	1.522879	1.520228	1.51774
1.547145	1.542515	1.538042	1.533677	1.529768	1.52648	1.523534	1.520793	1.518256
1.548446	1.543869	1.539354	1.53485	1.530782	1.527311	1.524206	1.521391	1.518826
1.549725	1.545197	1.540654	1.536049	1.531842	1.528175	1.524897	1.522019	1.519444
1.550976	1.546485	1.541929	1.537264	1.532953	1.52911	1.525662	1.522696	1.520072
1.552184	1.547723	1.543171	1.538491	1.534113	1.530122	1.526514	1.523424	1.520699
1.553405	1.548949	1.544404	1.539743	1.535325	1.531194	1.527423	1.524198	1.521353
1.554667	1.550165	1.545624	1.541027	1.536598	1.532319	1.528373	1.525009	1.522045
1.55596	1.5514	1.546862	1.542342	1.537914	1.533497	1.529379	1.525869	1.522773
1.557299	1.55272	1.548187	1.543704	1.539255	1.53472	1.530453	1.526784	1.523532
1.558674	1.554097	1.54957	1.545101	1.540621	1.535991	1.531594	1.527759	1.524331
1.560031	1.555463	1.550941	1.546478	1.541981	1.537309	1.532831	1.528835	1.525216
1.561369	1.556823	1.552308	1.547839	1.543335	1.538677	1.53417	1.530018	1.52619
1.562733	1.558187	1.553669	1.549197	1.544692	1.540061	1.535537	1.53124	1.527209
1.56413	1.559552	1.55502	1.550548	1.546049	1.541452	1.536919	1.532487	1.52826
1.565556	1.560933	1.556378	1.551908	1.547418	1.542853	1.538315	1.533767	1.529367
1.567019	1.562342	1.55776	1.553291	1.548807	1.544267	1.539722	1.535079	1.530537
1.568504	1.563768	1.559157	1.554688	1.550208	1.545685	1.541133	1.536423	1.531774
1.569979	1.565189	1.560552	1.556082	1.551606	1.547087	1.542532	1.537805	1.533115
1.571453	1.566613	1.56195	1.55748	1.553004	1.54848	1.543922	1.539221	1.534547
1.572952	1.568091	1.563406	1.558913	1.554415	1.549871	1.5453	1.540623	1.535967
1.574476	1.569628	1.564925	1.560381	1.555838	1.551256	1.546661	1.542005	1.537371
1.576036	1.5712	1.566475	1.561877	1.557284	1.552662	1.548041	1.543399	1.538782
1.577657	1.572818	1.568065	1.563411	1.558766	1.554103	1.549452	1.544808	1.540194
1.579291	1.574439	1.569654	1.564946	1.560256	1.555564	1.550893	1.546247	1.541633
1.518373	1.51415	1.571183	1.566429	1.561714	1.557031	1.552378	1.547747	1.543142
1.519743	1.515466	1.572689	1.567896	1.563168	1.558513	1.553895	1.549284	1.544689
1.521152	1.516829	1.574239	1.569419	1.564677	1.560029	1.555421	1.55081	1.546209
1.522597	1.518232	1.575823	1.570988	1.566234	1.561577	1.55696	1.552332	1.547712
1.524004	1.519617	1.577402	1.57255	1.567779	1.563104	1.558469	1.553829	1.5492
1.525352	1.520974	1.578962	1.574091	1.569298	1.564592	1.559932	1.555289	1.550665
1.480124	1.475796	1.518008	1.513719	1.570836	1.566094	1.561405	1.556755	1.552136
1.481624	1.477287	1.519381	1.5151	1.572424	1.567639	1.562909	1.558244	1.553624
1.483136	1.478789	1.520772	1.5165	1.574032	1.569208	1.564442	1.559758	1.555131
1.430049	1.425806	1.476061	1.471827	1.534898	1.530098	1.525367	1.561323	1.556672
1.431564	1.427324	1.477639	1.473365	1.536611	1.531825	1.52711	1.562924	1.558235
1.32584	1.321776	1.479171	1.474882	1.538348	1.533564	1.528849	1.524146	1.519473
1.327288	1.323137	1.48066	1.476377	1.540107	1.535321	1.53059	1.525836	1.521104

1.328707	1.324505	1.320431	1.316484	1.541874	1.537062	1.532303	1.52752	1.522762
1.330069	1.325881	1.321823	1.317897	1.543651	1.538777	1.533966	1.52919	1.524463
1.331449	1.327284	1.323234	1.319303	1.545423	1.540488	1.535633	1.530872	1.526184
1.3329	1.328745	1.324669	1.320675	1.478248	1.474022	1.469833	1.532591	1.527921
1.334392	1.330233	1.326114	1.32203	1.479703	1.475478	1.471312	1.534327	1.529667
1.266259	1.262111	1.327529	1.323378	1.319333	1.315442	1.311609	1.536053	1.531394
1.267809	1.263566	1.328927	1.324721	1.320654	1.316797	1.313004	1.537779	1.533111
1.269326	1.265034	1.33036	1.326114	1.322024	1.318166	1.314381	1.394359	1.389843
1.270805	1.266513	1.331827	1.32756	1.323444	1.319547	1.315737	1.396057	1.391505
1.272283	1.268007	1.33332	1.329037	1.324894	1.320952	1.317106	1.313312	1.309557
1.273766	1.269517	1.334838	1.330534	1.326364	1.322385	1.318505	1.314689	1.310924
1.275262	1.271046	1.336379	1.332064	1.327867	1.32384	1.319914	1.316069	1.31229
1.276795	1.272599	1.268393	1.264164	1.32943	1.325314	1.321304	1.31745	1.313692
1.278349	1.27417	1.269983	1.26578	1.331023	1.326808	1.322704	1.31884	1.315105
1.279888	1.275753	1.271581	1.267363	1.332577	1.328327	1.32418	1.320267	1.316486
1.281418	1.27735	1.273187	1.268923	1.334103	1.329869	1.325722	1.321727	1.317841
1.28295	1.278909	1.274761	1.270499	1.26627	1.262097	1.257996	1.323194	1.319224
1.284488	1.280419	1.276289	1.272098	1.267901	1.263683	1.259516	1.324661	1.32064
1.286012	1.281928	1.277821	1.273694	1.269527	1.26528	1.261064	1.326153	1.322077
1.287493	1.283469	1.279391	1.275271	1.271112	1.266889	1.262674	1.327687	1.323513
1.28898	1.285009	1.280959	1.276843	1.272693	1.268501	1.264296	1.329246	1.324989
1.290527	1.286509	1.282467	1.278418	1.274306	1.27009	1.265854	1.26163	1.257446
1.292117	1.287998	1.283949	1.279994	1.275936	1.271673	1.267393	1.263224	1.259111
1.209486	1.205724	1.28551	1.281579	1.27755	1.273319	1.269063	1.264899	1.260772
1.21102	1.207232	1.28715	1.283176	1.279154	1.275029	1.270862	1.266659	1.262439
1.212573	1.208728	1.288755	1.284742	1.280723	1.276685	1.272594	1.268367	1.264082
1.214136	1.210183	1.290293	1.28625	1.282236	1.278253	1.274215	1.269989	1.265682
1.215722	1.21165	1.291832	1.28777	1.283755	1.27979	1.275778	1.271576	1.26729
1.12715	1.123289	1.293389	1.289343	1.285328	1.281308	1.277263	1.273134	1.268955
1.12859	1.124621	1.294986	1.290949	1.286921	1.282824	1.278731	1.274686	1.270632
1.130009	1.126038	1.212287	1.208556	1.288502	1.284396	1.280314	1.276296	1.272279
1.131423	1.127522	1.213969	1.210075	1.290081	1.286007	1.281973	1.277943	1.273903
0.952928	0.949543	1.12525	1.121538	1.29167	1.287611	1.283607	1.27955	1.275473
0.954347	0.950907	1.126734	1.122894	1.293256	1.289204	1.28522	1.281111	1.276974
0.955731	0.952268	1.128242	1.124314	1.2148	1.210897	1.207051	1.282678	1.278515
0.957036	0.953613	1.129791	1.12584	1.216444	1.212442	1.20851	1.284269	1.280148
0.958307	0.954949	1.131334	1.127395	1.218128	1.214005	1.209964	1.285861	1.281798
0.810966	0.808274	1.132815	1.12892	1.21978	1.215534	1.211379	1.20742	1.203583
0.811988	0.80931	1.134272	1.130438	1.221433	1.217071	1.212805	1.208836	1.205031
0.812994	0.810356	1.135768	1.13196	1.128088	1.124067	1.120141	1.210364	1.206515
0.813987	0.811412	1.137291	1.133483	1.129612	1.125604	1.121668	1.211985	1.208028
0.814948	0.812416	0.809791	0.807077	1.131141	1.127158	1.123216	1.213626	1.209569
0.81586	0.813347	0.810749	0.808074	1.132688	1.128736	1.12479	1.215286	1.211131
0.69024	0.687918	0.811706	0.809064	1.134192	1.130281	1.12635	1.21696	1.212745
0.691182	0.688829	0.812694	0.810064	1.135609	1.131751	1.12787	1.218647	1.214443
0.692142	0.689762	0.813691	0.811066	1.136992	1.133188	1.129372	1.220338	1.216173
0.693032	0.690668	0.688267	0.685838	1.138379	1.134619	1.130859	1.127102	1.123347
0.693901	0.691567	0.68917	0.686719	1.139765	1.136044	1.132337	1.128626	1.124903
0.694854	0.69249	0.690072	0.687608	0.811423	0.808755	0.806078	1.130172	1.126453
0.695885	0.693434	0.690972	0.688505	0.812419	0.80981	0.807184	1.131741	1.127999

1.981658	1.97795	1.981832	1.978097	1.980628	1.977199	1.973812	1.977394	1.973848
1.981588	1.977881	1.981772	1.97804	1.980577	1.977146	1.973754	1.977334	1.973782
1.981519	1.977814	1.981713	1.977983	1.980526	1.977091	1.973695	1.977271	1.973713
1.981457	1.977753	1.981657	1.977928	1.974322	1.970912	1.967543	1.977212	1.97365
1.981398	1.977696	1.981605	1.977875	1.974267	1.970855	1.967482	1.977155	1.973589
1.981334	1.977635	1.981553	1.977829	1.974221	1.970802	1.96742	1.977095	1.973525
1.981265	1.977572	1.981502	1.977786	1.97418	1.97075	1.967356	1.97703	1.973456
1.981197	1.977509	1.98146	1.97777	1.974173	1.970722	1.967302	1.971194	1.967645
1.981127	1.977446	1.981432	1.977802	1.97423	1.970736	1.967264	1.971135	1.967579
1.981067	1.977391	1.981409	1.977833	1.974282	1.97075	1.967231	1.971082	1.967517
1.978974	1.975292	1.977625	1.974074	1.974266	1.970724	1.967192	1.963651	1.960128
1.978952	1.975269	1.977599	1.974036	1.974221	1.970682	1.967153	1.963607	1.960076
1.978943	1.975288	1.977624	1.974031	1.974193	1.970648	1.967122	1.963594	1.960084
1.978947	1.975343	1.977688	1.974047	1.974173	1.970618	1.967099	1.963606	1.960139
1.97256	1.968981	1.977706	1.97404	1.974152	1.97061	1.967108	1.963627	1.960165
1.972505	1.968927	1.977662	1.974001	1.974131	1.970633	1.967163	1.963661	1.960158
1.972482	1.968886	1.977622	1.973968	1.97039	1.966938	1.963507	1.963678	1.960143
1.972534	1.96889	1.977607	1.973947	1.970367	1.966913	1.963483	1.963653	1.960122
1.9726	1.968909	1.97761	1.973949	1.970364	1.966893	1.963448	1.96362	1.960101
1.972616	1.968919	1.971755	1.968127	1.970429	1.966925	1.963443	1.963603	1.960083
1.972614	1.968933	1.971797	1.96821	1.970528	1.966981	1.963453	1.963597	1.960075
1.972634	1.968982	1.969771	1.966173	1.970575	1.967008	1.963464	1.959944	1.956457
1.972672	1.969062	1.969855	1.96621	1.970579	1.967009	1.963478	1.959993	1.956544
1.972733	1.969134	1.969922	1.966245	1.964717	1.961169	1.957668	1.960051	1.956617
1.972827	1.969186	1.969956	1.966269	1.964746	1.961227	1.95775	1.960121	1.956657
1.97292	1.96925	1.970011	1.966322	1.964802	1.961298	1.957832	1.960194	1.956708
1.972986	1.969354	1.970137	1.966448	1.964916	1.961379	1.957887	1.960263	1.956804
1.97305	1.969465	1.970275	1.966588	1.965043	1.961467	1.957941	1.960335	1.956908
1.977642	1.974003	1.97034	1.966652	1.965112	1.961549	1.958032	1.954563	1.951125
1.977752	1.974057	1.970367	1.966677	1.96515	1.961629	1.958143	1.954649	1.951165
1.970088	1.966407	1.970453	1.966762	1.965233	1.961708	1.958219	1.954723	1.951238
1.970159	1.966527	1.9706	1.96691	1.965363	1.961786	1.958256	1.954783	1.951346
1.966949	1.963358	1.970716	1.967023	1.963371	1.959772	1.956228	1.954851	1.951437
1.96706	1.963428	1.970758	1.967057	1.963412	1.959848	1.956333	1.954937	1.951487
1.967172	1.963503	1.970808	1.967102	1.963466	1.959932	1.956439	1.955024	1.951541
1.967264	1.963616	1.963207	1.959525	1.963592	1.960025	1.956506	1.955097	1.951636
1.967356	1.963744	1.963358	1.959689	1.96375	1.960133	1.95657	1.955172	1.951743
1.967476	1.963848	1.963465	1.959826	1.956207	1.952599	1.949034	1.953197	1.949754
1.96762	1.963938	1.963546	1.95995	1.956365	1.952767	1.949197	1.953319	1.949821
1.967785	1.964073	1.963663	1.960064	1.956483	1.952906	1.94935	1.953459	1.94993
1.967982	1.964266	1.963824	1.960159	1.956548	1.953009	1.949498	1.953626	1.950093
1.968159	1.964451	1.960756	1.957069	1.956638	1.953111	1.949621	1.946115	1.942618
1.968279	1.964603	1.960923	1.957238	1.956792	1.953217	1.949691	1.946208	1.942755
1.968394	1.964739	1.961078	1.957414	1.956964	1.953336	1.949764	1.946293	1.942877
1.968547	1.964852	1.961188	1.957558	1.957129	1.95349	1.949897	1.946403	1.942965
1.968719	1.964957	1.961278	1.957685	1.957288	1.953661	1.950064	1.946529	1.943043
1.968897	1.96511	1.961412	1.957805	1.957405	1.953805	1.950232	1.946687	1.943178
1.969083	1.965304	1.961582	1.957914	1.957485	1.953929	1.950407	1.946875	1.943363
1.969237	1.965483	1.961759	1.958058	1.954434	1.950921	1.947445	1.947059	1.943551
1.96934	1.965636	1.961939	1.958247	1.954624	1.951102	1.947618	1.94724	1.943739

1.969465	1.965801	1.962129	1.958451	1.954827	1.951279	1.947772	1.947401	1.943918
1.969662	1.966003	1.962335	1.958661	1.955024	1.951425	1.947875	1.947517	1.944074
1.969885	1.966217	1.962545	1.958876	1.955225	1.951572	1.947977	1.94763	1.944227
1.970096	1.96642	1.962748	1.959087	1.955434	1.951762	1.948149	1.947798	1.944399
1.970304	1.966614	1.962942	1.959293	1.955649	1.951978	1.948362	1.947998	1.944585
1.970498	1.966774	1.963095	1.95947	1.955846	1.952183	1.94857	1.945096	1.941691
1.970676	1.966893	1.963203	1.959615	1.956025	1.952379	1.948777	1.945308	1.941902
1.970868	1.967042	1.963344	1.959781	1.956212	1.952579	1.948983	1.945518	1.942112
1.971086	1.967246	1.963542	1.959982	1.956416	1.952783	1.949188	1.945723	1.942319
1.971312	1.967466	1.963758	1.960195	1.956627	1.952992	1.949396	1.94593	1.942525
1.97154	1.967683	1.96397	1.96041	1.956845	1.95321	1.949613	1.946142	1.94273
1.971769	1.967903	1.964187	1.960629	1.957065	1.953431	1.949833	1.946354	1.942933
1.971996	1.968123	1.964402	1.96084	1.957274	1.95364	1.950041	1.946559	1.943133
1.972223	1.968344	1.964617	1.961047	1.957475	1.95384	1.95024	1.946758	1.943331
1.972454	1.968575	1.964842	1.961263	1.957683	1.95404	1.950435	1.94695	1.943522
1.972688	1.968809	1.965073	1.961487	1.957896	1.95424	1.950622	1.947133	1.943706
1.972941	1.969075	1.965336	1.961729	1.958119	1.954446	1.950816	1.947319	1.943889
1.973231	1.969404	1.965661	1.962008	1.958358	1.954665	1.951022	1.947511	1.944071
1.973522	1.969735	1.965989	1.962289	1.9586	1.954891	1.951235	1.94771	1.944258
1.973776	1.970004	1.966257	1.962538	1.958833	1.955114	1.951451	1.947917	1.944457
1.974014	1.970245	1.966497	1.962773	1.959063	1.955343	1.951678	1.948136	1.944666
1.974261	1.970488	1.966736	1.963007	1.9593	1.955602	1.951951	1.948393	1.944894
1.974511	1.970728	1.96697	1.963238	1.959542	1.955886	1.952267	1.948686	1.94514
1.974805	1.970993	1.967216	1.963476	1.959784	1.95615	1.952548	1.948949	1.945375
1.97516	1.971288	1.967475	1.963718	1.960015	1.956379	1.952778	1.949171	1.945592
1.975504	1.971591	1.967754	1.963987	1.960274	1.956621	1.953005	1.94939	1.94581
1.97579	1.971886	1.96806	1.96431	1.960595	1.956904	1.953248	1.949617	1.946032
1.976066	1.972191	1.968386	1.964652	1.960931	1.957199	1.9535	1.949857	1.946269
1.967378	1.963554	1.968735	1.964975	1.961229	1.957477	1.953765	1.950128	1.946554
1.967708	1.963918	1.969096	1.96529	1.96151	1.957751	1.954045	1.950424	1.946869
1.968011	1.964228	1.969406	1.965582	1.961797	1.958054	1.954362	1.950732	1.947156
1.968284	1.964484	1.969663	1.965851	1.962086	1.958386	1.95472	1.951055	1.947421
1.968595	1.96477	1.960968	1.957189	1.962396	1.958711	1.95505	1.951358	1.947685
1.968969	1.965106	1.961293	1.95753	1.962742	1.959025	1.95533	1.951625	1.94795
1.969355	1.965466	1.96164	1.957878	1.963082	1.959328	1.9556	1.951892	1.948227
1.96147	1.957638	1.962016	1.95821	1.954432	1.950679	1.946963	1.952177	1.948535
1.961828	1.958022	1.962396	1.958531	1.954712	1.95095	1.947239	1.952477	1.948854
1.9622	1.958367	1.962724	1.958828	1.954996	1.951255	1.947566	1.952799	1.949157
1.962579	1.958683	1.963018	1.959107	1.955281	1.951584	1.94793	1.95314	1.949453
1.962918	1.958991	1.9551	1.951236	1.955604	1.951917	1.94827	1.944573	1.940897
1.96321	1.959283	1.955408	1.951578	1.955971	1.952254	1.948581	1.944906	1.941266
1.963513	1.959599	1.955735	1.95192	1.95633	1.952596	1.948909	1.945246	1.941624
1.963843	1.959962	1.956097	1.952245	1.95665	1.952943	1.949278	1.945598	1.941945
1.964195	1.960334	1.956463	1.952574	1.956977	1.953292	1.949649	1.945951	1.942267
1.964575	1.960679	1.956799	1.952927	1.94914	1.945475	1.941854	1.946293	1.942625
1.964977	1.961024	1.957131	1.953293	1.949519	1.945818	1.942164	1.946633	1.942996
1.959248	1.955287	1.957511	1.953655	1.949867	1.94616	1.942503	1.946983	1.943344
1.959678	1.955727	1.957931	1.954017	1.950194	1.946503	1.942865	1.94734	1.943676
1.953517	1.949639	1.958357	1.954412	1.950564	1.946863	1.943218	1.939562	1.935937
1.953843	1.950031	1.958796	1.954858	1.950994	1.94724	1.943552	1.939911	1.936319

1.954163	1.950395	1.953064	1.949161	1.951427	1.947634	1.943909	1.940282	1.936719
1.954498	1.950702	1.953391	1.94954	1.951845	1.948053	1.944322	1.940699	1.93714
1.954836	1.951002	1.953705	1.949899	1.952248	1.948474	1.944751	1.941126	1.937558
1.955173	1.951358	1.954072	1.950253	1.946475	1.942739	1.939051	1.941516	1.937923
1.955517	1.951746	1.954469	1.950605	1.946805	1.943101	1.939442	1.941892	1.938264
1.955905	1.952155	1.954888	1.951006	1.947195	1.943501	1.93985	1.936195	1.932562
1.956337	1.952591	1.955335	1.951454	1.94764	1.943937	1.940277	1.936621	1.932986
1.956754	1.952991	1.955746	1.951885	1.948077	1.944349	1.940666	1.937019	1.933401
1.957139	1.953326	1.95609	1.95228	1.948493	1.944716	1.940988	1.937364	1.933793
1.957526	1.953673	1.956446	1.952677	1.948905	1.945084	1.941318	1.937712	1.934182
1.957944	1.95409	1.956875	1.953106	1.949328	1.945489	1.941707	1.938104	1.934585
1.958363	1.954528	1.957323	1.953533	1.94974	1.945899	1.942117	1.938507	1.934978
1.947573	1.943787	1.957728	1.953904	1.950093	1.946277	1.942515	1.938881	1.935316
1.947911	1.944155	1.958111	1.95424	1.950411	1.946636	1.94291	1.93924	1.935618
1.948293	1.944549	1.958525	1.954634	1.950797	1.947041	1.943329	1.939637	1.935983
1.948722	1.944971	1.95897	1.95509	1.951256	1.947493	1.943772	1.940074	1.936414
1.949166	1.945408	1.948692	1.944841	1.951725	1.947956	1.944226	1.940521	1.936854
1.94962	1.945864	1.949157	1.945296	1.952186	1.94842	1.944691	1.940974	1.937289
1.950082	1.946326	1.949627	1.945755	1.95265	1.948888	1.945159	1.941429	1.937726
1.950548	1.946779	1.950088	1.946217	1.953121	1.949354	1.945619	1.941876	1.93816
1.951017	1.947233	1.950548	1.94668	1.953594	1.94982	1.946075	1.942322	1.938595
1.947152	1.943394	1.951016	1.947142	1.943311	1.939547	1.935813	1.942772	1.939038
1.947639	1.943883	1.951491	1.947602	1.94376	1.939992	1.936256	1.943226	1.939488
1.948126	1.944368	1.951956	1.948056	1.944207	1.940439	1.936702	1.932951	1.929223
1.948616	1.944846	1.952412	1.948503	1.94465	1.940887	1.937152	1.933386	1.929638
1.934075	1.930301	1.952864	1.94895	1.945094	1.941332	1.937595	1.933813	1.930046
1.934551	1.930771	1.953313	1.949401	1.945541	1.941769	1.93802	1.934224	1.930443
1.935027	1.931241	1.953763	1.94985	1.945987	1.942204	1.938442	1.934633	1.93084
1.922566	1.918799	1.942876	1.938971	1.946422	1.94264	1.938878	1.935059	1.931252
1.923046	1.919275	1.94333	1.939408	1.946852	1.943077	1.939321	1.935492	1.93167
1.909704	1.905962	1.924737	1.920832	1.947288	1.943512	1.939754	1.935918	1.932087
1.910156	1.906411	1.925204	1.921298	1.947729	1.943945	1.940179	1.936337	1.932501
1.902167	1.898434	1.91246	1.908589	1.925221	1.92146	1.917718	1.93677	1.932936
1.902637	1.898904	1.912939	1.909062	1.925664	1.921897	1.918153	1.937217	1.933391
1.903142	1.899394	1.913436	1.909563	1.926142	1.922361	1.918607	1.937687	1.933876
1.903725	1.899918	1.913964	1.910124	1.923991	1.920164	1.91637	1.915318	1.911566
1.90433	1.900455	1.914504	1.910702	1.924567	1.920702	1.916871	1.915837	1.912113
1.888299	1.884443	1.907025	1.903245	1.912295	1.908442	1.904623	1.916312	1.912592
1.888792	1.884935	1.907533	1.903739	1.912756	1.908899	1.905074	1.916765	1.913033
1.889314	1.885466	1.892204	1.888417	1.905597	1.901766	1.897965	1.917267	1.913507
1.889867	1.886044	1.892782	1.888946	1.906081	1.902297	1.898539	1.917827	1.914017
1.890409	1.886598	1.893335	1.889464	1.901483	1.897745	1.894023	1.89526	1.891451
1.890915	1.887094	1.893829	1.889951	1.901976	1.898227	1.894496	1.895686	1.89188
1.891437	1.887586	1.894319	1.890449	1.90249	1.898714	1.894961	1.896113	1.892329
1.892029	1.888112	1.894839	1.891	1.887199	1.883432	1.879693	1.896603	1.892866
1.892646	1.888654	1.895375	1.891573	1.887787	1.883983	1.880212	1.897124	1.893438
1.893215	1.889209	1.885327	1.88157	1.888338	1.88454	1.880775	1.892838	1.889133
1.89375	1.889778	1.885894	1.882096	1.888862	1.885105	1.881376	1.89343	1.889676
1.869371	1.865512	1.886421	1.882597	1.889365	1.88563	1.881925	1.878208	1.874501
1.869863	1.865983	1.886894	1.883059	1.889833	1.886099	1.882408	1.878735	1.875079

1.852543	1.848675	1.887386	1.883552	1.890337	1.886588	1.882891	1.879247	1.875632
1.85309	1.849163	1.887922	1.884121	1.890926	1.887132	1.883389	1.879732	1.876123
1.853657	1.849681	1.888491	1.884713	1.891531	1.887696	1.883908	1.880223	1.876598
1.854208	1.850253	1.86431	1.860595	1.892085	1.888262	1.884481	1.88076	1.877088
1.854757	1.850855	1.864928	1.86114	1.892622	1.888832	1.885084	1.881324	1.877591
1.855315	1.851408	1.865504	1.861689	1.869721	1.866032	1.862387	1.881893	1.878162
1.855882	1.851919	1.866044	1.862241	1.870291	1.866573	1.862908	1.88247	1.878797
1.856444	1.85246	1.848594	1.844841	1.870853	1.867126	1.86346	1.859858	1.856303
1.856995	1.853056	1.84918	1.845362	1.871392	1.86769	1.864055	1.860453	1.856883
1.857548	1.853642	1.849761	1.845893	1.871953	1.868276	1.864664	1.861048	1.85745
1.858108	1.854168	1.850281	1.846439	1.872569	1.868901	1.865284	1.861634	1.85799
1.858673	1.854691	1.850804	1.847003	1.873209	1.869536	1.8659	1.86221	1.858522
1.859237	1.855288	1.851414	1.847609	1.856474	1.852802	1.849166	1.862763	1.85907
1.859805	1.85593	1.852077	1.848242	1.857077	1.853346	1.849662	1.863301	1.859627
1.828781	1.825238	1.852704	1.848835	1.857652	1.853901	1.850202	1.863867	1.860177
1.829368	1.825809	1.853299	1.849385	1.85819	1.854465	1.850787	1.864457	1.860716
1.829951	1.826368	1.853883	1.849957	1.858769	1.855054	1.85138	1.847642	1.843912
1.83052	1.826907	1.854451	1.850575	1.859423	1.855688	1.851986	1.848236	1.844496
1.831084	1.827451	1.855029	1.851203	1.860083	1.856315	1.852576	1.848824	1.845093
1.831636	1.82802	1.855648	1.851827	1.860705	1.856885	1.853103	1.849371	1.84568
1.832192	1.828604	1.856287	1.852447	1.861307	1.857434	1.853613	1.849909	1.846266
1.820319	1.81676	1.856922	1.853045	1.86189	1.858008	1.854188	1.85049	1.846855
1.820928	1.817361	1.857559	1.853626	1.862454	1.858598	1.854806	1.851101	1.847444
1.821526	1.817946	1.846774	1.842821	1.850328	1.846532	1.842801	1.851679	1.847998
1.822111	1.818517	1.847379	1.843418	1.850929	1.847112	1.843355	1.852215	1.848504
1.822683	1.819068	1.847963	1.844015	1.851545	1.847707	1.843923	1.852766	1.849029
1.823232	1.819583	1.848501	1.844605	1.852183	1.848338	1.844536	1.853372	1.84961
1.823779	1.820099	1.849041	1.845196	1.852821	1.848964	1.84514	1.853975	1.850202
1.824353	1.820655	1.84963	1.845791	1.841953	1.838066	1.834218	1.854515	1.850767
1.824937	1.821234	1.850248	1.846394	1.842527	1.838583	1.834692	1.855033	1.851327
1.8255	1.821814	1.818182	1.814614	1.843145	1.839171	1.835253	1.844764	1.841075
1.826044	1.822398	1.818787	1.815224	1.843799	1.839817	1.835887	1.845388	1.841674
1.826607	1.822982	1.819384	1.815828	1.844448	1.840461	1.83652	1.832731	1.829044
1.827192	1.823561	1.819965	1.81642	1.845083	1.841091	1.837138	1.833322	1.829605
1.812149	1.808532	1.820552	1.817019	1.845726	1.84173	1.837765	1.833924	1.830176
1.812766	1.809135	1.821161	1.817636	1.84639	1.84239	1.838415	1.834547	1.830769
1.81339	1.809744	1.821773	1.818257	1.847058	1.843055	1.839072	1.835182	1.831376
1.771204	1.767635	1.822366	1.818859	1.847703	1.843701	1.839717	1.835817	1.831996
1.771766	1.768186	1.822952	1.819453	1.848339	1.844341	1.840359	1.836458	1.83263
1.772352	1.768766	1.782834	1.779407	1.848997	1.845004	1.841026	1.837124	1.833291
1.772961	1.769378	1.783446	1.780003	1.849681	1.845692	1.841719	1.837819	1.833983
1.719745	1.716515	1.76647	1.763043	1.812522	1.8086	1.804706	1.838504	1.834667
1.720289	1.717049	1.767048	1.763607	1.813144	1.809222	1.80533	1.83916	1.835327
1.720847	1.717591	1.767632	1.764176	1.81377	1.809851	1.805964	1.839823	1.835992
1.721443	1.718163	1.768241	1.764766	1.814419	1.810505	1.806625	1.802826	1.799086
1.72206	1.718749	1.768862	1.765366	1.815082	1.811181	1.807314	1.803513	1.799764
1.722691	1.719333	1.71608	1.712934	1.762496	1.759058	1.755639	1.804252	1.800466
1.723334	1.719918	1.716632	1.713475	1.763111	1.759721	1.756344	1.805032	1.801189
1.723957	1.720504	1.717192	1.714013	1.763705	1.760343	1.756995	1.805776	1.801933
1.724556	1.721088	1.717753	1.714542	1.764271	1.760912	1.757583	1.806484	1.80271

1.72516	1.721691	1.718342	1.715101	1.71196	1.708946	1.705971	1.791101	1.787432
1.725786	1.722331	1.718978	1.715715	1.712548	1.7095	1.7065	1.791751	1.788092
1.726407	1.722974	1.719625	1.716348	1.713157	1.71007	1.707036	1.792391	1.788731
1.726994	1.723587	1.72025	1.716974	1.713771	1.710653	1.707588	1.793034	1.789383
1.727569	1.724188	1.720866	1.717601	1.714393	1.711247	1.708155	1.793684	1.790039
1.728182	1.724802	1.721481	1.718216	1.715005	1.711849	1.708746	1.757831	1.754498
1.728826	1.725426	1.722092	1.718822	1.715611	1.712461	1.70936	1.758502	1.755123
1.729488	1.726069	1.722715	1.719425	1.716199	1.713043	1.709939	1.70688	1.703857
1.730171	1.726731	1.723349	1.720017	1.716758	1.713579	1.710465	1.707421	1.704425
1.730859	1.727411	1.724005	1.720631	1.717336	1.714134	1.711007	1.70797	1.704989
1.731542	1.728119	1.724704	1.721288	1.717959	1.714742	1.711604	1.708543	1.705541
1.732222	1.728829	1.725414	1.721969	1.718613	1.715384	1.71223	1.709134	1.706094
1.732889	1.7295	1.726093	1.722663	1.719309	1.716062	1.712881	1.709745	1.706663
1.733551	1.73015	1.726756	1.723369	1.720036	1.716769	1.713553	1.710373	1.707244
1.734227	1.73081	1.727419	1.724059	1.720737	1.717453	1.714211	1.711007	1.707855
1.734915	1.731474	1.728078	1.724732	1.721415	1.718116	1.714858	1.71165	1.708499
1.735628	1.73217	1.728768	1.725427	1.722107	1.718786	1.715505	1.712285	1.709124
1.736376	1.73292	1.729515	1.726166	1.722826	1.719468	1.716147	1.712896	1.709707
1.73713	1.733667	1.730255	1.726899	1.723545	1.72016	1.716808	1.713526	1.710308
1.720621	1.717187	1.730935	1.727591	1.724248	1.720872	1.717521	1.714222	1.710974
1.721332	1.717843	1.731599	1.728268	1.724943	1.721587	1.718247	1.71494	1.711668
1.701968	1.698504	1.715151	1.711891	1.725634	1.72227	1.718924	1.715613	1.712338
1.7027	1.699228	1.715859	1.712578	1.726319	1.722932	1.71957	1.716262	1.713
1.703429	1.699924	1.716532	1.713228	1.726982	1.723595	1.720239	1.716934	1.713675
1.704153	1.70058	1.717157	1.71383	1.727614	1.724259	1.720935	1.717636	1.71437
1.704884	1.701274	1.697819	1.69452	1.711167	1.707903	1.704668	1.718324	1.715046
1.705626	1.702045	1.698585	1.695246	1.711865	1.708582	1.705329	1.718983	1.715681
1.706379	1.702839	1.699375	1.695985	1.712566	1.709252	1.705973	1.71963	1.716311
1.707158	1.703623	1.700128	1.696669	1.693276	1.689966	1.686702	1.720265	1.716966
1.707944	1.704403	1.70087	1.697338	1.693889	1.690557	1.68729	1.7209	1.717635
1.708676	1.705142	1.701598	1.69804	1.69457	1.691227	1.687955	1.684726	1.681545
1.709365	1.705845	1.702314	1.698767	1.695304	1.691962	1.688682	1.685431	1.682221
1.710104	1.7066	1.703076	1.69953	1.696059	1.692698	1.689394	1.686112	1.682869
1.710914	1.707427	1.703904	1.700341	1.696842	1.693438	1.690086	1.686758	1.683475
1.670371	1.667356	1.704723	1.701138	1.697607	1.694159	1.690764	1.687399	1.684091
1.671039	1.668016	1.705499	1.701874	1.698306	1.694825	1.691406	1.688037	1.684735
1.671689	1.668654	1.706252	1.7026	1.699006	1.695499	1.692062	1.688693	1.685402
1.672261	1.669231	1.706971	1.703359	1.699786	1.696263	1.6928	1.689412	1.686102
1.672791	1.669773	1.707673	1.704139	1.700614	1.697082	1.693591	1.690173	1.68683
1.673372	1.670365	1.708427	1.704947	1.701453	1.69791	1.694395	1.690953	1.687581
1.673998	1.671001	1.709229	1.705784	1.702308	1.698754	1.695218	1.691758	1.688361
1.617448	1.614807	1.668671	1.665715	1.703144	1.699577	1.696018	1.692529	1.689098
1.618015	1.615374	1.66934	1.666387	1.703956	1.70037	1.696781	1.693244	1.689759
1.618607	1.615951	1.670004	1.667045	1.704745	1.701142	1.69753	1.693958	1.690432
1.619245	1.616544	1.670658	1.667682	1.705506	1.701887	1.698267	1.694699	1.691179
1.619905	1.617143	1.671302	1.668302	1.706243	1.702619	1.699005	1.695457	1.691959
1.583373	1.58063	1.671931	1.668899	1.665847	1.66275	1.659645	1.69624	1.692735
1.583971	1.58117	1.672547	1.669476	1.666416	1.663361	1.660302	1.697045	1.693518
1.584544	1.581693	1.639981	1.636948	1.667005	1.663986	1.660965	1.697861	1.694326
1.585087	1.582196	1.640529	1.637501	1.667613	1.664626	1.661638	1.698692	1.695163

1.585638	1.582707	1.579916	1.577264	1.635097	1.63218	1.62926	1.659275	1.656217
1.586209	1.583234	1.580421	1.577769	1.635703	1.632794	1.629886	1.659956	1.656917
1.586783	1.583778	1.580948	1.578287	1.636318	1.633409	1.630504	1.660627	1.657607
1.543099	1.54033	1.581518	1.578826	1.63693	1.634016	1.631113	1.628172	1.625214
1.543574	1.540838	1.582112	1.579378	1.637545	1.63462	1.631715	1.628783	1.625839
1.544065	1.541336	1.582689	1.579927	1.638162	1.635227	1.632321	1.629399	1.626473
1.544571	1.54183	1.583257	1.580475	1.638781	1.635835	1.632928	1.630019	1.627113
1.545083	1.54234	1.539668	1.537058	1.578301	1.575691	1.573128	1.630627	1.627733
1.5456	1.542869	1.54018	1.537521	1.578812	1.576187	1.573618	1.631217	1.628327
1.546131	1.543408	1.540705	1.538009	1.579353	1.576705	1.574122	1.631811	1.628919
1.546686	1.543956	1.541243	1.538535	1.579943	1.577263	1.574649	1.632416	1.629512
1.547245	1.544512	1.541798	1.539088	1.580567	1.577849	1.575195	1.633035	1.630117
1.547787	1.545086	1.542385	1.539673	1.537027	1.53447	1.531976	1.600266	1.597534
1.548316	1.545664	1.542988	1.540278	1.537617	1.535018	1.532484	1.600892	1.598159
1.548813	1.546196	1.543543	1.540848	1.538187	1.535569	1.533013	1.601552	1.598812
1.54928	1.546686	1.544057	1.541388	1.538744	1.536127	1.533565	1.602249	1.599498
1.54975	1.547172	1.54456	1.541913	1.53928	1.536658	1.534086	1.531611	1.529201
1.550231	1.547659	1.545056	1.542422	1.539791	1.537151	1.534558	1.532073	1.529658
1.550714	1.548147	1.545549	1.542922	1.540291	1.537636	1.535025	1.532528	1.530103
1.551193	1.548636	1.546041	1.543413	1.540779	1.538123	1.535507	1.532991	1.530542
1.551682	1.549131	1.546537	1.543904	1.541268	1.538617	1.535999	1.533462	1.530982
1.515294	1.512844	1.547042	1.544416	1.541785	1.539134	1.53651	1.53395	1.531442
1.51578	1.513303	1.547557	1.544946	1.542323	1.539671	1.537038	1.534456	1.531919
1.516319	1.513821	1.511319	1.50882	1.5429	1.540246	1.537606	1.535006	1.532447
1.516921	1.514409	1.5119	1.509402	1.543525	1.540869	1.538222	1.53561	1.533035
1.517492	1.514965	1.512447	1.509946	1.544111	1.541455	1.538806	1.536185	1.533597
1.517994	1.515445	1.512915	1.510408	1.544616	1.541965	1.539321	1.536694	1.534096
1.518481	1.515916	1.513373	1.510858	1.545109	1.542462	1.539823	1.537192	1.534585
1.518972	1.516411	1.513863	1.511333	1.508802	1.506259	1.503722	1.537704	1.535089
1.519476	1.516926	1.514376	1.51183	1.509286	1.506738	1.504198	1.538231	1.535608
1.520052	1.517498	1.514944	1.512394	1.509845	1.507292	1.504744	1.502196	1.499668
1.520687	1.518118	1.515559	1.513013	1.510464	1.507904	1.505346	1.502787	1.500244
1.52128	1.518698	1.516129	1.513579	1.511024	1.508455	1.505888	1.503324	1.500777
1.52182	1.519225	1.516642	1.514077	1.511508	1.508924	1.506347	1.503788	1.501251
1.522353	1.519743	1.517145	1.514566	1.511988	1.509397	1.50682	1.504267	1.501741
1.522879	1.520252	1.517641	1.515053	1.512476	1.509902	1.507344	1.504803	1.502286
1.523424	1.520779	1.518157	1.515563	1.512989	1.510431	1.50789	1.505357	1.502841
1.524041	1.521384	1.518753	1.516155	1.513574	1.511005	1.50845	1.5059	1.503365
1.524696	1.522026	1.519388	1.516788	1.514198	1.511607	1.509024	1.506444	1.503881
1.525295	1.522612	1.519966	1.517368	1.514778	1.512186	1.509599	1.507006	1.504426
1.525847	1.523148	1.520497	1.517903	1.515323	1.512749	1.510177	1.507583	1.504995
1.526437	1.523718	1.521057	1.518466	1.515891	1.513324	1.510758	1.508164	1.505571
1.527097	1.52435	1.521672	1.51908	1.516501	1.513921	1.511342	1.508743	1.506146
1.527738	1.524967	1.522276	1.519683	1.517104	1.514519	1.511934	1.509333	1.506734
1.528281	1.525506	1.522816	1.520232	1.517667	1.515107	1.512545	1.509952	1.507352
1.528819	1.52604	1.52335	1.520771	1.518222	1.515692	1.513158	1.510576	1.507973
1.529476	1.526662	1.523947	1.521357	1.518803	1.516276	1.513745	1.511161	1.508554
1.53021	1.52734	1.524585	1.521971	1.519401	1.516859	1.514318	1.511727	1.509113
1.530955	1.52802	1.52522	1.522584	1.520002	1.51746	1.514921	1.512329	1.509711
1.531713	1.528706	1.52586	1.523203	1.520614	1.518084	1.51556	1.51297	1.510351

1.532494	1.529378	1.526466	1.523787	1.521193	1.518673	1.516166	1.51359	1.510984
1.533279	1.529998	1.526995	1.524298	1.521706	1.519194	1.516707	1.514167	1.511602
1.534126	1.530666	1.527559	1.524832	1.522229	1.519714	1.517239	1.51473	1.512205
1.535095	1.531475	1.52826	1.525476	1.522833	1.52029	1.517801	1.515293	1.51278
1.536144	1.532367	1.529036	1.526173	1.523471	1.520886	1.518372	1.515855	1.513344
1.537225	1.533294	1.529837	1.52687	1.524093	1.521471	1.518943	1.516434	1.513942
1.538347	1.534264	1.530671	1.527575	1.524709	1.522051	1.519516	1.517024	1.514562
1.539489	1.535244	1.531504	1.528265	1.5253	1.522611	1.520074	1.517589	1.515144
1.540643	1.536216	1.532312	1.528915	1.525848	1.523143	1.520616	1.518124	1.515681
1.541854	1.537279	1.533218	1.529645	1.526452	1.5237	1.521159	1.51865	1.516204
1.543156	1.538485	1.534278	1.530498	1.52714	1.524296	1.5217	1.519165	1.516719
1.544494	1.539774	1.535441	1.531451	1.527911	1.524944	1.522263	1.519684	1.517231
1.54582	1.541122	1.536704	1.532518	1.528794	1.525678	1.522879	1.520228	1.51774
1.547145	1.542515	1.538042	1.533677	1.529768	1.52648	1.523534	1.520793	1.518256
1.548446	1.543869	1.539354	1.53485	1.530782	1.527311	1.524206	1.521391	1.518826
1.549725	1.545197	1.540654	1.536049	1.531842	1.528175	1.524897	1.522019	1.519444
1.550976	1.546485	1.541929	1.537264	1.532953	1.52911	1.525662	1.522696	1.520072
1.552184	1.547723	1.543171	1.538491	1.534113	1.530122	1.526514	1.523424	1.520699
1.553405	1.548949	1.544404	1.539743	1.535325	1.531194	1.527423	1.524198	1.521353
1.554667	1.550165	1.545624	1.541027	1.536598	1.532319	1.528373	1.525009	1.522045
1.55596	1.5514	1.546862	1.542342	1.537914	1.533497	1.529379	1.525869	1.522773
1.557299	1.55272	1.548187	1.543704	1.539255	1.53472	1.530453	1.526784	1.523532
1.558674	1.554097	1.54957	1.545101	1.540621	1.535991	1.531594	1.527759	1.524331
1.560031	1.555463	1.550941	1.546478	1.541981	1.537309	1.532831	1.528835	1.525216
1.561369	1.556823	1.552308	1.547839	1.543335	1.538677	1.53417	1.530018	1.52619
1.562733	1.558187	1.553669	1.549197	1.544692	1.540061	1.535537	1.53124	1.527209
1.56413	1.559552	1.55502	1.550548	1.546049	1.541452	1.536919	1.532487	1.52826
1.565556	1.560933	1.556378	1.551908	1.547418	1.542853	1.538315	1.533767	1.529367
1.567019	1.562342	1.55776	1.553291	1.548807	1.544267	1.539722	1.535079	1.530537
1.568504	1.563768	1.559157	1.554688	1.550208	1.545685	1.541133	1.536423	1.531774
1.569979	1.565189	1.560552	1.556082	1.551606	1.547087	1.542532	1.537805	1.533115
1.571453	1.566613	1.56195	1.55748	1.553004	1.54848	1.543922	1.539221	1.534547
1.572952	1.568091	1.563406	1.558913	1.554415	1.549871	1.5453	1.540623	1.535967
1.574476	1.569628	1.564925	1.560381	1.555838	1.551256	1.546661	1.542005	1.537371
1.576036	1.5712	1.566475	1.561877	1.557284	1.552662	1.548041	1.543399	1.538782
1.577657	1.572818	1.568065	1.563411	1.558766	1.554103	1.549452	1.544808	1.540194
1.579291	1.574439	1.569654	1.564946	1.560256	1.555564	1.550893	1.546247	1.541633
1.518373	1.51415	1.571183	1.566429	1.561714	1.557031	1.552378	1.547747	1.543142
1.519743	1.515466	1.572689	1.567896	1.563168	1.558513	1.553895	1.549284	1.544689
1.521152	1.516829	1.574239	1.569419	1.564677	1.560029	1.555421	1.55081	1.546209
1.522597	1.518232	1.575823	1.570988	1.566234	1.561577	1.55696	1.552332	1.547712
1.524004	1.519617	1.577402	1.57255	1.567779	1.563104	1.558469	1.553829	1.5492
1.525352	1.520974	1.578962	1.574091	1.569298	1.564592	1.559932	1.555289	1.550665
1.480124	1.475796	1.518008	1.513719	1.570836	1.566094	1.561405	1.556755	1.552136
1.481624	1.477287	1.519381	1.5151	1.572424	1.567639	1.562909	1.558244	1.553624
1.483136	1.478789	1.520772	1.5165	1.574032	1.569208	1.564442	1.559758	1.555131
1.430049	1.425806	1.476061	1.471827	1.534898	1.530098	1.525367	1.561323	1.556672
1.431564	1.427324	1.477639	1.473365	1.536611	1.531825	1.52711	1.562924	1.558235
1.32584	1.321776	1.479171	1.474882	1.538348	1.533564	1.528849	1.524146	1.519473
1.327288	1.323137	1.48066	1.476377	1.540107	1.535321	1.53059	1.525836	1.521104

1.328707	1.324505	1.320431	1.316484	1.541874	1.537062	1.532303	1.52752	1.522762
1.330069	1.325881	1.321823	1.317897	1.543651	1.538777	1.533966	1.52919	1.524463
1.331449	1.327284	1.323234	1.319303	1.545423	1.540488	1.535633	1.530872	1.526184
1.3329	1.328745	1.324669	1.320675	1.478248	1.474022	1.469833	1.532591	1.527921
1.334392	1.330233	1.326114	1.32203	1.479703	1.475478	1.471312	1.534327	1.529667
1.266259	1.262111	1.327529	1.323378	1.319333	1.315442	1.311609	1.536053	1.531394
1.267809	1.263566	1.328927	1.324721	1.320654	1.316797	1.313004	1.537779	1.533111
1.269326	1.265034	1.33036	1.326114	1.322024	1.318166	1.314381	1.394359	1.389843
1.270805	1.266513	1.331827	1.32756	1.323444	1.319547	1.315737	1.396057	1.391505
1.272283	1.268007	1.33332	1.329037	1.324894	1.320952	1.317106	1.313312	1.309557
1.273766	1.269517	1.334838	1.330534	1.326364	1.322385	1.318505	1.314689	1.310924
1.275262	1.271046	1.336379	1.332064	1.327867	1.32384	1.319914	1.316069	1.31229
1.276795	1.272599	1.268393	1.264164	1.32943	1.325314	1.321304	1.31745	1.313692
1.278349	1.27417	1.269983	1.26578	1.331023	1.326808	1.322704	1.31884	1.315105
1.279888	1.275753	1.271581	1.267363	1.332577	1.328327	1.32418	1.320267	1.316486
1.281418	1.27735	1.273187	1.268923	1.334103	1.329869	1.325722	1.321727	1.317841
1.28295	1.278909	1.274761	1.270499	1.26627	1.262097	1.257996	1.323194	1.319224
1.284488	1.280419	1.276289	1.272098	1.267901	1.263683	1.259516	1.324661	1.32064
1.286012	1.281928	1.277821	1.273694	1.269527	1.26528	1.261064	1.326153	1.322077
1.287493	1.283469	1.279391	1.275271	1.271112	1.266889	1.262674	1.327687	1.323513
1.28898	1.285009	1.280959	1.276843	1.272693	1.268501	1.264296	1.329246	1.324989
1.290527	1.286509	1.282467	1.278418	1.274306	1.27009	1.265854	1.26163	1.257446
1.292117	1.287998	1.283949	1.279994	1.275936	1.271673	1.267393	1.263224	1.259111
1.209486	1.205724	1.28551	1.281579	1.27755	1.273319	1.269063	1.264899	1.260772
1.21102	1.207232	1.28715	1.283176	1.279154	1.275029	1.270862	1.266659	1.262439
1.212573	1.208728	1.288755	1.284742	1.280723	1.276685	1.272594	1.268367	1.264082
1.214136	1.210183	1.290293	1.28625	1.282236	1.278253	1.274215	1.269989	1.265682
1.215722	1.21165	1.291832	1.28777	1.283755	1.27979	1.275778	1.271576	1.26729
1.12715	1.123289	1.293389	1.289343	1.285328	1.281308	1.277263	1.273134	1.268955
1.12859	1.124621	1.294986	1.290949	1.286921	1.282824	1.278731	1.274686	1.270632
1.130009	1.126038	1.212287	1.208556	1.288502	1.284396	1.280314	1.276296	1.272279
1.131423	1.127522	1.213969	1.210075	1.290081	1.286007	1.281973	1.277943	1.273903
0.952928	0.949543	1.12525	1.121538	1.29167	1.287611	1.283607	1.27955	1.275473
0.954347	0.950907	1.126734	1.122894	1.293256	1.289204	1.28522	1.281111	1.276974
0.955731	0.952268	1.128242	1.124314	1.2148	1.210897	1.207051	1.282678	1.278515
0.957036	0.953613	1.129791	1.12584	1.216444	1.212442	1.20851	1.284269	1.280148
0.958307	0.954949	1.131334	1.127395	1.218128	1.214005	1.209964	1.285861	1.281798
0.810966	0.808274	1.132815	1.12892	1.21978	1.215534	1.211379	1.20742	1.203583
0.811988	0.80931	1.134272	1.130438	1.221433	1.217071	1.212805	1.208836	1.205031
0.812994	0.810356	1.135768	1.13196	1.128088	1.124067	1.120141	1.210364	1.206515
0.813987	0.811412	1.137291	1.133483	1.129612	1.125604	1.121668	1.211985	1.208028
0.814948	0.812416	0.809791	0.807077	1.131141	1.127158	1.123216	1.213626	1.209569
0.81586	0.813347	0.810749	0.808074	1.132688	1.128736	1.12479	1.215286	1.211131
0.69024	0.687918	0.811706	0.809064	1.134192	1.130281	1.12635	1.21696	1.212745
0.691182	0.688829	0.812694	0.810064	1.135609	1.131751	1.12787	1.218647	1.214443
0.692142	0.689762	0.813691	0.811066	1.136992	1.133188	1.129372	1.220338	1.216173
0.693032	0.690668	0.688267	0.685838	1.138379	1.134619	1.130859	1.127102	1.123347
0.693901	0.691567	0.68917	0.686719	1.139765	1.136044	1.132337	1.128626	1.124903
0.694854	0.69249	0.690072	0.687608	0.811423	0.808755	0.806078	1.130172	1.126453
0.695885	0.693434	0.690972	0.688505	0.812419	0.80981	0.807184	1.131741	1.127999

							0.717683	0.713478
							0.715457	0.711336
							0.713223	0.709206
							0.710951	0.707067
				0.476373	0.475088	0.47378	0.85079	0.847011
				0.47557	0.474313	0.473036	0.848978	0.845208
				0.474771	0.473532	0.472279	0.847192	0.843416
				0.857201	0.853118	0.849104	0.845226	0.841461
				0.855263	0.851148	0.847101	0.843212	0.839462
		0.475035	0.473832	0.853264	0.849204	0.845208	1.26645	1.261831
		0.474261	0.47301	0.85123	0.847284	0.843398	1.264212	1.259626
		0.857191	0.853213	0.849283	0.845376	0.841529	1.438408	1.43326
		0.855285	0.851363	0.847459	0.84348	0.839571	1.43578	1.430769
0.47451	0.47332	0.853362	0.849482	1.089439	1.084797	1.080238	1.606792	1.601511
0.473713	0.472508	0.851308	0.847453	1.087168	1.082604	1.078154	1.604193	1.599011
0.472913	0.471686	0.849273	0.845428	1.084898	1.080463	1.076173	1.601659	1.596557
0.855227	0.851293	0.847415	0.84358	1.302927	1.297962	1.293161	1.599204	1.594128
0.853397	0.849504	0.845652	0.841817	1.300519	1.295732	1.291126	1.596805	1.591727
0.851564	0.847699	1.087443	1.082873	1.609871	1.604577	1.599455	1.594366	1.589343
0.849747	0.845896	1.085313	1.08068	1.607104	1.60189	1.596875	1.591895	1.586979
0.847837	0.844004	1.303349	1.29809	1.604475	1.599323	1.594366	1.660171	1.654989
0.845764	0.841961	1.30076	1.295771	1.602043	1.596933	1.591963	1.657632	1.65246
0.843732	0.839954	1.298234	1.293531	1.599658	1.594577	1.589581	1.65509	1.649897
1.259243	1.254258	1.366599	1.36186	1.597238	1.59218	1.587173	1.705418	1.70012
1.256917	1.251994	1.364305	1.359691	1.594823	1.589781	1.58477	1.702803	1.697435
1.301118	1.296168	1.602531	1.597434	1.65029	1.645184	1.640114	1.798172	1.792513
1.298864	1.294063	1.600089	1.595012	1.647833	1.642744	1.637691	1.795496	1.789841
1.367494	1.3626	1.655666	1.650507	1.711284	1.705901	1.700556	1.829652	1.82387
1.365121	1.360368	1.65318	1.648082	1.708771	1.703418	1.69808	1.826977	1.821154
1.661123	1.655879	1.650728	1.645684	1.804586	1.798918	1.793256	1.824267	1.818398
1.658565	1.653401	1.648315	1.643324	1.801975	1.796283	1.790572	1.821493	1.815568
1.656067	1.650976	1.645936	1.640966	1.799354	1.793625	1.787861	1.818696	1.812739
1.653637	1.648624	1.709437	1.704111	1.796687	1.79092	1.785123	1.894782	1.888408
1.651261	1.646317	1.706996	1.701586	1.793989	1.788182	1.782363	1.891776	1.885477
1.648908	1.644	1.802833	1.797057	1.87653	1.870182	1.863864	1.999461	1.99258
1.646584	1.64168	1.800258	1.794376	1.873533	1.867169	1.86088	1.996198	1.989459
1.710167	1.70494	1.797625	1.791692	1.901974	1.895498	1.889135	2.061915	2.054844
1.707705	1.70238	1.794927	1.789008	1.899064	1.892579	1.886222	2.058488	2.051638
1.803676	1.797975	1.877606	1.871217	2.007554	2.000379	1.993369	2.082928	2.075681
1.801052	1.795245	1.874606	1.868244	2.004254	1.99721	1.990328	2.079505	2.072283
1.798418	1.792516	1.871613	1.865274	2.000964	1.994078	1.987352	2.076133	2.06888
1.862041	1.855955	1.89938	1.892935	2.066954	2.05969	2.052653	2.093373	2.086009
1.859354	1.853251	1.896471	1.89001	2.063444	2.056364	2.049522	2.089962	2.082501
1.875885	1.869415	2.016978	2.009711	2.108879	2.101383	2.094077	2.086607	2.079118
1.873059	1.866604	2.013745	2.00658	2.105417	2.097987	2.090728	2.0833	2.075846
1.900932	1.89437	2.069823	2.062325	2.101993	2.094623	2.087407	2.123268	2.115719
1.898061	1.891512	2.066385	2.059048	2.098603	2.09129	2.084115	2.119964	2.112498
2.043587	2.036066	2.09108	2.083387	2.095231	2.087974	2.080842	2.149124	2.141765
2.040343	2.032873	2.087567	2.079948	2.091859	2.084654	2.077571	2.145872	2.138584

2.03712	2.029723	2.084105	2.076539	2.088502	2.081351	2.074323	2.142637	2.135414
2.068402	2.060685	2.099999	2.092474	2.128849	2.121659	2.11459	2.13943	2.132252
2.065005	2.057456	2.096628	2.089135	2.125474	2.118356	2.111361	2.136248	2.129098
2.089611	2.081649	2.121916	2.114379	2.142636	2.135614	2.128705	2.170642	2.163514
2.086069	2.078195	2.118479	2.110994	2.139317	2.132381	2.12555	2.167501	2.160336
2.082576	2.074764	2.130079	2.122671	2.136036	2.129173	2.122404	2.174385	2.16722
2.079142	2.071349	2.126657	2.119336	2.132795	2.125989	2.11926	2.171234	2.164053
2.075748	2.067983	2.123288	2.116052	2.129591	2.122826	2.11612	2.168079	2.160898
2.103402	2.095637	2.140548	2.133395	2.167091	2.160325	2.153585	2.172347	2.165145
2.100024	2.092368	2.137361	2.130247	2.163955	2.157162	2.150391	2.169166	2.162015
2.096662	2.089183	2.134296	2.127179	2.171333	2.164518	2.157747	2.175215	2.168123
2.093321	2.086076	2.131338	2.12418	2.168252	2.161386	2.154588	2.172084	2.165115
2.121922	2.114832	2.128326	2.121175	2.182814	2.175799	2.168874	2.169	2.162184
2.118678	2.111689	2.12525	2.118158	2.179728	2.17267	2.165721	2.165964	2.159352
2.136036	2.129104	2.173176	2.166157	2.183732	2.176634	2.169649	2.162971	2.156508
2.132867	2.125939	2.170038	2.163136	2.180761	2.173675	2.16668	2.160025	2.153599
2.129762	2.122802	2.166902	2.160104	2.177788	2.170744	2.163768	2.157117	2.150681
2.126795	2.11976	2.181429	2.174536	2.174711	2.167753	2.160862	2.154234	2.147784
2.123898	2.116771	2.178294	2.171392	2.171592	2.164749	2.157987	2.151383	2.1449
2.153404	2.146134	2.175197	2.168296	2.168527	2.161808	2.155174	2.148581	2.142044
2.150274	2.143081	2.172134	2.165239	2.165503	2.158919	2.152423	2.145829	2.139214
2.157866	2.150732	2.169149	2.16227	2.162556	2.156046	2.149622	2.196093	2.189185
2.154856	2.147812	2.166266	2.15941	2.1597	2.15318	2.146746	2.193111	2.18629
2.177269	2.170288	2.170447	2.163619	2.156904	2.150342	2.143862	2.190129	2.183426
2.174365	2.167351	2.167529	2.160823	2.154189	2.147564	2.140998	2.18718	2.180611
2.17147	2.164417	2.164612	2.15803	2.15148	2.144795	2.138147	2.184249	2.177806
2.168454	2.161477	2.16172	2.155164	2.201789	2.194847	2.187935	2.2133	2.20645
2.165377	2.158543	2.15885	2.15226	2.198701	2.191834	2.185017	2.210287	2.203443
2.162395	2.155681	2.156041	2.149422	2.195699	2.188906	2.182185	2.207375	2.200534
2.159498	2.152891	2.153295	2.146645	2.192772	2.18606	2.179442	2.20457	2.197716
2.156681	2.150121	2.195868	2.189011	2.189907	2.183254	2.176708	2.201775	2.194949
2.153975	2.147359	2.192987	2.186189	2.187131	2.180491	2.173955	2.198966	2.192248
2.151287	2.144617	2.190125	2.183378	2.184365	2.177744	2.171227	2.196177	2.189551
2.148538	2.141908	2.195047	2.188246	2.214078	2.207078	2.200202	2.19798	2.191378
2.145778	2.139209	2.192259	2.185417	2.211113	2.204218	2.197463	2.195273	2.188629
2.189872	2.18313	2.189403	2.18256	2.212597	2.205775	2.19911	2.192457	2.185858
2.187092	2.180278	2.186507	2.179689	2.20963	2.20281	2.196154	2.189567	2.18307
2.198003	2.19114	2.221058	2.213846	2.206788	2.199957	2.19329	2.186755	2.180347
2.195146	2.188371	2.218277	2.211127	2.204099	2.197232	2.190519	2.184024	2.177697
2.192318	2.18561	2.215478	2.208379	2.201387	2.194533	2.187827	2.181355	2.175061
2.189557	2.182837	2.212572	2.205489	2.198548	2.191823	2.185246	2.178778	2.172416
2.186807	2.180079	2.209689	2.202627	2.195739	2.189142	2.18269	2.176218	2.169784
2.217251	2.210139	2.206958	2.199955	2.193105	2.186505	2.180041	2.173578	2.16717
2.214328	2.207361	2.204298	2.197379	2.190568	2.183914	2.177381	2.170923	2.164574
2.211493	2.204562	2.201565	2.194759	2.188037	2.181407	2.174867	2.168371	2.161975
2.208731	2.20175	2.198781	2.192116	2.185527	2.178979	2.172478	2.165905	2.159373
2.205948	2.19898	2.196054	2.189477	2.182977	2.176498	2.170054	2.1635	2.156973
2.203111	2.196263	2.193413	2.186844	2.180363	2.17394	2.167584	2.161174	2.154831
2.200337	2.193569	2.190773	2.18422	2.17776	2.171359	2.165061	2.15884	2.152766
2.197698	2.190858	2.188054	2.181597	2.175211	2.168754	2.162424	2.156417	2.150704

2.195125	2.188198	2.185372	2.178998	2.172677	2.166155	2.15979	2.154004	2.148671
2.192573	2.185706	2.182894	2.176464	2.170106	2.163594	2.157293	2.154362	2.149273
2.19004	2.183317	2.180538	2.173979	2.16753	2.161078	2.154906	2.152124	2.147236
2.187415	2.180835	2.178123	2.171518	2.167761	2.161405	2.155371	2.157844	2.153115
2.184692	2.178268	2.175664	2.169088	2.165388	2.159187	2.153342	2.155976	2.15141
2.182062	2.1757	2.175854	2.169331	2.163025	2.157021	2.151404	2.161006	2.156547
2.179572	2.173124	2.173264	2.166802	2.160629	2.154879	2.149547	2.159313	2.154994
2.177155	2.170602	2.170721	2.164331	2.158304	2.152808	2.147752	2.157665	2.153458
2.174812	2.16822	2.168342	2.162023	2.16333	2.158	2.153125	2.151595	2.147403
2.172498	2.165894	2.166038	2.159809	2.161213	2.156073	2.151394	2.149936	2.145794
2.172992	2.166407	2.163715	2.157635	2.159185	2.154225	2.149716	2.148341	2.144271
2.170534	2.163986	2.161396	2.155514	2.157245	2.152451	2.148085	2.146795	2.142811
2.168027	2.161546	2.166285	2.160591	2.158443	2.153707	2.149367	2.145236	2.141336
2.165447	2.15906	2.163935	2.158512	2.156575	2.151958	2.147695	2.143657	2.139844
2.170457	2.164171	2.161719	2.156536	2.154779	2.150275	2.146084	2.142115	2.13836
2.168109	2.161997	2.159712	2.154702	2.153062	2.148674	2.144568	2.14064	2.136891
2.165828	2.159923	2.157816	2.152961	2.151417	2.147133	2.143099	2.139189	2.135422
2.163544	2.157886	2.151804	2.147055	2.149855	2.145647	2.141647	2.13773	2.133925
2.161294	2.155897	2.15001	2.145438	2.148351	2.144203	2.140219	2.136281	2.132427
2.159096	2.153896	2.155438	2.151022	2.146831	2.142752	2.138795	2.134863	2.130993
2.156947	2.151894	2.153556	2.149337	2.145306	2.141302	2.13738	2.133472	2.129613
2.15091	2.145915	2.151796	2.147718	2.143802	2.139858	2.135968	2.146818	2.142953
2.149102	2.144221	2.150183	2.146178	2.142318	2.138417	2.134559	2.145408	2.141559
2.147361	2.142602	2.16342	2.159453	2.155611	2.151721	2.147865	2.144011	2.140185
2.145633	2.141027	2.161909	2.157982	2.154164	2.150297	2.146461	2.142633	2.138836
2.143942	2.139494	2.160436	2.156539	2.152734	2.148885	2.145067	2.14127	2.137512
2.158225	2.153895	2.158958	2.15509	2.151301	2.147473	2.143678	2.139925	2.136224
2.156577	2.152366	2.15749	2.153647	2.149873	2.146065	2.142296	2.138596	2.134962
2.161793	2.157628	2.156044	2.152221	2.14846	2.144669	2.140922	2.137269	2.133697
2.160277	2.156167	2.154618	2.150811	2.147062	2.143282	2.139553	2.135943	2.13243
2.158808	2.154742	2.15322	2.149424	2.145686	2.141925	2.13822	2.134647	2.131178
2.157386	2.153352	2.151847	2.148059	2.144332	2.140602	2.136934	2.133388	2.129943
2.155991	2.151998	2.150513	2.146724	2.143	2.139303	2.135672	2.13215	2.128721
2.154606	2.150695	2.149248	2.145439	2.141699	2.138028	2.134432	2.13093	2.127514
2.153234	2.14941	2.148001	2.144171	2.140413	2.136766	2.133204	2.12972	2.126316
2.151861	2.148072	2.146682	2.142858	2.139111	2.13549	2.13196	2.128501	2.125118
2.150496	2.146704	2.145319	2.14152	2.137802	2.13421	2.130709	2.127278	2.123923
2.149186	2.145376	2.143987	2.14021	2.136517	2.132948	2.12947	2.135217	2.131827
2.147941	2.144094	2.142688	2.138929	2.135258	2.131704	2.128242	2.133985	2.130616
2.149074	2.145196	2.14139	2.137654	2.134009	2.130474	2.127032	2.132783	2.129448
2.147748	2.143873	2.140082	2.136378	2.132766	2.129254	2.125839	2.131621	2.128352
2.146404	2.142539	2.138771	2.135103	2.131531	2.128052	2.124673	2.130484	2.127269
2.145055	2.141191	2.137443	2.133818	2.130297	2.12688	2.123558	2.129375	2.126154
2.143705	2.139845	2.13612	2.132542	2.129077	2.125727	2.122466	2.128275	2.125023
2.142382	2.138546	2.134856	2.131331	2.12791	2.124581	2.121331	2.127126	2.12387
2.14108	2.137286	2.133642	2.130174	2.126789	2.123444	2.120164	2.125939	2.122696
2.139841	2.136092	2.132481	2.129028	2.134813	2.131404	2.128046	2.124753	2.121513
2.138688	2.13499	2.13139	2.127897	2.133624	2.130214	2.126866	2.123566	2.120314
2.137517	2.133865	2.13028	2.126758	2.132439	2.129034	2.125698	2.132129	2.128637
2.136264	2.13265	2.129095	2.125591	2.131264	2.127871	2.124542	2.1309	2.127404

2.135001	2.131413	2.12789	2.124423	2.130107	2.126732	2.123415	2.129709	2.126212
2.133784	2.130195	2.126692	2.123276	2.128995	2.125655	2.122363	2.128607	2.125112
2.132599	2.128993	2.125504	2.122145	2.127909	2.124611	2.121349	2.12755	2.124061
2.131473	2.12787	2.133527	2.13013	2.1268	2.123513	2.120261	2.126403	2.122921
2.130411	2.126826	2.132467	2.129035	2.125672	2.122368	2.119107	2.125175	2.121704
2.129305	2.12574	2.13137	2.127925	2.134473	2.130921	2.127439	2.123954	2.120491
2.12812	2.124574	2.130199	2.126777	2.133269	2.12972	2.126227	2.122743	2.119285
2.126941	2.123417	2.129043	2.12565	2.132096	2.128553	2.125053	2.121569	2.118114
2.134994	2.131437	2.127966	2.124598	2.130999	2.127467	2.123966	2.127707	2.124185
2.13388	2.130355	2.126911	2.123563	2.12992	2.1264	2.122901	2.126635	2.123111
2.132733	2.129235	2.125809	2.122468	2.13616	2.132582	2.129021	2.129688	2.126145
2.131572	2.128098	2.124686	2.121341	2.134943	2.131368	2.127813	2.128487	2.124946
2.130448	2.127005	2.133495	2.129944	2.133793	2.130222	2.126669	2.127348	2.123806
2.129367	2.125962	2.132422	2.128879	2.13272	2.129151	2.125599	2.12628	2.122732
2.128252	2.124883	2.138781	2.135184	2.131609	2.128041	2.124488	2.135704	2.13204
2.127052	2.123717	2.137561	2.133975	2.130403	2.126837	2.123282	2.13447	2.130813
2.12587	2.122558	2.136342	2.132767	2.129199	2.125633	2.122076	2.133237	2.129589
2.132964	2.129576	2.135213	2.131646	2.128084	2.124513	2.120951	2.132088	2.128452
2.131938	2.128532	2.134109	2.13055	2.126993	2.123417	2.119848	2.130963	2.127342
2.14009	2.136503	2.132933	2.129376	2.125821	2.122244	2.118675	2.129766	2.126155
2.138862	2.135276	2.131705	2.128147	2.124591	2.121017	2.117454	2.128519	2.124917
2.137707	2.134127	2.130556	2.126991	2.134368	2.130663	2.126997	2.127355	2.123768
2.136646	2.133078	2.129507	2.125929	2.133256	2.129563	2.125912	2.126296	2.122733
2.135592	2.132035	2.128466	2.124876	2.132156	2.128471	2.124834	2.125247	2.121711
2.134526	2.13098	2.127415	2.123818	2.131056	2.127374	2.123744	2.124186	2.120685
2.133433	2.129894	2.126332	2.122731	2.129929	2.126252	2.122628	2.1231	2.119631
2.132251	2.128707	2.136137	2.132402	2.128703	2.125035	2.121423	2.126545	2.123053
2.131032	2.127472	2.134847	2.13112	2.127432	2.123777	2.120182	2.125305	2.121832
2.129914	2.126339	2.133663	2.12994	2.126262	2.122629	2.119056	2.124176	2.120711
2.128883	2.125291	2.132567	2.128846	2.125178	2.121573	2.11803	2.123143	2.119674
2.139021	2.135242	2.131497	2.127778	2.129034	2.125406	2.121841	2.122129	2.118656
2.137977	2.134197	2.130453	2.126738	2.127981	2.124375	2.120833	2.121132	2.117658
2.136929	2.133148	2.129406	2.125697	2.126927	2.123343	2.119823	2.120131	2.116652
2.13587	2.132082	2.128342	2.124642	2.12586	2.1223	2.118802	2.119117	2.115627
2.134778	2.130986	2.127249	2.123561	2.12477	2.121233	2.117754	2.118074	2.114569
2.133585	2.129798	2.126071	2.122401	2.123604	2.120083	2.116616	2.116941	2.113423
2.132327	2.128552	2.124841	2.121192	2.122393	2.118884	2.115422	2.115749	2.112221
2.131155	2.127391	2.128542	2.124873	2.121275	2.117769	2.114305	2.114632	2.111099
2.130068	2.126313	2.127462	2.123826	2.12025	2.11674	2.113262	2.113589	2.110057
2.129525	2.125772	2.127058	2.123448	2.119243	2.115729	2.112238	2.112564	2.109032
2.128461	2.124719	2.126002	2.122424	2.118243	2.114727	2.111226	2.111551	2.108015
2.12741	2.123679	2.124957	2.121408	2.117246	2.113729	2.110219	2.110545	2.107005
2.126372	2.122651	2.123922	2.120392	2.120942	2.11739	2.113841	2.11252	2.108931
2.125343	2.121635	2.122898	2.11938	2.11994	2.116384	2.112829	2.111483	2.107891
2.124316	2.120634	2.121893	2.118376	2.122113	2.118519	2.114919	2.118832	2.115106
2.123291	2.119645	2.120903	2.11738	2.12109	2.117484	2.113874	2.117744	2.114018
2.127155	2.123505	2.119909	2.11638	2.120065	2.116452	2.112834	2.116662	2.112935
2.126107	2.122491	2.118912	2.115376	2.119039	2.115421	2.1118	2.115584	2.111855
2.125058	2.121474	2.121257	2.11769	2.125736	2.122	2.11825	2.119597	2.115824
2.124002	2.120454	2.120233	2.116662	2.124657	2.120916	2.117168	2.118518	2.114768

2.122951	2.119432	2.119204	2.11563	2.123571	2.119828	2.116084	2.11744	2.113719
2.120778	2.117288	2.11817	2.114593	2.122484	2.118738	2.114999	2.116357	2.112661
2.11976	2.116274	2.117131	2.113552	2.121394	2.117647	2.113913	2.115274	2.111602
2.122186	2.11867	2.116089	2.112509	2.120305	2.11656	2.112833	2.114196	2.110547
2.121152	2.117629	2.115042	2.111462	2.119216	2.115476	2.111756	2.113123	2.109495
2.120146	2.11661	2.114011	2.110424	2.118132	2.114399	2.110692	2.112065	2.108461
2.119167	2.115607	2.112988	2.109387	2.117049	2.113329	2.109643	2.111027	2.107449
2.118228	2.11465	2.119798	2.116067	2.115988	2.112273	2.108604	2.109998	2.106447
2.117372	2.11379	2.118866	2.115082	2.114959	2.111232	2.107566	2.108969	2.105449
2.11652	2.112943	2.117958	2.114127	2.113963	2.110216	2.106545	2.107951	2.104461
2.115557	2.111989	2.116983	2.113176	2.118097	2.114284	2.110541	2.108531	2.105027
2.114525	2.11097	2.11597	2.112227	2.117176	2.113343	2.109566	2.10754	2.104055
2.113488	2.109939	2.114925	2.111223	2.116202	2.112393	2.108634	2.106611	2.103143
2.112437	2.10889	2.113844	2.110162	2.11518	2.11144	2.107753	2.105752	2.1023
2.111401	2.10784	2.112755	2.109085	2.115763	2.112042	2.108377	2.104848	2.101411
2.110381	2.106788	2.111655	2.107993	2.114666	2.110985	2.107365	2.103863	2.100444
2.109391	2.105757	2.11057	2.106908	2.113565	2.109913	2.106328	2.102844	2.099435
2.108465	2.104776	2.109518	2.105846	2.112477	2.108844	2.10528	2.101799	2.098385
2.107558	2.103821	2.108498	2.104814	2.111411	2.107784	2.104229	2.10074	2.097312
2.114312	2.110513	2.107539	2.103859	2.11042	2.106782	2.103211	2.099705	2.096258
2.113264	2.109548	2.10663	2.102965	2.109486	2.105823	2.102218	2.098686	2.095216
2.112219	2.108546	2.107589	2.103891	2.108508	2.104855	2.101255	2.097717	2.094232
2.111169	2.107489	2.106524	2.102835	2.107476	2.103878	2.100335	2.096814	2.093321
2.11016	2.106471	2.105488	2.101799	2.106448	2.102887	2.099385	2.095878	2.09238
2.109238	2.105554	2.104542	2.100822	2.10543	2.10186	2.098356	2.094848	2.091354
2.108316	2.104636	2.103601	2.099861	2.104434	2.100845	2.097326	2.093809	2.090313
2.107291	2.103617	2.107642	2.103904	2.103496	2.09991	2.096378	2.093762	2.090214
2.106225	2.102554	2.106598	2.102933	2.102581	2.099005	2.095461	2.092808	2.089216
2.107258	2.103512	2.105545	2.101915	2.101596	2.09803	2.094488	2.091833	2.088249
2.106288	2.102484	2.104479	2.100858	2.100554	2.096997	2.09347	2.090842	2.087311
2.105319	2.101503	2.103483	2.099866	2.100509	2.096936	2.093395	2.089867	2.086357
2.10434	2.10058	2.102584	2.098969	2.099598	2.096019	2.092466	2.088913	2.085372
2.103383	2.099681	2.101705	2.098081	2.098684	2.095096	2.091533	2.087964	2.084404
2.102484	2.098806	2.100824	2.097162	2.097716	2.094114	2.090552	2.087006	2.083486
2.10159	2.097925	2.099934	2.096237	2.096744	2.093131	2.089573	2.086056	2.082581
2.100626	2.096975	2.098995	2.095313	2.09582	2.092213	2.088656	2.085132	2.081646
2.099628	2.095986	2.098028	2.094386	2.094918	2.091328	2.087773	2.084226	2.0807
2.103921	2.100241	2.09709	2.093446	2.093968	2.090391	2.086851	2.083314	2.079793
2.103033	2.099347	2.096178	2.09249	2.092967	2.089401	2.085892	2.082395	2.078923
2.098217	2.094496	2.095258	2.091551	2.092004	2.088448	2.08496	2.081493	2.078058
2.097293	2.093544	2.094312	2.090635	2.091107	2.087563	2.084082	2.080625	2.077199
2.096362	2.092597	2.093378	2.089733	2.090224	2.086686	2.083203	2.079747	2.076321
2.095418	2.091675	2.093492	2.089842	2.089326	2.085774	2.082274	2.078809	2.075382
2.094477	2.090766	2.092606	2.088954	2.088425	2.084856	2.081338	2.077862	2.074431
2.093571	2.089869	2.091714	2.08805	2.08751	2.083952	2.080449	2.07698	2.07355
2.092691	2.088982	2.090821	2.087136	2.086586	2.083058	2.079594	2.076142	2.072718
2.091809	2.088075	2.086183	2.082488	2.0857	2.082197	2.078758	2.075316	2.071894
2.090926	2.087141	2.085235	2.081589	2.084857	2.081374	2.077945	2.074507	2.071081
2.09005	2.086229	2.084315	2.080714	2.084031	2.080559	2.07713	2.073689	2.070254
2.089197	2.085367	2.083455	2.079874	2.083218	2.079748	2.076311	2.072852	2.069391

2.088332	2.084513	2.082612	2.079048	2.082412	2.078932	2.075477	2.072001	2.068524
2.087407	2.083626	2.081179	2.077654	2.081603	2.078091	2.074599	2.071126	2.067675
2.086459	2.082732	2.080321	2.076824	2.080792	2.077241	2.073706	2.070245	2.06684
2.085556	2.081864	2.082899	2.079386	2.076307	2.072719	2.069146	2.069415	2.066034
2.084688	2.081017	2.082059	2.078554	2.075484	2.071902	2.068335	2.068625	2.065253
2.083833	2.080182	2.081222	2.077701	2.074627	2.071063	2.06752	2.067838	2.064474
2.082992	2.079361	2.080386	2.076817	2.073717	2.070188	2.066698	2.067049	2.063695
2.083364	2.079739	2.079558	2.075948	2.072827	2.069328	2.065883	2.062486	2.059134
2.082528	2.078925	2.078749	2.075124	2.071998	2.06851	2.065082	2.061693	2.058343
2.081693	2.078114	2.077947	2.07432	2.071195	2.067713	2.064291	2.060905	2.057555
2.080851	2.077295	2.07714	2.073515	2.070395	2.066921	2.063507	2.071473	2.068027
2.080007	2.076474	2.076333	2.072715	2.069604	2.066138	2.062732	2.070685	2.06724
2.082647	2.079098	2.075533	2.071921	2.068817	2.065357	2.061957	2.069896	2.066453
2.081823	2.078293	2.07474	2.071132	2.068034	2.064579	2.061184	2.069107	2.065666
2.081016	2.077497	2.073952	2.070347	2.067255	2.063804	2.060412	2.06832	2.06488
2.080229	2.076712	2.073169	2.069566	2.066479	2.063031	2.059642	2.067535	2.064095
2.079451	2.075933	2.07239	2.068789	2.065706	2.06226	2.058872	2.066749	2.063309
2.075231	2.071755	2.071617	2.068016	2.064934	2.061487	2.058099	2.06596	2.062521
2.074465	2.070992	2.070849	2.067249	2.064168	2.060719	2.057328	2.065172	2.061735
2.073694	2.070224	2.07008	2.066488	2.063415	2.05996	2.056562	2.064389	2.060955
2.07292	2.069455	2.069312	2.065736	2.062675	2.059211	2.0558	2.063611	2.060181
2.072157	2.068692	2.068556	2.065012	2.061972	2.058492	2.055054	2.06284	2.059412
2.071405	2.067937	2.067814	2.064322	2.061317	2.057809	2.054327	2.062078	2.058648
2.070658	2.067181	2.067063	2.063612	2.060634	2.057106	2.053592	2.061315	2.057885
2.069908	2.066417	2.06629	2.062855	2.059892	2.056359	2.052837	2.060546	2.057124
2.069174	2.065661	2.065515	2.062084	2.05913	2.055598	2.052079	2.059776	2.056361
2.068486	2.06493	2.064751	2.061315	2.058364	2.054835	2.05132	2.059	2.055584
2.067817	2.064213	2.063994	2.060547	2.057595	2.054075	2.050571	2.058233	2.054808
2.06711	2.063492	2.063254	2.059793	2.056841	2.053348	2.049874	2.05752	2.054069
2.066378	2.062771	2.062528	2.059052	2.056101	2.052649	2.049219	2.056853	2.053359
2.065662	2.062079	2.061838	2.058339	2.055376	2.051944	2.048541	2.05617	2.052671
2.06497	2.061429	2.061195	2.05766	2.054665	2.051225	2.047828	2.055468	2.052013
2.064274	2.060765	2.060541	2.056991	2.053981	2.050525	2.047119	2.054755	2.051336
2.063552	2.06005	2.059844	2.056333	2.053347	2.049869	2.046427	2.054023	2.0506
2.062836	2.059325	2.059132	2.055668	2.052716	2.049223	2.045749	2.053298	2.049853
2.062162	2.058624	2.058415	2.054957	2.052021	2.048553	2.045103	2.052622	2.049141
2.061513	2.057938	2.057697	2.054224	2.051292	2.047871	2.04447	2.051971	2.048448
2.060855	2.05728	2.057025	2.053522	2.050573	2.047167	2.043791	2.051287	2.047769
2.060191	2.056652	2.056395	2.052846	2.04986	2.046441	2.043069	2.050574	2.047109
2.062858	2.059306	2.056289	2.052707	2.060348	2.056805	2.053312	2.049847	2.046411
2.062221	2.058648	2.055625	2.052062	2.059703	2.056121	2.052579	2.049094	2.045654
2.06157	2.057985	2.054962	2.051418	2.059057	2.055448	2.051868	2.048356	2.044896
2.060877	2.057326	2.05432	2.050762	2.05837	2.054772	2.051204	2.05376	2.050035
2.060163	2.056659	2.053677	2.050102	2.057675	2.054099	2.050554	2.053056	2.049293
2.05944	2.055954	2.052993	2.049439	2.057011	2.053425	2.049866	2.05233	2.048581
2.05871	2.055226	2.052283	2.048773	2.056363	2.052752	2.049158	2.051597	2.047895
2.058044	2.054552	2.051613	2.048113	2.055692	2.052078	2.04848	2.050894	2.047208
2.057446	2.053939	2.050985	2.047457	2.054995	2.051402	2.047836	2.050224	2.04652
2.056846	2.053319	2.05035	2.046805	2.054311	2.050729	2.047179	2.049547	2.045843
2.05623	2.05267	2.049692	2.04616	2.053664	2.050061	2.046489	2.048844	2.045185

2.0556	2.052015	2.049031	2.045512	2.053012	2.049391	2.0458	2.048142	2.044526
2.054945	2.051365	2.048381	2.044848	2.052318	2.048711	2.045141	2.047461	2.043841
2.054275	2.050712	2.047733	2.04418	2.051612	2.04803	2.044492	2.046788	2.043151
2.053616	2.050043	2.047067	2.043526	2.050949	2.04736	2.043815	2.046099	2.042496
2.052963	2.049361	2.046386	2.042882	2.050319	2.046699	2.043118	2.045401	2.041866
2.052304	2.048703	2.063735	2.059887	2.05606	2.052189	2.048372	2.044743	2.041249
2.05163	2.04807	2.063092	2.05926	2.055442	2.051577	2.047763	2.044141	2.040652
2.050984	2.047455	2.062453	2.058617	2.054797	2.050948	2.04715	2.043532	2.040037
2.050398	2.046866	2.0618	2.05792	2.054076	2.050257	2.046501	2.042884	2.03937
2.049831	2.046281	2.061137	2.057216	2.05335	2.049559	2.045842	2.042228	2.0387
2.049242	2.045663	2.060444	2.056539	2.052688	2.048893	2.045172	2.041572	2.038069
2.048648	2.045035	2.059742	2.055882	2.052064	2.048252	2.044507	2.040926	2.037465
2.048072	2.044449	2.059105	2.05527	2.051474	2.047663	2.043914	2.040342	2.036895
2.047511	2.043901	2.058526	2.054702	2.050917	2.047124	2.04339	2.039817	2.036359
2.046926	2.043344	2.057947	2.054135	2.050362	2.046589	2.042873	2.039301	2.035831
2.046297	2.042767	2.057364	2.05357	2.049813	2.046061	2.042361	2.038794	2.035318
2.045682	2.042195	2.056773	2.052985	2.049236	2.045511	2.041839	2.038273	2.034783
2.045119	2.041639	2.056161	2.052345	2.048584	2.044903	2.04128	2.037706	2.034179
2.044582	2.041096	2.05555	2.051697	2.047921	2.044284	2.040714	2.037131	2.033562
2.044057	2.040573	2.054979	2.051112	2.047331	2.043719	2.040178	2.036593	2.033007
2.043547	2.040066	2.054434	2.050566	2.046788	2.043188	2.039659	2.036078	2.032489
2.05472	2.051134	2.053889	2.050026	2.046254	2.042665	2.039146	2.035567	2.031975
2.054192	2.05061	2.053344	2.049491	2.04573	2.04215	2.038639	2.035062	2.031468
2.053667	2.050089	2.052804	2.048962	2.045211	2.041639	2.038134	2.034558	2.030963
2.053147	2.049574	2.05227	2.048438	2.044694	2.04113	2.03763	2.034055	2.030457
2.052629	2.04906	2.05174	2.047917	2.044182	2.040622	2.037124	2.03355	2.029951
2.05211	2.048548	2.051213	2.047402	2.043673	2.040114	2.036614	2.033039	2.029441
2.051591	2.048036	2.050688	2.046888	2.043168	2.039606	2.036101	2.032525	2.028929
2.051074	2.047523	2.05016	2.046372	2.042659	2.039096	2.035586	2.03201	2.028414
2.050558	2.047008	2.049629	2.045853	2.042149	2.038585	2.035071	2.031493	2.027897
2.050042	2.046495	2.049099	2.045333	2.041636	2.03807	2.034553	2.030974	2.027378
2.049527	2.045983	2.04857	2.044811	2.041119	2.037552	2.034032	2.030454	2.026859
2.049011	2.045471	2.048041	2.044289	2.040601	2.037033	2.033509	2.029931	2.026338
2.048491	2.044956	2.04751	2.043768	2.040084	2.036512	2.032982	2.029403	2.025813
2.047974	2.044443	2.04698	2.043246	2.039567	2.035991	2.032454	2.028875	2.025286
2.046483	2.042949	2.046447	2.04272	2.039046	2.035468	2.031928	2.028346	2.024755
2.045991	2.042446	2.045917	2.042194	2.038524	2.034946	2.031404	2.027819	2.024224
2.049458	2.045859	2.045404	2.04168	2.038009	2.034427	2.030881	2.027297	2.023704
2.049057	2.045415	2.044907	2.041174	2.037499	2.033911	2.030361	2.026779	2.023193
2.048625	2.044957	2.04839	2.044644	2.037035	2.033429	2.029859	2.026284	2.022719
2.048143	2.044476	2.047913	2.044219	2.036641	2.033001	2.029385	2.025823	2.022302
2.047653	2.043992	2.046598	2.042951	2.036231	2.032564	2.028912	2.025361	2.021874
2.047169	2.043506	2.046092	2.042455	2.035742	2.032073	2.028418	2.024868	2.021386
2.046693	2.043033	2.045594	2.041948	2.03523	2.031572	2.02793	2.024378	2.020885
2.046239	2.042611	2.045162	2.041486	2.038657	2.034995	2.031355	2.023936	2.020416
2.045803	2.042221	2.044768	2.041048	2.038185	2.034553	2.030953	2.02352	2.019963
2.045386	2.041811	2.044332	2.040586	2.037704	2.034084	2.030503	2.023065	2.019492
2.044993	2.041382	2.043853	2.040101	2.037212	2.033587	2.030002	2.022567	2.019001
2.044573	2.04094	2.043381	2.039639	2.036754	2.033118	2.02952	2.025927	2.022351
2.044098	2.040476	2.042921	2.039216	2.036353	2.032697	2.029071	2.025491	2.021949

2.04362	2.040022	2.042478	2.038804	2.035959	2.03229	2.028645	2.025073	2.021553
2.043173	2.039612	2.042072	2.038385	2.034722	2.031074	2.027453	2.024674	2.021129
2.042744	2.039221	2.04168	2.037969	2.03429	2.030668	2.027077	2.02428	2.020691
2.042351	2.038821	2.041265	2.037566	2.033893	2.030266	2.026662	2.023845	2.020234
2.041986	2.038417	2.040837	2.037173	2.033522	2.029868	2.026223	2.023384	2.019762
2.041599	2.038024	2.040429	2.036771	2.033124	2.029468	2.025819	2.022974	2.019345
2.041184	2.037648	2.04005	2.036358	2.032692	2.029069	2.025463	2.022631	2.018997
2.040772	2.037261	2.033743	2.030203	2.026693	2.023251	2.019829	2.021467	2.017833
2.040379	2.036847	2.033327	2.029813	2.026319	2.022862	2.019416	2.021025	2.017392
2.039983	2.03643	2.032908	2.029422	2.025947	2.022473	2.019001	2.02058	2.016949
2.039562	2.036034	2.032522	2.029025	2.025544	2.022083	2.018627	2.020202	2.01657
2.039133	2.035644	2.032147	2.028627	2.025131	2.021692	2.018268	2.019849	2.016214
2.038727	2.035241	2.031749	2.028236	2.024745	2.021304	2.017875	2.019439	2.015803
2.038341	2.034828	2.031332	2.027852	2.024381	2.020917	2.017453	2.01898	2.015347
2.037944	2.034426	2.030931	2.027462	2.023999	2.020528	2.017056	2.018566	2.014938
2.037524	2.03404	2.030556	2.027061	2.023582	2.020135	2.016697	2.018219	2.014601
2.037125	2.033659	2.03018	2.026669	2.023181	2.019749	2.016332	2.012879	2.009413
2.036772	2.033267	2.029776	2.026295	2.022827	2.01937	2.015924	2.012486	2.009058
2.03644	2.032887	2.029382	2.025939	2.022496	2.019007	2.015519	2.012087	2.00869
2.036114	2.032555	2.029051	2.025619	2.022182	2.018681	2.015176	2.011722	2.008301
2.035792	2.032253	2.028758	2.025322	2.021878	2.018379	2.014869	2.011378	2.007902
2.035434	2.03193	2.028447	2.024991	2.021531	2.018044	2.014548	2.011047	2.007548
2.035036	2.031588	2.028123	2.024627	2.021138	2.017677	2.014221	2.010734	2.007239
2.033799	2.030366	2.027797	2.024276	2.020767	2.017308	2.013863	2.010394	2.006919
2.033485	2.030048	2.027479	2.023957	2.020437	2.016936	2.013451	2.009999	2.006572
2.03318	2.029719	2.027144	2.023635	2.020116	2.016576	2.013052	2.009612	2.006227
2.032852	2.029345	2.026761	2.023284	2.019793	2.016252	2.01272	2.009285	2.005911
2.03252	2.028961	2.026367	2.022925	2.01947	2.015944	2.012418	2.008981	2.005596
2.024177	2.020625	2.025095	2.021646	2.019146	2.015633	2.012115	2.008658	2.005237
2.023863	2.020311	2.024778	2.021321	2.018818	2.015322	2.011817	2.008327	2.004849
2.023554	2.020001	2.024459	2.020974	2.01846	2.014984	2.011505	2.008005	2.004499
2.023251	2.019695	2.024133	2.020598	2.018059	2.014612	2.011176	2.007693	2.004192
2.022953	2.019395	2.015846	2.0123	2.01671	2.013263	2.00984	2.007389	2.003897
2.022661	2.019101	2.015546	2.011986	2.016389	2.012946	2.009533	2.007092	2.003611
2.022372	2.018811	2.015254	2.011689	2.01609	2.012647	2.009234	2.0068	2.003329
2.022083	2.018523	2.014964	2.011396	2.015794	2.012352	2.008942	2.006512	2.003042
2.021797	2.018236	2.014678	2.011109	2.015507	2.012066	2.008658	2.006227	2.002754
2.021513	2.017949	2.014389	2.010821	2.015222	2.011785	2.008379	2.004908	2.001407
2.021231	2.017662	2.014099	2.010533	2.014938	2.011509	2.008107	2.004631	2.001117
2.019184	2.015624	2.013812	2.010249	2.006735	2.003331	1.999952	2.004353	2.000832
2.01891	2.015349	2.013527	2.009969	2.006458	2.003051	1.999668	2.004071	2.00055
2.01864	2.015078	2.013247	2.00969	2.006181	2.00277	1.999383	2.003789	2.000272
2.018373	2.01481	2.01297	2.009409	2.005896	2.002483	1.999097	2.003509	1.999999
2.018108	2.014545	2.012696	2.009132	2.005615	2.002199	1.998813	2.00323	1.999725
2.017844	2.014279	2.010722	2.007166	2.005345	2.001927	1.998537	1.995081	1.991596
2.017581	2.014014	2.010459	2.006913	2.005089	2.001668	1.998269	1.994803	1.991307
2.017318	2.013751	2.010208	2.006695	2.003209	1.99977	1.996341	1.994529	1.991021
2.017054	2.013489	2.009969	2.006512	2.003059	1.999589	1.996109	1.99426	1.990737
2.016802	2.013237	2.009731	2.006309	2.002877	1.999386	1.995871	1.992341	1.988801
2.016564	2.012994	2.009487	2.006067	2.002637	1.999141	1.99562	1.992084	1.98854

2.016338	2.012773	2.009263	2.005829	2.002386	1.99889	1.995372	1.991842	1.988304
2.016128	2.012598	2.009094	2.005626	2.002152	1.998646	1.995132	1.991624	1.988121
2.015925	2.012436	2.00894	2.005431	2.001922	1.998411	1.994906	1.991425	1.987958
2.0157	2.012226	2.008733	2.005209	2.001692	1.998202	1.994723	1.991246	1.98777
2.015463	2.011986	2.008493	2.00497	2.001464	1.998014	1.994572	1.991082	1.987566
2.015268	2.011769	2.008262	2.004736	2.001236	1.997803	1.994379	1.990883	1.987348
2.015123	2.011577	2.00804	2.004505	2.001	1.997565	1.99414	1.990645	1.987112
2.014966	2.011387	2.007831	2.004299	2.000797	1.997346	1.993905	1.990405	1.986877
2.014769	2.011185	2.007638	2.00414	2.000654	1.99717	1.993684	1.990168	1.986639
2.014567	2.010993	2.007462	2.003991	2.000516	1.997	1.993472	1.989946	1.986422
2.01438	2.010834	2.007313	2.003824	2.00033	1.996802	1.993269	1.989758	1.986261
2.014202	2.010693	2.007177	2.003651	2.000126	1.996597	1.993079	1.989593	1.98613
2.014051	2.010539	2.007015	2.003469	1.999932	1.996418	1.992919	1.989441	1.98598
2.013922	2.010379	2.006834	2.003279	1.999745	1.996257	1.992786	1.989304	1.985821
2.013786	2.01024	2.00669	2.00313	1.999593	1.996109	1.992644	1.989162	1.985677
2.013637	2.010131	2.006603	2.00304	1.99949	1.995977	1.992484	1.989011	1.985554
2.004749	2.001298	2.006491	2.002929	1.999374	1.995843	1.992334	2.002897	1.99935
2.004608	2.001123	2.006304	2.002748	1.999206	1.995701	1.992213	2.002757	1.999179
2.00446	2.000929	2.006093	2.002548	1.999027	1.995552	1.992091	2.002617	1.999006
1.995312	1.991822	2.005888	2.002369	1.998872	1.995398	1.991933	2.002457	1.998863
1.995101	1.991604	2.005689	2.002201	1.998725	1.995238	1.991757	2.002286	1.998732
1.994937	1.991442	1.996841	1.993371	1.998563	1.995078	1.9916	2.002117	1.998563
1.994818	1.991335	1.996737	1.993224	1.998386	1.994915	1.991461	2.001949	1.998356
1.994682	1.991221	1.996639	1.993101	1.998242	1.994766	1.991312	2.001775	1.998169
1.994514	1.991092	1.99655	1.993015	1.998151	1.994633	1.991135	2.001588	1.998018
1.994342	1.990947	1.996441	1.992924	1.998068	1.994515	1.990978	2.001414	1.997869
1.994178	1.990767	1.996275	1.992796	1.989307	1.985791	1.982279	2.001273	1.997694
1.994016	1.990579	1.996094	1.992652	1.989198	1.985701	1.982194	2.001154	1.997524
1.993845	1.990419	1.987006	1.983609	1.989048	1.985576	1.982097	2.001052	1.997406
1.993674	1.99028	1.986875	1.983446	1.98887	1.985429	1.981993	2.000964	1.997324
1.985025	1.981621	1.986763	1.983315	1.988734	1.985288	1.981856	1.992905	1.989326
1.984943	1.981538	1.986676	1.983223	1.988641	1.985151	1.981677	1.992723	1.989195
1.984859	1.981436	1.977996	1.974534	1.988549	1.985029	1.981523	1.992552	1.989044
1.984758	1.981286	1.97783	1.974395	1.988456	1.98494	1.981425	1.992408	1.988854
1.984653	1.981136	1.97766	1.974243	1.988346	1.984844	1.981335	1.992279	1.988676
1.979425	1.975904	1.977523	1.974075	1.979261	1.97584	1.972423	1.992172	1.988561
1.979317	1.975799	1.977404	1.973905	1.97907	1.975671	1.97229	1.992072	1.988475
1.979178	1.97569	1.972197	1.96869	1.970286	1.966846	1.963427	1.991932	1.988368
1.979014	1.97558	1.972116	1.968603	1.970181	1.966697	1.963238	1.991764	1.988252
1.97888	1.975473	1.972025	1.968514	1.965004	1.961507	1.958037	1.98353	1.980099
1.978784	1.975374	1.971927	1.968426	1.964921	1.961414	1.957932	1.983438	1.980012
1.978692	1.975259	1.971806	1.968323	1.964834	1.961327	1.957839	1.971181	1.967731
1.978587	1.975104	1.971636	1.968185	1.964729	1.961233	1.95775	1.971088	1.967634
1.978488	1.974952	1.971465	1.968045	1.964621	1.96114	1.957663	1.970992	1.967524
1.978411	1.974848	1.971349	1.967937	1.964524	1.961047	1.957568	1.970868	1.967366
1.978349	1.974773	1.971264	1.96785	1.964433	1.960955	1.957472	1.970733	1.967186
1.978294	1.974709	1.971194	1.967776	1.964356	1.960879	1.957392	1.970628	1.967052
1.978247	1.974659	1.971141	1.967717	1.964293	1.960816	1.957328	1.97055	1.966957
1.978196	1.974605	1.971083	1.967655	1.964228	1.96075	1.957261	1.970475	1.966872
1.978139	1.974545	1.971019	1.967587	1.964156	1.960677	1.957188	1.970398	1.966797

1.978078	1.97448	1.970951	1.967517	1.964085	1.960605	1.957116	1.970322	1.966723
1.978006	1.974407	1.970879	1.967447	1.964017	1.960538	1.95705	1.970243	1.966642
1.977931	1.974333	1.970806	1.967377	1.96395	1.960474	1.956987	1.970165	1.966563
1.977863	1.974263	1.970737	1.967311	1.963887	1.960414	1.95693	1.970096	1.966492
1.977797	1.974196	1.970671	1.967247	1.963827	1.960357	1.956877	1.970034	1.966431
1.974939	1.971342	1.970609	1.967184	1.963764	1.960297	1.956824	1.969991	1.966415
1.974871	1.971282	1.970547	1.967118	1.963697	1.960233	1.956772	1.969972	1.966454
1.974815	1.971247	1.970516	1.967072	1.963638	1.960171	1.956716	1.969935	1.966461
1.974779	1.971258	1.970539	1.967062	1.963593	1.960111	1.956648	1.969861	1.966403
1.974745	1.971267	1.970559	1.967051	1.963551	1.960054	1.956584	1.969781	1.966327
1.974692	1.971229	1.967747	1.964231	1.963498	1.959998	1.956527	1.969707	1.966249
1.974643	1.971177	1.967694	1.964179	1.963446	1.959953	1.956487	1.969645	1.966172
1.969157	1.965683	1.967656	1.964143	1.968449	1.964936	1.961453	1.973983	1.970449
1.9692	1.965675	1.967627	1.964117	1.968439	1.964967	1.961518	1.974024	1.970431
1.969219	1.965666	1.967614	1.964119	1.968463	1.965003	1.961559	1.974041	1.970419
1.969206	1.965651	1.967618	1.964157	1.968529	1.965046	1.96157	1.974027	1.970407
1.962195	1.958701	1.962169	1.958752	1.966033	1.962518	1.959002	1.974022	1.970419
1.962199	1.958745	1.962225	1.958785	1.966057	1.962529	1.959011	1.97404	1.970477
1.962205	1.958791	1.962282	1.958812	1.966072	1.962541	1.95903	1.974072	1.970541
1.96219	1.958788	1.96229	1.958815	1.966085	1.962576	1.959087	1.974118	1.970569
1.962174	1.958765	1.962277	1.95881	1.966101	1.962625	1.959163	1.974171	1.970579
1.956671	1.953279	1.962293	1.958844	1.960973	1.957521	1.954075	1.958287	1.954798
1.956753	1.953325	1.962331	1.958911	1.961068	1.957582	1.954094	1.958293	1.9548
1.956827	1.953388	1.962388	1.958971	1.961134	1.957623	1.95411	1.955743	1.952244
1.956878	1.953475	1.962476	1.959021	1.961155	1.95763	1.954119	1.955778	1.952317
1.953291	1.949931	1.955618	1.952166	1.961167	1.957643	1.954144	1.950697	1.947286
1.953375	1.949973	1.955632	1.952168	1.961181	1.957682	1.954209	1.950759	1.94733
1.95346	1.950001	1.955629	1.952169	1.961204	1.957733	1.954286	1.95083	1.947373
1.953502	1.950024	1.95565	1.95221	1.96127	1.957794	1.954339	1.95089	1.947448
1.953524	1.950045	1.955686	1.952276	1.961364	1.957864	1.954383	1.950947	1.947541
1.947763	1.944298	1.952398	1.949003	1.961442	1.957939	1.954455	1.951019	1.947613
1.947893	1.944432	1.952519	1.949079	1.961503	1.958018	1.954558	1.951105	1.947663
1.948001	1.944556	1.952645	1.949173	1.955002	1.951564	1.948156	1.951187	1.947728
1.948058	1.944651	1.952774	1.949304	1.955128	1.951652	1.948209	1.951255	1.947827
1.948113	1.944733	1.952885	1.949421	1.955246	1.951738	1.948265	1.951325	1.947923
1.948202	1.944796	1.947376	1.943917	1.952202	1.948729	1.945288	1.951408	1.947988
1.9483	1.944854	1.947407	1.94394	1.952236	1.948796	1.945387	1.951494	1.948044
1.948375	1.944937	1.94151	1.938089	1.947081	1.943635	1.940219	1.948753	1.945316
1.948436	1.945037	1.941639	1.938235	1.947227	1.943735	1.940271	1.948818	1.945409
1.948529	1.945127	1.941736	1.938356	1.94737	1.943859	1.94037	1.942193	1.938829
1.948659	1.945199	1.94179	1.938447	1.947512	1.944015	1.940526	1.94232	1.938904
1.946745	1.943246	1.941865	1.938534	1.947631	1.944159	1.940687	1.961641	1.957905
1.946899	1.943392	1.941988	1.938615	1.947694	1.944259	1.940835	1.961782	1.958034
1.947047	1.943551	1.942135	1.938712	1.947764	1.94436	1.940982	1.96193	1.958184
1.939523	1.936086	1.940254	1.936829	1.947912	1.94451	1.941136	1.962068	1.958328
1.939607	1.93622	1.940426	1.937011	1.948098	1.944681	1.941289	1.962197	1.958471
1.939742	1.936369	1.940591	1.937185	1.942661	1.939249	1.935861	1.962291	1.958593
1.939919	1.936533	1.940753	1.937354	1.942832	1.939373	1.935939	1.962352	1.958696
1.940092	1.936675	1.933284	1.929924	1.941209	1.937728	1.934268	1.962432	1.95878
1.940252	1.93678	1.93337	1.930045	1.941374	1.937899	1.93443	1.962537	1.958832

1.940415	1.9369	1.933475	1.930172	1.934436	1.931041	1.927631	1.957057	1.953331
1.940586	1.937069	1.933639	1.930328	1.934592	1.931206	1.927805	1.957215	1.95347
1.940761	1.937258	1.933825	1.930488	1.934738	1.931362	1.927979	1.957386	1.953636
1.940938	1.93744	1.933996	1.93062	1.927268	1.923927	1.920594	1.956347	1.952604
1.941119	1.937625	1.934165	1.930741	1.927357	1.924031	1.920729	1.9565	1.95277
1.941313	1.937819	1.934348	1.930897	1.927493	1.924173	1.920884	1.951452	1.947755
1.941522	1.938022	1.934545	1.931086	1.927676	1.924351	1.921059	1.951619	1.947918
1.941731	1.938228	1.963688	1.959857	1.956092	1.952439	1.948828	1.945158	1.94147
1.941939	1.938438	1.96388	1.960034	1.95626	1.952609	1.949002	1.945335	1.941645
1.942145	1.938647	1.964073	1.960217	1.956435	1.952783	1.949178	1.945513	1.941824
1.942348	1.938852	1.964261	1.960405	1.95662	1.952963	1.949351	1.945687	1.942001
1.942547	1.939052	1.964446	1.960593	1.956809	1.953143	1.949523	1.945859	1.942177
1.939672	1.936193	1.964622	1.960767	1.956983	1.953318	1.949699	1.946032	1.942348
1.939865	1.936377	1.964791	1.960932	1.957148	1.953488	1.949875	1.946205	1.942516
1.940055	1.936566	1.964972	1.961105	1.957315	1.953656	1.950046	1.946372	1.942678
1.940242	1.936759	1.965166	1.961283	1.957481	1.953819	1.950209	1.946532	1.942834
1.940427	1.936952	1.96306	1.959189	1.957658	1.95399	1.950375	1.946695	1.942995
1.940608	1.93714	1.963249	1.959374	1.957839	1.954164	1.950544	1.946863	1.943165
1.940792	1.937328	1.963442	1.95958	1.958049	1.954361	1.950724	1.947038	1.943342
1.940986	1.937521	1.963649	1.959838	1.958336	1.954616	1.950929	1.947224	1.943522
1.941188	1.937718	1.963864	1.960119	1.958657	1.954899	1.95115	1.94742	1.943712
1.941401	1.937923	1.96407	1.960354	1.956645	1.952896	1.949153	1.947642	1.943947
1.941623	1.938136	1.964269	1.960552	1.956845	1.953104	1.949373	1.947886	1.944222
1.941854	1.938381	1.964514	1.960775	1.957051	1.953309	1.949585	1.948113	1.944468
1.942095	1.938671	1.964828	1.961036	1.957264	1.953505	1.949778	1.948307	1.944671
1.942334	1.938947	1.965125	1.961295	1.957491	1.953726	1.949998	1.94852	1.944874
1.942552	1.939167	1.965352	1.961531	1.95774	1.953998	1.950287	1.948789	1.945097
1.942779	1.939377	1.96556	1.961769	1.958007	1.954291	1.950594	1.949071	1.945324
1.943053	1.939622	1.965792	1.96204	1.958305	1.954584	1.950867	1.947118	1.943363
1.943354	1.939885	1.966034	1.962326	1.958619	1.954877	1.951123	1.94735	1.943575
1.943624	1.940138	1.96627	1.962575	1.958875	1.955127	1.951364	1.947594	1.943828
1.943865	1.94038	1.9665	1.962786	1.959074	1.955332	1.951586	1.947848	1.944122
1.94412	1.940645	1.966759	1.963021	1.959294	1.955562	1.951837	1.948116	1.944406
1.944396	1.940946	1.967066	1.963295	1.959547	1.955835	1.952136	1.948406	1.944669
1.944686	1.941253	1.967378	1.963589	1.959829	1.956126	1.952438	1.948691	1.944923
1.944992	1.94154	1.967658	1.963903	1.96016	1.956432	1.952704	1.948935	1.945153
1.945306	1.941828	1.967937	1.964227	1.960507	1.956747	1.952972	1.94918	1.945387
1.945608	1.942144	1.968257	1.964532	1.960804	1.957055	1.953293	1.949498	1.945691
1.945907	1.942478	1.968603	1.964826	1.96107	1.95736	1.953651	1.949865	1.946041
1.946234	1.942801	1.968922	1.965138	1.961378	1.957671	1.953967	1.950181	1.946353
1.946596	1.943111	1.969205	1.965471	1.961737	1.957991	1.954233	1.950435	1.946618
1.938067	1.93457	1.969503	1.965794	1.962074	1.958306	1.954518	1.950714	1.946905
1.938382	1.934896	1.969835	1.96609	1.962352	1.958607	1.954852	1.951059	1.947248
1.938699	1.935226	1.970172	1.966389	1.962629	1.958908	1.955189	1.951407	1.947591
1.939049	1.935552	1.97049	1.966716	1.96296	1.959224	1.955485	1.951693	1.947874
1.939412	1.93588	1.970807	1.967056	1.963311	1.959547	1.955771	1.951964	1.948144
1.939757	1.93622	1.963322	1.959569	1.963633	1.959869	1.9561	1.9523	1.948488
1.940089	1.936571	1.963693	1.959894	1.963934	1.960192	1.956462	1.952686	1.948891
1.940444	1.936918	1.933432	1.929985	1.935398	1.932006	1.92864	1.953038	1.949275
1.940826	1.937251	1.933742	1.930313	1.935744	1.932321	1.928921	1.953338	1.949632

1.941227	1.937612	1.934079	1.93065	1.927247	1.923828	1.920431	1.953648	1.949974
1.94166	1.938036	1.934478	1.930997	1.927557	1.924149	1.920773	1.953996	1.950284
1.942092	1.938474	1.934897	1.93136	1.92788	1.924487	1.921137	1.954362	1.950599
1.934341	1.930776	1.935303	1.931758	1.928269	1.924869	1.921511	1.95475	1.950975
1.934681	1.931141	1.935702	1.932178	1.928697	1.925276	1.921892	1.95515	1.951387
1.935049	1.93149	1.936065	1.932567	1.929101	1.925655	1.92224	1.947715	1.943993
1.935441	1.931822	1.93639	1.932927	1.929485	1.926008	1.922554	1.948044	1.944377
1.929765	1.92612	1.936753	1.933304	1.929873	1.926377	1.922901	1.948401	1.944761
1.930177	1.926534	1.937179	1.933716	1.930273	1.92678	1.923306	1.948809	1.945154
1.930587	1.926953	1.93761	1.93412	1.930656	1.927172	1.923712	1.949214	1.945527
1.930991	1.927353	1.929845	1.926353	1.930984	1.927514	1.924074	1.949574	1.94584
1.931381	1.927744	1.930225	1.926689	1.931302	1.927846	1.924429	1.949927	1.946143
1.931723	1.92812	1.924551	1.921011	1.931676	1.928223	1.924813	1.950318	1.946517
1.932033	1.928485	1.924949	1.92141	1.932086	1.928629	1.925215	1.950732	1.946938
1.932397	1.928873	1.92535	1.921808	1.918315	1.914915	1.911559	1.922207	1.918793
1.932819	1.929284	1.925752	1.922207	1.918713	1.915319	1.911969	1.92263	1.919205
1.933249	1.929701	1.926161	1.922612	1.919118	1.915726	1.912376	1.923051	1.919615
1.933673	1.93012	1.926577	1.923028	1.919532	1.916133	1.912775	1.923463	1.920022
1.934101	1.930545	1.926999	1.923447	1.919946	1.916539	1.913173	1.923875	1.920432
1.934532	1.930979	1.927429	1.923863	1.920349	1.916937	1.913569	1.910175	1.906783
1.934965	1.931418	1.927864	1.924277	1.920747	1.917332	1.913965	1.910573	1.907184
1.935403	1.931851	1.928288	1.924685	1.921143	1.91773	1.914369	1.910972	1.907574
1.935843	1.932279	1.928704	1.925089	1.921539	1.918131	1.914777	1.911372	1.907957
1.936274	1.932702	1.929119	1.925496	1.92194	1.918533	1.915179	1.911766	1.908339
1.936693	1.933119	1.929533	1.925909	1.92235	1.918935	1.915572	1.912153	1.908721
1.937106	1.933534	1.929949	1.926322	1.922759	1.919336	1.915964	1.91254	1.909107
1.937513	1.933951	1.930369	1.926735	1.923164	1.919732	1.916353	1.91293	1.909499
1.93792	1.934366	1.930787	1.927149	1.923571	1.920131	1.916745	1.913321	1.909893
1.927595	1.924063	1.931195	1.927563	1.923987	1.920541	1.917145	1.913713	1.910277
1.928002	1.924458	1.931599	1.927978	1.924411	1.920959	1.917552	1.914107	1.910659
1.928412	1.924864	1.921309	1.917723	1.924838	1.921377	1.917957	1.914506	1.911053
1.928825	1.925279	1.921726	1.918144	1.925267	1.921794	1.918362	1.914907	1.911455
1.929255	1.9257	1.922149	1.918586	1.925732	1.922244	1.918792	1.915338	1.911898
1.929702	1.926125	1.92258	1.919064	1.926256	1.922746	1.919261	1.915818	1.912409
1.930174	1.926564	1.923014	1.919535	1.926767	1.923237	1.919723	1.916288	1.912904
1.930694	1.927026	1.923443	1.919968	1.916507	1.912992	1.909491	1.916703	1.913325
1.931225	1.927497	1.923875	1.920387	1.916918	1.913393	1.909884	1.917101	1.913719
1.908871	1.905158	1.924311	1.920807	1.917325	1.913794	1.910284	1.906866	1.903511
1.909309	1.9056	1.924755	1.921226	1.917725	1.914193	1.910688	1.907272	1.90392
1.909776	1.906076	1.902463	1.898956	1.918159	1.914634	1.911141	1.907716	1.904344
1.910274	1.906589	1.902973	1.899432	1.918626	1.915121	1.911651	1.908203	1.904783
1.910763	1.907084	1.903468	1.899919	1.896419	1.892969	1.889553	1.908682	1.905238
1.911214	1.907531	1.903927	1.900419	1.896941	1.893455	1.889992	1.909138	1.905718
1.911685	1.907982	1.90438	1.90091	1.897452	1.893926	1.890415	1.909582	1.90619
1.912229	1.908472	1.904834	1.901353	1.897888	1.894347	1.890824	1.898998	1.89564
1.912806	1.908987	1.9053	1.901785	1.898298	1.894755	1.891237	1.899411	1.896045
1.893656	1.889872	1.905831	1.902275	1.898759	1.895224	1.89172	1.888287	1.884911
1.894168	1.890434	1.906414	1.902809	1.899254	1.895738	1.892257	1.888789	1.885348
1.894703	1.890986	1.900124	1.8965	1.899767	1.896248	1.892767	1.88928	1.885809
1.895273	1.891524	1.900652	1.897037	1.900305	1.896753	1.893238	1.889753	1.886301

1.887686	1.883925	1.888373	1.884782	1.893992	1.89043	1.886905	1.89024	1.886796
1.888214	1.884497	1.888918	1.885287	1.894476	1.890938	1.887444	1.890764	1.88728
1.888723	1.88506	1.889462	1.885794	1.894961	1.89145	1.887988	1.891292	1.887765
1.873457	1.869869	1.889977	1.88632	1.882711	1.879204	1.875739	1.884965	1.881448
1.873945	1.870375	1.890481	1.886855	1.883259	1.879716	1.876206	1.88544	1.881945
1.874515	1.870941	1.876224	1.872629	1.883767	1.880218	1.876706	1.885943	1.882442
1.87516	1.871561	1.876773	1.873126	1.884239	1.880711	1.877233	1.886471	1.882941
1.875781	1.872152	1.868512	1.86484	1.884742	1.881216	1.877745	1.874238	1.870731
1.876361	1.87269	1.869037	1.865393	1.885297	1.881739	1.878229	1.874733	1.871262
1.876925	1.873228	1.869568	1.865943	1.871094	1.867566	1.864083	1.875244	1.871807
1.877475	1.873798	1.870135	1.866476	1.87157	1.868059	1.864605	1.875799	1.872374
1.878021	1.874368	1.870707	1.86701	1.872046	1.868561	1.865144	1.876368	1.872941
1.855433	1.851843	1.871237	1.867568	1.872597	1.869116	1.865694	1.862274	1.858873
1.855971	1.852336	1.871747	1.868144	1.873195	1.869704	1.86625	1.8628	1.859365
1.856511	1.85287	1.849299	1.845809	1.8652	1.86168	1.858188	1.863302	1.859869
1.857049	1.853445	1.849891	1.846394	1.865789	1.862223	1.858688	1.863775	1.860379
1.857618	1.854029	1.850476	1.846957	1.843461	1.839966	1.836506	1.864278	1.86091
1.858232	1.854621	1.851039	1.847479	1.843961	1.840486	1.837061	1.864843	1.861483
1.858857	1.855206	1.85159	1.848	1.844469	1.841016	1.837622	1.865406	1.86204
1.85947	1.855763	1.85212	1.848546	1.845029	1.841573	1.838167	1.844797	1.841476
1.860081	1.856314	1.852649	1.849106	1.845613	1.842139	1.838702	1.845264	1.841914
1.8607	1.856902	1.853218	1.849681	1.846185	1.842679	1.839206	1.84095	1.837603
1.861325	1.857514	1.853818	1.850269	1.846754	1.843203	1.839687	1.841383	1.83806
1.844287	1.840559	1.854436	1.850881	1.847352	1.843773	1.840228	1.841872	1.838553
1.844809	1.841137	1.855075	1.851522	1.847985	1.844397	1.840838	1.842429	1.839088
1.845347	1.84172	1.855714	1.852158	1.848614	1.845022	1.841455	1.838002	1.834631
1.845923	1.842305	1.856343	1.852781	1.849227	1.84563	1.842056	1.838584	1.835187
1.84651	1.842895	1.85697	1.85339	1.849821	1.846225	1.842653	1.839168	1.835746
1.847092	1.843488	1.857594	1.853973	1.850374	1.846792	1.843244	1.839759	1.836324
1.847676	1.844086	1.858217	1.854543	1.850908	1.847347	1.843834	1.840355	1.836913
1.84827	1.844688	1.858847	1.855145	1.851488	1.847933	1.844432	1.84095	1.837499
1.848874	1.845296	1.859483	1.855772	1.852106	1.848544	1.845036	1.841546	1.838084
1.849474	1.845904	1.842294	1.838609	1.852738	1.849171	1.845657	1.84216	1.83869
1.850063	1.846514	1.842915	1.839226	1.853385	1.849818	1.846303	1.8428	1.839323
1.839772	1.836244	1.843532	1.839844	1.854035	1.850467	1.846951	1.843445	1.83996
1.840365	1.836834	1.844136	1.840449	1.854677	1.851109	1.847589	1.844078	1.840587
1.840968	1.837424	1.844736	1.841053	1.85532	1.851751	1.848228	1.844714	1.841217
1.841583	1.838021	1.834425	1.830762	1.838034	1.834492	1.831001	1.845366	1.841868
1.842211	1.838624	1.835018	1.831367	1.838667	1.835127	1.831634	1.846027	1.842531
1.842864	1.839249	1.835627	1.831975	1.828366	1.824836	1.821351	1.846671	1.84318
1.843545	1.839897	1.836251	1.832585	1.828965	1.825426	1.821936	1.847298	1.843813
1.84422	1.840542	1.836874	1.833199	1.829573	1.826023	1.822524	1.82998	1.826542
1.844874	1.841169	1.837485	1.833808	1.830181	1.826623	1.823116	1.830584	1.82715
1.845535	1.841805	1.838109	1.834443	1.830822	1.827248	1.823722	1.831197	1.827765
1.832906	1.829194	1.83877	1.835145	1.831545	1.827936	1.824359	1.82087	1.817445
1.833594	1.829858	1.839448	1.835869	1.832296	1.828646	1.825012	1.821497	1.818062
1.796734	1.793053	1.840119	1.836546	1.832973	1.829306	1.82565	1.822117	1.818668
1.797421	1.793714	1.840788	1.837192	1.833604	1.829936	1.826282	1.822737	1.819267
1.798142	1.794389	1.805655	1.802105	1.834247	1.830599	1.826968	1.823408	1.819903
1.798908	1.795082	1.806312	1.802741	1.834906	1.831305	1.827722	1.824143	1.820579

1.799648	1.795776	1.806981	1.803382	1.799847	1.796347	1.792872	1.824864	1.82127
1.800322	1.796454	1.807658	1.804019	1.800455	1.796953	1.793491	1.825539	1.821981
1.800983	1.797145	1.808358	1.804673	1.801067	1.79755	1.794089	1.826195	1.822683
1.801671	1.797883	1.809113	1.805369	1.801708	1.798173	1.794708	1.791254	1.787823
1.802365	1.798638	1.809892	1.80609	1.802372	1.798814	1.795336	1.791863	1.788417
1.787038	1.783391	1.810622	1.806816	1.803081	1.799491	1.795977	1.792482	1.789023
1.787693	1.784051	1.811314	1.807549	1.803834	1.800203	1.796631	1.793107	1.789633
1.78837	1.784711	1.811988	1.808248	1.804551	1.800912	1.797323	1.79379	1.790309
1.78908	1.785367	1.812631	1.808894	1.805217	1.801618	1.798074	1.794561	1.791083
1.789789	1.786044	1.798275	1.79458	1.805882	1.802312	1.798802	1.795306	1.791832
1.79048	1.786776	1.799027	1.795289	1.80656	1.802966	1.799441	1.79594	1.79247
1.791161	1.787514	1.799797	1.796021	1.807259	1.803628	1.800073	1.796556	1.793082
1.743494	1.740165	1.800496	1.796751	1.808018	1.804375	1.800793	1.797247	1.79374
1.744101	1.74077	1.801163	1.797484	1.808812	1.805166	1.801559	1.797976	1.794424
1.704802	1.701839	1.785921	1.78229	1.809554	1.805897	1.802276	1.798689	1.795137
1.705439	1.702464	1.786692	1.783008	1.810245	1.806568	1.802947	1.799389	1.795884
1.706051	1.703058	1.73947	1.736089	1.810959	1.807273	1.803649	1.800097	1.796606
1.706608	1.703592	1.740064	1.736728	1.811728	1.80805	1.804412	1.800822	1.797275
1.707177	1.704129	1.74065	1.737357	1.812483	1.808816	1.805168	1.801543	1.797945
1.707803	1.704702	1.74125	1.737961	1.813162	1.809501	1.805861	1.802245	1.798655
1.708457	1.705303	1.741873	1.73857	1.813823	1.810167	1.806539	1.802941	1.799372
1.709119	1.705952	1.742574	1.739253	1.753955	1.750631	1.747333	1.80365	1.800048
1.709794	1.706641	1.743334	1.739986	1.754679	1.751344	1.748033	1.804366	1.800698
1.710471	1.70732	1.744077	1.740688	1.755364	1.752018	1.748705	1.805081	1.801398
1.711157	1.707992	1.744805	1.741354	1.756	1.752645	1.749342	1.805791	1.802154
1.711821	1.708645	1.705521	1.702448	1.738616	1.735332	1.732114	1.746704	1.743429
1.712442	1.709258	1.706135	1.703073	1.739314	1.736032	1.732811	1.747417	1.744136
1.713061	1.709872	1.706753	1.703707	1.740029	1.736742	1.733506	1.748123	1.744832
1.713724	1.710534	1.707409	1.704355	1.740743	1.73742	1.734147	1.748769	1.74548
1.714404	1.711213	1.708079	1.705007	1.741456	1.738087	1.73477	1.749396	1.746113
1.715075	1.711865	1.708715	1.705632	1.742135	1.738759	1.735438	1.750082	1.746804
1.715741	1.712497	1.709326	1.706234	1.742786	1.739432	1.736139	1.750814	1.747543
1.716395	1.713142	1.709965	1.706868	1.743482	1.740139	1.73686	1.751562	1.7483
1.717029	1.713801	1.710639	1.707543	1.744234	1.740888	1.737607	1.752335	1.749083
1.680894	1.677797	1.711329	1.708236	1.745007	1.741645	1.738348	1.753092	1.749845
1.681547	1.678465	1.712035	1.708946	1.745798	1.742407	1.739075	1.753823	1.750565
1.682221	1.679144	1.712748	1.709658	1.746588	1.743157	1.739778	1.754521	1.751245
1.682913	1.679837	1.676799	1.673799	1.707274	1.704205	1.701176	1.737111	1.733903
1.683622	1.680537	1.677483	1.674455	1.707917	1.704798	1.701721	1.73768	1.734464
1.68434	1.681219	1.678131	1.675074	1.689832	1.686729	1.683675	1.738335	1.7351
1.685072	1.681883	1.678745	1.675655	1.69043	1.687347	1.68432	1.73907	1.735803
1.685767	1.682526	1.679353	1.676249	1.673203	1.67021	1.667273	1.722764	1.719493
1.686392	1.683129	1.679951	1.676862	1.673832	1.670845	1.66791	1.72351	1.720223
1.687036	1.683754	1.680569	1.677492	1.674475	1.671492	1.668556	1.724268	1.720964
1.687765	1.684445	1.681234	1.678145	1.675122	1.672143	1.669214	1.708895	1.705615
1.688533	1.685174	1.68193	1.678817	1.67578	1.672803	1.66988	1.709655	1.706351
1.689295	1.685915	1.68265	1.679512	1.676457	1.673476	1.670553	1.667646	1.66477
1.690065	1.686676	1.683394	1.680228	1.677149	1.67416	1.671233	1.668317	1.665429
1.690866	1.687465	1.684163	1.680963	1.677853	1.674846	1.671906	1.668979	1.666081
1.691699	1.688286	1.684959	1.681718	1.678569	1.675533	1.672571	1.66963	1.666727

1.692538	1.689111	1.685758	1.682479	1.679291	1.676225	1.673239	1.670285	1.667377
1.693377	1.689931	1.686553	1.68324	1.68002	1.676924	1.673912	1.670946	1.668034
1.694211	1.690748	1.687347	1.684004	1.680753	1.677628	1.674592	1.671614	1.6687
1.655245	1.652192	1.688142	1.684767	1.681485	1.678335	1.67528	1.672294	1.669378
1.655913	1.652875	1.688936	1.685534	1.682224	1.679052	1.675979	1.672984	1.670064
1.623516	1.620534	1.689732	1.686318	1.682992	1.679794	1.676695	1.673683	1.670754
1.624164	1.621181	1.690527	1.687116	1.683784	1.680559	1.677426	1.674393	1.671448
1.624791	1.621811	1.651859	1.64883	1.684577	1.681328	1.678164	1.675108	1.672145
1.625391	1.622421	1.652509	1.649499	1.685373	1.682104	1.678911	1.67583	1.672843
1.625989	1.623032	1.620086	1.617146	1.647214	1.644305	1.641458	1.676554	1.673543
1.626588	1.623653	1.620722	1.617787	1.647876	1.644949	1.642078	1.677271	1.674235
1.627198	1.624283	1.621363	1.61843	1.64854	1.645598	1.642708	1.678	1.67494
1.627833	1.624921	1.622006	1.619079	1.616169	1.613265	1.610409	1.67876	1.675679
1.628492	1.625574	1.622658	1.619739	1.616832	1.613921	1.611052	1.679551	1.67645
1.629196	1.62628	1.623366	1.620452	1.617549	1.614641	1.611771	1.609004	1.606317
1.629948	1.62704	1.624132	1.62122	1.61832	1.615427	1.612566	1.609781	1.607058
1.59742	1.594714	1.624849	1.621935	1.619037	1.616156	1.613305	1.610505	1.607752
1.598032	1.595319	1.625491	1.622571	1.61967	1.616798	1.613956	1.611146	1.608374
1.598622	1.595898	1.626103	1.623178	1.620276	1.617409	1.614572	1.611753	1.608963
1.599188	1.596449	1.626694	1.62377	1.62087	1.618006	1.615167	1.612333	1.609519
1.599748	1.596992	1.627277	1.624356	1.621458	1.618592	1.615747	1.612899	1.610066
1.55859	1.556025	1.594836	1.592119	1.62206	1.619184	1.61633	1.613483	1.610656
1.559112	1.556551	1.595443	1.592715	1.622673	1.619783	1.616917	1.614081	1.611277
1.529973	1.527576	1.596096	1.593358	1.623346	1.620444	1.617568	1.614737	1.611946
1.530546	1.528133	1.596805	1.594056	1.624088	1.621177	1.618295	1.615463	1.612675
1.531092	1.528662	1.555765	1.553203	1.624798	1.62188	1.618991	1.616155	1.613363
1.531577	1.529127	1.556294	1.55373	1.625436	1.622513	1.619618	1.616772	1.613966
1.532053	1.529585	1.556813	1.554249	1.626057	1.623129	1.620227	1.617367	1.614546
1.532547	1.530069	1.557345	1.554777	1.587482	1.584721	1.581979	1.617958	1.615124
1.533058	1.530571	1.55789	1.555316	1.588047	1.585274	1.582519	1.618556	1.615712
1.533617	1.531114	1.528652	1.526238	1.553328	1.550758	1.548205	1.619235	1.616376
1.534216	1.53169	1.529212	1.526786	1.553936	1.551367	1.548814	1.619977	1.617103
1.534787	1.532248	1.529757	1.52732	1.554531	1.55196	1.549405	1.581667	1.578946
1.535319	1.532776	1.530278	1.527829	1.555103	1.552528	1.549968	1.582248	1.579516
1.499262	1.496826	1.530815	1.52836	1.555696	1.553113	1.550544	1.582848	1.580103
1.499807	1.497364	1.531396	1.528945	1.556339	1.553744	1.551158	1.583502	1.580743
1.50036	1.497909	1.531985	1.529537	1.556987	1.554379	1.551778	1.584163	1.58139
1.500874	1.498421	1.532544	1.530084	1.527641	1.525193	1.522755	1.549769	1.547188
1.501376	1.49892	1.533093	1.530612	1.528152	1.525698	1.523258	1.550336	1.547741
1.501909	1.499447	1.497024	1.494646	1.528699	1.526245	1.523807	1.550944	1.548337
1.50247	1.499996	1.497564	1.49518	1.529276	1.526829	1.524397	1.55159	1.548971
1.503033	1.500541	1.498095	1.495698	1.529839	1.527391	1.524961	1.552217	1.549596
1.503592	1.501073	1.498603	1.496185	1.530367	1.527909	1.525475	1.552805	1.550197
1.504163	1.501616	1.499123	1.496688	1.494295	1.491947	1.489627	1.553411	1.550809
1.504766	1.502191	1.499681	1.497245	1.494853	1.492506	1.490185	1.554075	1.551455
1.505373	1.50277	1.500246	1.497814	1.49543	1.493088	1.490767	1.554759	1.552116
1.505936	1.503305	1.500768	1.498345	1.495971	1.493635	1.491317	1.525406	1.522928
1.506477	1.50382	1.501271	1.498856	1.496493	1.494162	1.491849	1.525995	1.523529
1.507067	1.504399	1.501832	1.49939	1.497005	1.494668	1.492359	1.490031	1.487699
1.507709	1.505044	1.502453	1.499943	1.497501	1.495149	1.492841	1.490518	1.488195

1.508352	1.505693	1.503082	1.500514	1.498023	1.495653	1.493343	1.491021	1.488707
1.50899	1.506337	1.503716	1.501115	1.498593	1.496206	1.493885	1.491562	1.48925
1.509614	1.506968	1.504339	1.501714	1.499167	1.496764	1.494431	1.492106	1.489801
1.510199	1.507563	1.50493	1.502282	1.499712	1.497293	1.494951	1.492636	1.490352
1.510768	1.508141	1.505505	1.502839	1.500251	1.497815	1.495463	1.493159	1.490899
1.511366	1.508725	1.506078	1.503405	1.500808	1.498353	1.495983	1.493674	1.491419
1.511981	1.509311	1.506647	1.503978	1.501379	1.498903	1.496508	1.494182	1.491916
1.512573	1.509909	1.507247	1.504577	1.501967	1.499459	1.49703	1.494688	1.492419
1.513138	1.510526	1.507888	1.505209	1.502573	1.50002	1.497544	1.49519	1.492931
1.513689	1.511131	1.508523	1.505843	1.503189	1.500592	1.498068	1.495694	1.493434
1.514227	1.511713	1.509136	1.506474	1.503816	1.501179	1.498603	1.496195	1.493909
1.514754	1.51228	1.509735	1.507096	1.504443	1.501778	1.499161	1.496713	1.49439
1.515264	1.512828	1.510313	1.507695	1.505054	1.50239	1.499762	1.497282	1.49491
1.51577	1.513362	1.510874	1.508278	1.505656	1.503012	1.500391	1.497881	1.495455
1.516317	1.5139	1.51142	1.508857	1.506264	1.503633	1.501012	1.498478	1.496013
1.516902	1.51444	1.511952	1.509431	1.506878	1.504255	1.501629	1.499079	1.496586
1.517481	1.514977	1.51248	1.509993	1.507473	1.504863	1.502238	1.499672	1.49715
1.518041	1.515504	1.512998	1.510533	1.508039	1.505448	1.502833	1.500249	1.497691
1.518623	1.516053	1.513536	1.511084	1.508608	1.506036	1.503435	1.50084	1.498257
1.519244	1.516647	1.514117	1.511667	1.5092	1.506641	1.504053	1.501465	1.498883
1.519896	1.517272	1.514725	1.512266	1.5098	1.507255	1.504685	1.502109	1.499535
1.52057	1.517917	1.515347	1.512866	1.510394	1.507876	1.505341	1.50277	1.500183
1.521273	1.518588	1.51599	1.513473	1.510989	1.508506	1.506013	1.503444	1.500834
1.522043	1.5193	1.516657	1.514105	1.511604	1.509137	1.506671	1.504105	1.501485
1.522882	1.520055	1.517352	1.514761	1.51224	1.509772	1.507314	1.504753	1.502138
1.523757	1.520823	1.518047	1.515423	1.512887	1.510413	1.507957	1.505401	1.502794
1.524652	1.521584	1.518724	1.516081	1.513543	1.511061	1.508604	1.506053	1.503453
1.525603	1.522384	1.51943	1.516763	1.514217	1.511717	1.509247	1.506701	1.50412
1.526626	1.523249	1.52019	1.517488	1.51492	1.512377	1.509875	1.507343	1.504802
1.527726	1.52418	1.521003	1.518251	1.515645	1.513046	1.510501	1.507981	1.505487
1.528948	1.525216	1.521896	1.519062	1.516394	1.513734	1.511148	1.50862	1.506143
1.530277	1.526343	1.522861	1.519917	1.517164	1.51444	1.51181	1.509259	1.506781
1.531602	1.527481	1.523839	1.520767	1.517919	1.515145	1.512493	1.509926	1.507443
1.532918	1.528619	1.524816	1.5216	1.518649	1.515845	1.513197	1.510623	1.508136
1.534258	1.529819	1.525868	1.522491	1.519415	1.516572	1.51392	1.511331	1.50883
1.535623	1.531096	1.52702	1.523458	1.520229	1.517327	1.514653	1.512034	1.509509
1.537032	1.532449	1.52826	1.524503	1.521107	1.51813	1.515418	1.512758	1.510199
1.538524	1.533904	1.529612	1.525657	1.522083	1.519018	1.516252	1.513532	1.510923
1.540067	1.535428	1.531045	1.526896	1.523139	1.519967	1.517125	1.514336	1.511672
1.541581	1.536934	1.532477	1.528167	1.524245	1.520931	1.517972	1.515124	1.512443
1.543078	1.538434	1.533921	1.529479	1.525403	1.521916	1.518801	1.515902	1.513239
1.544561	1.539917	1.535361	1.530828	1.526633	1.522979	1.519702	1.516731	1.514054
1.546022	1.541368	1.536785	1.532213	1.52794	1.524134	1.520691	1.517625	1.514894
1.547493	1.542838	1.538239	1.533644	1.529304	1.525342	1.521722	1.518543	1.515736
1.548996	1.544369	1.539768	1.535144	1.530724	1.526585	1.522763	1.51945	1.516549
1.550512	1.545911	1.541313	1.536674	1.532186	1.527877	1.523856	1.520396	1.517381
1.552031	1.547408	1.542799	1.538177	1.533662	1.529228	1.525044	1.521437	1.518281
1.553553	1.548884	1.54426	1.539674	1.535161	1.530635	1.526318	1.522561	1.519243
1.555073	1.550399	1.545774	1.541198	1.536673	1.532106	1.527713	1.523814	1.520308
1.556587	1.551951	1.547341	1.542746	1.5382	1.533644	1.529235	1.525199	1.521479

1.518092	1.513496	1.548909	1.544297	1.539735	1.535196	1.53078	1.526614	1.522685
1.519797	1.51519	1.550463	1.545842	1.541273	1.536758	1.532339	1.528044	1.523904
1.521523	1.516898	1.552031	1.547404	1.542828	1.53832	1.533884	1.529481	1.525174
1.52326	1.51862	1.513998	1.509384	1.504825	1.500341	1.495929	1.530898	1.526514
1.525004	1.520354	1.515728	1.511128	1.506555	1.501993	1.497484	1.532323	1.527911
1.526738	1.522101	1.517469	1.512834	1.508222	1.503621	1.499075	1.494621	1.490273
1.528467	1.52386	1.519219	1.51452	1.509851	1.505236	1.500687	1.496233	1.491882
1.5302	1.525592	1.52094	1.516219	1.511525	1.506883	1.50231	1.497844	1.493484
1.531939	1.527289	1.522623	1.517929	1.513247	1.508566	1.503938	1.499448	1.495074
1.445848	1.441265	1.524342	1.519663	1.514984	1.510272	1.505603	1.501089	1.496694
1.447566	1.443026	1.526152	1.521441	1.516729	1.512002	1.50732	1.502787	1.498368
1.44929	1.444784	1.527956	1.523219	1.518481	1.513753	1.50907	1.504515	1.50006
1.393578	1.38909	1.529649	1.524936	1.520226	1.515517	1.510841	1.506251	1.501736
1.395225	1.390667	1.531305	1.526638	1.521979	1.517301	1.512638	1.508009	1.503422
1.312689	1.308903	1.445209	1.440646	1.523793	1.519134	1.514476	1.509819	1.505178
1.314027	1.31029	1.447012	1.442476	1.52566	1.521011	1.516354	1.511676	1.506996
1.315382	1.311661	1.391195	1.386762	1.439744	1.435183	1.430609	1.513505	1.508814
1.316759	1.313008	1.392812	1.388385	1.441464	1.436911	1.432345	1.515296	1.510633
1.318152	1.314367	1.310619	1.306925	1.443161	1.438622	1.434075	1.429523	1.424973
1.319546	1.31576	1.312005	1.308288	1.44484	1.440326	1.435818	1.431266	1.426695
1.320976	1.317165	1.313389	1.309645	1.446522	1.442027	1.437557	1.433004	1.428412
1.322491	1.31855	1.314714	1.311012	1.390485	1.386051	1.381651	1.434728	1.430159
1.324069	1.319941	1.316024	1.312391	1.392167	1.387694	1.383266	1.436448	1.431925
1.325666	1.321443	1.317467	1.313822	1.393889	1.389388	1.38495	1.380523	1.37611
1.327287	1.323055	1.319039	1.315305	1.395654	1.391132	1.386702	1.382262	1.377833
1.328901	1.324668	1.320615	1.316782	1.313027	1.309317	1.305616	1.383976	1.379532
1.33049	1.326264	1.322174	1.318236	1.314407	1.31072	1.30707	1.385641	1.381188
1.332095	1.327858	1.323728	1.319704	1.31581	1.312131	1.308511	1.38731	1.382853
1.264733	1.26047	1.325248	1.321192	1.317275	1.313577	1.309953	1.38901	1.384571
1.266417	1.26207	1.326784	1.322717	1.318788	1.315047	1.311386	1.39073	1.386306
1.268083	1.263743	1.328438	1.324336	1.320358	1.316534	1.312798	1.309069	1.305358
1.269737	1.265467	1.330173	1.32603	1.32198	1.318043	1.314199	1.31046	1.306798
1.271335	1.267146	1.262996	1.258886	1.323626	1.319604	1.315673	1.31191	1.308263
1.272864	1.268771	1.264682	1.260587	1.325305	1.321227	1.317226	1.313424	1.309756
1.274433	1.270414	1.266365	1.262265	1.326952	1.322842	1.3188	1.314959	1.311255
1.276098	1.272112	1.268057	1.263897	1.32852	1.324408	1.320374	1.316508	1.312764
1.277776	1.273801	1.269733	1.265524	1.330096	1.325992	1.321971	1.31807	1.314265
1.279405	1.275431	1.271372	1.267188	1.263028	1.258954	1.254943	1.31963	1.31572
1.281015	1.277026	1.272982	1.26886	1.264744	1.260669	1.256622	1.321205	1.317172
1.282617	1.278587	1.274546	1.270488	1.266429	1.26237	1.258313	1.254226	1.250146
1.284209	1.280114	1.276067	1.272083	1.268095	1.264068	1.260019	1.255908	1.251776
1.205587	1.201685	1.277647	1.273686	1.26973	1.265739	1.261718	1.257597	1.253429
1.207107	1.203204	1.279303	1.275296	1.271318	1.267371	1.263405	1.259292	1.255103
1.208683	1.204754	1.280978	1.276926	1.272925	1.269013	1.265096	1.260993	1.256791
1.210341	1.206328	1.282654	1.278587	1.274589	1.270697	1.266803	1.262704	1.258492
1.212033	1.207917	1.284338	1.280265	1.276275	1.272391	1.268507	1.264413	1.260199
1.213706	1.209496	1.205441	1.201572	1.27796	1.274072	1.270185	1.266099	1.261898
1.215378	1.211079	1.206971	1.203101	1.279651	1.275747	1.271848	1.267772	1.263595
1.217052	1.212698	1.208545	1.204643	1.281333	1.277412	1.273507	1.269447	1.265299
1.218727	1.214351	1.210161	1.206198	1.283008	1.279067	1.275162	1.271122	1.26701

1.978078	1.97448	1.970951	1.967517	1.964085	1.960605	1.957116	1.970322	1.966723
1.978006	1.974407	1.970879	1.967447	1.964017	1.960538	1.95705	1.970243	1.966642
1.977931	1.974333	1.970806	1.967377	1.96395	1.960474	1.956987	1.970165	1.966563
1.977863	1.974263	1.970737	1.967311	1.963887	1.960414	1.95693	1.970096	1.966492
1.977797	1.974196	1.970671	1.967247	1.963827	1.960357	1.956877	1.970034	1.966431
1.974939	1.971342	1.970609	1.967184	1.963764	1.960297	1.956824	1.969991	1.966415
1.974871	1.971282	1.970547	1.967118	1.963697	1.960233	1.956772	1.969972	1.966454
1.974815	1.971247	1.970516	1.967072	1.963638	1.960171	1.956716	1.969935	1.966461
1.974779	1.971258	1.970539	1.967062	1.963593	1.960111	1.956648	1.969861	1.966403
1.974745	1.971267	1.970559	1.967051	1.963551	1.960054	1.956584	1.969781	1.966327
1.974692	1.971229	1.967747	1.964231	1.963498	1.959998	1.956527	1.969707	1.966249
1.974643	1.971177	1.967694	1.964179	1.963446	1.959953	1.956487	1.969645	1.966172
1.969157	1.965683	1.967656	1.964143	1.968449	1.964936	1.961453	1.973983	1.970449
1.9692	1.965675	1.967627	1.964117	1.968439	1.964967	1.961518	1.974024	1.970431
1.969219	1.965666	1.967614	1.964119	1.968463	1.965003	1.961559	1.974041	1.970419
1.969206	1.965651	1.967618	1.964157	1.968529	1.965046	1.96157	1.974027	1.970407
1.962195	1.958701	1.962169	1.958752	1.966033	1.962518	1.959002	1.974022	1.970419
1.962199	1.958745	1.962225	1.958785	1.966057	1.962529	1.959011	1.97404	1.970477
1.962205	1.958791	1.962282	1.958812	1.966072	1.962541	1.95903	1.974072	1.970541
1.96219	1.958788	1.96229	1.958815	1.966085	1.962576	1.959087	1.974118	1.970569
1.962174	1.958765	1.962277	1.95881	1.966101	1.962625	1.959163	1.974171	1.970579
1.956671	1.953279	1.962293	1.958844	1.960973	1.957521	1.954075	1.958287	1.954798
1.956753	1.953325	1.962331	1.958911	1.961068	1.957582	1.954094	1.958293	1.9548
1.956827	1.953388	1.962388	1.958971	1.961134	1.957623	1.95411	1.955743	1.952244
1.956878	1.953475	1.962476	1.959021	1.961155	1.95763	1.954119	1.955778	1.952317
1.953291	1.949931	1.955618	1.952166	1.961167	1.957643	1.954144	1.950697	1.947286
1.953375	1.949973	1.955632	1.952168	1.961181	1.957682	1.954209	1.950759	1.94733
1.95346	1.950001	1.955629	1.952169	1.961204	1.957733	1.954286	1.95083	1.947373
1.953502	1.950024	1.95565	1.95221	1.96127	1.957794	1.954339	1.95089	1.947448
1.953524	1.950045	1.955686	1.952276	1.961364	1.957864	1.954383	1.950947	1.947541
1.947763	1.944298	1.952398	1.949003	1.961442	1.957939	1.954455	1.951019	1.947613
1.947893	1.944432	1.952519	1.949079	1.961503	1.958018	1.954558	1.951105	1.947663
1.948001	1.944556	1.952645	1.949173	1.955002	1.951564	1.948156	1.951187	1.947728
1.948058	1.944651	1.952774	1.949304	1.955128	1.951652	1.948209	1.951255	1.947827
1.948113	1.944733	1.952885	1.949421	1.955246	1.951738	1.948265	1.951325	1.947923
1.948202	1.944796	1.947376	1.943917	1.952202	1.948729	1.945288	1.951408	1.947988
1.9483	1.944854	1.947407	1.94394	1.952236	1.948796	1.945387	1.951494	1.948044
1.948375	1.944937	1.94151	1.938089	1.947081	1.943635	1.940219	1.948753	1.945316
1.948436	1.945037	1.941639	1.938235	1.947227	1.943735	1.940271	1.948818	1.945409
1.948529	1.945127	1.941736	1.938356	1.94737	1.943859	1.94037	1.942193	1.938829
1.948659	1.945199	1.94179	1.938447	1.947512	1.944015	1.940526	1.94232	1.938904
1.946745	1.943246	1.941865	1.938534	1.947631	1.944159	1.940687	1.961641	1.957905
1.946899	1.943392	1.941988	1.938615	1.947694	1.944259	1.940835	1.961782	1.958034
1.947047	1.943551	1.942135	1.938712	1.947764	1.94436	1.940982	1.96193	1.958184
1.939523	1.936086	1.940254	1.936829	1.947912	1.94451	1.941136	1.962068	1.958328
1.939607	1.93622	1.940426	1.937011	1.948098	1.944681	1.941289	1.962197	1.958471
1.939742	1.936369	1.940591	1.937185	1.942661	1.939249	1.935861	1.962291	1.958593
1.939919	1.936533	1.940753	1.937354	1.942832	1.939373	1.935939	1.962352	1.958696
1.940092	1.936675	1.933284	1.929924	1.941209	1.937728	1.934268	1.962432	1.95878
1.940252	1.93678	1.93337	1.930045	1.941374	1.937899	1.93443	1.962537	1.958832

1.940415	1.9369	1.933475	1.930172	1.934436	1.931041	1.927631	1.957057	1.953331
1.940586	1.937069	1.933639	1.930328	1.934592	1.931206	1.927805	1.957215	1.95347
1.940761	1.937258	1.933825	1.930488	1.934738	1.931362	1.927979	1.957386	1.953636
1.940938	1.93744	1.933996	1.93062	1.927268	1.923927	1.920594	1.956347	1.952604
1.941119	1.937625	1.934165	1.930741	1.927357	1.924031	1.920729	1.9565	1.95277
1.941313	1.937819	1.934348	1.930897	1.927493	1.924173	1.920884	1.951452	1.947755
1.941522	1.938022	1.934545	1.931086	1.927676	1.924351	1.921059	1.951619	1.947918
1.941731	1.938228	1.963688	1.959857	1.956092	1.952439	1.948828	1.945158	1.94147
1.941939	1.938438	1.96388	1.960034	1.95626	1.952609	1.949002	1.945335	1.941645
1.942145	1.938647	1.964073	1.960217	1.956435	1.952783	1.949178	1.945513	1.941824
1.942348	1.938852	1.964261	1.960405	1.95662	1.952963	1.949351	1.945687	1.942001
1.942547	1.939052	1.964446	1.960593	1.956809	1.953143	1.949523	1.945859	1.942177
1.939672	1.936193	1.964622	1.960767	1.956983	1.953318	1.949699	1.946032	1.942348
1.939865	1.936377	1.964791	1.960932	1.957148	1.953488	1.949875	1.946205	1.942516
1.940055	1.936566	1.964972	1.961105	1.957315	1.953656	1.950046	1.946372	1.942678
1.940242	1.936759	1.965166	1.961283	1.957481	1.953819	1.950209	1.946532	1.942834
1.940427	1.936952	1.96306	1.959189	1.957658	1.95399	1.950375	1.946695	1.942995
1.940608	1.93714	1.963249	1.959374	1.957839	1.954164	1.950544	1.946863	1.943165
1.940792	1.937328	1.963442	1.95958	1.958049	1.954361	1.950724	1.947038	1.943342
1.940986	1.937521	1.963649	1.959838	1.958336	1.954616	1.950929	1.947224	1.943522
1.941188	1.937718	1.963864	1.960119	1.958657	1.954899	1.95115	1.94742	1.943712
1.941401	1.937923	1.96407	1.960354	1.956645	1.952896	1.949153	1.947642	1.943947
1.941623	1.938136	1.964269	1.960552	1.956845	1.953104	1.949373	1.947886	1.944222
1.941854	1.938381	1.964514	1.960775	1.957051	1.953309	1.949585	1.948113	1.944468
1.942095	1.938671	1.964828	1.961036	1.957264	1.953505	1.949778	1.948307	1.944671
1.942334	1.938947	1.965125	1.961295	1.957491	1.953726	1.949998	1.94852	1.944874
1.942552	1.939167	1.965352	1.961531	1.95774	1.953998	1.950287	1.948789	1.945097
1.942779	1.939377	1.96556	1.961769	1.958007	1.954291	1.950594	1.949071	1.945324
1.943053	1.939622	1.965792	1.96204	1.958305	1.954584	1.950867	1.947118	1.943363
1.943354	1.939885	1.966034	1.962326	1.958619	1.954877	1.951123	1.94735	1.943575
1.943624	1.940138	1.96627	1.962575	1.958875	1.955127	1.951364	1.947594	1.943828
1.943865	1.94038	1.9665	1.962786	1.959074	1.955332	1.951586	1.947848	1.944122
1.94412	1.940645	1.966759	1.963021	1.959294	1.955562	1.951837	1.948116	1.944406
1.944396	1.940946	1.967066	1.963295	1.959547	1.955835	1.952136	1.948406	1.944669
1.944686	1.941253	1.967378	1.963589	1.959829	1.956126	1.952438	1.948691	1.944923
1.944992	1.94154	1.967658	1.963903	1.96016	1.956432	1.952704	1.948935	1.945153
1.945306	1.941828	1.967937	1.964227	1.960507	1.956747	1.952972	1.94918	1.945387
1.945608	1.942144	1.968257	1.964532	1.960804	1.957055	1.953293	1.949498	1.945691
1.945907	1.942478	1.968603	1.964826	1.96107	1.95736	1.953651	1.949865	1.946041
1.946234	1.942801	1.968922	1.965138	1.961378	1.957671	1.953967	1.950181	1.946353
1.946596	1.943111	1.969205	1.965471	1.961737	1.957991	1.954233	1.950435	1.946618
1.938067	1.93457	1.969503	1.965794	1.962074	1.958306	1.954518	1.950714	1.946905
1.938382	1.934896	1.969835	1.96609	1.962352	1.958607	1.954852	1.951059	1.947248
1.938699	1.935226	1.970172	1.966389	1.962629	1.958908	1.955189	1.951407	1.947591
1.939049	1.935552	1.97049	1.966716	1.96296	1.959224	1.955485	1.951693	1.947874
1.939412	1.93588	1.970807	1.967056	1.963311	1.959547	1.955771	1.951964	1.948144
1.939757	1.93622	1.963322	1.959569	1.963633	1.959869	1.9561	1.9523	1.948488
1.940089	1.936571	1.963693	1.959894	1.963934	1.960192	1.956462	1.952686	1.948891
1.940444	1.936918	1.933432	1.929985	1.935398	1.932006	1.92864	1.953038	1.949275
1.940826	1.937251	1.933742	1.930313	1.935744	1.932321	1.928921	1.953338	1.949632

1.941227	1.937612	1.934079	1.93065	1.927247	1.923828	1.920431	1.953648	1.949974
1.94166	1.938036	1.934478	1.930997	1.927557	1.924149	1.920773	1.953996	1.950284
1.942092	1.938474	1.934897	1.93136	1.92788	1.924487	1.921137	1.954362	1.950599
1.934341	1.930776	1.935303	1.931758	1.928269	1.924869	1.921511	1.95475	1.950975
1.934681	1.931141	1.935702	1.932178	1.928697	1.925276	1.921892	1.95515	1.951387
1.935049	1.93149	1.936065	1.932567	1.929101	1.925655	1.92224	1.947715	1.943993
1.935441	1.931822	1.93639	1.932927	1.929485	1.926008	1.922554	1.948044	1.944377
1.929765	1.92612	1.936753	1.933304	1.929873	1.926377	1.922901	1.948401	1.944761
1.930177	1.926534	1.937179	1.933716	1.930273	1.92678	1.923306	1.948809	1.945154
1.930587	1.926953	1.93761	1.93412	1.930656	1.927172	1.923712	1.949214	1.945527
1.930991	1.927353	1.929845	1.926353	1.930984	1.927514	1.924074	1.949574	1.94584
1.931381	1.927744	1.930225	1.926689	1.931302	1.927846	1.924429	1.949927	1.946143
1.931723	1.92812	1.924551	1.921011	1.931676	1.928223	1.924813	1.950318	1.946517
1.932033	1.928485	1.924949	1.92141	1.932086	1.928629	1.925215	1.950732	1.946938
1.932397	1.928873	1.92535	1.921808	1.918315	1.914915	1.911559	1.922207	1.918793
1.932819	1.929284	1.925752	1.922207	1.918713	1.915319	1.911969	1.92263	1.919205
1.933249	1.929701	1.926161	1.922612	1.919118	1.915726	1.912376	1.923051	1.919615
1.933673	1.93012	1.926577	1.923028	1.919532	1.916133	1.912775	1.923463	1.920022
1.934101	1.930545	1.926999	1.923447	1.919946	1.916539	1.913173	1.923875	1.920432
1.934532	1.930979	1.927429	1.923863	1.920349	1.916937	1.913569	1.910175	1.906783
1.934965	1.931418	1.927864	1.924277	1.920747	1.917332	1.913965	1.910573	1.907184
1.935403	1.931851	1.928288	1.924685	1.921143	1.91773	1.914369	1.910972	1.907574
1.935843	1.932279	1.928704	1.925089	1.921539	1.918131	1.914777	1.911372	1.907957
1.936274	1.932702	1.929119	1.925496	1.92194	1.918533	1.915179	1.911766	1.908339
1.936693	1.933119	1.929533	1.925909	1.92235	1.918935	1.915572	1.912153	1.908721
1.937106	1.933534	1.929949	1.926322	1.922759	1.919336	1.915964	1.91254	1.909107
1.937513	1.933951	1.930369	1.926735	1.923164	1.919732	1.916353	1.91293	1.909499
1.93792	1.934366	1.930787	1.927149	1.923571	1.920131	1.916745	1.913321	1.909893
1.927595	1.924063	1.931195	1.927563	1.923987	1.920541	1.917145	1.913713	1.910277
1.928002	1.924458	1.931599	1.927978	1.924411	1.920959	1.917552	1.914107	1.910659
1.928412	1.924864	1.921309	1.917723	1.924838	1.921377	1.917957	1.914506	1.911053
1.928825	1.925279	1.921726	1.918144	1.925267	1.921794	1.918362	1.914907	1.911455
1.929255	1.9257	1.922149	1.918586	1.925732	1.922244	1.918792	1.915338	1.911898
1.929702	1.926125	1.92258	1.919064	1.926256	1.922746	1.919261	1.915818	1.912409
1.930174	1.926564	1.923014	1.919535	1.926767	1.923237	1.919723	1.916288	1.912904
1.930694	1.927026	1.923443	1.919968	1.916507	1.912992	1.909491	1.916703	1.913325
1.931225	1.927497	1.923875	1.920387	1.916918	1.913393	1.909884	1.917101	1.913719
1.908871	1.905158	1.924311	1.920807	1.917325	1.913794	1.910284	1.906866	1.903511
1.909309	1.9056	1.924755	1.921226	1.917725	1.914193	1.910688	1.907272	1.90392
1.909776	1.906076	1.902463	1.898956	1.918159	1.914634	1.911141	1.907716	1.904344
1.910274	1.906589	1.902973	1.899432	1.918626	1.915121	1.911651	1.908203	1.904783
1.910763	1.907084	1.903468	1.899919	1.896419	1.892969	1.889553	1.908682	1.905238
1.911214	1.907531	1.903927	1.900419	1.896941	1.893455	1.889992	1.909138	1.905718
1.911685	1.907982	1.90438	1.90091	1.897452	1.893926	1.890415	1.909582	1.90619
1.912229	1.908472	1.904834	1.901353	1.897888	1.894347	1.890824	1.898998	1.89564
1.912806	1.908987	1.9053	1.901785	1.898298	1.894755	1.891237	1.899411	1.896045
1.893656	1.889872	1.905831	1.902275	1.898759	1.895224	1.89172	1.888287	1.884911
1.894168	1.890434	1.906414	1.902809	1.899254	1.895738	1.892257	1.888789	1.885348
1.894703	1.890986	1.900124	1.8965	1.899767	1.896248	1.892767	1.88928	1.885809
1.895273	1.891524	1.900652	1.897037	1.900305	1.896753	1.893238	1.889753	1.886301

1.887686	1.883925	1.888373	1.884782	1.893992	1.89043	1.886905	1.89024	1.886796
1.888214	1.884497	1.888918	1.885287	1.894476	1.890938	1.887444	1.890764	1.88728
1.888723	1.88506	1.889462	1.885794	1.894961	1.89145	1.887988	1.891292	1.887765
1.873457	1.869869	1.889977	1.88632	1.882711	1.879204	1.875739	1.884965	1.881448
1.873945	1.870375	1.890481	1.886855	1.883259	1.879716	1.876206	1.88544	1.881945
1.874515	1.870941	1.876224	1.872629	1.883767	1.880218	1.876706	1.885943	1.882442
1.87516	1.871561	1.876773	1.873126	1.884239	1.880711	1.877233	1.886471	1.882941
1.875781	1.872152	1.868512	1.86484	1.884742	1.881216	1.877745	1.874238	1.870731
1.876361	1.87269	1.869037	1.865393	1.885297	1.881739	1.878229	1.874733	1.871262
1.876925	1.873228	1.869568	1.865943	1.871094	1.867566	1.864083	1.875244	1.871807
1.877475	1.873798	1.870135	1.866476	1.87157	1.868059	1.864605	1.875799	1.872374
1.878021	1.874368	1.870707	1.86701	1.872046	1.868561	1.865144	1.876368	1.872941
1.855433	1.851843	1.871237	1.867568	1.872597	1.869116	1.865694	1.862274	1.858873
1.855971	1.852336	1.871747	1.868144	1.873195	1.869704	1.86625	1.8628	1.859365
1.856511	1.85287	1.849299	1.845809	1.8652	1.86168	1.858188	1.863302	1.859869
1.857049	1.853445	1.849891	1.846394	1.865789	1.862223	1.858688	1.863775	1.860379
1.857618	1.854029	1.850476	1.846957	1.843461	1.839966	1.836506	1.864278	1.86091
1.858232	1.854621	1.851039	1.847479	1.843961	1.840486	1.837061	1.864843	1.861483
1.858857	1.855206	1.85159	1.848	1.844469	1.841016	1.837622	1.865406	1.86204
1.85947	1.855763	1.85212	1.848546	1.845029	1.841573	1.838167	1.844797	1.841476
1.860081	1.856314	1.852649	1.849106	1.845613	1.842139	1.838702	1.845264	1.841914
1.8607	1.856902	1.853218	1.849681	1.846185	1.842679	1.839206	1.84095	1.837603
1.861325	1.857514	1.853818	1.850269	1.846754	1.843203	1.839687	1.841383	1.83806
1.844287	1.840559	1.854436	1.850881	1.847352	1.843773	1.840228	1.841872	1.838553
1.844809	1.841137	1.855075	1.851522	1.847985	1.844397	1.840838	1.842429	1.839088
1.845347	1.84172	1.855714	1.852158	1.848614	1.845022	1.841455	1.838002	1.834631
1.845923	1.842305	1.856343	1.852781	1.849227	1.84563	1.842056	1.838584	1.835187
1.84651	1.842895	1.85697	1.85339	1.849821	1.846225	1.842653	1.839168	1.835746
1.847092	1.843488	1.857594	1.853973	1.850374	1.846792	1.843244	1.839759	1.836324
1.847676	1.844086	1.858217	1.854543	1.850908	1.847347	1.843834	1.840355	1.836913
1.84827	1.844688	1.858847	1.855145	1.851488	1.847933	1.844432	1.84095	1.837499
1.848874	1.845296	1.859483	1.855772	1.852106	1.848544	1.845036	1.841546	1.838084
1.849474	1.845904	1.842294	1.838609	1.852738	1.849171	1.845657	1.84216	1.83869
1.850063	1.846514	1.842915	1.839226	1.853385	1.849818	1.846303	1.8428	1.839323
1.839772	1.836244	1.843532	1.839844	1.854035	1.850467	1.846951	1.843445	1.83996
1.840365	1.836834	1.844136	1.840449	1.854677	1.851109	1.847589	1.844078	1.840587
1.840968	1.837424	1.844736	1.841053	1.85532	1.851751	1.848228	1.844714	1.841217
1.841583	1.838021	1.834425	1.830762	1.838034	1.834492	1.831001	1.845366	1.841868
1.842211	1.838624	1.835018	1.831367	1.838667	1.835127	1.831634	1.846027	1.842531
1.842864	1.839249	1.835627	1.831975	1.828366	1.824836	1.821351	1.846671	1.84318
1.843545	1.839897	1.836251	1.832585	1.828965	1.825426	1.821936	1.847298	1.843813
1.84422	1.840542	1.836874	1.833199	1.829573	1.826023	1.822524	1.82998	1.826542
1.844874	1.841169	1.837485	1.833808	1.830181	1.826623	1.823116	1.830584	1.82715
1.845535	1.841805	1.838109	1.834443	1.830822	1.827248	1.823722	1.831197	1.827765
1.832906	1.829194	1.83877	1.835145	1.831545	1.827936	1.824359	1.82087	1.817445
1.833594	1.829858	1.839448	1.835869	1.832296	1.828646	1.825012	1.821497	1.818062
1.796734	1.793053	1.840119	1.836546	1.832973	1.829306	1.82565	1.822117	1.818668
1.797421	1.793714	1.840788	1.837192	1.833604	1.829936	1.826282	1.822737	1.819267
1.798142	1.794389	1.805655	1.802105	1.834247	1.830599	1.826968	1.823408	1.819903
1.798908	1.795082	1.806312	1.802741	1.834906	1.831305	1.827722	1.824143	1.820579

1.799648	1.795776	1.806981	1.803382	1.799847	1.796347	1.792872	1.824864	1.82127
1.800322	1.796454	1.807658	1.804019	1.800455	1.796953	1.793491	1.825539	1.821981
1.800983	1.797145	1.808358	1.804673	1.801067	1.79755	1.794089	1.826195	1.822683
1.801671	1.797883	1.809113	1.805369	1.801708	1.798173	1.794708	1.791254	1.787823
1.802365	1.798638	1.809892	1.80609	1.802372	1.798814	1.795336	1.791863	1.788417
1.787038	1.783391	1.810622	1.806816	1.803081	1.799491	1.795977	1.792482	1.789023
1.787693	1.784051	1.811314	1.807549	1.803834	1.800203	1.796631	1.793107	1.789633
1.78837	1.784711	1.811988	1.808248	1.804551	1.800912	1.797323	1.79379	1.790309
1.78908	1.785367	1.812631	1.808894	1.805217	1.801618	1.798074	1.794561	1.791083
1.789789	1.786044	1.798275	1.79458	1.805882	1.802312	1.798802	1.795306	1.791832
1.79048	1.786776	1.799027	1.795289	1.80656	1.802966	1.799441	1.79594	1.79247
1.791161	1.787514	1.799797	1.796021	1.807259	1.803628	1.800073	1.796556	1.793082
1.743494	1.740165	1.800496	1.796751	1.808018	1.804375	1.800793	1.797247	1.79374
1.744101	1.74077	1.801163	1.797484	1.808812	1.805166	1.801559	1.797976	1.794424
1.704802	1.701839	1.785921	1.78229	1.809554	1.805897	1.802276	1.798689	1.795137
1.705439	1.702464	1.786692	1.783008	1.810245	1.806568	1.802947	1.799389	1.795884
1.706051	1.703058	1.73947	1.736089	1.810959	1.807273	1.803649	1.800097	1.796606
1.706608	1.703592	1.740064	1.736728	1.811728	1.80805	1.804412	1.800822	1.797275
1.707177	1.704129	1.74065	1.737357	1.812483	1.808816	1.805168	1.801543	1.797945
1.707803	1.704702	1.74125	1.737961	1.813162	1.809501	1.805861	1.802245	1.798655
1.708457	1.705303	1.741873	1.73857	1.813823	1.810167	1.806539	1.802941	1.799372
1.709119	1.705952	1.742574	1.739253	1.753955	1.750631	1.747333	1.80365	1.800048
1.709794	1.706641	1.743334	1.739986	1.754679	1.751344	1.748033	1.804366	1.800698
1.710471	1.70732	1.744077	1.740688	1.755364	1.752018	1.748705	1.805081	1.801398
1.711157	1.707992	1.744805	1.741354	1.756	1.752645	1.749342	1.805791	1.802154
1.711821	1.708645	1.705521	1.702448	1.738616	1.735332	1.732114	1.746704	1.743429
1.712442	1.709258	1.706135	1.703073	1.739314	1.736032	1.732811	1.747417	1.744136
1.713061	1.709872	1.706753	1.703707	1.740029	1.736742	1.733506	1.748123	1.744832
1.713724	1.710534	1.707409	1.704355	1.740743	1.73742	1.734147	1.748769	1.74548
1.714404	1.711213	1.708079	1.705007	1.741456	1.738087	1.73477	1.749396	1.746113
1.715075	1.711865	1.708715	1.705632	1.742135	1.738759	1.735438	1.750082	1.746804
1.715741	1.712497	1.709326	1.706234	1.742786	1.739432	1.736139	1.750814	1.747543
1.716395	1.713142	1.709965	1.706868	1.743482	1.740139	1.73686	1.751562	1.7483
1.717029	1.713801	1.710639	1.707543	1.744234	1.740888	1.737607	1.752335	1.749083
1.680894	1.677797	1.711329	1.708236	1.745007	1.741645	1.738348	1.753092	1.749845
1.681547	1.678465	1.712035	1.708946	1.745798	1.742407	1.739075	1.753823	1.750565
1.682221	1.679144	1.712748	1.709658	1.746588	1.743157	1.739778	1.754521	1.751245
1.682913	1.679837	1.676799	1.673799	1.707274	1.704205	1.701176	1.737111	1.733903
1.683622	1.680537	1.677483	1.674455	1.707917	1.704798	1.701721	1.73768	1.734464
1.68434	1.681219	1.678131	1.675074	1.689832	1.686729	1.683675	1.738335	1.7351
1.685072	1.681883	1.678745	1.675655	1.69043	1.687347	1.68432	1.73907	1.735803
1.685767	1.682526	1.679353	1.676249	1.673203	1.67021	1.667273	1.722764	1.719493
1.686392	1.683129	1.679951	1.676862	1.673832	1.670845	1.66791	1.72351	1.720223
1.687036	1.683754	1.680569	1.677492	1.674475	1.671492	1.668556	1.724268	1.720964
1.687765	1.684445	1.681234	1.678145	1.675122	1.672143	1.669214	1.708895	1.705615
1.688533	1.685174	1.68193	1.678817	1.67578	1.672803	1.66988	1.709655	1.706351
1.689295	1.685915	1.68265	1.679512	1.676457	1.673476	1.670553	1.667646	1.66477
1.690065	1.686676	1.683394	1.680228	1.677149	1.67416	1.671233	1.668317	1.665429
1.690866	1.687465	1.684163	1.680963	1.677853	1.674846	1.671906	1.668979	1.666081
1.691699	1.688286	1.684959	1.681718	1.678569	1.675533	1.672571	1.66963	1.666727

1.692538	1.689111	1.685758	1.682479	1.679291	1.676225	1.673239	1.670285	1.667377
1.693377	1.689931	1.686553	1.68324	1.68002	1.676924	1.673912	1.670946	1.668034
1.694211	1.690748	1.687347	1.684004	1.680753	1.677628	1.674592	1.671614	1.6687
1.655245	1.652192	1.688142	1.684767	1.681485	1.678335	1.67528	1.672294	1.669378
1.655913	1.652875	1.688936	1.685534	1.682224	1.679052	1.675979	1.672984	1.670064
1.623516	1.620534	1.689732	1.686318	1.682992	1.679794	1.676695	1.673683	1.670754
1.624164	1.621181	1.690527	1.687116	1.683784	1.680559	1.677426	1.674393	1.671448
1.624791	1.621811	1.651859	1.64883	1.684577	1.681328	1.678164	1.675108	1.672145
1.625391	1.622421	1.652509	1.649499	1.685373	1.682104	1.678911	1.67583	1.672843
1.625989	1.623032	1.620086	1.617146	1.647214	1.644305	1.641458	1.676554	1.673543
1.626588	1.623653	1.620722	1.617787	1.647876	1.644949	1.642078	1.677271	1.674235
1.627198	1.624283	1.621363	1.61843	1.64854	1.645598	1.642708	1.678	1.67494
1.627833	1.624921	1.622006	1.619079	1.616169	1.613265	1.610409	1.67876	1.675679
1.628492	1.625574	1.622658	1.619739	1.616832	1.613921	1.611052	1.679551	1.67645
1.629196	1.62628	1.623366	1.620452	1.617549	1.614641	1.611771	1.609004	1.606317
1.629948	1.62704	1.624132	1.62122	1.61832	1.615427	1.612566	1.609781	1.607058
1.59742	1.594714	1.624849	1.621935	1.619037	1.616156	1.613305	1.610505	1.607752
1.598032	1.595319	1.625491	1.622571	1.61967	1.616798	1.613956	1.611146	1.608374
1.598622	1.595898	1.626103	1.623178	1.620276	1.617409	1.614572	1.611753	1.608963
1.599188	1.596449	1.626694	1.62377	1.62087	1.618006	1.615167	1.612333	1.609519
1.599748	1.596992	1.627277	1.624356	1.621458	1.618592	1.615747	1.612899	1.610066
1.55859	1.556025	1.594836	1.592119	1.62206	1.619184	1.61633	1.613483	1.610656
1.559112	1.556551	1.595443	1.592715	1.622673	1.619783	1.616917	1.614081	1.611277
1.529973	1.527576	1.596096	1.593358	1.623346	1.620444	1.617568	1.614737	1.611946
1.530546	1.528133	1.596805	1.594056	1.624088	1.621177	1.618295	1.615463	1.612675
1.531092	1.528662	1.555765	1.553203	1.624798	1.62188	1.618991	1.616155	1.613363
1.531577	1.529127	1.556294	1.55373	1.625436	1.622513	1.619618	1.616772	1.613966
1.532053	1.529585	1.556813	1.554249	1.626057	1.623129	1.620227	1.617367	1.614546
1.532547	1.530069	1.557345	1.554777	1.587482	1.584721	1.581979	1.617958	1.615124
1.533058	1.530571	1.55789	1.555316	1.588047	1.585274	1.582519	1.618556	1.615712
1.533617	1.531114	1.528652	1.526238	1.553328	1.550758	1.548205	1.619235	1.616376
1.534216	1.53169	1.529212	1.526786	1.553936	1.551367	1.548814	1.619977	1.617103
1.534787	1.532248	1.529757	1.52732	1.554531	1.55196	1.549405	1.581667	1.578946
1.535319	1.532776	1.530278	1.527829	1.555103	1.552528	1.549968	1.582248	1.579516
1.499262	1.496826	1.530815	1.52836	1.555696	1.553113	1.550544	1.582848	1.580103
1.499807	1.497364	1.531396	1.528945	1.556339	1.553744	1.551158	1.583502	1.580743
1.50036	1.497909	1.531985	1.529537	1.556987	1.554379	1.551778	1.584163	1.58139
1.500874	1.498421	1.532544	1.530084	1.527641	1.525193	1.522755	1.549769	1.547188
1.501376	1.49892	1.533093	1.530612	1.528152	1.525698	1.523258	1.550336	1.547741
1.501909	1.499447	1.497024	1.494646	1.528699	1.526245	1.523807	1.550944	1.548337
1.50247	1.499996	1.497564	1.49518	1.529276	1.526829	1.524397	1.55159	1.548971
1.503033	1.500541	1.498095	1.495698	1.529839	1.527391	1.524961	1.552217	1.549596
1.503592	1.501073	1.498603	1.496185	1.530367	1.527909	1.525475	1.552805	1.550197
1.504163	1.501616	1.499123	1.496688	1.494295	1.491947	1.489627	1.553411	1.550809
1.504766	1.502191	1.499681	1.497245	1.494853	1.492506	1.490185	1.554075	1.551455
1.505373	1.50277	1.500246	1.497814	1.49543	1.493088	1.490767	1.554759	1.552116
1.505936	1.503305	1.500768	1.498345	1.495971	1.493635	1.491317	1.525406	1.522928
1.506477	1.50382	1.501271	1.498856	1.496493	1.494162	1.491849	1.525995	1.523529
1.507067	1.504399	1.501832	1.49939	1.497005	1.494668	1.492359	1.490031	1.487699
1.507709	1.505044	1.502453	1.499943	1.497501	1.495149	1.492841	1.490518	1.488195

1.508352	1.505693	1.503082	1.500514	1.498023	1.495653	1.493343	1.491021	1.488707
1.50899	1.506337	1.503716	1.501115	1.498593	1.496206	1.493885	1.491562	1.48925
1.509614	1.506968	1.504339	1.501714	1.499167	1.496764	1.494431	1.492106	1.489801
1.510199	1.507563	1.50493	1.502282	1.499712	1.497293	1.494951	1.492636	1.490352
1.510768	1.508141	1.505505	1.502839	1.500251	1.497815	1.495463	1.493159	1.490899
1.511366	1.508725	1.506078	1.503405	1.500808	1.498353	1.495983	1.493674	1.491419
1.511981	1.509311	1.506647	1.503978	1.501379	1.498903	1.496508	1.494182	1.491916
1.512573	1.509909	1.507247	1.504577	1.501967	1.499459	1.49703	1.494688	1.492419
1.513138	1.510526	1.507888	1.505209	1.502573	1.50002	1.497544	1.49519	1.492931
1.513689	1.511131	1.508523	1.505843	1.503189	1.500592	1.498068	1.495694	1.493434
1.514227	1.511713	1.509136	1.506474	1.503816	1.501179	1.498603	1.496195	1.493909
1.514754	1.51228	1.509735	1.507096	1.504443	1.501778	1.499161	1.496713	1.49439
1.515264	1.512828	1.510313	1.507695	1.505054	1.50239	1.499762	1.497282	1.49491
1.51577	1.513362	1.510874	1.508278	1.505656	1.503012	1.500391	1.497881	1.495455
1.516317	1.5139	1.51142	1.508857	1.506264	1.503633	1.501012	1.498478	1.496013
1.516902	1.51444	1.511952	1.509431	1.506878	1.504255	1.501629	1.499079	1.496586
1.517481	1.514977	1.51248	1.509993	1.507473	1.504863	1.502238	1.499672	1.49715
1.518041	1.515504	1.512998	1.510533	1.508039	1.505448	1.502833	1.500249	1.497691
1.518623	1.516053	1.513536	1.511084	1.508608	1.506036	1.503435	1.50084	1.498257
1.519244	1.516647	1.514117	1.511667	1.5092	1.506641	1.504053	1.501465	1.498883
1.519896	1.517272	1.514725	1.512266	1.5098	1.507255	1.504685	1.502109	1.499535
1.52057	1.517917	1.515347	1.512866	1.510394	1.507876	1.505341	1.50277	1.500183
1.521273	1.518588	1.51599	1.513473	1.510989	1.508506	1.506013	1.503444	1.500834
1.522043	1.5193	1.516657	1.514105	1.511604	1.509137	1.506671	1.504105	1.501485
1.522882	1.520055	1.517352	1.514761	1.51224	1.509772	1.507314	1.504753	1.502138
1.523757	1.520823	1.518047	1.515423	1.512887	1.510413	1.507957	1.505401	1.502794
1.524652	1.521584	1.518724	1.516081	1.513543	1.511061	1.508604	1.506053	1.503453
1.525603	1.522384	1.51943	1.516763	1.514217	1.511717	1.509247	1.506701	1.50412
1.526626	1.523249	1.52019	1.517488	1.51492	1.512377	1.509875	1.507343	1.504802
1.527726	1.52418	1.521003	1.518251	1.515645	1.513046	1.510501	1.507981	1.505487
1.528948	1.525216	1.521896	1.519062	1.516394	1.513734	1.511148	1.50862	1.506143
1.530277	1.526343	1.522861	1.519917	1.517164	1.51444	1.51181	1.509259	1.506781
1.531602	1.527481	1.523839	1.520767	1.517919	1.515145	1.512493	1.509926	1.507443
1.532918	1.528619	1.524816	1.5216	1.518649	1.515845	1.513197	1.510623	1.508136
1.534258	1.529819	1.525868	1.522491	1.519415	1.516572	1.51392	1.511331	1.50883
1.535623	1.531096	1.52702	1.523458	1.520229	1.517327	1.514653	1.512034	1.509509
1.537032	1.532449	1.52826	1.524503	1.521107	1.51813	1.515418	1.512758	1.510199
1.538524	1.533904	1.529612	1.525657	1.522083	1.519018	1.516252	1.513532	1.510923
1.540067	1.535428	1.531045	1.526896	1.523139	1.519967	1.517125	1.514336	1.511672
1.541581	1.536934	1.532477	1.528167	1.524245	1.520931	1.517972	1.515124	1.512443
1.543078	1.538434	1.533921	1.529479	1.525403	1.521916	1.518801	1.515902	1.513239
1.544561	1.539917	1.535361	1.530828	1.526633	1.522979	1.519702	1.516731	1.514054
1.546022	1.541368	1.536785	1.532213	1.52794	1.524134	1.520691	1.517625	1.514894
1.547493	1.542838	1.538239	1.533644	1.529304	1.525342	1.521722	1.518543	1.515736
1.548996	1.544369	1.539768	1.535144	1.530724	1.526585	1.522763	1.51945	1.516549
1.550512	1.545911	1.541313	1.536674	1.532186	1.527877	1.523856	1.520396	1.517381
1.552031	1.547408	1.542799	1.538177	1.533662	1.529228	1.525044	1.521437	1.518281
1.553553	1.548884	1.54426	1.539674	1.535161	1.530635	1.526318	1.522561	1.519243
1.555073	1.550399	1.545774	1.541198	1.536673	1.532106	1.527713	1.523814	1.520308
1.556587	1.551951	1.547341	1.542746	1.5382	1.533644	1.529235	1.525199	1.521479

1.518092	1.513496	1.548909	1.544297	1.539735	1.535196	1.53078	1.526614	1.522685
1.519797	1.51519	1.550463	1.545842	1.541273	1.536758	1.532339	1.528044	1.523904
1.521523	1.516898	1.552031	1.547404	1.542828	1.53832	1.533884	1.529481	1.525174
1.52326	1.51862	1.513998	1.509384	1.504825	1.500341	1.495929	1.530898	1.526514
1.525004	1.520354	1.515728	1.511128	1.506555	1.501993	1.497484	1.532323	1.527911
1.526738	1.522101	1.517469	1.512834	1.508222	1.503621	1.499075	1.494621	1.490273
1.528467	1.52386	1.519219	1.51452	1.509851	1.505236	1.500687	1.496233	1.491882
1.5302	1.525592	1.52094	1.516219	1.511525	1.506883	1.50231	1.497844	1.493484
1.531939	1.527289	1.522623	1.517929	1.513247	1.508566	1.503938	1.499448	1.495074
1.445848	1.441265	1.524342	1.519663	1.514984	1.510272	1.505603	1.501089	1.496694
1.447566	1.443026	1.526152	1.521441	1.516729	1.512002	1.50732	1.502787	1.498368
1.44929	1.444784	1.527956	1.523219	1.518481	1.513753	1.50907	1.504515	1.50006
1.393578	1.38909	1.529649	1.524936	1.520226	1.515517	1.510841	1.506251	1.501736
1.395225	1.390667	1.531305	1.526638	1.521979	1.517301	1.512638	1.508009	1.503422
1.312689	1.308903	1.445209	1.440646	1.523793	1.519134	1.514476	1.509819	1.505178
1.314027	1.31029	1.447012	1.442476	1.52566	1.521011	1.516354	1.511676	1.506996
1.315382	1.311661	1.391195	1.386762	1.439744	1.435183	1.430609	1.513505	1.508814
1.316759	1.313008	1.392812	1.388385	1.441464	1.436911	1.432345	1.515296	1.510633
1.318152	1.314367	1.310619	1.306925	1.443161	1.438622	1.434075	1.429523	1.424973
1.319546	1.31576	1.312005	1.308288	1.44484	1.440326	1.435818	1.431266	1.426695
1.320976	1.317165	1.313389	1.309645	1.446522	1.442027	1.437557	1.433004	1.428412
1.322491	1.31855	1.314714	1.311012	1.390485	1.386051	1.381651	1.434728	1.430159
1.324069	1.319941	1.316024	1.312391	1.392167	1.387694	1.383266	1.436448	1.431925
1.325666	1.321443	1.317467	1.313822	1.393889	1.389388	1.38495	1.380523	1.37611
1.327287	1.323055	1.319039	1.315305	1.395654	1.391132	1.386702	1.382262	1.377833
1.328901	1.324668	1.320615	1.316782	1.313027	1.309317	1.305616	1.383976	1.379532
1.33049	1.326264	1.322174	1.318236	1.314407	1.31072	1.30707	1.385641	1.381188
1.332095	1.327858	1.323728	1.319704	1.31581	1.312131	1.308511	1.38731	1.382853
1.264733	1.26047	1.325248	1.321192	1.317275	1.313577	1.309953	1.38901	1.384571
1.266417	1.26207	1.326784	1.322717	1.318788	1.315047	1.311386	1.39073	1.386306
1.268083	1.263743	1.328438	1.324336	1.320358	1.316534	1.312798	1.309069	1.305358
1.269737	1.265467	1.330173	1.32603	1.32198	1.318043	1.314199	1.31046	1.306798
1.271335	1.267146	1.262996	1.258886	1.323626	1.319604	1.315673	1.31191	1.308263
1.272864	1.268771	1.264682	1.260587	1.325305	1.321227	1.317226	1.313424	1.309756
1.274433	1.270414	1.266365	1.262265	1.326952	1.322842	1.3188	1.314959	1.311255
1.276098	1.272112	1.268057	1.263897	1.32852	1.324408	1.320374	1.316508	1.312764
1.277776	1.273801	1.269733	1.265524	1.330096	1.325992	1.321971	1.31807	1.314265
1.279405	1.275431	1.271372	1.267188	1.263028	1.258954	1.254943	1.31963	1.31572
1.281015	1.277026	1.272982	1.26886	1.264744	1.260669	1.256622	1.321205	1.317172
1.282617	1.278587	1.274546	1.270488	1.266429	1.26237	1.258313	1.254226	1.250146
1.284209	1.280114	1.276067	1.272083	1.268095	1.264068	1.260019	1.255908	1.251776
1.205587	1.201685	1.277647	1.273686	1.26973	1.265739	1.261718	1.257597	1.253429
1.207107	1.203204	1.279303	1.275296	1.271318	1.267371	1.263405	1.259292	1.255103
1.208683	1.204754	1.280978	1.276926	1.272925	1.269013	1.265096	1.260993	1.256791
1.210341	1.206328	1.282654	1.278587	1.274589	1.270697	1.266803	1.262704	1.258492
1.212033	1.207917	1.284338	1.280265	1.276275	1.272391	1.268507	1.264413	1.260199
1.213706	1.209496	1.205441	1.201572	1.27796	1.274072	1.270185	1.266099	1.261898
1.215378	1.211079	1.206971	1.203101	1.279651	1.275747	1.271848	1.267772	1.263595
1.217052	1.212698	1.208545	1.204643	1.281333	1.277412	1.273507	1.269447	1.265299
1.218727	1.214351	1.210161	1.206198	1.283008	1.279067	1.275162	1.271122	1.26701

							0.478598	0.477374
							0.47778	0.476557
							0.714389	0.71027
							0.712263	0.708263
					0.477829	0.476619	0.852512	0.848692
					0.477033	0.475808	0.850495	0.846719
					0.714126	0.710092	1.206181	1.201984
					0.712026	0.70822	1.204099	1.199945
					0.709974	0.706375	1.202034	1.197915
		0.720038	0.71584	0.711772	0.850223	0.846657	1.268234	1.263712
		0.718125	0.713981	0.709941	0.848317	0.844644	1.265752	1.261256
		0.71603	0.711959	0.70798	1.203552	1.199393	1.335911	1.331188
		0.71377	0.709793	0.705909	1.201275	1.197138	1.333434	1.328833
0.719879	0.715651	0.853594	0.84975	0.845992	1.26742	1.262878	1.331023	1.326543
0.717601	0.71344	0.851476	0.847661	0.843921	1.264958	1.260444	1.328675	1.324321
0.715401	0.711324	0.849448	0.845651	0.841924	1.262582	1.258125	1.326427	1.322168
0.713406	0.709423	1.204951	1.200726	1.196576	1.333459	1.328797	1.599666	1.594711

0.711483	0.70759	1.202879	1.19868	1.194559	1.331279	1.32674	1.597215	1.592276
0.851386	0.847582	1.269392	1.264781	1.260287	1.60478	1.599632	1.665307	1.660047
0.84929	0.845498	1.266879	1.262294	1.257851	1.601859	1.59685	1.662444	1.657213
1.204494	1.200263	1.338155	1.333337	1.328662	1.599105	1.594178	1.706043	1.700629
1.202198	1.197991	1.335717	1.331029	1.326476	1.596521	1.591614	1.703216	1.697834
1.268776	1.264152	1.609454	1.604168	1.599018	1.664739	1.659504	1.700475	1.695128
1.266523	1.261915	1.60674	1.601567	1.596532	1.662159	1.656953	1.697867	1.69257
1.264293	1.259733	1.604093	1.599017	1.594075	1.659606	1.654426	1.695272	1.690004
1.438541	1.43345	1.601498	1.596483	1.591585	1.703267	1.697892	1.692555	1.68726
1.435829	1.430925	1.598959	1.593983	1.5891	1.700528	1.695168	1.689808	1.684454
1.606711	1.601532	1.596476	1.591539	1.586671	1.697883	1.692546	1.736881	1.731449
1.604072	1.599015	1.59405	1.589146	1.584288	1.695312	1.690002	1.734286	1.728795
1.601474	1.5965	1.708806	1.703373	1.697993	1.742387	1.737062	1.822763	1.816968
1.598913	1.593975	1.706025	1.700614	1.695249	1.739624	1.734306	1.819946	1.814163
1.596405	1.59149	1.703286	1.697893	1.692541	1.73689	1.731572	1.899189	1.892894
1.593968	1.589085	1.700663	1.69529	1.689949	1.734264	1.728936	1.896164	1.889888
1.591564	1.586697	1.698052	1.692696	1.687367	1.731645	1.726305	1.893145	1.886893
1.659765	1.654544	1.744997	1.739622	1.734274	1.820075	1.814433	1.943779	1.937063
1.65719	1.651922	1.742204	1.736839	1.731508	1.817234	1.811591	1.940493	1.93388
1.707688	1.702327	1.831362	1.825695	1.82007	1.873522	1.867413	2.091248	2.08374
1.705184	1.699786	1.828574	1.822894	1.817268	1.870517	1.864444	2.087646	2.080076
1.745323	1.739753	1.825795	1.820089	1.814449	1.973129	1.966528	2.100418	2.092817
1.742668	1.737087	1.82302	1.81726	1.811583	1.969791	1.963294	2.096781	2.089343
1.739985	1.734416	1.82026	1.814468	1.80877	1.966506	1.960087	2.093174	2.085908
1.829103	1.823306	1.877021	1.870738	1.864552	2.087923	2.080586	2.133476	2.126239
1.826291	1.820536	1.874079	1.867847	1.861698	2.084429	2.077072	2.129928	2.122769
1.823528	1.817754	1.977066	1.970335	1.963697	2.141177	2.133644	2.158572	2.151502
1.820797	1.814966	1.973728	1.96713	1.960632	2.137638	2.130047	2.154985	2.147939
1.896998	1.890706	2.064071	2.05701	2.050108	2.134059	2.126548	2.167162	2.160141
1.894069	1.887779	2.060413	2.05341	2.04661	2.130428	2.123155	2.163902	2.157012
1.97768	1.97075	2.149019	2.141472	2.134125	2.144071	2.136934	2.183461	2.176603
1.974344	1.967557	2.145468	2.137992	2.130685	2.140644	2.133541	2.180128	2.173402
1.971045	1.964409	2.141996	2.1346	2.127333	2.137284	2.130179	2.176786	2.170142
2.061015	2.054007	2.13852	2.131207	2.124001	2.164603	2.157575	2.173413	2.166718
2.057577	2.050703	2.135072	2.127834	2.120686	2.161324	2.154312	2.170045	2.163237
2.081972	2.074634	2.148853	2.141573	2.134392	2.180719	2.173713	2.166773	2.159904
2.078521	2.07123	2.145452	2.138106	2.130888	2.177301	2.170463	2.163577	2.156676
2.095773	2.088437	2.142016	2.134703	2.127533	2.173987	2.167225	2.159319	2.152441
2.092367	2.084949	2.13852	2.131365	2.124358	2.170801	2.164	2.156113	2.149277
2.149932	2.142417	2.159729	2.152632	2.14568	2.167633	2.16078	2.160654	2.153815
2.146562	2.139096	2.156415	2.149389	2.142497	2.164448	2.157561	2.157439	2.150645
2.143181	2.135817	2.15317	2.146191	2.139324	2.161257	2.154339	2.154216	2.147458
2.13972	2.132511	2.178711	2.171818	2.164977	2.156986	2.150072	2.16062	2.153885
2.136237	2.129212	2.175517	2.168628	2.161758	2.153765	2.14684	2.157372	2.150616
2.157421	2.150424	2.171209	2.164342	2.157473	2.158286	2.151286	2.16361	2.156904
2.154128	2.147175	2.168012	2.161139	2.154252	2.155009	2.147987	2.160388	2.15381
2.150395	2.143488	2.16482	2.157942	2.151051	2.161465	2.154498	2.166178	2.159705
2.147222	2.140327	2.161637	2.154751	2.147867	2.158328	2.151421	2.163138	2.15671
2.172235	2.165328	2.175903	2.169004	2.162135	2.164585	2.157753	2.160152	2.153755
2.169094	2.162148	2.17271	2.165861	2.159055	2.161547	2.154771	2.157202	2.150831

2.165952	2.158965	2.169525	2.162741	2.156012	2.15855	2.151829	2.154286	2.147937
2.162794	2.15578	2.166351	2.15963	2.152977	2.164557	2.157871	2.219851	2.212987
2.159625	2.152596	2.16319	2.156537	2.149959	2.161584	2.154943	2.21678	2.209958
2.173943	2.166925	2.169343	2.162758	2.156257	2.15863	2.152026	2.213703	2.206918
2.170768	2.163802	2.166259	2.159735	2.153292	2.155694	2.149118	2.210619	2.203868
2.176856	2.169937	2.163205	2.15674	2.15035	2.221629	2.214475	2.214282	2.207543
2.173681	2.166839	2.16018	2.153773	2.147432	2.218535	2.211419	2.211231	2.204486
2.170528	2.163766	2.157182	2.150828	2.144531	2.215451	2.208373	2.208189	2.201434
2.1674	2.16072	2.154207	2.1479	2.14164	2.21909	2.212036	2.205145	2.198388
2.16431	2.157705	2.151255	2.14499	2.138759	2.215992	2.208974	2.202101	2.195349
2.161298	2.154734	2.226953	2.220044	2.213176	2.212903	2.205906	2.199052	2.192315
2.158356	2.151804	2.223798	2.216922	2.210087	2.209821	2.202835	2.195999	2.189287
2.155499	2.148939	2.227414	2.220539	2.213712	2.206748	2.199773	2.192965	2.186293
2.152751	2.146156	2.224345	2.217463	2.210642	2.203681	2.196715	2.189944	2.183332
2.149973	2.14336	2.221282	2.214397	2.207585	2.200638	2.193698	2.186967	2.180411
2.147094	2.140503	2.218196	2.211327	2.204535	2.197621	2.190721	2.184033	2.177526
2.14419	2.137639	2.215124	2.208272	2.201501	2.194643	2.187813	2.181173	2.174695
2.22639	2.219166	2.212089	2.205238	2.198486	2.191737	2.185046	2.178455	2.171958
2.223263	2.216114	2.209093	2.202234	2.195495	2.188879	2.182358	2.175819	2.16928
2.220172	2.213133	2.206187	2.199312	2.192561	2.186018	2.179599	2.173107	2.166571
2.217115	2.210218	2.203367	2.196467	2.189679	2.18316	2.176791	2.170339	2.163843
2.2141	2.207285	2.200496	2.193613	2.186831	2.180327	2.173979	2.176014	2.169584
2.211128	2.20431	2.197537	2.190722	2.184007	2.17751	2.171155	2.173244	2.166923
2.208204	2.20136	2.194596	2.187876	2.181253	2.174757	2.168378	2.174884	2.168669
2.205355	2.198477	2.191728	2.185141	2.17864	2.172137	2.165698	2.172203	2.166021
2.202524	2.195626	2.188898	2.182438	2.176053	2.16955	2.163067	2.169582	2.163447
2.199607	2.192763	2.186084	2.179663	2.173321	2.17154	2.165134	2.167081	2.161109
2.196652	2.189911	2.183301	2.176862	2.170512	2.168815	2.162531	2.164663	2.158933
2.193793	2.187159	2.185218	2.178787	2.172436	2.166086	2.15986	2.16217	2.156726
2.191025	2.184513	2.182656	2.176168	2.16974	2.163338	2.157103	2.159587	2.154486
2.192862	2.186451	2.18004	2.173529	2.167083	2.168809	2.162638	2.157188	2.152376
2.190209	2.183748	2.177318	2.170853	2.164492	2.166348	2.16034	2.155069	2.150455
2.187548	2.181021	2.174566	2.168144	2.161884	2.163925	2.15815	2.153069	2.148617
2.184806	2.178262	2.171798	2.165349	2.159132	2.161406	2.155954	2.151095	2.146796
2.182039	2.175499	2.169041	2.162559	2.156383	2.158915	2.153824	2.149192	2.145024
2.179309	2.172804	2.166393	2.159955	2.153874	2.156628	2.151822	2.147375	2.143318
2.176603	2.170157	2.163826	2.157494	2.151552	2.154504	2.149932	2.145639	2.141674
2.173929	2.167484	2.169315	2.163185	2.157466	2.152521	2.148115	2.143952	2.140081
2.171291	2.164767	2.166654	2.160852	2.155482	2.150699	2.146375	2.142314	2.13855
2.168664	2.162108	2.164078	2.158582	2.153535	2.148921	2.144698	2.14863	2.144928
2.16602	2.159525	2.161619	2.156345	2.151541	2.147138	2.143095	2.147113	2.143399
2.163415	2.157044	2.159294	2.154213	2.14961	2.145399	2.141525	2.145616	2.141879
2.160909	2.154733	2.15717	2.152281	2.147844	2.14618	2.142397	2.145952	2.142207
2.158463	2.152542	2.155197	2.150484	2.146181	2.144582	2.140818	2.144407	2.140713
2.155983	2.150364	2.153271	2.148716	2.144525	2.138588	2.134835	2.142891	2.139195
2.153481	2.148215	2.151408	2.146994	2.142891	2.137032	2.133332	2.141399	2.137658
2.159253	2.154217	2.151966	2.147716	2.143743	2.142176	2.138489	2.139893	2.136142
2.157243	2.152291	2.150146	2.146065	2.142232	2.140667	2.136931	2.138359	2.134657
2.157761	2.152882	2.150779	2.146765	2.142987	2.144337	2.140559	2.136837	2.13317
2.155938	2.151238	2.149211	2.145208	2.141415	2.14281	2.139071	2.135349	2.131651

2.154162	2.149667	2.147731	2.143704	2.139858	2.141291	2.137594	2.133873	2.130145
2.15915	2.15483	2.146232	2.14225	2.138399	2.139819	2.136086	2.13238	2.128709
2.157276	2.15318	2.144738	2.140831	2.136998	2.138379	2.134571	2.130889	2.127319
2.151101	2.147088	2.143196	2.139355	2.13556	2.136952	2.133127	2.129461	2.125931
2.149449	2.145462	2.141602	2.137824	2.134092	2.135545	2.131755	2.128095	2.124544
2.147873	2.143912	2.140068	2.136306	2.132595	2.134105	2.130369	2.126739	2.1232
2.146397	2.142478	2.138635	2.134808	2.131042	2.1326	2.128945	2.125386	2.121919
2.144929	2.141054	2.137215	2.133333	2.12952	2.131116	2.127526	2.124038	2.120652
2.14338	2.13956	2.135763	2.131908	2.12811	2.129717	2.12613	2.12268	2.119361
2.141819	2.138042	2.134291	2.130493	2.126742	2.128359	2.12476	2.121334	2.118068
2.140324	2.136521	2.138112	2.134346	2.130637	2.127002	2.12345	2.129604	2.126291
2.138874	2.134995	2.136559	2.13284	2.129204	2.125654	2.122187	2.12834	2.124978
2.137432	2.133527	2.135086	2.131381	2.127781	2.124301	2.120919	2.140574	2.137186
2.135999	2.132125	2.133699	2.129969	2.126356	2.122934	2.119639	2.139321	2.135979
2.134563	2.130731	2.132339	2.128605	2.124994	2.135809	2.132555	2.138083	2.134794
2.133122	2.129334	2.130996	2.127306	2.123734	2.134549	2.131316	2.136868	2.133628
2.131668	2.12793	2.129659	2.126031	2.122512	2.133316	2.130083	2.135647	2.132456
2.130177	2.126488	2.142468	2.138893	2.135413	2.132057	2.128807	2.134375	2.131243
2.12868	2.125041	2.141074	2.137577	2.13416	2.130803	2.127527	2.133089	2.130007
2.142011	2.138405	2.139744	2.136303	2.132932	2.129592	2.126318	2.131852	2.128754
2.140631	2.137072	2.138467	2.135063	2.131725	2.128418	2.125168	2.130655	2.127484
2.139256	2.135726	2.137167	2.133809	2.130517	2.136008	2.132728	2.129478	2.126261
2.137873	2.134347	2.135824	2.132528	2.129302	2.134819	2.131566	2.128325	2.125098
2.136517	2.132999	2.13451	2.131266	2.128098	2.133633	2.130406	2.127177	2.123948
2.135198	2.131712	2.133263	2.130048	2.126909	2.132451	2.129244	2.126023	2.122793
2.133911	2.130461	2.13205	2.128856	2.125736	2.131274	2.128081	2.124868	2.12164
2.132662	2.129235	2.139674	2.136456	2.133297	2.130108	2.126919	2.123711	2.120486
2.131443	2.128033	2.138476	2.135277	2.132133	2.128948	2.125758	2.122553	2.119333
2.130221	2.126836	2.137287	2.134104	2.130971	2.127789	2.124598	2.130252	2.126771
2.128999	2.125646	2.136108	2.132937	2.129811	2.12663	2.123439	2.129014	2.125553
2.136957	2.133573	2.13493	2.13177	2.12865	2.125472	2.122281	2.12778	2.124341
2.135727	2.132368	2.13375	2.130601	2.127488	2.124315	2.121128	2.126553	2.123138
2.134503	2.131166	2.132569	2.129428	2.126321	2.123153	2.119972	2.125326	2.121935
2.133288	2.129964	2.131383	2.128245	2.125138	2.130928	2.127489	2.135476	2.131949
2.132081	2.128767	2.130201	2.127061	2.123954	2.129663	2.126238	2.134159	2.130679
2.130881	2.127582	2.129032	2.125892	2.122783	2.128417	2.125007	2.132879	2.129424
2.129689	2.126409	2.127878	2.124735	2.121624	2.127184	2.123791	2.131625	2.128179
2.12851	2.125271	2.126769	2.123613	2.120482	2.125961	2.12258	2.13038	2.126944
2.127342	2.124173	2.125715	2.12253	2.119362	2.124751	2.121378	2.129143	2.125722
2.126208	2.123074	2.133847	2.130388	2.126939	2.134897	2.131374	2.127907	2.124496
2.125131	2.121968	2.132637	2.129166	2.125715	2.1336	2.130114	2.126665	2.12326
2.124056	2.120851	2.131409	2.127935	2.124491	2.132313	2.128859	2.125427	2.122028
2.127714	2.124466	2.130187	2.126717	2.12328	2.131058	2.127613	2.124192	2.120807
2.126574	2.12332	2.12896	2.125502	2.122076	2.12982	2.126375	2.122967	2.119601
2.1347	2.131197	2.127723	2.124279	2.120869	2.128583	2.125147	2.127575	2.124187
2.133458	2.12995	2.126478	2.123051	2.119661	2.127348	2.123929	2.126385	2.123058
2.13221	2.128711	2.12525	2.121839	2.118467	2.126141	2.122754	2.134269	2.130751
2.130947	2.127474	2.124037	2.120636	2.11728	2.124963	2.121635	2.13308	2.129504
2.129708	2.126272	2.134139	2.130612	2.127163	2.129693	2.126333	2.131865	2.128233
2.128511	2.125133	2.133004	2.129507	2.126065	2.128557	2.125154	2.130586	2.126934

2.127349	2.124019	2.131893	2.12842	2.124976	2.127415	2.123953	2.129281	2.125622
2.126246	2.122894	2.136761	2.13322	2.129705	2.126208	2.122732	2.132415	2.128689
2.125177	2.121766	2.135526	2.131974	2.128458	2.124965	2.121496	2.131077	2.12736
2.130784	2.127284	2.134293	2.130736	2.127218	2.123726	2.120263	2.121501	2.118037
2.129564	2.12608	2.133057	2.129497	2.125973	2.122482	2.119029	2.120279	2.116875
2.138988	2.135412	2.131862	2.128312	2.12479	2.121305	2.117858	2.119094	2.115703
2.137756	2.134241	2.130739	2.127215	2.123708	2.120236	2.11679	2.117964	2.114508
2.136555	2.133073	2.129603	2.126106	2.122619	2.119158	2.115712	2.116828	2.11331
2.135408	2.131886	2.128392	2.124908	2.121439	2.122654	2.119145	2.115628	2.112104
2.134273	2.130689	2.127156	2.123686	2.120241	2.121439	2.117932	2.114423	2.110914
2.133073	2.129466	2.125933	2.122505	2.119108	2.120298	2.116798	2.113301	2.109804
2.131836	2.128229	2.124716	2.121347	2.118009	2.119197	2.115709	2.112226	2.108745
2.130662	2.127064	2.123559	2.120198	2.116867	2.118037	2.114558	2.11109	2.107627
2.129561	2.12598	2.12247	2.119061	2.115681	2.116813	2.113341	2.109885	2.106442
2.128427	2.124864	2.121353	2.117906	2.114489	2.115596	2.112143	2.108706	2.105286
2.12721	2.123661	2.120157	2.1167	2.11328	2.114397	2.110991	2.10759	2.1042
2.125999	2.122467	2.118976	2.11552	2.112104	2.113227	2.109853	2.10648	2.103114
2.124881	2.121374	2.122523	2.119018	2.115546	2.112112	2.108711	2.110694	2.107285
2.12379	2.120312	2.121456	2.117968	2.114498	2.11103	2.107579	2.109533	2.106116
2.12262	2.119163	2.120309	2.116856	2.113414	2.109952	2.106494	2.108434	2.105017
2.121394	2.117954	2.119108	2.115704	2.112311	2.108881	2.105445	2.107384	2.103969
2.124897	2.12141	2.117964	2.114564	2.111177	2.107759	2.104339	2.106276	2.102869
2.123849	2.120359	2.116892	2.113437	2.109998	2.106567	2.103153	2.105088	2.101693
2.122817	2.119322	2.115834	2.112332	2.108848	2.110799	2.107344	2.103925	2.100542
2.121781	2.118285	2.114791	2.111279	2.107786	2.109719	2.106262	2.102846	2.099471
2.12072	2.117222	2.113727	2.110217	2.106727	2.108645	2.105188	2.101779	2.098417
2.119557	2.116061	2.112566	2.109059	2.105572	2.107485	2.104036	2.100647	2.097315
2.118347	2.114852	2.11136	2.107858	2.10438	2.106292	2.102856	2.099495	2.096203
2.117227	2.113728	2.110234	2.106738	2.103268	2.105175	2.101749	2.105362	2.102033
2.116184	2.112673	2.109174	2.105685	2.102226	2.104123	2.100706	2.104317	2.101021
2.115161	2.111643	2.108139	2.104648	2.10119	2.110138	2.106651	2.118736	2.115254
2.114162	2.110643	2.107134	2.103631	2.100163	2.109069	2.105599	2.117632	2.114185
2.113154	2.109636	2.111541	2.107972	2.104441	2.110526	2.107045	2.116507	2.113085
2.112122	2.108607	2.11049	2.106885	2.103322	2.109355	2.105897	2.115313	2.111896
2.111058	2.107546	2.109411	2.105776	2.102191	2.10818	2.104746	2.114115	2.110693
2.109907	2.106394	2.117998	2.114261	2.11059	2.107078	2.103672	2.113913	2.110473
2.108701	2.105186	2.116748	2.113084	2.109489	2.106023	2.102648	2.112853	2.109404
2.110595	2.107039	2.115564	2.111945	2.108402	2.104979	2.101638	2.111808	2.108351
2.10953	2.105973	2.114445	2.110845	2.107329	2.103945	2.100646	2.110783	2.107317
2.120742	2.117016	2.113356	2.109775	2.106283	2.102929	2.099659	2.109757	2.106283
2.119657	2.115943	2.1123	2.108743	2.105274	2.101934	2.098673	2.108724	2.105241
2.118578	2.114879	2.111257	2.107727	2.104281	2.100949	2.097688	2.107689	2.104197
2.117492	2.113808	2.110207	2.106709	2.10329	2.112663	2.109249	2.10665	2.103151
2.116406	2.112737	2.109158	2.105694	2.102305	2.111643	2.108219	2.105607	2.102103
2.115318	2.111665	2.108108	2.104678	2.101318	2.110619	2.107184	2.105587	2.102045
2.114228	2.110592	2.107059	2.103663	2.100329	2.109593	2.106145	2.104523	2.100979
2.113143	2.109526	2.106016	2.102651	2.099342	2.110492	2.106987	2.103452	2.099896
2.112061	2.108464	2.104978	2.101643	2.098357	2.109447	2.105927	2.102376	2.098799
2.112154	2.108578	2.11687	2.113405	2.10999	2.108399	2.104861	2.101284	2.097673
2.111123	2.107577	2.115846	2.112388	2.108969	2.107345	2.103782	2.100162	2.096489

2.110104	2.106592	2.11484	2.11138	2.107947	2.106288	2.102704	2.099043	2.095312
2.109075	2.105601	2.115048	2.111547	2.108063	2.105233	2.101639	2.097962	2.09421
2.108046	2.10461	2.114022	2.110516	2.107018	2.104177	2.100581	2.096905	2.093157
2.107019	2.103618	2.114442	2.110925	2.107408	2.103116	2.099522	2.095867	2.092157
2.105993	2.102624	2.113411	2.109883	2.106352	2.10205	2.098464	2.09485	2.091211
2.106565	2.10318	2.112378	2.108843	2.105298	2.100983	2.097396	2.09381	2.090223
2.105554	2.102178	2.111345	2.107805	2.104251	2.099914	2.096316	2.092737	2.089172
2.104549	2.101177	2.110305	2.106761	2.103201	2.10036	2.096744	2.091655	2.088103
2.103547	2.100169	2.109255	2.105706	2.10214	2.099283	2.09566	2.090572	2.08703
2.102553	2.099163	2.108202	2.104646	2.101074	2.098202	2.094575	2.089488	2.085955
2.101567	2.098161	2.107151	2.103584	2.100005	2.09712	2.093495	2.088416	2.0849
2.100594	2.097167	2.106104	2.102522	2.098933	2.096036	2.092416	2.08735	2.083857
2.099674	2.096211	2.105821	2.1022	2.098578	2.094947	2.091332	2.087796	2.084329
2.098813	2.095294	2.104806	2.101142	2.097492	2.093851	2.090241	2.086728	2.0833
2.097922	2.094382	2.103825	2.100117	2.096427	2.092771	2.089161	2.085665	2.082271
2.096981	2.093481	2.102909	2.099144	2.095388	2.091703	2.088084	2.084598	2.081234
2.095999	2.092547	2.101967	2.09817	2.094373	2.09067	2.087042	2.083561	2.080216
2.094958	2.091528	2.100919	2.097165	2.093406	2.089713	2.086082	2.082595	2.07924
2.093884	2.090464	2.099816	2.096132	2.092444	2.08878	2.085155	2.081665	2.078292
2.092819	2.089394	2.098693	2.095041	2.091394	2.087771	2.084187	2.080724	2.077368
2.091758	2.088314	2.097545	2.093903	2.090282	2.086712	2.083193	2.07978	2.076464
2.091688	2.088198	2.096426	2.092782	2.089178	2.085646	2.082177	2.078801	2.075515
2.090742	2.087198	2.09533	2.091673	2.088074	2.084564	2.081131	2.07778	2.074512
2.089781	2.086214	2.094281	2.090617	2.087021	2.083524	2.080108	2.076768	2.073507
2.088768	2.085246	2.093316	2.089665	2.08607	2.082564	2.079132	2.075777	2.0725
2.08774	2.084275	2.092355	2.088723	2.085134	2.081626	2.078183	2.074814	2.071519
2.086711	2.083255	2.091305	2.08769	2.084124	2.08066	2.077265	2.073917	2.07062
2.085688	2.082222	2.090225	2.086624	2.083089	2.079685	2.076364	2.073051	2.069759
2.096475	2.092834	2.089225	2.085621	2.082089	2.078707	2.075414	2.084828	2.081233
2.095536	2.091903	2.08829	2.084669	2.081117	2.077723	2.074424	2.083767	2.080188
2.094557	2.090931	2.087327	2.083721	2.080182	2.076787	2.073483	2.082758	2.07919
2.093508	2.089888	2.086314	2.082774	2.079298	2.075923	2.072617	2.081835	2.078268
2.092484	2.088871	2.08532	2.081834	2.078408	2.075048	2.071739	2.080895	2.077328
2.091553	2.087945	2.084393	2.080904	2.077472	2.074105	2.07079	2.079868	2.076309
2.090639	2.087037	2.083482	2.07998	2.076533	2.073156	2.069834	2.078827	2.07527
2.089656	2.086062	2.082525	2.079056	2.075637	2.072268	2.06894	2.077847	2.074266
2.088643	2.085061	2.081551	2.078134	2.074764	2.071409	2.068076	2.076899	2.073281
2.087702	2.084131	2.080631	2.077218	2.07385	2.070499	2.067169	2.075937	2.072324
2.086831	2.083273	2.079764	2.076307	2.072896	2.069536	2.066217	2.07496	2.071395
2.085955	2.082402	2.078886	2.075408	2.071977	2.068613	2.0653	2.074008	2.07048
2.085064	2.081494	2.077974	2.074521	2.071119	2.067761	2.064448	2.073102	2.069586
2.084154	2.080582	2.077072	2.07365	2.070275	2.06692	2.063598	2.072191	2.068679
2.083197	2.07968	2.076212	2.072806	2.069431	2.066055	2.062701	2.071222	2.067716
2.082232	2.078787	2.075367	2.071967	2.068581	2.065182	2.061797	2.070246	2.066742
2.081331	2.077915	2.08764	2.083907	2.080202	2.076529	2.072895	2.069331	2.065833
2.080475	2.077059	2.086689	2.08294	2.079224	2.075584	2.071999	2.06846	2.064969
2.079619	2.076181	2.08572	2.081978	2.078274	2.074665	2.071117	2.067595	2.064105
2.078766	2.075273	2.084725	2.081012	2.077351	2.073773	2.070252	2.066741	2.063247
2.077904	2.074381	2.083764	2.08008	2.076454	2.072896	2.069388	2.071322	2.067608
2.077015	2.073517	2.082867	2.079203	2.075591	2.072037	2.068524	2.070388	2.06664

2.076133	2.072674	2.081998	2.078347	2.07474	2.071179	2.06765	2.069444	2.065675
2.07529	2.071854	2.081131	2.077489	2.073882	2.070301	2.066746	2.068493	2.064756
2.074472	2.071052	2.080274	2.076632	2.073019	2.069413	2.06583	2.067543	2.063862
2.073666	2.070255	2.079412	2.075754	2.072125	2.068517	2.06494	2.066628	2.06298
2.072874	2.069466	2.078549	2.07486	2.071205	2.067612	2.06407	2.065744	2.062113
2.072082	2.068673	2.077689	2.073985	2.070318	2.066736	2.063215	2.064863	2.06124
2.07129	2.067878	2.07684	2.073139	2.069476	2.065898	2.062379	2.063988	2.060363
2.070491	2.067063	2.075969	2.072283	2.068639	2.065067	2.061548	2.063107	2.059469
2.069672	2.066199	2.075038	2.07139	2.067789	2.06423	2.060708	2.062203	2.058533
2.068853	2.065334	2.074104	2.070497	2.066941	2.063396	2.059873	2.061301	2.057597
2.06806	2.064525	2.073239	2.069644	2.066103	2.067876	2.064155	2.060434	2.056715
2.067282	2.06375	2.072414	2.068816	2.065274	2.067012	2.063306	2.059589	2.055867
2.066504	2.06298	2.071596	2.067998	2.064456	2.06616	2.062464	2.05875	2.055025
2.065728	2.062216	2.070789	2.067193	2.06365	2.065318	2.06163	2.05792	2.054194
2.06141	2.057904	2.069983	2.066389	2.062846	2.064479	2.060799	2.057092	2.053368
2.06063	2.057133	2.069179	2.065587	2.062043	2.063642	2.059969	2.056267	2.052545
2.059848	2.05636	2.068373	2.064784	2.06124	2.062805	2.059141	2.055444	2.051725
2.059065	2.055584	2.067563	2.063977	2.060436	2.061969	2.058314	2.054623	2.050908
2.058282	2.054807	2.06675	2.063169	2.059631	2.061133	2.057488	2.053804	2.050092
2.057504	2.054029	2.062617	2.059023	2.055471	2.060297	2.05666	2.052981	2.049271
2.05673	2.053252	2.06179	2.0582	2.054653	2.05946	2.055832	2.052157	2.048448
2.055961	2.052484	2.066645	2.062849	2.05911	2.05863	2.055008	2.051337	2.047632
2.055197	2.051722	2.065797	2.062007	2.058272	2.057806	2.054186	2.050519	2.046819
2.054442	2.050984	2.064986	2.061191	2.057447	2.053773	2.05014	2.049719	2.046037
2.053706	2.050294	2.064244	2.060417	2.056638	2.052959	2.049336	2.048953	2.045316
2.052966	2.0496	2.063501	2.059647	2.055836	2.05215	2.048534	2.048188	2.044596
2.052198	2.04885	2.06269	2.05884	2.055027	2.051334	2.047712	2.047388	2.043819
2.05142	2.048076	2.061851	2.058021	2.054225	2.050525	2.046887	2.046575	2.043019
2.050665	2.047314	2.061029	2.057235	2.053469	2.049755	2.046085	2.045776	2.042226
2.049927	2.046559	2.060215	2.056473	2.052752	2.049019	2.045302	2.044985	2.041435
2.055318	2.051698	2.059394	2.055685	2.051996	2.048253	2.04451	2.040866	2.037305
2.054621	2.050942	2.058562	2.05486	2.051187	2.047444	2.043698	2.040061	2.036513
2.053905	2.050183	2.057736	2.054038	2.050375	2.046644	2.042913	2.039282	2.035735
2.053122	2.049399	2.056913	2.053226	2.049578	2.045882	2.04219	2.038558	2.034976
2.052328	2.048626	2.056113	2.052428	2.048786	2.045128	2.04148	2.03785	2.034237
2.051575	2.047909	2.044314	2.040811	2.037362	2.033947	2.030565	2.03712	2.03353
2.050846	2.047225	2.043651	2.040114	2.036626	2.0332	2.029822	2.03638	2.03284
2.050141	2.046527	2.042958	2.039426	2.035937	2.032495	2.029097	2.035624	2.032107
2.049464	2.045821	2.042241	2.03875	2.035296	2.03183	2.028385	2.034852	2.031336
2.048743	2.045088	2.041508	2.038046	2.03462	2.031155	2.027697	2.034112	2.030579
2.04795	2.044312	2.040748	2.037295	2.033883	2.030459	2.027046	2.033423	2.029847
2.047154	2.043547	2.040006	2.036549	2.033137	2.029751	2.026387	2.032736	2.029133
2.046394	2.042838	2.039325	2.03584	2.032397	2.029016	2.025671	2.03202	2.028446
2.045656	2.042152	2.038665	2.03515	2.031673	2.028285	2.024949	2.031299	2.02777
2.044954	2.041457	2.037979	2.034476	2.031009	2.027624	2.024287	2.030607	2.027077
2.044283	2.040765	2.037283	2.033817	2.030386	2.027007	2.023661	2.02993	2.026375
2.043605	2.040091	2.036611	2.03315	2.029722	2.026344	2.022997	2.029238	2.025685
2.042922	2.039438	2.035968	2.032473	2.02901	2.025627	2.022289	2.028527	2.025007
2.042251	2.03878	2.035315	2.031809	2.028333	2.024946	2.021609	2.02783	2.024328
2.041602	2.038103	2.034629	2.031161	2.027723	2.024339	2.020989	2.027163	2.023643

2.040955	2.037432	2.033955	2.030529	2.027132	2.023747	2.020376	2.0265	2.022964
2.040282	2.036789	2.033337	2.029927	2.026535	2.023128	2.019727	2.016346	2.012982
2.039607	2.036163	2.032743	2.029335	2.025931	2.0225	2.019067	2.015688	2.012353
2.038977	2.035552	2.032138	2.028713	2.02529	2.021857	2.018431	2.015051	2.01171
2.03838	2.034955	2.031528	2.028069	2.024619	2.021203	2.017809	2.014427	2.011058
2.037775	2.034332	2.030887	2.027417	2.02396	2.020549	2.017165	2.013786	2.010417
2.037161	2.033673	2.030204	2.026756	2.023323	2.019896	2.016487	2.01312	2.009792
2.036531	2.033017	2.029534	2.026097	2.022678	2.019245	2.015823	2.012467	2.009166
2.035866	2.032376	2.028903	2.025442	2.022001	2.018585	2.015194	2.01184	2.00852
2.035204	2.03175	2.028293	2.024803	2.021336	2.017941	2.014587	2.011234	2.007888
2.034594	2.031156	2.027707	2.024211	2.020738	2.017352	2.014013	2.010663	2.007313
2.034013	2.030581	2.027135	2.023646	2.020178	2.016795	2.013457	2.010112	2.006769
2.03344	2.029984	2.026529	2.023057	2.019607	2.016215	2.01286	2.009523	2.006207
2.032875	2.02937	2.02589	2.022449	2.01903	2.015616	2.012226	2.0089	2.005631
2.032327	2.028789	2.025293	2.021869	2.018469	2.015042	2.011629	2.008308	2.005067
2.031808	2.028263	2.024761	2.021338	2.017939	2.014508	2.011089	2.007767	2.004527
2.03127	2.027733	2.024235	2.020802	2.017393	2.013968	2.010558	2.007231	2.003975
2.030662	2.027157	2.023675	2.020219	2.016788	2.013383	2.010007	2.006671	2.003374
2.030043	2.026576	2.023115	2.01963	2.016174	2.012794	2.009457	2.006112	2.002768
2.029486	2.026036	2.022582	2.019086	2.015619	2.012249	2.008928	2.005579	2.002215
2.028968	2.025521	2.022069	2.018571	2.015103	2.011733	2.008413	2.005064	2.001697
2.028455	2.02501	2.02156	2.018063	2.014595	2.011224	2.007903	2.004551	2.001181
2.027947	2.024504	2.021057	2.017565	2.014099	2.010725	2.007397	2.004041	2.000669
2.027442	2.023999	2.020554	2.017066	2.013603	2.010227	2.006894	2.003534	2.000157
2.026936	2.023493	2.020048	2.016563	2.013102	2.009728	2.006396	2.003031	1.999645
2.026429	2.022987	2.019542	2.016057	2.012597	2.009223	2.005891	2.002522	1.999128
2.025921	2.022481	2.019038	2.015549	2.012084	2.008707	2.00537	2.002001	1.998609
2.02541	2.021975	2.018531	2.015037	2.011565	2.008178	2.004832	2.001463	1.998081
2.024896	2.021461	2.018016	2.014517	2.011034	2.007622	2.004248	2.000879	1.997519
2.024378	2.020942	2.017492	2.013989	2.010494	2.007045	2.003629	2.000259	1.996933
2.023858	2.020419	2.016967	2.013463	2.009967	2.006513	2.00309	1.999719	1.996396
2.023337	2.019891	2.016438	2.01294	2.009458	2.006039	2.002656	1.999283	1.995923
2.022814	2.019365	2.015911	2.012417	2.008946	2.00556	2.002215	1.998841	1.995449
2.022291	2.018842	2.015389	2.011892	2.008422	2.005049	2.001722	1.99835	1.994947
2.021765	2.018319	2.014866	2.011366	2.007895	2.004529	2.001211	1.997842	1.994438
2.021234	2.01779	2.014339	2.01084	2.007369	2.004007	2.000694	1.997328	1.993927
2.020702	2.017259	2.01381	2.010314	2.006847	2.003483	2.000169	1.996807	1.993413
2.020182	2.016733	2.013284	2.009795	2.006335	2.002969	1.999648	1.996285	1.992894
2.01967	2.016211	2.012758	2.009282	2.005833	2.002463	1.999131	1.995763	1.992371
2.019196	2.015716	2.012254	2.008799	2.005369	2.001988	1.998634	1.995255	1.99186
2.018782	2.01526	2.011779	2.008358	2.004961	2.001561	1.998166	1.994765	1.991358
2.018356	2.014804	2.011308	2.007914	2.004541	2.001125	1.997699	1.994286	1.99088
2.017871	2.014317	2.010821	2.00743	2.00406	2.000641	1.997214	1.99382	1.990453
2.017371	2.013833	2.010344	2.006943	2.003561	2.000143	1.996724	1.993358	1.990036
2.016903	2.013396	2.009922	2.006497	2.003087	1.999656	1.996232	1.992871	1.989565
2.01645	2.012985	2.00953	2.006073	2.002624	1.999176	1.995743	1.992376	1.989066
2.01598	2.012533	2.009088	2.005622	2.002163	1.998722	1.995301	1.991929	1.9886
2.01549	2.012037	2.008593	2.00514	2.001702	1.998296	1.994911	1.991534	1.988165
2.015033	2.011565	2.008116	2.00468	2.001264	1.997876	1.994505	1.991127	1.987744
2.014634	2.011135	2.007672	2.004259	2.000866	1.997463	1.994067	1.990695	1.987344

2.014239	2.010722	2.007248	2.003848	2.000464	1.99704	1.993613	1.990246	1.98693
2.017597	2.014078	2.00686	2.003438	2.000024	1.996579	1.993135	1.989764	1.986454
2.017157	2.01368	2.006479	2.003022	1.999566	1.996105	1.992655	1.989276	1.985957
2.016693	2.013228	2.006032	2.002567	1.999106	1.995657	1.992224	1.988838	1.985495
2.016212	2.012737	2.00554	2.002085	1.998642	1.995226	1.991827	1.988437	1.985056
2.01578	2.012282	2.008804	2.005335	2.001881	1.994806	1.991419	1.988027	1.984635
2.01541	2.011867	2.008366	2.004917	2.001486	1.9944	1.990993	1.987606	1.984238
2.01503	2.011465	2.00795	2.004508	2.001087	1.997635	1.994182	1.987193	1.983837
2.014603	2.011071	2.007571	2.004105	2.00066	1.997222	1.993794	1.986801	1.98342
2.014175	2.010678	2.007194	2.003705	2.000236	1.99681	1.993406	1.986407	1.983001
2.013786	2.010269	2.006776	2.0033	1.999844	1.996407	1.992984	1.985985	1.982596
2.013413	2.009856	2.006344	2.002894	1.999463	1.996006	1.99255	1.985551	1.982194
2.013005	2.009459	2.005951	2.002495	1.999055	1.995596	1.992139	1.988716	1.985321
2.012571	2.009074	2.00559	2.0021	1.998624	1.995176	1.991747	1.988319	1.984894
2.012179	2.008716	2.005247	2.001734	1.998231	1.994777	1.991348	1.987913	1.984476
2.011851	2.008399	2.004933	2.001413	1.997893	1.994402	1.99093	1.987488	1.984074
2.010734	2.00728	2.004597	2.001083	1.997561	1.994042	1.990533	1.98708	1.983674
2.010376	2.006893	2.004196	2.000711	1.997221	1.993707	1.990189	1.986709	1.98326
2.010001	2.00649	2.003781	2.000323	1.996865	1.99337	1.989859	1.986357	1.982857
2.009596	2.006103	2.003402	1.999929	1.996461	1.992986	1.989507	1.986013	1.982503
2.009176	2.005725	2.003044	1.999533	1.996028	1.992575	1.989142	1.985674	1.982179
2.008812	2.00538	2.002706	1.999174	1.995646	1.992183	1.988747	1.985292	1.981825
2.008503	2.005072	2.002395	1.998857	1.995313	1.991804	1.988316	1.984864	1.981446
2.008183	2.004737	2.001268	1.997741	1.994204	1.991441	1.987918	1.995486	1.992011
2.007832	2.004346	2.000858	1.997362	1.993859	1.991107	1.987578	1.995117	1.991587
2.007482	2.003957	2.000449	1.99697	1.993493	1.990762	1.987248	1.994767	1.991182
2.007156	2.003615	2.000085	1.996571	1.993071	1.990367	1.9869	1.994434	1.990839
2.00683	2.003288	1.999741	1.996177	1.992638	1.989962	1.98655	1.994111	1.990525
2.001815	1.998396	1.999405	1.995828	1.992278	1.989608	1.98621	1.993783	1.990207
2.001418	1.99804	1.999079	1.995515	1.991966	1.989288	1.985875	1.993451	1.989889
2.001064	1.997709	1.998761	1.995203	1.99165	1.988949	1.985506	1.993082	1.98955
2.00076	1.997406	1.998451	1.994891	1.991328	1.988585	1.985093	1.992664	1.989183
2.000469	1.997111	1.998145	1.994585	1.991014	1.987463	1.983934	1.992274	1.988829
2.000186	1.996823	1.997848	1.994288	1.990716	1.987155	1.983614	1.991949	1.988505
1.999905	1.996534	1.997547	1.993989	1.99042	1.986859	1.983315	1.991646	1.988194
1.999615	1.996235	1.997238	1.993683	1.990117	1.998051	1.994425	1.991344	1.98789
1.999319	1.995925	1.996916	1.993366	1.989809	1.997748	1.994127	1.991047	1.987593
1.999008	1.995575	1.996539	1.993013	1.989487	1.99744	1.993826	1.99075	1.987295
1.998683	1.995189	1.996115	1.992628	1.989152	1.997125	1.993522	1.990453	1.986998
1.998377	1.994847	1.991374	1.988004	1.984654	1.996812	1.993219	1.989703	1.986252
1.998093	1.994558	1.991079	1.987701	1.984345	1.996499	1.992916	1.989407	1.98596
1.996751	1.993218	1.990829	1.987432	1.984056	1.996196	1.992618	1.989111	1.985667
1.996501	1.993011	1.99064	1.987215	1.983803	1.995916	1.992331	1.988819	1.985371
1.996252	1.992811	1.990462	1.98701	1.98356	1.995643	1.992048	1.988527	1.985073
1.995977	1.992557	1.990221	1.986761	1.983298	1.991742	1.988227	1.98823	1.984773
1.995687	1.992274	1.989945	1.986489	1.983027	1.991458	1.987935	1.987933	1.984473
1.995397	1.991986	1.988562	1.985095	1.981621	1.991176	1.98765	1.987642	1.984177
1.995105	1.991691	1.988268	1.984809	1.981342	1.990895	1.987369	1.987356	1.983884
1.987004	1.983624	1.987978	1.984522	1.981064	1.990629	1.987122	1.983662	1.980243
1.986728	1.983343	1.987693	1.984234	1.980783	1.990387	1.986934	1.983475	1.980016

1.986478	1.983078	1.987418	1.983954	1.980508	1.990143	1.986737	1.98328	1.979787
1.984643	1.98119	1.999601	1.996037	1.99251	1.989064	1.985656	1.98302	1.979521
1.984478	1.980982	1.999342	1.995773	1.992246	1.988791	1.985373	1.982737	1.979244
1.984278	1.980766	1.998147	1.994598	1.991089	1.988571	1.985121	1.982471	1.978973
1.984058	1.98055	1.997928	1.994418	1.990938	1.988386	1.984888	1.982213	1.978706
1.98384	1.980355	1.990622	1.987157	1.98371	1.98724	1.983713	1.981135	1.977627
1.983626	1.980186	1.990471	1.986978	1.983494	1.987014	1.983482	1.980909	1.977446
1.983408	1.98	1.990294	1.986781	1.983274	1.986798	1.983276	1.979797	1.976354
1.983173	1.979771	1.990057	1.986546	1.983047	1.986603	1.983118	1.979637	1.976162
1.982954	1.979545	1.989813	1.986314	1.982834	1.986418	1.982963	1.979475	1.975962
1.982795	1.979358	1.989598	1.986124	1.982669	1.986246	1.98277	1.979269	1.975747
1.982664	1.979194	1.989402	1.985955	1.982524	1.986077	1.98256	1.979044	1.975528
1.982517	1.979049	1.989244	1.985784	1.982337	1.985877	1.982345	1.978835	1.975343
1.98236	1.978922	1.989119	1.985615	1.982121	1.985653	1.982124	1.978636	1.975184
1.982214	1.978777	1.988957	1.985425	1.981906	1.978404	1.974921	1.978458	1.975019
1.982087	1.978609	1.988746	1.985203	1.981688	1.978214	1.974767	1.978307	1.974843
1.995763	1.992129	1.988532	1.984991	1.98149	1.978036	1.974611	1.994778	1.991079
1.995571	1.991923	1.988325	1.984811	1.981334	1.977873	1.974431	1.994578	1.99091
1.995379	1.991725	1.988133	1.984644	1.981189	1.977715	1.974251	1.994378	1.990745
1.995243	1.991587	1.987987	1.984481	1.981014	1.977549	1.974102	1.994201	1.990549
1.995132	1.991477	1.987865	1.984324	1.980828	1.977381	1.973966	1.994033	1.990339
1.994963	1.991308	1.987697	1.984158	1.980664	1.980065	1.976626	1.990149	1.986445
1.994738	1.991084	1.987487	1.983985	1.980522	1.979911	1.97644	1.989929	1.98628
1.994548	1.990901	1.990238	1.986725	1.983249	1.979756	1.976273	1.989724	1.986098
1.99442	1.990787	1.990126	1.986578	1.983071	1.979593	1.976141	1.98955	1.985883
1.994293	1.990681	1.99003	1.986451	1.982914	1.979442	1.976008	1.989373	1.985672
1.99412	1.990553	1.989939	1.986365	1.982817	1.979314	1.975843	1.989161	1.985492
1.993943	1.990415	1.989841	1.986288	1.982743	1.979203	1.97568	1.988948	1.985321
1.99381	1.990262	1.989688	1.986166	1.982651	1.979111	1.975573	1.988777	1.985127
1.993705	1.990101	1.989501	1.986017	1.98255	1.979032	1.975502	1.988636	1.984924
1.993602	1.989974	1.989361	1.985873	1.982412	1.978916	1.975411	1.988504	1.984765
1.993506	1.98989	1.989272	1.985733	1.98223	1.978756	1.975303	1.98839	1.984656
1.993379	1.989789	1.989182	1.98561	1.982076	1.978607	1.975175	1.988248	1.984539
1.993191	1.989649	1.989086	1.985522	1.98198	1.978481	1.975011	1.988052	1.984402
1.993006	1.989498	1.988973	1.985435	1.981903	1.978373	1.974857	1.987853	1.984246
1.985003	1.981497	1.988812	1.985315	1.981825	1.978295	1.97476	1.987688	1.984049
1.9849	1.981341	1.988637	1.985186	1.981749	1.978233	1.974693	1.987542	1.98384
1.984802	1.981224	1.988522	1.985087	1.981669	1.978164	1.974629	1.98742	1.983687
1.984713	1.981138	1.98845	1.985008	1.981585	1.978092	1.974573	1.987319	1.983575
1.984629	1.981059	1.980778	1.977339	1.973924	1.977988	1.9745	1.987212	1.983466
1.984553	1.980986	1.980696	1.977206	1.973751	1.977841	1.974404	1.987096	1.98336
1.984475	1.980913	1.980621	1.977089	1.973601	1.977711	1.974313	1.986981	1.983256
1.984397	1.980842	1.980557	1.977012	1.973512	1.977632	1.974244	1.986869	1.983154
1.984305	1.980763	1.980494	1.976951	1.973449	1.977573	1.974181	1.986759	1.983054
1.972112	1.968676	1.980421	1.976888	1.973391	1.969985	1.96662	1.986659	1.982963
1.97194	1.968548	1.980344	1.976829	1.97334	1.969928	1.966551	1.986557	1.982871
1.967758	1.964323	1.97674	1.973248	1.969772	1.986842	1.983193	1.986398	1.982747
1.967662	1.96423	1.976664	1.973179	1.969697	1.986683	1.982975	1.986186	1.98259
1.967579	1.964148	1.976597	1.973115	1.969631	1.983555	1.979809	1.979156	1.97562
1.96751	1.964079	1.976542	1.973062	1.969578	1.983444	1.979677	1.979023	1.975497

1.967444	1.964011	1.960574	1.957103	1.953623	1.983346	1.979572	1.975936	1.972417
1.967367	1.963934	1.960498	1.957028	1.95355	1.983254	1.979483	1.975844	1.972319
1.967292	1.963857	1.96042	1.956951	1.953478	1.983176	1.979422	1.975781	1.972238
1.967223	1.963782	1.960343	1.956876	1.953412	1.983125	1.979417	1.975774	1.972189
1.967163	1.963712	1.960269	1.956803	1.953352	1.983095	1.979452	1.975806	1.972164
1.967155	1.963677	1.960212	1.956739	1.953291	1.976561	1.972916	1.9758	1.972126
1.967209	1.963682	1.960176	1.956685	1.95323	1.976485	1.972859	1.975756	1.972077
1.96723	1.96367	1.960134	1.956632	1.953174	1.976405	1.972788	1.975696	1.972016
1.967185	1.963619	1.960078	1.956574	1.953114	1.976316	1.972702	1.97562	1.971938
1.967116	1.963554	1.960019	1.956527	1.953075	1.976244	1.972618	1.975539	1.971856
1.967044	1.963482	1.959961	1.956511	1.953093	1.968449	1.964823	1.968866	1.965152
1.966967	1.963407	1.959904	1.956507	1.95313	1.968438	1.964763	1.968792	1.965067
1.966904	1.963341	1.979244	1.975633	1.972046	1.96838	1.964688	1.968716	1.964983
1.966854	1.963283	1.979127	1.975525	1.97195	1.968292	1.964603	1.968642	1.964903
1.966831	1.963272	1.979073	1.975458	1.971869	1.968209	1.964525	1.968586	1.964867
1.966838	1.963318	1.979098	1.975441	1.971805	1.968129	1.964447	1.968547	1.964884
1.966867	1.963367	1.979117	1.975424	1.971752	1.968073	1.964405	1.960776	1.957182
1.96693	1.963398	1.979091	1.975379	1.971703	1.968057	1.964433	1.960807	1.957182
1.966992	1.96342	1.979056	1.97534	1.971674	1.968059	1.964473	1.96084	1.957173
1.967006	1.963421	1.979027	1.975336	1.971694	1.96807	1.964461	1.960812	1.957131
1.966996	1.963414	1.979005	1.975353	1.971737	1.968084	1.964424	1.960755	1.957076
1.951334	1.947892	1.979021	1.975366	1.971739	1.968063	1.964378	1.960711	1.957057
1.951351	1.947946	1.979073	1.97538	1.971706	1.96801	1.964319	1.960674	1.957068
1.95139	1.947996	1.979101	1.975376	1.97167	1.967972	1.964292	1.960661	1.957074
1.951463	1.948039	1.97909	1.975341	1.971624	1.967951	1.964311	1.96068	1.957065
1.951532	1.948076	1.979074	1.975317	1.971605	1.967952	1.964337	1.960705	1.957067
1.951563	1.948095	1.979056	1.975334	1.971652	1.967988	1.964346	1.960717	1.957103
1.951593	1.948119	1.979051	1.975368	1.971718	1.968036	1.964359	1.960731	1.957147
1.97058	1.966802	1.979102	1.975404	1.971742	1.968065	1.964398	1.960768	1.95717
1.970676	1.9669	1.979186	1.975445	1.971747	1.968085	1.964453	1.960817	1.957183
1.970728	1.966952	1.979229	1.975484	1.971781	1.968117	1.964482	1.960846	1.957214
1.970733	1.966953	1.979225	1.975519	1.971848	1.96816	1.964483	1.960853	1.957264
1.970768	1.96699	1.977647	1.973938	1.97026	1.968198	1.964502	1.960873	1.957301
1.970867	1.967096	1.977748	1.973992	1.970271	1.968219	1.964557	1.960921	1.957311
1.970966	1.967213	1.977867	1.974066	1.970302	1.968252	1.964614	1.960969	1.957324
1.971012	1.967305	1.977993	1.974191	1.970409	1.968318	1.964637	1.960987	1.957368
1.971041	1.967385	1.978113	1.974332	1.970549	1.968406	1.964661	1.961004	1.957423
1.971095	1.967434	1.978167	1.974423	1.970672	1.966885	1.963096	1.961069	1.957462
1.971165	1.96746	1.978172	1.97448	1.970788	1.967023	1.963236	1.96117	1.957493
1.971227	1.96751	1.978216	1.974535	1.970859	1.967119	1.963358	1.961281	1.957567
1.971273	1.967585	1.978304	1.97458	1.970868	1.967164	1.963463	1.96141	1.957696
1.969319	1.96566	1.978417	1.974653	1.970905	1.967211	1.963542	1.959851	1.956144
1.969453	1.965808	1.97857	1.974786	1.97101	1.967275	1.963567	1.959886	1.956232
1.969607	1.965959	1.978715	1.974928	1.971138	1.967357	1.963594	1.959913	1.956303
1.969746	1.966053	1.978784	1.975028	1.97127	1.967478	1.963687	1.959981	1.956347
1.969882	1.966125	1.978821	1.975109	1.971402	1.967618	1.963815	1.960075	1.956383
1.954882	1.951153	1.977316	1.9736	1.969901	1.967718	1.963947	1.960203	1.956479
1.955006	1.95128	1.977434	1.97366	1.969919	1.967783	1.964088	1.960367	1.956625
1.955101	1.951397	1.977567	1.973755	1.969984	1.967877	1.964229	1.960524	1.956777
1.955148	1.951492	1.977712	1.973896	1.970115	1.968015	1.964375	1.960676	1.956934

1.955218	1.951602	1.947981	1.944312	1.940666	1.966574	1.962925	1.960804	1.957085
1.955357	1.951753	1.948138	1.944466	1.940807	1.966685	1.962988	1.960878	1.957214
1.955527	1.951923	1.948304	1.944631	1.940959	1.9668	1.963044	1.960946	1.95734
1.955698	1.952095	1.948476	1.944804	1.941129	1.966955	1.963166	1.961068	1.957485
1.955877	1.952271	1.948652	1.944984	1.94131	1.967136	1.963327	1.961225	1.957641
1.956049	1.952439	1.948817	1.945152	1.941481	1.954013	1.950233	1.959806	1.956229
1.956215	1.952601	1.948976	1.945312	1.941642	1.954173	1.950393	1.959983	1.956403
1.955377	1.951736	1.949094	1.945442	1.941789	1.954328	1.950557	1.960164	1.956579
1.955526	1.951814	1.949135	1.945518	1.941915	1.954478	1.950724	1.960349	1.956754
1.955683	1.951908	1.949195	1.945604	1.942042	1.954626	1.95089	1.960535	1.956933
1.94983	1.946051	1.949344	1.945746	1.942183	1.954773	1.951049	1.952016	1.948441
1.95002	1.946251	1.949548	1.945926	1.942338	1.954925	1.951208	1.952186	1.948618
1.950215	1.946486	1.942795	1.939155	1.935542	1.9389	1.935294	1.947737	1.944183
1.950415	1.946752	1.943094	1.939421	1.93576	1.939089	1.935461	1.947894	1.944338
1.950599	1.946979	1.943343	1.939653	1.935966	1.939274	1.935628	1.948051	1.944493
1.950765	1.947161	1.943537	1.939849	1.936158	1.939455	1.935792	1.948207	1.944646
1.939369	1.935824	1.943714	1.940031	1.936342	1.932661	1.929005	1.948376	1.944807
1.939538	1.935987	1.94388	1.9402	1.936514	1.93284	1.92919	1.948557	1.944975
1.939712	1.936153	1.944046	1.940365	1.936685	1.933028	1.929396	1.948763	1.945158
1.939888	1.93633	1.944228	1.940541	1.936862	1.933238	1.929648	1.927273	1.923741
1.940075	1.936518	1.944419	1.940723	1.937045	1.933461	1.929922	1.927543	1.923964
1.940307	1.936729	1.944618	1.940916	1.937239	1.933668	1.930147	1.926578	1.922979
1.940579	1.936962	1.944824	1.941118	1.937443	1.933864	1.930333	1.926768	1.923181
1.940825	1.937186	1.945041	1.94135	1.937686	1.934089	1.93053	1.926956	1.923376
1.941034	1.937395	1.945267	1.941619	1.937986	1.934359	1.930742	1.927141	1.923554
1.941239	1.937616	1.945512	1.941893	1.938277	1.934623	1.930964	1.927345	1.923757
1.941456	1.93787	1.945796	1.942158	1.938518	1.934855	1.931193	1.927585	1.924022
1.941673	1.938127	1.946081	1.942415	1.938743	1.935082	1.931436	1.927845	1.924303
1.941877	1.938344	1.946315	1.94264	1.938963	1.935324	1.93171	1.92812	1.924554
1.942079	1.938543	1.946522	1.942848	1.939182	1.935577	1.932001	1.928403	1.924792
1.94233	1.938771	1.946747	1.943093	1.939449	1.935847	1.932263	1.928653	1.925023
1.942629	1.939024	1.946984	1.943372	1.939766	1.936136	1.9325	1.92887	1.925245
1.940748	1.937141	1.9357	1.932161	1.928622	1.9364	1.932729	1.929094	1.925486
1.941009	1.937427	1.935997	1.932427	1.928853	1.93662	1.932943	1.929326	1.92576
1.941258	1.9377	1.936282	1.932678	1.92907	1.936839	1.933175	1.929578	1.926039
1.94148	1.937922	1.936504	1.932891	1.929282	1.937086	1.933461	1.929867	1.926304
1.941702	1.938129	1.936705	1.933094	1.929499	1.937347	1.933767	1.930172	1.926571
1.941981	1.938371	1.934788	1.931214	1.927662	1.937621	1.934042	1.930452	1.926858
1.9423	1.938636	1.935025	1.931475	1.927951	1.937908	1.934301	1.930719	1.92716
1.942601	1.938917	1.935293	1.931748	1.928231	1.938196	1.934585	1.931006	1.927455
1.94288	1.939217	1.935601	1.932034	1.928498	1.938483	1.934899	1.931317	1.927739
1.943172	1.939514	1.935898	1.932321	1.928776	1.925257	1.921758	1.931615	1.928027
1.943494	1.939791	1.936151	1.932599	1.929079	1.925543	1.922015	1.93188	1.928325
1.943818	1.940077	1.936418	1.932883	1.929381	1.925829	1.922273	1.932146	1.92862
1.944111	1.940396	1.936745	1.933181	1.929649	1.926104	1.922568	1.932444	1.928892
1.944403	1.940739	1.937107	1.933493	1.929912	1.92638	1.922879	1.932753	1.929158
1.944758	1.941109	1.937478	1.933842	1.930234	1.926683	1.923166	1.931419	1.927836
1.945164	1.941504	1.937862	1.934224	1.930605	1.927008	1.923436	1.931692	1.928148
1.945552	1.941867	1.93821	1.934585	1.930974	1.927353	1.923747	1.932008	1.92849
1.945917	1.942185	1.938506	1.934917	1.931347	1.92773	1.924114	1.932384	1.928875

1.946263	1.942508	1.938817	1.935244	1.931694	1.928087	1.924476	1.920948	1.917487
1.946571	1.942855	1.939179	1.935564	1.931975	1.928382	1.924804	1.921271	1.917778
1.946883	1.943218	1.939563	1.935895	1.932252	1.928667	1.925117	1.921578	1.918053
1.947261	1.943611	1.939958	1.936269	1.932599	1.928988	1.925416	1.921871	1.918354
1.947675	1.944019	1.940359	1.936668	1.932986	1.929333	1.92571	1.922157	1.918667
1.948076	1.944392	1.940716	1.937043	1.933377	1.929704	1.926049	1.922485	1.918999
1.948469	1.944731	1.941029	1.937397	1.933776	1.930105	1.926431	1.922852	1.919352
1.941065	1.937308	1.941376	1.937764	1.934167	1.930497	1.926816	1.92322	1.919692
1.941454	1.937706	1.941782	1.938159	1.934548	1.930878	1.927199	1.923578	1.920004
1.941822	1.938099	1.94219	1.938542	1.934906	1.931243	1.927579	1.923942	1.920327
1.942129	1.938447	1.942563	1.938879	1.935209	1.931571	1.927951	1.924322	1.920686
1.942427	1.938788	1.94293	1.939208	1.935505	1.931893	1.928322	1.924708	1.921064
1.942798	1.939167	1.935517	1.931797	1.928095	1.932266	1.928703	1.92509	1.921442
1.943215	1.939571	1.935915	1.932203	1.928509	1.932672	1.92909	1.92547	1.921824
1.943631	1.93998	1.93632	1.932613	1.928923	1.925304	1.921718	1.925863	1.922217
1.944048	1.940394	1.936734	1.933029	1.929339	1.925716	1.922124	1.92627	1.922622
1.944466	1.940815	1.937156	1.933447	1.929753	1.926125	1.922529	1.926675	1.923024
1.944885	1.941244	1.937588	1.933868	1.930161	1.926531	1.922935	1.927081	1.923421
1.945307	1.941675	1.938021	1.93429	1.930568	1.926929	1.923326	1.927471	1.923808
1.945737	1.942102	1.938443	1.934707	1.930974	1.927302	1.923661	1.920021	1.916387
1.94617	1.942525	1.938859	1.935123	1.931383	1.927673	1.923987	1.920342	1.916735
1.941781	1.938143	1.939274	1.935546	1.931813	1.928106	1.924417	1.920764	1.917144
1.942179	1.938544	1.939688	1.935974	1.932261	1.928585	1.924928	1.921265	1.9176
1.935179	1.931577	1.940106	1.936396	1.932692	1.929038	1.925404	1.921739	1.91805
1.935587	1.931984	1.940528	1.936811	1.933102	1.929454	1.925831	1.922174	1.918493
1.936004	1.932395	1.936147	1.932428	1.928721	1.929865	1.92625	1.922601	1.918928
1.936434	1.932808	1.93656	1.932835	1.929126	1.93028	1.926675	1.923028	1.91935
1.936865	1.933222	1.936973	1.933243	1.929534	1.930696	1.927096	1.923448	1.919766
1.906908	1.903608	1.929973	1.926275	1.9226	1.926321	1.922725	1.923859	1.920176
1.907281	1.903975	1.930374	1.926682	1.92301	1.926738	1.92313	1.924264	1.920583
1.90767	1.904359	1.930793	1.927098	1.923423	1.927158	1.923541	1.919895	1.91623
1.908071	1.904757	1.931226	1.927523	1.923839	1.927579	1.923955	1.920315	1.916665
1.908511	1.905176	1.93167	1.927974	1.924295	1.920659	1.91705	1.920758	1.917132
1.909012	1.905626	1.932129	1.928462	1.924815	1.921157	1.917509	1.921239	1.917647
1.909501	1.906071	1.932589	1.92895	1.92533	1.921652	1.917969	1.921718	1.918153
1.909919	1.906478	1.933032	1.929405	1.925796	1.922105	1.918401	1.922161	1.918603
1.910315	1.906881	1.933486	1.929858	1.926243	1.922546	1.918836	1.922604	1.919037
1.910738	1.907332	1.934005	1.930344	1.926696	1.923008	1.919319	1.923097	1.919505
1.911175	1.907809	1.934562	1.930849	1.927148	1.923478	1.919832	1.923621	1.919987
1.911604	1.908249	1.935056	1.931315	1.927586	1.923928	1.920306	1.916676	1.913045
1.912021	1.908646	1.935481	1.931734	1.928004	1.924352	1.920739	1.917104	1.913455
1.90183	1.898459	1.935924	1.932186	1.928465	1.924807	1.921182	1.917541	1.913892
1.902313	1.898924	1.936409	1.932701	1.929005	1.925317	1.921643	1.917997	1.914377
1.902798	1.899402	1.936916	1.933231	1.929554	1.92584	1.922126	1.918475	1.914878
1.903241	1.899871	1.907149	1.90381	1.900493	1.926347	1.922649	1.918993	1.915374
1.903667	1.900331	1.907646	1.904281	1.900938	1.926852	1.923191	1.919531	1.915874
1.893116	1.88981	1.897435	1.894112	1.890811	1.927372	1.923713	1.92005	1.916385
1.89352	1.890198	1.89783	1.894537	1.891268	1.927903	1.924222	1.920557	1.916905
1.882413	1.879093	1.887313	1.884054	1.880825	1.928415	1.924737	1.92107	1.91741
1.882898	1.879541	1.887736	1.884459	1.881223	1.928903	1.925265	1.921594	1.917893

1.883384	1.880004	1.876672	1.873392	1.870164	1.910206	1.906645	1.922107	1.918389
1.883853	1.880484	1.877147	1.873823	1.870554	1.910628	1.907085	1.922591	1.918919
1.884322	1.880967	1.87763	1.874271	1.870966	1.911067	1.907532	1.923079	1.919454
1.884806	1.881431	1.878086	1.874744	1.871456	1.901404	1.897879	1.914403	1.910777
1.885299	1.881891	1.878536	1.875234	1.871985	1.90199	1.89844	1.914953	1.911279
1.878994	1.875595	1.879023	1.875714	1.872456	1.869265	1.866115	1.895347	1.891722
1.879489	1.876116	1.879545	1.876187	1.872877	1.869669	1.866519	1.895821	1.892235
1.880004	1.876628	1.880051	1.876671	1.87334	1.870115	1.866954	1.896313	1.892747
1.880542	1.877115	1.880519	1.877166	1.873863	1.870627	1.867439	1.896834	1.893252
1.868374	1.864937	1.874219	1.870907	1.867646	1.87115	1.867929	1.897358	1.893768
1.868944	1.865504	1.874789	1.87147	1.868194	1.871664	1.8684	1.897869	1.894305
1.869515	1.866085	1.875379	1.87204	1.868735	1.872172	1.868872	1.898385	1.894855
1.870034	1.866613	1.86323	1.859894	1.856594	1.865899	1.862619	1.866151	1.863022
1.870521	1.86711	1.863725	1.860354	1.857025	1.866358	1.863111	1.866657	1.863522
1.871029	1.8676	1.864202	1.860829	1.857503	1.866859	1.863622	1.860435	1.857299
1.871554	1.868075	1.864653	1.861314	1.858026	1.867402	1.864158	1.860946	1.857767
1.857499	1.85404	1.865152	1.861835	1.858569	1.855306	1.852069	1.861442	1.85824
1.858076	1.854614	1.865737	1.862416	1.859134	1.855826	1.852533	1.861904	1.858721
1.858638	1.855193	1.866337	1.863006	1.859701	1.856349	1.853007	1.862378	1.859213
1.859127	1.85572	1.852357	1.849066	1.845796	1.85689	1.853534	1.850294	1.847153
1.859589	1.85623	1.852899	1.849603	1.846319	1.857432	1.854082	1.850832	1.847665
1.860081	1.856753	1.853435	1.850112	1.846803	1.843506	1.840234	1.851369	1.848192
1.860593	1.857282	1.853963	1.8506	1.847255	1.843976	1.840743	1.851911	1.848733
1.840048	1.836816	1.854488	1.851098	1.847732	1.844462	1.841251	1.852442	1.849256
1.840572	1.837325	1.855014	1.851614	1.84824	1.844969	1.841758	1.852959	1.849761
1.836301	1.833034	1.855514	1.852116	1.848748	1.845479	1.842268	1.83907	1.835892
1.836777	1.833467	1.855954	1.852576	1.849238	1.845985	1.842783	1.839567	1.836348
1.837257	1.833905	1.856401	1.853045	1.849736	1.846498	1.843301	1.840068	1.83681
1.837778	1.834412	1.835893	1.832613	1.829392	1.847029	1.843827	1.840583	1.83731
1.83832	1.834954	1.836439	1.833148	1.829914	1.847571	1.844356	1.841106	1.837831
1.838853	1.83548	1.832154	1.828875	1.825649	1.84811	1.844886	1.84163	1.838352
1.83938	1.835998	1.832665	1.829384	1.826155	1.848648	1.845417	1.842156	1.838876
1.83993	1.836533	1.833189	1.829906	1.826676	1.8283	1.825172	1.842692	1.839408
1.840513	1.837093	1.833733	1.830448	1.827217	1.828838	1.825696	1.843241	1.83995
1.8411	1.837654	1.834276	1.830988	1.827756	1.829375	1.826218	1.823049	1.819875
1.841669	1.838197	1.834801	1.831509	1.828277	1.829894	1.826724	1.823545	1.820364
1.842243	1.838745	1.835329	1.832033	1.828799	1.830416	1.827232	1.824043	1.820854
1.838361	1.834833	1.835888	1.832588	1.829351	1.826136	1.822949	1.824564	1.821366
1.839015	1.835467	1.836467	1.833162	1.82992	1.826693	1.823491	1.825099	1.821891
1.839659	1.836098	1.837043	1.833731	1.830484	1.827244	1.824028	1.825633	1.822417
1.840293	1.836725	1.837614	1.834296	1.831041	1.827789	1.824559	1.826165	1.822943
1.840936	1.837379	1.838225	1.834888	1.831611	1.828344	1.825103	1.821874	1.818658
1.841598	1.838082	1.838901	1.835523	1.8322	1.828913	1.825666	1.822445	1.819253
1.842261	1.838786	1.839583	1.836168	1.832801	1.829494	1.82624	1.823025	1.819853
1.824965	1.821552	1.840229	1.836799	1.833411	1.830089	1.82682	1.823599	1.820427
1.825591	1.822181	1.84086	1.837429	1.834035	1.830699	1.827412	1.824176	1.820992
1.815241	1.811843	1.837329	1.833911	1.830515	1.831334	1.828021	1.82476	1.821547
1.815831	1.812432	1.837977	1.834596	1.83123	1.831992	1.828648	1.825351	1.822096
1.816446	1.813038	1.80967	1.806349	1.803047	1.832645	1.829308	1.825989	1.822685
1.817086	1.813662	1.810284	1.806951	1.803651	1.833298	1.830013	1.826685	1.823323

1.81775	1.814301	1.8109	1.807544	1.804235	1.824038	1.820808	1.827358	1.823969
1.818454	1.814952	1.811504	1.808116	1.804788	1.824589	1.821383	1.827974	1.824618
1.81916	1.815619	1.812129	1.808704	1.805347	1.825132	1.821933	1.828565	1.825256
1.819826	1.816313	1.812828	1.809361	1.805956	1.807487	1.804309	1.819234	1.815957
1.820473	1.817017	1.813557	1.810053	1.806597	1.80806	1.804846	1.819782	1.816518
1.785612	1.782255	1.814228	1.81073	1.807266	1.808657	1.805389	1.820332	1.817073
1.786208	1.782836	1.814853	1.811399	1.807965	1.809278	1.805936	1.820883	1.817619
1.786864	1.783456	1.780074	1.776719	1.773385	1.809902	1.806526	1.803238	1.800028
1.787608	1.784131	1.780699	1.777326	1.773992	1.810532	1.80718	1.803902	1.800693
1.788337	1.784815	1.781342	1.777941	1.774592	1.776965	1.773718	1.804547	1.801337
1.788986	1.785488	1.782016	1.778574	1.775179	1.777487	1.774229	1.805096	1.801888
1.789614	1.786156	1.7827	1.779224	1.775785	1.77802	1.77474	1.805634	1.802418
1.790259	1.786806	1.783361	1.779905	1.77648	1.778669	1.775367	1.772121	1.768929
1.790915	1.787451	1.784016	1.780607	1.777225	1.779379	1.776057	1.772762	1.769494
1.791623	1.788148	1.784705	1.781291	1.777904	1.780009	1.776681	1.773376	1.770094
1.792388	1.788902	1.785429	1.781958	1.778519	1.78056	1.777241	1.773965	1.770731
1.793113	1.789623	1.786136	1.782637	1.77917	1.781157	1.777843	1.774579	1.771368
1.79376	1.790279	1.786812	1.783343	1.779904	1.781854	1.778533	1.775251	1.772008
1.794408	1.790934	1.787482	1.784036	1.780617	1.782527	1.7792	1.775898	1.772621
1.795117	1.791636	1.788164	1.784677	1.78122	1.783067	1.779743	1.776437	1.773156
1.795838	1.792344	1.788844	1.785303	1.781796	1.783576	1.780255	1.776952	1.773673
1.796497	1.793003	1.789507	1.785971	1.782468	1.784203	1.780882	1.777568	1.774271
1.797119	1.793632	1.790158	1.786664	1.783201	1.784903	1.781578	1.778247	1.774921
1.797811	1.794321	1.790849	1.787371	1.783917	1.78051	1.777136	1.778856	1.775522
1.798583	1.795081	1.791592	1.788097	1.784618	1.78116	1.777728	1.779378	1.776061
1.799352	1.795823	1.792307	1.788797	1.785301	1.781814	1.778352	1.779944	1.776639
1.800091	1.796502	1.792946	1.789428	1.785942	1.78248	1.779052	1.780611	1.7773
1.800816	1.79718	1.793591	1.79007	1.786596	1.783166	1.779778	1.781305	1.777986
1.801516	1.797895	1.794318	1.790798	1.787323	1.783892	1.780502	1.781979	1.778666
1.802211	1.798636	1.795089	1.791574	1.788091	1.784643	1.78123	1.782646	1.779338
1.74355	1.740321	1.795881	1.792365	1.788872	1.785401	1.781957	1.783293	1.779954
1.744296	1.741075	1.796696	1.793177	1.78967	1.786169	1.782682	1.783923	1.780522
1.745052	1.741822	1.738601	1.735382	1.732171	1.786872	1.783363	1.784539	1.781121
1.745827	1.742569	1.739308	1.736034	1.732773	1.787478	1.78397	1.785123	1.781756
1.746574	1.743282	1.739985	1.736675	1.733385	1.788121	1.78462	1.785749	1.78242
1.747266	1.743928	1.740608	1.737316	1.734054	1.788882	1.785386	1.786469	1.783117
1.747924	1.744554	1.741225	1.737967	1.734747	1.789684	1.786194	1.787225	1.783836
1.748544	1.745202	1.7419	1.738657	1.735448	1.732261	1.7291	1.787965	1.78456
1.749142	1.745863	1.742607	1.739372	1.736161	1.73298	1.729825	1.788708	1.785294
1.749796	1.746549	1.743318	1.74009	1.736883	1.733708	1.730558	1.770336	1.76694
1.750503	1.74726	1.744035	1.740814	1.737614	1.734446	1.7313	1.771142	1.76773
1.751227	1.747985	1.744763	1.741545	1.738347	1.735182	1.732038	1.728894	1.725753
1.751966	1.748728	1.745507	1.742285	1.739082	1.735915	1.732768	1.729619	1.726469
1.752714	1.749475	1.746252	1.743025	1.739816	1.736644	1.733494	1.730338	1.727181
1.737387	1.734165	1.746989	1.743756	1.740543	1.737368	1.734216	1.731058	1.7279
1.738141	1.734908	1.747723	1.744486	1.74127	1.73809	1.734932	1.731769	1.728606
1.719957	1.716778	1.732435	1.729223	1.726033	1.738817	1.735642	1.732454	1.729258
1.720719	1.717517	1.733175	1.729968	1.726779	1.739547	1.736347	1.73312	1.729873
1.705357	1.702182	1.733915	1.730707	1.727518	1.724327	1.721136	1.733813	1.730544
1.706099	1.702901	1.734655	1.73144	1.728248	1.725051	1.721855	1.734531	1.73127

1.706842	1.703618	1.720256	1.717085	1.71394	1.725781	1.722584	1.719375	1.716156
1.707583	1.704325	1.720958	1.717793	1.71466	1.726523	1.723329	1.720139	1.716953
1.708332	1.705042	1.72167	1.718508	1.715383	1.727266	1.724076	1.720904	1.717748
1.666491	1.663633	1.702569	1.699465	1.696403	1.728005	1.72481	1.721642	1.718496
1.66717	1.6643	1.703322	1.700196	1.697113	1.728741	1.725539	1.722367	1.71922
1.667849	1.664968	1.704073	1.700919	1.697812	1.714375	1.711219	1.723094	1.719942
1.66853	1.665639	1.704826	1.701638	1.698502	1.715077	1.71193	1.72382	1.720659
1.669211	1.666311	1.705582	1.702365	1.699203	1.715787	1.712643	1.709501	1.706369
1.669893	1.666983	1.706344	1.703102	1.699916	1.716507	1.713358	1.710205	1.707055
1.670576	1.667657	1.707109	1.703843	1.700633	1.697502	1.694423	1.710914	1.707752
1.671252	1.668326	1.707869	1.704578	1.701346	1.698209	1.695131	1.711637	1.708472
1.671942	1.669009	1.708641	1.705326	1.702071	1.698924	1.695844	1.712364	1.7092
1.672666	1.669727	1.666821	1.663933	1.661084	1.699657	1.696555	1.713081	1.709914
1.673422	1.670475	1.667556	1.66464	1.661763	1.700407	1.697271	1.713798	1.710628
1.67424	1.671274	1.66833	1.665379	1.662467	1.701174	1.69801	1.714538	1.711367
1.675122	1.672125	1.669146	1.66615	1.663197	1.701959	1.698769	1.715297	1.712127
1.605074	1.602474	1.669929	1.666915	1.663947	1.702785	1.699568	1.696413	1.693322
1.605681	1.603071	1.670662	1.667675	1.66473	1.703681	1.700431	1.69727	1.694193
1.606258	1.603641	1.671371	1.668413	1.665494	1.704558	1.701281	1.698112	1.69504
1.6068	1.604177	1.641356	1.638472	1.63563	1.663289	1.660417	1.698867	1.695775
1.607335	1.604707	1.642003	1.639117	1.636274	1.663961	1.661083	1.699599	1.696475
1.60792	1.605273	1.602677	1.600129	1.597614	1.66466	1.661779	1.700368	1.697217
1.60854	1.605868	1.603249	1.600691	1.59817	1.665375	1.662496	1.701161	1.697984
1.609204	1.606507	1.603868	1.601301	1.598773	1.596257	1.593767	1.702004	1.6988
1.609923	1.607203	1.604544	1.601969	1.599433	1.596901	1.594392	1.702906	1.699676
1.610599	1.607859	1.605185	1.602603	1.600063	1.597523	1.595004	1.631365	1.62859
1.611188	1.608431	1.605745	1.603158	1.600618	1.598085	1.595577	1.632054	1.629267
1.611756	1.608992	1.606302	1.603712	1.601173	1.598651	1.596156	1.632752	1.629953
1.612336	1.609588	1.606911	1.604325	1.601789	1.599273	1.596785	1.594301	1.591825
1.612928	1.610203	1.607543	1.604961	1.602425	1.599914	1.597429	1.594944	1.592463
1.613583	1.610854	1.608186	1.605587	1.603034	1.600514	1.598023	1.595533	1.593047
1.61429	1.611536	1.608841	1.606208	1.603625	1.601086	1.598583	1.596084	1.593594
1.614966	1.612192	1.609475	1.606816	1.604211	1.601666	1.599165	1.596665	1.594173
1.615596	1.612809	1.610078	1.607401	1.604785	1.602253	1.599776	1.597287	1.594798
1.616247	1.613447	1.610703	1.608009	1.605383	1.602855	1.60039	1.597905	1.595414
1.616958	1.614141	1.61138	1.608669	1.606024	1.603479	1.600996	1.598496	1.595991
1.617678	1.614845	1.612069	1.609348	1.60669	1.604119	1.601605	1.599084	1.596566
1.579262	1.576579	1.61273	1.610026	1.607378	1.60478	1.602227	1.59969	1.597175
1.579845	1.577154	1.613383	1.610707	1.608078	1.605453	1.602853	1.600302	1.597797
1.58048	1.57777	1.614062	1.611379	1.608741	1.606088	1.603455	1.600889	1.598382
1.581161	1.578422	1.614763	1.612042	1.609369	1.606688	1.604033	1.601451	1.598934
1.547014	1.544467	1.57639	1.573789	1.571234	1.607317	1.604648	1.602051	1.599521
1.547619	1.545068	1.577046	1.574422	1.571843	1.607994	1.605321	1.602711	1.600159
1.548231	1.545671	1.577694	1.575047	1.572444	1.608668	1.605995	1.603375	1.600808
1.548853	1.546265	1.578303	1.575626	1.573002	1.609296	1.606629	1.604013	1.601447
1.549488	1.546868	1.578916	1.57621	1.573565	1.609926	1.607262	1.604648	1.602085
1.550149	1.547512	1.544929	1.542413	1.539956	1.571612	1.569081	1.605295	1.602711
1.550829	1.548184	1.545588	1.543058	1.54058	1.57227	1.569703	1.605954	1.603333
1.551488	1.548846	1.546241	1.543684	1.541179	1.572916	1.570337	1.567809	1.565334
1.552126	1.549499	1.546891	1.544293	1.54175	1.573553	1.570985	1.568453	1.565963

1.518474	1.515963	1.54753	1.544905	1.542336	1.574194	1.571631	1.569095	1.566593
1.519066	1.516533	1.548145	1.545519	1.54295	1.574841	1.572274	1.56973	1.567219
1.519668	1.517112	1.548772	1.546148	1.543579	1.575494	1.572907	1.570353	1.567838
1.488012	1.485612	1.549444	1.546807	1.544217	1.541712	1.539274	1.570948	1.568445
1.488576	1.486189	1.550141	1.547487	1.54487	1.542322	1.539837	1.571535	1.569045
1.489111	1.486753	1.516473	1.513921	1.511388	1.542962	1.540425	1.572142	1.569639
1.489624	1.48731	1.517137	1.514589	1.512042	1.543627	1.541033	1.572766	1.570227
1.490141	1.487863	1.517792	1.515252	1.5127	1.510142	1.507607	1.539198	1.536825
1.490669	1.488413	1.518438	1.515912	1.513368	1.510811	1.508269	1.53988	1.537452
1.491179	1.488942	1.519063	1.516552	1.514021	1.511477	1.50894	1.540576	1.538093
1.491651	1.489435	1.519656	1.51716	1.514643	1.512124	1.509608	1.541276	1.538738
1.492118	1.489913	1.52023	1.517752	1.515256	1.512763	1.510269	1.541976	1.539391
1.492607	1.490385	1.488154	1.485888	1.483604	1.513393	1.5109	1.508398	1.505885
1.49311	1.490856	1.488619	1.48639	1.484146	1.514019	1.511517	1.50901	1.506494
1.493638	1.491361	1.489118	1.486911	1.484694	1.514651	1.512147	1.509639	1.507121
1.494191	1.491896	1.489646	1.48745	1.485247	1.515288	1.512787	1.510282	1.507764
1.494737	1.492433	1.490179	1.487985	1.485789	1.515918	1.513427	1.510924	1.508402
1.495262	1.492964	1.490714	1.488515	1.486315	1.516536	1.514065	1.511564	1.50903
1.495813	1.49351	1.491255	1.489045	1.486841	1.484598	1.48233	1.512205	1.50966
1.496422	1.494083	1.4918	1.489578	1.487372	1.485144	1.482898	1.512853	1.510299
1.497057	1.49467	1.492352	1.490115	1.487907	1.485691	1.483463	1.5135	1.51094
1.497681	1.495259	1.492912	1.490659	1.488444	1.486232	1.484014	1.514134	1.511576
1.498306	1.495853	1.49348	1.491211	1.488986	1.48677	1.484555	1.51476	1.512212
1.49894	1.496458	1.49406	1.491773	1.489535	1.48731	1.485091	1.482801	1.480454
1.499581	1.497073	1.49465	1.492345	1.490092	1.487849	1.485619	1.483347	1.481044
1.500231	1.497702	1.495258	1.492935	1.490664	1.488402	1.486156	1.483894	1.481621
1.500888	1.498344	1.495884	1.493545	1.491258	1.488975	1.486709	1.484442	1.482177
1.501558	1.499007	1.496531	1.49417	1.491858	1.489555	1.48727	1.484994	1.482726
1.502259	1.499706	1.497213	1.494808	1.492452	1.490129	1.487836	1.485549	1.483273
1.502969	1.500421	1.497912	1.495456	1.493049	1.490708	1.488411	1.486114	1.483826
1.503641	1.501112	1.498601	1.496108	1.493661	1.491305	1.489005	1.486707	1.484418
1.504289	1.501788	1.499287	1.496765	1.494285	1.491917	1.489616	1.48732	1.485039
1.504955	1.50247	1.499974	1.497436	1.494935	1.492548	1.490228	1.487925	1.485648
1.505645	1.503158	1.500661	1.498126	1.495619	1.4932	1.490839	1.488518	1.486241
1.506331	1.503843	1.501345	1.49881	1.496297	1.49385	1.491453	1.489112	1.486828
1.507	1.504516	1.502017	1.499472	1.496944	1.494482	1.492069	1.489709	1.487401
1.507673	1.505191	1.502693	1.500139	1.4976	1.495123	1.492692	1.490311	1.487979
1.508367	1.505878	1.503381	1.500841	1.498307	1.4958	1.493323	1.49092	1.488583
1.509081	1.506579	1.504082	1.501562	1.49904	1.496497	1.493965	1.491539	1.489203
1.509836	1.507319	1.504809	1.502283	1.499752	1.497189	1.494633	1.492187	1.489835
1.510629	1.508094	1.505561	1.503005	1.500448	1.497879	1.495324	1.492862	1.49048
1.511422	1.508857	1.506302	1.503733	1.501168	1.498594	1.496033	1.493545	1.491121
1.512212	1.5096	1.507021	1.504466	1.501919	1.499342	1.496765	1.494236	1.491748
1.512997	1.510347	1.507751	1.505208	1.502675	1.500092	1.497498	1.494934	1.492392
1.513758	1.511105	1.508509	1.505963	1.503424	1.500827	1.498214	1.495631	1.493071
1.514531	1.51188	1.509288	1.506728	1.504174	1.501562	1.498934	1.496339	1.49377
1.515354	1.512694	1.510093	1.507503	1.504924	1.502307	1.499682	1.497075	1.494482
1.516221	1.513539	1.510919	1.508287	1.505676	1.50306	1.50045	1.497832	1.495209
1.517155	1.514395	1.511724	1.509065	1.506442	1.503829	1.501229	1.498606	1.495966
1.518157	1.515264	1.512508	1.509834	1.507223	1.504614	1.50202	1.499398	1.496753

1.519193	1.516158	1.513311	1.510618	1.508015	1.505406	1.502817	1.500198	1.497558
1.520243	1.517072	1.514134	1.511419	1.508817	1.506203	1.503613	1.501006	1.498387
1.521358	1.518034	1.51499	1.512239	1.509631	1.507009	1.504421	1.501822	1.49922
1.522572	1.519064	1.515881	1.513077	1.510451	1.507826	1.505252	1.502651	1.500038
1.523863	1.520159	1.516821	1.513944	1.511283	1.508651	1.50609	1.503484	1.500853
1.486208	1.482409	1.517844	1.514866	1.512137	1.509473	1.506907	1.5043	1.501678
1.487741	1.483784	1.51894	1.515838	1.513011	1.510296	1.50771	1.505106	1.502507
1.489276	1.48519	1.481441	1.478144	1.475103	1.472212	1.469466	1.505919	1.503328
1.490807	1.486619	1.482755	1.479323	1.476166	1.473233	1.470484	1.506742	1.504139
1.492379	1.488114	1.484151	1.480577	1.477289	1.474292	1.471515	1.468725	1.465962
1.494015	1.489705	1.485658	1.481936	1.4785	1.475404	1.472558	1.469739	1.466983
1.495675	1.491338	1.487222	1.483354	1.479762	1.476553	1.473621	1.470761	1.468009
1.497313	1.49297	1.488801	1.48479	1.481045	1.477725	1.474708	1.471798	1.469029
1.498961	1.494619	1.490406	1.486264	1.482372	1.478937	1.475822	1.47285	1.470054
1.500671	1.49629	1.492019	1.48779	1.483792	1.480231	1.476984	1.473933	1.4711
1.502435	1.497984	1.493643	1.489364	1.485298	1.481599	1.478191	1.475044	1.472165
1.50422	1.499713	1.495317	1.491004	1.486881	1.483046	1.479471	1.476216	1.473275
1.506035	1.501492	1.49706	1.492732	1.488566	1.484592	1.480837	1.47746	1.474438
1.507834	1.503261	1.498797	1.494452	1.490243	1.486152	1.482243	1.478745	1.475622
1.509574	1.504978	1.500482	1.496099	1.491835	1.487662	1.483653	1.480045	1.476799
1.511307	1.506687	1.50216	1.497738	1.493426	1.489196	1.485118	1.481401	1.478012
1.425597	1.421023	1.503858	1.499425	1.495098	1.490838	1.48671	1.48288	1.479318
1.427358	1.422726	1.505578	1.501143	1.496816	1.492546	1.488387	1.484442	1.480692
1.429148	1.424511	1.507383	1.502903	1.498523	1.494217	1.490017	1.485974	1.482077
1.43097	1.42638	1.509276	1.504706	1.500222	1.495857	1.491607	1.487481	1.483473
1.375071	1.370592	1.423692	1.41915	1.414692	1.497533	1.493242	1.48905	1.484958
1.376741	1.372307	1.425483	1.420897	1.416386	1.499255	1.494941	1.49071	1.486566
1.378416	1.37401	1.427268	1.422666	1.418129	1.501017	1.49667	1.492401	1.488214
1.38014	1.375731	1.429071	1.42449	1.419955	1.415562	1.411274	1.494082	1.489845
1.381875	1.377446	1.43087	1.426333	1.42182	1.417367	1.412978	1.495784	1.491496
1.383564	1.379121	1.374688	1.370264	1.365849	1.419183	1.414735	1.49754	1.493201
1.385227	1.38077	1.376349	1.371981	1.367616	1.421012	1.416531	1.499337	1.494947
1.386933	1.382463	1.378045	1.3737	1.369355	1.364905	1.360438	1.413905	1.409601
1.38868	1.384201	1.379776	1.375418	1.371057	1.366587	1.362097	1.415622	1.411302
1.307491	1.303669	1.381528	1.377157	1.372785	1.368304	1.363801	1.35943	1.355164
1.30899	1.30519	1.383306	1.378935	1.37457	1.370093	1.365589	1.361187	1.356866
1.310472	1.306695	1.385086	1.380717	1.376364	1.371899	1.367403	1.362976	1.3586
1.311888	1.308143	1.386827	1.382466	1.378128	1.373683	1.369206	1.364773	1.360369
1.313291	1.30957	1.388566	1.384208	1.379886	1.375462	1.371009	1.366581	1.362167
1.314792	1.311051	1.390366	1.385987	1.381658	1.377242	1.372805	1.368384	1.363973
1.316371	1.312571	1.392216	1.387796	1.38344	1.379021	1.374595	1.370186	1.36579
1.317973	1.314114	1.310355	1.306733	1.30311	1.380804	1.376378	1.371978	1.367602
1.319597	1.315681	1.311881	1.308252	1.304631	1.382592	1.378153	1.373758	1.369405
1.252738	1.248819	1.313424	1.309777	1.306149	1.384383	1.379925	1.375533	1.371203
1.254406	1.250427	1.31498	1.311305	1.307661	1.386171	1.381691	1.377299	1.372992
1.256089	1.252059	1.316557	1.312845	1.309172	1.387975	1.383464	1.379066	1.374775
1.257779	1.253721	1.31817	1.314403	1.310682	1.30687	1.303042	1.380843	1.376554
1.25948	1.255409	1.319811	1.315978	1.312196	1.308365	1.304541	1.382627	1.378328
1.261193	1.257113	1.253125	1.249269	1.245473	1.309878	1.306043	1.384406	1.380092
1.262919	1.258836	1.254821	1.250902	1.247041	1.311406	1.307547	1.38618	1.381845

1.967444	1.964011	1.960574	1.957103	1.953623	1.983346	1.979572	1.975936	1.972417
1.967367	1.963934	1.960498	1.957028	1.95355	1.983254	1.979483	1.975844	1.972319
1.967292	1.963857	1.96042	1.956951	1.953478	1.983176	1.979422	1.975781	1.972238
1.967223	1.963782	1.960343	1.956876	1.953412	1.983125	1.979417	1.975774	1.972189
1.967163	1.963712	1.960269	1.956803	1.953352	1.983095	1.979452	1.975806	1.972164
1.967155	1.963677	1.960212	1.956739	1.953291	1.976561	1.972916	1.9758	1.972126
1.967209	1.963682	1.960176	1.956685	1.95323	1.976485	1.972859	1.975756	1.972077
1.96723	1.96367	1.960134	1.956632	1.953174	1.976405	1.972788	1.975696	1.972016
1.967185	1.963619	1.960078	1.956574	1.953114	1.976316	1.972702	1.97562	1.971938
1.967116	1.963554	1.960019	1.956527	1.953075	1.976244	1.972618	1.975539	1.971856
1.967044	1.963482	1.959961	1.956511	1.953093	1.968449	1.964823	1.968866	1.965152
1.966967	1.963407	1.959904	1.956507	1.95313	1.968438	1.964763	1.968792	1.965067
1.966904	1.963341	1.979244	1.975633	1.972046	1.96838	1.964688	1.968716	1.964983
1.966854	1.963283	1.979127	1.975525	1.97195	1.968292	1.964603	1.968642	1.964903
1.966831	1.963272	1.979073	1.975458	1.971869	1.968209	1.964525	1.968586	1.964867
1.966838	1.963318	1.979098	1.975441	1.971805	1.968129	1.964447	1.968547	1.964884
1.966867	1.963367	1.979117	1.975424	1.971752	1.968073	1.964405	1.960776	1.957182
1.96693	1.963398	1.979091	1.975379	1.971703	1.968057	1.964433	1.960807	1.957182
1.966992	1.96342	1.979056	1.97534	1.971674	1.968059	1.964473	1.96084	1.957173
1.967006	1.963421	1.979027	1.975336	1.971694	1.96807	1.964461	1.960812	1.957131
1.966996	1.963414	1.979005	1.975353	1.971737	1.968084	1.964424	1.960755	1.957076
1.951334	1.947892	1.979021	1.975366	1.971739	1.968063	1.964378	1.960711	1.957057
1.951351	1.947946	1.979073	1.97538	1.971706	1.96801	1.964319	1.960674	1.957068
1.95139	1.947996	1.979101	1.975376	1.97167	1.967972	1.964292	1.960661	1.957074
1.951463	1.948039	1.97909	1.975341	1.971624	1.967951	1.964311	1.96068	1.957065
1.951532	1.948076	1.979074	1.975317	1.971605	1.967952	1.964337	1.960705	1.957067
1.951563	1.948095	1.979056	1.975334	1.971652	1.967988	1.964346	1.960717	1.957103
1.951593	1.948119	1.979051	1.975368	1.971718	1.968036	1.964359	1.960731	1.957147
1.97058	1.966802	1.979102	1.975404	1.971742	1.968065	1.964398	1.960768	1.95717
1.970676	1.9669	1.979186	1.975445	1.971747	1.968085	1.964453	1.960817	1.957183
1.970728	1.966952	1.979229	1.975484	1.971781	1.968117	1.964482	1.960846	1.957214
1.970733	1.966953	1.979225	1.975519	1.971848	1.96816	1.964483	1.960853	1.957264
1.970768	1.96699	1.977647	1.973938	1.97026	1.968198	1.964502	1.960873	1.957301
1.970867	1.967096	1.977748	1.973992	1.970271	1.968219	1.964557	1.960921	1.957311
1.970966	1.967213	1.977867	1.974066	1.970302	1.968252	1.964614	1.960969	1.957324
1.971012	1.967305	1.977993	1.974191	1.970409	1.968318	1.964637	1.960987	1.957368
1.971041	1.967385	1.978113	1.974332	1.970549	1.968406	1.964661	1.961004	1.957423
1.971095	1.967434	1.978167	1.974423	1.970672	1.966885	1.963096	1.961069	1.957462
1.971165	1.96746	1.978172	1.97448	1.970788	1.967023	1.963236	1.96117	1.957493
1.971227	1.96751	1.978216	1.974535	1.970859	1.967119	1.963358	1.961281	1.957567
1.971273	1.967585	1.978304	1.97458	1.970868	1.967164	1.963463	1.96141	1.957696
1.969319	1.96566	1.978417	1.974653	1.970905	1.967211	1.963542	1.959851	1.956144
1.969453	1.965808	1.97857	1.974786	1.97101	1.967275	1.963567	1.959886	1.956232
1.969607	1.965959	1.978715	1.974928	1.971138	1.967357	1.963594	1.959913	1.956303
1.969746	1.966053	1.978784	1.975028	1.97127	1.967478	1.963687	1.959981	1.956347
1.969882	1.966125	1.978821	1.975109	1.971402	1.967618	1.963815	1.960075	1.956383
1.954882	1.951153	1.977316	1.9736	1.969901	1.967718	1.963947	1.960203	1.956479
1.955006	1.95128	1.977434	1.97366	1.969919	1.967783	1.964088	1.960367	1.956625
1.955101	1.951397	1.977567	1.973755	1.969984	1.967877	1.964229	1.960524	1.956777
1.955148	1.951492	1.977712	1.973896	1.970115	1.968015	1.964375	1.960676	1.956934

1.955218	1.951602	1.947981	1.944312	1.940666	1.966574	1.962925	1.960804	1.957085
1.955357	1.951753	1.948138	1.944466	1.940807	1.966685	1.962988	1.960878	1.957214
1.955527	1.951923	1.948304	1.944631	1.940959	1.9668	1.963044	1.960946	1.95734
1.955698	1.952095	1.948476	1.944804	1.941129	1.966955	1.963166	1.961068	1.957485
1.955877	1.952271	1.948652	1.944984	1.94131	1.967136	1.963327	1.961225	1.957641
1.956049	1.952439	1.948817	1.945152	1.941481	1.954013	1.950233	1.959806	1.956229
1.956215	1.952601	1.948976	1.945312	1.941642	1.954173	1.950393	1.959983	1.956403
1.955377	1.951736	1.949094	1.945442	1.941789	1.954328	1.950557	1.960164	1.956579
1.955526	1.951814	1.949135	1.945518	1.941915	1.954478	1.950724	1.960349	1.956754
1.955683	1.951908	1.949195	1.945604	1.942042	1.954626	1.95089	1.960535	1.956933
1.94983	1.946051	1.949344	1.945746	1.942183	1.954773	1.951049	1.952016	1.948441
1.95002	1.946251	1.949548	1.945926	1.942338	1.954925	1.951208	1.952186	1.948618
1.950215	1.946486	1.942795	1.939155	1.935542	1.9389	1.935294	1.947737	1.944183
1.950415	1.946752	1.943094	1.939421	1.93576	1.939089	1.935461	1.947894	1.944338
1.950599	1.946979	1.943343	1.939653	1.935966	1.939274	1.935628	1.948051	1.944493
1.950765	1.947161	1.943537	1.939849	1.936158	1.939455	1.935792	1.948207	1.944646
1.939369	1.935824	1.943714	1.940031	1.936342	1.932661	1.929005	1.948376	1.944807
1.939538	1.935987	1.94388	1.9402	1.936514	1.93284	1.92919	1.948557	1.944975
1.939712	1.936153	1.944046	1.940365	1.936685	1.933028	1.929396	1.948763	1.945158
1.939888	1.93633	1.944228	1.940541	1.936862	1.933238	1.929648	1.927273	1.923741
1.940075	1.936518	1.944419	1.940723	1.937045	1.933461	1.929922	1.927543	1.923964
1.940307	1.936729	1.944618	1.940916	1.937239	1.933668	1.930147	1.926578	1.922979
1.940579	1.936962	1.944824	1.941118	1.937443	1.933864	1.930333	1.926768	1.923181
1.940825	1.937186	1.945041	1.94135	1.937686	1.934089	1.93053	1.926956	1.923376
1.941034	1.937395	1.945267	1.941619	1.937986	1.934359	1.930742	1.927141	1.923554
1.941239	1.937616	1.945512	1.941893	1.938277	1.934623	1.930964	1.927345	1.923757
1.941456	1.93787	1.945796	1.942158	1.938518	1.934855	1.931193	1.927585	1.924022
1.941673	1.938127	1.946081	1.942415	1.938743	1.935082	1.931436	1.927845	1.924303
1.941877	1.938344	1.946315	1.94264	1.938963	1.935324	1.93171	1.92812	1.924554
1.942079	1.938543	1.946522	1.942848	1.939182	1.935577	1.932001	1.928403	1.924792
1.94233	1.938771	1.946747	1.943093	1.939449	1.935847	1.932263	1.928653	1.925023
1.942629	1.939024	1.946984	1.943372	1.939766	1.936136	1.9325	1.92887	1.925245
1.940748	1.937141	1.9357	1.932161	1.928622	1.9364	1.932729	1.929094	1.925486
1.941009	1.937427	1.935997	1.932427	1.928853	1.93662	1.932943	1.929326	1.92576
1.941258	1.9377	1.936282	1.932678	1.92907	1.936839	1.933175	1.929578	1.926039
1.94148	1.937922	1.936504	1.932891	1.929282	1.937086	1.933461	1.929867	1.926304
1.941702	1.938129	1.936705	1.933094	1.929499	1.937347	1.933767	1.930172	1.926571
1.941981	1.938371	1.934788	1.931214	1.927662	1.937621	1.934042	1.930452	1.926858
1.9423	1.938636	1.935025	1.931475	1.927951	1.937908	1.934301	1.930719	1.92716
1.942601	1.938917	1.935293	1.931748	1.928231	1.938196	1.934585	1.931006	1.927455
1.94288	1.939217	1.935601	1.932034	1.928498	1.938483	1.934899	1.931317	1.927739
1.943172	1.939514	1.935898	1.932321	1.928776	1.925257	1.921758	1.931615	1.928027
1.943494	1.939791	1.936151	1.932599	1.929079	1.925543	1.922015	1.93188	1.928325
1.943818	1.940077	1.936418	1.932883	1.929381	1.925829	1.922273	1.932146	1.92862
1.944111	1.940396	1.936745	1.933181	1.929649	1.926104	1.922568	1.932444	1.928892
1.944403	1.940739	1.937107	1.933493	1.929912	1.92638	1.922879	1.932753	1.929158
1.944758	1.941109	1.937478	1.933842	1.930234	1.926683	1.923166	1.931419	1.927836
1.945164	1.941504	1.937862	1.934224	1.930605	1.927008	1.923436	1.931692	1.928148
1.945552	1.941867	1.93821	1.934585	1.930974	1.927353	1.923747	1.932008	1.92849
1.945917	1.942185	1.938506	1.934917	1.931347	1.92773	1.924114	1.932384	1.928875

1.946263	1.942508	1.938817	1.935244	1.931694	1.928087	1.924476	1.920948	1.917487
1.946571	1.942855	1.939179	1.935564	1.931975	1.928382	1.924804	1.921271	1.917778
1.946883	1.943218	1.939563	1.935895	1.932252	1.928667	1.925117	1.921578	1.918053
1.947261	1.943611	1.939958	1.936269	1.932599	1.928988	1.925416	1.921871	1.918354
1.947675	1.944019	1.940359	1.936668	1.932986	1.929333	1.92571	1.922157	1.918667
1.948076	1.944392	1.940716	1.937043	1.933377	1.929704	1.926049	1.922485	1.918999
1.948469	1.944731	1.941029	1.937397	1.933776	1.930105	1.926431	1.922852	1.919352
1.941065	1.937308	1.941376	1.937764	1.934167	1.930497	1.926816	1.92322	1.919692
1.941454	1.937706	1.941782	1.938159	1.934548	1.930878	1.927199	1.923578	1.920004
1.941822	1.938099	1.94219	1.938542	1.934906	1.931243	1.927579	1.923942	1.920327
1.942129	1.938447	1.942563	1.938879	1.935209	1.931571	1.927951	1.924322	1.920686
1.942427	1.938788	1.94293	1.939208	1.935505	1.931893	1.928322	1.924708	1.921064
1.942798	1.939167	1.935517	1.931797	1.928095	1.932266	1.928703	1.92509	1.921442
1.943215	1.939571	1.935915	1.932203	1.928509	1.932672	1.92909	1.92547	1.921824
1.943631	1.93998	1.93632	1.932613	1.928923	1.925304	1.921718	1.925863	1.922217
1.944048	1.940394	1.936734	1.933029	1.929339	1.925716	1.922124	1.92627	1.922622
1.944466	1.940815	1.937156	1.933447	1.929753	1.926125	1.922529	1.926675	1.923024
1.944885	1.941244	1.937588	1.933868	1.930161	1.926531	1.922935	1.927081	1.923421
1.945307	1.941675	1.938021	1.93429	1.930568	1.926929	1.923326	1.927471	1.923808
1.945737	1.942102	1.938443	1.934707	1.930974	1.927302	1.923661	1.920021	1.916387
1.94617	1.942525	1.938859	1.935123	1.931383	1.927673	1.923987	1.920342	1.916735
1.941781	1.938143	1.939274	1.935546	1.931813	1.928106	1.924417	1.920764	1.917144
1.942179	1.938544	1.939688	1.935974	1.932261	1.928585	1.924928	1.921265	1.9176
1.935179	1.931577	1.940106	1.936396	1.932692	1.929038	1.925404	1.921739	1.91805
1.935587	1.931984	1.940528	1.936811	1.933102	1.929454	1.925831	1.922174	1.918493
1.936004	1.932395	1.936147	1.932428	1.928721	1.929865	1.92625	1.922601	1.918928
1.936434	1.932808	1.93656	1.932835	1.929126	1.93028	1.926675	1.923028	1.91935
1.936865	1.933222	1.936973	1.933243	1.929534	1.930696	1.927096	1.923448	1.919766
1.906908	1.903608	1.929973	1.926275	1.9226	1.926321	1.922725	1.923859	1.920176
1.907281	1.903975	1.930374	1.926682	1.92301	1.926738	1.92313	1.924264	1.920583
1.90767	1.904359	1.930793	1.927098	1.923423	1.927158	1.923541	1.919895	1.91623
1.908071	1.904757	1.931226	1.927523	1.923839	1.927579	1.923955	1.920315	1.916665
1.908511	1.905176	1.93167	1.927974	1.924295	1.920659	1.91705	1.920758	1.917132
1.909012	1.905626	1.932129	1.928462	1.924815	1.921157	1.917509	1.921239	1.917647
1.909501	1.906071	1.932589	1.92895	1.92533	1.921652	1.917969	1.921718	1.918153
1.909919	1.906478	1.933032	1.929405	1.925796	1.922105	1.918401	1.922161	1.918603
1.910315	1.906881	1.933486	1.929858	1.926243	1.922546	1.918836	1.922604	1.919037
1.910738	1.907332	1.934005	1.930344	1.926696	1.923008	1.919319	1.923097	1.919505
1.911175	1.907809	1.934562	1.930849	1.927148	1.923478	1.919832	1.923621	1.919987
1.911604	1.908249	1.935056	1.931315	1.927586	1.923928	1.920306	1.916676	1.913045
1.912021	1.908646	1.935481	1.931734	1.928004	1.924352	1.920739	1.917104	1.913455
1.90183	1.898459	1.935924	1.932186	1.928465	1.924807	1.921182	1.917541	1.913892
1.902313	1.898924	1.936409	1.932701	1.929005	1.925317	1.921643	1.917997	1.914377
1.902798	1.899402	1.936916	1.933231	1.929554	1.92584	1.922126	1.918475	1.914878
1.903241	1.899871	1.907149	1.90381	1.900493	1.926347	1.922649	1.918993	1.915374
1.903667	1.900331	1.907646	1.904281	1.900938	1.926852	1.923191	1.919531	1.915874
1.893116	1.88981	1.897435	1.894112	1.890811	1.927372	1.923713	1.92005	1.916385
1.89352	1.890198	1.89783	1.894537	1.891268	1.927903	1.924222	1.920557	1.916905
1.882413	1.879093	1.887313	1.884054	1.880825	1.928415	1.924737	1.92107	1.91741
1.882898	1.879541	1.887736	1.884459	1.881223	1.928903	1.925265	1.921594	1.917893

1.883384	1.880004	1.876672	1.873392	1.870164	1.910206	1.906645	1.922107	1.918389
1.883853	1.880484	1.877147	1.873823	1.870554	1.910628	1.907085	1.922591	1.918919
1.884322	1.880967	1.87763	1.874271	1.870966	1.911067	1.907532	1.923079	1.919454
1.884806	1.881431	1.878086	1.874744	1.871456	1.901404	1.897879	1.914403	1.910777
1.885299	1.881891	1.878536	1.875234	1.871985	1.90199	1.89844	1.914953	1.911279
1.878994	1.875595	1.879023	1.875714	1.872456	1.869265	1.866115	1.895347	1.891722
1.879489	1.876116	1.879545	1.876187	1.872877	1.869669	1.866519	1.895821	1.892235
1.880004	1.876628	1.880051	1.876671	1.87334	1.870115	1.866954	1.896313	1.892747
1.880542	1.877115	1.880519	1.877166	1.873863	1.870627	1.867439	1.896834	1.893252
1.868374	1.864937	1.874219	1.870907	1.867646	1.87115	1.867929	1.897358	1.893768
1.868944	1.865504	1.874789	1.87147	1.868194	1.871664	1.8684	1.897869	1.894305
1.869515	1.866085	1.875379	1.87204	1.868735	1.872172	1.868872	1.898385	1.894855
1.870034	1.866613	1.86323	1.859894	1.856594	1.865899	1.862619	1.866151	1.863022
1.870521	1.86711	1.863725	1.860354	1.857025	1.866358	1.863111	1.866657	1.863522
1.871029	1.8676	1.864202	1.860829	1.857503	1.866859	1.863622	1.860435	1.857299
1.871554	1.868075	1.864653	1.861314	1.858026	1.867402	1.864158	1.860946	1.857767
1.857499	1.85404	1.865152	1.861835	1.858569	1.855306	1.852069	1.861442	1.85824
1.858076	1.854614	1.865737	1.862416	1.859134	1.855826	1.852533	1.861904	1.858721
1.858638	1.855193	1.866337	1.863006	1.859701	1.856349	1.853007	1.862378	1.859213
1.859127	1.85572	1.852357	1.849066	1.845796	1.85689	1.853534	1.850294	1.847153
1.859589	1.85623	1.852899	1.849603	1.846319	1.857432	1.854082	1.850832	1.847665
1.860081	1.856753	1.853435	1.850112	1.846803	1.843506	1.840234	1.851369	1.848192
1.860593	1.857282	1.853963	1.8506	1.847255	1.843976	1.840743	1.851911	1.848733
1.840048	1.836816	1.854488	1.851098	1.847732	1.844462	1.841251	1.852442	1.849256
1.840572	1.837325	1.855014	1.851614	1.84824	1.844969	1.841758	1.852959	1.849761
1.836301	1.833034	1.855514	1.852116	1.848748	1.845479	1.842268	1.83907	1.835892
1.836777	1.833467	1.855954	1.852576	1.849238	1.845985	1.842783	1.839567	1.836348
1.837257	1.833905	1.856401	1.853045	1.849736	1.846498	1.843301	1.840068	1.83681
1.837778	1.834412	1.835893	1.832613	1.829392	1.847029	1.843827	1.840583	1.83731
1.83832	1.834954	1.836439	1.833148	1.829914	1.847571	1.844356	1.841106	1.837831
1.838853	1.83548	1.832154	1.828875	1.825649	1.84811	1.844886	1.84163	1.838352
1.83938	1.835998	1.832665	1.829384	1.826155	1.848648	1.845417	1.842156	1.838876
1.83993	1.836533	1.833189	1.829906	1.826676	1.8283	1.825172	1.842692	1.839408
1.840513	1.837093	1.833733	1.830448	1.827217	1.828838	1.825696	1.843241	1.83995
1.8411	1.837654	1.834276	1.830988	1.827756	1.829375	1.826218	1.823049	1.819875
1.841669	1.838197	1.834801	1.831509	1.828277	1.829894	1.826724	1.823545	1.820364
1.842243	1.838745	1.835329	1.832033	1.828799	1.830416	1.827232	1.824043	1.820854
1.838361	1.834833	1.835888	1.832588	1.829351	1.826136	1.822949	1.824564	1.821366
1.839015	1.835467	1.836467	1.833162	1.82992	1.826693	1.823491	1.825099	1.821891
1.839659	1.836098	1.837043	1.833731	1.830484	1.827244	1.824028	1.825633	1.822417
1.840293	1.836725	1.837614	1.834296	1.831041	1.827789	1.824559	1.826165	1.822943
1.840936	1.837379	1.838225	1.834888	1.831611	1.828344	1.825103	1.821874	1.818658
1.841598	1.838082	1.838901	1.835523	1.8322	1.828913	1.825666	1.822445	1.819253
1.842261	1.838786	1.839583	1.836168	1.832801	1.829494	1.82624	1.823025	1.819853
1.824965	1.821552	1.840229	1.836799	1.833411	1.830089	1.82682	1.823599	1.820427
1.825591	1.822181	1.84086	1.837429	1.834035	1.830699	1.827412	1.824176	1.820992
1.815241	1.811843	1.837329	1.833911	1.830515	1.831334	1.828021	1.82476	1.821547
1.815831	1.812432	1.837977	1.834596	1.83123	1.831992	1.828648	1.825351	1.822096
1.816446	1.813038	1.80967	1.806349	1.803047	1.832645	1.829308	1.825989	1.822685
1.817086	1.813662	1.810284	1.806951	1.803651	1.833298	1.830013	1.826685	1.823323

1.81775	1.814301	1.8109	1.807544	1.804235	1.824038	1.820808	1.827358	1.823969
1.818454	1.814952	1.811504	1.808116	1.804788	1.824589	1.821383	1.827974	1.824618
1.81916	1.815619	1.812129	1.808704	1.805347	1.825132	1.821933	1.828565	1.825256
1.819826	1.816313	1.812828	1.809361	1.805956	1.807487	1.804309	1.819234	1.815957
1.820473	1.817017	1.813557	1.810053	1.806597	1.80806	1.804846	1.819782	1.816518
1.785612	1.782255	1.814228	1.81073	1.807266	1.808657	1.805389	1.820332	1.817073
1.786208	1.782836	1.814853	1.811399	1.807965	1.809278	1.805936	1.820883	1.817619
1.786864	1.783456	1.780074	1.776719	1.773385	1.809902	1.806526	1.803238	1.800028
1.787608	1.784131	1.780699	1.777326	1.773992	1.810532	1.80718	1.803902	1.800693
1.788337	1.784815	1.781342	1.777941	1.774592	1.776965	1.773718	1.804547	1.801337
1.788986	1.785488	1.782016	1.778574	1.775179	1.777487	1.774229	1.805096	1.801888
1.789614	1.786156	1.7827	1.779224	1.775785	1.77802	1.77474	1.805634	1.802418
1.790259	1.786806	1.783361	1.779905	1.77648	1.778669	1.775367	1.772121	1.768929
1.790915	1.787451	1.784016	1.780607	1.777225	1.779379	1.776057	1.772762	1.769494
1.791623	1.788148	1.784705	1.781291	1.777904	1.780009	1.776681	1.773376	1.770094
1.792388	1.788902	1.785429	1.781958	1.778519	1.78056	1.777241	1.773965	1.770731
1.793113	1.789623	1.786136	1.782637	1.77917	1.781157	1.777843	1.774579	1.771368
1.79376	1.790279	1.786812	1.783343	1.779904	1.781854	1.778533	1.775251	1.772008
1.794408	1.790934	1.787482	1.784036	1.780617	1.782527	1.7792	1.775898	1.772621
1.795117	1.791636	1.788164	1.784677	1.78122	1.783067	1.779743	1.776437	1.773156
1.795838	1.792344	1.788844	1.785303	1.781796	1.783576	1.780255	1.776952	1.773673
1.796497	1.793003	1.789507	1.785971	1.782468	1.784203	1.780882	1.777568	1.774271
1.797119	1.793632	1.790158	1.786664	1.783201	1.784903	1.781578	1.778247	1.774921
1.797811	1.794321	1.790849	1.787371	1.783917	1.78051	1.777136	1.778856	1.775522
1.798583	1.795081	1.791592	1.788097	1.784618	1.78116	1.777728	1.779378	1.776061
1.799352	1.795823	1.792307	1.788797	1.785301	1.781814	1.778352	1.779944	1.776639
1.800091	1.796502	1.792946	1.789428	1.785942	1.78248	1.779052	1.780611	1.7773
1.800816	1.79718	1.793591	1.79007	1.786596	1.783166	1.779778	1.781305	1.777986
1.801516	1.797895	1.794318	1.790798	1.787323	1.783892	1.780502	1.781979	1.778666
1.802211	1.798636	1.795089	1.791574	1.788091	1.784643	1.78123	1.782646	1.779338
1.74355	1.740321	1.795881	1.792365	1.788872	1.785401	1.781957	1.783293	1.779954
1.744296	1.741075	1.796696	1.793177	1.78967	1.786169	1.782682	1.783923	1.780522
1.745052	1.741822	1.738601	1.735382	1.732171	1.786872	1.783363	1.784539	1.781121
1.745827	1.742569	1.739308	1.736034	1.732773	1.787478	1.78397	1.785123	1.781756
1.746574	1.743282	1.739985	1.736675	1.733385	1.788121	1.78462	1.785749	1.78242
1.747266	1.743928	1.740608	1.737316	1.734054	1.788882	1.785386	1.786469	1.783117
1.747924	1.744554	1.741225	1.737967	1.734747	1.789684	1.786194	1.787225	1.783836
1.748544	1.745202	1.7419	1.738657	1.735448	1.732261	1.7291	1.787965	1.78456
1.749142	1.745863	1.742607	1.739372	1.736161	1.73298	1.729825	1.788708	1.785294
1.749796	1.746549	1.743318	1.74009	1.736883	1.733708	1.730558	1.770336	1.76694
1.750503	1.74726	1.744035	1.740814	1.737614	1.734446	1.7313	1.771142	1.76773
1.751227	1.747985	1.744763	1.741545	1.738347	1.735182	1.732038	1.728894	1.725753
1.751966	1.748728	1.745507	1.742285	1.739082	1.735915	1.732768	1.729619	1.726469
1.752714	1.749475	1.746252	1.743025	1.739816	1.736644	1.733494	1.730338	1.727181
1.737387	1.734165	1.746989	1.743756	1.740543	1.737368	1.734216	1.731058	1.7279
1.738141	1.734908	1.747723	1.744486	1.74127	1.73809	1.734932	1.731769	1.728606
1.719957	1.716778	1.732435	1.729223	1.726033	1.738817	1.735642	1.732454	1.729258
1.720719	1.717517	1.733175	1.729968	1.726779	1.739547	1.736347	1.73312	1.729873
1.705357	1.702182	1.733915	1.730707	1.727518	1.724327	1.721136	1.733813	1.730544
1.706099	1.702901	1.734655	1.73144	1.728248	1.725051	1.721855	1.734531	1.73127

1.706842	1.703618	1.720256	1.717085	1.71394	1.725781	1.722584	1.719375	1.716156
1.707583	1.704325	1.720958	1.717793	1.71466	1.726523	1.723329	1.720139	1.716953
1.708332	1.705042	1.72167	1.718508	1.715383	1.727266	1.724076	1.720904	1.717748
1.666491	1.663633	1.702569	1.699465	1.696403	1.728005	1.72481	1.721642	1.718496
1.66717	1.6643	1.703322	1.700196	1.697113	1.728741	1.725539	1.722367	1.71922
1.667849	1.664968	1.704073	1.700919	1.697812	1.714375	1.711219	1.723094	1.719942
1.66853	1.665639	1.704826	1.701638	1.698502	1.715077	1.71193	1.72382	1.720659
1.669211	1.666311	1.705582	1.702365	1.699203	1.715787	1.712643	1.709501	1.706369
1.669893	1.666983	1.706344	1.703102	1.699916	1.716507	1.713358	1.710205	1.707055
1.670576	1.667657	1.707109	1.703843	1.700633	1.697502	1.694423	1.710914	1.707752
1.671252	1.668326	1.707869	1.704578	1.701346	1.698209	1.695131	1.711637	1.708472
1.671942	1.669009	1.708641	1.705326	1.702071	1.698924	1.695844	1.712364	1.7092
1.672666	1.669727	1.666821	1.663933	1.661084	1.699657	1.696555	1.713081	1.709914
1.673422	1.670475	1.667556	1.66464	1.661763	1.700407	1.697271	1.713798	1.710628
1.67424	1.671274	1.66833	1.665379	1.662467	1.701174	1.69801	1.714538	1.711367
1.675122	1.672125	1.669146	1.66615	1.663197	1.701959	1.698769	1.715297	1.712127
1.605074	1.602474	1.669929	1.666915	1.663947	1.702785	1.699568	1.696413	1.693322
1.605681	1.603071	1.670662	1.667675	1.66473	1.703681	1.700431	1.69727	1.694193
1.606258	1.603641	1.671371	1.668413	1.665494	1.704558	1.701281	1.698112	1.69504
1.6068	1.604177	1.641356	1.638472	1.63563	1.663289	1.660417	1.698867	1.695775
1.607335	1.604707	1.642003	1.639117	1.636274	1.663961	1.661083	1.699599	1.696475
1.60792	1.605273	1.602677	1.600129	1.597614	1.66466	1.661779	1.700368	1.697217
1.60854	1.605868	1.603249	1.600691	1.59817	1.665375	1.662496	1.701161	1.697984
1.609204	1.606507	1.603868	1.601301	1.598773	1.596257	1.593767	1.702004	1.6988
1.609923	1.607203	1.604544	1.601969	1.599433	1.596901	1.594392	1.702906	1.699676
1.610599	1.607859	1.605185	1.602603	1.600063	1.597523	1.595004	1.631365	1.62859
1.611188	1.608431	1.605745	1.603158	1.600618	1.598085	1.595577	1.632054	1.629267
1.611756	1.608992	1.606302	1.603712	1.601173	1.598651	1.596156	1.632752	1.629953
1.612336	1.609588	1.606911	1.604325	1.601789	1.599273	1.596785	1.594301	1.591825
1.612928	1.610203	1.607543	1.604961	1.602425	1.599914	1.597429	1.594944	1.592463
1.613583	1.610854	1.608186	1.605587	1.603034	1.600514	1.598023	1.595533	1.593047
1.61429	1.611536	1.608841	1.606208	1.603625	1.601086	1.598583	1.596084	1.593594
1.614966	1.612192	1.609475	1.606816	1.604211	1.601666	1.599165	1.596665	1.594173
1.615596	1.612809	1.610078	1.607401	1.604785	1.602253	1.599776	1.597287	1.594798
1.616247	1.613447	1.610703	1.608009	1.605383	1.602855	1.60039	1.597905	1.595414
1.616958	1.614141	1.61138	1.608669	1.606024	1.603479	1.600996	1.598496	1.595991
1.617678	1.614845	1.612069	1.609348	1.60669	1.604119	1.601605	1.599084	1.596566
1.579262	1.576579	1.61273	1.610026	1.607378	1.60478	1.602227	1.59969	1.597175
1.579845	1.577154	1.613383	1.610707	1.608078	1.605453	1.602853	1.600302	1.597797
1.58048	1.57777	1.614062	1.611379	1.608741	1.606088	1.603455	1.600889	1.598382
1.581161	1.578422	1.614763	1.612042	1.609369	1.606688	1.604033	1.601451	1.598934
1.547014	1.544467	1.57639	1.573789	1.571234	1.607317	1.604648	1.602051	1.599521
1.547619	1.545068	1.577046	1.574422	1.571843	1.607994	1.605321	1.602711	1.600159
1.548231	1.545671	1.577694	1.575047	1.572444	1.608668	1.605995	1.603375	1.600808
1.548853	1.546265	1.578303	1.575626	1.573002	1.609296	1.606629	1.604013	1.601447
1.549488	1.546868	1.578916	1.57621	1.573565	1.609926	1.607262	1.604648	1.602085
1.550149	1.547512	1.544929	1.542413	1.539956	1.571612	1.569081	1.605295	1.602711
1.550829	1.548184	1.545588	1.543058	1.54058	1.57227	1.569703	1.605954	1.603333
1.551488	1.548846	1.546241	1.543684	1.541179	1.572916	1.570337	1.567809	1.565334
1.552126	1.549499	1.546891	1.544293	1.54175	1.573553	1.570985	1.568453	1.565963

1.518474	1.515963	1.54753	1.544905	1.542336	1.574194	1.571631	1.569095	1.566593
1.519066	1.516533	1.548145	1.545519	1.54295	1.574841	1.572274	1.56973	1.567219
1.519668	1.517112	1.548772	1.546148	1.543579	1.575494	1.572907	1.570353	1.567838
1.488012	1.485612	1.549444	1.546807	1.544217	1.541712	1.539274	1.570948	1.568445
1.488576	1.486189	1.550141	1.547487	1.54487	1.542322	1.539837	1.571535	1.569045
1.489111	1.486753	1.516473	1.513921	1.511388	1.542962	1.540425	1.572142	1.569639
1.489624	1.48731	1.517137	1.514589	1.512042	1.543627	1.541033	1.572766	1.570227
1.490141	1.487863	1.517792	1.515252	1.5127	1.510142	1.507607	1.539198	1.536825
1.490669	1.488413	1.518438	1.515912	1.513368	1.510811	1.508269	1.53988	1.537452
1.491179	1.488942	1.519063	1.516552	1.514021	1.511477	1.50894	1.540576	1.538093
1.491651	1.489435	1.519656	1.51716	1.514643	1.512124	1.509608	1.541276	1.538738
1.492118	1.489913	1.52023	1.517752	1.515256	1.512763	1.510269	1.541976	1.539391
1.492607	1.490385	1.488154	1.485888	1.483604	1.513393	1.5109	1.508398	1.505885
1.49311	1.490856	1.488619	1.48639	1.484146	1.514019	1.511517	1.50901	1.506494
1.493638	1.491361	1.489118	1.486911	1.484694	1.514651	1.512147	1.509639	1.507121
1.494191	1.491896	1.489646	1.48745	1.485247	1.515288	1.512787	1.510282	1.507764
1.494737	1.492433	1.490179	1.487985	1.485789	1.515918	1.513427	1.510924	1.508402
1.495262	1.492964	1.490714	1.488515	1.486315	1.516536	1.514065	1.511564	1.50903
1.495813	1.49351	1.491255	1.489045	1.486841	1.484598	1.48233	1.512205	1.50966
1.496422	1.494083	1.4918	1.489578	1.487372	1.485144	1.482898	1.512853	1.510299
1.497057	1.49467	1.492352	1.490115	1.487907	1.485691	1.483463	1.5135	1.51094
1.497681	1.495259	1.492912	1.490659	1.488444	1.486232	1.484014	1.514134	1.511576
1.498306	1.495853	1.49348	1.491211	1.488986	1.48677	1.484555	1.51476	1.512212
1.49894	1.496458	1.49406	1.491773	1.489535	1.48731	1.485091	1.482801	1.480454
1.499581	1.497073	1.49465	1.492345	1.490092	1.487849	1.485619	1.483347	1.481044
1.500231	1.497702	1.495258	1.492935	1.490664	1.488402	1.486156	1.483894	1.481621
1.500888	1.498344	1.495884	1.493545	1.491258	1.488975	1.486709	1.484442	1.482177
1.501558	1.499007	1.496531	1.49417	1.491858	1.489555	1.48727	1.484994	1.482726
1.502259	1.499706	1.497213	1.494808	1.492452	1.490129	1.487836	1.485549	1.483273
1.502969	1.500421	1.497912	1.495456	1.493049	1.490708	1.488411	1.486114	1.483826
1.503641	1.501112	1.498601	1.496108	1.493661	1.491305	1.489005	1.486707	1.484418
1.504289	1.501788	1.499287	1.496765	1.494285	1.491917	1.489616	1.48732	1.485039
1.504955	1.50247	1.499974	1.497436	1.494935	1.492548	1.490228	1.487925	1.485648
1.505645	1.503158	1.500661	1.498126	1.495619	1.4932	1.490839	1.488518	1.486241
1.506331	1.503843	1.501345	1.49881	1.496297	1.49385	1.491453	1.489112	1.486828
1.507	1.504516	1.502017	1.499472	1.496944	1.494482	1.492069	1.489709	1.487401
1.507673	1.505191	1.502693	1.500139	1.4976	1.495123	1.492692	1.490311	1.487979
1.508367	1.505878	1.503381	1.500841	1.498307	1.4958	1.493323	1.49092	1.488583
1.509081	1.506579	1.504082	1.501562	1.49904	1.496497	1.493965	1.491539	1.489203
1.509836	1.507319	1.504809	1.502283	1.499752	1.497189	1.494633	1.492187	1.489835
1.510629	1.508094	1.505561	1.503005	1.500448	1.497879	1.495324	1.492862	1.49048
1.511422	1.508857	1.506302	1.503733	1.501168	1.498594	1.496033	1.493545	1.491121
1.512212	1.5096	1.507021	1.504466	1.501919	1.499342	1.496765	1.494236	1.491748
1.512997	1.510347	1.507751	1.505208	1.502675	1.500092	1.497498	1.494934	1.492392
1.513758	1.511105	1.508509	1.505963	1.503424	1.500827	1.498214	1.495631	1.493071
1.514531	1.51188	1.509288	1.506728	1.504174	1.501562	1.498934	1.496339	1.49377
1.515354	1.512694	1.510093	1.507503	1.504924	1.502307	1.499682	1.497075	1.494482
1.516221	1.513539	1.510919	1.508287	1.505676	1.50306	1.50045	1.497832	1.495209
1.517155	1.514395	1.511724	1.509065	1.506442	1.503829	1.501229	1.498606	1.495966
1.518157	1.515264	1.512508	1.509834	1.507223	1.504614	1.50202	1.499398	1.496753

1.519193	1.516158	1.513311	1.510618	1.508015	1.505406	1.502817	1.500198	1.497558
1.520243	1.517072	1.514134	1.511419	1.508817	1.506203	1.503613	1.501006	1.498387
1.521358	1.518034	1.51499	1.512239	1.509631	1.507009	1.504421	1.501822	1.49922
1.522572	1.519064	1.515881	1.513077	1.510451	1.507826	1.505252	1.502651	1.500038
1.523863	1.520159	1.516821	1.513944	1.511283	1.508651	1.50609	1.503484	1.500853
1.486208	1.482409	1.517844	1.514866	1.512137	1.509473	1.506907	1.5043	1.501678
1.487741	1.483784	1.51894	1.515838	1.513011	1.510296	1.50771	1.505106	1.502507
1.489276	1.48519	1.481441	1.478144	1.475103	1.472212	1.469466	1.505919	1.503328
1.490807	1.486619	1.482755	1.479323	1.476166	1.473233	1.470484	1.506742	1.504139
1.492379	1.488114	1.484151	1.480577	1.477289	1.474292	1.471515	1.468725	1.465962
1.494015	1.489705	1.485658	1.481936	1.4785	1.475404	1.472558	1.469739	1.466983
1.495675	1.491338	1.487222	1.483354	1.479762	1.476553	1.473621	1.470761	1.468009
1.497313	1.49297	1.488801	1.48479	1.481045	1.477725	1.474708	1.471798	1.469029
1.498961	1.494619	1.490406	1.486264	1.482372	1.478937	1.475822	1.47285	1.470054
1.500671	1.49629	1.492019	1.48779	1.483792	1.480231	1.476984	1.473933	1.4711
1.502435	1.497984	1.493643	1.489364	1.485298	1.481599	1.478191	1.475044	1.472165
1.50422	1.499713	1.495317	1.491004	1.486881	1.483046	1.479471	1.476216	1.473275
1.506035	1.501492	1.49706	1.492732	1.488566	1.484592	1.480837	1.47746	1.474438
1.507834	1.503261	1.498797	1.494452	1.490243	1.486152	1.482243	1.478745	1.475622
1.509574	1.504978	1.500482	1.496099	1.491835	1.487662	1.483653	1.480045	1.476799
1.511307	1.506687	1.50216	1.497738	1.493426	1.489196	1.485118	1.481401	1.478012
1.425597	1.421023	1.503858	1.499425	1.495098	1.490838	1.48671	1.48288	1.479318
1.427358	1.422726	1.505578	1.501143	1.496816	1.492546	1.488387	1.484442	1.480692
1.429148	1.424511	1.507383	1.502903	1.498523	1.494217	1.490017	1.485974	1.482077
1.43097	1.42638	1.509276	1.504706	1.500222	1.495857	1.491607	1.487481	1.483473
1.375071	1.370592	1.423692	1.41915	1.414692	1.497533	1.493242	1.48905	1.484958
1.376741	1.372307	1.425483	1.420897	1.416386	1.499255	1.494941	1.49071	1.486566
1.378416	1.37401	1.427268	1.422666	1.418129	1.501017	1.49667	1.492401	1.488214
1.38014	1.375731	1.429071	1.42449	1.419955	1.415562	1.411274	1.494082	1.489845
1.381875	1.377446	1.43087	1.426333	1.42182	1.417367	1.412978	1.495784	1.491496
1.383564	1.379121	1.374688	1.370264	1.365849	1.419183	1.414735	1.49754	1.493201
1.385227	1.38077	1.376349	1.371981	1.367616	1.421012	1.416531	1.499337	1.494947
1.386933	1.382463	1.378045	1.3737	1.369355	1.364905	1.360438	1.413905	1.409601
1.38868	1.384201	1.379776	1.375418	1.371057	1.366587	1.362097	1.415622	1.411302
1.307491	1.303669	1.381528	1.377157	1.372785	1.368304	1.363801	1.35943	1.355164
1.30899	1.30519	1.383306	1.378935	1.37457	1.370093	1.365589	1.361187	1.356866
1.310472	1.306695	1.385086	1.380717	1.376364	1.371899	1.367403	1.362976	1.3586
1.311888	1.308143	1.386827	1.382466	1.378128	1.373683	1.369206	1.364773	1.360369
1.313291	1.30957	1.388566	1.384208	1.379886	1.375462	1.371009	1.366581	1.362167
1.314792	1.311051	1.390366	1.385987	1.381658	1.377242	1.372805	1.368384	1.363973
1.316371	1.312571	1.392216	1.387796	1.38344	1.379021	1.374595	1.370186	1.36579
1.317973	1.314114	1.310355	1.306733	1.30311	1.380804	1.376378	1.371978	1.367602
1.319597	1.315681	1.311881	1.308252	1.304631	1.382592	1.378153	1.373758	1.369405
1.252738	1.248819	1.313424	1.309777	1.306149	1.384383	1.379925	1.375533	1.371203
1.254406	1.250427	1.31498	1.311305	1.307661	1.386171	1.381691	1.377299	1.372992
1.256089	1.252059	1.316557	1.312845	1.309172	1.387975	1.383464	1.379066	1.374775
1.257779	1.253721	1.31817	1.314403	1.310682	1.30687	1.303042	1.380843	1.376554
1.25948	1.255409	1.319811	1.315978	1.312196	1.308365	1.304541	1.382627	1.378328
1.261193	1.257113	1.253125	1.249269	1.245473	1.309878	1.306043	1.384406	1.380092
1.262919	1.258836	1.254821	1.250902	1.247041	1.311406	1.307547	1.38618	1.381845

							0.480934	0.479736
							0.47998	0.478846
							0.853932	0.850369
							0.851672	0.84839
					0.479865	0.478624	0.849595	0.846486
					0.47901	0.477777	0.847758	0.844682
					0.478162	0.47694	0.845993	0.842878
					0.851259	0.847631	1.202213	1.198448
					0.849283	0.845811	1.200066	1.1962
		0.716779	0.712403	0.708222	1.205818	1.201863	1.265859	1.261608
		0.714512	0.710346	0.706393	1.203686	1.199738	1.263558	1.259306
		0.855005	0.851096	0.847391	1.269842	1.265529	1.331923	1.3274
		0.852914	0.849135	0.845546	1.267522	1.263211	1.329574	1.325077
0.716615	0.712307	1.209479	1.205287	1.201261	1.265199	1.260899	1.468932	1.464035
0.714413	0.710282	1.207246	1.20311	1.199106	1.26287	1.258589	1.466395	1.461562
0.712275	0.708313	1.205054	1.200954	1.196948	1.260543	1.256294	1.46387	1.459124
0.852738	0.84895	1.271366	1.266887	1.262497	1.329231	1.32478	1.603862	1.598752
0.85077	0.847036	1.268973	1.264509	1.260133	1.326865	1.322484	1.60107	1.596062
1.206695	1.202529	1.266573	1.262164	1.257849	1.397867	1.393324	1.740728	1.735363
1.204452	1.200317	1.264167	1.259847	1.255633	1.395621	1.391098	1.737797	1.732388
1.270773	1.266219	1.333476	1.329025	1.324719	1.58796	1.582693	1.734972	1.729523
1.268377	1.263828	1.331141	1.326823	1.322662	1.58538	1.580172	1.732293	1.72681
1.266046	1.26153	1.402263	1.397841	1.393537	1.708034	1.702551	1.729579	1.724089
1.263847	1.259417	1.400048	1.395617	1.391289	1.705162	1.699685	1.726722	1.72128
1.261664	1.257344	1.397852	1.39339	1.389011	1.70225	1.696776	1.723826	1.718446
1.331445	1.326971	1.669113	1.663754	1.658468	1.731912	1.726403	1.768363	1.762959
1.329007	1.324649	1.666213	1.66088	1.655601	1.728969	1.723523	1.765481	1.760085
1.602886	1.597843	1.709855	1.704336	1.69887	1.726036	1.72064	1.762595	1.757215
1.600113	1.59511	1.706872	1.701378	1.695934	1.723105	1.717749	1.759703	1.754345
1.66822	1.662901	1.703942	1.698485	1.693079	1.767596	1.762217	1.778486	1.772788
1.665414	1.660117	1.701063	1.695665	1.69032	1.764795	1.759418	1.775476	1.769834
1.662688	1.657414	1.698268	1.692925	1.687636	1.76207	1.756693	1.772562	1.766973
1.706495	1.701034	1.728334	1.722928	1.717584	1.759468	1.754102	2.031568	2.024733

1.70385	1.698421	1.725731	1.720347	1.715024	1.756892	1.751541	2.028254	2.021509
1.701034	1.695654	1.770278	1.764871	1.759509	1.775548	1.769888	2.024786	2.018113
1.698115	1.692796	1.767425	1.762024	1.756654	1.77253	1.766915	2.021236	2.014617
1.695276	1.689998	1.764605	1.75921	1.75384	2.031368	2.024497	2.047101	2.039938
1.692509	1.687251	1.761807	1.756419	1.751057	2.027813	2.021038	2.043377	2.036221
1.739611	1.734327	1.901946	1.89583	1.889797	2.024352	2.017654	2.092778	2.085593
1.737049	1.731759	1.898929	1.892861	1.886876	2.021038	2.014388	2.089261	2.082086
1.73447	1.729167	1.895934	1.889908	1.883967	2.017738	2.011117	2.085743	2.078593
1.761826	1.756435	2.034826	2.02787	2.021038	2.043386	2.036206	2.118484	2.111378
1.758945	1.753546	2.031161	2.024291	2.017544	2.039603	2.032441	2.114705	2.107682
1.898767	1.892645	2.027562	2.020755	2.014073	2.088844	2.081656	2.124701	2.117812
1.895648	1.889521	2.02401	2.017248	2.010616	2.085063	2.077903	2.121022	2.114342
1.946521	1.939894	2.086768	2.079472	2.072278	2.117755	2.110657	2.155917	2.149263
1.943288	1.936733	2.083249	2.075982	2.06882	2.114316	2.107295	2.152583	2.146027
2.094465	2.087035	2.096107	2.088835	2.081672	2.150957	2.143959	2.149297	2.142799
2.090758	2.08335	2.092423	2.085177	2.078065	2.147395	2.140564	2.145976	2.139478
2.087062	2.079674	2.088748	2.081537	2.074489	2.143867	2.137208	2.142688	2.136171
2.083483	2.076135	2.161521	2.15431	2.147272	2.152593	2.146034	2.176253	2.16964
2.079962	2.072663	2.158031	2.150896	2.143934	2.149323	2.14284	2.173041	2.166458
2.136637	2.129259	2.154451	2.147433	2.140587	2.160889	2.154386	2.169716	2.163147
2.132851	2.125516	2.150793	2.143931	2.137243	2.157544	2.151008	2.166284	2.159718
2.161568	2.154299	2.162849	2.156067	2.14946	2.176152	2.16952	2.162924	2.156369
2.158141	2.150911	2.159472	2.152774	2.14625	2.172917	2.16628	2.159693	2.153166
2.15477	2.147593	2.156165	2.149509	2.143017	2.169651	2.16302	2.156449	2.149947
2.151384	2.144287	2.175629	2.168864	2.162245	2.166235	2.159632	2.162818	2.156331
2.148	2.141009	2.172335	2.165517	2.158818	2.1628	2.156228	2.159452	2.152976
2.16011	2.153246	2.179738	2.172918	2.166176	2.159522	2.152951	2.154766	2.148357
2.156611	2.149955	2.176567	2.169779	2.163013	2.156344	2.149753	2.151598	2.14525
2.176046	2.169375	2.173345	2.166598	2.159852	2.162926	2.156306	2.154974	2.148591
2.172907	2.166163	2.170077	2.163385	2.156708	2.159773	2.153077	2.151697	2.145314
2.169767	2.162928	2.166766	2.160101	2.153479	2.161775	2.155051	2.161518	2.15509
2.166597	2.159642	2.163378	2.156683	2.150078	2.158483	2.151907	2.158439	2.152025
2.163358	2.156333	2.160011	2.153292	2.1467	2.155218	2.1488	2.1554	2.14901
2.159936	2.152992	2.171837	2.165137	2.158552	2.152085	2.145726	2.20902	2.202107
2.156449	2.149652	2.168643	2.162008	2.15546	2.14902	2.142675	2.20577	2.198871
2.162438	2.155763	2.165485	2.158909	2.152403	2.184806	2.178347	2.209091	2.202283
2.159227	2.152597	2.162367	2.155849	2.149389	2.181706	2.175196	2.205806	2.199112
2.17244	2.165812	2.159259	2.152763	2.146321	2.216366	2.209412	2.202617	2.195992
2.169318	2.162711	2.156166	2.149638	2.143174	2.213065	2.206258	2.199545	2.19293
2.1662	2.1596	2.199374	2.192777	2.186256	2.209818	2.203142	2.196496	2.189881
2.16307	2.156442	2.196139	2.189605	2.183168	2.206676	2.200058	2.193445	2.186838
2.159921	2.153281	2.192924	2.186475	2.180137	2.203585	2.19699	2.190388	2.183777
2.156706	2.150144	2.220716	2.21389	2.207176	2.200518	2.193921	2.194061	2.187395
2.153471	2.147034	2.217525	2.210788	2.204126	2.197474	2.190844	2.190939	2.184221
2.196425	2.190029	2.214377	2.207714	2.201096	2.201174	2.194446	2.187758	2.181086
2.193361	2.187019	2.211278	2.204675	2.198093	2.198084	2.191212	2.184515	2.17798
2.221639	2.214881	2.20821	2.201631	2.195053	2.194996	2.18806	2.181379	2.174949
2.21852	2.211819	2.205181	2.198583	2.191975	2.191924	2.18503	2.178393	2.172009
2.215425	2.208773	2.208919	2.202258	2.195582	2.188821	2.182024	2.183961	2.177621
2.21234	2.205727	2.205865	2.199102	2.192324	2.185622	2.178986	2.181035	2.174765

2.209274	2.202686	2.202806	2.195938	2.18906	2.182426	2.175964	2.178136	2.17194
2.212991	2.206391	2.199747	2.192851	2.185956	2.179373	2.173005	2.175251	2.169106
2.209968	2.203346	2.196688	2.189815	2.18296	2.176417	2.170097	2.172388	2.166286
2.206937	2.200303	2.193646	2.186816	2.18002	2.173524	2.167248	2.169596	2.163565
2.203899	2.197262	2.19062	2.183857	2.177144	2.170701	2.164465	2.166875	2.160934
2.200857	2.194224	2.187605	2.18091	2.174278	2.17636	2.170157	2.164186	2.158415
2.197803	2.19118	2.184589	2.177958	2.1714	2.173511	2.167329	2.161508	2.156037
2.194756	2.188159	2.181608	2.175036	2.168545	2.170689	2.164539	2.158881	2.153725
2.191732	2.185189	2.178697	2.172178	2.165741	2.167922	2.161815	2.156302	2.151407
2.188723	2.18225	2.175828	2.169355	2.162964	2.165205	2.159182	2.153815	2.14914
2.185725	2.179319	2.181433	2.174984	2.168631	2.16256	2.156757	2.151559	2.146998
2.18274	2.176402	2.178579	2.172142	2.16583	2.159981	2.15451	2.149499	2.144966
2.179796	2.173517	2.175752	2.169343	2.163091	2.157452	2.152287	2.147487	2.143069
2.176893	2.170667	2.172954	2.166582	2.160397	2.154954	2.150075	2.145524	2.141334
2.182479	2.17629	2.170173	2.163897	2.157845	2.15259	2.14794	2.147987	2.14401
2.17963	2.173449	2.167394	2.161316	2.155502	2.150419	2.145905	2.146088	2.142231
2.176821	2.170634	2.164645	2.158806	2.153277	2.148362	2.14395	2.144237	2.140465
2.174069	2.167863	2.161932	2.156331	2.151091	2.150738	2.146464	2.147784	2.144079
2.171364	2.165141	2.159277	2.153922	2.148979	2.148809	2.144655	2.146076	2.142423
2.168685	2.16252	2.161159	2.156037	2.151352	2.146968	2.142906	2.144405	2.140789
2.166033	2.159991	2.158792	2.153905	2.149461	2.145213	2.14122	2.142776	2.139181
2.16783	2.161962	2.156563	2.151869	2.147608	2.143476	2.139552	2.141163	2.137585
2.165327	2.159684	2.154494	2.149939	2.145782	2.141745	2.137896	2.139563	2.136001
2.162853	2.157456	2.15249	2.148065	2.143997	2.145451	2.141658	2.137977	2.134425
2.160331	2.155193	2.150476	2.146208	2.142253	2.143797	2.14005	2.136396	2.132846
2.157889	2.153005	2.148526	2.144404	2.140551	2.142175	2.13847	2.134837	2.131279
2.155726	2.151041	2.146729	2.142696	2.138896	2.140594	2.136949	2.133335	2.129748
2.153757	2.149235	2.145043	2.141061	2.137284	2.139046	2.135467	2.13187	2.128242
2.151784	2.147427	2.148837	2.144907	2.141147	2.137491	2.133941	2.12806	2.124437
2.149814	2.145625	2.147164	2.143302	2.139573	2.135928	2.132369	2.126495	2.122911
2.147926	2.143869	2.145511	2.141719	2.138034	2.12983	2.126225	2.131385	2.12785
2.146152	2.14217	2.14388	2.14017	2.136554	2.128347	2.124715	2.129891	2.126424
2.14443	2.140506	2.142268	2.138623	2.135063	2.126861	2.123223	2.128423	2.12501
2.14828	2.144402	2.140668	2.13704	2.133491	2.1276	2.124044	2.12698	2.123572
2.146614	2.142796	2.139093	2.135457	2.131893	2.126045	2.122573	2.125548	2.122129
2.145001	2.141245	2.135287	2.131621	2.12802	2.130921	2.127495	2.133653	2.130186
2.143437	2.139743	2.133801	2.130124	2.126498	2.129396	2.125988	2.132146	2.128716
2.139638	2.135943	2.138708	2.135035	2.131411	2.127913	2.124511	2.13067	2.127286
2.138159	2.134435	2.137168	2.133535	2.129964	2.126476	2.123066	2.129223	2.125903
2.143081	2.139337	2.135653	2.132053	2.128523	2.13466	2.13119	2.127811	2.124528
2.14154	2.137842	2.134196	2.1306	2.127068	2.13321	2.129806	2.126453	2.123147
2.139999	2.136348	2.132744	2.129155	2.125626	2.131778	2.128437	2.125105	2.121774
2.138506	2.134842	2.131237	2.127695	2.124225	2.130381	2.127042	2.123722	2.12041
2.137031	2.133332	2.129713	2.126235	2.122842	2.129001	2.125639	2.122327	2.11906
2.135518	2.131833	2.137927	2.134406	2.13099	2.127588	2.12423	2.120948	2.117742
2.133974	2.130343	2.13645	2.132944	2.129534	2.12615	2.122817	2.119582	2.116454
2.132469	2.128879	2.134992	2.131499	2.128095	2.124741	2.121451	2.118258	2.115172
2.13102	2.127446	2.133548	2.130061	2.126668	2.123363	2.120145	2.116987	2.113893
2.139331	2.13569	2.132134	2.128669	2.125295	2.12203	2.118862	2.115728	2.112629
2.13777	2.134228	2.13077	2.127361	2.124022	2.120762	2.117577	2.114451	2.111388

2.136224	2.132783	2.129427	2.126085	2.122791	2.119533	2.116319	2.113193	2.110162
2.134803	2.131387	2.128061	2.124777	2.121542	2.11832	2.115125	2.111997	2.108937
2.133456	2.130027	2.126694	2.123461	2.120292	2.117123	2.113972	2.110842	2.107723
2.132085	2.128684	2.125378	2.122171	2.119031	2.115901	2.112796	2.123143	2.119978
2.13069	2.127361	2.124113	2.120907	2.117755	2.114655	2.1116	2.121962	2.118827
2.129346	2.126053	2.122837	2.119644	2.116503	2.126886	2.123828	2.120758	2.11767
2.128081	2.124755	2.121517	2.118373	2.115292	2.125651	2.122564	2.119513	2.116498
2.126838	2.123488	2.12023	2.117121	2.114084	2.124414	2.121304	2.118271	2.115317
2.125589	2.122282	2.132518	2.129351	2.126245	2.123147	2.120075	2.117065	2.114118
2.124337	2.121099	2.131346	2.128136	2.124969	2.121882	2.11886	2.115872	2.112917
2.136534	2.133332	2.130153	2.12695	2.123772	2.120675	2.117642	2.13413	2.130873
2.13519	2.132059	2.128951	2.125788	2.122631	2.119512	2.116429	2.132757	2.129596
2.133916	2.130798	2.127712	2.12459	2.121472	2.118362	2.11527	2.131474	2.12836
2.132716	2.129544	2.126424	2.123345	2.120296	2.117231	2.114173	2.130293	2.127173
2.131545	2.128329	2.125176	2.122124	2.119123	2.124397	2.121153	2.129117	2.125971
2.130403	2.127177	2.124013	2.120959	2.117963	2.123181	2.119993	2.127918	2.12472
2.129262	2.126044	2.122877	2.119809	2.1168	2.121957	2.118821	2.126709	2.123467
2.128092	2.124897	2.121737	2.118647	2.115613	2.120707	2.117619	2.125491	2.122257
2.126899	2.12374	2.120598	2.117487	2.114422	2.119449	2.116393	2.124255	2.121056
2.125655	2.122539	2.128048	2.124695	2.121393	2.129329	2.126149	2.137596	2.134125
2.124363	2.121299	2.126776	2.123479	2.120214	2.128084	2.124835	2.136144	2.132732
2.123125	2.120092	2.12552	2.122268	2.119036	2.126853	2.123562	2.134745	2.131356
2.121958	2.118929	2.124285	2.121061	2.117856	2.125641	2.122346	2.133418	2.130009
2.129571	2.126291	2.134364	2.131019	2.127732	2.124431	2.121139	2.132098	2.128645
2.12833	2.125072	2.133115	2.129791	2.126514	2.12322	2.119928	2.130759	2.127228
2.127093	2.12385	2.131869	2.128561	2.125292	2.122005	2.118713	2.129419	2.125808
2.125845	2.122583	2.130543	2.127273	2.124045	2.130235	2.126704	2.128085	2.124442
2.124596	2.121295	2.129182	2.125957	2.122785	2.128924	2.125395	2.126755	2.123106
2.1347	2.131235	2.127884	2.124684	2.12154	2.127618	2.124091	2.125427	2.12177
2.133433	2.129983	2.126643	2.123449	2.120308	2.126316	2.122793	2.124103	2.120437
2.132183	2.128747	2.125414	2.122223	2.11908	2.129975	2.126394	2.122776	2.1191
2.130951	2.127524	2.124193	2.121002	2.117857	2.128658	2.12507	2.121442	2.117751
2.129723	2.126305	2.122976	2.119784	2.116634	2.127337	2.123741	2.12011	2.116419
2.128487	2.125082	2.121756	2.118562	2.115406	2.126007	2.122405	2.118787	2.11513
2.127249	2.123859	2.120539	2.117341	2.114177	2.124673	2.121065	2.117468	2.113864
2.126013	2.122637	2.1288	2.125384	2.121997	2.123341	2.119728	2.120629	2.117023
2.124779	2.121418	2.127521	2.124098	2.1207	2.12201	2.118392	2.119311	2.115757
2.123554	2.120203	2.126235	2.122805	2.119395	2.12525	2.121567	2.137053	2.133306
2.122339	2.118989	2.124935	2.121499	2.118081	2.123885	2.12019	2.135613	2.131945
2.121127	2.117797	2.128788	2.125277	2.121779	2.122539	2.118853	2.134216	2.130601
2.119915	2.116646	2.127609	2.124055	2.120494	2.121232	2.117589	2.132897	2.129289
2.118706	2.115494	2.126425	2.122828	2.119209	2.119934	2.116345	2.131602	2.12799
2.12321	2.119946	2.129582	2.125911	2.122223	2.118622	2.115081	2.130293	2.126689
2.121991	2.11871	2.128225	2.124553	2.120877	2.117308	2.113815	2.128985	2.125393
2.12083	2.117514	2.126893	2.123208	2.119535	2.134997	2.13131	2.128884	2.125297
2.119716	2.116349	2.125575	2.121869	2.118195	2.133598	2.129967	2.127574	2.124004
2.131641	2.127946	2.124249	2.120547	2.116895	2.132242	2.128648	2.126286	2.122758
2.130323	2.126609	2.122913	2.119248	2.115642	2.130934	2.12735	2.12502	2.12157
2.128988	2.12526	2.121573	2.117953	2.114404	2.129657	2.126092	2.123789	2.120388
2.127644	2.1239	2.120218	2.116644	2.113159	2.128406	2.124909	2.122625	2.119197

2.1263	2.122546	2.118875	2.115354	2.111937	2.12718	2.123741	2.121465	2.117994
2.124968	2.121217	2.11757	2.114118	2.110778	2.12598	2.122524	2.120233	2.116756
2.123653	2.119918	2.116302	2.112924	2.109654	2.124794	2.121282	2.118961	2.115495
2.114573	2.11108	2.134166	2.130573	2.127091	2.124736	2.121189	2.117686	2.114228
2.113462	2.110016	2.133043	2.129395	2.125834	2.123446	2.119901	2.116402	2.112952
2.11231	2.108899	2.131863	2.128185	2.124575	2.122168	2.118631	2.115139	2.111694
2.111084	2.107691	2.130581	2.126916	2.12331	2.120904	2.117376	2.11389	2.110446
2.109855	2.106473	2.129282	2.125648	2.12207	2.119678	2.116167	2.11269	2.109246
2.108648	2.105268	2.127989	2.124411	2.120894	2.118539	2.115057	2.111595	2.108152
2.107468	2.104086	2.126714	2.123191	2.119736	2.117421	2.11397	2.110526	2.107086
2.111704	2.108285	2.125526	2.121991	2.118525	2.116215	2.112789	2.115285	2.111772
2.110645	2.107232	2.124395	2.120808	2.117285	2.114957	2.11155	2.11403	2.110529
2.109532	2.106127	2.116678	2.113187	2.10976	2.113723	2.110321	2.112794	2.109328
2.108358	2.104962	2.115455	2.11199	2.108589	2.112503	2.10909	2.111571	2.108171
2.121383	2.117793	2.115592	2.112144	2.108758	2.117301	2.113814	2.11038	2.107009
2.12026	2.116679	2.114482	2.111061	2.107702	2.116204	2.112708	2.109249	2.10583
2.119134	2.115563	2.113373	2.109977	2.106642	2.115106	2.111604	2.108115	2.10464
2.117921	2.114365	2.112187	2.108811	2.105494	2.113911	2.110408	2.106913	2.103425
2.116685	2.11315	2.110985	2.107625	2.104321	2.112687	2.109185	2.105693	2.102208
2.115537	2.112026	2.116189	2.112765	2.109389	2.111541	2.108037	2.104544	2.101061
2.114451	2.11097	2.115126	2.111703	2.108318	2.110449	2.106939	2.103445	2.099966
2.113306	2.109858	2.114007	2.110585	2.107192	2.109298	2.105787	2.10412	2.100635
2.112077	2.108668	2.112811	2.109391	2.105989	2.108062	2.104555	2.102871	2.099386
2.117417	2.113975	2.111638	2.108221	2.104812	2.108684	2.105162	2.101662	2.098181
2.116286	2.112873	2.110553	2.107137	2.103722	2.107551	2.104044	2.100558	2.097086
2.115175	2.111783	2.109472	2.106056	2.102638	2.106433	2.10295	2.099483	2.096025
2.115083	2.111681	2.110342	2.106882	2.103432	2.100026	2.096652	2.09839	2.094951
2.113892	2.110483	2.109107	2.105649	2.102209	2.09884	2.095519	2.097286	2.093869
2.114851	2.111396	2.107958	2.104498	2.101058	2.097686	2.094364	2.096124	2.092724
2.113779	2.110326	2.106883	2.103419	2.099967	2.096558	2.093183	2.094904	2.091518
2.112712	2.109258	2.105809	2.102344	2.098886	2.095453	2.092044	2.094525	2.091129
2.111655	2.108196	2.10474	2.101277	2.097823	2.094389	2.090977	2.09344	2.09007
2.11056	2.107095	2.103632	2.100171	2.096721	2.094266	2.090831	2.092341	2.089
2.109363	2.105889	2.102419	2.098956	2.095509	2.093049	2.089625	2.091141	2.087834
2.108146	2.104664	2.101187	2.097723	2.094278	2.091815	2.088408	2.089933	2.08666
2.107019	2.103537	2.100061	2.096596	2.093153	2.090685	2.087295	2.088824	2.085573
2.105947	2.10247	2.098999	2.095535	2.092094	2.089621	2.086248	2.087782	2.084543
2.104888	2.101412	2.098935	2.095445	2.091982	2.088577	2.085222	2.099343	2.095775
2.103848	2.100367	2.097879	2.094396	2.090946	2.087557	2.084222	2.09828	2.094711
2.103836	2.100319	2.096821	2.093347	2.089909	2.086538	2.083223	2.097219	2.093649
2.102773	2.099254	2.095757	2.09229	2.088864	2.085512	2.08222	2.096155	2.092585
2.101709	2.098186	2.094691	2.091231	2.087817	2.084482	2.08121	2.095077	2.091506
2.100644	2.097119	2.093625	2.090172	2.086768	2.083447	2.080191	2.089407	2.085854
2.099577	2.096049	2.092556	2.089111	2.085718	2.082405	2.079156	2.08827	2.084717
2.09851	2.094977	2.091483	2.08805	2.084668	2.094276	2.090666	2.087076	2.083512
2.097441	2.093902	2.090406	2.086986	2.083616	2.093103	2.089454	2.085836	2.082251
2.096344	2.092783	2.089275	2.085869	2.082518	2.091919	2.088269	2.084645	2.081051
2.095218	2.091611	2.088074	2.084679	2.081358	2.090716	2.087112	2.083514	2.079923
2.094065	2.090438	2.086895	2.083518	2.080228	2.089539	2.085976	2.082402	2.078816
2.092862	2.089273	2.085777	2.082432	2.079169	2.088414	2.084867	2.081303	2.077723

2.091671	2.088133	2.084692	2.081382	2.078143	2.087315	2.083773	2.080215	2.07664
2.090566	2.087051	2.083637	2.08035	2.077132	2.086227	2.082687	2.079132	2.075558
2.08952	2.086013	2.08261	2.079339	2.076136	2.085151	2.081609	2.078053	2.074478
2.088539	2.085023	2.094787	2.091165	2.08762	2.084065	2.080522	2.076966	2.073392
2.087624	2.084082	2.093724	2.090087	2.086533	2.082973	2.079428	2.075873	2.072301
2.086665	2.083121	2.092661	2.089012	2.085445	2.081878	2.07833	2.074777	2.07121
2.085638	2.082122	2.091588	2.087932	2.084351	2.080777	2.077227	2.073677	2.070121
2.084596	2.081129	2.090541	2.086876	2.083272	2.079686	2.076127	2.072578	2.069034
2.083558	2.080172	2.089555	2.085869	2.08222	2.078607	2.075029	2.071478	2.067947
2.08252	2.079217	2.088582	2.084883	2.081193	2.077551	2.073946	2.070385	2.066866
2.081492	2.078229	2.087562	2.08389	2.080212	2.076548	2.072897	2.074543	2.070821
2.08047	2.077221	2.086513	2.082894	2.07926	2.07558	2.071876	2.073437	2.069708
2.092593	2.089004	2.08544	2.081856	2.078257	2.074589	2.070882	2.072385	2.068634
2.091494	2.087902	2.084339	2.080775	2.077206	2.07358	2.069921	2.071389	2.067597
2.090394	2.0868	2.083236	2.079682	2.07613	2.072541	2.068933	2.070384	2.066591
2.089286	2.085698	2.082137	2.078577	2.075025	2.071463	2.0679	2.069361	2.065633
2.088187	2.084602	2.08104	2.07747	2.073911	2.070369	2.066844	2.068314	2.064663
2.088811	2.085215	2.079945	2.076367	2.072804	2.069271	2.065767	2.067225	2.063625
2.087754	2.084142	2.078859	2.07527	2.071701	2.068172	2.064679	2.066114	2.062555
2.086746	2.083106	2.077792	2.074186	2.070608	2.072295	2.068613	2.065005	2.061475
2.085778	2.0821	2.076745	2.073117	2.069527	2.071161	2.067489	2.063895	2.060385
2.084778	2.081103	2.077446	2.073814	2.070217	2.070098	2.066413	2.062817	2.059316
2.083742	2.080123	2.076511	2.072901	2.069308	2.06912	2.065389	2.061767	2.058261
2.082688	2.079111	2.075537	2.071953	2.068376	2.068146	2.064404	2.060774	2.057262
2.081595	2.078023	2.074466	2.070911	2.067373	2.067156	2.063482	2.059884	2.05637
2.080521	2.076936	2.073379	2.069846	2.066347	2.066149	2.062554	2.058996	2.055484
2.07954	2.07592	2.072335	2.068801	2.065313	2.066898	2.063333	2.057997	2.0545
2.078603	2.074941	2.071318	2.067772	2.064282	2.065822	2.062268	2.056948	2.053471
2.077621	2.07397	2.070355	2.066815	2.063329	2.064817	2.061251	2.057727	2.054248
2.076608	2.07301	2.069442	2.065924	2.062444	2.063874	2.06027	2.05672	2.053228
2.075625	2.072051	2.073954	2.070241	2.066566	2.062922	2.059313	2.05576	2.052265
2.074693	2.071089	2.072913	2.069208	2.065558	2.061951	2.058389	2.054867	2.051387
2.073747	2.07013	2.071892	2.068199	2.06457	2.06099	2.05746	2.05396	2.050494
2.072751	2.069182	2.070954	2.067281	2.063658	2.06006	2.056496	2.052979	2.049513
2.071739	2.068228	2.070022	2.066375	2.062755	2.059136	2.05553	2.051991	2.048522
2.070727	2.067233	2.069	2.065378	2.061784	2.058188	2.054604	2.051078	2.047611
2.069719	2.06622	2.067935	2.064338	2.060779	2.057229	2.053697	2.050199	2.046739
2.068766	2.06527	2.066944	2.06336	2.059818	2.056277	2.052751	2.049259	2.045803
2.06787	2.064384	2.066026	2.062446	2.058898	2.055331	2.051765	2.048255	2.044804
2.066975	2.063487	2.065081	2.061502	2.057954	2.054379	2.0508	2.047286	2.043838
2.066076	2.062559	2.064072	2.060491	2.056956	2.053412	2.049879	2.046389	2.042944
2.065166	2.061632	2.063078	2.059494	2.055966	2.052449	2.048954	2.045485	2.042042
2.069513	2.065812	2.062156	2.058573	2.055034	2.051499	2.047982	2.044504	2.041067
2.068516	2.064874	2.061263	2.057681	2.054121	2.050558	2.047005	2.043511	2.04008
2.067577	2.063956	2.060358	2.056765	2.053189	2.049628	2.046085	2.042601	2.039174
2.066678	2.063055	2.059449	2.055839	2.052249	2.048707	2.045207	2.041746	2.038325
2.065768	2.062133	2.058518	2.054908	2.051325	2.047803	2.04433	2.040884	2.037469
2.064851	2.061188	2.057558	2.05397	2.050422	2.04692	2.043459	2.040023	2.036614
2.063917	2.060244	2.056614	2.053046	2.049522	2.046026	2.042561	2.039125	2.035722
2.062943	2.059301	2.055702	2.052148	2.048625	2.045106	2.041603	2.038151	2.034755

2.06198	2.058375	2.054807	2.051256	2.047723	2.044181	2.040643	2.037178	2.033789
2.061082	2.057487	2.053918	2.050347	2.046793	2.043253	2.039733	2.03628	2.032897
2.06022	2.056623	2.05304	2.04944	2.045857	2.042332	2.038851	2.03542	2.032042
2.059359	2.055768	2.052184	2.048571	2.044976	2.041455	2.037991	2.034567	2.031188
2.058506	2.054926	2.051347	2.047736	2.044138	2.040617	2.037154	2.033726	2.03034
2.057635	2.054049	2.050469	2.046873	2.043294	2.039782	2.036319	2.032882	2.029474
2.056746	2.053126	2.04953	2.045969	2.042439	2.038947	2.035488	2.032033	2.028581
2.055837	2.052198	2.048598	2.04507	2.041583	2.03811	2.034655	2.031187	2.027701
2.054892	2.051275	2.047703	2.044196	2.040727	2.037268	2.033824	2.030359	2.026863
2.053949	2.050365	2.046826	2.043333	2.039871	2.036419	2.032979	2.02952	2.026033
2.053064	2.049493	2.045967	2.042478	2.039014	2.035546	2.032083	2.028625	2.025168
2.052214	2.048648	2.045123	2.041632	2.03816	2.034663	2.031159	2.0277	2.024287
2.051373	2.04781	2.044288	2.040798	2.037324	2.033812	2.030286	2.026828	2.023438
2.050542	2.046982	2.043463	2.039977	2.036506	2.032992	2.029461	2.026002	2.022618
2.049717	2.046159	2.042642	2.039159	2.03569	2.032176	2.028643	2.025185	2.021804
2.048896	2.04534	2.041824	2.03834	2.034871	2.031357	2.027825	2.024369	2.020992
2.048079	2.044525	2.04101	2.037528	2.03406	2.030548	2.027018	2.023564	2.020189
2.047266	2.043715	2.040205	2.036728	2.033264	2.029754	2.026225	2.022772	2.019396
2.046454	2.042909	2.039404	2.035933	2.032475	2.02897	2.025443	2.021989	2.018611
2.045637	2.042098	2.038601	2.035137	2.031687	2.028187	2.024664	2.021213	2.017833
2.044817	2.041286	2.037797	2.034341	2.030899	2.027407	2.023891	2.020442	2.017062
2.044005	2.040478	2.036994	2.033546	2.030112	2.026625	2.023114	2.019668	2.016287
2.043196	2.039671	2.036191	2.03275	2.029322	2.025841	2.022333	2.018888	2.015507
2.042422	2.03889	2.035402	2.031962	2.02854	2.025062	2.021559	2.018116	2.01473
2.041716	2.038154	2.034636	2.031189	2.027766	2.02429	2.020792	2.017347	2.013956
2.041011	2.037421	2.033875	2.03042	2.026999	2.023531	2.020045	2.0166	2.013194
2.040247	2.036651	2.033101	2.02965	2.026238	2.022798	2.019348	2.015907	2.012466
2.039457	2.035867	2.032324	2.028881	2.025482	2.022075	2.018671	2.015235	2.011754
2.038671	2.035088	2.031551	2.02811	2.024715	2.021325	2.017946	2.014514	2.011018
2.037884	2.034311	2.030781	2.027339	2.023941	2.020555	2.017183	2.013758	2.010265
2.037112	2.033568	2.030058	2.026607	2.023192	2.019799	2.016426	2.01301	2.009539
2.03636	2.032873	2.029401	2.025927	2.022469	2.019057	2.015676	2.012276	2.008854
2.035609	2.032168	2.028727	2.025246	2.021766	2.018334	2.014933	2.01154	2.008157
2.034843	2.031412	2.027993	2.024546	2.021096	2.017648	2.014204	2.010793	2.007419
2.034089	2.030652	2.027241	2.023833	2.020422	2.016963	2.013476	2.010044	2.006669
2.033392	2.029931	2.026498	2.023092	2.019689	2.016224	2.012725	2.00929	2.00592
2.032725	2.02923	2.02576	2.022337	2.018925	2.01546	2.011968	2.008539	2.005174
2.028601	2.025096	2.025055	2.021617	2.018189	2.014729	2.011258	2.007833	2.004454
2.027846	2.024387	2.024384	2.020929	2.017475	2.01403	2.010593	2.007171	2.003758
2.027091	2.023656	2.02368	2.020216	2.016747	2.013314	2.009903	2.006493	2.003075
2.02633	2.022889	2.022918	2.019452	2.015987	2.012562	2.009166	2.005784	2.002415
2.025592	2.022135	2.022161	2.018701	2.015246	2.011824	2.008429	2.005067	2.001744
2.024913	2.021432	2.017969	2.014511	2.011064	2.011133	2.007709	2.00434	2.001028
2.024255	2.020751	2.01727	2.013828	2.0104	2.010463	2.007007	2.00362	2.000302
2.023565	2.020071	2.016597	2.013153	2.009721	2.009794	2.006347	2.002952	1.999607
2.02286	2.019397	2.015947	2.012489	2.009038	2.009131	2.005717	2.002319	1.998931
2.022175	2.01872	2.015277	2.011821	2.008369	2.004935	2.001516	2.001675	1.998282
2.021511	2.018041	2.014587	2.011148	2.007717	2.004279	2.000843	2.001017	1.997663
2.020842	2.017368	2.013911	2.010478	2.007056	2.003618	2.000176	2.000366	1.997036
2.020155	2.016706	2.013266	2.009817	2.006372	2.002942	1.999526	1.999729	1.996384

2.019474	2.016047	2.012622	2.009157	2.005693	2.00227	1.998876	1.999093	1.995732
2.018818	2.015382	2.011952	2.008496	2.005043	2.001618	1.998214	1.994835	1.991479
2.018171	2.014717	2.011275	2.007836	2.004404	2.000974	1.99755	1.994174	1.990847
2.017504	2.014056	2.010618	2.007175	2.003739	2.000314	1.9969	1.993531	1.990206
2.016823	2.013398	2.009975	2.006515	2.003057	1.999643	1.99626	1.9929	1.98956
2.016163	2.012742	2.009323	2.005861	2.002402	1.998991	1.995615	1.992262	1.988929
2.01553	2.012087	2.008653	2.005212	2.001779	1.998359	1.994955	1.991606	1.988314
2.014893	2.01144	2.008001	2.004583	2.001176	1.99775	1.994324	1.990977	1.987714
2.014226	2.0108	2.007391	2.003995	2.000604	1.997186	1.993759	1.990409	1.987141
2.01357	2.010173	2.006794	2.003411	2.000029	1.996623	1.993206	1.989853	1.986568
2.012968	2.009571	2.006187	2.002789	1.999395	1.996003	1.992619	1.989271	1.985961
2.012399	2.008982	2.005575	2.002148	1.998731	1.995356	1.992012	1.988673	1.985339
2.011819	2.008375	2.004943	2.001512	1.998097	1.994723	1.991381	1.988049	1.984723
2.011232	2.007751	2.00429	2.000875	1.997484	1.994098	1.990726	1.987396	1.984112
2.010654	2.00715	2.003671	2.000266	1.996891	1.993498	1.990106	1.986779	1.983519
2.010093	2.006586	2.003105	1.999702	1.996329	1.992937	1.989544	1.986214	1.982953
2.000694	1.997366	2.002543	1.999133	1.995753	1.992367	1.988987	1.985659	1.982391
2.000078	1.996776	2.001951	1.998521	1.995116	1.991745	1.988404	1.985096	1.981826
1.999454	1.996181	2.00136	1.997904	1.994471	1.991116	1.987817	1.984527	1.981253
1.998892	1.995626	2.000796	1.997332	1.993886	1.990531	1.987237	1.983941	1.980643
1.99837	1.995099	2.000253	1.996788	1.99334	1.989974	1.986664	1.983343	1.980011
1.997851	1.994577	1.999718	1.996251	1.9928	1.989426	1.986105	1.982769	1.979415
1.997338	1.994065	1.999194	1.995725	1.992268	1.988889	1.985562	1.982218	1.978854
1.996824	1.993551	1.998669	1.995198	1.99174	1.988356	1.985022	1.981672	1.9783
1.996305	1.993034	1.998141	1.994672	1.991214	1.987826	1.984485	1.981128	1.97775
1.995784	1.99251	1.997606	1.99414	1.990684	1.987292	1.983943	1.980581	1.9772
1.995263	1.991982	1.997057	1.993593	1.99014	1.986747	1.983394	1.980028	1.976643
1.994736	1.991443	1.996494	1.993033	1.989586	1.98619	1.982831	1.979463	1.976079
1.994182	1.990872	1.98757	1.98426	1.980958	1.985602	1.982221	1.978851	1.975489
1.993608	1.990278	1.986959	1.983663	1.980377	1.984989	1.981574	1.978202	1.974878
1.993075	1.989747	1.986428	1.983127	1.979832	1.984416	1.980976	1.9776	1.974293
1.992591	1.989296	1.985999	1.982669	1.979332	1.983885	1.980432	1.977045	1.973733
1.992106	1.98884	1.98556	1.982209	1.97884	1.98338	1.979928	1.976534	1.973204
1.991601	1.988341	1.985069	1.981719	1.978353	1.982908	1.979485	1.976089	1.972722
1.99109	1.987829	1.984559	1.981216	1.977862	1.982438	1.979049	1.975654	1.97225
1.990579	1.987313	1.984039	1.980697	1.977347	1.981925	1.978551	1.975161	1.971747
1.990066	1.986791	1.98351	1.980166	1.976819	1.981389	1.978022	1.974637	1.971229
1.989549	1.986271	1.982987	1.97964	1.976289	1.973007	1.969765	1.974103	1.970696
1.989026	1.985751	1.982467	1.979115	1.975757	1.972471	1.969227	1.973555	1.970147
1.988512	1.985235	1.981949	1.978595	1.975235	1.971944	1.968694	1.973013	1.969607
1.988001	1.984719	1.98143	1.978077	1.974718	1.971422	1.968164	1.972476	1.969076
1.987519	1.984222	1.980924	1.977578	1.974229	1.970926	1.96765	1.971955	1.968568
1.987102	1.983768	1.980442	1.97712	1.973802	1.970484	1.967169	1.971466	1.968109
1.986697	1.983325	1.979971	1.976673	1.973387	1.970054	1.9667	1.970988	1.967663
1.986231	1.982848	1.979485	1.976192	1.972914	1.969579	1.966217	1.962926	1.95971
1.985733	1.982357	1.978997	1.975695	1.972408	1.969079	1.965731	1.962442	1.959212
1.985261	1.981901	1.978551	1.97523	1.97192	1.9686	1.96528	1.961993	1.958741
1.984813	1.981483	1.978151	1.974797	1.971447	1.968142	1.964867	1.961586	1.958296
1.984384	1.98106	1.977729	1.974349	1.970972	1.967675	1.964428	1.961153	1.957847
1.98399	1.980629	1.977262	1.973866	1.970482	1.967181	1.963935	1.960669	1.957378

1.983581	1.980184	1.976784	1.973387	1.97001	1.966702	1.963444	1.960184	1.956918
1.983107	1.979703	1.976305	1.972936	1.96959	1.966275	1.962989	1.985318	1.9818
1.98261	1.979213	1.975831	1.972501	1.969192	1.965872	1.962555	1.984834	1.981356
1.98214	1.978761	1.975397	1.972074	1.968769	1.965457	1.962148	1.984392	1.980899
1.981688	1.978335	1.974993	1.971656	1.968333	1.965037	1.961764	1.983981	1.980434
1.981263	1.977913	1.974575	1.971238	1.967915	1.974887	1.971527	1.983527	1.979948
1.98087	1.977496	1.97414	1.970821	1.967521	1.974487	1.971086	1.983023	1.979432
1.980472	1.977084	1.973718	1.970408	1.967117	1.974071	1.970633	1.982516	1.978932
1.980047	1.976682	1.97333	1.970001	1.966681	1.973614	1.970163	1.982013	1.978473
1.979622	1.976279	1.972943	1.969595	1.966248	1.973164	1.969707	1.981526	1.978026
1.979221	1.975865	1.983096	1.979667	1.976234	1.988367	1.984685	1.981085	1.97757
1.97883	1.975445	1.982649	1.979251	1.975861	1.987977	1.984287	1.980666	1.977114
1.978411	1.97503	1.982235	1.978835	1.975443	1.987519	1.983827	1.980209	1.976666
1.977971	1.974618	1.981845	1.978419	1.974989	1.987005	1.983318	1.979721	1.976223
1.981066	1.977691	1.981438	1.978004	1.974562	1.986537	1.982858	1.979269	1.975777
1.980672	1.977277	1.981001	1.97759	1.974182	1.986141	1.982472	1.978865	1.97532
1.991149	1.987656	1.984182	1.980754	1.97734	1.985738	1.982092	1.978476	1.974885
1.990714	1.987243	1.983787	1.98034	1.976904	1.985281	1.981691	1.978102	1.974508
1.990284	1.986835	1.983397	1.979927	1.976465	1.984813	1.981275	1.977719	1.974144
1.989914	1.986435	1.982976	1.979515	1.97607	1.984371	1.98082	1.977276	1.97374
1.989581	1.986044	1.982544	1.979109	1.975704	1.983945	1.980344	1.976798	1.973313
1.989241	1.985683	1.982167	1.978746	1.975358	1.987258	1.9836	1.976364	1.97288
1.988899	1.985355	1.981846	1.978427	1.975038	1.986903	1.983239	1.975972	1.972435
1.988524	1.985007	1.981516	1.978088	1.974683	1.986516	1.982866	1.979242	1.975637
1.988086	1.984615	1.98116	1.977705	1.974263	1.986069	1.982472	1.978876	1.975274
1.987661	1.984229	1.980802	1.977322	1.973846	1.985616	1.982059	1.978491	1.97491
1.987314	1.98389	1.980466	1.976974	1.97348	1.985185	1.981602	1.978041	1.974502
1.987	1.983562	1.980124	1.976632	1.973135	1.984768	1.981138	1.977574	1.974084
1.986671	1.983194	1.979728	1.976251	1.972779	1.984378	1.980734	1.97717	1.973698
1.98634	1.982802	1.979295	1.975845	1.972422	1.984011	1.980371	1.976808	1.97333
1.986008	1.98244	1.978911	1.975478	1.972082	1.98365	1.980013	1.976439	1.972931
1.985674	1.982111	1.978583	1.975154	1.971761	1.983297	1.97966	1.976059	1.972492
1.985346	1.981791	1.978266	1.974835	1.971436	1.982942	1.979308	1.975689	1.972076
1.985024	1.981473	1.97795	1.974512	1.971107	1.982588	1.978963	1.975341	1.971712
1.984708	1.98116	1.977635	1.97418	1.970757	1.982213	1.978608	1.974997	1.971367
1.984401	1.980851	1.992737	1.989035	1.985372	1.981771	1.978218	1.97464	1.971024
1.9841	1.980547	1.992381	1.988616	1.984889	1.981303	1.977814	1.974278	1.970686
1.9838	1.980244	1.992033	1.988237	1.984479	1.980897	1.977435	1.97392	1.970345
1.983502	1.979943	1.991693	1.98789	1.984125	1.980539	1.977076	1.973565	1.970001
1.983207	1.979645	1.991353	1.987549	1.983784	1.980197	1.976733	1.973223	1.969664
1.982916	1.979349	1.991014	1.987214	1.983453	1.979871	1.976409	1.972899	1.969335
1.982625	1.979053	1.990678	1.986885	1.983134	1.979555	1.976092	1.972579	1.969007
1.982328	1.978757	1.990345	1.98656	1.982816	1.979237	1.97577	1.972253	1.968677
1.982029	1.978462	1.990016	1.986248	1.982521	1.978939	1.975458	1.971931	1.968349
1.981732	1.978167	1.989693	1.985974	1.982293	1.978701	1.975174	1.971618	1.968023
1.981435	1.977873	1.989375	1.985721	1.982103	1.978497	1.974907	1.971311	1.9677
1.981139	1.977579	1.989051	1.985434	1.981852	1.978241	1.97462	1.971005	1.967384
1.980843	1.977283	1.988722	1.985116	1.981545	1.977938	1.974316	1.970699	1.967073
1.980103	1.976545	1.988401	1.984797	1.98123	1.977624	1.974004	1.970396	1.966791
1.979843	1.976272	1.98808	1.984474	1.980906	1.977299	1.97368	1.9701	1.966558

1.979591	1.97602	1.987789	1.984171	1.980591	1.976978	1.973359	1.969804	1.966318
1.994849	1.991141	1.987571	1.983926	1.980304	1.976673	1.97305	1.969498	1.966026
1.994556	1.990906	1.987372	1.983697	1.980026	1.97638	1.972758	1.969202	1.96572
1.994245	1.990619	1.987105	1.983421	1.979737	1.976107	1.972517	1.968962	1.965447
1.993924	1.990302	1.986794	1.983115	1.979441	1.97585	1.972312	1.96876	1.965192
1.990817	1.987238	1.986382	1.982706	1.979045	1.975575	1.972064	1.968514	1.96492
1.990616	1.986986	1.986079	1.982398	1.978745	1.975278	1.971767	1.968219	1.964627
1.990401	1.986733	1.985793	1.982119	1.978481	1.975008	1.971479	1.967924	1.964337
1.99014	1.986463	1.985526	1.981886	1.978282	1.974796	1.97122	1.96764	1.964047
1.989873	1.986203	1.985282	1.981671	1.97809	1.974589	1.970968	1.967367	1.963778
1.988637	1.984971	1.98237	1.978798	1.975241	1.974226	1.970596	1.967114	1.963559
1.988402	1.984772	1.982184	1.978582	1.974984	1.973958	1.970338	1.96688	1.96337
1.988197	1.984561	1.981571	1.977945	1.974326	1.973708	1.970118	1.966679	1.963172
1.98802	1.984342	1.981314	1.977678	1.974065	1.973472	1.969931	1.966508	1.962974
1.98782	1.984109	1.980416	1.976765	1.973151	1.970298	1.9668	1.967628	1.964075
1.987582	1.983849	1.980152	1.976537	1.972966	1.970115	1.966598	1.967427	1.963914
1.987366	1.983619	1.981782	1.978168	1.974608	1.971628	1.968081	1.967063	1.963564
1.987215	1.983467	1.981625	1.977989	1.974407	1.971437	1.967921	1.966905	1.963372
1.987071	1.983327	1.981481	1.977817	1.974208	1.971249	1.967763	1.966744	1.963163
1.9888	1.985019	1.981276	1.97763	1.974038	1.971077	1.967576	1.966538	1.962932
1.988564	1.984778	1.981045	1.977436	1.973882	1.970913	1.967374	1.96631	1.962692
1.988382	1.984597	1.980863	1.977252	1.973695	1.970136	1.966587	1.963623	1.960022
1.988251	1.984472	1.980728	1.977077	1.97348	1.969928	1.966416	1.963475	1.959887
1.988084	1.984308	1.98056	1.976894	1.97328	1.969729	1.966226	1.9633	1.959737
1.987847	1.984067	1.980328	1.976694	1.973113	1.969547	1.966005	1.963076	1.959558
1.987622	1.983842	1.980112	1.976502	1.972942	1.969362	1.965786	1.962852	1.959364
1.987466	1.983701	1.97997	1.976329	1.972733	1.969153	1.965594	1.962052	1.958533
1.987328	1.983585	1.979857	1.976173	1.972527	1.968944	1.96541	1.961866	1.958315
1.981465	1.97778	1.979739	1.976051	1.972384	1.968777	1.965213	1.961658	1.958116
1.981246	1.977612	1.979622	1.975951	1.972284	1.968638	1.965012	1.961439	1.957927
1.981073	1.977443	1.979465	1.975817	1.97217	1.968512	1.964852	1.961256	1.957731
1.980947	1.977266	1.97926	1.975646	1.972048	1.968404	1.964737	1.961111	1.957526
1.980831	1.977114	1.979084	1.975478	1.9719	1.968272	1.964619	1.960982	1.957351
1.980728	1.977012	1.978972	1.975321	1.971699	1.968086	1.964484	1.960868	1.95723
1.980604	1.976909	1.978875	1.975178	1.971505	1.967903	1.964349	1.960758	1.957127
1.980408	1.976759	1.973106	1.969427	1.965762	1.967774	1.964228	1.960646	1.957025
1.980184	1.976593	1.972994	1.969326	1.965658	1.967667	1.964106	1.960524	1.956924
1.98002	1.976449	1.972872	1.969215	1.965549	1.96754	1.96394	1.960356	1.956793
1.979899	1.976321	1.972741	1.969098	1.965441	1.967401	1.963742	1.96015	1.956638
1.979778	1.976171	1.972573	1.968952	1.965323	1.967274	1.963581	1.959985	1.956499
1.979654	1.975985	1.972349	1.968764	1.965192	1.967161	1.963467	1.959871	1.956382
1.979539	1.975819	1.97215	1.968593	1.965066	1.967056	1.963371	1.959769	1.956258
1.979436	1.975698	1.972016	1.968466	1.964952	1.966959	1.963286	1.959668	1.956102
1.97934	1.975599	1.971913	1.968362	1.96485	1.96687	1.96321	1.959573	1.955951
1.979254	1.975513	1.971826	1.968277	1.964767	1.961176	1.957544	1.959489	1.955843
1.979172	1.975437	1.971754	1.968205	1.964694	1.961102	1.957468	1.959413	1.95576
1.979072	1.975346	1.971668	1.968118	1.964603	1.961013	1.957384	1.959334	1.955678
1.978955	1.975244	1.971571	1.968018	1.964498	1.960912	1.957293	1.959253	1.955601
1.978841	1.975144	1.971473	1.967904	1.964364	1.960786	1.95719	1.959167	1.955522
1.978734	1.975046	1.971372	1.967759	1.964175	1.960611	1.957065	1.959071	1.955441

1.978633	1.974952	1.971277	1.967638	1.964028	1.960471	1.956954	1.953402	1.949804
1.97854	1.974863	1.971199	1.96759	1.964007	1.960442	1.956892	1.953324	1.949727
1.978456	1.974777	1.971125	1.967563	1.964027	1.960448	1.956848	1.953252	1.949652
1.968578	1.964912	1.971041	1.967499	1.963987	1.960406	1.956789	1.953184	1.94958
1.96851	1.964823	1.970951	1.967415	1.963915	1.96034	1.956725	1.953119	1.949511
1.968446	1.964744	1.96812	1.964593	1.961108	1.96027	1.956654	1.953049	1.949445
1.968389	1.964673	1.968043	1.964515	1.961032	1.960193	1.956575	1.952973	1.949378
1.968326	1.964604	1.960945	1.957441	1.953985	1.957383	1.953769	1.952911	1.949344
1.968247	1.964525	1.960867	1.957359	1.953901	1.957306	1.953701	1.952874	1.949367
1.968168	1.964461	1.960812	1.957295	1.953823	1.957234	1.953638	1.952842	1.949391
1.968102	1.964441	1.960822	1.957279	1.953767	1.957169	1.953576	1.950062	1.94664
1.968041	1.964436	1.960853	1.95728	1.953719	1.957113	1.953524	1.950013	1.946589
1.967973	1.964391	1.960824	1.957237	1.95366	1.950114	1.946595	1.950013	1.946562
1.967903	1.964319	1.960752	1.957164	1.953593	1.950085	1.94662	1.950052	1.946551
1.961186	1.957554	1.960683	1.957091	1.953526	1.950039	1.946607	1.950048	1.946517
1.961216	1.957531	1.960616	1.957013	1.953451	1.949968	1.946543	1.949991	1.946456
1.961238	1.957508	1.960561	1.956959	1.953408	1.94992	1.946481	1.943009	1.939502
1.961218	1.957465	1.960518	1.956949	1.953432	1.949926	1.94644	1.942944	1.939437
1.961188	1.957431	1.9605	1.95696	1.953471	1.949943	1.946406	1.942889	1.939389
1.961159	1.957439	1.953769	1.950202	1.946675	1.949928	1.946371	1.942858	1.93939
1.961133	1.957469	1.953834	1.950236	1.946661	1.949901	1.946345	1.942849	1.93942
1.961141	1.957482	1.953844	1.950228	1.946634	1.943062	1.939513	1.942877	1.939446
1.96118	1.957483	1.953809	1.950182	1.946594	1.943057	1.939563	1.94294	1.939469
1.953455	1.949777	1.953774	1.950153	1.946583	1.943067	1.939597	1.94299	1.939509
1.953423	1.949731	1.953734	1.950148	1.946618	1.943099	1.939602	1.943012	1.939576
1.95341	1.94971	1.953722	1.950159	1.946654	1.943131	1.939614	1.936142	1.932724
1.95346	1.949764	1.953779	1.950191	1.946661	1.943146	1.939658	1.936186	1.932738
1.953525	1.949834	1.953853	1.950231	1.946667	1.943161	1.939706	1.93623	1.932734
1.953538	1.949842	1.953868	1.950259	1.946706	1.943196	1.939724	1.93623	1.93271
1.953527	1.949822	1.953856	1.950281	1.946765	1.943242	1.939728	1.936208	1.932675
1.953558	1.949854	1.946198	1.942664	1.939185	1.943276	1.939764	1.936243	1.932707
1.953636	1.949942	1.94628	1.942707	1.939187	1.943298	1.939833	1.936338	1.932809
1.953687	1.949999	1.946337	1.942747	1.939208	1.943333	1.939888	1.936411	1.932903
1.953674	1.949986	1.946335	1.942777	1.93927	1.943393	1.939906	1.936431	1.932973
1.953668	1.94998	1.946341	1.942811	1.939331	1.943453	1.939928	1.93645	1.933028
1.95373	1.950048	1.946406	1.942854	1.93935	1.943489	1.939988	1.936509	1.933056
1.953812	1.950143	1.946497	1.942908	1.939364	1.943521	1.94006	1.936577	1.933075
1.953848	1.950213	1.946594	1.942996	1.939431	1.943583	1.940105	1.936616	1.93312
1.953856	1.95027	1.946698	1.94311	1.939536	1.943667	1.940133	1.936635	1.933182
1.953909	1.950324	1.94676	1.94319	1.939633	1.936093	1.932571	1.936687	1.933235
1.954015	1.950368	1.946764	1.943229	1.939724	1.936198	1.932669	1.936773	1.933271
1.954129	1.950436	1.946797	1.943272	1.939793	1.936285	1.932772	1.936879	1.933334
1.954246	1.950552	1.946898	1.943329	1.939809	1.936321	1.932864	1.937007	1.933449
1.954348	1.950677	1.947023	1.943406	1.939834	1.936361	1.932959	1.937141	1.933583
1.954392	1.950767	1.94715	1.943526	1.939936	1.936463	1.933071	1.937271	1.933716
1.954414	1.950846	1.947284	1.943673	1.94008	1.936597	1.933187	1.937394	1.933851
1.952827	1.949275	1.947417	1.943816	1.940222	1.936716	1.933267	1.937478	1.933966
1.952962	1.94941	1.947551	1.94396	1.940369	1.936825	1.933315	1.937525	1.934062
1.953102	1.949528	1.947653	1.94408	1.94051	1.936949	1.933401	1.937613	1.934173
1.953243	1.949616	1.947703	1.944162	1.94064	1.937094	1.933541	1.93776	1.93431

1.953391	1.949719	1.947772	1.944257	1.940777	1.937246	1.933696	1.930202	1.926767
1.953548	1.949863	1.9479	1.944395	1.94093	1.937404	1.933851	1.930335	1.926852
1.953711	1.950027	1.948056	1.944553	1.941088	1.937564	1.934011	1.930471	1.926935
1.953872	1.950198	1.948228	1.944719	1.941245	1.93772	1.934171	1.93062	1.927058
1.954035	1.950379	1.948414	1.944892	1.941398	1.937871	1.934329	1.930777	1.927203
1.954204	1.950555	1.946928	1.943356	1.939808	1.937987	1.934467	1.930926	1.927349
1.954378	1.950729	1.94709	1.943473	1.939879	1.938063	1.934583	1.931065	1.927497
1.954553	1.950905	1.947257	1.943607	1.93998	1.938163	1.93471	1.93121	1.927649
1.954726	1.951082	1.947435	1.943772	1.94013	1.938307	1.934857	1.93136	1.927805
1.954904	1.951261	1.947615	1.943947	1.940299	1.938468	1.935017	1.931519	1.927965
1.955092	1.951445	1.947793	1.944122	1.940472	1.938638	1.935191	1.931693	1.928132
1.955283	1.951629	1.94797	1.9443	1.940653	1.938817	1.935374	1.931873	1.928301
1.953871	1.950227	1.948147	1.94448	1.940836	1.937289	1.933806	1.932043	1.928465
1.954041	1.950397	1.948323	1.944662	1.941024	1.937475	1.933985	1.932208	1.928627
1.945526	1.941916	1.948513	1.944874	1.941257	1.9377	1.934183	1.932381	1.928797
1.945689	1.94209	1.948721	1.945127	1.941549	1.937974	1.934405	1.932565	1.928977
1.941244	1.937673	1.938693	1.935174	1.931667	1.938229	1.934621	1.93101	1.927386
1.941403	1.93783	1.938856	1.935348	1.931854	1.938435	1.934818	1.931203	1.927577
1.94157	1.93799	1.939018	1.935512	1.932022	1.938623	1.935008	1.931398	1.927782
1.941756	1.938162	1.93919	1.935685	1.9322	1.938812	1.935193	1.9316	1.928027
1.941955	1.93835	1.939377	1.935868	1.93238	1.938997	1.935374	1.931804	1.928289
1.937036	1.933463	1.935026	1.931513	1.928012	1.92901	1.925425	1.932001	1.928515
1.937259	1.933724	1.935313	1.931764	1.928212	1.929193	1.925601	1.932192	1.928712
1.936722	1.933209	1.935566	1.931994	1.928412	1.924842	1.921283	1.924214	1.920748
1.936902	1.933401	1.935769	1.93219	1.928603	1.925067	1.921562	1.924504	1.920985
1.920242	1.916823	1.930843	1.927285	1.923727	1.925308	1.921836	1.922869	1.919312
1.920508	1.917052	1.931044	1.927511	1.923989	1.925572	1.922071	1.923091	1.919518
1.920785	1.917285	1.931244	1.92774	1.924258	1.925836	1.92229	1.923298	1.919731
1.921014	1.917492	1.930697	1.927204	1.923739	1.926057	1.922497	1.918964	1.915453
1.92122	1.917689	1.93089	1.927395	1.923933	1.926254	1.922698	1.919192	1.91574
1.921442	1.917918	1.931129	1.927621	1.924144	1.921367	1.917849	1.919448	1.916004
1.921677	1.918179	1.931414	1.927879	1.924368	1.921601	1.918123	1.919735	1.916251
1.921933	1.918442	1.931686	1.928142	1.924619	1.921127	1.91766	1.919994	1.916481
1.92222	1.9187	1.931922	1.928404	1.924914	1.921404	1.91789	1.920202	1.916682
1.922507	1.918966	1.932171	1.928674	1.925207	1.921676	1.918115	1.920408	1.916895
1.922765	1.919248	1.915765	1.91234	1.908934	1.921905	1.918336	1.915531	1.912067
1.923021	1.919536	1.916074	1.91262	1.909172	1.92213	1.918566	1.915774	1.91234
1.923308	1.919812	1.916342	1.912891	1.909451	1.922416	1.91885	1.915344	1.9119
1.923617	1.920082	1.916584	1.913157	1.909754	1.92274	1.919171	1.915645	1.912161
1.92391	1.92036	1.916851	1.913428	1.910033	1.923025	1.91945	1.915921	1.912433
1.924184	1.920649	1.917149	1.913707	1.910285	1.92326	1.919677	1.916163	1.912721
1.924466	1.920932	1.91743	1.913979	1.910547	1.907077	1.903594	1.916424	1.913003
1.924769	1.921196	1.917666	1.914236	1.910837	1.907379	1.903895	1.916722	1.913263
1.925069	1.921462	1.917909	1.914495	1.911124	1.907684	1.904211	1.91704	1.913536
1.925334	1.92175	1.918211	1.914774	1.911378	1.907956	1.904528	1.917384	1.913863
1.925589	1.922048	1.918537	1.915062	1.911619	1.908215	1.904841	1.917734	1.914213
1.925883	1.922339	1.918824	1.915341	1.911889	1.908483	1.905112	1.901723	1.898315
1.926207	1.922627	1.919083	1.915613	1.912181	1.908756	1.905347	1.901959	1.898595
1.926555	1.922951	1.919383	1.915905	1.91247	1.909034	1.90561	1.90222	1.898864
1.926941	1.923327	1.919738	1.91622	1.912746	1.909309	1.905908	1.902511	1.899115

1.925692	1.922089	1.9201	1.916552	1.913044	1.909607	1.906224	1.90282	1.89939
1.925983	1.922414	1.920455	1.916906	1.913388	1.909949	1.906566	1.903159	1.899721
1.926257	1.922723	1.920798	1.917261	1.913746	1.910302	1.906909	1.903498	1.900068
1.926566	1.923024	1.921097	1.917581	1.914086	1.910629	1.907203	1.903788	1.900386
1.926892	1.923322	1.921374	1.917884	1.914421	1.910947	1.907477	1.904058	1.900694
1.927233	1.923653	1.921698	1.918216	1.914764	1.911285	1.907802	1.904379	1.901023
1.927591	1.924015	1.922063	1.918575	1.915115	1.911642	1.908173	1.904748	1.901373
1.91618	1.912679	1.920833	1.917337	1.91387	1.911983	1.908535	1.905109	1.901705
1.916475	1.912999	1.921179	1.917661	1.914166	1.912289	1.908874	1.905446	1.901999
1.916781	1.913327	1.921531	1.917994	1.914474	1.912606	1.909218	1.90579	1.90231
1.91713	1.91368	1.921897	1.918354	1.914827	1.912961	1.909582	1.906158	1.902678
1.917501	1.91405	1.922275	1.918731	1.915202	1.913337	1.909956	1.906538	1.903073
1.917879	1.914435	1.916434	1.912917	1.909413	1.912154	1.908791	1.906924	1.903466
1.918266	1.914833	1.91684	1.913317	1.909803	1.912545	1.90918	1.907316	1.903863
1.91866	1.915229	1.917239	1.913712	1.910193	1.912936	1.909568	1.907705	1.904252
1.919062	1.915624	1.917633	1.914103	1.910583	1.913329	1.90996	1.908095	1.904636
1.919459	1.916011	1.912573	1.909071	1.905575	1.907544	1.904178	1.906911	1.903481
1.919846	1.916388	1.912943	1.90945	1.905961	1.907898	1.904478	1.907201	1.903808
1.920226	1.91675	1.913295	1.909817	1.906341	1.908251	1.904781	1.907495	1.904138
1.920584	1.917075	1.913598	1.910142	1.9067	1.908617	1.905135	1.907849	1.9045
1.920941	1.917396	1.913895	1.910462	1.907056	1.908994	1.905522	1.908243	1.904886
1.913569	1.91004	1.914306	1.910862	1.907447	1.909401	1.905953	1.902552	1.899201
1.91401	1.910516	1.914807	1.911327	1.907866	1.909833	1.906424	1.903032	1.899658
1.914451	1.91097	1.915273	1.911769	1.908281	1.904865	1.901499	1.903483	1.900092
1.914892	1.911398	1.915693	1.912182	1.90869	1.905275	1.901912	1.903897	1.900497
1.915325	1.911815	1.908331	1.904831	1.901354	1.90568	1.902312	1.904294	1.900888
1.915741	1.91222	1.908728	1.905231	1.90176	1.906083	1.902698	1.904671	1.901263
1.916151	1.912621	1.909124	1.905631	1.902164	1.906483	1.903079	1.905043	1.901634
1.916559	1.91303	1.90953	1.906032	1.902557	1.899127	1.895732	1.905425	1.902017
1.916969	1.913443	1.909942	1.906434	1.902947	1.899511	1.896114	1.905815	1.902411
1.917397	1.913862	1.910353	1.906839	1.903348	1.899907	1.896507	1.90086	1.897495
1.917839	1.914287	1.910763	1.907247	1.903758	1.900314	1.896909	1.901287	1.89797
1.918314	1.914738	1.911194	1.907683	1.904205	1.900754	1.897334	1.893978	1.890697
1.918838	1.915227	1.911655	1.908163	1.904709	1.901245	1.897791	1.894409	1.891111
1.919354	1.915717	1.912126	1.908646	1.90521	1.901733	1.898248	1.89484	1.891514
1.915024	1.911398	1.91259	1.909108	1.905665	1.902178	1.89868	1.895255	1.891907
1.915461	1.911853	1.913063	1.909568	1.906106	1.902613	1.899113	1.895676	1.892304
1.915921	1.912338	1.913568	1.910051	1.90656	1.903067	1.899584	1.896141	1.892737
1.916391	1.912838	1.914091	1.910547	1.90702	1.903534	1.900079	1.896635	1.893196
1.916836	1.913297	1.909777	1.906245	1.902729	1.904015	1.900562	1.897113	1.893664
1.917251	1.913708	1.910197	1.906703	1.903227	1.904516	1.901035	1.897579	1.894145
1.917696	1.914138	1.910621	1.907151	1.903703	1.904994	1.90149	1.898025	1.894599
1.918198	1.914603	1.911052	1.907579	1.904135	1.905425	1.901908	1.898433	1.894996
1.918716	1.915091	1.91151	1.90802	1.904564	1.905852	1.902334	1.89885	1.895394
1.911778	1.908202	1.912036	1.90851	1.905013	1.901519	1.898036	1.899324	1.895832
1.912267	1.908723	1.912588	1.909023	1.905477	1.901986	1.898534	1.899823	1.896295
1.912771	1.909219	1.913095	1.909525	1.905976	1.902478	1.899018	1.900309	1.89679
1.913285	1.9097	1.913571	1.910024	1.906503	1.902992	1.899495	1.900791	1.897314
1.913782	1.910186	1.906628	1.903123	1.899646	1.903504	1.899996	1.896514	1.893057
1.91425	1.910678	1.90714	1.90362	1.900125	1.904012	1.90053	1.897044	1.893549

1.914739	1.911179	1.907648	1.904119	1.900611	1.904521	1.901053	1.897562	1.894041
1.915284	1.911693	1.908136	1.904614	1.901119	1.905032	1.901538	1.898042	1.894541
1.915835	1.912211	1.908624	1.905108	1.901626	1.90554	1.902018	1.898518	1.895041
1.916339	1.912721	1.909135	1.905602	1.902098	1.898603	1.895121	1.89902	1.895538
1.916826	1.913228	1.909656	1.906095	1.902559	1.899067	1.895611	1.899533	1.896036
1.908162	1.904577	1.910153	1.906592	1.903057	1.899561	1.896097	1.900036	1.896548
1.908702	1.905073	1.910627	1.90709	1.90359	1.900081	1.89658	1.900531	1.897073
1.909237	1.905594	1.90199	1.898479	1.895004	1.891498	1.887985	1.893616	1.890213
1.909756	1.906147	1.90256	1.899009	1.895483	1.89198	1.8885	1.894147	1.890752
1.890202	1.886667	1.903146	1.899557	1.895986	1.892483	1.889031	1.8856	1.882198
1.890759	1.887233	1.903731	1.900136	1.896554	1.893045	1.889587	1.886136	1.882693
1.891329	1.887799	1.90431	1.900724	1.897149	1.89363	1.89015	1.886668	1.883182
1.891887	1.888332	1.895191	1.891652	1.888129	1.894205	1.890685	1.887185	1.883703
1.892439	1.888847	1.895695	1.892195	1.888719	1.894779	1.891207	1.887695	1.884243
1.892973	1.889382	1.885823	1.882356	1.87891	1.885768	1.882194	1.888242	1.884801
1.893486	1.889937	1.886416	1.882945	1.879483	1.886351	1.882779	1.888828	1.885379
1.888313	1.884807	1.887	1.883513	1.880026	1.876503	1.872961	1.879857	1.876423
1.888864	1.88537	1.887563	1.88404	1.880516	1.877003	1.8735	1.880422	1.876981
1.889423	1.885926	1.88811	1.884559	1.88101	1.877509	1.874043	1.880985	1.877526
1.85656	1.853404	1.882935	1.879402	1.87588	1.878067	1.874612	1.881551	1.878046
1.85706	1.853873	1.883434	1.879926	1.876441	1.878646	1.87519	1.882116	1.878558
1.857586	1.854379	1.883978	1.880473	1.876997	1.87921	1.875756	1.872251	1.868683
1.858132	1.854914	1.884554	1.881037	1.877554	1.879769	1.876312	1.872803	1.86923
1.846077	1.842899	1.852238	1.849073	1.845927	1.874637	1.871171	1.87332	1.869762
1.846577	1.843404	1.85276	1.849587	1.846427	1.87516	1.871639	1.873787	1.87027
1.84706	1.843901	1.840757	1.837625	1.834495	1.875707	1.872139	1.868622	1.865155
1.847512	1.844381	1.841267	1.838146	1.835021	1.876302	1.872714	1.869189	1.865726
1.84797	1.844862	1.841775	1.838669	1.835554	1.876911	1.873313	1.869781	1.866313
1.834122	1.831049	1.842279	1.83918	1.836072	1.845354	1.84212	1.870359	1.866887
1.834637	1.831548	1.842782	1.839686	1.836585	1.845878	1.842638	1.870936	1.86746
1.835152	1.832051	1.828982	1.825931	1.822878	1.833935	1.83073	1.871513	1.868035
1.83567	1.832558	1.829482	1.826432	1.823383	1.834449	1.831236	1.87209	1.868611
1.836195	1.833073	1.829989	1.826942	1.823898	1.820775	1.817607	1.861566	1.858116
1.836731	1.833598	1.830508	1.827465	1.824427	1.821305	1.818132	1.862161	1.858712
1.837263	1.83412	1.831024	1.827984	1.824953	1.82183	1.818654	1.829649	1.826545
1.83778	1.834625	1.831521	1.828483	1.825457	1.822334	1.819155	1.830158	1.827055
1.838298	1.835131	1.832019	1.828981	1.825959	1.822836	1.819657	1.83067	1.827566
1.818228	1.81516	1.832544	1.829503	1.82648	1.82336	1.820188	1.817065	1.813991
1.818746	1.815672	1.833085	1.830038	1.82701	1.823898	1.820738	1.817619	1.814538
1.819264	1.816181	1.83362	1.830563	1.827532	1.824436	1.821305	1.818192	1.815091
1.819781	1.816684	1.834147	1.831079	1.828045	1.824977	1.821893	1.818788	1.815654
1.820331	1.817216	1.814153	1.811163	1.808208	1.825509	1.822457	1.819359	1.816206
1.820934	1.817789	1.814694	1.811673	1.808694	1.826019	1.822974	1.819881	1.816732
1.821543	1.818381	1.815261	1.812206	1.809194	1.826534	1.823488	1.820402	1.81727
1.822131	1.81899	1.815875	1.812782	1.809722	1.806722	1.803766	1.820959	1.81786
1.822705	1.819598	1.8165	1.813372	1.810263	1.807244	1.804286	1.821523	1.818461
1.81838	1.81527	1.817056	1.813913	1.810785	1.807752	1.804784	1.801819	1.798861
1.818899	1.81578	1.817565	1.814423	1.811298	1.808255	1.805271	1.8023	1.799346
1.819455	1.81632	1.818101	1.814973	1.811864	1.808812	1.805805	1.802818	1.799855
1.82005	1.816892	1.818669	1.81557	1.812496	1.809436	1.806394	1.803377	1.800386

1.820668	1.817482	1.814346	1.811244	1.808169	1.810049	1.806991	1.803958	1.800951
1.821319	1.818091	1.814915	1.8118	1.808726	1.81062	1.807589	1.804574	1.80158
1.821972	1.81871	1.815498	1.81236	1.809274	1.811177	1.808175	1.805183	1.802205
1.822587	1.819339	1.816127	1.812962	1.80984	1.811727	1.808718	1.805725	1.802752
1.823182	1.819968	1.816774	1.813582	1.810418	1.812276	1.809243	1.806238	1.803266
1.813846	1.810653	1.817361	1.814182	1.811025	1.807911	1.804833	1.806789	1.803811
1.814385	1.811176	1.8179	1.814766	1.81166	1.808543	1.805431	1.807366	1.804377
1.814978	1.811733	1.808541	1.80545	1.8024	1.80916	1.806041	1.80797	1.804972
1.815653	1.812336	1.809085	1.805984	1.802947	1.809751	1.806666	1.808607	1.805604
1.816314	1.812953	1.809664	1.80655	1.803514	1.800466	1.79743	1.804253	1.801232
1.816907	1.813579	1.810313	1.807197	1.804148	1.801086	1.798033	1.804868	1.801844
1.81748	1.814202	1.810976	1.807859	1.804792	1.80171	1.798634	1.805479	1.802453
1.799745	1.796515	1.811568	1.808444	1.80537	1.802296	1.799237	1.796199	1.793186
1.800291	1.797049	1.812129	1.808995	1.805917	1.802863	1.79984	1.796815	1.793793
1.766867	1.763695	1.812766	1.809613	1.806515	1.803455	1.800432	1.797402	1.794366
1.767533	1.764368	1.813479	1.810299	1.807165	1.804071	1.801014	1.797959	1.794905
1.768178	1.765003	1.761861	1.758757	1.755692	1.804659	1.801586	1.798521	1.795462
1.76878	1.765558	1.762374	1.759239	1.756156	1.805194	1.802146	1.799102	1.796063
1.76936	1.766105	1.762893	1.759734	1.756636	1.805735	1.802699	1.79967	1.796649
1.769906	1.766688	1.7635	1.760335	1.757213	1.774288	1.771263	1.800185	1.79716
1.770444	1.767275	1.764125	1.760961	1.757818	1.774876	1.771782	1.800695	1.797659
1.771022	1.767831	1.764663	1.761495	1.758352	1.775437	1.772342	1.769303	1.766321
1.771628	1.768369	1.76515	1.761974	1.758847	1.775986	1.772933	1.769921	1.766955
1.772218	1.768942	1.765713	1.762539	1.759419	1.77659	1.773537	1.770527	1.767564
1.772786	1.769555	1.766363	1.763204	1.760083	1.77726	1.774162	1.771123	1.768149
1.773383	1.770181	1.767011	1.763862	1.760735	1.757616	1.754515	1.771694	1.76871
1.774036	1.770821	1.767638	1.764483	1.761347	1.758187	1.755027	1.772212	1.769223
1.774703	1.771456	1.768244	1.76508	1.761935	1.758744	1.755541	1.772738	1.769743
1.775364	1.77206	1.768801	1.765627	1.762488	1.759301	1.756104	1.773329	1.770324
1.776015	1.772649	1.769339	1.766156	1.763027	1.759863	1.756699	1.773961	1.770944
1.776612	1.773241	1.76993	1.766744	1.763616	1.760468	1.757328	1.774629	1.771599
1.777161	1.773833	1.770563	1.767379	1.764245	1.761109	1.75799	1.775335	1.77229
1.777763	1.774468	1.771227	1.768044	1.764902	1.76177	1.758659	1.755597	1.752586
1.778436	1.775159	1.771927	1.768745	1.765597	1.762458	1.759336	1.756249	1.753198
1.779125	1.77586	1.772631	1.769444	1.766287	1.763138	1.760005	1.75689	1.753792
1.779809	1.776546	1.773317	1.770118	1.766946	1.763794	1.76066	1.757518	1.754359
1.780504	1.777237	1.774004	1.770788	1.767596	1.764433	1.761293	1.758127	1.754921
1.781217	1.777952	1.774714	1.771477	1.768256	1.765055	1.761873	1.758691	1.7555
1.781945	1.778679	1.775432	1.772169	1.768912	1.765661	1.762422	1.759233	1.756091
1.782672	1.779389	1.776118	1.772814	1.769515	1.766247	1.763007	1.759832	1.75672
1.783402	1.780086	1.776776	1.773412	1.770062	1.766808	1.763625	1.760484	1.757387
1.784123	1.780761	1.777409	1.774018	1.770654	1.767411	1.764257	1.761135	1.75805
1.784827	1.7814	1.778005	1.77464	1.771321	1.768079	1.764903	1.761774	1.758696
1.785535	1.782047	1.778613	1.775279	1.772007	1.768763	1.76556	1.762419	1.759346
1.772013	1.768524	1.779279	1.775945	1.772682	1.769435	1.766223	1.763081	1.760015
1.772742	1.769229	1.779968	1.776627	1.773363	1.77011	1.766892	1.763748	1.760685
1.73183	1.728595	1.766456	1.763168	1.759952	1.770798	1.767563	1.764406	1.761333
1.73238	1.729173	1.767125	1.763857	1.760652	1.771495	1.768237	1.765059	1.761967
1.732997	1.729793	1.76781	1.764542	1.761335	1.772182	1.768917	1.765728	1.762618
1.733682	1.730455	1.768507	1.765218	1.761996	1.772856	1.769605	1.766414	1.763288

1.734397	1.73115	1.727961	1.724882	1.721863	1.759449	1.756246	1.7671	1.763953
1.73515	1.731884	1.728672	1.725571	1.72253	1.76015	1.756928	1.767771	1.764594
1.735904	1.732637	1.729413	1.726291	1.723225	1.760877	1.757631	1.768457	1.76525
1.736623	1.733398	1.7302	1.727062	1.723962	1.761653	1.758374	1.755137	1.751943
1.737321	1.734156	1.731	1.727849	1.724716	1.762455	1.759145	1.755884	1.752672
1.716807	1.713691	1.731715	1.728559	1.725416	1.722326	1.719278	1.756687	1.753466
1.717517	1.714398	1.732364	1.729209	1.726076	1.72302	1.720021	1.757539	1.75432
1.718221	1.7151	1.717762	1.714627	1.711517	1.723686	1.720708	1.758313	1.755101
1.718915	1.715796	1.718411	1.715275	1.712159	1.724316	1.721322	1.758983	1.755788
1.71962	1.716497	1.719057	1.715922	1.712804	1.709756	1.70676	1.718944	1.715996
1.72035	1.717211	1.719697	1.716569	1.71346	1.710401	1.707385	1.719557	1.716608
1.721089	1.71793	1.720339	1.717219	1.71412	1.711051	1.708012	1.72017	1.717215
1.706769	1.703647	1.72099	1.717874	1.714777	1.711694	1.708631	1.70563	1.702697
1.707479	1.70435	1.721651	1.718535	1.715436	1.712338	1.709253	1.706241	1.703307
1.708213	1.705074	1.707867	1.704798	1.701743	1.713004	1.709909	1.706888	1.703945
1.708969	1.705815	1.708538	1.705465	1.702409	1.713688	1.710594	1.707568	1.704609
1.709778	1.706612	1.709259	1.706183	1.703125	1.714424	1.711332	1.708299	1.705323
1.710677	1.707503	1.710065	1.706985	1.703929	1.715248	1.712149	1.709104	1.706111
1.711547	1.708369	1.71085	1.707766	1.704706	1.716051	1.712956	1.709904	1.706896
1.712289	1.709115	1.711535	1.708439	1.705368	1.702317	1.699286	1.71063	1.707613
1.712988	1.70982	1.712183	1.709076	1.705993	1.702959	1.699963	1.711344	1.708322
1.713727	1.710555	1.712847	1.709734	1.706654	1.703649	1.7007	1.697729	1.694735
1.714488	1.711306	1.713519	1.710405	1.707335	1.704366	1.701468	1.698522	1.695524
1.715306	1.712123	1.714266	1.711147	1.708072	1.705097	1.702195	1.699246	1.696246
1.71619	1.713016	1.7151	1.711972	1.708873	1.705848	1.702878	1.699895	1.696898
1.697349	1.694251	1.715902	1.712765	1.709646	1.70658	1.703555	1.700544	1.697547
1.698135	1.695027	1.716623	1.713474	1.710345	1.707266	1.704227	1.701204	1.698197
1.698935	1.695811	1.717343	1.714185	1.711049	1.707962	1.704914	1.701883	1.69887
1.671446	1.668334	1.718134	1.71497	1.711833	1.708727	1.705653	1.702614	1.699614
1.672336	1.669199	1.718943	1.715773	1.712638	1.709513	1.706409	1.703364	1.700383
1.637001	1.634143	1.693391	1.690235	1.687113	1.710226	1.707109	1.704059	1.701081
1.637622	1.634752	1.694051	1.690884	1.687752	1.710889	1.707771	1.704716	1.701728
1.591704	1.589256	1.668313	1.665202	1.662121	1.711614	1.708492	1.705429	1.70243
1.592323	1.589858	1.669148	1.666035	1.662956	1.712429	1.709296	1.706221	1.703206
1.592932	1.590453	1.633985	1.631158	1.628364	1.686894	1.683779	1.706993	1.703972
1.593498	1.59101	1.634615	1.631769	1.628959	1.687545	1.684432	1.707684	1.704691
1.594062	1.59157	1.635247	1.632378	1.629547	1.688191	1.685086	1.708375	1.705399
1.594671	1.592174	1.589699	1.58725	1.584832	1.655987	1.653158	1.682796	1.679799
1.595298	1.592798	1.590317	1.587858	1.585429	1.656665	1.653851	1.683573	1.680491
1.595884	1.593385	1.590907	1.588456	1.586034	1.657361	1.654542	1.684356	1.681239
1.596433	1.593939	1.591474	1.589049	1.586652	1.658082	1.65524	1.68515	1.682043
1.597014	1.594526	1.592067	1.589645	1.587247	1.584859	1.582484	1.653034	1.650191
1.597642	1.595162	1.592701	1.590244	1.587804	1.585394	1.583009	1.65358	1.650738
1.598282	1.595806	1.593341	1.590853	1.588377	1.585947	1.583552	1.654148	1.651302
1.598922	1.596445	1.593978	1.591487	1.589006	1.586565	1.584154	1.654815	1.651947
1.599559	1.597076	1.594606	1.592126	1.589654	1.587208	1.584781	1.655523	1.652633
1.600168	1.597673	1.5952	1.59274	1.590293	1.587846	1.585403	1.656236	1.65335
1.600764	1.598248	1.595771	1.593339	1.590927	1.588483	1.586027	1.656959	1.654092
1.601402	1.598858	1.596361	1.593927	1.591521	1.589084	1.586639	1.591514	1.589143
1.602085	1.5995	1.596966	1.594497	1.592069	1.589648	1.587242	1.592058	1.589674

1.602777	1.600164	1.597599	1.595095	1.592636	1.590217	1.587833	1.592589	1.590196
1.603475	1.600859	1.598281	1.595739	1.593239	1.590796	1.588402	1.593097	1.59071
1.604172	1.601553	1.598964	1.596395	1.593862	1.591393	1.588977	1.593607	1.591224
1.565963	1.563495	1.599604	1.597036	1.594508	1.592024	1.589582	1.594138	1.591747
1.566561	1.564065	1.600233	1.597678	1.595168	1.592673	1.590203	1.594681	1.592278
1.567147	1.564649	1.56219	1.559797	1.557442	1.593314	1.590833	1.595234	1.592822
1.567722	1.565242	1.562797	1.560389	1.558011	1.593953	1.591473	1.595796	1.593379
1.568334	1.565861	1.56342	1.560991	1.558588	1.594597	1.592116	1.596369	1.593943
1.568988	1.56651	1.564063	1.561607	1.559175	1.595248	1.592765	1.596953	1.594518
1.569652	1.567157	1.564695	1.562217	1.559764	1.557376	1.555039	1.597534	1.59509
1.570308	1.567781	1.565295	1.562804	1.560343	1.557943	1.555593	1.598095	1.595648
1.570974	1.568409	1.565895	1.563397	1.560937	1.558524	1.556154	1.598662	1.596212
1.537538	1.535146	1.566526	1.564031	1.561578	1.559147	1.556746	1.561874	1.559562
1.538186	1.53576	1.567177	1.564688	1.562245	1.559796	1.557361	1.562421	1.560101
1.504676	1.502311	1.567841	1.565349	1.562909	1.560447	1.55799	1.562978	1.560634
1.505308	1.502913	1.568519	1.566016	1.563572	1.561103	1.558636	1.563546	1.561165
1.505933	1.503514	1.535259	1.53293	1.530662	1.561757	1.559288	1.564129	1.561708
1.506546	1.504109	1.535889	1.533541	1.531258	1.56241	1.559947	1.56473	1.562265
1.507161	1.504706	1.502325	1.500055	1.497855	1.563061	1.560601	1.565332	1.562834
1.507782	1.5053	1.502894	1.500611	1.498402	1.563704	1.561238	1.565922	1.563421
1.508409	1.505901	1.503472	1.501176	1.498957	1.564349	1.561872	1.56651	1.564016
1.509042	1.506525	1.504082	1.501771	1.499535	1.565016	1.562518	1.567095	1.564606
1.509683	1.507167	1.504718	1.502388	1.500128	1.565697	1.563171	1.567681	1.565196
1.510348	1.507834	1.505374	1.503011	1.500712	1.534358	1.531919	1.568283	1.565795
1.511041	1.50853	1.506054	1.503642	1.501285	1.534969	1.532531	1.568899	1.566402
1.511716	1.509214	1.50673	1.504275	1.501869	1.499565	1.497343	1.569524	1.567022
1.512358	1.509873	1.507392	1.50491	1.502467	1.500143	1.49791	1.570156	1.567661
1.512991	1.510524	1.508052	1.50555	1.503077	1.500731	1.498478	1.57079	1.568304
1.48099	1.478702	1.508714	1.506198	1.503699	1.501319	1.49903	1.540277	1.53791
1.481544	1.479278	1.509375	1.506854	1.504338	1.501923	1.499587	1.540836	1.538485
1.482135	1.479864	1.510033	1.507524	1.505016	1.502573	1.500186	1.541443	1.539082
1.482753	1.480459	1.51069	1.508206	1.505726	1.503261	1.50082	1.542089	1.539698
1.48336	1.481056	1.511362	1.508894	1.506432	1.503956	1.501488	1.507781	1.50557
1.483958	1.48166	1.512056	1.509592	1.507134	1.504663	1.502197	1.50842	1.506172
1.484542	1.482249	1.479959	1.477674	1.47539	1.505366	1.502913	1.509072	1.506781
1.4851	1.482799	1.48051	1.478241	1.475978	1.506062	1.503628	1.509733	1.507388
1.485662	1.483355	1.481066	1.478805	1.476556	1.506748	1.504335	1.510391	1.507996
1.486265	1.483964	1.481677	1.479401	1.477135	1.507412	1.505011	1.511023	1.508598
1.48689	1.484599	1.482313	1.480011	1.477716	1.508068	1.505671	1.511645	1.509205
1.48751	1.48521	1.482919	1.480612	1.478314	1.508749	1.506347	1.512288	1.50985
1.488129	1.485806	1.483502	1.481208	1.478929	1.50945	1.507034	1.512947	1.510529
1.48874	1.486407	1.484102	1.481815	1.479547	1.477291	1.475049	1.513614	1.511217
1.489335	1.487015	1.484727	1.48244	1.480167	1.477906	1.47566	1.51429	1.511918
1.48995	1.487635	1.485355	1.483066	1.48079	1.478527	1.47628	1.514967	1.512602
1.490609	1.488271	1.485975	1.483682	1.48141	1.479157	1.476922	1.515643	1.513243
1.49129	1.488918	1.486596	1.484302	1.482038	1.479793	1.477566	1.516316	1.513869
1.491973	1.489565	1.487219	1.484938	1.482693	1.480437	1.478183	1.516979	1.514505
1.492669	1.49022	1.48785	1.485584	1.483363	1.481085	1.478785	1.517638	1.515146
1.493404	1.490927	1.488529	1.48624	1.483999	1.481707	1.479397	1.486176	1.48388
1.494178	1.491684	1.489255	1.486903	1.4846	1.4823	1.480016	1.486772	1.484466

1.494973	1.49245	1.489984	1.487585	1.485238	1.482927	1.480652	1.487379	1.485061
1.495792	1.493212	1.490694	1.488284	1.485937	1.483607	1.481305	1.48799	1.485664
1.496617	1.493992	1.491433	1.48901	1.486656	1.484306	1.48198	1.48862	1.486282
1.497429	1.494812	1.492246	1.489777	1.48737	1.485004	1.482683	1.489285	1.486926
1.498242	1.495649	1.49309	1.49057	1.488099	1.485718	1.483406	1.489969	1.487588
1.499068	1.496469	1.4939	1.491365	1.488876	1.486471	1.484132	1.481792	1.47945
1.499904	1.497282	1.494694	1.492167	1.489687	1.487254	1.484866	1.482499	1.480156
1.500726	1.498097	1.495499	1.492966	1.490478	1.488027	1.485614	1.483232	1.480884
1.501533	1.498913	1.496318	1.493761	1.491245	1.488787	1.486379	1.483996	1.481639
1.46322	1.460493	1.497133	1.494558	1.492019	1.489551	1.487141	1.484753	1.48239
1.464235	1.461479	1.497935	1.495355	1.492813	1.490325	1.487885	1.485482	1.483118
1.465254	1.46247	1.498742	1.496158	1.493612	1.491104	1.488635	1.486215	1.483848
1.466264	1.463475	1.460725	1.458035	1.455393	1.452798	1.450248	1.486978	1.484593
1.467274	1.464491	1.46174	1.45902	1.456344	1.453745	1.451208	1.487761	1.485352
1.4683	1.465515	1.462761	1.460021	1.457323	1.454716	1.452179	1.44965	1.447134
1.469338	1.466548	1.463792	1.461038	1.458326	1.455707	1.453161	1.450634	1.44813
1.470409	1.46761	1.464848	1.462075	1.459341	1.456703	1.45414	1.451606	1.449108
1.471522	1.468711	1.46594	1.463136	1.460366	1.457698	1.455108	1.45256	1.450061
1.472642	1.469811	1.46703	1.464205	1.461412	1.458713	1.456088	1.453517	1.451009
1.473737	1.47088	1.468091	1.465271	1.462484	1.459757	1.457083	1.45448	1.451953
1.474852	1.471957	1.469155	1.466347	1.463576	1.460823	1.4581	1.455461	1.452909
1.476037	1.473088	1.470261	1.467452	1.464689	1.461917	1.459158	1.456489	1.453911
1.477272	1.474255	1.47139	1.468572	1.465815	1.463028	1.460246	1.457551	1.454942
1.478535	1.475423	1.472494	1.469659	1.466906	1.464113	1.461321	1.458609	1.455974
1.47982	1.476589	1.473571	1.470711	1.467962	1.465171	1.462386	1.459666	1.457009
1.4812	1.477829	1.474699	1.4718	1.469048	1.466257	1.463481	1.460749	1.458056
1.48271	1.479168	1.475893	1.472944	1.470182	1.46739	1.464626	1.461871	1.459115
1.484266	1.480555	1.477134	1.474118	1.471328	1.468527	1.465774	1.463001	1.460197
1.48581	1.481956	1.478404	1.475307	1.472463	1.469641	1.466893	1.464122	1.461322
1.487381	1.483401	1.479727	1.476526	1.473601	1.470745	1.467999	1.46524	1.462466
1.48901	1.484929	1.481138	1.477795	1.474739	1.471835	1.469099	1.46635	1.463592
1.490689	1.486527	1.482628	1.479111	1.475883	1.472918	1.470193	1.467454	1.46471
1.49239	1.488178	1.484194	1.480509	1.477103	1.474053	1.471304	1.468556	1.465825
1.494113	1.489886	1.485842	1.481991	1.478395	1.475237	1.47243	1.469656	1.466938
1.408806	1.404688	1.487523	1.48353	1.479766	1.476485	1.473581	1.470759	1.468046
1.410532	1.40638	1.489214	1.485115	1.481222	1.477802	1.474756	1.47186	1.469147
1.412291	1.4081	1.490939	1.486758	1.48276	1.479192	1.47597	1.472975	1.470248
1.356081	1.351926	1.492709	1.488477	1.4844	1.480667	1.477227	1.474104	1.471338
1.357852	1.353652	1.494515	1.490248	1.48611	1.482212	1.478541	1.475271	1.472442
1.359639	1.355395	1.35122	1.347066	1.343028	1.483787	1.479959	1.476562	1.473633
1.361446	1.357157	1.352924	1.348712	1.344605	1.485399	1.481472	1.477962	1.474898
1.363254	1.35893	1.354651	1.350388	1.346219	1.342238	1.338442	1.47944	1.476209
1.365061	1.360713	1.356398	1.352094	1.347877	1.343855	1.340014	1.481004	1.477564
1.366866	1.362507	1.358169	1.353825	1.34956	1.345499	1.341616	1.43234	1.428761
1.36867	1.364319	1.359969	1.355585	1.351261	1.347148	1.343208	1.433892	1.430159
1.370469	1.366134	1.361783	1.357361	1.352983	1.348816	1.344816	1.435474	1.431624
1.372255	1.367937	1.36359	1.359139	1.354715	1.350502	1.346448	1.342532	1.338728
1.374031	1.369734	1.365397	1.360926	1.356466	1.352209	1.348102	1.344136	1.340294
1.375796	1.371521	1.367202	1.362735	1.358263	1.35396	1.349783	1.34576	1.341885
1.377548	1.373297	1.369004	1.364563	1.360102	1.355751	1.351487	1.347406	1.343509

1.978633	1.974952	1.971277	1.967638	1.964028	1.960471	1.956954	1.953402	1.949804
1.97854	1.974863	1.971199	1.96759	1.964007	1.960442	1.956892	1.953324	1.949727
1.978456	1.974777	1.971125	1.967563	1.964027	1.960448	1.956848	1.953252	1.949652
1.968578	1.964912	1.971041	1.967499	1.963987	1.960406	1.956789	1.953184	1.94958
1.96851	1.964823	1.970951	1.967415	1.963915	1.96034	1.956725	1.953119	1.949511
1.968446	1.964744	1.96812	1.964593	1.961108	1.96027	1.956654	1.953049	1.949445
1.968389	1.964673	1.968043	1.964515	1.961032	1.960193	1.956575	1.952973	1.949378
1.968326	1.964604	1.960945	1.957441	1.953985	1.957383	1.953769	1.952911	1.949344
1.968247	1.964525	1.960867	1.957359	1.953901	1.957306	1.953701	1.952874	1.949367
1.968168	1.964461	1.960812	1.957295	1.953823	1.957234	1.953638	1.952842	1.949391
1.968102	1.964441	1.960822	1.957279	1.953767	1.957169	1.953576	1.950062	1.94664
1.968041	1.964436	1.960853	1.95728	1.953719	1.957113	1.953524	1.950013	1.946589
1.967973	1.964391	1.960824	1.957237	1.95366	1.950114	1.946595	1.950013	1.946562
1.967903	1.964319	1.960752	1.957164	1.953593	1.950085	1.94662	1.950052	1.946551
1.961186	1.957554	1.960683	1.957091	1.953526	1.950039	1.946607	1.950048	1.946517
1.961216	1.957531	1.960616	1.957013	1.953451	1.949968	1.946543	1.949991	1.946456
1.961238	1.957508	1.960561	1.956959	1.953408	1.94992	1.946481	1.943009	1.939502
1.961218	1.957465	1.960518	1.956949	1.953432	1.949926	1.94644	1.942944	1.939437
1.961188	1.957431	1.9605	1.95696	1.953471	1.949943	1.946406	1.942889	1.939389
1.961159	1.957439	1.953769	1.950202	1.946675	1.949928	1.946371	1.942858	1.93939
1.961133	1.957469	1.953834	1.950236	1.946661	1.949901	1.946345	1.942849	1.93942
1.961141	1.957482	1.953844	1.950228	1.946634	1.943062	1.939513	1.942877	1.939446
1.96118	1.957483	1.953809	1.950182	1.946594	1.943057	1.939563	1.94294	1.939469
1.953455	1.949777	1.953774	1.950153	1.946583	1.943067	1.939597	1.94299	1.939509
1.953423	1.949731	1.953734	1.950148	1.946618	1.943099	1.939602	1.943012	1.939576
1.95341	1.94971	1.953722	1.950159	1.946654	1.943131	1.939614	1.936142	1.932724
1.95346	1.949764	1.953779	1.950191	1.946661	1.943146	1.939658	1.936186	1.932738
1.953525	1.949834	1.953853	1.950231	1.946667	1.943161	1.939706	1.93623	1.932734
1.953538	1.949842	1.953868	1.950259	1.946706	1.943196	1.939724	1.93623	1.93271
1.953527	1.949822	1.953856	1.950281	1.946765	1.943242	1.939728	1.936208	1.932675
1.953558	1.949854	1.946198	1.942664	1.939185	1.943276	1.939764	1.936243	1.932707
1.953636	1.949942	1.94628	1.942707	1.939187	1.943298	1.939833	1.936338	1.932809
1.953687	1.949999	1.946337	1.942747	1.939208	1.943333	1.939888	1.936411	1.932903
1.953674	1.949986	1.946335	1.942777	1.93927	1.943393	1.939906	1.936431	1.932973
1.953668	1.94998	1.946341	1.942811	1.939331	1.943453	1.939928	1.93645	1.933028
1.95373	1.950048	1.946406	1.942854	1.93935	1.943489	1.939988	1.936509	1.933056
1.953812	1.950143	1.946497	1.942908	1.939364	1.943521	1.94006	1.936577	1.933075
1.953848	1.950213	1.946594	1.942996	1.939431	1.943583	1.940105	1.936616	1.93312
1.953856	1.95027	1.946698	1.94311	1.939536	1.943667	1.940133	1.936635	1.933182
1.953909	1.950324	1.94676	1.94319	1.939633	1.936093	1.932571	1.936687	1.933235
1.954015	1.950368	1.946764	1.943229	1.939724	1.936198	1.932669	1.936773	1.933271
1.954129	1.950436	1.946797	1.943272	1.939793	1.936285	1.932772	1.936879	1.933334
1.954246	1.950552	1.946898	1.943329	1.939809	1.936321	1.932864	1.937007	1.933449
1.954348	1.950677	1.947023	1.943406	1.939834	1.936361	1.932959	1.937141	1.933583
1.954392	1.950767	1.94715	1.943526	1.939936	1.936463	1.933071	1.937271	1.933716
1.954414	1.950846	1.947284	1.943673	1.94008	1.936597	1.933187	1.937394	1.933851
1.952827	1.949275	1.947417	1.943816	1.940222	1.936716	1.933267	1.937478	1.933966
1.952962	1.94941	1.947551	1.94396	1.940369	1.936825	1.933315	1.937525	1.934062
1.953102	1.949528	1.947653	1.94408	1.94051	1.936949	1.933401	1.937613	1.934173
1.953243	1.949616	1.947703	1.944162	1.94064	1.937094	1.933541	1.93776	1.93431

1.953391	1.949719	1.947772	1.944257	1.940777	1.937246	1.933696	1.930202	1.926767
1.953548	1.949863	1.9479	1.944395	1.94093	1.937404	1.933851	1.930335	1.926852
1.953711	1.950027	1.948056	1.944553	1.941088	1.937564	1.934011	1.930471	1.926935
1.953872	1.950198	1.948228	1.944719	1.941245	1.93772	1.934171	1.93062	1.927058
1.954035	1.950379	1.948414	1.944892	1.941398	1.937871	1.934329	1.930777	1.927203
1.954204	1.950555	1.946928	1.943356	1.939808	1.937987	1.934467	1.930926	1.927349
1.954378	1.950729	1.94709	1.943473	1.939879	1.938063	1.934583	1.931065	1.927497
1.954553	1.950905	1.947257	1.943607	1.93998	1.938163	1.93471	1.93121	1.927649
1.954726	1.951082	1.947435	1.943772	1.94013	1.938307	1.934857	1.93136	1.927805
1.954904	1.951261	1.947615	1.943947	1.940299	1.938468	1.935017	1.931519	1.927965
1.955092	1.951445	1.947793	1.944122	1.940472	1.938638	1.935191	1.931693	1.928132
1.955283	1.951629	1.94797	1.9443	1.940653	1.938817	1.935374	1.931873	1.928301
1.953871	1.950227	1.948147	1.94448	1.940836	1.937289	1.933806	1.932043	1.928465
1.954041	1.950397	1.948323	1.944662	1.941024	1.937475	1.933985	1.932208	1.928627
1.945526	1.941916	1.948513	1.944874	1.941257	1.9377	1.934183	1.932381	1.928797
1.945689	1.94209	1.948721	1.945127	1.941549	1.937974	1.934405	1.932565	1.928977
1.941244	1.937673	1.938693	1.935174	1.931667	1.938229	1.934621	1.93101	1.927386
1.941403	1.93783	1.938856	1.935348	1.931854	1.938435	1.934818	1.931203	1.927577
1.94157	1.93799	1.939018	1.935512	1.932022	1.938623	1.935008	1.931398	1.927782
1.941756	1.938162	1.93919	1.935685	1.9322	1.938812	1.935193	1.9316	1.928027
1.941955	1.93835	1.939377	1.935868	1.93238	1.938997	1.935374	1.931804	1.928289
1.937036	1.933463	1.935026	1.931513	1.928012	1.92901	1.925425	1.932001	1.928515
1.937259	1.933724	1.935313	1.931764	1.928212	1.929193	1.925601	1.932192	1.928712
1.936722	1.933209	1.935566	1.931994	1.928412	1.924842	1.921283	1.924214	1.920748
1.936902	1.933401	1.935769	1.93219	1.928603	1.925067	1.921562	1.924504	1.920985
1.920242	1.916823	1.930843	1.927285	1.923727	1.925308	1.921836	1.922869	1.919312
1.920508	1.917052	1.931044	1.927511	1.923989	1.925572	1.922071	1.923091	1.919518
1.920785	1.917285	1.931244	1.92774	1.924258	1.925836	1.92229	1.923298	1.919731
1.921014	1.917492	1.930697	1.927204	1.923739	1.926057	1.922497	1.918964	1.915453
1.92122	1.917689	1.93089	1.927395	1.923933	1.926254	1.922698	1.919192	1.91574
1.921442	1.917918	1.931129	1.927621	1.924144	1.921367	1.917849	1.919448	1.916004
1.921677	1.918179	1.931414	1.927879	1.924368	1.921601	1.918123	1.919735	1.916251
1.921933	1.918442	1.931686	1.928142	1.924619	1.921127	1.91766	1.919994	1.916481
1.92222	1.9187	1.931922	1.928404	1.924914	1.921404	1.91789	1.920202	1.916682
1.922507	1.918966	1.932171	1.928674	1.925207	1.921676	1.918115	1.920408	1.916895
1.922765	1.919248	1.915765	1.91234	1.908934	1.921905	1.918336	1.915531	1.912067
1.923021	1.919536	1.916074	1.91262	1.909172	1.92213	1.918566	1.915774	1.91234
1.923308	1.919812	1.916342	1.912891	1.909451	1.922416	1.91885	1.915344	1.9119
1.923617	1.920082	1.916584	1.913157	1.909754	1.92274	1.919171	1.915645	1.912161
1.92391	1.92036	1.916851	1.913428	1.910033	1.923025	1.91945	1.915921	1.912433
1.924184	1.920649	1.917149	1.913707	1.910285	1.92326	1.919677	1.916163	1.912721
1.924466	1.920932	1.91743	1.913979	1.910547	1.907077	1.903594	1.916424	1.913003
1.924769	1.921196	1.917666	1.914236	1.910837	1.907379	1.903895	1.916722	1.913263
1.925069	1.921462	1.917909	1.914495	1.911124	1.907684	1.904211	1.91704	1.913536
1.925334	1.92175	1.918211	1.914774	1.911378	1.907956	1.904528	1.917384	1.913863
1.925589	1.922048	1.918537	1.915062	1.911619	1.908215	1.904841	1.917734	1.914213
1.925883	1.922339	1.918824	1.915341	1.911889	1.908483	1.905112	1.901723	1.898315
1.926207	1.922627	1.919083	1.915613	1.912181	1.908756	1.905347	1.901959	1.898595
1.926555	1.922951	1.919383	1.915905	1.91247	1.909034	1.90561	1.90222	1.898864
1.926941	1.923327	1.919738	1.91622	1.912746	1.909309	1.905908	1.902511	1.899115

1.925692	1.922089	1.9201	1.916552	1.913044	1.909607	1.906224	1.90282	1.89939
1.925983	1.922414	1.920455	1.916906	1.913388	1.909949	1.906566	1.903159	1.899721
1.926257	1.922723	1.920798	1.917261	1.913746	1.910302	1.906909	1.903498	1.900068
1.926566	1.923024	1.921097	1.917581	1.914086	1.910629	1.907203	1.903788	1.900386
1.926892	1.923322	1.921374	1.917884	1.914421	1.910947	1.907477	1.904058	1.900694
1.927233	1.923653	1.921698	1.918216	1.914764	1.911285	1.907802	1.904379	1.901023
1.927591	1.924015	1.922063	1.918575	1.915115	1.911642	1.908173	1.904748	1.901373
1.91618	1.912679	1.920833	1.917337	1.91387	1.911983	1.908535	1.905109	1.901705
1.916475	1.912999	1.921179	1.917661	1.914166	1.912289	1.908874	1.905446	1.901999
1.916781	1.913327	1.921531	1.917994	1.914474	1.912606	1.909218	1.90579	1.90231
1.91713	1.91368	1.921897	1.918354	1.914827	1.912961	1.909582	1.906158	1.902678
1.917501	1.91405	1.922275	1.918731	1.915202	1.913337	1.909956	1.906538	1.903073
1.917879	1.914435	1.916434	1.912917	1.909413	1.912154	1.908791	1.906924	1.903466
1.918266	1.914833	1.91684	1.913317	1.909803	1.912545	1.90918	1.907316	1.903863
1.91866	1.915229	1.917239	1.913712	1.910193	1.912936	1.909568	1.907705	1.904252
1.919062	1.915624	1.917633	1.914103	1.910583	1.913329	1.90996	1.908095	1.904636
1.919459	1.916011	1.912573	1.909071	1.905575	1.907544	1.904178	1.906911	1.903481
1.919846	1.916388	1.912943	1.90945	1.905961	1.907898	1.904478	1.907201	1.903808
1.920226	1.91675	1.913295	1.909817	1.906341	1.908251	1.904781	1.907495	1.904138
1.920584	1.917075	1.913598	1.910142	1.9067	1.908617	1.905135	1.907849	1.9045
1.920941	1.917396	1.913895	1.910462	1.907056	1.908994	1.905522	1.908243	1.904886
1.913569	1.91004	1.914306	1.910862	1.907447	1.909401	1.905953	1.902552	1.899201
1.91401	1.910516	1.914807	1.911327	1.907866	1.909833	1.906424	1.903032	1.899658
1.914451	1.91097	1.915273	1.911769	1.908281	1.904865	1.901499	1.903483	1.900092
1.914892	1.911398	1.915693	1.912182	1.90869	1.905275	1.901912	1.903897	1.900497
1.915325	1.911815	1.908331	1.904831	1.901354	1.90568	1.902312	1.904294	1.900888
1.915741	1.91222	1.908728	1.905231	1.90176	1.906083	1.902698	1.904671	1.901263
1.916151	1.912621	1.909124	1.905631	1.902164	1.906483	1.903079	1.905043	1.901634
1.916559	1.91303	1.90953	1.906032	1.902557	1.899127	1.895732	1.905425	1.902017
1.916969	1.913443	1.909942	1.906434	1.902947	1.899511	1.896114	1.905815	1.902411
1.917397	1.913862	1.910353	1.906839	1.903348	1.899907	1.896507	1.90086	1.897495
1.917839	1.914287	1.910763	1.907247	1.903758	1.900314	1.896909	1.901287	1.89797
1.918314	1.914738	1.911194	1.907683	1.904205	1.900754	1.897334	1.893978	1.890697
1.918838	1.915227	1.911655	1.908163	1.904709	1.901245	1.897791	1.894409	1.891111
1.919354	1.915717	1.912126	1.908646	1.90521	1.901733	1.898248	1.89484	1.891514
1.915024	1.911398	1.91259	1.909108	1.905665	1.902178	1.89868	1.895255	1.891907
1.915461	1.911853	1.913063	1.909568	1.906106	1.902613	1.899113	1.895676	1.892304
1.915921	1.912338	1.913568	1.910051	1.90656	1.903067	1.899584	1.896141	1.892737
1.916391	1.912838	1.914091	1.910547	1.90702	1.903534	1.900079	1.896635	1.893196
1.916836	1.913297	1.909777	1.906245	1.902729	1.904015	1.900562	1.897113	1.893664
1.917251	1.913708	1.910197	1.906703	1.903227	1.904516	1.901035	1.897579	1.894145
1.917696	1.914138	1.910621	1.907151	1.903703	1.904994	1.90149	1.898025	1.894599
1.918198	1.914603	1.911052	1.907579	1.904135	1.905425	1.901908	1.898433	1.894996
1.918716	1.915091	1.91151	1.90802	1.904564	1.905852	1.902334	1.89885	1.895394
1.911778	1.908202	1.912036	1.90851	1.905013	1.901519	1.898036	1.899324	1.895832
1.912267	1.908723	1.912588	1.909023	1.905477	1.901986	1.898534	1.899823	1.896295
1.912771	1.909219	1.913095	1.909525	1.905976	1.902478	1.899018	1.900309	1.89679
1.913285	1.9097	1.913571	1.910024	1.906503	1.902992	1.899495	1.900791	1.897314
1.913782	1.910186	1.906628	1.903123	1.899646	1.903504	1.899996	1.896514	1.893057
1.91425	1.910678	1.90714	1.90362	1.900125	1.904012	1.90053	1.897044	1.893549

1.914739	1.911179	1.907648	1.904119	1.900611	1.904521	1.901053	1.897562	1.894041
1.915284	1.911693	1.908136	1.904614	1.901119	1.905032	1.901538	1.898042	1.894541
1.915835	1.912211	1.908624	1.905108	1.901626	1.90554	1.902018	1.898518	1.895041
1.916339	1.912721	1.909135	1.905602	1.902098	1.898603	1.895121	1.89902	1.895538
1.916826	1.913228	1.909656	1.906095	1.902559	1.899067	1.895611	1.899533	1.896036
1.908162	1.904577	1.910153	1.906592	1.903057	1.899561	1.896097	1.900036	1.896548
1.908702	1.905073	1.910627	1.90709	1.90359	1.900081	1.89658	1.900531	1.897073
1.909237	1.905594	1.90199	1.898479	1.895004	1.891498	1.887985	1.893616	1.890213
1.909756	1.906147	1.90256	1.899009	1.895483	1.89198	1.8885	1.894147	1.890752
1.890202	1.886667	1.903146	1.899557	1.895986	1.892483	1.889031	1.8856	1.882198
1.890759	1.887233	1.903731	1.900136	1.896554	1.893045	1.889587	1.886136	1.882693
1.891329	1.887799	1.90431	1.900724	1.897149	1.89363	1.89015	1.886668	1.883182
1.891887	1.888332	1.895191	1.891652	1.888129	1.894205	1.890685	1.887185	1.883703
1.892439	1.888847	1.895695	1.892195	1.888719	1.894779	1.891207	1.887695	1.884243
1.892973	1.889382	1.885823	1.882356	1.87891	1.885768	1.882194	1.888242	1.884801
1.893486	1.889937	1.886416	1.882945	1.879483	1.886351	1.882779	1.888828	1.885379
1.888313	1.884807	1.887	1.883513	1.880026	1.876503	1.872961	1.879857	1.876423
1.888864	1.88537	1.887563	1.88404	1.880516	1.877003	1.8735	1.880422	1.876981
1.889423	1.885926	1.88811	1.884559	1.88101	1.877509	1.874043	1.880985	1.877526
1.85656	1.853404	1.882935	1.879402	1.87588	1.878067	1.874612	1.881551	1.878046
1.85706	1.853873	1.883434	1.879926	1.876441	1.878646	1.87519	1.882116	1.878558
1.857586	1.854379	1.883978	1.880473	1.876997	1.87921	1.875756	1.872251	1.868683
1.858132	1.854914	1.884554	1.881037	1.877554	1.879769	1.876312	1.872803	1.86923
1.846077	1.842899	1.852238	1.849073	1.845927	1.874637	1.871171	1.87332	1.869762
1.846577	1.843404	1.85276	1.849587	1.846427	1.87516	1.871639	1.873787	1.87027
1.84706	1.843901	1.840757	1.837625	1.834495	1.875707	1.872139	1.868622	1.865155
1.847512	1.844381	1.841267	1.838146	1.835021	1.876302	1.872714	1.869189	1.865726
1.84797	1.844862	1.841775	1.838669	1.835554	1.876911	1.873313	1.869781	1.866313
1.834122	1.831049	1.842279	1.83918	1.836072	1.845354	1.84212	1.870359	1.866887
1.834637	1.831548	1.842782	1.839686	1.836585	1.845878	1.842638	1.870936	1.86746
1.835152	1.832051	1.828982	1.825931	1.822878	1.833935	1.83073	1.871513	1.868035
1.83567	1.832558	1.829482	1.826432	1.823383	1.834449	1.831236	1.87209	1.868611
1.836195	1.833073	1.829989	1.826942	1.823898	1.820775	1.817607	1.861566	1.858116
1.836731	1.833598	1.830508	1.827465	1.824427	1.821305	1.818132	1.862161	1.858712
1.837263	1.83412	1.831024	1.827984	1.824953	1.82183	1.818654	1.829649	1.826545
1.83778	1.834625	1.831521	1.828483	1.825457	1.822334	1.819155	1.830158	1.827055
1.838298	1.835131	1.832019	1.828981	1.825959	1.822836	1.819657	1.83067	1.827566
1.818228	1.81516	1.832544	1.829503	1.82648	1.82336	1.820188	1.817065	1.813991
1.818746	1.815672	1.833085	1.830038	1.82701	1.823898	1.820738	1.817619	1.814538
1.819264	1.816181	1.83362	1.830563	1.827532	1.824436	1.821305	1.818192	1.815091
1.819781	1.816684	1.834147	1.831079	1.828045	1.824977	1.821893	1.818788	1.815654
1.820331	1.817216	1.814153	1.811163	1.808208	1.825509	1.822457	1.819359	1.816206
1.820934	1.817789	1.814694	1.811673	1.808694	1.826019	1.822974	1.819881	1.816732
1.821543	1.818381	1.815261	1.812206	1.809194	1.826534	1.823488	1.820402	1.81727
1.822131	1.81899	1.815875	1.812782	1.809722	1.806722	1.803766	1.820959	1.81786
1.822705	1.819598	1.8165	1.813372	1.810263	1.807244	1.804286	1.821523	1.818461
1.81838	1.81527	1.817056	1.813913	1.810785	1.807752	1.804784	1.801819	1.798861
1.818899	1.81578	1.817565	1.814423	1.811298	1.808255	1.805271	1.8023	1.799346
1.819455	1.81632	1.818101	1.814973	1.811864	1.808812	1.805805	1.802818	1.799855
1.82005	1.816892	1.818669	1.81557	1.812496	1.809436	1.806394	1.803377	1.800386

1.820668	1.817482	1.814346	1.811244	1.808169	1.810049	1.806991	1.803958	1.800951
1.821319	1.818091	1.814915	1.8118	1.808726	1.81062	1.807589	1.804574	1.80158
1.821972	1.81871	1.815498	1.81236	1.809274	1.811177	1.808175	1.805183	1.802205
1.822587	1.819339	1.816127	1.812962	1.80984	1.811727	1.808718	1.805725	1.802752
1.823182	1.819968	1.816774	1.813582	1.810418	1.812276	1.809243	1.806238	1.803266
1.813846	1.810653	1.817361	1.814182	1.811025	1.807911	1.804833	1.806789	1.803811
1.814385	1.811176	1.8179	1.814766	1.81166	1.808543	1.805431	1.807366	1.804377
1.814978	1.811733	1.808541	1.80545	1.8024	1.80916	1.806041	1.80797	1.804972
1.815653	1.812336	1.809085	1.805984	1.802947	1.809751	1.806666	1.808607	1.805604
1.816314	1.812953	1.809664	1.80655	1.803514	1.800466	1.79743	1.804253	1.801232
1.816907	1.813579	1.810313	1.807197	1.804148	1.801086	1.798033	1.804868	1.801844
1.81748	1.814202	1.810976	1.807859	1.804792	1.80171	1.798634	1.805479	1.802453
1.799745	1.796515	1.811568	1.808444	1.80537	1.802296	1.799237	1.796199	1.793186
1.800291	1.797049	1.812129	1.808995	1.805917	1.802863	1.79984	1.796815	1.793793
1.766867	1.763695	1.812766	1.809613	1.806515	1.803455	1.800432	1.797402	1.794366
1.767533	1.764368	1.813479	1.810299	1.807165	1.804071	1.801014	1.797959	1.794905
1.768178	1.765003	1.761861	1.758757	1.755692	1.804659	1.801586	1.798521	1.795462
1.76878	1.765558	1.762374	1.759239	1.756156	1.805194	1.802146	1.799102	1.796063
1.76936	1.766105	1.762893	1.759734	1.756636	1.805735	1.802699	1.79967	1.796649
1.769906	1.766688	1.7635	1.760335	1.757213	1.774288	1.771263	1.800185	1.79716
1.770444	1.767275	1.764125	1.760961	1.757818	1.774876	1.771782	1.800695	1.797659
1.771022	1.767831	1.764663	1.761495	1.758352	1.775437	1.772342	1.769303	1.766321
1.771628	1.768369	1.76515	1.761974	1.758847	1.775986	1.772933	1.769921	1.766955
1.772218	1.768942	1.765713	1.762539	1.759419	1.77659	1.773537	1.770527	1.767564
1.772786	1.769555	1.766363	1.763204	1.760083	1.77726	1.774162	1.771123	1.768149
1.773383	1.770181	1.767011	1.763862	1.760735	1.757616	1.754515	1.771694	1.76871
1.774036	1.770821	1.767638	1.764483	1.761347	1.758187	1.755027	1.772212	1.769223
1.774703	1.771456	1.768244	1.76508	1.761935	1.758744	1.755541	1.772738	1.769743
1.775364	1.77206	1.768801	1.765627	1.762488	1.759301	1.756104	1.773329	1.770324
1.776015	1.772649	1.769339	1.766156	1.763027	1.759863	1.756699	1.773961	1.770944
1.776612	1.773241	1.76993	1.766744	1.763616	1.760468	1.757328	1.774629	1.771599
1.777161	1.773833	1.770563	1.767379	1.764245	1.761109	1.75799	1.775335	1.77229
1.777763	1.774468	1.771227	1.768044	1.764902	1.76177	1.758659	1.755597	1.752586
1.778436	1.775159	1.771927	1.768745	1.765597	1.762458	1.759336	1.756249	1.753198
1.779125	1.77586	1.772631	1.769444	1.766287	1.763138	1.760005	1.75689	1.753792
1.779809	1.776546	1.773317	1.770118	1.766946	1.763794	1.76066	1.757518	1.754359
1.780504	1.777237	1.774004	1.770788	1.767596	1.764433	1.761293	1.758127	1.754921
1.781217	1.777952	1.774714	1.771477	1.768256	1.765055	1.761873	1.758691	1.7555
1.781945	1.778679	1.775432	1.772169	1.768912	1.765661	1.762422	1.759233	1.756091
1.782672	1.779389	1.776118	1.772814	1.769515	1.766247	1.763007	1.759832	1.75672
1.783402	1.780086	1.776776	1.773412	1.770062	1.766808	1.763625	1.760484	1.757387
1.784123	1.780761	1.777409	1.774018	1.770654	1.767411	1.764257	1.761135	1.75805
1.784827	1.7814	1.778005	1.77464	1.771321	1.768079	1.764903	1.761774	1.758696
1.785535	1.782047	1.778613	1.775279	1.772007	1.768763	1.76556	1.762419	1.759346
1.772013	1.768524	1.779279	1.775945	1.772682	1.769435	1.766223	1.763081	1.760015
1.772742	1.769229	1.779968	1.776627	1.773363	1.77011	1.766892	1.763748	1.760685
1.73183	1.728595	1.766456	1.763168	1.759952	1.770798	1.767563	1.764406	1.761333
1.73238	1.729173	1.767125	1.763857	1.760652	1.771495	1.768237	1.765059	1.761967
1.732997	1.729793	1.76781	1.764542	1.761335	1.772182	1.768917	1.765728	1.762618
1.733682	1.730455	1.768507	1.765218	1.761996	1.772856	1.769605	1.766414	1.763288

1.734397	1.73115	1.727961	1.724882	1.721863	1.759449	1.756246	1.7671	1.763953
1.73515	1.731884	1.728672	1.725571	1.72253	1.76015	1.756928	1.767771	1.764594
1.735904	1.732637	1.729413	1.726291	1.723225	1.760877	1.757631	1.768457	1.76525
1.736623	1.733398	1.7302	1.727062	1.723962	1.761653	1.758374	1.755137	1.751943
1.737321	1.734156	1.731	1.727849	1.724716	1.762455	1.759145	1.755884	1.752672
1.716807	1.713691	1.731715	1.728559	1.725416	1.722326	1.719278	1.756687	1.753466
1.717517	1.714398	1.732364	1.729209	1.726076	1.72302	1.720021	1.757539	1.75432
1.718221	1.7151	1.717762	1.714627	1.711517	1.723686	1.720708	1.758313	1.755101
1.718915	1.715796	1.718411	1.715275	1.712159	1.724316	1.721322	1.758983	1.755788
1.71962	1.716497	1.719057	1.715922	1.712804	1.709756	1.70676	1.718944	1.715996
1.72035	1.717211	1.719697	1.716569	1.71346	1.710401	1.707385	1.719557	1.716608
1.721089	1.71793	1.720339	1.717219	1.71412	1.711051	1.708012	1.72017	1.717215
1.706769	1.703647	1.72099	1.717874	1.714777	1.711694	1.708631	1.70563	1.702697
1.707479	1.70435	1.721651	1.718535	1.715436	1.712338	1.709253	1.706241	1.703307
1.708213	1.705074	1.707867	1.704798	1.701743	1.713004	1.709909	1.706888	1.703945
1.708969	1.705815	1.708538	1.705465	1.702409	1.713688	1.710594	1.707568	1.704609
1.709778	1.706612	1.709259	1.706183	1.703125	1.714424	1.711332	1.708299	1.705323
1.710677	1.707503	1.710065	1.706985	1.703929	1.715248	1.712149	1.709104	1.706111
1.711547	1.708369	1.71085	1.707766	1.704706	1.716051	1.712956	1.709904	1.706896
1.712289	1.709115	1.711535	1.708439	1.705368	1.702317	1.699286	1.71063	1.707613
1.712988	1.70982	1.712183	1.709076	1.705993	1.702959	1.699963	1.711344	1.708322
1.713727	1.710555	1.712847	1.709734	1.706654	1.703649	1.7007	1.697729	1.694735
1.714488	1.711306	1.713519	1.710405	1.707335	1.704366	1.701468	1.698522	1.695524
1.715306	1.712123	1.714266	1.711147	1.708072	1.705097	1.702195	1.699246	1.696246
1.71619	1.713016	1.7151	1.711972	1.708873	1.705848	1.702878	1.699895	1.696898
1.697349	1.694251	1.715902	1.712765	1.709646	1.70658	1.703555	1.700544	1.697547
1.698135	1.695027	1.716623	1.713474	1.710345	1.707266	1.704227	1.701204	1.698197
1.698935	1.695811	1.717343	1.714185	1.711049	1.707962	1.704914	1.701883	1.69887
1.671446	1.668334	1.718134	1.71497	1.711833	1.708727	1.705653	1.702614	1.699614
1.672336	1.669199	1.718943	1.715773	1.712638	1.709513	1.706409	1.703364	1.700383
1.637001	1.634143	1.693391	1.690235	1.687113	1.710226	1.707109	1.704059	1.701081
1.637622	1.634752	1.694051	1.690884	1.687752	1.710889	1.707771	1.704716	1.701728
1.591704	1.589256	1.668313	1.665202	1.662121	1.711614	1.708492	1.705429	1.70243
1.592323	1.589858	1.669148	1.666035	1.662956	1.712429	1.709296	1.706221	1.703206
1.592932	1.590453	1.633985	1.631158	1.628364	1.686894	1.683779	1.706993	1.703972
1.593498	1.59101	1.634615	1.631769	1.628959	1.687545	1.684432	1.707684	1.704691
1.594062	1.59157	1.635247	1.632378	1.629547	1.688191	1.685086	1.708375	1.705399
1.594671	1.592174	1.589699	1.58725	1.584832	1.655987	1.653158	1.682796	1.679799
1.595298	1.592798	1.590317	1.587858	1.585429	1.656665	1.653851	1.683573	1.680491
1.595884	1.593385	1.590907	1.588456	1.586034	1.657361	1.654542	1.684356	1.681239
1.596433	1.593939	1.591474	1.589049	1.586652	1.658082	1.65524	1.68515	1.682043
1.597014	1.594526	1.592067	1.589645	1.587247	1.584859	1.582484	1.653034	1.650191
1.597642	1.595162	1.592701	1.590244	1.587804	1.585394	1.583009	1.65358	1.650738
1.598282	1.595806	1.593341	1.590853	1.588377	1.585947	1.583552	1.654148	1.651302
1.598922	1.596445	1.593978	1.591487	1.589006	1.586565	1.584154	1.654815	1.651947
1.599559	1.597076	1.594606	1.592126	1.589654	1.587208	1.584781	1.655523	1.652633
1.600168	1.597673	1.5952	1.59274	1.590293	1.587846	1.585403	1.656236	1.65335
1.600764	1.598248	1.595771	1.593339	1.590927	1.588483	1.586027	1.656959	1.654092
1.601402	1.598858	1.596361	1.593927	1.591521	1.589084	1.586639	1.591514	1.589143
1.602085	1.5995	1.596966	1.594497	1.592069	1.589648	1.587242	1.592058	1.589674

1.602777	1.600164	1.597599	1.595095	1.592636	1.590217	1.587833	1.592589	1.590196
1.603475	1.600859	1.598281	1.595739	1.593239	1.590796	1.588402	1.593097	1.59071
1.604172	1.601553	1.598964	1.596395	1.593862	1.591393	1.588977	1.593607	1.591224
1.565963	1.563495	1.599604	1.597036	1.594508	1.592024	1.589582	1.594138	1.591747
1.566561	1.564065	1.600233	1.597678	1.595168	1.592673	1.590203	1.594681	1.592278
1.567147	1.564649	1.56219	1.559797	1.557442	1.593314	1.590833	1.595234	1.592822
1.567722	1.565242	1.562797	1.560389	1.558011	1.593953	1.591473	1.595796	1.593379
1.568334	1.565861	1.56342	1.560991	1.558588	1.594597	1.592116	1.596369	1.593943
1.568988	1.56651	1.564063	1.561607	1.559175	1.595248	1.592765	1.596953	1.594518
1.569652	1.567157	1.564695	1.562217	1.559764	1.557376	1.555039	1.597534	1.59509
1.570308	1.567781	1.565295	1.562804	1.560343	1.557943	1.555593	1.598095	1.595648
1.570974	1.568409	1.565895	1.563397	1.560937	1.558524	1.556154	1.598662	1.596212
1.537538	1.535146	1.566526	1.564031	1.561578	1.559147	1.556746	1.561874	1.559562
1.538186	1.53576	1.567177	1.564688	1.562245	1.559796	1.557361	1.562421	1.560101
1.504676	1.502311	1.567841	1.565349	1.562909	1.560447	1.55799	1.562978	1.560634
1.505308	1.502913	1.568519	1.566016	1.563572	1.561103	1.558636	1.563546	1.561165
1.505933	1.503514	1.535259	1.53293	1.530662	1.561757	1.559288	1.564129	1.561708
1.506546	1.504109	1.535889	1.533541	1.531258	1.56241	1.559947	1.56473	1.562265
1.507161	1.504706	1.502325	1.500055	1.497855	1.563061	1.560601	1.565332	1.562834
1.507782	1.5053	1.502894	1.500611	1.498402	1.563704	1.561238	1.565922	1.563421
1.508409	1.505901	1.503472	1.501176	1.498957	1.564349	1.561872	1.56651	1.564016
1.509042	1.506525	1.504082	1.501771	1.499535	1.565016	1.562518	1.567095	1.564606
1.509683	1.507167	1.504718	1.502388	1.500128	1.565697	1.563171	1.567681	1.565196
1.510348	1.507834	1.505374	1.503011	1.500712	1.534358	1.531919	1.568283	1.565795
1.511041	1.50853	1.506054	1.503642	1.501285	1.534969	1.532531	1.568899	1.566402
1.511716	1.509214	1.50673	1.504275	1.501869	1.499565	1.497343	1.569524	1.567022
1.512358	1.509873	1.507392	1.50491	1.502467	1.500143	1.49791	1.570156	1.567661
1.512991	1.510524	1.508052	1.50555	1.503077	1.500731	1.498478	1.57079	1.568304
1.48099	1.478702	1.508714	1.506198	1.503699	1.501319	1.49903	1.540277	1.53791
1.481544	1.479278	1.509375	1.506854	1.504338	1.501923	1.499587	1.540836	1.538485
1.482135	1.479864	1.510033	1.507524	1.505016	1.502573	1.500186	1.541443	1.539082
1.482753	1.480459	1.51069	1.508206	1.505726	1.503261	1.50082	1.542089	1.539698
1.48336	1.481056	1.511362	1.508894	1.506432	1.503956	1.501488	1.507781	1.50557
1.483958	1.48166	1.512056	1.509592	1.507134	1.504663	1.502197	1.50842	1.506172
1.484542	1.482249	1.479959	1.477674	1.47539	1.505366	1.502913	1.509072	1.506781
1.4851	1.482799	1.48051	1.478241	1.475978	1.506062	1.503628	1.509733	1.507388
1.485662	1.483355	1.481066	1.478805	1.476556	1.506748	1.504335	1.510391	1.507996
1.486265	1.483964	1.481677	1.479401	1.477135	1.507412	1.505011	1.511023	1.508598
1.48689	1.484599	1.482313	1.480011	1.477716	1.508068	1.505671	1.511645	1.509205
1.48751	1.48521	1.482919	1.480612	1.478314	1.508749	1.506347	1.512288	1.50985
1.488129	1.485806	1.483502	1.481208	1.478929	1.50945	1.507034	1.512947	1.510529
1.48874	1.486407	1.484102	1.481815	1.479547	1.477291	1.475049	1.513614	1.511217
1.489335	1.487015	1.484727	1.48244	1.480167	1.477906	1.47566	1.51429	1.511918
1.48995	1.487635	1.485355	1.483066	1.48079	1.478527	1.47628	1.514967	1.512602
1.490609	1.488271	1.485975	1.483682	1.48141	1.479157	1.476922	1.515643	1.513243
1.49129	1.488918	1.486596	1.484302	1.482038	1.479793	1.477566	1.516316	1.513869
1.491973	1.489565	1.487219	1.484938	1.482693	1.480437	1.478183	1.516979	1.514505
1.492669	1.49022	1.48785	1.485584	1.483363	1.481085	1.478785	1.517638	1.515146
1.493404	1.490927	1.488529	1.48624	1.483999	1.481707	1.479397	1.486176	1.48388
1.494178	1.491684	1.489255	1.486903	1.4846	1.4823	1.480016	1.486772	1.484466

1.494973	1.49245	1.489984	1.487585	1.485238	1.482927	1.480652	1.487379	1.485061
1.495792	1.493212	1.490694	1.488284	1.485937	1.483607	1.481305	1.48799	1.485664
1.496617	1.493992	1.491433	1.48901	1.486656	1.484306	1.48198	1.48862	1.486282
1.497429	1.494812	1.492246	1.489777	1.48737	1.485004	1.482683	1.489285	1.486926
1.498242	1.495649	1.49309	1.49057	1.488099	1.485718	1.483406	1.489969	1.487588
1.499068	1.496469	1.4939	1.491365	1.488876	1.486471	1.484132	1.481792	1.47945
1.499904	1.497282	1.494694	1.492167	1.489687	1.487254	1.484866	1.482499	1.480156
1.500726	1.498097	1.495499	1.492966	1.490478	1.488027	1.485614	1.483232	1.480884
1.501533	1.498913	1.496318	1.493761	1.491245	1.488787	1.486379	1.483996	1.481639
1.46322	1.460493	1.497133	1.494558	1.492019	1.489551	1.487141	1.484753	1.48239
1.464235	1.461479	1.497935	1.495355	1.492813	1.490325	1.487885	1.485482	1.483118
1.465254	1.46247	1.498742	1.496158	1.493612	1.491104	1.488635	1.486215	1.483848
1.466264	1.463475	1.460725	1.458035	1.455393	1.452798	1.450248	1.486978	1.484593
1.467274	1.464491	1.46174	1.45902	1.456344	1.453745	1.451208	1.487761	1.485352
1.4683	1.465515	1.462761	1.460021	1.457323	1.454716	1.452179	1.44965	1.447134
1.469338	1.466548	1.463792	1.461038	1.458326	1.455707	1.453161	1.450634	1.44813
1.470409	1.46761	1.464848	1.462075	1.459341	1.456703	1.45414	1.451606	1.449108
1.471522	1.468711	1.46594	1.463136	1.460366	1.457698	1.455108	1.45256	1.450061
1.472642	1.469811	1.46703	1.464205	1.461412	1.458713	1.456088	1.453517	1.451009
1.473737	1.47088	1.468091	1.465271	1.462484	1.459757	1.457083	1.45448	1.451953
1.474852	1.471957	1.469155	1.466347	1.463576	1.460823	1.4581	1.455461	1.452909
1.476037	1.473088	1.470261	1.467452	1.464689	1.461917	1.459158	1.456489	1.453911
1.477272	1.474255	1.47139	1.468572	1.465815	1.463028	1.460246	1.457551	1.454942
1.478535	1.475423	1.472494	1.469659	1.466906	1.464113	1.461321	1.458609	1.455974
1.47982	1.476589	1.473571	1.470711	1.467962	1.465171	1.462386	1.459666	1.457009
1.4812	1.477829	1.474699	1.4718	1.469048	1.466257	1.463481	1.460749	1.458056
1.48271	1.479168	1.475893	1.472944	1.470182	1.46739	1.464626	1.461871	1.459115
1.484266	1.480555	1.477134	1.474118	1.471328	1.468527	1.465774	1.463001	1.460197
1.48581	1.481956	1.478404	1.475307	1.472463	1.469641	1.466893	1.464122	1.461322
1.487381	1.483401	1.479727	1.476526	1.473601	1.470745	1.467999	1.46524	1.462466
1.48901	1.484929	1.481138	1.477795	1.474739	1.471835	1.469099	1.46635	1.463592
1.490689	1.486527	1.482628	1.479111	1.475883	1.472918	1.470193	1.467454	1.46471
1.49239	1.488178	1.484194	1.480509	1.477103	1.474053	1.471304	1.468556	1.465825
1.494113	1.489886	1.485842	1.481991	1.478395	1.475237	1.47243	1.469656	1.466938
1.408806	1.404688	1.487523	1.48353	1.479766	1.476485	1.473581	1.470759	1.468046
1.410532	1.40638	1.489214	1.485115	1.481222	1.477802	1.474756	1.47186	1.469147
1.412291	1.4081	1.490939	1.486758	1.48276	1.479192	1.47597	1.472975	1.470248
1.356081	1.351926	1.492709	1.488477	1.4844	1.480667	1.477227	1.474104	1.471338
1.357852	1.353652	1.494515	1.490248	1.48611	1.482212	1.478541	1.475271	1.472442
1.359639	1.355395	1.35122	1.347066	1.343028	1.483787	1.479959	1.476562	1.473633
1.361446	1.357157	1.352924	1.348712	1.344605	1.485399	1.481472	1.477962	1.474898
1.363254	1.35893	1.354651	1.350388	1.346219	1.342238	1.338442	1.47944	1.476209
1.365061	1.360713	1.356398	1.352094	1.347877	1.343855	1.340014	1.481004	1.477564
1.366866	1.362507	1.358169	1.353825	1.34956	1.345499	1.341616	1.43234	1.428761
1.36867	1.364319	1.359969	1.355585	1.351261	1.347148	1.343208	1.433892	1.430159
1.370469	1.366134	1.361783	1.357361	1.352983	1.348816	1.344816	1.435474	1.431624
1.372255	1.367937	1.36359	1.359139	1.354715	1.350502	1.346448	1.342532	1.338728
1.374031	1.369734	1.365397	1.360926	1.356466	1.352209	1.348102	1.344136	1.340294
1.375796	1.371521	1.367202	1.362735	1.358263	1.35396	1.349783	1.34576	1.341885
1.377548	1.373297	1.369004	1.364563	1.360102	1.355751	1.351487	1.347406	1.343509

							0.483953	0.482822
							0.483146	0.481945
					0.484673	0.483511	0.881518	0.877224
					0.483761	0.482645	0.879008	0.875036
					0.48286	0.48176	0.99092	0.987183
					0.481993	0.480841	0.988638	0.985055
					0.481139	0.479931	0.986468	0.982983
			0.482715	0.481499	0.87778	0.873953	1.084407	1.080589
			0.481874	0.480648	0.875601	0.871965	1.08237	1.07853
			0.710774	0.706676	0.987674	0.984015	1.410759	1.406211
			0.708336	0.704851	0.985727	0.982055	1.408269	1.403615
0.482817	0.481577	0.480356	0.849754	0.846565	1.352469	1.348147	1.405784	1.401061
0.481961	0.480728	0.479509	0.847736	0.844579	1.349834	1.345645	1.403314	1.398571
0.714063	0.709748	0.705763	0.986506	0.982907	1.409663	1.405308	1.650345	1.645014
0.711625	0.707648	0.703992	0.984509	0.980873	1.407113	1.402728	1.647422	1.642136
0.709293	0.705647	0.7023	0.982574	0.978896	1.404596	1.400123	1.64443	1.639196
0.850651	0.847186	0.84391	1.348643	1.344202	1.402063	1.397472	1.680069	1.674792
0.848784	0.845394	0.84212	1.346219	1.341775	1.399531	1.394798	1.676957	1.671657
1.205669	1.201785	1.197941	1.4065	1.401776	1.64614	1.640652	1.706331	1.700762
1.203505	1.199674	1.195836	1.404017	1.399275	1.643272	1.637783	1.703305	1.697606
1.269474	1.265367	1.261228	1.468509	1.463624	1.640395	1.634905	1.750499	1.744735
1.267131	1.263053	1.258937	1.46595	1.461029	1.637502	1.631988	1.747475	1.741833
1.264759	1.260711	1.256629	1.463375	1.458427	1.634613	1.629097	1.744491	1.738999
1.40782	1.403301	1.398756	1.642742	1.637222	1.666196	1.660509	1.801014	1.795525
1.405132	1.400663	1.396188	1.639804	1.634314	1.66322	1.657603	1.798057	1.792658
1.469497	1.464928	1.460355	1.636884	1.631419	1.746929	1.74132	1.795186	1.789845
1.466925	1.462346	1.45777	1.633975	1.62854	1.743991	1.738522	1.792387	1.78708
1.607342	1.602301	1.597265	1.665481	1.65992	1.800449	1.794976	1.831101	1.825713
1.604482	1.599437	1.594419	1.662561	1.657087	1.797552	1.792146	1.82828	1.822915
1.675718	1.670355	1.665057	1.728126	1.722674	1.794691	1.789335	1.825465	1.820121
1.672831	1.667425	1.662101	1.725174	1.719851	1.791869	1.786542	1.82265	1.817328
1.670002	1.66456	1.659209	1.722274	1.717059	1.789066	1.78376	1.819841	1.814539
1.735715	1.730203	1.724758	1.796875	1.791559	1.82776	1.822373	1.968349	1.961676
1.732915	1.727443	1.722016	1.794079	1.788746	1.824914	1.819552	1.964756	1.958157
1.730016	1.724614	1.719251	1.791283	1.785946	1.82209	1.816755	2.018045	2.010852
1.727041	1.721739	1.716482	1.788497	1.783161	1.819286	1.81398	2.014248	2.00708
1.771609	1.766323	1.761088	1.827247	1.821832	1.967547	1.960902	2.063588	2.056374
1.768833	1.76352	1.758269	1.824401	1.819014	1.96397	1.957415	2.05978	2.052572
1.766067	1.760725	1.755451	1.839729	1.834072	2.04902	2.041882	2.089557	2.08238
1.763266	1.757922	1.752644	1.836741	1.831134	2.045259	2.038125	2.085771	2.078582
1.760449	1.755114	1.74984	1.833774	1.828215	2.041512	2.034377	2.081999	2.074826
1.829316	1.823863	1.818469	1.963203	1.956682	2.056078	2.048954	2.078233	2.071174
1.826427	1.821004	1.815644	1.959673	1.953238	2.052297	2.045171	2.07449	2.067604
1.841737	1.836055	1.830458	2.04439	2.03731	2.084905	2.077773	2.07083	2.064096
1.838702	1.833068	1.827513	2.040621	2.033551	2.081153	2.074088	2.067242	2.060648
2.028209	2.021505	2.01492	2.055184	2.048129	2.07746	2.070534	2.128491	2.12193
2.02467	2.018035	2.011501	2.051442	2.044387	2.073821	2.067158	2.125216	2.118754
2.021247	2.014656	2.008149	2.047788	2.040741	2.070265	2.063828	2.121972	2.11559
2.047386	2.040248	2.033167	2.044325	2.037292	2.131502	2.125023	2.141522	2.135023

2.043957	2.036824	2.029748	2.040928	2.033938	2.128099	2.121633	2.138066	2.13159
2.058582	2.05144	2.044377	2.122484	2.115563	2.147729	2.141127	2.134608	2.128162
2.054801	2.047656	2.040624	2.118824	2.112088	2.144229	2.137645	2.131147	2.124734
2.085868	2.078743	2.071761	2.11523	2.108655	2.140769	2.134212	2.151816	2.145407
2.08214	2.07506	2.068146	2.111681	2.105252	2.137337	2.130818	2.148426	2.142043
2.128727	2.121695	2.114831	2.132228	2.125731	2.147655	2.14117	2.179017	2.17255
2.125243	2.118355	2.11164	2.129031	2.122556	2.144493	2.138051	2.17585	2.169384
2.121786	2.115058	2.108498	2.125864	2.119393	2.141332	2.134924	2.172673	2.166208
2.142311	2.135611	2.129044	2.15094	2.144443	2.182287	2.175775	2.169296	2.162845
2.138655	2.132059	2.125587	2.147498	2.141021	2.178812	2.172301	2.165831	2.159395
2.149444	2.142899	2.136465	2.144085	2.13763	2.175363	2.16885	2.162378	2.155936
2.146018	2.139498	2.13306	2.140684	2.134254	2.171924	2.165403	2.158921	2.152456
2.142707	2.136211	2.129775	2.181644	2.175112	2.16859	2.162079	2.168776	2.162263
2.139545	2.133082	2.126661	2.178455	2.171915	2.165406	2.158928	2.165623	2.159082
2.173106	2.166559	2.160042	2.175203	2.168659	2.162167	2.155723	2.192086	2.185116
2.169699	2.163137	2.156607	2.171754	2.165221	2.158746	2.152322	2.188615	2.181867
2.166286	2.159691	2.153133	2.168259	2.161741	2.155288	2.148893	2.185128	2.178628
2.163027	2.15637	2.149749	2.164824	2.158316	2.163208	2.156848	2.209022	2.202515
2.159837	2.15311	2.146415	2.161429	2.154931	2.159844	2.153567	2.205593	2.199139
2.166238	2.159526	2.152853	2.15654	2.150081	2.2157	2.20895	2.202353	2.195933
2.162857	2.156237	2.149663	2.15338	2.146963	2.212459	2.205826	2.199302	2.192891
2.159546	2.152977	2.14646	2.168662	2.162213	2.209182	2.202641	2.196175	2.189775
2.156357	2.149758	2.143218	2.165384	2.158993	2.205801	2.199317	2.192892	2.18651
2.15316	2.146549	2.140004	2.162151	2.15583	2.202479	2.196032	2.189635	2.183272
2.154987	2.148425	2.141942	2.212682	2.205939	2.199349	2.192917	2.186533	2.180188
2.151665	2.145199	2.138826	2.20944	2.202805	2.196267	2.189836	2.183459	2.177133
2.1615	2.155024	2.148636	2.206103	2.199526	2.19302	2.186602	2.180241	2.173937
2.158393	2.151903	2.145498	2.202732	2.196177	2.18969	2.183287	2.176946	2.170665
2.211823	2.204824	2.19792	2.199522	2.192977	2.192846	2.186478	2.194813	2.18858
2.208349	2.201545	2.194833	2.196486	2.189931	2.189807	2.183443	2.191792	2.185574
2.204964	2.19831	2.191749	2.199754	2.19323	2.186781	2.1804	2.188743	2.182523
2.201745	2.19515	2.188647	2.196706	2.190219	2.183751	2.177302	2.185593	2.179331
2.198564	2.191985	2.1855	2.193604	2.187163	2.180696	2.174208	2.18247	2.176177
2.201945	2.195363	2.188884	2.190344	2.183954	2.192171	2.185773	2.179472	2.173191
2.198711	2.192087	2.185574	2.187034	2.180694	2.188995	2.182731	2.176542	2.170316
2.195565	2.188942	2.182425	2.190309	2.183997	2.185919	2.179723	2.173634	2.167545
2.192488	2.185898	2.179394	2.187276	2.180978	2.182916	2.176744	2.170755	2.164877
2.196206	2.18964	2.183149	2.184246	2.177933	2.179872	2.17372	2.16786	2.162273
2.193149	2.186627	2.180166	2.181218	2.174846	2.176751	2.170595	2.164896	2.159712
2.190092	2.183596	2.177157	2.186348	2.179954	2.173731	2.16765	2.162134	2.157297
2.187034	2.180511	2.174062	2.18327	2.176965	2.170895	2.165032	2.159729	2.155094
2.183958	2.177416	2.170972	2.18022	2.174035	2.168167	2.162588	2.15751	2.153015
2.180812	2.174331	2.167972	2.177267	2.171162	2.165494	2.160223	2.155374	2.150995
2.177641	2.171262	2.165031	2.17439	2.168365	2.162905	2.157955	2.153334	2.149045
2.183088	2.176784	2.170646	2.171607	2.165733	2.160455	2.15572	2.151276	2.147116
2.180107	2.173859	2.167794	2.168918	2.163259	2.15814	2.153523	2.14921	2.145213
2.177175	2.17098	2.164995	2.16629	2.160859	2.155914	2.15143	2.152221	2.148341
2.174285	2.168135	2.162233	2.163705	2.158516	2.153771	2.14946	2.150393	2.146599
2.171448	2.165367	2.15957	2.161223	2.156269	2.151715	2.147564	2.148624	2.144899
2.168678	2.162711	2.157056	2.158889	2.154147	2.149755	2.145728	2.14689	2.143226

2.165952	2.160134	2.154649	2.156663	2.152116	2.147869	2.143946	2.145193	2.141576
2.163219	2.157588	2.152313	2.154507	2.150136	2.15107	2.147228	2.143513	2.139934
2.160505	2.155095	2.15006	2.152431	2.148216	2.14928	2.145517	2.141855	2.138299
2.157906	2.152707	2.147896	2.143489	2.139432	2.14753	2.143837	2.140209	2.136641
2.155409	2.150419	2.14582	2.14157	2.137637	2.145824	2.142191	2.138577	2.134954
2.15307	2.148261	2.143831	2.139711	2.135881	2.144139	2.140556	2.140943	2.137306
2.150944	2.146271	2.141937	2.137911	2.134157	2.14247	2.138929	2.139347	2.135705
2.148895	2.144356	2.140109	2.136162	2.132467	2.140819	2.137307	2.137752	2.134122
2.146814	2.142448	2.138334	2.139907	2.136238	2.14315	2.139671	2.136154	2.132545
2.144755	2.140568	2.136597	2.138255	2.134615	2.141539	2.138052	2.134543	2.130969
2.142727	2.138679	2.134832	2.14078	2.13722	2.139911	2.136385	2.132876	2.12936
2.140735	2.136794	2.133057	2.139113	2.135604	2.138273	2.134683	2.131166	2.127729
2.14859	2.144736	2.141063	2.137489	2.134007	2.130463	2.126879	2.129524	2.126142
2.147014	2.143184	2.139492	2.135917	2.132431	2.128889	2.125309	2.127955	2.124602
2.145437	2.141634	2.137932	2.134356	2.130865	2.127333	2.123776	2.121682	2.118334
2.143765	2.140022	2.136357	2.132795	2.129307	2.125797	2.122277	2.12019	2.116857
2.142085	2.1384	2.134778	2.131233	2.127749	2.124273	2.120809	2.11874	2.115415
2.140443	2.136791	2.133189	2.129652	2.126174	2.118092	2.114708	2.133115	2.129709
2.138821	2.135187	2.131596	2.128065	2.124595	2.11657	2.113289	2.131724	2.128315
2.137211	2.133592	2.130012	2.1265	2.123055	2.115067	2.111844	2.132014	2.128653
2.135616	2.132004	2.128435	2.124953	2.121548	2.113583	2.110377	2.130535	2.127215
2.134031	2.130426	2.126868	2.118772	2.11539	2.130444	2.127206	2.129063	2.125784
2.132457	2.128854	2.125302	2.11723	2.113884	2.128936	2.125728	2.127604	2.124364
2.12628	2.122685	2.119146	2.12455	2.121228	2.127445	2.124261	2.126153	2.122953
2.124715	2.121191	2.117717	2.123125	2.119788	2.125977	2.122806	2.124707	2.121545
2.12316	2.119716	2.116317	2.121728	2.118372	2.124531	2.121367	2.123275	2.120154
2.123954	2.120567	2.117235	2.129781	2.126378	2.123108	2.119956	2.121876	2.118812
2.122426	2.119057	2.115756	2.128306	2.124949	2.121707	2.118569	2.120504	2.117501
2.127344	2.123978	2.12069	2.126847	2.123552	2.120341	2.117211	2.114162	2.111222
2.125847	2.122484	2.119206	2.1254	2.122189	2.119015	2.115884	2.112831	2.109885
2.133958	2.130533	2.127203	2.12397	2.120816	2.117675	2.114555	2.111507	2.108551
2.132558	2.129127	2.12578	2.122552	2.119413	2.116296	2.11321	2.11018	2.107214
2.131166	2.127743	2.124391	2.121162	2.118024	2.114928	2.111882	2.108874	2.105898
2.129717	2.126348	2.123045	2.119827	2.116684	2.113609	2.110605	2.107619	2.104631
2.128253	2.124949	2.121713	2.118512	2.115366	2.112312	2.109346	2.10638	2.103387
2.126801	2.123524	2.120316	2.117145	2.114029	2.110997	2.108047	2.105096	2.102121
2.125357	2.12208	2.118877	2.115747	2.112685	2.109676	2.106724	2.103785	2.100846
2.123963	2.120699	2.117507	2.114412	2.111391	2.108397	2.105438	2.111167	2.108084
2.122632	2.11939	2.116213	2.113148	2.110157	2.107168	2.104191	2.109832	2.106821
2.121295	2.11809	2.114947	2.1119	2.108921	2.10594	2.102969	2.120573	2.117472
2.119918	2.116784	2.113709	2.110665	2.107661	2.104702	2.101785	2.119318	2.116191
2.118544	2.115476	2.112465	2.10942	2.106392	2.103455	2.100599	2.118078	2.114915
2.117201	2.114152	2.111158	2.108121	2.105099	2.102181	2.099354	2.116747	2.113607
2.115882	2.112827	2.109825	2.106803	2.103806	2.100903	2.098088	2.115381	2.112292
2.114604	2.111543	2.108531	2.105537	2.10258	2.120191	2.117113	2.124214	2.120881
2.113365	2.110292	2.107265	2.104312	2.101413	2.118949	2.115826	2.122799	2.119531
2.112115	2.109051	2.106032	2.103106	2.10024	2.117699	2.114573	2.154367	2.150775
2.11084	2.107818	2.104841	2.101922	2.09905	2.116423	2.113361	2.152933	2.149253
2.109582	2.106594	2.103652	2.1335	2.130267	2.136727	2.133429	2.151485	2.147735
2.108367	2.105377	2.102434	2.132131	2.128983	2.13538	2.132049	2.149955	2.146237

2.107163	2.104168	2.101224	2.130786	2.127716	2.134042	2.130661	2.148412	2.14474
2.119388	2.116392	2.113447	2.129496	2.126424	2.132655	2.129298	2.146933	2.14322
2.118113	2.115172	2.112288	2.128243	2.125126	2.131247	2.127944	2.14548	2.141686
2.136619	2.133315	2.130134	2.136625	2.133239	2.129878	2.126551	2.143957	2.140157
2.13529	2.131984	2.128806	2.135245	2.13193	2.128548	2.125118	2.142363	2.138633
2.13401	2.130713	2.127533	2.133888	2.130595	2.148359	2.144564	2.140805	2.137097
2.132792	2.129531	2.12635	2.132567	2.1292	2.146818	2.143073	2.139322	2.135539
2.131569	2.128353	2.12518	2.131262	2.127816	2.145288	2.141579	2.137833	2.133991
2.130267	2.127111	2.12398	2.12998	2.126502	2.143818	2.140036	2.136265	2.132467
2.128948	2.125847	2.12276	2.128694	2.125208	2.142376	2.138495	2.134694	2.130973
2.137475	2.134108	2.130761	2.132376	2.128873	2.140943	2.13704	2.133236	2.12954
2.136172	2.132756	2.129345	2.130951	2.12749	2.139517	2.135645	2.131858	2.128158
2.134878	2.131438	2.127987	2.129549	2.126071	2.138025	2.134198	2.135318	2.131637
2.133603	2.130165	2.126698	2.128171	2.124603	2.136447	2.13269	2.133884	2.1303
2.137387	2.13391	2.130401	2.126805	2.123176	2.134915	2.131198	2.132446	2.128953
2.135973	2.132521	2.129047	2.125466	2.121842	2.133484	2.129733	2.130987	2.127573
2.134561	2.131116	2.127663	2.124111	2.120519	2.132086	2.12831	2.129558	2.126191
2.133229	2.129722	2.126217	2.13822	2.134436	2.135574	2.131823	2.129766	2.126357
2.131931	2.128336	2.124749	2.136668	2.132956	2.134168	2.130513	2.128481	2.124999
2.130606	2.126989	2.123369	2.135169	2.131439	2.132697	2.129158	2.127186	2.12369
2.129264	2.125675	2.122058	2.133709	2.129885	2.131172	2.127774	2.125892	2.122426
2.127912	2.124344	2.12074	2.137215	2.133333	2.13129	2.127956	2.124593	2.121156
2.126556	2.122995	2.119413	2.135847	2.132021	2.129994	2.126656	2.123296	2.119881
2.125168	2.121619	2.118069	2.134482	2.130731	2.128713	2.12534	2.121968	2.118579
2.123709	2.120188	2.116693	2.133089	2.129413	2.127392	2.12396	2.120571	2.11723
2.122245	2.118752	2.115313	2.131695	2.128095	2.12607	2.122579	2.119166	2.11586
2.120861	2.117377	2.113963	2.130317	2.126784	2.124773	2.121265	2.120708	2.117333
2.119523	2.116036	2.11263	2.128947	2.125475	2.123488	2.119989	2.119404	2.115957
2.138483	2.134726	2.131072	2.129087	2.12566	2.125147	2.121621	2.11812	2.114639
2.137049	2.133283	2.129635	2.127683	2.124321	2.123846	2.120346	2.116859	2.113376
2.13562	2.131869	2.128245	2.129347	2.125985	2.122552	2.119075	2.115602	2.112122
2.134185	2.130487	2.12692	2.128034	2.124683	2.121267	2.11781	2.114348	2.110869
2.13278	2.129136	2.125622	2.126741	2.123393	2.119988	2.116548	2.113095	2.109619
2.134843	2.131181	2.127644	2.125448	2.122108	2.118713	2.115284	2.11184	2.108366
2.133535	2.129888	2.126357	2.124163	2.120831	2.117444	2.114023	2.110585	2.107114
2.13223	2.128598	2.125075	2.122887	2.119566	2.116186	2.11277	2.109334	2.10586
2.130936	2.127318	2.1238	2.121618	2.11831	2.114937	2.111522	2.108086	2.104607
2.130866	2.127253	2.12373	2.12035	2.117052	2.113685	2.110271	2.10684	2.103372
2.12956	2.125968	2.122453	2.119084	2.115793	2.11243	2.109017	2.105597	2.102159
2.128253	2.124678	2.121173	2.117814	2.114532	2.111173	2.107761	2.111175	2.107414
2.12694	2.123376	2.119881	2.116534	2.113264	2.109914	2.106505	2.109828	2.106107
2.125632	2.122078	2.118597	2.115259	2.111998	2.108653	2.105248	2.108482	2.104799
2.124333	2.120796	2.11733	2.113996	2.110733	2.107389	2.103986	2.107127	2.103482
2.123044	2.119532	2.116085	2.11275	2.109475	2.106128	2.102725	2.105775	2.102163
2.12177	2.11831	2.114903	2.111554	2.108241	2.110728	2.107263	2.104455	2.100858
2.120511	2.117126	2.113777	2.110402	2.107027	2.109468	2.106016	2.103162	2.099564
2.1193	2.115945	2.112611	2.109214	2.105799	2.108204	2.104766	2.10187	2.098273
2.118154	2.114772	2.111396	2.107979	2.104552	2.106933	2.103511	2.100579	2.096984
2.116994	2.113581	2.110165	2.112615	2.109113	2.105663	2.102258	2.099289	2.095701
2.115777	2.112351	2.108925	2.111337	2.107832	2.10439	2.101001	2.097992	2.094411

2.114538	2.111103	2.107676	2.11006	2.106562	2.103129	2.099754	2.096706	2.093144
2.113294	2.10985	2.106421	2.108791	2.105317	2.102586	2.098997	2.11919	2.115247
2.112042	2.108591	2.105161	2.107531	2.104095	2.10133	2.097747	2.117861	2.114001
2.116747	2.113221	2.109728	2.106304	2.102925	2.100145	2.096568	2.116592	2.112755
2.11545	2.111935	2.108467	2.105107	2.101807	2.099034	2.095461	2.115387	2.111517
2.114177	2.11069	2.107255	2.10393	2.100666	2.09787	2.094299	2.114129	2.110249
2.112928	2.1095	2.106116	2.102784	2.099487	2.096623	2.093046	2.112781	2.108936
2.111719	2.108331	2.104977	2.101626	2.098287	2.095351	2.091776	2.111423	2.107614
2.110585	2.107174	2.103792	2.100419	2.097061	2.09407	2.090514	2.121884	2.117772
2.109472	2.106017	2.102592	2.099194	2.095826	2.092791	2.089267	2.120473	2.116366
2.108281	2.104819	2.101384	2.097975	2.094602	2.091548	2.088092	2.120184	2.116112
2.107049	2.103598	2.100173	2.096762	2.093388	2.090333	2.086965	2.118938	2.114899
2.105877	2.10244	2.099023	2.095612	2.092235	2.090857	2.087518	2.11761	2.11359
2.104773	2.101356	2.097945	2.094535	2.091152	2.089697	2.086327	2.116192	2.112182
2.103634	2.100227	2.096823	2.095335	2.091946	2.100943	2.097218	2.114777	2.110767
2.102421	2.099007	2.095609	2.094123	2.090764	2.099617	2.095899	2.11337	2.109348
2.10119	2.097764	2.094372	2.0929	2.089589	2.098316	2.094613	2.112	2.107967
2.100977	2.097319	2.093716	2.091848	2.088403	2.097104	2.093417	2.11063	2.10658
2.099708	2.096043	2.092451	2.090636	2.087284	2.095925	2.092258	2.10939	2.105352
2.10014	2.096469	2.092877	2.089452	2.086127	2.095003	2.091322	2.108068	2.104039
2.098999	2.095332	2.091737	2.088295	2.084948	2.093705	2.090034	2.106691	2.10267
2.097796	2.094144	2.090566	2.099476	2.095811	2.092456	2.088794	2.105363	2.101357
2.096499	2.092879	2.089343	2.098206	2.094605	2.091274	2.087623	2.104106	2.100123
2.095252	2.091663	2.088165	2.097291	2.093702	2.090075	2.08643	2.102828	2.098865
2.09413	2.090567	2.087083	2.096073	2.092451	2.088808	2.085157	2.101467	2.097518
2.09305	2.089512	2.08604	2.094882	2.091212	2.087542	2.083881	2.10011	2.096171
2.092549	2.089019	2.085544	2.093729	2.09004	2.086359	2.08269	2.098853	2.09492
2.091442	2.087949	2.084504	2.092591	2.088897	2.085209	2.081531	2.097639	2.09371
2.102624	2.09883	2.09509	2.091369	2.087673	2.08398	2.080292	2.096341	2.092423
2.101272	2.0975	2.09378	2.090068	2.086376	2.08268	2.078982	2.094967	2.091068
2.099995	2.096243	2.092536	2.08883	2.085138	2.08144	2.077737	2.09764	2.093576
2.098842	2.095103	2.091401	2.087694	2.083996	2.080295	2.076594	2.096393	2.092336
2.09768	2.093963	2.090275	2.08657	2.082866	2.079166	2.075468	2.09517	2.091122
2.096418	2.092758	2.089117	2.085429	2.081727	2.078031	2.074336	2.093949	2.089918
2.09514	2.091532	2.087934	2.084264	2.080564	2.076873	2.073184	2.092709	2.088701
2.093929	2.090305	2.086687	2.083006	2.079301	2.095471	2.09151	2.091366	2.087382
2.092764	2.089079	2.085402	2.081692	2.077978	2.094082	2.090143	2.089963	2.086004
2.091633	2.087915	2.084203	2.080474	2.076751	2.092789	2.088863	2.08865	2.084718
2.090543	2.086816	2.083092	2.079353	2.075621	2.091589	2.08767	2.087429	2.083526
2.089454	2.085721	2.081989	2.078244	2.074508	2.094311	2.090223	2.08623	2.082349
2.088362	2.08462	2.08088	2.077135	2.073403	2.093107	2.089032	2.085053	2.081189
2.087255	2.083507	2.079763	2.076022	2.072298	2.091903	2.087832	2.083859	2.080006
2.086128	2.08238	2.078639	2.074907	2.071192	2.090676	2.086573	2.082582	2.078726
2.084969	2.081224	2.077489	2.07377	2.07007	2.089431	2.085296	2.081284	2.077423
2.084484	2.080899	2.077331	2.073793	2.07028	2.088147	2.084059	2.080077	2.076222
2.083207	2.079618	2.076057	2.07254	2.069061	2.086831	2.08285	2.078937	2.075095
2.082001	2.078412	2.074858	2.076056	2.072411	2.085566	2.081664	2.077804	2.073979
2.080878	2.077294	2.073747	2.074909	2.071282	2.084356	2.080502	2.076678	2.072873
2.079782	2.076207	2.072669	2.073799	2.070189	2.083187	2.079361	2.075562	2.071774
2.078701	2.075141	2.07162	2.072722	2.06913	2.082069	2.078249	2.074455	2.070675

2.077632	2.074088	2.070586	2.071662	2.068088	2.080974	2.077149	2.073355	2.069582
2.076559	2.07303	2.069544	2.070591	2.067035	2.079868	2.076049	2.072262	2.0685
2.075487	2.071972	2.068502	2.069521	2.065982	2.078763	2.074952	2.071175	2.067425
2.079374	2.075675	2.07203	2.068453	2.06493	2.077665	2.07386	2.070089	2.066349
2.078246	2.074568	2.070945	2.067387	2.063881	2.076573	2.072772	2.069005	2.065271
2.077119	2.073461	2.069858	2.066317	2.062824	2.075472	2.071678	2.067918	2.064192
2.075994	2.072353	2.068767	2.065239	2.061758	2.074357	2.070576	2.066827	2.063114
2.074874	2.07125	2.06768	2.064163	2.060691	2.073241	2.069472	2.065735	2.062032
2.07376	2.070155	2.066603	2.063096	2.05963	2.072132	2.068373	2.064642	2.060942
2.07265	2.069065	2.065532	2.062033	2.05857	2.071024	2.067274	2.063549	2.059849
2.067174	2.063616	2.060115	2.060971	2.057513	2.072473	2.068813	2.062456	2.05876
2.066066	2.062524	2.059042	2.05991	2.056459	2.071372	2.067718	2.061361	2.057671
2.064979	2.061445	2.057973	2.058851	2.055405	2.070271	2.066624	2.060267	2.056581
2.06391	2.060373	2.056907	2.05779	2.054349	2.06917	2.065529	2.059174	2.055488
2.062895	2.059346	2.055866	2.05675	2.053308	2.068078	2.064437	2.058079	2.054394
2.061967	2.058383	2.054854	2.055723	2.052273	2.066986	2.06334	2.056977	2.053298
2.061041	2.057438	2.053876	2.054739	2.051279	2.06593	2.062265	2.055887	2.052207
2.060052	2.056494	2.052965	2.053848	2.050382	2.064957	2.061236	2.054814	2.051117
2.059031	2.055539	2.052069	2.052985	2.049519	2.064018	2.060238	2.053769	2.050045
2.057983	2.054523	2.051081	2.05203	2.048581	2.063043	2.059274	2.052798	2.049041
2.056909	2.053458	2.050029	2.051012	2.047593	2.062045	2.058338	2.051886	2.048091
2.055848	2.052398	2.048973	2.049981	2.046581	2.061005	2.057344	2.050928	2.047152
2.054791	2.051336	2.047909	2.04893	2.045537	2.059916	2.056284	2.04992	2.046234
2.053784	2.050312	2.04687	2.047897	2.044505	2.058829	2.055214	2.04889	2.045281
2.052871	2.049355	2.045868	2.046889	2.043491	2.057752	2.054142	2.047832	2.044257
2.051971	2.048422	2.044901	2.04592	2.042516	2.056709	2.053089	2.046776	2.043219
2.051006	2.047489	2.043996	2.040572	2.037192	2.055757	2.052093	2.045749	2.042202
2.050011	2.046555	2.043119	2.039719	2.036341	2.054854	2.051137	2.044755	2.041209
2.049003	2.045576	2.042171	2.038788	2.035421	2.053919	2.050222	2.046619	2.043141
2.047978	2.044557	2.041163	2.037794	2.034447	2.052965	2.049348	2.045792	2.042319
2.047015	2.043598	2.040209	2.036836	2.033485	2.051992	2.04843	2.044908	2.041441
2.046145	2.042733	2.039338	2.035933	2.03254	2.050985	2.047438	2.043932	2.040474
2.047053	2.043643	2.040244	2.035031	2.031614	2.050012	2.046474	2.042972	2.039507
2.046078	2.042675	2.03929	2.034109	2.030733	2.049142	2.045615	2.042099	2.038582
2.045089	2.041687	2.038315	2.033173	2.02985	2.048286	2.044769	2.041238	2.037671
2.044171	2.040742	2.037344	2.033998	2.030689	2.047332	2.04383	2.040316	2.036768
2.043286	2.039817	2.036377	2.033011	2.029697	2.046326	2.04284	2.039357	2.035867
2.042355	2.03889	2.035453	2.032087	2.028768	2.041227	2.037733	2.038379	2.034913
2.041378	2.037965	2.034577	2.031228	2.027904	2.0403	2.036747	2.037375	2.033902
2.040423	2.037034	2.033671	2.030332	2.02701	2.041439	2.037862	2.036412	2.032935
2.039517	2.036095	2.032701	2.029358	2.02605	2.040467	2.036929	2.035518	2.032049
2.038607	2.035158	2.031739	2.028395	2.025099	2.039506	2.03601	2.034638	2.031167
2.037646	2.034228	2.030839	2.027509	2.024218	2.038599	2.035113	2.033746	2.030239
2.036681	2.033309	2.029965	2.026648	2.023355	2.0377	2.034212	2.032842	2.029296
2.035783	2.032426	2.02909	2.025757	2.022435	2.03673	2.033239	2.034059	2.030535
2.034932	2.03157	2.028219	2.024848	2.021479	2.035712	2.032217	2.03307	2.029606
2.034072	2.030693	2.027321	2.023934	2.020553	2.034745	2.031243	2.027779	2.024358
2.033209	2.029792	2.026388	2.023012	2.01966	2.033833	2.030327	2.026865	2.02346
2.032317	2.028886	2.02547	2.022107	2.018777	2.032925	2.029405	2.025934	2.022534
2.031369	2.027974	2.024592	2.021241	2.017909	2.03201	2.028448	2.02495	2.021541

2.030422	2.027067	2.023723	2.020376	2.017031	2.031085	2.027492	2.023974	2.020554
2.029536	2.026188	2.022843	2.019476	2.016104	2.012749	2.009406	2.02306	2.019644
2.028678	2.02532	2.021958	2.018561	2.015158	2.011815	2.008522	2.022181	2.018773
2.027814	2.024434	2.021051	2.017649	2.014252	2.010929	2.007669	2.021317	2.017912
2.02695	2.023534	2.020124	2.016737	2.013376	2.01008	2.006845	2.02047	2.017065
2.026067	2.022638	2.019226	2.015856	2.012521	2.009243	2.006019	2.019613	2.016192
2.02515	2.021744	2.018364	2.015011	2.011689	2.008417	2.005193	2.018744	2.015283
2.024253	2.02087	2.01752	2.014182	2.010868	2.007593	2.004356	2.017856	2.014371
2.023412	2.020035	2.016694	2.013363	2.010053	2.006761	2.003488	2.016928	2.013461
2.022589	2.019216	2.015878	2.012552	2.009244	2.005934	2.002623	2.016002	2.012567
2.021749	2.018389	2.015058	2.011743	2.008444	2.005132	2.001809	2.01514	2.011719
2.020906	2.01756	2.014239	2.010935	2.007646	2.00434	2.001021	2.014315	2.010903
2.020077	2.016737	2.013416	2.010103	2.006801	2.020489	2.016953	2.013481	2.010085
2.019259	2.015918	2.012589	2.009246	2.005909	2.019558	2.016073	2.012642	2.009268
2.018446	2.015106	2.011772	2.008409	2.005049	2.018657	2.015208	2.011806	2.008451
2.017639	2.014302	2.010969	2.007604	2.00424	2.017808	2.014367	2.010974	2.007633
2.016838	2.013503	2.010173	2.006811	2.003449	2.016979	2.013539	2.010149	2.006821
2.016044	2.012709	2.009379	2.00602	2.002663	2.016158	2.01272	2.009337	2.006022
2.015257	2.01192	2.008591	2.005236	2.001885	2.015347	2.01191	2.008532	2.005231
2.014479	2.011144	2.007817	2.004462	2.001112	2.014535	2.011098	2.007725	2.004436
2.013708	2.010378	2.007054	2.003697	2.000343	2.013725	2.010287	2.006918	2.003642
2.012933	2.009607	2.006288	2.002933	1.999578	2.012921	2.009486	2.006121	2.00285
2.012154	2.008834	2.005521	2.002169	1.998817	2.012124	2.008695	2.005333	2.00206
2.011377	2.00806	2.004752	2.001405	1.998057	2.011327	2.007905	2.004546	2.001271
2.010599	2.007285	2.003982	2.000638	1.997293	2.010526	2.007113	2.003759	2.000483
2.009827	2.006514	2.003214	1.999874	1.996534	2.009729	2.006324	2.002974	1.999697
2.009071	2.00575	2.00245	1.999113	1.995779	2.008936	2.00554	2.002196	1.998918
2.008325	2.004993	2.001691	1.998359	1.995035	2.008155	2.004765	2.001422	1.998141
2.007575	2.004238	2.000939	1.997629	1.994333	2.007418	2.004007	2.00065	1.997364
2.006826	2.003487	2.000194	1.996919	1.993666	2.006721	2.003266	1.999881	1.996585
2.006112	2.002761	1.999455	1.996195	1.992967	2.005989	2.002515	1.999114	1.995808
2.00545	2.002068	1.998722	1.995451	1.992223	2.005209	2.001745	1.998345	1.995027
2.004774	2.001369	1.997995	1.994711	1.991478	2.004435	2.000994	1.997602	1.994264
2.004045	2.000641	1.997266	1.993979	1.990745	2.003689	2.000291	1.99691	1.993534
2.003301	1.999912	1.996552	1.993264	1.990024	2.002954	1.999599	1.996229	1.992815
2.002569	1.999217	1.995887	1.992592	1.989328	2.002219	1.998875	1.995508	1.992083
2.00184	1.998536	1.995248	1.991947	1.988652	2.001492	1.99814	1.994769	1.991351
2.001116	1.997834	1.99457	1.991284	1.987999	2.000806	1.997428	1.99405	1.990653
2.000395	1.99711	1.993858	1.990609	1.987371	2.000161	1.996739	1.993346	1.98999
1.999707	1.996411	1.993154	1.989925	1.986715	1.999484	1.996035	1.992635	1.989305
1.999071	1.995752	1.992463	1.989223	1.986009	1.998751	1.9953	1.991903	1.988579
1.998427	1.995099	1.99179	1.988535	1.985308	1.998021	1.994581	1.99119	1.987859
1.997732	1.994438	1.991157	1.987894	1.984643	1.997326	1.993917	1.990533	1.98717
1.997019	1.99377	1.990532	1.987267	1.983996	1.996651	1.993275	1.989896	1.986499
1.996309	1.993075	1.989858	1.986613	1.983361	1.995998	1.992616	1.989235	1.985843
1.995602	1.992362	1.989155	1.985944	1.982737	1.995363	1.991952	1.988562	1.985201
1.994943	1.991685	1.988466	1.985269	1.982087	1.994705	1.991299	1.987912	1.984551
1.99434	1.991046	1.987787	1.984581	1.981407	1.99402	1.990658	1.987285	1.983894
1.993725	1.990415	1.987137	1.983926	1.980754	1.993353	1.990013	1.986648	1.983238
1.99307	1.98979	1.986542	1.98334	1.980166	1.992727	1.98936	1.985983	1.98259

1.992416	1.989164	1.985946	1.982752	1.979575	1.992098	1.988704	1.985317	1.98194
1.991793	1.988525	1.985289	1.982092	1.978918	1.991427	1.988051	1.984671	1.981282
1.99118	1.987884	1.984616	1.981411	1.978241	1.99074	1.987396	1.984028	1.980619
1.990549	1.987261	1.984	1.980792	1.977615	1.99008	1.986725	1.983354	1.979955
1.989906	1.986653	1.983425	1.980218	1.977025	1.989438	1.986041	1.982656	1.979289
1.985634	1.982393	1.979173	1.979634	1.976419	1.988785	1.98537	1.981978	1.978619
1.985051	1.98182	1.978597	1.979039	1.975789	1.988117	1.984718	1.981328	1.977941
1.984471	1.981237	1.978001	1.97843	1.975159	1.987452	1.984061	1.98067	1.977266
1.983884	1.980622	1.977361	1.977796	1.974537	1.986799	1.983383	1.979983	1.9766
1.983289	1.979998	1.976715	1.977159	1.973914	1.986147	1.982705	1.979299	1.975939
1.982666	1.97938	1.976102	1.972824	1.969553	1.985473	1.982049	1.978648	1.975274
1.98203	1.978765	1.975506	1.972209	1.968905	1.984796	1.981408	1.978019	1.974616
1.981425	1.978174	1.974924	1.97162	1.968301	1.984165	1.980792	1.97741	1.973994
1.980847	1.977605	1.974358	1.971056	1.967738	1.983577	1.980204	1.976822	1.973409
1.98027	1.97702	1.973762	1.97047	1.967167	1.979695	1.976294	1.976204	1.972807
1.979691	1.976404	1.973117	1.969843	1.966578	1.979075	1.975631	1.975533	1.972176
1.979113	1.975792	1.97248	1.969221	1.965987	1.978456	1.974975	1.974874	1.971548
1.978542	1.975212	1.971891	1.968634	1.965406	1.977849	1.974364	1.974268	1.970948
1.977964	1.974636	1.971317	1.968054	1.964819	1.977237	1.973765	1.97368	1.970352
1.977342	1.974025	1.970718	1.967444	1.964191	1.976583	1.973145	1.973079	1.969725
1.976693	1.973396	1.970109	1.96682	1.963541	1.975906	1.972518	1.972473	1.969081
1.976085	1.972798	1.969523	1.966226	1.962935	1.97527	1.971909	1.968534	1.965121
1.975517	1.972231	1.968959	1.965663	1.962372	1.974676	1.971319	1.967949	1.964542
1.974959	1.971673	1.968404	1.965111	1.961823	1.974095	1.970738	1.975392	1.971919
1.974407	1.971126	1.967862	1.96457	1.961282	1.973526	1.970169	1.974828	1.971356
1.973856	1.970577	1.967317	1.964029	1.960745	1.97296	1.9696	1.97426	1.97079
1.973297	1.970019	1.966761	1.979303	1.975814	1.972391	1.969022	1.973682	1.970214
1.972733	1.96945	1.96619	1.978715	1.975239	1.971814	1.968433	1.973089	1.969627
1.972154	1.968854	1.965578	1.978085	1.974637	1.971211	1.967806	1.97245	1.969008
1.971563	1.968236	1.964934	1.977421	1.974012	1.970587	1.967148	1.971774	1.968363
1.970986	1.967644	1.964327	1.976786	1.973401	1.978128	1.974605	1.971134	1.967741
1.970424	1.96708	1.963762	1.976184	1.972803	1.97753	1.974005	1.970534	1.967146
1.969882	1.966538	1.963223	1.975603	1.972218	1.976943	1.973422	1.969954	1.966565
1.969371	1.966023	1.962707	1.97504	1.971645	1.976365	1.972848	1.969383	1.965996
1.968872	1.965526	1.962217	1.974501	1.97109	1.975804	1.972298	1.968837	1.965445
1.96837	1.96505	1.961764	1.974007	1.970567	1.97528	1.971803	1.96835	1.96493
1.967867	1.964585	1.961329	1.973534	1.970061	1.974775	1.971335	1.967891	1.964434
1.967341	1.964079	1.960842	1.981275	1.977719	1.974236	1.970815	1.967378	1.963907
1.966794	1.963539	1.96031	1.98071	1.977152	1.97367	1.970252	1.966821	1.963354
1.982226	1.978784	1.97536	1.980134	1.976575	1.973095	1.969682	1.966255	1.962791
1.981663	1.978213	1.974781	1.979547	1.975986	1.972509	1.969104	1.965683	1.962216
1.981129	1.977664	1.974214	1.978968	1.975402	1.971926	1.968528	1.96511	1.961643
1.980661	1.977168	1.973681	1.97841	1.97483	1.971348	1.967953	1.96454	1.961078
1.980206	1.976684	1.97316	1.977867	1.974277	1.970788	1.967389	1.963978	1.960528
1.988136	1.984534	1.980932	1.977345	1.973779	1.970286	1.966859	1.963441	1.960022
1.987581	1.983979	1.980389	1.976838	1.973317	1.969821	1.966351	1.962923	1.959546
1.987042	1.983432	1.979845	1.976313	1.972817	1.969323	1.965834	1.962401	1.959047
1.986512	1.982888	1.979296	1.975768	1.972281	1.968791	1.965305	1.961876	1.958526
1.985997	1.982374	1.978787	1.975255	1.971764	1.968279	1.964805	1.961379	1.958017
1.985497	1.981908	1.978348	1.974802	1.97128	1.967799	1.964355	1.960934	1.957533

1.985016	1.981454	1.977913	1.974359	1.970817	1.967337	1.963912	1.960489	1.95705
1.98457	1.980992	1.977433	1.973898	1.970386	1.966903	1.963448	1.959999	1.956541
1.984134	1.980523	1.976935	1.973427	1.96996	1.966473	1.962974	1.959495	1.95603
1.9774	1.973856	1.970337	1.972922	1.96947	1.965985	1.962479	1.959001	1.955554
1.976893	1.973339	1.969818	1.972393	1.968934	1.965455	1.961967	1.958513	1.955102
1.976387	1.972846	1.969339	1.9719	1.968422	1.964949	1.961485	1.95805	1.95465
1.975878	1.97238	1.96891	1.97145	1.967935	1.964466	1.96104	1.957621	1.954197
1.975394	1.971921	1.968472	1.971003	1.967471	1.964004	1.960596	1.957184	1.953749
1.97496	1.971466	1.967995	1.970546	1.967043	1.963574	1.960138	1.956717	1.953308
1.974534	1.971015	1.967519	1.970092	1.966622	1.963151	1.959684	1.956253	1.952868
1.974072	1.970567	1.967084	1.963645	1.960236	1.962705	1.959249	1.955821	1.952426
1.973599	1.97012	1.966664	1.963211	1.959773	1.962252	1.958823	1.955403	1.951985
1.973155	1.96967	1.966209	1.962762	1.959333	1.961816	1.95838	1.954959	1.951548
1.972734	1.969218	1.965725	1.9623	1.958912	1.961395	1.957925	1.954494	1.951117
1.97229	1.968765	1.965265	1.961847	1.958472	1.96096	1.95748	1.954047	1.950681
1.971803	1.968307	1.964839	1.961405	1.958	1.960498	1.957051	1.953629	1.950237
1.971341	1.967861	1.964413	1.960969	1.957543	1.960049	1.956623	1.953208	1.949796
1.970949	1.967436	1.96396	1.960527	1.957127	1.959631	1.956181	1.952756	1.949362
1.970582	1.967028	1.963516	1.960099	1.956735	1.959235	1.955748	1.952309	1.948933
1.970205	1.966649	1.963129	1.95972	1.956366	1.952965	1.949528	1.951907	1.948507
1.969823	1.966288	1.962779	1.959371	1.956013	1.95261	1.949176	1.951535	1.948088
1.969401	1.965896	1.962413	1.958996	1.955613	1.952213	1.948801	1.951166	1.9477
1.968933	1.965473	1.962035	1.958595	1.955166	1.951771	1.948407	1.950807	1.947347
1.968501	1.96507	1.961661	1.958208	1.95475	1.951349	1.947997	1.950416	1.946974
1.968134	1.964711	1.961308	1.957857	1.954395	1.950965	1.947559	1.949965	1.946563
1.967776	1.964345	1.960933	1.957492	1.954043	1.950591	1.947132	1.949522	1.946146
1.970985	1.967489	1.964012	1.957071	1.953664	1.950224	1.946758	1.943347	1.940012
1.970593	1.967063	1.963552	1.956629	1.953265	1.949852	1.946401	1.942982	1.9396
1.970215	1.966674	1.963146	1.959695	1.956283	1.949428	1.946018	1.942619	1.939225
1.969843	1.966307	1.962776	1.959282	1.955816	1.948968	1.945621	1.942264	1.938878
1.969431	1.965919	1.96241	1.958899	1.955402	1.951985	1.94864	1.941915	1.938537
1.968966	1.965501	1.96205	1.95855	1.955047	1.951634	1.948297	1.941575	1.938204
1.96853	1.965102	1.961695	1.958209	1.954707	1.951295	1.947961	1.944603	1.941205
1.968158	1.964738	1.961344	1.957866	1.954371	1.950965	1.947636	1.94428	1.940884
1.967812	1.964392	1.960998	1.957527	1.954039	1.950633	1.947296	1.943939	1.940549
1.967476	1.964057	1.960661	1.957191	1.953703	1.950277	1.9469	1.943531	1.940171
1.967152	1.963732	1.96033	1.956859	1.953368	1.949912	1.946481	1.943096	1.939774
1.966821	1.963403	1.96	1.95653	1.953038	1.949568	1.946111	1.942716	1.939411
1.966487	1.963073	1.959672	1.956205	1.952713	1.94924	1.945778	1.94238	1.939071
1.966153	1.962743	1.959345	1.955881	1.952392	1.948919	1.945454	1.942052	1.938741
1.965821	1.962413	1.959018	1.955559	1.952077	1.948605	1.945137	1.941733	1.93842
1.96549	1.962082	1.958691	1.955236	1.951759	1.948289	1.94482	1.941414	1.938099
1.965157	1.96175	1.95836	1.954908	1.951433	1.947963	1.944492	1.941085	1.937769
1.964825	1.961419	1.958031	1.954579	1.951104	1.947634	1.944164	1.940758	1.937441
1.964493	1.96109	1.957707	1.954254	1.950775	1.947306	1.943841	1.940436	1.937119
1.964163	1.960763	1.957386	1.95393	1.950447	1.946981	1.943524	1.940124	1.936805
1.963842	1.96044	1.957063	1.953605	1.950122	1.946672	1.943248	1.939859	1.93652
1.963526	1.960119	1.956738	1.953279	1.9498	1.946382	1.943014	1.939643	1.936264
1.963252	1.959827	1.956425	1.952956	1.949476	1.946079	1.942751	1.939394	1.935989
1.963049	1.959584	1.956131	1.952638	1.949147	1.94575	1.942436	1.939087	1.93568

1.962836	1.959337	1.955837	1.952325	1.948823	1.945422	1.94211	1.938764	1.935359
1.96255	1.959043	1.955532	1.952012	1.948506	1.945102	1.941789	1.938442	1.935032
1.962238	1.958732	1.955225	1.951709	1.948206	1.944798	1.941475	1.93812	1.934703
1.961939	1.958427	1.954925	1.951432	1.947958	1.944545	1.941189	1.937811	1.934383
1.961645	1.958125	1.954632	1.951177	1.947749	1.94433	1.940924	1.937511	1.934072
1.961358	1.957854	1.954383	1.950942	1.947523	1.944091	1.940653	1.937224	1.933797
1.96108	1.957621	1.954186	1.950734	1.94728	1.943827	1.940374	1.93695	1.933563
1.960804	1.957376	1.953967	1.950503	1.947021	1.943559	1.940112	1.936699	1.93333
1.960516	1.957095	1.953693	1.950224	1.946736	1.943294	1.939889	1.93649	1.933093
1.960248	1.956818	1.953407	1.949931	1.946442	1.943023	1.939662	1.936276	1.932845
1.960043	1.956582	1.953132	1.94964	1.946144	1.943903	1.94054	1.937146	1.933693
1.959869	1.956364	1.952862	1.949352	1.945853	1.943608	1.94024	1.936849	1.933408
1.959663	1.956128	1.9526	1.950315	1.946815	1.94337	1.939977	1.93658	1.933162
1.959438	1.955882	1.952345	1.950088	1.946628	1.943177	1.93974	1.936329	1.932947
1.960532	1.956945	1.953378	1.949883	1.946428	1.942974	1.939526	1.936108	1.932733
1.960401	1.956816	1.953236	1.949705	1.946208	1.942752	1.939339	1.93593	1.93252
1.960245	1.956663	1.953077	1.949523	1.945998	1.942541	1.93915	1.935741	1.932296
1.960017	1.956429	1.952848	1.949318	1.945821	1.942359	1.938935	1.9355	1.932037
1.959763	1.956174	1.952607	1.949107	1.945645	1.942181	1.938721	1.93526	1.931786
1.959446	1.955882	1.952342	1.948908	1.945434	1.941976	1.938538	1.935088	1.93161
1.95921	1.95568	1.952171	1.948712	1.945206	1.94176	1.938369	1.934947	1.93147
1.958914	1.955376	1.951861	1.948451	1.944947	1.941562	1.938169	1.934745	1.931266
1.958768	1.955184	1.951629	1.948239	1.944778	1.941381	1.937941	1.934487	1.931002
1.958623	1.955012	1.951428	1.94804	1.944592	1.941182	1.937724	1.934259	1.930776
1.958479	1.954878	1.951288	1.947868	1.944374	1.940952	1.93753	1.934092	1.93062
1.958311	1.954723	1.951133	1.947688	1.944159	1.940724	1.937331	1.933918	1.930467
1.955623	1.952042	1.948465	1.947473	1.943969	1.940502	1.937074	1.933688	1.930279
1.95538	1.951789	1.948215	1.947252	1.943784	1.940316	1.936853	1.933448	1.930078
1.955189	1.951605	1.948033	1.946943	1.943461	1.940108	1.936662	1.933241	1.929854
1.955028	1.951461	1.947892	1.946768	1.94324	1.939891	1.936491	1.933085	1.929657
1.954204	1.950649	1.947091	1.944197	1.940661	1.939723	1.936345	1.932945	1.929498
1.953969	1.950451	1.946943	1.944068	1.94054	1.939607	1.93623	1.932829	1.929384
1.953767	1.950258	1.946771	1.94392	1.940417	1.939487	1.936094	1.932687	1.929256
1.953624	1.950069	1.946548	1.943725	1.940274	1.939343	1.935907	1.932488	1.929095
1.953508	1.949902	1.946337	1.943536	1.940132	1.9392	1.935723	1.93229	1.928922
1.953408	1.949784	1.946202	1.942736	1.93933	1.936569	1.933094	1.932139	1.928737
1.95332	1.949694	1.946104	1.942628	1.93921	1.936448	1.932982	1.93201	1.928554
1.953217	1.9496	1.946005	1.942502	1.939049	1.936287	1.932852	1.93189	1.928418
1.953106	1.949508	1.945913	1.942367	1.938856	1.936095	1.932711	1.931781	1.928319
1.952991	1.949411	1.945823	1.942254	1.938708	1.935945	1.932585	1.929428	1.925967
1.952878	1.949313	1.945738	1.94217	1.938618	1.935849	1.932483	1.929329	1.925878
1.952744	1.949199	1.945648	1.942089	1.938538	1.935756	1.932366	1.929205	1.925772
1.952562	1.949052	1.945546	1.942003	1.938453	1.935644	1.9322	1.929022	1.925624
1.952381	1.948904	1.945442	1.941917	1.938371	1.935534	1.932035	1.928836	1.925472
1.95226	1.948794	1.945347	1.941831	1.93829	1.934753	1.931214	1.928464	1.925113
1.952173	1.948705	1.945258	1.941747	1.938212	1.934674	1.931127	1.928371	1.925017
1.95209	1.948621	1.945174	1.941666	1.938135	1.934596	1.931044	1.928283	1.924927
1.952013	1.948544	1.945097	1.941589	1.938059	1.93452	1.930968	1.928205	1.924847
1.951939	1.948471	1.945023	1.941516	1.937986	1.934447	1.930895	1.927415	1.92404
1.951867	1.948402	1.944955	1.941448	1.937918	1.934378	1.930825	1.927343	1.923967

1.951795	1.948334	1.944888	1.941383	1.937852	1.934311	1.930755	1.927272	1.923895
1.951723	1.948265	1.944822	1.941317	1.937785	1.934242	1.930685	1.927199	1.92382
1.951651	1.948195	1.944756	1.941252	1.937719	1.934175	1.930614	1.927126	1.923745
1.95158	1.948127	1.944691	1.941187	1.937654	1.934107	1.930542	1.927051	1.923669
1.951509	1.948059	1.944626	1.941123	1.937589	1.934039	1.93047	1.926976	1.923593
1.9459	1.942466	1.939046	1.941059	1.937525	1.933976	1.930408	1.926913	1.923523
1.94584	1.942404	1.93898	1.940995	1.937463	1.933916	1.930352	1.926856	1.923458
1.94582	1.942372	1.938931	1.94094	1.937404	1.933867	1.930324	1.926834	1.923417
1.945874	1.942394	1.938908	1.940897	1.937348	1.933832	1.930344	1.926872	1.923417
1.945925	1.942415	1.938889	1.940861	1.9373	1.933804	1.930365	1.926909	1.923418
1.945906	1.942391	1.938856	1.935307	1.931759	1.933773	1.930346	1.926889	1.92338
1.945856	1.942345	1.938815	1.935275	1.931737	1.93375	1.930312	1.926846	1.923329
1.943096	1.939584	1.936066	1.935257	1.931754	1.933768	1.930298	1.926812	1.92329
1.943044	1.939522	1.936012	1.935249	1.931803	1.933818	1.930297	1.926779	1.923257
1.942988	1.939461	1.935958	1.932526	1.929125	1.928334	1.924803	1.926759	1.923253
1.942924	1.939397	1.935898	1.932473	1.92908	1.928298	1.924773	1.926748	1.923285
1.942869	1.939356	1.935867	1.932437	1.929034	1.925579	1.922084	1.924398	1.920967
1.942825	1.939354	1.9359	1.932449	1.929004	1.925559	1.922114	1.924451	1.920997
1.942802	1.939368	1.935943	1.932467	1.928979	1.925541	1.922145	1.924502	1.921017
1.942829	1.939385	1.93594	1.932448	1.928945	1.925507	1.922125	1.918713	1.915244
1.942884	1.939404	1.935917	1.932415	1.928915	1.925474	1.922084	1.918673	1.915216
1.936004	1.932526	1.929047	1.932407	1.928929	1.925485	1.922073	1.918657	1.915225
1.935995	1.932503	1.929025	1.932418	1.928983	1.925537	1.922085	1.918659	1.915265
1.936024	1.932523	1.92904	1.932448	1.929026	1.925581	1.922118	1.918688	1.915303
1.936119	1.932614	1.929113	1.932501	1.929048	1.925608	1.922181	1.918758	1.915336
1.936196	1.932691	1.92918	1.925711	1.922267	1.925641	1.922242	1.918822	1.915361
1.936196	1.932697	1.929201	1.925749	1.922322	1.925699	1.922278	1.918839	1.91536
1.936168	1.932682	1.929211	1.925784	1.922382	1.925762	1.922311	1.918851	1.915364
1.929208	1.925737	1.922287	1.925826	1.922419	1.919013	1.915611	1.918914	1.915427
1.929179	1.925745	1.92233	1.92587	1.922444	1.919047	1.915675	1.919005	1.915524
1.929208	1.925773	1.922358	1.92591	1.922489	1.919095	1.915725	1.919058	1.915575
1.929299	1.925821	1.922369	1.925944	1.922556	1.919159	1.915759	1.919072	1.915578
1.929397	1.925888	1.922403	1.918985	1.915601	1.919211	1.9158	1.912374	1.908913
1.929501	1.925989	1.922485	1.919034	1.915614	1.919237	1.915861	1.912458	1.909005
1.929581	1.926071	1.922558	1.91908	1.915627	1.919263	1.915918	1.91254	1.909105
1.929587	1.926073	1.922564	1.919101	1.915667	1.919309	1.915946	1.912573	1.909181
1.929569	1.926052	1.922554	1.919116	1.915714	1.919362	1.915968	1.912593	1.909247
1.929619	1.926101	1.922599	1.919152	1.91574	1.912338	1.908952	1.912653	1.909303
1.929713	1.926197	1.922684	1.919204	1.915753	1.912357	1.909015	1.912741	1.909351
1.929775	1.926279	1.922781	1.919288	1.915811	1.912418	1.909101	1.912842	1.909431
1.929791	1.92634	1.922892	1.919408	1.915923	1.91253	1.909218	1.912965	1.909552
1.929835	1.926417	1.923009	1.919535	1.916048	1.912646	1.909319	1.905963	1.902554
1.929942	1.926534	1.923138	1.919668	1.916177	1.912748	1.909371	1.905999	1.902626
1.93007	1.926655	1.923253	1.919788	1.916304	1.912852	1.909424	1.906032	1.902687
1.930194	1.92674	1.923307	1.919861	1.916415	1.912969	1.909522	1.90611	1.90274
1.930322	1.926814	1.923335	1.919909	1.916512	1.913087	1.909643	1.906214	1.902793
1.930452	1.926922	1.923415	1.919976	1.916575	1.913164	1.909751	1.906335	1.902895
1.930585	1.927059	1.923537	1.920056	1.916606	1.913205	1.909852	1.906474	1.903039
1.930716	1.927199	1.923676	1.920167	1.91668	1.913286	1.909975	1.906624	1.903193
1.930849	1.927346	1.923835	1.920318	1.916816	1.913424	1.91013	1.906788	1.903358

1.930964	1.92748	1.923992	1.920478	1.91697	1.913578	1.910288	1.906949	1.903524
1.931032	1.927577	1.924129	1.920629	1.917126	1.913736	1.910445	1.907106	1.903687
1.931095	1.92767	1.924263	1.920782	1.917288	1.913895	1.91059	1.907246	1.903838
1.931213	1.927798	1.924408	1.920936	1.917445	1.914033	1.910685	1.907329	1.903952
1.931363	1.927949	1.92456	1.921092	1.917602	1.914159	1.910753	1.907378	1.904043
1.93152	1.92811	1.924725	1.921258	1.917766	1.914304	1.910863	1.907477	1.904166
1.931687	1.928285	1.924905	1.921436	1.917937	1.914466	1.911012	1.907621	1.904319
1.931857	1.928461	1.925084	1.921613	1.918109	1.914632	1.911172	1.907778	1.904481
1.932029	1.928635	1.925258	1.921786	1.91828	1.914801	1.911339	1.907946	1.90465
1.932203	1.928808	1.92543	1.921958	1.918452	1.914973	1.91151	1.908116	1.90482
1.92464	1.921289	1.917957	1.922131	1.91863	1.915147	1.911674	1.908274	1.904979
1.924801	1.921447	1.918113	1.922304	1.918809	1.915323	1.911839	1.908432	1.905136
1.92496	1.921603	1.918268	1.922473	1.918983	1.915498	1.912011	1.9086	1.905298
1.925119	1.921758	1.918422	1.92264	1.919154	1.915672	1.912189	1.908777	1.905467
1.925286	1.921918	1.918577	1.922808	1.919327	1.915861	1.912403	1.908997	1.905663
1.925462	1.922082	1.918732	1.922975	1.919502	1.916066	1.912661	1.90927	1.905894
1.925644	1.92225	1.918889	1.923142	1.919674	1.916263	1.912902	1.909524	1.906116
1.925821	1.922417	1.919045	1.923305	1.919837	1.916437	1.913099	1.90973	1.90631
1.926018	1.9226	1.919213	1.923476	1.920005	1.916609	1.91328	1.909915	1.906492
1.926274	1.922829	1.919408	1.915969	1.912543	1.916785	1.913454	1.910089	1.906661
1.926555	1.923082	1.919619	1.916165	1.912731	1.916972	1.913627	1.910254	1.906824
1.925029	1.921512	1.918005	1.916383	1.912966	1.917209	1.91383	1.910434	1.906997
1.925233	1.92172	1.918222	1.916623	1.913244	1.917491	1.914058	1.910624	1.907177
1.925442	1.921943	1.918464	1.915039	1.911642	1.91775	1.914284	1.910833	1.907396
1.925658	1.92219	1.918745	1.915307	1.911878	1.91797	1.914497	1.911059	1.907667
1.925878	1.922432	1.919013	1.915563	1.912108	1.910531	1.907122	1.91131	1.907947
1.92609	1.922646	1.919234	1.915787	1.912337	1.910769	1.90739	1.911604	1.908218
1.926318	1.922862	1.91944	1.916003	1.912572	1.91102	1.907668	1.911905	1.908484
1.918417	1.914957	1.91153	1.916238	1.912842	1.909443	1.906046	1.912159	1.908722
1.918734	1.915223	1.911746	1.916481	1.913129	1.909728	1.906293	1.912389	1.908948
1.917079	1.913548	1.910051	1.908593	1.905296	1.909975	1.906525	1.910948	1.907482
1.917299	1.913755	1.910251	1.908792	1.905491	1.910184	1.906735	1.911194	1.907773
1.912973	1.909455	1.905979	1.909021	1.905701	1.910407	1.906976	1.903574	1.900216
1.913186	1.909712	1.906273	1.909298	1.905935	1.910654	1.907266	1.903883	1.900502
1.913417	1.90998	1.906571	1.909588	1.906192	1.910921	1.907566	1.904196	1.900794
1.913697	1.91025	1.906828	1.907947	1.904571	1.903128	1.899788	1.904475	1.901092
1.913994	1.910523	1.907079	1.908223	1.90488	1.903433	1.90006	1.904748	1.901392
1.914256	1.9108	1.907369	1.903996	1.900659	1.903711	1.900347	1.896995	1.893661
1.914499	1.911079	1.907684	1.904289	1.900908	1.903974	1.900644	1.897302	1.893934
1.908987	1.905593	1.902222	1.904568	1.901182	1.899948	1.896638	1.897582	1.894212
1.909296	1.905869	1.902464	1.904832	1.901487	1.900249	1.896895	1.897832	1.894497
1.909587	1.90614	1.902716	1.905097	1.901773	1.898418	1.895041	1.898091	1.894776
1.909826	1.9064	1.902997	1.905371	1.902013	1.89866	1.895313	1.898381	1.895042
1.910072	1.906663	1.90328	1.905645	1.902255	1.898903	1.895586	1.898671	1.895307
1.910376	1.906938	1.903531	1.900152	1.896798	1.899183	1.895841	1.89463	1.8913
1.910709	1.907225	1.90378	1.900416	1.897099	1.899484	1.896098	1.894875	1.891572
1.911036	1.907546	1.904092	1.900732	1.897421	1.894067	1.890682	1.893045	1.889729
1.911362	1.907897	1.904456	1.901091	1.897765	1.894411	1.891036	1.893389	1.890014
1.911652	1.908214	1.904795	1.90142	1.89807	1.894718	1.891366	1.893728	1.890321
1.911884	1.908484	1.905104	1.901708	1.898319	1.894973	1.891666	1.894063	1.89066

1.896004	1.8927	1.889417	1.901985	1.898576	1.895234	1.89195	1.88864	1.885278
1.896319	1.89298	1.889667	1.90225	1.898875	1.895528	1.892209	1.888891	1.885568
1.896658	1.893273	1.889922	1.902522	1.899191	1.895838	1.892472	1.88914	1.885857
1.896994	1.893599	1.890235	1.902844	1.899523	1.896166	1.892785	1.889452	1.886187
1.897332	1.893946	1.890582	1.903195	1.899863	1.896504	1.893128	1.889798	1.886536
1.897669	1.89429	1.890928	1.887619	1.884342	1.896806	1.89345	1.890127	1.886849
1.898007	1.894635	1.891276	1.887934	1.884612	1.897074	1.893755	1.890442	1.887129
1.898323	1.89497	1.891628	1.888267	1.884913	1.897377	1.894081	1.890774	1.887436
1.898598	1.895283	1.891983	1.888628	1.885264	1.897733	1.894436	1.891127	1.887785
1.898894	1.895608	1.892342	1.888996	1.885631	1.898111	1.894809	1.891496	1.888152
1.899262	1.895973	1.892706	1.889364	1.886006	1.882723	1.879501	1.891887	1.888532
1.899662	1.896356	1.893072	1.889733	1.886386	1.88311	1.879893	1.892285	1.888919
1.900055	1.896736	1.893438	1.8901	1.886759	1.883484	1.880266	1.892662	1.88929
1.90045	1.897116	1.893803	1.890463	1.887126	1.88385	1.880626	1.893024	1.889651
1.900831	1.897467	1.894129	1.890796	1.88748	1.884199	1.880952	1.877698	1.87443
1.901196	1.897785	1.894408	1.891093	1.887817	1.884529	1.881236	1.877967	1.874731
1.901559	1.898117	1.894715	1.891412	1.888162	1.884867	1.88154	1.87826	1.87505
1.901919	1.898482	1.895077	1.891774	1.888521	1.88522	1.881885	1.878603	1.875403
1.902283	1.898862	1.895463	1.892155	1.88889	1.885585	1.88225	1.87897	1.875773
1.901147	1.89775	1.89437	1.892542	1.889271	1.885962	1.882627	1.879346	1.876149
1.901531	1.898145	1.894775	1.892944	1.889663	1.886354	1.883023	1.879743	1.876537
1.901949	1.898577	1.89522	1.893376	1.890074	1.886768	1.883464	1.88019	1.876959
1.902396	1.89904	1.895699	1.893836	1.890501	1.887204	1.883942	1.88068	1.877408
1.902823	1.899474	1.896141	1.892791	1.889449	1.887626	1.884385	1.881128	1.877831
1.903225	1.899871	1.896535	1.89319	1.889855	1.888031	1.884784	1.881521	1.878222
1.89751	1.894176	1.890862	1.89358	1.890249	1.886961	1.883711	1.8819	1.8786
1.897885	1.894557	1.891249	1.893965	1.890625	1.887332	1.884082	1.882269	1.878967
1.89826	1.894938	1.891635	1.89435	1.890997	1.8877	1.88445	1.882636	1.879335
1.898644	1.895324	1.89202	1.894737	1.891381	1.88808	1.884828	1.883013	1.879719
1.89904	1.895716	1.892409	1.895131	1.891776	1.888473	1.885215	1.883399	1.880117
1.899487	1.896145	1.892818	1.889496	1.886187	1.888906	1.885633	1.882371	1.879116
1.899984	1.896609	1.893246	1.889934	1.886652	1.889379	1.886081	1.88281	1.879575
1.900448	1.897054	1.893671	1.890363	1.887094	1.889828	1.886515	1.883238	1.880013
1.90086	1.897466	1.894084	1.890771	1.887495	1.890232	1.886921	1.883644	1.880416
1.901258	1.897876	1.894508	1.891187	1.887895	1.890635	1.887333	1.884057	1.880816
1.894238	1.8909	1.887575	1.891634	1.888316	1.885033	1.881783	1.884505	1.881233
1.89463	1.891326	1.888033	1.892086	1.888739	1.885454	1.882222	1.884954	1.881648
1.889381	1.886096	1.882826	1.892501	1.889138	1.885847	1.882617	1.885355	1.882036
1.889809	1.886504	1.883221	1.892894	1.889524	1.886225	1.882987	1.885726	1.882409
1.890261	1.886934	1.883631	1.885909	1.882558	1.886641	1.883384	1.88013	1.876863
1.890745	1.887387	1.884054	1.886342	1.883013	1.887094	1.883807	1.880545	1.87731
1.891202	1.887835	1.884494	1.881177	1.877884	1.88017	1.876886	1.880989	1.877767
1.891604	1.88827	1.884961	1.881639	1.878327	1.880623	1.877361	1.881476	1.878227
1.891999	1.888698	1.885423	1.882101	1.878777	1.881083	1.877842	1.881966	1.878689
1.892412	1.88911	1.885846	1.882548	1.879253	1.881561	1.878302	1.88242	1.879149
1.892843	1.889523	1.886254	1.882986	1.879734	1.882043	1.878755	1.882863	1.879609
1.893333	1.889975	1.886672	1.883406	1.880173	1.876926	1.873671	1.875942	1.872693
1.89387	1.890459	1.887096	1.883812	1.88058	1.877349	1.874125	1.876408	1.873141
1.89438	1.890942	1.887551	1.884257	1.881024	1.877794	1.874575	1.871352	1.868116
1.894852	1.891423	1.888046	1.884754	1.881517	1.878268	1.875017	1.871785	1.868578

1.895331	1.891903	1.888532	1.885241	1.882002	1.878742	1.875471	1.872231	1.86904
1.895839	1.892379	1.888973	1.885677	1.882447	1.8792	1.875948	1.872711	1.869497
1.896354	1.892862	1.889422	1.886119	1.882895	1.87966	1.876429	1.873195	1.869953
1.89207	1.888596	1.885173	1.886625	1.88339	1.88014	1.87689	1.873647	1.870409
1.892564	1.889114	1.885713	1.887164	1.883906	1.880629	1.877345	1.874086	1.870865
1.893085	1.889645	1.886247	1.887684	1.884395	1.881103	1.877813	1.874549	1.87132
1.893631	1.890192	1.886781	1.88819	1.884863	1.881562	1.878291	1.87503	1.871773
1.894179	1.890727	1.887297	1.888693	1.885348	1.882039	1.878768	1.875505	1.872242
1.894731	1.891244	1.88778	1.889187	1.885857	1.882536	1.879232	1.875961	1.872729
1.895268	1.891761	1.88828	1.884912	1.881606	1.883054	1.879721	1.876441	1.873233
1.895764	1.892282	1.888833	1.885472	1.882161	1.883609	1.880271	1.876984	1.873765
1.896257	1.892812	1.889404	1.886044	1.882719	1.884169	1.880839	1.877547	1.874304
1.88026	1.876869	1.873513	1.886578	1.883219	1.884675	1.881369	1.878082	1.87481
1.88082	1.877421	1.874043	1.88709	1.883686	1.885149	1.881881	1.878604	1.875299
1.881378	1.877953	1.874546	1.871152	1.867787	1.885651	1.882401	1.879132	1.875814
1.881936	1.878465	1.875017	1.871624	1.868279	1.886177	1.882933	1.879667	1.876354
1.88249	1.878987	1.875512	1.87212	1.868788	1.873263	1.870035	1.880186	1.876889
1.883042	1.879533	1.876052	1.872657	1.869322	1.873791	1.870521	1.880672	1.877408
1.88358	1.880079	1.876605	1.873208	1.869862	1.874326	1.871014	1.881161	1.877928
1.874562	1.871106	1.867684	1.87376	1.870403	1.867075	1.863777	1.881687	1.878459
1.875047	1.871622	1.868235	1.874311	1.870938	1.867598	1.864292	1.882227	1.878997
1.875571	1.872156	1.868776	1.874834	1.871428	1.868081	1.864785	1.869319	1.866125
1.876124	1.872703	1.869311	1.875336	1.871887	1.868534	1.865266	1.869838	1.866652
1.866257	1.862854	1.859475	1.866397	1.862939	1.869029	1.865774	1.862567	1.859419
1.866797	1.863401	1.86002	1.866949	1.863482	1.869571	1.866313	1.863107	1.859968
1.867353	1.863961	1.860578	1.867518	1.864046	1.860695	1.857446	1.863647	1.860512
1.867935	1.864544	1.86116	1.86811	1.864636	1.861267	1.857988	1.864175	1.861039
1.868525	1.865131	1.861744	1.868706	1.865231	1.861844	1.858531	1.864701	1.861561
1.863454	1.860071	1.856695	1.858862	1.855409	1.862404	1.859065	1.855818	1.852695
1.864021	1.860628	1.857243	1.859415	1.855961	1.862957	1.859598	1.856337	1.85321
1.864591	1.861188	1.857793	1.854359	1.850922	1.85312	1.849769	1.856878	1.85374
1.865163	1.861748	1.858342	1.854906	1.85147	1.853674	1.850324	1.857438	1.854283
1.865749	1.862327	1.858914	1.855473	1.852034	1.848652	1.845318	1.858015	1.854843
1.866356	1.862933	1.859518	1.856068	1.852619	1.849235	1.845905	1.858615	1.855426
1.855888	1.852497	1.84911	1.856669	1.853211	1.849824	1.846497	1.848815	1.845632
1.856466	1.853077	1.849692	1.857259	1.853797	1.850407	1.847077	1.849394	1.846193
1.857045	1.853659	1.850276	1.857858	1.854399	1.851005	1.847664	1.849974	1.846753
1.857648	1.854263	1.850886	1.858494	1.855052	1.851649	1.848277	1.84497	1.841736
1.858264	1.854879	1.85151	1.859151	1.855738	1.852324	1.848908	1.845562	1.842295
1.812024	1.809007	1.80601	1.848742	1.845375	1.852972	1.849538	1.84617	1.842875
1.812555	1.809523	1.806519	1.849331	1.845979	1.853594	1.850169	1.846795	1.843473
1.813088	1.810041	1.807028	1.849914	1.84657	1.843206	1.839826	1.847442	1.844094
1.813615	1.810557	1.807534	1.850488	1.847145	1.843808	1.840481	1.848124	1.844745
1.814162	1.811095	1.808059	1.851074	1.847726	1.84441	1.841127	1.8488	1.845407
1.814768	1.811688	1.80863	1.80561	1.802622	1.845016	1.841736	1.83844	1.835102
1.815392	1.812303	1.809224	1.806186	1.803177	1.845625	1.84233	1.839043	1.835758
1.81596	1.81289	1.809823	1.806771	1.803736	1.833671	1.830396	1.839646	1.836387
1.81649	1.813461	1.810429	1.807367	1.804301	1.83428	1.830989	1.840244	1.836994
1.796901	1.793962	1.791016	1.807948	1.80488	1.801855	1.798864	1.84085	1.8376
1.797415	1.794468	1.791526	1.808509	1.805477	1.802448	1.799421	1.841456	1.838195

1.797965	1.795	1.792049	1.789101	1.786157	1.803038	1.799995	1.829553	1.826315
1.798589	1.795593	1.792607	1.789646	1.7867	1.803616	1.800598	1.830257	1.827015
1.799216	1.796193	1.793176	1.790195	1.787236	1.804185	1.801198	1.830966	1.827724
1.799769	1.796754	1.79374	1.790745	1.787766	1.80473	1.801751	1.831611	1.828374
1.80029	1.797298	1.794302	1.791296	1.788295	1.805269	1.802289	1.832234	1.829001
1.800834	1.797846	1.794855	1.791851	1.788851	1.785896	1.782981	1.799917	1.796978
1.801392	1.798394	1.795399	1.792409	1.78943	1.786486	1.783575	1.80053	1.797554
1.801974	1.798958	1.795948	1.792962	1.789995	1.787057	1.784147	1.781242	1.778333
1.802589	1.799539	1.796498	1.793502	1.790537	1.787598	1.784687	1.781789	1.778899
1.803209	1.800134	1.797068	1.794061	1.79109	1.788144	1.785225	1.782328	1.779453
1.803821	1.800748	1.797681	1.794664	1.791678	1.788711	1.785765	1.78285	1.779966
1.804432	1.801369	1.798308	1.795281	1.792276	1.789288	1.786317	1.783382	1.780484
1.805051	1.801982	1.798914	1.795877	1.792861	1.78987	1.786903	1.78397	1.781069
1.80567	1.802591	1.799513	1.796466	1.793443	1.79046	1.787515	1.784594	1.781694
1.79674	1.793695	1.790653	1.79708	1.794051	1.791071	1.788137	1.785218	1.782308
1.79728	1.794269	1.791264	1.797719	1.794684	1.791705	1.788774	1.785849	1.782917
1.792429	1.789438	1.786458	1.788866	1.785843	1.79232	1.789384	1.786454	1.783517
1.793046	1.79007	1.787092	1.789481	1.786418	1.792891	1.78994	1.787012	1.784103
1.793647	1.790679	1.787703	1.790085	1.787	1.793477	1.790509	1.78758	1.784695
1.794161	1.791198	1.788235	1.790658	1.787617	1.794129	1.791154	1.788215	1.785321
1.794659	1.791696	1.788748	1.791224	1.788246	1.794802	1.791819	1.788865	1.785953
1.795249	1.792283	1.789333	1.78638	1.783429	1.785894	1.782907	1.789457	1.786537
1.7959	1.792925	1.789962	1.786998	1.784035	1.786487	1.783462	1.790013	1.787092
1.764615	1.761669	1.758737	1.787568	1.784609	1.787073	1.784048	1.781076	1.778172
1.76518	1.762185	1.759211	1.788077	1.785144	1.787649	1.784666	1.781717	1.778807
1.765728	1.762705	1.759709	1.756804	1.753952	1.782776	1.779855	1.782365	1.779452
1.766246	1.763239	1.760262	1.757368	1.754524	1.783381	1.780478	1.783013	1.780107
1.766774	1.763793	1.760845	1.757962	1.755121	1.783999	1.781103	1.783652	1.780744
1.767354	1.76439	1.761459	1.758584	1.755743	1.752888	1.750028	1.784266	1.781319
1.767967	1.765014	1.762095	1.759221	1.756374	1.753504	1.750617	1.784862	1.781863
1.768609	1.765647	1.762719	1.759828	1.756961	1.754071	1.751165	1.748246	1.745301
1.769279	1.766293	1.763336	1.760413	1.757513	1.754599	1.751676	1.748758	1.745837
1.769925	1.766907	1.763918	1.760967	1.758043	1.755118	1.752197	1.749287	1.746385
1.770538	1.767476	1.764448	1.761473	1.758537	1.75562	1.752725	1.749836	1.746943
1.771135	1.768044	1.764994	1.762003	1.759059	1.75615	1.753279	1.750407	1.747519
1.771708	1.768623	1.76559	1.762596	1.759645	1.756739	1.753879	1.751011	1.748121
1.772286	1.76922	1.766212	1.763221	1.760265	1.757357	1.754497	1.751628	1.748735
1.752387	1.7494	1.746467	1.763864	1.760899	1.757981	1.755108	1.752233	1.749347
1.753016	1.75003	1.747088	1.764525	1.761553	1.758618	1.755721	1.752836	1.749962
1.753663	1.750668	1.747711	1.765187	1.762212	1.759267	1.756353	1.753457	1.75058
1.754332	1.751316	1.748338	1.765851	1.762879	1.759929	1.757003	1.754094	1.751202
1.754995	1.751965	1.74897	1.746021	1.743109	1.760588	1.75765	1.754729	1.751826
1.755646	1.752611	1.749608	1.746653	1.743733	1.761238	1.758284	1.755354	1.752452
1.756299	1.753258	1.750248	1.747285	1.744357	1.76189	1.758923	1.755985	1.753082
1.756967	1.753912	1.750886	1.747916	1.744986	1.762558	1.759586	1.756639	1.753718
1.757635	1.754565	1.751522	1.748545	1.745611	1.763225	1.760253	1.757297	1.754355
1.758276	1.755203	1.752157	1.749167	1.746217	1.743287	1.740381	1.757932	1.754987
1.758898	1.755831	1.752789	1.749783	1.746808	1.743867	1.740959	1.758554	1.755616
1.759535	1.756462	1.753415	1.750394	1.747403	1.744456	1.741553	1.759188	1.756245
1.760187	1.757097	1.754033	1.751	1.748001	1.745056	1.742164	1.759836	1.756873

1.760833	1.757731	1.754656	1.751609	1.748595	1.745648	1.742766	1.739878	1.73697
1.761455	1.758355	1.755284	1.752215	1.749172	1.746216	1.743339	1.74046	1.737564
1.76209	1.758993	1.755924	1.752836	1.749767	1.746798	1.743918	1.741042	1.738158
1.762766	1.759656	1.756577	1.753483	1.750409	1.747428	1.744532	1.741643	1.738749
1.76348	1.76035	1.757257	1.75416	1.751088	1.748092	1.745167	1.742254	1.739343
1.764274	1.76113	1.758021	1.754913	1.751827	1.748795	1.745813	1.742865	1.739952
1.765138	1.761983	1.758859	1.755733	1.752621	1.749535	1.746471	1.743478	1.740577
1.751919	1.748783	1.745668	1.756488	1.753368	1.750256	1.747153	1.74414	1.741244
1.752616	1.749464	1.746335	1.757162	1.754054	1.750958	1.747873	1.74487	1.741974
1.753288	1.750124	1.746982	1.743879	1.740807	1.751638	1.748569	1.745574	1.742674
1.753952	1.750778	1.747629	1.744527	1.741459	1.752286	1.7492	1.746191	1.743285
1.754608	1.751428	1.748274	1.745172	1.742104	1.752923	1.749817	1.746791	1.743872
1.714879	1.711932	1.709008	1.745816	1.742745	1.753566	1.750462	1.747427	1.744477
1.715491	1.712548	1.709628	1.746472	1.743395	1.75422	1.751129	1.748086	1.745097
1.716133	1.713214	1.710317	1.747216	1.744128	1.741052	1.737986	1.748806	1.745785
1.716803	1.713927	1.711073	1.748047	1.744946	1.741857	1.738779	1.749588	1.746543
1.702405	1.699569	1.696755	1.708925	1.706038	1.7426	1.739516	1.736457	1.733416
1.703167	1.70029	1.697432	1.709571	1.706653	1.743237	1.740161	1.737099	1.734037
1.703925	1.701002	1.698098	1.710206	1.707261	1.743865	1.740792	1.737728	1.734653
1.704628	1.701683	1.698755	1.695814	1.692881	1.704981	1.702117	1.738368	1.735295
1.705326	1.702368	1.699425	1.696474	1.693532	1.705623	1.702746	1.739028	1.735963
1.706089	1.703123	1.700172	1.697212	1.694259	1.70634	1.703446	1.739768	1.736699
1.706887	1.703921	1.700968	1.698	1.695038	1.707108	1.704194	1.740561	1.737484
1.707626	1.704649	1.701688	1.698721	1.695763	1.692833	1.689928	1.701991	1.699131
1.708301	1.705304	1.702326	1.699368	1.696428	1.693492	1.690561	1.702617	1.699767
1.694564	1.691589	1.688637	1.700033	1.697113	1.694178	1.69123	1.688338	1.685523
1.69521	1.692241	1.689301	1.70074	1.697848	1.694924	1.691974	1.68908	1.686263
1.695882	1.692919	1.68999	1.701465	1.69859	1.695675	1.69273	1.689833	1.687005
1.696635	1.693667	1.690727	1.68784	1.684983	1.69639	1.693445	1.69054	1.687688
1.697415	1.694438	1.69148	1.688567	1.68568	1.697095	1.694152	1.691237	1.688355
1.698114	1.69513	1.692169	1.689263	1.686388	1.683498	1.6806	1.691972	1.68907
1.698755	1.695767	1.692811	1.689936	1.687102	1.684234	1.681343	1.692734	1.689818
1.699449	1.696461	1.693507	1.690629	1.687794	1.684929	1.682045	1.679164	1.676277
1.700213	1.697229	1.69427	1.691347	1.688451	1.685563	1.682686	1.679805	1.676904
1.700976	1.697997	1.695034	1.692075	1.68913	1.686223	1.683352	1.680474	1.67757
1.701717	1.698746	1.695785	1.692824	1.689875	1.686965	1.684089	1.681217	1.678335
1.702439	1.699473	1.696516	1.693563	1.690623	1.687718	1.684843	1.68198	1.679126
1.703113	1.700143	1.697191	1.694238	1.691299	1.688412	1.685571	1.682734	1.679894
1.70377	1.700789	1.697841	1.694885	1.691948	1.689084	1.686286	1.683477	1.680645
1.704516	1.701519	1.698563	1.6956	1.692658	1.689784	1.686973	1.684158	1.681328
1.705343	1.702327	1.699347	1.696373	1.693421	1.690508	1.687632	1.684777	1.681944
1.679735	1.676748	1.673796	1.697138	1.694186	1.69125	1.688328	1.685444	1.682607
1.680387	1.677424	1.674499	1.697888	1.694953	1.692016	1.68908	1.686185	1.683344
1.64851	1.645793	1.643113	1.67229	1.669407	1.69278	1.689841	1.686941	1.684093
1.649133	1.646384	1.643684	1.672966	1.670114	1.693528	1.690596	1.687696	1.684837
1.649791	1.646999	1.644263	1.673637	1.670807	1.694259	1.691337	1.68844	1.685572
1.650499	1.647676	1.644903	1.642216	1.639583	1.668599	1.665708	1.689126	1.686275
1.651244	1.648396	1.645584	1.642846	1.640157	1.669227	1.666325	1.689777	1.686953
1.651952	1.649095	1.646267	1.643495	1.640765	1.669906	1.666995	1.690468	1.687631
1.652623	1.649776	1.646954	1.644165	1.641407	1.670638	1.667717	1.691197	1.6883

1.653285	1.650452	1.647641	1.64484	1.642061	1.63933	1.636643	1.665548	1.662639
1.653943	1.651128	1.648328	1.64552	1.642723	1.639983	1.637291	1.666293	1.663374
1.654594	1.651795	1.649009	1.6462	1.643395	1.640647	1.637948	1.667048	1.664129
1.655231	1.65245	1.649686	1.646885	1.644081	1.641324	1.638604	1.635912	1.633243
1.655869	1.653095	1.650349	1.647563	1.644771	1.642006	1.639264	1.636557	1.633891
1.590441	1.588093	1.585768	1.648189	1.645427	1.642673	1.639926	1.637214	1.634548
1.590984	1.588603	1.58625	1.648778	1.646061	1.643329	1.640591	1.637882	1.635211
1.591538	1.589141	1.586774	1.649417	1.646719	1.643994	1.641256	1.638544	1.635866
1.592107	1.589714	1.58735	1.650116	1.647402	1.644666	1.641919	1.639199	1.636511
1.592676	1.590287	1.587927	1.650831	1.648105	1.645355	1.642594	1.639861	1.637161
1.593234	1.590845	1.588488	1.651555	1.648836	1.646076	1.643288	1.640533	1.637821
1.593796	1.591404	1.589048	1.652283	1.649573	1.646804	1.643993	1.641218	1.638489
1.594381	1.591977	1.58961	1.587308	1.585041	1.647514	1.644708	1.641924	1.639164
1.594978	1.592557	1.590175	1.587863	1.585589	1.648218	1.645431	1.642645	1.639847
1.558328	1.556045	1.553803	1.588432	1.586148	1.648925	1.646152	1.643363	1.640539
1.558836	1.556561	1.55433	1.589012	1.586713	1.649634	1.646872	1.644082	1.641238
1.559352	1.557078	1.554854	1.589596	1.587288	1.584977	1.582668	1.644801	1.641949
1.559875	1.557593	1.555369	1.590179	1.587874	1.585559	1.583241	1.645521	1.642677
1.560418	1.558123	1.555893	1.553702	1.551544	1.586149	1.583814	1.646237	1.643403
1.560996	1.558681	1.556434	1.554242	1.552089	1.586745	1.584387	1.646941	1.644112
1.56159	1.559256	1.556988	1.554791	1.552639	1.587345	1.584963	1.647648	1.644821
1.562181	1.559839	1.557558	1.555346	1.553176	1.550967	1.54873	1.583174	1.580802
1.562774	1.560431	1.558143	1.555907	1.553709	1.551492	1.549266	1.583772	1.581392
1.563372	1.561028	1.558735	1.556474	1.554245	1.552016	1.549793	1.547568	1.545333
1.563971	1.56163	1.559335	1.557048	1.554783	1.552538	1.550312	1.548102	1.545914
1.564586	1.562239	1.559934	1.557626	1.555336	1.553077	1.550848	1.548648	1.54649
1.565223	1.562857	1.56053	1.558208	1.555908	1.553644	1.551413	1.549211	1.547051
1.565866	1.563476	1.561122	1.55879	1.556486	1.55422	1.551991	1.549784	1.547609
1.5665	1.564084	1.561703	1.55936	1.557054	1.554793	1.552575	1.550366	1.548166
1.567135	1.564694	1.562286	1.559934	1.557629	1.555373	1.553166	1.550954	1.548724
1.536745	1.53439	1.532068	1.560543	1.558237	1.555977	1.553763	1.551539	1.54929
1.537339	1.534973	1.532646	1.561181	1.558873	1.5566	1.554365	1.552124	1.549863
1.537955	1.535589	1.533264	1.56184	1.559508	1.557214	1.554959	1.55271	1.550457
1.538594	1.536245	1.533931	1.562525	1.56014	1.557813	1.555541	1.553294	1.551075
1.539242	1.5369	1.534592	1.532255	1.529928	1.558431	1.556141	1.55389	1.551691
1.539897	1.537543	1.535227	1.532875	1.530531	1.559079	1.556774	1.554508	1.552292
1.540555	1.538179	1.535848	1.533492	1.53115	1.559746	1.557422	1.555134	1.552892
1.506267	1.504082	1.501956	1.534106	1.531794	1.529508	1.527247	1.555757	1.553497
1.506853	1.504634	1.502484	1.534722	1.532451	1.530158	1.527851	1.556383	1.554108
1.507489	1.505238	1.503054	1.535359	1.533085	1.530778	1.528451	1.557022	1.554734
1.50817	1.505885	1.50366	1.536015	1.533701	1.531376	1.529046	1.557673	1.555373
1.508858	1.50653	1.504256	1.502071	1.499946	1.53198	1.529662	1.527391	1.525177
1.509555	1.507167	1.504829	1.502621	1.500494	1.532595	1.530308	1.528041	1.525786
1.51023	1.507802	1.50542	1.50319	1.501051	1.533219	1.530948	1.52868	1.526399
1.510849	1.508428	1.506054	1.503802	1.50163	1.533855	1.531556	1.529278	1.527012
1.511451	1.509055	1.506705	1.504432	1.502219	1.534497	1.532161	1.529867	1.527621
1.512073	1.509692	1.507355	1.505058	1.502807	1.500617	1.498487	1.530483	1.528212
1.512706	1.510337	1.508008	1.505687	1.503399	1.501193	1.499064	1.531113	1.528795
1.513357	1.510988	1.508657	1.506321	1.50401	1.501788	1.499646	1.53174	1.529402
1.514025	1.511644	1.509303	1.506959	1.50464	1.502401	1.500233	1.532364	1.530033

1.514713	1.512314	1.509955	1.507605	1.505285	1.50303	1.500834	1.498668	1.496527
1.515425	1.512999	1.510614	1.508263	1.50595	1.503682	1.501458	1.499268	1.497113
1.516148	1.513693	1.511281	1.508925	1.506615	1.504332	1.502079	1.499868	1.497701
1.484598	1.482309	1.480049	1.50958	1.507253	1.504949	1.502671	1.500445	1.498282
1.485234	1.482917	1.480631	1.51024	1.507888	1.505561	1.503262	1.501026	1.498868
1.485887	1.483547	1.481238	1.510921	1.508546	1.506203	1.503893	1.501646	1.499477
1.486556	1.484197	1.481867	1.51162	1.509222	1.506867	1.504555	1.502299	1.500106
1.487249	1.484875	1.482528	1.480222	1.477951	1.507563	1.505244	1.502972	1.500753
1.48797	1.485586	1.483229	1.48091	1.478626	1.508299	1.505965	1.503672	1.50142
1.488687	1.486293	1.483922	1.481591	1.479293	1.509035	1.506682	1.504367	1.502085
1.489387	1.486974	1.484582	1.482236	1.479926	1.509753	1.507376	1.505037	1.50273
1.490085	1.487657	1.485247	1.482885	1.480559	1.510478	1.508075	1.505713	1.503385
1.490786	1.488355	1.485941	1.483562	1.481215	1.511222	1.508799	1.506418	1.504072
1.491498	1.489069	1.486658	1.484263	1.481893	1.511984	1.509545	1.507146	1.504784
1.454709	1.452136	1.449578	1.48501	1.482623	1.480292	1.478011	1.507912	1.505531
1.455677	1.453117	1.450565	1.485797	1.4834	1.481051	1.478746	1.508713	1.506311
1.446612	1.444099	1.441593	1.448985	1.446458	1.481806	1.479472	1.509508	1.50709
1.447565	1.445052	1.442547	1.449912	1.447391	1.482548	1.48018	1.510286	1.507864
1.448511	1.446003	1.443506	1.450844	1.448331	1.44582	1.44331	1.44086	1.438485
1.449451	1.446965	1.444491	1.451801	1.449293	1.446779	1.444261	1.441794	1.439392
1.4504	1.447933	1.445484	1.452765	1.45026	1.447748	1.44523	1.44275	1.440313
1.451384	1.448913	1.446467	1.444005	1.441545	1.448707	1.446197	1.44371	1.441245
1.452391	1.449904	1.447447	1.444984	1.442529	1.449664	1.447167	1.444677	1.442187
1.453401	1.450902	1.448437	1.445973	1.44352	1.450622	1.44813	1.445636	1.443128
1.454417	1.451908	1.449438	1.446972	1.444519	1.45158	1.449085	1.446584	1.444067
1.455434	1.452906	1.450426	1.447958	1.445509	1.443059	1.440612	1.44755	1.44503
1.456439	1.453881	1.451385	1.448918	1.446483	1.444048	1.44162	1.448544	1.446032
1.457475	1.454882	1.452359	1.449886	1.447457	1.445036	1.442627	1.449539	1.447037
1.458588	1.455952	1.453384	1.450885	1.448444	1.446022	1.443626	1.450516	1.448028
1.459735	1.457053	1.454432	1.4519	1.449435	1.447006	1.444617	1.451483	1.449008
1.460859	1.458139	1.455474	1.452913	1.450426	1.447983	1.445591	1.452435	1.449964
1.461975	1.459223	1.456519	1.453928	1.451417	1.448956	1.446551	1.453374	1.4509
1.463095	1.460328	1.457597	1.45497	1.452415	1.449924	1.4475	1.454294	1.451821
1.46422	1.461457	1.458717	1.45604	1.453419	1.450884	1.448431	1.455191	1.452722
1.465337	1.462576	1.459826	1.45711	1.454434	1.45186	1.44938	1.456106	1.453635
1.466442	1.463671	1.460906	1.458165	1.45546	1.452862	1.450361	1.457054	1.454567
1.467539	1.464758	1.461983	1.459226	1.4565	1.453881	1.451359	1.45802	1.455516
1.468616	1.465839	1.463073	1.460302	1.457552	1.454911	1.452366	1.449868	1.447414
1.469692	1.466923	1.464174	1.461393	1.458624	1.455961	1.45339	1.450874	1.448412
1.47082	1.468041	1.4653	1.462518	1.459745	1.457061	1.454455	1.45191	1.449431
1.471989	1.469186	1.466448	1.463671	1.460909	1.458206	1.455555	1.452973	1.450471
1.473179	1.470344	1.467603	1.46482	1.462054	1.45933	1.456644	1.454032	1.451506
1.474381	1.471511	1.468769	1.465962	1.463172	1.460424	1.457715	1.45508	1.452532
1.475639	1.472705	1.46993	1.467096	1.46429	1.461524	1.458796	1.45614	1.453564
1.476966	1.47392	1.471064	1.468208	1.465414	1.462642	1.459896	1.457214	1.4546
1.478367	1.47518	1.472209	1.469326	1.466551	1.463776	1.461017	1.458311	1.455657
1.429529	1.426226	1.423169	1.470477	1.467719	1.464941	1.462173	1.459449	1.456766
1.431067	1.427571	1.424331	1.47166	1.468908	1.466127	1.463353	1.460616	1.457911
1.338202	1.334683	1.331438	1.422599	1.419881	1.467295	1.464516	1.461774	1.459062
1.339764	1.336106	1.332706	1.423837	1.421013	1.468449	1.465665	1.462926	1.460224

1.951795	1.948334	1.944888	1.941383	1.937852	1.934311	1.930755	1.927272	1.923895
1.951723	1.948265	1.944822	1.941317	1.937785	1.934242	1.930685	1.927199	1.92382
1.951651	1.948195	1.944756	1.941252	1.937719	1.934175	1.930614	1.927126	1.923745
1.95158	1.948127	1.944691	1.941187	1.937654	1.934107	1.930542	1.927051	1.923669
1.951509	1.948059	1.944626	1.941123	1.937589	1.934039	1.93047	1.926976	1.923593
1.9459	1.942466	1.939046	1.941059	1.937525	1.933976	1.930408	1.926913	1.923523
1.94584	1.942404	1.93898	1.940995	1.937463	1.933916	1.930352	1.926856	1.923458
1.94582	1.942372	1.938931	1.94094	1.937404	1.933867	1.930324	1.926834	1.923417
1.945874	1.942394	1.938908	1.940897	1.937348	1.933832	1.930344	1.926872	1.923417
1.945925	1.942415	1.938889	1.940861	1.9373	1.933804	1.930365	1.926909	1.923418
1.945906	1.942391	1.938856	1.935307	1.931759	1.933773	1.930346	1.926889	1.92338
1.945856	1.942345	1.938815	1.935275	1.931737	1.93375	1.930312	1.926846	1.923329
1.943096	1.939584	1.936066	1.935257	1.931754	1.933768	1.930298	1.926812	1.92329
1.943044	1.939522	1.936012	1.935249	1.931803	1.933818	1.930297	1.926779	1.923257
1.942988	1.939461	1.935958	1.932526	1.929125	1.928334	1.924803	1.926759	1.923253
1.942924	1.939397	1.935898	1.932473	1.92908	1.928298	1.924773	1.926748	1.923285
1.942869	1.939356	1.935867	1.932437	1.929034	1.925579	1.922084	1.924398	1.920967
1.942825	1.939354	1.9359	1.932449	1.929004	1.925559	1.922114	1.924451	1.920997
1.942802	1.939368	1.935943	1.932467	1.928979	1.925541	1.922145	1.924502	1.921017
1.942829	1.939385	1.93594	1.932448	1.928945	1.925507	1.922125	1.918713	1.915244
1.942884	1.939404	1.935917	1.932415	1.928915	1.925474	1.922084	1.918673	1.915216
1.936004	1.932526	1.929047	1.932407	1.928929	1.925485	1.922073	1.918657	1.915225
1.935995	1.932503	1.929025	1.932418	1.928983	1.925537	1.922085	1.918659	1.915265
1.936024	1.932523	1.92904	1.932448	1.929026	1.925581	1.922118	1.918688	1.915303
1.936119	1.932614	1.929113	1.932501	1.929048	1.925608	1.922181	1.918758	1.915336
1.936196	1.932691	1.92918	1.925711	1.922267	1.925641	1.922242	1.918822	1.915361
1.936196	1.932697	1.929201	1.925749	1.922322	1.925699	1.922278	1.918839	1.91536
1.936168	1.932682	1.929211	1.925784	1.922382	1.925762	1.922311	1.918851	1.915364
1.929208	1.925737	1.922287	1.925826	1.922419	1.919013	1.915611	1.918914	1.915427
1.929179	1.925745	1.92233	1.92587	1.922444	1.919047	1.915675	1.919005	1.915524
1.929208	1.925773	1.922358	1.92591	1.922489	1.919095	1.915725	1.919058	1.915575
1.929299	1.925821	1.922369	1.925944	1.922556	1.919159	1.915759	1.919072	1.915578
1.929397	1.925888	1.922403	1.918985	1.915601	1.919211	1.9158	1.912374	1.908913
1.929501	1.925989	1.922485	1.919034	1.915614	1.919237	1.915861	1.912458	1.909005
1.929581	1.926071	1.922558	1.91908	1.915627	1.919263	1.915918	1.91254	1.909105
1.929587	1.926073	1.922564	1.919101	1.915667	1.919309	1.915946	1.912573	1.909181
1.929569	1.926052	1.922554	1.919116	1.915714	1.919362	1.915968	1.912593	1.909247
1.929619	1.926101	1.922599	1.919152	1.91574	1.912338	1.908952	1.912653	1.909303
1.929713	1.926197	1.922684	1.919204	1.915753	1.912357	1.909015	1.912741	1.909351
1.929775	1.926279	1.922781	1.919288	1.915811	1.912418	1.909101	1.912842	1.909431
1.929791	1.92634	1.922892	1.919408	1.915923	1.91253	1.909218	1.912965	1.909552
1.929835	1.926417	1.923009	1.919535	1.916048	1.912646	1.909319	1.905963	1.902554
1.929942	1.926534	1.923138	1.919668	1.916177	1.912748	1.909371	1.905999	1.902626
1.93007	1.926655	1.923253	1.919788	1.916304	1.912852	1.909424	1.906032	1.902687
1.930194	1.92674	1.923307	1.919861	1.916415	1.912969	1.909522	1.90611	1.90274
1.930322	1.926814	1.923335	1.919909	1.916512	1.913087	1.909643	1.906214	1.902793
1.930452	1.926922	1.923415	1.919976	1.916575	1.913164	1.909751	1.906335	1.902895
1.930585	1.927059	1.923537	1.920056	1.916606	1.913205	1.909852	1.906474	1.903039
1.930716	1.927199	1.923676	1.920167	1.91668	1.913286	1.909975	1.906624	1.903193
1.930849	1.927346	1.923835	1.920318	1.916816	1.913424	1.91013	1.906788	1.903358

1.930964	1.92748	1.923992	1.920478	1.91697	1.913578	1.910288	1.906949	1.903524
1.931032	1.927577	1.924129	1.920629	1.917126	1.913736	1.910445	1.907106	1.903687
1.931095	1.92767	1.924263	1.920782	1.917288	1.913895	1.91059	1.907246	1.903838
1.931213	1.927798	1.924408	1.920936	1.917445	1.914033	1.910685	1.907329	1.903952
1.931363	1.927949	1.92456	1.921092	1.917602	1.914159	1.910753	1.907378	1.904043
1.93152	1.92811	1.924725	1.921258	1.917766	1.914304	1.910863	1.907477	1.904166
1.931687	1.928285	1.924905	1.921436	1.917937	1.914466	1.911012	1.907621	1.904319
1.931857	1.928461	1.925084	1.921613	1.918109	1.914632	1.911172	1.907778	1.904481
1.932029	1.928635	1.925258	1.921786	1.91828	1.914801	1.911339	1.907946	1.90465
1.932203	1.928808	1.92543	1.921958	1.918452	1.914973	1.91151	1.908116	1.90482
1.92464	1.921289	1.917957	1.922131	1.91863	1.915147	1.911674	1.908274	1.904979
1.924801	1.921447	1.918113	1.922304	1.918809	1.915323	1.911839	1.908432	1.905136
1.92496	1.921603	1.918268	1.922473	1.918983	1.915498	1.912011	1.9086	1.905298
1.925119	1.921758	1.918422	1.92264	1.919154	1.915672	1.912189	1.908777	1.905467
1.925286	1.921918	1.918577	1.922808	1.919327	1.915861	1.912403	1.908997	1.905663
1.925462	1.922082	1.918732	1.922975	1.919502	1.916066	1.912661	1.90927	1.905894
1.925644	1.92225	1.918889	1.923142	1.919674	1.916263	1.912902	1.909524	1.906116
1.925821	1.922417	1.919045	1.923305	1.919837	1.916437	1.913099	1.90973	1.90631
1.926018	1.9226	1.919213	1.923476	1.920005	1.916609	1.91328	1.909915	1.906492
1.926274	1.922829	1.919408	1.915969	1.912543	1.916785	1.913454	1.910089	1.906661
1.926555	1.923082	1.919619	1.916165	1.912731	1.916972	1.913627	1.910254	1.906824
1.925029	1.921512	1.918005	1.916383	1.912966	1.917209	1.91383	1.910434	1.906997
1.925233	1.92172	1.918222	1.916623	1.913244	1.917491	1.914058	1.910624	1.907177
1.925442	1.921943	1.918464	1.915039	1.911642	1.91775	1.914284	1.910833	1.907396
1.925658	1.92219	1.918745	1.915307	1.911878	1.91797	1.914497	1.911059	1.907667
1.925878	1.922432	1.919013	1.915563	1.912108	1.910531	1.907122	1.91131	1.907947
1.92609	1.922646	1.919234	1.915787	1.912337	1.910769	1.90739	1.911604	1.908218
1.926318	1.922862	1.91944	1.916003	1.912572	1.91102	1.907668	1.911905	1.908484
1.918417	1.914957	1.91153	1.916238	1.912842	1.909443	1.906046	1.912159	1.908722
1.918734	1.915223	1.911746	1.916481	1.913129	1.909728	1.906293	1.912389	1.908948
1.917079	1.913548	1.910051	1.908593	1.905296	1.909975	1.906525	1.910948	1.907482
1.917299	1.913755	1.910251	1.908792	1.905491	1.910184	1.906735	1.911194	1.907773
1.912973	1.909455	1.905979	1.909021	1.905701	1.910407	1.906976	1.903574	1.900216
1.913186	1.909712	1.906273	1.909298	1.905935	1.910654	1.907266	1.903883	1.900502
1.913417	1.90998	1.906571	1.909588	1.906192	1.910921	1.907566	1.904196	1.900794
1.913697	1.91025	1.906828	1.907947	1.904571	1.903128	1.899788	1.904475	1.901092
1.913994	1.910523	1.907079	1.908223	1.90488	1.903433	1.90006	1.904748	1.901392
1.914256	1.9108	1.907369	1.903996	1.900659	1.903711	1.900347	1.896995	1.893661
1.914499	1.911079	1.907684	1.904289	1.900908	1.903974	1.900644	1.897302	1.893934
1.908987	1.905593	1.902222	1.904568	1.901182	1.899948	1.896638	1.897582	1.894212
1.909296	1.905869	1.902464	1.904832	1.901487	1.900249	1.896895	1.897832	1.894497
1.909587	1.90614	1.902716	1.905097	1.901773	1.898418	1.895041	1.898091	1.894776
1.909826	1.9064	1.902997	1.905371	1.902013	1.89866	1.895313	1.898381	1.895042
1.910072	1.906663	1.90328	1.905645	1.902255	1.898903	1.895586	1.898671	1.895307
1.910376	1.906938	1.903531	1.900152	1.896798	1.899183	1.895841	1.89463	1.8913
1.910709	1.907225	1.90378	1.900416	1.897099	1.899484	1.896098	1.894875	1.891572
1.911036	1.907546	1.904092	1.900732	1.897421	1.894067	1.890682	1.893045	1.889729
1.911362	1.907897	1.904456	1.901091	1.897765	1.894411	1.891036	1.893389	1.890014
1.911652	1.908214	1.904795	1.90142	1.89807	1.894718	1.891366	1.893728	1.890321
1.911884	1.908484	1.905104	1.901708	1.898319	1.894973	1.891666	1.894063	1.89066

1.896004	1.8927	1.889417	1.901985	1.898576	1.895234	1.89195	1.88864	1.885278
1.896319	1.89298	1.889667	1.90225	1.898875	1.895528	1.892209	1.888891	1.885568
1.896658	1.893273	1.889922	1.902522	1.899191	1.895838	1.892472	1.88914	1.885857
1.896994	1.893599	1.890235	1.902844	1.899523	1.896166	1.892785	1.889452	1.886187
1.897332	1.893946	1.890582	1.903195	1.899863	1.896504	1.893128	1.889798	1.886536
1.897669	1.89429	1.890928	1.887619	1.884342	1.896806	1.89345	1.890127	1.886849
1.898007	1.894635	1.891276	1.887934	1.884612	1.897074	1.893755	1.890442	1.887129
1.898323	1.89497	1.891628	1.888267	1.884913	1.897377	1.894081	1.890774	1.887436
1.898598	1.895283	1.891983	1.888628	1.885264	1.897733	1.894436	1.891127	1.887785
1.898894	1.895608	1.892342	1.888996	1.885631	1.898111	1.894809	1.891496	1.888152
1.899262	1.895973	1.892706	1.889364	1.886006	1.882723	1.879501	1.891887	1.888532
1.899662	1.896356	1.893072	1.889733	1.886386	1.88311	1.879893	1.892285	1.888919
1.900055	1.896736	1.893438	1.8901	1.886759	1.883484	1.880266	1.892662	1.88929
1.90045	1.897116	1.893803	1.890463	1.887126	1.88385	1.880626	1.893024	1.889651
1.900831	1.897467	1.894129	1.890796	1.88748	1.884199	1.880952	1.877698	1.87443
1.901196	1.897785	1.894408	1.891093	1.887817	1.884529	1.881236	1.877967	1.874731
1.901559	1.898117	1.894715	1.891412	1.888162	1.884867	1.88154	1.87826	1.87505
1.901919	1.898482	1.895077	1.891774	1.888521	1.88522	1.881885	1.878603	1.875403
1.902283	1.898862	1.895463	1.892155	1.88889	1.885585	1.88225	1.87897	1.875773
1.901147	1.89775	1.89437	1.892542	1.889271	1.885962	1.882627	1.879346	1.876149
1.901531	1.898145	1.894775	1.892944	1.889663	1.886354	1.883023	1.879743	1.876537
1.901949	1.898577	1.89522	1.893376	1.890074	1.886768	1.883464	1.88019	1.876959
1.902396	1.89904	1.895699	1.893836	1.890501	1.887204	1.883942	1.88068	1.877408
1.902823	1.899474	1.896141	1.892791	1.889449	1.887626	1.884385	1.881128	1.877831
1.903225	1.899871	1.896535	1.89319	1.889855	1.888031	1.884784	1.881521	1.878222
1.89751	1.894176	1.890862	1.89358	1.890249	1.886961	1.883711	1.8819	1.8786
1.897885	1.894557	1.891249	1.893965	1.890625	1.887332	1.884082	1.882269	1.878967
1.89826	1.894938	1.891635	1.89435	1.890997	1.8877	1.88445	1.882636	1.879335
1.898644	1.895324	1.89202	1.894737	1.891381	1.88808	1.884828	1.883013	1.879719
1.89904	1.895716	1.892409	1.895131	1.891776	1.888473	1.885215	1.883399	1.880117
1.899487	1.896145	1.892818	1.889496	1.886187	1.888906	1.885633	1.882371	1.879116
1.899984	1.896609	1.893246	1.889934	1.886652	1.889379	1.886081	1.88281	1.879575
1.900448	1.897054	1.893671	1.890363	1.887094	1.889828	1.886515	1.883238	1.880013
1.90086	1.897466	1.894084	1.890771	1.887495	1.890232	1.886921	1.883644	1.880416
1.901258	1.897876	1.894508	1.891187	1.887895	1.890635	1.887333	1.884057	1.880816
1.894238	1.8909	1.887575	1.891634	1.888316	1.885033	1.881783	1.884505	1.881233
1.89463	1.891326	1.888033	1.892086	1.888739	1.885454	1.882222	1.884954	1.881648
1.889381	1.886096	1.882826	1.892501	1.889138	1.885847	1.882617	1.885355	1.882036
1.889809	1.886504	1.883221	1.892894	1.889524	1.886225	1.882987	1.885726	1.882409
1.890261	1.886934	1.883631	1.885909	1.882558	1.886641	1.883384	1.88013	1.876863
1.890745	1.887387	1.884054	1.886342	1.883013	1.887094	1.883807	1.880545	1.87731
1.891202	1.887835	1.884494	1.881177	1.877884	1.88017	1.876886	1.880989	1.877767
1.891604	1.88827	1.884961	1.881639	1.878327	1.880623	1.877361	1.881476	1.878227
1.891999	1.888698	1.885423	1.882101	1.878777	1.881083	1.877842	1.881966	1.878689
1.892412	1.88911	1.885846	1.882548	1.879253	1.881561	1.878302	1.88242	1.879149
1.892843	1.889523	1.886254	1.882986	1.879734	1.882043	1.878755	1.882863	1.879609
1.893333	1.889975	1.886672	1.883406	1.880173	1.876926	1.873671	1.875942	1.872693
1.89387	1.890459	1.887096	1.883812	1.88058	1.877349	1.874125	1.876408	1.873141
1.89438	1.890942	1.887551	1.884257	1.881024	1.877794	1.874575	1.871352	1.868116
1.894852	1.891423	1.888046	1.884754	1.881517	1.878268	1.875017	1.871785	1.868578

1.895331	1.891903	1.888532	1.885241	1.882002	1.878742	1.875471	1.872231	1.86904
1.895839	1.892379	1.888973	1.885677	1.882447	1.8792	1.875948	1.872711	1.869497
1.896354	1.892862	1.889422	1.886119	1.882895	1.87966	1.876429	1.873195	1.869953
1.89207	1.888596	1.885173	1.886625	1.88339	1.88014	1.87689	1.873647	1.870409
1.892564	1.889114	1.885713	1.887164	1.883906	1.880629	1.877345	1.874086	1.870865
1.893085	1.889645	1.886247	1.887684	1.884395	1.881103	1.877813	1.874549	1.87132
1.893631	1.890192	1.886781	1.88819	1.884863	1.881562	1.878291	1.87503	1.871773
1.894179	1.890727	1.887297	1.888693	1.885348	1.882039	1.878768	1.875505	1.872242
1.894731	1.891244	1.88778	1.889187	1.885857	1.882536	1.879232	1.875961	1.872729
1.895268	1.891761	1.88828	1.884912	1.881606	1.883054	1.879721	1.876441	1.873233
1.895764	1.892282	1.888833	1.885472	1.882161	1.883609	1.880271	1.876984	1.873765
1.896257	1.892812	1.889404	1.886044	1.882719	1.884169	1.880839	1.877547	1.874304
1.88026	1.876869	1.873513	1.886578	1.883219	1.884675	1.881369	1.878082	1.87481
1.88082	1.877421	1.874043	1.88709	1.883686	1.885149	1.881881	1.878604	1.875299
1.881378	1.877953	1.874546	1.871152	1.867787	1.885651	1.882401	1.879132	1.875814
1.881936	1.878465	1.875017	1.871624	1.868279	1.886177	1.882933	1.879667	1.876354
1.88249	1.878987	1.875512	1.87212	1.868788	1.873263	1.870035	1.880186	1.876889
1.883042	1.879533	1.876052	1.872657	1.869322	1.873791	1.870521	1.880672	1.877408
1.88358	1.880079	1.876605	1.873208	1.869862	1.874326	1.871014	1.881161	1.877928
1.874562	1.871106	1.867684	1.87376	1.870403	1.867075	1.863777	1.881687	1.878459
1.875047	1.871622	1.868235	1.874311	1.870938	1.867598	1.864292	1.882227	1.878997
1.875571	1.872156	1.868776	1.874834	1.871428	1.868081	1.864785	1.869319	1.866125
1.876124	1.872703	1.869311	1.875336	1.871887	1.868534	1.865266	1.869838	1.866652
1.866257	1.862854	1.859475	1.866397	1.862939	1.869029	1.865774	1.862567	1.859419
1.866797	1.863401	1.86002	1.866949	1.863482	1.869571	1.866313	1.863107	1.859968
1.867353	1.863961	1.860578	1.867518	1.864046	1.860695	1.857446	1.863647	1.860512
1.867935	1.864544	1.86116	1.86811	1.864636	1.861267	1.857988	1.864175	1.861039
1.868525	1.865131	1.861744	1.868706	1.865231	1.861844	1.858531	1.864701	1.861561
1.863454	1.860071	1.856695	1.858862	1.855409	1.862404	1.859065	1.855818	1.852695
1.864021	1.860628	1.857243	1.859415	1.855961	1.862957	1.859598	1.856337	1.85321
1.864591	1.861188	1.857793	1.854359	1.850922	1.85312	1.849769	1.856878	1.85374
1.865163	1.861748	1.858342	1.854906	1.85147	1.853674	1.850324	1.857438	1.854283
1.865749	1.862327	1.858914	1.855473	1.852034	1.848652	1.845318	1.858015	1.854843
1.866356	1.862933	1.859518	1.856068	1.852619	1.849235	1.845905	1.858615	1.855426
1.855888	1.852497	1.84911	1.856669	1.853211	1.849824	1.846497	1.848815	1.845632
1.856466	1.853077	1.849692	1.857259	1.853797	1.850407	1.847077	1.849394	1.846193
1.857045	1.853659	1.850276	1.857858	1.854399	1.851005	1.847664	1.849974	1.846753
1.857648	1.854263	1.850886	1.858494	1.855052	1.851649	1.848277	1.84497	1.841736
1.858264	1.854879	1.85151	1.859151	1.855738	1.852324	1.848908	1.845562	1.842295
1.812024	1.809007	1.80601	1.848742	1.845375	1.852972	1.849538	1.84617	1.842875
1.812555	1.809523	1.806519	1.849331	1.845979	1.853594	1.850169	1.846795	1.843473
1.813088	1.810041	1.807028	1.849914	1.84657	1.843206	1.839826	1.847442	1.844094
1.813615	1.810557	1.807534	1.850488	1.847145	1.843808	1.840481	1.848124	1.844745
1.814162	1.811095	1.808059	1.851074	1.847726	1.84441	1.841127	1.8488	1.845407
1.814768	1.811688	1.80863	1.80561	1.802622	1.845016	1.841736	1.83844	1.835102
1.815392	1.812303	1.809224	1.806186	1.803177	1.845625	1.84233	1.839043	1.835758
1.81596	1.81289	1.809823	1.806771	1.803736	1.833671	1.830396	1.839646	1.836387
1.81649	1.813461	1.810429	1.807367	1.804301	1.83428	1.830989	1.840244	1.836994
1.796901	1.793962	1.791016	1.807948	1.80488	1.801855	1.798864	1.84085	1.8376
1.797415	1.794468	1.791526	1.808509	1.805477	1.802448	1.799421	1.841456	1.838195

1.797965	1.795	1.792049	1.789101	1.786157	1.803038	1.799995	1.829553	1.826315
1.798589	1.795593	1.792607	1.789646	1.7867	1.803616	1.800598	1.830257	1.827015
1.799216	1.796193	1.793176	1.790195	1.787236	1.804185	1.801198	1.830966	1.827724
1.799769	1.796754	1.79374	1.790745	1.787766	1.80473	1.801751	1.831611	1.828374
1.80029	1.797298	1.794302	1.791296	1.788295	1.805269	1.802289	1.832234	1.829001
1.800834	1.797846	1.794855	1.791851	1.788851	1.785896	1.782981	1.799917	1.796978
1.801392	1.798394	1.795399	1.792409	1.78943	1.786486	1.783575	1.80053	1.797554
1.801974	1.798958	1.795948	1.792962	1.789995	1.787057	1.784147	1.781242	1.778333
1.802589	1.799539	1.796498	1.793502	1.790537	1.787598	1.784687	1.781789	1.778899
1.803209	1.800134	1.797068	1.794061	1.79109	1.788144	1.785225	1.782328	1.779453
1.803821	1.800748	1.797681	1.794664	1.791678	1.788711	1.785765	1.78285	1.779966
1.804432	1.801369	1.798308	1.795281	1.792276	1.789288	1.786317	1.783382	1.780484
1.805051	1.801982	1.798914	1.795877	1.792861	1.78987	1.786903	1.78397	1.781069
1.80567	1.802591	1.799513	1.796466	1.793443	1.79046	1.787515	1.784594	1.781694
1.79674	1.793695	1.790653	1.79708	1.794051	1.791071	1.788137	1.785218	1.782308
1.79728	1.794269	1.791264	1.797719	1.794684	1.791705	1.788774	1.785849	1.782917
1.792429	1.789438	1.786458	1.788866	1.785843	1.79232	1.789384	1.786454	1.783517
1.793046	1.79007	1.787092	1.789481	1.786418	1.792891	1.78994	1.787012	1.784103
1.793647	1.790679	1.787703	1.790085	1.787	1.793477	1.790509	1.78758	1.784695
1.794161	1.791198	1.788235	1.790658	1.787617	1.794129	1.791154	1.788215	1.785321
1.794659	1.791696	1.788748	1.791224	1.788246	1.794802	1.791819	1.788865	1.785953
1.795249	1.792283	1.789333	1.78638	1.783429	1.785894	1.782907	1.789457	1.786537
1.7959	1.792925	1.789962	1.786998	1.784035	1.786487	1.783462	1.790013	1.787092
1.764615	1.761669	1.758737	1.787568	1.784609	1.787073	1.784048	1.781076	1.778172
1.76518	1.762185	1.759211	1.788077	1.785144	1.787649	1.784666	1.781717	1.778807
1.765728	1.762705	1.759709	1.756804	1.753952	1.782776	1.779855	1.782365	1.779452
1.766246	1.763239	1.760262	1.757368	1.754524	1.783381	1.780478	1.783013	1.780107
1.766774	1.763793	1.760845	1.757962	1.755121	1.783999	1.781103	1.783652	1.780744
1.767354	1.76439	1.761459	1.758584	1.755743	1.752888	1.750028	1.784266	1.781319
1.767967	1.765014	1.762095	1.759221	1.756374	1.753504	1.750617	1.784862	1.781863
1.768609	1.765647	1.762719	1.759828	1.756961	1.754071	1.751165	1.748246	1.745301
1.769279	1.766293	1.763336	1.760413	1.757513	1.754599	1.751676	1.748758	1.745837
1.769925	1.766907	1.763918	1.760967	1.758043	1.755118	1.752197	1.749287	1.746385
1.770538	1.767476	1.764448	1.761473	1.758537	1.75562	1.752725	1.749836	1.746943
1.771135	1.768044	1.764994	1.762003	1.759059	1.75615	1.753279	1.750407	1.747519
1.771708	1.768623	1.76559	1.762596	1.759645	1.756739	1.753879	1.751011	1.748121
1.772286	1.76922	1.766212	1.763221	1.760265	1.757357	1.754497	1.751628	1.748735
1.752387	1.7494	1.746467	1.763864	1.760899	1.757981	1.755108	1.752233	1.749347
1.753016	1.75003	1.747088	1.764525	1.761553	1.758618	1.755721	1.752836	1.749962
1.753663	1.750668	1.747711	1.765187	1.762212	1.759267	1.756353	1.753457	1.75058
1.754332	1.751316	1.748338	1.765851	1.762879	1.759929	1.757003	1.754094	1.751202
1.754995	1.751965	1.74897	1.746021	1.743109	1.760588	1.75765	1.754729	1.751826
1.755646	1.752611	1.749608	1.746653	1.743733	1.761238	1.758284	1.755354	1.752452
1.756299	1.753258	1.750248	1.747285	1.744357	1.76189	1.758923	1.755985	1.753082
1.756967	1.753912	1.750886	1.747916	1.744986	1.762558	1.759586	1.756639	1.753718
1.757635	1.754565	1.751522	1.748545	1.745611	1.763225	1.760253	1.757297	1.754355
1.758276	1.755203	1.752157	1.749167	1.746217	1.743287	1.740381	1.757932	1.754987
1.758898	1.755831	1.752789	1.749783	1.746808	1.743867	1.740959	1.758554	1.755616
1.759535	1.756462	1.753415	1.750394	1.747403	1.744456	1.741553	1.759188	1.756245
1.760187	1.757097	1.754033	1.751	1.748001	1.745056	1.742164	1.759836	1.756873

1.760833	1.757731	1.754656	1.751609	1.748595	1.745648	1.742766	1.739878	1.73697
1.761455	1.758355	1.755284	1.752215	1.749172	1.746216	1.743339	1.74046	1.737564
1.76209	1.758993	1.755924	1.752836	1.749767	1.746798	1.743918	1.741042	1.738158
1.762766	1.759656	1.756577	1.753483	1.750409	1.747428	1.744532	1.741643	1.738749
1.76348	1.76035	1.757257	1.75416	1.751088	1.748092	1.745167	1.742254	1.739343
1.764274	1.76113	1.758021	1.754913	1.751827	1.748795	1.745813	1.742865	1.739952
1.765138	1.761983	1.758859	1.755733	1.752621	1.749535	1.746471	1.743478	1.740577
1.751919	1.748783	1.745668	1.756488	1.753368	1.750256	1.747153	1.74414	1.741244
1.752616	1.749464	1.746335	1.757162	1.754054	1.750958	1.747873	1.74487	1.741974
1.753288	1.750124	1.746982	1.743879	1.740807	1.751638	1.748569	1.745574	1.742674
1.753952	1.750778	1.747629	1.744527	1.741459	1.752286	1.7492	1.746191	1.743285
1.754608	1.751428	1.748274	1.745172	1.742104	1.752923	1.749817	1.746791	1.743872
1.714879	1.711932	1.709008	1.745816	1.742745	1.753566	1.750462	1.747427	1.744477
1.715491	1.712548	1.709628	1.746472	1.743395	1.75422	1.751129	1.748086	1.745097
1.716133	1.713214	1.710317	1.747216	1.744128	1.741052	1.737986	1.748806	1.745785
1.716803	1.713927	1.711073	1.748047	1.744946	1.741857	1.738779	1.749588	1.746543
1.702405	1.699569	1.696755	1.708925	1.706038	1.7426	1.739516	1.736457	1.733416
1.703167	1.70029	1.697432	1.709571	1.706653	1.743237	1.740161	1.737099	1.734037
1.703925	1.701002	1.698098	1.710206	1.707261	1.743865	1.740792	1.737728	1.734653
1.704628	1.701683	1.698755	1.695814	1.692881	1.704981	1.702117	1.738368	1.735295
1.705326	1.702368	1.699425	1.696474	1.693532	1.705623	1.702746	1.739028	1.735963
1.706089	1.703123	1.700172	1.697212	1.694259	1.70634	1.703446	1.739768	1.736699
1.706887	1.703921	1.700968	1.698	1.695038	1.707108	1.704194	1.740561	1.737484
1.707626	1.704649	1.701688	1.698721	1.695763	1.692833	1.689928	1.701991	1.699131
1.708301	1.705304	1.702326	1.699368	1.696428	1.693492	1.690561	1.702617	1.699767
1.694564	1.691589	1.688637	1.700033	1.697113	1.694178	1.69123	1.688338	1.685523
1.69521	1.692241	1.689301	1.70074	1.697848	1.694924	1.691974	1.68908	1.686263
1.695882	1.692919	1.68999	1.701465	1.69859	1.695675	1.69273	1.689833	1.687005
1.696635	1.693667	1.690727	1.68784	1.684983	1.69639	1.693445	1.69054	1.687688
1.697415	1.694438	1.69148	1.688567	1.68568	1.697095	1.694152	1.691237	1.688355
1.698114	1.69513	1.692169	1.689263	1.686388	1.683498	1.6806	1.691972	1.68907
1.698755	1.695767	1.692811	1.689936	1.687102	1.684234	1.681343	1.692734	1.689818
1.699449	1.696461	1.693507	1.690629	1.687794	1.684929	1.682045	1.679164	1.676277
1.700213	1.697229	1.69427	1.691347	1.688451	1.685563	1.682686	1.679805	1.676904
1.700976	1.697997	1.695034	1.692075	1.68913	1.686223	1.683352	1.680474	1.67757
1.701717	1.698746	1.695785	1.692824	1.689875	1.686965	1.684089	1.681217	1.678335
1.702439	1.699473	1.696516	1.693563	1.690623	1.687718	1.684843	1.68198	1.679126
1.703113	1.700143	1.697191	1.694238	1.691299	1.688412	1.685571	1.682734	1.679894
1.70377	1.700789	1.697841	1.694885	1.691948	1.689084	1.686286	1.683477	1.680645
1.704516	1.701519	1.698563	1.6956	1.692658	1.689784	1.686973	1.684158	1.681328
1.705343	1.702327	1.699347	1.696373	1.693421	1.690508	1.687632	1.684777	1.681944
1.679735	1.676748	1.673796	1.697138	1.694186	1.69125	1.688328	1.685444	1.682607
1.680387	1.677424	1.674499	1.697888	1.694953	1.692016	1.68908	1.686185	1.683344
1.64851	1.645793	1.643113	1.67229	1.669407	1.69278	1.689841	1.686941	1.684093
1.649133	1.646384	1.643684	1.672966	1.670114	1.693528	1.690596	1.687696	1.684837
1.649791	1.646999	1.644263	1.673637	1.670807	1.694259	1.691337	1.68844	1.685572
1.650499	1.647676	1.644903	1.642216	1.639583	1.668599	1.665708	1.689126	1.686275
1.651244	1.648396	1.645584	1.642846	1.640157	1.669227	1.666325	1.689777	1.686953
1.651952	1.649095	1.646267	1.643495	1.640765	1.669906	1.666995	1.690468	1.687631
1.652623	1.649776	1.646954	1.644165	1.641407	1.670638	1.667717	1.691197	1.6883

1.653285	1.650452	1.647641	1.64484	1.642061	1.63933	1.636643	1.665548	1.662639
1.653943	1.651128	1.648328	1.64552	1.642723	1.639983	1.637291	1.666293	1.663374
1.654594	1.651795	1.649009	1.6462	1.643395	1.640647	1.637948	1.667048	1.664129
1.655231	1.65245	1.649686	1.646885	1.644081	1.641324	1.638604	1.635912	1.633243
1.655869	1.653095	1.650349	1.647563	1.644771	1.642006	1.639264	1.636557	1.633891
1.590441	1.588093	1.585768	1.648189	1.645427	1.642673	1.639926	1.637214	1.634548
1.590984	1.588603	1.58625	1.648778	1.646061	1.643329	1.640591	1.637882	1.635211
1.591538	1.589141	1.586774	1.649417	1.646719	1.643994	1.641256	1.638544	1.635866
1.592107	1.589714	1.58735	1.650116	1.647402	1.644666	1.641919	1.639199	1.636511
1.592676	1.590287	1.587927	1.650831	1.648105	1.645355	1.642594	1.639861	1.637161
1.593234	1.590845	1.588488	1.651555	1.648836	1.646076	1.643288	1.640533	1.637821
1.593796	1.591404	1.589048	1.652283	1.649573	1.646804	1.643993	1.641218	1.638489
1.594381	1.591977	1.58961	1.587308	1.585041	1.647514	1.644708	1.641924	1.639164
1.594978	1.592557	1.590175	1.587863	1.585589	1.648218	1.645431	1.642645	1.639847
1.558328	1.556045	1.553803	1.588432	1.586148	1.648925	1.646152	1.643363	1.640539
1.558836	1.556561	1.55433	1.589012	1.586713	1.649634	1.646872	1.644082	1.641238
1.559352	1.557078	1.554854	1.589596	1.587288	1.584977	1.582668	1.644801	1.641949
1.559875	1.557593	1.555369	1.590179	1.587874	1.585559	1.583241	1.645521	1.642677
1.560418	1.558123	1.555893	1.553702	1.551544	1.586149	1.583814	1.646237	1.643403
1.560996	1.558681	1.556434	1.554242	1.552089	1.586745	1.584387	1.646941	1.644112
1.56159	1.559256	1.556988	1.554791	1.552639	1.587345	1.584963	1.647648	1.644821
1.562181	1.559839	1.557558	1.555346	1.553176	1.550967	1.54873	1.583174	1.580802
1.562774	1.560431	1.558143	1.555907	1.553709	1.551492	1.549266	1.583772	1.581392
1.563372	1.561028	1.558735	1.556474	1.554245	1.552016	1.549793	1.547568	1.545333
1.563971	1.56163	1.559335	1.557048	1.554783	1.552538	1.550312	1.548102	1.545914
1.564586	1.562239	1.559934	1.557626	1.555336	1.553077	1.550848	1.548648	1.54649
1.565223	1.562857	1.56053	1.558208	1.555908	1.553644	1.551413	1.549211	1.547051
1.565866	1.563476	1.561122	1.55879	1.556486	1.55422	1.551991	1.549784	1.547609
1.5665	1.564084	1.561703	1.55936	1.557054	1.554793	1.552575	1.550366	1.548166
1.567135	1.564694	1.562286	1.559934	1.557629	1.555373	1.553166	1.550954	1.548724
1.536745	1.53439	1.532068	1.560543	1.558237	1.555977	1.553763	1.551539	1.54929
1.537339	1.534973	1.532646	1.561181	1.558873	1.5566	1.554365	1.552124	1.549863
1.537955	1.535589	1.533264	1.56184	1.559508	1.557214	1.554959	1.55271	1.550457
1.538594	1.536245	1.533931	1.562525	1.56014	1.557813	1.555541	1.553294	1.551075
1.539242	1.5369	1.534592	1.532255	1.529928	1.558431	1.556141	1.55389	1.551691
1.539897	1.537543	1.535227	1.532875	1.530531	1.559079	1.556774	1.554508	1.552292
1.540555	1.538179	1.535848	1.533492	1.53115	1.559746	1.557422	1.555134	1.552892
1.506267	1.504082	1.501956	1.534106	1.531794	1.529508	1.527247	1.555757	1.553497
1.506853	1.504634	1.502484	1.534722	1.532451	1.530158	1.527851	1.556383	1.554108
1.507489	1.505238	1.503054	1.535359	1.533085	1.530778	1.528451	1.557022	1.554734
1.50817	1.505885	1.50366	1.536015	1.533701	1.531376	1.529046	1.557673	1.555373
1.508858	1.50653	1.504256	1.502071	1.499946	1.53198	1.529662	1.527391	1.525177
1.509555	1.507167	1.504829	1.502621	1.500494	1.532595	1.530308	1.528041	1.525786
1.51023	1.507802	1.50542	1.50319	1.501051	1.533219	1.530948	1.52868	1.526399
1.510849	1.508428	1.506054	1.503802	1.50163	1.533855	1.531556	1.529278	1.527012
1.511451	1.509055	1.506705	1.504432	1.502219	1.534497	1.532161	1.529867	1.527621
1.512073	1.509692	1.507355	1.505058	1.502807	1.500617	1.498487	1.530483	1.528212
1.512706	1.510337	1.508008	1.505687	1.503399	1.501193	1.499064	1.531113	1.528795
1.513357	1.510988	1.508657	1.506321	1.50401	1.501788	1.499646	1.53174	1.529402
1.514025	1.511644	1.509303	1.506959	1.50464	1.502401	1.500233	1.532364	1.530033

1.514713	1.512314	1.509955	1.507605	1.505285	1.50303	1.500834	1.498668	1.496527
1.515425	1.512999	1.510614	1.508263	1.50595	1.503682	1.501458	1.499268	1.497113
1.516148	1.513693	1.511281	1.508925	1.506615	1.504332	1.502079	1.499868	1.497701
1.484598	1.482309	1.480049	1.50958	1.507253	1.504949	1.502671	1.500445	1.498282
1.485234	1.482917	1.480631	1.51024	1.507888	1.505561	1.503262	1.501026	1.498868
1.485887	1.483547	1.481238	1.510921	1.508546	1.506203	1.503893	1.501646	1.499477
1.486556	1.484197	1.481867	1.51162	1.509222	1.506867	1.504555	1.502299	1.500106
1.487249	1.484875	1.482528	1.480222	1.477951	1.507563	1.505244	1.502972	1.500753
1.48797	1.485586	1.483229	1.48091	1.478626	1.508299	1.505965	1.503672	1.50142
1.488687	1.486293	1.483922	1.481591	1.479293	1.509035	1.506682	1.504367	1.502085
1.489387	1.486974	1.484582	1.482236	1.479926	1.509753	1.507376	1.505037	1.50273
1.490085	1.487657	1.485247	1.482885	1.480559	1.510478	1.508075	1.505713	1.503385
1.490786	1.488355	1.485941	1.483562	1.481215	1.511222	1.508799	1.506418	1.504072
1.491498	1.489069	1.486658	1.484263	1.481893	1.511984	1.509545	1.507146	1.504784
1.454709	1.452136	1.449578	1.48501	1.482623	1.480292	1.478011	1.507912	1.505531
1.455677	1.453117	1.450565	1.485797	1.4834	1.481051	1.478746	1.508713	1.506311
1.446612	1.444099	1.441593	1.448985	1.446458	1.481806	1.479472	1.509508	1.50709
1.447565	1.445052	1.442547	1.449912	1.447391	1.482548	1.48018	1.510286	1.507864
1.448511	1.446003	1.443506	1.450844	1.448331	1.44582	1.44331	1.44086	1.438485
1.449451	1.446965	1.444491	1.451801	1.449293	1.446779	1.444261	1.441794	1.439392
1.4504	1.447933	1.445484	1.452765	1.45026	1.447748	1.44523	1.44275	1.440313
1.451384	1.448913	1.446467	1.444005	1.441545	1.448707	1.446197	1.44371	1.441245
1.452391	1.449904	1.447447	1.444984	1.442529	1.449664	1.447167	1.444677	1.442187
1.453401	1.450902	1.448437	1.445973	1.44352	1.450622	1.44813	1.445636	1.443128
1.454417	1.451908	1.449438	1.446972	1.444519	1.45158	1.449085	1.446584	1.444067
1.455434	1.452906	1.450426	1.447958	1.445509	1.443059	1.440612	1.44755	1.44503
1.456439	1.453881	1.451385	1.448918	1.446483	1.444048	1.44162	1.448544	1.446032
1.457475	1.454882	1.452359	1.449886	1.447457	1.445036	1.442627	1.449539	1.447037
1.458588	1.455952	1.453384	1.450885	1.448444	1.446022	1.443626	1.450516	1.448028
1.459735	1.457053	1.454432	1.4519	1.449435	1.447006	1.444617	1.451483	1.449008
1.460859	1.458139	1.455474	1.452913	1.450426	1.447983	1.445591	1.452435	1.449964
1.461975	1.459223	1.456519	1.453928	1.451417	1.448956	1.446551	1.453374	1.4509
1.463095	1.460328	1.457597	1.45497	1.452415	1.449924	1.4475	1.454294	1.451821
1.46422	1.461457	1.458717	1.45604	1.453419	1.450884	1.448431	1.455191	1.452722
1.465337	1.462576	1.459826	1.45711	1.454434	1.45186	1.44938	1.456106	1.453635
1.466442	1.463671	1.460906	1.458165	1.45546	1.452862	1.450361	1.457054	1.454567
1.467539	1.464758	1.461983	1.459226	1.4565	1.453881	1.451359	1.45802	1.455516
1.468616	1.465839	1.463073	1.460302	1.457552	1.454911	1.452366	1.449868	1.447414
1.469692	1.466923	1.464174	1.461393	1.458624	1.455961	1.45339	1.450874	1.448412
1.47082	1.468041	1.4653	1.462518	1.459745	1.457061	1.454455	1.45191	1.449431
1.471989	1.469186	1.466448	1.463671	1.460909	1.458206	1.455555	1.452973	1.450471
1.473179	1.470344	1.467603	1.46482	1.462054	1.45933	1.456644	1.454032	1.451506
1.474381	1.471511	1.468769	1.465962	1.463172	1.460424	1.457715	1.45508	1.452532
1.475639	1.472705	1.46993	1.467096	1.46429	1.461524	1.458796	1.45614	1.453564
1.476966	1.47392	1.471064	1.468208	1.465414	1.462642	1.459896	1.457214	1.4546
1.478367	1.47518	1.472209	1.469326	1.466551	1.463776	1.461017	1.458311	1.455657
1.429529	1.426226	1.423169	1.470477	1.467719	1.464941	1.462173	1.459449	1.456766
1.431067	1.427571	1.424331	1.47166	1.468908	1.466127	1.463353	1.460616	1.457911
1.338202	1.334683	1.331438	1.422599	1.419881	1.467295	1.464516	1.461774	1.459062
1.339764	1.336106	1.332706	1.423837	1.421013	1.468449	1.465665	1.462926	1.460224

							0.725346	0.723004
							0.723814	0.721485
							0.722269	0.719997
							0.72069	0.718547
					0.723801	0.721445	1.090893	1.086582
					0.722269	0.72003	1.088468	1.084164
					0.720725	0.718637	1.086164	1.081818
					0.719323	0.717213	1.26982	1.265256
					0.717996	0.715779	1.267261	1.262804
			0.72082	0.718729	1.087398	1.083197	1.331248	1.326713
			0.719416	0.717229	1.084987	1.08086	1.328781	1.324264
0.486166	0.48498	0.483757	0.992182	0.988135	1.199031	1.194815	1.326307	1.32185
0.485213	0.484068	0.482911	0.989896	0.98599	1.196694	1.19239	1.323769	1.319469
0.484265	0.483144	0.482036	0.987675	0.983895	1.194401	1.190033	1.32131	1.317132
0.720396	0.718232	0.71611	1.084288	1.080203	1.26157	1.257125	1.632644	1.627467

0.719033	0.716818	0.714618	1.081972	1.077928	1.259238	1.25493	1.629967	1.624673
0.992258	0.988372	0.984709	1.195742	1.191597	1.504069	1.499311	1.678017	1.67251
0.989847	0.986124	0.982609	1.193412	1.189231	1.501337	1.496642	1.675091	1.669653
1.087286	1.083202	1.079279	1.457479	1.452761	1.631701	1.62659	1.720406	1.714989
1.085167	1.081031	1.076984	1.454781	1.450192	1.628928	1.623677	1.717235	1.711813
1.198996	1.194702	1.190445	1.502892	1.498312	1.678034	1.672641	1.791287	1.785614
1.196766	1.19252	1.188277	1.500338	1.495726	1.675135	1.66978	1.7882	1.782493
1.194545	1.190366	1.186168	1.497816	1.49312	1.672195	1.666906	1.785142	1.779439
1.458909	1.454157	1.449353	1.627964	1.622699	1.751861	1.746367	1.781984	1.776418
1.456329	1.451621	1.446865	1.625171	1.619746	1.748647	1.743171	1.778799	1.773432
1.504571	1.499808	1.49503	1.674062	1.668536	1.786744	1.78113	1.85528	1.8499
1.501835	1.497087	1.492363	1.671101	1.66564	1.783652	1.778034	1.852149	1.846824
1.683937	1.678661	1.673408	1.750621	1.745029	1.780573	1.775031	1.892969	1.887504
1.681073	1.675655	1.670262	1.747336	1.741877	1.777499	1.772148	1.889993	1.884471
1.710846	1.705101	1.699379	1.744115	1.738767	1.868989	1.86363	1.978082	1.971444
1.707871	1.702082	1.696304	1.741026	1.735731	1.865804	1.860548	1.974406	1.967897
1.704863	1.699094	1.69333	1.738051	1.732787	1.862664	1.85746	1.970688	1.96435
1.751971	1.746301	1.740653	1.794804	1.789462	1.859677	1.854432	1.966933	1.960712
1.748727	1.74326	1.737854	1.792034	1.78673	1.856796	1.85145	1.963164	1.95703
1.745499	1.740202	1.735018	1.864892	1.85938	1.9726	1.965978	2.035428	2.028679
1.742263	1.737118	1.732153	1.861934	1.856503	1.969088	1.962493	2.03161	2.024796
1.798717	1.793551	1.788588	1.858973	1.853626	1.965598	1.959034	2.046225	2.039325
1.795827	1.79067	1.7857	1.856017	1.850757	1.962135	1.955594	2.042391	2.035362
1.793016	1.787842	1.782825	1.853043	1.847866	1.958669	1.952157	2.038582	2.031472
1.831634	1.826335	1.821154	1.944076	1.937924	2.030639	2.023493	2.034815	2.027752
1.828789	1.823453	1.818216	1.940389	1.934334	2.026797	2.019698	2.031084	2.024141
1.82597	1.82063	1.815378	2.01669	2.010024	2.041365	2.034325	2.075744	2.068762
1.823174	1.817853	1.812613	2.01288	2.006192	2.037553	2.030564	2.071994	2.065207
1.949288	1.9427	1.936215	2.028817	2.022136	2.082221	2.075203	2.068332	2.06171
1.945818	1.939234	1.932741	2.024984	2.018278	2.07837	2.071488	2.064759	2.058272
2.0228	2.015511	2.008337	2.039525	2.032775	2.074497	2.067809	2.061258	2.054884
2.018953	2.011698	2.004533	2.035653	2.028781	2.070524	2.06414	2.057839	2.051555
2.015124	2.007902	2.000752	2.031829	2.024878	2.066639	2.060537	2.054464	2.048256
2.011305	2.004114	1.997001	2.076569	2.069566	2.063041	2.057061	2.087907	2.081771
2.007501	2.000341	1.993279	2.072889	2.066039	2.059614	2.053672	2.08451	2.078443
2.090425	2.083227	2.076115	2.06923	2.062548	2.056224	2.050314	2.104535	2.098483
2.08663	2.079439	2.072357	2.065601	2.059112	2.052895	2.046996	2.101176	2.095141
2.082848	2.075718	2.068716	2.062066	2.0557	2.09727	2.091356	2.13601	2.129848
2.079078	2.072074	2.065218	2.058639	2.052301	2.093872	2.088013	2.132574	2.126416
2.075326	2.068462	2.061768	2.102957	2.096619	2.141224	2.135198	2.129154	2.122999
2.07156	2.064842	2.058324	2.099557	2.093278	2.1378	2.131785	2.125751	2.119604
2.067857	2.061277	2.054919	2.09621	2.090007	2.134433	2.128411	2.12237	2.116227
2.112022	2.105473	2.099148	2.132718	2.12644	2.131186	2.125113	2.119028	2.112873
2.10854	2.102073	2.095817	2.129383	2.123244	2.128015	2.121865	2.115715	2.109538
2.122209	2.115833	2.109638	2.137057	2.130941	2.124787	2.118594	2.134146	2.127882
2.118828	2.112582	2.106489	2.133833	2.127687	2.121513	2.115306	2.130805	2.124526
2.138366	2.132036	2.125833	2.130576	2.124406	2.152316	2.146004	2.195902	2.189052
2.135158	2.128804	2.122546	2.127263	2.12109	2.148931	2.142607	2.192327	2.185567
2.131941	2.125557	2.119244	2.123935	2.117758	2.145533	2.139206	2.188764	2.182106
2.139561	2.133191	2.126875	2.133705	2.127479	2.154804	2.148456	2.185218	2.178646

2.136162	2.129831	2.123552	2.130359	2.124109	2.151375	2.145054	2.181703	2.175207
2.145861	2.13953	2.133245	2.160601	2.154247	2.191653	2.184889	2.178259	2.17181
2.142431	2.136105	2.129823	2.157118	2.150778	2.188057	2.181412	2.174875	2.168451
2.17285	2.166429	2.16005	2.153696	2.147379	2.184534	2.17799	2.17609	2.169657
2.169443	2.163029	2.156656	2.150324	2.14404	2.181069	2.174608	2.172754	2.166366
2.166122	2.159717	2.153356	2.191086	2.184341	2.182361	2.175914	2.169538	2.163193
2.162959	2.156559	2.150215	2.187864	2.181227	2.179297	2.172877	2.166529	2.160227
2.159786	2.1534	2.147081	2.184664	2.17814	2.17626	2.16986	2.163532	2.157269
2.156444	2.150088	2.14381	2.181307	2.174874	2.173037	2.166667	2.189812	2.183594
2.15302	2.14671	2.140485	2.177894	2.171535	2.169736	2.163398	2.186533	2.18033
2.194339	2.187599	2.180984	2.179126	2.172759	2.186808	2.180521	2.18324	2.177046
2.190692	2.184066	2.177587	2.175764	2.169405	2.183445	2.177187	2.179923	2.17373
2.189394	2.182824	2.176418	2.19277	2.186426	2.189072	2.182837	2.176638	2.170436
2.186046	2.179498	2.173128	2.189447	2.183136	2.185801	2.179569	2.173373	2.167145
2.205628	2.19902	2.192596	2.186228	2.179943	2.182621	2.176389	2.1702	2.163968
2.202484	2.195947	2.189544	2.183201	2.176931	2.179618	2.173387	2.167216	2.160997
2.199374	2.192916	2.186532	2.180208	2.173945	2.176633	2.170398	2.164257	2.15811
2.196103	2.189709	2.183363	2.185972	2.179705	2.173455	2.167193	2.161112	2.155178
2.192773	2.186433	2.180131	2.182747	2.176463	2.170196	2.163896	2.157895	2.152266
2.189592	2.183283	2.177008	2.179638	2.173358	2.167085	2.160745	2.161439	2.15612
2.186548	2.180247	2.173983	2.176634	2.17038	2.164103	2.157709	2.158476	2.153429
2.183431	2.177134	2.170875	2.173533	2.167294	2.173418	2.167187	2.161517	2.156713
2.180174	2.173875	2.167611	2.170254	2.164009	2.17032	2.164482	2.159158	2.154556
2.176948	2.170651	2.164389	2.167016	2.160765	2.167272	2.16186	2.156902	2.15249
2.18279	2.176515	2.17027	2.17623	2.169968	2.164296	2.159224	2.154576	2.150369
2.179757	2.173506	2.167287	2.173252	2.166997	2.161465	2.156689	2.152311	2.148297
2.188765	2.182548	2.176393	2.170286	2.164286	2.158988	2.154429	2.150235	2.146363
2.185523	2.179302	2.173194	2.167359	2.161786	2.156781	2.152378	2.1483	2.14453
2.182408	2.176206	2.17016	2.164556	2.159329	2.154577	2.150331	2.14637	2.142677
2.179432	2.173263	2.167287	2.161869	2.156913	2.152375	2.148291	2.144443	2.1408
2.176418	2.170353	2.16453	2.159322	2.154617	2.150275	2.146331	2.142576	2.138973
2.173268	2.167413	2.161875	2.156924	2.15247	2.148313	2.144484	2.140806	2.137238
2.170172	2.164568	2.159354	2.15466	2.15043	2.146429	2.142683	2.139055	2.135508
2.167265	2.161918	2.157019	2.152554	2.148502	2.144604	2.14088	2.141981	2.138416
2.164498	2.159411	2.154811	2.150551	2.146644	2.14282	2.139091	2.140182	2.136602
2.161889	2.157001	2.152606	2.148503	2.144719	2.140985	2.137309	2.138438	2.134861
2.159434	2.154698	2.150431	2.146443	2.142759	2.139119	2.135535	2.136738	2.133172
2.157114	2.152555	2.148415	2.144523	2.1409	2.142115	2.138572	2.135039	2.131469
2.154939	2.150595	2.146588	2.142772	2.139168	2.140408	2.136881	2.133348	2.129741
2.152849	2.148698	2.144813	2.145899	2.142276	2.138695	2.135168	2.131636	2.128031
2.150826	2.146829	2.143058	2.144181	2.140525	2.136921	2.133374	2.129859	2.126346
2.14886	2.144981	2.141303	2.142459	2.138778	2.135156	2.131589	2.128097	2.124691
2.151831	2.148002	2.144354	2.140688	2.137056	2.133464	2.129905	2.126435	2.123086
2.149936	2.146135	2.142507	2.138902	2.135354	2.131818	2.128282	2.124836	2.121519
2.14811	2.14436	2.140761	2.137195	2.133686	2.130189	2.126693	2.123275	2.119963
2.146349	2.142668	2.139103	2.135556	2.132052	2.128582	2.125143	2.121757	2.118422
2.144628	2.141003	2.137465	2.133933	2.13043	2.126977	2.123573	2.130347	2.12691
2.142946	2.139361	2.135844	2.132324	2.128822	2.12537	2.121964	2.128704	2.125323
2.14129	2.137732	2.134228	2.130714	2.127212	2.123764	2.120366	2.130848	2.127546
2.139654	2.136113	2.13261	2.129091	2.125578	2.122149	2.118801	2.129304	2.126044

2.13803	2.134491	2.13098	2.127458	2.123947	2.120548	2.117265	2.127799	2.124578
2.136401	2.132838	2.129296	2.129848	2.126407	2.123075	2.119857	2.121393	2.11821
2.134766	2.131163	2.127583	2.128184	2.124837	2.121575	2.118399	2.119936	2.116814
2.137104	2.133525	2.12998	2.126584	2.123302	2.120091	2.116952	2.118485	2.115399
2.135391	2.131863	2.128395	2.125048	2.121803	2.118626	2.115519	2.117044	2.113965
2.133721	2.130239	2.12684	2.123538	2.120327	2.121914	2.118725	2.121689	2.118615
2.132121	2.12867	2.125312	2.122048	2.118874	2.120444	2.117248	2.12021	2.11718
2.130553	2.12713	2.123806	2.120577	2.117437	2.118992	2.115793	2.118754	2.115765
2.129001	2.125609	2.122318	2.114327	2.111202	2.123632	2.120461	2.134321	2.131108
2.127464	2.124105	2.120846	2.112879	2.109781	2.122211	2.119081	2.13286	2.129633
2.121163	2.117832	2.114593	2.127061	2.123916	2.120797	2.117701	2.132778	2.129556
2.119612	2.11633	2.11313	2.125596	2.122482	2.119392	2.116325	2.131316	2.128083
2.11808	2.114843	2.111681	2.124134	2.121031	2.119796	2.116777	2.129831	2.126609
2.116568	2.11337	2.110245	2.122672	2.119553	2.118333	2.115348	2.128318	2.125133
2.130795	2.127556	2.124387	2.123041	2.119933	2.129976	2.126787	2.134668	2.131281
2.129297	2.126088	2.122951	2.121635	2.118543	2.128486	2.125279	2.133013	2.129723
2.127828	2.124644	2.121535	2.120248	2.117174	2.127019	2.123788	2.131386	2.128189
2.128167	2.125023	2.121957	2.118874	2.115801	2.125545	2.122317	2.129826	2.126689
2.126771	2.123641	2.120592	2.117512	2.114435	2.124074	2.120866	2.128315	2.125221
2.12537	2.122259	2.119224	2.11615	2.113072	2.125762	2.122565	2.126844	2.123782
2.123967	2.120878	2.117857	2.11479	2.111713	2.124289	2.121137	2.125413	2.122372
2.122572	2.119499	2.116488	2.113439	2.110386	2.122865	2.119742	2.123999	2.120974
2.121185	2.118119	2.115113	2.112102	2.109104	2.121508	2.118388	2.1226	2.119588
2.119806	2.116746	2.113741	2.123367	2.120182	2.127573	2.124348	2.147645	2.14416
2.118429	2.115375	2.112371	2.121915	2.118791	2.12617	2.122962	2.146066	2.14257
2.117072	2.11402	2.111011	2.120467	2.117398	2.124777	2.121586	2.144491	2.14097
2.115771	2.11271	2.109677	2.119029	2.116008	2.123375	2.120208	2.147752	2.144158
2.114504	2.111429	2.108364	2.117605	2.114623	2.121973	2.118833	2.146144	2.142515
2.113196	2.110132	2.107071	2.123877	2.120822	2.12058	2.117459	2.144542	2.140876
2.111849	2.108819	2.105799	2.122525	2.119448	2.119193	2.116087	2.142942	2.139238
2.123061	2.119861	2.116698	2.121204	2.118102	2.148724	2.145061	2.141372	2.137623
2.121592	2.118479	2.11542	2.11992	2.116792	2.147165	2.14354	2.139861	2.136054
2.12015	2.11711	2.114139	2.118629	2.115487	2.145609	2.142005	2.138329	2.134477
2.126605	2.123436	2.120393	2.117284	2.114159	2.144027	2.140396	2.136701	2.132838
2.125198	2.122039	2.119002	2.115918	2.112824	2.142445	2.138762	2.135036	2.131186
2.123806	2.120662	2.117626	2.148263	2.144611	2.140913	2.137165	2.133405	2.129594
2.12244	2.119308	2.116258	2.146686	2.143106	2.139413	2.135589	2.131793	2.128044
2.121116	2.117993	2.114921	2.145102	2.141542	2.144918	2.14093	2.14993	2.146102
2.119844	2.116723	2.113619	2.143502	2.139905	2.143231	2.139253	2.148261	2.144518
2.151068	2.147348	2.143629	2.141936	2.138278	2.150258	2.146301	2.146643	2.142969
2.149501	2.145835	2.142179	2.140446	2.136693	2.148596	2.14471	2.145113	2.14148
2.147918	2.144302	2.140713	2.13895	2.135114	2.146947	2.14314	2.143607	2.140007
2.146346	2.142726	2.139131	2.146533	2.142662	2.149509	2.145726	2.142054	2.138503
2.144786	2.141135	2.137495	2.144872	2.141027	2.147865	2.144125	2.140498	2.137001
2.152837	2.149129	2.145413	2.143258	2.139464	2.146304	2.142598	2.139008	2.135565
2.151399	2.147624	2.143818	2.141676	2.137963	2.144816	2.141134	2.137573	2.134187
2.147153	2.143375	2.139562	2.140135	2.136444	2.143302	2.139688	2.136179	2.132814
2.145562	2.141837	2.1381	2.138647	2.134878	2.141727	2.138254	2.134839	2.131443
2.14399	2.140299	2.136618	2.141625	2.137808	2.140211	2.136838	2.133487	2.130069
2.142512	2.138772	2.135044	2.140103	2.136415	2.138844	2.13544	2.132069	2.128682

2.141045	2.137246	2.13346	2.138588	2.135061	2.137519	2.134055	2.130644	2.12729
2.139492	2.135703	2.131948	2.137112	2.133649	2.136119	2.132681	2.129276	2.125901
2.1379	2.134155	2.130477	2.135663	2.132219	2.134689	2.131313	2.127933	2.124508
2.136376	2.132654	2.129009	2.134224	2.13083	2.133293	2.129918	2.126528	2.123077
2.13492	2.131198	2.127544	2.132793	2.129481	2.131931	2.128497	2.125062	2.121605
2.13828	2.134573	2.130937	2.131401	2.128124	2.130542	2.127063	2.123602	2.120161
2.136686	2.133087	2.129587	2.130067	2.126742	2.129097	2.125598	2.122142	2.118761
2.135114	2.131616	2.128241	2.128734	2.125366	2.12767	2.12417	2.120727	2.117387
2.133635	2.130177	2.12684	2.127357	2.124022	2.126329	2.122852	2.119412	2.116033
2.132207	2.128764	2.125431	2.125969	2.122684	2.125012	2.121576	2.118143	2.114704
2.130796	2.127383	2.124076	2.124605	2.121308	2.123634	2.120252	2.143138	2.139158
2.129411	2.12603	2.122754	2.123257	2.119907	2.122219	2.118901	2.141704	2.137764
2.129621	2.126234	2.12294	2.121887	2.118535	2.120837	2.11753	2.140196	2.13631
2.128339	2.124906	2.121539	2.120488	2.117196	2.119496	2.116132	2.138598	2.134789
2.12705	2.123593	2.120175	2.116852	2.113601	2.118135	2.114737	2.138049	2.134283
2.12574	2.122309	2.118891	2.115533	2.11222	2.116717	2.113356	2.136563	2.132779
2.124404	2.121014	2.117621	2.114233	2.110853	2.115306	2.111976	2.135075	2.131282
2.123008	2.119658	2.11632	2.112945	2.109553	2.136676	2.132845	2.128421	2.124511
2.121591	2.118276	2.115	2.111659	2.108285	2.135232	2.131304	2.126753	2.12297
2.120255	2.116921	2.113632	2.113024	2.109671	2.133812	2.129871	2.129947	2.126161
2.118984	2.115584	2.112223	2.111633	2.108354	2.132418	2.128528	2.128558	2.124767
2.117718	2.114279	2.11087	2.110286	2.107043	2.130589	2.126491	2.127165	2.123346
2.116459	2.113014	2.109592	2.109001	2.105748	2.129097	2.125077	2.12576	2.121878
2.117992	2.114526	2.111076	2.107721	2.104436	2.132278	2.128292	2.12435	2.120426
2.116696	2.113252	2.109807	2.106417	2.103071	2.130667	2.126796	2.122941	2.119024
2.11537	2.11196	2.108534	2.105118	2.101716	2.129064	2.125287	2.121511	2.117631
2.113981	2.110612	2.107243	2.129874	2.125696	2.127574	2.123776	2.120007	2.1162
2.112559	2.109234	2.105942	2.128435	2.124314	2.126154	2.12227	2.118467	2.114754
2.111217	2.107905	2.104644	2.131823	2.12772	2.12476	2.120835	2.117016	2.113352
2.109948	2.10662	2.103348	2.13033	2.126316	2.123399	2.11947	2.115647	2.111991
2.108687	2.105333	2.102041	2.128821	2.124901	2.122032	2.118105	2.114274	2.110603
2.107424	2.104031	2.100701	2.127275	2.123465	2.120652	2.116724	2.112874	2.109159
2.106167	2.102747	2.099393	2.125763	2.122036	2.119267	2.115349	2.11149	2.107734
2.111639	2.107879	2.104213	2.12435	2.120631	2.116836	2.112953	2.110145	2.106384
2.110289	2.106571	2.102948	2.12298	2.119238	2.115448	2.111603	2.108821	2.105068
2.133625	2.129491	2.125473	2.1216	2.117849	2.114072	2.110264	2.118955	2.11492
2.132146	2.128054	2.124081	2.120222	2.116466	2.112704	2.108934	2.117538	2.113501
2.13071	2.126643	2.122691	2.11884	2.115085	2.111338	2.107601	2.116118	2.112085
2.129321	2.12526	2.121302	2.117453	2.113703	2.109971	2.106263	2.114692	2.110672
2.127935	2.12388	2.119915	2.116074	2.112337	2.108622	2.104933	2.11327	2.109265
2.126542	2.122495	2.118533	2.114709	2.110997	2.107297	2.103613	2.11185	2.107863
2.125147	2.121108	2.117148	2.113345	2.109666	2.105985	2.102303	2.110438	2.106467
2.123751	2.119714	2.115748	2.111969	2.108331	2.116185	2.112218	2.109044	2.105078
2.122354	2.118317	2.114347	2.110594	2.106999	2.114769	2.11082	2.107657	2.103692
2.120952	2.116927	2.112968	2.10924	2.105677	2.113355	2.109415	2.10626	2.102302
2.119545	2.115541	2.111609	2.107905	2.104366	2.111942	2.108006	2.104855	2.100907
2.118149	2.1142	2.110329	2.106638	2.103082	2.110538	2.106605	2.103457	2.099512
2.116764	2.112918	2.109156	2.105464	2.101837	2.109146	2.105213	2.102067	2.098117
2.115388	2.111631	2.107963	2.115697	2.111716	2.107757	2.103823	2.101816	2.097878
2.114001	2.110293	2.106677	2.114312	2.11033	2.106369	2.102431	2.100414	2.09648

2.112647	2.108963	2.105368	2.112904	2.108936	2.104981	2.101039	2.099012	2.095089
2.111397	2.107704	2.104083	2.111501	2.107544	2.104787	2.100849	2.101518	2.097425
2.110207	2.106484	2.102804	2.110096	2.106151	2.103388	2.099446	2.100065	2.096003
2.12073	2.116681	2.112678	2.109854	2.10592	2.101989	2.098056	2.09832	2.094292
2.119342	2.115294	2.111299	2.10846	2.104516	2.10059	2.096677	2.096915	2.092949
2.117947	2.113903	2.109915	2.107068	2.103125	2.099104	2.09521	2.095551	2.091615
2.116547	2.112509	2.108518	2.105671	2.101744	2.097747	2.093913	2.094248	2.090295
2.115145	2.111116	2.10713	2.104292	2.100392	2.096416	2.092623	2.092935	2.08896
2.114785	2.110788	2.10684	2.102945	2.099099	2.095128	2.091314	2.091557	2.087581
2.113376	2.109397	2.105475	2.101621	2.097824	2.093848	2.089991	2.090153	2.086183
2.112091	2.108113	2.104183	2.10018	2.096349	2.096329	2.092282	2.088741	2.084784
2.110886	2.106897	2.102939	2.098879	2.094992	2.094883	2.090844	2.087322	2.083387
2.109491	2.105481	2.101494	2.097547	2.093635	2.09346	2.089436	2.085941	2.082052
2.108091	2.104094	2.100119	2.096179	2.092272	2.09205	2.08805	2.084595	2.080784
2.106677	2.102693	2.098737	2.094823	2.090946	2.090698	2.086725	2.083298	2.079527
2.105243	2.101273	2.097346	2.093488	2.089687	2.08945	2.085506	2.082083	2.078274
2.103846	2.099886	2.095984	2.092173	2.088436	2.088202	2.084286	2.080866	2.077013
2.102573	2.098614	2.09471	2.094681	2.090763	2.086858	2.082965	2.079101	2.075264
2.10135	2.097389	2.093475	2.09336	2.089396	2.085477	2.081604	2.077768	2.073959
2.100045	2.096096	2.092191	2.092014	2.088036	2.084118	2.080261	2.076452	2.072691
2.098688	2.094761	2.090878	2.090652	2.086681	2.082772	2.078925	2.075146	2.071451
2.097392	2.093483	2.089619	2.089358	2.085404	2.081507	2.077664	2.073895	2.070226
2.096181	2.092285	2.088433	2.088156	2.084233	2.08035	2.076501	2.072718	2.069022
2.09895	2.094889	2.090878	2.086929	2.083036	2.079167	2.075319	2.071528	2.067809
2.097559	2.093519	2.089531	2.085602	2.081729	2.077879	2.074047	2.070269	2.066553
2.096164	2.092147	2.088182	2.084272	2.080414	2.076583	2.072773	2.069015	2.065313
2.094875	2.090877	2.086933	2.08304	2.079196	2.075379	2.071586	2.067846	2.064163
2.093631	2.08965	2.085726	2.081847	2.078015	2.074212	2.070435	2.066712	2.063059
2.092305	2.088342	2.084437	2.080571	2.076749	2.072962	2.069207	2.065513	2.061907
2.090908	2.086963	2.083074	2.07922	2.075407	2.071638	2.067909	2.064255	2.060717
2.089577	2.085652	2.081783	2.077941	2.074137	2.070382	2.066672	2.063039	2.059525
2.088346	2.084444	2.080599	2.076773	2.072976	2.069228	2.065525	2.061884	2.058329
2.087144	2.083261	2.079437	2.075625	2.071837	2.068097	2.064403	2.060752	2.057152
2.085958	2.082085	2.078271	2.074471	2.070697	2.066971	2.063294	2.059649	2.056036
2.084761	2.080896	2.077087	2.073298	2.06954	2.06583	2.062171	2.058537	2.05492
2.083463	2.079611	2.075811	2.072035	2.06829	2.064593	2.060947	2.057322	2.053706
2.082107	2.078269	2.074481	2.070717	2.066986	2.0633	2.059663	2.056047	2.052436
2.080845	2.077019	2.073239	2.06949	2.065774	2.0621	2.058469	2.054857	2.051252
2.079678	2.075862	2.072087	2.068353	2.064658	2.060994	2.057364	2.053755	2.050155
2.078523	2.074716	2.070948	2.067227	2.063549	2.059895	2.056266	2.05266	2.049069
2.077378	2.073582	2.069823	2.066112	2.062444	2.058797	2.055172	2.051573	2.047995
2.076205	2.072419	2.06867	2.064968	2.061308	2.057668	2.054046	2.050454	2.046893
2.074931	2.071152	2.067413	2.063722	2.060073	2.056439	2.052818	2.049231	2.045688
2.073629	2.069858	2.066131	2.062449	2.05881	2.055182	2.05156	2.047979	2.044455
2.07243	2.068671	2.064956	2.061281	2.057645	2.05402	2.050401	2.046828	2.043317
2.071306	2.067562	2.06386	2.060189	2.05655	2.052927	2.049314	2.045748	2.042248
2.070197	2.066467	2.062776	2.059109	2.05547	2.051851	2.048249	2.044694	2.041201
2.069107	2.065387	2.061703	2.058041	2.054405	2.050795	2.047209	2.043667	2.04018
2.068021	2.064312	2.060636	2.056979	2.053345	2.049744	2.046175	2.042647	2.039165
2.066931	2.063234	2.059569	2.055917	2.052285	2.048692	2.045138	2.041623	2.038149

2.065846	2.062158	2.058504	2.054856	2.051225	2.04764	2.044102	2.0406	2.037134
2.064773	2.061089	2.057435	2.053789	2.050159	2.046583	2.043063	2.039576	2.036116
2.063708	2.060023	2.056366	2.05272	2.049092	2.045525	2.042023	2.038552	2.035097
2.062638	2.058957	2.055302	2.051661	2.048042	2.044483	2.040989	2.037524	2.034075
2.061566	2.05789	2.054241	2.050611	2.047007	2.043455	2.03996	2.036494	2.033049
2.060495	2.056823	2.053178	2.04956	2.045971	2.042429	2.038937	2.035473	2.032031
2.059427	2.055757	2.052115	2.048508	2.044934	2.041405	2.037923	2.034465	2.031026
2.058352	2.054686	2.051046	2.047451	2.043894	2.040379	2.036907	2.033455	2.030024
2.057265	2.0536	2.049965	2.046383	2.042847	2.039346	2.035881	2.032437	2.029019
2.056173	2.052511	2.048881	2.045314	2.041798	2.03831	2.034851	2.031415	2.028013
2.055087	2.05143	2.047806	2.044249	2.040748	2.037272	2.03382	2.030393	2.027004
2.054004	2.050353	2.046736	2.043189	2.0397	2.036233	2.032789	2.029371	2.025994
2.052919	2.049279	2.045674	2.042138	2.038659	2.035202	2.031767	2.028357	2.024991
2.051831	2.048207	2.044621	2.041098	2.037628	2.034179	2.030754	2.027352	2.023992
2.050744	2.047137	2.043572	2.040061	2.036598	2.033159	2.029743	2.026351	2.022997
2.049658	2.04607	2.042526	2.039027	2.03557	2.032138	2.028732	2.025352	2.022008
2.048574	2.045004	2.041482	2.037994	2.034543	2.031119	2.027724	2.024354	2.021019
2.047481	2.043928	2.040431	2.036958	2.033515	2.030102	2.026718	2.023358	2.020022
2.0464	2.042859	2.039385	2.035924	2.03249	2.029086	2.025714	2.022361	2.019023
2.045367	2.041824	2.038357	2.034899	2.031467	2.028069	2.024705	2.021359	2.018022
2.044376	2.040818	2.037346	2.033883	2.030449	2.027052	2.023693	2.020354	2.017019
2.043446	2.039889	2.036409	2.032929	2.029471	2.026058	2.022694	2.019353	2.016018
2.042598	2.039058	2.035564	2.032049	2.028535	2.025085	2.021703	2.018352	2.015013
2.04171	2.038191	2.034696	2.031168	2.027629	2.024154	2.020744	2.017374	2.01403
2.040722	2.037229	2.033759	2.030268	2.026771	2.023296	2.019835	2.016423	2.01307
2.039707	2.036234	2.03279	2.029345	2.025906	2.022444	2.018947	2.015505	2.01215
2.038704	2.035234	2.031793	2.028368	2.02496	2.021531	2.01807	2.014655	2.011316
2.037715	2.034233	2.030781	2.027362	2.023973	2.020585	2.017194	2.013834	2.010521
2.036805	2.033292	2.029805	2.026374	2.02299	2.019619	2.016261	2.012931	2.009642
2.035966	2.032402	2.028856	2.025397	2.022003	2.01863	2.015276	2.011961	2.008697
2.035086	2.031512	2.027951	2.024483	2.021083	2.017698	2.014325	2.011003	2.007751
2.034145	2.030622	2.027114	2.023665	2.02026	2.016848	2.013419	2.010055	2.006796
2.033191	2.029712	2.026254	2.022825	2.01942	2.015994	2.012535	2.009147	2.005883
2.032228	2.028746	2.025303	2.021884	2.01849	2.015092	2.011681	2.008326	2.00507
2.031277	2.027782	2.02434	2.020923	2.017536	2.014171	2.010827	2.007522	2.004276
2.030396	2.026908	2.023463	2.020024	2.016603	2.013234	2.009915	2.006633	2.003395
2.029551	2.026079	2.022628	2.019157	2.015683	2.012289	2.008976	2.005705	2.00247
2.028632	2.025178	2.021739	2.018273	2.0148	2.011409	2.008106	2.004834	2.001578
2.027638	2.024204	2.020797	2.017377	2.013959	2.010599	2.007306	2.004021	2.000715
2.029496	2.026097	2.022725	2.016478	2.013086	2.009745	2.006463	2.003178	1.999854
2.028611	2.025215	2.021839	2.015579	2.012152	2.008801	2.005533	2.002275	1.998991
2.027722	2.024323	2.020941	2.014673	2.011219	2.00786	2.004606	2.00137	1.998122
2.026762	2.023354	2.019977	2.013746	2.010333	2.006997	2.003749	2.000507	1.997236
2.025787	2.022368	2.018996	2.012814	2.009458	2.006152	2.002906	1.999652	1.996346
2.024871	2.021453	2.018075	2.014707	2.011358	2.005246	2.002005	1.998761	1.995473
2.023993	2.020582	2.017192	2.013798	2.010413	2.004303	2.001068	1.997846	1.994613
2.025283	2.021865	2.018455	2.012885	2.009502	2.003415	2.000181	1.996972	1.993772
2.024403	2.020955	2.017515	2.011963	2.00863	2.00259	1.999356	1.996146	1.992956
2.023493	2.020036	2.016589	2.011059	2.007768	2.004495	2.001242	1.9953	1.992113
2.022519	2.019096	2.015693	2.010191	2.006917	2.003634	2.000339	1.994394	1.991204

2.02155	2.018167	2.014811	2.00933	2.006057	2.002761	1.999432	1.993491	1.990296
2.020657	2.01729	2.013947	2.010627	2.007329	2.001854	1.998545	1.992646	1.989455
2.019802	2.016437	2.01309	2.009746	2.006412	2.000935	1.997669	1.991829	1.988643
2.018947	2.015566	2.012201	2.008853	2.005525	2.002235	1.998988	1.991009	1.987803
2.0181	2.014684	2.011285	2.00795	2.004662	2.001392	1.998146	1.990191	1.986945
2.017222	2.0138	2.010401	2.007081	2.003819	2.000554	1.99729	1.991839	1.988534
2.016297	2.012907	2.009558	2.006258	2.003	1.999718	1.996407	1.99093	1.987652
2.015385	2.012032	2.00873	2.00545	2.002192	1.998894	1.995546	1.992231	1.988981
2.014513	2.011188	2.007913	2.004648	2.001397	1.998098	1.994738	1.991418	1.988177
2.013667	2.010364	2.007106	2.003848	2.000596	1.997299	1.993945	1.99063	1.987397
2.008358	2.005075	2.00183	2.00303	1.999746	1.996447	1.993124	1.989836	1.986616
2.00755	2.004271	2.001025	2.002204	1.998871	1.995568	1.992292	1.989044	1.985841
2.006742	2.003469	2.00022	2.001395	1.998038	1.994732	1.991479	1.988251	1.98506
2.005939	2.00267	1.99942	2.000605	1.997241	1.993933	1.99068	1.987457	1.984275
2.005132	2.001853	1.998587	1.999787	1.996439	1.993139	1.98989	1.986664	1.983473
2.004315	2.001	1.997695	1.99892	1.995618	1.992346	1.989105	1.985872	1.982641
2.003504	2.000156	1.996815	1.998062	1.994803	1.991557	1.988328	1.985089	1.981819
2.002714	1.999354	1.995997	1.997256	1.994008	1.990774	1.98756	1.984326	1.981042
2.001932	1.99857	1.995208	1.996475	1.993225	1.989996	1.986797	1.983573	1.980284
2.001148	1.997787	1.994421	1.9957	1.99245	1.989225	1.986036	1.982817	1.979525
2.000364	1.997006	1.993642	1.994934	1.991683	1.98846	1.985276	1.982061	1.978768
1.999576	1.99622	1.992857	1.989567	1.986326	1.987689	1.984512	1.981301	1.978007
1.998785	1.995428	1.992065	1.988776	1.985538	1.986913	1.983744	1.980538	1.977243
1.997993	1.994636	1.991272	1.987984	1.984749	1.986135	1.982973	1.979772	1.976475
1.997203	1.993843	1.990477	1.98719	1.983957	1.985355	1.9822	1.979002	1.975704
1.996415	1.993055	1.989689	1.986402	1.98317	1.984578	1.981427	1.978232	1.974933
1.995634	1.992275	1.988912	1.985627	1.982394	1.983809	1.980659	1.977464	1.974163
1.994857	1.991506	1.988152	1.984867	1.98163	1.983047	1.979894	1.976697	1.973396
1.994084	1.990759	1.987434	1.984146	1.980887	1.982295	1.979133	1.975932	1.97263
1.993314	1.990033	1.986754	1.983461	1.980163	1.981553	1.978376	1.975168	1.971868
1.992537	1.989284	1.986038	1.982746	1.979429	1.976174	1.972991	1.97441	1.971128
1.991749	1.988506	1.985275	1.98199	1.978677	1.975419	1.972225	1.973658	1.970421
1.990968	1.987725	1.984504	1.981235	1.977942	1.974687	1.971473	1.97291	1.969706
1.990201	1.986952	1.983739	1.980501	1.977253	1.974003	1.970749	1.972163	1.96896
1.989447	1.986186	1.982976	1.979768	1.976569	1.973328	1.970033	1.971422	1.968208
1.988702	1.985429	1.982212	1.979017	1.975846	1.972612	1.969304	1.970687	1.967463
1.987972	1.984685	1.981451	1.978259	1.975101	1.971876	1.968573	1.969963	1.966726
1.987289	1.983981	1.980717	1.977513	1.974356	1.971143	1.967867	1.964598	1.961343
1.986653	1.983316	1.980006	1.976777	1.973606	1.970408	1.967186	1.963944	1.960654
1.98599	1.98265	1.979326	1.976075	1.972875	1.969676	1.966483	1.963257	1.959946
1.98528	1.981981	1.978695	1.975425	1.97217	1.968938	1.965738	1.96251	1.9592
1.984563	1.9813	1.978052	1.974774	1.971482	1.968222	1.965001	1.961763	1.958466
1.983846	1.980584	1.977349	1.974091	1.970824	1.967561	1.964302	1.961045	1.957774
1.983139	1.979862	1.976625	1.973392	1.970168	1.966914	1.963621	1.960346	1.957104
1.982481	1.979177	1.975911	1.972675	1.969467	1.966227	1.96295	1.959684	1.956442
1.981856	1.978516	1.9752	1.971946	1.968737	1.965517	1.962286	1.959048	1.955789
1.981203	1.977854	1.974524	1.97125	1.968018	1.964795	1.961585	1.958368	1.955124
1.98052	1.977192	1.973888	1.970591	1.967308	1.964058	1.960843	1.957642	1.95445
1.97985	1.976531	1.973239	1.96993	1.966619	1.963346	1.960111	1.956909	1.953753
1.979212	1.975872	1.972555	1.969252	1.965967	1.962685	1.959401	1.956167	1.953015

1.978574	1.975214	1.971872	1.968578	1.965318	1.962033	1.958709	1.955443	1.95228
1.977905	1.974552	1.97122	1.967919	1.964644	1.961355	1.958043	1.961955	1.958709
1.977227	1.97389	1.970579	1.967267	1.963964	1.960673	1.957394	1.961312	1.958032
1.976572	1.973233	1.969917	1.966607	1.963307	1.960018	1.956735	1.960643	1.957366
1.975933	1.97258	1.969242	1.965941	1.962668	1.959381	1.956071	1.959961	1.956715
1.975271	1.971919	1.968582	1.965284	1.962017	1.958734	1.955428	1.959314	1.956071
1.974575	1.971248	1.967942	1.964639	1.961345	1.958068	1.954807	1.958712	1.95543
1.973891	1.970578	1.967293	1.963991	1.960685	1.964679	1.961401	1.958124	1.954812
1.973241	1.969914	1.966611	1.963322	1.960051	1.964069	1.960816	1.957553	1.954244
1.972597	1.969254	1.965932	1.962661	1.959432	1.963472	1.960229	1.956973	1.953679
1.971925	1.968596	1.965296	1.962043	1.958828	1.962864	1.959599	1.956334	1.953063
1.971246	1.967946	1.964687	1.961449	1.958233	1.96225	1.958947	1.955664	1.952418
1.970617	1.967331	1.964088	1.968204	1.964903	1.961603	1.958302	1.955018	1.951769
1.970033	1.966748	1.963501	1.967583	1.964235	1.960926	1.957659	1.954394	1.951113
1.969444	1.966152	1.962889	1.96695	1.963586	1.960271	1.957012	1.95375	1.950457
1.968843	1.965529	1.962234	1.966292	1.962966	1.959652	1.956352	1.953067	1.9498
1.975775	1.972371	1.968979	1.965647	1.962357	1.959047	1.955708	1.952404	1.949161
1.975182	1.971773	1.968374	1.965046	1.961766	1.958458	1.955112	1.951802	1.948563
1.974582	1.971177	1.967784	1.964455	1.961171	1.957864	1.954528	1.951223	1.947982
1.973932	1.970544	1.967175	1.963837	1.960523	1.957215	1.95391	1.950628	1.947393
1.973253	1.969892	1.96656	1.963205	1.959844	1.956532	1.953273	1.950029	1.9468
1.972628	1.969279	1.965963	1.962597	1.959212	1.955896	1.952658	1.949426	1.946183
1.972059	1.968706	1.965384	1.962014	1.958626	1.955306	1.952063	1.948817	1.945537
1.971499	1.968143	1.964815	1.961442	1.958052	1.954727	1.951478	1.94822	1.944911
1.970943	1.967589	1.964261	1.960885	1.957486	1.954156	1.950905	1.947643	1.944326
1.970384	1.967031	1.963703	1.960324	1.956921	1.953587	1.950332	1.947067	1.943749
1.966776	1.963416	1.960081	1.959748	1.956348	1.953011	1.949749	1.946481	1.943165
1.966192	1.962823	1.959478	1.959156	1.955765	1.952428	1.949154	1.945879	1.942574
1.965586	1.962195	1.958823	1.958516	1.955155	1.951821	1.948515	1.945224	1.941943
1.964961	1.961537	1.958124	1.957839	1.954524	1.951194	1.947842	1.944527	1.941283
1.964352	1.960909	1.957473	1.957203	1.953914	1.950587	1.947208	1.94388	1.940658
1.96376	1.960315	1.956876	1.956615	1.95333	1.950003	1.946621	1.943291	1.940074
1.963179	1.959736	1.956297	1.95295	1.949661	1.949431	1.946052	1.942724	1.939508
1.96261	1.959165	1.955724	1.952377	1.949091	1.948869	1.945495	1.942171	1.938954
1.962053	1.958605	1.955164	1.951816	1.94853	1.948317	1.944952	1.941633	1.938413
1.961511	1.958056	1.954613	1.951263	1.947976	1.947771	1.944413	1.941099	1.937876
1.96098	1.957518	1.954079	1.950725	1.947432	1.947234	1.943887	1.940579	1.937352
1.960445	1.957007	1.953594	1.950234	1.946914	1.946719	1.9434	1.940107	1.936863
1.959909	1.95652	1.953158	1.949787	1.946422	1.946226	1.94295	1.939682	1.936408
1.959358	1.956005	1.95268	1.949306	1.945912	1.942586	1.939336	1.939218	1.935926
1.958783	1.955447	1.95214	1.948772	1.945376	1.942051	1.938809	1.9387	1.935404
1.958212	1.954884	1.951587	1.948227	1.944837	1.941516	1.938271	1.938165	1.934868
1.957652	1.954327	1.951033	1.947681	1.9443	1.940981	1.93773	1.937622	1.934322
1.957112	1.953783	1.950484	1.94714	1.943777	1.940459	1.937193	1.937078	1.933773
1.95663	1.953284	1.94996	1.94663	1.943302	1.939989	1.936693	1.936557	1.933242
1.956184	1.952813	1.949454	1.94614	1.942856	1.939549	1.936215	1.936051	1.932723
1.955702	1.952316	1.948938	1.945628	1.942365	1.93906	1.935707	1.932367	1.929054
1.955183	1.951793	1.948409	1.945095	1.941827	1.938522	1.935172	1.931846	1.928574
1.954664	1.951283	1.947908	1.944585	1.941295	1.937982	1.934636	1.931323	1.928079
1.954154	1.9508	1.947462	1.944122	1.940783	1.937445	1.9341	1.930791	1.927547

1.953644	1.950316	1.947011	1.943656	1.940274	1.936917	1.933582	1.930279	1.927025
1.953124	1.949799	1.946504	1.943144	1.939752	1.936405	1.933106	1.929823	1.926546
1.952611	1.949278	1.945977	1.94262	1.939236	1.935911	1.932652	1.929389	1.92609
1.952144	1.948796	1.945474	1.942123	1.938762	1.935449	1.93219	1.928928	1.925636
1.951714	1.948341	1.944985	1.941645	1.93832	1.935013	1.931726	1.92845	1.925189
1.951267	1.947889	1.944525	1.941186	1.937869	1.934565	1.931274	1.927995	1.924736
1.950797	1.947439	1.944102	1.940753	1.937404	1.934097	1.930837	1.927572	1.92428
1.95034	1.946993	1.943669	1.940315	1.936951	1.93364	1.930392	1.927708	1.924365
1.949917	1.94655	1.943201	1.939859	1.936528	1.933217	1.92993	1.927216	1.923865
1.949496	1.946109	1.942733	1.939402	1.936102	1.932787	1.929455	1.926711	1.923365
1.949046	1.945667	1.9423	1.93896	1.935641	1.932309	1.92896	1.926215	1.922895
1.948586	1.945225	1.941879	1.938522	1.935162	1.931808	1.928457	1.925726	1.922441
1.948155	1.944788	1.941432	1.938058	1.934676	1.93132	1.927987	1.925275	1.921995
1.947747	1.944357	1.940963	1.937571	1.934181	1.930837	1.927543	1.924859	1.921559
1.94732	1.943925	1.940523	1.937127	1.933737	1.930396	1.927108	1.924432	1.921126
1.946859	1.94349	1.940126	1.936747	1.933363	1.930006	1.926677	1.923979	1.920696
1.946406	1.943057	1.939723	1.936366	1.932997	1.929634	1.926269	1.92355	1.920277
1.945987	1.942627	1.939281	1.935945	1.932618	1.929274	1.925907	1.923178	1.919869
1.945574	1.942198	1.938834	1.935515	1.932227	1.928909	1.925557	1.922828	1.919476
1.945131	1.941762	1.938412	1.935093	1.931798	1.928493	1.925179	1.922478	1.919117
1.944678	1.941326	1.938004	1.934674	1.931348	1.928049	1.924784	1.922127	1.918778
1.94427	1.940907	1.937579	1.934247	1.930925	1.928297	1.92502	1.921729	1.918402
1.943907	1.940505	1.937135	1.933811	1.930526	1.927905	1.924589	1.92128	1.91799
1.943543	1.94012	1.936724	1.933407	1.930146	1.927532	1.924187	1.92086	1.917579
1.943173	1.939761	1.936367	1.933055	1.929799	1.92719	1.923839	1.920496	1.917173
1.942787	1.939396	1.936018	1.932707	1.929442	1.926834	1.923491	1.920141	1.916779
1.942359	1.938992	1.935645	1.932324	1.929025	1.926416	1.923105	1.91978	1.916417
1.941923	1.938581	1.935269	1.931935	1.928595	1.92598	1.922702	1.919407	1.916061
1.941542	1.93821	1.93491	1.931571	1.928216	1.925586	1.922285	1.918984	1.915664
1.941201	1.937866	1.934561	1.931223	1.92787	1.92522	1.921859	1.918525	1.915241
1.940862	1.937506	1.934177	1.930843	1.927512	1.924863	1.921471	1.91812	1.914856
1.940525	1.937123	1.933746	1.930422	1.927137	1.924518	1.921127	1.917776	1.914516
1.940193	1.936755	1.933341	1.930023	1.926772	1.924176	1.920793	1.917443	1.914181
1.93987	1.93642	1.932992	1.929671	1.926422	1.923835	1.920457	1.917111	1.913847
1.939544	1.936094	1.932664	1.929338	1.926083	1.923499	1.920126	1.91678	1.913504
1.936802	1.933384	1.929974	1.929011	1.925754	1.923171	1.919797	1.91644	1.913128
1.936447	1.93305	1.929653	1.928693	1.925433	1.92285	1.919471	1.916097	1.91274
1.936103	1.932721	1.929335	1.932466	1.929154	1.922528	1.919148	1.915765	1.912384
1.935765	1.932394	1.929021	1.93216	1.928841	1.922207	1.918827	1.915442	1.912053
1.935434	1.93207	1.928705	1.931853	1.928528	1.925159	1.92174	1.915119	1.911725
1.935113	1.93175	1.928387	1.931544	1.928216	1.924843	1.921421	1.914793	1.911398
1.93479	1.931428	1.928066	1.92573	1.922456	1.924527	1.921103	1.917676	1.914245
1.934461	1.9311	1.92774	1.925408	1.922134	1.924212	1.920786	1.917358	1.913927
1.934131	1.930771	1.927411	1.925084	1.921809	1.923896	1.920471	1.917043	1.91361
1.933808	1.930444	1.927081	1.924757	1.921484	1.92358	1.920158	1.916732	1.913296
1.93349	1.93012	1.92675	1.924429	1.921157	1.923263	1.919848	1.916425	1.912984
1.933181	1.929799	1.92642	1.924101	1.920829	1.922947	1.919537	1.916117	1.912677
1.932882	1.929481	1.926092	1.923771	1.9205	1.922631	1.919223	1.915808	1.912373
1.932579	1.929166	1.925772	1.92345	1.920175	1.922315	1.91891	1.915503	1.91209
1.932266	1.928852	1.925456	1.923132	1.91985	1.921997	1.918594	1.915204	1.911841

1.931949	1.928548	1.925163	1.922838	1.91954	1.921687	1.918285	1.914909	1.91159
1.932689	1.929308	1.925949	1.9226	1.919263	1.91592	1.912565	1.914608	1.911302
1.932372	1.92904	1.925732	1.922377	1.918997	1.915631	1.912273	1.914319	1.911009
1.932057	1.928744	1.925459	1.922105	1.918715	1.915354	1.912023	1.914084	1.910754
1.931747	1.928433	1.925151	1.921803	1.918423	1.915087	1.911803	1.913887	1.910522
1.931486	1.928157	1.924855	1.921502	1.918126	1.914804	1.911546	1.913647	1.910264
1.931284	1.927922	1.92457	1.921198	1.917819	1.914498	1.911246	1.913358	1.909976
1.931066	1.927678	1.924291	1.920912	1.917541	1.914219	1.910955	1.907675	1.904347
1.930803	1.9274	1.92401	1.920651	1.917317	1.913997	1.910693	1.907383	1.904048
1.930528	1.927125	1.923747	1.920409	1.917102	1.913778	1.910435	1.907097	1.903763
1.930257	1.926883	1.923537	1.920193	1.916857	1.913513	1.910159	1.906823	1.903519
1.929992	1.926659	1.923349	1.919987	1.916599	1.913231	1.909881	1.906562	1.903297
1.929768	1.926436	1.923118	1.919739	1.916327	1.912959	1.909634	1.906335	1.903071
1.929578	1.926217	1.922856	1.919459	1.916043	1.912692	1.909412	1.906136	1.902847
1.929363	1.925978	1.922588	1.919189	1.915788	1.912451	1.909188	1.905919	1.902609
1.929115	1.925708	1.922309	1.918931	1.915572	1.912246	1.90896	1.905668	1.902346
1.92886	1.925445	1.922051	1.91869	1.915356	1.912038	1.908739	1.905437	1.902109
1.928595	1.925205	1.921841	1.918476	1.915118	1.911802	1.908537	1.905261	1.901943
1.928345	1.92498	1.921644	1.91827	1.914883	1.911568	1.908338	1.905091	1.901786
1.928158	1.924775	1.92142	1.918049	1.914677	1.911365	1.908124	1.90487	1.901563
1.928005	1.924583	1.921189	1.917823	1.914487	1.911179	1.907905	1.904625	1.901311
1.927799	1.924376	1.920981	1.917616	1.914278	1.910971	1.907698	1.904419	1.901105
1.927543	1.924155	1.920797	1.917425	1.914054	1.910743	1.907503	1.904249	1.900943
1.927323	1.923945	1.920598	1.917222	1.91384	1.910528	1.907298	1.904053	1.900749
1.927164	1.92375	1.920363	1.916994	1.913646	1.910337	1.907075	1.903805	1.900496
1.927016	1.923573	1.920148	1.916779	1.913453	1.910143	1.906855	1.903565	1.900255
1.926866	1.92343	1.919998	1.916604	1.913239	1.909919	1.906652	1.903381	1.900081
1.926706	1.923294	1.919872	1.91645	1.913032	1.909697	1.906455	1.90321	1.899927
1.926496	1.923113	1.919723	1.916306	1.912875	1.909521	1.906253	1.902997	1.899739
1.926254	1.922902	1.919563	1.91617	1.91275	1.909377	1.906051	1.902763	1.899534
1.92607	1.922707	1.919363	1.915996	1.912604	1.909235	1.905886	1.902578	1.899339
1.925946	1.922536	1.919145	1.915776	1.912436	1.9091	1.905766	1.902448	1.899152
1.925823	1.922383	1.918957	1.915588	1.912262	1.90894	1.90563	1.902316	1.898982
1.925698	1.922266	1.918833	1.915438	1.91207	1.908732	1.90546	1.902175	1.898844
1.925554	1.922144	1.918723	1.915304	1.911889	1.908527	1.905277	1.902015	1.898705
1.925352	1.921973	1.918595	1.915186	1.911761	1.908393	1.90509	1.901807	1.89853
1.925132	1.921784	1.918457	1.915072	1.911656	1.90827	1.904909	1.901585	1.898345
1.924976	1.921613	1.918277	1.914911	1.91153	1.908154	1.904774	1.901439	1.89819
1.924869	1.921454	1.918064	1.914713	1.911391	1.908048	1.904674	1.901338	1.898081
1.92477	1.921324	1.917901	1.914558	1.911272	1.907949	1.904581	1.90124	1.897958
1.924683	1.921231	1.917799	1.914457	1.911177	1.907858	1.904496	1.901144	1.897811
1.92459	1.921143	1.91771	1.914367	1.911084	1.907767	1.904412	1.901052	1.897679
1.924476	1.921045	1.917621	1.91428	1.910994	1.907678	1.90433	1.900971	1.897583
1.924359	1.920948	1.917535	1.914191	1.91089	1.907572	1.904238	1.900888	1.897499
1.922003	1.918575	1.915134	1.914075	1.910735	1.907411	1.90411	1.900788	1.897408
1.921908	1.918485	1.915045	1.913948	1.910556	1.907224	1.903964	1.900679	1.897317
1.92182	1.918402	1.914962	1.913845	1.910425	1.907087	1.903846	1.900578	1.897227
1.921741	1.918324	1.914886	1.913762	1.910334	1.906992	1.90375	1.900484	1.89714
1.921665	1.918249	1.914813	1.911362	1.907904	1.90691	1.903667	1.9004	1.89706
1.921592	1.918178	1.914743	1.911294	1.907837	1.90684	1.903596	1.90033	1.89699

1.921266	1.917857	1.914429	1.911226	1.907772	1.906773	1.903528	1.900261	1.89692
1.921189	1.917779	1.914352	1.911151	1.907697	1.906694	1.903452	1.900186	1.896847
1.921111	1.917701	1.914274	1.911077	1.907624	1.906617	1.903376	1.900111	1.896773
1.920293	1.916866	1.913423	1.911008	1.907561	1.904197	1.900929	1.900038	1.8967
1.920217	1.916791	1.913351	1.910945	1.907509	1.904146	1.900871	1.899967	1.896628
1.920144	1.916718	1.913283	1.910201	1.906781	1.904138	1.90084	1.897526	1.894159
1.920072	1.916645	1.913219	1.910177	1.906815	1.90418	1.90084	1.89749	1.894106
1.920012	1.916577	1.913153	1.91014	1.906822	1.904194	1.900826	1.897452	1.894057
1.919971	1.916516	1.913081	1.910076	1.906774	1.904151	1.900783	1.897406	1.894009
1.919932	1.916459	1.913014	1.910009	1.906711	1.90409	1.900728	1.897358	1.893973
1.919878	1.9164	1.912956	1.909595	1.906296	1.903311	1.899933	1.897307	1.893963
1.919823	1.916351	1.912913	1.909546	1.906233	1.903245	1.899868	1.897257	1.893963
1.919788	1.916336	1.91292	1.909543	1.9062	1.903198	1.899818	1.897209	1.893936
1.919768	1.916351	1.912968	1.909577	1.906191	1.903166	1.89978	1.897166	1.893891
1.919783	1.916378	1.913001	1.909597	1.906183	1.903151	1.899779	1.896432	1.893121
1.919844	1.916424	1.913016	1.909594	1.90617	1.903152	1.899824	1.896497	1.893148
1.919893	1.916457	1.913025	1.909599	1.906182	1.90282	1.899521	1.896551	1.893172
1.9199	1.916454	1.913022	1.90962	1.906244	1.902887	1.899555	1.896557	1.893165
1.919894	1.916447	1.913027	1.909653	1.906314	1.902958	1.899583	1.896554	1.893162
1.917533	1.91412	1.910738	1.909704	1.90635	1.90298	1.899592	1.896563	1.893192
1.917527	1.914153	1.910806	1.909762	1.906368	1.90298	1.899595	1.896579	1.89324
1.911818	1.90847	1.905142	1.907454	1.904052	1.902987	1.899624	1.896625	1.893284
1.911885	1.908511	1.905142	1.907444	1.904042	1.902998	1.899675	1.896699	1.893328
1.911925	1.90853	1.905138	1.907445	1.904059	1.900713	1.897417	1.894098	1.890714
1.911922	1.908516	1.905127	1.907459	1.904114	1.900779	1.89746	1.894116	1.890713
1.911914	1.908508	1.905133	1.901805	1.898515	1.900847	1.897509	1.894149	1.890736
1.911909	1.908529	1.905188	1.901861	1.898554	1.900891	1.897576	1.894234	1.890829
1.911917	1.908566	1.905256	1.901925	1.898593	1.900933	1.897646	1.894328	1.890936
1.911976	1.908614	1.905294	1.901969	1.898651	1.900996	1.897698	1.894371	1.890979
1.912066	1.908673	1.905319	1.902003	1.89872	1.90107	1.89774	1.894388	1.890988
1.912115	1.90872	1.905366	1.90205	1.898769	1.895473	1.892165	1.894442	1.891043
1.912119	1.908755	1.905434	1.902111	1.898798	1.895501	1.892228	1.894535	1.891147
1.912155	1.908802	1.905494	1.902167	1.898839	1.895542	1.892285	1.894608	1.891227
1.912249	1.908866	1.905525	1.902203	1.898904	1.89561	1.892327	1.894635	1.891249
1.912362	1.90895	1.905573	1.90225	1.898972	1.895678	1.892372	1.894668	1.89128
1.91248	1.90907	1.90568	1.902333	1.89902	1.895719	1.892434	1.889132	1.885783
1.912594	1.909202	1.905812	1.902435	1.899072	1.895758	1.892504	1.889231	1.885904
1.905961	1.902622	1.899285	1.902549	1.899171	1.895841	1.892568	1.889292	1.885995
1.905981	1.902672	1.899382	1.902675	1.899303	1.895956	1.892631	1.88933	1.886068
1.906042	1.902735	1.899456	1.902769	1.899419	1.896073	1.892724	1.889406	1.886145
1.906153	1.902811	1.899498	1.902824	1.899518	1.896196	1.892854	1.889526	1.886223
1.906268	1.902901	1.899559	1.896262	1.892999	1.896298	1.89298	1.889658	1.886324
1.906379	1.903018	1.899668	1.896346	1.893046	1.89635	1.893083	1.889798	1.886465
1.906477	1.903135	1.899793	1.896447	1.893102	1.896405	1.893185	1.88994	1.886619
1.899395	1.89608	1.892777	1.896574	1.893214	1.889937	1.886758	1.890074	1.886766
1.899419	1.896141	1.892894	1.896719	1.893358	1.890075	1.886882	1.8902	1.886908
1.899506	1.896242	1.893017	1.89686	1.893506	1.890212	1.886984	1.890285	1.887019
1.899646	1.896377	1.893147	1.897002	1.893665	1.890354	1.887065	1.890334	1.887103
1.899794	1.896502	1.893248	1.889957	1.886649	1.890496	1.88717	1.883891	1.88071
1.899946	1.896606	1.893304	1.890023	1.886763	1.890639	1.887306	1.88402	1.880834

1.900103	1.896722	1.893376	1.890102	1.886882	1.883617	1.880296	1.884161	1.880954
1.900266	1.896869	1.893504	1.890229	1.887019	1.88376	1.880441	1.8843	1.881049
1.900428	1.897028	1.893656	1.890378	1.887167	1.883907	1.880592	1.884445	1.881145
1.900574	1.897182	1.89381	1.890534	1.887323	1.884062	1.880747	1.884598	1.881276
1.90071	1.897336	1.893972	1.890699	1.887486	1.884223	1.880906	1.884759	1.881428
1.900859	1.897495	1.894134	1.890863	1.887648	1.884385	1.881069	1.87775	1.874423
1.90102	1.897657	1.894296	1.891025	1.88781	1.884549	1.881236	1.877918	1.874586
1.901186	1.897823	1.894461	1.891189	1.887974	1.884713	1.881403	1.878084	1.874749
1.901358	1.897995	1.894632	1.891358	1.888141	1.884878	1.881568	1.878248	1.874909
1.901527	1.898163	1.894799	1.891524	1.888306	1.885042	1.881731	1.87841	1.875069
1.901687	1.898321	1.894955	1.89168	1.888464	1.885202	1.881891	1.878569	1.875227
1.901844	1.898475	1.895107	1.891833	1.88862	1.885359	1.882049	1.878728	1.875386
1.902003	1.898635	1.895268	1.891993	1.888778	1.885517	1.882209	1.87889	1.875552
1.902164	1.898797	1.895434	1.892157	1.888936	1.885674	1.882369	1.879055	1.875724
1.902336	1.898962	1.895597	1.892317	1.889093	1.885832	1.882529	1.879226	1.875924
1.902523	1.899129	1.895754	1.892469	1.889247	1.88599	1.882691	1.879408	1.876161
1.902709	1.8993	1.895917	1.892628	1.889405	1.886149	1.882853	1.879584	1.876382
1.902889	1.899473	1.896083	1.89279	1.889565	1.886308	1.88301	1.879745	1.87656
1.903067	1.899654	1.896269	1.892971	1.889736	1.886472	1.883172	1.879907	1.876727
1.903241	1.899859	1.896505	1.893198	1.88993	1.886647	1.883344	1.880079	1.876898
1.903415	1.900074	1.896763	1.893448	1.890138	1.886834	1.88353	1.880265	1.877075
1.90359	1.900266	1.896976	1.893663	1.890345	1.887044	1.883759	1.880501	1.877287
1.903767	1.900441	1.897154	1.893853	1.890553	1.887277	1.884027	1.880782	1.877532
1.903994	1.900651	1.897344	1.894053	1.890783	1.887525	1.884283	1.881035	1.877759
1.904292	1.90091	1.897552	1.894271	1.891045	1.887796	1.884522	1.881245	1.877956
1.904585	1.901169	1.897767	1.894489	1.891295	1.888049	1.884748	1.881449	1.878161
1.90483	1.901395	1.897977	1.894693	1.891499	1.888251	1.884945	1.881652	1.878395
1.905061	1.90162	1.898205	1.894909	1.891696	1.888443	1.885144	1.881865	1.87864
1.905295	1.901875	1.898484	1.895175	1.891927	1.888665	1.885387	1.882122	1.878886
1.905531	1.902149	1.898796	1.895473	1.892179	1.888905	1.885656	1.882403	1.879133
1.905804	1.902433	1.899086	1.89576	1.892457	1.889175	1.885921	1.882656	1.879361
1.906117	1.902729	1.899355	1.896039	1.892765	1.88948	1.886184	1.882884	1.87957
1.906418	1.903028	1.899645	1.896328	1.893055	1.889757	1.88643	1.883108	1.879791
1.90669	1.903327	1.899976	1.89664	1.893315	1.889985	1.886643	1.883322	1.880034
1.90697	1.903628	1.900302	1.896942	1.893565	1.890206	1.886863	1.883552	1.880287
1.905575	1.902178	1.898786	1.897198	1.893798	1.890438	1.887121	1.883826	1.880547
1.905915	1.902495	1.899067	1.897449	1.894038	1.890686	1.887398	1.884117	1.880813
1.906216	1.902805	1.899389	1.895947	1.892491	1.890982	1.887675	1.884382	1.881083
1.906494	1.903111	1.899736	1.896311	1.892857	1.891316	1.88796	1.884637	1.881357
1.898872	1.895538	1.892214	1.89664	1.893209	1.891658	1.88828	1.884937	1.881646
1.899189	1.89583	1.892475	1.89693	1.893549	1.892013	1.888641	1.885285	1.88195
1.891461	1.888113	1.884765	1.897222	1.89387	1.890498	1.887102	1.88564	1.882275
1.89171	1.88838	1.885057	1.897534	1.89416	1.890795	1.887441	1.885998	1.88264
1.891958	1.888645	1.885347	1.897843	1.894444	1.891082	1.887762	1.886339	1.883003
1.892242	1.888917	1.885602	1.890674	1.887322	1.891391	1.888057	1.884722	1.881377
1.892539	1.889192	1.885848	1.89094	1.887622	1.891705	1.888337	1.884989	1.881685
1.88854	1.885215	1.881899	1.891234	1.887904	1.884577	1.88125	1.885288	1.881985
1.888789	1.885488	1.88221	1.891551	1.888172	1.884835	1.881542	1.885609	1.882275
1.889079	1.885794	1.88254	1.883457	1.880104	1.88513	1.881838	1.885916	1.882575
1.889423	1.886147	1.882899	1.883822	1.880456	1.885471	1.882128	1.886194	1.882881

1.887632	1.884364	1.88112	1.87993	1.876594	1.885831	1.882447	1.879113	1.875865
1.887956	1.884666	1.88139	1.88022	1.876926	1.886208	1.882819	1.879479	1.87623
1.888281	1.884964	1.881653	1.880505	1.877256	1.886591	1.883212	1.879871	1.876606
1.882927	1.879626	1.876326	1.880834	1.877598	1.88302	1.87967	1.880247	1.876944
1.883277	1.879976	1.876674	1.881186	1.877942	1.88338	1.880041	1.880619	1.877268
1.88358	1.880291	1.877005	1.879381	1.876122	1.874984	1.871719	1.88099	1.877618
1.883833	1.880572	1.877323	1.879691	1.87639	1.875255	1.872032	1.881358	1.877992
1.884122	1.880878	1.877653	1.874378	1.871079	1.875564	1.872365	1.877795	1.874455
1.884469	1.881225	1.878003	1.874733	1.87144	1.875937	1.872733	1.878173	1.874834
1.884833	1.881582	1.878355	1.875091	1.871811	1.876322	1.873106	1.878549	1.875213
1.885202	1.881941	1.878706	1.875444	1.872173	1.868933	1.865732	1.878927	1.87559
1.885574	1.882297	1.879047	1.875786	1.872527	1.86929	1.866081	1.879292	1.875958
1.885933	1.882627	1.879349	1.876091	1.872854	1.869618	1.866384	1.870886	1.867621
1.886284	1.882941	1.879625	1.876371	1.873162	1.869927	1.866659	1.871152	1.867925
1.88663	1.883271	1.879934	1.876683	1.87349	1.870256	1.866971	1.863715	1.86053
1.886968	1.883616	1.880278	1.877028	1.873838	1.870606	1.867323	1.864067	1.860881
1.887317	1.883971	1.880632	1.877383	1.874194	1.870965	1.867689	1.864436	1.861245
1.887687	1.884337	1.88099	1.87774	1.874555	1.871332	1.868066	1.864819	1.861622
1.888067	1.884711	1.881358	1.878107	1.874922	1.871704	1.86845	1.865208	1.862005
1.888448	1.885091	1.881736	1.87848	1.87529	1.872073	1.868824	1.865586	1.862379
1.888836	1.885482	1.882131	1.878869	1.875668	1.872447	1.869204	1.86597	1.862758
1.873735	1.870494	1.867261	1.879307	1.876081	1.872854	1.869627	1.8664	1.863171
1.874153	1.870932	1.867729	1.879787	1.876522	1.873286	1.870084	1.866869	1.863611
1.874557	1.871346	1.86816	1.880227	1.876939	1.873695	1.870507	1.867297	1.864023
1.874944	1.871732	1.868546	1.880617	1.877323	1.874076	1.870886	1.867675	1.864398
1.875324	1.87211	1.868921	1.865708	1.862488	1.874446	1.871252	1.86804	1.864766
1.87569	1.872482	1.869298	1.866079	1.862848	1.874806	1.871611	1.8684	1.86513
1.876059	1.872855	1.869673	1.866449	1.863206	1.875166	1.871969	1.868761	1.865503
1.876448	1.873239	1.870049	1.86682	1.863574	1.875537	1.872336	1.869137	1.865917
1.876853	1.873633	1.870428	1.867195	1.863951	1.875921	1.872713	1.869523	1.866351
1.877291	1.874054	1.870827	1.867595	1.864366	1.861175	1.858021	1.869919	1.86676
1.877764	1.874502	1.871245	1.86802	1.86482	1.86163	1.858447	1.870325	1.867148
1.87679	1.873529	1.87027	1.868437	1.865251	1.86206	1.858859	1.870727	1.867532
1.87719	1.873931	1.870673	1.868833	1.865638	1.862443	1.859243	1.871114	1.86791
1.87758	1.87433	1.871085	1.86924	1.86603	1.862831	1.85964	1.87152	1.868302
1.877976	1.874744	1.871526	1.869681	1.866455	1.863254	1.860079	1.856918	1.853763
1.878368	1.875157	1.871972	1.870132	1.866895	1.863694	1.860534	1.857376	1.854201
1.878749	1.87555	1.872384	1.86919	1.865987	1.864151	1.860982	1.857818	1.85465
1.879127	1.875931	1.872775	1.869608	1.866444	1.864619	1.861428	1.858252	1.855108
1.879552	1.876344	1.873171	1.87001	1.866865	1.863707	1.860532	1.858697	1.855556
1.880031	1.876789	1.873568	1.870391	1.867247	1.864106	1.86097	1.859156	1.855994
1.874554	1.871321	1.868099	1.870793	1.867637	1.864499	1.861387	1.859587	1.856413
1.874991	1.871765	1.868554	1.871238	1.868044	1.864885	1.861766	1.859964	1.856799
1.87543	1.872212	1.869015	1.871693	1.868468	1.865285	1.862149	1.860339	1.857189
1.875893	1.872662	1.869448	1.872132	1.868919	1.865733	1.862575	1.859435	1.856309
1.876365	1.873114	1.869877	1.872571	1.869385	1.866203	1.863023	1.859871	1.856758
1.876815	1.873567	1.870334	1.867136	1.863966	1.866657	1.863474	1.860319	1.8572
1.877253	1.874022	1.870812	1.867608	1.864416	1.867103	1.863932	1.86078	1.85764
1.870344	1.867124	1.863924	1.868063	1.864866	1.861692	1.85854	1.861228	1.858087
1.870825	1.867583	1.864351	1.868494	1.865316	1.862144	1.858972	1.861657	1.858542

1.871298	1.868038	1.864784	1.861586	1.858429	1.862591	1.859413	1.862104	1.859011
1.871741	1.868483	1.865235	1.862028	1.858853	1.863021	1.859871	1.862592	1.859505
1.872181	1.868927	1.865689	1.862473	1.859281	1.863458	1.860342	1.863095	1.860004
1.867178	1.863944	1.860723	1.862916	1.859738	1.856601	1.853509	1.857742	1.854664
1.867646	1.864396	1.861156	1.863363	1.860213	1.857091	1.854002	1.858232	1.855134
1.868097	1.864852	1.861621	1.858456	1.855342	1.857585	1.854483	1.851387	1.848292
1.868533	1.86531	1.862113	1.858964	1.855854	1.858087	1.854955	1.851836	1.848739
1.869002	1.865797	1.86262	1.85947	1.856345	1.858566	1.855422	1.852294	1.849187
1.869519	1.866326	1.863148	1.859972	1.856802	1.859009	1.855884	1.852763	1.849633
1.870045	1.866852	1.863663	1.860462	1.857258	1.854111	1.851029	1.853242	1.850093
1.870562	1.867344	1.864129	1.860923	1.857727	1.854589	1.851516	1.853736	1.850588
1.871073	1.867824	1.864585	1.861381	1.858207	1.85508	1.852007	1.854231	1.851092
1.871553	1.868305	1.865076	1.861878	1.85871	1.855575	1.852476	1.854689	1.851566
1.872018	1.868788	1.865588	1.862401	1.859231	1.856077	1.852937	1.855129	1.852027
1.872522	1.869301	1.866116	1.862935	1.859766	1.856602	1.853437	1.850308	1.847239
1.873066	1.869846	1.866661	1.863483	1.86032	1.857154	1.853979	1.850841	1.847769
1.873617	1.870402	1.867216	1.864031	1.860854	1.857681	1.854506	1.851368	1.848293
1.874169	1.87097	1.867785	1.864572	1.861345	1.858154	1.854995	1.851871	1.848801
1.87472	1.871534	1.86835	1.86511	1.861838	1.858629	1.855484	1.852372	1.849299
1.87526	1.872079	1.868897	1.865652	1.86237	1.859152	1.856	1.852876	1.849774
1.875796	1.872611	1.869428	1.866188	1.862915	1.859694	1.856529	1.853384	1.850246
1.876331	1.873126	1.869921	1.866687	1.863438	1.860228	1.857062	1.853909	1.850752
1.876865	1.873625	1.870383	1.867159	1.863948	1.860761	1.8576	1.854447	1.851285
1.869143	1.865895	1.862641	1.867672	1.864484	1.86131	1.858153	1.854999	1.851833
1.869719	1.866464	1.863202	1.868235	1.865051	1.861879	1.858722	1.855568	1.852399
1.865156	1.861911	1.858658	1.86056	1.857388	1.862449	1.859292	1.856137	1.852964
1.865704	1.862461	1.859211	1.86112	1.857949	1.863014	1.859857	1.856699	1.853519
1.866246	1.863007	1.859761	1.861678	1.858508	1.863576	1.860421	1.85726	1.854071
1.858945	1.855735	1.852519	1.86223	1.859061	1.855904	1.852763	1.857821	1.854624
1.859464	1.856255	1.853041	1.862779	1.85961	1.856455	1.853318	1.858379	1.855177
1.850617	1.847424	1.844229	1.858198	1.855044	1.857	1.853868	1.850718	1.847514
1.851143	1.847942	1.844742	1.858732	1.855576	1.85754	1.854411	1.851263	1.848053
1.851688	1.848481	1.84528	1.842136	1.839035	1.858093	1.854967	1.85182	1.848608
1.852256	1.849047	1.845846	1.842697	1.839587	1.858666	1.855542	1.852396	1.849184
1.852828	1.849626	1.846434	1.843277	1.840148	1.850705	1.847598	1.852972	1.849769
1.853394	1.850217	1.847054	1.843882	1.840711	1.851271	1.848157	1.853541	1.850367
1.853957	1.850809	1.847678	1.844492	1.841277	1.85184	1.848715	1.854105	1.850964
1.844125	1.841012	1.837925	1.845067	1.841838	1.844482	1.841379	1.846154	1.843047
1.844669	1.84155	1.838466	1.845626	1.842401	1.845046	1.841922	1.846701	1.843598
1.839653	1.836542	1.833477	1.846208	1.843006	1.839836	1.836695	1.847272	1.844159
1.840226	1.837103	1.834038	1.846815	1.843658	1.84049	1.837303	1.847865	1.844729
1.840819	1.837675	1.834596	1.831509	1.828429	1.841113	1.837897	1.840518	1.837401
1.841429	1.83825	1.835141	1.832045	1.82897	1.841676	1.838455	1.841078	1.837952
1.842064	1.838848	1.835699	1.832583	1.829502	1.84223	1.839019	1.841655	1.838521
1.842747	1.83949	1.836283	1.83314	1.83005	1.826937	1.823796	1.836461	1.833328
1.843446	1.840155	1.836893	1.833719	1.830613	1.827504	1.824396	1.8371	1.833954
1.833137	1.82987	1.826622	1.834355	1.831217	1.828093	1.824985	1.832584	1.82947
1.833761	1.830526	1.827306	1.835034	1.831856	1.828702	1.825571	1.833178	1.8301
1.834374	1.831151	1.827944	1.824762	1.821601	1.829335	1.826182	1.823075	1.820032
1.834965	1.831734	1.828527	1.825369	1.822247	1.829999	1.826821	1.823692	1.820644

1.835591	1.832342	1.82912	1.825972	1.822878	1.819756	1.816596	1.824329	1.821276
1.836293	1.833005	1.82974	1.82657	1.82347	1.820361	1.817237	1.825	1.821949
1.837005	1.833687	1.830384	1.827185	1.824062	1.820958	1.817873	1.825669	1.822618
1.825145	1.821883	1.818631	1.827855	1.824686	1.82156	1.818479	1.815424	1.812392
1.825782	1.822553	1.819332	1.828552	1.825328	1.822169	1.819078	1.816017	1.812976
1.826406	1.823188	1.819986	1.82922	1.825986	1.822821	1.81973	1.816662	1.813597
1.827015	1.823792	1.820604	1.829868	1.82666	1.823511	1.820426	1.817348	1.814247
1.827662	1.824436	1.82125	1.818065	1.814895	1.824172	1.821091	1.818012	1.814896
1.828368	1.82514	1.821939	1.818727	1.81552	1.824783	1.821705	1.818637	1.815543
1.776595	1.773756	1.770927	1.819397	1.816163	1.813007	1.809938	1.819267	1.816186
1.77711	1.774288	1.771482	1.820077	1.816862	1.813705	1.81061	1.819922	1.816813
1.777625	1.774819	1.772039	1.82076	1.817569	1.814411	1.81129	1.820583	1.817442
1.778206	1.775397	1.772613	1.82143	1.81822	1.81505	1.811924	1.821227	1.818098
1.778824	1.776002	1.773197	1.822094	1.818844	1.815658	1.812538	1.821864	1.818771
1.779426	1.776589	1.773772	1.770928	1.768073	1.816313	1.813186	1.810092	1.807031
1.780012	1.777162	1.774337	1.771499	1.768659	1.817013	1.813868	1.810759	1.807698
1.780607	1.777746	1.774912	1.77208	1.769255	1.766441	1.763633	1.811405	1.808338
1.781216	1.778352	1.775508	1.772681	1.769869	1.767042	1.764192	1.812006	1.808925
1.781828	1.778957	1.776098	1.773272	1.770467	1.767632	1.764755	1.812627	1.809534
1.78244	1.779541	1.776653	1.773813	1.771007	1.768182	1.765332	1.813323	1.81023
1.783048	1.780121	1.777207	1.77435	1.771535	1.768726	1.76592	1.814053	1.810962
1.783631	1.780714	1.777811	1.774953	1.772129	1.76932	1.766526	1.763751	1.761005
1.784198	1.781316	1.778451	1.775602	1.772767	1.769948	1.767144	1.764358	1.761596
1.784804	1.781941	1.779098	1.776251	1.773407	1.770573	1.767748	1.764941	1.762159
1.785453	1.782594	1.779756	1.776907	1.774056	1.771201	1.768337	1.765495	1.762685
1.776565	1.773723	1.770901	1.777538	1.774673	1.771801	1.768915	1.766054	1.763226
1.777216	1.774353	1.771502	1.778128	1.775233	1.772349	1.769474	1.766623	1.7638
1.777843	1.774958	1.772079	1.778697	1.77578	1.772893	1.770036	1.767203	1.764389
1.778385	1.775487	1.772606	1.769724	1.766848	1.773472	1.770623	1.767796	1.764988
1.778888	1.775982	1.773112	1.770251	1.767405	1.77407	1.771226	1.768402	1.7656
1.77944	1.776527	1.773655	1.770803	1.767975	1.774676	1.771843	1.769027	1.766231
1.780025	1.777107	1.774226	1.771376	1.768557	1.775293	1.772474	1.76967	1.766879
1.749988	1.747132	1.744313	1.741524	1.738768	1.766345	1.763558	1.7703	1.767513
1.750568	1.747726	1.744916	1.742122	1.739348	1.766941	1.764152	1.770912	1.768125
1.75116	1.748325	1.745519	1.74272	1.739934	1.737177	1.734451	1.761971	1.759197
1.751768	1.74892	1.746101	1.743301	1.740523	1.737769	1.73504	1.762586	1.7598
1.752387	1.749521	1.746683	1.743881	1.741113	1.738362	1.735631	1.763202	1.760406
1.746474	1.743622	1.740791	1.744473	1.741696	1.738936	1.736198	1.733467	1.730748
1.747098	1.744239	1.741395	1.745071	1.742275	1.739502	1.736754	1.734021	1.731315
1.747713	1.744844	1.741988	1.745666	1.742864	1.740084	1.737325	1.734584	1.731878
1.74832	1.745436	1.74257	1.746258	1.743463	1.74068	1.737908	1.735156	1.732437
1.748935	1.746043	1.74317	1.746863	1.744063	1.741274	1.738497	1.735739	1.733011
1.749564	1.746675	1.743801	1.747489	1.744662	1.741862	1.739093	1.736339	1.733608
1.750194	1.747307	1.744432	1.748118	1.745266	1.742456	1.739691	1.736941	1.734205
1.750814	1.747921	1.745045	1.74219	1.739356	1.743065	1.74029	1.73753	1.734783
1.751432	1.748532	1.745652	1.742797	1.739965	1.743679	1.74089	1.738118	1.735358
1.752061	1.749154	1.746268	1.743403	1.74056	1.744279	1.741492	1.73872	1.73595
1.752694	1.749783	1.746888	1.744008	1.741147	1.744872	1.742096	1.73933	1.736552
1.753319	1.750408	1.747513	1.744629	1.741761	1.738942	1.736178	1.739934	1.737154
1.753931	1.751026	1.748145	1.745269	1.742406	1.739577	1.736785	1.740532	1.737757

1.754552	1.75165	1.748774	1.745902	1.743043	1.740206	1.737393	1.741136	1.738361
1.755194	1.752284	1.749395	1.746518	1.743656	1.740817	1.738001	1.74175	1.738965
1.755837	1.752918	1.750016	1.747133	1.744269	1.741428	1.73861	1.742364	1.73957
1.75646	1.75354	1.75064	1.747759	1.744898	1.742049	1.739212	1.736399	1.733621
1.757082	1.754163	1.751269	1.748393	1.745534	1.742678	1.739817	1.736989	1.734219
1.737067	1.734204	1.731364	1.749025	1.746167	1.743308	1.740443	1.737611	1.734835
1.737705	1.73483	1.731971	1.74966	1.746798	1.743941	1.741088	1.738259	1.735466
1.73838	1.735499	1.732632	1.750355	1.747484	1.744625	1.741776	1.738946	1.736141
1.73911	1.736236	1.733374	1.751138	1.748254	1.745384	1.742529	1.739693	1.736883
1.739806	1.736934	1.734076	1.731232	1.7284	1.746111	1.74325	1.740408	1.73759
1.740411	1.737532	1.734666	1.73182	1.728991	1.746746	1.743886	1.741038	1.738199
1.740986	1.738096	1.735224	1.732376	1.72955	1.747348	1.744491	1.741639	1.738781
1.741566	1.738673	1.735804	1.73295	1.730114	1.747948	1.745093	1.742242	1.739381
1.742151	1.739257	1.736397	1.733535	1.730684	1.74855	1.745696	1.742849	1.739994
1.742813	1.739911	1.737049	1.734183	1.731327	1.749237	1.746383	1.743535	1.740677
1.743549	1.740632	1.737761	1.734894	1.732043	1.750008	1.747152	1.744299	1.741429
1.744238	1.741307	1.738426	1.735557	1.73271	1.750723	1.747862	1.745003	1.742133
1.744843	1.741901	1.739014	1.736143	1.733296	1.751345	1.748469	1.745605	1.74275
1.745447	1.742491	1.739591	1.736713	1.733864	1.751949	1.749063	1.746198	1.743364
1.746088	1.743107	1.740175	1.737281	1.734424	1.731592	1.728783	1.746826	1.744021
1.746758	1.743749	1.740782	1.737868	1.735001	1.732168	1.729368	1.747471	1.744692
1.733662	1.730656	1.727689	1.738551	1.735676	1.73283	1.730013	1.74815	1.745359
1.734438	1.731419	1.728439	1.739298	1.736414	1.733548	1.730697	1.748853	1.746026
1.735171	1.732142	1.729147	1.739997	1.737098	1.734215	1.731345	1.74953	1.746678
1.735854	1.732818	1.729807	1.740639	1.737718	1.734821	1.731949	1.750175	1.747308
1.697613	1.694774	1.691953	1.72754	1.724617	1.73547	1.732592	1.729752	1.72695
1.698349	1.695504	1.692674	1.728312	1.72537	1.736211	1.733317	1.730463	1.727653
1.699082	1.696232	1.693395	1.729088	1.726127	1.736955	1.734047	1.731181	1.728367
1.684856	1.682026	1.679212	1.691244	1.688454	1.737618	1.734706	1.731843	1.729042
1.685499	1.682669	1.679861	1.691878	1.689067	1.738266	1.735354	1.732497	1.729708
1.686196	1.683366	1.680561	1.692573	1.689752	1.68699	1.684287	1.733212	1.730398
1.686932	1.6841	1.681297	1.693311	1.690492	1.687724	1.685009	1.733974	1.731106
1.687631	1.684799	1.682002	1.679206	1.676423	1.688456	1.685729	1.69844	1.695734
1.688272	1.685443	1.682658	1.67989	1.677144	1.689187	1.68645	1.699157	1.696443
1.674702	1.671909	1.669162	1.680582	1.677855	1.675129	1.672399	1.684426	1.681745
1.675478	1.672664	1.669884	1.681296	1.678542	1.675792	1.673042	1.685063	1.6824
1.676284	1.673449	1.670631	1.682027	1.679236	1.676459	1.673688	1.685702	1.683049
1.677057	1.674215	1.671385	1.668592	1.665828	1.677193	1.674407	1.671671	1.669014
1.677809	1.67497	1.672139	1.669347	1.666583	1.677963	1.675166	1.672404	1.66969
1.678496	1.675655	1.672825	1.670048	1.667308	1.664564	1.661811	1.673155	1.670415
1.679119	1.676274	1.673447	1.670701	1.668011	1.665298	1.662557	1.673929	1.671186
1.679783	1.676936	1.67411	1.67137	1.668693	1.665994	1.663272	1.66056	1.657866
1.680516	1.677668	1.674837	1.672065	1.66934	1.666631	1.663944	1.661257	1.658555
1.68126	1.678416	1.675583	1.672779	1.670001	1.667273	1.664601	1.661929	1.659223
1.681995	1.67916	1.676337	1.673519	1.67071	1.667953	1.66525	1.662561	1.659864
1.682726	1.679904	1.677094	1.67427	1.671442	1.668654	1.665905	1.663186	1.660496
1.683444	1.680631	1.677827	1.675007	1.67218	1.669379	1.666604	1.66386	1.661146
1.68415	1.681346	1.678547	1.675738	1.672926	1.670125	1.667334	1.664565	1.661811
1.684824	1.682043	1.679273	1.676478	1.673669	1.670868	1.668074	1.665292	1.66251
1.685459	1.682716	1.680006	1.677227	1.674411	1.671609	1.668824	1.666042	1.663245

1.686135	1.683407	1.680728	1.677963	1.675152	1.672357	1.669577	1.666795	1.663992
1.686885	1.684136	1.681439	1.678683	1.6759	1.673117	1.670333	1.667546	1.664751
1.687659	1.684869	1.682131	1.679382	1.676636	1.673871	1.671083	1.668294	1.665509
1.662004	1.659187	1.656418	1.680034	1.677338	1.674601	1.67182	1.669033	1.66625
1.662748	1.659865	1.657027	1.680675	1.678026	1.675318	1.672548	1.669766	1.666982
1.631899	1.629239	1.626607	1.655	1.652384	1.676032	1.673271	1.670497	1.667713
1.632557	1.6299	1.627268	1.655734	1.653079	1.676742	1.673991	1.671226	1.668441
1.633206	1.630549	1.627917	1.625351	1.622835	1.651091	1.648383	1.67195	1.669163
1.633843	1.631182	1.628549	1.625988	1.62348	1.651806	1.649085	1.672669	1.669876
1.634483	1.631815	1.629178	1.626617	1.624112	1.652514	1.649786	1.647048	1.644289
1.635133	1.632451	1.6298	1.627225	1.624708	1.653195	1.650488	1.647761	1.64499
1.635786	1.63309	1.630427	1.627836	1.625302	1.653878	1.651187	1.648471	1.645692
1.636434	1.633737	1.631077	1.628476	1.625924	1.623376	1.620839	1.649155	1.646386
1.637085	1.634389	1.631738	1.62913	1.626562	1.623996	1.621438	1.649825	1.647076
1.637749	1.635039	1.632382	1.629771	1.627204	1.624629	1.622047	1.619463	1.616875
1.638425	1.635689	1.63301	1.630402	1.627852	1.625273	1.622663	1.620061	1.617476
1.639123	1.63636	1.633654	1.631039	1.628495	1.625913	1.623287	1.620674	1.618087
1.639849	1.637055	1.634307	1.631674	1.629126	1.626542	1.623919	1.621305	1.618707
1.640581	1.637771	1.635	1.632344	1.629772	1.627177	1.624556	1.621944	1.619345
1.641303	1.63852	1.63577	1.633086	1.630453	1.627822	1.625191	1.622586	1.620018
1.642026	1.63928	1.636563	1.63385	1.63115	1.628478	1.625833	1.623232	1.6207
1.642746	1.640009	1.637302	1.634575	1.631846	1.62915	1.626483	1.623872	1.621352
1.643471	1.640727	1.638015	1.635282	1.632546	1.629836	1.627146	1.624517	1.621991
1.644263	1.641497	1.638752	1.636	1.633251	1.630539	1.627859	1.625229	1.622673
1.645127	1.642321	1.639512	1.636725	1.633959	1.631257	1.628626	1.626013	1.623396
1.613459	1.610726	1.607973	1.637476	1.6347	1.632001	1.629392	1.626781	1.624126
1.61418	1.611451	1.608709	1.638269	1.635494	1.632783	1.630144	1.627511	1.624851
1.614883	1.612164	1.609444	1.639066	1.636298	1.633572	1.630893	1.628234	1.625583
1.545966	1.543755	1.541544	1.639835	1.637077	1.634345	1.631638	1.628965	1.626336
1.546501	1.544296	1.542102	1.6406	1.637851	1.635113	1.63238	1.629695	1.62709
1.547054	1.544856	1.542674	1.608938	1.606271	1.635875	1.633108	1.630402	1.627805
1.547622	1.545429	1.543256	1.609707	1.607014	1.636632	1.633827	1.631095	1.628493
1.548221	1.546017	1.543827	1.541633	1.53944	1.605042	1.602333	1.631858	1.629231
1.548859	1.546621	1.544384	1.542177	1.539991	1.605772	1.603115	1.632708	1.630027
1.549486	1.547224	1.544955	1.542737	1.540555	1.606514	1.603892	1.633549	1.630835
1.550076	1.547816	1.545554	1.543332	1.54114	1.607272	1.604632	1.634336	1.631649
1.55066	1.548411	1.546168	1.543945	1.541739	1.608036	1.605373	1.635123	1.632462
1.551258	1.549024	1.546798	1.54457	1.542346	1.540154	1.537997	1.603523	1.600931
1.551865	1.549645	1.547437	1.545205	1.542963	1.540763	1.538611	1.604314	1.601665
1.552483	1.55026	1.548054	1.545829	1.543597	1.541393	1.539219	1.605067	1.602384
1.55311	1.550871	1.548653	1.546446	1.544249	1.542042	1.539823	1.60579	1.603088
1.553746	1.551495	1.549271	1.547066	1.544877	1.542666	1.540427	1.538205	1.53601
1.554389	1.552137	1.549917	1.547691	1.54547	1.543249	1.541028	1.538819	1.536623
1.524157	1.522	1.519877	1.548319	1.546072	1.543844	1.541637	1.539439	1.537245
1.524779	1.522595	1.520439	1.548947	1.546714	1.544485	1.54226	1.540052	1.537868
1.525401	1.523189	1.520999	1.549579	1.547365	1.545139	1.542893	1.540672	1.538497
1.525974	1.523764	1.521582	1.51943	1.517303	1.545774	1.543539	1.541323	1.539137
1.526522	1.524328	1.522176	1.520017	1.517865	1.546403	1.544194	1.541991	1.539785
1.527112	1.524906	1.522746	1.520587	1.518441	1.547045	1.544838	1.542635	1.540427
1.527742	1.525496	1.523292	1.521139	1.51903	1.547699	1.545471	1.543257	1.541062

1.528374	1.526096	1.523858	1.521701	1.519604	1.517485	1.515338	1.543888	1.541698
1.528997	1.526708	1.524462	1.522281	1.520154	1.518033	1.515919	1.544543	1.542338
1.529629	1.527328	1.525072	1.522863	1.520701	1.518574	1.516492	1.545194	1.542977
1.53028	1.527952	1.525661	1.523432	1.521257	1.519124	1.517043	1.514958	1.512845
1.530943	1.528586	1.526257	1.524006	1.521819	1.519677	1.517584	1.515501	1.513417
1.497334	1.495178	1.493045	1.524618	1.522393	1.520227	1.518126	1.516045	1.513973
1.497942	1.495782	1.493645	1.52526	1.522978	1.520779	1.518669	1.516586	1.514517
1.498566	1.496403	1.494265	1.52593	1.523606	1.521376	1.519245	1.517149	1.515073
1.499209	1.497045	1.494911	1.526634	1.524281	1.522021	1.519858	1.517736	1.515641
1.49985	1.497681	1.495549	1.493396	1.491244	1.522684	1.520482	1.518327	1.516214
1.500475	1.498295	1.496156	1.494005	1.491859	1.523358	1.521105	1.518908	1.516784
1.501111	1.498915	1.496766	1.494617	1.492483	1.524045	1.521742	1.519504	1.517368
1.501781	1.499567	1.497401	1.49525	1.49312	1.524745	1.522407	1.520138	1.517983
1.502476	1.500239	1.498052	1.495894	1.493765	1.525454	1.523092	1.520798	1.518619
1.5032	1.500931	1.498713	1.496542	1.494414	1.492269	1.490095	1.521473	1.519253
1.503953	1.501643	1.499384	1.497196	1.495067	1.492916	1.490733	1.522163	1.519889
1.504715	1.502377	1.500088	1.497875	1.495723	1.493559	1.491379	1.52287	1.52055
1.505485	1.50314	1.500836	1.498585	1.49638	1.494196	1.492036	1.523604	1.521243
1.506262	1.503908	1.501591	1.499302	1.497044	1.494836	1.492685	1.490538	1.488351
1.50704	1.504669	1.502333	1.500011	1.497711	1.495471	1.493297	1.49114	1.488969
1.507829	1.505436	1.503079	1.500729	1.498397	1.496125	1.493917	1.491744	1.489596
1.474265	1.471722	1.469217	1.501471	1.49913	1.49684	1.494601	1.492403	1.490243
1.475269	1.472694	1.47016	1.502233	1.499898	1.497596	1.495327	1.493098	1.490909
1.440649	1.438225	1.435838	1.468614	1.466155	1.498364	1.496068	1.493813	1.491606
1.441576	1.439139	1.436739	1.469583	1.467128	1.499146	1.496826	1.494551	1.492335
1.442532	1.440076	1.437654	1.470566	1.468105	1.465641	1.463168	1.495301	1.493077
1.443528	1.441044	1.438587	1.471564	1.469085	1.466607	1.464126	1.496063	1.493831
1.444532	1.442021	1.43953	1.472569	1.470062	1.467568	1.465085	1.496833	1.494591
1.445527	1.442997	1.440477	1.473569	1.471021	1.46851	1.466035	1.463604	1.461228
1.446513	1.443973	1.441433	1.474577	1.471983	1.469451	1.466987	1.464563	1.46218
1.447475	1.444945	1.442409	1.439914	1.437451	1.470422	1.467953	1.465526	1.463137
1.448417	1.445911	1.4434	1.440894	1.438398	1.471415	1.46893	1.466494	1.464098
1.449347	1.446863	1.444374	1.441866	1.43935	1.472428	1.469921	1.467468	1.465066
1.450261	1.447797	1.44533	1.442827	1.440306	1.473462	1.470924	1.468448	1.466042
1.451179	1.448732	1.446283	1.443792	1.441277	1.438818	1.436412	1.469443	1.467029
1.452105	1.449668	1.447237	1.444763	1.442266	1.439804	1.43737	1.470458	1.468032
1.453044	1.450612	1.448194	1.44574	1.443266	1.440804	1.438342	1.471488	1.469043
1.454007	1.451576	1.449165	1.44673	1.444284	1.441821	1.439329	1.472531	1.470055
1.454988	1.452552	1.450142	1.447724	1.445305	1.442844	1.440326	1.473588	1.471071
1.45598	1.45353	1.45111	1.448695	1.446288	1.443833	1.441317	1.438846	1.436454
1.456984	1.454511	1.452071	1.449648	1.447241	1.444797	1.442306	1.439839	1.437402
1.457983	1.455485	1.453024	1.450591	1.448182	1.445746	1.44328	1.44082	1.438353
1.458972	1.456448	1.453963	1.451516	1.449103	1.446675	1.444232	1.441783	1.439302
1.459964	1.457419	1.454916	1.452458	1.450039	1.447616	1.445191	1.442753	1.440269
1.460953	1.458399	1.455896	1.453433	1.45101	1.448588	1.446173	1.443743	1.441269
1.461962	1.4594	1.456897	1.454428	1.451997	1.449573	1.447161	1.444739	1.442282
1.46303	1.460444	1.457921	1.455433	1.452984	1.450552	1.448139	1.445725	1.443294
1.464137	1.461522	1.458967	1.456452	1.45398	1.451534	1.449117	1.446709	1.444306
1.456406	1.453823	1.451299	1.457496	1.454998	1.452535	1.45011	1.4477	1.445303
1.45757	1.454965	1.452408	1.458565	1.456036	1.453551	1.451117	1.448698	1.446286

1.921266	1.917857	1.914429	1.911226	1.907772	1.906773	1.903528	1.900261	1.89692
1.921189	1.917779	1.914352	1.911151	1.907697	1.906694	1.903452	1.900186	1.896847
1.921111	1.917701	1.914274	1.911077	1.907624	1.906617	1.903376	1.900111	1.896773
1.920293	1.916866	1.913423	1.911008	1.907561	1.904197	1.900929	1.900038	1.8967
1.920217	1.916791	1.913351	1.910945	1.907509	1.904146	1.900871	1.899967	1.896628
1.920144	1.916718	1.913283	1.910201	1.906781	1.904138	1.90084	1.897526	1.894159
1.920072	1.916645	1.913219	1.910177	1.906815	1.90418	1.90084	1.89749	1.894106
1.920012	1.916577	1.913153	1.91014	1.906822	1.904194	1.900826	1.897452	1.894057
1.919971	1.916516	1.913081	1.910076	1.906774	1.904151	1.900783	1.897406	1.894009
1.919932	1.916459	1.913014	1.910009	1.906711	1.90409	1.900728	1.897358	1.893973
1.919878	1.9164	1.912956	1.909595	1.906296	1.903311	1.899933	1.897307	1.893963
1.919823	1.916351	1.912913	1.909546	1.906233	1.903245	1.899868	1.897257	1.893963
1.919788	1.916336	1.91292	1.909543	1.9062	1.903198	1.899818	1.897209	1.893936
1.919768	1.916351	1.912968	1.909577	1.906191	1.903166	1.89978	1.897166	1.893891
1.919783	1.916378	1.913001	1.909597	1.906183	1.903151	1.899779	1.896432	1.893121
1.919844	1.916424	1.913016	1.909594	1.90617	1.903152	1.899824	1.896497	1.893148
1.919893	1.916457	1.913025	1.909599	1.906182	1.90282	1.899521	1.896551	1.893172
1.9199	1.916454	1.913022	1.90962	1.906244	1.902887	1.899555	1.896557	1.893165
1.919894	1.916447	1.913027	1.909653	1.906314	1.902958	1.899583	1.896554	1.893162
1.917533	1.91412	1.910738	1.909704	1.90635	1.90298	1.899592	1.896563	1.893192
1.917527	1.914153	1.910806	1.909762	1.906368	1.90298	1.899595	1.896579	1.89324
1.911818	1.90847	1.905142	1.907454	1.904052	1.902987	1.899624	1.896625	1.893284
1.911885	1.908511	1.905142	1.907444	1.904042	1.902998	1.899675	1.896699	1.893328
1.911925	1.90853	1.905138	1.907445	1.904059	1.900713	1.897417	1.894098	1.890714
1.911922	1.908516	1.905127	1.907459	1.904114	1.900779	1.89746	1.894116	1.890713
1.911914	1.908508	1.905133	1.901805	1.898515	1.900847	1.897509	1.894149	1.890736
1.911909	1.908529	1.905188	1.901861	1.898554	1.900891	1.897576	1.894234	1.890829
1.911917	1.908566	1.905256	1.901925	1.898593	1.900933	1.897646	1.894328	1.890936
1.911976	1.908614	1.905294	1.901969	1.898651	1.900996	1.897698	1.894371	1.890979
1.912066	1.908673	1.905319	1.902003	1.89872	1.90107	1.89774	1.894388	1.890988
1.912115	1.90872	1.905366	1.90205	1.898769	1.895473	1.892165	1.894442	1.891043
1.912119	1.908755	1.905434	1.902111	1.898798	1.895501	1.892228	1.894535	1.891147
1.912155	1.908802	1.905494	1.902167	1.898839	1.895542	1.892285	1.894608	1.891227
1.912249	1.908866	1.905525	1.902203	1.898904	1.89561	1.892327	1.894635	1.891249
1.912362	1.90895	1.905573	1.90225	1.898972	1.895678	1.892372	1.894668	1.89128
1.91248	1.90907	1.90568	1.902333	1.89902	1.895719	1.892434	1.889132	1.885783
1.912594	1.909202	1.905812	1.902435	1.899072	1.895758	1.892504	1.889231	1.885904
1.905961	1.902622	1.899285	1.902549	1.899171	1.895841	1.892568	1.889292	1.885995
1.905981	1.902672	1.899382	1.902675	1.899303	1.895956	1.892631	1.88933	1.886068
1.906042	1.902735	1.899456	1.902769	1.899419	1.896073	1.892724	1.889406	1.886145
1.906153	1.902811	1.899498	1.902824	1.899518	1.896196	1.892854	1.889526	1.886223
1.906268	1.902901	1.899559	1.896262	1.892999	1.896298	1.89298	1.889658	1.886324
1.906379	1.903018	1.899668	1.896346	1.893046	1.89635	1.893083	1.889798	1.886465
1.906477	1.903135	1.899793	1.896447	1.893102	1.896405	1.893185	1.88994	1.886619
1.899395	1.89608	1.892777	1.896574	1.893214	1.889937	1.886758	1.890074	1.886766
1.899419	1.896141	1.892894	1.896719	1.893358	1.890075	1.886882	1.8902	1.886908
1.899506	1.896242	1.893017	1.89686	1.893506	1.890212	1.886984	1.890285	1.887019
1.899646	1.896377	1.893147	1.897002	1.893665	1.890354	1.887065	1.890334	1.887103
1.899794	1.896502	1.893248	1.889957	1.886649	1.890496	1.88717	1.883891	1.88071
1.899946	1.896606	1.893304	1.890023	1.886763	1.890639	1.887306	1.88402	1.880834

1.900103	1.896722	1.893376	1.890102	1.886882	1.883617	1.880296	1.884161	1.880954
1.900266	1.896869	1.893504	1.890229	1.887019	1.88376	1.880441	1.8843	1.881049
1.900428	1.897028	1.893656	1.890378	1.887167	1.883907	1.880592	1.884445	1.881145
1.900574	1.897182	1.89381	1.890534	1.887323	1.884062	1.880747	1.884598	1.881276
1.90071	1.897336	1.893972	1.890699	1.887486	1.884223	1.880906	1.884759	1.881428
1.900859	1.897495	1.894134	1.890863	1.887648	1.884385	1.881069	1.87775	1.874423
1.90102	1.897657	1.894296	1.891025	1.88781	1.884549	1.881236	1.877918	1.874586
1.901186	1.897823	1.894461	1.891189	1.887974	1.884713	1.881403	1.878084	1.874749
1.901358	1.897995	1.894632	1.891358	1.888141	1.884878	1.881568	1.878248	1.874909
1.901527	1.898163	1.894799	1.891524	1.888306	1.885042	1.881731	1.87841	1.875069
1.901687	1.898321	1.894955	1.89168	1.888464	1.885202	1.881891	1.878569	1.875227
1.901844	1.898475	1.895107	1.891833	1.88862	1.885359	1.882049	1.878728	1.875386
1.902003	1.898635	1.895268	1.891993	1.888778	1.885517	1.882209	1.87889	1.875552
1.902164	1.898797	1.895434	1.892157	1.888936	1.885674	1.882369	1.879055	1.875724
1.902336	1.898962	1.895597	1.892317	1.889093	1.885832	1.882529	1.879226	1.875924
1.902523	1.899129	1.895754	1.892469	1.889247	1.88599	1.882691	1.879408	1.876161
1.902709	1.8993	1.895917	1.892628	1.889405	1.886149	1.882853	1.879584	1.876382
1.902889	1.899473	1.896083	1.89279	1.889565	1.886308	1.88301	1.879745	1.87656
1.903067	1.899654	1.896269	1.892971	1.889736	1.886472	1.883172	1.879907	1.876727
1.903241	1.899859	1.896505	1.893198	1.88993	1.886647	1.883344	1.880079	1.876898
1.903415	1.900074	1.896763	1.893448	1.890138	1.886834	1.88353	1.880265	1.877075
1.90359	1.900266	1.896976	1.893663	1.890345	1.887044	1.883759	1.880501	1.877287
1.903767	1.900441	1.897154	1.893853	1.890553	1.887277	1.884027	1.880782	1.877532
1.903994	1.900651	1.897344	1.894053	1.890783	1.887525	1.884283	1.881035	1.877759
1.904292	1.90091	1.897552	1.894271	1.891045	1.887796	1.884522	1.881245	1.877956
1.904585	1.901169	1.897767	1.894489	1.891295	1.888049	1.884748	1.881449	1.878161
1.90483	1.901395	1.897977	1.894693	1.891499	1.888251	1.884945	1.881652	1.878395
1.905061	1.90162	1.898205	1.894909	1.891696	1.888443	1.885144	1.881865	1.87864
1.905295	1.901875	1.898484	1.895175	1.891927	1.888665	1.885387	1.882122	1.878886
1.905531	1.902149	1.898796	1.895473	1.892179	1.888905	1.885656	1.882403	1.879133
1.905804	1.902433	1.899086	1.89576	1.892457	1.889175	1.885921	1.882656	1.879361
1.906117	1.902729	1.899355	1.896039	1.892765	1.88948	1.886184	1.882884	1.87957
1.906418	1.903028	1.899645	1.896328	1.893055	1.889757	1.88643	1.883108	1.879791
1.90669	1.903327	1.899976	1.89664	1.893315	1.889985	1.886643	1.883322	1.880034
1.90697	1.903628	1.900302	1.896942	1.893565	1.890206	1.886863	1.883552	1.880287
1.905575	1.902178	1.898786	1.897198	1.893798	1.890438	1.887121	1.883826	1.880547
1.905915	1.902495	1.899067	1.897449	1.894038	1.890686	1.887398	1.884117	1.880813
1.906216	1.902805	1.899389	1.895947	1.892491	1.890982	1.887675	1.884382	1.881083
1.906494	1.903111	1.899736	1.896311	1.892857	1.891316	1.88796	1.884637	1.881357
1.898872	1.895538	1.892214	1.89664	1.893209	1.891658	1.88828	1.884937	1.881646
1.899189	1.89583	1.892475	1.89693	1.893549	1.892013	1.888641	1.885285	1.88195
1.891461	1.888113	1.884765	1.897222	1.89387	1.890498	1.887102	1.88564	1.882275
1.89171	1.88838	1.885057	1.897534	1.89416	1.890795	1.887441	1.885998	1.88264
1.891958	1.888645	1.885347	1.897843	1.894444	1.891082	1.887762	1.886339	1.883003
1.892242	1.888917	1.885602	1.890674	1.887322	1.891391	1.888057	1.884722	1.881377
1.892539	1.889192	1.885848	1.89094	1.887622	1.891705	1.888337	1.884989	1.881685
1.88854	1.885215	1.881899	1.891234	1.887904	1.884577	1.88125	1.885288	1.881985
1.888789	1.885488	1.88221	1.891551	1.888172	1.884835	1.881542	1.885609	1.882275
1.889079	1.885794	1.88254	1.883457	1.880104	1.88513	1.881838	1.885916	1.882575
1.889423	1.886147	1.882899	1.883822	1.880456	1.885471	1.882128	1.886194	1.882881

1.887632	1.884364	1.88112	1.87993	1.876594	1.885831	1.882447	1.879113	1.875865
1.887956	1.884666	1.88139	1.88022	1.876926	1.886208	1.882819	1.879479	1.87623
1.888281	1.884964	1.881653	1.880505	1.877256	1.886591	1.883212	1.879871	1.876606
1.882927	1.879626	1.876326	1.880834	1.877598	1.88302	1.87967	1.880247	1.876944
1.883277	1.879976	1.876674	1.881186	1.877942	1.88338	1.880041	1.880619	1.877268
1.88358	1.880291	1.877005	1.879381	1.876122	1.874984	1.871719	1.88099	1.877618
1.883833	1.880572	1.877323	1.879691	1.87639	1.875255	1.872032	1.881358	1.877992
1.884122	1.880878	1.877653	1.874378	1.871079	1.875564	1.872365	1.877795	1.874455
1.884469	1.881225	1.878003	1.874733	1.87144	1.875937	1.872733	1.878173	1.874834
1.884833	1.881582	1.878355	1.875091	1.871811	1.876322	1.873106	1.878549	1.875213
1.885202	1.881941	1.878706	1.875444	1.872173	1.868933	1.865732	1.878927	1.87559
1.885574	1.882297	1.879047	1.875786	1.872527	1.86929	1.866081	1.879292	1.875958
1.885933	1.882627	1.879349	1.876091	1.872854	1.869618	1.866384	1.870886	1.867621
1.886284	1.882941	1.879625	1.876371	1.873162	1.869927	1.866659	1.871152	1.867925
1.88663	1.883271	1.879934	1.876683	1.87349	1.870256	1.866971	1.863715	1.86053
1.886968	1.883616	1.880278	1.877028	1.873838	1.870606	1.867323	1.864067	1.860881
1.887317	1.883971	1.880632	1.877383	1.874194	1.870965	1.867689	1.864436	1.861245
1.887687	1.884337	1.88099	1.87774	1.874555	1.871332	1.868066	1.864819	1.861622
1.888067	1.884711	1.881358	1.878107	1.874922	1.871704	1.86845	1.865208	1.862005
1.888448	1.885091	1.881736	1.87848	1.87529	1.872073	1.868824	1.865586	1.862379
1.888836	1.885482	1.882131	1.878869	1.875668	1.872447	1.869204	1.86597	1.862758
1.873735	1.870494	1.867261	1.879307	1.876081	1.872854	1.869627	1.8664	1.863171
1.874153	1.870932	1.867729	1.879787	1.876522	1.873286	1.870084	1.866869	1.863611
1.874557	1.871346	1.86816	1.880227	1.876939	1.873695	1.870507	1.867297	1.864023
1.874944	1.871732	1.868546	1.880617	1.877323	1.874076	1.870886	1.867675	1.864398
1.875324	1.87211	1.868921	1.865708	1.862488	1.874446	1.871252	1.86804	1.864766
1.87569	1.872482	1.869298	1.866079	1.862848	1.874806	1.871611	1.8684	1.86513
1.876059	1.872855	1.869673	1.866449	1.863206	1.875166	1.871969	1.868761	1.865503
1.876448	1.873239	1.870049	1.86682	1.863574	1.875537	1.872336	1.869137	1.865917
1.876853	1.873633	1.870428	1.867195	1.863951	1.875921	1.872713	1.869523	1.866351
1.877291	1.874054	1.870827	1.867595	1.864366	1.861175	1.858021	1.869919	1.86676
1.877764	1.874502	1.871245	1.86802	1.86482	1.86163	1.858447	1.870325	1.867148
1.87679	1.873529	1.87027	1.868437	1.865251	1.86206	1.858859	1.870727	1.867532
1.87719	1.873931	1.870673	1.868833	1.865638	1.862443	1.859243	1.871114	1.86791
1.87758	1.87433	1.871085	1.86924	1.86603	1.862831	1.85964	1.87152	1.868302
1.877976	1.874744	1.871526	1.869681	1.866455	1.863254	1.860079	1.856918	1.853763
1.878368	1.875157	1.871972	1.870132	1.866895	1.863694	1.860534	1.857376	1.854201
1.878749	1.87555	1.872384	1.86919	1.865987	1.864151	1.860982	1.857818	1.85465
1.879127	1.875931	1.872775	1.869608	1.866444	1.864619	1.861428	1.858252	1.855108
1.879552	1.876344	1.873171	1.87001	1.866865	1.863707	1.860532	1.858697	1.855556
1.880031	1.876789	1.873568	1.870391	1.867247	1.864106	1.86097	1.859156	1.855994
1.874554	1.871321	1.868099	1.870793	1.867637	1.864499	1.861387	1.859587	1.856413
1.874991	1.871765	1.868554	1.871238	1.868044	1.864885	1.861766	1.859964	1.856799
1.87543	1.872212	1.869015	1.871693	1.868468	1.865285	1.862149	1.860339	1.857189
1.875893	1.872662	1.869448	1.872132	1.868919	1.865733	1.862575	1.859435	1.856309
1.876365	1.873114	1.869877	1.872571	1.869385	1.866203	1.863023	1.859871	1.856758
1.876815	1.873567	1.870334	1.867136	1.863966	1.866657	1.863474	1.860319	1.8572
1.877253	1.874022	1.870812	1.867608	1.864416	1.867103	1.863932	1.86078	1.85764
1.870344	1.867124	1.863924	1.868063	1.864866	1.861692	1.85854	1.861228	1.858087
1.870825	1.867583	1.864351	1.868494	1.865316	1.862144	1.858972	1.861657	1.858542

1.871298	1.868038	1.864784	1.861586	1.858429	1.862591	1.859413	1.862104	1.859011
1.871741	1.868483	1.865235	1.862028	1.858853	1.863021	1.859871	1.862592	1.859505
1.872181	1.868927	1.865689	1.862473	1.859281	1.863458	1.860342	1.863095	1.860004
1.867178	1.863944	1.860723	1.862916	1.859738	1.856601	1.853509	1.857742	1.854664
1.867646	1.864396	1.861156	1.863363	1.860213	1.857091	1.854002	1.858232	1.855134
1.868097	1.864852	1.861621	1.858456	1.855342	1.857585	1.854483	1.851387	1.848292
1.868533	1.86531	1.862113	1.858964	1.855854	1.858087	1.854955	1.851836	1.848739
1.869002	1.865797	1.86262	1.85947	1.856345	1.858566	1.855422	1.852294	1.849187
1.869519	1.866326	1.863148	1.859972	1.856802	1.859009	1.855884	1.852763	1.849633
1.870045	1.866852	1.863663	1.860462	1.857258	1.854111	1.851029	1.853242	1.850093
1.870562	1.867344	1.864129	1.860923	1.857727	1.854589	1.851516	1.853736	1.850588
1.871073	1.867824	1.864585	1.861381	1.858207	1.85508	1.852007	1.854231	1.851092
1.871553	1.868305	1.865076	1.861878	1.85871	1.855575	1.852476	1.854689	1.851566
1.872018	1.868788	1.865588	1.862401	1.859231	1.856077	1.852937	1.855129	1.852027
1.872522	1.869301	1.866116	1.862935	1.859766	1.856602	1.853437	1.850308	1.847239
1.873066	1.869846	1.866661	1.863483	1.86032	1.857154	1.853979	1.850841	1.847769
1.873617	1.870402	1.867216	1.864031	1.860854	1.857681	1.854506	1.851368	1.848293
1.874169	1.87097	1.867785	1.864572	1.861345	1.858154	1.854995	1.851871	1.848801
1.87472	1.871534	1.86835	1.86511	1.861838	1.858629	1.855484	1.852372	1.849299
1.87526	1.872079	1.868897	1.865652	1.86237	1.859152	1.856	1.852876	1.849774
1.875796	1.872611	1.869428	1.866188	1.862915	1.859694	1.856529	1.853384	1.850246
1.876331	1.873126	1.869921	1.866687	1.863438	1.860228	1.857062	1.853909	1.850752
1.876865	1.873625	1.870383	1.867159	1.863948	1.860761	1.8576	1.854447	1.851285
1.869143	1.865895	1.862641	1.867672	1.864484	1.86131	1.858153	1.854999	1.851833
1.869719	1.866464	1.863202	1.868235	1.865051	1.861879	1.858722	1.855568	1.852399
1.865156	1.861911	1.858658	1.86056	1.857388	1.862449	1.859292	1.856137	1.852964
1.865704	1.862461	1.859211	1.86112	1.857949	1.863014	1.859857	1.856699	1.853519
1.866246	1.863007	1.859761	1.861678	1.858508	1.863576	1.860421	1.85726	1.854071
1.858945	1.855735	1.852519	1.86223	1.859061	1.855904	1.852763	1.857821	1.854624
1.859464	1.856255	1.853041	1.862779	1.85961	1.856455	1.853318	1.858379	1.855177
1.850617	1.847424	1.844229	1.858198	1.855044	1.857	1.853868	1.850718	1.847514
1.851143	1.847942	1.844742	1.858732	1.855576	1.85754	1.854411	1.851263	1.848053
1.851688	1.848481	1.84528	1.842136	1.839035	1.858093	1.854967	1.85182	1.848608
1.852256	1.849047	1.845846	1.842697	1.839587	1.858666	1.855542	1.852396	1.849184
1.852828	1.849626	1.846434	1.843277	1.840148	1.850705	1.847598	1.852972	1.849769
1.853394	1.850217	1.847054	1.843882	1.840711	1.851271	1.848157	1.853541	1.850367
1.853957	1.850809	1.847678	1.844492	1.841277	1.85184	1.848715	1.854105	1.850964
1.844125	1.841012	1.837925	1.845067	1.841838	1.844482	1.841379	1.846154	1.843047
1.844669	1.84155	1.838466	1.845626	1.842401	1.845046	1.841922	1.846701	1.843598
1.839653	1.836542	1.833477	1.846208	1.843006	1.839836	1.836695	1.847272	1.844159
1.840226	1.837103	1.834038	1.846815	1.843658	1.84049	1.837303	1.847865	1.844729
1.840819	1.837675	1.834596	1.831509	1.828429	1.841113	1.837897	1.840518	1.837401
1.841429	1.83825	1.835141	1.832045	1.82897	1.841676	1.838455	1.841078	1.837952
1.842064	1.838848	1.835699	1.832583	1.829502	1.84223	1.839019	1.841655	1.838521
1.842747	1.83949	1.836283	1.83314	1.83005	1.826937	1.823796	1.836461	1.833328
1.843446	1.840155	1.836893	1.833719	1.830613	1.827504	1.824396	1.8371	1.833954
1.833137	1.82987	1.826622	1.834355	1.831217	1.828093	1.824985	1.832584	1.82947
1.833761	1.830526	1.827306	1.835034	1.831856	1.828702	1.825571	1.833178	1.8301
1.834374	1.831151	1.827944	1.824762	1.821601	1.829335	1.826182	1.823075	1.820032
1.834965	1.831734	1.828527	1.825369	1.822247	1.829999	1.826821	1.823692	1.820644

1.835591	1.832342	1.82912	1.825972	1.822878	1.819756	1.816596	1.824329	1.821276
1.836293	1.833005	1.82974	1.82657	1.82347	1.820361	1.817237	1.825	1.821949
1.837005	1.833687	1.830384	1.827185	1.824062	1.820958	1.817873	1.825669	1.822618
1.825145	1.821883	1.818631	1.827855	1.824686	1.82156	1.818479	1.815424	1.812392
1.825782	1.822553	1.819332	1.828552	1.825328	1.822169	1.819078	1.816017	1.812976
1.826406	1.823188	1.819986	1.82922	1.825986	1.822821	1.81973	1.816662	1.813597
1.827015	1.823792	1.820604	1.829868	1.82666	1.823511	1.820426	1.817348	1.814247
1.827662	1.824436	1.82125	1.818065	1.814895	1.824172	1.821091	1.818012	1.814896
1.828368	1.82514	1.821939	1.818727	1.81552	1.824783	1.821705	1.818637	1.815543
1.776595	1.773756	1.770927	1.819397	1.816163	1.813007	1.809938	1.819267	1.816186
1.77711	1.774288	1.771482	1.820077	1.816862	1.813705	1.81061	1.819922	1.816813
1.777625	1.774819	1.772039	1.82076	1.817569	1.814411	1.81129	1.820583	1.817442
1.778206	1.775397	1.772613	1.82143	1.81822	1.81505	1.811924	1.821227	1.818098
1.778824	1.776002	1.773197	1.822094	1.818844	1.815658	1.812538	1.821864	1.818771
1.779426	1.776589	1.773772	1.770928	1.768073	1.816313	1.813186	1.810092	1.807031
1.780012	1.777162	1.774337	1.771499	1.768659	1.817013	1.813868	1.810759	1.807698
1.780607	1.777746	1.774912	1.77208	1.769255	1.766441	1.763633	1.811405	1.808338
1.781216	1.778352	1.775508	1.772681	1.769869	1.767042	1.764192	1.812006	1.808925
1.781828	1.778957	1.776098	1.773272	1.770467	1.767632	1.764755	1.812627	1.809534
1.78244	1.779541	1.776653	1.773813	1.771007	1.768182	1.765332	1.813323	1.81023
1.783048	1.780121	1.777207	1.77435	1.771535	1.768726	1.76592	1.814053	1.810962
1.783631	1.780714	1.777811	1.774953	1.772129	1.76932	1.766526	1.763751	1.761005
1.784198	1.781316	1.778451	1.775602	1.772767	1.769948	1.767144	1.764358	1.761596
1.784804	1.781941	1.779098	1.776251	1.773407	1.770573	1.767748	1.764941	1.762159
1.785453	1.782594	1.779756	1.776907	1.774056	1.771201	1.768337	1.765495	1.762685
1.776565	1.773723	1.770901	1.777538	1.774673	1.771801	1.768915	1.766054	1.763226
1.777216	1.774353	1.771502	1.778128	1.775233	1.772349	1.769474	1.766623	1.7638
1.777843	1.774958	1.772079	1.778697	1.77578	1.772893	1.770036	1.767203	1.764389
1.778385	1.775487	1.772606	1.769724	1.766848	1.773472	1.770623	1.767796	1.764988
1.778888	1.775982	1.773112	1.770251	1.767405	1.77407	1.771226	1.768402	1.7656
1.77944	1.776527	1.773655	1.770803	1.767975	1.774676	1.771843	1.769027	1.766231
1.780025	1.777107	1.774226	1.771376	1.768557	1.775293	1.772474	1.76967	1.766879
1.749988	1.747132	1.744313	1.741524	1.738768	1.766345	1.763558	1.7703	1.767513
1.750568	1.747726	1.744916	1.742122	1.739348	1.766941	1.764152	1.770912	1.768125
1.75116	1.748325	1.745519	1.74272	1.739934	1.737177	1.734451	1.761971	1.759197
1.751768	1.74892	1.746101	1.743301	1.740523	1.737769	1.73504	1.762586	1.7598
1.752387	1.749521	1.746683	1.743881	1.741113	1.738362	1.735631	1.763202	1.760406
1.746474	1.743622	1.740791	1.744473	1.741696	1.738936	1.736198	1.733467	1.730748
1.747098	1.744239	1.741395	1.745071	1.742275	1.739502	1.736754	1.734021	1.731315
1.747713	1.744844	1.741988	1.745666	1.742864	1.740084	1.737325	1.734584	1.731878
1.74832	1.745436	1.74257	1.746258	1.743463	1.74068	1.737908	1.735156	1.732437
1.748935	1.746043	1.74317	1.746863	1.744063	1.741274	1.738497	1.735739	1.733011
1.749564	1.746675	1.743801	1.747489	1.744662	1.741862	1.739093	1.736339	1.733608
1.750194	1.747307	1.744432	1.748118	1.745266	1.742456	1.739691	1.736941	1.734205
1.750814	1.747921	1.745045	1.74219	1.739356	1.743065	1.74029	1.73753	1.734783
1.751432	1.748532	1.745652	1.742797	1.739965	1.743679	1.74089	1.738118	1.735358
1.752061	1.749154	1.746268	1.743403	1.74056	1.744279	1.741492	1.73872	1.73595
1.752694	1.749783	1.746888	1.744008	1.741147	1.744872	1.742096	1.73933	1.736552
1.753319	1.750408	1.747513	1.744629	1.741761	1.738942	1.736178	1.739934	1.737154
1.753931	1.751026	1.748145	1.745269	1.742406	1.739577	1.736785	1.740532	1.737757

1.754552	1.75165	1.748774	1.745902	1.743043	1.740206	1.737393	1.741136	1.738361
1.755194	1.752284	1.749395	1.746518	1.743656	1.740817	1.738001	1.74175	1.738965
1.755837	1.752918	1.750016	1.747133	1.744269	1.741428	1.73861	1.742364	1.73957
1.75646	1.75354	1.75064	1.747759	1.744898	1.742049	1.739212	1.736399	1.733621
1.757082	1.754163	1.751269	1.748393	1.745534	1.742678	1.739817	1.736989	1.734219
1.737067	1.734204	1.731364	1.749025	1.746167	1.743308	1.740443	1.737611	1.734835
1.737705	1.73483	1.731971	1.74966	1.746798	1.743941	1.741088	1.738259	1.735466
1.73838	1.735499	1.732632	1.750355	1.747484	1.744625	1.741776	1.738946	1.736141
1.73911	1.736236	1.733374	1.751138	1.748254	1.745384	1.742529	1.739693	1.736883
1.739806	1.736934	1.734076	1.731232	1.7284	1.746111	1.74325	1.740408	1.73759
1.740411	1.737532	1.734666	1.73182	1.728991	1.746746	1.743886	1.741038	1.738199
1.740986	1.738096	1.735224	1.732376	1.72955	1.747348	1.744491	1.741639	1.738781
1.741566	1.738673	1.735804	1.73295	1.730114	1.747948	1.745093	1.742242	1.739381
1.742151	1.739257	1.736397	1.733535	1.730684	1.74855	1.745696	1.742849	1.739994
1.742813	1.739911	1.737049	1.734183	1.731327	1.749237	1.746383	1.743535	1.740677
1.743549	1.740632	1.737761	1.734894	1.732043	1.750008	1.747152	1.744299	1.741429
1.744238	1.741307	1.738426	1.735557	1.73271	1.750723	1.747862	1.745003	1.742133
1.744843	1.741901	1.739014	1.736143	1.733296	1.751345	1.748469	1.745605	1.74275
1.745447	1.742491	1.739591	1.736713	1.733864	1.751949	1.749063	1.746198	1.743364
1.746088	1.743107	1.740175	1.737281	1.734424	1.731592	1.728783	1.746826	1.744021
1.746758	1.743749	1.740782	1.737868	1.735001	1.732168	1.729368	1.747471	1.744692
1.733662	1.730656	1.727689	1.738551	1.735676	1.73283	1.730013	1.74815	1.745359
1.734438	1.731419	1.728439	1.739298	1.736414	1.733548	1.730697	1.748853	1.746026
1.735171	1.732142	1.729147	1.739997	1.737098	1.734215	1.731345	1.74953	1.746678
1.735854	1.732818	1.729807	1.740639	1.737718	1.734821	1.731949	1.750175	1.747308
1.697613	1.694774	1.691953	1.72754	1.724617	1.73547	1.732592	1.729752	1.72695
1.698349	1.695504	1.692674	1.728312	1.72537	1.736211	1.733317	1.730463	1.727653
1.699082	1.696232	1.693395	1.729088	1.726127	1.736955	1.734047	1.731181	1.728367
1.684856	1.682026	1.679212	1.691244	1.688454	1.737618	1.734706	1.731843	1.729042
1.685499	1.682669	1.679861	1.691878	1.689067	1.738266	1.735354	1.732497	1.729708
1.686196	1.683366	1.680561	1.692573	1.689752	1.68699	1.684287	1.733212	1.730398
1.686932	1.6841	1.681297	1.693311	1.690492	1.687724	1.685009	1.733974	1.731106
1.687631	1.684799	1.682002	1.679206	1.676423	1.688456	1.685729	1.69844	1.695734
1.688272	1.685443	1.682658	1.67989	1.677144	1.689187	1.68645	1.699157	1.696443
1.674702	1.671909	1.669162	1.680582	1.677855	1.675129	1.672399	1.684426	1.681745
1.675478	1.672664	1.669884	1.681296	1.678542	1.675792	1.673042	1.685063	1.6824
1.676284	1.673449	1.670631	1.682027	1.679236	1.676459	1.673688	1.685702	1.683049
1.677057	1.674215	1.671385	1.668592	1.665828	1.677193	1.674407	1.671671	1.669014
1.677809	1.67497	1.672139	1.669347	1.666583	1.677963	1.675166	1.672404	1.66969
1.678496	1.675655	1.672825	1.670048	1.667308	1.664564	1.661811	1.673155	1.670415
1.679119	1.676274	1.673447	1.670701	1.668011	1.665298	1.662557	1.673929	1.671186
1.679783	1.676936	1.67411	1.67137	1.668693	1.665994	1.663272	1.66056	1.657866
1.680516	1.677668	1.674837	1.672065	1.66934	1.666631	1.663944	1.661257	1.658555
1.68126	1.678416	1.675583	1.672779	1.670001	1.667273	1.664601	1.661929	1.659223
1.681995	1.67916	1.676337	1.673519	1.67071	1.667953	1.66525	1.662561	1.659864
1.682726	1.679904	1.677094	1.67427	1.671442	1.668654	1.665905	1.663186	1.660496
1.683444	1.680631	1.677827	1.675007	1.67218	1.669379	1.666604	1.66386	1.661146
1.68415	1.681346	1.678547	1.675738	1.672926	1.670125	1.667334	1.664565	1.661811
1.684824	1.682043	1.679273	1.676478	1.673669	1.670868	1.668074	1.665292	1.66251
1.685459	1.682716	1.680006	1.677227	1.674411	1.671609	1.668824	1.666042	1.663245

1.686135	1.683407	1.680728	1.677963	1.675152	1.672357	1.669577	1.666795	1.663992
1.686885	1.684136	1.681439	1.678683	1.6759	1.673117	1.670333	1.667546	1.664751
1.687659	1.684869	1.682131	1.679382	1.676636	1.673871	1.671083	1.668294	1.665509
1.662004	1.659187	1.656418	1.680034	1.677338	1.674601	1.67182	1.669033	1.66625
1.662748	1.659865	1.657027	1.680675	1.678026	1.675318	1.672548	1.669766	1.666982
1.631899	1.629239	1.626607	1.655	1.652384	1.676032	1.673271	1.670497	1.667713
1.632557	1.6299	1.627268	1.655734	1.653079	1.676742	1.673991	1.671226	1.668441
1.633206	1.630549	1.627917	1.625351	1.622835	1.651091	1.648383	1.67195	1.669163
1.633843	1.631182	1.628549	1.625988	1.62348	1.651806	1.649085	1.672669	1.669876
1.634483	1.631815	1.629178	1.626617	1.624112	1.652514	1.649786	1.647048	1.644289
1.635133	1.632451	1.6298	1.627225	1.624708	1.653195	1.650488	1.647761	1.64499
1.635786	1.63309	1.630427	1.627836	1.625302	1.653878	1.651187	1.648471	1.645692
1.636434	1.633737	1.631077	1.628476	1.625924	1.623376	1.620839	1.649155	1.646386
1.637085	1.634389	1.631738	1.62913	1.626562	1.623996	1.621438	1.649825	1.647076
1.637749	1.635039	1.632382	1.629771	1.627204	1.624629	1.622047	1.619463	1.616875
1.638425	1.635689	1.63301	1.630402	1.627852	1.625273	1.622663	1.620061	1.617476
1.639123	1.63636	1.633654	1.631039	1.628495	1.625913	1.623287	1.620674	1.618087
1.639849	1.637055	1.634307	1.631674	1.629126	1.626542	1.623919	1.621305	1.618707
1.640581	1.637771	1.635	1.632344	1.629772	1.627177	1.624556	1.621944	1.619345
1.641303	1.63852	1.63577	1.633086	1.630453	1.627822	1.625191	1.622586	1.620018
1.642026	1.63928	1.636563	1.63385	1.63115	1.628478	1.625833	1.623232	1.6207
1.642746	1.640009	1.637302	1.634575	1.631846	1.62915	1.626483	1.623872	1.621352
1.643471	1.640727	1.638015	1.635282	1.632546	1.629836	1.627146	1.624517	1.621991
1.644263	1.641497	1.638752	1.636	1.633251	1.630539	1.627859	1.625229	1.622673
1.645127	1.642321	1.639512	1.636725	1.633959	1.631257	1.628626	1.626013	1.623396
1.613459	1.610726	1.607973	1.637476	1.6347	1.632001	1.629392	1.626781	1.624126
1.61418	1.611451	1.608709	1.638269	1.635494	1.632783	1.630144	1.627511	1.624851
1.614883	1.612164	1.609444	1.639066	1.636298	1.633572	1.630893	1.628234	1.625583
1.545966	1.543755	1.541544	1.639835	1.637077	1.634345	1.631638	1.628965	1.626336
1.546501	1.544296	1.542102	1.6406	1.637851	1.635113	1.63238	1.629695	1.62709
1.547054	1.544856	1.542674	1.608938	1.606271	1.635875	1.633108	1.630402	1.627805
1.547622	1.545429	1.543256	1.609707	1.607014	1.636632	1.633827	1.631095	1.628493
1.548221	1.546017	1.543827	1.541633	1.53944	1.605042	1.602333	1.631858	1.629231
1.548859	1.546621	1.544384	1.542177	1.539991	1.605772	1.603115	1.632708	1.630027
1.549486	1.547224	1.544955	1.542737	1.540555	1.606514	1.603892	1.633549	1.630835
1.550076	1.547816	1.545554	1.543332	1.54114	1.607272	1.604632	1.634336	1.631649
1.55066	1.548411	1.546168	1.543945	1.541739	1.608036	1.605373	1.635123	1.632462
1.551258	1.549024	1.546798	1.54457	1.542346	1.540154	1.537997	1.603523	1.600931
1.551865	1.549645	1.547437	1.545205	1.542963	1.540763	1.538611	1.604314	1.601665
1.552483	1.55026	1.548054	1.545829	1.543597	1.541393	1.539219	1.605067	1.602384
1.55311	1.550871	1.548653	1.546446	1.544249	1.542042	1.539823	1.60579	1.603088
1.553746	1.551495	1.549271	1.547066	1.544877	1.542666	1.540427	1.538205	1.53601
1.554389	1.552137	1.549917	1.547691	1.54547	1.543249	1.541028	1.538819	1.536623
1.524157	1.522	1.519877	1.548319	1.546072	1.543844	1.541637	1.539439	1.537245
1.524779	1.522595	1.520439	1.548947	1.546714	1.544485	1.54226	1.540052	1.537868
1.525401	1.523189	1.520999	1.549579	1.547365	1.545139	1.542893	1.540672	1.538497
1.525974	1.523764	1.521582	1.51943	1.517303	1.545774	1.543539	1.541323	1.539137
1.526522	1.524328	1.522176	1.520017	1.517865	1.546403	1.544194	1.541991	1.539785
1.527112	1.524906	1.522746	1.520587	1.518441	1.547045	1.544838	1.542635	1.540427
1.527742	1.525496	1.523292	1.521139	1.51903	1.547699	1.545471	1.543257	1.541062

1.528374	1.526096	1.523858	1.521701	1.519604	1.517485	1.515338	1.543888	1.541698
1.528997	1.526708	1.524462	1.522281	1.520154	1.518033	1.515919	1.544543	1.542338
1.529629	1.527328	1.525072	1.522863	1.520701	1.518574	1.516492	1.545194	1.542977
1.53028	1.527952	1.525661	1.523432	1.521257	1.519124	1.517043	1.514958	1.512845
1.530943	1.528586	1.526257	1.524006	1.521819	1.519677	1.517584	1.515501	1.513417
1.497334	1.495178	1.493045	1.524618	1.522393	1.520227	1.518126	1.516045	1.513973
1.497942	1.495782	1.493645	1.52526	1.522978	1.520779	1.518669	1.516586	1.514517
1.498566	1.496403	1.494265	1.52593	1.523606	1.521376	1.519245	1.517149	1.515073
1.499209	1.497045	1.494911	1.526634	1.524281	1.522021	1.519858	1.517736	1.515641
1.49985	1.497681	1.495549	1.493396	1.491244	1.522684	1.520482	1.518327	1.516214
1.500475	1.498295	1.496156	1.494005	1.491859	1.523358	1.521105	1.518908	1.516784
1.501111	1.498915	1.496766	1.494617	1.492483	1.524045	1.521742	1.519504	1.517368
1.501781	1.499567	1.497401	1.49525	1.49312	1.524745	1.522407	1.520138	1.517983
1.502476	1.500239	1.498052	1.495894	1.493765	1.525454	1.523092	1.520798	1.518619
1.5032	1.500931	1.498713	1.496542	1.494414	1.492269	1.490095	1.521473	1.519253
1.503953	1.501643	1.499384	1.497196	1.495067	1.492916	1.490733	1.522163	1.519889
1.504715	1.502377	1.500088	1.497875	1.495723	1.493559	1.491379	1.52287	1.52055
1.505485	1.50314	1.500836	1.498585	1.49638	1.494196	1.492036	1.523604	1.521243
1.506262	1.503908	1.501591	1.499302	1.497044	1.494836	1.492685	1.490538	1.488351
1.50704	1.504669	1.502333	1.500011	1.497711	1.495471	1.493297	1.49114	1.488969
1.507829	1.505436	1.503079	1.500729	1.498397	1.496125	1.493917	1.491744	1.489596
1.474265	1.471722	1.469217	1.501471	1.49913	1.49684	1.494601	1.492403	1.490243
1.475269	1.472694	1.47016	1.502233	1.499898	1.497596	1.495327	1.493098	1.490909
1.440649	1.438225	1.435838	1.468614	1.466155	1.498364	1.496068	1.493813	1.491606
1.441576	1.439139	1.436739	1.469583	1.467128	1.499146	1.496826	1.494551	1.492335
1.442532	1.440076	1.437654	1.470566	1.468105	1.465641	1.463168	1.495301	1.493077
1.443528	1.441044	1.438587	1.471564	1.469085	1.466607	1.464126	1.496063	1.493831
1.444532	1.442021	1.43953	1.472569	1.470062	1.467568	1.465085	1.496833	1.494591
1.445527	1.442997	1.440477	1.473569	1.471021	1.46851	1.466035	1.463604	1.461228
1.446513	1.443973	1.441433	1.474577	1.471983	1.469451	1.466987	1.464563	1.46218
1.447475	1.444945	1.442409	1.439914	1.437451	1.470422	1.467953	1.465526	1.463137
1.448417	1.445911	1.4434	1.440894	1.438398	1.471415	1.46893	1.466494	1.464098
1.449347	1.446863	1.444374	1.441866	1.43935	1.472428	1.469921	1.467468	1.465066
1.450261	1.447797	1.44533	1.442827	1.440306	1.473462	1.470924	1.468448	1.466042
1.451179	1.448732	1.446283	1.443792	1.441277	1.438818	1.436412	1.469443	1.467029
1.452105	1.449668	1.447237	1.444763	1.442266	1.439804	1.43737	1.470458	1.468032
1.453044	1.450612	1.448194	1.44574	1.443266	1.440804	1.438342	1.471488	1.469043
1.454007	1.451576	1.449165	1.44673	1.444284	1.441821	1.439329	1.472531	1.470055
1.454988	1.452552	1.450142	1.447724	1.445305	1.442844	1.440326	1.473588	1.471071
1.45598	1.45353	1.45111	1.448695	1.446288	1.443833	1.441317	1.438846	1.436454
1.456984	1.454511	1.452071	1.449648	1.447241	1.444797	1.442306	1.439839	1.437402
1.457983	1.455485	1.453024	1.450591	1.448182	1.445746	1.44328	1.44082	1.438353
1.458972	1.456448	1.453963	1.451516	1.449103	1.446675	1.444232	1.441783	1.439302
1.459964	1.457419	1.454916	1.452458	1.450039	1.447616	1.445191	1.442753	1.440269
1.460953	1.458399	1.455896	1.453433	1.45101	1.448588	1.446173	1.443743	1.441269
1.461962	1.4594	1.456897	1.454428	1.451997	1.449573	1.447161	1.444739	1.442282
1.46303	1.460444	1.457921	1.455433	1.452984	1.450552	1.448139	1.445725	1.443294
1.464137	1.461522	1.458967	1.456452	1.45398	1.451534	1.449117	1.446709	1.444306
1.456406	1.453823	1.451299	1.457496	1.454998	1.452535	1.45011	1.4477	1.445303
1.45757	1.454965	1.452408	1.458565	1.456036	1.453551	1.451117	1.448698	1.446286

								0.724074
								0.722533
								0.72099
					0.726203	0.72386	0.721566	0.874127
					0.724647	0.722334	0.720069	0.871534
					0.723109	0.720881	0.718684	1.197217
					0.721575	0.719481	0.717386	1.194676
			0.724613	0.722274	0.876144	0.872321	0.868543	1.326469
			0.72309	0.720752	0.873591	0.869808	0.866087	1.323806
	0.725918	0.723782	0.879646	0.875297	1.334895	1.330256	1.325699	1.466319
	0.724475	0.72223	0.876995	0.872959	1.332361	1.327628	1.323006	1.463471
	0.723048	0.720714	0.874446	0.870684	1.32984	1.325054	1.320402	1.460695
	0.721571	0.719325	1.270127	1.265578	1.327195	1.32252	1.317985	1.458084
	0.720075	0.717995	1.267655	1.262956	1.324504	1.320026	1.315685	1.455578
0.720682	0.875441	0.871688	1.33135	1.326506	1.466854	1.462157	1.457588	1.603748
0.719174	0.872922	0.869298	1.328641	1.32385	1.464017	1.459415	1.454954	1.600901
0.717743	1.268754	1.264305	1.325989	1.321321	1.461333	1.456786	1.452372	1.666859
0.716428	1.266243	1.261687	1.323398	1.318954	1.458851	1.454302	1.449851	1.66376
1.082417	1.330098	1.325371	1.465622	1.460985	1.676478	1.671165	1.665899	1.693234
1.079991	1.327534	1.322925	1.463002	1.458427	1.67347	1.668211	1.662978	1.690132
1.077581	1.325007	1.32056	1.460464	1.455911	1.670473	1.665264	1.660069	1.687071
1.260767	1.32259	1.318223	1.608602	1.60367	1.667529	1.662287	1.657069	1.851062
1.258416	1.320253	1.31593	1.605806	1.60093	1.664615	1.659295	1.654029	1.847823
1.322265	1.462148	1.457534	1.672061	1.666862	1.740072	1.734505	1.728995	1.879581
1.319839	1.459581	1.455124	1.669133	1.663836	1.736861	1.731379	1.725949	1.8764
1.317491	1.676646	1.671447	1.666169	1.660831	1.854907	1.849358	1.843866	1.901873
1.315272	1.673689	1.668413	1.663138	1.657866	1.851685	1.846147	1.840681	1.898615
1.313056	1.67072	1.665369	1.660103	1.654909	1.848488	1.842963	1.837526	1.895396
1.622343	1.667663	1.662346	1.782345	1.776862	1.908917	1.903269	1.897702	1.892243

1.619408	1.664594	1.659334	1.779129	1.773689	1.905615	1.900028	1.894531	1.889141
1.667017	1.787027	1.781418	1.855329	1.849856	1.902378	1.896832	1.891387	2.019042
1.664194	1.783961	1.778245	1.852113	1.846722	1.89919	1.893678	1.888277	2.015183
1.709563	1.78087	1.775188	1.907066	1.901502	1.994688	1.988161	1.981766	2.011399
1.706439	1.777743	1.77227	1.904011	1.898338	1.990813	1.984469	1.978238	2.007708
1.78004	1.854102	1.848699	1.913747	1.907989	2.02351	2.01693	2.010482	2.013407
1.776898	1.850967	1.845661	1.910654	1.905021	2.019829	2.013169	2.006654	2.00953
1.773848	1.847895	1.842631	1.907555	1.902089	2.01624	2.009482	2.00287	2.005688
1.770952	1.888815	1.883331	1.989405	1.983075	2.012599	2.005913	1.999316	2.027846
1.768148	1.885801	1.880228	1.985628	1.979421	2.008939	2.002394	1.995863	2.024115
1.8446	1.973102	1.966462	2.018186	2.011654	2.040786	2.033973	2.027217	2.062156
1.841608	1.969537	1.963072	2.014579	2.007871	2.036704	2.029999	2.023415	2.058548
1.882148	1.987941	1.981584	2.020596	2.0136	2.032778	2.026107	2.019613	2.098432
1.879044	1.984213	1.977982	2.016858	2.00996	2.02907	2.02231	2.015772	2.094619
1.964884	2.016607	2.010155	2.039146	2.032215	2.066926	2.060175	2.05364	2.090884
1.961414	2.012873	2.006285	2.035065	2.028269	2.063115	2.056586	2.050206	2.087317
1.957997	2.009209	2.002433	2.030981	2.024311	2.059311	2.053054	2.046867	2.083839
1.954481	2.041161	2.034	2.027125	2.02049	2.099182	2.092889	2.086687	2.093298
1.95092	2.037349	2.030256	2.023414	2.016772	2.095374	2.089213	2.083157	2.089931
2.022011	2.03351	2.026518	2.105025	2.09826	2.091767	2.085623	2.079598	2.086485
2.018074	2.029646	2.022791	2.101336	2.094769	2.08836	2.082113	2.075998	2.082961
2.032547	2.07408	2.067212	2.097716	2.091323	2.097885	2.0916	2.085433	2.079484
2.028503	2.070124	2.063537	2.094184	2.087907	2.094565	2.088326	2.08215	2.076076
2.024568	2.066244	2.059898	2.090669	2.084501	2.091256	2.085081	2.078912	2.072717
2.020894	2.099588	2.093277	2.087092	2.081065	2.08793	2.081799	2.075643	2.092716
2.017386	2.095956	2.089646	2.08353	2.077631	2.084595	2.078506	2.072372	2.089428
2.06195	2.092481	2.086182	2.093035	2.087177	2.081247	2.075192	2.069086	2.102909
2.058584	2.089146	2.082849	2.089702	2.083818	2.077883	2.071857	2.065787	2.099584
2.055237	2.085832	2.079549	2.086377	2.080416	2.097808	2.091763	2.085699	2.119898
2.051912	2.082545	2.076288	2.083059	2.076957	2.094249	2.088293	2.082342	2.116568
2.048607	2.09222	2.085982	2.112776	2.106475	2.131166	2.125211	2.119286	2.164447
2.045316	2.088939	2.08272	2.109449	2.103115	2.127744	2.121756	2.115846	2.160976
2.042044	2.085657	2.079449	2.106133	2.099794	2.124367	2.118328	2.112414	2.157507
2.075607	2.092756	2.086526	2.118206	2.111858	2.154034	2.147474	2.141093	2.154007
2.072333	2.089476	2.083224	2.114852	2.108534	2.15052	2.143918	2.13753	2.150515
2.092376	2.124163	2.117807	2.126853	2.12053	2.166128	2.159539	2.153164	2.147139
2.089047	2.120762	2.114419	2.123466	2.117167	2.162619	2.156165	2.149897	2.143862
2.123645	2.117362	2.111042	2.17263	2.165811	2.159202	2.152845	2.146641	2.161733
2.120219	2.11396	2.107678	2.169115	2.16242	2.155899	2.149587	2.143389	2.158378
2.116807	2.132304	2.125994	2.165619	2.159056	2.173803	2.167494	2.161265	2.165626
2.113422	2.12889	2.122599	2.162113	2.155687	2.170495	2.164204	2.157975	2.162339
2.110056	2.125502	2.119238	2.158648	2.152337	2.167194	2.160914	2.15469	2.159076
2.106712	2.168496	2.161779	2.176445	2.170149	2.163887	2.157629	2.151421	2.155814
2.103387	2.165041	2.158446	2.173139	2.166838	2.160581	2.154348	2.148165	2.152562
2.121668	2.18274	2.176229	2.169823	2.163528	2.167828	2.16163	2.155477	2.175942
2.118314	2.179254	2.172855	2.166505	2.160219	2.164534	2.158362	2.152237	2.172628
2.182314	2.175824	2.169497	2.16319	2.156923	2.16534	2.159164	2.153045	2.169338
2.178924	2.172472	2.166159	2.159876	2.153637	2.162062	2.155901	2.149808	2.166051
2.175563	2.169145	2.162833	2.156571	2.150369	2.158808	2.152665	2.146615	2.162858
2.17219	2.165808	2.15951	2.167893	2.161709	2.178014	2.171832	2.165775	2.159822

2.168825	2.16248	2.156204	2.164604	2.158463	2.174754	2.168599	2.162632	2.156916
2.165467	2.163645	2.157379	2.183766	2.177623	2.16879	2.162725	2.156906	2.151462
2.162121	2.160324	2.154115	2.180498	2.174391	2.165632	2.159665	2.154001	2.148831
2.163312	2.167198	2.161044	2.174489	2.168462	2.16256	2.15671	2.151204	2.146274
2.160058	2.163983	2.157872	2.171325	2.16538	2.159583	2.15386	2.148511	2.143788
2.156924	2.180331	2.174229	2.168228	2.162319	2.156623	2.151094	2.145955	2.141424
2.153996	2.177386	2.171284	2.165241	2.159243	2.153614	2.148395	2.143571	2.13922
2.151074	2.174429	2.168322	2.16226	2.156223	2.150695	2.145793	2.141281	2.137099
2.177426	2.171259	2.165144	2.159172	2.153312	2.147984	2.143313	2.139016	2.14182
2.174172	2.167991	2.161876	2.156056	2.150491	2.145422	2.140944	2.136811	2.13977
2.170898	2.164724	2.158641	2.153005	2.147765	2.149715	2.145456	2.141498	2.137768
2.167591	2.161443	2.155421	2.150004	2.145133	2.147384	2.143309	2.139476	2.135813
2.16432	2.158281	2.152431	2.15395	2.149391	2.14515	2.141233	2.137509	2.133892
2.161059	2.155232	2.149689	2.151453	2.147085	2.143004	2.139222	2.13559	2.131998
2.15794	2.15902	2.153834	2.149143	2.14494	2.140989	2.137306	2.13374	2.130165
2.155043	2.156376	2.151515	2.147056	2.143014	2.139175	2.135545	2.132004	2.128448
2.152287	2.153874	2.149314	2.145068	2.14117	2.137421	2.133819	2.130284	2.126743
2.149615	2.151432	2.147085	2.143003	2.139226	2.141405	2.137857	2.134357	2.130855
2.147047	2.149077	2.144887	2.140934	2.137257	2.139512	2.13598	2.132484	2.128986
2.151241	2.152573	2.14857	2.144757	2.141172	2.137633	2.134116	2.130626	2.131607
2.148861	2.150408	2.146556	2.142834	2.139279	2.135758	2.132254	2.128772	2.129761
2.152398	2.148369	2.144642	2.140994	2.137461	2.133954	2.13046	2.126989	2.128002
2.150388	2.14649	2.142863	2.139273	2.135754	2.132259	2.128773	2.125319	2.126379
2.148444	2.144651	2.141095	2.137547	2.134041	2.130556	2.127077	2.123639	2.124749
2.146472	2.142762	2.139237	2.135716	2.132225	2.1333	2.129805	2.126361	2.127873
2.144538	2.140891	2.137375	2.13387	2.130394	2.131487	2.127997	2.124566	2.126099
2.142706	2.139108	2.135606	2.132116	2.128652	2.129774	2.126324	2.122923	2.134145
2.140947	2.137385	2.133891	2.130414	2.126961	2.128118	2.12473	2.121372	2.132509
2.139141	2.135607	2.132122	2.133263	2.129797	2.126387	2.123049	2.119738	2.130839
2.137284	2.133767	2.13029	2.131439	2.127965	2.124568	2.12127	2.118017	2.129133
2.135469	2.136624	2.133138	2.129665	2.126205	2.132981	2.129592	2.126255	2.12743
2.133747	2.13492	2.131441	2.127998	2.124591	2.131345	2.127917	2.124547	2.125716
2.132024	2.133212	2.129741	2.126335	2.12299	2.129726	2.126272	2.122881	2.124051
2.134909	2.131409	2.127946	2.124572	2.121282	2.132497	2.129082	2.125738	2.157432
2.133084	2.129587	2.126138	2.122795	2.119554	2.130762	2.127427	2.12417	2.155671
2.131341	2.127871	2.124461	2.131279	2.127955	2.129116	2.125798	2.12257	2.153821
2.129657	2.126233	2.122884	2.12969	2.126383	2.127536	2.124193	2.120955	2.151914
2.127947	2.124556	2.121257	2.128045	2.124759	2.14063	2.137169	2.133801	2.150095
2.126187	2.122812	2.119553	2.126315	2.123057	2.138899	2.135564	2.132303	2.148389
2.124485	2.131266	2.127926	2.124637	2.121403	2.137212	2.133994	2.130828	2.146688
2.122898	2.129675	2.12635	2.123075	2.119852	2.135618	2.132474	2.12936	2.144948
2.121359	2.128134	2.124825	2.121567	2.118357	2.134063	2.130963	2.127878	2.143199
2.119816	2.12659	2.123314	2.139103	2.135747	2.155031	2.151437	2.147861	2.144903
2.118282	2.125051	2.121817	2.137555	2.134204	2.153223	2.149606	2.146039	2.143145
2.116728	2.123462	2.120251	2.139179	2.135914	2.146134	2.142499	2.138935	2.146012
2.115156	2.121823	2.118621	2.137534	2.134389	2.144462	2.140822	2.137263	2.144368
2.123537	2.124069	2.120919	2.130808	2.127723	2.147612	2.143942	2.14036	2.142691
2.122023	2.12264	2.119565	2.129363	2.126279	2.145919	2.142204	2.138597	2.140941
2.124339	2.134311	2.131101	2.151486	2.147851	2.144205	2.140489	2.136896	2.139243
2.122879	2.132818	2.129652	2.149783	2.146081	2.142434	2.138829	2.135342	2.137688

2.121448	2.131335	2.128196	2.148063	2.144295	2.140664	2.137206	2.133852	2.136194
2.115106	2.135302	2.132126	2.146303	2.142552	2.144726	2.141308	2.137977	2.13469
2.113756	2.13381	2.130577	2.144521	2.140839	2.143063	2.139718	2.136446	2.13319
2.112369	2.132292	2.129052	2.142807	2.139206	2.141467	2.138165	2.134918	2.132557
2.110943	2.130744	2.127548	2.14116	2.137654	2.139943	2.136654	2.133396	2.131008
2.115594	2.129218	2.126055	2.140528	2.137136	2.139283	2.136004	2.132743	2.153077
2.114208	2.127728	2.124566	2.138905	2.135617	2.137782	2.134463	2.13116	2.151286
2.112835	2.126255	2.123097	2.137319	2.134111	2.136282	2.132931	2.129593	2.149519
2.127945	2.142664	2.139146	2.135804	2.132626	2.134782	2.131445	2.12811	2.147827
2.126459	2.14105	2.137627	2.134326	2.131145	2.133272	2.129968	2.126653	2.14617
2.126391	2.139445	2.136112	2.138268	2.134988	2.155813	2.152022	2.148245	2.14909
2.12491	2.137853	2.134607	2.136746	2.133414	2.153976	2.150245	2.146533	2.147398
2.123448	2.136293	2.133103	2.135226	2.131879	2.152214	2.148505	2.14482	2.145696
2.122006	2.13477	2.131602	2.133715	2.130397	2.150544	2.146811	2.143106	2.143984
2.127996	2.133258	2.130086	2.156453	2.152649	2.148868	2.145116	2.141394	2.142266
2.126531	2.131746	2.128526	2.154631	2.150887	2.147148	2.143406	2.139685	2.140541
2.125083	2.130247	2.126976	2.152813	2.149118	2.145416	2.141689	2.137977	2.138818
2.123638	2.134121	2.130774	2.151099	2.147392	2.148283	2.144546	2.140827	2.137106
2.122202	2.132642	2.12931	2.149437	2.145686	2.146546	2.142811	2.139107	2.135407
2.12078	2.15539	2.151552	2.14775	2.143975	2.144816	2.141079	2.13739	2.133743
2.119369	2.153711	2.149861	2.146049	2.142265	2.143094	2.139348	2.135676	2.13211
2.117968	2.152041	2.148171	2.144347	2.140552	2.141381	2.137655	2.13402	2.130521
2.116581	2.15038	2.146474	2.142631	2.138831	2.139675	2.136008	2.132435	2.128982
2.140672	2.148732	2.144814	2.140953	2.137128	2.13798	2.134389	2.130891	2.127473
2.139071	2.147118	2.143249	2.139367	2.135458	2.136286	2.132787	2.12938	2.125991
2.137454	2.145498	2.141692	2.137798	2.13381	2.134619	2.131212	2.127894	2.124529
2.140578	2.143808	2.140022	2.140717	2.136732	2.133012	2.129674	2.126415	2.123072
2.138897	2.142074	2.138286	2.13902	2.135111	2.131457	2.128168	2.124947	2.121627
2.137219	2.140344	2.136563	2.137347	2.133536	2.129954	2.126695	2.123493	2.128567
2.135541	2.138613	2.134843	2.135691	2.132008	2.128507	2.125255	2.122055	2.127149
2.133881	2.136905	2.133152	2.134064	2.130503	2.135438	2.13217	2.128947	2.125712
2.13225	2.135222	2.131486	2.132456	2.129006	2.133983	2.130723	2.127488	2.124238
2.130631	2.133575	2.129878	2.130908	2.127555	2.13256	2.129292	2.126031	2.122752
2.129003	2.136553	2.132929	2.137805	2.134504	2.131207	2.127893	2.124575	2.121262
2.127395	2.134992	2.131479	2.136407	2.133164	2.129876	2.126501	2.123117	2.119769
2.125865	2.141789	2.138324	2.134978	2.131764	2.128477	2.125064	2.121641	2.143673
2.124397	2.140309	2.136869	2.133537	2.130327	2.127033	2.123596	2.120153	2.142007
2.14239	2.138842	2.135424	2.132097	2.128875	2.125576	2.122153	2.118718	2.140391
2.140892	2.137385	2.133983	2.130652	2.127402	2.124107	2.120743	2.117351	2.138837
2.139413	2.135951	2.132577	2.129238	2.125944	2.148882	2.145064	2.141235	2.137271
2.13796	2.134558	2.131242	2.127889	2.124516	2.147205	2.143402	2.139599	2.135647
2.136517	2.133166	2.129901	2.12654	2.123106	2.145575	2.141749	2.137944	2.134033
2.135054	2.131723	2.128466	2.125127	2.121725	2.14405	2.140162	2.136321	2.132498
2.133595	2.130262	2.126986	2.123678	2.120351	2.142572	2.138605	2.134711	2.131001
2.132199	2.128848	2.125526	2.148639	2.144844	2.140993	2.137009	2.133112	2.129472
2.130859	2.127471	2.124075	2.146942	2.143161	2.139334	2.135383	2.131524	2.12792
2.129489	2.126087	2.122664	2.145325	2.141521	2.137705	2.133818	2.130016	2.124716
2.128069	2.124691	2.121314	2.143821	2.139939	2.136111	2.132334	2.128622	2.123229
2.12666	2.123296	2.119951	2.142314	2.138388	2.134561	2.130866	2.127223	2.121754
2.125302	2.121916	2.11853	2.140741	2.136877	2.13309	2.129394	2.125754	2.120266

2.123945	2.120526	2.117087	2.139145	2.135371	2.131634	2.127915	2.124262	2.118767
2.122526	2.145329	2.14137	2.13753	2.133798	2.12882	2.124859	2.120973	2.121885
2.121073	2.143687	2.139737	2.135908	2.132193	2.127159	2.123243	2.119395	2.12029
2.119622	2.142084	2.138176	2.134366	2.13065	2.12556	2.121704	2.117902	2.118759
2.118166	2.140517	2.13669	2.132904	2.12916	2.124013	2.120237	2.116495	2.117289
2.116753	2.138971	2.135182	2.130385	2.126403	2.127254	2.123453	2.119675	2.115825
2.11541	2.13745	2.133605	2.128774	2.124933	2.125825	2.121965	2.11814	2.114354
2.114074	2.135942	2.132066	2.1272	2.123471	2.124389	2.120479	2.116617	2.112891
2.112689	2.134435	2.130654	2.125752	2.121977	2.123078	2.119177	2.11533	2.111599
2.111307	2.132943	2.129288	2.124346	2.120482	2.121535	2.117699	2.113912	2.11017
2.135239	2.130634	2.126744	2.12788	2.123949	2.120069	2.11624	2.112465	2.108751
2.133856	2.129147	2.125207	2.126347	2.122492	2.118651	2.114797	2.111005	2.107341
2.13245	2.128318	2.124335	2.124853	2.121011	2.117189	2.113359	2.109589	2.11688
2.131024	2.126882	2.122926	2.123401	2.119497	2.115668	2.111929	2.108231	2.115365
2.130568	2.129873	2.125919	2.12197	2.118024	2.114185	2.110497	2.10684	2.113837
2.129053	2.128338	2.124482	2.120577	2.116633	2.112779	2.109047	2.105361	2.112279
2.127551	2.126801	2.123021	2.119167	2.115259	2.111404	2.107616	2.103889	2.110728
2.120681	2.125302	2.121484	2.117664	2.113848	2.110049	2.106255	2.102508	2.109214
2.119271	2.123827	2.119922	2.116124	2.112421	2.108698	2.104925	2.101172	2.107728
2.122454	2.122385	2.118452	2.114651	2.110964	2.118255	2.114245	2.110254	2.106285
2.121044	2.120973	2.117054	2.113228	2.109478	2.116652	2.112733	2.108827	2.104883
2.11959	2.119532	2.115662	2.111841	2.108058	2.115103	2.111209	2.107338	2.103435
2.118065	2.118046	2.114279	2.110504	2.106727	2.113627	2.109664	2.105756	2.101919
2.116567	2.116571	2.112874	2.120239	2.116194	2.11217	2.108151	2.104206	2.10041
2.115148	2.115124	2.111405	2.118684	2.11471	2.11073	2.106714	2.102756	2.098932
2.113767	2.113706	2.109939	2.117114	2.113207	2.10928	2.105297	2.101349	2.097481
2.112409	2.112347	2.108556	2.115595	2.111666	2.107751	2.103839	2.099951	2.09608
2.111071	2.111029	2.107225	2.114106	2.110108	2.106186	2.102368	2.098567	2.094715
2.109725	2.120791	2.116707	2.112658	2.108632	2.10471	2.100935	2.097178	2.093342
2.108377	2.119345	2.115286	2.111253	2.107234	2.103316	2.09954	2.095787	2.091969
2.106983	2.117862	2.113851	2.109844	2.105835	2.101915	2.098117	2.094354	2.093826
2.105508	2.116319	2.112392	2.108425	2.104421	2.100486	2.096637	2.092848	2.092328
2.104053	2.114783	2.110927	2.106997	2.103006	2.099063	2.09517	2.091357	2.090826
2.102695	2.113307	2.10947	2.105561	2.101596	2.097665	2.093767	2.089942	2.089337
2.101378	2.111851	2.108005	2.104111	2.100183	2.096278	2.092391	2.088561	2.087864
2.110942	2.110368	2.106491	2.102616	2.098751	2.098393	2.094331	2.090329	2.086431
2.109504	2.108874	2.104947	2.101092	2.097309	2.096949	2.092903	2.088911	2.085031
2.108083	2.107421	2.103471	2.099629	2.095891	2.095515	2.091498	2.087525	2.083653
2.106679	2.106013	2.102069	2.098231	2.094498	2.094089	2.090117	2.086177	2.082301
2.105285	2.104615	2.100677	2.100489	2.096581	2.092673	2.088739	2.084828	2.080598
2.103899	2.10322	2.099281	2.099045	2.095157	2.09127	2.087358	2.083466	2.079236
2.102516	2.10183	2.097892	2.097608	2.093739	2.08987	2.085977	2.082101	2.077876
2.101131	2.100443	2.096515	2.096182	2.092318	2.089065	2.085188	2.081329	2.077488
2.099746	2.099057	2.095143	2.094762	2.090901	2.087642	2.083786	2.079946	2.076121
2.098362	2.101383	2.097302	2.094316	2.090487	2.086239	2.082398	2.078571	2.074756
2.096978	2.099939	2.095865	2.09289	2.089089	2.084854	2.081021	2.077201	2.073393
2.095589	2.099417	2.095368	2.091041	2.087257	2.083467	2.079644	2.075832	2.072036
2.094191	2.097946	2.093907	2.089594	2.085844	2.082078	2.078265	2.074463	2.070688
2.093966	2.096076	2.09204	2.088162	2.084434	2.080687	2.076885	2.073094	2.069339
2.092577	2.094628	2.090612	2.086746	2.083024	2.079286	2.0755	2.071725	2.067983

2.091202	2.093208	2.08922	2.085362	2.08163	2.077891	2.074119	2.07036	2.066629
2.093391	2.091837	2.087907	2.084056	2.080287	2.076525	2.072756	2.069007	2.065287
2.092011	2.090501	2.086642	2.082796	2.078972	2.075175	2.071403	2.067662	2.063952
2.090334	2.089177	2.085333	2.081482	2.077633	2.073822	2.070053	2.066321	2.062624
2.089041	2.087868	2.083991	2.080126	2.076274	2.072466	2.068704	2.064985	2.061303
2.087726	2.08653	2.082627	2.078753	2.074907	2.071108	2.067359	2.063656	2.059987
2.086375	2.085147	2.081235	2.077362	2.073524	2.069739	2.066014	2.062331	2.058669
2.085009	2.083754	2.079842	2.075975	2.072153	2.068387	2.064684	2.061022	2.057376
2.083634	2.082365	2.078461	2.074609	2.070806	2.067059	2.063369	2.059728	2.056131
2.082254	2.080979	2.077091	2.073262	2.06949	2.065767	2.062087	2.058464	2.054916
2.080875	2.079601	2.075733	2.071947	2.06824	2.06456	2.060895	2.057274	2.053721
2.079504	2.078235	2.074392	2.070661	2.067039	2.063414	2.059765	2.056135	2.052544
2.078216	2.076954	2.073125	2.069415	2.065817	2.062209	2.058565	2.054933	2.051329
2.077021	2.075771	2.071944	2.068215	2.064577	2.060942	2.057292	2.053665	2.050074
2.075801	2.074554	2.070737	2.066996	2.063324	2.059668	2.056016	2.052395	2.048819
2.074508	2.073251	2.069464	2.065732	2.062053	2.058393	2.054741	2.051125	2.047562
2.073203	2.071933	2.068175	2.064458	2.060781	2.057127	2.053489	2.049889	2.04634
2.071469	2.068161	2.064462	2.063176	2.059509	2.055883	2.052301	2.04875	2.045216
2.070189	2.06689	2.063195	2.061907	2.058254	2.054661	2.051145	2.047648	2.044129
2.06897	2.0657	2.062025	2.060733	2.057078	2.053484	2.049972	2.046479	2.04296
2.0678	2.064577	2.060932	2.059634	2.055965	2.052347	2.048792	2.045264	2.041735
2.066604	2.063405	2.059781	2.058479	2.054808	2.051177	2.047594	2.044041	2.040503
2.065375	2.062167	2.058548	2.057243	2.053585	2.049957	2.046363	2.0428	2.039257
2.064142	2.060936	2.057335	2.056033	2.052389	2.048767	2.045169	2.0416	2.03805
2.062897	2.059733	2.056195	2.054914	2.051279	2.047661	2.044067	2.040495	2.036931
2.061675	2.058552	2.055067	2.053802	2.050175	2.046563	2.042973	2.039401	2.035829
2.060538	2.056992	2.053496	2.052608	2.04899	2.045385	2.041791	2.03822	2.034661
2.059449	2.055865	2.052307	2.051381	2.047774	2.044174	2.040574	2.037004	2.033472
2.058336	2.05475	2.051169	2.049956	2.046374	2.043022	2.03942	2.035855	2.032346
2.05721	2.053651	2.050076	2.048855	2.045273	2.041921	2.038322	2.034765	2.031275
2.056041	2.052495	2.048927	2.047701	2.044123	2.040556	2.036985	2.03346	2.030168
2.054803	2.051246	2.047684	2.046455	2.042888	2.039329	2.035762	2.032249	2.028988
2.05358	2.050012	2.046456	2.045225	2.041668	2.038121	2.034565	2.031067	2.027688
2.052447	2.048877	2.045327	2.044092	2.04054	2.037006	2.033475	2.030001	2.026635
2.051326	2.047759	2.044215	2.042976	2.039429	2.035911	2.032412	2.028966	2.025606
2.050112	2.046552	2.043018	2.041781	2.038246	2.034745	2.031277	2.027856	2.024503
2.048849	2.045299	2.041779	2.040548	2.037028	2.033547	2.030108	2.026712	2.023365
2.047672	2.044133	2.040627	2.039398	2.035888	2.032425	2.029015	2.025641	2.022299
2.046582	2.043054	2.039561	2.038334	2.034828	2.031378	2.027997	2.024647	2.021308
2.045506	2.04199	2.038508	2.037283	2.033788	2.030351	2.02699	2.023656	2.020319
2.044448	2.040941	2.037468	2.036245	2.032772	2.029351	2.025995	2.022664	2.019329
2.043363	2.039867	2.036402	2.035185	2.031735	2.028328	2.024975	2.021643	2.01831
2.042179	2.038698	2.035247	2.034035	2.030606	2.027213	2.023864	2.020536	2.017206
2.040967	2.037502	2.034066	2.032861	2.029449	2.026069	2.022726	2.0194	2.016076
2.039845	2.036392	2.032966	2.031767	2.028376	2.025009	2.021667	2.018343	2.015026
2.038786	2.035341	2.031924	2.03073	2.02736	2.024006	2.020664	2.01734	2.014032
2.037747	2.03431	2.030902	2.029709	2.026352	2.023007	2.019665	2.016342	2.013045
2.03673	2.033301	2.029902	2.028705	2.025354	2.022012	2.018668	2.015348	2.012063
2.035719	2.032299	2.028909	2.027707	2.024359	2.021018	2.017675	2.014357	2.011085
2.03471	2.031299	2.02792	2.026711	2.023366	2.020028	2.016687	2.013373	2.010106

2.033701	2.0303	2.026931	2.025714	2.022368	2.019032	2.015695	2.012386	2.009124
2.032689	2.029297	2.025938	2.024709	2.021359	2.018023	2.014696	2.011397	2.008143
2.031674	2.028291	2.02494	2.023695	2.020334	2.016997	2.013682	2.010395	2.007151
2.030655	2.027279	2.023933	2.022657	2.01926	2.015903	2.012596	2.009323	2.006091
2.029633	2.026261	2.022916	2.021597	2.018139	2.014747	2.011444	2.008186	2.004967
2.028615	2.025232	2.021868	2.020517	2.017039	2.01364	2.010346	2.007104	2.003896
2.027603	2.024183	2.020771	2.019404	2.015967	2.012602	2.009326	2.006097	2.0029
2.026597	2.023144	2.01969	2.018313	2.014921	2.011592	2.008334	2.005119	2.001933
2.025601	2.02214	2.01867	2.017284	2.013912	2.010601	2.007358	2.004156	2.000981
2.024609	2.021149	2.017675	2.016282	2.012924	2.009626	2.006397	2.003208	2.000043
2.023613	2.020153	2.016675	2.015278	2.011938	2.008658	2.005442	2.002264	1.999107
2.022614	2.019157	2.01568	2.01428	2.01096	2.007697	2.004494	2.001325	1.998174
2.021619	2.018165	2.01469	2.013286	2.009981	2.006732	2.003539	2.000378	1.997233
2.020625	2.017174	2.013701	2.012292	2.009	2.005761	2.002577	1.999422	1.996282
2.01964	2.016207	2.012756	2.00941	2.006155	2.004807	2.001622	1.998468	1.995331
2.018668	2.015282	2.011885	2.008545	2.005254	2.003866	2.00067	1.997511	1.994375
2.017695	2.014356	2.011016	2.007691	2.004377	2.002957	1.999742	1.996568	1.993428
2.016704	2.013395	2.010097	2.006811	2.003535	2.002115	1.998863	1.99565	1.992491
2.015707	2.012419	2.009155	2.005915	2.0027	2.001297	1.998012	1.994757	1.991573
2.01471	2.011439	2.008201	2.004991	2.001812	2.000425	1.997161	1.993911	1.990701
2.013712	2.010454	2.007233	2.004042	2.000884	1.999518	1.996315	1.993103	1.989866
2.012715	2.009469	2.006265	2.003089	1.999943	1.998589	1.995428	1.99225	1.989021
2.011713	2.008481	2.005298	2.002132	1.998988	1.997634	1.994494	1.991347	1.98817
2.010732	2.007507	2.004335	2.001175	1.998031	1.996674	1.993547	1.990423	1.987291
2.009773	2.006546	2.003374	2.000214	1.997072	1.99571	1.992591	1.989479	1.986366
2.008854	2.005617	2.002432	1.999265	1.996123	1.994753	1.991634	1.988526	1.985422
2.008021	2.004759	2.001533	1.998344	1.995194	1.993812	1.990687	1.987577	1.984478
2.007231	2.00394	2.000665	1.99745	1.994289	1.992891	1.989752	1.986633	1.983533
2.006372	2.003094	1.999827	1.996613	1.993447	1.992031	1.988863	1.985716	1.982603
2.005462	2.002233	1.999019	1.99583	1.992666	1.991229	1.988015	1.984823	1.981687
2.004538	2.001337	1.998157	1.994987	1.991832	1.99039	1.987169	1.983967	1.980821
2.003588	2.00039	1.997216	1.994058	1.99092	1.9895	1.986331	1.983168	1.980031
2.002672	1.999463	1.996276	1.99312	1.989993	1.988591	1.985468	1.982342	1.979214
2.001849	1.998606	1.995371	1.992191	1.98906	1.987654	1.984542	1.981428	1.978304
2.001045	1.99777	1.994489	1.991284	1.988144	1.986725	1.983605	1.980488	1.977362
2.00017	1.996912	1.993651	1.990454	1.987314	1.985875	1.982723	1.979575	1.97643
1.999257	1.996043	1.992839	1.989669	1.986531	1.985069	1.981871	1.978677	1.975505
1.998348	1.995147	1.991972	1.988818	1.985687	1.984218	1.981019	1.97782	1.97464
1.997435	1.99422	1.991051	1.987904	1.984784	1.983328	1.980172	1.977009	1.973839
1.996553	1.993334	1.990167	1.987017	1.983891	1.982431	1.979301	1.976161	1.972999
1.995727	1.992525	1.989359	1.98619	1.983022	1.981524	1.978383	1.97524	1.97208
1.994892	1.991705	1.988543	1.985361	1.982163	1.980633	1.977475	1.974325	1.971165
1.993984	1.990797	1.987648	1.984486	1.981318	1.979795	1.976636	1.97348	1.970321
1.99306	1.989869	1.986733	1.983599	1.980474	1.978968	1.97581	1.972649	1.969491
1.992206	1.989019	1.98588	1.982739	1.979604	1.978077	1.974911	1.971746	1.968588
1.991397	1.98822	1.985066	1.981897	1.978716	1.977146	1.973966	1.970798	1.96764
1.990584	1.987402	1.984227	1.981043	1.977852	1.97468	1.971532	1.968399	1.966744
1.989772	1.986566	1.983355	1.980172	1.977013	1.973861	1.970714	1.967577	1.965911
1.988932	1.985718	1.982497	1.979322	1.976188	1.973048	1.96989	1.966741	1.965063
1.988034	1.98485	1.981668	1.978513	1.975384	1.972232	1.969035	1.965852	1.964158

1.987135	1.983986	1.98085	1.977712	1.974574	1.971403	1.968174	1.964966	1.96326
1.986299	1.983164	1.980044	1.976894	1.97372	1.970533	1.967319	1.964131	1.962426
1.985484	1.982353	1.979235	1.976064	1.972846	1.969647	1.96647	1.963322	1.961621
1.984621	1.981484	1.978368	1.975201	1.971989	1.968801	1.965646	1.962511	1.960795
1.98372	1.980567	1.977453	1.974309	1.971144	1.967987	1.964842	1.961705	1.959956
1.985248	1.979702	1.976586	1.973455	1.970316	1.967172	1.964019	1.960871	1.959114
1.984403	1.978902	1.975785	1.972653	1.969515	1.966358	1.963165	1.959994	1.958264
1.985766	1.978125	1.975003	1.971862	1.968709	1.965535	1.962318	1.959139	1.957437
1.984973	1.977361	1.974234	1.97107	1.967875	1.964685	1.961491	1.958342	1.956657
1.984195	1.976602	1.973462	1.970273	1.967039	1.963841	1.960683	1.957574	1.955901
1.983416	1.978081	1.974903	1.969445	1.966222	1.96304	1.959908	1.95682	1.95515
1.98264	1.977298	1.974068	1.968603	1.965418	1.962268	1.959158	1.956082	1.954406
1.981862	1.976532	1.973273	1.970066	1.9669	1.961505	1.958413	1.955345	1.953646
1.981086	1.975784	1.972517	1.969308	1.966144	1.960754	1.957676	1.954611	1.952871
1.980276	1.977012	1.973721	1.968553	1.965392	1.960005	1.95694	1.953878	1.952109
1.979408	1.976162	1.972914	1.967797	1.964641	1.95926	1.956206	1.953153	1.951377
1.978552	1.975321	1.972113	1.967043	1.963894	1.958513	1.955459	1.952407	1.950634
1.97776	1.974532	1.971333	1.966294	1.963147	1.960029	1.956944	1.953874	1.949845
1.976996	1.973764	1.970564	1.965548	1.962405	1.959275	1.956152	1.953057	1.949046
1.976231	1.972997	1.969797	1.964812	1.961676	1.958544	1.955403	1.952295	1.950622
1.975472	1.972236	1.969034	1.964081	1.960957	1.95783	1.954683	1.951569	1.949889
1.974709	1.971471	1.968267	1.965089	1.96194	1.957115	1.953968	1.950852	1.949164
1.973941	1.970702	1.967498	1.964326	1.961188	1.956402	1.95326	1.950147	1.94845
1.973171	1.96993	1.966726	1.963561	1.960435	1.955686	1.952551	1.94944	1.947734
1.972398	1.969156	1.965952	1.962793	1.959681	1.954966	1.951835	1.948726	1.947012
1.971625	1.968381	1.965178	1.962026	1.958927	1.954244	1.951119	1.948012	1.946289
1.970854	1.967609	1.964405	1.961261	1.958177	1.955066	1.951901	1.948751	1.945565
1.970085	1.966839	1.963635	1.960498	1.957428	1.954327	1.951167	1.948018	1.944841
1.969322	1.966077	1.962874	1.959742	1.956682	1.95359	1.950437	1.947291	1.949989
1.968564	1.965321	1.96212	1.958992	1.955938	1.952853	1.94971	1.946569	1.949262
1.967844	1.964597	1.961383	1.958251	1.955199	1.952124	1.949006	1.945881	1.950437
1.967177	1.963918	1.960668	1.95752	1.954465	1.951406	1.948336	1.945243	1.949763
1.966495	1.963231	1.959958	1.956797	1.953735	1.957328	1.954232	1.951109	1.949073
1.965759	1.962503	1.959238	1.956076	1.953004	1.956581	1.953497	1.950383	1.948333
1.965007	1.961769	1.958531	1.955372	1.952284	1.955835	1.952748	1.949633	1.947576
1.964264	1.96106	1.957869	1.954714	1.951592	1.955104	1.952007	1.948887	1.94682
1.963523	1.960362	1.957229	1.954079	1.950917	1.954382	1.951267	1.948138	1.946066
1.958123	1.959645	1.956546	1.960115	1.956868	1.953664	1.950535	1.947402	1.945344
1.957389	1.958911	1.955824	1.959387	1.956148	1.952948	1.949808	1.946676	1.944655
1.956656	1.958176	1.955094	1.958655	1.955444	1.952255	1.949097	1.945953	1.943947
1.955914	1.957436	1.954353	1.957926	1.95477	1.951601	1.948405	1.945223	1.943198
1.955194	1.956715	1.95362	1.957195	1.954084	1.950934	1.947719	1.944511	1.942458
1.95453	1.956038	1.952905	1.956452	1.953347	1.950214	1.947035	1.94385	1.941763
1.953887	1.955383	1.952209	1.955718	1.952597	1.949473	1.946345	1.943196	1.941081
1.953222	1.96146	1.958231	1.955036	1.951877	1.948734	1.945616	1.942485	1.939299
1.952548	1.960793	1.957599	1.954391	1.951177	1.947995	1.944863	1.941736	1.938594
1.951895	1.955802	1.952618	1.953731	1.9505	1.947299	1.944138	1.940996	1.93787
1.951268	1.95516	1.951941	1.953053	1.949851	1.94665	1.943443	1.940257	1.937119
1.95061	1.954497	1.951267	1.952385	1.949197	1.945996	1.942764	1.939551	1.936392
1.949892	1.953799	1.95061	1.951741	1.948527	1.945315	1.942103	1.9389	1.93571

1.949162	1.953085	1.949941	1.951088	1.947854	1.944636	1.941447	1.938258	1.935044
1.95551	1.952351	1.949219	1.95039	1.947187	1.943983	1.940781	1.937581	1.934377
1.954792	1.951617	1.948484	1.949677	1.946519	1.943336	1.940114	1.936896	1.933714
1.95413	1.950954	1.947817	1.944664	1.941505	1.942662	1.939447	1.936236	1.933054
1.953514	1.950345	1.9472	1.944023	1.940821	1.941967	1.938781	1.935596	1.932397
1.95287	1.949698	1.946547	1.943362	1.940147	1.941293	1.93811	1.934929	1.93173
1.952181	1.948993	1.945842	1.942671	1.939487	1.94065	1.937432	1.934225	1.93105
1.951523	1.948318	1.94516	1.941992	1.938823	1.939995	1.936755	1.933531	1.930371
1.950948	1.947725	1.944542	1.941345	1.938141	1.939304	1.936081	1.932872	1.929697
1.95039	1.947151	1.943936	1.940705	1.937461	1.938611	1.935409	1.932219	1.929026
1.949795	1.946538	1.94329	1.940044	1.936801	1.937954	1.934736	1.931535	1.928357
1.949178	1.945907	1.942628	1.939377	1.93615	1.937313	1.934064	1.930843	1.927693
1.948526	1.945258	1.941983	1.938727	1.935488	1.932255	1.929019	1.925815	1.927061
1.947837	1.944588	1.941352	1.938093	1.934814	1.931569	1.928363	1.925192	1.92646
1.947174	1.943942	1.940731	1.937472	1.934169	1.930915	1.927728	1.924574	1.925846
1.946551	1.943335	1.940131	1.936874	1.933568	1.93031	1.927124	1.923964	1.925207
1.945943	1.942728	1.939516	1.936261	1.932967	1.929713	1.926519	1.923343	1.924565
1.945344	1.942101	1.938851	1.935597	1.932338	1.929098	1.925882	1.922687	1.92392
1.944751	1.941471	1.938178	1.934924	1.931704	1.928482	1.925243	1.922029	1.923283
1.944158	1.940867	1.937557	1.934302	1.931094	1.927879	1.924637	1.921421	1.922689
1.943563	1.940274	1.936967	1.933709	1.930495	1.92728	1.924051	1.920845	1.922121
1.942931	1.939651	1.936361	1.933101	1.929866	1.926889	1.923664	1.920458	1.921591
1.942255	1.938993	1.935739	1.932476	1.929203	1.926217	1.923034	1.919862	1.920979
1.941608	1.938358	1.935133	1.931868	1.928569	1.925579	1.922425	1.919276	1.916064
1.941015	1.93777	1.934554	1.931292	1.92799	1.925002	1.921854	1.91871	1.915495
1.940439	1.937195	1.933982	1.930724	1.927427	1.924445	1.921294	1.918147	1.914934
1.93986	1.936621	1.933413	1.930158	1.926865	1.923888	1.920734	1.917585	1.914373
1.939281	1.936045	1.932836	1.929586	1.926302	1.923331	1.920168	1.917012	1.913807
1.938677	1.935434	1.932205	1.92926	1.925989	1.922752	1.919564	1.916388	1.9132
1.938056	1.934797	1.931533	1.928598	1.925375	1.922155	1.91893	1.915724	1.912562
1.937454	1.934186	1.930901	1.927975	1.924783	1.921576	1.918327	1.915102	1.911954
1.936876	1.933606	1.930316	1.927398	1.924219	1.921015	1.91776	1.914528	1.911382
1.936309	1.933039	1.929749	1.926837	1.923663	1.920462	1.917204	1.913969	1.910821
1.935754	1.932482	1.929191	1.926283	1.923112	1.919914	1.916655	1.913419	1.910268
1.935208	1.931935	1.928645	1.925738	1.922568	1.919369	1.91611	1.912873	1.90972
1.934668	1.931394	1.928103	1.925196	1.922023	1.918822	1.915561	1.912322	1.909167
1.934137	1.930864	1.927575	1.924665	1.921485	1.918278	1.915014	1.911772	1.908615
1.933629	1.930364	1.927094	1.924182	1.920976	1.917751	1.914481	1.911236	1.908073
1.933143	1.929895	1.926659	1.923743	1.920494	1.917238	1.913962	1.910713	1.907539
1.932641	1.929404	1.92619	1.923273	1.919996	1.916727	1.913462	1.910222	1.907032
1.932115	1.92888	1.92567	1.922756	1.919476	1.916219	1.912995	1.909784	1.906569
1.931579	1.928703	1.925477	1.922222	1.918944	1.915701	1.912514	1.909327	1.906093
1.93103	1.928157	1.924932	1.921677	1.918397	1.915156	1.911982	1.908804	1.905566
1.93048	1.927608	1.924384	1.921129	1.917846	1.914605	1.911431	1.908255	1.90502
1.929944	1.92707	1.923849	1.920592	1.917307	1.914064	1.910888	1.907712	1.904478
1.92942	1.926544	1.923322	1.920064	1.916778	1.913531	1.910348	1.907167	1.903936
1.925764	1.926054	1.922813	1.919557	1.916288	1.913045	1.909841	1.906642	1.903427
1.92532	1.925604	1.922325	1.919072	1.915843	1.912612	1.909367	1.906139	1.902952
1.924848	1.925132	1.921829	1.918579	1.915375	1.912151	1.908883	1.905637	1.902469
1.924317	1.92461	1.92131	1.918061	1.914859	1.911636	1.908368	1.905123	1.901962

1.923785	1.924091	1.920805	1.917556	1.914339	1.911112	1.907857	1.904626	1.901461
1.923282	1.923609	1.920354	1.917098	1.913844	1.910606	1.907383	1.904178	1.900996
1.922799	1.923146	1.919921	1.916656	1.913357	1.910103	1.906918	1.903743	1.90054
1.922351	1.922699	1.919459	1.916176	1.912854	1.909591	1.90642	1.903258	1.900049
1.92193	1.922265	1.918977	1.91567	1.912342	1.909076	1.9059	1.902738	1.899535
1.921476	1.918704	1.915392	1.915177	1.911864	1.908602	1.905411	1.902238	1.899049
1.920982	1.918197	1.914884	1.914694	1.911427	1.908182	1.904957	1.901756	1.898592
1.921017	1.917697	1.914397	1.914229	1.91099	1.907756	1.904515	1.901301	1.902679
1.920521	1.917223	1.913951	1.913791	1.910533	1.907297	1.904083	1.90089	1.902259
1.920039	1.916762	1.913515	1.91336	1.910079	1.906839	1.903655	1.900486	1.901845
1.919596	1.916319	1.913059	1.912911	1.909646	1.906412	1.903221	1.900046	1.901422
1.919175	1.915887	1.9126	1.912458	1.909222	1.905999	1.902786	1.899595	1.900998
1.918731	1.915448	1.912165	1.912027	1.908787	1.905564	1.902356	1.899169	1.900577
1.918271	1.915005	1.911752	1.911616	1.908345	1.905112	1.901931	1.898765	1.900157
1.917833	1.914571	1.911325	1.911193	1.907914	1.904678	1.901504	1.898343	1.899733
1.917429	1.914151	1.910876	1.910749	1.907499	1.904273	1.901073	1.897892	1.899302
1.917022	1.91373	1.910433	1.907171	1.903942	1.903863	1.900644	1.897448	1.898872
1.916581	1.913293	1.910014	1.906751	1.903501	1.90342	1.90022	1.897037	1.898448
1.916146	1.91286	1.909603	1.906335	1.903062	1.902977	1.899798	1.896631	1.898025
1.915766	1.912457	1.909174	1.905903	1.902645	1.90257	1.899376	1.896194	1.897595
1.915421	1.912074	1.908742	1.905467	1.902246	1.902187	1.898958	1.895747	1.897164
1.915062	1.911707	1.908355	1.905079	1.901873	1.901822	1.898576	1.895345	1.896751
1.914696	1.91136	1.908015	1.904741	1.901531	1.90148	1.898232	1.894989	1.896354
1.914303	1.910989	1.907665	1.904393	1.901165	1.90111	1.897874	1.894635	1.895977
1.913864	1.91057	1.907288	1.904015	1.90075	1.900688	1.897488	1.894282	1.895632
1.913435	1.910156	1.906911	1.903637	1.900339	1.90027	1.897093	1.893912	1.89528
1.913067	1.909793	1.906556	1.903281	1.899976	1.899896	1.896687	1.893488	1.894883
1.912721	1.909445	1.906198	1.902923	1.899624	1.899539	1.896281	1.89305	1.894476
1.91235	1.909062	1.905789	1.902516	1.899244	1.895979	1.89271	1.889471	1.89411
1.911967	1.908658	1.905343	1.902074	1.898845	1.89561	1.892354	1.889119	1.893771
1.911603	1.908282	1.904939	1.901659	1.898432	1.895207	1.891974	1.888755	1.893408
1.911263	1.907935	1.90458	1.901271	1.897998	1.894758	1.891563	1.888375	1.893015
1.910924	1.907595	1.904236	1.900904	1.897592	1.894338	1.89117	1.888005	1.892638
1.910587	1.90726	1.903904	1.900567	1.897241	1.89398	1.890815	1.887653	1.892294
1.910234	1.906913	1.90357	1.900239	1.89691	1.893646	1.890477	1.887311	1.891965
1.909828	1.906519	1.903208	1.899895	1.896575	1.893316	1.890147	1.886978	1.888569
1.909402	1.906107	1.902836	1.899547	1.896244	1.892993	1.889824	1.886651	1.888247
1.909026	1.905735	1.902482	1.899205	1.89591	1.897541	1.894355	1.891165	1.887918
1.908688	1.905395	1.902144	1.898868	1.895576	1.897211	1.89402	1.890827	1.887585
1.908356	1.905063	1.901812	1.898536	1.895243	1.896875	1.893659	1.89045	1.88722
1.908029	1.904738	1.901488	1.89821	1.894912	1.896528	1.893259	1.890014	1.88681
1.910841	1.904416	1.901168	1.897888	1.894585	1.896188	1.892874	1.889598	1.886413
1.910522	1.904094	1.900848	1.897568	1.894264	1.895865	1.892533	1.889243	1.886062
1.910205	1.903774	1.90053	1.897251	1.893946	1.895551	1.892214	1.88892	1.885734
1.909887	1.906567	1.903295	1.896937	1.893631	1.895242	1.891905	1.88861	1.88542
1.90957	1.906253	1.902986	1.896625	1.89332	1.89494	1.891611	1.888321	1.885125
1.909263	1.905946	1.902678	1.896314	1.893013	1.894651	1.891349	1.888075	1.884864
1.908964	1.905644	1.902372	1.896004	1.892711	1.894375	1.891121	1.887878	1.884642
1.908699	1.905372	1.902081	1.89877	1.895443	1.894089	1.890869	1.88765	1.884398
1.908491	1.90515	1.901816	1.898479	1.895138	1.893782	1.890575	1.887368	1.884116

1.908276	1.904922	1.901551	1.898192	1.894837	1.893475	1.890271	1.887069	1.88382
1.908003	1.904648	1.901269	1.897902	1.894541	1.896263	1.893019	1.88978	1.883515
1.907708	1.904354	1.90098	1.897616	1.894257	1.895982	1.892726	1.889478	1.883207
1.907428	1.904064	1.900687	1.902408	1.899069	1.895755	1.892472	1.889196	1.885903
1.907153	1.903771	1.900391	1.902143	1.898861	1.895567	1.892247	1.888931	1.885623
1.906872	1.903481	1.9001	1.901872	1.898619	1.895338	1.892002	1.888671	1.885371
1.906583	1.903191	1.899809	1.901587	1.898339	1.895063	1.891734	1.888416	1.885152
1.901008	1.902917	1.899548	1.901329	1.898067	1.894791	1.891479	1.888183	1.884938
1.900718	1.902665	1.899337	1.901125	1.897823	1.894534	1.89126	1.887993	1.88473
1.900448	1.902429	1.899135	1.900924	1.897582	1.89428	1.891039	1.887797	1.88451
1.900232	1.902216	1.898903	1.90068	1.897324	1.894014	1.890778	1.887542	1.88425
1.900041	1.902013	1.898661	1.900423	1.897065	1.893752	1.890505	1.887262	1.883974
1.899811	1.901778	1.898416	1.900185	1.896847	1.893538	1.890268	1.887007	1.883733
1.899553	1.90152	1.898168	1.899961	1.896661	1.893364	1.890059	1.88677	1.883517
1.899295	1.895996	1.892709	1.899757	1.896468	1.893174	1.88986	1.886562	1.883308
1.899031	1.895761	1.892515	1.899578	1.896261	1.892959	1.889676	1.886397	1.883111
1.898796	1.895544	1.892321	1.899395	1.896061	1.892753	1.889486	1.886215	1.882899
1.898629	1.895356	1.892106	1.899188	1.895883	1.89258	1.889284	1.88598	1.882646
1.898471	1.895175	1.891892	1.898981	1.895706	1.892407	1.889069	1.885727	1.882385
1.898251	1.894969	1.891701	1.898801	1.895507	1.892189	1.888829	1.885475	1.882149
1.898005	1.894751	1.891521	1.898631	1.895299	1.891952	1.888578	1.885225	1.881928
1.897799	1.894544	1.891312	1.893429	1.890147	1.891754	1.888377	1.885021	1.881722
1.897631	1.894348	1.891079	1.8932	1.889955	1.891594	1.888224	1.884861	1.88153
1.897437	1.894141	1.890856	1.892983	1.88975	1.891404	1.888038	1.884674	1.881333
1.897189	1.893915	1.890658	1.892789	1.889522	1.891157	1.887786	1.88443	1.881121
1.896955	1.893696	1.890457	1.892592	1.889298	1.890921	1.887549	1.884202	1.880915
1.896782	1.8935	1.890228	1.892366	1.889098	1.890747	1.887385	1.884037	1.880729
1.896632	1.89332	1.890003	1.892141	1.888905	1.89059	1.887245	1.883899	1.880559
1.896472	1.893161	1.889832	1.891959	1.888698	1.890388	1.887075	1.883758	1.880417
1.896309	1.893018	1.889697	1.891806	1.888486	1.890162	1.88689	1.883617	1.880296
1.896112	1.892839	1.889543	1.891658	1.888312	1.885016	1.881796	1.878585	1.880141
1.895873	1.892617	1.889372	1.89152	1.888182	1.884872	1.881601	1.878355	1.879948
1.895663	1.892408	1.889182	1.891356	1.888041	1.884732	1.881428	1.878156	1.879764
1.895514	1.892224	1.888955	1.891138	1.887869	1.884593	1.881296	1.878017	1.879595
1.895383	1.89206	1.888741	1.890922	1.887687	1.88444	1.881168	1.877897	1.879445
1.895245	1.891924	1.888591	1.890757	1.887489	1.884238	1.881008	1.877775	1.879328
1.895108	1.891806	1.888474	1.890619	1.887293	1.884023	1.880839	1.877656	1.87923
1.894953	1.891683	1.888361	1.890502	1.887148	1.883868	1.8807	1.877535	1.879125
1.894837	1.89156	1.888256	1.890406	1.887046	1.883758	1.880583	1.877413	1.879018
1.894691	1.891411	1.888136	1.890305	1.886946	1.883649	1.880446	1.877257	1.878885
1.894501	1.891222	1.887997	1.890198	1.886848	1.883536	1.880279	1.877051	1.878715
1.894333	1.891087	1.887888	1.890085	1.886748	1.883428	1.880127	1.876868	1.873691
1.894223	1.890973	1.887777	1.889971	1.886647	1.88333	1.880017	1.876747	1.873573
1.894133	1.890869	1.887657	1.889842	1.886537	1.883233	1.879922	1.876649	1.873471
1.894038	1.890746	1.887494	1.889877	1.886602	1.883122	1.87982	1.87655	1.873369
1.893947	1.890617	1.887313	1.889696	1.886467	1.883005	1.879718	1.876455	1.873267
1.893861	1.890512	1.887181	1.889565	1.886357	1.88312	1.879827	1.876544	1.873138
1.893779	1.890428	1.88709	1.889477	1.886268	1.883032	1.879742	1.87645	1.872983
1.893703	1.890351	1.88701	1.8894	1.886189	1.882951	1.879663	1.876364	1.873049
1.893633	1.89028	1.886939	1.889333	1.886121	1.88288	1.879592	1.876289	1.872959

1.893563	1.890212	1.886872	1.88927	1.886055	1.882813	1.879524	1.87622	1.872886
1.893491	1.890139	1.886799	1.8892	1.885986	1.882745	1.879458	1.876157	1.872826
1.893418	1.890068	1.886729	1.889132	1.885919	1.882679	1.879395	1.876099	1.872782
1.893347	1.890002	1.886669	1.889074	1.885855	1.882614	1.879332	1.876046	1.872765
1.893278	1.889941	1.886619	1.889025	1.885797	1.882552	1.879269	1.875998	1.872766
1.89079	1.889899	1.886607	1.889018	1.885764	1.882501	1.879211	1.875943	1.87274
1.890736	1.889878	1.88664	1.889059	1.88576	1.882461	1.879155	1.87588	1.872684
1.890689	1.889851	1.88665	1.889075	1.885744	1.882422	1.879105	1.875825	1.87263
1.890641	1.889801	1.886609	1.889038	1.885703	1.882377	1.879058	1.875777	1.872578
1.890613	1.889762	1.88656	1.888987	1.885653	1.882333	1.879025	1.875751	1.872543
1.890638	1.88736	1.884105	1.888942	1.8856	1.882289	1.879024	1.87578	1.872555
1.890681	1.887387	1.884089	1.888897	1.885544	1.882247	1.879034	1.875829	1.872583
1.890672	1.887373	1.884058	1.886599	1.883218	1.882202	1.879011	1.875823	1.87257
1.890628	1.88733	1.884016	1.88656	1.883179	1.882157	1.878965	1.875778	1.872529
1.889819	1.887292	1.883982	1.886538	1.883176	1.882152	1.878943	1.87574	1.872489
1.889801	1.887258	1.88395	1.886531	1.883217	1.882201	1.878953	1.87571	1.872449
1.889791	1.887242	1.883944	1.886545	1.883256	1.879966	1.876665	1.873371	1.872427
1.889784	1.887255	1.883989	1.886596	1.883278	1.879966	1.876653	1.87336	1.872438
1.889793	1.887282	1.884042	1.886648	1.883291	1.879955	1.876645	1.873365	1.872464
1.889844	1.886541	1.883263	1.886656	1.883285	1.879952	1.87667	1.873412	1.872495
1.889915	1.886592	1.883271	1.886642	1.883273	1.879954	1.876711	1.873478	1.872528
1.88995	1.88661	1.883272	1.886642	1.883294	1.879988	1.876745	1.873506	1.870229
1.889955	1.886601	1.883262	1.886649	1.883344	1.880052	1.876775	1.873501	1.870215
1.887328	1.886605	1.883279	1.886045	1.882749	1.880099	1.876794	1.873498	1.870217
1.887319	1.886625	1.883337	1.886113	1.882789	1.880111	1.876791	1.873493	1.870241
1.887338	1.884491	1.881233	1.886181	1.882836	1.88014	1.876811	1.873513	1.870281
1.88743	1.884564	1.881275	1.886225	1.882906	1.880224	1.8769	1.873596	1.870342
1.887538	1.884647	1.881318	1.886268	1.882982	1.88032	1.877002	1.873694	1.87041
1.887583	1.884702	1.881382	1.884358	1.881065	1.880363	1.877042	1.873735	1.870463
1.887597	1.884745	1.88146	1.884441	1.88111	1.880382	1.877051	1.873749	1.87051
1.887654	1.884338	1.88106	1.884507	1.881175	1.877847	1.874514	1.871211	1.868732
1.887757	1.884417	1.881106	1.884556	1.881262	1.877962	1.874643	1.871338	1.868816
1.887838	1.884488	1.881162	1.884618	1.881342	1.87806	1.874763	1.871467	1.868919
1.887864	1.884536	1.881241	1.884706	1.8814	1.878114	1.874855	1.871596	1.869056
1.887902	1.884593	1.881321	1.884794	1.881461	1.878168	1.874936	1.871706	1.869184
1.882438	1.884682	1.881381	1.884852	1.881541	1.878255	1.875003	1.871763	1.868529
1.882568	1.88479	1.881444	1.88491	1.881629	1.878352	1.875066	1.871803	1.868595
1.882689	1.884917	1.881561	1.884708	1.88141	1.878423	1.875145	1.871889	1.868674
1.882808	1.885062	1.881719	1.884848	1.881491	1.87848	1.875236	1.872008	1.868759
1.882895	1.879616	1.876324	1.884988	1.881604	1.878582	1.875355	1.872139	1.868873
1.882938	1.879681	1.876437	1.885133	1.881756	1.878738	1.87551	1.87229	1.869025
1.883007	1.879754	1.876534	1.880165	1.876839	1.878898	1.875654	1.872419	1.869161
1.883134	1.879852	1.876596	1.880237	1.876962	1.879043	1.875759	1.872491	1.869253
1.883284	1.879968	1.876666	1.880309	1.877083	1.879184	1.875861	1.87256	1.869335
1.883439	1.880111	1.876789	1.880433	1.877218	1.879017	1.875692	1.872383	1.869424
1.883598	1.880271	1.876942	1.880581	1.877352	1.879149	1.875832	1.87252	1.869518
1.883741	1.880423	1.877091	1.880716	1.877451	1.874188	1.870921	1.867656	1.869641
1.883871	1.88057	1.87724	1.880843	1.877518	1.874235	1.87101	1.867789	1.869787
1.877537	1.88071	1.877388	1.88098	1.877618	1.874321	1.871123	1.867929	1.869942
1.877659	1.880847	1.877537	1.881135	1.877764	1.874462	1.871269	1.868079	1.870105

1.877762	1.874528	1.871273	1.88129	1.87792	1.874617	1.87142	1.868227	1.865518
1.877819	1.874601	1.871388	1.881435	1.878072	1.874771	1.87157	1.868374	1.865675
1.877874	1.874671	1.8715	1.881579	1.878228	1.874928	1.871712	1.868505	1.86582
1.877984	1.874785	1.87163	1.881729	1.878389	1.875081	1.871822	1.868584	1.865368
1.878129	1.874926	1.87177	1.881883	1.878554	1.875232	1.871913	1.868632	1.865442
1.871127	1.875075	1.871917	1.875929	1.872628	1.875389	1.872035	1.868729	1.865551
1.871283	1.875232	1.872072	1.876088	1.872791	1.875552	1.872186	1.86887	1.865692
1.871441	1.868235	1.86508	1.876248	1.872953	1.869642	1.866294	1.862997	1.86585
1.871598	1.868389	1.865231	1.876407	1.873113	1.869805	1.866462	1.863168	1.86602
1.871756	1.868545	1.865384	1.876566	1.873274	1.869973	1.86665	1.863369	1.866213
1.871912	1.868699	1.865538	1.876727	1.873438	1.870155	1.866878	1.863627	1.866451
1.872072	1.868857	1.865695	1.87689	1.873603	1.870341	1.867118	1.863903	1.866704
1.872239	1.869023	1.865856	1.87039	1.867111	1.870515	1.867317	1.86412	1.860891
1.872417	1.869198	1.866024	1.870558	1.867274	1.870681	1.86749	1.864299	1.861071
1.87264	1.869411	1.86621	1.870733	1.867441	1.86419	1.861001	1.857816	1.861242
1.872923	1.869673	1.866418	1.870915	1.86761	1.864351	1.861159	1.857974	1.861403
1.873181	1.869916	1.866621	1.8711	1.867786	1.86452	1.861322	1.858134	1.861568
1.873375	1.870109	1.866805	1.871282	1.867965	1.864695	1.861492	1.858302	1.861741
1.873548	1.870286	1.866986	1.871474	1.868163	1.864892	1.861678	1.858479	1.861927
1.87372	1.870462	1.86717	1.871684	1.868409	1.865148	1.861901	1.858674	1.855477
1.87389	1.870639	1.867363	1.87191	1.86868	1.865432	1.862143	1.858882	1.855709
1.874082	1.870844	1.867597	1.872163	1.868932	1.865675	1.862364	1.859086	1.85592
1.874291	1.871073	1.867868	1.872442	1.869173	1.865888	1.86257	1.859289	1.856114
1.874494	1.871287	1.868105	1.872686	1.869396	1.866102	1.862797	1.859524	1.856331
1.874684	1.871474	1.868294	1.872881	1.869593	1.866316	1.863054	1.859806	1.856581
1.874893	1.871673	1.868478	1.873071	1.869801	1.866545	1.863313	1.86008	1.856827
1.875152	1.871911	1.868677	1.873278	1.87005	1.866814	1.863567	1.860314	1.857042
1.87542	1.872156	1.86888	1.873487	1.870302	1.867084	1.863812	1.860532	1.857254
1.875649	1.87237	1.869082	1.873689	1.87051	1.867296	1.864021	1.860744	1.857485
1.875857	1.872571	1.869288	1.873891	1.870697	1.867479	1.864212	1.860952	1.857729
1.876065	1.872787	1.869525	1.87413	1.870909	1.867682	1.864436	1.861199	1.857984
1.876268	1.873017	1.869795	1.874406	1.871142	1.867901	1.864692	1.861486	1.858253
1.876497	1.873261	1.870057	1.874675	1.871395	1.868148	1.864954	1.86176	1.858521
1.876768	1.873524	1.870298	1.874923	1.871676	1.868439	1.865218	1.862002	1.858782
1.877042	1.87379	1.870545	1.875179	1.871958	1.86873	1.865486	1.862252	1.859048
1.877286	1.874046	1.870823	1.875462	1.872217	1.868982	1.865758	1.862541	1.859323
1.877528	1.874303	1.871109	1.875753	1.872473	1.869227	1.866033	1.862839	1.859602
1.877803	1.874574	1.871372	1.87602	1.87275	1.869507	1.866306	1.863106	1.859877
1.878101	1.874854	1.871621	1.876275	1.873041	1.86981	1.866578	1.863355	1.860151
1.878384	1.875131	1.871894	1.876554	1.873323	1.870094	1.866858	1.863634	1.860433
1.878645	1.875402	1.872194	1.87686	1.873595	1.870353	1.867149	1.863948	1.860725
1.878939	1.875692	1.872492	1.877161	1.873877	1.870628	1.867439	1.86425	1.861016
1.879295	1.876014	1.872771	1.877436	1.874178	1.870938	1.867722	1.864513	1.861298
1.879664	1.876353	1.873062	1.877721	1.874487	1.871254	1.868012	1.864783	1.861586
1.878031	1.876709	1.873401	1.870127	1.866883	1.871547	1.868319	1.865101	1.861889
1.878384	1.877069	1.873762	1.87047	1.867187	1.87184	1.868641	1.865446	1.862206
1.87869	1.877386	1.874106	1.870819	1.867523	1.872177	1.86899	1.865804	1.862552
1.878954	1.877668	1.874439	1.871177	1.867886	1.872551	1.869364	1.866176	1.862926
1.879249	1.87597	1.872714	1.871504	1.868237	1.872919	1.869716	1.866515	1.863276
1.879585	1.876287	1.872995	1.871786	1.868569	1.873278	1.870036	1.866803	1.863593

1.872638	1.876621	1.873295	1.870026	1.866806	1.873637	1.870363	1.867105	1.863919
1.873	1.87699	1.873654	1.870385	1.867174	1.874006	1.870724	1.86746	1.864283
1.873358	1.877361	1.87403	1.870757	1.867537	1.874363	1.871087	1.867828	1.86465
1.873663	1.870387	1.867124	1.871106	1.867856	1.874664	1.871414	1.868177	1.864979
1.873943	1.870682	1.867459	1.871448	1.868156	1.874937	1.871726	1.868519	1.865292
1.874271	1.871019	1.867816	1.871813	1.868497	1.873397	1.870168	1.866939	1.865633
1.874642	1.871393	1.868193	1.872199	1.868877	1.873776	1.870548	1.867321	1.866001
1.871126	1.871774	1.868574	1.865325	1.862039	1.86598	1.862761	1.859551	1.864426
1.871507	1.872162	1.868958	1.865708	1.862422	1.866372	1.863145	1.85993	1.864812
1.871889	1.872547	1.869336	1.866084	1.862802	1.86676	1.863516	1.860288	1.865181
1.872266	1.869023	1.865821	1.866452	1.863174	1.859909	1.856657	1.85343	1.865502
1.872637	1.869384	1.866166	1.866803	1.863535	1.860262	1.856967	1.85371	1.865813
1.864365	1.869721	1.866464	1.867108	1.863872	1.860617	1.857316	1.854052	1.866172
1.864704	1.870042	1.86673	1.867382	1.864191	1.860971	1.85769	1.854436	1.866563
1.85735	1.870386	1.867044	1.863805	1.860657	1.86133	1.858064	1.854819	1.860313
1.857699	1.870755	1.867408	1.864165	1.861012	1.861694	1.858439	1.855204	1.860702
1.858057	1.866407	1.863074	1.864536	1.861374	1.858196	1.854975	1.851771	1.86109
1.858425	1.866789	1.863452	1.864909	1.861741	1.85856	1.855343	1.852142	1.861473
1.858798	1.867179	1.86384	1.865293	1.862118	1.858934	1.855718	1.852519	1.86186
1.859169	1.855909	1.852637	1.865588	1.862406	1.859323	1.856106	1.852905	1.862254
1.859544	1.856289	1.853026	1.866007	1.862818	1.859725	1.856509	1.853307	1.862661
1.859941	1.856698	1.853459	1.861859	1.858654	1.860139	1.856944	1.853759	1.859546
1.860354	1.857132	1.853929	1.86235	1.859101	1.860566	1.857408	1.854252	1.860022
1.860751	1.857543	1.854366	1.860587	1.857321	1.856305	1.853173	1.850038	1.851485
1.861125	1.857927	1.854762	1.860996	1.857716	1.856697	1.853565	1.850433	1.851879
1.861498	1.858305	1.855146	1.851946	1.848713	1.85709	1.853953	1.850818	1.852183
1.861866	1.858675	1.855517	1.852318	1.849085	1.857482	1.854341	1.851204	1.852576
1.862251	1.859058	1.855891	1.852693	1.849468	1.857891	1.854739	1.851594	1.85298
1.862698	1.859492	1.856293	1.853095	1.849897	1.85612	1.852953	1.849803	1.848839
1.863174	1.859951	1.85671	1.853512	1.850349	1.856604	1.8534	1.850222	1.849295
1.863598	1.860369	1.857114	1.853917	1.850769	1.857046	1.853826	1.850636	1.849727
1.863976	1.86075	1.857506	1.854312	1.851161	1.857452	1.854234	1.851045	1.85014
1.864351	1.861138	1.857918	1.854726	1.851557	1.8484	1.845249	1.84212	1.848356
1.864726	1.861543	1.858365	1.855173	1.851967	1.848799	1.845686	1.842586	1.848813
1.865109	1.861947	1.858801	1.855608	1.852371	1.849195	1.846112	1.843038	1.849267
1.850623	1.862334	1.859188	1.855992	1.852751	1.849573	1.846492	1.843428	1.849698
1.851035	1.862727	1.859564	1.856365	1.853135	1.84996	1.846865	1.843797	1.850116
1.851489	1.863166	1.859972	1.856773	1.853572	1.850404	1.847277	1.844181	1.850513
1.85197	1.863635	1.860401	1.857205	1.854044	1.850885	1.847715	1.844574	1.850896
1.852419	1.849262	1.846099	1.857661	1.854504	1.851343	1.848161	1.845003	1.851315
1.852836	1.849702	1.84658	1.858147	1.854953	1.851776	1.848617	1.845474	1.851773
1.853244	1.850131	1.847041	1.858615	1.855405	1.85222	1.849071	1.845935	1.8428
1.853643	1.850537	1.847455	1.859042	1.855865	1.852692	1.849519	1.846363	1.843241
1.854053	1.850953	1.847873	1.859471	1.856325	1.853163	1.849968	1.846793	1.843684
1.8532	1.851416	1.848342	1.845245	1.842131	1.853608	1.850425	1.847258	1.844134
1.853668	1.851895	1.848825	1.84572	1.842583	1.854045	1.850884	1.847733	1.844585
1.854106	1.851035	1.847978	1.846172	1.843042	1.839914	1.836789	1.833676	1.845033
1.854523	1.851449	1.8484	1.846607	1.843505	1.840385	1.837236	1.834105	1.845478
1.85497	1.851895	1.848844	1.845772	1.842684	1.840838	1.837687	1.834554	1.845927
1.855453	1.85238	1.849319	1.84623	1.843119	1.841263	1.838142	1.83503	1.846382

1.855942	1.852865	1.849788	1.846685	1.843558	1.840445	1.837351	1.834263	1.832389
1.856431	1.853335	1.85023	1.847125	1.844017	1.840909	1.837797	1.834696	1.832836
1.856917	1.853799	1.850671	1.847564	1.844475	1.841374	1.838243	1.835131	1.833291
1.85159	1.854262	1.851135	1.84802	1.844913	1.841807	1.838694	1.835602	1.832556
1.852041	1.854721	1.851607	1.848481	1.845342	1.84223	1.839152	1.836093	1.833054
1.845203	1.849401	1.846314	1.848924	1.845789	1.842685	1.839626	1.83658	1.833534
1.84565	1.849837	1.846729	1.849352	1.846249	1.843168	1.840116	1.837068	1.834001
1.84609	1.842988	1.839893	1.849802	1.846719	1.843649	1.840592	1.837536	1.834463
1.846513	1.843421	1.840349	1.850284	1.847204	1.844126	1.841044	1.837973	1.834919
1.846956	1.843874	1.840823	1.837775	1.834732	1.844595	1.841496	1.838417	1.835382
1.847449	1.84437	1.841321	1.838257	1.835183	1.845038	1.84195	1.838887	1.835863
1.847961	1.844883	1.84183	1.838747	1.835638	1.845485	1.842417	1.839375	1.836353
1.848456	1.845385	1.842332	1.839246	1.836133	1.833057	1.830037	1.827036	1.836841
1.848946	1.845883	1.84283	1.839752	1.836653	1.833584	1.830561	1.827548	1.837334
1.844194	1.846379	1.84331	1.840235	1.837153	1.834097	1.831079	1.828065	1.825025
1.844716	1.846872	1.84377	1.840691	1.837633	1.834598	1.831593	1.828587	1.825544
1.845232	1.847369	1.844243	1.841163	1.838123	1.835101	1.832104	1.829103	1.826061
1.845742	1.847876	1.844746	1.841666	1.83863	1.835611	1.832614	1.829612	1.826576
1.846235	1.848375	1.845251	1.842175	1.839142	1.836121	1.833113	1.830106	1.827077
1.846688	1.843601	1.840525	1.842675	1.83965	1.836624	1.833588	1.83056	1.827543
1.84713	1.844054	1.841007	1.843176	1.840161	1.837128	1.834058	1.831004	1.828
1.847621	1.844544	1.841507	1.843685	1.840679	1.837645	1.83456	1.831493	1.828491
1.848146	1.845064	1.842021	1.844201	1.841203	1.838174	1.835085	1.832013	1.829007
1.848688	1.845601	1.842554	1.839544	1.836574	1.838713	1.835622	1.832548	1.829539
1.849251	1.84616	1.84311	1.840098	1.837127	1.839265	1.836176	1.833103	1.830091
1.849811	1.846717	1.843664	1.840649	1.837676	1.834671	1.831607	1.828558	1.830641
1.850358	1.84726	1.844205	1.841188	1.838211	1.835205	1.832144	1.829096	1.831177
1.850901	1.8478	1.844744	1.841725	1.838744	1.835736	1.832676	1.829629	1.831709
1.851447	1.848343	1.845287	1.842265	1.839282	1.836271	1.833209	1.83016	1.832235
1.851992	1.848886	1.845831	1.842808	1.839822	1.836808	1.833743	1.830691	1.83276
1.844327	1.849427	1.846374	1.843351	1.840364	1.83735	1.834287	1.831235	1.828226
1.844861	1.849964	1.846914	1.843893	1.840907	1.837896	1.834838	1.831788	1.828772
1.845413	1.842315	1.839276	1.844448	1.841463	1.838457	1.835414	1.832372	1.829343
1.845988	1.842886	1.839843	1.845018	1.842036	1.839042	1.836025	1.832998	1.829948
1.84658	1.843473	1.840418	1.837404	1.834434	1.83146	1.828479	1.825478	1.830555
1.847204	1.844084	1.840997	1.837966	1.834988	1.832015	1.82905	1.826063	1.831147
1.847831	1.844698	1.84158	1.838528	1.835537	1.832561	1.829606	1.826632	1.831734
1.83995	1.845283	1.842156	1.839093	1.836087	1.833105	1.830154	1.827193	1.824184
1.840509	1.845851	1.84273	1.839662	1.83664	1.833649	1.830695	1.827743	1.824771
1.84107	1.83798	1.834903	1.84026	1.837221	1.834215	1.831249	1.828293	1.825337
1.841632	1.838564	1.835522	1.84089	1.83783	1.834801	1.831812	1.82884	1.825881
1.834331	1.839141	1.836125	1.833097	1.830064	1.835414	1.832399	1.829404	1.826432
1.834875	1.839704	1.836695	1.833692	1.830702	1.83607	1.833024	1.829989	1.82699
1.835434	1.840275	1.837261	1.834277	1.831328	1.83672	1.833656	1.830596	1.82757
1.830233	1.832969	1.829971	1.834843	1.831904	1.828938	1.825928	1.82291	1.828198
1.830836	1.833554	1.830534	1.835414	1.832466	1.829511	1.826544	1.82356	1.828844
1.826384	1.8342	1.831168	1.83605	1.833069	1.830096	1.827133	1.824159	1.821148
1.827054	1.834892	1.831855	1.836735	1.833701	1.830688	1.827703	1.824726	1.821744
1.817022	1.829769	1.826745	1.829483	1.826458	1.831308	1.828297	1.825305	1.822333
1.817634	1.830395	1.827369	1.830117	1.827098	1.831962	1.828917	1.825894	1.822909

1.818258	1.815245	1.812248	1.824973	1.821976	1.824714	1.821694	1.818696	1.823514
1.81892	1.815881	1.812849	1.825574	1.822568	1.825315	1.822319	1.81934	1.824178
1.819578	1.816516	1.813454	1.82618	1.823155	1.825908	1.822942	1.819989	1.824848
1.809374	1.817133	1.814083	1.821727	1.81869	1.826492	1.823529	1.820584	1.825472
1.809956	1.817741	1.814722	1.822374	1.819292	1.827078	1.824106	1.821162	1.82608
1.810555	1.807552	1.804572	1.812303	1.809237	1.816896	1.813956	1.811045	1.818844
1.811166	1.808149	1.805168	1.812919	1.809881	1.817568	1.814619	1.811696	1.819499
1.8118	1.808777	1.805795	1.802801	1.7998	1.807536	1.804591	1.801669	1.809414
1.812468	1.809453	1.806469	1.803464	1.800441	1.808162	1.805194	1.802257	1.810004
1.813125	1.810113	1.807127	1.804116	1.801084	1.808801	1.805818	1.802867	1.799953
1.813728	1.810706	1.807721	1.804727	1.801729	1.809476	1.806498	1.803543	1.800618
1.814331	1.811294	1.808305	1.805331	1.802377	1.810161	1.80719	1.804231	1.801296
1.814998	1.81195	1.808942	1.80597	1.803034	1.800092	1.797131	1.794182	1.801924
1.815701	1.812643	1.809606	1.806624	1.803693	1.800745	1.797755	1.794782	1.802531
1.803986	1.813312	1.810243	1.807249	1.804322	1.801378	1.79839	1.795421	1.792509
1.804651	1.813961	1.810855	1.807845	1.804919	1.801989	1.799034	1.796095	1.793194
1.805289	1.802192	1.799095	1.808464	1.805538	1.802619	1.799694	1.796781	1.793886
1.805879	1.802799	1.799728	1.809123	1.806196	1.803284	1.800384	1.797488	1.794591
1.806488	1.803425	1.80038	1.809796	1.806863	1.803951	1.80106	1.798172	1.795273
1.807179	1.804114	1.801066	1.810483	1.807531	1.804599	1.801685	1.798781	1.795885
1.8079	1.804825	1.801761	1.811169	1.808193	1.805235	1.802288	1.799364	1.796475
1.758267	1.805502	1.802416	1.799387	1.79641	1.805848	1.802904	1.799988	1.797112
1.758843	1.806159	1.803044	1.799993	1.796999	1.806445	1.803525	1.800637	1.797777
1.759389	1.756616	1.753849	1.800621	1.797612	1.794663	1.791787	1.788945	1.798456
1.759899	1.757135	1.754394	1.80127	1.798246	1.795296	1.792443	1.789622	1.799151
1.76043	1.757676	1.754955	1.752239	1.749529	1.795957	1.793113	1.790299	1.799841
1.761007	1.758259	1.755545	1.752833	1.750126	1.796655	1.793798	1.790971	1.800525
1.761604	1.758861	1.756148	1.75344	1.750739	1.797373	1.79449	1.791638	1.801192
1.762208	1.759469	1.75676	1.754059	1.751369	1.798107	1.795187	1.792294	1.789419
1.762824	1.760087	1.757378	1.754682	1.752003	1.798846	1.795882	1.79294	1.790021
1.763456	1.760711	1.757989	1.755285	1.7526	1.749909	1.747201	1.744505	1.790628
1.764103	1.761341	1.758595	1.755871	1.753168	1.750462	1.747743	1.745039	1.791236
1.764734	1.761958	1.759191	1.756445	1.753719	1.750998	1.748275	1.745575	1.742912
1.765344	1.762559	1.759777	1.757007	1.754248	1.751511	1.748795	1.74611	1.743469
1.756425	1.763149	1.760346	1.757557	1.754779	1.752035	1.749328	1.746658	1.744035
1.75701	1.763718	1.760878	1.758081	1.755322	1.752593	1.749893	1.747228	1.74461
1.757598	1.76429	1.761411	1.758609	1.755874	1.753165	1.750473	1.747813	1.745194
1.728018	1.755339	1.752452	1.759182	1.756441	1.753734	1.751058	1.74841	1.745787
1.728598	1.755954	1.753053	1.759783	1.75702	1.754306	1.751649	1.749016	1.746386
1.729163	1.726375	1.723562	1.750831	1.748061	1.754882	1.752231	1.749608	1.746979
1.729715	1.726936	1.724137	1.751438	1.74864	1.75546	1.752807	1.750186	1.747564
1.730281	1.727507	1.724716	1.721962	1.719233	1.746491	1.743853	1.741251	1.748151
1.730873	1.728091	1.725293	1.72253	1.719795	1.747084	1.744442	1.741835	1.74874
1.731465	1.728684	1.725888	1.723122	1.720379	1.747695	1.745043	1.742427	1.749339
1.732041	1.729289	1.726533	1.723771	1.720999	1.718303	1.715711	1.713157	1.710611
1.732614	1.729896	1.727189	1.724436	1.72164	1.71892	1.716303	1.713732	1.711191
1.733198	1.730485	1.727792	1.725059	1.722293	1.719583	1.71695	1.71436	1.711815
1.73379	1.731064	1.728361	1.725657	1.722954	1.720279	1.717634	1.715024	1.712469
1.734389	1.73165	1.728934	1.72624	1.723568	1.72091	1.718259	1.715639	1.713078
1.734998	1.732246	1.729509	1.726804	1.724128	1.721469	1.71882	1.716202	1.713638

1.735603	1.732841	1.730089	1.727374	1.724691	1.722027	1.719373	1.716752	1.714187
1.736198	1.733432	1.730678	1.727962	1.72528	1.722608	1.719931	1.717292	1.714728
1.736794	1.734024	1.731269	1.728551	1.725867	1.723187	1.72049	1.717834	1.715268
1.730857	1.73462	1.731852	1.729122	1.726428	1.723743	1.721051	1.718396	1.715813
1.731463	1.735221	1.732438	1.729694	1.726984	1.724294	1.721616	1.718969	1.716364
1.732074	1.735835	1.73305	1.730296	1.72757	1.724869	1.722193	1.719547	1.716931
1.732688	1.736459	1.733685	1.730925	1.728178	1.725463	1.722781	1.720131	1.717512
1.733351	1.730568	1.727795	1.731591	1.728834	1.726109	1.723422	1.720765	1.718137
1.734088	1.731297	1.728515	1.732315	1.729559	1.726832	1.724138	1.72147	1.718822
1.734786	1.731989	1.729203	1.733006	1.730249	1.727521	1.724824	1.722151	1.719488
1.735377	1.732582	1.729807	1.73361	1.730836	1.728101	1.725416	1.722753	1.720086
1.73594	1.733148	1.730383	1.734187	1.731398	1.728657	1.725983	1.723329	1.720663
1.736536	1.733738	1.730968	1.728186	1.725395	1.729254	1.72657	1.723909	1.721249
1.737153	1.734345	1.731559	1.728779	1.726006	1.729873	1.727169	1.724492	1.721841
1.737831	1.735015	1.732221	1.729438	1.726667	1.730537	1.727822	1.725137	1.722489
1.738571	1.73575	1.732953	1.730162	1.727379	1.731247	1.728529	1.725843	1.723191
1.739273	1.736447	1.733642	1.730846	1.72806	1.7253	1.722565	1.719866	1.723854
1.739906	1.737071	1.73425	1.731453	1.728678	1.725916	1.723153	1.720433	1.724441
1.740541	1.737705	1.734872	1.732074	1.729308	1.726543	1.72376	1.721022	1.725043
1.741228	1.738406	1.735579	1.732779	1.730004	1.727235	1.724457	1.72172	1.725729
1.741925	1.739119	1.736302	1.733501	1.730713	1.72794	1.725175	1.722443	1.726431
1.742584	1.739774	1.736955	1.734153	1.731365	1.72859	1.725824	1.723087	1.720394
1.74322	1.740395	1.73757	1.734768	1.731985	1.729211	1.726437	1.72369	1.720987
1.743854	1.741034	1.738228	1.735432	1.732647	1.729871	1.7271	1.72435	1.72163
1.744478	1.741689	1.73893	1.73615	1.733356	1.730577	1.72782	1.725075	1.722328
1.724186	1.742368	1.739628	1.736851	1.734045	1.731261	1.72851	1.725767	1.723005
1.724877	1.74308	1.7403	1.737499	1.734681	1.731887	1.729123	1.726375	1.723622
1.725584	1.743805	1.740982	1.738156	1.735327	1.732523	1.729747	1.726993	1.724252
1.726271	1.744538	1.741715	1.738883	1.736045	1.733235	1.73046	1.727707	1.724964
1.726952	1.745271	1.74246	1.739631	1.736786	1.733973	1.731206	1.728457	1.725712
1.727616	1.724844	1.722085	1.740306	1.737464	1.734659	1.731905	1.729168	1.726435
1.728269	1.72546	1.722676	1.740929	1.738099	1.73531	1.732573	1.729855	1.727146
1.693052	1.726134	1.723332	1.741619	1.73879	1.767781	1.76493	1.762106	1.759304
1.693754	1.726885	1.724072	1.742395	1.739548	1.768519	1.765615	1.762747	1.759925
1.679089	1.691787	1.689138	1.722009	1.719225	1.769278	1.766333	1.763427	1.760582
1.679764	1.692469	1.689809	1.722729	1.719954	1.770064	1.767113	1.764193	1.761324
1.680427	1.693136	1.690465	1.723436	1.720677	1.770846	1.767903	1.76498	1.76209
1.666392	1.693757	1.691083	1.688454	1.685867	1.751801	1.748931	1.746074	1.762833
1.667012	1.694362	1.691686	1.689063	1.686493	1.752472	1.749607	1.746756	1.763563
1.667706	1.665034	1.662399	1.689704	1.687119	1.753117	1.750251	1.747402	1.764257
1.668466	1.665766	1.663094	1.690369	1.687739	1.753732	1.750859	1.748012	1.764913
1.655186	1.666497	1.663809	1.661163	1.658555	1.721566	1.718838	1.71613	1.745837
1.655865	1.667221	1.664552	1.6619	1.659264	1.722258	1.719516	1.716795	1.746506
1.656531	1.653899	1.651299	1.66264	1.65999	1.695747	1.693042	1.690356	1.714746
1.657189	1.654568	1.65198	1.663359	1.660721	1.696479	1.69376	1.691057	1.715443
1.657838	1.655224	1.652641	1.664065	1.66145	1.697217	1.69449	1.691774	1.716162
1.658472	1.655857	1.653284	1.650713	1.648151	1.697925	1.695213	1.692503	1.689789
1.6591	1.656475	1.653909	1.651349	1.648802	1.698613	1.695933	1.693241	1.690516
1.659767	1.657112	1.654519	1.651961	1.649443	1.686595	1.683965	1.681316	1.691236
1.660474	1.657764	1.655108	1.652543	1.650067	1.687252	1.684627	1.681988	1.691951

1.661204	1.658448	1.655735	1.653157	1.650706	1.687911	1.685291	1.682659	1.680017
1.661964	1.659178	1.656423	1.653824	1.651369	1.688573	1.685963	1.683339	1.680701
1.662726	1.659924	1.657142	1.654519	1.652042	1.689236	1.686632	1.684015	1.681377
1.663467	1.660668	1.657885	1.65524	1.65272	1.689897	1.687291	1.684678	1.682044
1.664199	1.661412	1.658643	1.655976	1.653403	1.690561	1.687946	1.685335	1.682704
1.664928	1.662143	1.659375	1.656693	1.654087	1.691236	1.688608	1.68599	1.683355
1.665653	1.662865	1.660091	1.657396	1.654772	1.69192	1.689276	1.686644	1.684
1.666371	1.663581	1.660804	1.6581	1.655462	1.692608	1.689951	1.687308	1.684652
1.667081	1.664289	1.661512	1.658803	1.656156	1.693299	1.690637	1.687985	1.685316
1.641527	1.664997	1.662219	1.659507	1.656855	1.693994	1.691318	1.688651	1.685973
1.642212	1.665703	1.662924	1.66021	1.657557	1.694693	1.691985	1.689292	1.686614
1.642903	1.666416	1.663635	1.66092	1.658265	1.695396	1.692649	1.689926	1.68725
1.64361	1.640841	1.638085	1.661639	1.658981	1.696097	1.693314	1.690561	1.687882
1.644326	1.641562	1.638801	1.662374	1.659707	1.696801	1.693982	1.6912	1.688517
1.614286	1.642295	1.639565	1.636864	1.634191	1.69752	1.694674	1.691873	1.689185
1.614905	1.643042	1.640375	1.637673	1.634943	1.698254	1.695386	1.692577	1.689885
1.615523	1.643771	1.641144	1.638445	1.635685	1.67462	1.671762	1.668972	1.690598
1.616133	1.644467	1.64185	1.639161	1.636413	1.67536	1.672495	1.669689	1.691324
1.616769	1.614248	1.611756	1.639868	1.637135	1.676097	1.673241	1.67043	1.667716
1.617469	1.614934	1.612407	1.640582	1.637853	1.676818	1.674007	1.671216	1.668452
1.618185	1.615632	1.613065	1.6413	1.63857	1.677535	1.674771	1.672005	1.669193
1.61885	1.616296	1.613721	1.611139	1.608546	1.678251	1.67549	1.672728	1.669904
1.619489	1.616943	1.614378	1.61179	1.609176	1.678971	1.676186	1.673414	1.670598
1.62014	1.617589	1.615032	1.612456	1.60986	1.679745	1.676922	1.674118	1.6713
1.620797	1.618229	1.615681	1.613138	1.610602	1.680578	1.677697	1.674836	1.672005
1.621484	1.618903	1.61636	1.613833	1.611328	1.652635	1.649872	1.647141	1.672746
1.62221	1.619629	1.617085	1.614544	1.612013	1.653295	1.650554	1.647838	1.673544
1.622957	1.620375	1.617819	1.615256	1.61269	1.653948	1.651239	1.648547	1.674351
1.623729	1.621127	1.618536	1.615949	1.613366	1.654623	1.651931	1.649252	1.646584
1.624506	1.621883	1.61925	1.616642	1.614051	1.655314	1.652627	1.649954	1.64727
1.625234	1.622609	1.61997	1.617363	1.614783	1.656041	1.653328	1.65063	1.647931
1.625928	1.623314	1.620693	1.618107	1.615551	1.656796	1.654033	1.651289	1.648575
1.626645	1.62404	1.62144	1.61886	1.616299	1.657533	1.654743	1.651975	1.649251
1.627382	1.624785	1.622218	1.619629	1.617025	1.658246	1.655456	1.652691	1.649962
1.628152	1.625548	1.622984	1.620379	1.617742	1.65897	1.65618	1.65342	1.650691
1.628985	1.626342	1.623715	1.62108	1.61844	1.659714	1.656917	1.654163	1.651441
1.629817	1.627133	1.624442	1.621782	1.619149	1.660478	1.657668	1.654913	1.652199
1.598351	1.627881	1.625191	1.622535	1.619908	1.661275	1.65843	1.655651	1.652944
1.599028	1.62861	1.625953	1.623313	1.620693	1.662096	1.659209	1.656395	1.653692
1.599721	1.597136	1.594595	1.624093	1.62147	1.662929	1.660028	1.657198	1.654474
1.600424	1.597849	1.595328	1.624879	1.622244	1.663773	1.660883	1.658053	1.655285
1.533842	1.598582	1.596047	1.625649	1.623025	1.664616	1.661737	1.658904	1.656106
1.534445	1.599334	1.596737	1.626391	1.623814	1.665457	1.66259	1.659747	1.656941
1.535063	1.600105	1.597457	1.594909	1.592452	1.666287	1.663428	1.660581	1.657771
1.535697	1.600898	1.598255	1.595681	1.593168	1.667082	1.66423	1.661398	1.658582
1.536337	1.601702	1.599079	1.596476	1.593892	1.667884	1.66503	1.662209	1.659389
1.536965	1.534792	1.532625	1.597259	1.59466	1.6389	1.636096	1.633336	1.660201
1.53759	1.535417	1.533259	1.598042	1.595457	1.639766	1.636906	1.634102	1.661019
1.538228	1.536052	1.533892	1.598847	1.596274	1.640655	1.637761	1.634925	1.66188
1.538878	1.536697	1.534526	1.599675	1.597117	1.641574	1.638665	1.635808	1.662786

1.53952	1.537337	1.53516	1.533016	1.530902	1.642459	1.63954	1.636671	1.633873
1.540142	1.537963	1.535797	1.533643	1.531501	1.643277	1.640358	1.637494	1.634672
1.540766	1.538589	1.536431	1.534267	1.532099	1.644099	1.64117	1.638303	1.635467
1.510732	1.539227	1.537051	1.534881	1.532717	1.644974	1.641995	1.63909	1.636266
1.511329	1.539868	1.53767	1.535498	1.533349	1.645876	1.64284	1.639886	1.637077
1.5119	1.5098	1.507691	1.536147	1.534004	1.646804	1.643753	1.640772	1.637931
1.512455	1.510374	1.508289	1.536819	1.534677	1.647754	1.644718	1.641724	1.63882
1.513012	1.510948	1.508886	1.537482	1.535332	1.586239	1.58364	1.581047	1.639716
1.513572	1.511524	1.509489	1.538141	1.535967	1.587004	1.584426	1.581843	1.640617
1.514136	1.512091	1.510067	1.508007	1.505916	1.534438	1.532308	1.530175	1.594723
1.514702	1.512639	1.510596	1.508545	1.506489	1.535098	1.532973	1.530841	1.595562
1.515278	1.513191	1.511122	1.509079	1.50706	1.535758	1.533634	1.531501	1.596403
1.515878	1.513772	1.511686	1.509637	1.507626	1.505613	1.503594	1.501565	1.530002
1.516492	1.514367	1.512267	1.510209	1.508193	1.506178	1.504162	1.50214	1.530657
1.517088	1.514953	1.512856	1.510796	1.508775	1.506754	1.504727	1.502702	1.531307
1.517672	1.515533	1.513454	1.511398	1.509372	1.507339	1.505288	1.503251	1.531952
1.518285	1.516134	1.514059	1.511997	1.509957	1.507913	1.50585	1.50381	1.501835
1.518932	1.516758	1.514669	1.512586	1.510522	1.508466	1.506411	1.504381	1.50241
1.486207	1.517406	1.515293	1.513187	1.5111	1.509034	1.506986	1.504965	1.50299
1.486838	1.518087	1.515936	1.513808	1.51171	1.509637	1.507588	1.505565	1.503581
1.487486	1.518786	1.516596	1.514446	1.512337	1.510257	1.508204	1.506176	1.504177
1.488124	1.519481	1.517278	1.515111	1.51298	1.510884	1.508827	1.506791	1.50477
1.488766	1.520182	1.517978	1.515795	1.513637	1.511521	1.509458	1.507413	1.505364
1.489446	1.48734	1.485283	1.516479	1.514297	1.512165	1.510098	1.508048	1.505983
1.490164	1.488028	1.485932	1.517164	1.514961	1.512816	1.510747	1.508697	1.506624
1.490895	1.488731	1.486599	1.517853	1.515628	1.513469	1.511398	1.509347	1.507271
1.491636	1.489449	1.487286	1.518541	1.516289	1.514116	1.512046	1.509999	1.507921
1.492384	1.490178	1.487992	1.519252	1.516973	1.514781	1.512702	1.510651	1.508572
1.458882	1.490918	1.488718	1.48655	1.484409	1.515479	1.513369	1.511297	1.509216
1.459825	1.491667	1.489461	1.487277	1.485114	1.516205	1.514053	1.511948	1.509861
1.460775	1.458421	1.456085	1.453772	1.451478	1.483742	1.481726	1.479767	1.510516
1.461732	1.459375	1.457035	1.454718	1.45242	1.484479	1.482423	1.48042	1.51118
1.462698	1.460335	1.457991	1.455671	1.453374	1.451123	1.448918	1.446767	1.511882
1.463674	1.461304	1.45895	1.45663	1.454341	1.452078	1.449833	1.447641	1.512624
1.464658	1.462282	1.459923	1.4576	1.455312	1.453039	1.450766	1.448543	1.446414
1.465655	1.463277	1.460917	1.458588	1.456288	1.454003	1.45172	1.449482	1.447324
1.466653	1.464272	1.461916	1.459579	1.457263	1.454967	1.452682	1.450435	1.448245
1.467639	1.465254	1.462903	1.460561	1.458232	1.455925	1.45364	1.451384	1.449156
1.468621	1.466228	1.463885	1.461539	1.459197	1.456882	1.454597	1.452333	1.450067
1.434121	1.467201	1.464863	1.462516	1.460168	1.457845	1.455552	1.453275	1.450989
1.435022	1.46817	1.465837	1.46349	1.461142	1.458813	1.456504	1.454212	1.451921
1.435937	1.469151	1.466806	1.464455	1.462114	1.459782	1.457459	1.455152	1.452857
1.436863	1.47014	1.467763	1.465406	1.463079	1.460753	1.458414	1.456092	1.453795
1.437818	1.435486	1.433229	1.466369	1.464052	1.461728	1.459378	1.457044	1.454744
1.438818	1.436459	1.434164	1.467362	1.465039	1.46271	1.460355	1.458014	1.455705
1.43984	1.437455	1.435115	1.468369	1.466032	1.463695	1.461339	1.458996	1.456678
1.440868	1.438453	1.436062	1.469374	1.467021	1.464678	1.462329	1.459992	1.457668
1.441902	1.439458	1.437016	1.470387	1.468013	1.465661	1.463324	1.460998	1.45867
1.442905	1.440458	1.438007	1.43566	1.433404	1.466652	1.464319	1.461999	1.459662
1.443879	1.441455	1.439035	1.436673	1.43436	1.467649	1.465313	1.462995	1.460644

1.893563	1.890212	1.886872	1.88927	1.886055	1.882813	1.879524	1.87622	1.872886
1.893491	1.890139	1.886799	1.8892	1.885986	1.882745	1.879458	1.876157	1.872826
1.893418	1.890068	1.886729	1.889132	1.885919	1.882679	1.879395	1.876099	1.872782
1.893347	1.890002	1.886669	1.889074	1.885855	1.882614	1.879332	1.876046	1.872765
1.893278	1.889941	1.886619	1.889025	1.885797	1.882552	1.879269	1.875998	1.872766
1.89079	1.889899	1.886607	1.889018	1.885764	1.882501	1.879211	1.875943	1.87274
1.890736	1.889878	1.88664	1.889059	1.88576	1.882461	1.879155	1.87588	1.872684
1.890689	1.889851	1.88665	1.889075	1.885744	1.882422	1.879105	1.875825	1.87263
1.890641	1.889801	1.886609	1.889038	1.885703	1.882377	1.879058	1.875777	1.872578
1.890613	1.889762	1.88656	1.888987	1.885653	1.882333	1.879025	1.875751	1.872543
1.890638	1.88736	1.884105	1.888942	1.8856	1.882289	1.879024	1.87578	1.872555
1.890681	1.887387	1.884089	1.888897	1.885544	1.882247	1.879034	1.875829	1.872583
1.890672	1.887373	1.884058	1.886599	1.883218	1.882202	1.879011	1.875823	1.87257
1.890628	1.88733	1.884016	1.88656	1.883179	1.882157	1.878965	1.875778	1.872529
1.889819	1.887292	1.883982	1.886538	1.883176	1.882152	1.878943	1.87574	1.872489
1.889801	1.887258	1.88395	1.886531	1.883217	1.882201	1.878953	1.87571	1.872449
1.889791	1.887242	1.883944	1.886545	1.883256	1.879966	1.876665	1.873371	1.872427
1.889784	1.887255	1.883989	1.886596	1.883278	1.879966	1.876653	1.87336	1.872438
1.889793	1.887282	1.884042	1.886648	1.883291	1.879955	1.876645	1.873365	1.872464
1.889844	1.886541	1.883263	1.886656	1.883285	1.879952	1.87667	1.873412	1.872495
1.889915	1.886592	1.883271	1.886642	1.883273	1.879954	1.876711	1.873478	1.872528
1.88995	1.88661	1.883272	1.886642	1.883294	1.879988	1.876745	1.873506	1.870229
1.889955	1.886601	1.883262	1.886649	1.883344	1.880052	1.876775	1.873501	1.870215
1.887328	1.886605	1.883279	1.886045	1.882749	1.880099	1.876794	1.873498	1.870217
1.887319	1.886625	1.883337	1.886113	1.882789	1.880111	1.876791	1.873493	1.870241
1.887338	1.884491	1.881233	1.886181	1.882836	1.88014	1.876811	1.873513	1.870281
1.88743	1.884564	1.881275	1.886225	1.882906	1.880224	1.8769	1.873596	1.870342
1.887538	1.884647	1.881318	1.886268	1.882982	1.88032	1.877002	1.873694	1.87041
1.887583	1.884702	1.881382	1.884358	1.881065	1.880363	1.877042	1.873735	1.870463
1.887597	1.884745	1.88146	1.884441	1.88111	1.880382	1.877051	1.873749	1.87051
1.887654	1.884338	1.88106	1.884507	1.881175	1.877847	1.874514	1.871211	1.868732
1.887757	1.884417	1.881106	1.884556	1.881262	1.877962	1.874643	1.871338	1.868816
1.887838	1.884488	1.881162	1.884618	1.881342	1.87806	1.874763	1.871467	1.868919
1.887864	1.884536	1.881241	1.884706	1.8814	1.878114	1.874855	1.871596	1.869056
1.887902	1.884593	1.881321	1.884794	1.881461	1.878168	1.874936	1.871706	1.869184
1.882438	1.884682	1.881381	1.884852	1.881541	1.878255	1.875003	1.871763	1.868529
1.882568	1.88479	1.881444	1.88491	1.881629	1.878352	1.875066	1.871803	1.868595
1.882689	1.884917	1.881561	1.884708	1.88141	1.878423	1.875145	1.871889	1.868674
1.882808	1.885062	1.881719	1.884848	1.881491	1.87848	1.875236	1.872008	1.868759
1.882895	1.879616	1.876324	1.884988	1.881604	1.878582	1.875355	1.872139	1.868873
1.882938	1.879681	1.876437	1.885133	1.881756	1.878738	1.87551	1.87229	1.869025
1.883007	1.879754	1.876534	1.880165	1.876839	1.878898	1.875654	1.872419	1.869161
1.883134	1.879852	1.876596	1.880237	1.876962	1.879043	1.875759	1.872491	1.869253
1.883284	1.879968	1.876666	1.880309	1.877083	1.879184	1.875861	1.87256	1.869335
1.883439	1.880111	1.876789	1.880433	1.877218	1.879017	1.875692	1.872383	1.869424
1.883598	1.880271	1.876942	1.880581	1.877352	1.879149	1.875832	1.87252	1.869518
1.883741	1.880423	1.877091	1.880716	1.877451	1.874188	1.870921	1.867656	1.869641
1.883871	1.88057	1.87724	1.880843	1.877518	1.874235	1.87101	1.867789	1.869787
1.877537	1.88071	1.877388	1.88098	1.877618	1.874321	1.871123	1.867929	1.869942
1.877659	1.880847	1.877537	1.881135	1.877764	1.874462	1.871269	1.868079	1.870105

1.877762	1.874528	1.871273	1.88129	1.87792	1.874617	1.87142	1.868227	1.865518
1.877819	1.874601	1.871388	1.881435	1.878072	1.874771	1.87157	1.868374	1.865675
1.877874	1.874671	1.8715	1.881579	1.878228	1.874928	1.871712	1.868505	1.86582
1.877984	1.874785	1.87163	1.881729	1.878389	1.875081	1.871822	1.868584	1.865368
1.878129	1.874926	1.87177	1.881883	1.878554	1.875232	1.871913	1.868632	1.865442
1.871127	1.875075	1.871917	1.875929	1.872628	1.875389	1.872035	1.868729	1.865551
1.871283	1.875232	1.872072	1.876088	1.872791	1.875552	1.872186	1.86887	1.865692
1.871441	1.868235	1.86508	1.876248	1.872953	1.869642	1.866294	1.862997	1.86585
1.871598	1.868389	1.865231	1.876407	1.873113	1.869805	1.866462	1.863168	1.86602
1.871756	1.868545	1.865384	1.876566	1.873274	1.869973	1.86665	1.863369	1.866213
1.871912	1.868699	1.865538	1.876727	1.873438	1.870155	1.866878	1.863627	1.866451
1.872072	1.868857	1.865695	1.87689	1.873603	1.870341	1.867118	1.863903	1.866704
1.872239	1.869023	1.865856	1.87039	1.867111	1.870515	1.867317	1.86412	1.860891
1.872417	1.869198	1.866024	1.870558	1.867274	1.870681	1.86749	1.864299	1.861071
1.87264	1.869411	1.86621	1.870733	1.867441	1.86419	1.861001	1.857816	1.861242
1.872923	1.869673	1.866418	1.870915	1.86761	1.864351	1.861159	1.857974	1.861403
1.873181	1.869916	1.866621	1.8711	1.867786	1.86452	1.861322	1.858134	1.861568
1.873375	1.870109	1.866805	1.871282	1.867965	1.864695	1.861492	1.858302	1.861741
1.873548	1.870286	1.866986	1.871474	1.868163	1.864892	1.861678	1.858479	1.861927
1.87372	1.870462	1.86717	1.871684	1.868409	1.865148	1.861901	1.858674	1.855477
1.87389	1.870639	1.867363	1.87191	1.86868	1.865432	1.862143	1.858882	1.855709
1.874082	1.870844	1.867597	1.872163	1.868932	1.865675	1.862364	1.859086	1.85592
1.874291	1.871073	1.867868	1.872442	1.869173	1.865888	1.86257	1.859289	1.856114
1.874494	1.871287	1.868105	1.872686	1.869396	1.866102	1.862797	1.859524	1.856331
1.874684	1.871474	1.868294	1.872881	1.869593	1.866316	1.863054	1.859806	1.856581
1.874893	1.871673	1.868478	1.873071	1.869801	1.866545	1.863313	1.86008	1.856827
1.875152	1.871911	1.868677	1.873278	1.87005	1.866814	1.863567	1.860314	1.857042
1.87542	1.872156	1.86888	1.873487	1.870302	1.867084	1.863812	1.860532	1.857254
1.875649	1.87237	1.869082	1.873689	1.87051	1.867296	1.864021	1.860744	1.857485
1.875857	1.872571	1.869288	1.873891	1.870697	1.867479	1.864212	1.860952	1.857729
1.876065	1.872787	1.869525	1.87413	1.870909	1.867682	1.864436	1.861199	1.857984
1.876268	1.873017	1.869795	1.874406	1.871142	1.867901	1.864692	1.861486	1.858253
1.876497	1.873261	1.870057	1.874675	1.871395	1.868148	1.864954	1.86176	1.858521
1.876768	1.873524	1.870298	1.874923	1.871676	1.868439	1.865218	1.862002	1.858782
1.877042	1.87379	1.870545	1.875179	1.871958	1.86873	1.865486	1.862252	1.859048
1.877286	1.874046	1.870823	1.875462	1.872217	1.868982	1.865758	1.862541	1.859323
1.877528	1.874303	1.871109	1.875753	1.872473	1.869227	1.866033	1.862839	1.859602
1.877803	1.874574	1.871372	1.87602	1.87275	1.869507	1.866306	1.863106	1.859877
1.878101	1.874854	1.871621	1.876275	1.873041	1.86981	1.866578	1.863355	1.860151
1.878384	1.875131	1.871894	1.876554	1.873323	1.870094	1.866858	1.863634	1.860433
1.878645	1.875402	1.872194	1.87686	1.873595	1.870353	1.867149	1.863948	1.860725
1.878939	1.875692	1.872492	1.877161	1.873877	1.870628	1.867439	1.86425	1.861016
1.879295	1.876014	1.872771	1.877436	1.874178	1.870938	1.867722	1.864513	1.861298
1.879664	1.876353	1.873062	1.877721	1.874487	1.871254	1.868012	1.864783	1.861586
1.878031	1.876709	1.873401	1.870127	1.866883	1.871547	1.868319	1.865101	1.861889
1.878384	1.877069	1.873762	1.87047	1.867187	1.87184	1.868641	1.865446	1.862206
1.87869	1.877386	1.874106	1.870819	1.867523	1.872177	1.86899	1.865804	1.862552
1.878954	1.877668	1.874439	1.871177	1.867886	1.872551	1.869364	1.866176	1.862926
1.879249	1.87597	1.872714	1.871504	1.868237	1.872919	1.869716	1.866515	1.863276
1.879585	1.876287	1.872995	1.871786	1.868569	1.873278	1.870036	1.866803	1.863593

1.872638	1.876621	1.873295	1.870026	1.866806	1.873637	1.870363	1.867105	1.863919
1.873	1.87699	1.873654	1.870385	1.867174	1.874006	1.870724	1.86746	1.864283
1.873358	1.877361	1.87403	1.870757	1.867537	1.874363	1.871087	1.867828	1.86465
1.873663	1.870387	1.867124	1.871106	1.867856	1.874664	1.871414	1.868177	1.864979
1.873943	1.870682	1.867459	1.871448	1.868156	1.874937	1.871726	1.868519	1.865292
1.874271	1.871019	1.867816	1.871813	1.868497	1.873397	1.870168	1.866939	1.865633
1.874642	1.871393	1.868193	1.872199	1.868877	1.873776	1.870548	1.867321	1.866001
1.871126	1.871774	1.868574	1.865325	1.862039	1.86598	1.862761	1.859551	1.864426
1.871507	1.872162	1.868958	1.865708	1.862422	1.866372	1.863145	1.85993	1.864812
1.871889	1.872547	1.869336	1.866084	1.862802	1.86676	1.863516	1.860288	1.865181
1.872266	1.869023	1.865821	1.866452	1.863174	1.859909	1.856657	1.85343	1.865502
1.872637	1.869384	1.866166	1.866803	1.863535	1.860262	1.856967	1.85371	1.865813
1.864365	1.869721	1.866464	1.867108	1.863872	1.860617	1.857316	1.854052	1.866172
1.864704	1.870042	1.86673	1.867382	1.864191	1.860971	1.85769	1.854436	1.866563
1.85735	1.870386	1.867044	1.863805	1.860657	1.86133	1.858064	1.854819	1.860313
1.857699	1.870755	1.867408	1.864165	1.861012	1.861694	1.858439	1.855204	1.860702
1.858057	1.866407	1.863074	1.864536	1.861374	1.858196	1.854975	1.851771	1.86109
1.858425	1.866789	1.863452	1.864909	1.861741	1.85856	1.855343	1.852142	1.861473
1.858798	1.867179	1.86384	1.865293	1.862118	1.858934	1.855718	1.852519	1.86186
1.859169	1.855909	1.852637	1.865588	1.862406	1.859323	1.856106	1.852905	1.862254
1.859544	1.856289	1.853026	1.866007	1.862818	1.859725	1.856509	1.853307	1.862661
1.859941	1.856698	1.853459	1.861859	1.858654	1.860139	1.856944	1.853759	1.859546
1.860354	1.857132	1.853929	1.86235	1.859101	1.860566	1.857408	1.854252	1.860022
1.860751	1.857543	1.854366	1.860587	1.857321	1.856305	1.853173	1.850038	1.851485
1.861125	1.857927	1.854762	1.860996	1.857716	1.856697	1.853565	1.850433	1.851879
1.861498	1.858305	1.855146	1.851946	1.848713	1.85709	1.853953	1.850818	1.852183
1.861866	1.858675	1.855517	1.852318	1.849085	1.857482	1.854341	1.851204	1.852576
1.862251	1.859058	1.855891	1.852693	1.849468	1.857891	1.854739	1.851594	1.85298
1.862698	1.859492	1.856293	1.853095	1.849897	1.85612	1.852953	1.849803	1.848839
1.863174	1.859951	1.85671	1.853512	1.850349	1.856604	1.8534	1.850222	1.849295
1.863598	1.860369	1.857114	1.853917	1.850769	1.857046	1.853826	1.850636	1.849727
1.863976	1.86075	1.857506	1.854312	1.851161	1.857452	1.854234	1.851045	1.85014
1.864351	1.861138	1.857918	1.854726	1.851557	1.8484	1.845249	1.84212	1.848356
1.864726	1.861543	1.858365	1.855173	1.851967	1.848799	1.845686	1.842586	1.848813
1.865109	1.861947	1.858801	1.855608	1.852371	1.849195	1.846112	1.843038	1.849267
1.850623	1.862334	1.859188	1.855992	1.852751	1.849573	1.846492	1.843428	1.849698
1.851035	1.862727	1.859564	1.856365	1.853135	1.84996	1.846865	1.843797	1.850116
1.851489	1.863166	1.859972	1.856773	1.853572	1.850404	1.847277	1.844181	1.850513
1.85197	1.863635	1.860401	1.857205	1.854044	1.850885	1.847715	1.844574	1.850896
1.852419	1.849262	1.846099	1.857661	1.854504	1.851343	1.848161	1.845003	1.851315
1.852836	1.849702	1.84658	1.858147	1.854953	1.851776	1.848617	1.845474	1.851773
1.853244	1.850131	1.847041	1.858615	1.855405	1.85222	1.849071	1.845935	1.8428
1.853643	1.850537	1.847455	1.859042	1.855865	1.852692	1.849519	1.846363	1.843241
1.854053	1.850953	1.847873	1.859471	1.856325	1.853163	1.849968	1.846793	1.843684
1.8532	1.851416	1.848342	1.845245	1.842131	1.853608	1.850425	1.847258	1.844134
1.853668	1.851895	1.848825	1.84572	1.842583	1.854045	1.850884	1.847733	1.844585
1.854106	1.851035	1.847978	1.846172	1.843042	1.839914	1.836789	1.833676	1.845033
1.854523	1.851449	1.8484	1.846607	1.843505	1.840385	1.837236	1.834105	1.845478
1.85497	1.851895	1.848844	1.845772	1.842684	1.840838	1.837687	1.834554	1.845927
1.855453	1.85238	1.849319	1.84623	1.843119	1.841263	1.838142	1.83503	1.846382

1.855942	1.852865	1.849788	1.846685	1.843558	1.840445	1.837351	1.834263	1.832389
1.856431	1.853335	1.85023	1.847125	1.844017	1.840909	1.837797	1.834696	1.832836
1.856917	1.853799	1.850671	1.847564	1.844475	1.841374	1.838243	1.835131	1.833291
1.85159	1.854262	1.851135	1.84802	1.844913	1.841807	1.838694	1.835602	1.832556
1.852041	1.854721	1.851607	1.848481	1.845342	1.84223	1.839152	1.836093	1.833054
1.845203	1.849401	1.846314	1.848924	1.845789	1.842685	1.839626	1.83658	1.833534
1.84565	1.849837	1.846729	1.849352	1.846249	1.843168	1.840116	1.837068	1.834001
1.84609	1.842988	1.839893	1.849802	1.846719	1.843649	1.840592	1.837536	1.834463
1.846513	1.843421	1.840349	1.850284	1.847204	1.844126	1.841044	1.837973	1.834919
1.846956	1.843874	1.840823	1.837775	1.834732	1.844595	1.841496	1.838417	1.835382
1.847449	1.84437	1.841321	1.838257	1.835183	1.845038	1.84195	1.838887	1.835863
1.847961	1.844883	1.84183	1.838747	1.835638	1.845485	1.842417	1.839375	1.836353
1.848456	1.845385	1.842332	1.839246	1.836133	1.833057	1.830037	1.827036	1.836841
1.848946	1.845883	1.84283	1.839752	1.836653	1.833584	1.830561	1.827548	1.837334
1.844194	1.846379	1.84331	1.840235	1.837153	1.834097	1.831079	1.828065	1.825025
1.844716	1.846872	1.84377	1.840691	1.837633	1.834598	1.831593	1.828587	1.825544
1.845232	1.847369	1.844243	1.841163	1.838123	1.835101	1.832104	1.829103	1.826061
1.845742	1.847876	1.844746	1.841666	1.83863	1.835611	1.832614	1.829612	1.826576
1.846235	1.848375	1.845251	1.842175	1.839142	1.836121	1.833113	1.830106	1.827077
1.846688	1.843601	1.840525	1.842675	1.83965	1.836624	1.833588	1.83056	1.827543
1.84713	1.844054	1.841007	1.843176	1.840161	1.837128	1.834058	1.831004	1.828
1.847621	1.844544	1.841507	1.843685	1.840679	1.837645	1.83456	1.831493	1.828491
1.848146	1.845064	1.842021	1.844201	1.841203	1.838174	1.835085	1.832013	1.829007
1.848688	1.845601	1.842554	1.839544	1.836574	1.838713	1.835622	1.832548	1.829539
1.849251	1.84616	1.84311	1.840098	1.837127	1.839265	1.836176	1.833103	1.830091
1.849811	1.846717	1.843664	1.840649	1.837676	1.834671	1.831607	1.828558	1.830641
1.850358	1.84726	1.844205	1.841188	1.838211	1.835205	1.832144	1.829096	1.831177
1.850901	1.8478	1.844744	1.841725	1.838744	1.835736	1.832676	1.829629	1.831709
1.851447	1.848343	1.845287	1.842265	1.839282	1.836271	1.833209	1.83016	1.832235
1.851992	1.848886	1.845831	1.842808	1.839822	1.836808	1.833743	1.830691	1.83276
1.844327	1.849427	1.846374	1.843351	1.840364	1.83735	1.834287	1.831235	1.828226
1.844861	1.849964	1.846914	1.843893	1.840907	1.837896	1.834838	1.831788	1.828772
1.845413	1.842315	1.839276	1.844448	1.841463	1.838457	1.835414	1.832372	1.829343
1.845988	1.842886	1.839843	1.845018	1.842036	1.839042	1.836025	1.832998	1.829948
1.84658	1.843473	1.840418	1.837404	1.834434	1.83146	1.828479	1.825478	1.830555
1.847204	1.844084	1.840997	1.837966	1.834988	1.832015	1.82905	1.826063	1.831147
1.847831	1.844698	1.84158	1.838528	1.835537	1.832561	1.829606	1.826632	1.831734
1.83995	1.845283	1.842156	1.839093	1.836087	1.833105	1.830154	1.827193	1.824184
1.840509	1.845851	1.84273	1.839662	1.83664	1.833649	1.830695	1.827743	1.824771
1.84107	1.83798	1.834903	1.84026	1.837221	1.834215	1.831249	1.828293	1.825337
1.841632	1.838564	1.835522	1.84089	1.83783	1.834801	1.831812	1.82884	1.825881
1.834331	1.839141	1.836125	1.833097	1.830064	1.835414	1.832399	1.829404	1.826432
1.834875	1.839704	1.836695	1.833692	1.830702	1.83607	1.833024	1.829989	1.82699
1.835434	1.840275	1.837261	1.834277	1.831328	1.83672	1.833656	1.830596	1.82757
1.830233	1.832969	1.829971	1.834843	1.831904	1.828938	1.825928	1.82291	1.828198
1.830836	1.833554	1.830534	1.835414	1.832466	1.829511	1.826544	1.82356	1.828844
1.826384	1.8342	1.831168	1.83605	1.833069	1.830096	1.827133	1.824159	1.821148
1.827054	1.834892	1.831855	1.836735	1.833701	1.830688	1.827703	1.824726	1.821744
1.817022	1.829769	1.826745	1.829483	1.826458	1.831308	1.828297	1.825305	1.822333
1.817634	1.830395	1.827369	1.830117	1.827098	1.831962	1.828917	1.825894	1.822909

1.818258	1.815245	1.812248	1.824973	1.821976	1.824714	1.821694	1.818696	1.823514
1.81892	1.815881	1.812849	1.825574	1.822568	1.825315	1.822319	1.81934	1.824178
1.819578	1.816516	1.813454	1.82618	1.823155	1.825908	1.822942	1.819989	1.824848
1.809374	1.817133	1.814083	1.821727	1.81869	1.826492	1.823529	1.820584	1.825472
1.809956	1.817741	1.814722	1.822374	1.819292	1.827078	1.824106	1.821162	1.82608
1.810555	1.807552	1.804572	1.812303	1.809237	1.816896	1.813956	1.811045	1.818844
1.811166	1.808149	1.805168	1.812919	1.809881	1.817568	1.814619	1.811696	1.819499
1.8118	1.808777	1.805795	1.802801	1.7998	1.807536	1.804591	1.801669	1.809414
1.812468	1.809453	1.806469	1.803464	1.800441	1.808162	1.805194	1.802257	1.810004
1.813125	1.810113	1.807127	1.804116	1.801084	1.808801	1.805818	1.802867	1.799953
1.813728	1.810706	1.807721	1.804727	1.801729	1.809476	1.806498	1.803543	1.800618
1.814331	1.811294	1.808305	1.805331	1.802377	1.810161	1.80719	1.804231	1.801296
1.814998	1.81195	1.808942	1.80597	1.803034	1.800092	1.797131	1.794182	1.801924
1.815701	1.812643	1.809606	1.806624	1.803693	1.800745	1.797755	1.794782	1.802531
1.803986	1.813312	1.810243	1.807249	1.804322	1.801378	1.79839	1.795421	1.792509
1.804651	1.813961	1.810855	1.807845	1.804919	1.801989	1.799034	1.796095	1.793194
1.805289	1.802192	1.799095	1.808464	1.805538	1.802619	1.799694	1.796781	1.793886
1.805879	1.802799	1.799728	1.809123	1.806196	1.803284	1.800384	1.797488	1.794591
1.806488	1.803425	1.80038	1.809796	1.806863	1.803951	1.80106	1.798172	1.795273
1.807179	1.804114	1.801066	1.810483	1.807531	1.804599	1.801685	1.798781	1.795885
1.8079	1.804825	1.801761	1.811169	1.808193	1.805235	1.802288	1.799364	1.796475
1.758267	1.805502	1.802416	1.799387	1.79641	1.805848	1.802904	1.799988	1.797112
1.758843	1.806159	1.803044	1.799993	1.796999	1.806445	1.803525	1.800637	1.797777
1.759389	1.756616	1.753849	1.800621	1.797612	1.794663	1.791787	1.788945	1.798456
1.759899	1.757135	1.754394	1.80127	1.798246	1.795296	1.792443	1.789622	1.799151
1.76043	1.757676	1.754955	1.752239	1.749529	1.795957	1.793113	1.790299	1.799841
1.761007	1.758259	1.755545	1.752833	1.750126	1.796655	1.793798	1.790971	1.800525
1.761604	1.758861	1.756148	1.75344	1.750739	1.797373	1.79449	1.791638	1.801192
1.762208	1.759469	1.75676	1.754059	1.751369	1.798107	1.795187	1.792294	1.789419
1.762824	1.760087	1.757378	1.754682	1.752003	1.798846	1.795882	1.79294	1.790021
1.763456	1.760711	1.757989	1.755285	1.7526	1.749909	1.747201	1.744505	1.790628
1.764103	1.761341	1.758595	1.755871	1.753168	1.750462	1.747743	1.745039	1.791236
1.764734	1.761958	1.759191	1.756445	1.753719	1.750998	1.748275	1.745575	1.742912
1.765344	1.762559	1.759777	1.757007	1.754248	1.751511	1.748795	1.74611	1.743469
1.756425	1.763149	1.760346	1.757557	1.754779	1.752035	1.749328	1.746658	1.744035
1.75701	1.763718	1.760878	1.758081	1.755322	1.752593	1.749893	1.747228	1.74461
1.757598	1.76429	1.761411	1.758609	1.755874	1.753165	1.750473	1.747813	1.745194
1.728018	1.755339	1.752452	1.759182	1.756441	1.753734	1.751058	1.74841	1.745787
1.728598	1.755954	1.753053	1.759783	1.75702	1.754306	1.751649	1.749016	1.746386
1.729163	1.726375	1.723562	1.750831	1.748061	1.754882	1.752231	1.749608	1.746979
1.729715	1.726936	1.724137	1.751438	1.74864	1.75546	1.752807	1.750186	1.747564
1.730281	1.727507	1.724716	1.721962	1.719233	1.746491	1.743853	1.741251	1.748151
1.730873	1.728091	1.725293	1.72253	1.719795	1.747084	1.744442	1.741835	1.74874
1.731465	1.728684	1.725888	1.723122	1.720379	1.747695	1.745043	1.742427	1.749339
1.732041	1.729289	1.726533	1.723771	1.720999	1.718303	1.715711	1.713157	1.710611
1.732614	1.729896	1.727189	1.724436	1.72164	1.71892	1.716303	1.713732	1.711191
1.733198	1.730485	1.727792	1.725059	1.722293	1.719583	1.71695	1.71436	1.711815
1.73379	1.731064	1.728361	1.725657	1.722954	1.720279	1.717634	1.715024	1.712469
1.734389	1.73165	1.728934	1.72624	1.723568	1.72091	1.718259	1.715639	1.713078
1.734998	1.732246	1.729509	1.726804	1.724128	1.721469	1.71882	1.716202	1.713638

1.735603	1.732841	1.730089	1.727374	1.724691	1.722027	1.719373	1.716752	1.714187
1.736198	1.733432	1.730678	1.727962	1.72528	1.722608	1.719931	1.717292	1.714728
1.736794	1.734024	1.731269	1.728551	1.725867	1.723187	1.72049	1.717834	1.715268
1.730857	1.73462	1.731852	1.729122	1.726428	1.723743	1.721051	1.718396	1.715813
1.731463	1.735221	1.732438	1.729694	1.726984	1.724294	1.721616	1.718969	1.716364
1.732074	1.735835	1.73305	1.730296	1.72757	1.724869	1.722193	1.719547	1.716931
1.732688	1.736459	1.733685	1.730925	1.728178	1.725463	1.722781	1.720131	1.717512
1.733351	1.730568	1.727795	1.731591	1.728834	1.726109	1.723422	1.720765	1.718137
1.734088	1.731297	1.728515	1.732315	1.729559	1.726832	1.724138	1.72147	1.718822
1.734786	1.731989	1.729203	1.733006	1.730249	1.727521	1.724824	1.722151	1.719488
1.735377	1.732582	1.729807	1.73361	1.730836	1.728101	1.725416	1.722753	1.720086
1.73594	1.733148	1.730383	1.734187	1.731398	1.728657	1.725983	1.723329	1.720663
1.736536	1.733738	1.730968	1.728186	1.725395	1.729254	1.72657	1.723909	1.721249
1.737153	1.734345	1.731559	1.728779	1.726006	1.729873	1.727169	1.724492	1.721841
1.737831	1.735015	1.732221	1.729438	1.726667	1.730537	1.727822	1.725137	1.722489
1.738571	1.73575	1.732953	1.730162	1.727379	1.731247	1.728529	1.725843	1.723191
1.739273	1.736447	1.733642	1.730846	1.72806	1.7253	1.722565	1.719866	1.723854
1.739906	1.737071	1.73425	1.731453	1.728678	1.725916	1.723153	1.720433	1.724441
1.740541	1.737705	1.734872	1.732074	1.729308	1.726543	1.72376	1.721022	1.725043
1.741228	1.738406	1.735579	1.732779	1.730004	1.727235	1.724457	1.72172	1.725729
1.741925	1.739119	1.736302	1.733501	1.730713	1.72794	1.725175	1.722443	1.726431
1.742584	1.739774	1.736955	1.734153	1.731365	1.72859	1.725824	1.723087	1.720394
1.74322	1.740395	1.73757	1.734768	1.731985	1.729211	1.726437	1.72369	1.720987
1.743854	1.741034	1.738228	1.735432	1.732647	1.729871	1.7271	1.72435	1.72163
1.744478	1.741689	1.73893	1.73615	1.733356	1.730577	1.72782	1.725075	1.722328
1.724186	1.742368	1.739628	1.736851	1.734045	1.731261	1.72851	1.725767	1.723005
1.724877	1.74308	1.7403	1.737499	1.734681	1.731887	1.729123	1.726375	1.723622
1.725584	1.743805	1.740982	1.738156	1.735327	1.732523	1.729747	1.726993	1.724252
1.726271	1.744538	1.741715	1.738883	1.736045	1.733235	1.73046	1.727707	1.724964
1.726952	1.745271	1.74246	1.739631	1.736786	1.733973	1.731206	1.728457	1.725712
1.727616	1.724844	1.722085	1.740306	1.737464	1.734659	1.731905	1.729168	1.726435
1.728269	1.72546	1.722676	1.740929	1.738099	1.73531	1.732573	1.729855	1.727146
1.693052	1.726134	1.723332	1.741619	1.73879	1.767781	1.76493	1.762106	1.759304
1.693754	1.726885	1.724072	1.742395	1.739548	1.768519	1.765615	1.762747	1.759925
1.679089	1.691787	1.689138	1.722009	1.719225	1.769278	1.766333	1.763427	1.760582
1.679764	1.692469	1.689809	1.722729	1.719954	1.770064	1.767113	1.764193	1.761324
1.680427	1.693136	1.690465	1.723436	1.720677	1.770846	1.767903	1.76498	1.76209
1.666392	1.693757	1.691083	1.688454	1.685867	1.751801	1.748931	1.746074	1.762833
1.667012	1.694362	1.691686	1.689063	1.686493	1.752472	1.749607	1.746756	1.763563
1.667706	1.665034	1.662399	1.689704	1.687119	1.753117	1.750251	1.747402	1.764257
1.668466	1.665766	1.663094	1.690369	1.687739	1.753732	1.750859	1.748012	1.764913
1.655186	1.666497	1.663809	1.661163	1.658555	1.721566	1.718838	1.71613	1.745837
1.655865	1.667221	1.664552	1.6619	1.659264	1.722258	1.719516	1.716795	1.746506
1.656531	1.653899	1.651299	1.66264	1.65999	1.695747	1.693042	1.690356	1.714746
1.657189	1.654568	1.65198	1.663359	1.660721	1.696479	1.69376	1.691057	1.715443
1.657838	1.655224	1.652641	1.664065	1.66145	1.697217	1.69449	1.691774	1.716162
1.658472	1.655857	1.653284	1.650713	1.648151	1.697925	1.695213	1.692503	1.689789
1.6591	1.656475	1.653909	1.651349	1.648802	1.698613	1.695933	1.693241	1.690516
1.659767	1.657112	1.654519	1.651961	1.649443	1.686595	1.683965	1.681316	1.691236
1.660474	1.657764	1.655108	1.652543	1.650067	1.687252	1.684627	1.681988	1.691951

1.661204	1.658448	1.655735	1.653157	1.650706	1.687911	1.685291	1.682659	1.680017
1.661964	1.659178	1.656423	1.653824	1.651369	1.688573	1.685963	1.683339	1.680701
1.662726	1.659924	1.657142	1.654519	1.652042	1.689236	1.686632	1.684015	1.681377
1.663467	1.660668	1.657885	1.65524	1.65272	1.689897	1.687291	1.684678	1.682044
1.664199	1.661412	1.658643	1.655976	1.653403	1.690561	1.687946	1.685335	1.682704
1.664928	1.662143	1.659375	1.656693	1.654087	1.691236	1.688608	1.68599	1.683355
1.665653	1.662865	1.660091	1.657396	1.654772	1.69192	1.689276	1.686644	1.684
1.666371	1.663581	1.660804	1.6581	1.655462	1.692608	1.689951	1.687308	1.684652
1.667081	1.664289	1.661512	1.658803	1.656156	1.693299	1.690637	1.687985	1.685316
1.641527	1.664997	1.662219	1.659507	1.656855	1.693994	1.691318	1.688651	1.685973
1.642212	1.665703	1.662924	1.66021	1.657557	1.694693	1.691985	1.689292	1.686614
1.642903	1.666416	1.663635	1.66092	1.658265	1.695396	1.692649	1.689926	1.68725
1.64361	1.640841	1.638085	1.661639	1.658981	1.696097	1.693314	1.690561	1.687882
1.644326	1.641562	1.638801	1.662374	1.659707	1.696801	1.693982	1.6912	1.688517
1.614286	1.642295	1.639565	1.636864	1.634191	1.69752	1.694674	1.691873	1.689185
1.614905	1.643042	1.640375	1.637673	1.634943	1.698254	1.695386	1.692577	1.689885
1.615523	1.643771	1.641144	1.638445	1.635685	1.67462	1.671762	1.668972	1.690598
1.616133	1.644467	1.64185	1.639161	1.636413	1.67536	1.672495	1.669689	1.691324
1.616769	1.614248	1.611756	1.639868	1.637135	1.676097	1.673241	1.67043	1.667716
1.617469	1.614934	1.612407	1.640582	1.637853	1.676818	1.674007	1.671216	1.668452
1.618185	1.615632	1.613065	1.6413	1.63857	1.677535	1.674771	1.672005	1.669193
1.61885	1.616296	1.613721	1.611139	1.608546	1.678251	1.67549	1.672728	1.669904
1.619489	1.616943	1.614378	1.61179	1.609176	1.678971	1.676186	1.673414	1.670598
1.62014	1.617589	1.615032	1.612456	1.60986	1.679745	1.676922	1.674118	1.6713
1.620797	1.618229	1.615681	1.613138	1.610602	1.680578	1.677697	1.674836	1.672005
1.621484	1.618903	1.61636	1.613833	1.611328	1.652635	1.649872	1.647141	1.672746
1.62221	1.619629	1.617085	1.614544	1.612013	1.653295	1.650554	1.647838	1.673544
1.622957	1.620375	1.617819	1.615256	1.61269	1.653948	1.651239	1.648547	1.674351
1.623729	1.621127	1.618536	1.615949	1.613366	1.654623	1.651931	1.649252	1.646584
1.624506	1.621883	1.61925	1.616642	1.614051	1.655314	1.652627	1.649954	1.64727
1.625234	1.622609	1.61997	1.617363	1.614783	1.656041	1.653328	1.65063	1.647931
1.625928	1.623314	1.620693	1.618107	1.615551	1.656796	1.654033	1.651289	1.648575
1.626645	1.62404	1.62144	1.61886	1.616299	1.657533	1.654743	1.651975	1.649251
1.627382	1.624785	1.622218	1.619629	1.617025	1.658246	1.655456	1.652691	1.649962
1.628152	1.625548	1.622984	1.620379	1.617742	1.65897	1.65618	1.65342	1.650691
1.628985	1.626342	1.623715	1.62108	1.61844	1.659714	1.656917	1.654163	1.651441
1.629817	1.627133	1.624442	1.621782	1.619149	1.660478	1.657668	1.654913	1.652199
1.598351	1.627881	1.625191	1.622535	1.619908	1.661275	1.65843	1.655651	1.652944
1.599028	1.62861	1.625953	1.623313	1.620693	1.662096	1.659209	1.656395	1.653692
1.599721	1.597136	1.594595	1.624093	1.62147	1.662929	1.660028	1.657198	1.654474
1.600424	1.597849	1.595328	1.624879	1.622244	1.663773	1.660883	1.658053	1.655285
1.533842	1.598582	1.596047	1.625649	1.623025	1.664616	1.661737	1.658904	1.656106
1.534445	1.599334	1.596737	1.626391	1.623814	1.665457	1.66259	1.659747	1.656941
1.535063	1.600105	1.597457	1.594909	1.592452	1.666287	1.663428	1.660581	1.657771
1.535697	1.600898	1.598255	1.595681	1.593168	1.667082	1.66423	1.661398	1.658582
1.536337	1.601702	1.599079	1.596476	1.593892	1.667884	1.66503	1.662209	1.659389
1.536965	1.534792	1.532625	1.597259	1.59466	1.6389	1.636096	1.633336	1.660201
1.53759	1.535417	1.533259	1.598042	1.595457	1.639766	1.636906	1.634102	1.661019
1.538228	1.536052	1.533892	1.598847	1.596274	1.640655	1.637761	1.634925	1.66188
1.538878	1.536697	1.534526	1.599675	1.597117	1.641574	1.638665	1.635808	1.662786

1.53952	1.537337	1.53516	1.533016	1.530902	1.642459	1.63954	1.636671	1.633873
1.540142	1.537963	1.535797	1.533643	1.531501	1.643277	1.640358	1.637494	1.634672
1.540766	1.538589	1.536431	1.534267	1.532099	1.644099	1.64117	1.638303	1.635467
1.510732	1.539227	1.537051	1.534881	1.532717	1.644974	1.641995	1.63909	1.636266
1.511329	1.539868	1.53767	1.535498	1.533349	1.645876	1.64284	1.639886	1.637077
1.5119	1.5098	1.507691	1.536147	1.534004	1.646804	1.643753	1.640772	1.637931
1.512455	1.510374	1.508289	1.536819	1.534677	1.647754	1.644718	1.641724	1.63882
1.513012	1.510948	1.508886	1.537482	1.535332	1.586239	1.58364	1.581047	1.639716
1.513572	1.511524	1.509489	1.538141	1.535967	1.587004	1.584426	1.581843	1.640617
1.514136	1.512091	1.510067	1.508007	1.505916	1.534438	1.532308	1.530175	1.594723
1.514702	1.512639	1.510596	1.508545	1.506489	1.535098	1.532973	1.530841	1.595562
1.515278	1.513191	1.511122	1.509079	1.50706	1.535758	1.533634	1.531501	1.596403
1.515878	1.513772	1.511686	1.509637	1.507626	1.505613	1.503594	1.501565	1.530002
1.516492	1.514367	1.512267	1.510209	1.508193	1.506178	1.504162	1.50214	1.530657
1.517088	1.514953	1.512856	1.510796	1.508775	1.506754	1.504727	1.502702	1.531307
1.517672	1.515533	1.513454	1.511398	1.509372	1.507339	1.505288	1.503251	1.531952
1.518285	1.516134	1.514059	1.511997	1.509957	1.507913	1.50585	1.50381	1.501835
1.518932	1.516758	1.514669	1.512586	1.510522	1.508466	1.506411	1.504381	1.50241
1.486207	1.517406	1.515293	1.513187	1.5111	1.509034	1.506986	1.504965	1.50299
1.486838	1.518087	1.515936	1.513808	1.51171	1.509637	1.507588	1.505565	1.503581
1.487486	1.518786	1.516596	1.514446	1.512337	1.510257	1.508204	1.506176	1.504177
1.488124	1.519481	1.517278	1.515111	1.51298	1.510884	1.508827	1.506791	1.50477
1.488766	1.520182	1.517978	1.515795	1.513637	1.511521	1.509458	1.507413	1.505364
1.489446	1.48734	1.485283	1.516479	1.514297	1.512165	1.510098	1.508048	1.505983
1.490164	1.488028	1.485932	1.517164	1.514961	1.512816	1.510747	1.508697	1.506624
1.490895	1.488731	1.486599	1.517853	1.515628	1.513469	1.511398	1.509347	1.507271
1.491636	1.489449	1.487286	1.518541	1.516289	1.514116	1.512046	1.509999	1.507921
1.492384	1.490178	1.487992	1.519252	1.516973	1.514781	1.512702	1.510651	1.508572
1.458882	1.490918	1.488718	1.48655	1.484409	1.515479	1.513369	1.511297	1.509216
1.459825	1.491667	1.489461	1.487277	1.485114	1.516205	1.514053	1.511948	1.509861
1.460775	1.458421	1.456085	1.453772	1.451478	1.483742	1.481726	1.479767	1.510516
1.461732	1.459375	1.457035	1.454718	1.45242	1.484479	1.482423	1.48042	1.51118
1.462698	1.460335	1.457991	1.455671	1.453374	1.451123	1.448918	1.446767	1.511882
1.463674	1.461304	1.45895	1.45663	1.454341	1.452078	1.449833	1.447641	1.512624
1.464658	1.462282	1.459923	1.4576	1.455312	1.453039	1.450766	1.448543	1.446414
1.465655	1.463277	1.460917	1.458588	1.456288	1.454003	1.45172	1.449482	1.447324
1.466653	1.464272	1.461916	1.459579	1.457263	1.454967	1.452682	1.450435	1.448245
1.467639	1.465254	1.462903	1.460561	1.458232	1.455925	1.45364	1.451384	1.449156
1.468621	1.466228	1.463885	1.461539	1.459197	1.456882	1.454597	1.452333	1.450067
1.434121	1.467201	1.464863	1.462516	1.460168	1.457845	1.455552	1.453275	1.450989
1.435022	1.46817	1.465837	1.46349	1.461142	1.458813	1.456504	1.454212	1.451921
1.435937	1.469151	1.466806	1.464455	1.462114	1.459782	1.457459	1.455152	1.452857
1.436863	1.47014	1.467763	1.465406	1.463079	1.460753	1.458414	1.456092	1.453795
1.437818	1.435486	1.433229	1.466369	1.464052	1.461728	1.459378	1.457044	1.454744
1.438818	1.436459	1.434164	1.467362	1.465039	1.46271	1.460355	1.458014	1.455705
1.43984	1.437455	1.435115	1.468369	1.466032	1.463695	1.461339	1.458996	1.456678
1.440868	1.438453	1.436062	1.469374	1.467021	1.464678	1.462329	1.459992	1.457668
1.441902	1.439458	1.437016	1.470387	1.468013	1.465661	1.463324	1.460998	1.45867
1.442905	1.440458	1.438007	1.43566	1.433404	1.466652	1.464319	1.461999	1.459662
1.443879	1.441455	1.439035	1.436673	1.43436	1.467649	1.465313	1.462995	1.460644

								0.728739
								0.727242
								0.725734
								0.724226
								0.722715
					0.727412	0.725322	0.723275	1.082298
					0.725899	0.723798	0.721743	1.079708
					0.72444	0.722312	0.720225	1.077118
					0.723068	0.720886	0.71873	1.074524
			0.725948	0.723829	1.085387	1.080809	1.076249	1.281472
			0.724411	0.722324	1.082791	1.078204	1.073637	1.278571
			0.722868	0.720798	1.080121	1.075556	1.071027	1.275706
	0.725536	0.723439	1.085984	1.081764	1.195133	1.19048	1.185875	1.458935
	0.724016	0.721932	1.083318	1.079084	1.192349	1.187715	1.183177	1.45614
	0.722552	0.720437	1.08065	1.076416	1.468256	1.463163	1.458156	1.499232
	0.72115	0.718954	1.077975	1.073752	1.465276	1.460416	1.455639	1.496583
0.721933	1.084294	1.079734	1.326335	1.321761	1.462331	1.457681	1.453117	1.606717
0.720441	1.081679	1.077144	1.323631	1.319023	1.459391	1.454912	1.450532	1.603944
0.718935	1.079066	1.074607	1.320999	1.31639	1.456558	1.452196	1.447946	1.60118
0.870322	1.327789	1.323088	1.463527	1.458624	1.499556	1.494977	1.49051	1.68887
0.867782	1.325085	1.320519	1.460869	1.456077	1.496931	1.492295	1.487772	1.685987
1.192819	1.467725	1.462895	1.45817	1.453553	1.697803	1.692769	1.687829	1.713736
1.190172	1.464824	1.460054	1.455458	1.451068	1.695049	1.689973	1.684942	1.710652
1.321707	1.462039	1.457317	1.498239	1.493749	1.723262	1.71795	1.71265	1.80507
1.319194	1.459387	1.454697	1.495487	1.491003	1.720123	1.714874	1.709634	1.801819
1.461507	1.456776	1.452127	1.695934	1.691104	1.774363	1.768936	1.763529	1.875711
1.458805	1.454166	1.449581	1.69302	1.688162	1.771052	1.765762	1.76049	1.872514
1.456147	1.451595	1.447082	1.690153	1.685256	1.767784	1.762606	1.757448	1.869318
1.453571	1.5995	1.594622	1.775458	1.769985	1.80507	1.799795	1.79455	1.866017
1.451062	1.59674	1.591917	1.77239	1.766912	1.801928	1.796563	1.791248	1.862689
1.598861	1.695262	1.689847	1.809728	1.804258	1.875822	1.87031	1.864851	1.859506
1.59614	1.692189	1.68667	1.806448	1.801102	1.872679	1.867158	1.861695	1.856445
1.66184	1.814372	1.808685	1.880202	1.874814	1.869444	1.863999	1.85862	2.004907
1.658681	1.810986	1.8054	1.876889	1.871422	1.866054	1.860811	1.855651	2.001147
1.687849	1.8496	1.84411	1.873615	1.86809	2.016928	2.010362	2.003926	1.997388
1.684712	1.846358	1.840922	1.87041	1.864916	2.012777	2.006306	1.999985	1.993531
1.681649	1.84318	1.837778	1.86725	1.861832	2.008777	2.002335	1.996051	1.989681
1.845506	1.874962	1.869499	1.892603	1.887159	2.004996	1.998564	1.992267	2.040977
1.842368	1.871806	1.866342	1.88941	1.884136	2.00138	1.994936	1.988583	2.037294
1.874113	1.897222	1.89167	2.010628	2.004118	2.006511	2.000141	1.993843	2.041152
1.87092	1.893988	1.888558	2.006832	2.000342	2.002775	1.996497	1.990284	2.037521
1.896265	2.016384	2.009663	2.011822	2.005412	2.052856	2.046469	2.040171	2.057236
1.893092	2.012474	2.00583	2.008083	2.001788	2.049153	2.042737	2.036405	2.053504
1.88997	2.00863	2.002059	2.004394	1.998176	2.045461	2.03903	2.032678	2.049795
1.886891	2.013522	2.007129	2.00079	1.994506	2.065125	2.058705	2.052376	2.046153

1.883859	2.009749	2.00351	1.997212	1.99083	2.061265	2.054998	2.048812	2.042539
2.012457	2.006057	1.99982	2.046992	2.040542	2.057539	2.051333	2.045185	2.073289
2.008812	2.002431	1.99609	2.043152	2.036853	2.053907	2.047705	2.041529	2.069634
2.005111	2.007997	2.001466	2.062778	2.056483	2.050255	2.044074	2.037909	2.105484
2.001339	2.004131	1.997711	2.058997	2.052752	2.046571	2.040438	2.03433	2.101992
2.006782	2.068017	2.061579	2.055258	2.049063	2.116919	2.110738	2.104615	2.098499
2.003033	2.064221	2.057819	2.051552	2.045439	2.113297	2.107135	2.10102	2.094941
1.999308	2.060462	2.054106	2.04789	2.041837	2.109698	2.103565	2.09747	2.091422
2.021271	2.05664	2.050439	2.100377	2.094219	2.106058	2.100027	2.094041	2.088059
2.017415	2.052839	2.046814	2.09679	2.090592	2.102425	2.096519	2.090672	2.084765
2.055539	2.092631	2.086456	2.111081	2.104941	2.098906	2.093035	2.087222	2.08137
2.052147	2.089118	2.082802	2.107418	2.10142	2.095485	2.089576	2.083713	2.077901
2.092028	2.098481	2.092144	2.103891	2.097941	2.092039	2.086119	2.080244	2.07445
2.088422	2.095031	2.08879	2.100547	2.094502	2.088536	2.082657	2.076837	2.071028
2.084811	2.091559	2.085442	2.097228	2.09111	2.085086	2.079216	2.073419	2.122643
2.081167	2.08799	2.082018	2.093884	2.087803	2.081768	2.0758	2.069915	2.118982
2.077575	2.084434	2.078571	2.090508	2.0845	2.078484	2.07242	2.066445	2.115359
2.087086	2.081032	2.0751	2.087019	2.081081	2.097367	2.091294	2.085287	2.111888
2.083829	2.077722	2.071623	2.08347	2.077595	2.093948	2.088004	2.082098	2.108524
2.080489	2.092244	2.086074	2.102294	2.096333	2.123942	2.117653	2.111419	2.12619
2.077082	2.088893	2.082771	2.098924	2.092806	2.120168	2.114047	2.108029	2.122893
2.073624	2.10779	2.101696	2.09558	2.0894	2.137607	2.131531	2.125585	2.138566
2.070077	2.104209	2.098255	2.092254	2.086171	2.134258	2.128098	2.122071	2.135087
2.066575	2.100657	2.094812	2.088931	2.082997	2.131003	2.124756	2.118629	2.13164
2.086471	2.097244	2.091394	2.140293	2.134022	2.146829	2.140543	2.134339	2.140041
2.083184	2.093901	2.088002	2.136799	2.13069	2.143602	2.137367	2.131165	2.136794
2.096643	2.1459	2.139523	2.15239	2.146347	2.140299	2.134141	2.127992	2.15383
2.093338	2.142428	2.136035	2.148888	2.142909	2.136943	2.130891	2.124838	2.150669
2.113681	2.158121	2.151709	2.145488	2.139485	2.165484	2.1595	2.153519	2.147472
2.110426	2.154775	2.148435	2.142198	2.136061	2.161949	2.156056	2.150209	2.144241
2.157882	2.151479	2.145195	2.160224	2.153966	2.158513	2.152642	2.146859	2.140975
2.154577	2.148242	2.141962	2.156972	2.150738	2.155268	2.149293	2.143431	2.137614
2.151287	2.145022	2.138747	2.153748	2.147564	2.15209	2.145995	2.140041	2.134316
2.147952	2.163012	2.156756	2.161178	2.155013	2.148897	2.142759	2.136804	2.135187
2.144602	2.159723	2.153533	2.15799	2.151829	2.145722	2.139581	2.133674	2.132237
2.14127	2.167036	2.160926	2.154804	2.148654	2.146568	2.140432	2.134576	2.151935
2.137951	2.163724	2.157694	2.151623	2.145487	2.143422	2.137342	2.131593	2.149066
2.155741	2.160367	2.154419	2.148408	2.142297	2.162951	2.156954	2.151318	2.152766
2.152301	2.15694	2.151082	2.145142	2.13906	2.159739	2.154072	2.148791	2.150435
2.159494	2.153555	2.147764	2.168549	2.162412	2.156602	2.151286	2.146377	2.14822
2.15621	2.150279	2.144505	2.165241	2.159172	2.153522	2.148536	2.14398	2.146046
2.152972	2.14705	2.141278	2.161985	2.156055	2.150607	2.145922	2.141673	2.143941
2.149722	2.170401	2.164532	2.158821	2.153208	2.148058	2.143587	2.139507	2.139258
2.146482	2.167095	2.161251	2.155743	2.15056	2.145767	2.141451	2.137454	2.137264
2.169791	2.163838	2.158075	2.152775	2.147959	2.140929	2.136809	2.13294	2.135314
2.166495	2.160621	2.154989	2.149905	2.145414	2.138668	2.134738	2.130999	2.133412
2.16325	2.157525	2.152121	2.144673	2.140454	2.136493	2.132709	2.129069	2.131509
2.160023	2.154545	2.149521	2.142341	2.138272	2.13442	2.130721	2.127138	2.129595
2.156951	2.151735	2.147076	2.14014	2.136187	2.132412	2.128768	2.125219	2.127685
2.154145	2.146511	2.142104	2.137973	2.134148	2.136499	2.132878	2.129326	2.13241

2.151528	2.144105	2.139869	2.135878	2.132163	2.134569	2.130968	2.127417	2.130514
2.146385	2.141829	2.137735	2.13385	2.130204	2.139226	2.135661	2.132131	2.150222
2.144056	2.139684	2.135697	2.131885	2.128275	2.137308	2.133756	2.130233	2.148211
2.141758	2.137559	2.133685	2.136823	2.133257	2.135391	2.13185	2.128337	2.146209
2.139489	2.135453	2.131703	2.134921	2.131363	2.133478	2.129943	2.126441	2.144216
2.137302	2.140247	2.136618	2.133024	2.129472	2.131568	2.128038	2.124548	2.170066
2.135221	2.138266	2.134687	2.131124	2.12758	2.129661	2.126129	2.122646	2.167913
2.133205	2.136327	2.132773	2.129228	2.125691	2.127757	2.124233	2.120765	2.165792
2.13808	2.134421	2.130876	2.132932	2.129375	2.130176	2.126665	2.123221	2.163736
2.136148	2.132541	2.128993	2.131028	2.127466	2.128284	2.12482	2.121432	2.161731
2.134214	2.130646	2.12711	2.133498	2.129918	2.171385	2.167401	2.16352	2.159777
2.132285	2.128746	2.12523	2.131615	2.128039	2.169243	2.165317	2.161517	2.157874
2.130378	2.126871	2.123394	2.129804	2.126263	2.171361	2.167515	2.163793	2.170142
2.128482	2.125021	2.121614	2.128088	2.124623	2.169556	2.165749	2.162043	2.168254
2.126647	2.138567	2.135192	2.131828	2.128466	2.167731	2.163963	2.160275	2.166355
2.124931	2.136819	2.133393	2.130027	2.12672	2.165784	2.162059	2.15841	2.164407
2.123229	2.135082	2.131596	2.128217	2.124955	2.163807	2.160123	2.156517	2.16244
2.127377	2.133281	2.129815	2.16238	2.158661	2.171875	2.167997	2.164185	2.160459
2.125517	2.131452	2.128046	2.160414	2.156714	2.169809	2.165953	2.162164	2.158475
2.128129	2.129634	2.126287	2.158464	2.154792	2.167788	2.163969	2.160215	2.156549
2.126291	2.127818	2.124532	2.156528	2.15289	2.165806	2.162039	2.158329	2.154674
2.124556	2.135819	2.132465	2.171569	2.167703	2.163901	2.160178	2.156508	2.152862
2.12298	2.134224	2.130887	2.169735	2.165887	2.162108	2.158421	2.154784	2.151148
2.121402	2.13262	2.129298	2.167889	2.164065	2.160311	2.15665	2.153037	2.149414
2.124568	2.130896	2.127594	2.165933	2.162149	2.158426	2.154776	2.151171	2.147562
2.122837	2.12914	2.125858	2.163944	2.160206	2.156516	2.152869	2.149265	2.145679
2.130752	2.163119	2.159302	2.161979	2.158278	2.154618	2.150976	2.14738	2.143842
2.129066	2.161143	2.15734	2.16003	2.156363	2.15273	2.149093	2.145511	2.142037
2.127415	2.159257	2.15548	2.158185	2.154539	2.150927	2.147305	2.143745	2.140324
2.125805	2.157476	2.15374	2.156458	2.152821	2.149221	2.145621	2.142091	2.138714
2.124182	2.155655	2.151962	2.154695	2.151065	2.147481	2.143922	2.140436	2.137093
2.122497	2.15372	2.150074	2.152828	2.149202	2.14564	2.142151	2.138734	2.135414
2.120864	2.151808	2.148201	2.150972	2.147354	2.143819	2.140403	2.137056	2.133747
2.153706	2.156449	2.152829	2.149225	2.145623	2.142115	2.138761	2.135466	2.12677
2.151979	2.154726	2.151107	2.147517	2.143941	2.140467	2.137164	2.133909	2.125201
2.150159	2.15291	2.149296	2.145736	2.142221	2.135996	2.132748	2.129539	2.134791
2.14828	2.151034	2.147426	2.143908	2.140482	2.134338	2.131097	2.127886	2.13307
2.146476	2.149231	2.145635	2.139324	2.136	2.132729	2.129502	2.126291	2.15589
2.144769	2.14752	2.143942	2.137678	2.13441	2.131184	2.127984	2.124778	2.154098
2.143053	2.142937	2.139427	2.136049	2.13282	2.142671	2.139245	2.135798	2.152295
2.141268	2.14118	2.137738	2.134401	2.131183	2.140911	2.137482	2.134036	2.150443
2.139491	2.139441	2.136073	2.132774	2.129558	2.139154	2.135713	2.132265	2.148591
2.141248	2.142336	2.139003	2.135718	2.132491	2.158008	2.154271	2.150538	2.146784
2.139588	2.140721	2.13743	2.134172	2.130955	2.156268	2.152539	2.1488	2.144998
2.142515	2.139128	2.135833	2.14105	2.137617	2.154434	2.150712	2.146984	2.143592
2.140948	2.137562	2.134219	2.139294	2.135848	2.152519	2.148804	2.145101	2.141784
2.13934	2.135979	2.132633	2.158152	2.154399	2.151006	2.14732	2.143662	2.14002
2.137655	2.134361	2.131087	2.156427	2.15267	2.14928	2.145589	2.141934	2.138323
2.136001	2.132761	2.129549	2.155014	2.151288	2.147575	2.143866	2.140202	2.136625
2.134449	2.131219	2.128015	2.153298	2.149587	2.145869	2.142102	2.138391	2.13486

2.132942	2.129697	2.126471	2.151559	2.147865	2.144148	2.140332	2.136587	2.133107
2.131439	2.152588	2.148843	2.149736	2.146038	2.14234	2.138572	2.134888	2.131472
2.129944	2.150858	2.147025	2.147861	2.144149	2.140484	2.136826	2.133265	2.129919
2.129318	2.153717	2.149856	2.14607	2.142356	2.138721	2.135161	2.131701	2.128403
2.127727	2.151897	2.148104	2.144361	2.140655	2.137051	2.133579	2.130206	2.126934
2.149261	2.150113	2.146378	2.142674	2.138984	2.135411	2.132016	2.128712	2.123425
2.147527	2.148397	2.144682	2.140998	2.137325	2.133784	2.130458	2.12721	2.12187
2.145819	2.146702	2.142998	2.139337	2.1357	2.132193	2.128906	2.125683	2.120291
2.144124	2.145005	2.141315	2.137694	2.134136	2.130674	2.127356	2.12409	2.118673
2.142444	2.143315	2.139635	2.136058	2.132592	2.129179	2.125806	2.122474	2.117043
2.145348	2.141633	2.137951	2.134395	2.130977	2.127603	2.124241	2.120914	2.115464
2.143672	2.139958	2.136265	2.132713	2.129314	2.125973	2.122666	2.119391	2.11392
2.141987	2.138286	2.134604	2.13107	2.127697	2.124385	2.121115	2.117869	2.112378
2.140295	2.13662	2.132969	2.129467	2.126132	2.122847	2.119589	2.116348	2.110838
2.138591	2.134953	2.131357	2.127899	2.124595	2.119268	2.115937	2.112619	2.135075
2.136863	2.133278	2.129774	2.126375	2.123093	2.117746	2.114417	2.111087	2.133296
2.135139	2.131613	2.128208	2.124868	2.121604	2.116234	2.112903	2.109557	2.131522
2.133443	2.129967	2.126637	2.123344	2.120099	2.11471	2.111378	2.108027	2.129784
2.131775	2.128346	2.125075	2.12182	2.11859	2.113182	2.109844	2.106488	2.128058
2.130176	2.12679	2.123549	2.120311	2.117083	2.111641	2.108272	2.104892	2.126276
2.128639	2.125294	2.122056	2.118814	2.115578	2.110089	2.106665	2.103244	2.124444
2.127121	2.123806	2.120571	2.117324	2.114076	2.134483	2.130482	2.126516	2.129685
2.125618	2.122323	2.119093	2.115843	2.11258	2.132767	2.128755	2.124792	2.128008
2.124129	2.120843	2.117612	2.114357	2.111082	2.138025	2.13401	2.130055	2.131347
2.12265	2.11936	2.116114	2.112852	2.109579	2.136299	2.13228	2.128336	2.129679
2.121184	2.117881	2.114612	2.11134	2.108073	2.134579	2.130574	2.126655	2.128042
2.119731	2.124792	2.121481	2.143209	2.139446	2.132868	2.128924	2.125072	2.123583
2.118293	2.123307	2.11996	2.141485	2.137723	2.131166	2.127317	2.123559	2.122027
2.125236	2.121864	2.118464	2.139773	2.136011	2.129474	2.125712	2.12204	2.120463
2.123864	2.120468	2.116996	2.138074	2.134306	2.127787	2.124107	2.12052	2.118897
2.122449	2.144325	2.140292	2.136396	2.132641	2.123955	2.120111	2.116376	2.11733
2.120957	2.142658	2.138625	2.134747	2.13103	2.122308	2.118513	2.114816	2.115752
2.119442	2.140975	2.136957	2.133112	2.129449	2.120708	2.116958	2.11329	2.114195
2.11793	2.139286	2.135278	2.131474	2.127889	2.123825	2.12009	2.116406	2.112706
2.116415	2.137598	2.13361	2.129853	2.126351	2.122288	2.118617	2.114967	2.111235
2.139855	2.135942	2.131992	2.128279	2.124828	2.12072	2.117077	2.113439	2.109699
2.13819	2.13431	2.130417	2.126746	2.123323	2.119136	2.115492	2.11185	2.108121
2.136546	2.132716	2.12892	2.123182	2.119534	2.117557	2.113909	2.110269	2.106552
2.134926	2.131165	2.12752	2.121783	2.118059	2.115982	2.112326	2.108693	2.104988
2.133323	2.129626	2.126105	2.125075	2.121241	2.114412	2.11075	2.107128	2.114934
2.131717	2.128071	2.124625	2.123547	2.119692	2.112838	2.109178	2.105577	2.113319
2.130161	2.126553	2.123133	2.121993	2.118145	2.111286	2.107627	2.104036	2.111712
2.128741	2.120348	2.116613	2.117424	2.113581	2.109802	2.106121	2.102506	2.110077
2.127386	2.118929	2.115086	2.115856	2.112081	2.108348	2.10464	2.100987	2.108433
2.125938	2.11742	2.113531	2.114275	2.110547	2.118534	2.114547	2.110615	2.106798
2.12442	2.115841	2.111952	2.112683	2.108984	2.116874	2.112896	2.108972	2.105169
2.120972	2.118858	2.114943	2.111137	2.107453	2.115243	2.111298	2.107403	2.103604
2.119421	2.117298	2.113434	2.109654	2.105959	2.113645	2.10977	2.105938	2.102127
2.117908	2.115772	2.111948	2.119965	2.115988	2.11208	2.108255	2.104469	2.100196
2.116475	2.114316	2.110482	2.118392	2.114457	2.110576	2.106738	2.102938	2.098714

2.11505	2.11287	2.109019	2.11682	2.112933	2.109079	2.105212	2.101383	2.097212
2.118134	2.111343	2.107513	2.11521	2.11135	2.107118	2.103247	2.099406	2.095661
2.116541	2.109775	2.105989	2.113584	2.109736	2.105522	2.101655	2.097818	2.094082
2.114988	2.108248	2.104521	2.111632	2.107777	2.103945	2.100105	2.096299	2.092568
2.113465	2.106753	2.103108	2.110136	2.106232	2.102378	2.098592	2.094847	2.091113
2.111997	2.116415	2.112527	2.108631	2.104716	2.10086	2.097104	2.093391	2.092835
2.110612	2.114919	2.110987	2.107093	2.103238	2.099422	2.095645	2.091907	2.091303
2.109228	2.113429	2.109459	2.105568	2.101765	2.097986	2.094192	2.090434	2.089783
2.107931	2.111898	2.107969	2.104093	2.10027	2.096484	2.092726	2.089006	2.088294
2.106479	2.110358	2.106489	2.10263	2.098772	2.094971	2.09126	2.087589	2.086812
2.105088	2.108848	2.104967	2.101116	2.097292	2.096901	2.092993	2.089134	2.085286
2.103736	2.107355	2.103419	2.099572	2.095825	2.095408	2.091471	2.08757	2.083736
2.112999	2.10584	2.101906	2.098073	2.094353	2.093885	2.089964	2.086078	2.082262
2.11143	2.104295	2.100435	2.096627	2.092871	2.09232	2.088476	2.084671	2.080879
2.109877	2.102765	2.098961	2.098659	2.09469	2.090791	2.086997	2.083245	2.079467
2.108368	2.101267	2.097463	2.097112	2.093207	2.089346	2.08553	2.081744	2.077962
2.106867	2.099778	2.09597	2.095568	2.091725	2.087902	2.084066	2.080249	2.076463
2.105343	2.098272	2.094504	2.094047	2.090196	2.08638	2.082596	2.078833	2.075066
2.103822	2.096769	2.093049	2.092533	2.088649	2.084832	2.081124	2.077446	2.073709
2.102363	2.095302	2.091574	2.090996	2.087135	2.083341	2.079652	2.075995	2.072296
2.100957	2.093867	2.090082	2.089439	2.085644	2.081895	2.078179	2.074496	2.070841
2.099544	2.099134	2.095144	2.091263	2.087504	2.080432	2.076712	2.073027	2.069395
2.098126	2.097696	2.09371	2.089817	2.086021	2.078937	2.075252	2.071605	2.067961
2.096684	2.096235	2.09228	2.088391	2.084565	2.077468	2.073802	2.070179	2.066532
2.09518	2.094699	2.090823	2.086978	2.083157	2.076055	2.072358	2.068711	2.065095
2.093676	2.093153	2.089356	2.085562	2.081767	2.074674	2.070939	2.067258	2.063678
2.092252	2.09166	2.087865	2.084098	2.080363	2.073319	2.069581	2.06589	2.061946
2.090875	2.0902	2.086368	2.082616	2.078958	2.071985	2.068265	2.064575	2.060624
2.089509	2.088779	2.084941	2.081202	2.077581	2.070647	2.066943	2.063259	2.059284
2.088159	2.087399	2.083581	2.079853	2.076233	2.069311	2.065623	2.061948	2.057932
2.089888	2.086023	2.082221	2.078492	2.07485	2.067543	2.063874	2.060218	2.056595
2.088486	2.084656	2.080851	2.077099	2.073402	2.066076	2.062475	2.058897	2.055293
2.087059	2.083266	2.079475	2.075712	2.071968	2.064622	2.061067	2.057548	2.053975
2.085561	2.081805	2.078083	2.073992	2.070233	2.063246	2.059663	2.056127	2.053501
2.084051	2.080328	2.076685	2.072634	2.068891	2.061905	2.05827	2.054692	2.052104
2.082617	2.078569	2.074943	2.071273	2.067546	2.064967	2.061328	2.057753	2.050764
2.081235	2.077184	2.073565	2.069908	2.066202	2.063635	2.060003	2.056431	2.04946
2.079856	2.07578	2.07214	2.069563	2.065923	2.0623	2.058679	2.055109	2.04814
2.07848	2.074352	2.070655	2.068076	2.064518	2.060961	2.05736	2.053786	2.046799
2.076744	2.073975	2.070253	2.066632	2.063132	2.059349	2.055787	2.052235	2.045478
2.075388	2.072614	2.068893	2.065276	2.061784	2.058013	2.054464	2.050927	2.044189
2.074041	2.071271	2.067561	2.063949	2.060453	2.056684	2.053151	2.049632	2.042918
2.073679	2.069924	2.066222	2.062619	2.059132	2.055376	2.051862	2.04836	2.041658
2.072324	2.068581	2.064889	2.061296	2.05782	2.054078	2.050586	2.047102	2.040409
2.070968	2.067236	2.063555	2.059973	2.056506	2.052777	2.049306	2.045842	2.0424
2.069612	2.06589	2.062222	2.058651	2.055191	2.051475	2.048026	2.044584	2.041155
2.068269	2.064557	2.060897	2.057334	2.053882	2.050178	2.04675	2.043329	2.03991
2.066942	2.06324	2.059581	2.056023	2.052577	2.048884	2.045477	2.042076	2.038662
2.065613	2.061576	2.057943	2.054411	2.050994	2.047603	2.044211	2.040824	2.037417
2.06427	2.06025	2.056624	2.053108	2.049717	2.046345	2.042957	2.039571	2.036173

2.062926	2.058924	2.05531	2.051812	2.048449	2.045096	2.041708	2.03832	2.034932
2.061596	2.05761	2.054003	2.05052	2.047179	2.043845	2.040462	2.037077	2.0337
2.060276	2.056307	2.052711	2.049241	2.045917	2.042596	2.039218	2.035838	2.032473
2.058967	2.055036	2.051483	2.04803	2.044693	2.041358	2.037974	2.034592	2.031237
2.05767	2.053798	2.050319	2.046886	2.043508	2.040132	2.036728	2.033338	2.029993
2.056372	2.052549	2.049129	2.045714	2.042306	2.038903	2.03549	2.032099	2.028762
2.05506	2.051268	2.047888	2.044492	2.041076	2.037668	2.034263	2.030881	2.027551
2.05378	2.050009	2.046644	2.043256	2.039838	2.036431	2.033037	2.029669	2.026349
2.052585	2.048819	2.045425	2.042019	2.038597	2.035191	2.031806	2.02845	2.025143
2.051418	2.047648	2.044209	2.040778	2.037351	2.03395	2.030576	2.027237	2.023948
2.050212	2.046433	2.042977	2.039535	2.0361	2.032702	2.02935	2.026042	2.022789
2.048986	2.045193	2.041737	2.03829	2.034846	2.031454	2.028132	2.024865	2.021657
2.047754	2.043958	2.040499	2.037055	2.033617	2.030241	2.026956	2.023726	2.020535
2.046515	2.042725	2.039259	2.035823	2.032408	2.029062	2.025825	2.022633	2.019427
2.045281	2.04151	2.038059	2.034641	2.031248	2.027923	2.024707	2.021525	2.018301
2.044046	2.040322	2.03693	2.033551	2.030175	2.026849	2.023603	2.020384	2.017141
2.04284	2.039153	2.035806	2.032457	2.0291	2.025771	2.022488	2.019227	2.015969
2.041714	2.038009	2.034627	2.031269	2.027937	2.024624	2.021331	2.018055	2.014794
2.040616	2.036874	2.033427	2.030046	2.026738	2.023447	2.020153	2.016876	2.013623
2.039446	2.035951	2.032475	2.029074	2.025757	2.02227	2.018978	2.015708	2.012484
2.038225	2.034743	2.03129	2.027898	2.024573	2.021088	2.017801	2.014549	2.011371
2.036998	2.033545	2.030139	2.026773	2.02345	2.01997	2.016688	2.013442	2.010277
2.035758	2.032356	2.029032	2.025718	2.022412	2.018942	2.015661	2.012405	2.009208
2.034551	2.031183	2.027916	2.024638	2.021346	2.017887	2.014613	2.011354	2.008132
2.033414	2.030036	2.026765	2.023485	2.020195	2.016746	2.013488	2.010243	2.007021
2.032298	2.028899	2.025603	2.022309	2.019017	2.015581	2.012347	2.009128	2.005919
2.031146	2.027746	2.024436	2.021136	2.017844	2.014427	2.011235	2.008064	2.00488
2.029989	2.026595	2.023274	2.01997	2.016677	2.013281	2.010138	2.007021	2.00387
2.028889	2.025504	2.022181	2.018877	2.015583	2.012192	2.009053	2.005943	2.002806
2.027841	2.024469	2.021153	2.017852	2.014559	2.01116	2.007982	2.004835	2.001696
2.026762	2.023402	2.020089	2.01679	2.013502	2.010101	2.006899	2.003731	2.000604
2.025623	2.022275	2.018955	2.015656	2.012377	2.008987	2.005784	2.002623	1.999536
2.024371	2.021164	2.017839	2.014543	2.011276	2.007902	2.004704	2.001553	1.998493
2.023327	2.020126	2.016806	2.013518	2.010264	2.006903	2.003714	2.000562	1.997482
2.022296	2.019101	2.015789	2.012514	2.009278	2.005936	2.002759	1.999608	1.996494
2.021193	2.01789	2.014605	2.011448	2.008254	2.004948	2.00179	1.998647	1.995527
2.020056	2.016759	2.013484	2.010352	2.00721	2.003951	2.000816	1.997686	1.994572
2.018988	2.015695	2.012427	2.009228	2.006108	2.002916	1.999792	1.99667	1.993559
2.017992	2.0147	2.011435	2.00822	2.00506	2.001839	1.998711	1.995595	1.992483
2.017	2.013711	2.01045	2.007224	2.004034	2.00083	1.997717	1.994622	1.991432
2.016011	2.012727	2.009475	2.006252	2.003058	1.999853	1.996736	1.993639	1.990447
2.014995	2.011717	2.008472	2.005255	2.002066	1.998866	1.995749	1.99265	1.989457
2.013894	2.010621	2.007379	2.004168	2.000985	1.997795	1.994683	1.991589	1.988498
2.01277	2.0095	2.006261	2.003055	1.999882	1.996704	1.993601	1.990514	1.987425
2.011728	2.008465	2.005234	2.002034	1.998868	1.995699	1.992599	1.989514	1.986427
2.010746	2.007493	2.004271	2.001079	1.997919	1.994755	1.991656	1.98857	1.985483
2.009772	2.00653	2.003318	2.000135	1.996983	1.993828	1.990729	1.987644	1.984558
2.008808	2.00558	2.002379	1.999207	1.996067	1.992922	1.989826	1.986741	1.983658
2.007844	2.004625	2.001432	1.998269	1.995137	1.992004	1.98891	1.985826	1.982746
2.006872	2.003659	2.000471	1.997313	1.994189	1.991064	1.987972	1.984889	1.981812

2.005894	2.002682	1.99949	1.996331	1.993208	1.990092	1.987001	1.983919	1.980845
2.004916	2.001686	1.998462	1.995284	1.992155	1.989042	1.98595	1.982869	1.979796
2.003927	2.000674	1.99741	1.994206	1.991067	1.987956	1.984862	1.981781	1.978707
2.002879	1.999639	1.996388	1.99319	1.99005	1.98693	1.98382	1.980724	1.977691
2.001776	1.99858	1.995389	1.992224	1.98909	1.98597	1.982859	1.97976	1.976734
2.000721	1.997558	1.99441	1.991271	1.988142	1.985023	1.981913	1.978813	1.975793
1.999735	1.996585	1.993455	1.990328	1.987205	1.984089	1.980981	1.977882	1.974868
1.998775	1.995634	1.992512	1.989392	1.986273	1.98316	1.980053	1.976955	1.973946
1.997832	1.994697	1.991578	1.988459	1.985342	1.982229	1.979121	1.976022	1.973017
1.996902	1.99377	1.990648	1.987529	1.984412	1.981299	1.978189	1.975087	1.972087
1.995971	1.99284	1.989718	1.986599	1.98348	1.980366	1.977255	1.974151	1.971155
1.995041	1.991912	1.988788	1.985667	1.982548	1.979433	1.976319	1.973215	1.970222
1.994103	1.990976	1.987854	1.984733	1.981614	1.978498	1.975384	1.972279	1.96929
1.993158	1.990034	1.986914	1.983796	1.980678	1.977564	1.97445	1.971344	1.96836
1.99221	1.989089	1.985972	1.982856	1.97974	1.976626	1.973513	1.970408	1.967431
1.99126	1.988142	1.985026	1.981911	1.978796	1.975684	1.972573	1.969471	1.966507
1.990312	1.987194	1.98408	1.980966	1.97785	1.974739	1.971631	1.968533	1.965581
1.989365	1.986244	1.983134	1.980021	1.976906	1.973795	1.970688	1.967593	1.964654
1.988428	1.985299	1.98219	1.979078	1.975962	1.972851	1.969744	1.96665	1.963724
1.987519	1.984371	1.981253	1.978135	1.975017	1.971907	1.968798	1.965706	1.962791
1.986633	1.983457	1.980322	1.977194	1.974074	1.970962	1.967852	1.96476	1.961856
1.985782	1.982586	1.979421	1.976274	1.973146	1.970029	1.966914	1.96382	1.960923
1.984978	1.981771	1.978552	1.97537	1.972228	1.969102	1.965981	1.962884	1.959992
1.984144	1.980942	1.977702	1.974504	1.971352	1.968216	1.96508	1.961969	1.959072
1.983244	1.98008	1.976885	1.973711	1.970561	1.96741	1.964235	1.96108	1.958163
1.982318	1.979193	1.976055	1.972913	1.96977	1.96661	1.963406	1.960215	1.957276
1.981384	1.978274	1.975154	1.972027	1.968895	1.965748	1.96257	1.959394	1.956435
1.980441	1.977333	1.974214	1.971095	1.967974	1.96485	1.961724	1.95859	1.955617
1.979506	1.976393	1.973272	1.970152	1.967032	1.963919	1.960822	1.95772	1.954768
1.978576	1.97545	1.972322	1.969194	1.966065	1.962953	1.959871	1.956796	1.953895
1.977696	1.97455	1.971395	1.968249	1.965111	1.961996	1.958919	1.955858	1.952995
1.976893	1.97371	1.970496	1.967315	1.964164	1.961044	1.957961	1.9549	1.952054
1.976069	1.972864	1.969616	1.966414	1.963258	1.960132	1.957033	1.95396	1.951121
1.975164	1.971982	1.968768	1.965587	1.962441	1.959307	1.956174	1.953063	1.950216
1.974228	1.971078	1.967915	1.964767	1.961637	1.958502	1.95534	1.952195	1.949339
1.973287	1.97014	1.966991	1.963859	1.960747	1.957632	1.954494	1.95137	1.948529
1.972343	1.969183	1.966027	1.962901	1.959808	1.956725	1.953643	1.950571	1.947758
1.971466	1.968283	1.965099	1.961961	1.958875	1.955808	1.952752	1.949708	1.946916
1.970657	1.96744	1.9642	1.961031	1.957939	1.954873	1.951817	1.94878	1.946006
1.969818	1.966589	1.963328	1.960147	1.957053	1.953983	1.950916	1.947868	1.945102
1.968909	1.965711	1.962496	1.959341	1.956253	1.953173	1.950071	1.946985	1.944209
1.968004	1.964836	1.961665	1.958536	1.955455	1.952368	1.949242	1.946129	1.943345
1.96716	1.963984	1.960801	1.957666	1.954586	1.95151	1.948409	1.945322	1.942552
1.966327	1.963136	1.959928	1.956782	1.953704	1.950643	1.947581	1.944533	1.941782
1.965431	1.962259	1.959079	1.955951	1.952881	1.949821	1.946753	1.943697	1.940946
1.964495	1.961363	1.958243	1.955152	1.952093	1.949027	1.945926	1.942832	1.940071
1.963604	1.960485	1.957386	1.954309	1.951258	1.948196	1.945094	1.941997	1.939239
1.962766	1.959628	1.956501	1.95341	1.950362	1.947317	1.944255	1.941197	1.93846
1.961918	1.958773	1.955635	1.952541	1.949495	1.946459	1.943418	1.940381	1.937358
1.961025	1.957908	1.954811	1.95174	1.9487	1.945655	1.942584	1.939516	1.936484

1.960139	1.957055	1.954006	1.950958	1.947917	1.944859	1.941756	1.938656	1.935613
1.959315	1.956246	1.953217	1.950169	1.947102	1.944026	1.940935	1.937853	1.934812
1.958515	1.955455	1.952433	1.949376	1.946275	1.943185	1.940124	1.937077	1.93404
1.957675	1.954613	1.951597	1.948548	1.945459	1.942382	1.939338	1.936301	1.933247
1.956805	1.953731	1.950716	1.947689	1.94465	1.941609	1.938574	1.935528	1.932442
1.955968	1.952896	1.949882	1.946869	1.943859	1.940835	1.93779	1.934726	1.936861
1.955171	1.952122	1.949112	1.946102	1.943095	1.940062	1.936979	1.93388	1.936015
1.954393	1.951364	1.948358	1.945342	1.942322	1.939271	1.936162	1.933045	1.935578
1.953633	1.950615	1.947612	1.94458	1.941515	1.938437	1.93534	1.932248	1.934792
1.952882	1.949866	1.94686	1.943807	1.940699	1.937599	1.934526	1.931469	1.934025
1.95213	1.949096	1.946058	1.942993	1.939894	1.942457	1.939374	1.936309	1.933264
1.951377	1.948313	1.945231	1.942158	1.939095	1.941688	1.938616	1.935554	1.932507
1.950591	1.947515	1.944427	1.941361	1.938319	1.940926	1.937861	1.934797	1.931731
1.949773	1.946698	1.943642	1.940597	1.937567	1.940175	1.937113	1.934041	1.930938
1.948984	1.945912	1.942879	1.939848	1.936823	1.939425	1.936348	1.933255	1.93013
1.948246	1.945174	1.942143	1.939112	1.936089	1.938675	1.935553	1.932419	1.929298
1.947515	1.944447	1.941415	1.938382	1.935357	1.937924	1.934761	1.931591	1.928478
1.946764	1.943713	1.940683	1.943325	1.940272	1.937179	1.934002	1.930818	1.927704
1.946012	1.942978	1.939952	1.942587	1.939524	1.936423	1.933247	1.930064	1.92695
1.947605	1.942244	1.939219	1.941833	1.938733	1.93561	1.93245	1.929293	1.926187
1.946872	1.94151	1.938484	1.94107	1.937912	1.934759	1.931629	1.928514	1.925422
1.946145	1.94312	1.940095	1.940322	1.93713	1.933956	1.93084	1.927746	1.924661
1.945426	1.942399	1.939374	1.939591	1.936388	1.933206	1.930085	1.926988	1.923902
1.944705	1.947508	1.944457	1.93886	1.935653	1.932465	1.92934	1.926239	1.92315
1.943981	1.946776	1.943724	1.938125	1.934916	1.931728	1.928603	1.9255	1.92241
1.943254	1.946042	1.942987	1.937387	1.934179	1.930991	1.927866	1.924764	1.921672
1.942525	1.945305	1.942248	1.939123	1.935915	1.930245	1.927121	1.92402	1.92093
1.941795	1.944566	1.941505	1.938377	1.935167	1.929496	1.926372	1.923272	1.920186
1.946901	1.943825	1.94076	1.937629	1.934417	1.928745	1.925621	1.922523	1.919439
1.946165	1.943082	1.940012	1.936878	1.933664	1.927991	1.924868	1.921772	1.918691
1.947274	1.942345	1.939266	1.936128	1.932916	1.929729	1.926605	1.923508	1.917945
1.946551	1.941614	1.938522	1.935379	1.93217	1.928985	1.925861	1.922763	1.9172
1.945822	1.940881	1.937779	1.934637	1.931445	1.928271	1.925138	1.922027	1.918944
1.945072	1.94014	1.937033	1.933909	1.930767	1.927619	1.924457	1.921307	1.918209
1.944315	1.939398	1.936287	1.933183	1.930094	1.926975	1.923785	1.920595	1.917478
1.943558	1.940364	1.937212	1.93244	1.929375	1.926273	1.923079	1.919877	1.916748
1.942808	1.939609	1.93645	1.931691	1.928635	1.925542	1.922359	1.919164	1.916025
1.942113	1.938909	1.935724	1.930965	1.927905	1.924818	1.921663	1.918491	1.915336
1.941474	1.938262	1.935031	1.930256	1.92718	1.924099	1.920989	1.917859	1.914682
1.940798	1.937594	1.934354	1.931164	1.928028	1.92338	1.920297	1.917193	1.914003
1.940056	1.936888	1.933704	1.930523	1.927341	1.922664	1.919575	1.916473	1.91328
1.939306	1.936165	1.933035	1.929865	1.926646	1.921945	1.918842	1.915737	1.912552
1.938569	1.93542	1.9323	1.929134	1.925908	1.921211	1.918098	1.914993	1.911844
1.937844	1.934674	1.931545	1.928379	1.925163	1.920484	1.917357	1.914246	1.911137
1.936099	1.933979	1.930823	1.927653	1.924464	1.921286	1.918122	1.914978	1.910409
1.935445	1.933316	1.93012	1.926944	1.923793	1.920633	1.917436	1.914251	1.909672
1.934747	1.932624	1.929431	1.926258	1.92311	1.919951	1.916752	1.91356	1.908972
1.934001	1.931903	1.928762	1.925602	1.922418	1.919238	1.916073	1.912914	1.908312
1.933262	1.931173	1.928069	1.924917	1.921707	1.918514	1.915376	1.912247	1.909075
1.932539	1.930429	1.927326	1.924174	1.92096	1.917769	1.914639	1.911523	1.908382

1.931837	1.929698	1.92658	1.923424	1.92022	1.917039	1.913913	1.910805	1.907694
1.931181	1.929019	1.925864	1.922701	1.919527	1.916373	1.913259	1.910156	1.907034
1.930545	1.928364	1.925166	1.921996	1.918855	1.91573	1.912628	1.909526	1.906383
1.929881	1.927694	1.924497	1.921322	1.918172	1.91504	1.911936	1.908835	1.905703
1.929201	1.927018	1.923852	1.920676	1.917484	1.914318	1.911199	1.9081	1.905002
1.928534	1.926351	1.923193	1.920015	1.91681	1.913628	1.910486	1.907372	1.904291
1.927889	1.925699	1.922514	1.919332	1.916154	1.912981	1.909803	1.906648	1.90356
1.927233	1.925037	1.921838	1.918657	1.915497	1.912331	1.909134	1.905958	1.902863
1.926538	1.924347	1.92118	1.918007	1.914825	1.91165	1.908475	1.905325	1.90224
1.925845	1.923658	1.920522	1.917358	1.914155	1.910973	1.907832	1.904718	1.901641
1.925194	1.922997	1.919843	1.916679	1.913499	1.91034	1.907218	1.904116	1.901023
1.924569	1.922355	1.919158	1.915991	1.912855	1.909732	1.906622	1.903516	1.9004
1.923958	1.921737	1.918523	1.915353	1.912235	1.909122	1.906001	1.902881	1.899759
1.923364	1.921144	1.917932	1.914762	1.911641	1.908517	1.905362	1.902212	1.8991
1.92274	1.92052	1.917324	1.914156	1.911022	1.907882	1.904715	1.901557	1.898448
1.922064	1.919845	1.916688	1.913527	1.91036	1.907202	1.904061	1.90093	1.897809
1.921394	1.919172	1.916039	1.912883	1.909696	1.906402	1.903267	1.900145	1.897002
1.920763	1.918525	1.915365	1.912207	1.909049	1.905775	1.902622	1.89948	1.896351
1.920153	1.917895	1.914696	1.911533	1.908415	1.90517	1.901989	1.898819	1.895711
1.919564	1.917234	1.914004	1.910827	1.907712	1.904585	1.901399	1.898221	1.895122
1.918992	1.91666	1.913433	1.910256	1.90714	1.904013	1.900835	1.897666	1.894568
1.918427	1.916054	1.912847	1.909672	1.906536	1.9034	1.900242	1.897097	1.894007
1.917778	1.915412	1.912246	1.909075	1.905896	1.902739	1.899617	1.896516	1.893441
1.912845	1.91399	1.910861	1.908493	1.905283	1.902109	1.899011	1.895939	1.892862
1.912271	1.913426	1.910305	1.907935	1.904719	1.901539	1.898443	1.895368	1.892258
1.911713	1.912876	1.909755	1.907383	1.904169	1.900989	1.89789	1.894804	1.89166
1.911156	1.912326	1.909204	1.90683	1.90362	1.900442	1.897346	1.894257	1.891099
1.910598	1.911773	1.908645	1.906268	1.903068	1.899899	1.896805	1.893715	1.890553
1.910016	1.911193	1.90804	1.905658	1.902484	1.899336	1.896248	1.89316	1.889998
1.909416	1.910592	1.9074	1.905011	1.901874	1.898758	1.89568	1.892594	1.889437
1.90883	1.91001	1.906798	1.904402	1.901286	1.898183	1.895091	1.89199	1.888842
1.908261	1.909451	1.90624	1.90384	1.900725	1.89761	1.894473	1.891334	1.8882
1.907699	1.908899	1.905692	1.903289	1.900172	1.897043	1.893869	1.890697	1.887573
1.907144	1.908352	1.905146	1.902739	1.899621	1.896488	1.8933	1.890115	1.88699
1.906592	1.907807	1.904602	1.902191	1.899072	1.895936	1.892744	1.889554	1.886426
1.906037	1.907259	1.904053	1.900891	1.897784	1.895382	1.892187	1.888993	1.885864
1.905484	1.90671	1.903502	1.90034	1.897234	1.894828	1.891632	1.888439	1.88531
1.904936	1.906168	1.902961	1.8998	1.896694	1.894281	1.891081	1.887883	1.884754
1.904392	1.905629	1.902427	1.899267	1.896161	1.893739	1.890531	1.887326	1.884197
1.903866	1.905112	1.901928	1.898771	1.89565	1.893208	1.889992	1.886782	1.88365
1.903366	1.904629	1.901484	1.898331	1.895172	1.892692	1.889463	1.886246	1.88311
1.902861	1.904138	1.901026	1.897877	1.894686	1.891485	1.88827	1.885071	1.882592
1.902326	1.903612	1.900509	1.897363	1.894167	1.890971	1.88779	1.884621	1.882123
1.901783	1.903073	1.899969	1.896824	1.893632	1.890452	1.887313	1.884179	1.881661
1.901242	1.902535	1.899427	1.89628	1.893087	1.889913	1.886792	1.883674	1.881147
1.900706	1.901999	1.898881	1.895729	1.892535	1.889361	1.886244	1.883132	1.880603
1.900217	1.901508	1.898362	1.895195	1.891997	1.888822	1.885704	1.882592	1.880062
1.899781	1.901065	1.897872	1.894675	1.89147	1.888291	1.885166	1.882051	1.87952
1.899325	1.900607	1.897383	1.894173	1.890975	1.887797	1.884656	1.881525	1.878997
1.898825	1.900112	1.896885	1.893689	1.890527	1.887368	1.884192	1.881024	1.878508

1.898319	1.899615	1.896402	1.893224	1.89009	1.88694	1.883731	1.880531	1.878022
1.897828	1.899141	1.895964	1.892795	1.889639	1.886464	1.883238	1.880024	1.877508
1.89734	1.898671	1.895539	1.892376	1.889179	1.885971	1.882738	1.879524	1.876994
1.89684	1.898181	1.895062	1.891902	1.888693	1.885481	1.882268	1.879072	1.87593
1.896334	1.897677	1.894551	1.89139	1.888187	1.884991	1.881818	1.878655	1.875496
1.895865	1.897209	1.894063	1.890898	1.887711	1.88453	1.881366	1.87821	1.87505
1.895443	1.896783	1.893598	1.890425	1.88727	1.884107	1.880916	1.877731	1.874592
1.899542	1.89636	1.893153	1.889974	1.886829	1.883667	1.880449	1.87724	1.874116
1.899097	1.895921	1.89274	1.889558	1.886378	1.883184	1.87995	1.876732	1.873604
1.898655	1.895487	1.892332	1.889144	1.885919	1.882689	1.879449	1.876233	1.873096
1.898248	1.895073	1.891896	1.888686	1.885437	1.882197	1.87898	1.875787	1.872636
1.897855	1.894667	1.89145	1.888215	1.884951	1.881713	1.878529	1.875367	1.872202
1.89743	1.894243	1.891028	1.88779	1.884521	1.881277	1.878088	1.874922	1.871758
1.896979	1.893803	1.890624	1.887406	1.884139	1.880885	1.877658	1.874459	1.871308
1.896545	1.89337	1.8902	1.886987	1.88372	1.880462	1.877225	1.874018	1.87152
1.89614	1.892952	1.889745	1.88651	1.883237	1.879987	1.876785	1.873611	1.871105
1.895726	1.892529	1.889298	1.886049	1.882774	1.879531	1.876354	1.873201	1.870689
1.895278	1.892089	1.888886	1.885656	1.882388	1.879141	1.875936	1.872758	1.870258
1.894826	1.891648	1.88848	1.885275	1.882024	1.878776	1.875535	1.872321	1.869832
1.894406	1.891223	1.888045	1.884848	1.881632	1.878408	1.875168	1.871943	1.869437
1.894001	1.890807	1.887594	1.884399	1.881227	1.878041	1.87482	1.871599	1.869062
1.893577	1.890376	1.887163	1.883971	1.880804	1.877632	1.87444	1.871241	1.8687
1.893132	1.889931	1.886749	1.883561	1.880363	1.877179	1.874026	1.870876	1.868354
1.892719	1.889509	1.886333	1.883144	1.879935	1.876749	1.873612	1.870483	1.867984
1.892361	1.88912	1.8859	1.882701	1.879525	1.876359	1.873196	1.870042	1.867567
1.892015	1.888751	1.885488	1.882279	1.879137	1.875989	1.872798	1.869614	1.867152
1.891658	1.888403	1.885131	1.881921	1.878786	1.875643	1.872446	1.869251	1.866769
1.891301	1.888067	1.8848	1.881591	1.87845	1.875301	1.872112	1.868917	1.866404
1.890953	1.887729	1.884468	1.881248	1.878076	1.874911	1.871744	1.868576	1.866062
1.890608	1.88739	1.884138	1.880902	1.877679	1.874489	1.871357	1.868235	1.865739
1.890227	1.887017	1.883789	1.880557	1.87731	1.874107	1.870992	1.867895	1.865413
1.889796	1.8866	1.883419	1.880214	1.876973	1.873771	1.870656	1.867559	1.865088
1.88939	1.886202	1.883052	1.879868	1.876639	1.87344	1.87031	1.867198	1.864742
1.889039	1.885851	1.882704	1.879526	1.876307	1.873103	1.869934	1.866787	1.864353
1.888709	1.88551	1.882348	1.879168	1.875966	1.872766	1.869559	1.866377	1.863963
1.885308	1.885152	1.881949	1.878762	1.875595	1.872422	1.869213	1.866025	1.863619
1.884979	1.884793	1.881535	1.878337	1.875212	1.872078	1.868883	1.865701	1.863296
1.88465	1.884454	1.881169	1.877965	1.87486	1.871743	1.868558	1.865376	1.862954
1.884323	1.884132	1.880841	1.877635	1.874531	1.871416	1.868238	1.865055	1.862601
1.883981	1.883805	1.880517	1.877312	1.87421	1.871096	1.867924	1.864741	1.862267
1.883617	1.883471	1.880194	1.876997	1.873896	1.870784	1.867616	1.864435	1.861959
1.883258	1.880035	1.876777	1.876684	1.873586	1.870476	1.86731	1.864132	1.861658
1.882917	1.879702	1.876451	1.87637	1.873276	1.870168	1.867005	1.863828	1.861358
1.882587	1.879374	1.876127	1.876057	1.872968	1.869864	1.866702	1.863526	1.861059
1.882269	1.879055	1.875811	1.875749	1.872662	1.86956	1.8664	1.863225	1.860757
1.881963	1.878745	1.875499	1.875444	1.872359	1.869258	1.8661	1.862925	1.860455
1.881676	1.878444	1.87519	1.875139	1.872056	1.868959	1.865803	1.862629	1.860161
1.881408	1.878151	1.874884	1.874834	1.871754	1.868661	1.865508	1.862338	1.859875
1.881134	1.877862	1.874586	1.874538	1.871457	1.868366	1.865214	1.862049	1.859603
1.880848	1.877572	1.874293	1.874248	1.871162	1.868069	1.864918	1.861762	1.859355

1.880556	1.877289	1.874021	1.873983	1.870884	1.867781	1.864629	1.861481	1.859107
1.880258	1.87702	1.873795	1.870616	1.867491	1.867513	1.864351	1.8612	1.858835
1.879959	1.876754	1.873582	1.870415	1.867256	1.867255	1.864085	1.860933	1.858563
1.88262	1.87647	1.873318	1.870159	1.866989	1.86383	1.860691	1.857564	1.858334
1.882335	1.876177	1.873024	1.869868	1.866703	1.86356	1.860465	1.857375	1.858133
1.882095	1.878856	1.875649	1.869583	1.866418	1.863284	1.860213	1.857143	1.857897
1.88191	1.878657	1.875403	1.869304	1.866132	1.862996	1.859928	1.856862	1.857622
1.881709	1.878442	1.875154	1.87191	1.86871	1.862735	1.859655	1.856576	1.857342
1.881466	1.878183	1.874889	1.871657	1.868494	1.862528	1.859416	1.856297	1.857058
1.88121	1.877918	1.874634	1.871419	1.868286	1.862323	1.859177	1.856021	1.856783
1.880947	1.877673	1.87442	1.871213	1.868063	1.864905	1.861708	1.858508	1.856535
1.880684	1.877437	1.874222	1.871019	1.867831	1.864644	1.861442	1.858252	1.856305
1.880464	1.877221	1.873999	1.870786	1.86758	1.864386	1.861203	1.858033	1.85893
1.880278	1.877022	1.873758	1.870522	1.867313	1.864129	1.860983	1.857846	1.858733
1.880068	1.876796	1.873508	1.870265	1.867069	1.863899	1.860764	1.857631	1.855373
1.879826	1.876534	1.873241	1.870012	1.866856	1.863707	1.860546	1.857379	1.855113
1.879575	1.876274	1.872992	1.869779	1.866648	1.863508	1.86032	1.857124	1.85486
1.879313	1.876031	1.872786	1.869583	1.866432	1.863272	1.860069	1.856868	1.854627
1.87906	1.875801	1.872592	1.869397	1.86622	1.863038	1.859826	1.856627	1.854409
1.878849	1.875594	1.872377	1.869185	1.866025	1.862855	1.859646	1.856443	1.854217
1.878662	1.875401	1.872155	1.868963	1.865838	1.862696	1.859496	1.85629	1.85404
1.878455	1.875197	1.871956	1.868765	1.865635	1.862491	1.859292	1.856089	1.853844
1.878229	1.874981	1.871778	1.868588	1.865417	1.862241	1.859038	1.855844	1.853629
1.878021	1.874777	1.871589	1.8684	1.865211	1.862022	1.858821	1.855634	1.853429
1.877842	1.874595	1.871379	1.868187	1.865027	1.861862	1.858671	1.855483	1.853253
1.877662	1.874411	1.871172	1.867979	1.86484	1.861697	1.858522	1.85534	1.853088
1.87745	1.874202	1.870986	1.867796	1.864636	1.861483	1.858338	1.855188	1.852941
1.87724	1.873995	1.870808	1.867619	1.864427	1.861259	1.85814	1.855022	1.852788
1.877086	1.873825	1.870615	1.867418	1.864233	1.861067	1.857937	1.854807	1.852572
1.876969	1.87368	1.870419	1.867205	1.864047	1.860893	1.857727	1.854557	1.852316
1.876838	1.873544	1.870259	1.867027	1.86386	1.860698	1.857522	1.854345	1.852108
1.876698	1.873423	1.870138	1.866886	1.863667	1.860476	1.857319	1.854174	1.851952
1.876537	1.873283	1.870017	1.866759	1.863504	1.861229	1.858074	1.854941	1.851789
1.876334	1.873097	1.869881	1.866647	1.863387	1.861095	1.8579	1.854732	1.851591
1.876142	1.872917	1.869748	1.86654	1.863287	1.860986	1.857749	1.85454	1.851404
1.876016	1.872794	1.869634	1.866435	1.86319	1.860891	1.857639	1.854412	1.851274
1.875921	1.872694	1.869522	1.866323	1.863093	1.860805	1.857551	1.854317	1.851168
1.875818	1.872572	1.869368	1.866163	1.86296	1.860696	1.857451	1.854217	1.851045
1.875713	1.872437	1.869182	1.865966	1.862798	1.860569	1.857345	1.854118	1.850912
1.8756	1.872313	1.869027	1.865796	1.86263	1.860412	1.857214	1.854009	1.850791
1.875474	1.872202	1.868913	1.86566	1.862446	1.86021	1.857046	1.853886	1.850682
1.870545	1.872097	1.868812	1.865544	1.862288	1.859074	1.855937	1.852812	1.85058
1.870434	1.872	1.868721	1.865451	1.862183	1.858961	1.85583	1.852712	1.850486
1.870327	1.871904	1.868634	1.865368	1.862099	1.858875	1.855742	1.852622	1.850397
1.87022	1.871808	1.868544	1.865282	1.862014	1.85879	1.855657	1.852537	1.850314
1.870108	1.871705	1.86845	1.865196	1.861933	1.858713	1.85558	1.852458	1.850236
1.869948	1.87156	1.868333	1.865098	1.861846	1.858632	1.855501	1.852379	1.850158
1.869744	1.871373	1.868193	1.86499	1.861754	1.858551	1.855423	1.852301	1.85008
1.869753	1.871217	1.868066	1.864885	1.861663	1.858469	1.855344	1.852222	1.850001
1.869647	1.871104	1.867959	1.864785	1.861573	1.858386	1.855265	1.852143	1.849923

1.86957	1.866377	1.863272	1.864692	1.861485	1.858303	1.855183	1.852061	1.849841
1.869511	1.866315	1.863204	1.864608	1.861401	1.858219	1.855097	1.851973	1.849752
1.869481	1.866281	1.863155	1.864535	1.861321	1.858134	1.855008	1.851882	1.849661
1.8695	1.866294	1.863134	1.864476	1.861249	1.858052	1.854922	1.851793	1.849571
1.869553	1.866341	1.863133	1.864429	1.861183	1.857973	1.854836	1.851704	1.849481
1.869559	1.866345	1.863113	1.86474	1.861467	1.857893	1.854751	1.851617	1.849393
1.869516	1.866306	1.863073	1.864698	1.861412	1.857812	1.854666	1.85153	1.849307
1.869465	1.866257	1.863026	1.859797	1.85656	1.858113	1.854949	1.851797	1.849221
1.86941	1.866201	1.86297	1.85974	1.856504	1.858058	1.854886	1.851728	1.849133
1.869362	1.866148	1.862917	1.859692	1.856467	1.858028	1.854842	1.851668	1.849052
1.869341	1.866112	1.862878	1.859666	1.856477	1.858058	1.854843	1.851632	1.850468
1.86933	1.866088	1.862854	1.859658	1.856509	1.858112	1.854863	1.851608	1.850428
1.869306	1.866076	1.86287	1.859685	1.856529	1.858122	1.85485	1.851576	1.850405
1.869274	1.866077	1.862917	1.85974	1.856544	1.858104	1.854815	1.85154	1.850398
1.869241	1.86606	1.862926	1.859751	1.856531	1.858075	1.854778	1.851502	1.850375
1.869199	1.866018	1.862885	1.859709	1.856484	1.858031	1.854732	1.851457	1.850328
1.869184	1.865996	1.862847	1.859662	1.856436	1.857993	1.854704	1.851436	1.850298
1.869227	1.866025	1.862833	1.859623	1.85639	1.857961	1.854704	1.851465	1.850306
1.869279	1.866061	1.862825	1.859592	1.856358	1.857948	1.854722	1.851506	1.850325
1.869291	1.866057	1.862806	1.859576	1.856369	1.853176	1.850002	1.846828	1.849352
1.869284	1.866037	1.862788	1.859576	1.856406	1.853232	1.850037	1.846832	1.849348
1.866947	1.866032	1.862805	1.859604	1.856435	1.853262	1.85007	1.846868	1.84939
1.866936	1.866039	1.862852	1.859661	1.856462	1.853272	1.850104	1.846932	1.849475
1.866953	1.866064	1.862893	1.859706	1.856498	1.853302	1.850135	1.846967	1.849524
1.867014	1.866114	1.862916	1.859726	1.856548	1.853363	1.850164	1.846959	1.849513
1.867076	1.863844	1.860601	1.859756	1.856597	1.854754	1.851486	1.84821	1.8495
1.867113	1.863887	1.860669	1.859817	1.856635	1.854769	1.851478	1.848189	1.849508
1.86715	1.86393	1.860742	1.859885	1.856672	1.85478	1.851473	1.848179	1.849529
1.867216	1.863994	1.860794	1.857579	1.854351	1.854831	1.851526	1.848229	1.849575
1.8673	1.86407	1.860836	1.857614	1.854411	1.854905	1.851611	1.848315	1.84964
1.865526	1.864132	1.860895	1.85767	1.854465	1.854946	1.851652	1.848358	1.845108
1.865565	1.864176	1.860972	1.85775	1.854509	1.854952	1.851647	1.848355	1.845131
1.865637	1.86241	1.85922	1.85782	1.854562	1.852751	1.849424	1.846118	1.84517
1.86577	1.862512	1.859271	1.85786	1.854629	1.852842	1.849526	1.84622	1.845237
1.865911	1.862628	1.859339	1.857913	1.854701	1.852933	1.849634	1.846335	1.84532
1.865293	1.862757	1.859461	1.85619	1.852946	1.852979	1.849706	1.84644	1.843172
1.8654	1.862886	1.859604	1.856316	1.853016	1.85302	1.849768	1.846537	1.84329
1.865471	1.862968	1.859717	1.85644	1.853125	1.851408	1.848141	1.844901	1.841674
1.865514	1.863017	1.859812	1.856565	1.853264	1.851537	1.848218	1.84493	1.841719
1.865603	1.864219	1.860975	1.858308	1.854987	1.851659	1.848316	1.845	1.841782
1.865748	1.864327	1.861031	1.858349	1.855071	1.851779	1.848444	1.845119	1.841864
1.865892	1.864448	1.861109	1.857804	1.854541	1.851876	1.848559	1.845245	1.841965
1.866021	1.864587	1.861241	1.857915	1.854609	1.851919	1.848638	1.845365	1.842091
1.866132	1.864719	1.861386	1.858045	1.854688	1.851966	1.848716	1.845486	1.842227
1.866195	1.8648	1.861508	1.858184	1.854812	1.852076	1.848833	1.845613	1.842364
1.86624	1.864861	1.861624	1.85833	1.854962	1.852219	1.848966	1.845738	1.842498
1.866341	1.864968	1.861755	1.858475	1.855113	1.851771	1.848483	1.845223	1.842595
1.866487	1.865114	1.861897	1.858619	1.85527	1.851922	1.848582	1.845274	1.842661
1.866642	1.865255	1.862014	1.858733	1.855408	1.85207	1.8487	1.845361	1.842152
1.866803	1.865385	1.862088	1.858796	1.855514	1.852211	1.848843	1.845498	1.842287

1.86224	1.865524	1.862179	1.858877	1.85563	1.852357	1.848998	1.845652	1.842426
1.862402	1.865684	1.862323	1.859016	1.855779	1.852513	1.849159	1.845808	1.842546
1.86256	1.865852	1.862489	1.859181	1.855943	1.852677	1.849327	1.845971	1.842668
1.862157	1.86601	1.862654	1.85935	1.856113	1.852846	1.849498	1.84614	1.842816
1.862281	1.866166	1.862824	1.859527	1.856289	1.853021	1.849673	1.846313	1.842981
1.862416	1.861881	1.858565	1.859703	1.856465	1.853196	1.849848	1.846486	1.843147
1.862561	1.86204	1.858732	1.859877	1.85664	1.853371	1.850023	1.846658	1.843313
1.862716	1.862204	1.8589	1.855636	1.852424	1.853546	1.850198	1.846833	1.843482
1.86288	1.862373	1.85907	1.855807	1.852596	1.853722	1.850374	1.847008	1.843652
1.863055	1.862547	1.859242	1.855978	1.852767	1.853897	1.850551	1.847185	1.843827
1.863251	1.862283	1.858981	1.856145	1.852936	1.854072	1.850729	1.847365	1.844009
1.863454	1.862466	1.859154	1.856315	1.853106	1.854248	1.850908	1.847548	1.8442
1.857652	1.862651	1.859335	1.856495	1.853281	1.85004	1.846722	1.843393	1.844412
1.857831	1.862837	1.859524	1.856683	1.853461	1.850216	1.846897	1.843576	1.844639
1.858005	1.857261	1.854004	1.856911	1.853666	1.850403	1.847081	1.843767	1.840534
1.858171	1.857465	1.854261	1.857189	1.853903	1.850604	1.847275	1.843962	1.840729
1.858344	1.857668	1.854501	1.857005	1.853698	1.850806	1.847486	1.844186	1.840945
1.85852	1.857855	1.854697	1.857205	1.853889	1.851004	1.847729	1.844467	1.841207
1.858716	1.858052	1.854885	1.85739	1.854073	1.851203	1.847972	1.844748	1.841469
1.852301	1.858295	1.855093	1.851852	1.848561	1.851397	1.84818	1.844969	1.841683
1.852569	1.858554	1.855306	1.852042	1.848754	1.851597	1.848376	1.845162	1.841874
1.852791	1.858775	1.855505	1.852241	1.84898	1.84632	1.843102	1.839884	1.842071
1.852977	1.858965	1.855692	1.852447	1.849236	1.846606	1.843353	1.84009	1.842269
1.853169	1.852821	1.849565	1.852649	1.849467	1.846265	1.843007	1.839734	1.842492
1.853369	1.853004	1.849745	1.852837	1.849659	1.846463	1.843212	1.839952	1.842746
1.853574	1.853201	1.84995	1.853049	1.849862	1.846665	1.843429	1.840191	1.837545
1.853777	1.853424	1.850207	1.853315	1.8501	1.846889	1.843682	1.840473	1.837825
1.853992	1.853666	1.850483	1.853599	1.850356	1.84713	1.843947	1.840763	1.838103
1.854247	1.851062	1.84792	1.844764	1.84159	1.847408	1.844213	1.841013	1.837766
1.854526	1.851338	1.848166	1.845008	1.841868	1.847708	1.844479	1.84124	1.837985
1.854784	1.8516	1.84843	1.845271	1.842128	1.847965	1.844718	1.841466	1.838221
1.855025	1.85185	1.848713	1.845554	1.84237	1.848184	1.84493	1.841685	1.83847
1.855282	1.852111	1.848985	1.845819	1.842602	1.842111	1.838886	1.835683	1.838733
1.855571	1.852391	1.849234	1.846045	1.842813	1.842326	1.839134	1.835966	1.839015
1.855857	1.852671	1.849489	1.846283	1.843043	1.842562	1.839396	1.836253	1.8393
1.856113	1.852932	1.849771	1.846578	1.843339	1.842855	1.839671	1.836513	1.839577
1.856364	1.853191	1.850062	1.846886	1.843652	1.843166	1.839953	1.83677	1.839854
1.856649	1.853472	1.850329	1.847146	1.843911	1.843434	1.840228	1.837052	1.833902
1.856956	1.853768	1.850584	1.847378	1.844139	1.843677	1.840501	1.83735	1.834185
1.857243	1.854052	1.850862	1.847654	1.844417	1.843963	1.840785	1.837633	1.834466
1.857505	1.854324	1.851169	1.847981	1.844753	1.844295	1.84108	1.837894	1.83474
1.85778	1.854604	1.851466	1.848295	1.845085	1.841881	1.838687	1.835521	1.835039
1.858089	1.854903	1.851734	1.848565	1.845396	1.842224	1.839032	1.835861	1.835388
1.858402	1.855206	1.852008	1.848836	1.845698	1.842554	1.839377	1.836212	1.835741
1.858685	1.855494	1.852315	1.849145	1.845985	1.842832	1.839679	1.836539	1.83606
1.858965	1.855784	1.852643	1.849475	1.846272	1.843092	1.839964	1.836851	1.836363
1.859297	1.856119	1.852992	1.849823	1.846602	1.843403	1.840259	1.837135	1.836654
1.859671	1.856491	1.853361	1.850189	1.846968	1.843754	1.840562	1.837393	1.836932
1.860038	1.856852	1.8537	1.850522	1.847314	1.844103	1.840884	1.83769	1.837242
1.860395	1.857194	1.853994	1.850806	1.847634	1.844454	1.841237	1.838038	1.837595

1.860753	1.85754	1.8543	1.851099	1.847943	1.844782	1.84158	1.838391	1.837947
1.861128	1.85791	1.854653	1.851427	1.848233	1.845053	1.841883	1.838726	1.838271
1.861491	1.858273	1.855018	1.851772	1.848528	1.845322	1.842184	1.839064	1.838597
1.861794	1.858585	1.855361	1.852127	1.848869	1.845655	1.84253	1.839422	1.836253
1.862066	1.858872	1.855699	1.852492	1.849239	1.846024	1.8429	1.839791	1.836619
1.862388	1.859203	1.856056	1.852864	1.849614	1.846398	1.84327	1.840157	1.836983
1.862755	1.859573	1.856432	1.853244	1.849997	1.846781	1.843645	1.840523	1.837349
1.86115	1.859947	1.856806	1.85362	1.850377	1.847157	1.844	1.840857	1.837687
1.86154	1.860325	1.857183	1.853996	1.850755	1.847523	1.844319	1.841134	1.837977
1.861923	1.860692	1.857539	1.854349	1.85112	1.847885	1.844643	1.841422	1.838276
1.862278	1.861025	1.857837	1.854645	1.851452	1.848244	1.844998	1.841767	1.838623
1.862626	1.86135	1.858122	1.854928	1.851777	1.848605	1.845368	1.842137	1.838992
1.862996	1.859755	1.856479	1.855266	1.852136	1.848982	1.845747	1.842514	1.839367
1.863381	1.860138	1.856863	1.85564	1.852519	1.849369	1.846136	1.842901	1.83975
1.857149	1.860527	1.857253	1.854033	1.850882	1.849757	1.846524	1.843287	1.840132
1.857536	1.860924	1.857654	1.854435	1.851284	1.850146	1.84691	1.84367	1.840511
1.857923	1.854706	1.851463	1.854833	1.851682	1.848501	1.845232	1.841958	1.840896
1.858303	1.855084	1.851838	1.855218	1.85207	1.848892	1.845627	1.842356	1.841281
1.858687	1.855465	1.852216	1.855605	1.852458	1.849285	1.846034	1.842775	1.841685
1.859077	1.855853	1.852603	1.852596	1.849465	1.849687	1.846475	1.843252	1.842138
1.859476	1.856251	1.853005	1.853001	1.84986	1.850094	1.84693	1.84375	1.842609
1.856348	1.85668	1.853462	1.850268	1.847097	1.847127	1.844	1.840862	1.840956
1.856783	1.857135	1.853966	1.850773	1.847547	1.847533	1.844395	1.841257	1.841358
1.848259	1.854022	1.850892	1.851233	1.847973	1.844744	1.841603	1.838469	1.83843
1.848653	1.854421	1.851289	1.851634	1.848367	1.845132	1.841985	1.838849	1.838818
1.848958	1.854817	1.851676	1.852025	1.848758	1.845523	1.84237	1.83923	1.839217
1.849356	1.855213	1.852066	1.852417	1.849149	1.845913	1.842755	1.839619	1.839644
1.849772	1.855625	1.852463	1.852815	1.849554	1.846322	1.843153	1.840012	1.840075
1.845677	1.856213	1.853008	1.849683	1.846465	1.846789	1.843589	1.840415	1.837292
1.846175	1.856714	1.853458	1.850117	1.846938	1.847286	1.844046	1.840823	1.837685
1.846623	1.852903	1.849643	1.850666	1.847499	1.844193	1.84095	1.837718	1.838079
1.847029	1.853317	1.850061	1.851085	1.847908	1.844588	1.841345	1.838116	1.838472
1.845246	1.844273	1.841075	1.847292	1.844108	1.844996	1.841763	1.838545	1.838897
1.845667	1.844713	1.841549	1.847781	1.84456	1.845422	1.842214	1.839024	1.839368
1.846101	1.845161	1.842019	1.848265	1.845022	1.84587	1.842677	1.839503	1.839847
1.846564	1.845627	1.84246	1.84871	1.845497	1.846511	1.843293	1.84009	1.836777
1.847026	1.846088	1.842885	1.849136	1.845966	1.847014	1.843767	1.840532	1.837231
1.847434	1.844282	1.841085	1.849547	1.846388	1.843223	1.84002	1.836823	1.837868
1.847807	1.844656	1.841468	1.849948	1.84678	1.843624	1.840477	1.837331	1.838367
1.848205	1.845057	1.84189	1.840946	1.837788	1.844034	1.840907	1.837787	1.834635
1.848623	1.845483	1.842354	1.841418	1.838226	1.844445	1.841305	1.838186	1.835066
1.839675	1.845928	1.842815	1.839666	1.836469	1.844893	1.841727	1.838592	1.835488
1.840137	1.846396	1.843256	1.840108	1.836951	1.845405	1.842197	1.839011	1.835893
1.840599	1.846868	1.843704	1.840558	1.837436	1.845922	1.842684	1.839459	1.836322
1.84103	1.847318	1.844181	1.841043	1.837904	1.846392	1.843173	1.839968	1.836829
1.841453	1.847764	1.844667	1.841537	1.838365	1.846849	1.843669	1.840508	1.83737
1.841906	1.848228	1.845122	1.84199	1.838826	1.845358	1.842211	1.839076	1.837881
1.842382	1.848705	1.845554	1.842414	1.839285	1.845862	1.84273	1.839596	1.838376
1.842834	1.839722	1.836604	1.842863	1.83974	1.846332	1.843205	1.840075	1.838854
1.843252	1.840147	1.837063	1.843342	1.840183	1.846752	1.84362	1.840497	1.839309

1.829292	1.840591	1.837537	1.843837	1.840651	1.837487	1.834379	1.831288	1.837805
1.829766	1.841081	1.838037	1.844358	1.841173	1.838006	1.83489	1.831785	1.838286
1.830253	1.841581	1.838534	1.844873	1.841702	1.83854	1.835414	1.83229	1.838773
1.829534	1.827715	1.824693	1.845336	1.842191	1.839044	1.835898	1.832753	1.839248
1.830025	1.828185	1.825131	1.845773	1.842659	1.839531	1.836364	1.8332	1.839724
1.830492	1.827447	1.824403	1.836777	1.833693	1.839989	1.83683	1.833678	1.840228
1.830935	1.827896	1.824877	1.837251	1.834129	1.840416	1.837291	1.834181	1.840758
1.831394	1.828365	1.825363	1.822329	1.819255	1.840884	1.83778	1.834696	1.841284
1.83188	1.828865	1.825867	1.822837	1.819765	1.84141	1.838309	1.835222	1.841797
1.832371	1.829364	1.826364	1.823338	1.82028	1.827655	1.824605	1.821559	1.842318
1.832858	1.829845	1.82683	1.823809	1.82078	1.828196	1.825155	1.82211	1.842872
1.833343	1.830319	1.827289	1.824275	1.82128	1.828737	1.825701	1.822655	1.843426
1.833812	1.830786	1.827765	1.824764	1.821787	1.829266	1.826212	1.823148	1.833723
1.834274	1.83125	1.828252	1.825266	1.822298	1.829788	1.826703	1.823611	1.834193
1.821989	1.831742	1.828755	1.825776	1.822813	1.830317	1.827217	1.824113	1.821062
1.822501	1.832263	1.829274	1.826295	1.823333	1.830856	1.827757	1.824655	1.821604
1.823018	1.820027	1.817073	1.826811	1.823837	1.820845	1.817808	1.814769	1.822148
1.823537	1.820546	1.817587	1.82732	1.82431	1.8213	1.818286	1.81528	1.822689
1.82405	1.821063	1.818103	1.827833	1.824788	1.82176	1.818769	1.815793	1.823231
1.82454	1.821568	1.818618	1.815635	1.81261	1.822269	1.819283	1.816316	1.823778
1.825023	1.822069	1.819134	1.816159	1.813134	1.8228	1.819812	1.816846	1.824328
1.825524	1.822578	1.819652	1.816683	1.813658	1.810652	1.807694	1.804758	1.824883
1.826037	1.823093	1.820173	1.817208	1.814186	1.811182	1.808226	1.80529	1.825443
1.826566	1.823622	1.820705	1.817742	1.814722	1.811719	1.808761	1.805823	1.814136
1.827115	1.82417	1.821251	1.818288	1.81527	1.812266	1.809301	1.806357	1.814683
1.827661	1.824713	1.821792	1.818828	1.815809	1.812804	1.809836	1.806888	1.803968
1.828195	1.825244	1.822319	1.81935	1.816327	1.813321	1.810358	1.807417	1.804501
1.828724	1.825769	1.822839	1.819867	1.816842	1.813836	1.810877	1.807942	1.805032
1.829248	1.826291	1.823359	1.820387	1.817363	1.814358	1.811396	1.808459	1.80555
1.829769	1.82681	1.823877	1.820907	1.817891	1.814889	1.811919	1.808973	1.806063
1.825246	1.827333	1.824401	1.82144	1.818446	1.815455	1.812469	1.809502	1.806582
1.825781	1.82786	1.824928	1.821983	1.819026	1.816053	1.813045	1.810046	1.807107
1.826332	1.823371	1.820447	1.822518	1.819585	1.816626	1.813608	1.81059	1.807636
1.826902	1.823918	1.820978	1.82304	1.820114	1.817162	1.814147	1.81113	1.808166
1.827484	1.824478	1.821519	1.823566	1.820638	1.817691	1.81469	1.811684	1.808713
1.828072	1.825055	1.822076	1.824105	1.821168	1.818224	1.815252	1.812273	1.809295
1.828671	1.825646	1.822647	1.824654	1.821703	1.818758	1.815817	1.81287	1.809891
1.821157	1.826261	1.823235	1.820244	1.81729	1.819302	1.816372	1.813441	1.810476
1.821784	1.826889	1.823839	1.820827	1.817855	1.819853	1.816922	1.813998	1.811056
1.822374	1.819367	1.816331	1.821433	1.81844	1.820415	1.81747	1.814542	1.811613
1.822927	1.819948	1.816953	1.822066	1.819046	1.820986	1.818015	1.815068	1.812147
1.823474	1.820512	1.817549	1.814563	1.811546	1.816645	1.813662	1.810707	1.812689
1.824011	1.821047	1.818096	1.815133	1.812156	1.817276	1.814277	1.811292	1.813245
1.824565	1.82159	1.818641	1.815695	1.812756	1.817903	1.814899	1.811896	1.813818
1.825164	1.822169	1.819205	1.816254	1.813322	1.810381	1.807411	1.804439	1.814424
1.825787	1.822773	1.819788	1.816824	1.813883	1.810947	1.808012	1.805071	1.815045
1.81813	1.823411	1.820428	1.817454	1.814491	1.811538	1.808601	1.805666	1.810779
1.818767	1.824077	1.821109	1.818129	1.815132	1.812146	1.809184	1.806235	1.81137
1.819377	1.81643	1.813493	1.818762	1.81576	1.812765	1.809785	1.806821	1.803888
1.819949	1.817	1.814063	1.819346	1.816376	1.813399	1.810407	1.807423	1.804478

1.820543	1.817583	1.814636	1.811699	1.808776	1.814024	1.811032	1.808042	1.805091
1.821191	1.818206	1.815227	1.812276	1.809359	1.81462	1.811652	1.808688	1.805751
1.821848	1.818842	1.815834	1.812868	1.809949	1.815219	1.812276	1.809337	1.806414
1.822477	1.819474	1.816468	1.813502	1.810584	1.807669	1.80474	1.801814	1.807032
1.8231	1.82011	1.817118	1.81416	1.811241	1.808318	1.805368	1.802422	1.807634
1.815911	1.820761	1.817762	1.814799	1.811878	1.808954	1.806002	1.803057	1.800146
1.816561	1.821425	1.818402	1.815425	1.812501	1.809581	1.806643	1.803712	1.800806
1.806507	1.814226	1.811242	1.81607	1.813144	1.810224	1.80729	1.804361	1.801452
1.807097	1.814846	1.811882	1.816744	1.813817	1.81089	1.807941	1.804995	1.80207
1.797062	1.810753	1.80782	1.809597	1.806709	1.811551	1.808597	1.805644	1.802709
1.797709	1.811393	1.80843	1.810202	1.807313	1.812183	1.809253	1.806326	1.803405
1.798369	1.812044	1.809048	1.810813	1.807922	1.812822	1.80992	1.807025	1.804121
1.798999	1.806721	1.803756	1.811492	1.808595	1.813513	1.810618	1.807731	1.80483
1.799619	1.807377	1.804449	1.812213	1.809307	1.814237	1.811335	1.808441	1.805537
1.789627	1.797374	1.794483	1.802253	1.799383	1.807119	1.804229	1.801345	1.806203
1.790314	1.798068	1.795177	1.802957	1.800087	1.807823	1.804892	1.801966	1.806827
1.791002	1.798753	1.795846	1.792961	1.790106	1.803799	1.800868	1.79794	1.799706
1.791691	1.799419	1.796471	1.793563	1.790698	1.804414	1.801517	1.798624	1.800403
1.792364	1.800077	1.797099	1.794171	1.791296	1.805035	1.802175	1.799323	1.801117
1.792989	1.790064	1.787122	1.79483	1.791949	1.789098	1.78629	1.783489	1.797161
1.7936	1.790701	1.787788	1.795518	1.79263	1.789775	1.786975	1.784182	1.79787
1.794254	1.791375	1.788486	1.785608	1.782736	1.790473	1.787661	1.784854	1.782006
1.794934	1.792075	1.789209	1.786339	1.783462	1.791189	1.788349	1.78551	1.782631
1.79562	1.79277	1.789913	1.787047	1.784168	1.781297	1.778446	1.775595	1.783248
1.796313	1.793461	1.790601	1.787733	1.784858	1.781976	1.779081	1.776189	1.783852
1.796998	1.794134	1.791257	1.788381	1.785508	1.782622	1.779706	1.776797	1.773936
1.797675	1.794782	1.79186	1.788959	1.786083	1.783207	1.780318	1.777438	1.774591
1.798331	1.795411	1.792455	1.789535	1.786655	1.78379	1.780937	1.778096	1.775263
1.786554	1.796016	1.793083	1.790174	1.787292	1.784429	1.781589	1.778762	1.775943
1.787123	1.79661	1.793726	1.790844	1.787963	1.785099	1.782261	1.77944	1.776632
1.787722	1.784852	1.782014	1.791511	1.788645	1.78579	1.782952	1.780127	1.777322
1.788343	1.785481	1.782652	1.79218	1.789342	1.786505	1.783661	1.780825	1.778013
1.740278	1.78613	1.783303	1.792855	1.790033	1.787209	1.784368	1.781529	1.801577
1.740858	1.786804	1.783971	1.793536	1.790716	1.787897	1.78507	1.782243	1.802284
1.741443	1.738853	1.73627	1.781828	1.779028	1.788581	1.785765	1.78295	1.80299
1.742022	1.73944	1.736868	1.782514	1.779702	1.789262	1.786449	1.783641	1.803688
1.742603	1.740026	1.737464	1.7832	1.780377	1.789942	1.787129	1.784324	1.804381
1.743186	1.740605	1.738043	1.735475	1.732902	1.77824	1.775446	1.77266	1.805065
1.743773	1.741182	1.738611	1.736046	1.73349	1.778919	1.77612	1.773327	1.805745
1.744362	1.741765	1.739185	1.736617	1.734067	1.77959	1.776794	1.774003	1.794884
1.744955	1.742353	1.739762	1.737187	1.734632	1.780254	1.777468	1.774687	1.795569
1.745544	1.74294	1.740344	1.737765	1.735204	1.732649	1.730098	1.727543	1.796251
1.746121	1.743519	1.740933	1.738355	1.735785	1.733223	1.730671	1.728119	1.796926
1.746712	1.744109	1.741527	1.738948	1.736371	1.733802	1.731245	1.728692	1.797599
1.708082	1.744721	1.74212	1.739531	1.736956	1.761653	1.759033	1.756415	1.753792
1.708672	1.745353	1.742724	1.740122	1.737548	1.76225	1.759629	1.757009	1.754381
1.709295	1.706748	1.704191	1.740752	1.738163	1.762867	1.760248	1.757632	1.755004
1.70994	1.707383	1.704814	1.741411	1.738796	1.763501	1.760886	1.75828	1.755653
1.710544	1.707989	1.705425	1.702874	1.700337	1.76417	1.761557	1.758953	1.75633
1.711104	1.708563	1.706027	1.703503	1.700992	1.764885	1.762269	1.75966	1.757046

1.711653	1.709124	1.706606	1.7041	1.701609	1.729362	1.72682	1.724279	1.757727
1.712203	1.709673	1.707149	1.704641	1.702151	1.729909	1.727374	1.724841	1.758346
1.712746	1.710211	1.707679	1.705165	1.70267	1.730427	1.727898	1.725371	1.758934
1.71327	1.710734	1.708211	1.736058	1.733494	1.730942	1.728407	1.725877	1.723335
1.713792	1.711253	1.708743	1.736589	1.734017	1.731455	1.728909	1.726371	1.723831
1.714345	1.711799	1.709287	1.737138	1.734564	1.731999	1.729446	1.726902	1.724364
1.714923	1.712367	1.709841	1.737702	1.735131	1.732568	1.730011	1.727462	1.724926
1.715538	1.712976	1.710445	1.738309	1.735736	1.733171	1.730613	1.728064	1.725527
1.716205	1.71364	1.711119	1.73898	1.736393	1.73382	1.731266	1.728722	1.726179
1.716854	1.714283	1.711764	1.739623	1.737032	1.734456	1.731905	1.729364	1.726817
1.717441	1.714856	1.71232	1.740178	1.737606	1.735044	1.732488	1.729939	1.727398
1.718014	1.715414	1.712855	1.740715	1.738165	1.73562	1.733059	1.730503	1.727969
1.718608	1.716	1.713423	1.741292	1.738755	1.736221	1.733662	1.731108	1.728579
1.719215	1.716603	1.71401	1.741892	1.739358	1.736829	1.734277	1.731729	1.729203
1.719867	1.71725	1.714645	1.742524	1.739967	1.737421	1.734863	1.732313	1.729786
1.720565	1.717942	1.71533	1.743188	1.740584	1.738	1.735423	1.732865	1.730333
1.721231	1.749208	1.746506	1.743835	1.741195	1.738579	1.735985	1.733417	1.730885
1.721841	1.749834	1.747128	1.744447	1.74179	1.739157	1.736546	1.733967	1.731443
1.722455	1.750458	1.747751	1.745063	1.742393	1.739748	1.737126	1.73454	1.732017
1.72312	1.751115	1.748412	1.745718	1.74303	1.740372	1.737752	1.735167	1.732625
1.723791	1.75177	1.749072	1.746374	1.743669	1.741001	1.73839	1.735811	1.733247
1.717734	1.752371	1.749668	1.746965	1.744253	1.741583	1.738984	1.736418	1.733855
1.718316	1.75294	1.750228	1.747519	1.744805	1.742139	1.739554	1.737005	1.734457
1.718938	1.753556	1.750848	1.748138	1.745418	1.742745	1.740149	1.737591	1.735041
1.719603	1.754222	1.751532	1.748827	1.746096	1.743402	1.740771	1.738176	1.735601
1.720263	1.74898	1.746306	1.749505	1.746771	1.744063	1.741398	1.738768	1.736178
1.720894	1.749608	1.746938	1.750152	1.74743	1.744724	1.742036	1.739382	1.736793
1.721541	1.750247	1.747568	1.750785	1.748073	1.745368	1.742661	1.739987	1.737402
1.722247	1.750927	1.748206	1.7514	1.748672	1.745953	1.743227	1.74054	1.737958
1.722981	1.751628	1.748853	1.752017	1.749264	1.746525	1.743783	1.741085	1.7385
1.723712	1.752334	1.749536	1.746759	1.744005	1.747181	1.744421	1.741702	1.739089
1.724442	1.753048	1.750248	1.747472	1.744721	1.747901	1.745122	1.742373	1.739713
1.756517	1.753728	1.750942	1.748172	1.745422	1.748609	1.745827	1.743067	1.740375
1.757136	1.754365	1.751613	1.748861	1.746107	1.749303	1.746537	1.74379	1.741084
1.75778	1.755012	1.752277	1.749531	1.746772	1.749978	1.747233	1.744504	1.741794
1.758493	1.755694	1.752927	1.750162	1.747395	1.750615	1.747893	1.745189	1.742481
1.759228	1.756395	1.753588	1.750799	1.748023	1.751253	1.748548	1.745865	1.743163
1.759958	1.757116	1.754303	1.751502	1.748709	1.745952	1.74325	1.740573	1.743842
1.760687	1.757851	1.755047	1.752242	1.74943	1.746652	1.743926	1.741233	1.744521
1.761387	1.758562	1.755771	1.752974	1.750162	1.747377	1.744634	1.741923	1.745211
1.762061	1.759253	1.756482	1.753702	1.750907	1.748129	1.745373	1.74264	1.74591
1.743049	1.759941	1.757187	1.754422	1.751645	1.748874	1.746113	1.743364	1.746626
1.743684	1.76062	1.757883	1.755134	1.752371	1.74961	1.746848	1.744095	1.747367
1.71204	1.741598	1.738931	1.755838	1.753091	1.750341	1.747581	1.744826	1.742089
1.712726	1.742294	1.739596	1.756539	1.753806	1.751069	1.748307	1.745548	1.74281
1.713435	1.743007	1.740261	1.757233	1.754514	1.75179	1.749029	1.746268	1.74353
1.68709	1.743703	1.740905	1.757905	1.7552	1.752494	1.749746	1.746995	1.744259
1.687789	1.744395	1.741541	1.758569	1.755875	1.753187	1.750459	1.747727	1.744993
1.688505	1.685795	1.683104	1.739465	1.736838	1.753895	1.751177	1.748455	1.745717
1.689242	1.686529	1.683817	1.740192	1.737532	1.754617	1.7519	1.749181	1.746428

1.677363	1.687257	1.684534	1.681906	1.679383	1.75535	1.752628	1.749908	1.747145
1.678048	1.687966	1.685248	1.682612	1.680067	1.75609	1.753361	1.750639	1.747873
1.678724	1.688668	1.685957	1.683317	1.680754	1.756842	1.754101	1.751373	1.748604
1.679397	1.676728	1.674046	1.684003	1.681434	1.713829	1.711161	1.708503	1.749335
1.680062	1.677395	1.674711	1.684684	1.682114	1.714554	1.711872	1.709199	1.750066
1.680714	1.678055	1.675385	1.67277	1.670216	1.680249	1.67771	1.675179	1.72859
1.681355	1.678709	1.676064	1.673459	1.670898	1.680933	1.678376	1.675831	1.729332
1.682	1.679357	1.676725	1.674129	1.671576	1.669029	1.666471	1.663921	1.673939
1.682649	1.679998	1.677362	1.674775	1.672249	1.669716	1.667137	1.664561	1.674588
1.6833	1.680646	1.67801	1.67543	1.672921	1.670395	1.667801	1.665208	1.662673
1.683953	1.681309	1.678684	1.676107	1.67359	1.671057	1.668461	1.66587	1.663339
1.684605	1.681978	1.679372	1.676797	1.674262	1.671715	1.669121	1.666536	1.664012
1.685249	1.682644	1.68007	1.677504	1.67495	1.672386	1.669784	1.667197	1.664685
1.685892	1.683312	1.680775	1.678219	1.675644	1.67306	1.670449	1.667859	1.66536
1.686565	1.683991	1.681462	1.678903	1.676309	1.673714	1.67111	1.66853	1.666026
1.687263	1.684679	1.682134	1.67956	1.67695	1.674349	1.671768	1.669209	1.666686
1.687966	1.685369	1.682811	1.680226	1.677607	1.675005	1.67244	1.669894	1.667353
1.688673	1.686065	1.683499	1.68091	1.67829	1.675689	1.673132	1.670589	1.668036
1.665072	1.68675	1.684174	1.681585	1.678978	1.676386	1.673827	1.671276	1.668711
1.665735	1.687405	1.684813	1.682234	1.679671	1.677105	1.674522	1.671939	1.669364
1.666402	1.688057	1.685444	1.682873	1.680356	1.677817	1.675208	1.67259	1.67001
1.667092	1.688743	1.686094	1.683508	1.681001	1.678471	1.675858	1.673238	1.67067
1.667797	1.689451	1.686758	1.684143	1.681622	1.679089	1.676485	1.67388	1.671335
1.668505	1.665776	1.663105	1.684823	1.682275	1.679723	1.67712	1.674523	1.671983
1.669212	1.666485	1.663818	1.68555	1.682957	1.680369	1.677759	1.675161	1.67261
1.669952	1.667214	1.664529	1.661901	1.659338	1.681029	1.678422	1.675833	1.67327
1.670745	1.667969	1.665221	1.662554	1.659975	1.681706	1.679126	1.67656	1.673988
1.671542	1.66873	1.665925	1.663221	1.660624	1.682393	1.679835	1.677292	1.674717
1.643932	1.669479	1.666657	1.663927	1.661295	1.683081	1.680519	1.677983	1.675432
1.644596	1.670228	1.667409	1.664662	1.661987	1.683779	1.681204	1.678667	1.676143
1.645249	1.670986	1.668187	1.665432	1.662712	1.660074	1.657558	1.655094	1.676851
1.645894	1.671751	1.668988	1.666232	1.663467	1.66079	1.658269	1.655801	1.677558
1.646567	1.643944	1.641373	1.66702	1.664243	1.661549	1.659003	1.656507	1.678274
1.64727	1.644632	1.642043	1.667789	1.665046	1.66236	1.659763	1.657208	1.678999
1.647996	1.645345	1.642736	1.640161	1.637625	1.663167	1.660527	1.657925	1.655365
1.648753	1.646095	1.643468	1.640874	1.63831	1.663937	1.661279	1.658667	1.656109
1.649522	1.64686	1.644219	1.641604	1.639011	1.664717	1.662044	1.659424	1.656864
1.650286	1.647625	1.644976	1.642354	1.639758	1.637195	1.634675	1.632181	1.657592
1.65105	1.648393	1.64574	1.643118	1.64053	1.637959	1.635392	1.632849	1.658321
1.651812	1.649149	1.646502	1.643882	1.64129	1.638711	1.636132	1.633575	1.631061
1.652571	1.649897	1.647265	1.644648	1.642043	1.639459	1.636895	1.634351	1.631825
1.653354	1.650665	1.64803	1.645407	1.642792	1.640202	1.637641	1.635101	1.63257
1.654172	1.651458	1.648794	1.646153	1.643535	1.640939	1.638362	1.635809	1.633287
1.654991	1.65226	1.64957	1.646911	1.644284	1.641677	1.639083	1.636517	1.633998
1.655793	1.653059	1.65037	1.647698	1.645042	1.642412	1.63981	1.637239	1.634704
1.656597	1.653864	1.65118	1.648498	1.645815	1.643162	1.640551	1.637971	1.635414
1.657418	1.654679	1.651979	1.649294	1.646621	1.643968	1.64133	1.638719	1.636148
1.658253	1.655503	1.652777	1.650091	1.647451	1.64481	1.642137	1.639482	1.636901
1.659108	1.656341	1.653596	1.650903	1.648272	1.645636	1.642952	1.640282	1.637689
1.659984	1.657197	1.654436	1.651731	1.649091	1.646453	1.643782	1.641124	1.638516

1.631124	1.658035	1.655261	1.65254	1.64988	1.647232	1.644575	1.64193	1.639308
1.631895	1.658838	1.656053	1.653311	1.650616	1.647947	1.645304	1.642675	1.640035
1.632674	1.65965	1.656849	1.654086	1.651359	1.648669	1.646029	1.643406	1.640754
1.633498	1.630727	1.62797	1.654881	1.652149	1.64945	1.646784	1.644138	1.641486
1.634338	1.631554	1.628758	1.655699	1.652968	1.650261	1.647559	1.644881	1.642234
1.635161	1.632369	1.629582	1.626849	1.624176	1.651091	1.648371	1.64568	1.643036
1.635976	1.63318	1.630434	1.627707	1.625	1.651939	1.649214	1.646522	1.643879
1.636805	1.633995	1.631265	1.628534	1.625795	1.623094	1.620447	1.617839	1.64472
1.637642	1.634806	1.632072	1.629326	1.626555	1.623838	1.621209	1.618625	1.645561
1.592117	1.635655	1.632895	1.630125	1.627332	1.624602	1.621979	1.619403	1.61683
1.59295	1.63657	1.63375	1.630943	1.628142	1.625403	1.62276	1.62016	1.617561
1.593802	1.637505	1.634617	1.631773	1.628971	1.626226	1.623558	1.620928	1.618296
1.527849	1.638419	1.635473	1.632602	1.629811	1.627071	1.624388	1.621738	1.619079
1.528513	1.639335	1.63634	1.633448	1.630668	1.627935	1.625242	1.622573	1.619888
1.529188	1.640288	1.637269	1.634357	1.631561	1.628812	1.626103	1.623417	1.62071
1.52987	1.641272	1.638253	1.635324	1.632487	1.629702	1.626974	1.624273	1.621547
1.499877	1.580417	1.577782	1.63628	1.633415	1.6306	1.627839	1.625108	1.622367
1.500455	1.581202	1.578575	1.637224	1.634349	1.631509	1.628695	1.625914	1.623161
1.50103	1.553924	1.551389	1.63815	1.635271	1.632412	1.629551	1.626724	1.623965
1.50161	1.554609	1.552083	1.639041	1.636164	1.633299	1.630412	1.627562	1.624806
1.502189	1.55529	1.552771	1.639922	1.637048	1.634181	1.631274	1.628405	1.625654
1.502758	1.500736	1.498709	1.579036	1.576505	1.635063	1.632126	1.629228	1.626474
1.503325	1.501305	1.499299	1.579803	1.577273	1.635949	1.632978	1.630047	1.627285
1.503926	1.501902	1.499903	1.552432	1.549978	1.636861	1.633872	1.63092	1.628135
1.504559	1.502527	1.50052	1.553173	1.550722	1.637799	1.634807	1.631843	1.629023
1.505201	1.503161	1.501146	1.499143	1.49716	1.549006	1.546471	1.543962	1.629918
1.505848	1.503803	1.501779	1.499772	1.497789	1.549752	1.547209	1.544687	1.630809
1.506499	1.504449	1.502418	1.500407	1.498423	1.550503	1.547956	1.545427	1.631719
1.507149	1.505098	1.503063	1.501048	1.499062	1.551257	1.548718	1.546195	1.632663
1.507799	1.505748	1.503711	1.501694	1.499706	1.55202	1.549493	1.546981	1.633632
1.508436	1.506383	1.504357	1.502345	1.500355	1.498347	1.496296	1.494243	1.545271
1.509067	1.50701	1.505002	1.503	1.501007	1.499004	1.49698	1.494947	1.546068
1.509747	1.507672	1.505652	1.503641	1.501644	1.499645	1.497641	1.495624	1.546848
1.510478	1.50837	1.506303	1.504265	1.502262	1.500266	1.498277	1.496273	1.547609
1.444342	1.509084	1.506972	1.504907	1.502896	1.500898	1.49891	1.496911	1.548373
1.445218	1.509809	1.507663	1.505576	1.503557	1.50155	1.499543	1.49753	1.549141
1.4461	1.510546	1.508377	1.506272	1.504239	1.502217	1.500186	1.498156	1.549923
1.446967	1.480408	1.478133	1.475905	1.473728	1.502897	1.500856	1.498823	1.496795
1.447835	1.481337	1.47908	1.476845	1.474632	1.503595	1.501546	1.499513	1.497478
1.448735	1.446587	1.444522	1.477776	1.475553	1.473349	1.471161	1.468987	1.466819
1.449662	1.447493	1.445397	1.478705	1.47649	1.474284	1.47207	1.469865	1.46768
1.450593	1.448398	1.44626	1.479616	1.477407	1.475204	1.472982	1.470765	1.468562
1.451527	1.449294	1.447099	1.480498	1.478293	1.476103	1.4739	1.471696	1.469472
1.452468	1.450206	1.447964	1.445826	1.443806	1.476994	1.474809	1.47262	1.470387
1.453418	1.451142	1.448884	1.446718	1.444651	1.477876	1.475692	1.473517	1.4713
1.454379	1.452095	1.44983	1.447635	1.445511	1.478761	1.476573	1.474407	1.472209
1.455358	1.453061	1.45078	1.448557	1.446389	1.479667	1.477468	1.4753	1.473104
1.45635	1.45404	1.451742	1.449493	1.447287	1.480593	1.478376	1.476196	1.473991
1.45733	1.455017	1.452721	1.450459	1.448223	1.446055	1.443992	1.441972	1.474896
1.458299	1.455993	1.453719	1.451457	1.449198	1.447	1.444898	1.442843	1.475814

1.86957	1.866377	1.863272	1.864692	1.861485	1.858303	1.855183	1.852061	1.849841
1.869511	1.866315	1.863204	1.864608	1.861401	1.858219	1.855097	1.851973	1.849752
1.869481	1.866281	1.863155	1.864535	1.861321	1.858134	1.855008	1.851882	1.849661
1.8695	1.866294	1.863134	1.864476	1.861249	1.858052	1.854922	1.851793	1.849571
1.869553	1.866341	1.863133	1.864429	1.861183	1.857973	1.854836	1.851704	1.849481
1.869559	1.866345	1.863113	1.86474	1.861467	1.857893	1.854751	1.851617	1.849393
1.869516	1.866306	1.863073	1.864698	1.861412	1.857812	1.854666	1.85153	1.849307
1.869465	1.866257	1.863026	1.859797	1.85656	1.858113	1.854949	1.851797	1.849221
1.86941	1.866201	1.86297	1.85974	1.856504	1.858058	1.854886	1.851728	1.849133
1.869362	1.866148	1.862917	1.859692	1.856467	1.858028	1.854842	1.851668	1.849052
1.869341	1.866112	1.862878	1.859666	1.856477	1.858058	1.854843	1.851632	1.850468
1.86933	1.866088	1.862854	1.859658	1.856509	1.858112	1.854863	1.851608	1.850428
1.869306	1.866076	1.86287	1.859685	1.856529	1.858122	1.85485	1.851576	1.850405
1.869274	1.866077	1.862917	1.85974	1.856544	1.858104	1.854815	1.85154	1.850398
1.869241	1.86606	1.862926	1.859751	1.856531	1.858075	1.854778	1.851502	1.850375
1.869199	1.866018	1.862885	1.859709	1.856484	1.858031	1.854732	1.851457	1.850328
1.869184	1.865996	1.862847	1.859662	1.856436	1.857993	1.854704	1.851436	1.850298
1.869227	1.866025	1.862833	1.859623	1.85639	1.857961	1.854704	1.851465	1.850306
1.869279	1.866061	1.862825	1.859592	1.856358	1.857948	1.854722	1.851506	1.850325
1.869291	1.866057	1.862806	1.859576	1.856369	1.853176	1.850002	1.846828	1.849352
1.869284	1.866037	1.862788	1.859576	1.856406	1.853232	1.850037	1.846832	1.849348
1.866947	1.866032	1.862805	1.859604	1.856435	1.853262	1.85007	1.846868	1.84939
1.866936	1.866039	1.862852	1.859661	1.856462	1.853272	1.850104	1.846932	1.849475
1.866953	1.866064	1.862893	1.859706	1.856498	1.853302	1.850135	1.846967	1.849524
1.867014	1.866114	1.862916	1.859726	1.856548	1.853363	1.850164	1.846959	1.849513
1.867076	1.863844	1.860601	1.859756	1.856597	1.854754	1.851486	1.84821	1.8495
1.867113	1.863887	1.860669	1.859817	1.856635	1.854769	1.851478	1.848189	1.849508
1.86715	1.86393	1.860742	1.859885	1.856672	1.85478	1.851473	1.848179	1.849529
1.867216	1.863994	1.860794	1.857579	1.854351	1.854831	1.851526	1.848229	1.849575
1.8673	1.86407	1.860836	1.857614	1.854411	1.854905	1.851611	1.848315	1.84964
1.865526	1.864132	1.860895	1.85767	1.854465	1.854946	1.851652	1.848358	1.845108
1.865565	1.864176	1.860972	1.85775	1.854509	1.854952	1.851647	1.848355	1.845131
1.865637	1.86241	1.85922	1.85782	1.854562	1.852751	1.849424	1.846118	1.84517
1.86577	1.862512	1.859271	1.85786	1.854629	1.852842	1.849526	1.84622	1.845237
1.865911	1.862628	1.859339	1.857913	1.854701	1.852933	1.849634	1.846335	1.84532
1.865293	1.862757	1.859461	1.85619	1.852946	1.852979	1.849706	1.84644	1.843172
1.8654	1.862886	1.859604	1.856316	1.853016	1.85302	1.849768	1.846537	1.84329
1.865471	1.862968	1.859717	1.85644	1.853125	1.851408	1.848141	1.844901	1.841674
1.865514	1.863017	1.859812	1.856565	1.853264	1.851537	1.848218	1.84493	1.841719
1.865603	1.864219	1.860975	1.858308	1.854987	1.851659	1.848316	1.845	1.841782
1.865748	1.864327	1.861031	1.858349	1.855071	1.851779	1.848444	1.845119	1.841864
1.865892	1.864448	1.861109	1.857804	1.854541	1.851876	1.848559	1.845245	1.841965
1.866021	1.864587	1.861241	1.857915	1.854609	1.851919	1.848638	1.845365	1.842091
1.866132	1.864719	1.861386	1.858045	1.854688	1.851966	1.848716	1.845486	1.842227
1.866195	1.8648	1.861508	1.858184	1.854812	1.852076	1.848833	1.845613	1.842364
1.86624	1.864861	1.861624	1.85833	1.854962	1.852219	1.848966	1.845738	1.842498
1.866341	1.864968	1.861755	1.858475	1.855113	1.851771	1.848483	1.845223	1.842595
1.866487	1.865114	1.861897	1.858619	1.85527	1.851922	1.848582	1.845274	1.842661
1.866642	1.865255	1.862014	1.858733	1.855408	1.85207	1.8487	1.845361	1.842152
1.866803	1.865385	1.862088	1.858796	1.855514	1.852211	1.848843	1.845498	1.842287

1.86224	1.865524	1.862179	1.858877	1.85563	1.852357	1.848998	1.845652	1.842426
1.862402	1.865684	1.862323	1.859016	1.855779	1.852513	1.849159	1.845808	1.842546
1.86256	1.865852	1.862489	1.859181	1.855943	1.852677	1.849327	1.845971	1.842668
1.862157	1.86601	1.862654	1.85935	1.856113	1.852846	1.849498	1.84614	1.842816
1.862281	1.866166	1.862824	1.859527	1.856289	1.853021	1.849673	1.846313	1.842981
1.862416	1.861881	1.858565	1.859703	1.856465	1.853196	1.849848	1.846486	1.843147
1.862561	1.86204	1.858732	1.859877	1.85664	1.853371	1.850023	1.846658	1.843313
1.862716	1.862204	1.8589	1.855636	1.852424	1.853546	1.850198	1.846833	1.843482
1.86288	1.862373	1.85907	1.855807	1.852596	1.853722	1.850374	1.847008	1.843652
1.863055	1.862547	1.859242	1.855978	1.852767	1.853897	1.850551	1.847185	1.843827
1.863251	1.862283	1.858981	1.856145	1.852936	1.854072	1.850729	1.847365	1.844009
1.863454	1.862466	1.859154	1.856315	1.853106	1.854248	1.850908	1.847548	1.8442
1.857652	1.862651	1.859335	1.856495	1.853281	1.85004	1.846722	1.843393	1.844412
1.857831	1.862837	1.859524	1.856683	1.853461	1.850216	1.846897	1.843576	1.844639
1.858005	1.857261	1.854004	1.856911	1.853666	1.850403	1.847081	1.843767	1.840534
1.858171	1.857465	1.854261	1.857189	1.853903	1.850604	1.847275	1.843962	1.840729
1.858344	1.857668	1.854501	1.857005	1.853698	1.850806	1.847486	1.844186	1.840945
1.85852	1.857855	1.854697	1.857205	1.853889	1.851004	1.847729	1.844467	1.841207
1.858716	1.858052	1.854885	1.85739	1.854073	1.851203	1.847972	1.844748	1.841469
1.852301	1.858295	1.855093	1.851852	1.848561	1.851397	1.84818	1.844969	1.841683
1.852569	1.858554	1.855306	1.852042	1.848754	1.851597	1.848376	1.845162	1.841874
1.852791	1.858775	1.855505	1.852241	1.84898	1.84632	1.843102	1.839884	1.842071
1.852977	1.858965	1.855692	1.852447	1.849236	1.846606	1.843353	1.84009	1.842269
1.853169	1.852821	1.849565	1.852649	1.849467	1.846265	1.843007	1.839734	1.842492
1.853369	1.853004	1.849745	1.852837	1.849659	1.846463	1.843212	1.839952	1.842746
1.853574	1.853201	1.84995	1.853049	1.849862	1.846665	1.843429	1.840191	1.837545
1.853777	1.853424	1.850207	1.853315	1.8501	1.846889	1.843682	1.840473	1.837825
1.853992	1.853666	1.850483	1.853599	1.850356	1.84713	1.843947	1.840763	1.838103
1.854247	1.851062	1.84792	1.844764	1.84159	1.847408	1.844213	1.841013	1.837766
1.854526	1.851338	1.848166	1.845008	1.841868	1.847708	1.844479	1.84124	1.837985
1.854784	1.8516	1.84843	1.845271	1.842128	1.847965	1.844718	1.841466	1.838221
1.855025	1.85185	1.848713	1.845554	1.84237	1.848184	1.84493	1.841685	1.83847
1.855282	1.852111	1.848985	1.845819	1.842602	1.842111	1.838886	1.835683	1.838733
1.855571	1.852391	1.849234	1.846045	1.842813	1.842326	1.839134	1.835966	1.839015
1.855857	1.852671	1.849489	1.846283	1.843043	1.842562	1.839396	1.836253	1.8393
1.856113	1.852932	1.849771	1.846578	1.843339	1.842855	1.839671	1.836513	1.839577
1.856364	1.853191	1.850062	1.846886	1.843652	1.843166	1.839953	1.83677	1.839854
1.856649	1.853472	1.850329	1.847146	1.843911	1.843434	1.840228	1.837052	1.833902
1.856956	1.853768	1.850584	1.847378	1.844139	1.843677	1.840501	1.83735	1.834185
1.857243	1.854052	1.850862	1.847654	1.844417	1.843963	1.840785	1.837633	1.834466
1.857505	1.854324	1.851169	1.847981	1.844753	1.844295	1.84108	1.837894	1.83474
1.85778	1.854604	1.851466	1.848295	1.845085	1.841881	1.838687	1.835521	1.835039
1.858089	1.854903	1.851734	1.848565	1.845396	1.842224	1.839032	1.835861	1.835388
1.858402	1.855206	1.852008	1.848836	1.845698	1.842554	1.839377	1.836212	1.835741
1.858685	1.855494	1.852315	1.849145	1.845985	1.842832	1.839679	1.836539	1.83606
1.858965	1.855784	1.852643	1.849475	1.846272	1.843092	1.839964	1.836851	1.836363
1.859297	1.856119	1.852992	1.849823	1.846602	1.843403	1.840259	1.837135	1.836654
1.859671	1.856491	1.853361	1.850189	1.846968	1.843754	1.840562	1.837393	1.836932
1.860038	1.856852	1.8537	1.850522	1.847314	1.844103	1.840884	1.83769	1.837242
1.860395	1.857194	1.853994	1.850806	1.847634	1.844454	1.841237	1.838038	1.837595

1.860753	1.85754	1.8543	1.851099	1.847943	1.844782	1.84158	1.838391	1.837947
1.861128	1.85791	1.854653	1.851427	1.848233	1.845053	1.841883	1.838726	1.838271
1.861491	1.858273	1.855018	1.851772	1.848528	1.845322	1.842184	1.839064	1.838597
1.861794	1.858585	1.855361	1.852127	1.848869	1.845655	1.84253	1.839422	1.836253
1.862066	1.858872	1.855699	1.852492	1.849239	1.846024	1.8429	1.839791	1.836619
1.862388	1.859203	1.856056	1.852864	1.849614	1.846398	1.84327	1.840157	1.836983
1.862755	1.859573	1.856432	1.853244	1.849997	1.846781	1.843645	1.840523	1.837349
1.86115	1.859947	1.856806	1.85362	1.850377	1.847157	1.844	1.840857	1.837687
1.86154	1.860325	1.857183	1.853996	1.850755	1.847523	1.844319	1.841134	1.837977
1.861923	1.860692	1.857539	1.854349	1.85112	1.847885	1.844643	1.841422	1.838276
1.862278	1.861025	1.857837	1.854645	1.851452	1.848244	1.844998	1.841767	1.838623
1.862626	1.86135	1.858122	1.854928	1.851777	1.848605	1.845368	1.842137	1.838992
1.862996	1.859755	1.856479	1.855266	1.852136	1.848982	1.845747	1.842514	1.839367
1.863381	1.860138	1.856863	1.85564	1.852519	1.849369	1.846136	1.842901	1.83975
1.857149	1.860527	1.857253	1.854033	1.850882	1.849757	1.846524	1.843287	1.840132
1.857536	1.860924	1.857654	1.854435	1.851284	1.850146	1.84691	1.84367	1.840511
1.857923	1.854706	1.851463	1.854833	1.851682	1.848501	1.845232	1.841958	1.840896
1.858303	1.855084	1.851838	1.855218	1.85207	1.848892	1.845627	1.842356	1.841281
1.858687	1.855465	1.852216	1.855605	1.852458	1.849285	1.846034	1.842775	1.841685
1.859077	1.855853	1.852603	1.852596	1.849465	1.849687	1.846475	1.843252	1.842138
1.859476	1.856251	1.853005	1.853001	1.84986	1.850094	1.84693	1.84375	1.842609
1.856348	1.85668	1.853462	1.850268	1.847097	1.847127	1.844	1.840862	1.840956
1.856783	1.857135	1.853966	1.850773	1.847547	1.847533	1.844395	1.841257	1.841358
1.848259	1.854022	1.850892	1.851233	1.847973	1.844744	1.841603	1.838469	1.83843
1.848653	1.854421	1.851289	1.851634	1.848367	1.845132	1.841985	1.838849	1.838818
1.848958	1.854817	1.851676	1.852025	1.848758	1.845523	1.84237	1.83923	1.839217
1.849356	1.855213	1.852066	1.852417	1.849149	1.845913	1.842755	1.839619	1.839644
1.849772	1.855625	1.852463	1.852815	1.849554	1.846322	1.843153	1.840012	1.840075
1.845677	1.856213	1.853008	1.849683	1.846465	1.846789	1.843589	1.840415	1.837292
1.846175	1.856714	1.853458	1.850117	1.846938	1.847286	1.844046	1.840823	1.837685
1.846623	1.852903	1.849643	1.850666	1.847499	1.844193	1.84095	1.837718	1.838079
1.847029	1.853317	1.850061	1.851085	1.847908	1.844588	1.841345	1.838116	1.838472
1.845246	1.844273	1.841075	1.847292	1.844108	1.844996	1.841763	1.838545	1.838897
1.845667	1.844713	1.841549	1.847781	1.84456	1.845422	1.842214	1.839024	1.839368
1.846101	1.845161	1.842019	1.848265	1.845022	1.84587	1.842677	1.839503	1.839847
1.846564	1.845627	1.84246	1.84871	1.845497	1.846511	1.843293	1.84009	1.836777
1.847026	1.846088	1.842885	1.849136	1.845966	1.847014	1.843767	1.840532	1.837231
1.847434	1.844282	1.841085	1.849547	1.846388	1.843223	1.84002	1.836823	1.837868
1.847807	1.844656	1.841468	1.849948	1.84678	1.843624	1.840477	1.837331	1.838367
1.848205	1.845057	1.84189	1.840946	1.837788	1.844034	1.840907	1.837787	1.834635
1.848623	1.845483	1.842354	1.841418	1.838226	1.844445	1.841305	1.838186	1.835066
1.839675	1.845928	1.842815	1.839666	1.836469	1.844893	1.841727	1.838592	1.835488
1.840137	1.846396	1.843256	1.840108	1.836951	1.845405	1.842197	1.839011	1.835893
1.840599	1.846868	1.843704	1.840558	1.837436	1.845922	1.842684	1.839459	1.836322
1.84103	1.847318	1.844181	1.841043	1.837904	1.846392	1.843173	1.839968	1.836829
1.841453	1.847764	1.844667	1.841537	1.838365	1.846849	1.843669	1.840508	1.83737
1.841906	1.848228	1.845122	1.84199	1.838826	1.845358	1.842211	1.839076	1.837881
1.842382	1.848705	1.845554	1.842414	1.839285	1.845862	1.84273	1.839596	1.838376
1.842834	1.839722	1.836604	1.842863	1.83974	1.846332	1.843205	1.840075	1.838854
1.843252	1.840147	1.837063	1.843342	1.840183	1.846752	1.84362	1.840497	1.839309

1.829292	1.840591	1.837537	1.843837	1.840651	1.837487	1.834379	1.831288	1.837805
1.829766	1.841081	1.838037	1.844358	1.841173	1.838006	1.83489	1.831785	1.838286
1.830253	1.841581	1.838534	1.844873	1.841702	1.83854	1.835414	1.83229	1.838773
1.829534	1.827715	1.824693	1.845336	1.842191	1.839044	1.835898	1.832753	1.839248
1.830025	1.828185	1.825131	1.845773	1.842659	1.839531	1.836364	1.8332	1.839724
1.830492	1.827447	1.824403	1.836777	1.833693	1.839989	1.83683	1.833678	1.840228
1.830935	1.827896	1.824877	1.837251	1.834129	1.840416	1.837291	1.834181	1.840758
1.831394	1.828365	1.825363	1.822329	1.819255	1.840884	1.83778	1.834696	1.841284
1.83188	1.828865	1.825867	1.822837	1.819765	1.84141	1.838309	1.835222	1.841797
1.832371	1.829364	1.826364	1.823338	1.82028	1.827655	1.824605	1.821559	1.842318
1.832858	1.829845	1.82683	1.823809	1.82078	1.828196	1.825155	1.82211	1.842872
1.833343	1.830319	1.827289	1.824275	1.82128	1.828737	1.825701	1.822655	1.843426
1.833812	1.830786	1.827765	1.824764	1.821787	1.829266	1.826212	1.823148	1.833723
1.834274	1.83125	1.828252	1.825266	1.822298	1.829788	1.826703	1.823611	1.834193
1.821989	1.831742	1.828755	1.825776	1.822813	1.830317	1.827217	1.824113	1.821062
1.822501	1.832263	1.829274	1.826295	1.823333	1.830856	1.827757	1.824655	1.821604
1.823018	1.820027	1.817073	1.826811	1.823837	1.820845	1.817808	1.814769	1.822148
1.823537	1.820546	1.817587	1.82732	1.82431	1.8213	1.818286	1.81528	1.822689
1.82405	1.821063	1.818103	1.827833	1.824788	1.82176	1.818769	1.815793	1.823231
1.82454	1.821568	1.818618	1.815635	1.81261	1.822269	1.819283	1.816316	1.823778
1.825023	1.822069	1.819134	1.816159	1.813134	1.8228	1.819812	1.816846	1.824328
1.825524	1.822578	1.819652	1.816683	1.813658	1.810652	1.807694	1.804758	1.824883
1.826037	1.823093	1.820173	1.817208	1.814186	1.811182	1.808226	1.80529	1.825443
1.826566	1.823622	1.820705	1.817742	1.814722	1.811719	1.808761	1.805823	1.814136
1.827115	1.82417	1.821251	1.818288	1.81527	1.812266	1.809301	1.806357	1.814683
1.827661	1.824713	1.821792	1.818828	1.815809	1.812804	1.809836	1.806888	1.803968
1.828195	1.825244	1.822319	1.81935	1.816327	1.813321	1.810358	1.807417	1.804501
1.828724	1.825769	1.822839	1.819867	1.816842	1.813836	1.810877	1.807942	1.805032
1.829248	1.826291	1.823359	1.820387	1.817363	1.814358	1.811396	1.808459	1.80555
1.829769	1.82681	1.823877	1.820907	1.817891	1.814889	1.811919	1.808973	1.806063
1.825246	1.827333	1.824401	1.82144	1.818446	1.815455	1.812469	1.809502	1.806582
1.825781	1.82786	1.824928	1.821983	1.819026	1.816053	1.813045	1.810046	1.807107
1.826332	1.823371	1.820447	1.822518	1.819585	1.816626	1.813608	1.81059	1.807636
1.826902	1.823918	1.820978	1.82304	1.820114	1.817162	1.814147	1.81113	1.808166
1.827484	1.824478	1.821519	1.823566	1.820638	1.817691	1.81469	1.811684	1.808713
1.828072	1.825055	1.822076	1.824105	1.821168	1.818224	1.815252	1.812273	1.809295
1.828671	1.825646	1.822647	1.824654	1.821703	1.818758	1.815817	1.81287	1.809891
1.821157	1.826261	1.823235	1.820244	1.81729	1.819302	1.816372	1.813441	1.810476
1.821784	1.826889	1.823839	1.820827	1.817855	1.819853	1.816922	1.813998	1.811056
1.822374	1.819367	1.816331	1.821433	1.81844	1.820415	1.81747	1.814542	1.811613
1.822927	1.819948	1.816953	1.822066	1.819046	1.820986	1.818015	1.815068	1.812147
1.823474	1.820512	1.817549	1.814563	1.811546	1.816645	1.813662	1.810707	1.812689
1.824011	1.821047	1.818096	1.815133	1.812156	1.817276	1.814277	1.811292	1.813245
1.824565	1.82159	1.818641	1.815695	1.812756	1.817903	1.814899	1.811896	1.813818
1.825164	1.822169	1.819205	1.816254	1.813322	1.810381	1.807411	1.804439	1.814424
1.825787	1.822773	1.819788	1.816824	1.813883	1.810947	1.808012	1.805071	1.815045
1.81813	1.823411	1.820428	1.817454	1.814491	1.811538	1.808601	1.805666	1.810779
1.818767	1.824077	1.821109	1.818129	1.815132	1.812146	1.809184	1.806235	1.81137
1.819377	1.81643	1.813493	1.818762	1.81576	1.812765	1.809785	1.806821	1.803888
1.819949	1.817	1.814063	1.819346	1.816376	1.813399	1.810407	1.807423	1.804478

1.820543	1.817583	1.814636	1.811699	1.808776	1.814024	1.811032	1.808042	1.805091
1.821191	1.818206	1.815227	1.812276	1.809359	1.81462	1.811652	1.808688	1.805751
1.821848	1.818842	1.815834	1.812868	1.809949	1.815219	1.812276	1.809337	1.806414
1.822477	1.819474	1.816468	1.813502	1.810584	1.807669	1.80474	1.801814	1.807032
1.8231	1.82011	1.817118	1.81416	1.811241	1.808318	1.805368	1.802422	1.807634
1.815911	1.820761	1.817762	1.814799	1.811878	1.808954	1.806002	1.803057	1.800146
1.816561	1.821425	1.818402	1.815425	1.812501	1.809581	1.806643	1.803712	1.800806
1.806507	1.814226	1.811242	1.81607	1.813144	1.810224	1.80729	1.804361	1.801452
1.807097	1.814846	1.811882	1.816744	1.813817	1.81089	1.807941	1.804995	1.80207
1.797062	1.810753	1.80782	1.809597	1.806709	1.811551	1.808597	1.805644	1.802709
1.797709	1.811393	1.80843	1.810202	1.807313	1.812183	1.809253	1.806326	1.803405
1.798369	1.812044	1.809048	1.810813	1.807922	1.812822	1.80992	1.807025	1.804121
1.798999	1.806721	1.803756	1.811492	1.808595	1.813513	1.810618	1.807731	1.80483
1.799619	1.807377	1.804449	1.812213	1.809307	1.814237	1.811335	1.808441	1.805537
1.789627	1.797374	1.794483	1.802253	1.799383	1.807119	1.804229	1.801345	1.806203
1.790314	1.798068	1.795177	1.802957	1.800087	1.807823	1.804892	1.801966	1.806827
1.791002	1.798753	1.795846	1.792961	1.790106	1.803799	1.800868	1.79794	1.799706
1.791691	1.799419	1.796471	1.793563	1.790698	1.804414	1.801517	1.798624	1.800403
1.792364	1.800077	1.797099	1.794171	1.791296	1.805035	1.802175	1.799323	1.801117
1.792989	1.790064	1.787122	1.79483	1.791949	1.789098	1.78629	1.783489	1.797161
1.7936	1.790701	1.787788	1.795518	1.79263	1.789775	1.786975	1.784182	1.79787
1.794254	1.791375	1.788486	1.785608	1.782736	1.790473	1.787661	1.784854	1.782006
1.794934	1.792075	1.789209	1.786339	1.783462	1.791189	1.788349	1.78551	1.782631
1.79562	1.79277	1.789913	1.787047	1.784168	1.781297	1.778446	1.775595	1.783248
1.796313	1.793461	1.790601	1.787733	1.784858	1.781976	1.779081	1.776189	1.783852
1.796998	1.794134	1.791257	1.788381	1.785508	1.782622	1.779706	1.776797	1.773936
1.797675	1.794782	1.79186	1.788959	1.786083	1.783207	1.780318	1.777438	1.774591
1.798331	1.795411	1.792455	1.789535	1.786655	1.78379	1.780937	1.778096	1.775263
1.786554	1.796016	1.793083	1.790174	1.787292	1.784429	1.781589	1.778762	1.775943
1.787123	1.79661	1.793726	1.790844	1.787963	1.785099	1.782261	1.77944	1.776632
1.787722	1.784852	1.782014	1.791511	1.788645	1.78579	1.782952	1.780127	1.777322
1.788343	1.785481	1.782652	1.79218	1.789342	1.786505	1.783661	1.780825	1.778013
1.740278	1.78613	1.783303	1.792855	1.790033	1.787209	1.784368	1.781529	1.801577
1.740858	1.786804	1.783971	1.793536	1.790716	1.787897	1.78507	1.782243	1.802284
1.741443	1.738853	1.73627	1.781828	1.779028	1.788581	1.785765	1.78295	1.80299
1.742022	1.73944	1.736868	1.782514	1.779702	1.789262	1.786449	1.783641	1.803688
1.742603	1.740026	1.737464	1.7832	1.780377	1.789942	1.787129	1.784324	1.804381
1.743186	1.740605	1.738043	1.735475	1.732902	1.77824	1.775446	1.77266	1.805065
1.743773	1.741182	1.738611	1.736046	1.73349	1.778919	1.77612	1.773327	1.805745
1.744362	1.741765	1.739185	1.736617	1.734067	1.77959	1.776794	1.774003	1.794884
1.744955	1.742353	1.739762	1.737187	1.734632	1.780254	1.777468	1.774687	1.795569
1.745544	1.74294	1.740344	1.737765	1.735204	1.732649	1.730098	1.727543	1.796251
1.746121	1.743519	1.740933	1.738355	1.735785	1.733223	1.730671	1.728119	1.796926
1.746712	1.744109	1.741527	1.738948	1.736371	1.733802	1.731245	1.728692	1.797599
1.708082	1.744721	1.74212	1.739531	1.736956	1.761653	1.759033	1.756415	1.753792
1.708672	1.745353	1.742724	1.740122	1.737548	1.76225	1.759629	1.757009	1.754381
1.709295	1.706748	1.704191	1.740752	1.738163	1.762867	1.760248	1.757632	1.755004
1.70994	1.707383	1.704814	1.741411	1.738796	1.763501	1.760886	1.75828	1.755653
1.710544	1.707989	1.705425	1.702874	1.700337	1.76417	1.761557	1.758953	1.75633
1.711104	1.708563	1.706027	1.703503	1.700992	1.764885	1.762269	1.75966	1.757046

1.711653	1.709124	1.706606	1.7041	1.701609	1.729362	1.72682	1.724279	1.757727
1.712203	1.709673	1.707149	1.704641	1.702151	1.729909	1.727374	1.724841	1.758346
1.712746	1.710211	1.707679	1.705165	1.70267	1.730427	1.727898	1.725371	1.758934
1.71327	1.710734	1.708211	1.736058	1.733494	1.730942	1.728407	1.725877	1.723335
1.713792	1.711253	1.708743	1.736589	1.734017	1.731455	1.728909	1.726371	1.723831
1.714345	1.711799	1.709287	1.737138	1.734564	1.731999	1.729446	1.726902	1.724364
1.714923	1.712367	1.709841	1.737702	1.735131	1.732568	1.730011	1.727462	1.724926
1.715538	1.712976	1.710445	1.738309	1.735736	1.733171	1.730613	1.728064	1.725527
1.716205	1.71364	1.711119	1.73898	1.736393	1.73382	1.731266	1.728722	1.726179
1.716854	1.714283	1.711764	1.739623	1.737032	1.734456	1.731905	1.729364	1.726817
1.717441	1.714856	1.71232	1.740178	1.737606	1.735044	1.732488	1.729939	1.727398
1.718014	1.715414	1.712855	1.740715	1.738165	1.73562	1.733059	1.730503	1.727969
1.718608	1.716	1.713423	1.741292	1.738755	1.736221	1.733662	1.731108	1.728579
1.719215	1.716603	1.71401	1.741892	1.739358	1.736829	1.734277	1.731729	1.729203
1.719867	1.71725	1.714645	1.742524	1.739967	1.737421	1.734863	1.732313	1.729786
1.720565	1.717942	1.71533	1.743188	1.740584	1.738	1.735423	1.732865	1.730333
1.721231	1.749208	1.746506	1.743835	1.741195	1.738579	1.735985	1.733417	1.730885
1.721841	1.749834	1.747128	1.744447	1.74179	1.739157	1.736546	1.733967	1.731443
1.722455	1.750458	1.747751	1.745063	1.742393	1.739748	1.737126	1.73454	1.732017
1.72312	1.751115	1.748412	1.745718	1.74303	1.740372	1.737752	1.735167	1.732625
1.723791	1.75177	1.749072	1.746374	1.743669	1.741001	1.73839	1.735811	1.733247
1.717734	1.752371	1.749668	1.746965	1.744253	1.741583	1.738984	1.736418	1.733855
1.718316	1.75294	1.750228	1.747519	1.744805	1.742139	1.739554	1.737005	1.734457
1.718938	1.753556	1.750848	1.748138	1.745418	1.742745	1.740149	1.737591	1.735041
1.719603	1.754222	1.751532	1.748827	1.746096	1.743402	1.740771	1.738176	1.735601
1.720263	1.74898	1.746306	1.749505	1.746771	1.744063	1.741398	1.738768	1.736178
1.720894	1.749608	1.746938	1.750152	1.74743	1.744724	1.742036	1.739382	1.736793
1.721541	1.750247	1.747568	1.750785	1.748073	1.745368	1.742661	1.739987	1.737402
1.722247	1.750927	1.748206	1.7514	1.748672	1.745953	1.743227	1.74054	1.737958
1.722981	1.751628	1.748853	1.752017	1.749264	1.746525	1.743783	1.741085	1.7385
1.723712	1.752334	1.749536	1.746759	1.744005	1.747181	1.744421	1.741702	1.739089
1.724442	1.753048	1.750248	1.747472	1.744721	1.747901	1.745122	1.742373	1.739713
1.756517	1.753728	1.750942	1.748172	1.745422	1.748609	1.745827	1.743067	1.740375
1.757136	1.754365	1.751613	1.748861	1.746107	1.749303	1.746537	1.74379	1.741084
1.75778	1.755012	1.752277	1.749531	1.746772	1.749978	1.747233	1.744504	1.741794
1.758493	1.755694	1.752927	1.750162	1.747395	1.750615	1.747893	1.745189	1.742481
1.759228	1.756395	1.753588	1.750799	1.748023	1.751253	1.748548	1.745865	1.743163
1.759958	1.757116	1.754303	1.751502	1.748709	1.745952	1.74325	1.740573	1.743842
1.760687	1.757851	1.755047	1.752242	1.74943	1.746652	1.743926	1.741233	1.744521
1.761387	1.758562	1.755771	1.752974	1.750162	1.747377	1.744634	1.741923	1.745211
1.762061	1.759253	1.756482	1.753702	1.750907	1.748129	1.745373	1.74264	1.74591
1.743049	1.759941	1.757187	1.754422	1.751645	1.748874	1.746113	1.743364	1.746626
1.743684	1.76062	1.757883	1.755134	1.752371	1.74961	1.746848	1.744095	1.747367
1.71204	1.741598	1.738931	1.755838	1.753091	1.750341	1.747581	1.744826	1.742089
1.712726	1.742294	1.739596	1.756539	1.753806	1.751069	1.748307	1.745548	1.74281
1.713435	1.743007	1.740261	1.757233	1.754514	1.75179	1.749029	1.746268	1.74353
1.68709	1.743703	1.740905	1.757905	1.7552	1.752494	1.749746	1.746995	1.744259
1.687789	1.744395	1.741541	1.758569	1.755875	1.753187	1.750459	1.747727	1.744993
1.688505	1.685795	1.683104	1.739465	1.736838	1.753895	1.751177	1.748455	1.745717
1.689242	1.686529	1.683817	1.740192	1.737532	1.754617	1.7519	1.749181	1.746428

1.677363	1.687257	1.684534	1.681906	1.679383	1.75535	1.752628	1.749908	1.747145
1.678048	1.687966	1.685248	1.682612	1.680067	1.75609	1.753361	1.750639	1.747873
1.678724	1.688668	1.685957	1.683317	1.680754	1.756842	1.754101	1.751373	1.748604
1.679397	1.676728	1.674046	1.684003	1.681434	1.713829	1.711161	1.708503	1.749335
1.680062	1.677395	1.674711	1.684684	1.682114	1.714554	1.711872	1.709199	1.750066
1.680714	1.678055	1.675385	1.67277	1.670216	1.680249	1.67771	1.675179	1.72859
1.681355	1.678709	1.676064	1.673459	1.670898	1.680933	1.678376	1.675831	1.729332
1.682	1.679357	1.676725	1.674129	1.671576	1.669029	1.666471	1.663921	1.673939
1.682649	1.679998	1.677362	1.674775	1.672249	1.669716	1.667137	1.664561	1.674588
1.6833	1.680646	1.67801	1.67543	1.672921	1.670395	1.667801	1.665208	1.662673
1.683953	1.681309	1.678684	1.676107	1.67359	1.671057	1.668461	1.66587	1.663339
1.684605	1.681978	1.679372	1.676797	1.674262	1.671715	1.669121	1.666536	1.664012
1.685249	1.682644	1.68007	1.677504	1.67495	1.672386	1.669784	1.667197	1.664685
1.685892	1.683312	1.680775	1.678219	1.675644	1.67306	1.670449	1.667859	1.66536
1.686565	1.683991	1.681462	1.678903	1.676309	1.673714	1.67111	1.66853	1.666026
1.687263	1.684679	1.682134	1.67956	1.67695	1.674349	1.671768	1.669209	1.666686
1.687966	1.685369	1.682811	1.680226	1.677607	1.675005	1.67244	1.669894	1.667353
1.688673	1.686065	1.683499	1.68091	1.67829	1.675689	1.673132	1.670589	1.668036
1.665072	1.68675	1.684174	1.681585	1.678978	1.676386	1.673827	1.671276	1.668711
1.665735	1.687405	1.684813	1.682234	1.679671	1.677105	1.674522	1.671939	1.669364
1.666402	1.688057	1.685444	1.682873	1.680356	1.677817	1.675208	1.67259	1.67001
1.667092	1.688743	1.686094	1.683508	1.681001	1.678471	1.675858	1.673238	1.67067
1.667797	1.689451	1.686758	1.684143	1.681622	1.679089	1.676485	1.67388	1.671335
1.668505	1.665776	1.663105	1.684823	1.682275	1.679723	1.67712	1.674523	1.671983
1.669212	1.666485	1.663818	1.68555	1.682957	1.680369	1.677759	1.675161	1.67261
1.669952	1.667214	1.664529	1.661901	1.659338	1.681029	1.678422	1.675833	1.67327
1.670745	1.667969	1.665221	1.662554	1.659975	1.681706	1.679126	1.67656	1.673988
1.671542	1.66873	1.665925	1.663221	1.660624	1.682393	1.679835	1.677292	1.674717
1.643932	1.669479	1.666657	1.663927	1.661295	1.683081	1.680519	1.677983	1.675432
1.644596	1.670228	1.667409	1.664662	1.661987	1.683779	1.681204	1.678667	1.676143
1.645249	1.670986	1.668187	1.665432	1.662712	1.660074	1.657558	1.655094	1.676851
1.645894	1.671751	1.668988	1.666232	1.663467	1.66079	1.658269	1.655801	1.677558
1.646567	1.643944	1.641373	1.66702	1.664243	1.661549	1.659003	1.656507	1.678274
1.64727	1.644632	1.642043	1.667789	1.665046	1.66236	1.659763	1.657208	1.678999
1.647996	1.645345	1.642736	1.640161	1.637625	1.663167	1.660527	1.657925	1.655365
1.648753	1.646095	1.643468	1.640874	1.63831	1.663937	1.661279	1.658667	1.656109
1.649522	1.64686	1.644219	1.641604	1.639011	1.664717	1.662044	1.659424	1.656864
1.650286	1.647625	1.644976	1.642354	1.639758	1.637195	1.634675	1.632181	1.657592
1.65105	1.648393	1.64574	1.643118	1.64053	1.637959	1.635392	1.632849	1.658321
1.651812	1.649149	1.646502	1.643882	1.64129	1.638711	1.636132	1.633575	1.631061
1.652571	1.649897	1.647265	1.644648	1.642043	1.639459	1.636895	1.634351	1.631825
1.653354	1.650665	1.64803	1.645407	1.642792	1.640202	1.637641	1.635101	1.63257
1.654172	1.651458	1.648794	1.646153	1.643535	1.640939	1.638362	1.635809	1.633287
1.654991	1.65226	1.64957	1.646911	1.644284	1.641677	1.639083	1.636517	1.633998
1.655793	1.653059	1.65037	1.647698	1.645042	1.642412	1.63981	1.637239	1.634704
1.656597	1.653864	1.65118	1.648498	1.645815	1.643162	1.640551	1.637971	1.635414
1.657418	1.654679	1.651979	1.649294	1.646621	1.643968	1.64133	1.638719	1.636148
1.658253	1.655503	1.652777	1.650091	1.647451	1.64481	1.642137	1.639482	1.636901
1.659108	1.656341	1.653596	1.650903	1.648272	1.645636	1.642952	1.640282	1.637689
1.659984	1.657197	1.654436	1.651731	1.649091	1.646453	1.643782	1.641124	1.638516

1.631124	1.658035	1.655261	1.65254	1.64988	1.647232	1.644575	1.64193	1.639308
1.631895	1.658838	1.656053	1.653311	1.650616	1.647947	1.645304	1.642675	1.640035
1.632674	1.65965	1.656849	1.654086	1.651359	1.648669	1.646029	1.643406	1.640754
1.633498	1.630727	1.62797	1.654881	1.652149	1.64945	1.646784	1.644138	1.641486
1.634338	1.631554	1.628758	1.655699	1.652968	1.650261	1.647559	1.644881	1.642234
1.635161	1.632369	1.629582	1.626849	1.624176	1.651091	1.648371	1.64568	1.643036
1.635976	1.63318	1.630434	1.627707	1.625	1.651939	1.649214	1.646522	1.643879
1.636805	1.633995	1.631265	1.628534	1.625795	1.623094	1.620447	1.617839	1.64472
1.637642	1.634806	1.632072	1.629326	1.626555	1.623838	1.621209	1.618625	1.645561
1.592117	1.635655	1.632895	1.630125	1.627332	1.624602	1.621979	1.619403	1.61683
1.59295	1.63657	1.63375	1.630943	1.628142	1.625403	1.62276	1.62016	1.617561
1.593802	1.637505	1.634617	1.631773	1.628971	1.626226	1.623558	1.620928	1.618296
1.527849	1.638419	1.635473	1.632602	1.629811	1.627071	1.624388	1.621738	1.619079
1.528513	1.639335	1.63634	1.633448	1.630668	1.627935	1.625242	1.622573	1.619888
1.529188	1.640288	1.637269	1.634357	1.631561	1.628812	1.626103	1.623417	1.62071
1.52987	1.641272	1.638253	1.635324	1.632487	1.629702	1.626974	1.624273	1.621547
1.499877	1.580417	1.577782	1.63628	1.633415	1.6306	1.627839	1.625108	1.622367
1.500455	1.581202	1.578575	1.637224	1.634349	1.631509	1.628695	1.625914	1.623161
1.50103	1.553924	1.551389	1.63815	1.635271	1.632412	1.629551	1.626724	1.623965
1.50161	1.554609	1.552083	1.639041	1.636164	1.633299	1.630412	1.627562	1.624806
1.502189	1.55529	1.552771	1.639922	1.637048	1.634181	1.631274	1.628405	1.625654
1.502758	1.500736	1.498709	1.579036	1.576505	1.635063	1.632126	1.629228	1.626474
1.503325	1.501305	1.499299	1.579803	1.577273	1.635949	1.632978	1.630047	1.627285
1.503926	1.501902	1.499903	1.552432	1.549978	1.636861	1.633872	1.63092	1.628135
1.504559	1.502527	1.50052	1.553173	1.550722	1.637799	1.634807	1.631843	1.629023
1.505201	1.503161	1.501146	1.499143	1.49716	1.549006	1.546471	1.543962	1.629918
1.505848	1.503803	1.501779	1.499772	1.497789	1.549752	1.547209	1.544687	1.630809
1.506499	1.504449	1.502418	1.500407	1.498423	1.550503	1.547956	1.545427	1.631719
1.507149	1.505098	1.503063	1.501048	1.499062	1.551257	1.548718	1.546195	1.632663
1.507799	1.505748	1.503711	1.501694	1.499706	1.55202	1.549493	1.546981	1.633632
1.508436	1.506383	1.504357	1.502345	1.500355	1.498347	1.496296	1.494243	1.545271
1.509067	1.50701	1.505002	1.503	1.501007	1.499004	1.49698	1.494947	1.546068
1.509747	1.507672	1.505652	1.503641	1.501644	1.499645	1.497641	1.495624	1.546848
1.510478	1.50837	1.506303	1.504265	1.502262	1.500266	1.498277	1.496273	1.547609
1.444342	1.509084	1.506972	1.504907	1.502896	1.500898	1.49891	1.496911	1.548373
1.445218	1.509809	1.507663	1.505576	1.503557	1.50155	1.499543	1.49753	1.549141
1.4461	1.510546	1.508377	1.506272	1.504239	1.502217	1.500186	1.498156	1.549923
1.446967	1.480408	1.478133	1.475905	1.473728	1.502897	1.500856	1.498823	1.496795
1.447835	1.481337	1.47908	1.476845	1.474632	1.503595	1.501546	1.499513	1.497478
1.448735	1.446587	1.444522	1.477776	1.475553	1.473349	1.471161	1.468987	1.466819
1.449662	1.447493	1.445397	1.478705	1.47649	1.474284	1.47207	1.469865	1.46768
1.450593	1.448398	1.44626	1.479616	1.477407	1.475204	1.472982	1.470765	1.468562
1.451527	1.449294	1.447099	1.480498	1.478293	1.476103	1.4739	1.471696	1.469472
1.452468	1.450206	1.447964	1.445826	1.443806	1.476994	1.474809	1.47262	1.470387
1.453418	1.451142	1.448884	1.446718	1.444651	1.477876	1.475692	1.473517	1.4713
1.454379	1.452095	1.44983	1.447635	1.445511	1.478761	1.476573	1.474407	1.472209
1.455358	1.453061	1.45078	1.448557	1.446389	1.479667	1.477468	1.4753	1.473104
1.45635	1.45404	1.451742	1.449493	1.447287	1.480593	1.478376	1.476196	1.473991
1.45733	1.455017	1.452721	1.450459	1.448223	1.446055	1.443992	1.441972	1.474896
1.458299	1.455993	1.453719	1.451457	1.449198	1.447	1.444898	1.442843	1.475814

					0.729762
					0.728298
					0.726814
					0.725315
			0.72786	0.725839	1.098658
			0.726353	0.724316	1.095943
			0.724841	0.722784	1.093278
0.72943	0.727431	0.72541	0.723345	0.72125	1.177981

			0.727955	0.72596	0.723937	0.721847	0.719713	1.175231
	0.730549	0.728517	0.726484	0.724454	0.722404	1.181515	1.1771	1.335621
	0.729095	0.727067	0.725013	0.722921	0.720824	1.17883	1.174386	1.332804
	0.727618	0.725583	1.083365	1.079189	1.075062	1.339389	1.334652	1.438989
	0.726115	0.724059	1.080817	1.076701	1.072594	1.336433	1.331558	1.435824
0.726678	0.724608	0.722531	1.184236	1.179739	1.175229	1.442806	1.437602	1.550339
0.725187	0.723109	0.72101	1.181507	1.176996	1.17249	1.43983	1.43472	1.547392
0.723687	0.721605	0.719492	1.17878	1.174233	1.169731	1.436876	1.43193	1.544536
0.722166	1.078987	1.074845	1.339286	1.33446	1.329718	1.497292	1.492515	1.541675
0.720633	1.07647	1.072303	1.336398	1.331497	1.326744	1.49424	1.489667	1.538838
1.078104	1.283781	1.279126	1.506291	1.501108	1.49606	1.545169	1.540579	1.594625
1.07552	1.280897	1.276232	1.503263	1.498257	1.493356	1.542366	1.537721	1.591776
1.072939	1.395837	1.39104	1.554192	1.549278	1.544435	1.598437	1.593628	1.588995
1.070357	1.392945	1.38827	1.551271	1.546527	1.541813	1.595803	1.590944	1.586307
1.276839	1.504143	1.499374	1.607478	1.602771	1.598056	1.593174	1.588288	1.704537
1.273962	1.501153	1.496525	1.604633	1.599986	1.595316	1.590477	1.585624	1.701585
1.271157	1.498222	1.493724	1.601818	1.597201	1.592554	1.587763	1.582958	1.698642
1.454277	1.549014	1.544484	1.599008	1.594446	1.589841	1.705858	1.700652	1.834405
1.451652	1.546214	1.541684	1.596212	1.59171	1.587152	1.702849	1.697648	1.83118
1.494545	1.602581	1.597984	1.714807	1.709914	1.704973	1.699826	1.694644	1.863561
1.491827	1.599772	1.595209	1.711706	1.706827	1.701905	1.696788	1.691638	1.86028
1.601755	1.718572	1.713564	1.708616	1.70375	1.698849	1.867798	1.862323	1.971991
1.599021	1.715515	1.710488	1.705536	1.700692	1.695818	1.864508	1.859077	1.968061
1.596335	1.71251	1.707444	1.702472	1.697639	1.692792	1.861249	1.855897	1.96424
1.683998	1.709612	1.704457	1.8737	1.868454	1.863262	1.973339	1.966924	2.045308
1.681272	1.706748	1.7015	1.870434	1.865136	1.859945	1.969496	1.963365	2.041603
1.708843	1.801056	1.795519	1.867184	1.861858	1.856666	2.050558	2.04426	2.037773
1.705749	1.797782	1.792331	1.863957	1.858618	1.853422	2.046448	2.040266	2.033874
1.79982	1.871569	1.866155	1.976727	1.970214	1.963897	2.04243	2.036304	2.030021
1.796511	1.868232	1.862965	1.972993	1.966516	1.960206	2.038511	2.032374	2.026221
1.870231	1.864921	1.859775	2.054376	2.047615	2.040996	2.034651	2.028486	2.022442
1.866953	1.861642	1.856557	2.050325	2.043694	2.03717	2.030817	2.02462	2.018647
1.863717	1.858407	1.853357	2.046289	2.039808	2.033404	2.027064	2.020844	2.01491
1.860491	2.00711	2.000704	2.042322	2.036015	2.02976	2.023475	2.017263	2.057862
1.857308	2.003091	1.996737	2.038416	2.032277	2.026173	2.019977	2.013807	2.054235
1.854259	2.047257	2.040872	2.034618	2.028504	2.022452	2.023905	2.017873	2.056539
1.851336	2.043642	2.037232	2.030924	2.024707	2.018616	2.020249	2.014441	2.053047
1.998507	2.039959	2.033588	2.034756	2.02853	2.022443	2.061422	2.055556	2.069584
1.994687	2.036159	2.029933	2.031218	2.025034	2.018948	2.057741	2.05182	2.06585
1.990873	2.039773	2.033697	2.058451	2.052167	2.045949	2.054096	2.048087	2.062136
1.987084	2.035969	2.029891	2.054591	2.048487	2.042402	2.050513	2.044413	2.058516
1.983333	2.032198	2.026082	2.050708	2.044775	2.038829	2.046942	2.040781	2.054943
2.034717	2.059368	2.05309	2.061238	2.055301	2.049382	2.063303	2.057207	2.05139
2.031095	2.055676	2.049431	2.057576	2.051544	2.045573	2.059594	2.053665	2.047871
2.034907	2.052	2.045809	2.074002	2.067901	2.061888	2.056002	2.050203	2.044433
2.031256	2.048344	2.042234	2.070476	2.064346	2.058321	2.05252	2.046829	2.041079
2.050921	2.059076	2.053009	2.066935	2.060842	2.054855	2.0491	2.043452	2.076557
2.047334	2.055549	2.049416	2.063335	2.057395	2.051536	2.045757	2.040041	2.073144
2.043729	2.052006	2.045841	2.059769	2.053961	2.048212	2.042419	2.036653	2.069755
2.040019	2.06831	2.062269	2.056326	2.050503	2.044746	2.07798	2.072197	2.079294

2.036278	2.064622	2.058762	2.052918	2.047035	2.041233	2.074536	2.068869	2.075983
2.066989	2.100305	2.094456	2.088597	2.082656	2.076779	2.071067	2.065429	2.103766
2.063338	2.096657	2.090824	2.085	2.079127	2.073303	2.067578	2.061916	2.100148
2.099199	2.093139	2.087289	2.081473	2.075659	2.069888	2.097266	2.091163	2.130187
2.095815	2.089774	2.083858	2.078018	2.072257	2.066542	2.093799	2.087702	2.126685
2.092416	2.086408	2.080468	2.074622	2.068891	2.063212	2.110651	2.104495	2.135712
2.08891	2.082969	2.077113	2.071314	2.065564	2.059872	2.10717	2.101096	2.1323
2.085429	2.079545	2.073768	2.068014	2.062252	2.056553	2.103704	2.097705	2.128905
2.082103	2.07621	2.070373	2.098354	2.092207	2.086136	2.110943	2.104935	2.125472
2.078845	2.07292	2.066979	2.094751	2.088727	2.082772	2.107498	2.101436	2.122039
2.075513	2.103991	2.097577	2.111582	2.105477	2.099442	2.117116	2.111107	2.118676
2.072127	2.10057	2.094267	2.108252	2.102033	2.095912	2.11365	2.107777	2.115372
2.068715	2.117392	2.11115	2.10492	2.098667	2.092515	2.110275	2.104464	2.146838
2.06526	2.113761	2.107681	2.101581	2.095407	2.089295	2.10701	2.10115	2.143493
2.116314	2.110169	2.104198	2.121857	2.115804	2.109777	2.140282	2.134323	2.140217
2.112801	2.106706	2.100703	2.118371	2.112449	2.106555	2.137098	2.131188	2.137052
2.109338	2.103286	2.097225	2.114882	2.109079	2.10332	2.133922	2.128097	2.133962
2.105847	2.099818	2.09382	2.148049	2.142207	2.136423	2.142372	2.136603	2.130923
2.102369	2.09635	2.090467	2.144712	2.138837	2.133034	2.13902	2.133361	2.12794
2.119985	2.144668	2.138742	2.141339	2.135491	2.129728	2.135732	2.130152	2.143265
2.116758	2.141401	2.135376	2.13793	2.132167	2.126501	2.132494	2.12695	2.140292
2.132478	2.138151	2.132085	2.153972	2.148193	2.142551	2.147648	2.142237	2.140775
2.129145	2.13491	2.128904	2.150755	2.144854	2.139195	2.144601	2.139627	2.138368
2.125801	2.131653	2.125743	2.147599	2.141618	2.135975	2.141708	2.137168	2.136095
2.13416	2.156088	2.150258	2.144462	2.138562	2.133038	2.138993	2.134712	2.133832
2.130809	2.152701	2.147001	2.141366	2.135654	2.130326	2.136467	2.132364	2.13165
2.147757	2.149444	2.143791	2.149108	2.143678	2.13863	2.137393	2.133374	2.150346
2.144598	2.146282	2.140622	2.146083	2.141085	2.136444	2.135356	2.131405	2.148304
2.141436	2.143158	2.137544	2.14642	2.141749	2.137415	2.154141	2.150163	2.157066
2.138272	2.140068	2.134538	2.14358	2.139222	2.135193	2.152012	2.148185	2.155142
2.135119	2.140687	2.135323	2.16188	2.157645	2.153714	2.149886	2.146198	2.153207
2.131926	2.137737	2.132748	2.159466	2.155314	2.15144	2.147735	2.144174	2.151236
2.128838	2.134922	2.130331	2.157207	2.153107	2.149247	2.145616	2.142139	2.149232
2.129921	2.154067	2.149659	2.154993	2.151011	2.147214	2.143584	2.140081	2.157072
2.127253	2.151531	2.147351	2.152858	2.149009	2.145283	2.141612	2.138029	2.15485
2.14713	2.149078	2.145121	2.150784	2.147033	2.143357	2.160399	2.156498	2.169219
2.144545	2.14672	2.142975	2.148775	2.145092	2.141452	2.158374	2.154488	2.167003
2.148461	2.154022	2.150383	2.144051	2.140451	2.136871	2.156358	2.152489	2.164842
2.146259	2.151913	2.148349	2.142076	2.138526	2.134987	2.154346	2.150497	2.162743
2.144164	2.147108	2.143584	2.153723	2.150004	2.146296	2.168772	2.16467	2.165855
2.142148	2.14513	2.141566	2.151572	2.147925	2.144291	2.166607	2.162528	2.16378
2.14019	2.143195	2.139574	2.149439	2.145852	2.142287	2.164453	2.160413	2.16173
2.135611	2.141263	2.137641	2.147397	2.14383	2.140296	2.167508	2.163573	2.159746
2.13367	2.139347	2.13575	2.145411	2.141838	2.13831	2.165421	2.161591	2.157802
2.13175	2.150915	2.147124	2.174828	2.170906	2.167059	2.163285	2.159564	2.166372
2.12985	2.14891	2.14514	2.172662	2.168675	2.164796	2.161101	2.1575	2.164244
2.127953	2.15278	2.149051	2.170501	2.166496	2.162616	2.158978	2.155457	2.164626
2.126053	2.150779	2.147061	2.168341	2.164389	2.160561	2.156948	2.153446	2.162533
2.124158	2.14878	2.145075	2.166197	2.162311	2.158542	2.154948	2.151457	2.160464
2.128912	2.146786	2.143101	2.164079	2.160235	2.156509	2.16587	2.162153	2.16495

2.127027	2.144797	2.141139	2.161985	2.158178	2.15449	2.163758	2.160078	2.16293
2.146489	2.167734	2.163782	2.162795	2.159013	2.15535	2.161681	2.158049	2.160943
2.144486	2.16558	2.161694	2.160767	2.157014	2.153384	2.159635	2.156062	2.158986
2.142504	2.166365	2.162524	2.169092	2.165105	2.161252	2.157618	2.154109	2.157055
2.140543	2.164289	2.160488	2.166964	2.163009	2.159193	2.155629	2.152193	2.155154
2.166101	2.16224	2.158477	2.164868	2.16096	2.157191	2.15368	2.150291	2.153256
2.164017	2.16021	2.15649	2.162807	2.15897	2.15526	2.151772	2.14839	2.151347
2.161967	2.158214	2.154534	2.160782	2.157032	2.153391	2.149908	2.146506	2.149449
2.159965	2.166464	2.162563	2.158785	2.155155	2.151605	2.148107	2.144653	2.151346
2.158012	2.164437	2.160562	2.156838	2.153324	2.14986	2.146335	2.142817	2.149491
2.156145	2.166442	2.162645	2.158974	2.155487	2.152033	2.154667	2.15111	2.147635
2.154355	2.164648	2.160924	2.157269	2.153709	2.15018	2.152784	2.149228	2.14578
2.166467	2.162789	2.159125	2.15549	2.151884	2.148312	2.150911	2.14738	2.14396
2.164523	2.160843	2.157223	2.153617	2.150002	2.146428	2.149053	2.145572	2.142181
2.162582	2.158897	2.155304	2.151718	2.148104	2.144542	2.147201	2.143778	2.140417
2.160661	2.156988	2.153393	2.149807	2.146194	2.142649	2.145346	2.141985	2.138658
2.158742	2.155086	2.151478	2.14789	2.144295	2.140779	2.143512	2.140205	2.136909
2.156803	2.153165	2.149553	2.145983	2.142444	2.138983	2.141731	2.138445	2.14355
2.15486	2.151242	2.147629	2.144088	2.140629	2.137239	2.139991	2.136705	2.141703
2.15295	2.149348	2.145744	2.142234	2.13884	2.135509	2.138269	2.134992	2.155613
2.151068	2.147474	2.143891	2.140413	2.137075	2.133795	2.136565	2.133303	2.153692
2.149253	2.14568	2.142137	2.144808	2.141477	2.138198	2.159328	2.155562	2.151852
2.147541	2.144006	2.140534	2.143234	2.139919	2.136646	2.157576	2.153816	2.150132
2.145817	2.142324	2.138925	2.141657	2.138353	2.135081	2.155808	2.152064	2.148403
2.143986	2.140534	2.137198	2.139965	2.13668	2.133418	2.153926	2.150214	2.14657
2.142137	2.138728	2.135444	2.13824	2.134974	2.131721	2.152007	2.148333	2.144703
2.140364	2.136997	2.133738	2.161319	2.157535	2.15379	2.150104	2.146462	2.137217
2.138645	2.13532	2.132065	2.159372	2.1556	2.151872	2.148213	2.1446	2.135368
2.136999	2.13371	2.130464	2.151903	2.14815	2.144441	2.140799	2.137203	2.133614
2.135439	2.132182	2.128952	2.150181	2.146455	2.142768	2.139133	2.135541	2.131968
2.133846	2.155911	2.152138	2.148401	2.144701	2.141039	2.139418	2.135853	2.132331
2.132165	2.153976	2.150211	2.14649	2.142819	2.139183	2.137576	2.134017	2.130545
2.130481	2.152019	2.148263	2.144557	2.14091	2.137298	2.135711	2.132169	2.128751
2.123468	2.151997	2.148284	2.14462	2.141008	2.137435	2.137431	2.133921	2.130544
2.121857	2.15011	2.146412	2.142761	2.13915	2.135589	2.135615	2.132155	2.128819
2.131241	2.148311	2.14463	2.144563	2.140939	2.137382	2.133908	2.13051	2.125086
2.129588	2.146584	2.14292	2.14285	2.139234	2.135698	2.132288	2.128965	2.123538
2.152106	2.14838	2.144706	2.14108	2.137486	2.133983	2.13063	2.127366	2.122424
2.150258	2.146508	2.142835	2.139228	2.135671	2.132212	2.128907	2.125688	2.120757
2.148428	2.144668	2.140998	2.137414	2.133909	2.130501	2.12563	2.122384	2.119114
2.146647	2.142923	2.13926	2.135705	2.132273	2.12893	2.124057	2.120816	2.11755
2.144868	2.141188	2.13754	2.13402	2.130665	2.127386	2.122505	2.119256	2.115991
2.143035	2.139362	2.135749	2.132762	2.129492	2.126287	2.12087	2.117619	2.114353
2.141185	2.137508	2.133945	2.131021	2.127799	2.124631	2.119206	2.115952	2.112685
2.139828	2.136221	2.132752	2.127377	2.124094	2.120859	2.117598	2.114341	2.111078
2.138107	2.134583	2.131196	2.125835	2.122553	2.119306	2.116041	2.11278	2.109524
2.136418	2.132957	2.129626	2.124264	2.120979	2.117721	2.11445	2.111185	2.132826
2.134768	2.131332	2.128011	2.122632	2.11934	2.116072	2.112796	2.109528	2.130962
2.133124	2.127756	2.124363	2.121018	2.11772	2.114444	2.111163	2.107892	2.129126
2.131451	2.126121	2.122782	2.119469	2.116174	2.112893	2.109611	2.106338	2.127389

2.129795	2.124507	2.121223	2.117943	2.114655	2.11137	2.108083	2.104803	2.125676
2.128224	2.122945	2.119679	2.116411	2.11313	2.109841	2.131409	2.12762	2.123888
2.126714	2.121419	2.118146	2.114873	2.111601	2.108309	2.129651	2.125799	2.122067
2.125214	2.119879	2.116571	2.113273	2.109997	2.106708	2.127859	2.124015	2.120321
2.123734	2.118331	2.114951	2.111605	2.108311	2.105031	2.126028	2.122265	2.118649
2.120119	2.116755	2.113338	2.135519	2.131722	2.127983	2.124238	2.120548	2.117004
2.118503	2.115127	2.111741	2.133747	2.129958	2.126228	2.122519	2.118876	2.115389
2.116878	2.113503	2.110161	2.13201	2.128229	2.124505	2.120825	2.117219	2.113776
2.115283	2.11193	2.10861	2.130278	2.126507	2.122792	2.119131	2.115546	2.113985
2.113704	2.11038	2.107075	2.128556	2.124792	2.121085	2.117445	2.113884	2.112216
2.112142	2.134397	2.130607	2.126834	2.123049	2.119337	2.115757	2.112273	2.110544
2.110595	2.132657	2.128886	2.125113	2.121288	2.117565	2.114071	2.110704	2.108945
2.109052	2.130907	2.12711	2.123339	2.119557	2.115891	2.114527	2.110954	2.107368
2.107516	2.129148	2.125262	2.12149	2.117855	2.114334	2.112949	2.109388	2.10582
2.131242	2.127366	2.123441	2.125078	2.121521	2.118069	2.117718	2.114159	2.110594
2.129399	2.125528	2.121673	2.123365	2.119898	2.11652	2.116132	2.112579	2.108984
2.127556	2.123709	2.119961	2.121722	2.118321	2.114986	2.114541	2.11098	2.107353
2.125797	2.121991	2.118341	2.123598	2.120029	2.116505	2.112915	2.109308	2.105677
2.124082	2.120335	2.116787	2.122015	2.118461	2.114922	2.111275	2.107606	2.103983
2.122358	2.130687	2.127177	2.120438	2.116901	2.113353	2.109674	2.105967	2.113921
2.120631	2.129011	2.125599	2.118875	2.115353	2.111799	2.108107	2.10438	2.112199
2.125972	2.124363	2.120824	2.117304	2.113788	2.110229	2.10653	2.102795	2.110485
2.124395	2.122763	2.119236	2.115721	2.112207	2.108647	2.104946	2.101209	2.108778
2.127776	2.121189	2.117658	2.114129	2.110594	2.10702	2.114929	2.110951	2.107078
2.126208	2.119618	2.116082	2.112523	2.108917	2.105296	2.113118	2.109212	2.105383
2.124655	2.118054	2.114511	2.110917	2.10723	2.103557	2.111298	2.107473	2.103696
2.120047	2.116487	2.112941	2.109332	2.105611	2.101915	2.109545	2.105756	2.102023
2.118481	2.114919	2.111371	2.10776	2.104031	2.10033	2.107833	2.104055	2.100363
2.116907	2.113342	2.109793	2.117942	2.113942	2.109979	2.106135	2.102375	2.09872
2.115327	2.111757	2.108204	2.116224	2.112231	2.10828	2.104451	2.100714	2.097095
2.113745	2.110169	2.106617	2.11451	2.110527	2.106591	2.10279	2.099084	2.095496
2.112154	2.108577	2.105031	2.112802	2.108828	2.104912	2.101155	2.097499	2.093934
2.110574	2.106989	2.103448	2.111101	2.10714	2.103247	2.099534	2.095927	2.092382
2.109022	2.117112	2.113272	2.109405	2.105455	2.101581	2.097907	2.094343	2.090822
2.107483	2.115418	2.11158	2.107725	2.103795	2.099948	2.096303	2.092767	2.089265
2.105929	2.113733	2.10991	2.106089	2.102227	2.098433	2.094786	2.091227	2.087715
2.104367	2.112058	2.108258	2.104487	2.100728	2.097009	2.093337	2.089715	2.086172
2.102816	2.110391	2.106604	2.102868	2.099185	2.095525	2.091847	2.088193	2.087702
2.10127	2.108723	2.10494	2.101223	2.097588	2.093973	2.09031	2.086661	2.086113
2.110974	2.107104	2.103312	2.099602	2.095998	2.092413	2.088765	2.085126	2.083634
2.109427	2.105575	2.101753	2.098026	2.094431	2.090859	2.087211	2.083572	2.082046
2.107892	2.104059	2.100214	2.096471	2.092876	2.089309	2.085671	2.082046	2.080489
2.106294	2.102483	2.098648	2.094913	2.091325	2.087775	2.086398	2.082667	2.079033
2.104671	2.100886	2.097083	2.093372	2.089785	2.086249	2.084904	2.081258	2.077625
2.103058	2.099314	2.095574	2.091309	2.087685	2.08412	2.083366	2.079778	2.076158
2.10145	2.097762	2.094112	2.089904	2.086223	2.082604	2.081796	2.078246	2.074648
2.099879	2.095697	2.092086	2.091543	2.087661	2.083854	2.080243	2.076717	2.073138
2.098353	2.094165	2.090556	2.089946	2.086085	2.0823	2.078704	2.075193	2.071624
2.096377	2.092657	2.089029	2.088348	2.084531	2.080789	2.077203	2.07369	2.070135
2.094936	2.091211	2.087535	2.086783	2.08304	2.079363	2.075768	2.072229	2.0687

2.093493	2.08977	2.086043	2.085225	2.081565	2.07796	2.074356	2.070784	2.067281
2.091971	2.091344	2.087427	2.083643	2.080049	2.076502	2.072905	2.069324	2.065829
2.090407	2.089713	2.085814	2.082061	2.07852	2.075019	2.071441	2.067866	2.064365
2.088888	2.088147	2.084304	2.080588	2.07705	2.073552	2.070003	2.066457	2.062944
2.087406	2.086642	2.082896	2.079224	2.075636	2.072095	2.068589	2.065101	2.061567
2.088944	2.085162	2.081481	2.077841	2.07423	2.070665	2.067172	2.063705	2.060165
2.087467	2.083711	2.080033	2.076404	2.072826	2.069279	2.065752	2.062241	2.058723
2.085994	2.08226	2.078584	2.074964	2.071415	2.067882	2.064313	2.060753	2.057258
2.084496	2.080784	2.077155	2.073557	2.069989	2.066428	2.062831	2.059246	2.055758
2.082991	2.079296	2.075721	2.072145	2.068546	2.064948	2.061333	2.05774	2.054255
2.081479	2.077798	2.074241	2.070672	2.067061	2.063457	2.059864	2.056299	2.052817
2.079964	2.076294	2.072727	2.069154	2.065545	2.061956	2.058413	2.054903	2.051422
2.078505	2.074829	2.071236	2.067653	2.064063	2.0605	2.056985	2.053503	2.05004
2.077115	2.073412	2.069766	2.066167	2.062617	2.059097	2.055582	2.052094	2.048673
2.075698	2.071983	2.068314	2.064709	2.061184	2.05769	2.054165	2.050702	2.046835
2.074202	2.07051	2.066882	2.063298	2.059755	2.056251	2.052765	2.049348	2.045483
2.072713	2.069047	2.06546	2.061897	2.058337	2.054823	2.051373	2.047998	2.044132
2.071315	2.067638	2.064027	2.06046	2.056931	2.053446	2.049521	2.046118	2.04276
2.06996	2.066261	2.062602	2.059021	2.055537	2.052094	2.048145	2.044722	2.041384
2.068588	2.064902	2.06123	2.057582	2.054114	2.05069	2.046775	2.043374	2.040041
2.067209	2.063564	2.059904	2.056252	2.052743	2.049293	2.045411	2.042066	2.038727
2.065798	2.062189	2.058571	2.05448	2.050933	2.047446	2.044064	2.040738	2.037396
2.064337	2.060761	2.057231	2.053185	2.049643	2.046143	2.042731	2.039373	2.036036
2.062898	2.058975	2.055482	2.051866	2.048349	2.044858	2.041423	2.038033	2.034694
2.061516	2.057593	2.05408	2.050475	2.047021	2.043585	2.040158	2.036754	2.033395
2.060166	2.056236	2.052682	2.049076	2.045682	2.042306	2.038901	2.0355	2.032121
2.058444	2.054764	2.05122	2.047737	2.044343	2.040976	2.037606	2.034246	2.032131
2.057127	2.053456	2.049922	2.046434	2.043006	2.039621	2.036292	2.032992	2.0309
2.055769	2.052112	2.048614	2.045146	2.041708	2.038312	2.036184	2.032904	2.029619
2.054373	2.050733	2.047302	2.043879	2.04045	2.037044	2.034862	2.031537	2.028289
2.053007	2.049373	2.045972	2.043731	2.040338	2.036955	2.03357	2.030218	2.026984
2.051695	2.048042	2.044605	2.042355	2.03902	2.035695	2.032329	2.028977	2.025719
2.050394	2.046733	2.043261	2.040994	2.037697	2.034415	2.031081	2.027749	2.024473
2.050023	2.046558	2.043104	2.039704	2.036373	2.033072	2.02978	2.026507	2.023247
2.048709	2.045291	2.041856	2.038446	2.035059	2.031719	2.028464	2.025254	2.022024
2.04741	2.04402	2.040605	2.0372	2.033794	2.030435	2.027173	2.023964	2.020753
2.046119	2.042747	2.039357	2.035967	2.032565	2.029198	2.025897	2.022648	2.019454
2.044789	2.041433	2.038084	2.034724	2.031336	2.027971	2.024651	2.021379	2.018192
2.04341	2.040068	2.036781	2.03347	2.030108	2.026757	2.023438	2.020158	2.016968
2.042062	2.038731	2.035485	2.032212	2.028878	2.025545	2.022233	2.018952	2.015754
2.040779	2.037454	2.034217	2.030957	2.027645	2.024333	2.021032	2.017754	2.014541
2.039516	2.03619	2.032941	2.029684	2.026401	2.023118	2.019833	2.016563	2.013339
2.038248	2.034902	2.031617	2.028357	2.025126	2.021896	2.018631	2.015368	2.012154
2.036988	2.033613	2.030282	2.027016	2.023842	2.020673	2.01743	2.014178	2.010981
2.038978	2.032361	2.029011	2.025744	2.022598	2.019459	2.016237	2.013	2.009818
2.037742	2.031138	2.02779	2.024527	2.021385	2.018255	2.01505	2.011834	2.008664
2.036501	2.029914	2.026576	2.023322	2.020187	2.017066	2.013878	2.010679	2.00752
2.035253	2.028684	2.025366	2.022128	2.019006	2.015897	2.012724	2.00954	2.006387
2.03401	2.027462	2.024163	2.020942	2.017829	2.01473	2.011572	2.008402	2.005255
2.032779	2.026252	2.022969	2.019759	2.016649	2.013554	2.010412	2.007261	2.004123

2.031554	2.02505	2.021781	2.01858	2.015473	2.012382	2.009253	2.006118	2.002989
2.030337	2.023854	2.020596	2.017406	2.01431	2.011229	2.008106	2.004974	2.001847
2.029125	2.022664	2.019413	2.016234	2.013153	2.010084	2.006963	2.003829	2.000702
2.027906	2.024615	2.021358	2.015054	2.011985	2.008926	2.005811	2.002682	1.999562
2.026682	2.023403	2.020153	2.013867	2.010805	2.007755	2.004651	2.001535	1.998427
2.025466	2.022197	2.018954	2.012687	2.00963	2.006585	2.003489	2.000382	1.997284
2.024261	2.020995	2.017754	2.011505	2.008455	2.005418	2.002324	1.999218	1.996131
2.023066	2.019811	2.016582	2.010355	2.0073	2.004256	2.00116	1.998055	1.994975
2.021877	2.018654	2.015474	2.009277	2.006188	2.003106	1.999999	1.996892	1.993818
2.020706	2.017519	2.01439	2.008221	2.005089	2.001962	1.998841	1.995734	1.992664
2.019583	2.016411	2.013278	2.007114	2.003963	2.000819	1.997694	1.994589	1.991522
2.018495	2.015324	2.01215	2.005977	2.00282	1.999677	1.996554	1.993454	1.99039
2.017376	2.014196	2.011	2.007813	2.004634	2.001475	1.995408	1.992318	1.98926
2.016228	2.013027	2.009825	2.006635	2.003451	2.000293	1.994253	1.991179	1.988133
2.015068	2.01185	2.008645	2.005455	2.002273	1.999122	1.993109	1.990051	1.987022
2.013895	2.010669	2.007459	2.004269	2.001095	1.997955	1.991967	1.988926	1.985933
2.012722	2.009493	2.006281	2.003098	1.999943	1.996824	1.990861	1.987832	1.984864
2.01155	2.008325	2.005117	2.001955	1.998848	1.995773	1.98984	1.986813	1.983832
2.010392	2.007177	2.003977	2.000839	1.997787	1.994759	1.988854	1.985824	1.982813
2.009292	2.0061	2.00291	1.999779	1.99673	1.993704	1.99066	1.98762	1.981752
2.008239	2.005081	2.001903	1.998765	1.995683	1.992623	1.989566	1.986522	1.980662
2.007161	2.004016	2.000848	1.997709	1.99461	1.991532	1.988469	1.985423	1.979575
2.00605	2.002889	1.99973	1.996599	1.993504	1.990429	1.987366	1.984321	1.978487
2.004936	2.001763	1.998612	1.995489	1.992398	1.98933	1.986278	1.983246	1.980228
2.00382	2.000648	1.997505	1.994388	1.991296	1.988236	1.98522	1.98223	1.979236
2.002724	1.999559	1.996423	1.993312	1.990218	1.987165	1.984178	1.981226	1.978254
2.0017	1.998546	1.995417	1.992308	1.989215	1.986155	1.983154	1.980185	1.977209
2.000712	1.997573	1.99445	1.991342	1.988249	1.985179	1.98214	1.979126	1.976133
1.999666	1.99654	1.993426	1.990323	1.987229	1.984152	1.981096	1.978063	1.975062
1.998571	1.995457	1.992355	1.989259	1.986163	1.983082	1.980026	1.976993	1.973989
1.997507	1.994411	1.991318	1.988226	1.985129	1.982048	1.978994	1.975964	1.97296
1.996496	1.993428	1.990341	1.987251	1.984156	1.981075	1.978026	1.975001	1.971999
1.995487	1.992437	1.989352	1.986264	1.98317	1.980092	1.977048	1.974028	1.971029
1.994446	1.991381	1.988295	1.985209	1.982118	1.979044	1.976004	1.972988	1.969991
1.993408	1.990318	1.987229	1.984144	1.981056	1.977987	1.974951	1.971939	1.968944
1.992423	1.989323	1.986228	1.983141	1.980059	1.976996	1.973963	1.970953	1.967961
1.991468	1.988365	1.985261	1.982171	1.979097	1.976041	1.973011	1.970003	1.967014
1.990455	1.987349	1.984241	1.981151	1.978083	1.975034	1.972008	1.969002	1.966017
1.989377	1.98627	1.983162	1.980075	1.977012	1.97397	1.970948	1.967946	1.964965
1.988326	1.985221	1.982117	1.979036	1.975981	1.972946	1.969929	1.966931	1.963954
1.987343	1.984243	1.981148	1.978075	1.975029	1.972001	1.968989	1.965994	1.963022
1.986354	1.98326	1.980175	1.977115	1.974085	1.971072	1.968068	1.965078	1.96211
1.985416	1.982203	1.979125	1.976085	1.973099	1.970126	1.967141	1.964159	1.961197
1.984344	1.98113	1.978057	1.975038	1.972098	1.969168	1.966203	1.963232	1.960274
1.983346	1.980283	1.977236	1.974043	1.971096	1.968162	1.965201	1.962236	1.959282
1.982402	1.97934	1.976294	1.973085	1.970092	1.967118	1.964149	1.96119	1.958236
1.981479	1.978418	1.975373	1.97235	1.96935	1.966372	1.963156	1.960199	1.957245
1.980582	1.977523	1.974478	1.971452	1.968443	1.965457	1.962236	1.959278	1.956323
1.979673	1.976616	1.973573	1.970547	1.967536	1.964547	1.961321	1.958362	1.955407
1.978744	1.975691	1.972653	1.96963	1.966621	1.963634	1.960406	1.957445	1.95449

1.977781	1.974732	1.971698	1.968681	1.965678	1.962695	1.959465	1.956504	1.953549
1.976733	1.973687	1.970656	1.967644	1.964649	1.961672	1.958441	1.955482	1.952527
1.975645	1.9726	1.969572	1.966564	1.963578	1.960611	1.957378	1.954422	1.951468
1.974629	1.971584	1.968556	1.965551	1.962574	1.959615	1.956381	1.953427	1.950473
1.973672	1.970626	1.967594	1.964591	1.961623	1.958673	1.955437	1.952482	1.949529
1.972731	1.969685	1.966652	1.963651	1.960689	1.957744	1.954507	1.951553	1.948601
1.971806	1.968761	1.965731	1.962732	1.959771	1.956827	1.95359	1.950638	1.947687
1.970884	1.967841	1.964817	1.961821	1.95886	1.955917	1.952678	1.949729	1.946779
1.969957	1.966918	1.9639	1.960908	1.957949	1.955007	1.951766	1.948818	1.945868
1.969028	1.965994	1.962984	1.959998	1.957041	1.954099	1.950856	1.947907	1.944956
1.968096	1.965067	1.962065	1.959086	1.956131	1.95319	1.949943	1.946993	1.944041
1.967162	1.964137	1.961146	1.958174	1.955223	1.952282	1.949029	1.946076	1.943124
1.966229	1.963209	1.960228	1.957263	1.954314	1.951373	1.948116	1.94516	1.942208
1.965298	1.962282	1.959311	1.956353	1.953405	1.950465	1.947203	1.944246	1.941292
1.964372	1.96136	1.958395	1.955441	1.952496	1.949556	1.946291	1.943333	1.940377
1.963455	1.960447	1.957482	1.954529	1.951585	1.948647	1.945381	1.942423	1.939463
1.962539	1.959535	1.956568	1.953614	1.950671	1.947734	1.944467	1.941508	1.938546
1.961621	1.958619	1.95565	1.952694	1.949751	1.946814	1.943543	1.940584	1.937622
1.9607	1.957702	1.95473	1.951771	1.948827	1.945889	1.942613	1.939654	1.936693
1.959776	1.956781	1.953808	1.950848	1.947901	1.94496	1.94168	1.938721	1.935762
1.958848	1.955857	1.952884	1.949922	1.946971	1.944027	1.940744	1.937785	1.934828
1.957919	1.954929	1.951956	1.948992	1.946039	1.943093	1.939807	1.936849	1.933894
1.956989	1.953998	1.951022	1.948057	1.945104	1.942158	1.93887	1.935912	1.932959
1.956062	1.953066	1.950087	1.94712	1.944168	1.941224	1.937933	1.934976	1.932024
1.955138	1.952132	1.949151	1.946184	1.943233	1.94029	1.936998	1.934042	1.931087
1.954226	1.951205	1.948218	1.945249	1.942298	1.939357	1.936064	1.933108	1.93015
1.953339	1.950292	1.947291	1.944315	1.941363	1.938423	1.935127	1.932171	1.929212
1.952473	1.949395	1.946376	1.943387	1.940432	1.937491	1.934191	1.931233	1.928273
1.95163	1.948542	1.945495	1.942485	1.939519	1.936569	1.933259	1.930295	1.927334
1.950811	1.94773	1.944647	1.941605	1.93862	1.935656	1.932331	1.929358	1.926393
1.949952	1.94689	1.943808	1.940763	1.93777	1.934793	1.931446	1.92845	1.925473
1.949033	1.94601	1.942986	1.939977	1.936994	1.934006	1.930621	1.927576	1.924572
1.948112	1.945119	1.942144	1.939169	1.936196	1.933206	1.929801	1.926728	1.923708
1.947203	1.944213	1.941247	1.938281	1.935316	1.932339	1.928964	1.925924	1.922917
1.946319	1.943319	1.940341	1.937371	1.934409	1.931448	1.928112	1.92512	1.922133
1.94551	1.942492	1.939479	1.936484	1.933513	1.93055	1.92722	1.924241	1.921262
1.944744	1.941705	1.938644	1.935612	1.932624	1.929651	1.926306	1.923321	1.920343
1.943913	1.940881	1.93782	1.934469	1.931458	1.928456	1.925441	1.922429	1.924609
1.943021	1.940023	1.937012	1.933693	1.930691	1.927678	1.924622	1.921558	1.923704
1.942127	1.939151	1.936177	1.932881	1.929884	1.926866	1.923796	1.920713	1.922842
1.941229	1.938253	1.935284	1.931992	1.928997	1.925989	1.922953	1.919909	1.922055
1.940358	1.937377	1.934407	1.93111	1.928105	1.925099	1.922097	1.919095	1.92126
1.939565	1.936584	1.933613	1.930302	1.927258	1.924218	1.926389	1.923384	1.92038
1.938795	1.935813	1.932839	1.929511	1.926425	1.923346	1.925497	1.922488	1.919478
1.937955	1.934969	1.931992	1.928666	1.92559	1.922521	1.924672	1.92166	1.918632
1.937071	1.934079	1.931098	1.927784	1.924755	1.921728	1.923892	1.920875	1.91782
1.936234	1.93294	1.929917	1.932094	1.929077	1.926066	1.923058	1.920044	1.916986
1.935454	1.932134	1.929059	1.931197	1.928173	1.925163	1.922158	1.919153	1.916126
1.934337	1.93132	1.92822	1.930336	1.927303	1.92429	1.921285	1.918287	1.915275
1.933469	1.930471	1.927404	1.929541	1.926511	1.923494	1.92049	1.917487	1.914452

1.932601	1.929627	1.926603	1.928765	1.925731	1.922704	1.919697	1.916687	1.913626
1.931795	1.934031	1.931013	1.927979	1.924913	1.921854	1.918833	1.915821	1.91277
1.931012	1.933245	1.930231	1.927185	1.924081	1.920985	1.917948	1.914932	1.911903
1.93019	1.932408	1.929391	1.926347	1.92325	1.920161	1.917121	1.9141	1.911065
1.929339	1.93153	1.928502	1.925469	1.922419	1.919371	1.916342	1.913312	1.910249
1.933701	1.930693	1.92766	1.924634	1.921611	1.918585	1.915549	1.912501	1.909424
1.932896	1.929913	1.926883	1.923857	1.920839	1.917807	1.914738	1.911654	1.908581
1.932517	1.929149	1.926121	1.92309	1.920059	1.917011	1.91392	1.910816	1.907754
1.931749	1.928395	1.925367	1.922322	1.91925	1.916172	1.913091	1.910015	1.906967
1.93099	1.927641	1.924605	1.921542	1.918431	1.915326	1.912266	1.909226	1.90619
1.93023	1.927357	1.924303	1.920714	1.917609	1.914517	1.91147	1.90844	1.905393
1.929465	1.926571	1.923469	1.91986	1.916788	1.913729	1.910691	1.907655	1.904583
1.928662	1.925757	1.922651	1.919571	1.916534	1.913505	1.909894	1.906834	1.903753
1.927819	1.924914	1.921842	1.918788	1.915764	1.912732	1.909082	1.905976	1.902901
1.92699	1.923894	1.920835	1.918012	1.914995	1.91196	1.908857	1.90574	1.902078
1.926186	1.923105	1.920054	1.917241	1.914229	1.911195	1.908088	1.904963	1.901303
1.925397	1.922331	1.919282	1.916471	1.913451	1.910411	1.907309	1.904194	1.900541
1.924629	1.921565	1.918519	1.9157	1.912637	1.909568	1.906485	1.903408	1.900377
1.923872	1.920807	1.91776	1.914928	1.911816	1.908711	1.905653	1.902621	1.899612
1.92311	1.920046	1.916998	1.914164	1.911031	1.907912	1.904862	1.901846	1.898847
1.922348	1.919284	1.916233	1.913403	1.910269	1.90715	1.904098	1.90108	1.898083
1.921586	1.91851	1.915434	1.912599	1.909482	1.90638	1.903336	1.90032	1.89731
1.920824	1.917719	1.914589	1.911742	1.908663	1.905604	1.902578	1.899565	1.896527
1.920069	1.916942	1.913772	1.910916	1.907863	1.904833	1.901822	1.898814	1.895755
1.919325	1.916191	1.913011	1.910154	1.907099	1.90407	1.901068	1.898069	1.895005
1.918585	1.915451	1.912269	1.909413	1.906349	1.903315	1.900318	1.897328	1.894264
1.917845	1.914711	1.911527	1.908672	1.905604	1.902568	1.899576	1.896593	1.893531
1.917106	1.913973	1.91079	1.907936	1.904865	1.901828	1.898839	1.895861	1.892802
1.916363	1.91323	1.910045	1.907193	1.904123	1.901088	1.898101	1.895124	1.892065
1.915617	1.912484	1.909295	1.906445	1.903379	1.900348	1.897362	1.894385	1.891324
1.914873	1.911741	1.908552	1.905704	1.90264	1.89961	1.896622	1.893643	1.890581
1.91413	1.911	1.907811	1.904966	1.901901	1.898869	1.89588	1.892899	1.889834
1.915874	1.910266	1.907092	1.904255	1.901181	1.898139	1.895143	1.892157	1.88909
1.915136	1.909549	1.906423	1.903606	1.900503	1.897427	1.894416	1.891421	1.888351
1.914398	1.908831	1.905756	1.902961	1.89983	1.896719	1.893692	1.890688	1.88762
1.913657	1.908093	1.905036	1.902253	1.899115	1.895995	1.892961	1.889954	1.886908
1.912915	1.907343	1.904286	1.901509	1.898378	1.895264	1.892227	1.889221	1.886206
1.912192	1.90909	1.906029	1.900765	1.897641	1.894535	1.891494	1.888484	1.885484
1.911486	1.908354	1.90528	1.900014	1.896901	1.893806	1.890759	1.887741	1.884744
1.910775	1.907624	1.904539	1.90181	1.898721	1.895644	1.890055	1.887018	1.884013
1.910048	1.906894	1.903806	1.901093	1.898051	1.895007	1.889397	1.886318	1.883293
1.909337	1.906186	1.903091	1.900387	1.89738	1.894363	1.888743	1.88564	1.882594
1.908679	1.905535	1.902407	1.899684	1.896673	1.893664	1.890605	1.887532	1.884472
1.90803	1.904899	1.901742	1.898996	1.895964	1.892948	1.889926	1.886898	1.883829
1.907326	1.904218	1.90109	1.898352	1.895293	1.892252	1.889235	1.886218	1.883143
1.906587	1.903511	1.900447	1.897738	1.894648	1.891568	1.888535	1.88551	1.882431
1.905866	1.902796	1.899764	1.896711	1.893616	1.890521	1.887858	1.884812	1.881738
1.905157	1.902066	1.899036	1.896002	1.892955	1.889894	1.887209	1.884126	1.881066
1.905896	1.901366	1.898322	1.89529	1.892274	1.889238	1.88654	1.883432	1.880382
1.905238	1.90072	1.897637	1.894577	1.891555	1.888519	1.885823	1.882714	1.87967

1.904585	1.900084	1.896971	1.893883	1.890841	1.887792	1.885107	1.882011	1.878966
1.903908	1.899423	1.896328	1.893241	1.890167	1.887094	1.884431	1.881363	1.87831
1.903225	1.898757	1.895697	1.892622	1.889513	1.886413	1.883775	1.880739	1.877676
1.902561	1.899456	1.896384	1.89197	1.888864	1.885766	1.883131	1.880093	1.877031
1.90191	1.898813	1.895711	1.891298	1.888222	1.885149	1.882499	1.879434	1.876382
1.901232	1.898148	1.895045	1.890647	1.887578	1.884511	1.881861	1.878791	1.875743
1.900511	1.897453	1.894391	1.890024	1.886932	1.883843	1.881216	1.878173	1.875119
1.899818	1.896772	1.893734	1.890668	1.887554	1.884443	1.880572	1.877544	1.874486
1.899194	1.896134	1.893067	1.88999	1.886897	1.883804	1.879933	1.876883	1.873829
1.898586	1.895505	1.892402	1.88931	1.886239	1.883165	1.879294	1.876217	1.873169
1.897941	1.894851	1.891754	1.888662	1.885577	1.882493	1.878645	1.875579	1.872527
1.897282	1.894187	1.891113	1.888027	1.884913	1.881806	1.877992	1.874953	1.871892
1.896638	1.893538	1.890456	1.887364	1.884251	1.881147	1.878307	1.875248	1.870777
1.896008	1.892901	1.889782	1.886676	1.883591	1.880511	1.877658	1.874571	1.870125
1.895364	1.89205	1.888902	1.885777	1.882689	1.879606	1.877009	1.873918	1.8695
1.894697	1.891387	1.888271	1.885155	1.882034	1.878927	1.876353	1.873301	1.868916
1.893855	1.89074	1.887656	1.88455	1.881399	1.878268	1.875715	1.872696	1.869687
1.893235	1.890139	1.887065	1.883963	1.880807	1.877671	1.875121	1.872102	1.869075
1.892637	1.889558	1.88648	1.883377	1.88023	1.877099	1.874542	1.871508	1.868454
1.892061	1.888972	1.885862	1.882747	1.879626	1.876515	1.873939	1.870876	1.867818
1.891504	1.888388	1.885227	1.882093	1.879009	1.875929	1.873326	1.870224	1.867173
1.890941	1.887812	1.884624	1.88148	1.878413	1.875347	1.872733	1.869614	1.866566
1.890376	1.887247	1.884057	1.88091	1.87784	1.874773	1.872163	1.869048	1.865999
1.889783	1.886655	1.883479	1.880336	1.87725	1.874172	1.871575	1.868476	1.865424
1.889136	1.886007	1.88287	1.879742	1.876622	1.873517	1.870946	1.867883	1.864819
1.888495	1.885365	1.882266	1.879151	1.876001	1.872871	1.870324	1.867293	1.864219
1.887916	1.884783	1.881694	1.878584	1.875425	1.872287	1.869748	1.866726	1.863648
1.887363	1.884226	1.881136	1.878026	1.874869	1.871733	1.869193	1.866169	1.86309
1.886805	1.883667	1.880575	1.877464	1.87431	1.871176	1.868634	1.865605	1.862526
1.886248	1.883109	1.880011	1.876899	1.873751	1.870621	1.868075	1.865038	1.86196
1.885671	1.882531	1.879418	1.876805	1.873677	1.870564	1.86749	1.864433	1.861359
1.885067	1.881928	1.878783	1.876161	1.873062	1.869971	1.866871	1.863777	1.860712
1.88447	1.881332	1.878163	1.875534	1.872459	1.869385	1.866265	1.863141	1.860082
1.883893	1.880756	1.877585	1.874958	1.871889	1.868818	1.865691	1.86256	1.859501
1.883325	1.88019	1.877023	1.874401	1.87133	1.868259	1.865132	1.862	1.85894
1.882765	1.879631	1.876465	1.873845	1.870774	1.867701	1.864574	1.861443	1.858383
1.882212	1.879078	1.875913	1.873295	1.870222	1.867148	1.864023	1.860895	1.857834
1.881658	1.878525	1.87536	1.872744	1.86967	1.866596	1.863473	1.860347	1.857287
1.881104	1.877972	1.874806	1.872192	1.869119	1.866047	1.862926	1.859802	1.856743
1.880557	1.877425	1.87426	1.87165	1.868575	1.8655	1.86238	1.859257	1.8562
1.880014	1.876882	1.873719	1.871111	1.868032	1.864954	1.861833	1.85871	1.855654
1.879484	1.876352	1.873202	1.870599	1.867509	1.864419	1.861295	1.858173	1.855115
1.87898	1.875851	1.872737	1.870148	1.867029	1.863907	1.860771	1.857645	1.854583
1.878481	1.875355	1.872281	1.869708	1.866557	1.863402	1.86026	1.857135	1.854066
1.877954	1.87483	1.87177	1.869206	1.866045	1.862887	1.859768	1.85667	1.853595
1.877413	1.874289	1.871228	1.868668	1.865511	1.862366	1.859288	1.856233	1.853152
1.876876	1.873753	1.870688	1.86813	1.864978	1.861843	1.858786	1.855752	1.852664
1.87634	1.873217	1.870146	1.867588	1.864442	1.861313	1.858259	1.855225	1.852133
1.875835	1.872711	1.869622	1.86706	1.86393	1.860815	1.857753	1.854705	1.851605
1.875385	1.872259	1.869129	1.866557	1.863464	1.860377	1.857292	1.854204	1.851085

1.874933	1.871803	1.868639	1.866057	1.862996	1.859934	1.85683	1.853707	1.850578
1.874425	1.871294	1.868123	1.865541	1.862488	1.859435	1.856329	1.853206	1.850095
1.873901	1.87077	1.867607	1.865029	1.86197	1.858914	1.855817	1.852711	1.849629
1.872819	1.870274	1.867134	1.864565	1.861483	1.85841	1.855333	1.852256	1.84918
1.872346	1.869797	1.866694	1.864137	1.861017	1.857915	1.854865	1.851829	1.848746
1.871896	1.86934	1.866241	1.863679	1.860536	1.857417	1.85438	1.851364	1.84828
1.871477	1.868914	1.865775	1.863187	1.860033	1.85691	1.853869	1.850852	1.847771
1.871031	1.869015	1.865847	1.86269	1.859544	1.856429	1.853378	1.85035	1.847274
1.870522	1.868502	1.865332	1.862188	1.859084	1.855999	1.85293	1.849872	1.846806
1.870002	1.86798	1.864821	1.861699	1.858635	1.855579	1.852497	1.849414	1.846356
1.869515	1.867491	1.86436	1.861254	1.858185	1.855126	1.852058	1.848992	1.845931
1.869047	1.867022	1.863924	1.860833	1.857741	1.854664	1.851621	1.848588	1.84552
1.868606	1.866579	1.863481	1.860391	1.857306	1.854234	1.851191	1.848156	1.84509
1.868191	1.866163	1.863034	1.859935	1.856879	1.85383	1.850767	1.847702	1.844646
1.868414	1.865293	1.862163	1.859501	1.856454	1.853412	1.850342	1.847267	1.844212
1.86797	1.864853	1.861753	1.8591	1.856028	1.852966	1.849911	1.846859	1.843797
1.867535	1.86442	1.861342	1.858696	1.855608	1.852531	1.849486	1.846448	1.843381
1.867128	1.864014	1.860917	1.858263	1.855195	1.852134	1.849073	1.846013	1.842953
1.866727	1.863613	1.860493	1.857827	1.854784	1.851743	1.848664	1.845578	1.842526
1.866308	1.863189	1.860082	1.85742	1.854367	1.851318	1.848247	1.845172	1.842118
1.865883	1.862756	1.85968	1.857027	1.853949	1.850879	1.847828	1.844782	1.841719
1.8655	1.862361	1.859276	1.856202	1.85313	1.850067	1.847421	1.844372	1.841307
1.86516	1.862001	1.858871	1.855777	1.852736	1.849697	1.847025	1.84394	1.84088
1.864809	1.861646	1.858489	1.855385	1.852365	1.849343	1.846652	1.843541	1.840475
1.864435	1.861295	1.858146	1.855047	1.852033	1.849013	1.846317	1.843198	1.840103
1.86405	1.860934	1.857807	1.854719	1.851698	1.84867	1.845977	1.842863	1.839744
1.863641	1.860532	1.857442	1.85437	1.851322	1.848272	1.845593	1.84251	1.839399
1.863232	1.860124	1.857075	1.854019	1.850938	1.847861	1.845195	1.842145	1.839055
1.862877	1.85977	1.856736	1.853685	1.850593	1.847501	1.844433	1.84137	1.838671
1.862559	1.859453	1.856415	1.85336	1.850273	1.847174	1.844063	1.840954	1.838261
1.862241	1.859128	1.856066	1.853002	1.849931	1.846846	1.843716	1.840582	1.837886
1.861926	1.858792	1.855681	1.852599	1.849564	1.846515	1.843394	1.840258	1.837555
1.8616	1.858459	1.855314	1.852219	1.849206	1.846184	1.843075	1.839942	1.837224
1.861255	1.858133	1.854987	1.85189	1.848879	1.845858	1.842756	1.839626	1.836878
1.860906	1.857807	1.854672	1.851576	1.848549	1.845516	1.842423	1.839306	1.836533
1.860574	1.857484	1.854356	1.85125	1.848183	1.84512	1.842048	1.838968	1.8362
1.860243	1.857155	1.854039	1.850924	1.847804	1.844704	1.841657	1.838623	1.835874
1.859875	1.856788	1.853701	1.850597	1.84746	1.844344	1.841309	1.838295	1.83521
1.859475	1.856388	1.853343	1.850271	1.847142	1.844028	1.840994	1.837979	1.834899
1.859111	1.856024	1.853004	1.849951	1.84683	1.84372	1.840687	1.837671	1.834593
1.858796	1.855706	1.852689	1.84964	1.846523	1.843418	1.840387	1.837371	1.834296
1.858493	1.855402	1.852382	1.849333	1.846221	1.843119	1.840089	1.837073	1.833999
1.858193	1.855102	1.852081	1.849031	1.84592	1.84282	1.839789	1.836775	1.833702
1.857895	1.854804	1.851782	1.848732	1.84562	1.842519	1.839488	1.836473	1.833401
1.857589	1.854497	1.851478	1.848429	1.845316	1.842214	1.839179	1.836161	1.833091
1.857281	1.854188	1.851173	1.848126	1.845011	1.841906	1.838867	1.835844	1.832775
1.856986	1.853891	1.850873	1.847823	1.844706	1.841599	1.838557	1.835531	1.832462
1.856701	1.853603	1.850576	1.84752	1.844402	1.841294	1.838249	1.835221	1.83215
1.856448	1.853347	1.850299	1.847227	1.844102	1.840989	1.837943	1.834914	1.831841
1.856246	1.853145	1.850056	1.84695	1.843807	1.840684	1.837634	1.834607	1.831534

1.856037	1.852937	1.849811	1.846675	1.843517	1.840385	1.837331	1.834303	1.831229
1.855775	1.852674	1.849538	1.846393	1.84323	1.840092	1.837031	1.833998	1.830921
1.855496	1.852391	1.849254	1.84611	1.84295	1.839814	1.836744	1.833699	1.830615
1.855237	1.852113	1.848967	1.845828	1.842699	1.839585	1.836499	1.833423	1.830323
1.854987	1.851836	1.848675	1.845546	1.842467	1.83939	1.836283	1.833165	1.83004
1.854724	1.851555	1.848384	1.845258	1.842206	1.839151	1.836032	1.832894	1.829769
1.854445	1.851269	1.848088	1.844958	1.841911	1.838862	1.835744	1.832609	1.829513
1.854167	1.850995	1.847821	1.844691	1.841633	1.838574	1.835455	1.832326	1.829836
1.853886	1.850736	1.847605	1.844489	1.841396	1.8383	1.83517	1.832039	1.829547
1.853621	1.850494	1.8474	1.844294	1.841165	1.838033	1.834897	1.83177	1.829268
1.853406	1.850286	1.847176	1.844055	1.84091	1.837771	1.834655	1.831553	1.829038
1.853212	1.85009	1.846944	1.843798	1.84065	1.837519	1.834431	1.831359	1.828827
1.8558	1.849857	1.846696	1.843547	1.840416	1.837301	1.834212	1.83113	1.828582
1.855564	1.849596	1.846434	1.843297	1.840203	1.837112	1.833999	1.830878	1.828312
1.852187	1.85214	1.848964	1.843075	1.839991	1.836901	1.833769	1.830624	1.828054
1.851929	1.851905	1.848773	1.842892	1.839777	1.836653	1.833508	1.830361	1.827809
1.851688	1.851687	1.848584	1.845457	1.842293	1.839118	1.833886	1.830712	1.827582
1.851489	1.8515	1.848378	1.845248	1.842107	1.838954	1.833716	1.830537	1.82739
1.851302	1.851321	1.848175	1.845038	1.841922	1.838794	1.833551	1.830368	1.827203
1.851096	1.85112	1.847991	1.844858	1.841724	1.838577	1.836041	1.832825	1.826982
1.850884	1.850911	1.847816	1.84469	1.841516	1.838329	1.83578	1.832568	1.826744
1.850689	1.847563	1.844469	1.844485	1.841285	1.838079	1.835537	1.832343	1.826521
1.85051	1.847383	1.844253	1.844245	1.841029	1.837823	1.835304	1.832145	1.826311
1.85032	1.84719	1.844047	1.844032	1.840814	1.837609	1.835095	1.831944	1.828786
1.850106	1.846974	1.84386	1.84387	1.840665	1.83746	1.834912	1.831726	1.828571
1.849903	1.846764	1.843671	1.843704	1.840521	1.837325	1.834749	1.831529	1.828373
1.849745	1.846587	1.843462	1.843491	1.840343	1.837183	1.834614	1.831389	1.828211
1.849593	1.846416	1.843251	1.843268	1.840155	1.837033	1.834484	1.831269	1.828068
1.84938	1.84621	1.843056	1.843082	1.839962	1.836839	1.834315	1.831131	1.827936
1.849131	1.845985	1.842874	1.84292	1.839765	1.836617	1.834121	1.830983	1.827811
1.848931	1.845801	1.842714	1.839603	1.836443	1.833288	1.83394	1.830808	1.827651
1.848784	1.845662	1.842582	1.839475	1.836319	1.833157	1.83377	1.830597	1.827452
1.848637	1.845518	1.842431	1.839323	1.836182	1.833027	1.833615	1.830412	1.827265
1.848471	1.845351	1.842235	1.83912	1.836012	1.83289	1.833489	1.830281	1.82711
1.848311	1.845188	1.842042	1.838917	1.835832	1.832738	1.833357	1.83016	1.826967
1.848183	1.845053	1.841892	1.838751	1.835645	1.83254	1.833184	1.830022	1.826839
1.848064	1.844926	1.841762	1.838606	1.835458	1.832326	1.832993	1.829874	1.826714
1.847908	1.844765	1.841622	1.838473	1.835308	1.832158	1.829767	1.826651	1.826551
1.847724	1.844579	1.841478	1.838354	1.835188	1.832023	1.829594	1.826444	1.826358
1.847574	1.844427	1.841345	1.838235	1.835073	1.831903	1.829448	1.826273	1.826196
1.847466	1.844317	1.841231	1.838121	1.834966	1.831801	1.829336	1.826147	1.826073
1.847369	1.844212	1.841105	1.837986	1.834847	1.831697	1.829232	1.826035	1.822909
1.847273	1.844093	1.840938	1.837802	1.834693	1.831577	1.829126	1.825929	1.822775
1.847183	1.843978	1.840773	1.837617	1.834538	1.831457	1.829023	1.82583	1.822648
1.8471	1.843883	1.840656	1.838275	1.83518	1.832086	1.828924	1.825737	1.822544
1.847021	1.8438	1.840566	1.838177	1.835078	1.831985	1.828829	1.82565	1.822456
1.846941	1.843716	1.840478	1.838083	1.834983	1.83189	1.828738	1.825563	1.822369
1.846861	1.843635	1.840395	1.837999	1.834896	1.831802	1.828651	1.825479	1.822286
1.84678	1.843552	1.840311	1.837913	1.834809	1.831714	1.828564	1.825393	1.822202
1.846699	1.843469	1.840226	1.837826	1.834721	1.831626	1.828477	1.825307	1.822117

1.846615	1.844239	1.840953	1.837737	1.834632	1.831536	1.828387	1.825218	1.822029
1.846525	1.844148	1.840861	1.837644	1.834538	1.831441	1.828292	1.825122	1.821934
1.846433	1.844055	1.840767	1.83755	1.834443	1.831345	1.828195	1.825024	1.821836
1.846342	1.843963	1.840674	1.837456	1.834348	1.83125	1.828098	1.824927	1.821738
1.846251	1.843871	1.840581	1.837362	1.834254	1.831155	1.828002	1.824829	1.82164
1.846163	1.843783	1.840496	1.837278	1.834167	1.831064	1.827909	1.824735	1.821547
1.846076	1.843699	1.840417	1.8372	1.834085	1.830978	1.827819	1.824644	1.821458
1.845992	1.843624	1.840362	1.837153	1.834023	1.8309	1.827734	1.824555	1.821379
1.845908	1.843564	1.840351	1.83716	1.833998	1.830838	1.827655	1.82447	1.821315
1.845829	1.843508	1.840339	1.837164	1.833972	1.830778	1.827577	1.824386	1.821251
1.847184	1.843437	1.840283	1.837114	1.833912	1.830705	1.827492	1.824292	1.821165
1.84714	1.84337	1.840213	1.837042	1.833838	1.830629	1.827412	1.824207	1.82108
1.847141	1.843912	1.840719	1.83698	1.833767	1.830558	1.827359	1.824178	1.821046
1.847178	1.843947	1.840711	1.836922	1.833696	1.830489	1.827329	1.824192	1.821052
1.84718	1.843949	1.84069	1.836873	1.833656	1.830464	1.827318	1.824191	1.82104
1.847137	1.843909	1.84065	1.83683	1.833656	1.830495	1.827332	1.824171	1.821002
1.847095	1.843869	1.840626	1.837414	1.834253	1.831094	1.827335	1.824145	1.82097
1.847067	1.843846	1.840642	1.837449	1.83427	1.831088	1.827305	1.824111	1.820953
1.847046	1.843826	1.840662	1.837484	1.834278	1.831069	1.827272	1.824087	1.820948
1.846047	1.843795	1.840639	1.837467	1.834257	1.831049	1.827262	1.824104	1.820965
1.846031	1.843768	1.840601	1.837425	1.834222	1.831031	1.827266	1.824141	1.820993
1.846069	1.843792	1.840595	1.837409	1.834223	1.831049	1.827902	1.82476	1.820988
1.846159	1.843861	1.840618	1.837411	1.834253	1.831102	1.827933	1.824756	1.820955
1.846215	1.843912	1.840652	1.837436	1.834282	1.831133	1.827946	1.824747	1.820932
1.846212	1.843928	1.840702	1.837494	1.83431	1.831129	1.827928	1.824728	1.82092
1.846213	1.843948	1.84075	1.837548	1.834331	1.831119	1.827916	1.824725	1.821571
1.846254	1.843995	1.840779	1.83756	1.834325	1.831105	1.827924	1.824763	1.821612
1.846306	1.844047	1.840809	1.837572	1.834327	1.831107	1.827948	1.824816	1.821662
1.846342	1.844087	1.840863	1.837636	1.834394	1.831172	1.828	1.824853	1.821697
1.846372	1.844121	1.840931	1.83773	1.834502	1.831278	1.828073	1.824884	1.821725
1.841888	1.844168	1.840982	1.83779	1.834584	1.831374	1.828153	1.824938	1.821768
1.841948	1.84423	1.841014	1.837815	1.834643	1.831465	1.828246	1.825018	1.821825
1.842001	1.844285	1.841055	1.837852	1.834693	1.831527	1.828307	1.825073	1.821868
1.84203	1.844319	1.841113	1.837916	1.834733	1.831541	1.828308	1.82507	1.82188
1.842076	1.844364	1.841175	1.83798	1.834771	1.831554	1.828314	1.825077	1.8219
1.839912	1.844449	1.841228	1.838016	1.834815	1.831611	1.828381	1.82515	1.82196
1.840043	1.844554	1.841286	1.838051	1.834864	1.831682	1.828468	1.825249	1.822041
1.83846	1.840294	1.837064	1.83813	1.83492	1.831724	1.828527	1.825334	1.822131
1.838551	1.840403	1.837186	1.838241	1.834983	1.83175	1.82857	1.825414	1.822231
1.838618	1.836768	1.833567	1.838351	1.83507	1.831814	1.828627	1.825473	1.822305
1.838653	1.836806	1.833648	1.838464	1.835189	1.831923	1.828699	1.825503	1.822342
1.838713	1.836858	1.833719	1.835142	1.831876	1.828606	1.828789	1.825558	1.822401
1.838827	1.836949	1.833771	1.835195	1.831965	1.828731	1.828908	1.825667	1.82251
1.838963	1.83706	1.833833	1.83525	1.832055	1.828858	1.829038	1.825798	1.822633
1.839106	1.837192	1.833939	1.835354	1.832171	1.828989	1.827871	1.824637	1.821459
1.839256	1.83734	1.834072	1.835484	1.832297	1.829116	1.827997	1.82477	1.821565
1.839386	1.83748	1.83421	1.835617	1.832403	1.829202	1.825986	1.822767	1.821677
1.839501	1.837616	1.834355	1.835756	1.83249	1.829251	1.826058	1.822883	1.821795
1.83901	1.837748	1.834499	1.831253	1.828005	1.824791	1.826159	1.823012	1.819804
1.839144	1.837881	1.834643	1.831401	1.828144	1.824921	1.8263	1.823157	1.819951

1.839261	1.837993	1.834775	1.831546	1.82829	1.825066	1.826454	1.82331	1.820105
1.839332	1.838058	1.834876	1.831676	1.828432	1.825215	1.826613	1.823469	1.820264
1.8394	1.838117	1.834973	1.831804	1.828575	1.825368	1.826778	1.823632	1.820426
1.839521	1.837729	1.834592	1.831941	1.828721	1.825521	1.826942	1.823795	1.820588
1.839676	1.837877	1.83474	1.832083	1.828869	1.825674	1.827106	1.823958	1.82075
1.839831	1.838027	1.834892	1.831734	1.828518	1.825321	1.827267	1.824117	1.82091
1.839989	1.838182	1.83505	1.831895	1.82868	1.825483	1.827428	1.824273	1.821067
1.840151	1.838339	1.835208	1.832054	1.82884	1.825643	1.827586	1.824429	1.821223
1.840313	1.838495	1.835365	1.832211	1.828996	1.825799	1.827742	1.824584	1.821378
1.840485	1.83866	1.835525	1.832369	1.829152	1.825953	1.827898	1.82474	1.821534
1.840668	1.838834	1.835692	1.832529	1.829308	1.826106	1.82771	1.824556	1.821695
1.840868	1.839026	1.83587	1.832695	1.829467	1.826263	1.827874	1.824722	1.821858
1.841112	1.839264	1.836077	1.832879	1.829641	1.826431	1.823305	1.820207	1.821685
1.841391	1.839539	1.836308	1.833076	1.829828	1.826611	1.823475	1.820369	1.821851
1.837342	1.839775	1.836518	1.833271	1.830033	1.826823	1.823673	1.820548	1.82203
1.837539	1.839967	1.836703	1.833464	1.830261	1.827077	1.823909	1.820748	1.822222
1.837737	1.840157	1.836889	1.833656	1.830479	1.827312	1.824128	1.820944	1.822422
1.837953	1.840355	1.837077	1.83384	1.830662	1.827496	1.824307	1.821121	1.822635
1.838169	1.840557	1.837281	1.83404	1.830848	1.82767	1.824477	1.821297	1.822848
1.838368	1.836716	1.833501	1.834295	1.83107	1.827859	1.824656	1.821474	1.823037
1.838561	1.836928	1.833761	1.834574	1.831309	1.828055	1.824842	1.82166	1.823223
1.83876	1.837132	1.833985	1.830802	1.827546	1.824303	1.825063	1.821896	1.823457
1.838961	1.83733	1.834182	1.831002	1.827752	1.824522	1.825314	1.822176	1.82373
1.839202	1.837564	1.834396	1.831211	1.827981	1.824772	1.825576	1.822439	1.823987
1.839497	1.837849	1.834635	1.831435	1.828246	1.82507	1.825854	1.822678	1.824215
1.834338	1.838126	1.834875	1.831661	1.828504	1.825353	1.822175	1.81899	1.8197
1.834592	1.838361	1.835097	1.831877	1.828724	1.825578	1.822394	1.819204	1.819931
1.834833	1.838586	1.835325	1.832105	1.828942	1.825789	1.822607	1.819427	1.820176
1.8345	1.833559	1.83035	1.832382	1.829196	1.826024	1.822856	1.819698	1.816554
1.834726	1.833806	1.830637	1.832689	1.829473	1.826274	1.823125	1.819997	1.816845
1.834985	1.834076	1.830916	1.832976	1.82976	1.826559	1.823409	1.820278	1.817125
1.835275	1.834372	1.831188	1.833243	1.830058	1.826882	1.823709	1.820544	1.817393
1.835559	1.834196	1.831015	1.828295	1.825147	1.822002	1.824006	1.820825	1.817673
1.835824	1.834464	1.831309	1.828605	1.825437	1.822275	1.824296	1.821138	1.817974
1.836095	1.834736	1.831604	1.828912	1.825727	1.822552	1.824586	1.821444	1.818273
1.836395	1.835032	1.831886	1.829192	1.826024	1.82286	1.819712	1.816569	1.818547
1.8367	1.835333	1.832163	1.829463	1.826319	1.823173	1.820002	1.816829	1.818815
1.830773	1.835603	1.832443	1.829289	1.826145	1.822999	1.820281	1.817113	1.819101
1.831023	1.835856	1.832724	1.829581	1.826414	1.82325	1.820553	1.817412	1.819396
1.831302	1.836135	1.833001	1.829855	1.826686	1.823521	1.820829	1.817691	1.814547
1.831609	1.836441	1.83327	1.830108	1.82696	1.823813	1.821108	1.817944	1.814808
1.831934	1.834987	1.831779	1.830381	1.827239	1.824099	1.821391	1.818217	1.815085
1.832287	1.835335	1.832114	1.830693	1.82752	1.82436	1.821672	1.818527	1.815387
1.832632	1.835675	1.832455	1.831019	1.827814	1.824632	1.821969	1.818855	1.815707
1.83292	1.829765	1.826598	1.831348	1.828141	1.824956	1.821837	1.818739	1.816052
1.833185	1.830032	1.826903	1.831676	1.828482	1.825305	1.822187	1.819086	1.816405
1.833479	1.830324	1.827189	1.831965	1.828801	1.825646	1.822514	1.81939	1.816718
1.833794	1.830634	1.827457	1.832222	1.829102	1.825983	1.822825	1.81966	1.816998
1.834125	1.83096	1.827756	1.832515	1.82942	1.82632	1.823147	1.819958	1.817293
1.83448	1.831313	1.8281	1.832857	1.829762	1.826663	1.823491	1.820297	1.817609

1.834818	1.831649	1.828444	1.825282	1.822192	1.819098	1.82382	1.820638	1.81746
1.835103	1.831934	1.828765	1.825614	1.822488	1.819366	1.824109	1.820966	1.817797
1.835385	1.832216	1.829088	1.82595	1.822783	1.819629	1.824394	1.821291	1.818137
1.833068	1.832551	1.82944	1.826306	1.82312	1.819949	1.824712	1.821618	1.818466
1.83343	1.832917	1.829809	1.826673	1.823479	1.820299	1.825047	1.82194	1.818787
1.833793	1.833286	1.830176	1.827037	1.82384	1.820651	1.819529	1.816416	1.819077
1.834161	1.833662	1.830545	1.827401	1.824207	1.821008	1.819835	1.816671	1.819337
1.834514	1.834022	1.830885	1.827733	1.824553	1.821362	1.820154	1.816955	1.819624
1.83484	1.834354	1.831175	1.828008	1.824864	1.821708	1.820492	1.817285	1.819956
1.83517	1.834689	1.831473	1.828293	1.82518	1.822056	1.820841	1.817632	1.820305
1.835523	1.832378	1.829194	1.828635	1.82553	1.822416	1.819215	1.815997	1.814858
1.835888	1.832741	1.829557	1.829003	1.825897	1.822782	1.819584	1.816369	1.81522
1.836259	1.83311	1.829925	1.829378	1.826272	1.823156	1.819957	1.816741	1.815582
1.836637	1.833486	1.830302	1.829765	1.826656	1.823537	1.820334	1.817115	1.815946
1.837013	1.83386	1.830675	1.830149	1.827038	1.823917	1.820712	1.81749	1.816312
1.837388	1.834232	1.831045	1.830528	1.827418	1.824297	1.82109	1.817865	1.816681
1.837768	1.834609	1.831423	1.830914	1.827803	1.824681	1.821475	1.818251	1.81706
1.838149	1.834989	1.831804	1.831304	1.82819	1.825067	1.821863	1.818643	1.817446
1.838542	1.835382	1.832207	1.831719	1.828594	1.825462	1.822266	1.819059	1.817854
1.838961	1.835805	1.832668	1.832201	1.82904	1.825879	1.822704	1.819529	1.818311
1.839392	1.836241	1.83315	1.832707	1.829503	1.826306	1.823156	1.820021	1.818786
1.837687	1.83665	1.833578	1.83047	1.827288	1.824117	1.823576	1.82046	1.81729
1.838089	1.837039	1.833968	1.830859	1.827673	1.824501	1.823972	1.820861	1.817691
1.835186	1.835307	1.832195	1.831243	1.82806	1.824889	1.824367	1.821256	1.818088
1.835583	1.835708	1.832589	1.831624	1.828444	1.825276	1.824759	1.821642	1.818477
1.836003	1.83284	1.829724	1.829841	1.826638	1.823444	1.82517	1.822038	1.818878
1.836478	1.833317	1.83016	1.830267	1.827098	1.82393	1.825626	1.822456	1.819306
1.836957	1.833799	1.830603	1.8307	1.827564	1.824423	1.826089	1.822881	1.819741
1.834196	1.834226	1.831022	1.827853	1.824749	1.821636	1.824418	1.821158	1.820154
1.834588	1.834627	1.831435	1.82827	1.825157	1.822036	1.82484	1.82158	1.820565
1.834978	1.831848	1.828698	1.828726	1.825589	1.822449	1.819282	1.816115	1.818871
1.83536	1.832254	1.829161	1.829218	1.826042	1.82287	1.819735	1.816616	1.819375
1.835767	1.832667	1.829603	1.829679	1.826479	1.823288	1.820173	1.817084	1.819864
1.836204	1.833084	1.830008	1.830083	1.826883	1.823695	1.820581	1.817499	1.820329
1.836664	1.833522	1.83042	1.830487	1.8273	1.824124	1.821002	1.81791	1.820782
1.833638	1.834004	1.830862	1.827719	1.824569	1.821425	1.821467	1.818339	1.818181
1.83413	1.834501	1.831322	1.828165	1.825045	1.821924	1.821951	1.818785	1.818614
1.834745	1.831455	1.828302	1.828648	1.825519	1.82239	1.822431	1.819273	1.816153
1.835197	1.831901	1.82879	1.829158	1.825997	1.822842	1.822913	1.819792	1.816657
1.831474	1.832364	1.82926	1.82964	1.826478	1.823321	1.820197	1.817082	1.817143
1.83195	1.832847	1.829712	1.830092	1.826964	1.823833	1.820686	1.817538	1.817611
1.832405	1.829295	1.826159	1.827041	1.823947	1.820847	1.821172	1.818007	1.81488
1.832815	1.829726	1.826631	1.827531	1.824415	1.821294	1.821647	1.81851	1.815375
1.833237	1.830161	1.827101	1.828018	1.82488	1.821741	1.822121	1.819008	1.815866
1.83373	1.830638	1.827558	1.824467	1.821359	1.818247	1.819107	1.815988	1.816328
1.834249	1.831129	1.828011	1.824907	1.821827	1.818739	1.819577	1.816423	1.81678
1.834729	1.831596	1.828478	1.825373	1.822287	1.819194	1.816066	1.81293	1.813769
1.835185	1.832046	1.828956	1.825858	1.82274	1.819623	1.816517	1.813413	1.814247
1.835653	1.832511	1.829422	1.826324	1.823204	1.820085	1.816982	1.813882	1.814717
1.836141	1.832996	1.829871	1.826762	1.823678	1.820587	1.817461	1.814328	1.815172

1.834669	1.833477	1.830333	1.827222	1.824168	1.821102	1.81796	1.814801	1.811702
1.835119	1.833934	1.830821	1.827734	1.824692	1.821632	1.818494	1.815334	1.812233
1.835574	1.8344	1.831324	1.828262	1.825218	1.822155	1.81903	1.815885	1.812779
1.836071	1.834915	1.831846	1.828778	1.825708	1.822629	1.819526	1.816412	1.813302
1.836591	1.835456	1.832376	1.829289	1.826183	1.823081	1.820006	1.816934	1.813819
1.837116	1.834011	1.830916	1.829772	1.826671	1.823581	1.820525	1.81747	1.814354
1.837647	1.83452	1.83138	1.830228	1.827168	1.824122	1.821076	1.818022	1.814908
1.83816	1.83502	1.831863	1.830715	1.82768	1.824654	1.821604	1.818538	1.81543
1.83864	1.835501	1.832375	1.831251	1.828214	1.825176	1.822095	1.818998	1.815903
1.839134	1.835996	1.832899	1.829824	1.826781	1.823732	1.822576	1.819462	1.816381
1.839675	1.836529	1.83343	1.830341	1.82726	1.824183	1.823045	1.819961	1.816899
1.840229	1.837079	1.83397	1.830861	1.827736	1.824631	1.823521	1.820477	1.817433
1.830598	1.837624	1.83452	1.831406	1.828263	1.825143	1.824043	1.821008	1.817971
1.831112	1.83817	1.835078	1.831969	1.828822	1.825695	1.824595	1.821554	1.818514
1.818038	1.838717	1.835632	1.832525	1.829377	1.826247	1.82515	1.822104	1.819047
1.81858	1.839265	1.836184	1.833081	1.829933	1.826802	1.825711	1.822658	1.819569
1.819125	1.826734	1.823677	1.833604	1.830468	1.827348	1.82429	1.821247	1.820105
1.819669	1.827269	1.82417	1.834069	1.830961	1.827876	1.824834	1.821801	1.820663
1.820218	1.827809	1.82467	1.83454	1.831457	1.828402	1.825377	1.822356	1.821228
1.820773	1.817715	1.814606	1.834841	1.831762	1.828716	1.825923	1.822911	1.821793
1.821333	1.818278	1.815166	1.835403	1.832314	1.829261	1.826471	1.823467	1.822359
1.821894	1.818841	1.815726	1.823237	1.820176	1.817151	1.826807	1.823816	1.822922
1.822457	1.819405	1.816292	1.823809	1.820742	1.817712	1.827364	1.824374	1.823484
1.811177	1.819966	1.816854	1.824378	1.821307	1.818273	1.82792	1.824927	1.821854
1.811722	1.820522	1.81741	1.824941	1.82187	1.818834	1.828473	1.825476	1.822399
1.801058	1.809251	1.806178	1.814894	1.811857	1.808853	1.816414	1.813445	1.822945
1.801597	1.809812	1.806746	1.815473	1.812426	1.809411	1.816969	1.813994	1.823489
1.802133	1.810376	1.807325	1.816067	1.813008	1.809978	1.817529	1.814547	1.824042
1.802658	1.810939	1.807924	1.816696	1.813618	1.810565	1.818108	1.815118	1.824624
1.803176	1.8115	1.808529	1.817339	1.814243	1.811165	1.818696	1.815696	1.825219
1.803691	1.812044	1.809098	1.806111	1.803053	1.800005	1.808745	1.805769	1.813267
1.804204	1.812574	1.809637	1.806661	1.803616	1.800576	1.809316	1.806322	1.813825
1.804719	1.801818	1.798937	1.807209	1.80419	1.801167	1.798162	1.795174	1.814385
1.805233	1.802327	1.799451	1.807759	1.804785	1.801792	1.798769	1.795745	1.814948
1.805763	1.802849	1.799969	1.808306	1.805368	1.802403	1.799369	1.796319	1.793341
1.806322	1.803389	1.800491	1.808841	1.805916	1.802965	1.799937	1.796887	1.793899
1.8069	1.803947	1.801027	1.809381	1.806455	1.80351	1.800496	1.797462	1.794469
1.807496	1.804536	1.801591	1.809945	1.807007	1.804061	1.801072	1.798068	1.795073
1.808105	1.805147	1.802179	1.810527	1.807568	1.804615	1.801657	1.798693	1.795698
1.808687	1.805743	1.802783	1.799834	1.796902	1.793983	1.802238	1.799291	1.796306
1.809244	1.806329	1.803408	1.800473	1.797512	1.794561	1.802813	1.799862	1.796896
1.809791	1.806894	1.804004	1.801085	1.798121	1.795158	1.803409	1.800441	1.797483
1.810325	1.807424	1.804545	1.801649	1.798725	1.79579	1.804042	1.801035	1.798058
1.810869	1.807955	1.805078	1.802198	1.799315	1.796412	1.804676	1.801644	1.798648
1.811447	1.808514	1.805624	1.802741	1.799867	1.796981	1.805289	1.802285	1.799281
1.812045	1.809093	1.806184	1.80329	1.80041	1.79753	1.805892	1.802934	1.799931
1.807802	1.809709	1.806797	1.803893	1.800992	1.798098	1.806483	1.803546	1.817536
1.808431	1.810353	1.807448	1.804535	1.8016	1.798676	1.807065	1.804135	1.818162
1.800979	1.810965	1.808061	1.805146	1.802205	1.799276	1.79639	1.793517	1.818799
1.801559	1.811534	1.808626	1.805719	1.802808	1.799906	1.797028	1.794155	1.81945

1.802164	1.799249	1.796348	1.806292	1.803402	1.800521	1.797652	1.79478	1.820095
1.802828	1.799895	1.796953	1.806866	1.80397	1.801091	1.798227	1.795366	1.820724
1.803497	1.800551	1.797577	1.807462	1.804552	1.801668	1.798804	1.795952	1.82135
1.804118	1.801183	1.798228	1.800389	1.797485	1.794604	1.799435	1.796576	1.821985
1.804722	1.801809	1.798898	1.801076	1.798161	1.795262	1.800088	1.797215	1.822622
1.797257	1.802457	1.799566	1.796666	1.793745	1.790837	1.811289	1.808368	1.812556
1.797917	1.803124	1.800234	1.797328	1.794386	1.791457	1.811891	1.808962	1.813171
1.798557	1.795674	1.792804	1.797967	1.795026	1.792094	1.812518	1.809582	1.806665
1.799159	1.796269	1.793398	1.798573	1.795661	1.792757	1.813182	1.810243	1.807324
1.799788	1.79689	1.794013	1.791142	1.788274	1.785412	1.809294	1.806357	1.807972
1.800489	1.797584	1.79469	1.791807	1.788939	1.786074	1.809936	1.806978	1.808578
1.801213	1.798302	1.795384	1.792482	1.789604	1.786728	1.81057	1.807594	1.80918
1.801923	1.798997	1.796051	1.812634	1.809672	1.806721	1.803769	1.800829	1.809826
1.802625	1.799682	1.796707	1.813272	1.81029	1.807331	1.8044	1.801492	1.810495
1.803288	1.800343	1.79737	1.81394	1.810953	1.807992	1.805065	1.802162	1.799271
1.803908	1.800977	1.798036	1.814636	1.811661	1.808702	1.805762	1.80284	1.79994
1.796821	1.801639	1.798717	1.815333	1.812365	1.809406	1.806449	1.803504	1.800591
1.797511	1.802346	1.799423	1.816031	1.813061	1.810094	1.807115	1.804142	1.80121
1.798219	1.803066	1.800125	1.816717	1.813741	1.810766	1.807773	1.804784	1.801837
1.794276	1.803783	1.800801	1.81737	1.81438	1.811401	1.808418	1.805446	1.802504
1.794985	1.804496	1.80147	1.818017	1.815012	1.812032	1.809068	1.806123	1.803193
1.779133	1.813443	1.810414	1.818712	1.8157	1.812715	1.809759	1.806825	1.803903
1.779731	1.814076	1.81109	1.819438	1.816427	1.813437	1.810481	1.807549	1.804633
1.780346	1.799469	1.796541	1.820171	1.817167	1.81418	1.811221	1.808284	1.805367
1.780978	1.800123	1.797212	1.820909	1.817923	1.814946	1.811982	1.809031	1.806105
1.771103	1.80079	1.797891	1.800659	1.797747	1.79484	1.812745	1.809786	1.80685
1.771764	1.801472	1.798579	1.801361	1.798454	1.795551	1.813506	1.81055	1.807606
1.77244	1.802165	1.799275	1.802067	1.799162	1.79626	1.814266	1.811314	1.808365
1.773132	1.793066	1.79019	1.797087	1.794197	1.791313	1.801996	1.79907	1.809114
1.773835	1.793772	1.79089	1.797791	1.794901	1.792015	1.802731	1.799803	1.809861
1.774528	1.794468	1.791591	1.788717	1.785846	1.782982	1.789841	1.786965	1.797603
1.775214	1.795158	1.792293	1.789424	1.786548	1.783677	1.790543	1.787664	1.798339
1.798713	1.795851	1.79299	1.790125	1.787249	1.784377	1.781519	1.778667	1.791128
1.799413	1.796545	1.793681	1.790816	1.787949	1.785084	1.782222	1.779362	1.791835
1.800116	1.797244	1.794374	1.791507	1.788647	1.785787	1.782924	1.78006	1.777206
1.800824	1.797953	1.795075	1.792203	1.789338	1.786478	1.78362	1.780765	1.777912
1.801528	1.79866	1.795779	1.792901	1.79003	1.787166	1.784316	1.781471	1.778619
1.802213	1.79935	1.796479	1.793606	1.79073	1.787861	1.78501	1.782167	1.779314
1.802887	1.80003	1.797176	1.794313	1.791434	1.78856	1.785704	1.782857	1.780003
1.792039	1.800713	1.797865	1.795008	1.792135	1.789264	1.786404	1.783549	1.780696
1.792716	1.801397	1.798545	1.795691	1.792834	1.789974	1.787108	1.784244	1.781393
1.793397	1.790556	1.787725	1.796376	1.793527	1.790675	1.787807	1.784937	1.782088
1.794084	1.791243	1.788402	1.797063	1.794214	1.791362	1.788495	1.785626	1.782778
1.794767	1.791928	1.789081	1.797754	1.794901	1.792047	1.789183	1.786319	1.783471
1.751167	1.792595	1.789755	1.786922	1.784101	1.781283	1.789876	1.787019	1.784171
1.751751	1.793264	1.790433	1.787606	1.784782	1.781962	1.790576	1.787727	1.784878
1.752371	1.793961	1.791128	1.788301	1.785483	1.782668	1.779853	1.777039	1.785599
1.753018	1.794681	1.791838	1.78901	1.786202	1.783397	1.780576	1.777753	1.786334
1.7537	1.75107	1.748439	1.789762	1.786953	1.784147	1.781325	1.778503	1.775699
1.754428	1.751799	1.749158	1.790568	1.787743	1.784926	1.78211	1.779298	1.776493

1.755117	1.75249	1.749846	1.74721	1.744589	1.741974	1.782866	1.780061	1.777255
1.755731	1.753106	1.750472	1.747838	1.745204	1.742579	1.78356	1.780757	1.777947
1.756314	1.753692	1.751068	1.748439	1.745802	1.743172	1.784241	1.781435	1.778622
1.720788	1.727991	1.725413	1.749017	1.746399	1.743785	1.741175	1.738568	1.779304
1.721292	1.72852	1.725941	1.749584	1.746997	1.744407	1.741794	1.739176	1.779993
1.721831	1.719289	1.716739	1.723957	1.721434	1.718908	1.742435	1.739818	1.754421
1.722399	1.719864	1.717322	1.724572	1.722056	1.719538	1.743096	1.740489	1.755135
1.722998	1.720466	1.717932	1.715411	1.712913	1.710413	1.717604	1.715046	1.738538
1.723639	1.721106	1.718579	1.716051	1.713522	1.71099	1.718188	1.715621	1.739136
1.724271	1.721737	1.719214	1.716679	1.714126	1.71157	1.70902	1.706472	1.713627
1.724863	1.72233	1.7198	1.717262	1.714711	1.712157	1.7096	1.707043	1.714215
1.725448	1.722918	1.720382	1.717842	1.715301	1.712754	1.710195	1.707631	1.714823
1.726064	1.72354	1.721011	1.718474	1.715929	1.713379	1.710825	1.708268	1.705707
1.726689	1.724173	1.721657	1.719126	1.716573	1.714017	1.711472	1.708928	1.706363
1.727274	1.724759	1.722247	1.719719	1.717168	1.714614	1.712073	1.709531	1.706967
1.727821	1.725303	1.722784	1.720256	1.717717	1.715173	1.71263	1.710083	1.707522
1.728378	1.725863	1.723345	1.720821	1.718291	1.715755	1.71321	1.710661	1.708105
1.728955	1.726455	1.72395	1.721436	1.718913	1.716381	1.713838	1.711289	1.708739
1.729537	1.727046	1.724551	1.722046	1.719527	1.716998	1.714458	1.711912	1.709368
1.730117	1.727613	1.725118	1.722613	1.72009	1.717561	1.715026	1.712488	1.709948
1.730703	1.72818	1.72568	1.72317	1.720644	1.718113	1.715585	1.713057	1.710524
1.731307	1.72878	1.726273	1.723761	1.721237	1.718712	1.716192	1.713674	1.711151
1.731923	1.729398	1.726883	1.724369	1.721851	1.719336	1.716826	1.714318	1.711809
1.732506	1.729978	1.727458	1.724942	1.722427	1.719919	1.717424	1.714934	1.712439
1.733051	1.730513	1.727991	1.725474	1.722959	1.720457	1.717983	1.71552	1.713042
1.73362	1.731077	1.728552	1.726034	1.723519	1.721021	1.718557	1.716105	1.713635
1.734248	1.731707	1.729174	1.726652	1.724139	1.721639	1.719158	1.716687	1.714208
1.734873	1.732338	1.729803	1.727281	1.724775	1.722276	1.719778	1.717286	1.714796
1.735442	1.73292	1.730401	1.727895	1.725408	1.722923	1.720429	1.717935	1.715444
1.735989	1.73348	1.730983	1.728501	1.726037	1.723573	1.721092	1.718605	1.716117
1.736549	1.734033	1.731547	1.729079	1.726634	1.724189	1.721724	1.719249	1.716771
1.737118	1.734577	1.732094	1.729636	1.727206	1.724779	1.722333	1.719877	1.717414
1.737738	1.735173	1.732683	1.730221	1.727788	1.725362	1.72292	1.720472	1.718022
1.73842	1.735832	1.733321	1.730837	1.728379	1.725931	1.723475	1.721023	1.718585
1.739111	1.736507	1.733979	1.731475	1.728992	1.726522	1.724052	1.721593	1.719157
1.739789	1.737181	1.734651	1.732141	1.729643	1.727158	1.724682	1.722216	1.719761
1.740471	1.737863	1.735333	1.732818	1.73031	1.727815	1.725333	1.72286	1.720382
1.741164	1.738557	1.736016	1.733492	1.73098	1.728479	1.725991	1.723509	1.721017
1.741863	1.739256	1.736699	1.734164	1.731652	1.729151	1.726656	1.724165	1.721665
1.742544	1.739926	1.737356	1.734815	1.732307	1.729812	1.727315	1.72482	1.722319
1.743208	1.740569	1.737989	1.735447	1.732948	1.730463	1.727969	1.725476	1.72298
1.743901	1.741238	1.738634	1.73608	1.733589	1.731114	1.728622	1.726128	1.723636
1.744636	1.741945	1.739289	1.736706	1.734223	1.731761	1.729269	1.726773	1.724282
1.739369	1.742667	1.739971	1.737363	1.734878	1.732419	1.729923	1.727423	1.724928
1.740089	1.743399	1.740702	1.738086	1.735582	1.733103	1.730594	1.728084	1.725581
1.74081	1.744138	1.741455	1.738836	1.736306	1.733799	1.731274	1.728752	1.726238
1.741536	1.744876	1.742195	1.739571	1.737022	1.734497	1.731959	1.729426	1.7269
1.742265	1.745613	1.742931	1.7403	1.737738	1.735197	1.732648	1.730105	1.727564
1.742975	1.746331	1.74365	1.741015	1.738441	1.735888	1.733328	1.730775	1.728226
1.743666	1.747028	1.74435	1.741715	1.739133	1.73657	1.733999	1.731436	1.728885

1.744368	1.74165	1.738981	1.74241	1.739824	1.737256	1.734675	1.7321	1.729546
1.745094	1.742364	1.739674	1.743102	1.740518	1.737951	1.735361	1.732775	1.730213
1.745826	1.743083	1.74037	1.743795	1.741212	1.738646	1.736047	1.733448	1.730878
1.746553	1.7438	1.74107	1.744494	1.741907	1.739335	1.73672	1.734105	1.731531
1.747281	1.744519	1.741774	1.745199	1.742604	1.740024	1.737393	1.734763	1.732185
1.725824	1.745242	1.742481	1.739791	1.737203	1.734628	1.738086	1.73545	1.732863
1.726564	1.745969	1.743194	1.740493	1.7379	1.735324	1.738792	1.736158	1.73356
1.67141	1.724557	1.721813	1.74124	1.738627	1.73603	1.739502	1.736865	1.734255
1.672066	1.725328	1.722616	1.742046	1.739392	1.736751	1.740217	1.737567	1.734947
1.660173	1.726103	1.723414	1.720737	1.718069	1.715415	1.734794	1.732141	1.735643
1.660844	1.726873	1.724178	1.721487	1.718791	1.716113	1.735486	1.732833	1.736344
1.661525	1.727652	1.724943	1.722233	1.719508	1.716807	1.736175	1.733527	1.737048
1.662217	1.659745	1.657278	1.723011	1.720237	1.717495	1.714837	1.712229	1.737753
1.662911	1.660446	1.657975	1.723798	1.720973	1.718188	1.715526	1.712928	1.738463
1.663567	1.661094	1.658616	1.656105	1.653529	1.650986	1.716265	1.713666	1.711073
1.664188	1.661694	1.659207	1.656704	1.654162	1.651651	1.71705	1.71444	1.711824
1.664824	1.66231	1.659813	1.657315	1.654804	1.652316	1.64988	1.647474	1.712562
1.665484	1.662955	1.660445	1.657947	1.655458	1.652982	1.650512	1.648062	1.713277
1.666146	1.663606	1.661087	1.658586	1.656111	1.65364	1.651141	1.648657	1.703734
1.666797	1.664256	1.661738	1.659237	1.656758	1.654282	1.651777	1.649287	1.704467
1.66745	1.664911	1.662391	1.659886	1.657398	1.654915	1.652415	1.649933	1.705212
1.668127	1.665579	1.663028	1.660503	1.658014	1.655539	1.653059	1.650596	1.648188
1.668815	1.666254	1.663656	1.661103	1.658617	1.656157	1.653706	1.651269	1.648841
1.669468	1.666908	1.664305	1.661748	1.659256	1.656792	1.654341	1.651903	1.649461
1.670083	1.667537	1.664974	1.662436	1.659933	1.657446	1.654965	1.652498	1.650045
1.670727	1.668188	1.665656	1.663128	1.660599	1.658083	1.655584	1.653105	1.650652
1.671419	1.668874	1.666354	1.663816	1.661238	1.658682	1.656191	1.653738	1.6513
1.672135	1.669581	1.667054	1.664499	1.661879	1.659293	1.656811	1.654384	1.651961
1.672878	1.670315	1.667744	1.665157	1.662533	1.659947	1.657464	1.655036	1.652619
1.673627	1.671054	1.668429	1.665813	1.663203	1.660634	1.658142	1.655701	1.653284
1.674322	1.671741	1.669114	1.666504	1.663917	1.661362	1.658855	1.656387	1.653964
[truncated: 505,789 more chars]
